# Supplementary figures and images for: Strong Components of Epigenetic Memory in Cultured Human Fibroblasts Related to Site of Origin and Donor Age
Source: PLoS Genet. 2016 Feb 25;12(2):e1005819. doi: 10.1371/journal.pgen.1005819 (PMC4767228; doi:10.1371/journal.pgen.1005819)

0.1yrs

85yrs

# Aging

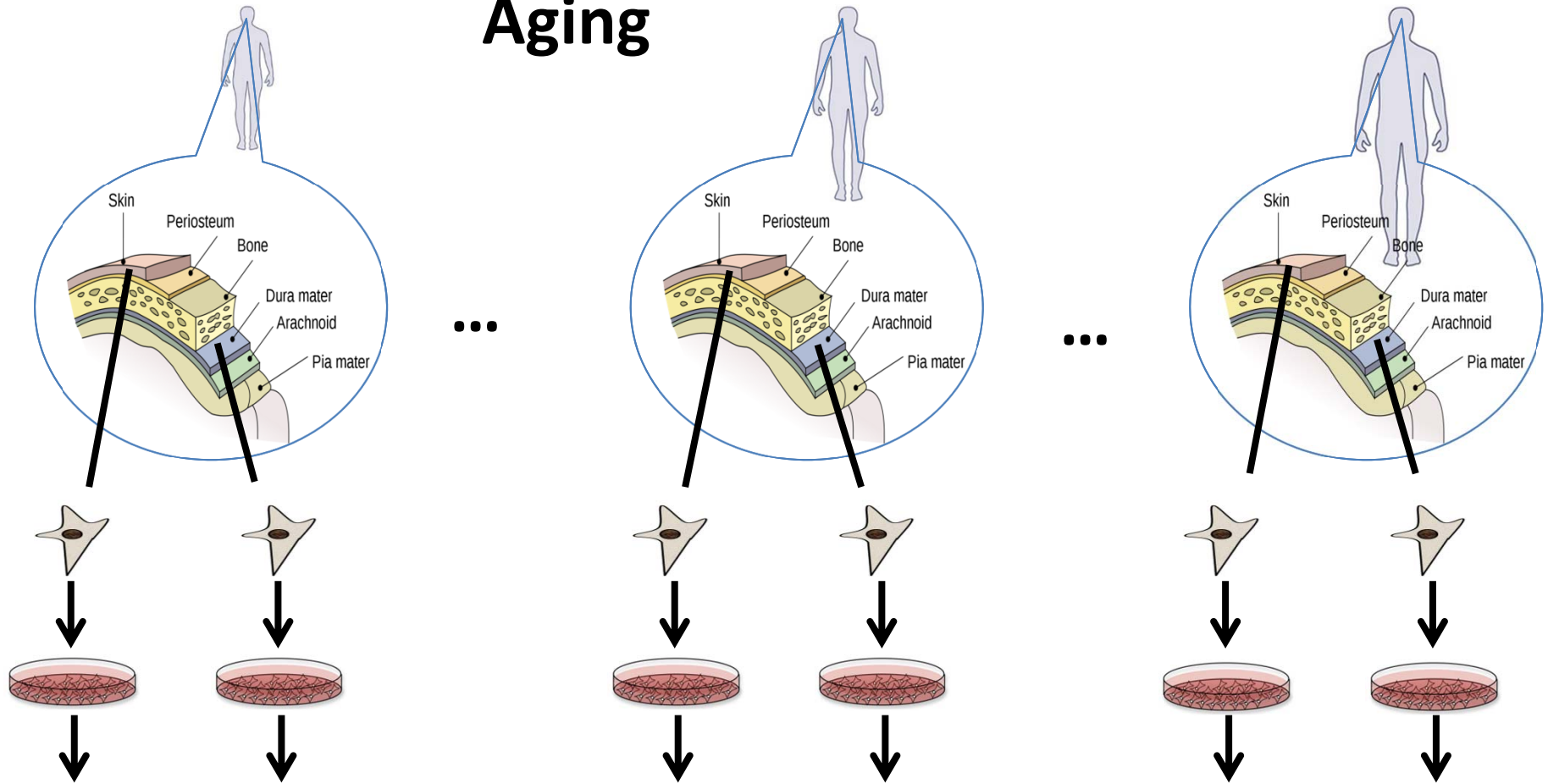

**DNA Methylation and RNA sequencing on 11 pairs of samples**

Supplement: S1 Fig — We took dura and scalp samples from 11 donors ranging from 0.1 to 85 years of age. We then extracted and cultured fibroblasts from these samples, and performed genome-wide DNA methylation and RNA sequencing procedures on these fibroblasts. (PDF) [file pgen.1005819.s001.pdf]

PC1: Explains 35.4% of Variation

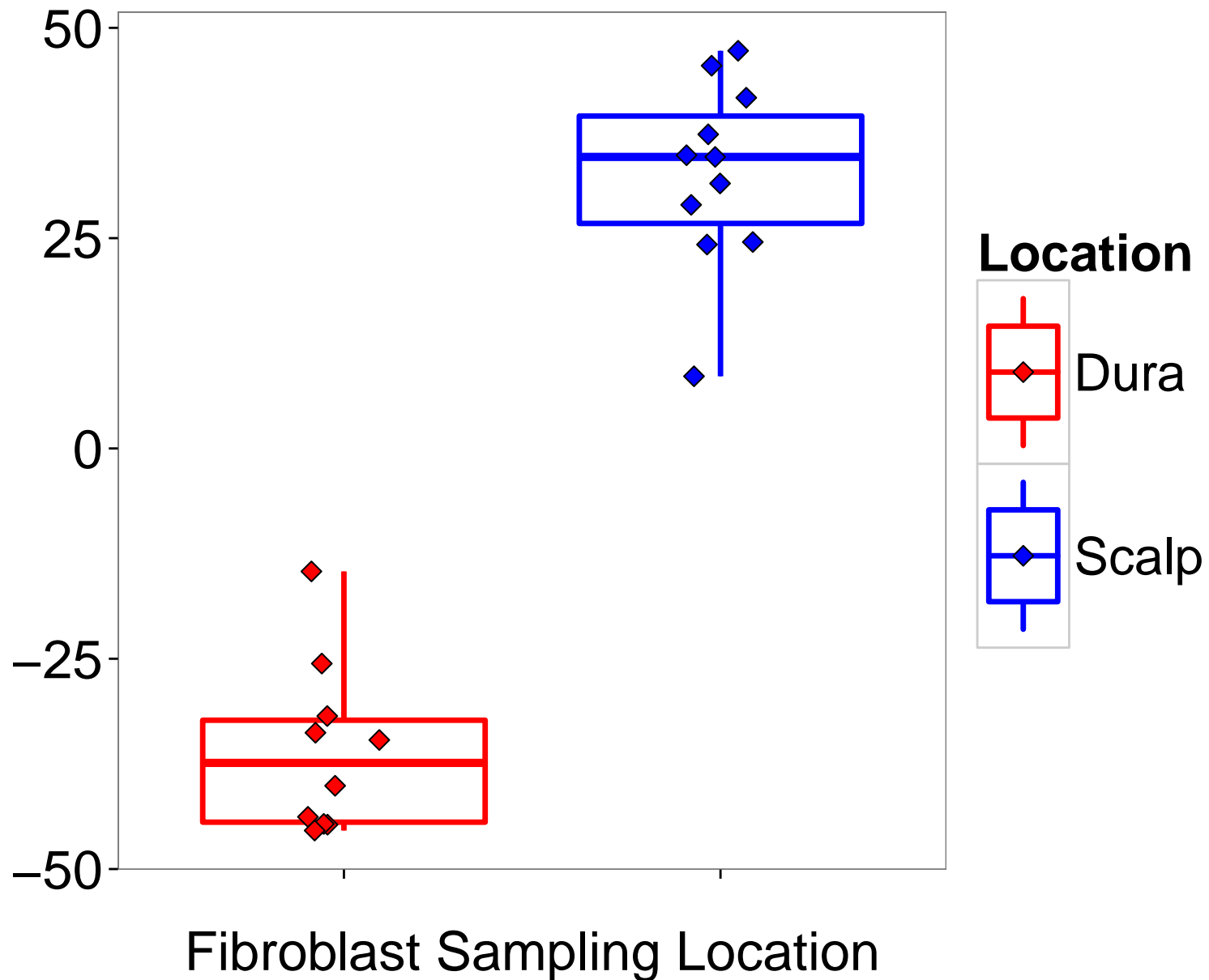

Supplement: S4 Fig — The first principal component (PC1) of the gene expression data plotted against fibroblast sampling location (scalp versus dura). The first PC of the gene expression data mimics the first PC of the DNAm data; both represent sampling location. (PDF) [file pgen.1005819.s004.pdf]

Cluster Dendrogram

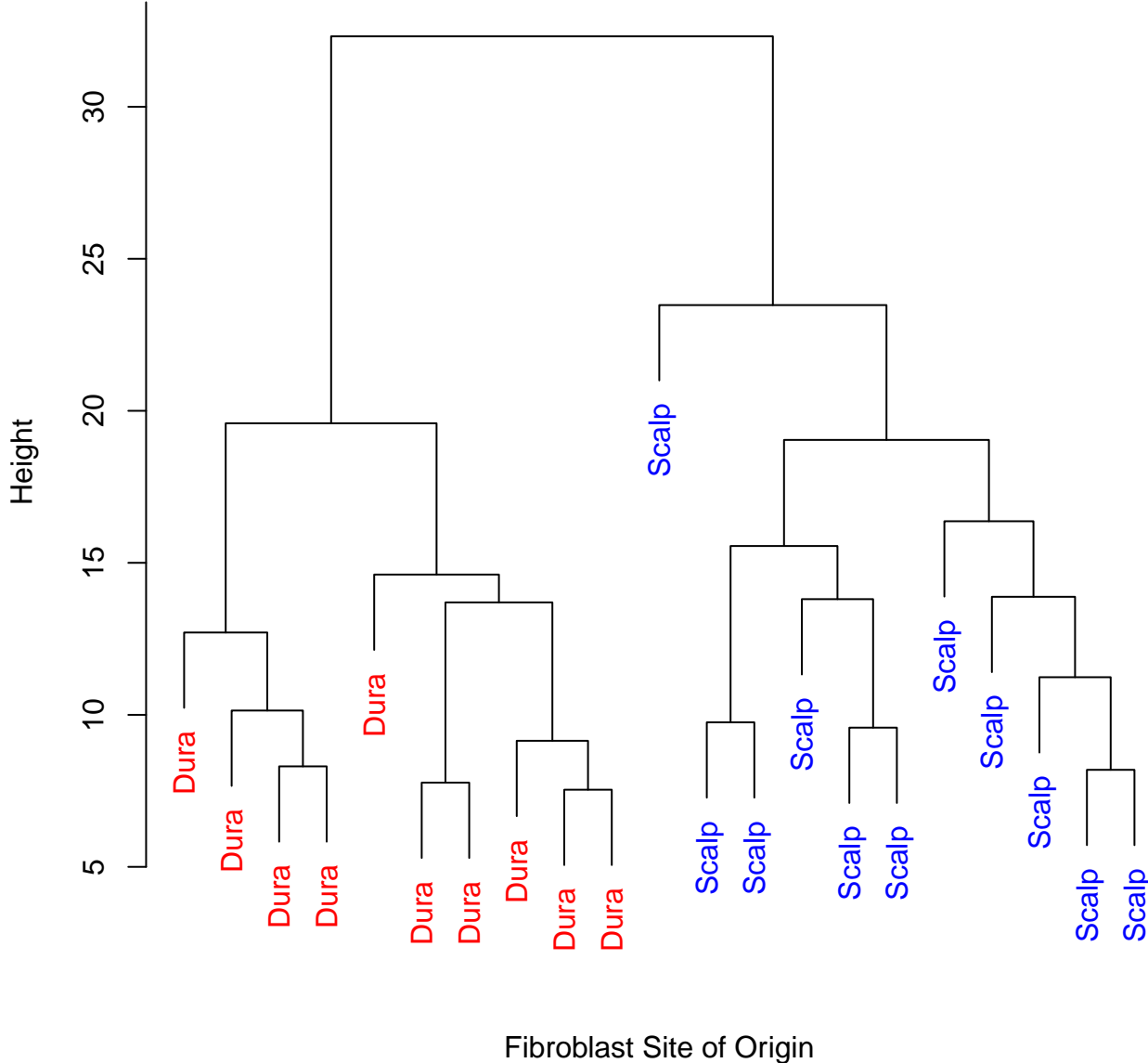

Supplement: S5 Fig — When we analyzed the expression of 210 genes (which were found to demarcate fibroblasts by anatomical site of origin (17)), our samples separated into categories by their sampling location. When these 210 genes were used, the mean Scalp-Dura Euclidean distance was 27.53; when we performed 1000 iterations taking random subsets of genes, the range of mean Scalp-Dura Euclidean distances was 7.58–14.67. (PDF) [file pgen.1005819.s005.pdf]

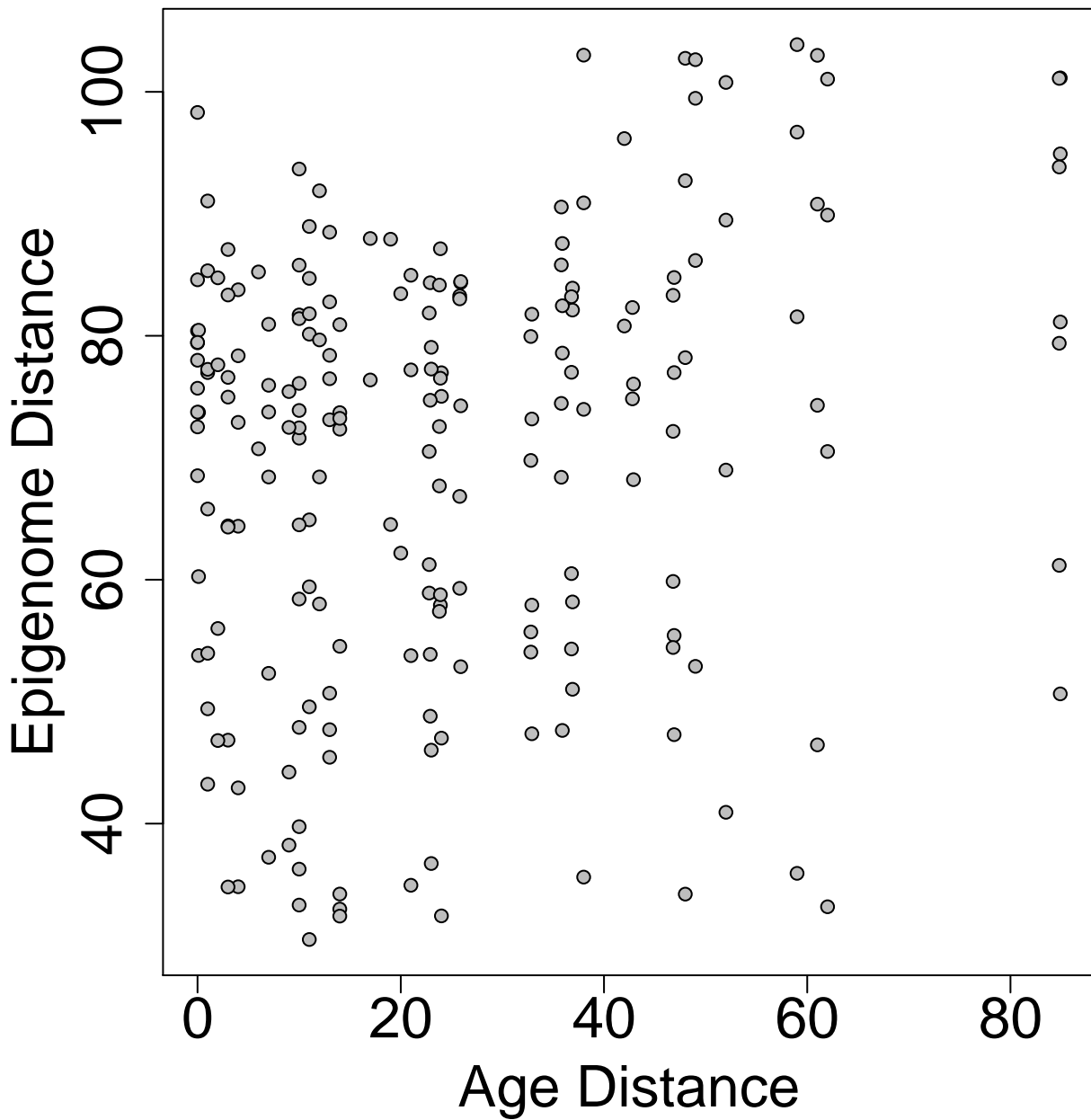

Supplement: S7 Fig — (PDF) [file pgen.1005819.s007.pdf]

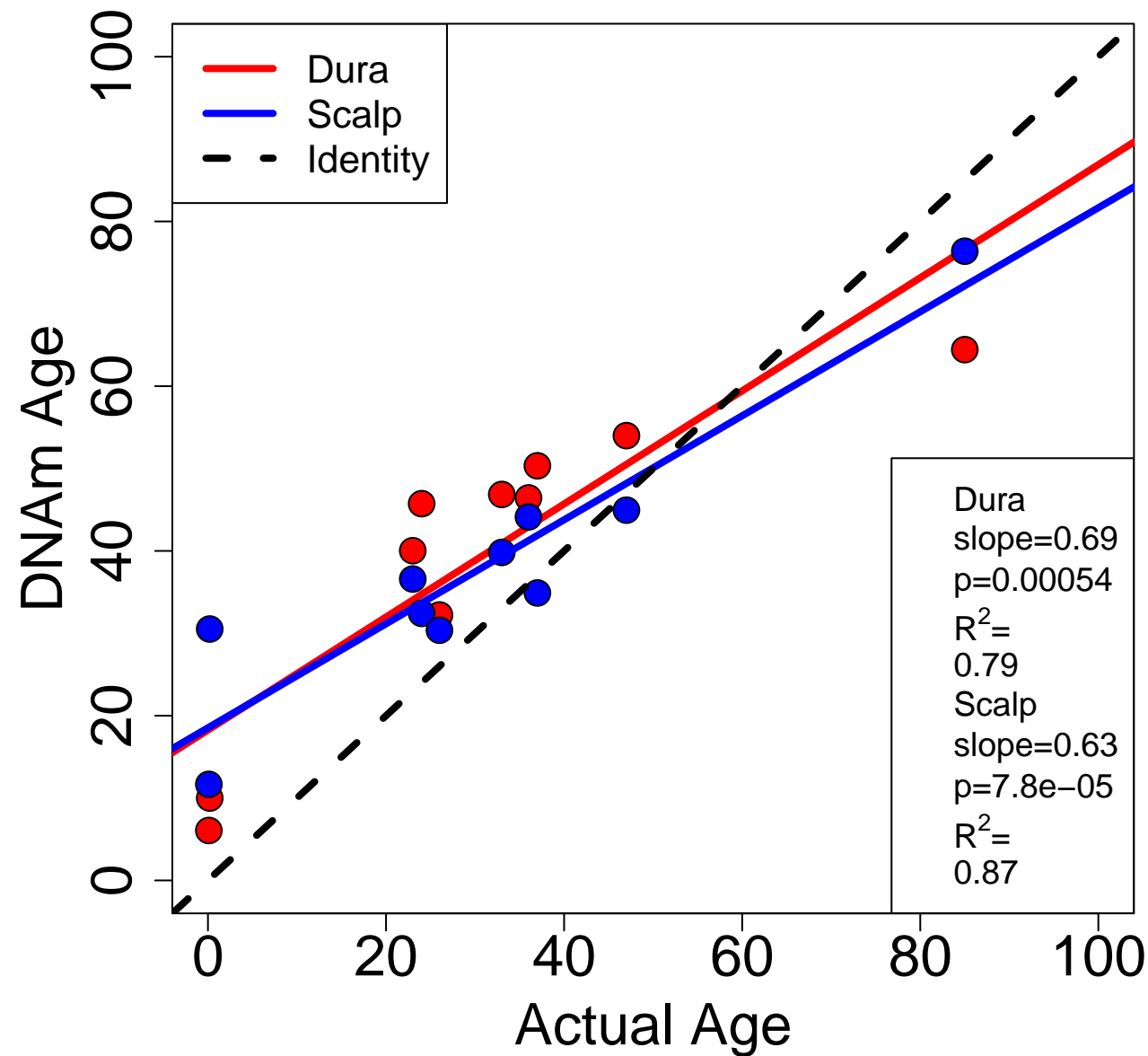

Supplement: S9 Fig — (PDF) [file pgen.1005819.s009.pdf]

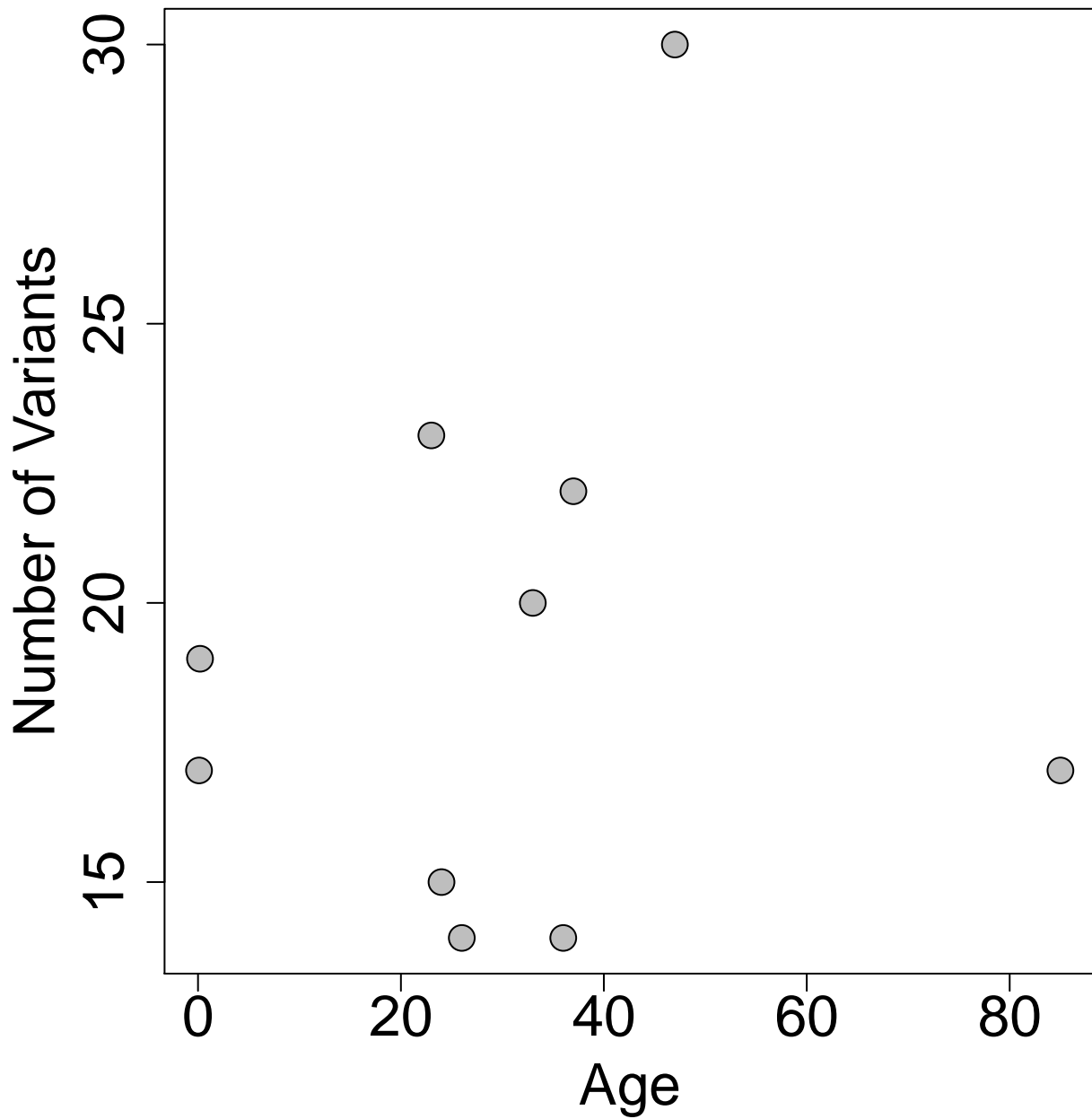

Supplement: S10 Fig — (PDF) [file pgen.1005819.s010.pdf]
