# Supplementary material for: Strong Components of Epigenetic Memory in Cultured Human Fibroblasts Related to Site of Origin and Donor Age
Source: PLoS Genet. 2016 Feb 25;12(2):e1005819. doi: 10.1371/journal.pgen.1005819 (PMC4767228; doi:10.1371/journal.pgen.1005819)
Supplement: S3 Fig — DNA methylation levels (proportion methylation) of all 243 significant differentially methylated blocks (FWER < 10%). (PDF) [file pgen.1005819.s003.pdf]

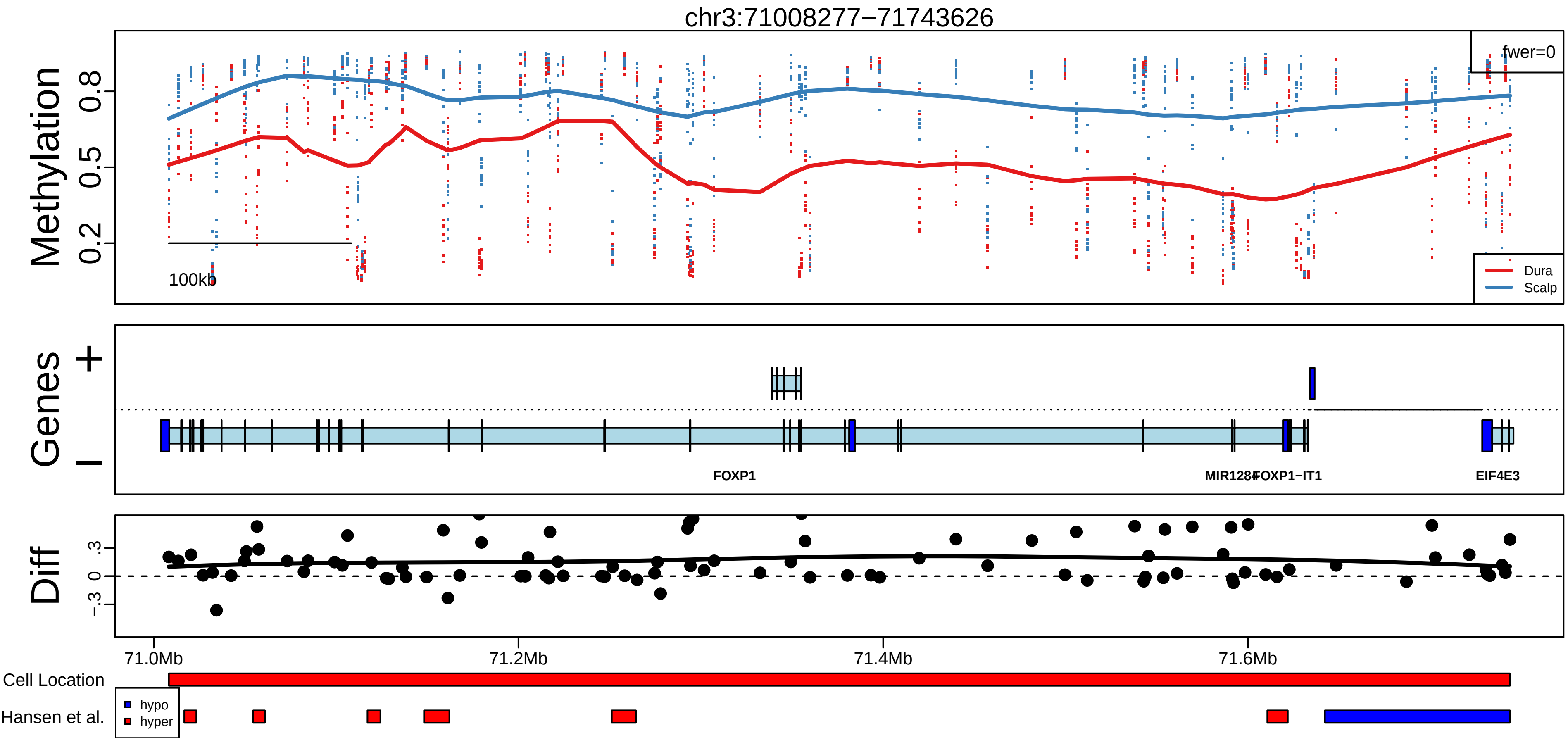

chr3:71008277−71743626
Methylation
0.2
0.5
0.8
fwer=0
100kb
Dura
Scalp
Genes
+
−
FOXP1
MIR1284
FOXP1−IT1
EIF4E3
Diff
−.3
0
.3
71.0Mb
71.2Mb
71.4Mb
71.6Mb
Cell Location
Hansen et al.
hypo
hyper

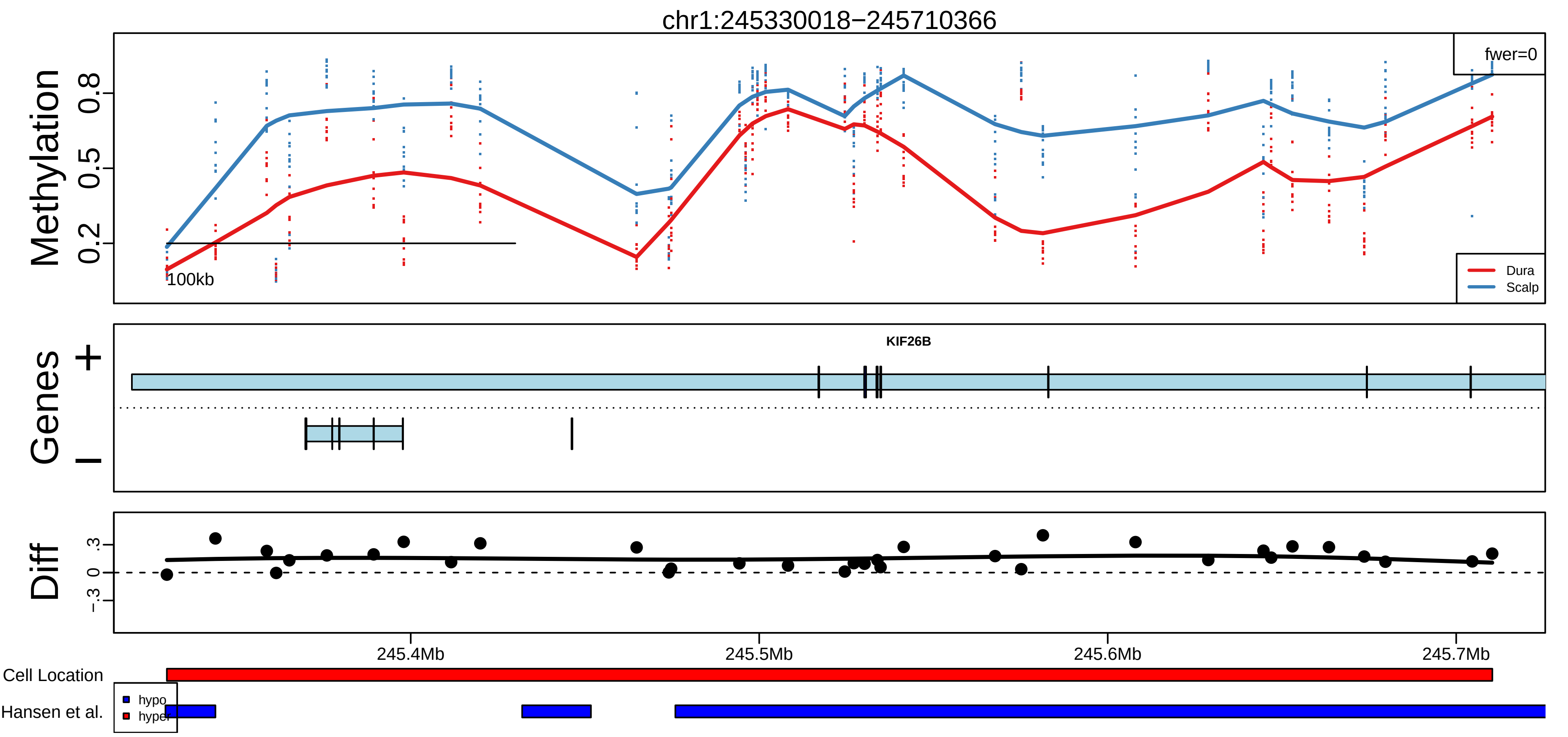

chr1:245330018−245710366
fwer=0
Methylation
0.2
0.5
0.8
100kb
Dura
Scalp
Genes
+
−
KIF26B
Diff
−.3
0
.3
245.4Mb
245.5Mb
245.6Mb
245.7Mb
Cell Location
Hansen et al.
hypo
hyper

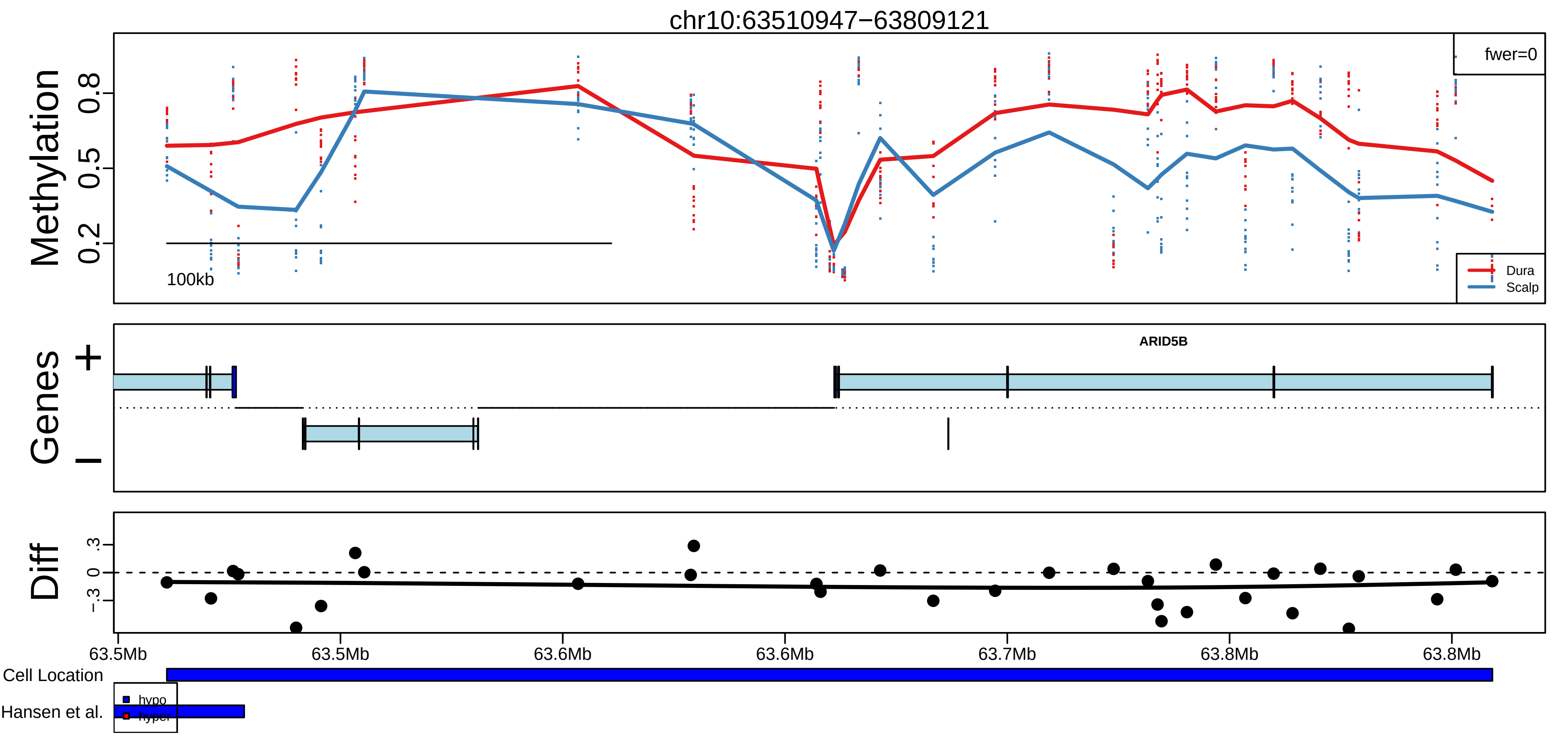

chr10:63510947−63809121
Methylation
0.2
0.5
0.8
fwer=0
100kb
Dura
Scalp
Genes
+
−
ARID5B
Diff
−.3
0
.3
63.5Mb
63.5Mb
63.6Mb
63.6Mb
63.7Mb
63.8Mb
63.8Mb
Cell Location
Hansen et al.
hypo
hyper

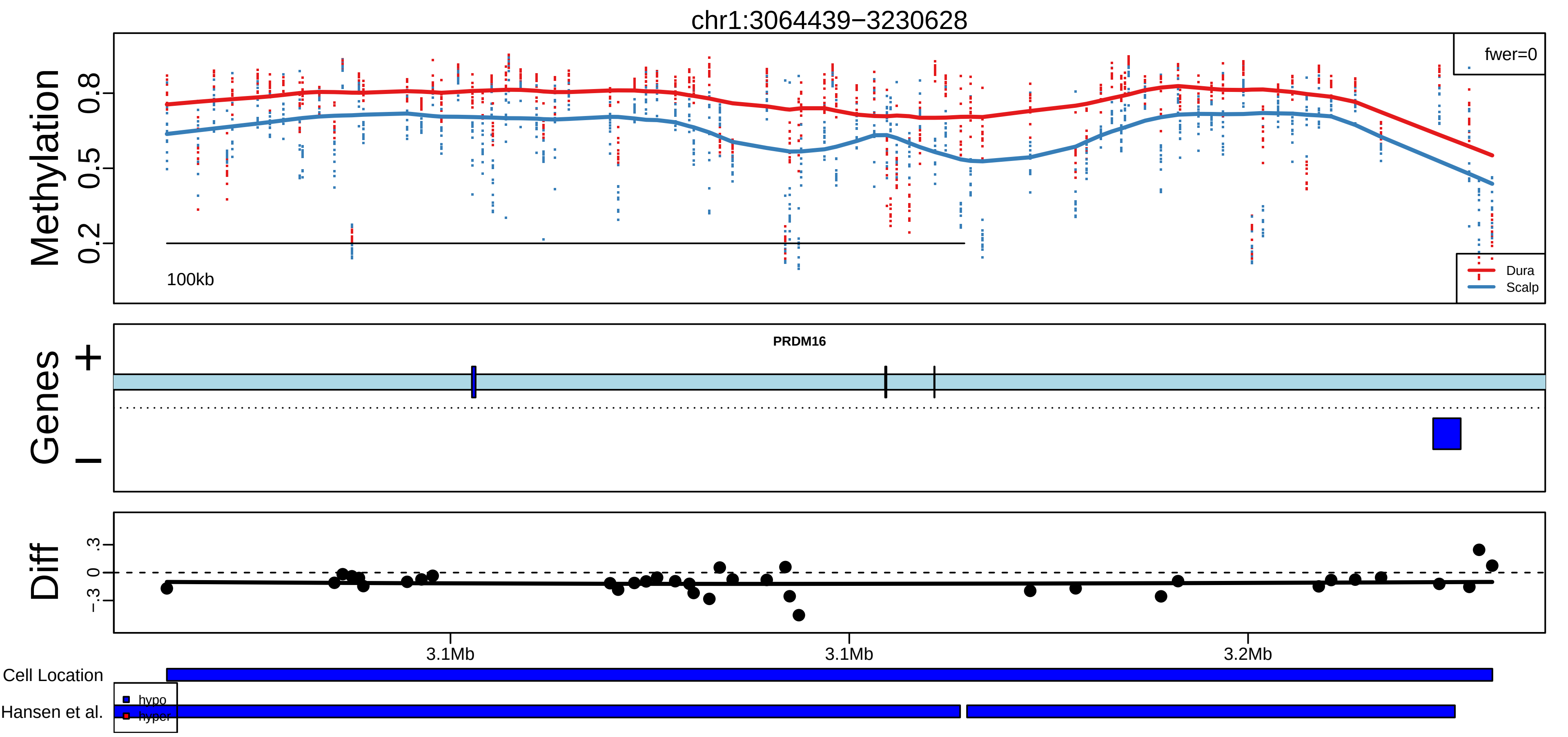

chr1:3064439−3230628
Methylation
0.2
0.5
0.8
fwer=0
100kb
Dura
Scalp
Genes
+
−
PRDM16
Diff
−.3
0
.3
3.1Mb
3.1Mb
3.2Mb
Cell Location
Hansen et al.
hypo
hyper

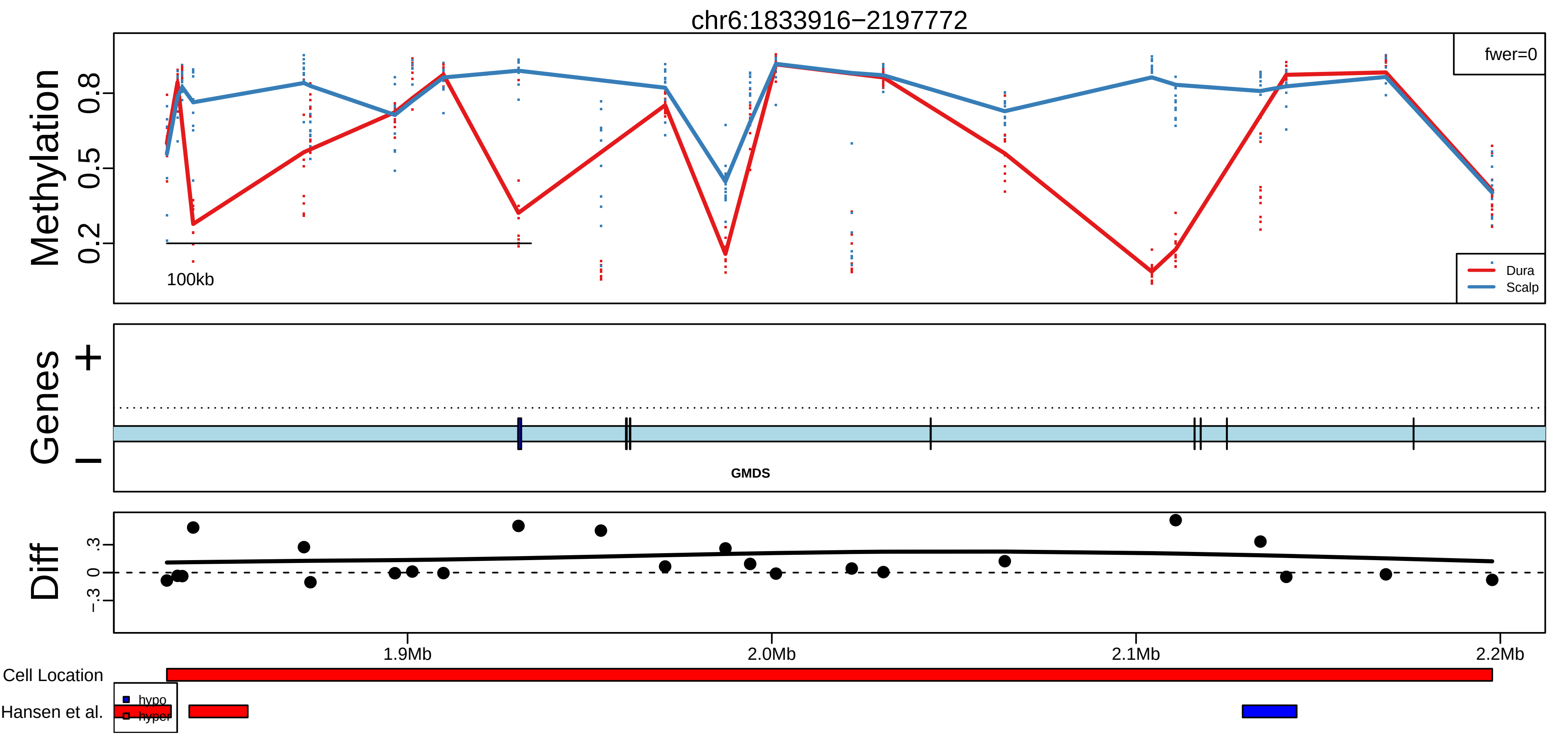
chr6:1833916−2197772
Methylation
0.2
0.5
0.8
fwer=0
100kb
Dura
Scalp
Genes
+
−
GMDS
Diff
−.3
0
.3
1.9Mb
2.0Mb
2.1Mb
2.2Mb
Cell Location
Hansen et al.
hypo
hyper

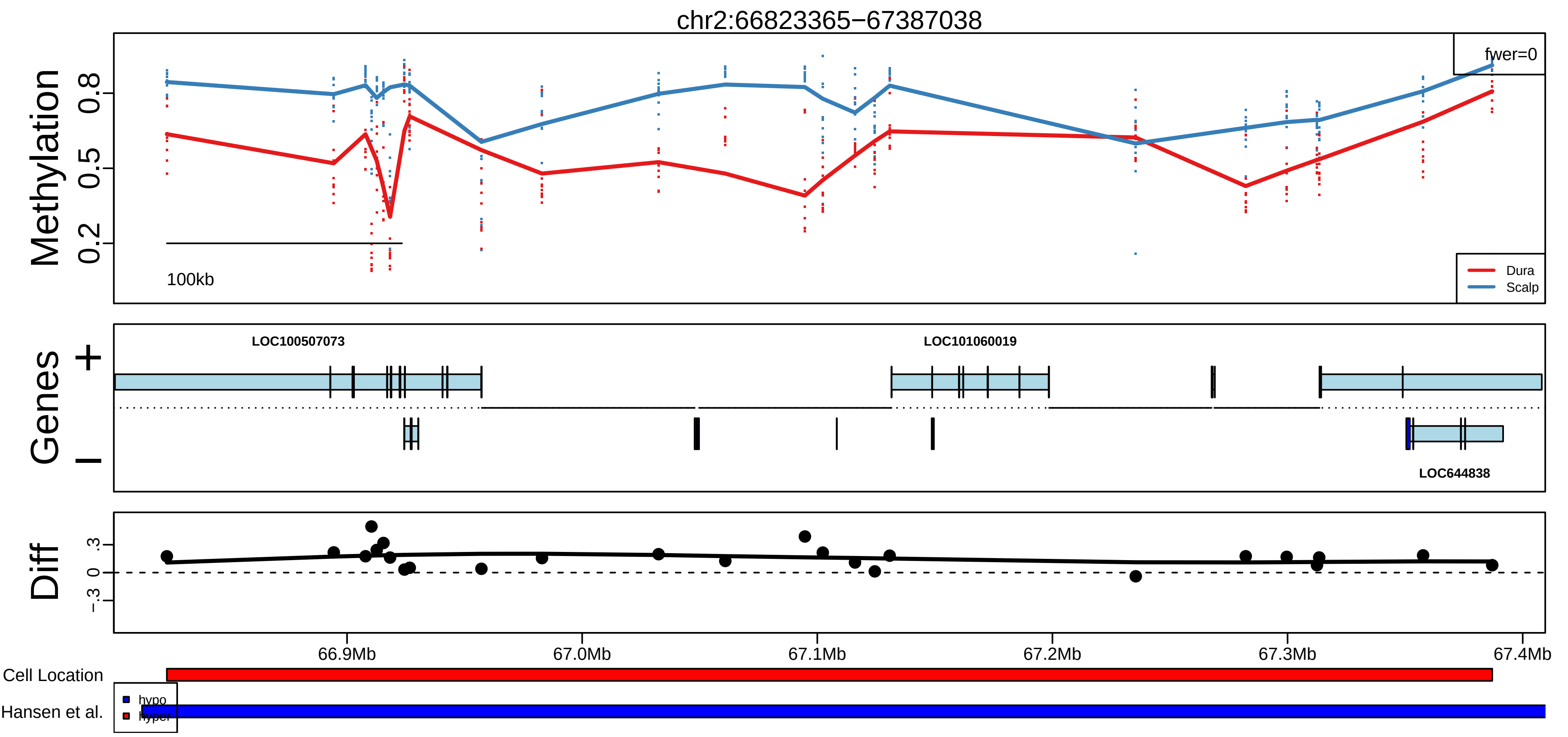
chr2:66823365−67387038
fwer=0
Methylation
0.2
0.5
0.8
100kb
Dura
Scalp
Genes
+
−
LOC100507073
LOC101060019
LOC644838
Diff
.3
0
−.3
66.9Mb
67.0Mb
67.1Mb
67.2Mb
67.3Mb
67.4Mb
Cell Location
Hansen et al.
hypo
hyper

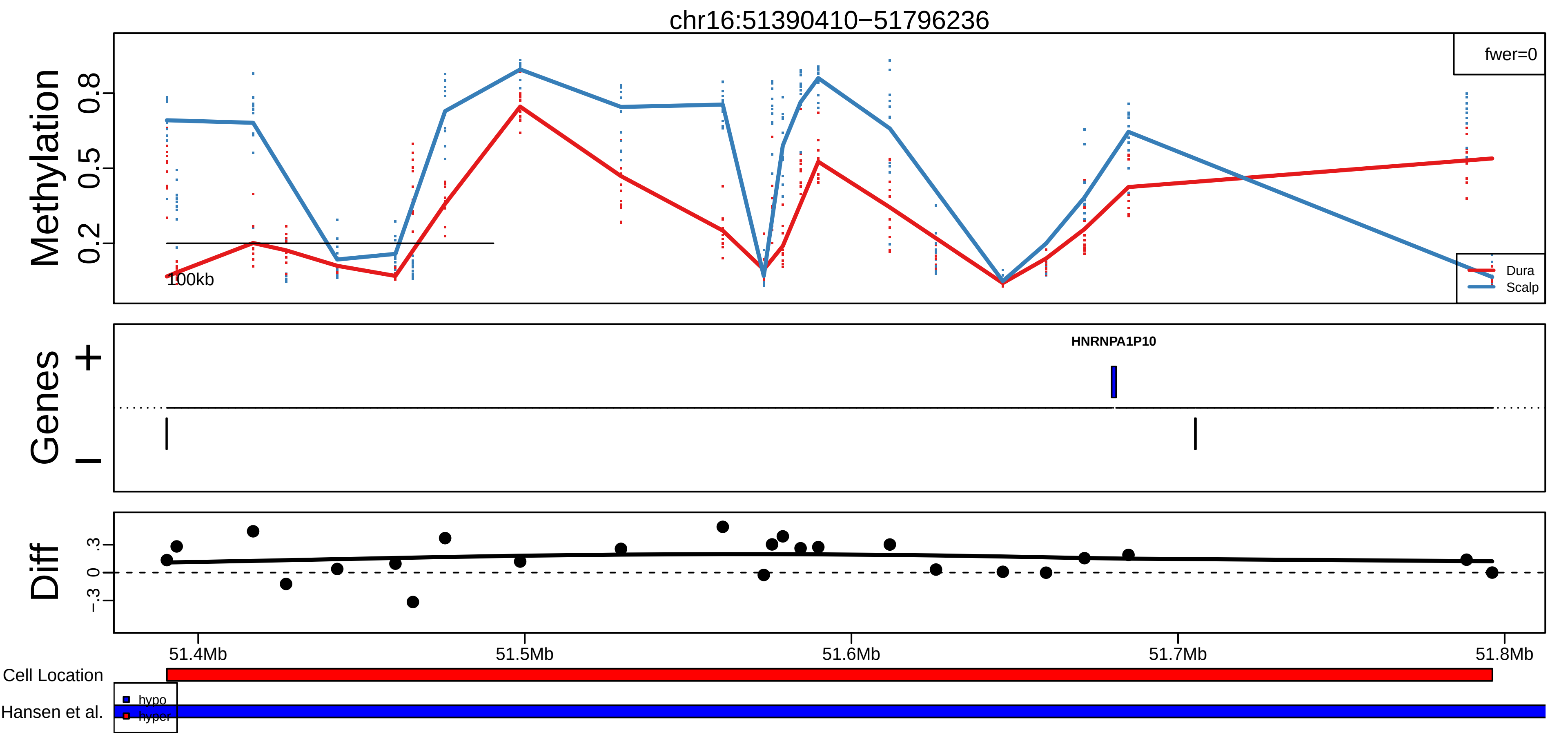
chr16:51390410−51796236
fwer=0
Methylation
0.2
0.5
0.8
100kb
Dura
Scalp
Genes
+
−
HNRNPA1P10
Diff
−.3
0
.3
51.4Mb
51.5Mb
51.6Mb
51.7Mb
51.8Mb
Cell Location
Hansen et al.
hypo
hyper

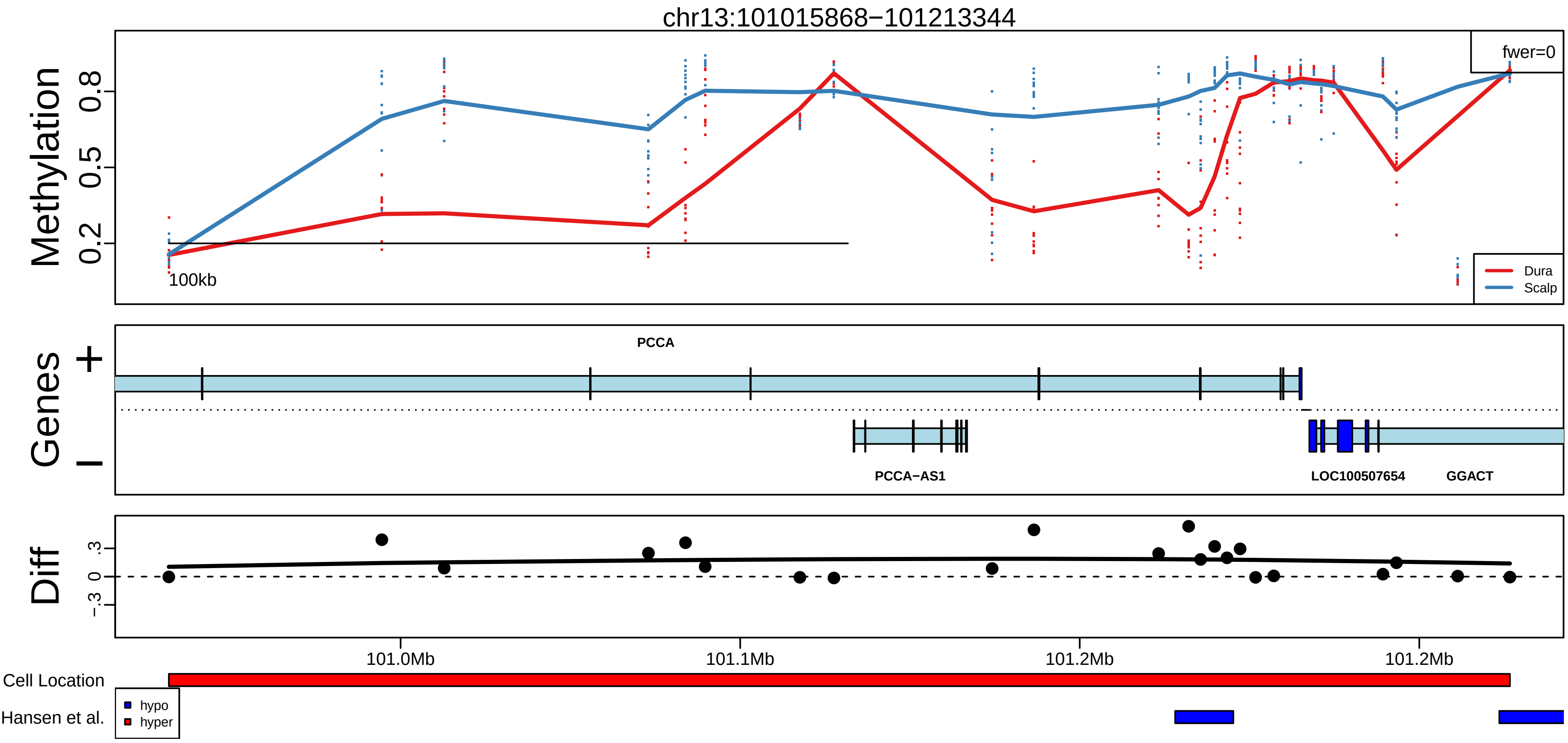
chr13:101015868−101213344
fwer=0
Methylation
0.2
0.5
0.8
100kb
Dura
Scalp
Genes
+
−
PCCA
PCCA−AS1
LOC100507654
GGACT
Diff
.3
0
−.3
101.0Mb
101.1Mb
101.2Mb
101.2Mb
Cell Location
Hansen et al.
hypo
hyper

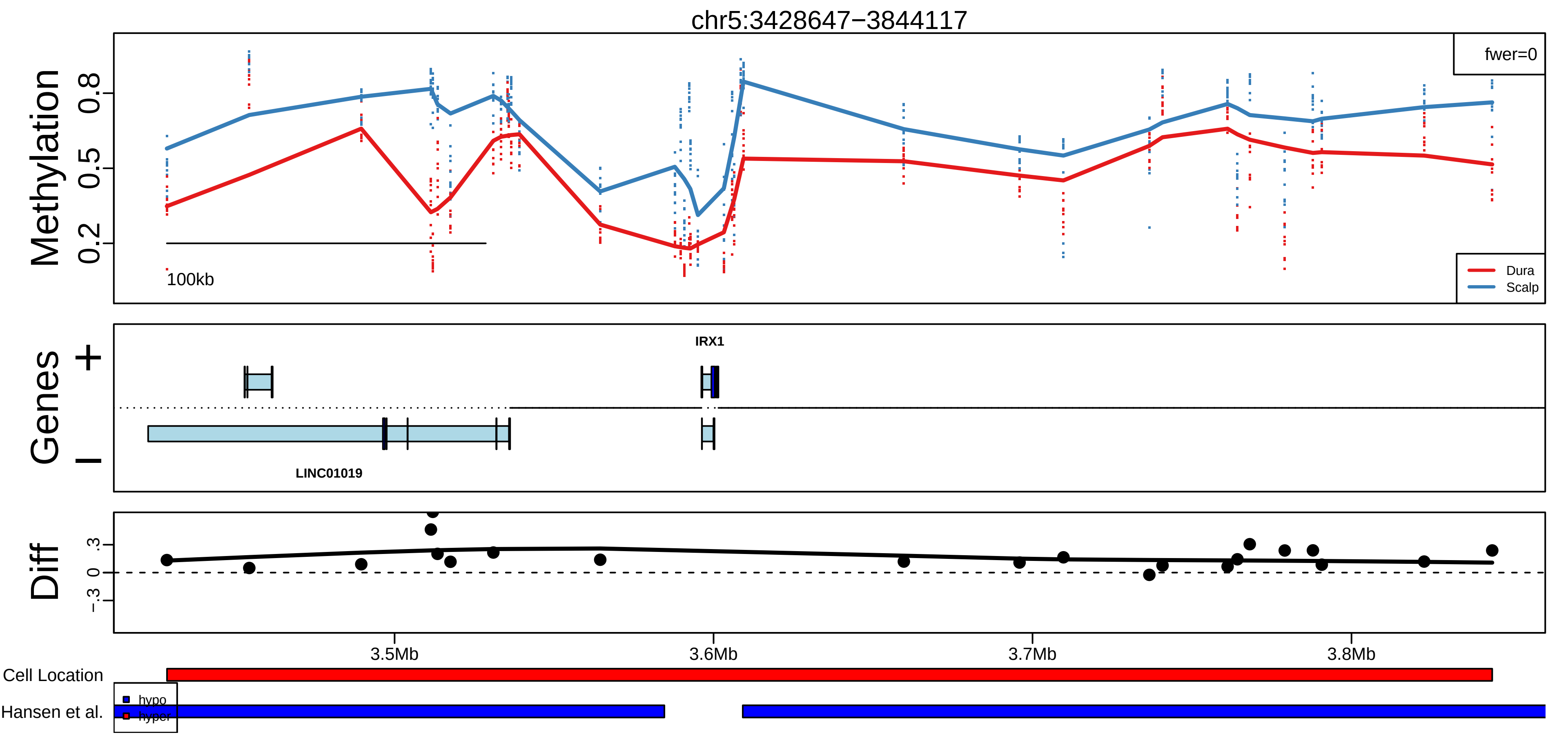
chr5:3428647−3844117
Methylation
0.2
0.5
0.8
fwer=0
100kb
Dura
Scalp
Genes
+
−
IRX1
LINC01019
Diff
.3
0
−.3
3.5Mb
3.6Mb
3.7Mb
3.8Mb
Cell Location
Hansen et al.
hypo
hyper

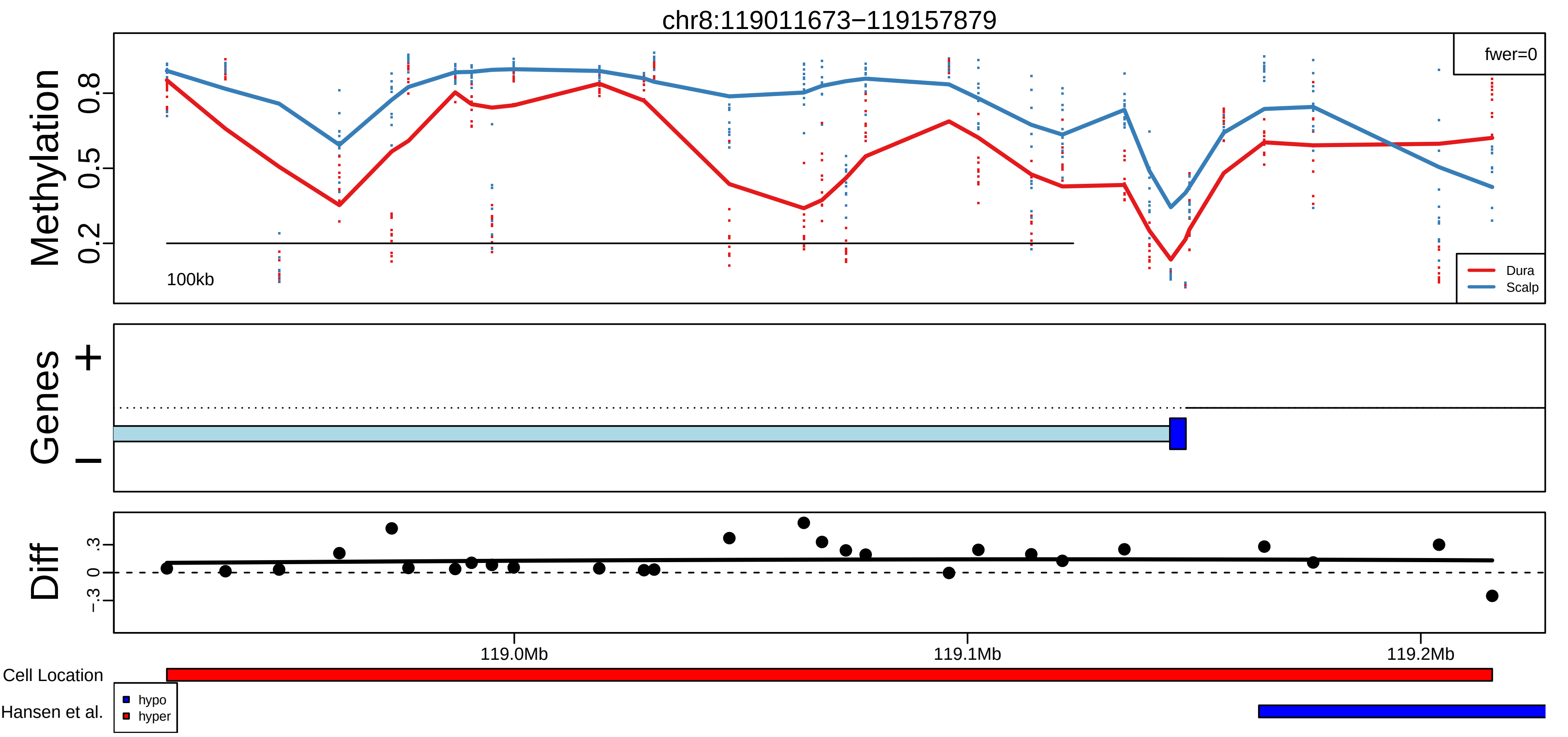

chr8:119011673−119157879
Methylation
0.2
0.5
0.8
fwer=0
100kb
Dura
Scalp
Genes
+
−
Diff
.3
0
−.3
119.0Mb
119.1Mb
119.2Mb
Cell Location
Hansen et al.
hypo
hyper

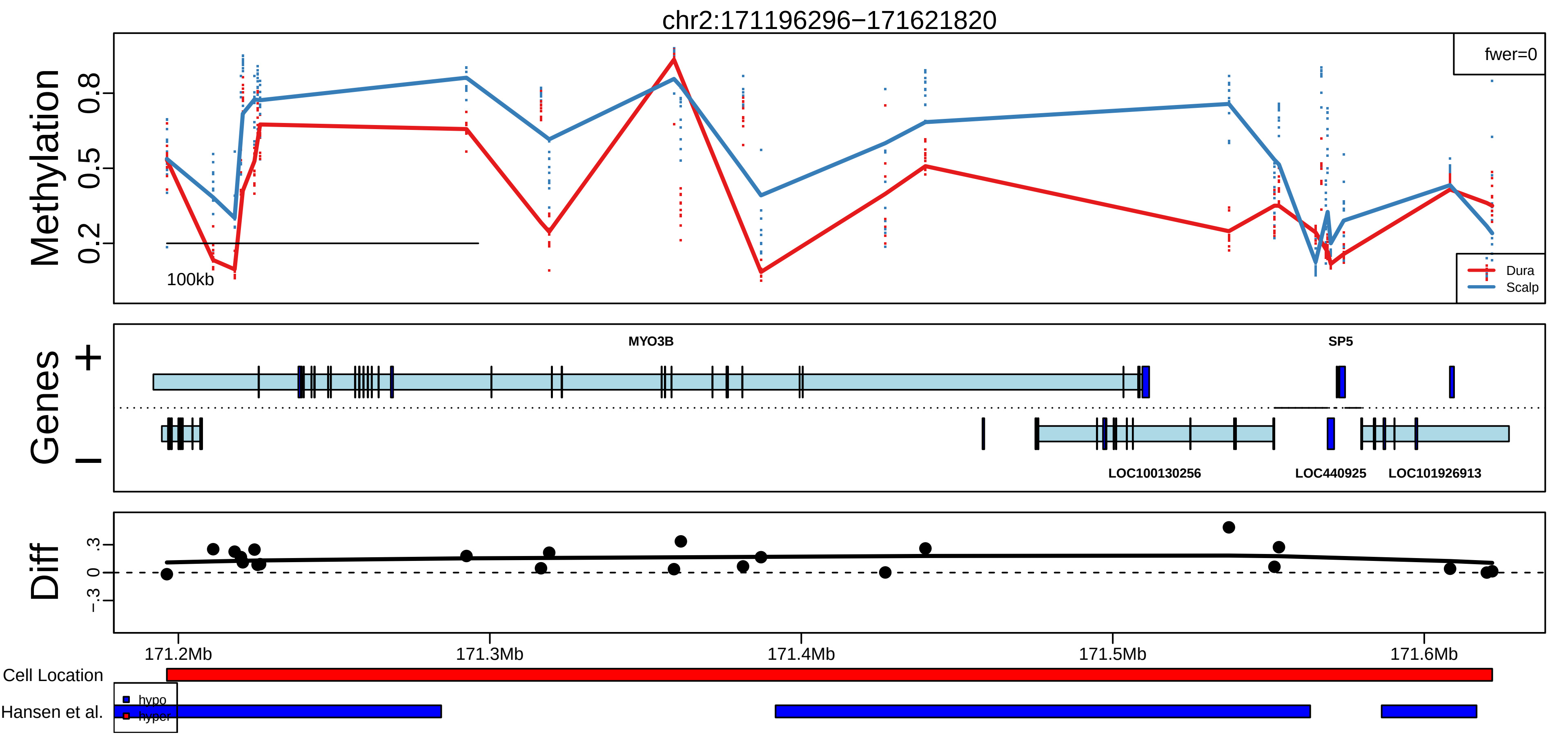

chr2:171196296−171621820
Methylation
0.2
0.5
0.8
fwer=0
100kb
Dura
Scalp
Genes
+
−
MYO3B
SP5
LOC100130256
LOC440925
LOC101926913
Diff
−.3
0
.3
171.2Mb
171.3Mb
171.4Mb
171.5Mb
171.6Mb
Cell Location
Hansen et al.
hypo
hyper

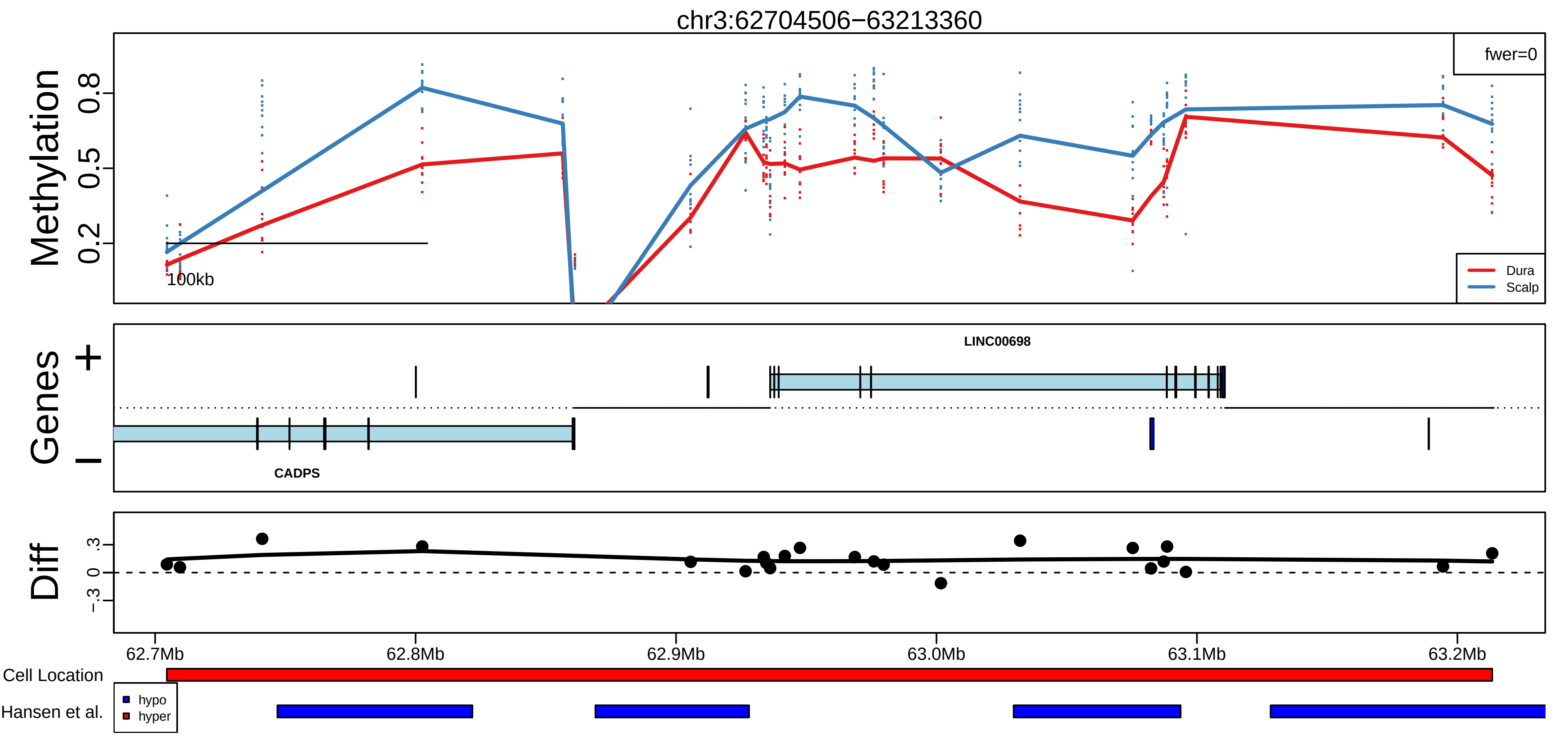
chr3:62704506−63213360
fwer=0
Methylation
0.2
0.5
0.8
100kb
Dura
Scalp
Genes
+
−
LINC00698
CADPS
Diff
−.3
0
.3
62.7Mb
62.8Mb
62.9Mb
63.0Mb
63.1Mb
63.2Mb
Cell Location
Hansen et al.
hypo
hyper

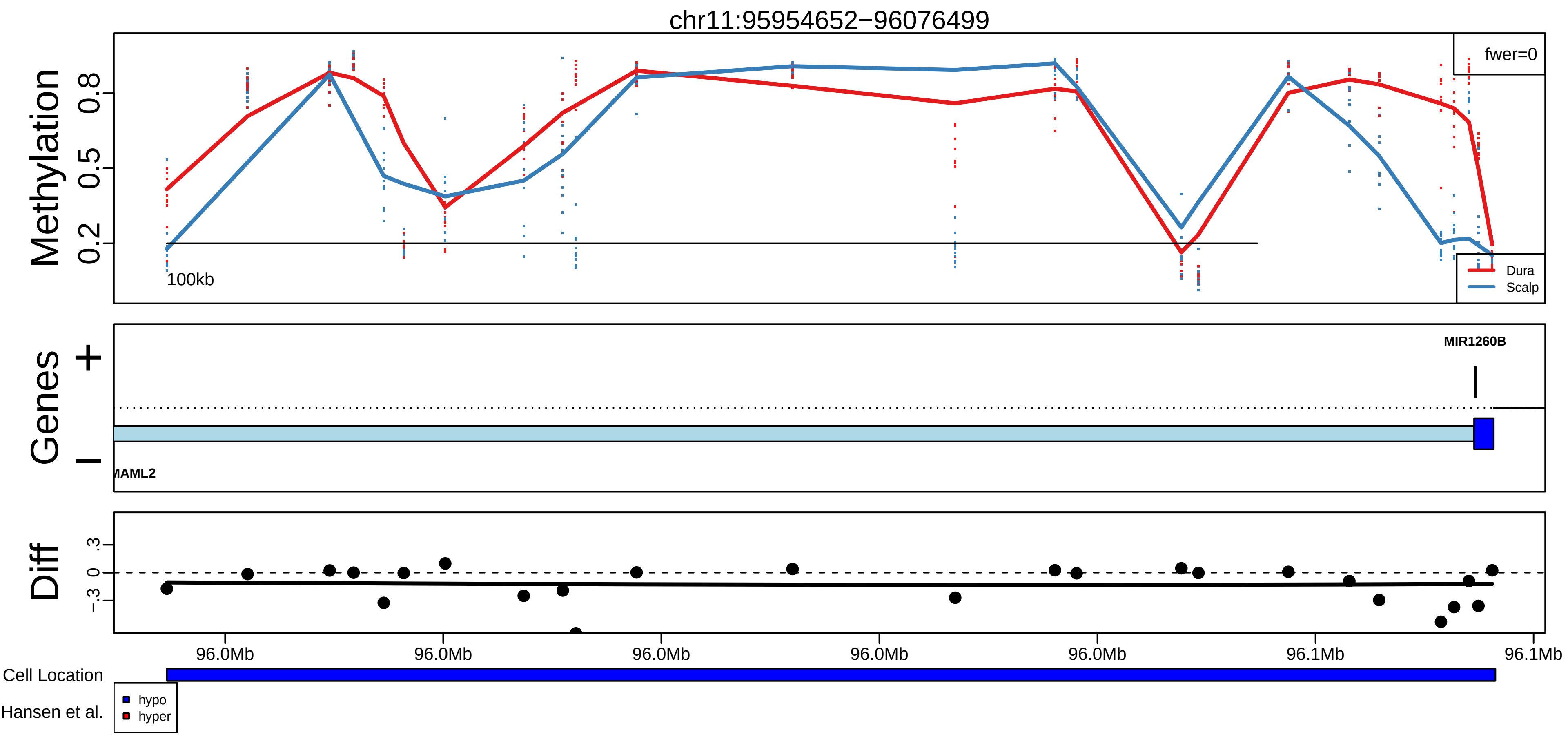

chr11:95954652−96076499
Methylation
0.2
0.5
0.8
fwer=0
100kb
Dura
Scalp
Genes
+
−
MIR1260B
MAML2
Diff
−.3
0
.3
96.0Mb
96.0Mb
96.0Mb
96.0Mb
96.0Mb
96.1Mb
96.1Mb
Cell Location
Hansen et al.
hypo
hyper

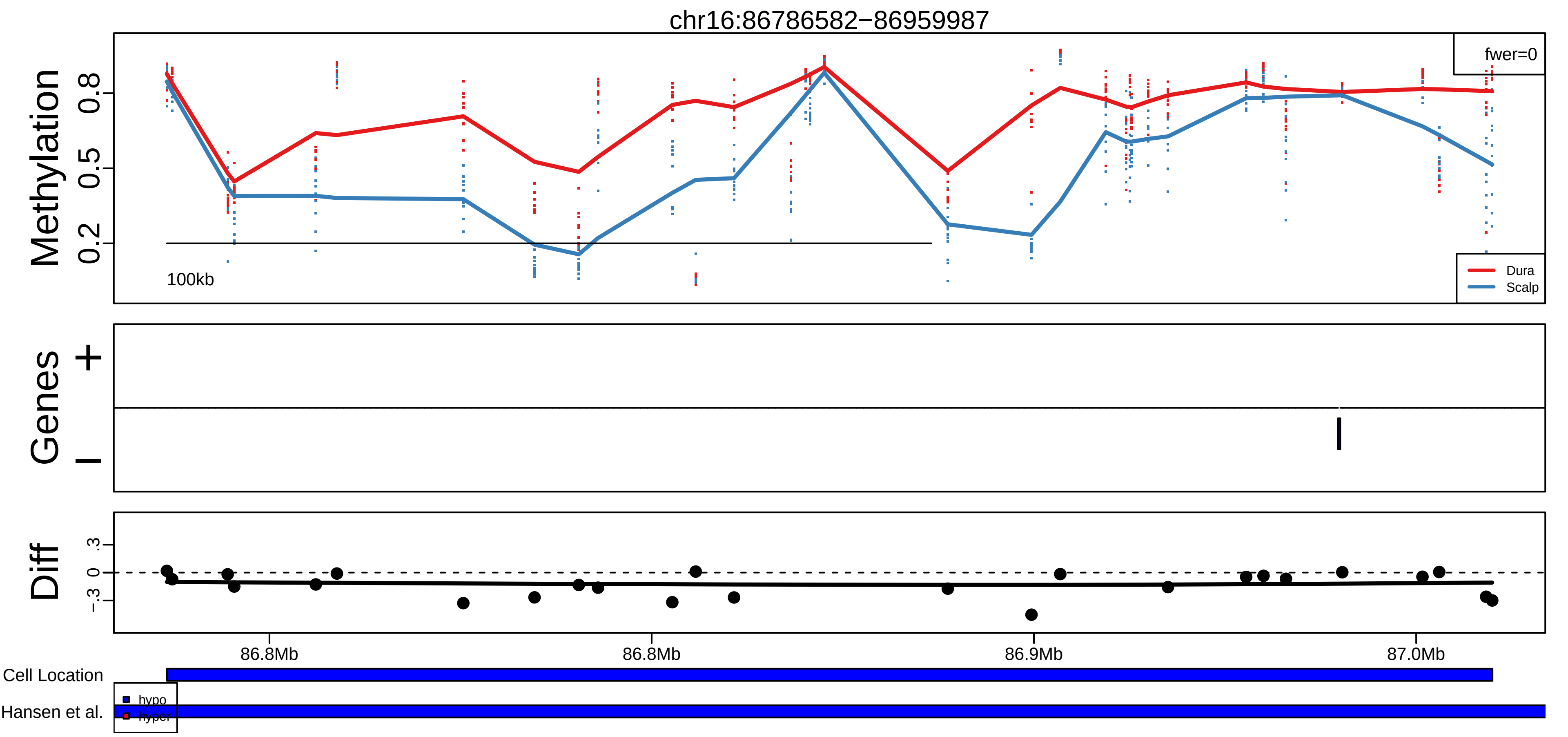

chr16:86786582−86959987
Methylation
0.2
0.5
0.8
fwer=0
100kb
Dura
Scalp
Genes
+
−
Diff
−.3
0
.3
86.8Mb
86.8Mb
86.9Mb
87.0Mb
Cell Location
Hansen et al.
hypo
hyper

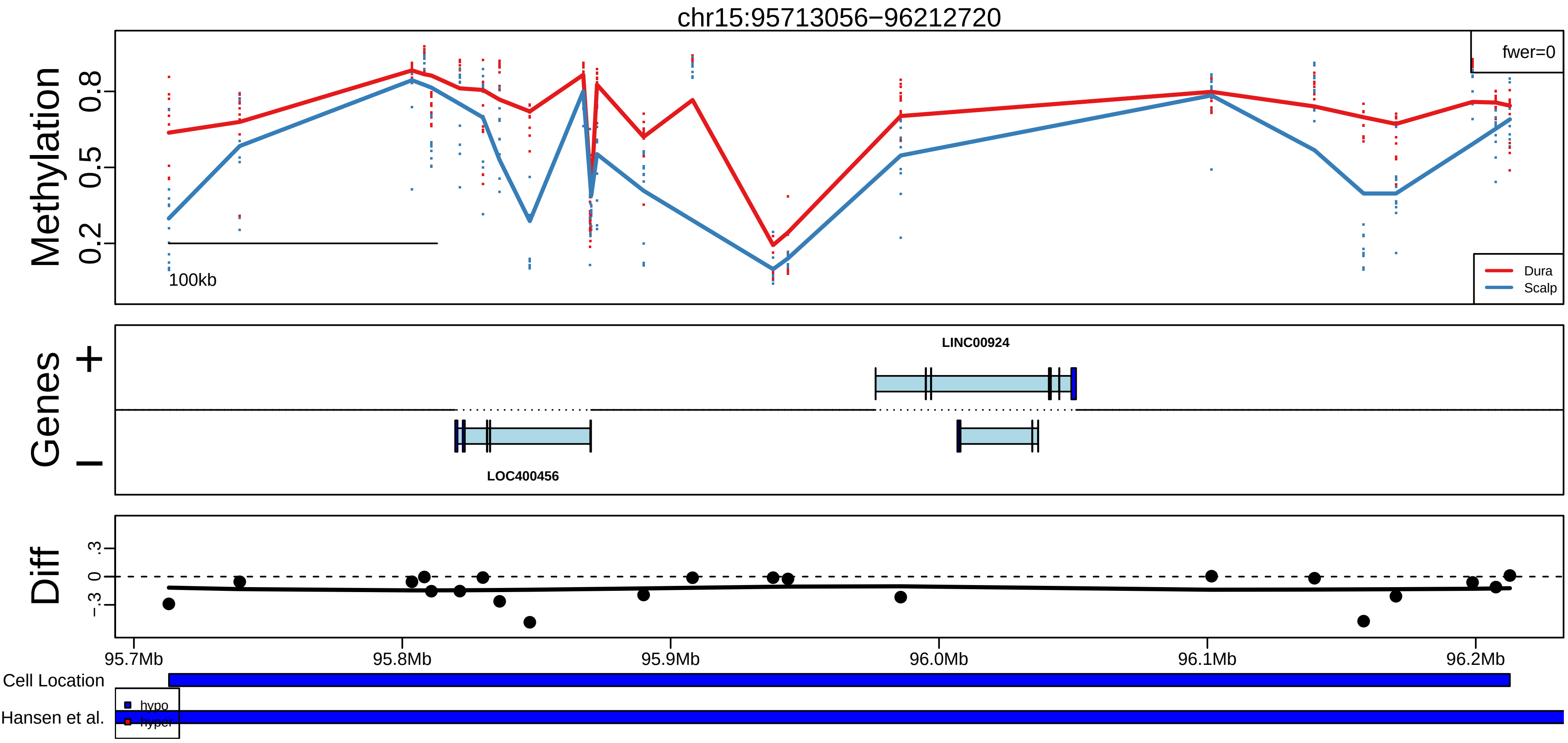

chr15:95713056−96212720
fwer=0
Methylation
0.2
0.5
0.8
100kb
Dura
Scalp
Genes
+
−
LINC00924
LOC400456
Diff
−.3
0
.3
95.7Mb
95.8Mb
95.9Mb
96.0Mb
96.1Mb
96.2Mb
Cell Location
Hansen et al.
hypo
hyper

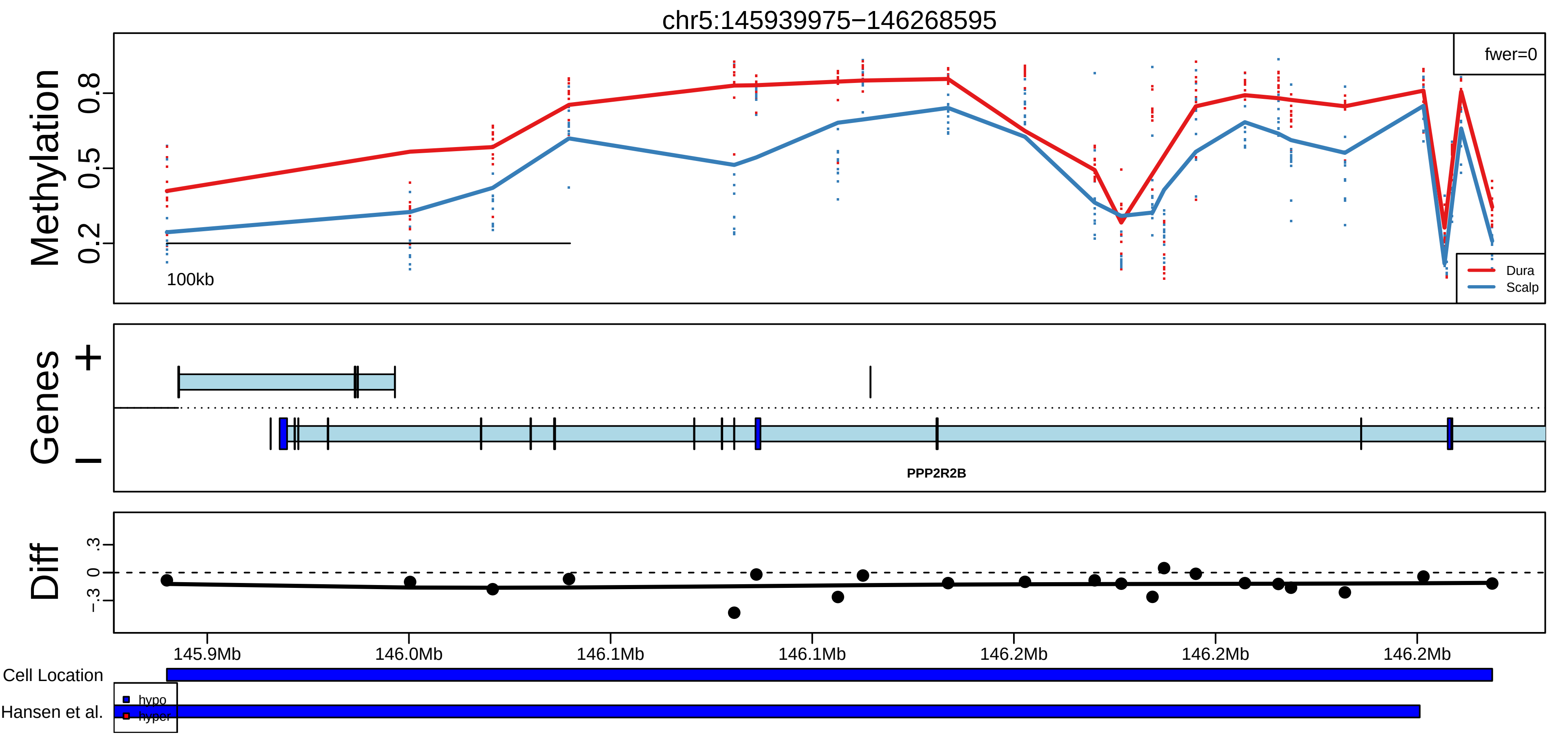

chr5:145939975−146268595
Methylation
0.2
0.5
0.8
fwer=0
100kb
Dura
Scalp
Genes
+
−
PPP2R2B
Diff
−.3
0
.3
145.9Mb
146.0Mb
146.1Mb
146.1Mb
146.2Mb
146.2Mb
146.2Mb
Cell Location
Hansen et al.
hypo
hyper

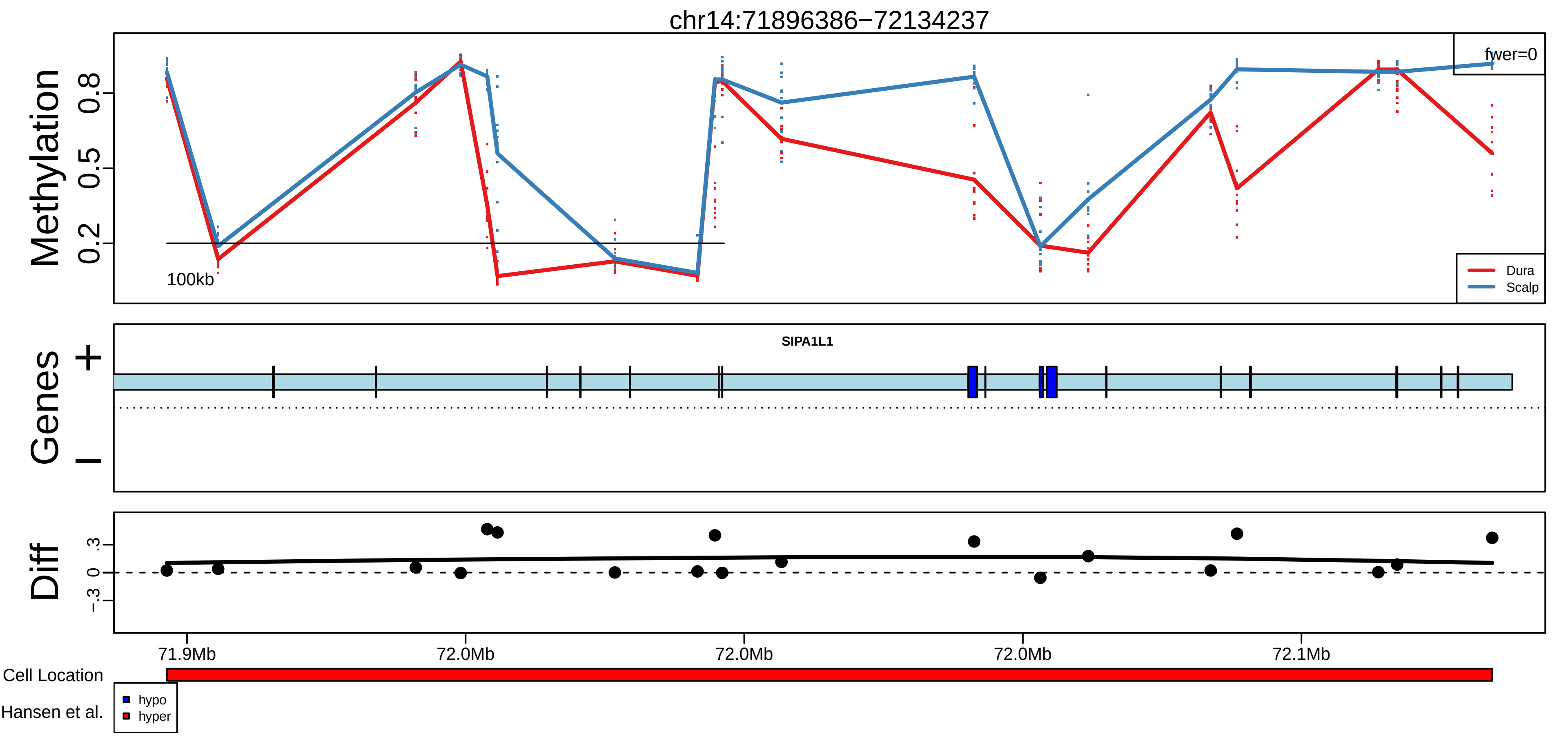

chr14:71896386−72134237
Methylation
0.2
0.5
0.8
fwer=0
100kb
Dura
Scalp
Genes
+
−
SIPA1L1
Diff
−.3
0
.3
71.9Mb
72.0Mb
72.0Mb
72.0Mb
72.1Mb
Cell Location
Hansen et al.
hypo
hyper

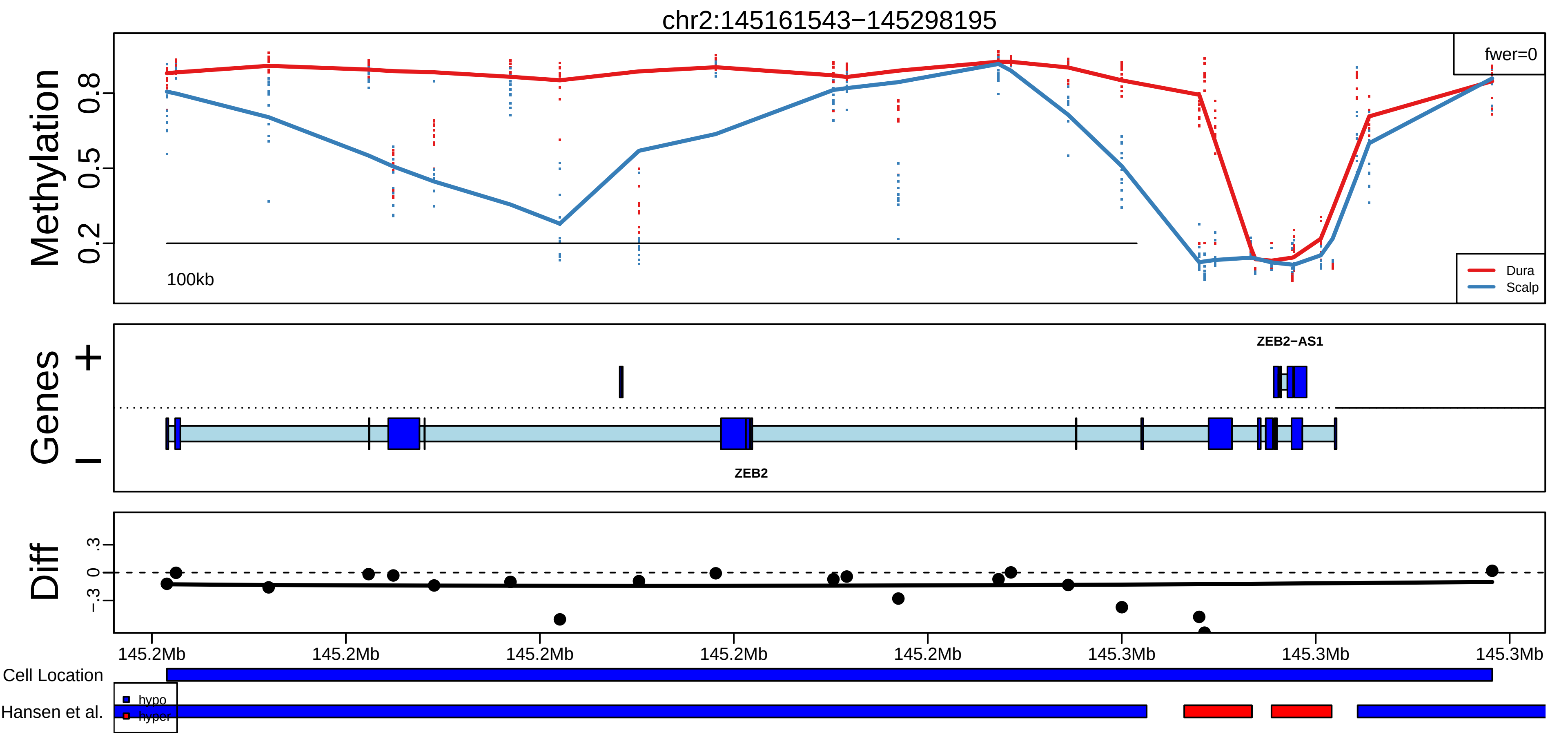
chr2:145161543−145298195
fwer=0
Methylation
0.2
0.5
0.8
100kb
Dura
Scalp
Genes
+
−
ZEB2−AS1
ZEB2
Diff
−.3
0
.3
145.2Mb
145.2Mb
145.2Mb
145.2Mb
145.2Mb
145.3Mb
145.3Mb
145.3Mb
Cell Location
Hansen et al.
hypo
hyper

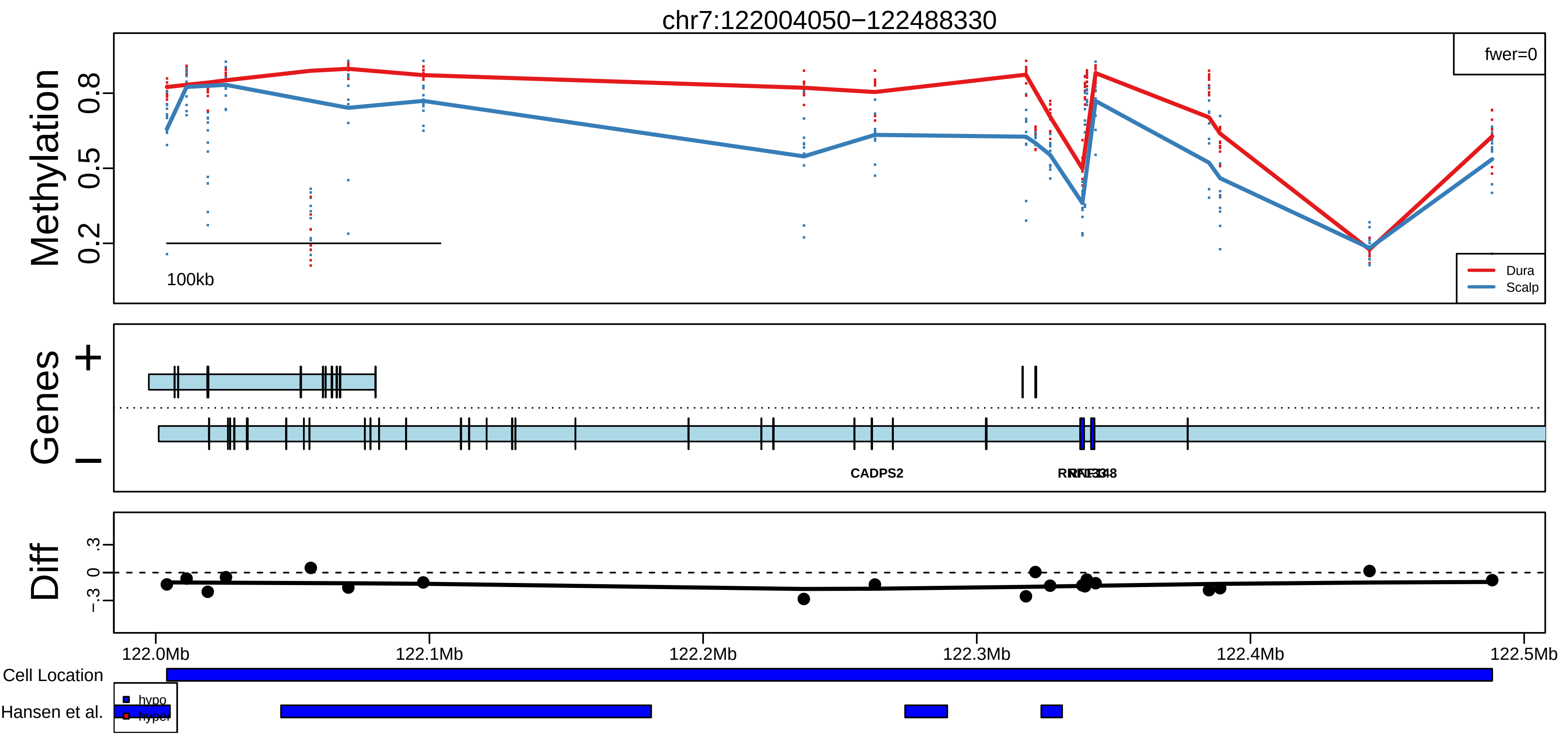

chr7:122004050−122488330
fwer=0
Methylation
0.2
0.5
0.8
100kb
Dura
Scalp
Genes
+
−
CADPS2
RNF133
RNF148
Diff
−.3
0
.3
122.0Mb
122.1Mb
122.2Mb
122.3Mb
122.4Mb
122.5Mb
Cell Location
Hansen et al.
hypo
hyper

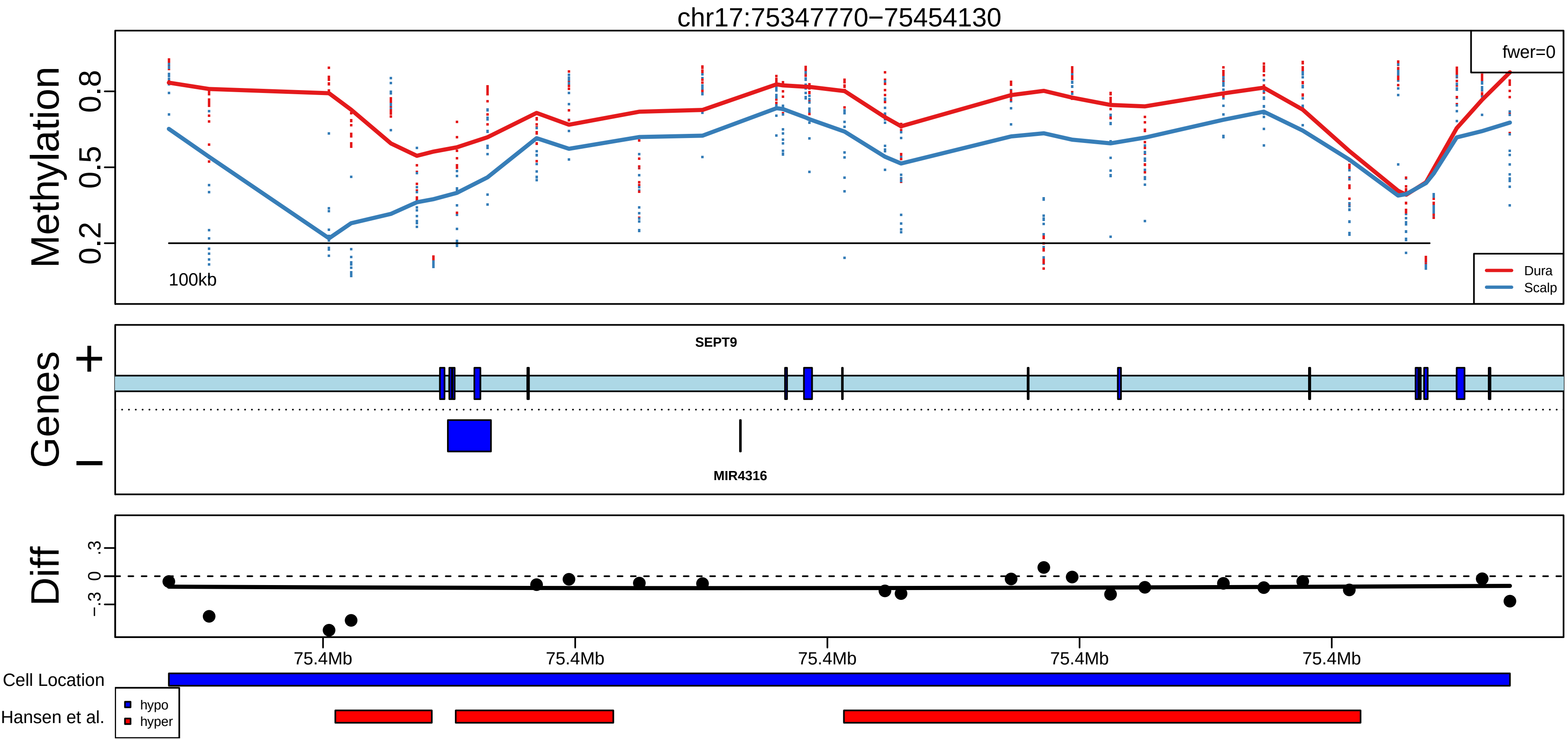
chr17:75347770−75454130
fwer=0
Methylation
0.2
0.5
0.8
100kb
Dura
Scalp
Genes
+
−
SEPT9
MIR4316
Diff
.3
0
−.3
75.4Mb
75.4Mb
75.4Mb
75.4Mb
75.4Mb
Cell Location
Hansen et al.
hypo
hyper

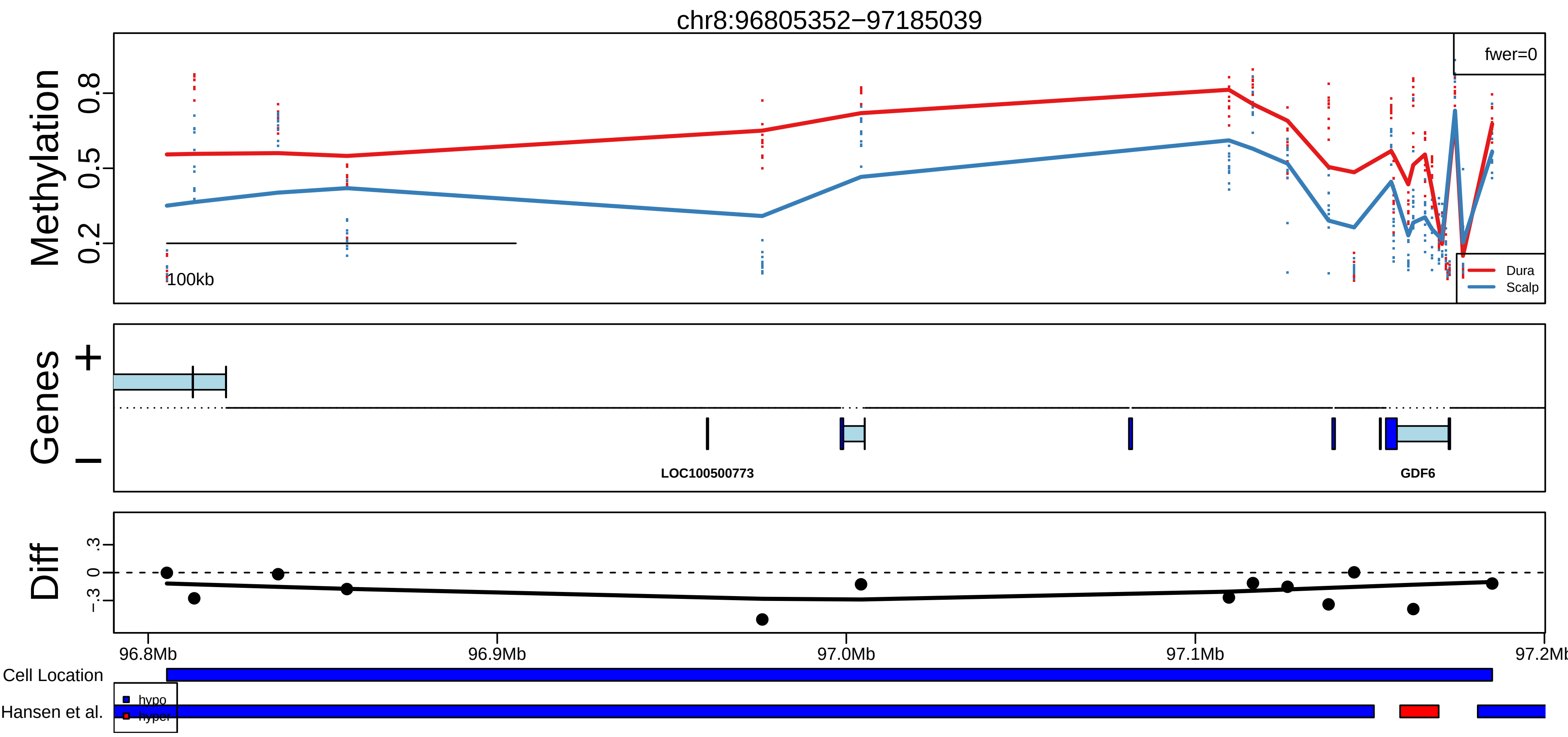

chr8:96805352−97185039
fwer=0
Methylation
0.2
0.5
0.8
100kb
Dura
Scalp
Genes
+
−
LOC100500773
GDF6
Diff
−.3
0
.3
96.8Mb
96.9Mb
97.0Mb
97.1Mb
97.2Mb
Cell Location
Hansen et al.
hypo
hyper

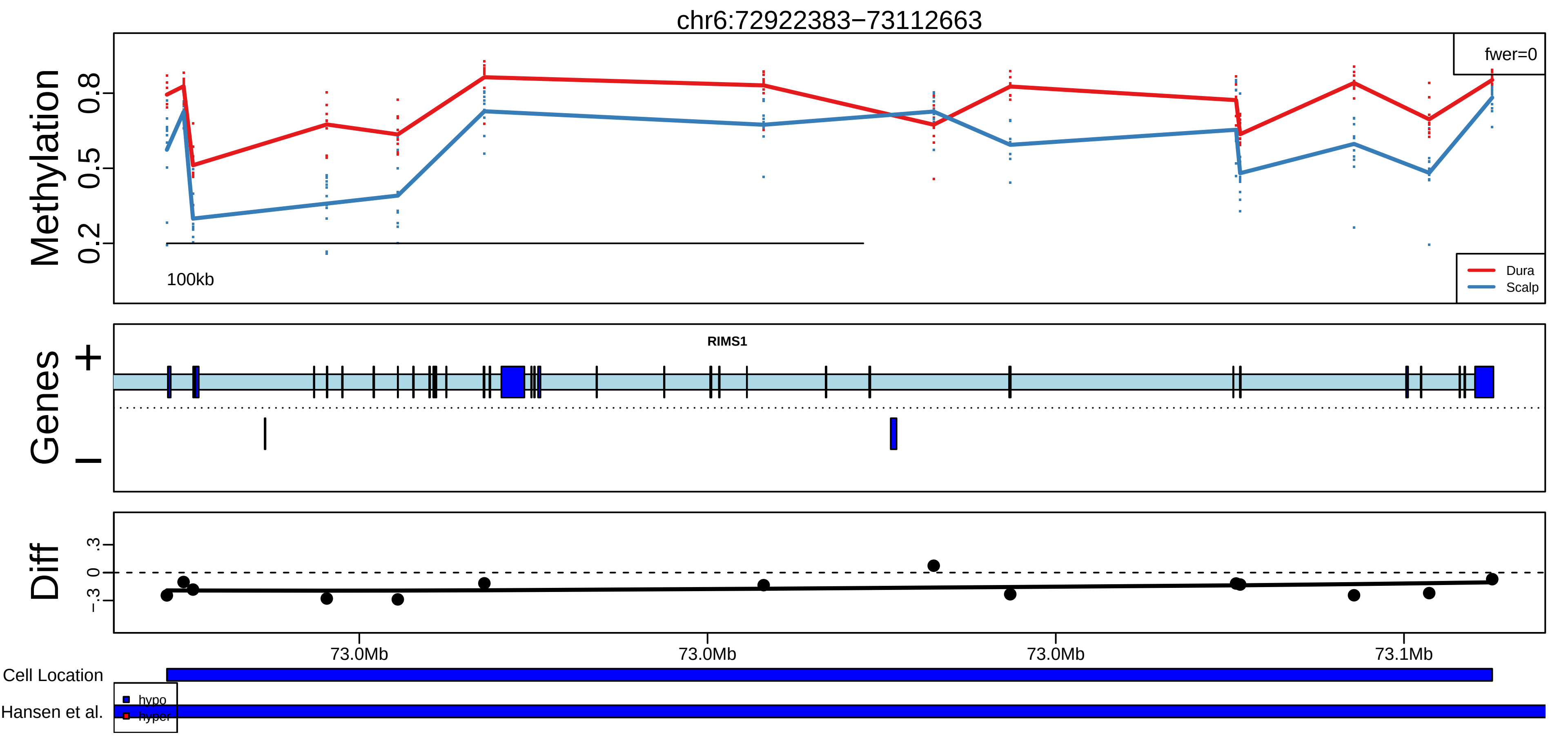

chr6:72922383−73112663
fwer=0
Methylation
0.2
0.5
0.8
100kb
Dura
Scalp
Genes
+
−
RIMS1
Diff
−.3
0
.3
73.0Mb
73.0Mb
73.0Mb
73.1Mb
Cell Location
Hansen et al.
hypo
hyper

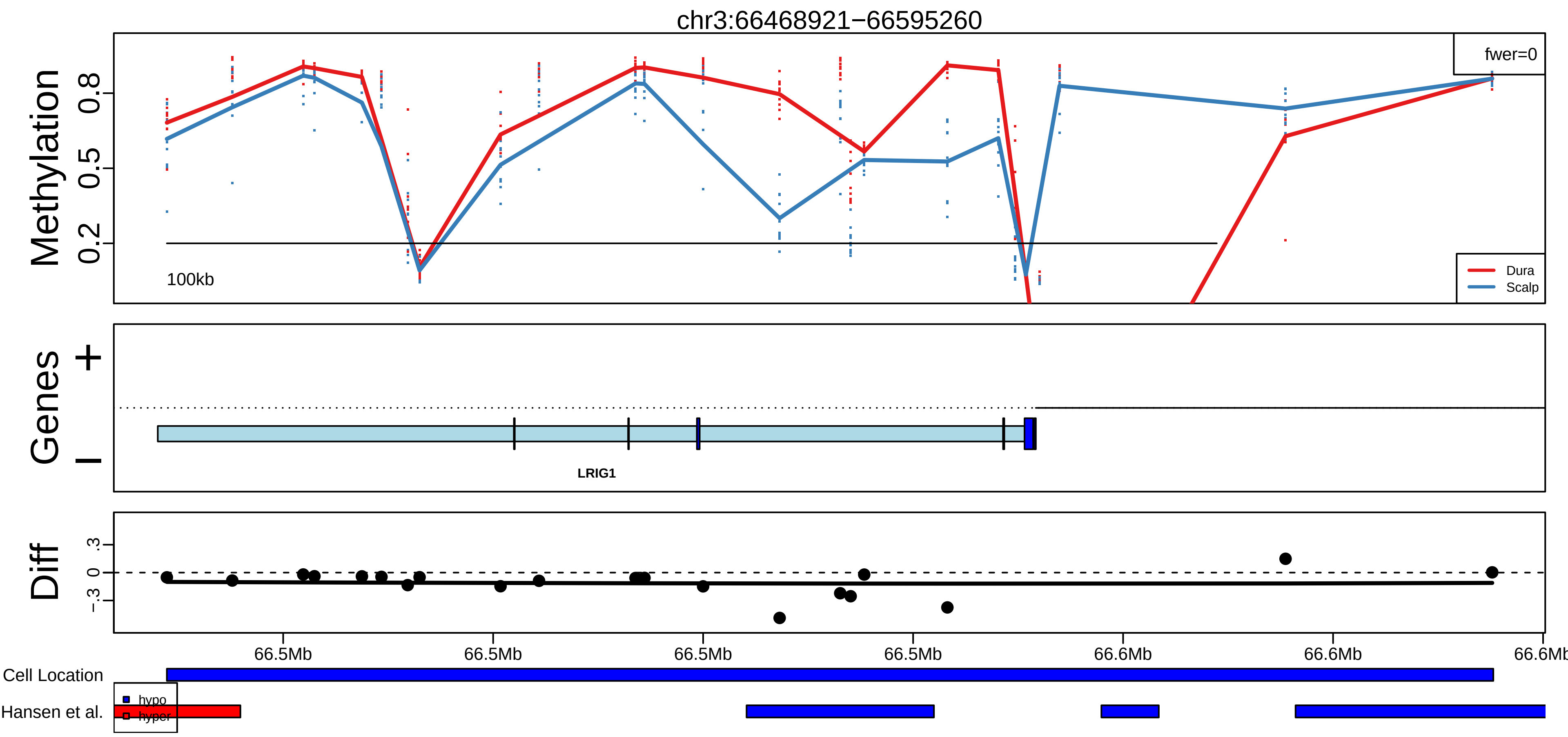
chr3:66468921−66595260
Methylation
0.2
0.5
0.8
fwer=0
100kb
Dura
Scalp
Genes
+
−
LRIG1
Diff
−.3
0
.3
66.5Mb
66.5Mb
66.5Mb
66.5Mb
66.6Mb
66.6Mb
66.6Mb
Cell Location
Hansen et al.
hypo
hyper

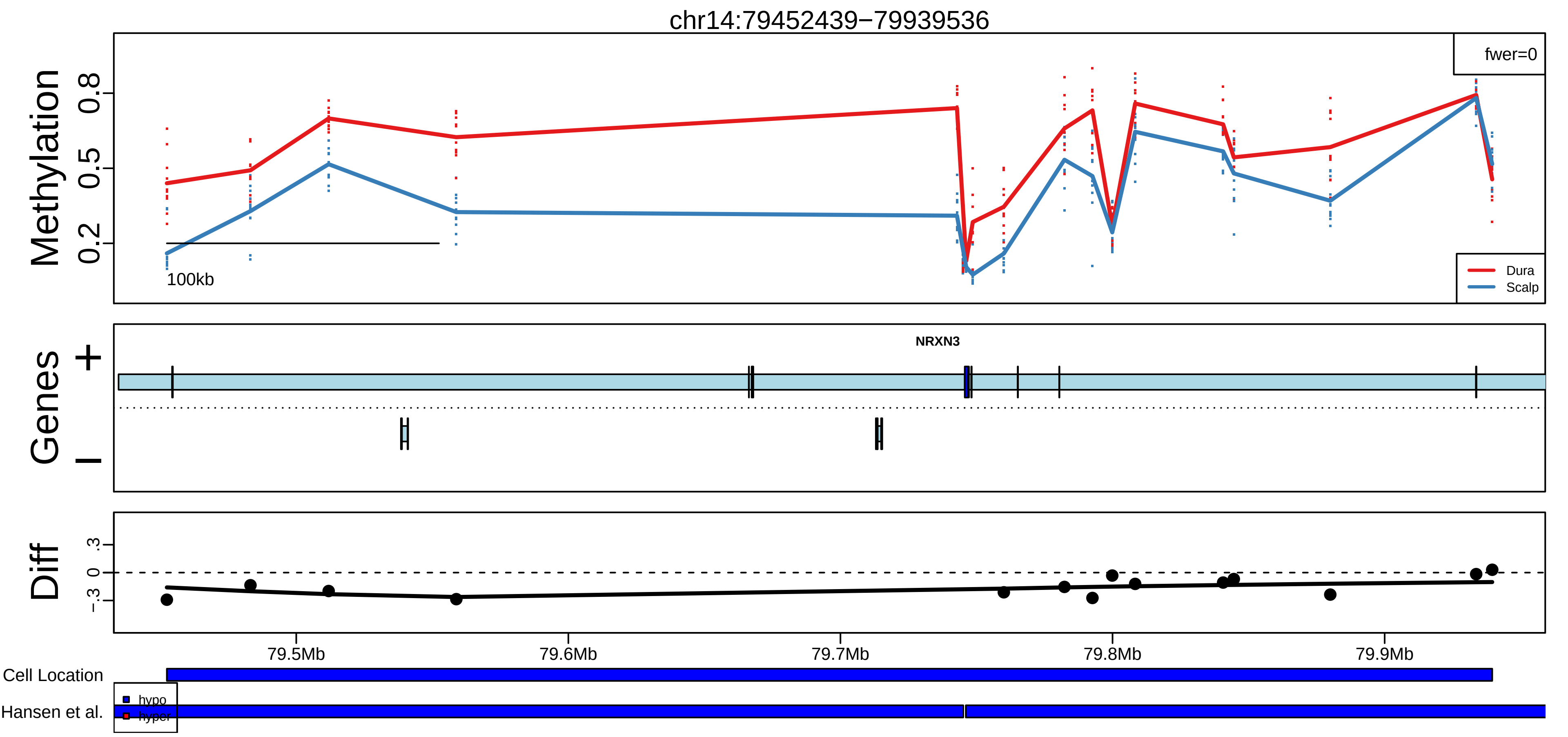

chr14:79452439−79939536
fwer=0
Methylation
0.2
0.5
0.8
100kb
Dura
Scalp
Genes
+
−
NRXN3
Diff
−.3
0
.3
79.5Mb
79.6Mb
79.7Mb
79.8Mb
79.9Mb
Cell Location
Hansen et al.
hypo
hyper

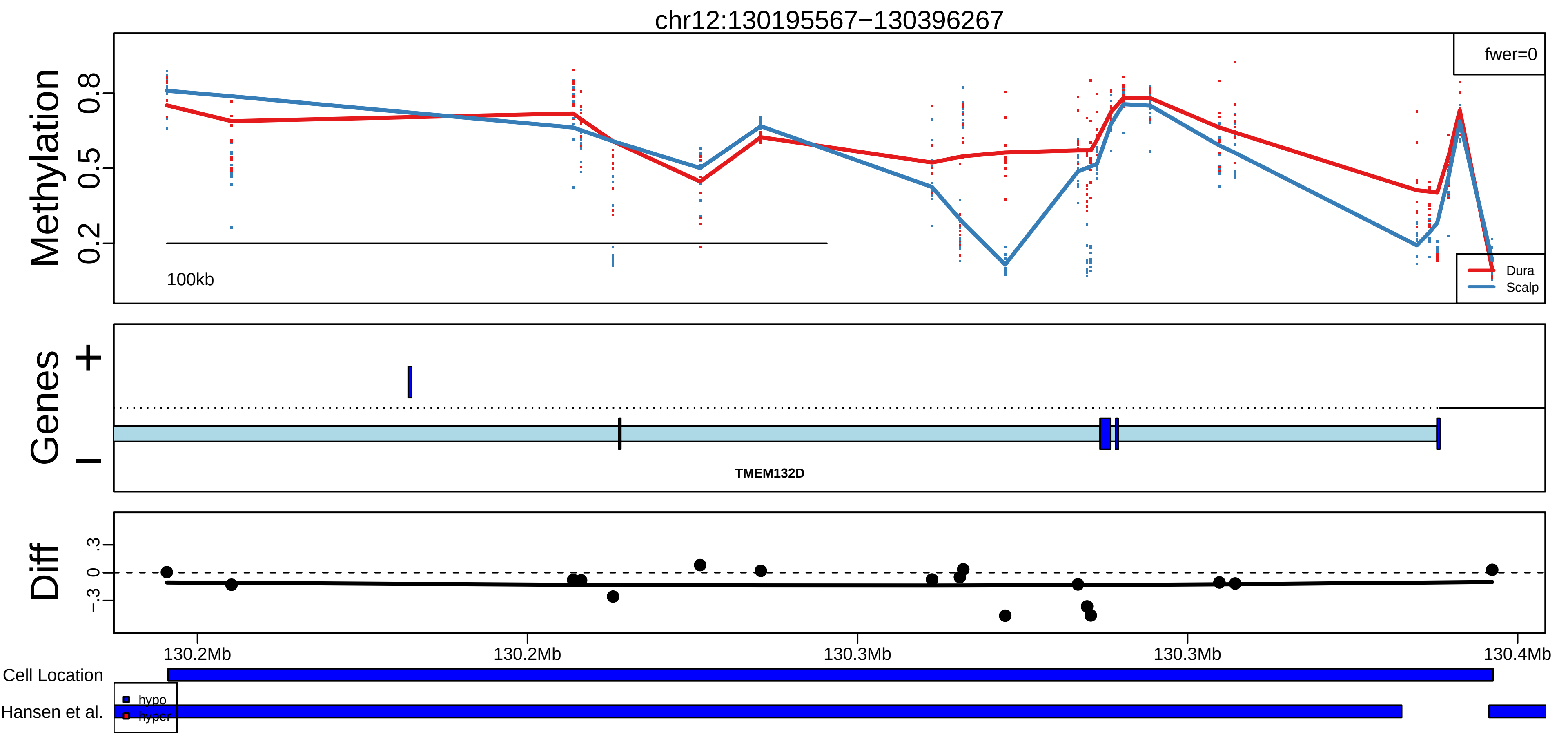

chr12:130195567−130396267
fwer=0
Methylation
0.2
0.5
0.8
100kb
Dura
Scalp
Genes
+
−
TMEM132D
Diff
−.3
0
.3
130.2Mb
130.2Mb
130.3Mb
130.3Mb
130.4Mb
Cell Location
Hansen et al.
hypo
hyper

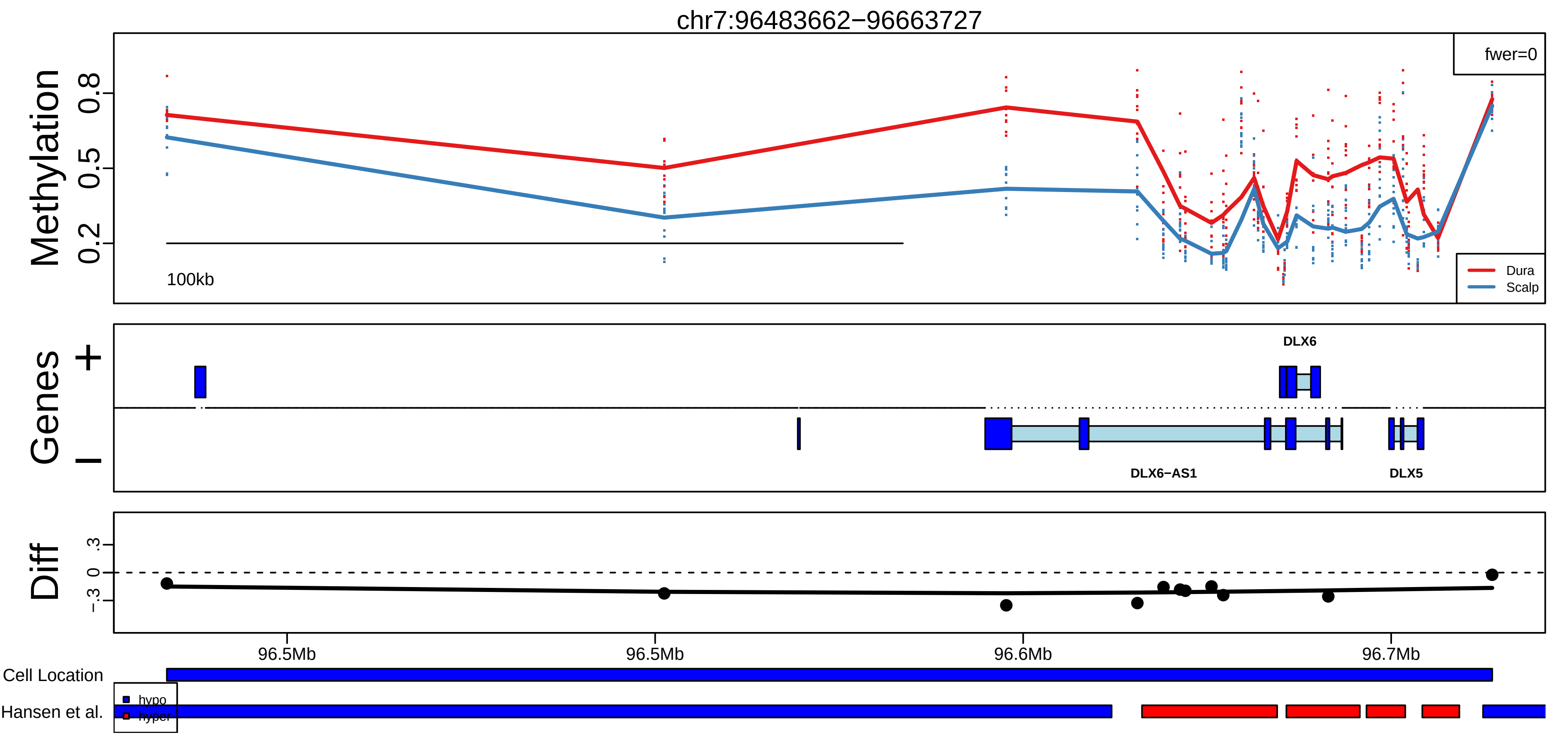

chr7:96483662−96663727
Methylation
0.2
0.5
0.8
fwer=0
100kb
Dura
Scalp
Genes
+
−
DLX6
DLX6−AS1
DLX5
Diff
−.3
0
.3
96.5Mb
96.5Mb
96.6Mb
96.7Mb
Cell Location
Hansen et al.
hypo
hyper

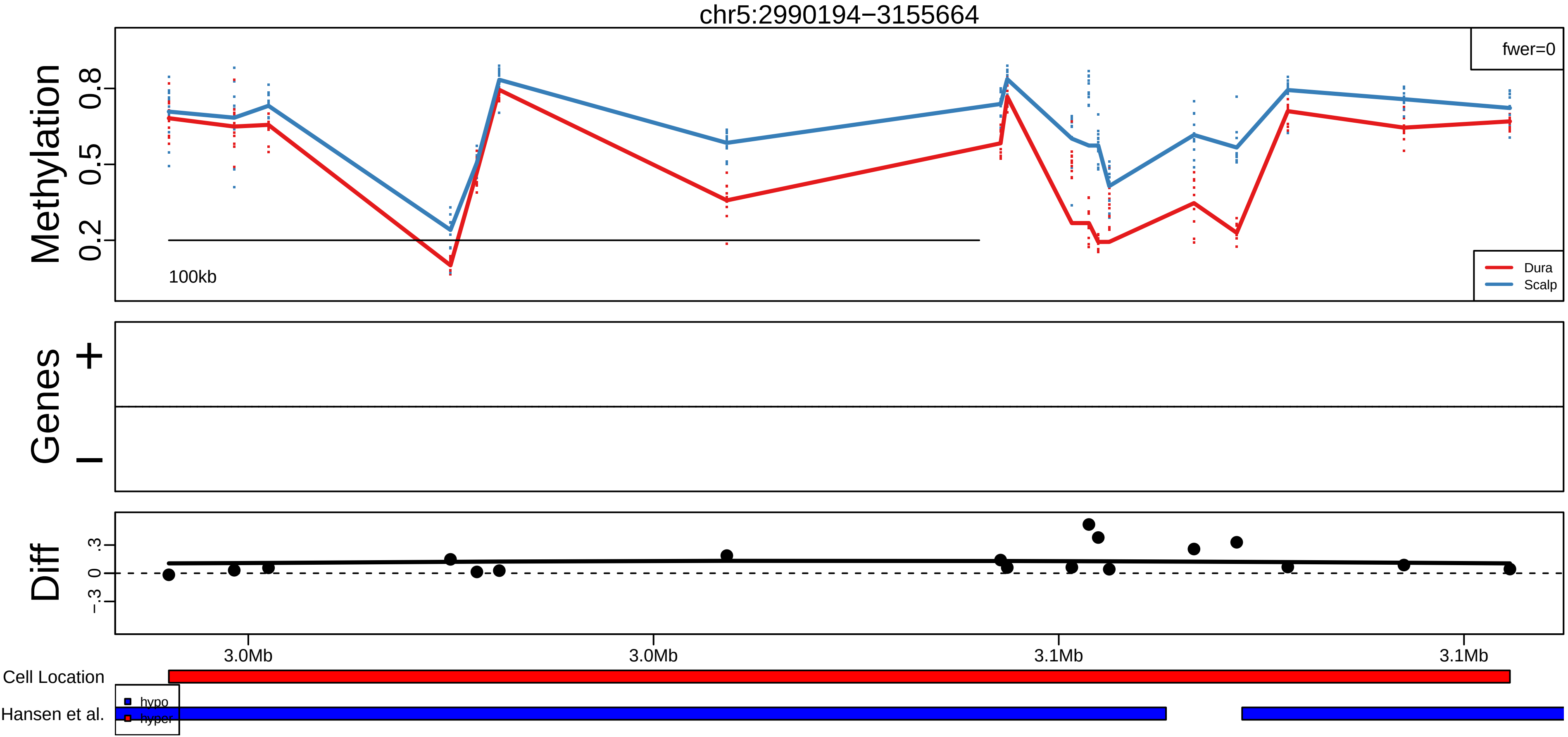

chr5:2990194−3155664
Methylation
0.2
0.5
0.8
fwer=0
100kb
Dura
Scalp
Genes
+
−
Diff
−.3
0
.3
3.0Mb
3.0Mb
3.1Mb
3.1Mb
Cell Location
Hansen et al.
hypo
hyper

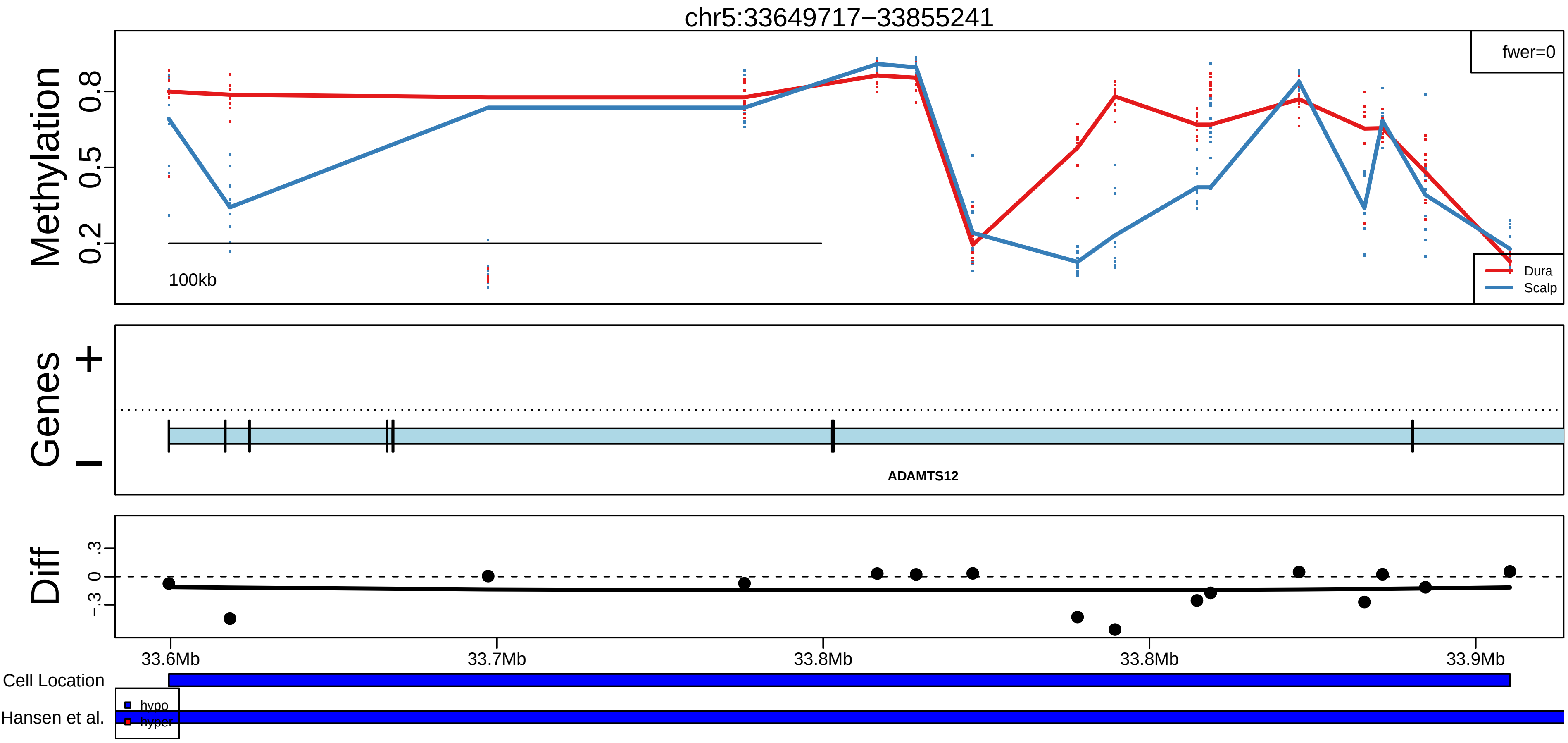

chr5:33649717−33855241
fwer=0
Methylation
0.2
0.5
0.8
100kb
Dura
Scalp
Genes
+
−
ADAMTS12
Diff
−.3
0
.3
33.6Mb
33.7Mb
33.8Mb
33.8Mb
33.9Mb
Cell Location
hypo
hyper
Hansen et al.

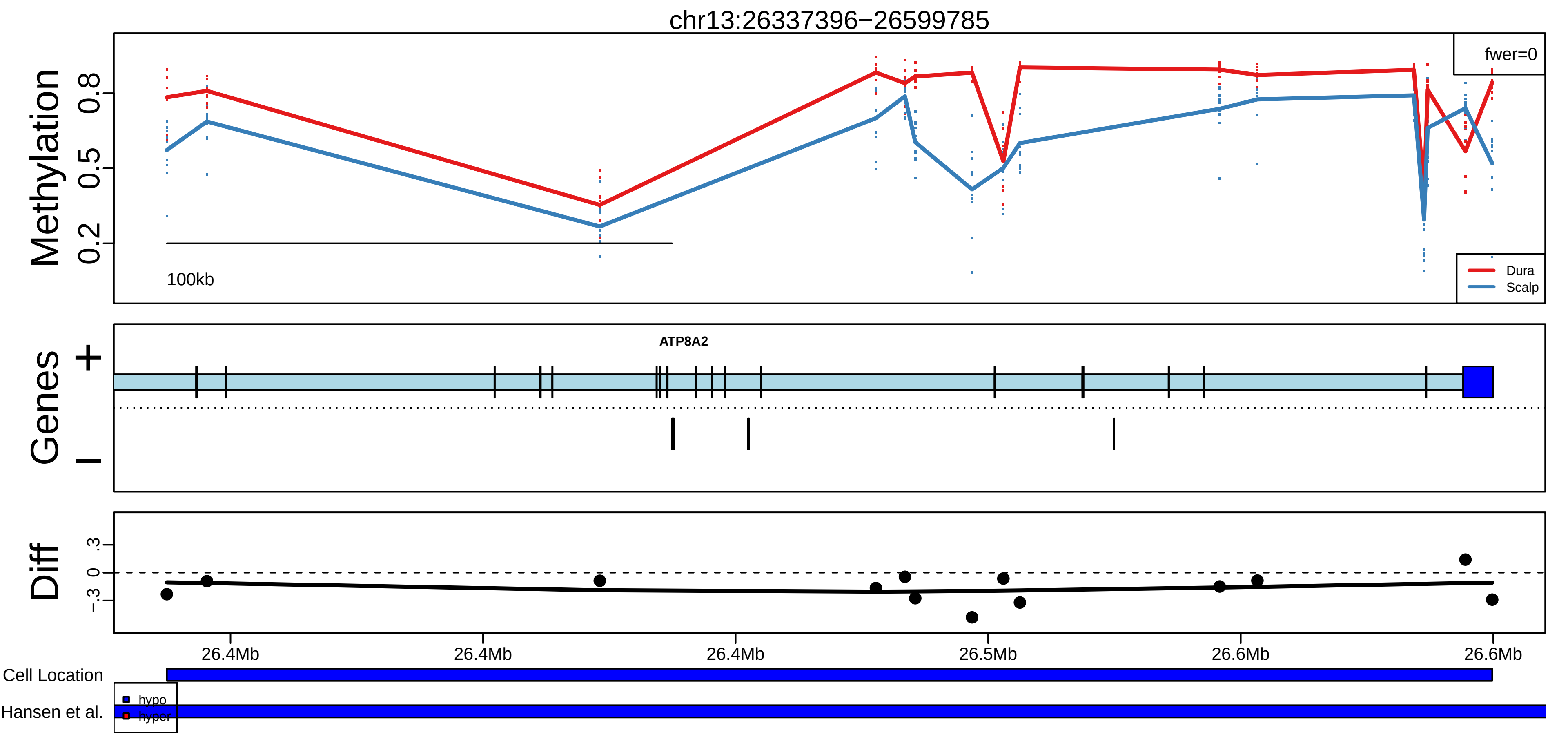
chr13:26337396−26599785
Methylation
0.2
0.5
0.8
fwer=0
100kb
Dura
Scalp
Genes
+
−
ATP8A2
Diff
−.3
0
.3
26.4Mb
26.4Mb
26.4Mb
26.5Mb
26.6Mb
26.6Mb
Cell Location
Hansen et al.
hypo
hyper

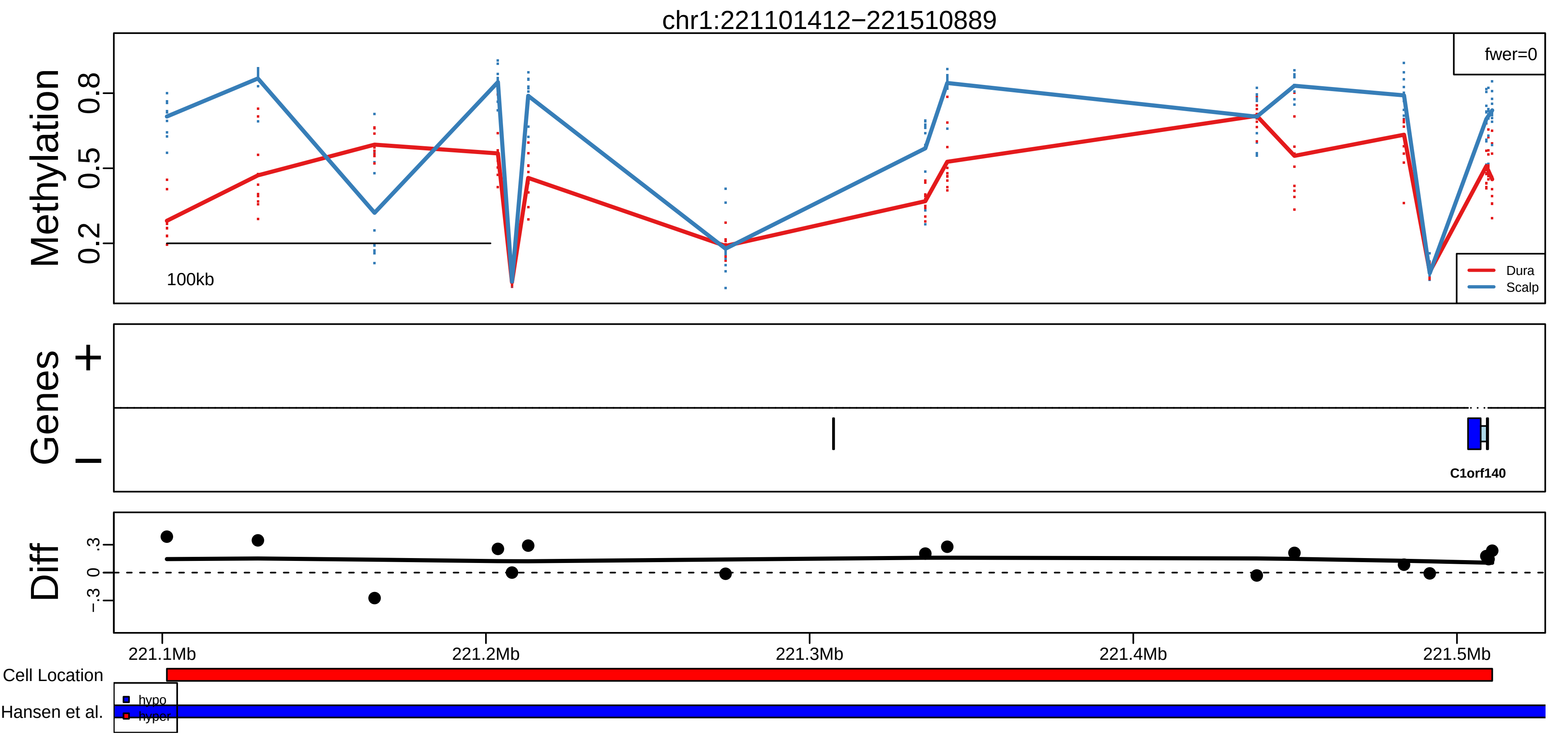
chr1:221101412−221510889
fwer=0
Methylation
0.2
0.5
0.8
100kb
Dura
Scalp
Genes
+
−
C1orf140
Diff
−.3
0
.3
221.1Mb
221.2Mb
221.3Mb
221.4Mb
221.5Mb
Cell Location
Hansen et al.
hypo
hyper

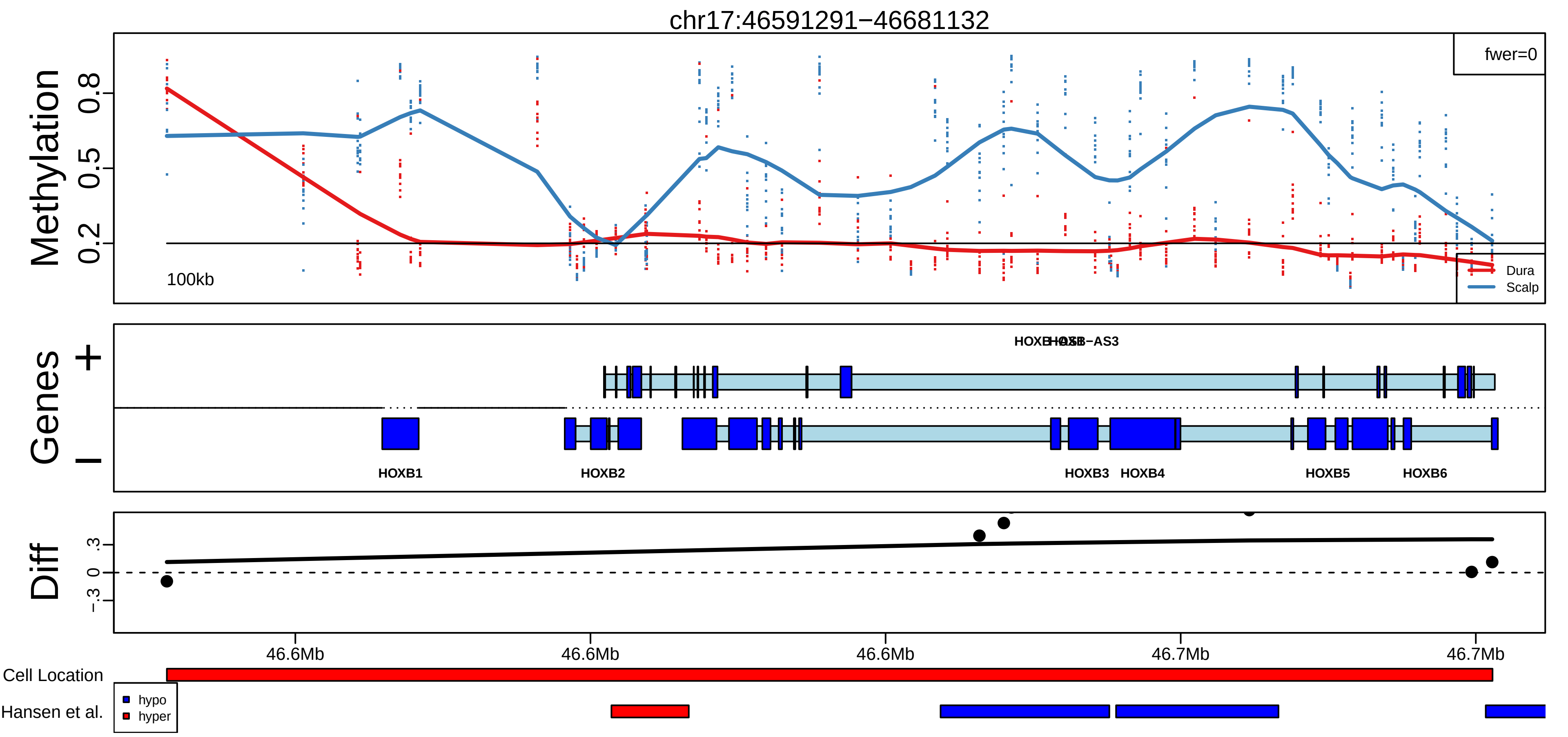

chr17:46591291−46681132
Methylation
0.2
0.5
0.8
fwer=0
100kb
Dura
Scalp
Genes
+
−
HOXB-AS3
HOXB1
HOXB2
HOXB3
HOXB4
HOXB5
HOXB6
Diff
−.3
0
.3
46.6Mb
46.6Mb
46.6Mb
46.7Mb
46.7Mb
Cell Location
Hansen et al.
hypo
hyper

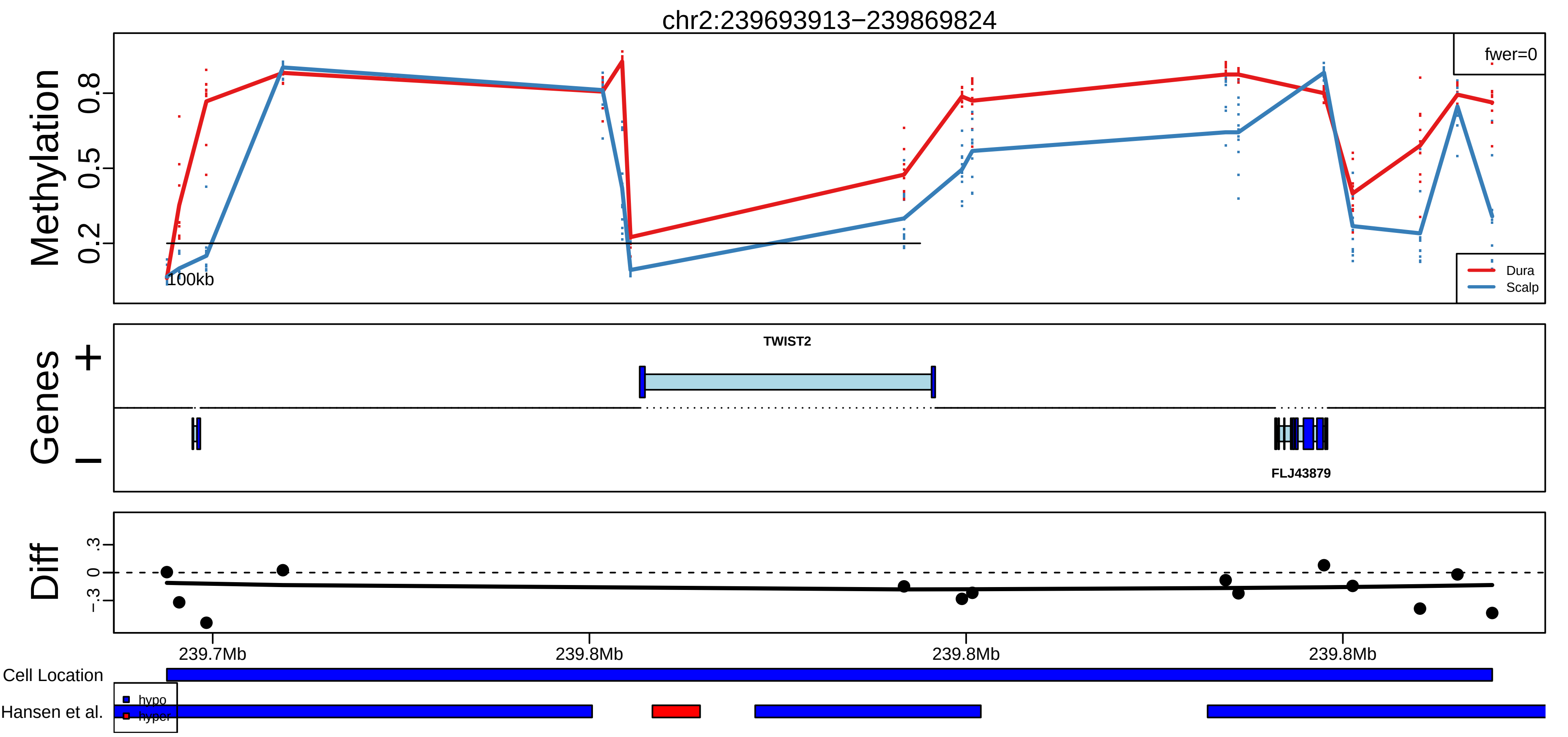

chr2:239693913−239869824
fwer=0
Methylation
0.2
0.5
0.8
100kb
Dura
Scalp
Genes
+
−
TWIST2
FLJ43879
Diff
.3
0
−.3
239.7Mb
239.8Mb
239.8Mb
239.8Mb
Cell Location
Hansen et al.
hypo
hyper

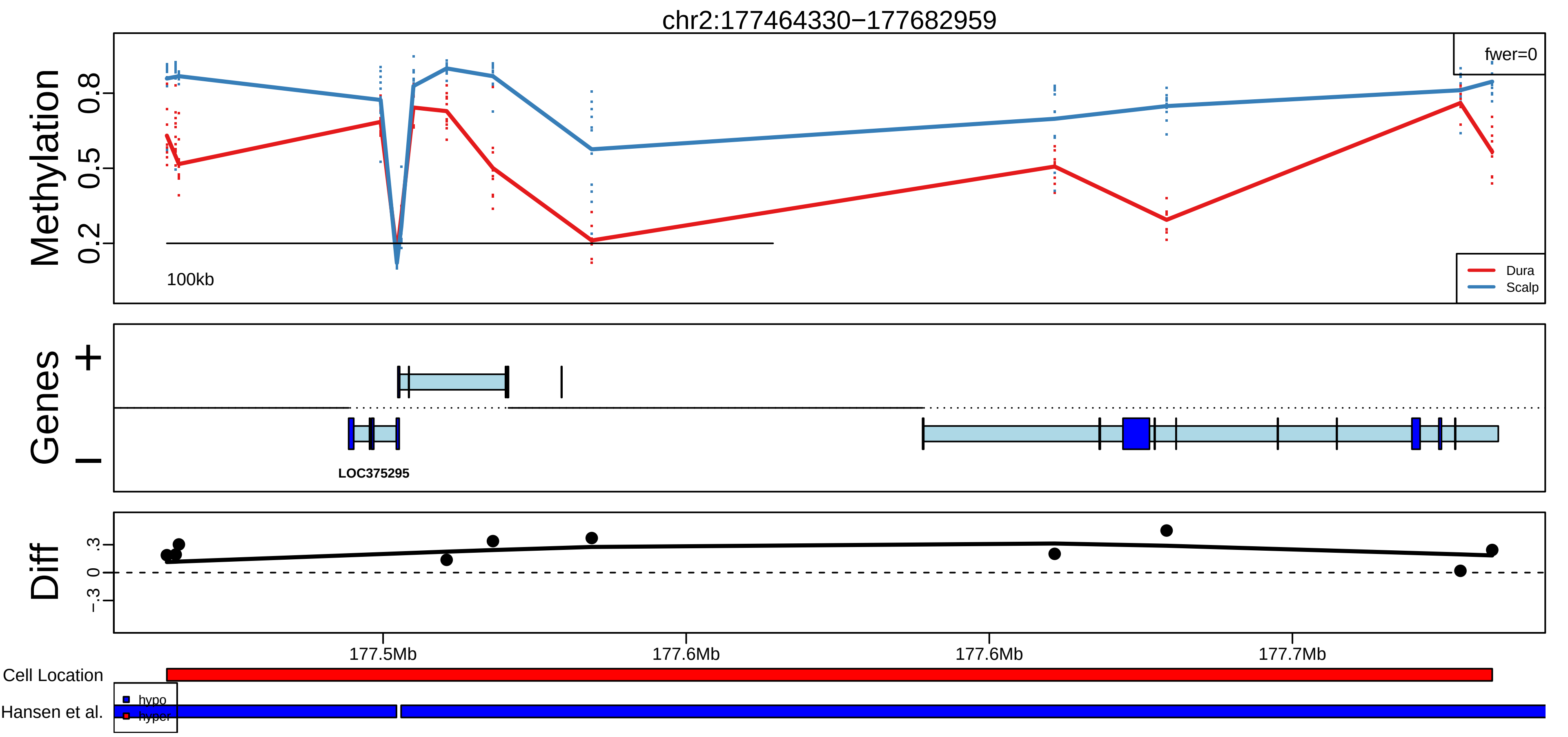

chr2:177464330−177682959
Methylation
0.2
0.5
0.8
fwer=0
100kb
Dura
Scalp
Genes
+
−
LOC375295
Diff
−.3
0
.3
177.5Mb
177.6Mb
177.6Mb
177.7Mb
Cell Location
Hansen et al.
hypo
hyper

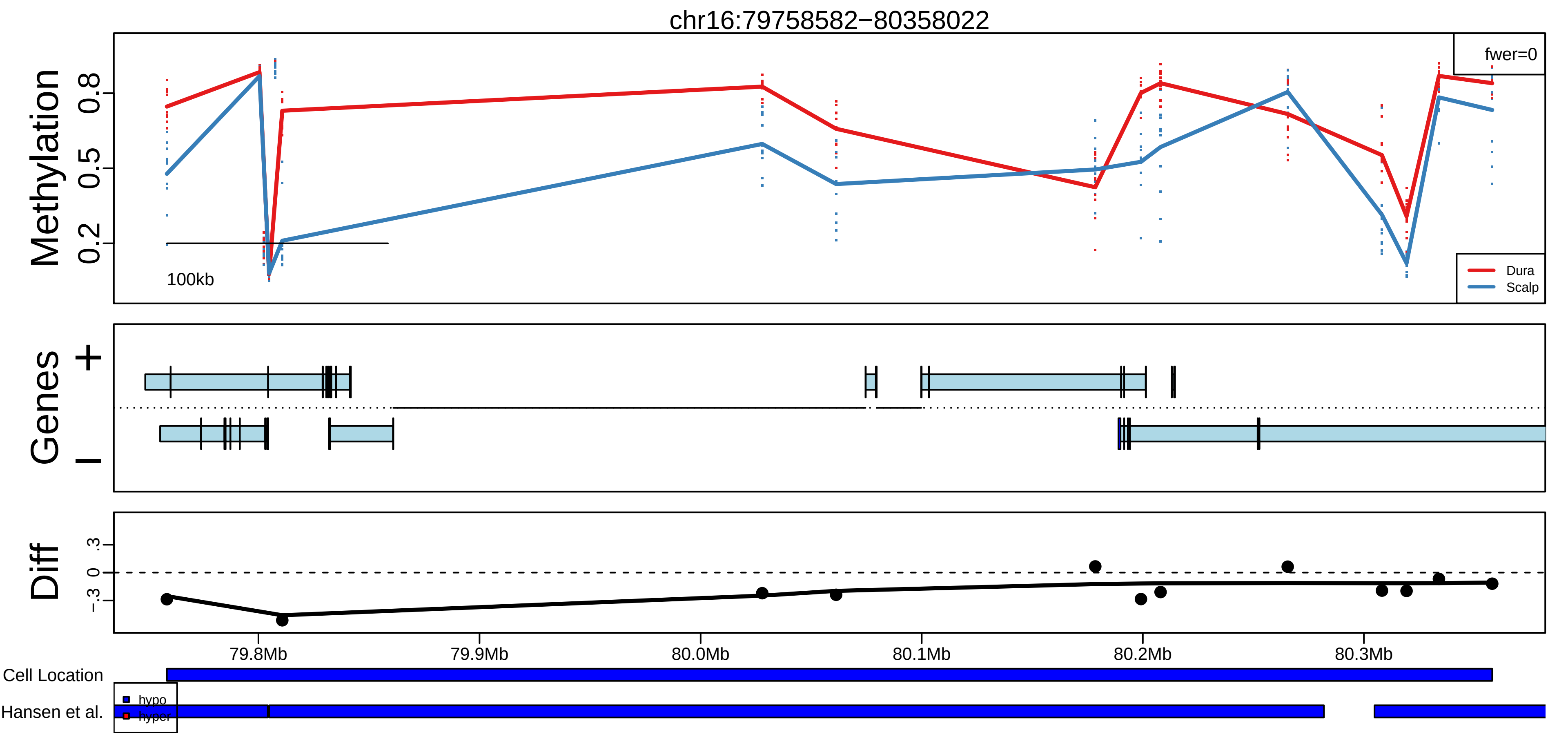

chr16:79758582−80358022
Methylation
0.2
0.5
0.8
fwer=0
100kb
Dura
Scalp
Genes
+
−
Diff
−.3
0
.3
79.8Mb
79.9Mb
80.0Mb
80.1Mb
80.2Mb
80.3Mb
Cell Location
Hansen et al.
hypo
hyper

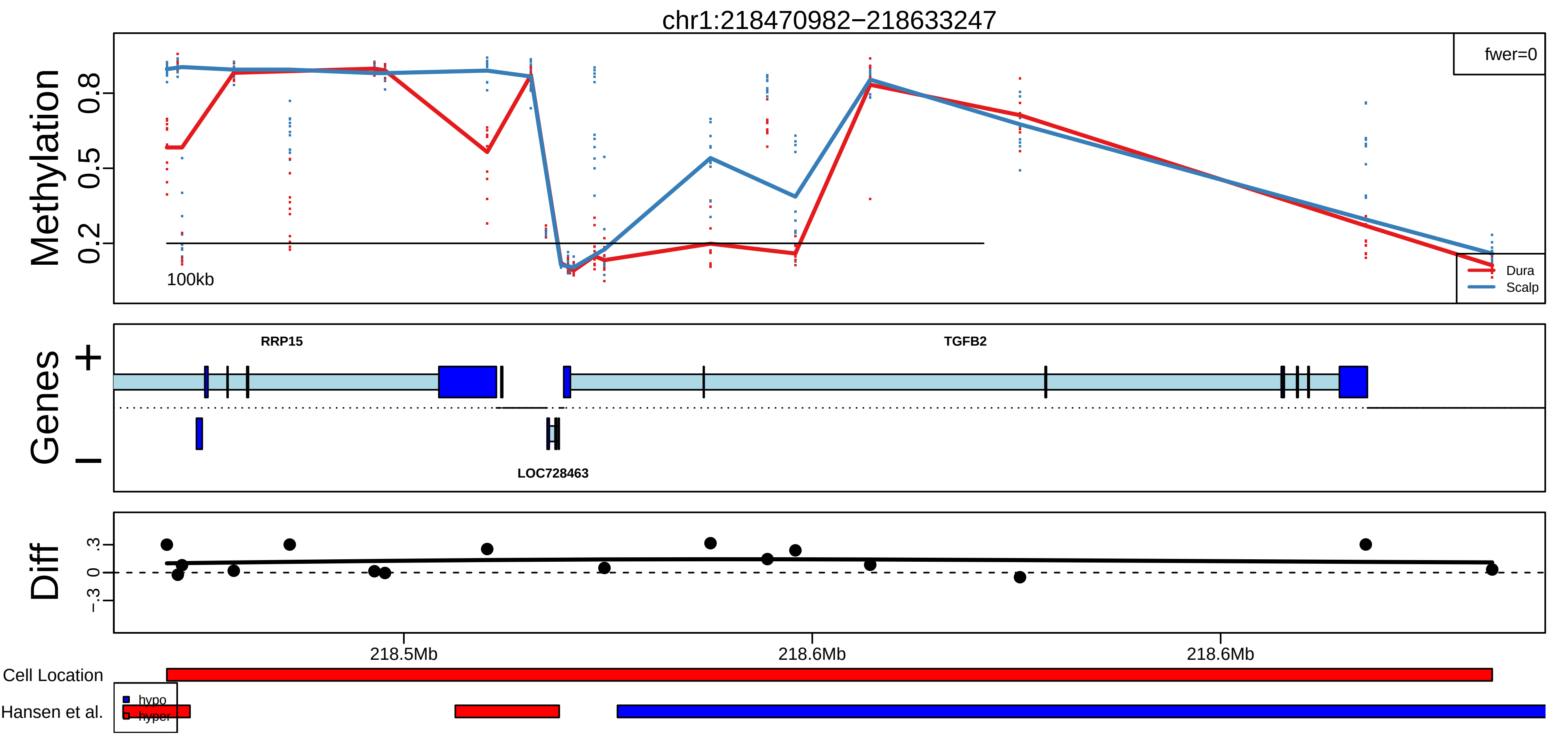

chr1:218470982−218633247
fwer=0
Methylation
0.2
0.5
0.8
100kb
Dura
Scalp
Genes
+
−
RRP15
TGFB2
LOC728463
Diff
−.3
0
.3
218.5Mb
218.6Mb
218.6Mb
Cell Location
Hansen et al.
hypo
hyper

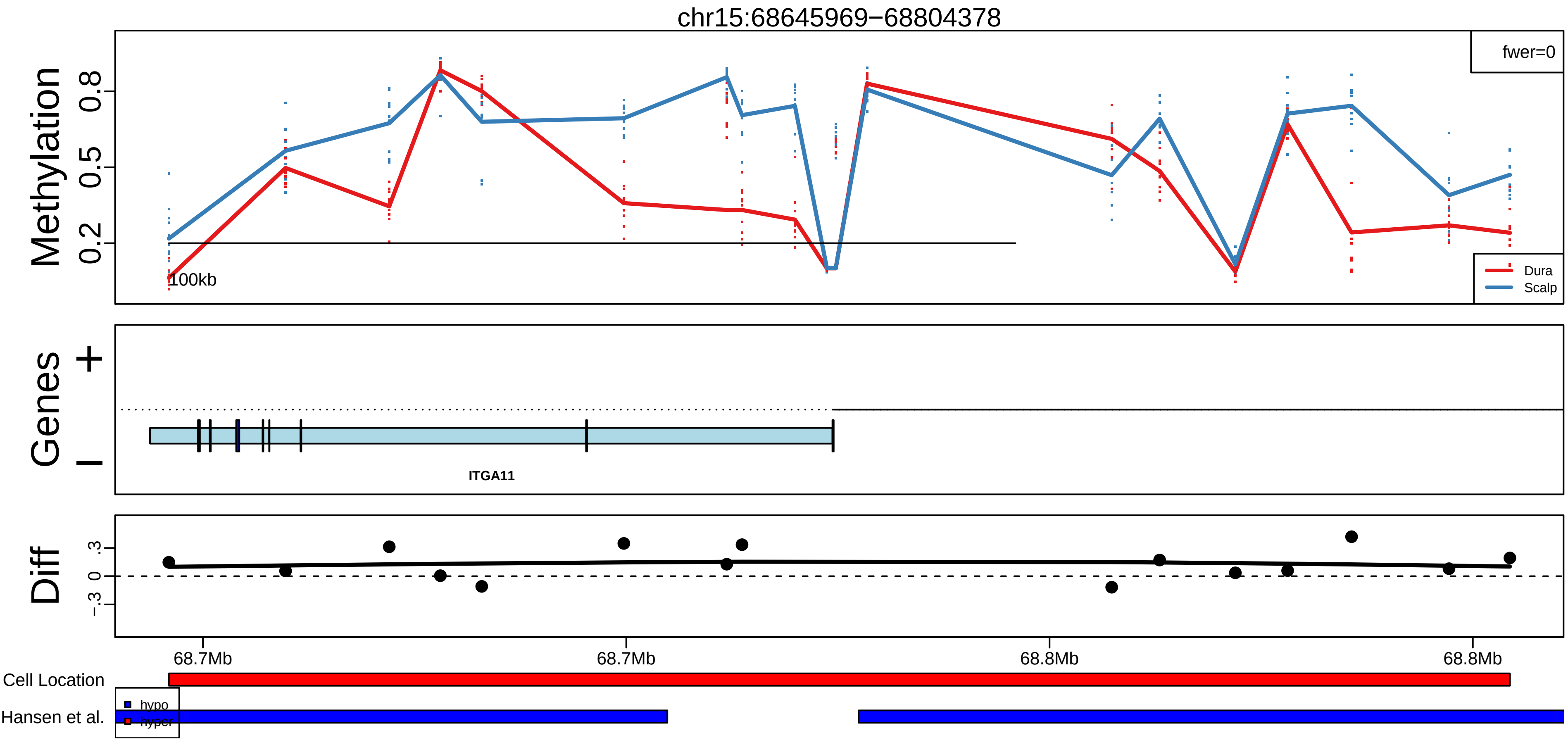

chr15:68645969−68804378
fwer=0
Methylation
0.2
0.5
0.8
100kb
Dura
Scalp
Genes
+
−
ITGA11
Diff
−.3
0
.3
68.7Mb
68.7Mb
68.8Mb
68.8Mb
Cell Location
Hansen et al.
hypo
hyper

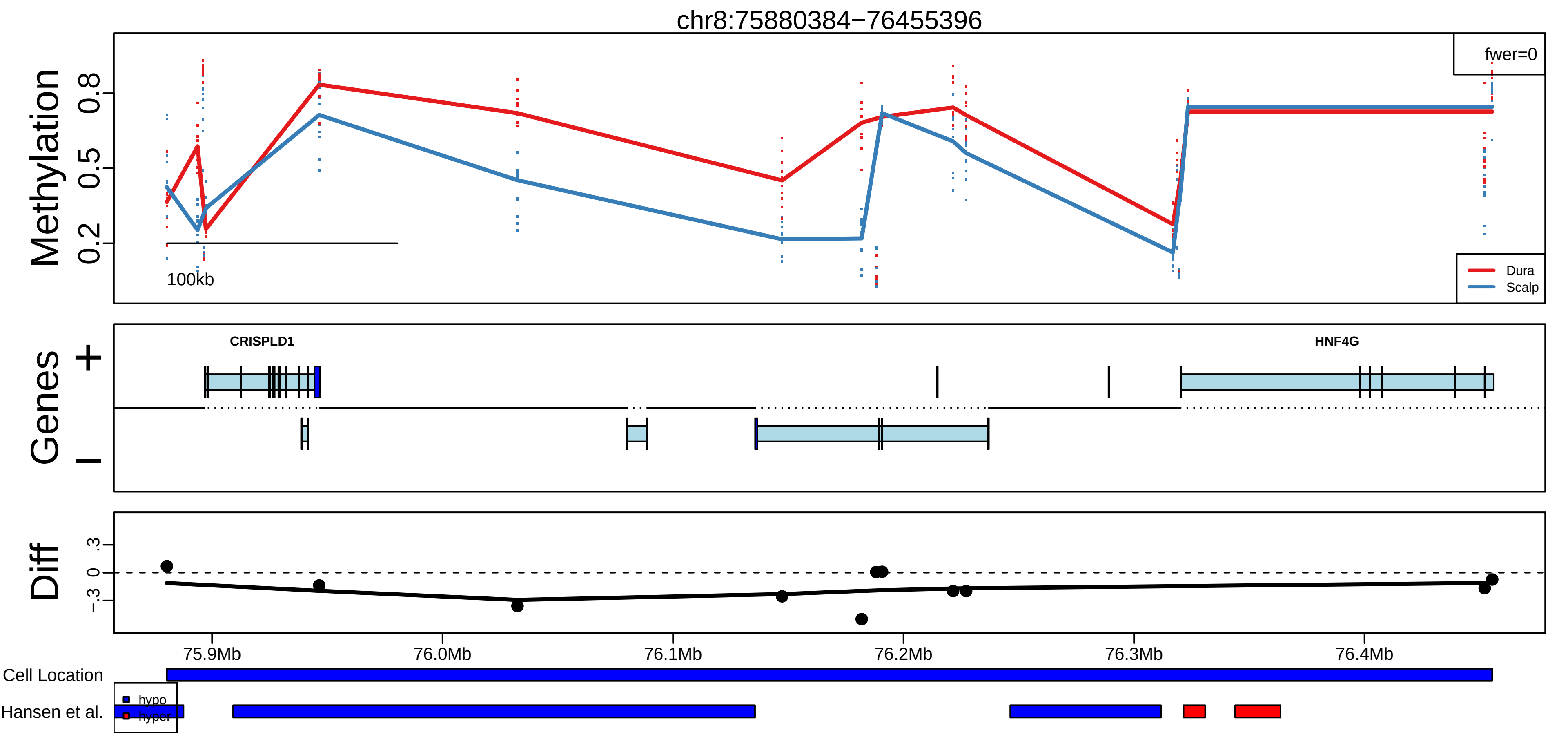
chr8:75880384−76455396
fwer=0
Methylation
0.2
0.5
0.8
100kb
Dura
Scalp
Genes
+
−
CRISPLD1
HNF4G
Diff
−.3
0
.3
75.9Mb
76.0Mb
76.1Mb
76.2Mb
76.3Mb
76.4Mb
Cell Location
Hansen et al.
hypo
hyper

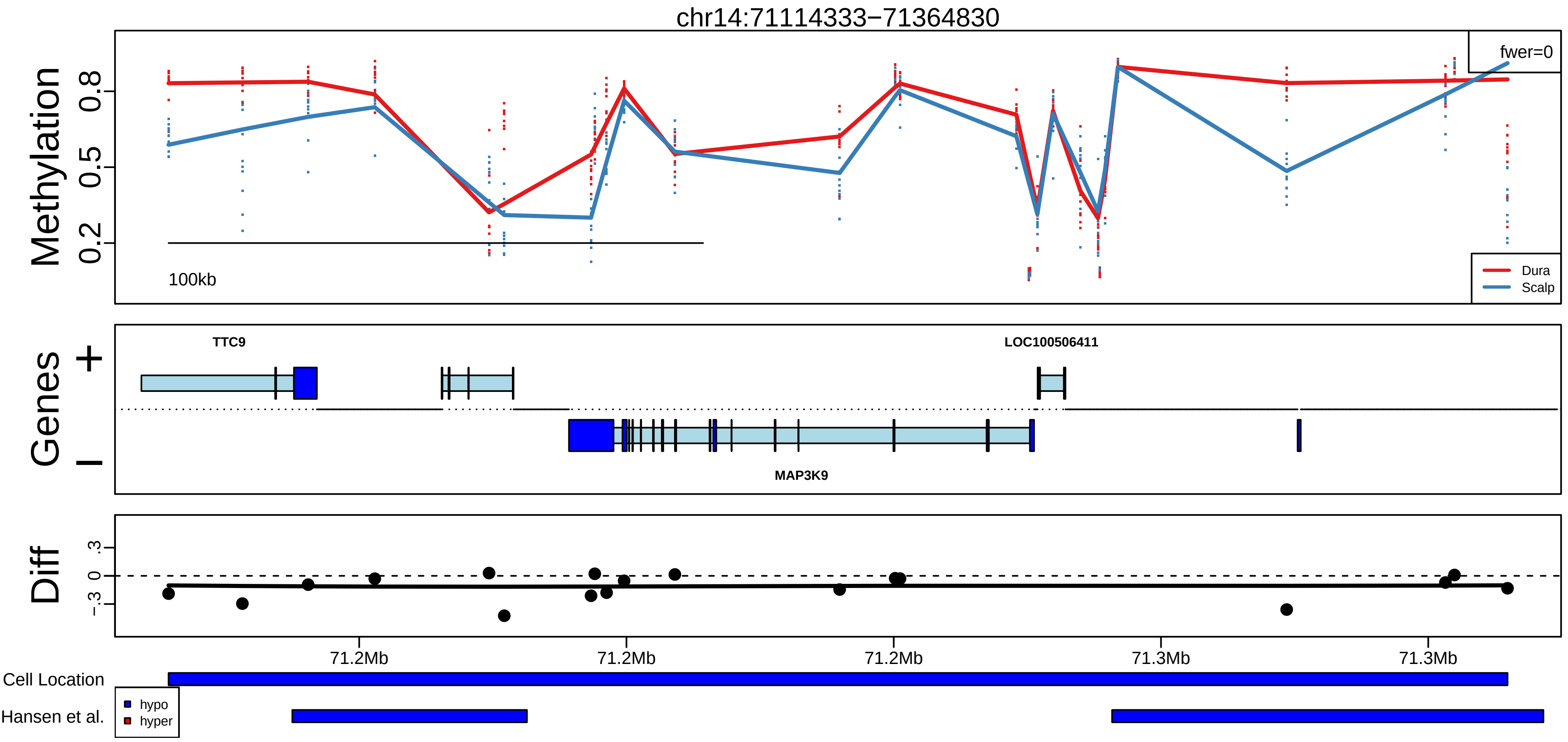
chr14:71114333−71364830
Methylation
0.2
0.5
0.8
fwer=0
100kb
Dura
Scalp
Genes
+
−
TTC9
LOC100506411
MAP3K9
Diff
.3
0
−.3
71.2Mb
71.2Mb
71.2Mb
71.3Mb
71.3Mb
Cell Location
Hansen et al.
hypo
hyper

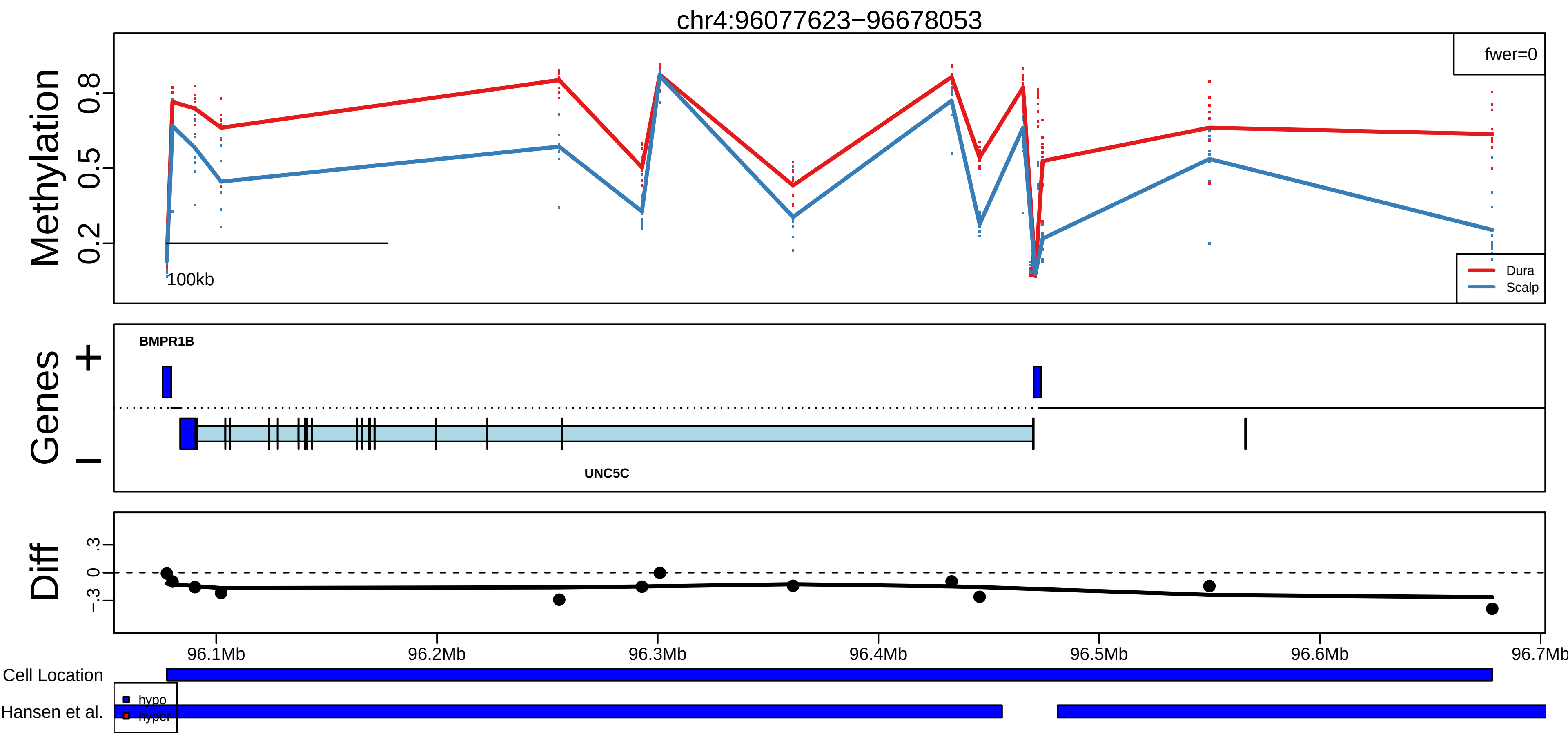

chr4:96077623−96678053
fwer=0
Methylation
0.8
0.5
0.2
100kb
Dura
Scalp
Genes
+
−
BMPR1B
UNC5C
Diff
.3
0
−.3
96.1Mb
96.2Mb
96.3Mb
96.4Mb
96.5Mb
96.6Mb
96.7Mb
Cell Location
Hansen et al.
hypo
hyper

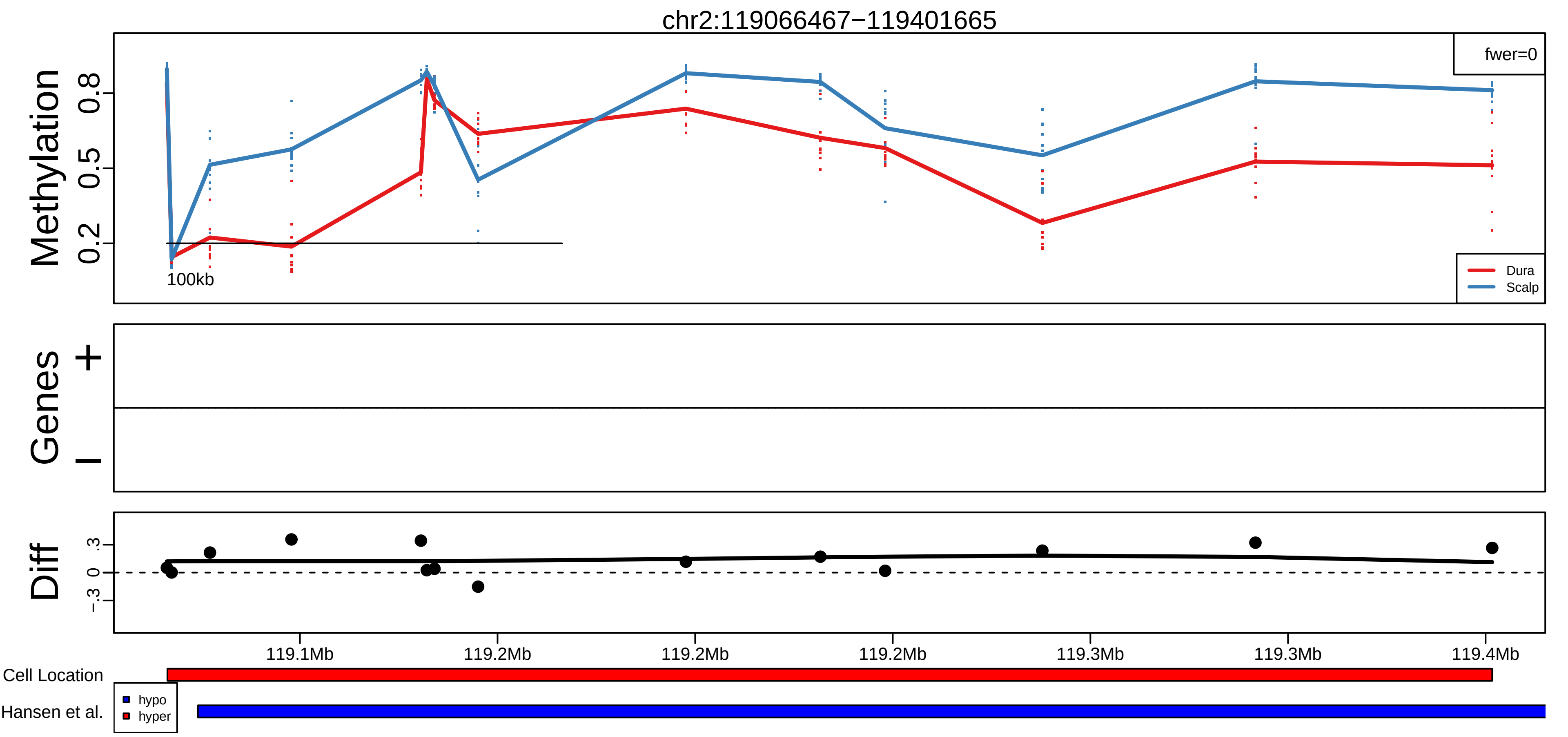
chr2:119066467−119401665
fwer=0
Methylation
0.2
0.5
0.8
100kb
Dura
Scalp
Genes
+
−
Diff
−.3
0
.3
119.1Mb
119.2Mb
119.2Mb
119.2Mb
119.3Mb
119.3Mb
119.4Mb
Cell Location
Hansen et al.
hypo
hyper

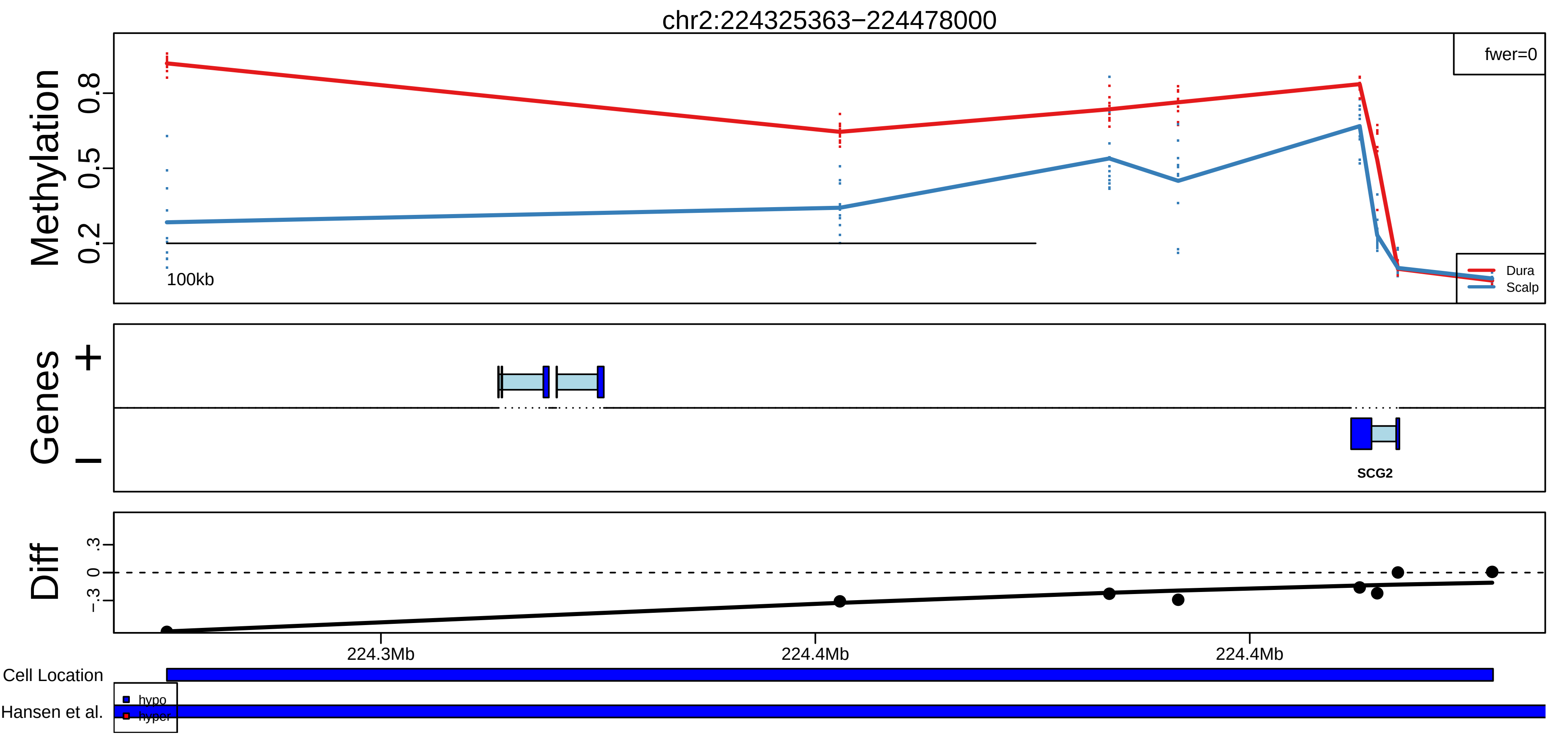
chr2:224325363−224478000
Methylation
0.8
0.5
0.2
fwer=0
100kb
Dura
Scalp
Genes
+
−
SCG2
Diff
.3
0
−.3
224.3Mb
224.4Mb
224.4Mb
Cell Location
Hansen et al.
hypo
hyper

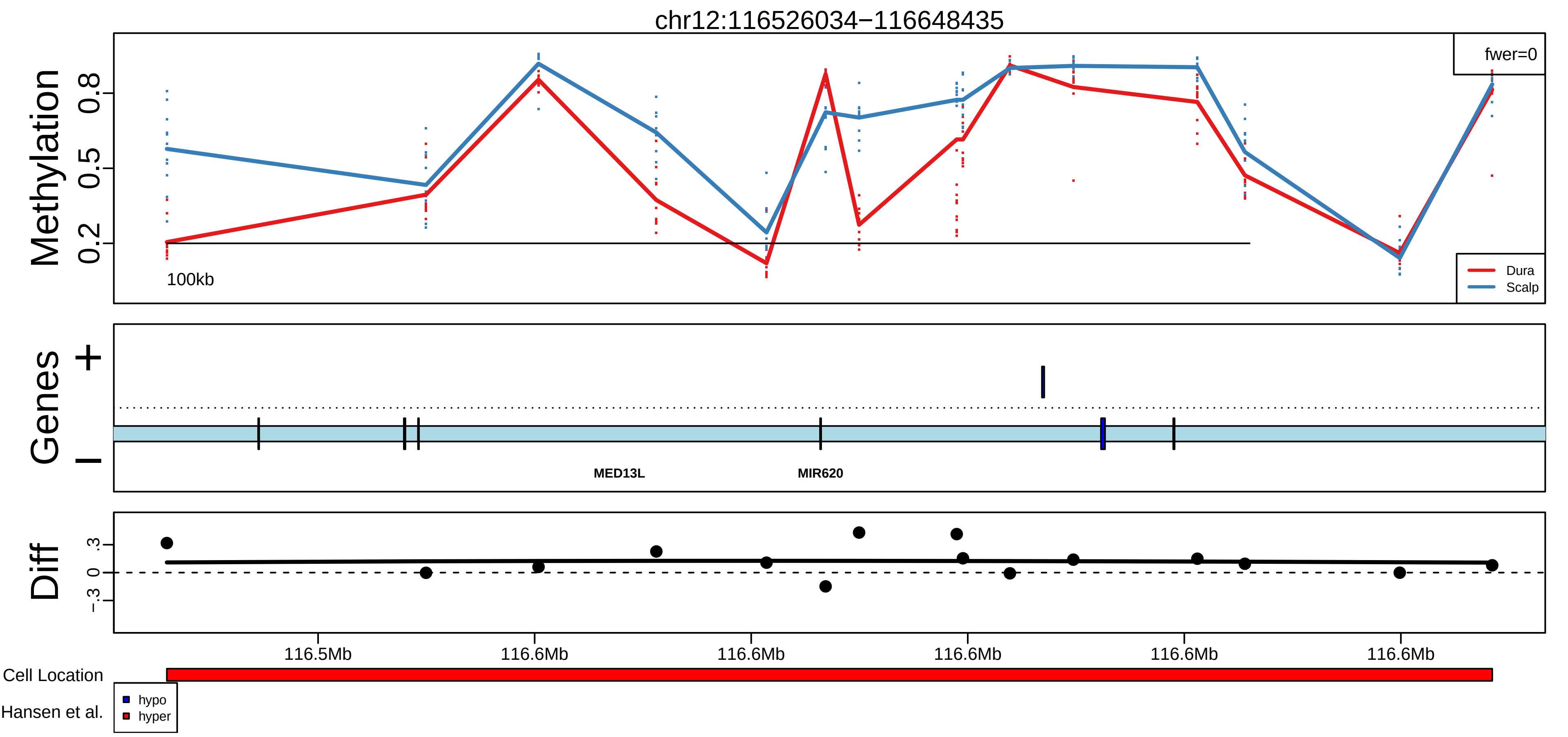

chr12:116526034−116648435
Methylation
0.2
0.5
0.8
fwer=0
100kb
Dura
Scalp
Genes
+
−
MED13L
MIR620
Diff
−.3
0
.3
116.5Mb
116.6Mb
116.6Mb
116.6Mb
116.6Mb
116.6Mb
Cell Location
Hansen et al.
hypo
hyper

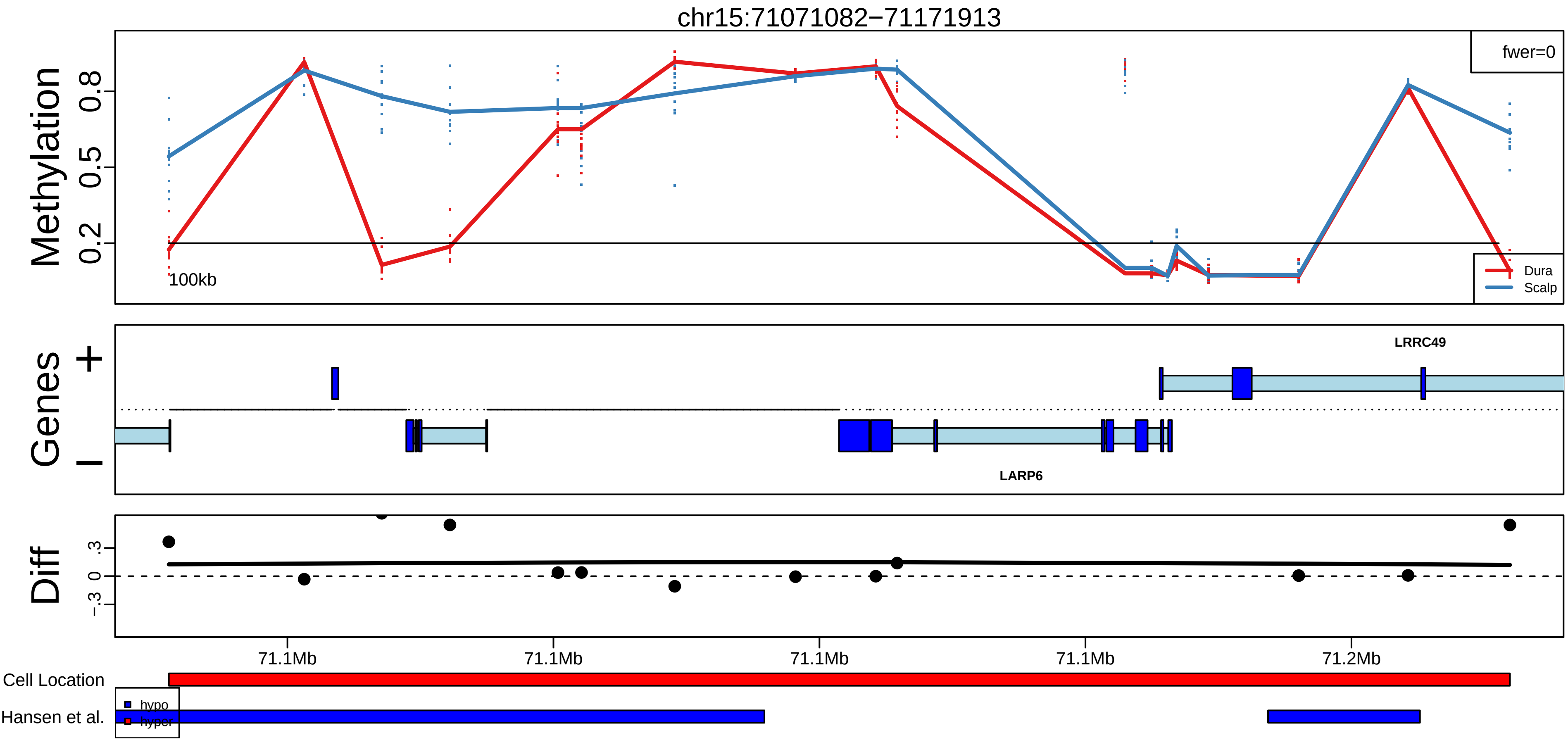
chr15:71071082−71171913
Methylation
0.2
0.5
0.8
fwer=0
100kb
Dura
Scalp
Genes
+
−
LRRC49
LARP6
Diff
−.3
0
.3
71.1Mb
71.1Mb
71.1Mb
71.1Mb
71.2Mb
Cell Location
Hansen et al.
hypo
hyper

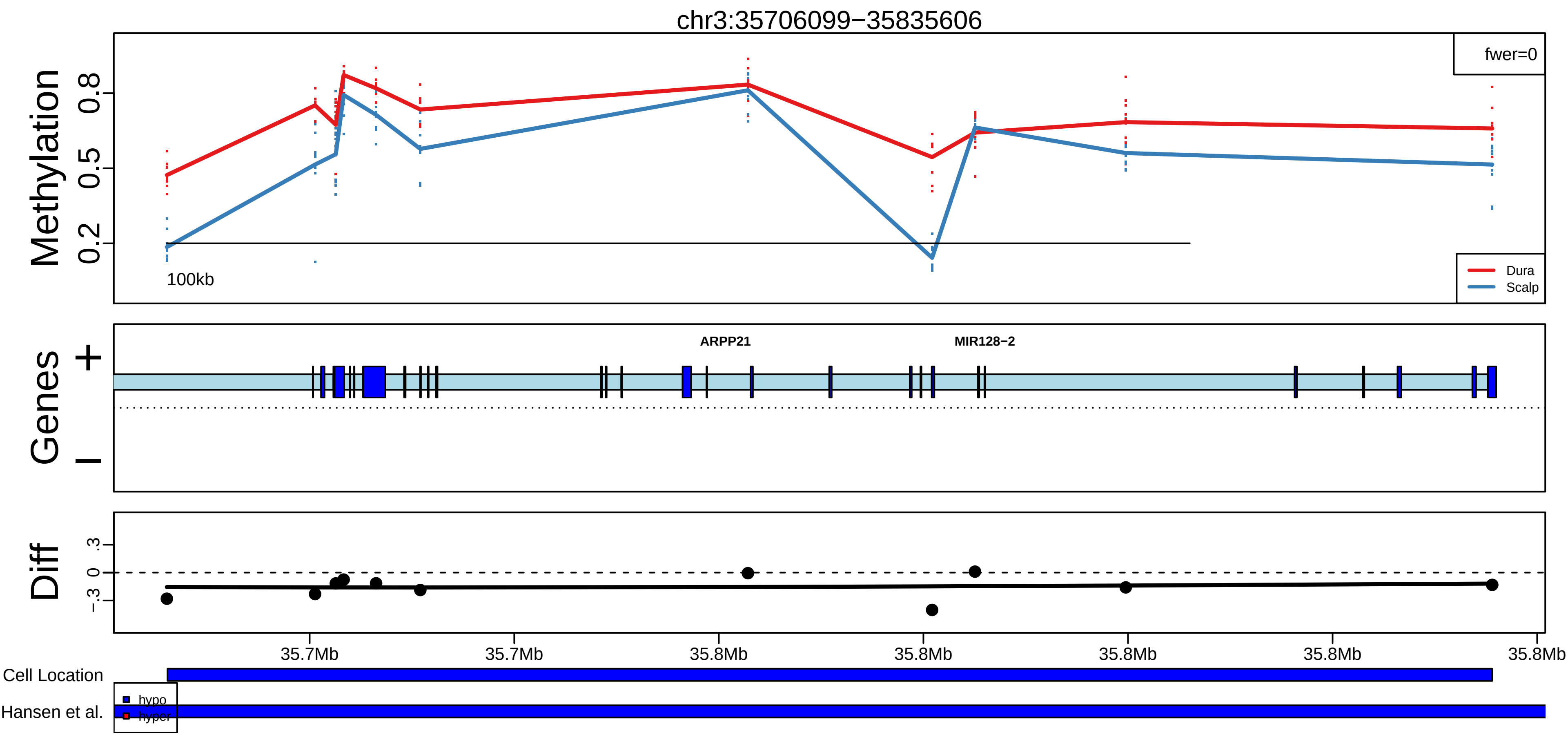

chr3:35706099−35835606
Methylation
0.2
0.5
0.8
fwer=0
100kb
Dura
Scalp
Genes
+
−
ARPP21
MIR128−2
Diff
−.3
0
.3
35.7Mb
35.7Mb
35.8Mb
35.8Mb
35.8Mb
35.8Mb
35.8Mb
Cell Location
Hansen et al.
hypo
hyper

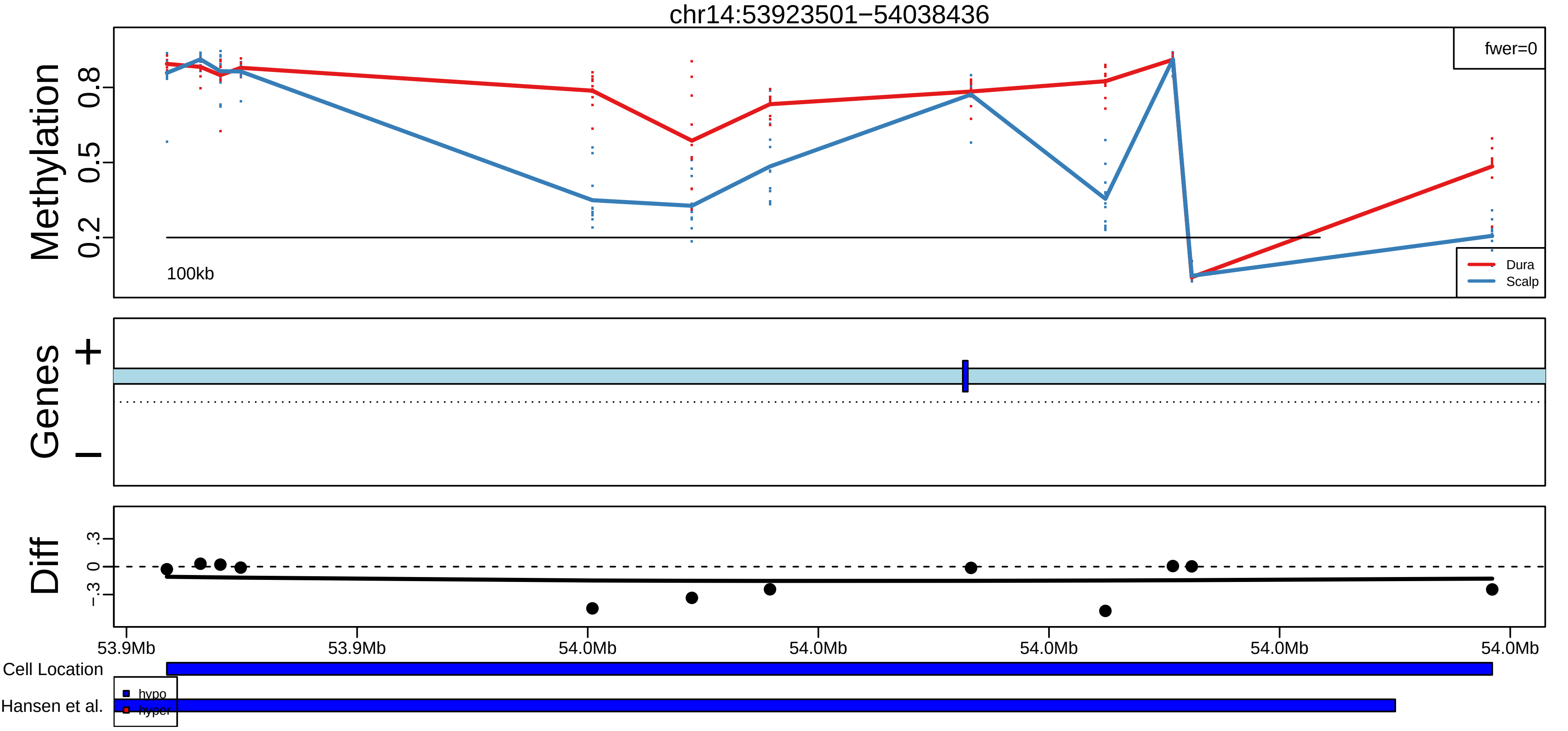
chr14:53923501−54038436
fwer=0
Methylation
0.8
0.5
0.2
100kb
Dura
Scalp
Genes
+
−
Diff
.3
0
−.3
53.9Mb
53.9Mb
54.0Mb
54.0Mb
54.0Mb
54.0Mb
54.0Mb
Cell Location
Hansen et al.
hypo
hyper

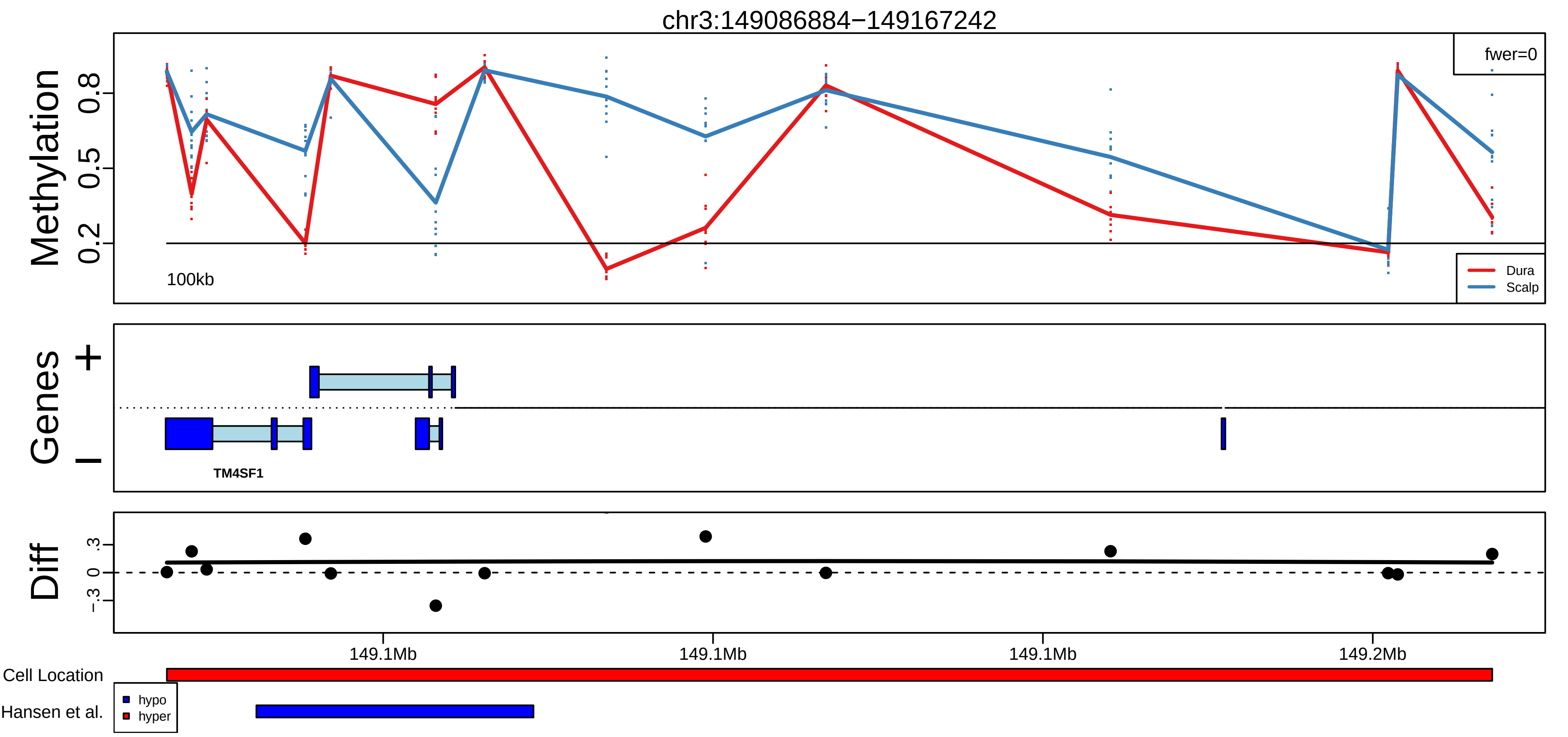

chr3:149086884−149167242
fwer=0
Methylation
0.2
0.5
0.8
100kb
Dura
Scalp
Genes
+
−
TM4SF1
Diff
.3
0
−.3
149.1Mb
149.1Mb
149.1Mb
149.2Mb
Cell Location
Hansen et al.
hypo
hyper

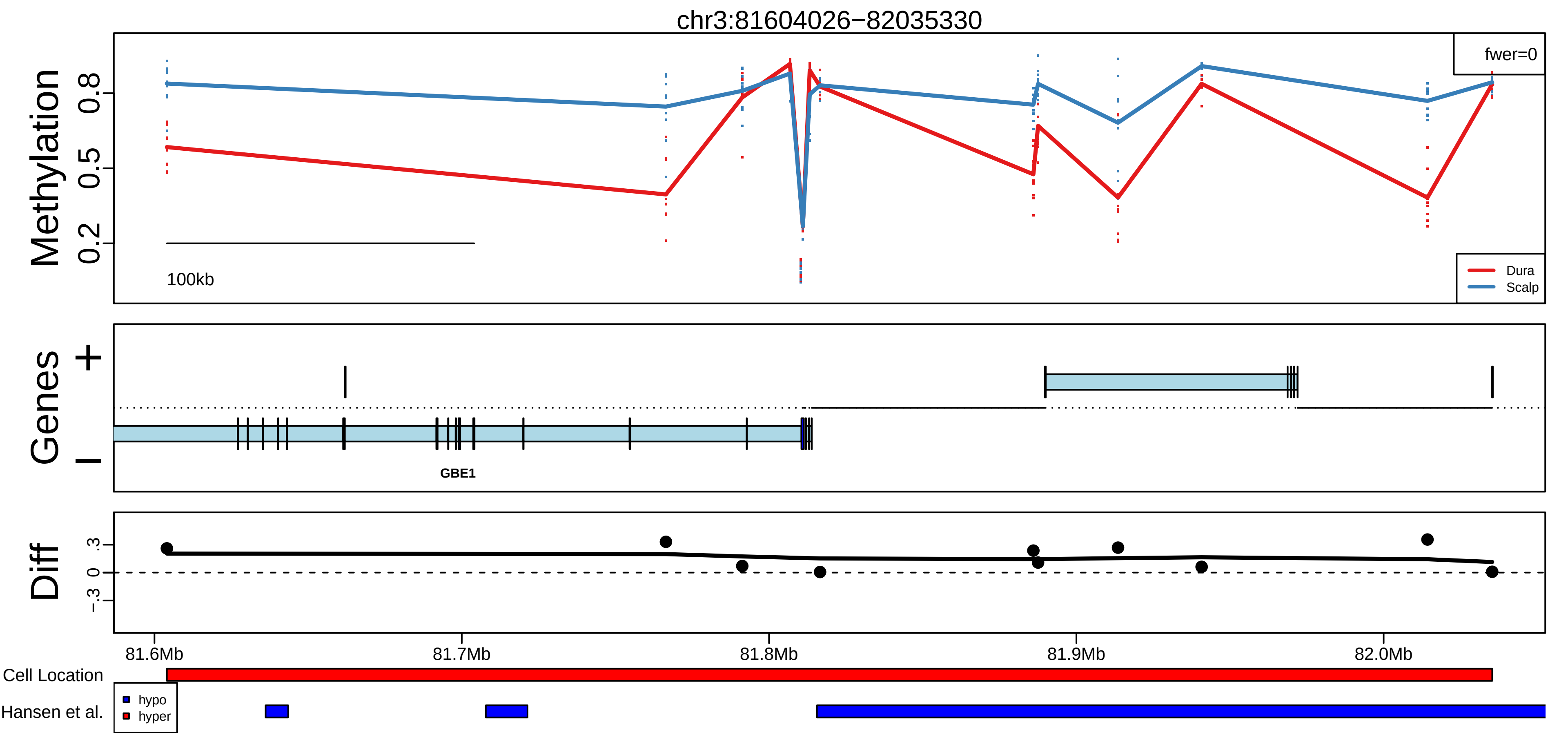

chr3:81604026−82035330
Methylation
0.2
0.5
0.8
fwer=0
100kb
Dura
Scalp
Genes
+
−
GBE1
Diff
.3
0
−.3
81.6Mb
81.7Mb
81.8Mb
81.9Mb
82.0Mb
Cell Location
Hansen et al.
hypo
hyper

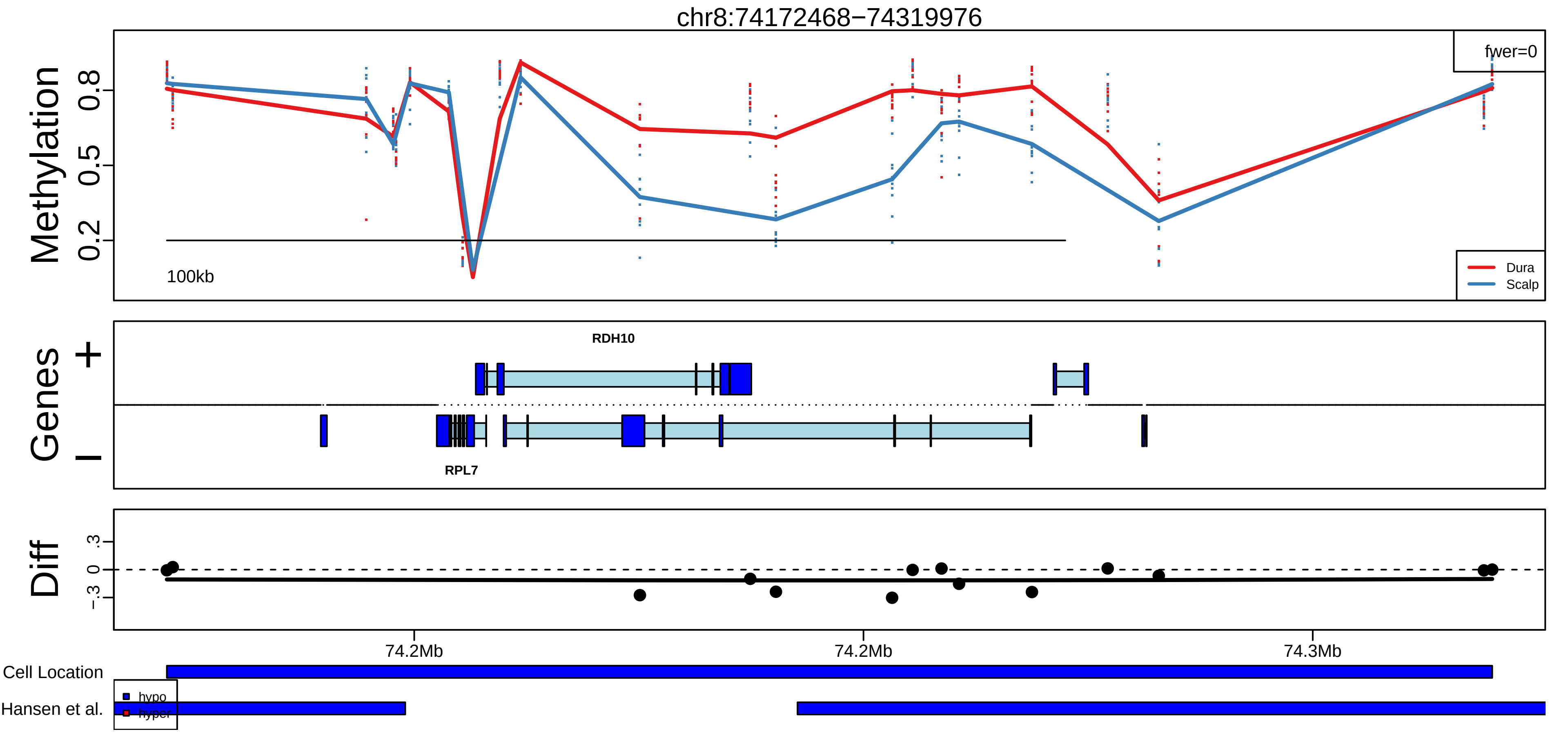
chr8:74172468−74319976
Methylation
0.2
0.5
0.8
fwer=0
100kb
Dura
Scalp
Genes
+
−
RDH10
RPL7
Diff
−.3
0
.3
74.2Mb
74.2Mb
74.3Mb
Cell Location
Hansen et al.
hypo
hyper

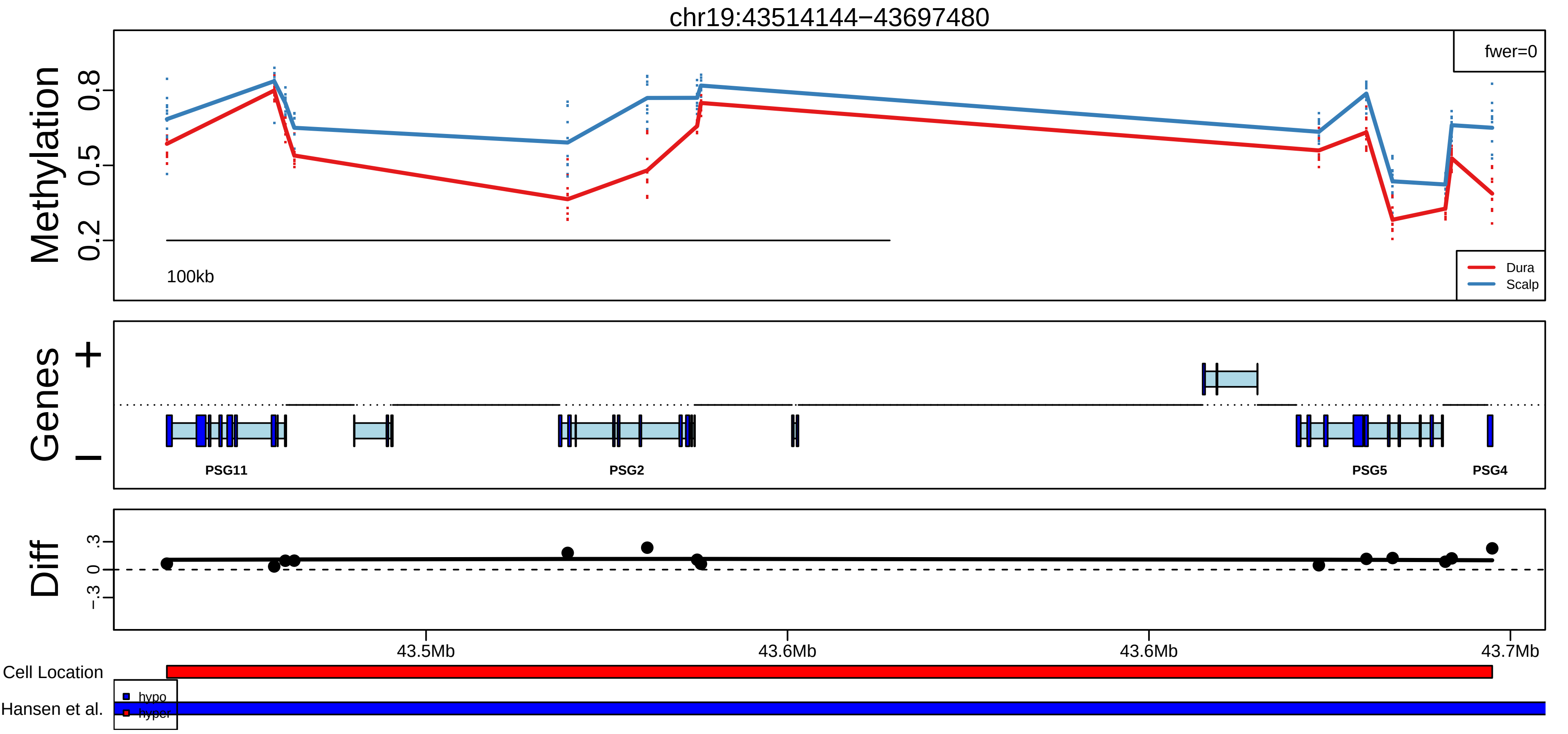

chr19:43514144−43697480
Methylation
0.2
0.5
0.8
fwer=0
100kb
Dura
Scalp
Genes
+
−
PSG11
PSG2
PSG5
PSG4
Diff
−.3
0
.3
43.5Mb
43.6Mb
43.6Mb
43.7Mb
Cell Location
Hansen et al.
hypo
hyper

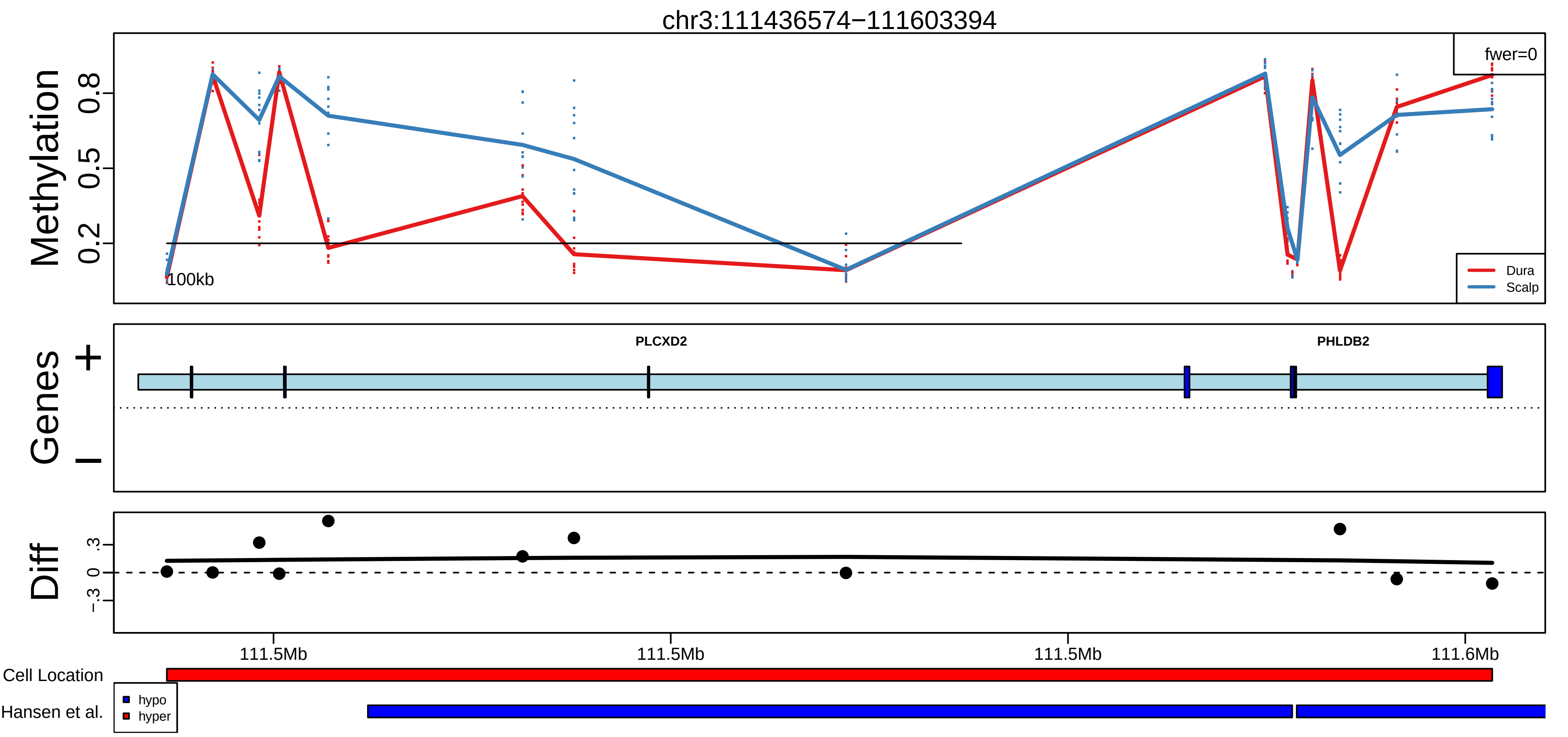

chr3:111436574−111603394
fwer=0
Methylation
0.2
0.5
0.8
100kb
Dura
Scalp
Genes
+
−
PLCXD2
PHLDB2
Diff
−.3
0
.3
111.5Mb
111.5Mb
111.5Mb
111.6Mb
Cell Location
Hansen et al.
hypo
hyper

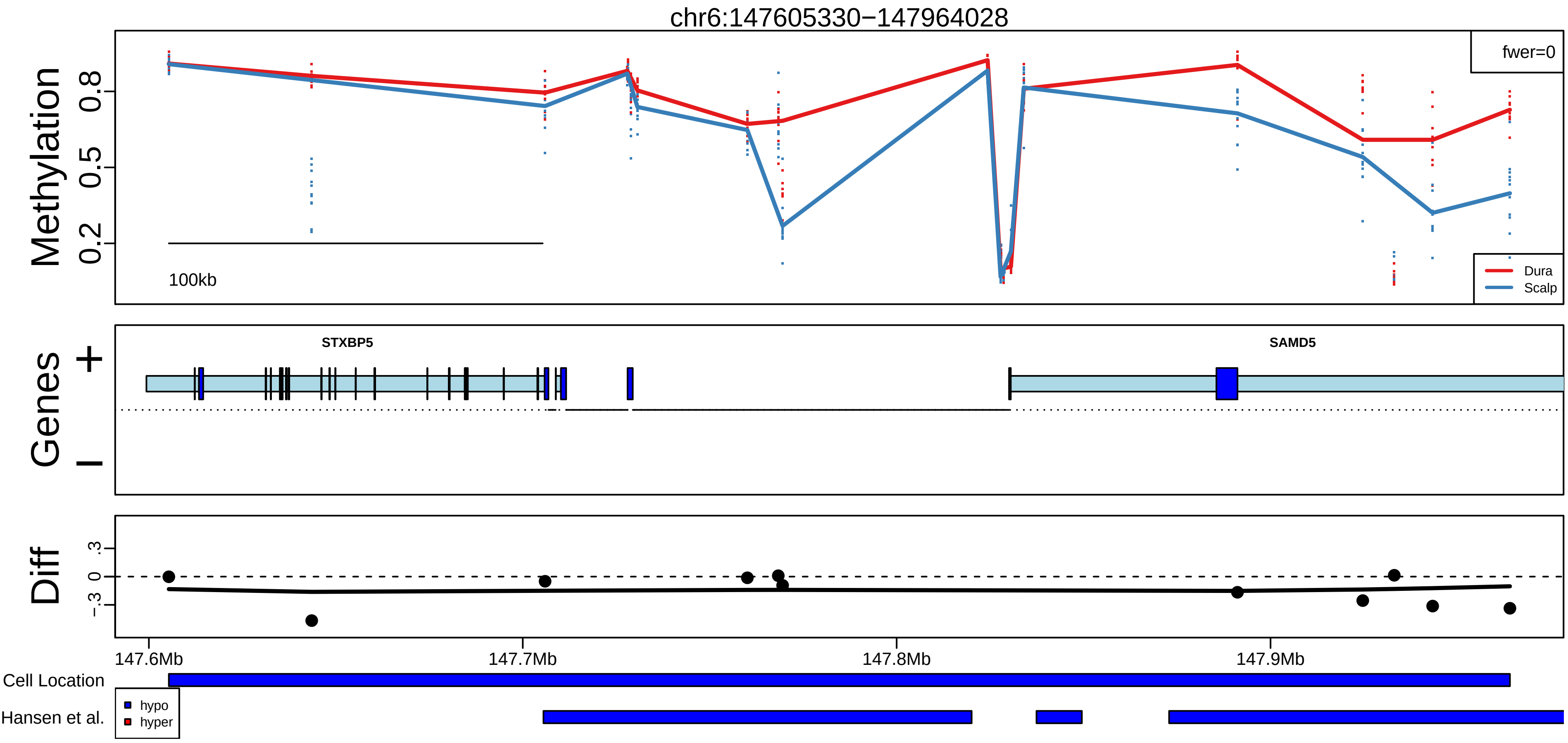

chr6:147605330−147964028
Methylation
0.2
0.5
0.8
fwer=0
100kb
Dura
Scalp
Genes
+
−
STXBP5
SAMD5
Diff
−.3
0
.3
147.6Mb
147.7Mb
147.8Mb
147.9Mb
Cell Location
Hansen et al.
hypo
hyper

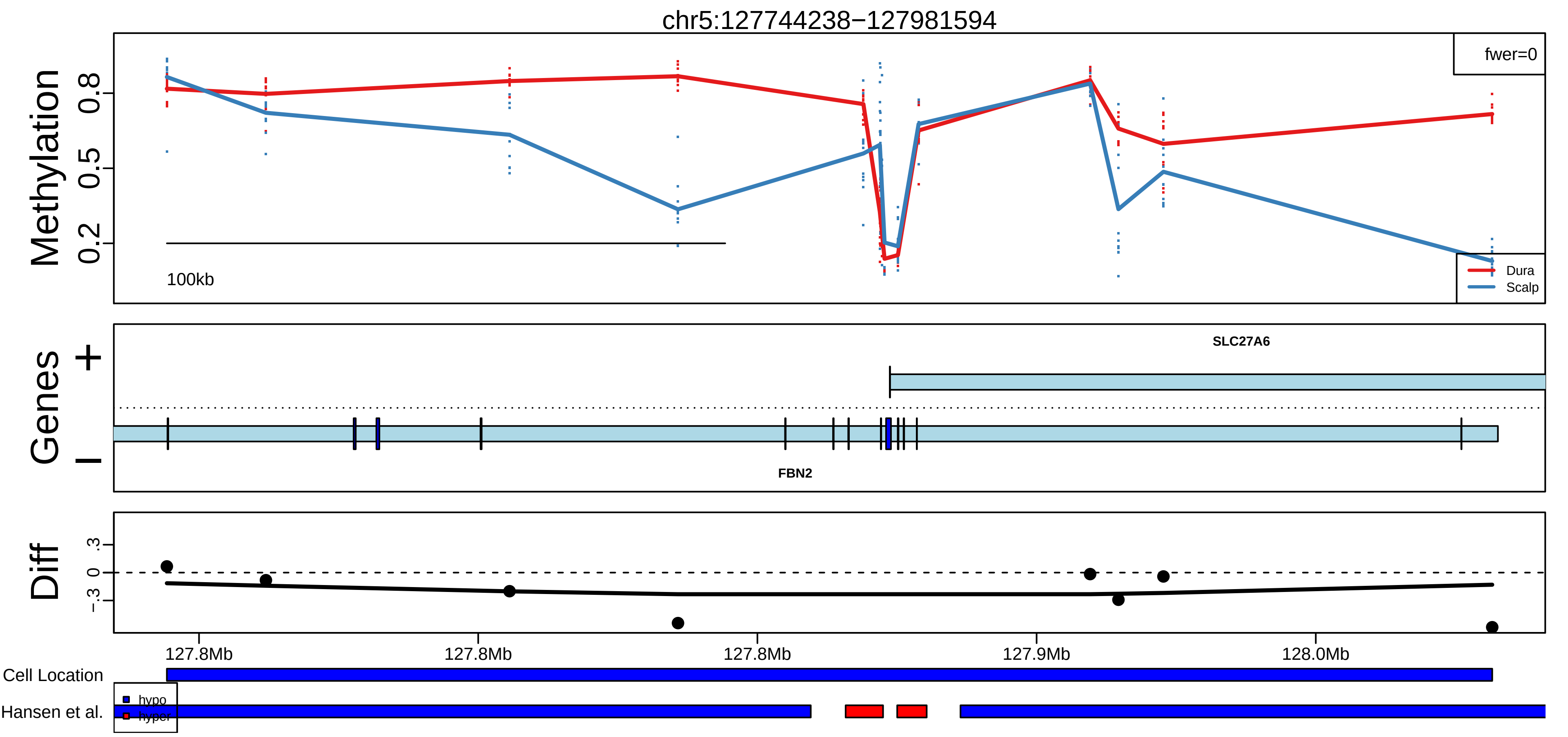
chr5:127744238−127981594
Methylation
0.2
0.5
0.8
fwer=0
100kb
Dura
Scalp
Genes
+
−
SLC27A6
FBN2
Diff
−.3
0
.3
127.8Mb
127.8Mb
127.8Mb
127.9Mb
128.0Mb
Cell Location
Hansen et al.
hypo
hyper

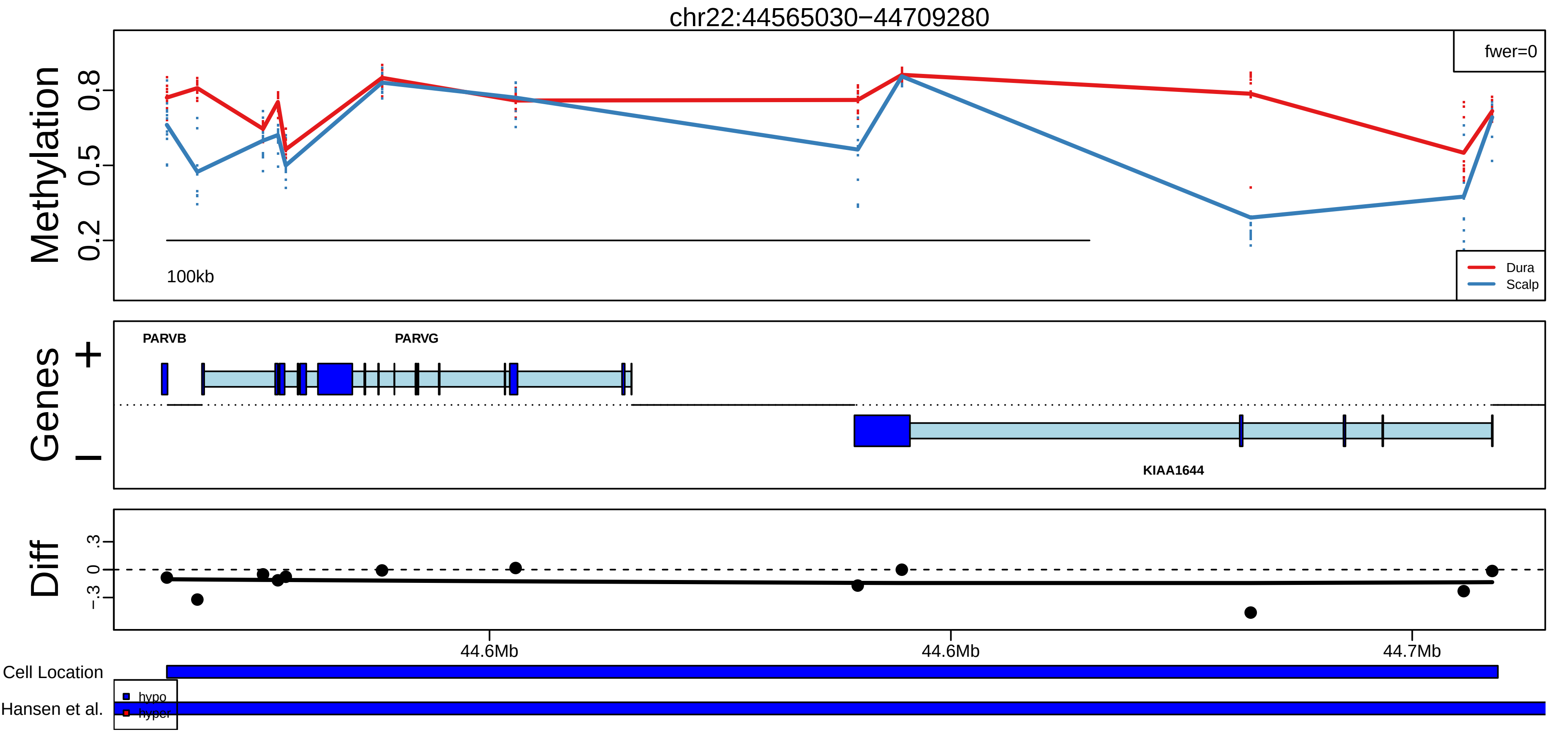

chr22:44565030−44709280
fwer=0
Methylation
0.2
0.5
0.8
100kb
Dura
Scalp
Genes
+
−
PARVB
PARVG
KIAA1644
Diff
−.3
0
.3
44.6Mb
44.6Mb
44.7Mb
Cell Location
Hansen et al.
hypo
hyper

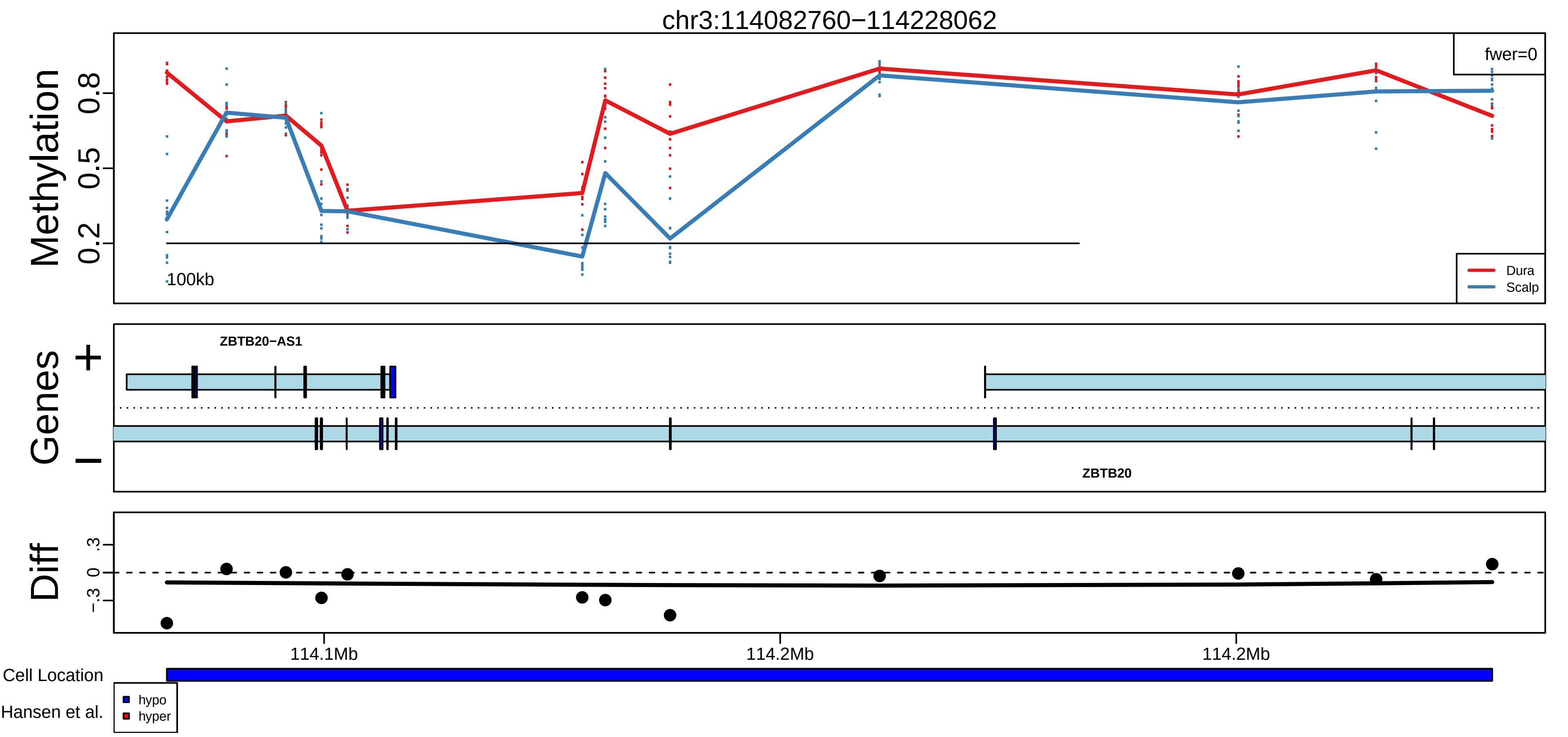

chr3:114082760−114228062
Methylation
0.2
0.5
0.8
fwer=0
100kb
Dura
Scalp
Genes
+
−
ZBTB20−AS1
ZBTB20
Diff
.3
0
−.3
114.1Mb
114.2Mb
114.2Mb
Cell Location
Hansen et al.
hypo
hyper

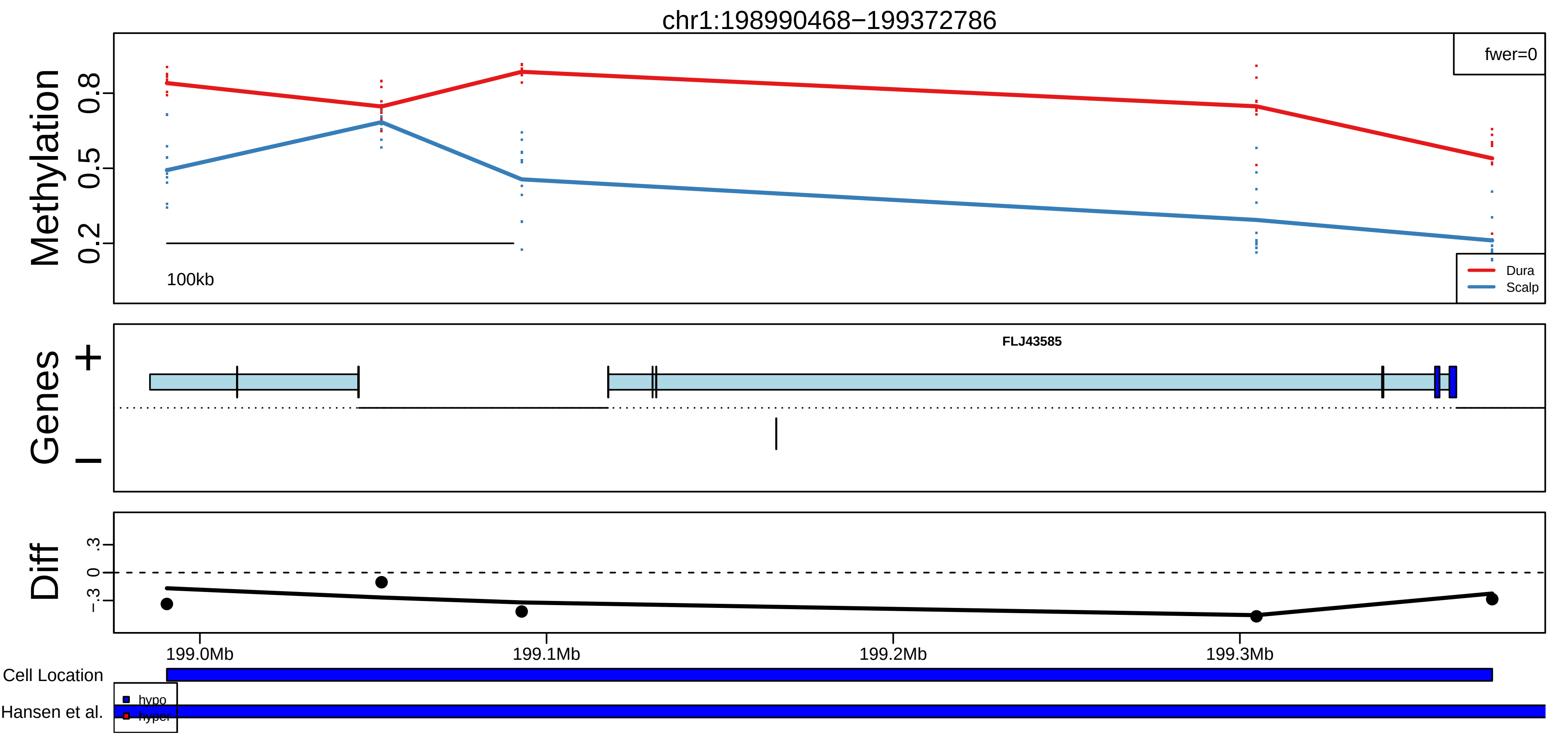
chr1:198990468−199372786
fwer=0
Methylation
0.2
0.5
0.8
100kb
Dura
Scalp
Genes
+
−
FLJ43585
Diff
−.3
0
.3
199.0Mb
199.1Mb
199.2Mb
199.3Mb
Cell Location
Hansen et al.
hypo
hyper

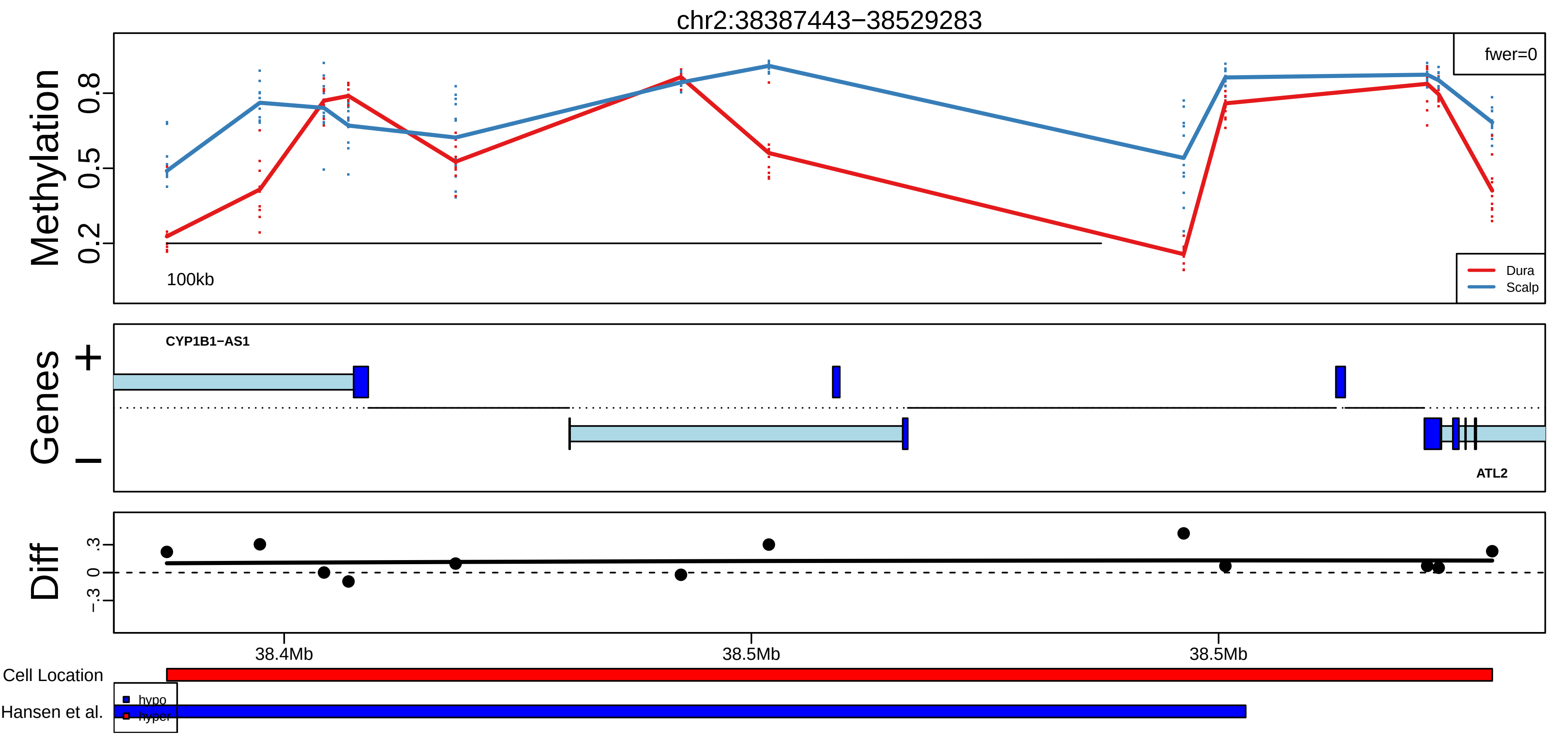

chr2:38387443−38529283
fwer=0
Methylation
0.2
0.5
0.8
100kb
Dura
Scalp
Genes
+
−
CYP1B1−AS1
ATL2
Diff
−.3
0
.3
38.4Mb
38.5Mb
38.5Mb
Cell Location
Hansen et al.
hypo
hyper

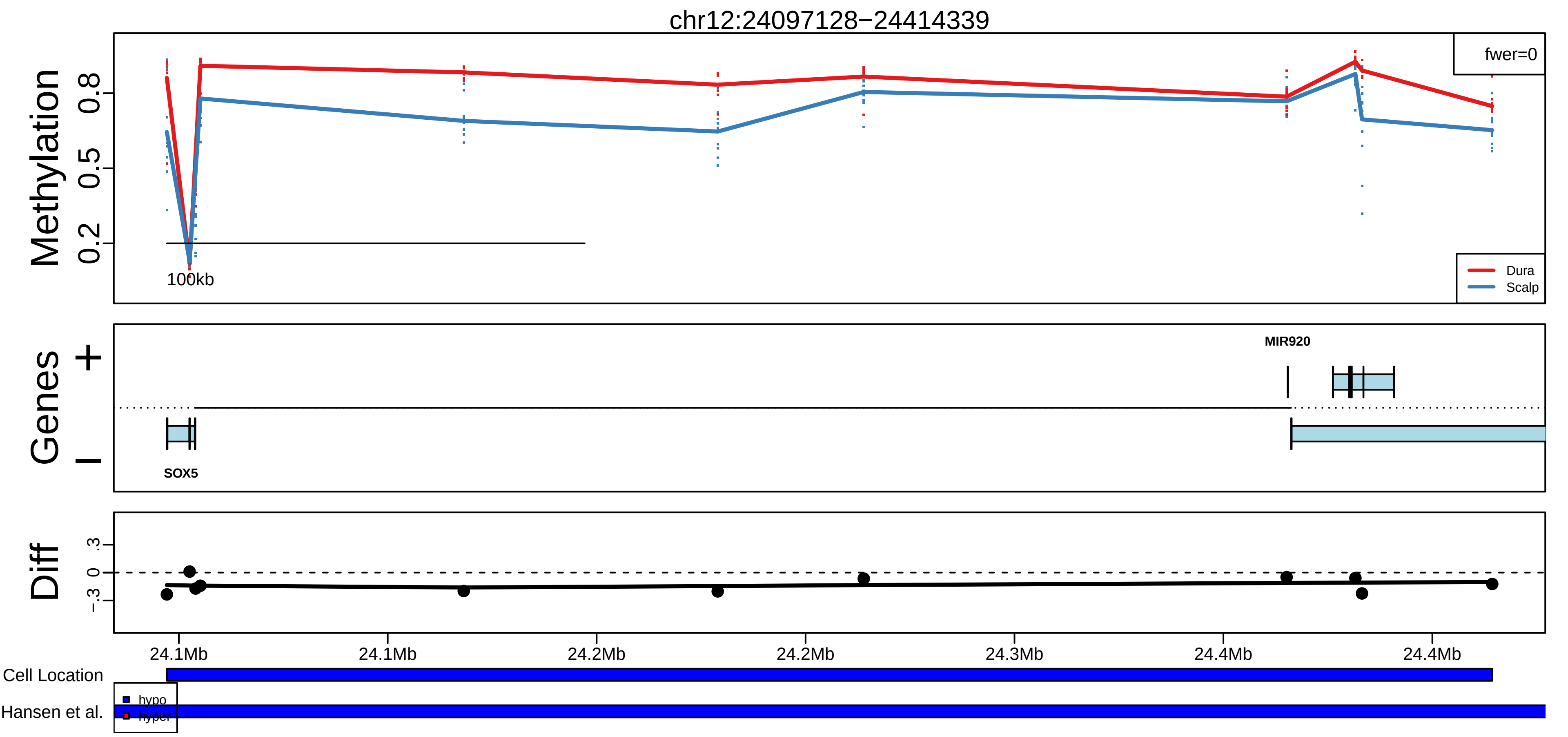

chr12:24097128−24414339
fwer=0
Methylation
0.2
0.5
0.8
100kb
Dura
Scalp
Genes
+
−
MIR920
SOX5
Diff
−.3
0
.3
24.1Mb
24.1Mb
24.2Mb
24.2Mb
24.3Mb
24.4Mb
24.4Mb
Cell Location
Hansen et al.
hypo
hyper

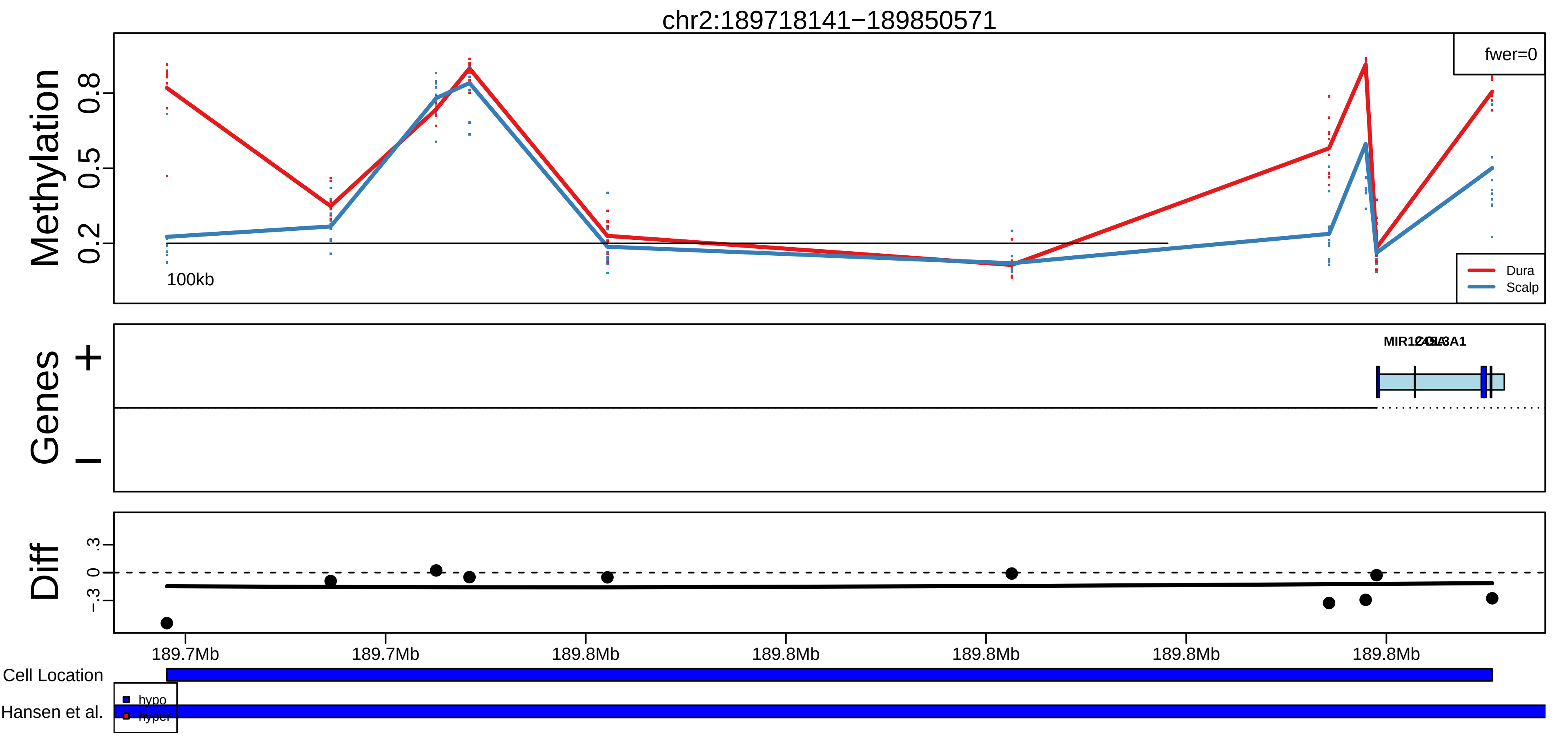

chr2:189718141−189850571
Methylation
0.2
0.5
0.8
fwer=0
100kb
Dura
Scalp
Genes
+
−
MIR1245A
COL3A1
Diff
−.3
0
.3
189.7Mb
189.7Mb
189.8Mb
189.8Mb
189.8Mb
189.8Mb
189.8Mb
Cell Location
Hansen et al.
hypo
hyper

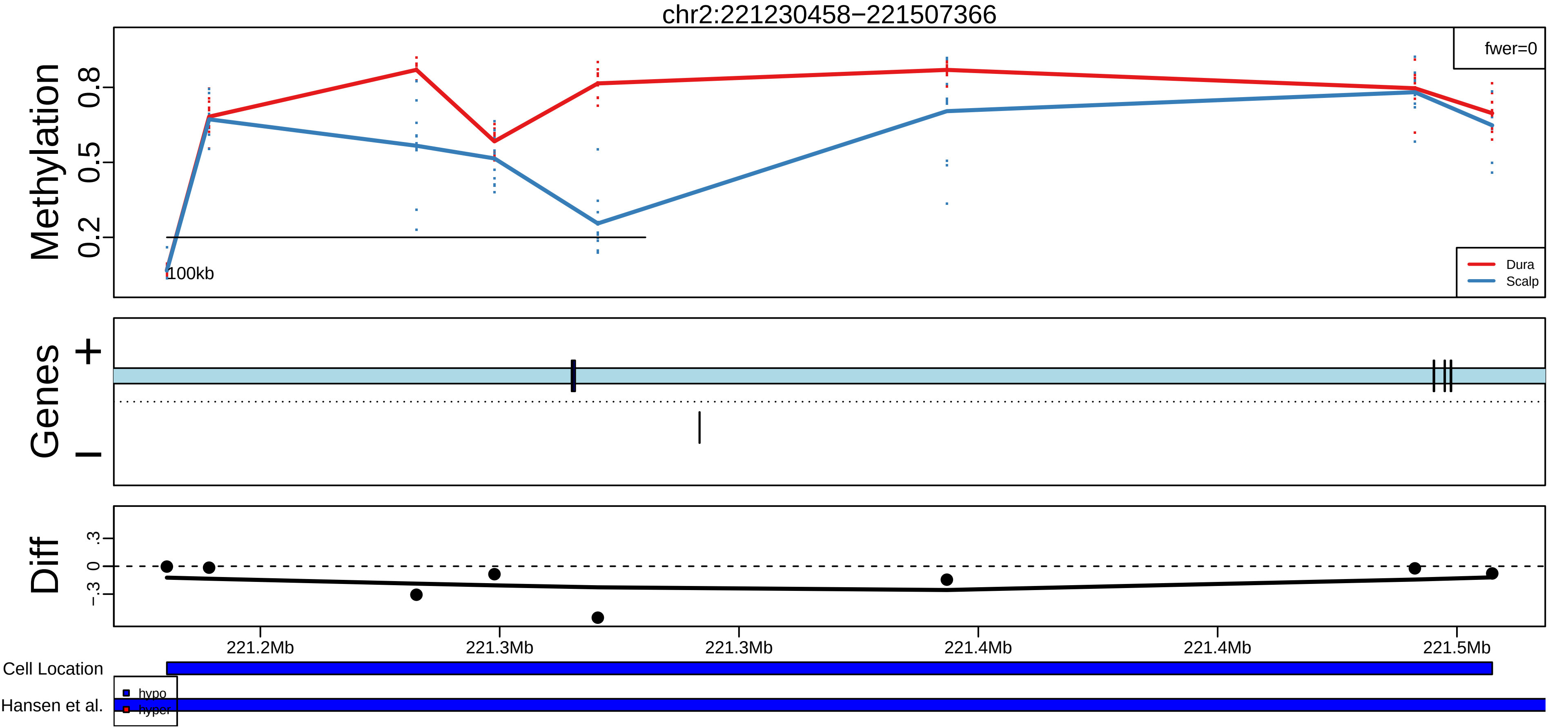

chr2:221230458−221507366
Methylation
0.2
0.5
0.8
fwer=0
100kb
Dura
Scalp
Genes
+
−
Diff
.3
0
−.3
221.2Mb
221.3Mb
221.3Mb
221.4Mb
221.4Mb
221.5Mb
Cell Location
Hansen et al.
hypo
hyper

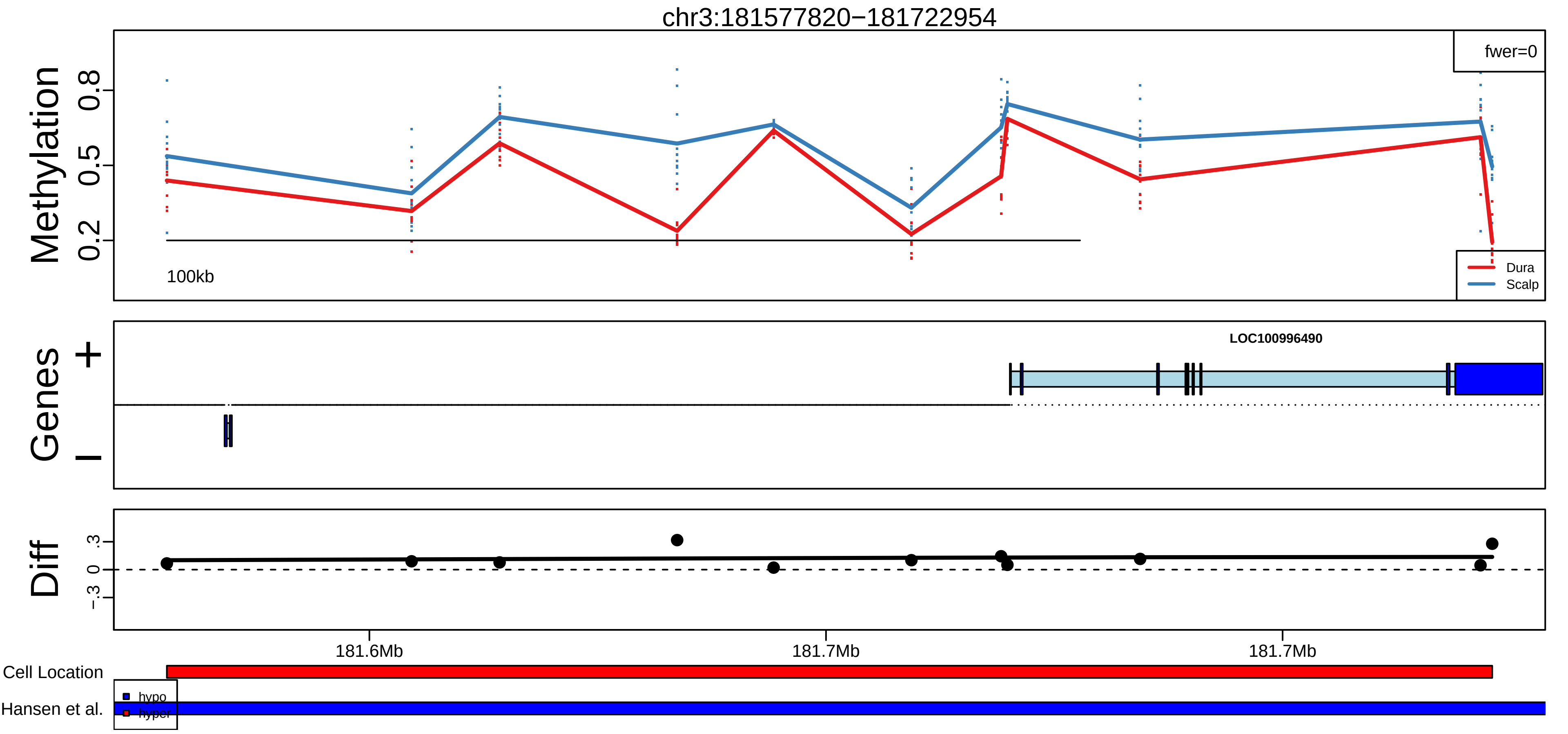

chr3:181577820−181722954
fwer=0
Methylation
0.2
0.5
0.8
100kb
Dura
Scalp
Genes
+
−
LOC100996490
Diff
−.3
0
.3
181.6Mb
181.7Mb
181.7Mb
Cell Location
Hansen et al.
hypo
hyper

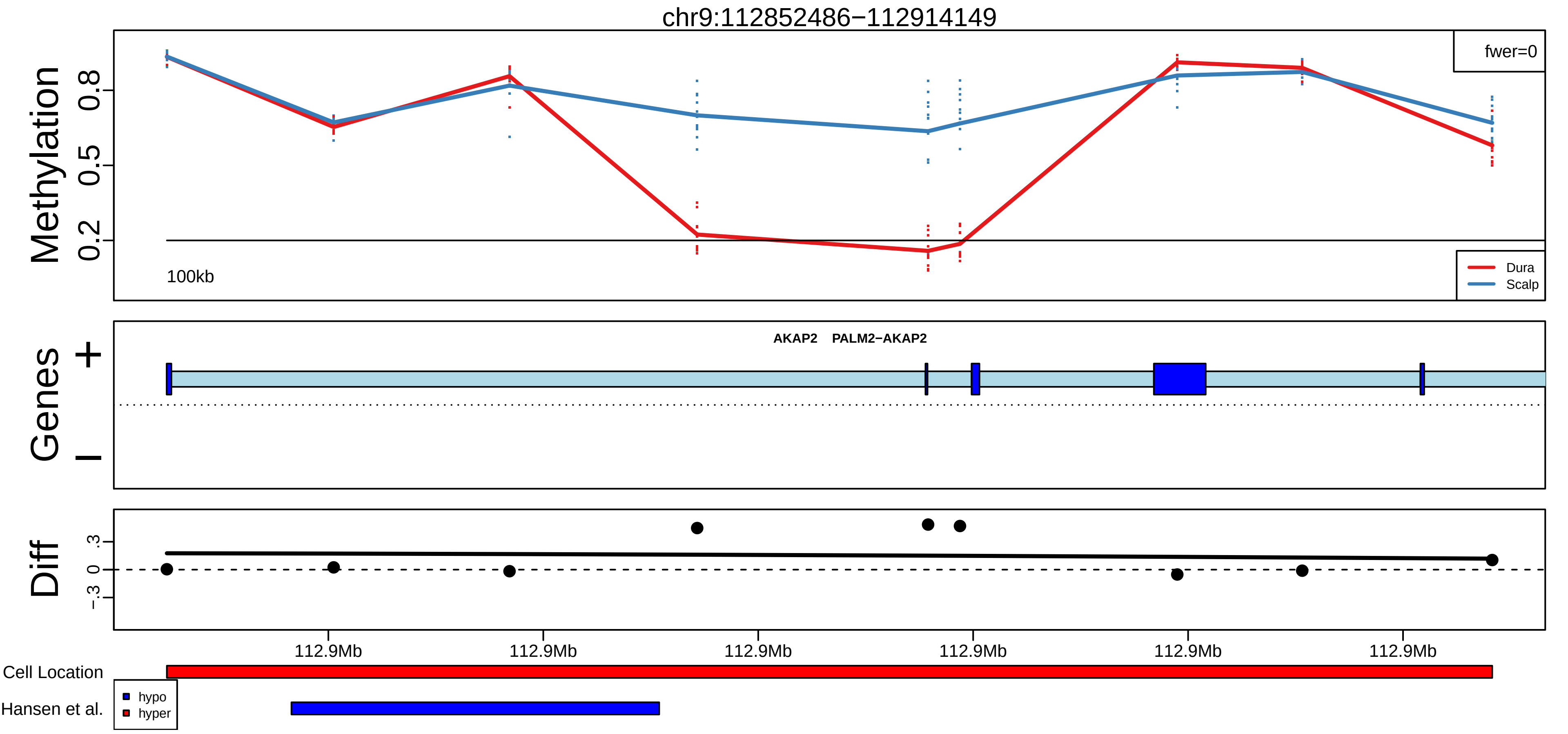
chr9:112852486−112914149
Methylation
0.2
0.5
0.8
fwer=0
100kb
Dura
Scalp
Genes
+
−
AKAP2
PALM2−AKAP2
Diff
−.3
0
.3
112.9Mb
112.9Mb
112.9Mb
112.9Mb
112.9Mb
112.9Mb
Cell Location
Hansen et al.
hypo
hyper

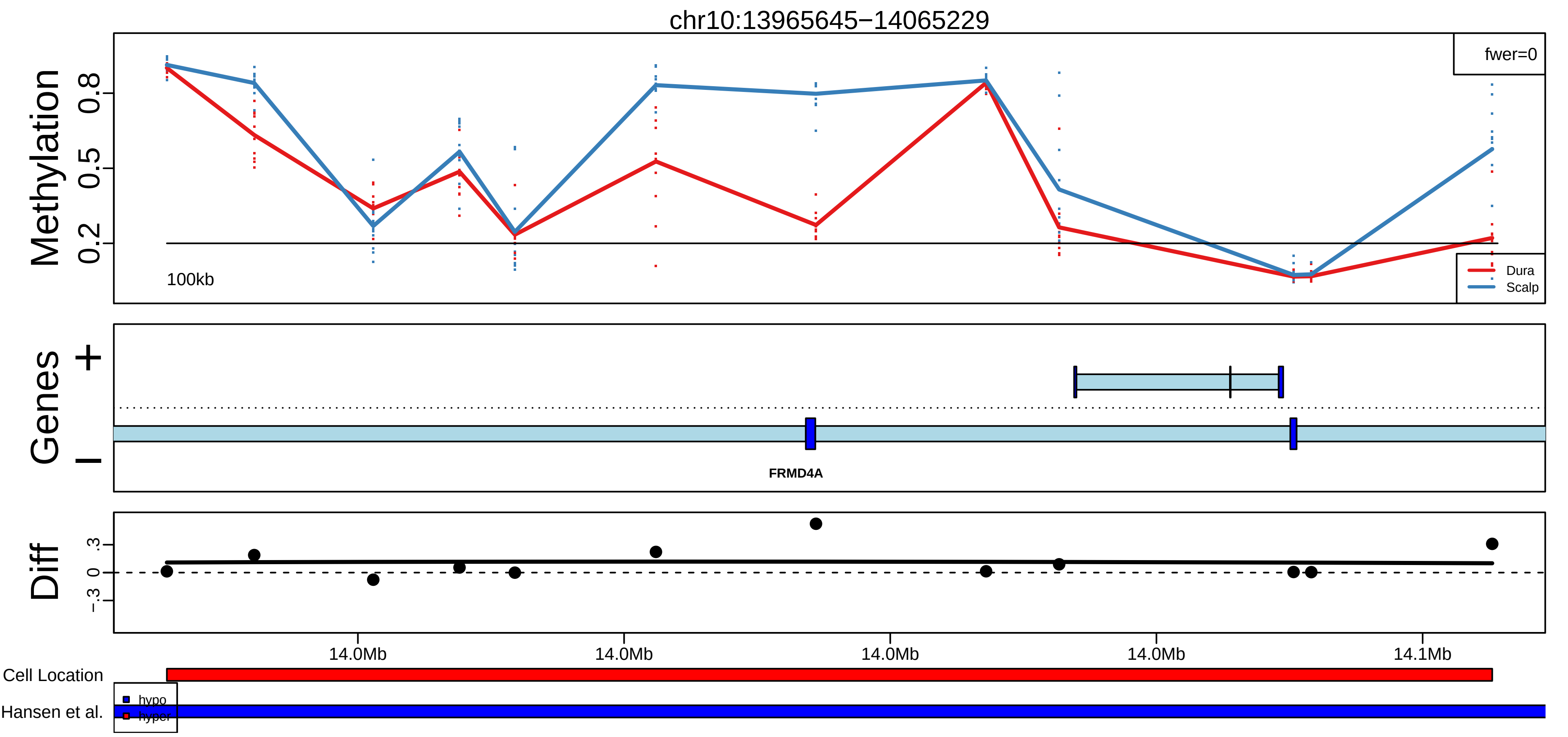
chr10:13965645−14065229
Methylation
0.2
0.5
0.8
fwer=0
100kb
Dura
Scalp
Genes
+
−
FRMD4A
Diff
−.3
0
.3
14.0Mb
14.0Mb
14.0Mb
14.0Mb
14.1Mb
Cell Location
Hansen et al.
hypo
hyper

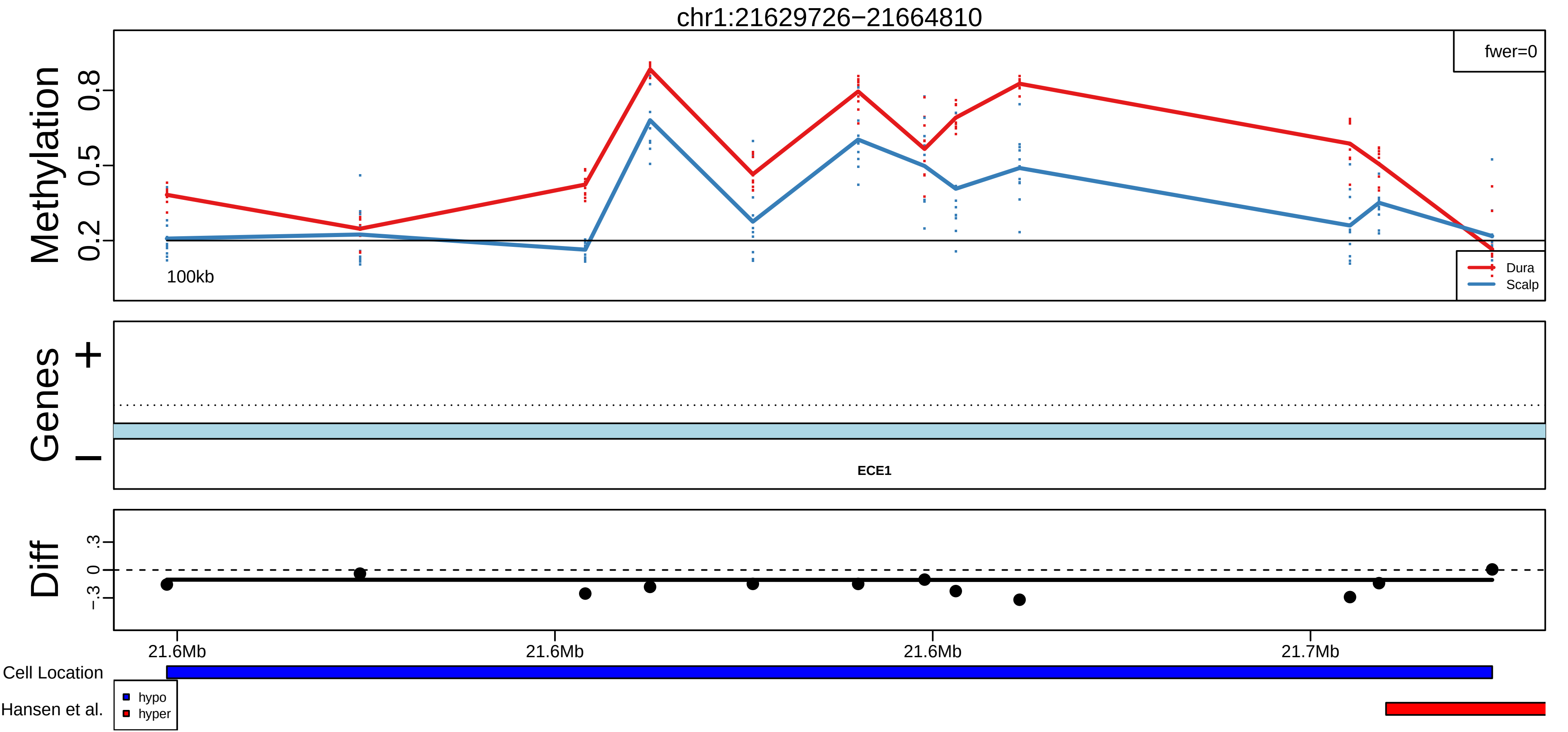
chr1:21629726−21664810
fwer=0
Methylation
0.2
0.5
0.8
100kb
Dura
Scalp
Genes
+
−
ECE1
Diff
−.3
0
.3
21.6Mb
21.6Mb
21.6Mb
21.7Mb
Cell Location
Hansen et al.
hypo
hyper

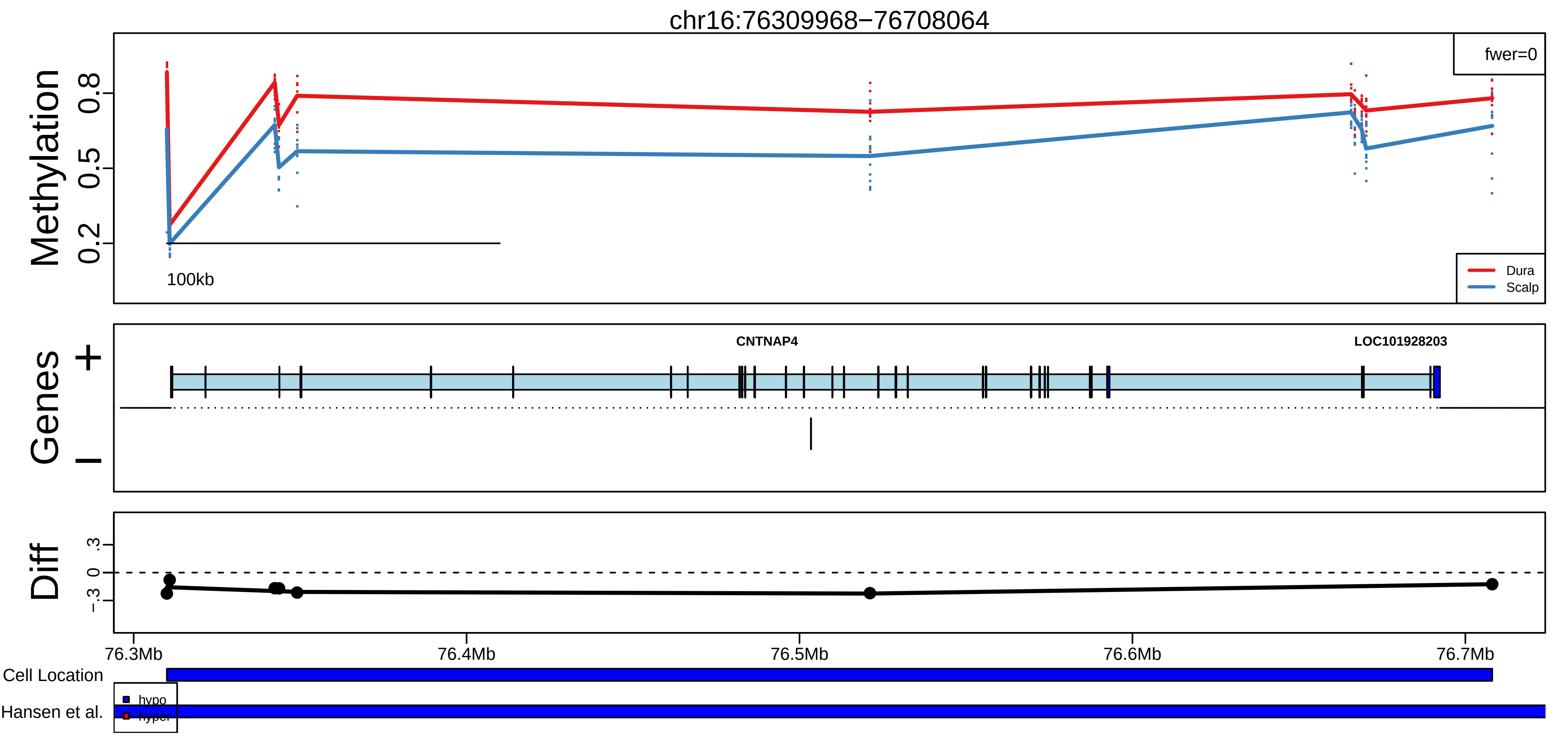

chr16:76309968−76708064
fwer=0
Methylation
0.2
0.5
0.8
100kb
Dura
Scalp
Genes
+
−
CNTNAP4
LOC101928203
Diff
−.3
0
.3
76.3Mb
76.4Mb
76.5Mb
76.6Mb
76.7Mb
Cell Location
Hansen et al.
hypo
hyper

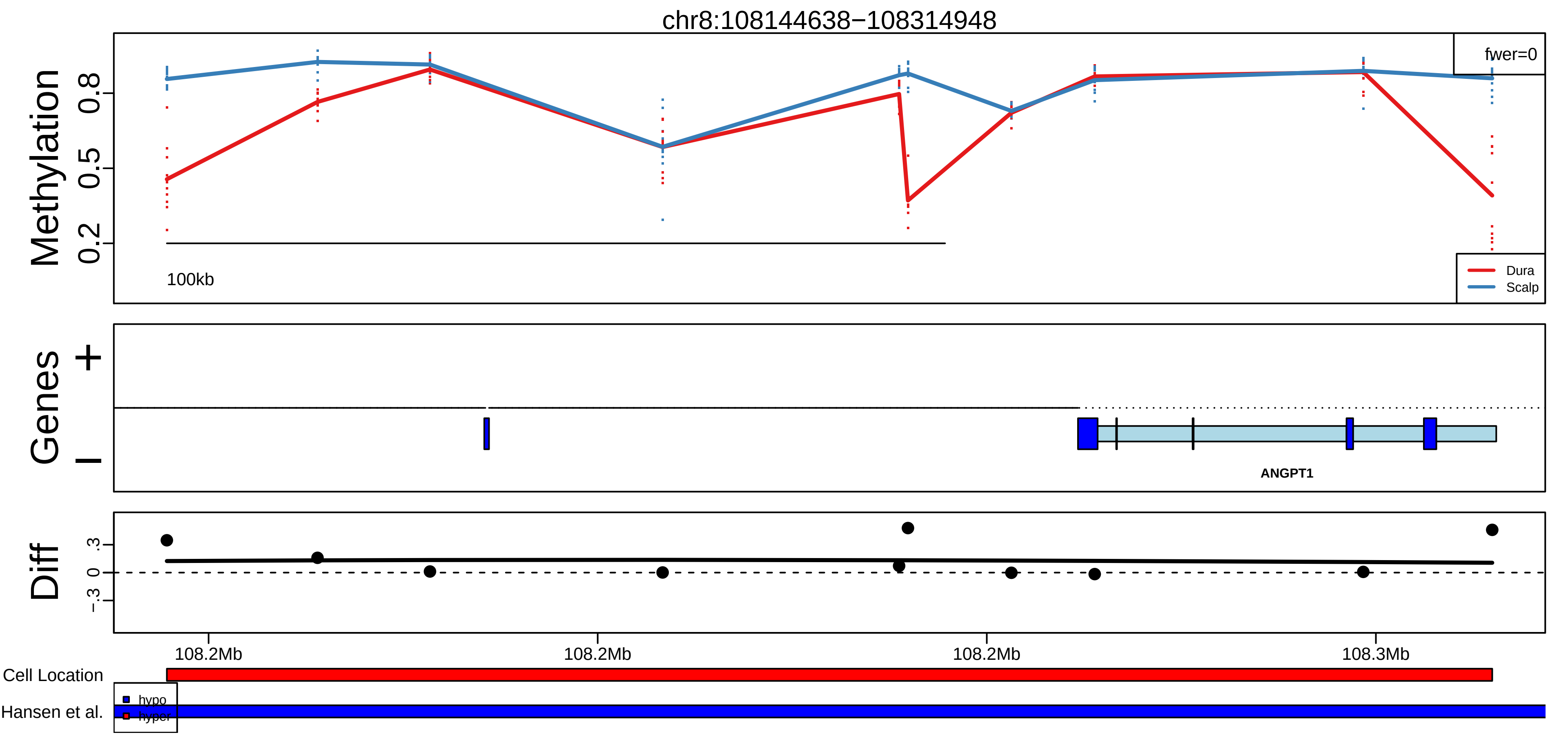

chr8:108144638−108314948
fwer=0
Methylation
0.2
0.5
0.8
100kb
Dura
Scalp
Genes
+
−
ANGPT1
Diff
−.3
0
.3
108.2Mb
108.2Mb
108.2Mb
108.3Mb
Cell Location
Hansen et al.
hypo
hyper

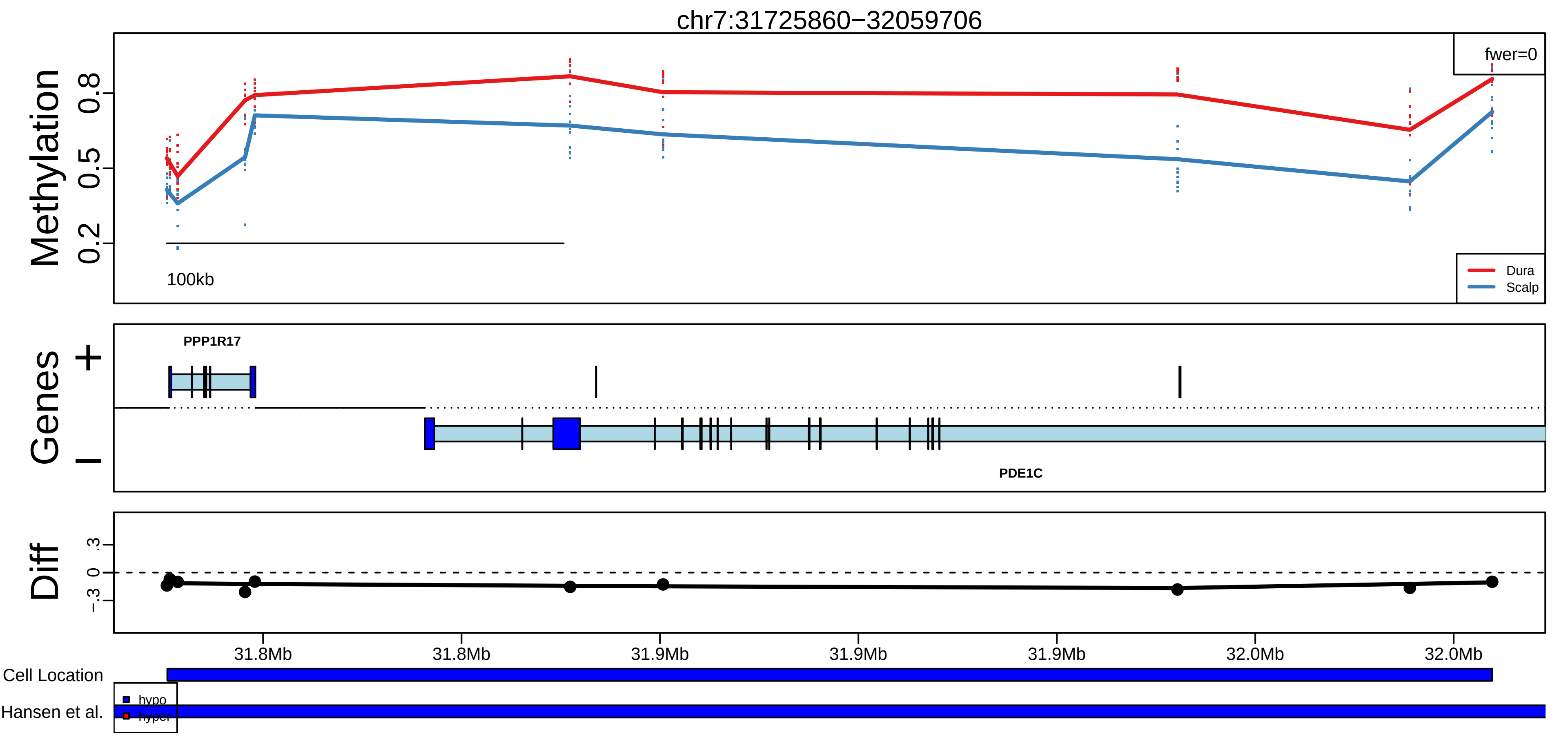

chr7:31725860−32059706
fwer=0
Methylation
0.8
0.5
0.2
100kb
Dura
Scalp
Genes
+
−
PPP1R17
PDE1C
Diff
.3
0
−.3
31.8Mb
31.8Mb
31.9Mb
31.9Mb
31.9Mb
32.0Mb
32.0Mb
Cell Location
hypo
hyper
Hansen et al.

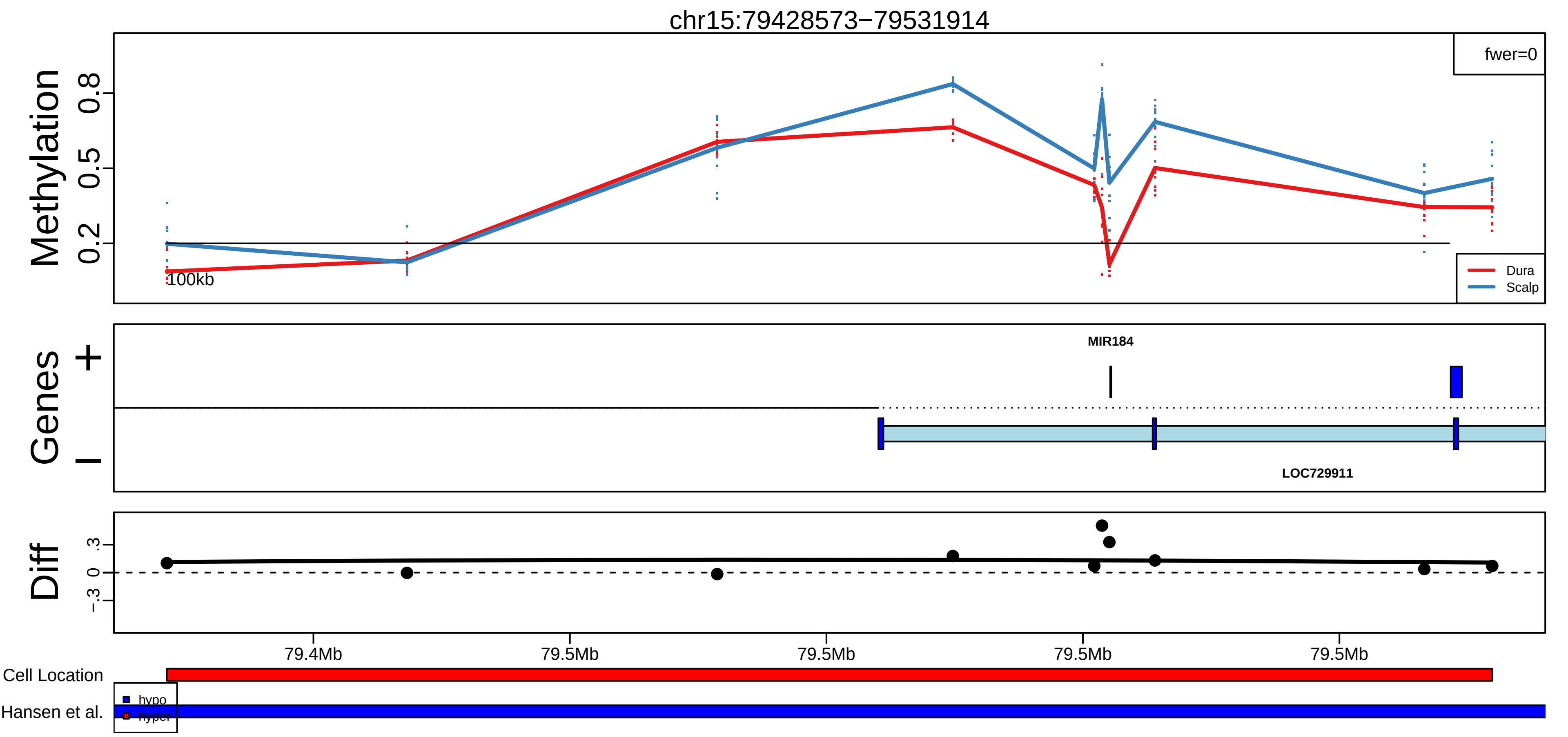
chr15:79428573−79531914
fwer=0
Methylation
0.2
0.5
0.8
100kb
Dura
Scalp
Genes
+
−
MIR184
LOC729911
Diff
−.3
0
.3
79.4Mb
79.5Mb
79.5Mb
79.5Mb
79.5Mb
Cell Location
Hansen et al.
hypo
hyper

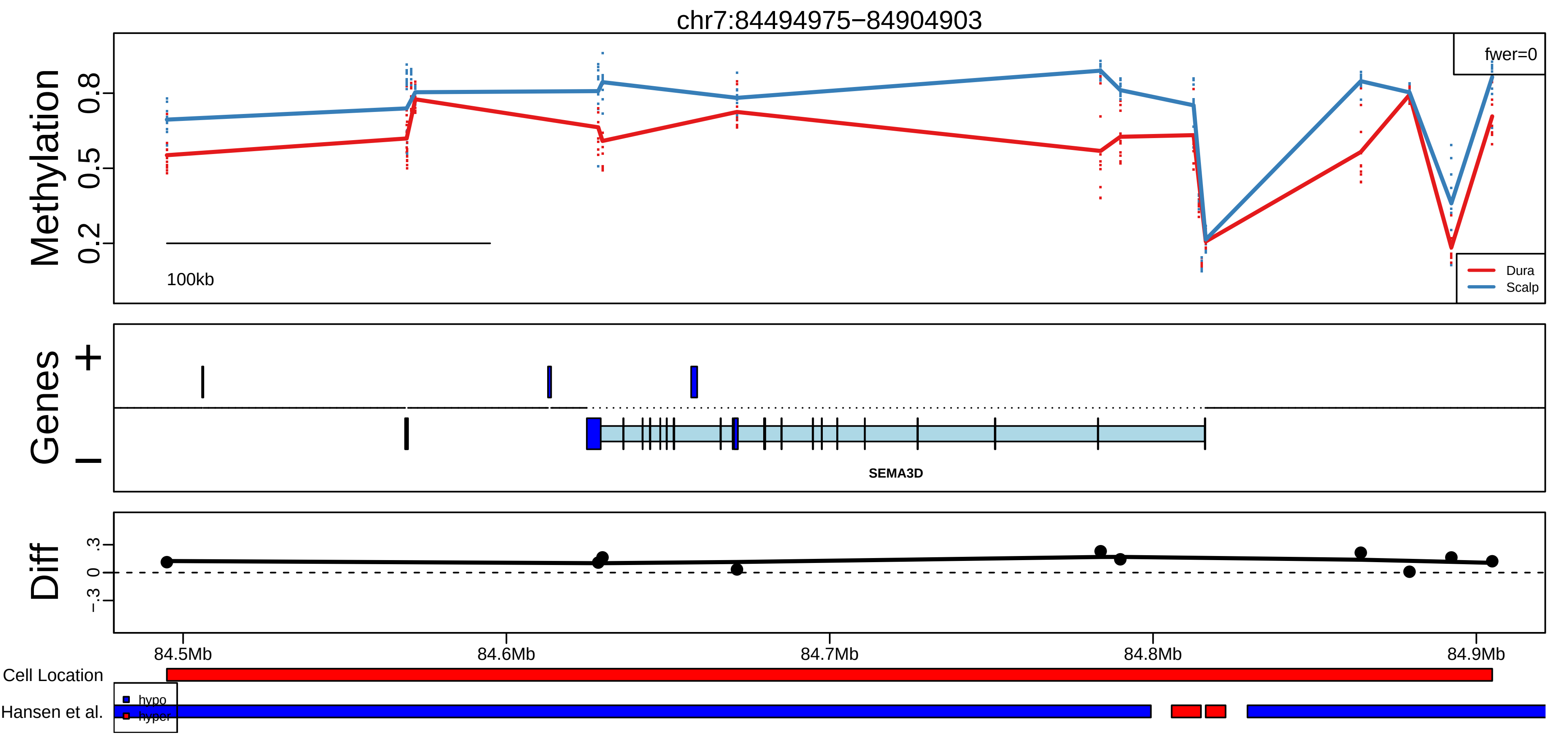

chr7:84494975−84904903
fwer=0
Methylation
0.2
0.5
0.8
100kb
Dura
Scalp
Genes
+
−
SEMA3D
Diff
−.3
0
.3
84.5Mb
84.6Mb
84.7Mb
84.8Mb
84.9Mb
Cell Location
Hansen et al.
hypo
hyper

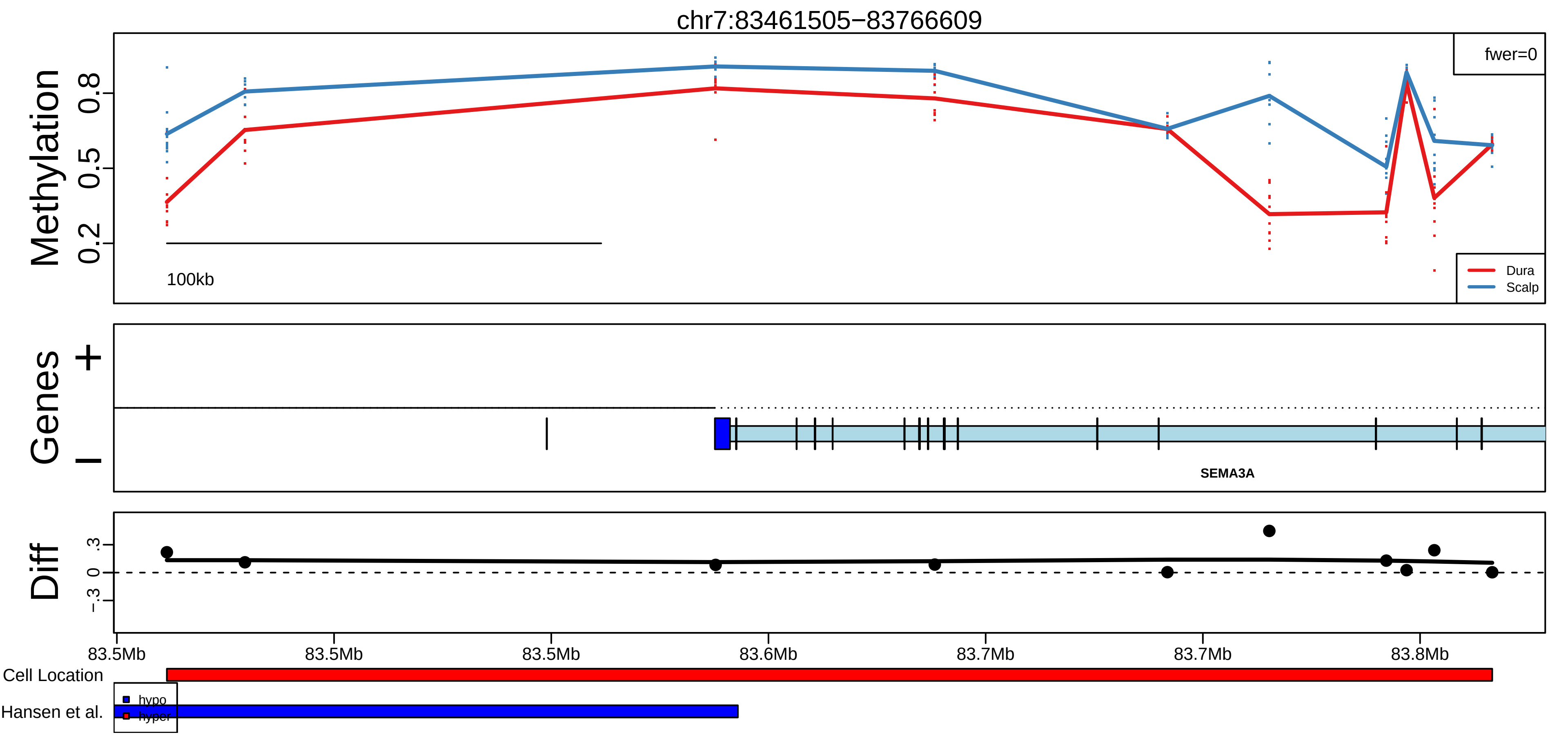
chr7:83461505−83766609
Methylation
0.2
0.5
0.8
fwer=0
100kb
Dura
Scalp
Genes
+
−
SEMA3A
Diff
.3
0
−.3
83.5Mb
83.5Mb
83.5Mb
83.6Mb
83.7Mb
83.7Mb
83.8Mb
Cell Location
Hansen et al.
hypo
hyper

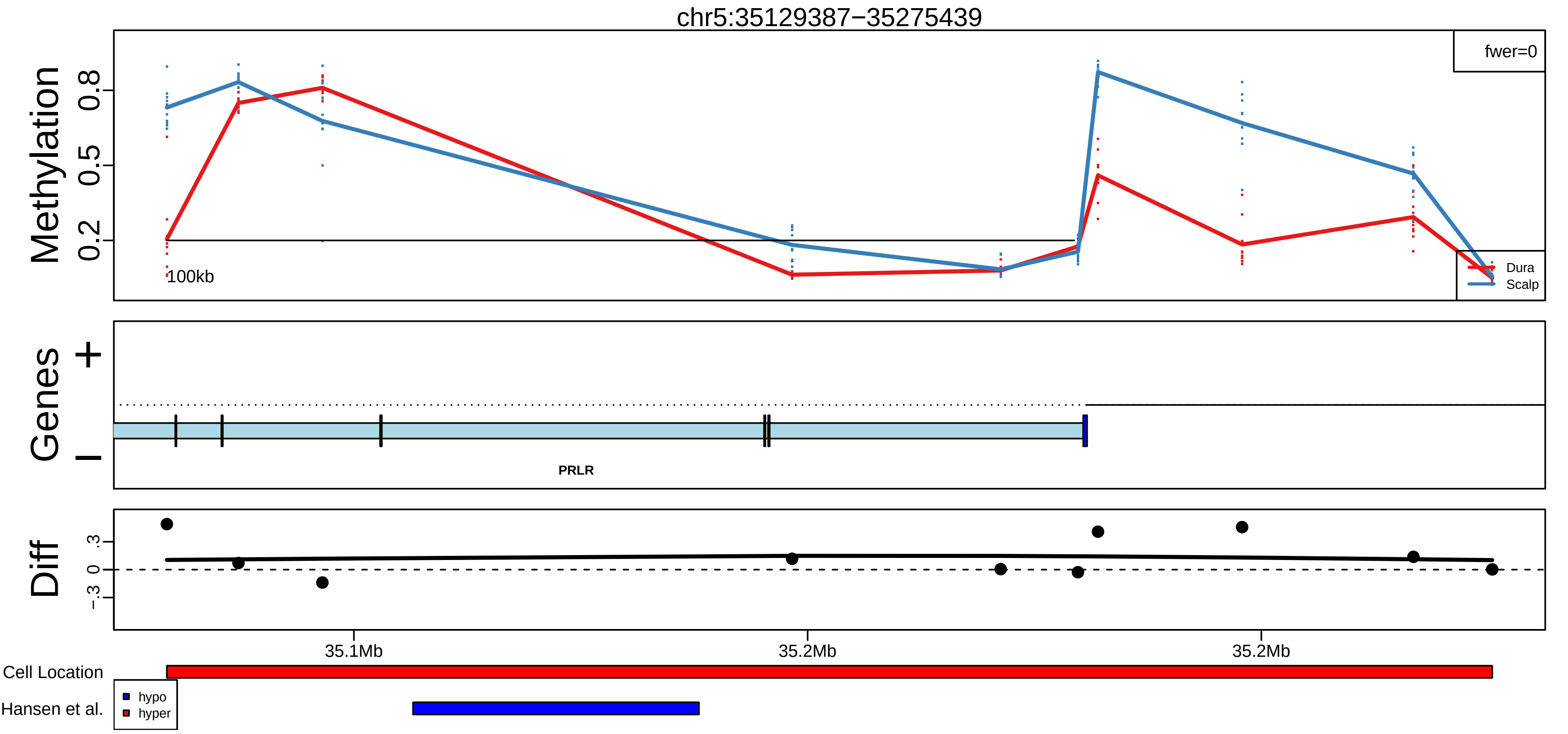

chr5:35129387−35275439
fwer=0
Methylation
0.2
0.5
0.8
100kb
Dura
Scalp
Genes
+
−
PRLR
Diff
−.3
0
.3
35.1Mb
35.2Mb
35.2Mb
Cell Location
Hansen et al.
hypo
hyper

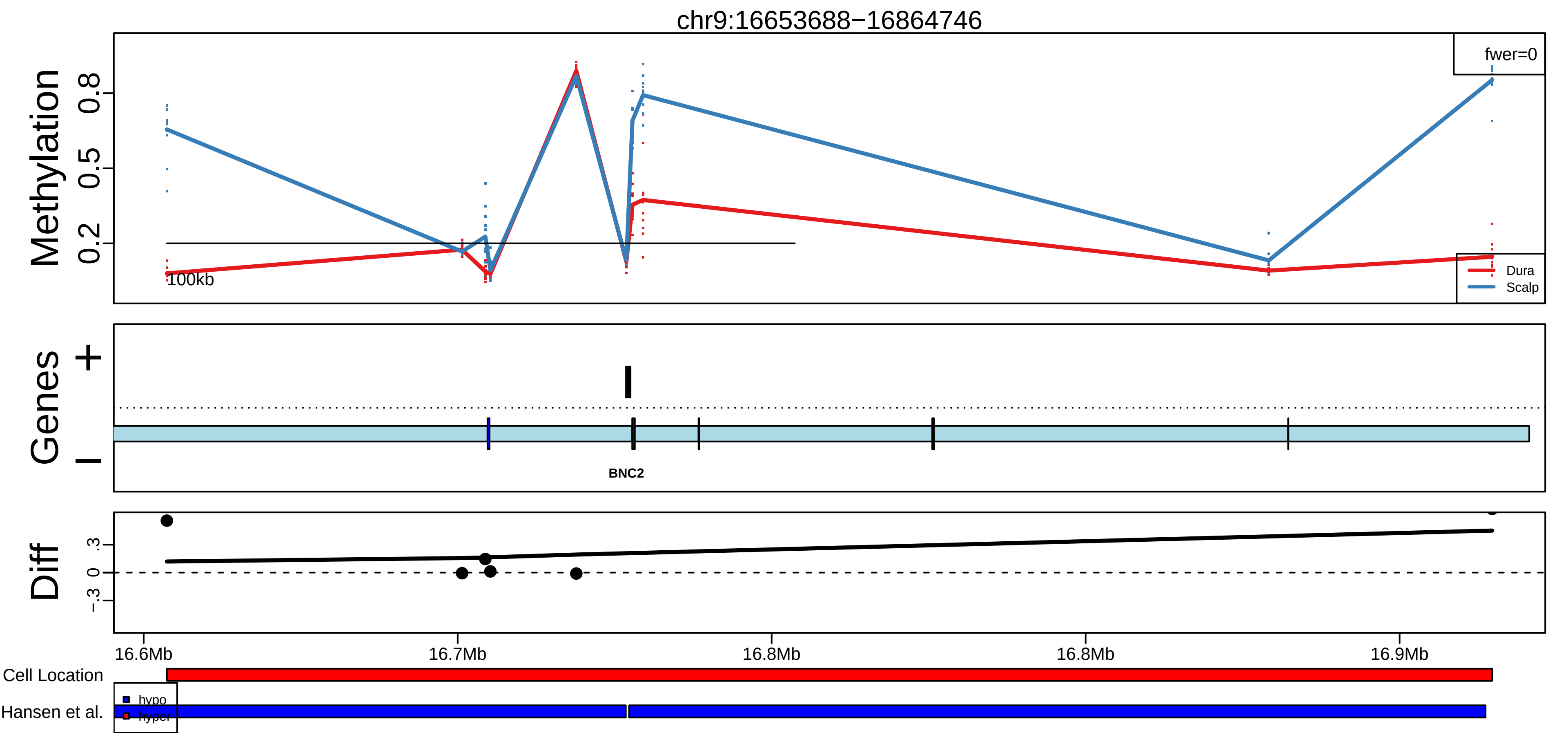

chr9:16653688−16864746
Methylation
0.2
0.5
0.8
fwer=0
100kb
Dura
Scalp
Genes
+
−
BNC2
Diff
−.3
0
.3
16.6Mb
16.7Mb
16.8Mb
16.9Mb
Cell Location
Hansen et al.
hypo
hyper

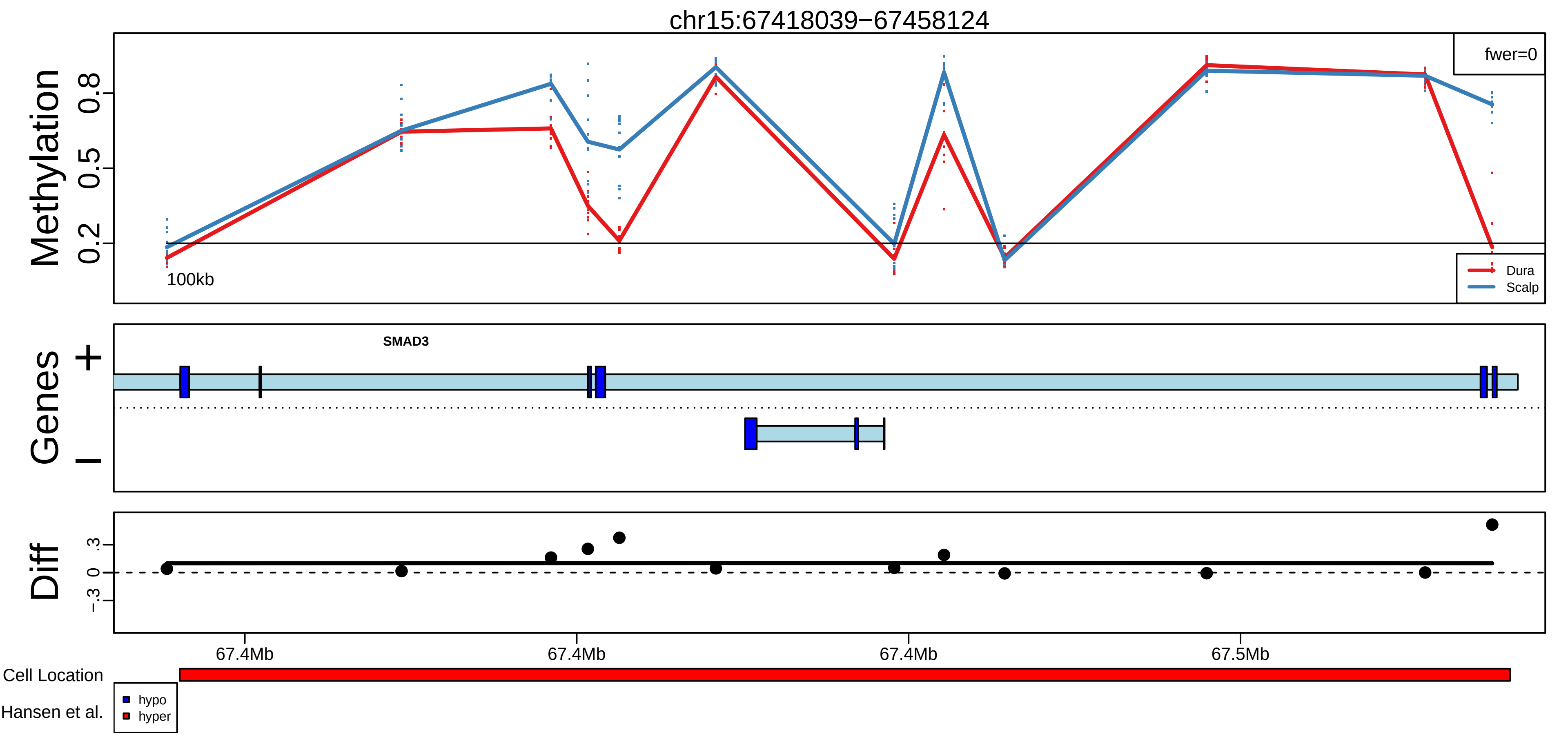
chr15:67418039−67458124
fwer=0
Methylation
0.8
0.5
0.2
100kb
Dura
Scalp
Genes
+
−
SMAD3
Diff
.3
0
−.3
67.4Mb
67.4Mb
67.4Mb
67.5Mb
Cell Location
Hansen et al.
hypo
hyper

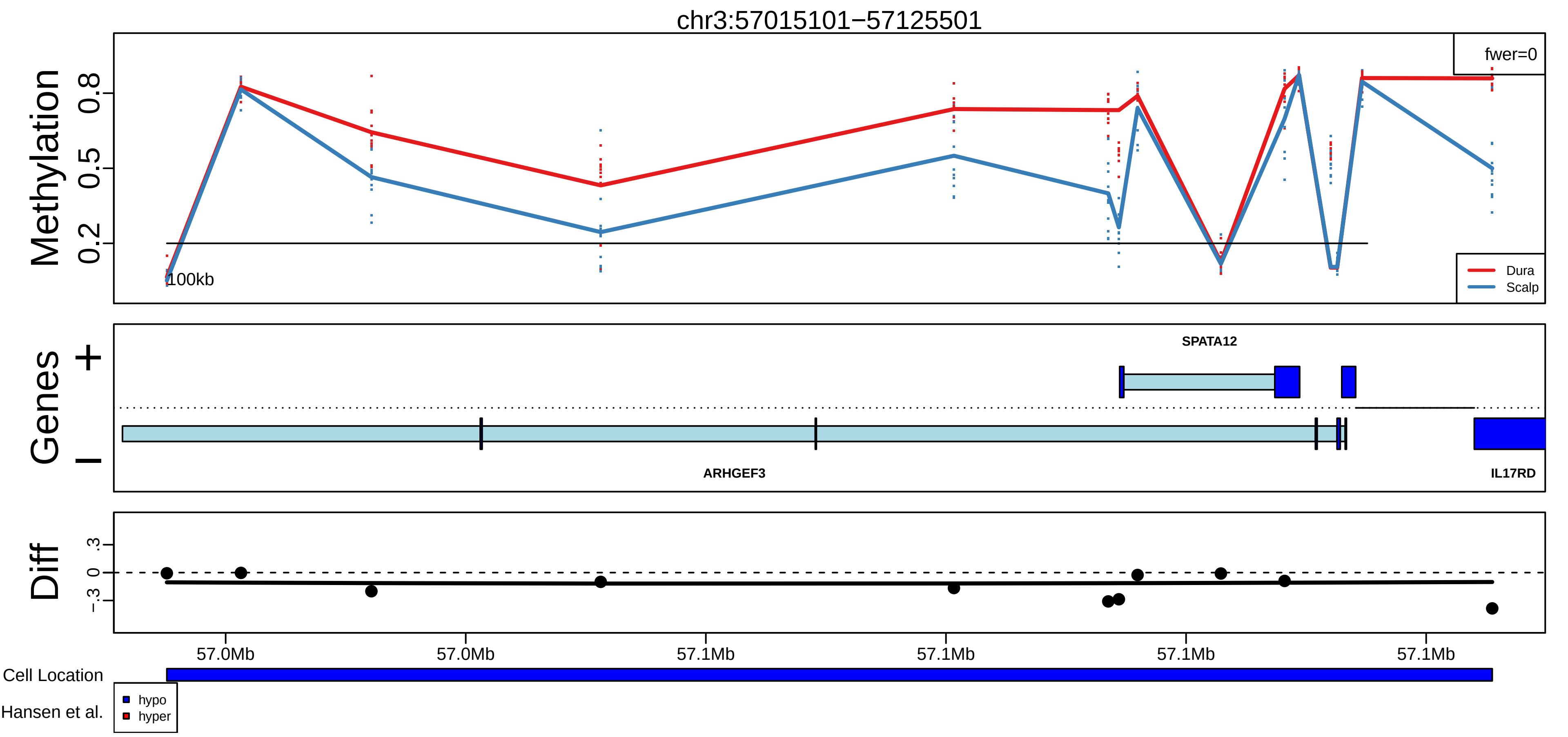

chr3:57015101−57125501
fwer=0
Methylation
0.2
0.5
0.8
100kb
Dura
Scalp
Genes
+
−
SPATA12
ARHGEF3
IL17RD
Diff
.3
0
−.3
57.0Mb
57.0Mb
57.1Mb
57.1Mb
57.1Mb
57.1Mb
Cell Location
Hansen et al.
hypo
hyper

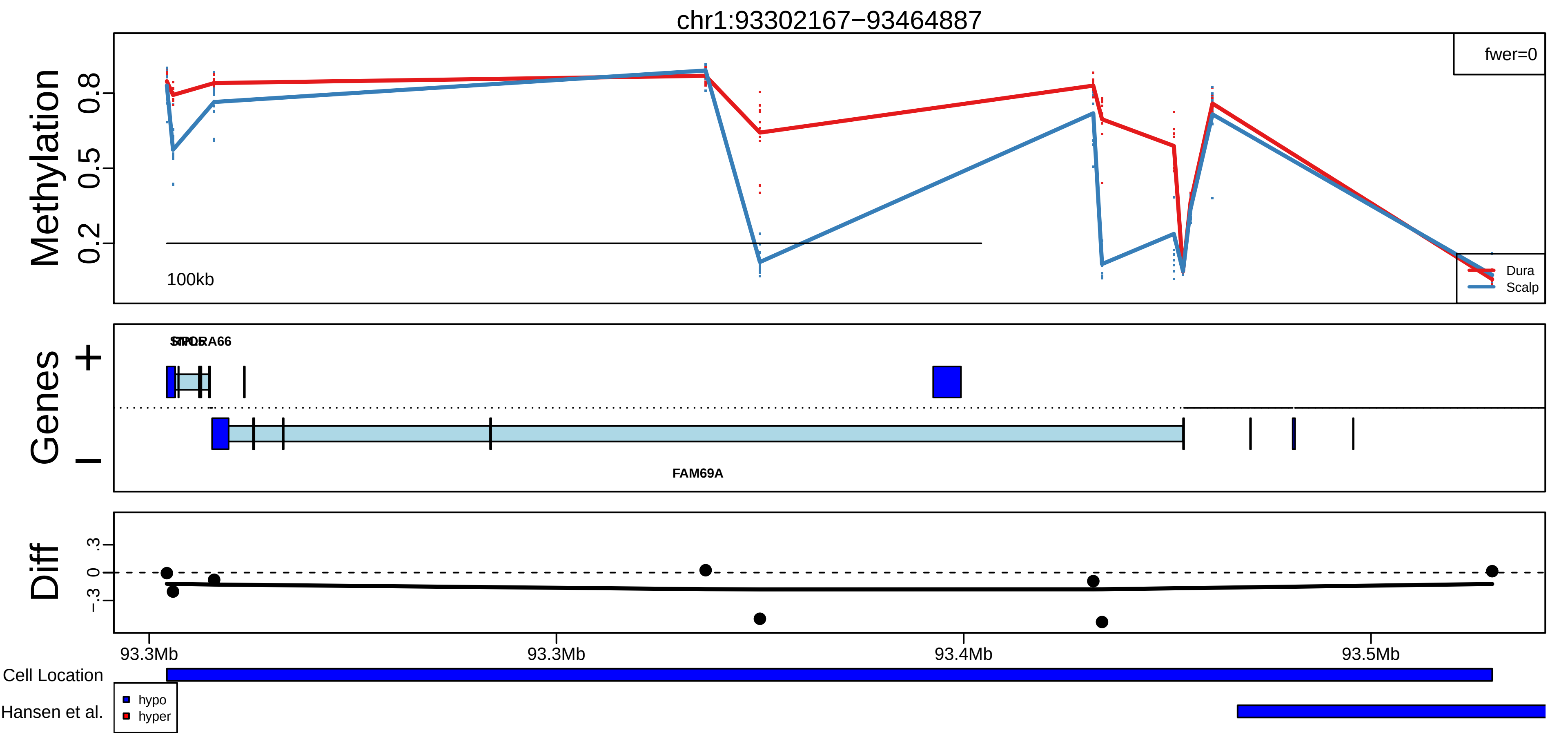
chr1:93302167−93464887
fwer=0
Methylation
0.8
0.5
0.2
100kb
Dura
Scalp
Genes
+
−
STRDR66
FAM69A
Diff
.3
0
−.3
93.3Mb
93.3Mb
93.4Mb
93.5Mb
Cell Location
Hansen et al.
hypo
hyper

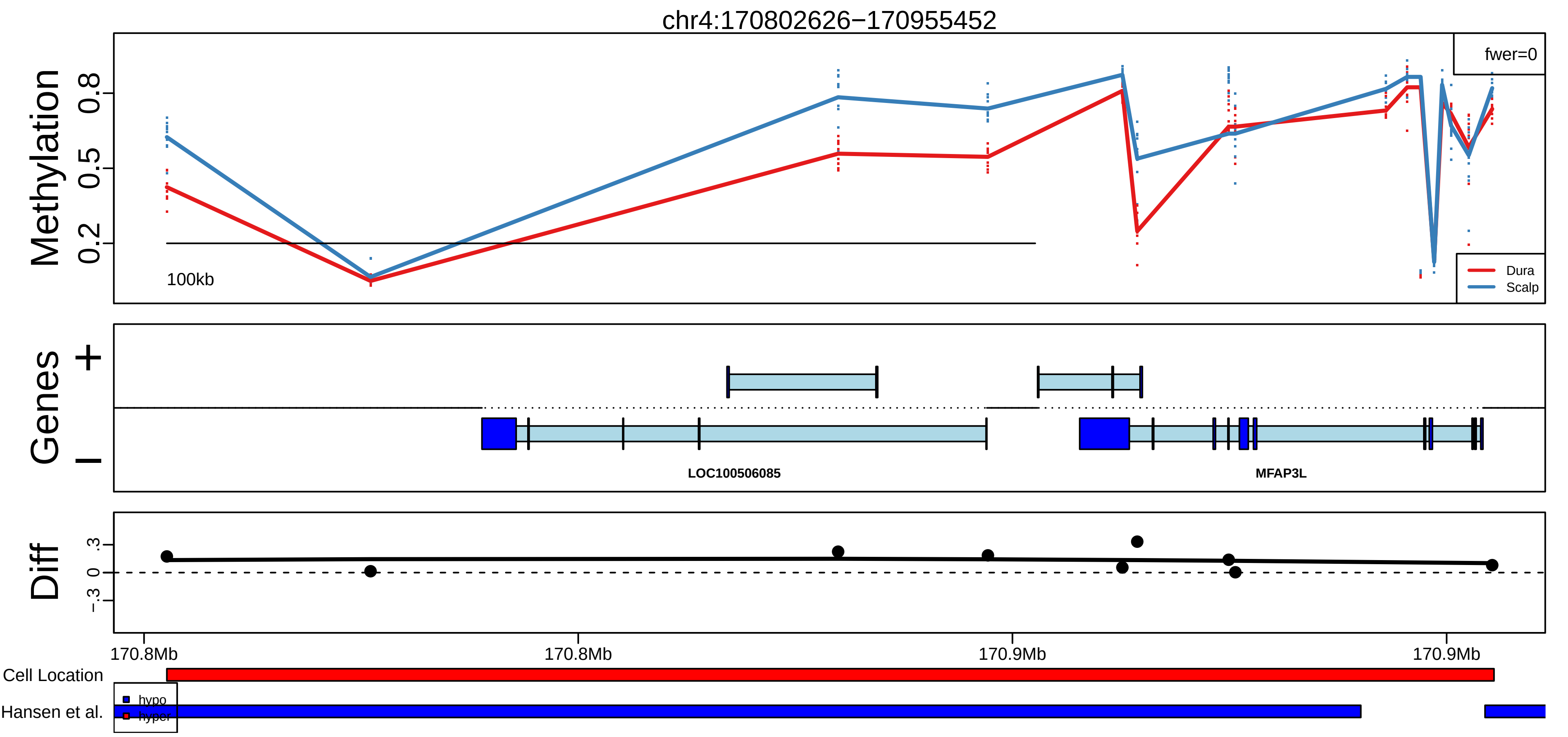

chr4:170802626−170955452
fwer=0
Methylation
0.2
0.5
0.8
100kb
Dura
Scalp
Genes
+
−
LOC100506085
MFAP3L
Diff
.3
0
−.3
170.8Mb
170.8Mb
170.9Mb
170.9Mb
Cell Location
hypo
hyper
Hansen et al.

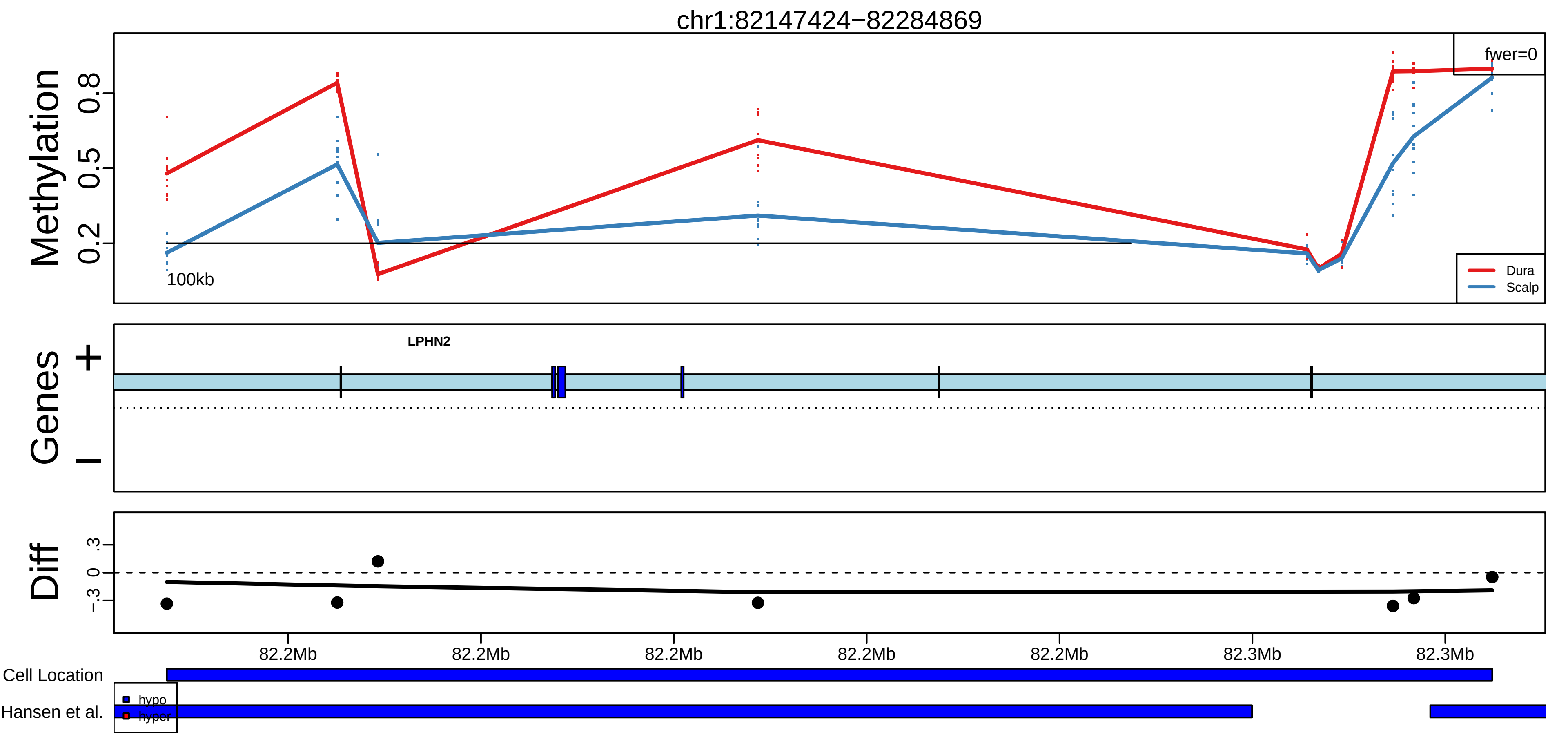

chr1:82147424−82284869
Methylation
0.2
0.5
0.8
fwer=0
100kb
Dura
Scalp
Genes
+
−
LPHN2
Diff
−.3
0
.3
82.2Mb
82.2Mb
82.2Mb
82.2Mb
82.2Mb
82.3Mb
82.3Mb
Cell Location
Hansen et al.
hypo
hyper

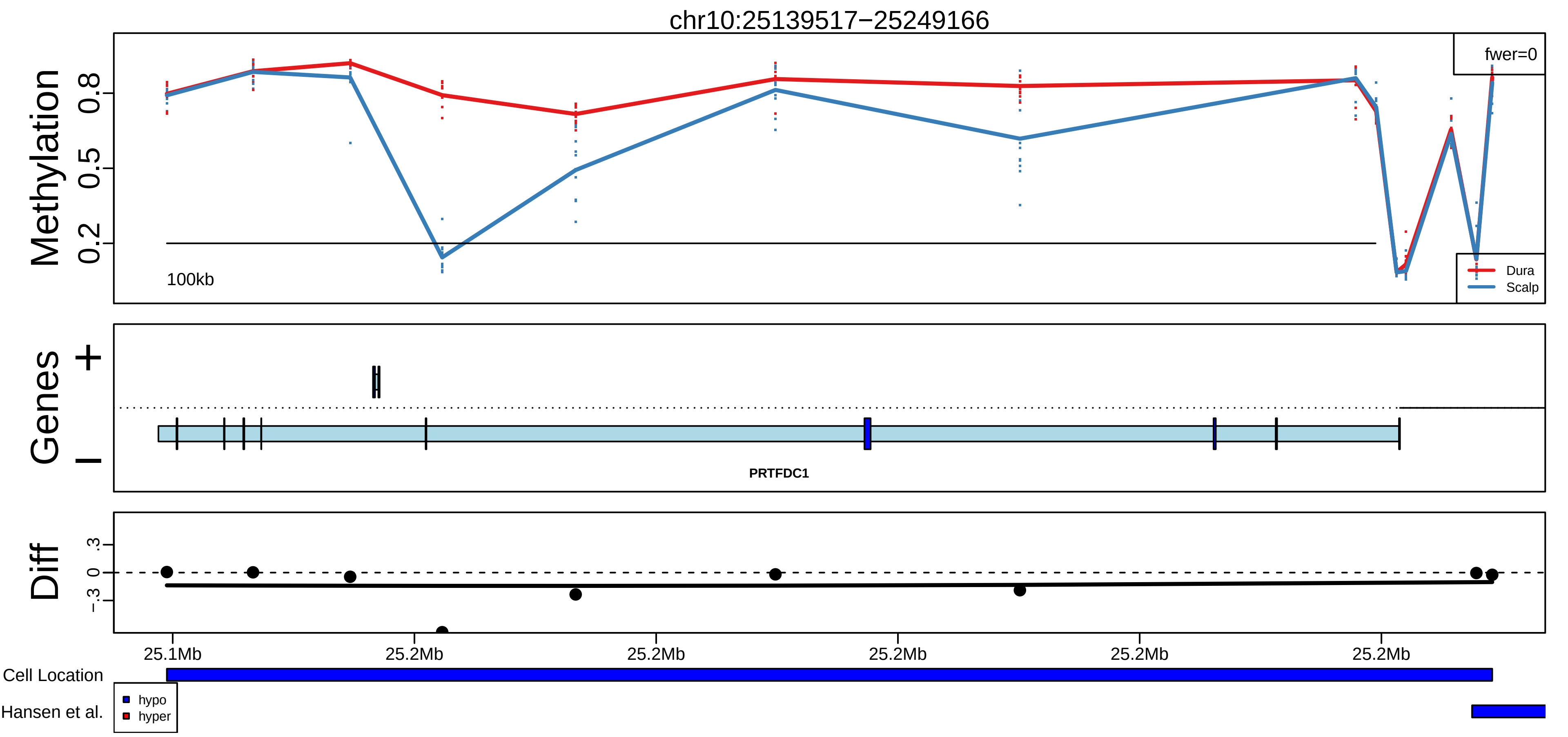

chr10:25139517−25249166
fwer=0
Methylation
0.2
0.5
0.8
100kb
Dura
Scalp
Genes
+
−
PRTFDC1
Diff
−.3
0
.3
25.1Mb
25.2Mb
25.2Mb
25.2Mb
25.2Mb
25.2Mb
Cell Location
Hansen et al.
hypo
hyper

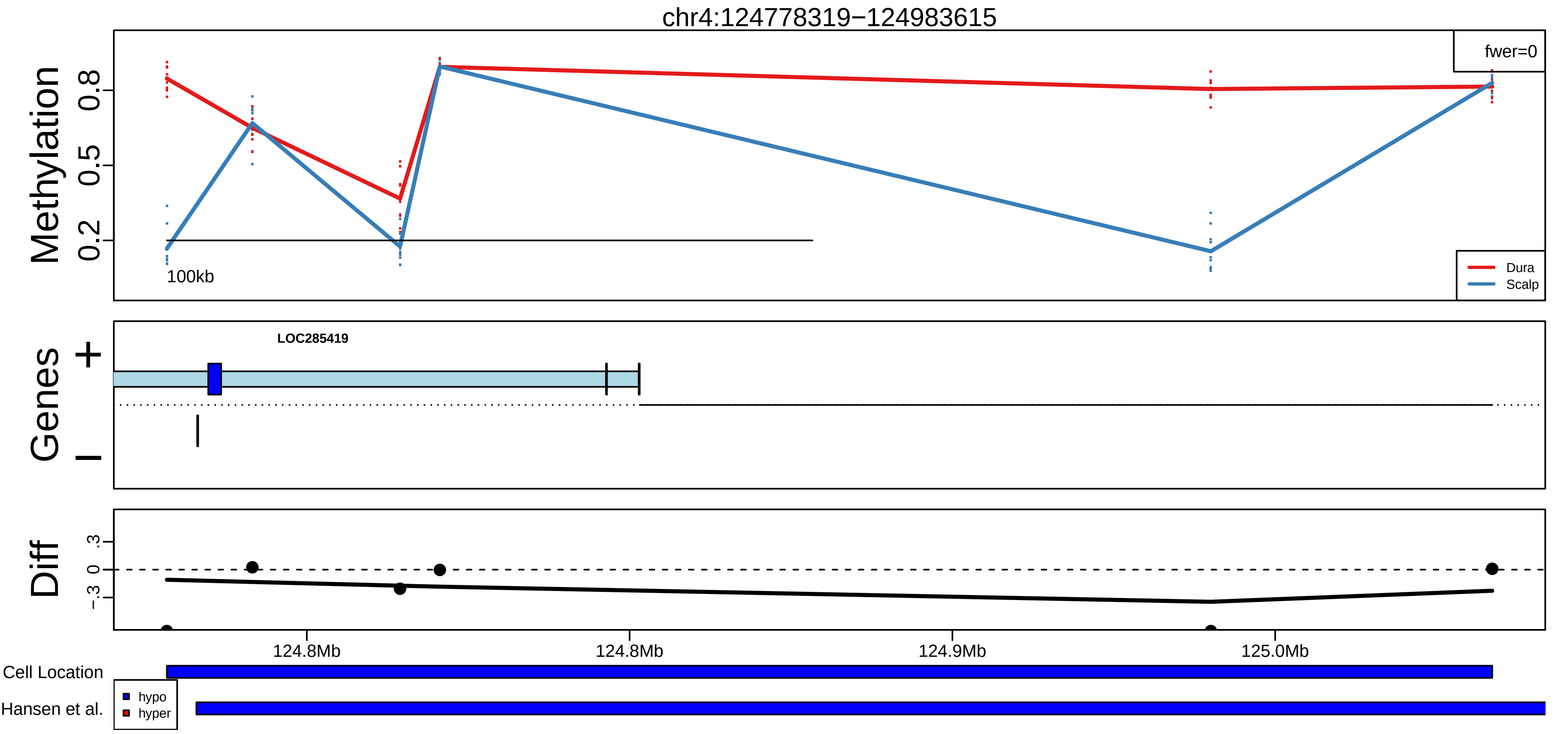

chr4:124778319−124983615
fwer=0
Methylation
0.2
0.5
0.8
100kb
Dura
Scalp
Genes
+
−
LOC285419
Diff
−.3
0
.3
124.8Mb
124.8Mb
124.9Mb
125.0Mb
Cell Location
Hansen et al.
hypo
hyper

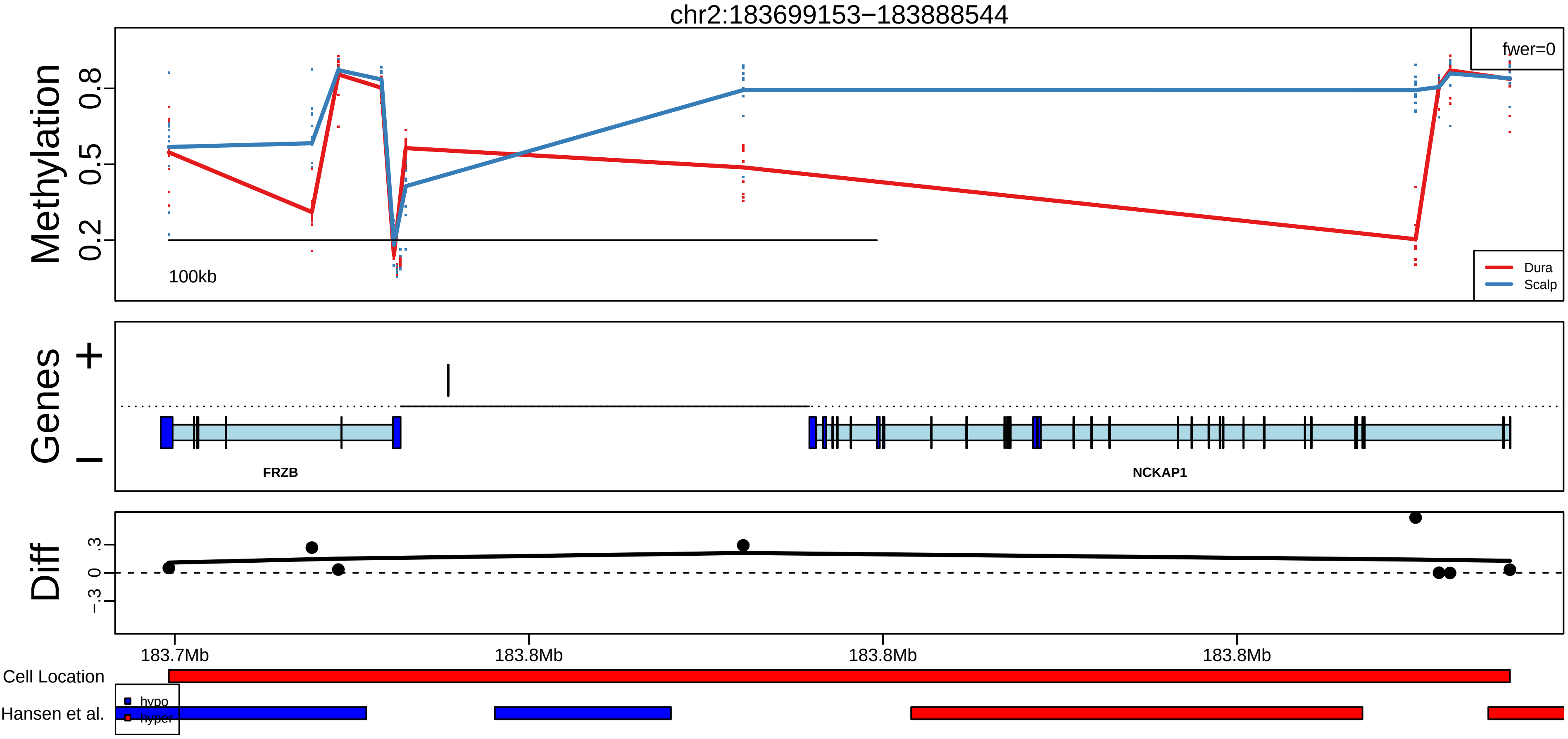
chr2:183699153−183888544
fwer=0
Methylation
0.2
0.5
0.8
100kb
Dura
Scalp
Genes
+
−
FRZB
NCKAP1
Diff
−.3
0
.3
183.7Mb
183.8Mb
183.8Mb
183.8Mb
Cell Location
Hansen et al.
hypo
hyper

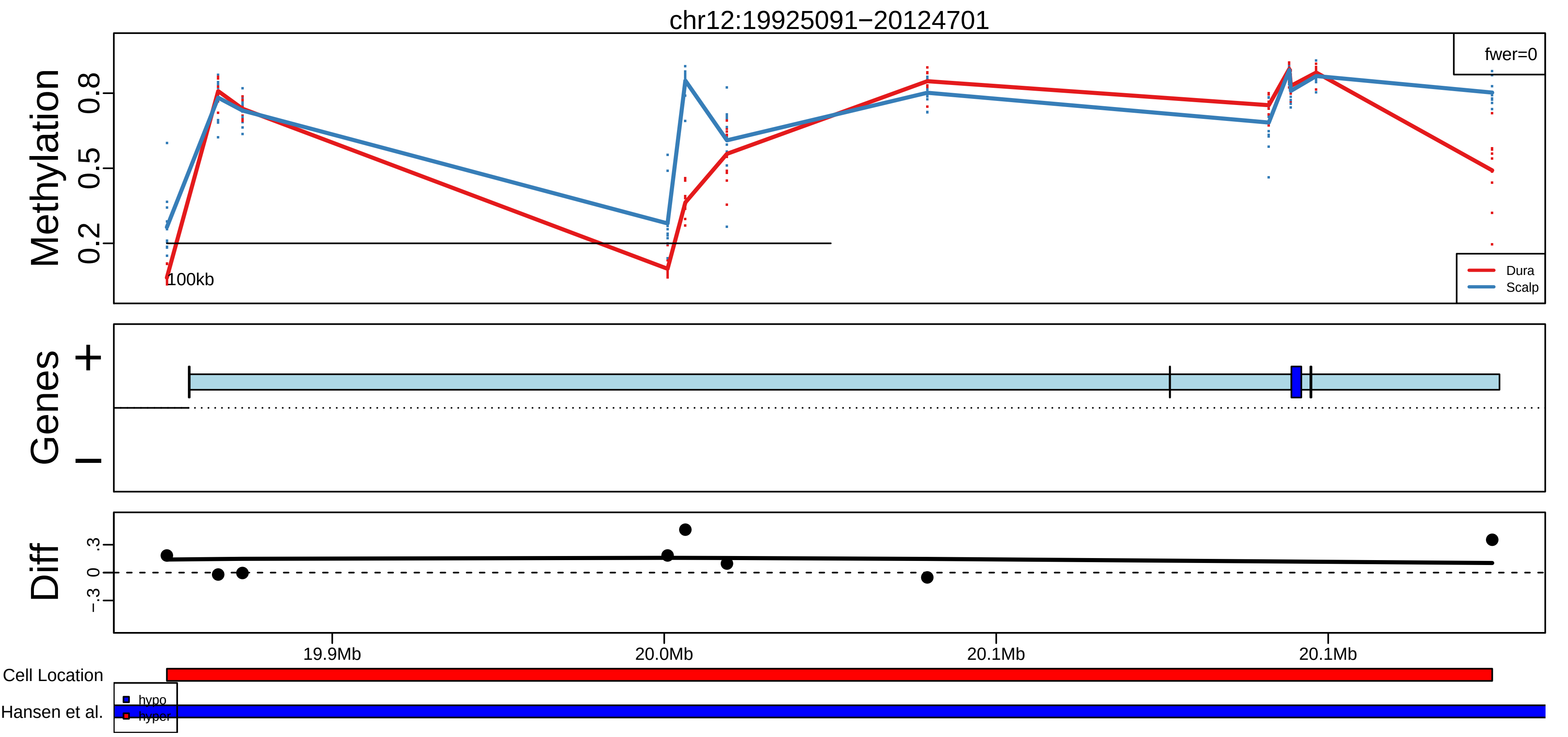

chr12:19925091−20124701
Methylation
0.2
0.5
0.8
fwer=0
100kb
Dura
Scalp
Genes
+
−
Diff
.3
0
−.3
19.9Mb
20.0Mb
20.1Mb
20.1Mb
Cell Location
Hansen et al.
hypo
hyper

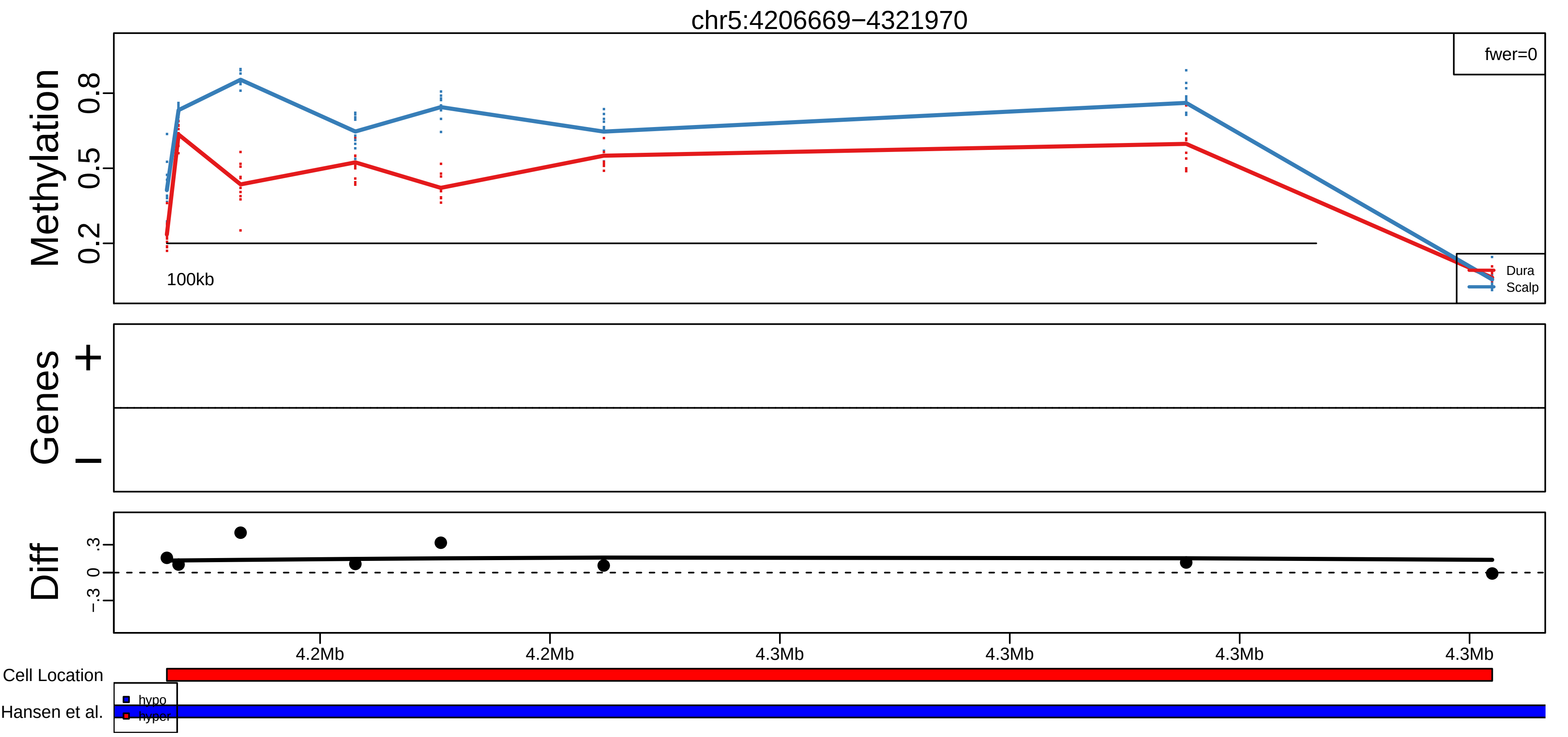
chr5:4206669−4321970
fwer=0
Methylation
0.2
0.5
0.8
100kb
Dura
Scalp
Genes
+
−
Diff
.3
0
−.3
4.2Mb
4.2Mb
4.3Mb
4.3Mb
4.3Mb
4.3Mb
Cell Location
Hansen et al.
hypo
hyper

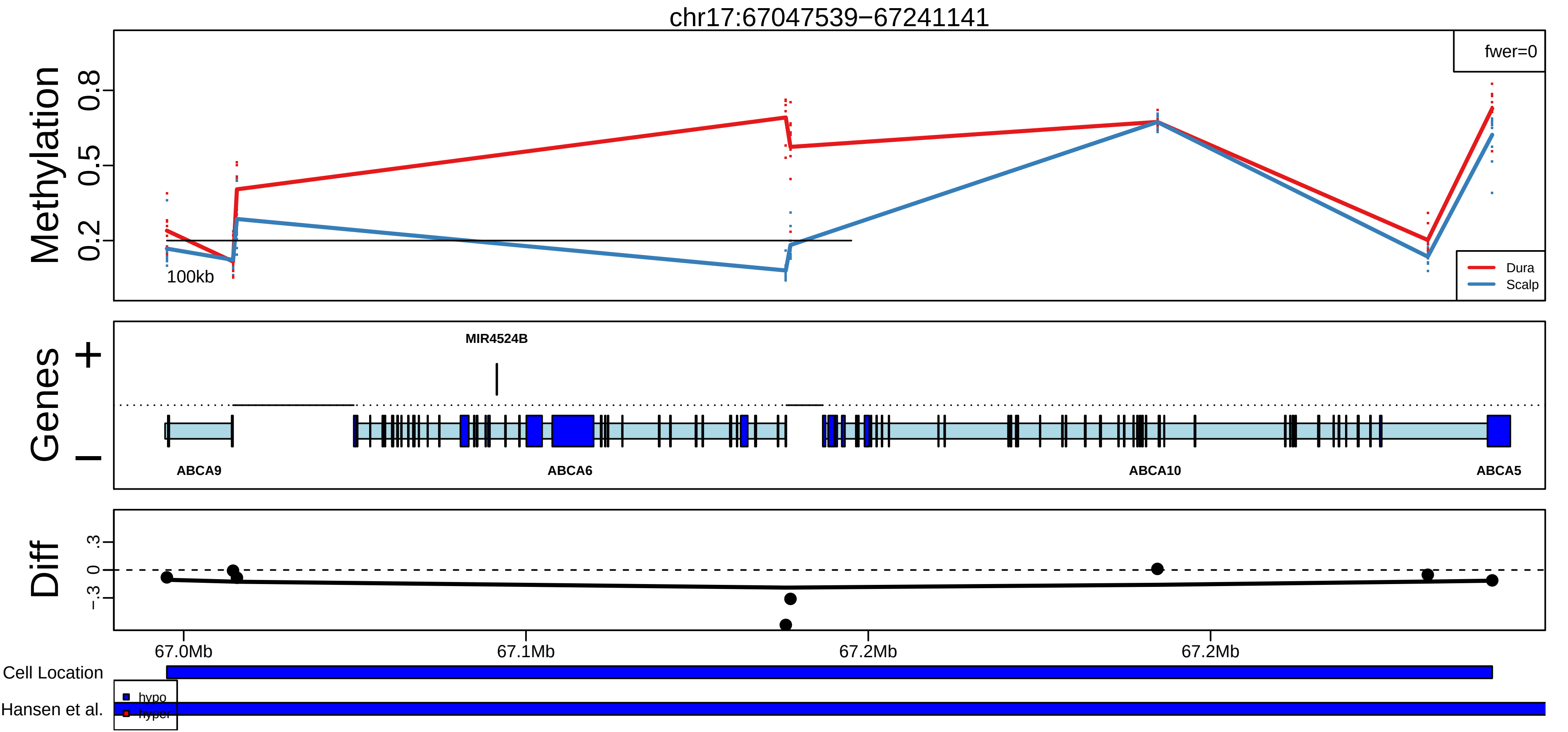

chr17:67047539−67241141
Methylation
0.2
0.5
0.8
fwer=0
100kb
Dura
Scalp
Genes
+
−
MIR4524B
ABCA9
ABCA6
ABCA10
ABCA5
Diff
−.3
0
.3
67.0Mb
67.1Mb
67.2Mb
67.2Mb
Cell Location
Hansen et al.
hypo
hyper

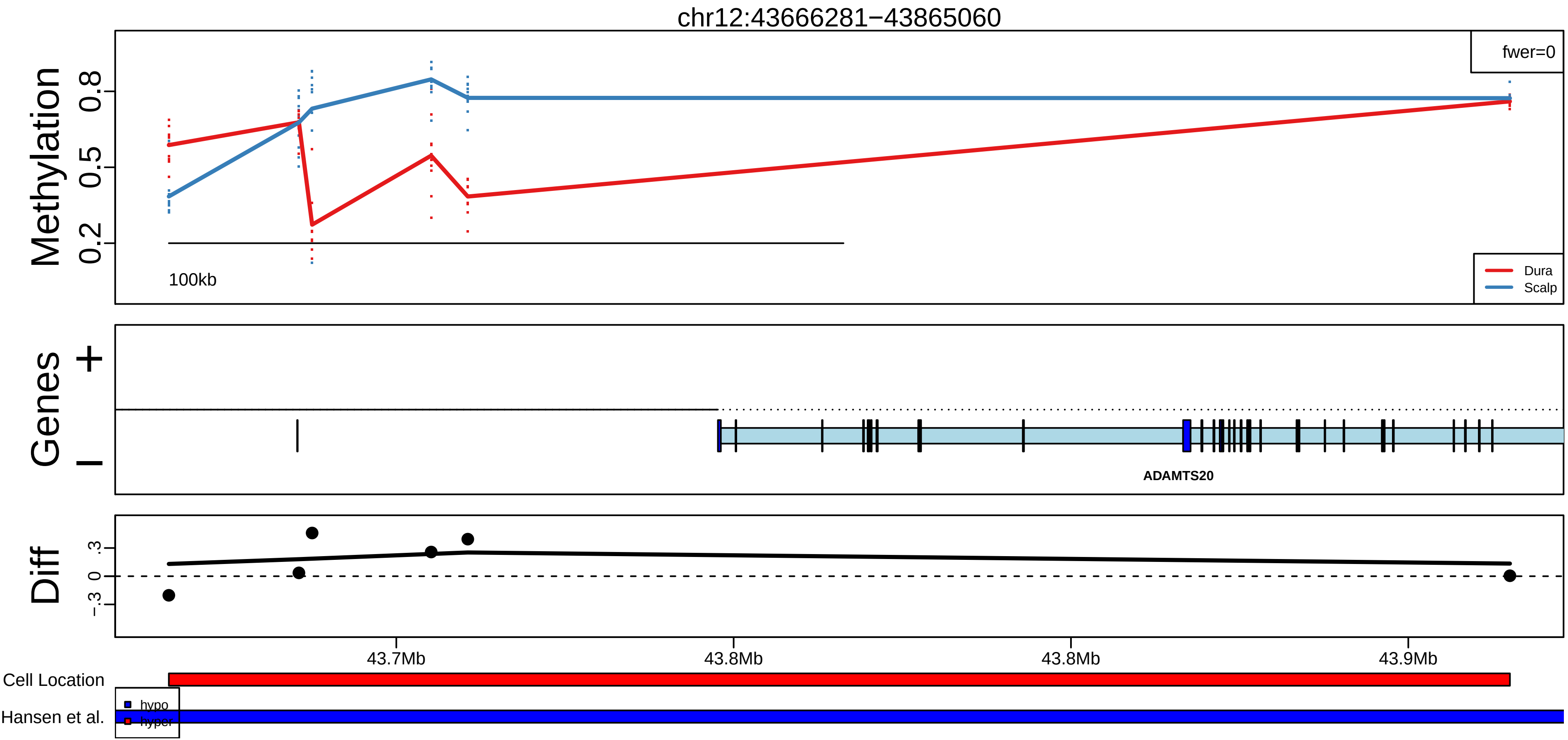

chr12:43666281−43865060
fwer=0
Methylation
0.2
0.5
0.8
100kb
Dura
Scalp
Genes
+
−
ADAMTS20
Diff
−.3
0
.3
43.7Mb
43.8Mb
43.8Mb
43.9Mb
Cell Location
Hansen et al.
hypo
hyper

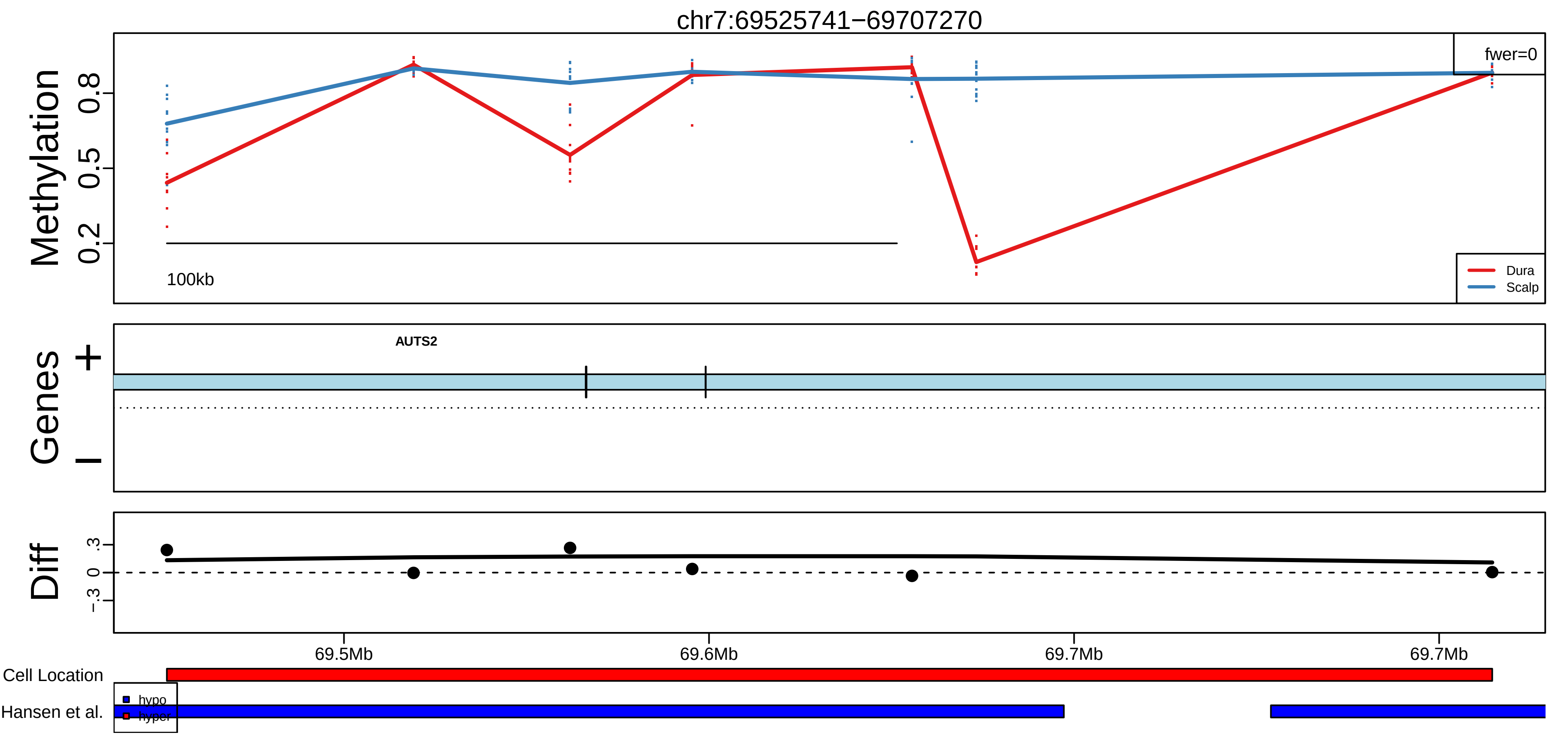

chr7:69525741−69707270
Methylation
0.2
0.5
0.8
fwer=0
100kb
Dura
Scalp
Genes
+
−
AUTS2
Diff
−.3
0
.3
69.5Mb
69.6Mb
69.7Mb
69.7Mb
Cell Location
Hansen et al.
hypo
hyper

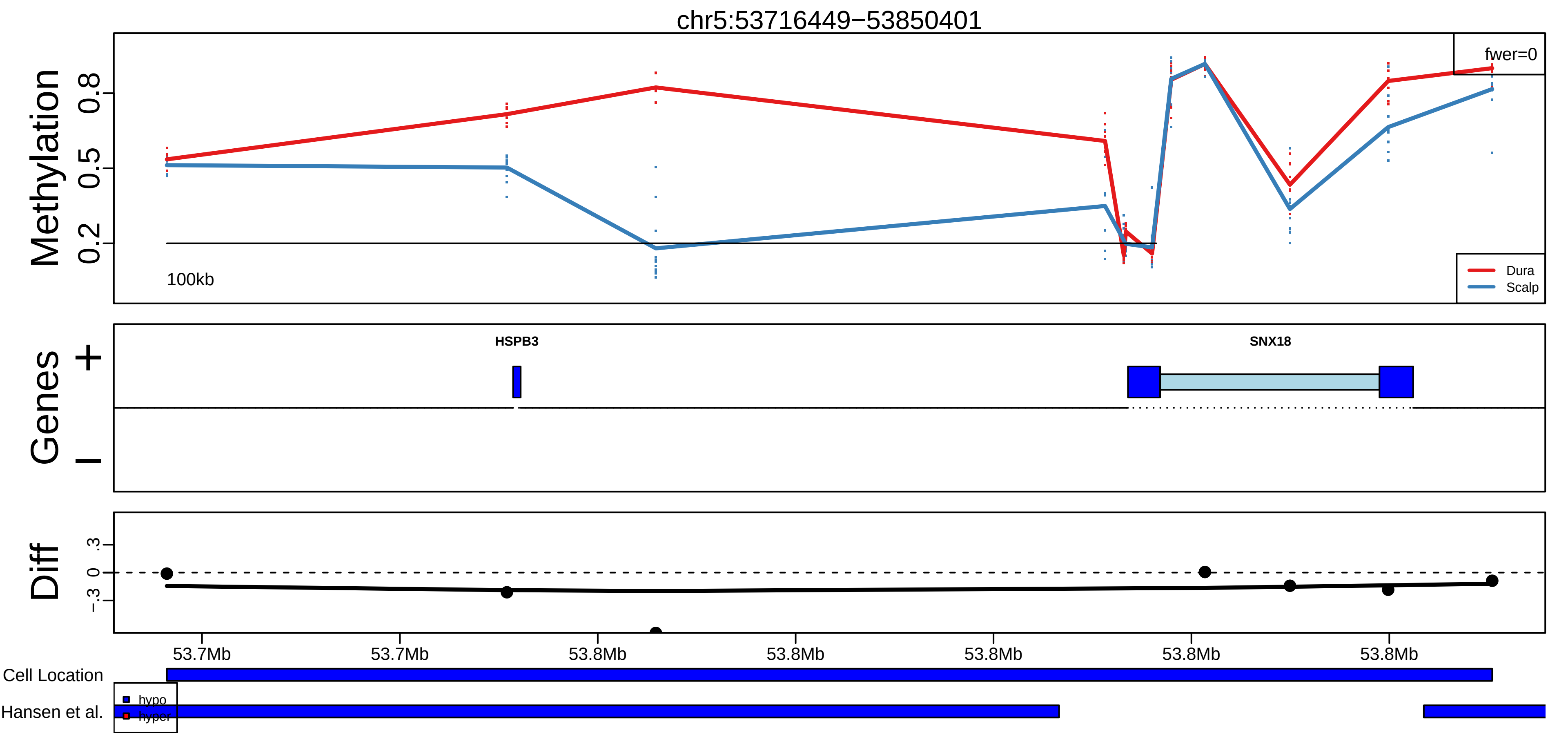
chr5:53716449−53850401
Methylation
0.2
0.5
0.8
fwer=0
100kb
Dura
Scalp
Genes
+
−
HSPB3
SNX18
Diff
−.3
0
.3
53.7Mb
53.7Mb
53.8Mb
53.8Mb
53.8Mb
53.8Mb
53.8Mb
Cell Location
Hansen et al.
hypo
hyper

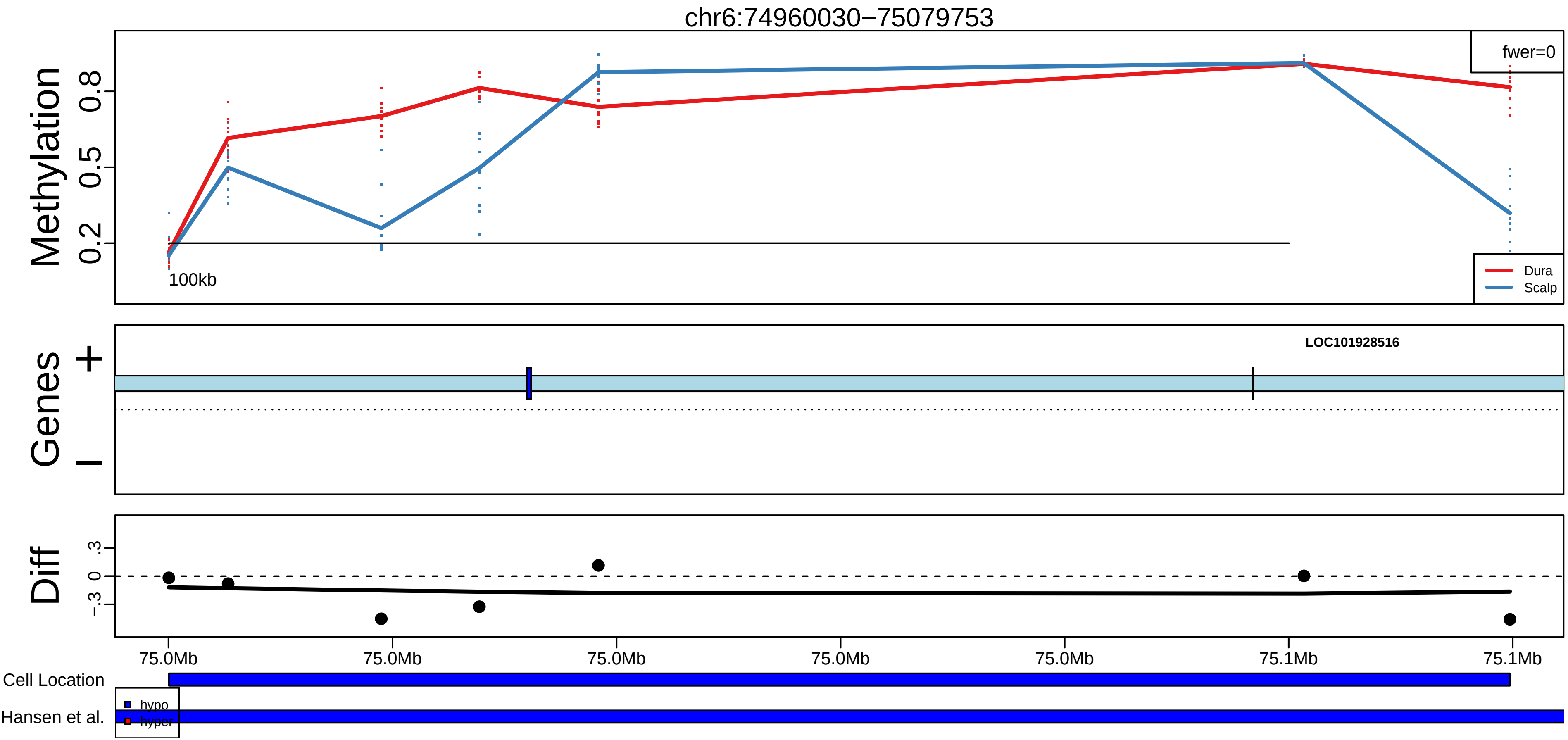

chr6:74960030−75079753
Methylation
0.2
0.5
0.8
fwer=0
100kb
Dura
Scalp
Genes
+
−
LOC101928516
Diff
−.3
0
.3
75.0Mb
75.0Mb
75.0Mb
75.0Mb
75.0Mb
75.1Mb
75.1Mb
Cell Location
Hansen et al.
hypo
hyper

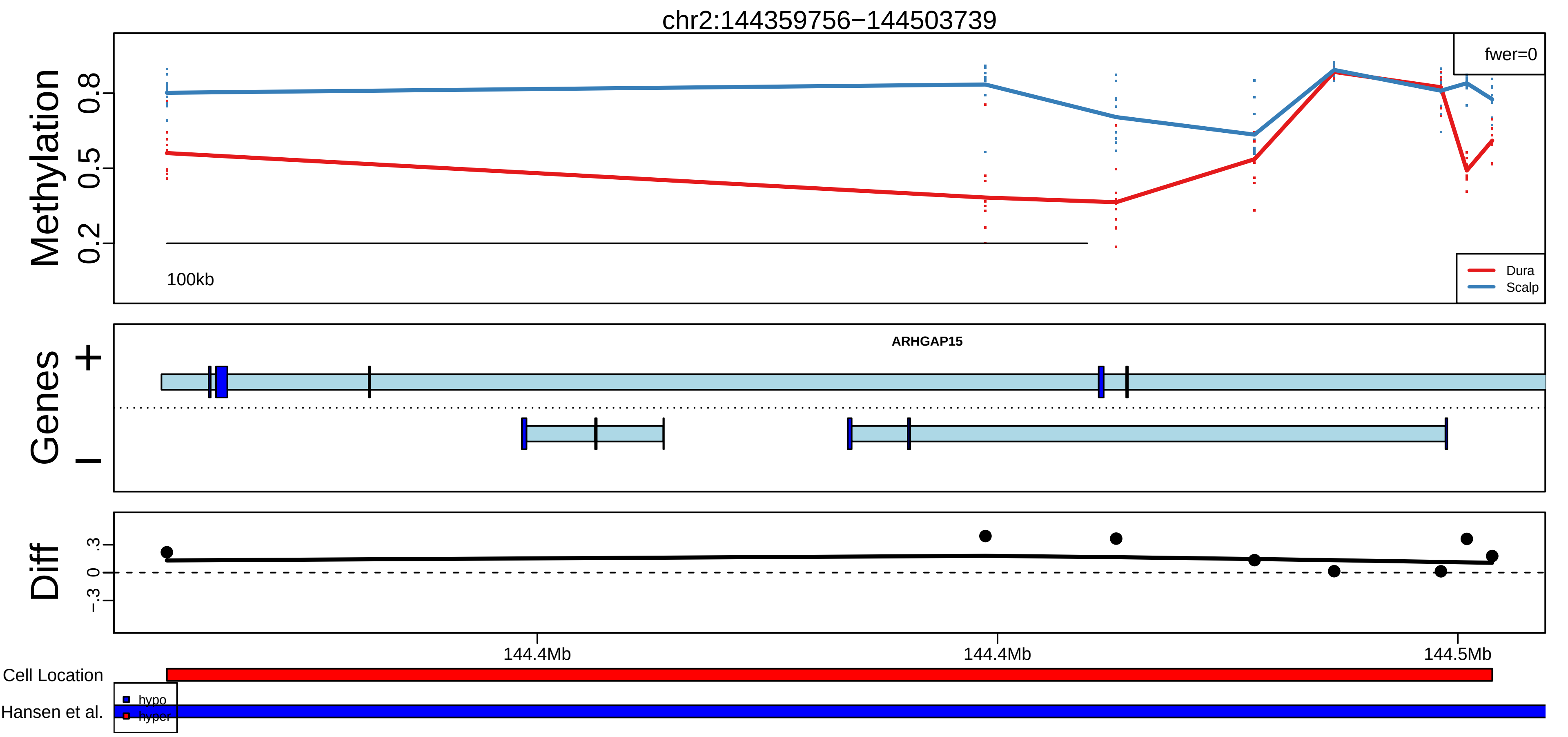
chr2:144359756−144503739
fwer=0
Methylation
0.2
0.5
0.8
100kb
Dura
Scalp
Genes
+
−
ARHGAP15
Diff
−.3
0
.3
144.4Mb
144.4Mb
144.5Mb
Cell Location
Hansen et al.
hypo
hyper

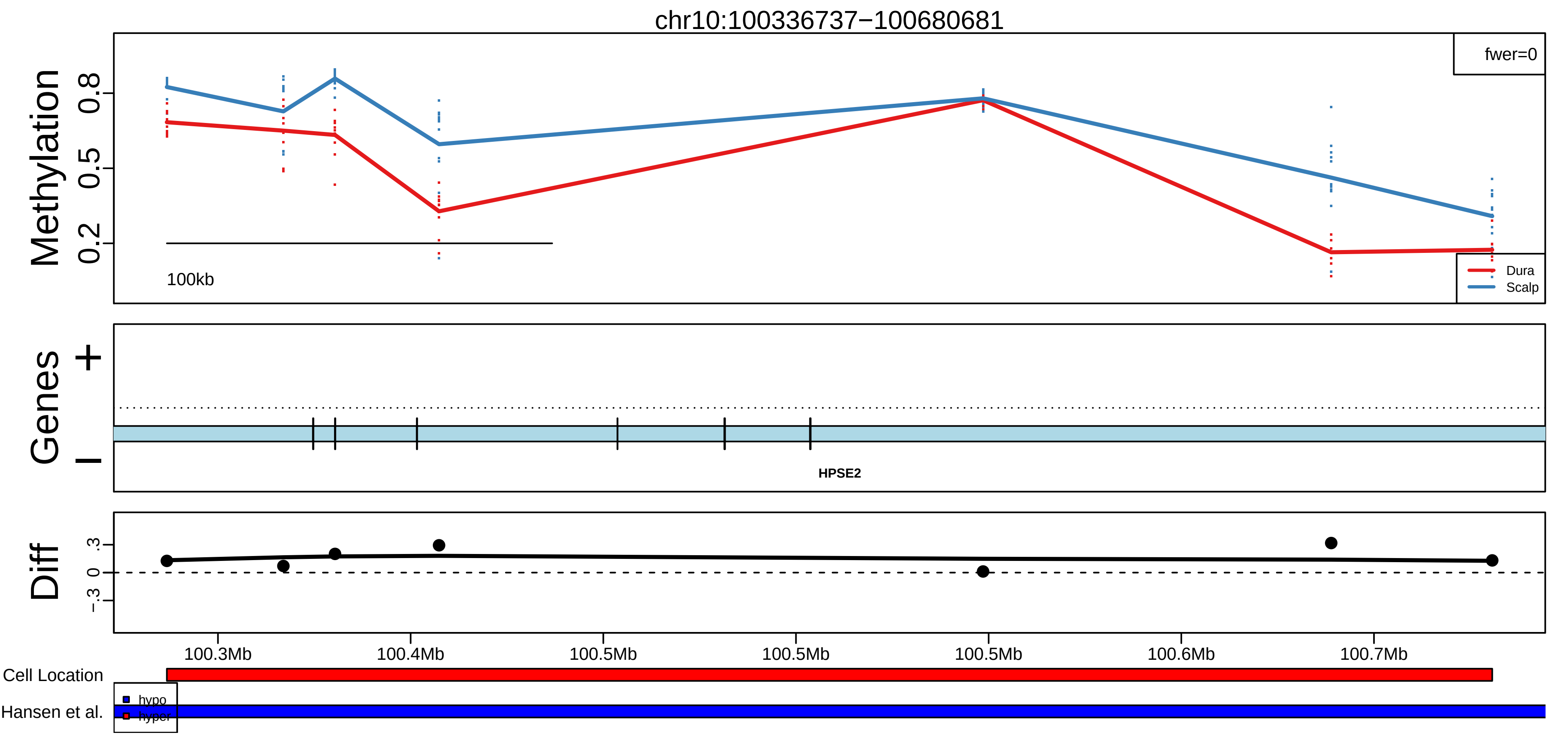
chr10:100336737−100680681
fwer=0
Methylation
0.2
0.5
0.8
100kb
Dura
Scalp
Genes
+
−
HPSE2
Diff
−.3
0
.3
100.3Mb
100.4Mb
100.5Mb
100.5Mb
100.5Mb
100.6Mb
100.7Mb
Cell Location
Hansen et al.
hypo
hyper

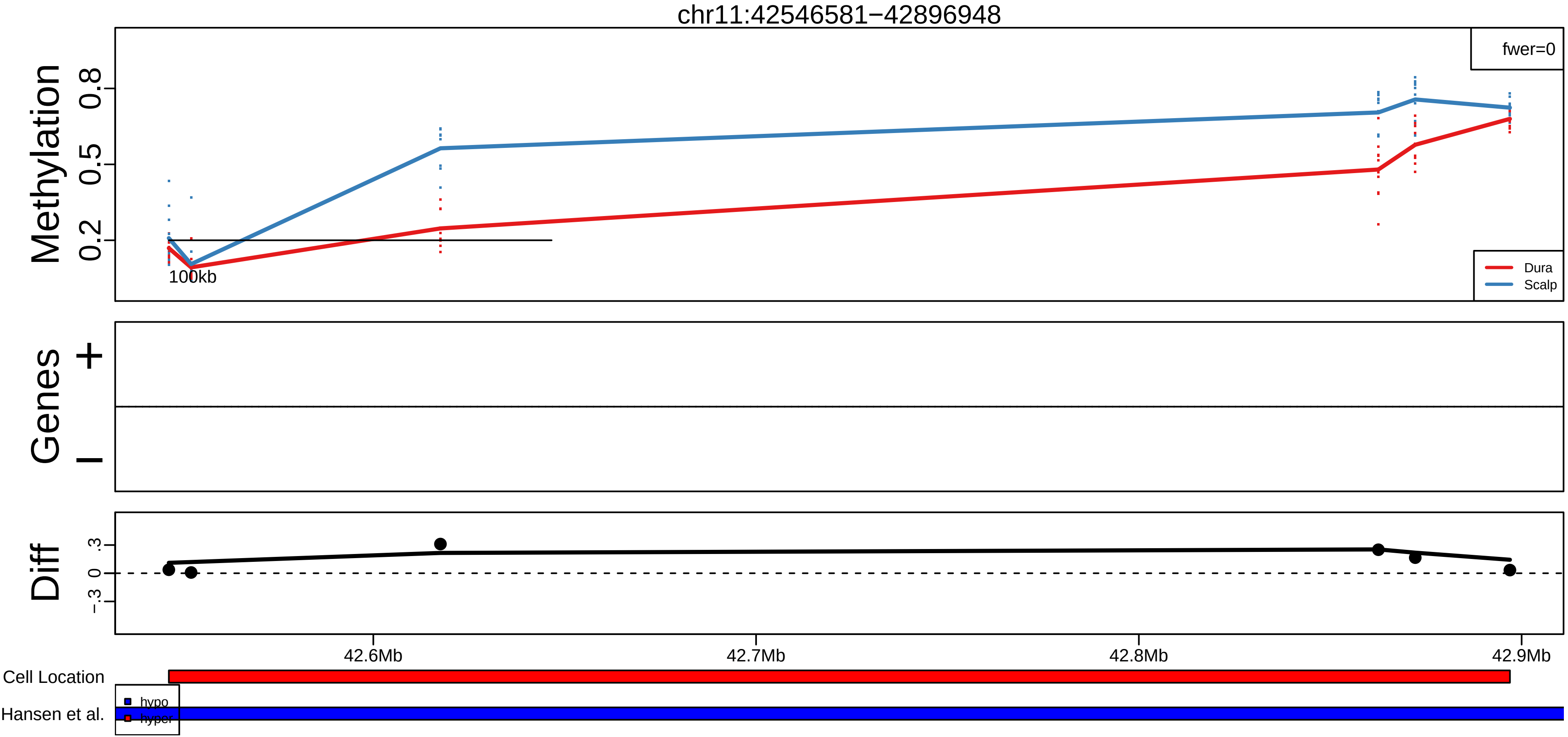
chr11:42546581−42896948
Methylation
0.2
0.5
0.8
fwer=0
100kb
Dura
Scalp
Genes
+
−
Diff
.3
0
−.3
42.6Mb
42.7Mb
42.8Mb
42.9Mb
Cell Location
Hansen et al.
hypo
hyper

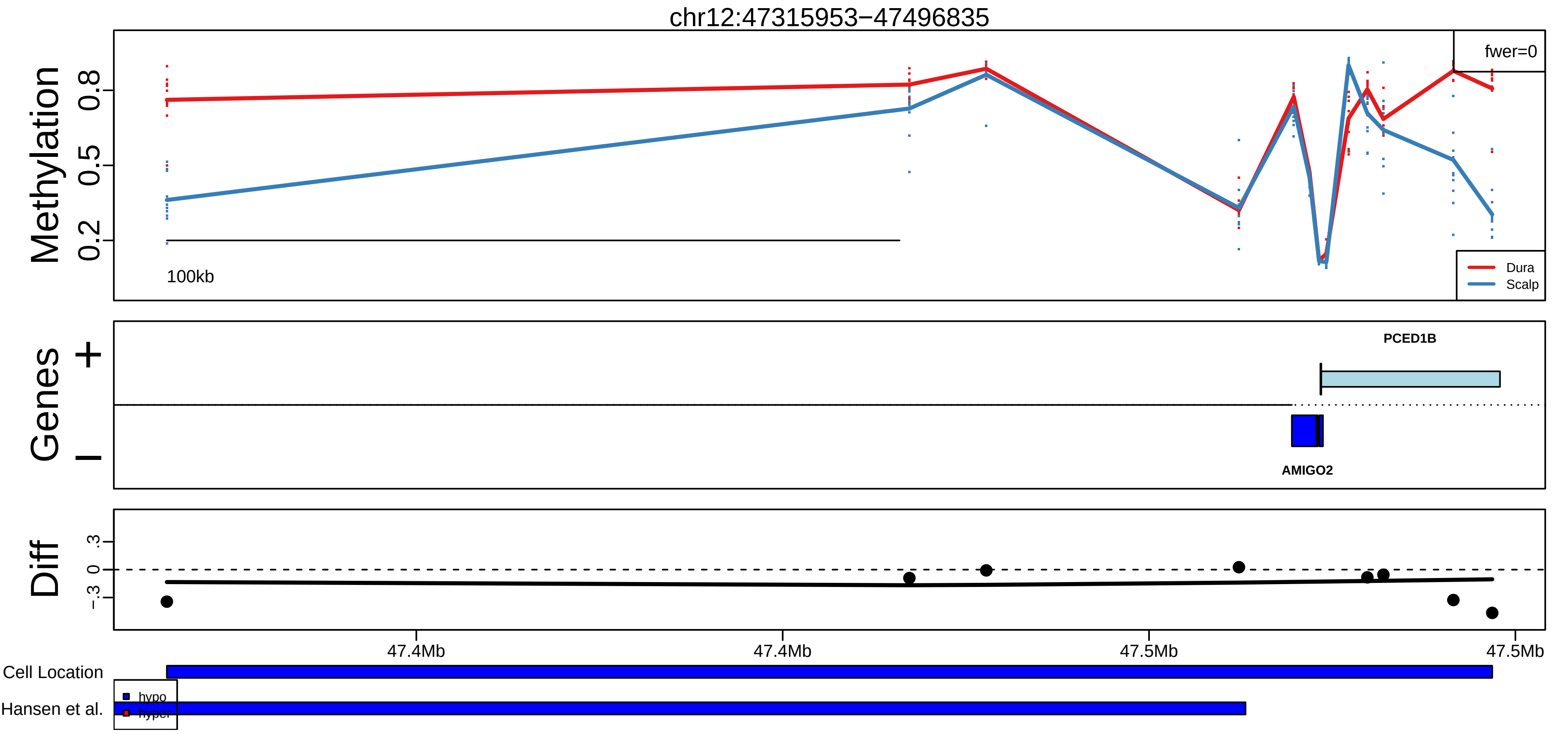

chr12:47315953−47496835
Methylation
0.2
0.5
0.8
fwer=0
100kb
Dura
Scalp
Genes
+
−
PCED1B
AMIGO2
Diff
.3
0
−.3
47.4Mb
47.4Mb
47.5Mb
47.5Mb
Cell Location
Hansen et al.
hypo
hyper

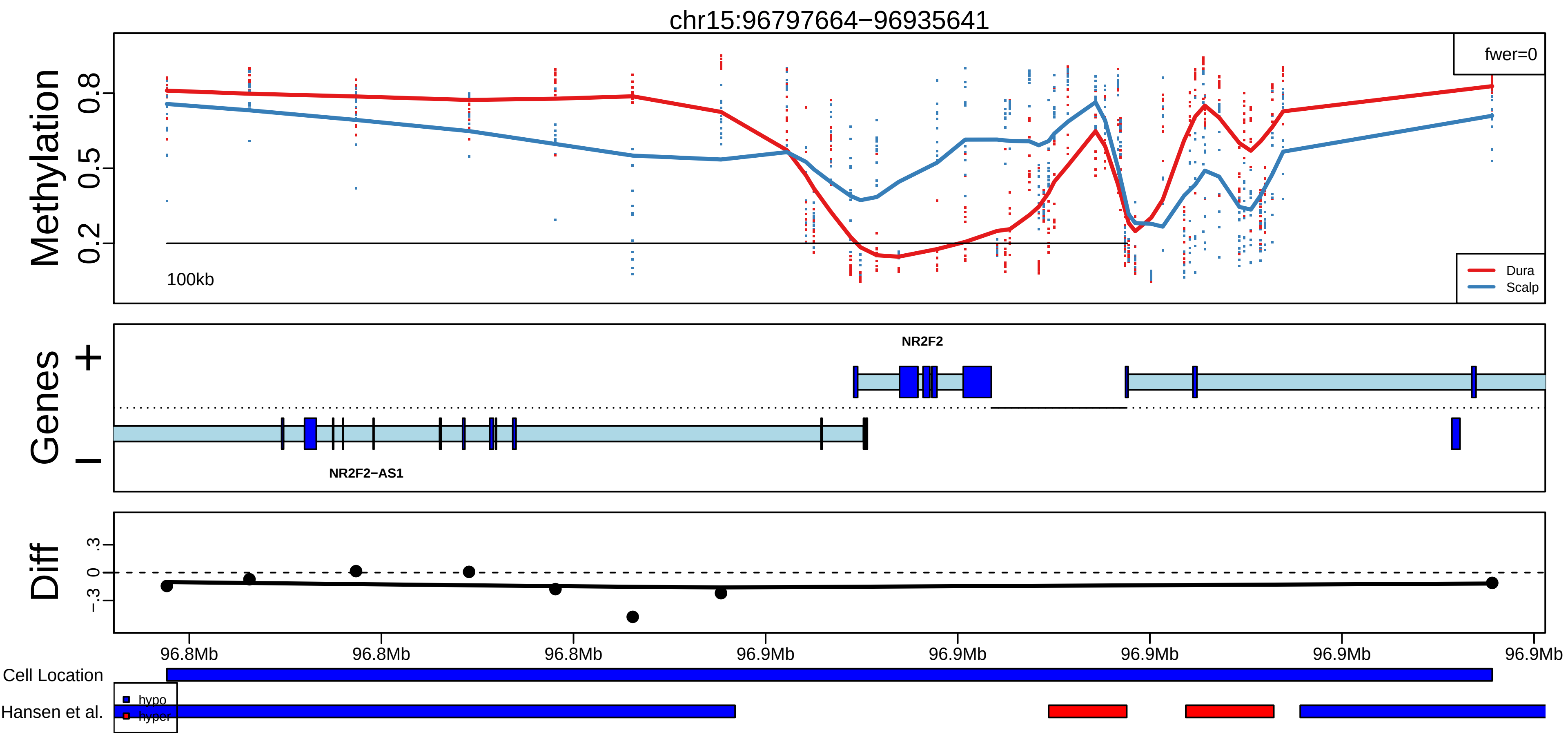
chr15:96797664−96935641
Methylation
0.2
0.5
0.8
fwer=0
100kb
Dura
Scalp
Genes
+
−
NR2F2
NR2F2−AS1
Diff
.3
0
−.3
96.8Mb
96.8Mb
96.8Mb
96.9Mb
96.9Mb
96.9Mb
96.9Mb
96.9Mb
Cell Location
Hansen et al.
hypo
hyper

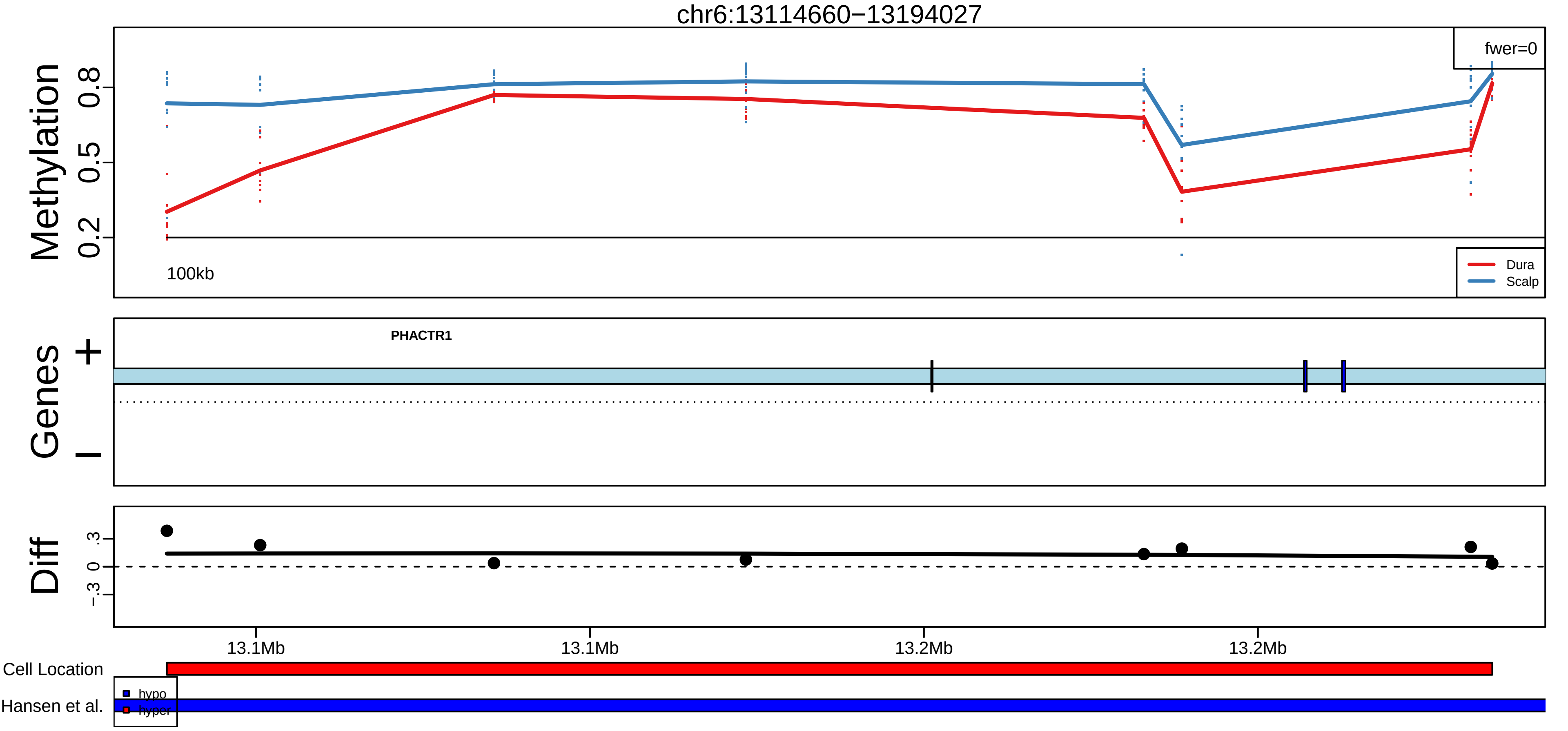
chr6:13114660−13194027
fwer=0
Methylation
0.2
0.5
0.8
100kb
Dura
Scalp
Genes
+
−
PHACTR1
Diff
−.3
0
.3
13.1Mb
13.1Mb
13.2Mb
13.2Mb
Cell Location
Hansen et al.
hypo
hyper

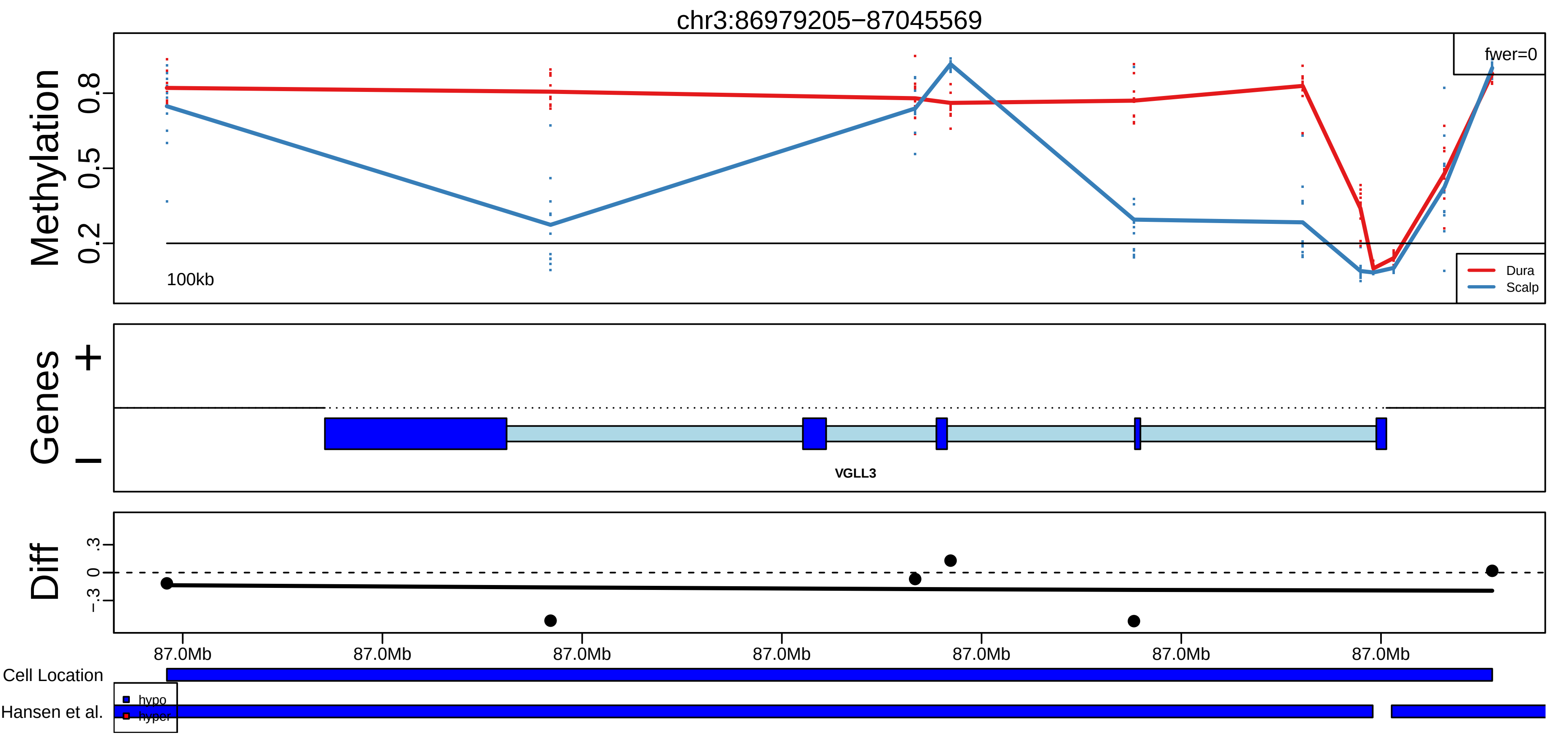
chr3:86979205−87045569
Methylation
0.2
0.5
0.8
fwer=0
100kb
Dura
Scalp
Genes
+
−
VGLL3
Diff
−.3
0
.3
87.0Mb
87.0Mb
87.0Mb
87.0Mb
87.0Mb
87.0Mb
87.0Mb
Cell Location
Hansen et al.
hypo
hyper

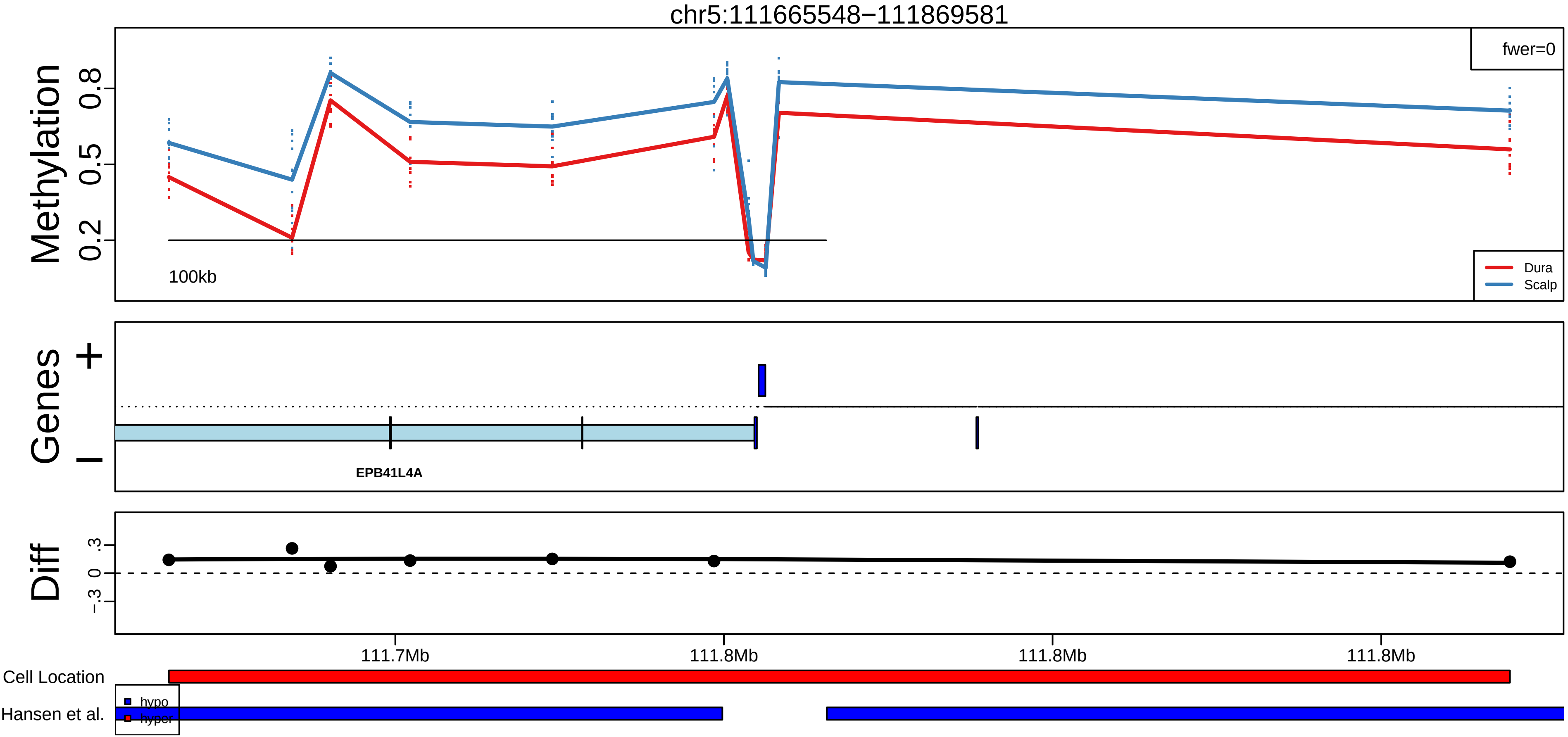

chr5:111665548−111869581
fwer=0
Methylation
0.2
0.5
0.8
100kb
Dura
Scalp
Genes
+
−
EPB41L4A
Diff
−.3
0
.3
111.7Mb
111.8Mb
111.8Mb
111.8Mb
Cell Location
Hansen et al.
hypo
hyper

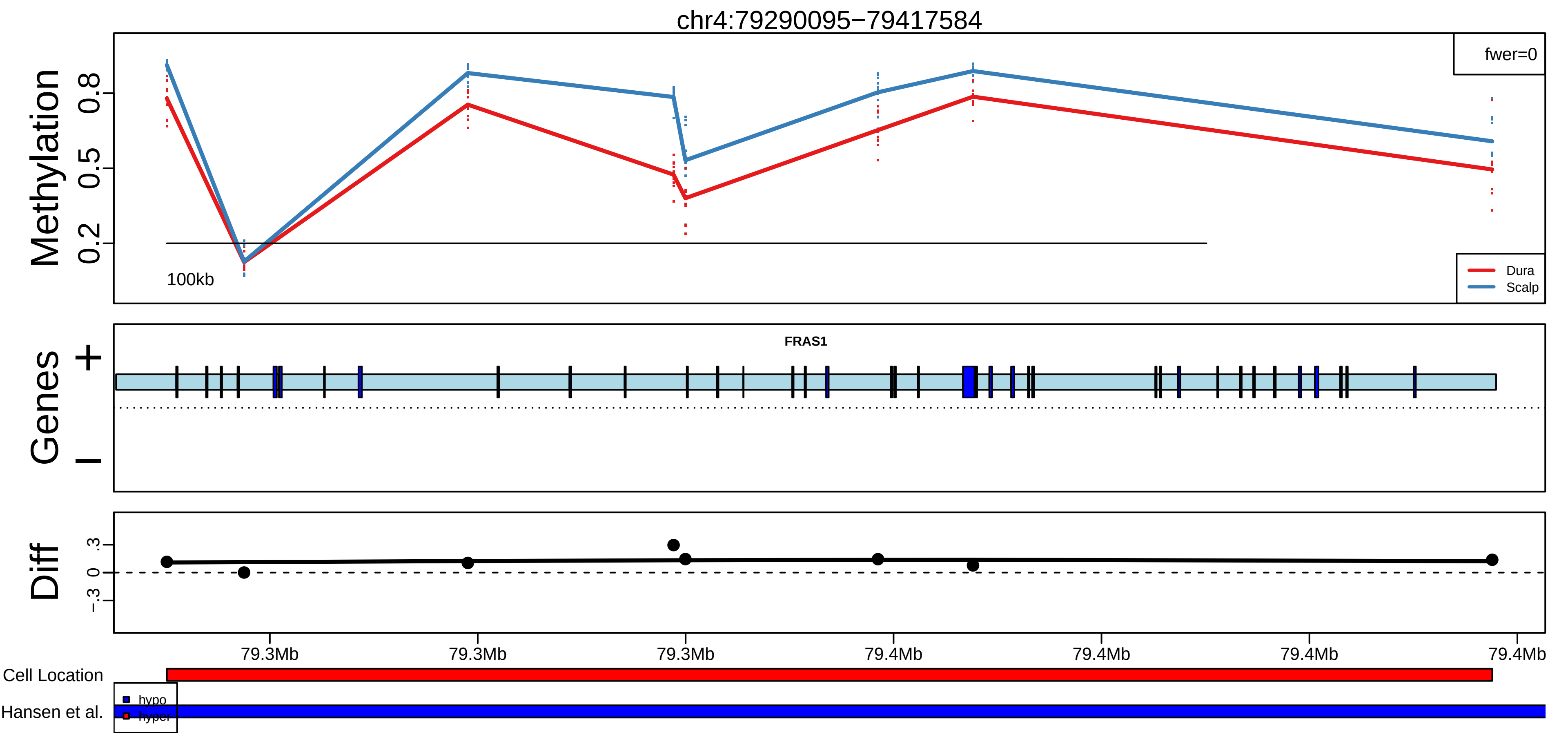

chr4:79290095−79417584
Methylation
0.2
0.5
0.8
fwer=0
100kb
Dura
Scalp
Genes
+
−
FRAS1
Diff
−.3
0
.3
79.3Mb
79.3Mb
79.3Mb
79.4Mb
79.4Mb
79.4Mb
79.4Mb
Cell Location
Hansen et al.
hypo
hyper

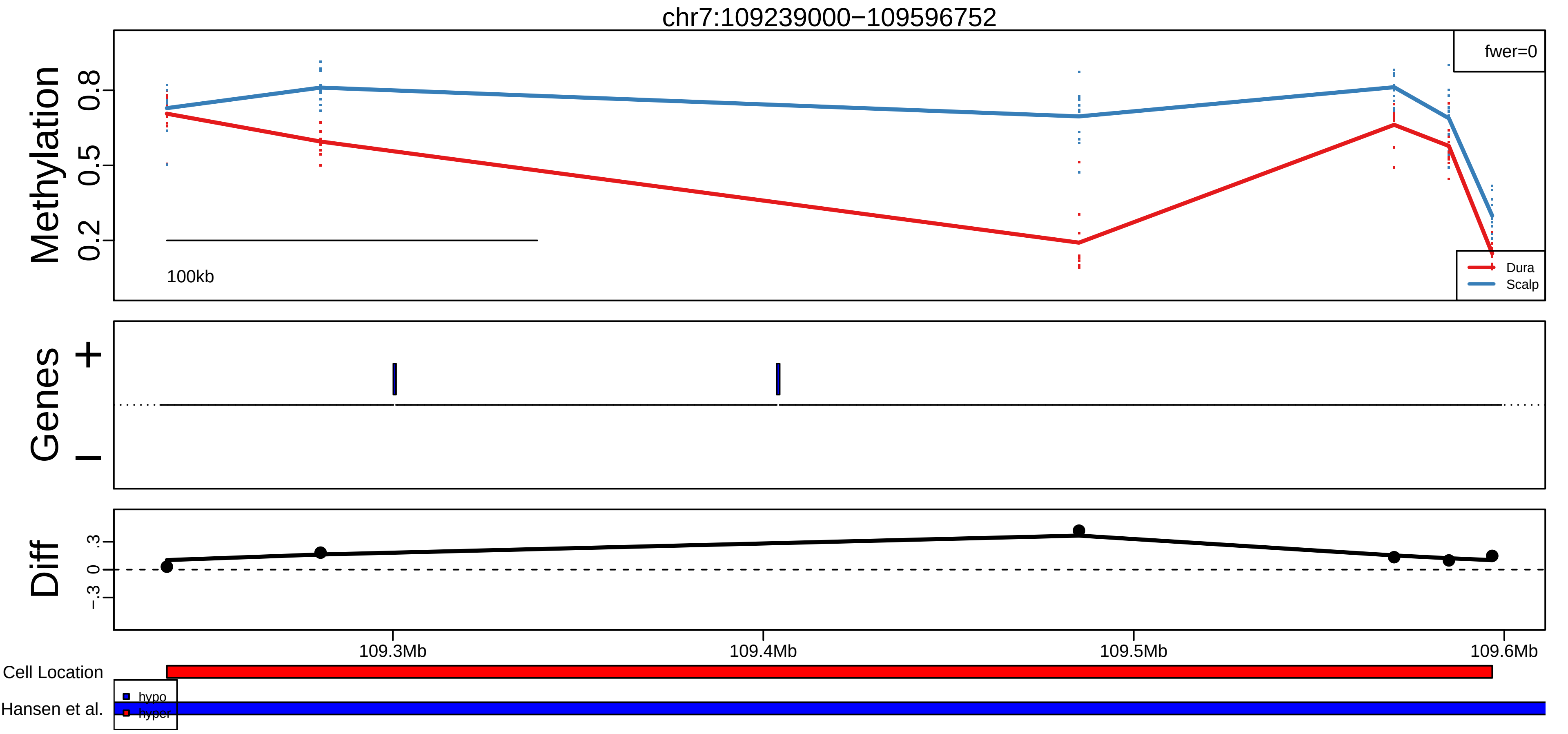

chr7:109239000−109596752
Methylation
0.2
0.5
0.8
fwer=0
100kb
Dura
Scalp
Genes
+
−
Diff
.3
0
−.3
109.3Mb
109.4Mb
109.5Mb
109.6Mb
Cell Location
Hansen et al.
hypo
hyper

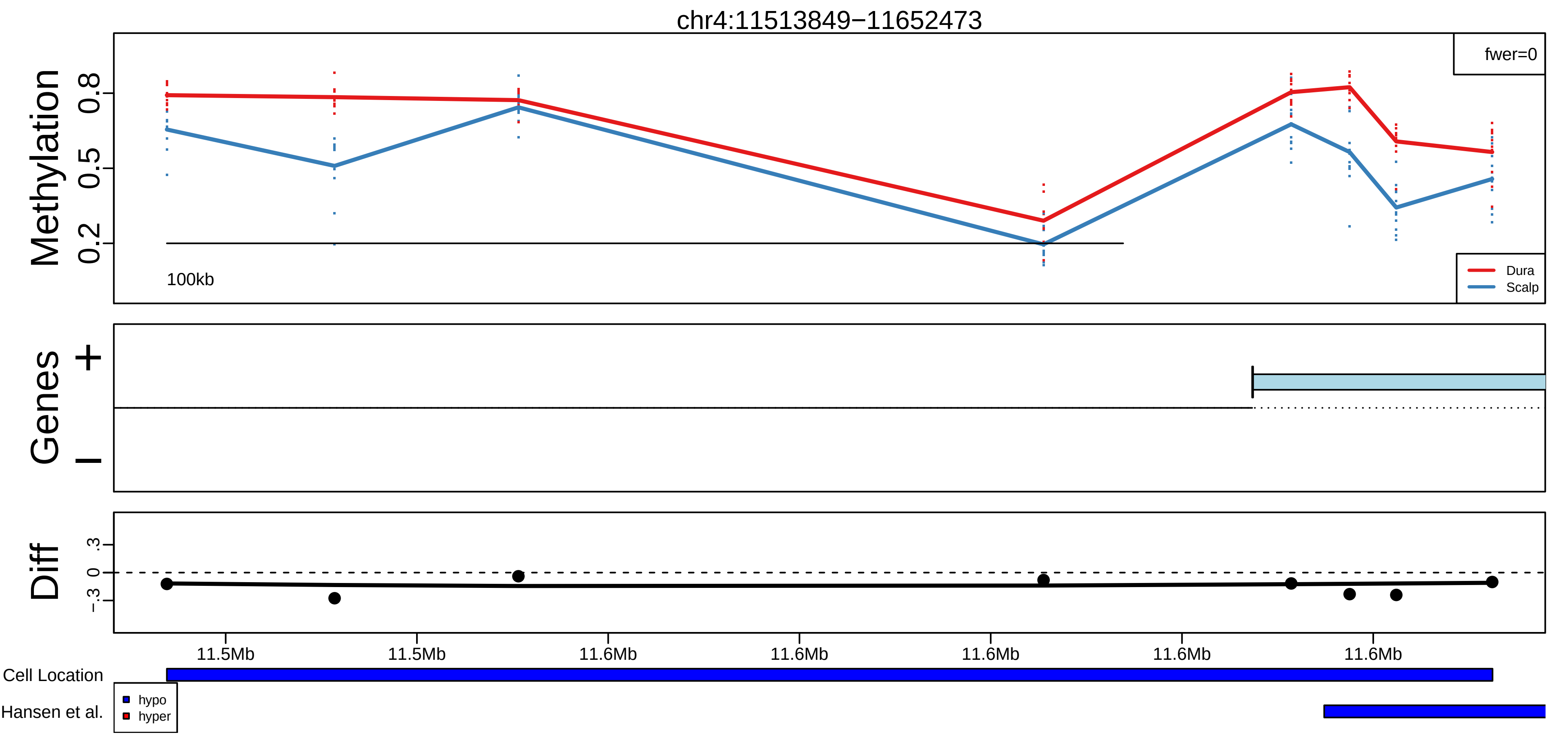
chr4:11513849−11652473
fwer=0
Methylation
0.2
0.5
0.8
100kb
Dura
Scalp
Genes
+
−
Diff
−.3
0
.3
11.5Mb
11.5Mb
11.6Mb
11.6Mb
11.6Mb
11.6Mb
11.6Mb
Cell Location
Hansen et al.
hypo
hyper

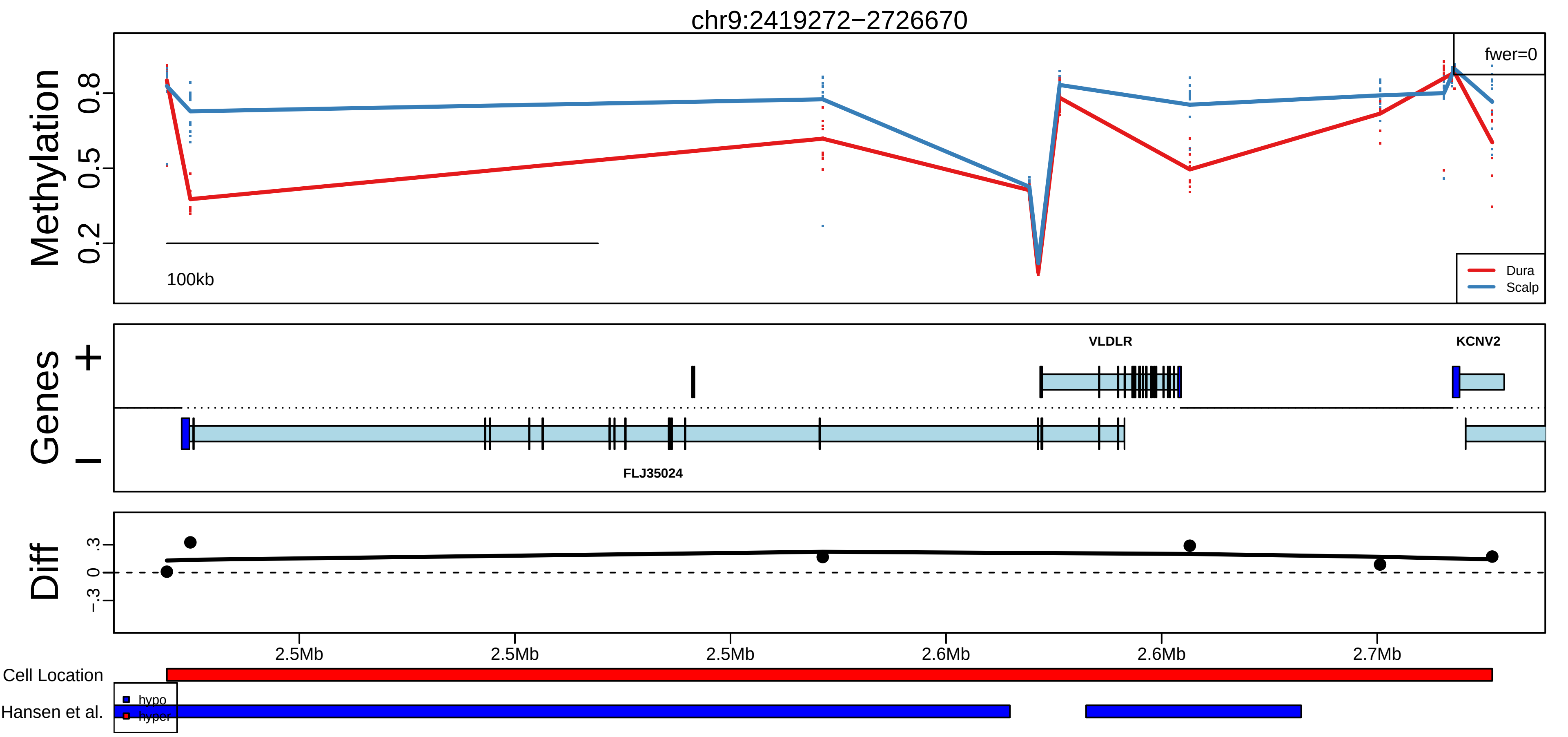

chr9:2419272−2726670
Methylation
0.2
0.5
0.8
fwer=0
100kb
Dura
Scalp
Genes
+
−
VLDLR
KCNV2
FLJ35024
Diff
−.3
0
.3
2.5Mb
2.5Mb
2.5Mb
2.6Mb
2.6Mb
2.7Mb
Cell Location
Hansen et al.
hypo
hyper

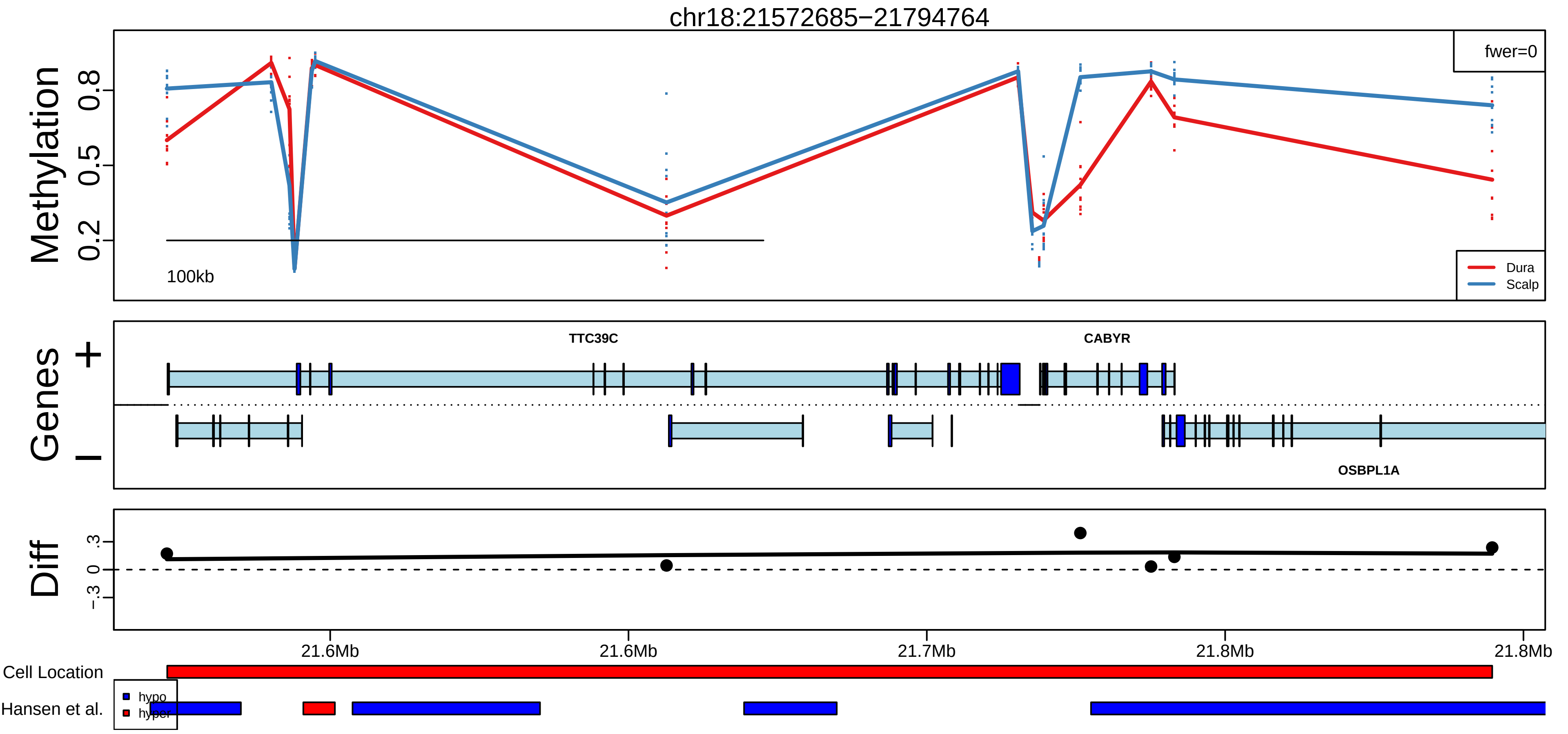

chr18:21572685−21794764
Methylation
0.2
0.5
0.8
fwer=0
100kb
Dura
Scalp
Genes
+
−
TTC39C
CABYR
OSBPL1A
Diff
−.3
0
.3
21.6Mb
21.6Mb
21.7Mb
21.8Mb
21.8Mb
Cell Location
Hansen et al.
hypo
hyper

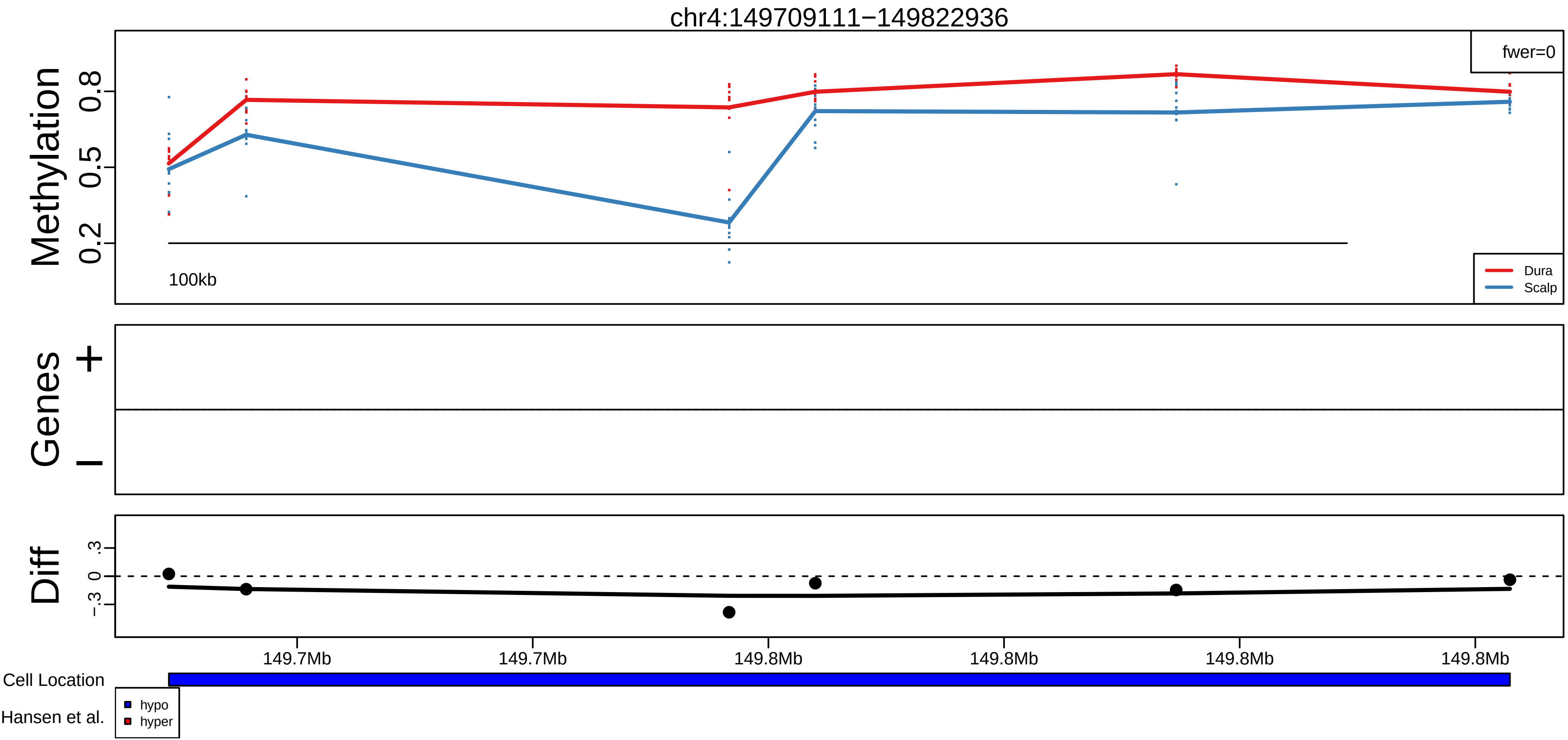
chr4:149709111−149822936
Methylation
0.2
0.5
0.8
fwer=0
100kb
Dura
Scalp
Genes
+
−
Diff
−.3
0
.3
149.7Mb
149.7Mb
149.8Mb
149.8Mb
149.8Mb
149.8Mb
Cell Location
Hansen et al.
hypo
hyper

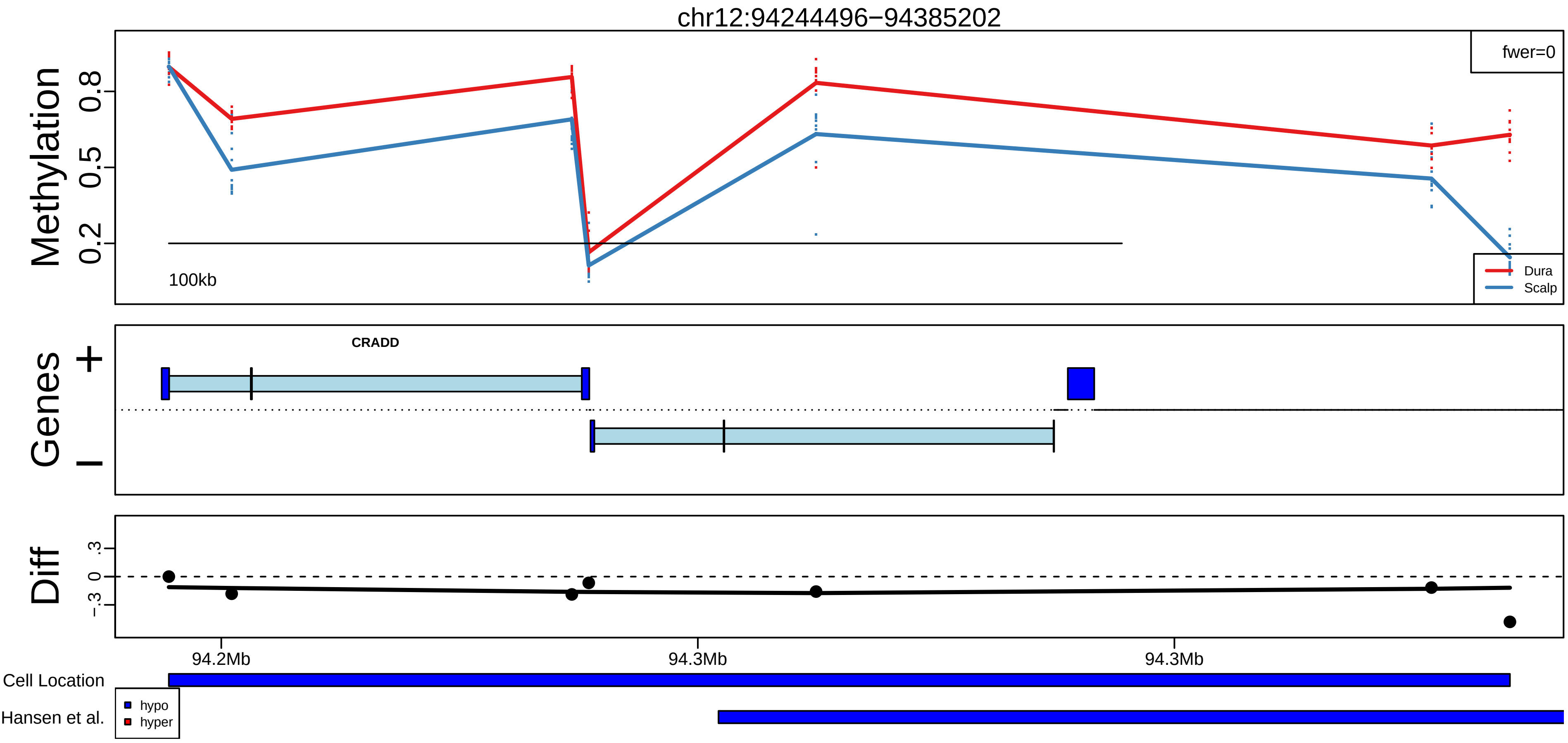
chr12:94244496−94385202
fwer=0
Methylation
0.2
0.5
0.8
100kb
Dura
Scalp
Genes
+
−
CRADD
Diff
−.3
0
.3
94.2Mb
94.3Mb
94.3Mb
Cell Location
Hansen et al.
hypo
hyper

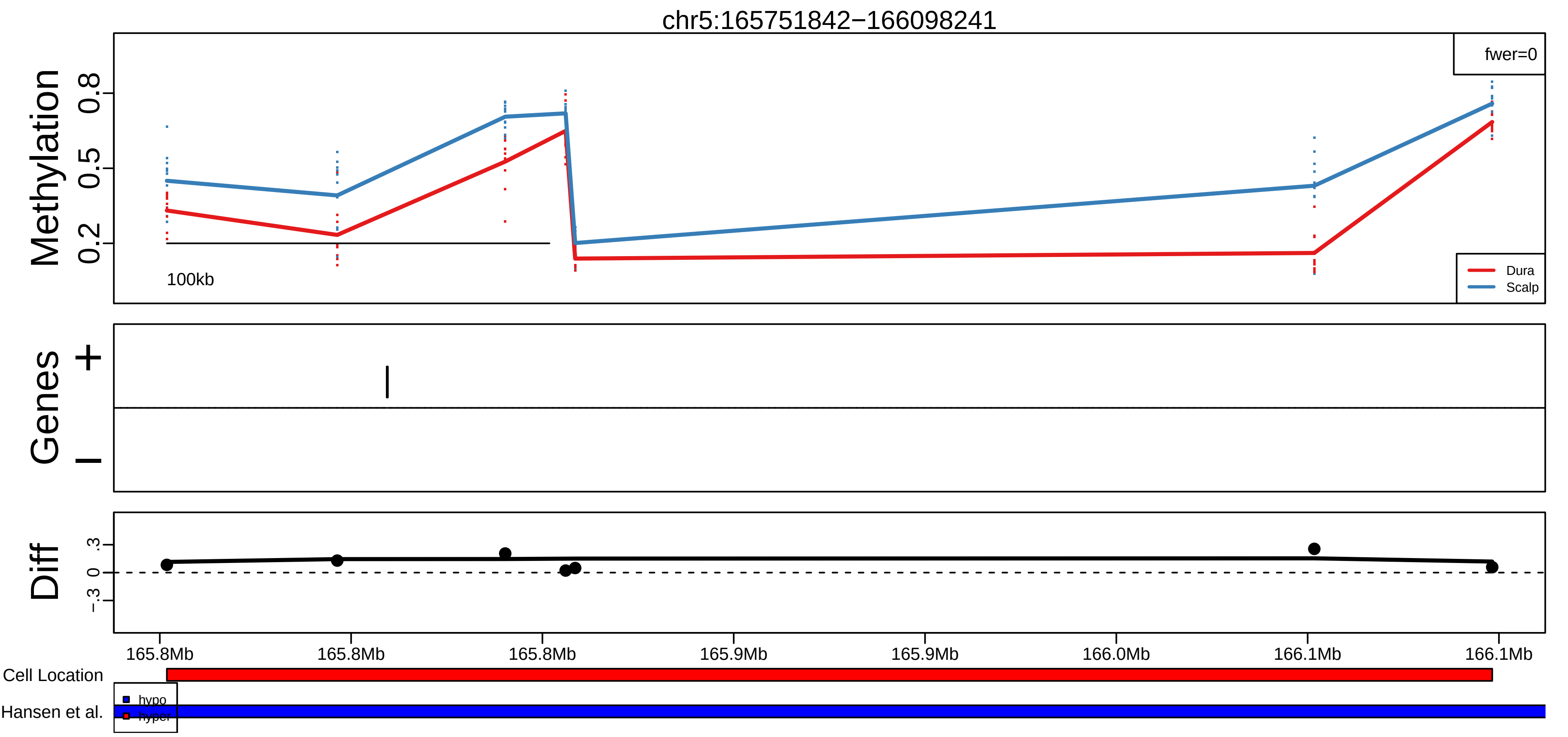

chr5:165751842−166098241
Methylation
0.2
0.5
0.8
fwer=0
100kb
Dura
Scalp
Genes
+
−
Diff
−.3
0
.3
165.8Mb
165.8Mb
165.8Mb
165.9Mb
165.9Mb
166.0Mb
166.1Mb
166.1Mb
Cell Location
Hansen et al.
hypo
hyper

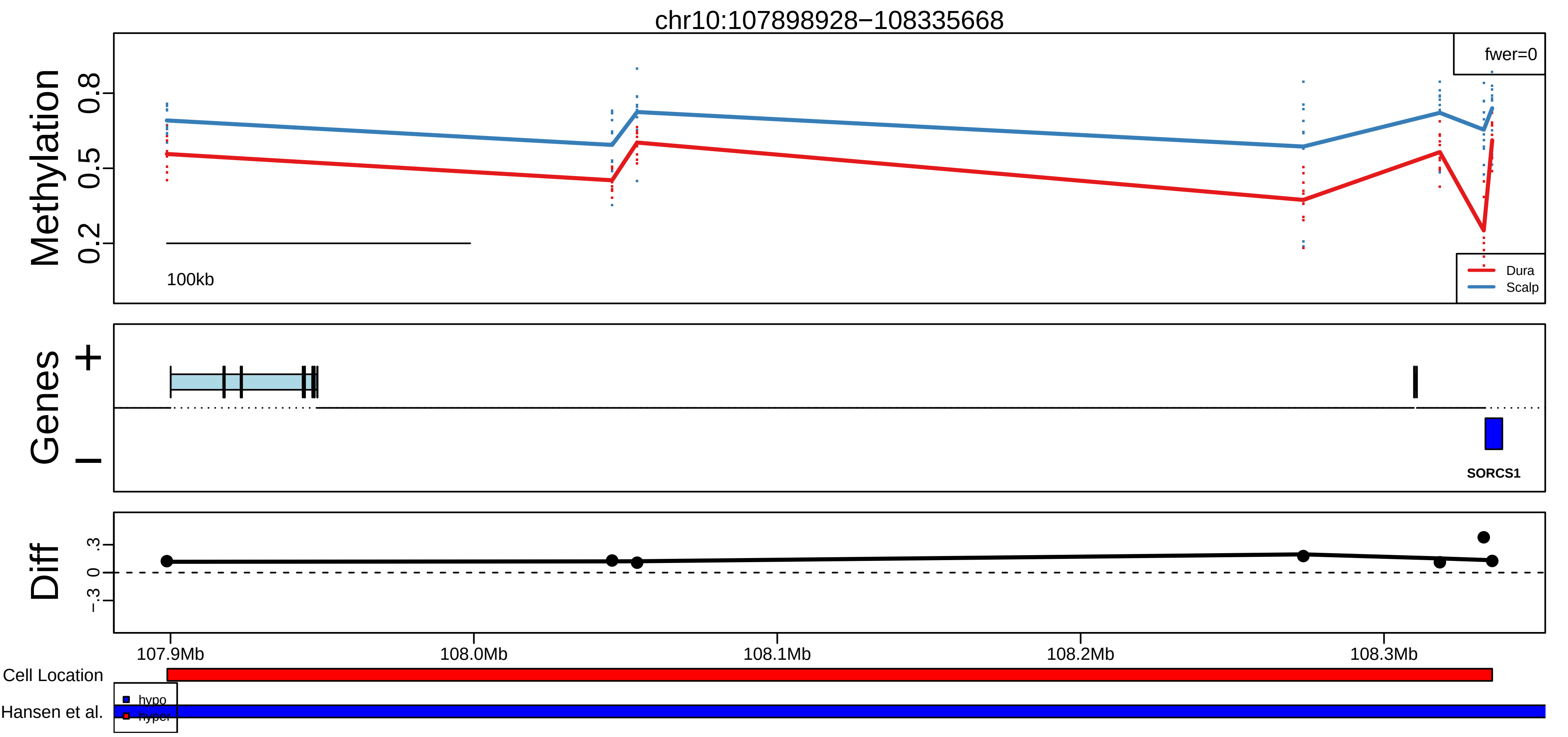

chr10:107898928−108335668
Methylation
0.2
0.5
0.8
fwer=0
100kb
Dura
Scalp
Genes
+
−
SORCS1
Diff
−.3
0
.3
107.9Mb
108.0Mb
108.1Mb
108.2Mb
108.3Mb
Cell Location
Hansen et al.
hypo
hyper

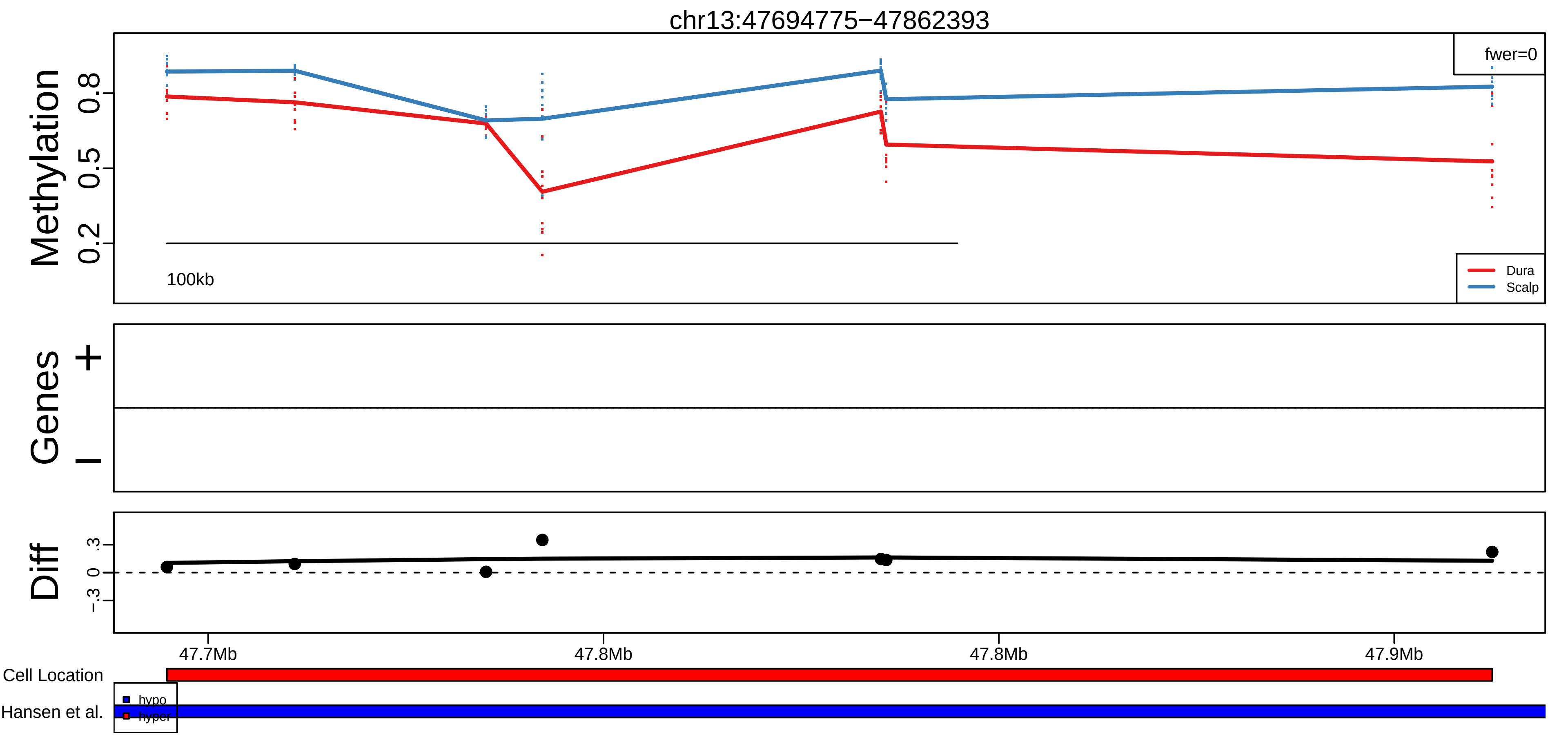

chr13:47694775−47862393
Methylation
0.2
0.5
0.8
fwer=0
100kb
Dura
Scalp
Genes
+
−
Diff
−.3
0
.3
47.7Mb
47.8Mb
47.8Mb
47.9Mb
Cell Location
Hansen et al.
hypo
hyper

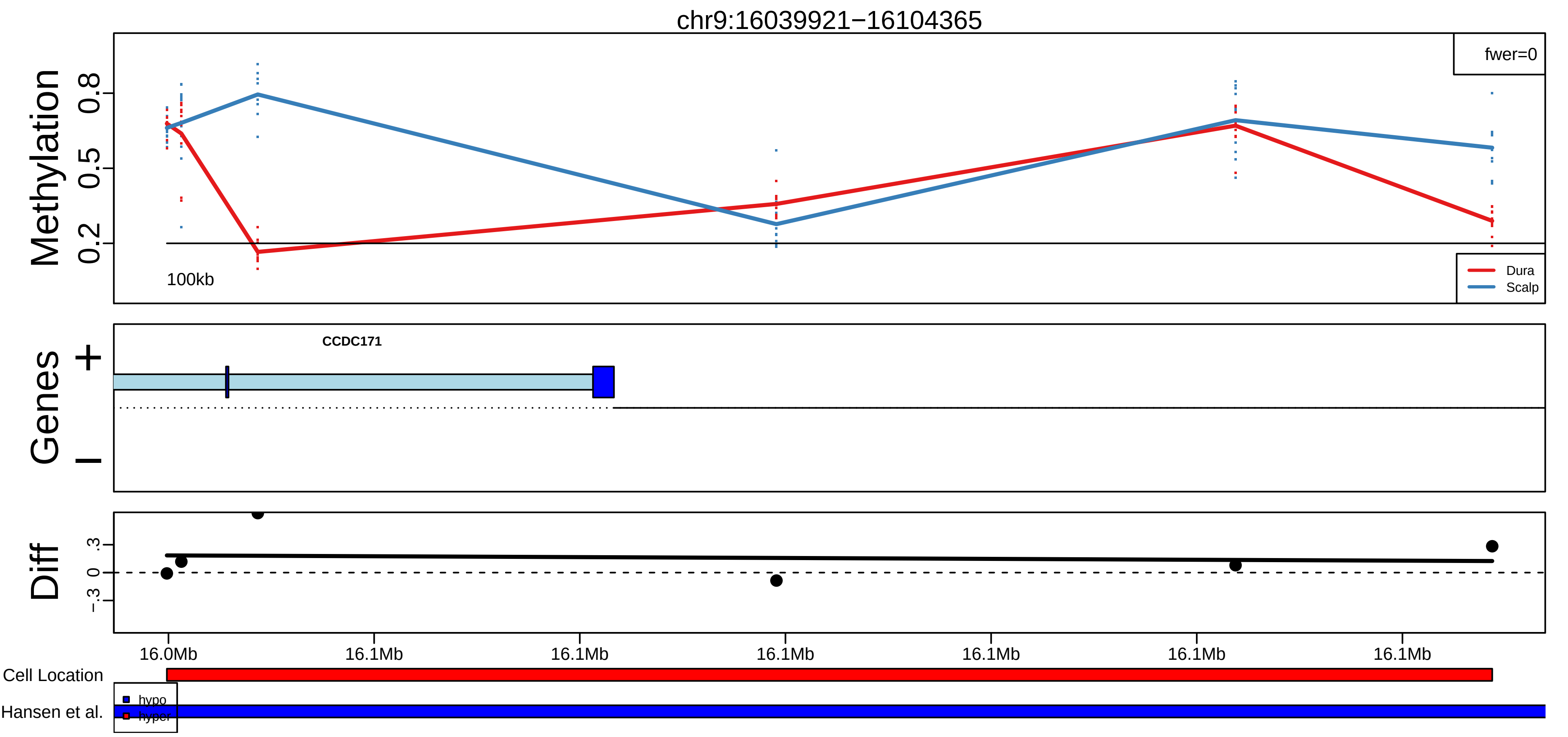

chr9:16039921−16104365
fwer=0
Methylation
0.2
0.5
0.8
100kb
Dura
Scalp
Genes
+
−
CCDC171
Diff
−.3
0
.3
16.0Mb
16.1Mb
16.1Mb
16.1Mb
16.1Mb
16.1Mb
16.1Mb
Cell Location
Hansen et al.
hypo
hyper

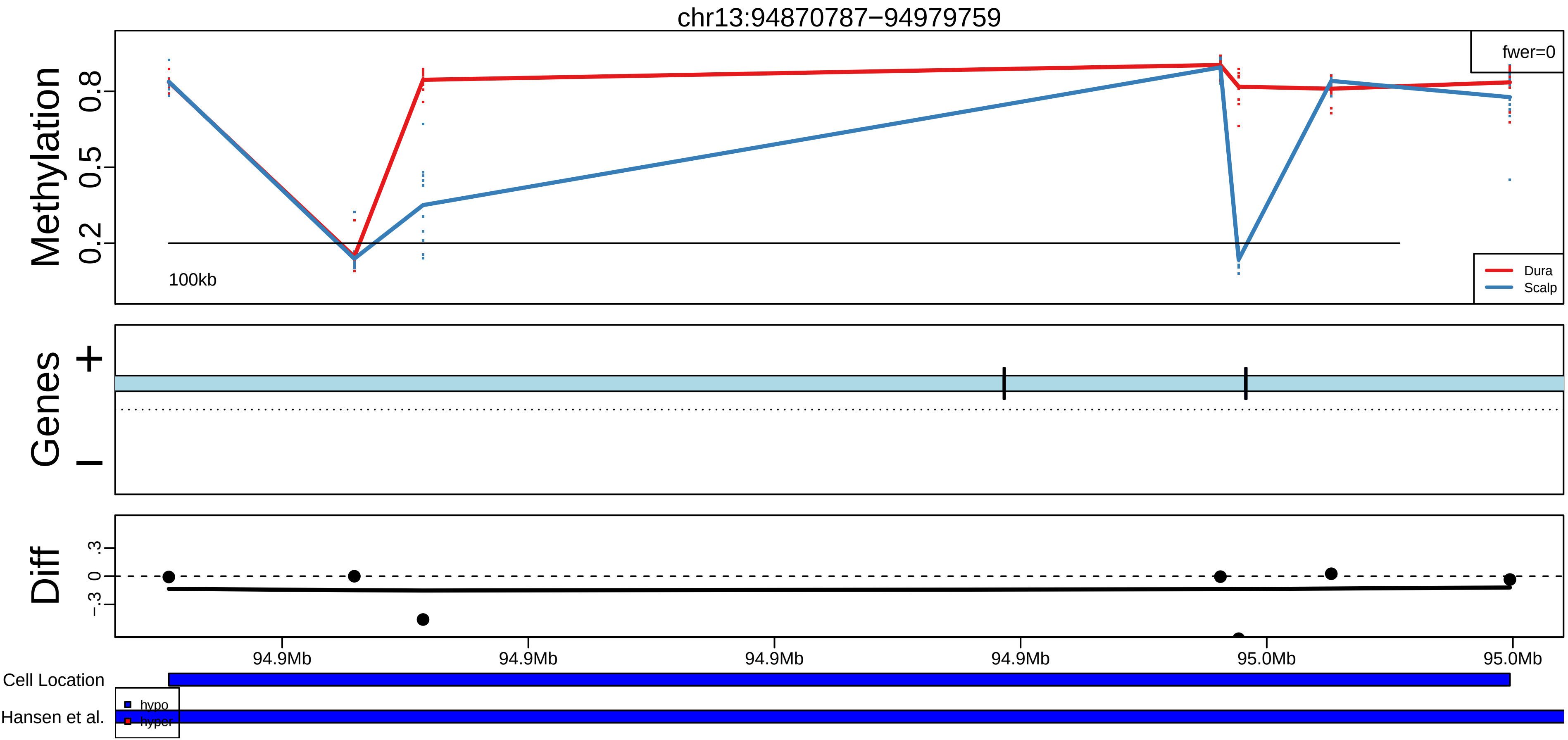

chr13:94870787−94979759
fwer=0
Methylation
0.2
0.5
0.8
100kb
Dura
Scalp
Genes
+
−
Diff
.3
0
−.3
94.9Mb
94.9Mb
94.9Mb
94.9Mb
95.0Mb
95.0Mb
Cell Location
Hansen et al.
hypo
hyper

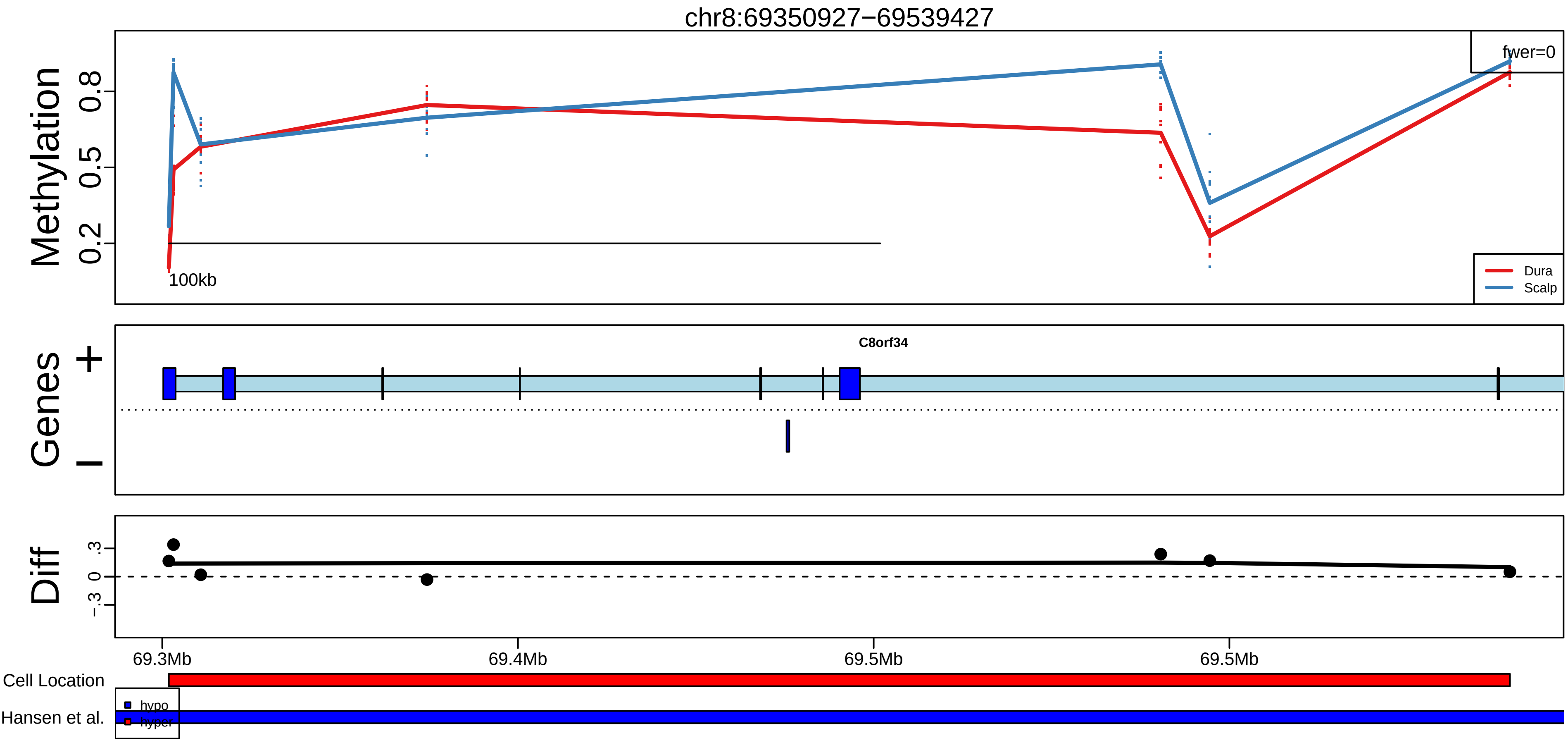
chr8:69350927−69539427
fwer=0
Methylation
0.2
0.5
0.8
100kb
Dura
Scalp
Genes
+
−
C8orf34
Diff
−.3
0
.3
69.3Mb
69.4Mb
69.5Mb
69.5Mb
Cell Location
Hansen et al.
hypo
hyper

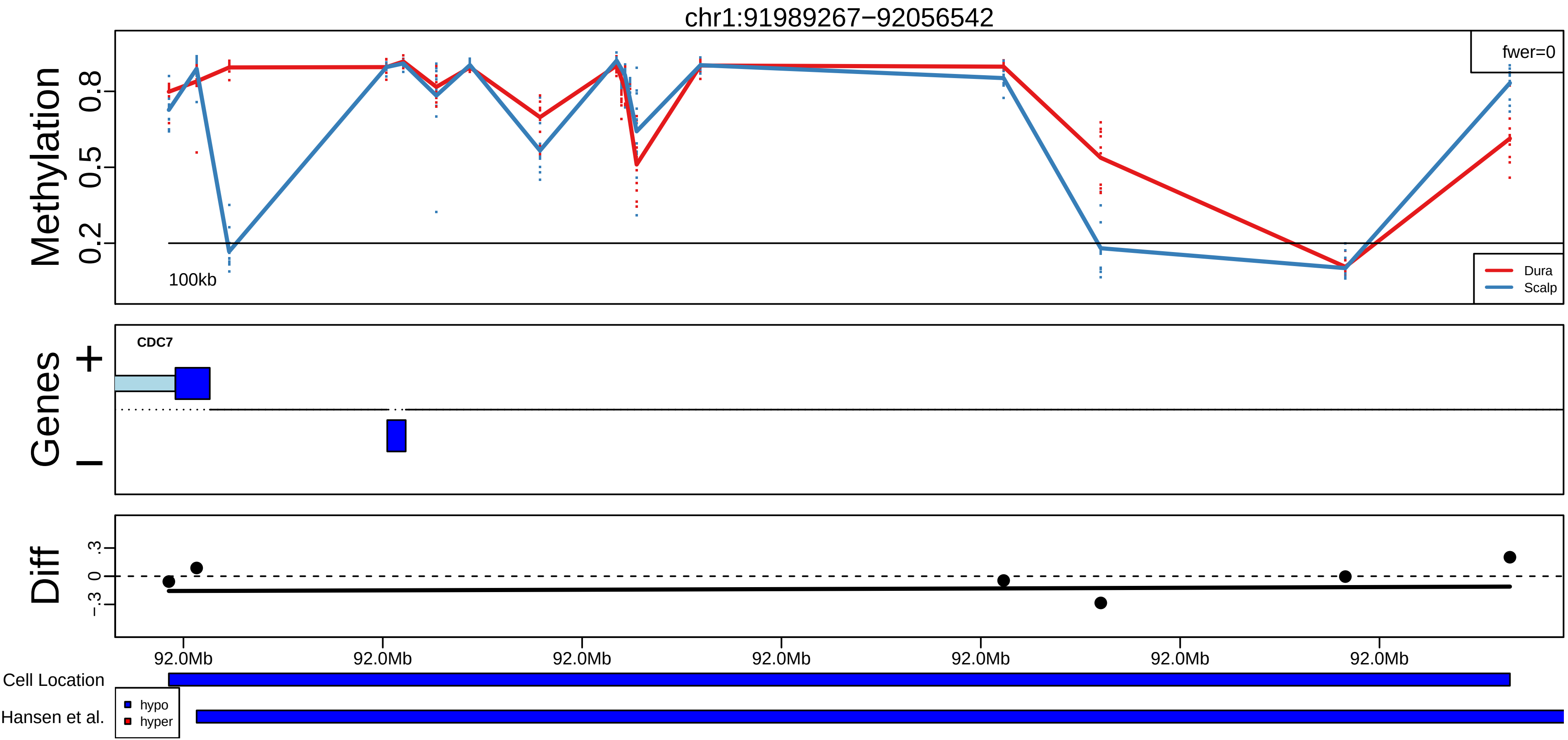
chr1:91989267−92056542
Methylation
0.2
0.5
0.8
fwer=0
100kb
Dura
Scalp
Genes
+
−
CDC7
Diff
−.3
0
.3
92.0Mb
92.0Mb
92.0Mb
92.0Mb
92.0Mb
92.0Mb
92.0Mb
Cell Location
Hansen et al.
hypo
hyper

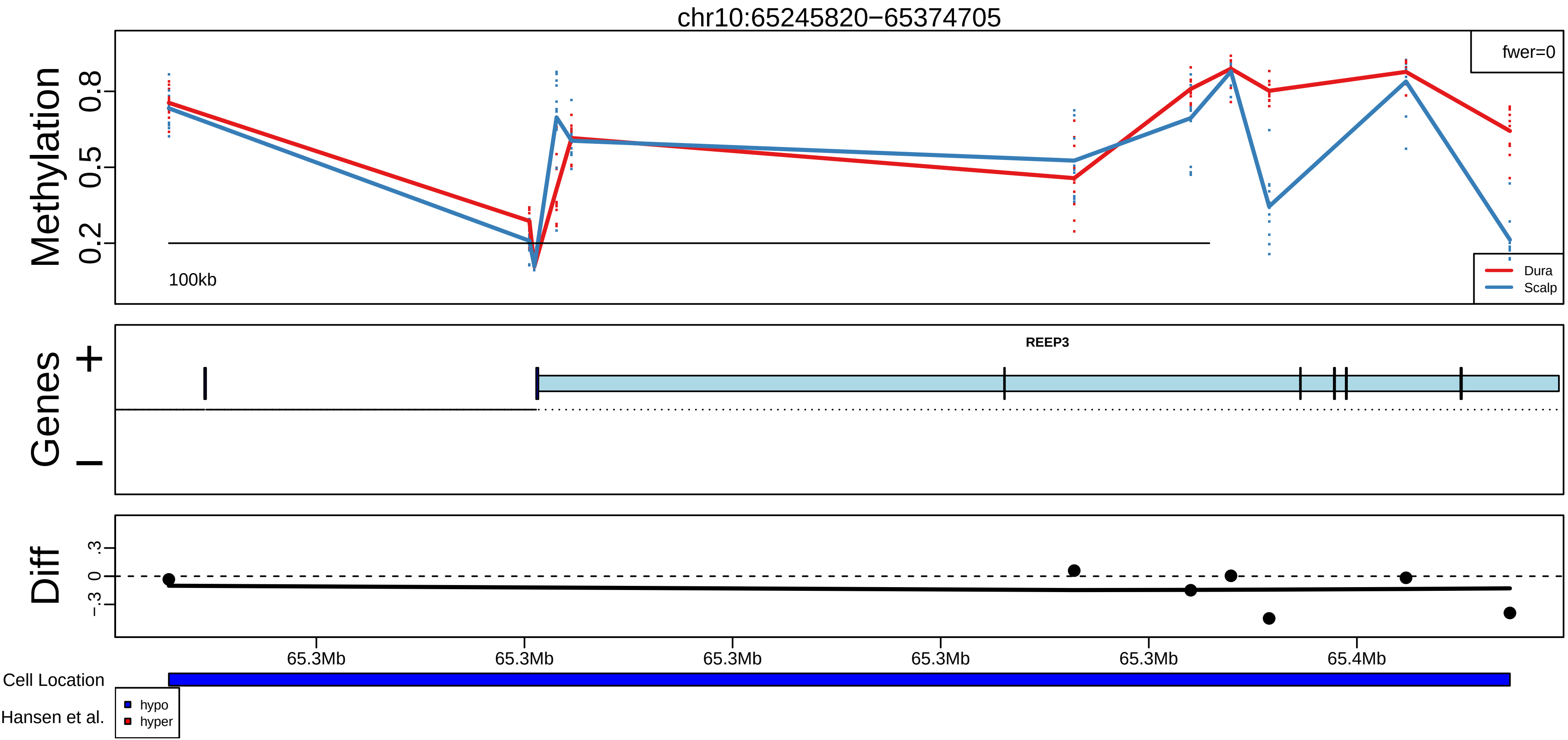

chr10:65245820−65374705
fwer=0
Methylation
0.2
0.5
0.8
100kb
Dura
Scalp
Genes
+
−
REEP3
Diff
−.3
0
.3
65.3Mb
65.3Mb
65.3Mb
65.3Mb
65.3Mb
65.4Mb
Cell Location
Hansen et al.
hypo
hyper

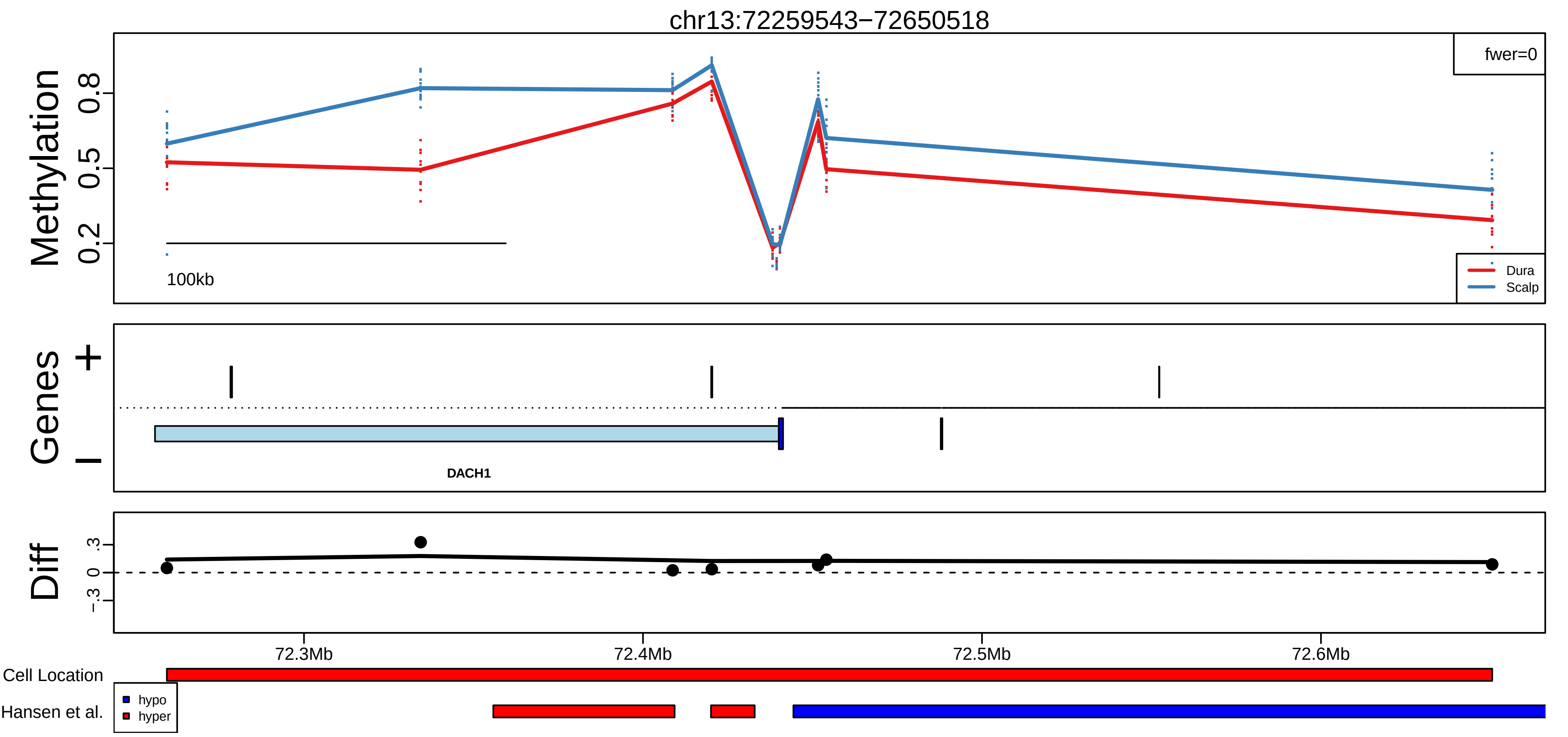

chr13:72259543−72650518
fwer=0
Methylation
0.2
0.5
0.8
100kb
Dura
Scalp
Genes
+
−
DACH1
Diff
−.3
0
.3
72.3Mb
72.4Mb
72.5Mb
72.6Mb
Cell Location
Hansen et al.
hypo
hyper

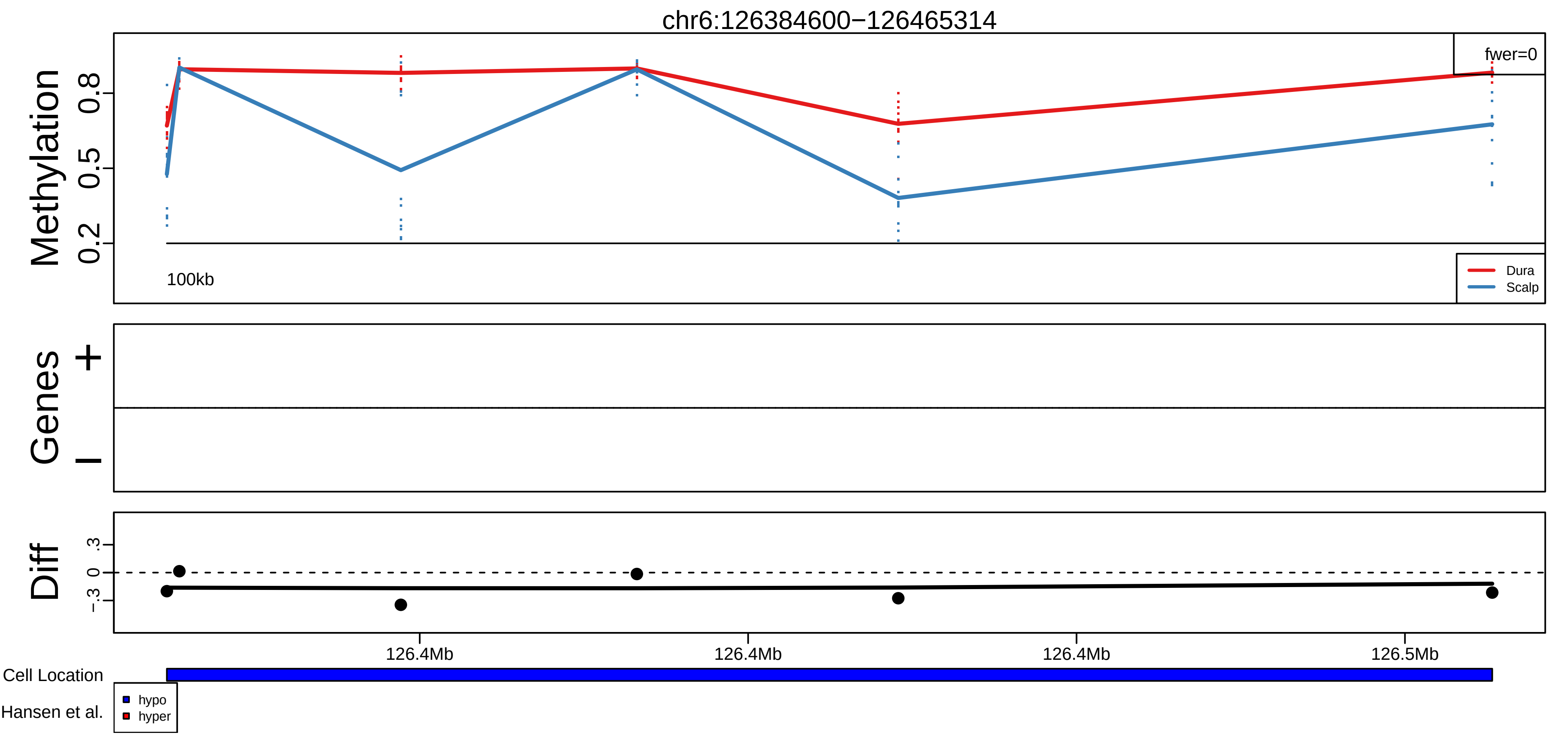
chr6:126384600−126465314
Methylation
0.2
0.5
0.8
fwer=0
100kb
Dura
Scalp
Genes
+
−
Diff
.3
0
−.3
126.4Mb
126.4Mb
126.4Mb
126.5Mb
Cell Location
Hansen et al.
hypo
hyper

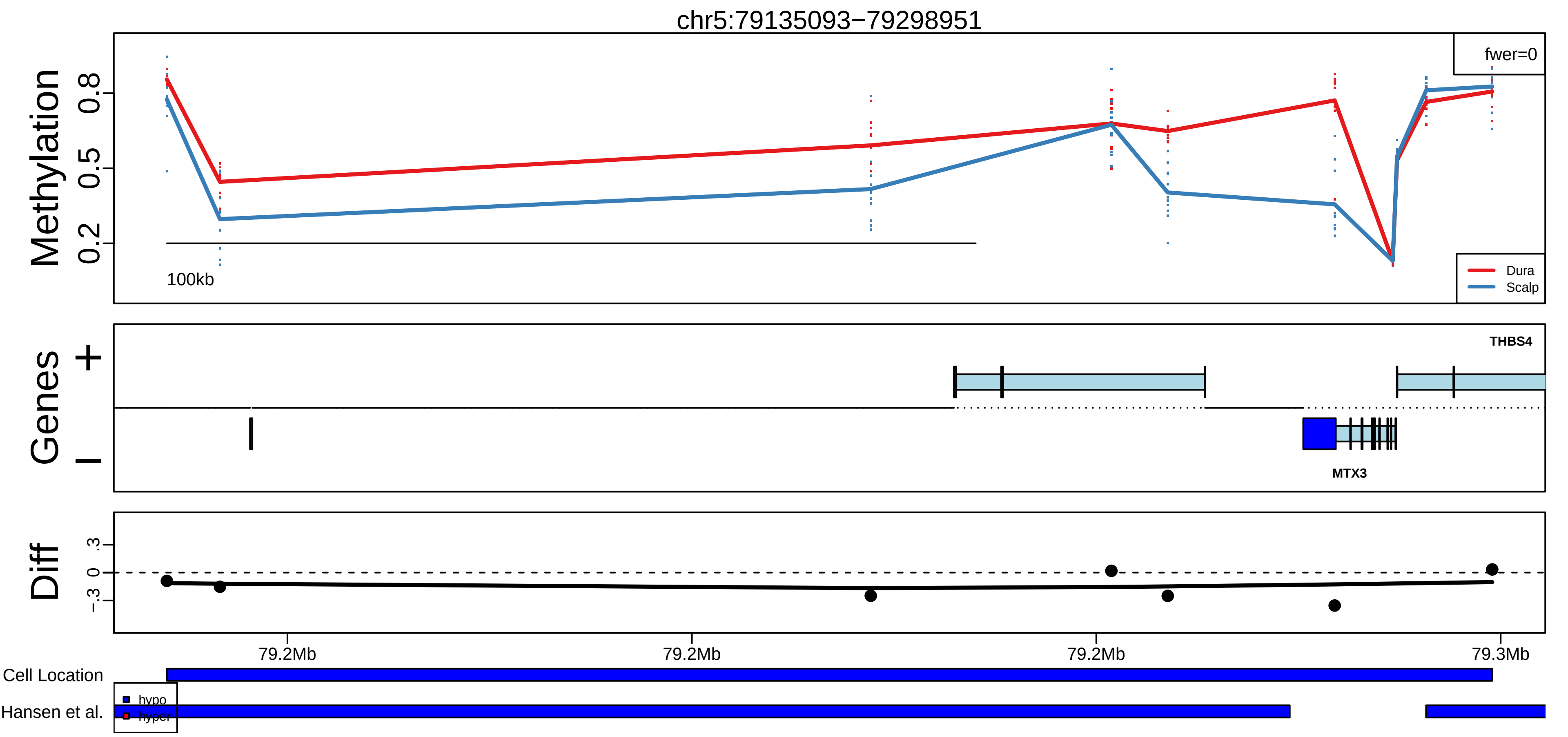

chr5:79135093−79298951
Methylation
0.2
0.5
0.8
fwer=0
100kb
Dura
Scalp
Genes
+
−
THBS4
MTX3
Diff
−.3
0
.3
79.2Mb
79.2Mb
79.2Mb
79.3Mb
Cell Location
Hansen et al.
hypo
hyper

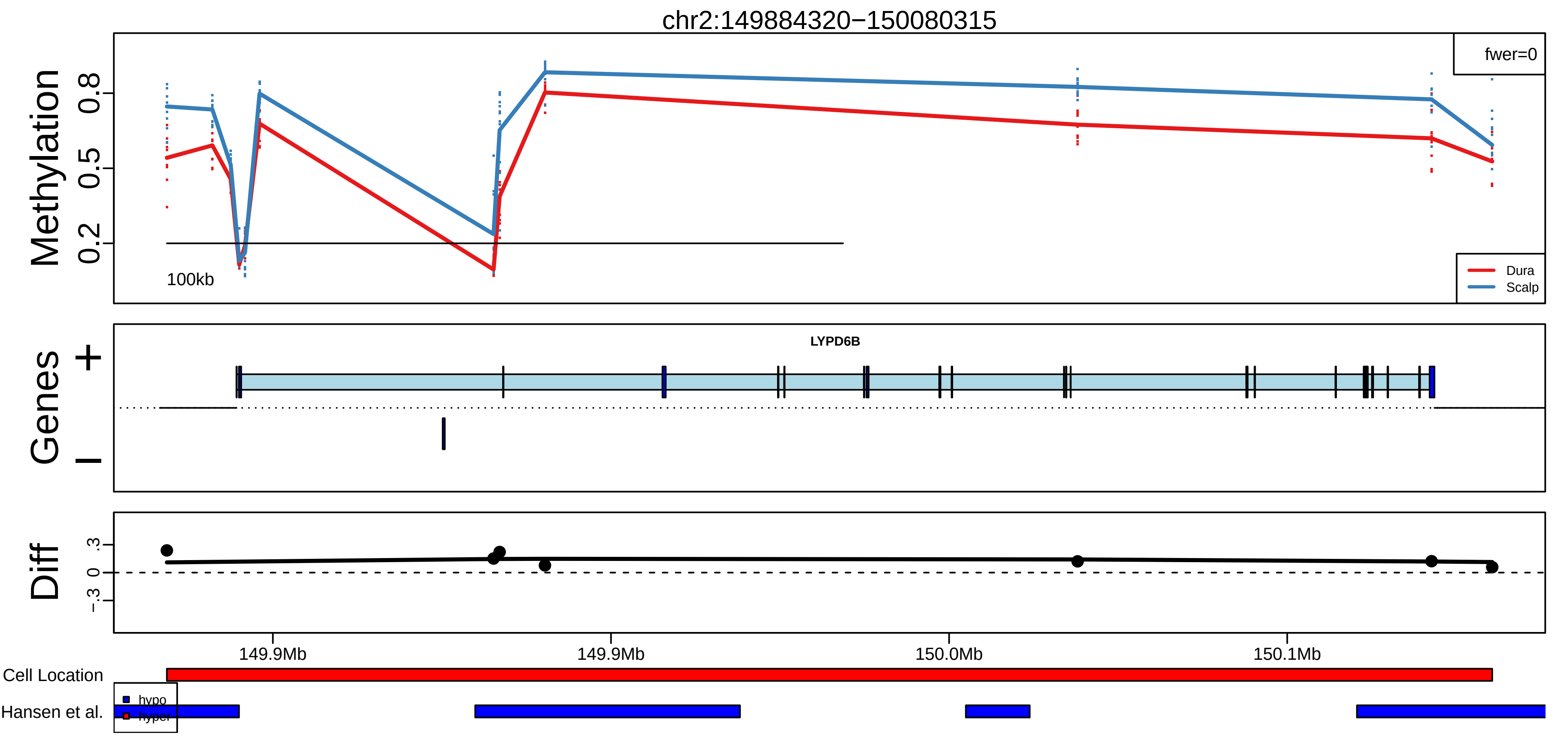
chr2:149884320−150080315
Methylation
0.2
0.5
0.8
fwer=0
100kb
Dura
Scalp
Genes
+
−
LYPD6B
Diff
−.3
0
.3
149.9Mb
149.9Mb
150.0Mb
150.1Mb
Cell Location
Hansen et al.
hypo
hyper

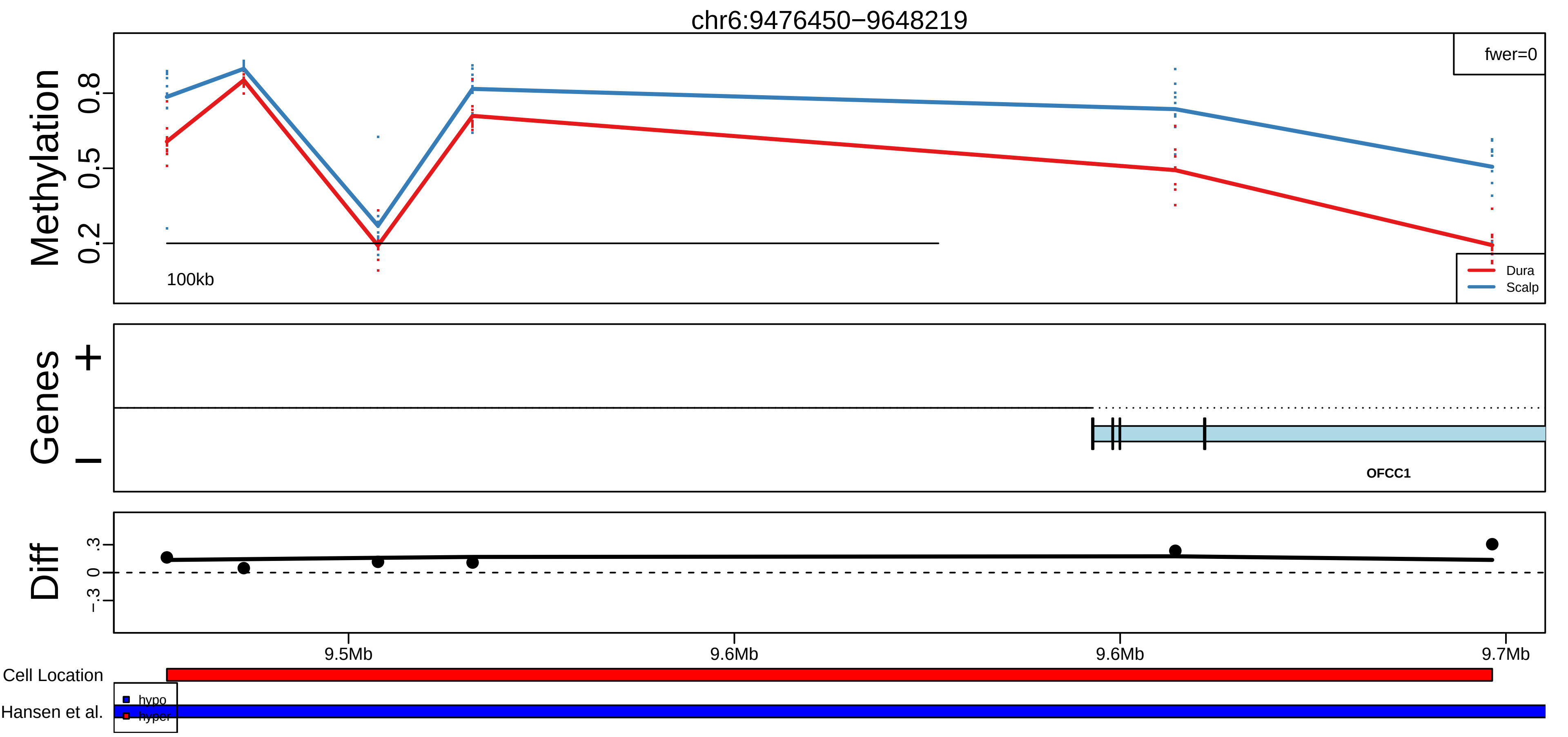

chr6:9476450−9648219
Methylation
0.2
0.5
0.8
fwer=0
100kb
Dura
Scalp
Genes
+
−
OFCC1
Diff
−.3
0
.3
9.5Mb
9.6Mb
9.6Mb
9.7Mb
Cell Location
Hansen et al.
hypo
hyper

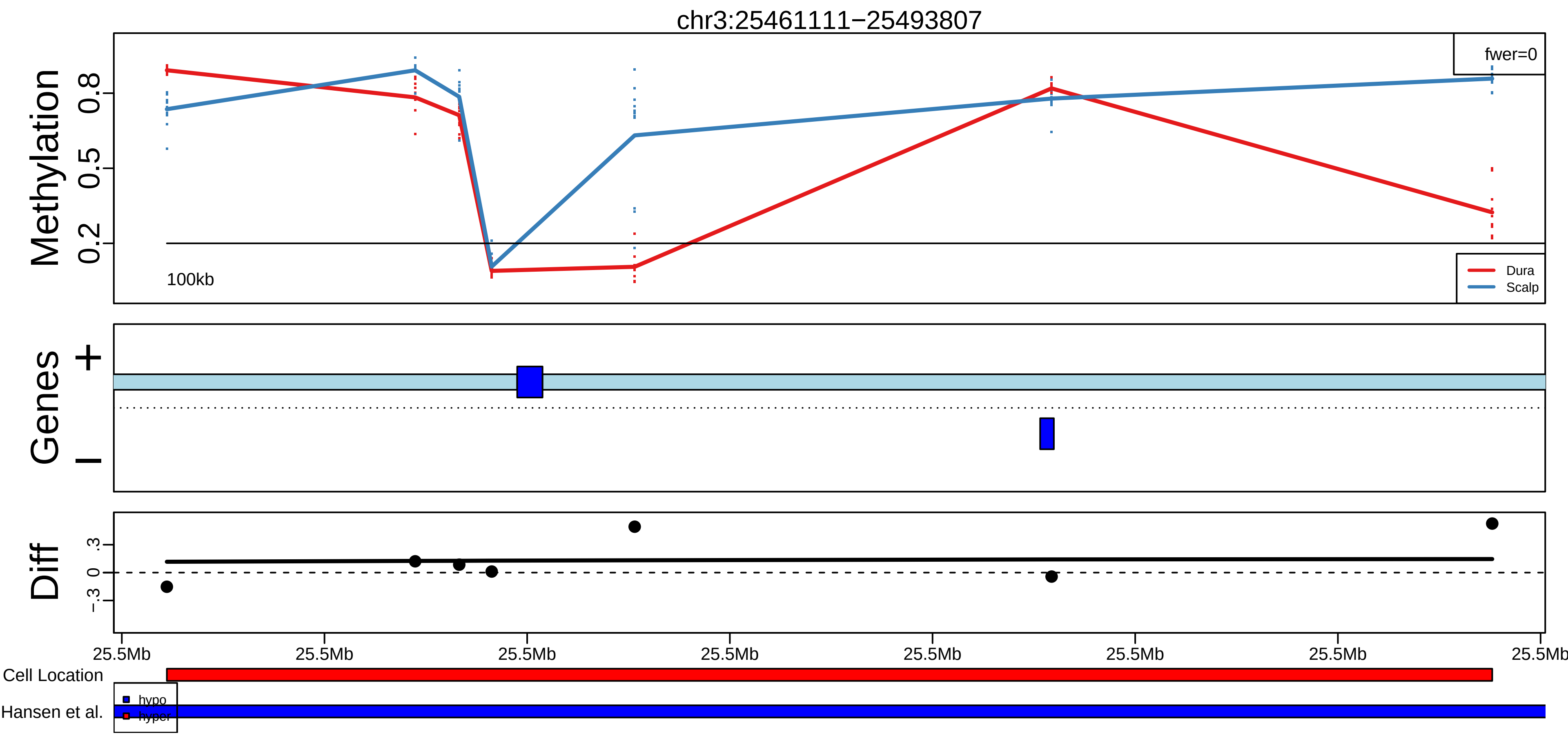
chr3:25461111−25493807
fwer=0
Methylation
0.2
0.5
0.8
100kb
Dura
Scalp
Genes
+
−
Diff
−.3
0
.3
25.5Mb
25.5Mb
25.5Mb
25.5Mb
25.5Mb
25.5Mb
25.5Mb
25.5Mb
Cell Location
Hansen et al.
hypo
hyper

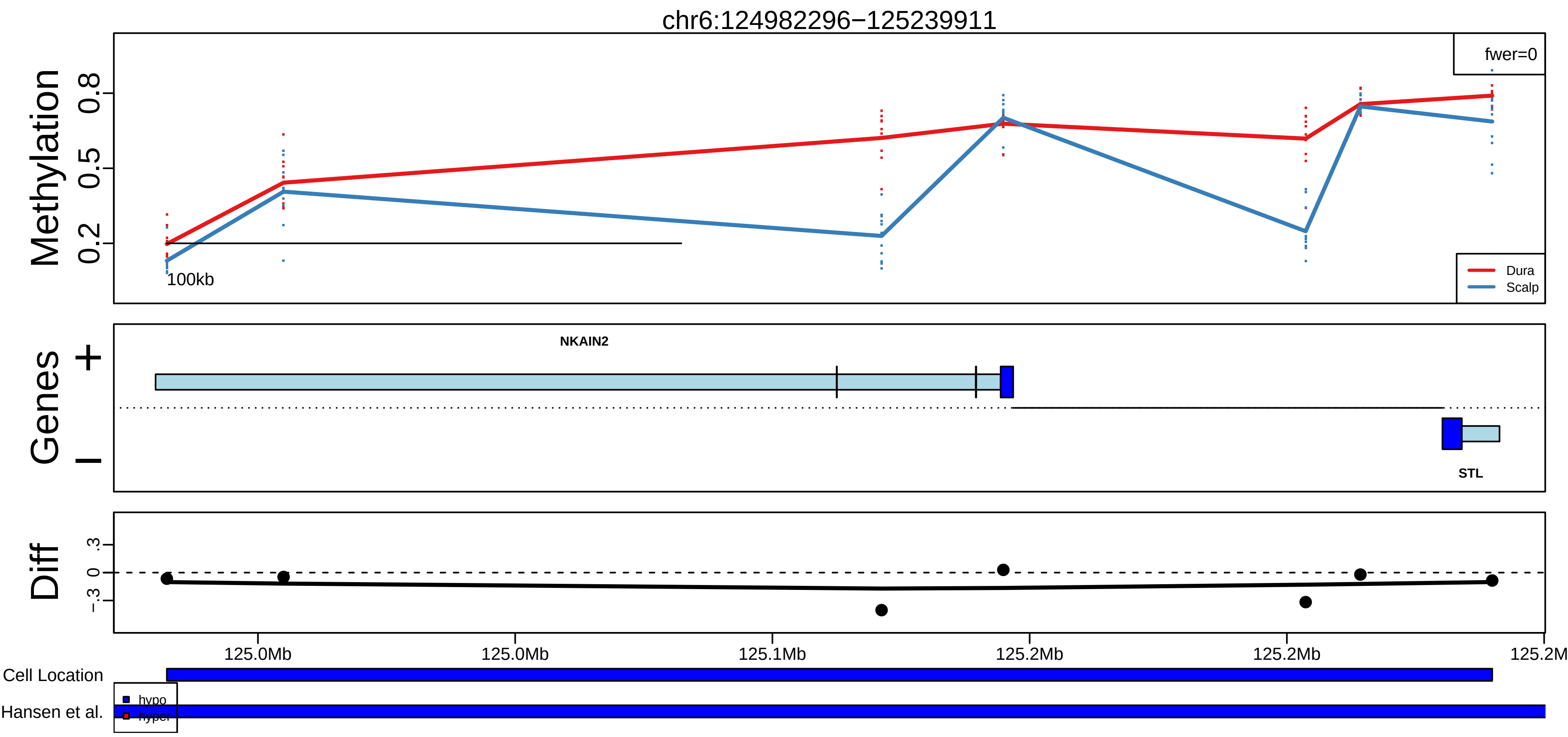

chr6:124982296−125239911
fwer=0
Methylation
0.2
0.5
0.8
100kb
Dura
Scalp
Genes
+
−
NKAIN2
STL
Diff
−.3
0
.3
125.0Mb
125.0Mb
125.1Mb
125.2Mb
125.2Mb
125.2M
Cell Location
Hansen et al.
hypo
hyper

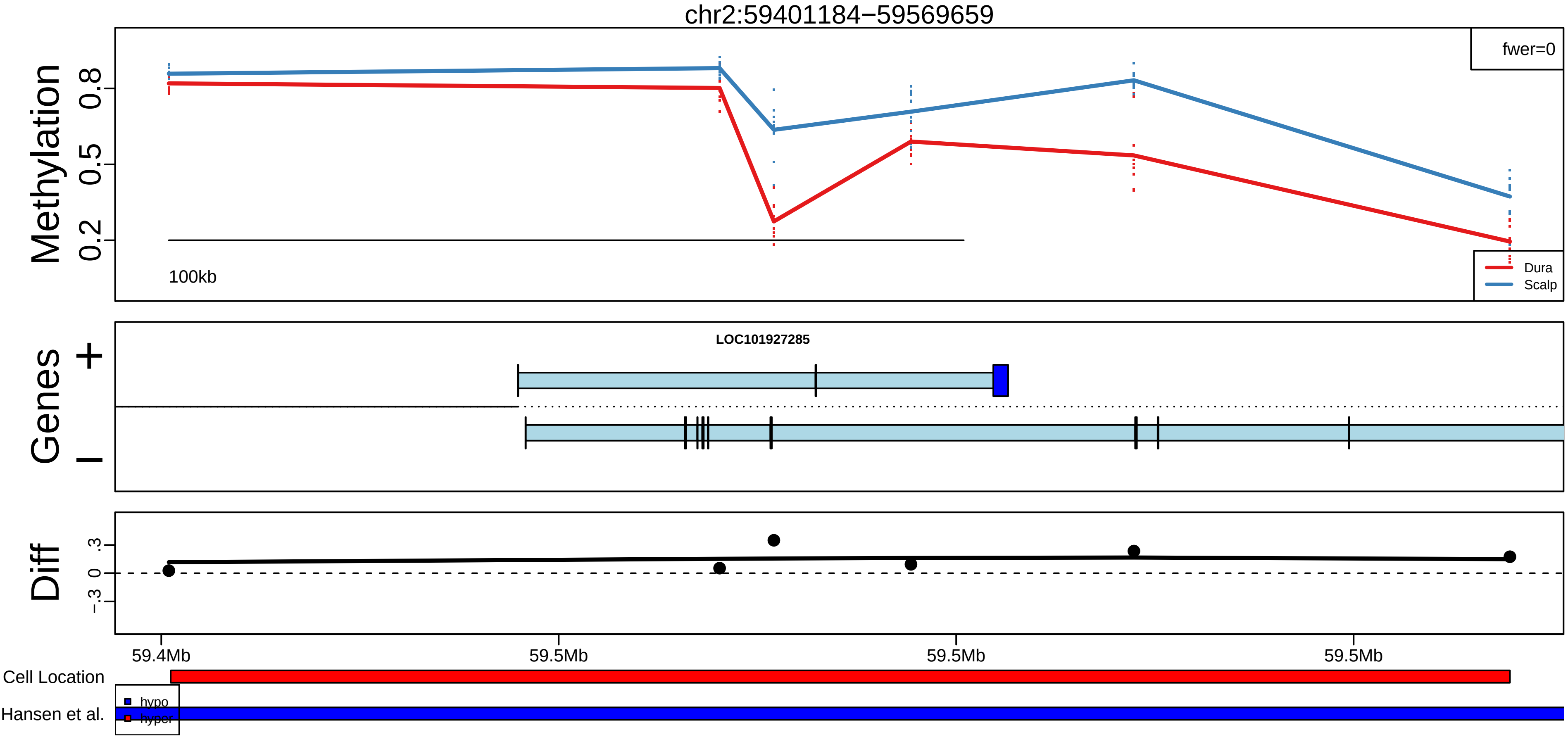
chr2:59401184−59569659
fwer=0
Methylation
0.2
0.5
0.8
100kb
Dura
Scalp
Genes
+
−
LOC101927285
Diff
−.3
0
.3
59.4Mb
59.5Mb
59.5Mb
59.5Mb
Cell Location
Hansen et al.
hypo
hyper

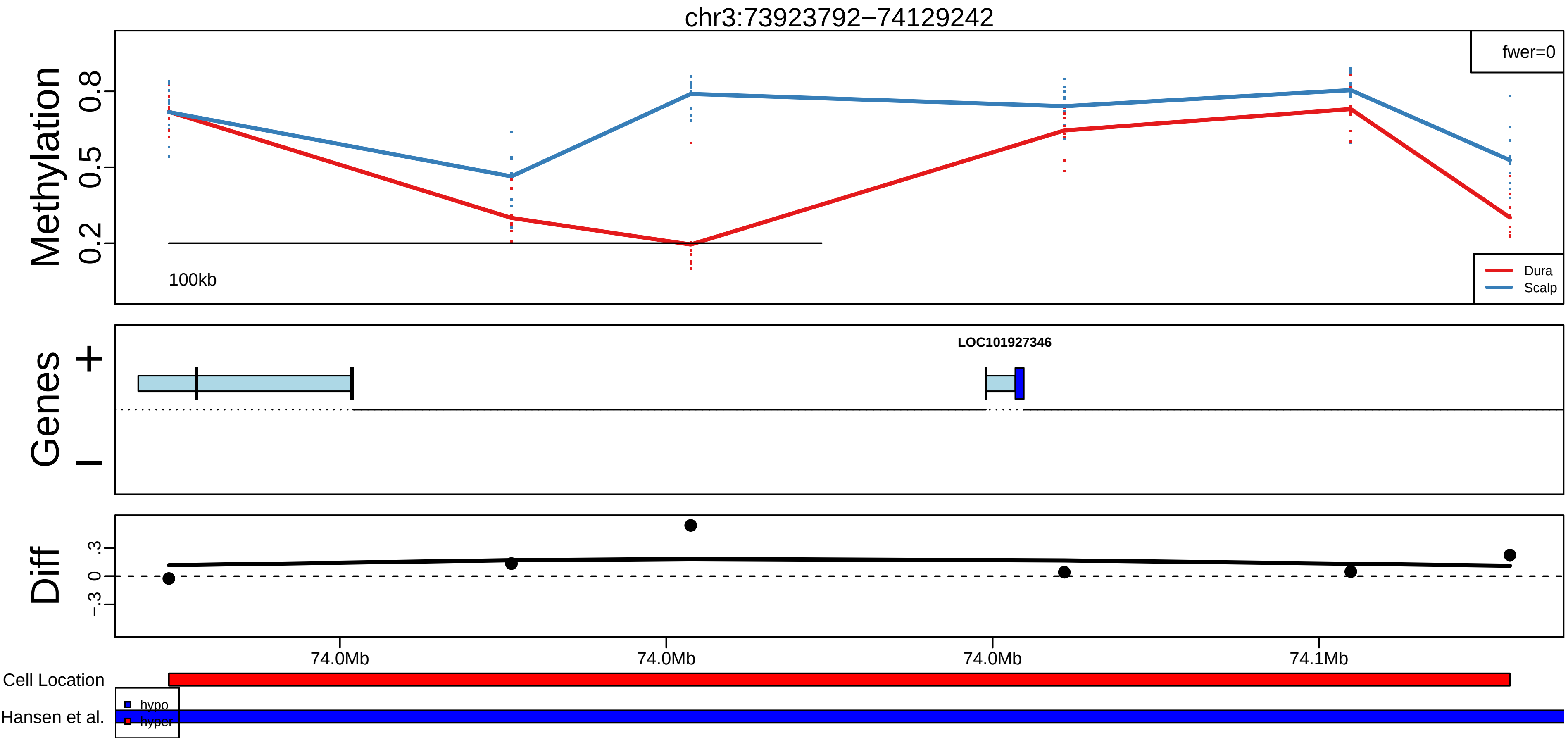

chr3:73923792−74129242
Methylation
0.2
0.5
0.8
fwer=0
100kb
Dura
Scalp
Genes
+
−
LOC101927346
Diff
−.3
0
.3
74.0Mb
74.0Mb
74.0Mb
74.1Mb
Cell Location
Hansen et al.
hypo
hyper

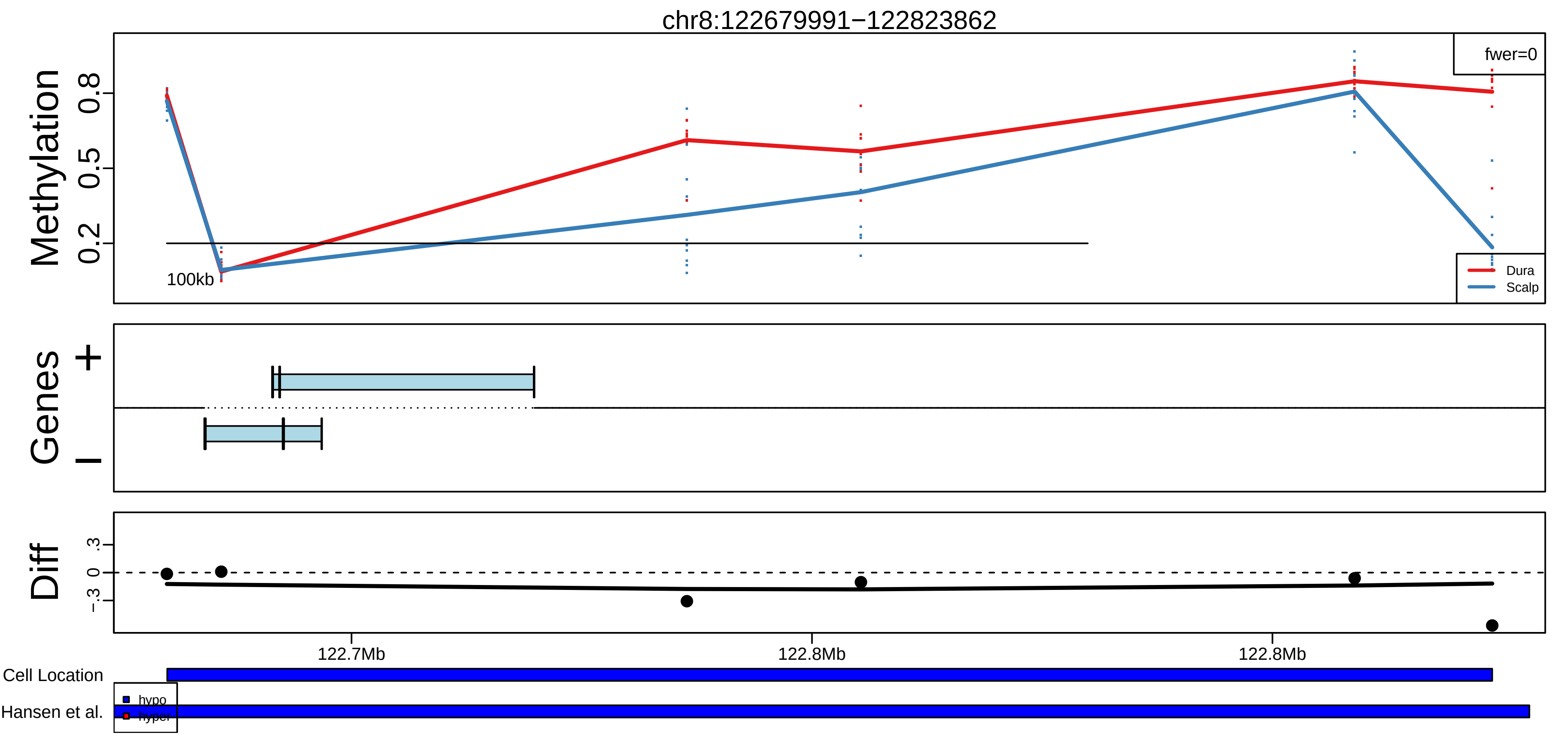

chr8:122679991−122823862
Methylation
0.2
0.5
0.8
fwer=0
100kb
Dura
Scalp
Genes
+
−
Diff
−.3
0
.3
122.7Mb
122.8Mb
122.8Mb
Cell Location
Hansen et al.
hypo
hyper

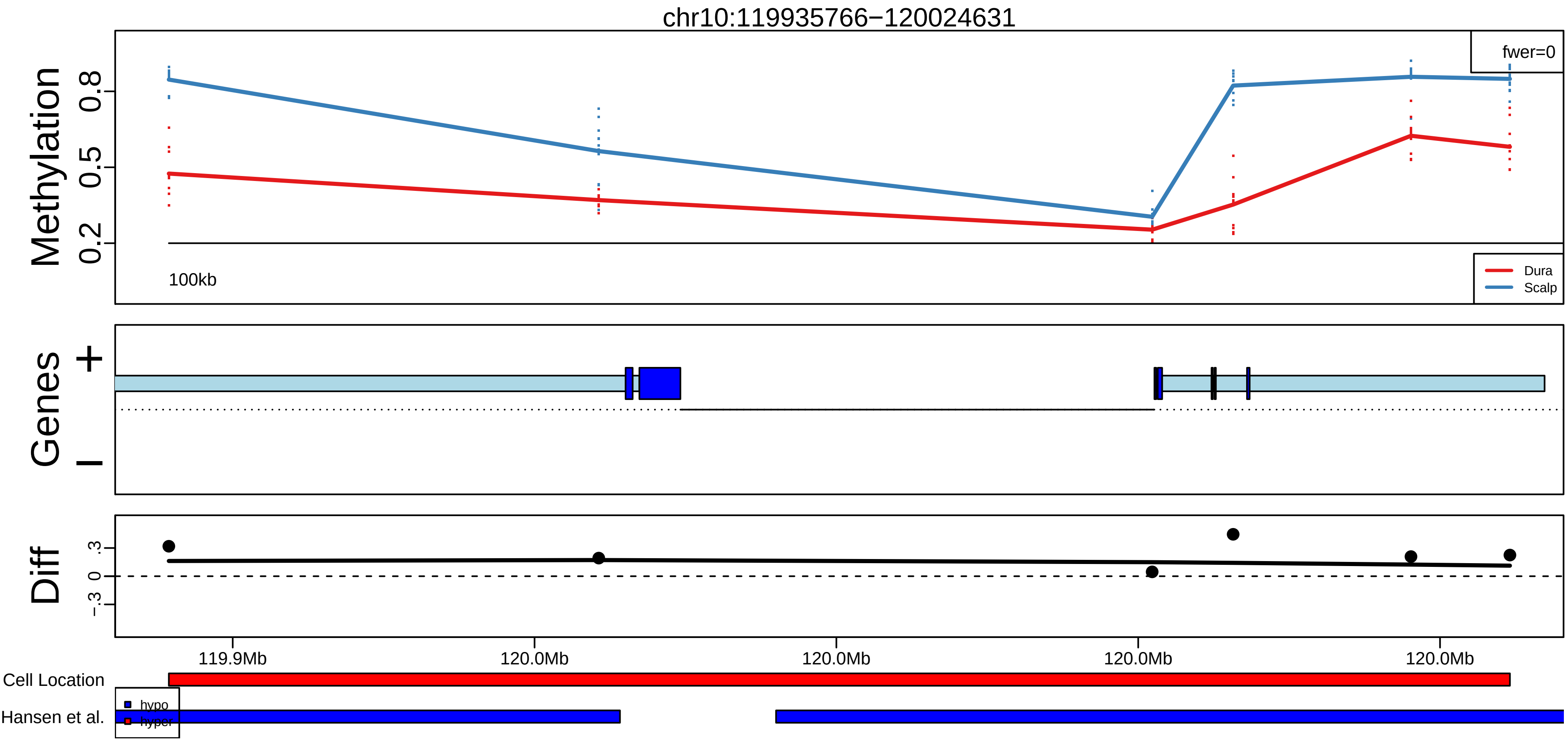
chr10:119935766−120024631
fwer=0
Methylation
0.2
0.5
0.8
100kb
Dura
Scalp
Genes
+
−
Diff
−.3
0
.3
119.9Mb
120.0Mb
120.0Mb
120.0Mb
120.0Mb
Cell Location
Hansen et al.
hypo
hyper

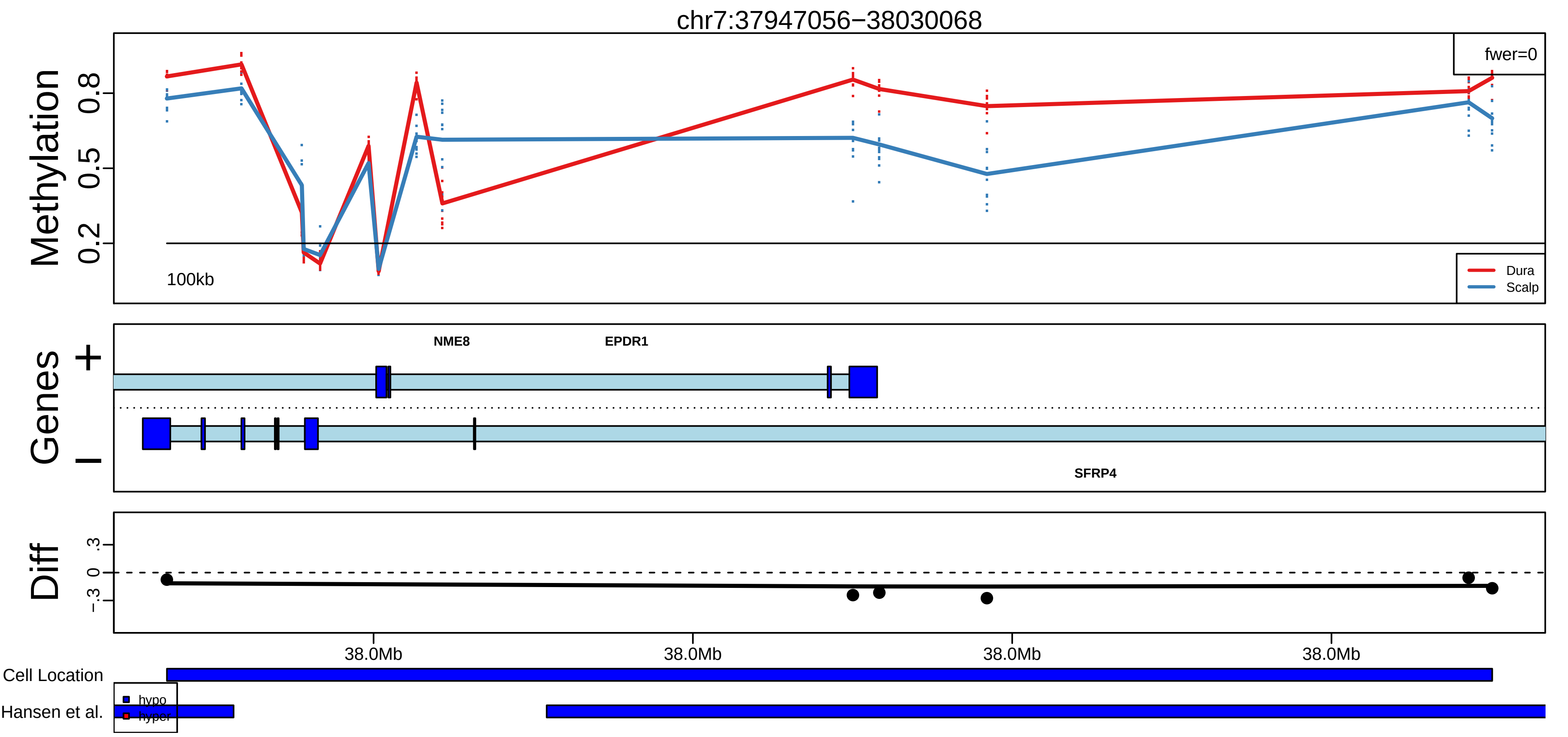

chr7:37947056−38030068
Methylation
0.2
0.5
0.8
fwer=0
100kb
Dura
Scalp
Genes
+
−
NME8
EPDR1
SFRP4
Diff
−.3
0
.3
38.0Mb
38.0Mb
38.0Mb
38.0Mb
Cell Location
Hansen et al.
hypo
hyper

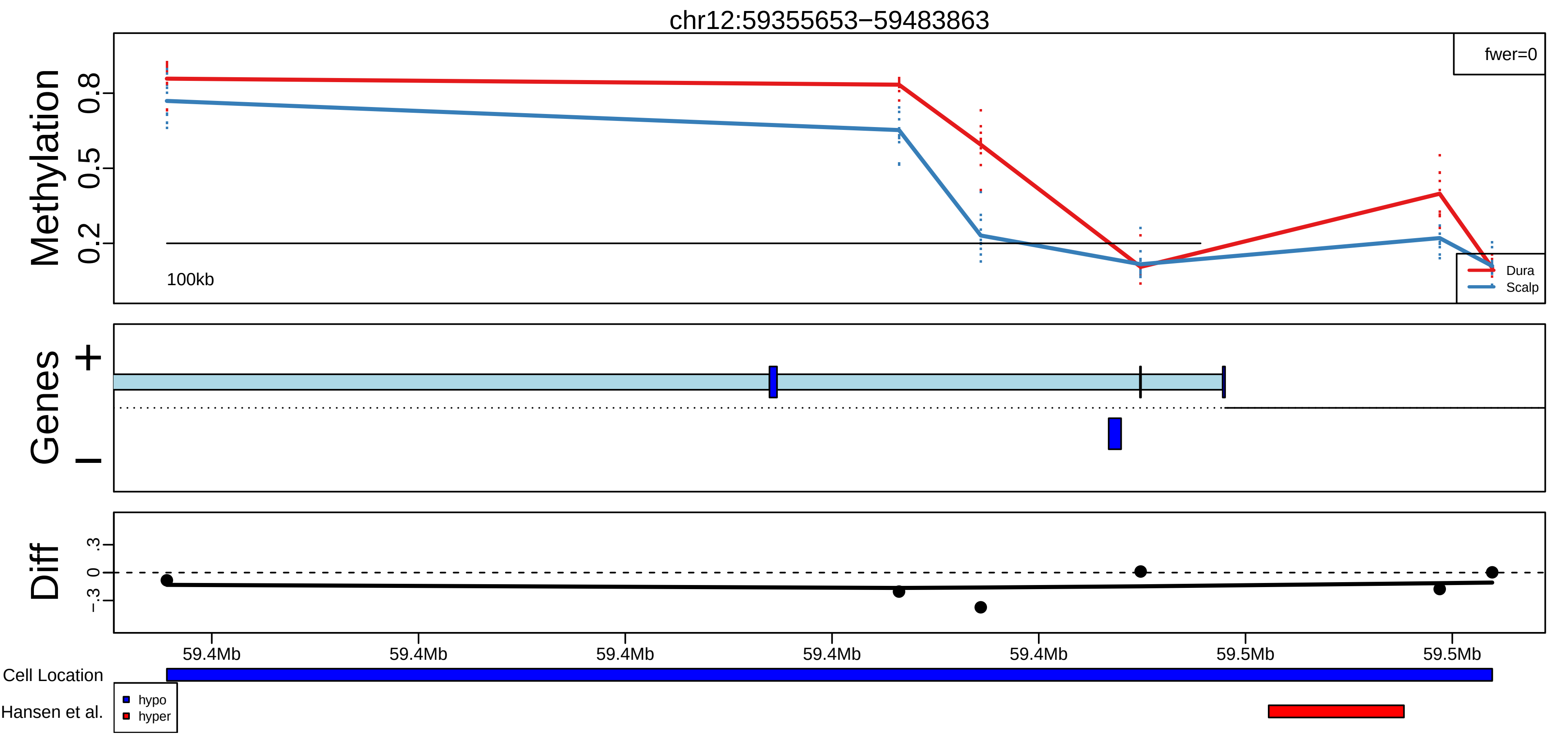

chr12:59355653−59483863
fwer=0
Methylation
0.8
0.5
0.2
100kb
Dura
Scalp
Genes
+
−
Diff
.3
0
−.3
59.4Mb
59.4Mb
59.4Mb
59.4Mb
59.4Mb
59.5Mb
59.5Mb
Cell Location
Hansen et al.
hypo
hyper

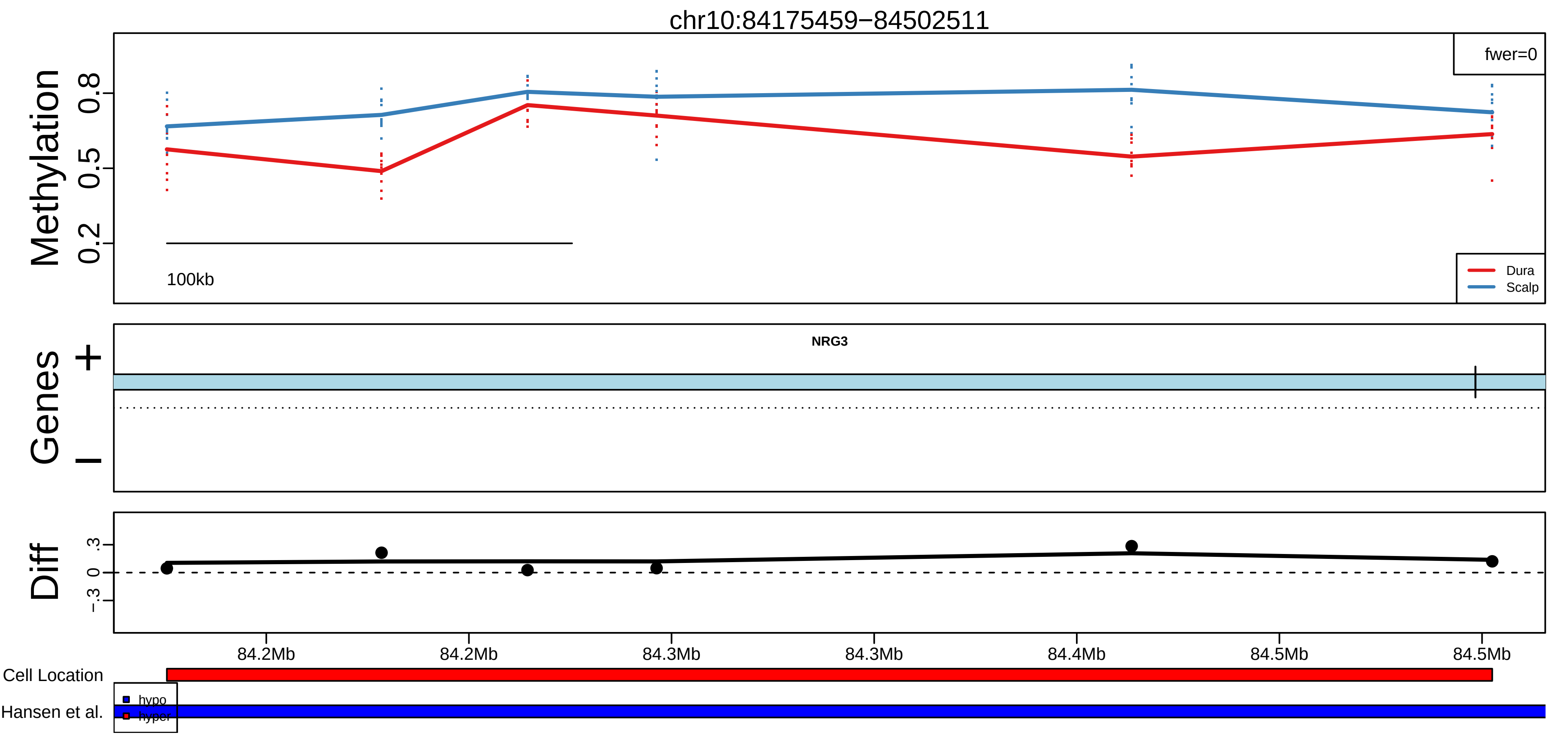
chr10:84175459−84502511
fwer=0
Methylation
0.2
0.5
0.8
100kb
Dura
Scalp
Genes
+
−
NRG3
Diff
−.3
0
.3
84.2Mb
84.2Mb
84.3Mb
84.3Mb
84.4Mb
84.5Mb
84.5Mb
Cell Location
Hansen et al.
hypo
hyper

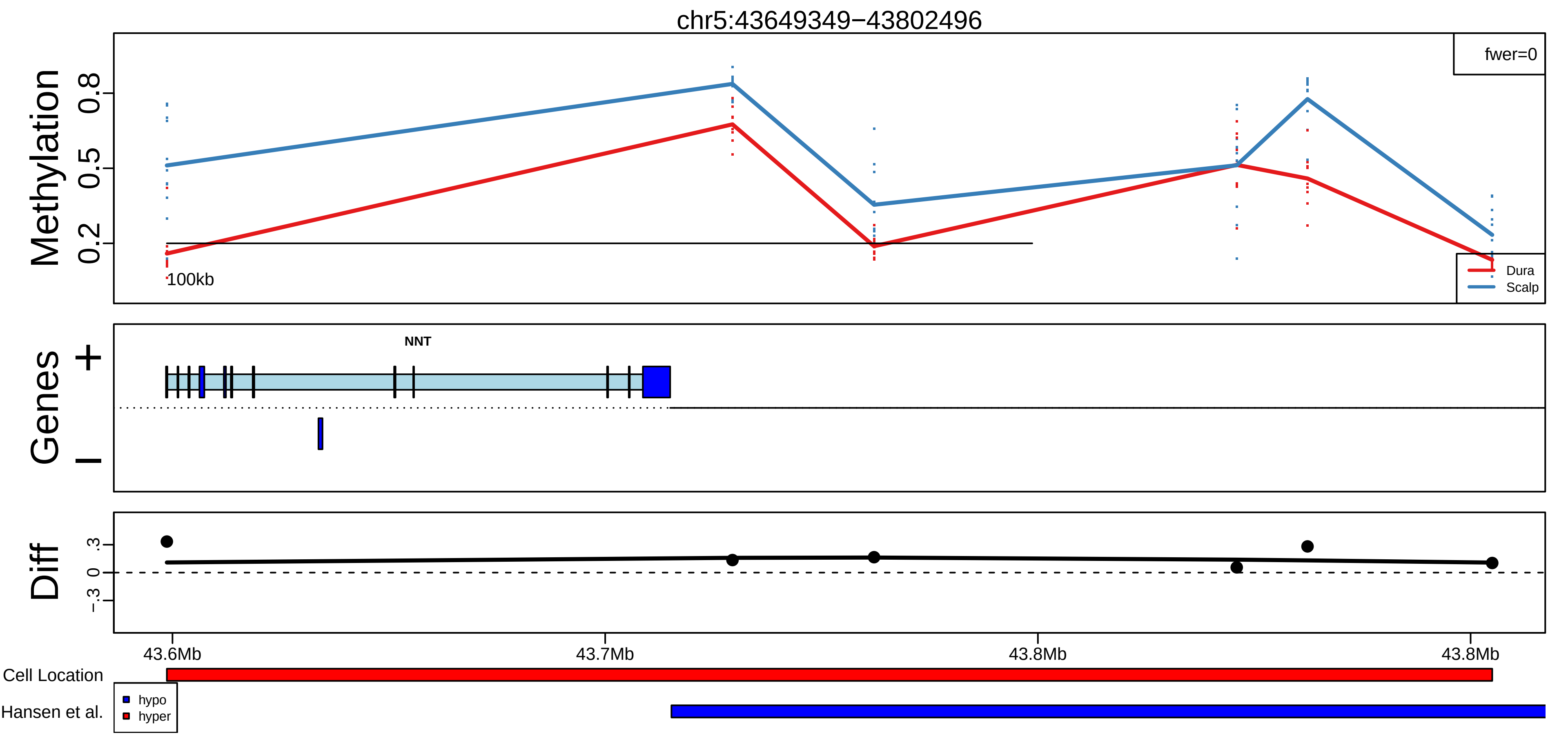
chr5:43649349−43802496
fwer=0
Methylation
0.8
0.5
0.2
100kb
Dura
Scalp
Genes
+
−
NNT
Diff
.3
0
−.3
43.6Mb
43.7Mb
43.8Mb
Cell Location
Hansen et al.
hypo
hyper

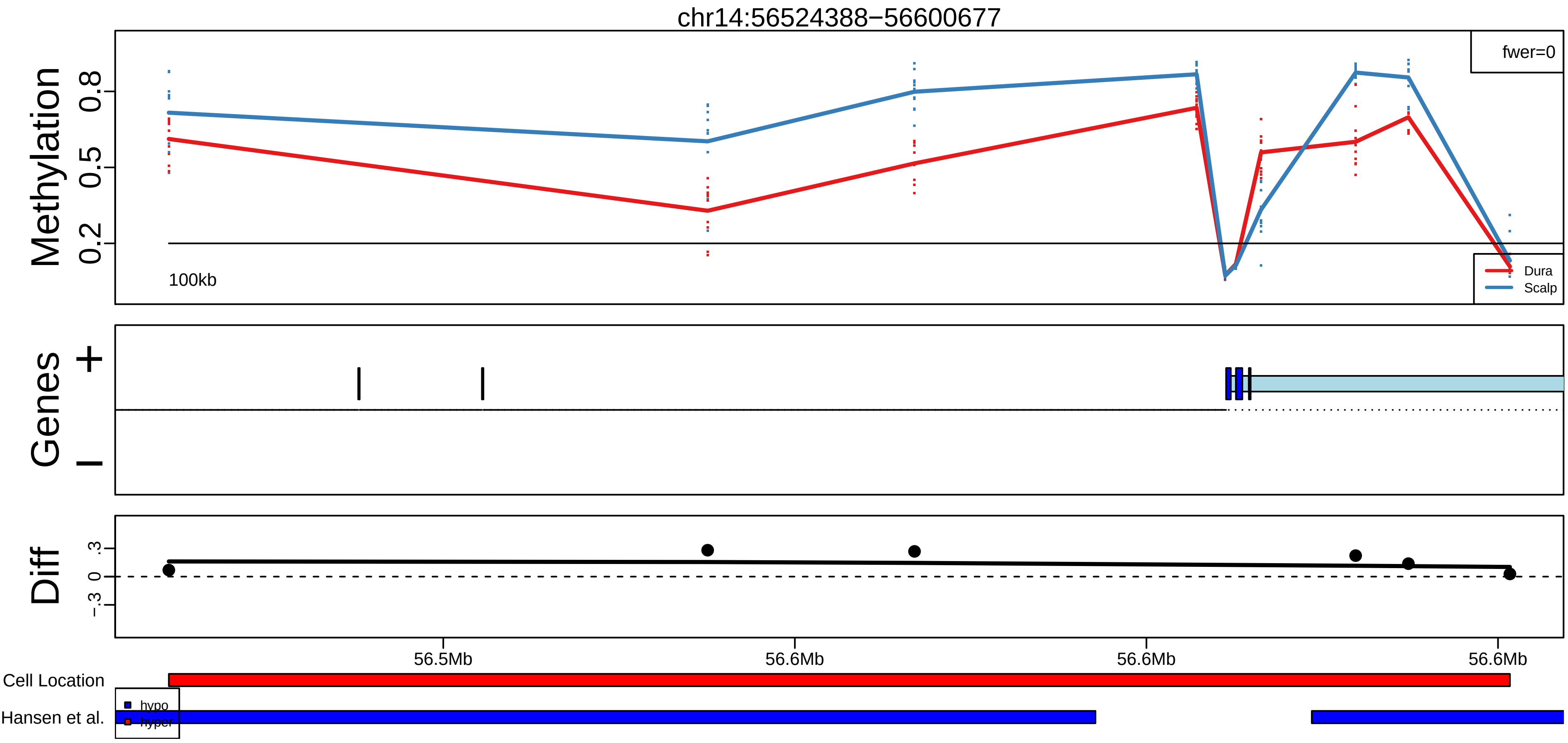
chr14:56524388−56600677
fwer=0
Methylation
0.2
0.5
0.8
100kb
Dura
Scalp
Genes
+
−
Diff
−.3
0
.3
56.5Mb
56.6Mb
56.6Mb
56.6Mb
Cell Location
Hansen et al.
hypo
hyper

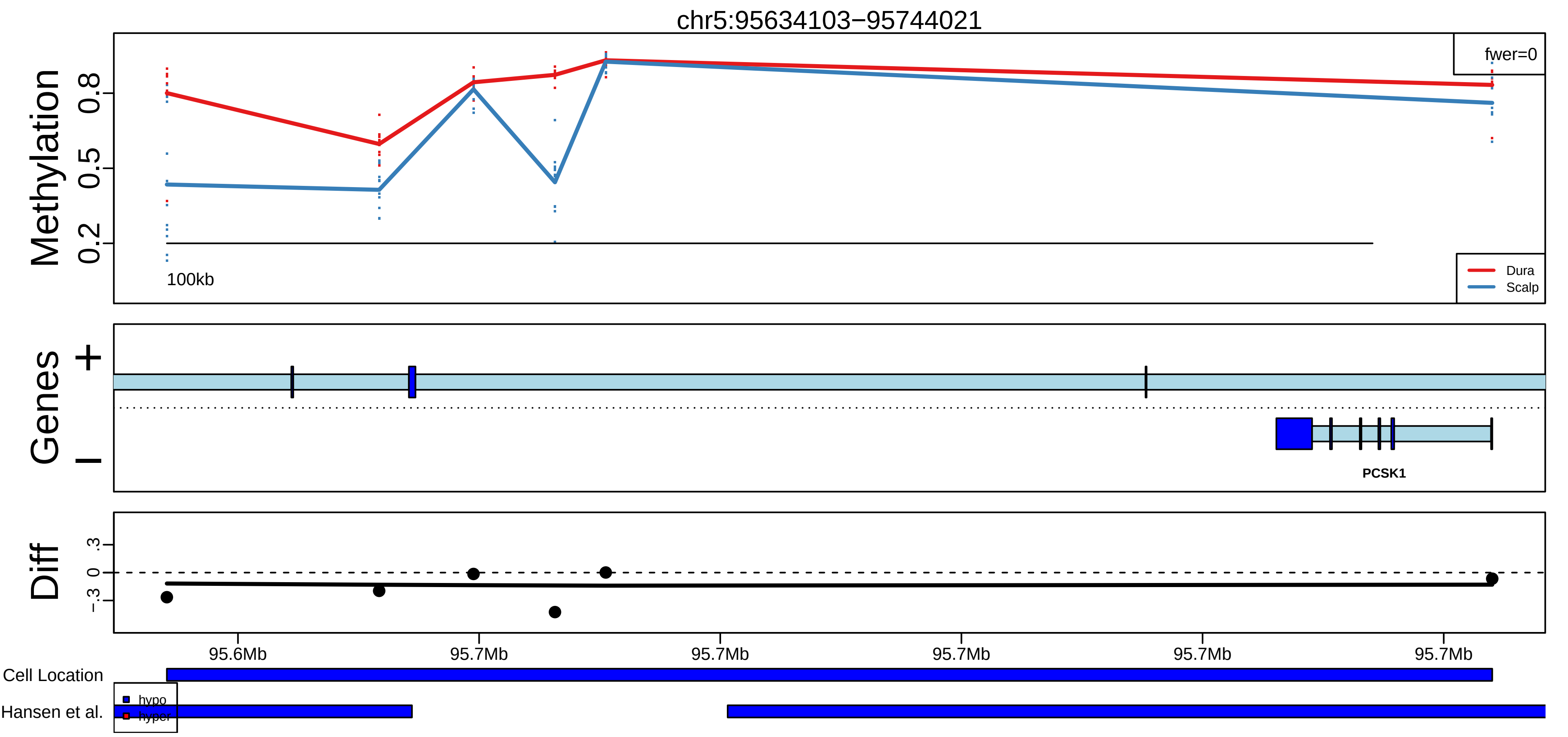
chr5:95634103−95744021
fwer=0
Methylation
0.2
0.5
0.8
100kb
Dura
Scalp
Genes
+
−
PCSK1
Diff
.3
0
−.3
95.6Mb
95.7Mb
95.7Mb
95.7Mb
95.7Mb
95.7Mb
Cell Location
hypo
hyper
Hansen et al.

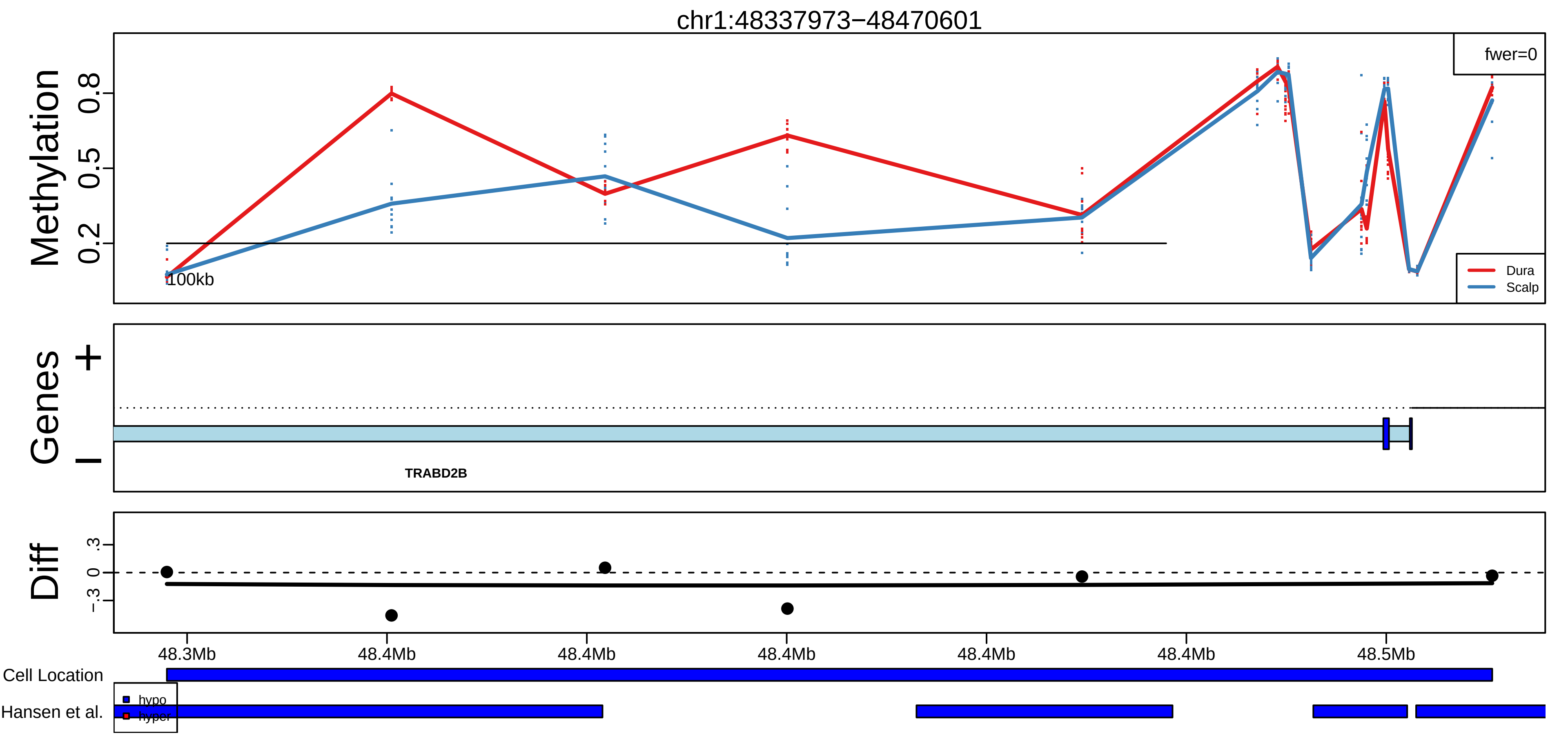

chr1:48337973−48470601
Methylation
0.2
0.5
0.8
fwer=0
100kb
Dura
Scalp
Genes
+
−
TRABD2B
Diff
−.3
0
.3
48.3Mb
48.4Mb
48.4Mb
48.4Mb
48.4Mb
48.4Mb
48.5Mb
Cell Location
Hansen et al.
hypo
hyper

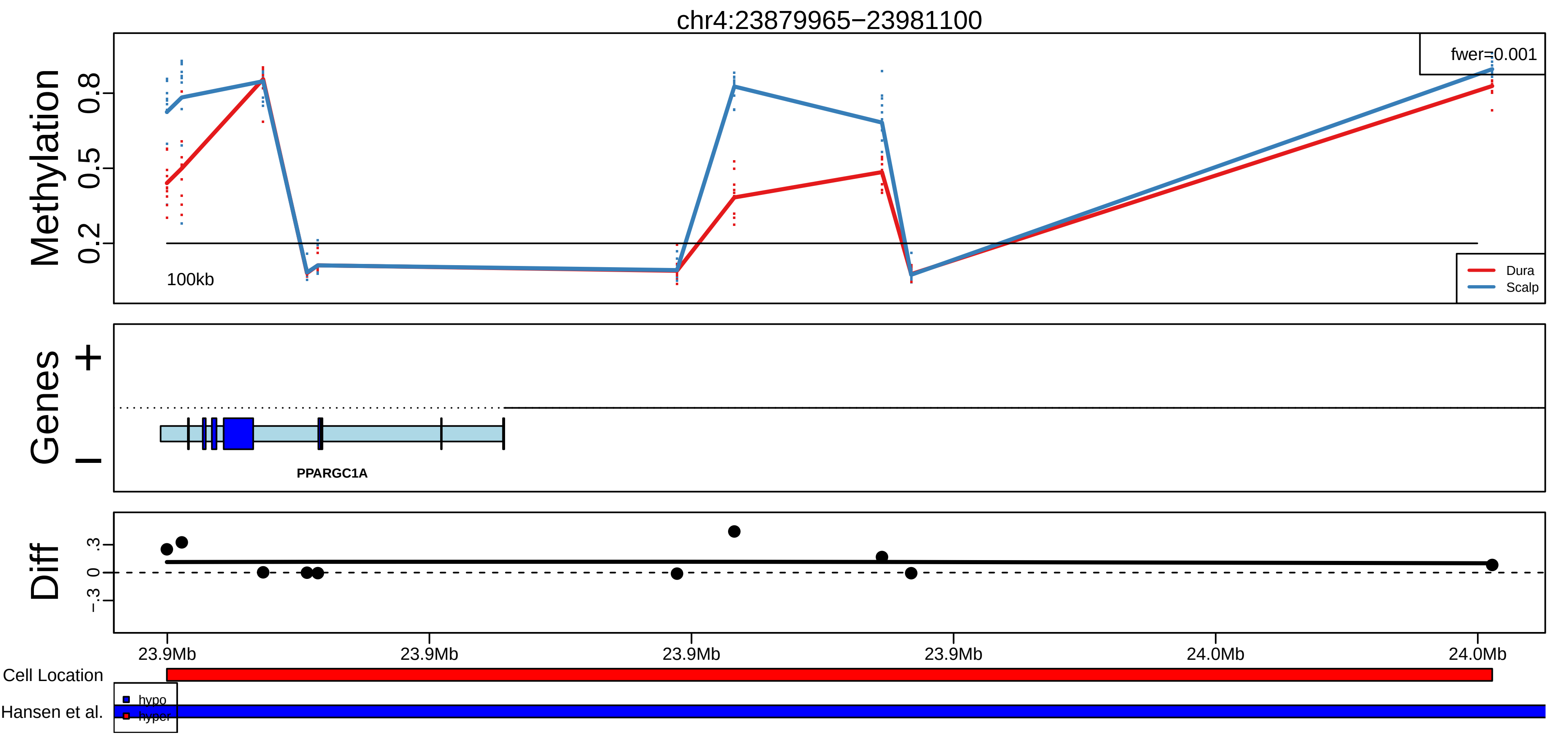

chr4:23879965−23981100
fwer=0.001
Methylation
0.2
0.5
0.8
100kb
Dura
Scalp
Genes
+
−
PPARGC1A
Diff
−.3
0
.3
23.9Mb
23.9Mb
23.9Mb
23.9Mb
24.0Mb
24.0Mb
Cell Location
Hansen et al.
hypo
hyper

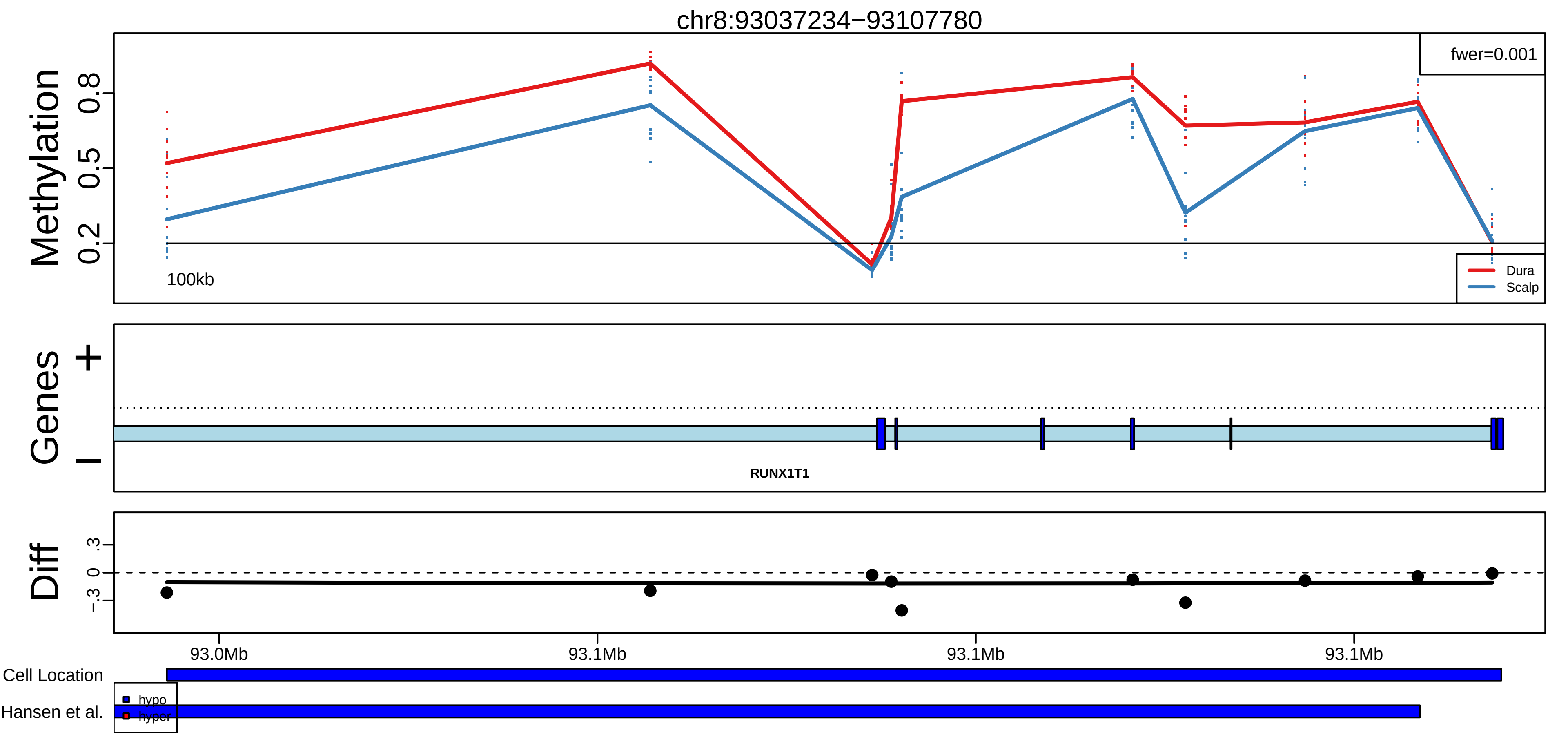
chr8:93037234−93107780
fwer=0.001
Methylation
0.2
0.5
0.8
100kb
Dura
Scalp
Genes
+
−
RUNX1T1
Diff
−.3
0
.3
93.0Mb
93.1Mb
93.1Mb
93.1Mb
Cell Location
Hansen et al.
hypo
hyper

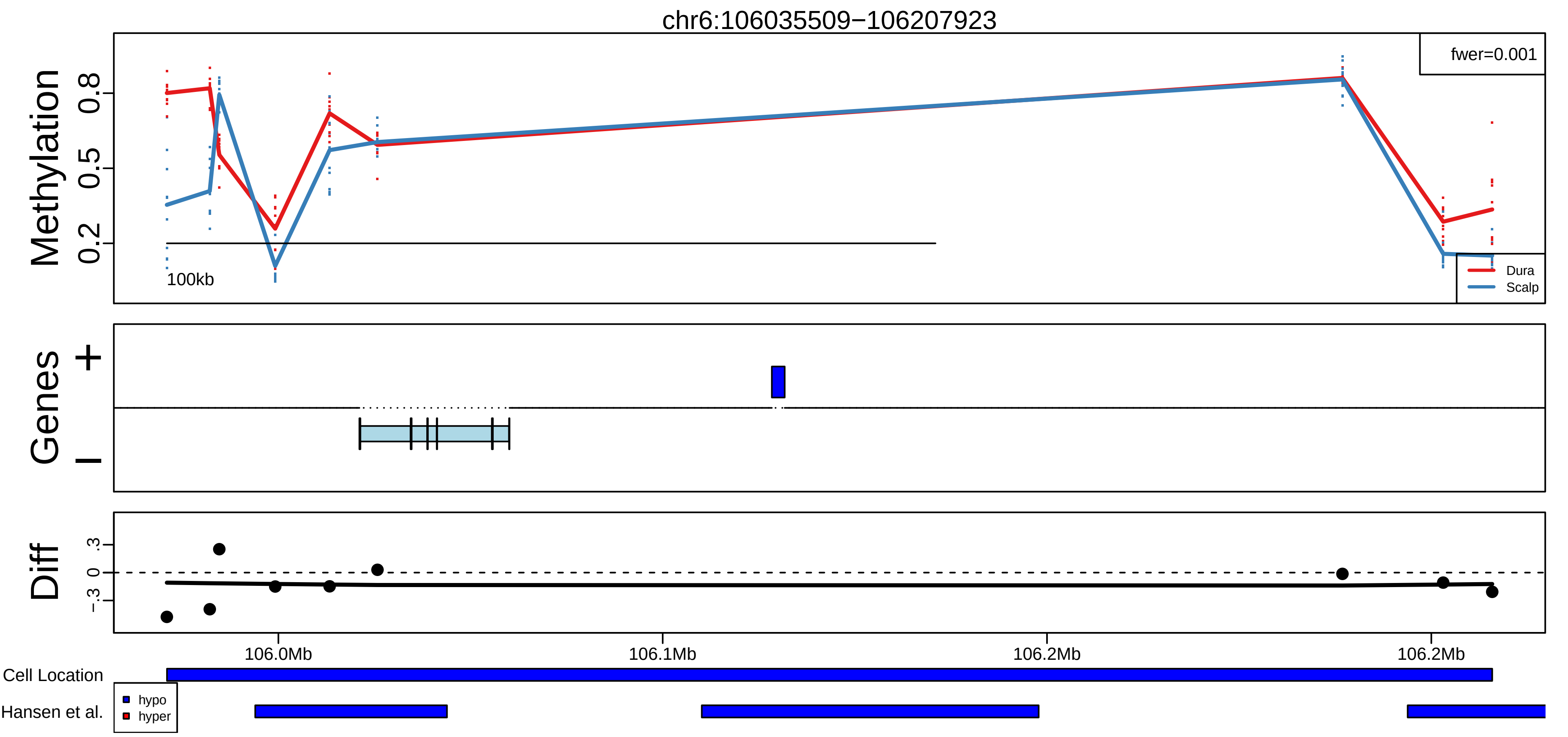

chr6:106035509−106207923
fwer=0.001
Methylation
0.2
0.5
0.8
100kb
Dura
Scalp
Genes
+
−
Diff
−.3
0
.3
106.0Mb
106.1Mb
106.2Mb
106.2Mb
Cell Location
Hansen et al.
hypo
hyper

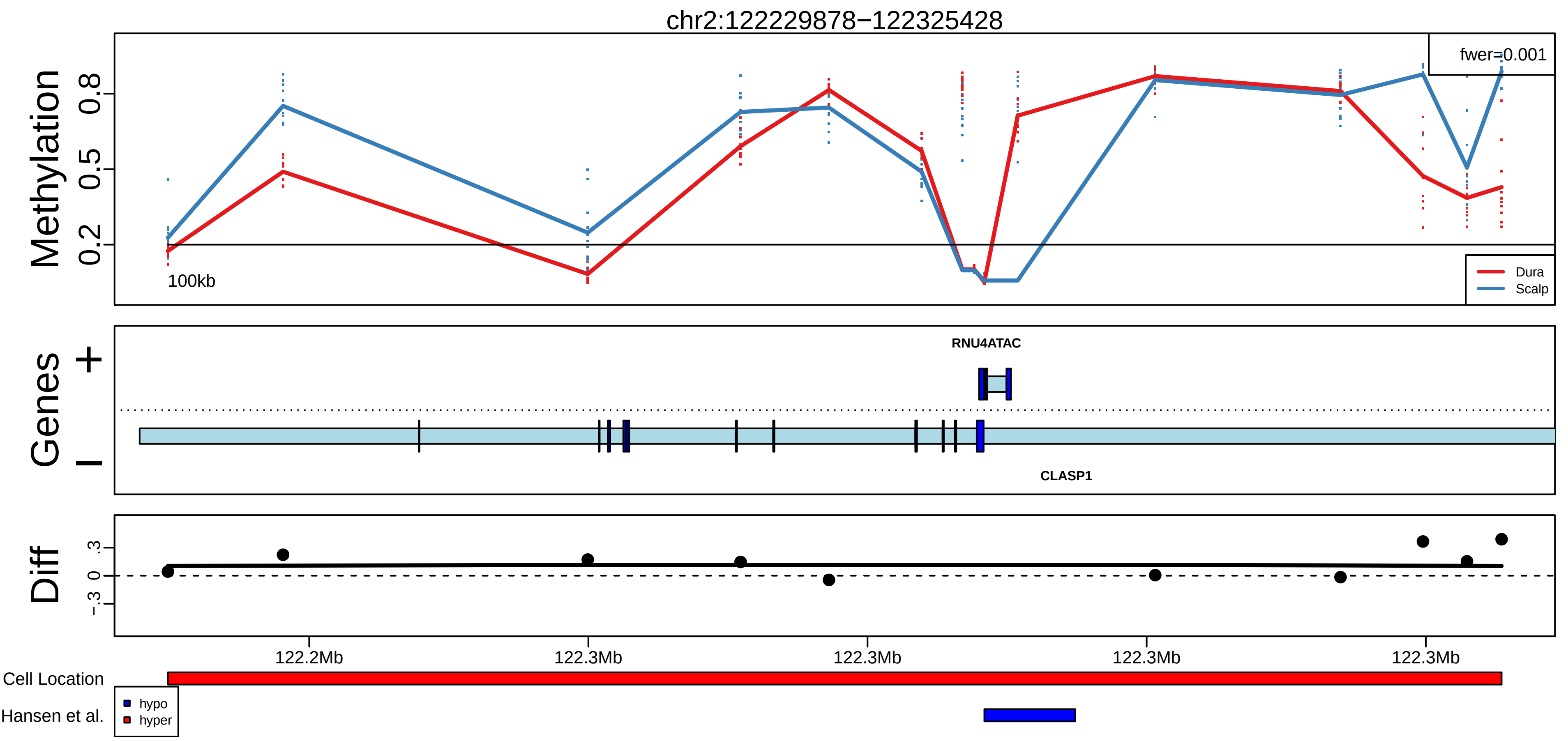
chr2:122229878−122325428
fwer=0.001
Methylation
0.2
0.5
0.8
100kb
Dura
Scalp
Genes
+
−
RNU4ATAC
CLASP1
Diff
−.3
0
.3
122.2Mb
122.3Mb
122.3Mb
122.3Mb
122.3Mb
Cell Location
Hansen et al.
hypo
hyper

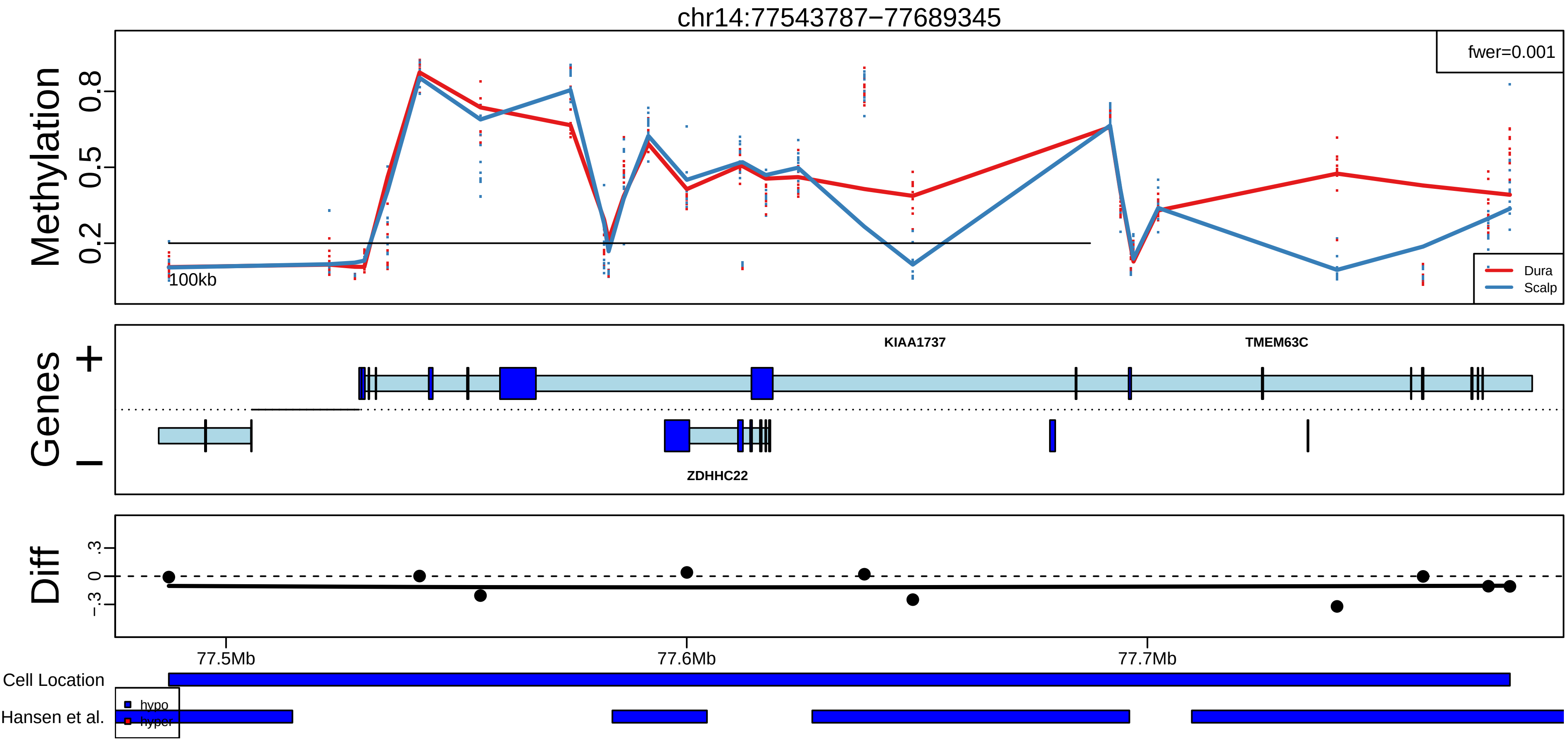

chr14:77543787−77689345
fwer=0.001
Methylation
0.2
0.5
0.8
100kb
Dura
Scalp
Genes
+
−
KIAA1737
TMEM63C
ZDHHC22
Diff
.3
0
−.3
77.5Mb
77.6Mb
77.7Mb
Cell Location
Hansen et al.
hypo
hyper

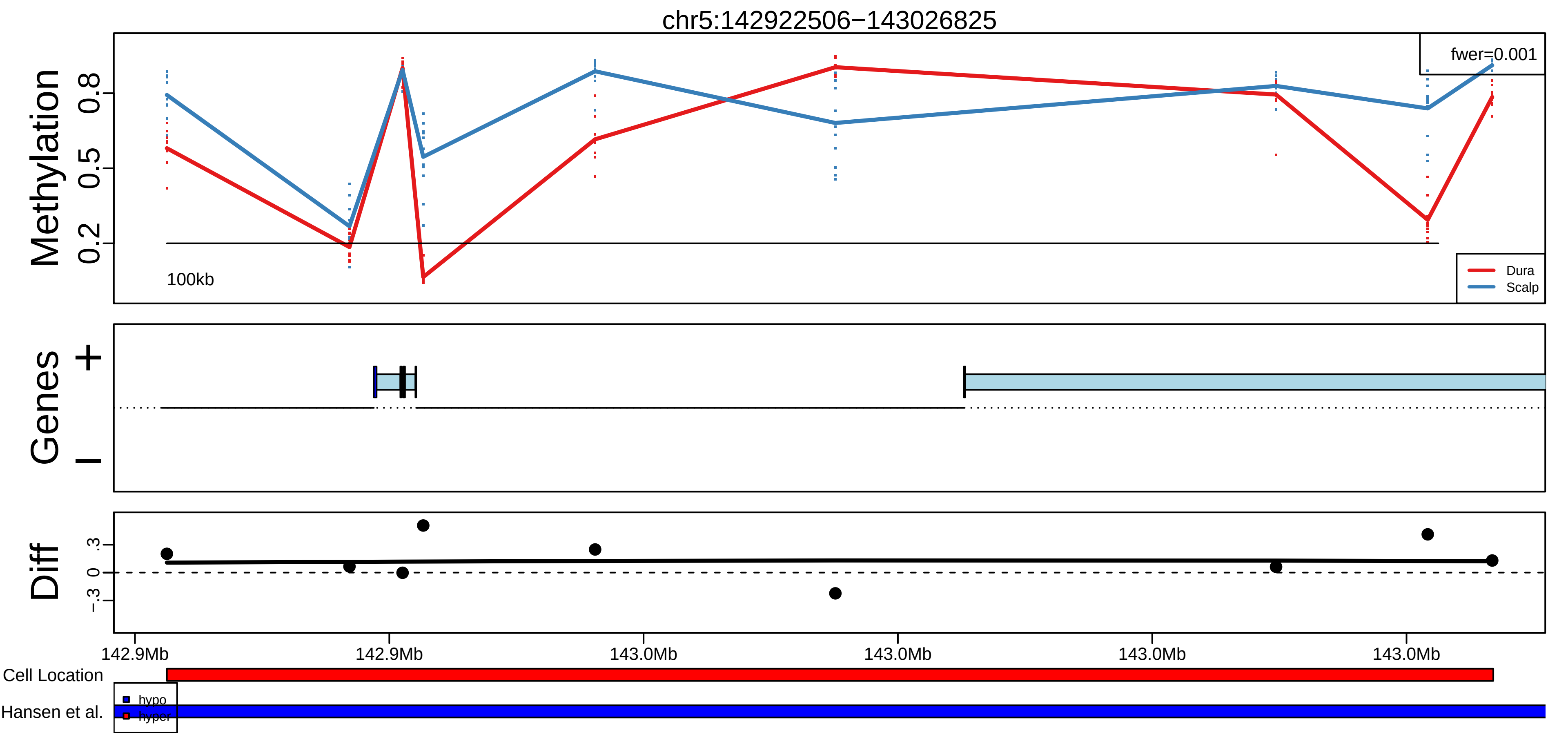
chr5:142922506−143026825
fwer=0.001
Methylation
0.2
0.5
0.8
100kb
Dura
Scalp
Genes
+
−
Diff
−.3
0
.3
142.9Mb
142.9Mb
143.0Mb
143.0Mb
143.0Mb
143.0Mb
Cell Location
Hansen et al.
hypo
hyper

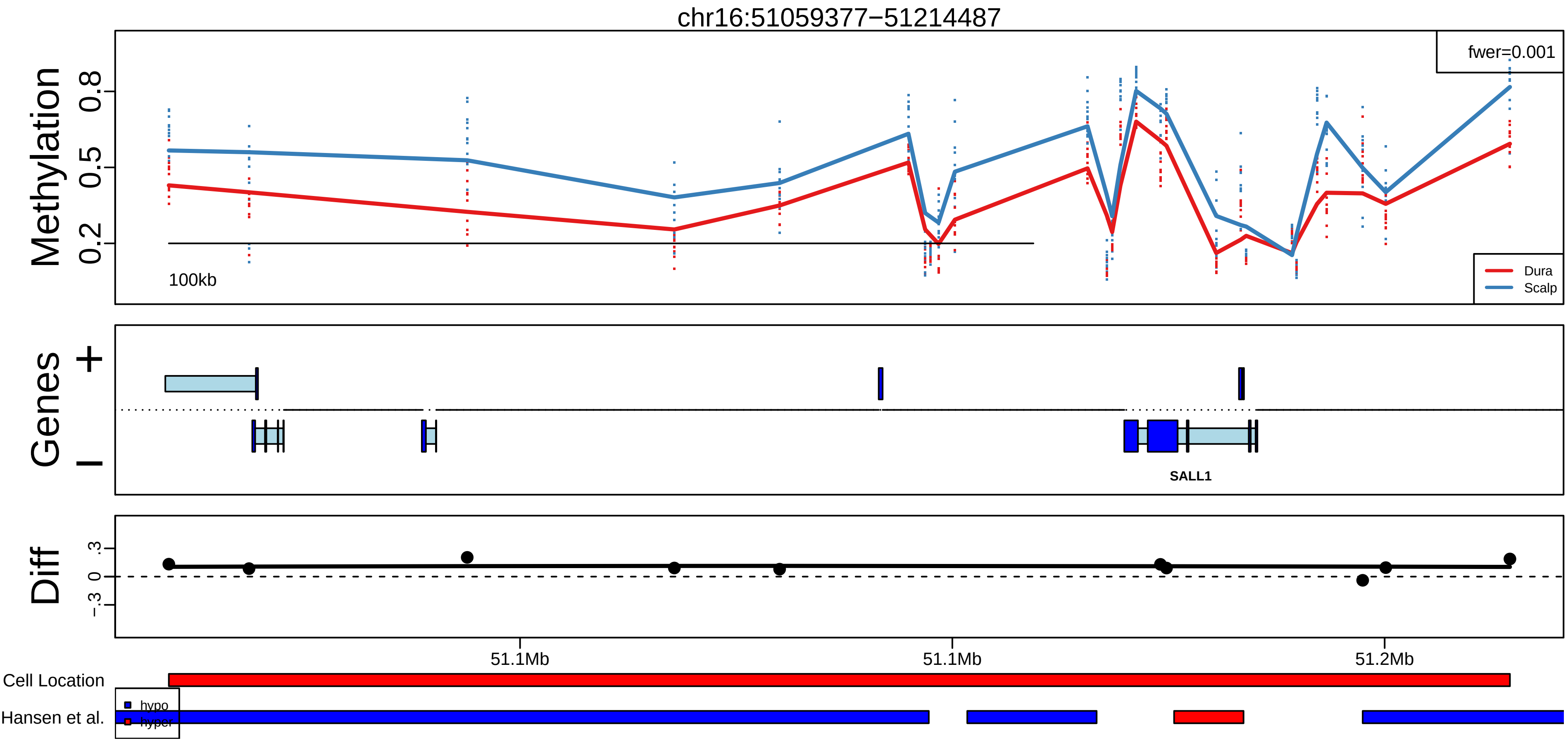

chr16:51059377−51214487
fwer=0.001
Methylation
0.2
0.5
0.8
100kb
Dura
Scalp
Genes
+
−
SALL1
Diff
.3
0
−.3
51.1Mb
51.1Mb
51.2Mb
Cell Location
Hansen et al.
hypo
hyper

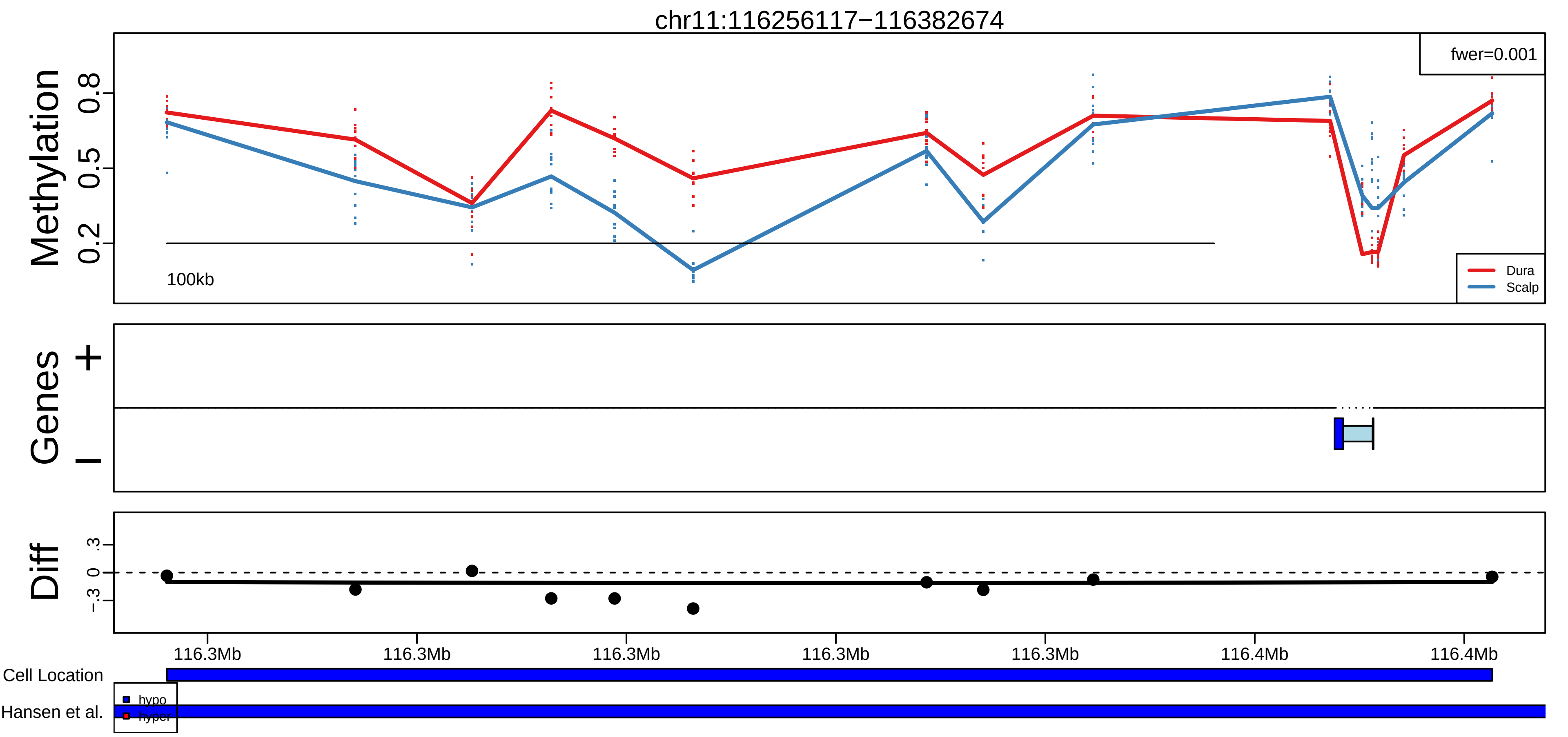
chr11:116256117−116382674
fwer=0.001
Methylation
0.2
0.5
0.8
100kb
Dura
Scalp
Genes
+
−
Diff
.3
0
−.3
116.3Mb
116.3Mb
116.3Mb
116.3Mb
116.3Mb
116.4Mb
116.4Mb
Cell Location
Hansen et al.
hypo
hyper

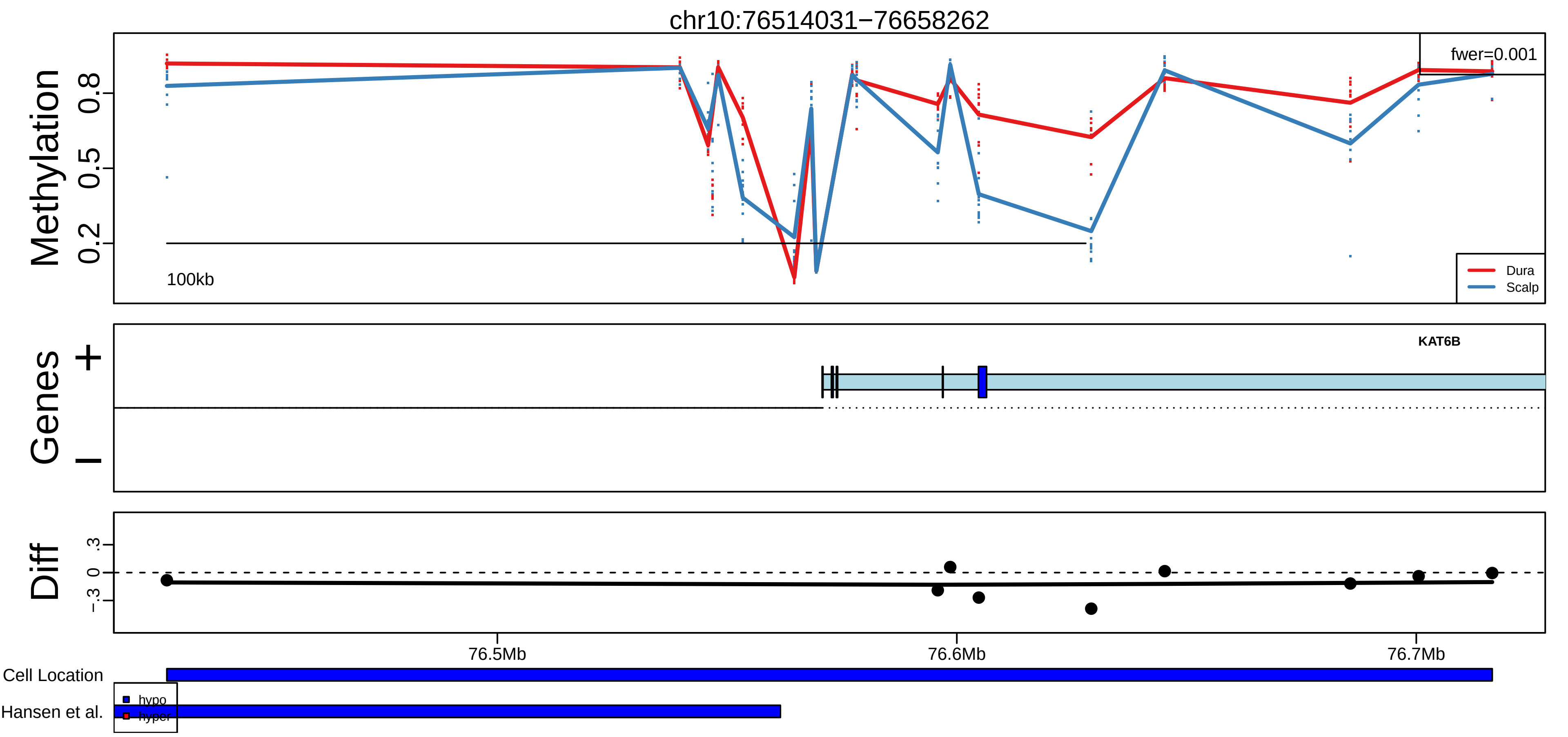

chr10:76514031−76658262
Methylation
0.2
0.5
0.8
fwer=0.001
100kb
Dura
Scalp
Genes
+
−
KAT6B
Diff
−.3
0
.3
76.5Mb
76.6Mb
76.7Mb
Cell Location
Hansen et al.
hypo
hyper

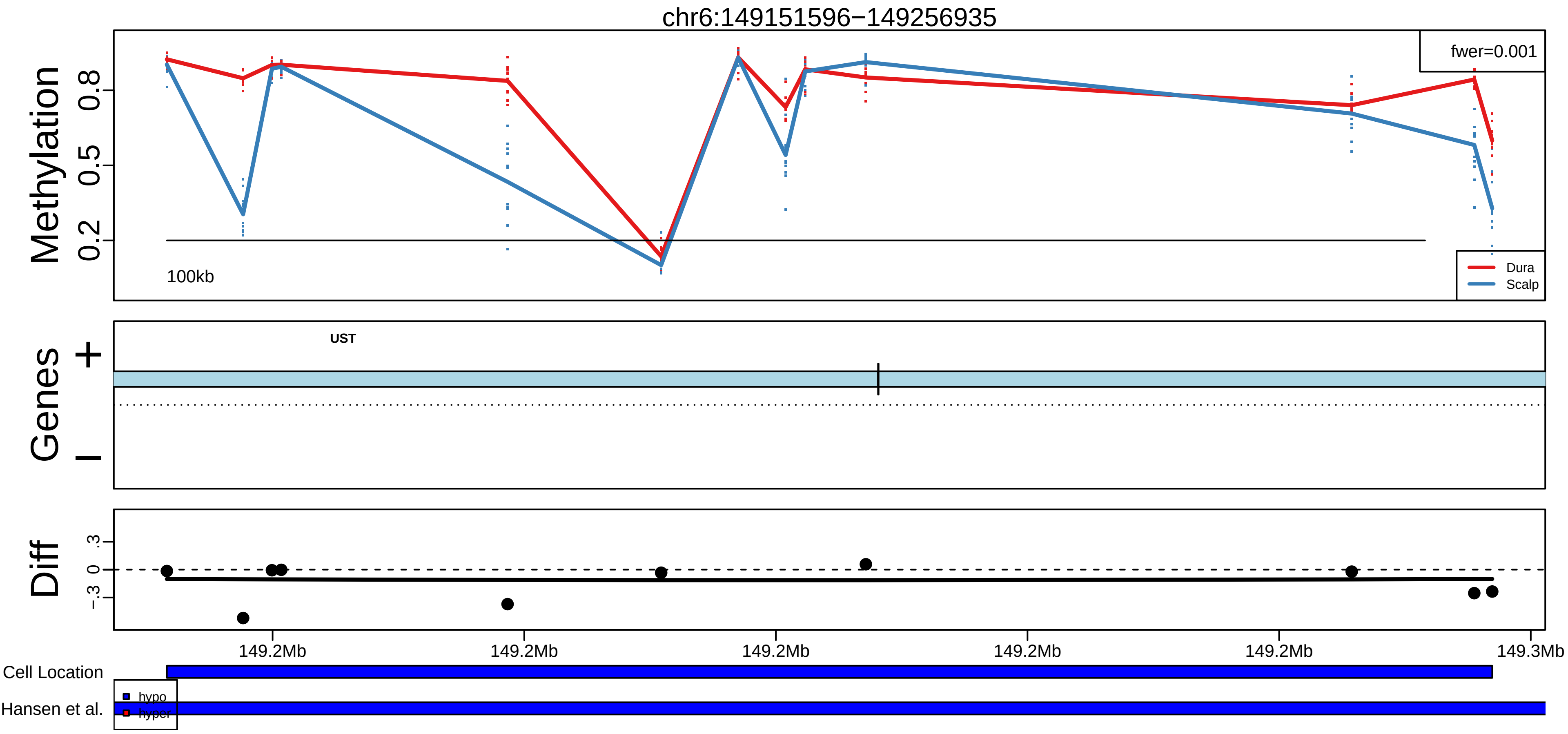

chr6:149151596−149256935
fwer=0.001
Methylation
0.2
0.5
0.8
100kb
Dura
Scalp
Genes
+
−
UST
Diff
−.3
0
.3
149.2Mb
149.2Mb
149.2Mb
149.2Mb
149.2Mb
149.3Mb
Cell Location
Hansen et al.
hypo
hyper

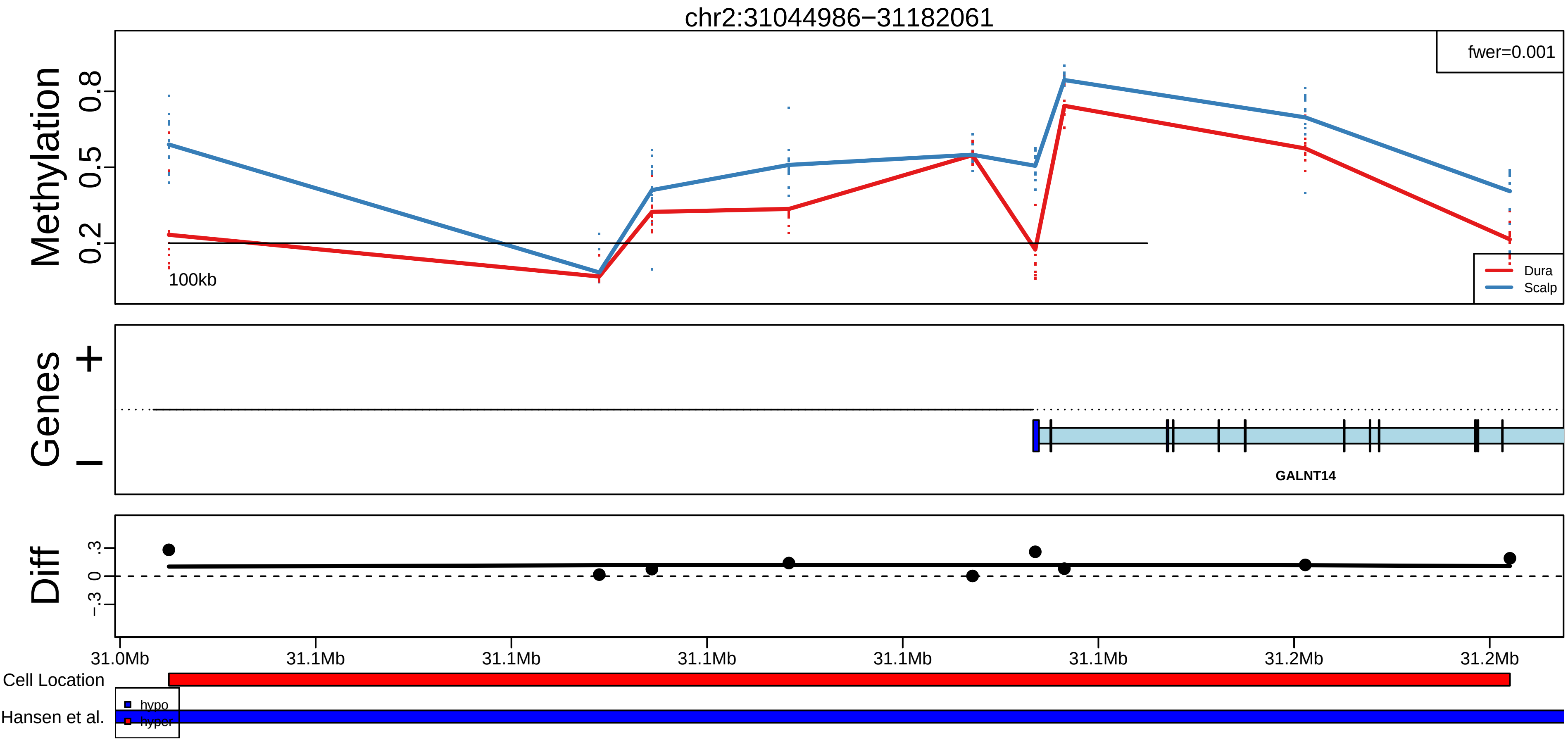

chr2:31044986−31182061
fwer=0.001
Methylation
0.2
0.5
0.8
100kb
Dura
Scalp
Genes
+
−
GALNT14
Diff
−.3
0
.3
31.0Mb
31.1Mb
31.1Mb
31.1Mb
31.1Mb
31.1Mb
31.2Mb
31.2Mb
Cell Location
Hansen et al.
hypo
hyper

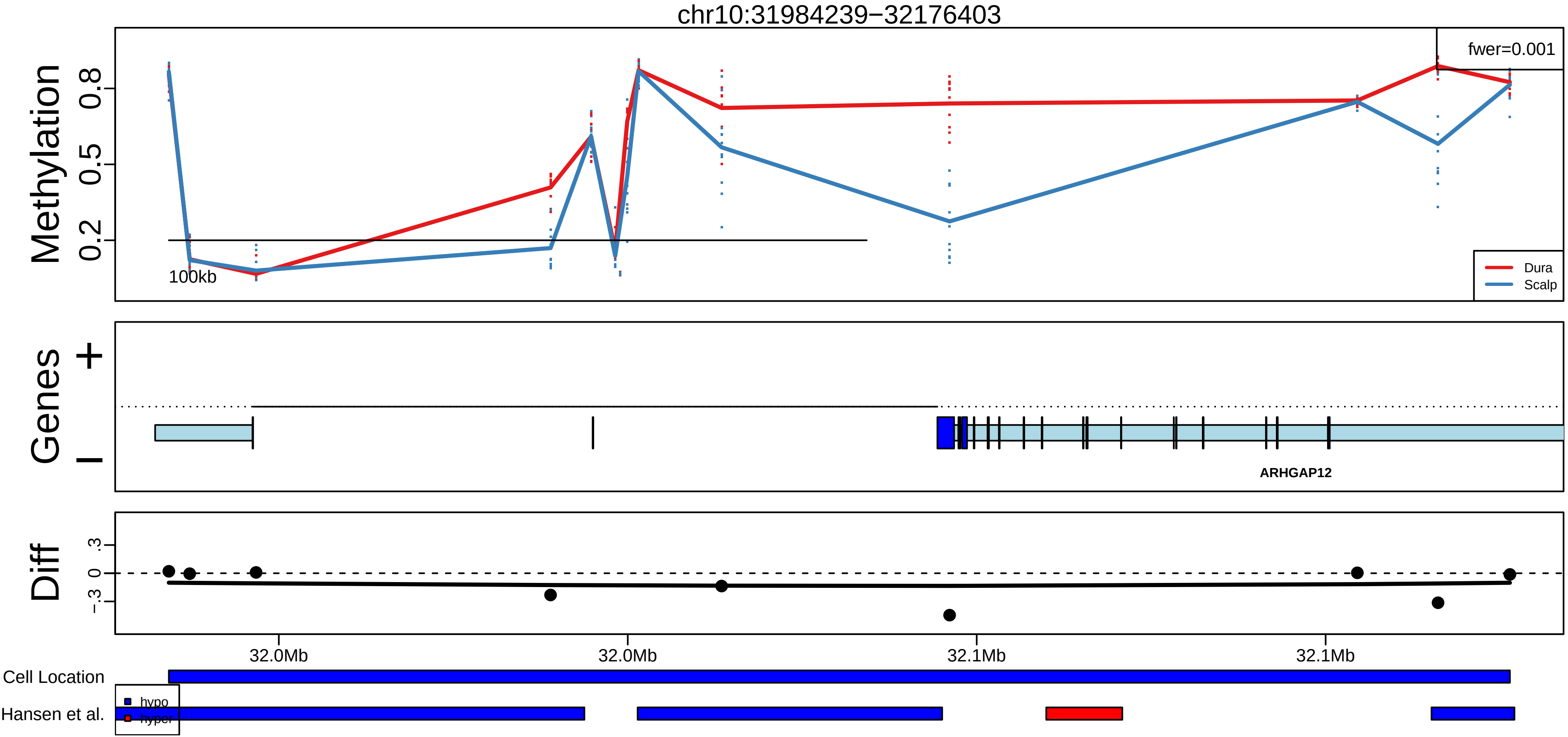
chr10:31984239−32176403
fwer=0.001
Methylation
0.2
0.5
0.8
100kb
Dura
Scalp
Genes
+
−
ARHGAP12
Diff
.3
0
−.3
32.0Mb
32.0Mb
32.1Mb
32.1Mb
Cell Location
Hansen et al.
hypo
hyper

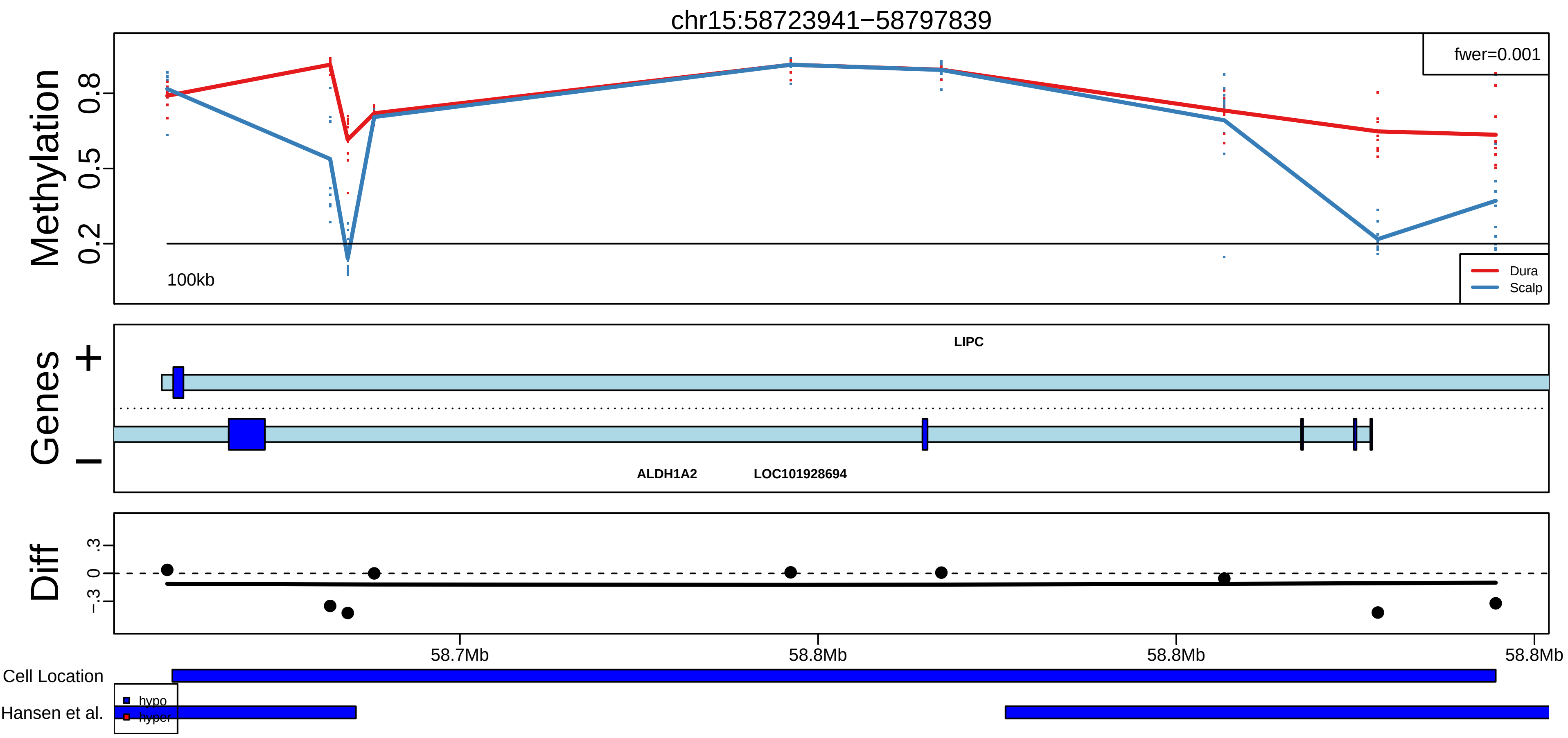

chr15:58723941−58797839
fwer=0.001
Methylation
0.2
0.5
0.8
100kb
Dura
Scalp
Genes
+
−
LIPC
ALDH1A2
LOC101928694
Diff
−.3
0
.3
58.7Mb
58.8Mb
58.8Mb
58.8Mb
Cell Location
Hansen et al.
hypo
hyper

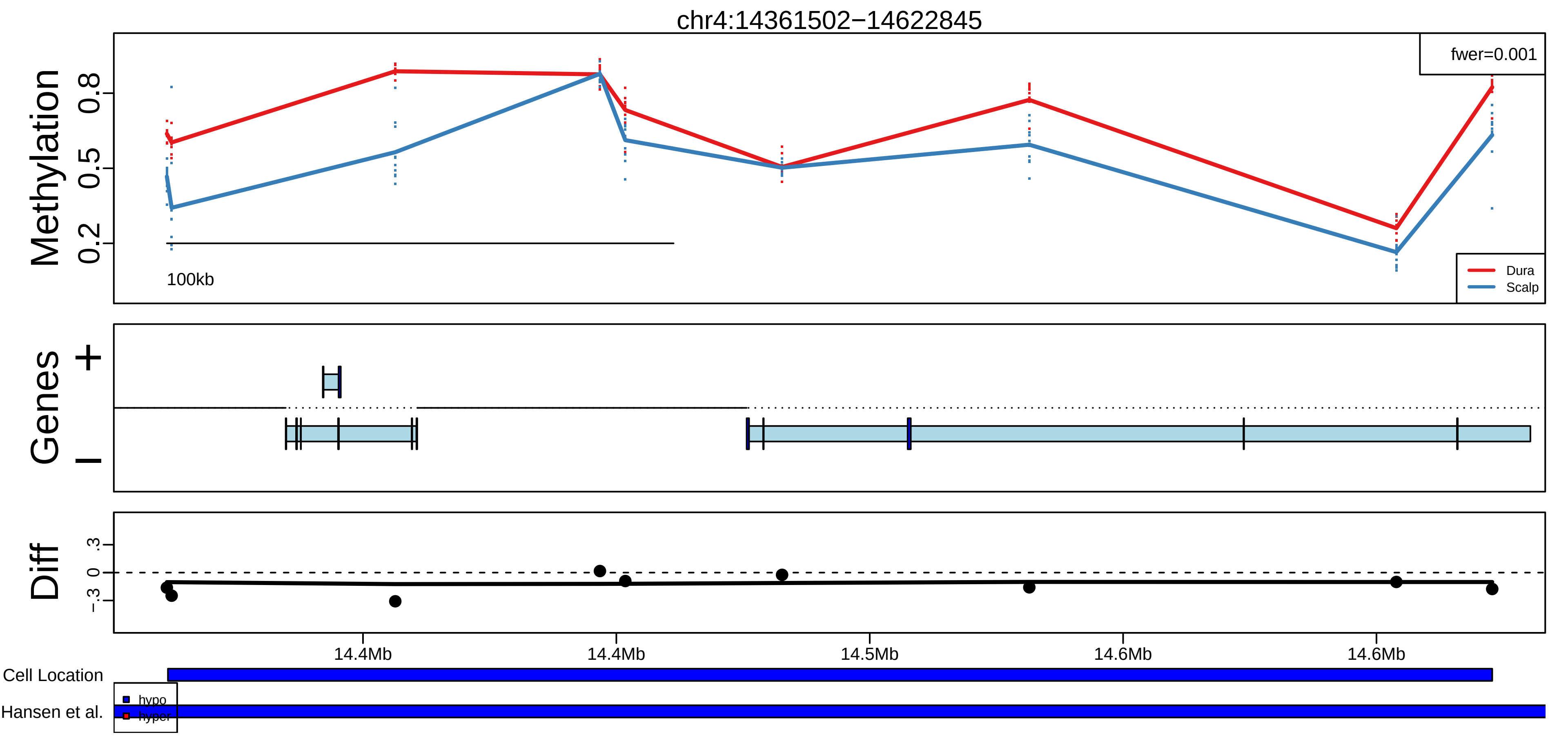
chr4:14361502−14622845
fwer=0.001
Methylation
0.8
0.5
0.2
100kb
Dura
Scalp
Genes
+
−
Diff
.3
0
−.3
14.4Mb
14.4Mb
14.5Mb
14.6Mb
14.6Mb
Cell Location
Hansen et al.
hypo
hyper

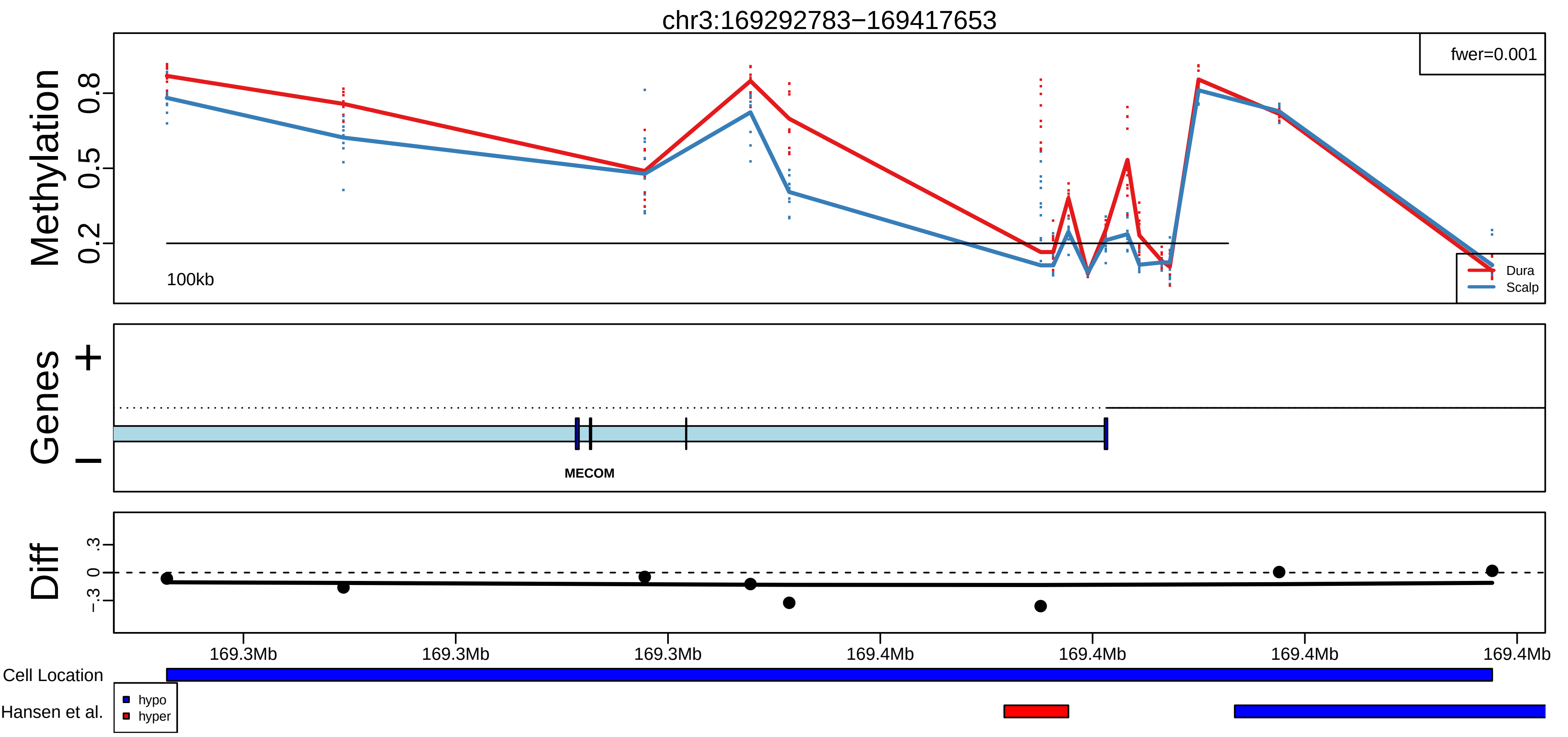
chr3:169292783−169417653
fwer=0.001
Methylation
0.2
0.5
0.8
100kb
Dura
Scalp
Genes
+
−
MECOM
Diff
−.3
0
.3
169.3Mb
169.3Mb
169.3Mb
169.4Mb
169.4Mb
169.4Mb
169.4Mb
Cell Location
Hansen et al.
hypo
hyper

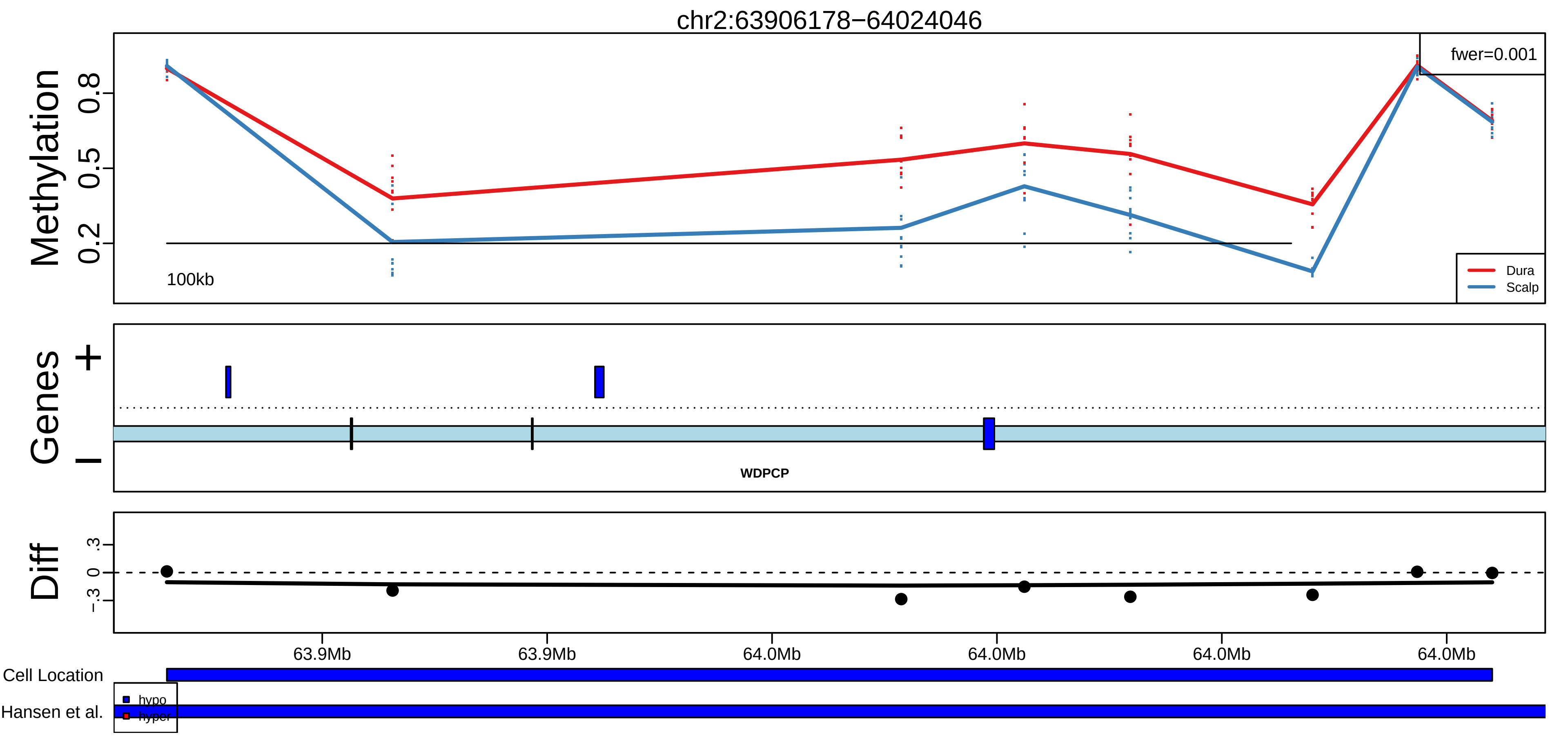

chr2:63906178−64024046
Methylation
0.2
0.5
0.8
fwer=0.001
100kb
Dura
Scalp
Genes
+
−
WDPCP
Diff
−.3
0
.3
63.9Mb
63.9Mb
64.0Mb
64.0Mb
64.0Mb
64.0Mb
Cell Location
Hansen et al.
hypo
hyper

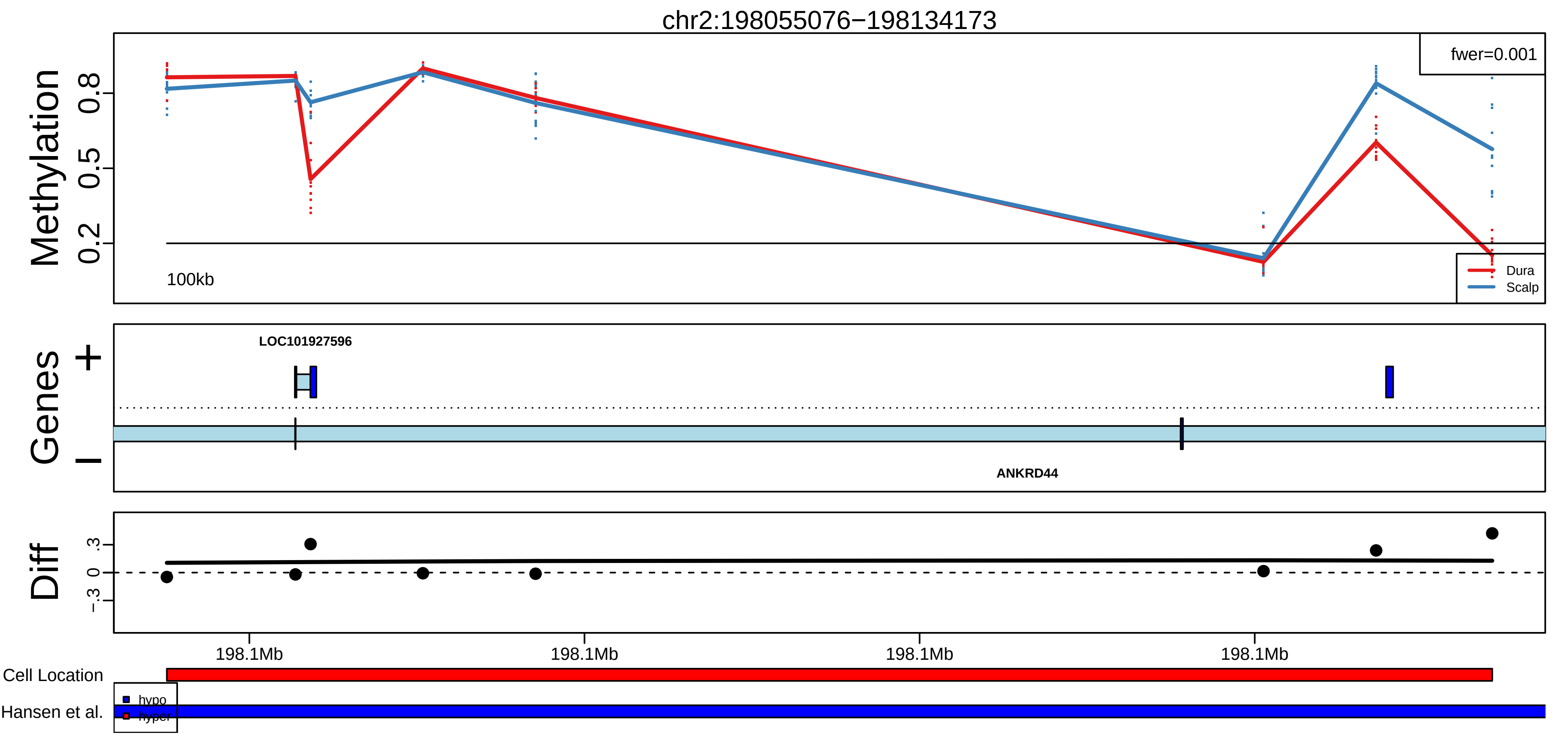

chr2:198055076−198134173
fwer=0.001
Methylation
0.2
0.5
0.8
100kb
Dura
Scalp
Genes
+
−
LOC101927596
ANKRD44
Diff
−.3
0
.3
198.1Mb
198.1Mb
198.1Mb
198.1Mb
Cell Location
Hansen et al.
hypo
hyper

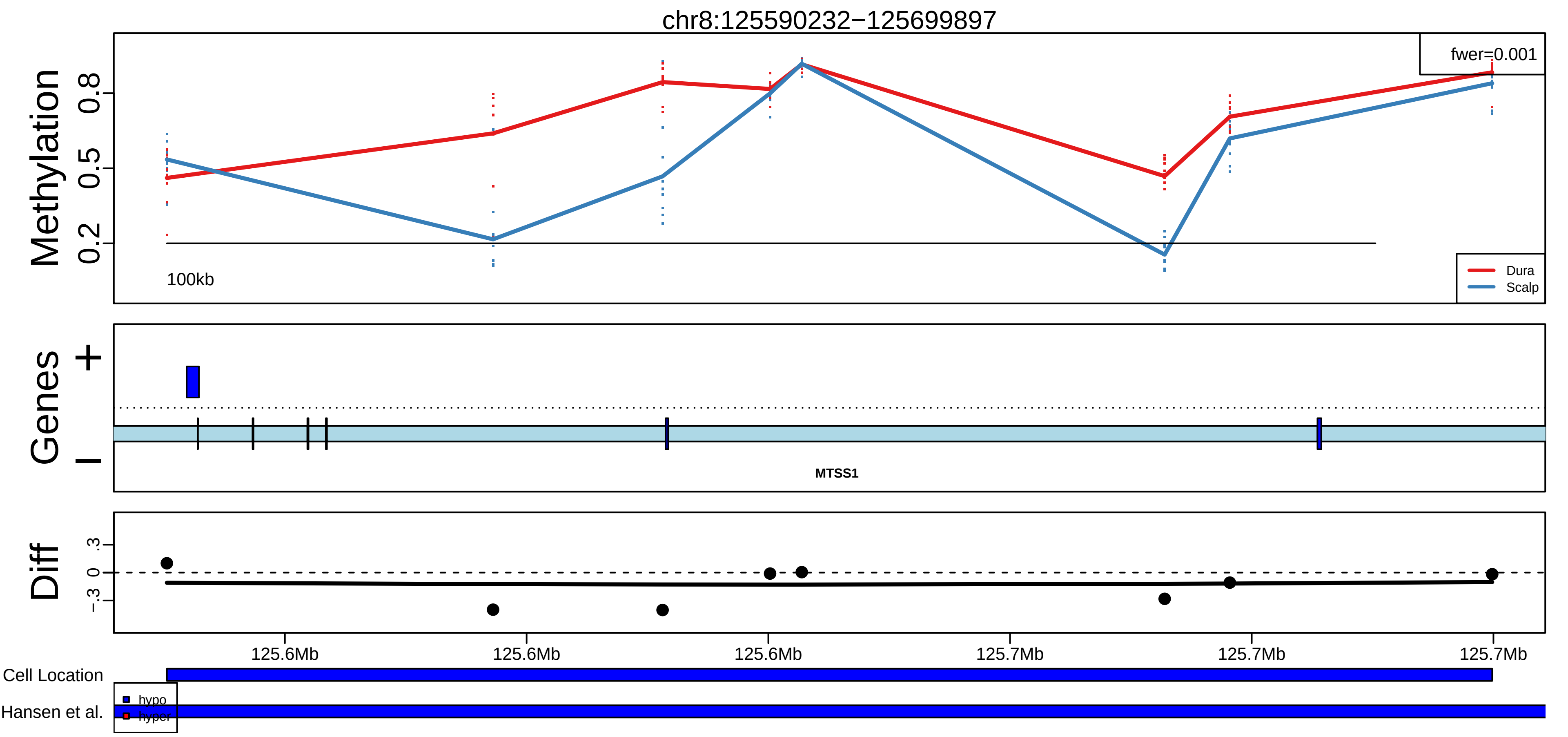

chr8:125590232−125699897
Methylation
0.2
0.5
0.8
fwer=0.001
100kb
Dura
Scalp
Genes
+
−
MTSS1
Diff
−.3
0
.3
125.6Mb
125.6Mb
125.6Mb
125.7Mb
125.7Mb
125.7Mb
Cell Location
Hansen et al.
hypo
hyper

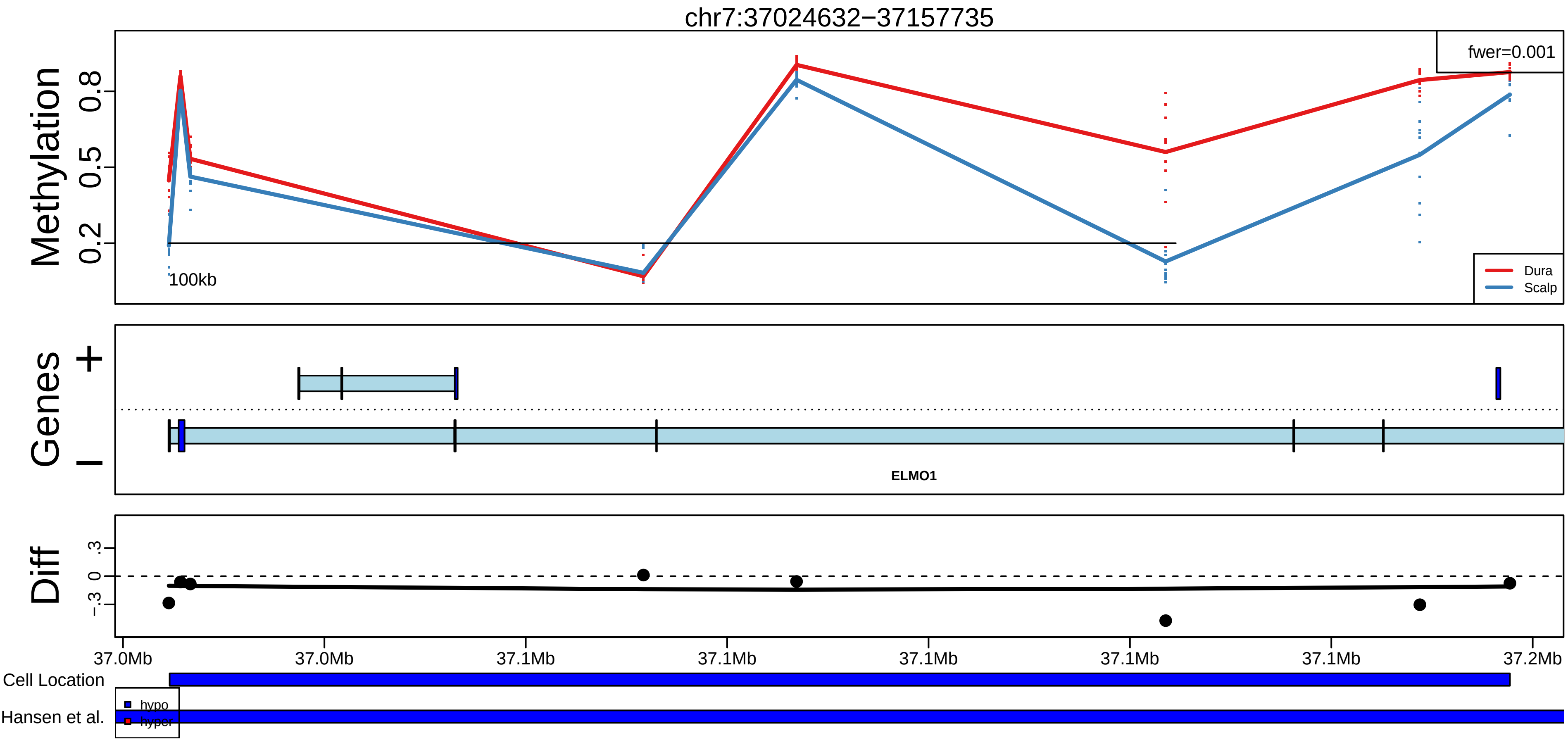

chr7:37024632−37157735
Methylation
0.2
0.5
0.8
fwer=0.001
100kb
Dura
Scalp
Genes
+
−
ELMO1
Diff
−.3
0
.3
37.0Mb
37.0Mb
37.1Mb
37.1Mb
37.1Mb
37.1Mb
37.1Mb
37.2Mb
Cell Location
Hansen et al.
hypo
hyper

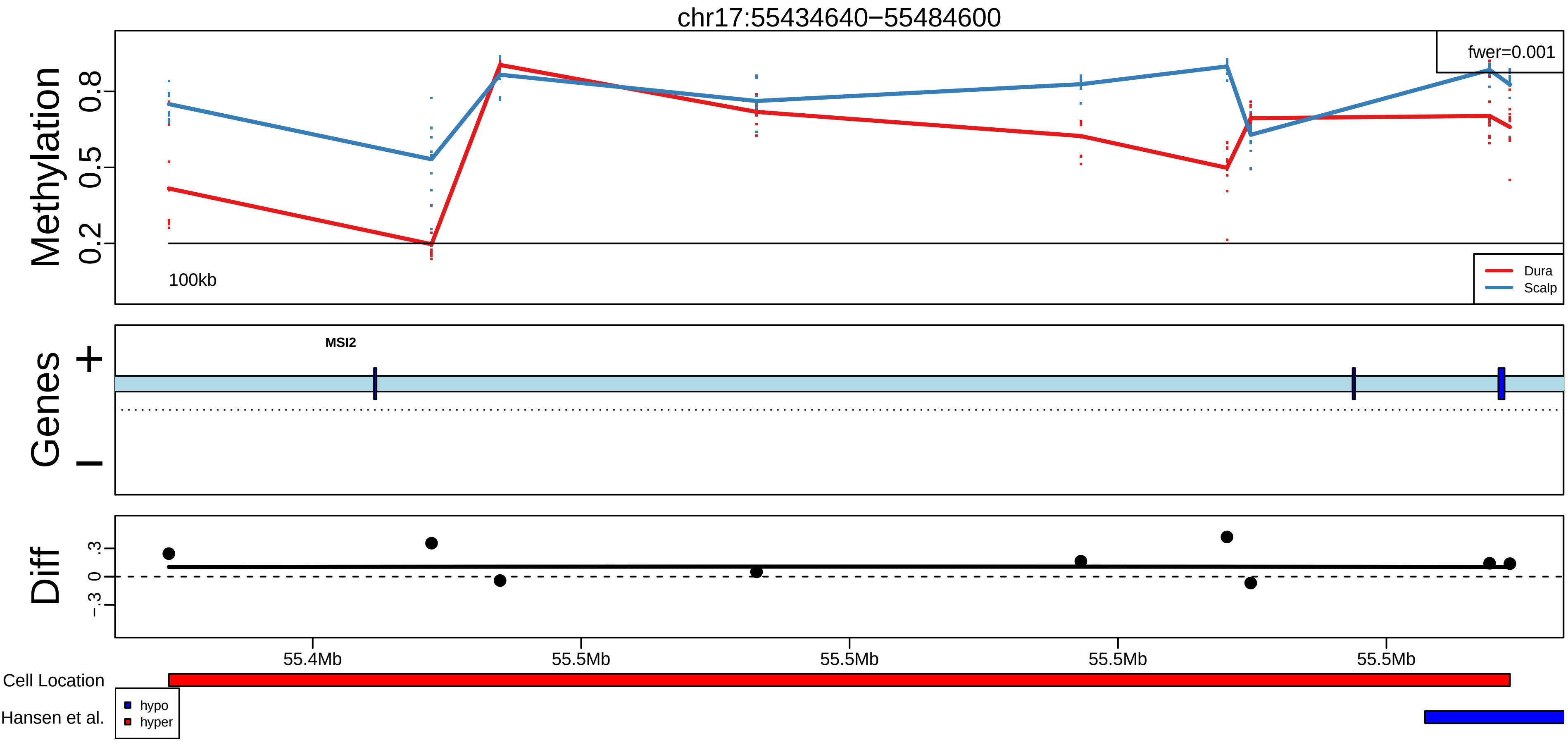

chr17:55434640−55484600
Methylation
0.2
0.5
0.8
fwer=0.001
100kb
Dura
Scalp
Genes
+
−
MSI2
Diff
−.3
0
.3
55.4Mb
55.5Mb
55.5Mb
55.5Mb
55.5Mb
Cell Location
Hansen et al.
hypo
hyper

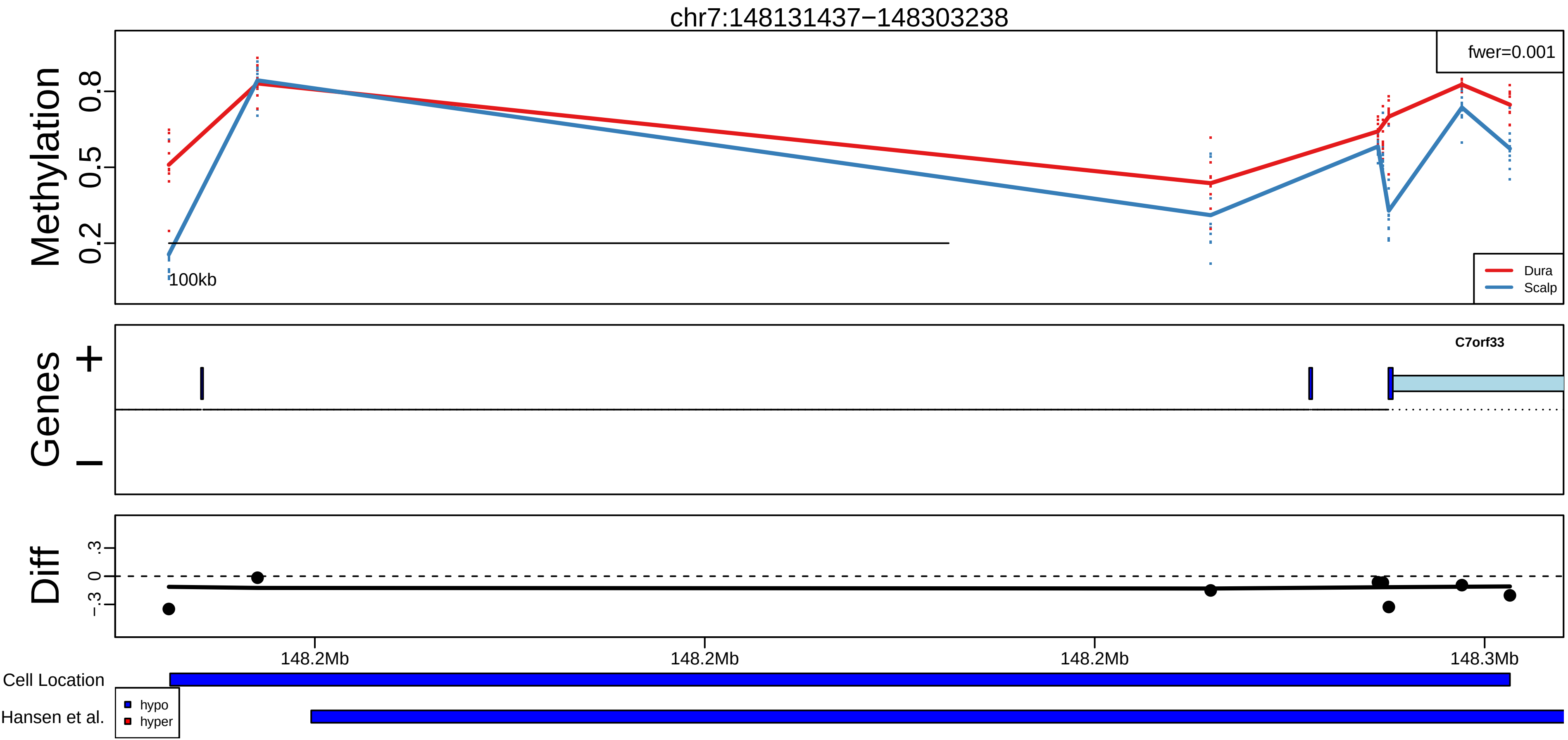

chr7:148131437−148303238
fwer=0.001
Methylation
0.2
0.5
0.8
100kb
Dura
Scalp
Genes
+
−
C7orf33
Diff
.3
0
−.3
148.2Mb
148.2Mb
148.2Mb
148.3Mb
Cell Location
Hansen et al.
hypo
hyper

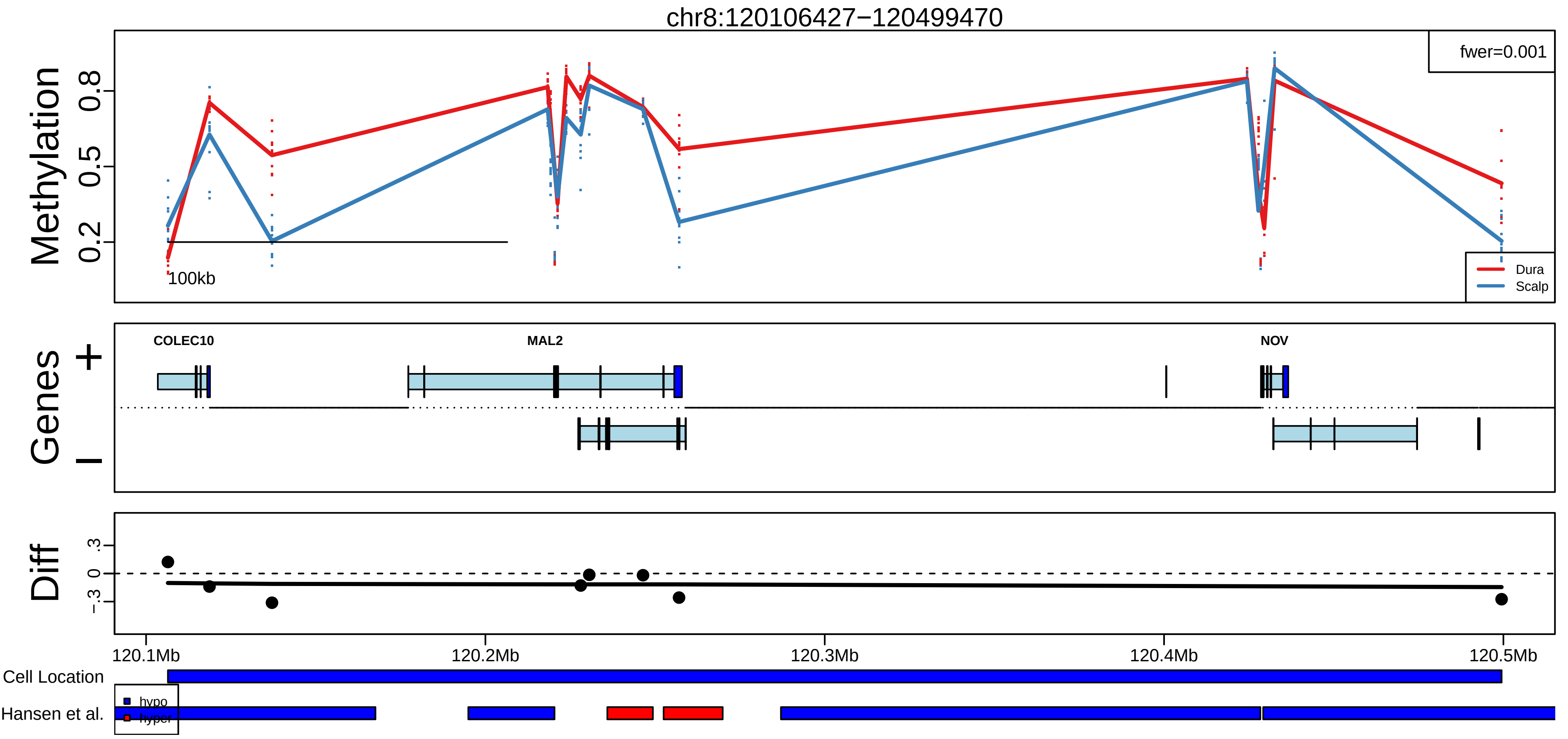
chr8:120106427−120499470
fwer=0.001
Methylation
0.2
0.5
0.8
100kb
Dura
Scalp
Genes
+
−
COLEC10
MAL2
NOV
Diff
−.3
0
.3
120.1Mb
120.2Mb
120.3Mb
120.4Mb
120.5Mb
Cell Location
Hansen et al.
hypo
hyper

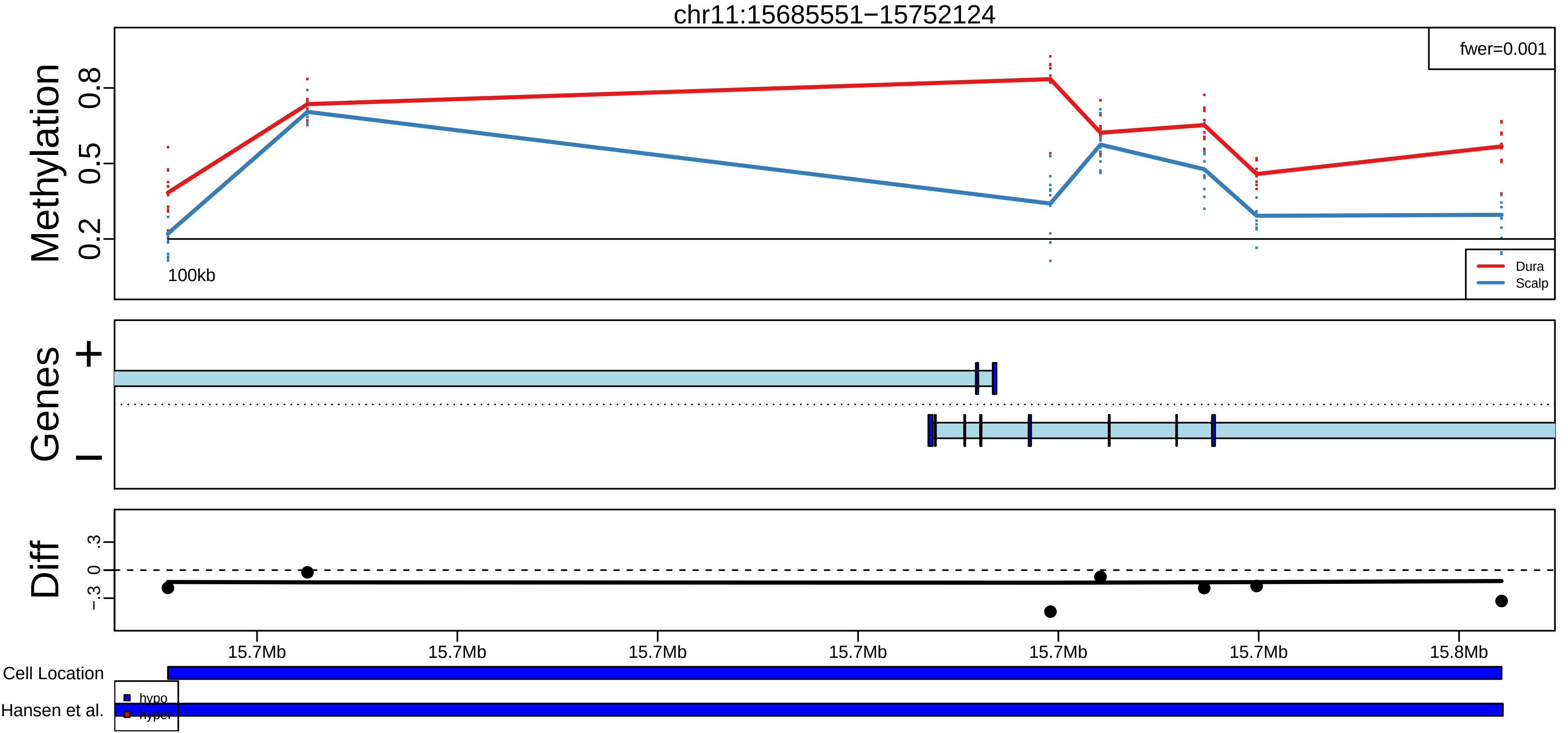
chr11:15685551−15752124
fwer=0.001
Methylation
0.2
0.5
0.8
100kb
Dura
Scalp
Genes
+
−
Diff
.3
0
−.3
15.7Mb
15.7Mb
15.7Mb
15.7Mb
15.7Mb
15.7Mb
15.8Mb
Cell Location
Hansen et al.
hypo
hyper

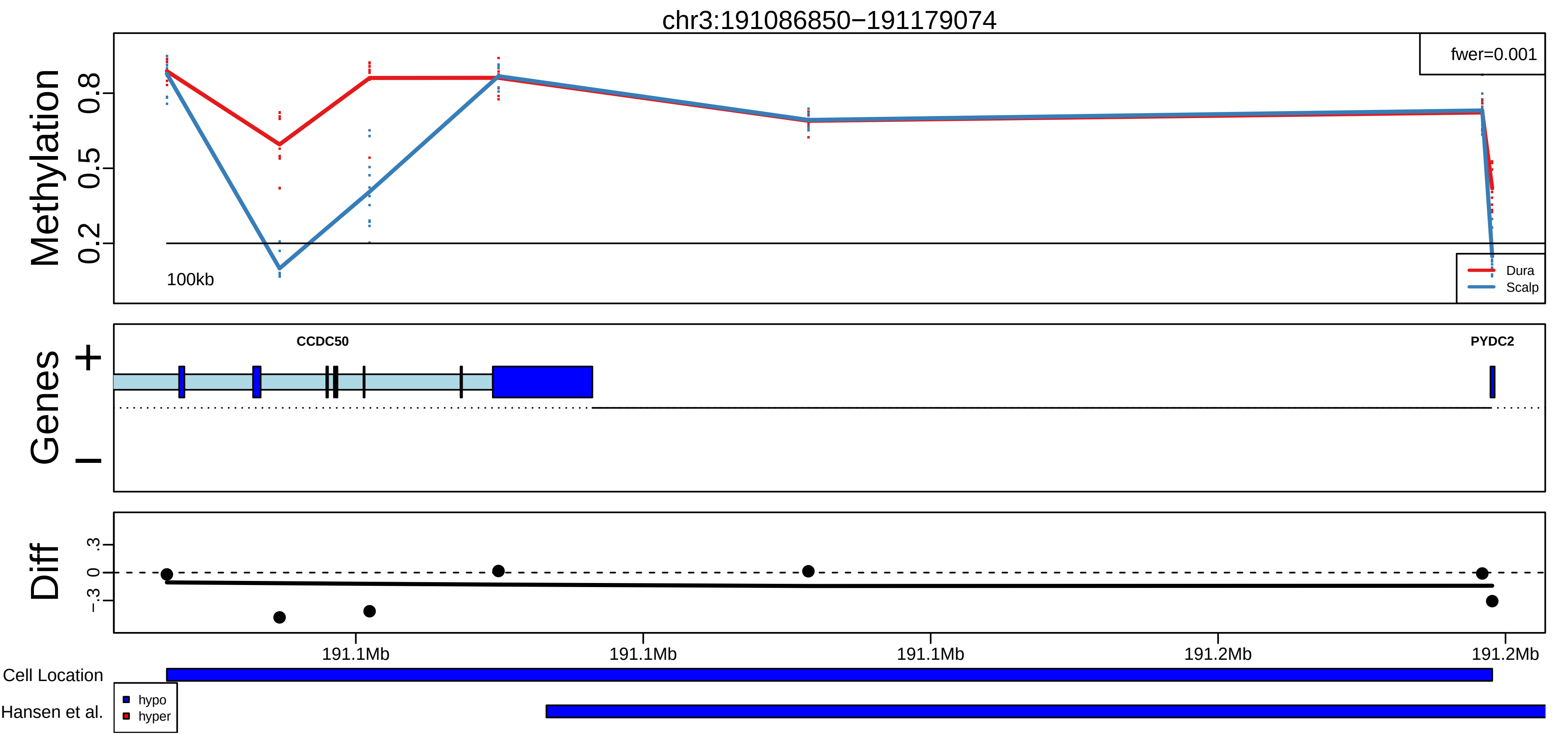
chr3:191086850−191179074
fwer=0.001
Methylation
0.8
0.5
0.2
100kb
Dura
Scalp
Genes
+
−
CCDC50
PYDC2
Diff
.3
0
−.3
191.1Mb
191.1Mb
191.1Mb
191.2Mb
191.2Mb
Cell Location
Hansen et al.
hypo
hyper

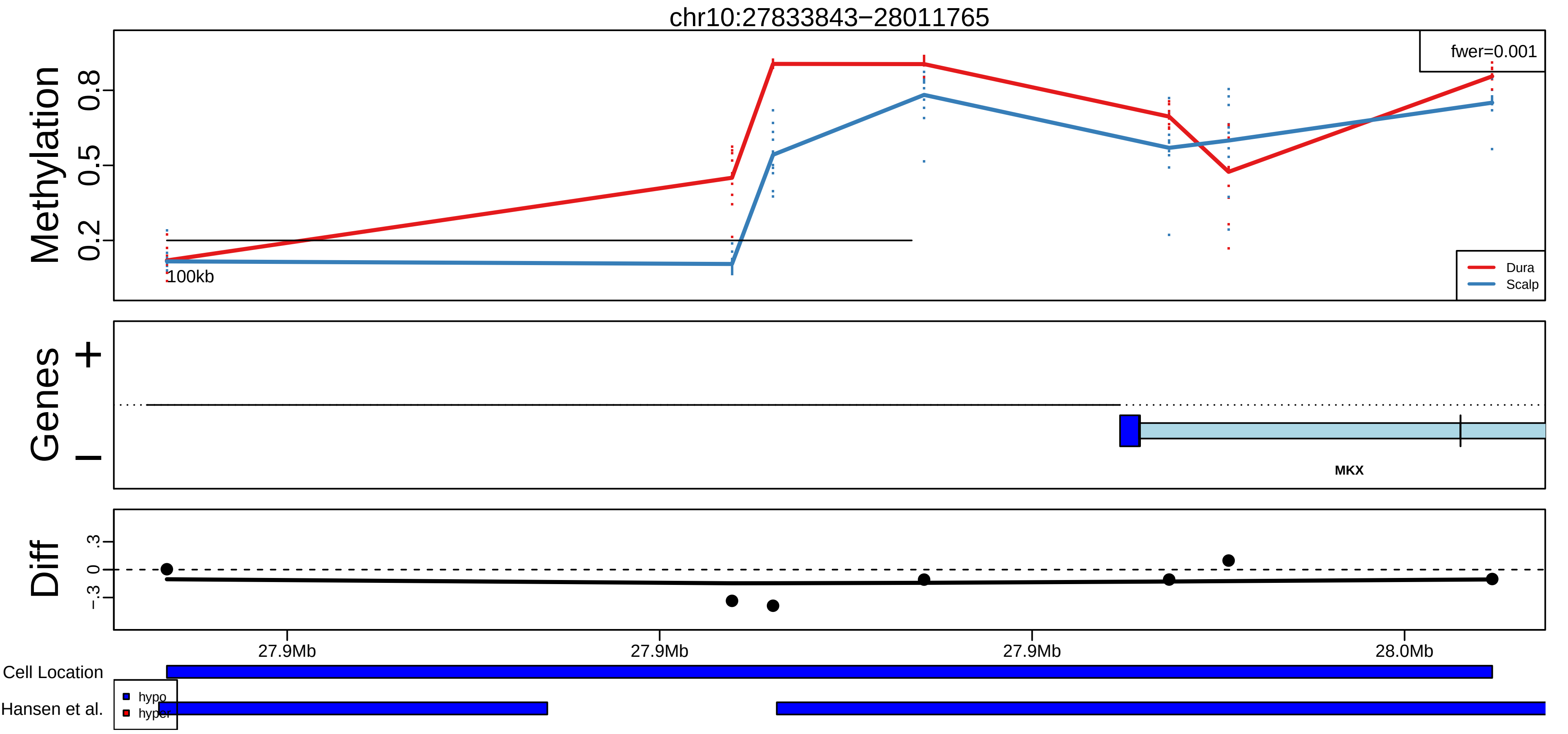

chr10:27833843−28011765
fwer=0.001
Methylation
0.2
0.5
0.8
100kb
Dura
Scalp
Genes
+
−
MKX
Diff
−.3
0
.3
27.9Mb
27.9Mb
27.9Mb
28.0Mb
Cell Location
Hansen et al.
hypo
hyper

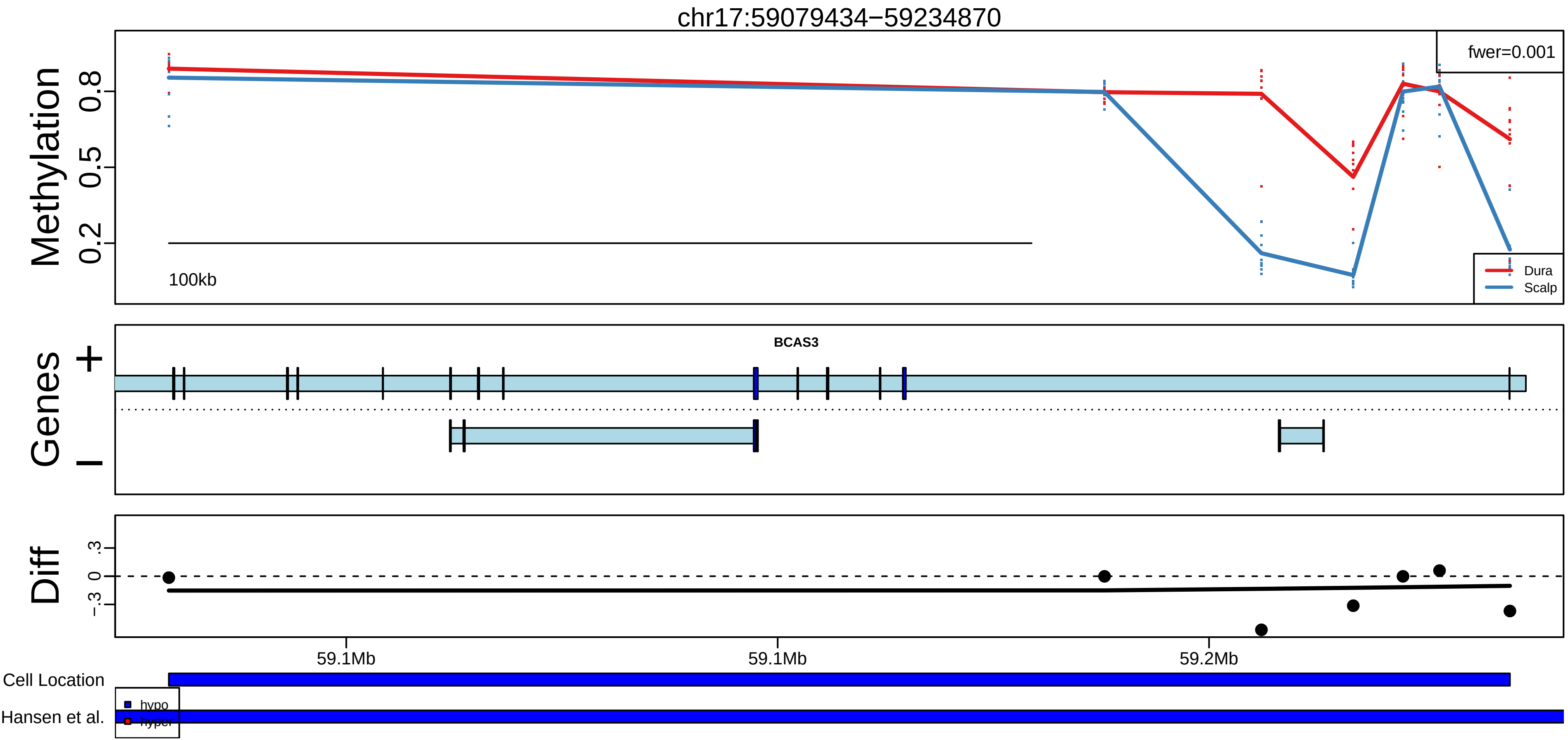
chr17:59079434−59234870
Methylation
0.2
0.5
0.8
fwer=0.001
100kb
Dura
Scalp
Genes
+
−
BCAS3
Diff
.3
0
−.3
59.1Mb
59.1Mb
59.2Mb
Cell Location
Hansen et al.
hypo
hyper

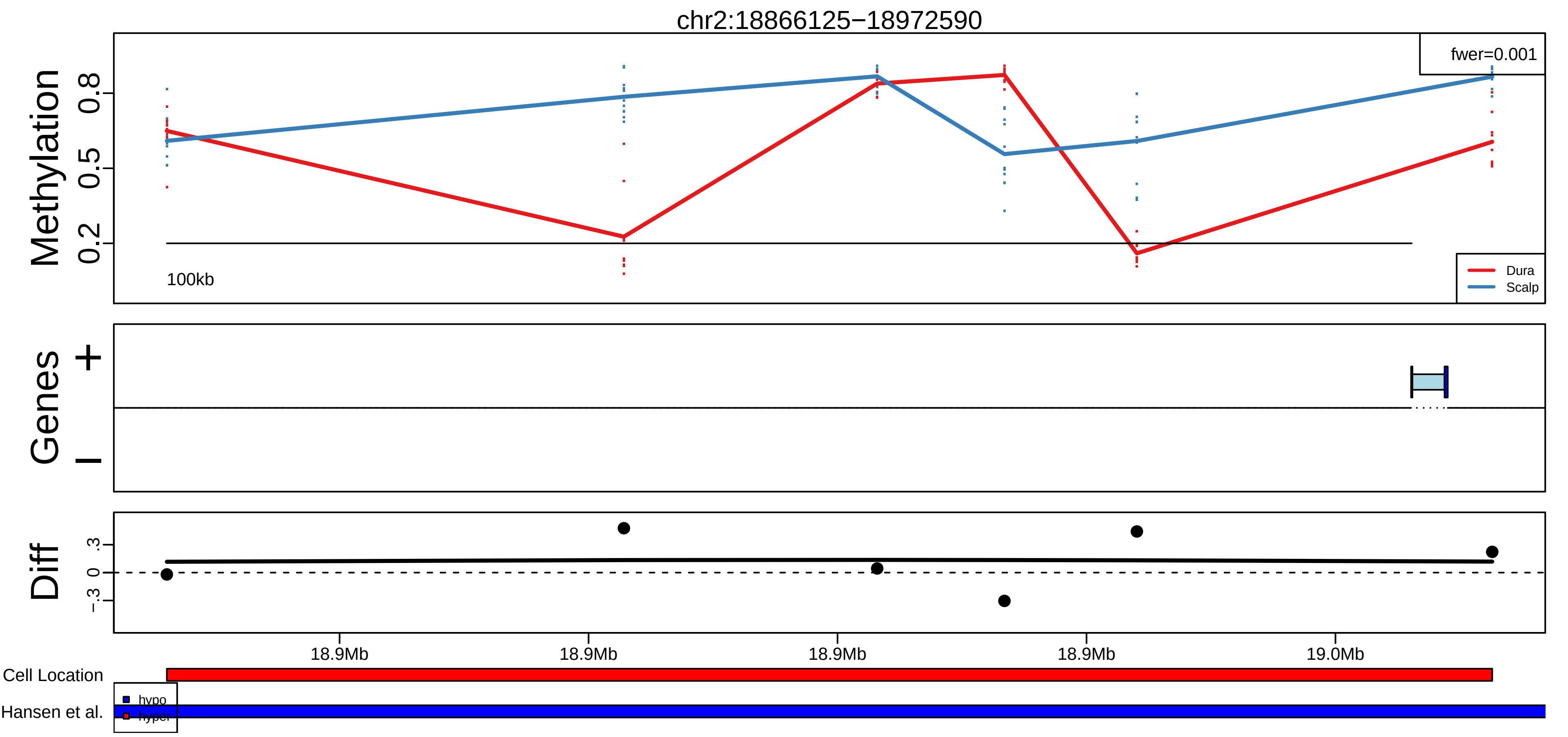

chr2:18866125−18972590
fwer=0.001
Methylation
0.2
0.5
0.8
100kb
Dura
Scalp
Genes
+
−
Diff
−.3
0
.3
18.9Mb
18.9Mb
18.9Mb
18.9Mb
19.0Mb
Cell Location
Hansen et al.
hypo
hyper

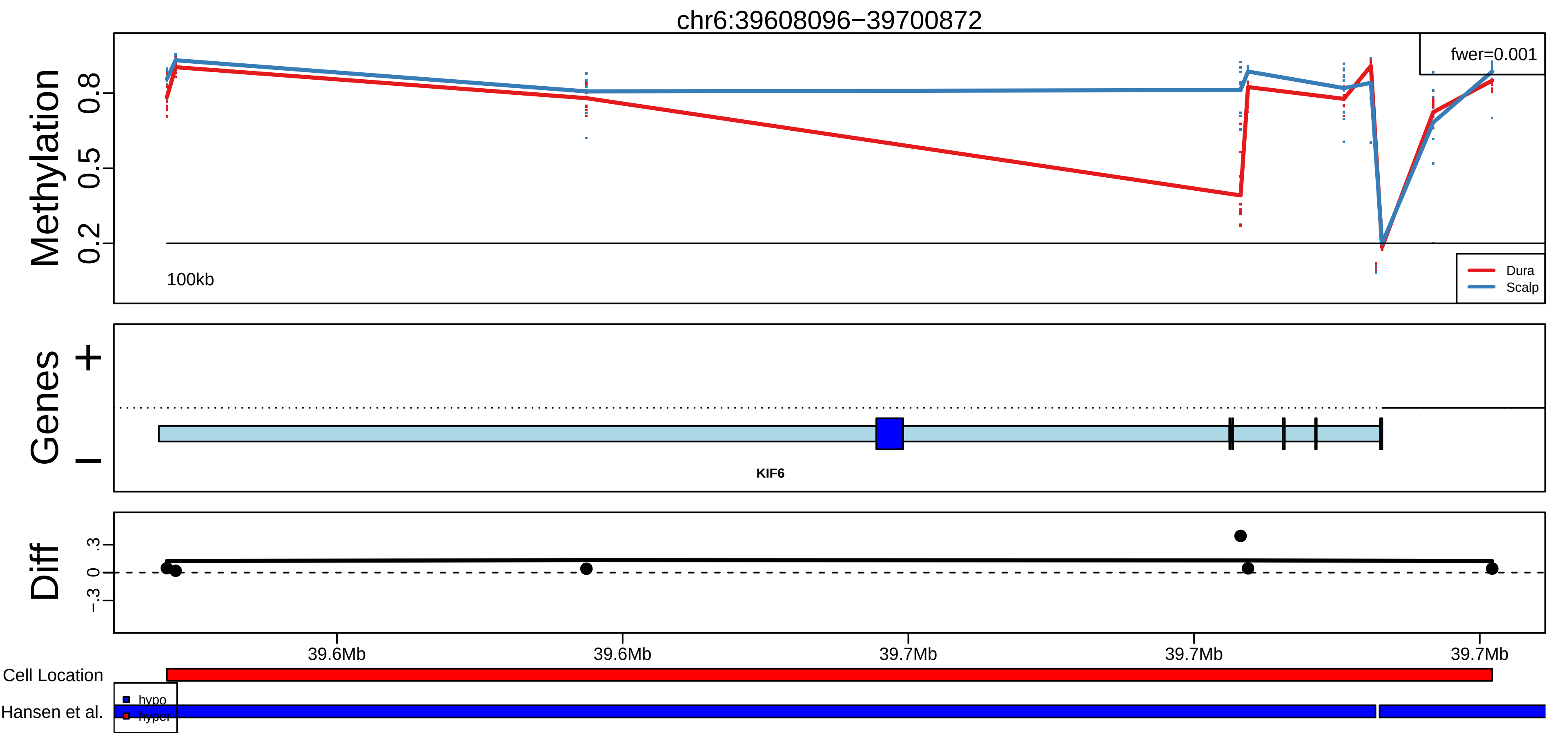

chr6:39608096−39700872
fwer=0.001
Methylation
0.2
0.5
0.8
100kb
Dura
Scalp
Genes
+
−
KIF6
Diff
.3
0
−.3
39.6Mb
39.6Mb
39.7Mb
39.7Mb
39.7Mb
Cell Location
Hansen et al.
hypo
hyper

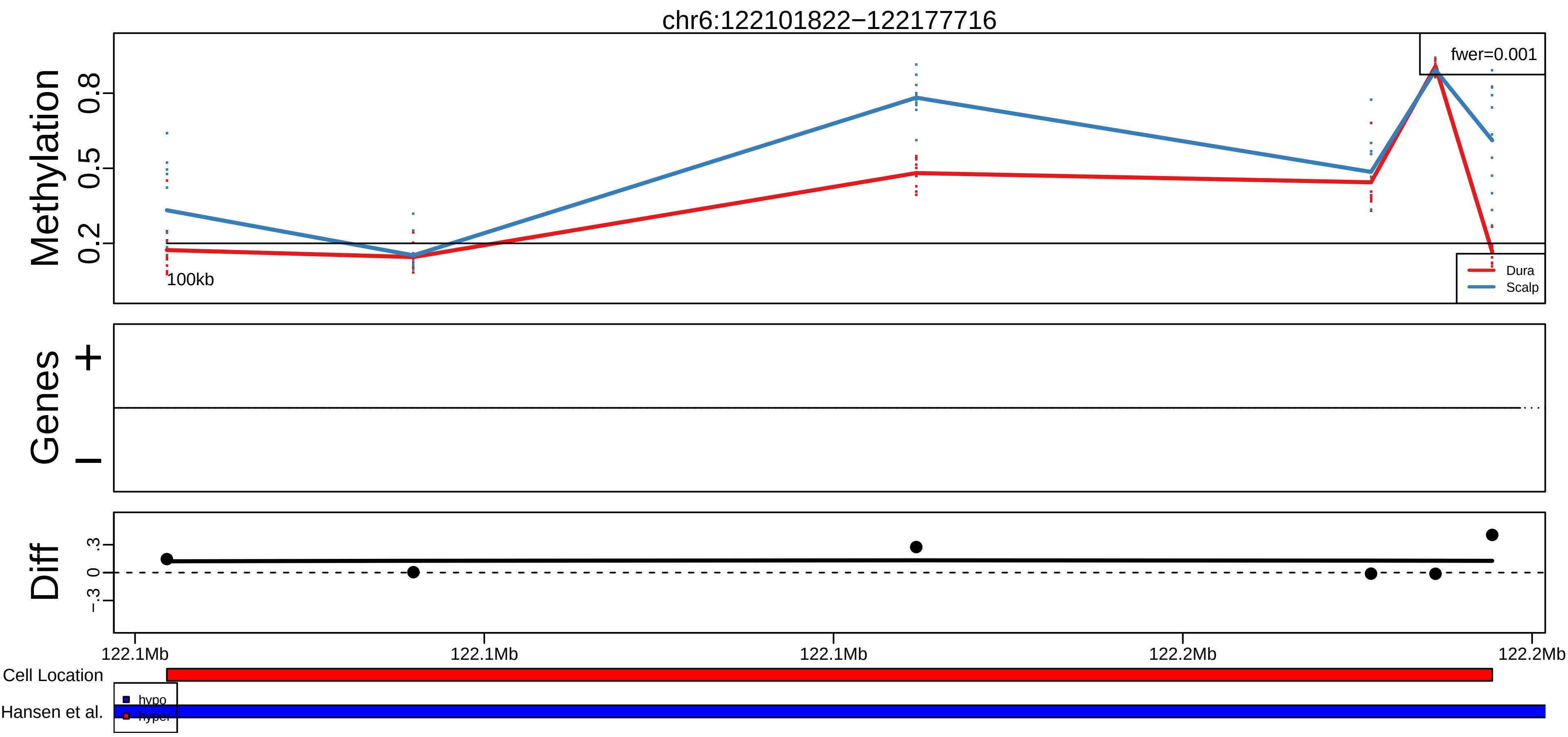
chr6:122101822−122177716
fwer=0.001
Methylation
0.2
0.5
0.8
100kb
Dura
Scalp
Genes
+
−
Diff
−.3
0
.3
122.1Mb
122.1Mb
122.1Mb
122.2Mb
122.2Mb
Cell Location
Hansen et al.
hypo
hyper

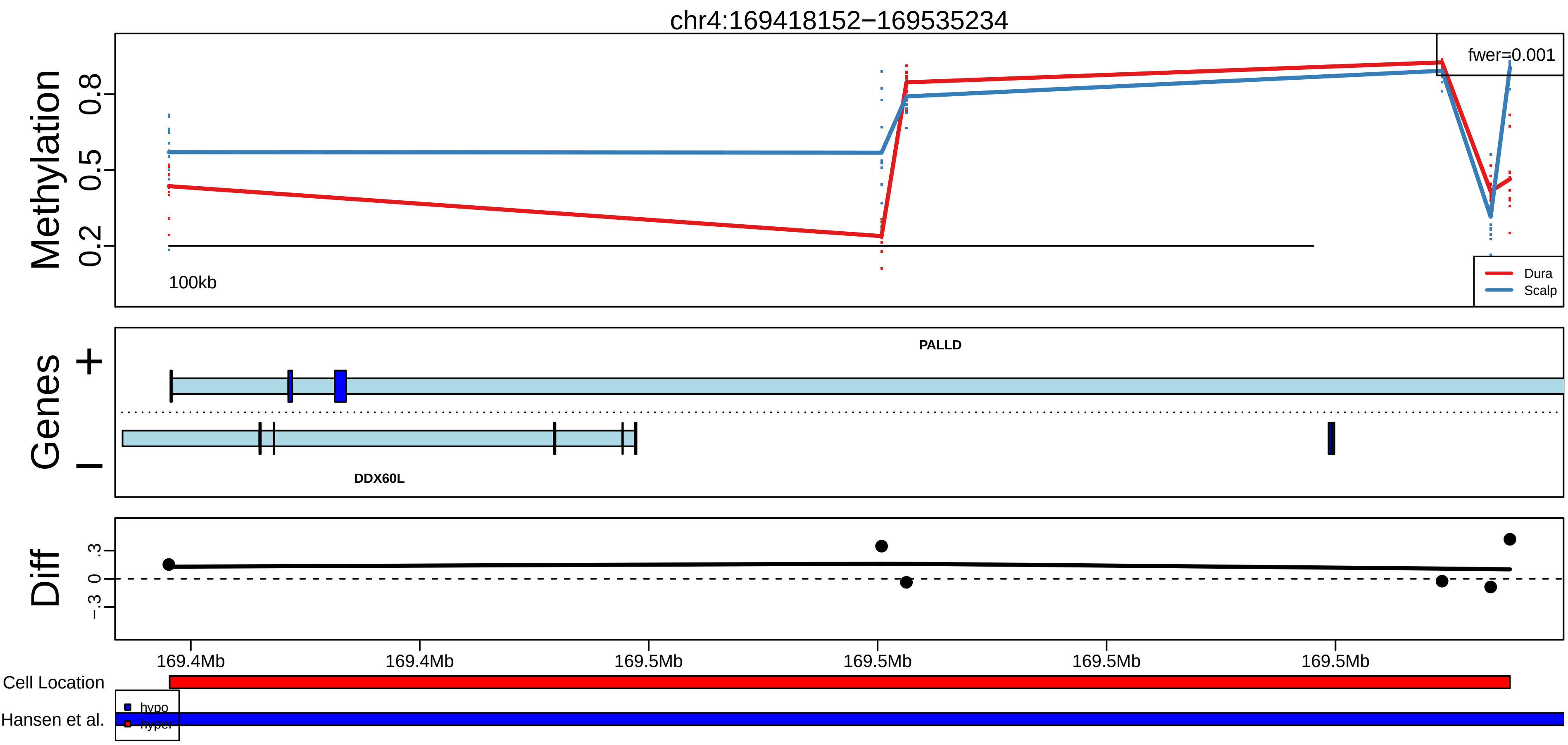
chr4:169418152−169535234
fwer=0.001
Methylation
0.2
0.5
0.8
100kb
Dura
Scalp
Genes
+
−
PALLD
DDX60L
Diff
−.3
0
.3
169.4Mb
169.4Mb
169.5Mb
169.5Mb
169.5Mb
169.5Mb
Cell Location
Hansen et al.
hypo
hyper

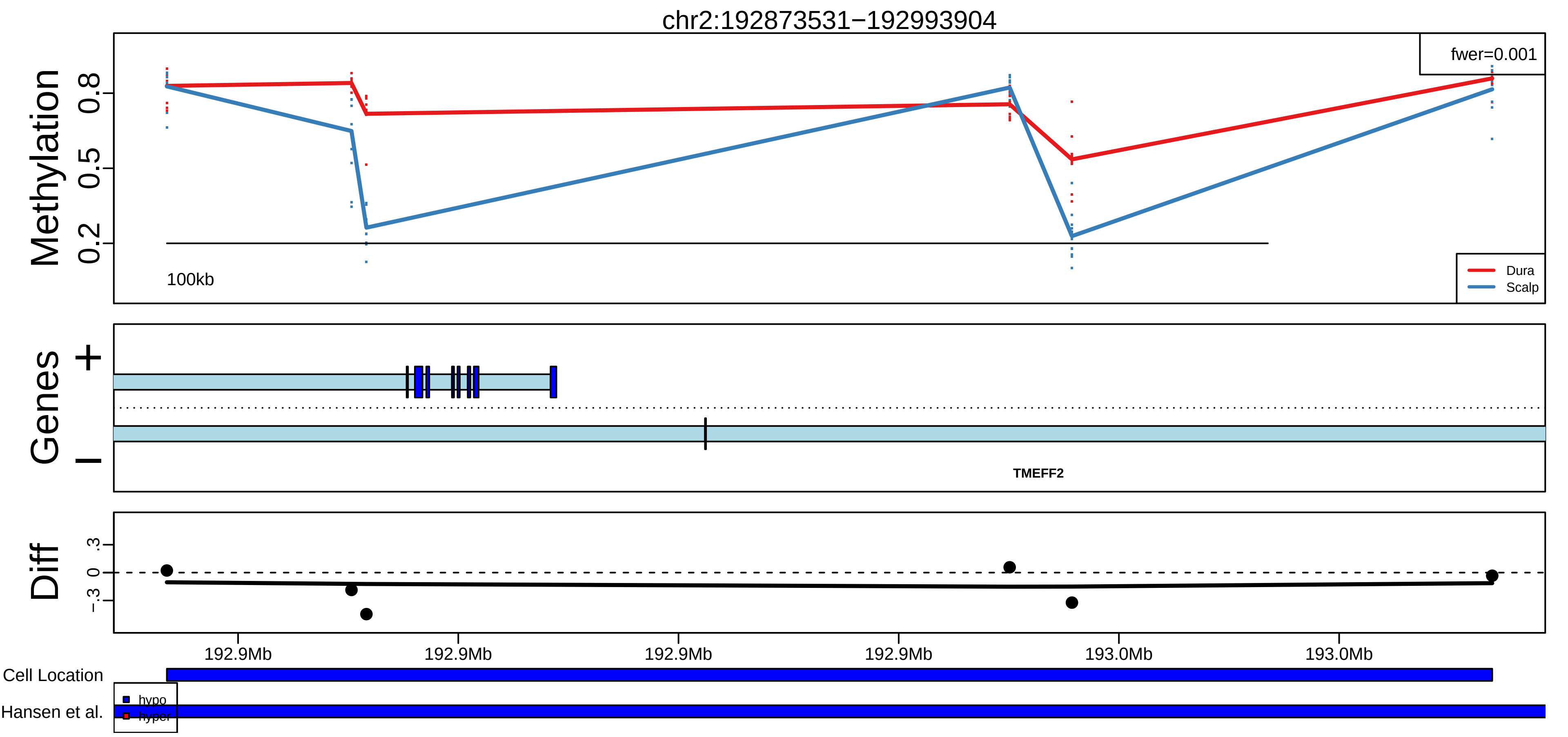
chr2:192873531−192993904
fwer=0.001
Methylation
0.2
0.5
0.8
100kb
Dura
Scalp
Genes
+
−
TMEFF2
Diff
−.3
0
.3
192.9Mb
192.9Mb
192.9Mb
192.9Mb
193.0Mb
193.0Mb
Cell Location
Hansen et al.
hypo
hyper

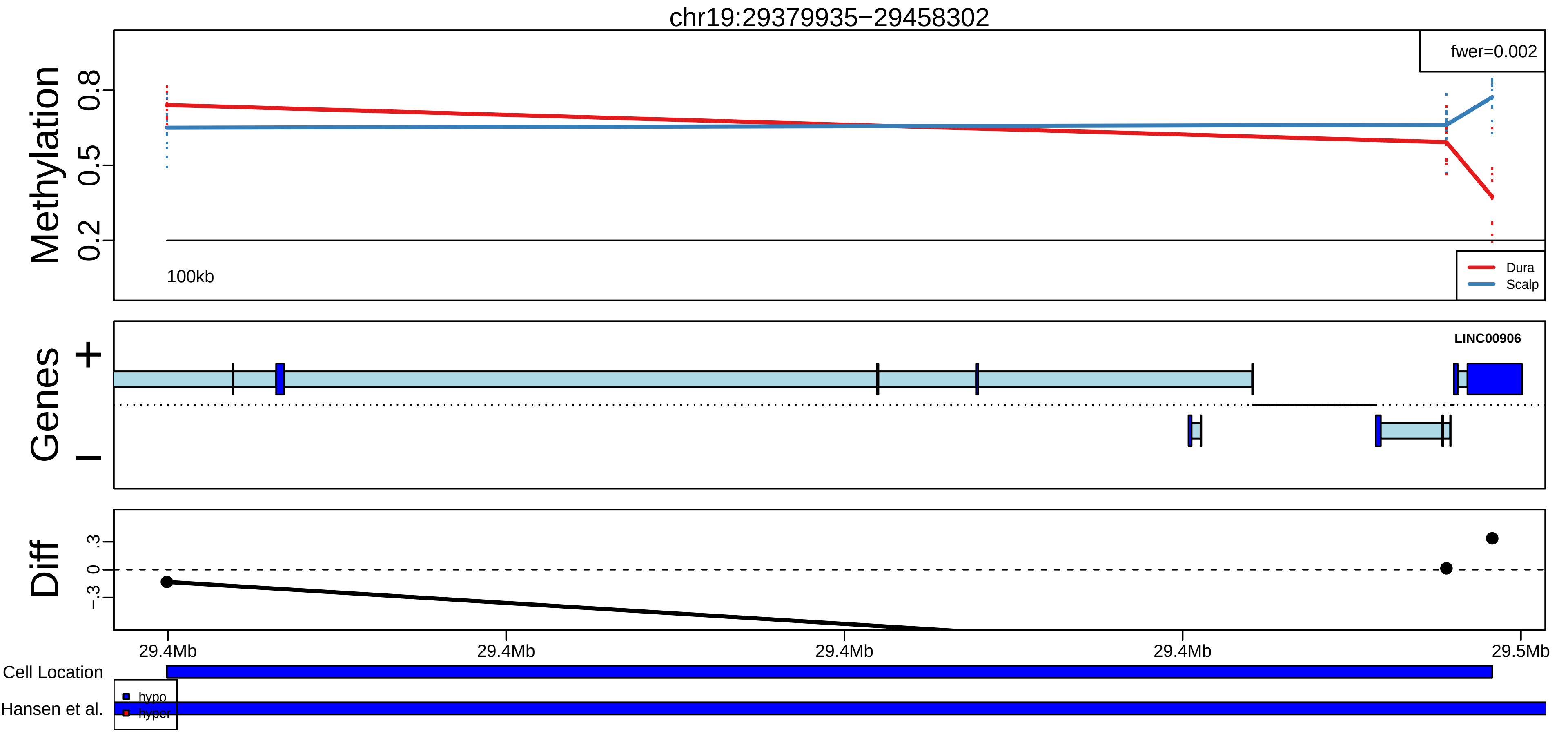
chr19:29379935−29458302
fwer=0.002
Methylation
0.2
0.5
0.8
100kb
Dura
Scalp
Genes
+
−
LINC00906
Diff
−.3
0
.3
29.4Mb
29.4Mb
29.4Mb
29.4Mb
29.5Mb
Cell Location
Hansen et al.
hypo
hyper

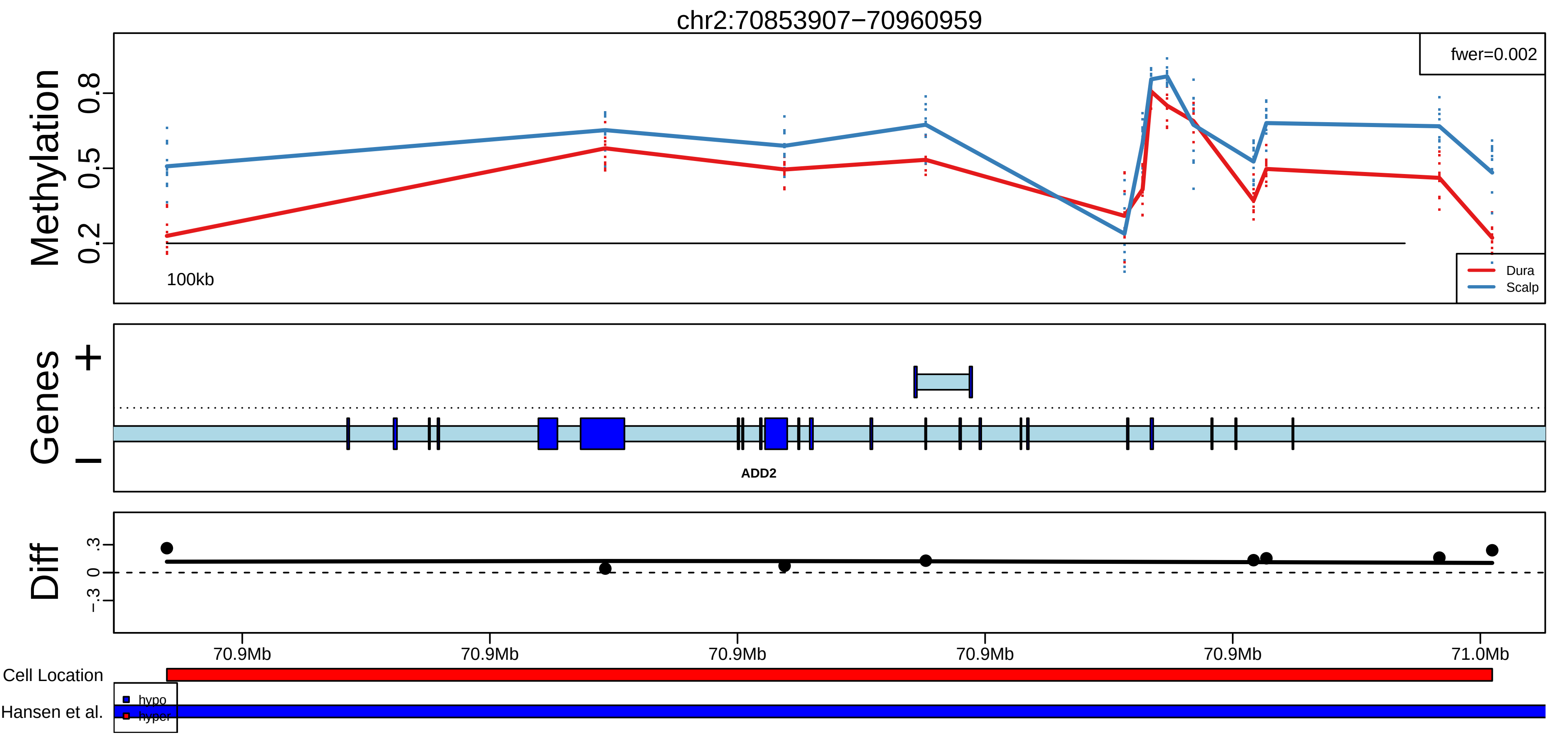
chr2:70853907−70960959
fwer=0.002
Methylation
0.2
0.5
0.8
100kb
Dura
Scalp
Genes
+
−
ADD2
Diff
−.3
0
.3
70.9Mb
70.9Mb
70.9Mb
70.9Mb
70.9Mb
71.0Mb
Cell Location
Hansen et al.
hypo
hyper

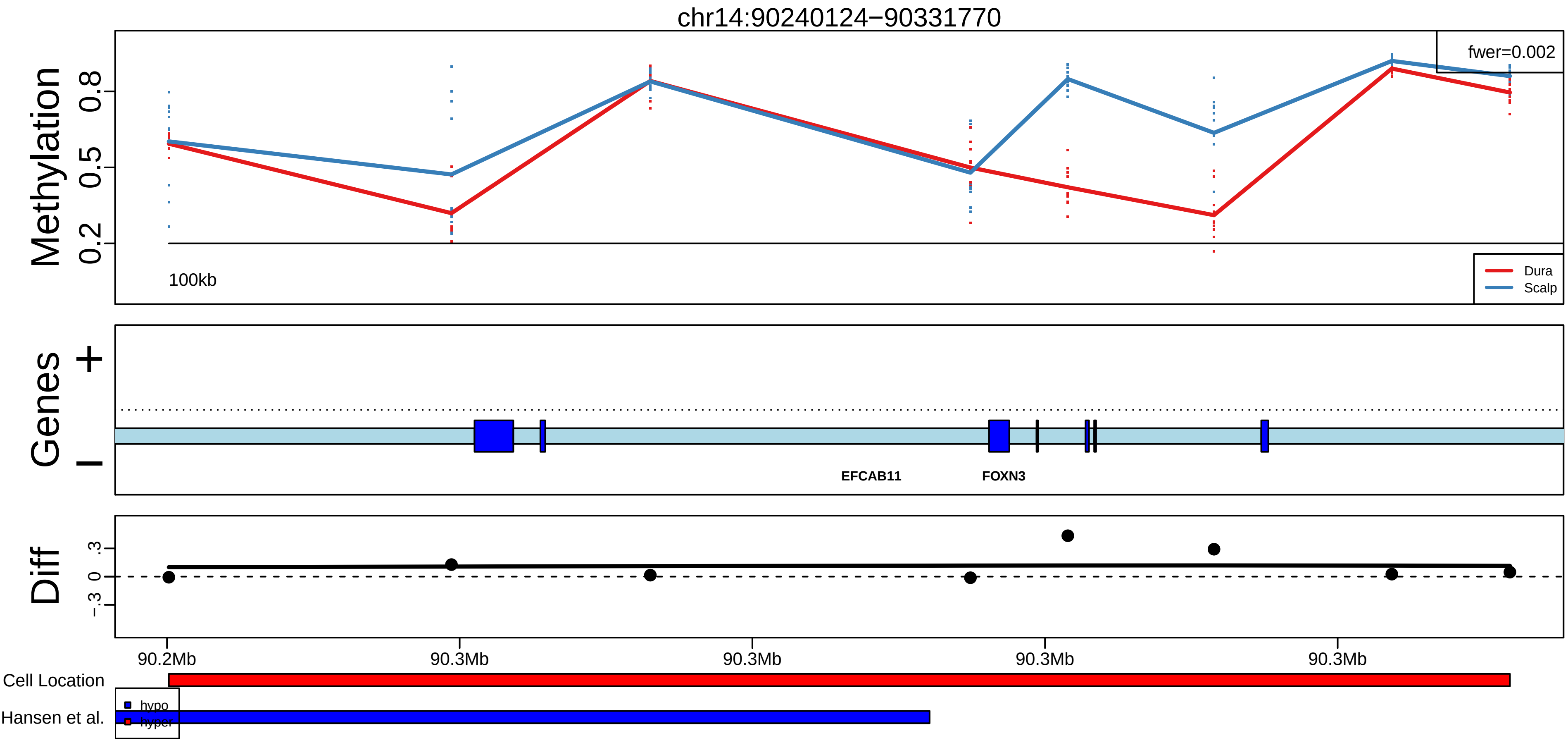
chr14:90240124−90331770
fwer=0.002
Methylation
0.2
0.5
0.8
100kb
Dura
Scalp
Genes
+
−
EFCAB11
FOXN3
Diff
−.3
0
.3
90.2Mb
90.3Mb
90.3Mb
90.3Mb
90.3Mb
Cell Location
Hansen et al.
hypo
hyper

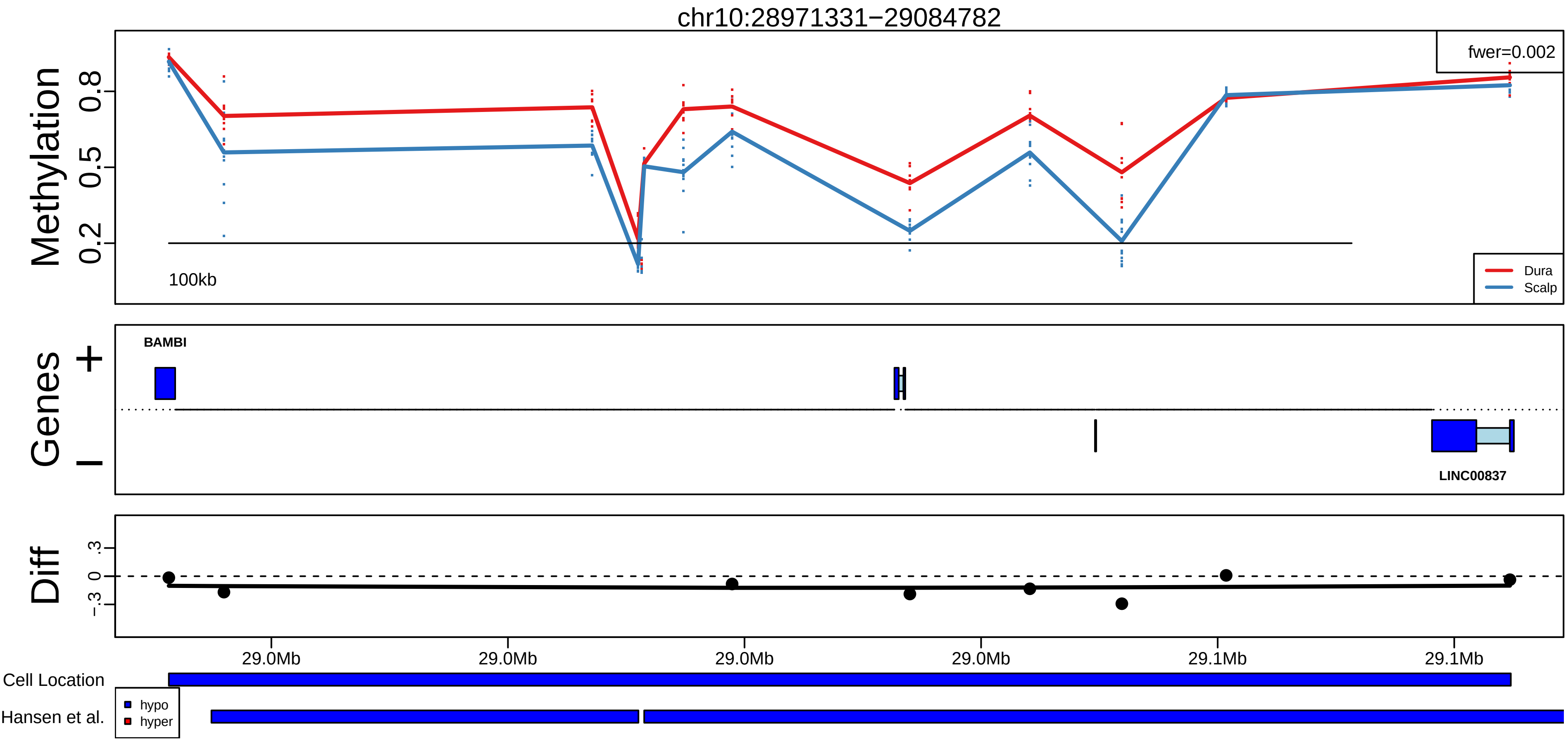
chr10:28971331−29084782
Methylation
0.2
0.5
0.8
fwer=0.002
100kb
Dura
Scalp
Genes
+
−
BAMBI
LINC00837
Diff
.3
0
−.3
29.0Mb
29.0Mb
29.0Mb
29.0Mb
29.1Mb
29.1Mb
Cell Location
Hansen et al.
hypo
hyper

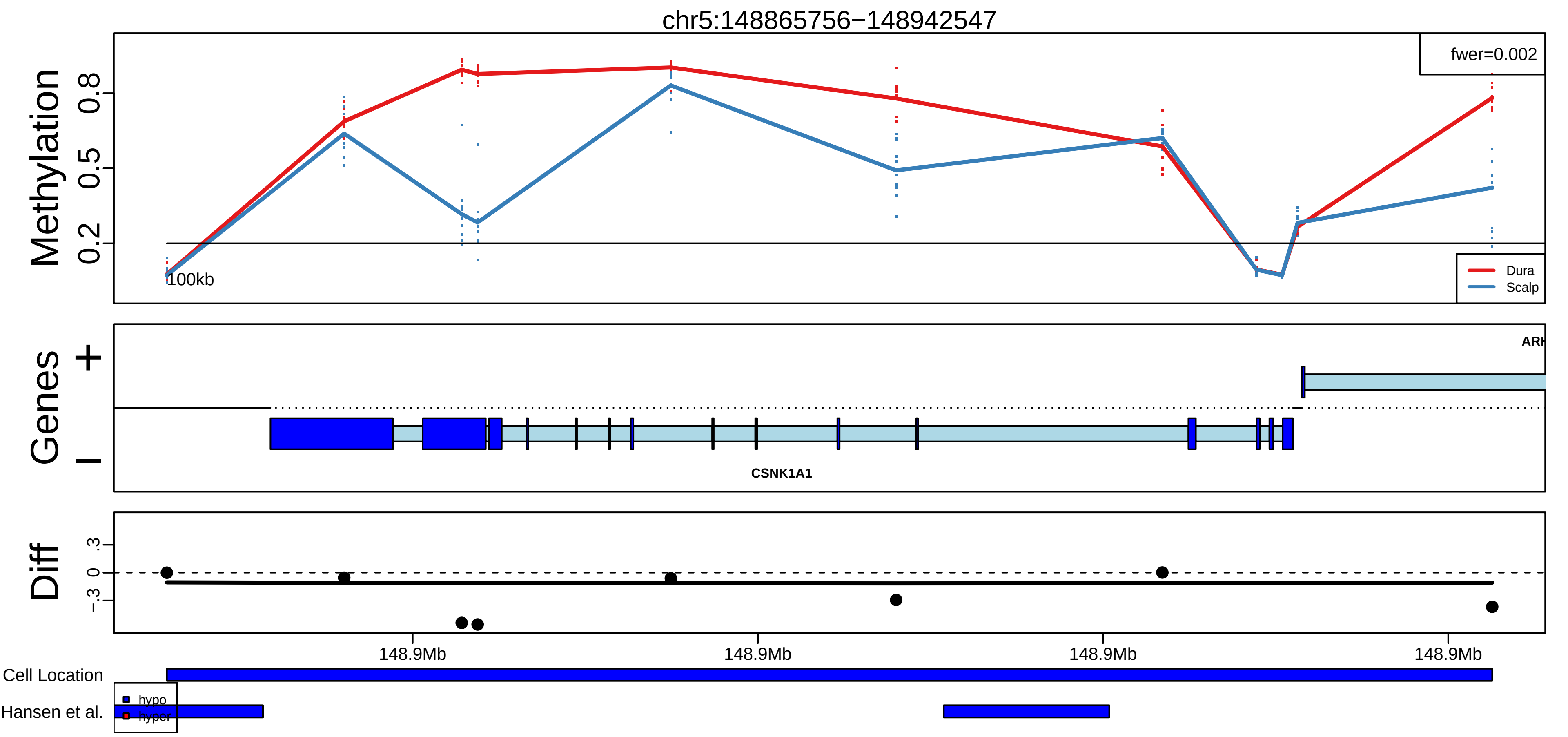

chr5:148865756−148942547
fwer=0.002
Methylation
0.2
0.5
0.8
100kb
Dura
Scalp
Genes
+
−
ARH
CSNK1A1
Diff
.3
0
−.3
148.9Mb
148.9Mb
148.9Mb
148.9Mb
Cell Location
Hansen et al.
hypo
hyper

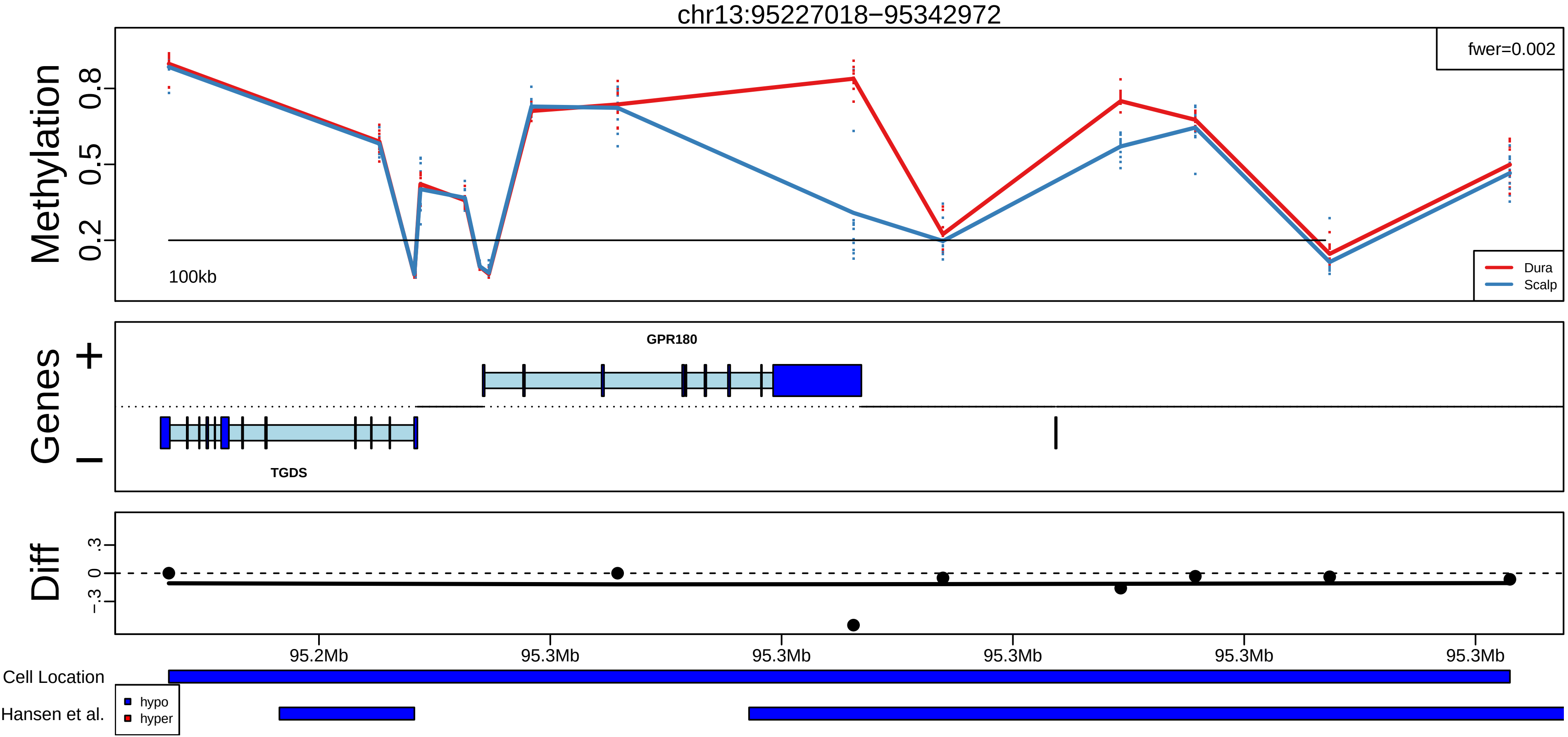

chr13:95227018−95342972
fwer=0.002
Methylation
0.2
0.5
0.8
100kb
Dura
Scalp
Genes
+
−
GPR180
TGDS
Diff
.3
0
−.3
95.2Mb
95.3Mb
95.3Mb
95.3Mb
95.3Mb
95.3Mb
Cell Location
Hansen et al.
hypo
hyper

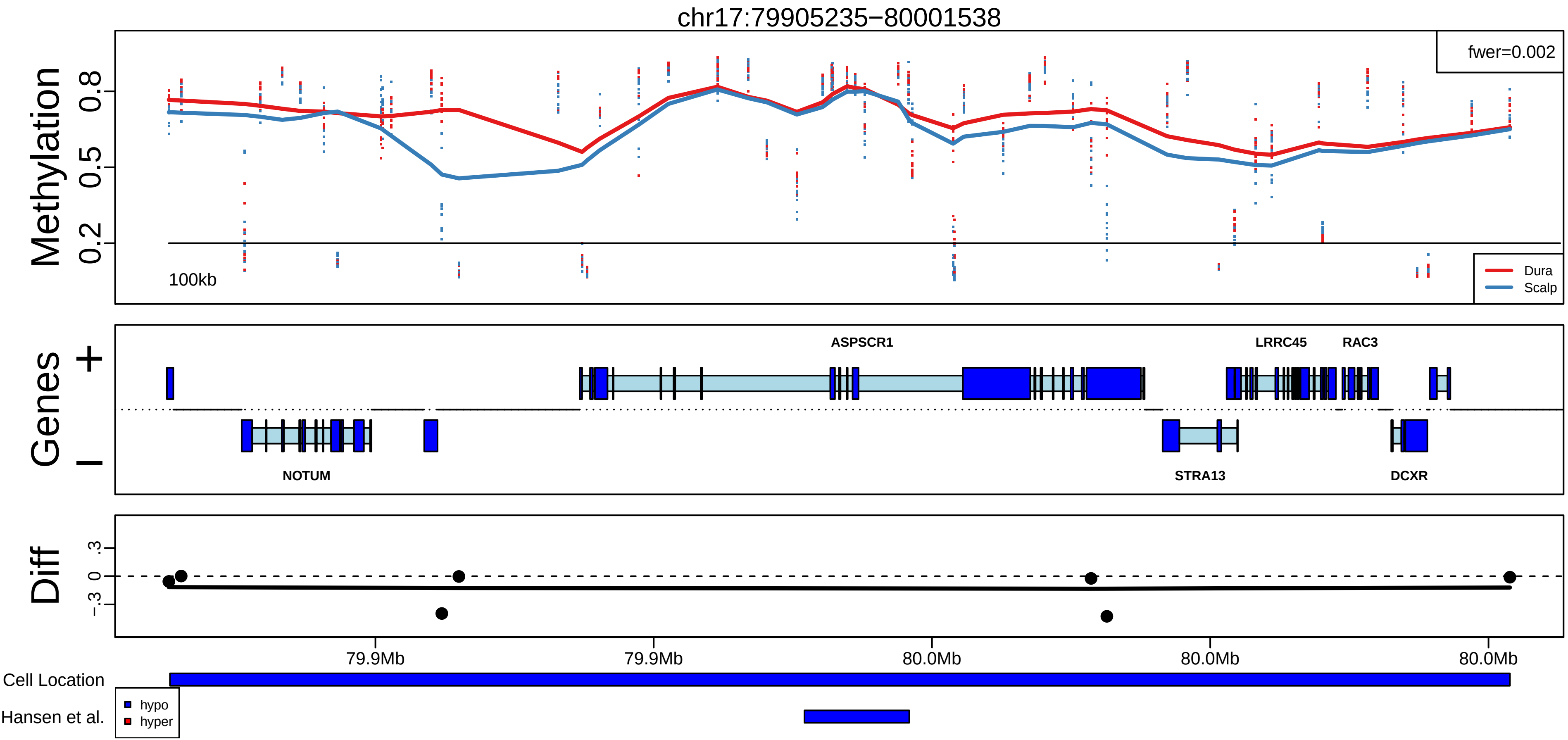

chr17:79905235−80001538
fwer=0.002
Methylation
0.2
0.5
0.8
100kb
Dura
Scalp
Genes
+
−
ASPSCR1
LRRC45
RAC3
NOTUM
STRA13
DCXR
Diff
−.3
0
.3
79.9Mb
79.9Mb
80.0Mb
80.0Mb
80.0Mb
Cell Location
Hansen et al.
hypo
hyper

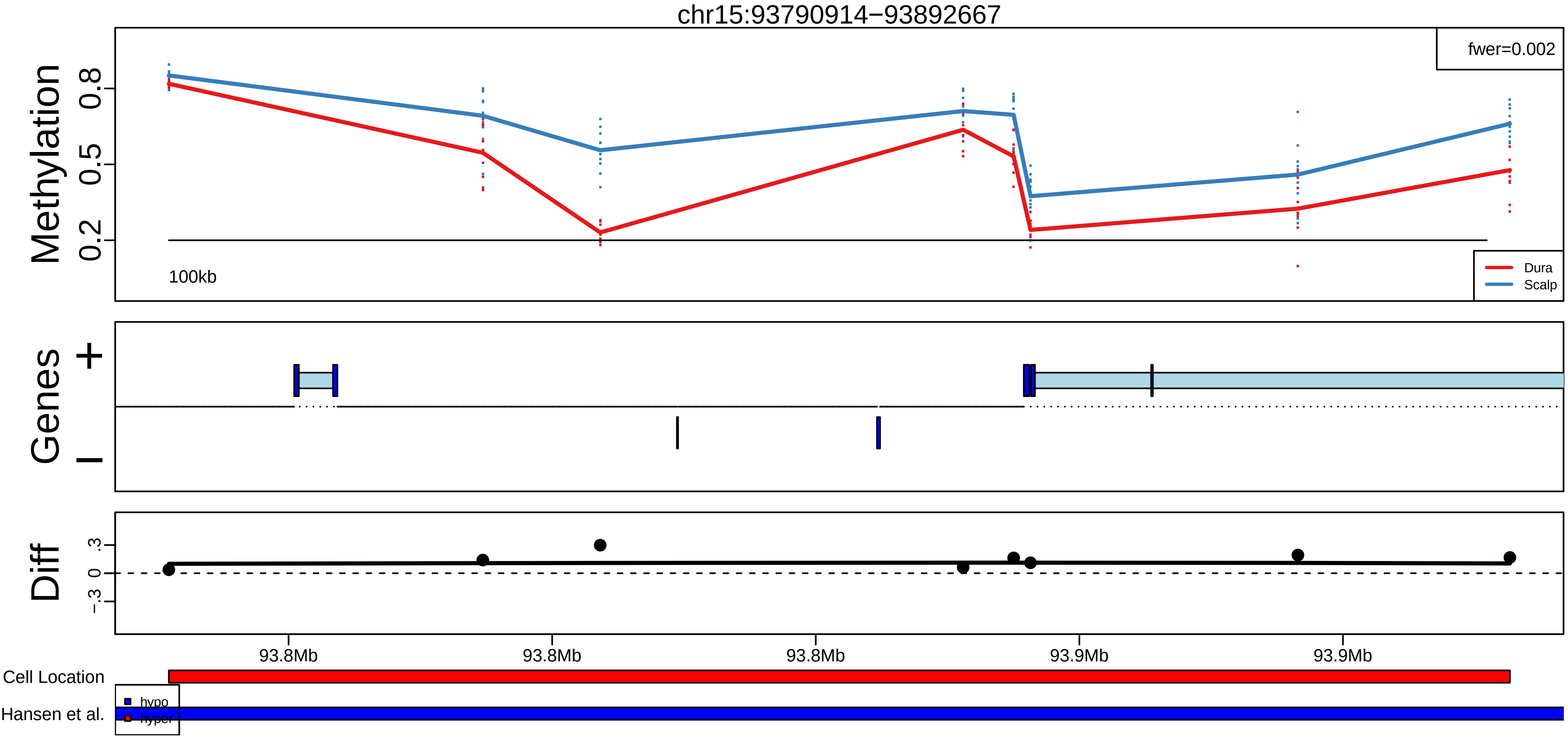

chr15:93790914−93892667
fwer=0.002
Methylation
0.2
0.5
0.8
100kb
Dura
Scalp
Genes
+
−
Diff
−.3
0
.3
93.8Mb
93.8Mb
93.8Mb
93.9Mb
93.9Mb
Cell Location
Hansen et al.
hypo
hyper

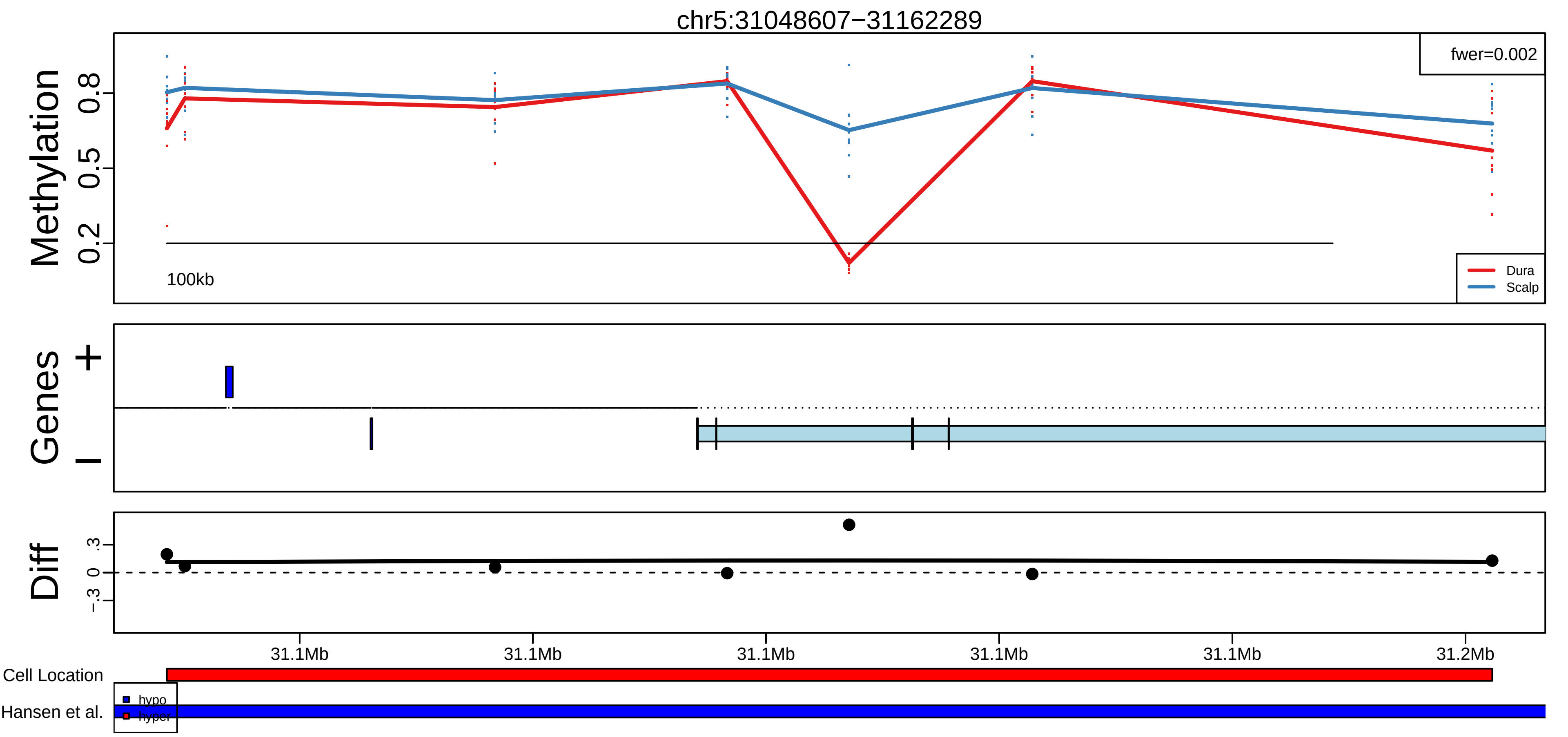

chr5:31048607−31162289
fwer=0.002
Methylation
0.2
0.5
0.8
100kb
Dura
Scalp
Genes
+
−
Diff
−.3
0
.3
31.1Mb
31.1Mb
31.1Mb
31.1Mb
31.1Mb
31.2Mb
Cell Location
Hansen et al.
hypo
hyper

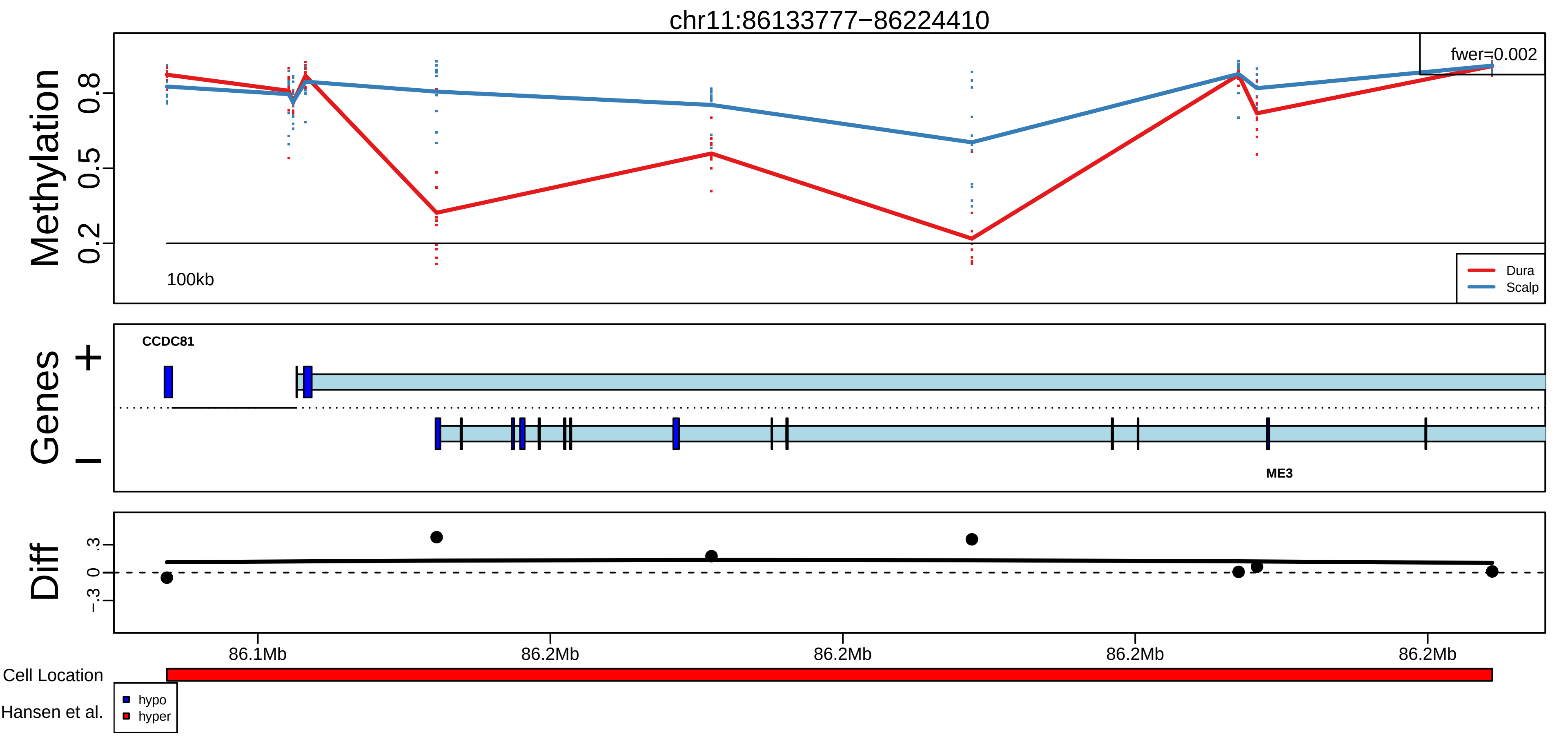

chr11:86133777−86224410
fwer=0.002
Methylation
0.2
0.5
0.8
100kb
Dura
Scalp
Genes
+
−
CCDC81
ME3
Diff
−.3
0
.3
86.1Mb
86.2Mb
86.2Mb
86.2Mb
86.2Mb
Cell Location
Hansen et al.
hypo
hyper

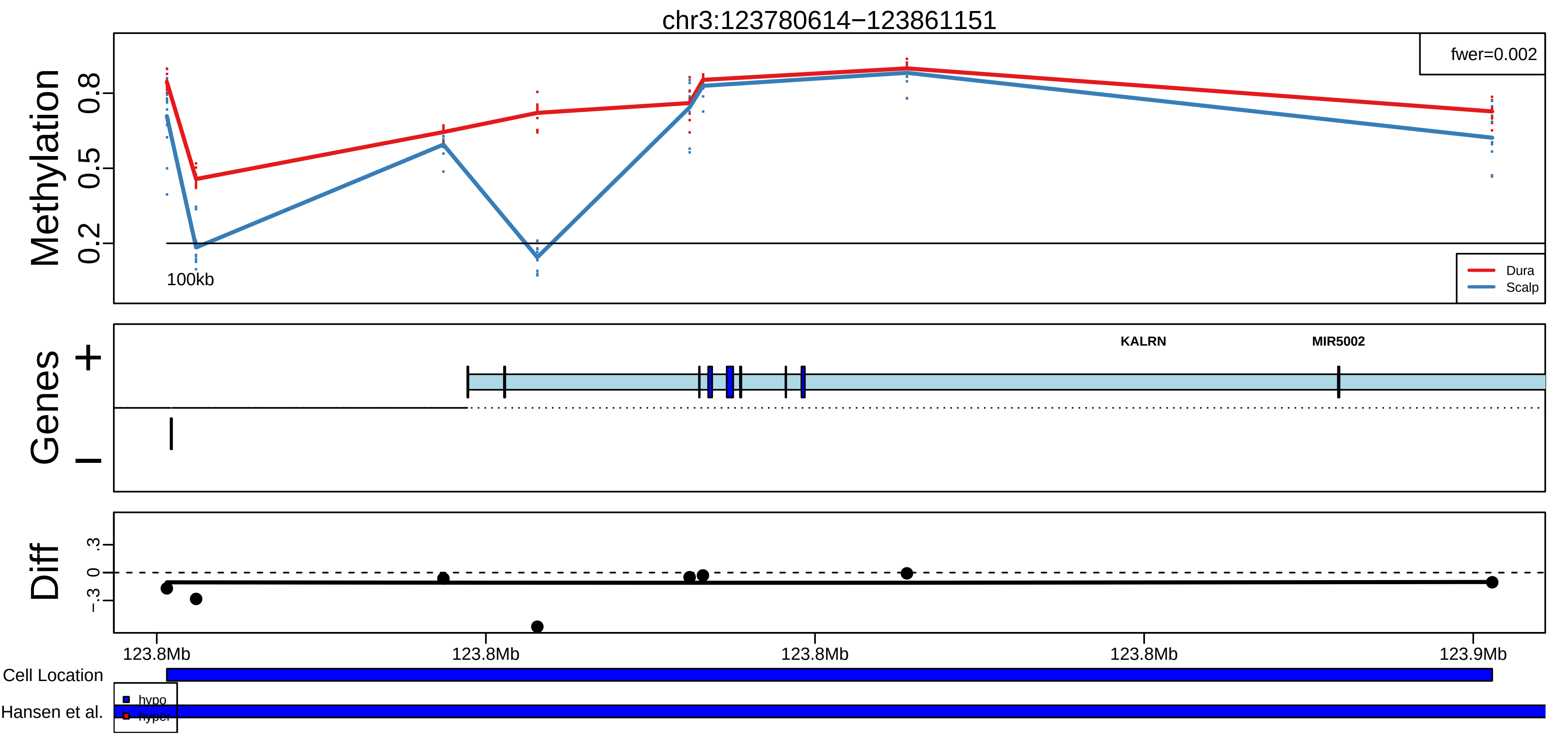

chr3:123780614−123861151
fwer=0.002
Methylation
0.2
0.5
0.8
100kb
Dura
Scalp
Genes
+
−
KALRN
MIR5002
Diff
−.3
0
.3
123.8Mb
123.8Mb
123.8Mb
123.8Mb
123.9Mb
Cell Location
Hansen et al.
hypo
hyper

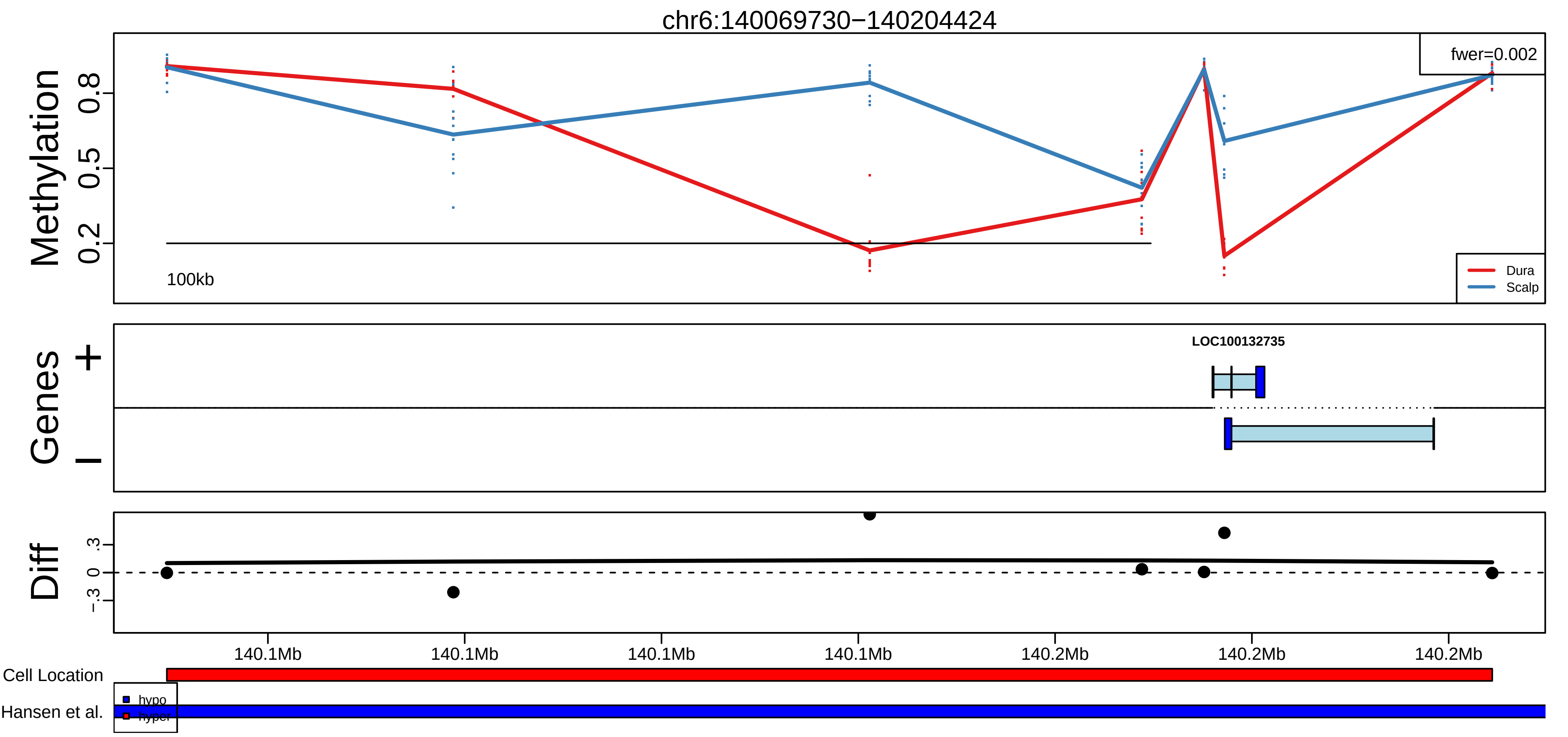

chr6:140069730−140204424
fwer=0.002
Methylation
0.2
0.5
0.8
100kb
Dura
Scalp
Genes
+
−
LOC100132735
Diff
-.3
0
.3
140.1Mb
140.1Mb
140.1Mb
140.1Mb
140.2Mb
140.2Mb
140.2Mb
Cell Location
Hansen et al.
hypo
hyper

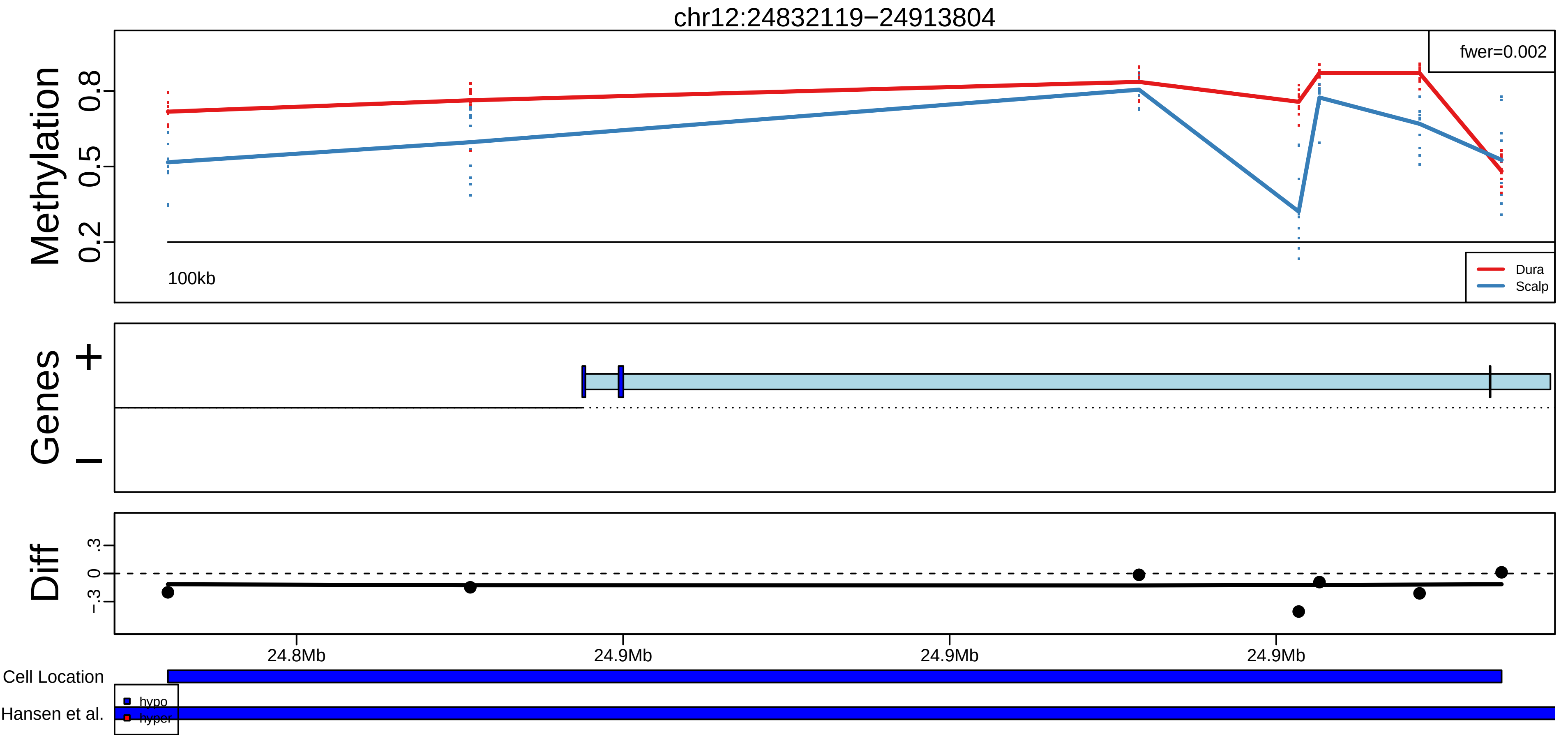

chr12:24832119−24913804
fwer=0.002
Methylation
0.2
0.5
0.8
100kb
Dura
Scalp
Genes
+
−
Diff
−.3
0
.3
24.8Mb
24.9Mb
24.9Mb
24.9Mb
Cell Location
Hansen et al.
hypo
hyper

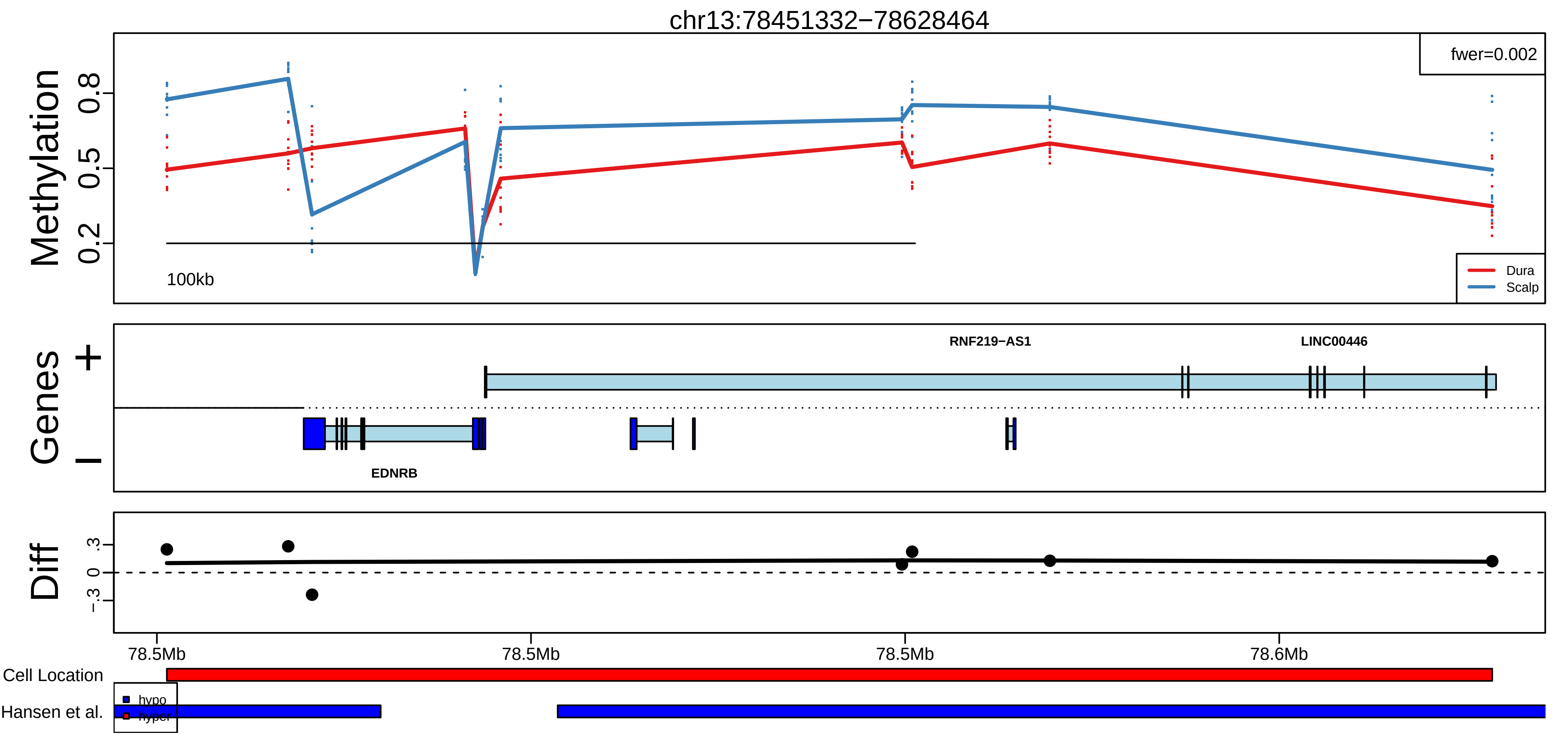
chr13:78451332−78628464
fwer=0.002
Methylation
0.2
0.5
0.8
100kb
Dura
Scalp
Genes
+
−
RNF219−AS1
LINC00446
EDNRB
Diff
−.3
0
.3
78.5Mb
78.5Mb
78.5Mb
78.6Mb
Cell Location
Hansen et al.
hypo
hyper

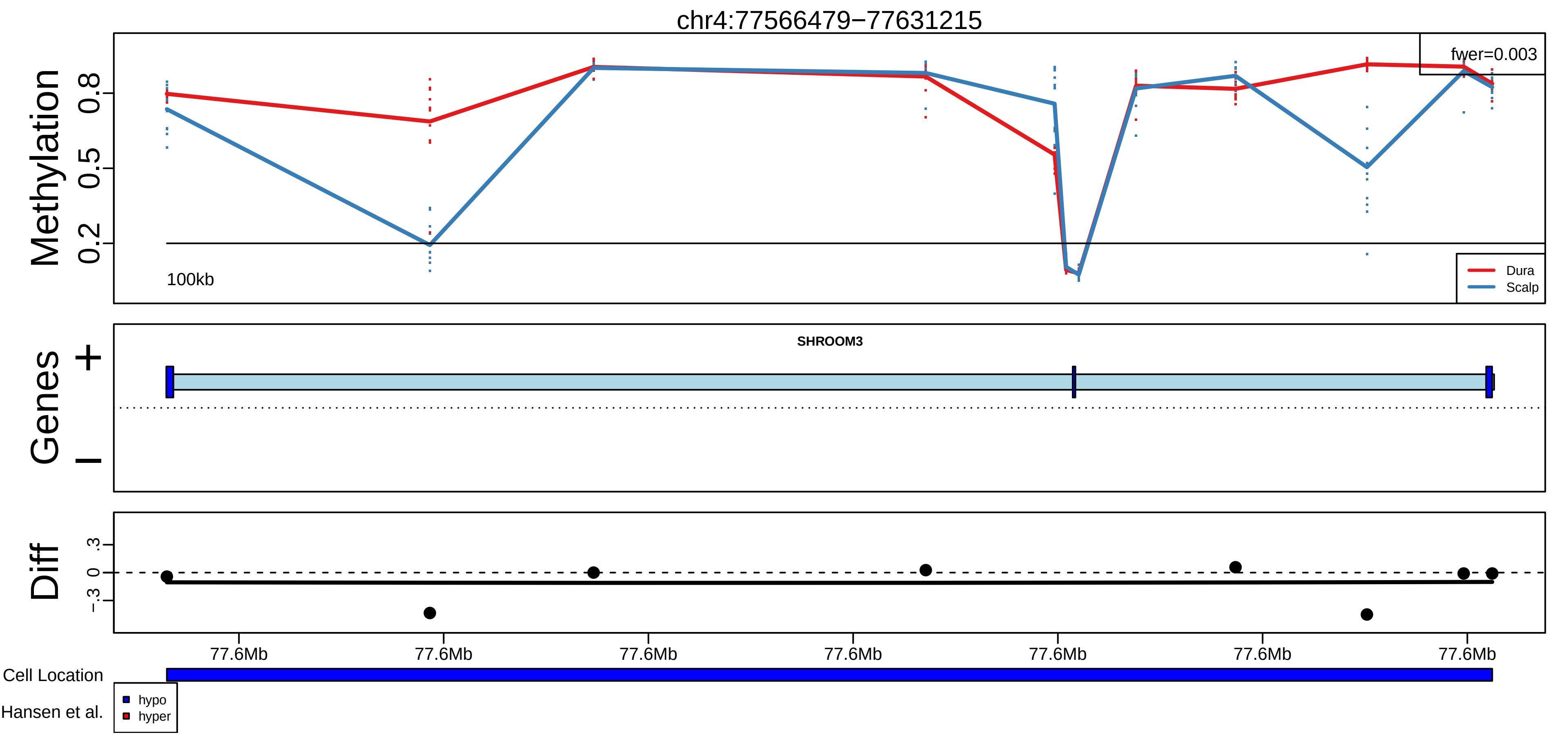

chr4:77566479−77631215
fwer=0.003
Methylation
0.2
0.5
0.8
100kb
Dura
Scalp
Genes
+
−
SHROOM3
Diff
−.3
0
.3
77.6Mb
77.6Mb
77.6Mb
77.6Mb
77.6Mb
77.6Mb
77.6Mb
Cell Location
Hansen et al.
hypo
hyper

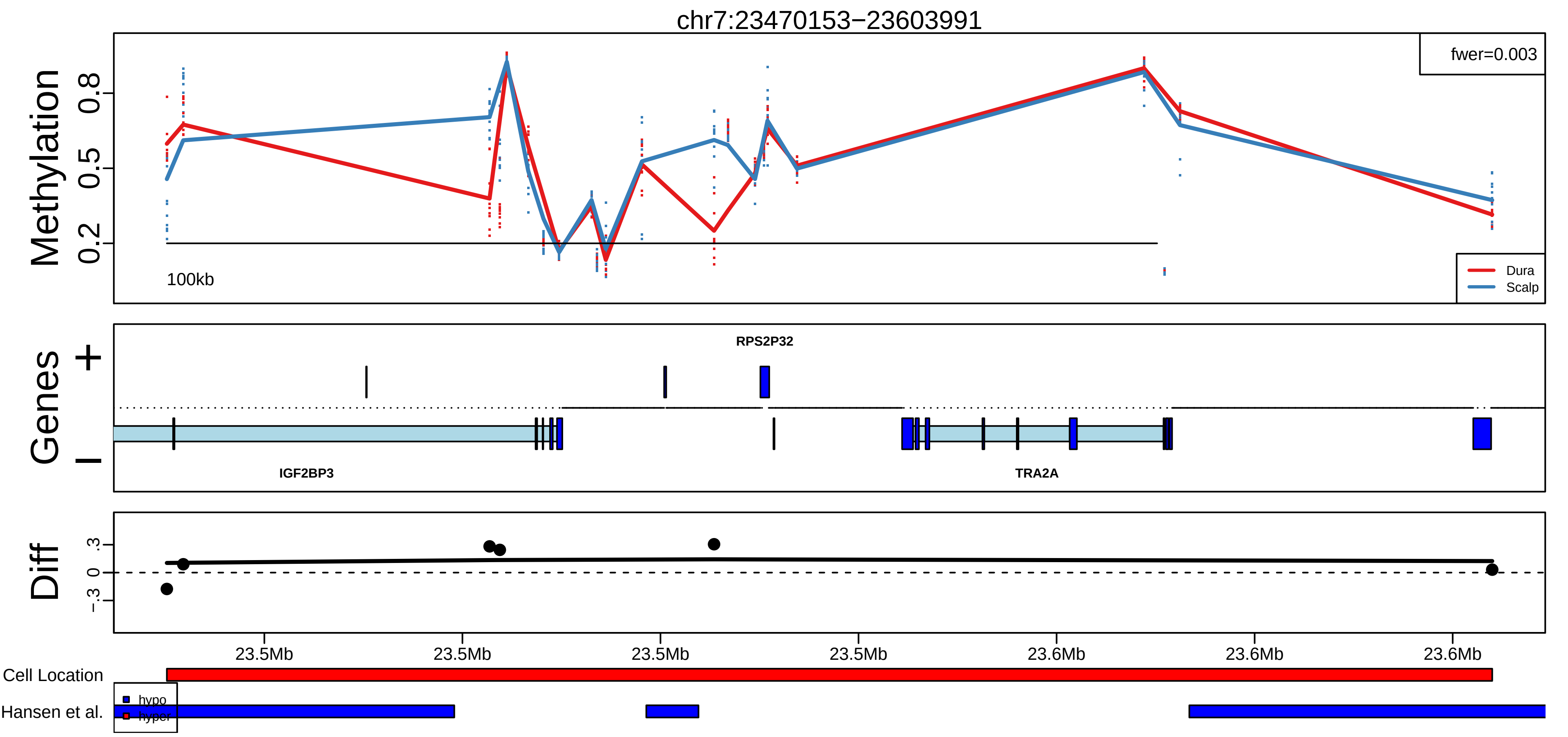
chr7:23470153−23603991
fwer=0.003
Methylation
0.2
0.5
0.8
100kb
Dura
Scalp
Genes
+
−
RPS2P32
IGF2BP3
TRA2A
Diff
.3
0
−.3
23.5Mb
23.5Mb
23.5Mb
23.5Mb
23.6Mb
23.6Mb
23.6Mb
Cell Location
Hansen et al.
hypo
hyper

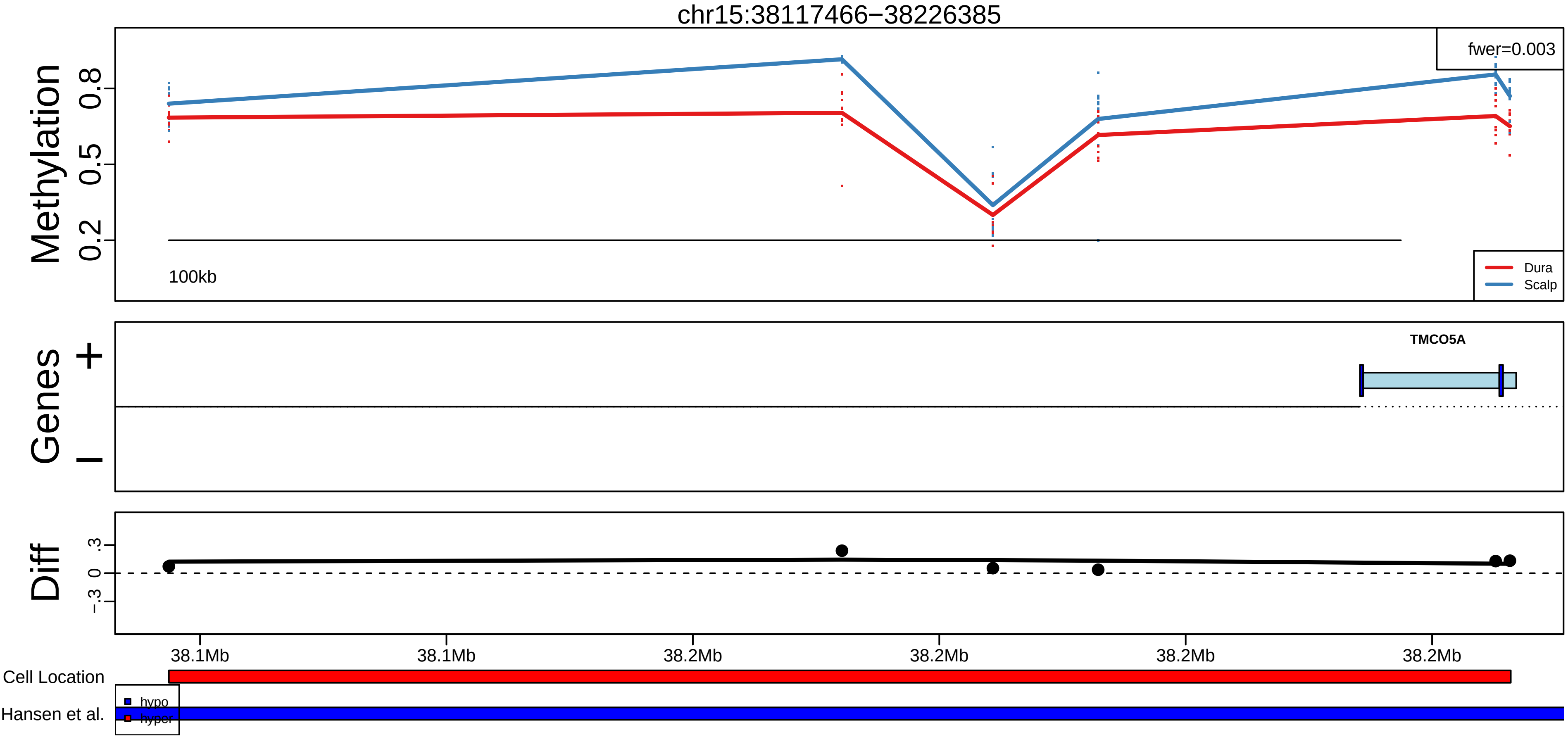

chr15:38117466−38226385
fwer=0.003
Methylation
0.2
0.5
0.8
100kb
Dura
Scalp
Genes
+
−
TMCO5A
Diff
−.3
0
.3
38.1Mb
38.1Mb
38.2Mb
38.2Mb
38.2Mb
38.2Mb
Cell Location
Hansen et al.
hypo
hyper

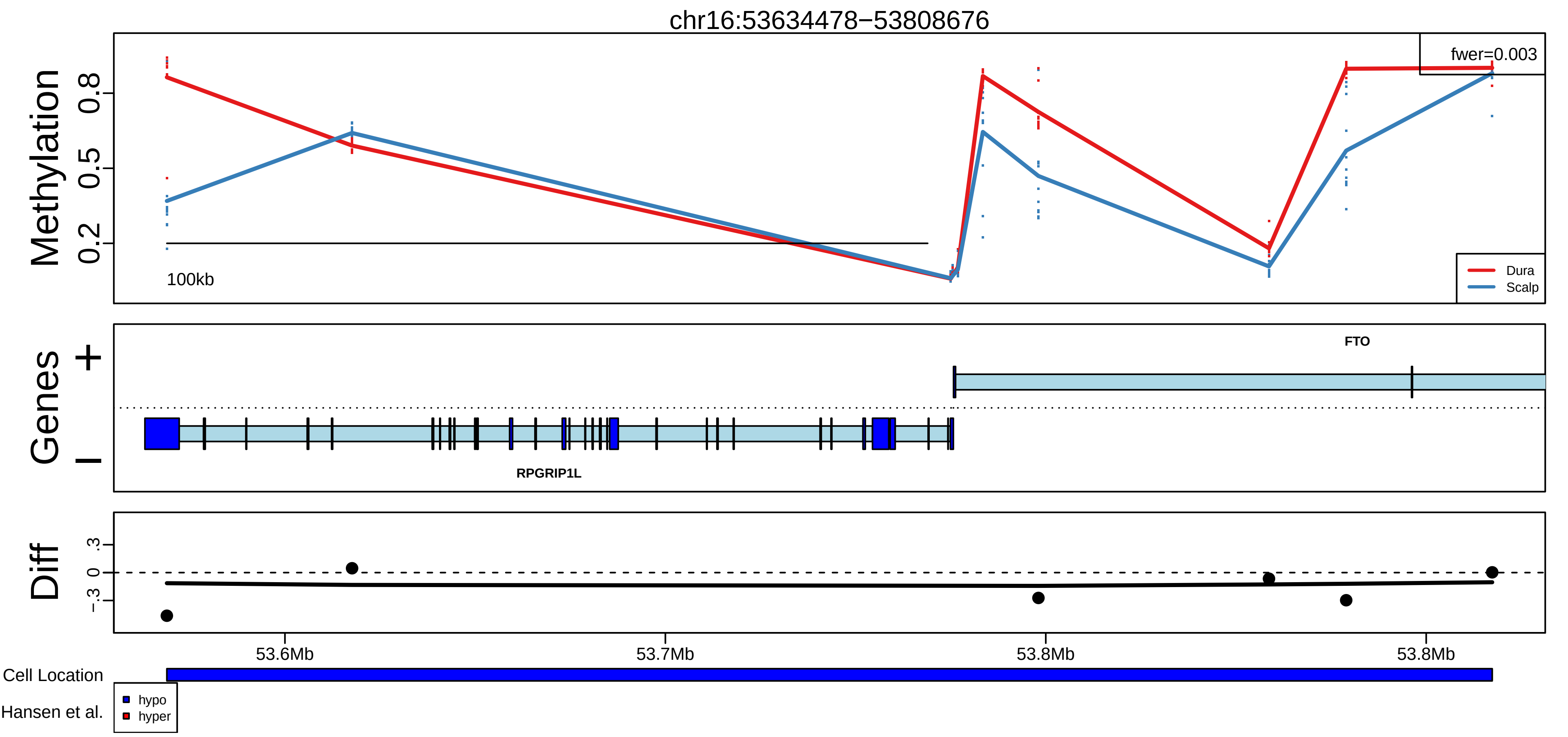
chr16:53634478−53808676
fwer=0.003
Methylation
0.2
0.5
0.8
100kb
Dura
Scalp
Genes
+
−
FTO
RPGRIP1L
Diff
−.3
0
.3
53.6Mb
53.7Mb
53.8Mb
53.8Mb
Cell Location
Hansen et al.
hypo
hyper

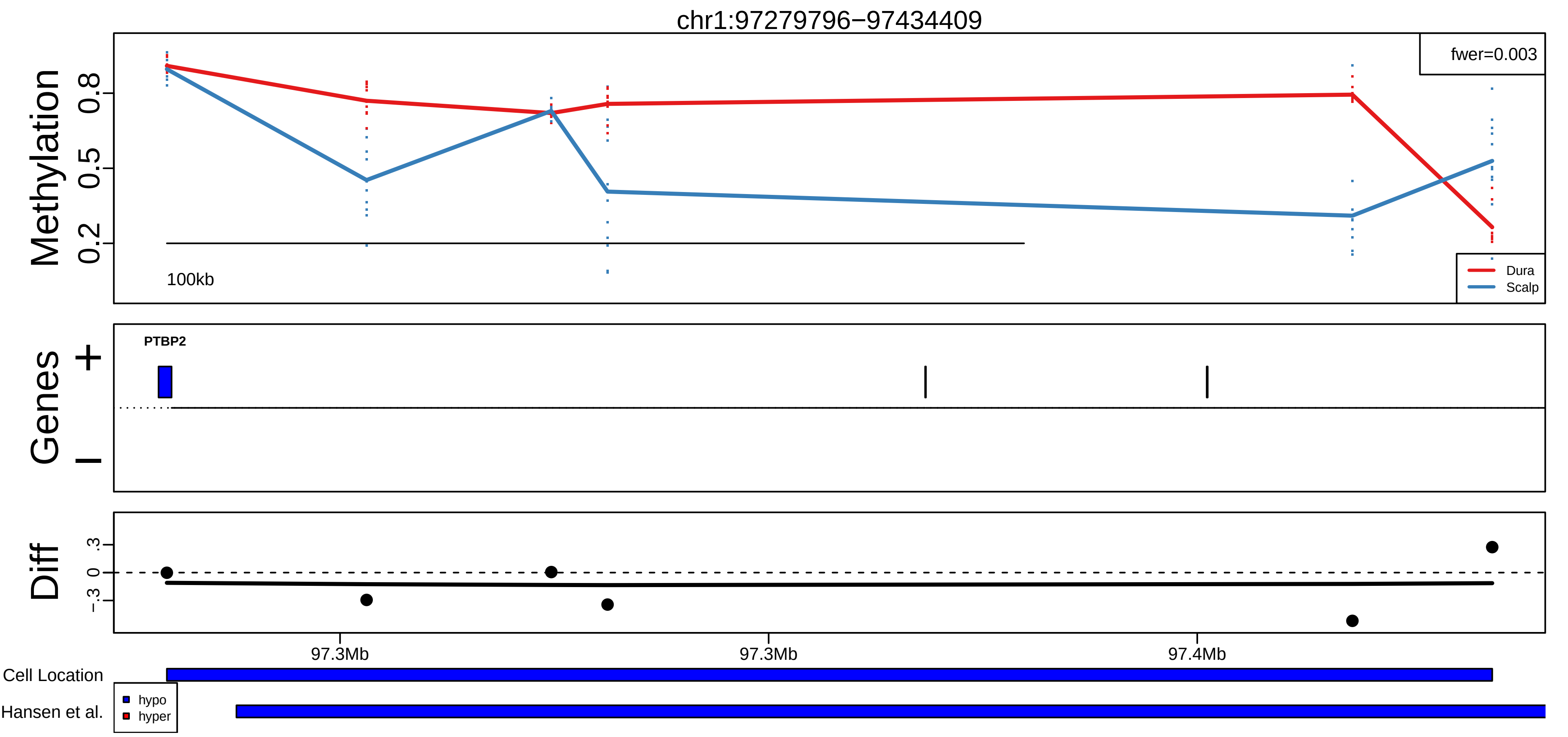

chr1:97279796−97434409
fwer=0.003
Methylation
0.2
0.5
0.8
100kb
Dura
Scalp
Genes
+
−
PTBP2
Diff
−.3
0
.3
97.3Mb
97.3Mb
97.4Mb
Cell Location
Hansen et al.
hypo
hyper

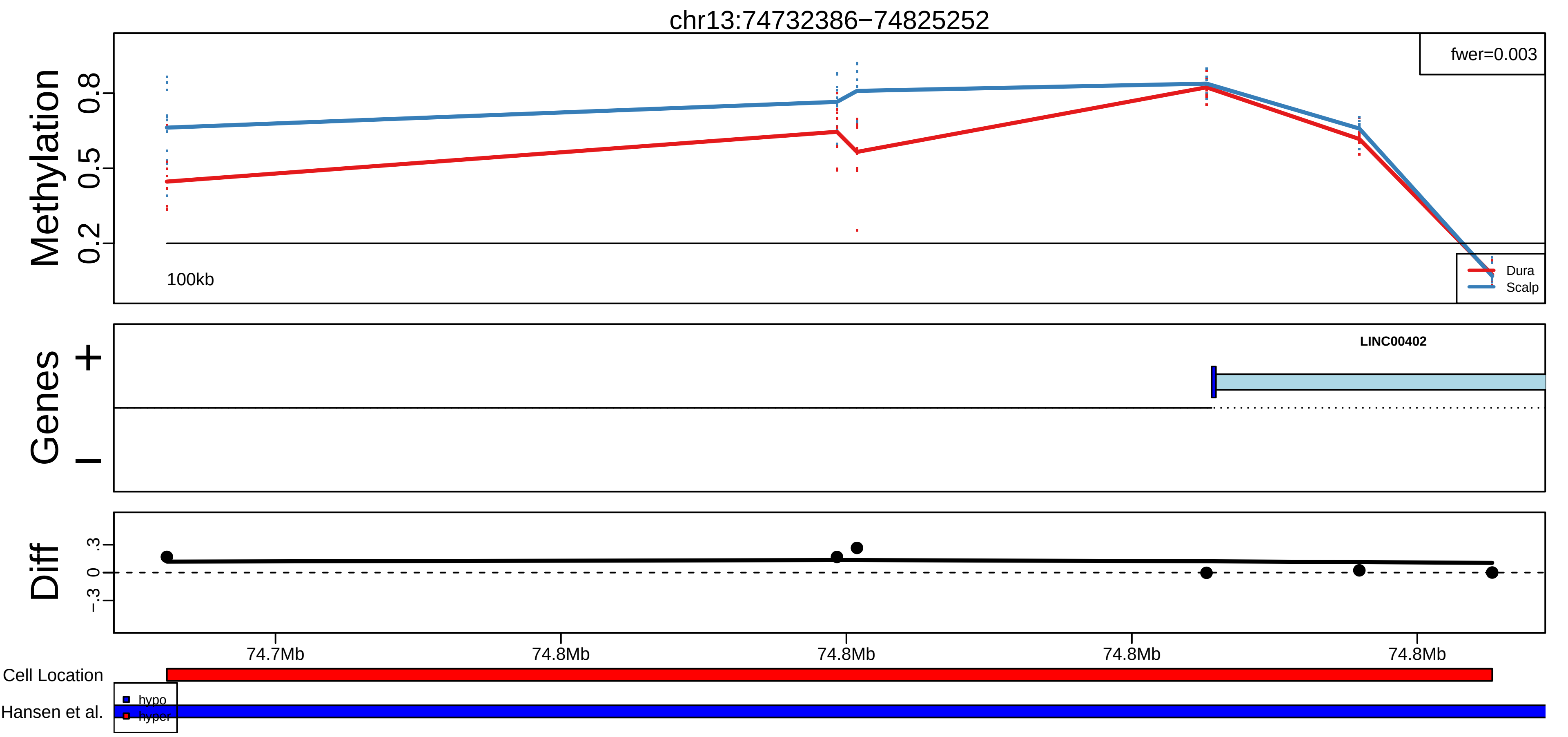

chr13:74732386−74825252
fwer=0.003
Methylation
0.2
0.5
0.8
100kb
Dura
Scalp
Genes
+
−
LINC00402
Diff
−.3
0
.3
74.7Mb
74.8Mb
74.8Mb
74.8Mb
74.8Mb
Cell Location
Hansen et al.
hypo
hyper

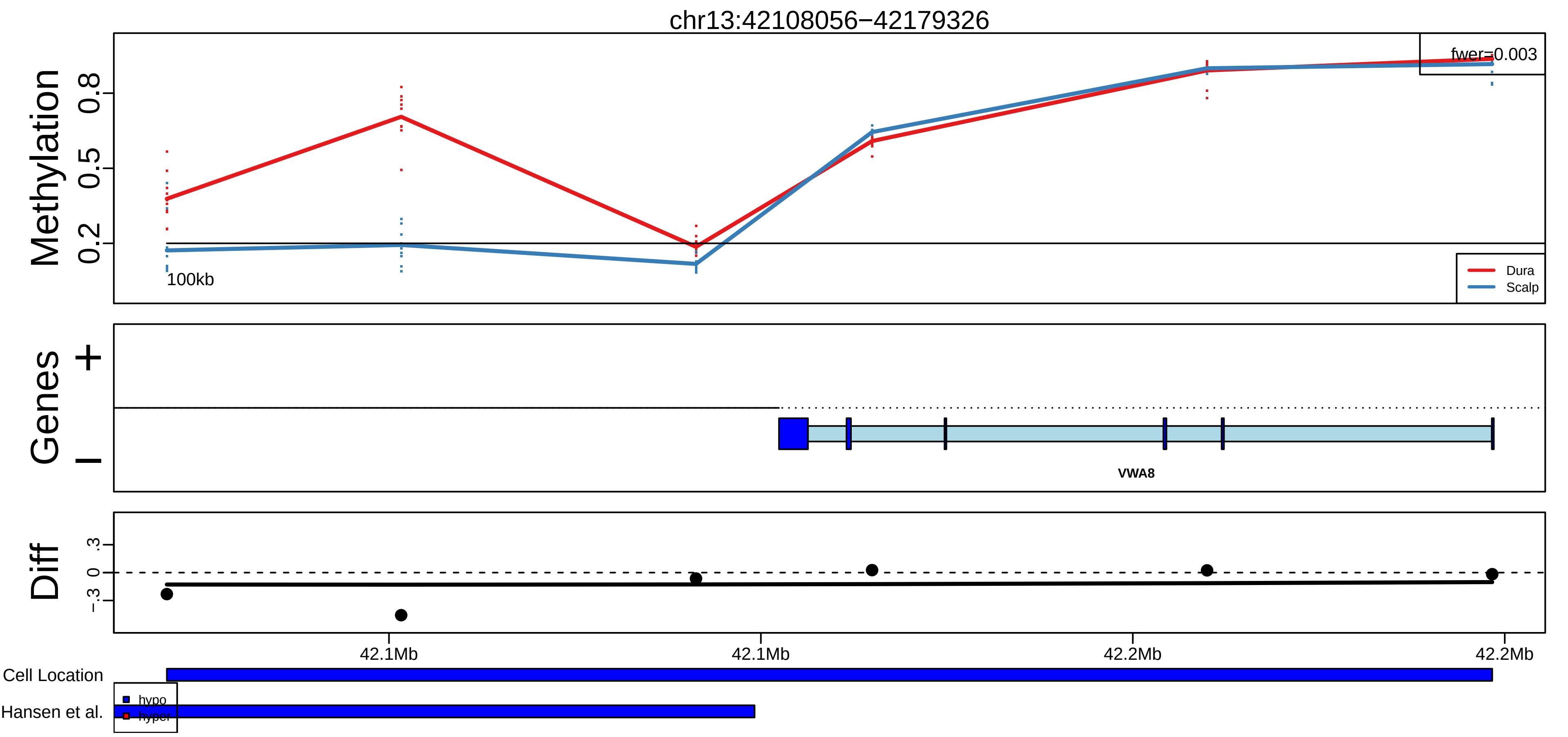
chr13:42108056−42179326
Methylation
0.2
0.5
0.8
fwer=0.003
100kb
Dura
Scalp
Genes
+
−
VWA8
Diff
−.3
0
.3
42.1Mb
42.1Mb
42.2Mb
42.2Mb
Cell Location
Hansen et al.
hypo
hyper

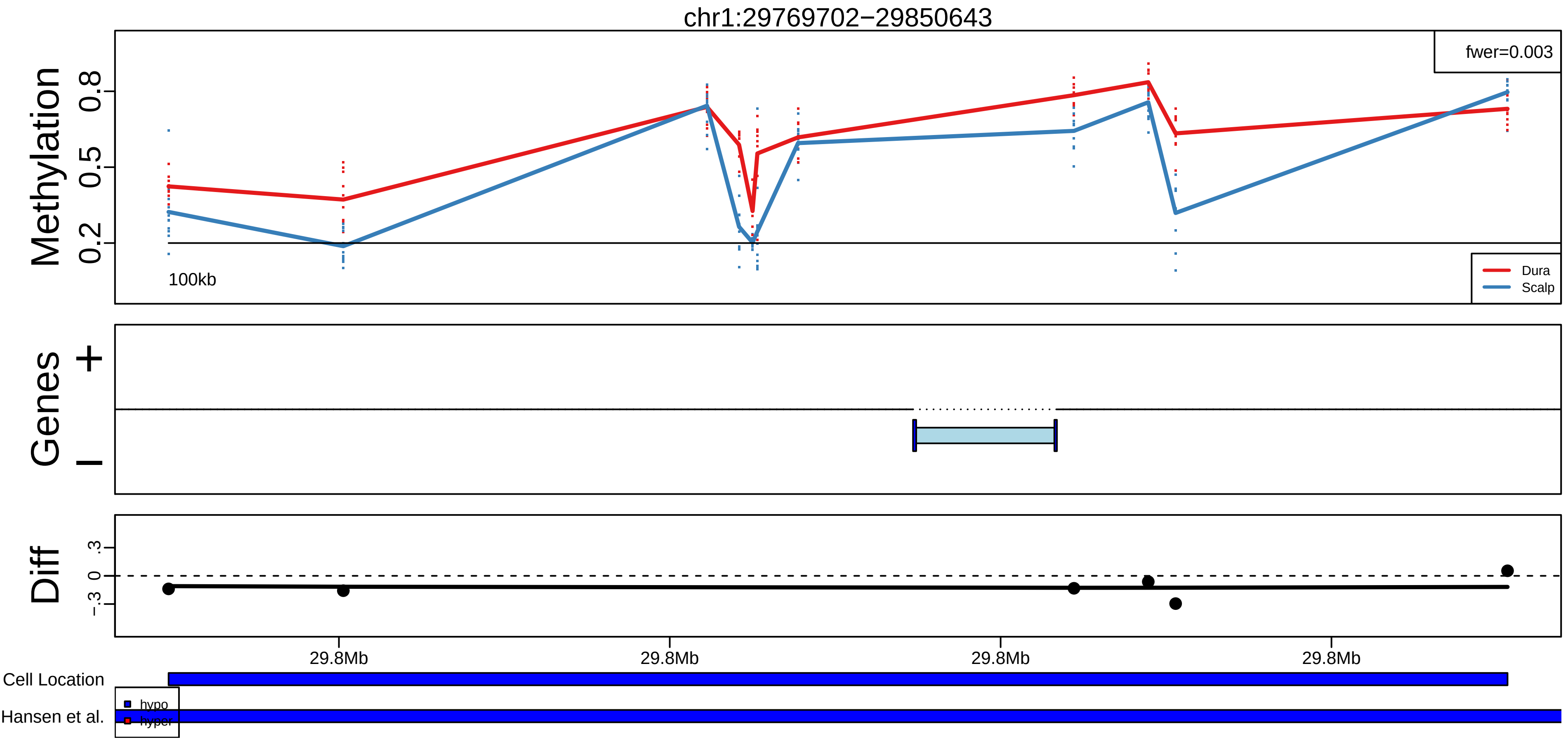
chr1:29769702−29850643
fwer=0.003
Methylation
0.2
0.5
0.8
100kb
Dura
Scalp
Genes
+
−
Diff
−.3
0
.3
29.8Mb
29.8Mb
29.8Mb
29.8Mb
Cell Location
Hansen et al.
hypo
hyper

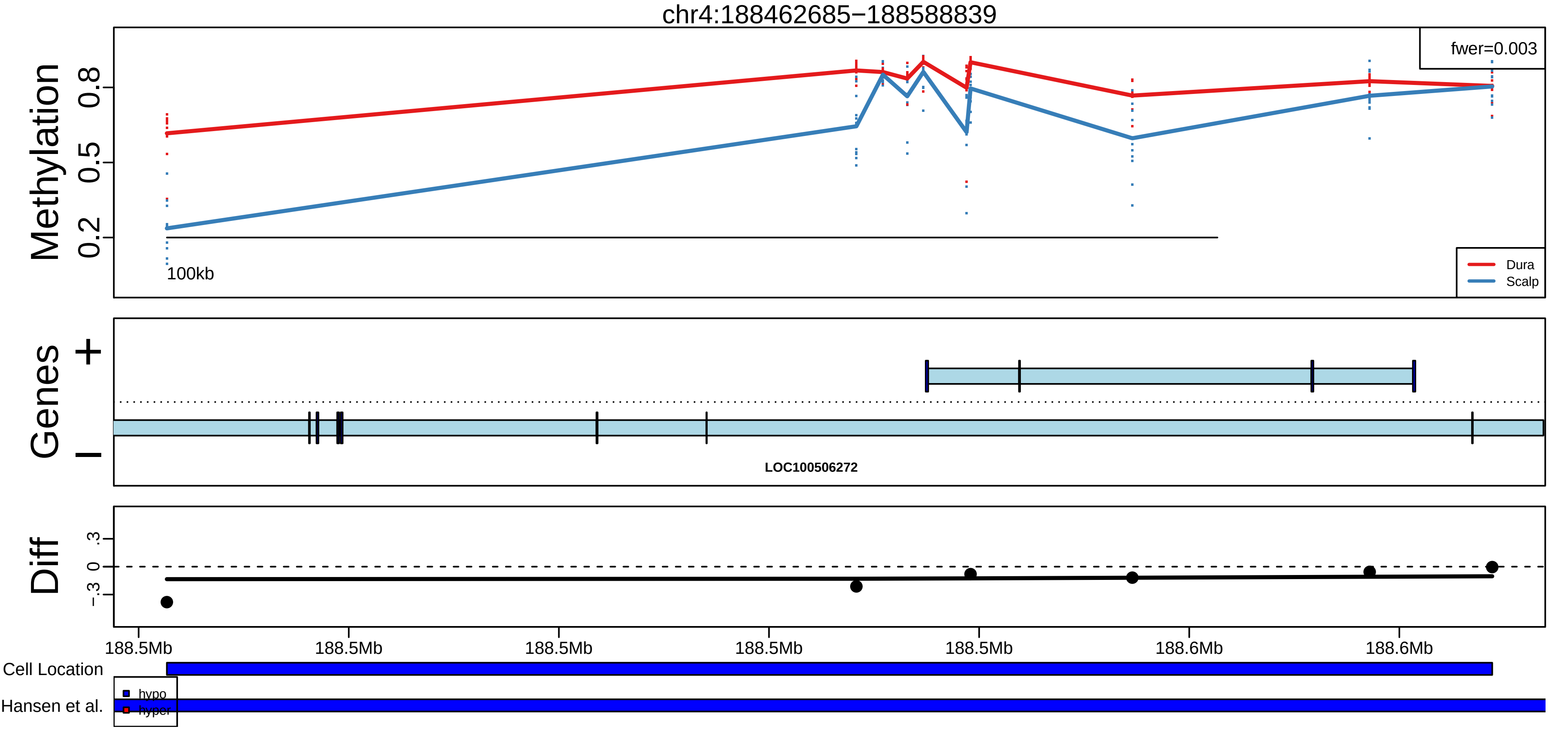

chr4:188462685−188588839
fwer=0.003
Methylation
0.2
0.5
0.8
100kb
Dura
Scalp
Genes
+
−
LOC100506272
Diff
−.3
0
.3
188.5Mb
188.5Mb
188.5Mb
188.5Mb
188.5Mb
188.6Mb
188.6Mb
Cell Location
Hansen et al.
hypo
hyper

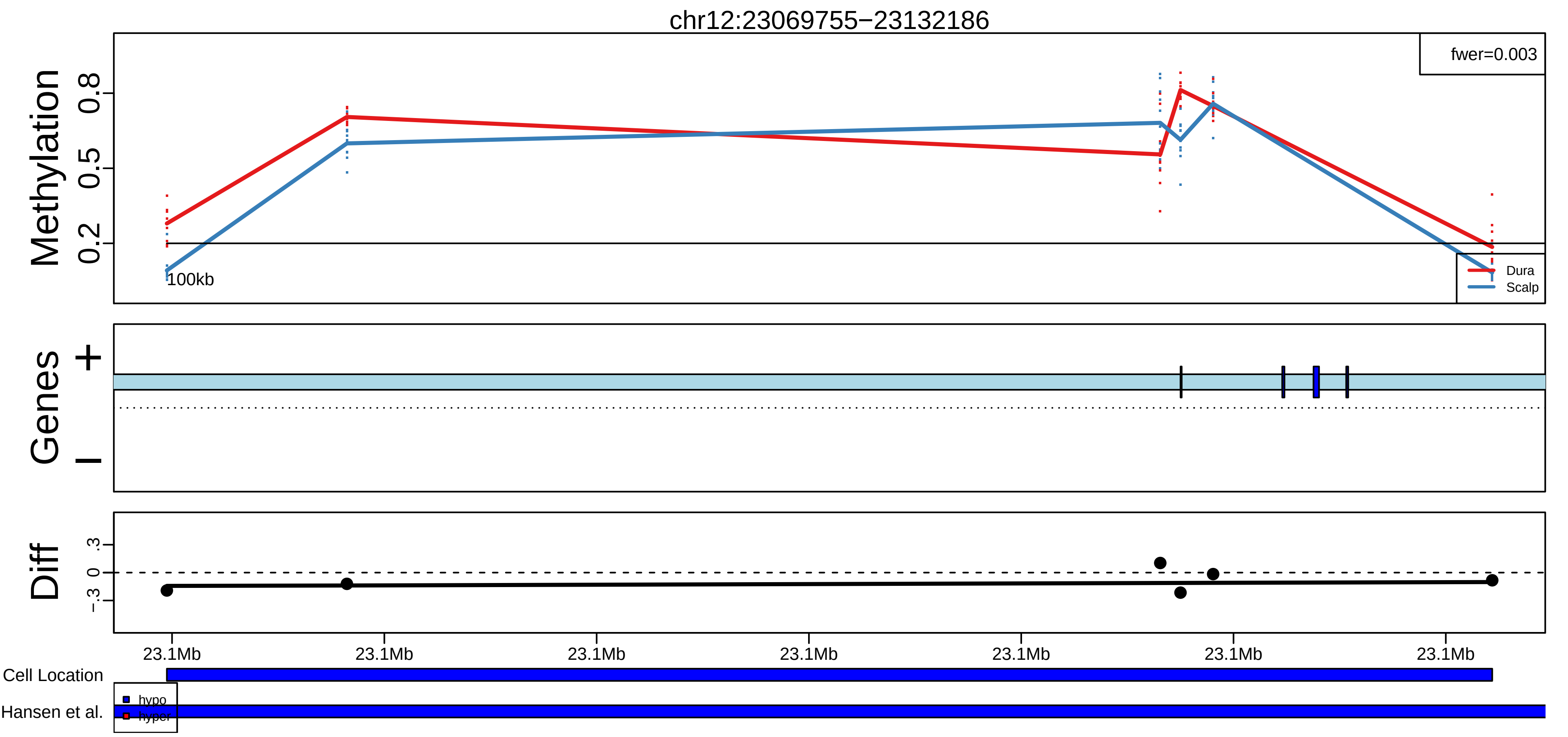

chr12:23069755−23132186
fwer=0.003
Methylation
0.2
0.5
0.8
100kb
Dura
Scalp
Genes
+
−
Diff
−.3
0
.3
23.1Mb
23.1Mb
23.1Mb
23.1Mb
23.1Mb
23.1Mb
23.1Mb
Cell Location
Hansen et al.
hypo
hyper

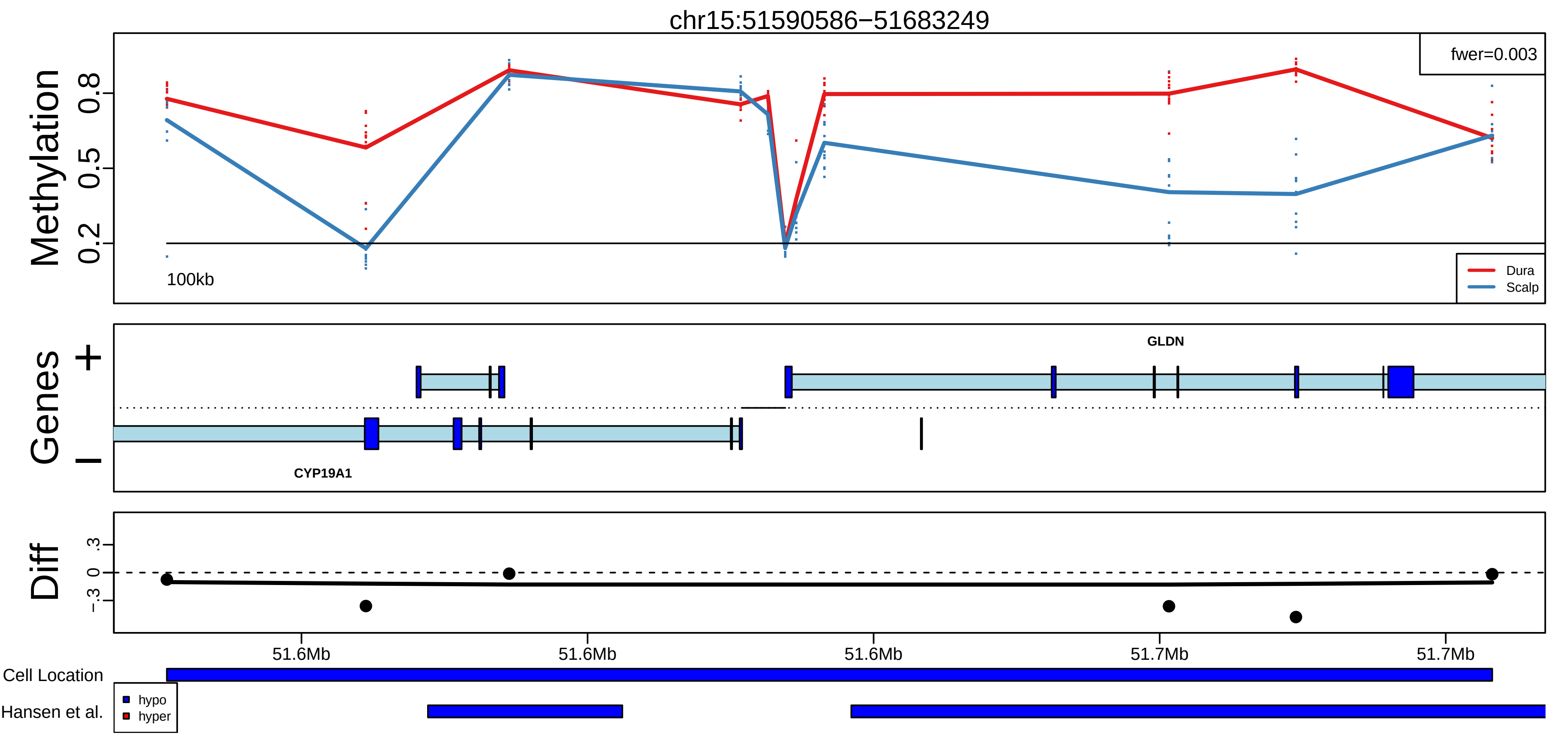

chr15:51590586−51683249
fwer=0.003
Methylation
0.2
0.5
0.8
100kb
Dura
Scalp
Genes
+
−
GLDN
CYP19A1
Diff
−.3
0
.3
51.6Mb
51.6Mb
51.6Mb
51.7Mb
51.7Mb
Cell Location
Hansen et al.
hypo
hyper

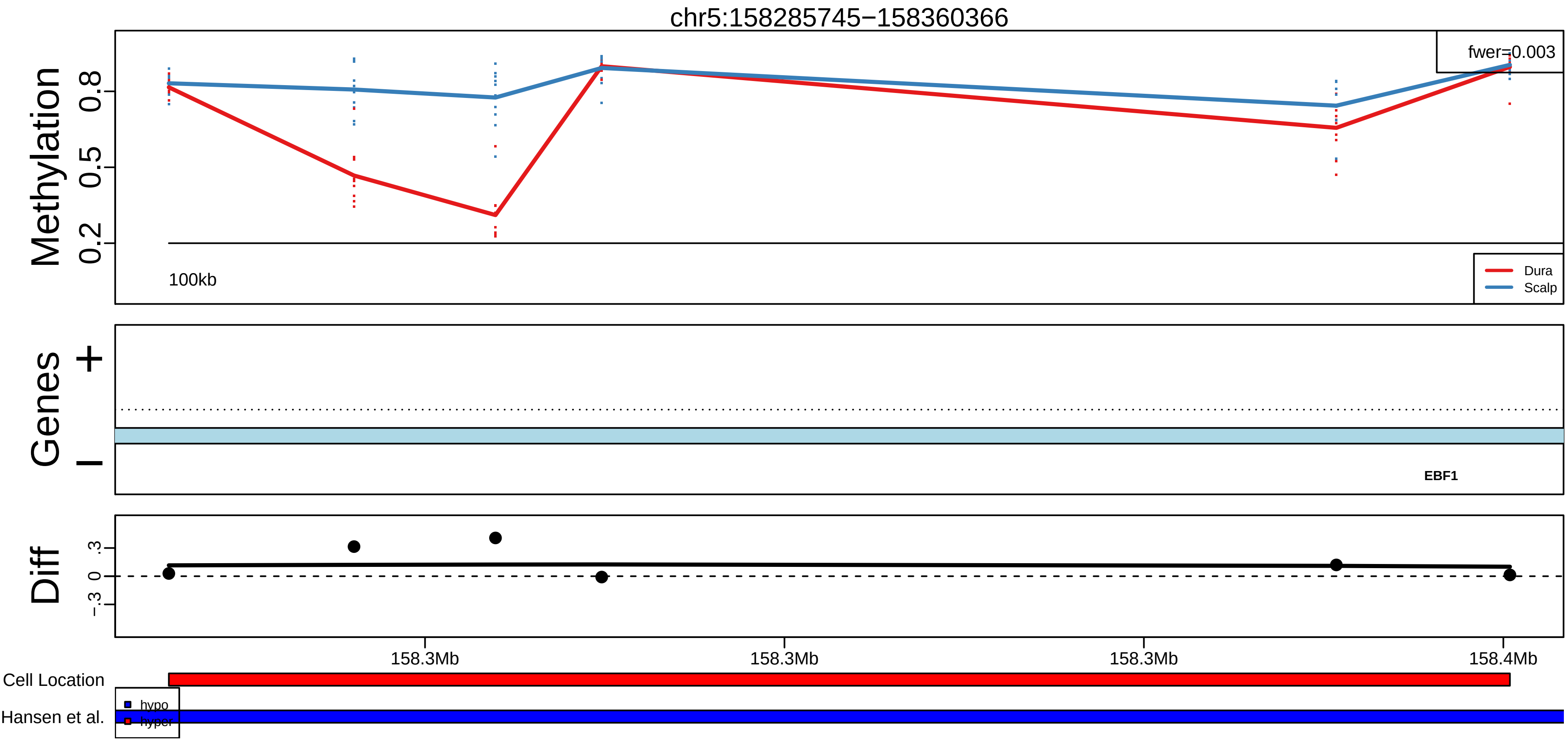

chr5:158285745−158360366
Methylation
0.2
0.5
0.8
fwer=0.003
100kb
Dura
Scalp
Genes
+
−
EBF1
Diff
−.3
0
.3
158.3Mb
158.3Mb
158.3Mb
158.4Mb
Cell Location
Hansen et al.
hypo
hyper

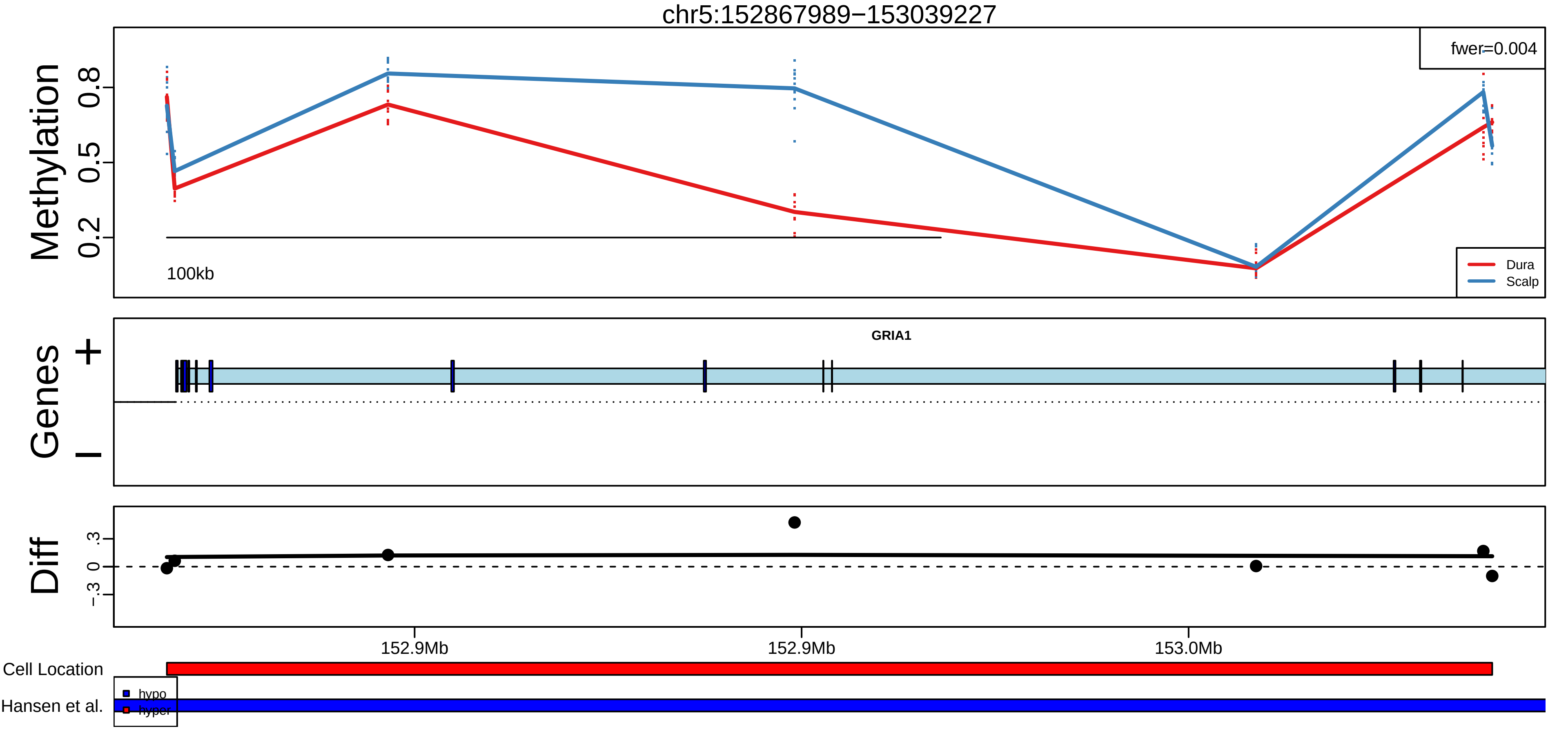
chr5:152867989−153039227
fwer=0.004
Methylation
0.2
0.5
0.8
100kb
Dura
Scalp
Genes
+
−
GRIA1
Diff
−.3
0
.3
152.9Mb
152.9Mb
153.0Mb
Cell Location
Hansen et al.
hypo
hyper

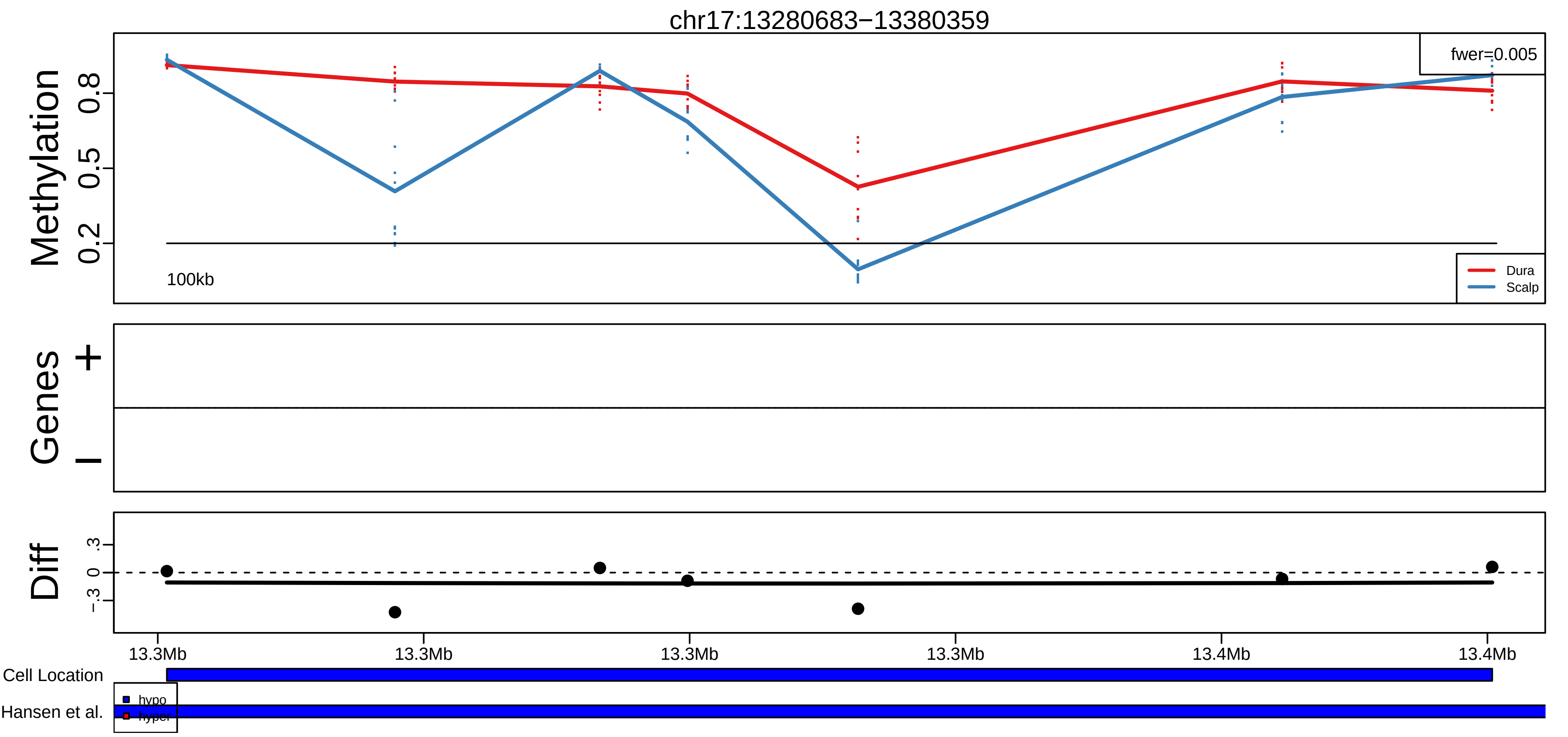
chr17:13280683−13380359
fwer=0.005
Methylation
0.2
0.5
0.8
100kb
Dura
Scalp
Genes
+
−
Diff
−.3
0
.3
13.3Mb
13.3Mb
13.3Mb
13.3Mb
13.4Mb
13.4Mb
Cell Location
Hansen et al.
hypo
hyper

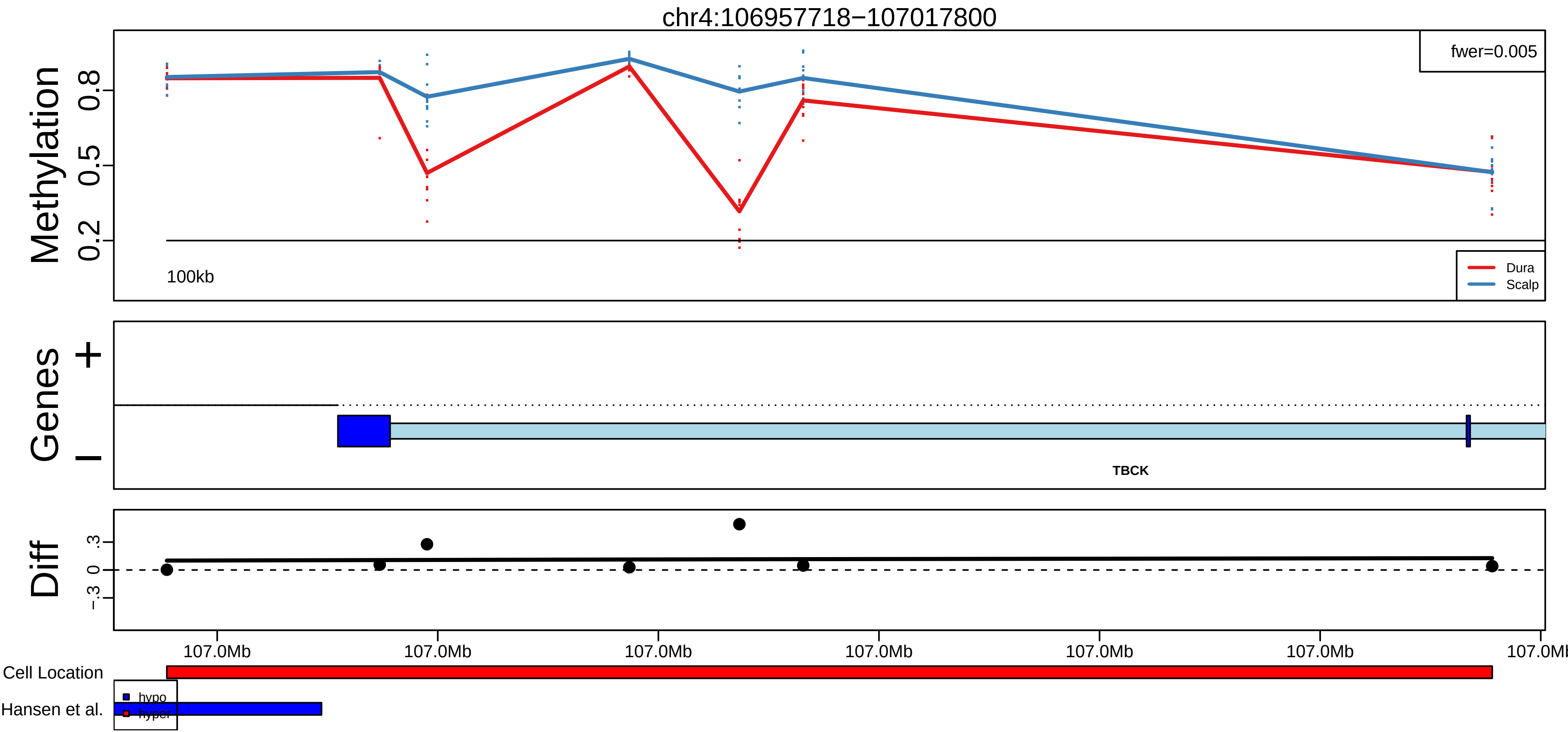

chr4:106957718−107017800
fwer=0.005
Methylation
0.2
0.5
0.8
100kb
Dura
Scalp
Genes
+
−
TBCK
Diff
−.3
0
.3
107.0Mb
107.0Mb
107.0Mb
107.0Mb
107.0Mb
107.0Mb
Cell Location
Hansen et al.
hypo
hyper

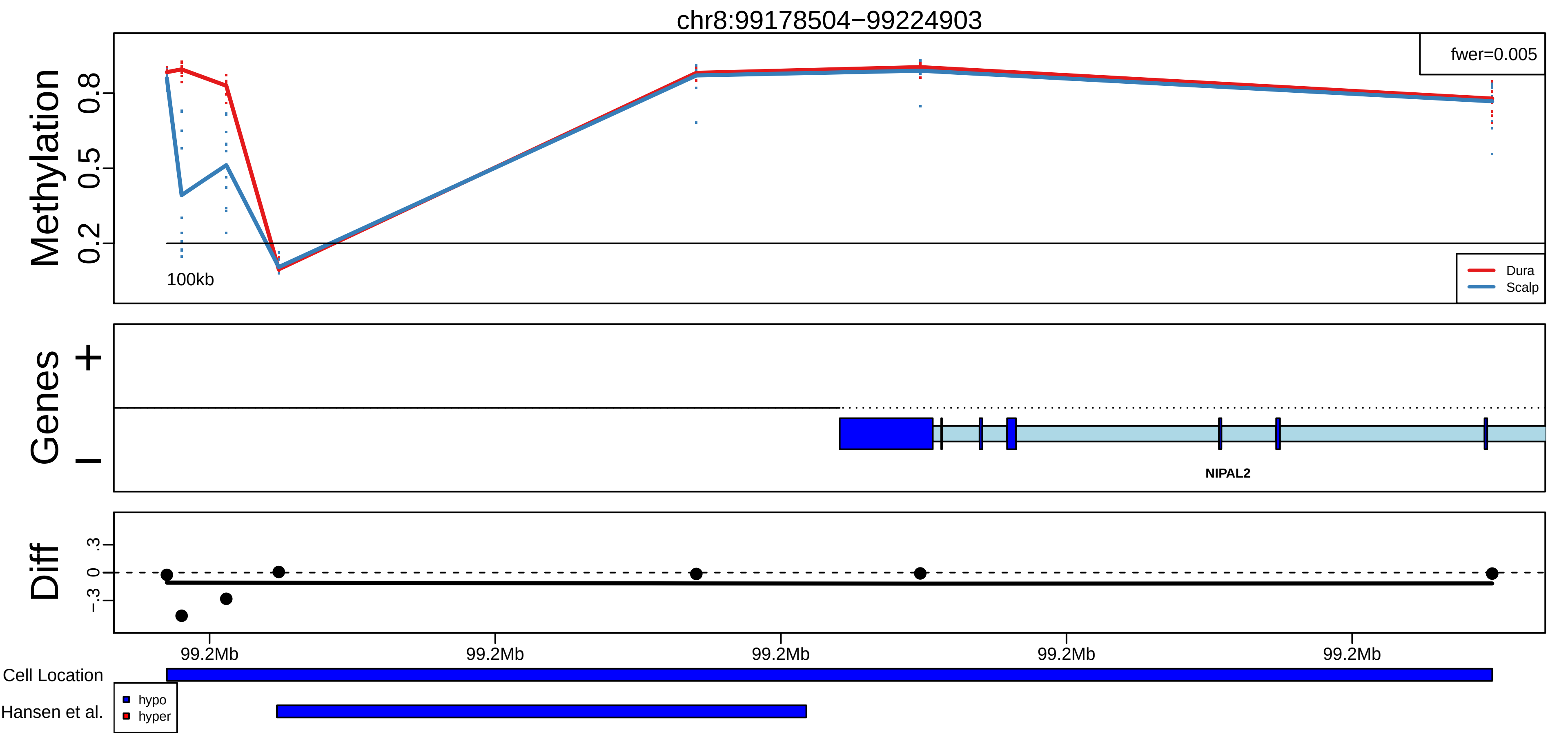
chr8:99178504−99224903
fwer=0.005
Methylation
0.2
0.5
0.8
100kb
Dura
Scalp
Genes
+
−
NIPAL2
Diff
.3
0
−.3
99.2Mb
99.2Mb
99.2Mb
99.2Mb
99.2Mb
Cell Location
Hansen et al.
hypo
hyper

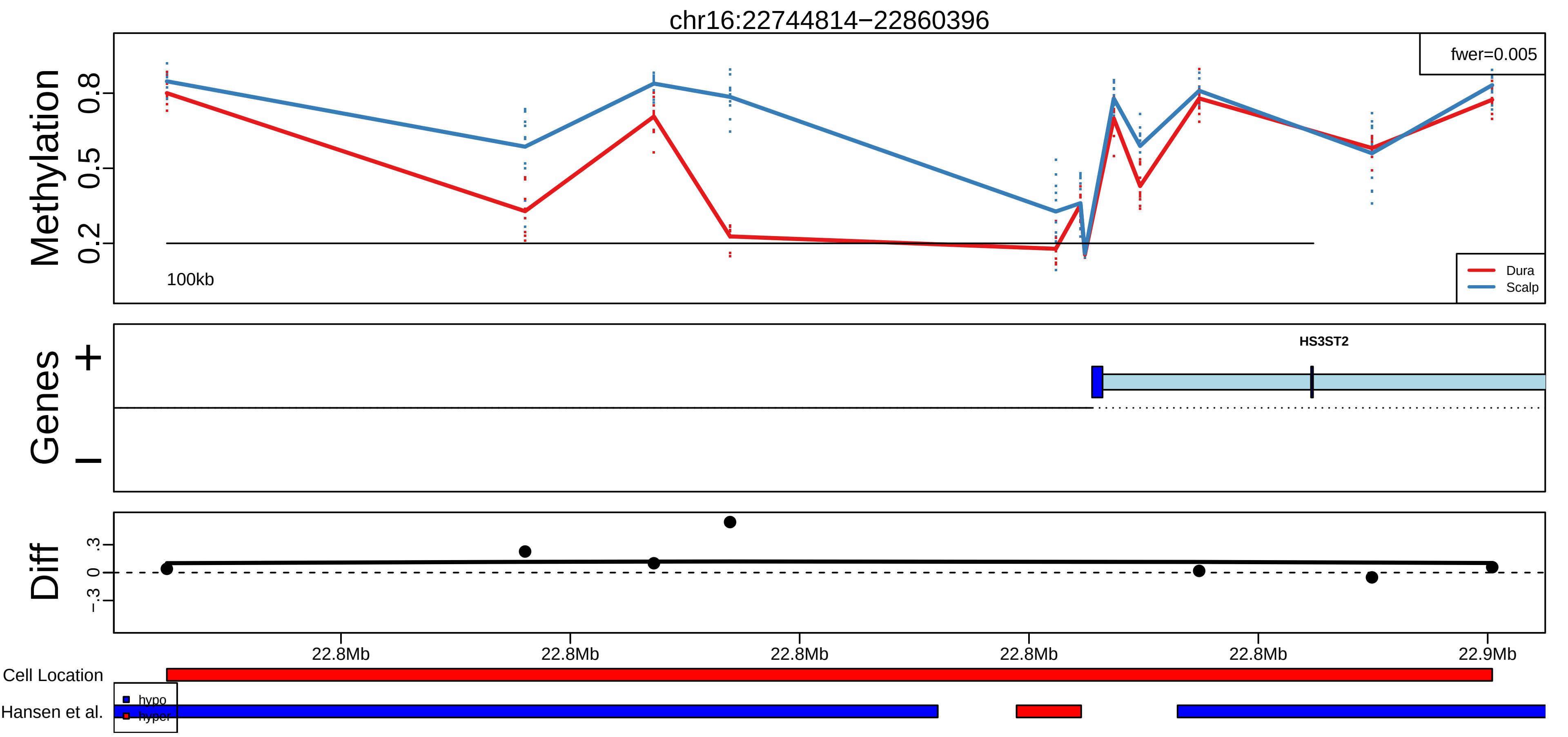

chr16:22744814−22860396
fwer=0.005
Methylation
0.2
0.5
0.8
100kb
Dura
Scalp
Genes
+
−
HS3ST2
Diff
−.3
0
.3
22.8Mb
22.8Mb
22.8Mb
22.8Mb
22.8Mb
22.9Mb
Cell Location
Hansen et al.
hypo
hyper

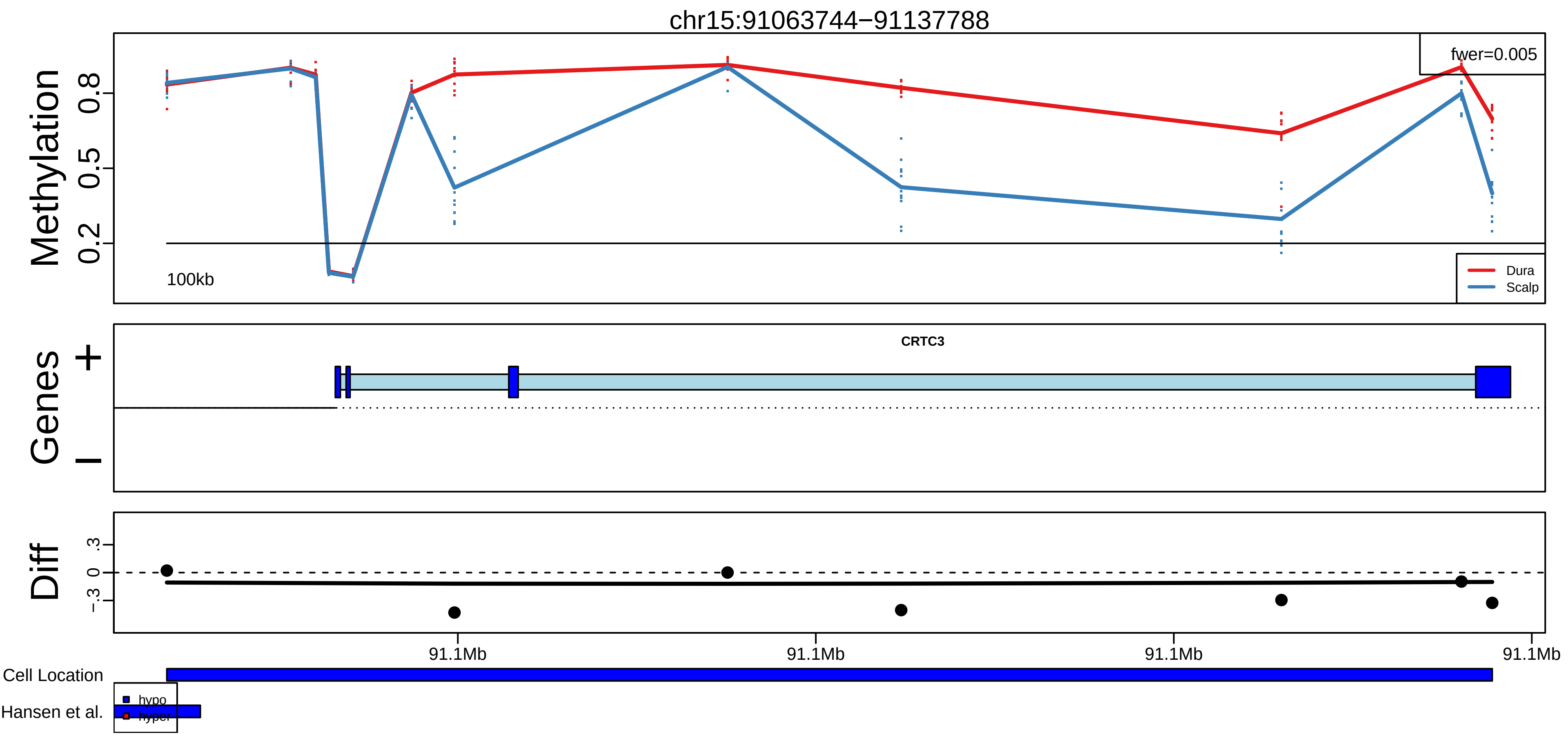

chr15:91063744−91137788
Methylation
0.2
0.5
0.8
fwer=0.005
100kb
Dura
Scalp
Genes
+
−
CRTC3
Diff
−.3
0
.3
91.1Mb
91.1Mb
91.1Mb
91.1Mb
Cell Location
Hansen et al.
hypo
hyper

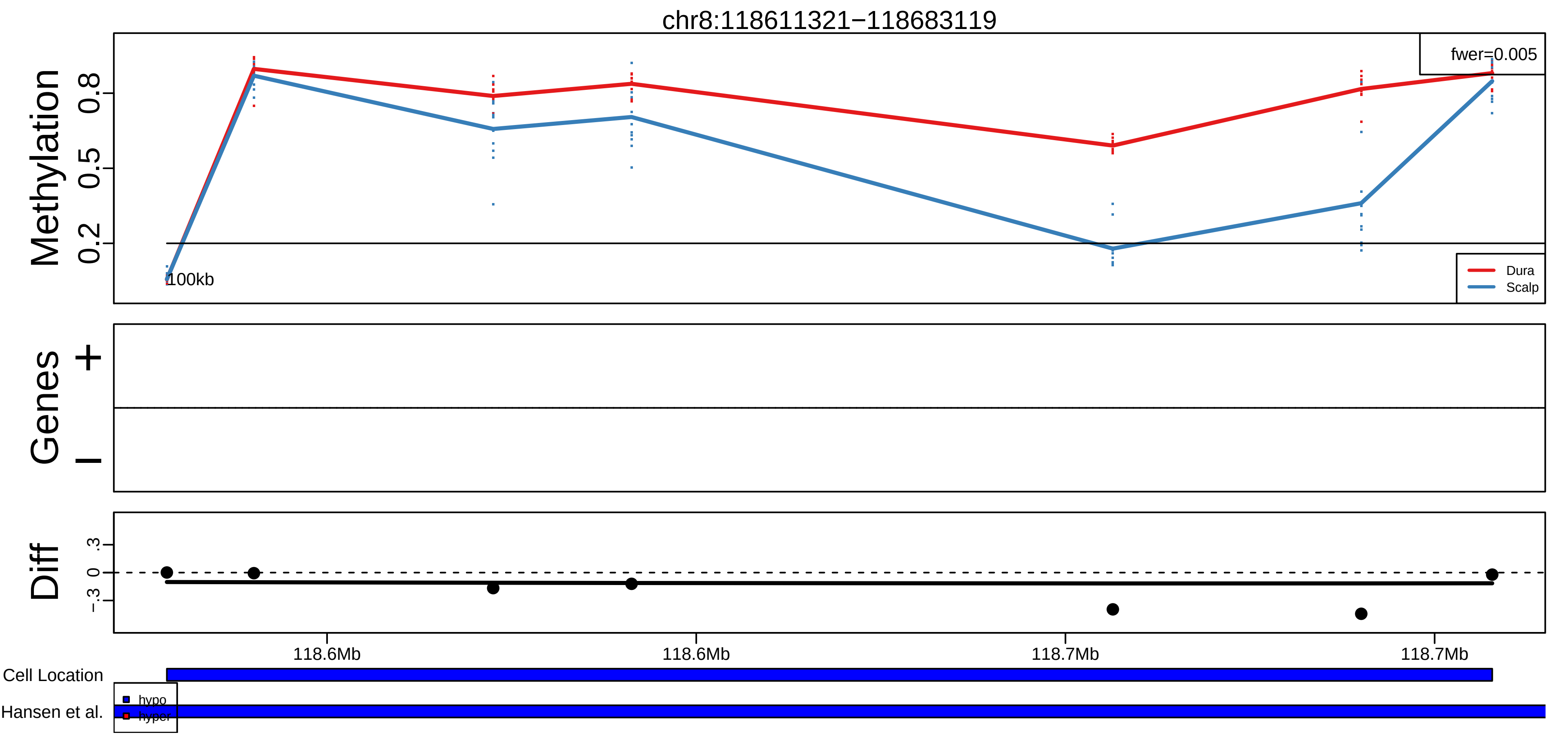

chr8:118611321−118683119
fwer=0.005
Methylation
0.8
0.5
0.2
100kb
Dura
Scalp
Genes
+
−
Diff
.3
0
−.3
118.6Mb
118.6Mb
118.7Mb
118.7Mb
Cell Location
Hansen et al.
hypo
hyper

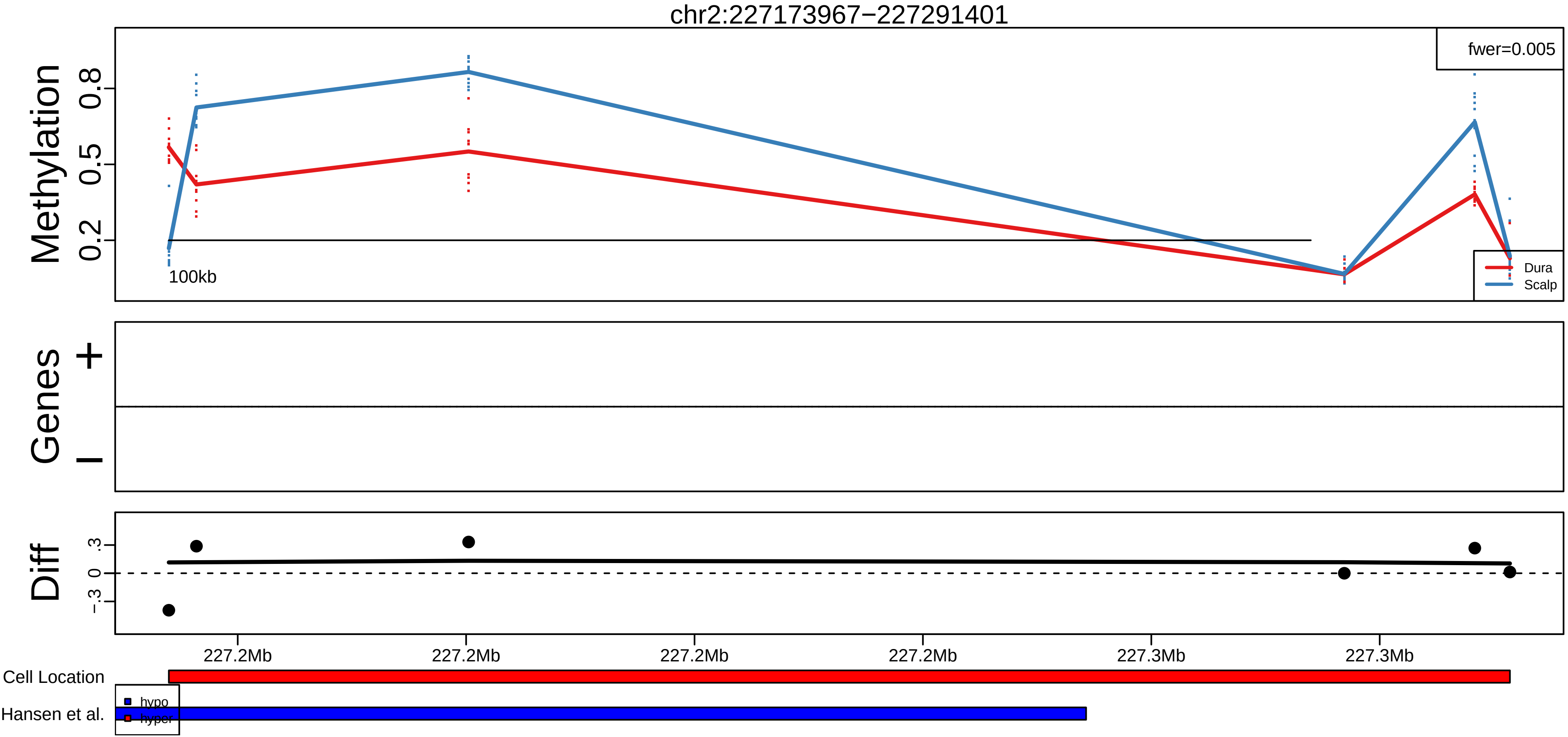

chr2:227173967−227291401
fwer=0.005
Methylation
0.2
0.5
0.8
100kb
Dura
Scalp
Genes
+
−
Diff
−.3
0
.3
227.2Mb
227.2Mb
227.2Mb
227.2Mb
227.3Mb
227.3Mb
Cell Location
Hansen et al.
hypo
hyper

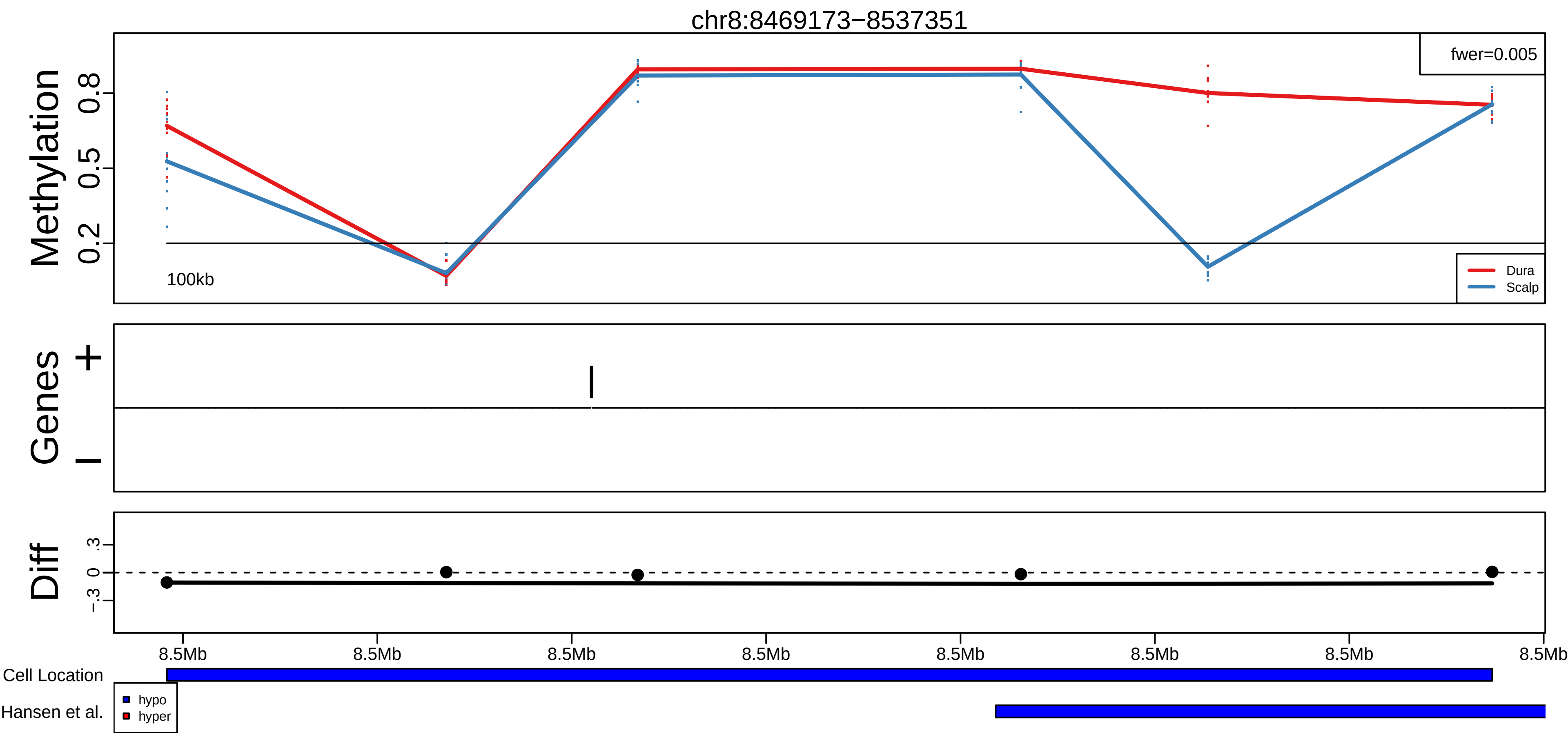

chr8:8469173−8537351
fwer=0.005
Methylation
0.8
0.5
0.2
100kb
Dura
Scalp
Genes
+
−
Diff
.3
0
−.3
8.5Mb
8.5Mb
8.5Mb
8.5Mb
8.5Mb
8.5Mb
8.5Mb
8.5Mb
Cell Location
Hansen et al.
hypo
hyper

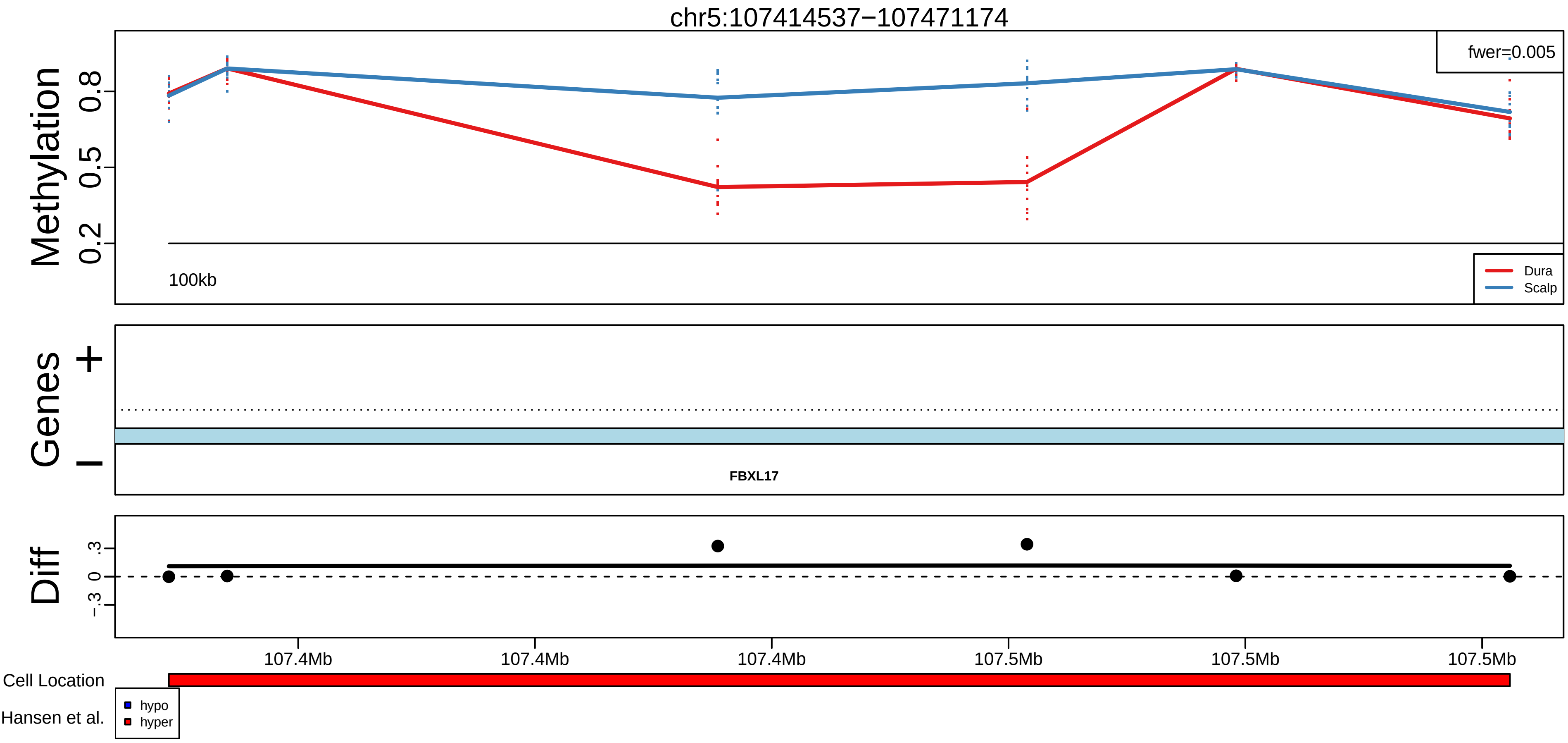

chr5:107414537−107471174
fwer=0.005
Methylation
0.2
0.5
0.8
100kb
Dura
Scalp
Genes
+
−
FBXL17
Diff
−.3
0
.3
107.4Mb
107.4Mb
107.4Mb
107.5Mb
107.5Mb
107.5Mb
Cell Location
Hansen et al.
hypo
hyper

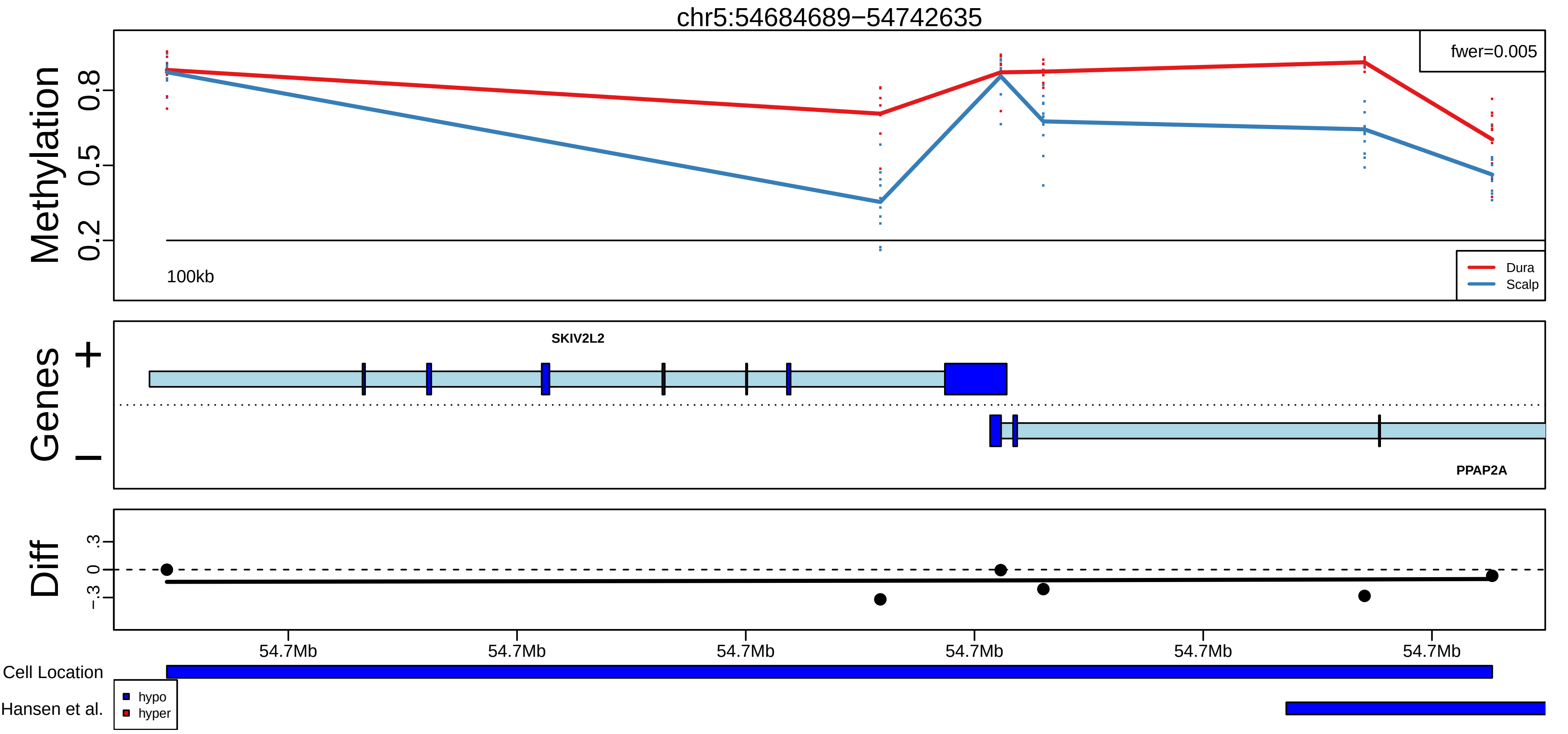
chr5:54684689−54742635
fwer=0.005
Methylation
0.2
0.5
0.8
100kb
Dura
Scalp
Genes
+
−
SKIV2L2
PPAP2A
Diff
.3
0
−.3
54.7Mb
54.7Mb
54.7Mb
54.7Mb
54.7Mb
54.7Mb
Cell Location
Hansen et al.
hypo
hyper

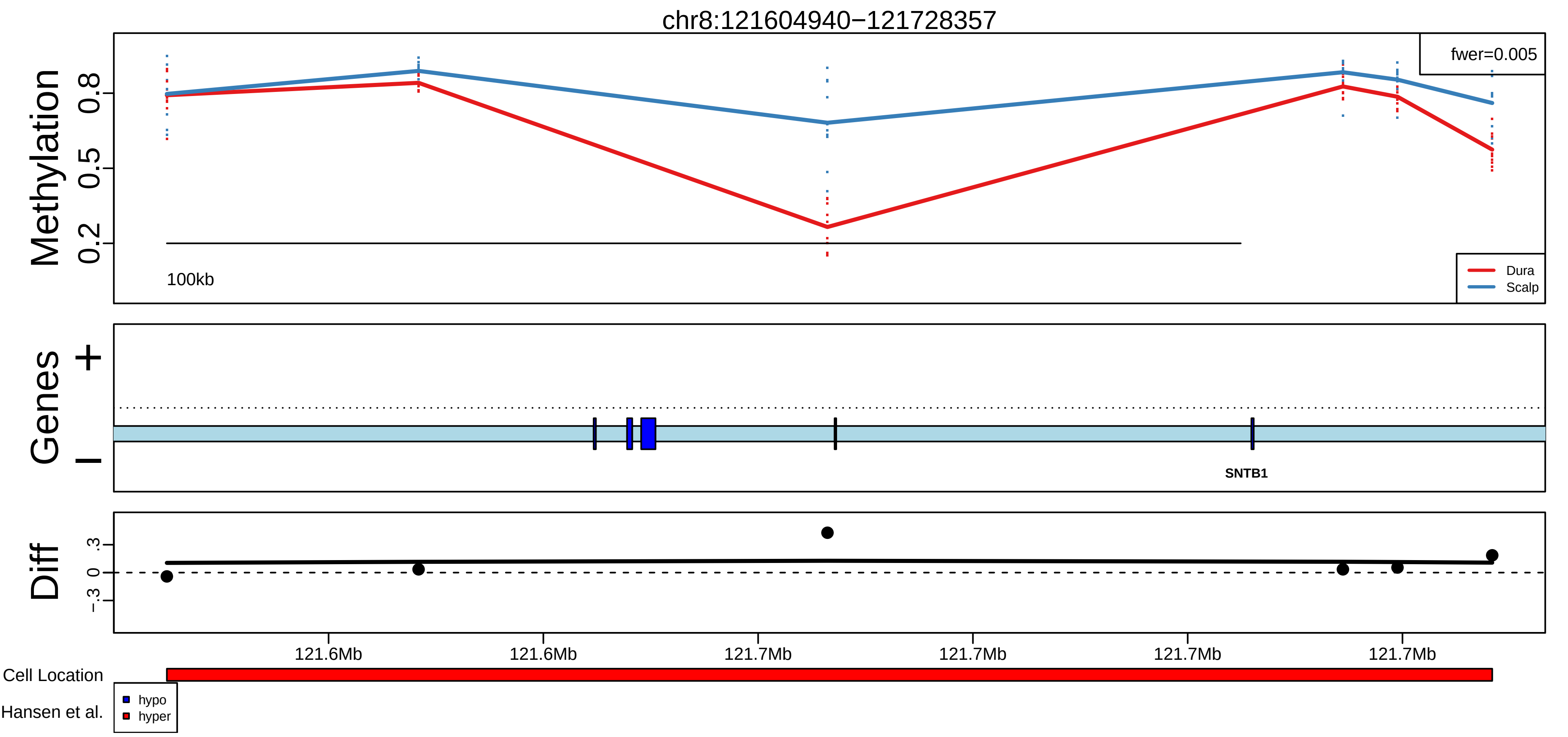
chr8:121604940−121728357
Methylation
0.2
0.5
0.8
fwer=0.005
100kb
Dura
Scalp
Genes
+
−
SNTB1
Diff
−.3
0
.3
121.6Mb
121.6Mb
121.7Mb
121.7Mb
121.7Mb
121.7Mb
Cell Location
Hansen et al.
hypo
hyper

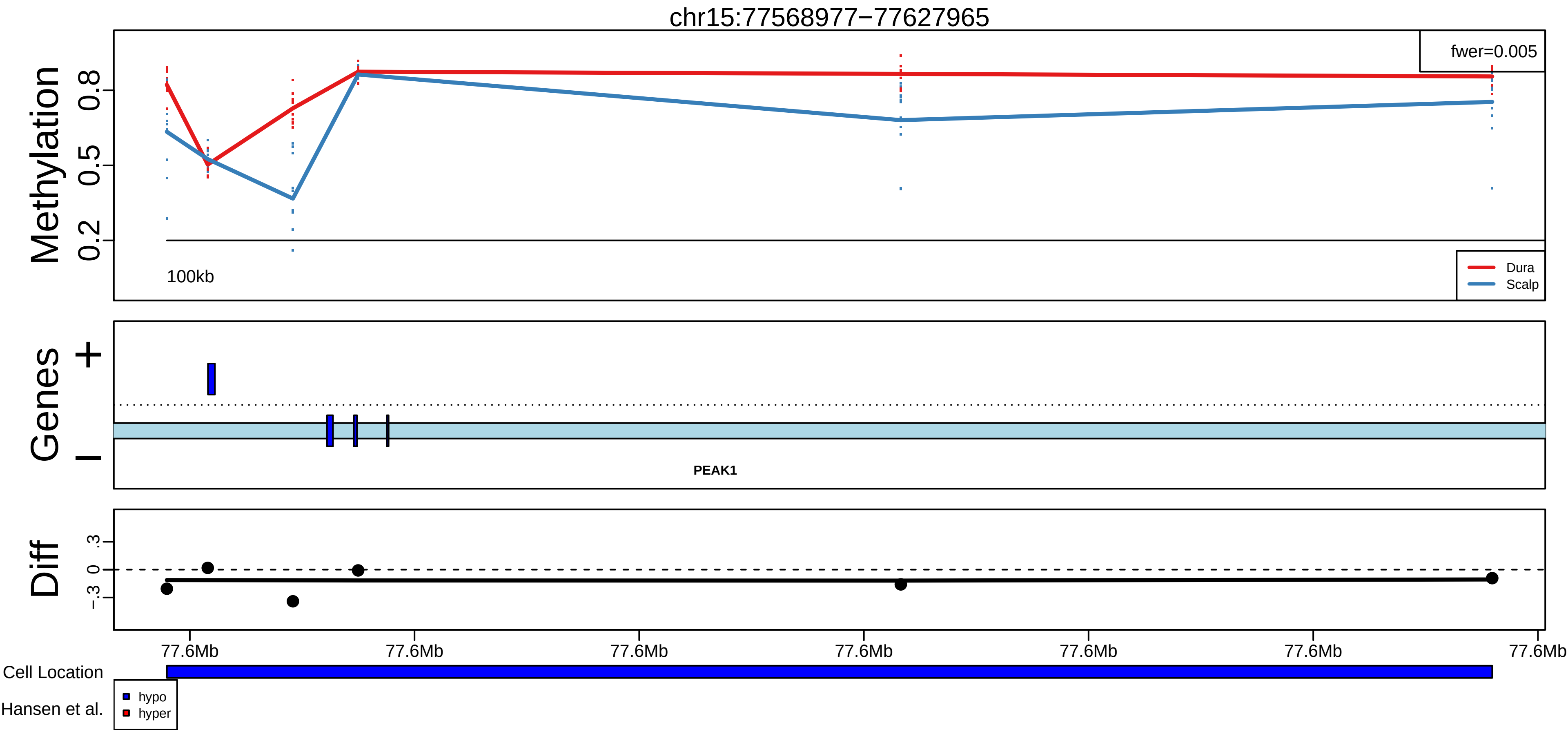

chr15:77568977−77627965
fwer=0.005
Methylation
0.2
0.5
0.8
100kb
Dura
Scalp
Genes
+
−
PEAK1
Diff
−.3
0
.3
77.6Mb
77.6Mb
77.6Mb
77.6Mb
77.6Mb
77.6Mb
77.6Mb
Cell Location
Hansen et al.
hypo
hyper

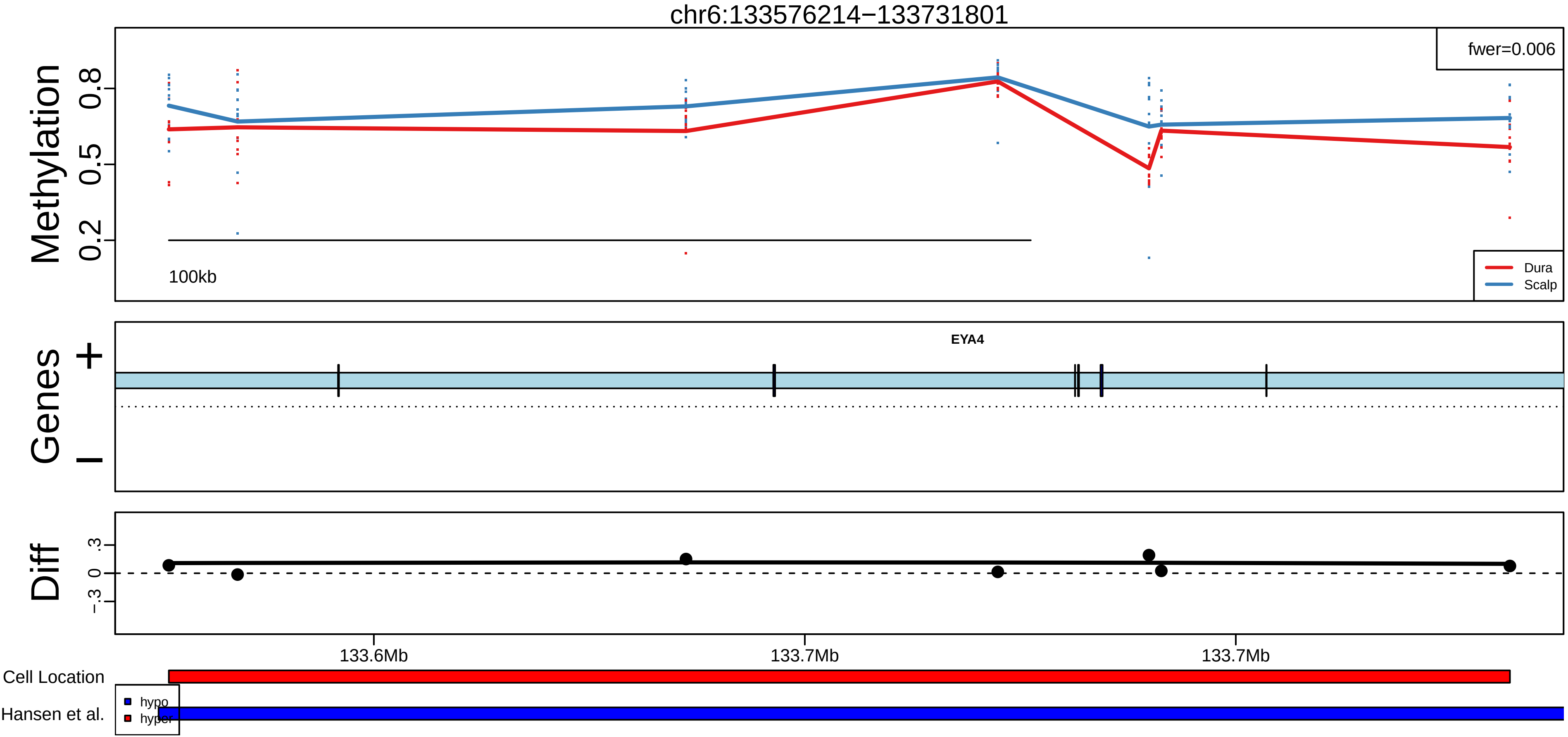
chr6:133576214−133731801
fwer=0.006
Methylation
0.2
0.5
0.8
100kb
Dura
Scalp
Genes
+
−
EYA4
Diff
−.3
0
.3
133.6Mb
133.7Mb
133.7Mb
Cell Location
Hansen et al.
hypo
hyper

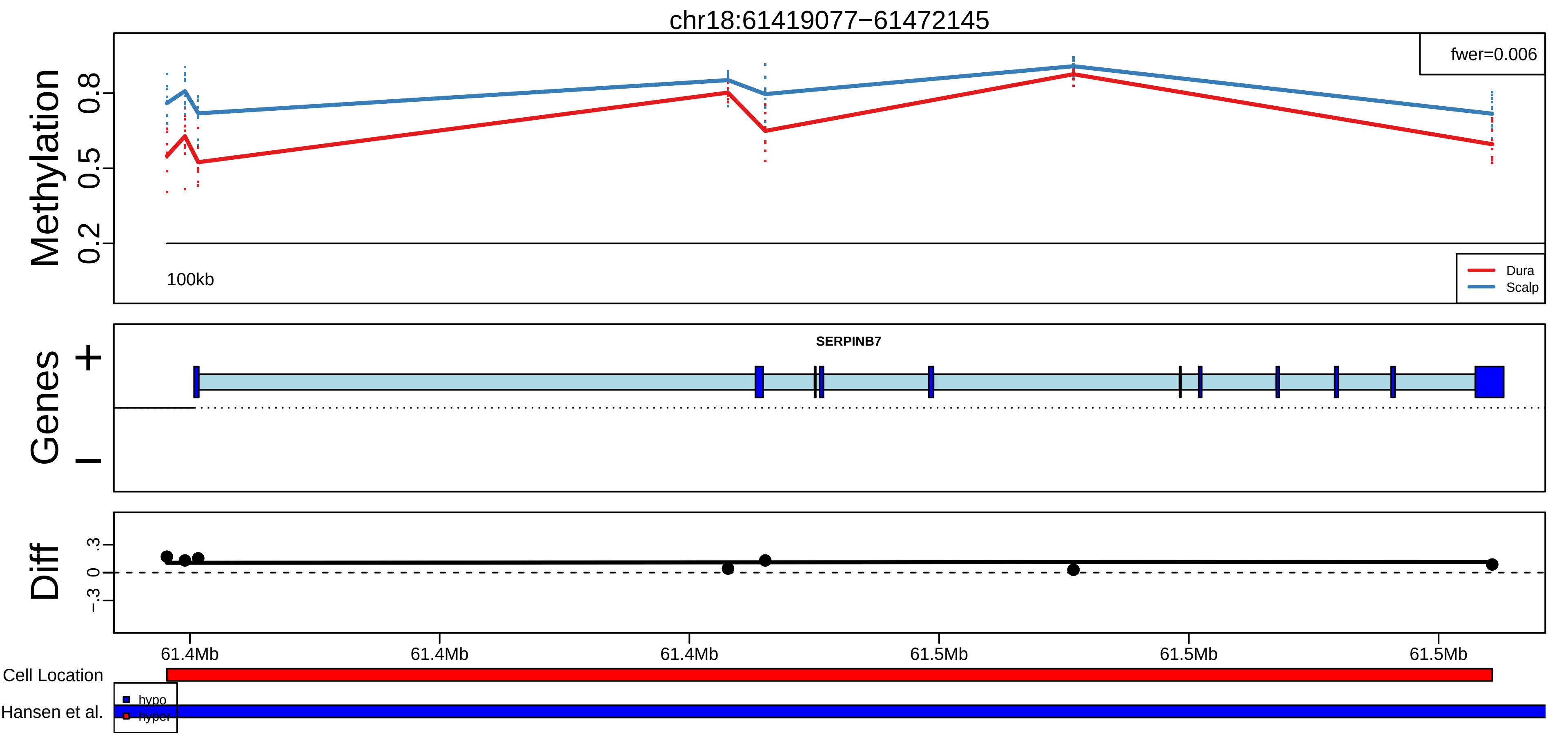

chr18:61419077−61472145
fwer=0.006
Methylation
0.2
0.5
0.8
100kb
Dura
Scalp
Genes
+
−
SERPINB7
Diff
−.3
0
.3
61.4Mb
61.4Mb
61.4Mb
61.5Mb
61.5Mb
61.5Mb
Cell Location
Hansen et al.
hypo
hyper

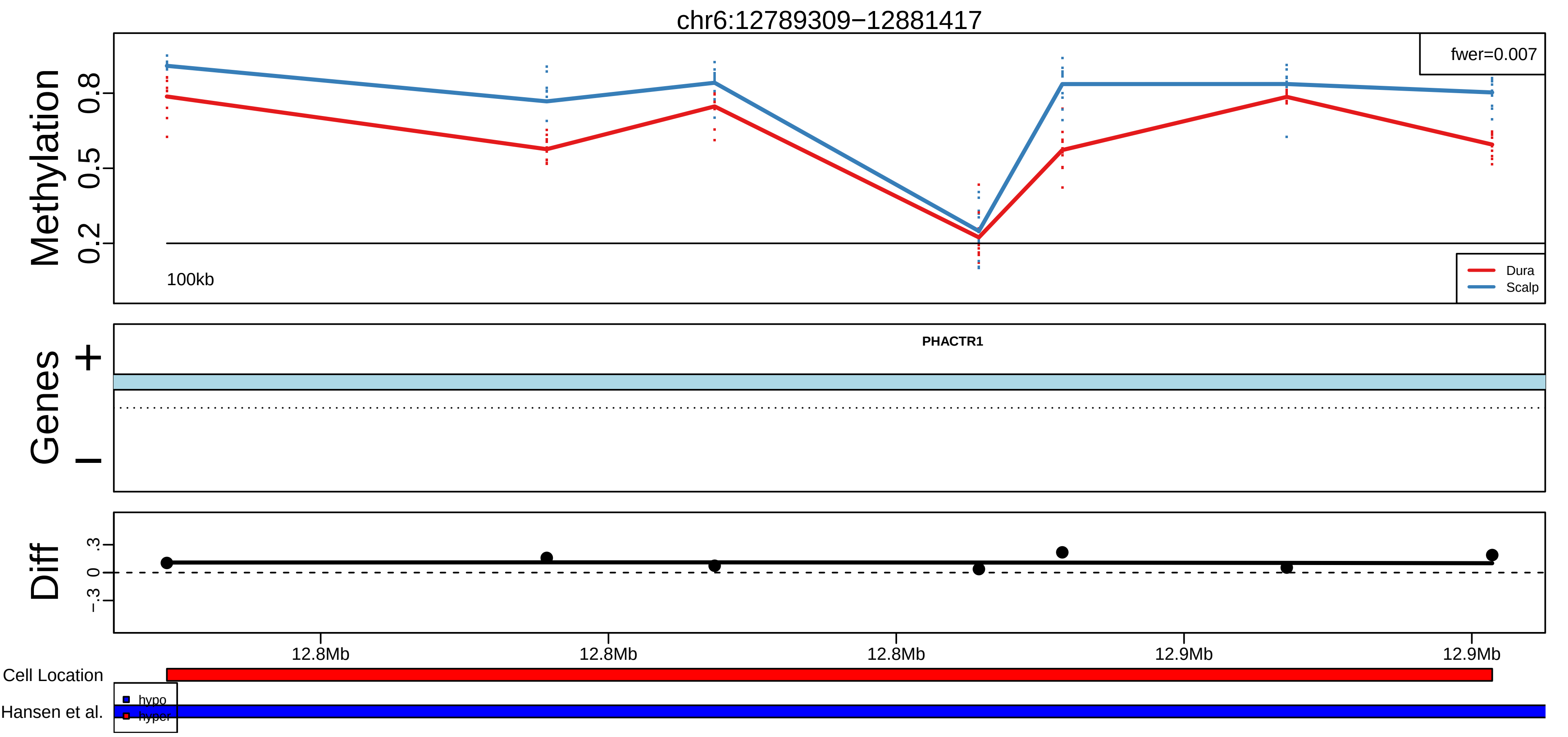
chr6:12789309−12881417
Methylation
0.2
0.5
0.8
fwer=0.007
100kb
Dura
Scalp
Genes
+
−
PHACTR1
Diff
−.3
0
.3
12.8Mb
12.8Mb
12.8Mb
12.9Mb
12.9Mb
Cell Location
Hansen et al.
hypo
hyper

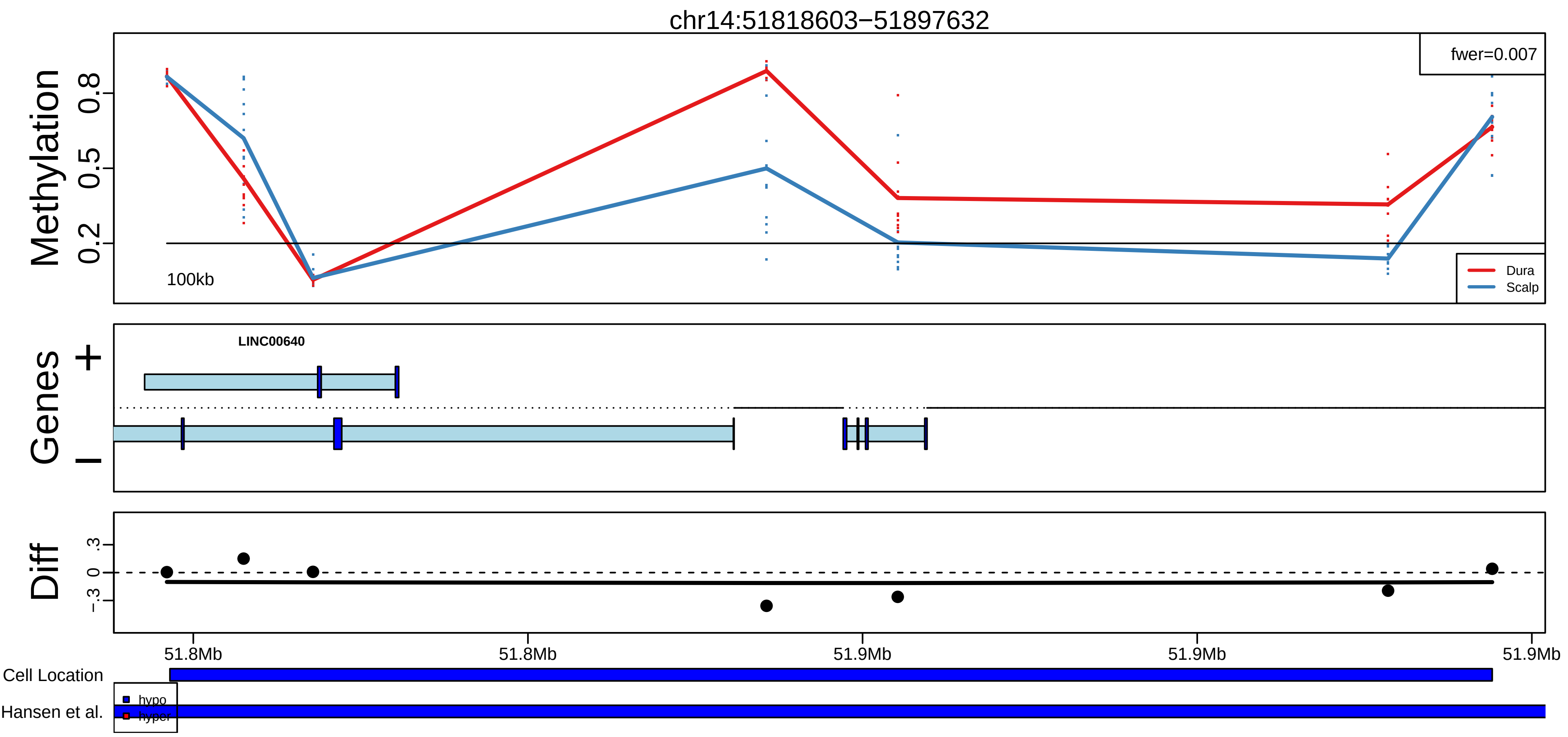
chr14:51818603−51897632
fwer=0.007
Methylation
0.2
0.5
0.8
100kb
Dura
Scalp
Genes
+
−
LINC00640
Diff
−.3
0
.3
51.8Mb
51.8Mb
51.9Mb
51.9Mb
51.9Mb
Cell Location
Hansen et al.
hypo
hyper

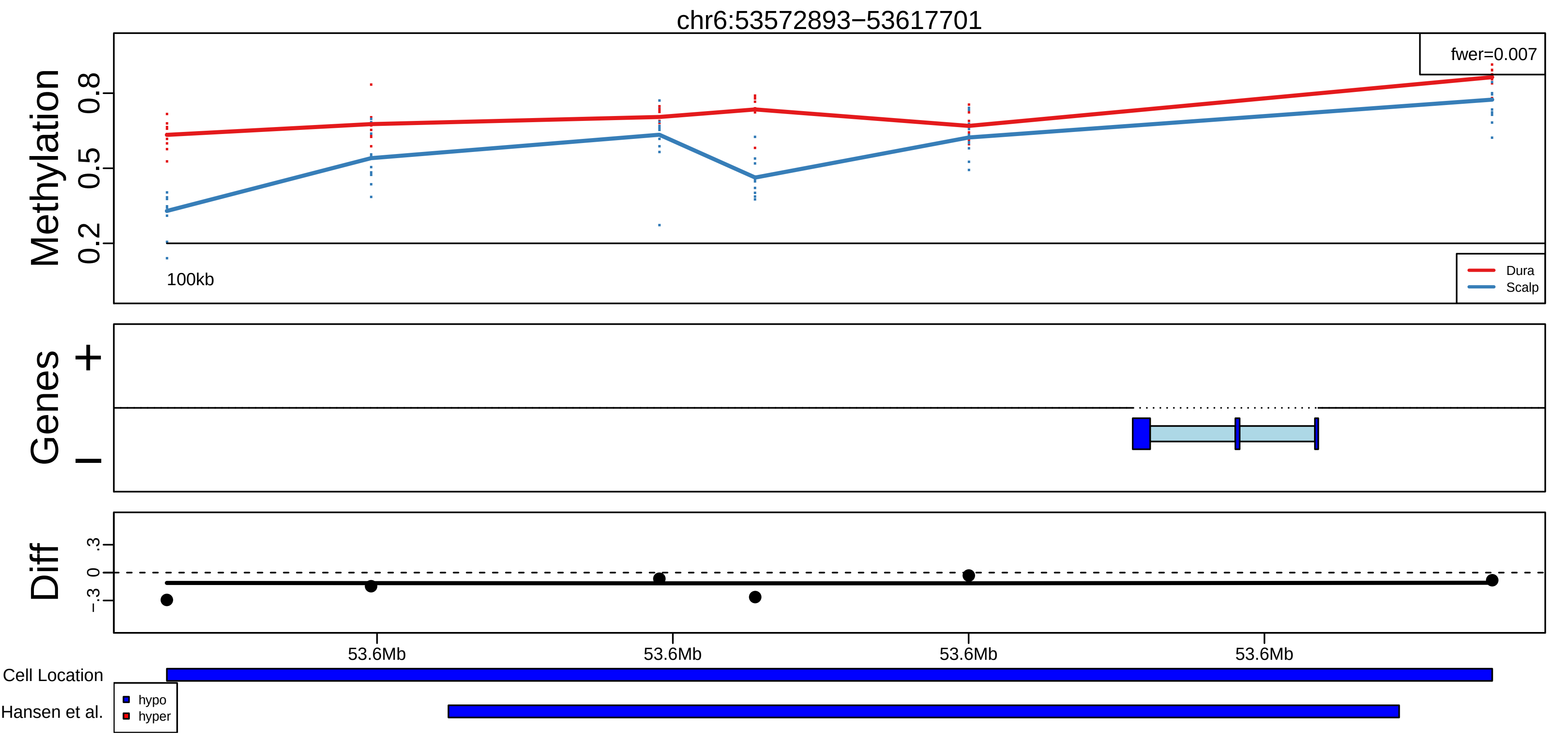

chr6:53572893−53617701
fwer=0.007
Methylation
0.2
0.5
0.8
100kb
Dura
Scalp
Genes
+
−
Diff
−.3
0
.3
53.6Mb
53.6Mb
53.6Mb
53.6Mb
Cell Location
Hansen et al.
hypo
hyper

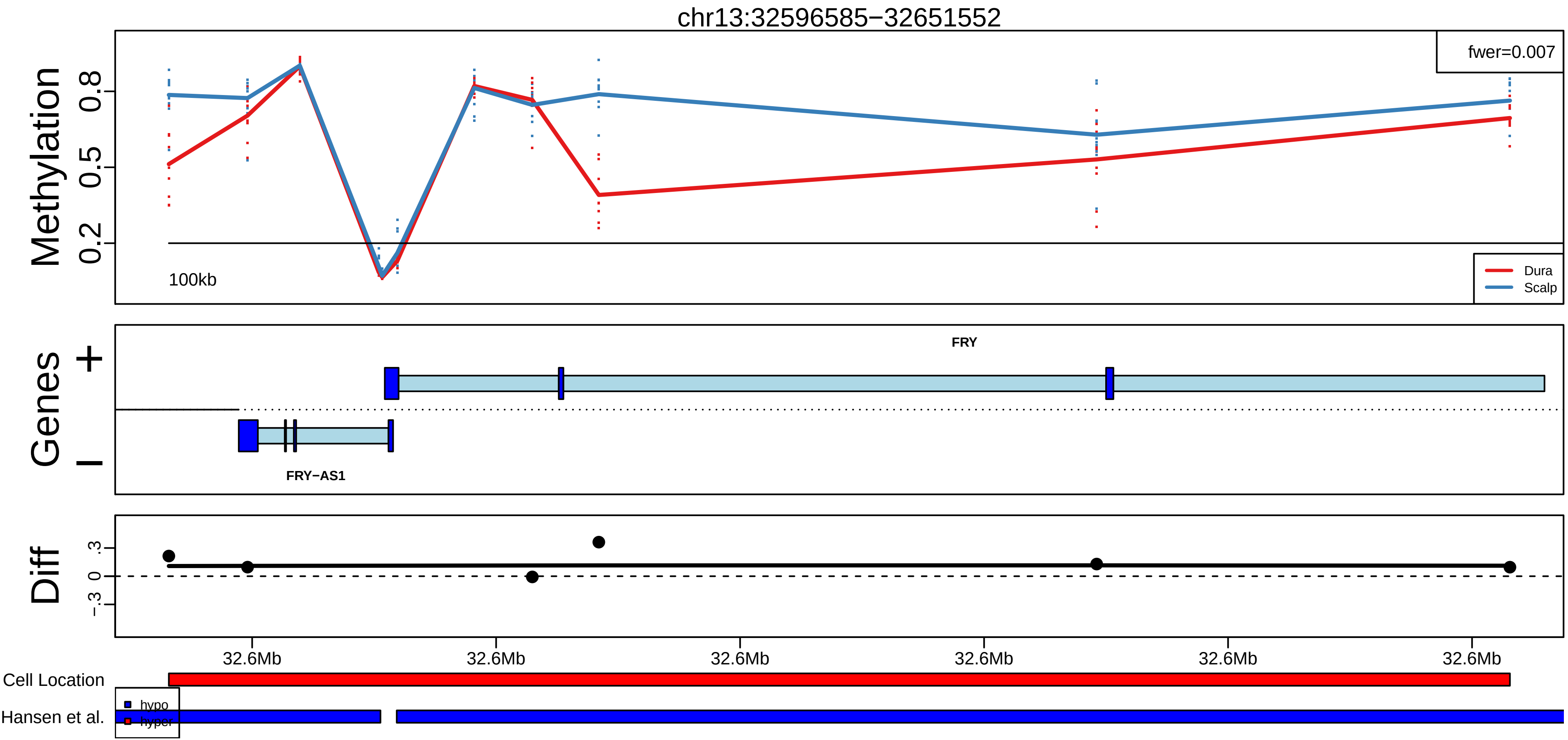

chr13:32596585−32651552
fwer=0.007
Methylation
0.2
0.5
0.8
100kb
Dura
Scalp
Genes
+
−
FRY
FRY−AS1
Diff
−.3
0
.3
32.6Mb
32.6Mb
32.6Mb
32.6Mb
32.6Mb
32.6Mb
Cell Location
Hansen et al.
hypo
hyper

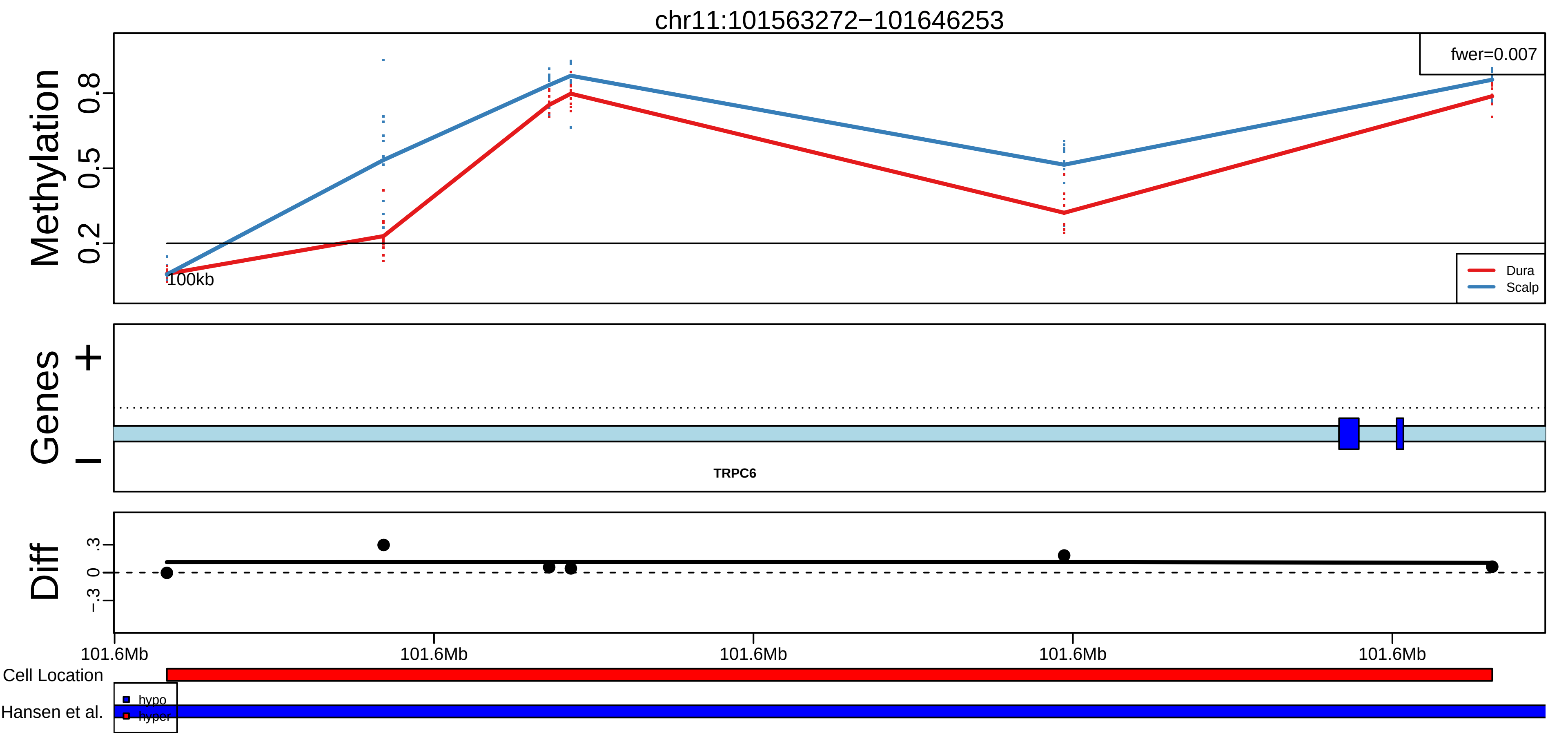

chr11:101563272−101646253
Methylation
0.2
0.5
0.8
fwer=0.007
100kb
Dura
Scalp
Genes
+
−
TRPC6
Diff
−.3
0
.3
101.6Mb
101.6Mb
101.6Mb
101.6Mb
101.6Mb
Cell Location
Hansen et al.
hypo
hyper

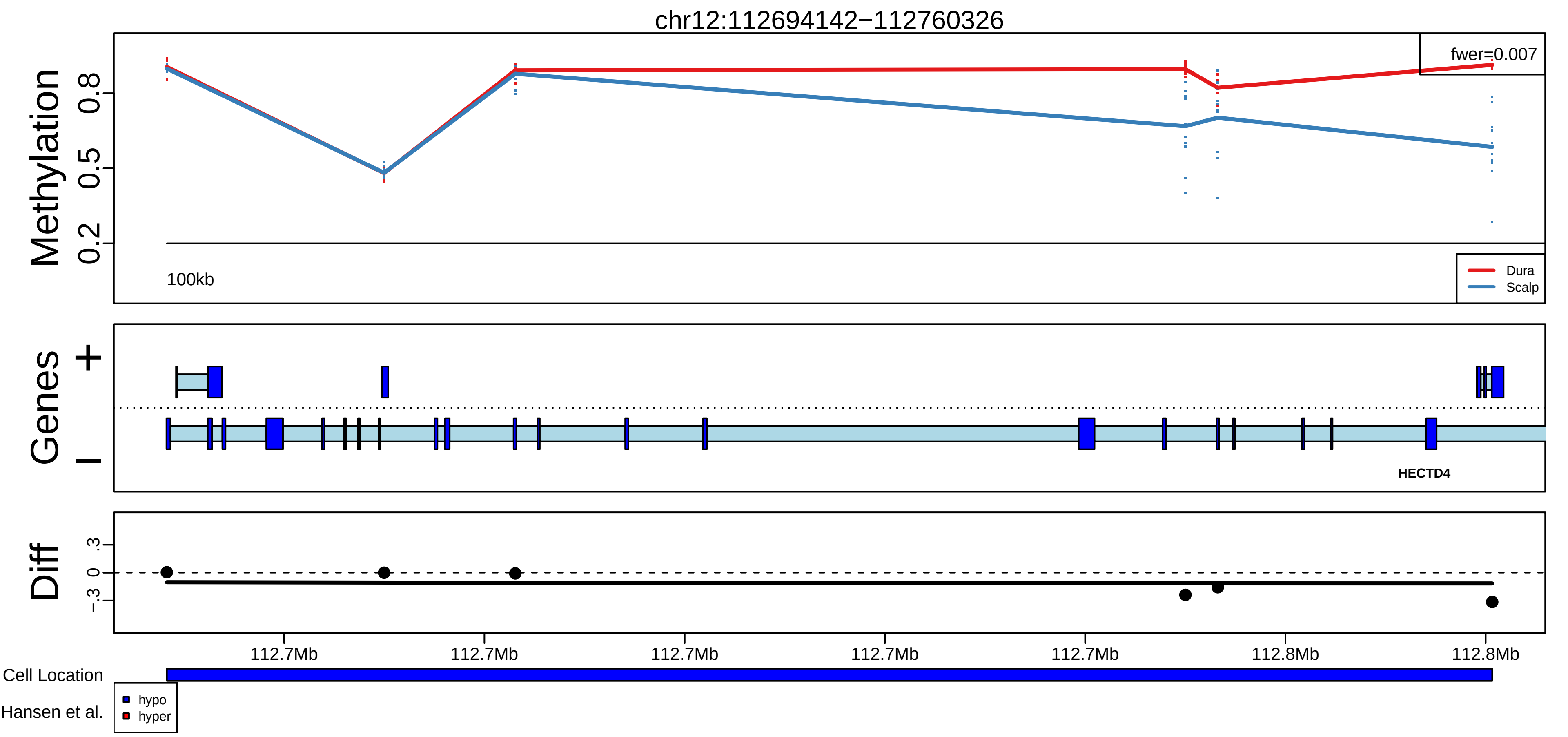
chr12:112694142−112760326
fwer=0.007
Methylation
0.2
0.5
0.8
100kb
Dura
Scalp
Genes
+
−
HECTD4
Diff
−.3
0
.3
112.7Mb
112.7Mb
112.7Mb
112.7Mb
112.7Mb
112.8Mb
112.8Mb
Cell Location
Hansen et al.
hypo
hyper

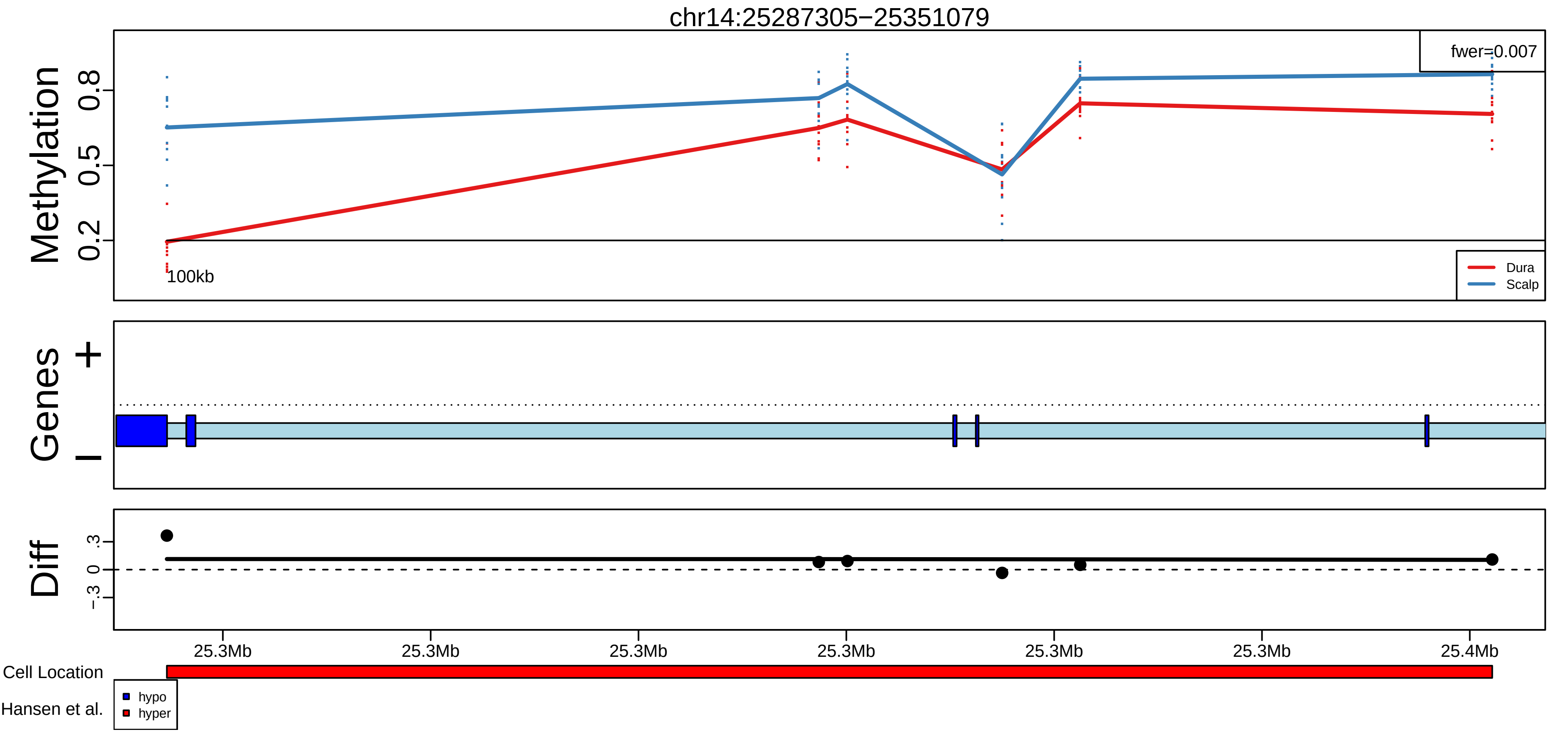

chr14:25287305−25351079
fwer=0.007
Methylation
0.2
0.5
0.8
100kb
Dura
Scalp
Genes
+
−
Diff
−.3
0
.3
25.3Mb
25.3Mb
25.3Mb
25.3Mb
25.3Mb
25.3Mb
25.4Mb
Cell Location
Hansen et al.
hypo
hyper

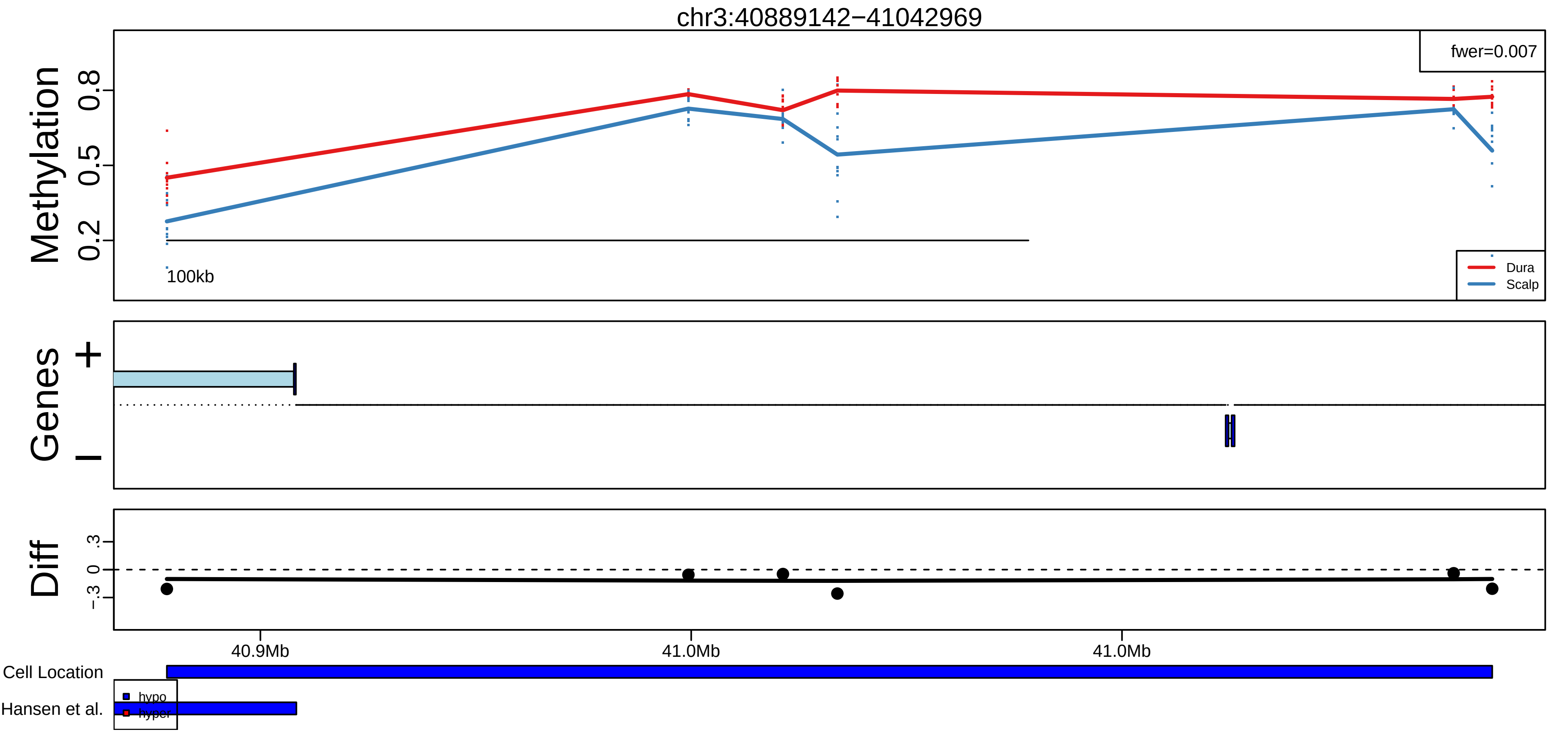

chr3:40889142−41042969
fwer=0.007
Methylation
0.2
0.5
0.8
100kb
Dura
Scalp
Genes
+
−
Diff
.3
0
−.3
40.9Mb
41.0Mb
41.0Mb
Cell Location
Hansen et al.
hypo
hyper

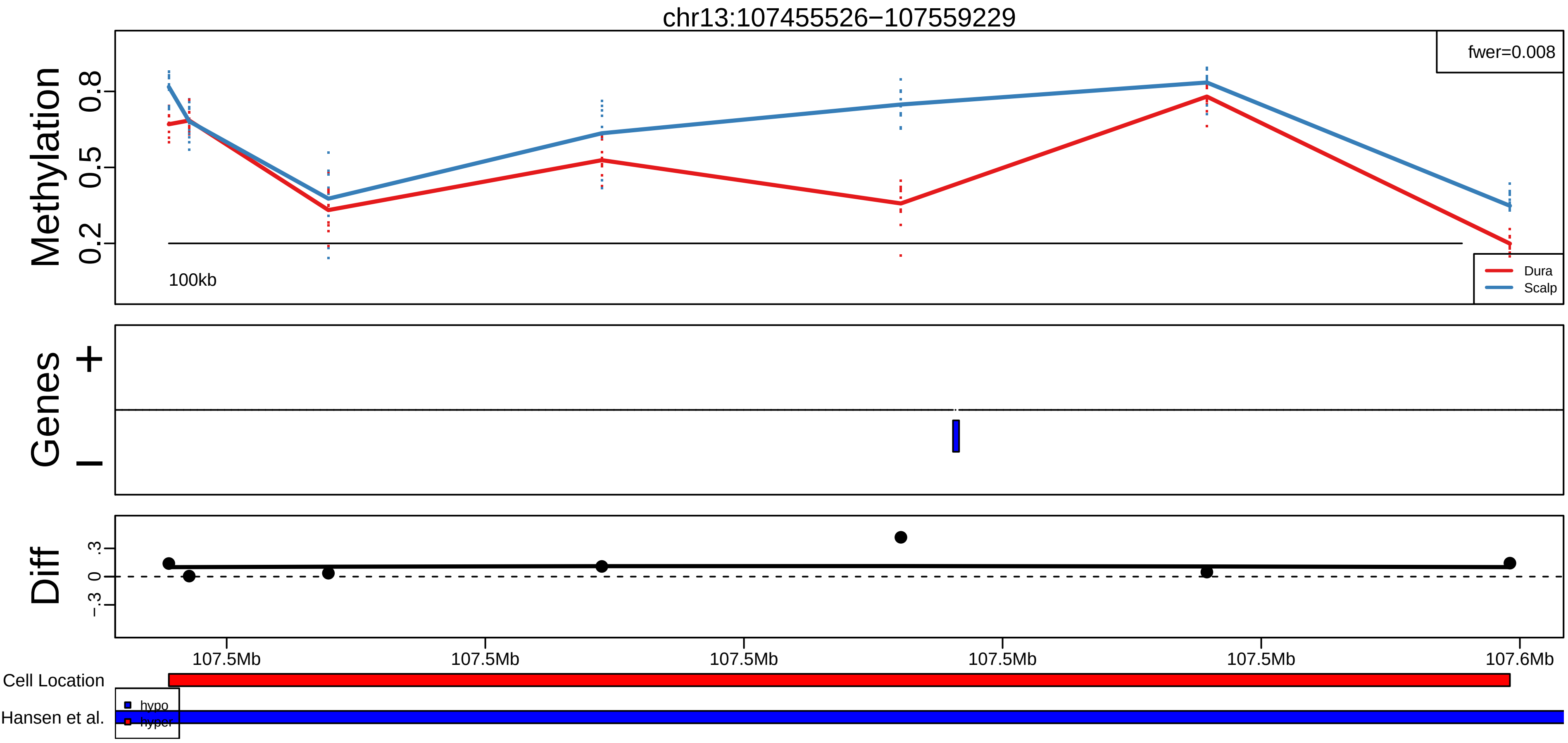

chr13:107455526−107559229
fwer=0.008
Methylation
0.2
0.5
0.8
100kb
Dura
Scalp
Genes
+
−
Diff
.3
0
−.3
107.5Mb
107.5Mb
107.5Mb
107.5Mb
107.5Mb
107.6Mb
Cell Location
Hansen et al.
hypo
hyper

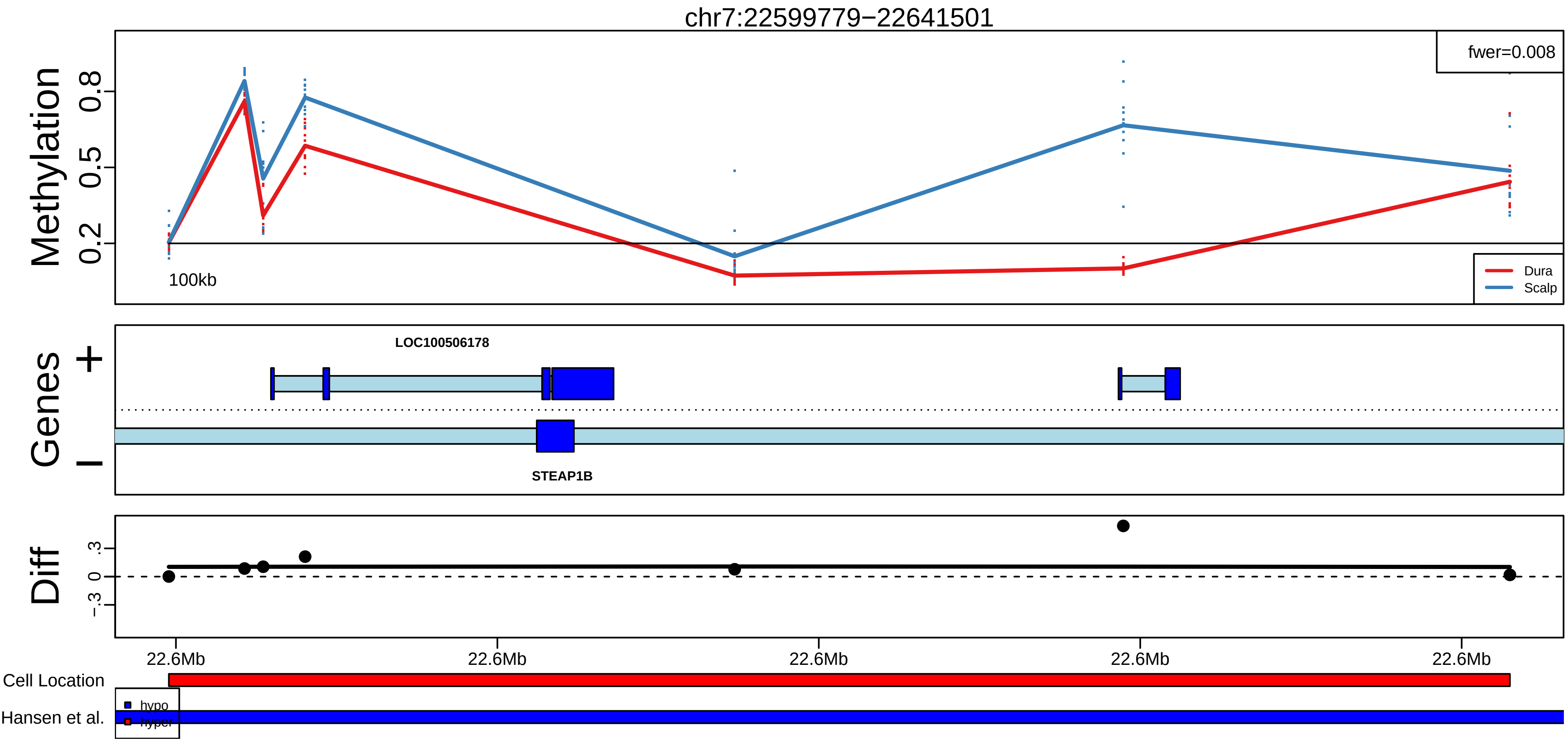
chr7:22599779−22641501
fwer=0.008
Methylation
0.2
0.5
0.8
100kb
Dura
Scalp
Genes
+
−
LOC100506178
STEAP1B
Diff
.3
0
−.3
22.6Mb
22.6Mb
22.6Mb
22.6Mb
22.6Mb
Cell Location
Hansen et al.
hypo
hyper

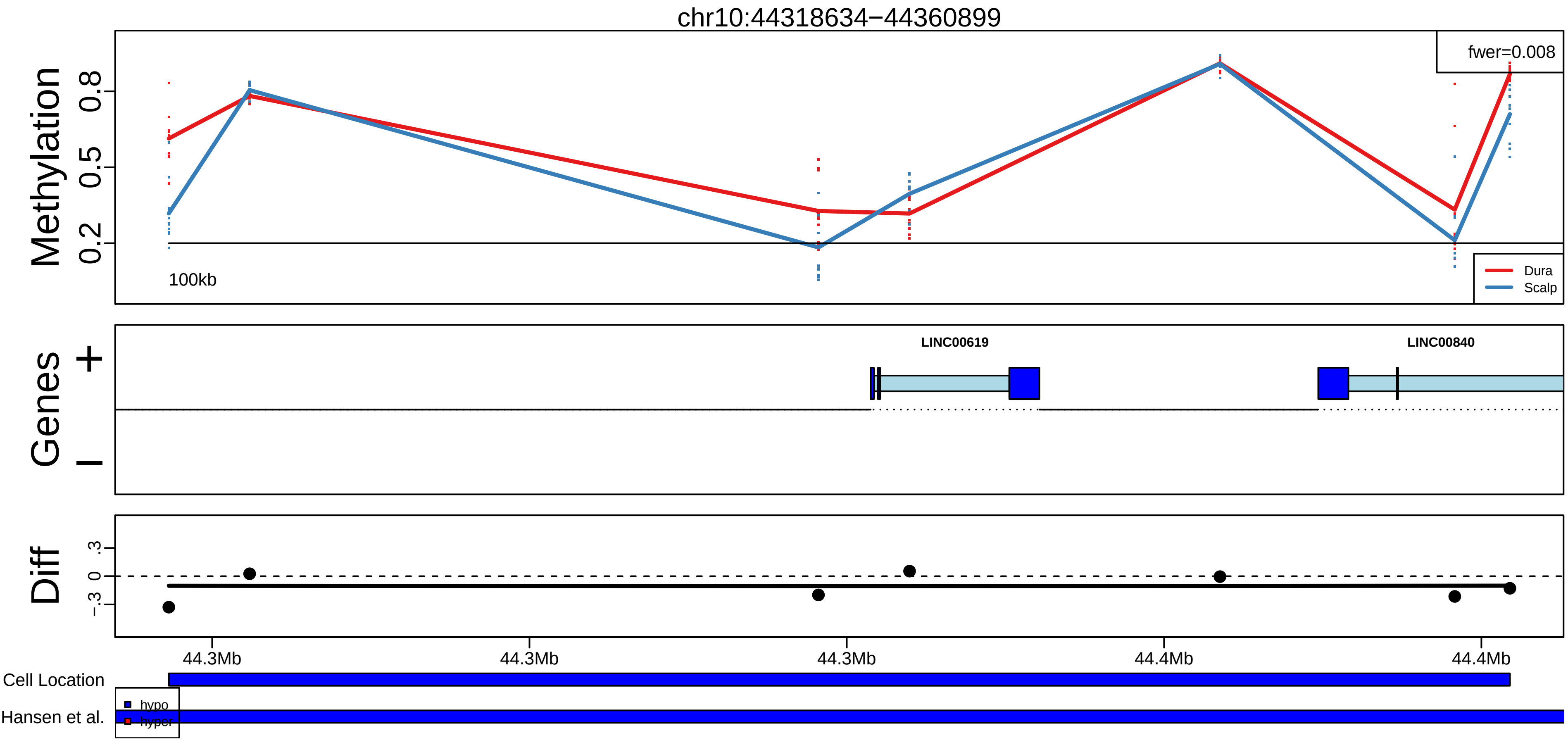
chr10:44318634−44360899
fwer=0.008
Methylation
0.2
0.5
0.8
100kb
Dura
Scalp
Genes
+
−
LINC00619
LINC00840
Diff
−.3
0
.3
44.3Mb
44.3Mb
44.3Mb
44.4Mb
44.4Mb
Cell Location
Hansen et al.
hypo
hyper

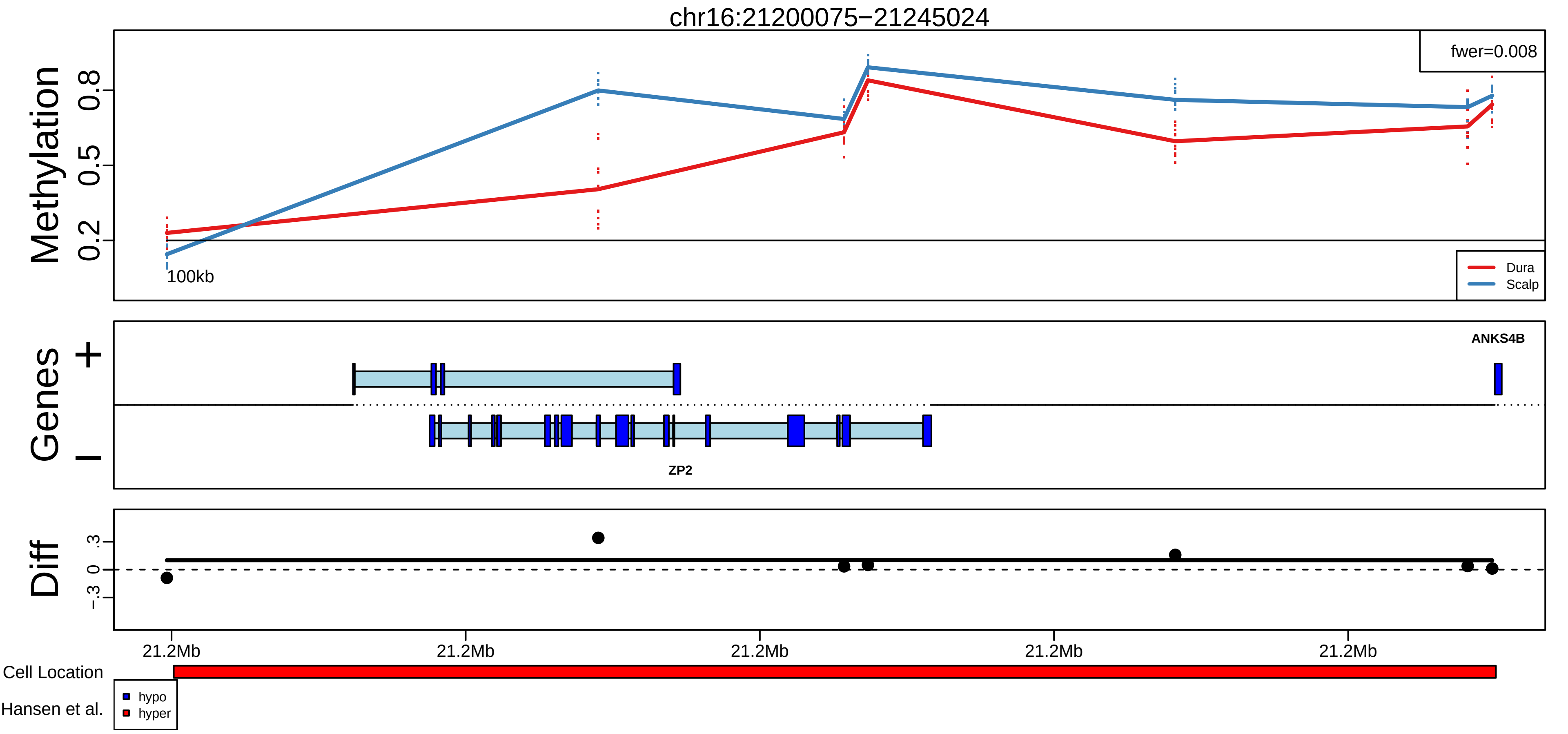

chr16:21200075−21245024
fwer=0.008
Methylation
0.2
0.5
0.8
100kb
Dura
Scalp
Genes
+
−
ANKS4B
ZP2
Diff
−.3
0
.3
21.2Mb
21.2Mb
21.2Mb
21.2Mb
21.2Mb
Cell Location
Hansen et al.
hypo
hyper

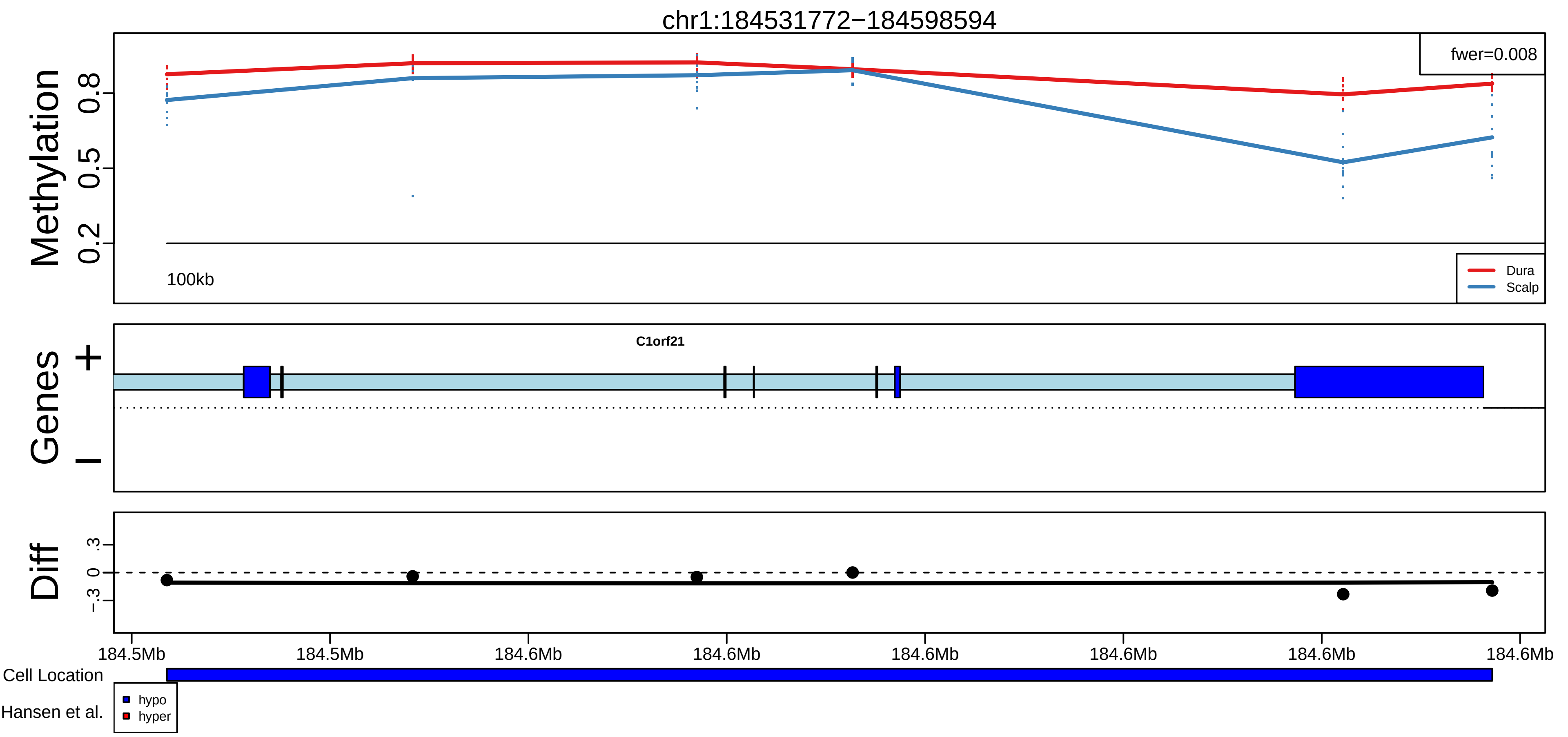

chr1:184531772−184598594
Methylation
0.2
0.5
0.8
fwer=0.008
100kb
Dura
Scalp
Genes
+
−
C1orf21
Diff
−.3
0
.3
184.5Mb
184.5Mb
184.6Mb
184.6Mb
184.6Mb
184.6Mb
184.6Mb
184.6Mb
Cell Location
Hansen et al.
hypo
hyper

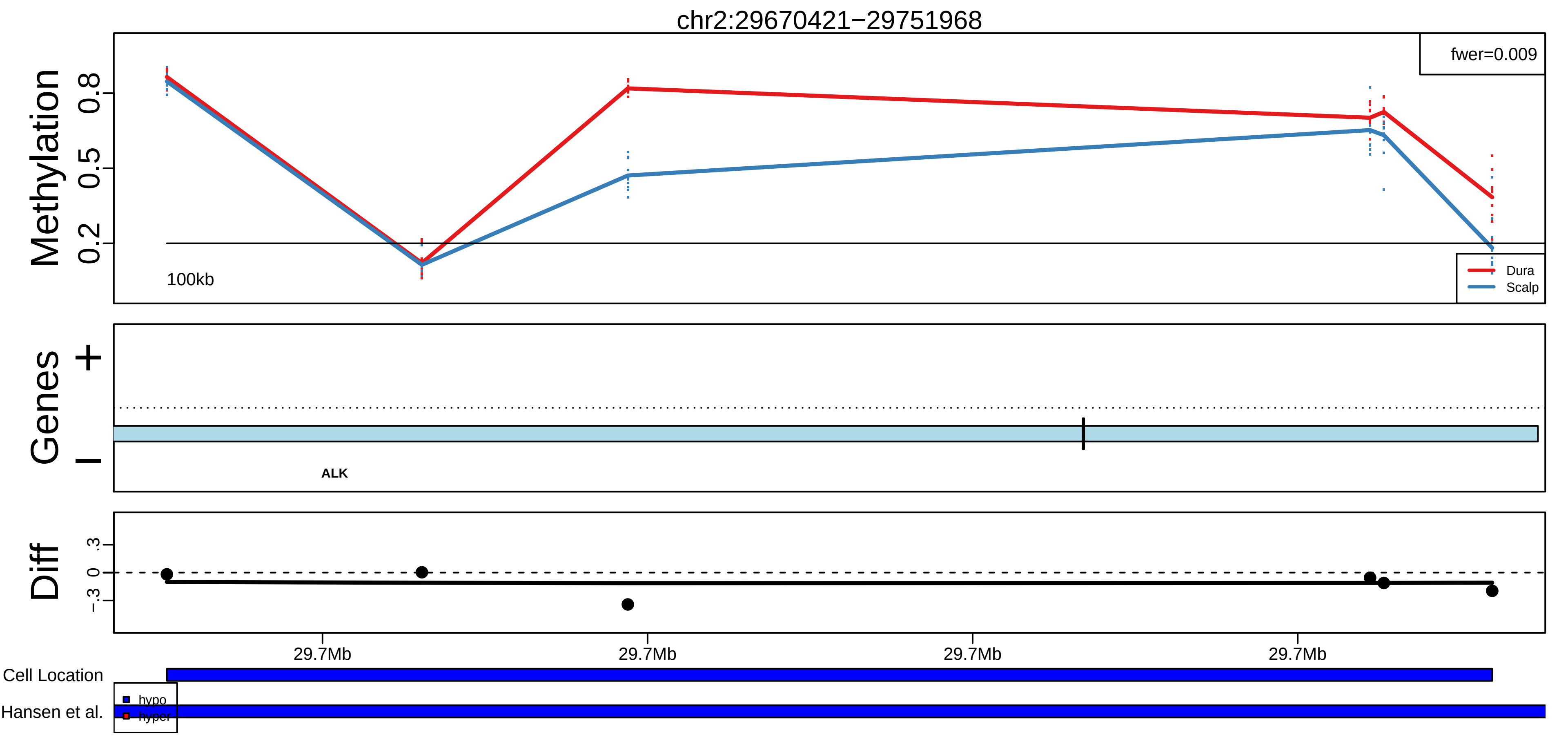
chr2:29670421−29751968
fwer=0.009
Methylation
0.2
0.5
0.8
100kb
Dura
Scalp
Genes
+
ALK
Diff
−.3
0
.3
29.7Mb
29.7Mb
29.7Mb
29.7Mb
Cell Location
Hansen et al.
hypo
hyper

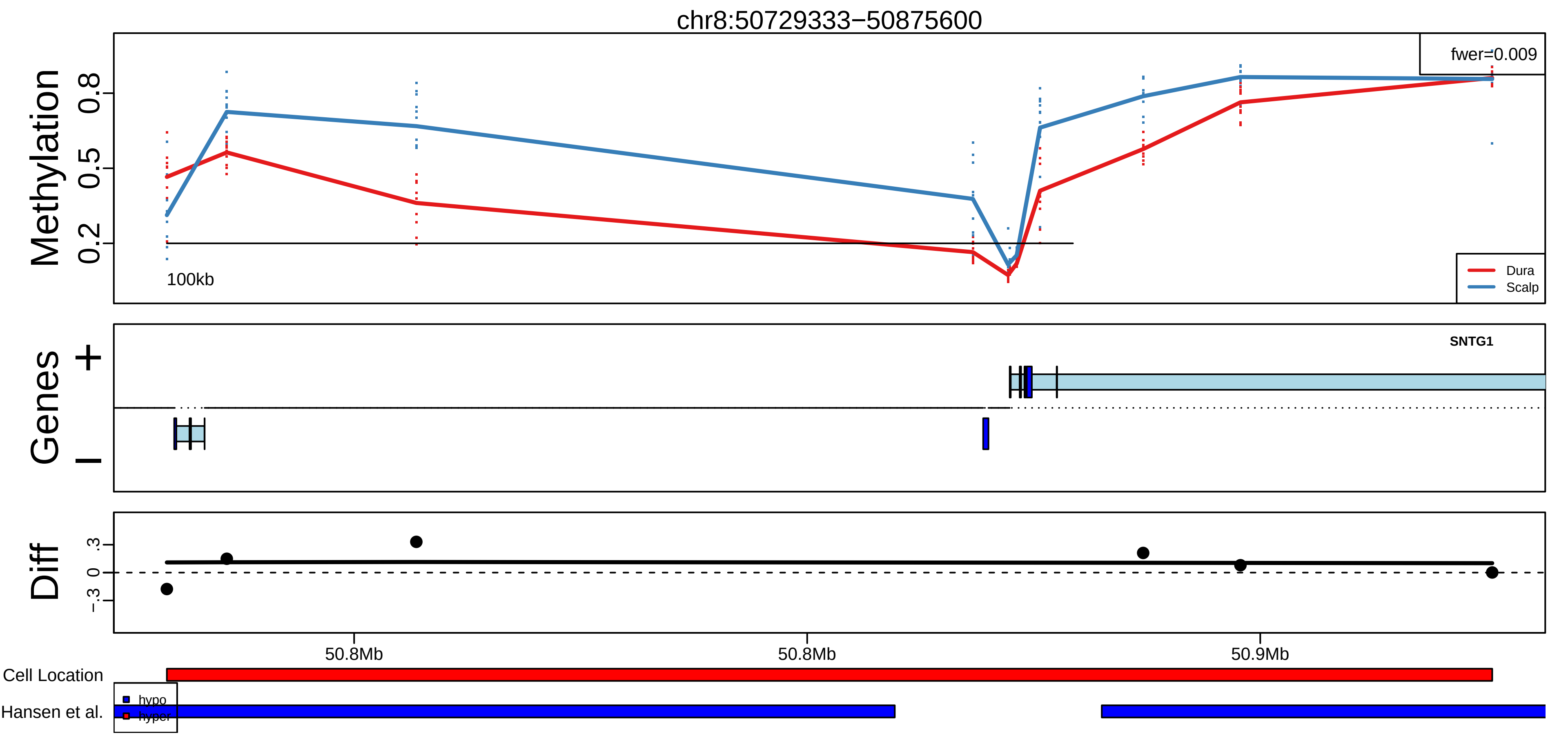

chr8:50729333−50875600
fwer=0.009
Methylation
0.2
0.5
0.8
100kb
Dura
Scalp
Genes
+
−
SNTG1
Diff
−.3
0
.3
50.8Mb
50.8Mb
50.9Mb
Cell Location
Hansen et al.
hypo
hyper

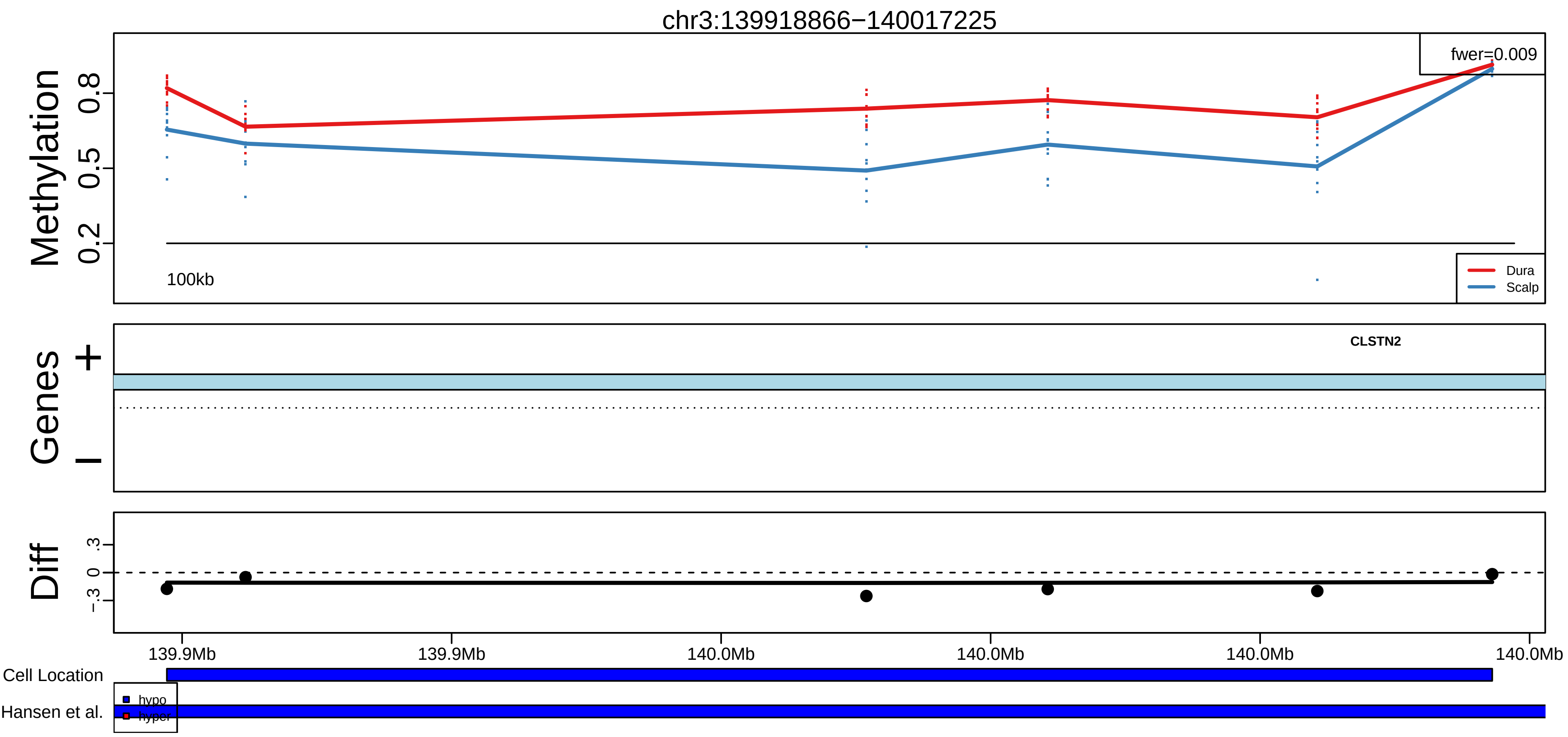

chr3:139918866−140017225
fwer=0.009
Methylation
0.2
0.5
0.8
100kb
Dura
Scalp
Genes
+
−
CLSTN2
Diff
.3
0
−.3
139.9Mb
139.9Mb
140.0Mb
140.0Mb
140.0Mb
140.0Mb
Cell Location
Hansen et al.
hypo
hyper

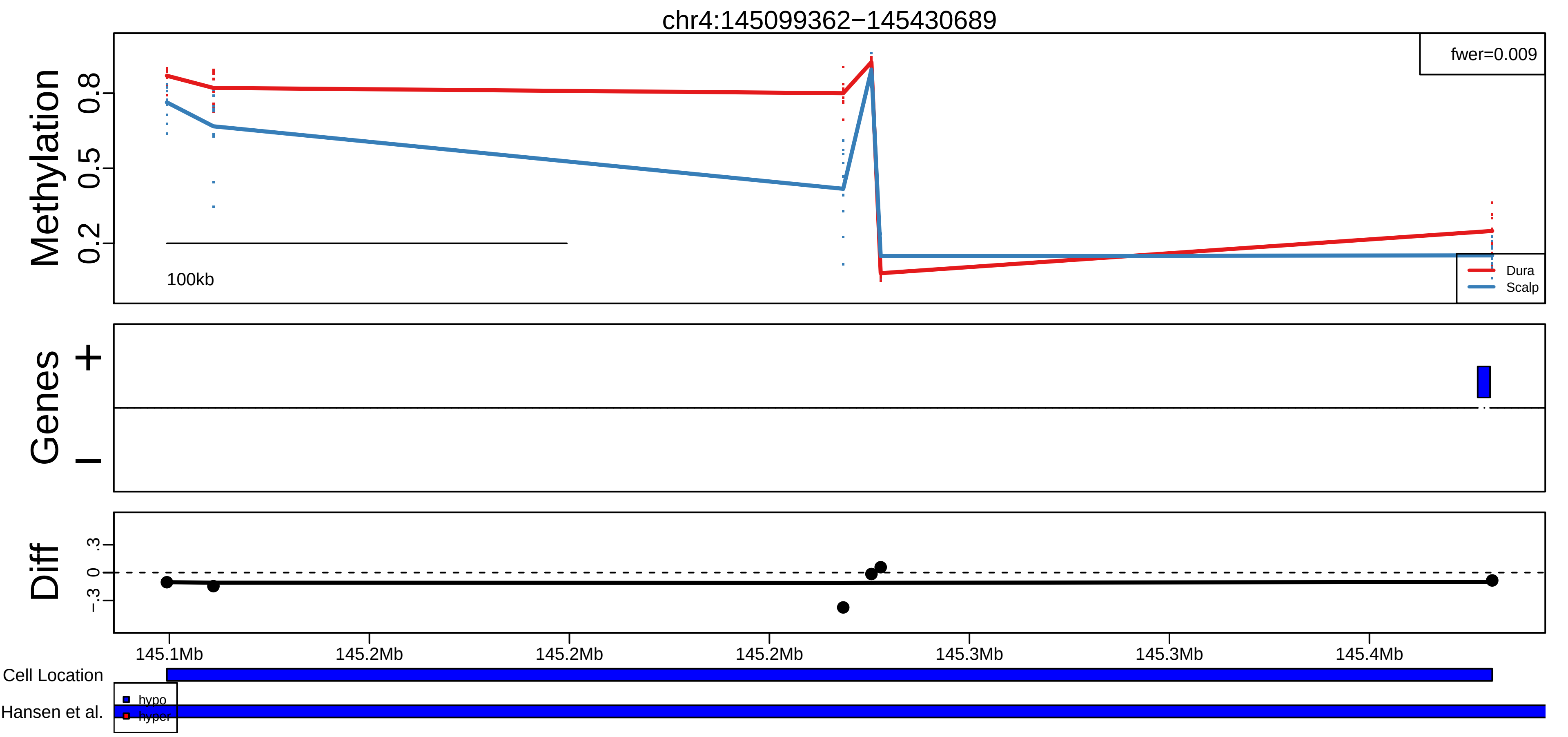

chr4:145099362−145430689
fwer=0.009
Methylation
0.2
0.5
0.8
100kb
Dura
Scalp
Genes
+
−
Diff
−.3
0
.3
145.1Mb
145.2Mb
145.2Mb
145.2Mb
145.3Mb
145.3Mb
145.4Mb
Cell Location
Hansen et al.
hypo
hyper

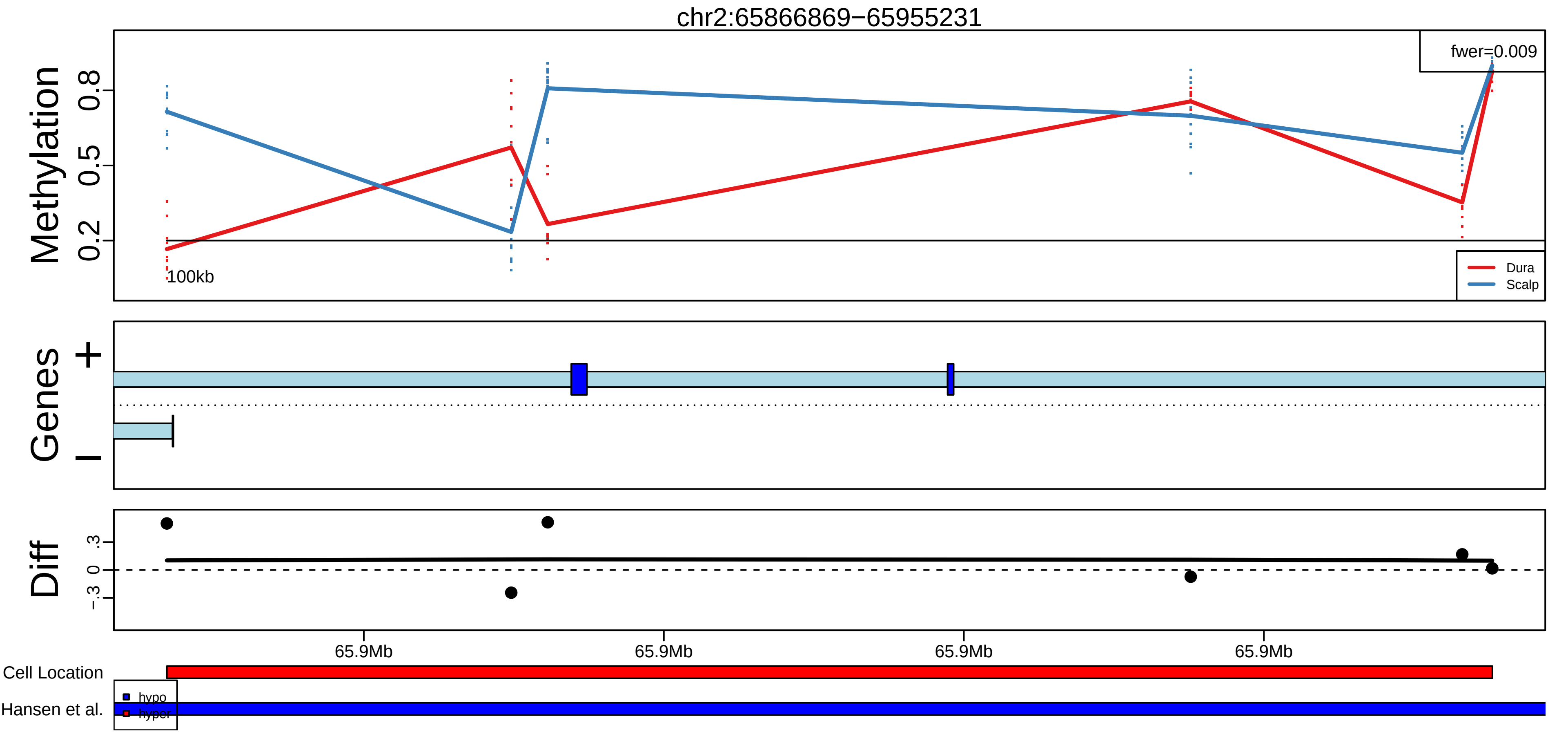
chr2:65866869−65955231
fwer=0.009
Methylation
0.2
0.5
0.8
100kb
Dura
Scalp
Genes
+
−
Diff
.3
0
−.3
65.9Mb
65.9Mb
65.9Mb
65.9Mb
Cell Location
Hansen et al.
hypo
hyper

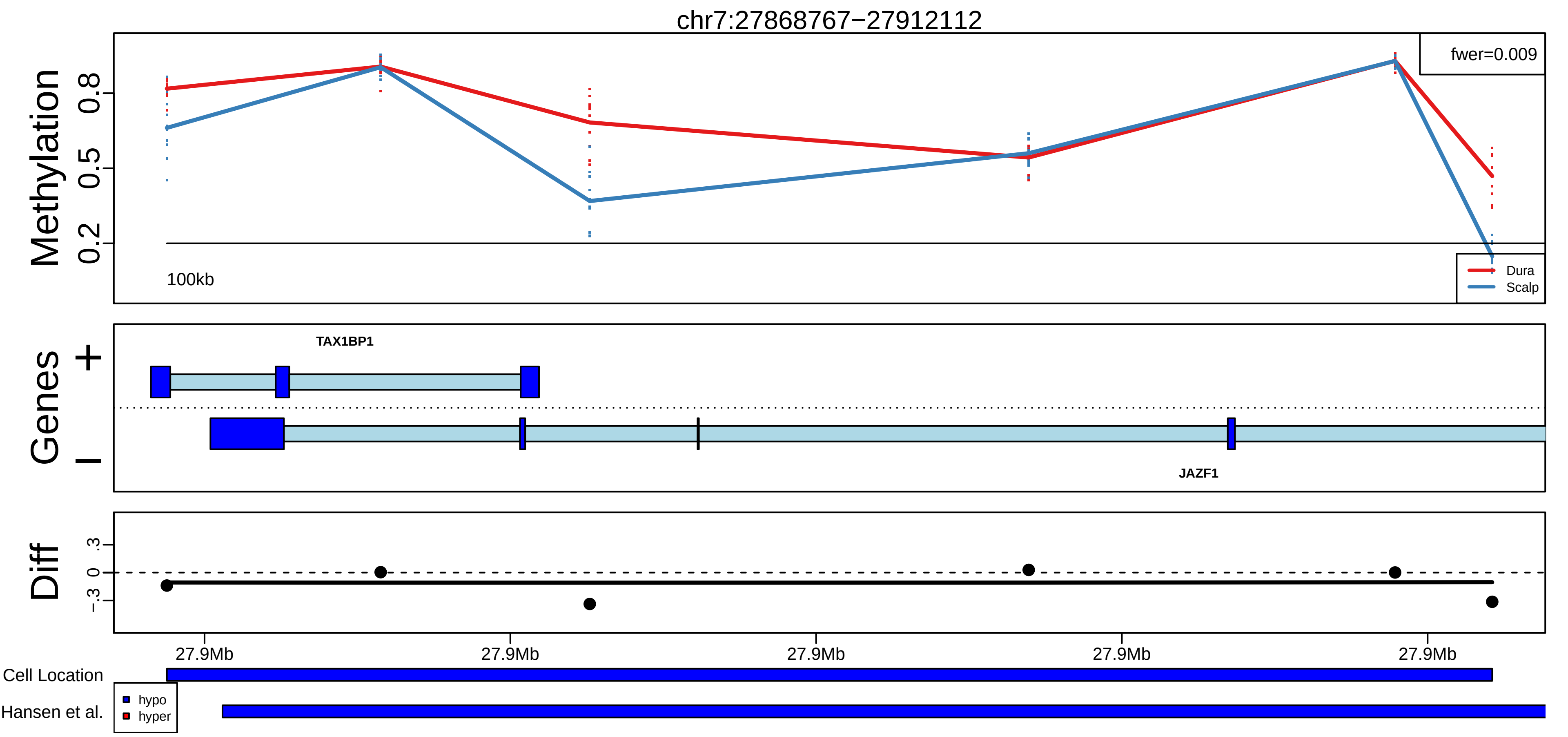
chr7:27868767−27912112
fwer=0.009
Methylation
0.2
0.5
0.8
100kb
Dura
Scalp
Genes
+
−
TAX1BP1
JAZF1
Diff
−.3
0
.3
27.9Mb
27.9Mb
27.9Mb
27.9Mb
27.9Mb
Cell Location
Hansen et al.
hypo
hyper

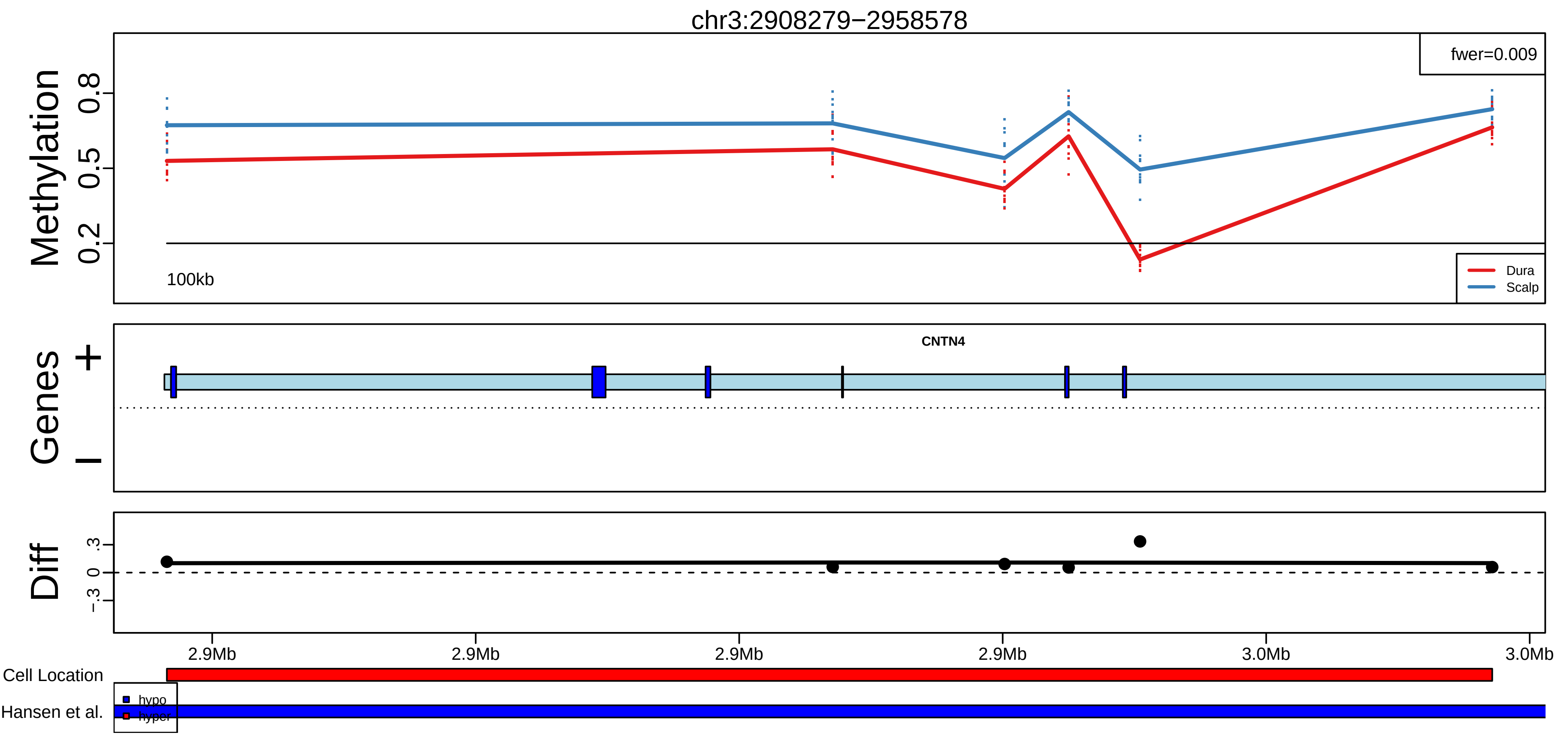
chr3:2908279−2958578
fwer=0.009
Methylation
0.8
0.5
0.2
100kb
Dura
Scalp
Genes
+
−
CNTN4
Diff
.3
0
−.3
2.9Mb
2.9Mb
2.9Mb
2.9Mb
3.0Mb
3.0Mb
Cell Location
Hansen et al.
hypo
hyper

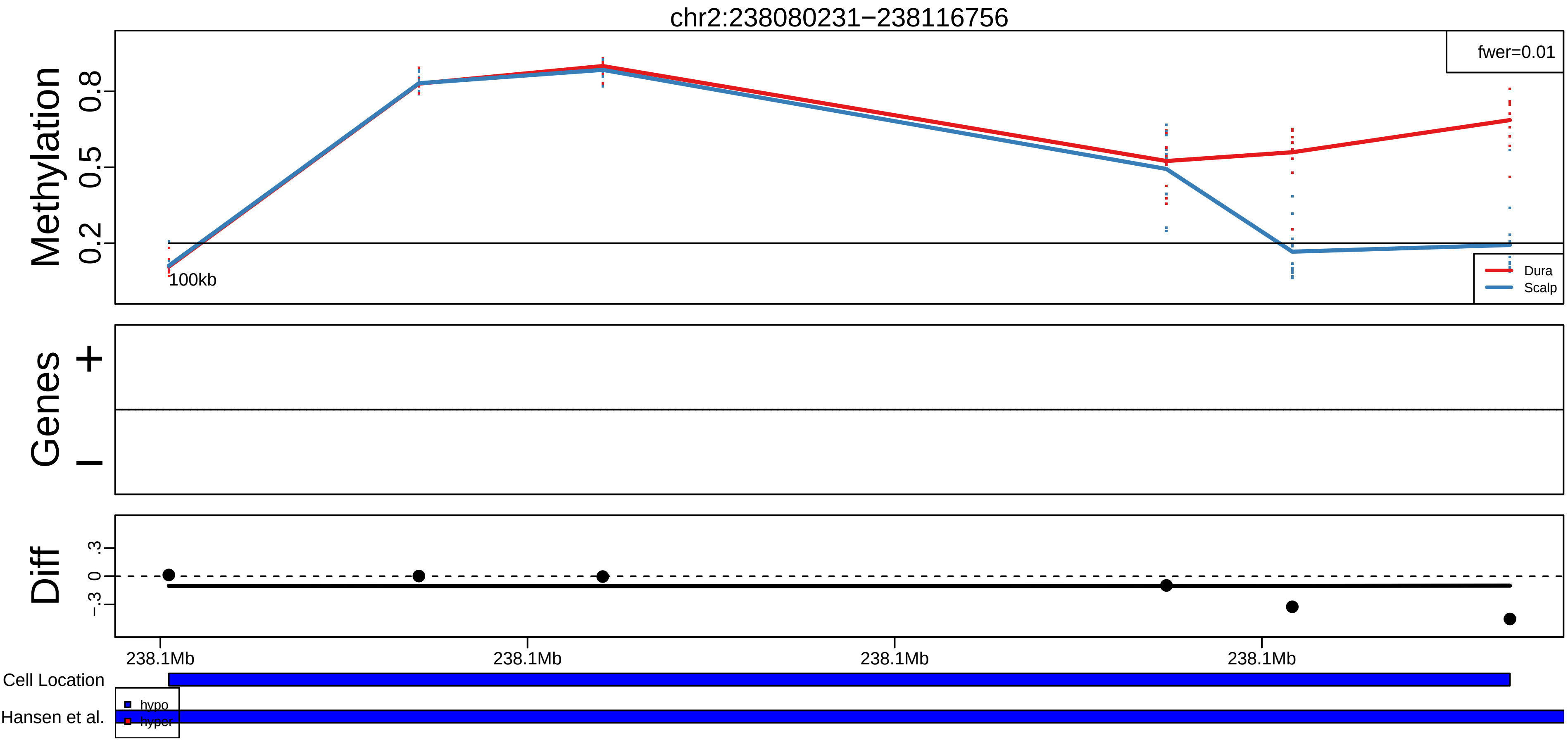

chr2:238080231−238116756
Methylation
0.2
0.5
0.8
100kb
fwer=0.01
Dura
Scalp
Genes
+
−
Diff
−.3
0
.3
238.1Mb
238.1Mb
238.1Mb
238.1Mb
Cell Location
hypo
hyper
Hansen et al.

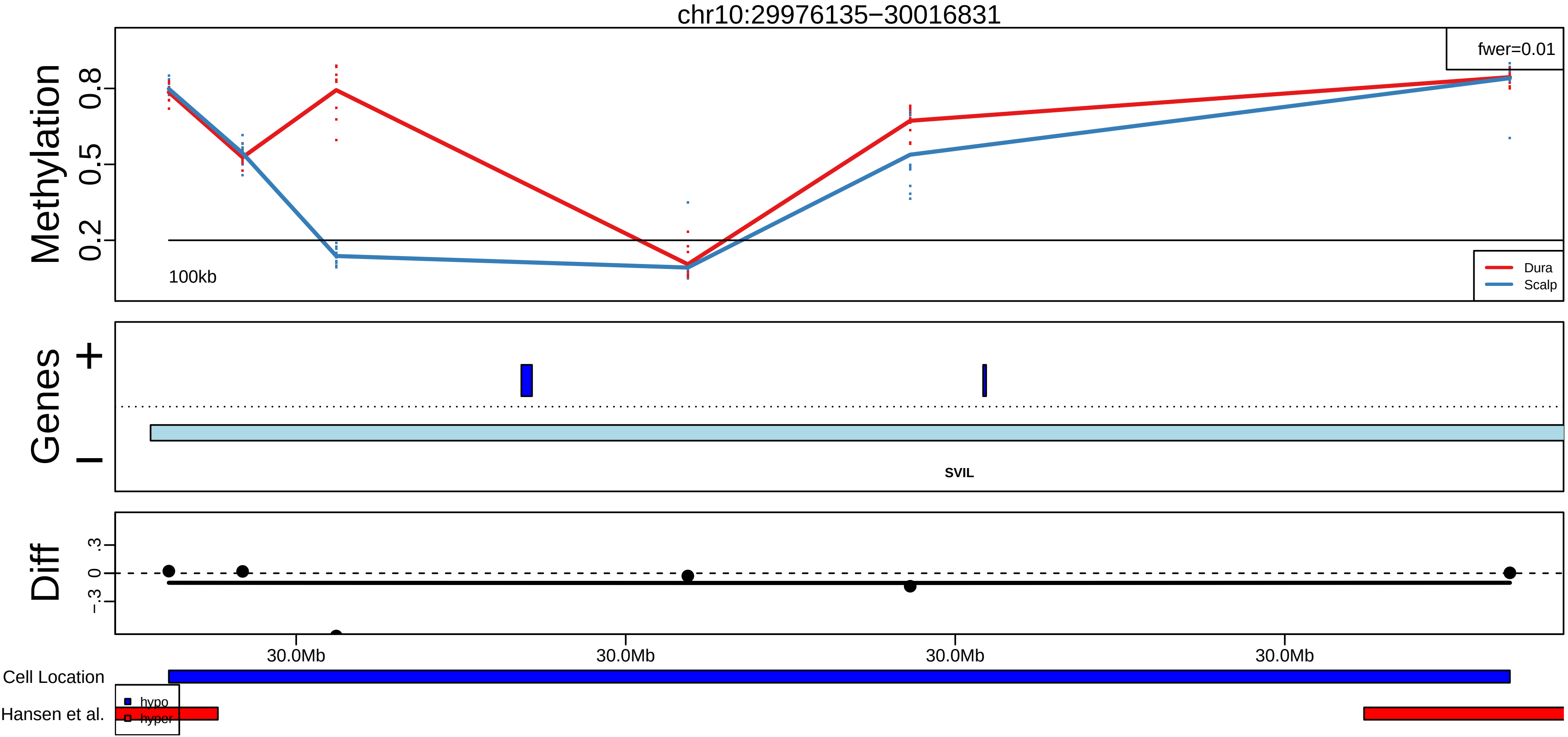
chr10:29976135−30016831
fwer=0.01
Methylation
0.2
0.5
0.8
100kb
Dura
Scalp
Genes
+
−
SVIL
Diff
−.3
0
.3
30.0Mb
30.0Mb
30.0Mb
30.0Mb
Cell Location
Hansen et al.
hypo
hyper

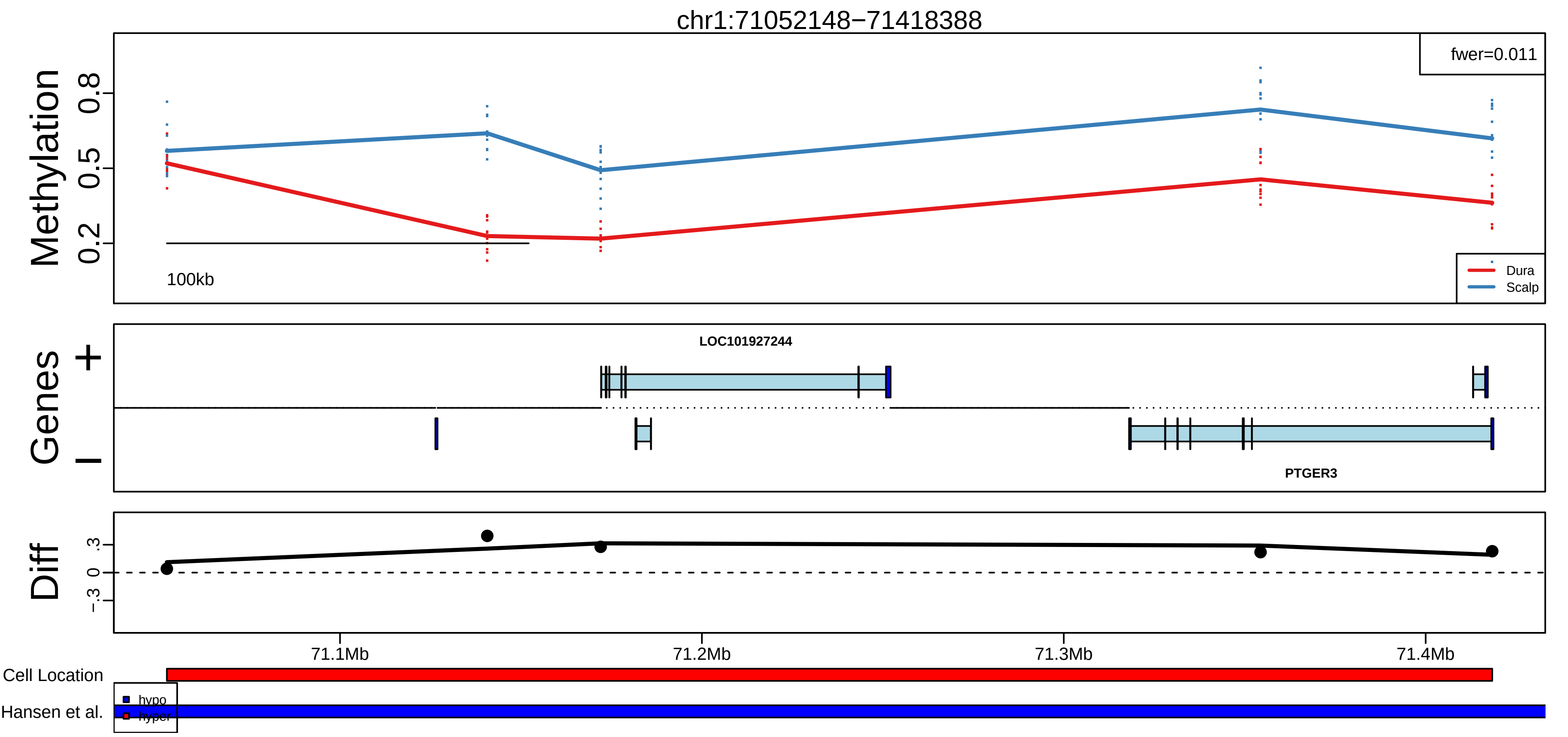
chr1:71052148−71418388
fwer=0.011
Methylation
0.2
0.5
0.8
100kb
Dura
Scalp
Genes
+
−
LOC101927244
PTGER3
Diff
−.3
0
.3
71.1Mb
71.2Mb
71.3Mb
71.4Mb
Cell Location
Hansen et al.
hypo
hyper

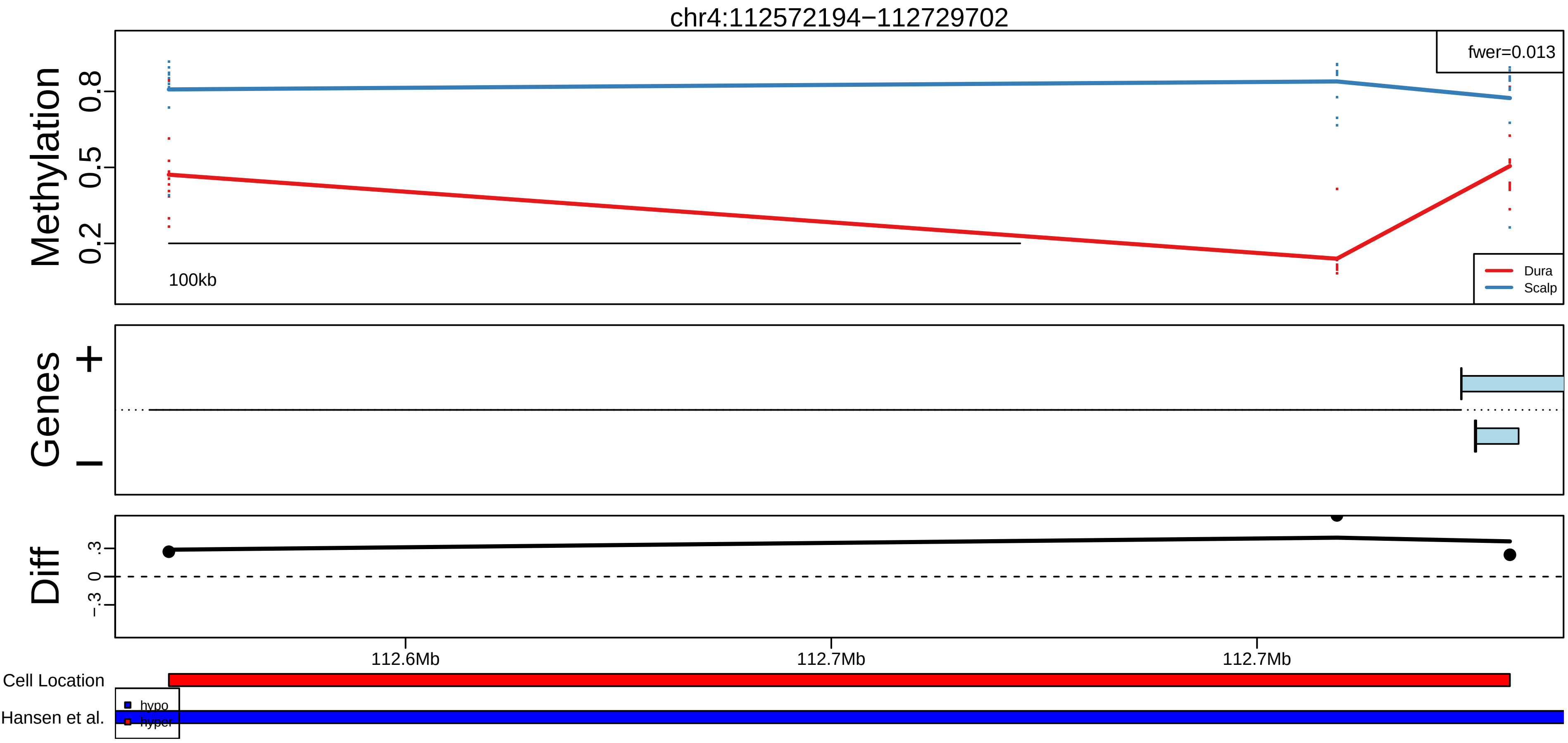

chr4:112572194−112729702
fwer=0.013
Methylation
0.8
0.5
0.2
100kb
Dura
Scalp
Genes
+
−
Diff
.3
0
−.3
112.6Mb
112.7Mb
112.7Mb
Cell Location
Hansen et al.
hypo
hyper

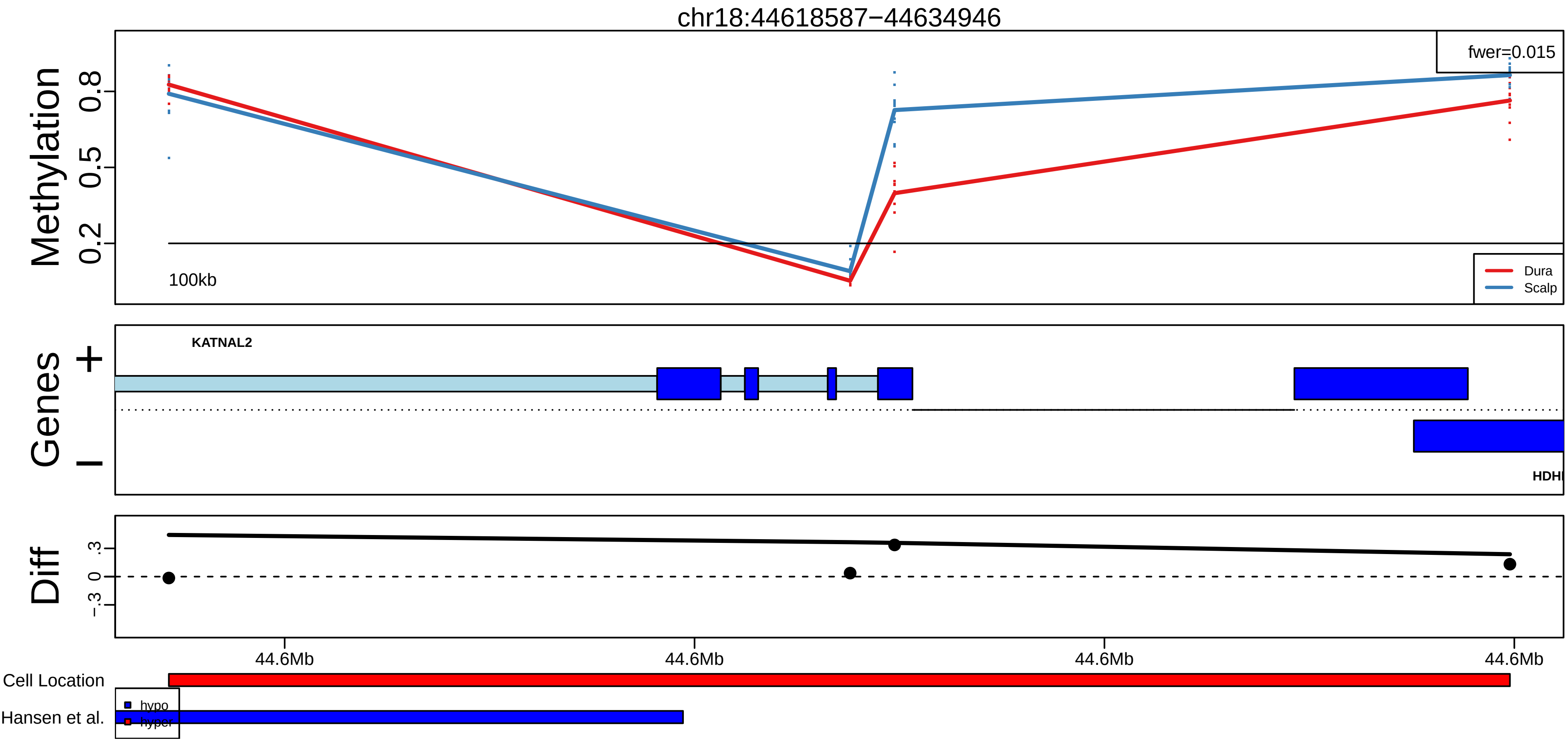

chr18:44618587−44634946
fwer=0.015
Methylation
0.2
0.5
0.8
100kb
Dura
Scalp
Genes
+
−
KATNAL2
HDHI
Diff
.3
0
−.3
44.6Mb
44.6Mb
44.6Mb
44.6Mb
Cell Location
Hansen et al.
hypo
hyper

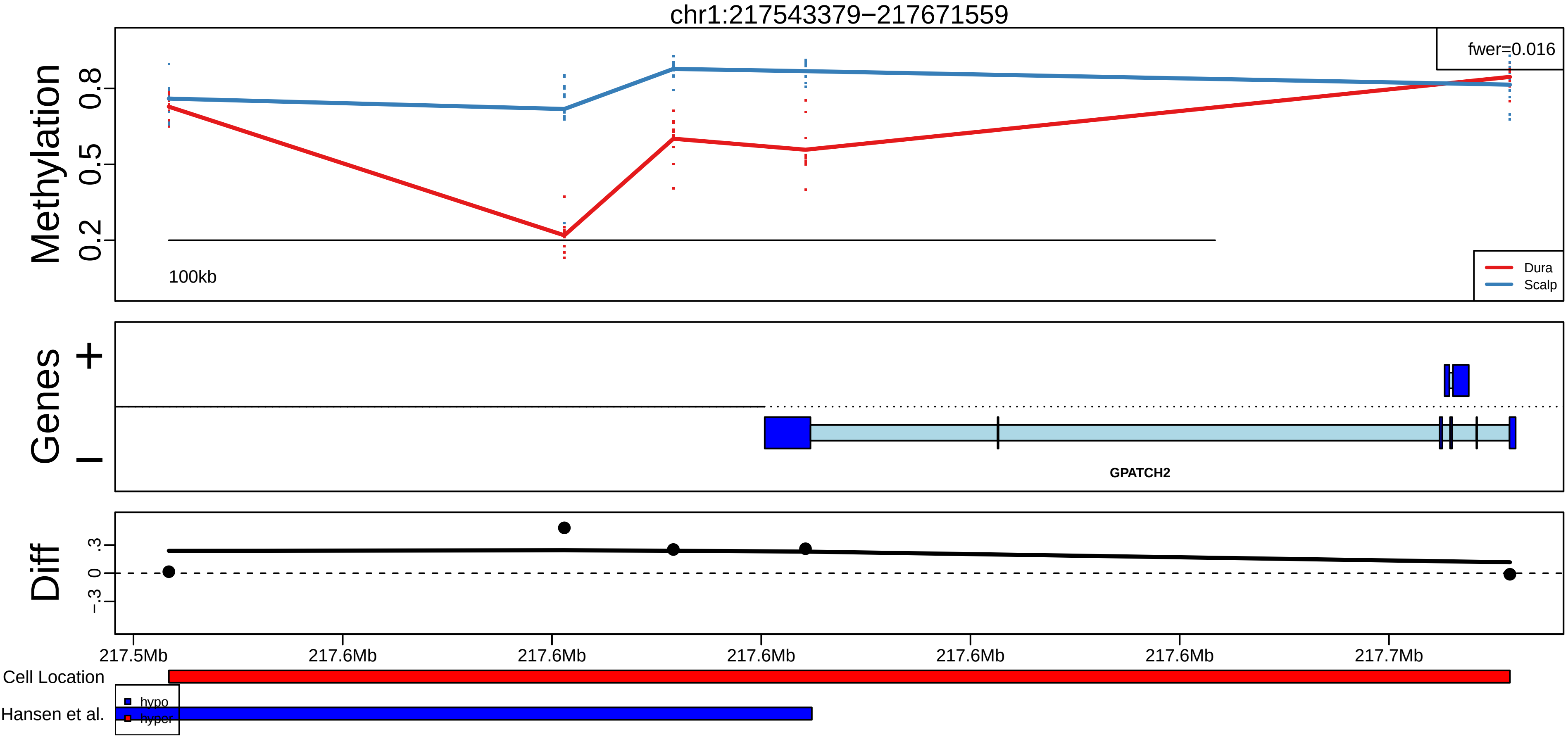
chr1:217543379−217671559
fwer=0.016
Methylation
0.2
0.5
0.8
100kb
Dura
Scalp
Genes
+
−
GPATCH2
Diff
.3
0
−.3
217.5Mb
217.6Mb
217.6Mb
217.6Mb
217.6Mb
217.6Mb
217.7Mb
Cell Location
Hansen et al.
hypo
hyper

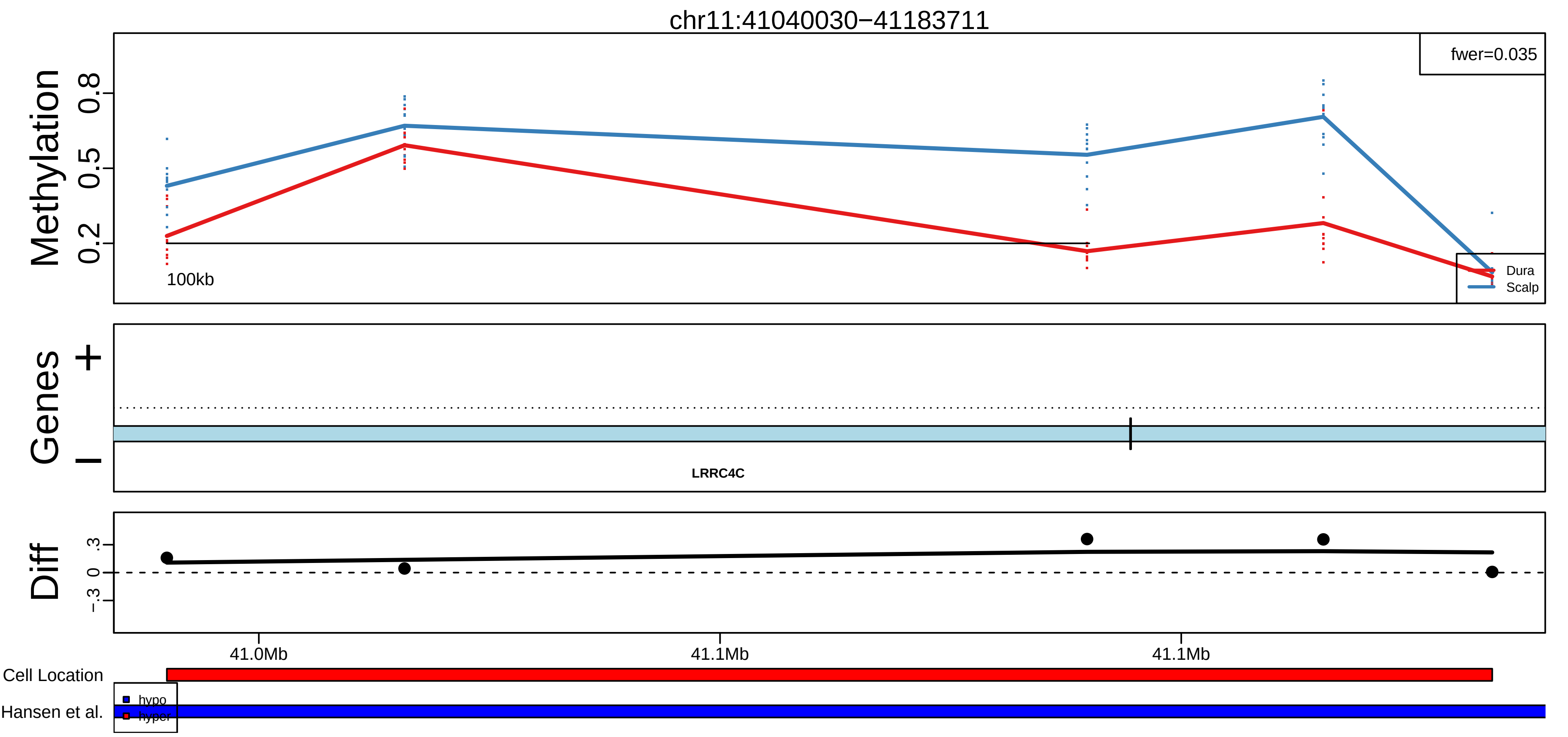
chr11:41040030−41183711
fwer=0.035
Methylation
0.2
0.5
0.8
100kb
Dura
Scalp
Genes
+
−
LRRC4C
Diff
−.3
0
.3
41.0Mb
41.1Mb
41.1Mb
Cell Location
Hansen et al.
hypo
hyper

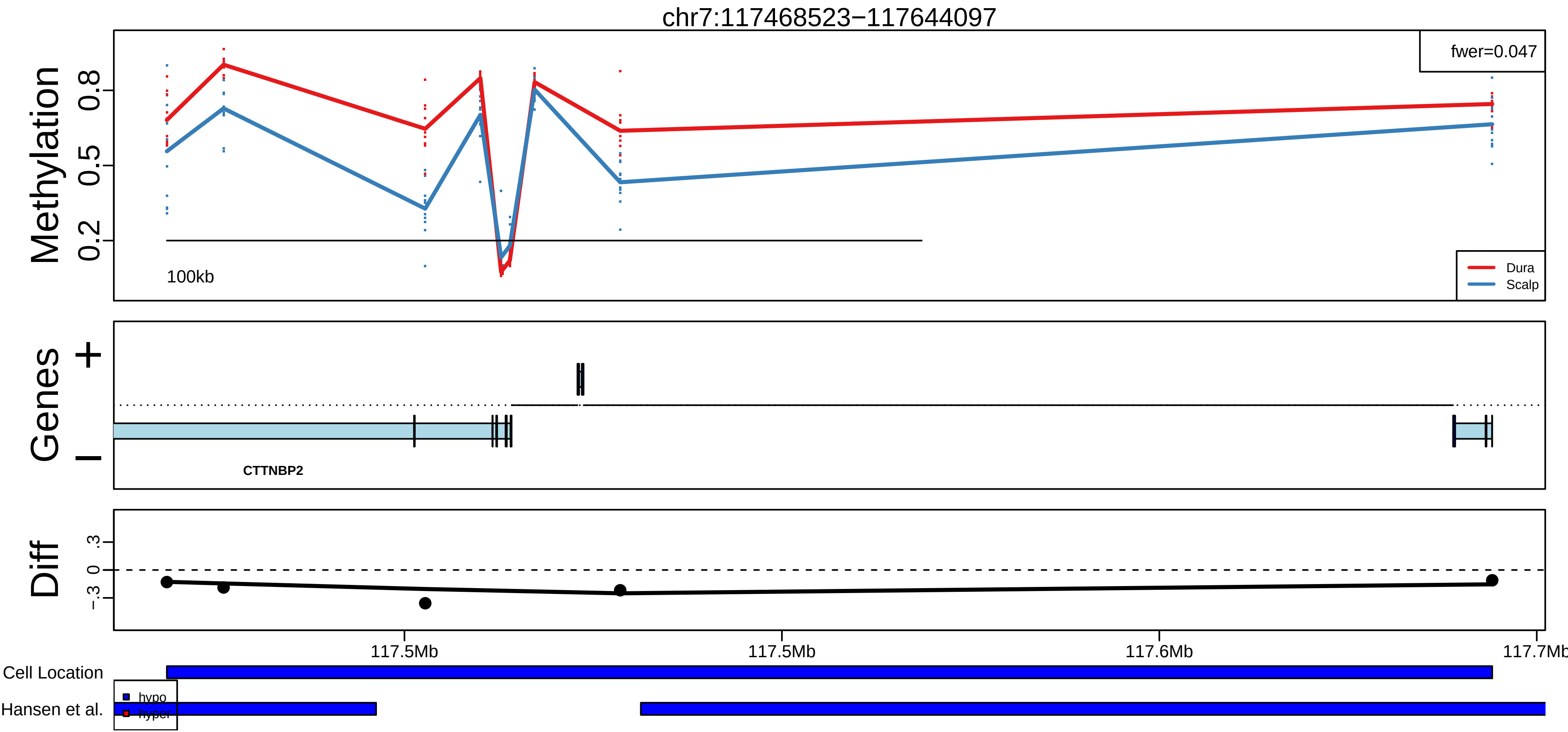
chr7:117468523−117644097
Methylation
0.8
0.5
0.2
fwer=0.047
100kb
Dura
Scalp
Genes
+
−
CTTNBP2
Diff
.3
0
−.3
117.5Mb
117.5Mb
117.6Mb
117.7Mb
Cell Location
Hansen et al.
hypo
hyper

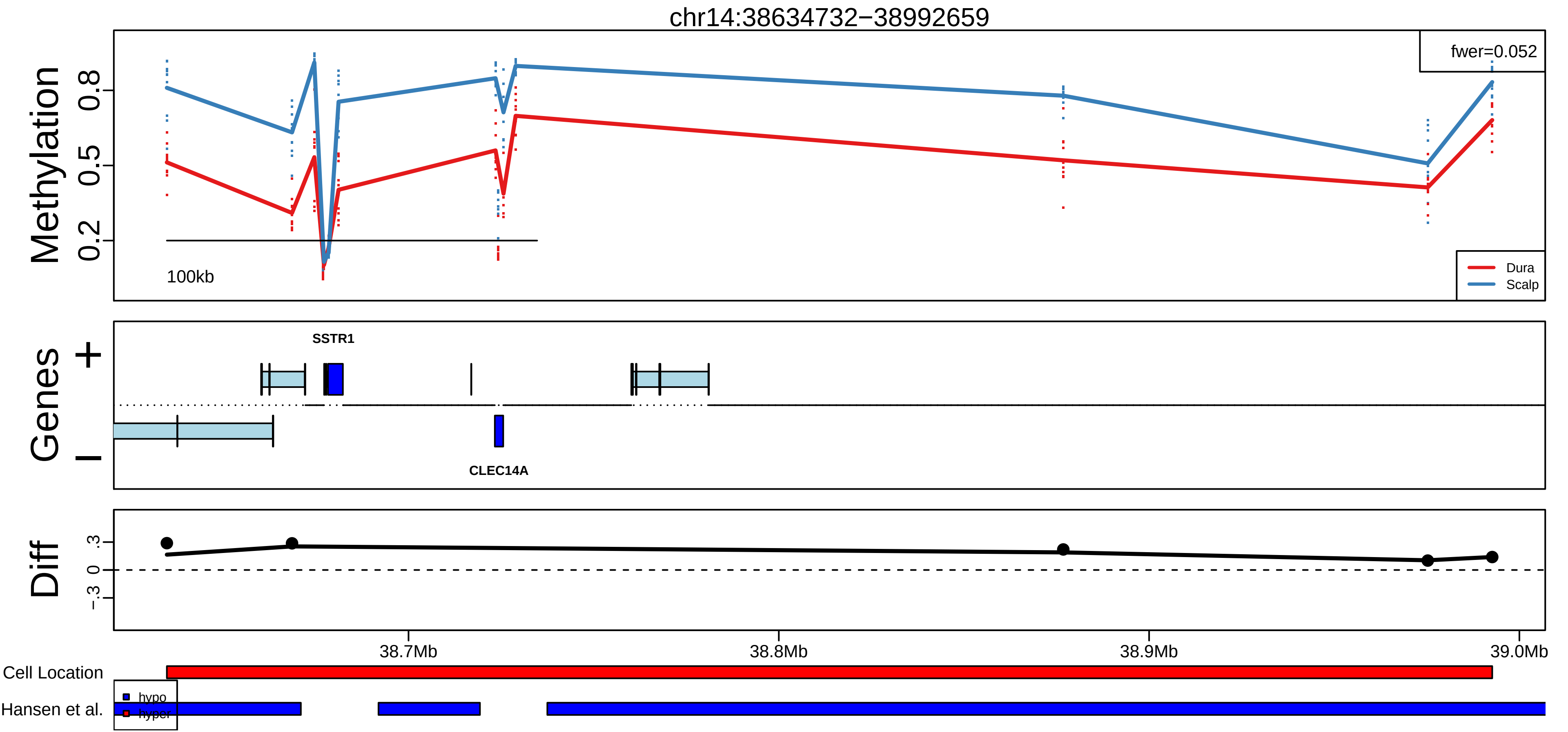

chr14:38634732−38992659
fwer=0.052
Methylation
0.2
0.5
0.8
100kb
Dura
Scalp
Genes
+
−
SSTR1
CLEC14A
Diff
−.3
0
.3
38.7Mb
38.8Mb
38.9Mb
39.0Mb
Cell Location
Hansen et al.
hypo
hyper

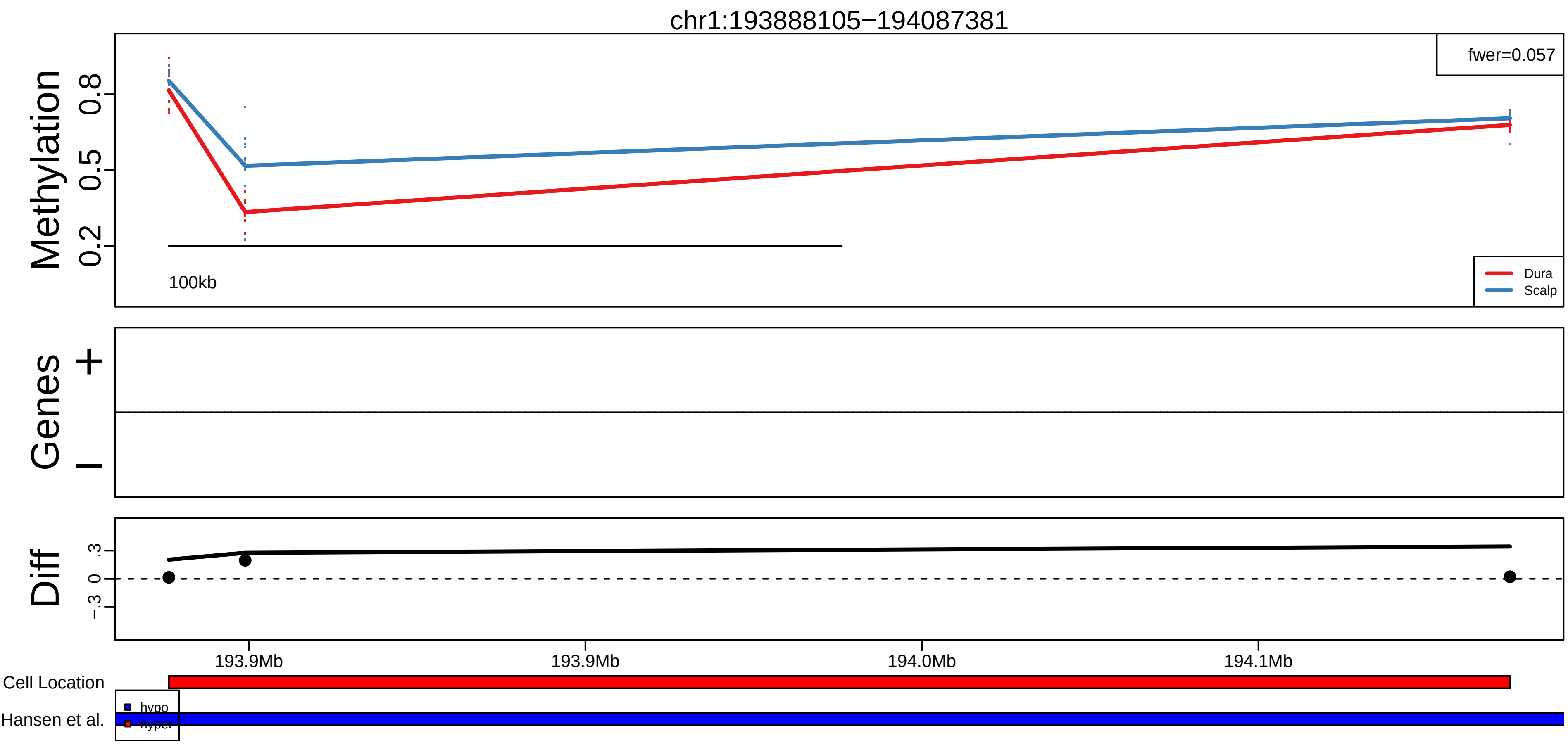
chr1:193888105−194087381
fwer=0.057
Methylation
0.8
0.5
0.2
100kb
Dura
Scalp
Genes
+
−
Diff
.3
0
−.3
193.9Mb
193.9Mb
194.0Mb
194.1Mb
Cell Location
Hansen et al.
hypo
hyper

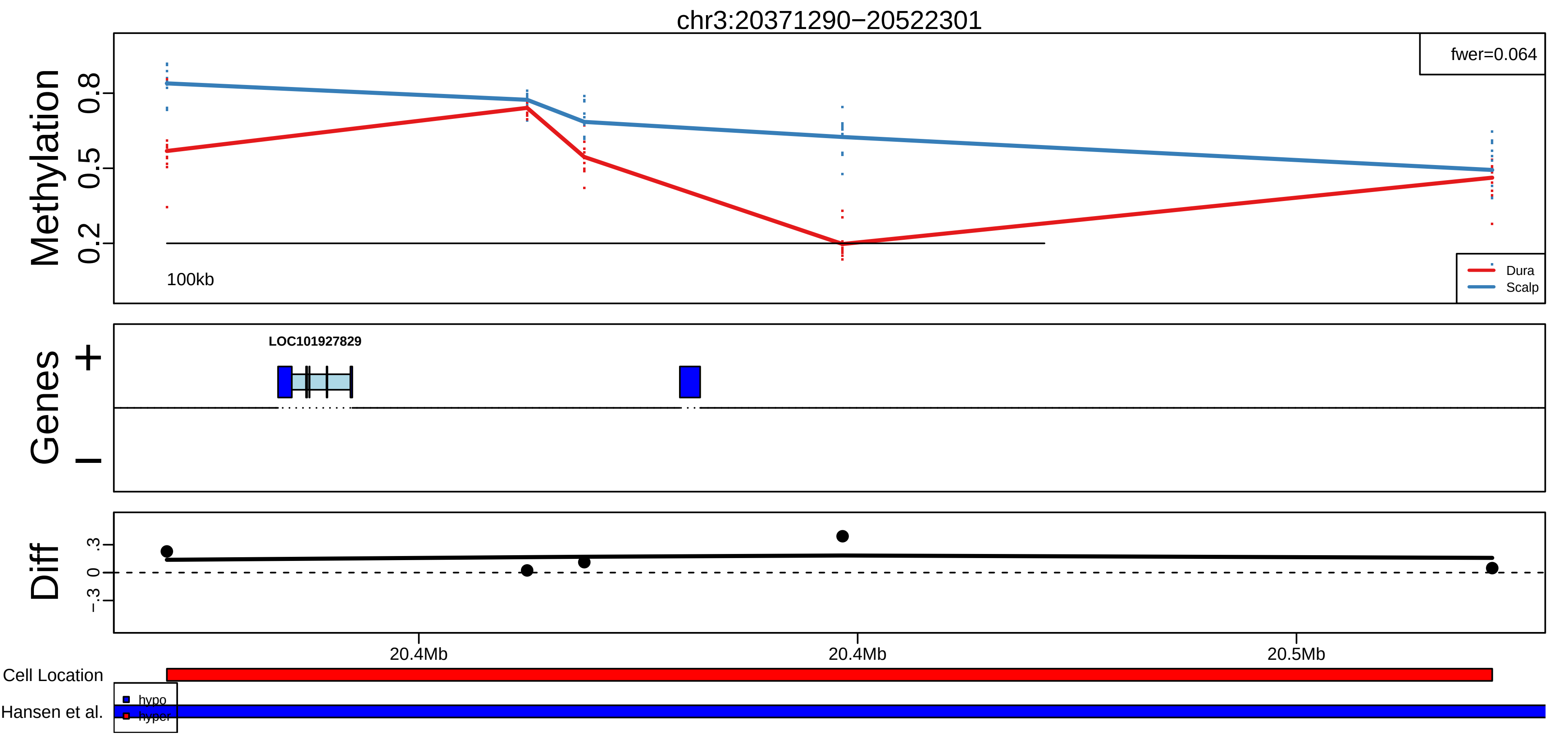
chr3:20371290−20522301
fwer=0.064
Methylation
0.2
0.5
0.8
100kb
Dura
Scalp
Genes
+
−
LOC101927829
Diff
−.3
0
.3
20.4Mb
20.4Mb
20.5Mb
Cell Location
Hansen et al.
hypo
hyper

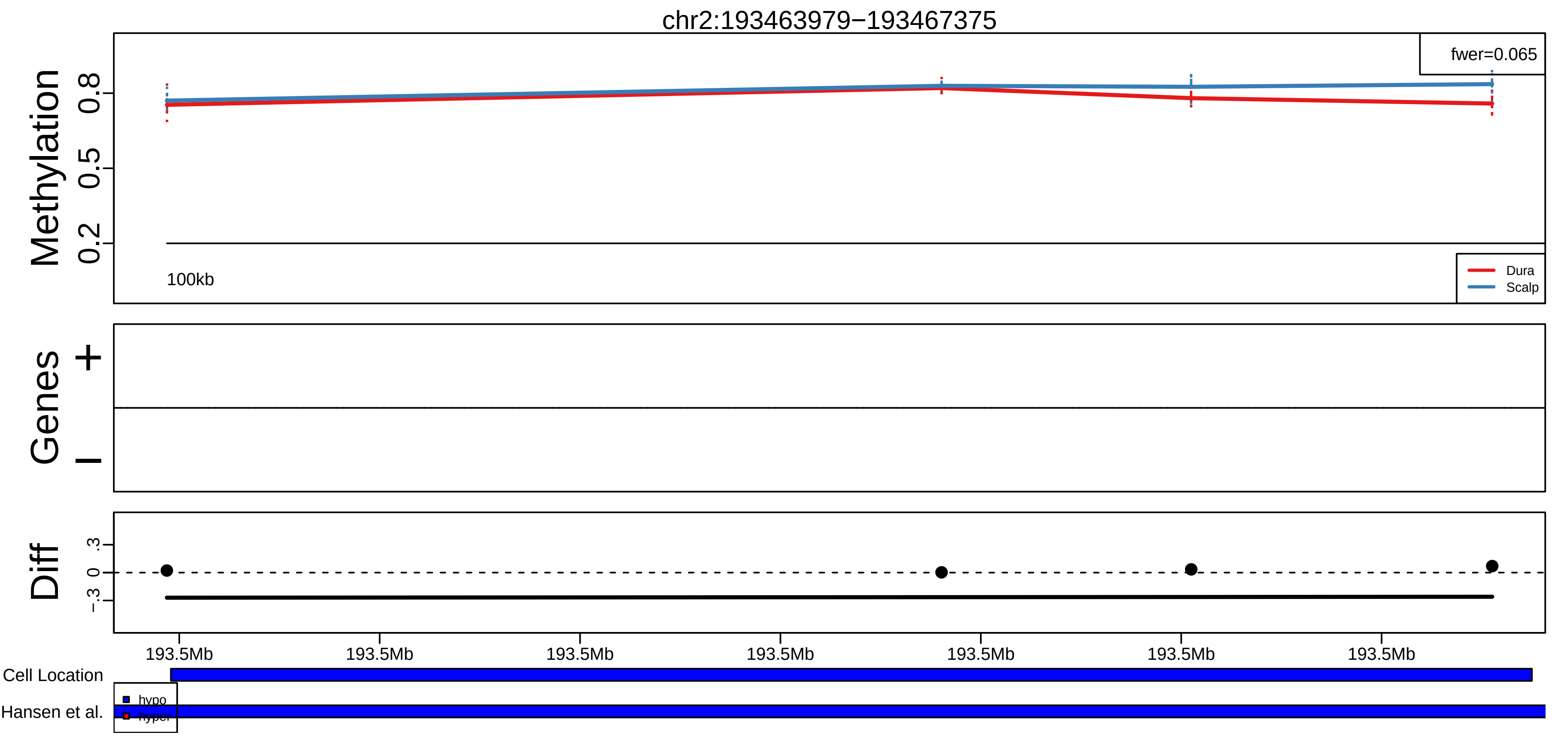

chr2:193463979−193467375
fwer=0.065
Methylation
0.8
0.5
0.2
100kb
Dura
Scalp
Genes
+
−
Diff
.3
0
−.3
193.5Mb
193.5Mb
193.5Mb
193.5Mb
193.5Mb
193.5Mb
193.5Mb
Cell Location
Hansen et al.
hypo
hyper

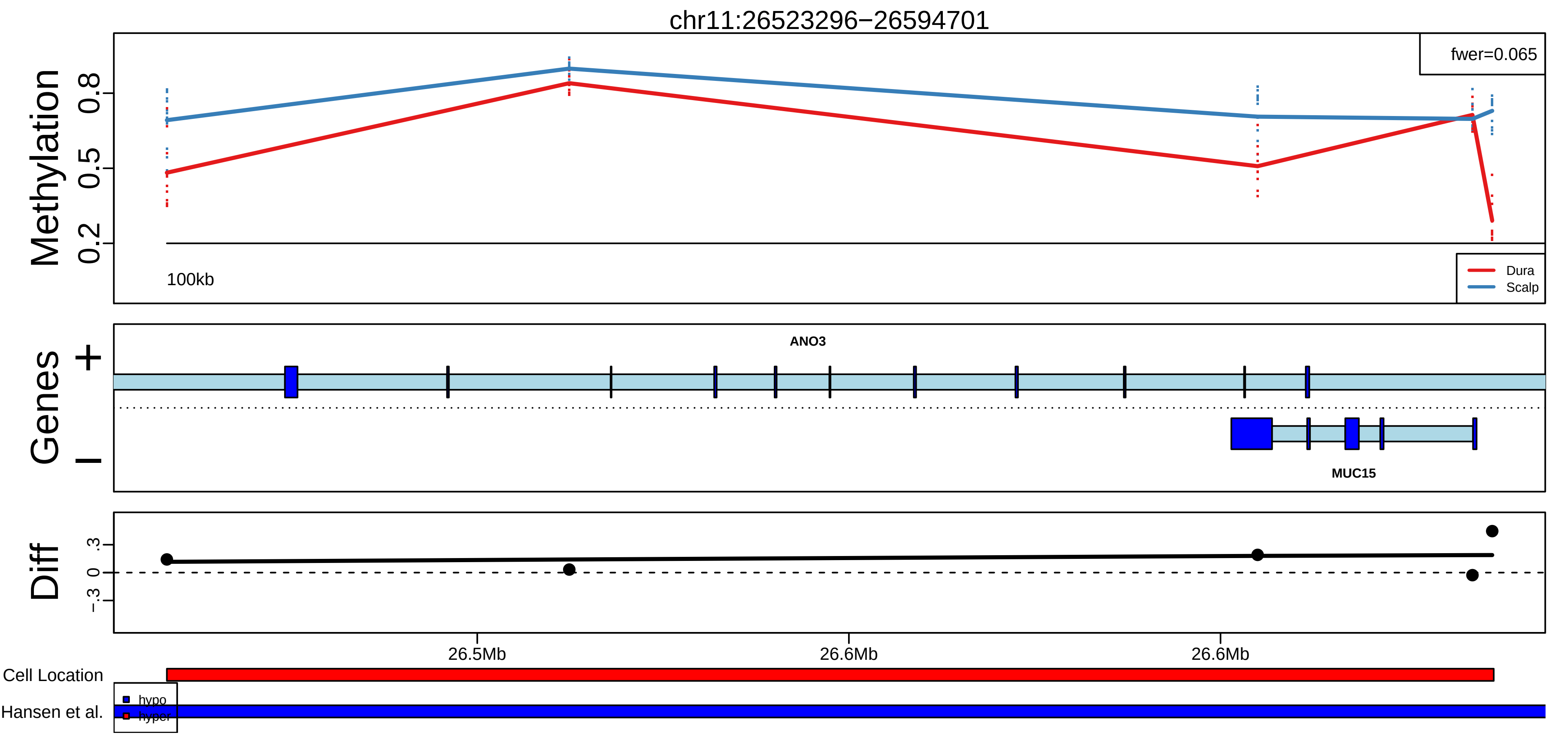
chr11:26523296−26594701
fwer=0.065
Methylation
0.2
0.5
0.8
100kb
Dura
Scalp
Genes
+
−
ANO3
MUC15
Diff
−.3
0
.3
26.5Mb
26.6Mb
26.6Mb
Cell Location
Hansen et al.
hypo
hyper

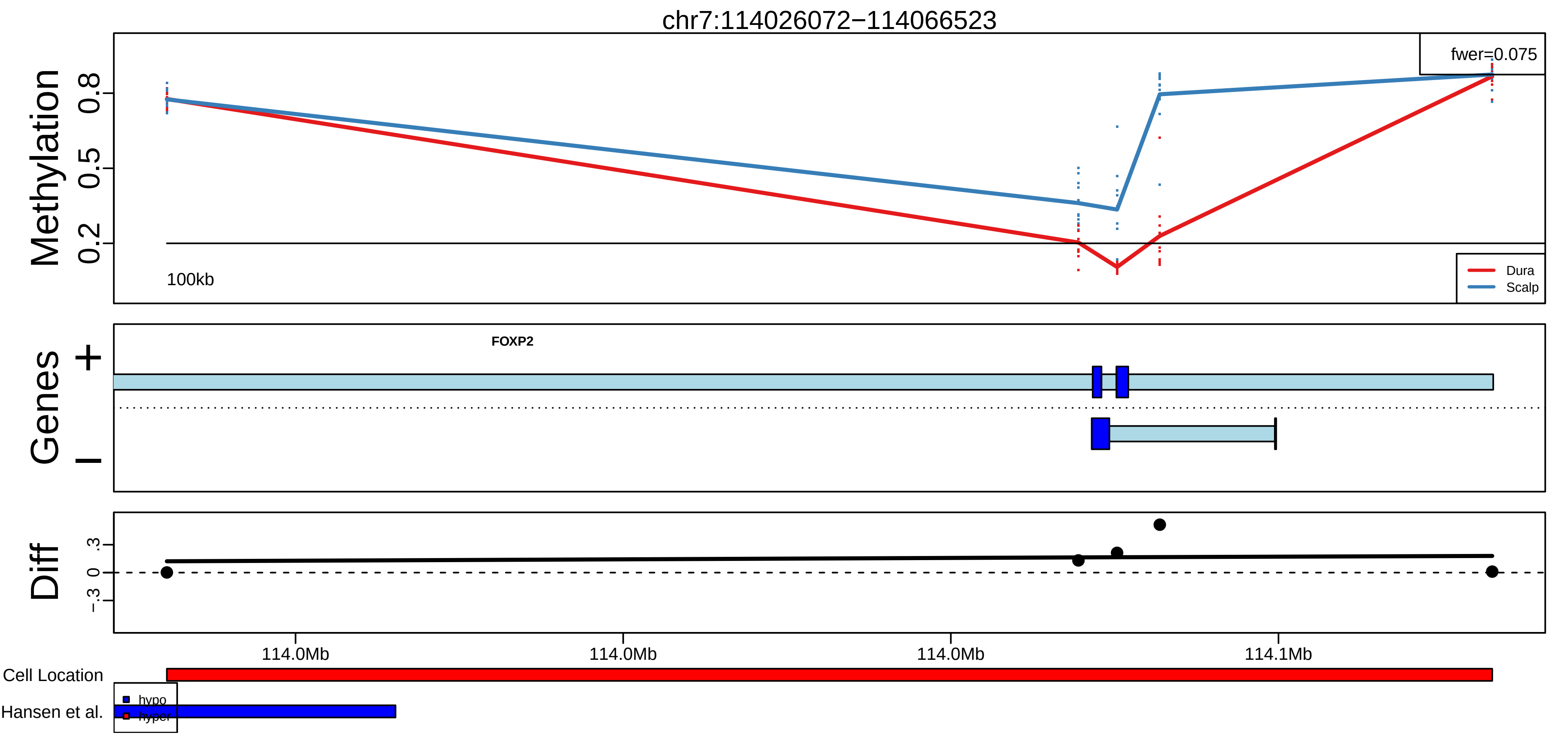

chr7:114026072−114066523
fwer=0.075
Methylation
0.8
0.5
0.2
100kb
Dura
Scalp
Genes
+
−
FOXP2
Diff
.3
0
−.3
114.0Mb
114.0Mb
114.0Mb
114.1Mb
Cell Location
Hansen et al.
hypo
hyper

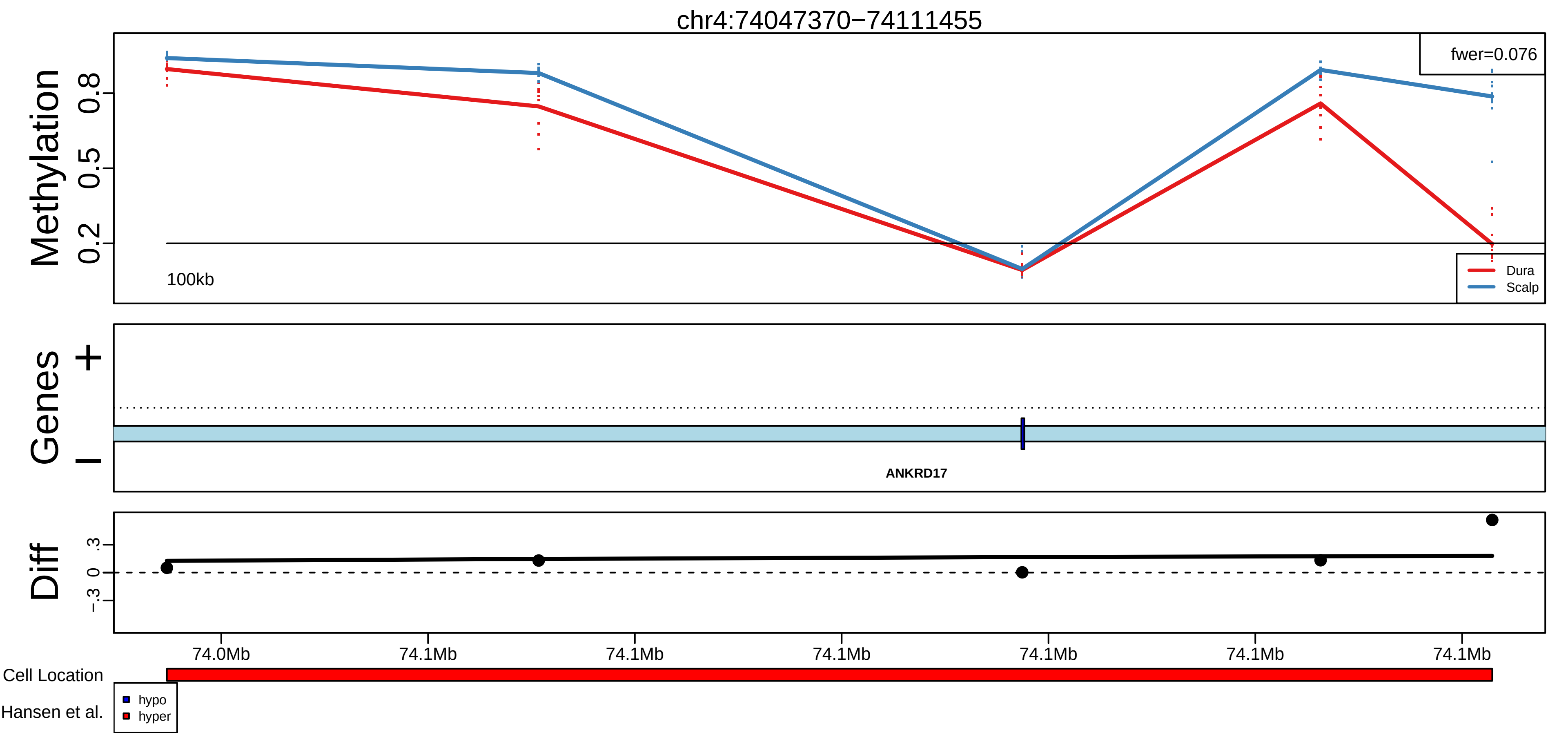

chr4:74047370−74111455
Methylation
0.2
0.5
0.8
fwer=0.076
100kb
Dura
Scalp
Genes
+
−
ANKRD17
Diff
−.3
0
.3
74.0Mb
74.1Mb
74.1Mb
74.1Mb
74.1Mb
74.1Mb
74.1Mb
Cell Location
Hansen et al.
hypo
hyper

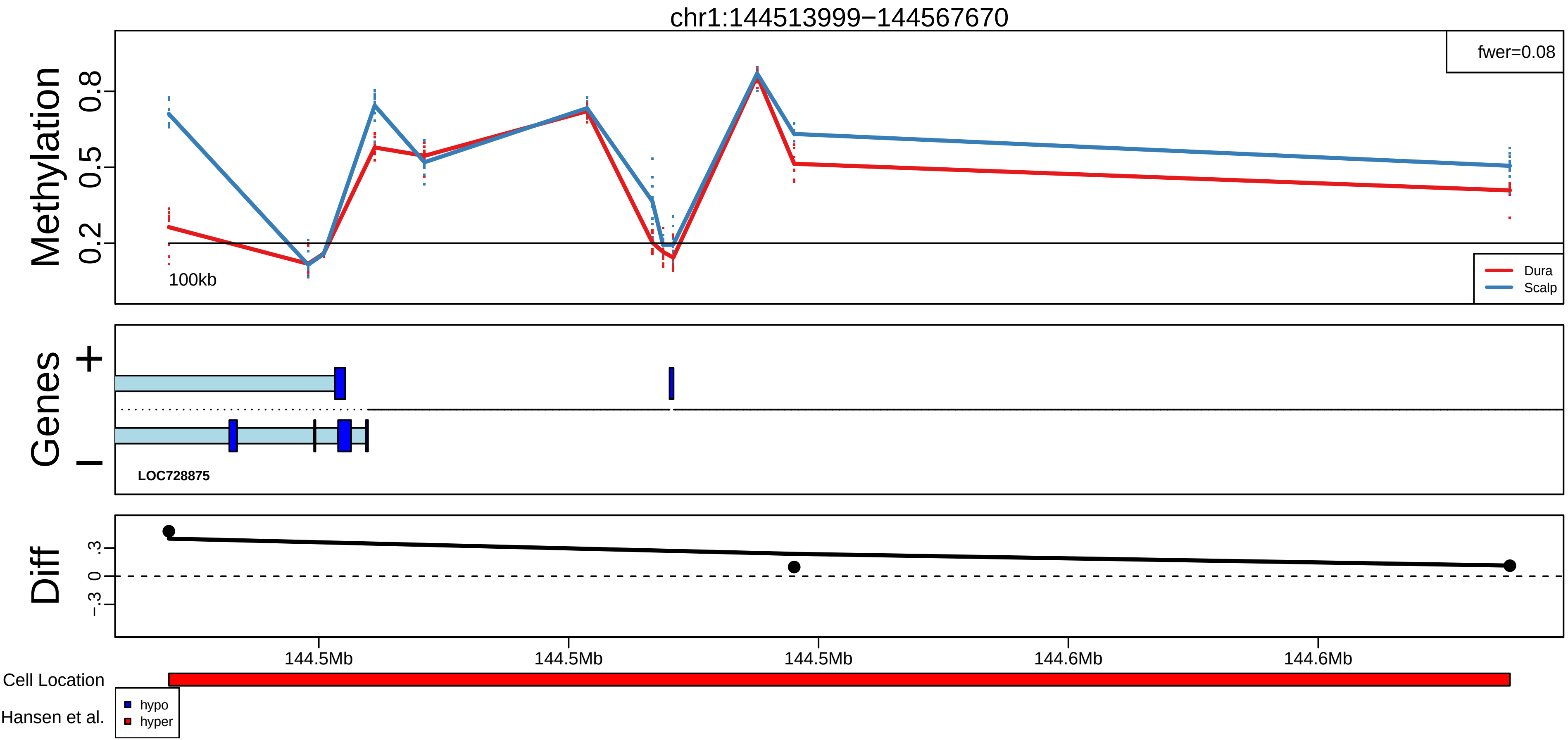

chr1:144513999−144567670
fwer=0.08
Methylation
0.2
0.5
0.8
100kb
Dura
Scalp
Genes
+
−
LOC728875
Diff
−.3
0
.3
144.5Mb
144.5Mb
144.5Mb
144.6Mb
144.6Mb
Cell Location
Hansen et al.
hypo
hyper

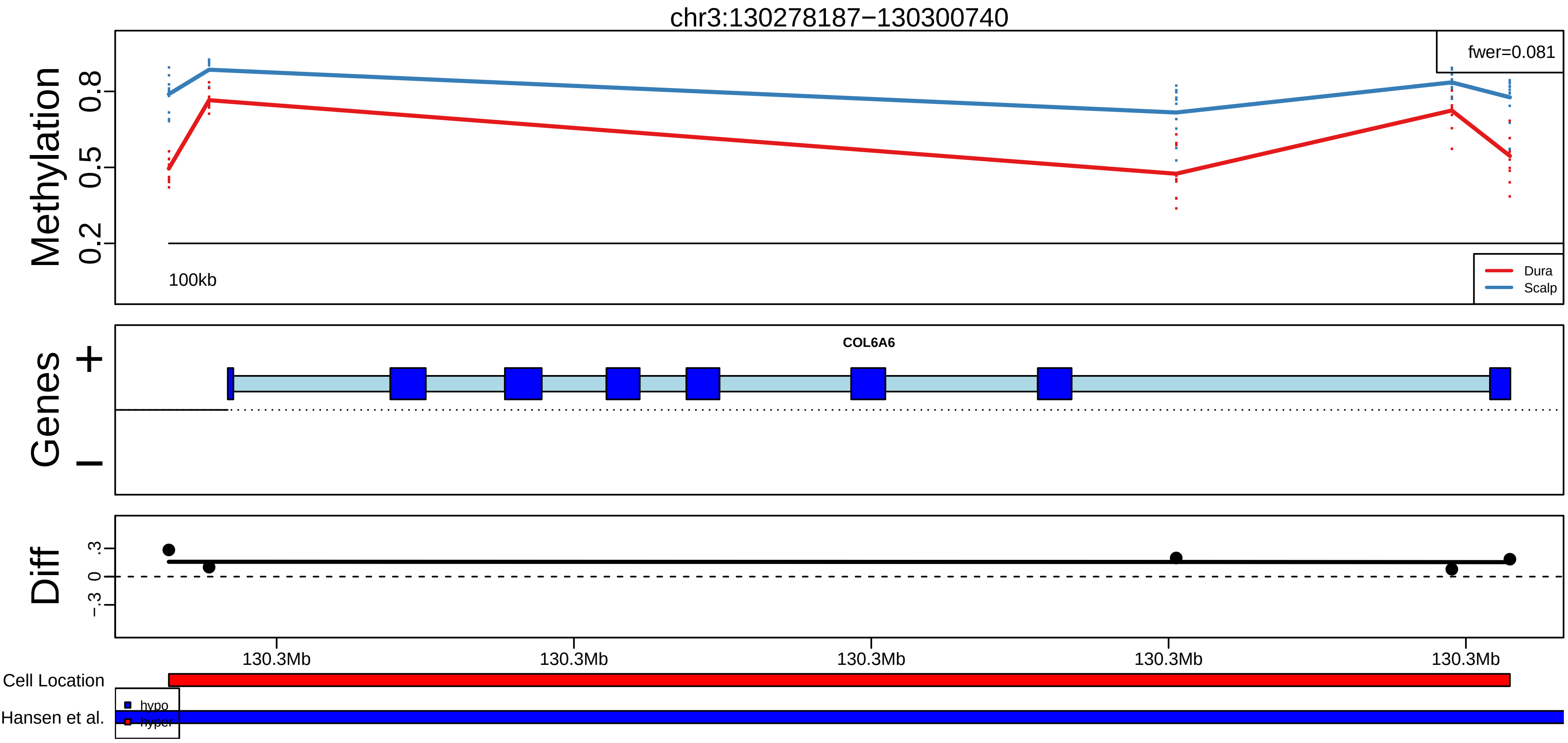

chr3:130278187−130300740
Methylation
0.2
0.5
0.8
fwer=0.081
100kb
Dura
Scalp
Genes
+
−
COL6A6
Diff
−.3
0
.3
130.3Mb
130.3Mb
130.3Mb
130.3Mb
130.3Mb
Cell Location
Hansen et al.
hypo
hyper

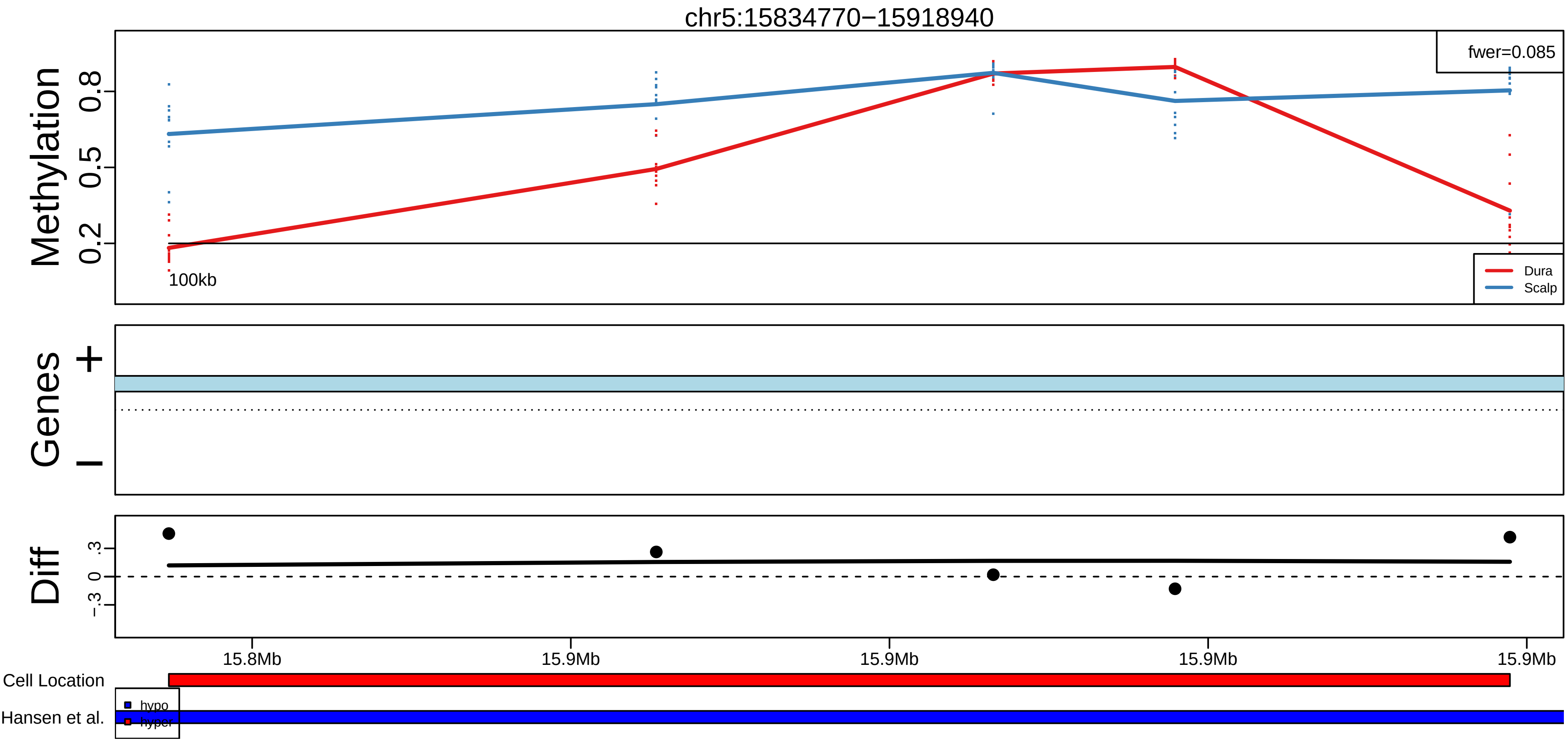

chr5:15834770−15918940
fwer=0.085
Methylation
0.2
0.5
0.8
100kb
Dura
Scalp
Genes
+
−
Diff
.3
0
−.3
15.8Mb
15.9Mb
15.9Mb
15.9Mb
15.9Mb
Cell Location
Hansen et al.
hypo
hyper

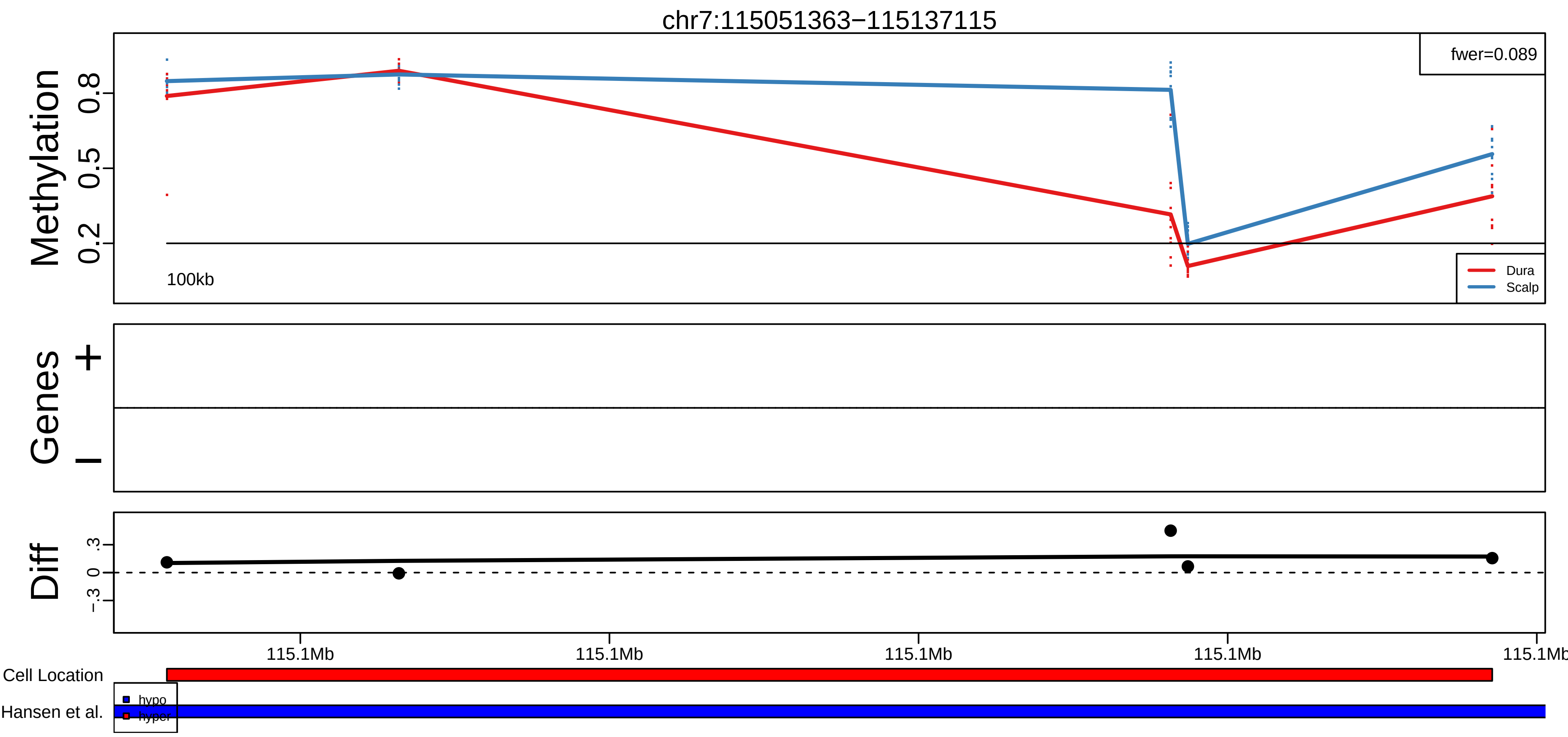

chr7:115051363−115137115
fwer=0.089
Methylation
0.2
0.5
0.8
100kb
Dura
Scalp
Genes
+
−
Diff
−.3
0
.3
115.1Mb
115.1Mb
115.1Mb
115.1Mb
115.1Mb
Cell Location
Hansen et al.
hypo
hyper

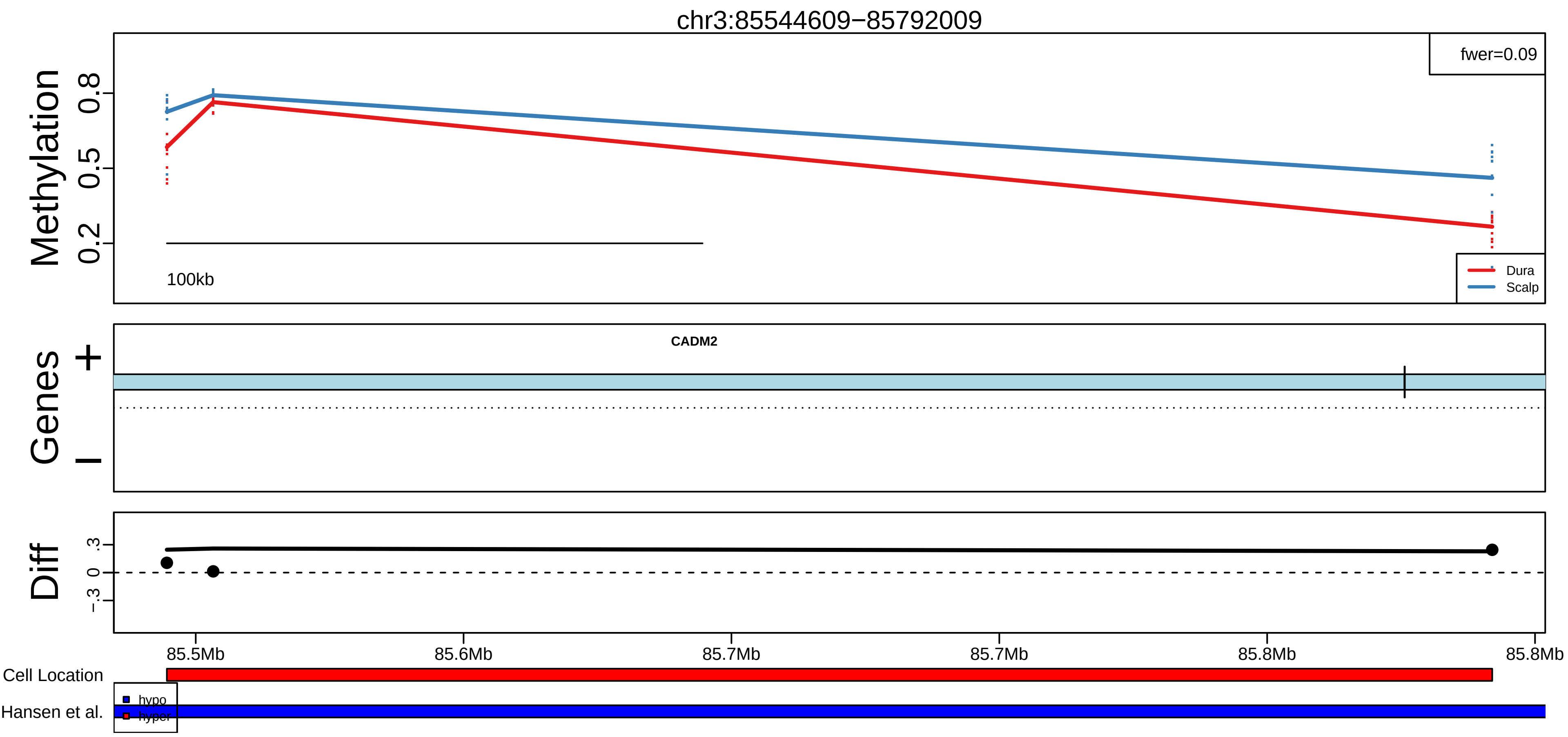
chr3:85544609−85792009
fwer=0.09
Methylation
0.2
0.5
0.8
100kb
Dura
Scalp
CADM2
Genes
+
−
Diff
−.3
0
.3
85.5Mb
85.6Mb
85.7Mb
85.7Mb
85.8Mb
85.8Mb
Cell Location
Hansen et al.
hypo
hyper

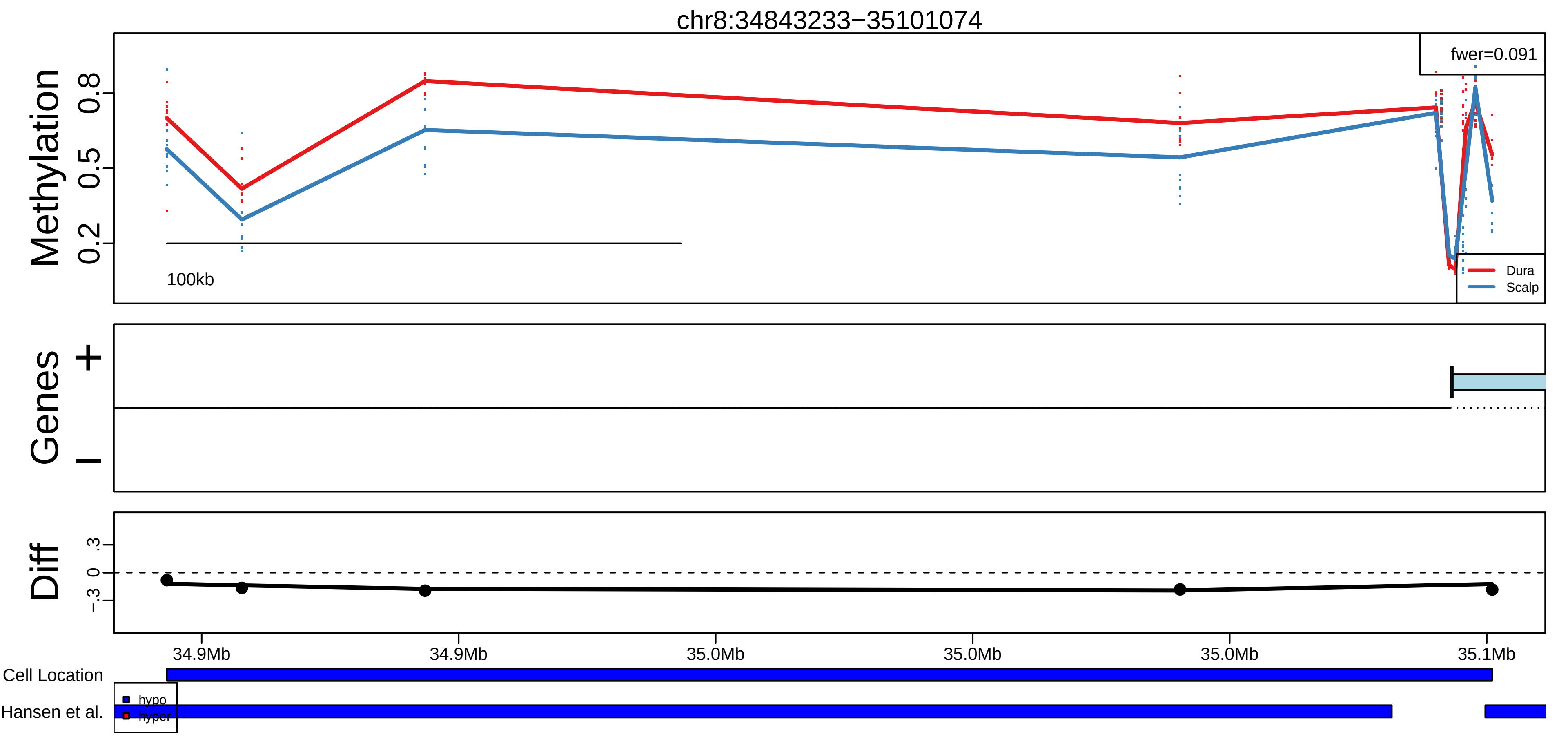

chr8:34843233−35101074
fwer=0.091
Methylation
0.2
0.5
0.8
100kb
Dura
Scalp
Genes
+
−
Diff
.3
0
−.3
34.9Mb
34.9Mb
35.0Mb
35.0Mb
35.0Mb
35.1Mb
Cell Location
Hansen et al.
hypo
hyper

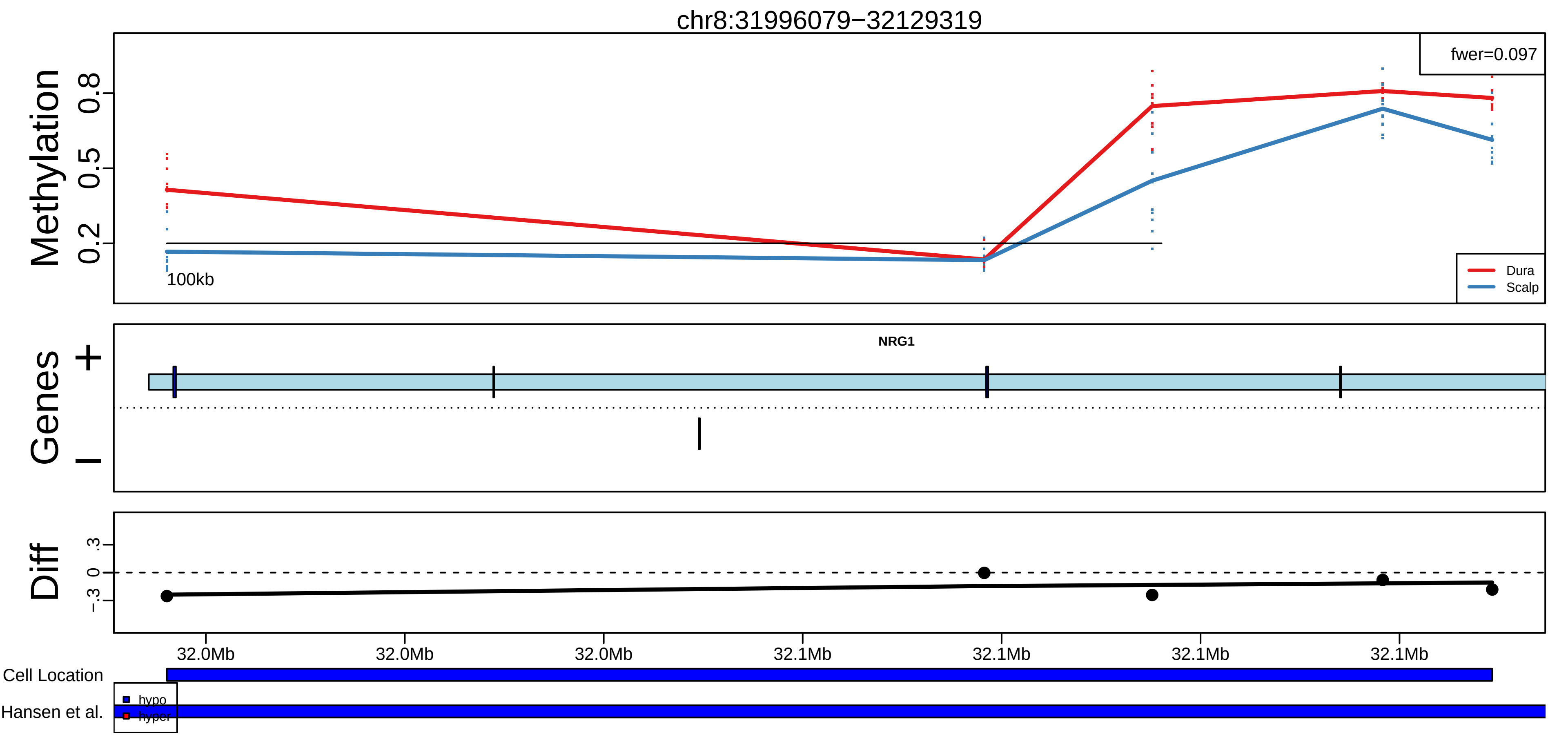
chr8:31996079−32129319
fwer=0.097
Methylation
0.2
0.5
0.8
100kb
Dura
Scalp
Genes
+
−
NRG1
Diff
−.3
0
.3
32.0Mb
32.0Mb
32.0Mb
32.1Mb
32.1Mb
32.1Mb
32.1Mb
Cell Location
Hansen et al.
hypo
hyper

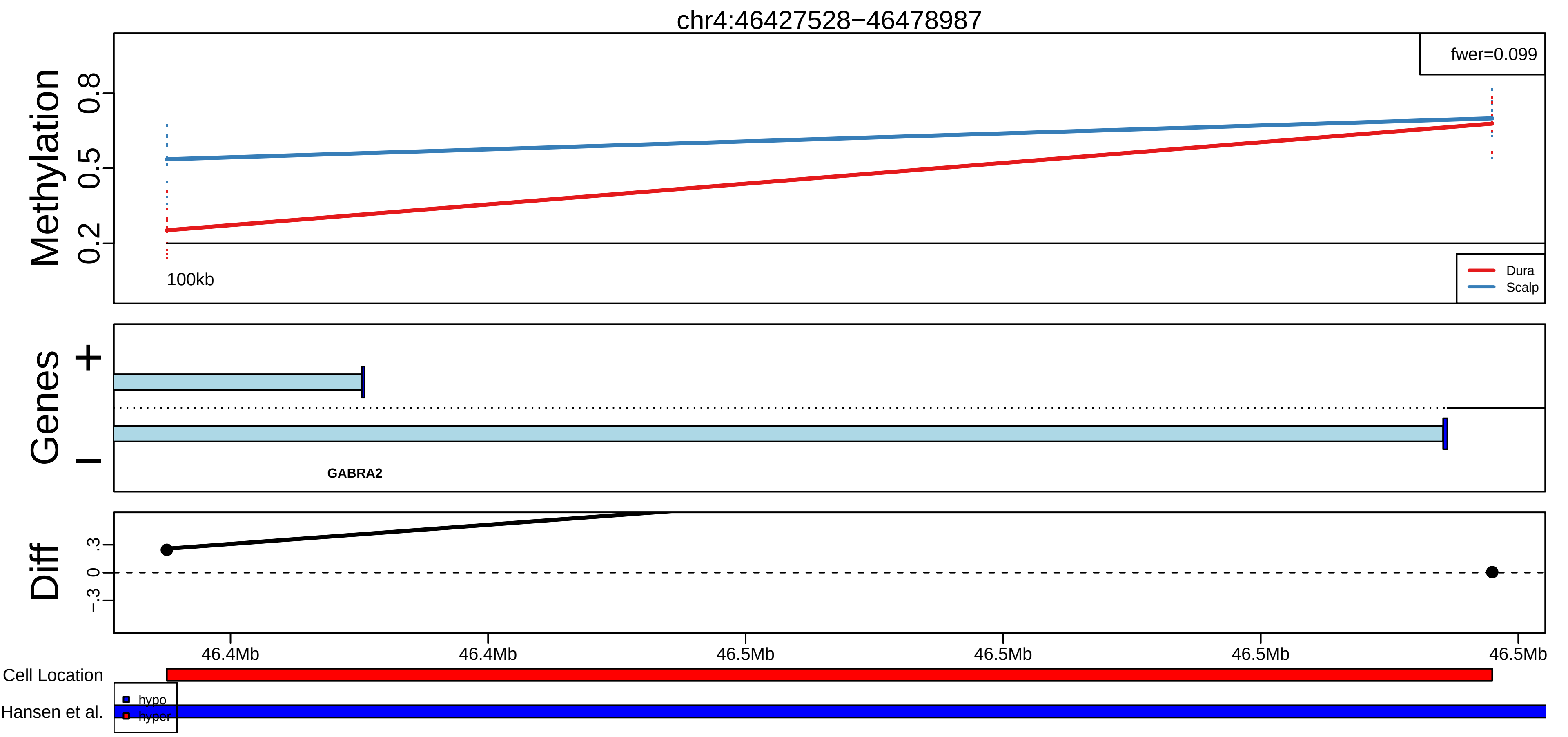

chr4:46427528−46478987
fwer=0.099
Methylation
0.2
0.5
0.8
100kb
Dura
Scalp
Genes
+
−
GABRA2
Diff
−.3
0
.3
46.4Mb
46.4Mb
46.5Mb
46.5Mb
46.5Mb
46.5Mb
Cell Location
Hansen et al.
hypo
hyper

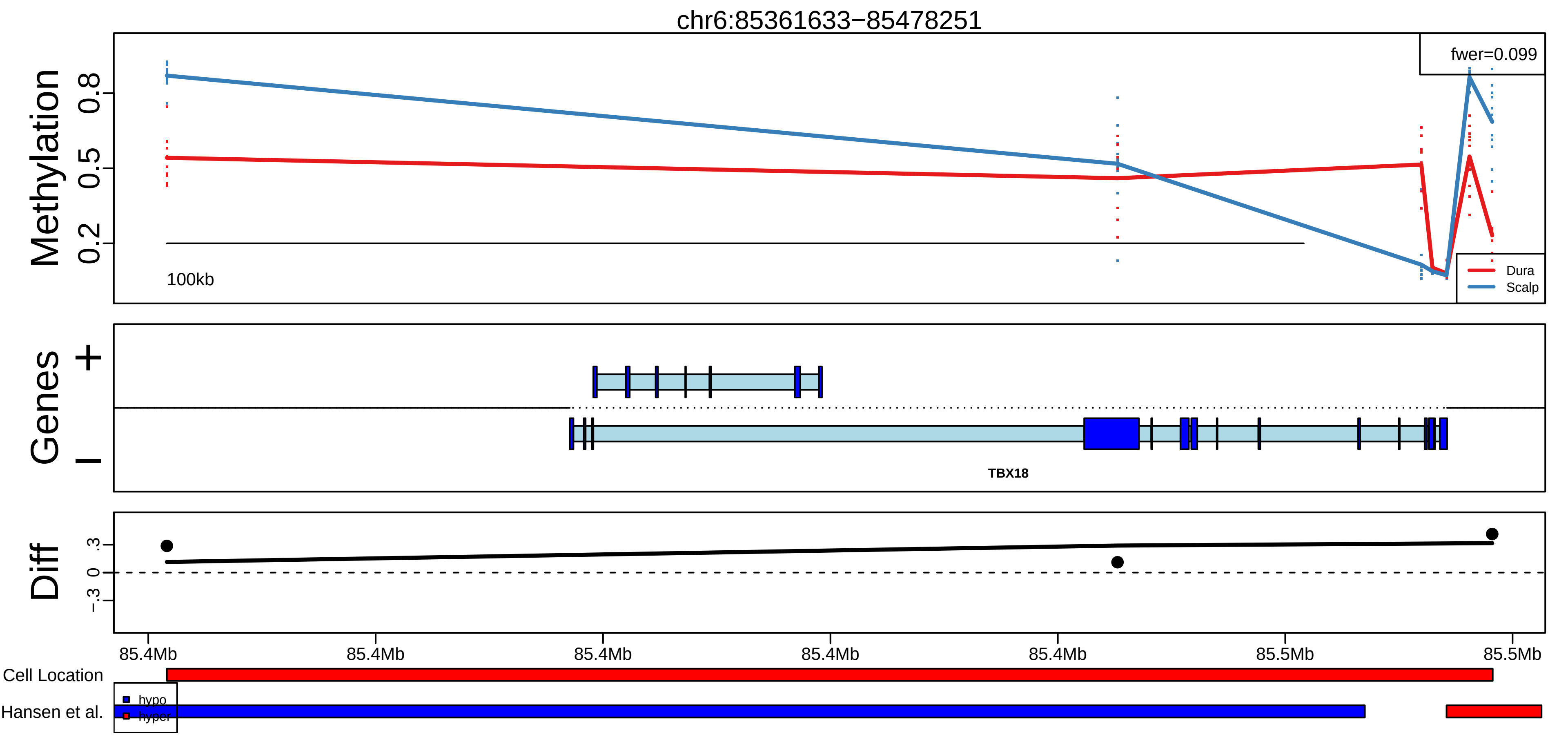
chr6:85361633−85478251
Methylation
0.2
0.5
0.8
fwer=0.099
100kb
Dura
Scalp
Genes
+
−
TBX18
Diff
−.3
0
.3
85.4Mb
85.4Mb
85.4Mb
85.4Mb
85.4Mb
85.5Mb
85.5Mb
Cell Location
Hansen et al.
hypo
hyper
