# Supplementary material for: Strong Components of Epigenetic Memory in Cultured Human Fibroblasts Related to Site of Origin and Donor Age
Source: PLoS Genet. 2016 Feb 25;12(2):e1005819. doi: 10.1371/journal.pgen.1005819 (PMC4767228; doi:10.1371/journal.pgen.1005819)
Supplement: S6 Fig — (A) PC1 with respect to PC2 of the DNAm data from the following cells: various cells of the blood; neuronal (NeuN+) and glial (NeuN-) cells from the DLPFC; cultured fibroblasts derived from skin, dura mater, and scalp; cells from a primary solid skin tumor. (B) Cluster dendrogram constructed from the DNAm data from the cells in panel A. (PDF) [file pgen.1005819.s006.pdf]

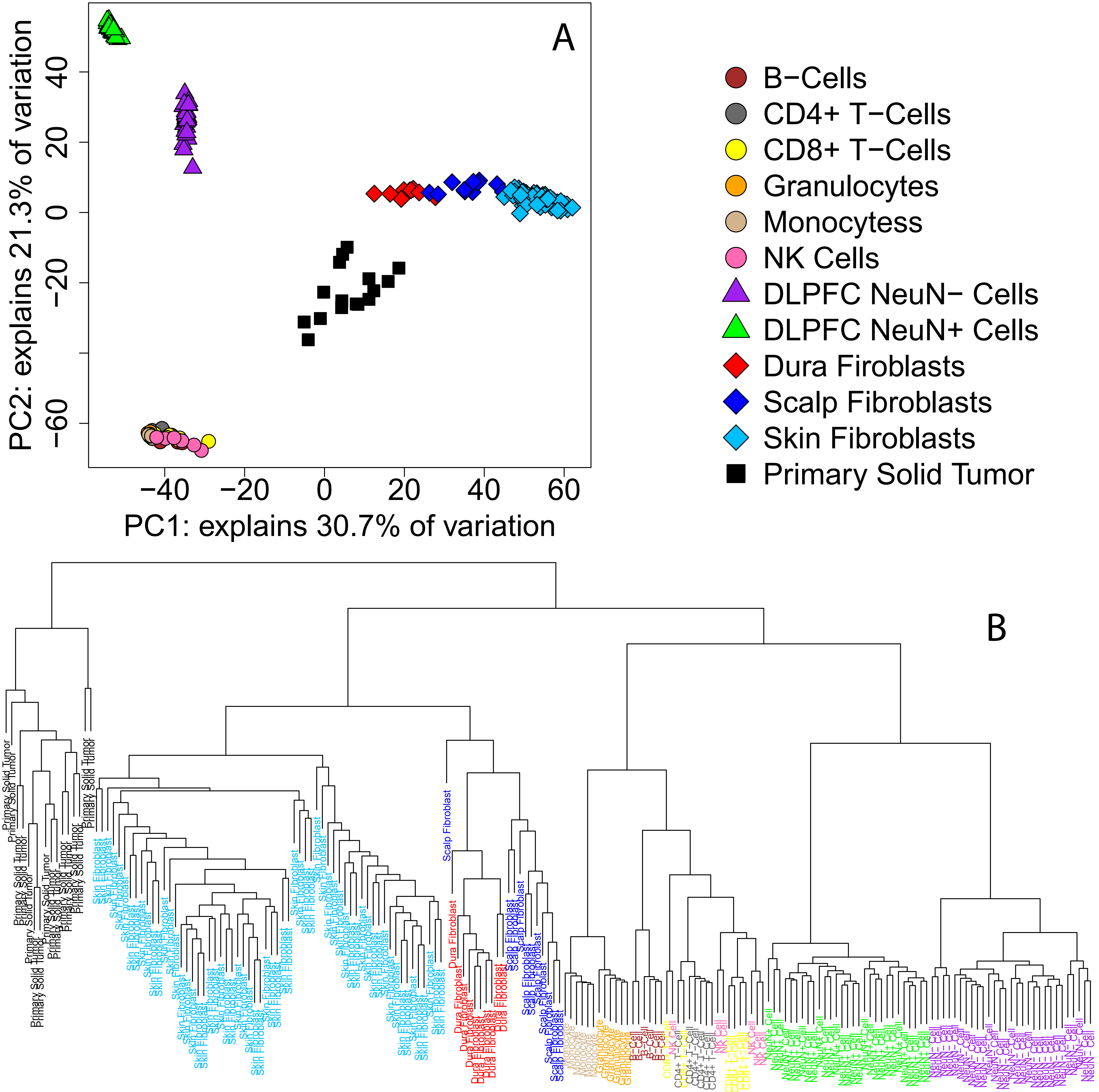
A
PC2: explains 21.3% of variation
40
20
0
−20
−60
−40
−20
0
20
40
60
PC1: explains 30.7% of variation
B−Cells
CD4+ T−Cells
CD8+ T−Cells
Granulocytes
Monocytess
NK Cells
DLPFC NeuN− Cells
DLPFC NeuN+ Cells
Dura Firoblasts
Scalp Fibroblasts
Skin Fibroblasts
Primary Solid Tumor
B
