# Supplementary material for: Strong Components of Epigenetic Memory in Cultured Human Fibroblasts Related to Site of Origin and Donor Age
Source: PLoS Genet. 2016 Feb 25;12(2):e1005819. doi: 10.1371/journal.pgen.1005819 (PMC4767228; doi:10.1371/journal.pgen.1005819)
Supplement: S2 Fig — DNA methylation levels (proportion methylation) of all 697 significant DMRs (FWER < 10%). (PDF) [file pgen.1005819.s002.pdf]

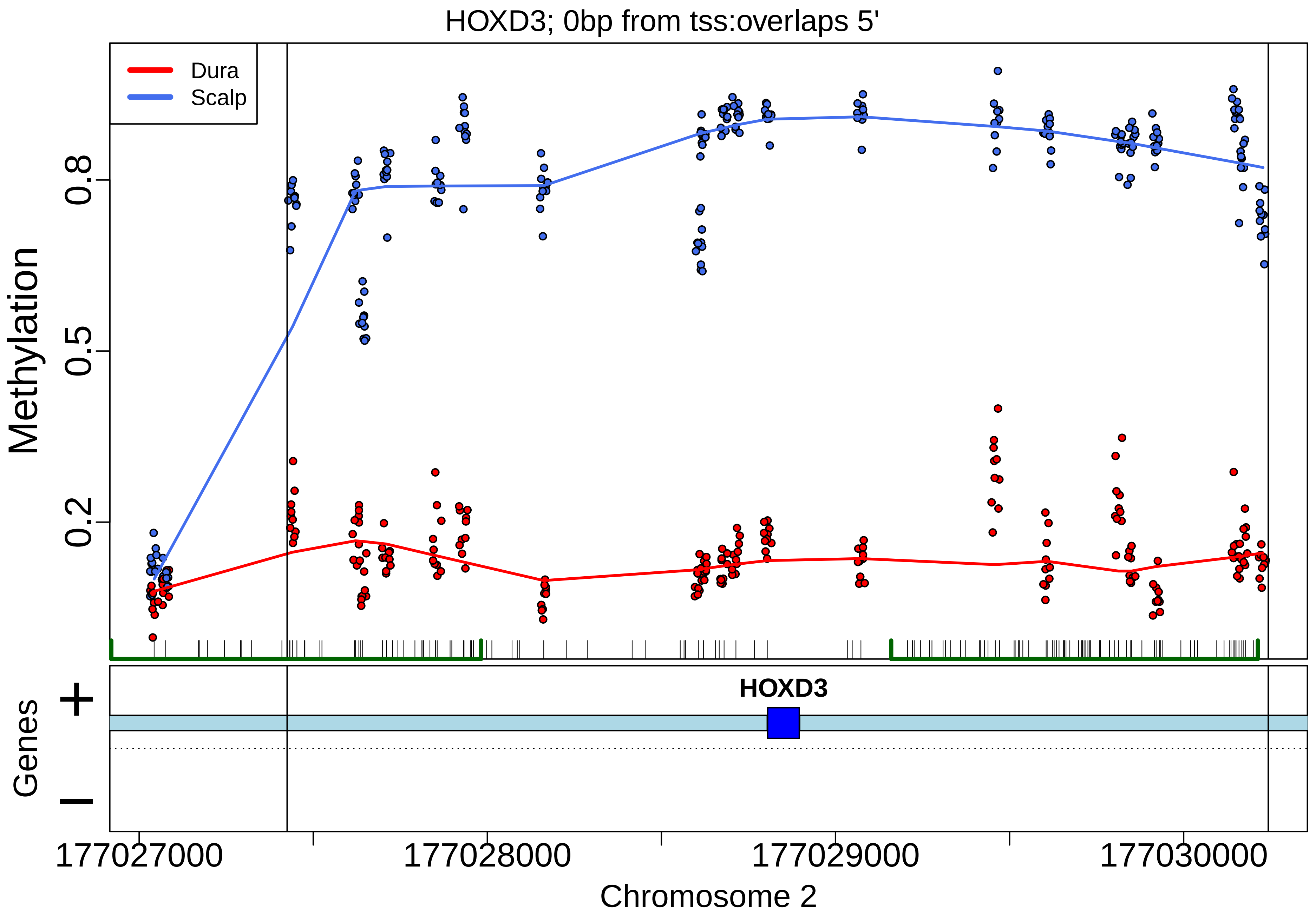

HOXD3; 0bp from tss:overlaps 5'
Dura
Scalp
Methylation
0.2
0.5
0.8
Genes
+
−
HOXD3
177027000
177028000
177029000
177030000
Chromosome 2

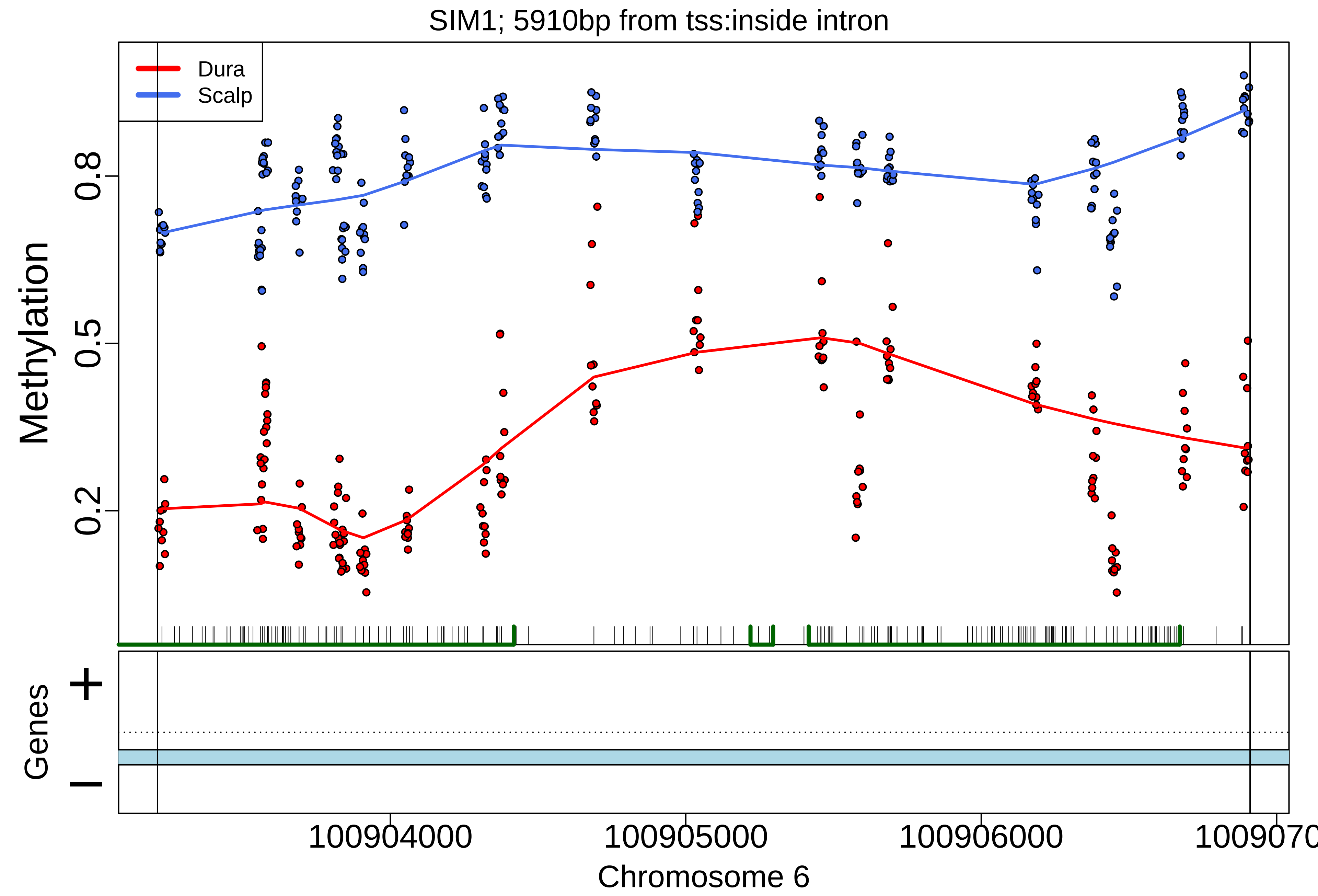

SIM1; 5910bp from tss:inside intron
Dura
Scalp
Methylation
0.2
0.5
0.8
Genes
+
−
100904000
100905000
100906000
1009070
Chromosome 6

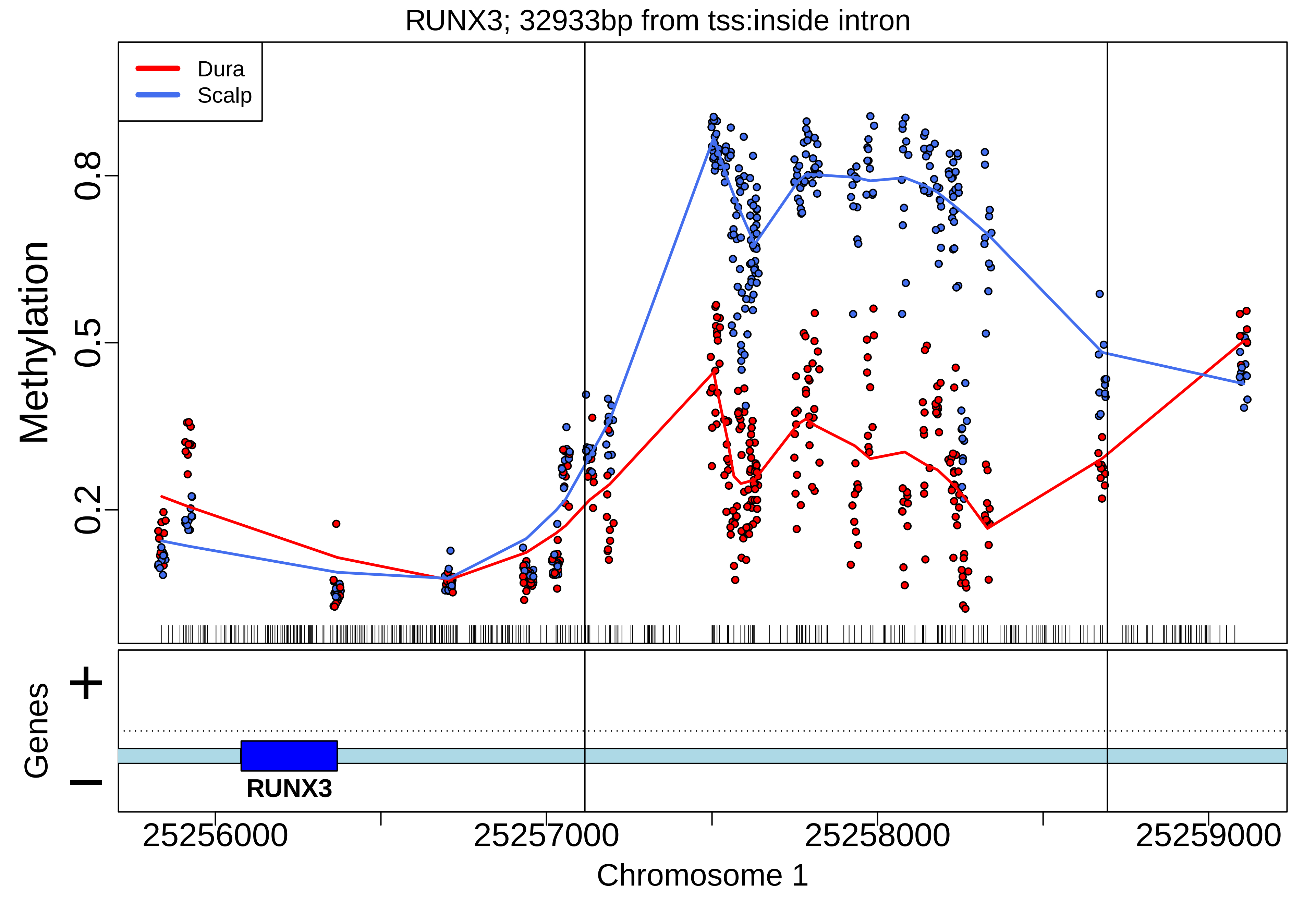

RUNX3; 32933bp from tss:inside intron
Dura
Scalp
Methylation
0.2
0.5
0.8
Genes
+
-
RUNX3
25256000
25257000
25258000
25259000
Chromosome 1

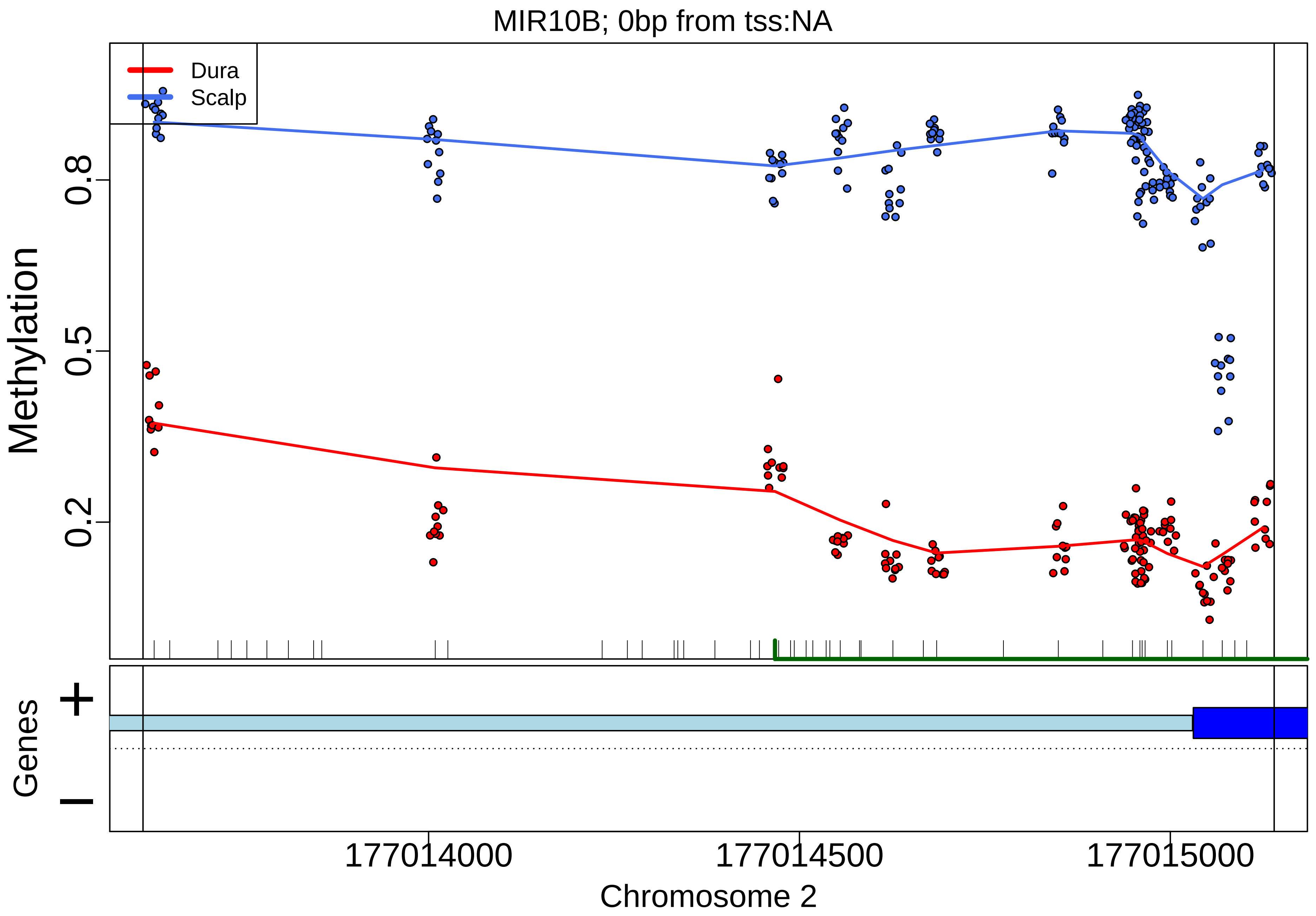

MIR10B; 0bp from tss:NA
Dura
Scalp
Methylation
0.2
0.5
0.8
Genes
+
-
177014000
177014500
177015000
Chromosome 2

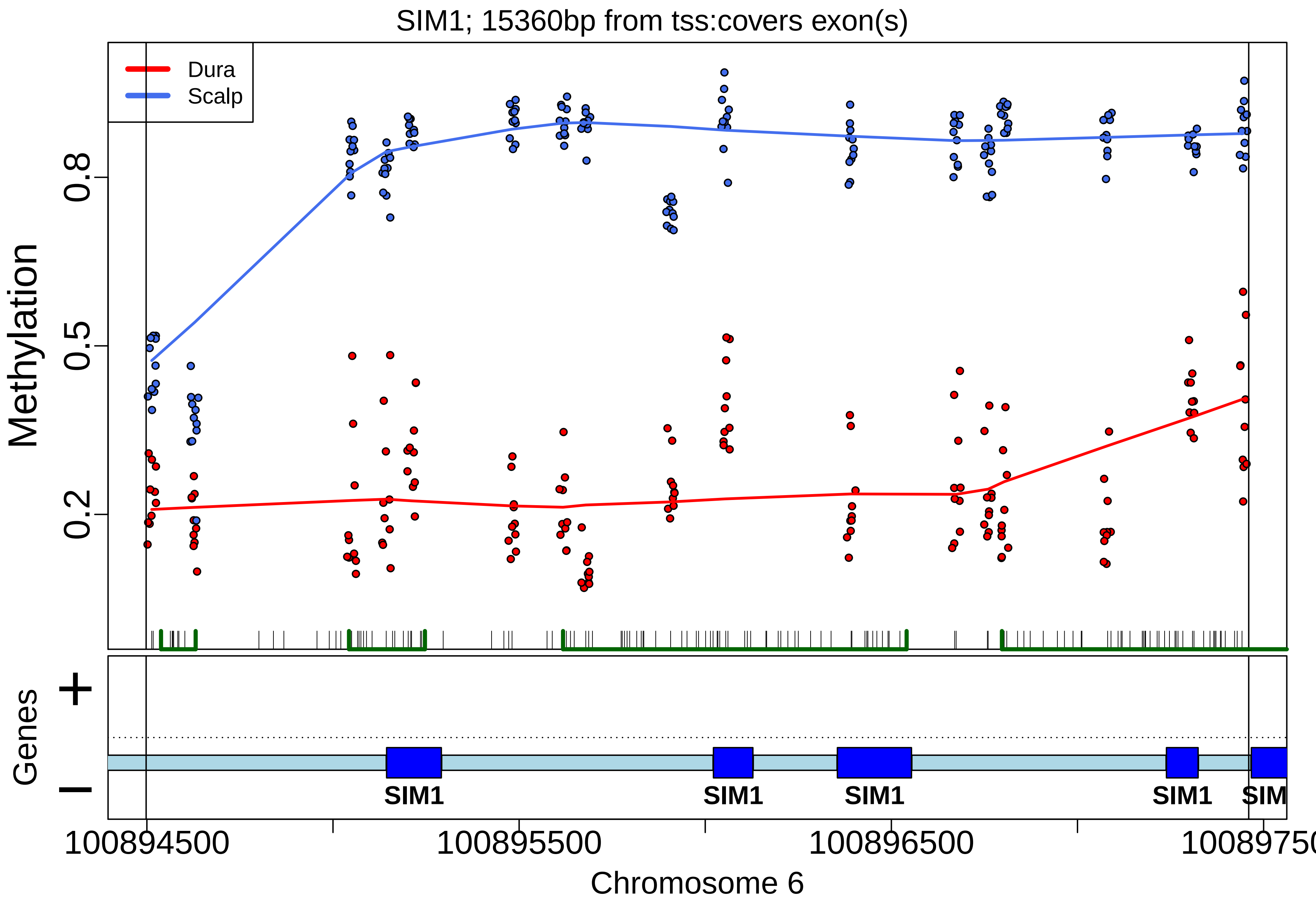
SIM1; 15360bp from tss:covers exon(s)
Dura
Scalp
Methylation
0.2
0.5
0.8
Genes
+
−
SIM1
SIM1
SIM1
SIM1
SIM
100894500
100895500
100896500
Chromosome 6

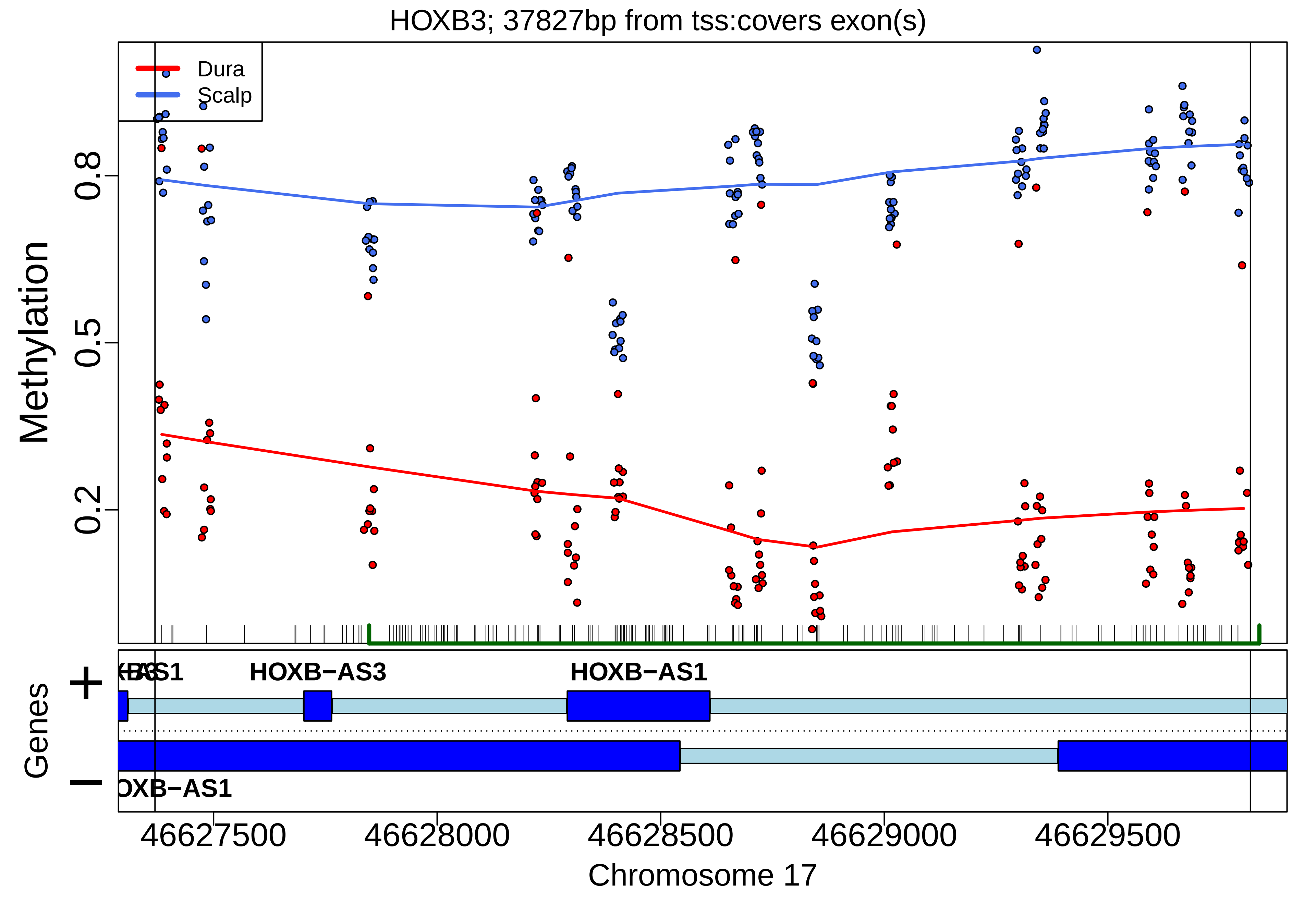
HOXB3; 37827bp from tss:covers exon(s)
Dura
Scalp
Methylation
0.2
0.5
0.8
Genes
+
−
HOXB−AS3
HOXB−AS1
46627500
46628000
46628500
46629000
46629500
Chromosome 17

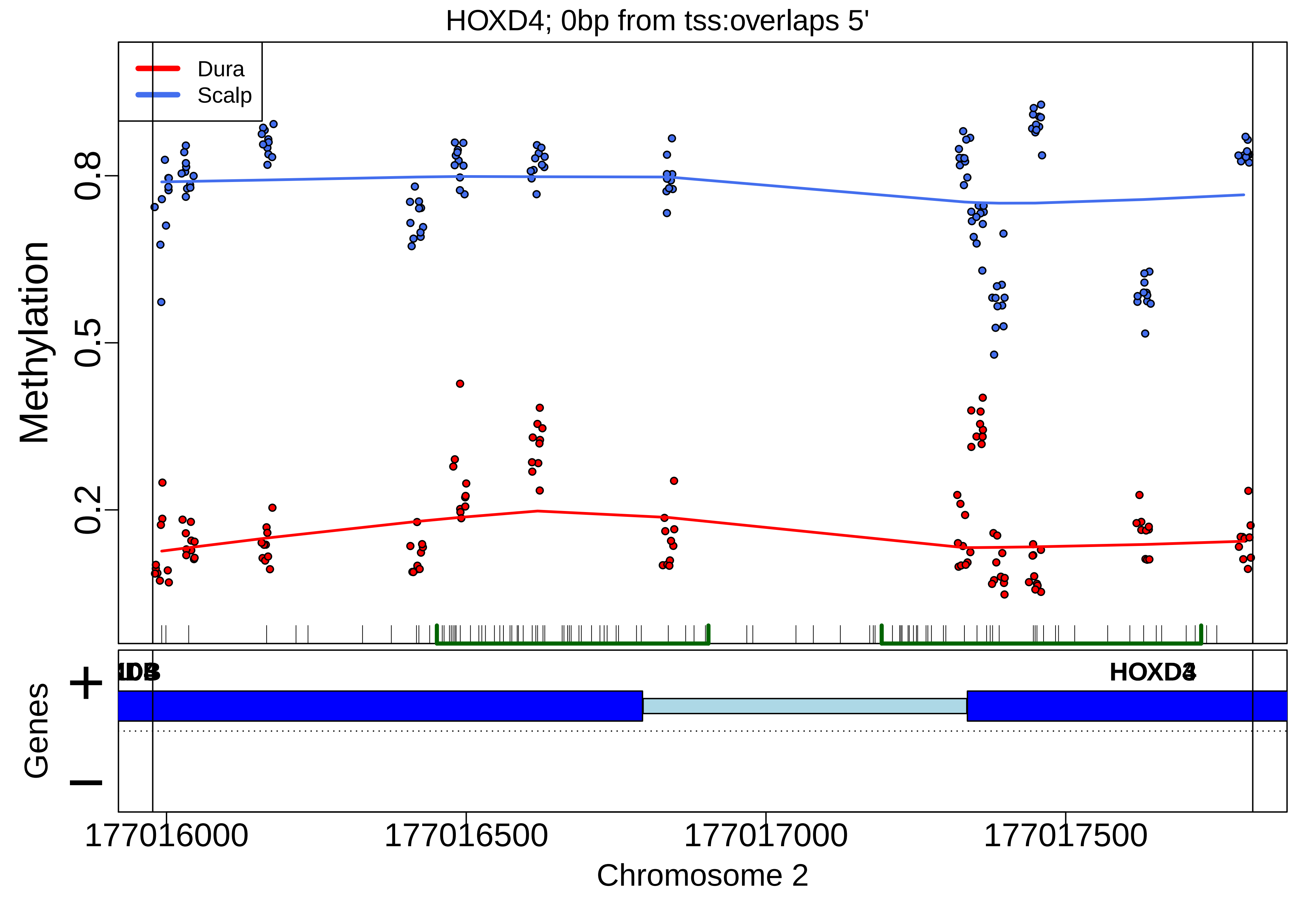
HOXD4; 0bp from tss:overlaps 5'
Dura
Scalp
Methylation
0.2
0.5
0.8
Genes
+
−
HOXD4
177016000
177016500
177017000
177017500
Chromosome 2

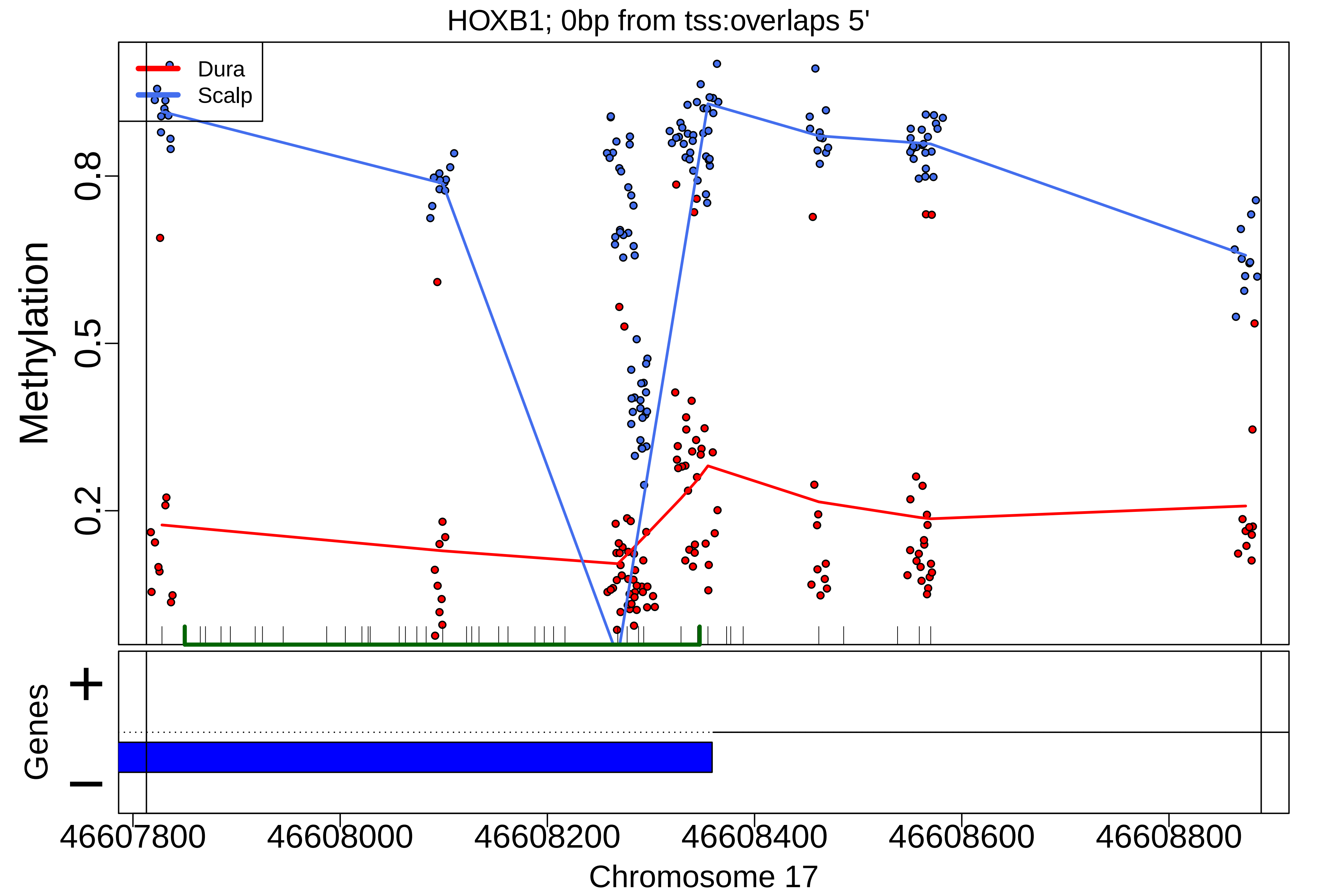
HOXB1; 0bp from tss:overlaps 5'
Dura
Scalp
Methylation
0.2
0.5
0.8
Genes
+
−
46607800
46608000
46608200
46608400
46608600
46608800
Chromosome 17

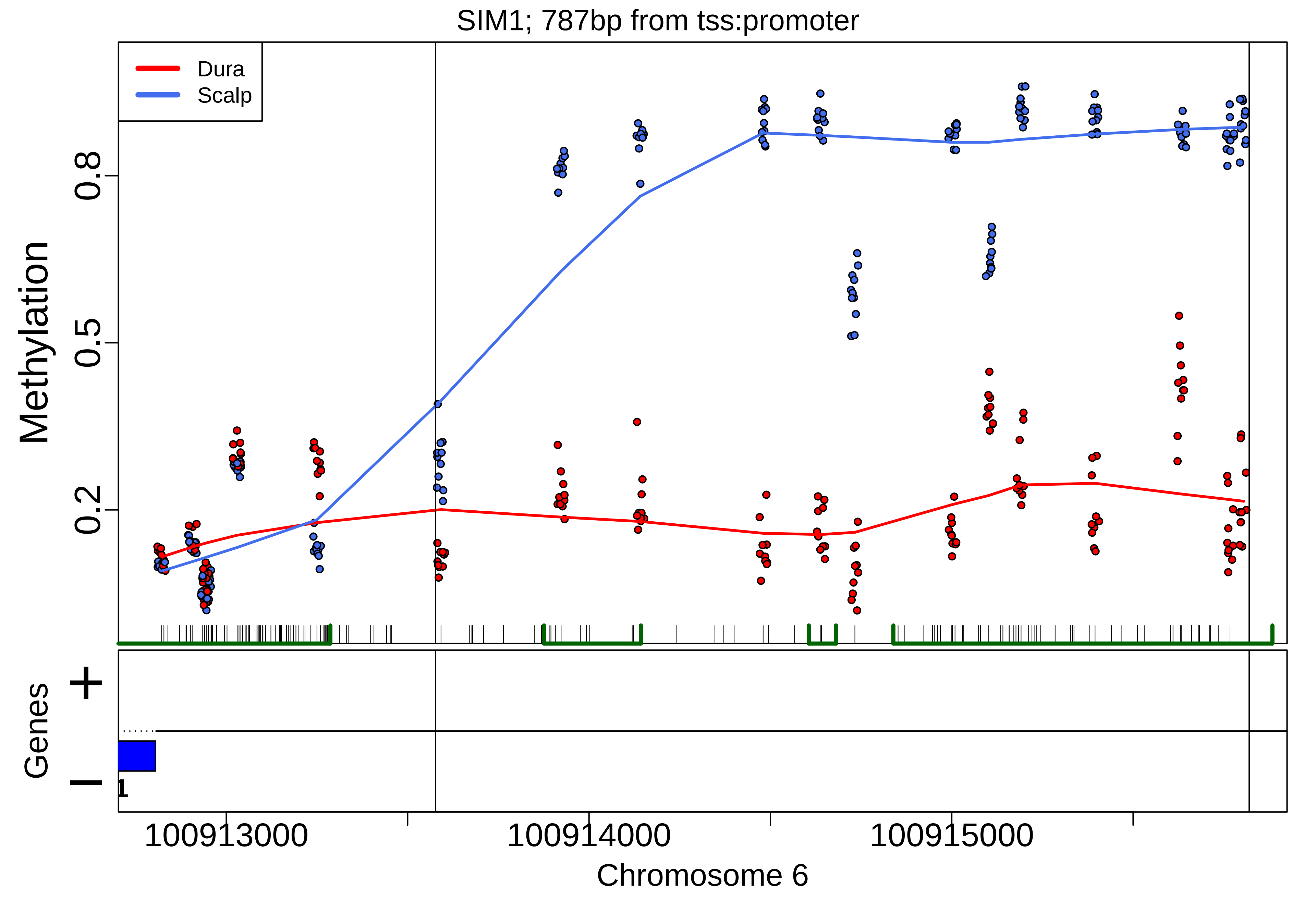
SIM1; 787bp from tss:promoter
Dura
Scalp
Methylation
0.2
0.5
0.8
Genes
+
−
1
100913000
100914000
100915000
Chromosome 6

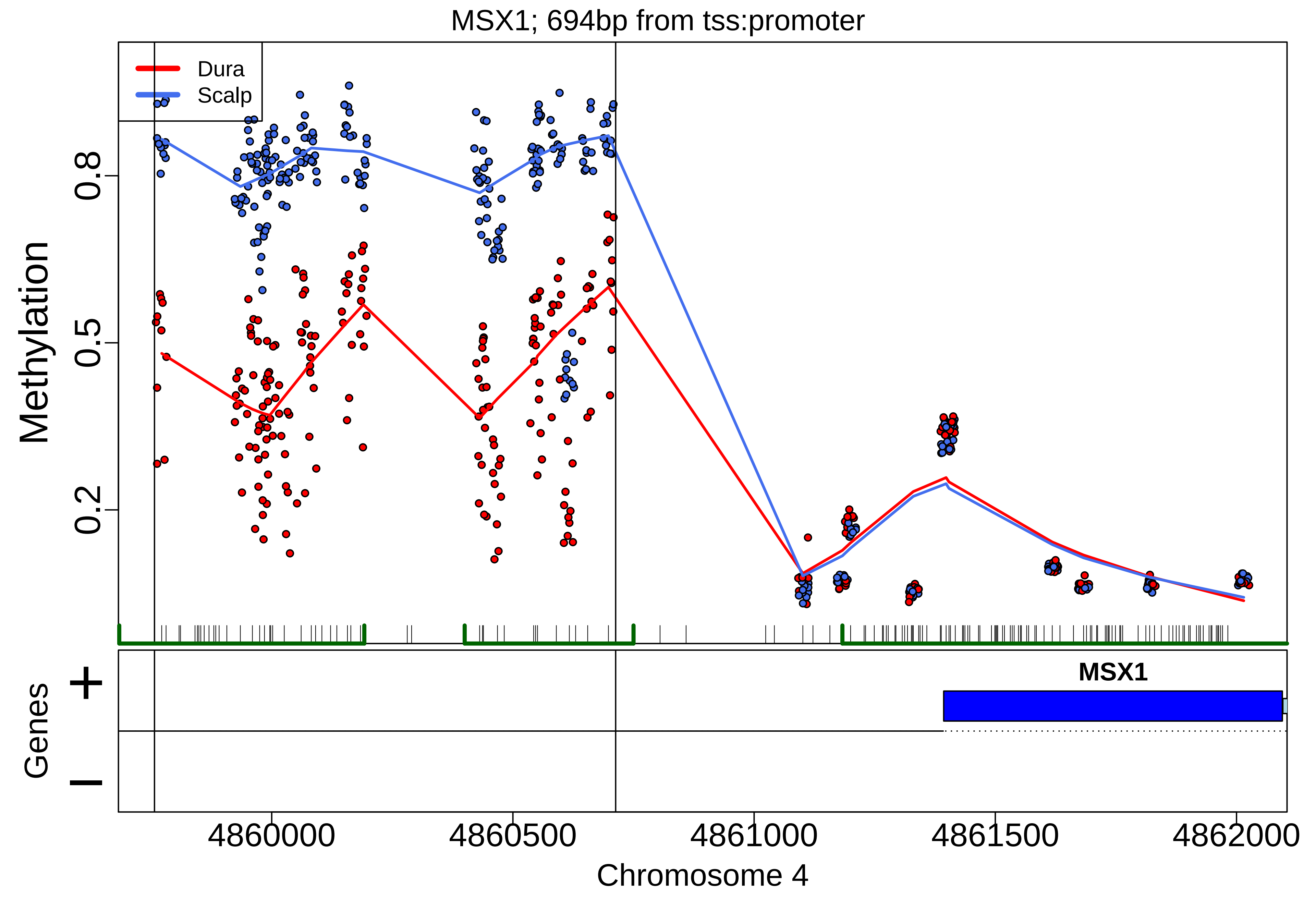

MSX1; 694bp from tss:promoter
Dura
Scalp
Methylation
0.2
0.5
0.8
Genes
+
–
MSX1
4860000
4860500
4861000
4861500
4862000
Chromosome 4

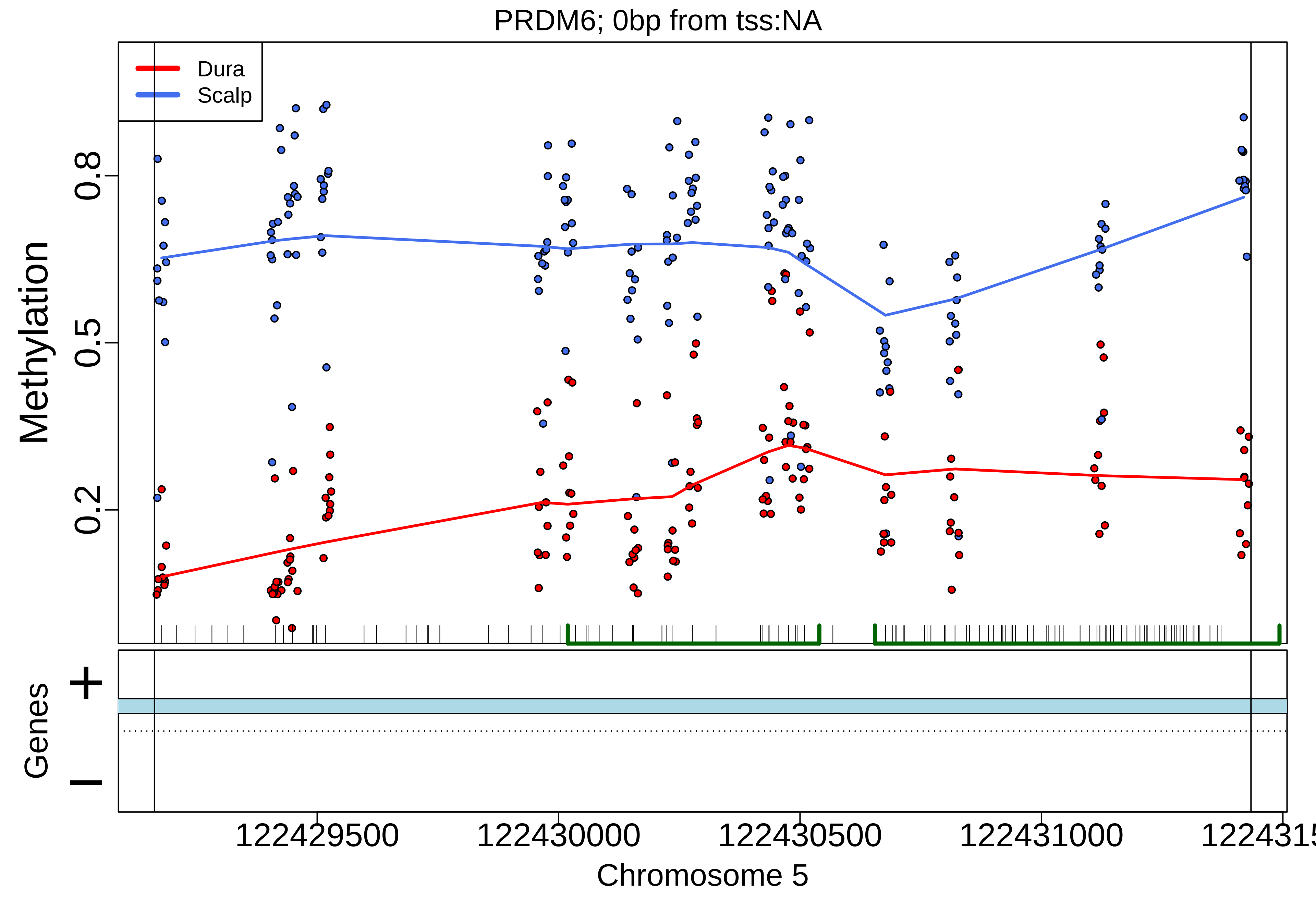

PRDM6; 0bp from tss:NA
Dura
Scalp
Methylation
0.2
0.5
0.8
Genes
+
−
122429500
122430000
122430500
122431000
Chromosome 5

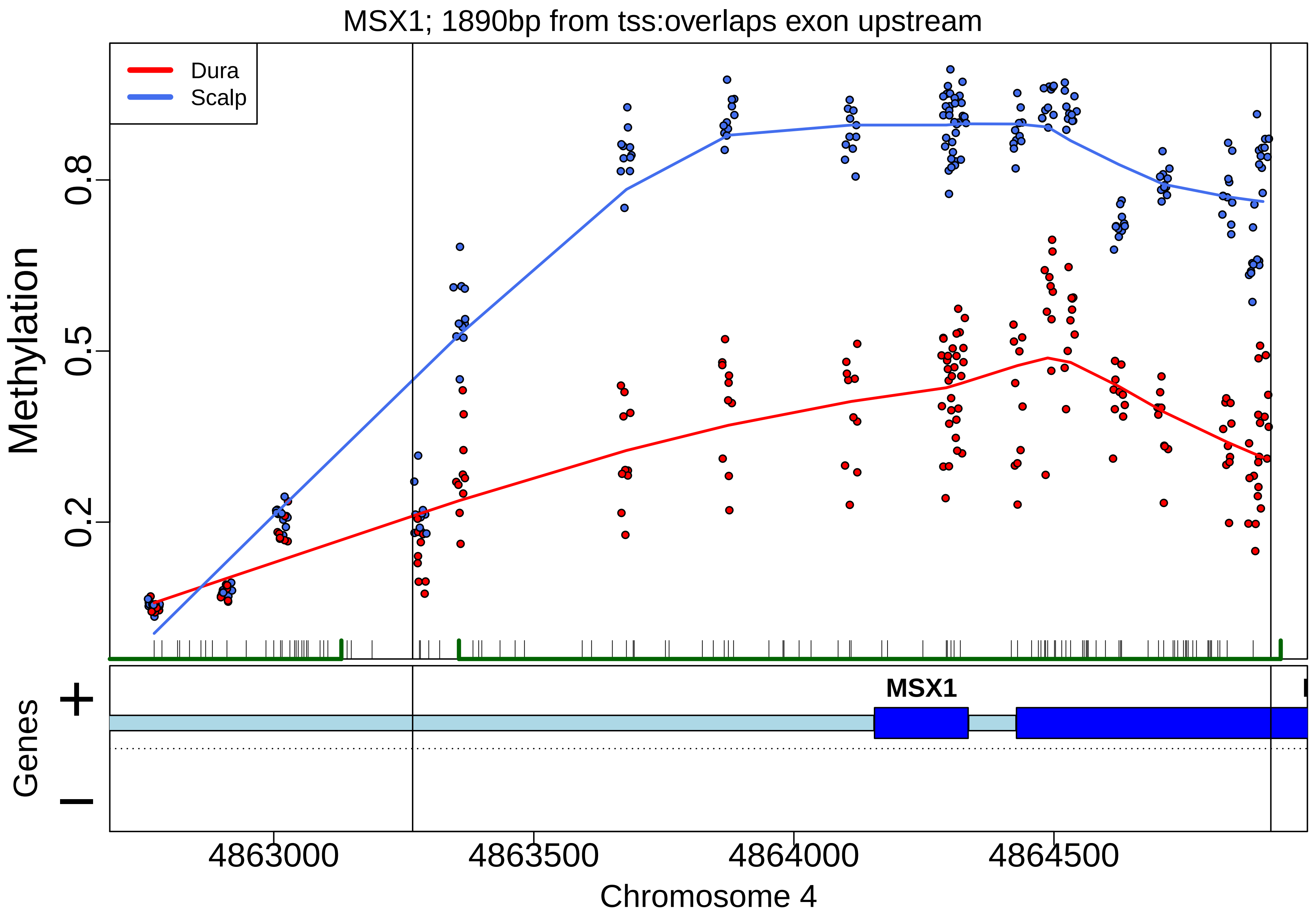
MSX1; 1890bp from tss:overlaps exon upstream
Dura
Scalp
Methylation
0.2
0.5
0.8
Genes
+
−
MSX1
4863000
4863500
4864000
4864500
Chromosome 4

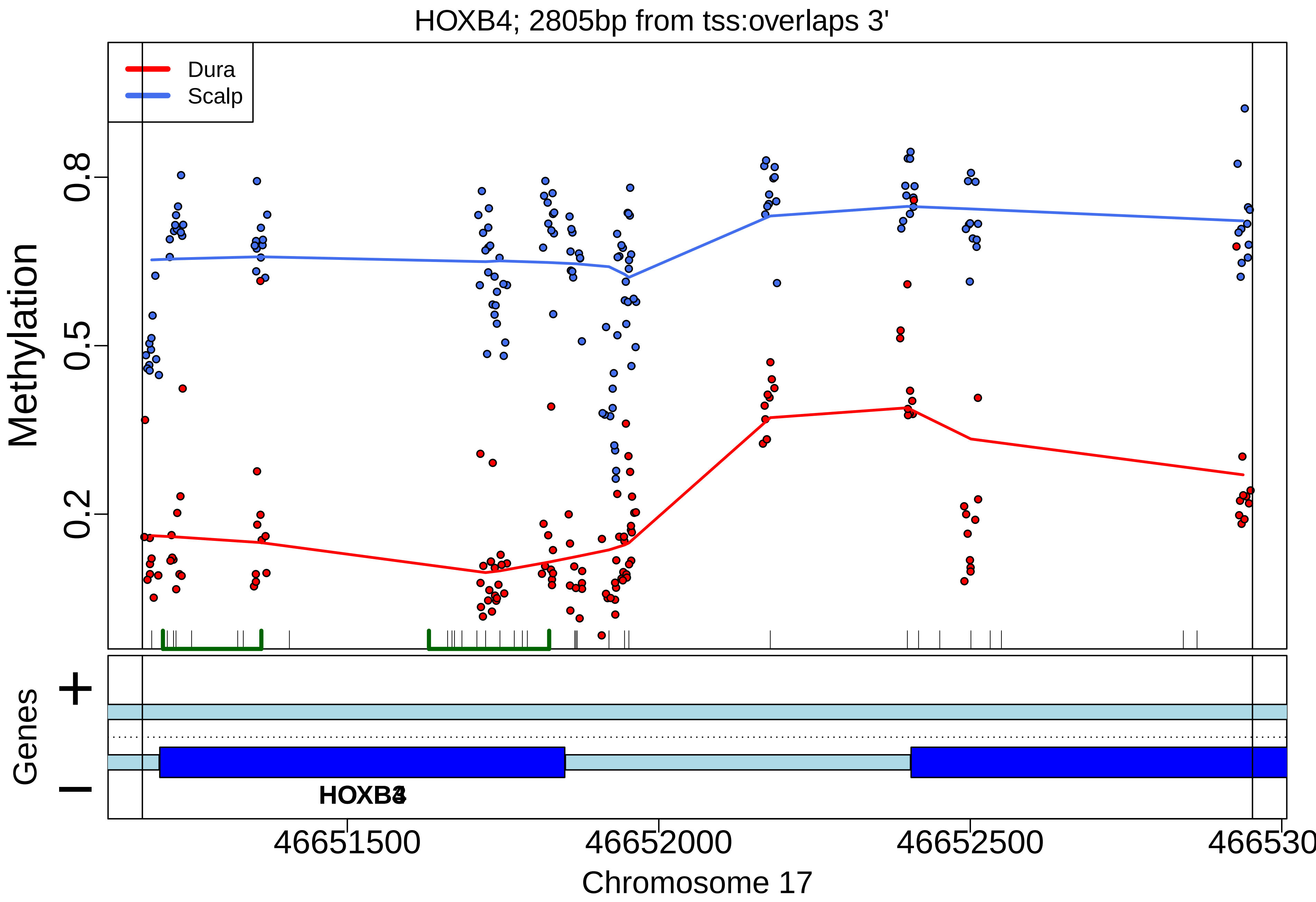

HOXB4; 2805bp from tss:overlaps 3'
Dura
Scalp
Methylation
0.2
0.5
0.8
Genes
+
–
HOXB4
46651500
46652000
46652500
466530
Chromosome 17

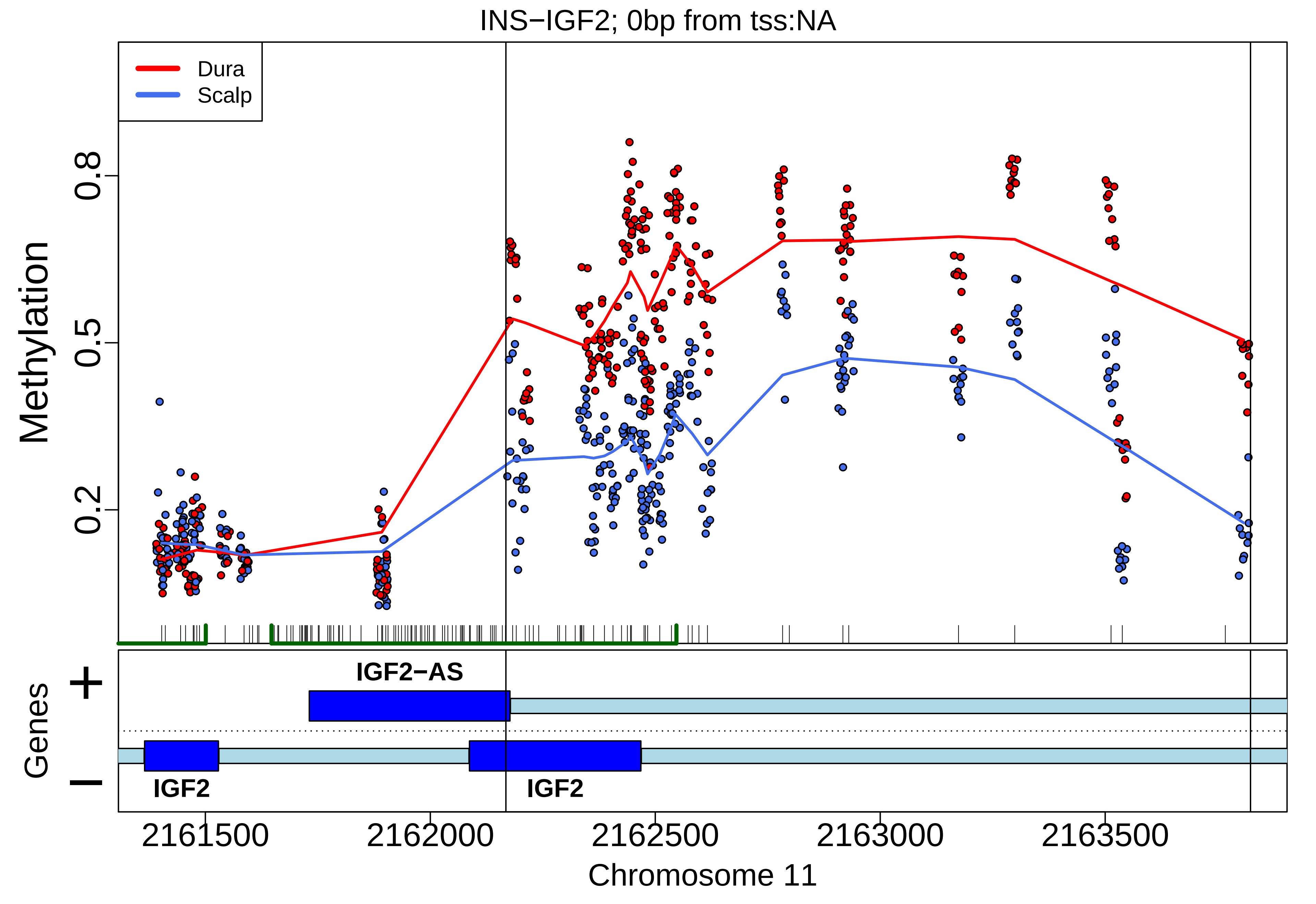

INS−IGF2; 0bp from tss:NA
Dura
Scalp
Methylation
0.2
0.5
0.8
Genes
+
−
IGF2−AS
IGF2
IGF2
2161500
2162000
2162500
2163000
2163500
Chromosome 11

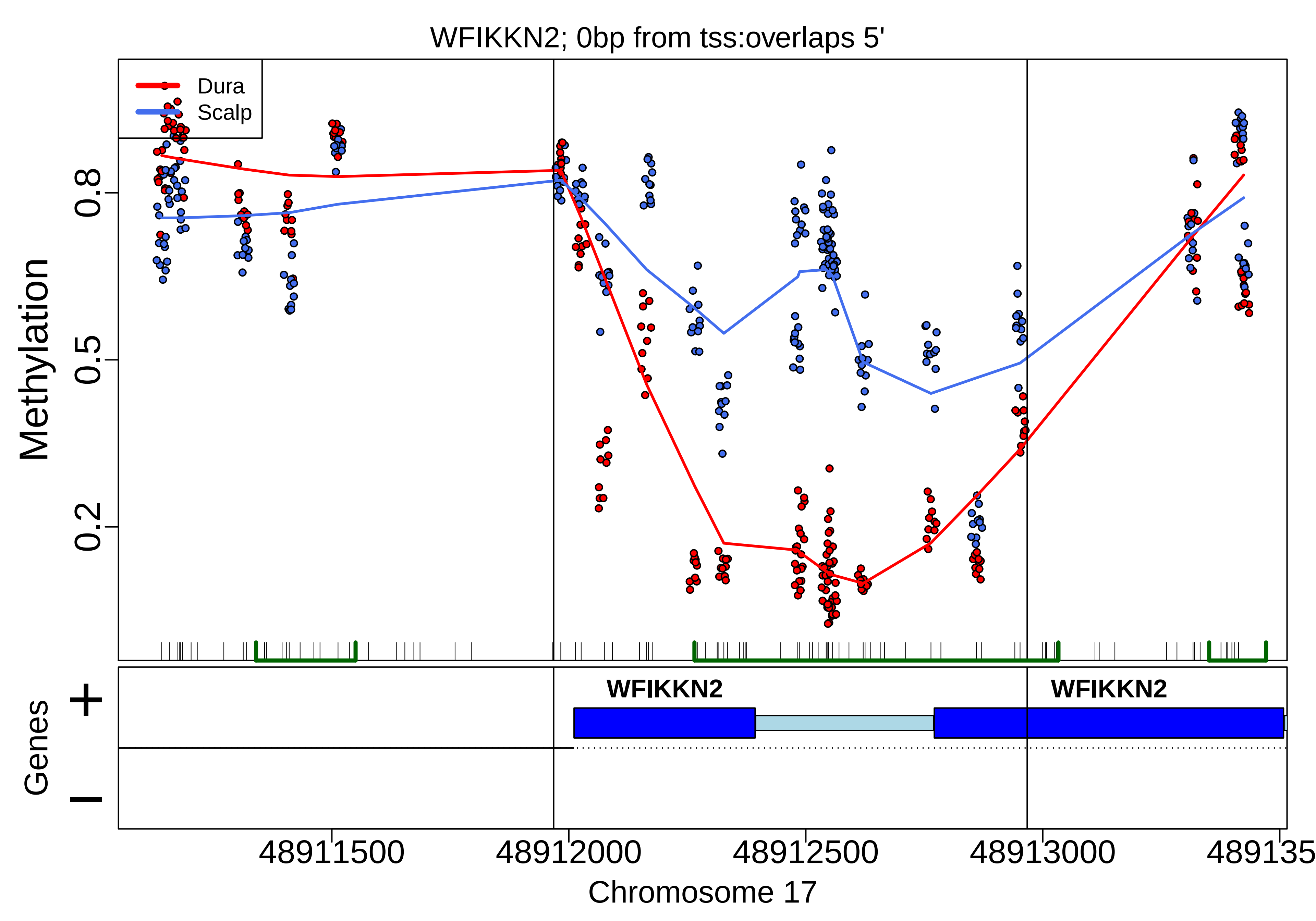

WFIKKN2; 0bp from tss:overlaps 5'
Dura
Scalp
Methylation
0.2
0.5
0.8
Genes
+
-
WFIKKN2
WFIKKN2
48911500
48912000
48912500
48913000
489135
Chromosome 17

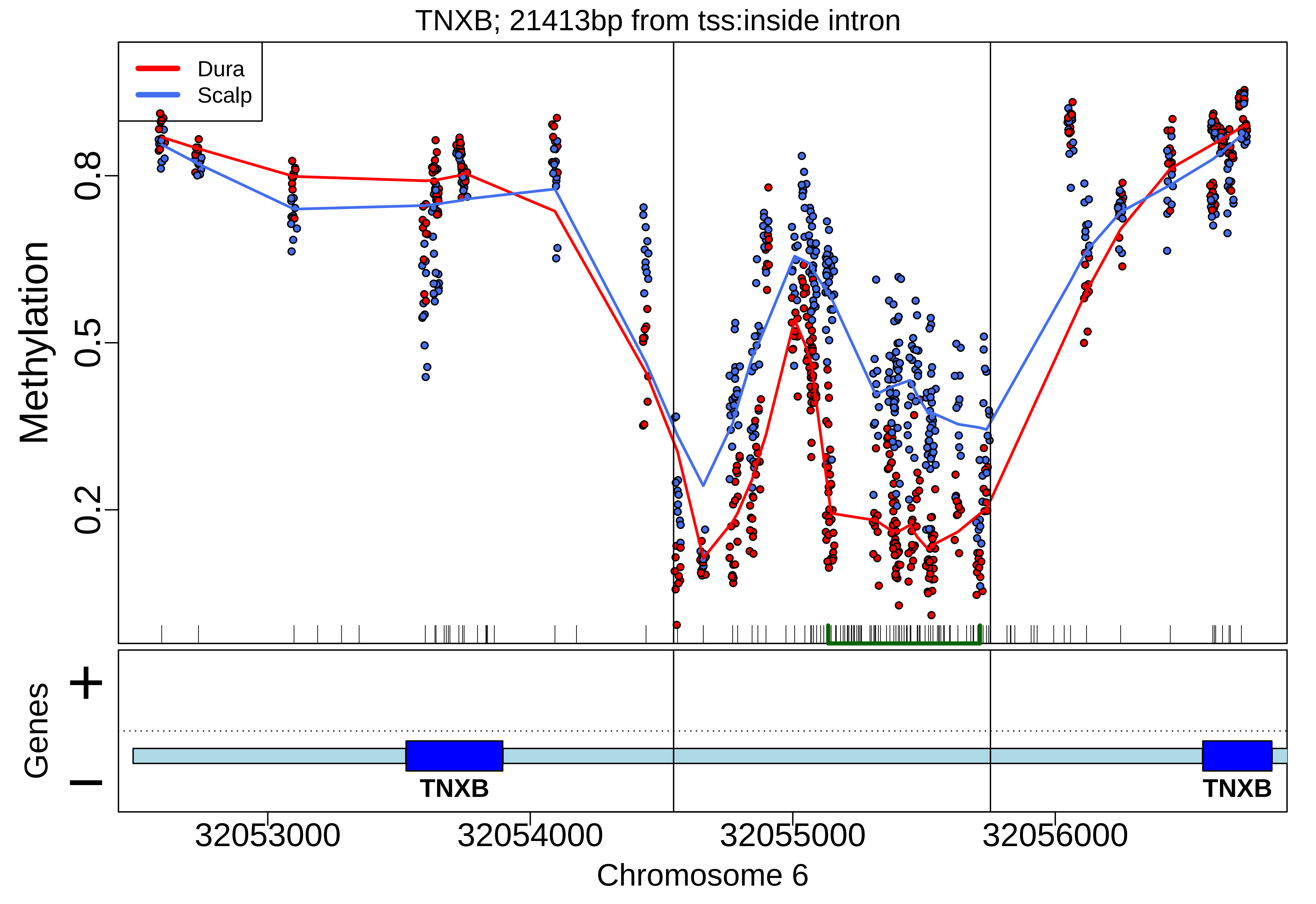

TNXB; 21413bp from tss:inside intron
Dura
Scalp
Methylation
0.2
0.5
0.8
Genes
+
-
TNXB
TNXB
32053000
32054000
32055000
32056000
Chromosome 6

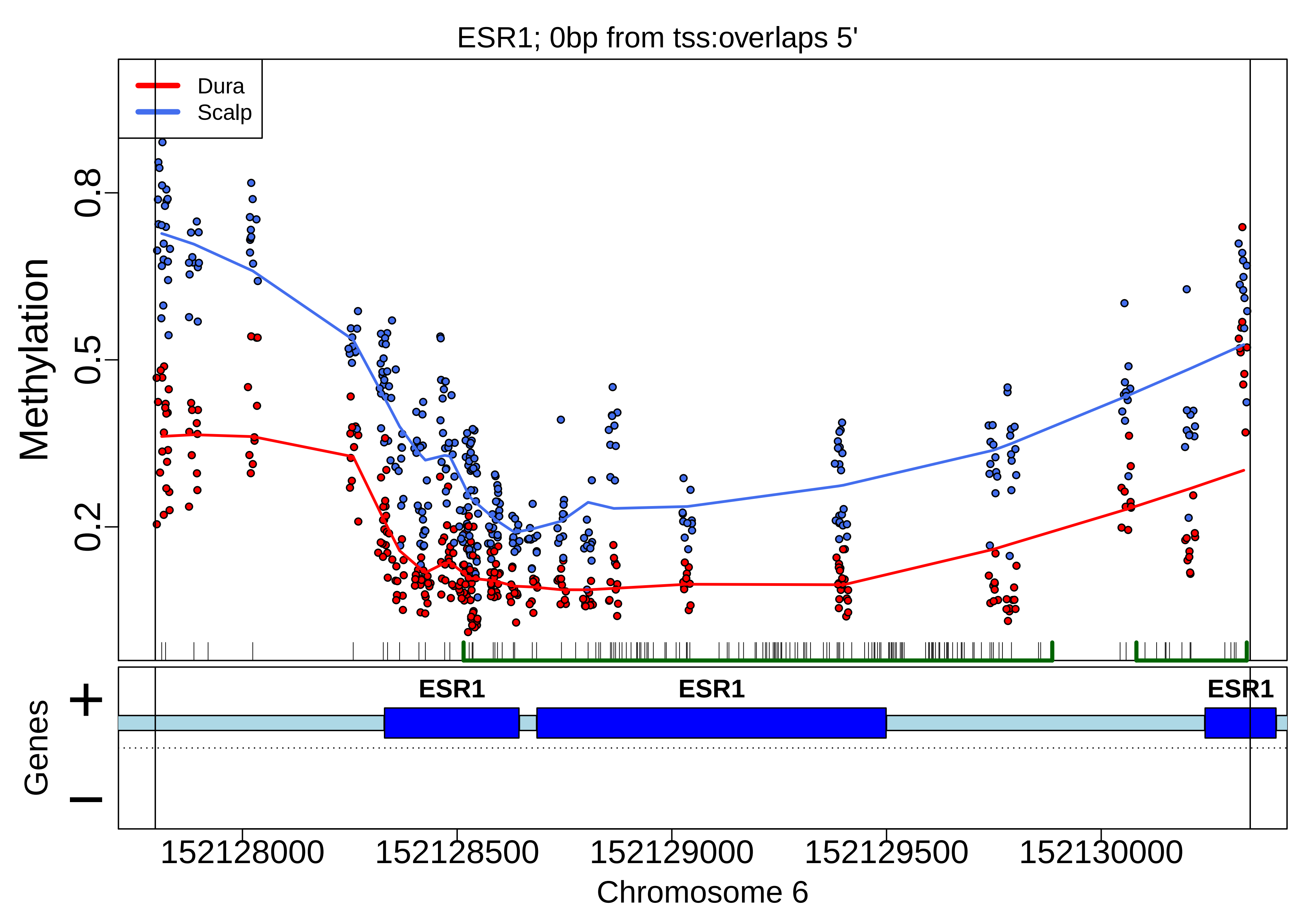

ESR1; 0bp from tss:overlaps 5'
Dura
Scalp
Methylation
0.2
0.5
0.8
Genes
+
−
ESR1
ESR1
ESR1
152128000
152128500
152129000
152129500
152130000
Chromosome 6

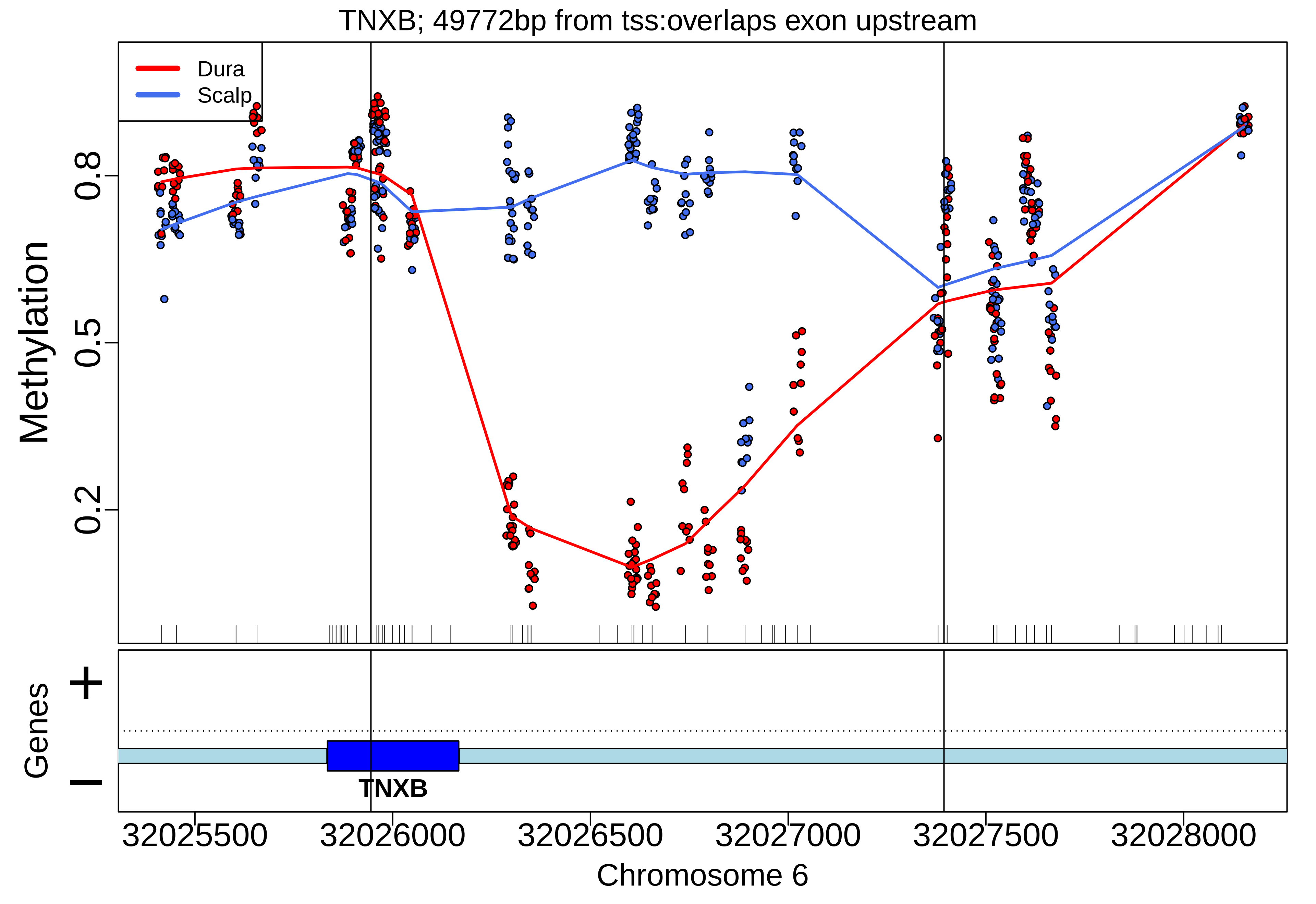
TNXB; 49772bp from tss:overlaps exon upstream
Dura
Scalp
Methylation
0.8
0.5
0.2
Genes
+
−
TNXB
32025500
32026000
32026500
32027000
32027500
32028000
Chromosome 6

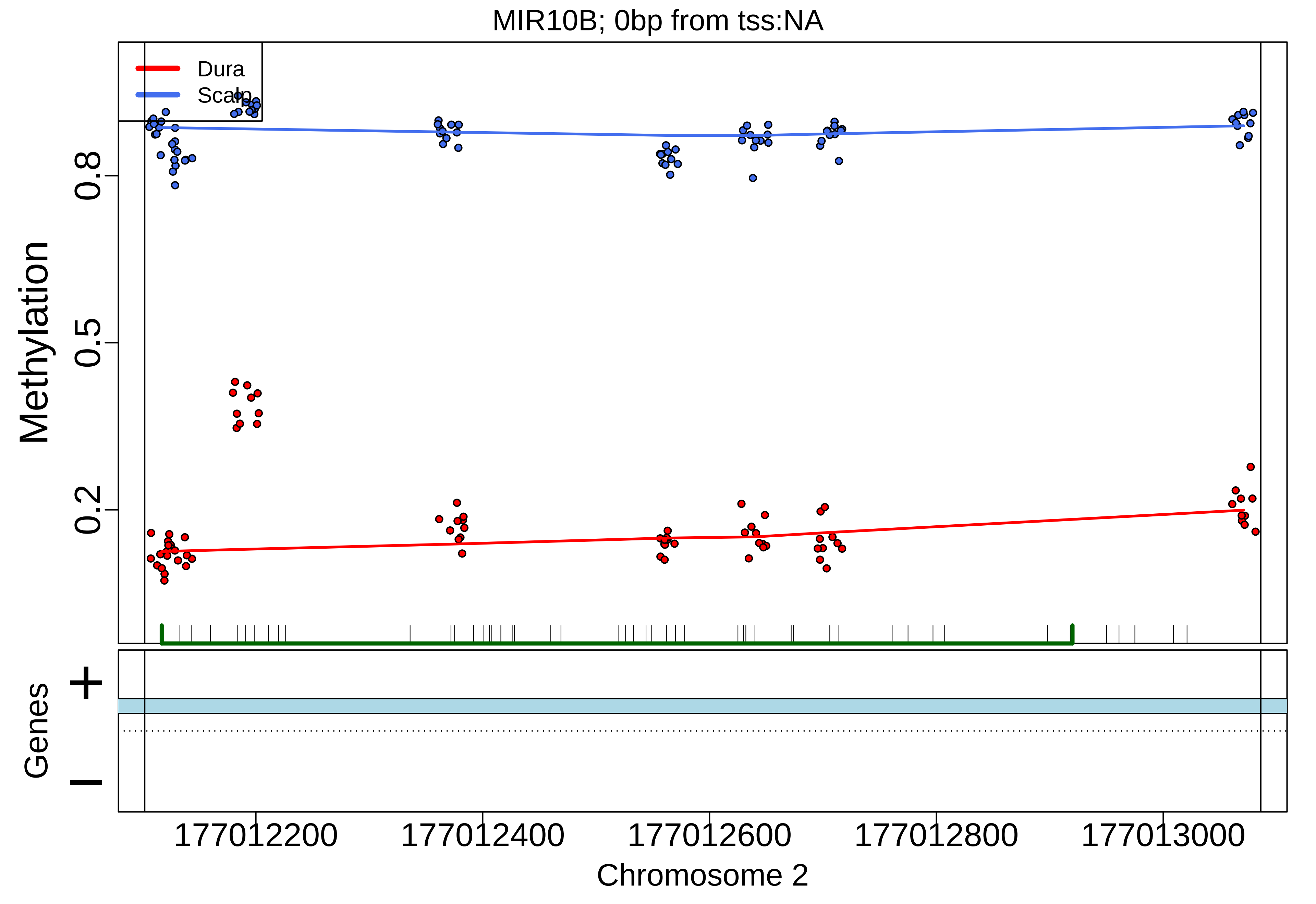

MIR10B; 0bp from tss:NA
Dura
Scalp
Methylation
0.2
0.5
0.8
Genes
+
-
177012200
177012400
177012600
177012800
177013000
Chromosome 2

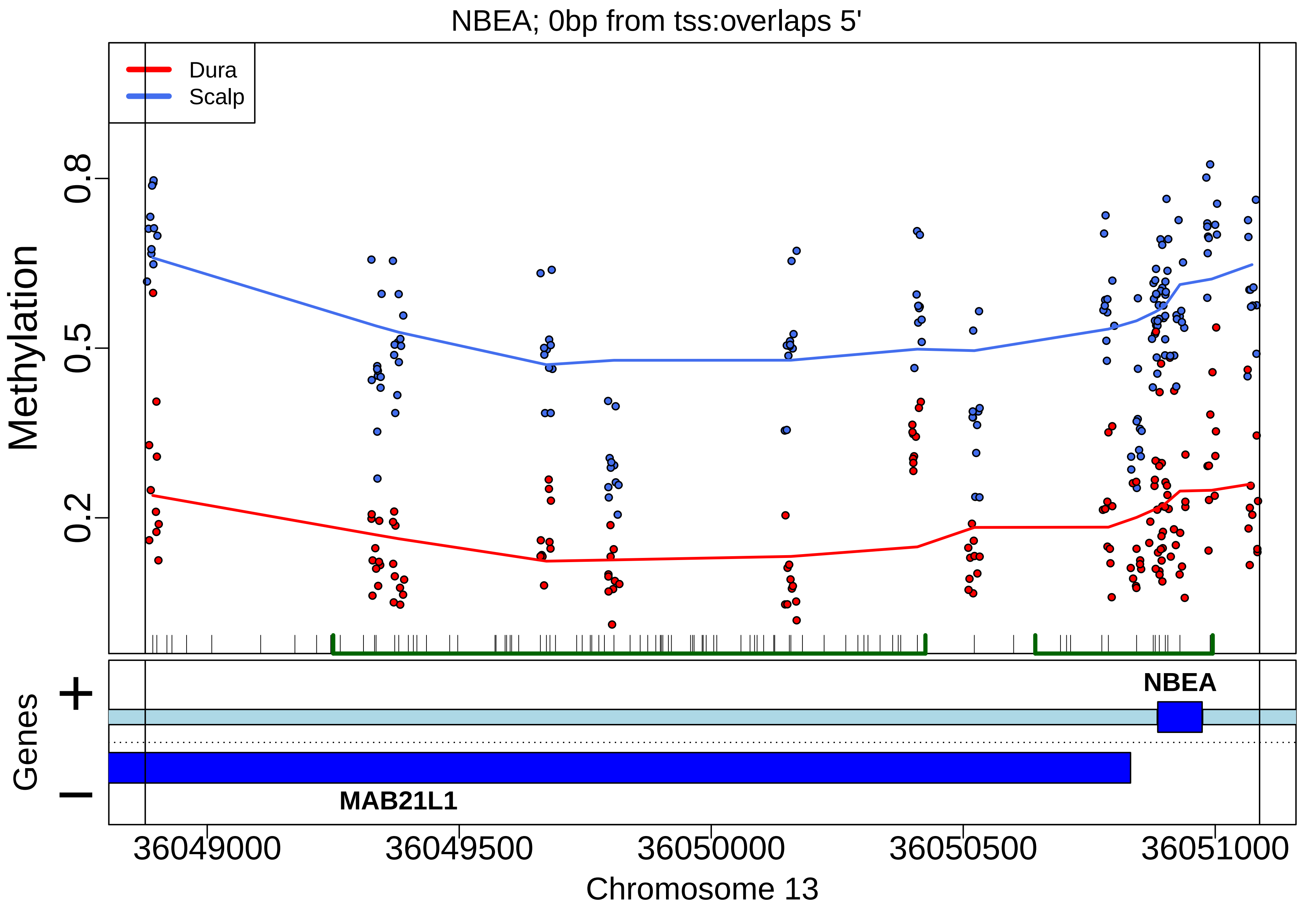

NBEA; 0bp from tss:overlaps 5'
Dura
Scalp
Methylation
0.2
0.5
0.8
Genes
+
−
NBEA
MAB21L1
36049000
36049500
36050000
36050500
36051000
Chromosome 13

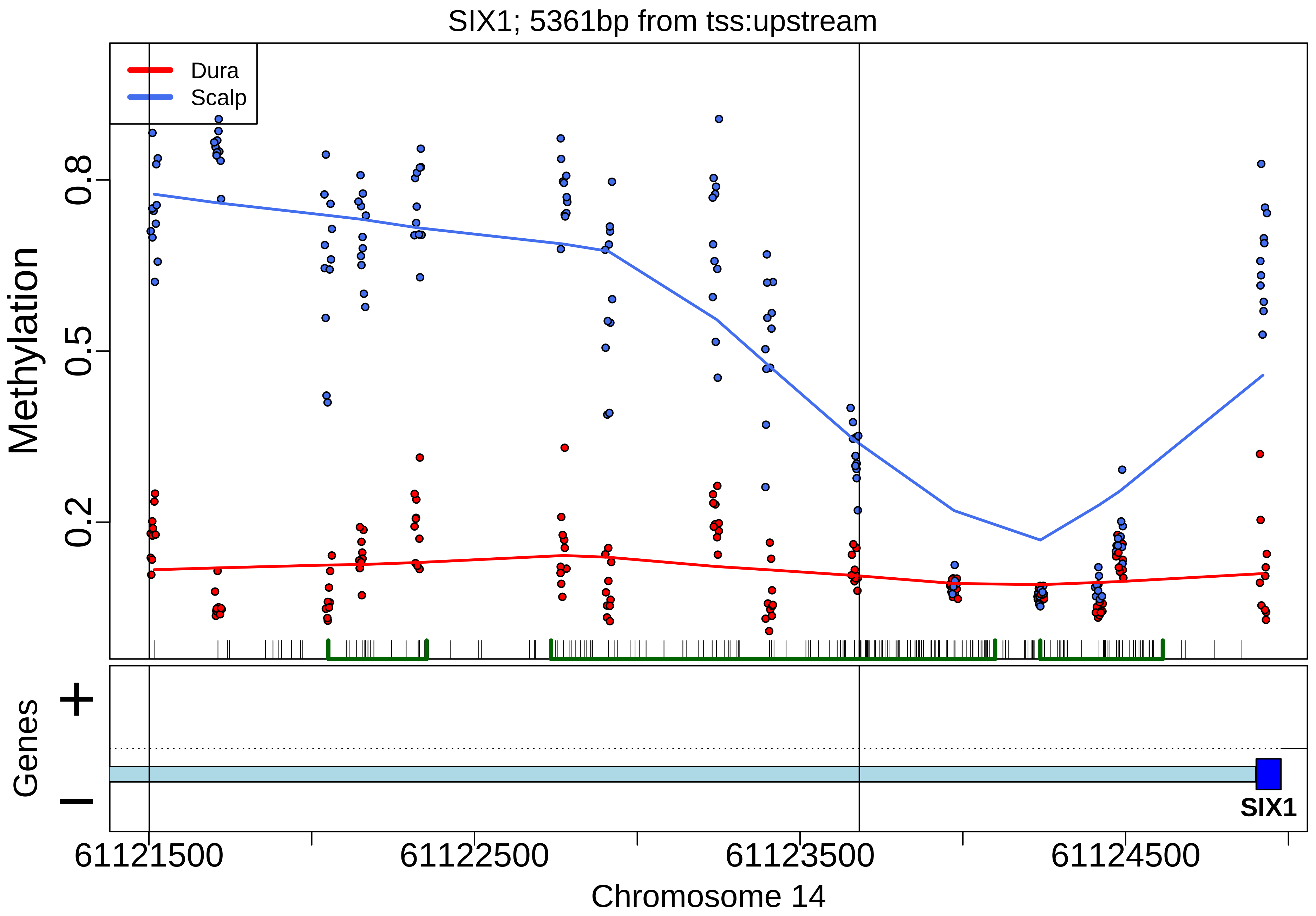

SIX1; 5361bp from tss:upstream
Dura
Scalp
Methylation
0.8
0.5
0.2
Genes
+
-
SIX1
61121500
61122500
61123500
61124500
Chromosome 14

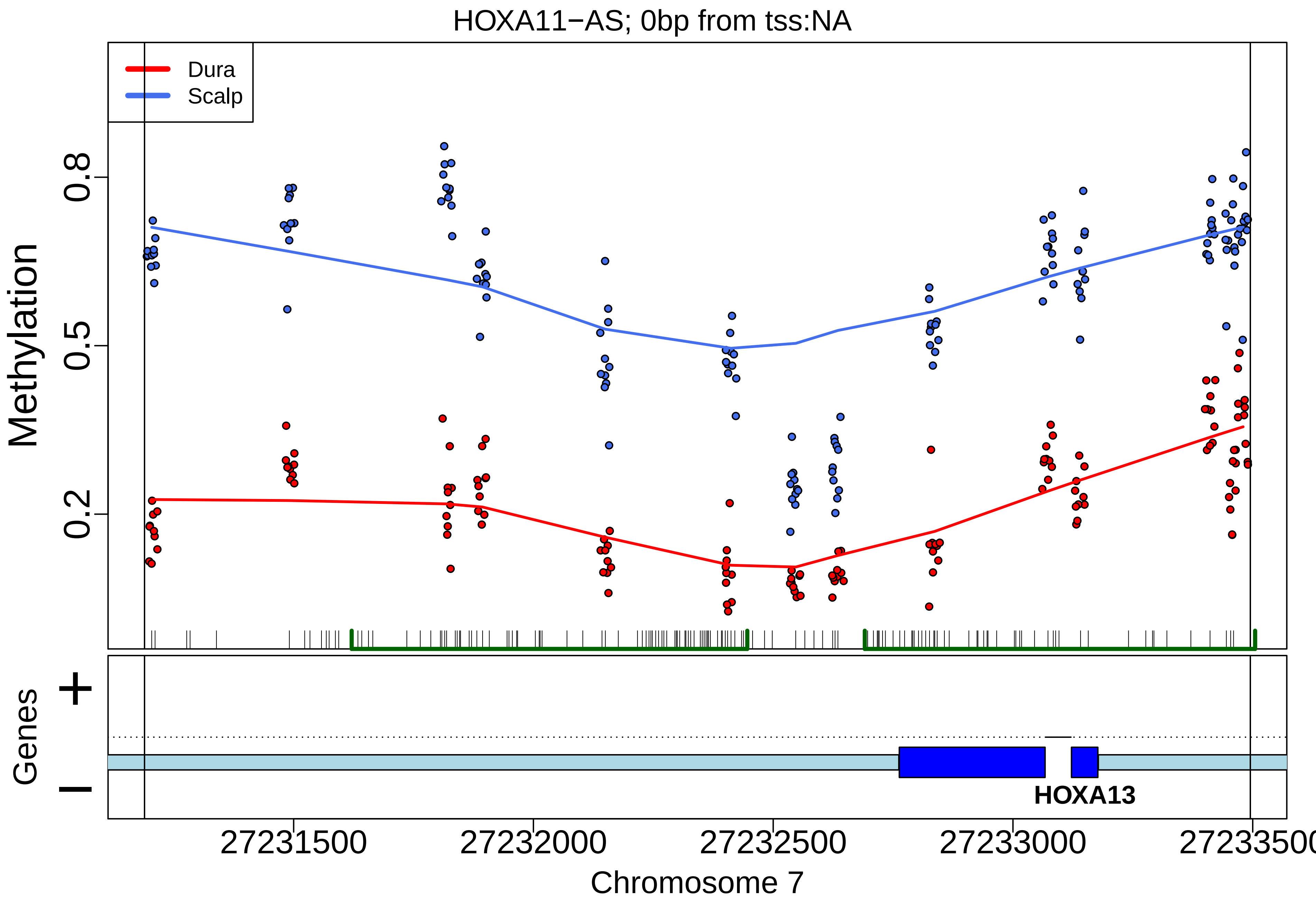

HOXA11−AS; 0bp from tss:NA
Dura
Scalp
Methylation
0.8
0.5
0.2
Genes
+
−
HOXA13
27231500
27232000
27232500
27233000
27233500
Chromosome 7

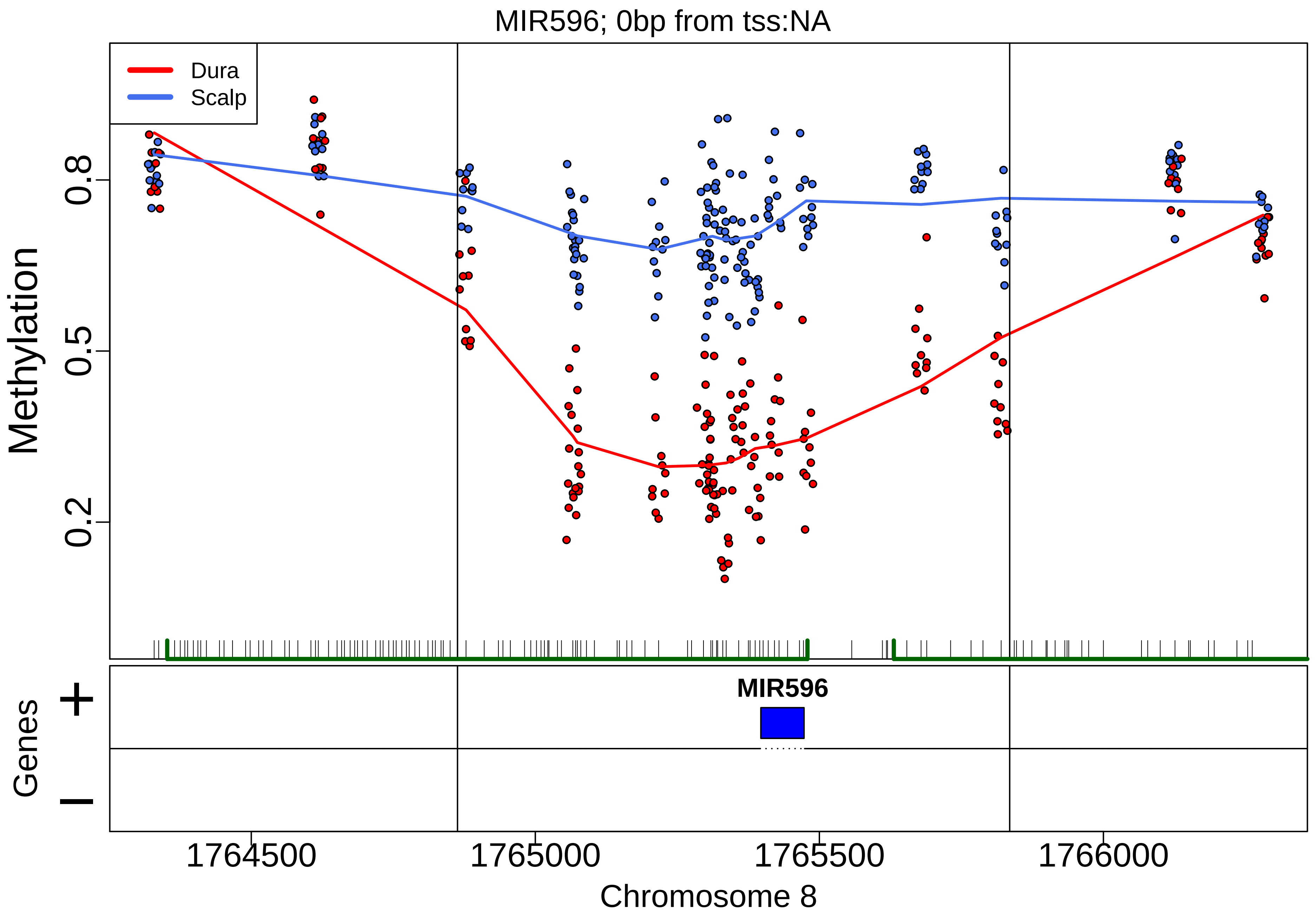

MIR596; 0bp from tss:NA
Dura
Scalp
Methylation
0.2
0.5
0.8
Genes
+
−
MIR596
1764500
1765000
1765500
1766000
Chromosome 8

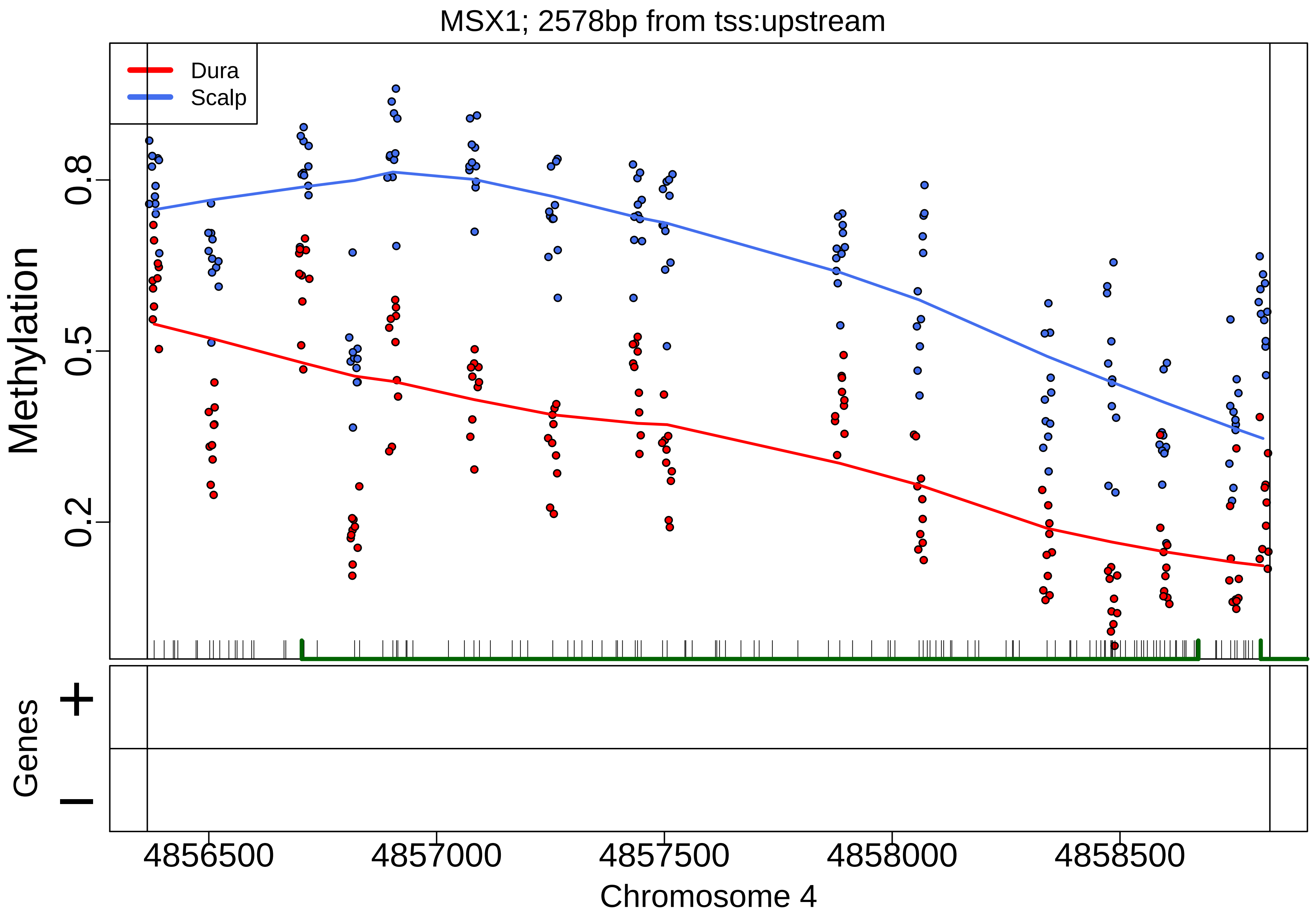

MSX1; 2578bp from tss:upstream
Dura
Scalp
Methylation
0.2
0.5
0.8
Genes
+
-
4856500
4857000
4857500
4858000
4858500
Chromosome 4

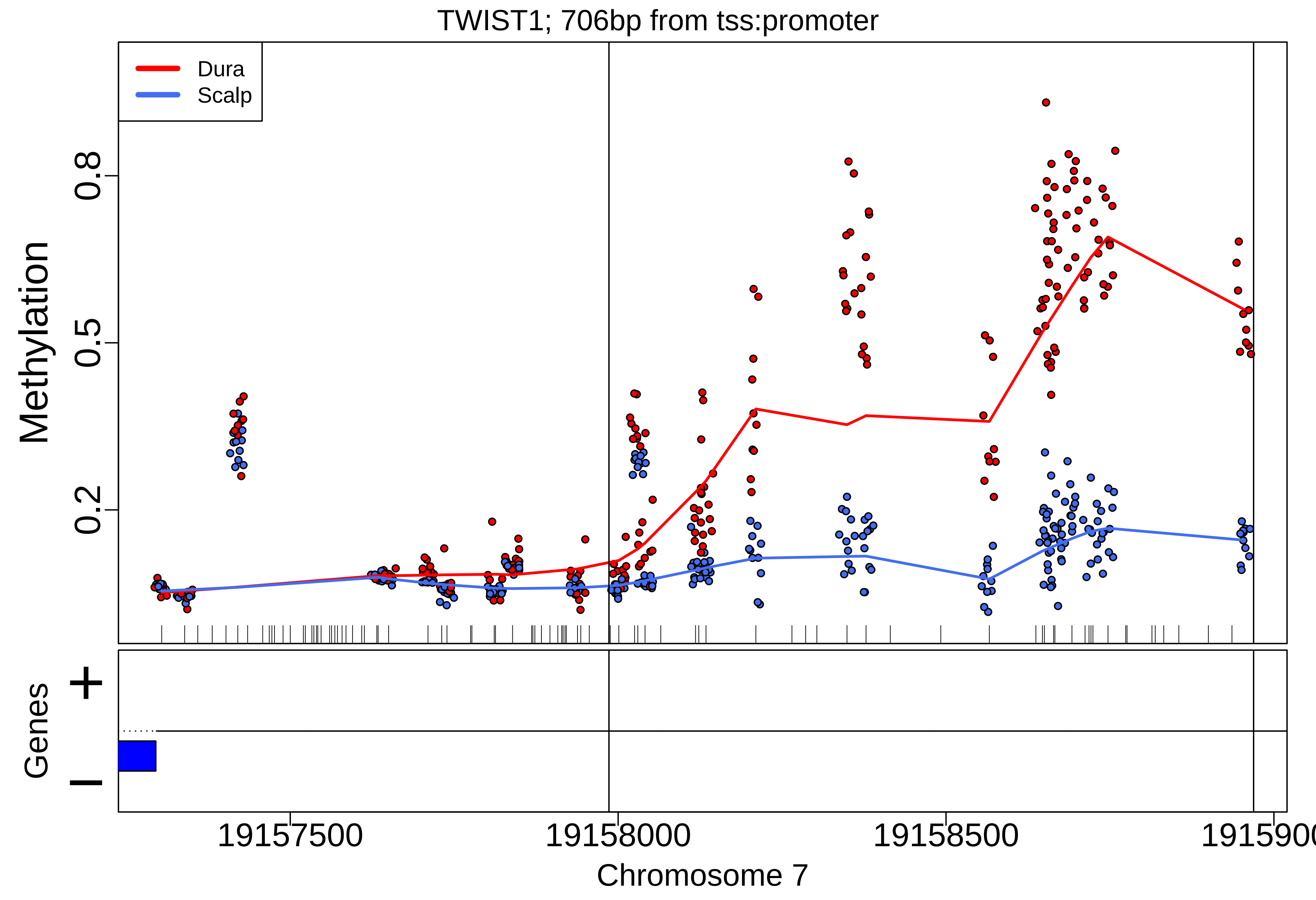
TWIST1; 706bp from tss:promoter
Dura
Scalp
Methylation
0.2
0.5
0.8
Genes
+
-
19157500
19158000
19158500
1915900
Chromosome 7

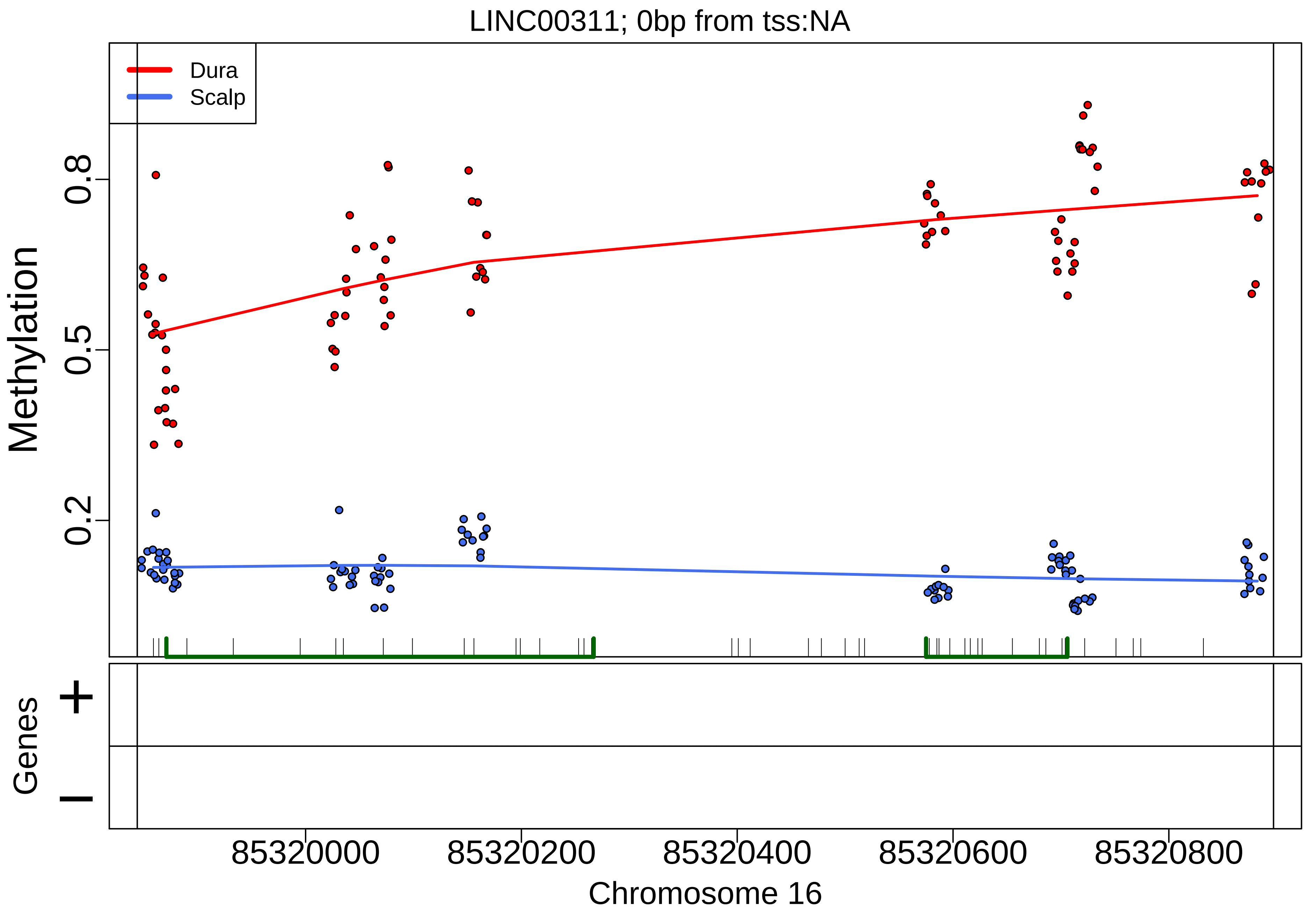

LINC00311; 0bp from tss:NA
Dura
Scalp
Methylation
0.2
0.5
0.8
Genes
+
−
85320000
85320200
85320400
85320600
85320800
Chromosome 16

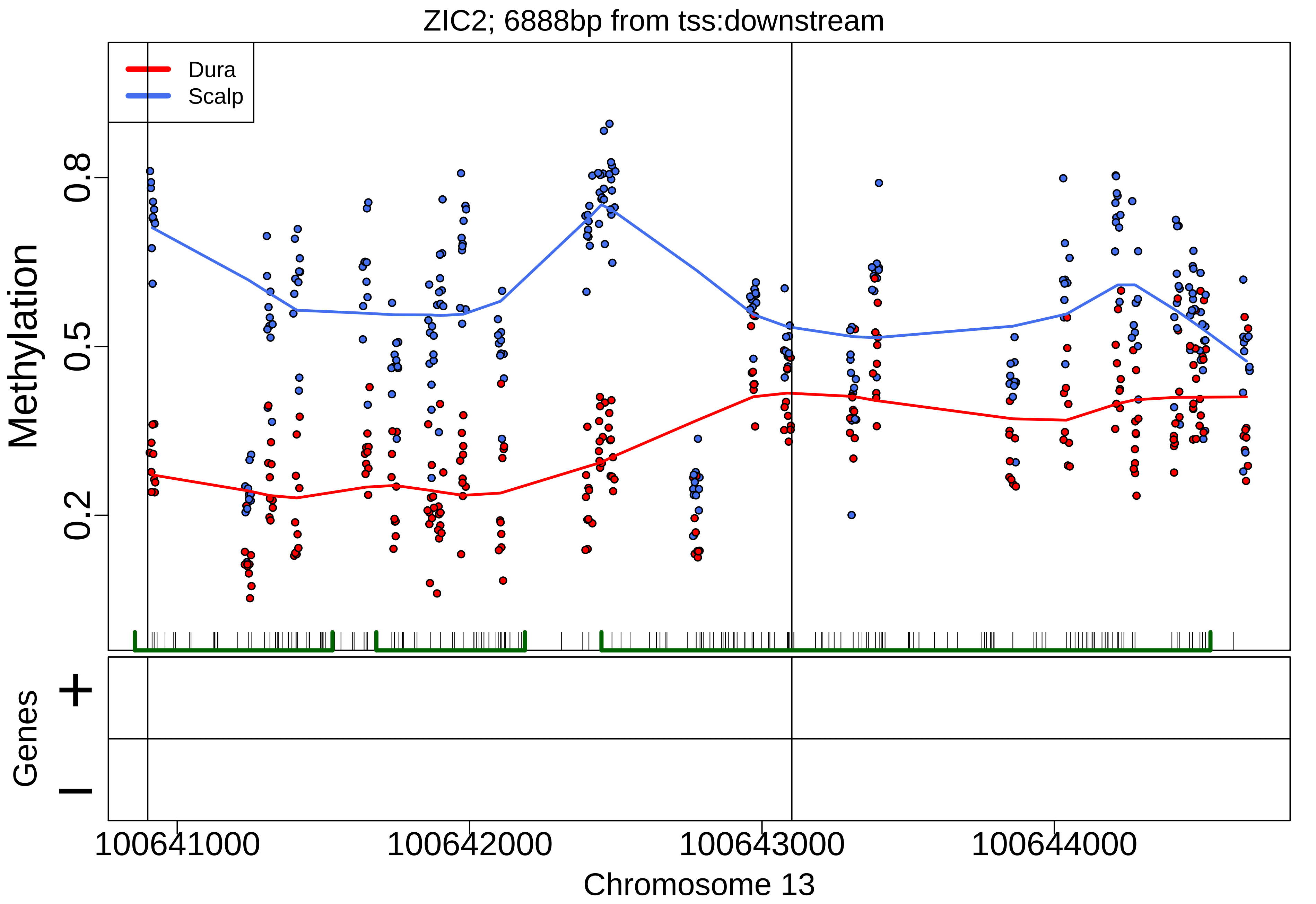
ZIC2; 6888bp from tss:downstream
Dura
Scalp
Methylation
0.2
0.5
0.8
Genes
+
-
100641000
100642000
100643000
100644000
Chromosome 13

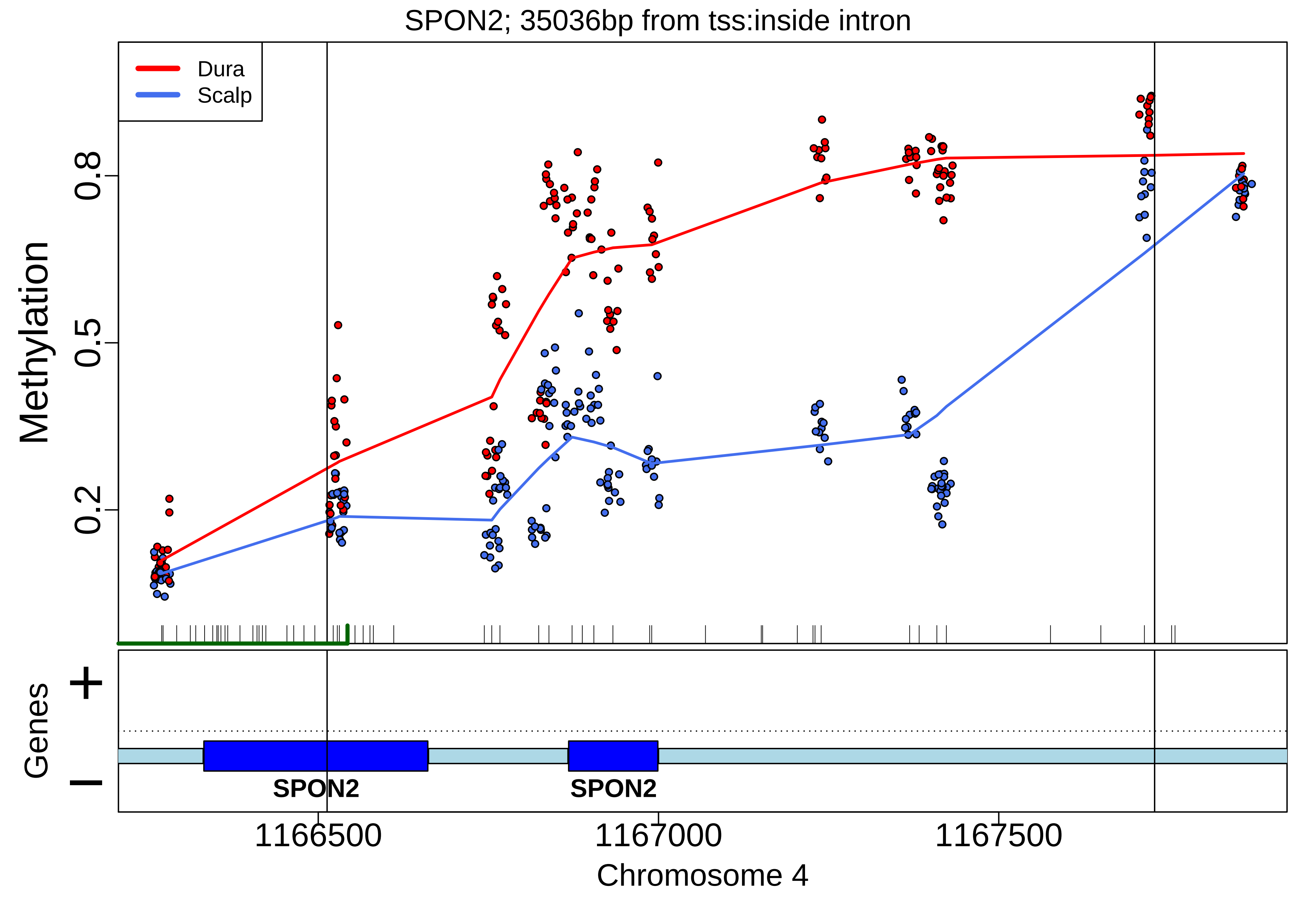

SPON2; 35036bp from tss:inside intron
Dura
Scalp
Methylation
0.2
0.5
0.8
Genes
+
-
SPON2
SPON2
1166500
1167000
1167500
Chromosome 4

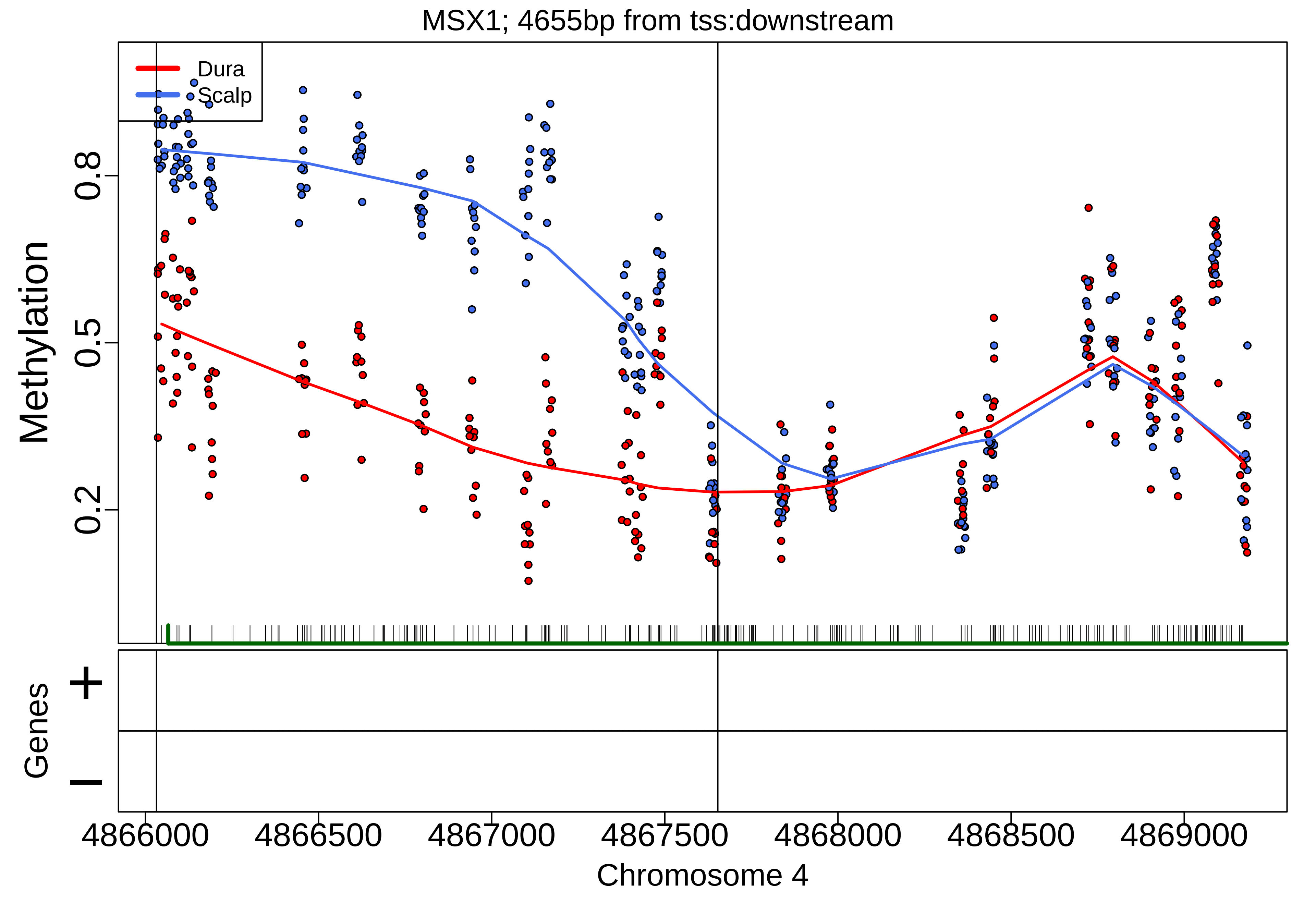
MSX1; 4655bp from tss:downstream
Dura
Scalp
Methylation
0.2
0.5
0.8
Genes
+
–
4866000
4866500
4867000
4867500
4868000
4868500
4869000
Chromosome 4

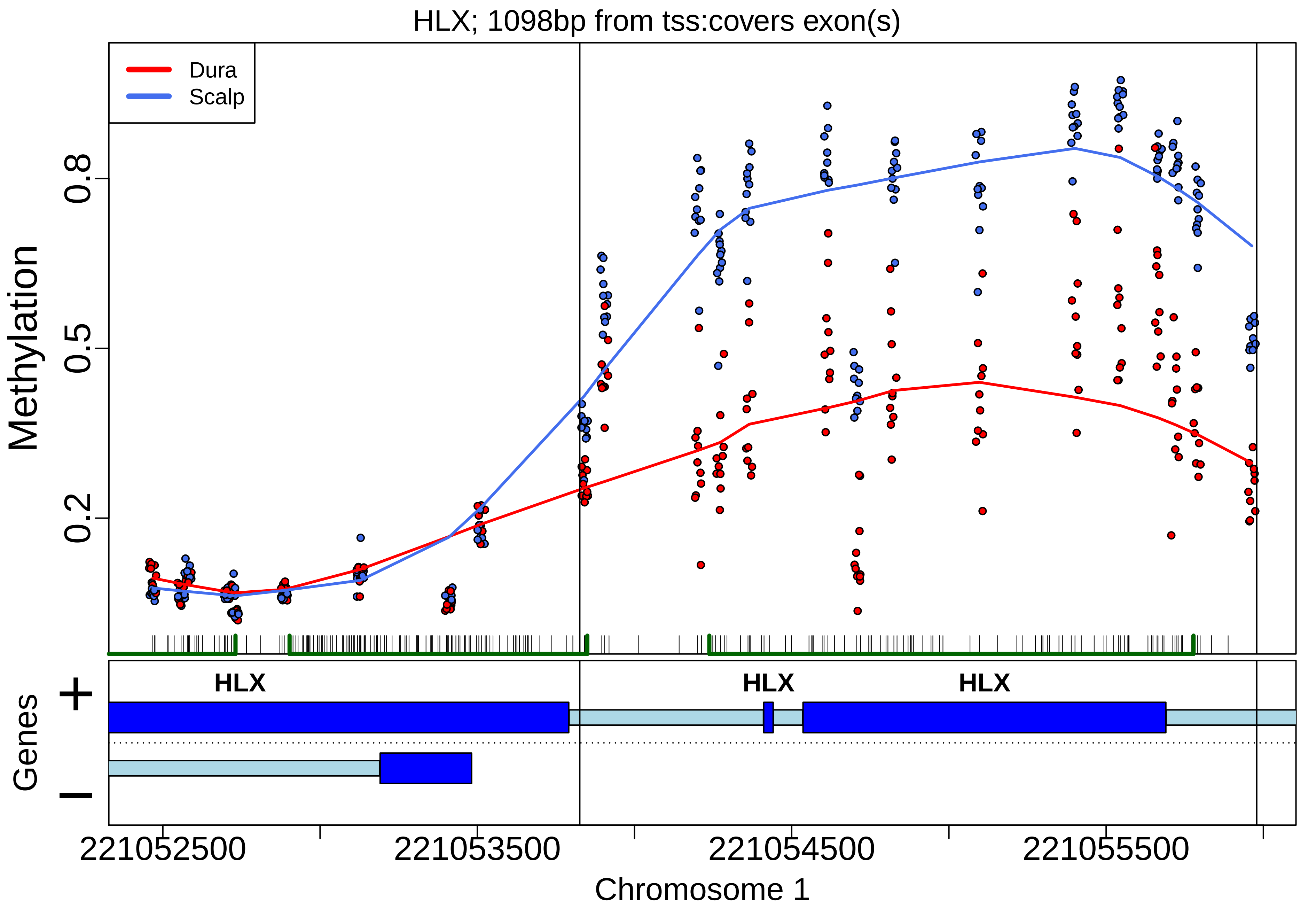
HLX; 1098bp from tss:covers exon(s)
Dura
Scalp
Methylation
0.2
0.5
0.8
Genes
+
-
HLX
HLX
HLX
221052500
221053500
221054500
221055500
Chromosome 1

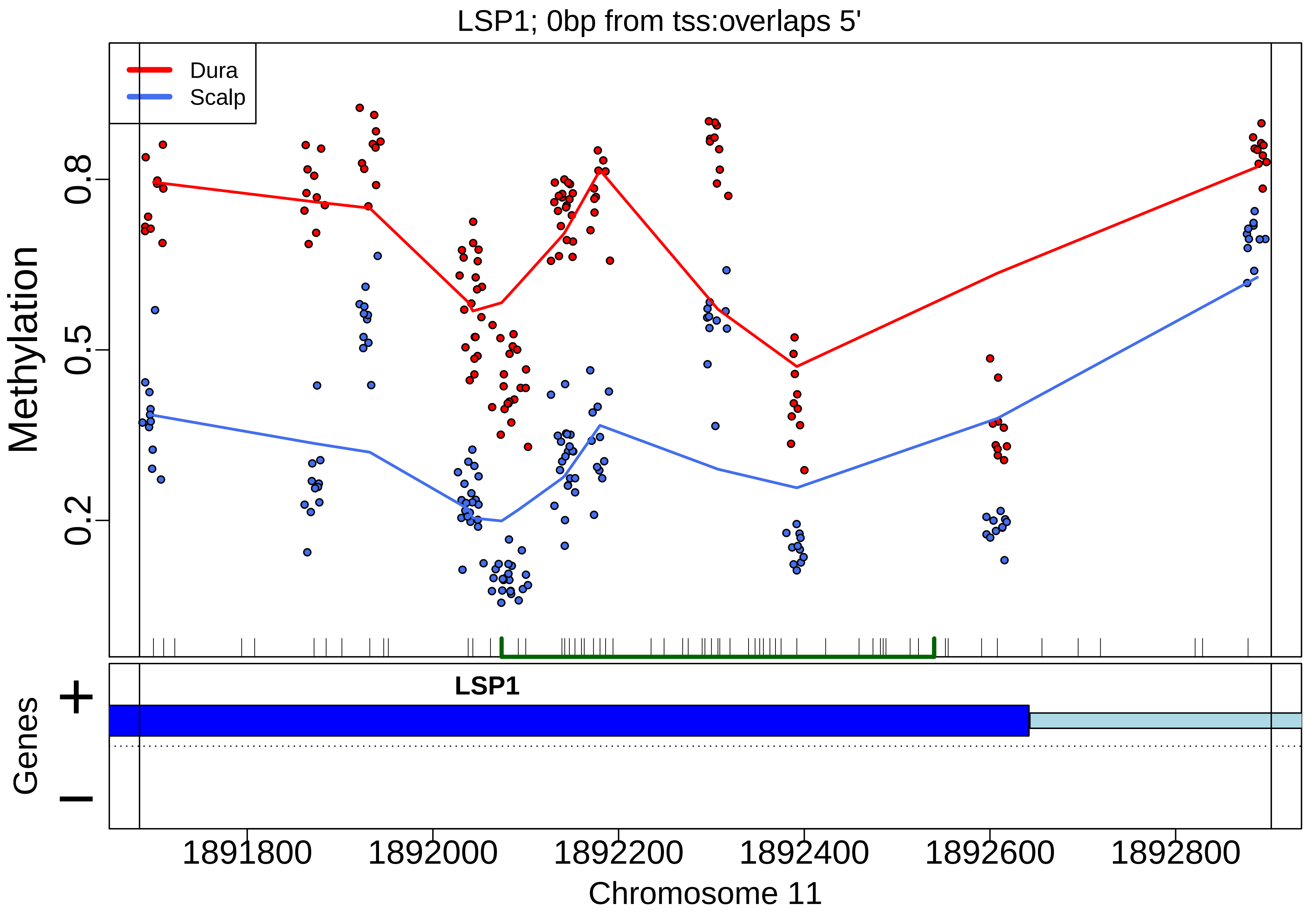
LSP1; 0bp from tss:overlaps 5'
Dura
Scalp
Methylation
0.2
0.5
0.8
Genes
+
-
LSP1
1891800
1892000
1892200
1892400
1892600
1892800
Chromosome 11

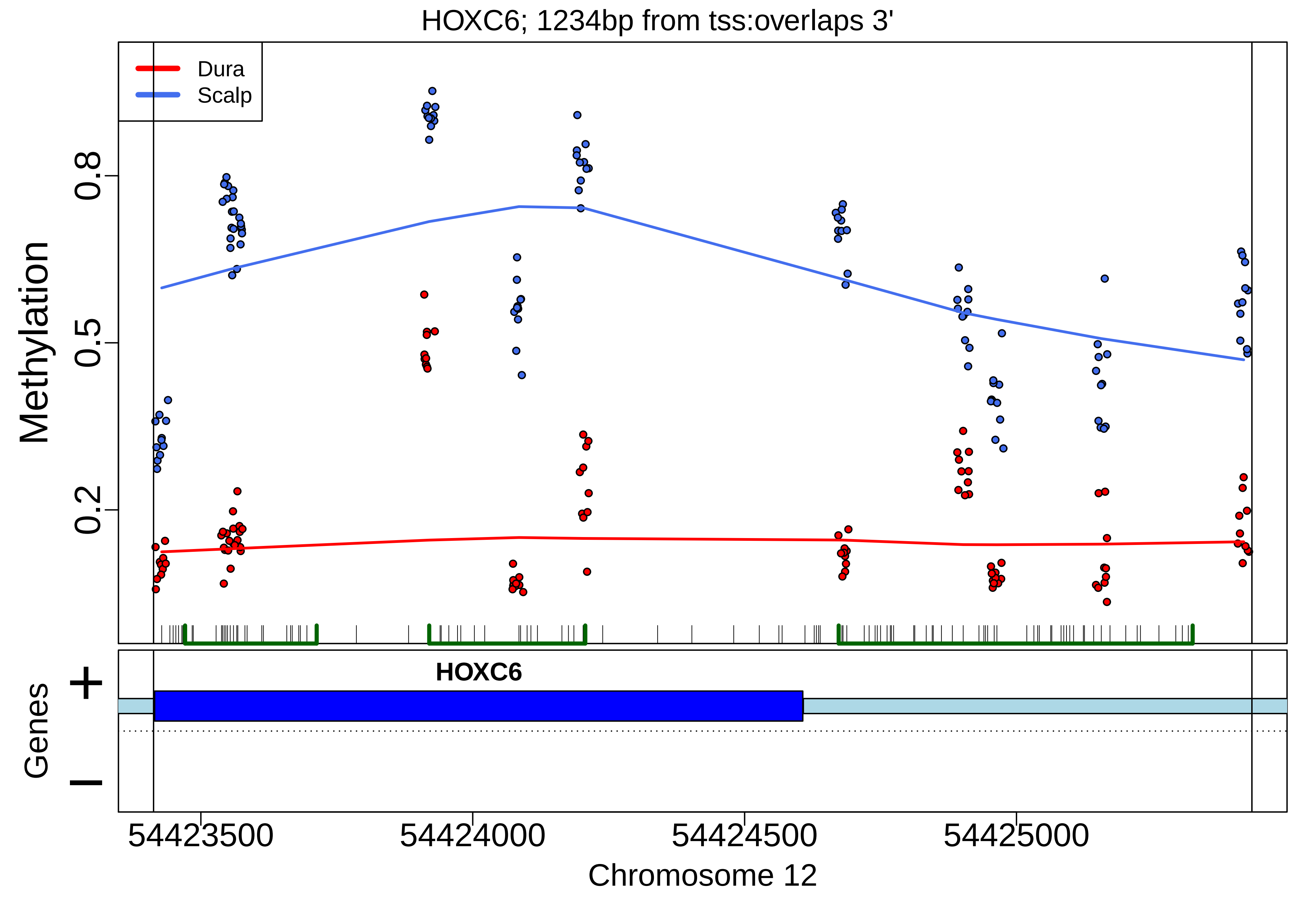

HOXC6; 1234bp from tss:overlaps 3'
Dura
Scalp
Methylation
0.2
0.5
0.8
Genes
+
HOXC6
54423500
54424000
54424500
54425000
Chromosome 12

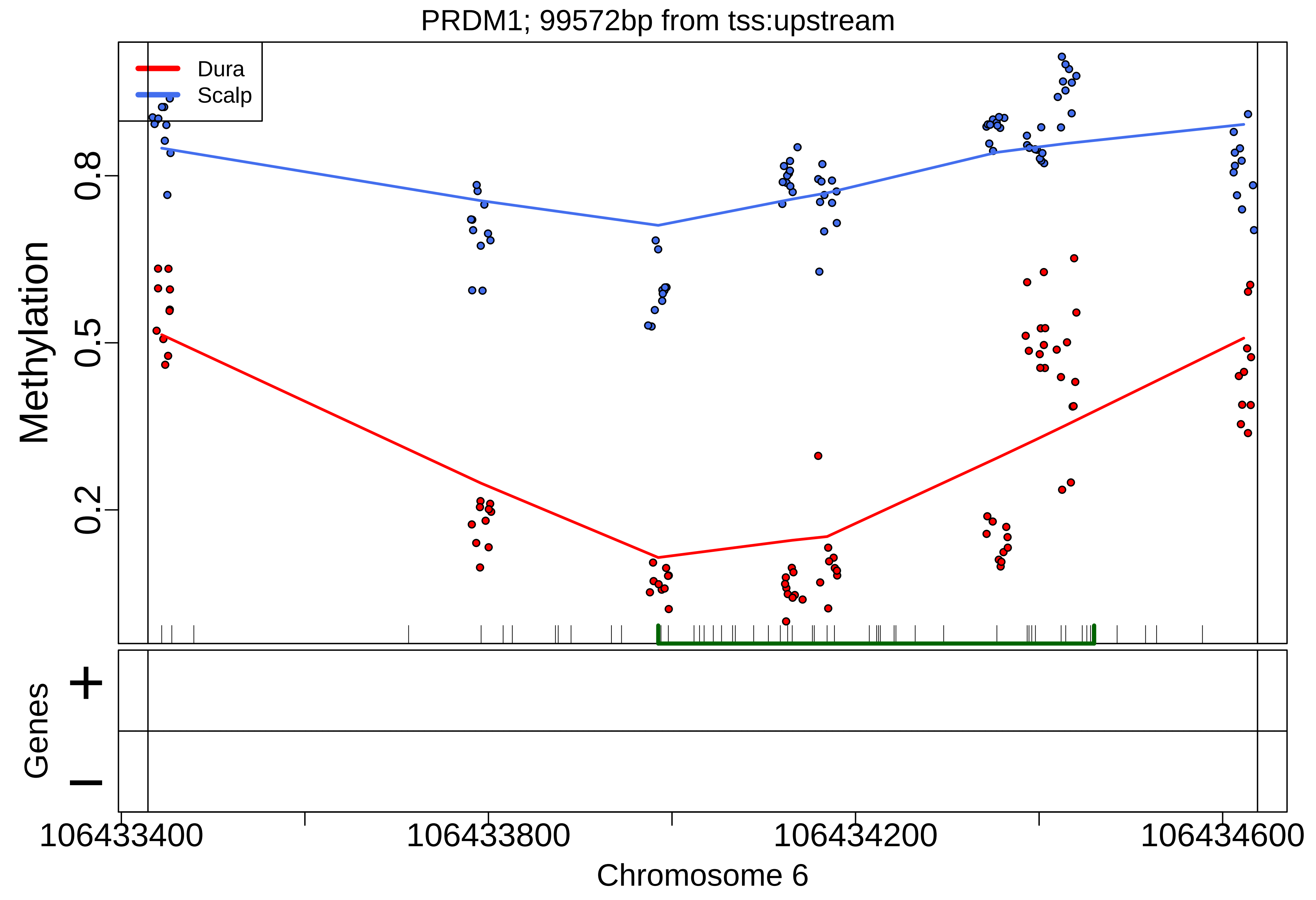

PRDM1; 99572bp from tss:upstream
Dura
Scalp
Methylation
0.2
0.5
0.8
Genes
+
-
106433400
106433800
106434200
106434600
Chromosome 6

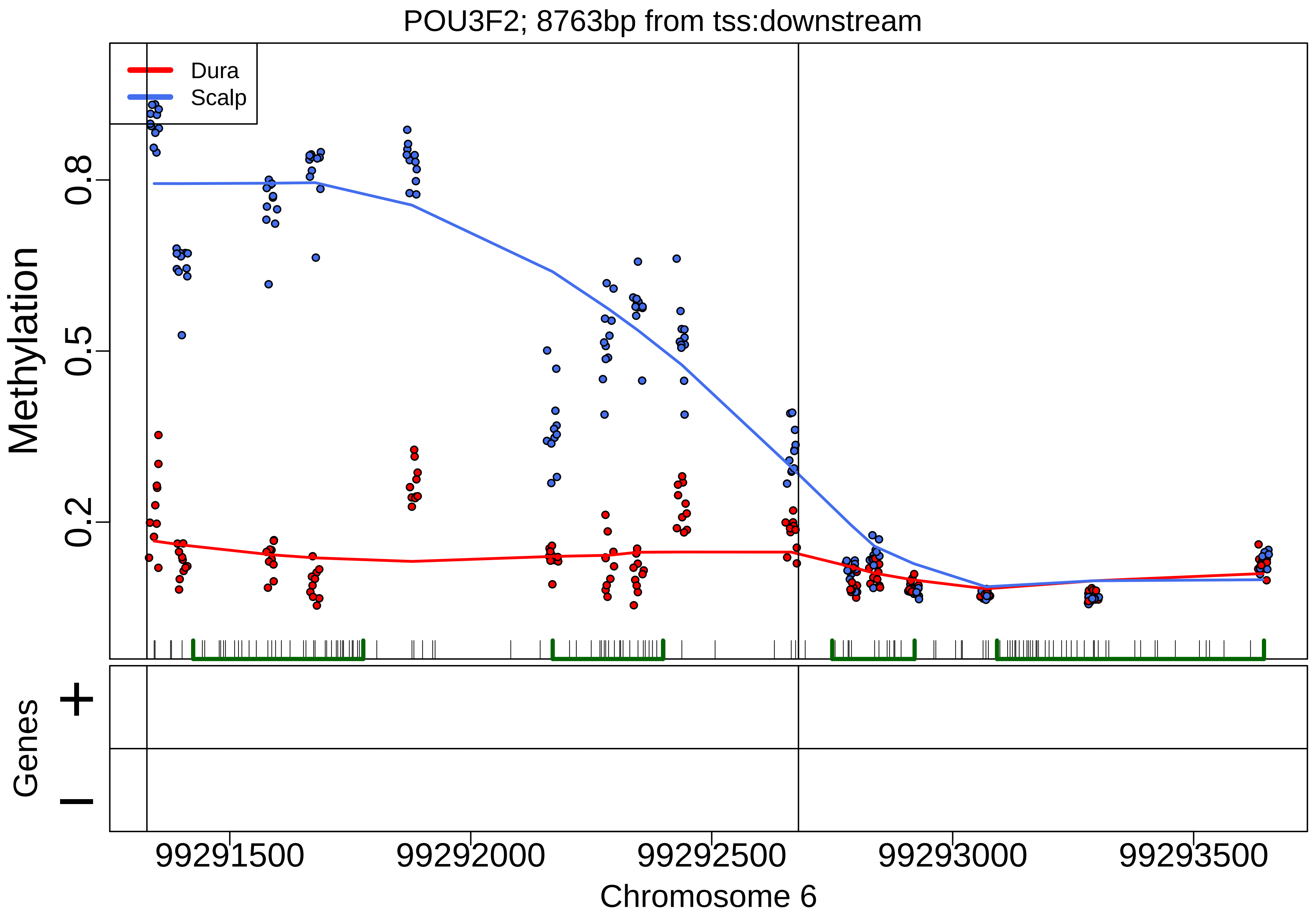

POU3F2; 8763bp from tss:downstream
Dura
Scalp
Methylation
0.2
0.5
0.8
Genes
+
−
99291500
99292000
99292500
99293000
99293500
Chromosome 6

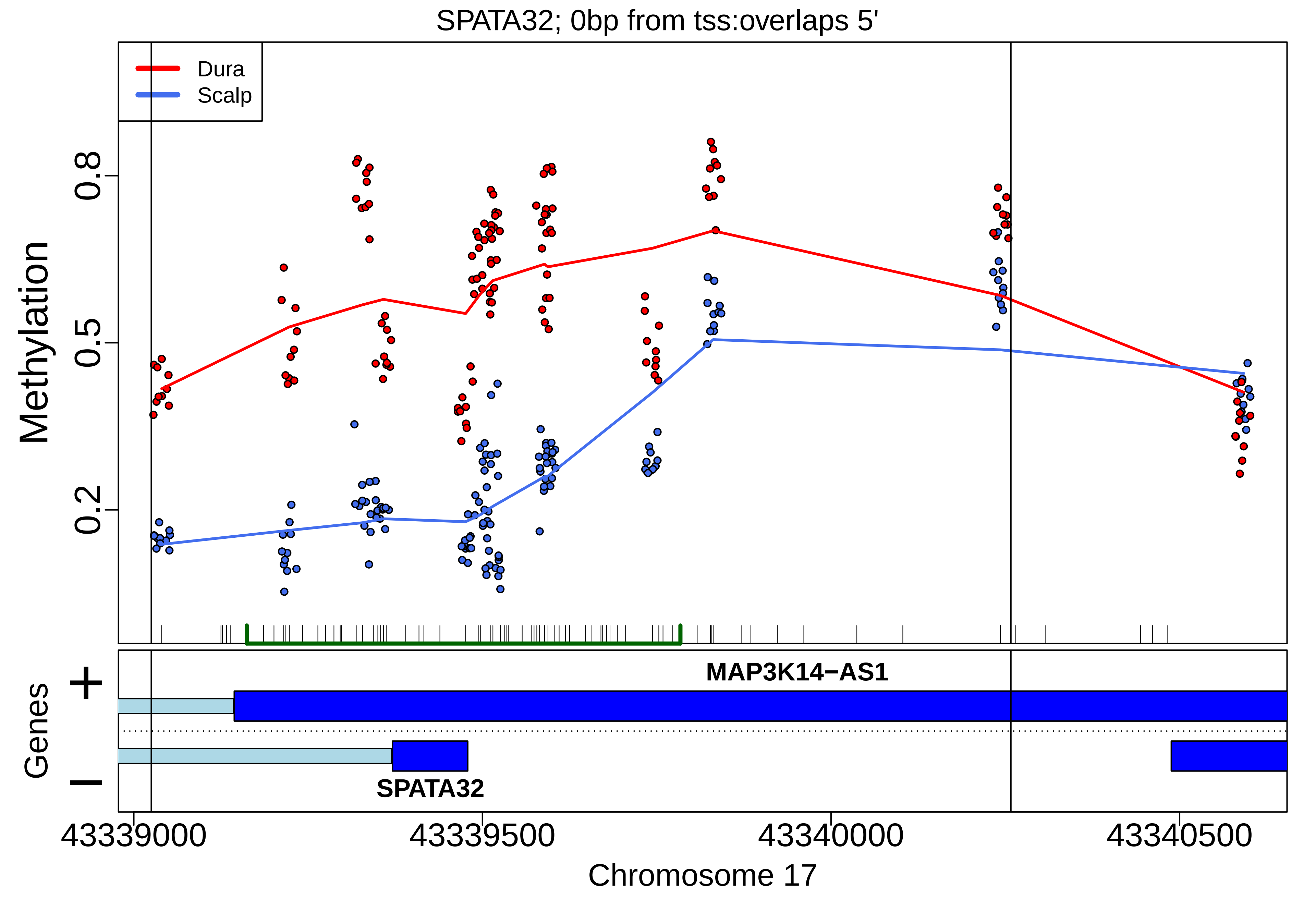
SPATA32; 0bp from tss:overlaps 5'
Dura
Scalp
Methylation
0.2
0.5
0.8
Genes
+
−
MAP3K14−AS1
SPATA32
43339000
43339500
43340000
43340500
Chromosome 17

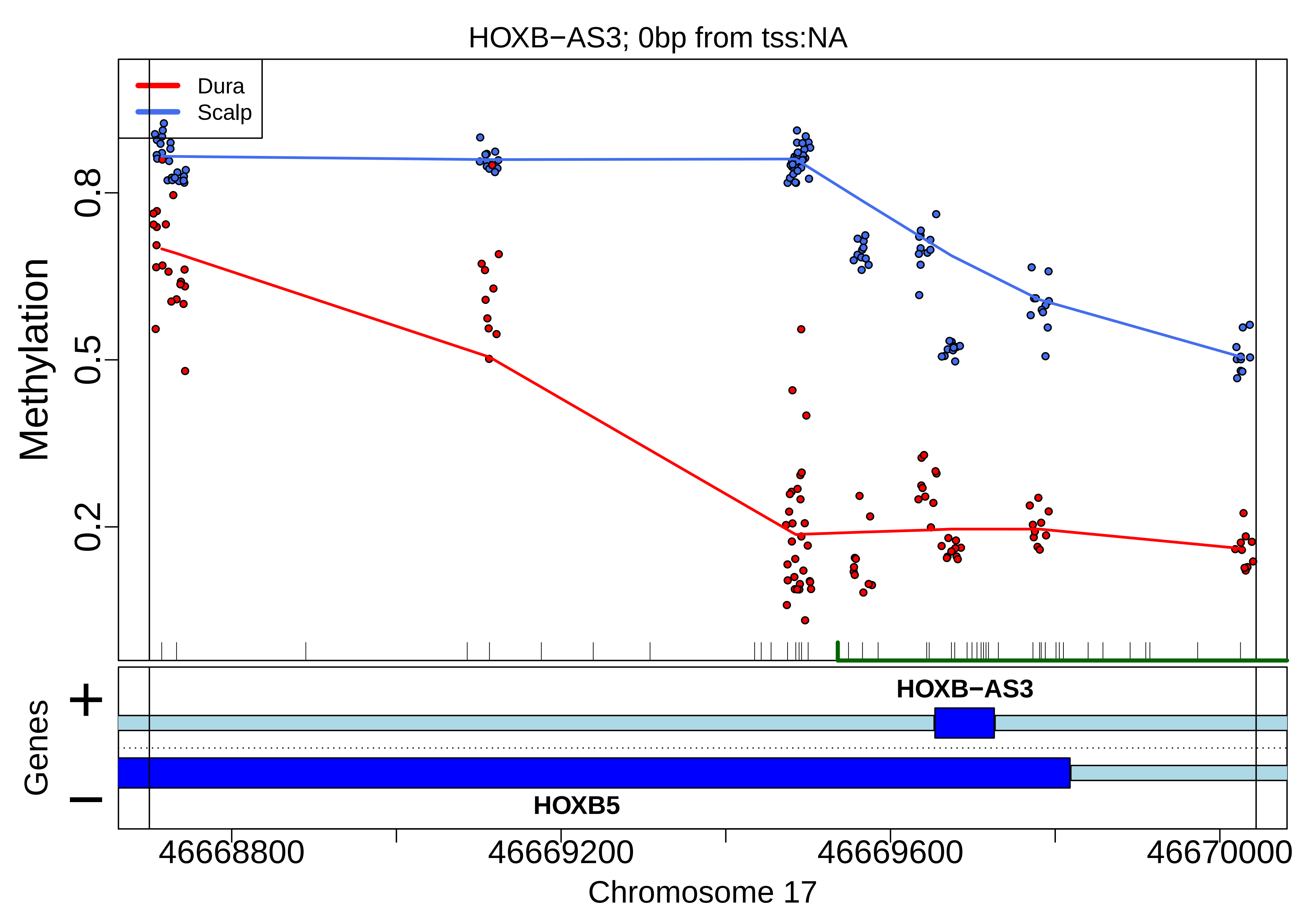
HOXB−AS3; 0bp from tss:NA
Dura
Scalp
Methylation
0.8
0.5
0.2
Genes
+
−
HOXB−AS3
HOXB5
46668800
46669200
46669600
46670000
Chromosome 17

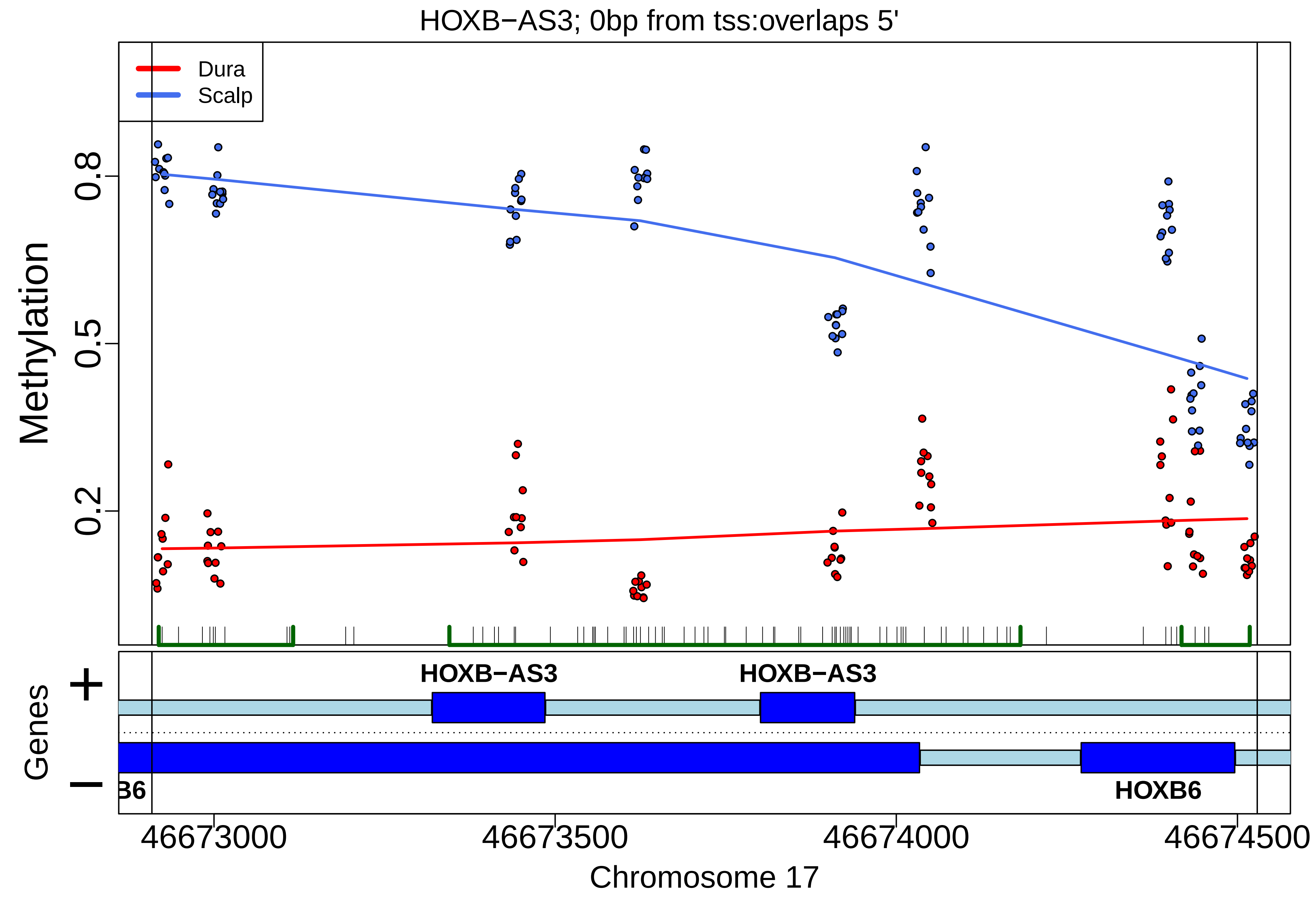

HOXB−AS3; 0bp from tss:overlaps 5'
Dura
Scalp
Methylation
0.2
0.5
0.8
Genes
+
−
HOXB−AS3
HOXB−AS3
B6
HOXB6
46673000
46673500
46674000
46674500
Chromosome 17

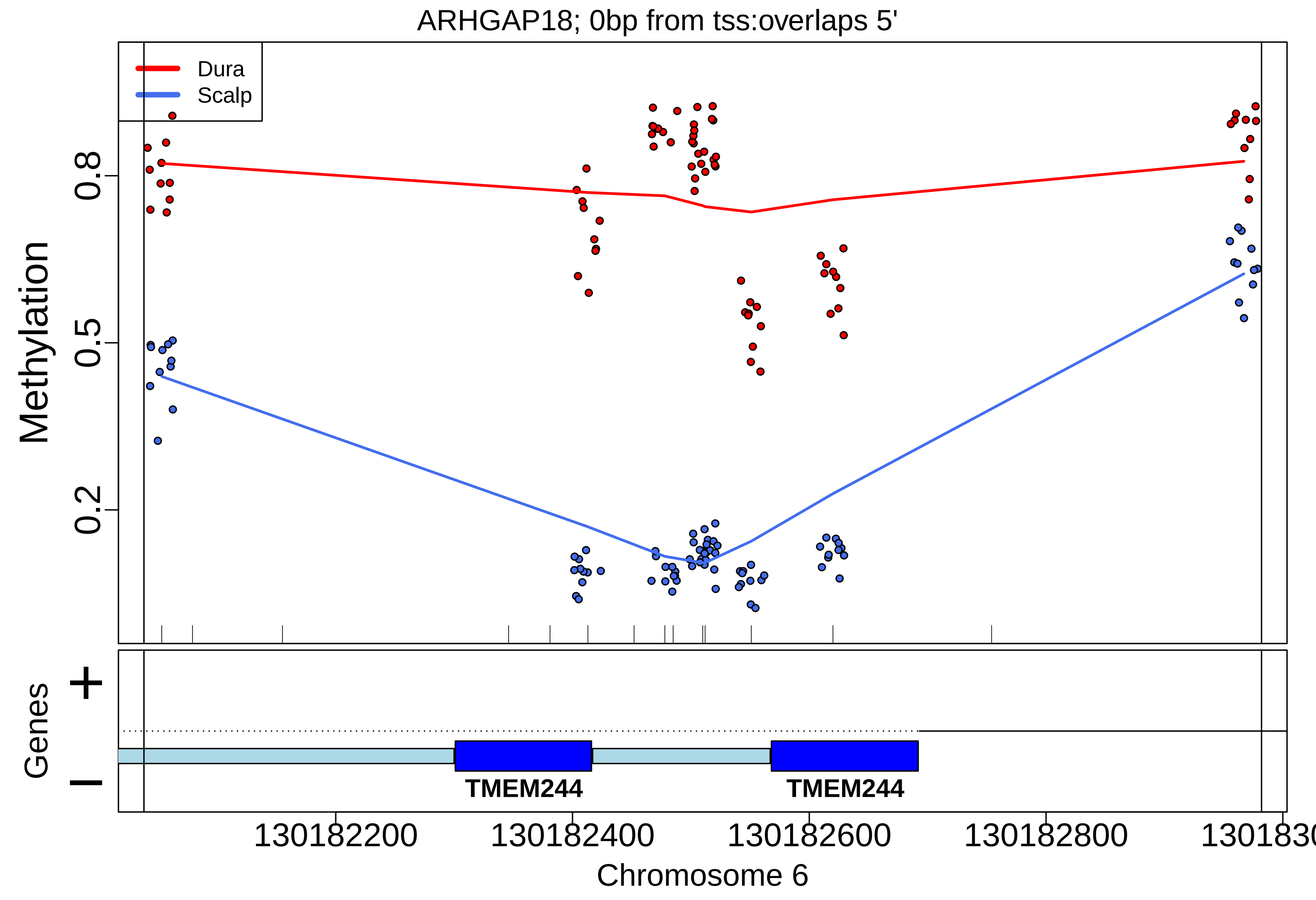

ARHGAP18; 0bp from tss:overlaps 5'
Dura
Scalp
Methylation
0.2
0.5
0.8
Genes
+
-
TMEM244
TMEM244
130182200
130182400
130182600
130182800
Chromosome 6

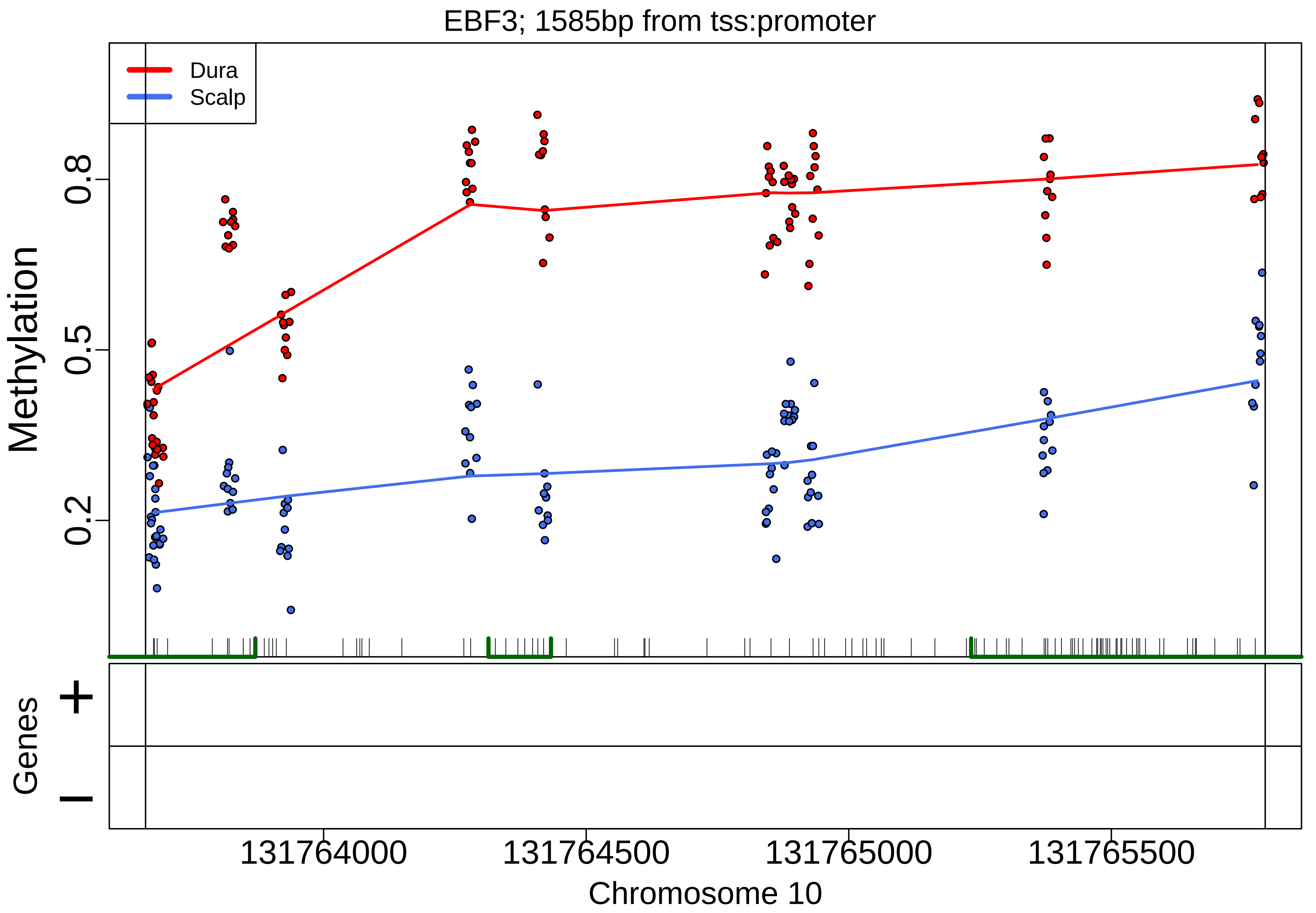
EBF3; 1585bp from tss:promoter
Dura
Scalp
Methylation
0.2
0.5
0.8
Genes
+
−
131764000
131764500
131765000
131765500
Chromosome 10

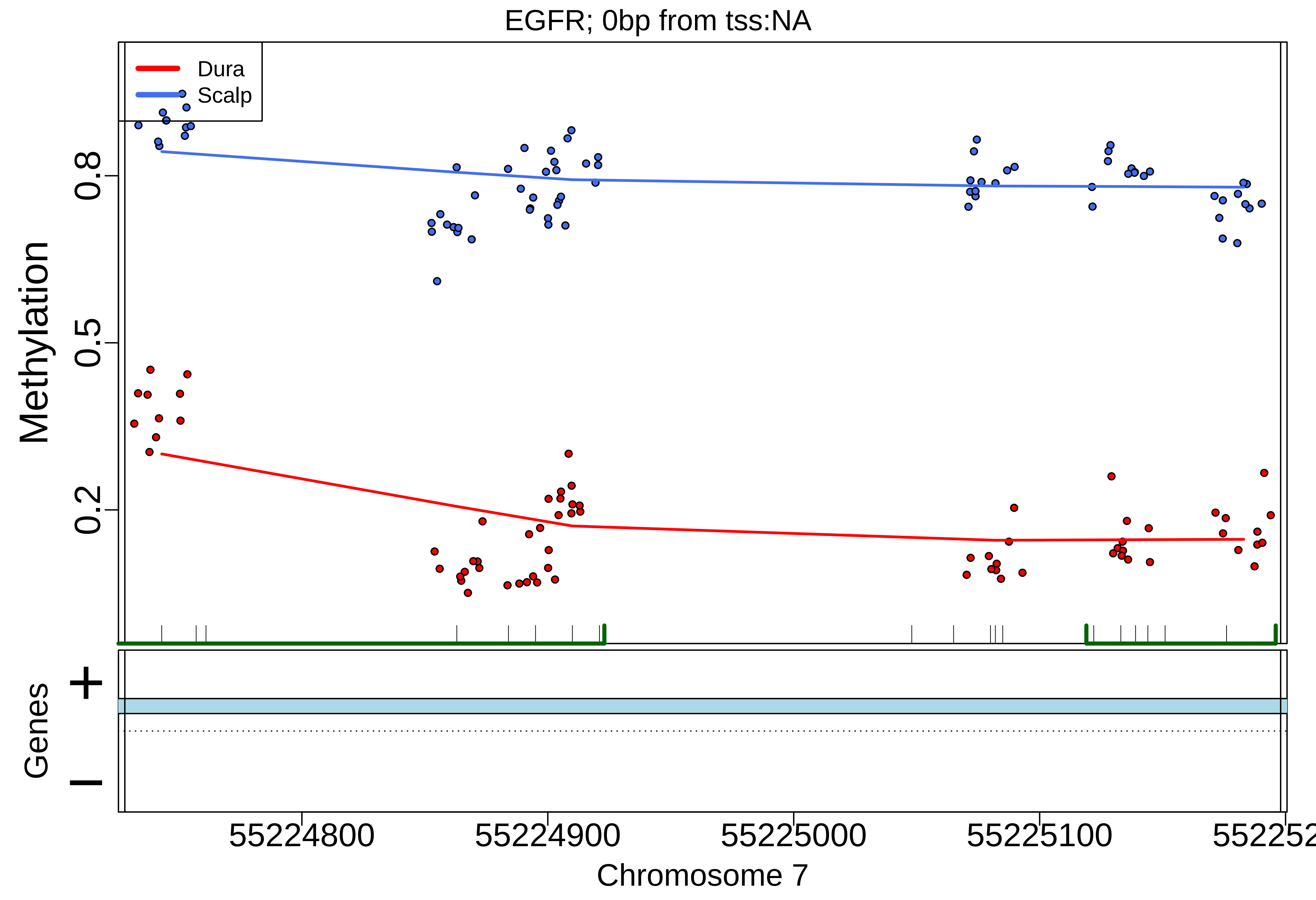
EGFR; 0bp from tss:NA
Dura
Scalp
Methylation
0.2
0.5
0.8
Genes
+
−
55224800
55224900
55225000
55225100
552252
Chromosome 7

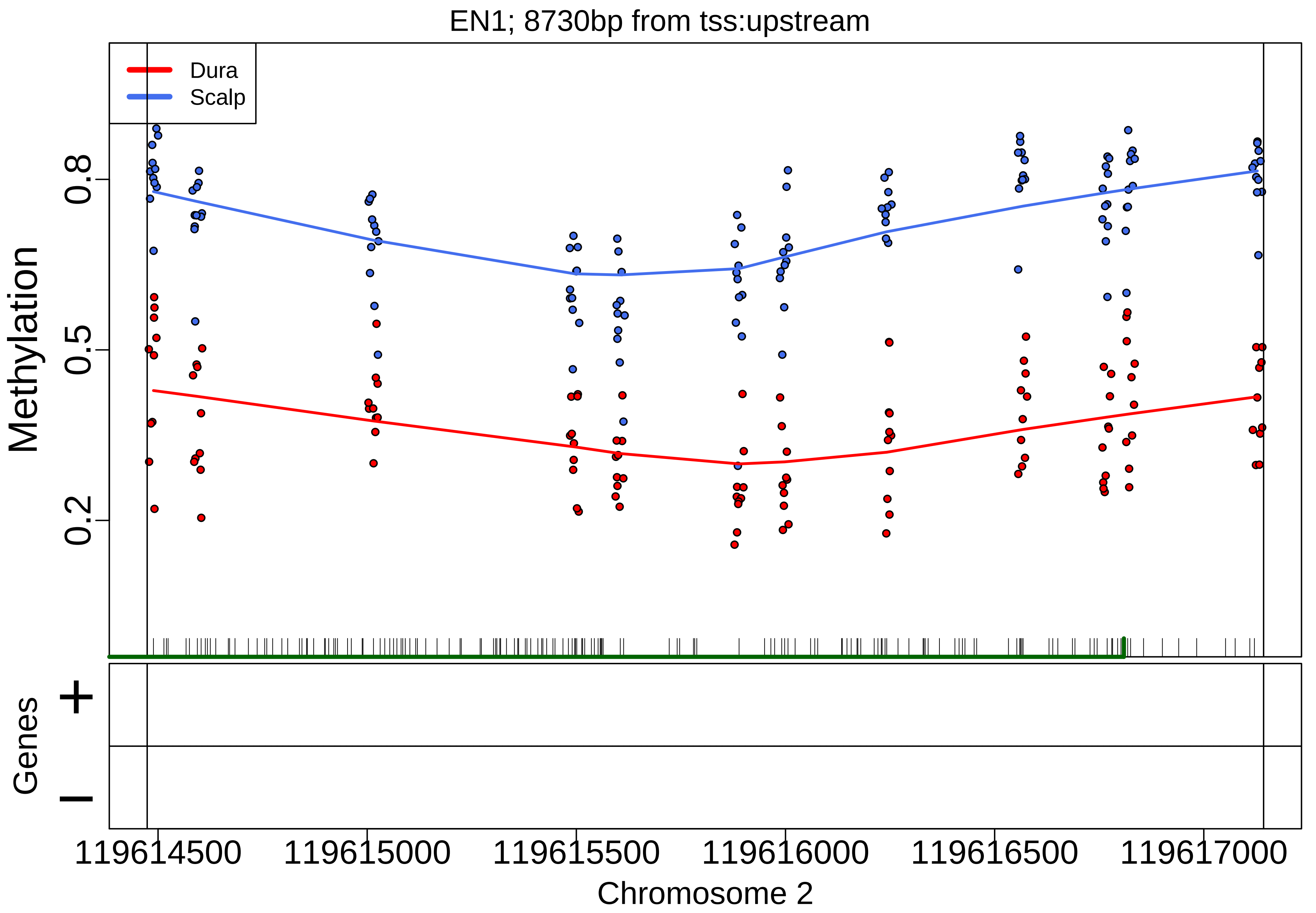
EN1; 8730bp from tss:upstream
Dura
Scalp
Methylation
0.2
0.5
0.8
Genes
+
−
119614500
119615000
119615500
119616000
119616500
119617000
Chromosome 2

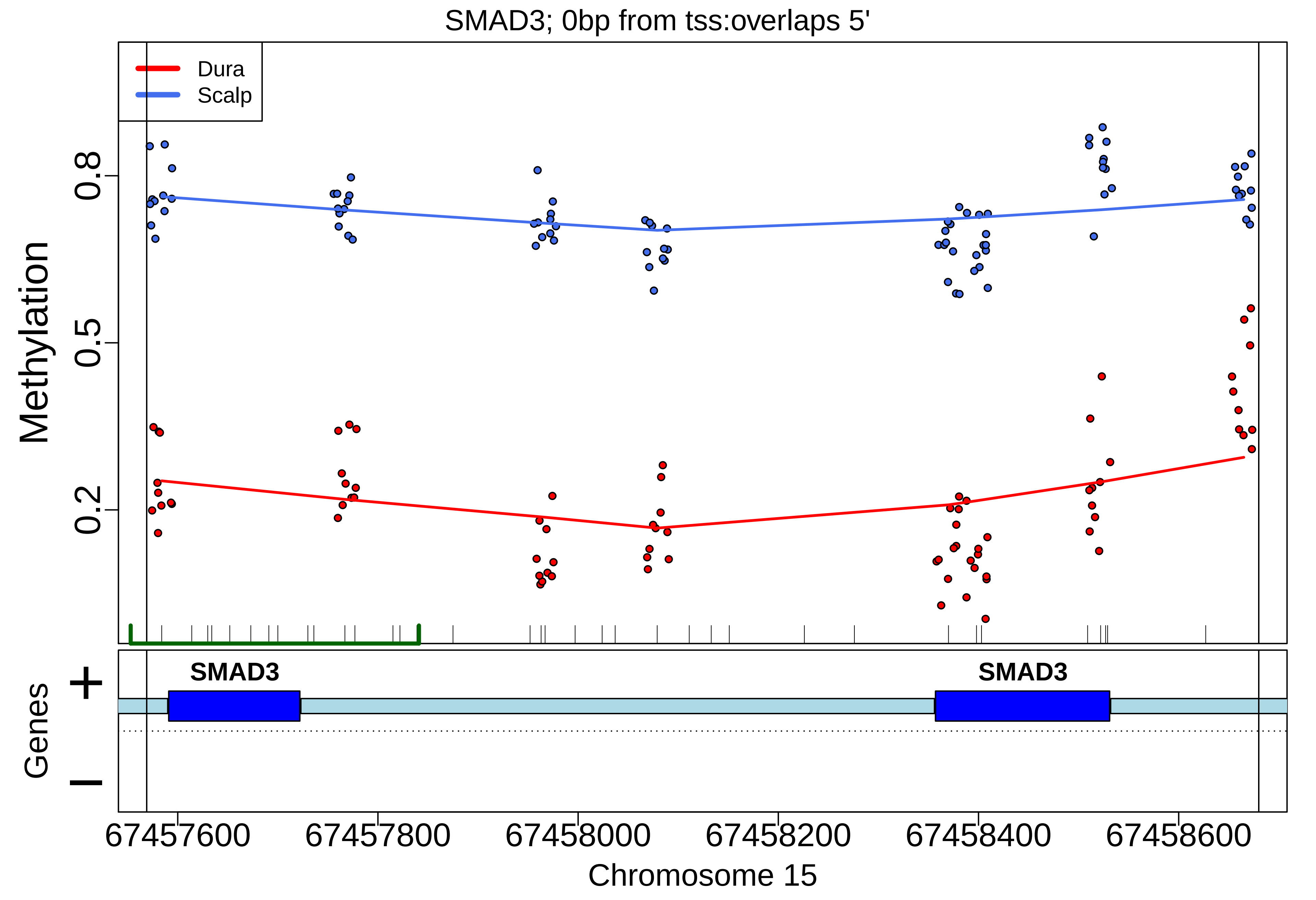
SMAD3; 0bp from tss:overlaps 5'
Dura
Scalp
Methylation
0.2
0.5
0.8
Genes
+
-
SMAD3
SMAD3
67457600
67457800
67458000
67458200
67458400
67458600
Chromosome 15

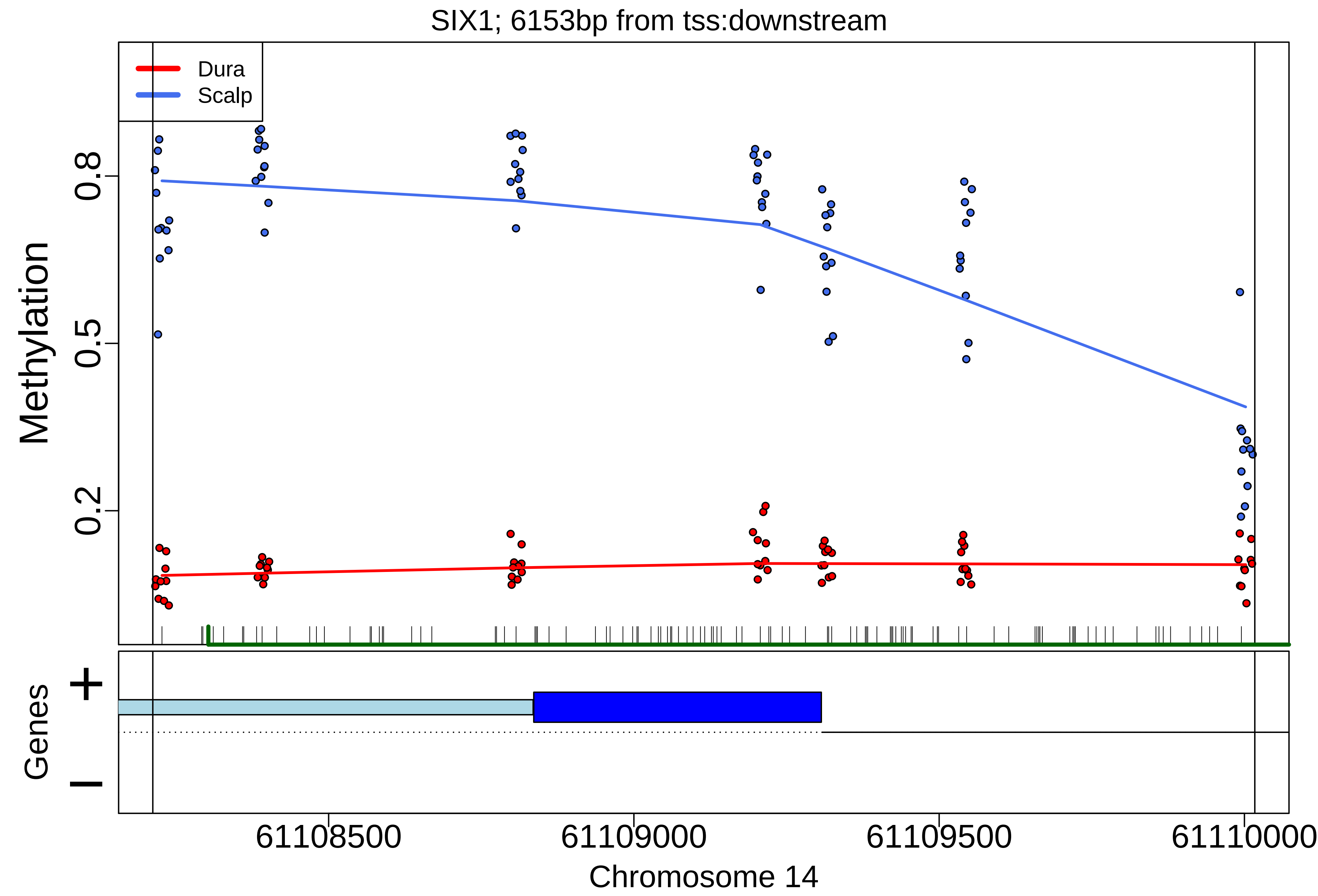
SIX1; 6153bp from tss:downstream
Dura
Scalp
Methylation
0.2
0.5
0.8
Genes
+
–
61108500
61109000
61109500
61110000
Chromosome 14

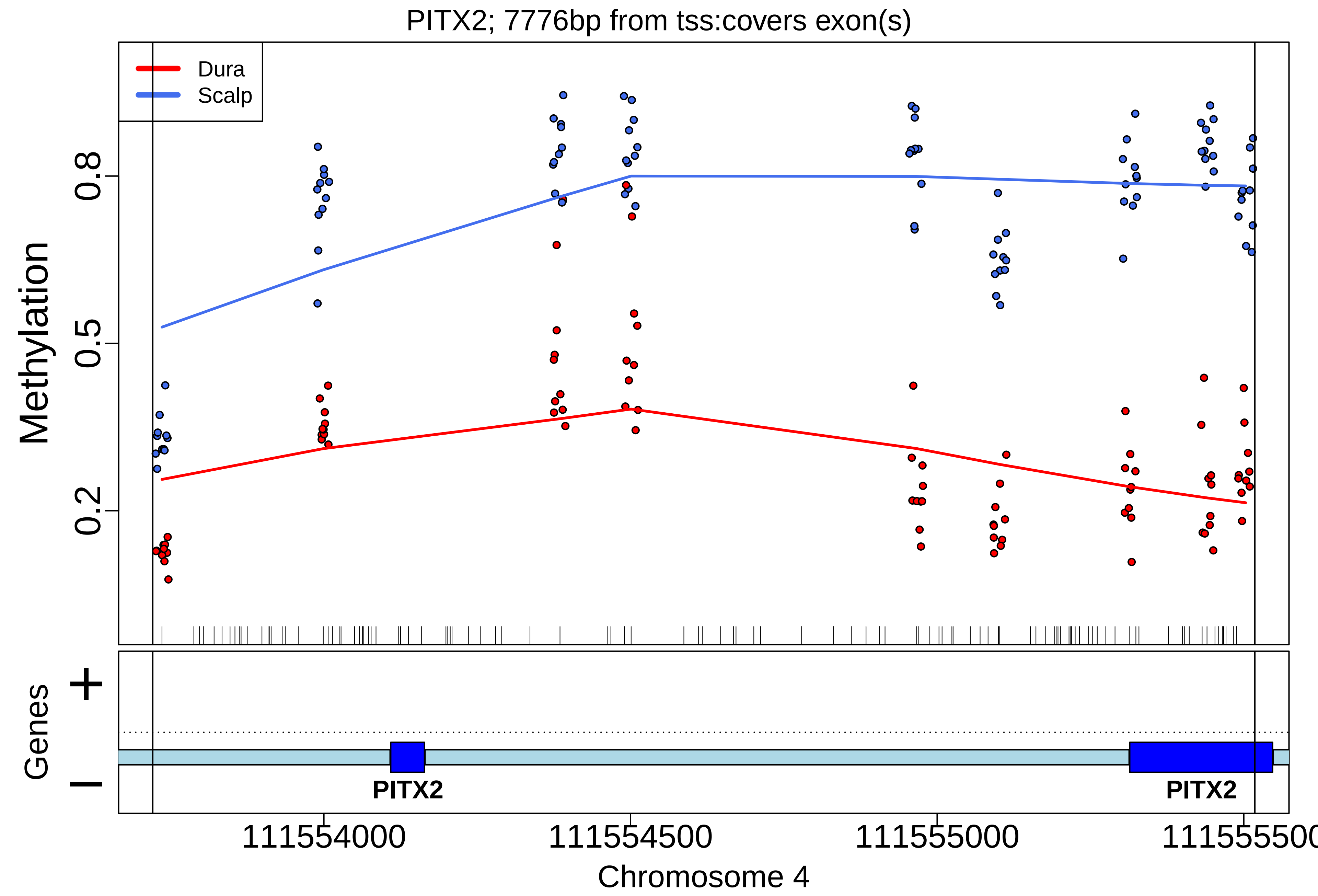
PITX2; 7776bp from tss:covers exon(s)
Dura
Scalp
Methylation
0.2
0.5
0.8
Genes
+
−
PITX2
PITX2
111554000
111554500
111555000
111555500
Chromosome 4

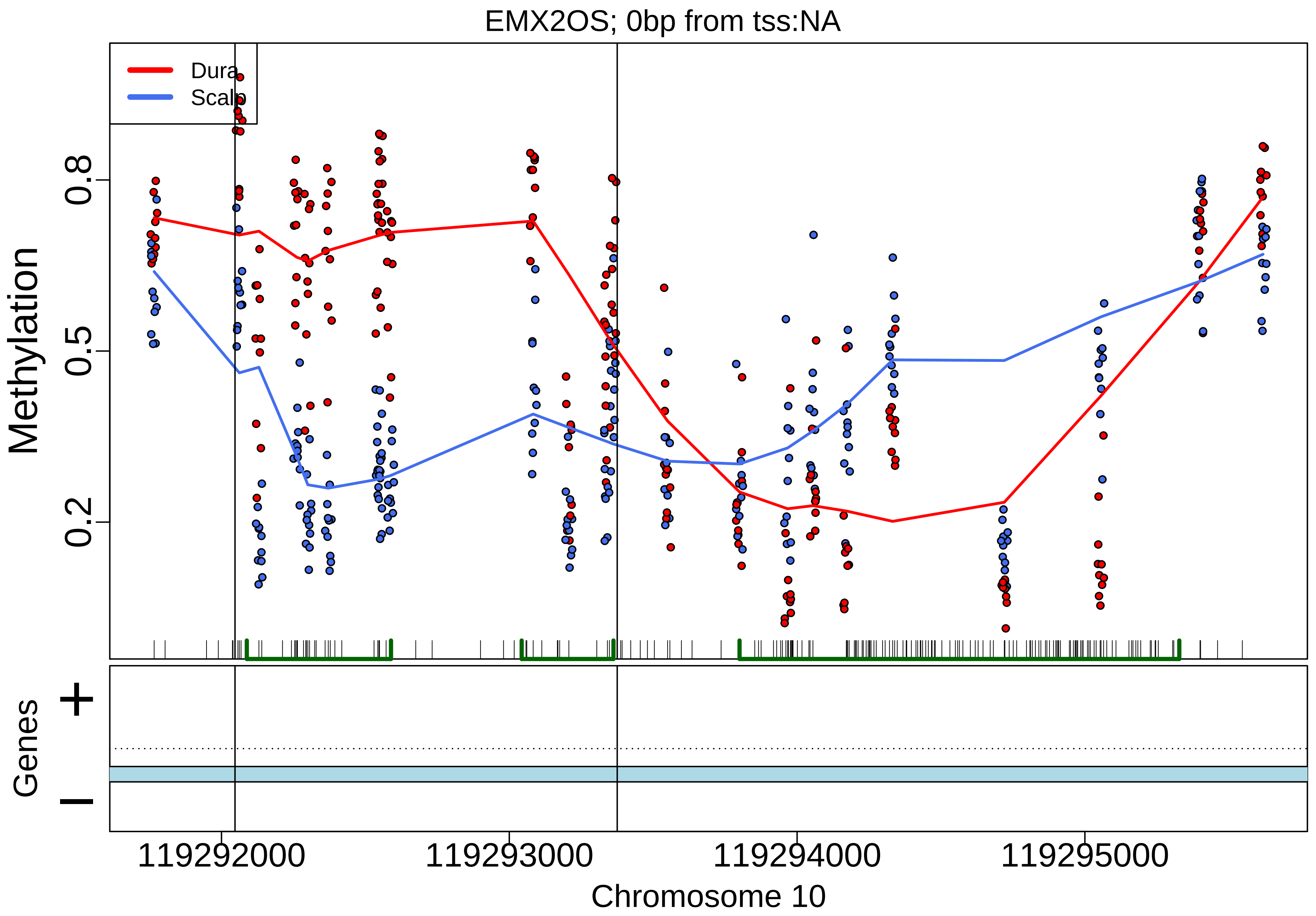

EMX2OS; 0bp from tss:NA
Dura
Scalp
Methylation
0.2
0.5
0.8
Genes
+
-
119292000
119293000
119294000
119295000
Chromosome 10

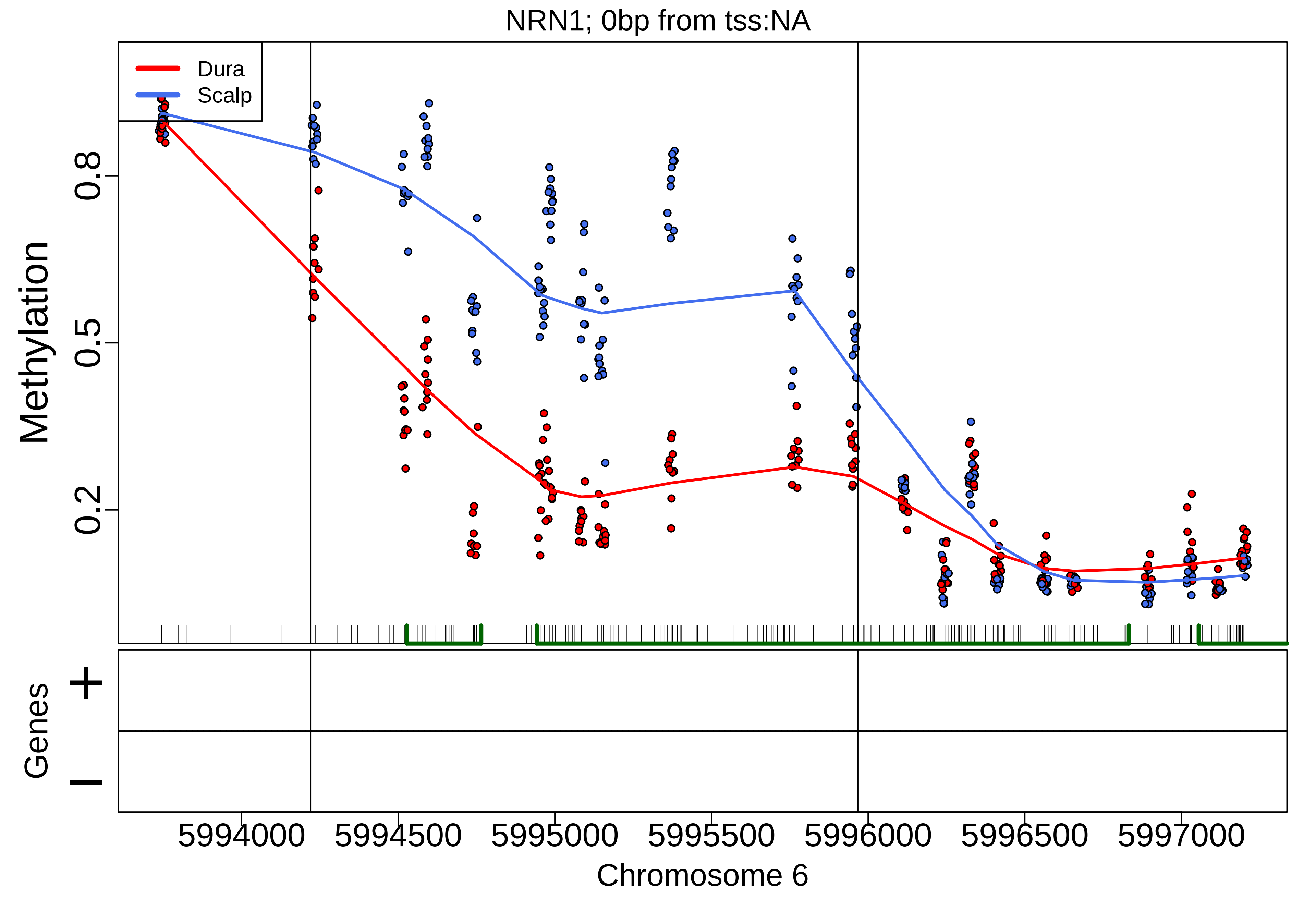

NRN1; 0bp from tss:NA
Dura
Scalp
Methylation
0.2
0.5
0.8
Genes
+
−
5994000
5994500
5995000
5995500
5996000
5996500
5997000
Chromosome 6

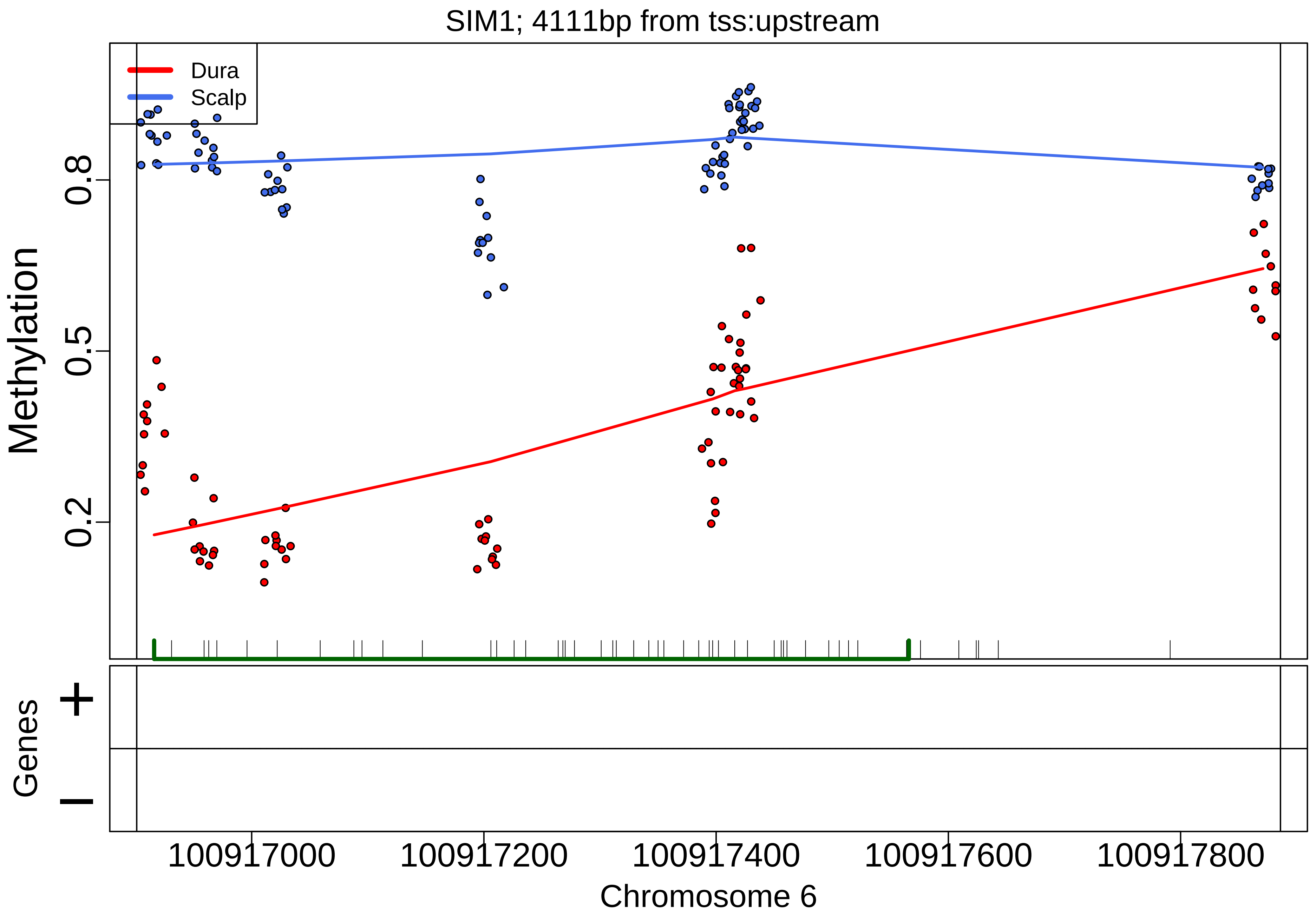

SIM1; 4111bp from tss:upstream
Dura
Scalp
Methylation
0.2
0.5
0.8
Genes
+
-
100917000
100917200
100917400
100917600
100917800
Chromosome 6

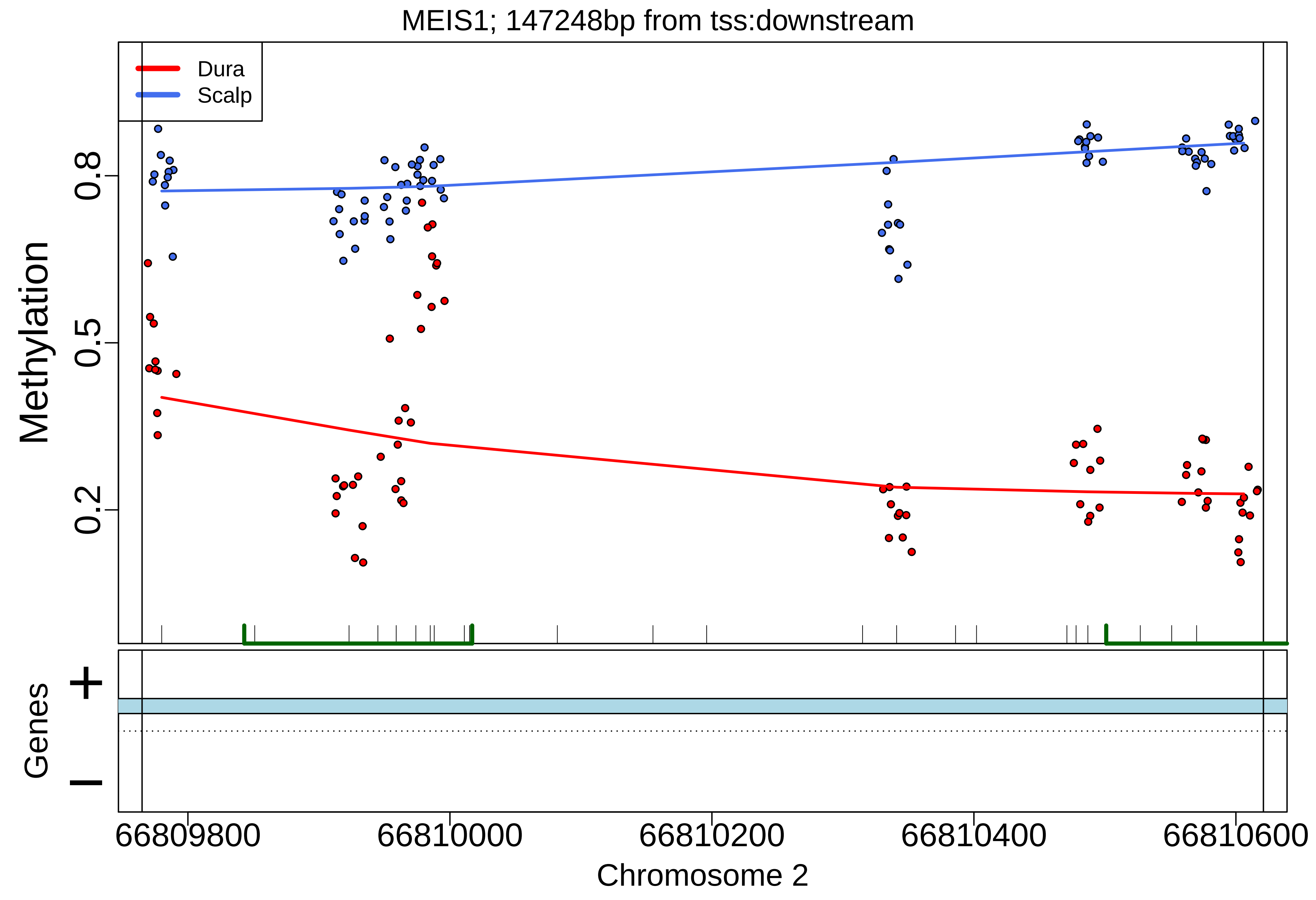
MEIS1; 147248bp from tss:downstream
Dura
Scalp
Methylation
0.2
0.5
0.8
Genes
+
-
66809800
66810000
66810200
66810400
66810600
Chromosome 2

BLCAP; 6878bp from tss:inside intron

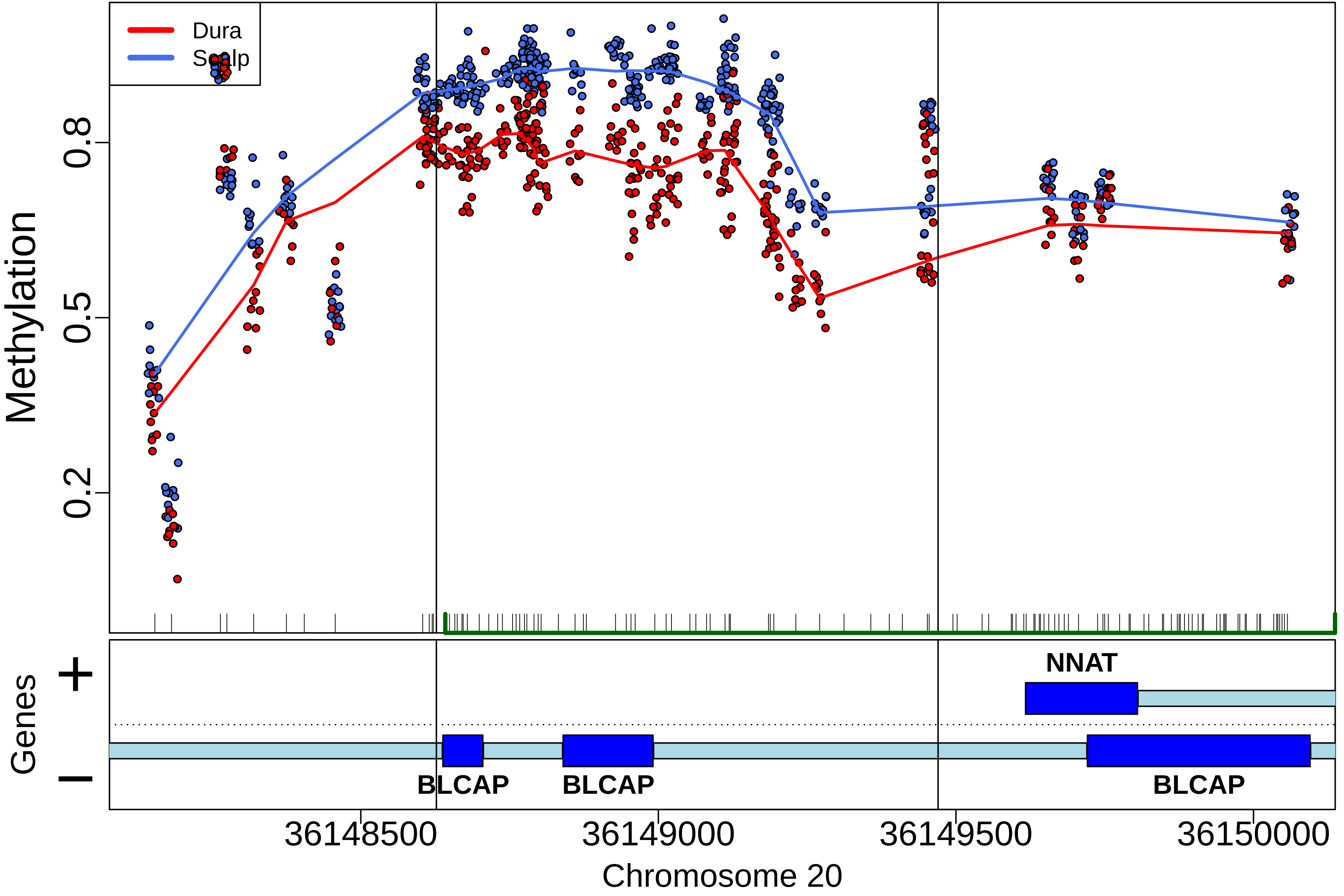

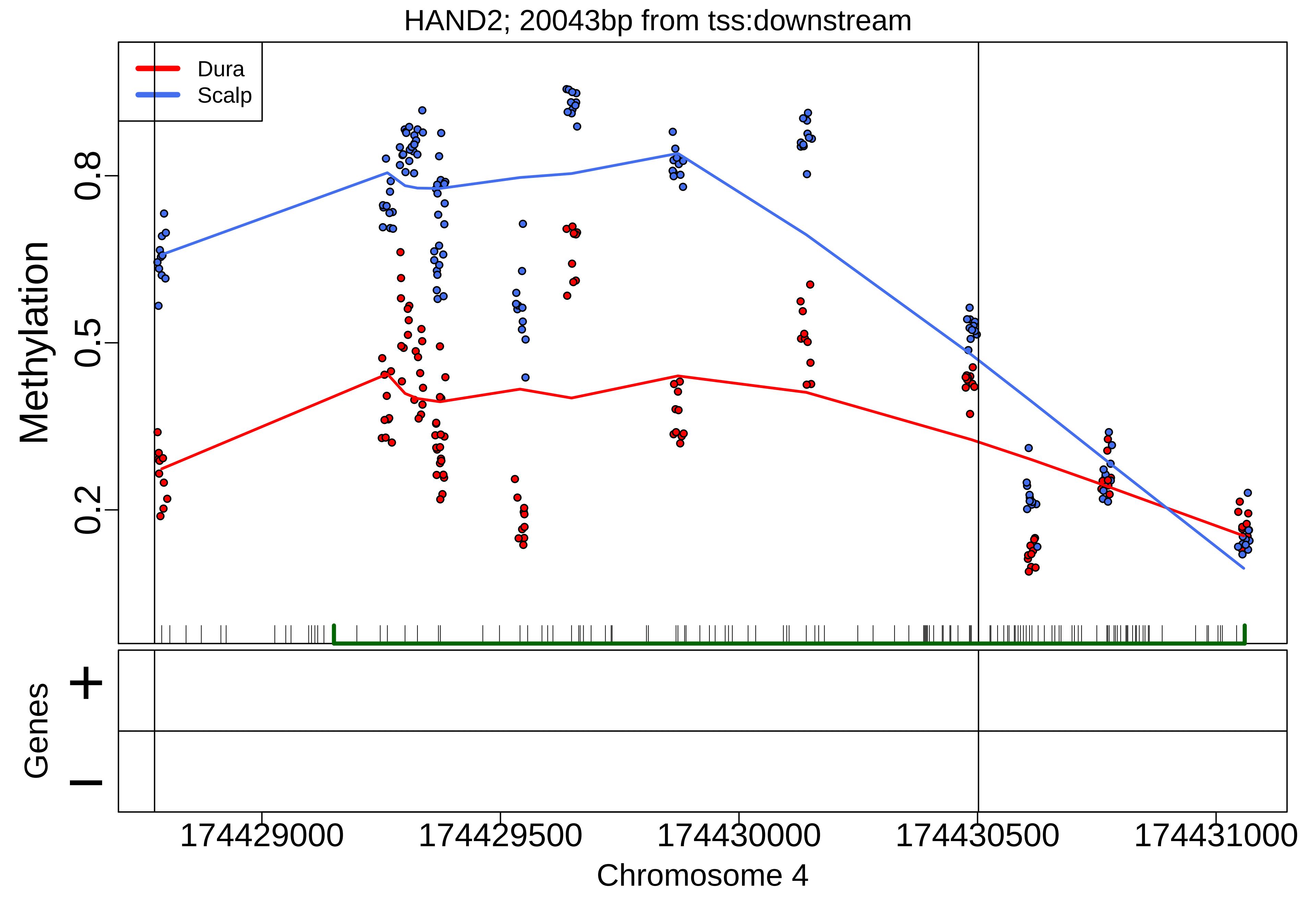

HAND2; 20043bp from tss:downstream
Dura
Scalp
Methylation
0.2
0.5
0.8
Genes
+
−
174429000
174429500
174430000
174430500
174431000
Chromosome 4

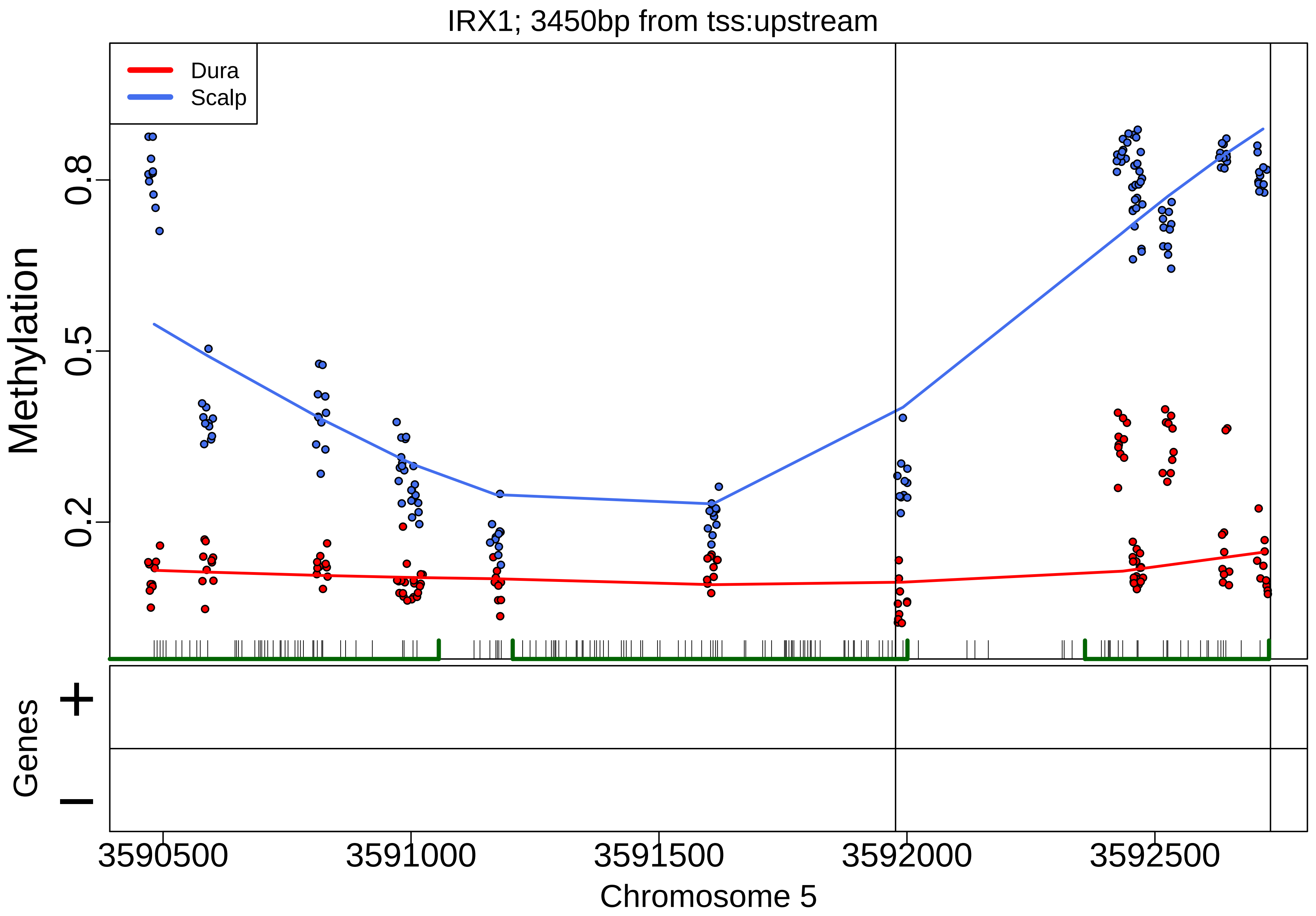

IRX1; 3450bp from tss:upstream
Dura
Scalp
Methylation
0.2
0.5
0.8
Genes
+
−
3590500
3591000
3591500
3592000
3592500
Chromosome 5

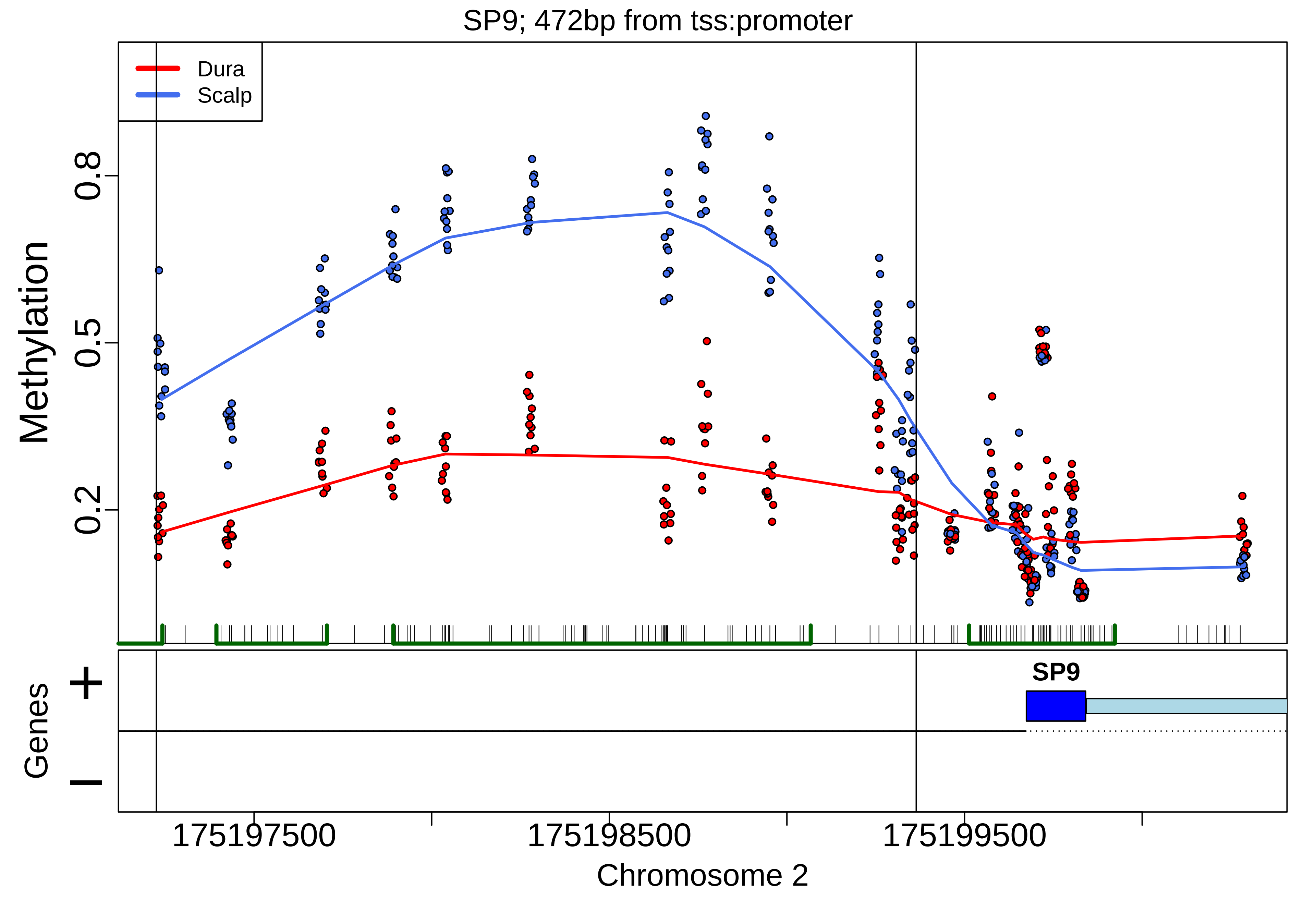

SP9; 472bp from tss:promoter
Dura
Scalp
Methylation
0.2
0.5
0.8
Genes
+
-
SP9
175197500
175198500
175199500
Chromosome 2

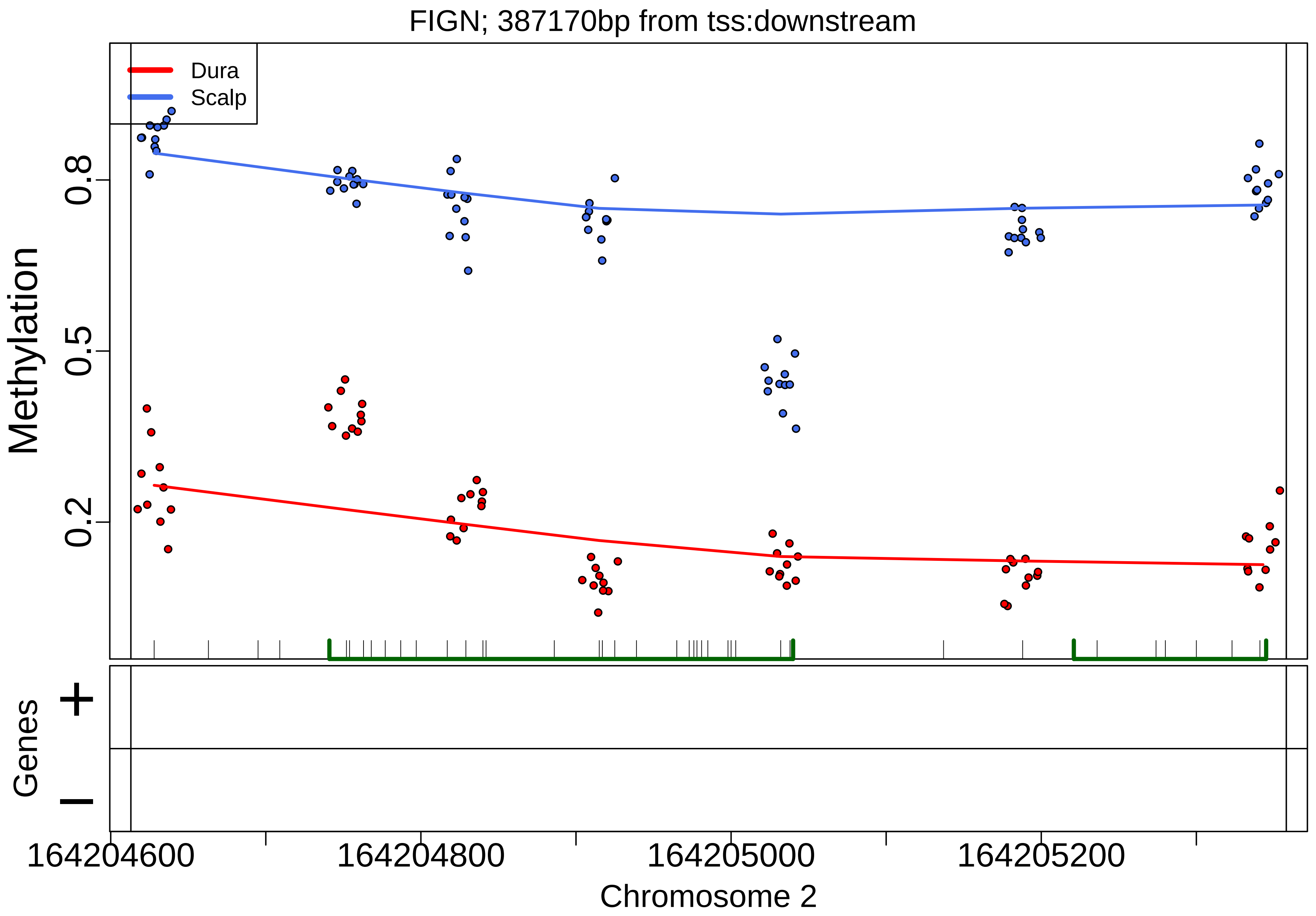
FIGN; 387170bp from tss:downstream
Dura
Scalp
Methylation
0.2
0.5
0.8
Genes
+
−
164204600
164204800
164205000
164205200
Chromosome 2

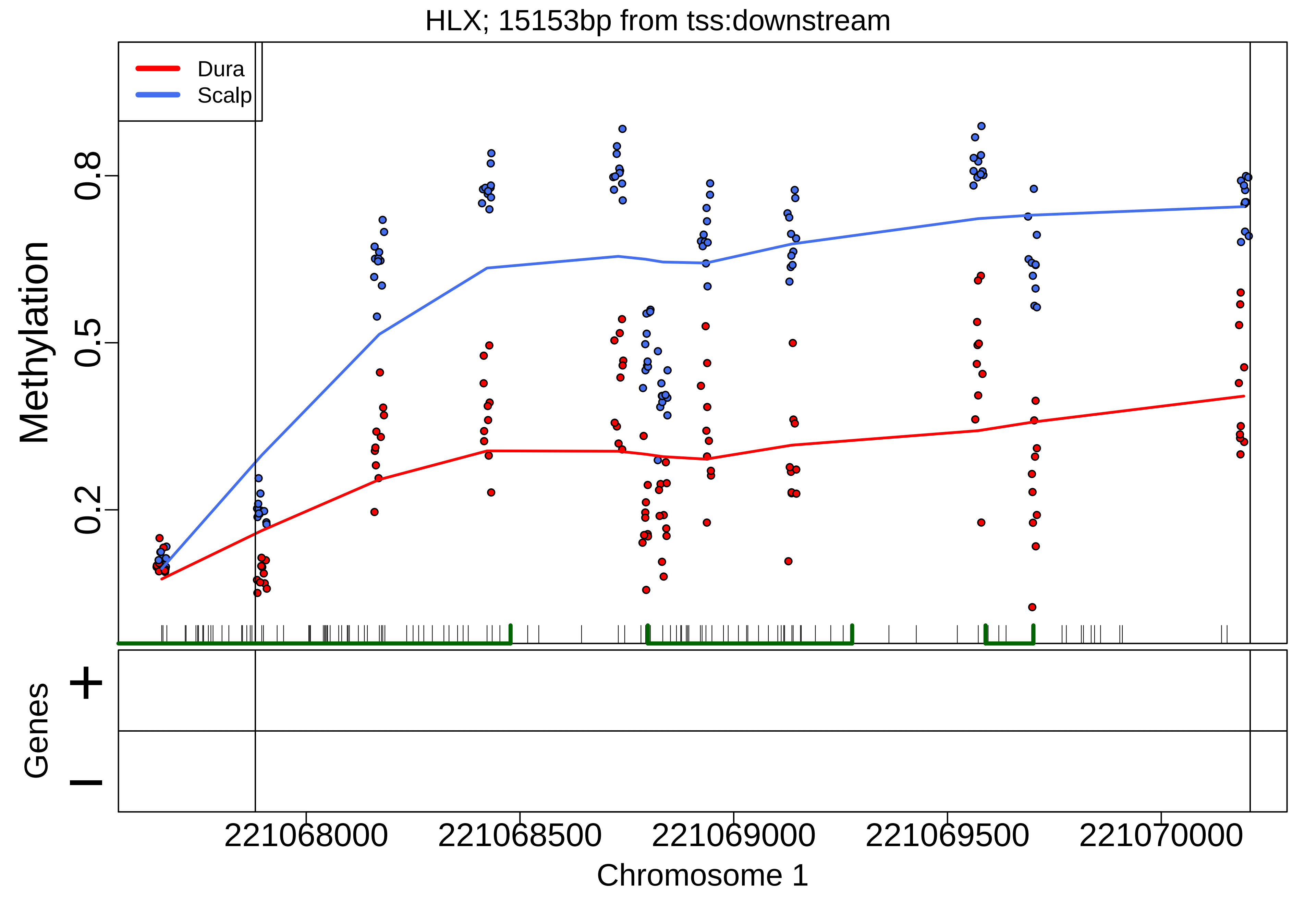

HLX; 15153bp from tss:downstream
Dura
Scalp
Methylation
0.2
0.5
0.8
Genes
+
-
221068000
221068500
221069000
221069500
221070000
Chromosome 1

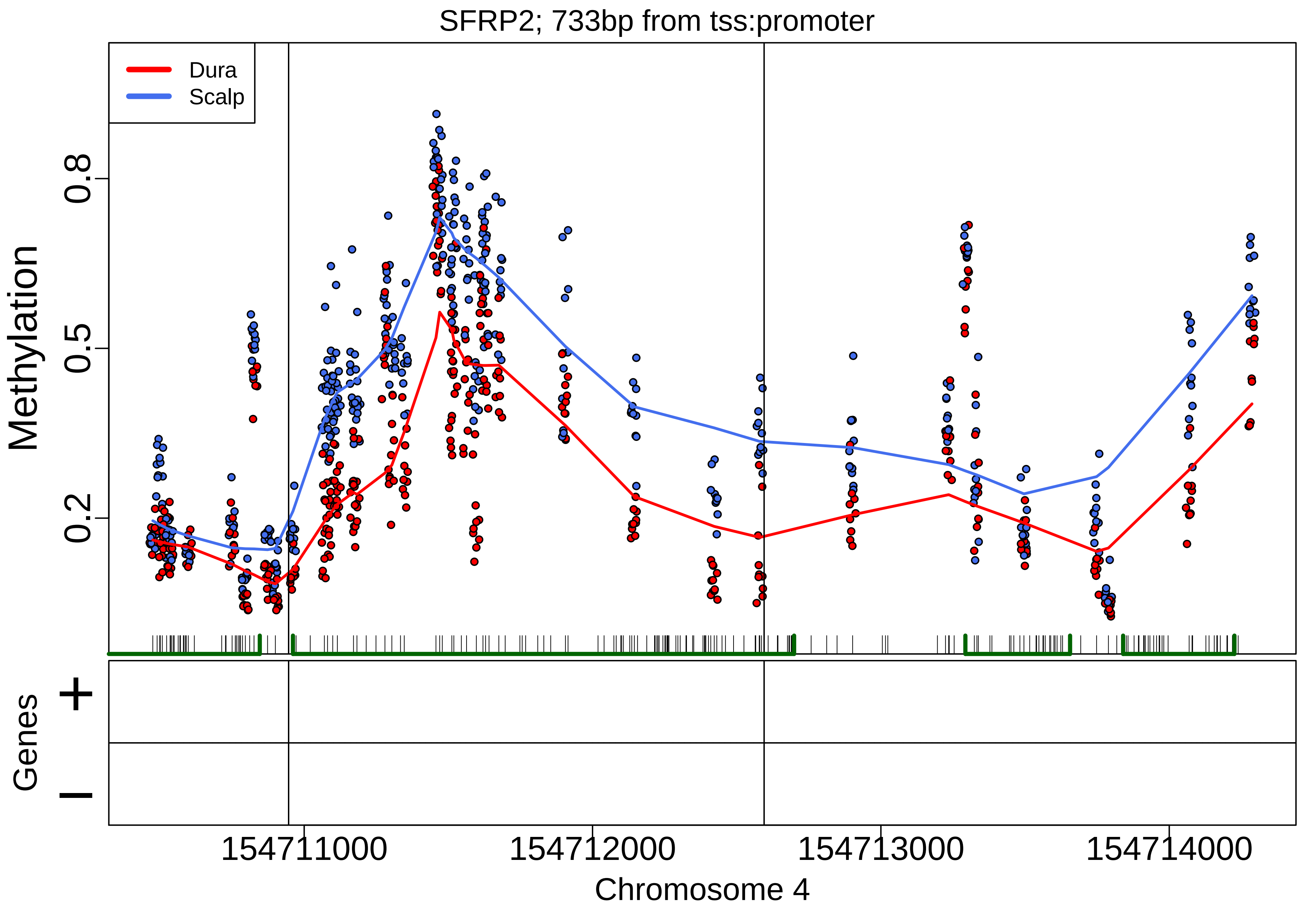

SFRP2; 733bp from tss:promoter
Dura
Scalp
Methylation
0.2
0.5
0.8
Genes
+
-
154711000
154712000
154713000
154714000
Chromosome 4

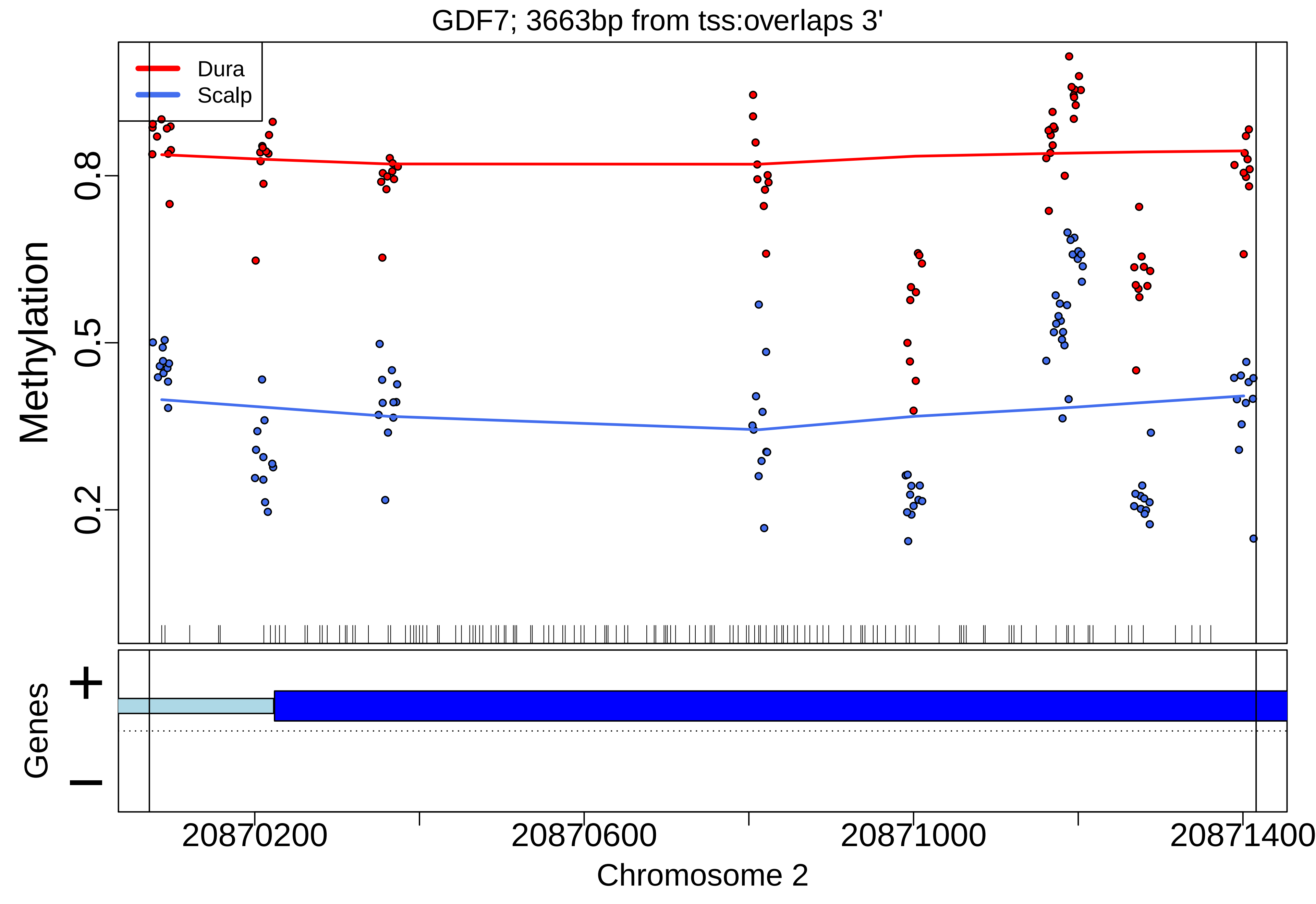

GDF7; 3663bp from tss:overlaps 3'
Dura
Scalp
Methylation
0.2
0.5
0.8
Genes
+
-
20870200
20870600
20871000
20871400
Chromosome 2

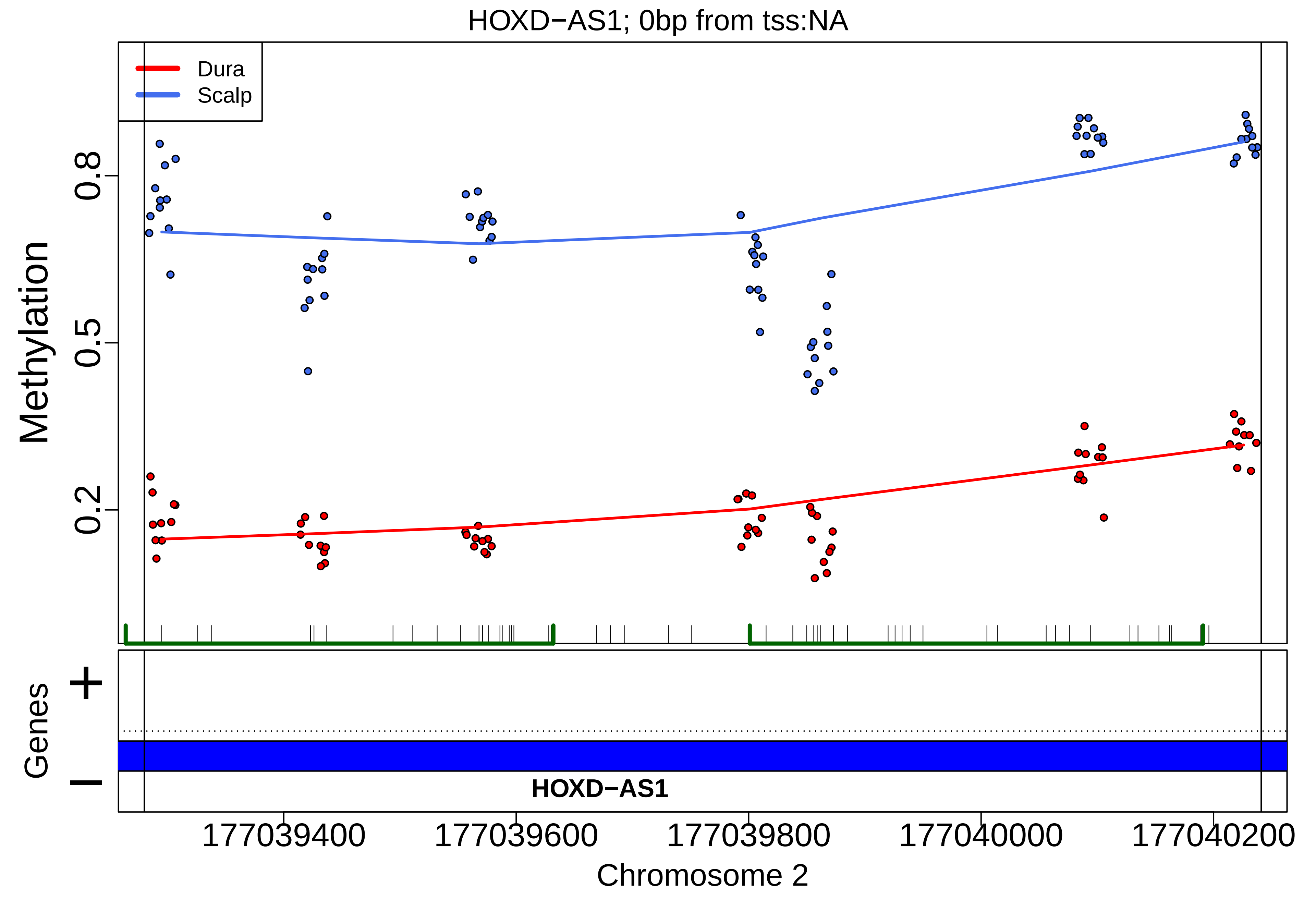

HOXD−AS1; 0bp from tss:NA
Dura
Scalp
Methylation
0.2
0.5
0.8
Genes
+
−
HOXD−AS1
177039400
177039600
177039800
177040000
177040200
Chromosome 2

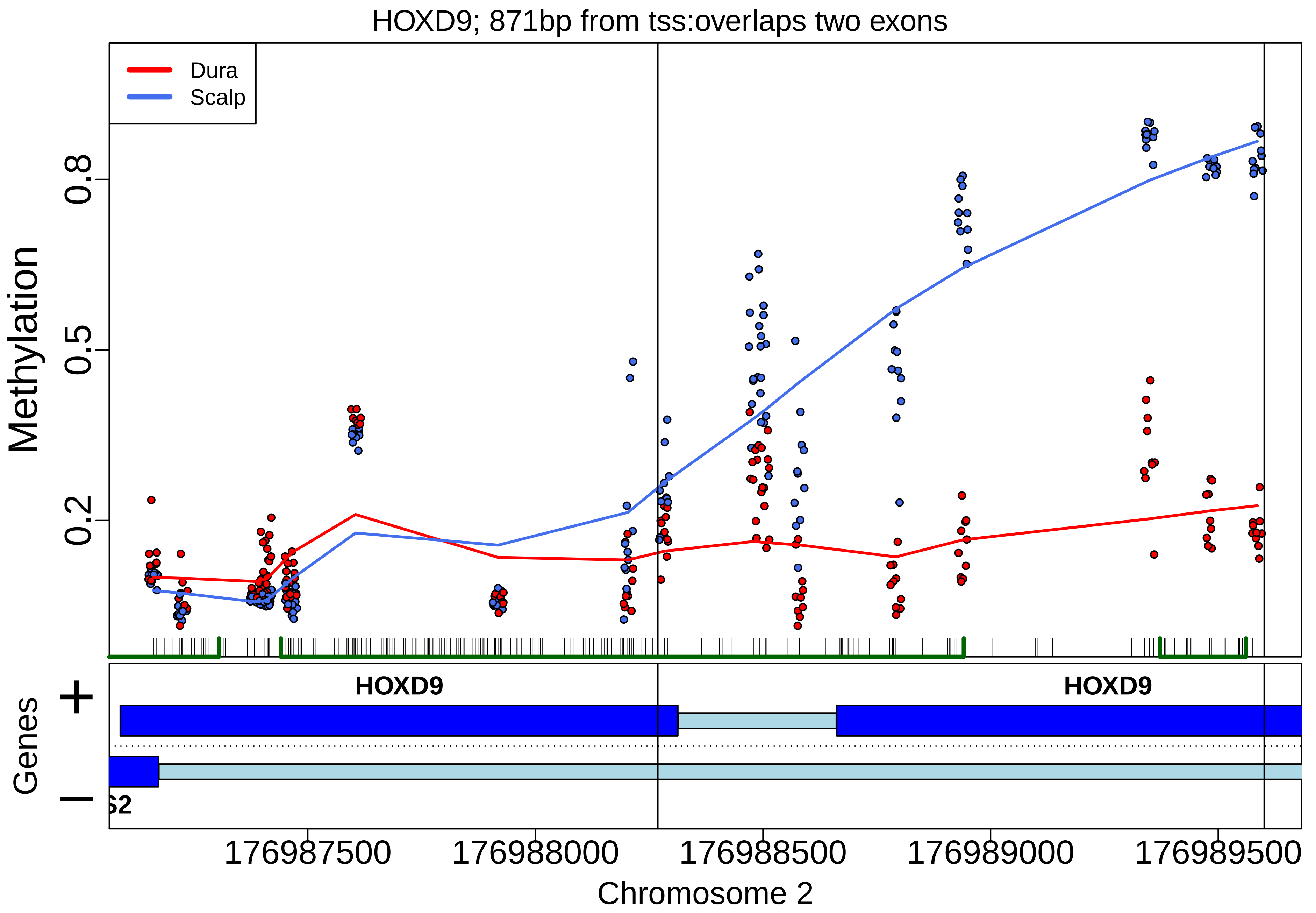
HOXD9; 871bp from tss:overlaps two exons
Dura
Scalp
Methylation
0.2
0.5
0.8
Genes
+
−
HOXD9
HOXD9
S2
176987500
176988000
176988500
176989000
176989500
Chromosome 2

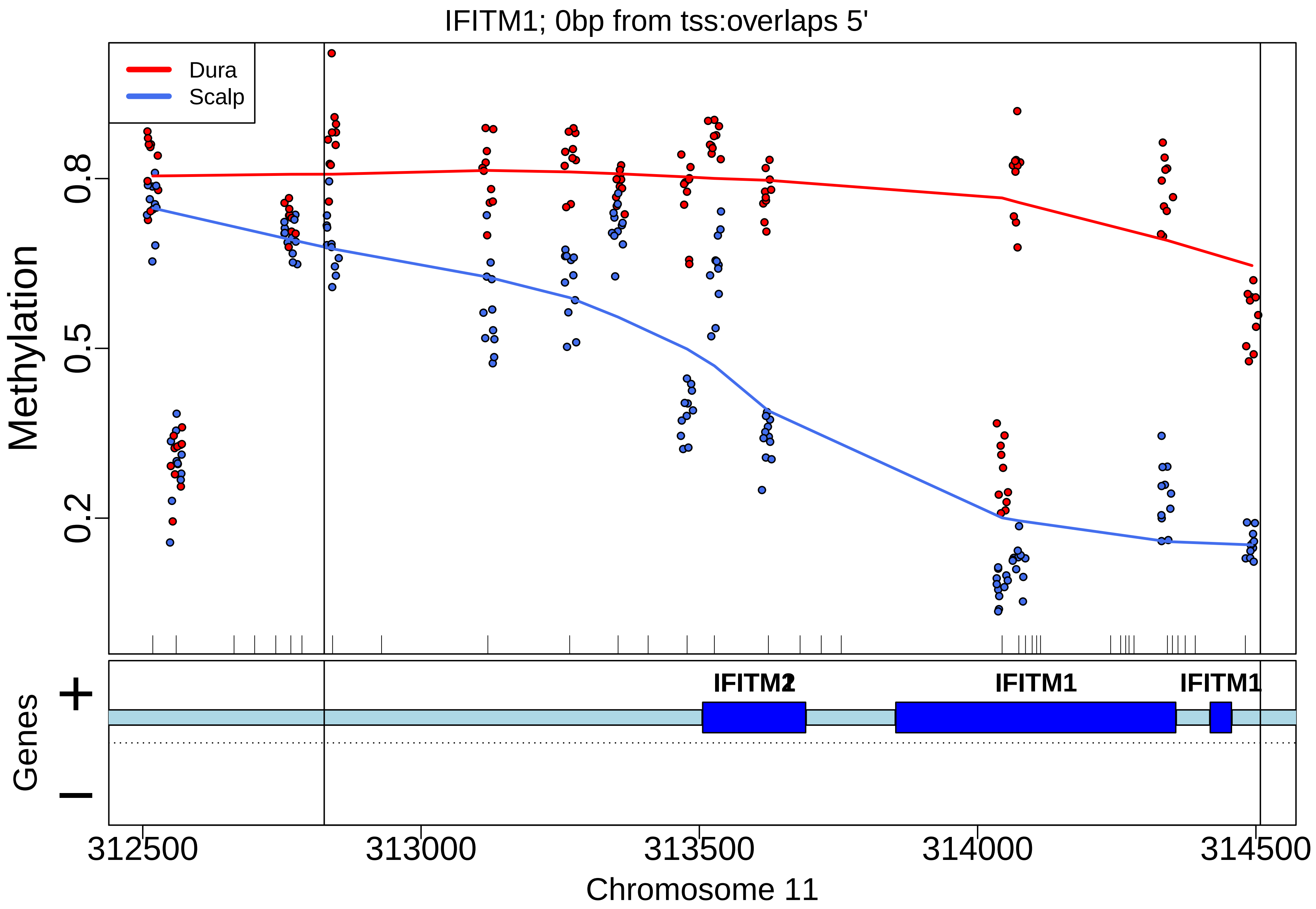
IFITM1; 0bp from tss:overlaps 5'
Dura
Scalp
Methylation
0.2
0.5
0.8
Genes
+
-
IFITM2
IFITM1
IFITM1
312500
313000
313500
314000
314500
Chromosome 11

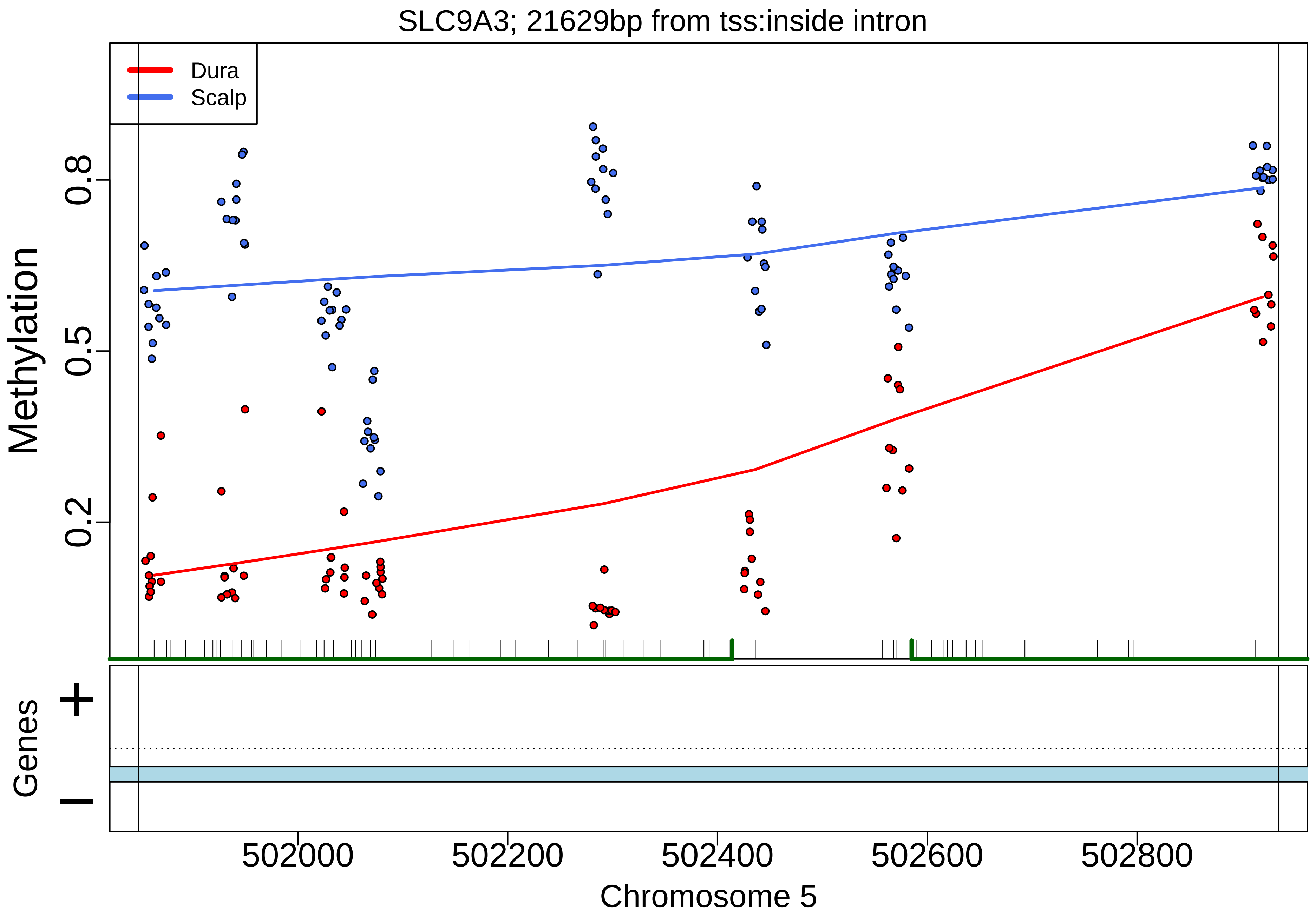

SLC9A3; 21629bp from tss:inside intron
Dura
Scalp
Methylation
0.2
0.5
0.8
Genes
+
−
502000
502200
502400
502600
502800
Chromosome 5

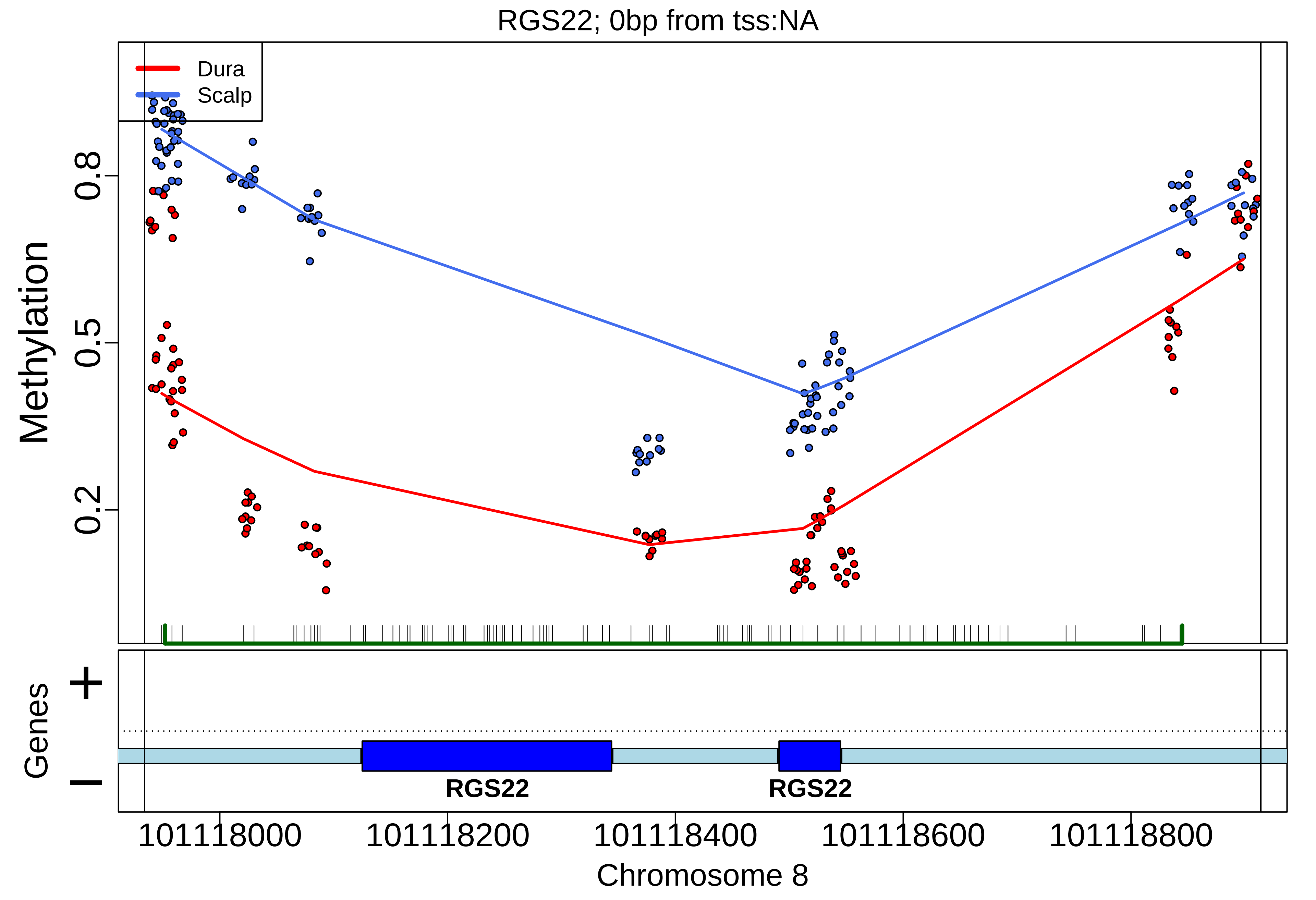
RGS22; 0bp from tss:NA
Dura
Scalp
Methylation
0.2
0.5
0.8
Genes
+
−
RGS22
RGS22
101118000
101118200
101118400
101118600
101118800
Chromosome 8

SIX6; 1413bp from tss:promoter

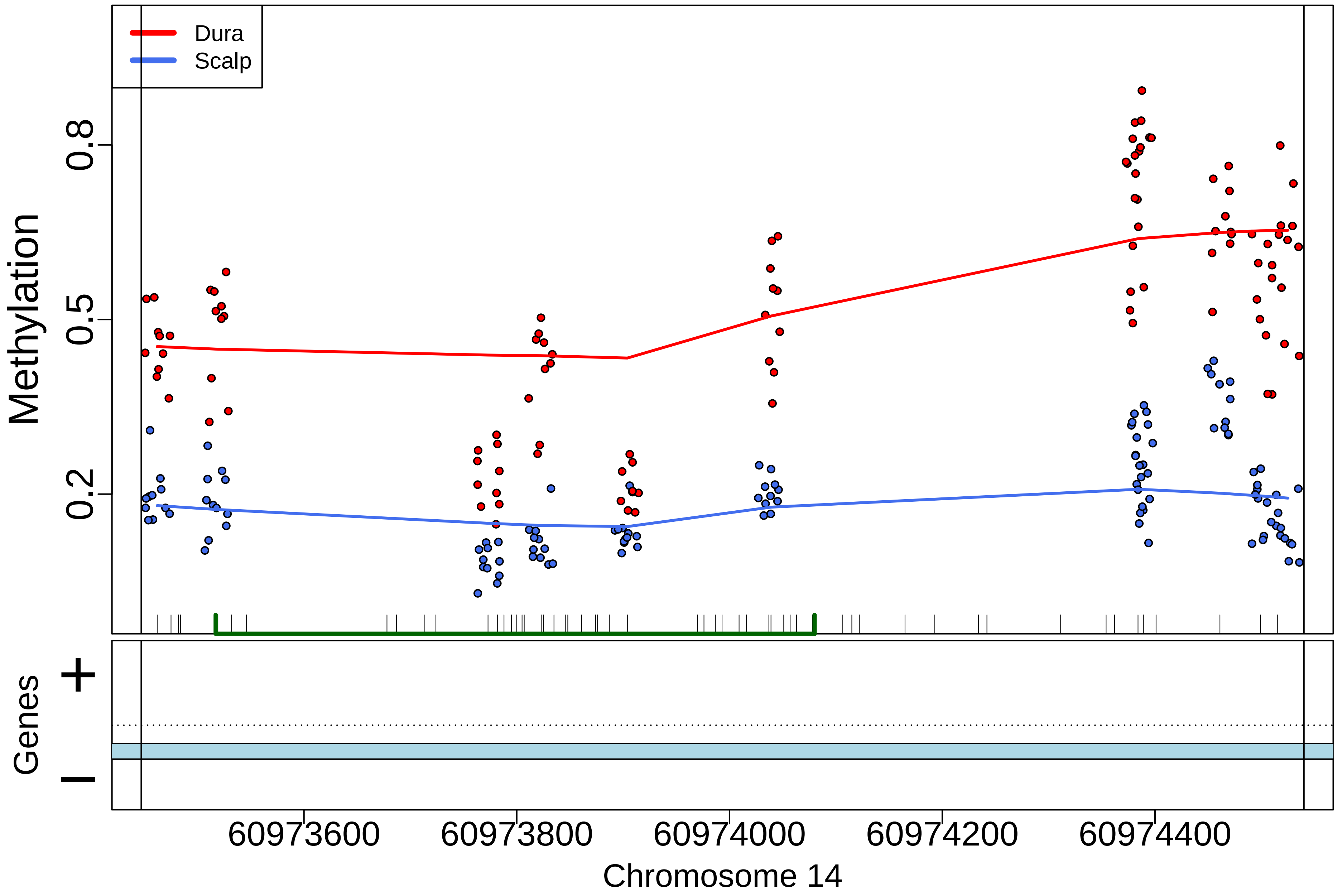

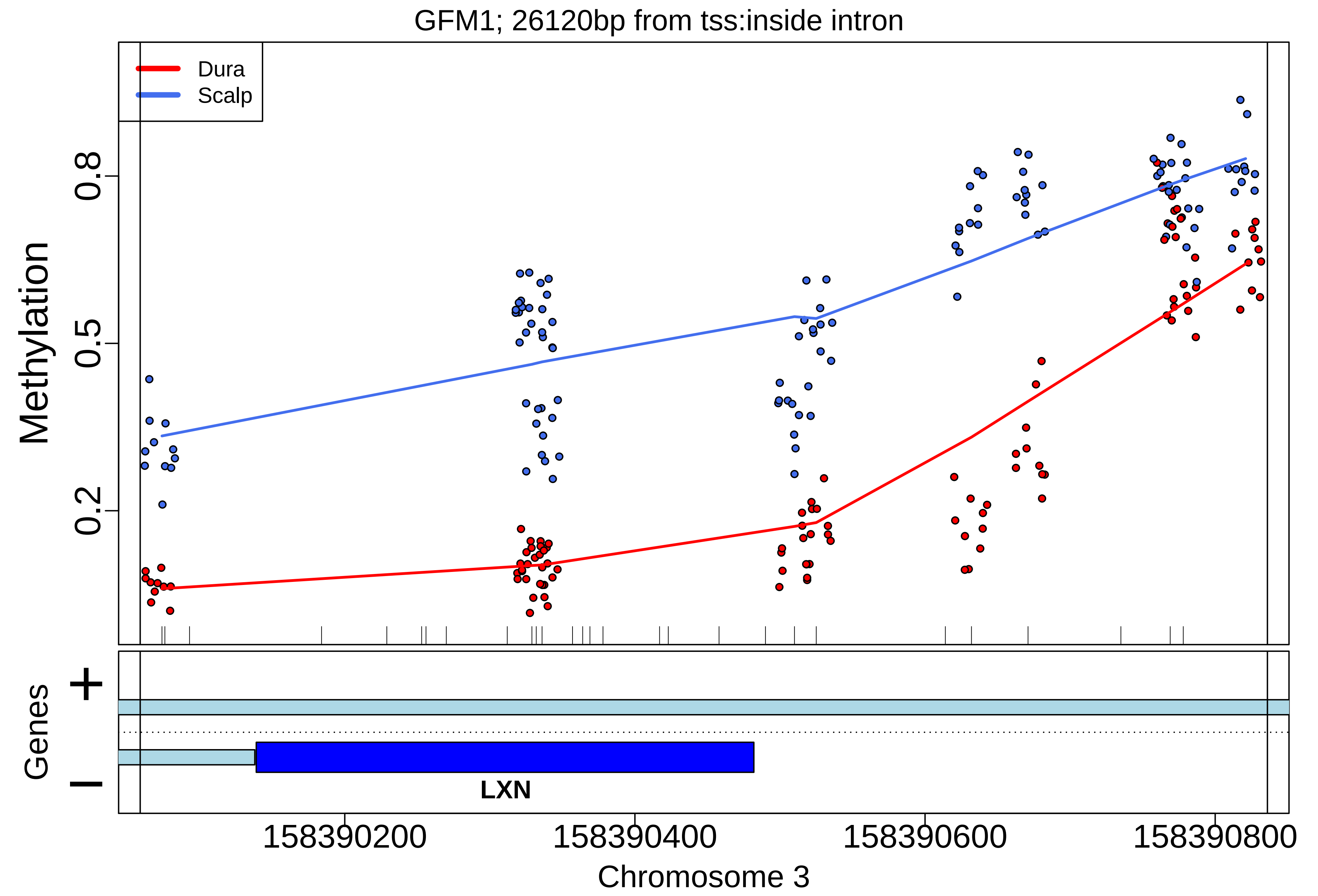

GFM1; 26120bp from tss:inside intron
Dura
Scalp
Methylation
0.2
0.5
0.8
Genes
+
-
LXN
158390200
158390400
158390600
158390800
Chromosome 3

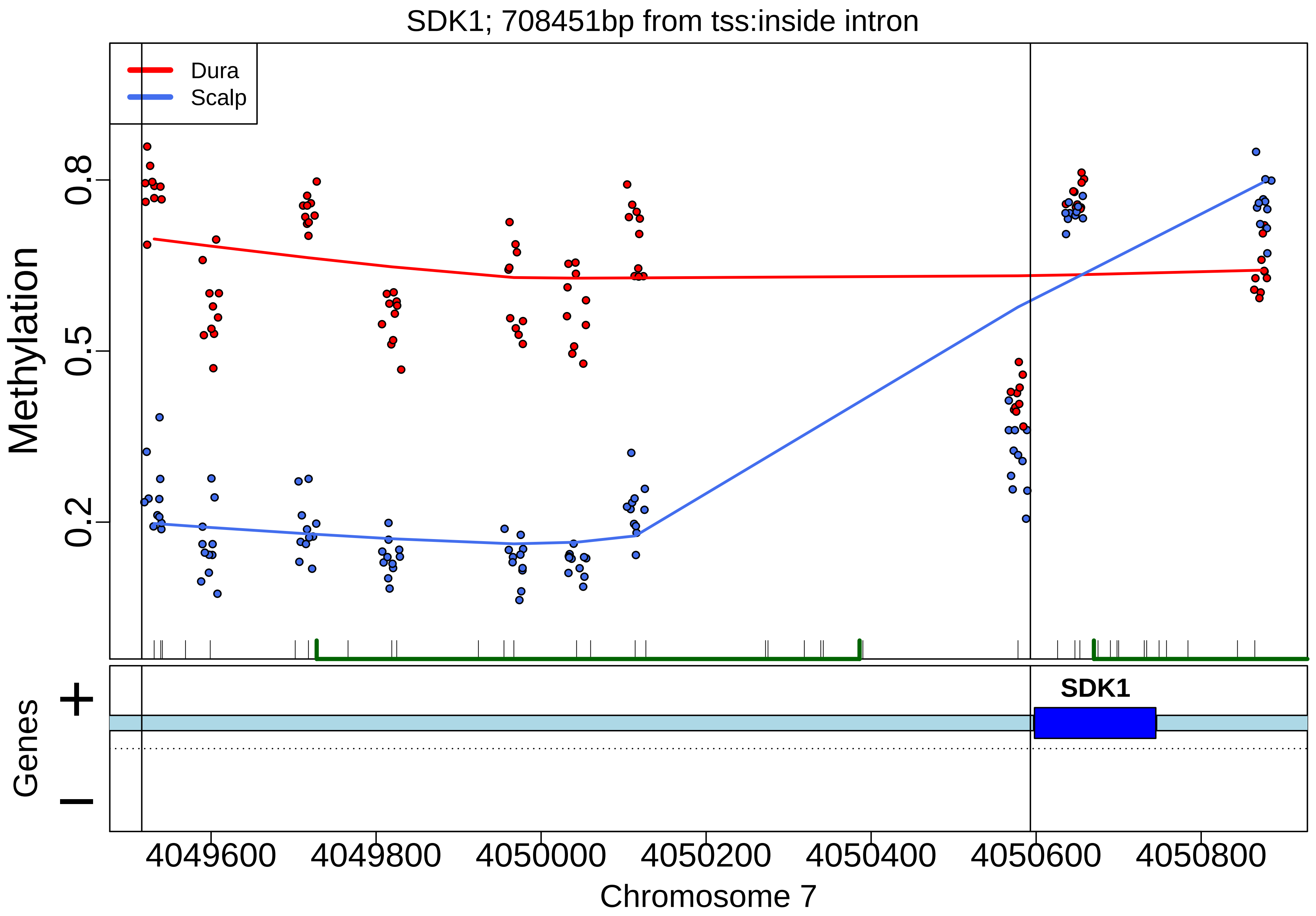

SDK1; 708451bp from tss:inside intron
Dura
Scalp
Methylation
0.2
0.5
0.8
Genes
+
−
SDK1
4049600
4049800
4050000
4050200
4050400
4050600
4050800
Chromosome 7

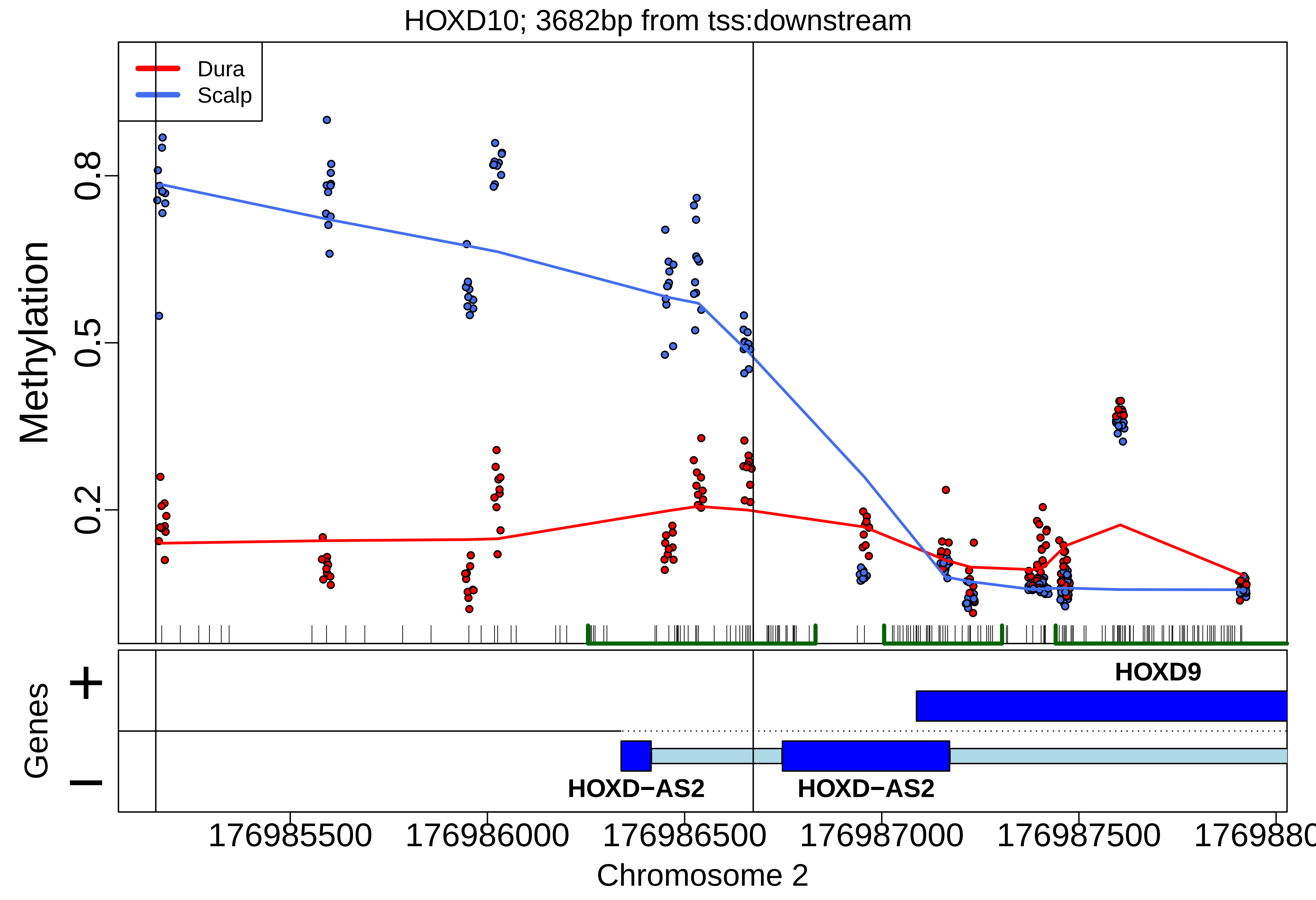
HOXD10; 3682bp from tss:downstream
Dura
Scalp
Methylation
0.8
0.5
0.2
Genes
+
−
HOXD9
HOXD−AS2
HOXD−AS2
176985500
176986000
176986500
176987000
176987500
Chromosome 2

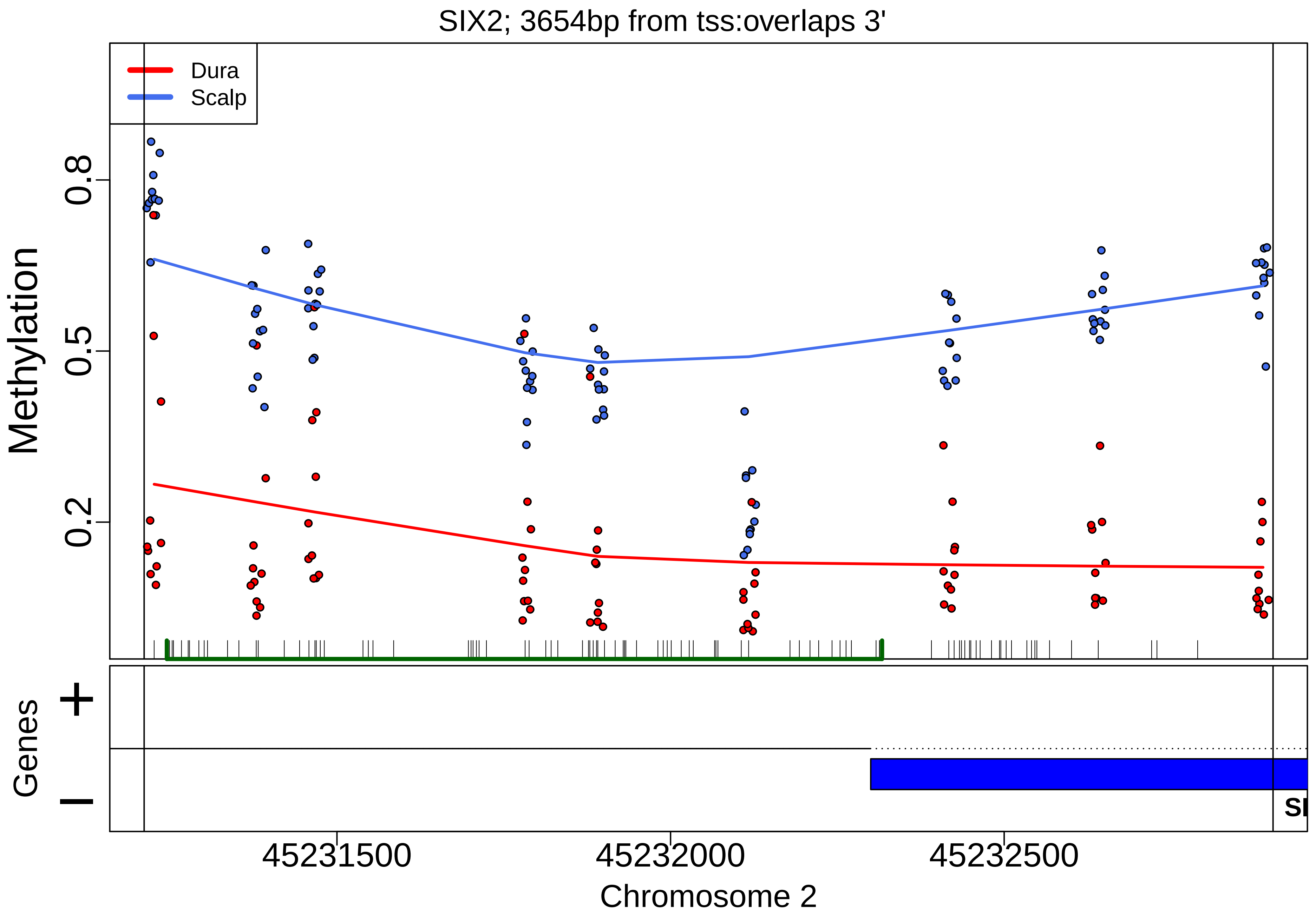

SIX2; 3654bp from tss:overlaps 3'
Dura
Scalp
Methylation
0.2
0.5
0.8
Genes
+
−
SI
45231500
45232000
45232500
Chromosome 2

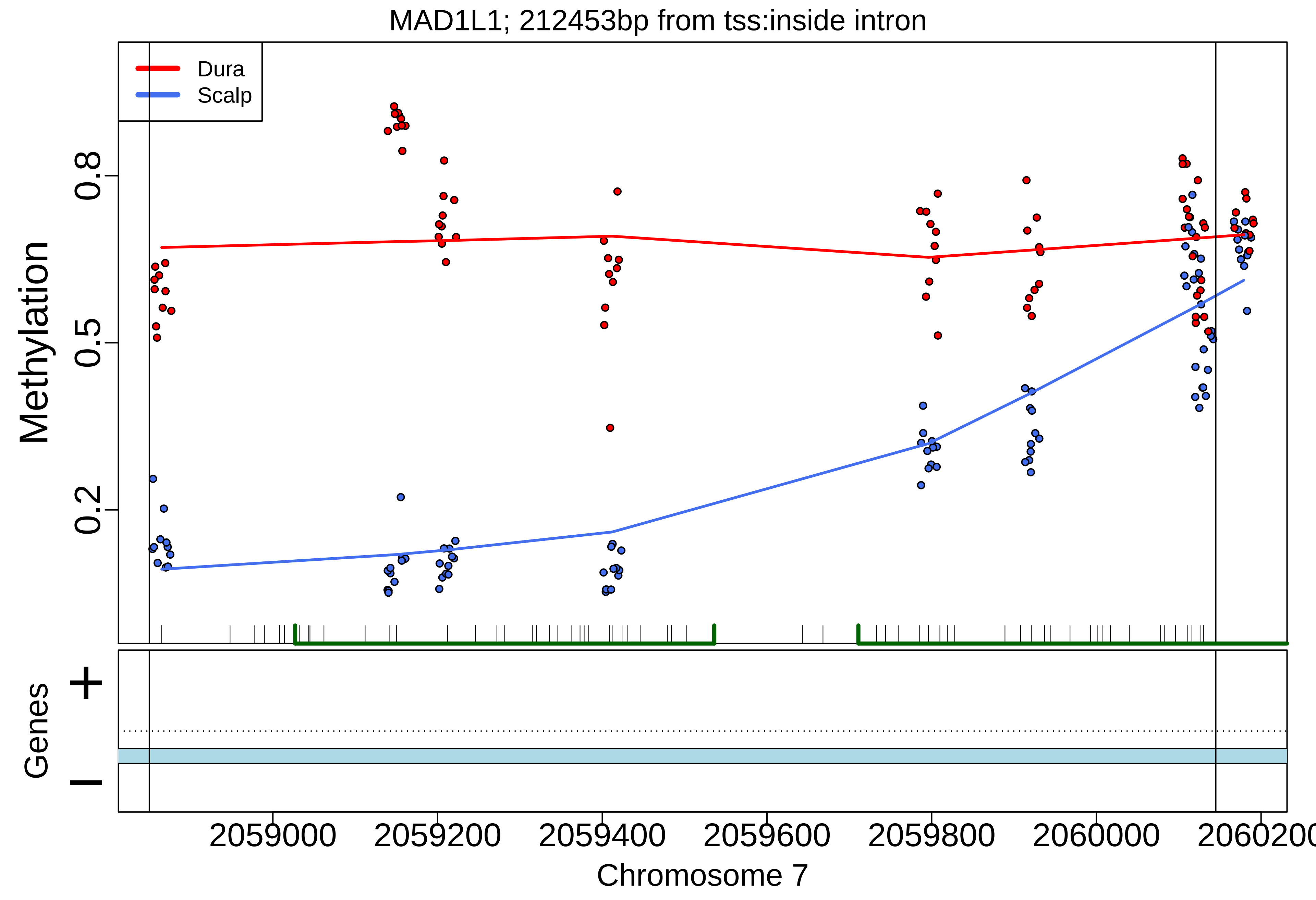

MAD1L1; 212453bp from tss:inside intron
Dura
Scalp
Methylation
0.2
0.5
0.8
Genes
+
-
2059000
2059200
2059400
2059600
2059800
2060000
2060200
Chromosome 7

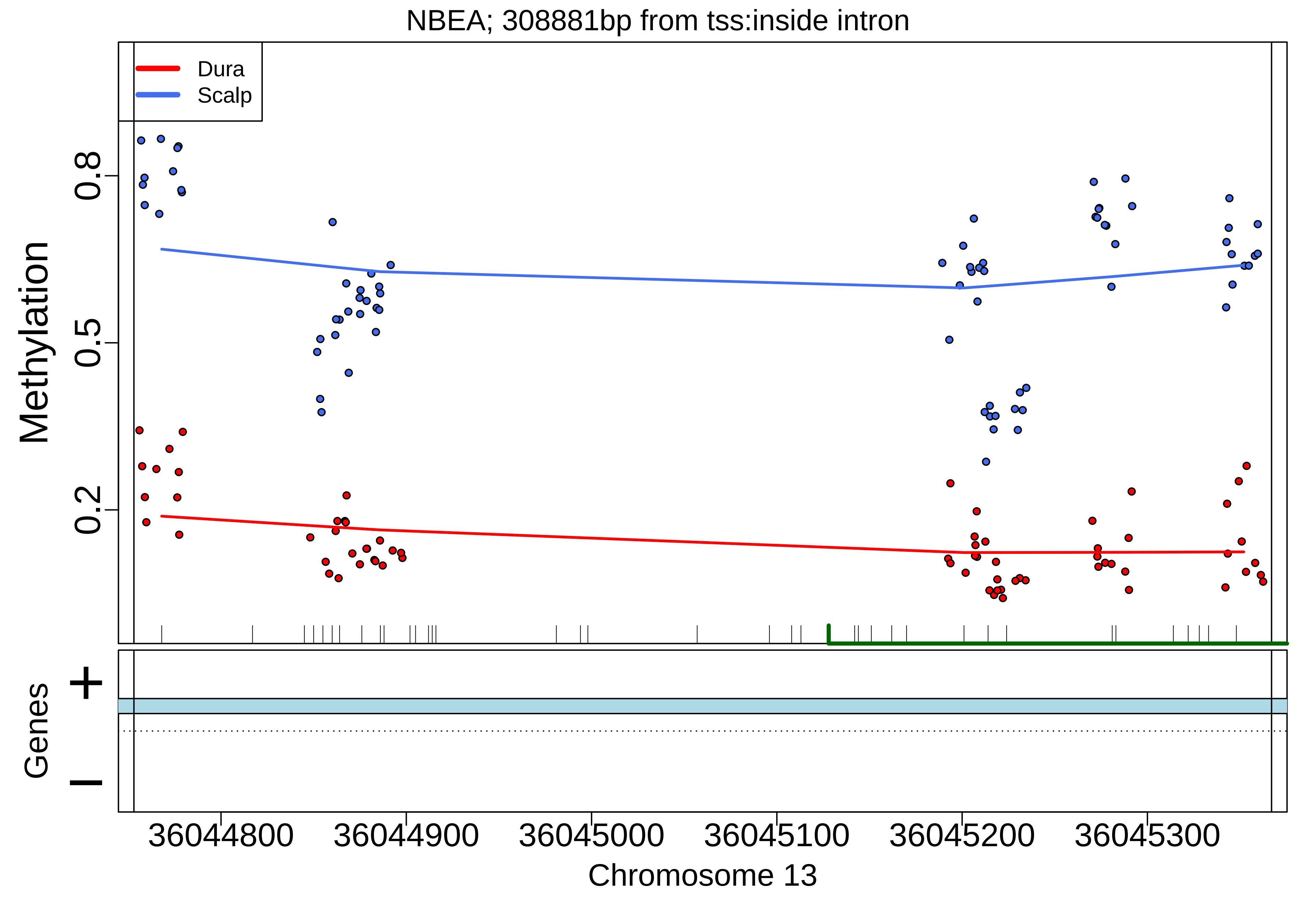
NBEA; 308881bp from tss:inside intron
Dura
Scalp
Methylation
0.2
0.5
0.8
Genes
+
−
36044800
36044900
36045000
36045100
36045200
36045300
Chromosome 13

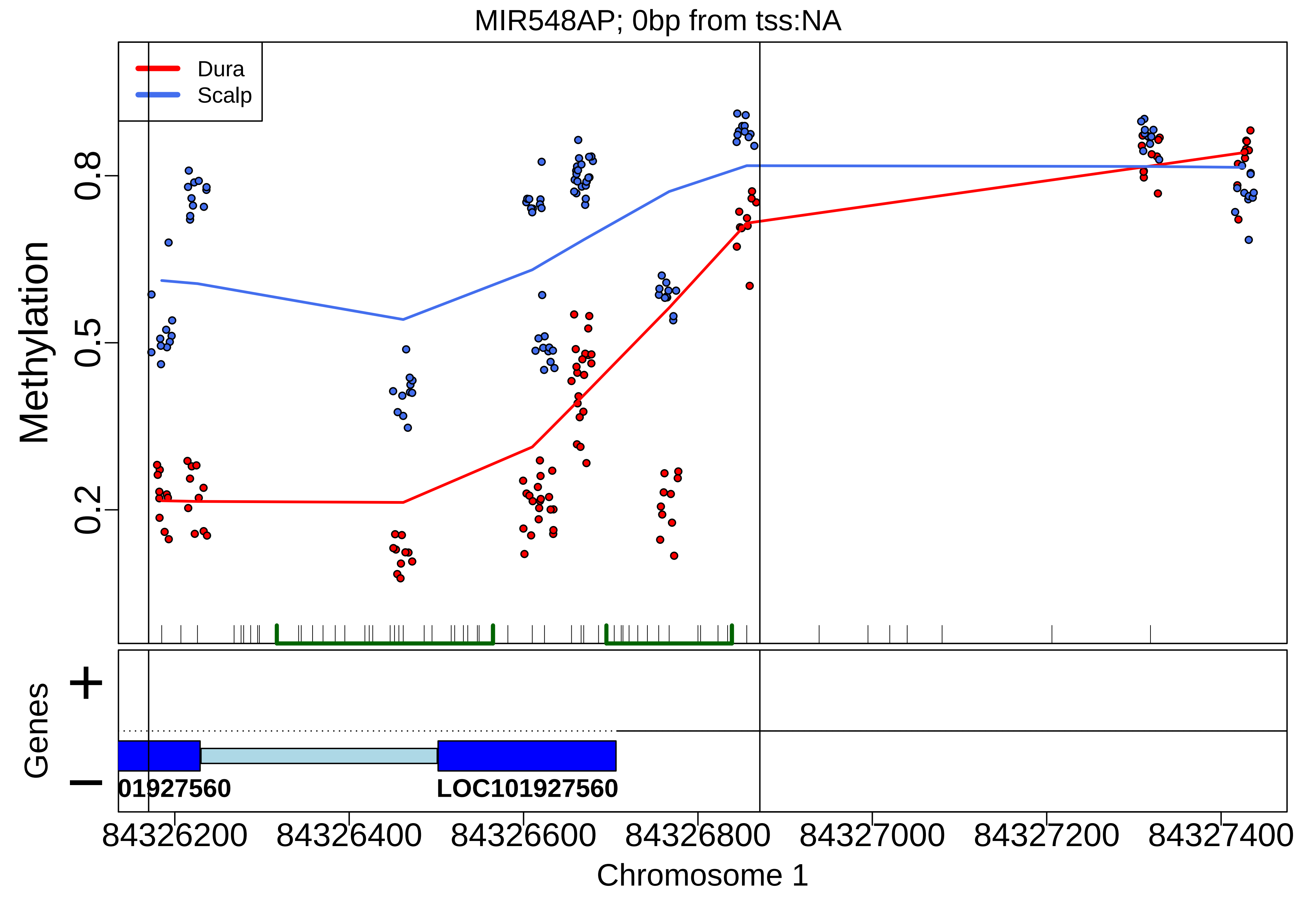

MIR548AP; 0bp from tss:NA
Dura
Scalp
Methylation
0.2
0.5
0.8
Genes
+
−
01927560
LOC101927560
84326200
84326400
84326600
84326800
84327000
84327200
84327400
Chromosome 1

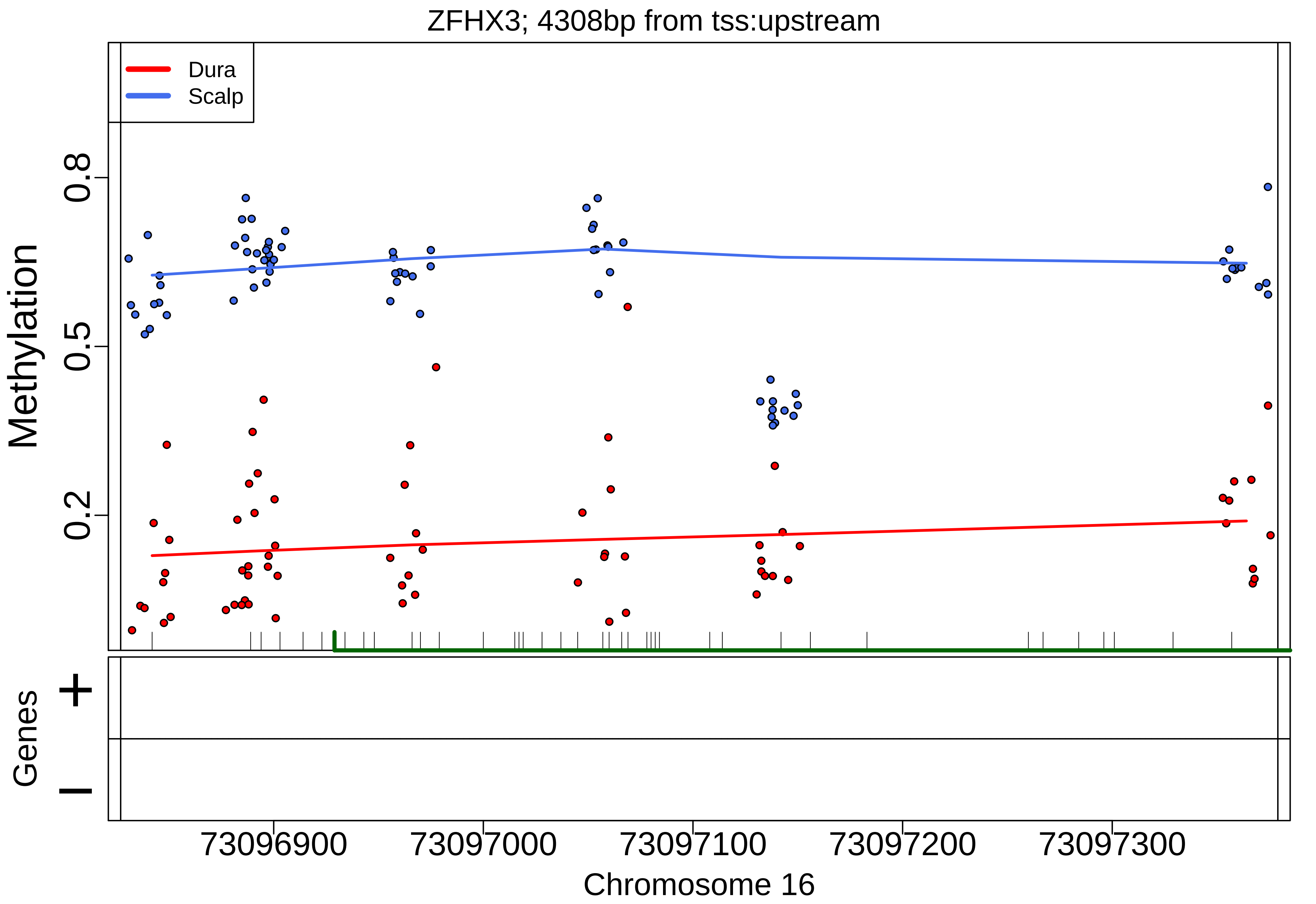
ZFHX3; 4308bp from tss:upstream
Dura
Scalp
Methylation
0.2
0.5
0.8
Genes
+
-
73096900
73097000
73097100
73097200
73097300
Chromosome 16

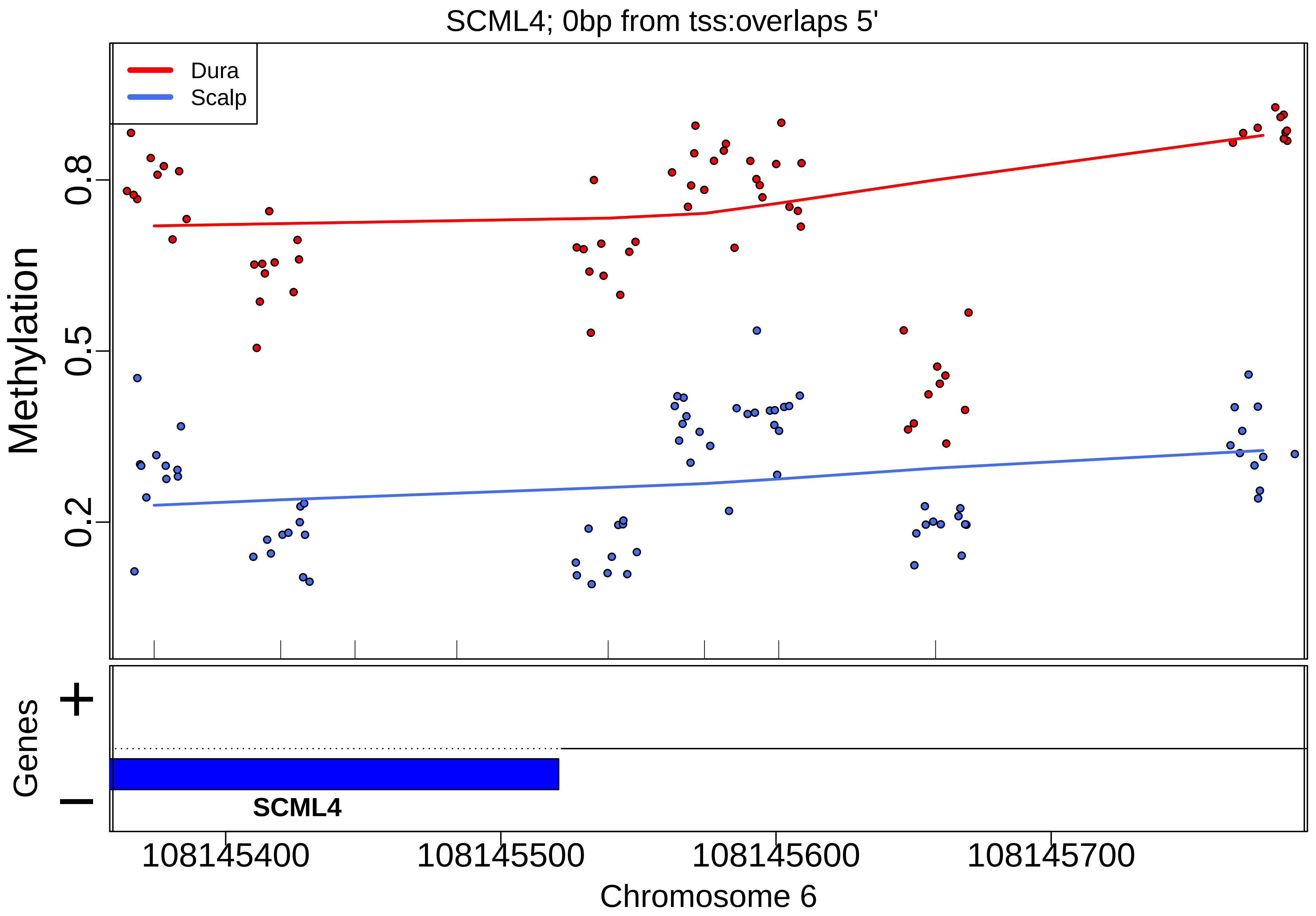
SCML4; 0bp from tss:overlaps 5'
Dura
Scalp
Methylation
0.8
0.5
0.2
Genes
+
-
SCML4
108145400
108145500
108145600
108145700
Chromosome 6

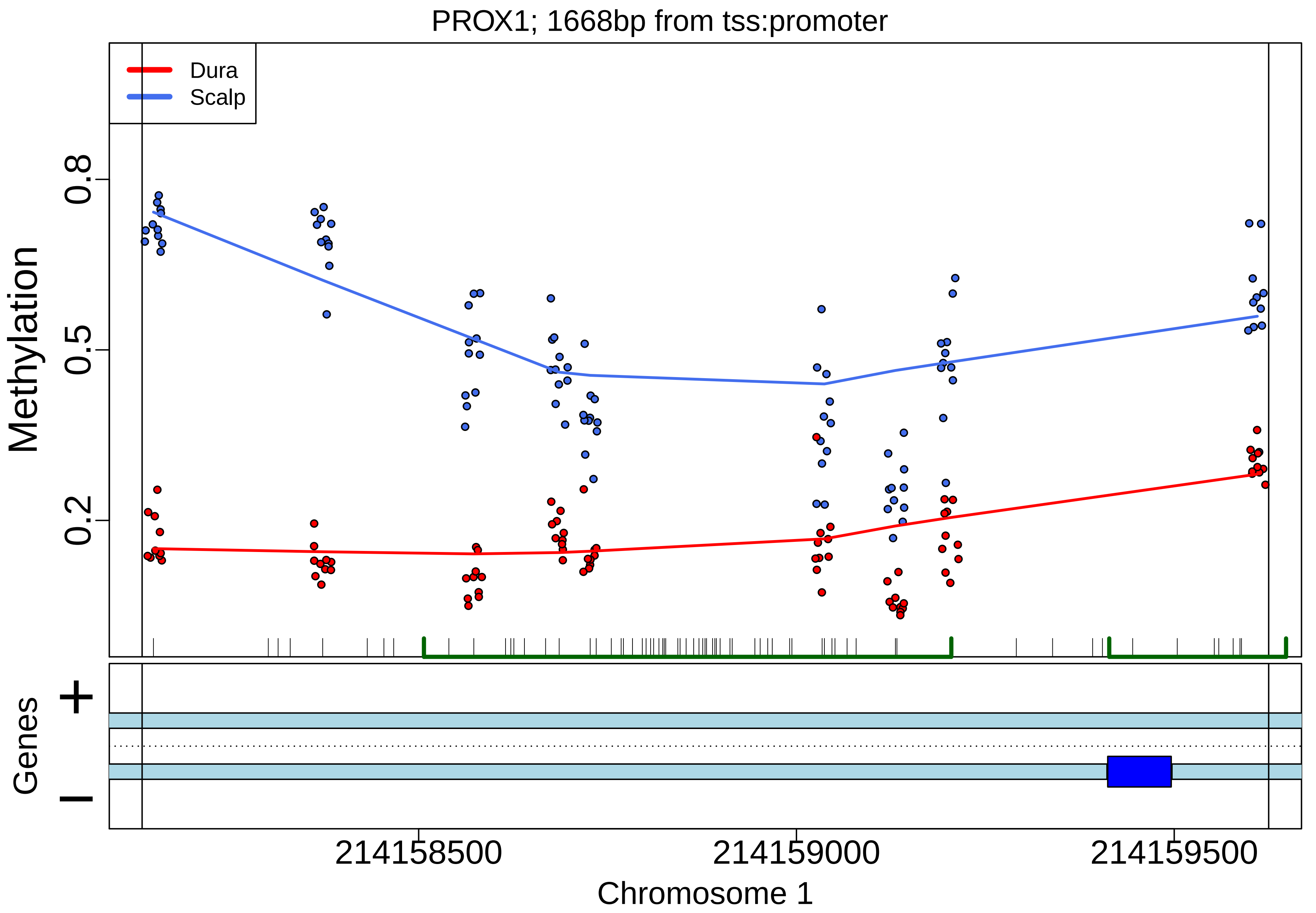

PROX1; 1668bp from tss:promoter
Dura
Scalp
Methylation
0.2
0.5
0.8
Genes
+
−
214158500
214159000
214159500
Chromosome 1

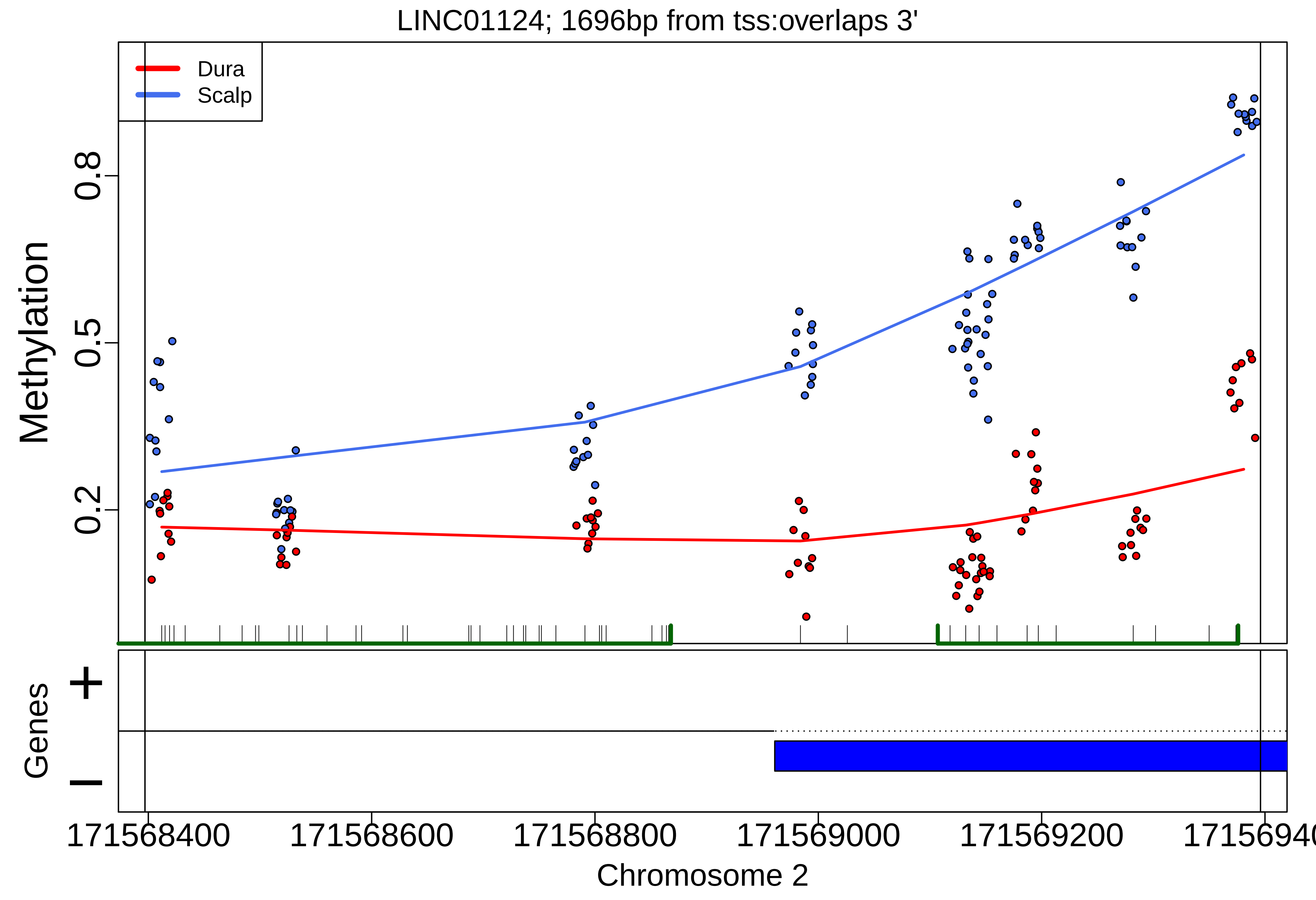

LINC01124; 1696bp from tss:overlaps 3'
Dura
Scalp
Methylation
0.2
0.5
0.8
Genes
+
−
171568400
171568600
171568800
171569000
171569200
Chromosome 2

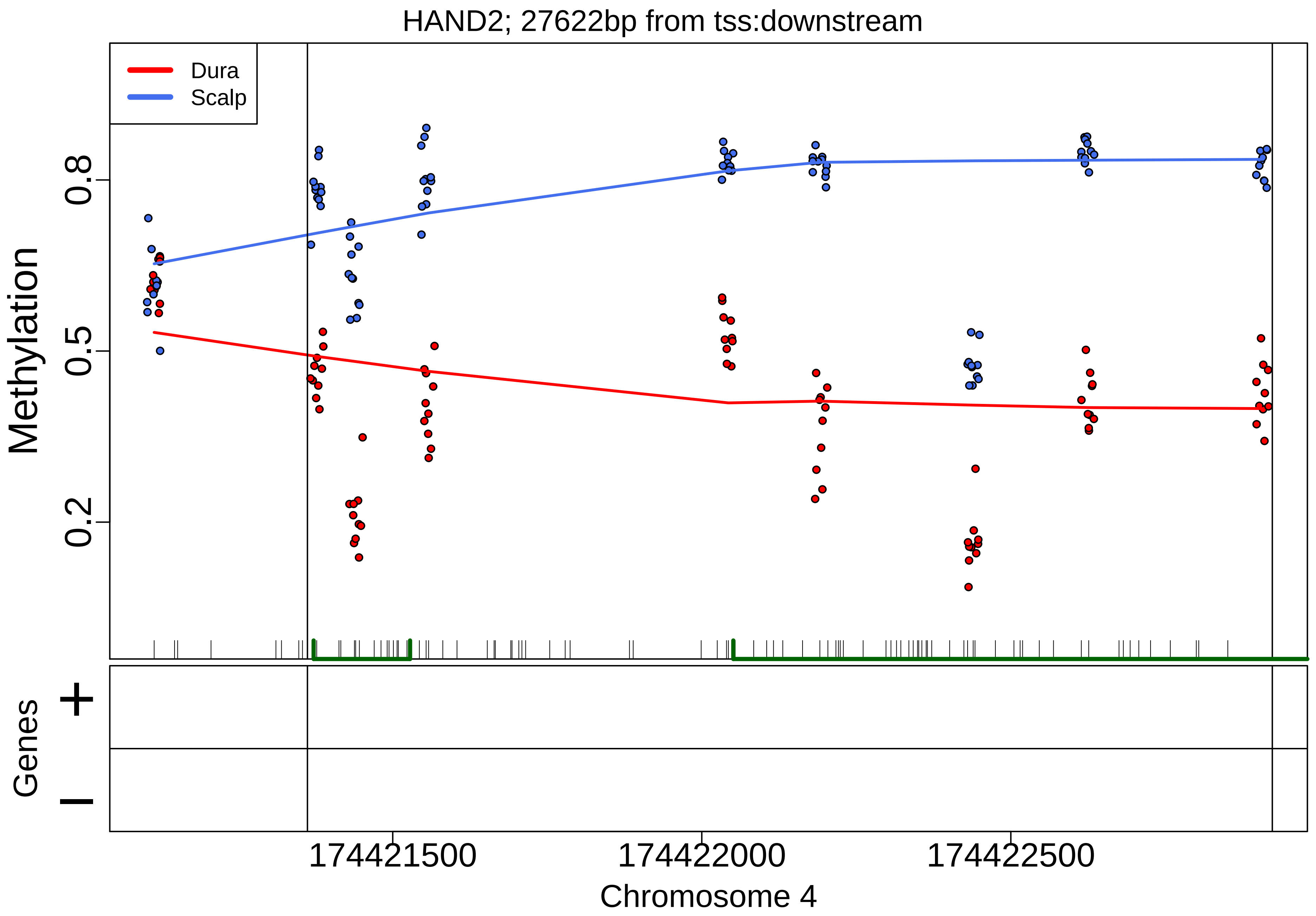
HAND2; 27622bp from tss:downstream
Dura
Scalp
Methylation
0.2
0.5
0.8
Genes
+
−
174421500
174422000
174422500
Chromosome 4

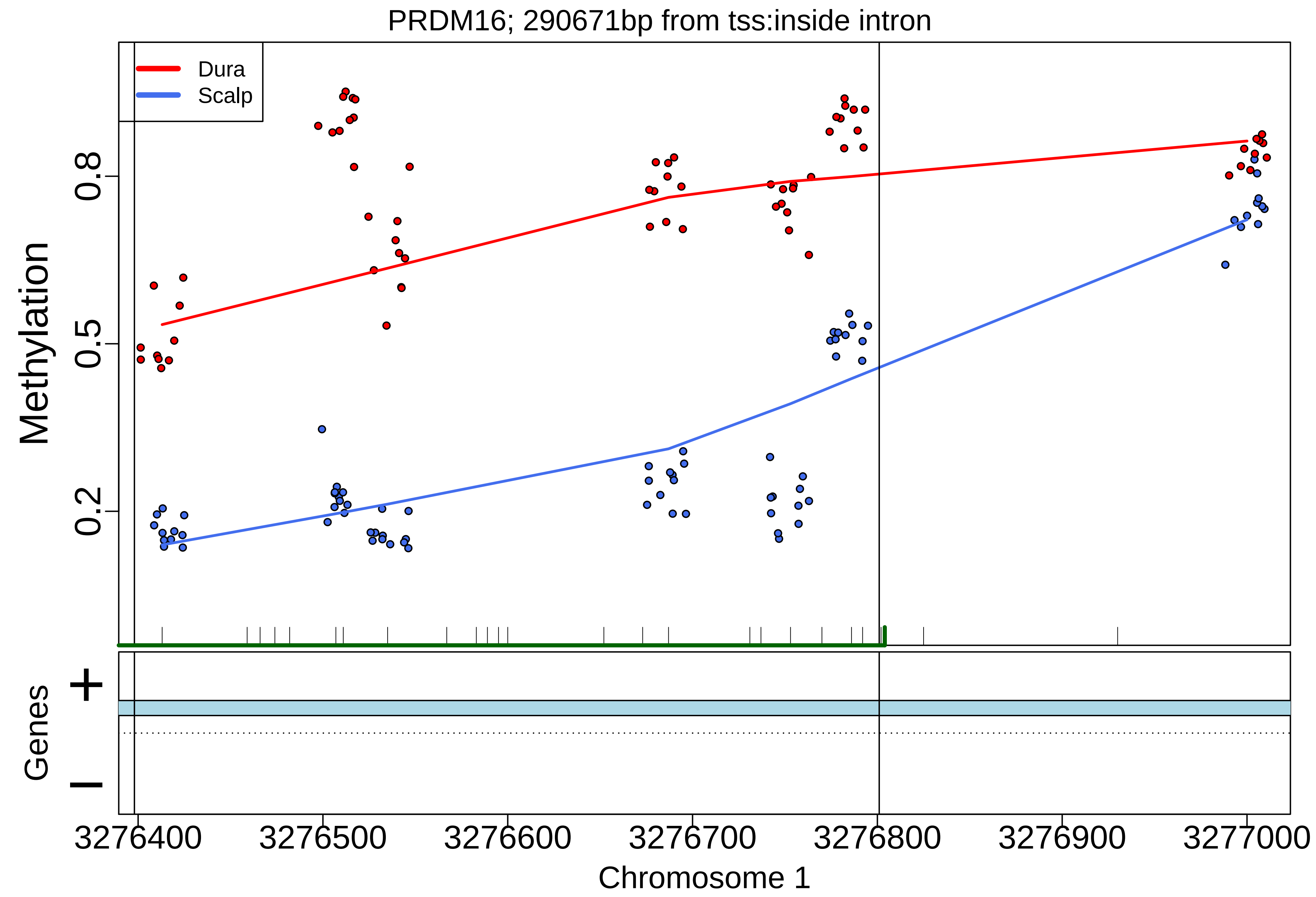
PRDM16; 290671bp from tss:inside intron
Dura
Scalp
Methylation
0.2
0.5
0.8
Genes
+
−
3276400
3276500
3276600
3276700
3276800
3276900
3277000
Chromosome 1

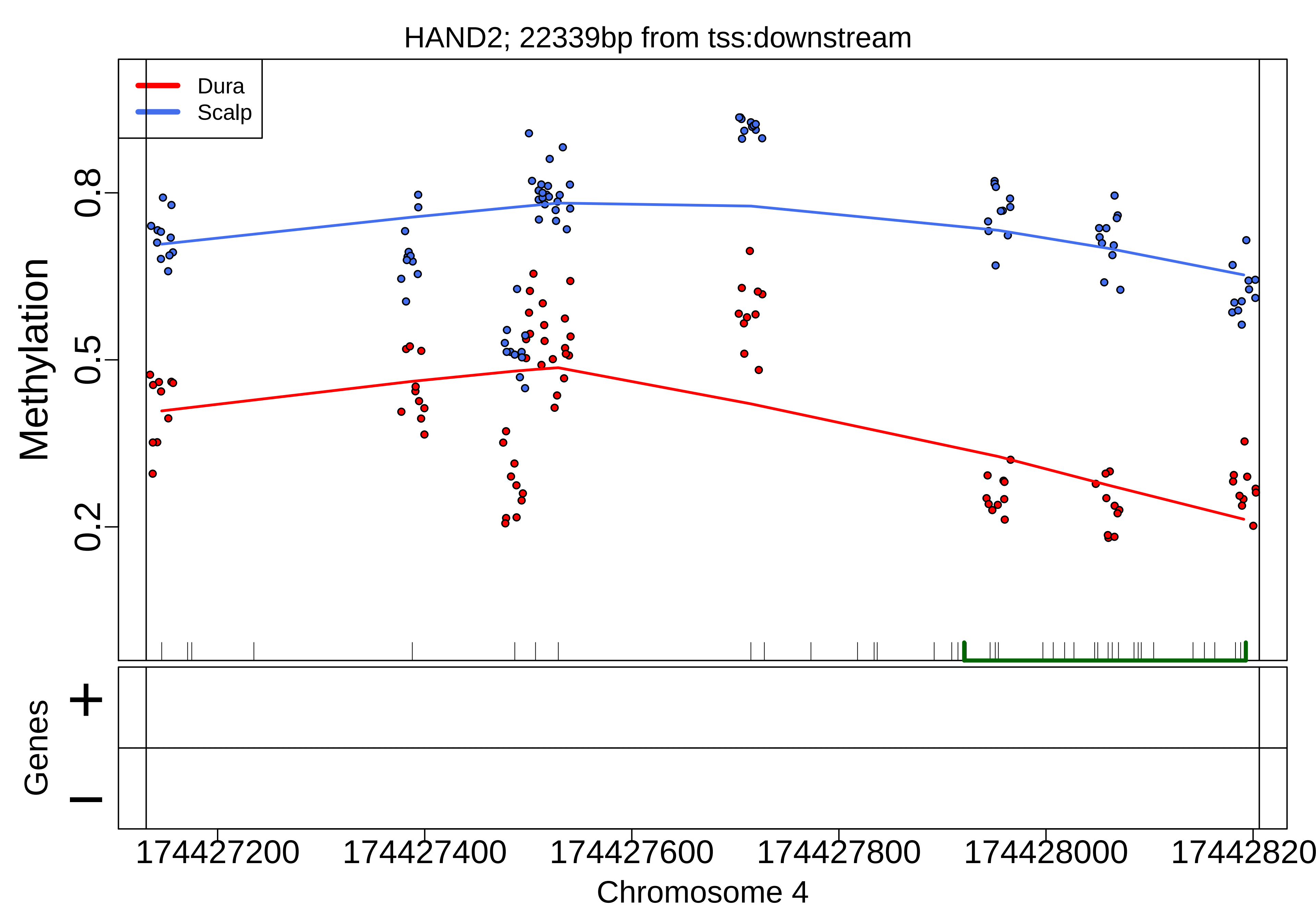
HAND2; 22339bp from tss:downstream
Dura
Scalp
Methylation
0.2
0.5
0.8
Genes
+
-
174427200
174427400
174427600
174427800
174428000
17442820
Chromosome 4

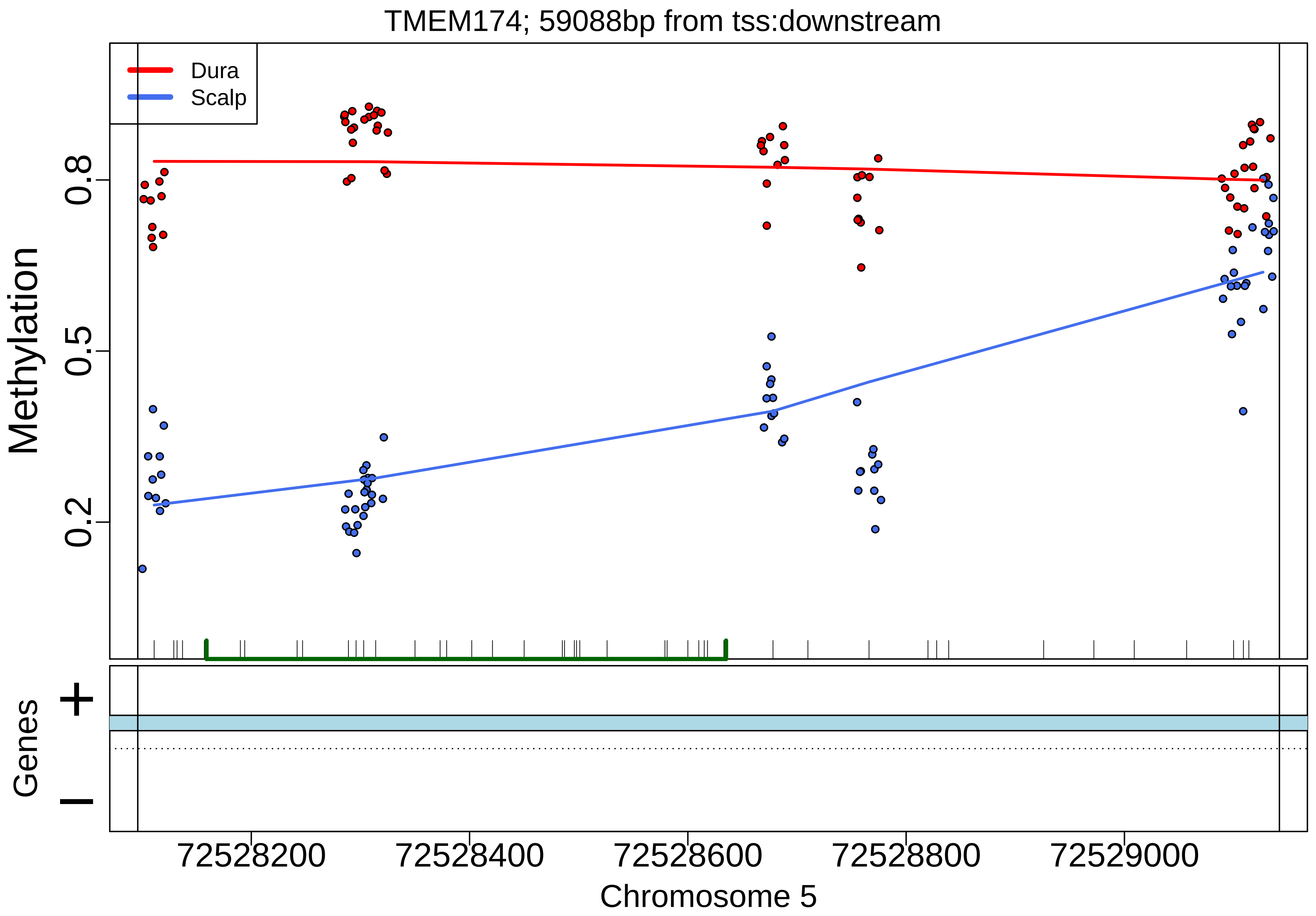

TMEM174; 59088bp from tss:downstream
Dura
Scalp
Methylation
0.2
0.5
0.8
Genes
+
−
72528200
72528400
72528600
72528800
72529000
Chromosome 5

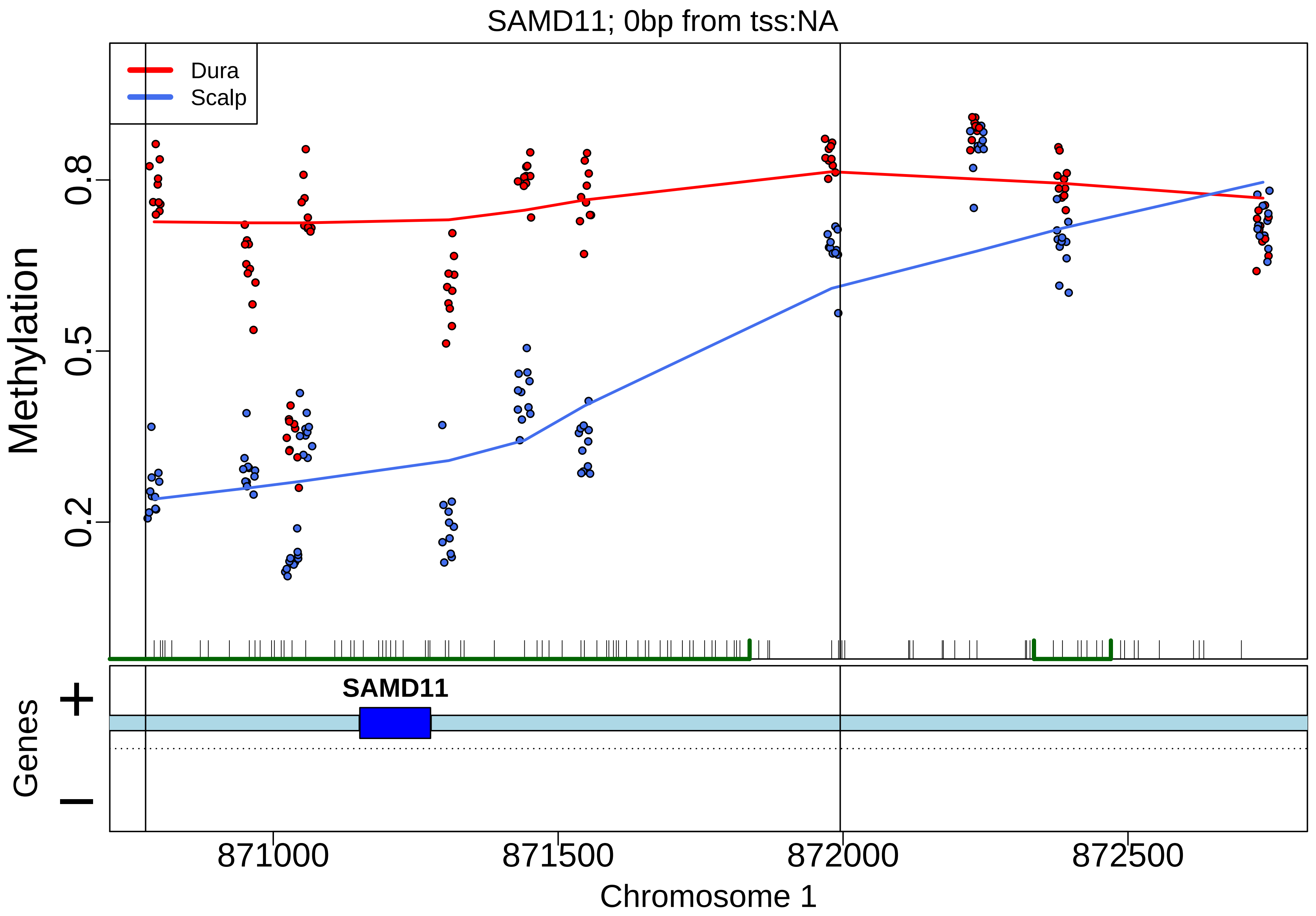

SAMD11; 0bp from tss:NA
Dura
Scalp
Methylation
0.2
0.5
0.8
Genes
+
–
SAMD11
871000
871500
872000
872500
Chromosome 1

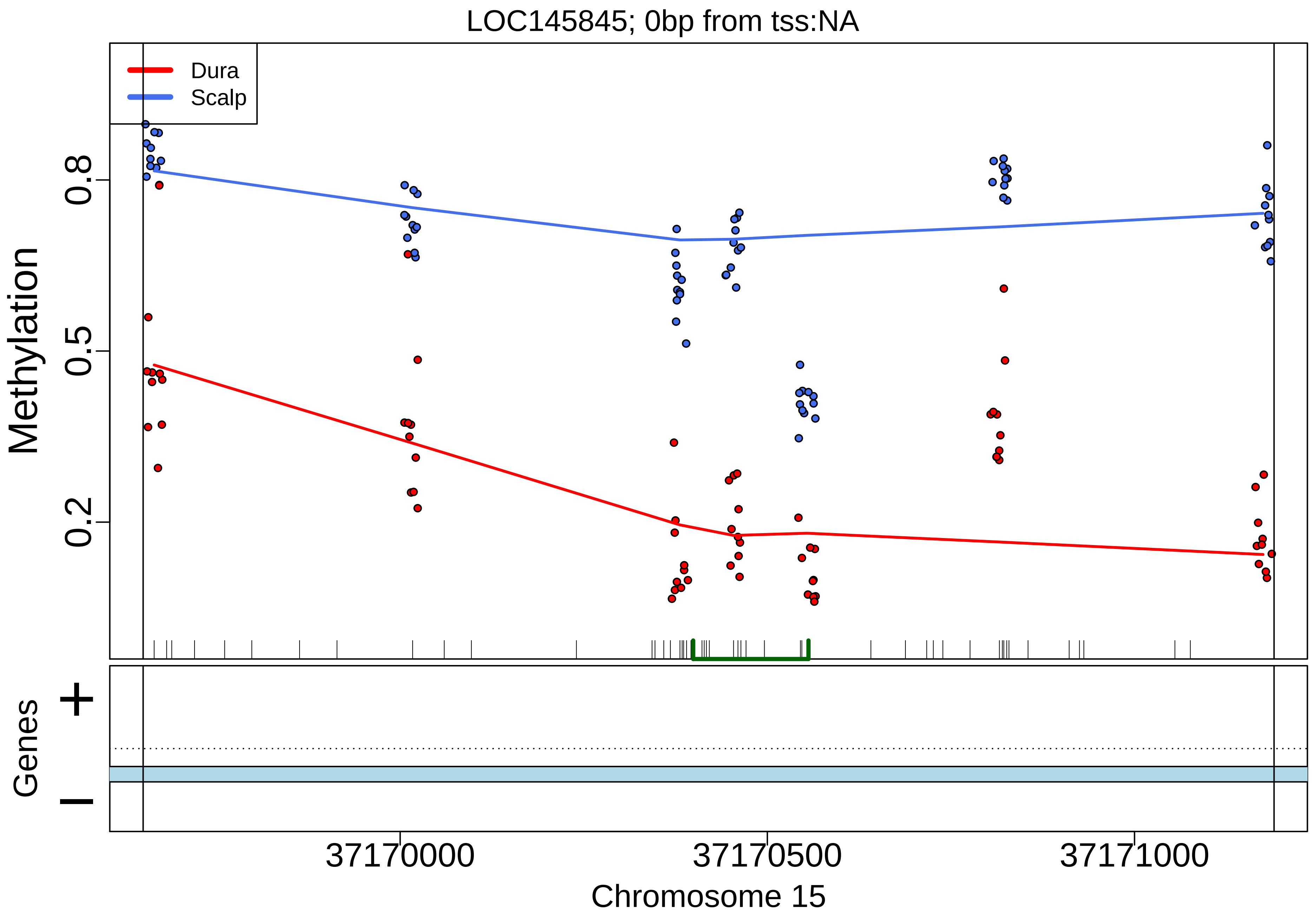

LOC145845; 0bp from tss:NA
Dura
Scalp
Methylation
0.2
0.5
0.8
Genes
+
-
37170000
37170500
37171000
Chromosome 15

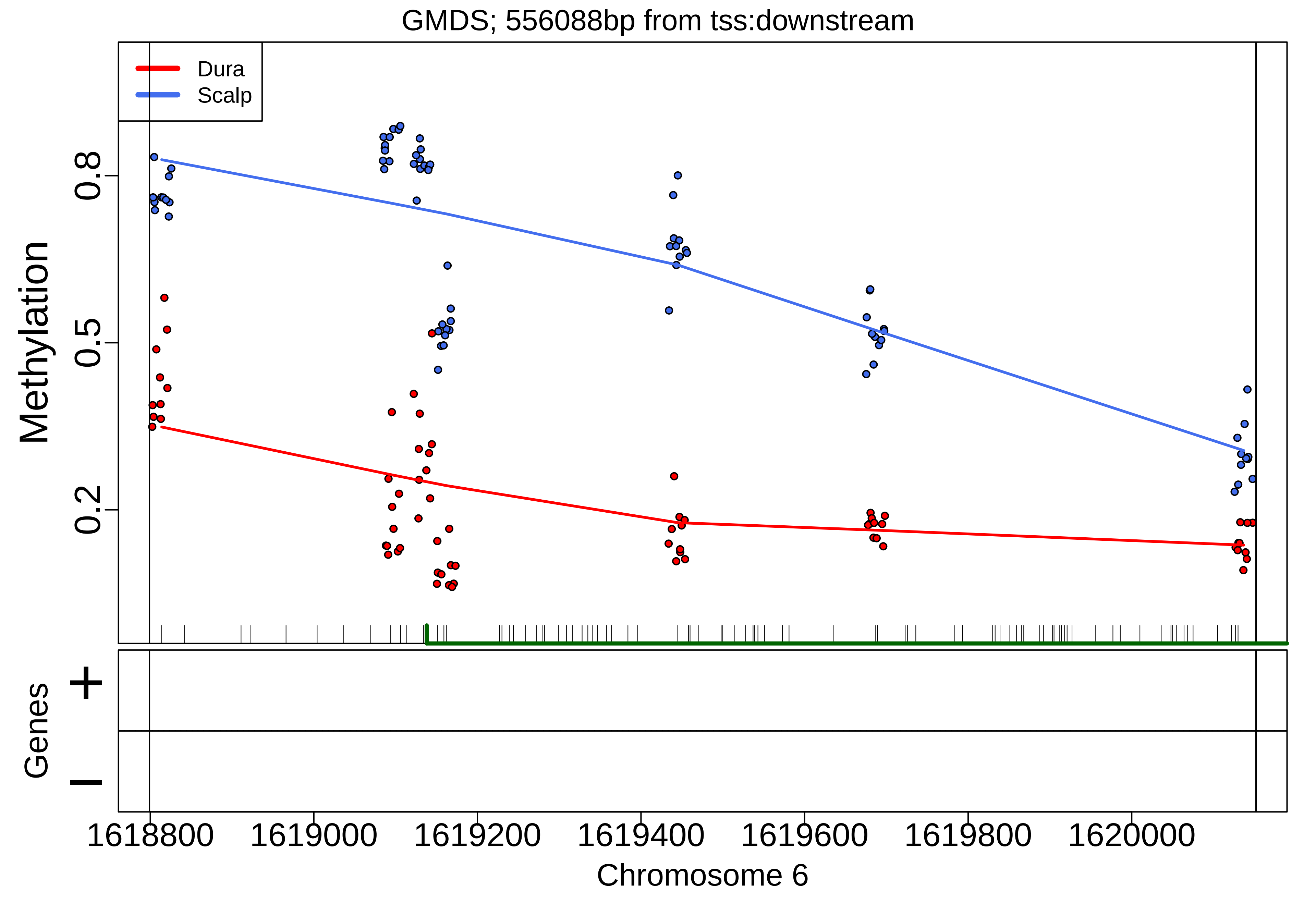

GMDS; 556088bp from tss:downstream
Dura
Scalp
Methylation
0.2
0.5
0.8
Genes
+
-
1618800
1619000
1619200
1619400
1619600
1619800
1620000
Chromosome 6

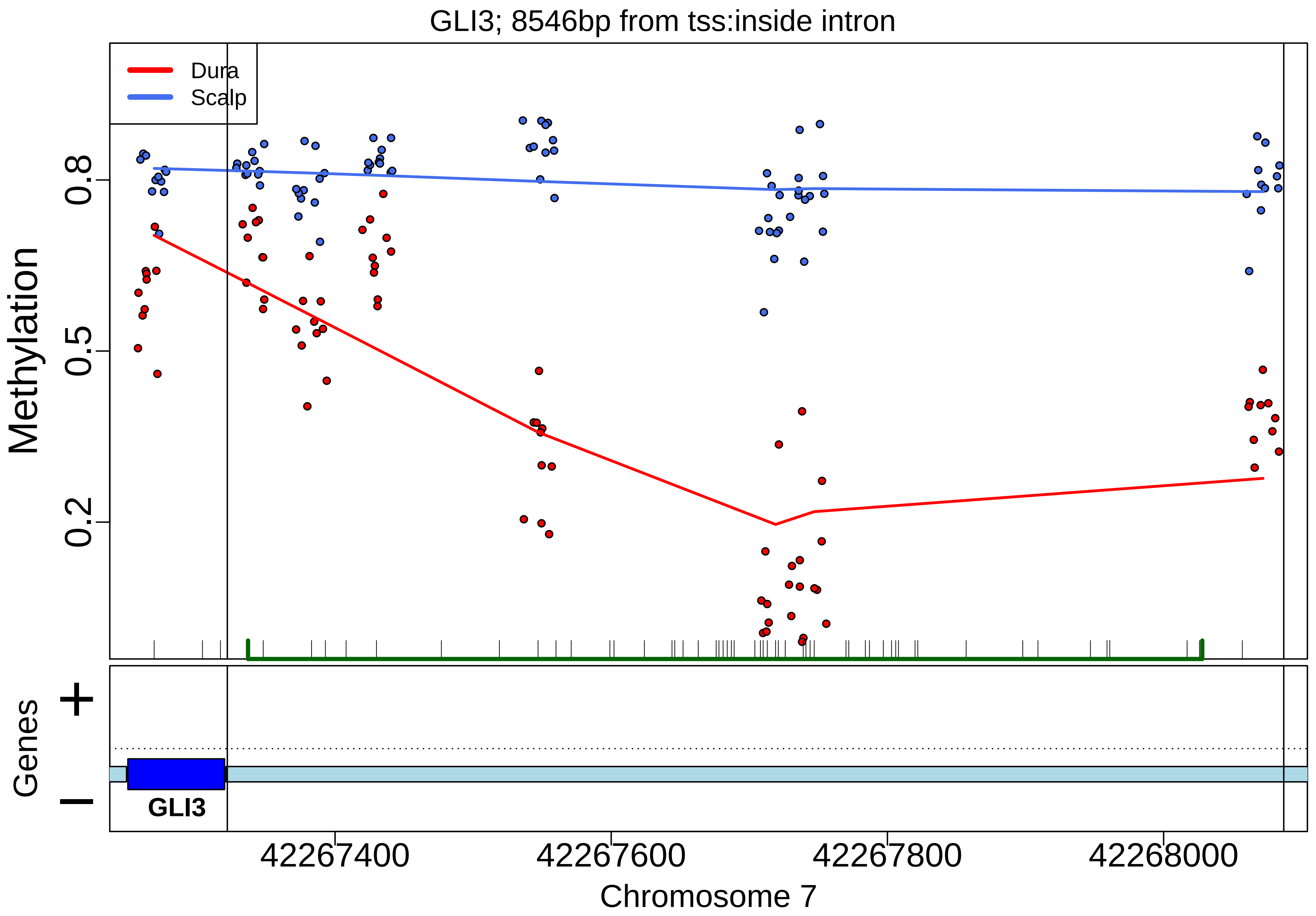

GLI3; 8546bp from tss:inside intron
Dura
Scalp
Methylation
0.2
0.5
0.8
Genes
+
-
GLI3
42267400
42267600
42267800
42268000
Chromosome 7

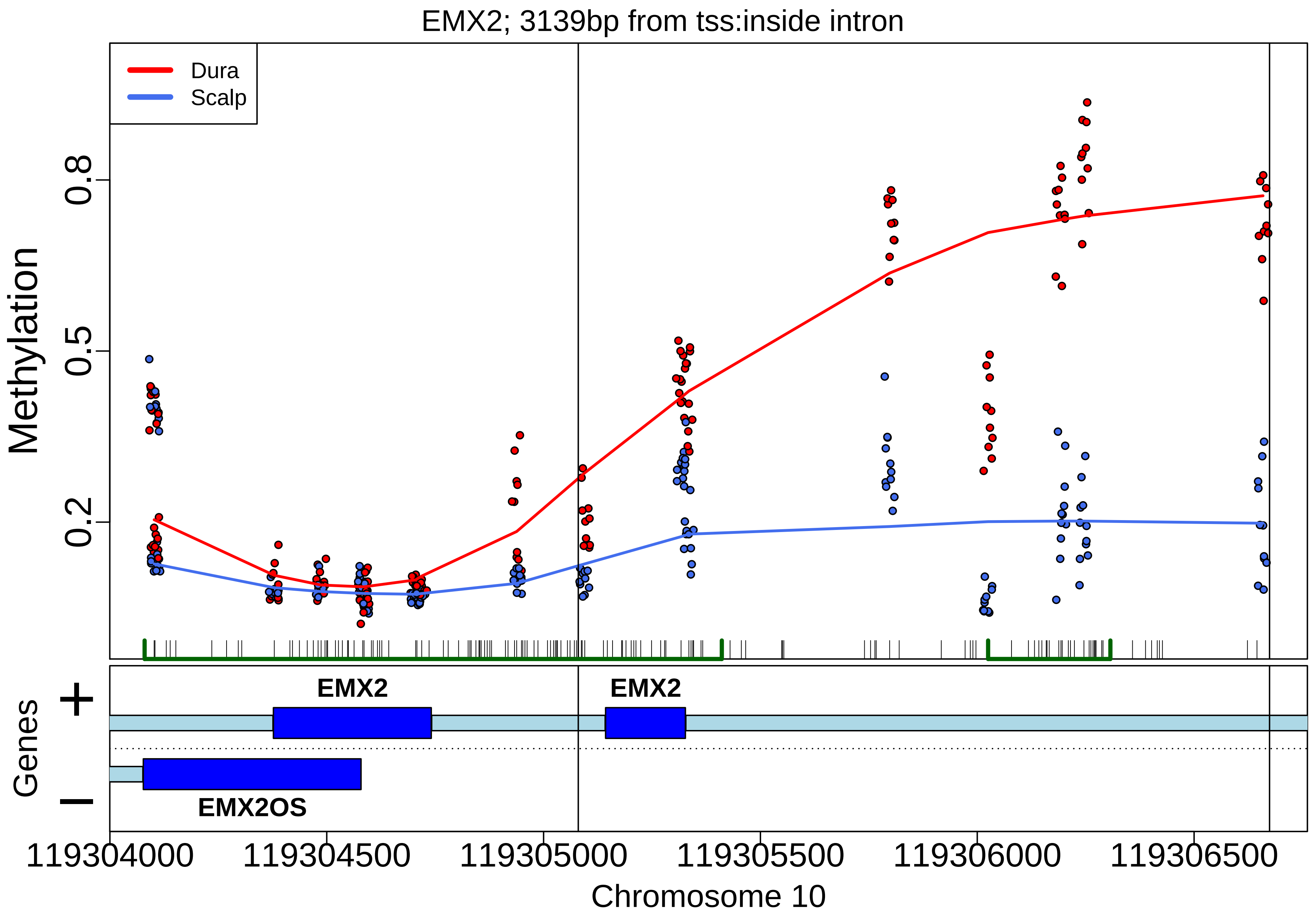
EMX2; 3139bp from tss:inside intron
Dura
Scalp
Methylation
0.2
0.5
0.8
Genes
+
−
EMX2
EMX2
EMX2OS
119304000
119304500
119305000
119305500
119306000
119306500
Chromosome 10

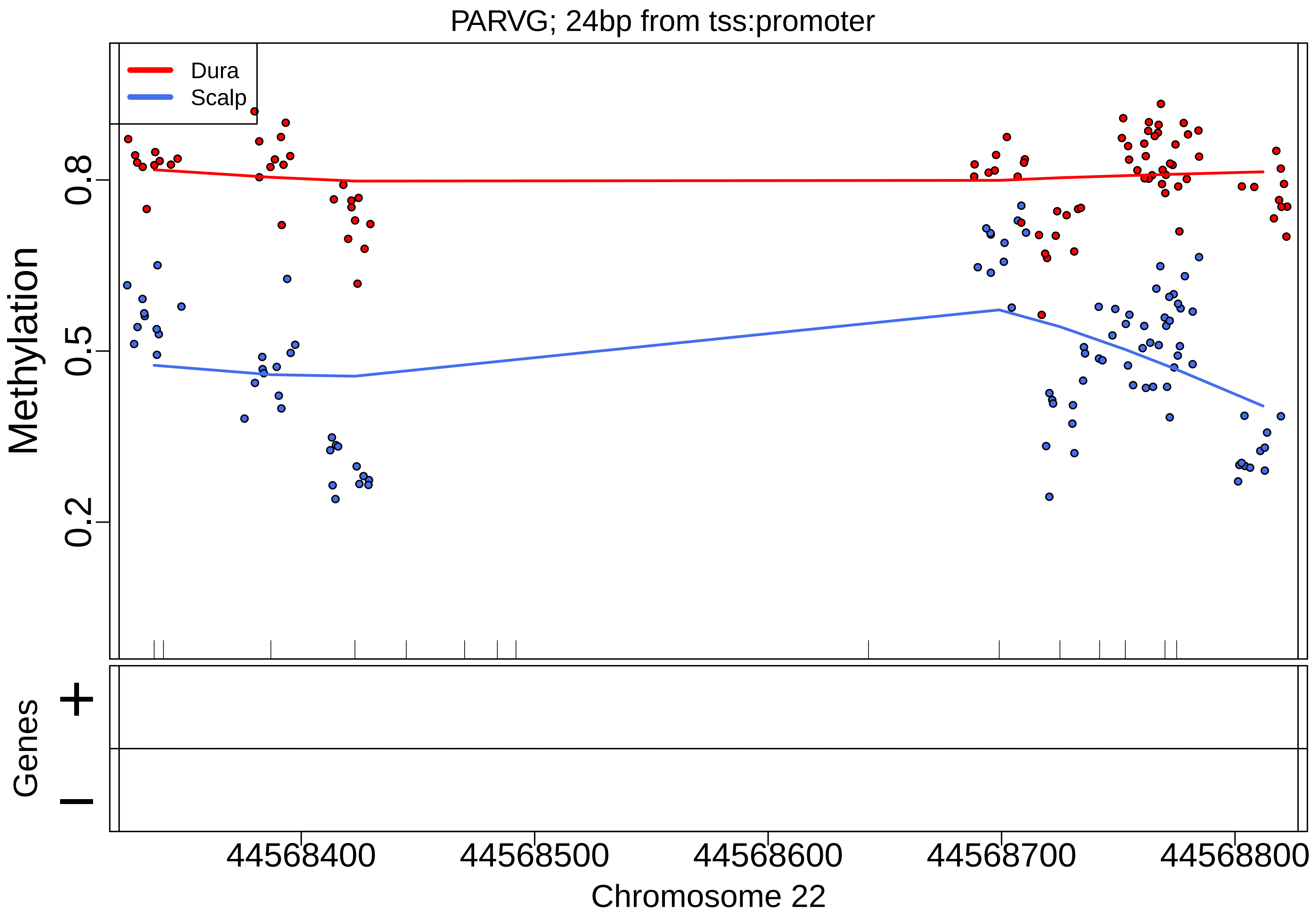
PARVG; 24bp from tss:promoter
Dura
Scalp
Methylation
0.2
0.5
0.8
Genes
+
-
44568400
44568500
44568600
44568700
44568800
Chromosome 22

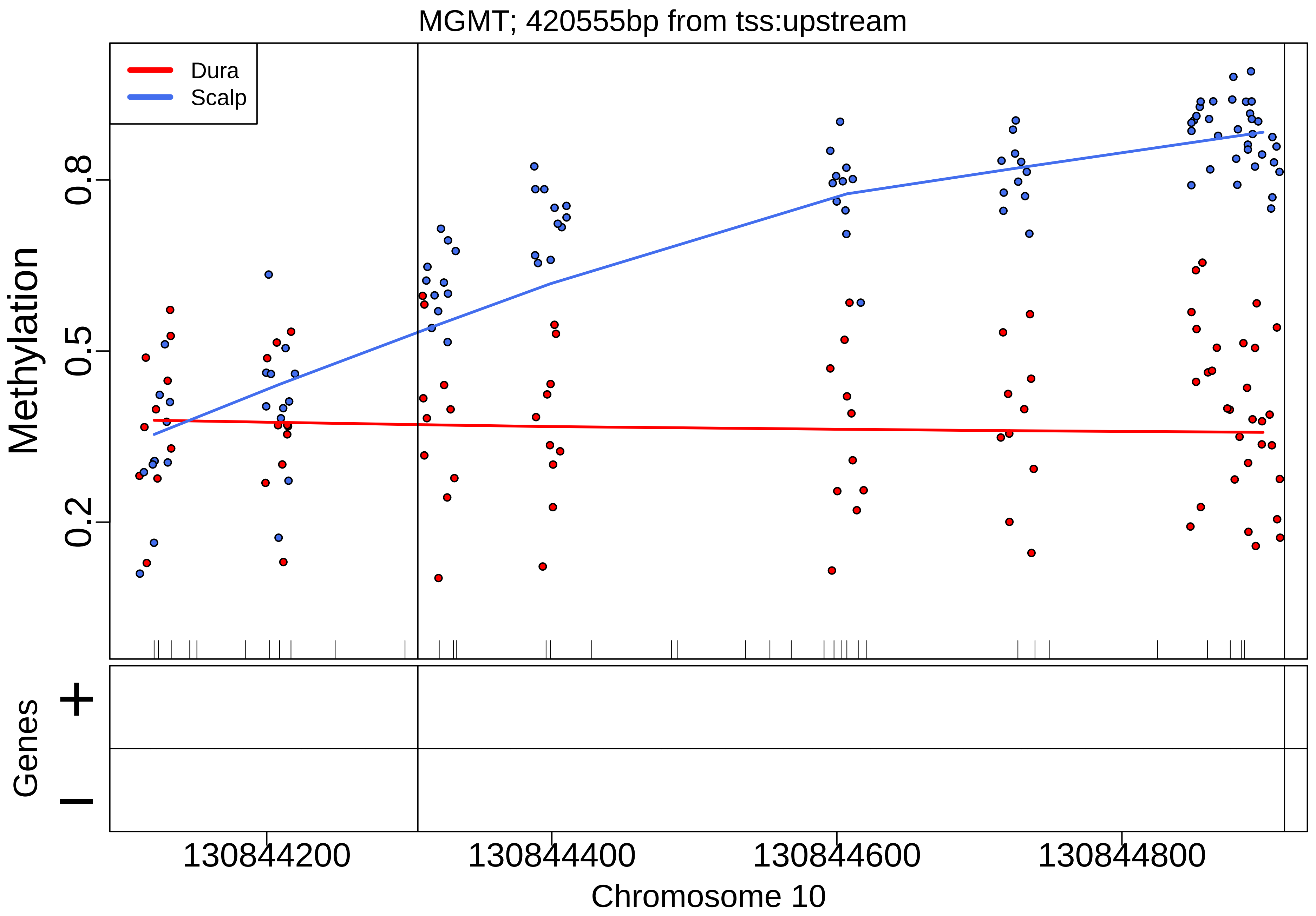
MGMT; 420555bp from tss:upstream
Dura
Scalp
Methylation
0.2
0.5
0.8
Genes
+
−
130844200
130844400
130844600
130844800
Chromosome 10

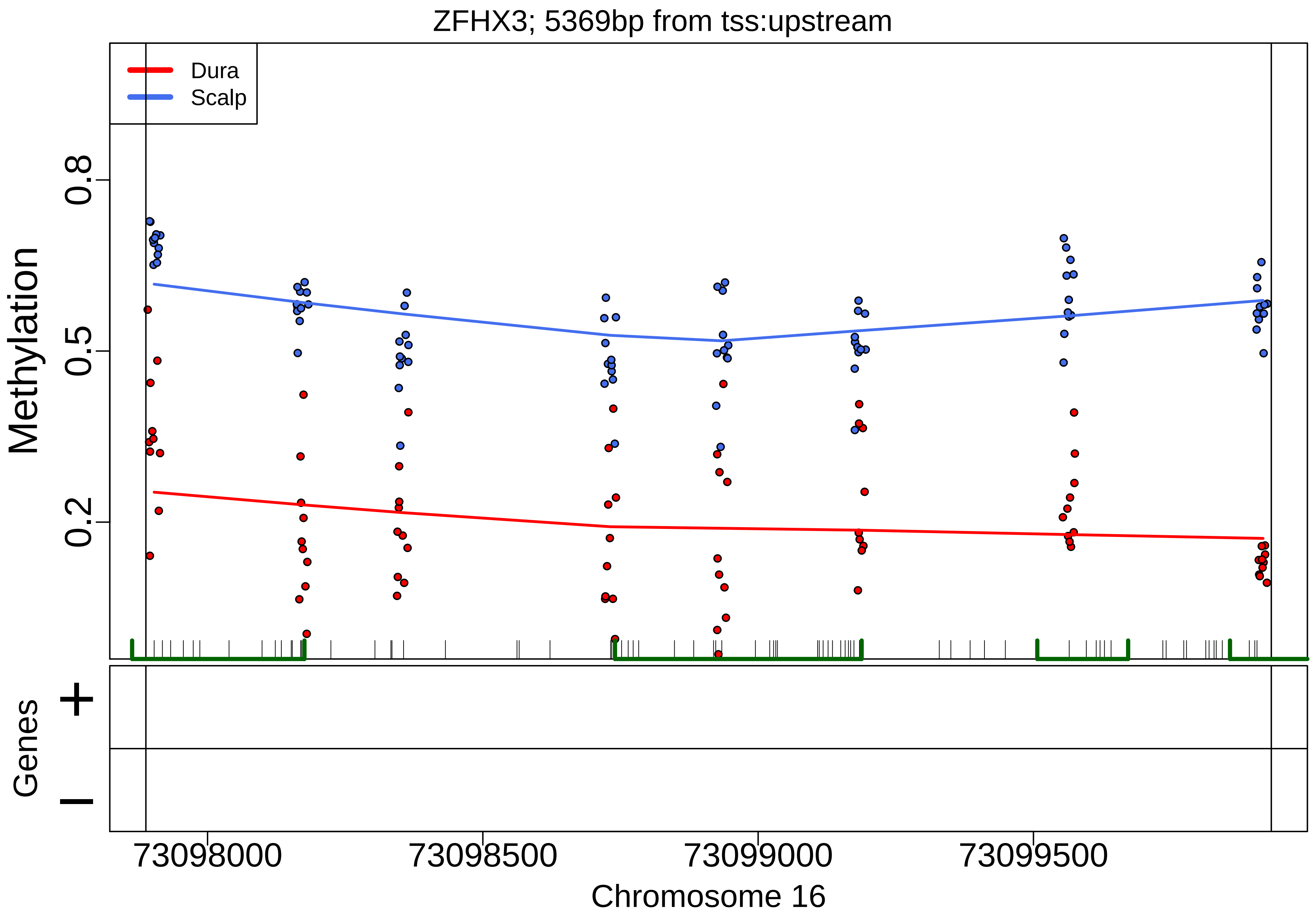

ZFHX3; 5369bp from tss:upstream
Dura
Scalp
Methylation
0.2
0.5
0.8
Genes
+
-
73098000
73098500
73099000
73099500
Chromosome 16

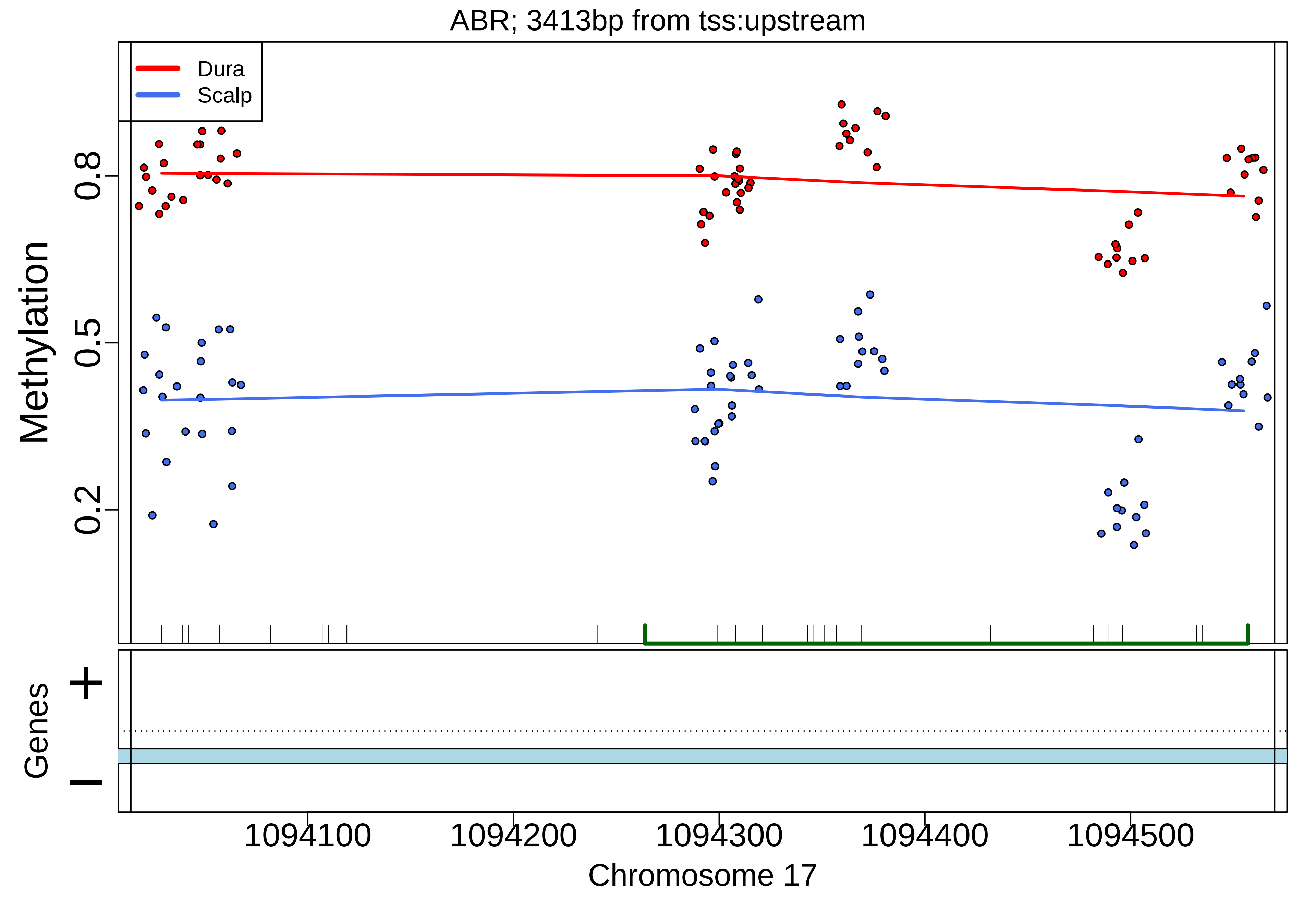

ABR; 3413bp from tss:upstream
Dura
Scalp
Methylation
0.2
0.5
0.8
Genes
+
-
1094100
1094200
1094300
1094400
1094500
Chromosome 17

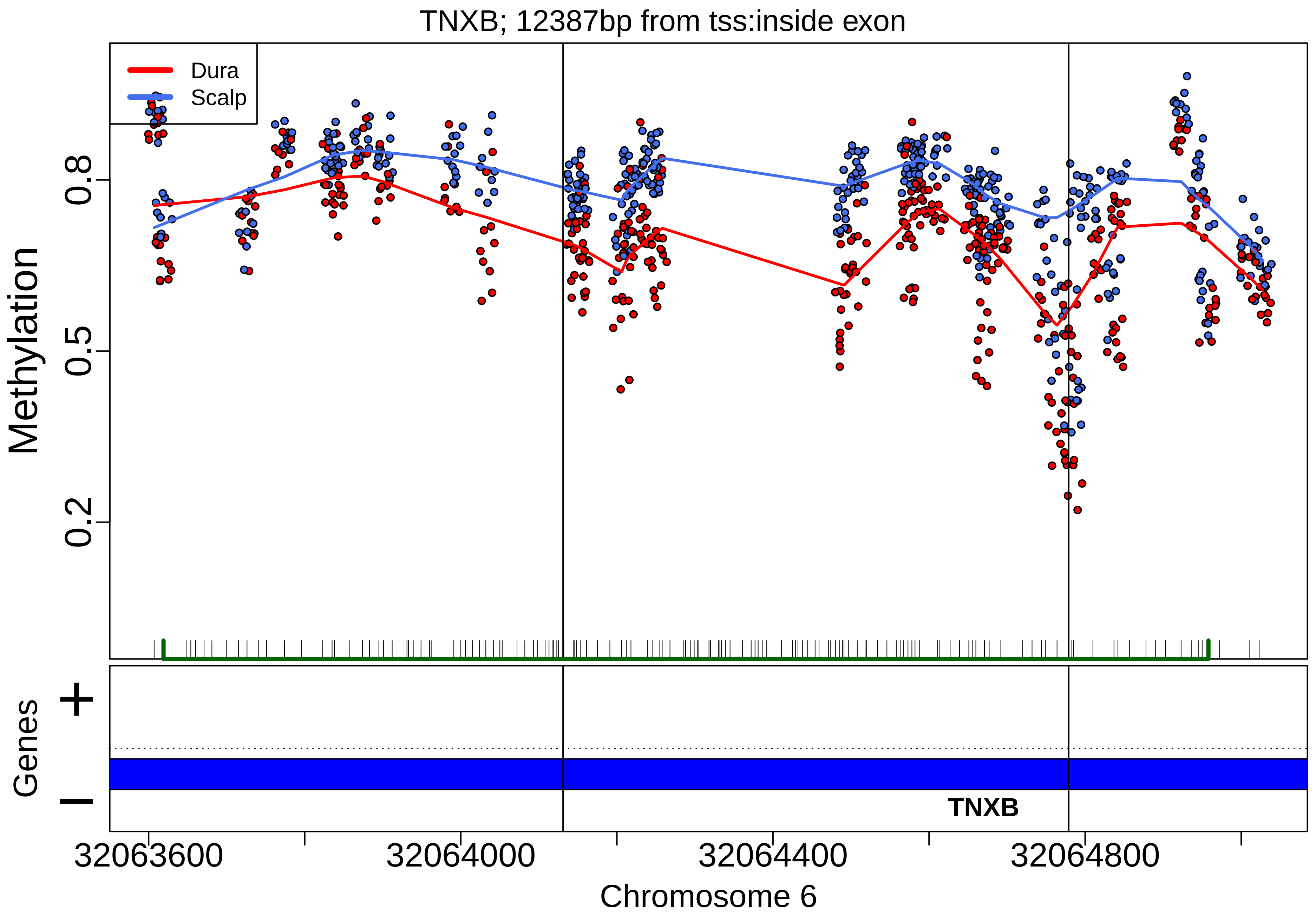

TNXB; 12387bp from tss:inside exon
Dura
Scalp
Methylation
0.2
0.5
0.8
Genes
+
−
TNXB
32063600
32064000
32064400
32064800
Chromosome 6

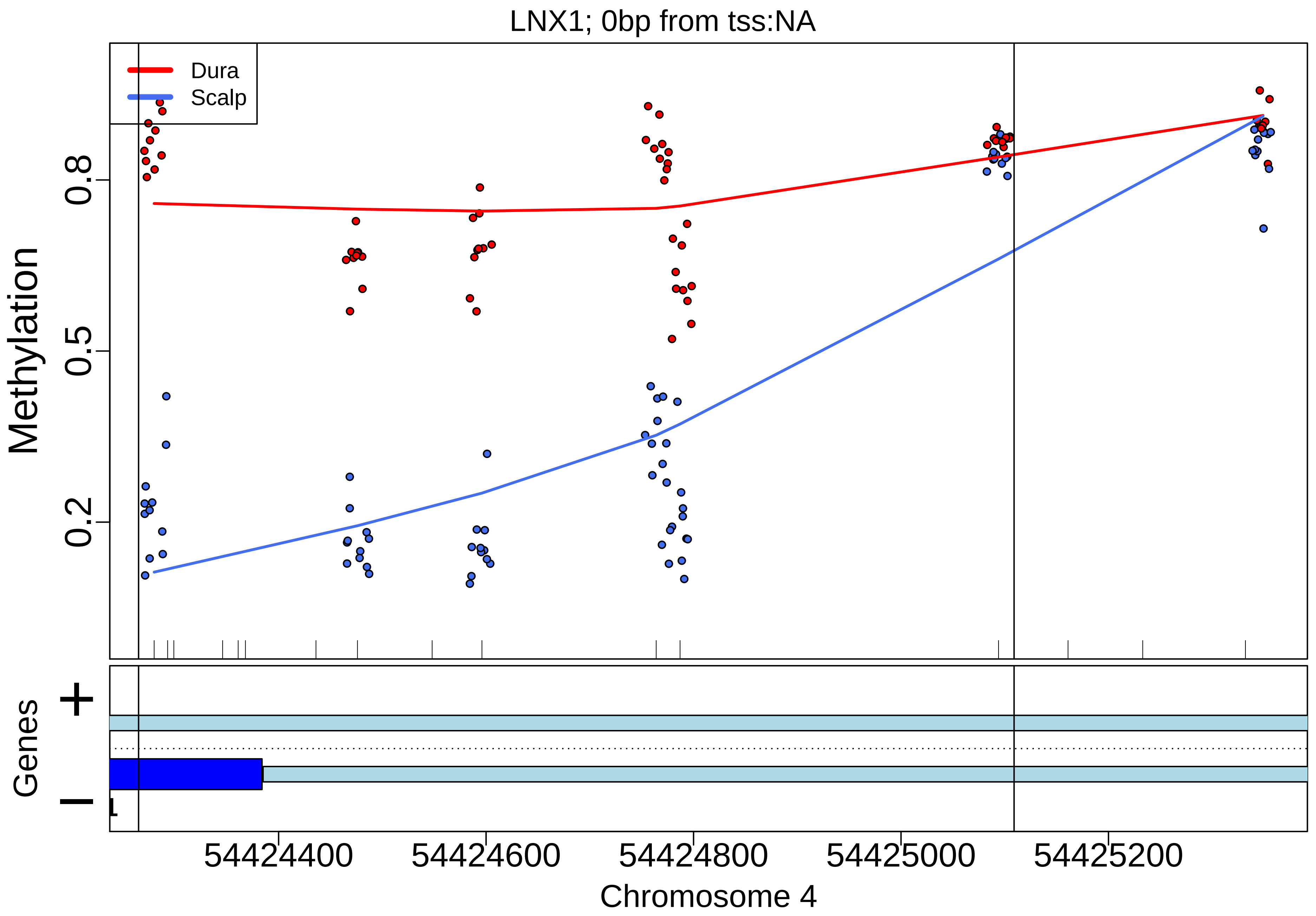
LNX1; 0bp from tss:NA
Dura
Scalp
Methylation
0.2
0.5
0.8
Genes
+
−
54424400
54424600
54424800
54425000
54425200
Chromosome 4

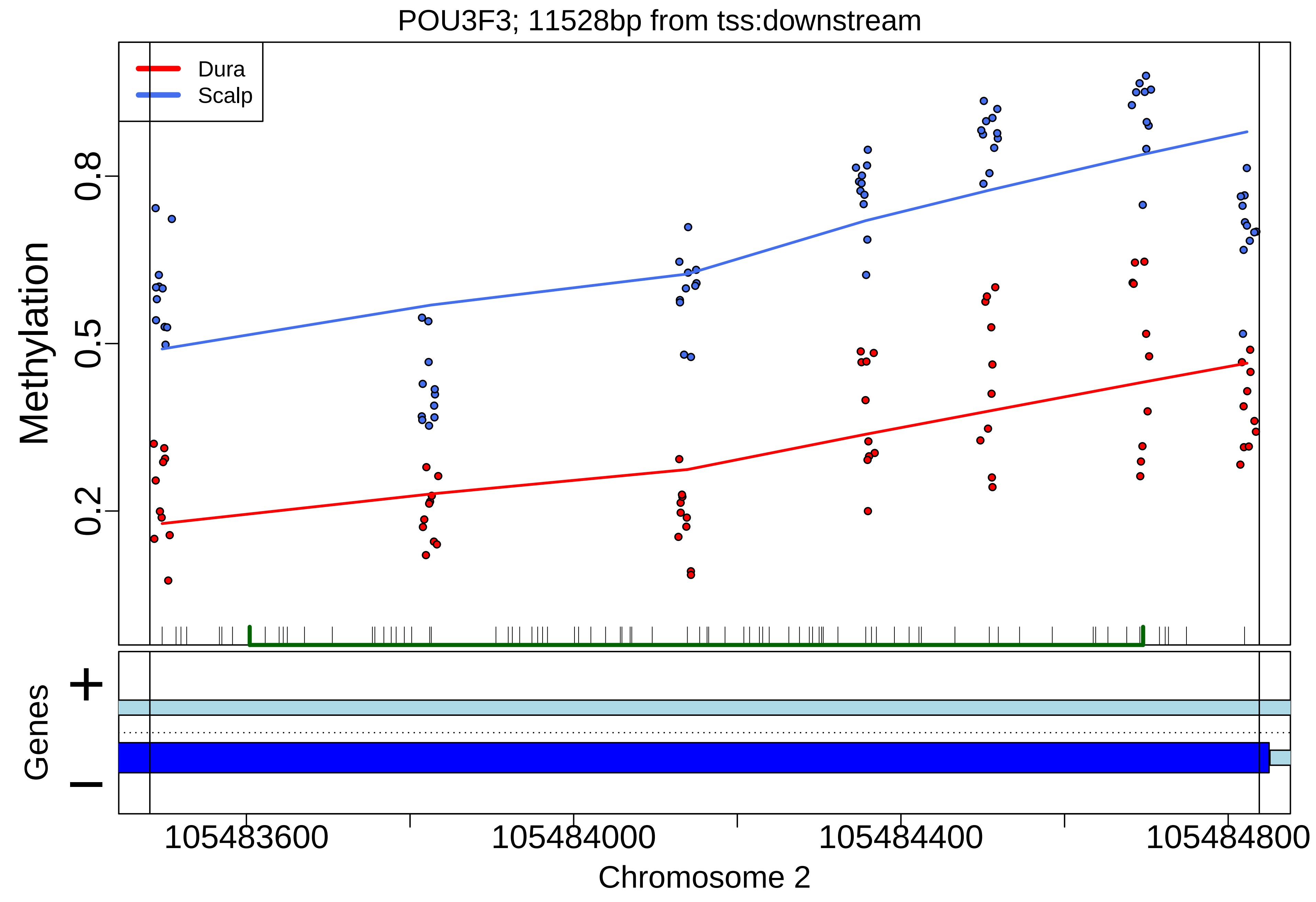
POU3F3; 11528bp from tss:downstream
Dura
Scalp
Methylation
0.2
0.5
0.8
Genes
+
-
105483600
105484000
105484400
105484800
Chromosome 2

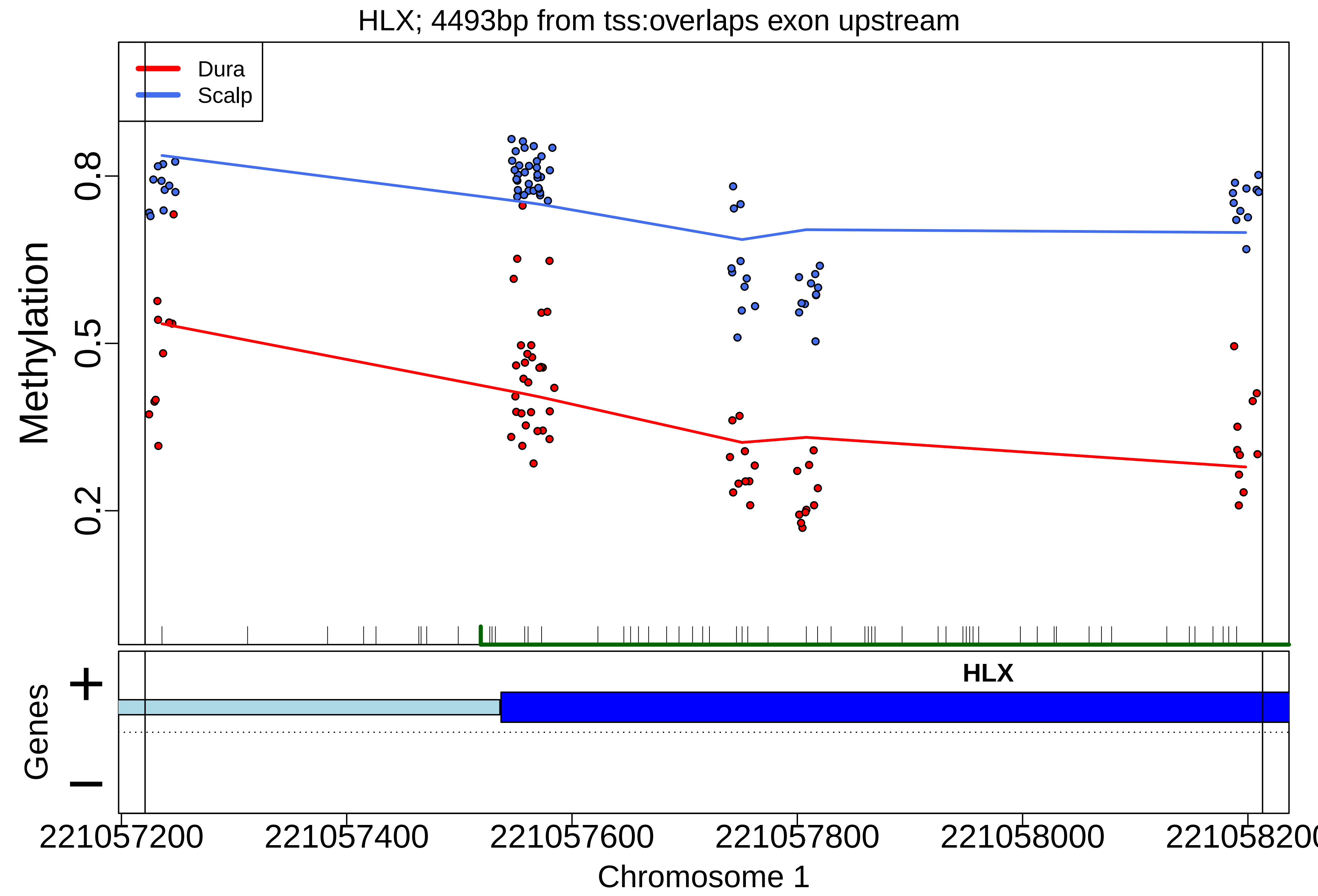
HLX; 4493bp from tss:overlaps exon upstream
Dura
Scalp
Methylation
0.2
0.5
0.8
Genes
+
-
HLX
221057200
221057400
221057600
221057800
221058000
221058200
Chromosome 1

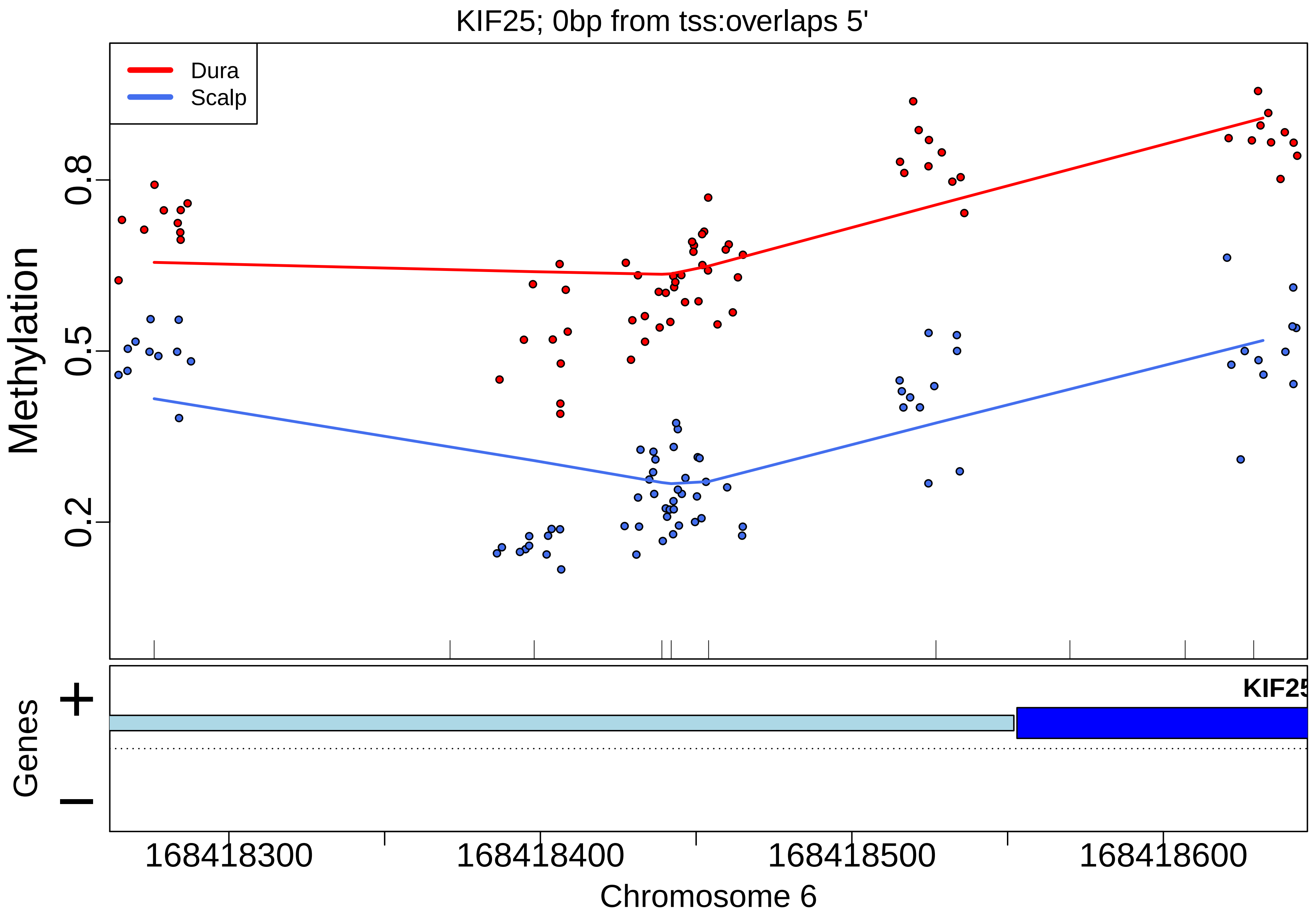

KIF25; 0bp from tss:overlaps 5'
Dura
Scalp
Methylation
0.2
0.5
0.8
Genes
+
−
KIF25
168418300
168418400
168418500
168418600
Chromosome 6

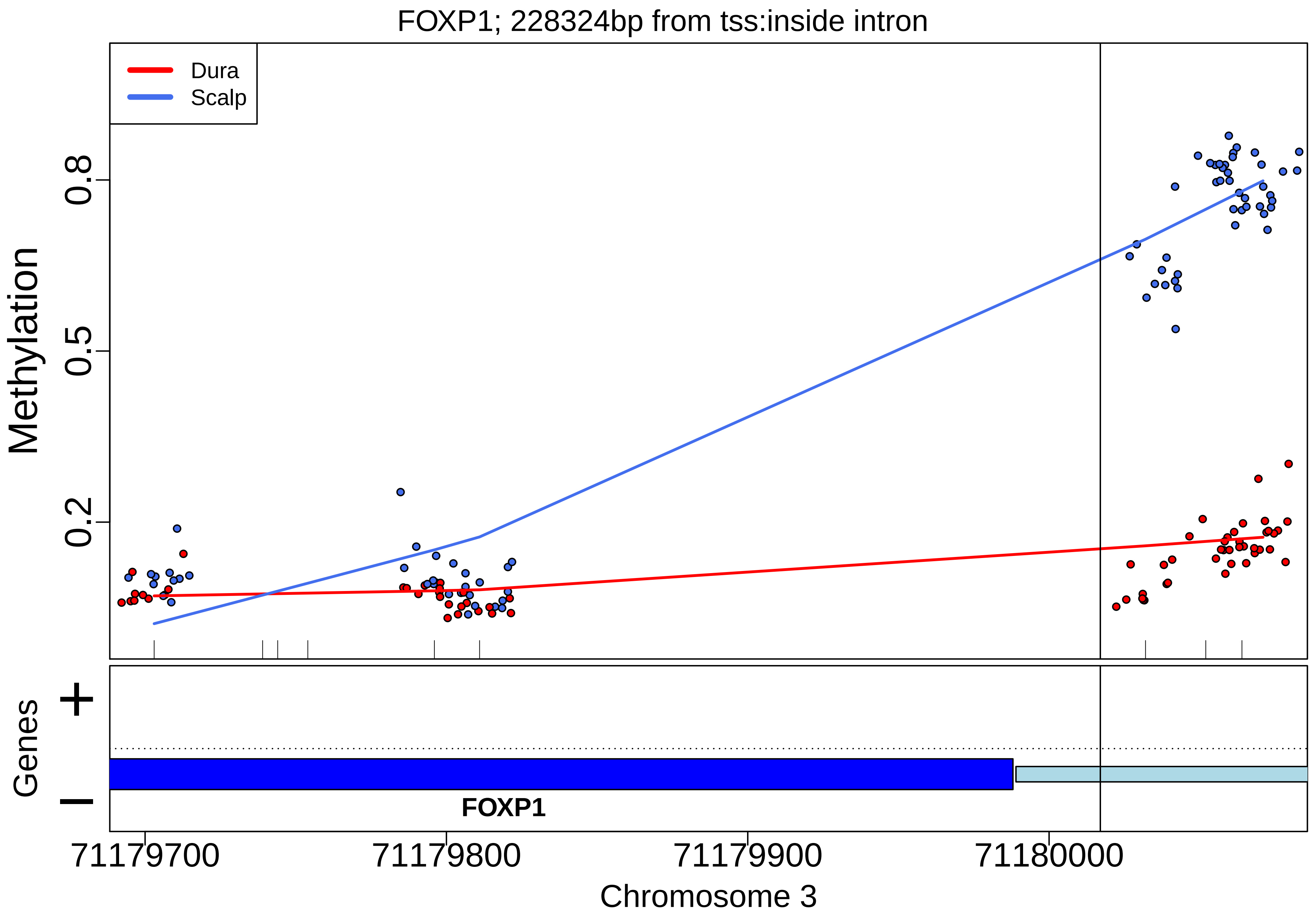
FOXP1; 228324bp from tss:inside intron
Dura
Scalp
Methylation
0.2
0.5
0.8
Genes
+
−
FOXP1
71179700
71179800
71179900
71180000
Chromosome 3

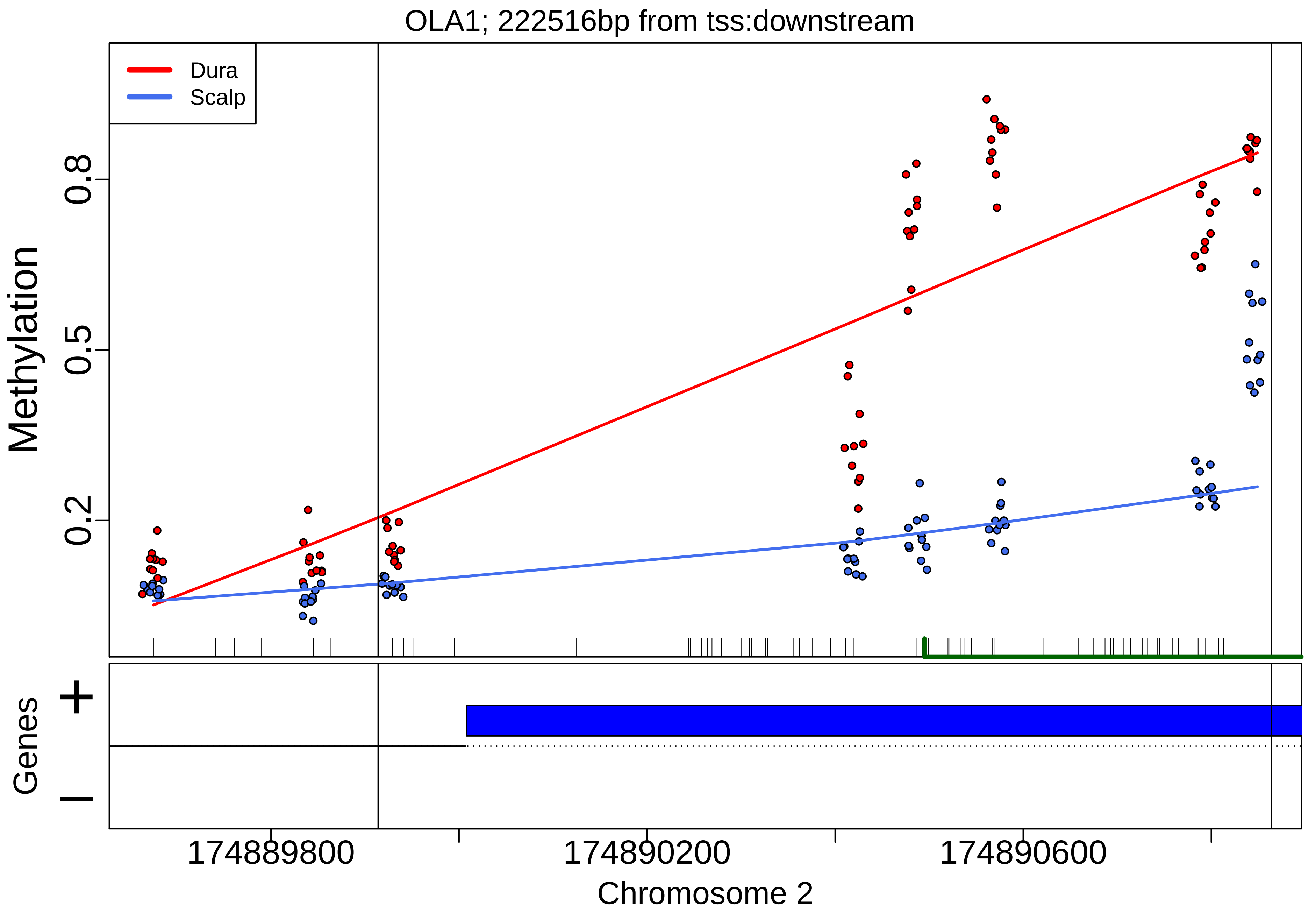

OLA1; 222516bp from tss:downstream
Dura
Scalp
Methylation
0.2
0.5
0.8
Genes
+
−
174889800
174890200
174890600
Chromosome 2

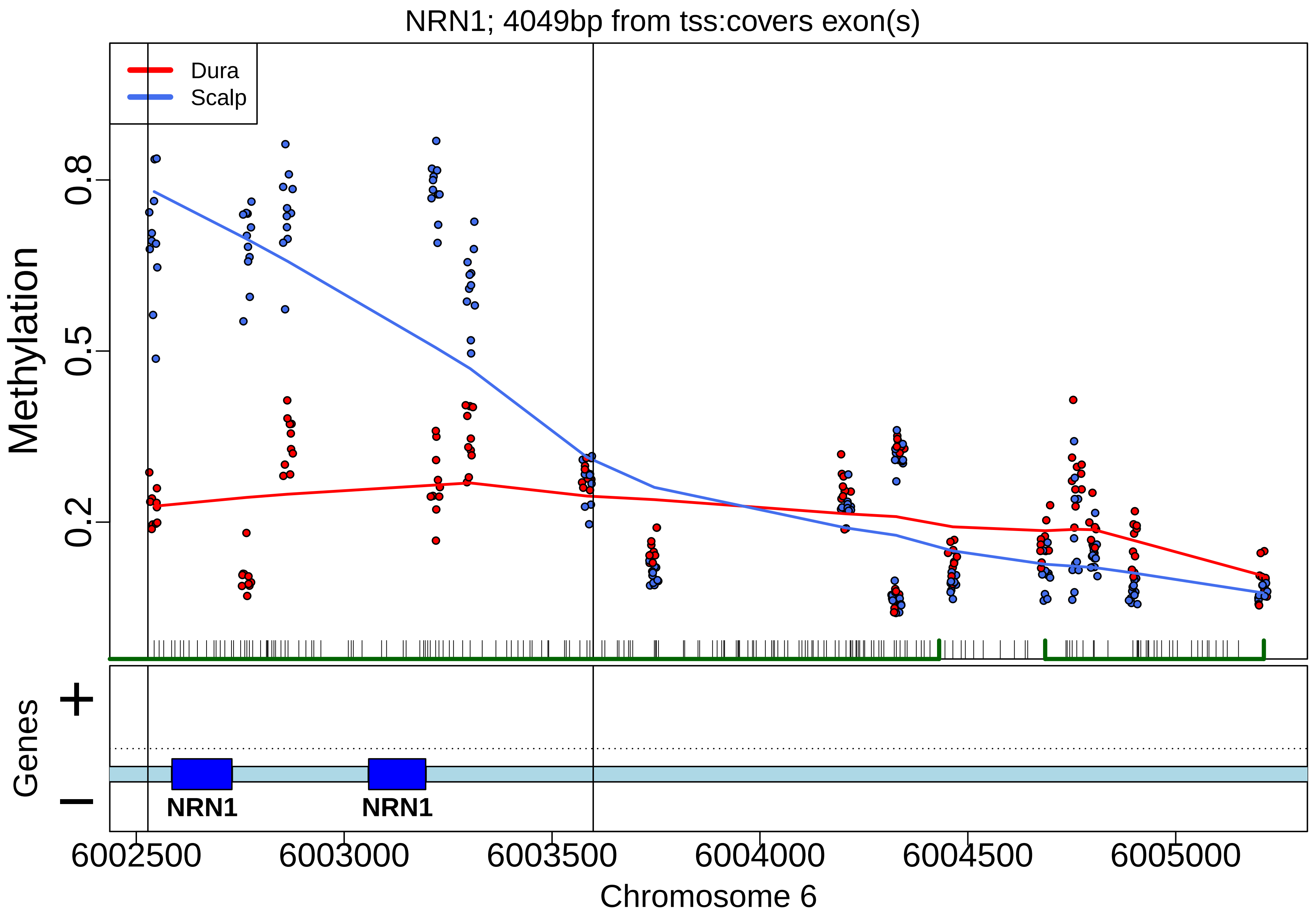

NRN1; 4049bp from tss:covers exon(s)
Dura
Scalp
Methylation
0.2
0.5
0.8
Genes
+
−
NRN1
NRN1
6002500
6003000
6003500
6004000
6004500
6005000
Chromosome 6

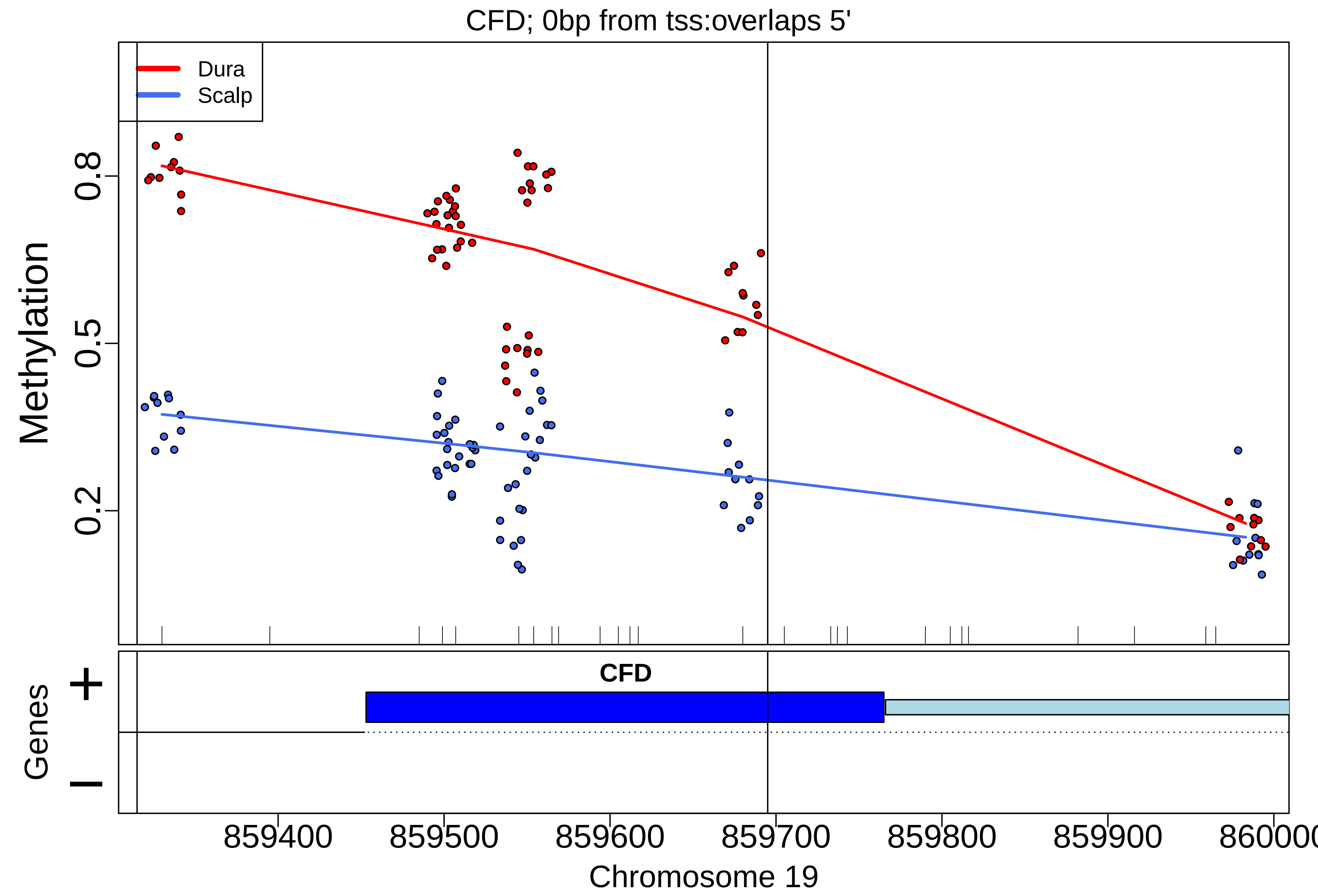

CFD; 0bp from tss:overlaps 5'
Dura
Scalp
Methylation
0.2
0.5
0.8
Genes
+
-
CFD
859400
859500
859600
859700
859800
859900
Chromosome 19

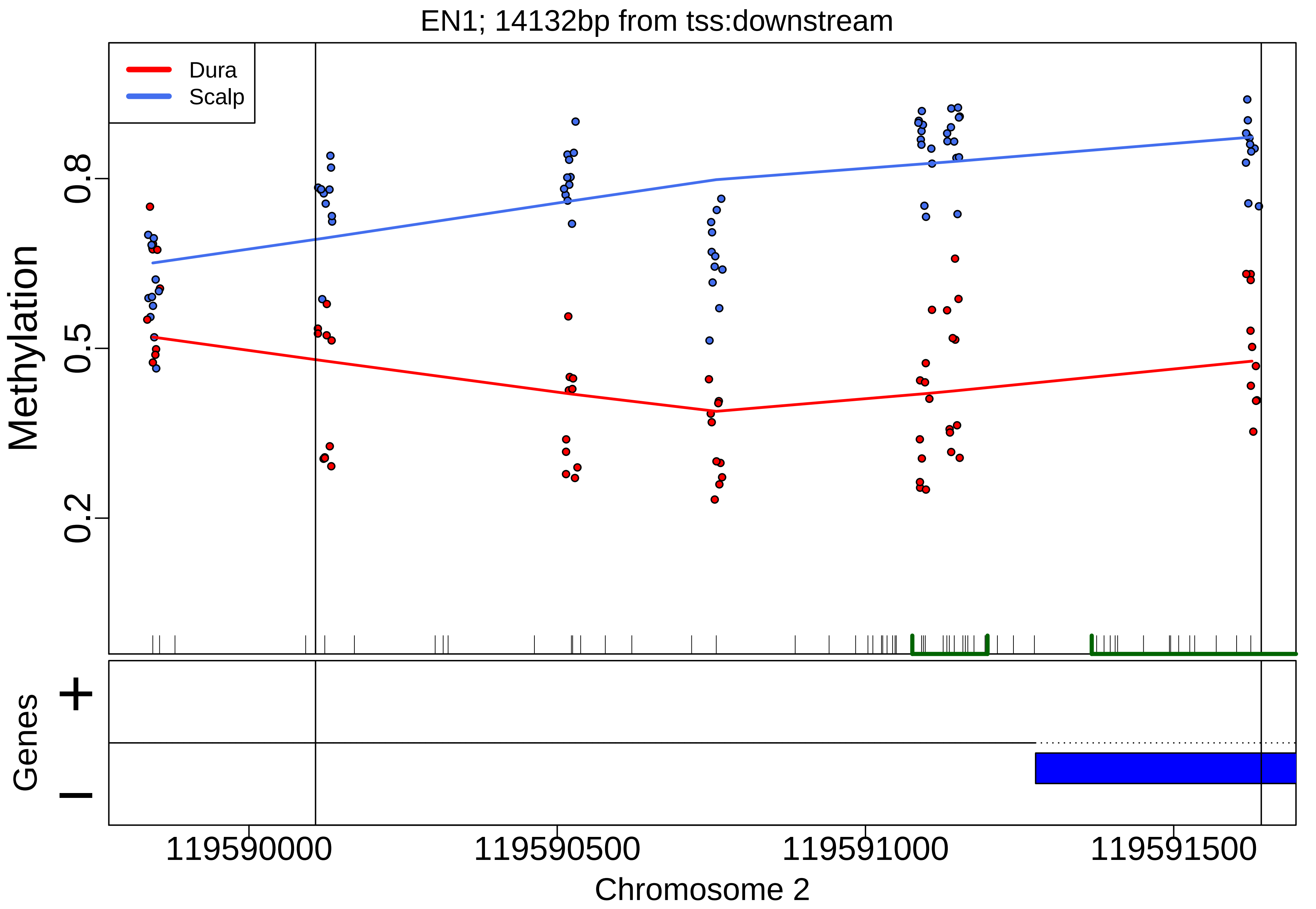
EN1; 14132bp from tss:downstream
Dura
Scalp
Methylation
0.2
0.5
0.8
Genes
+
-
119590000
119590500
119591000
119591500
Chromosome 2

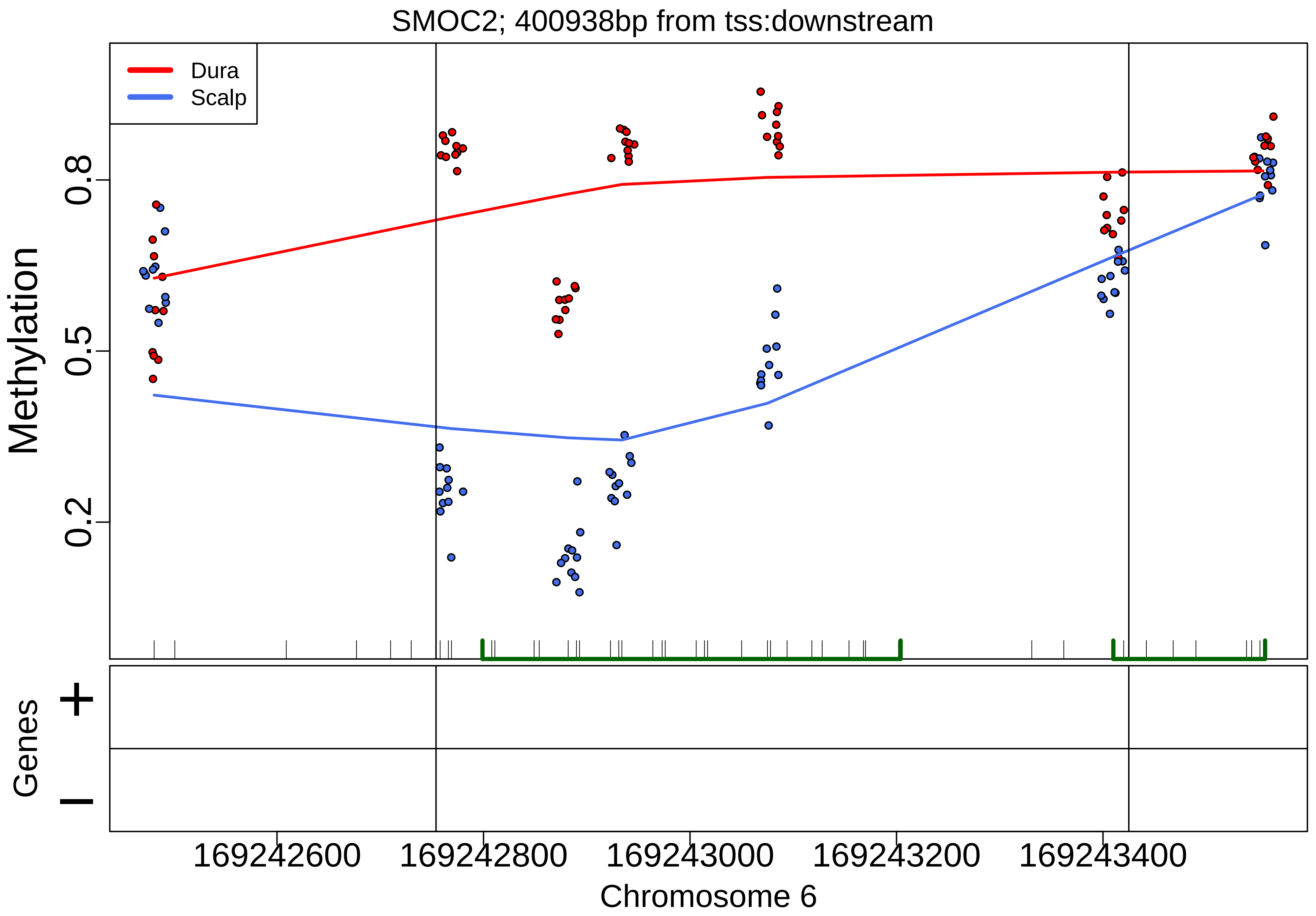
SMOC2; 400938bp from tss:downstream
Dura
Scalp
Methylation
0.2
0.5
0.8
Genes
+
–
169242600
169242800
169243000
169243200
169243400
Chromosome 6

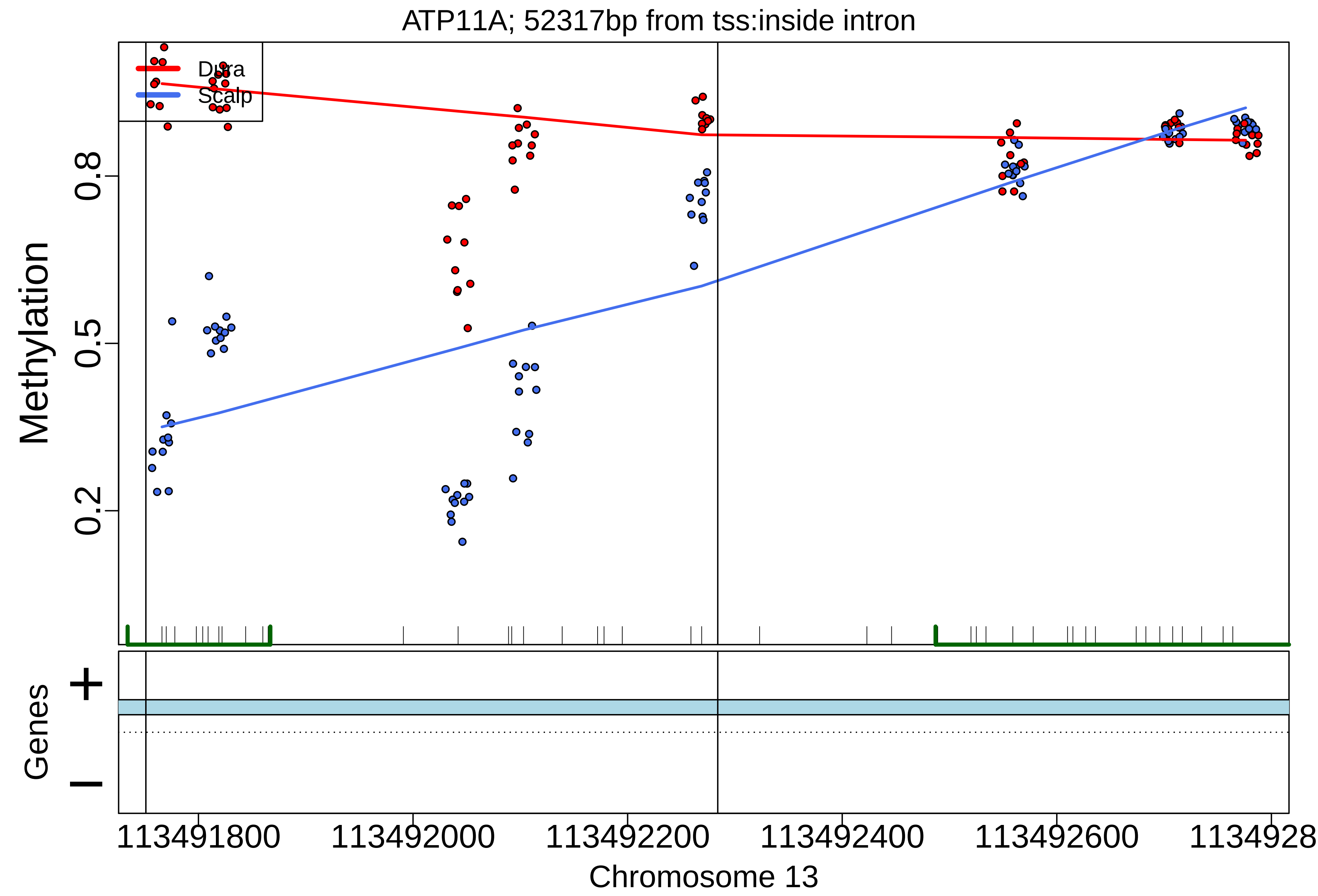

ATP11A; 52317bp from tss:inside intron
Dura
Scalp
Methylation
0.2
0.5
0.8
Genes
+
−
113491800
113492000
113492200
113492400
113492600
1134928
Chromosome 13

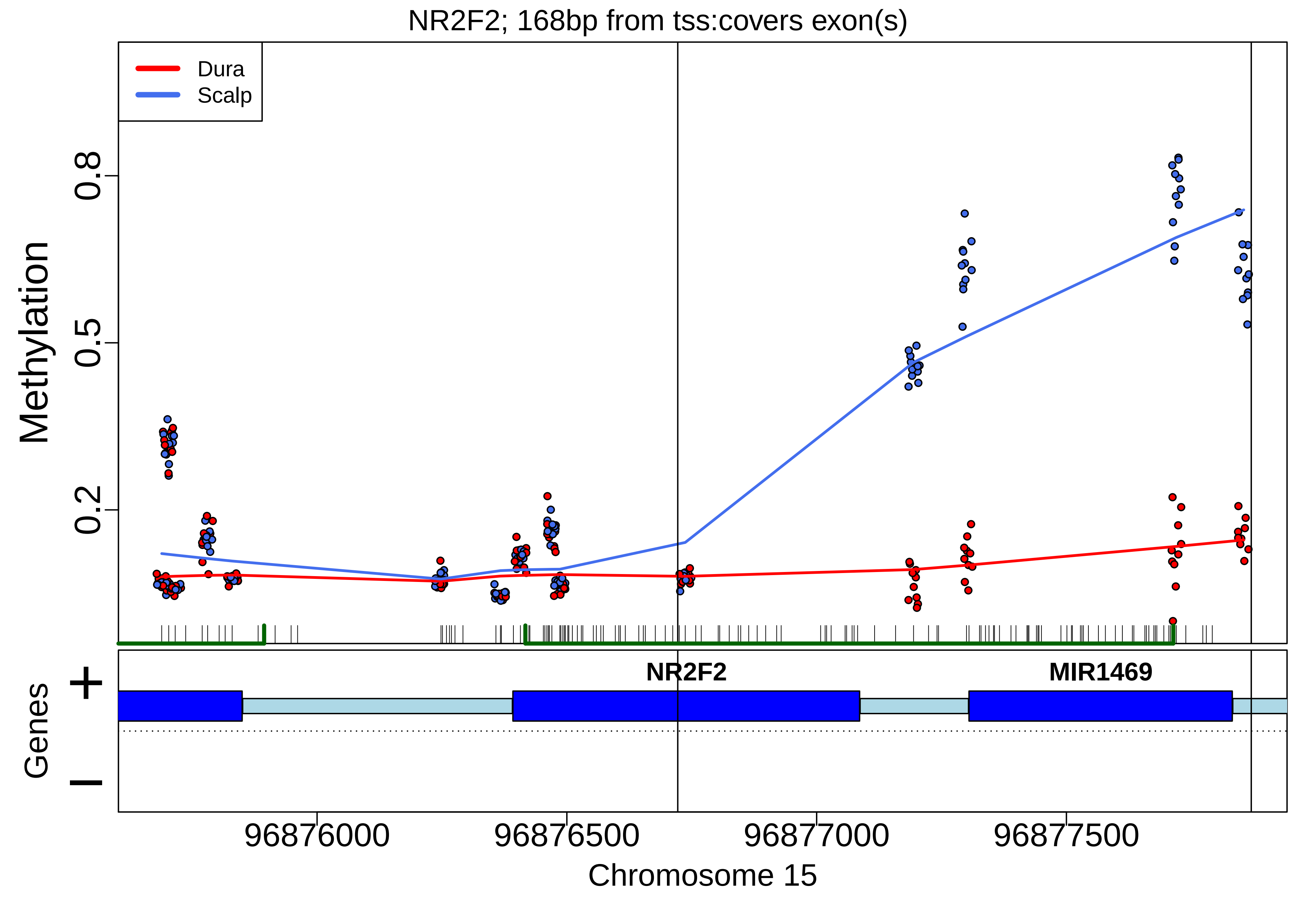
NR2F2; 168bp from tss:covers exon(s)
Dura
Scalp
Methylation
0.2
0.5
0.8
Genes
+
-
NR2F2
MIR1469
96876000
96876500
96877000
96877500
Chromosome 15

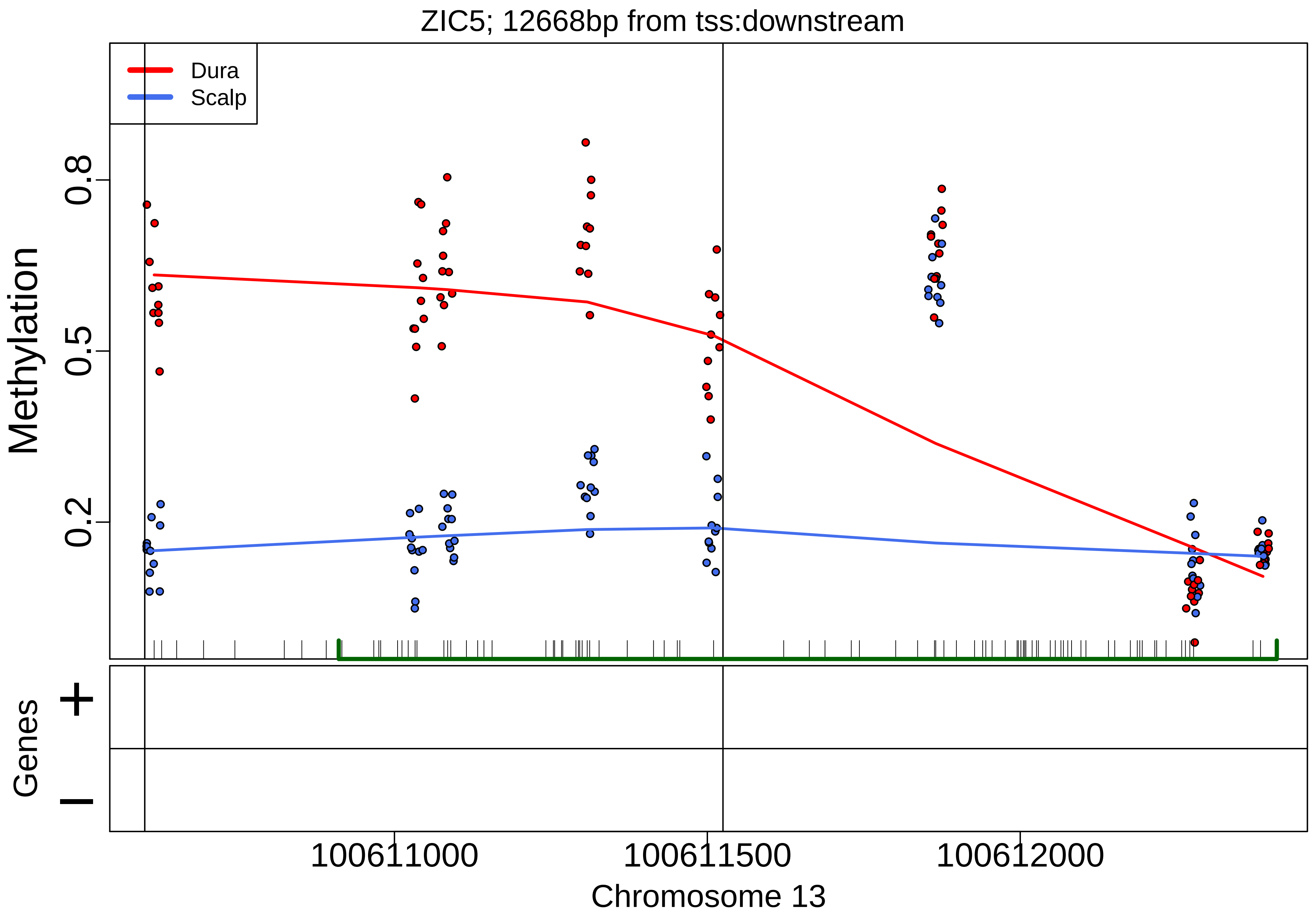

ZIC5; 12668bp from tss:downstream
Dura
Scalp
Methylation
0.2
0.5
0.8
Genes
+
–
100611000
100611500
100612000
Chromosome 13

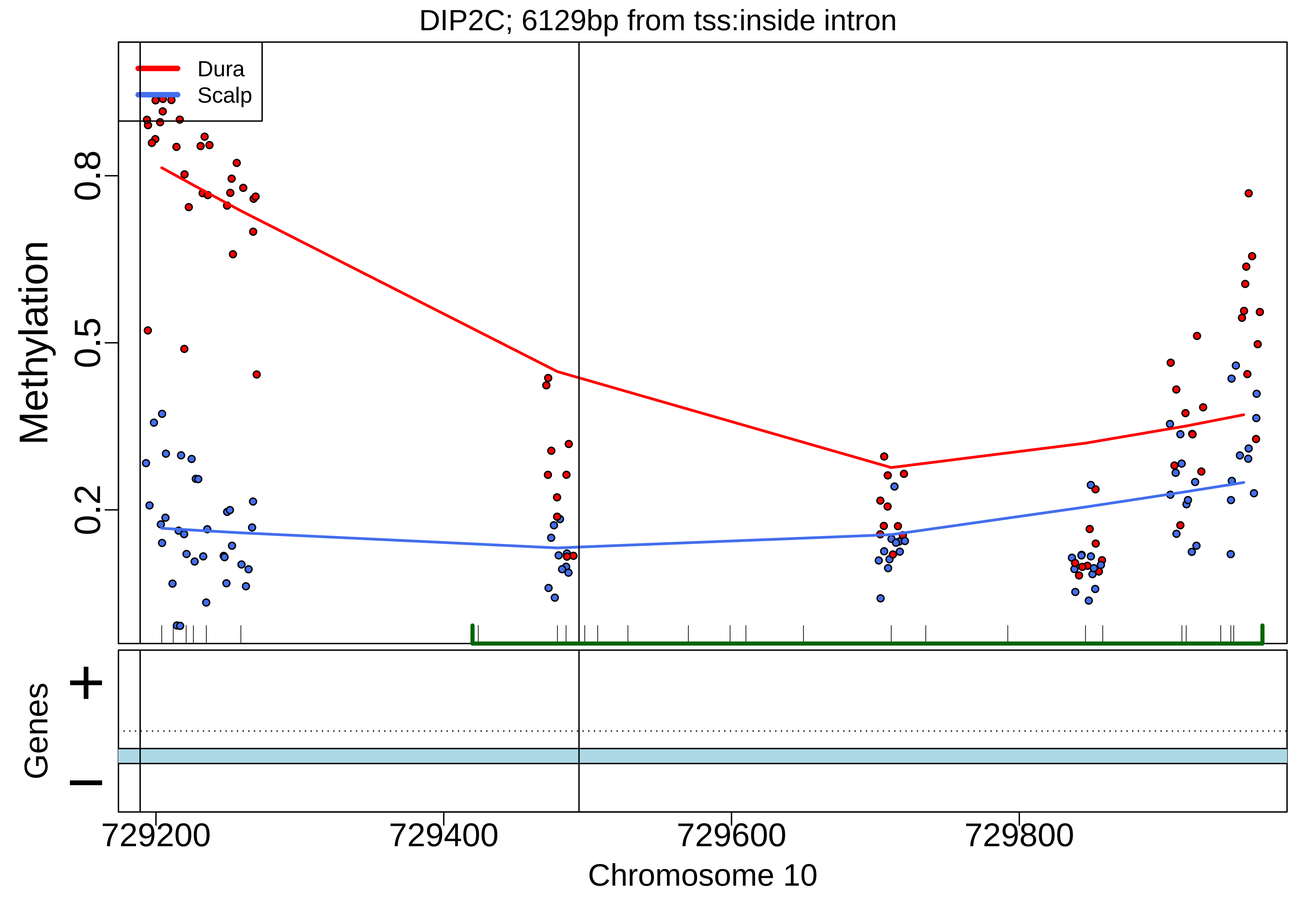
DIP2C; 6129bp from tss:inside intron
Dura
Scalp
Methylation
0.2
0.5
0.8
Genes
+
-
729200
729400
729600
729800
Chromosome 10

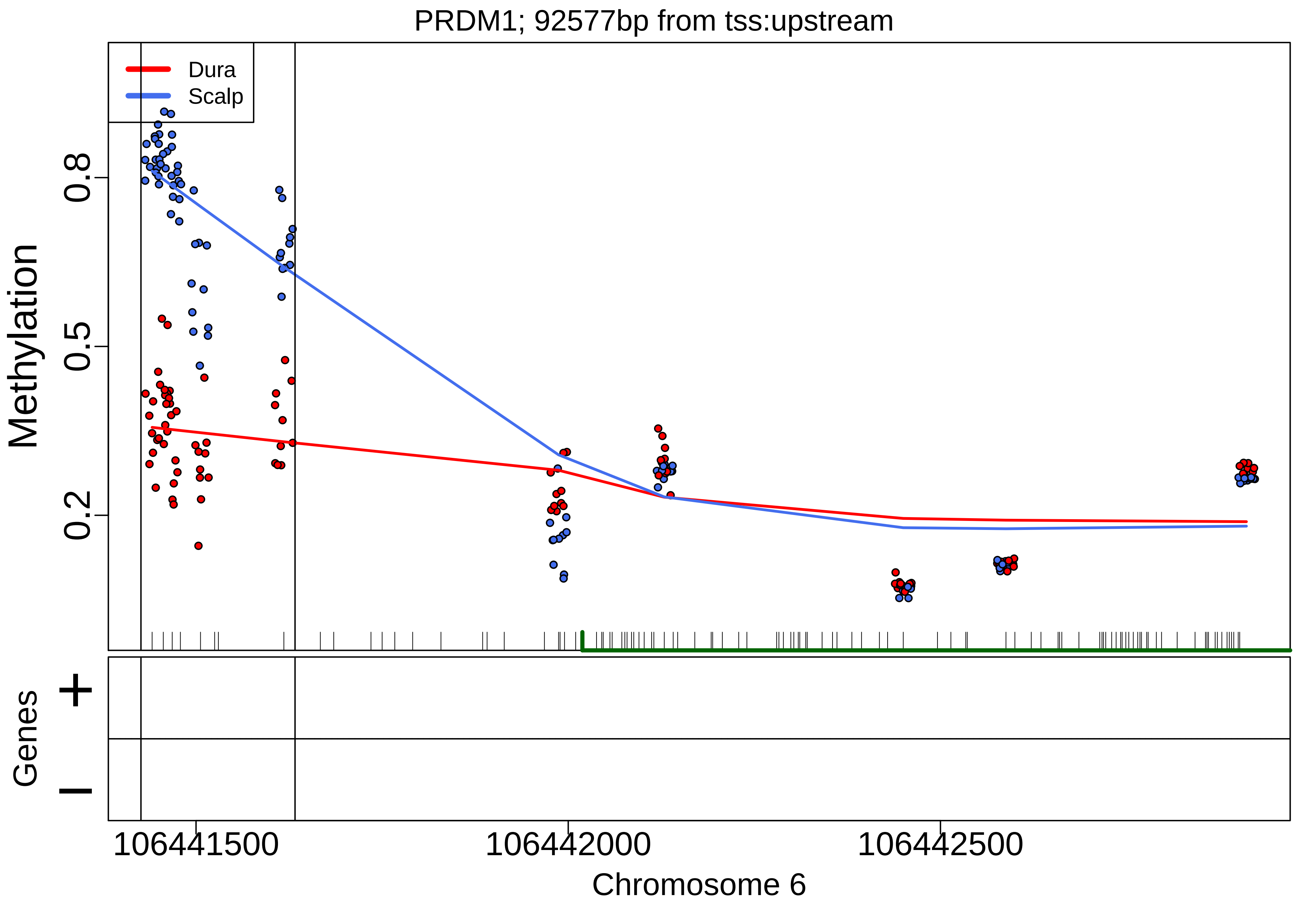

PRDM1; 92577bp from tss:upstream
Dura
Scalp
Methylation
0.2
0.5
0.8
Genes
+
−
106441500
106442000
106442500
Chromosome 6

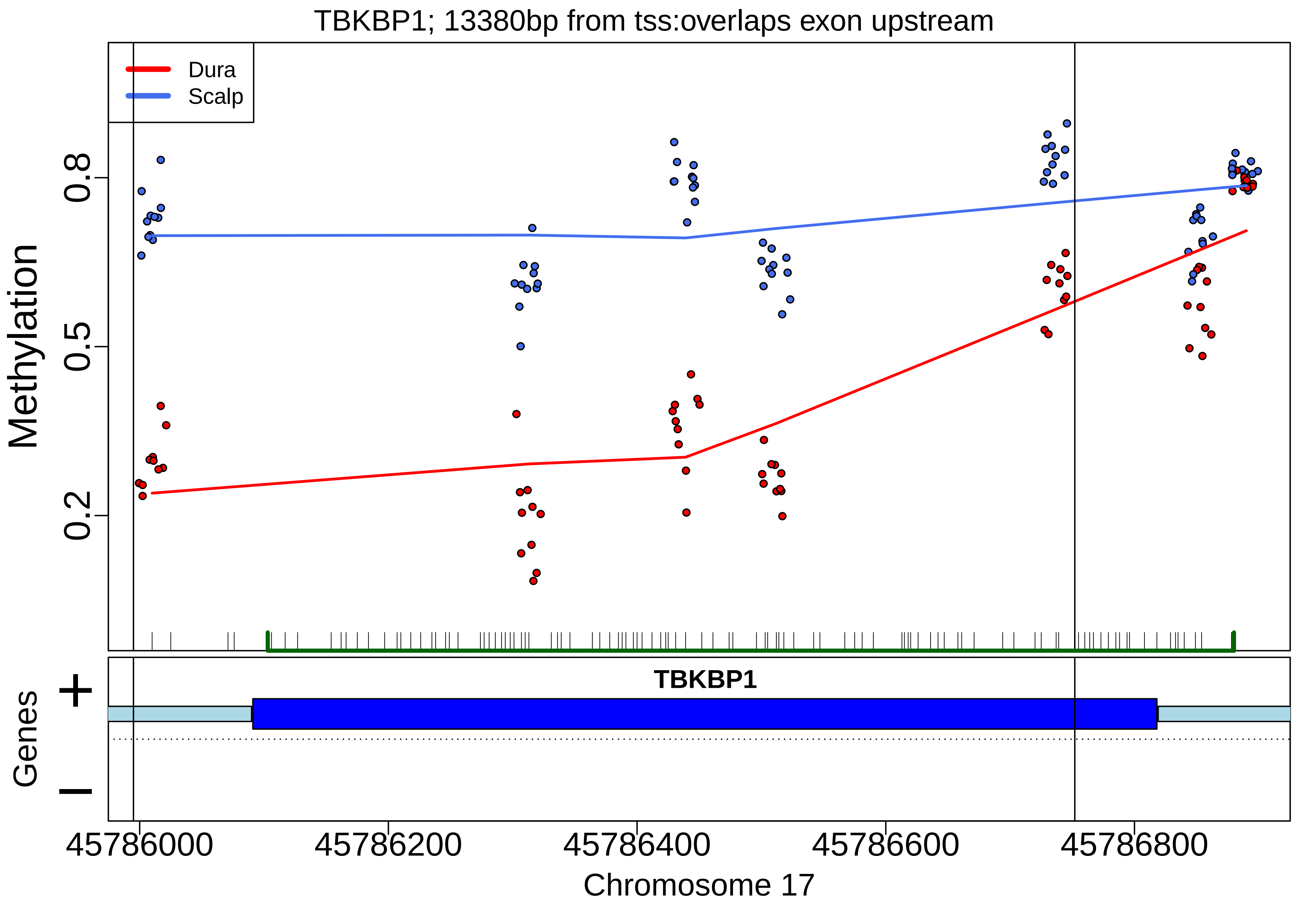

TBKBP1; 13380bp from tss:overlaps exon upstream
Dura
Scalp
Methylation
0.2
0.5
0.8
Genes
+
-
TBKBP1
45786000
45786200
45786400
45786600
45786800
Chromosome 17

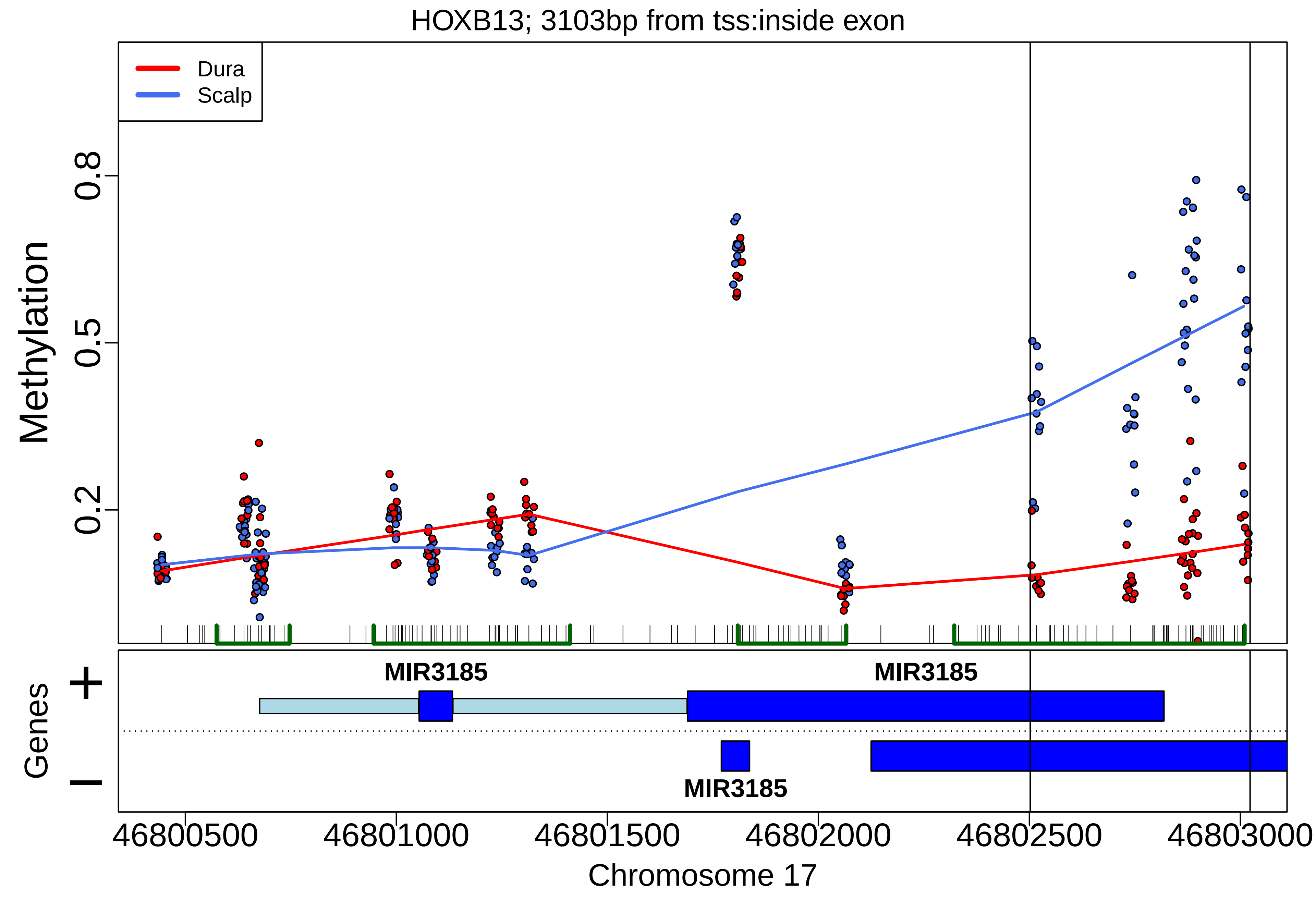

HOXB13; 3103bp from tss:inside exon
Dura
Scalp
Methylation
0.2
0.5
0.8
Genes
+
−
MIR3185
MIR3185
MIR3185
46800500
46801000
46801500
46802000
46802500
46803000
Chromosome 17

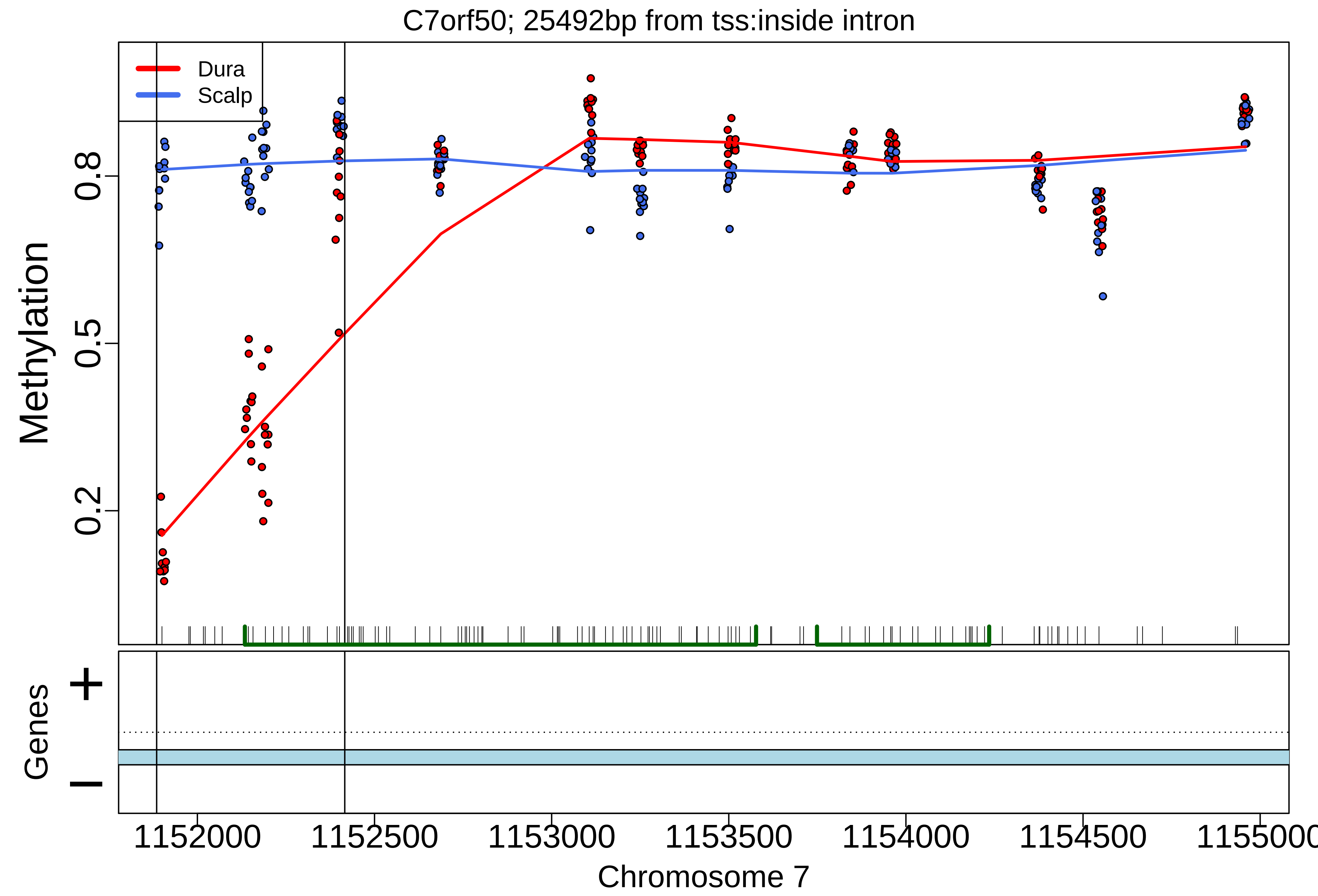
C7orf50; 25492bp from tss:inside intron
Dura
Scalp
Methylation
0.2
0.5
0.8
Genes
+
-
1152000
1152500
1153000
1153500
1154000
1154500
1155000
Chromosome 7

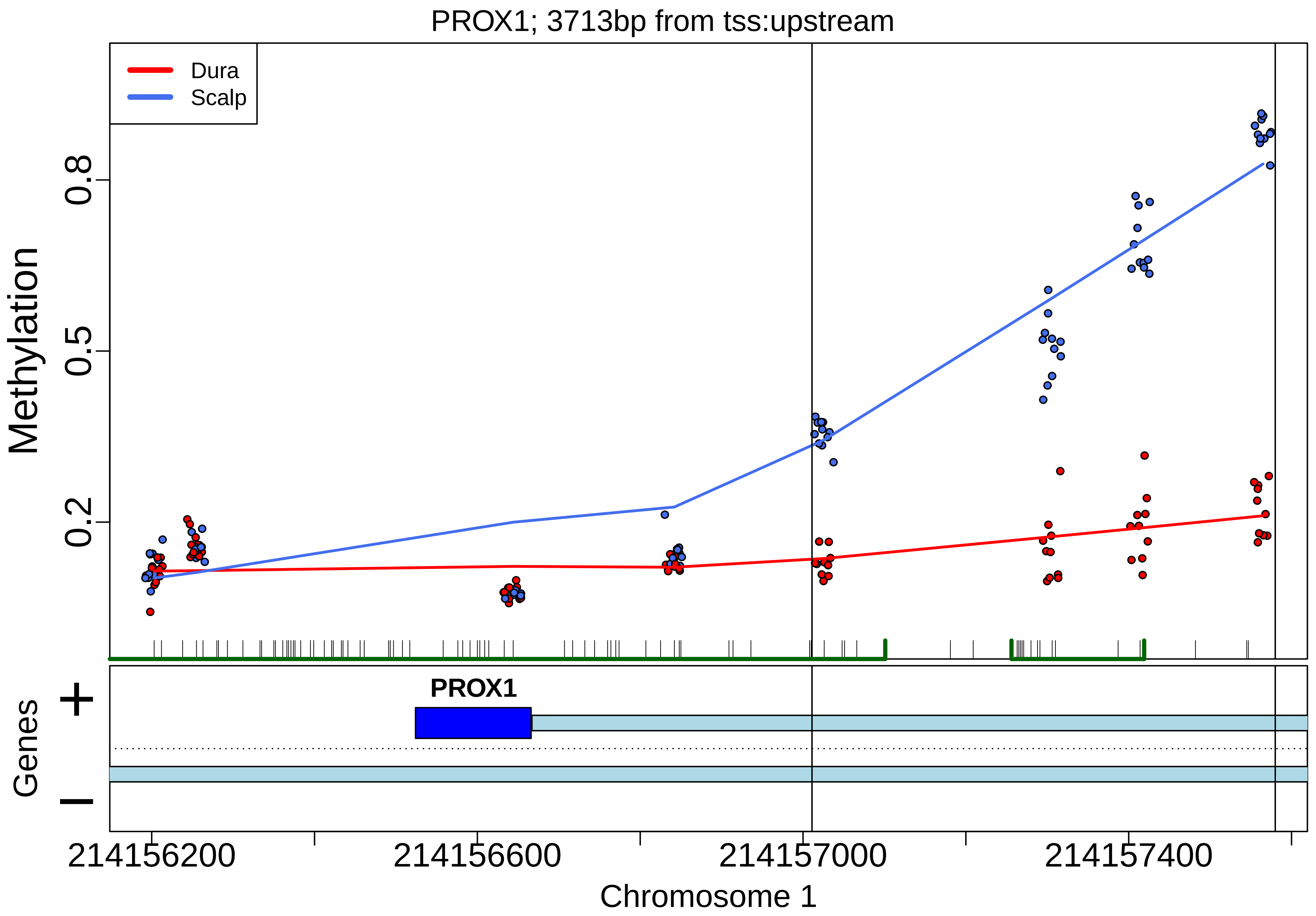

PROX1; 3713bp from tss:upstream
Dura
Scalp
Methylation
0.2
0.5
0.8
Genes
+
−
PROX1
214156200
214156600
214157000
214157400
Chromosome 1

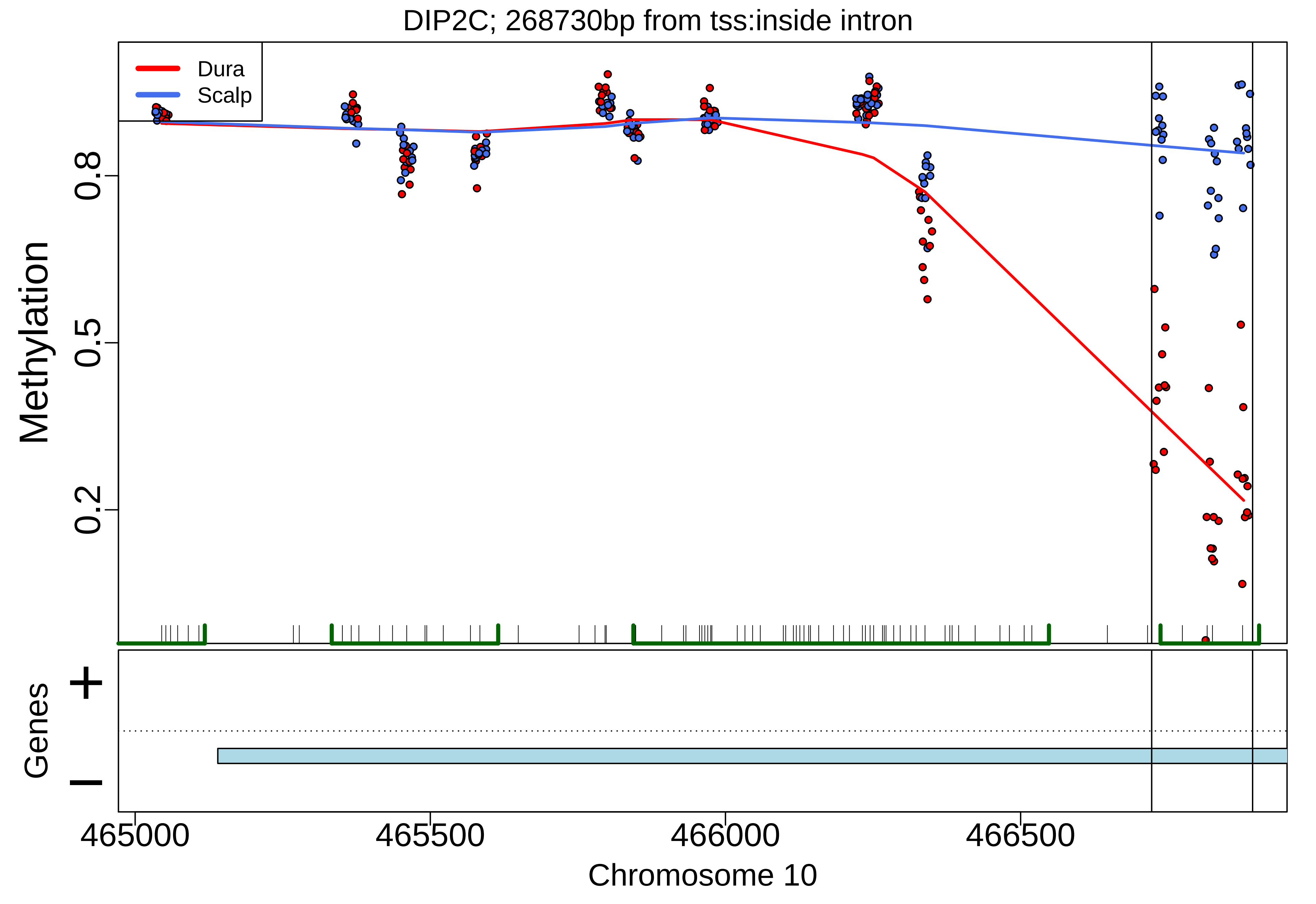
DIP2C; 268730bp from tss:inside intron
Dura
Scalp
Methylation
0.2
0.5
0.8
Genes
+
-
465000
465500
466000
466500
Chromosome 10

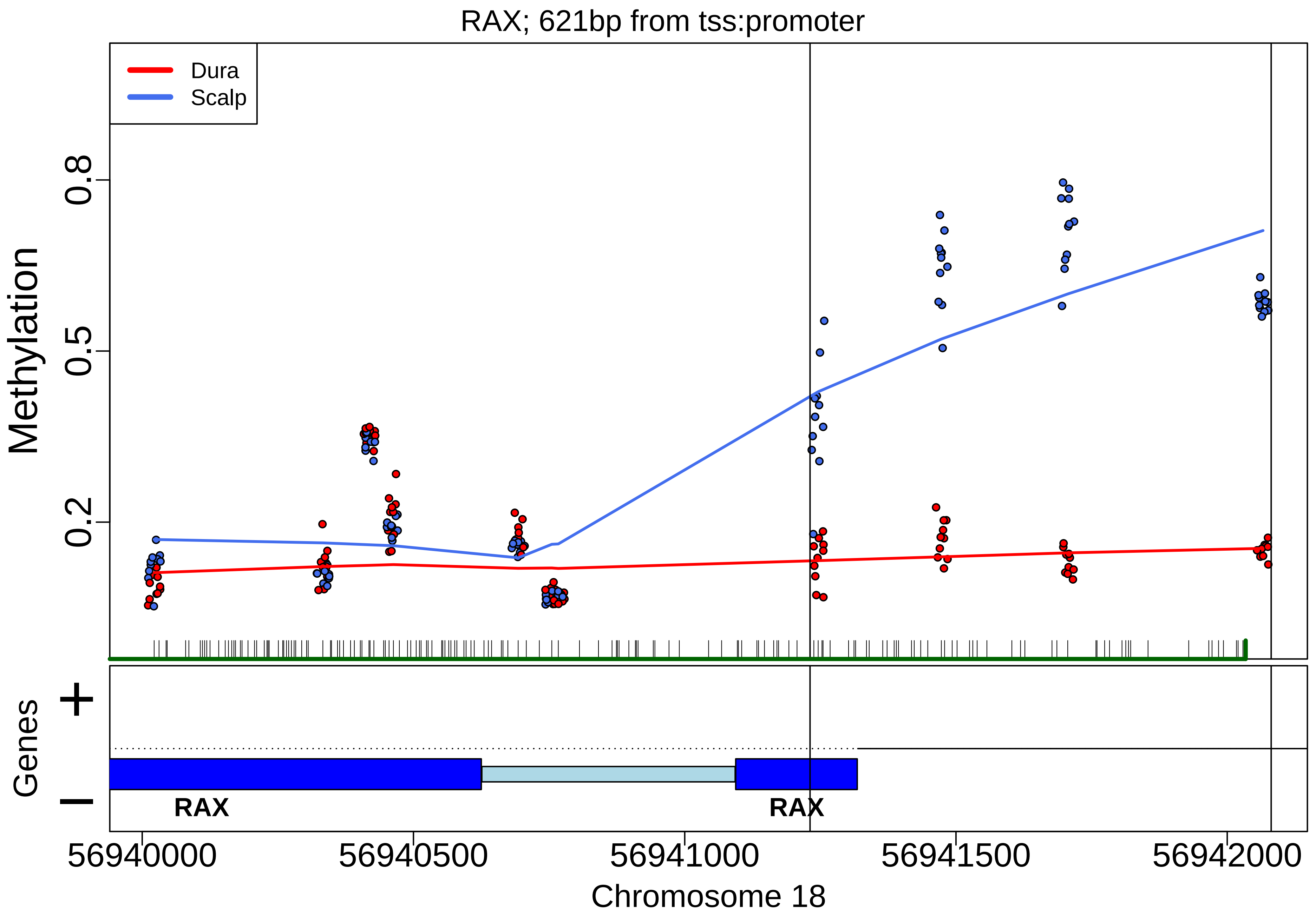

RAX; 621bp from tss:promoter
Dura
Scalp
Methylation
0.2
0.5
0.8
Genes
+
−
RAX
RAX
56940000
56940500
56941000
56941500
56942000
Chromosome 18

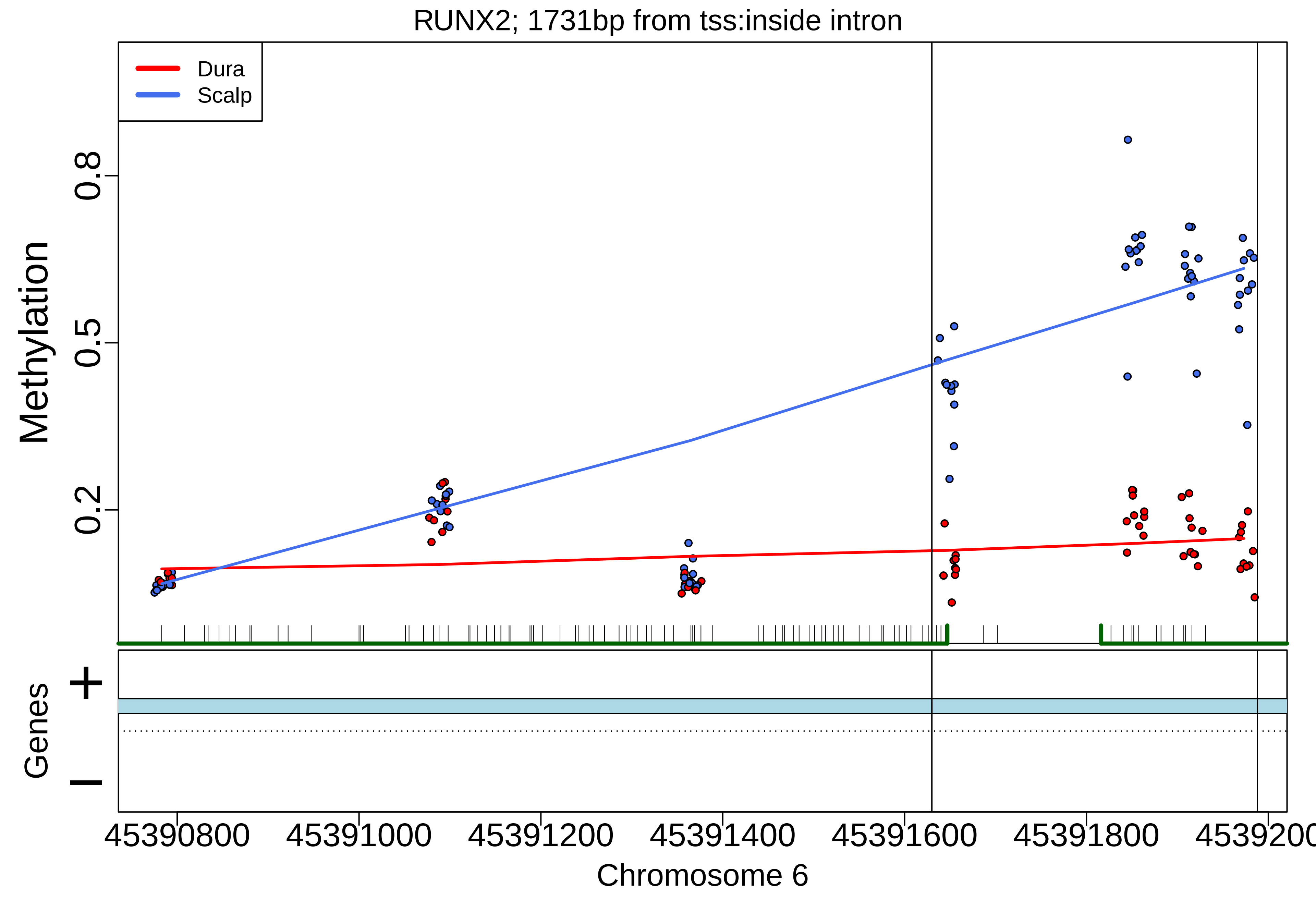

RUNX2; 1731bp from tss:inside intron
Dura
Scalp
Methylation
0.2
0.5
0.8
Genes
+
−
45390800
45391000
45391200
45391400
45391600
45391800
4539200
Chromosome 6

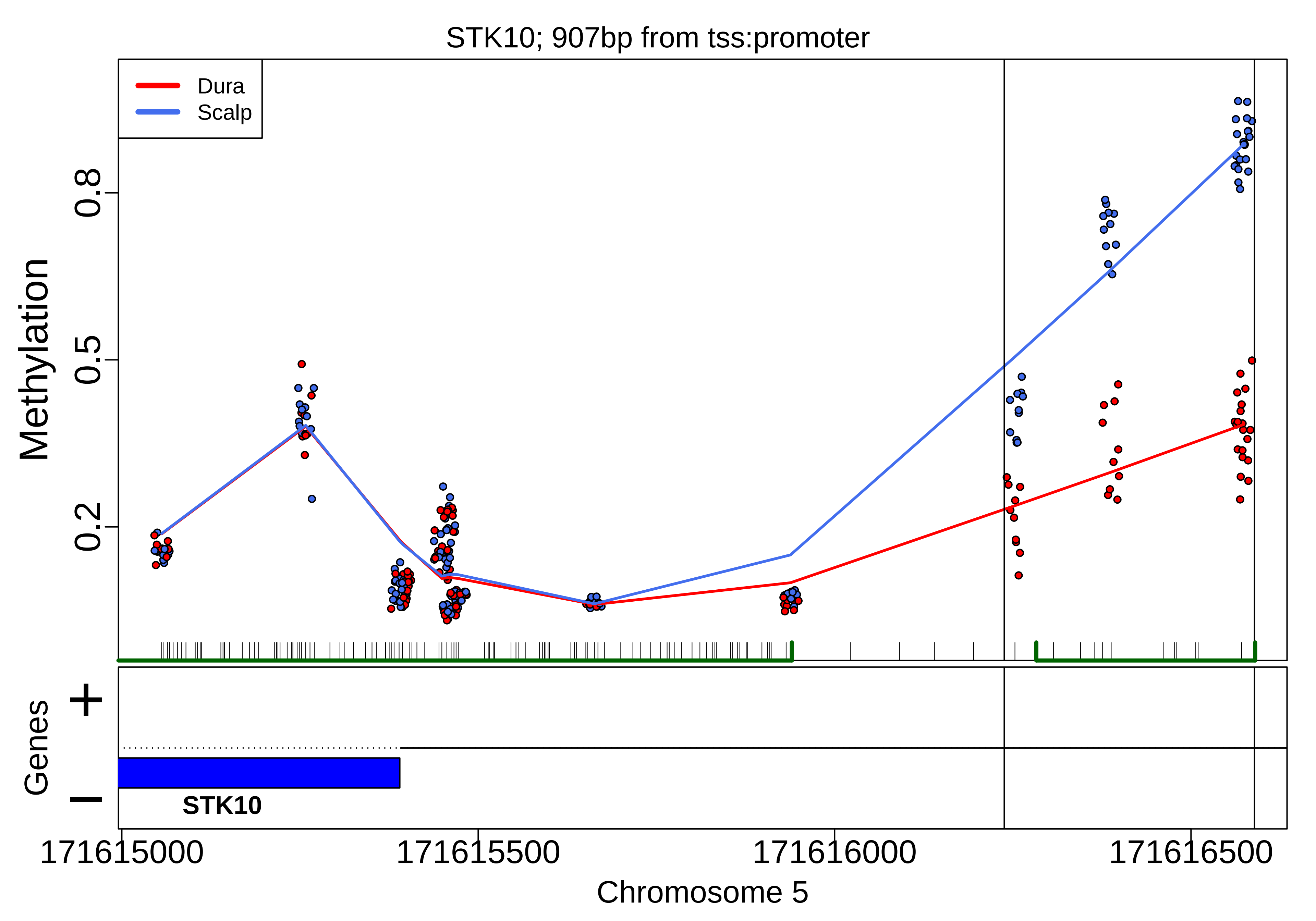
STK10; 907bp from tss:promoter
Dura
Scalp
Methylation
0.2
0.5
0.8
Genes
+
−
STK10
171615000
171615500
171616000
171616500
Chromosome 5

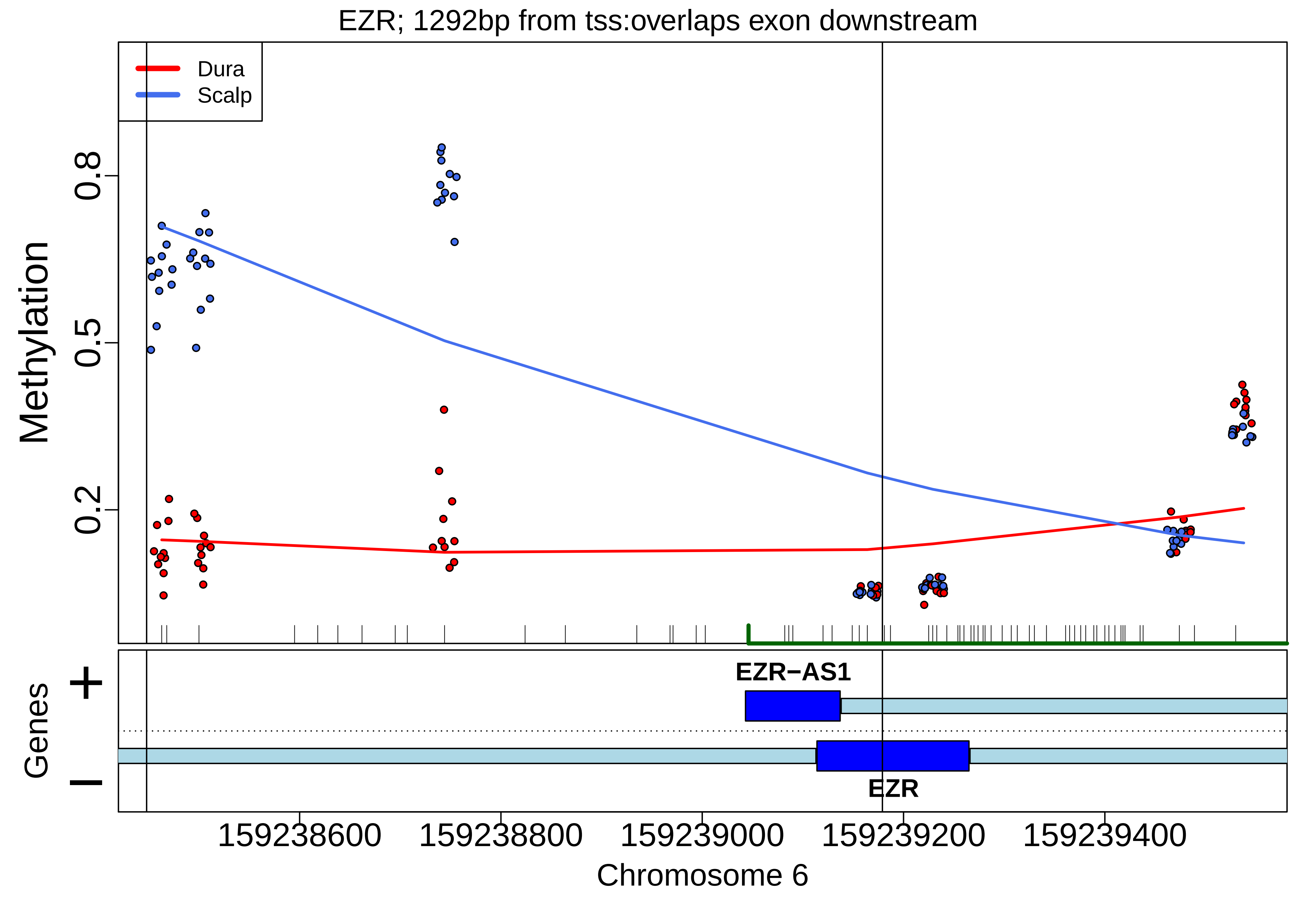
EZR; 1292bp from tss:overlaps exon downstream
Dura
Scalp
Methylation
0.2
0.5
0.8
Genes
+
−
EZR−AS1
EZR
159238600
159238800
159239000
159239200
159239400
Chromosome 6

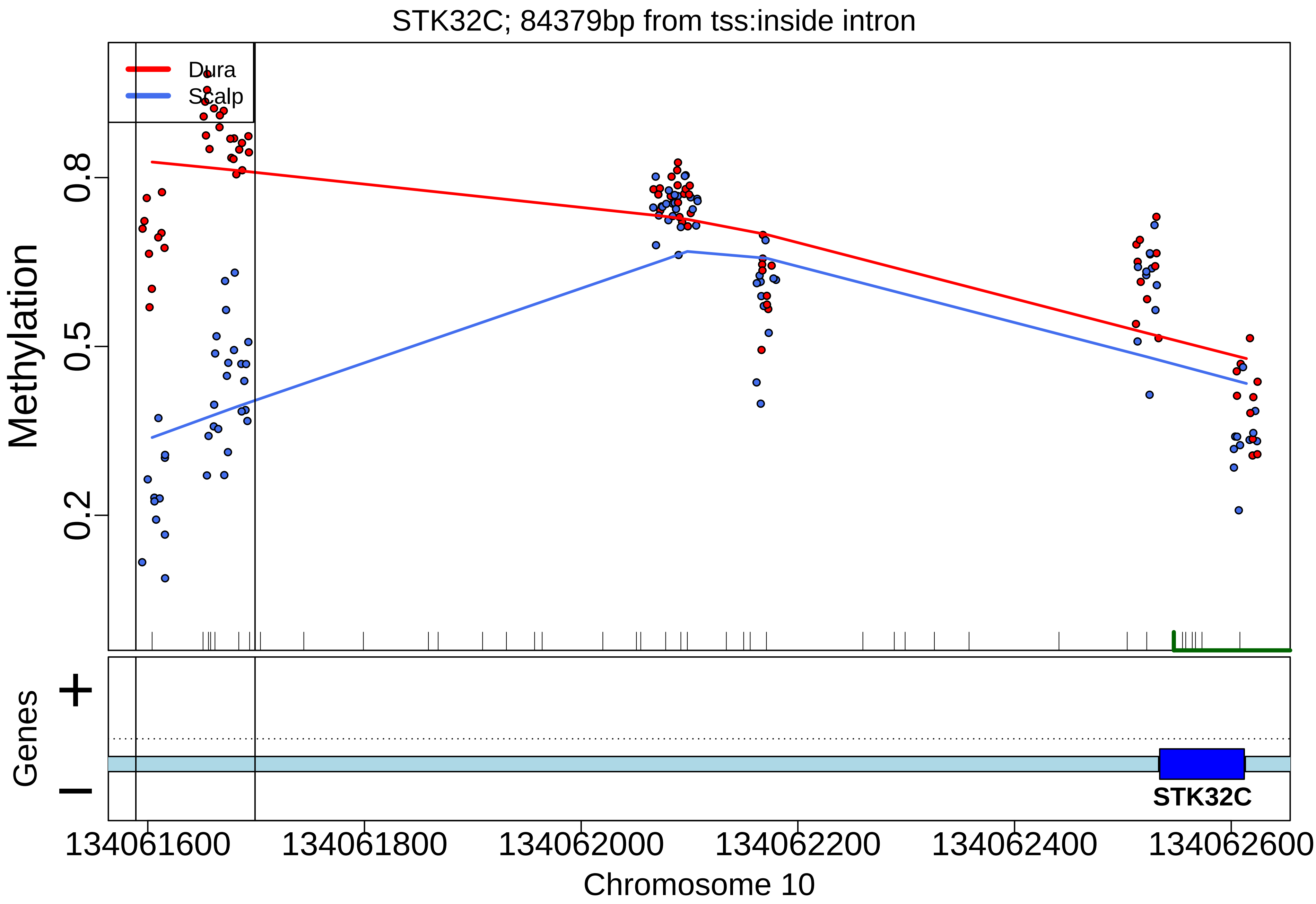

STK32C; 84379bp from tss:inside intron
Dura
Scalp
Methylation
0.2
0.5
0.8
Genes
+
−
STK32C
134061600
134061800
134062000
134062200
134062400
134062600
Chromosome 10

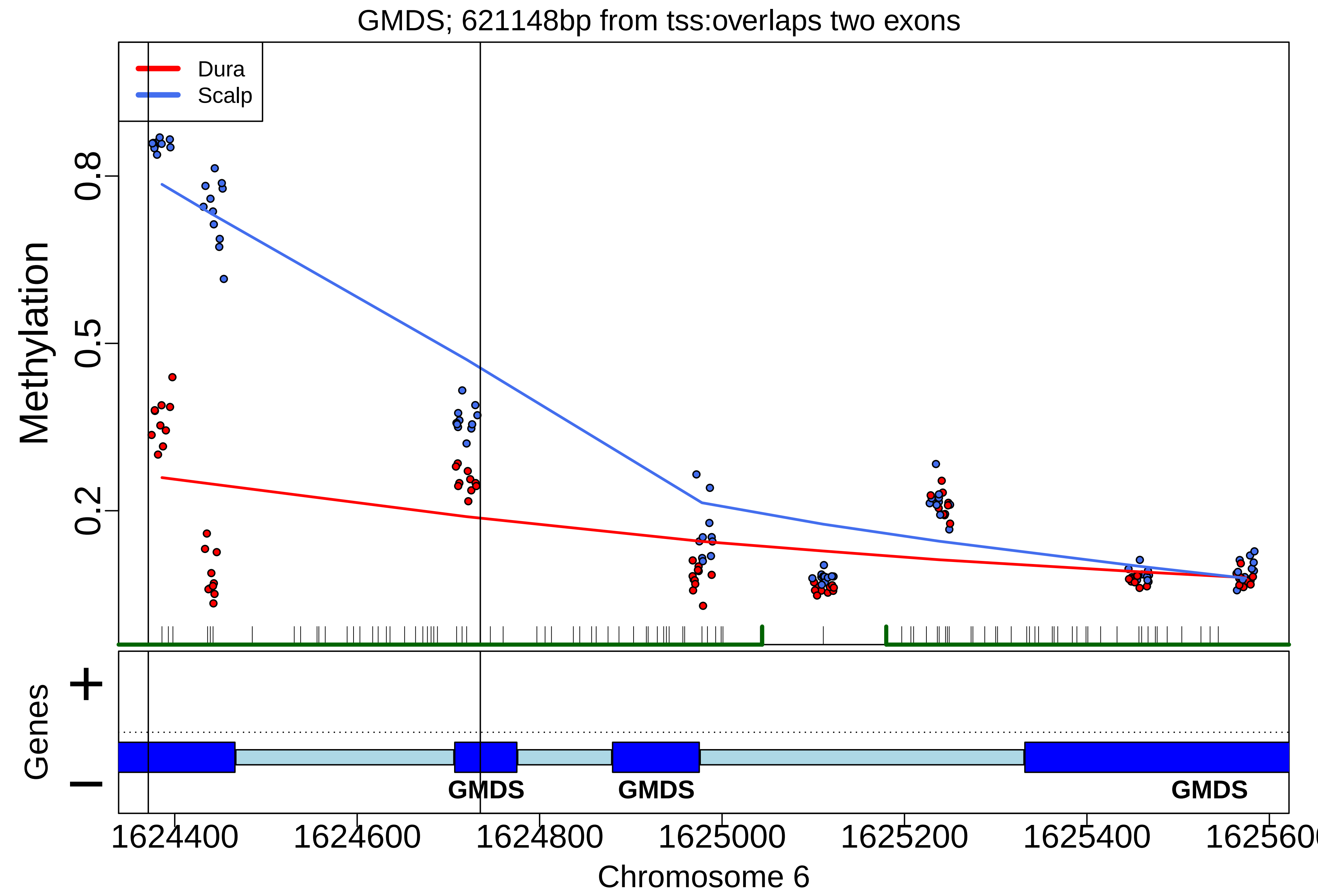
GMDS; 621148bp from tss:overlaps two exons
Dura
Scalp
Methylation
0.8
0.5
0.2
Genes
+
−
GMDS
GMDS
GMDS
1624400
1624600
1624800
1625000
1625200
1625400
162560
Chromosome 6

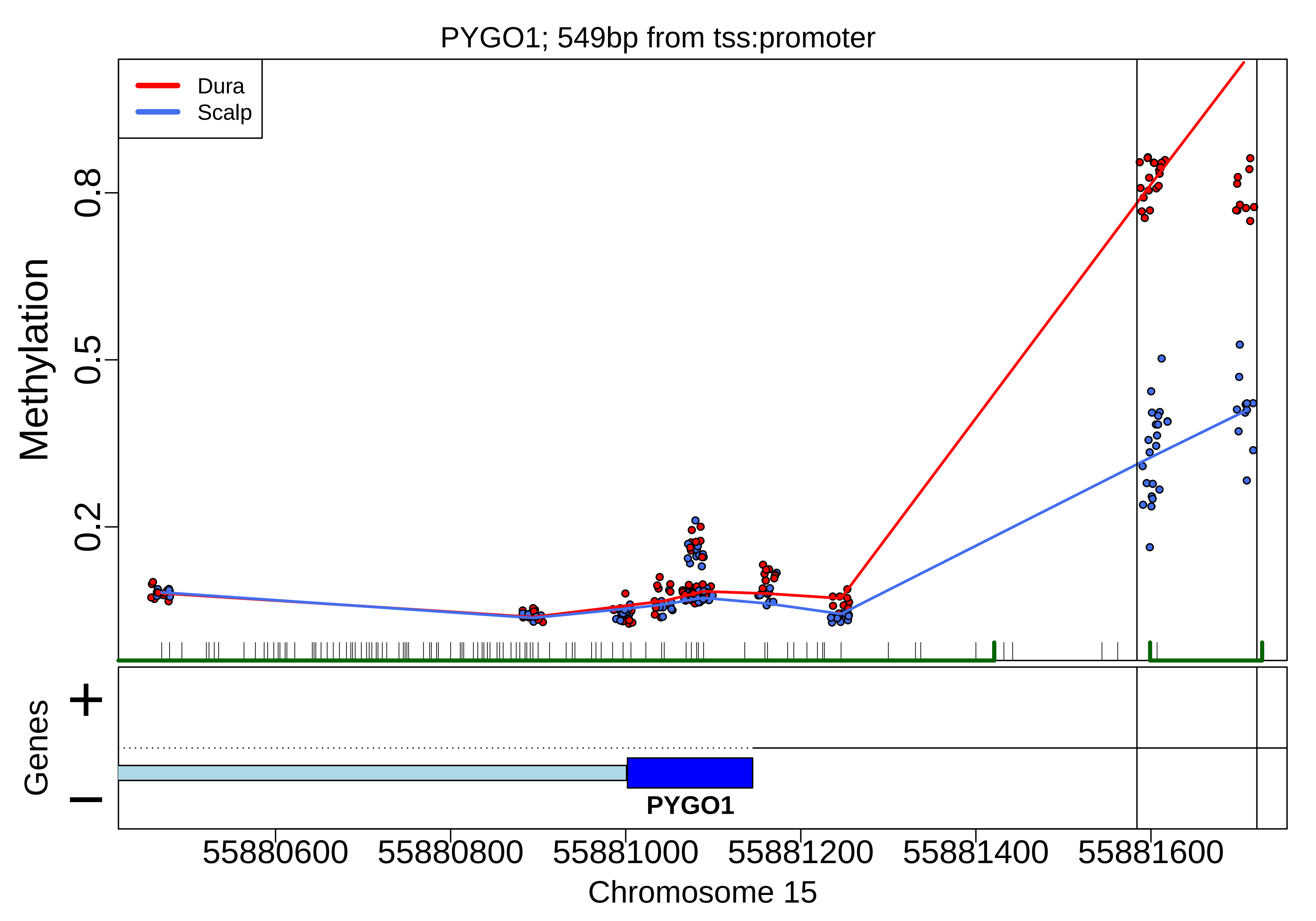

PYGO1; 549bp from tss:promoter
Dura
Scalp
Methylation
0.2
0.5
0.8
Genes
+
-
PYGO1
55880600
55880800
55881000
55881200
55881400
55881600
Chromosome 15

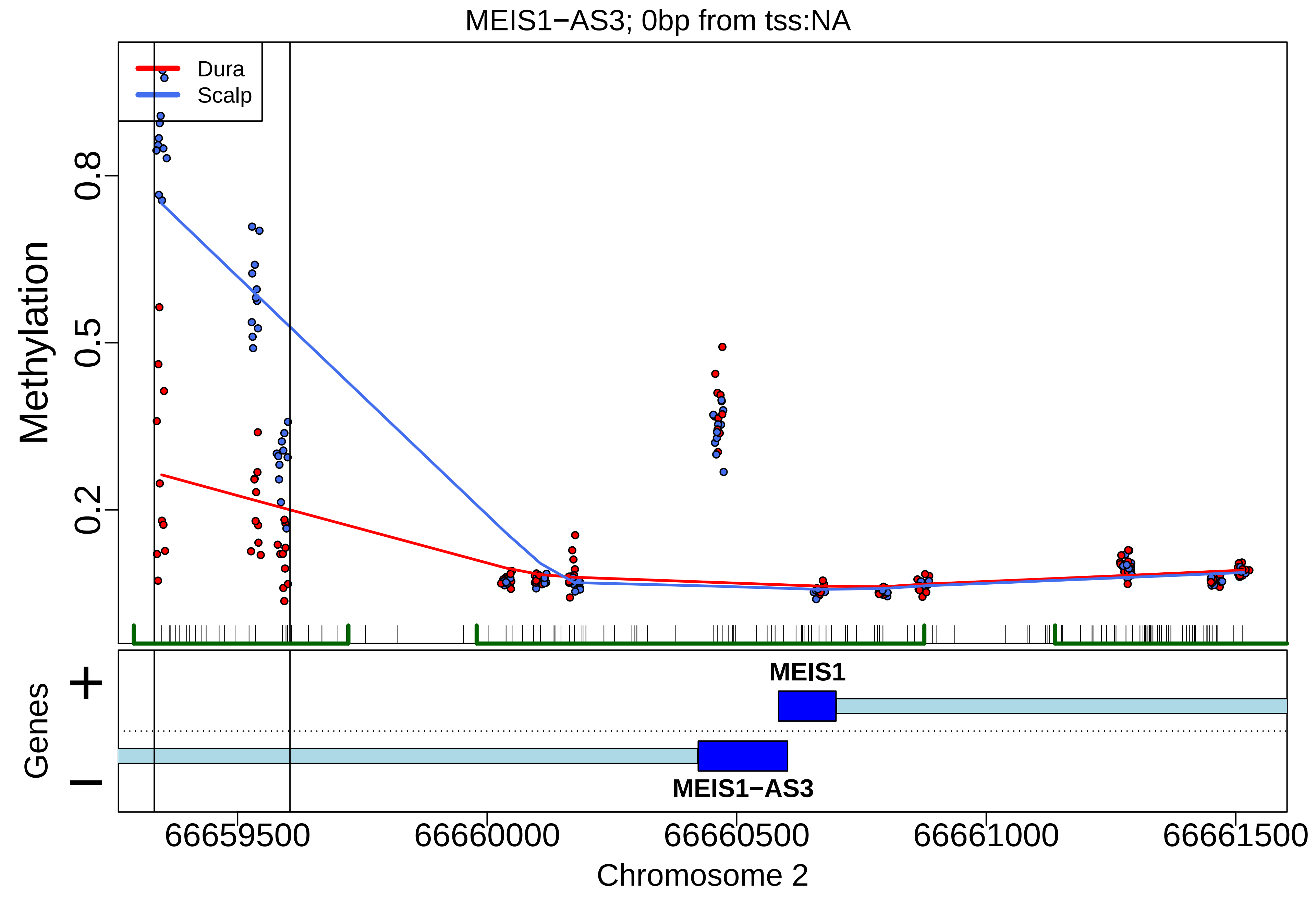
MEIS1−AS3; 0bp from tss:NA
Dura
Scalp
Methylation
0.8
0.5
0.2
Genes
+
−
MEIS1
MEIS1−AS3
66659500
66660000
66660500
66661000
66661500
Chromosome 2

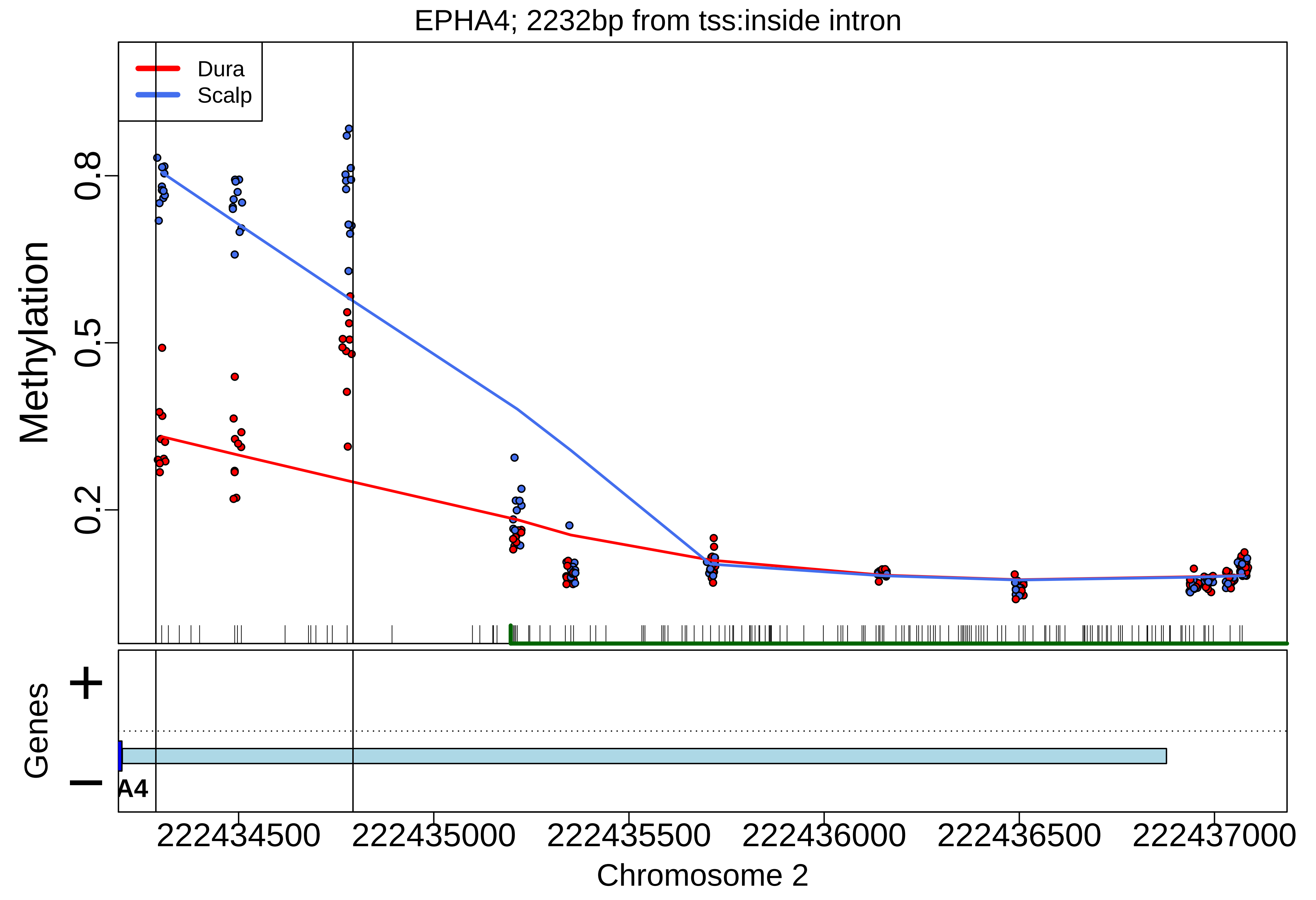
EPHA4; 2232bp from tss:inside intron
Dura
Scalp
Methylation
0.2
0.5
0.8
Genes
+
−
A4
222434500
222435000
222435500
222436000
222436500
222437000
Chromosome 2

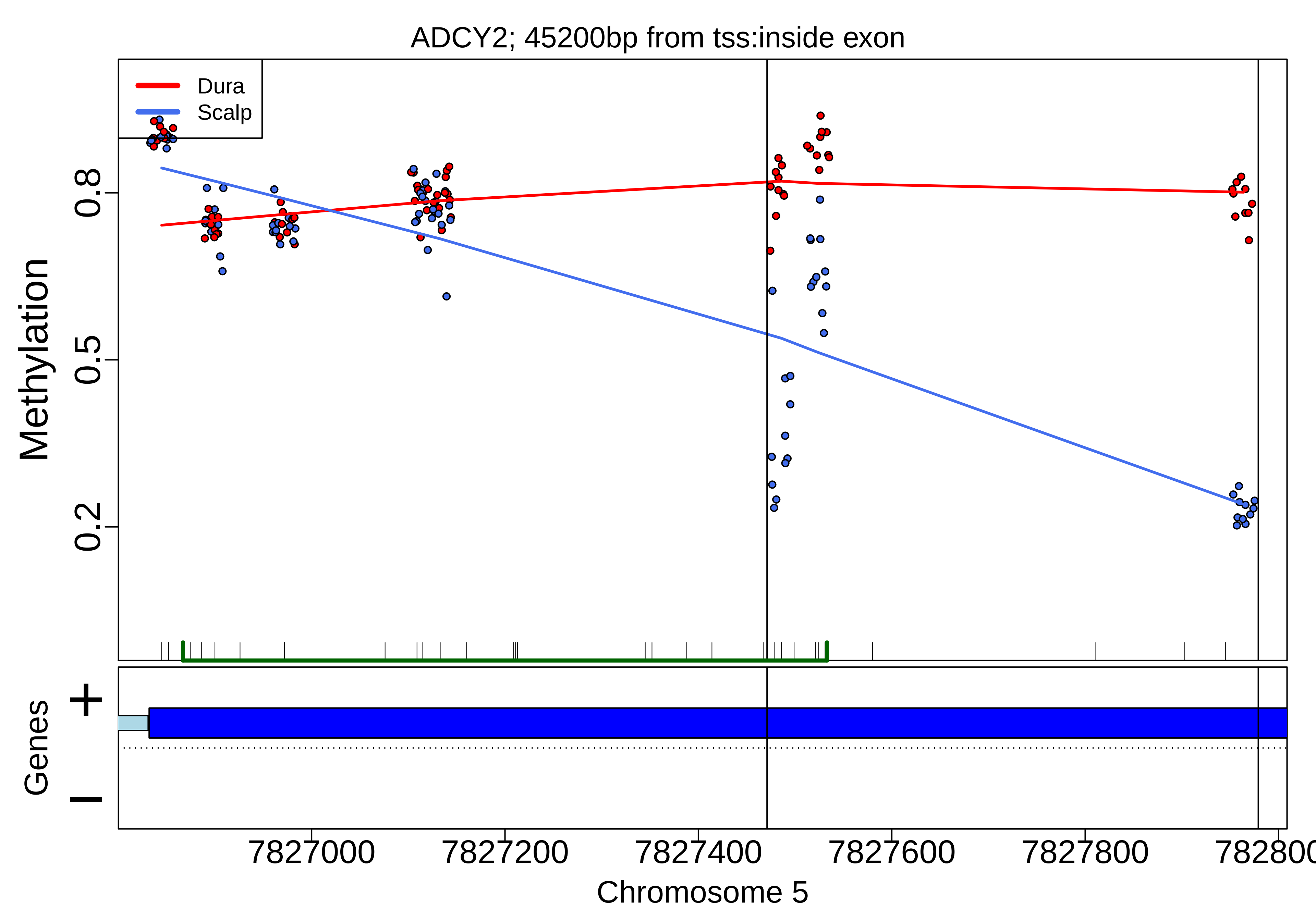

ADCY2; 45200bp from tss:inside exon
Dura
Scalp
Methylation
0.2
0.5
0.8
Genes
+
-
7827000
7827200
7827400
7827600
7827800
782800
Chromosome 5

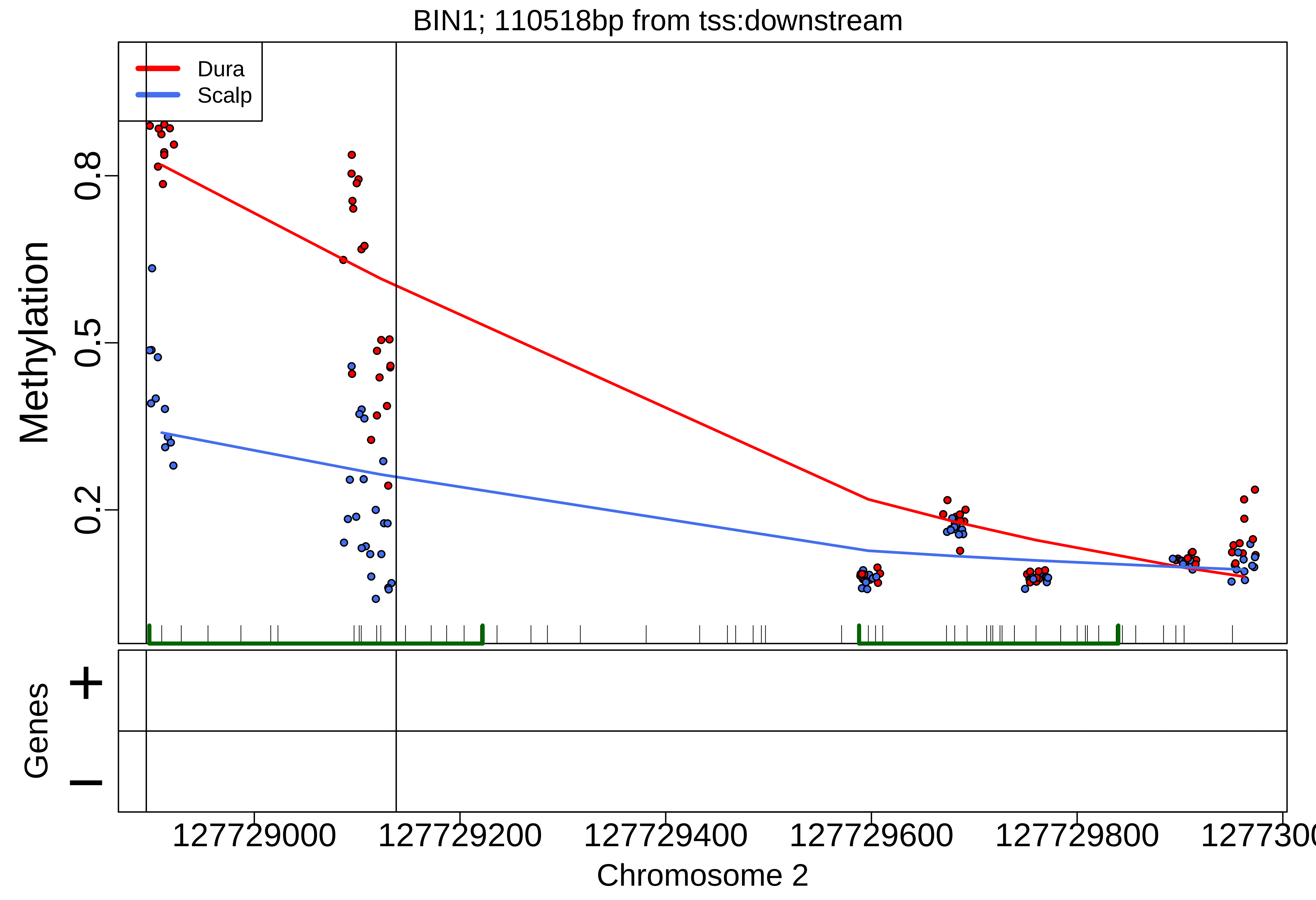
BIN1; 110518bp from tss:downstream
Dura
Scalp
Methylation
0.2
0.5
0.8
Genes
+
−
127729000
127729200
127729400
127729600
127729800
Chromosome 2

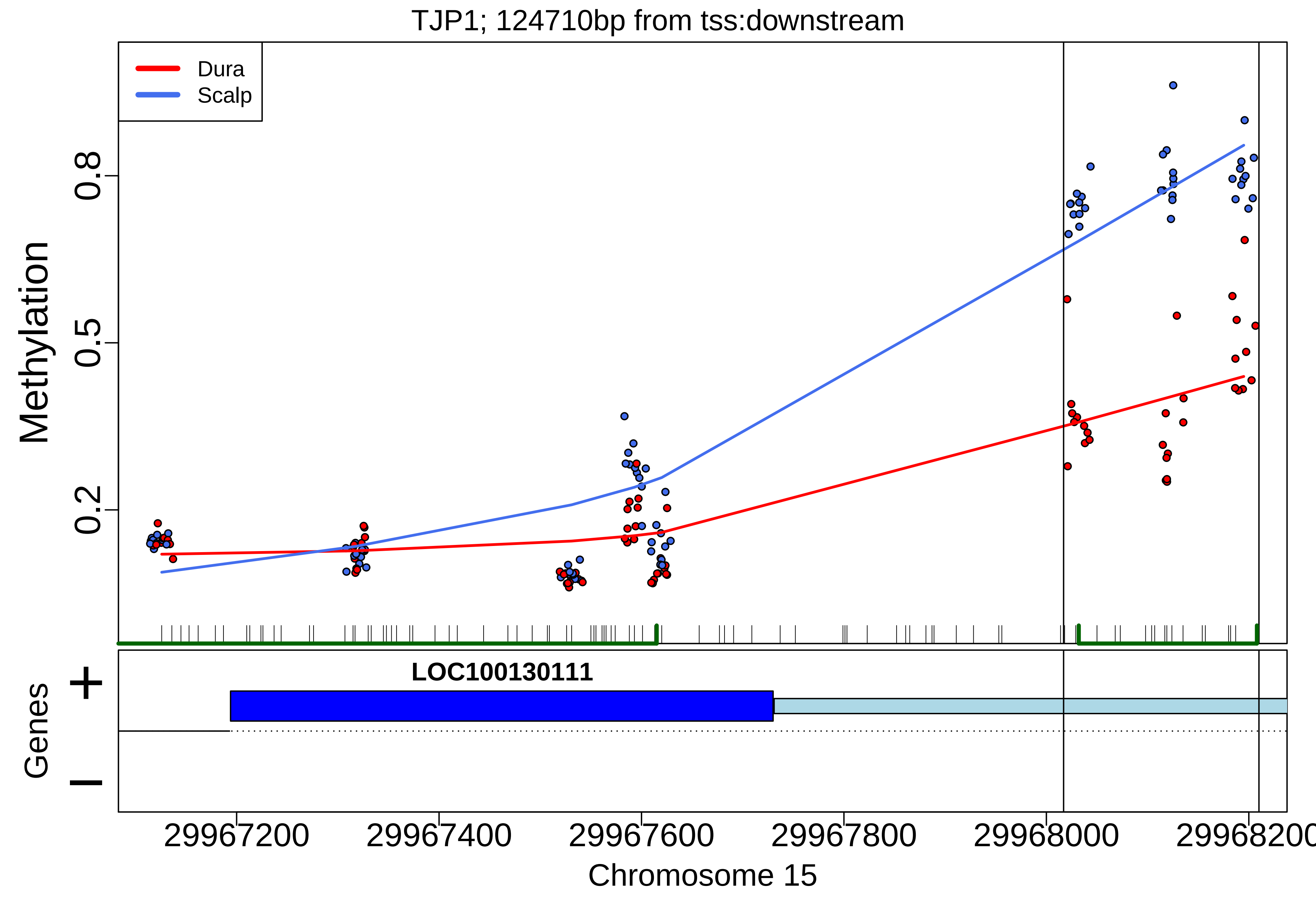

TJP1; 124710bp from tss:downstream
Dura
Scalp
Methylation
0.2
0.5
0.8
Genes
+
-
LOC100130111
29967200
29967400
29967600
29967800
29968000
29968200
Chromosome 15

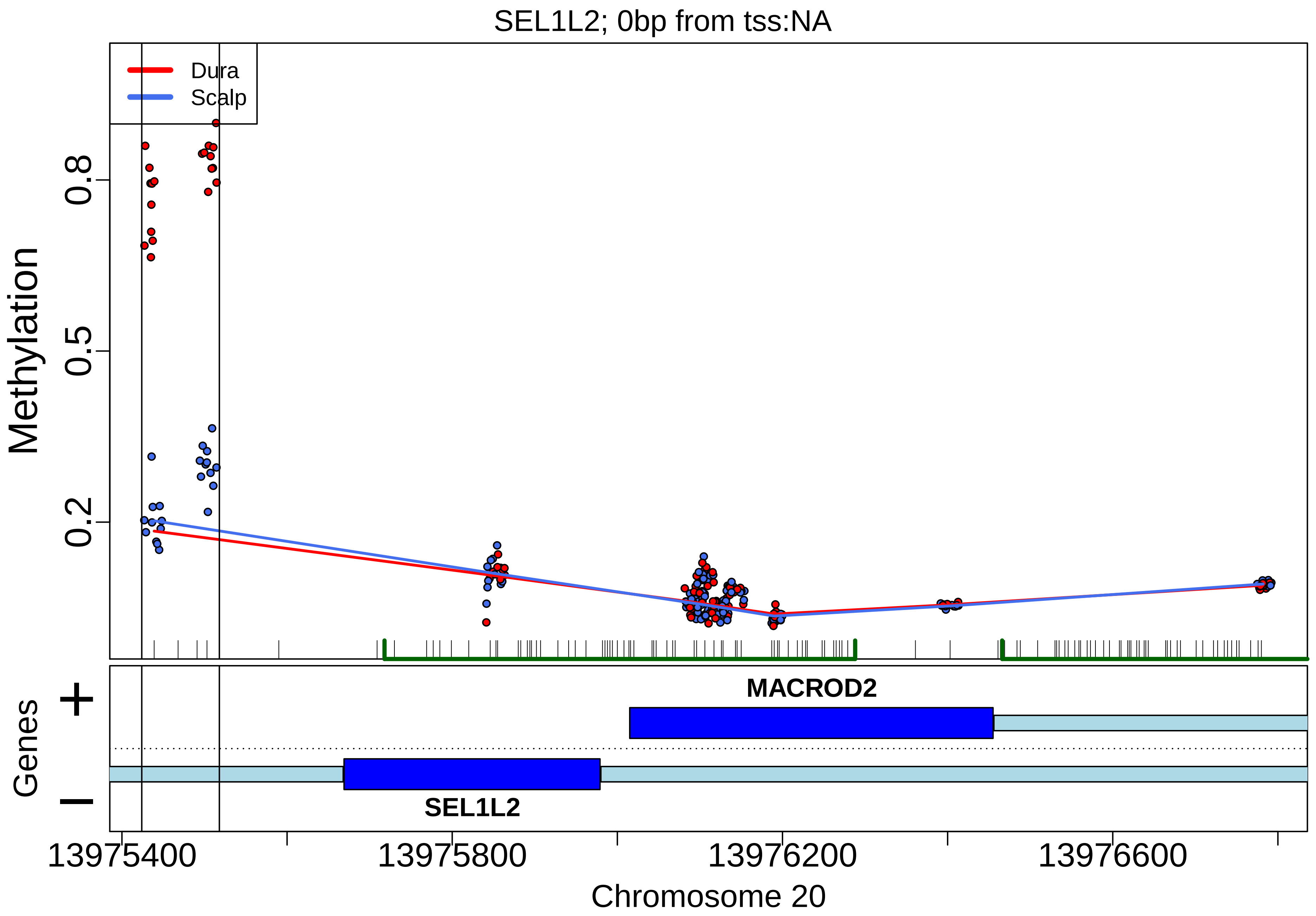

SEL1L2; 0bp from tss:NA
Dura
Scalp
Methylation
0.2
0.5
0.8
Genes
+
−
MACROD2
SEL1L2
13975400
13975800
13976200
13976600
Chromosome 20

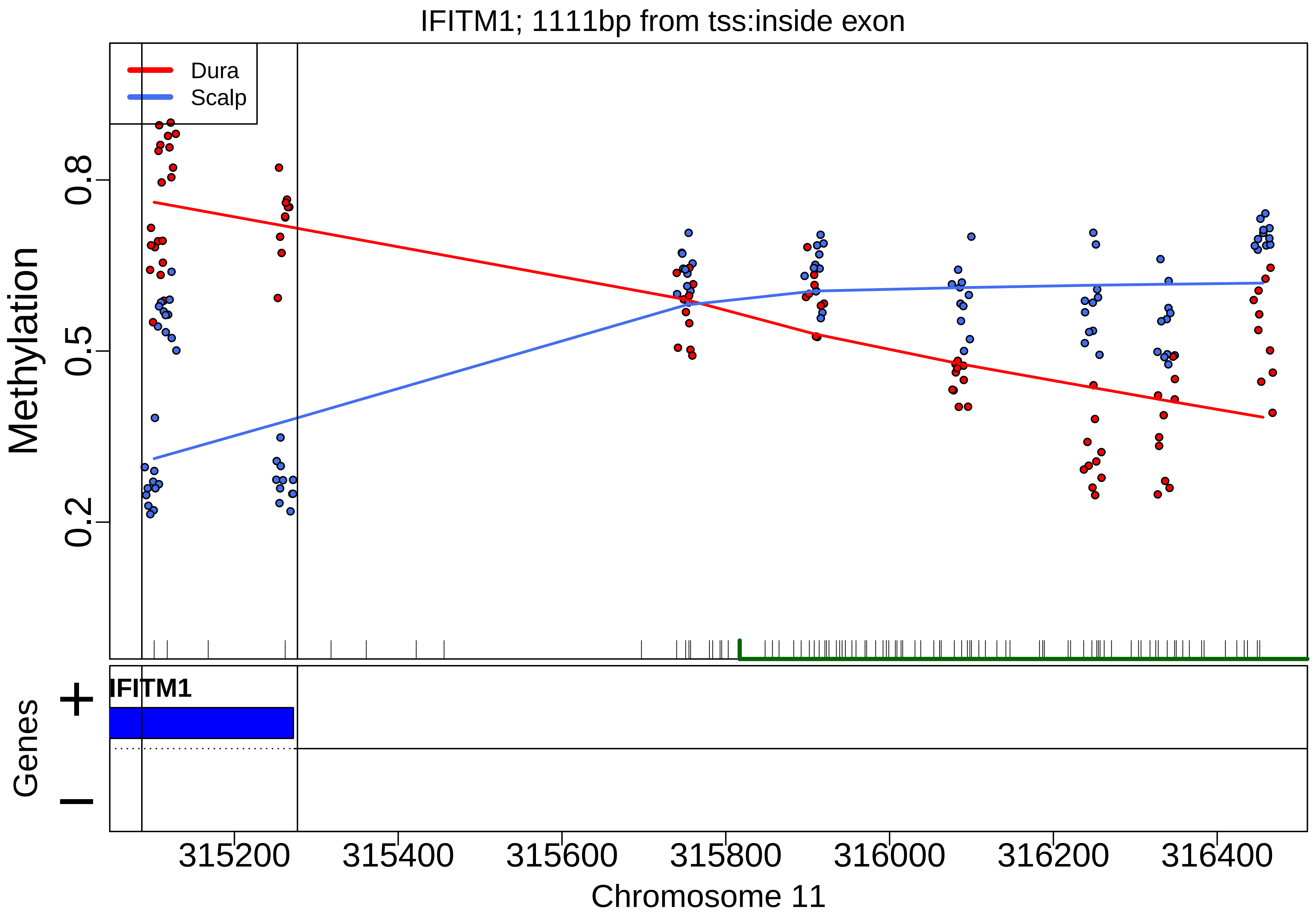

IFITM1; 1111bp from tss:inside exon
Dura
Scalp
Methylation
0.2
0.5
0.8
Genes
+
-
IFITM1
315200
315400
315600
315800
316000
316200
316400
Chromosome 11

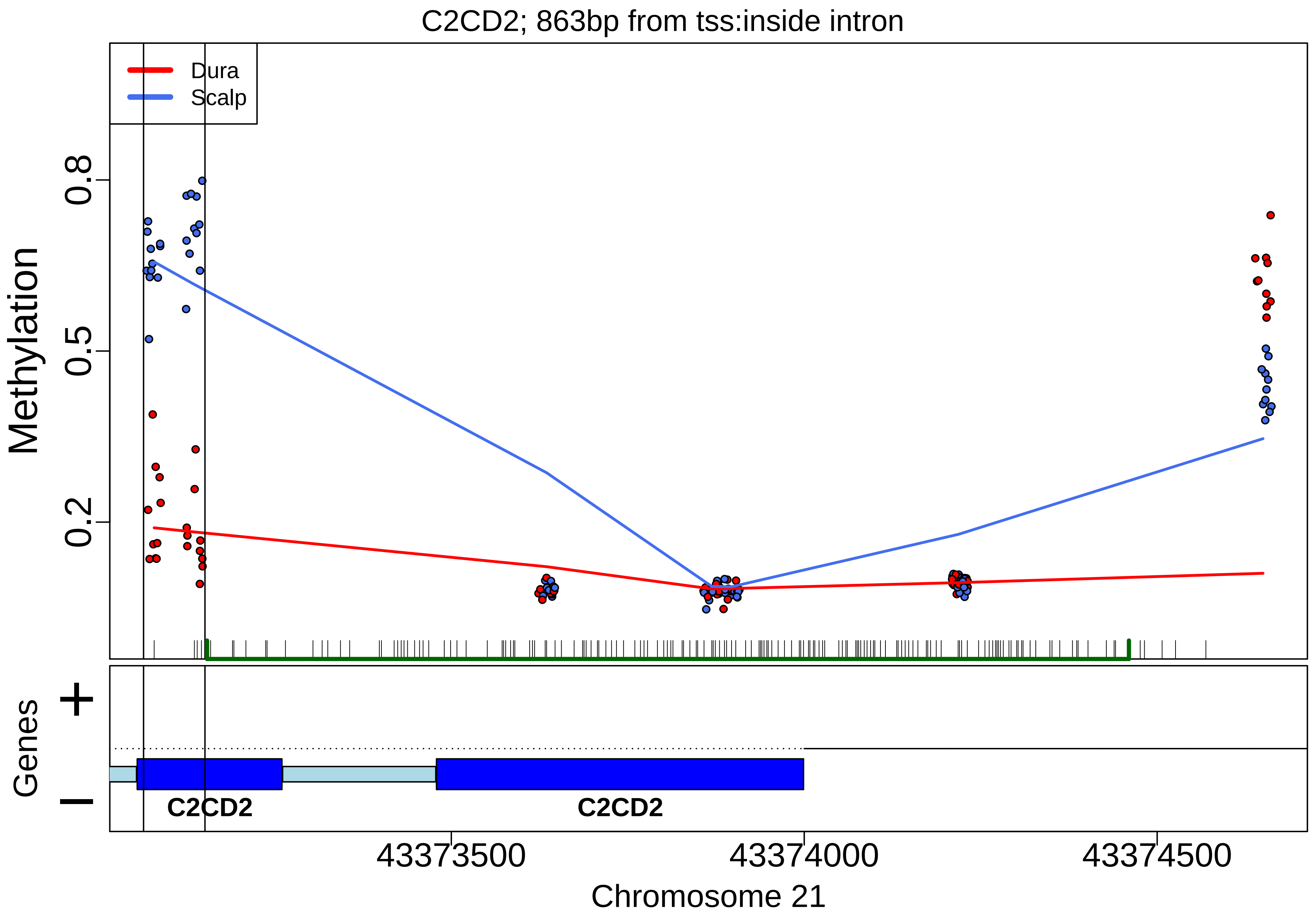

C2CD2; 863bp from tss:inside intron
Dura
Scalp
Methylation
0.8
0.5
0.2
Genes
+
−
C2CD2
C2CD2
43373500
43374000
43374500
Chromosome 21

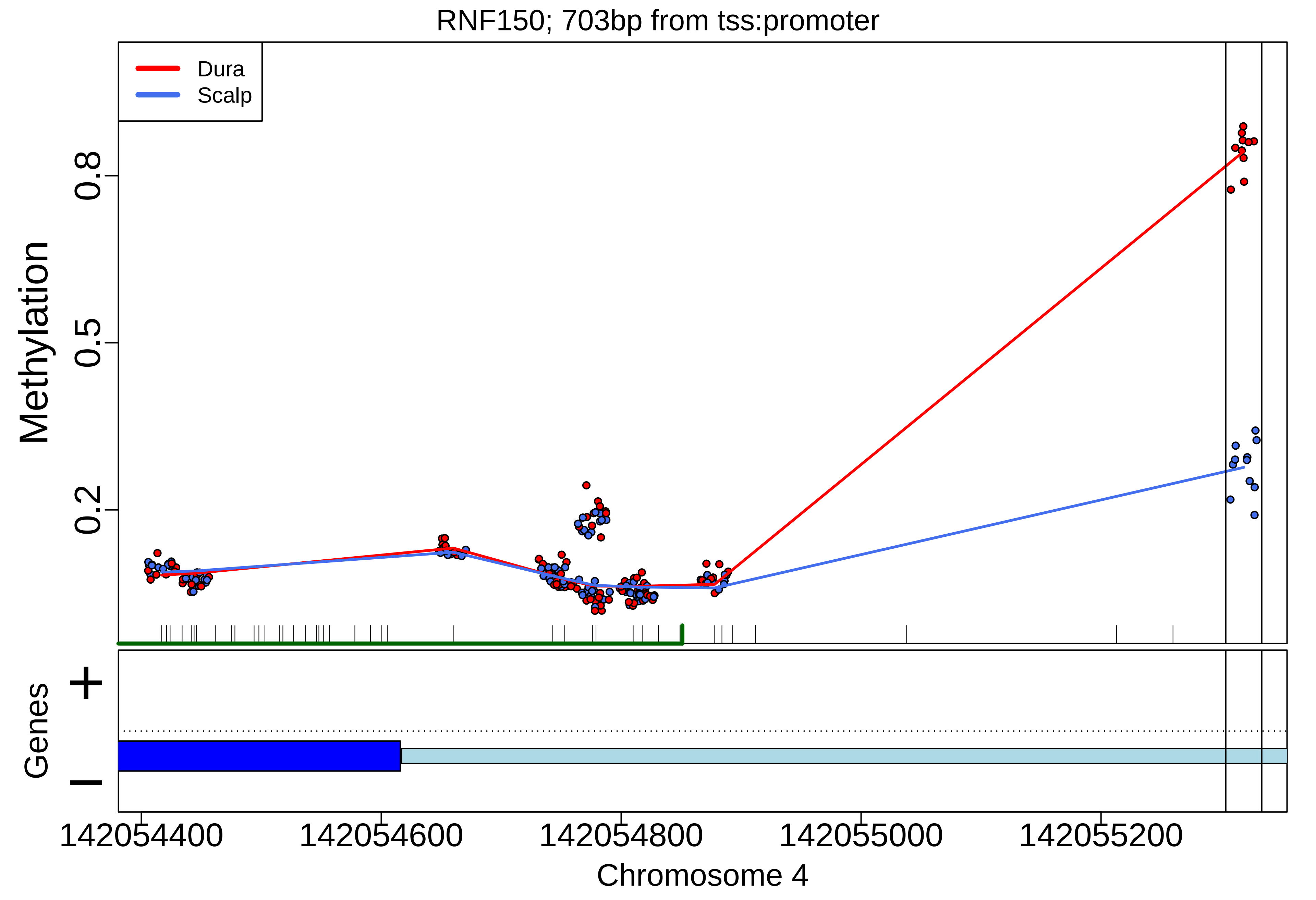
RNF150; 703bp from tss:promoter
Dura
Scalp
Methylation
0.2
0.5
0.8
Genes
+
-
142054400
142054600
142054800
142055000
142055200
Chromosome 4

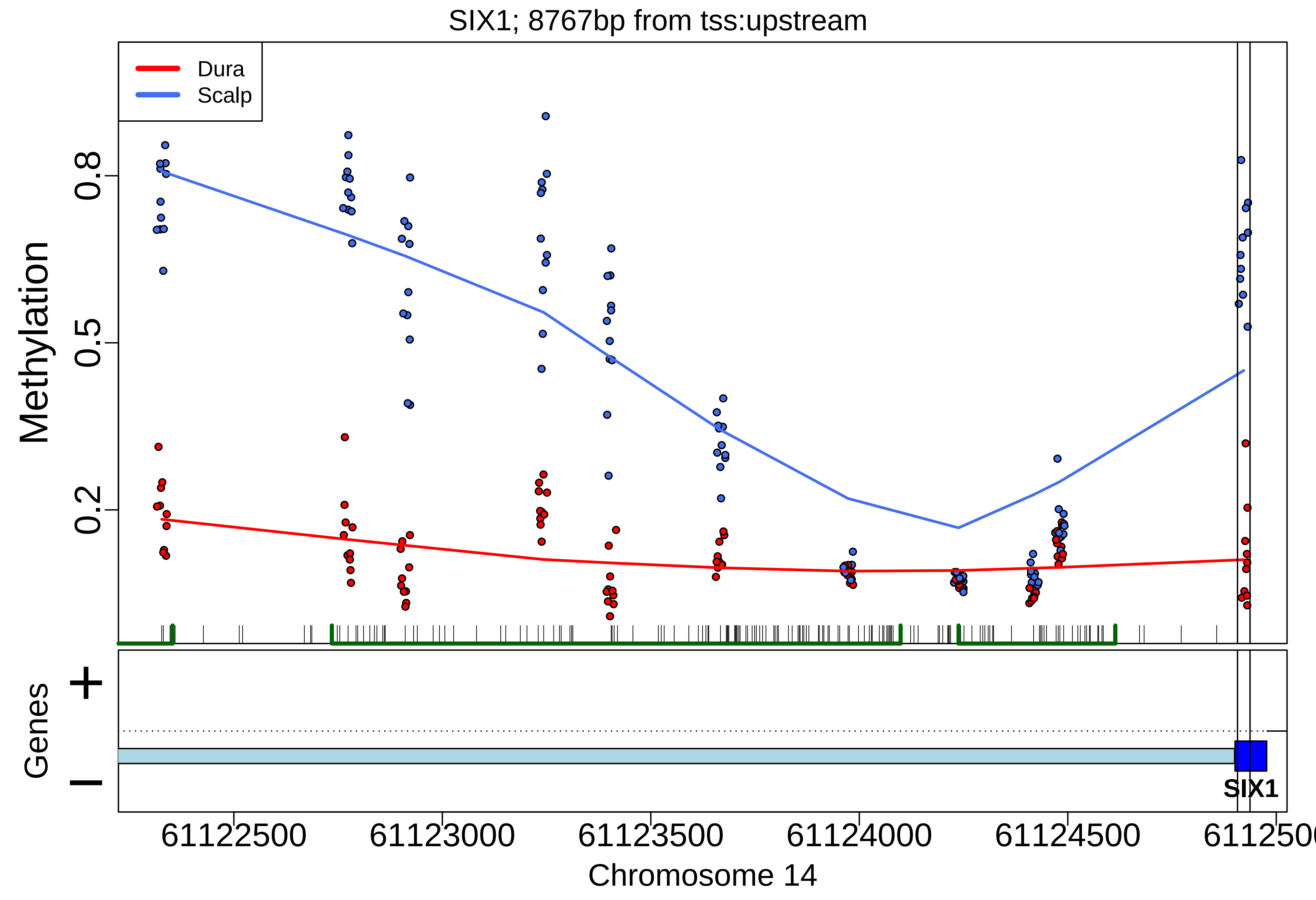
SIX1; 8767bp from tss:upstream
Dura
Scalp
Methylation
0.8
0.5
0.2
Genes
+
-
SIX1
61122500
61123000
61123500
61124000
61124500
Chromosome 14

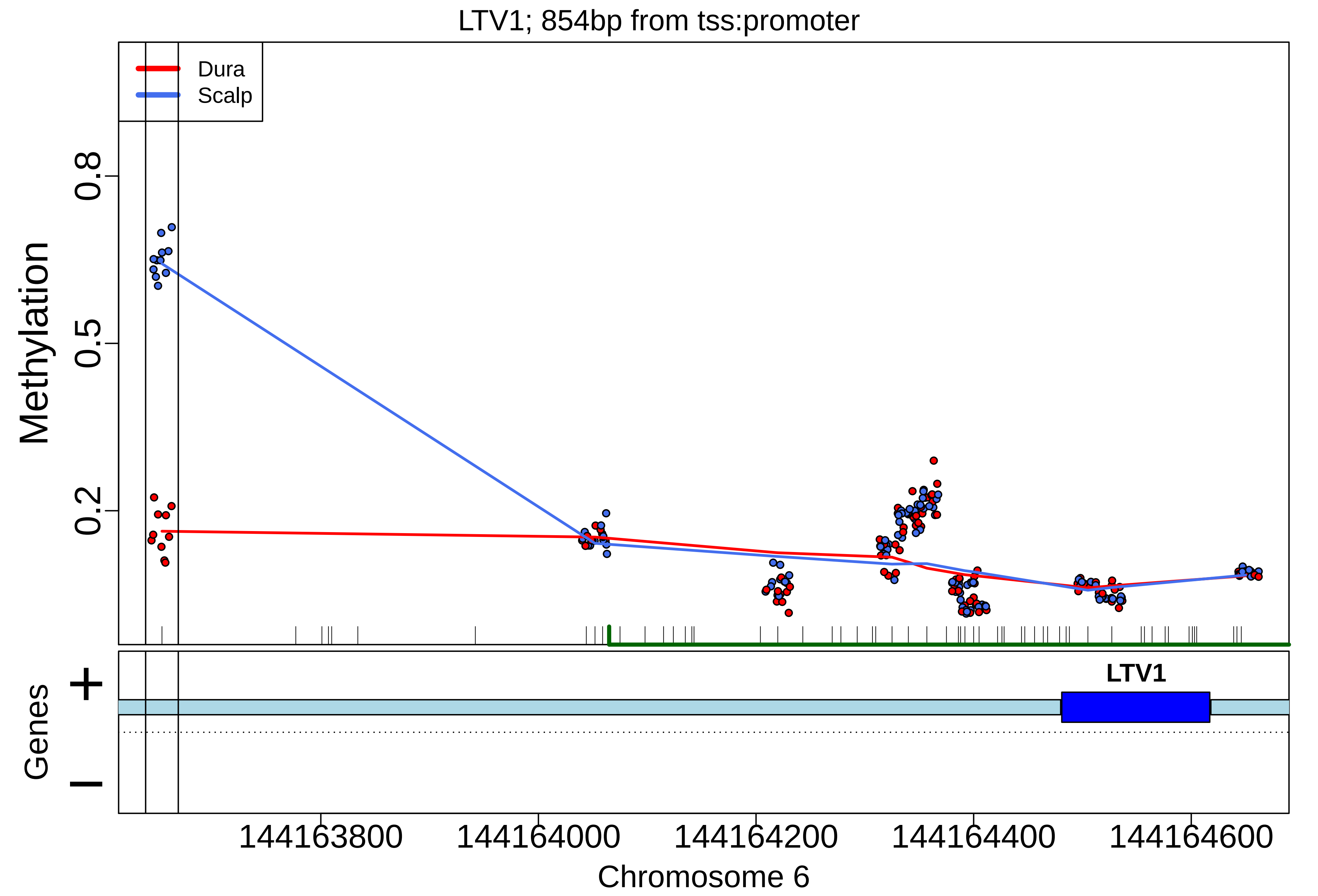

LTV1; 854bp from tss:promoter
Dura
Scalp
Methylation
0.2
0.5
0.8
Genes
+
−
LTV1
144163800
144164000
144164200
144164400
144164600
Chromosome 6

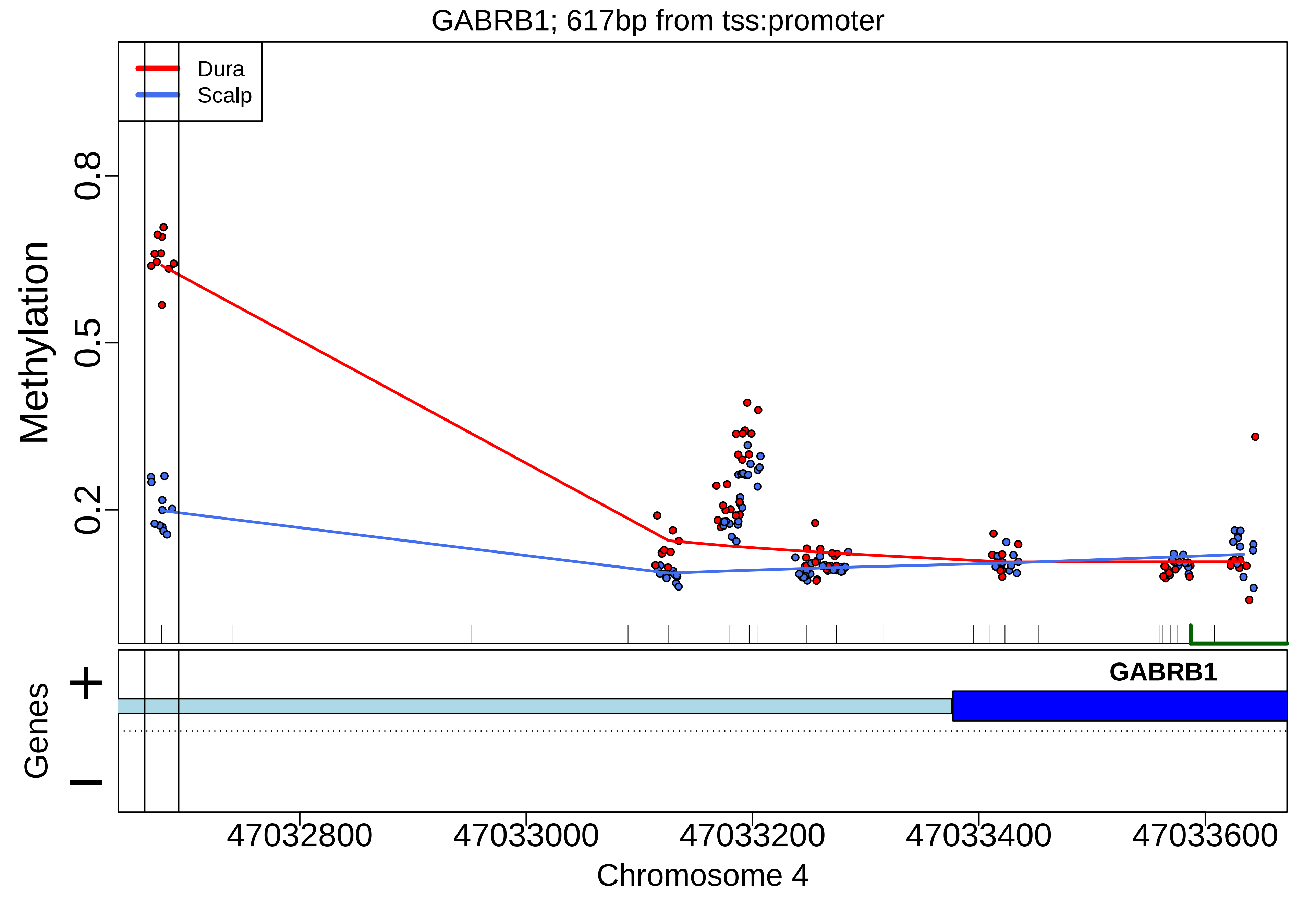
GABRB1; 617bp from tss:promoter
Dura
Scalp
Methylation
0.2
0.5
0.8
Genes
+
-
GABRB1
47032800
47033000
47033200
47033400
47033600
Chromosome 4

FBXO4; 564bp from tss:promoter

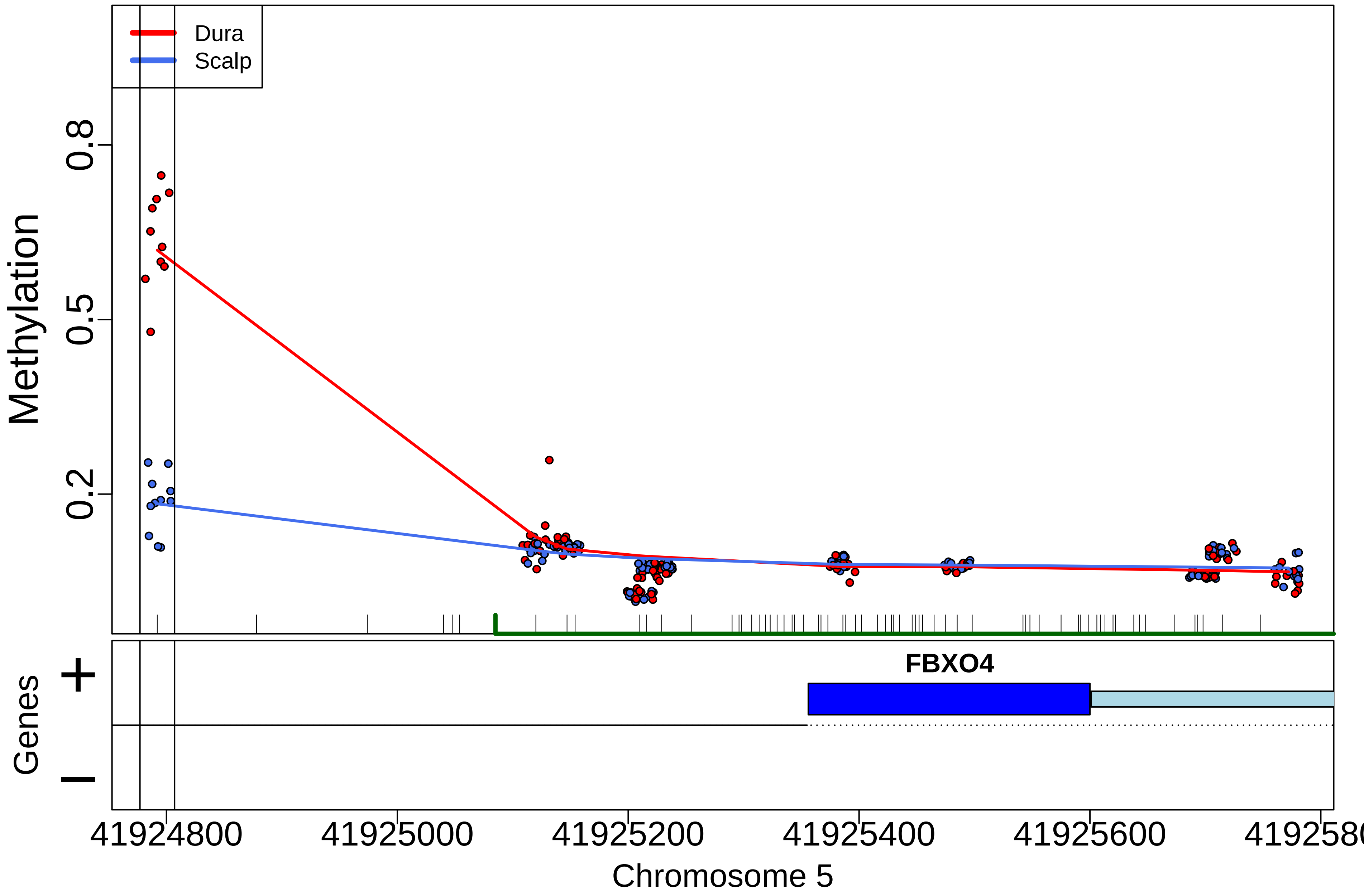

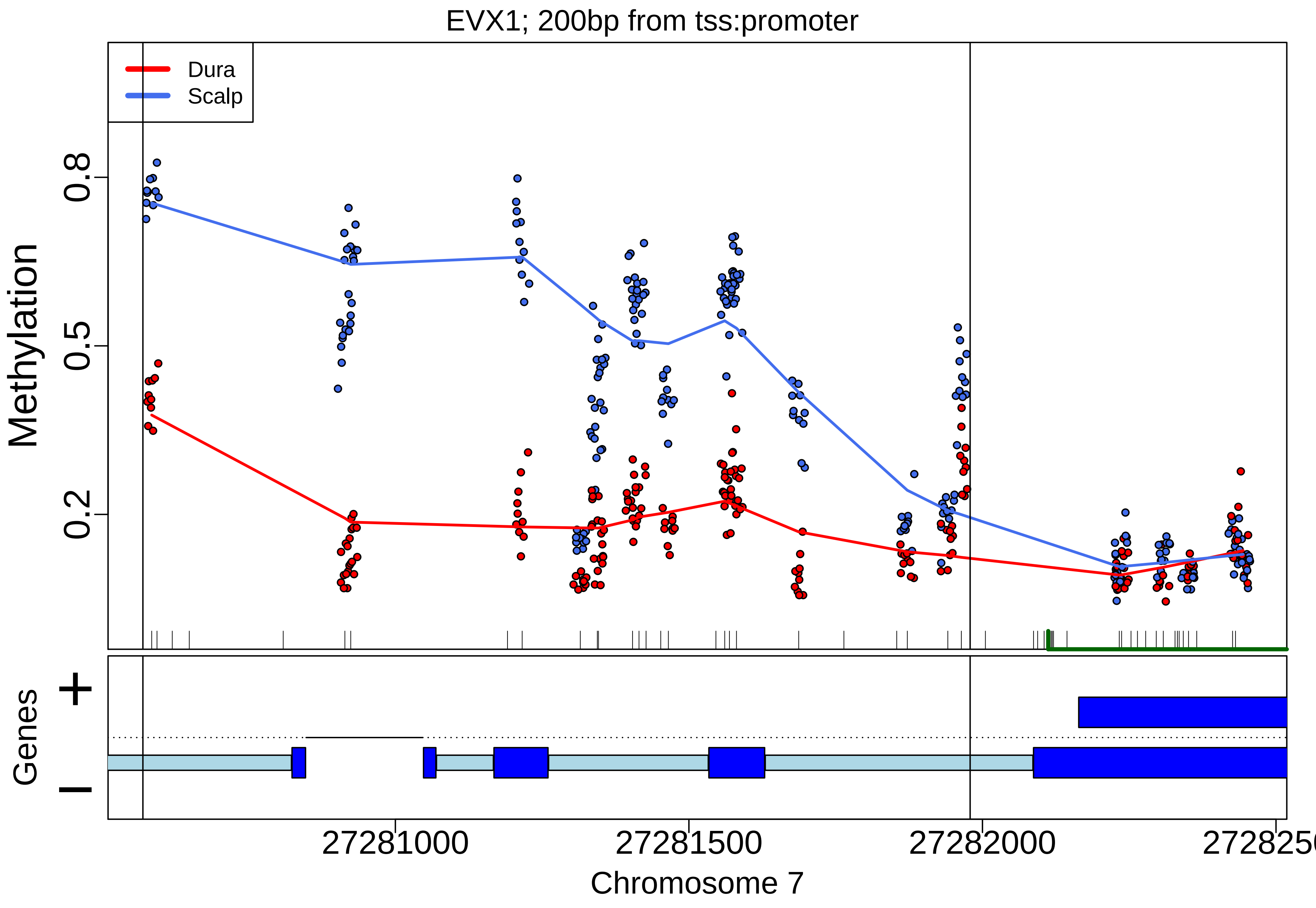
EVX1; 200bp from tss:promoter
Dura
Scalp
Methylation
0.2
0.5
0.8
Genes
+
-
27281000
27281500
27282000
Chromosome 7

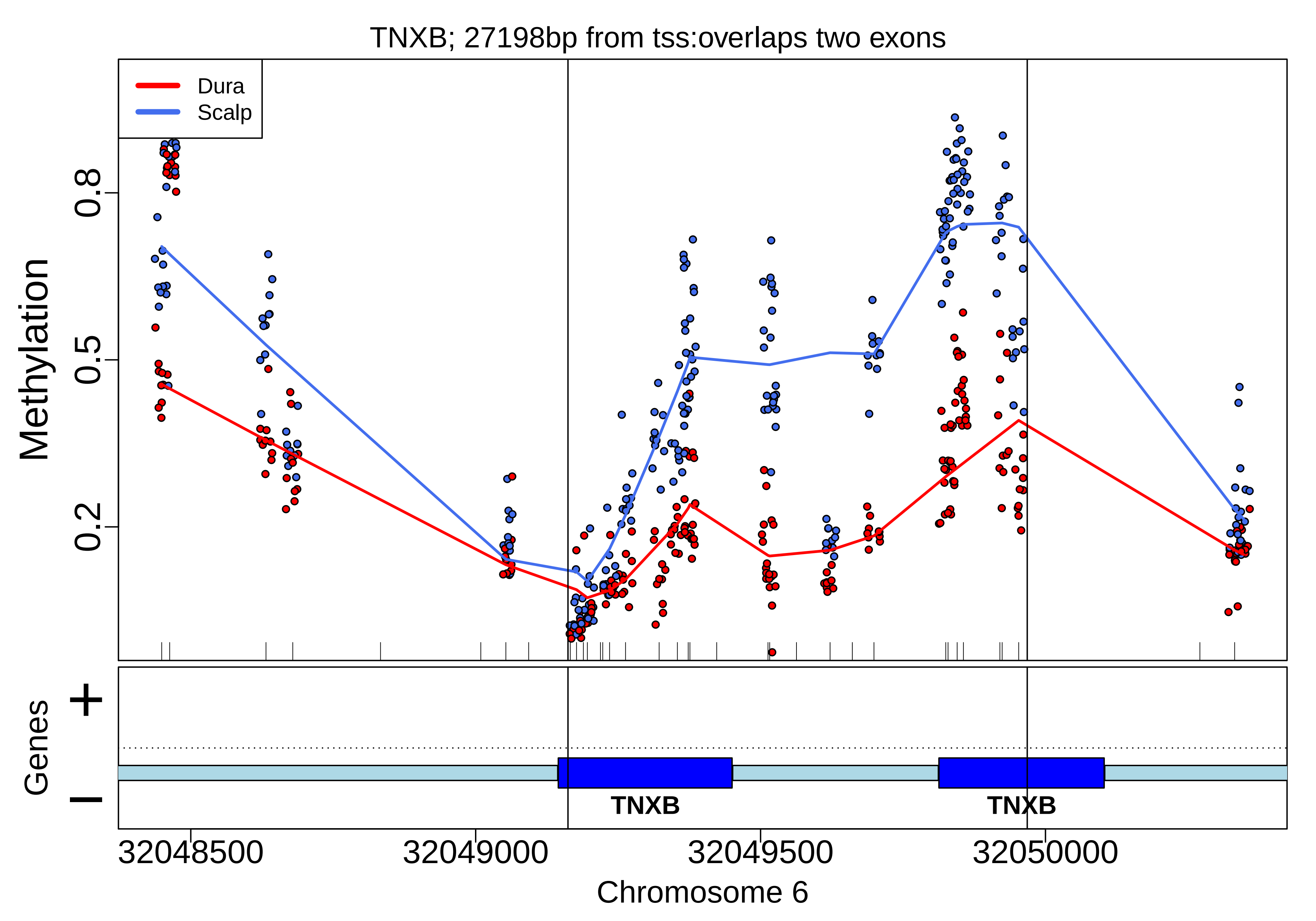

TNXB; 27198bp from tss:overlaps two exons
Dura
Scalp
Methylation
0.8
0.5
0.2
Genes
+
−
TNXB
TNXB
32048500
32049000
32049500
32050000
Chromosome 6

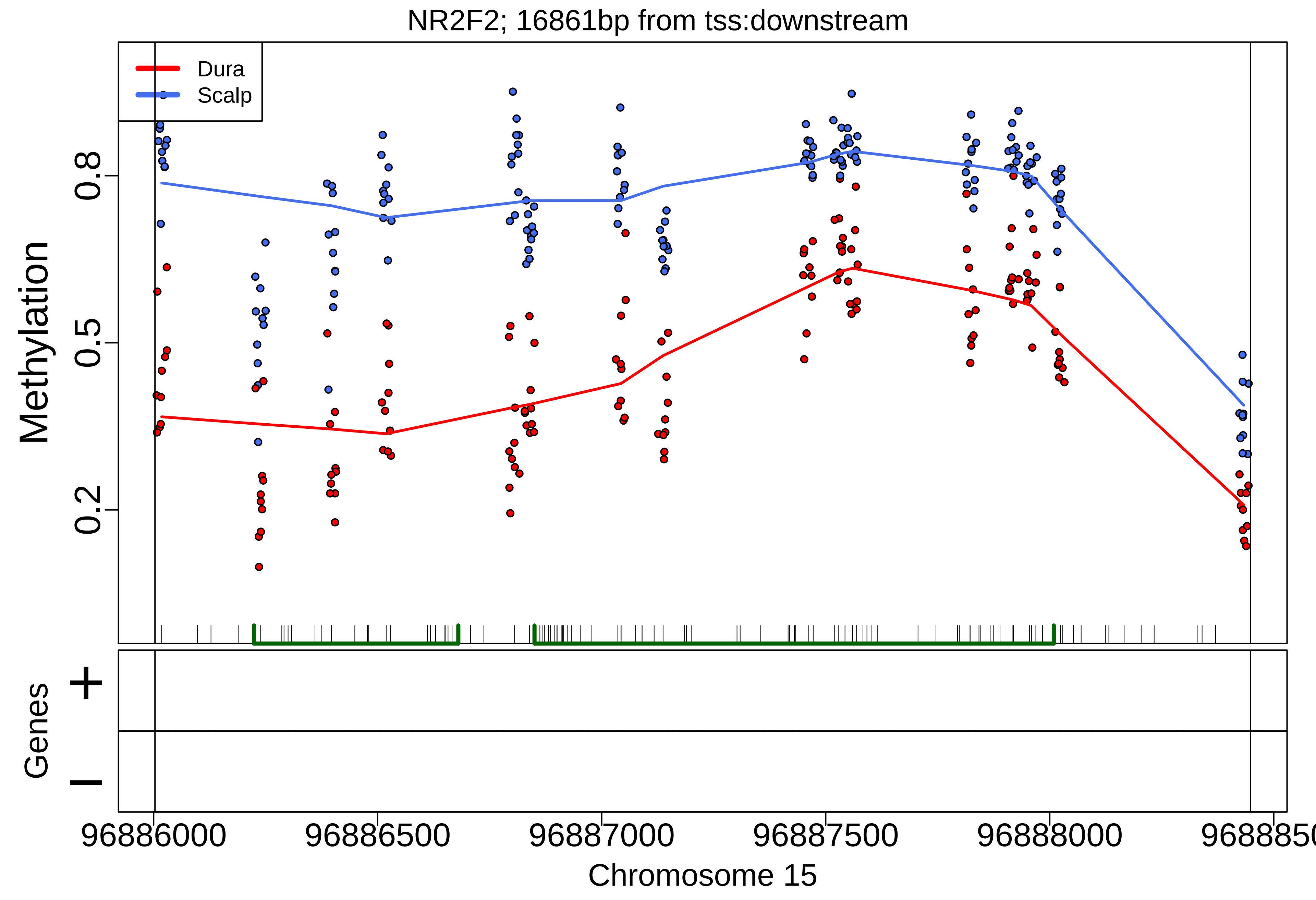

NR2F2; 16861bp from tss:downstream
Dura
Scalp
Methylation
0.2
0.5
0.8
Genes
+
-
96886000
96886500
96887000
96887500
96888000
Chromosome 15

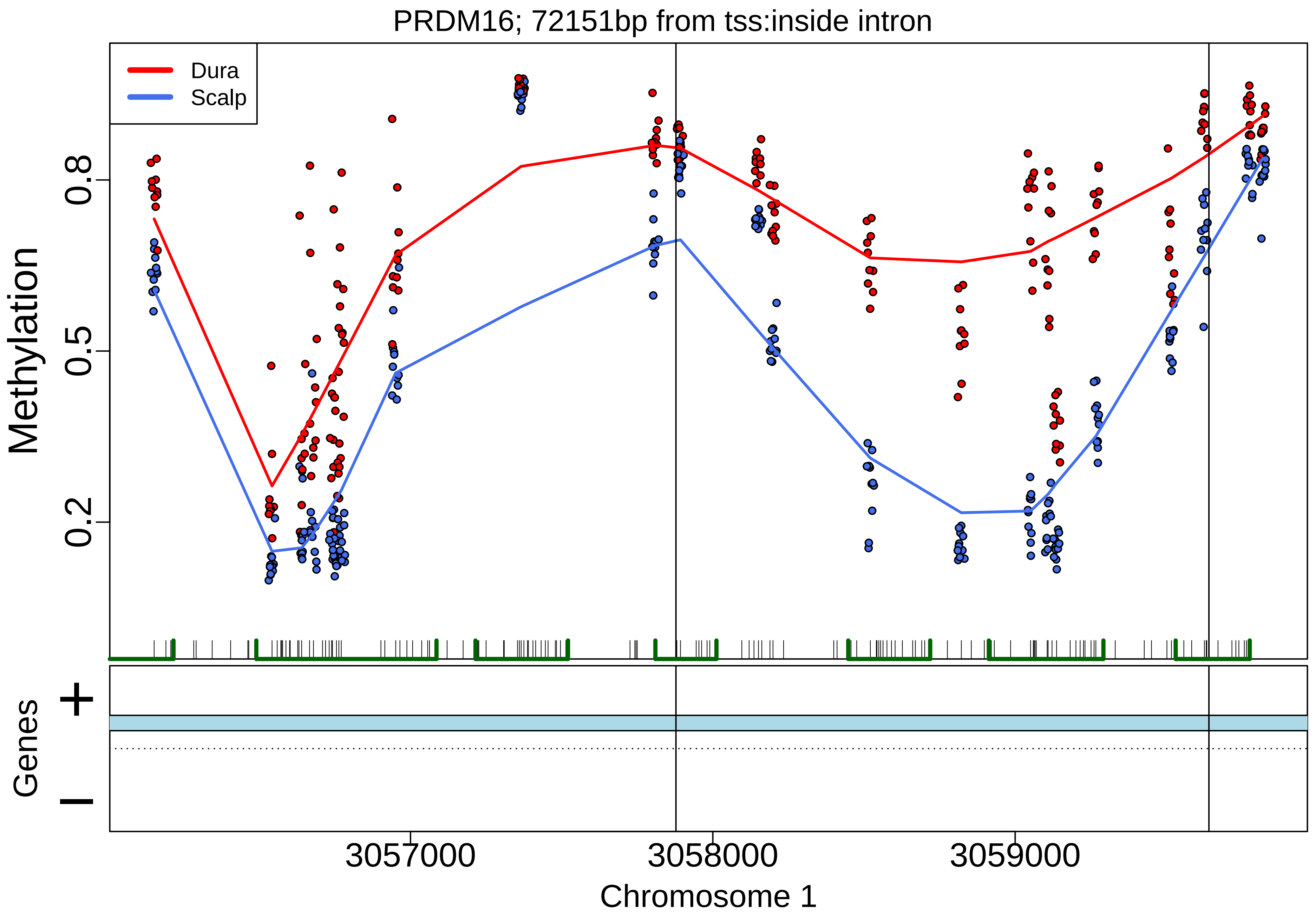

PRDM16; 72151bp from tss:inside intron
Dura
Scalp
Methylation
0.2
0.5
0.8
Genes
+
-
3057000
3058000
3059000
Chromosome 1

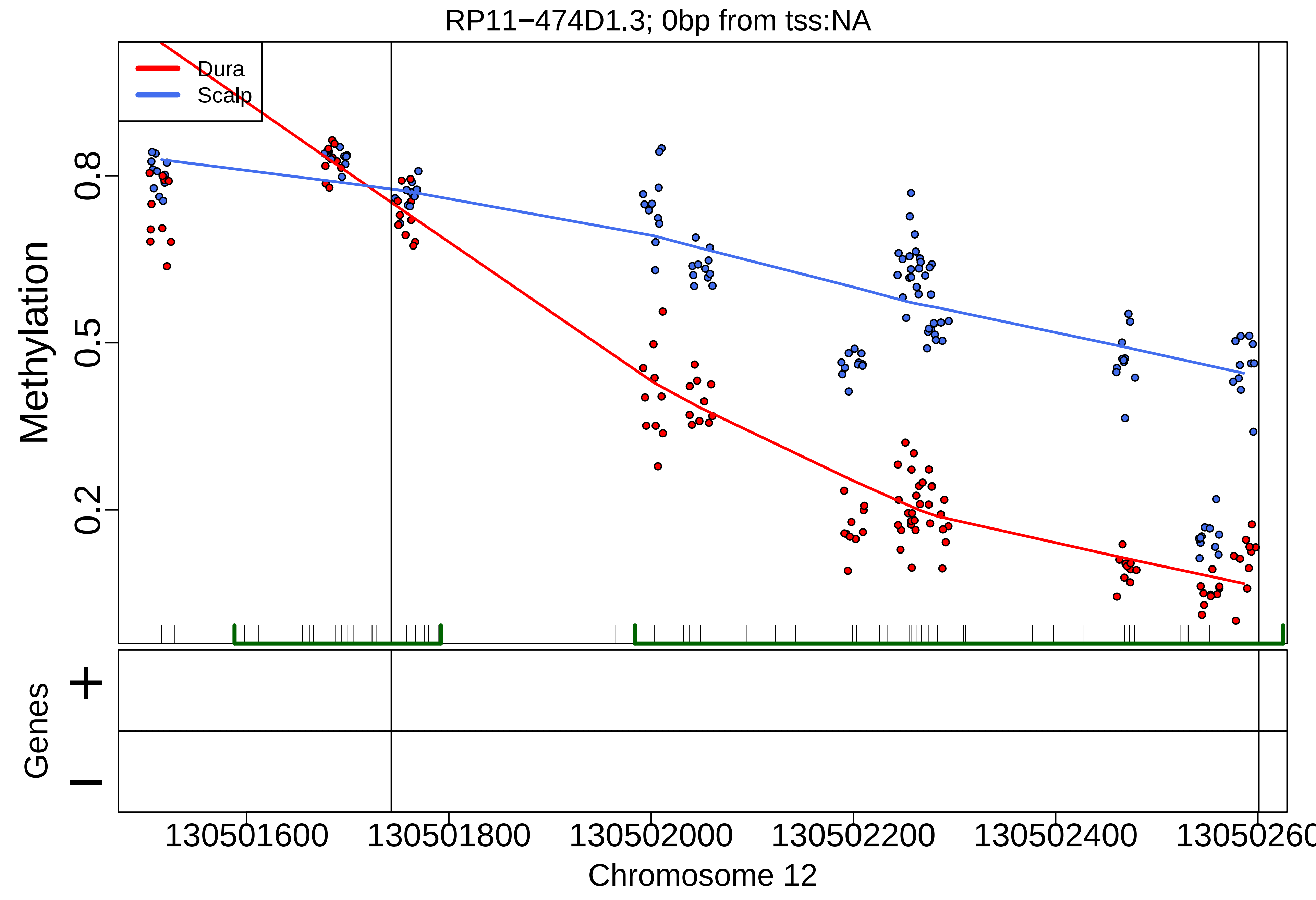

RP11−474D1.3; 0bp from tss:NA
Dura
Scalp
Methylation
0.8
0.5
0.2
Genes
+
−
130501600
130501800
130502000
130502200
130502400
13050260
Chromosome 12

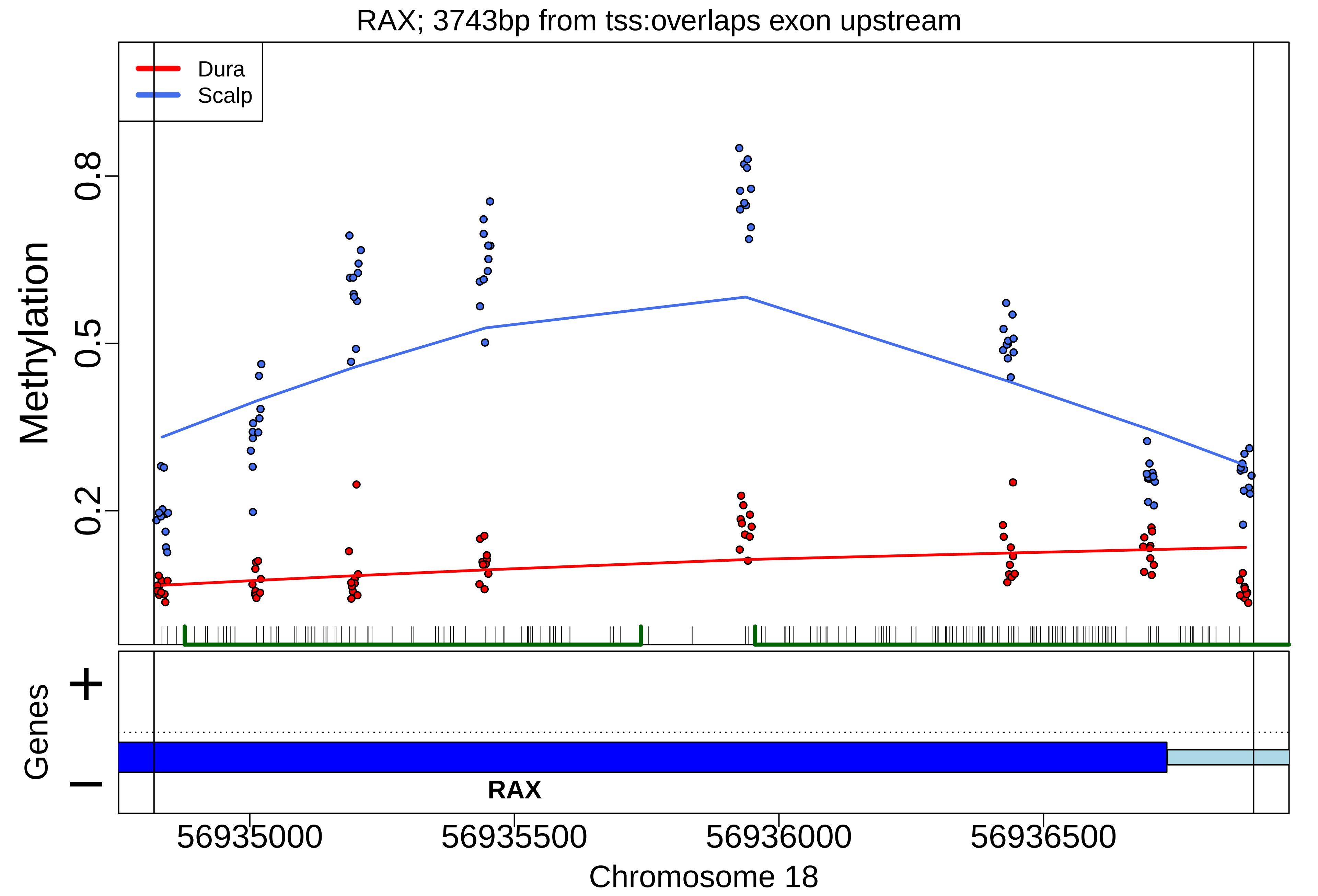

RAX; 3743bp from tss:overlaps exon upstream
Dura
Scalp
Methylation
0.2
0.5
0.8
Genes
+
-
RAX
56935000
56935500
56936000
56936500
Chromosome 18

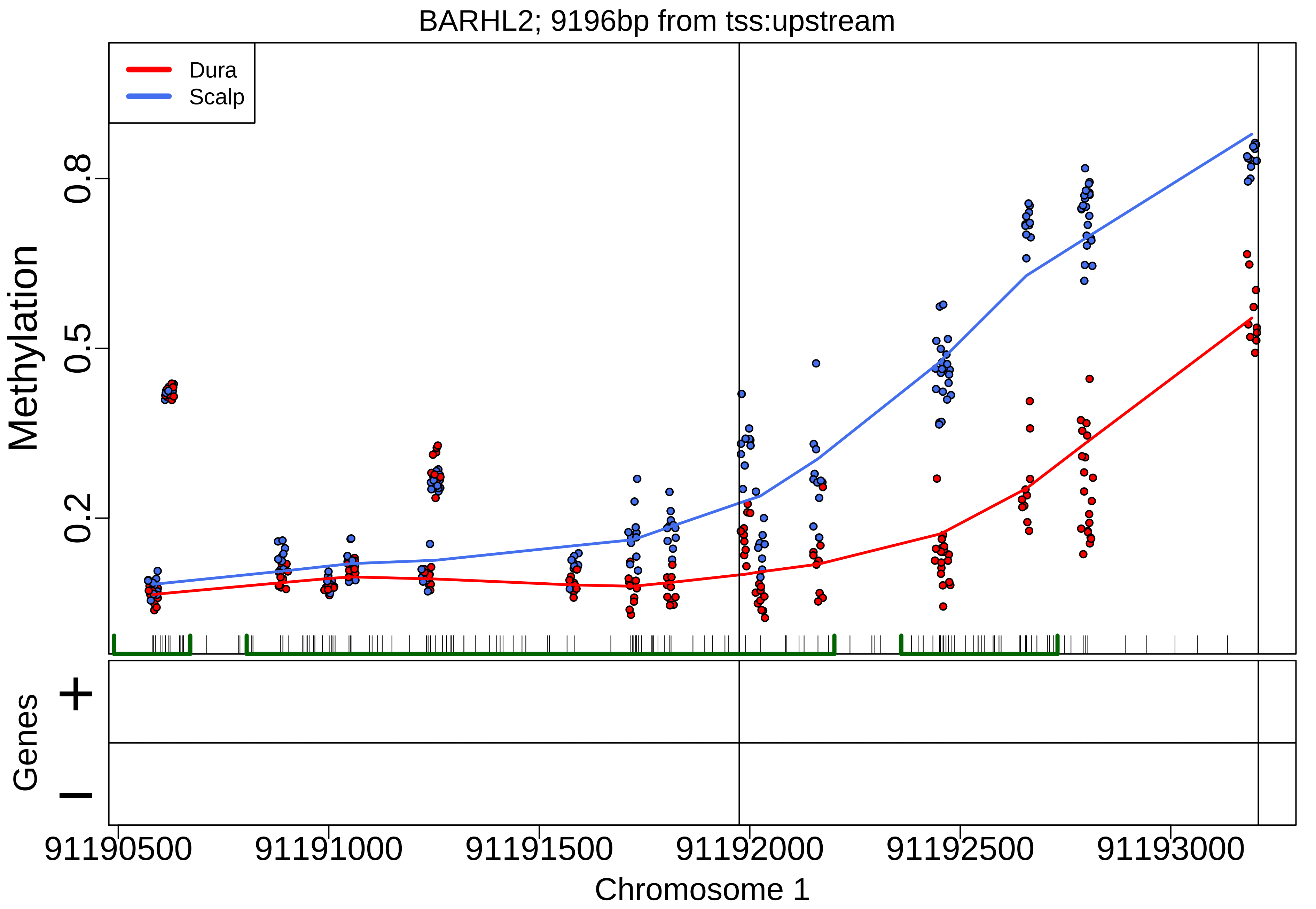

BARHL2; 9196bp from tss:upstream
Dura
Scalp
Methylation
0.2
0.5
0.8
Genes
+
−
91190500
91191000
91191500
91192000
91192500
91193000
Chromosome 1

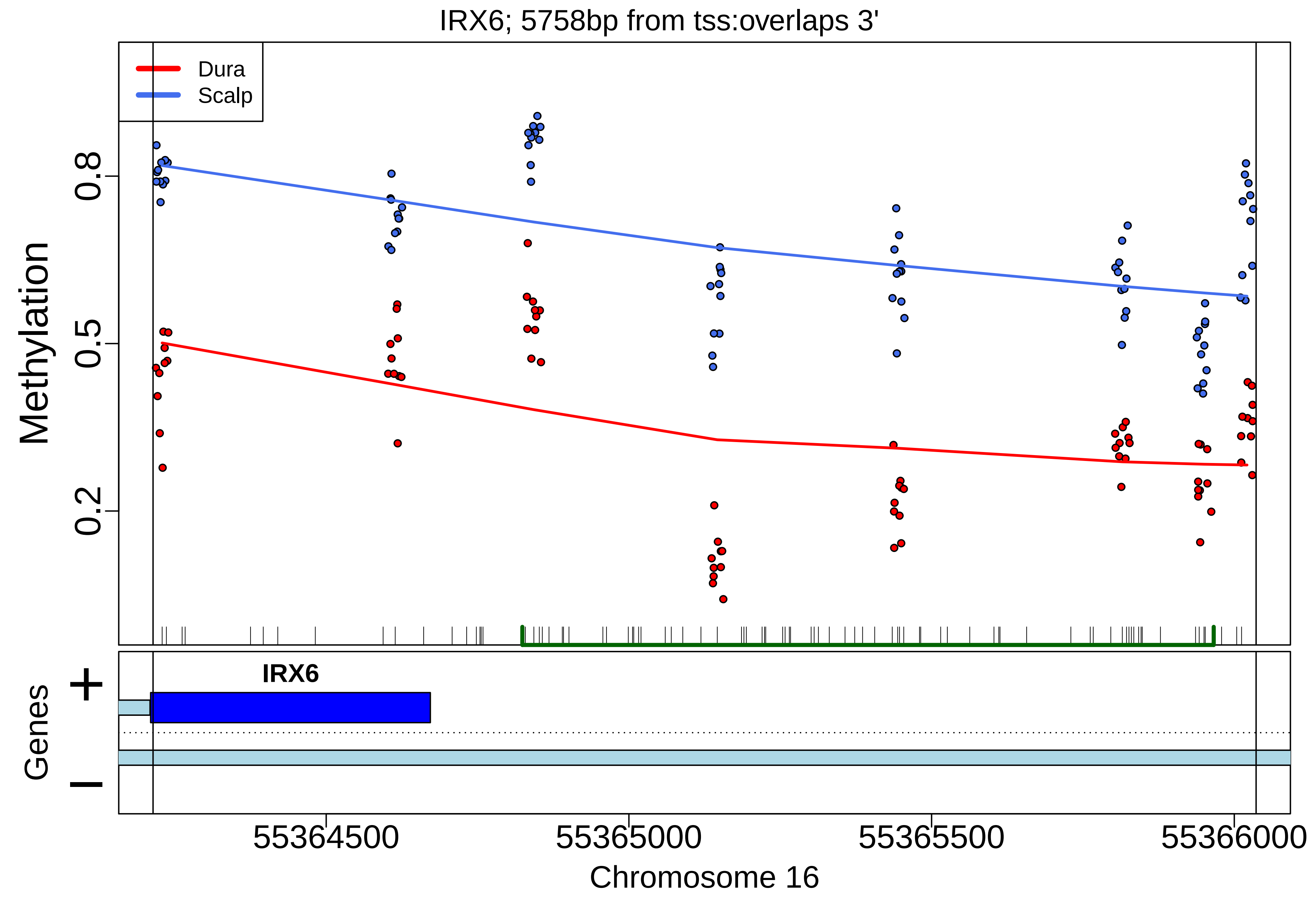

IRX6; 5758bp from tss:overlaps 3'
Dura
Scalp
Methylation
0.2
0.5
0.8
Genes
+
-
IRX6
55364500
55365000
55365500
55366000
Chromosome 16

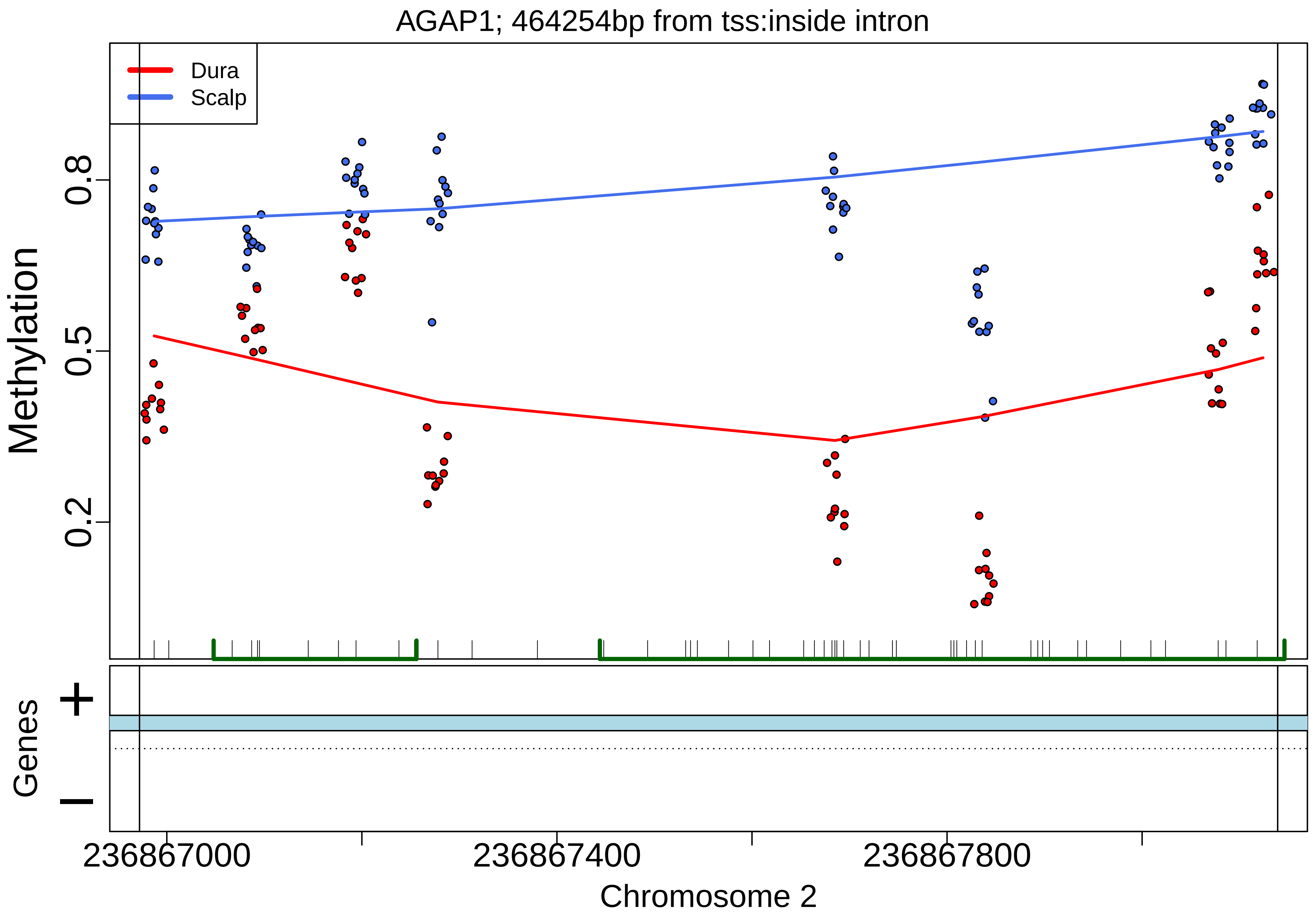

AGAP1; 464254bp from tss:inside intron
Dura
Scalp
Methylation
0.2
0.5
0.8
Genes
+
-
236867000
236867400
236867800
Chromosome 2

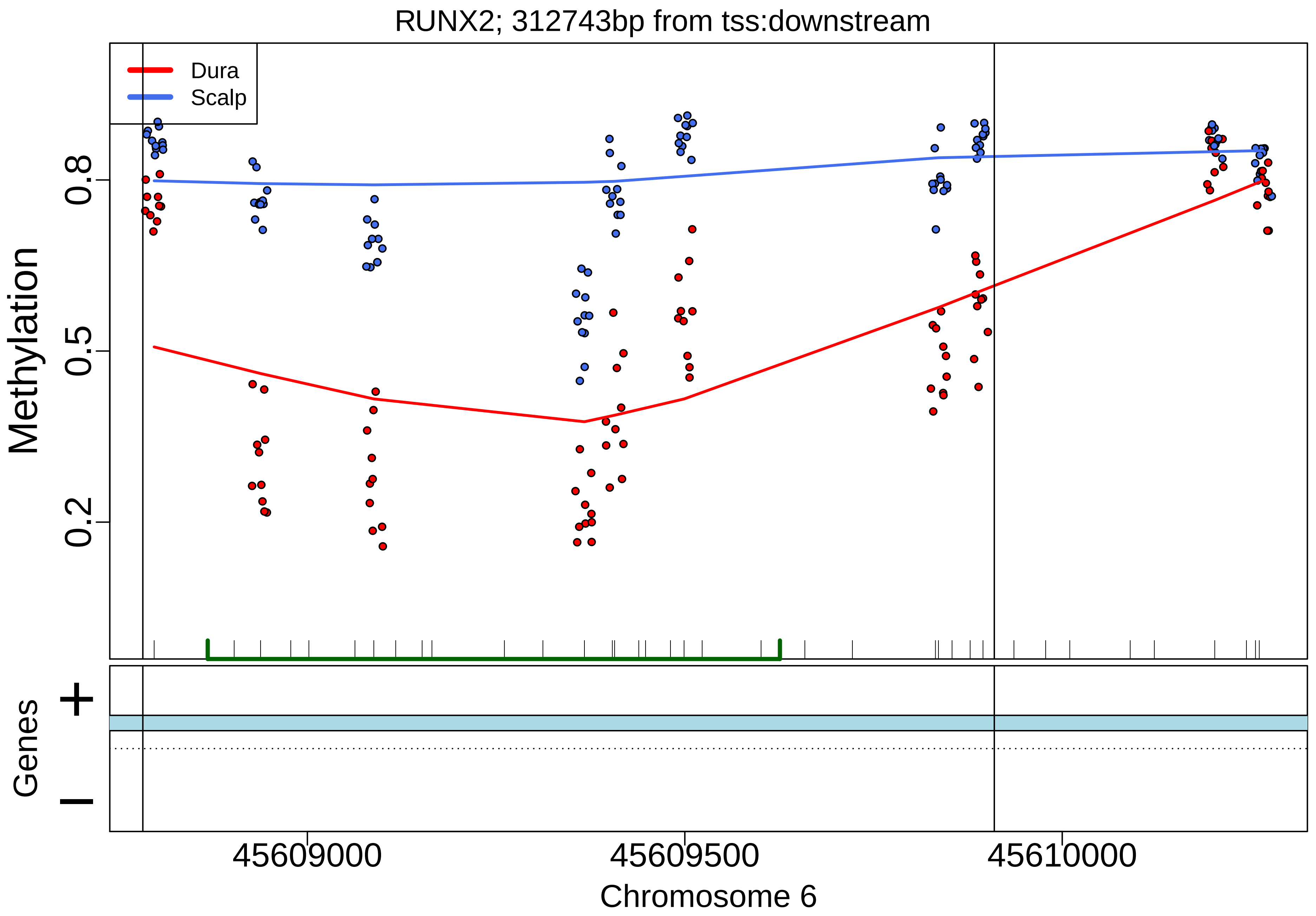

RUNX2; 312743bp from tss:downstream
Dura
Scalp
Methylation
0.8
0.5
0.2
Genes
+
−
45609000
45609500
45610000
Chromosome 6

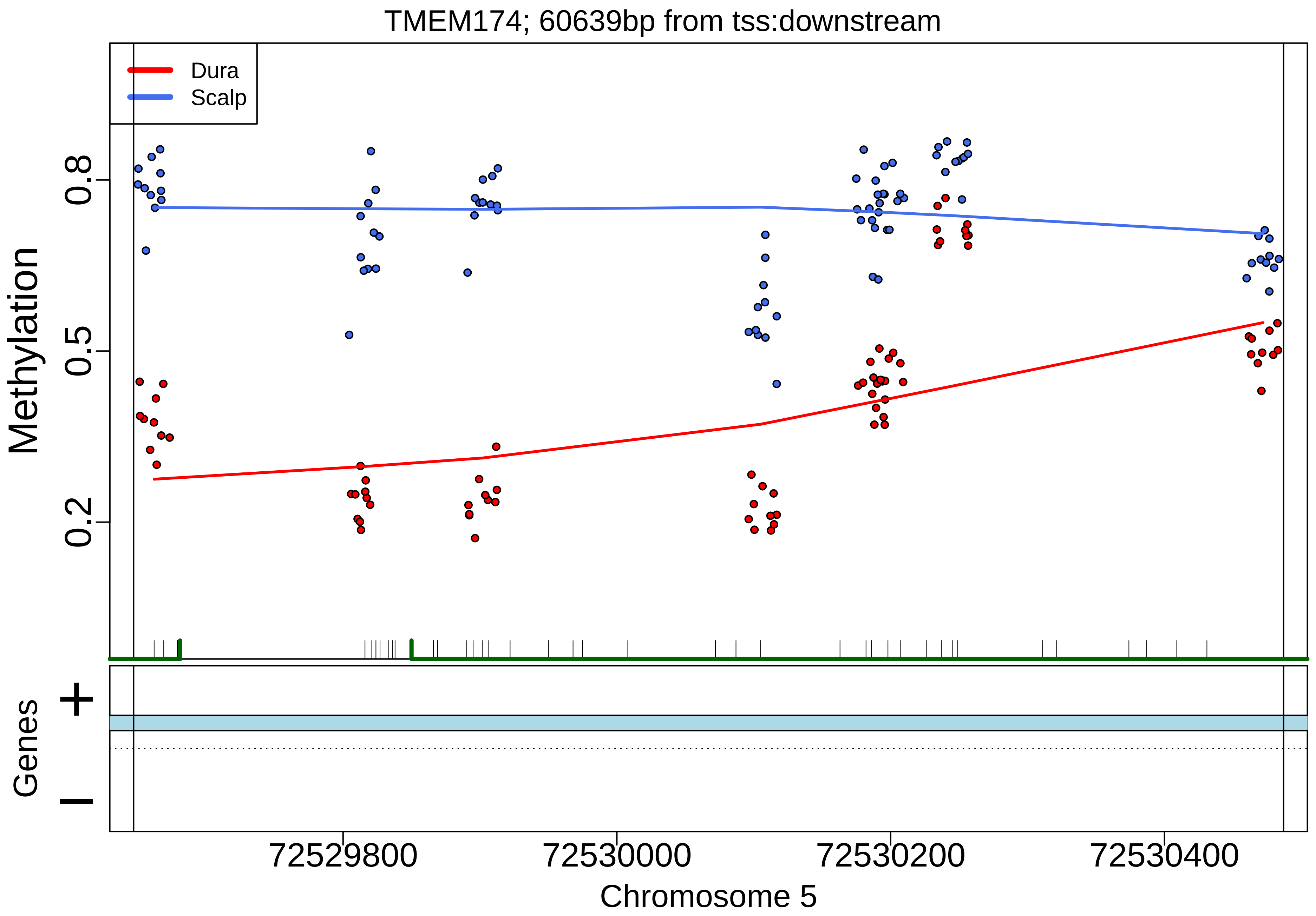

TMEM174; 60639bp from tss:downstream
Dura
Scalp
Methylation
0.2
0.5
0.8
Genes
+
-
72529800
72530000
72530200
72530400
Chromosome 5

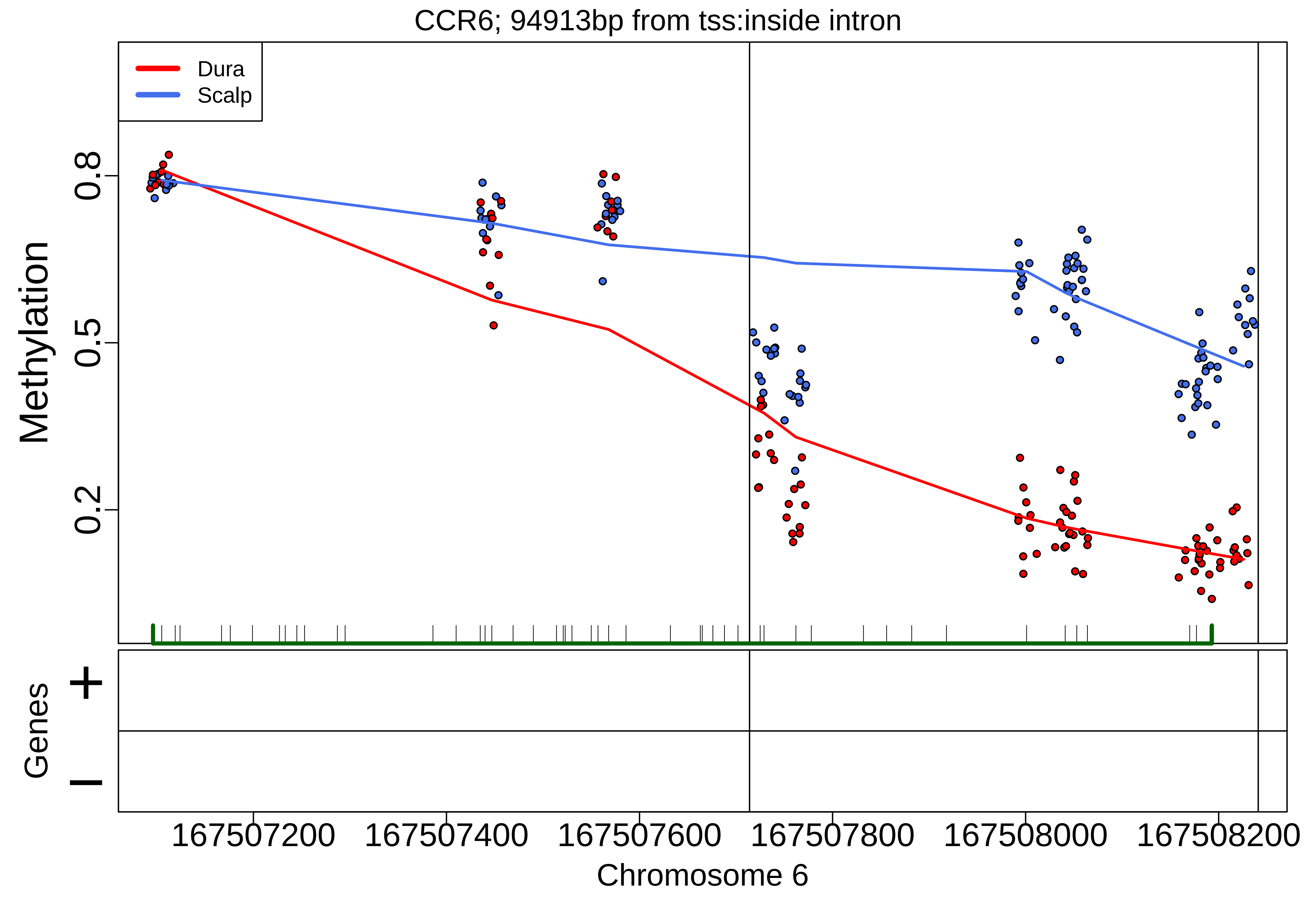
CCR6; 94913bp from tss:inside intron
Dura
Scalp
Methylation
0.2
0.5
0.8
Genes
+
-
167507200
167507400
167507600
167507800
167508000
167508200
Chromosome 6

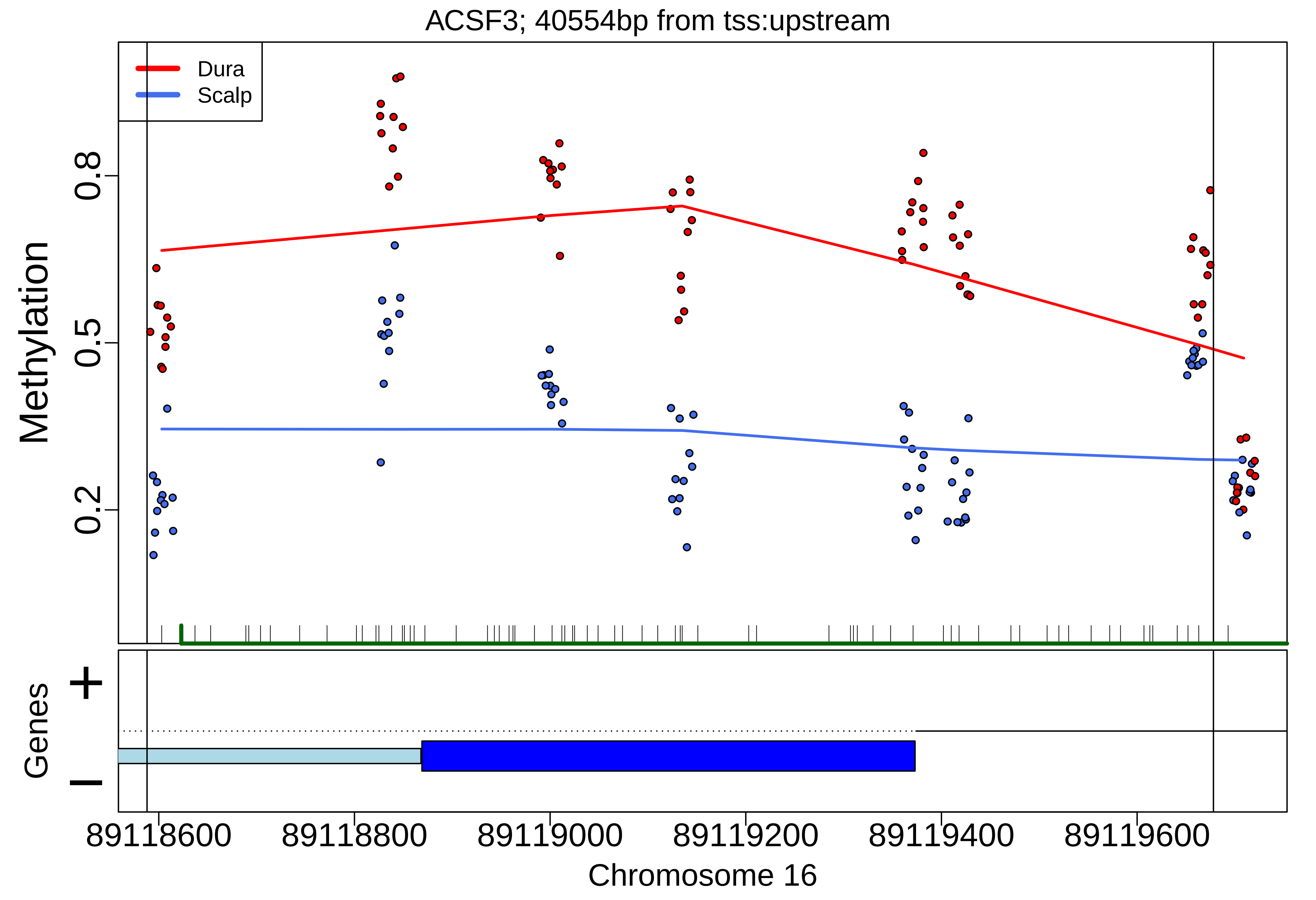

ACSF3; 40554bp from tss:upstream
Dura
Scalp
Methylation
0.2
0.5
0.8
Genes
+
−
89118600
89118800
89119000
89119200
89119400
89119600
Chromosome 16

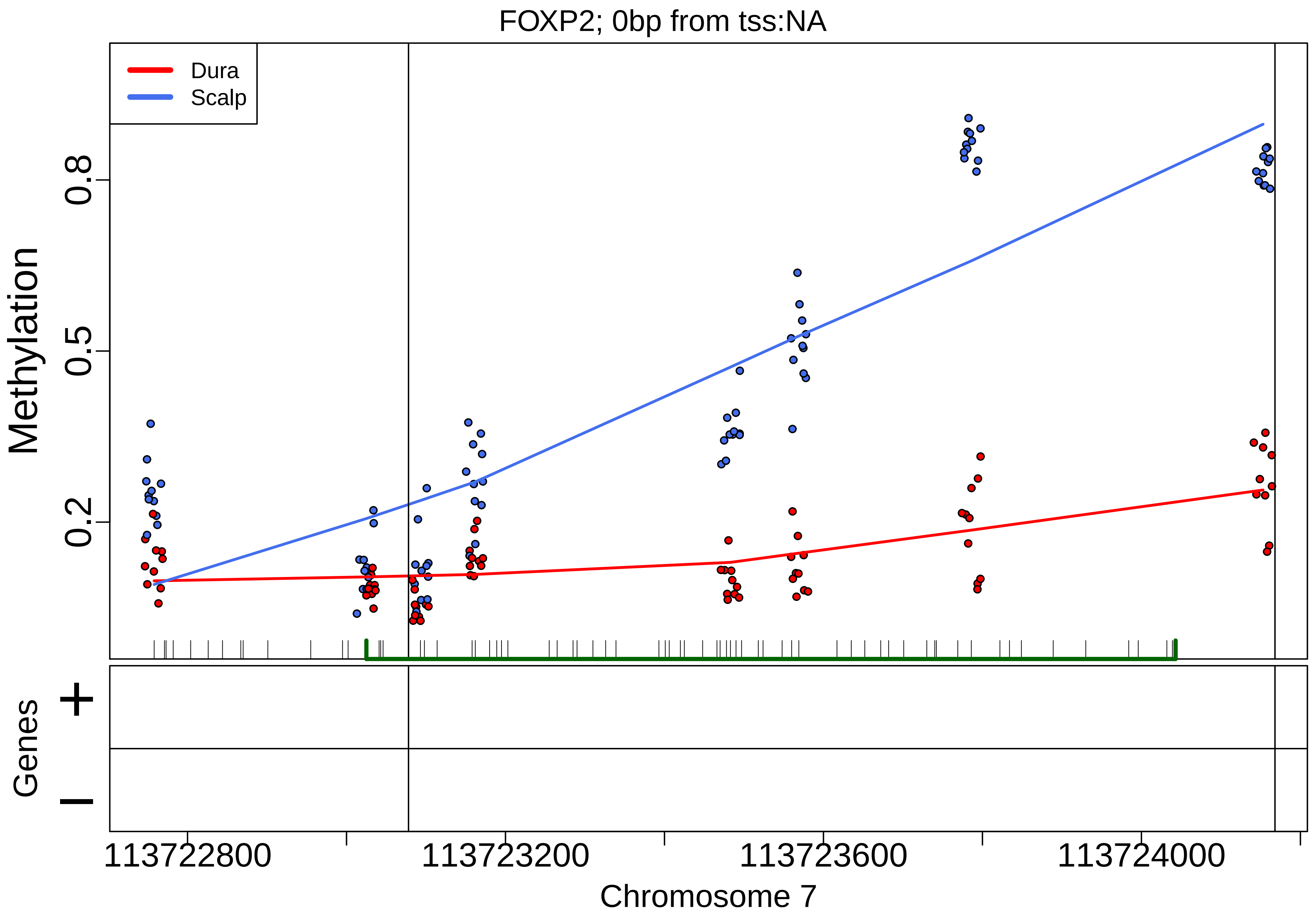
FOXP2; 0bp from tss:NA
Dura
Scalp
Methylation
0.2
0.5
0.8
Genes
+
−
113722800
113723200
113723600
113724000
Chromosome 7

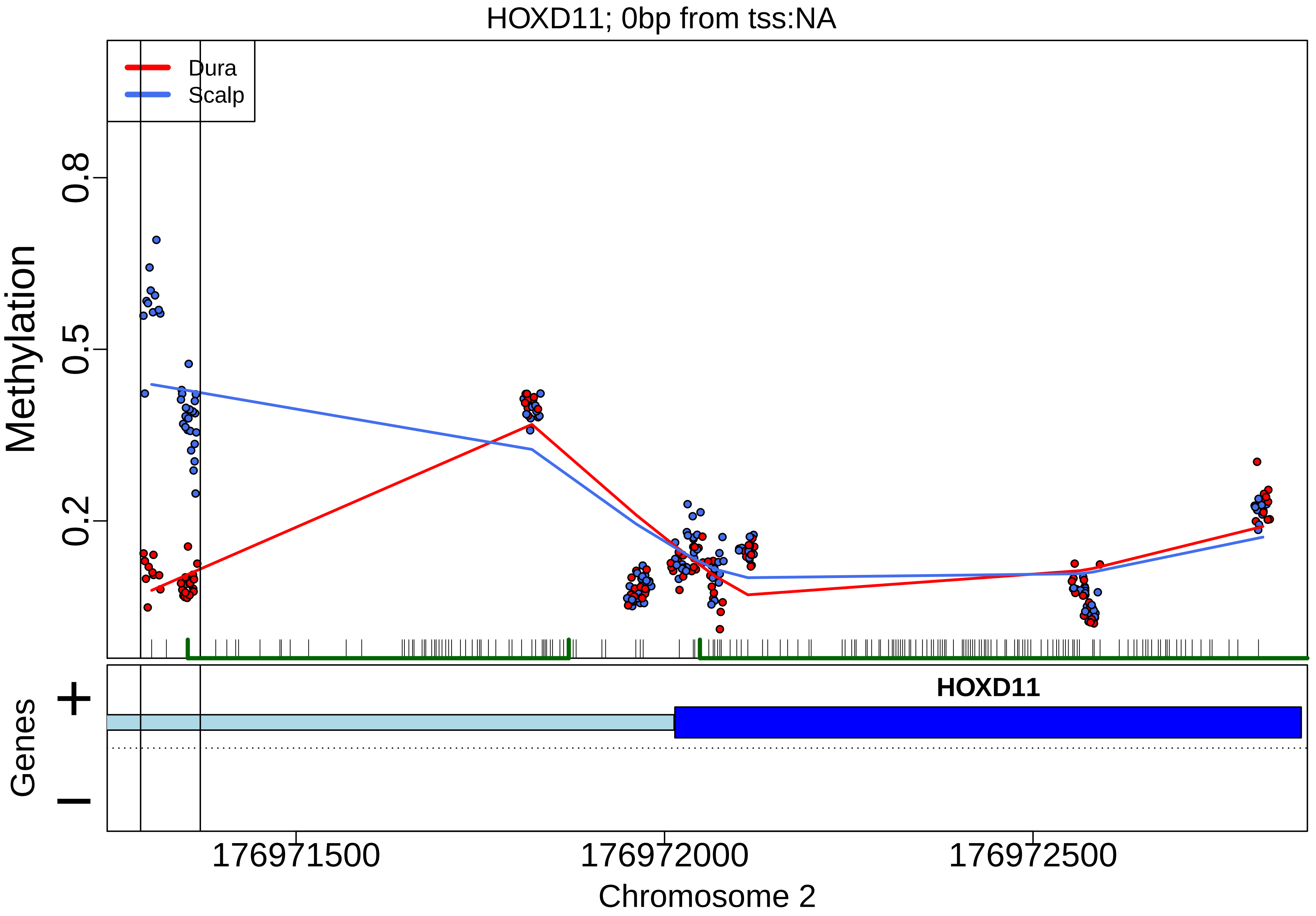

HOXD11; 0bp from tss:NA
Dura
Scalp
Methylation
0.2
0.5
0.8
Genes
+
−
HOXD11
176971500
176972000
176972500
Chromosome 2

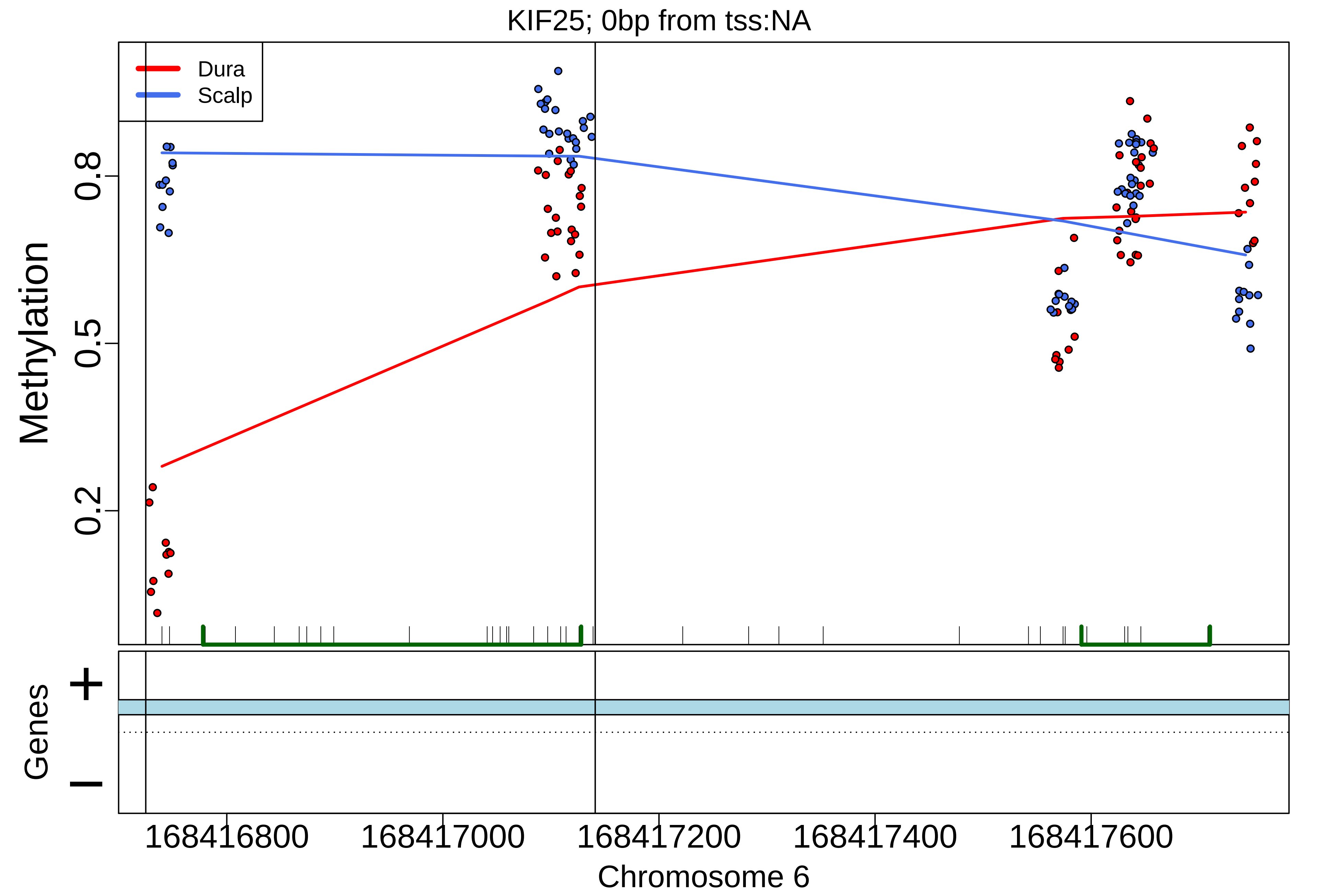

KIF25; 0bp from tss:NA
Dura
Scalp
Methylation
0.2
0.5
0.8
Genes
+
-
168416800
168417000
168417200
168417400
168417600
Chromosome 6

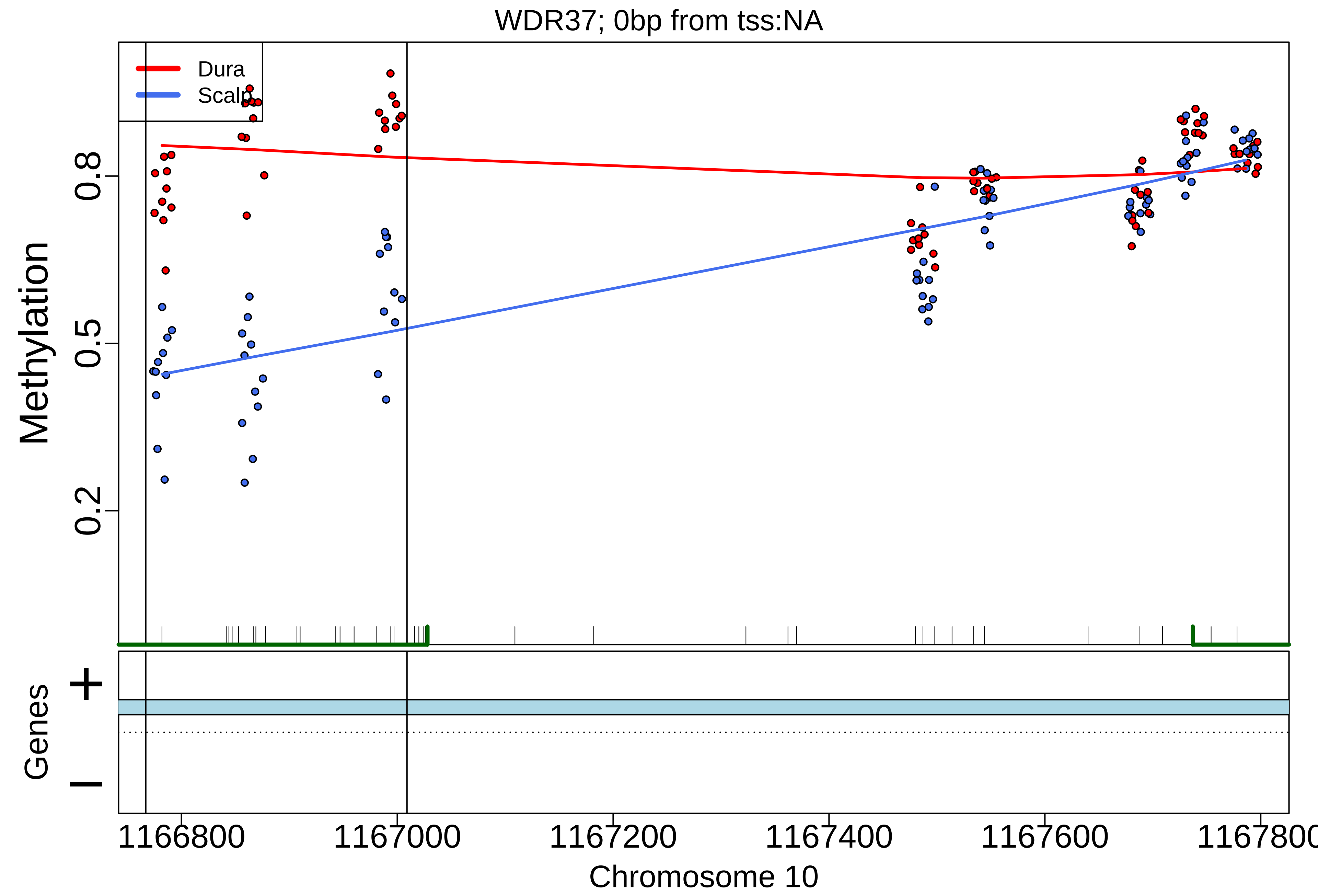
WDR37; 0bp from tss:NA
Dura
Scalp
Methylation
0.2
0.5
0.8
Genes
+
-
1166800
1167000
1167200
1167400
1167600
1167800
Chromosome 10

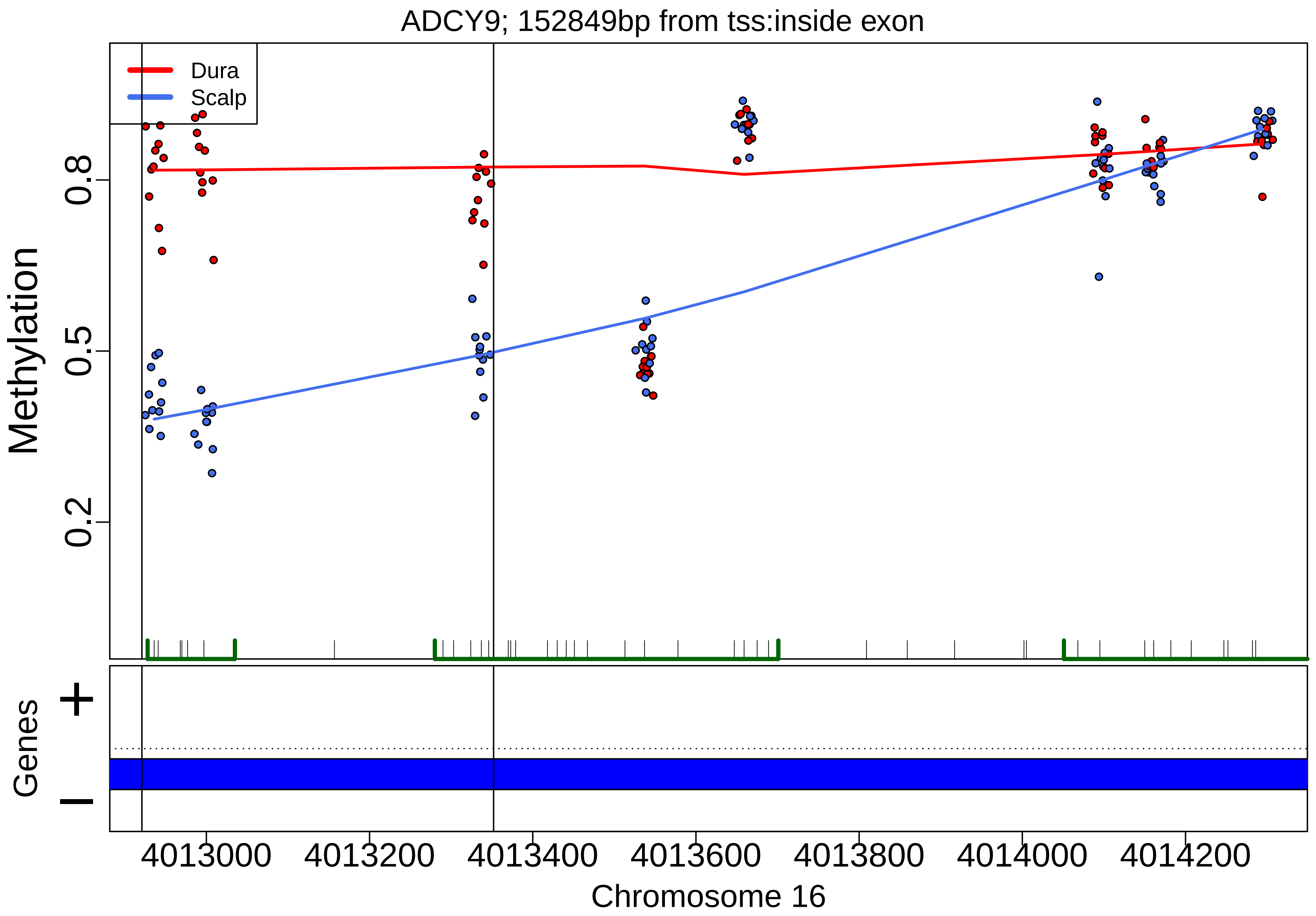

ADCY9; 152849bp from tss:inside exon
Dura
Scalp
Methylation
0.2
0.5
0.8
Genes
+
−
4013000
4013200
4013400
4013600
4013800
4014000
4014200
Chromosome 16

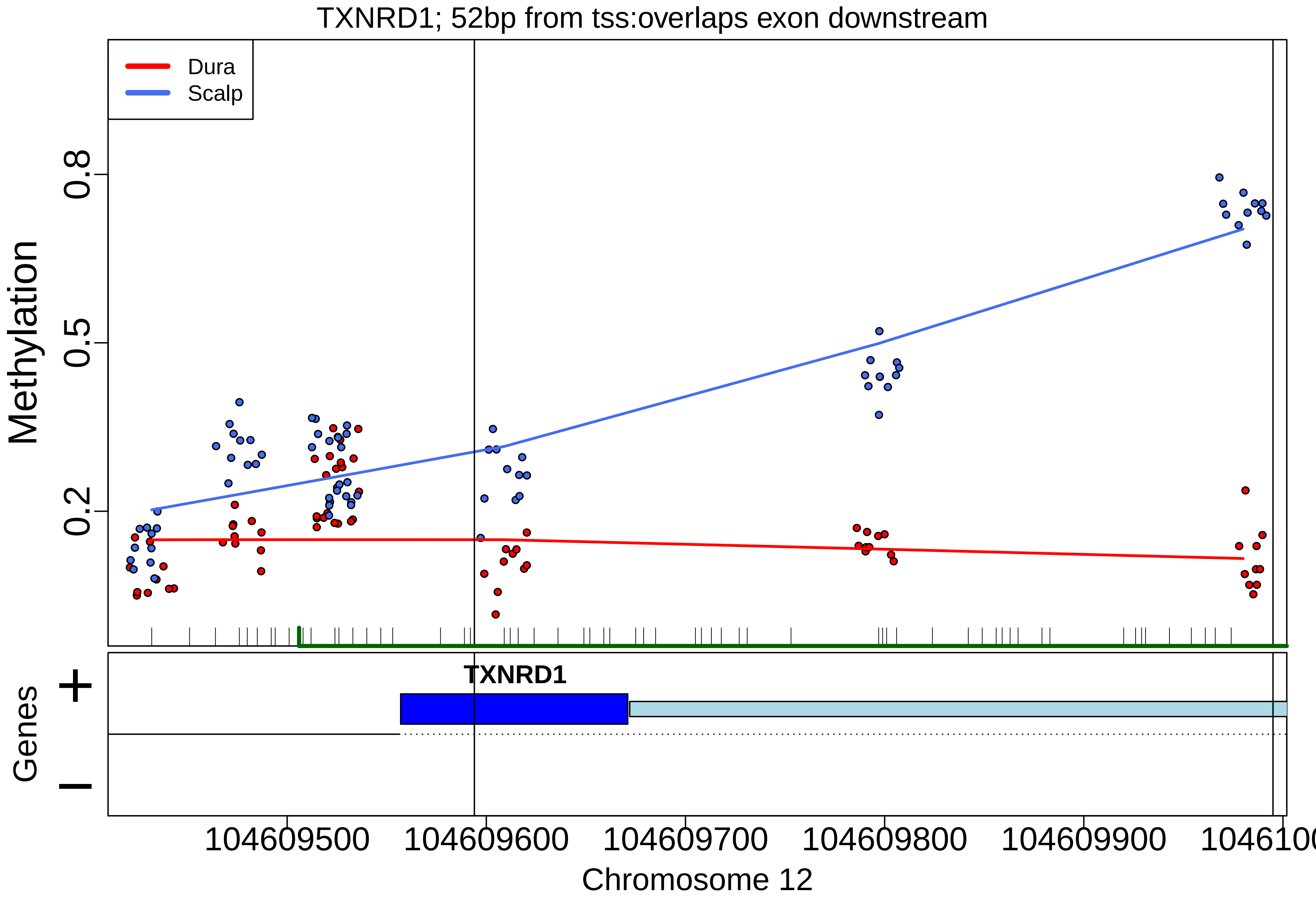
TXNRD1; 52bp from tss:overlaps exon downstream
Dura
Scalp
Methylation
0.2
0.5
0.8
Genes
+
-
TXNRD1
104609500
104609600
104609700
104609800
104609900
Chromosome 12

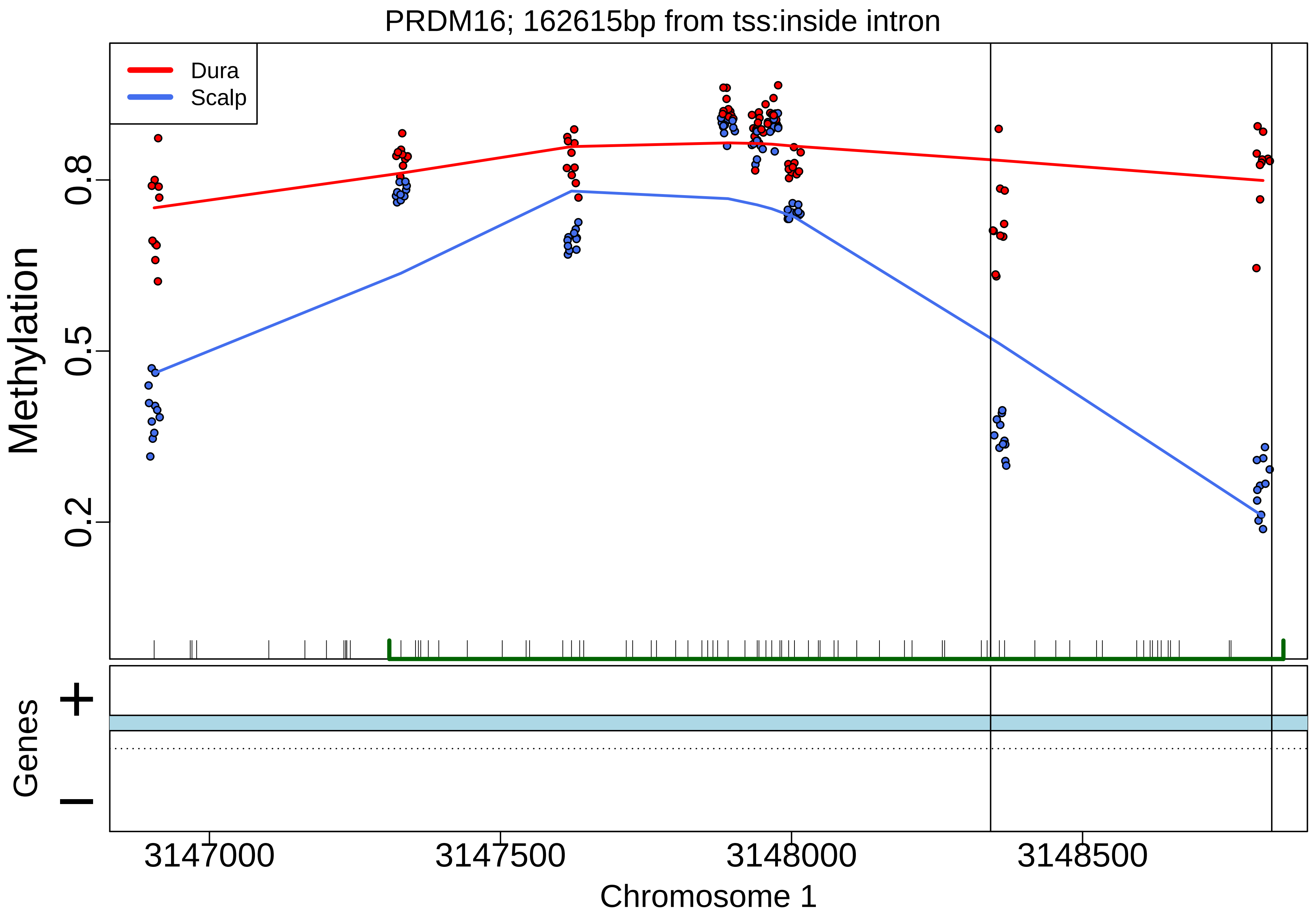
PRDM16; 162615bp from tss:inside intron
Dura
Scalp
Methylation
0.2
0.5
0.8
Genes
+
−
3147000
3147500
3148000
3148500
Chromosome 1

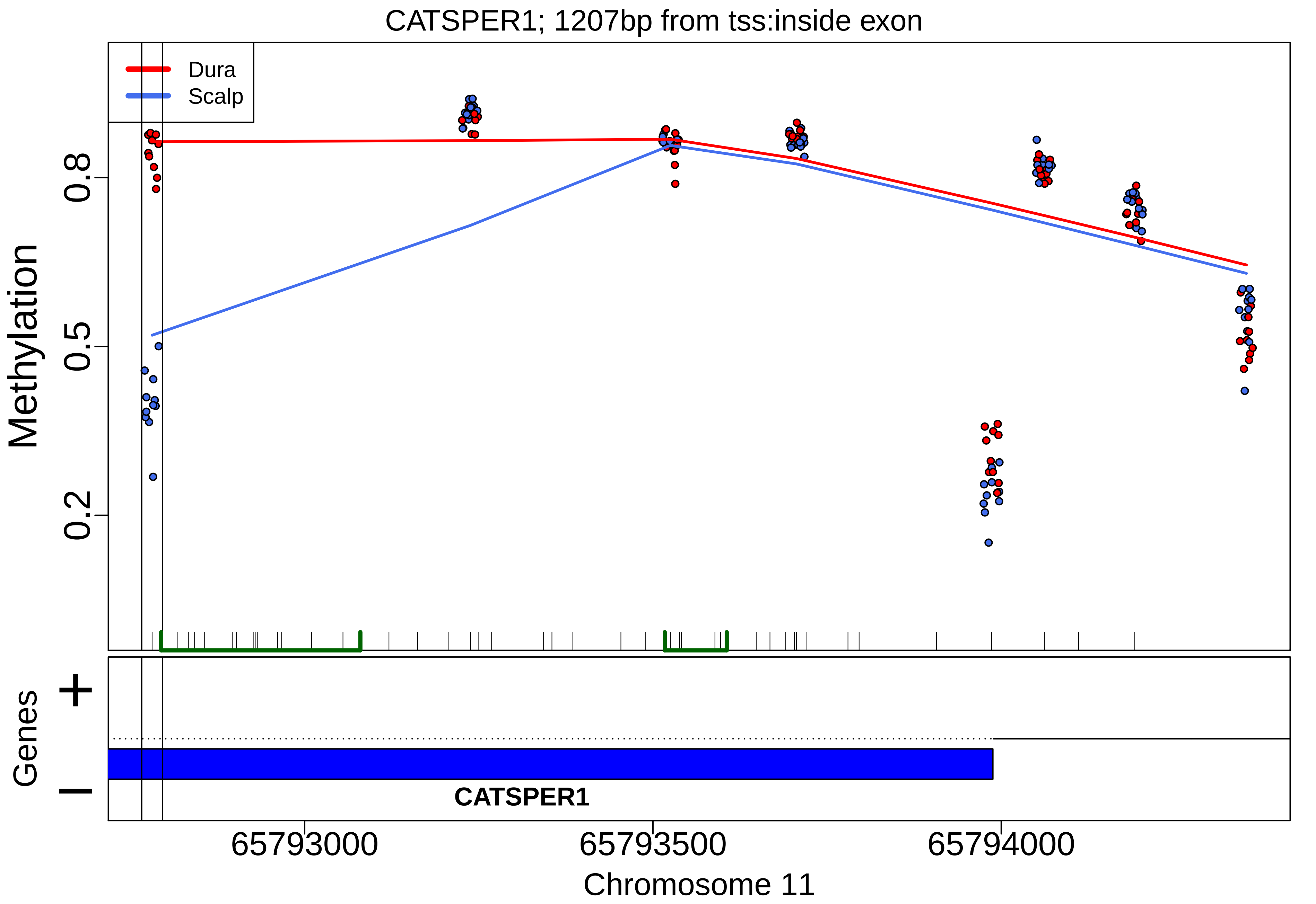
CATSPER1; 1207bp from tss:inside exon
Dura
Scalp
Methylation
0.2
0.5
0.8
Genes
+
-
CATSPER1
65793000
65793500
65794000
Chromosome 11

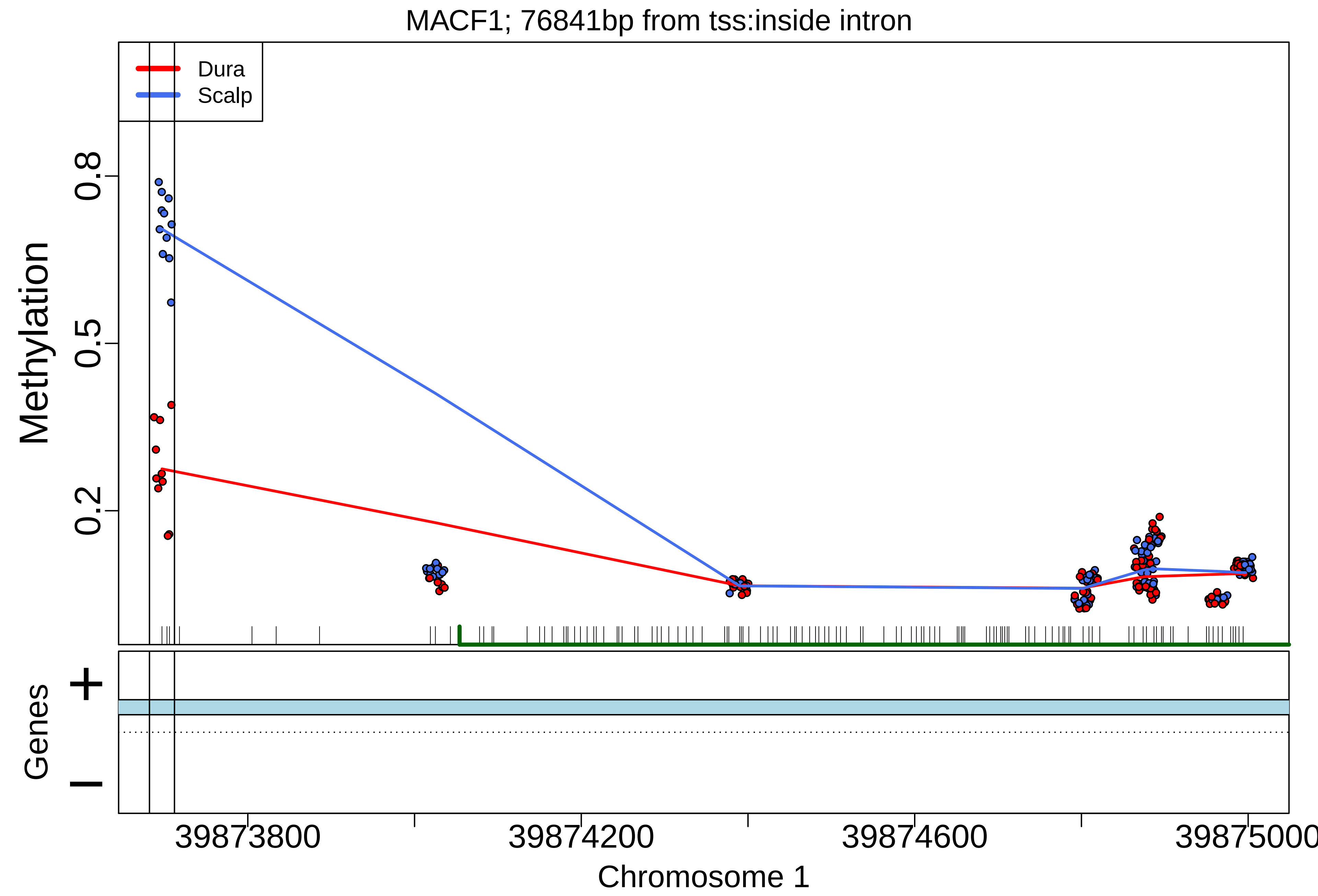
MACF1; 76841bp from tss:inside intron
Dura
Scalp
Methylation
0.2
0.5
0.8
Genes
+
-
39873800
39874200
39874600
39875000
Chromosome 1

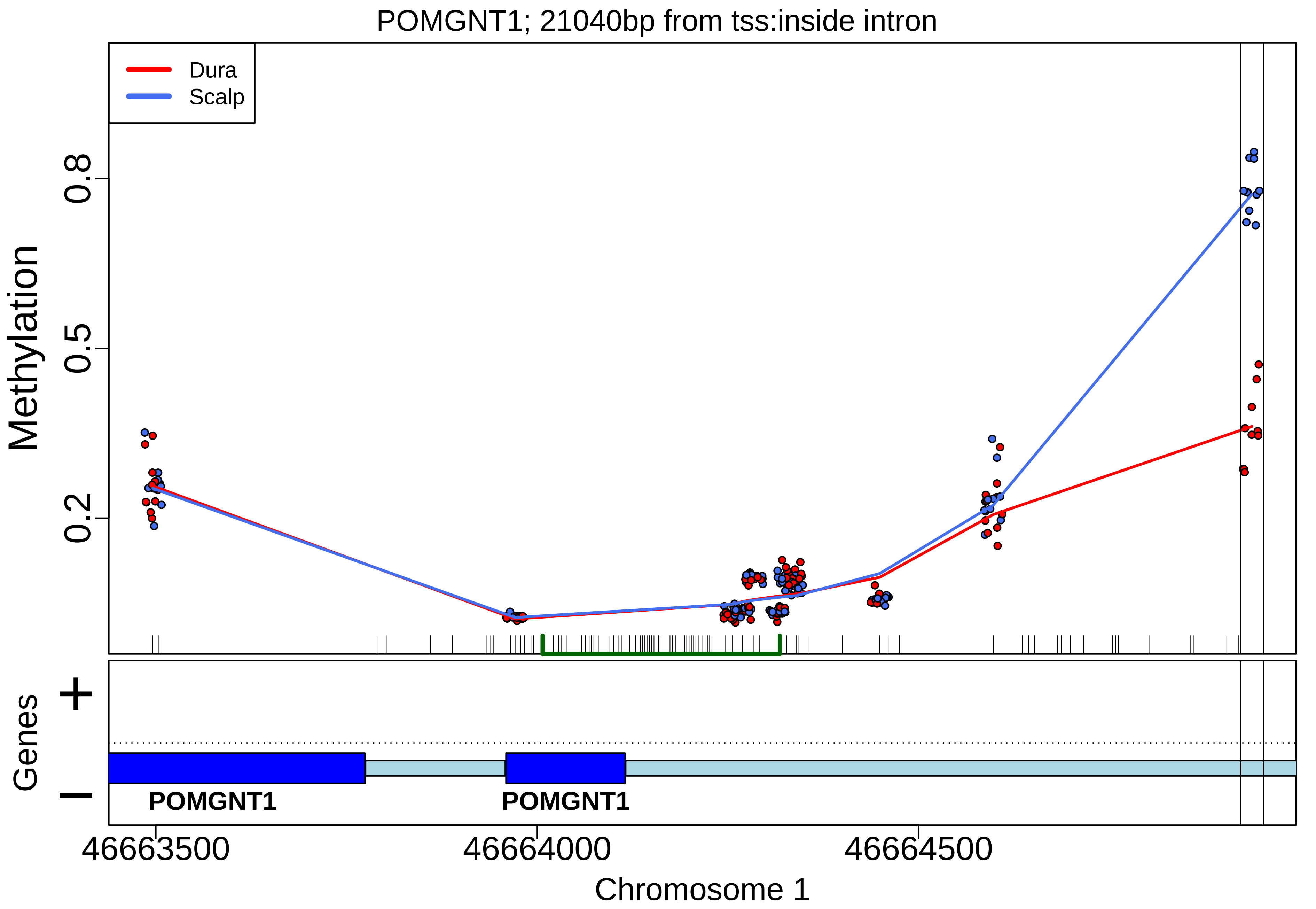
POMGNT1; 21040bp from tss:inside intron
Dura
Scalp
Methylation
0.2
0.5
0.8
Genes
+
-
POMGNT1
POMGNT1
46663500
46664000
46664500
Chromosome 1

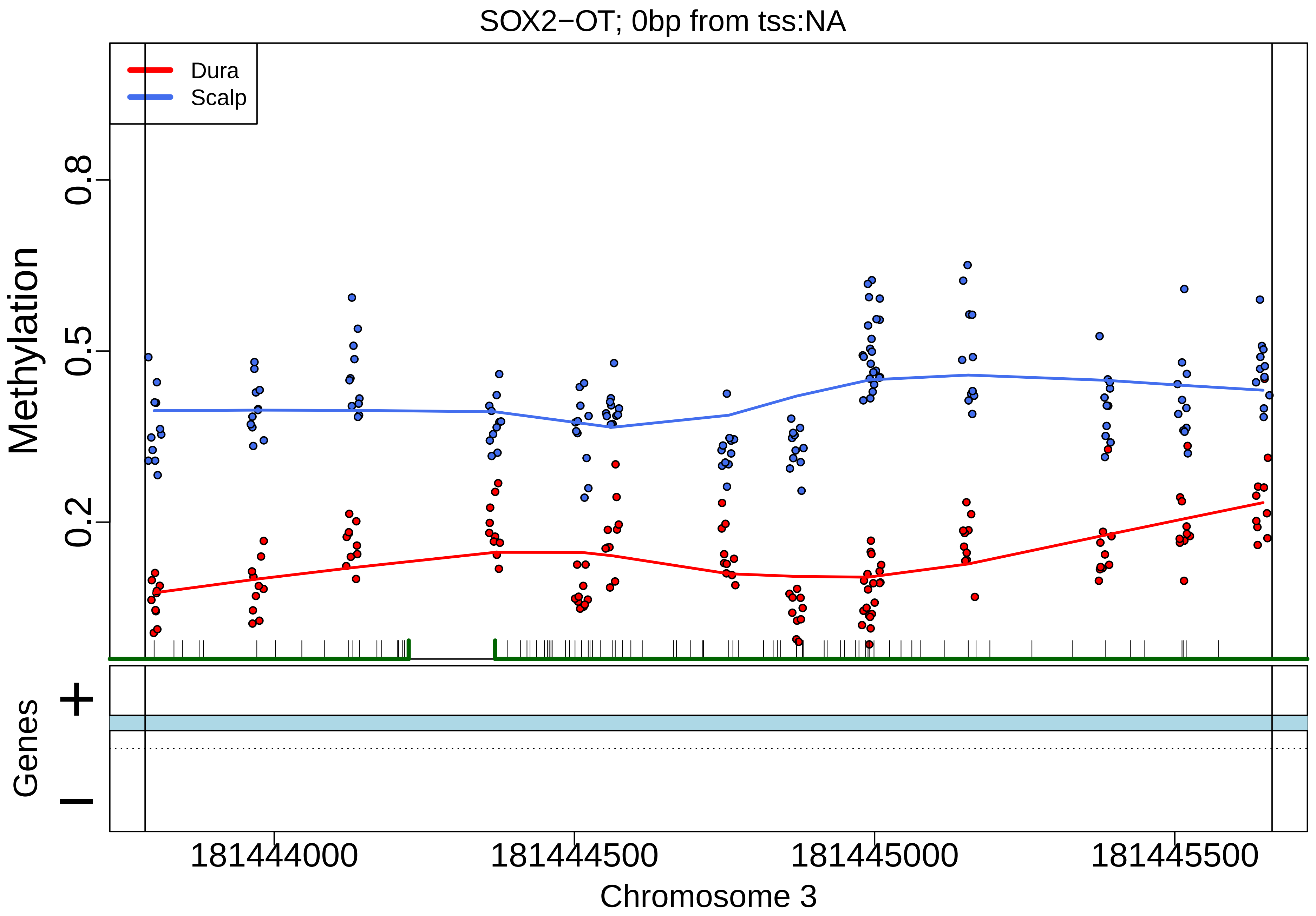
SOX2−OT; 0bp from tss:NA
Dura
Scalp
Methylation
0.2
0.5
0.8
Genes
+
−
181444000
181444500
181445000
181445500
Chromosome 3

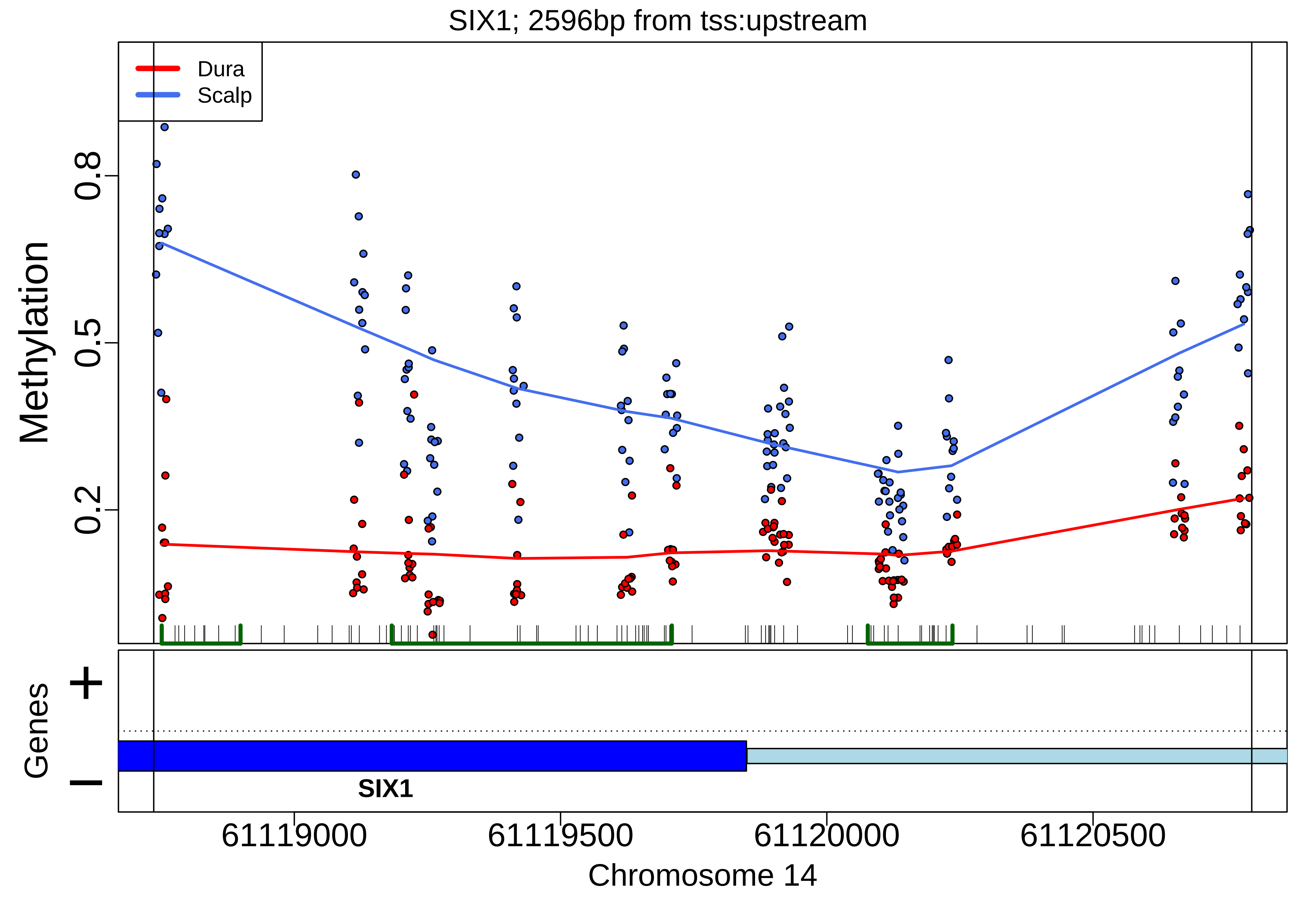

SIX1; 2596bp from tss:upstream
Dura
Scalp
Methylation
0.2
0.5
0.8
Genes
+
-
SIX1
61119000
61119500
61120000
61120500
Chromosome 14

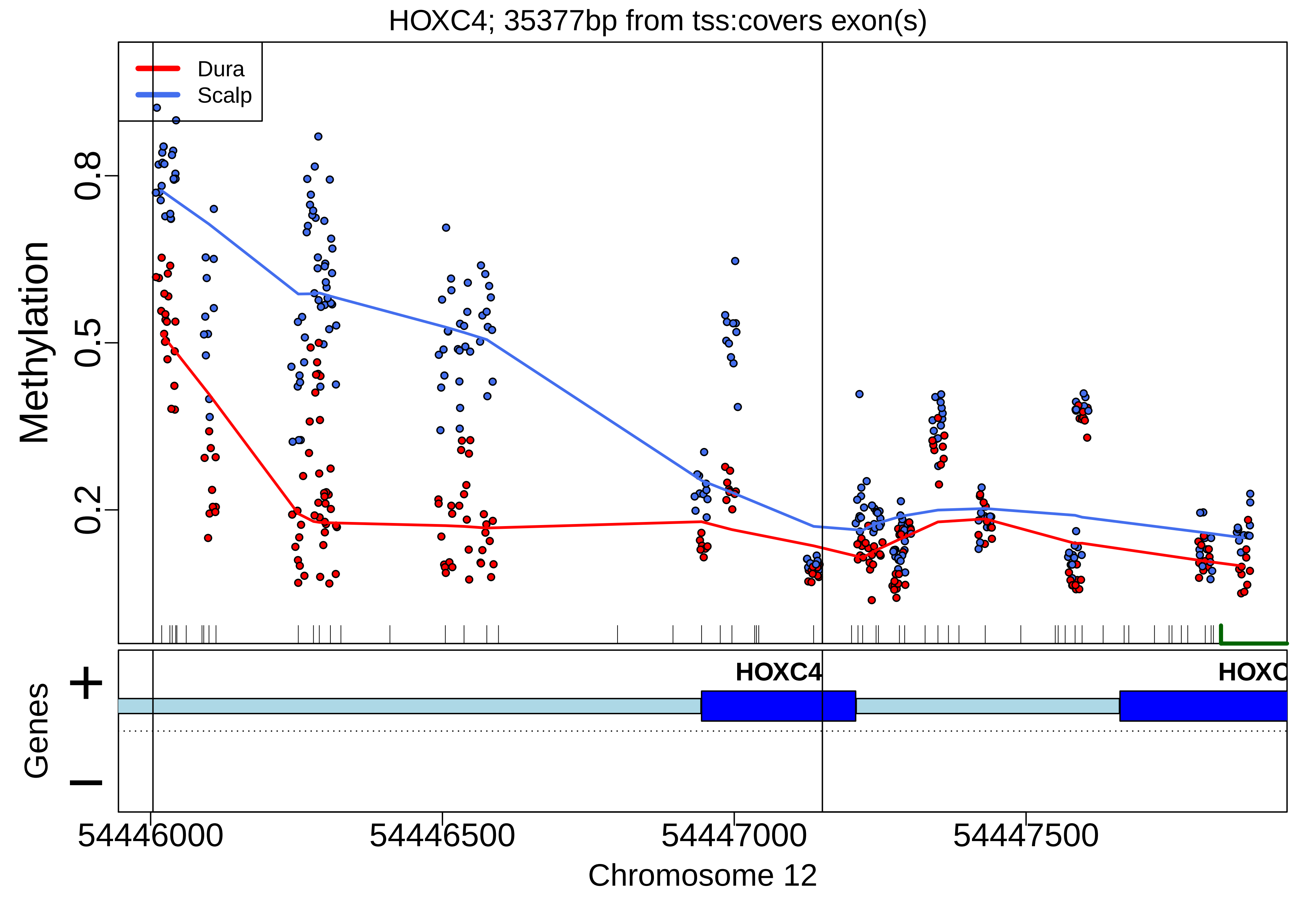

HOXC4; 35377bp from tss:covers exon(s)
Dura
Scalp
Methylation
0.2
0.5
0.8
Genes
+
−
HOXC4
HOXC
54446000
54446500
54447000
54447500
Chromosome 12

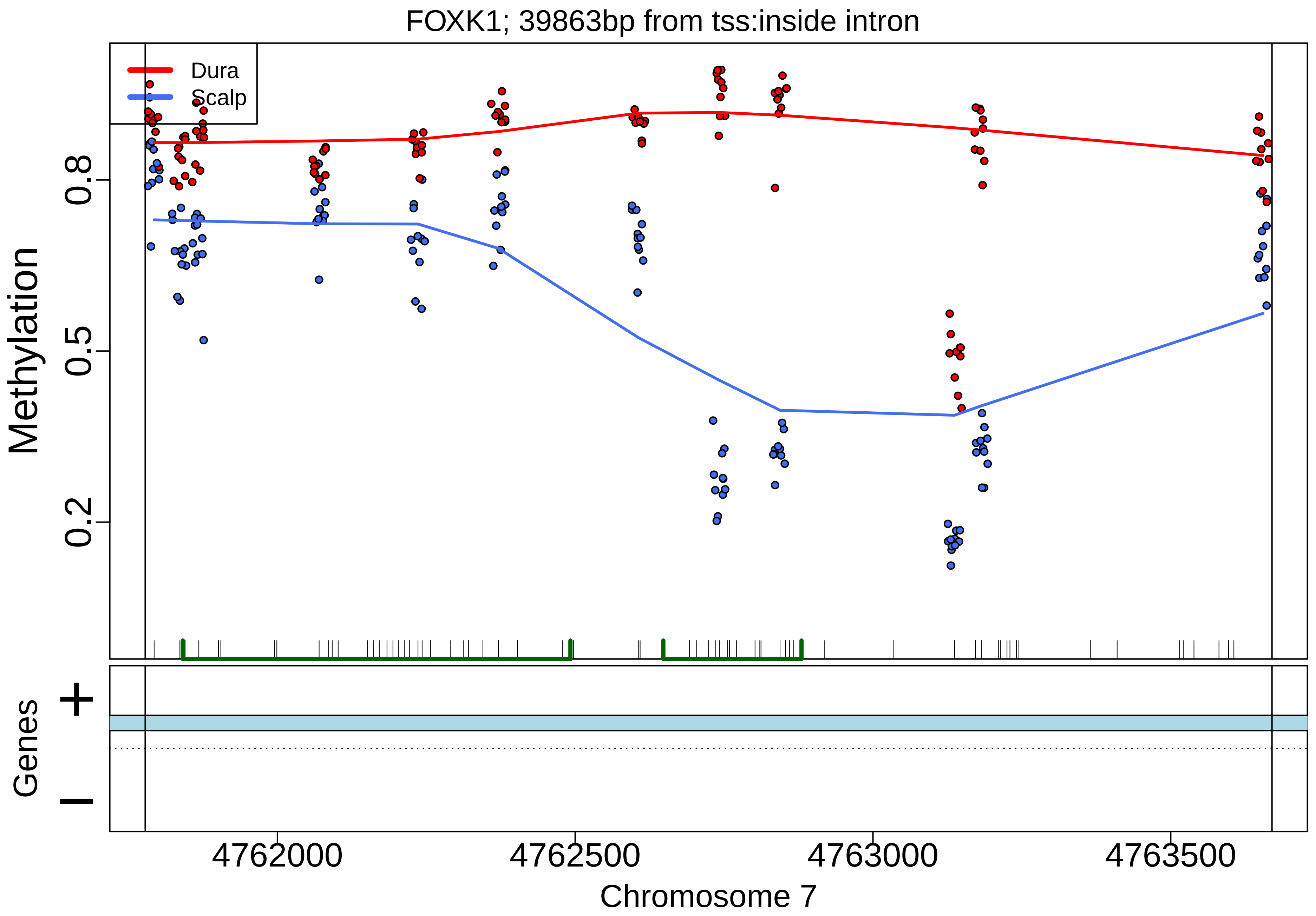

FOXK1; 39863bp from tss:inside intron
Dura
Scalp
Methylation
0.2
0.5
0.8
Genes
+
–
4762000
4762500
4763000
4763500
Chromosome 7

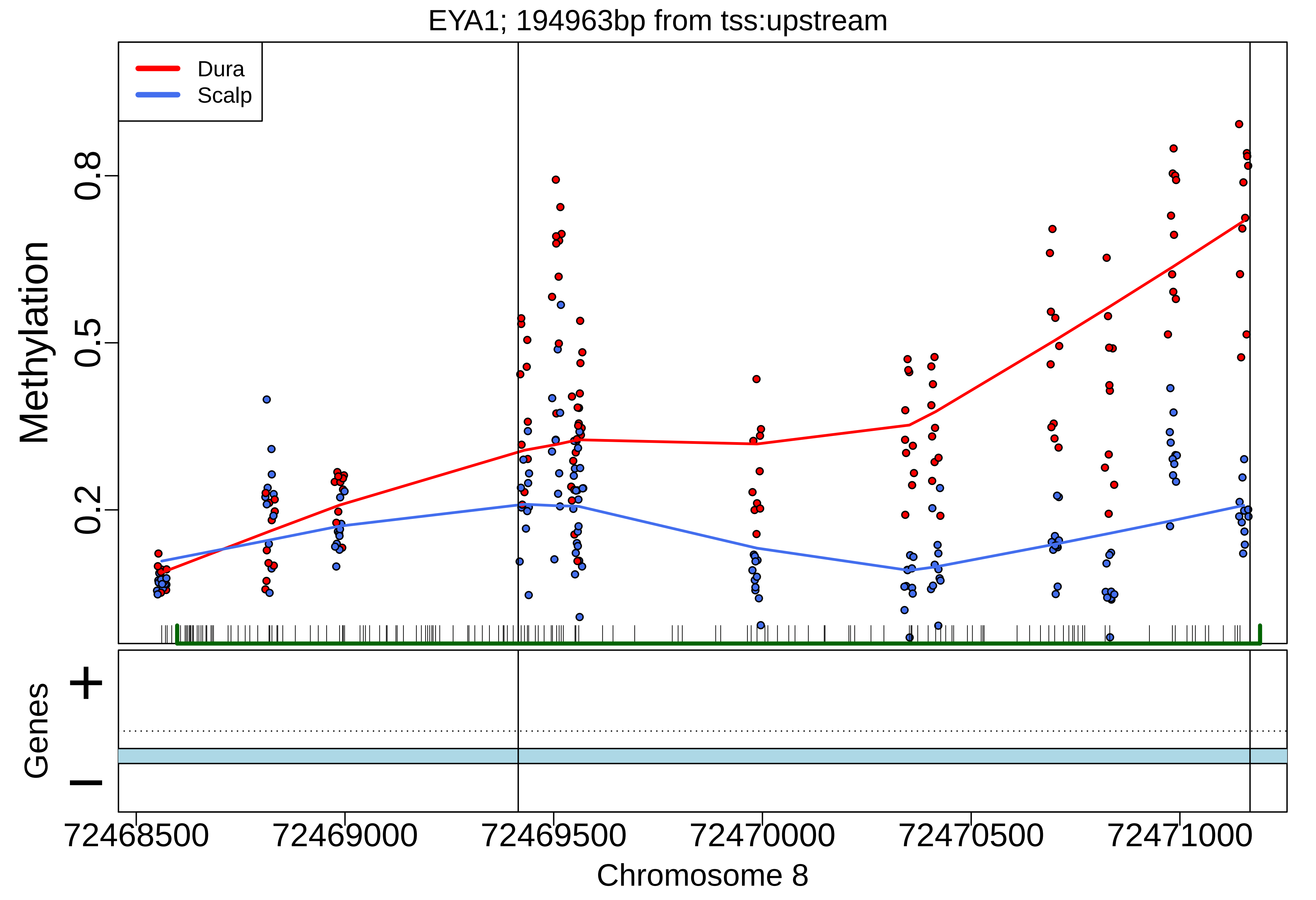
EYA1; 194963bp from tss:upstream
Dura
Scalp
Methylation
0.2
0.5
0.8
Genes
+
−
72468500
72469000
72469500
72470000
72470500
72471000
Chromosome 8

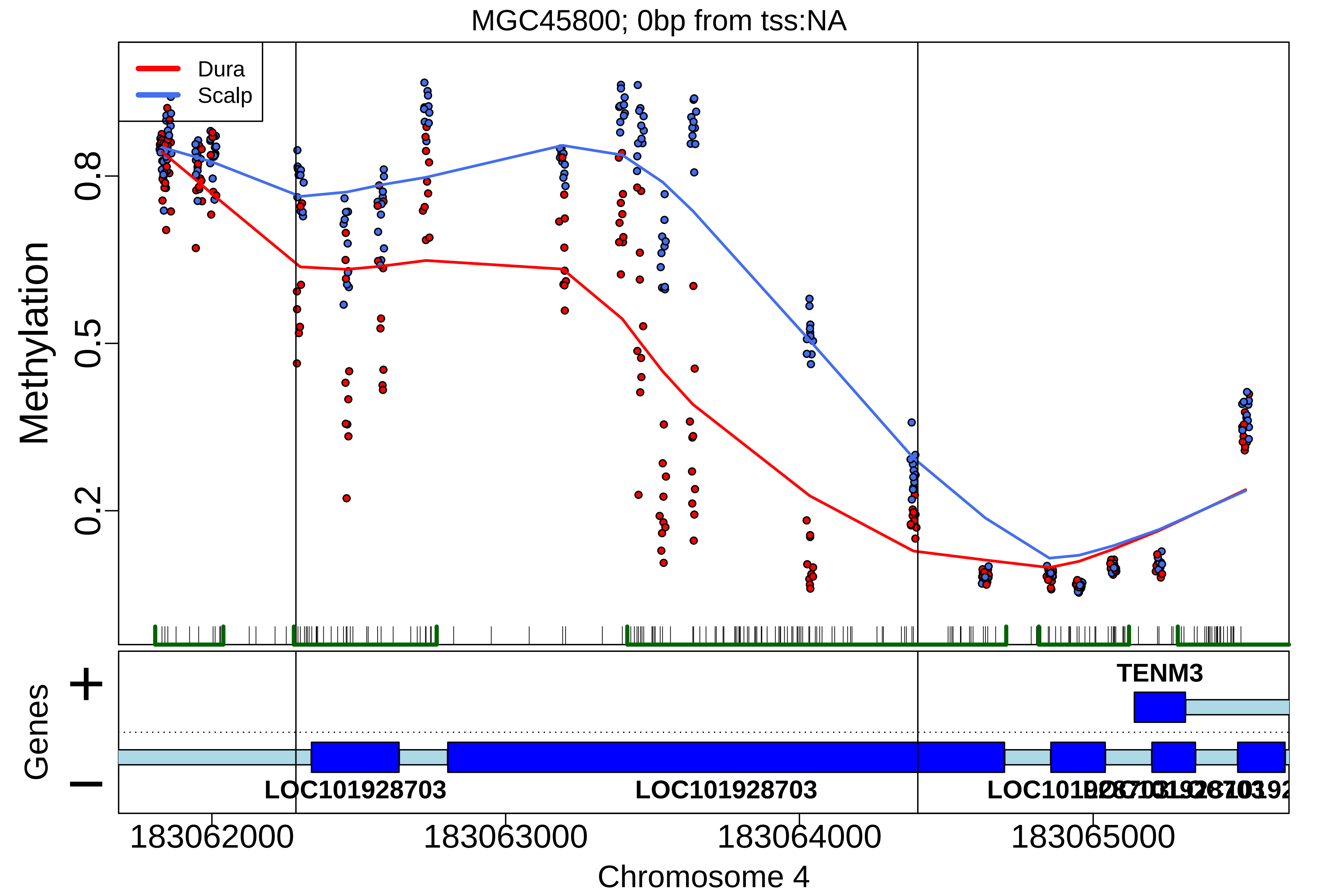
MGC45800; 0bp from tss:NA
Dura
Scalp
Methylation
0.2
0.5
0.8
Genes
+
−
TENM3
LOC101928703
LOC101928703
183062000
183063000
183064000
183065000
Chromosome 4

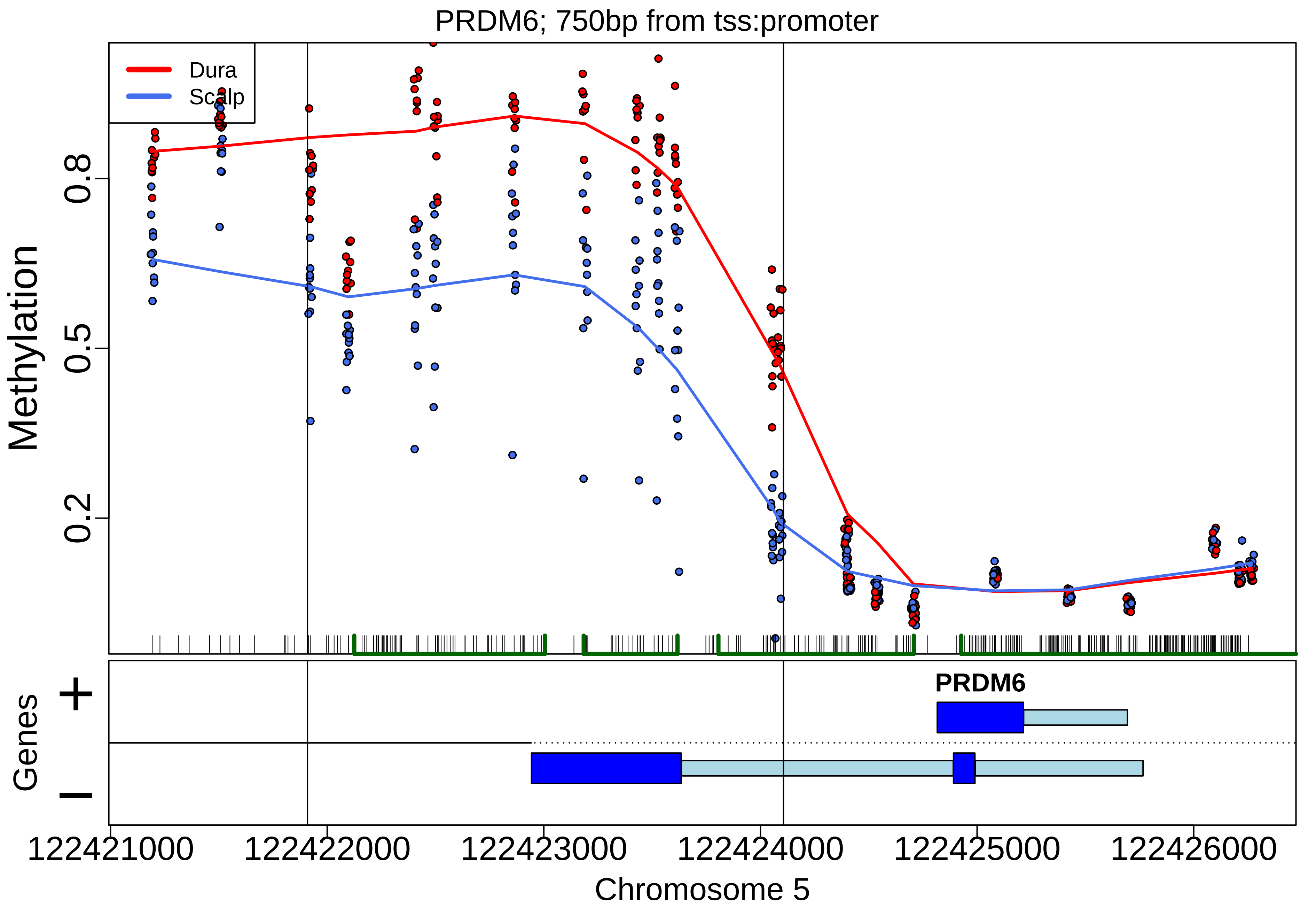

PRDM6; 750bp from tss:promoter
Dura
Scalp
Methylation
0.2
0.5
0.8
Genes
+
−
PRDM6
122421000
122422000
122423000
122424000
122425000
122426000
Chromosome 5

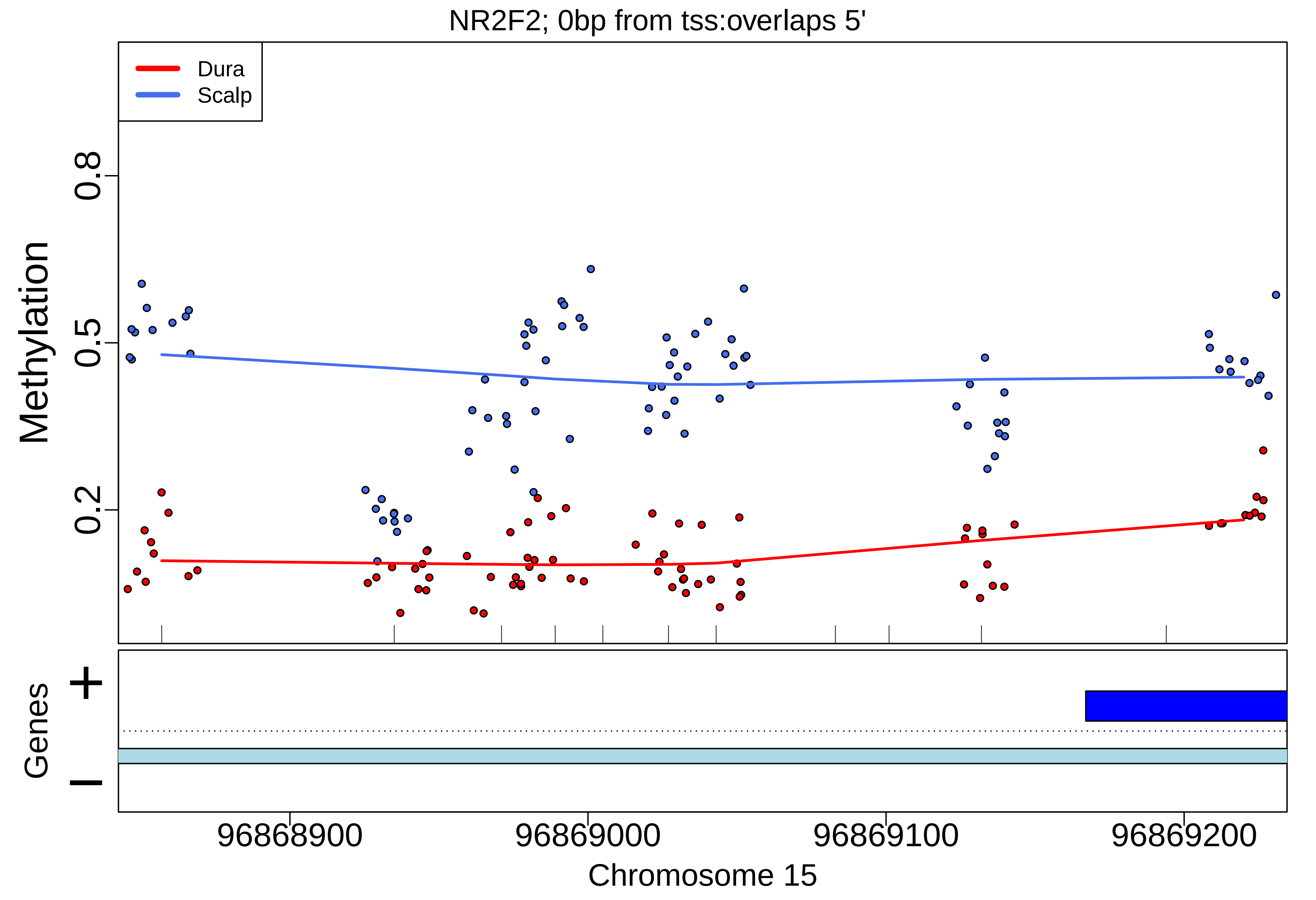
NR2F2; 0bp from tss:overlaps 5'
Dura
Scalp
Methylation
0.2
0.5
0.8
Genes
+
-
96868900
96869000
96869100
96869200
Chromosome 15

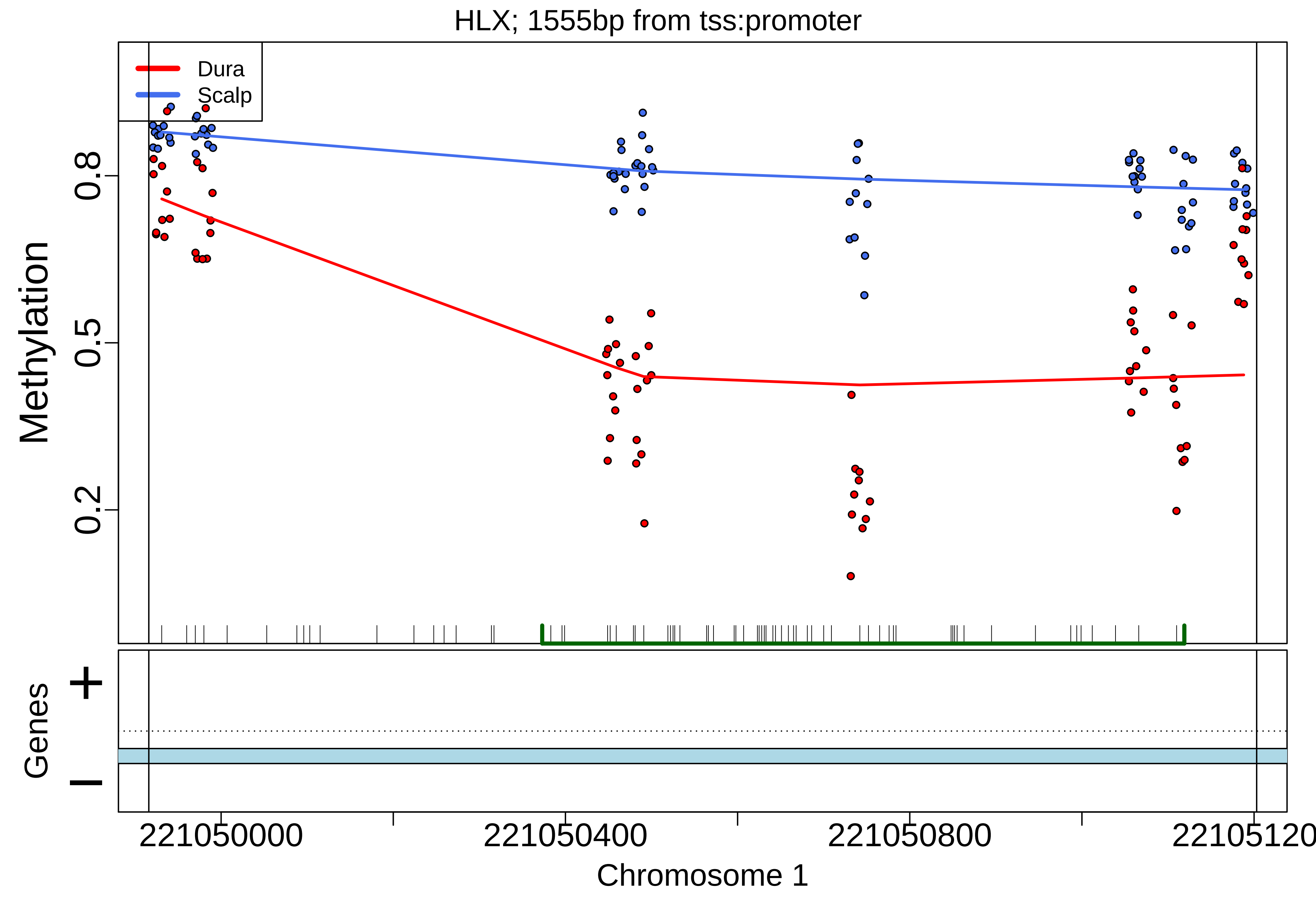
HLX; 1555bp from tss:promoter
Dura
Scalp
Methylation
0.2
0.5
0.8
Genes
+
-
221050000
221050400
221050800
22105120
Chromosome 1

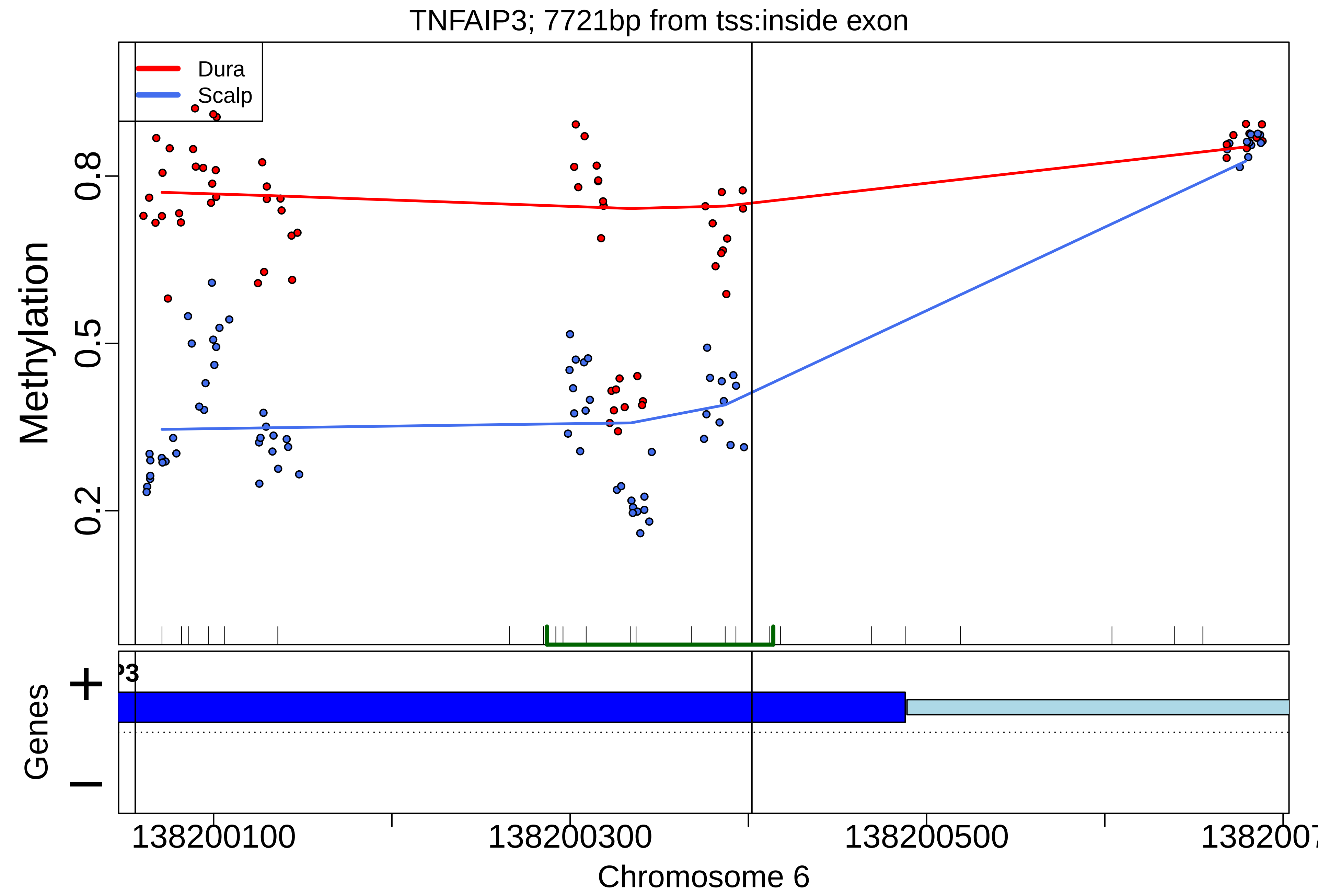
TNFAIP3; 7721bp from tss:inside exon
Dura
Scalp
Methylation
0.2
0.5
0.8
Genes
+
-
P3
138200100
138200300
138200500
Chromosome 6

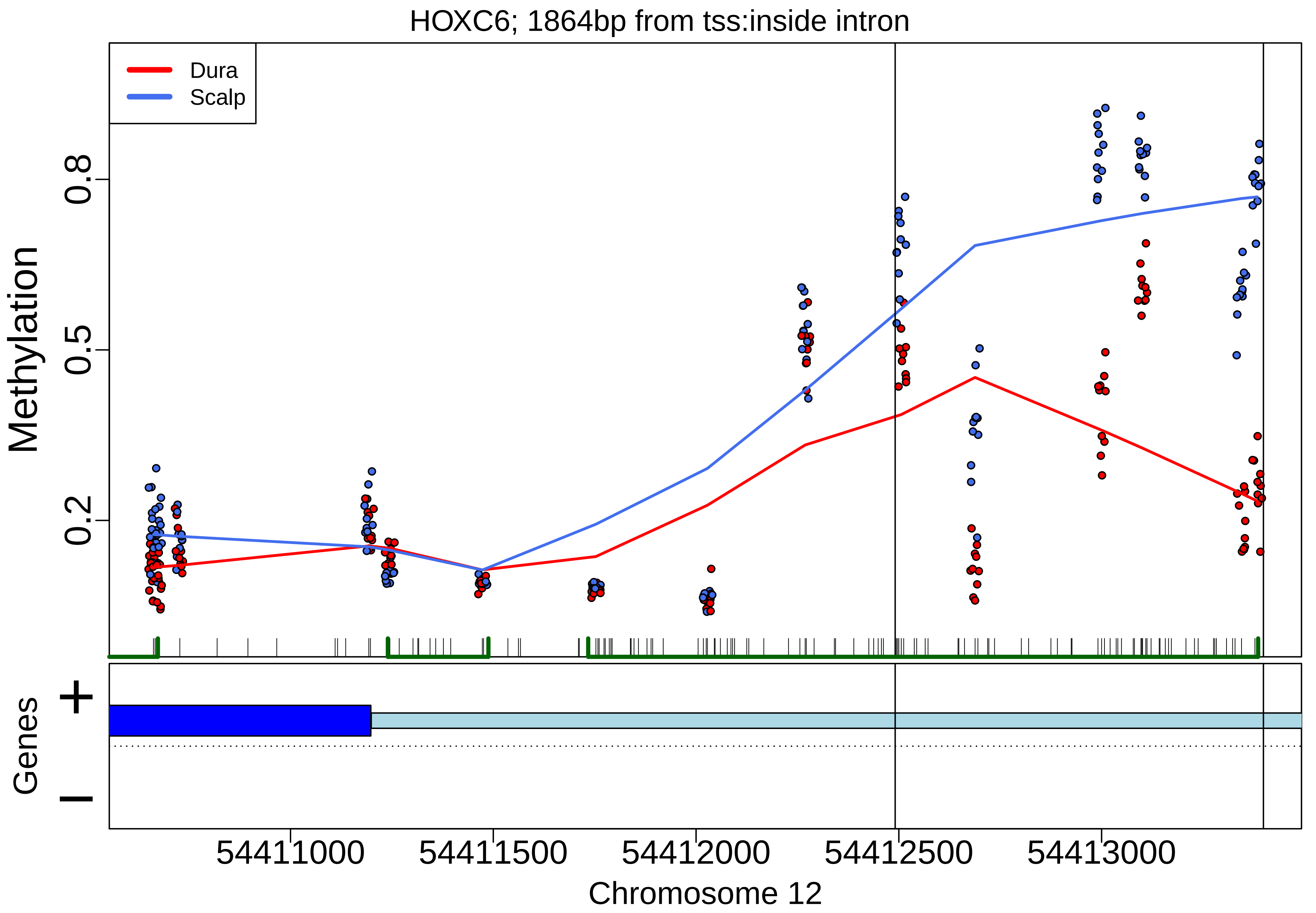
HOXC6; 1864bp from tss:inside intron
Dura
Scalp
Methylation
0.2
0.5
0.8
Genes
+
−
54411000
54411500
54412000
54412500
54413000
Chromosome 12

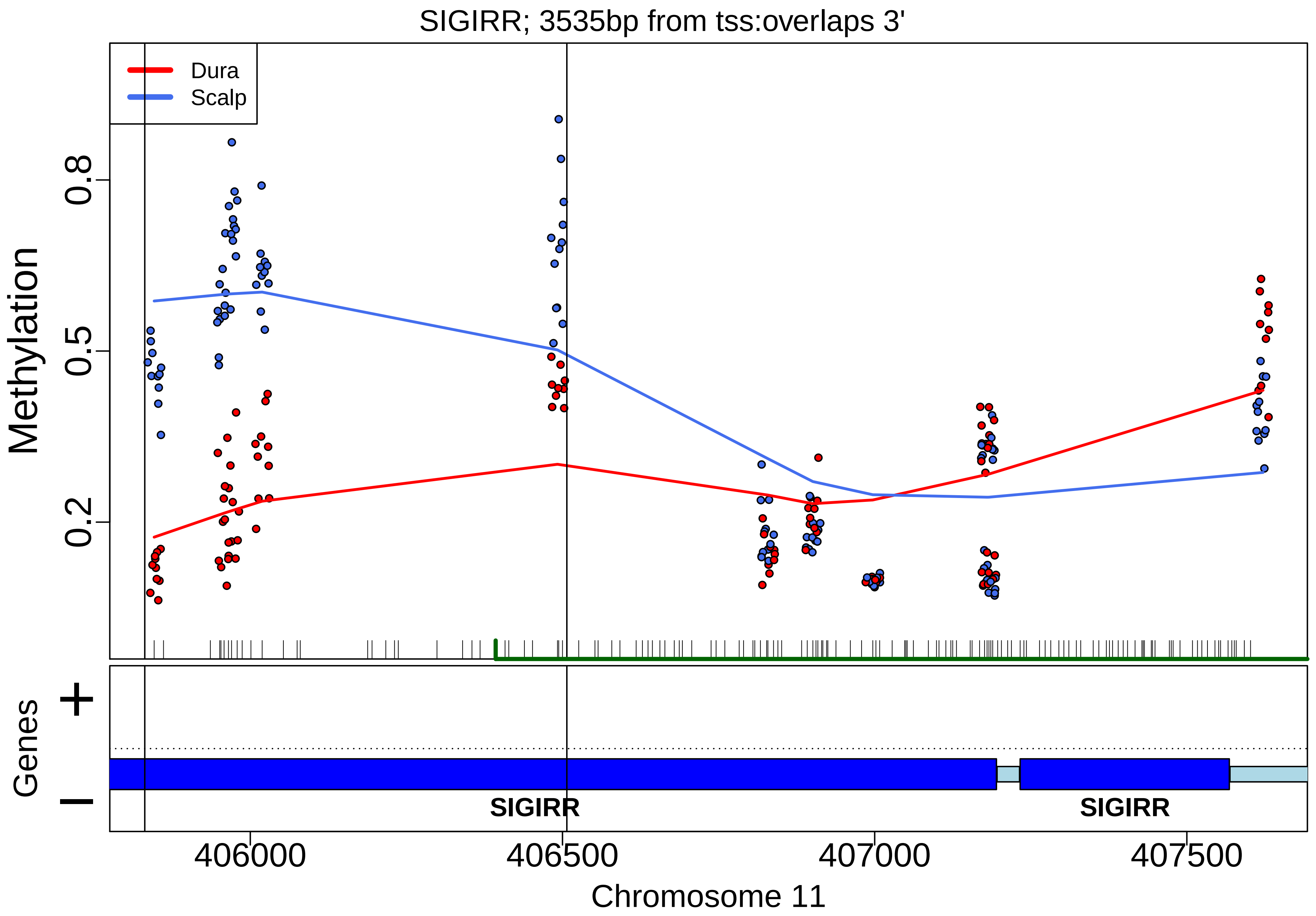

SIGIRR; 3535bp from tss:overlaps 3'
Dura
Scalp
Methylation
0.2
0.5
0.8
Genes
+
−
SIGIRR
SIGIRR
406000
406500
407000
407500
Chromosome 11

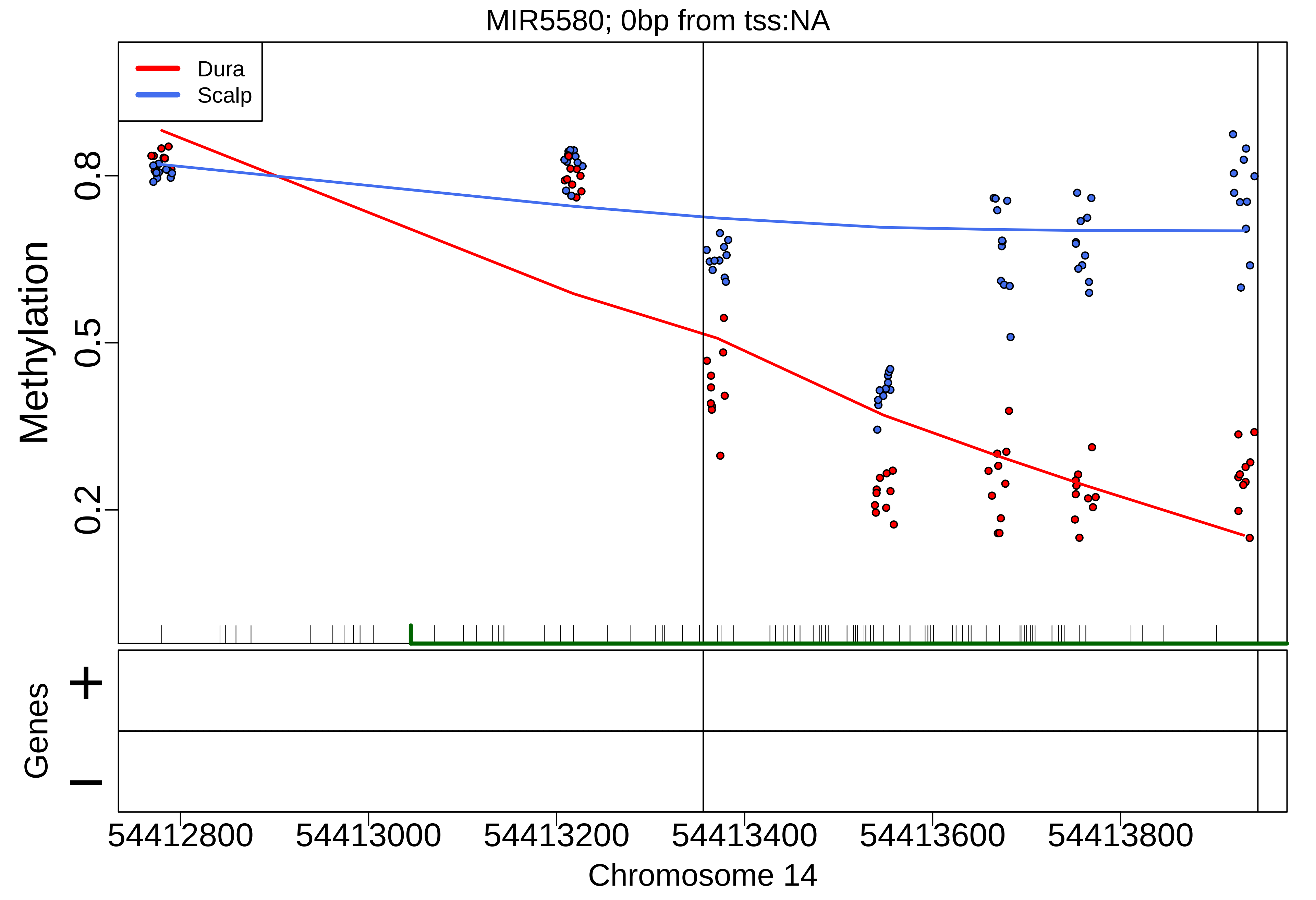

MIR5580; 0bp from tss:NA
Dura
Scalp
Methylation
0.2
0.5
0.8
Genes
+
-
54412800
54413000
54413200
54413400
54413600
54413800
Chromosome 14

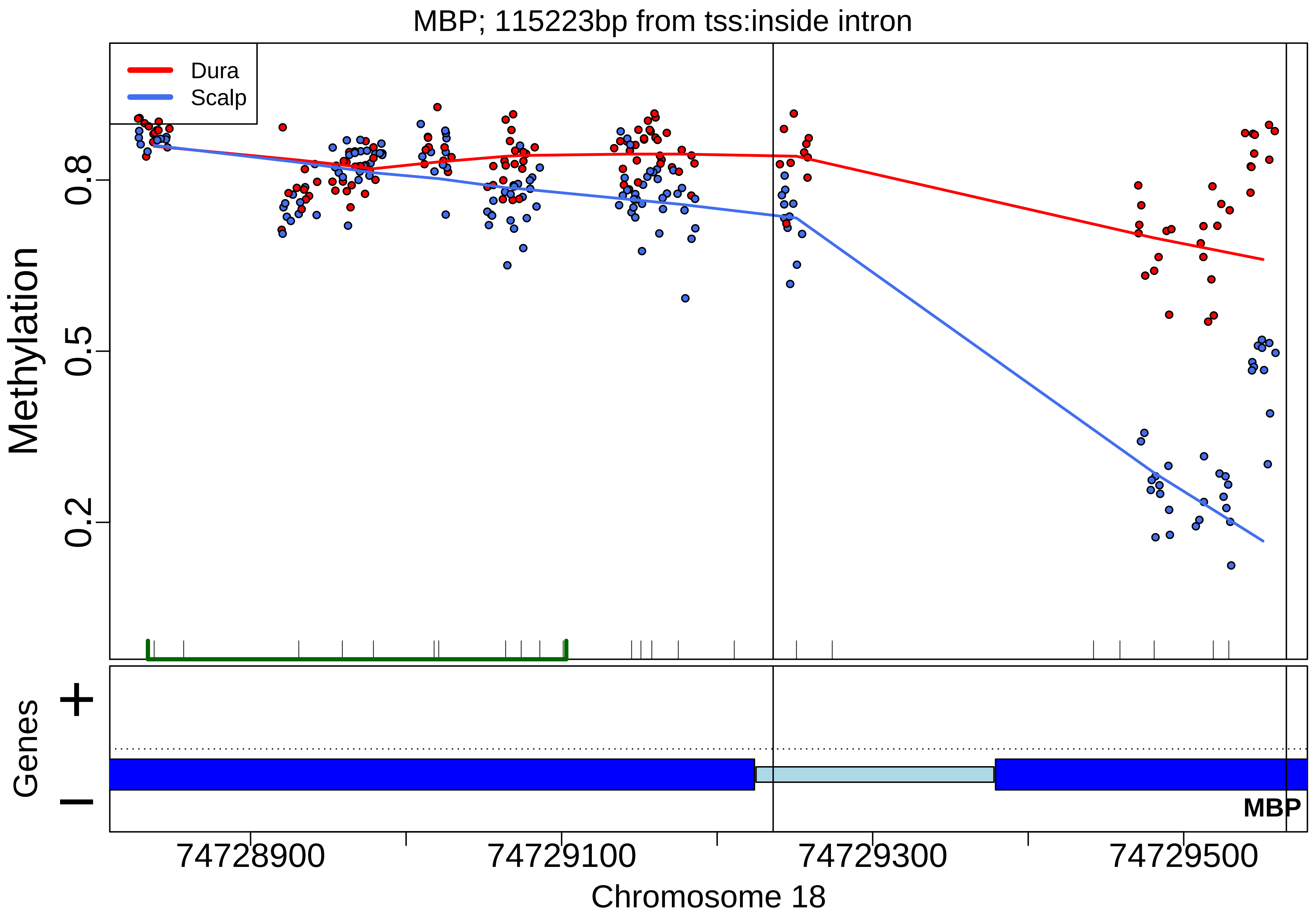
MBP; 115223bp from tss:inside intron
Dura
Scalp
Methylation
0.8
0.5
0.2
Genes
+
−
MBP
74728900
74729100
74729300
74729500
Chromosome 18

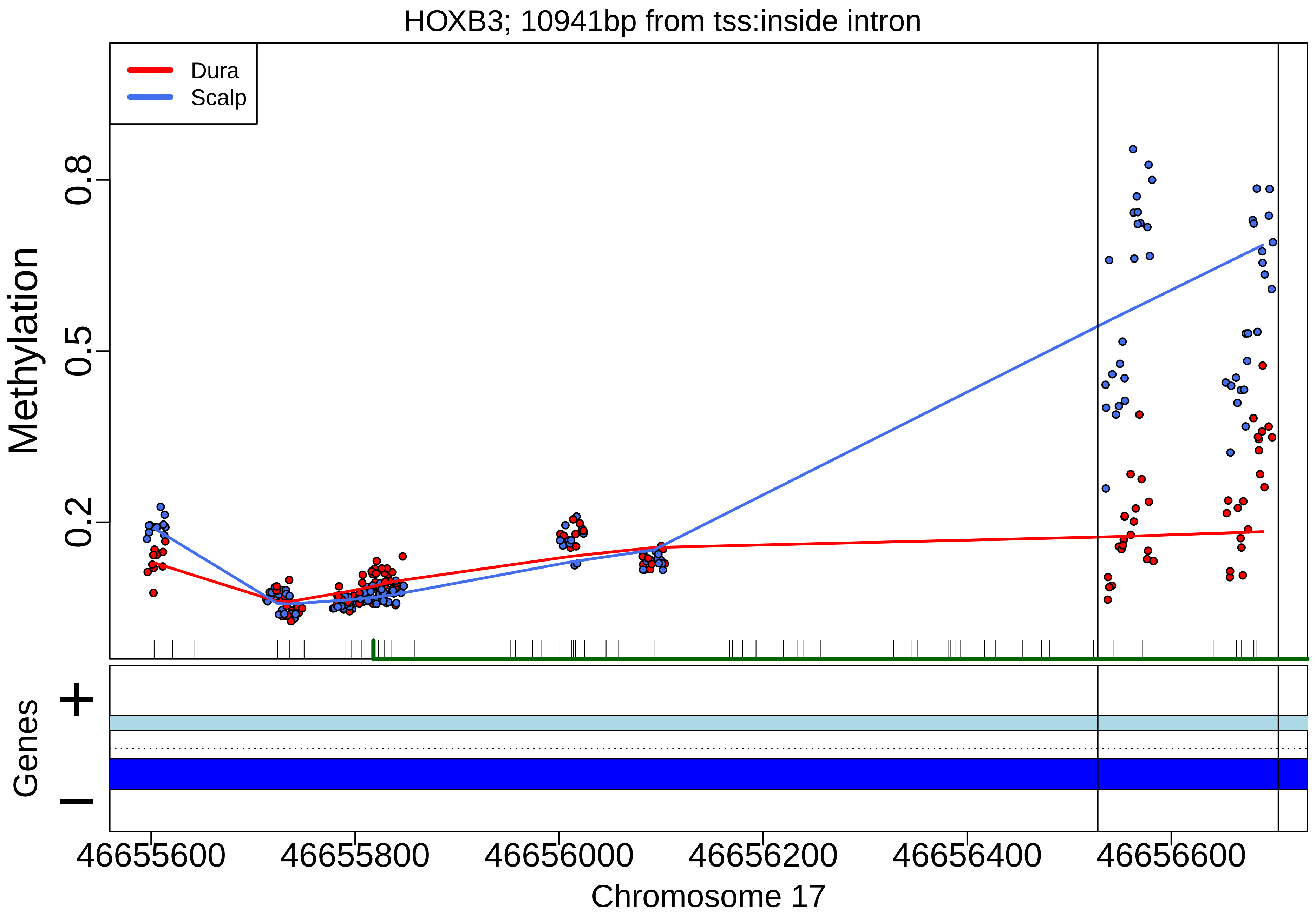

HOXB3; 10941bp from tss:inside intron
Dura
Scalp
Methylation
0.2
0.5
0.8
Genes
+
−
46655600
46655800
46656000
46656200
46656400
46656600
Chromosome 17

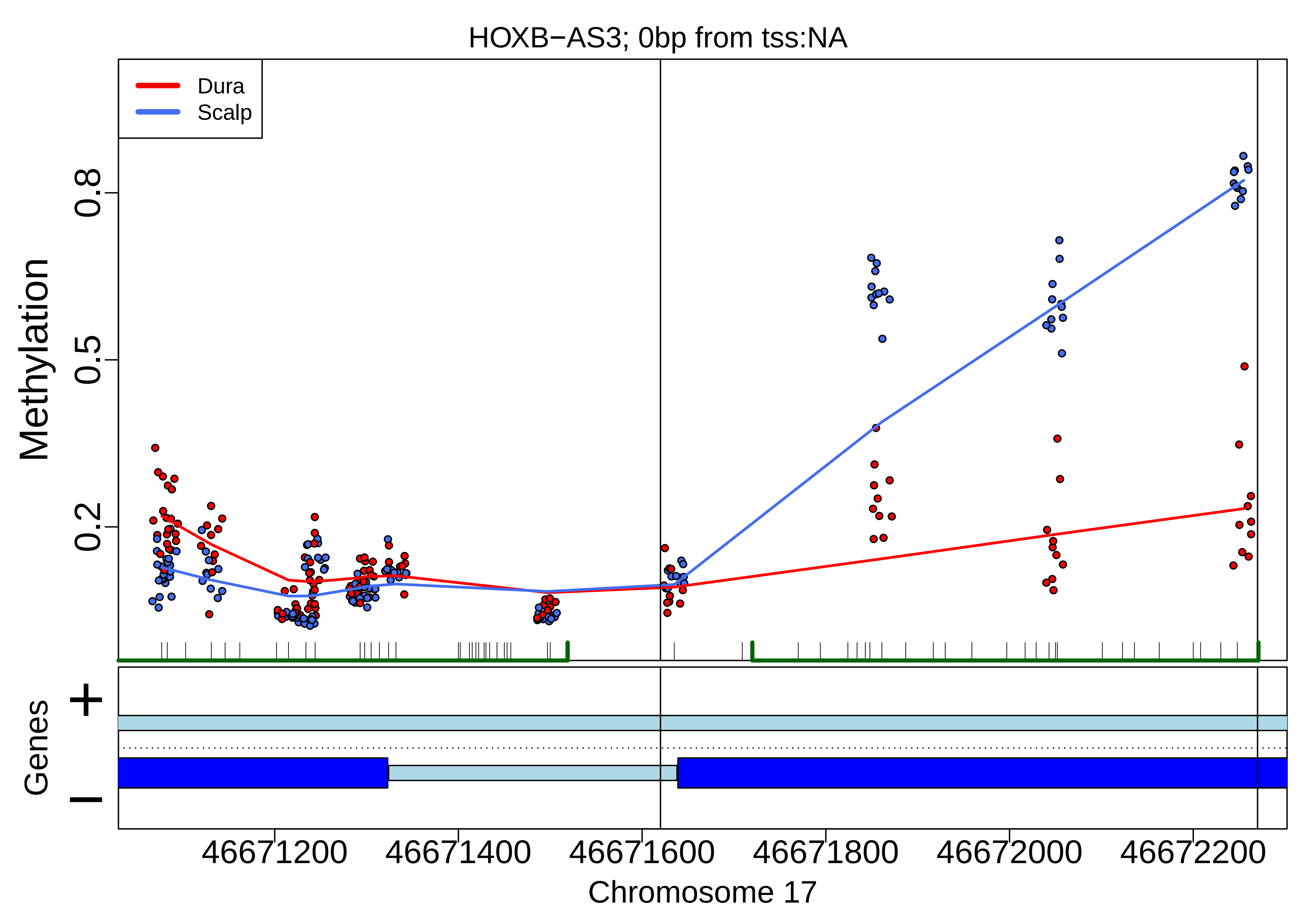
HOXB−AS3; 0bp from tss:NA
Dura
Scalp
Methylation
0.2
0.5
0.8
Genes
+
−
46671200
46671400
46671600
46671800
46672000
46672200
Chromosome 17

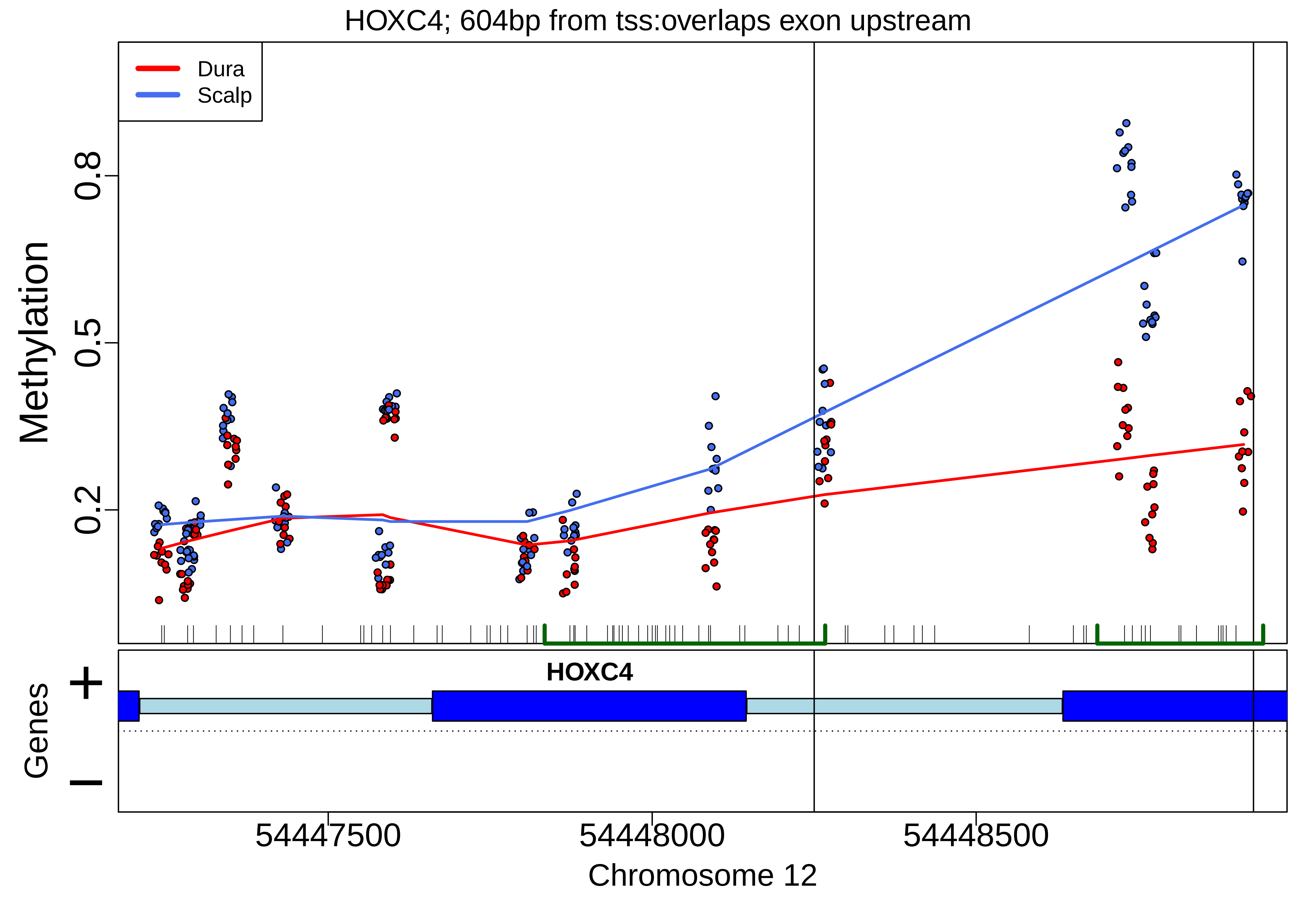
HOXC4; 604bp from tss:overlaps exon upstream
Dura
Scalp
Methylation
0.2
0.5
0.8
Genes
+
−
HOXC4
54447500
54448000
54448500
Chromosome 12

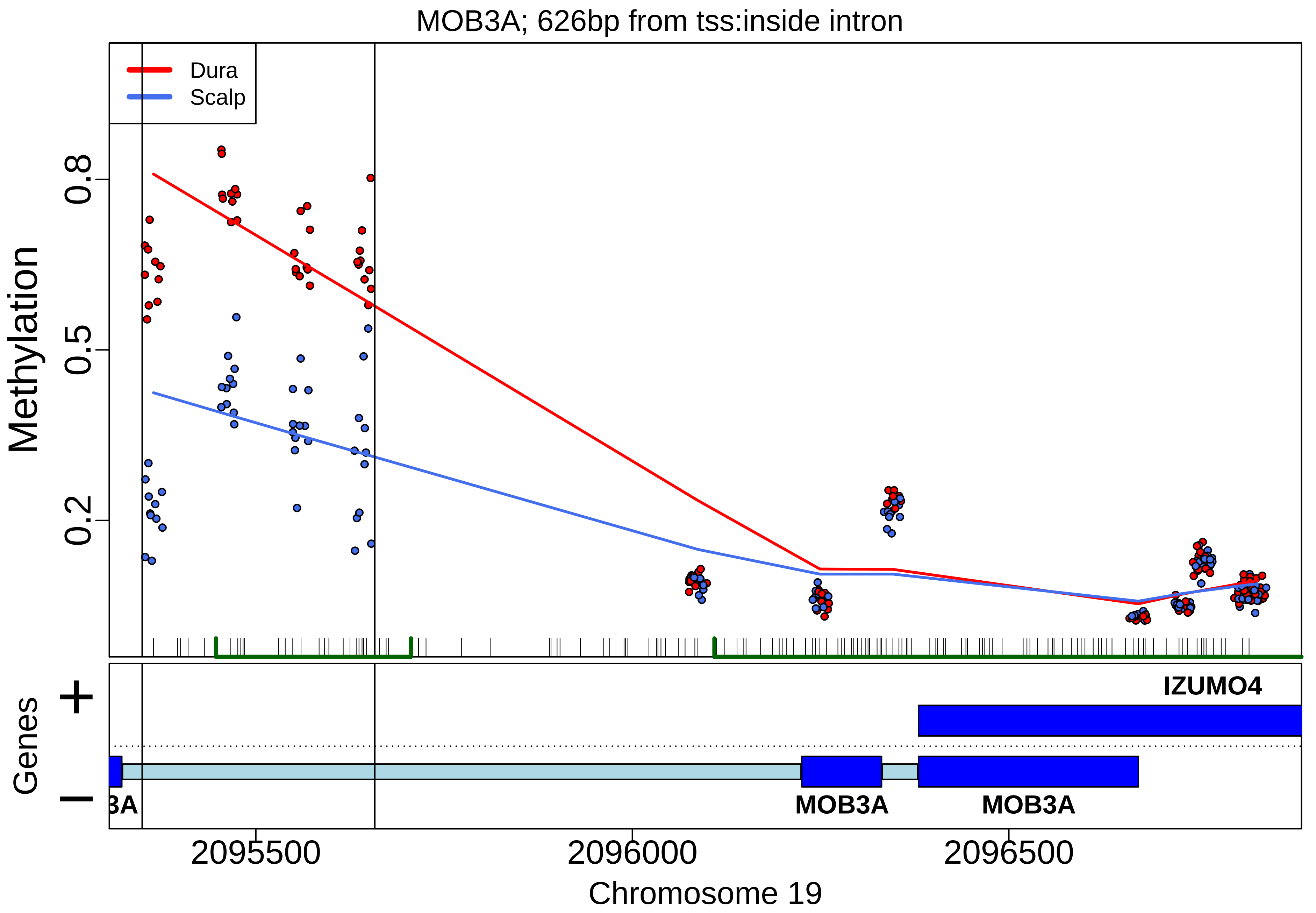
MOB3A; 626bp from tss:inside intron
Dura
Scalp
Methylation
0.8
0.5
0.2
Genes
+
IZUMO4
3A
MOB3A
MOB3A
2095500
2096000
2096500
Chromosome 19

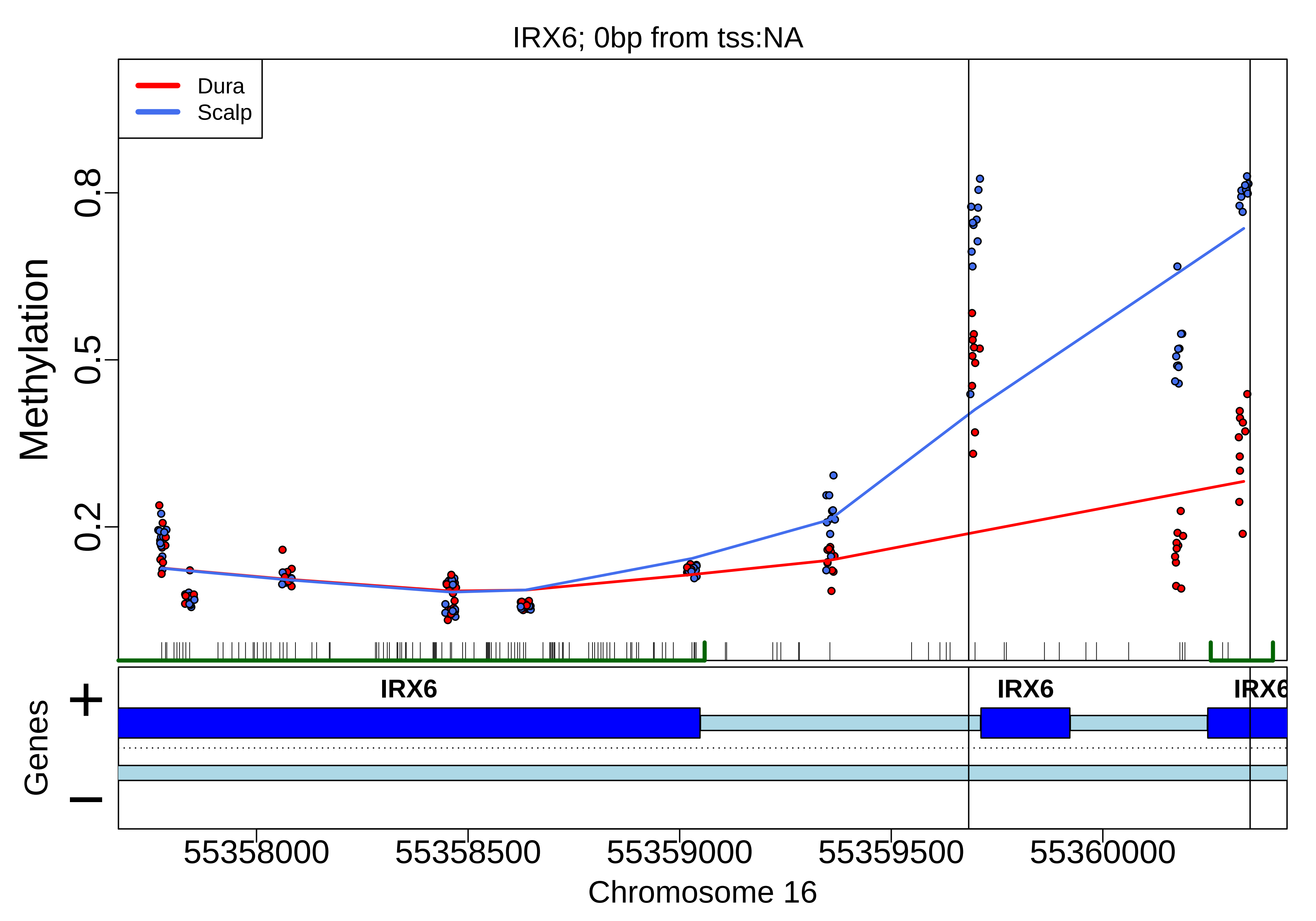
IRX6; 0bp from tss:NA
Dura
Scalp
Methylation
0.2
0.5
0.8
Genes
+
-
IRX6
IRX6
IRX6
55358000
55358500
55359000
55359500
55360000
Chromosome 16

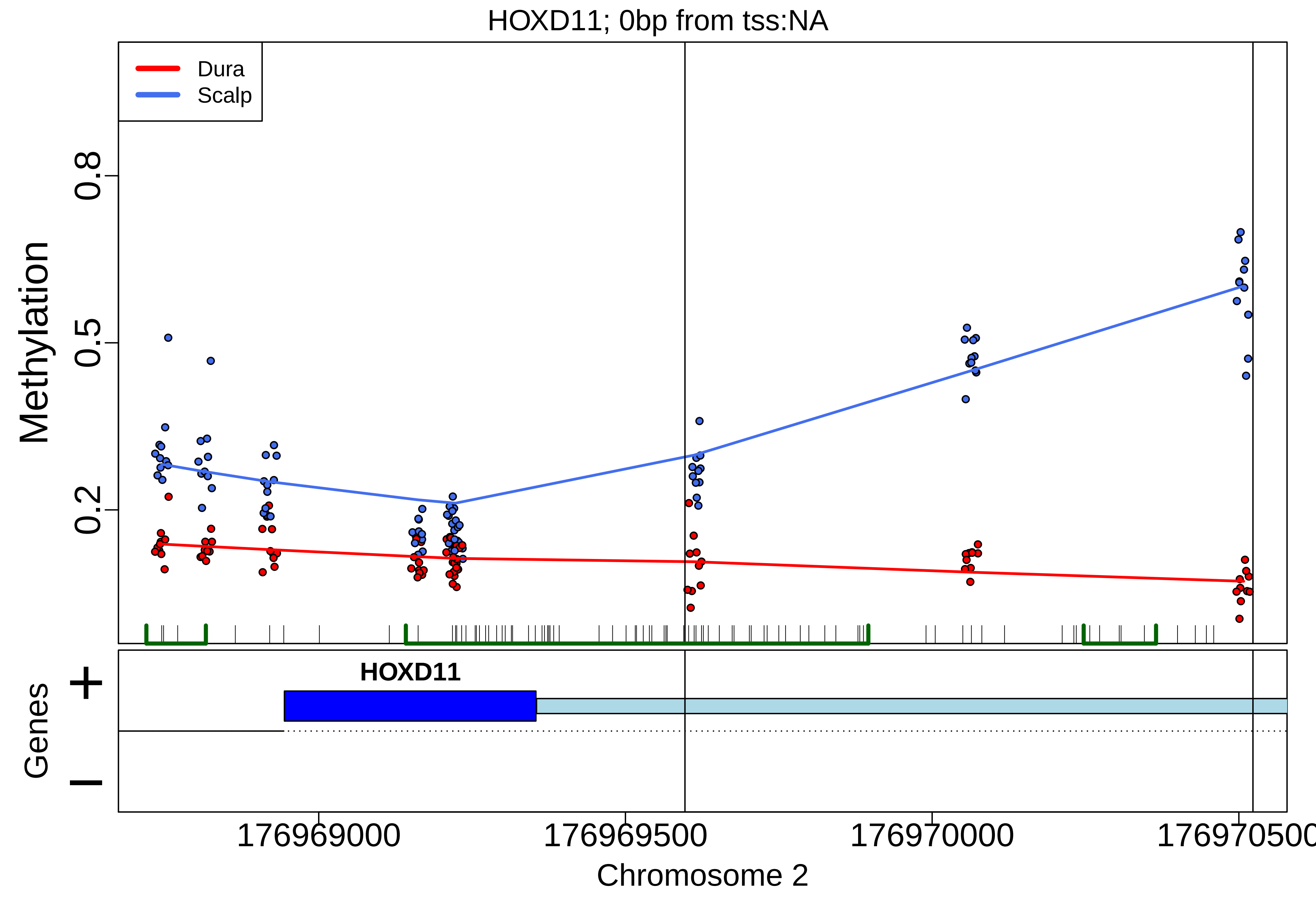
HOXD11; 0bp from tss:NA
Dura
Scalp
Methylation
0.2
0.5
0.8
Genes
+
-
HOXD11
176969000
176969500
176970000
176970500
Chromosome 2

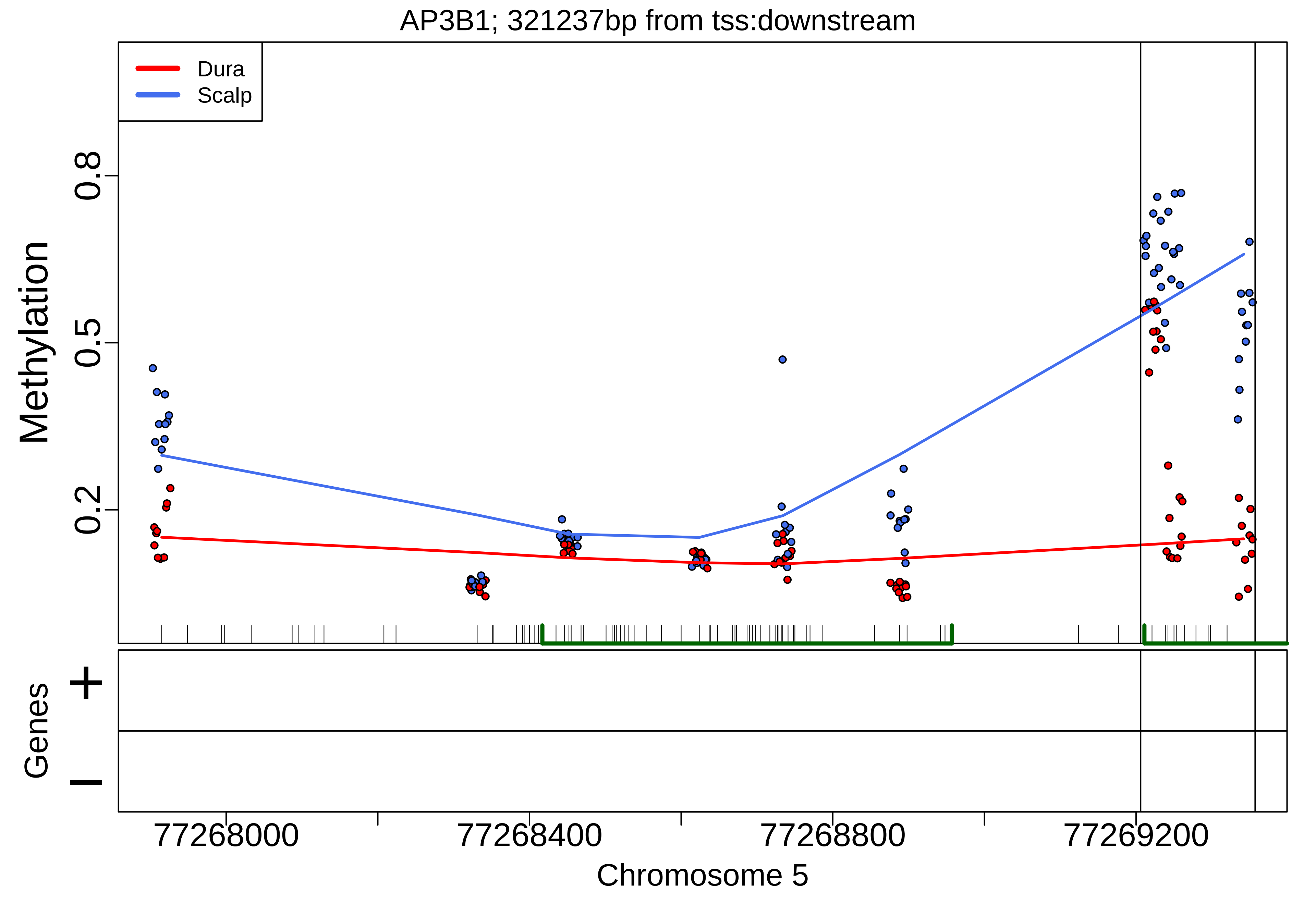

AP3B1; 321237bp from tss:downstream
Dura
Scalp
Methylation
0.2
0.5
0.8
Genes
+
-
77268000
77268400
77268800
77269200
Chromosome 5

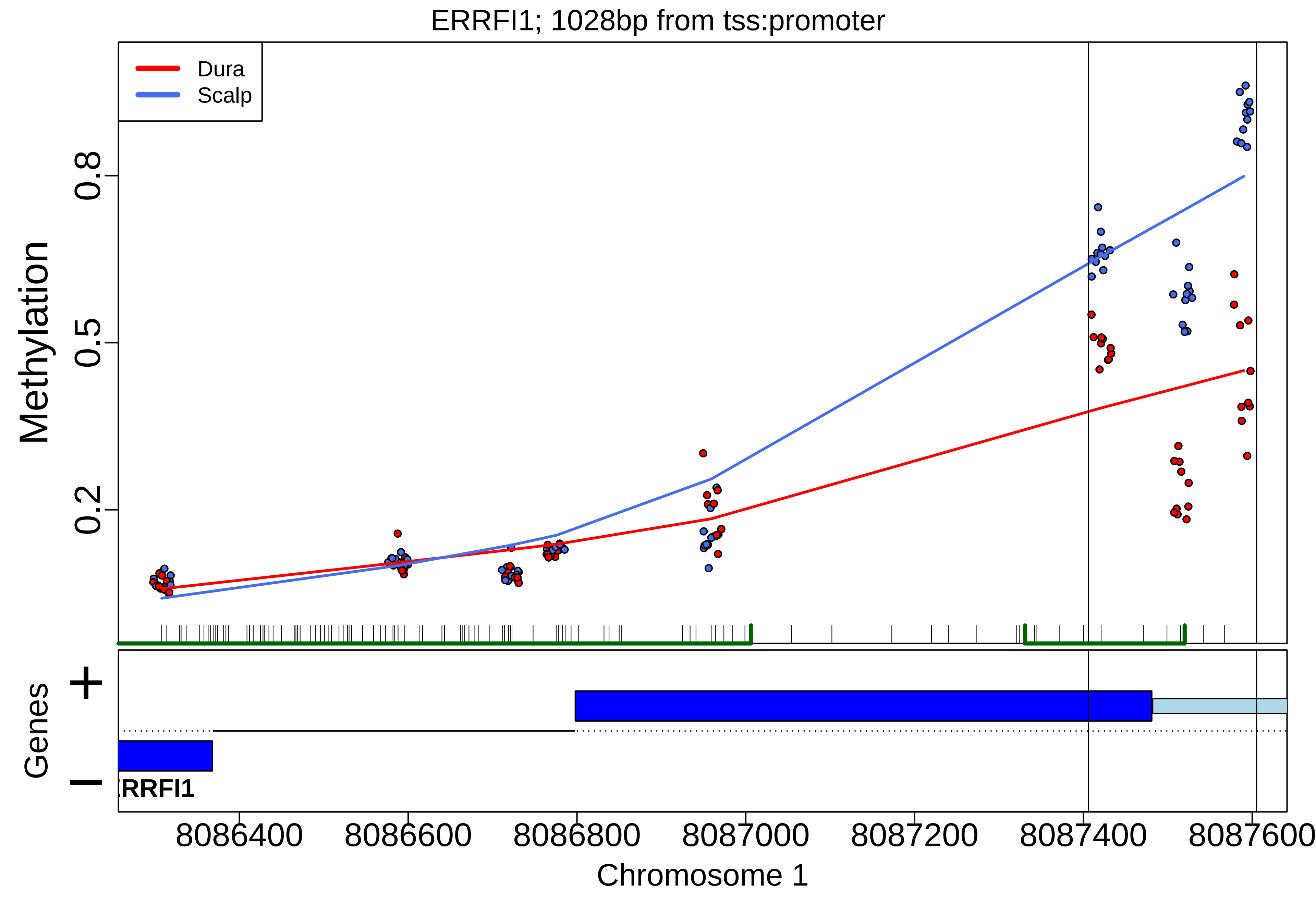

ERRFI1; 1028bp from tss:promoter
Dura
Scalp
Methylation
0.2
0.5
0.8
Genes
+
−
ERRFI1
8086400
8086600
8086800
8087000
8087200
8087400
8087600
Chromosome 1

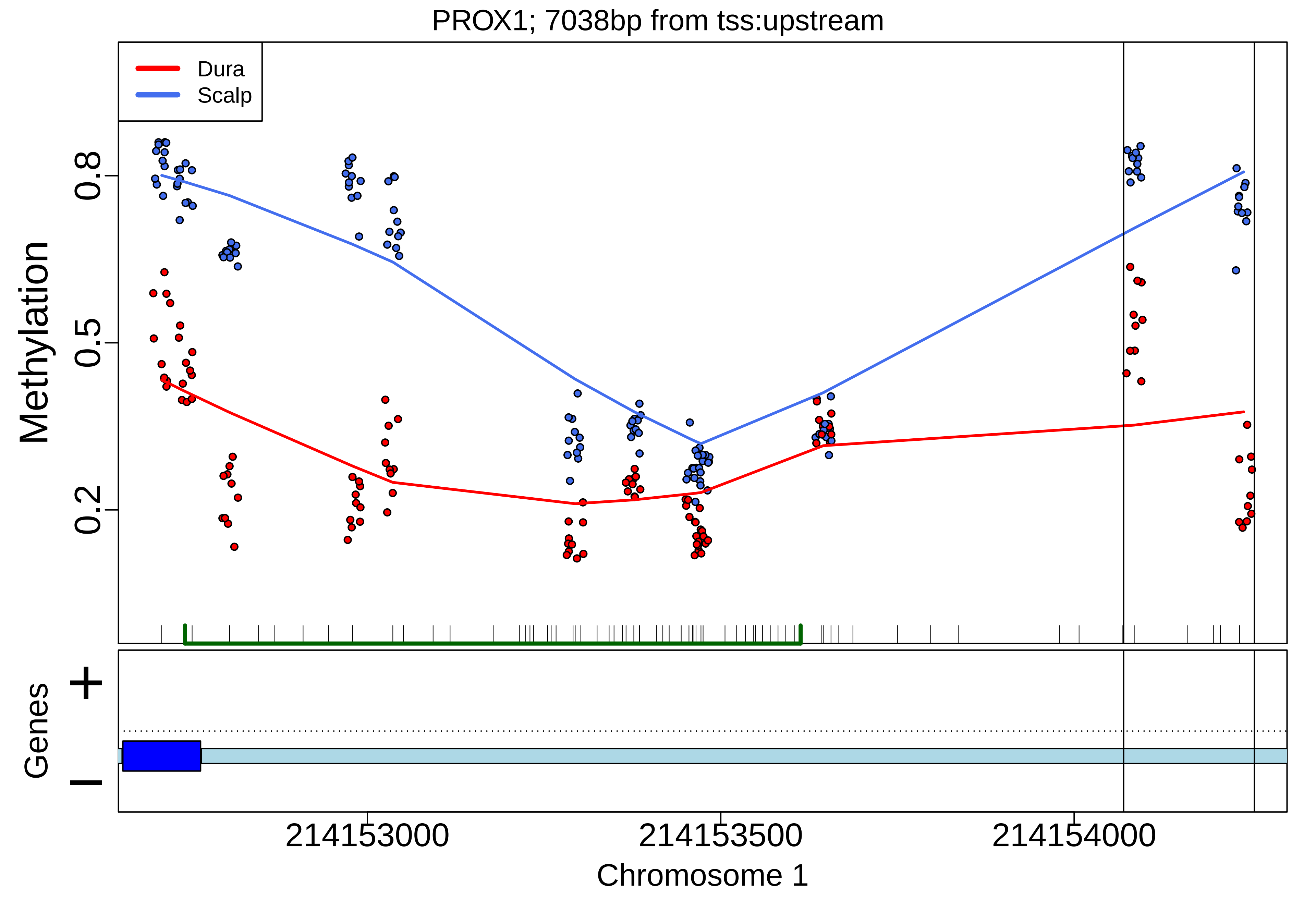

PROX1; 7038bp from tss:upstream
Dura
Scalp
Methylation
0.2
0.5
0.8
Genes
+
−
214153000
214153500
214154000
Chromosome 1

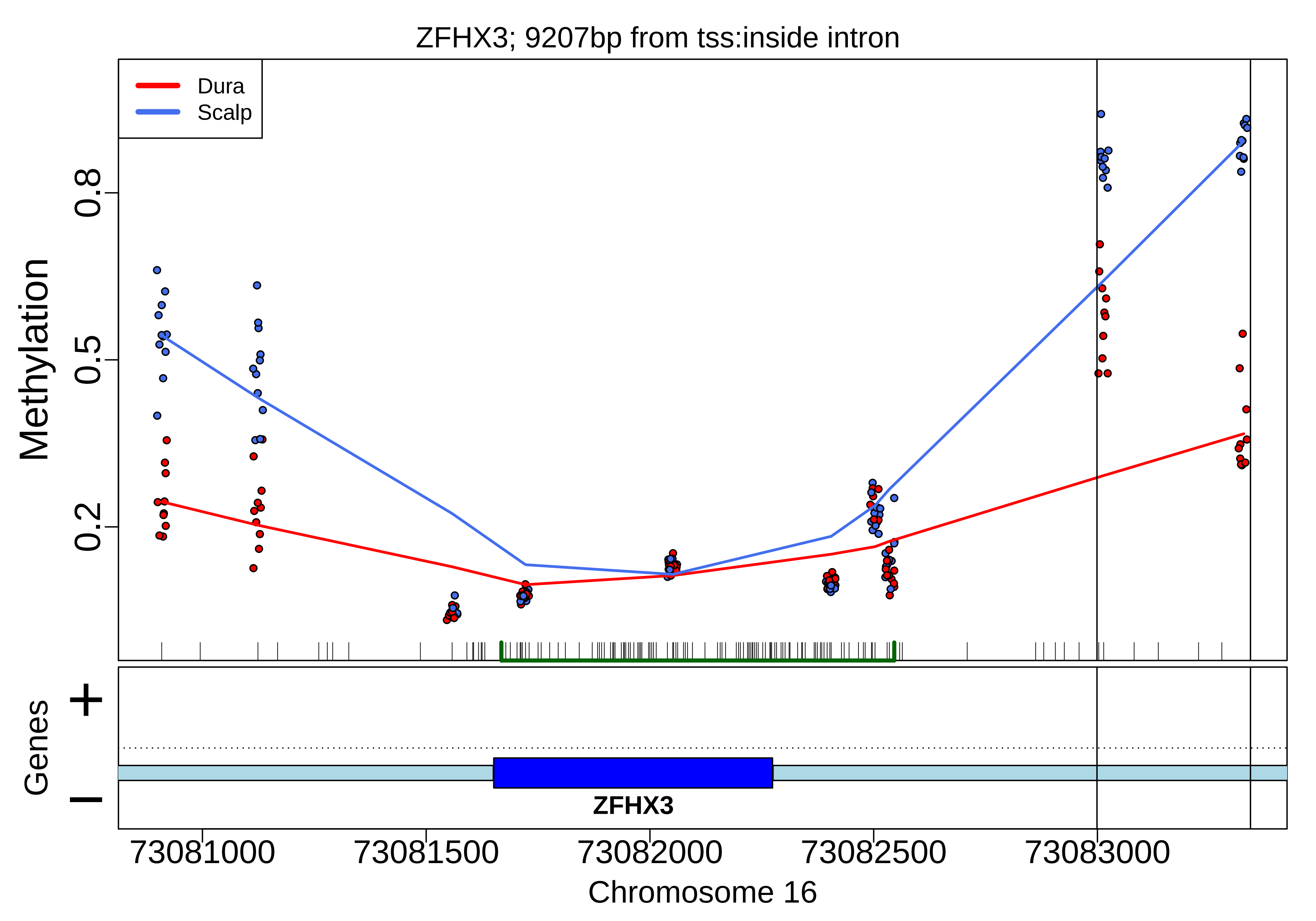
ZFHX3; 9207bp from tss:inside intron
Dura
Scalp
Methylation
0.2
0.5
0.8
Genes
+
−
ZFHX3
73081000
73081500
73082000
73082500
73083000
Chromosome 16

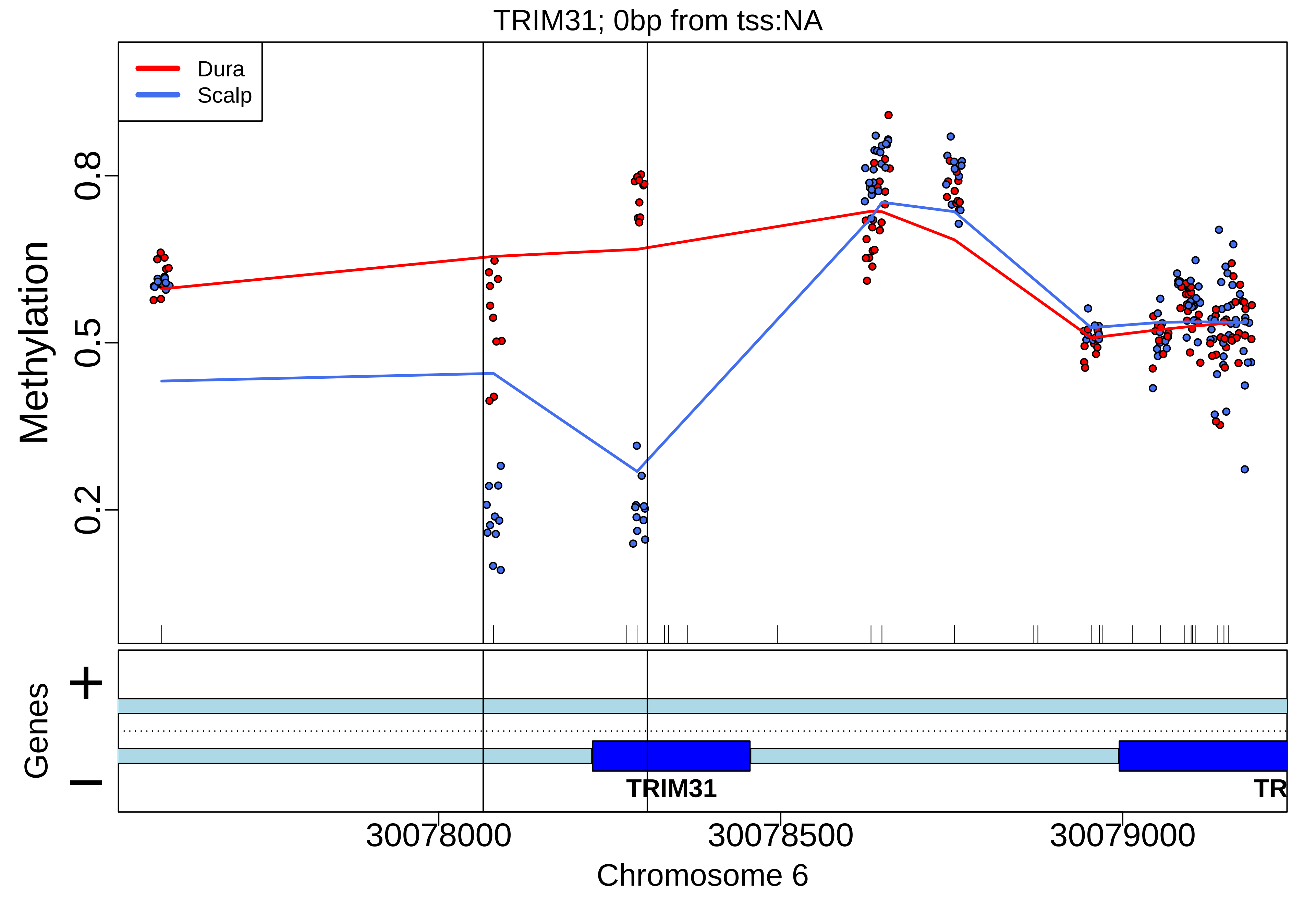
TRIM31; 0bp from tss:NA
Dura
Scalp
Methylation
0.2
0.5
0.8
Genes
+
−
TRIM31
TR
30078000
30078500
30079000
Chromosome 6

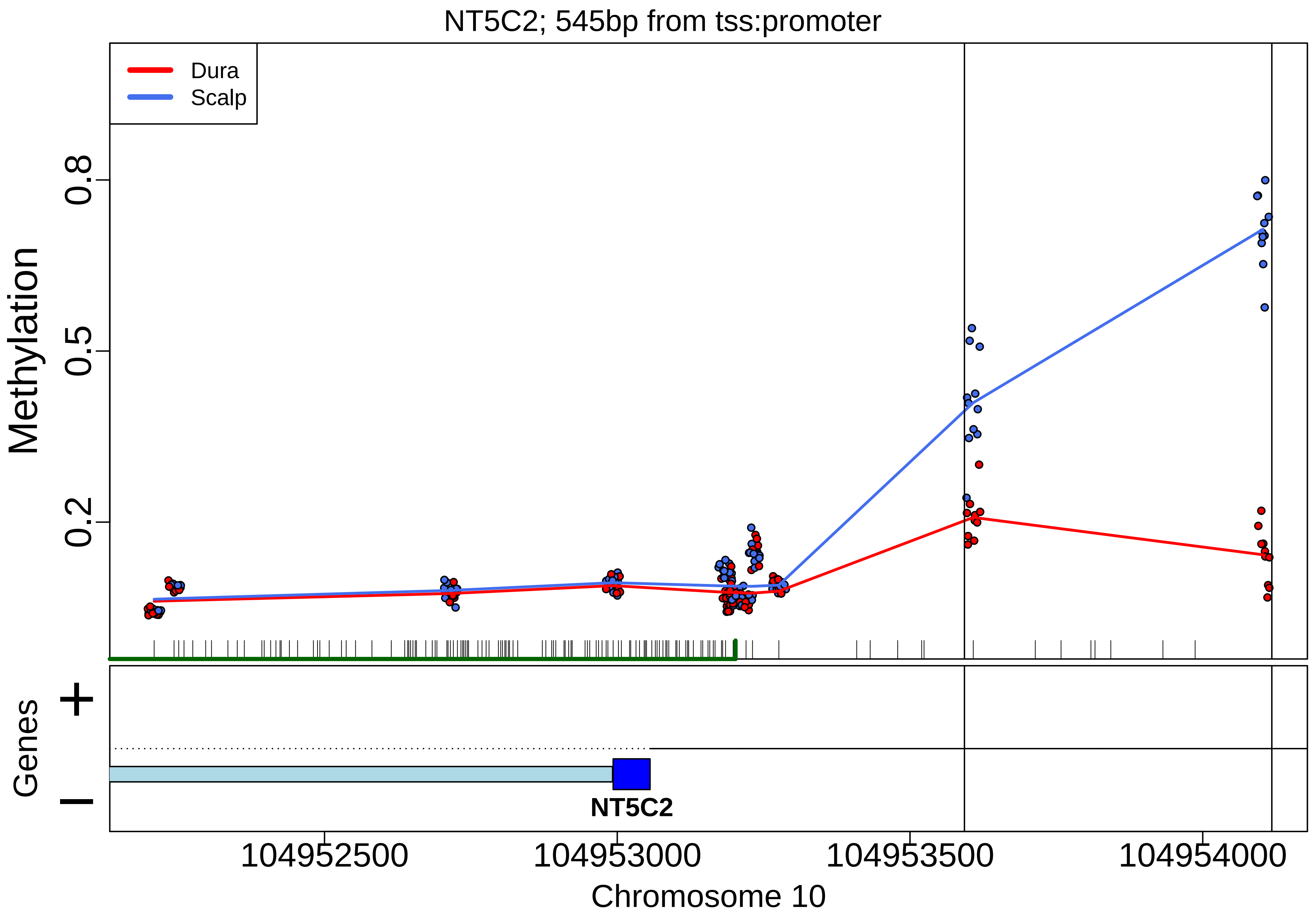
NT5C2; 545bp from tss:promoter
Dura
Scalp
Methylation
0.2
0.5
0.8
Genes
+
-
NT5C2
104952500
104953000
104953500
104954000
Chromosome 10

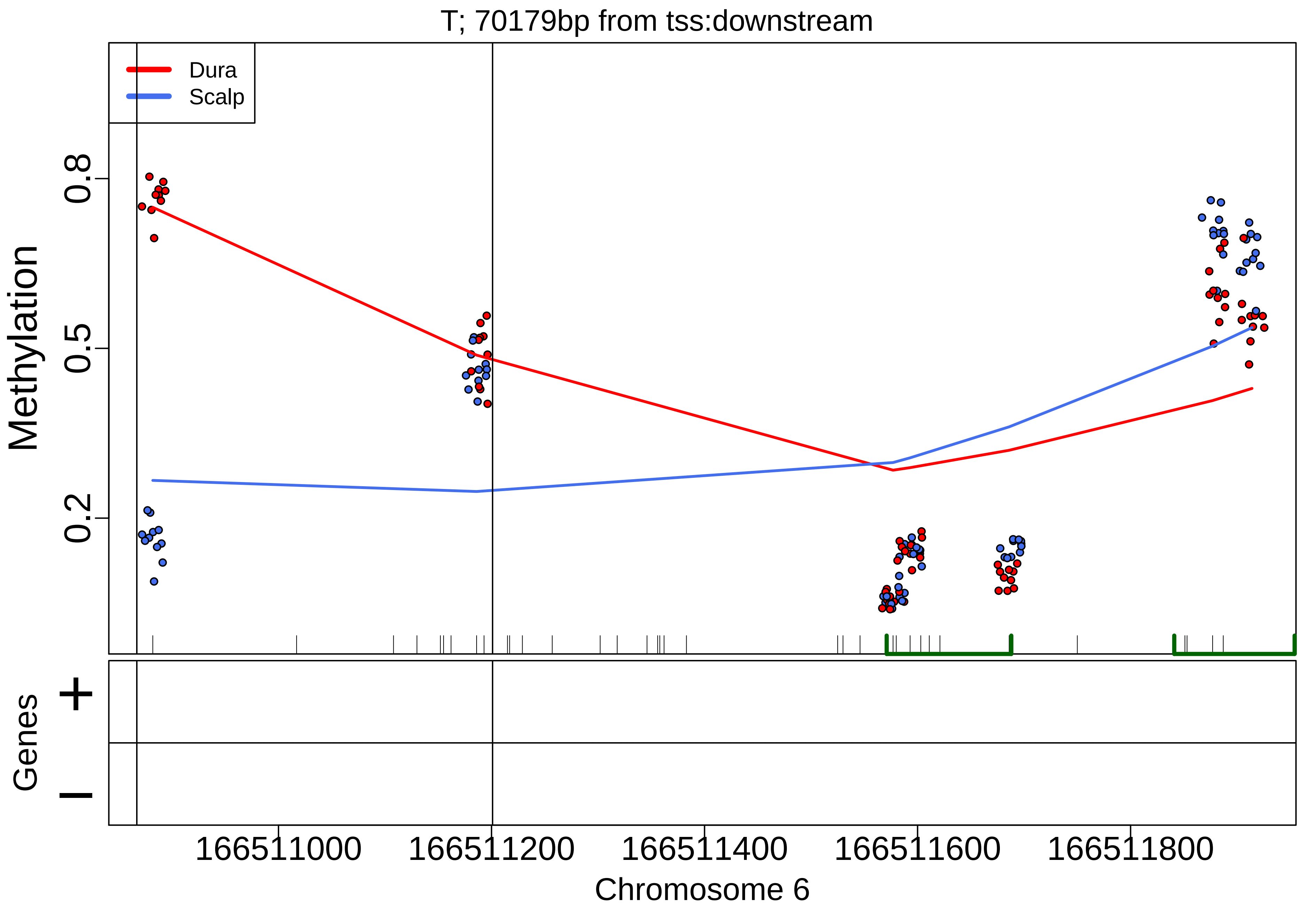
T; 70179bp from tss:downstream
Dura
Scalp
Methylation
0.2
0.5
0.8
Genes
+
-
166511000
166511200
166511400
166511600
166511800
Chromosome 6

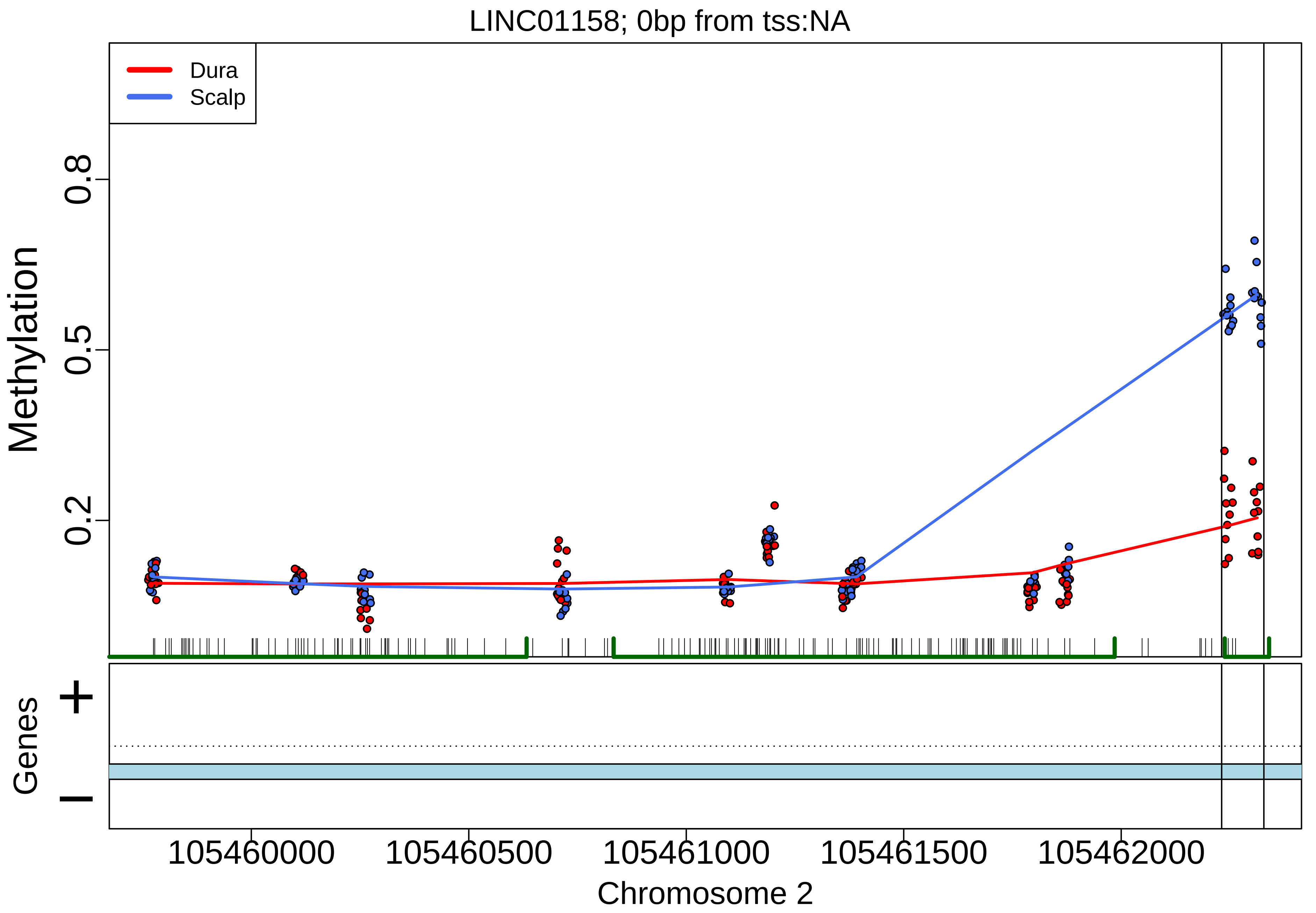

LINC01158; 0bp from tss:NA
Dura
Scalp
Methylation
0.2
0.5
0.8
Genes
+
-
105460000
105460500
105461000
105461500
105462000
Chromosome 2

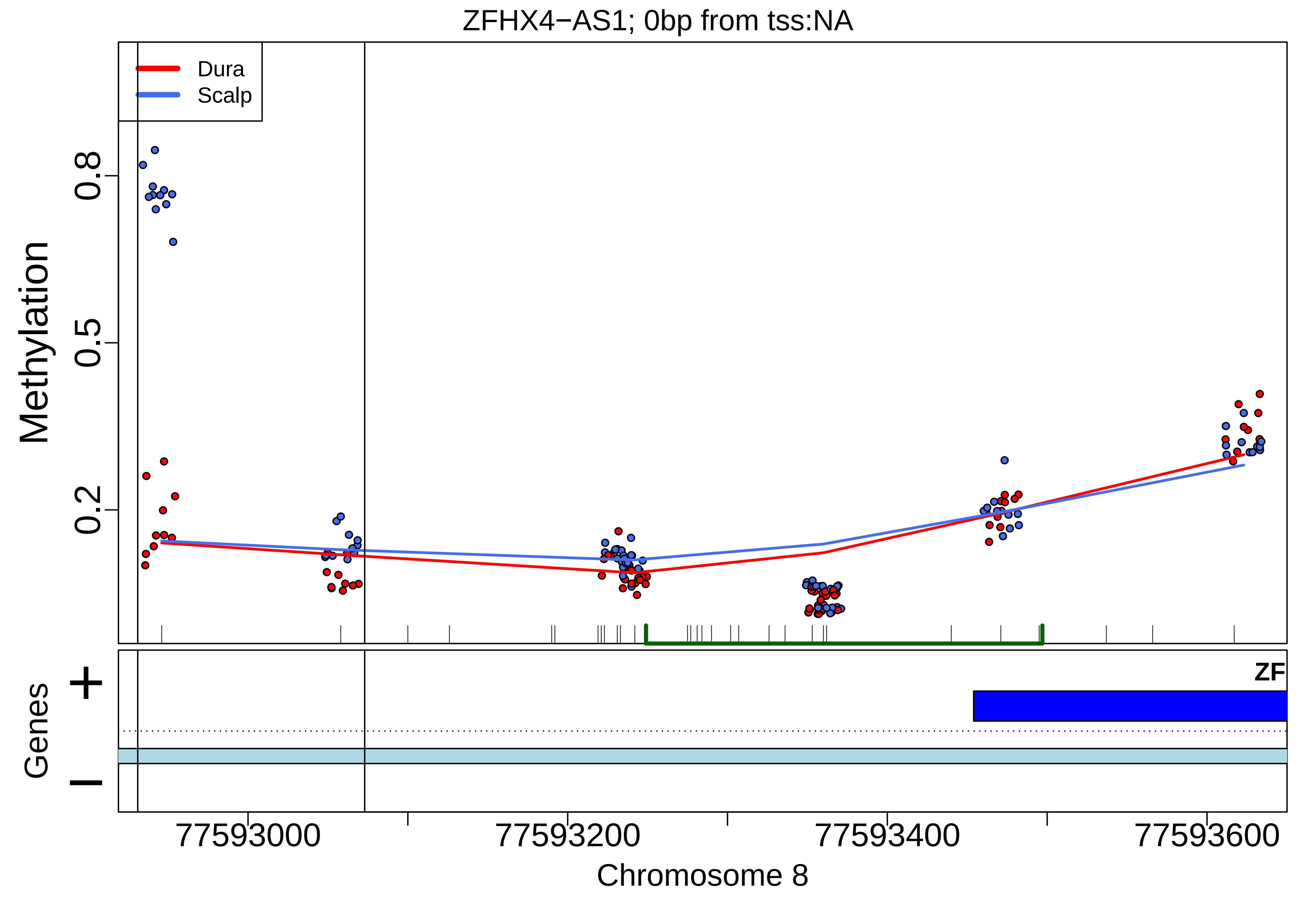
ZFHX4−AS1; 0bp from tss:NA
Dura
Scalp
Methylation
0.2
0.5
0.8
Genes
+
−
ZF
77593000
77593200
77593400
77593600
Chromosome 8

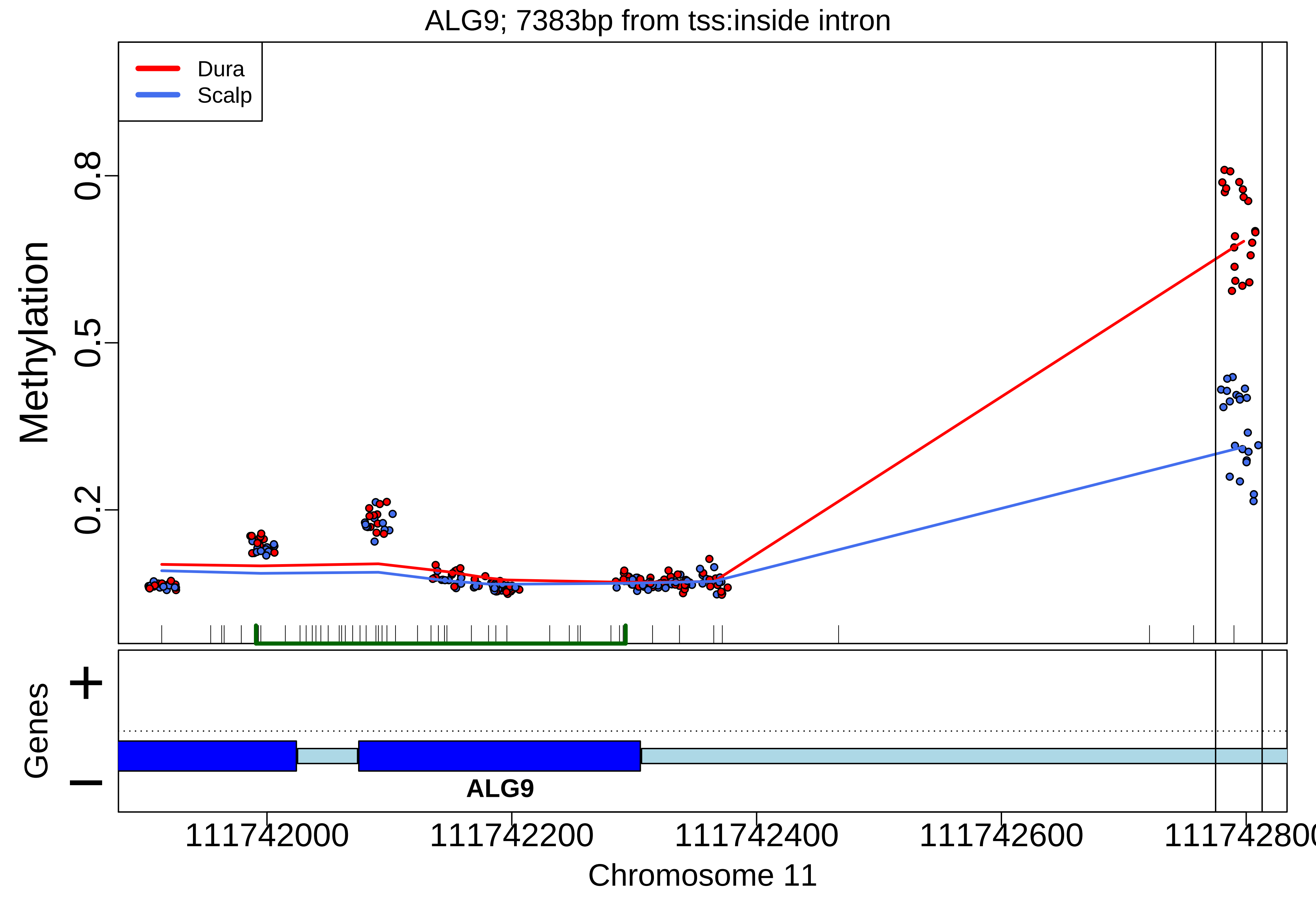
ALG9; 7383bp from tss:inside intron
Dura
Scalp
Methylation
0.2
0.5
0.8
Genes
+
−
ALG9
111742000
111742200
111742400
111742600
111742800
Chromosome 11

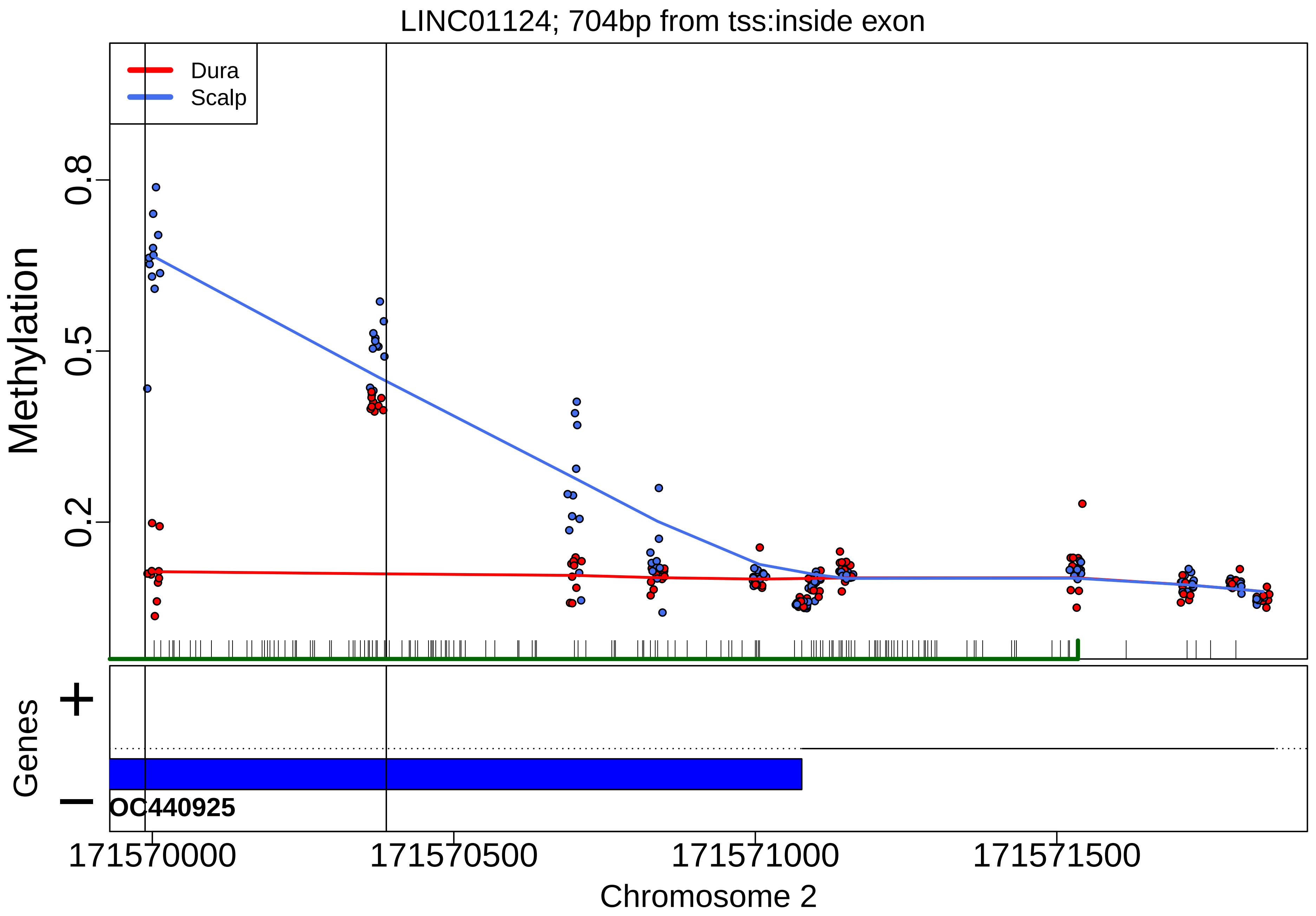

LINC01124; 704bp from tss:inside exon
Dura
Scalp
Methylation
0.8
0.5
0.2
Genes
+
−
OC440925
171570000
171570500
171571000
171571500
Chromosome 2

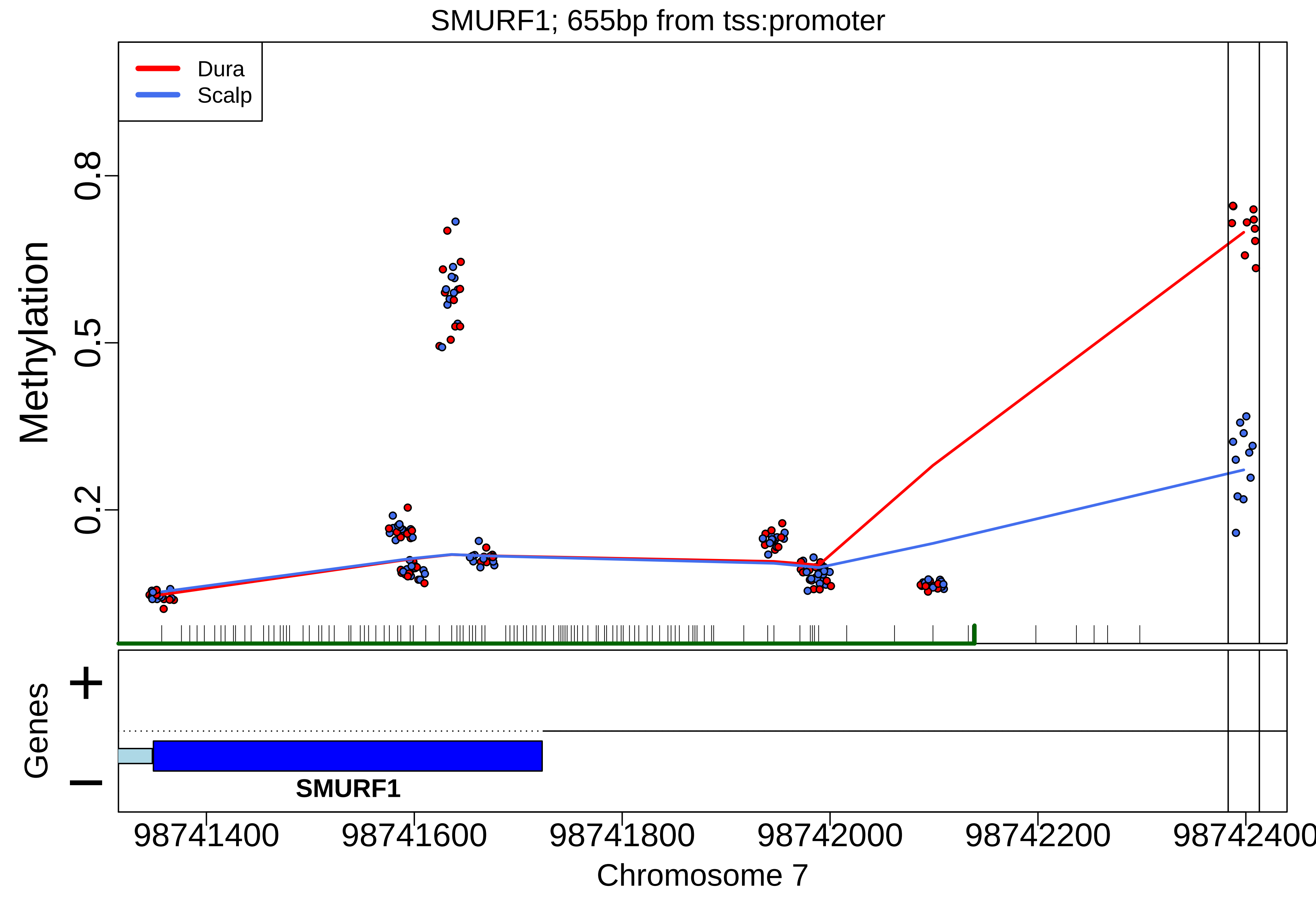

SMURF1; 655bp from tss:promoter
Dura
Scalp
Methylation
0.2
0.5
0.8
Genes
+
−
SMURF1
98741400
98741600
98741800
98742000
98742200
98742400
Chromosome 7

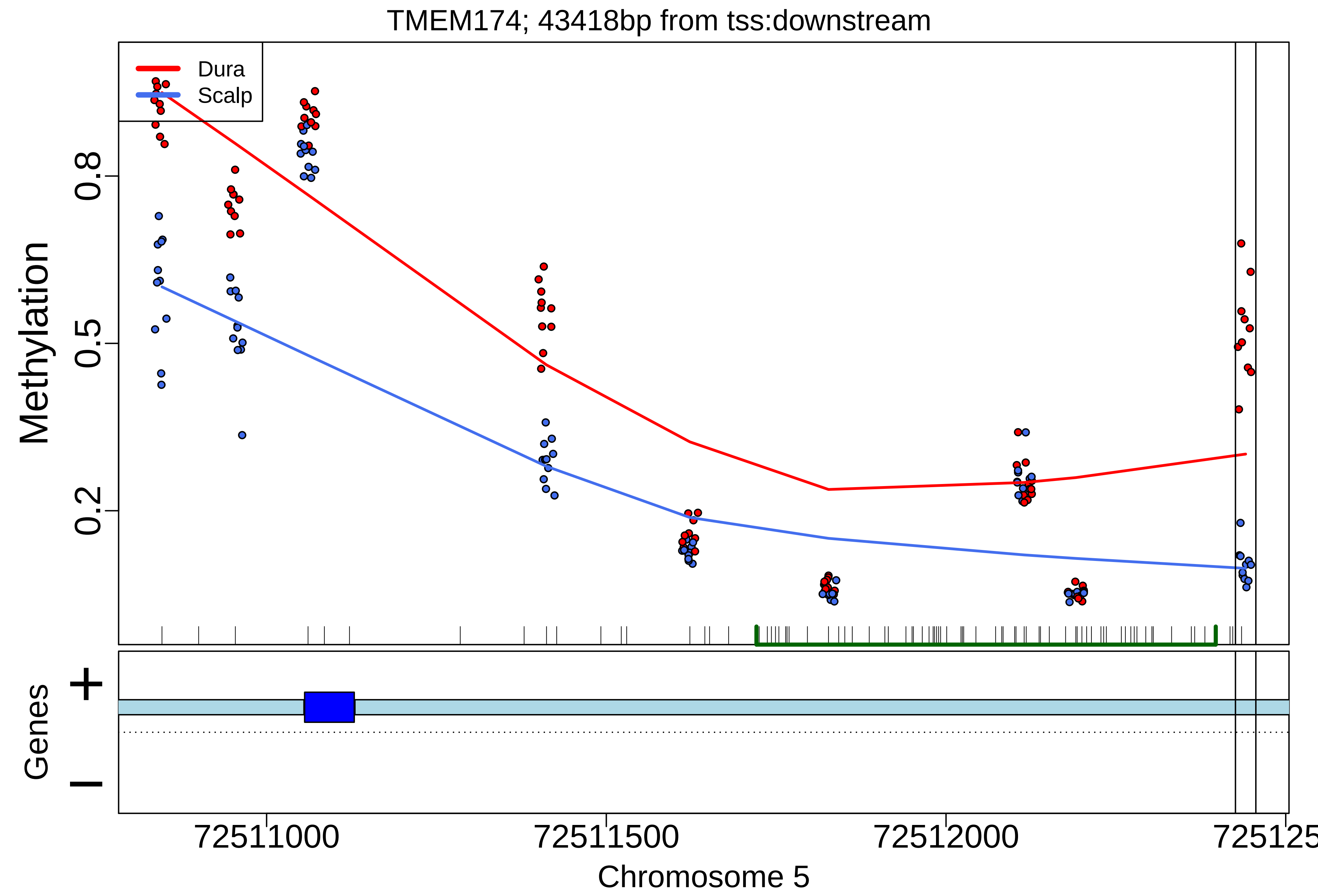

TMEM174; 43418bp from tss:downstream
Dura
Scalp
Methylation
0.2
0.5
0.8
Genes
+
−
72511000
72511500
72512000
725125
Chromosome 5

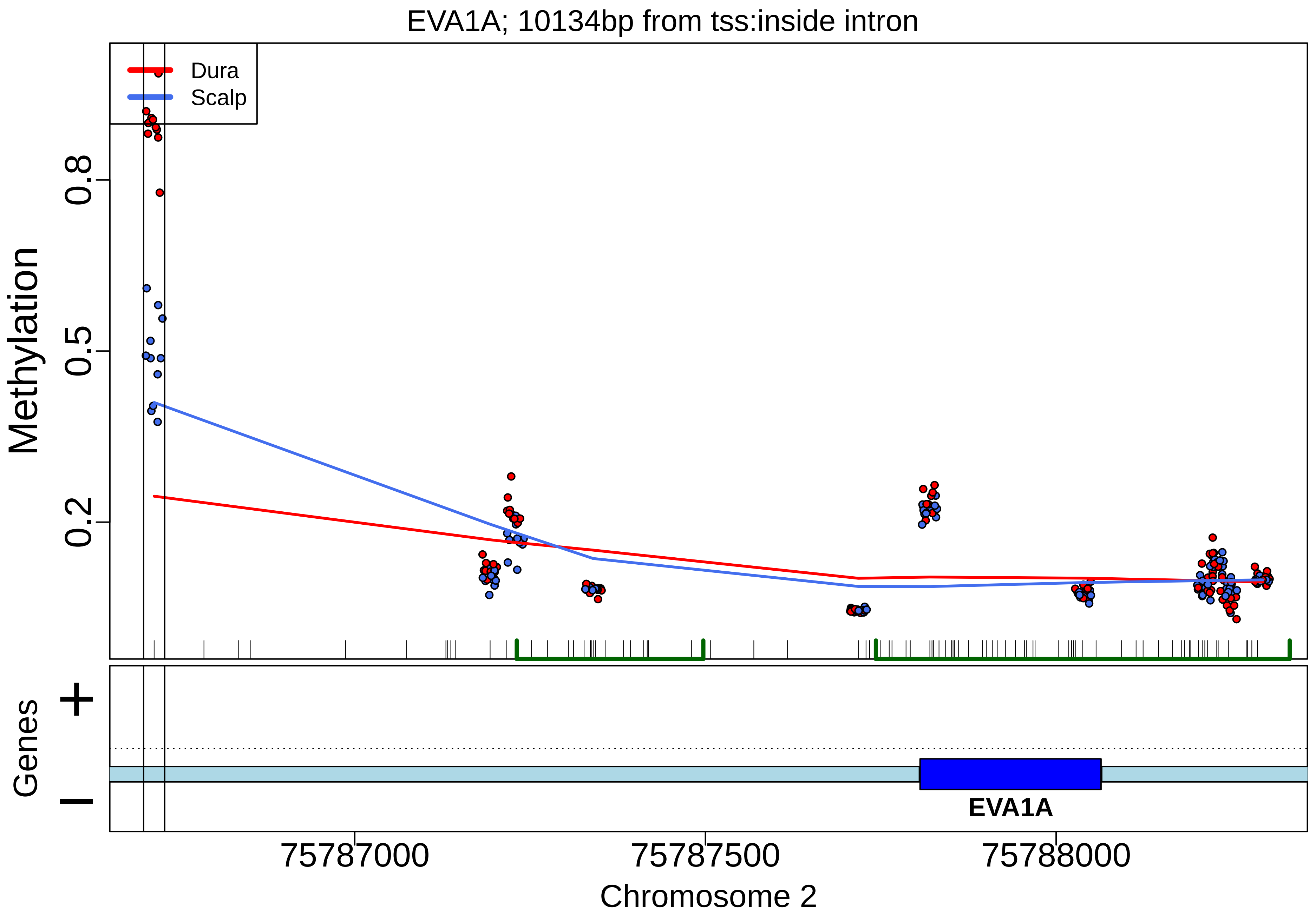
EVA1A; 10134bp from tss:inside intron
Dura
Scalp
Methylation
0.8
0.5
0.2
Genes
+
-
EVA1A
75787000
75787500
75788000
Chromosome 2

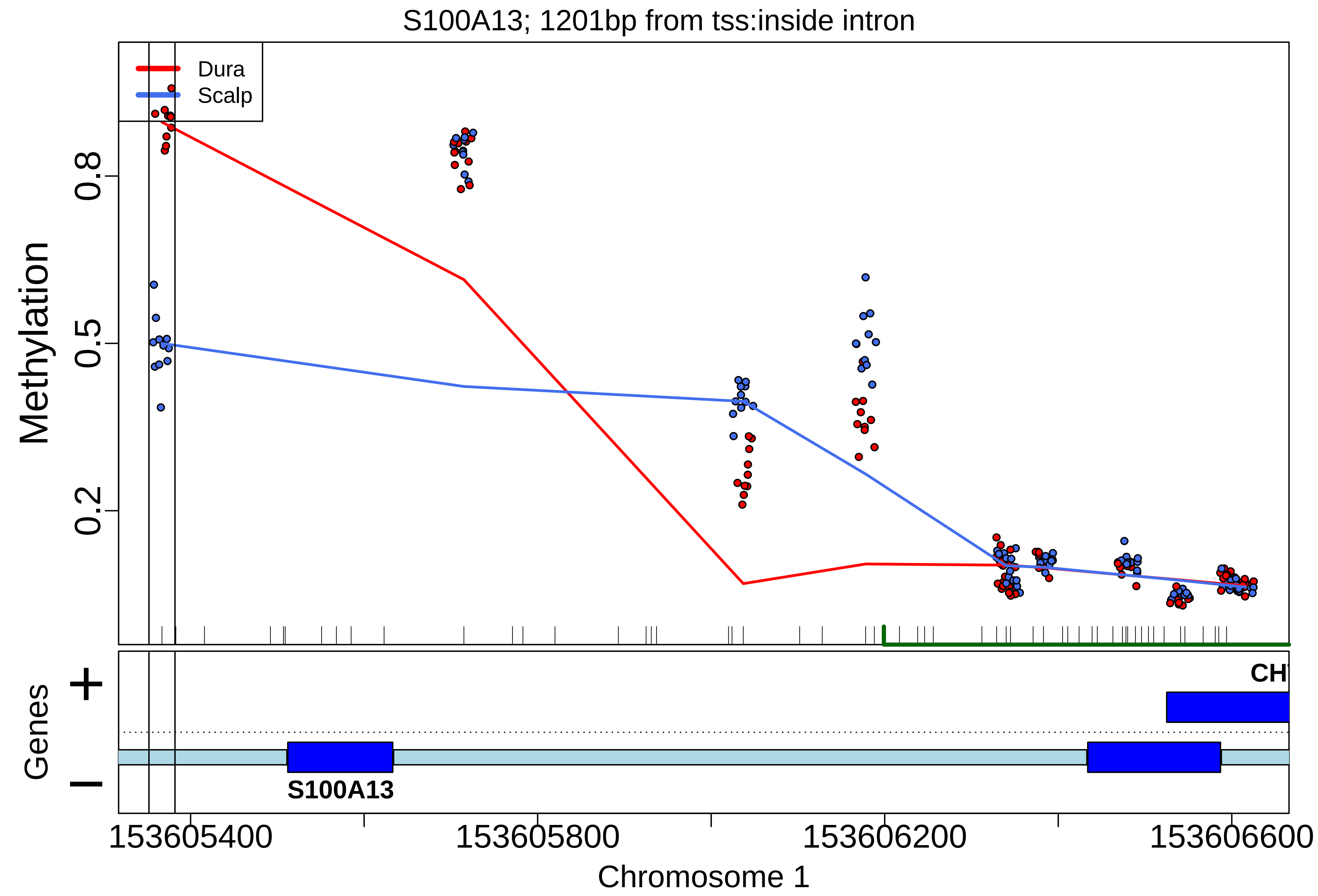

S100A13; 1201bp from tss:inside intron
Dura
Scalp
Methylation
0.2
0.5
0.8
Genes
+
−
S100A13
CH
153605400
153605800
153606200
153606600
Chromosome 1

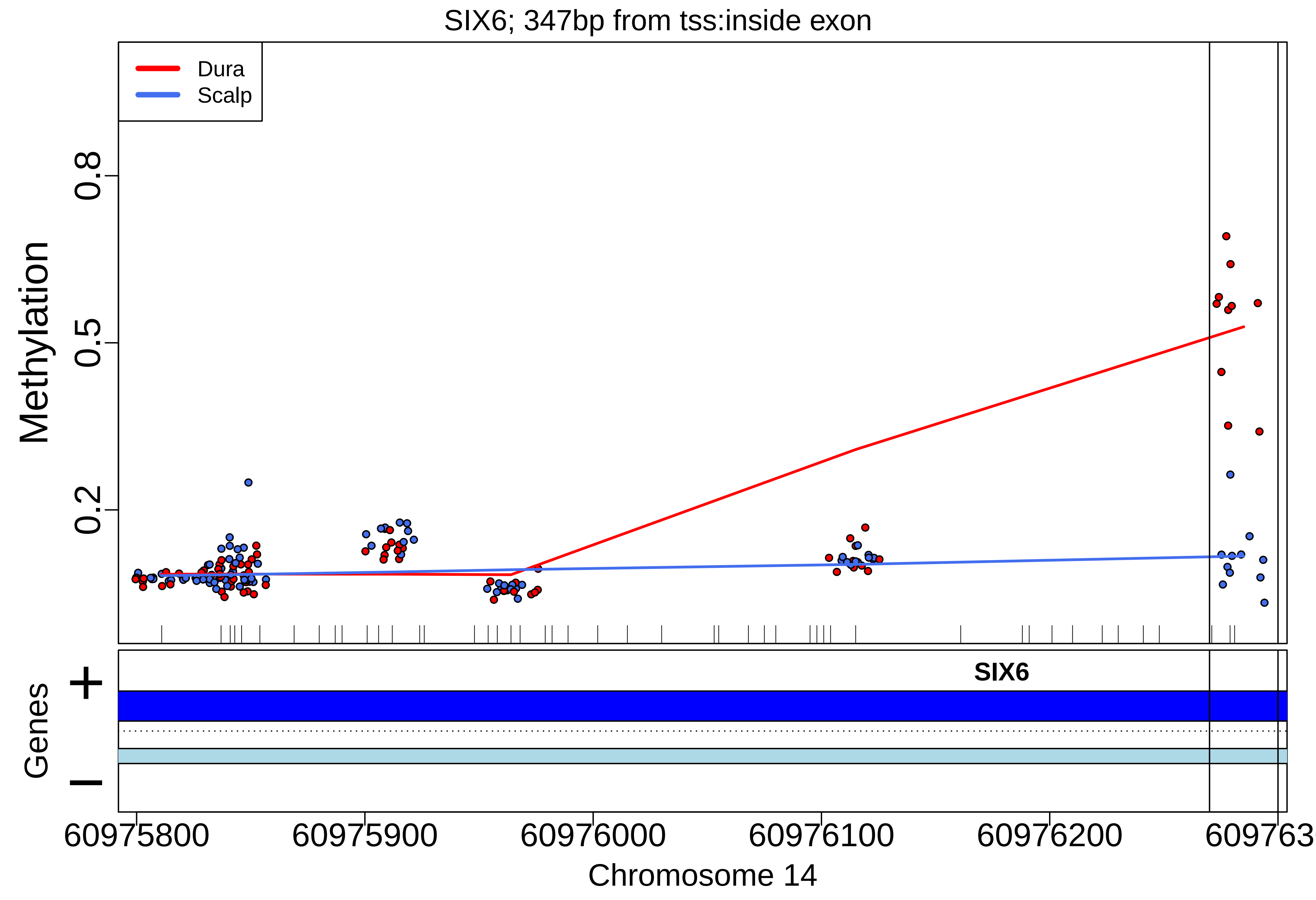
SIX6; 347bp from tss:inside exon
Dura
Scalp
Methylation
0.2
0.5
0.8
Genes
+
−
SIX6
60975800
60975900
60976000
60976100
60976200
609763
Chromosome 14

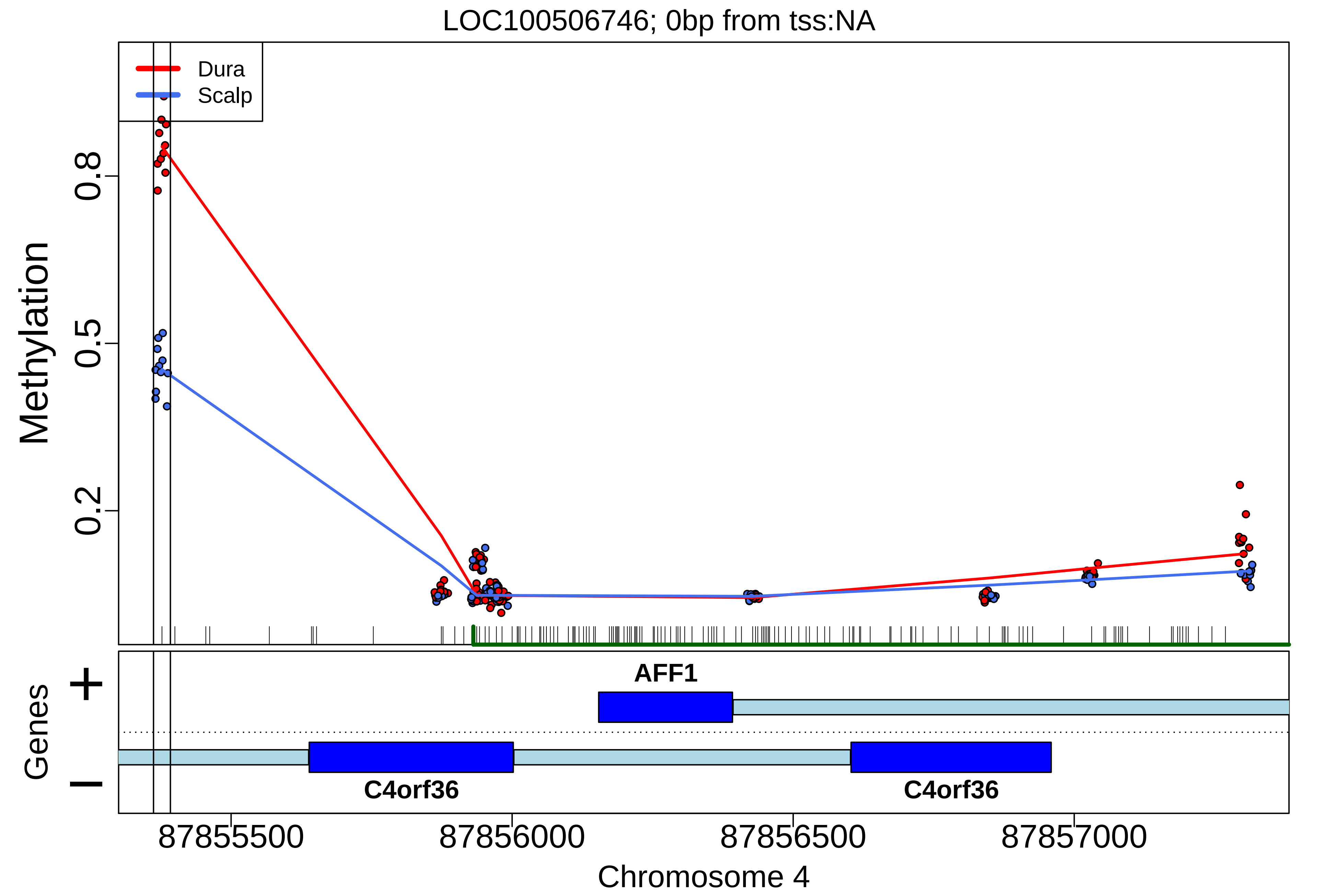

LOC100506746; 0bp from tss:NA
Dura
Scalp
Methylation
0.8
0.5
0.2
Genes
+
-
AFF1
C4orf36
C4orf36
87855500
87856000
87856500
87857000
Chromosome 4

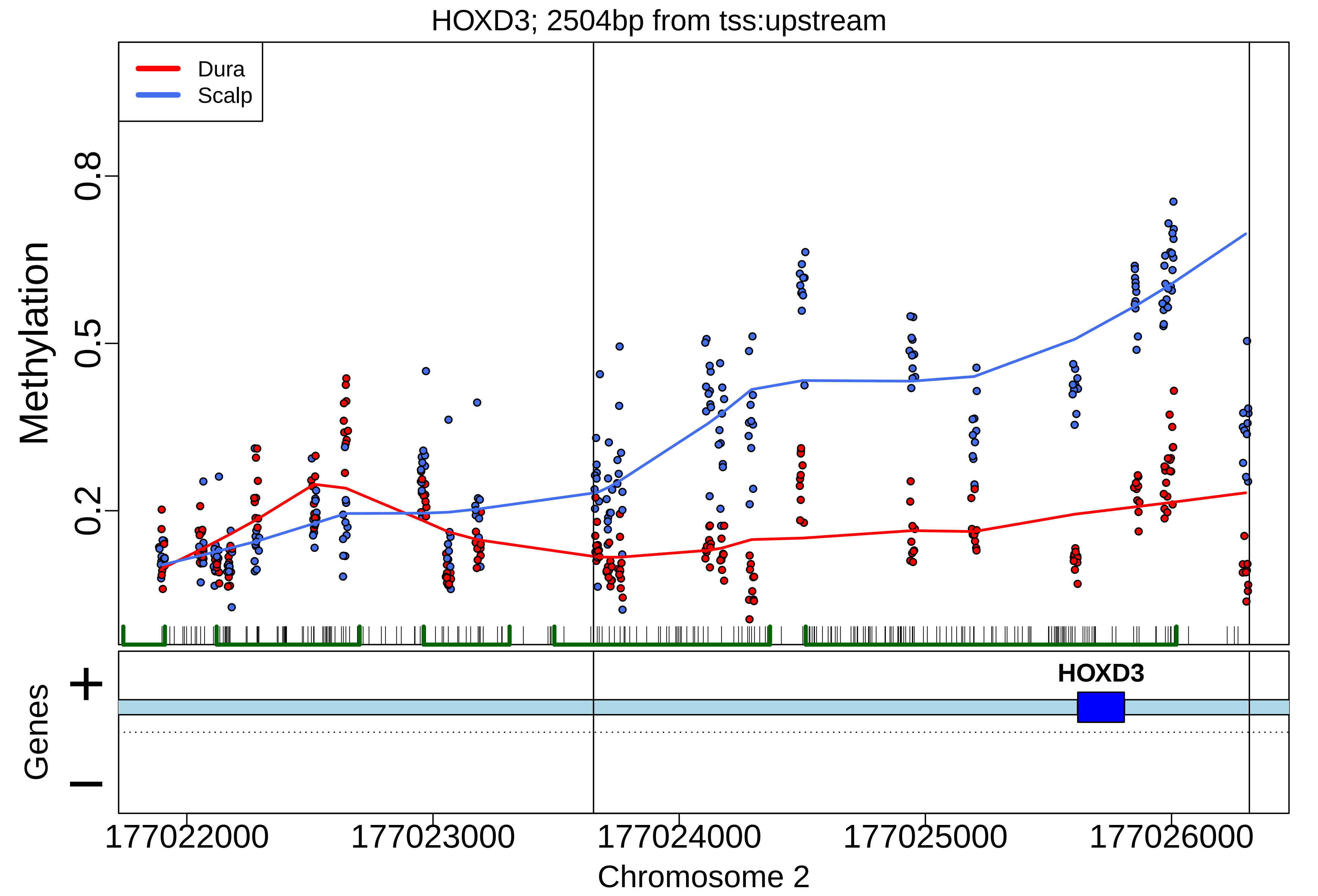

HOXD3; 2504bp from tss:upstream
Dura
Scalp
Methylation
0.2
0.5
0.8
Genes
+
-
HOXD3
177022000
177023000
177024000
177025000
177026000
Chromosome 2

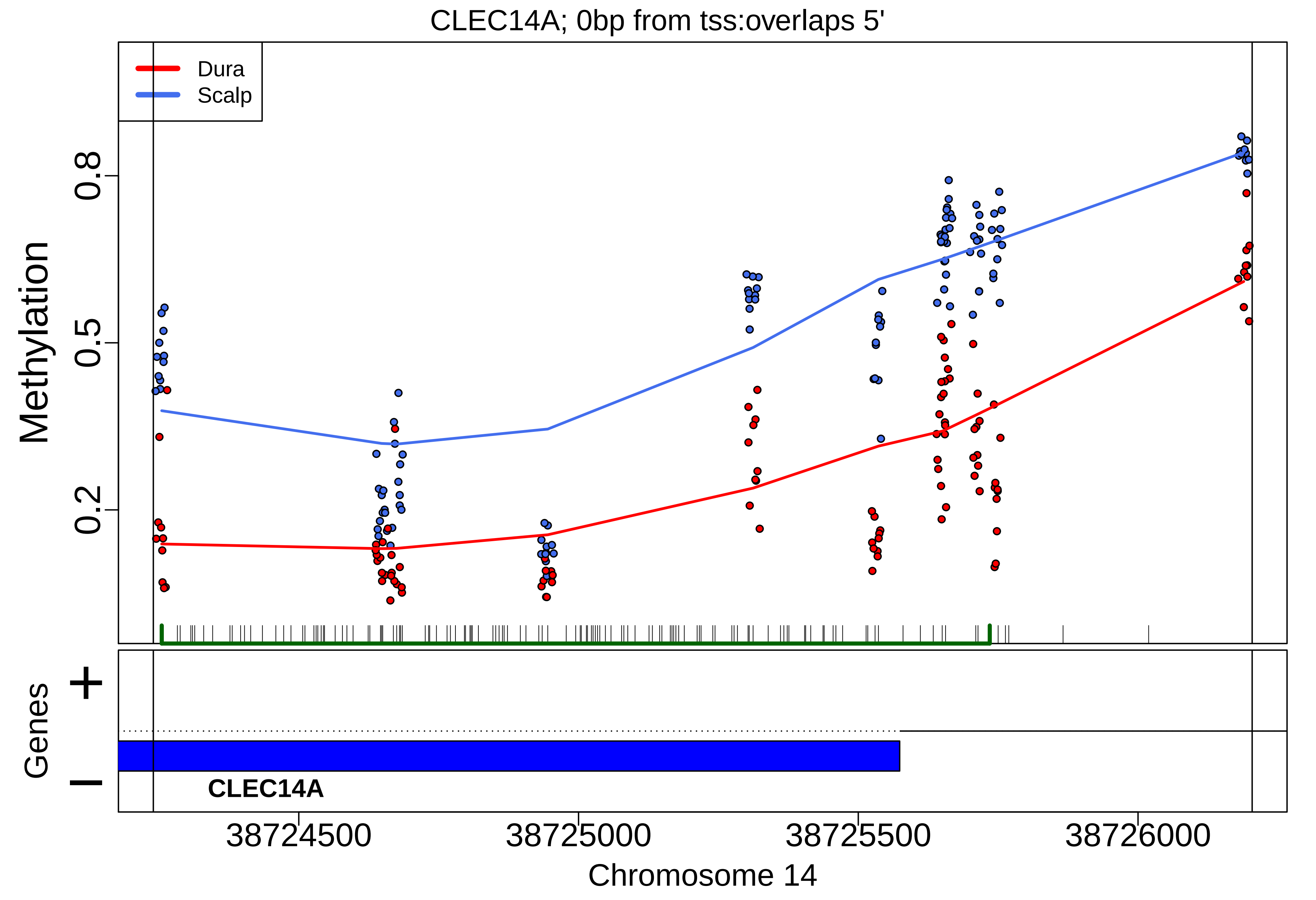

CLEC14A; 0bp from tss:overlaps 5'
Dura
Scalp
Methylation
0.2
0.5
0.8
Genes
+
-
CLEC14A
38724500
38725000
38725500
38726000
Chromosome 14

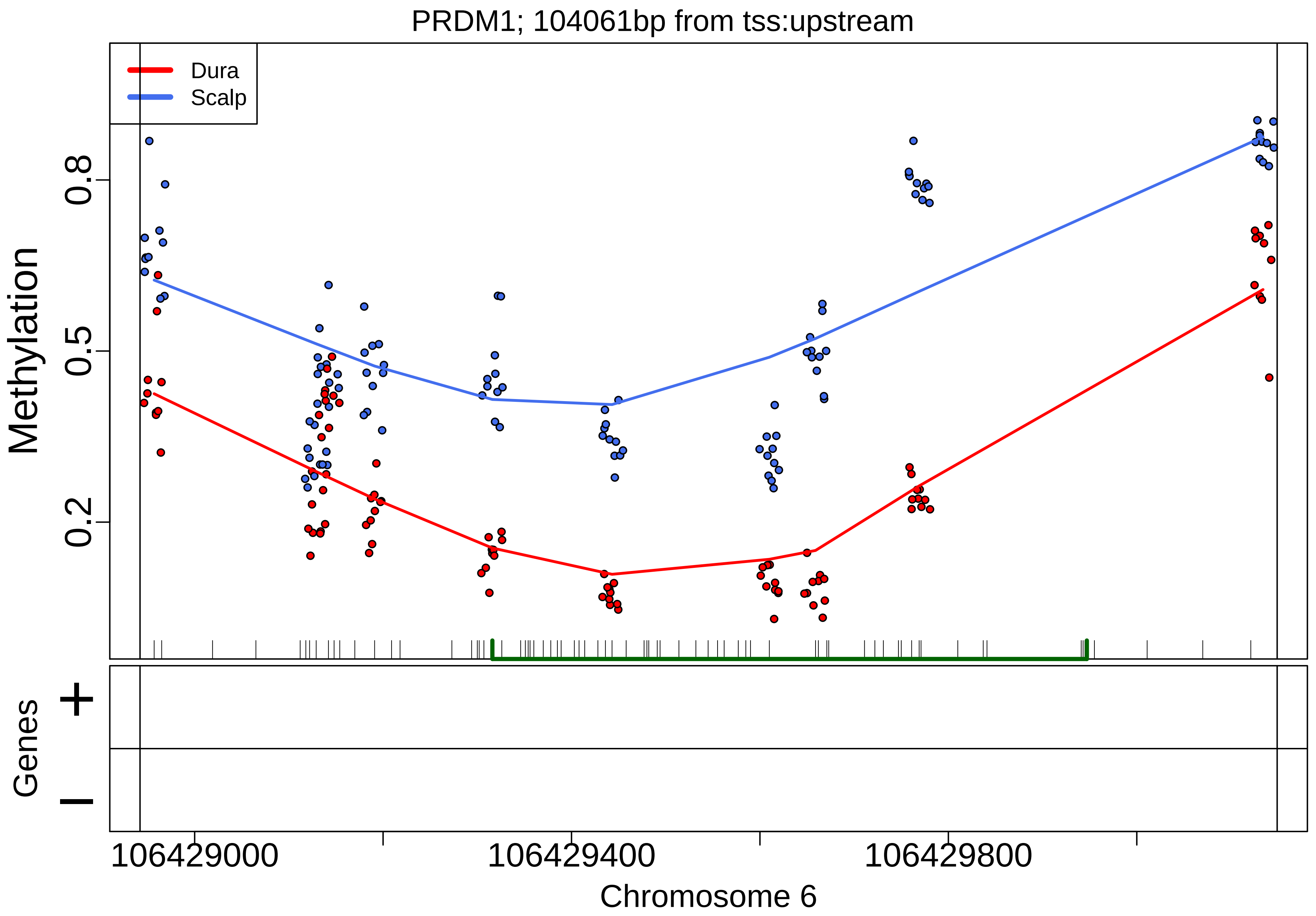

PRDM1; 104061bp from tss:upstream
Dura
Scalp
Methylation
0.2
0.5
0.8
Genes
+
−
106429000
106429400
106429800
Chromosome 6

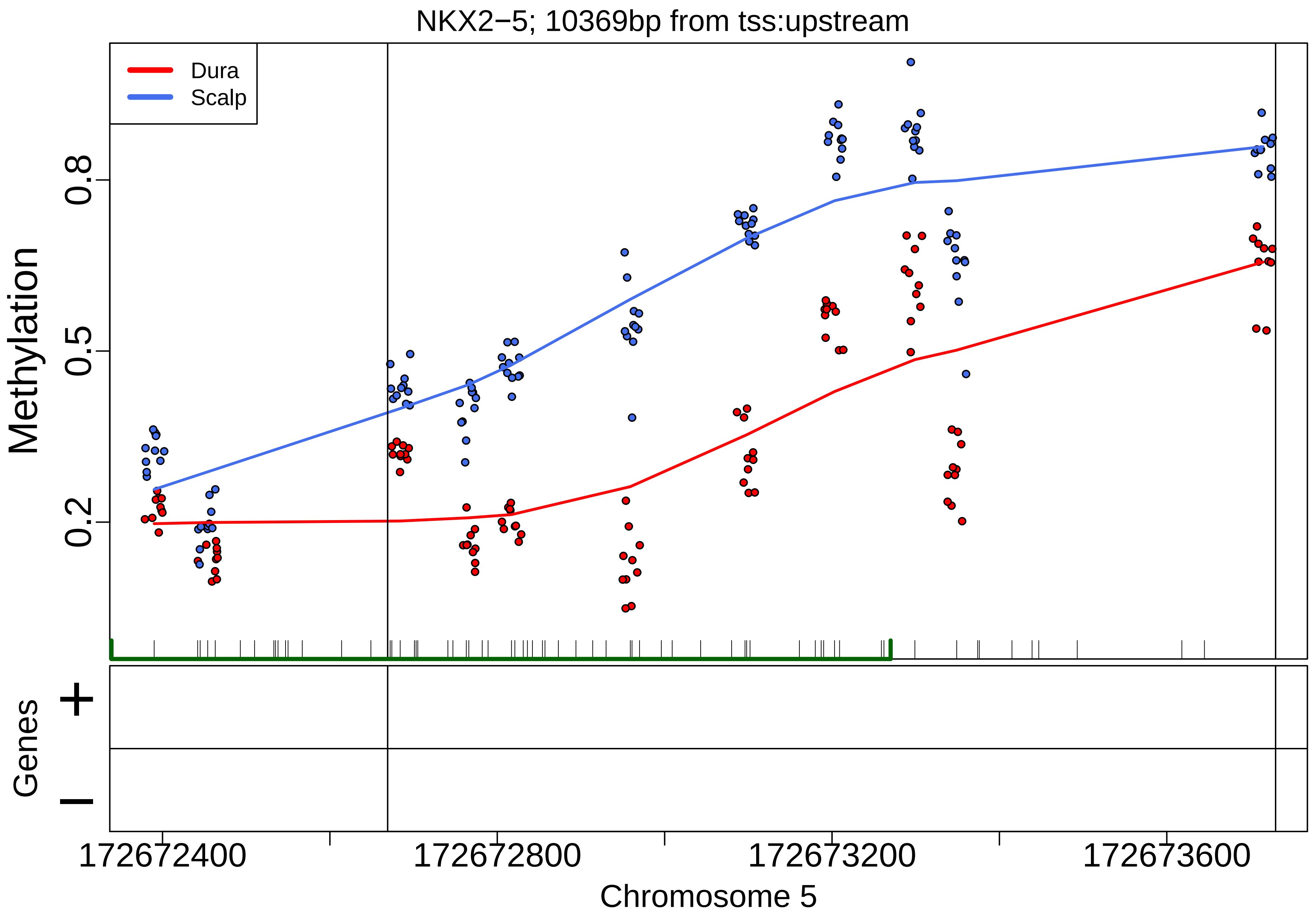

NKX2−5; 10369bp from tss:upstream
Dura
Scalp
Methylation
0.2
0.5
0.8
Genes
+
−
172672400
172672800
172673200
172673600
Chromosome 5

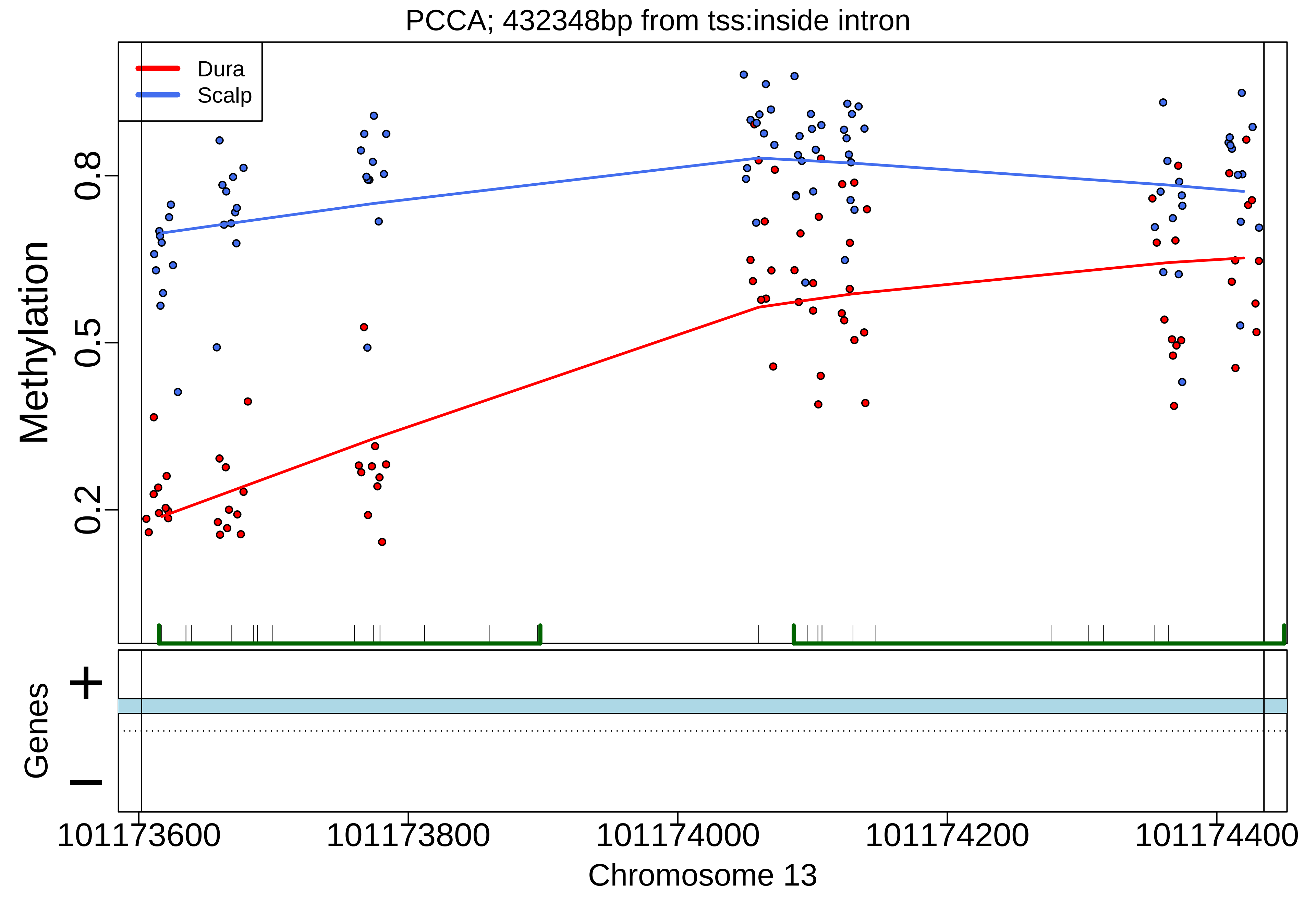
PCCA; 432348bp from tss:inside intron
Dura
Scalp
Methylation
0.2
0.5
0.8
Genes
+
-
101173600
101173800
101174000
101174200
101174400
Chromosome 13

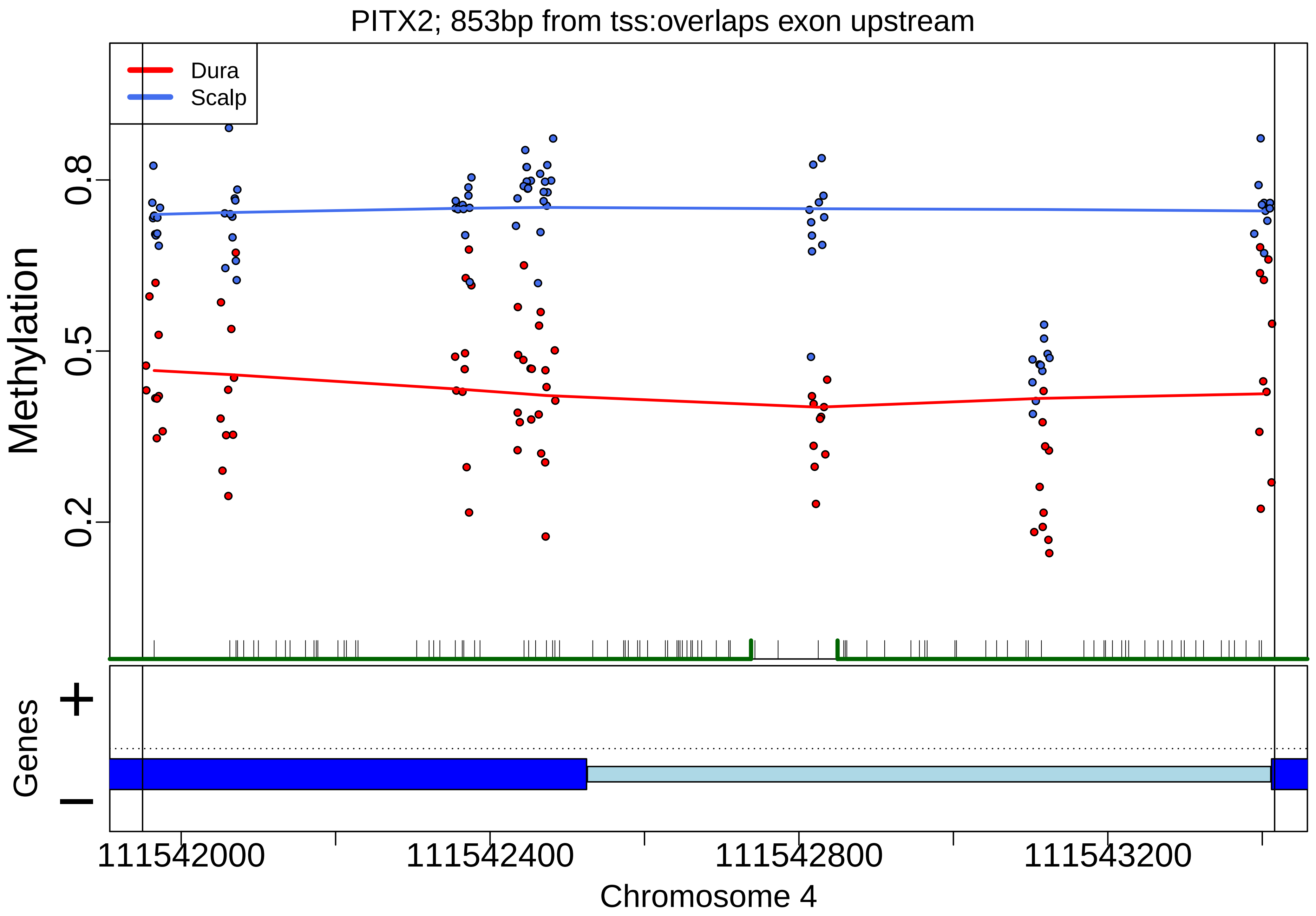
PITX2; 853bp from tss:overlaps exon upstream
Dura
Scalp
Methylation
0.2
0.5
0.8
Genes
+
−
111542000
111542400
111542800
111543200
Chromosome 4

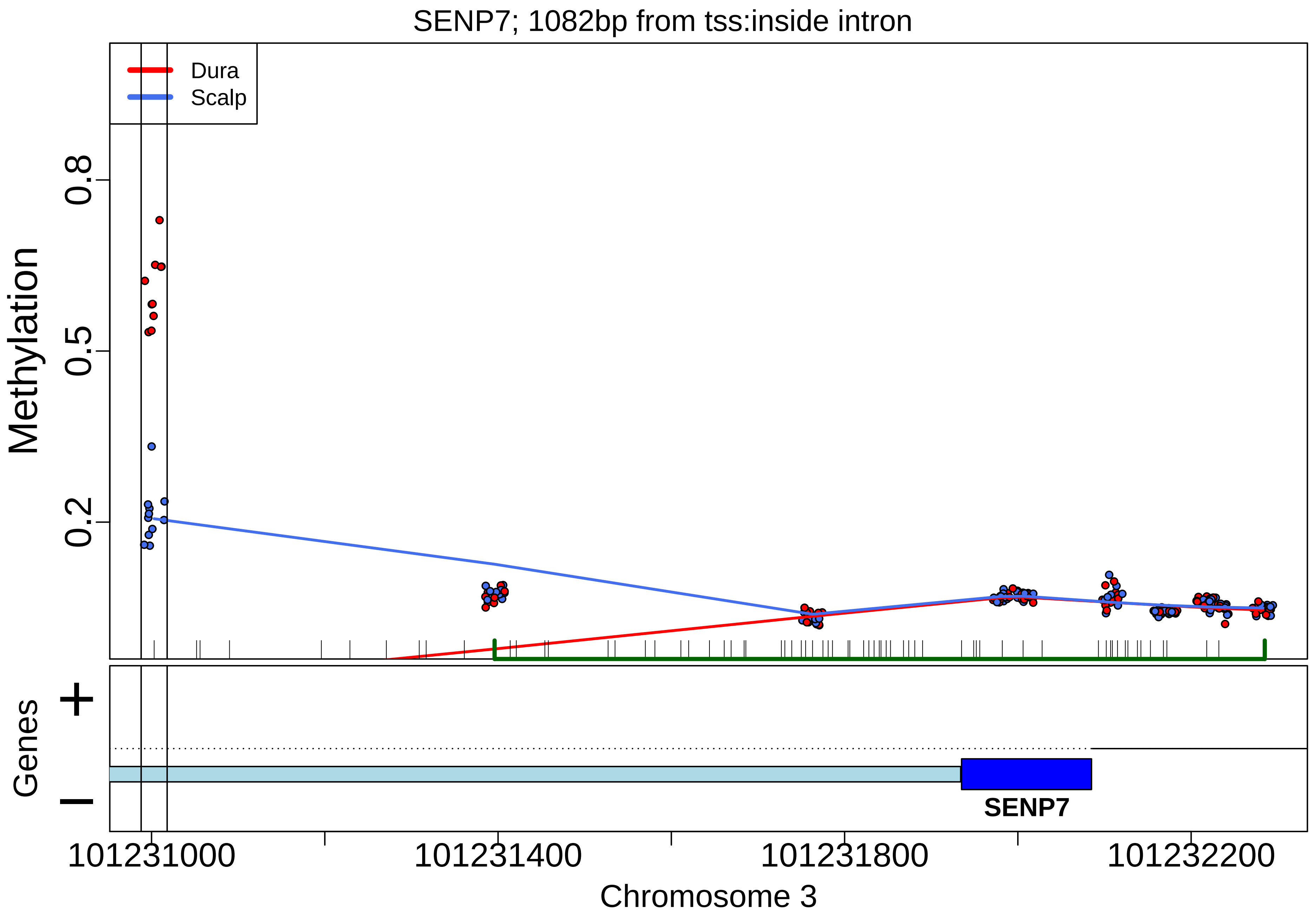
SENP7; 1082bp from tss:inside intron
Dura
Scalp
Methylation
0.8
0.5
0.2
Genes
+
-
SENP7
101231000
101231400
101231800
101232200
Chromosome 3

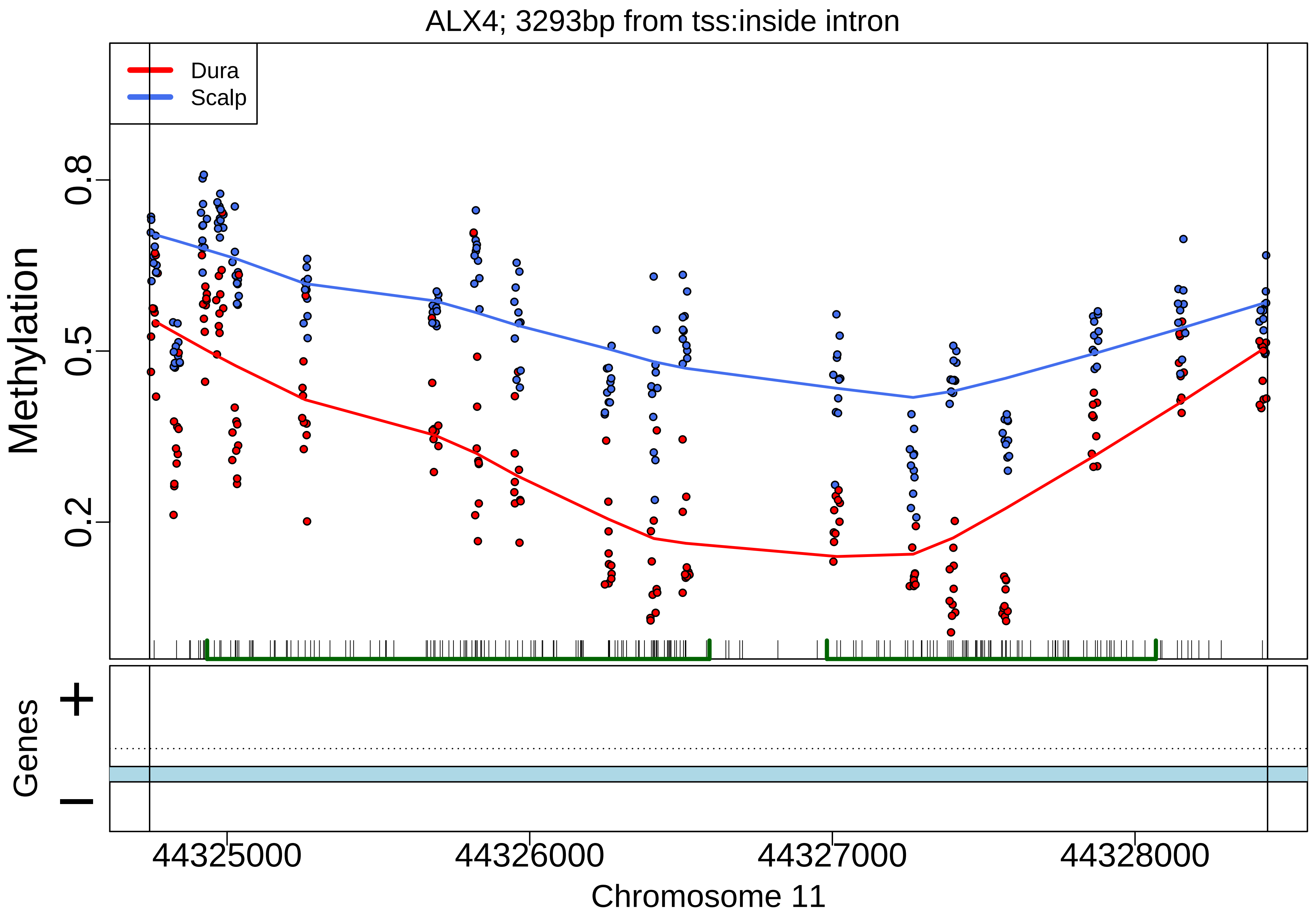

ALX4; 3293bp from tss:inside intron
Dura
Scalp
Methylation
0.2
0.5
0.8
Genes
+
−
44325000
44326000
44327000
44328000
Chromosome 11

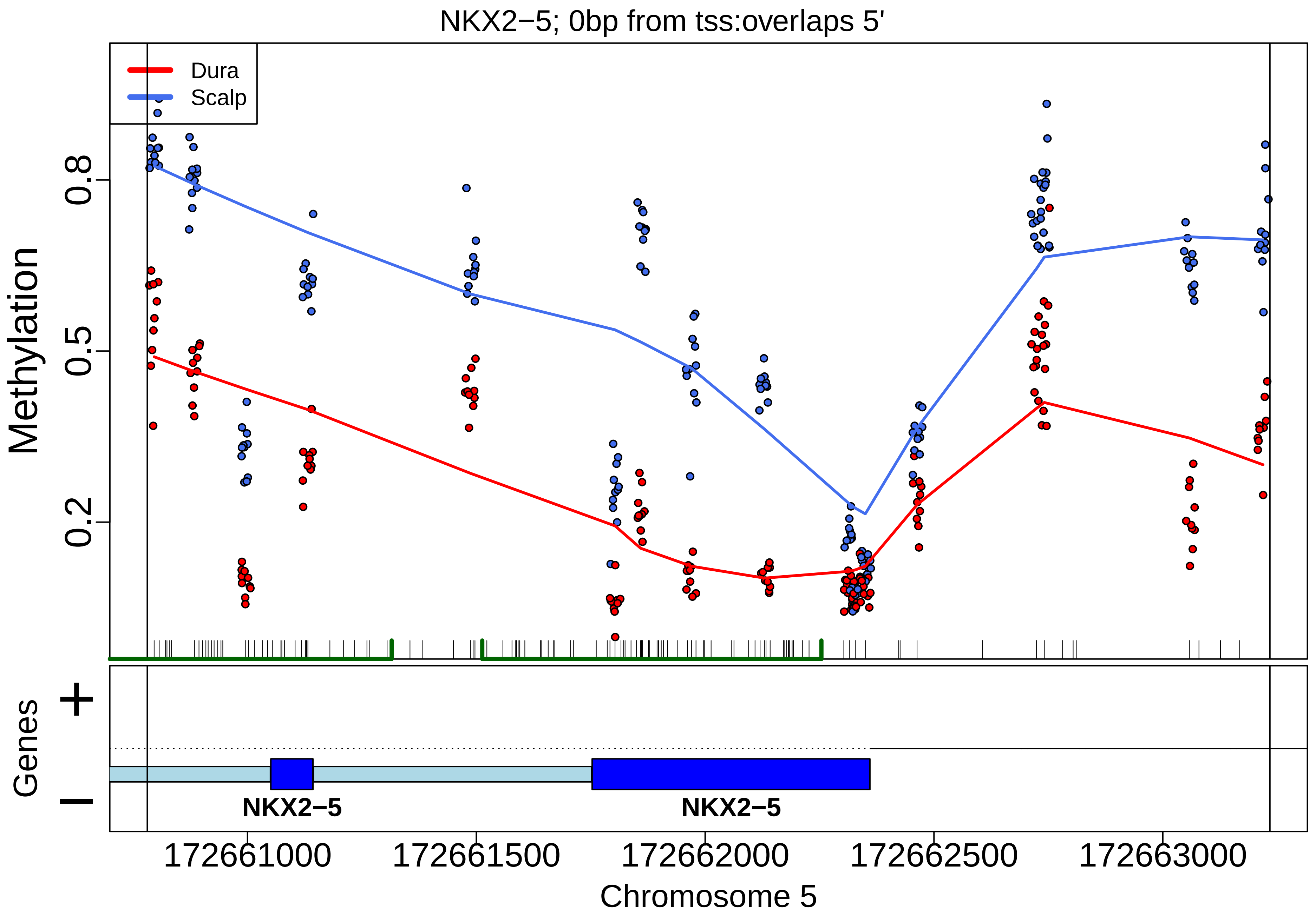
NKX2−5; 0bp from tss:overlaps 5'
Dura
Scalp
Methylation
0.2
0.5
0.8
Genes
+
−
NKX2−5
NKX2−5
172661000
172661500
172662000
172662500
172663000
Chromosome 5

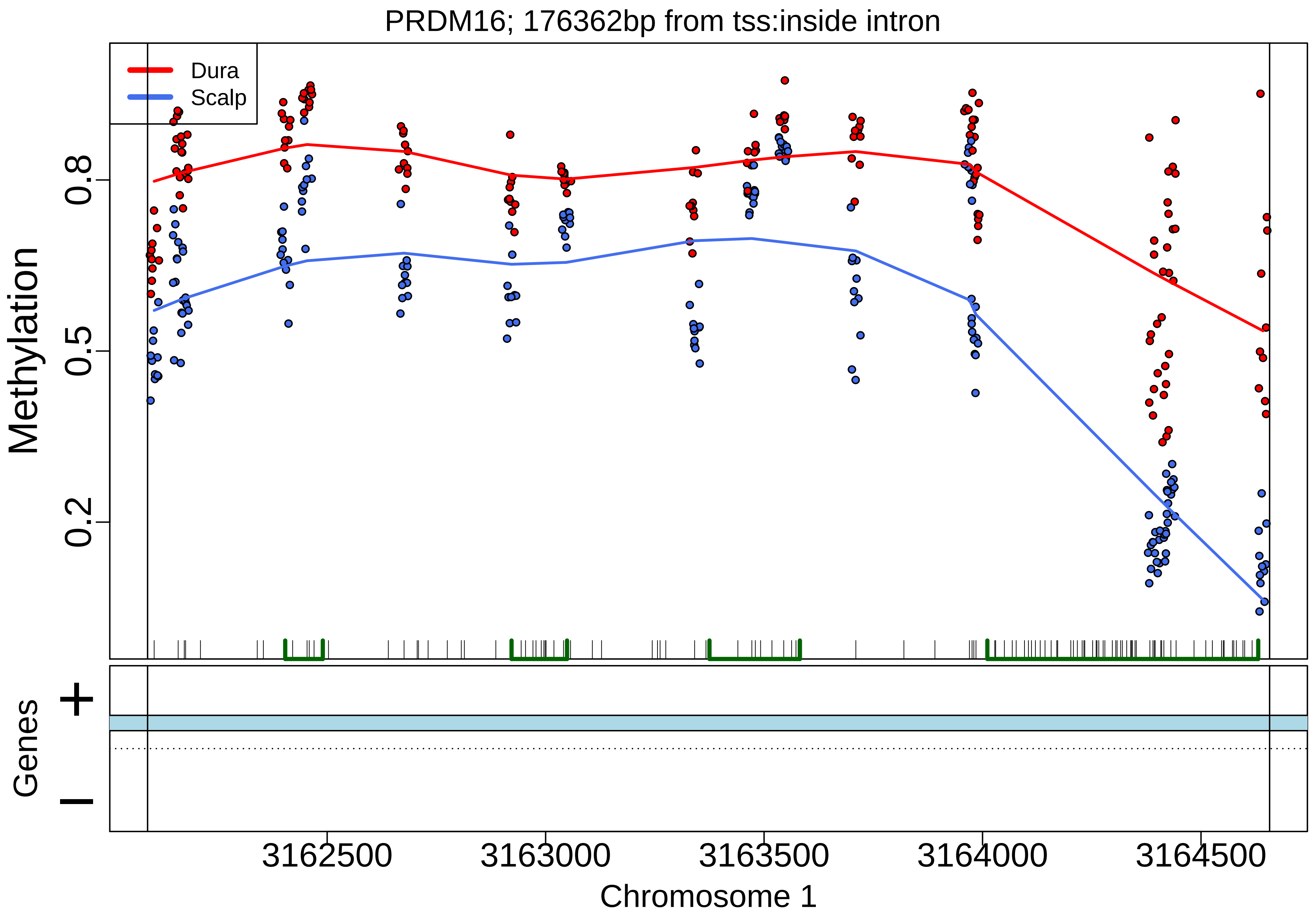

PRDM16; 176362bp from tss:inside intron
Dura
Scalp
Methylation
0.2
0.5
0.8
Genes
+
-
3162500
3163000
3163500
3164000
3164500
Chromosome 1

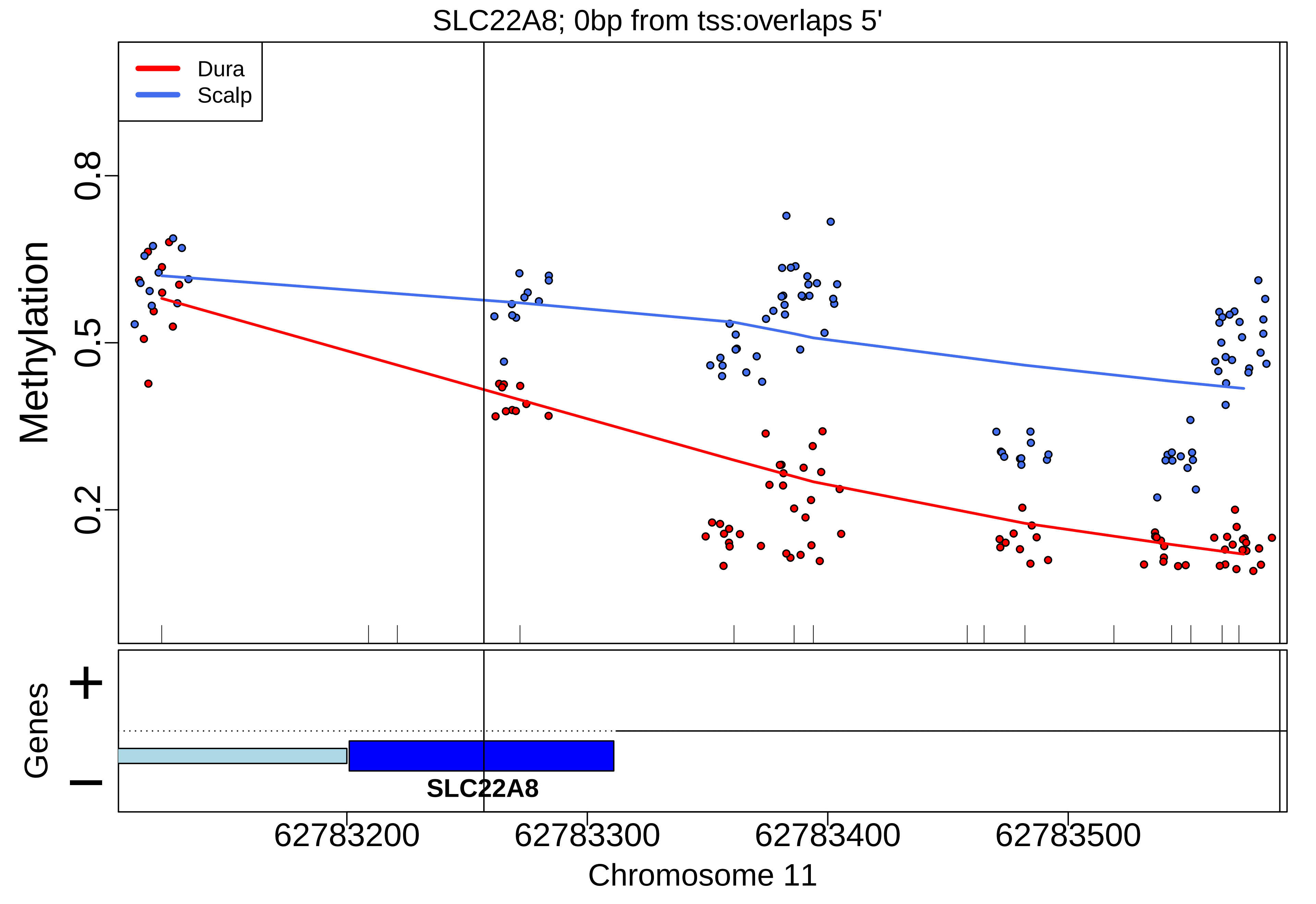
SLC22A8; 0bp from tss:overlaps 5'
Dura
Scalp
Methylation
0.2
0.5
0.8
Genes
+
-
SLC22A8
62783200
62783300
62783400
62783500
Chromosome 11

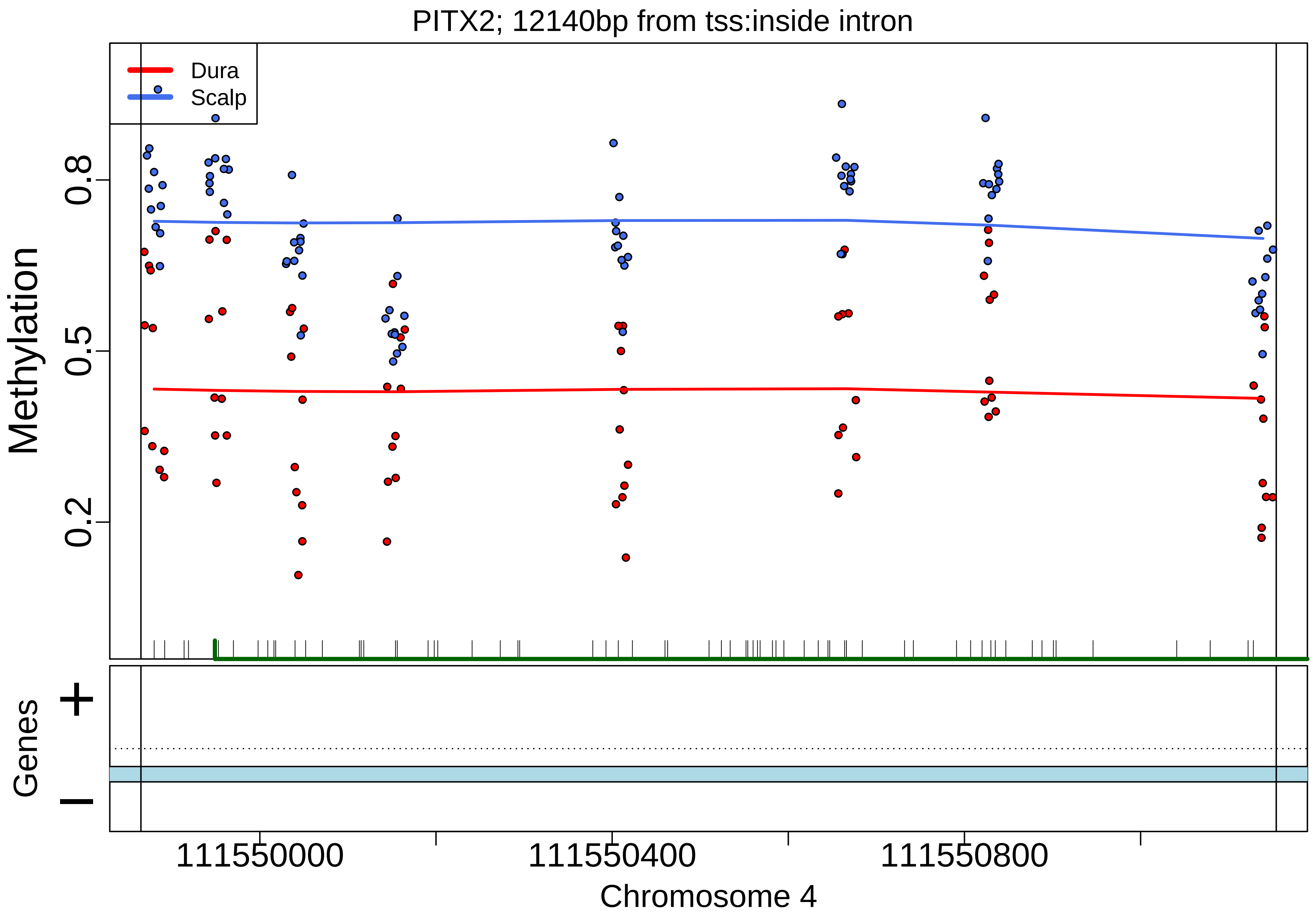
PITX2; 12140bp from tss:inside intron
Dura
Scalp
Methylation
0.2
0.5
0.8
Genes
+
−
111550000
111550400
111550800
Chromosome 4

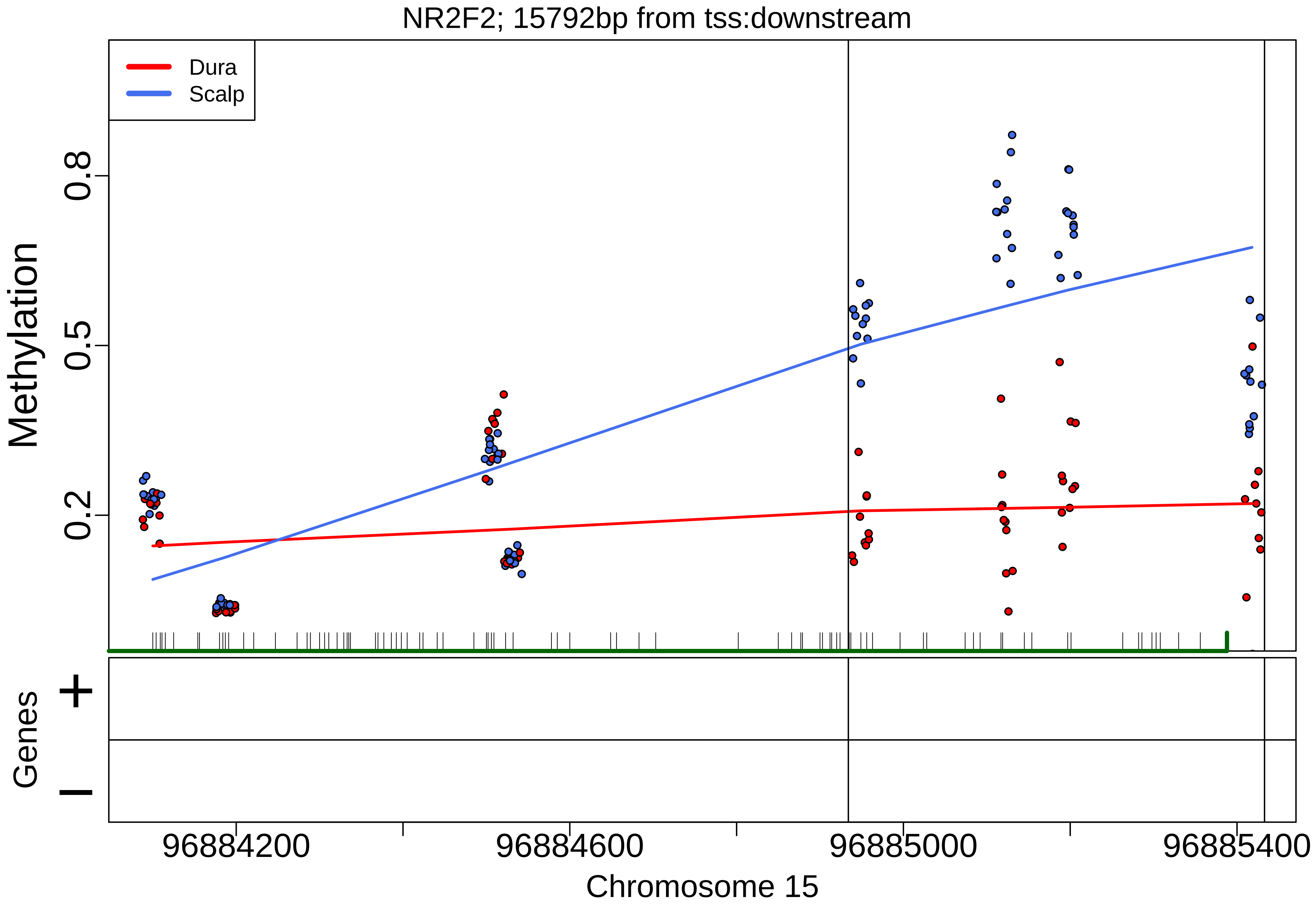
NR2F2; 15792bp from tss:downstream
Dura
Scalp
Methylation
0.2
0.5
0.8
Genes
+
−
96884200
96884600
96885000
96885400
Chromosome 15

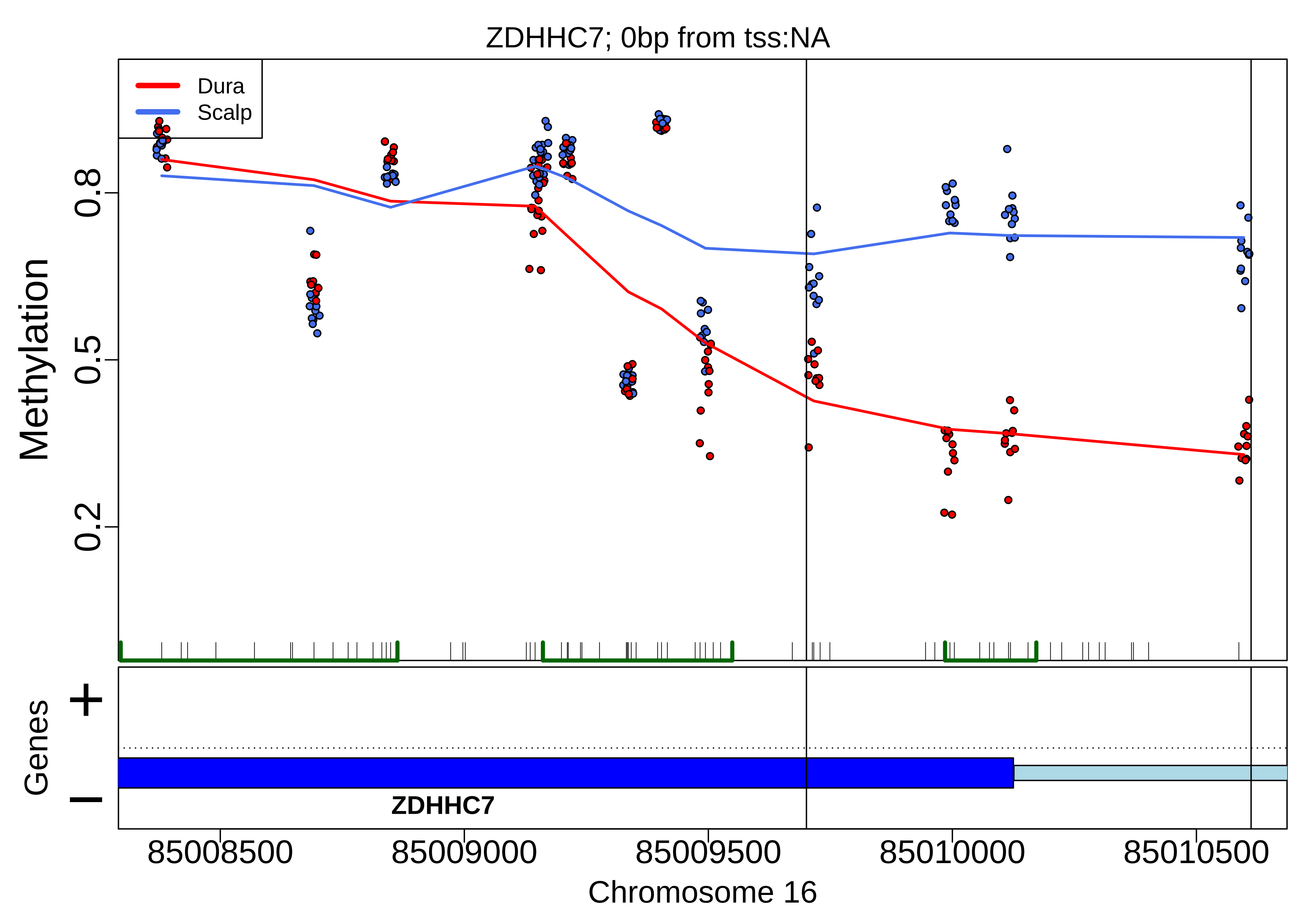
ZDHHC7; 0bp from tss:NA
Dura
Scalp
Methylation
0.2
0.5
0.8
Genes
+
−
ZDHHC7
85008500
85009000
85009500
85010000
85010500
Chromosome 16

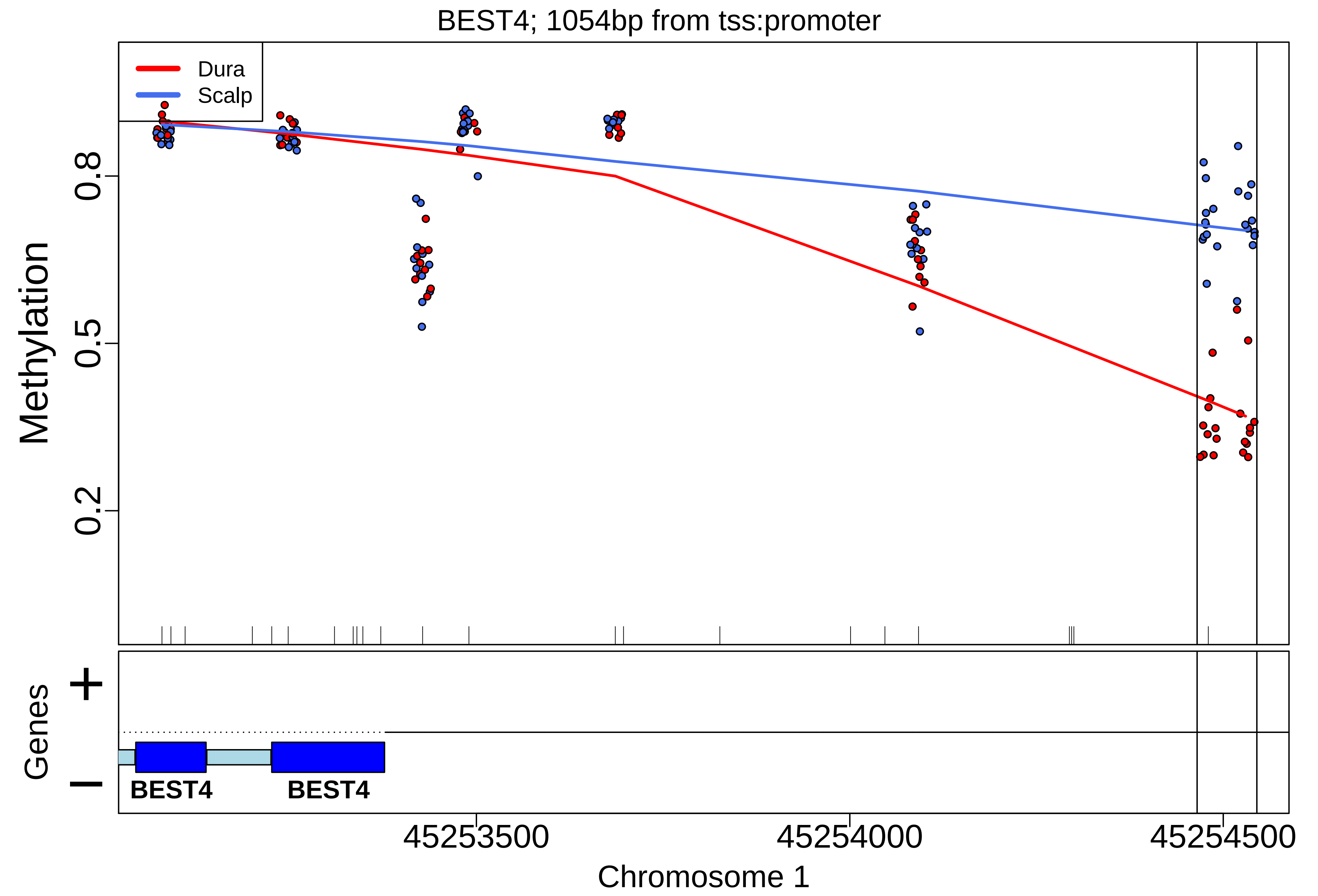
BEST4; 1054bp from tss:promoter
Dura
Scalp
Methylation
0.2
0.5
0.8
Genes
+
-
BEST4
BEST4
45253500
45254000
45254500
Chromosome 1

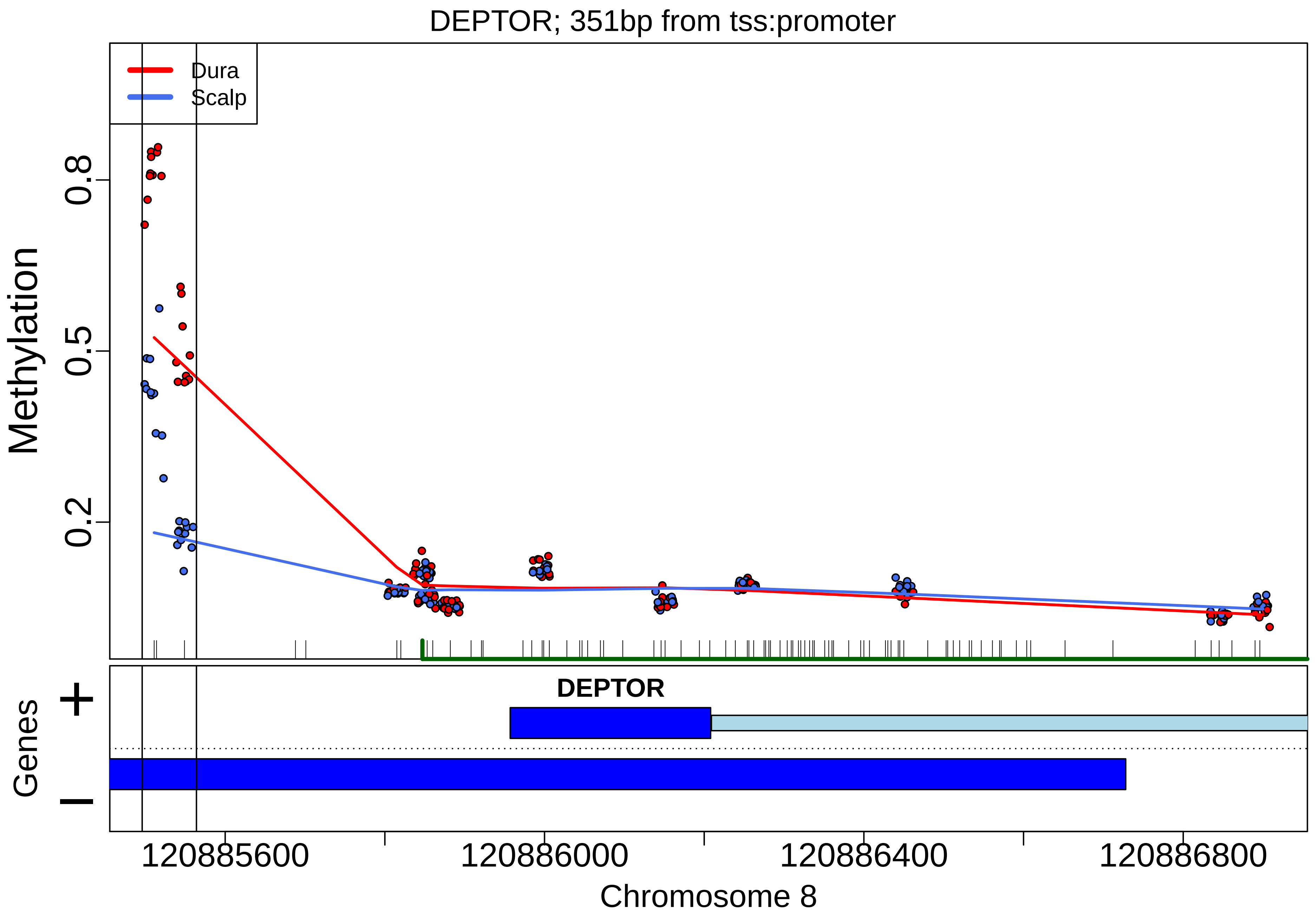
DEPTOR; 351bp from tss:promoter
Dura
Scalp
Methylation
0.2
0.5
0.8
Genes
+
−
DEPTOR
120885600
120886000
120886400
120886800
Chromosome 8

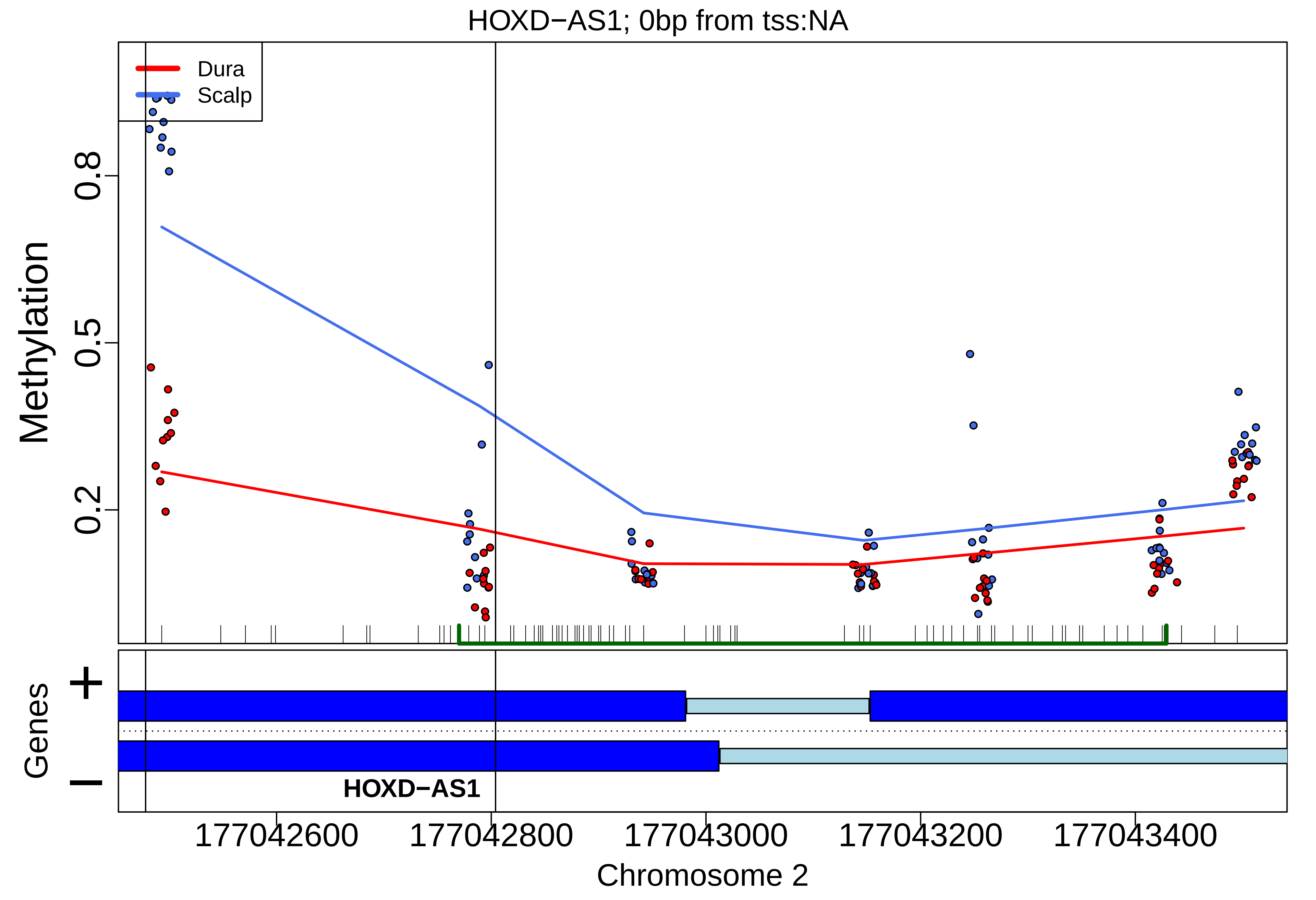
HOXD−AS1; 0bp from tss:NA
Dura
Scalp
Methylation
0.2
0.5
0.8
Genes
+
−
HOXD−AS1
177042600
177042800
177043000
177043200
177043400
Chromosome 2

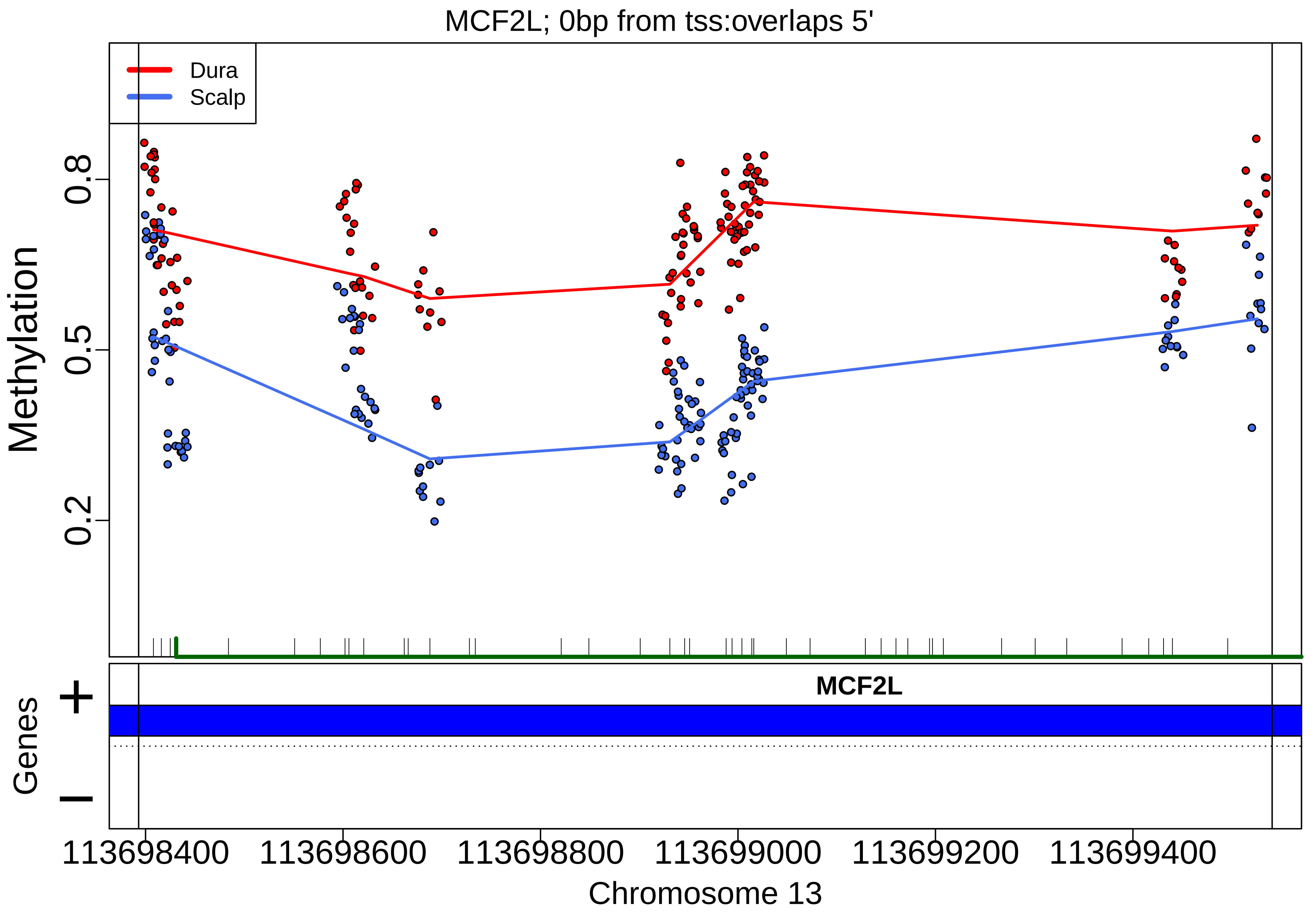

MCF2L; 0bp from tss:overlaps 5'
Dura
Scalp
Methylation
0.2
0.5
0.8
Genes
+
-
MCF2L
113698400
113698600
113698800
113699000
113699200
113699400
Chromosome 13

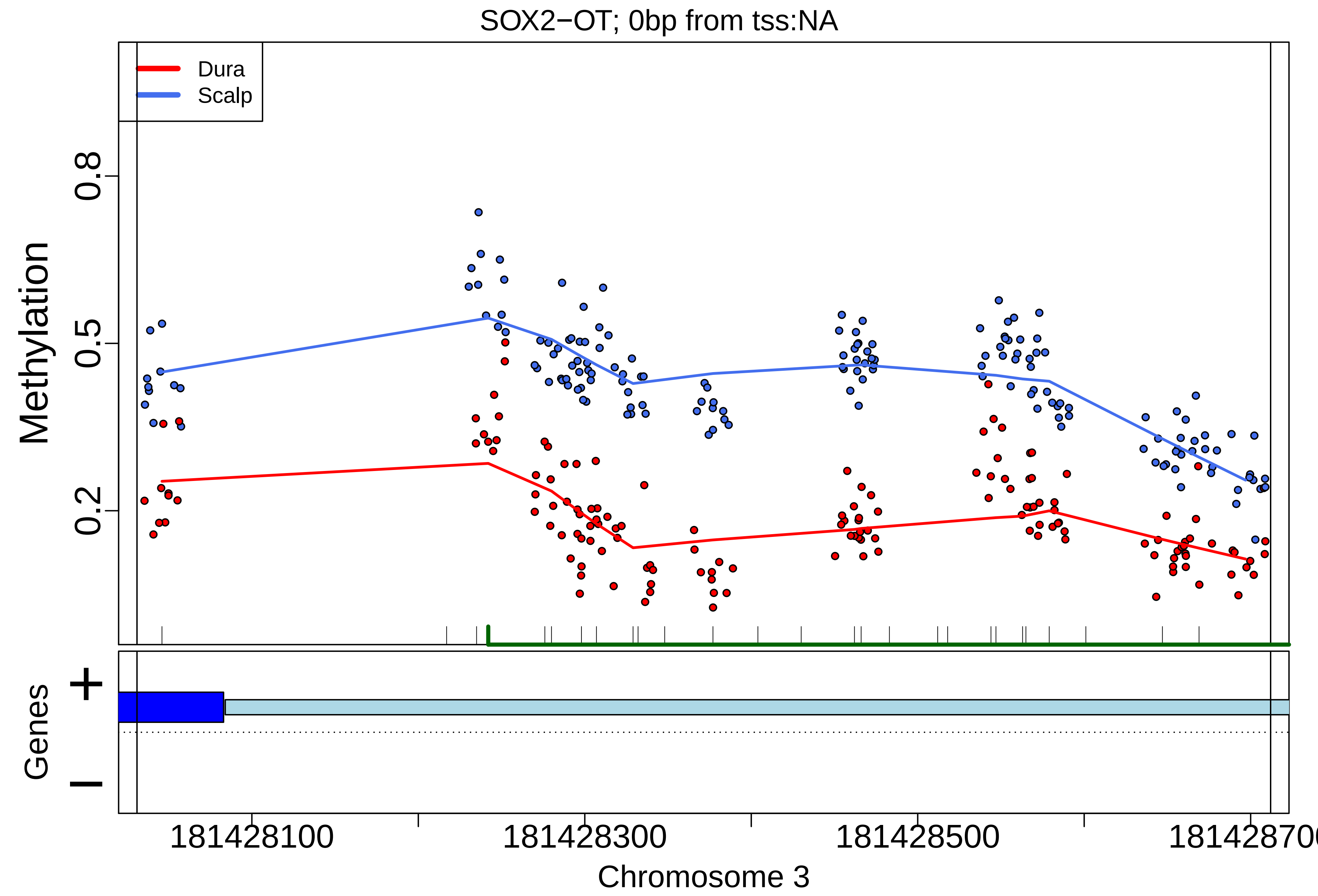

SOX2−OT; 0bp from tss:NA
Dura
Scalp
Methylation
0.2
0.5
0.8
Genes
+
−
181428100
181428300
181428500
181428700
Chromosome 3

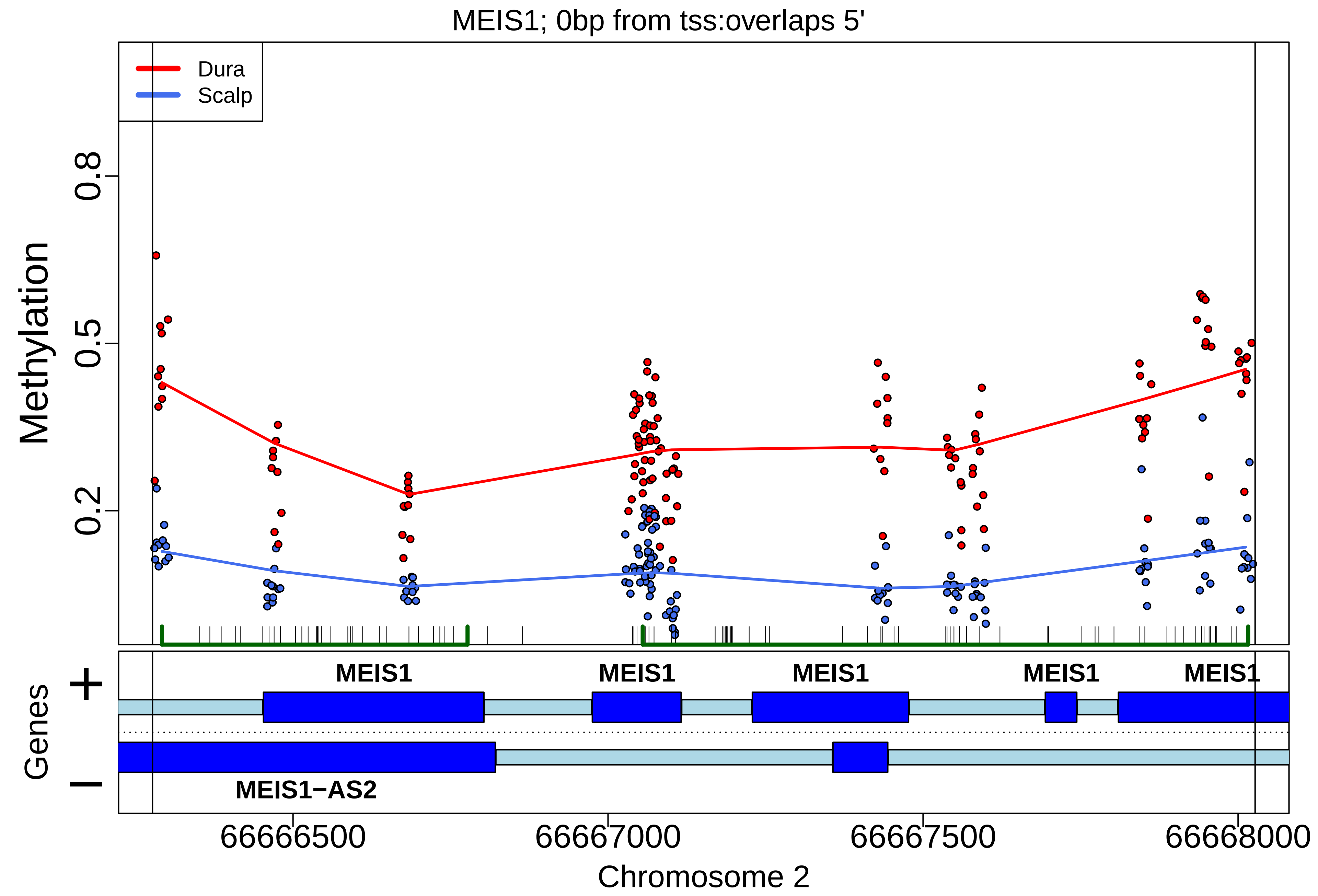

MEIS1; 0bp from tss:overlaps 5'
Dura
Scalp
Methylation
0.2
0.5
0.8
Genes
+
−
MEIS1
MEIS1
MEIS1
MEIS1
MEIS1
MEIS1−AS2
66666500
66667000
66667500
66668000
Chromosome 2

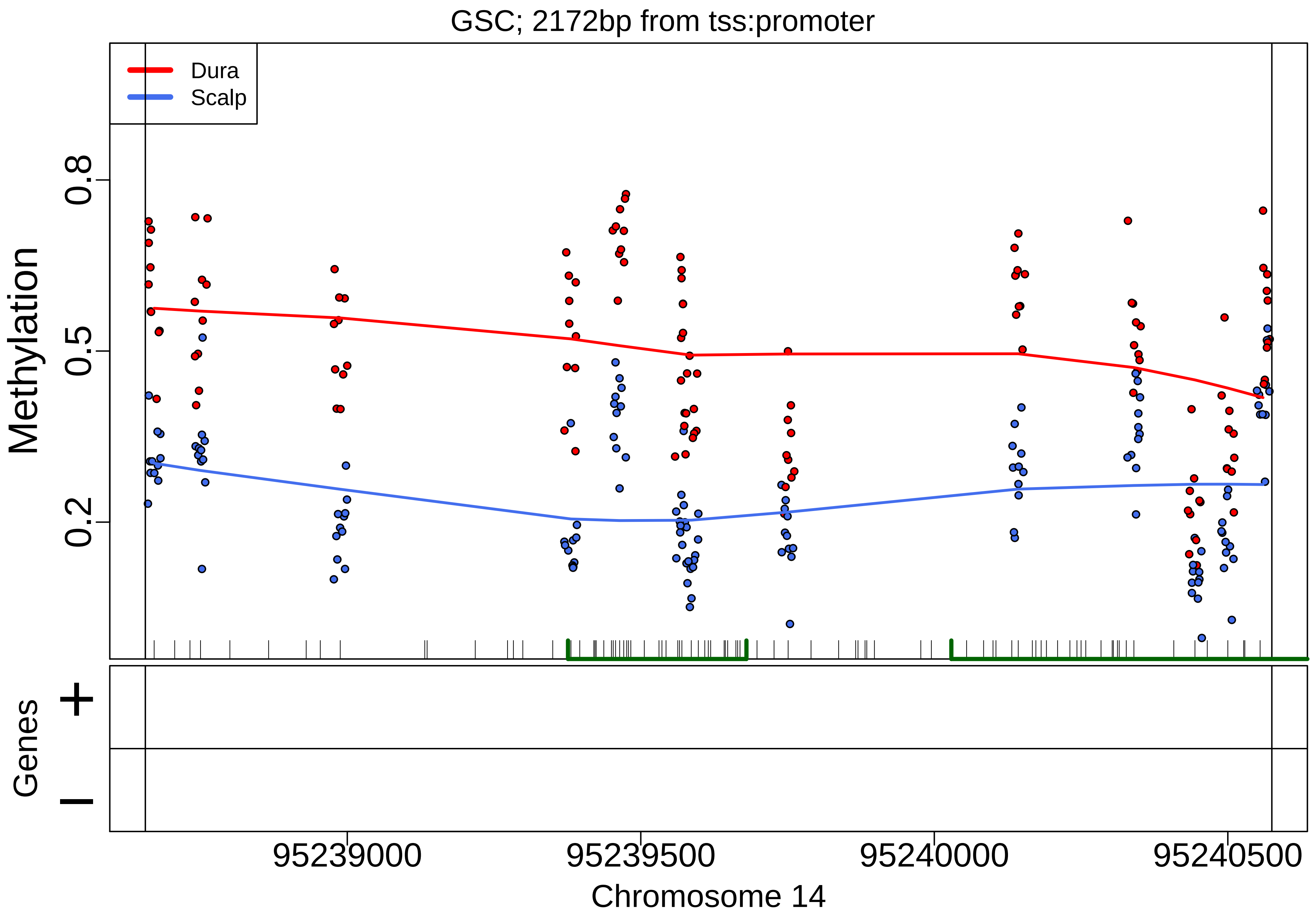

GSC; 2172bp from tss:promoter
Dura
Scalp
Methylation
0.2
0.5
0.8
Genes
+
–
95239000
95239500
95240000
95240500
Chromosome 14

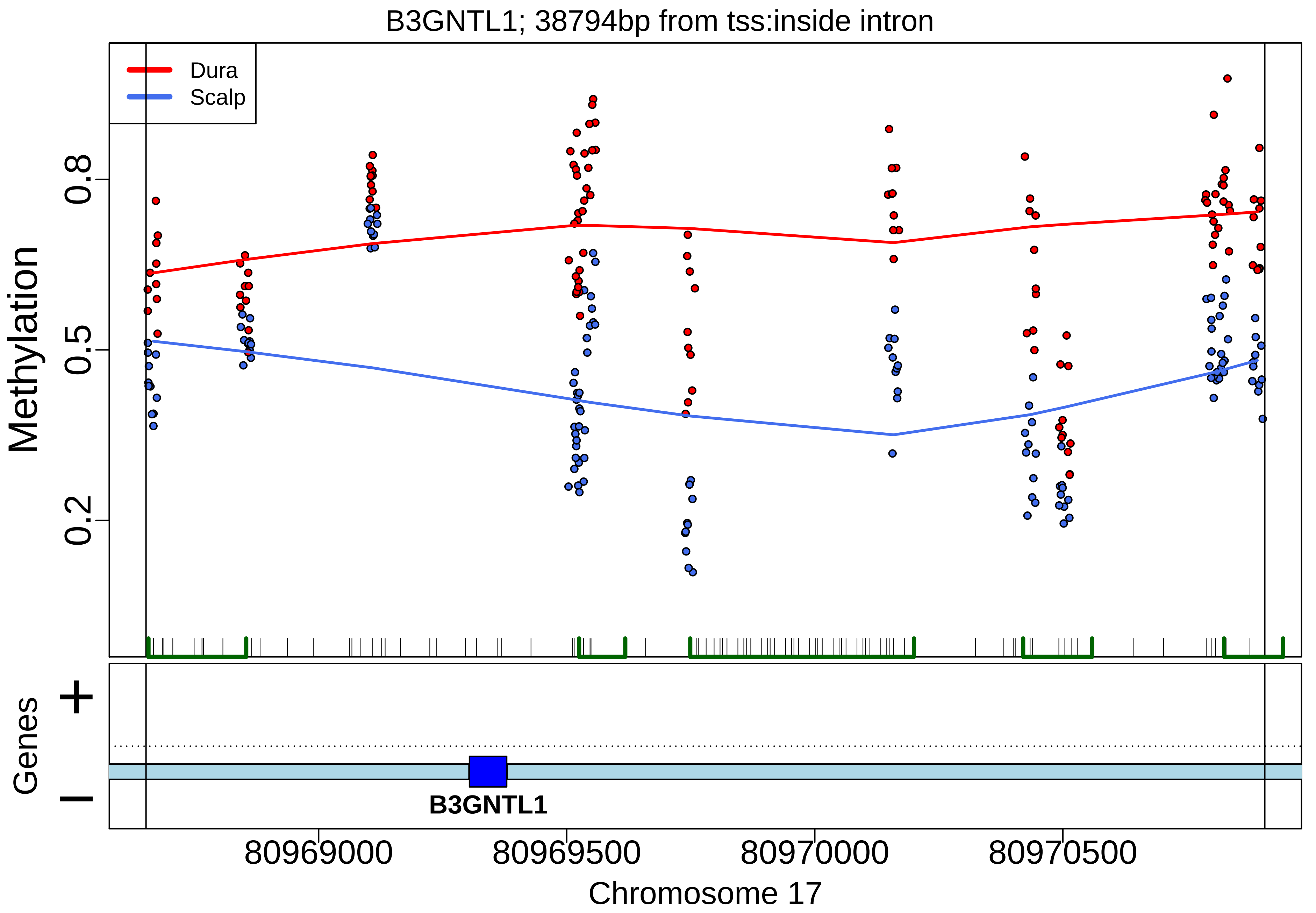
B3GNTL1; 38794bp from tss:inside intron
Dura
Scalp
Methylation
0.8
0.5
0.2
Genes
+
-
B3GNTL1
80969000
80969500
80970000
80970500
Chromosome 17

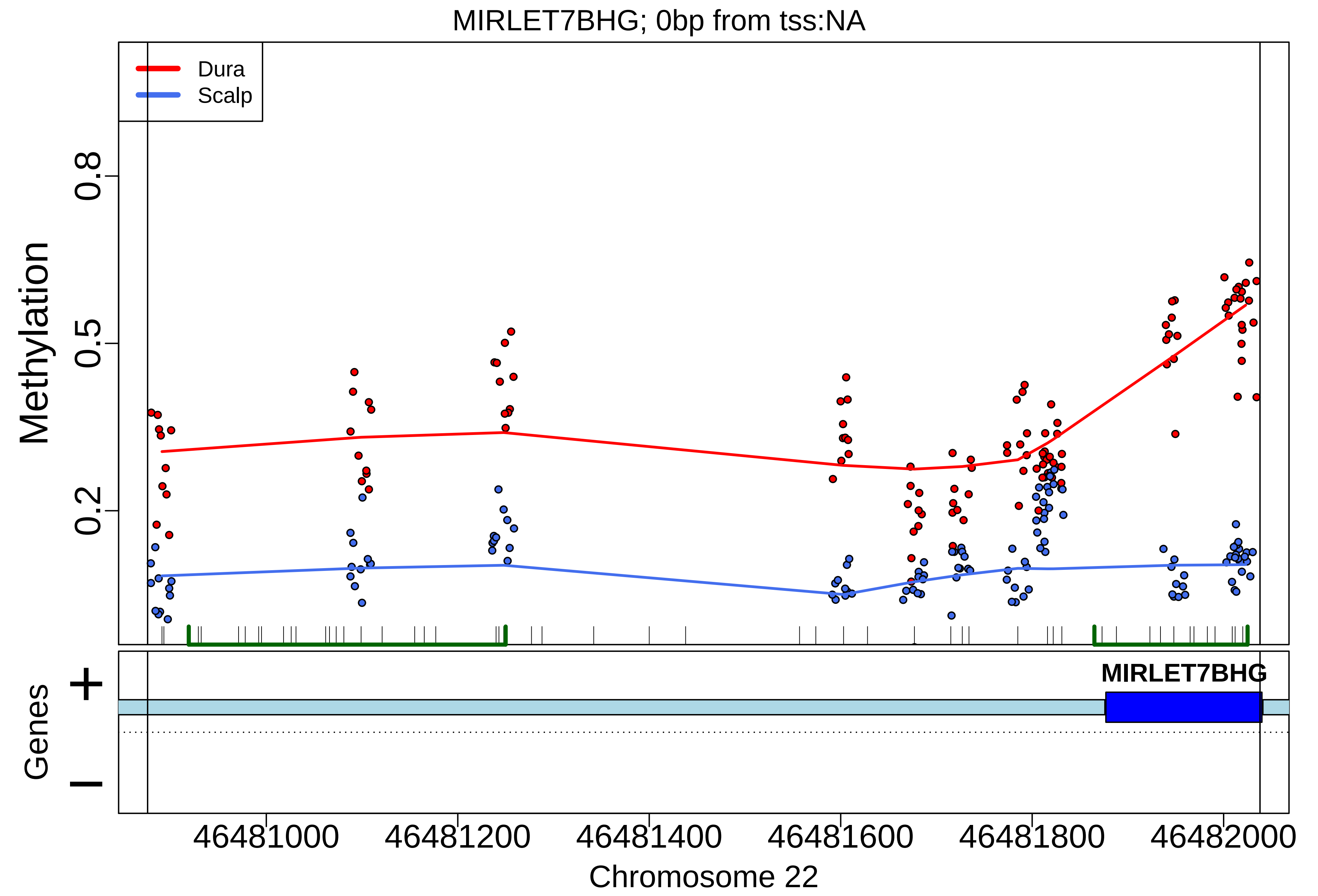

MIRLET7BHG; 0bp from tss:NA
Dura
Scalp
Methylation
0.2
0.5
0.8
Genes
+
-
MIRLET7BHG
46481000
46481200
46481400
46481600
46481800
46482000
Chromosome 22

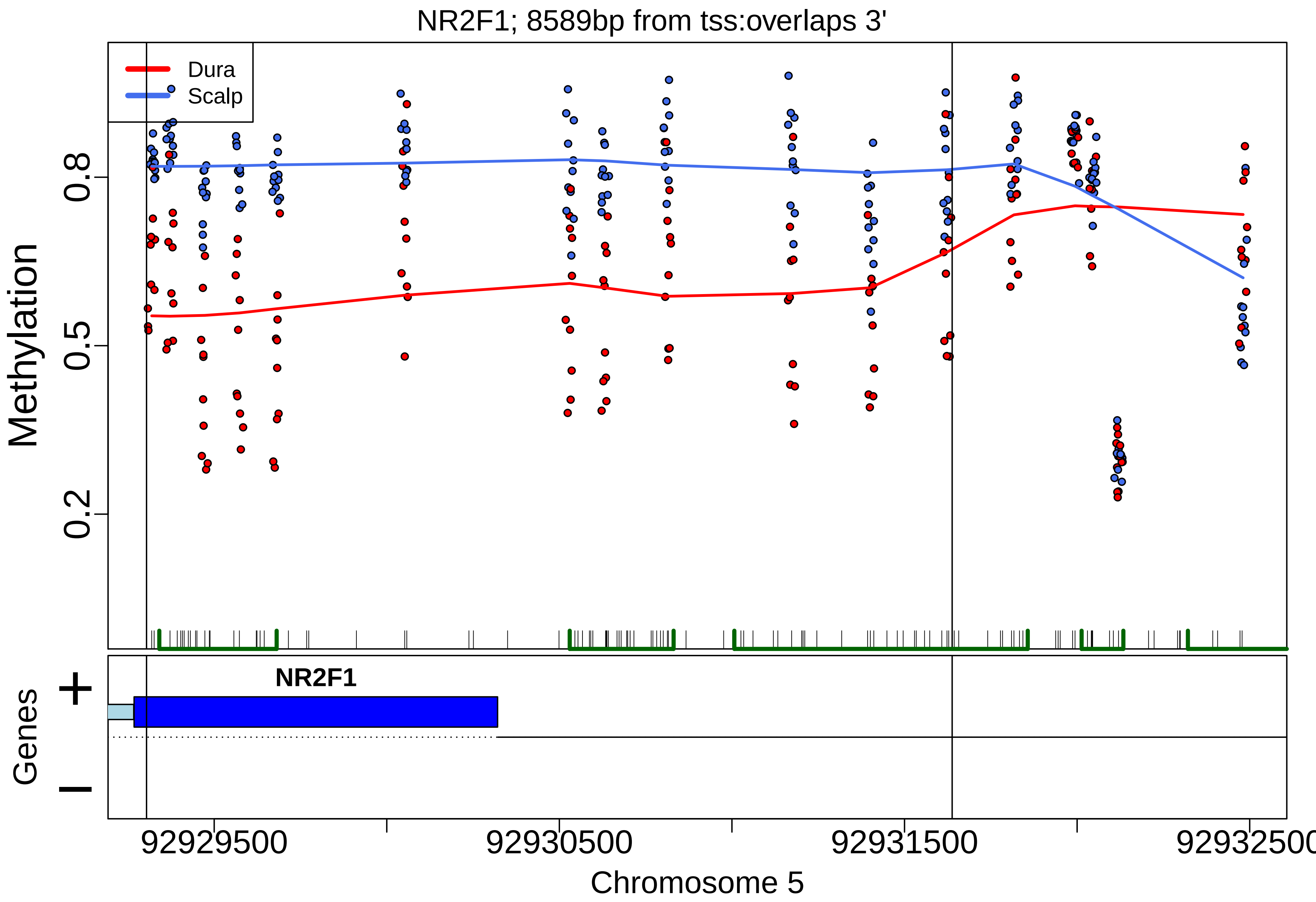
NR2F1; 8589bp from tss:overlaps 3'
Dura
Scalp
Methylation
0.2
0.5
0.8
Genes
+
-
NR2F1
92929500
92930500
92931500
92932500
Chromosome 5

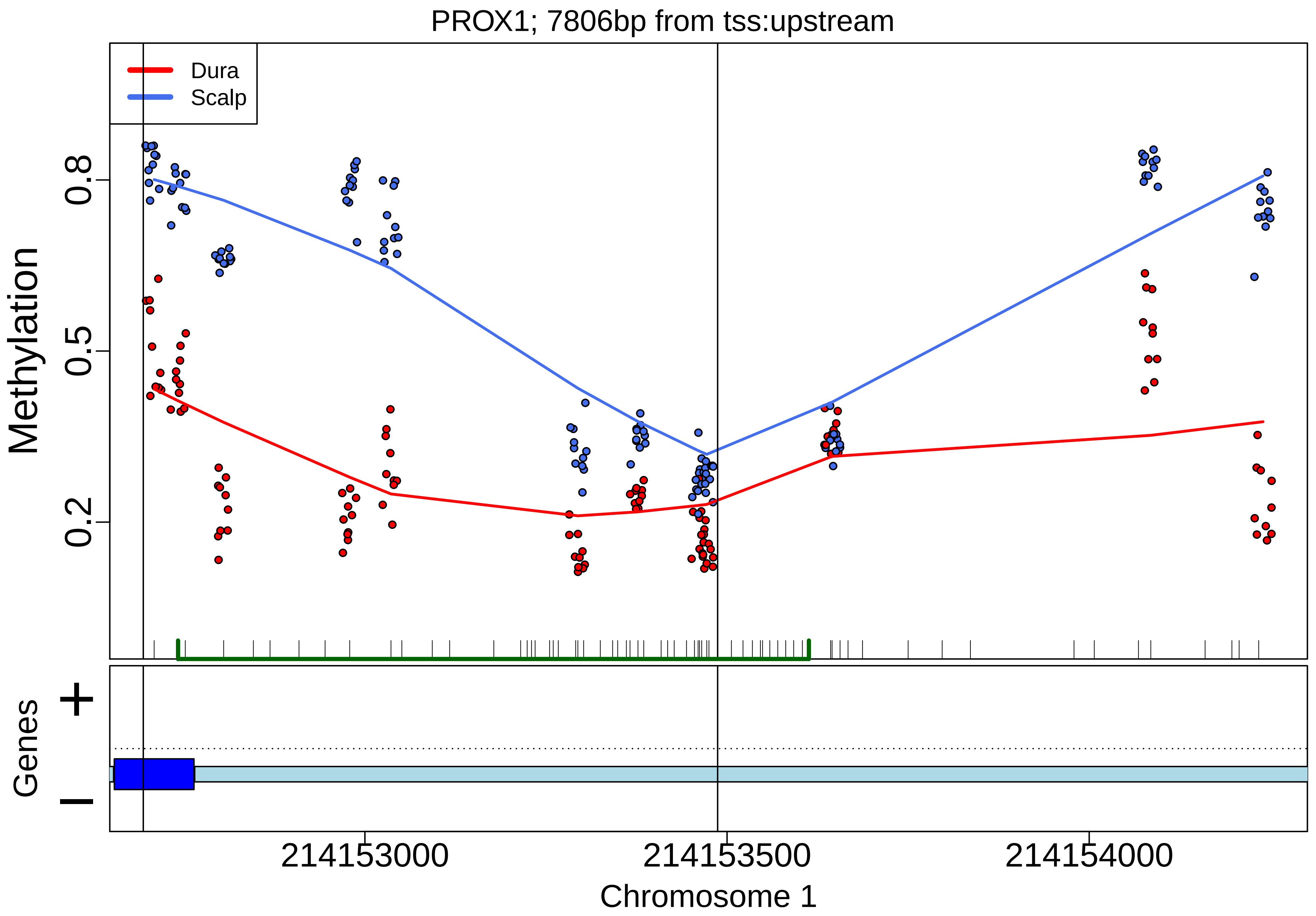
PROX1; 7806bp from tss:upstream
Dura
Scalp
Methylation
0.2
0.5
0.8
Genes
+
-
214153000
214153500
214154000
Chromosome 1

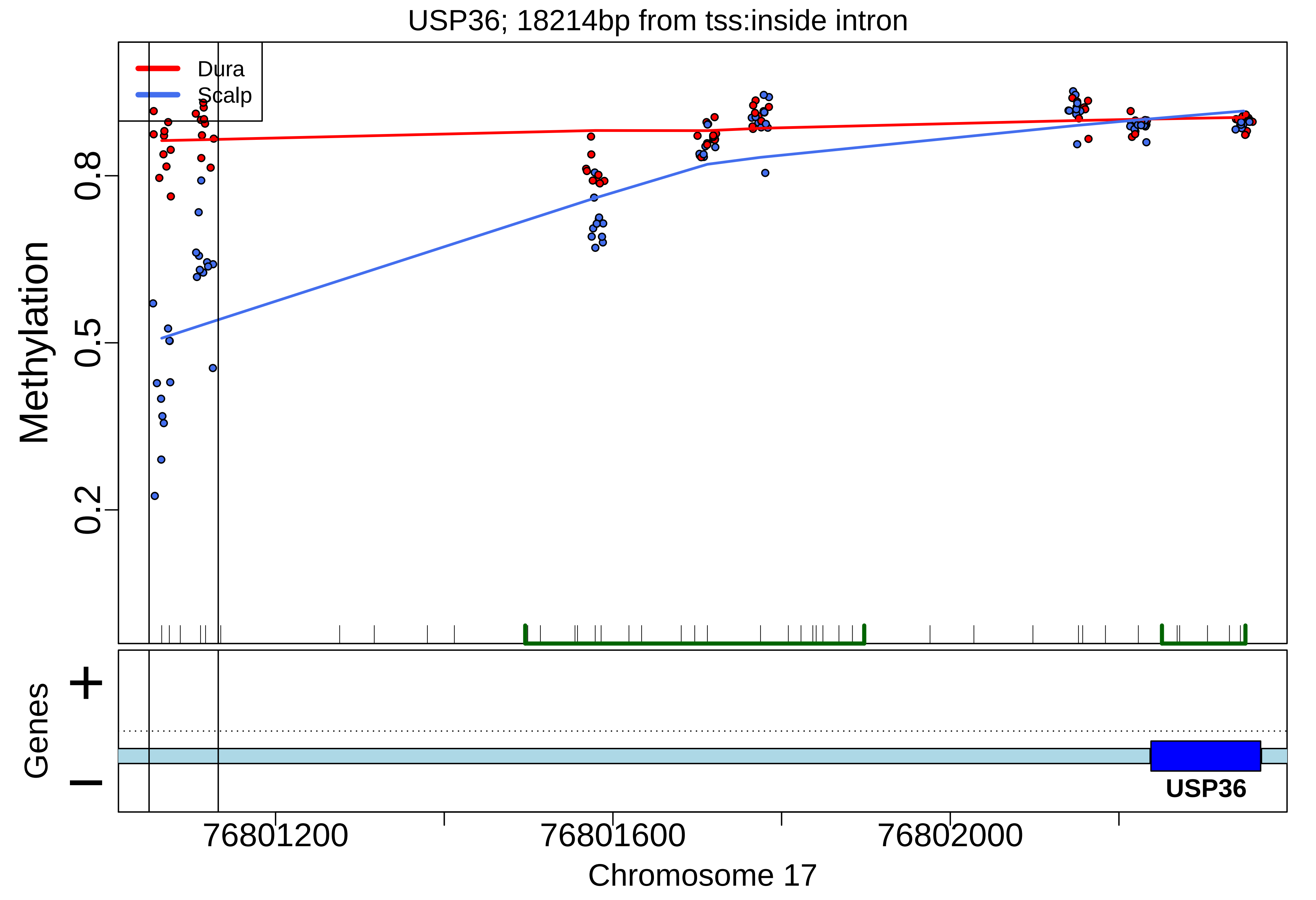
USP36; 18214bp from tss:inside intron
Dura
Scalp
Methylation
0.2
0.5
0.8
Genes
+
-
USP36
76801200
76801600
76802000
Chromosome 17

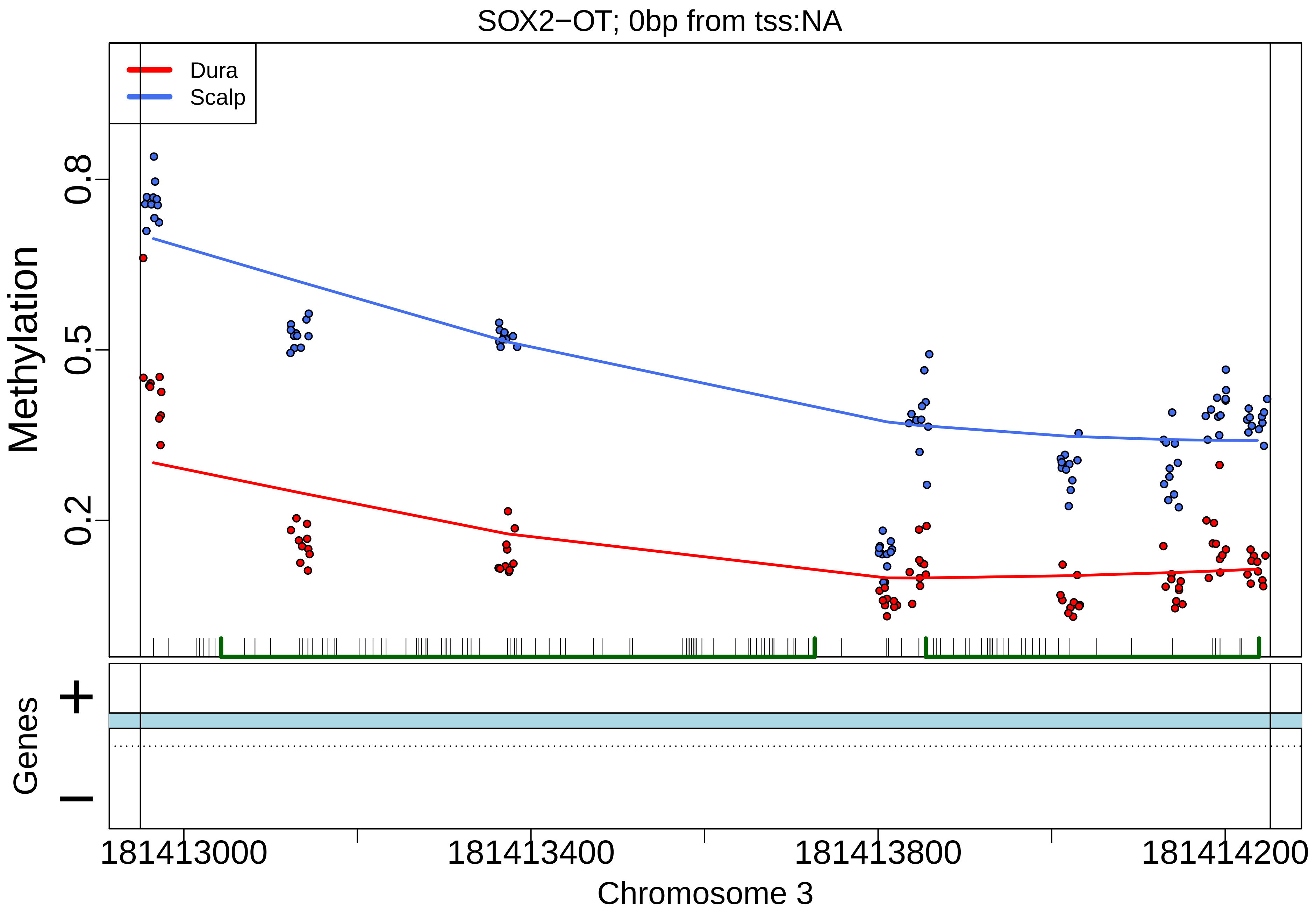

SOX2−OT; 0bp from tss:NA
Dura
Scalp
Methylation
0.2
0.5
0.8
Genes
+
−
181413000
181413400
181413800
181414200
Chromosome 3

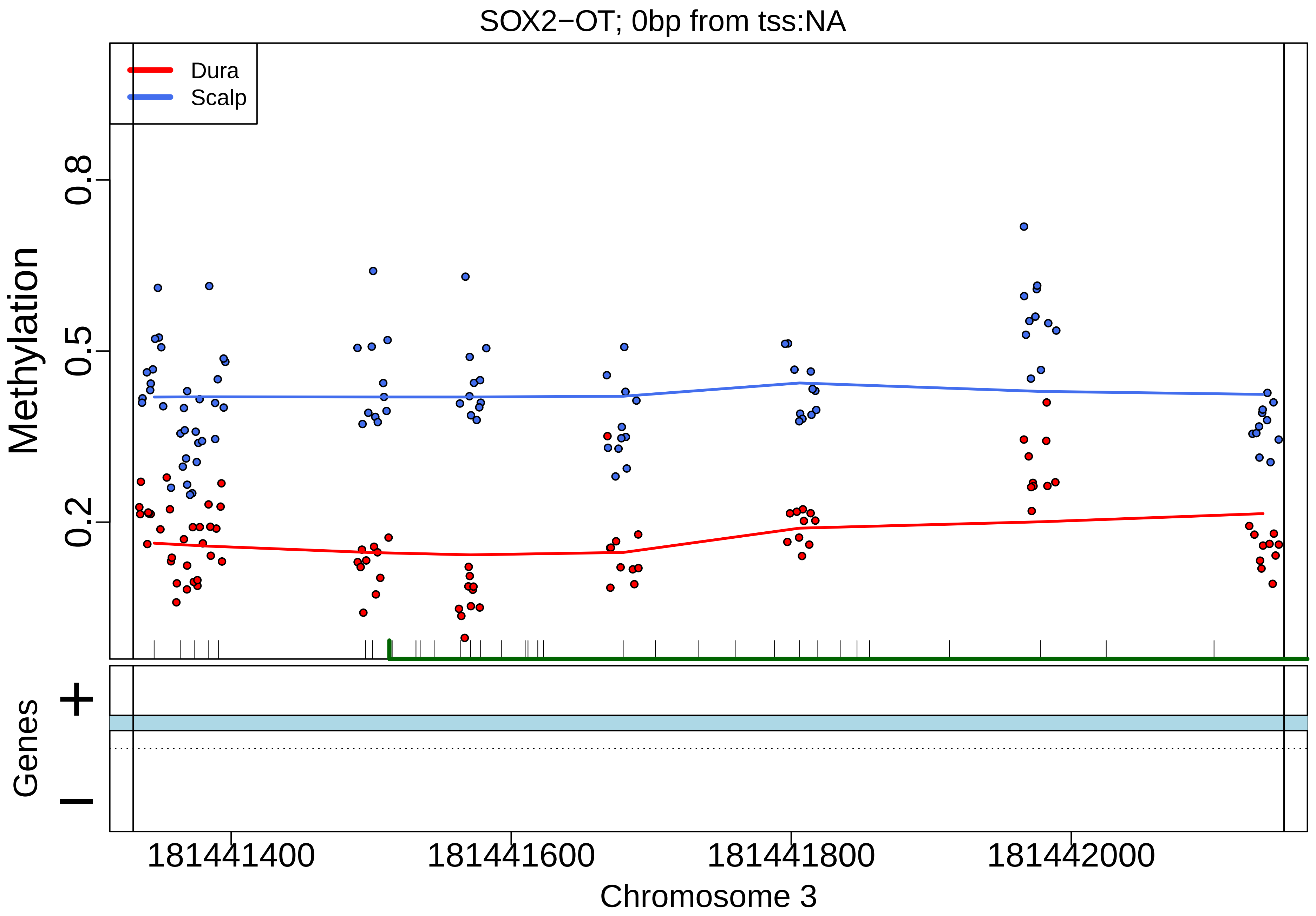

SOX2−OT; 0bp from tss:NA
Dura
Scalp
Methylation
0.2
0.5
0.8
Genes
+
−
181441400
181441600
181441800
181442000
Chromosome 3

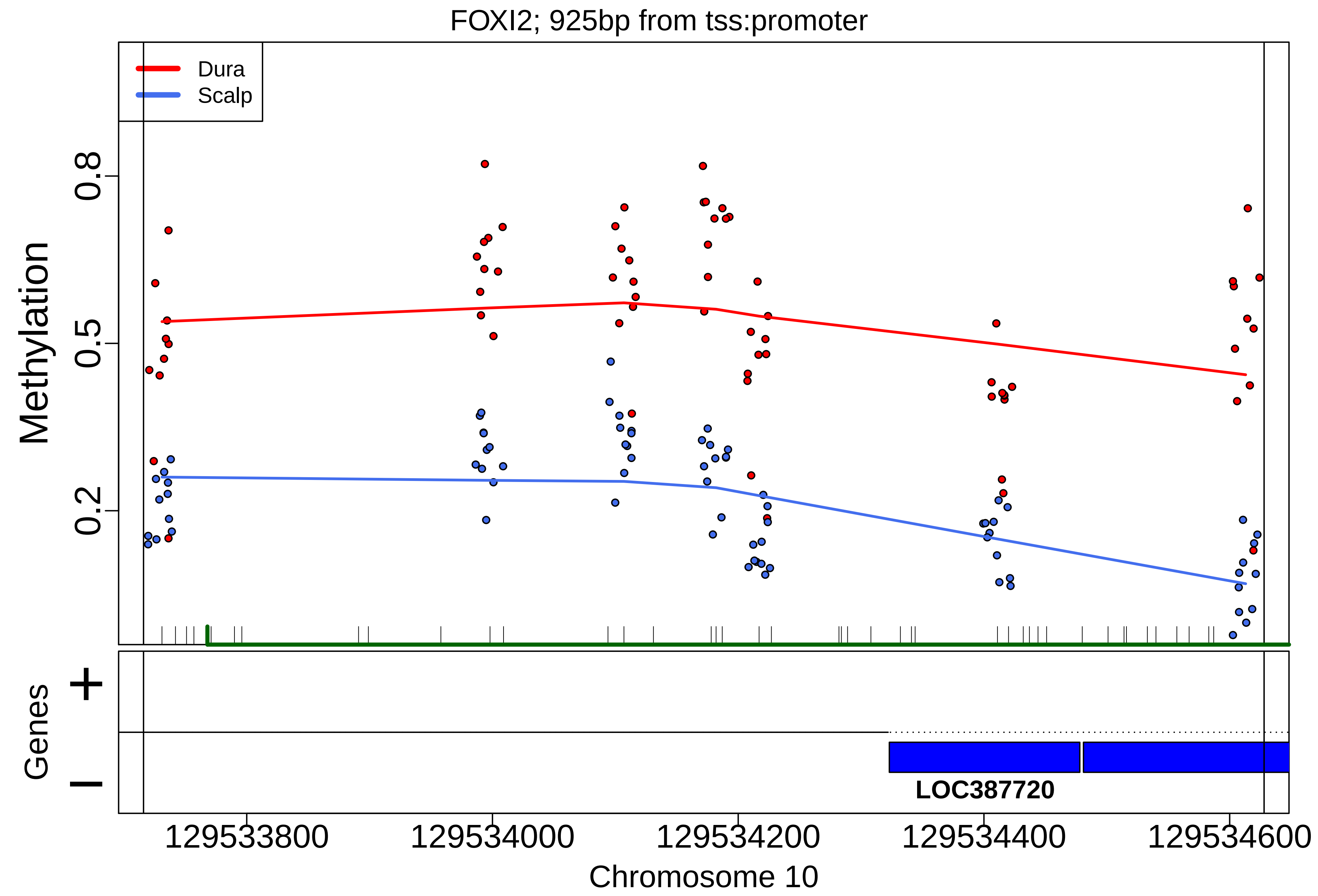

FOXI2; 925bp from tss:promoter
Dura
Scalp
Methylation
0.2
0.5
0.8
Genes
+
−
LOC387720
129533800
129534000
129534200
129534400
129534600
Chromosome 10

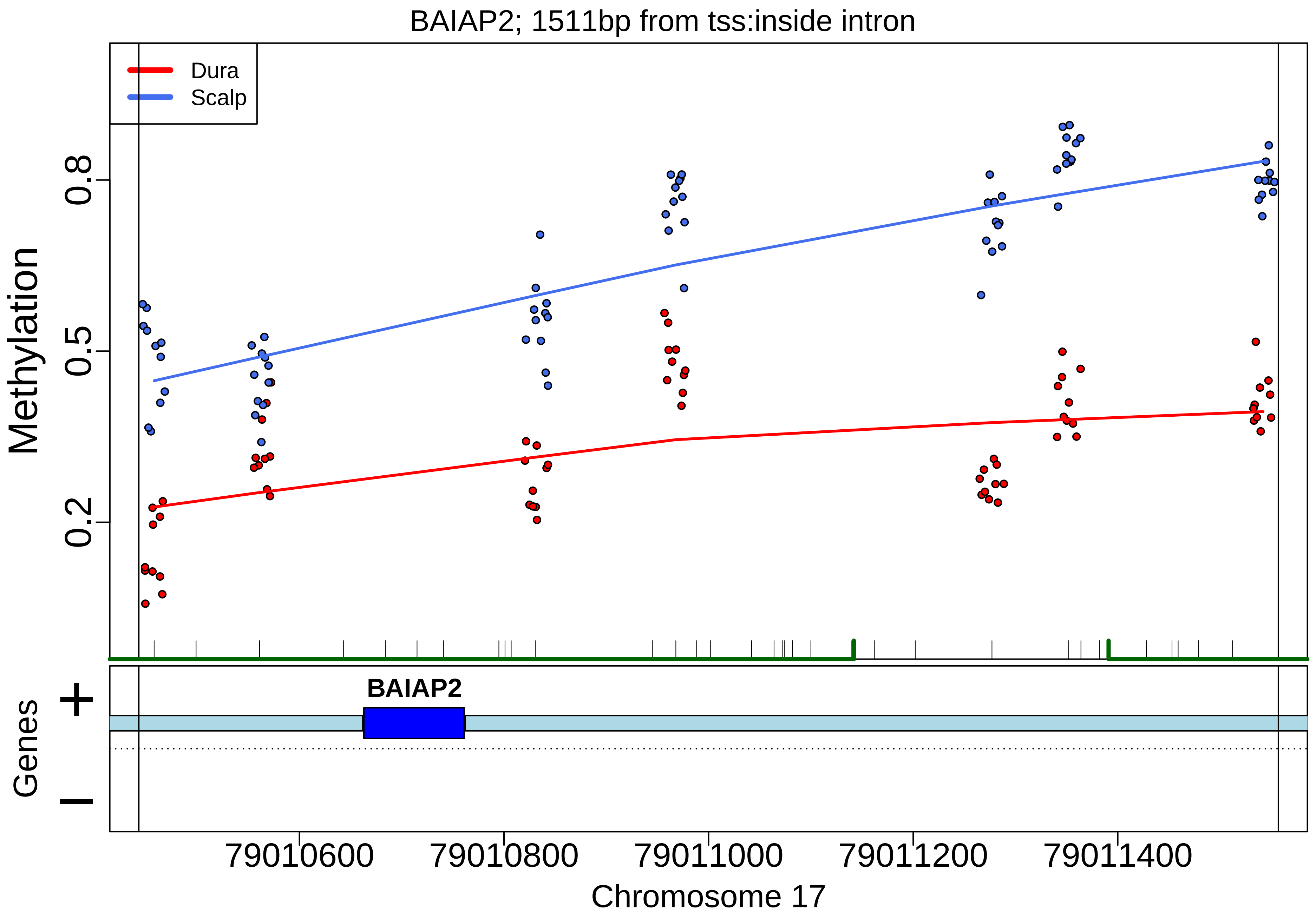

BAIAP2; 1511bp from tss:inside intron
Dura
Scalp
Methylation
0.2
0.5
0.8
Genes
+
-
BAIAP2
79010600
79010800
79011000
79011200
79011400
Chromosome 17

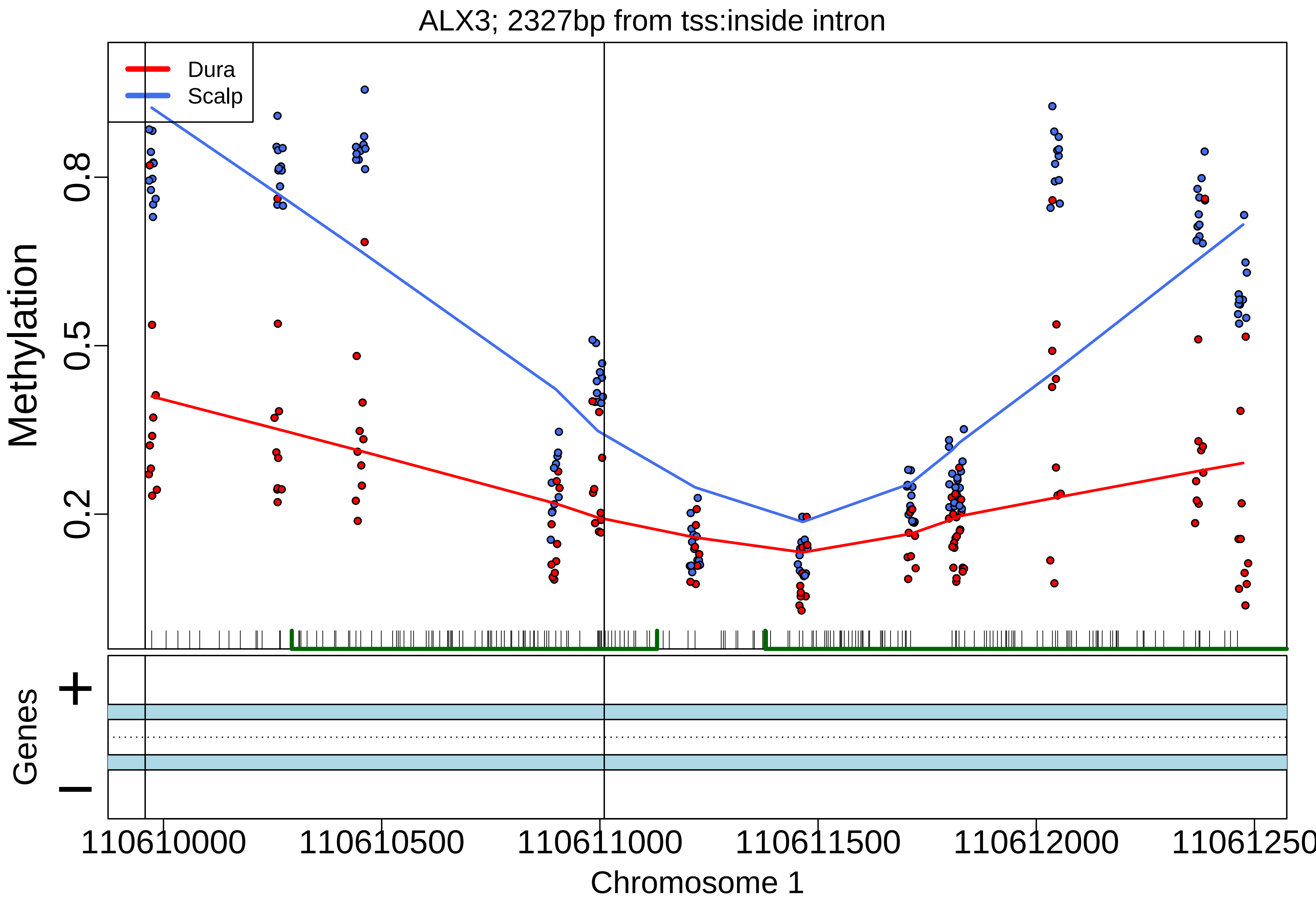

ALX3; 2327bp from tss:inside intron
Dura
Scalp
Methylation
0.2
0.5
0.8
Genes
+
−
110610000
110610500
110611000
110611500
110612000
11061250
Chromosome 1

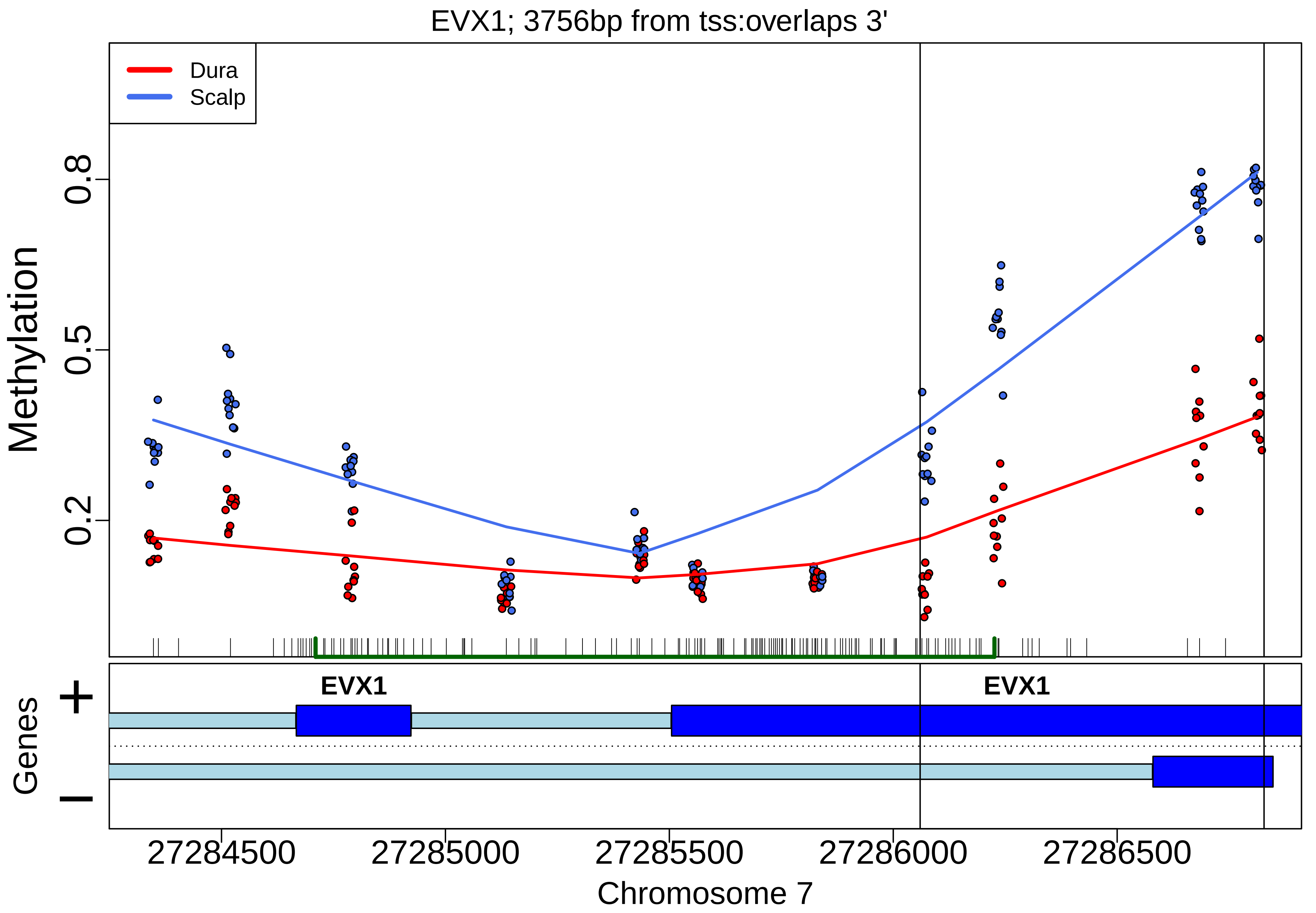

EVX1; 3756bp from tss:overlaps 3'
Dura
Scalp
Methylation
0.2
0.5
0.8
Genes
+
−
EVX1
EVX1
27284500
27285000
27285500
27286000
27286500
Chromosome 7

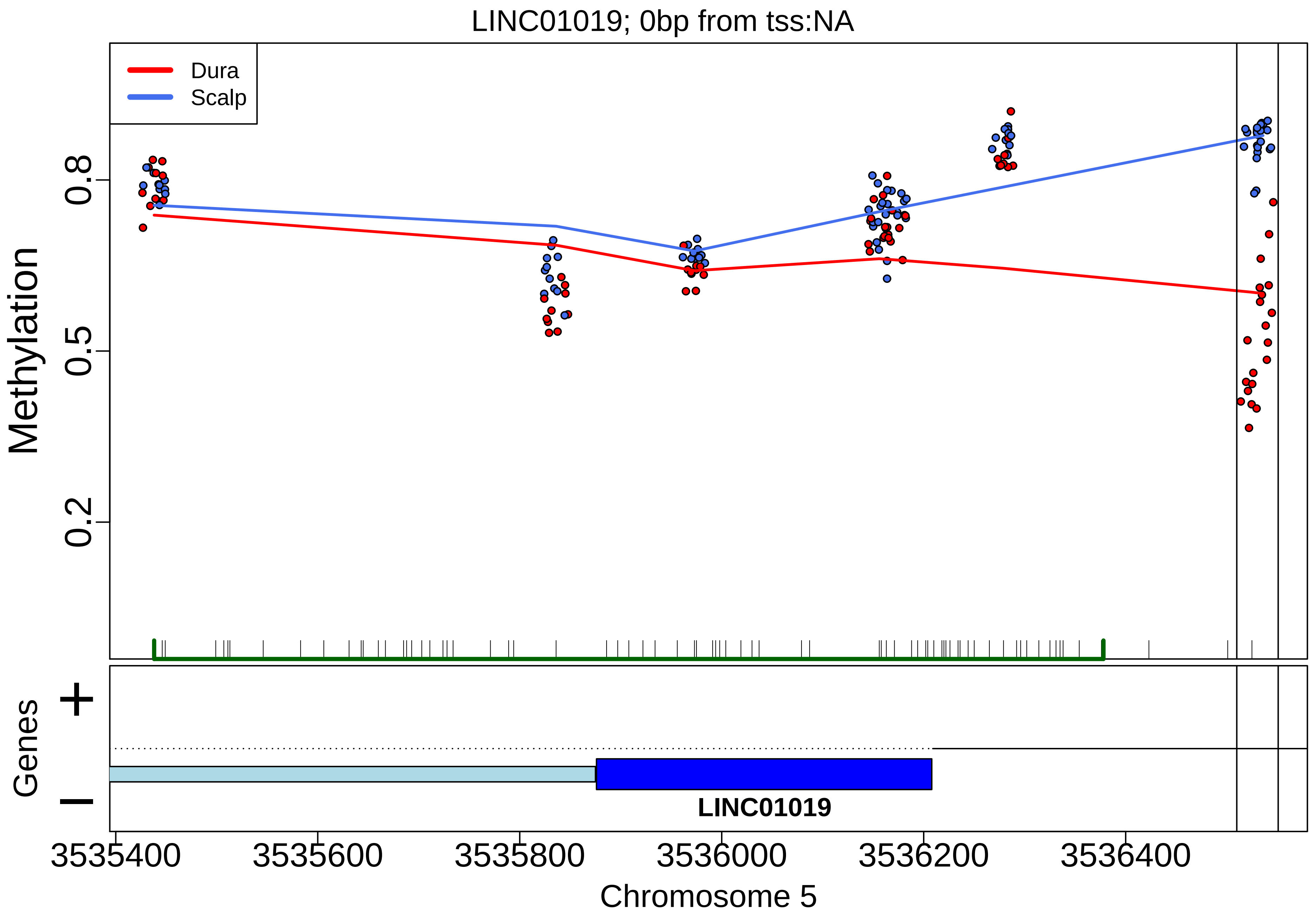
LINC01019; 0bp from tss:NA
Dura
Scalp
Methylation
0.8
0.5
0.2
Genes
+
-
LINC01019
3535400
3535600
3535800
3536000
3536200
3536400
Chromosome 5

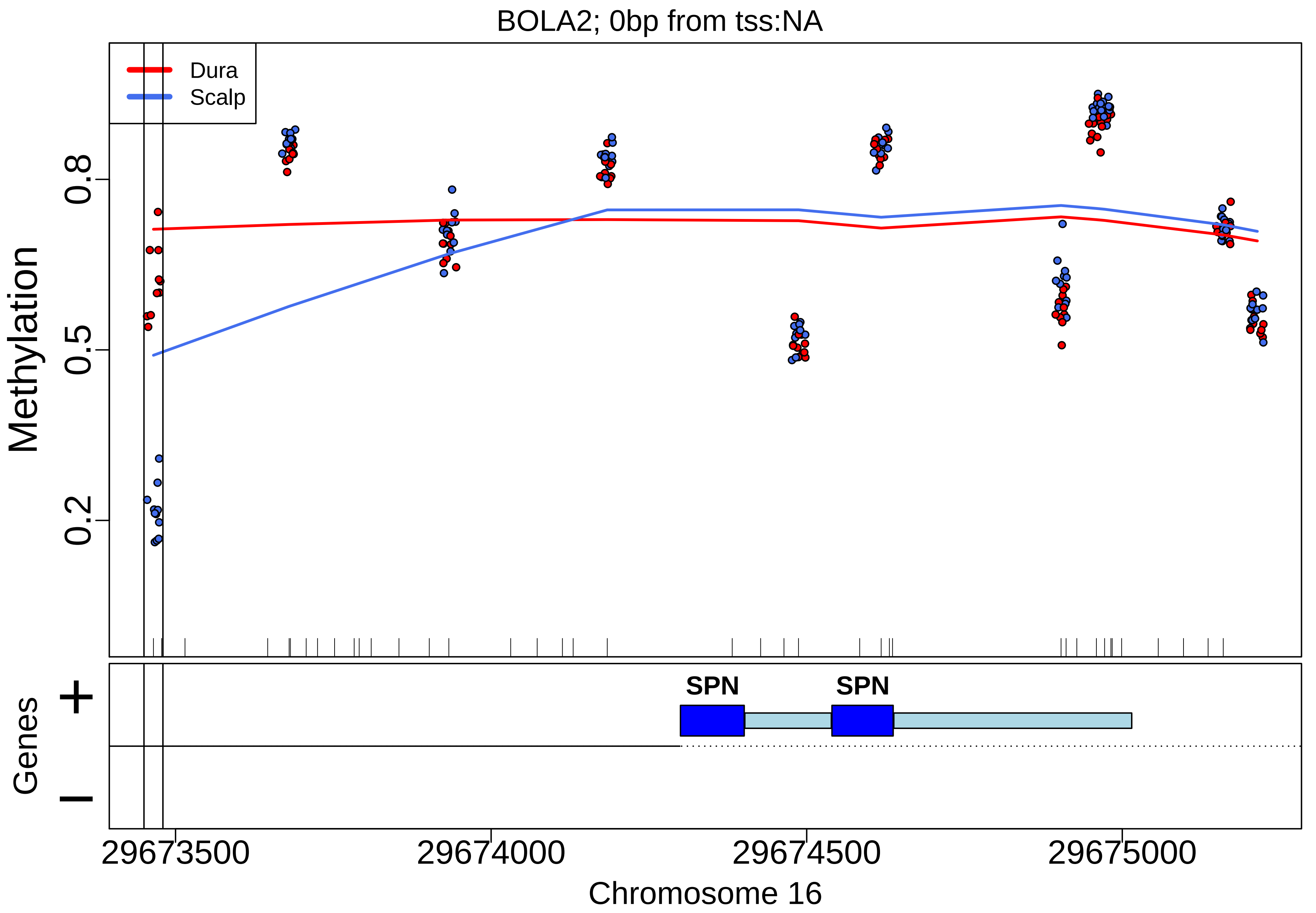

BOLA2; 0bp from tss:NA
Dura
Scalp
Methylation
0.2
0.5
0.8
Genes
+
-
SPN
SPN
29673500
29674000
29674500
29675000
Chromosome 16

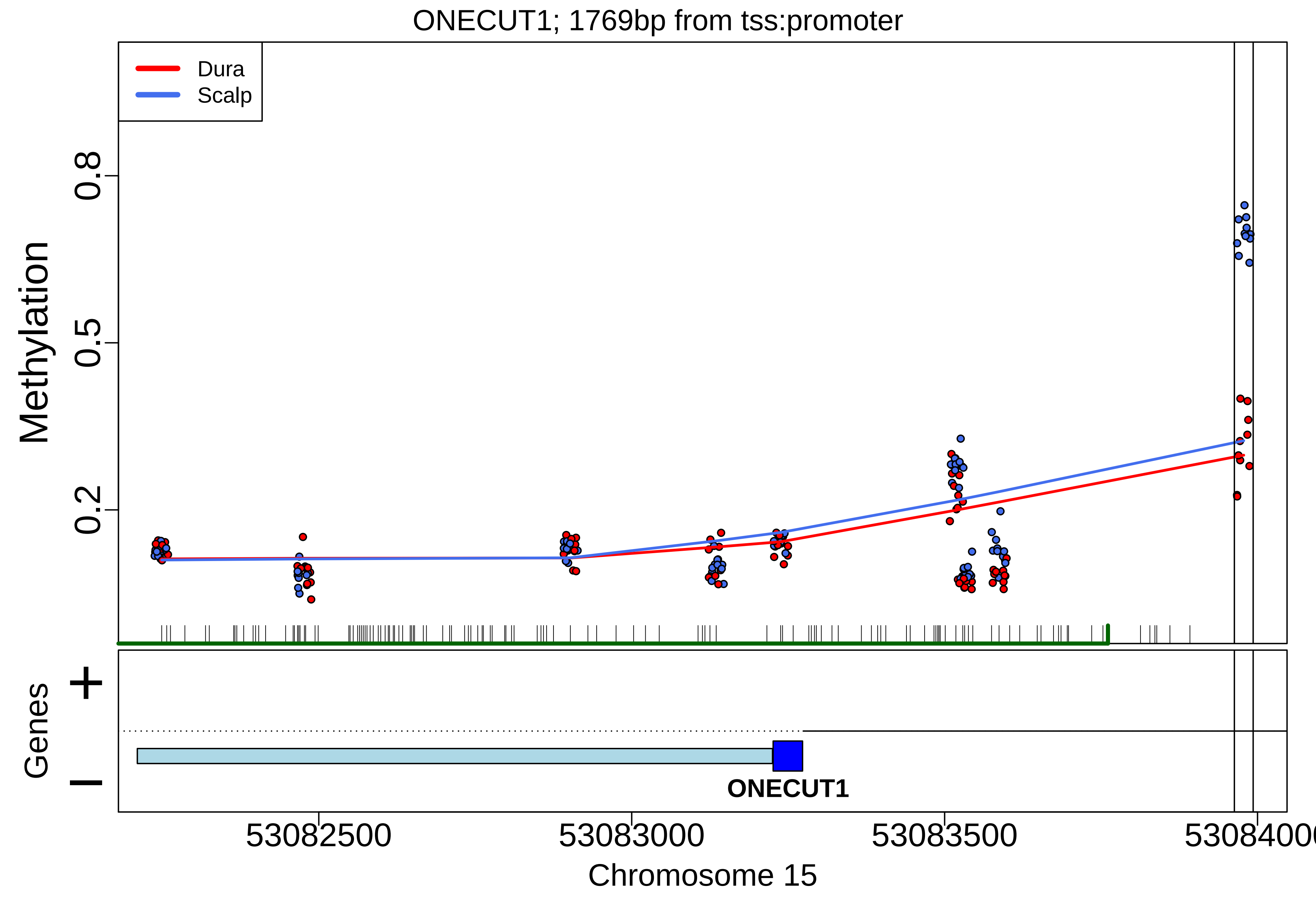

ONECUT1; 1769bp from tss:promoter
Dura
Scalp
Methylation
0.2
0.5
0.8
Genes
+
-
ONECUT1
53082500
53083000
53083500
5308400
Chromosome 15

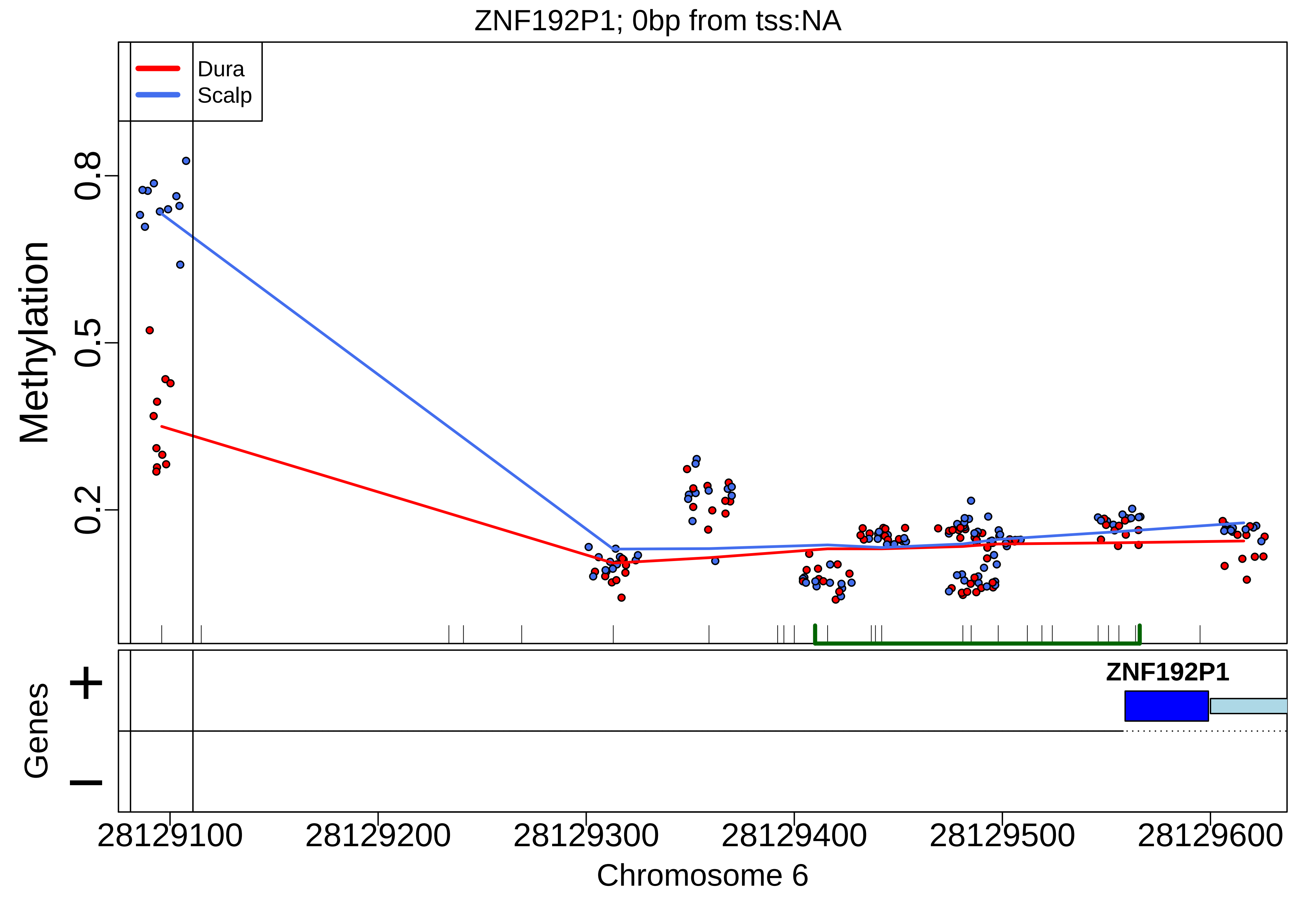

ZNF192P1; 0bp from tss:NA
Dura
Scalp
Methylation
0.2
0.5
0.8
Genes
+
-
ZNF192P1
28129100
28129200
28129300
28129400
28129500
28129600
Chromosome 6

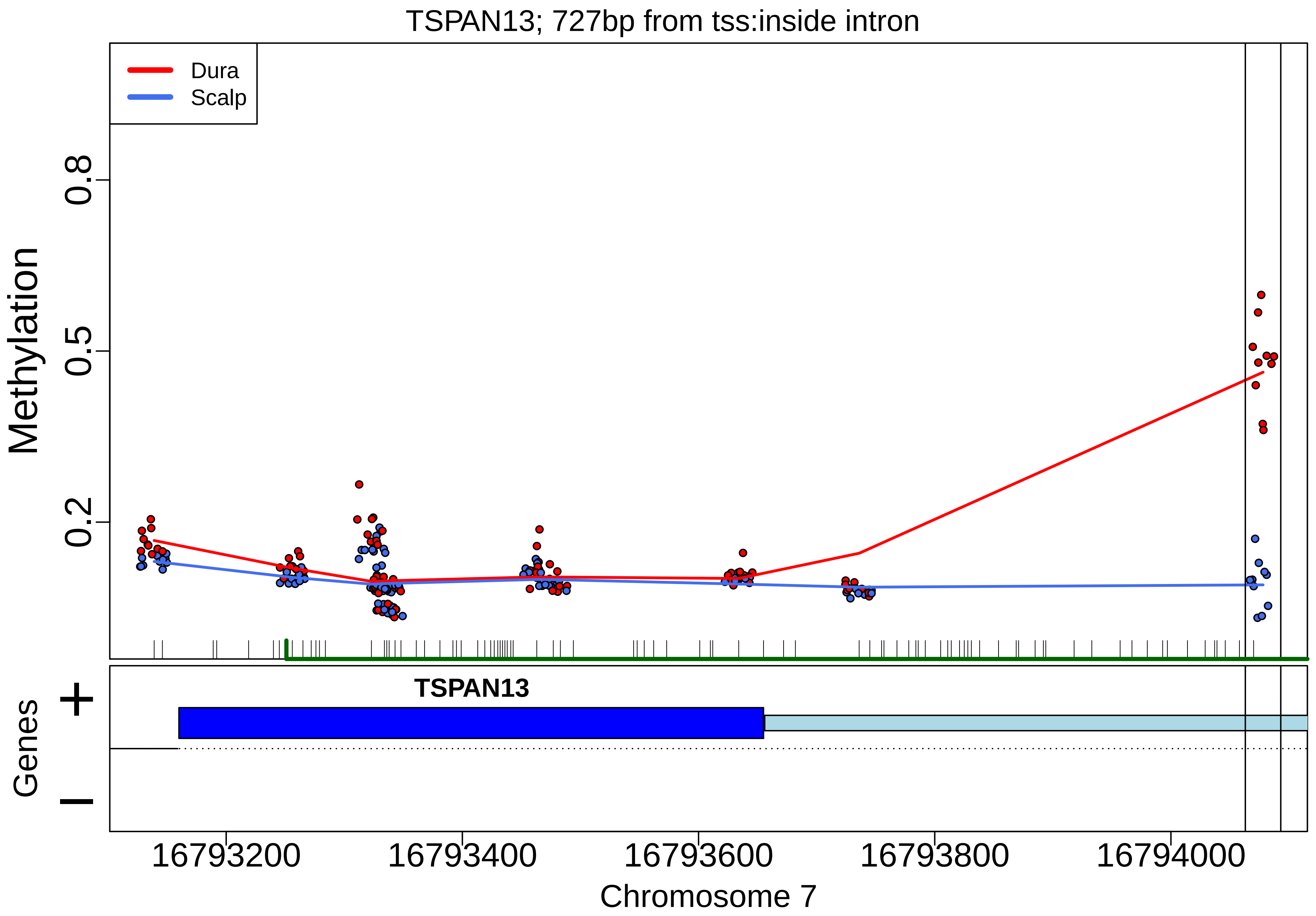

TSPAN13; 727bp from tss:inside intron
Dura
Scalp
Methylation
0.2
0.5
0.8
Genes
+
-
TSPAN13
16793200
16793400
16793600
16793800
16794000
Chromosome 7

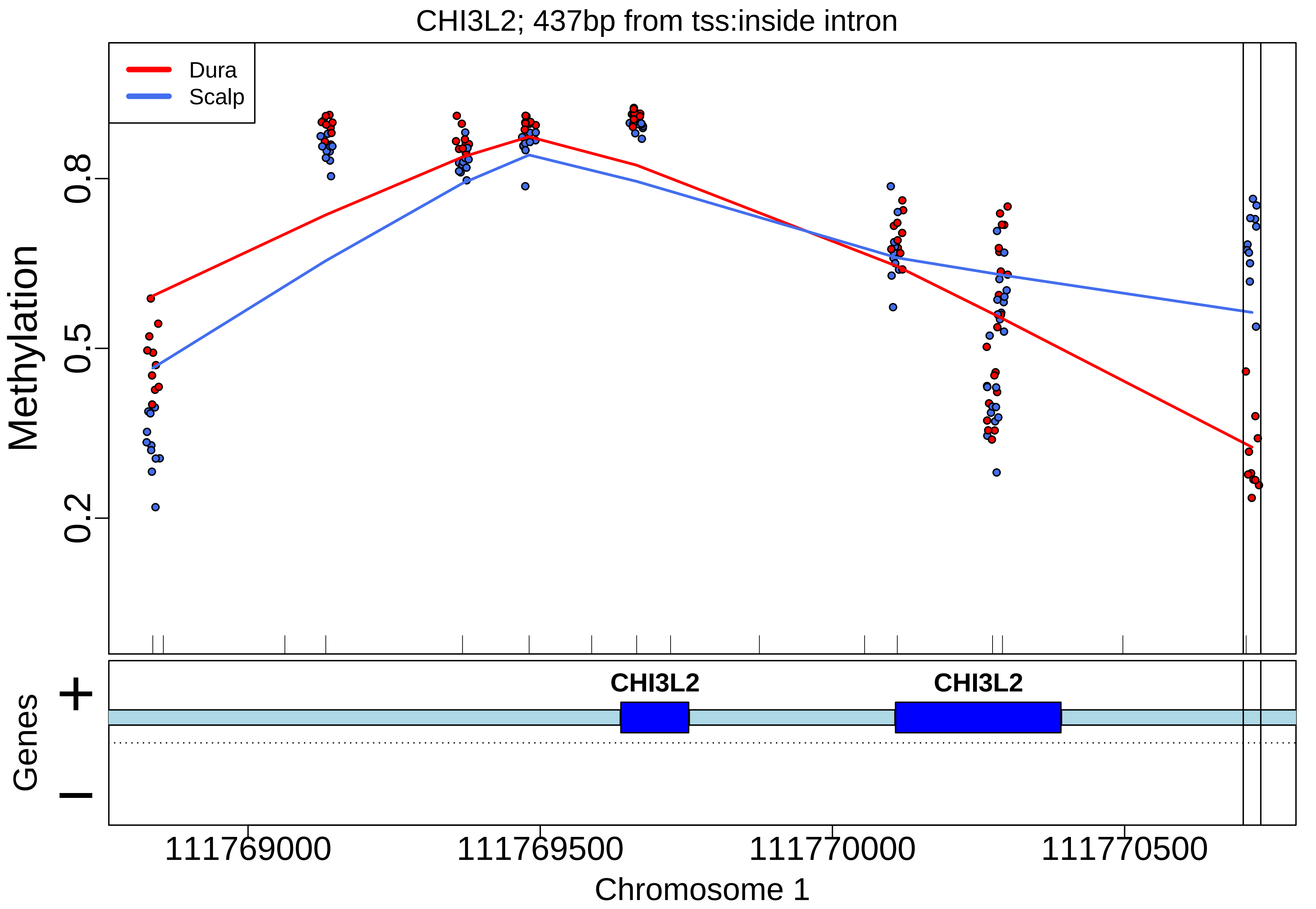

CHI3L2; 437bp from tss:inside intron
Dura
Scalp
Methylation
0.2
0.5
0.8
Genes
+
-
CHI3L2
CHI3L2
111769000
111769500
111770000
111770500
Chromosome 1

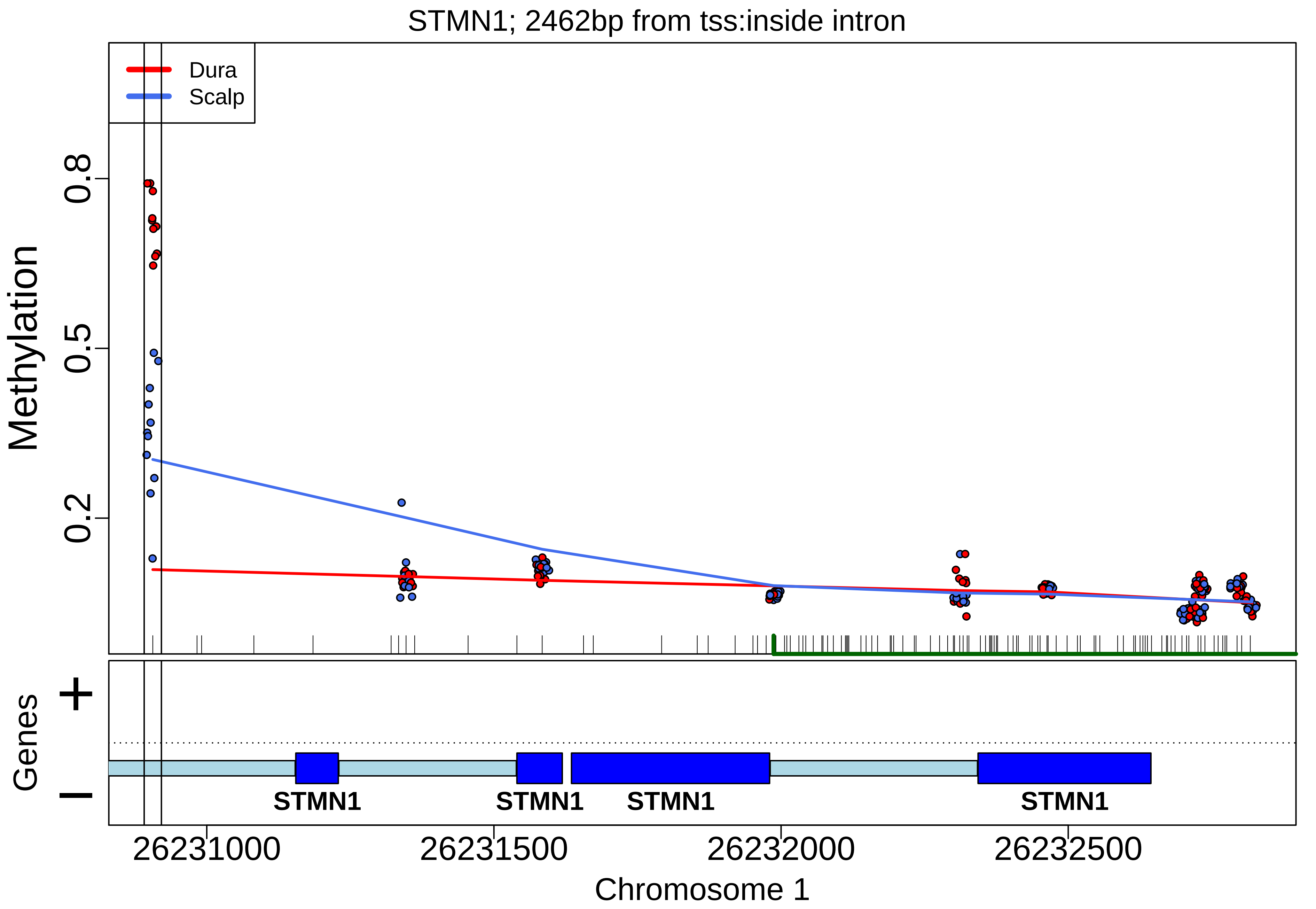

STMN1; 2462bp from tss:inside intron
Dura
Scalp
Methylation
0.8
0.5
0.2
Genes
+
-
STMN1
STMN1
STMN1
STMN1
26231000
26231500
26232000
26232500
Chromosome 1

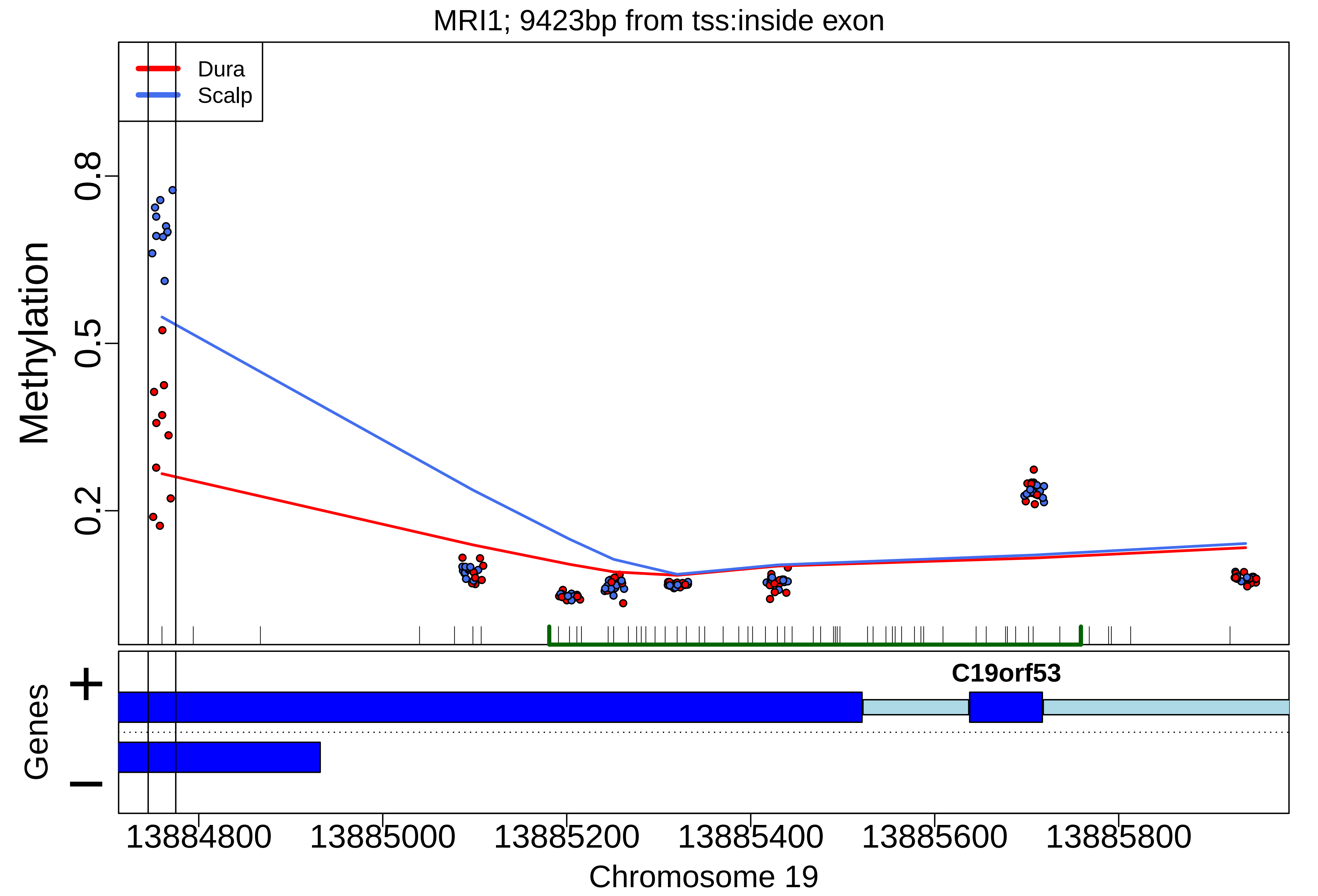

MRI1; 9423bp from tss:inside exon
Dura
Scalp
Methylation
0.2
0.5
0.8
Genes
+
C19orf53
13884800
13885000
13885200
13885400
13885600
13885800
Chromosome 19

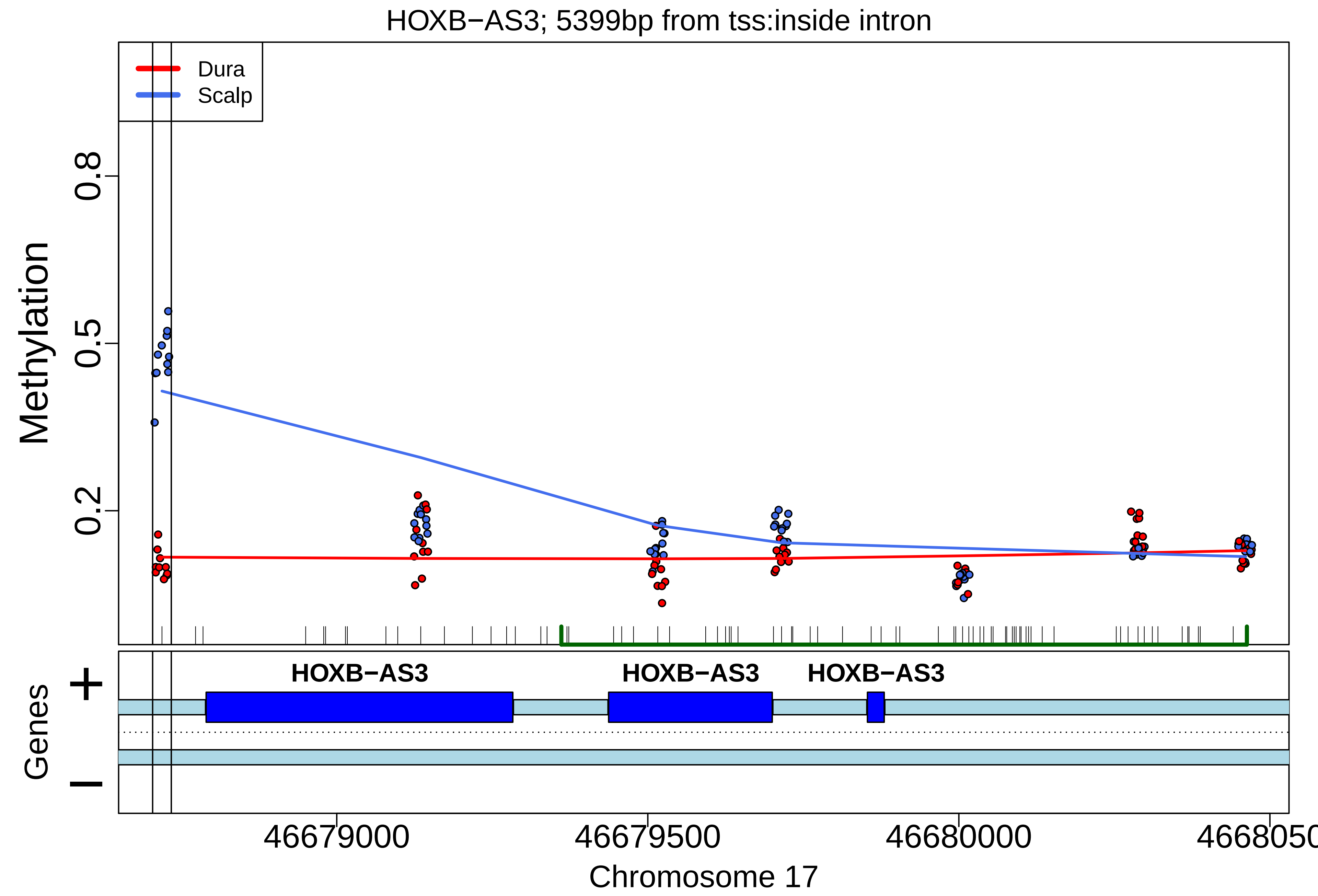

HOXB−AS3; 5399bp from tss:inside intron
Dura
Scalp
Methylation
0.8
0.5
0.2
Genes
+
−
HOXB−AS3
HOXB−AS3
HOXB−AS3
46679000
46679500
46680000
4668050
Chromosome 17

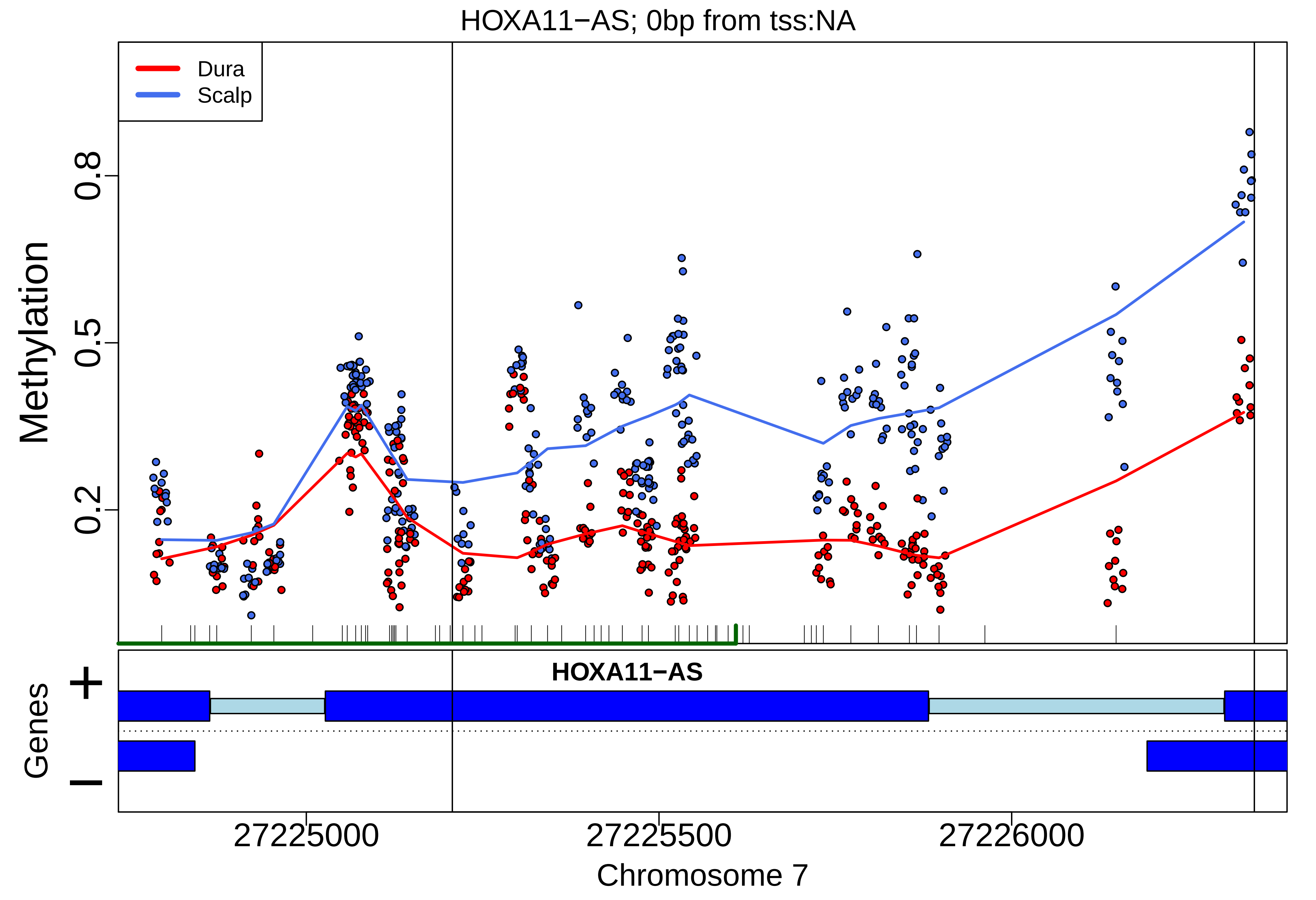

HOXA11−AS; 0bp from tss:NA
Dura
Scalp
Methylation
0.2
0.5
0.8
Genes
+
−
HOXA11−AS
27225000
27225500
27226000
Chromosome 7

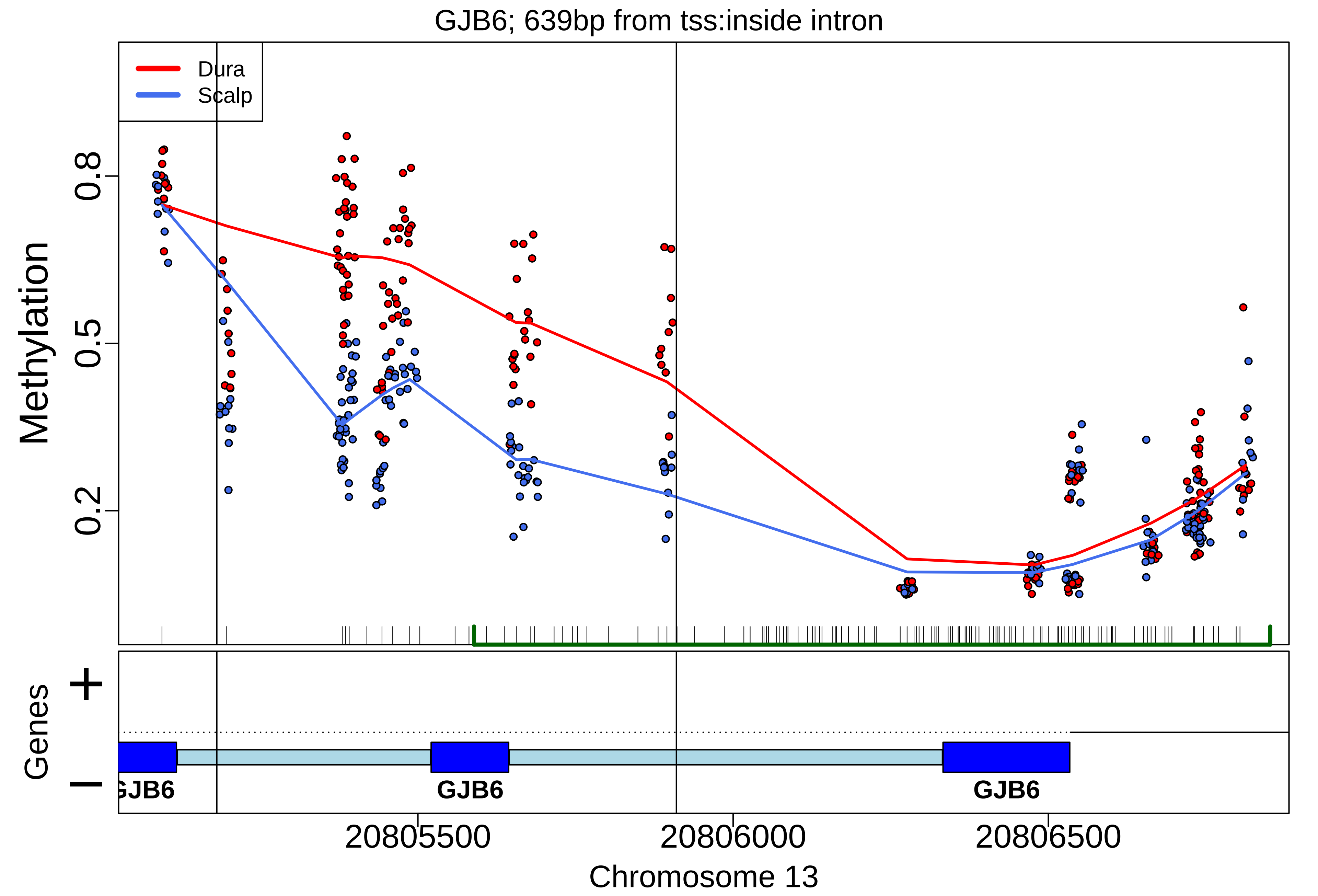

GJB6; 639bp from tss:inside intron
Dura
Scalp
Methylation
0.2
0.5
0.8
Genes
+
-
GJB6
GJB6
GJB6
20805500
20806000
20806500
Chromosome 13

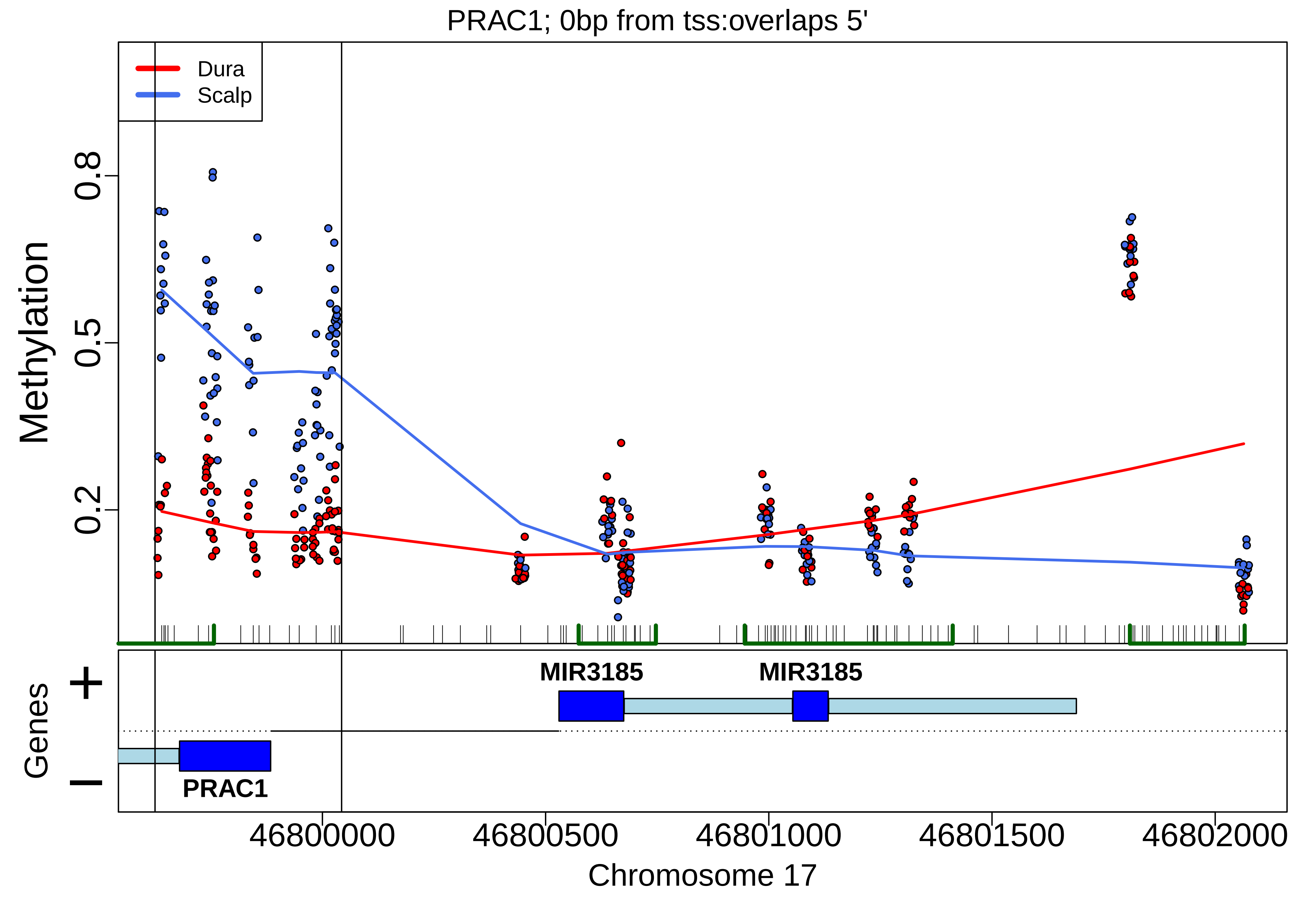
PRAC1; 0bp from tss:overlaps 5'
Dura
Scalp
Methylation
0.2
0.5
0.8
Genes
+
-
MIR3185
MIR3185
PRAC1
46800000
46800500
46801000
46801500
46802000
Chromosome 17

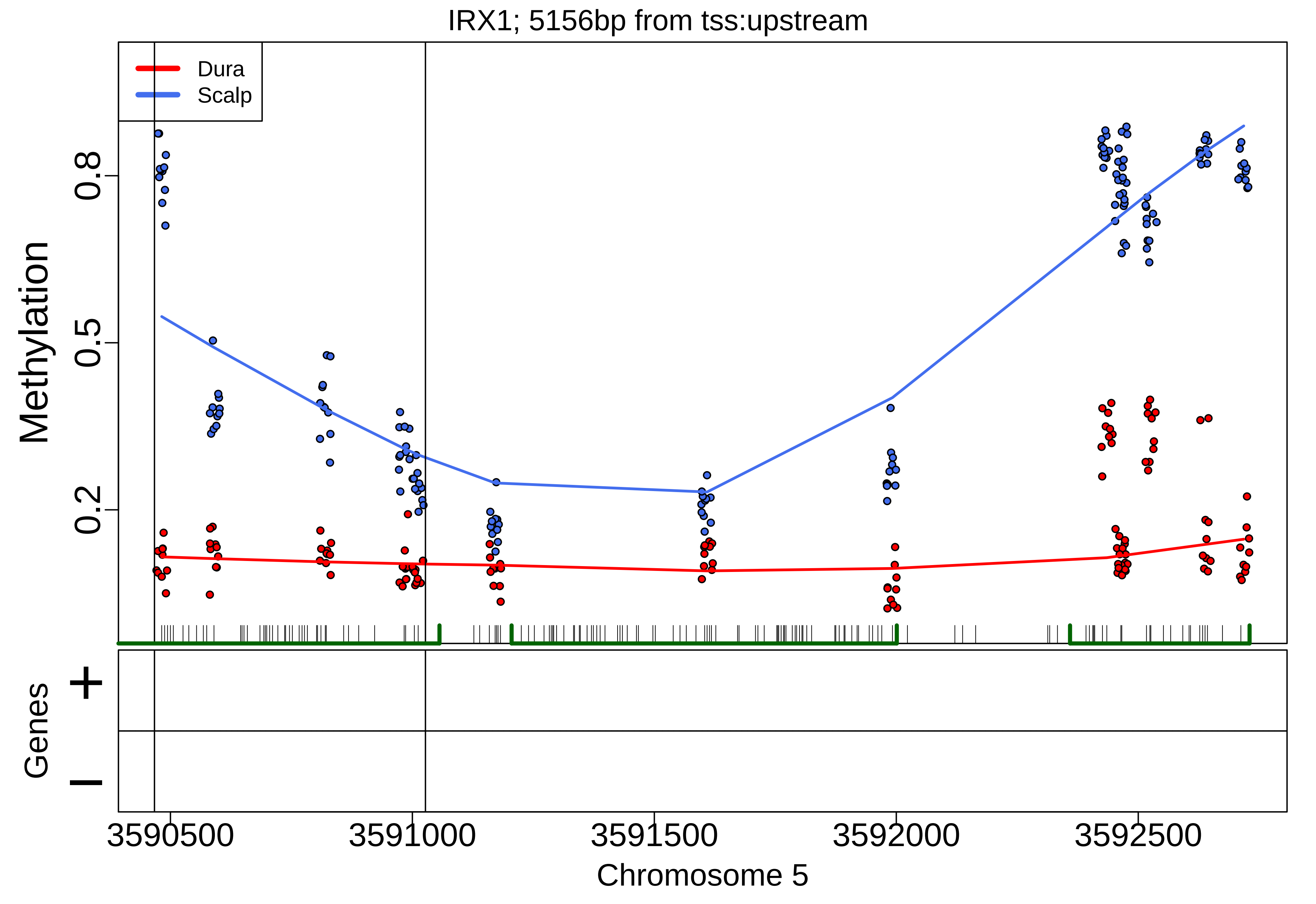

IRX1; 5156bp from tss:upstream
Dura
Scalp
Methylation
0.2
0.5
0.8
Genes
+
−
3590500
3591000
3591500
3592000
3592500
Chromosome 5

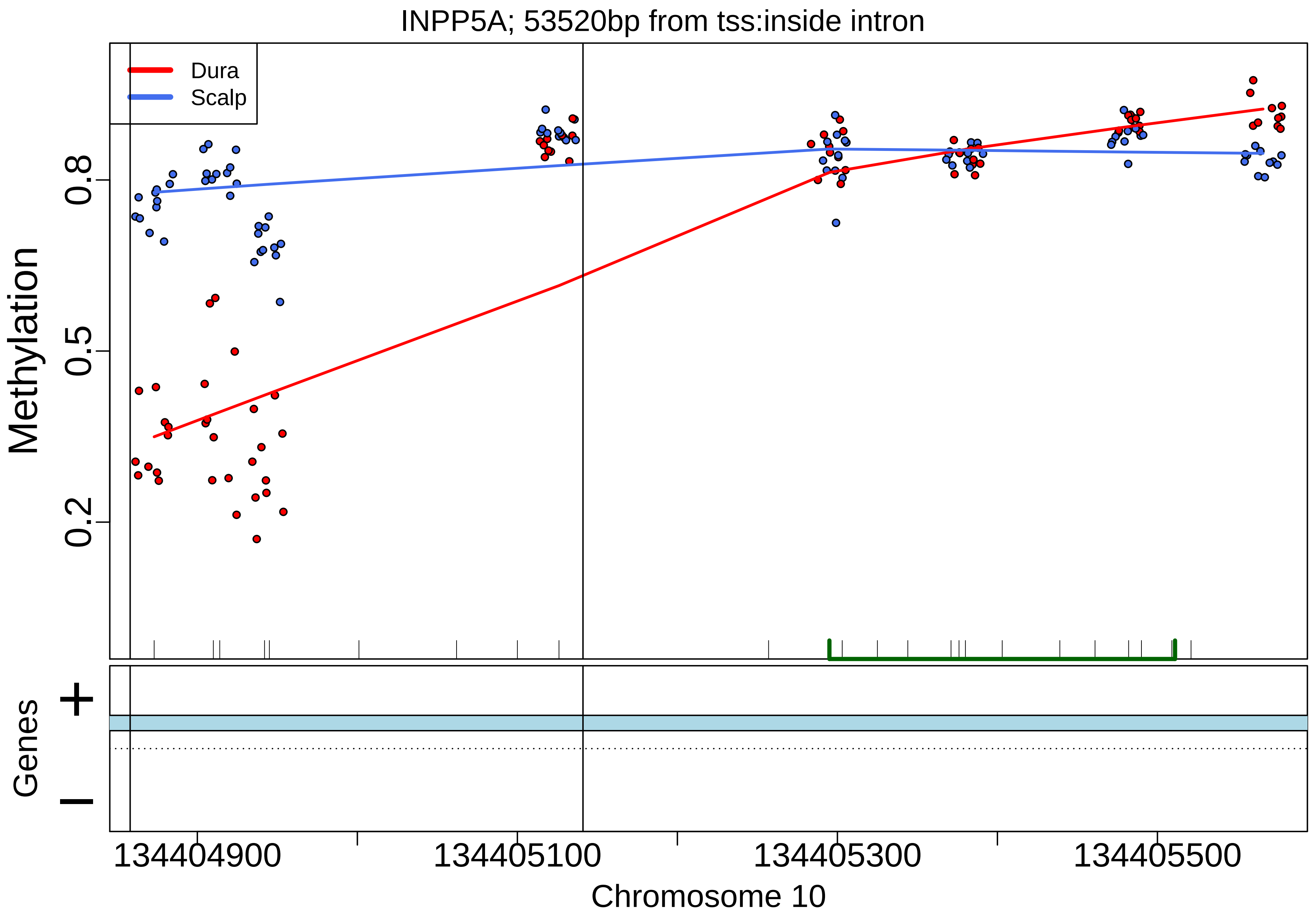

INPP5A; 53520bp from tss:inside intron
Dura
Scalp
Methylation
0.2
0.5
0.8
Genes
+
−
134404900
134405100
134405300
134405500
Chromosome 10

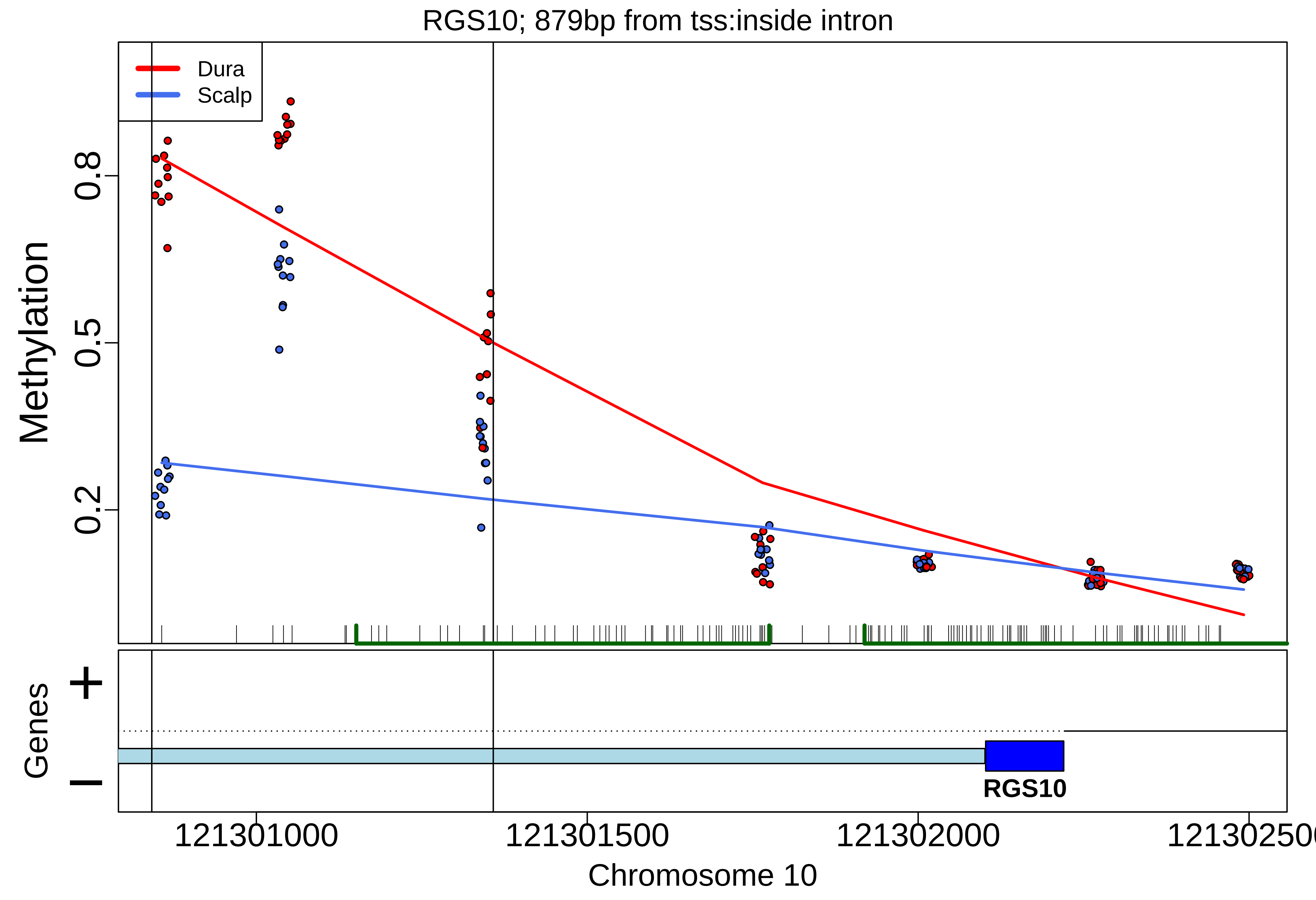
RGS10; 879bp from tss:inside intron
Dura
Scalp
Methylation
0.8
0.5
0.2
Genes
+
-
RGS10
121301000
121301500
121302000
12130250
Chromosome 10

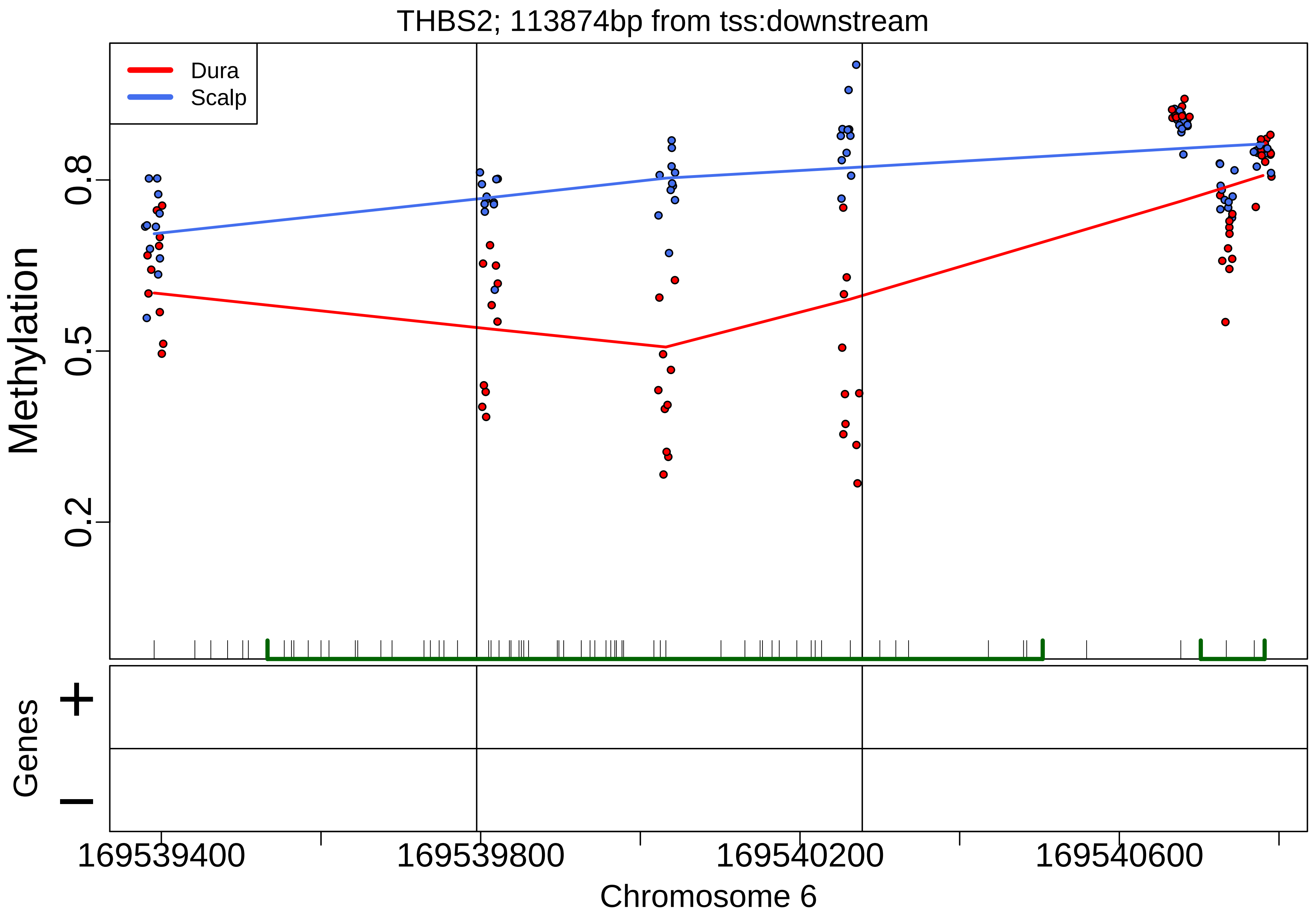
THBS2; 113874bp from tss:downstream
Dura
Scalp
Methylation
0.2
0.5
0.8
Genes
+
−
169539400
169539800
169540200
169540600
Chromosome 6

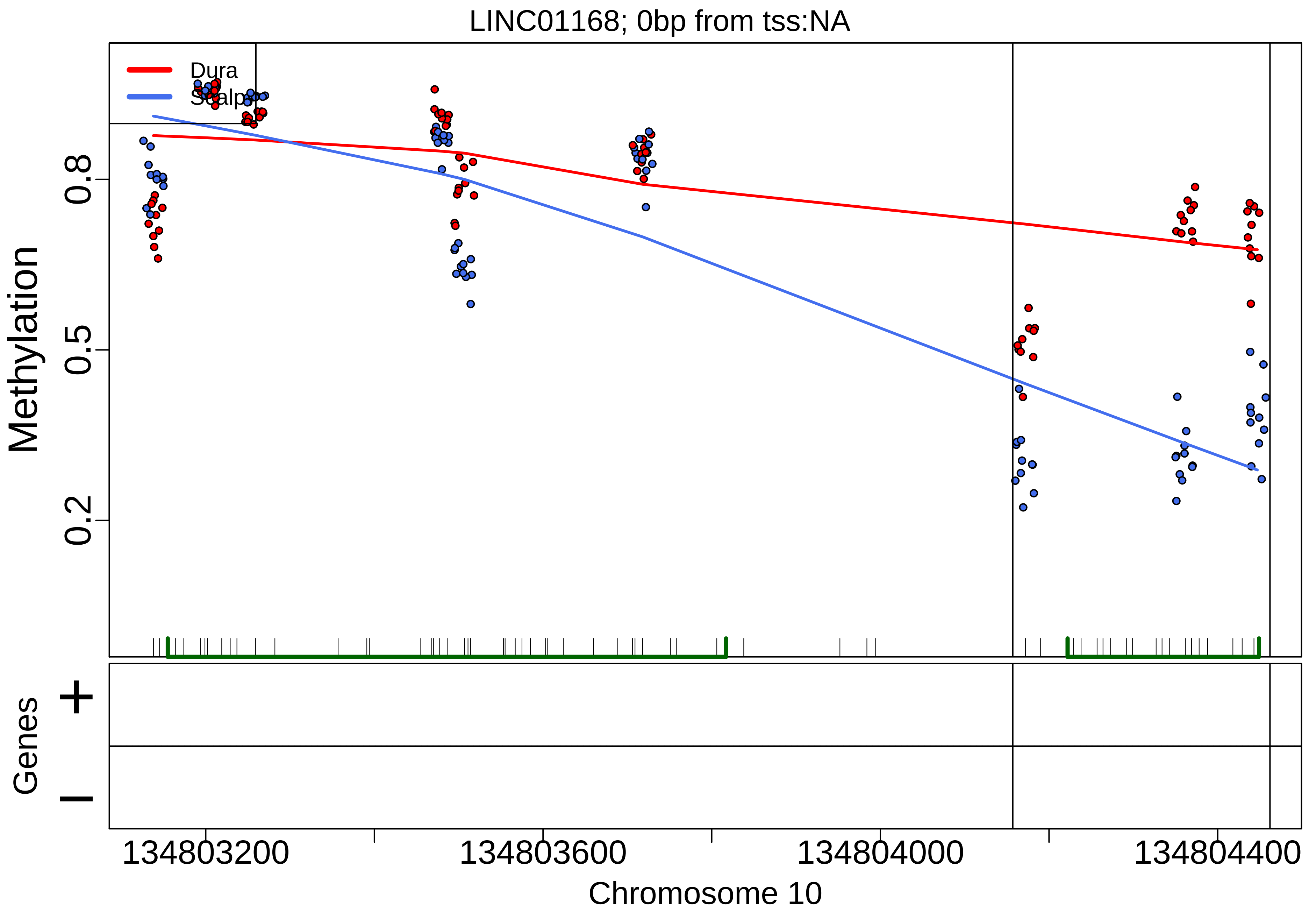
LINC01168; 0bp from tss:NA
Dura
Scalp
Methylation
0.2
0.5
0.8
Genes
+
-
134803200
134803600
134804000
134804400
Chromosome 10

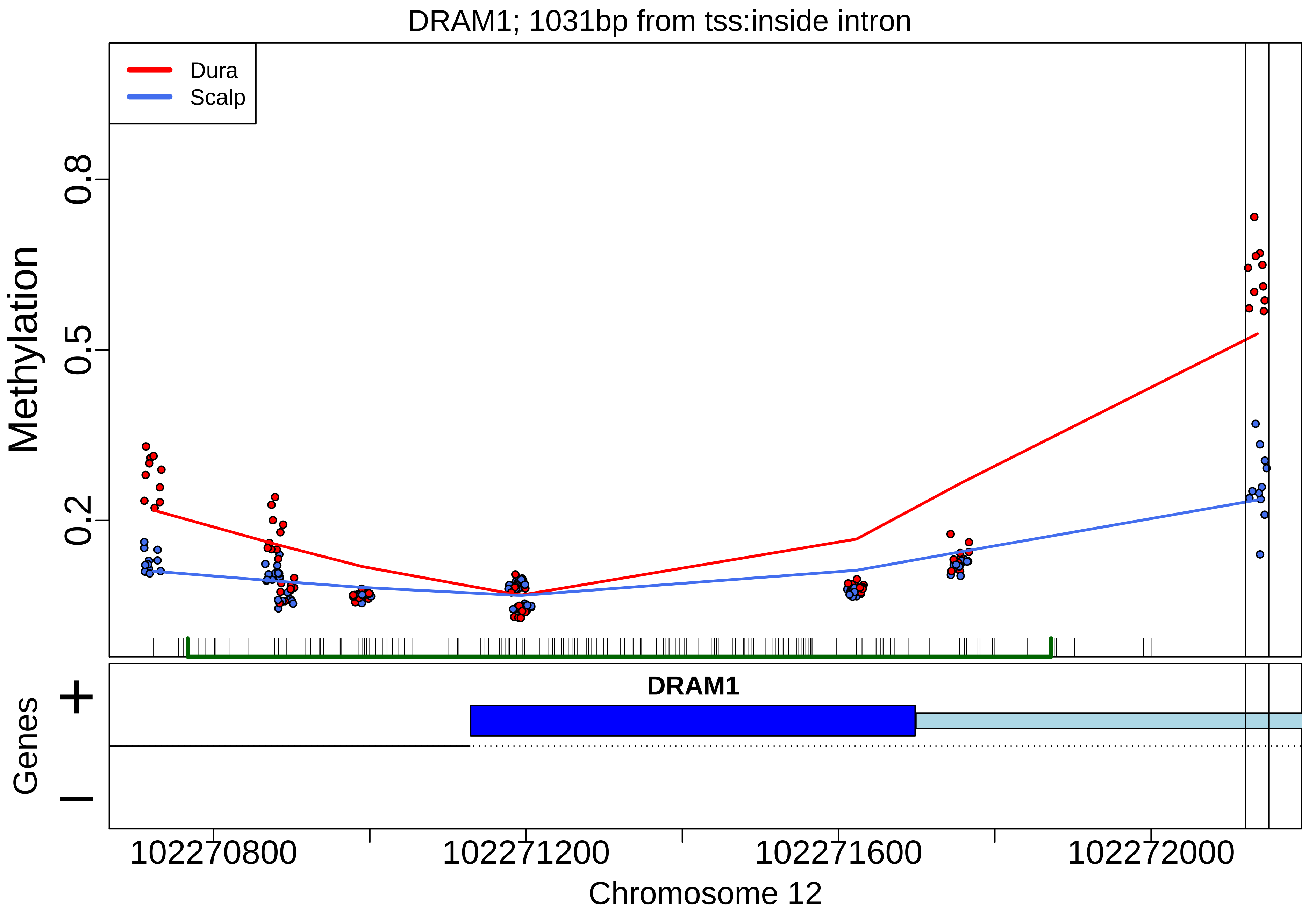

DRAM1; 1031bp from tss:inside intron
Dura
Scalp
Methylation
0.2
0.5
0.8
Genes
+
-
DRAM1
102270800
102271200
102271600
102272000
Chromosome 12

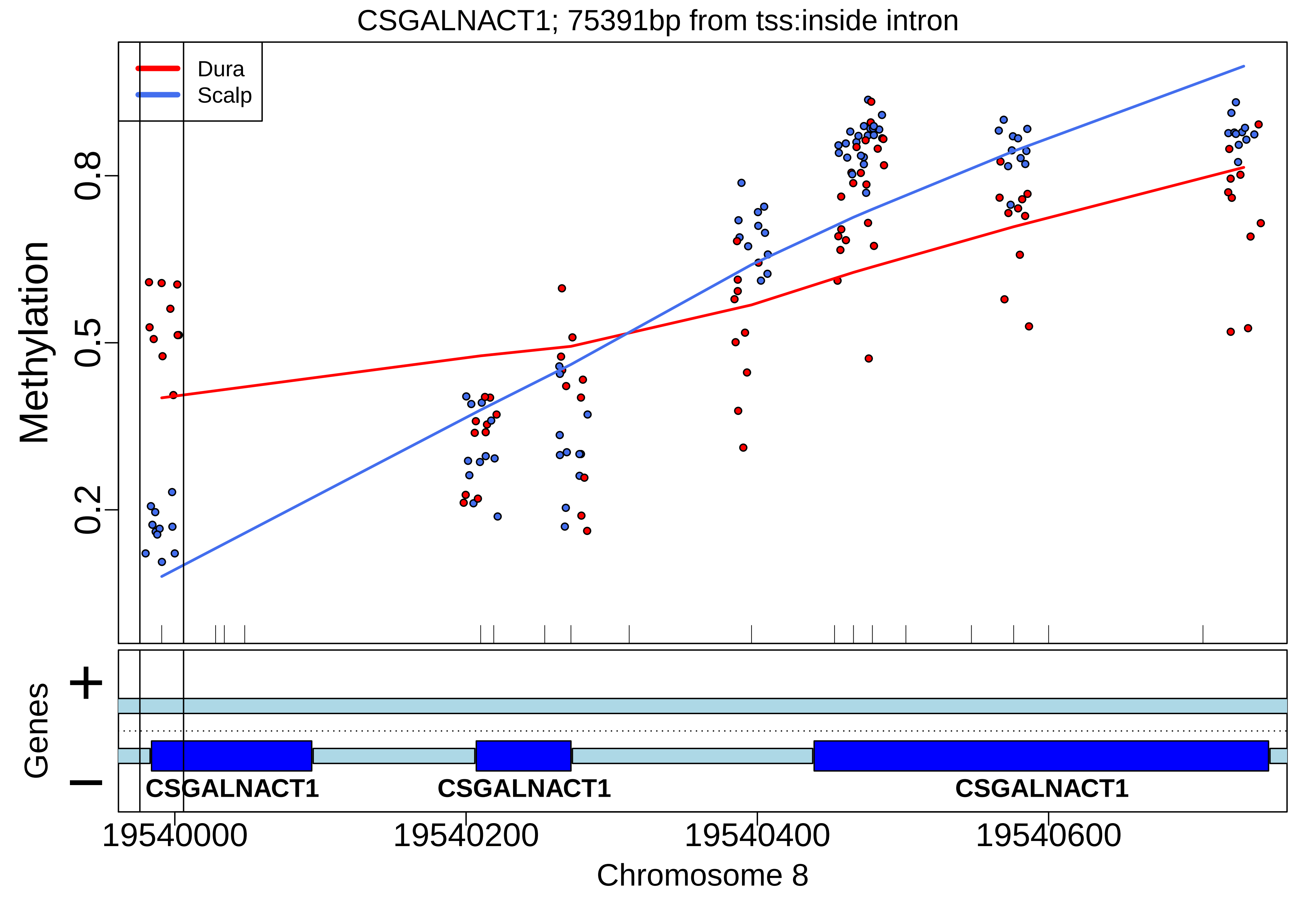

CSGALNACT1; 75391bp from tss:inside intron
Dura
Scalp
Methylation
0.2
0.5
0.8
Genes
+
−
CSGALNACT1
CSGALNACT1
CSGALNACT1
19540000
19540200
19540400
19540600
Chromosome 8

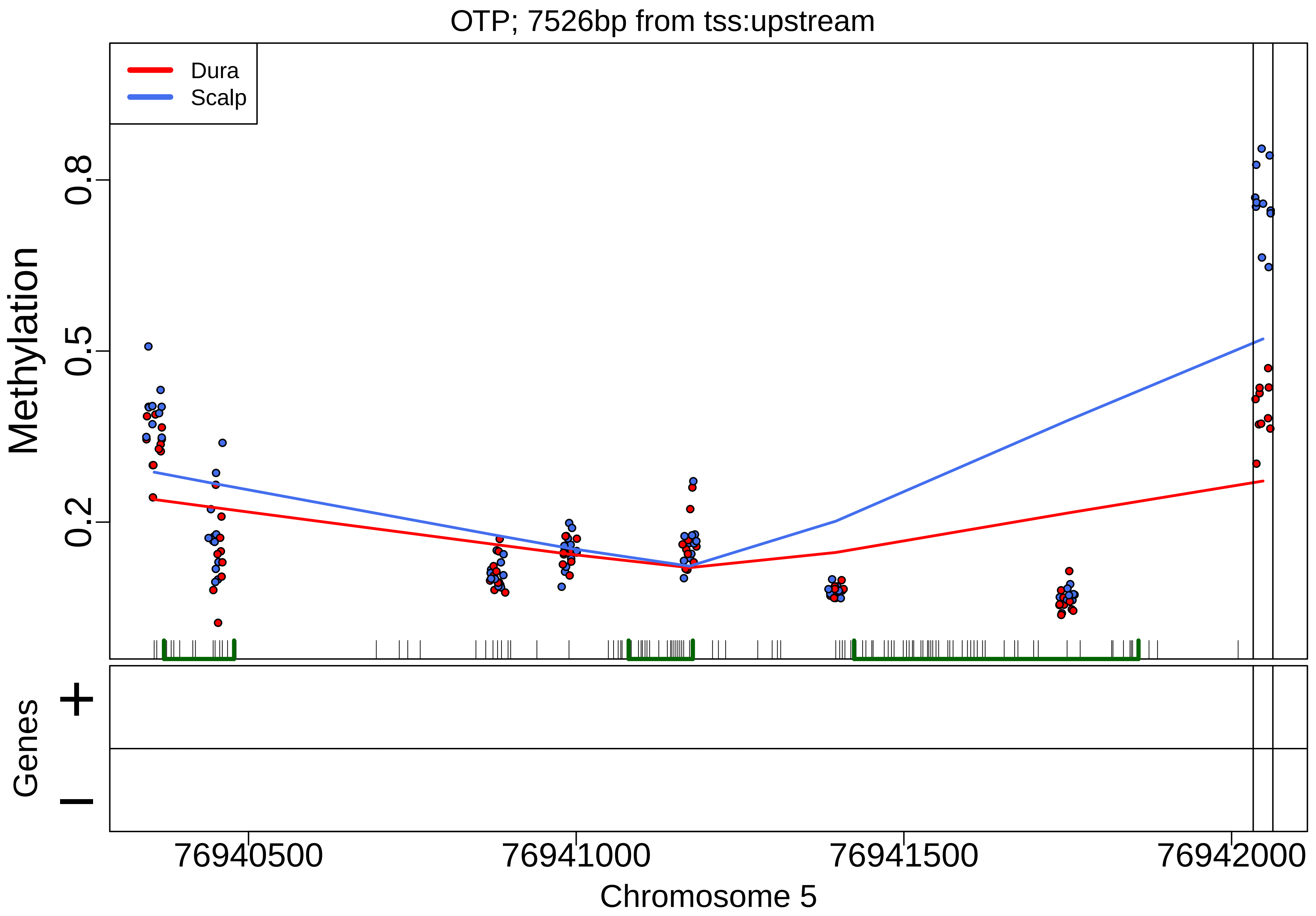
OTP; 7526bp from tss:upstream
Dura
Scalp
Methylation
0.2
0.5
0.8
Genes
+
-
76940500
76941000
76941500
76942000
Chromosome 5

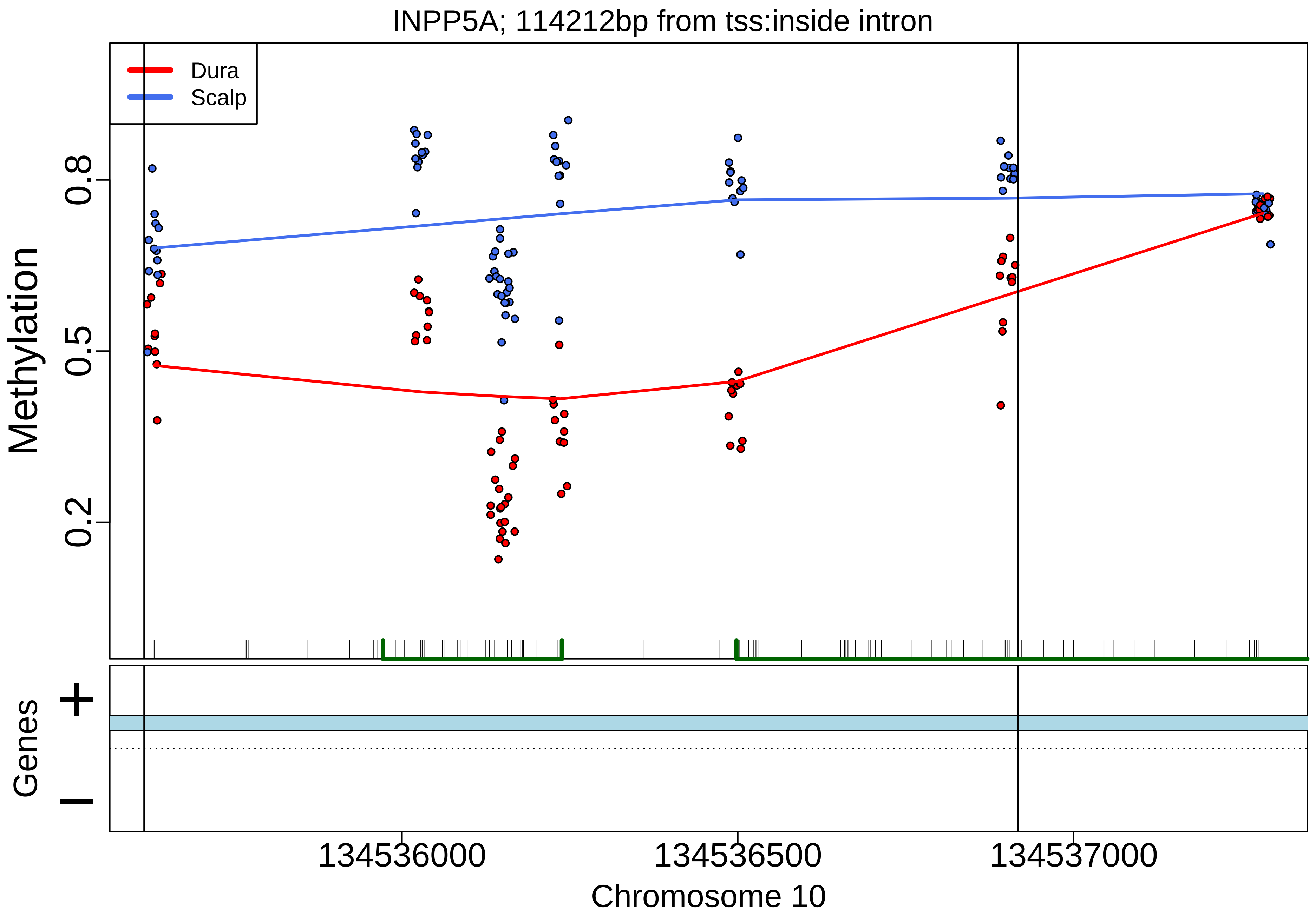
INPP5A; 114212bp from tss:inside intron
Dura
Scalp
Methylation
0.2
0.5
0.8
Genes
+
-
134536000
134536500
134537000
Chromosome 10

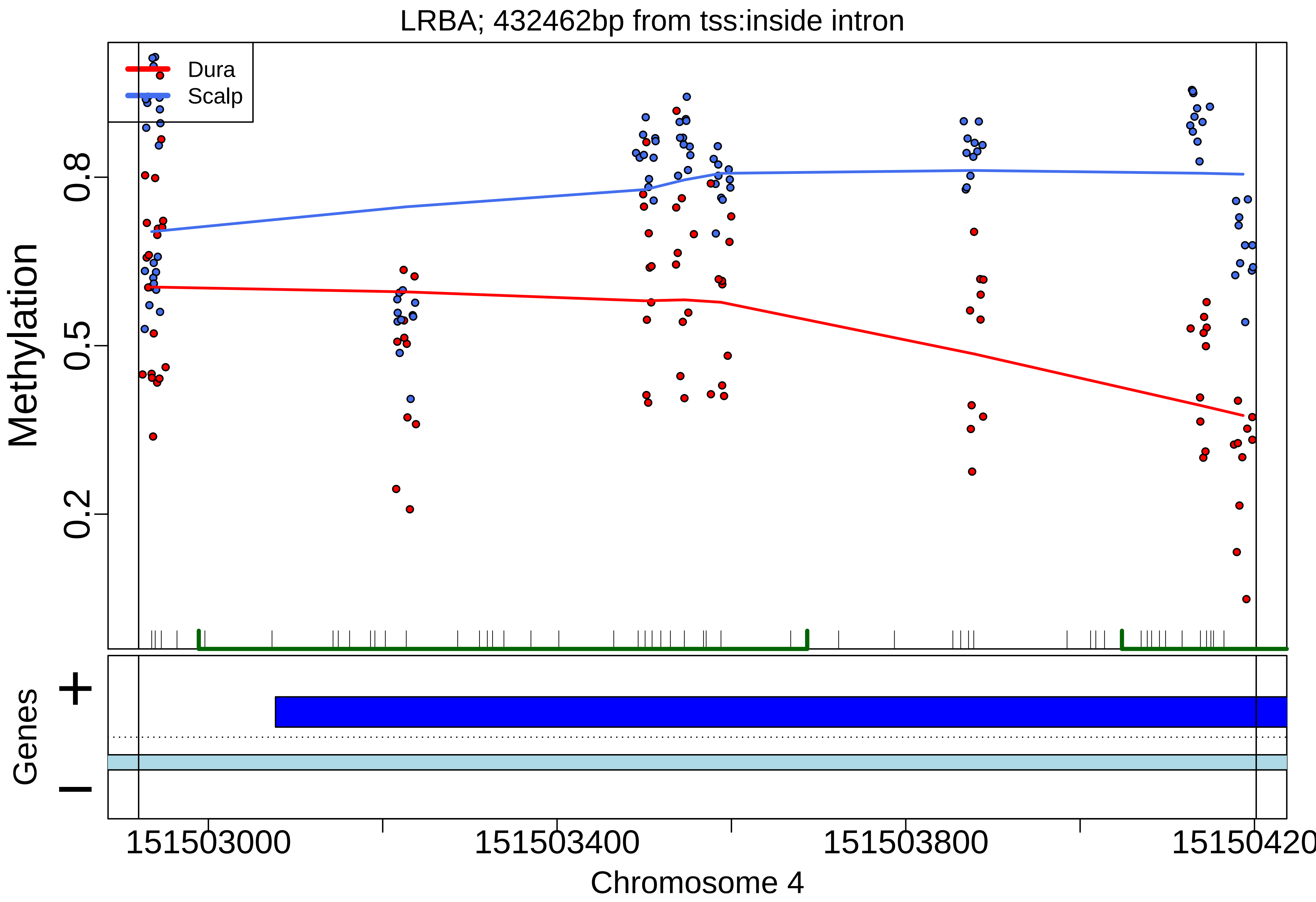

LRBA; 432462bp from tss:inside intron
Dura
Scalp
Methylation
0.2
0.5
0.8
Genes
+
-
151503000
151503400
151503800
15150420
Chromosome 4

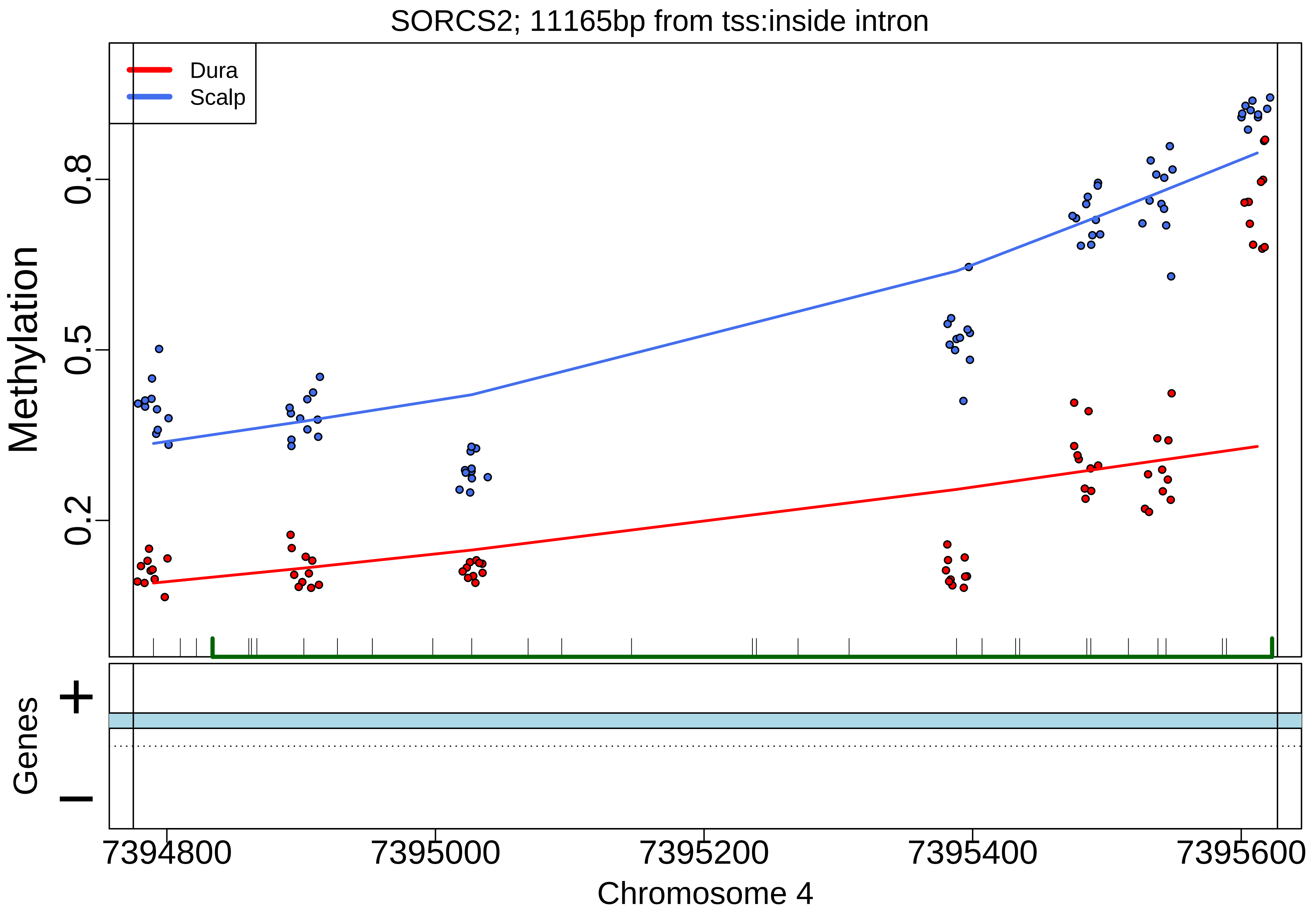

SORCS2; 11165bp from tss:inside intron
Dura
Scalp
Methylation
0.2
0.5
0.8
Genes
+
-
7394800
7395000
7395200
7395400
7395600
Chromosome 4

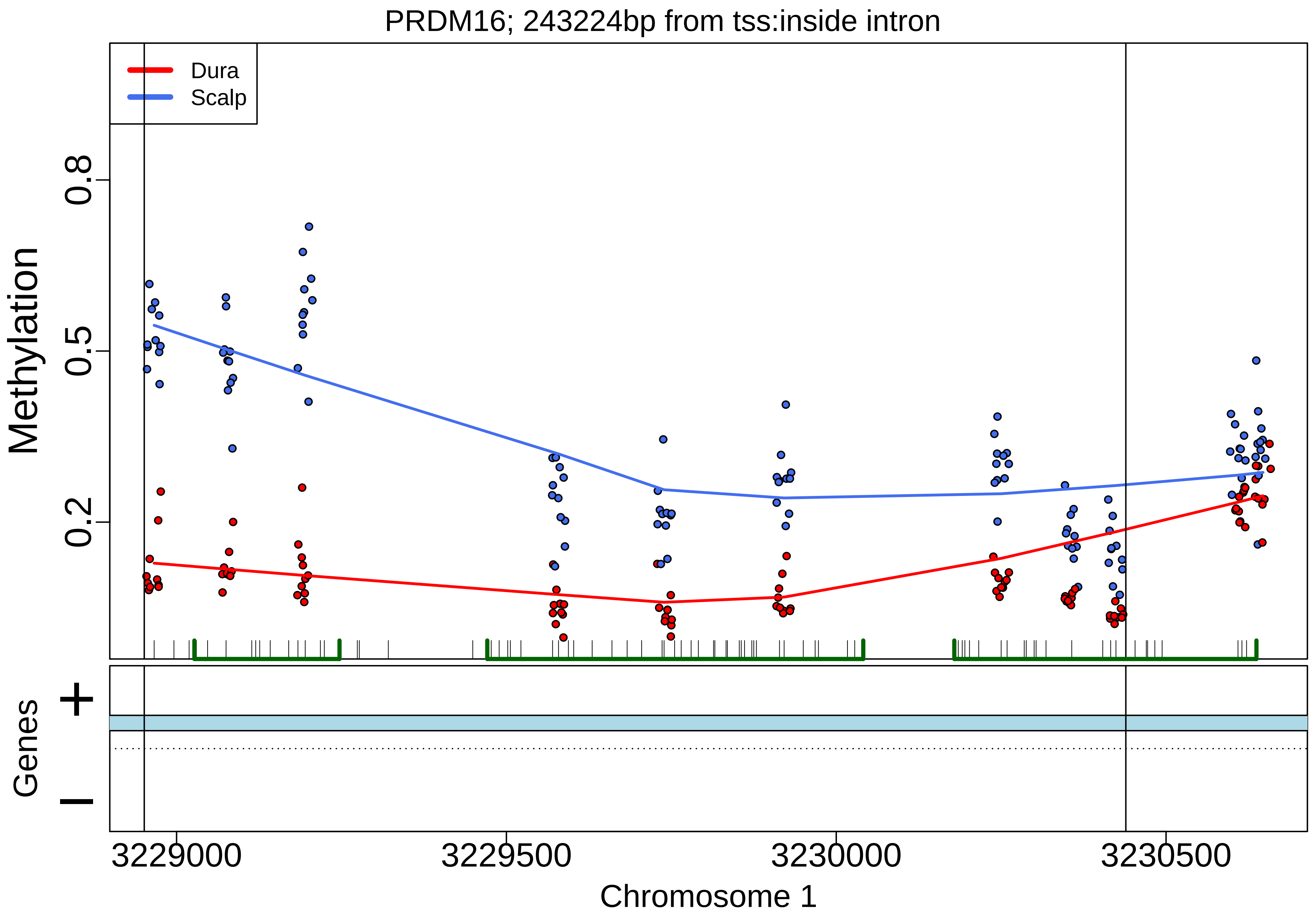
PRDM16; 243224bp from tss:inside intron
Dura
Scalp
Methylation
0.2
0.5
0.8
Genes
+
−
3229000
3229500
3230000
3230500
Chromosome 1

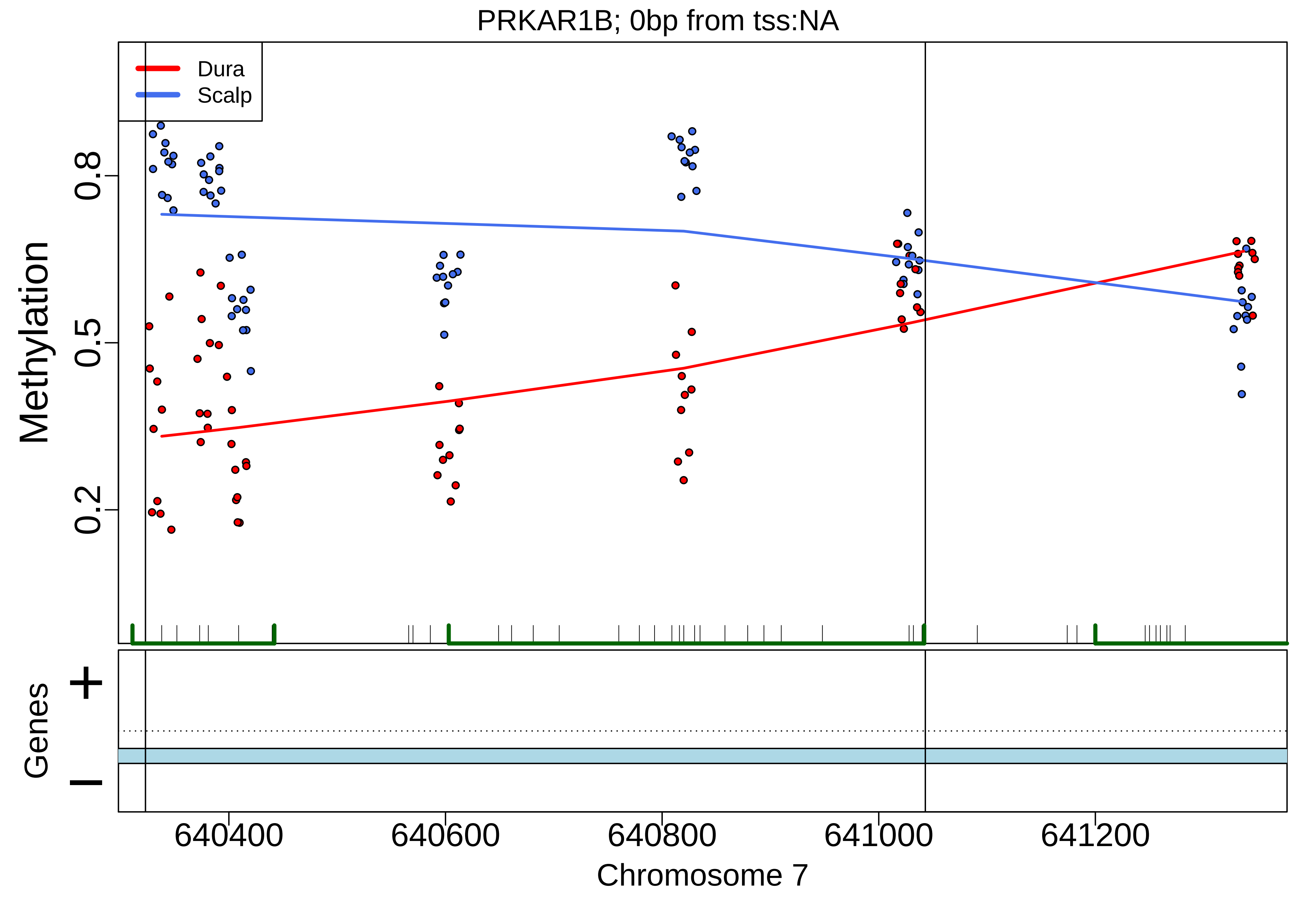
PRKAR1B; 0bp from tss:NA
Dura
Scalp
Methylation
0.2
0.5
0.8
Genes
+
-
640400
640600
640800
641000
641200
Chromosome 7

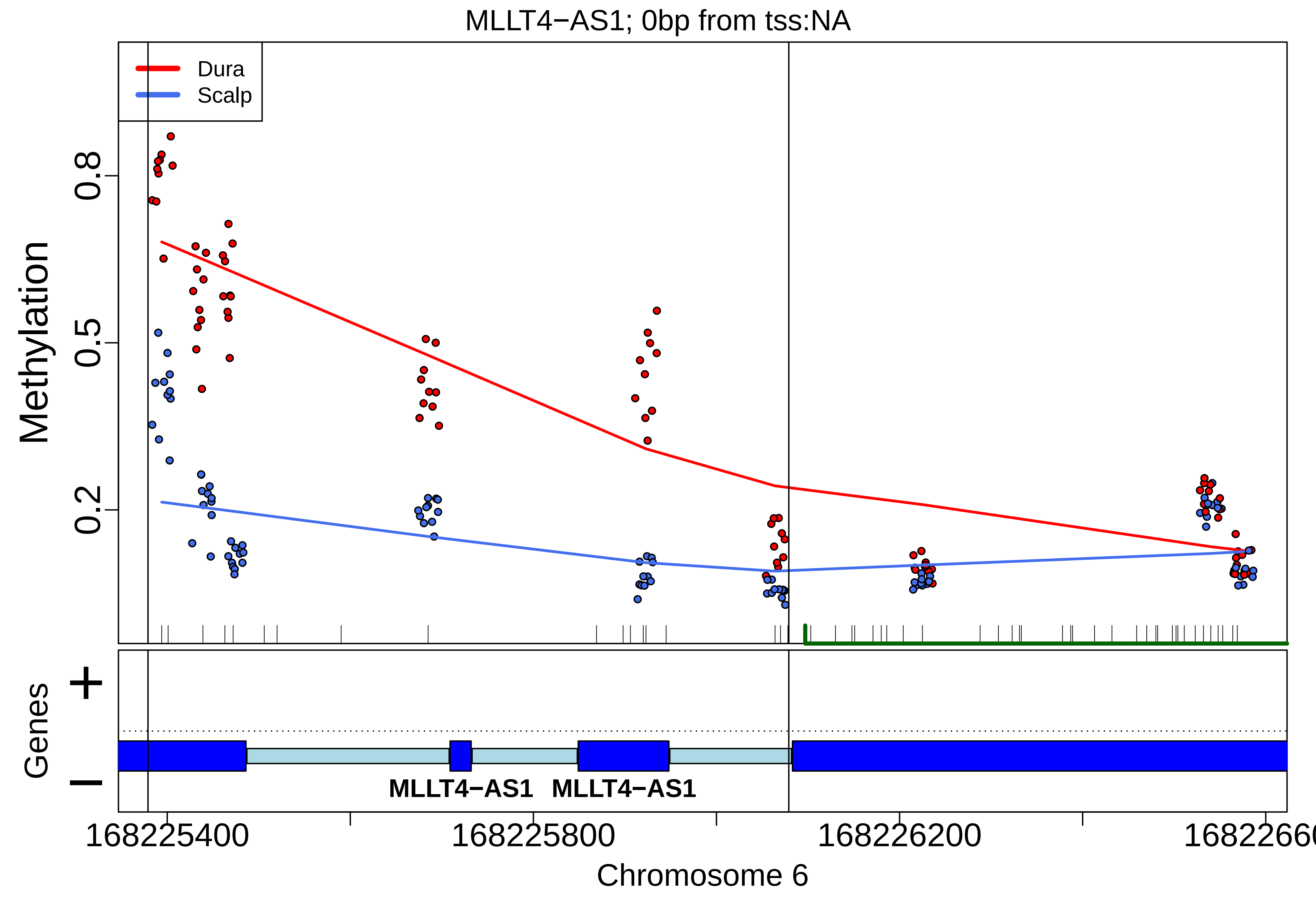
MLLT4−AS1; 0bp from tss:NA
Dura
Scalp
Methylation
0.8
0.5
0.2
Genes
+
−
MLLT4−AS1
MLLT4−AS1
168225400
168225800
168226200
Chromosome 6

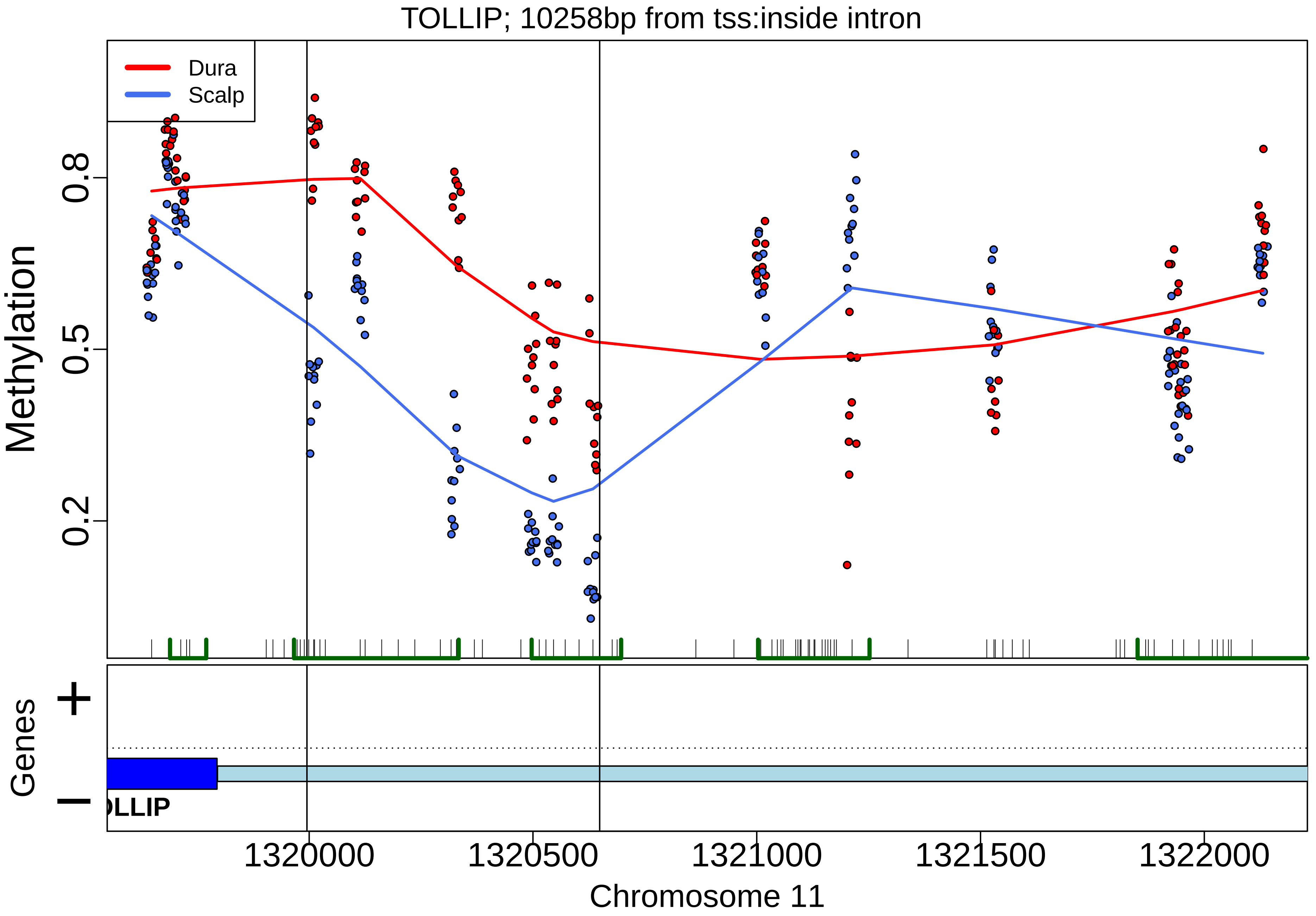

TOLLIP; 10258bp from tss:inside intron
Dura
Scalp
Methylation
0.2
0.5
0.8
Genes
+
-
LLIP
1320000
1320500
1321000
1321500
1322000
Chromosome 11

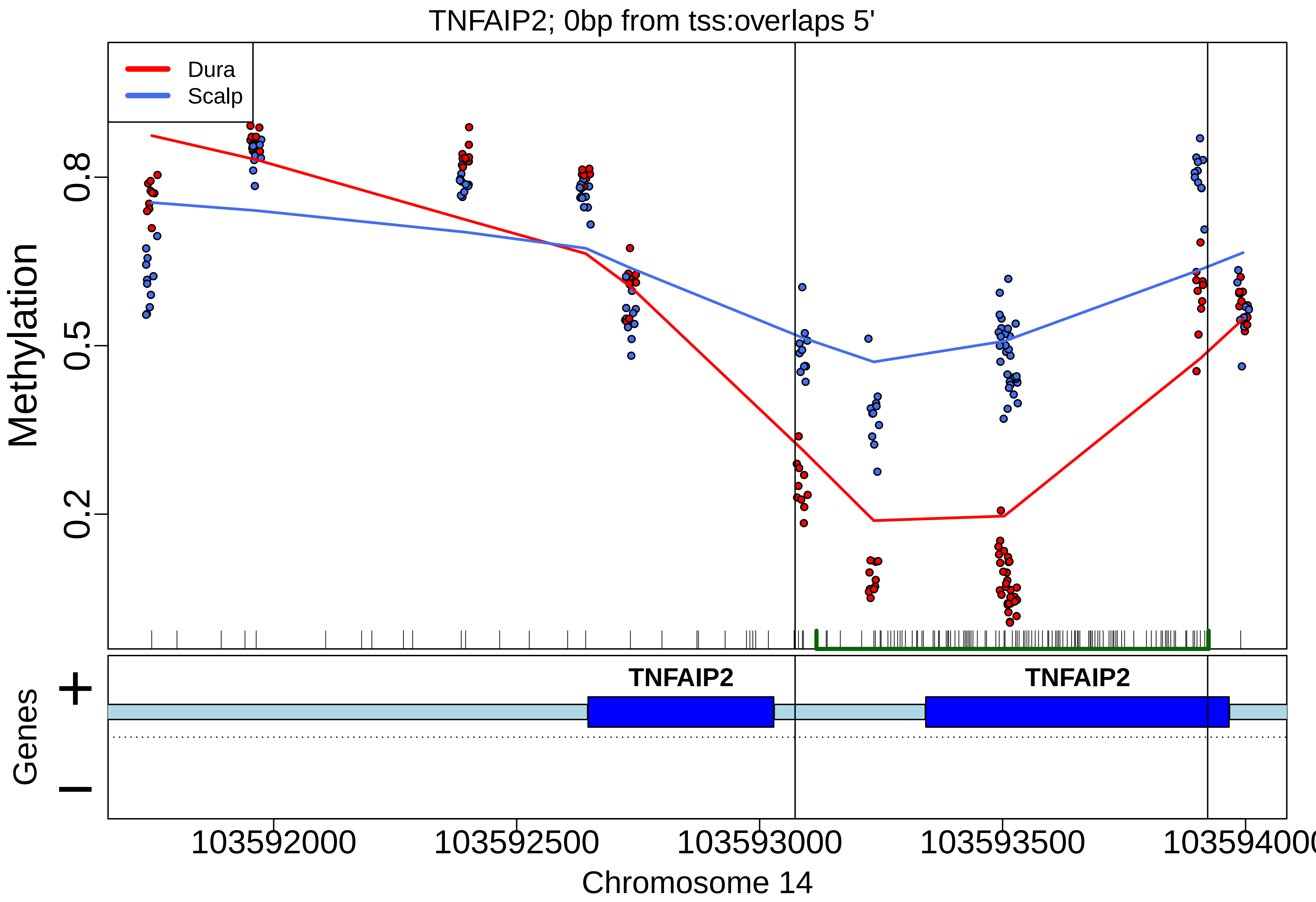

TNFAIP2; 0bp from tss:overlaps 5'
Dura
Scalp
Methylation
0.8
0.5
0.2
Genes
+
-
TNFAIP2
TNFAIP2
103592000
103592500
103593000
103593500
103594000
Chromosome 14

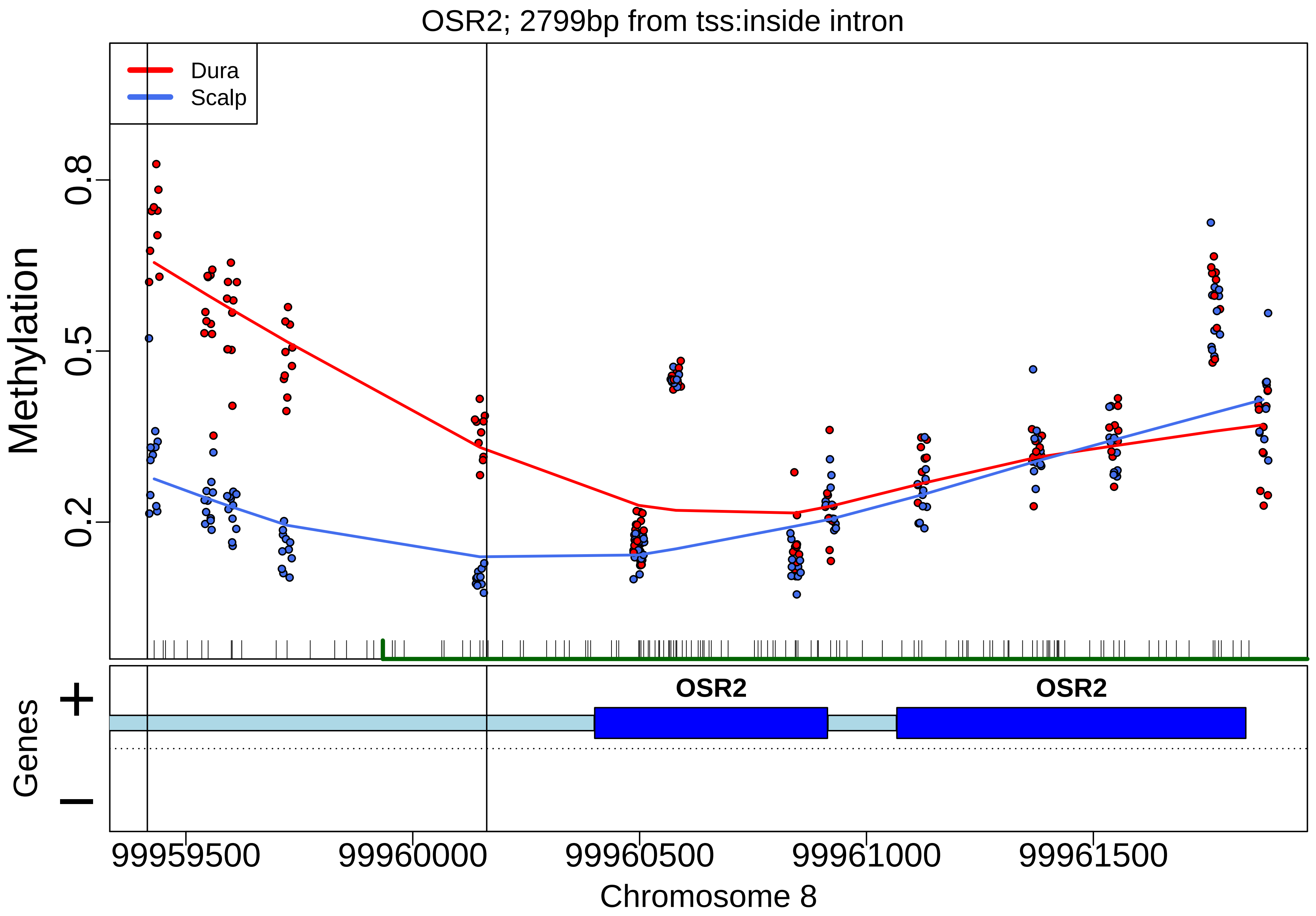

OSR2; 2799bp from tss:inside intron
Dura
Scalp
Methylation
0.2
0.5
0.8
Genes
+
-
OSR2
OSR2
99959500
99960000
99960500
99961000
99961500
Chromosome 8

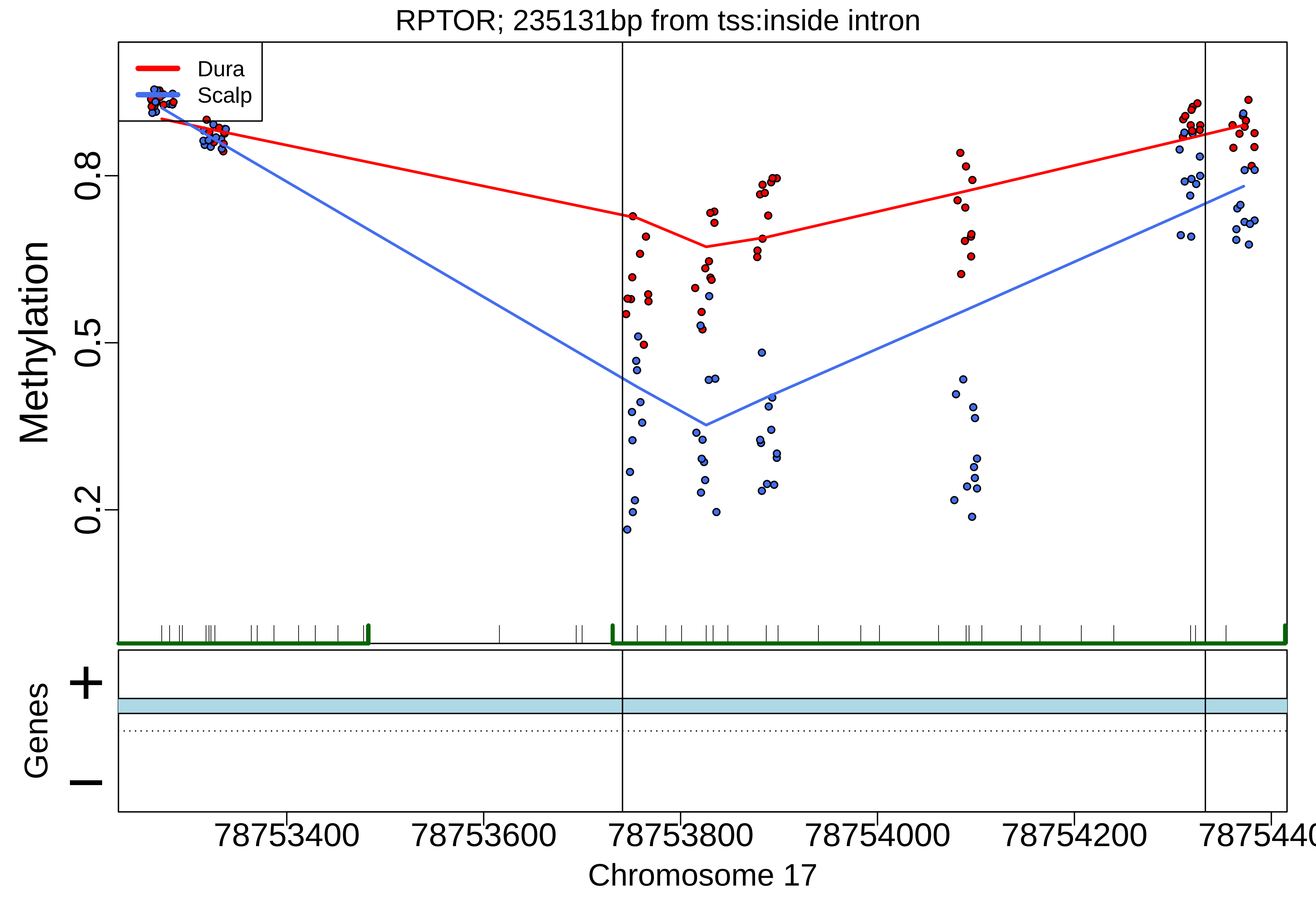
RPTOR; 235131bp from tss:inside intron
Dura
Scalp
Methylation
0.2
0.5
0.8
Genes
+
-
78753400
78753600
78753800
78754000
78754200
7875440
Chromosome 17

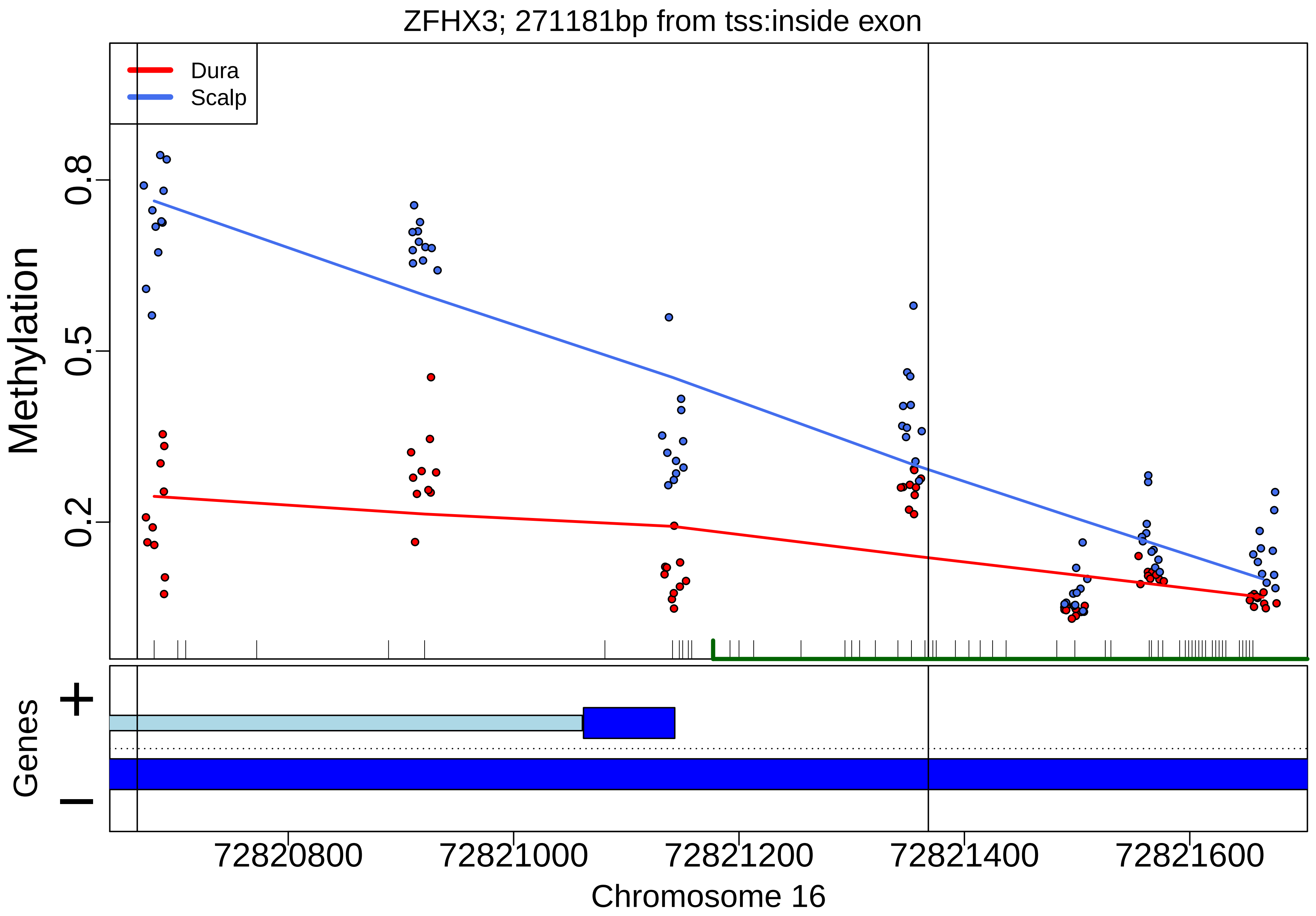
ZFHX3; 271181bp from tss:inside exon
Dura
Scalp
Methylation
0.2
0.5
0.8
Genes
+
−
72820800
72821000
72821200
72821400
72821600
Chromosome 16

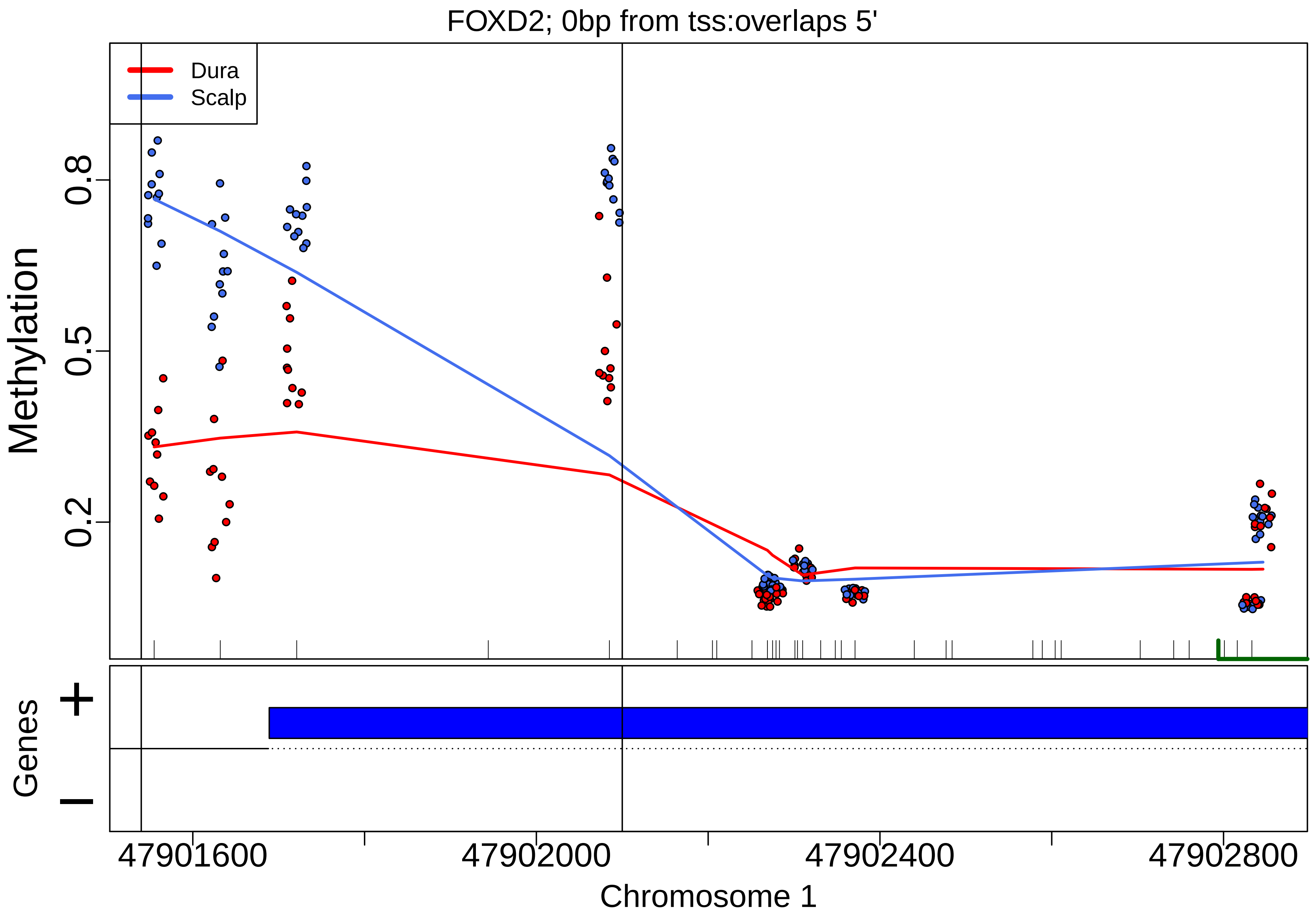

FOXD2; 0bp from tss:overlaps 5'
Dura
Scalp
Methylation
0.2
0.5
0.8
Genes
+
-
47901600
47902000
47902400
47902800
Chromosome 1

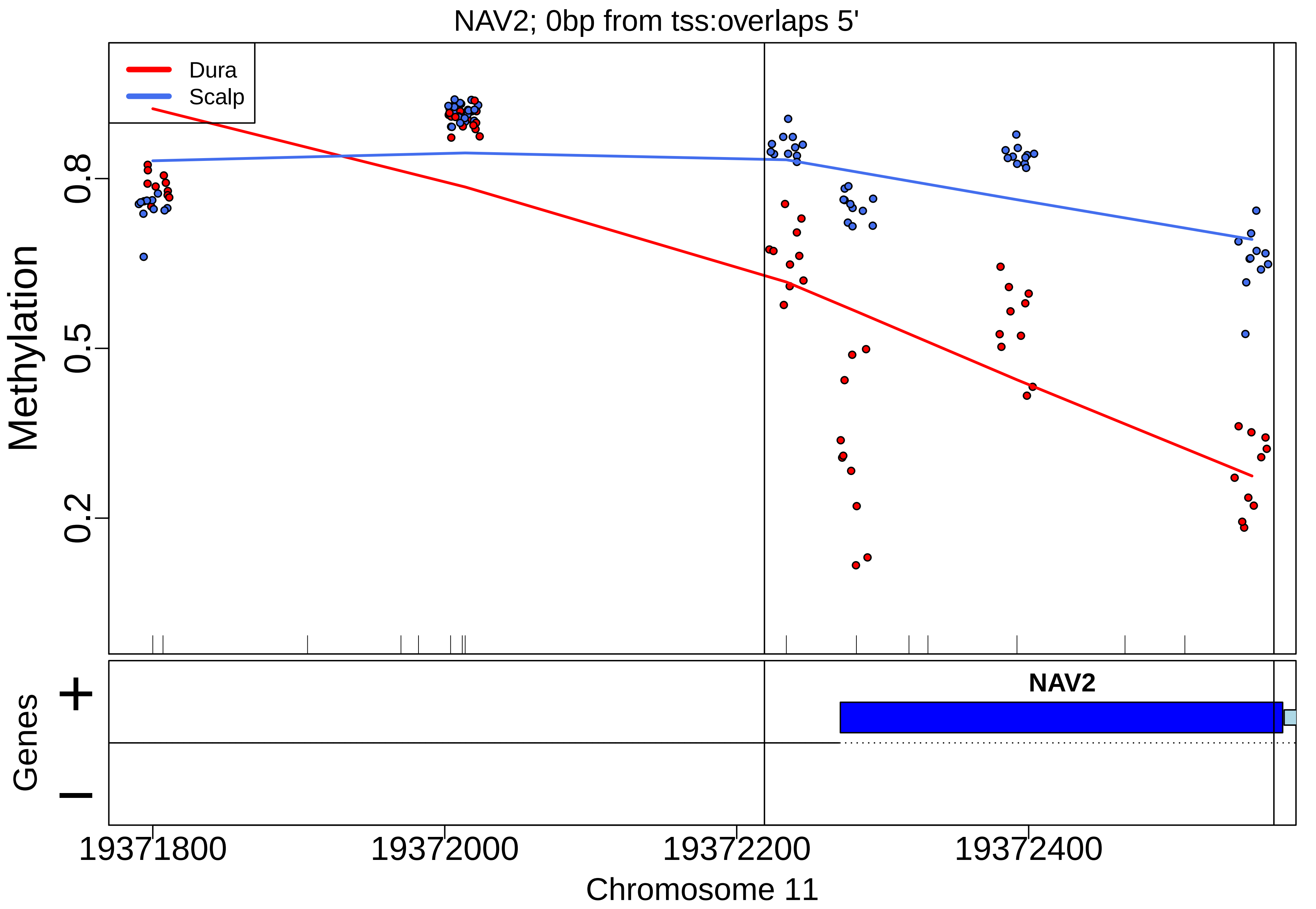

NAV2; 0bp from tss:overlaps 5'
Dura
Scalp
Methylation
0.2
0.5
0.8
Genes
+
-
NAV2
19371800
19372000
19372200
19372400
Chromosome 11

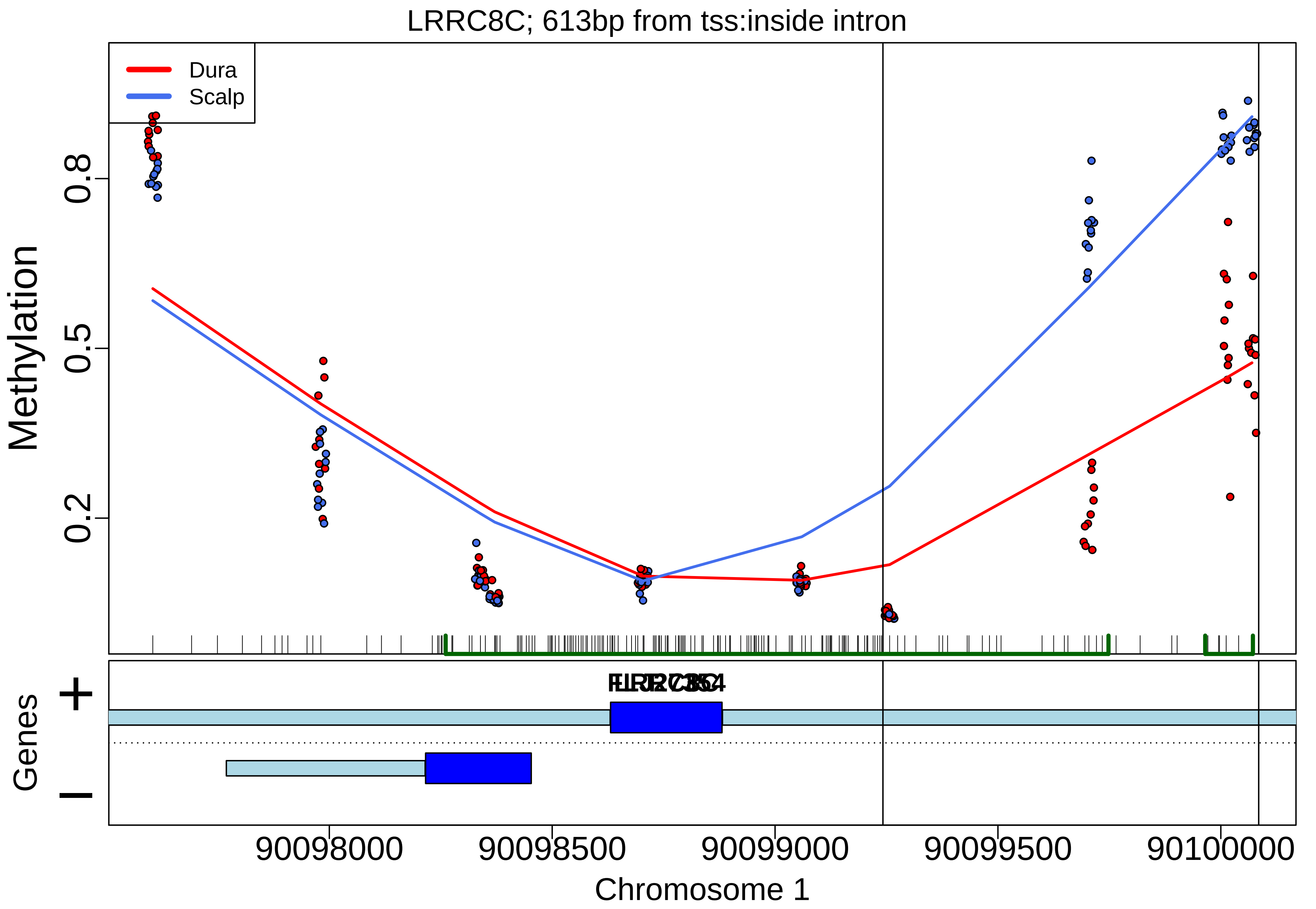
LRRC8C; 613bp from tss:inside intron
Dura
Scalp
Methylation
0.2
0.5
0.8
Genes
+
−
90098000
90098500
90099000
90099500
90100000
Chromosome 1

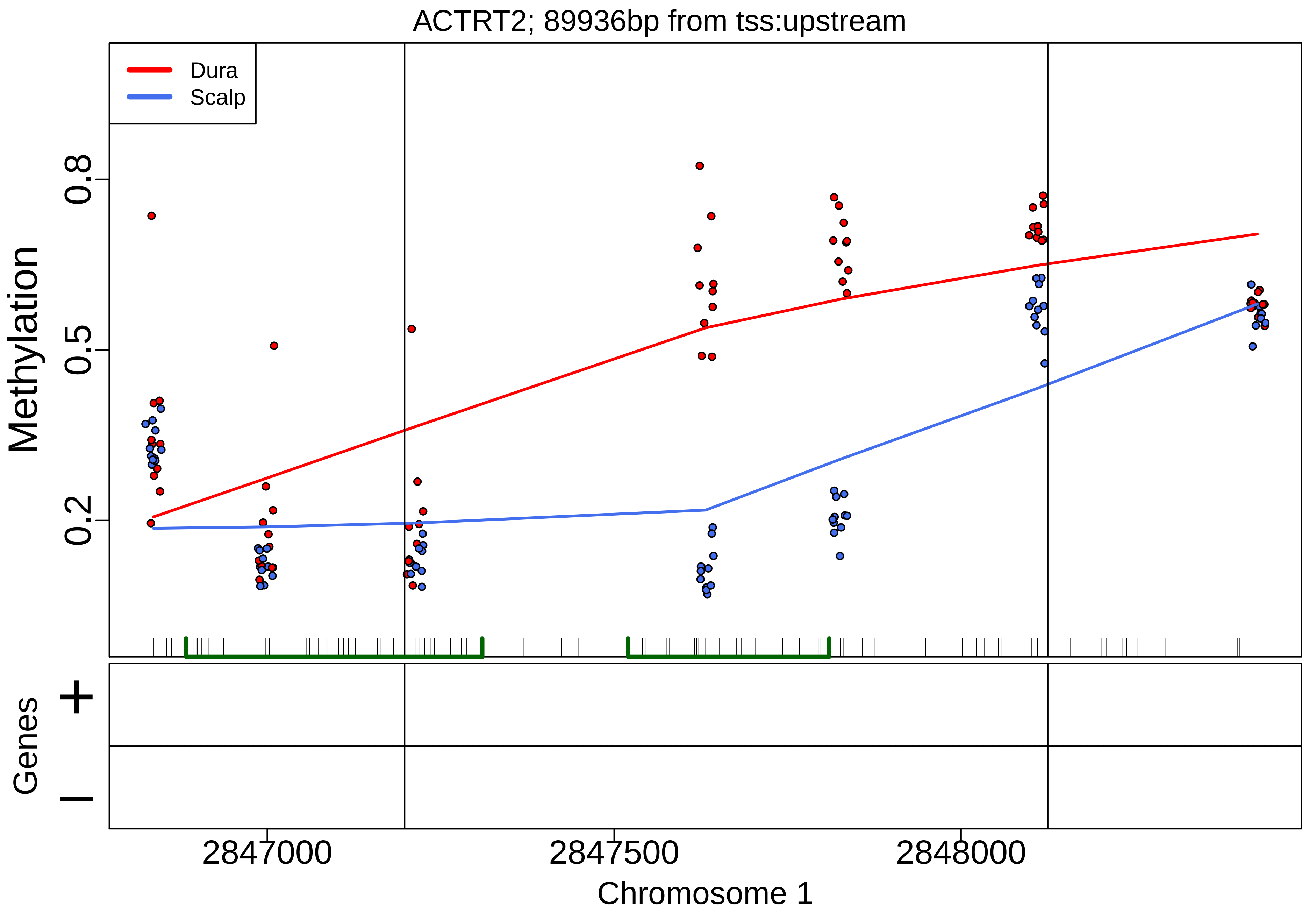
ACTRT2; 89936bp from tss:upstream
Dura
Scalp
Methylation
0.2
0.5
0.8
Genes
+
−
2847000
2847500
2848000
Chromosome 1

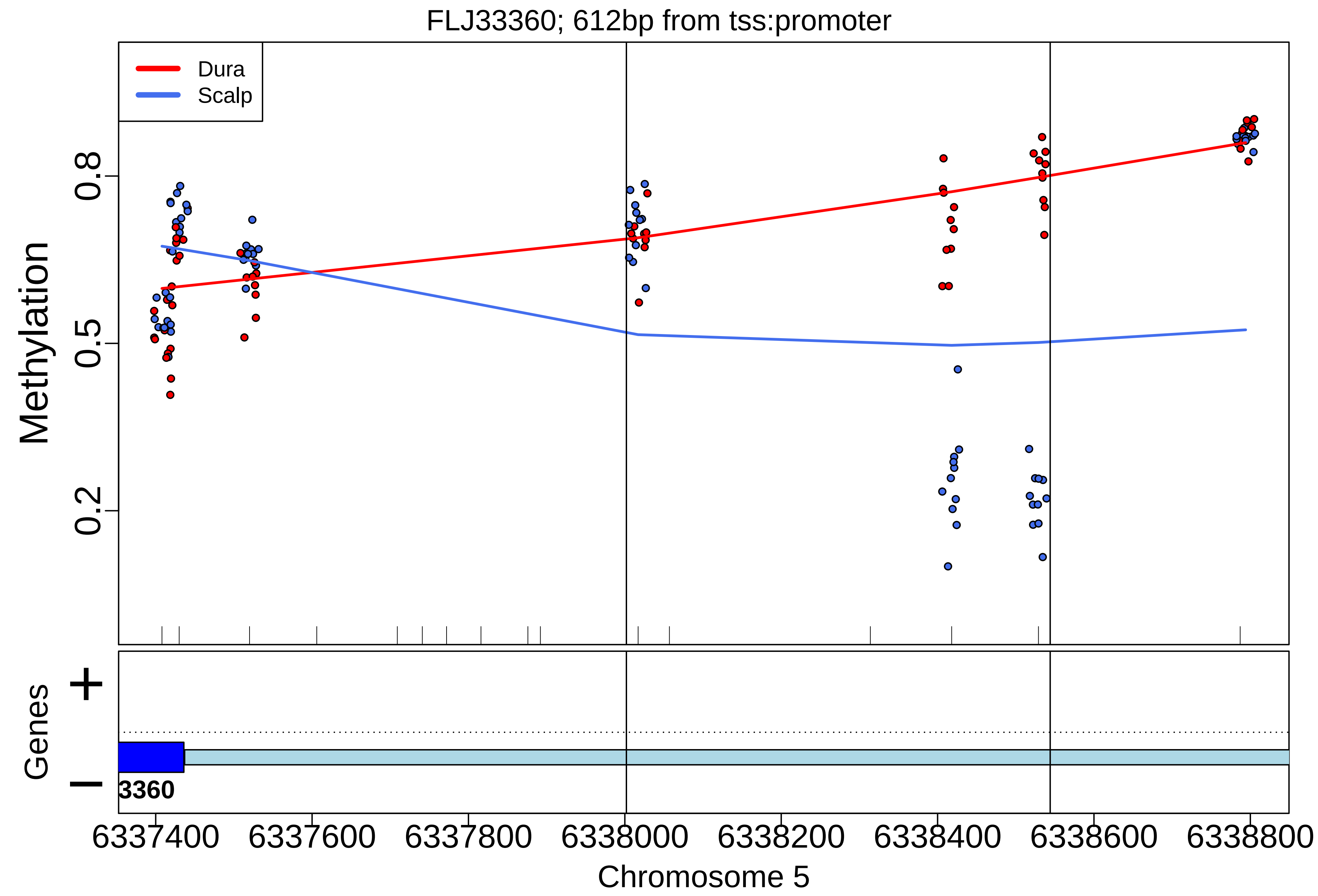

FLJ33360; 612bp from tss:promoter
Dura
Scalp
Methylation
0.2
0.5
0.8
Genes
+
-
3360
6337400
6337600
6337800
6338000
6338200
6338400
6338600
6338800
Chromosome 5

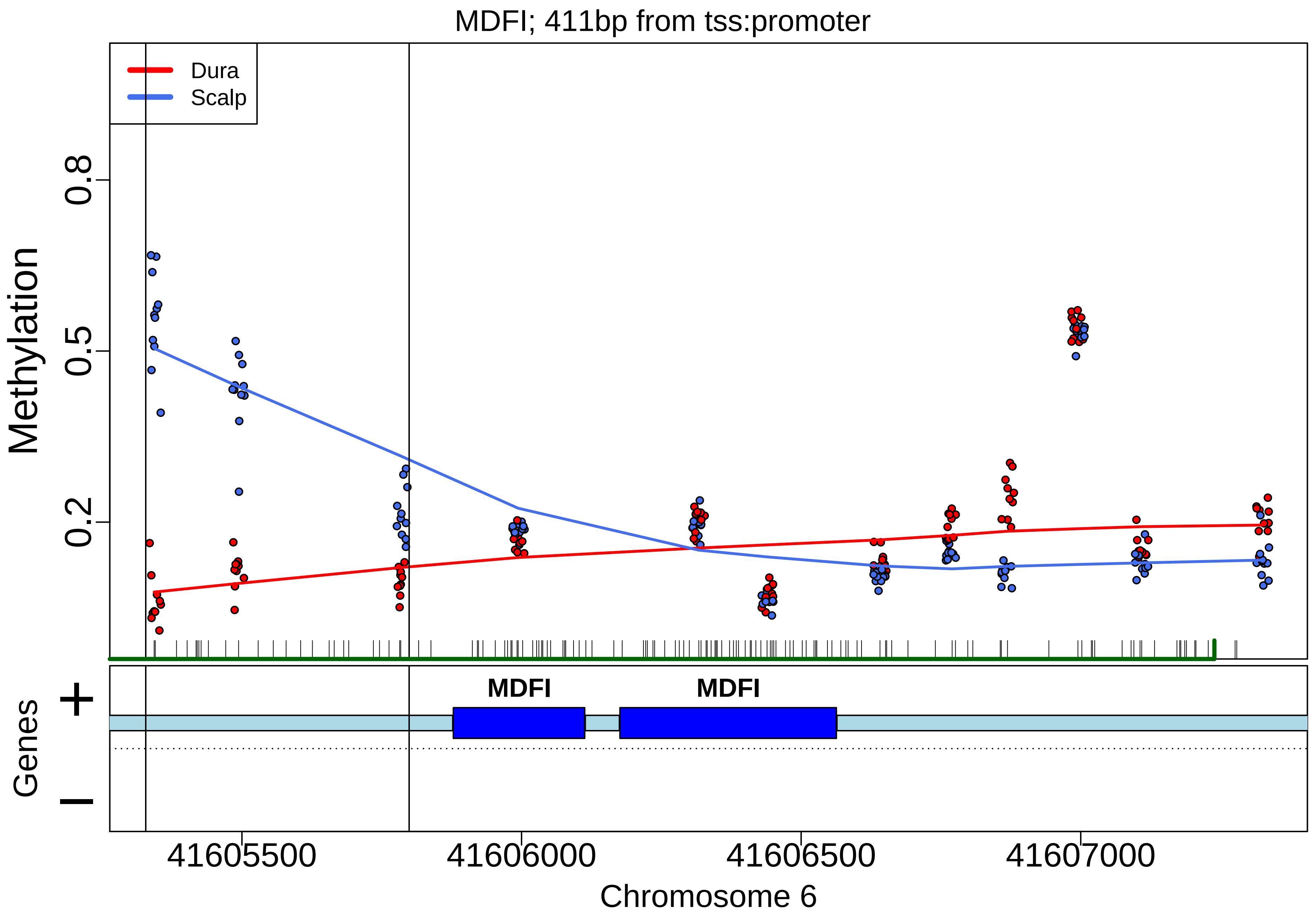
MDFI; 411bp from tss:promoter
Dura
Scalp
Methylation
0.2
0.5
0.8
Genes
+
MDFI
MDFI
41605500
41606000
41606500
41607000
Chromosome 6

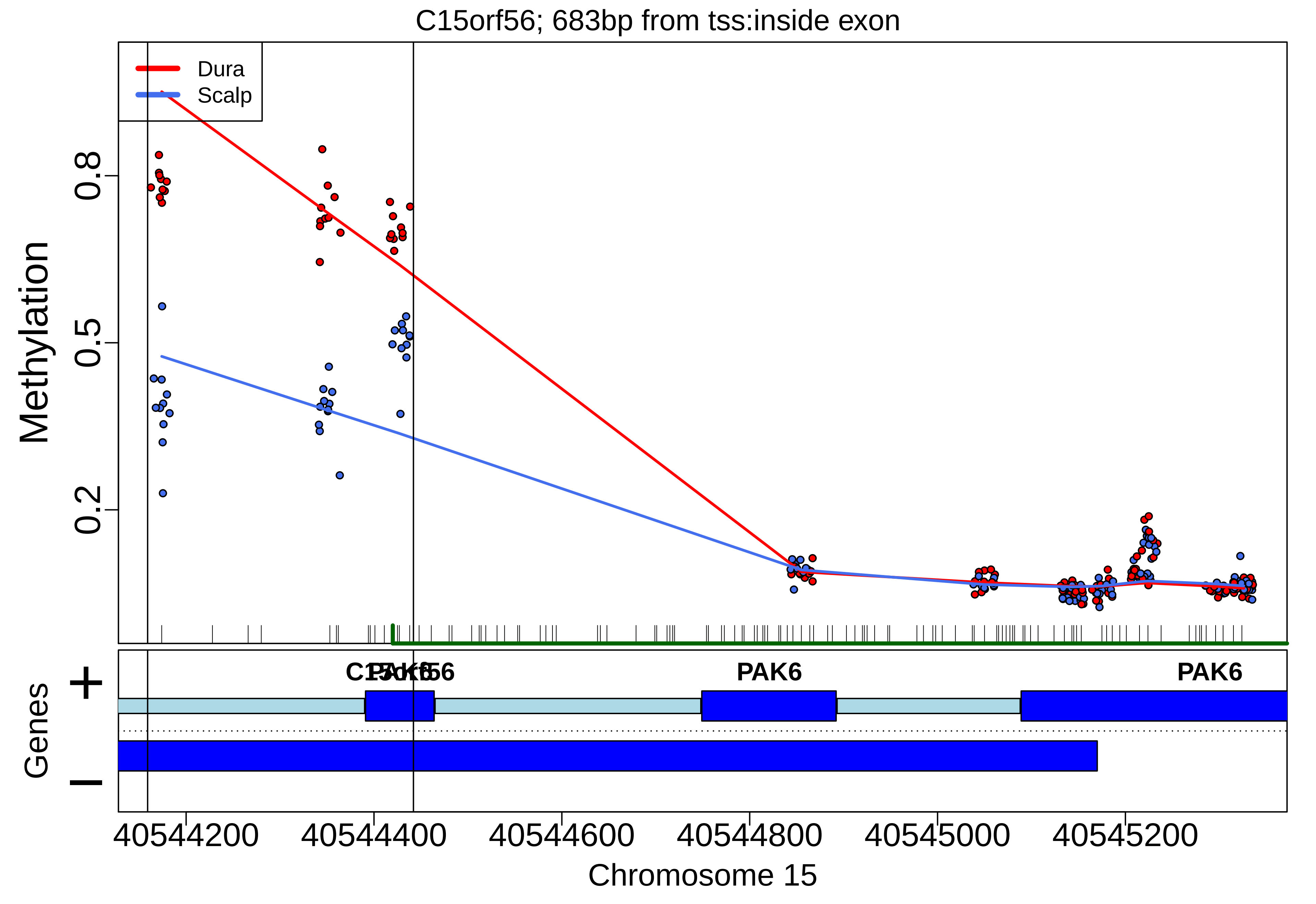
C15orf56; 683bp from tss:inside exon
Dura
Scalp
Methylation
0.8
0.5
0.2
Genes
+
-
C15orf56
PAK6
PAK6
PAK6
40544200
40544400
40544600
40544800
40545000
40545200
Chromosome 15

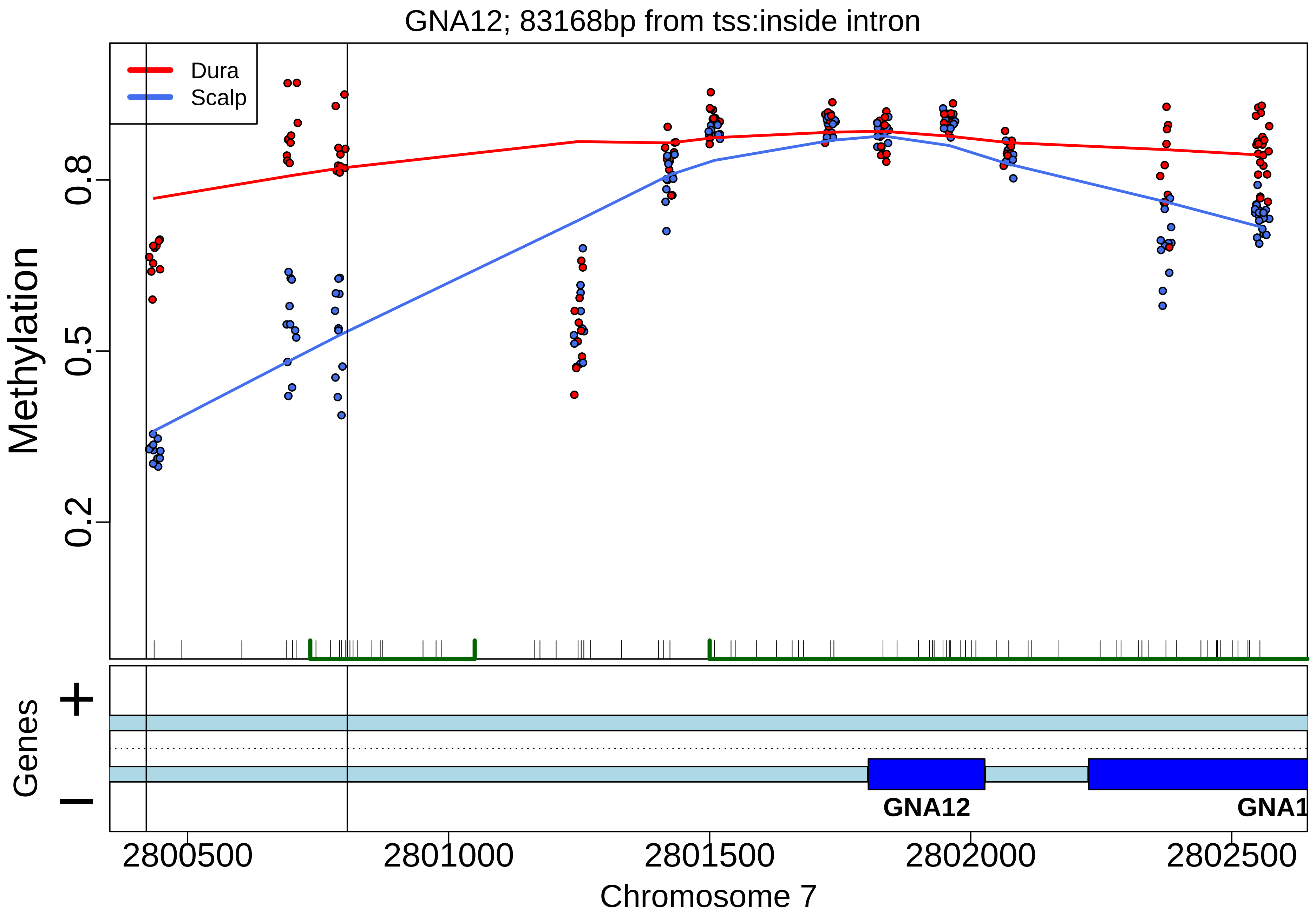

GNA12; 83168bp from tss:inside intron
Dura
Scalp
Methylation
0.2
0.5
0.8
Genes
+
−
GNA12
GNA1
2800500
2801000
2801500
2802000
2802500
Chromosome 7

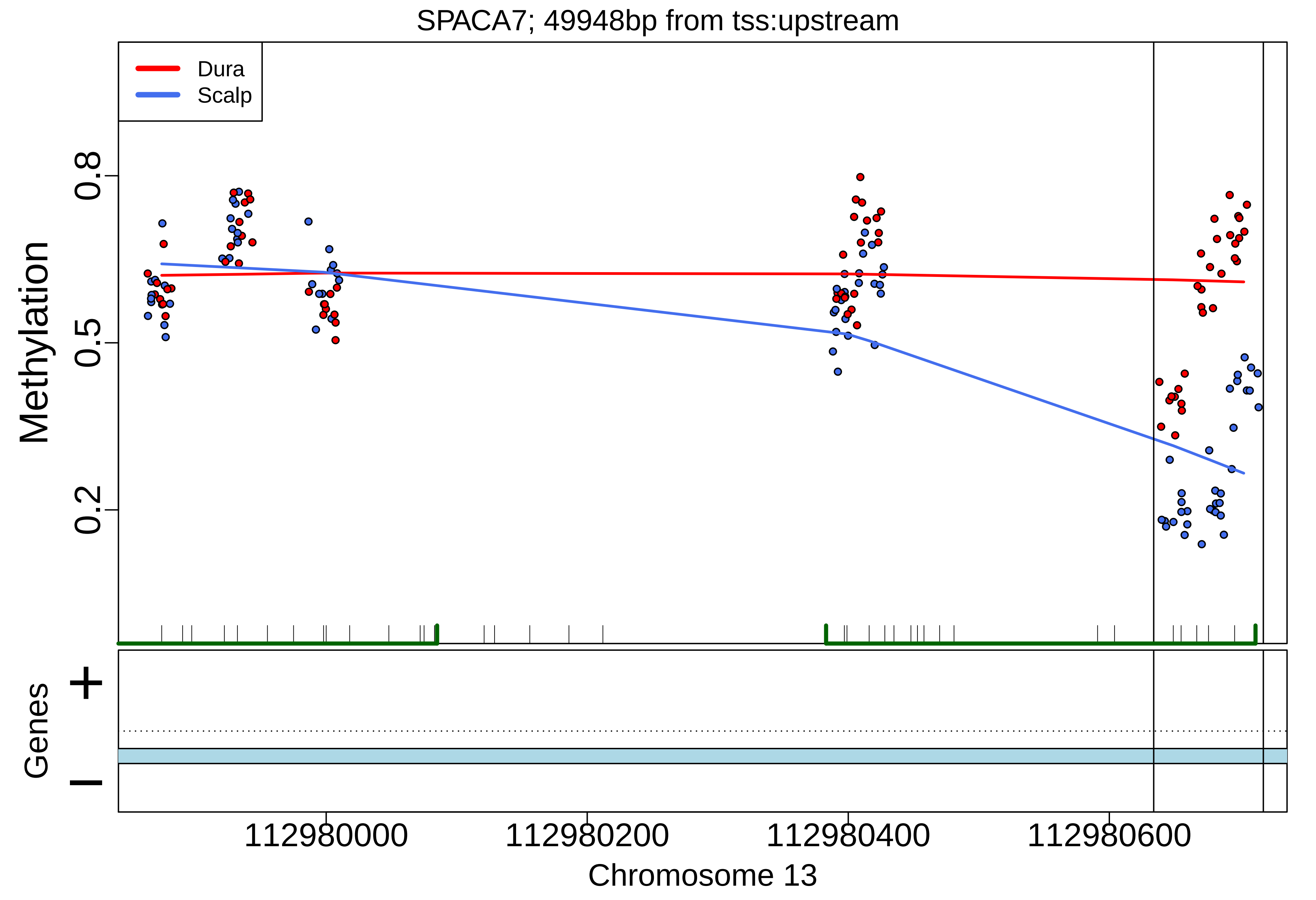
SPACA7; 49948bp from tss:upstream
Dura
Scalp
Methylation
0.2
0.5
0.8
Genes
+
−
112980000
112980200
112980400
112980600
Chromosome 13

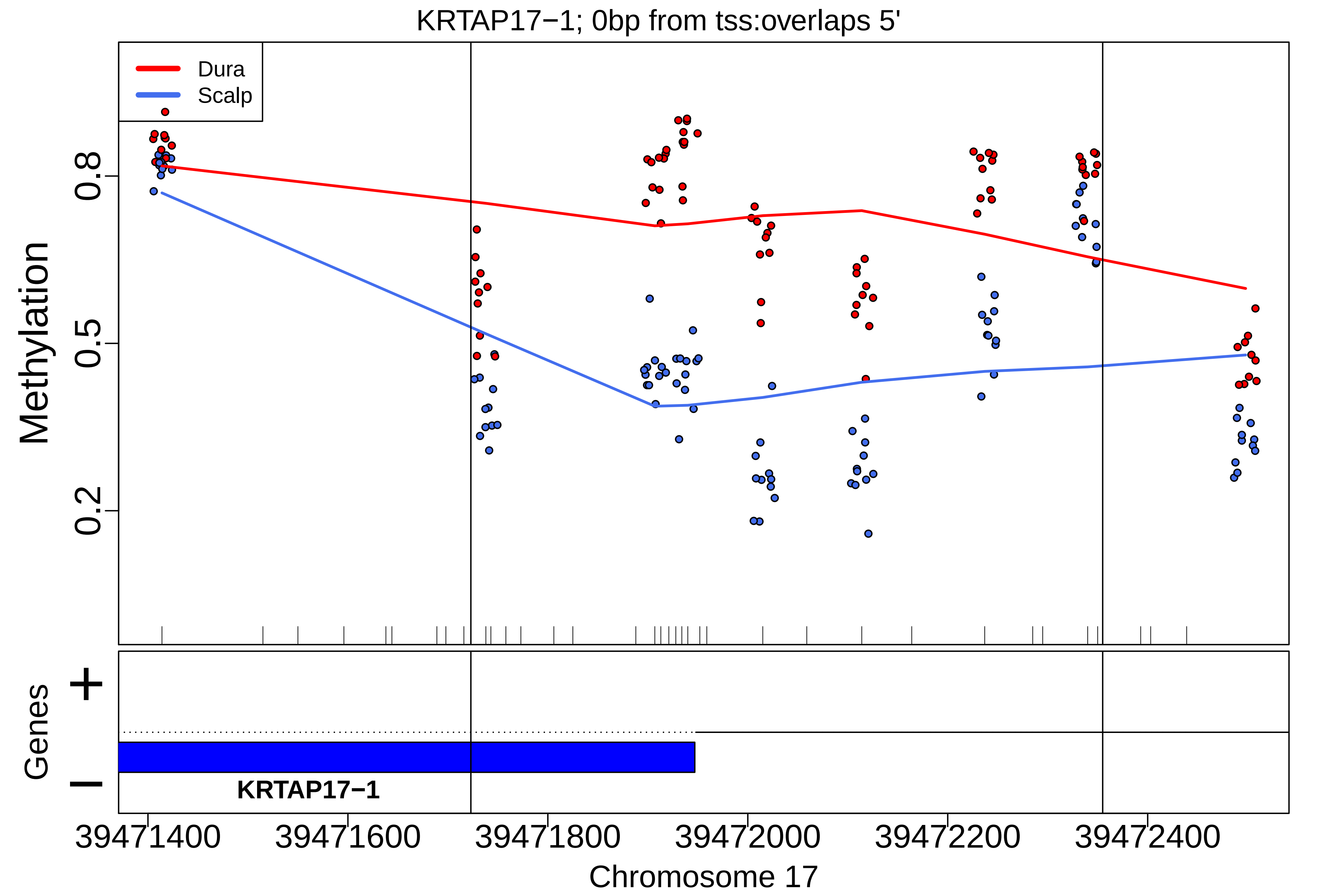
KRTAP17−1; 0bp from tss:overlaps 5'
Dura
Scalp
Methylation
0.2
0.5
0.8
Genes
+
−
KRTAP17−1
39471400
39471600
39471800
39472000
39472200
39472400
Chromosome 17

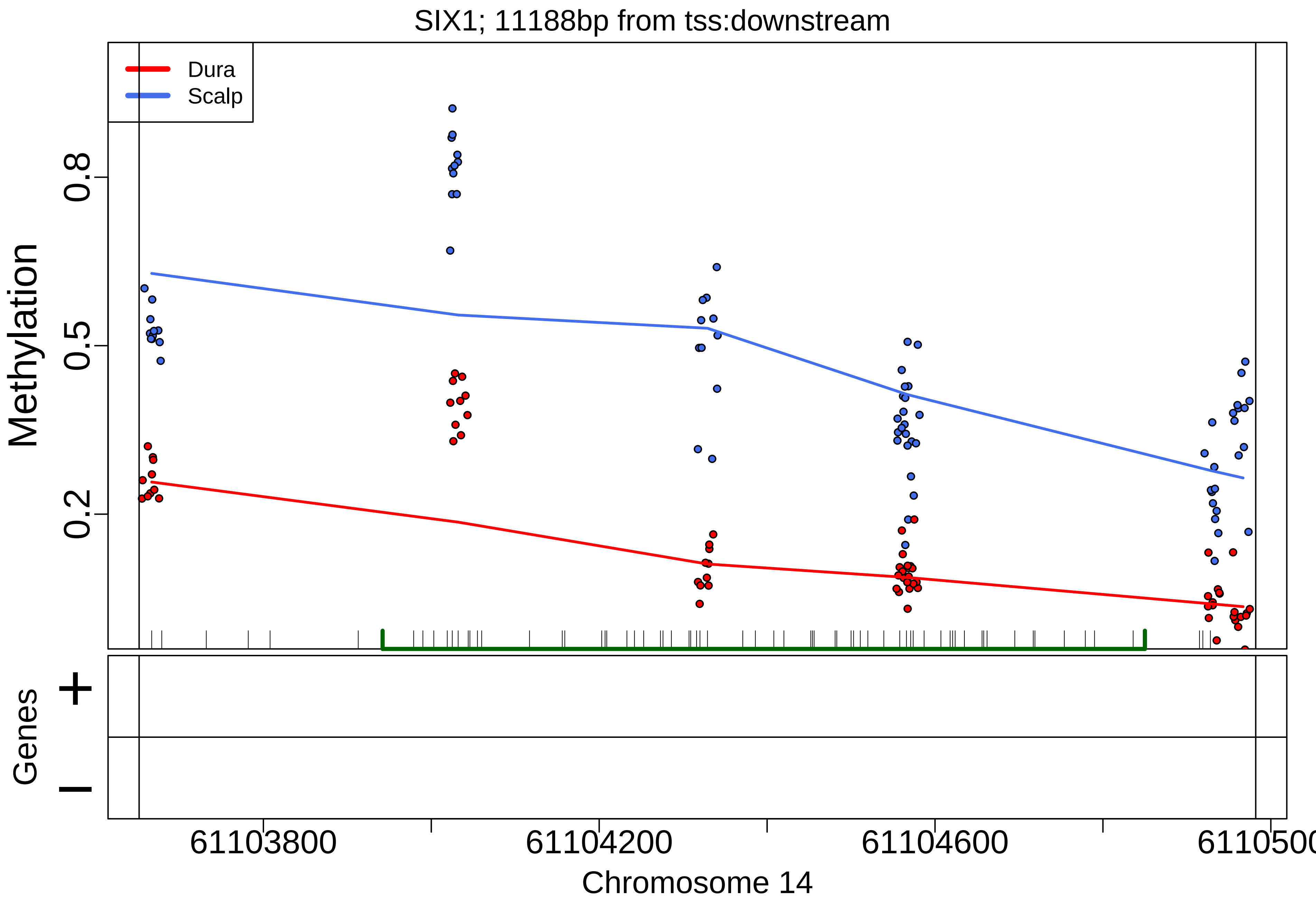
SIX1; 11188bp from tss:downstream
Dura
Scalp
Methylation
0.2
0.5
0.8
Genes
+
-
61103800
61104200
61104600
6110500
Chromosome 14

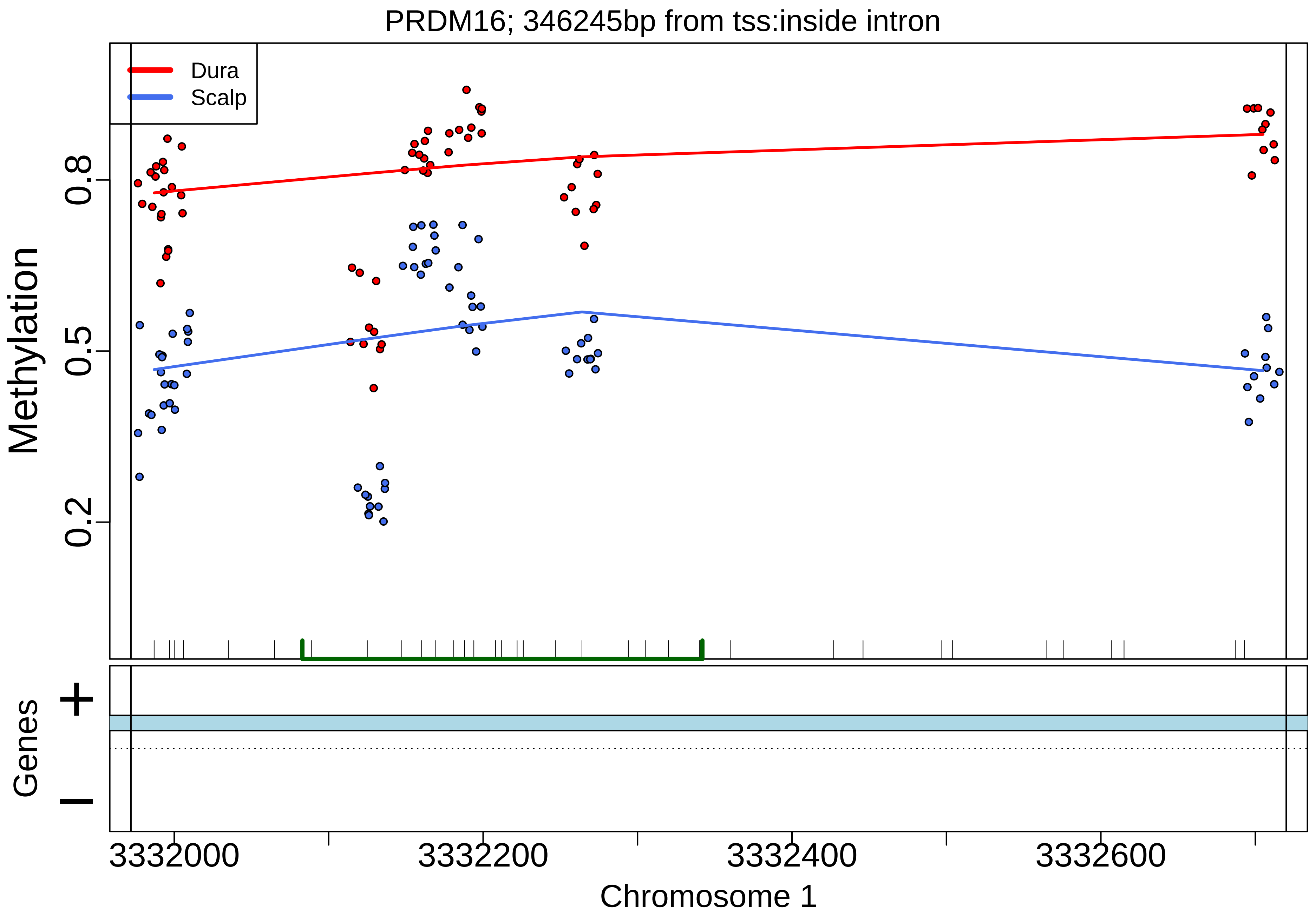
PRDM16; 346245bp from tss:inside intron
Dura
Scalp
Methylation
0.2
0.5
0.8
Genes
+
-
3332000
3332200
3332400
3332600
Chromosome 1

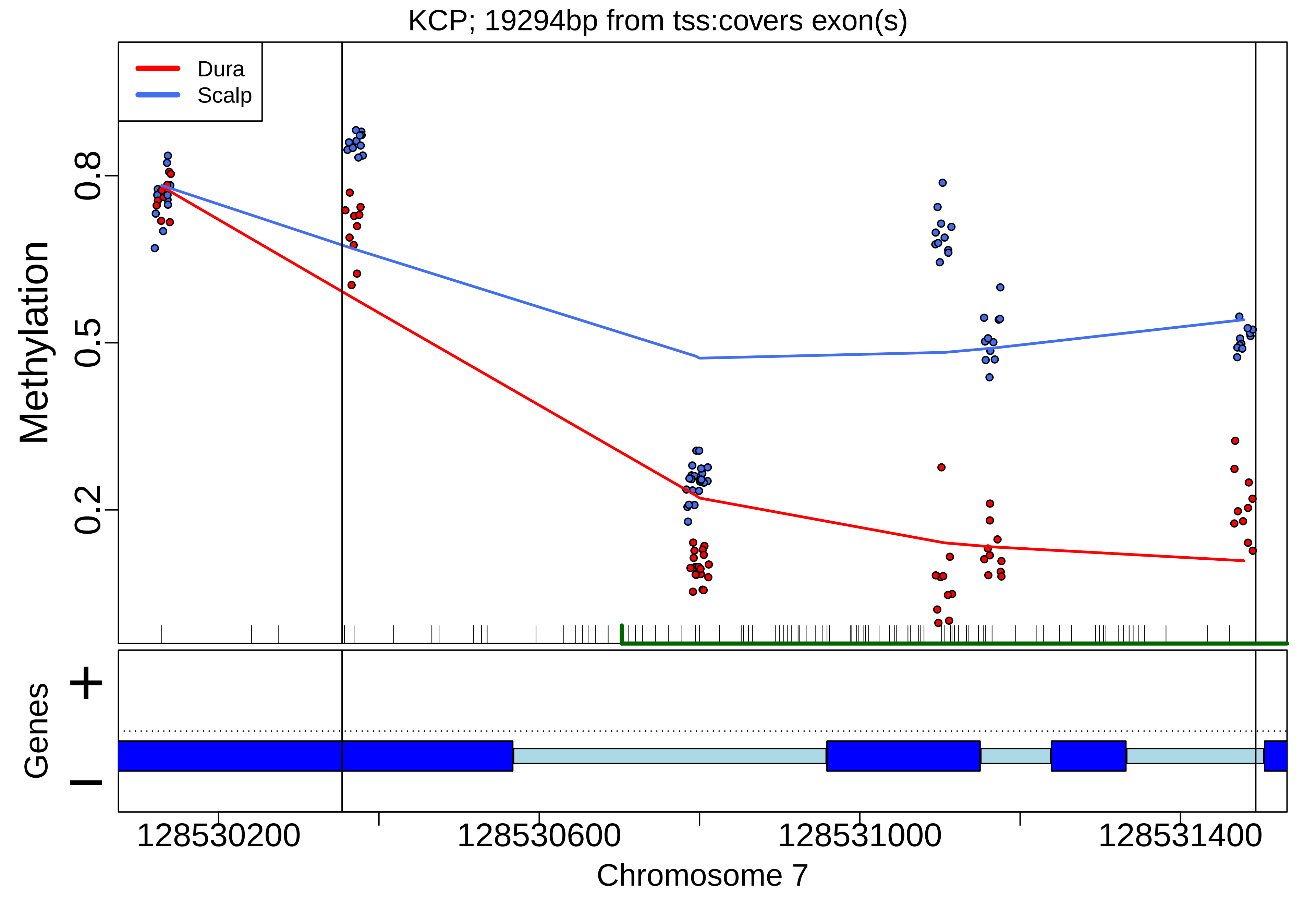
KCP; 19294bp from tss:covers exon(s)
Dura
Scalp
Methylation
0.2
0.5
0.8
Genes
+
−
128530200
128530600
128531000
128531400
Chromosome 7

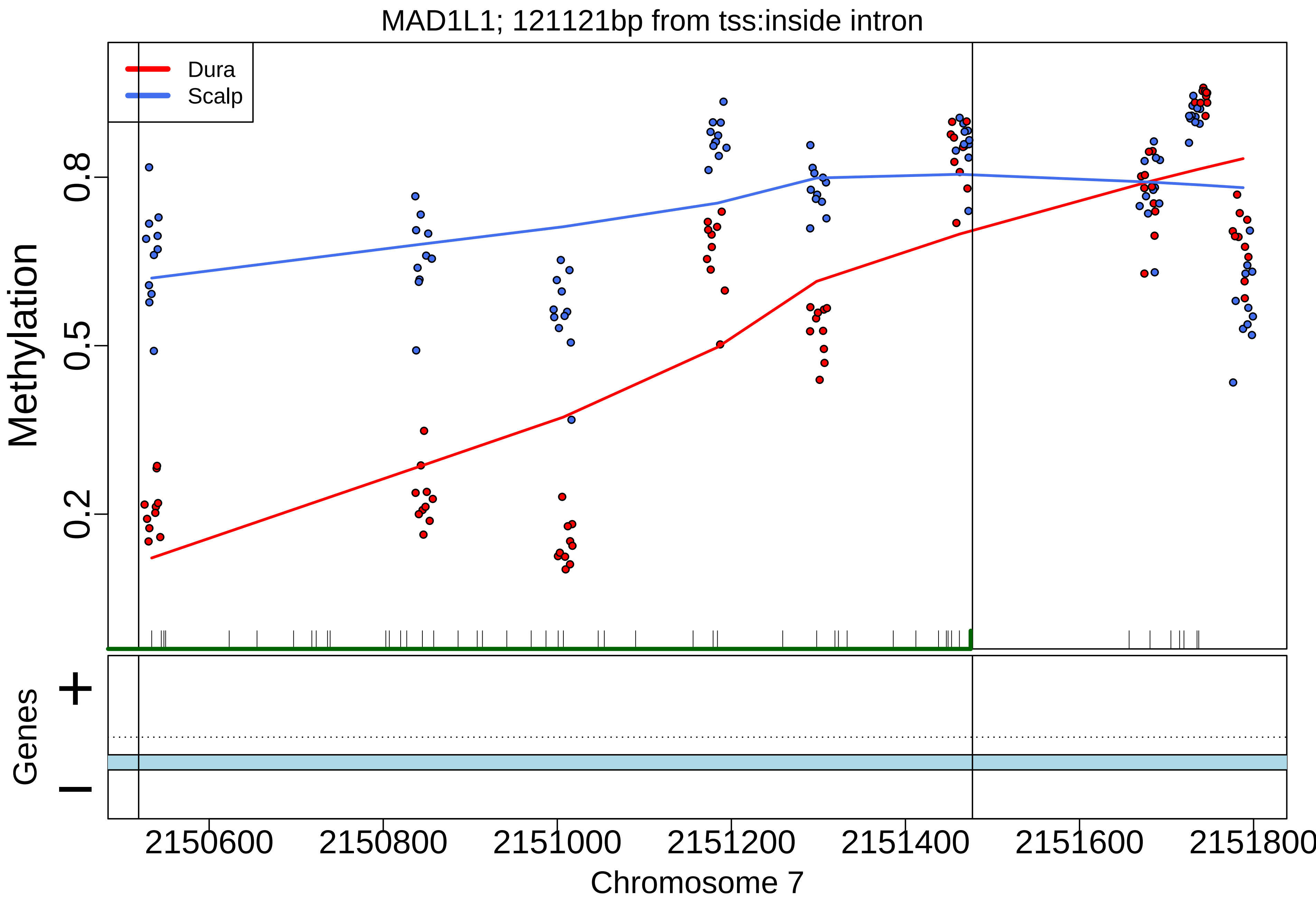
MAD1L1; 121121bp from tss:inside intron
Dura
Scalp
Methylation
0.2
0.5
0.8
Genes
+
−
2150600
2150800
2151000
2151200
2151400
2151600
2151800
Chromosome 7

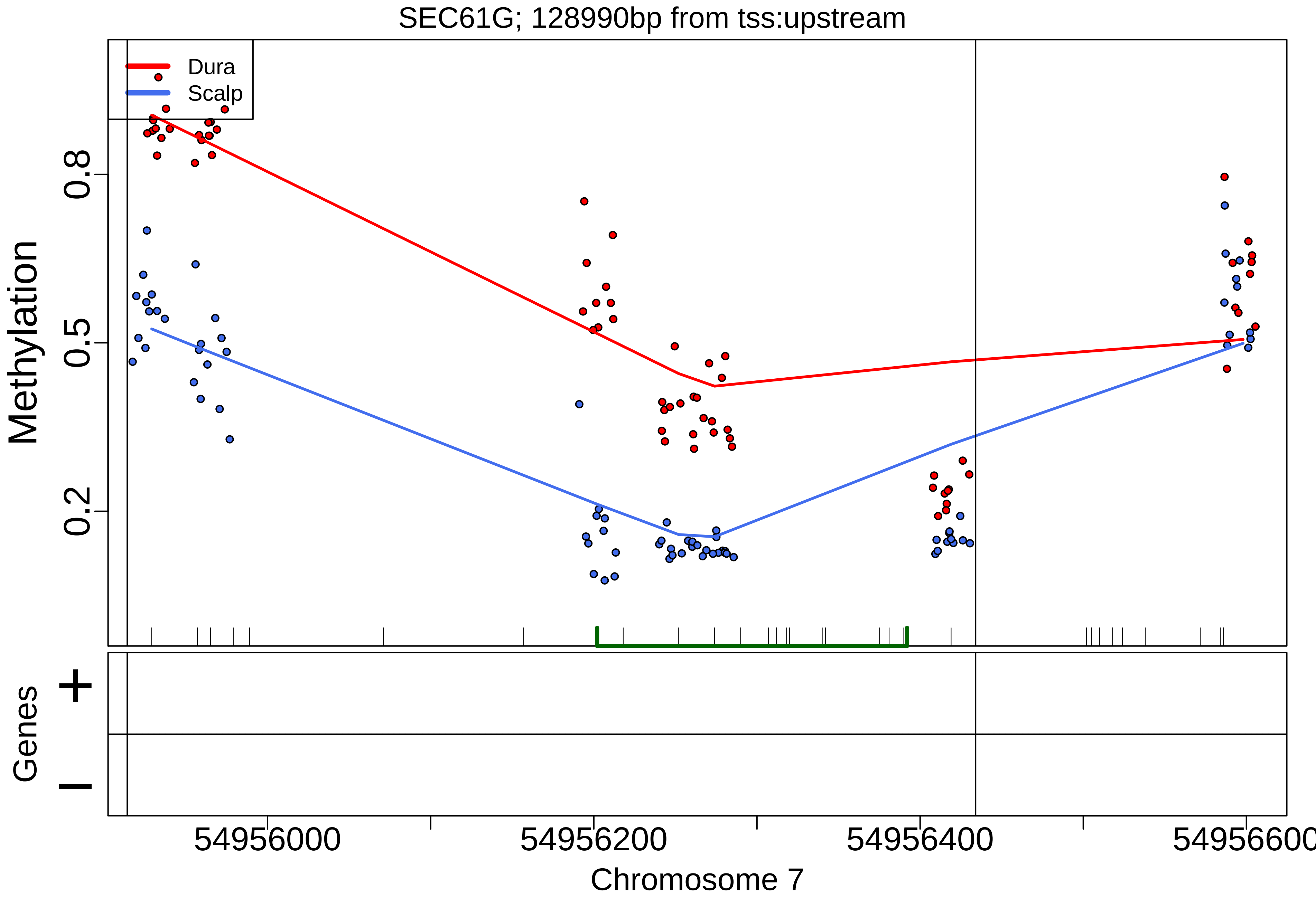

SEC61G; 128990bp from tss:upstream
Dura
Scalp
Methylation
0.2
0.5
0.8
Genes
+
-
54956000
54956200
54956400
54956600
Chromosome 7

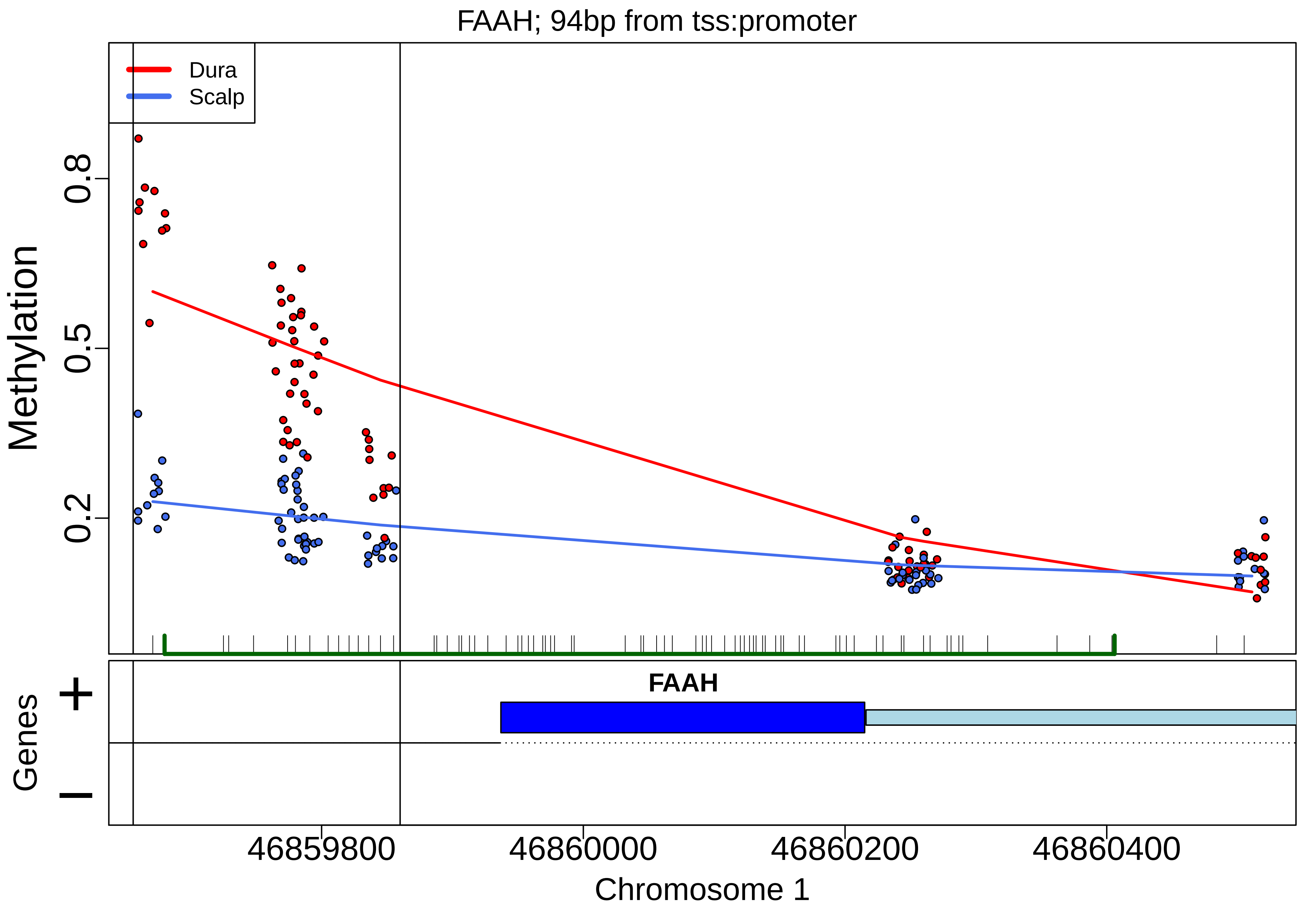

FAAH; 94bp from tss:promoter
Dura
Scalp
Methylation
0.2
0.5
0.8
Genes
+
-
FAAH
46859800
46860000
46860200
46860400
Chromosome 1

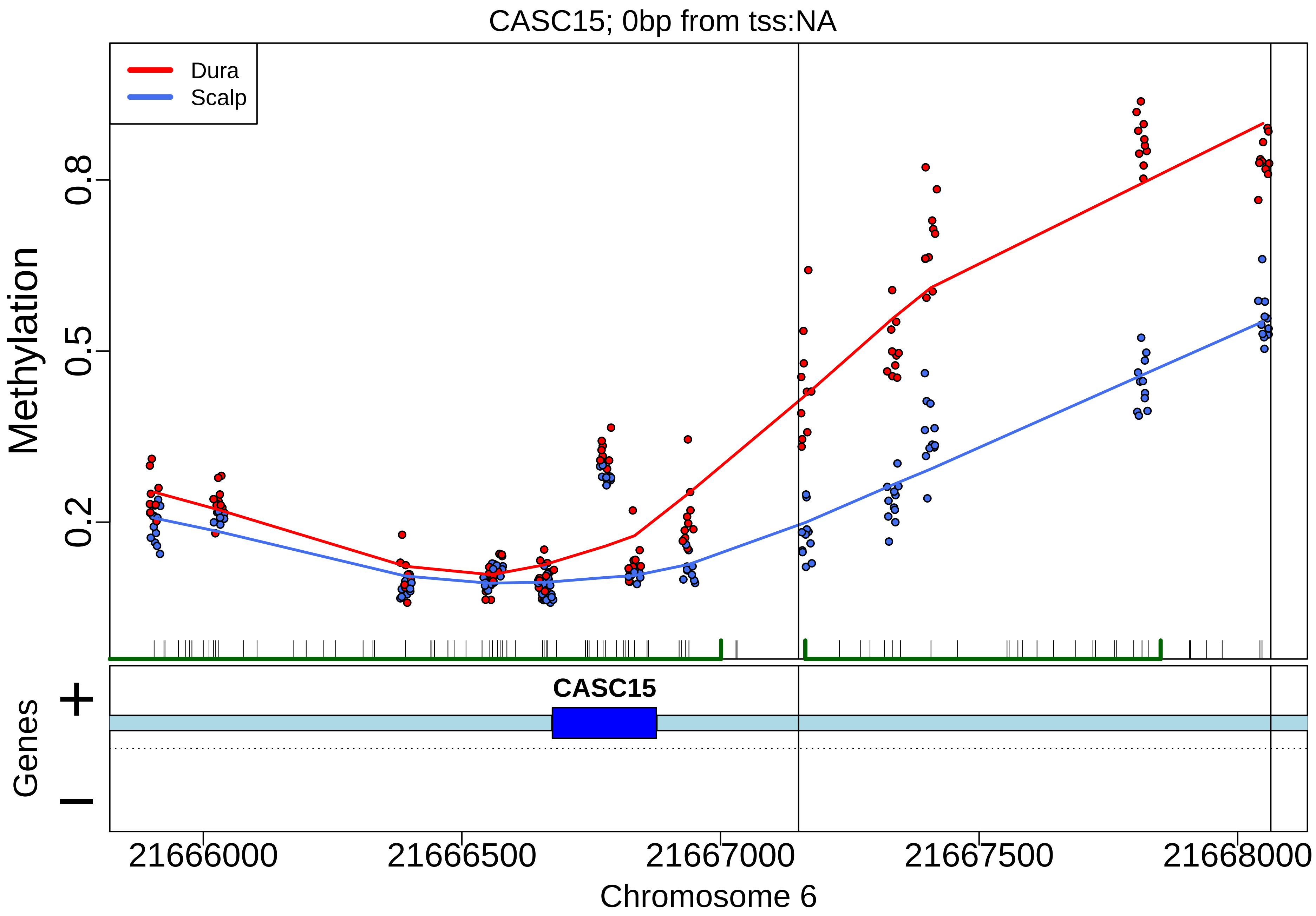

CASC15; 0bp from tss:NA
Dura
Scalp
Methylation
0.2
0.5
0.8
Genes
+
-
CASC15
21666000
21666500
21667000
21667500
21668000
Chromosome 6

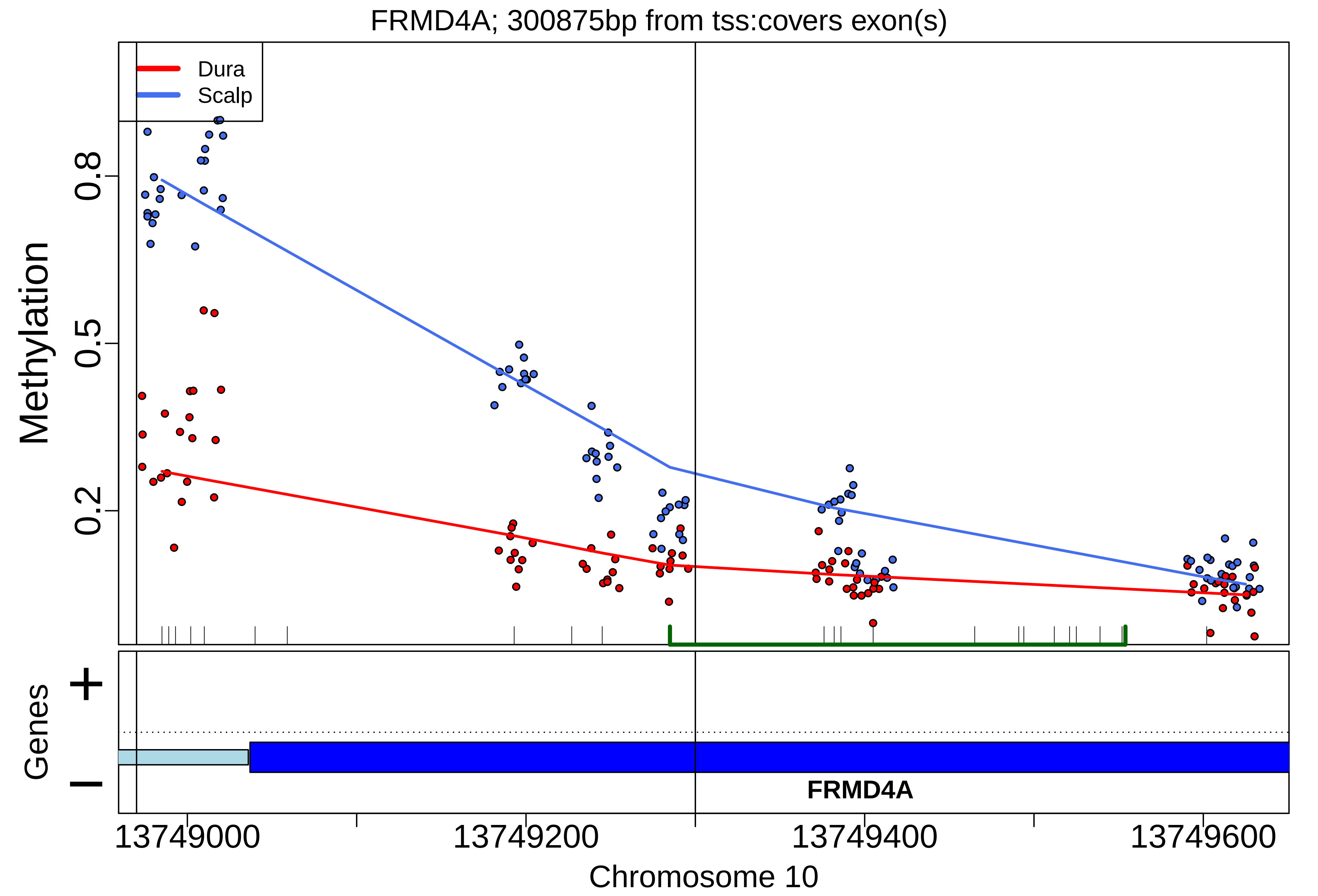

FRMD4A; 300875bp from tss:covers exon(s)
Dura
Scalp
Methylation
0.2
0.5
0.8
Genes
+
-
FRMD4A
13749000
13749200
13749400
13749600
Chromosome 10

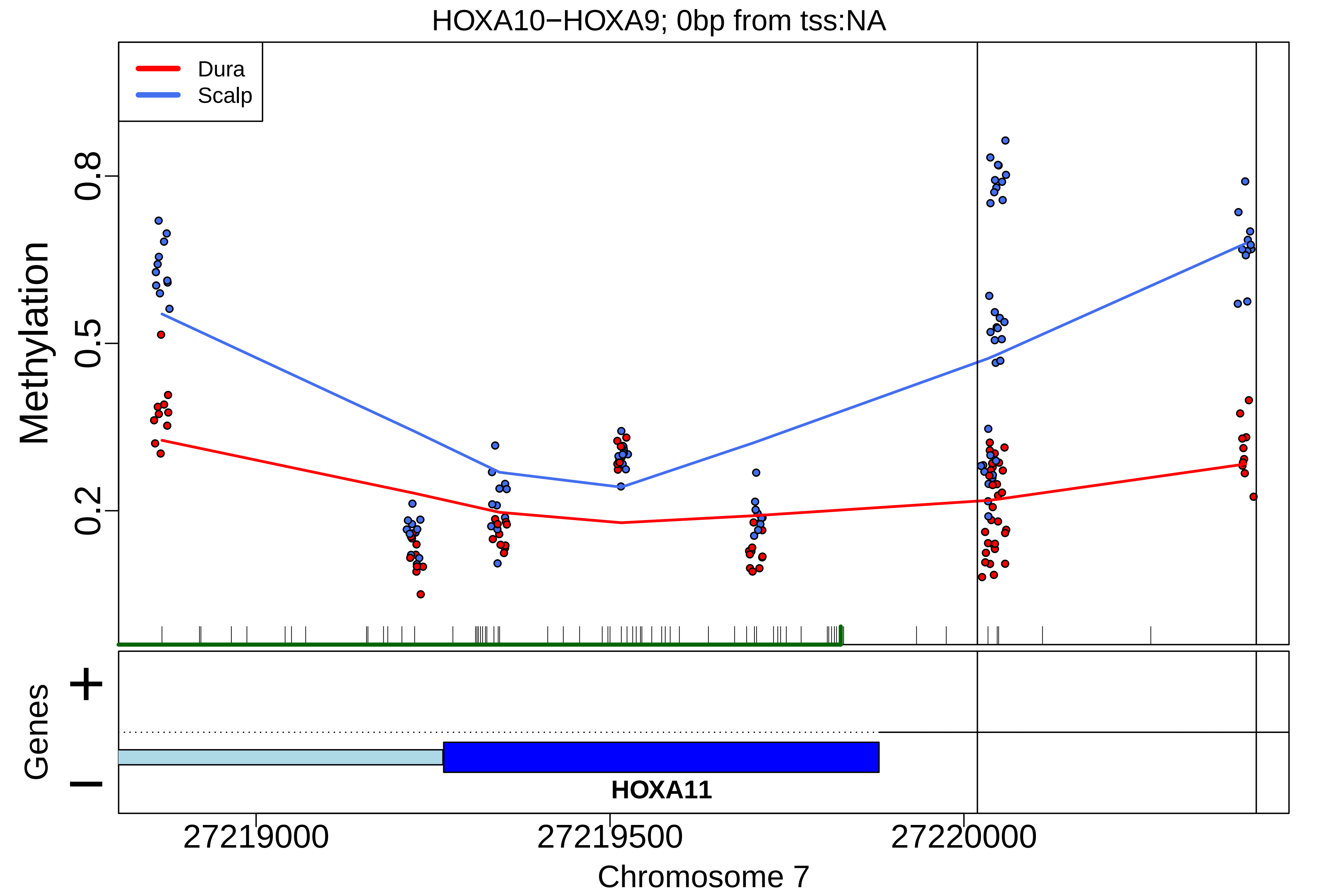
HOXA10−HOXA9; 0bp from tss:NA
Dura
Scalp
Methylation
0.2
0.5
0.8
Genes
+
−
HOXA11
27219000
27219500
27220000
Chromosome 7

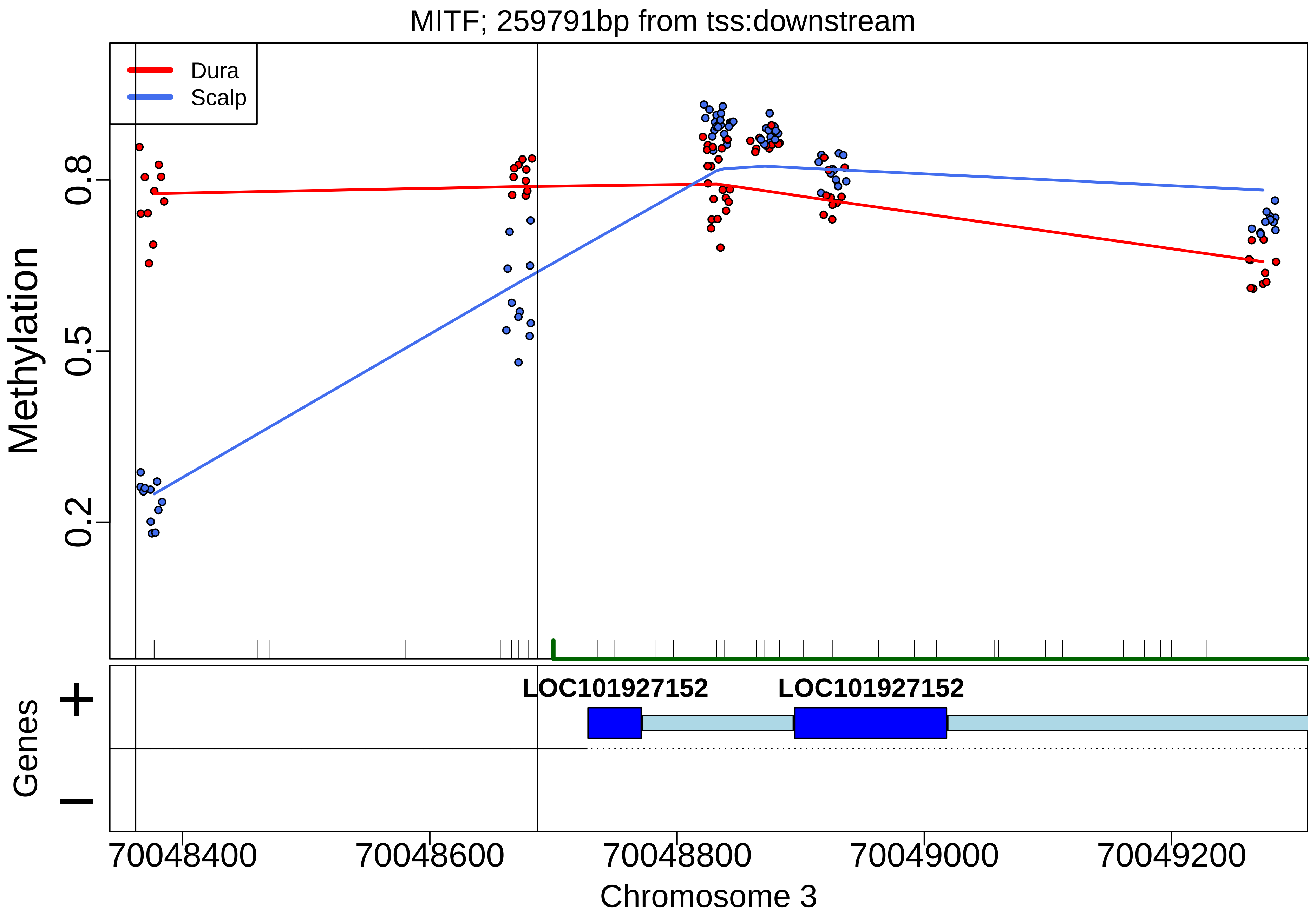
MITF; 259791bp from tss:downstream
Dura
Scalp
Methylation
0.2
0.5
0.8
Genes
+
-
LOC101927152
LOC101927152
70048400
70048600
70048800
70049000
70049200
Chromosome 3

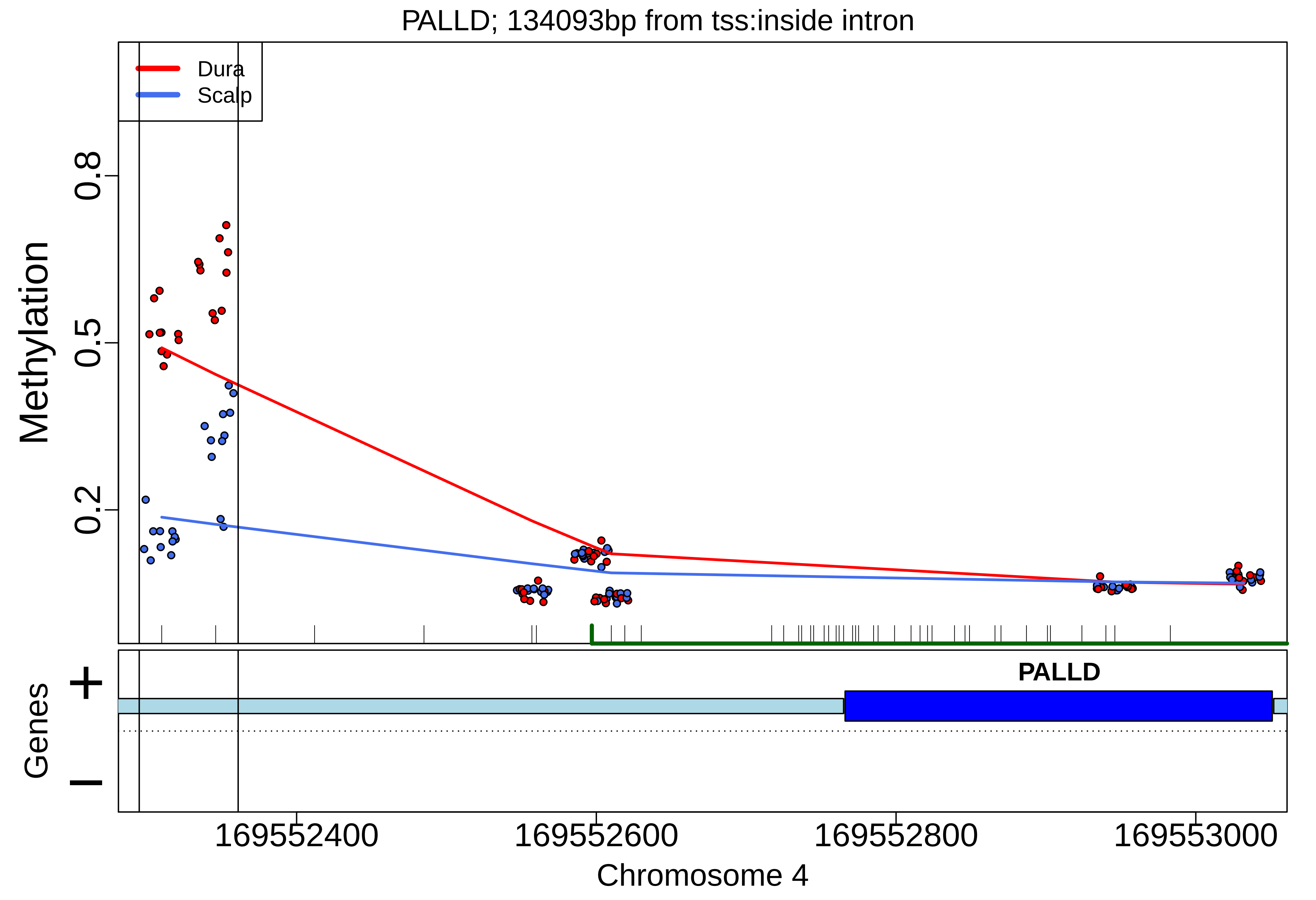
PALLD; 134093bp from tss:inside intron
Dura
Scalp
Methylation
0.2
0.5
0.8
Genes
+
−
PALLD
169552400
169552600
169552800
169553000
Chromosome 4

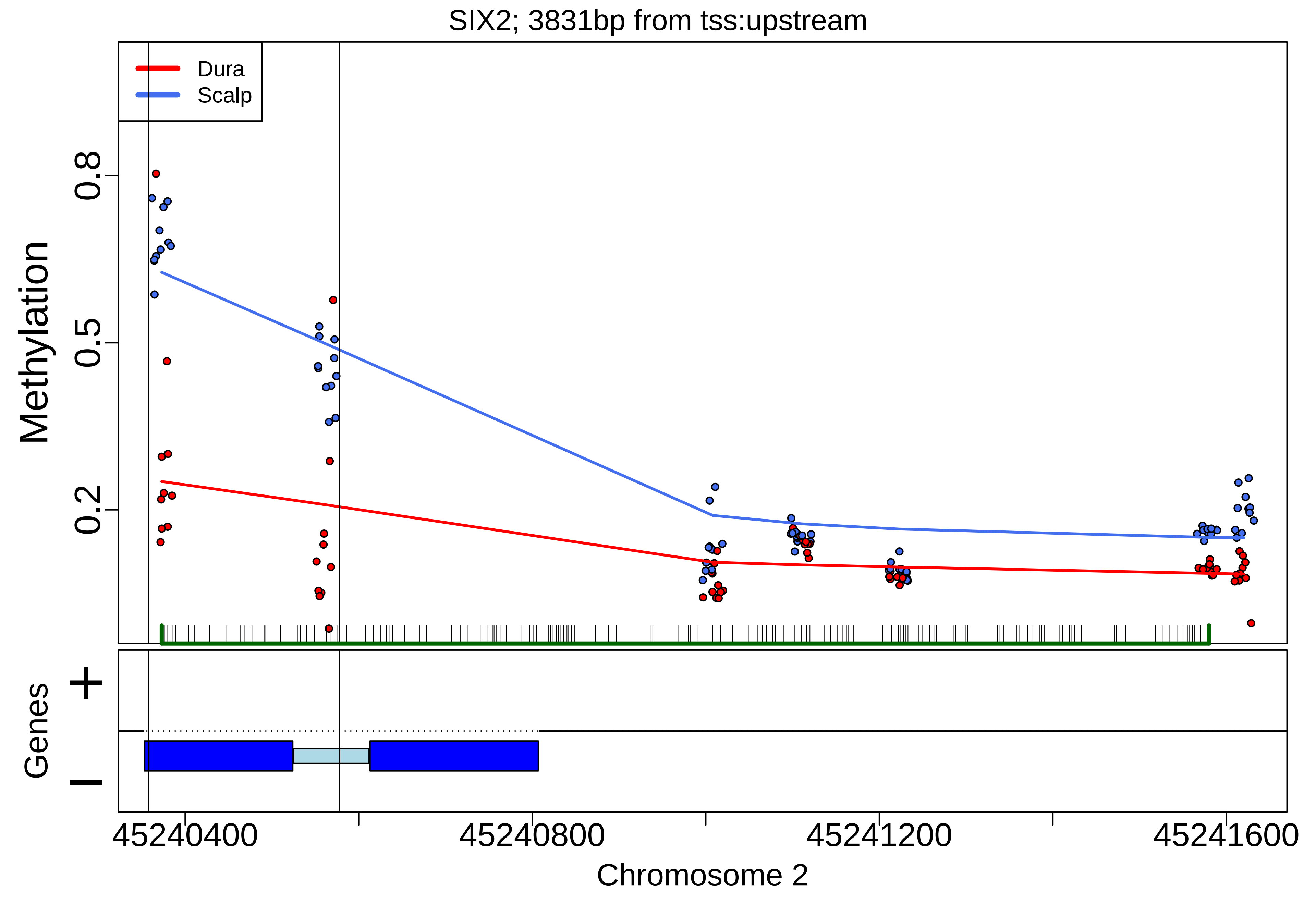
SIX2; 3831bp from tss:upstream
Dura
Scalp
Methylation
0.8
0.5
0.2
Genes
+
-
45240400
45240800
45241200
45241600
Chromosome 2

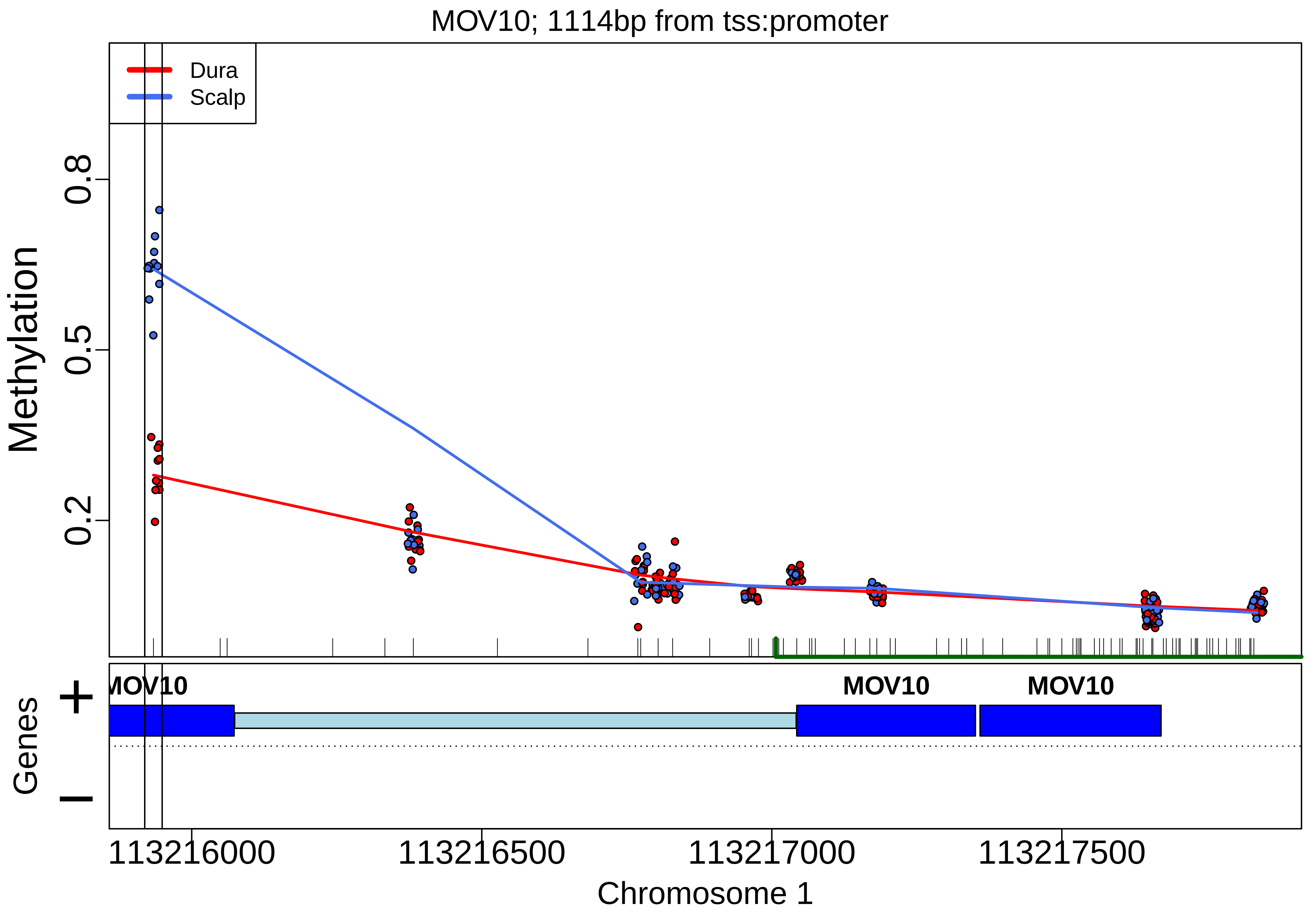
MOV10; 1114bp from tss:promoter
Dura
Scalp
Methylation
0.2
0.5
0.8
Genes
+
−
MOV10
MOV10
MOV10
113216000
113216500
113217000
113217500
Chromosome 1

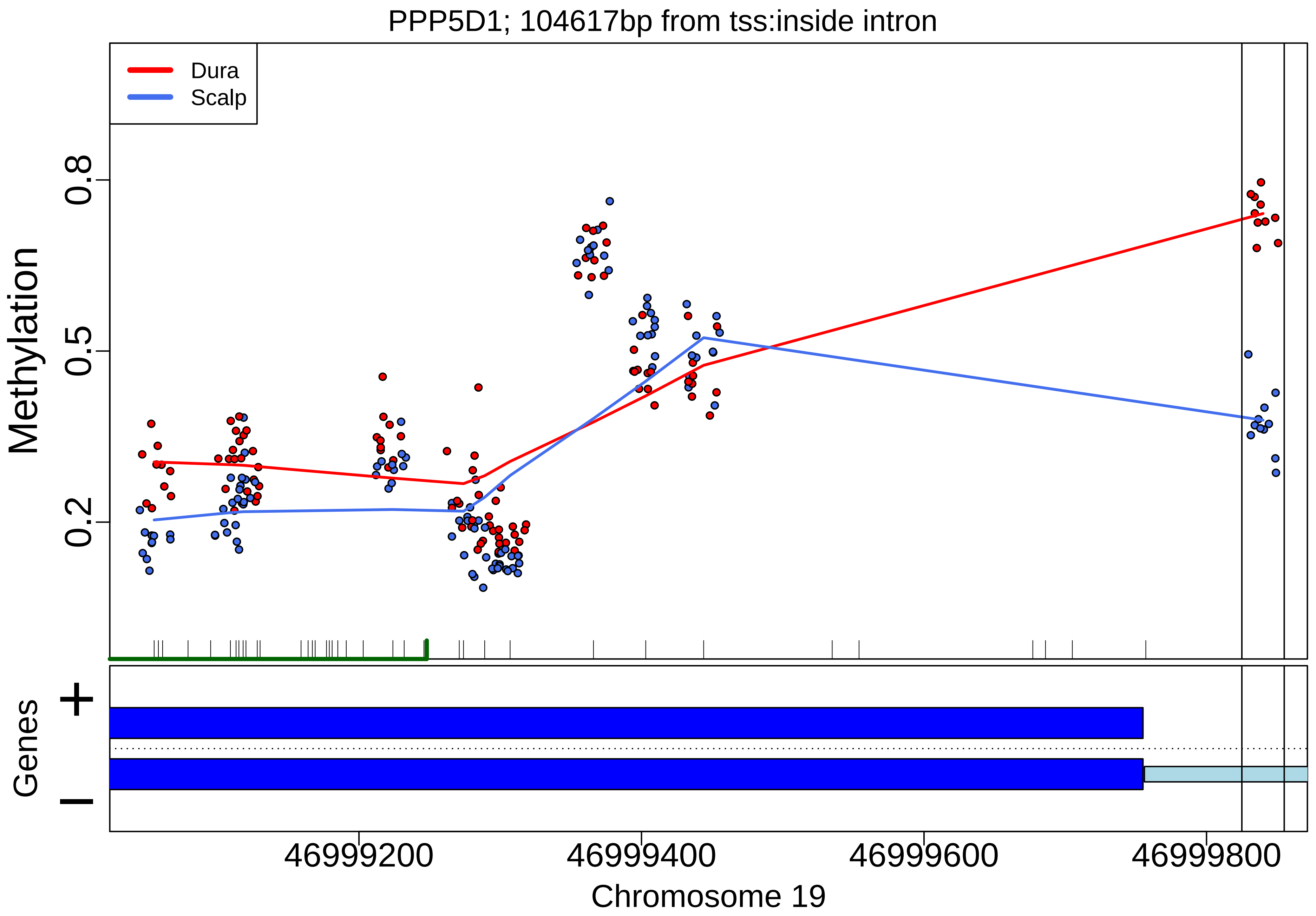
PPP5D1; 104617bp from tss:inside intron
Dura
Scalp
Methylation
0.2
0.5
0.8
Genes
+
−
46999200
46999400
46999600
46999800
Chromosome 19

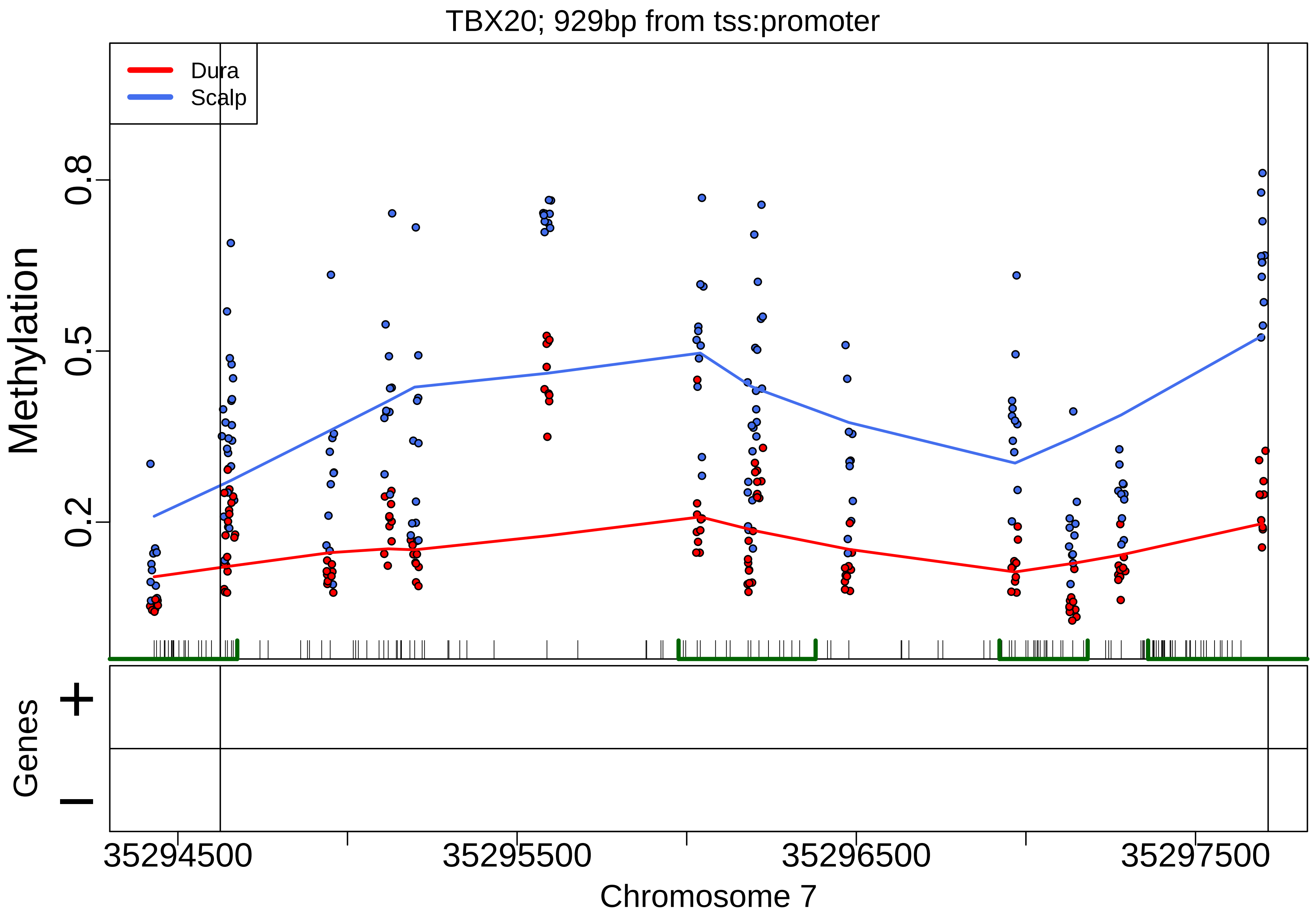

TBX20; 929bp from tss:promoter
Dura
Scalp
Methylation
0.2
0.5
0.8
Genes
+
-
35294500
35295500
35296500
35297500
Chromosome 7

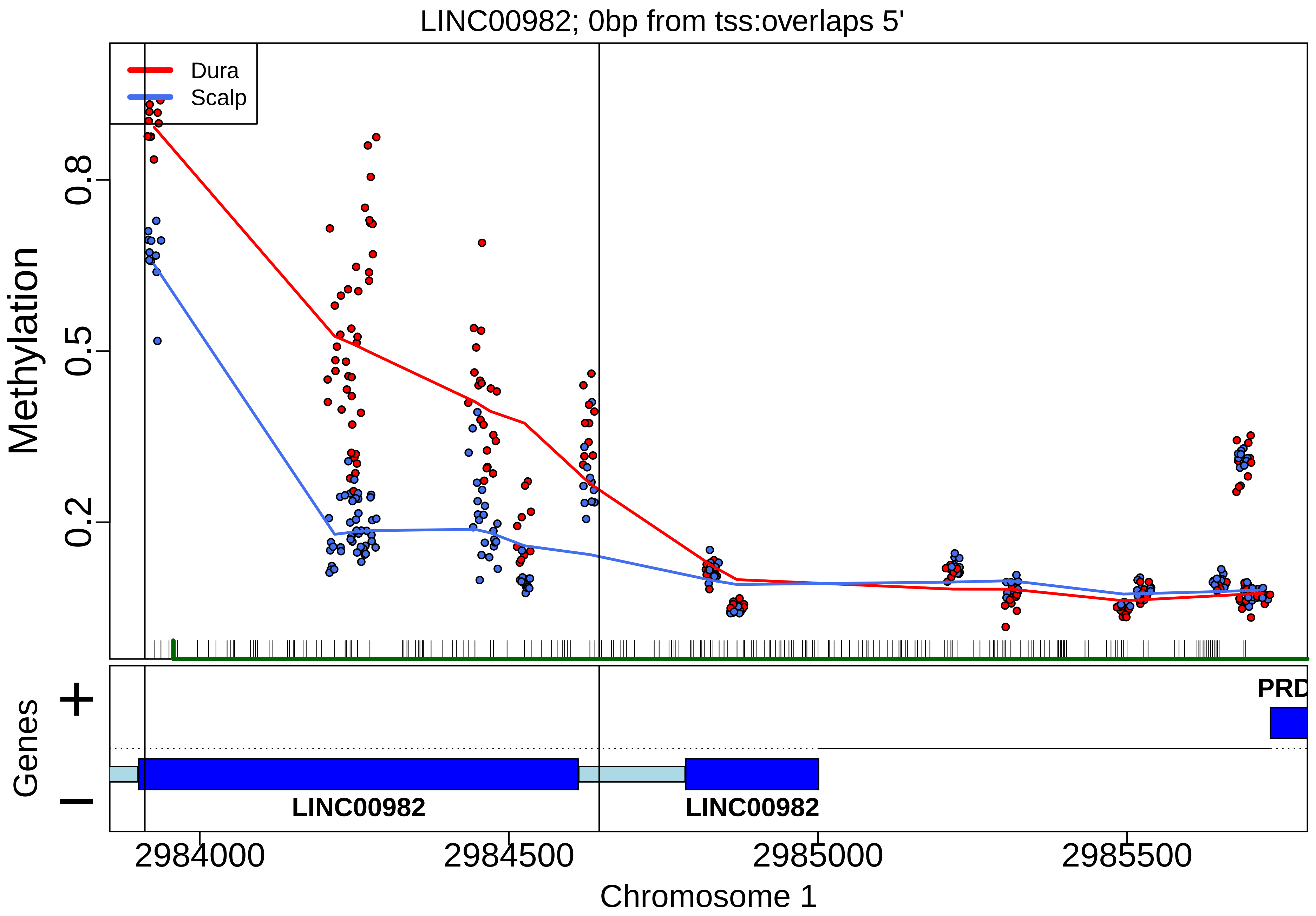

LINC00982; 0bp from tss:overlaps 5'
Dura
Scalp
Methylation
0.2
0.5
0.8
Genes
+
−
PRD
LINC00982
LINC00982
2984000
2984500
2985000
2985500
Chromosome 1

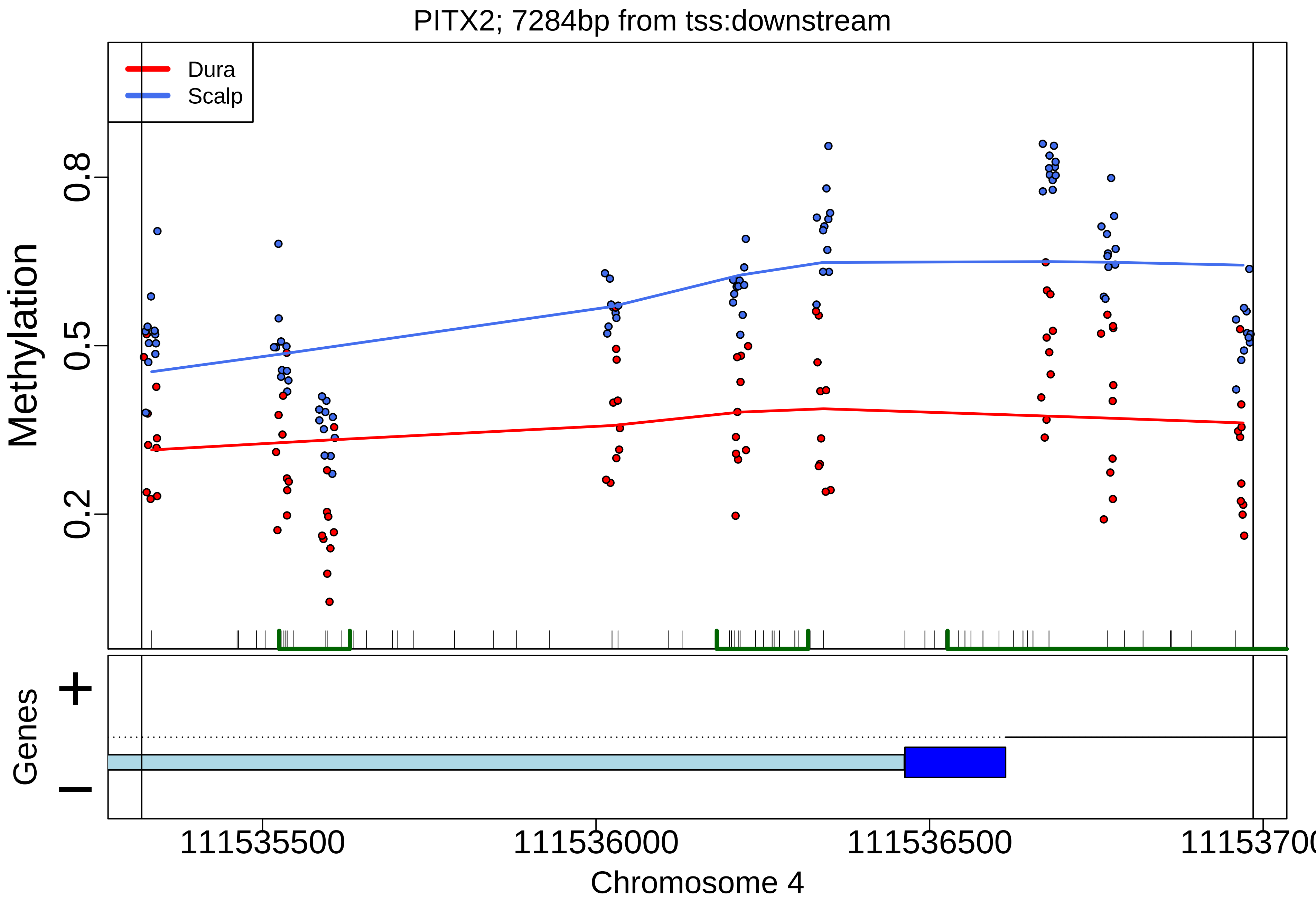

PITX2; 7284bp from tss:downstream
Dura
Scalp
Methylation
0.2
0.5
0.8
Genes
+
-
111535500
111536000
111536500
11153700
Chromosome 4

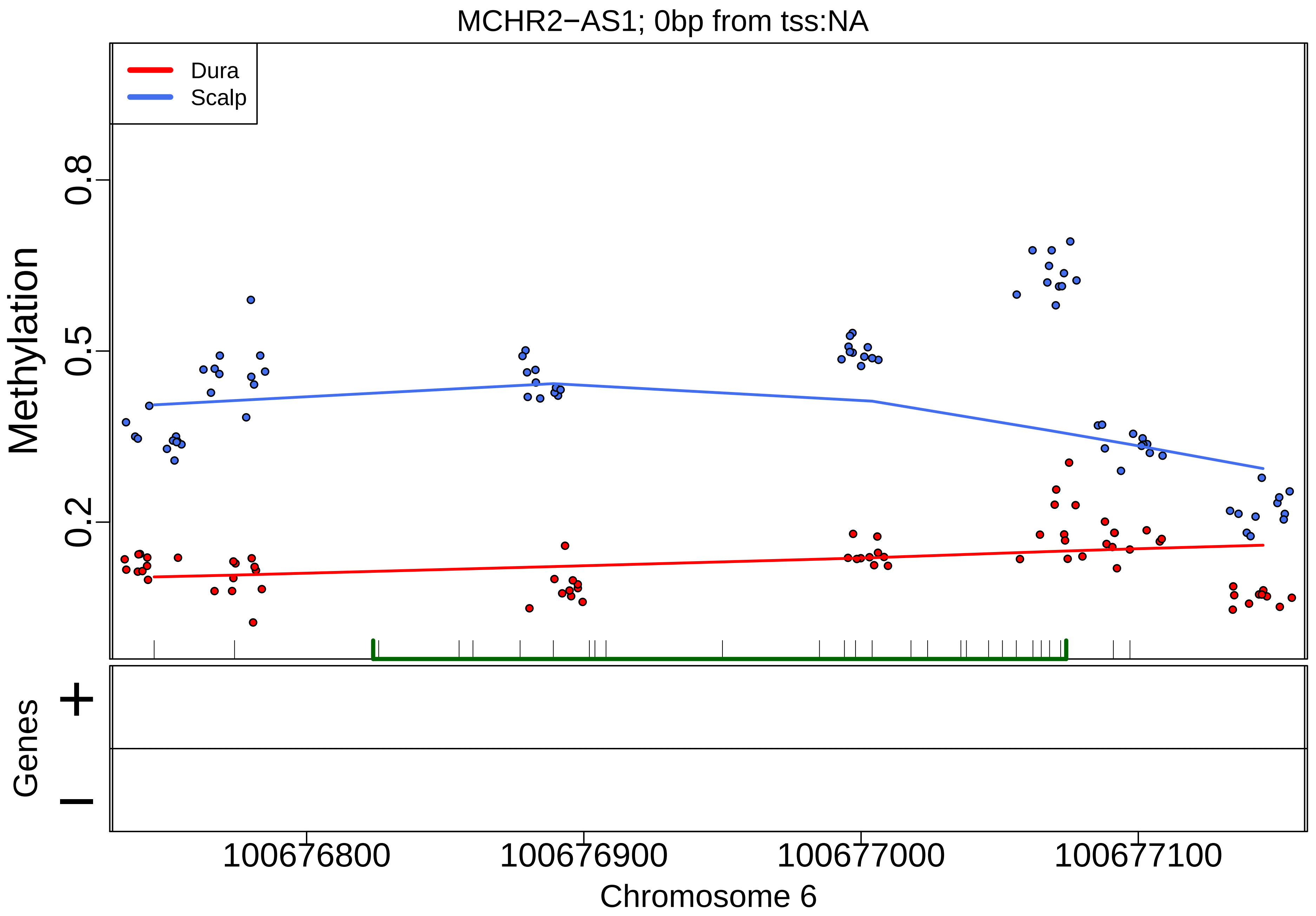

MCHR2−AS1; 0bp from tss:NA
Dura
Scalp
Methylation
0.2
0.5
0.8
Genes
+
−
100676800
100676900
100677000
100677100
Chromosome 6

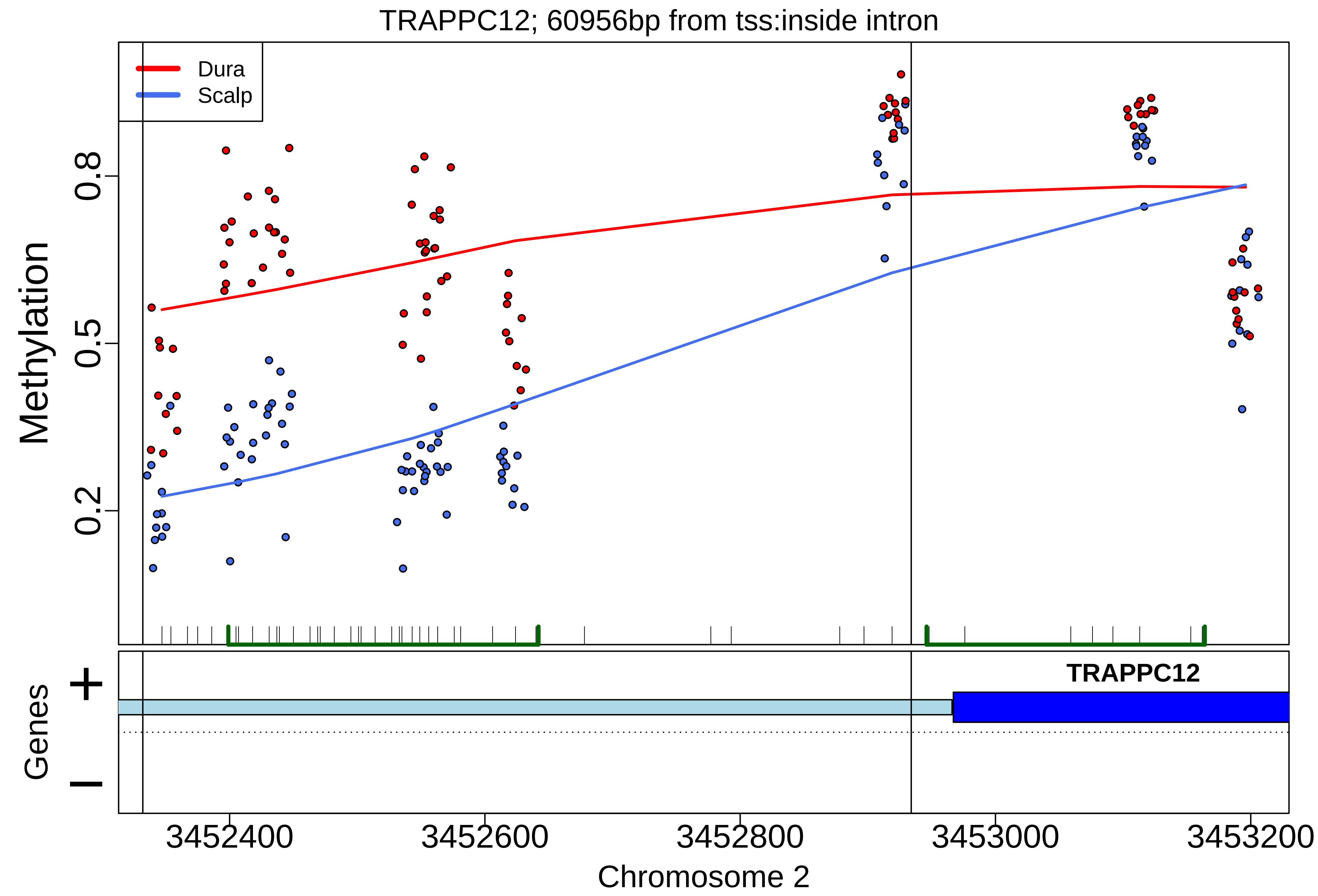
TRAPPC12; 60956bp from tss:inside intron
Dura
Scalp
Methylation
0.2
0.5
0.8
Genes
+
-
TRAPPC12
3452400
3452600
3452800
3453000
3453200
Chromosome 2

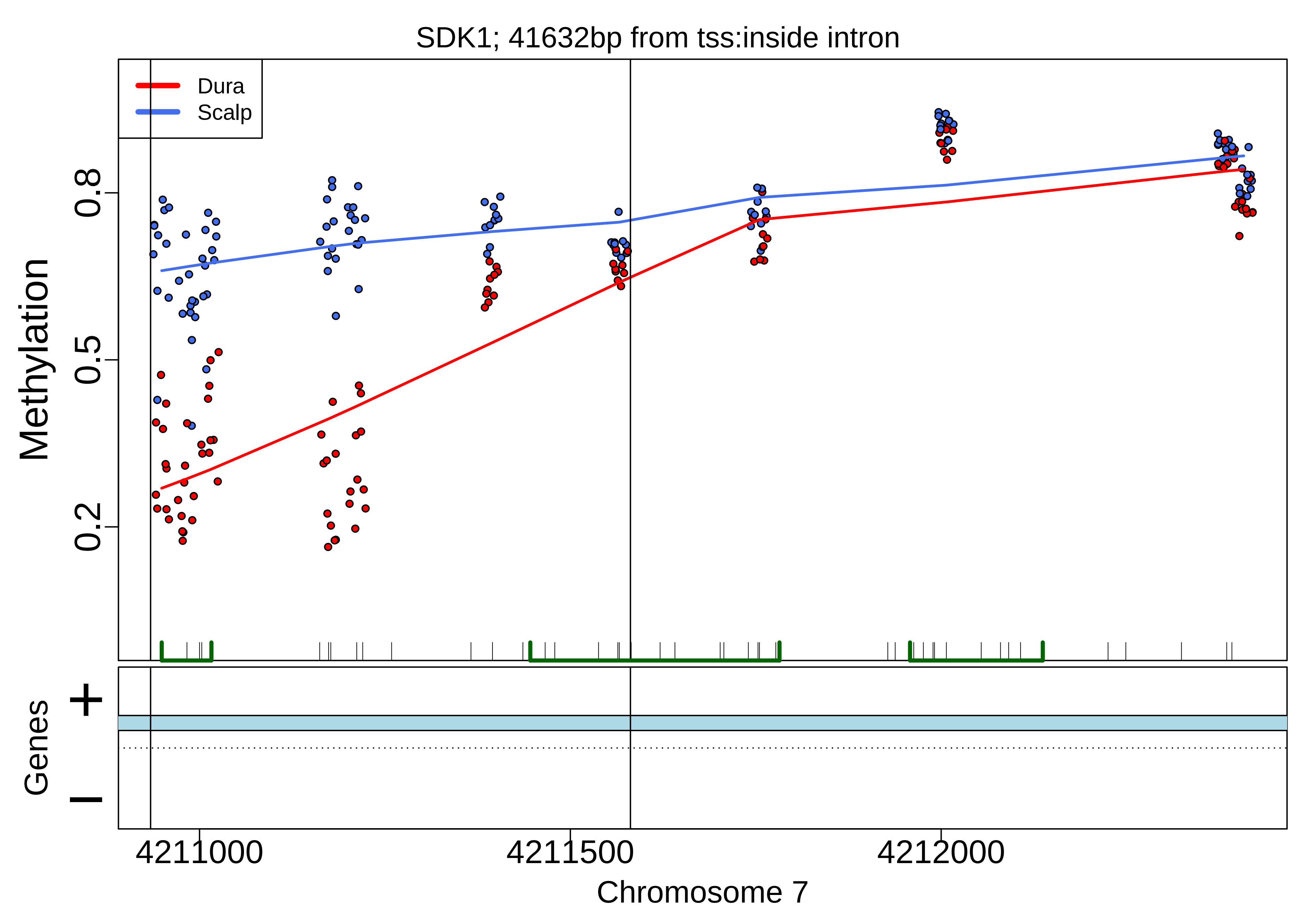
SDK1; 41632bp from tss:inside intron
Dura
Scalp
Methylation
0.2
0.5
0.8
Genes
+
-
4211000
4211500
4212000
Chromosome 7

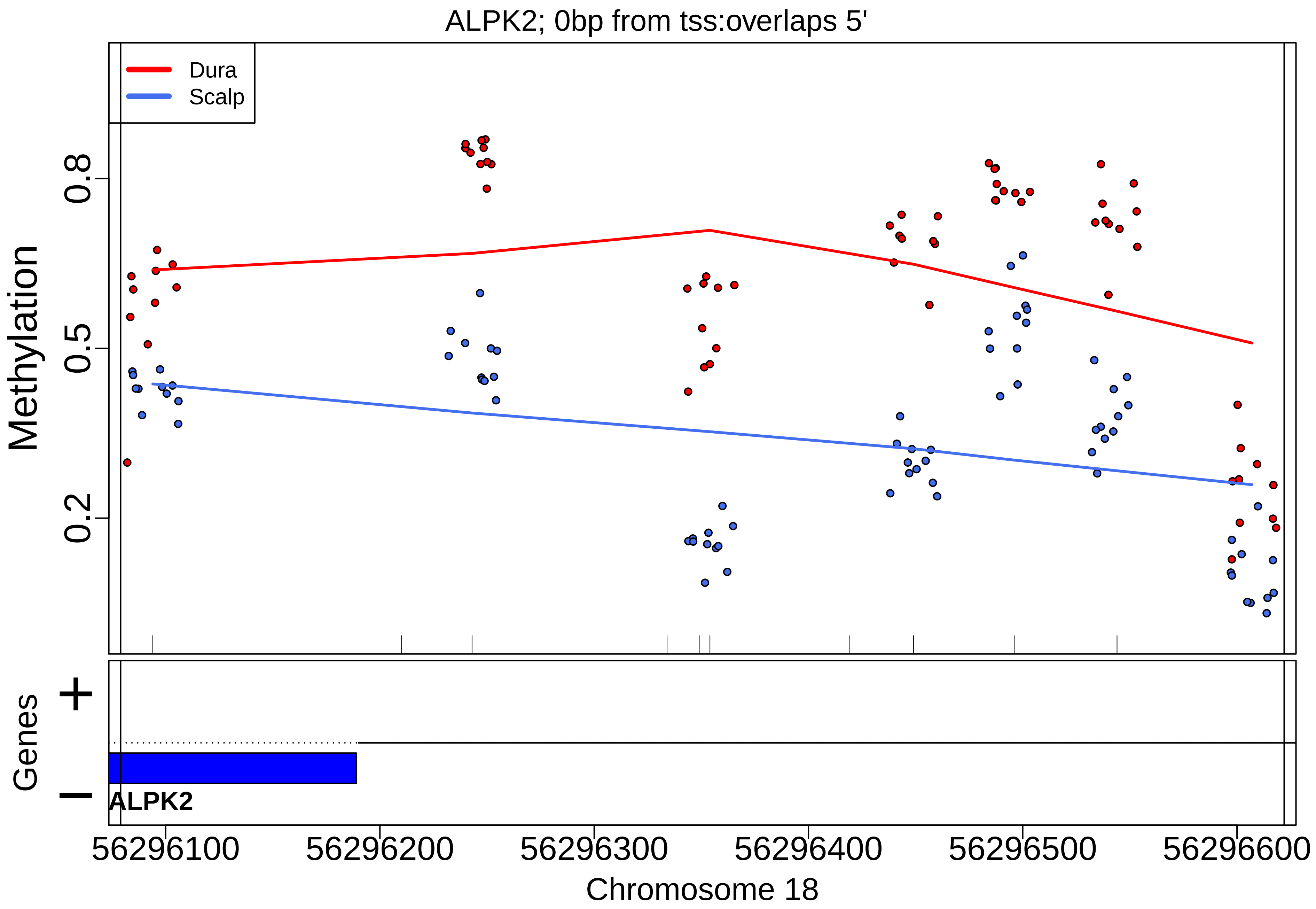
ALPK2; 0bp from tss:overlaps 5'
Dura
Scalp
Methylation
0.2
0.5
0.8
Genes
+
−
ALPK2
56296100
56296200
56296300
56296400
56296500
56296600
Chromosome 18

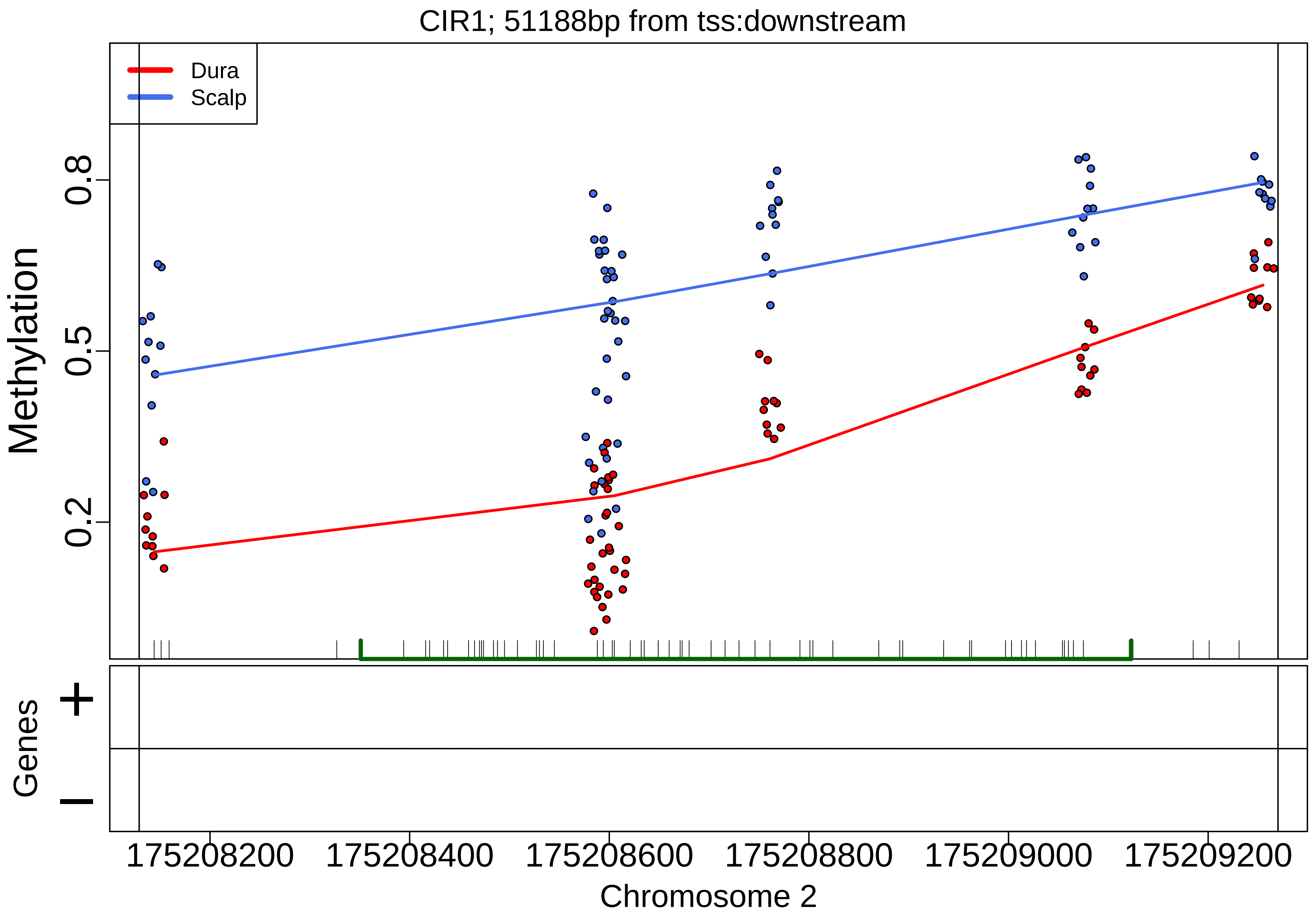

CIR1; 51188bp from tss:downstream
Dura
Scalp
Methylation
0.2
0.5
0.8
Genes
+
−
175208200
175208400
175208600
175208800
175209000
175209200
Chromosome 2

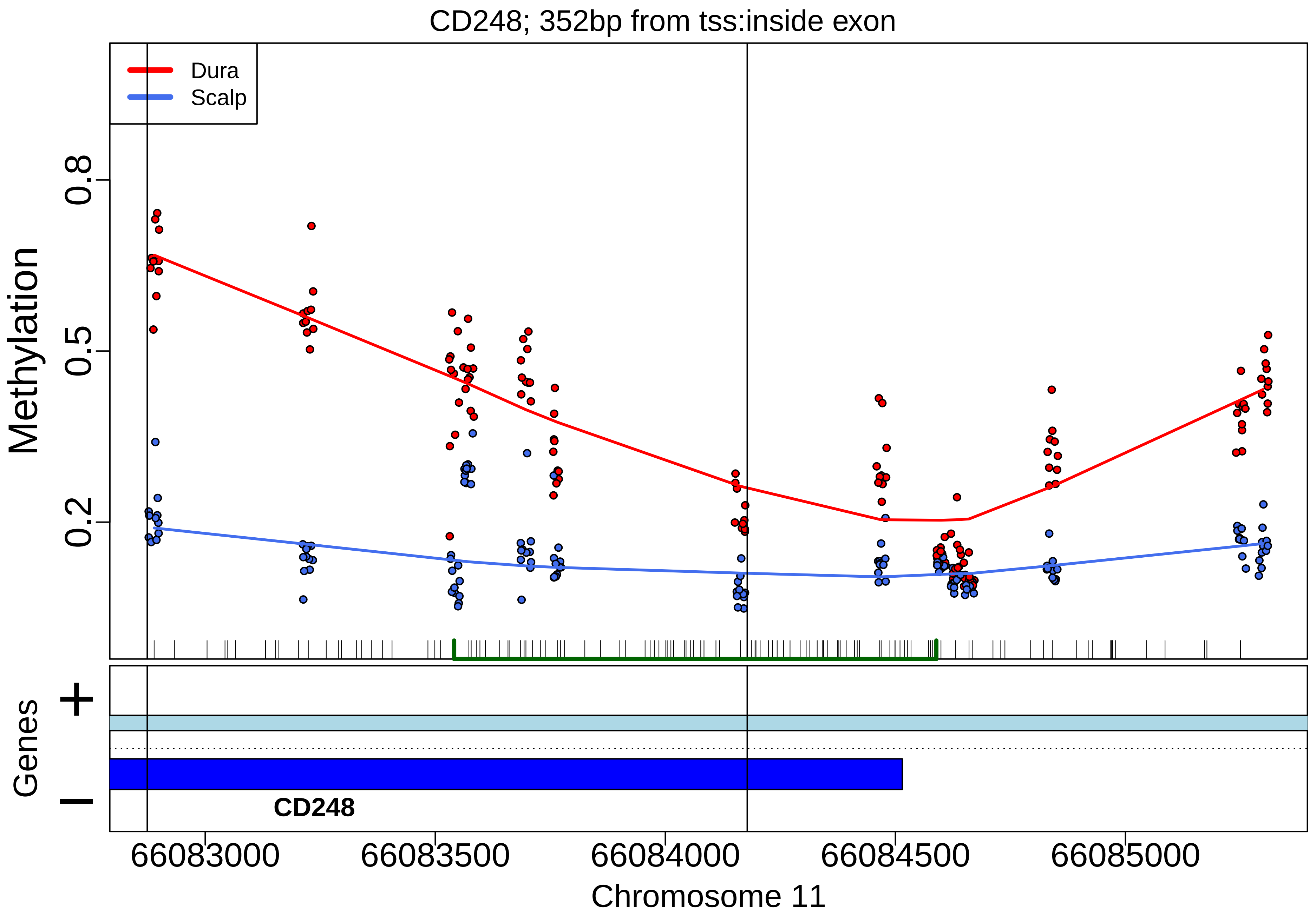

CD248; 352bp from tss:inside exon
Dura
Scalp
Methylation
0.2
0.5
0.8
Genes
+
-
CD248
66083000
66083500
66084000
66084500
66085000
Chromosome 11

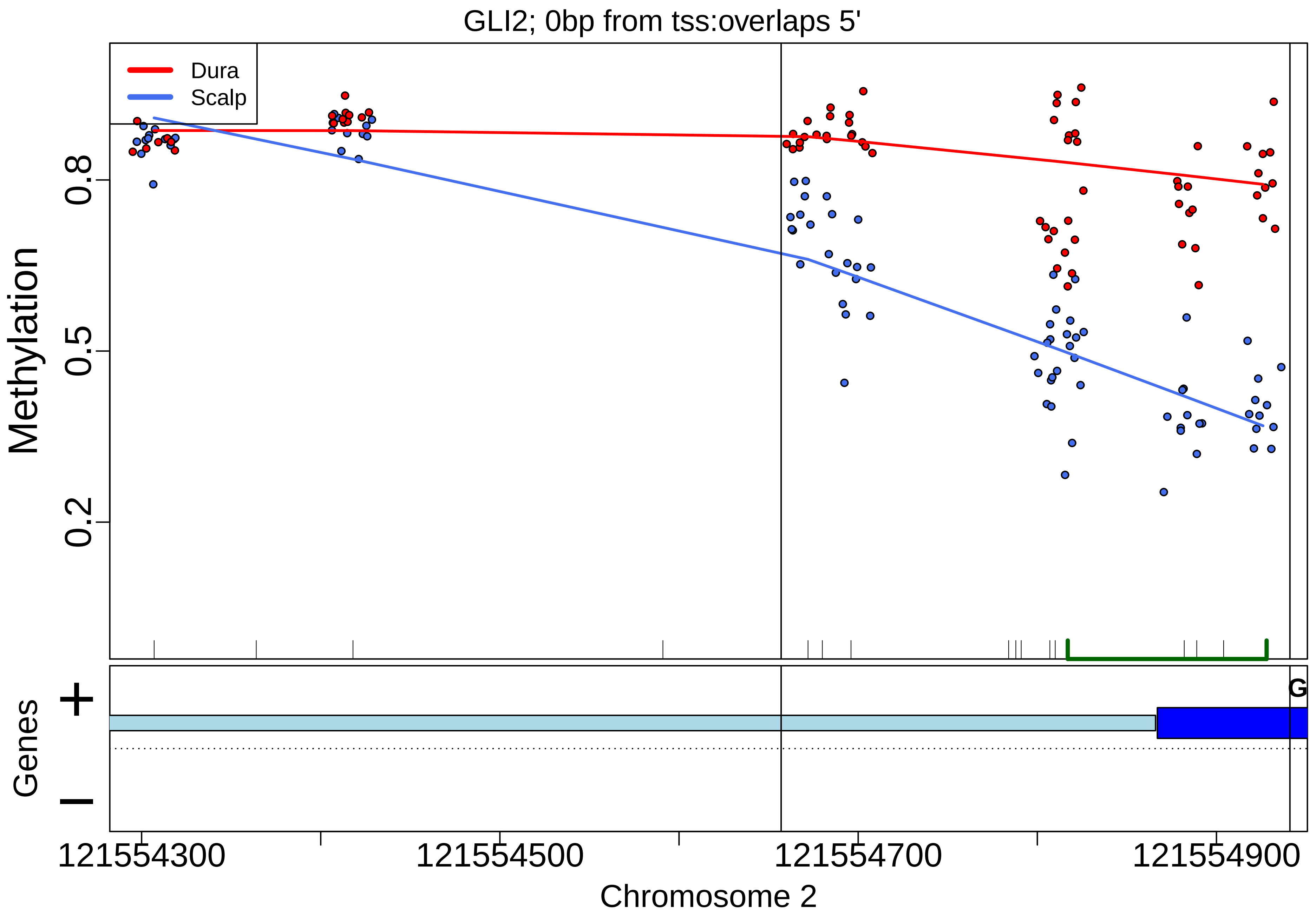

GLI2; 0bp from tss:overlaps 5'
Dura
Scalp
Methylation
0.2
0.5
0.8
Genes
+
-
G
121554300
121554500
121554700
121554900
Chromosome 2

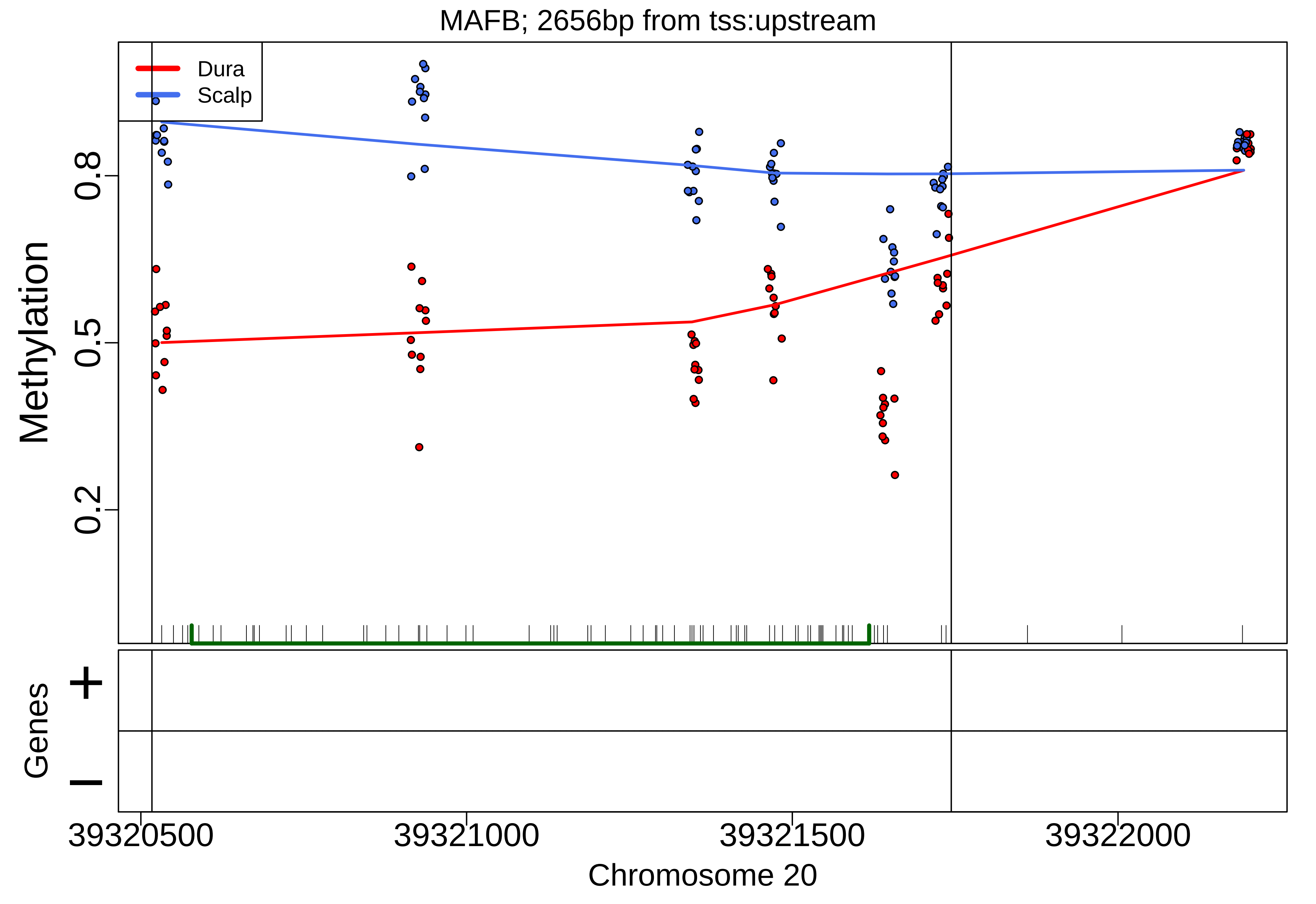
MAFB; 2656bp from tss:upstream
Dura
Scalp
Methylation
0.2
0.5
0.8
Genes
+
-
39320500
39321000
39321500
39322000
Chromosome 20

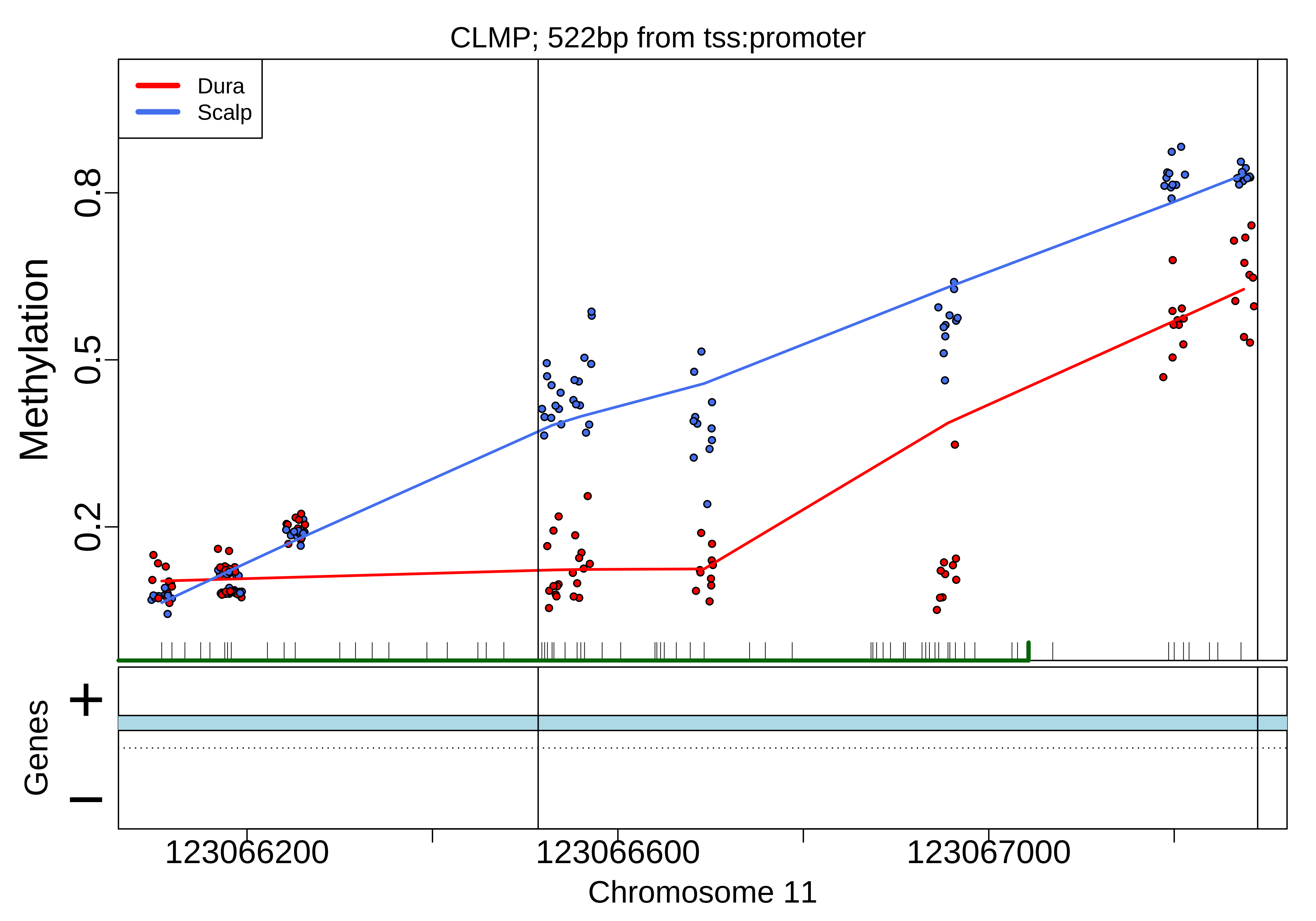
CLMP; 522bp from tss:promoter
Dura
Scalp
Methylation
0.2
0.5
0.8
Genes
+
−
123066200
123066600
123067000
Chromosome 11

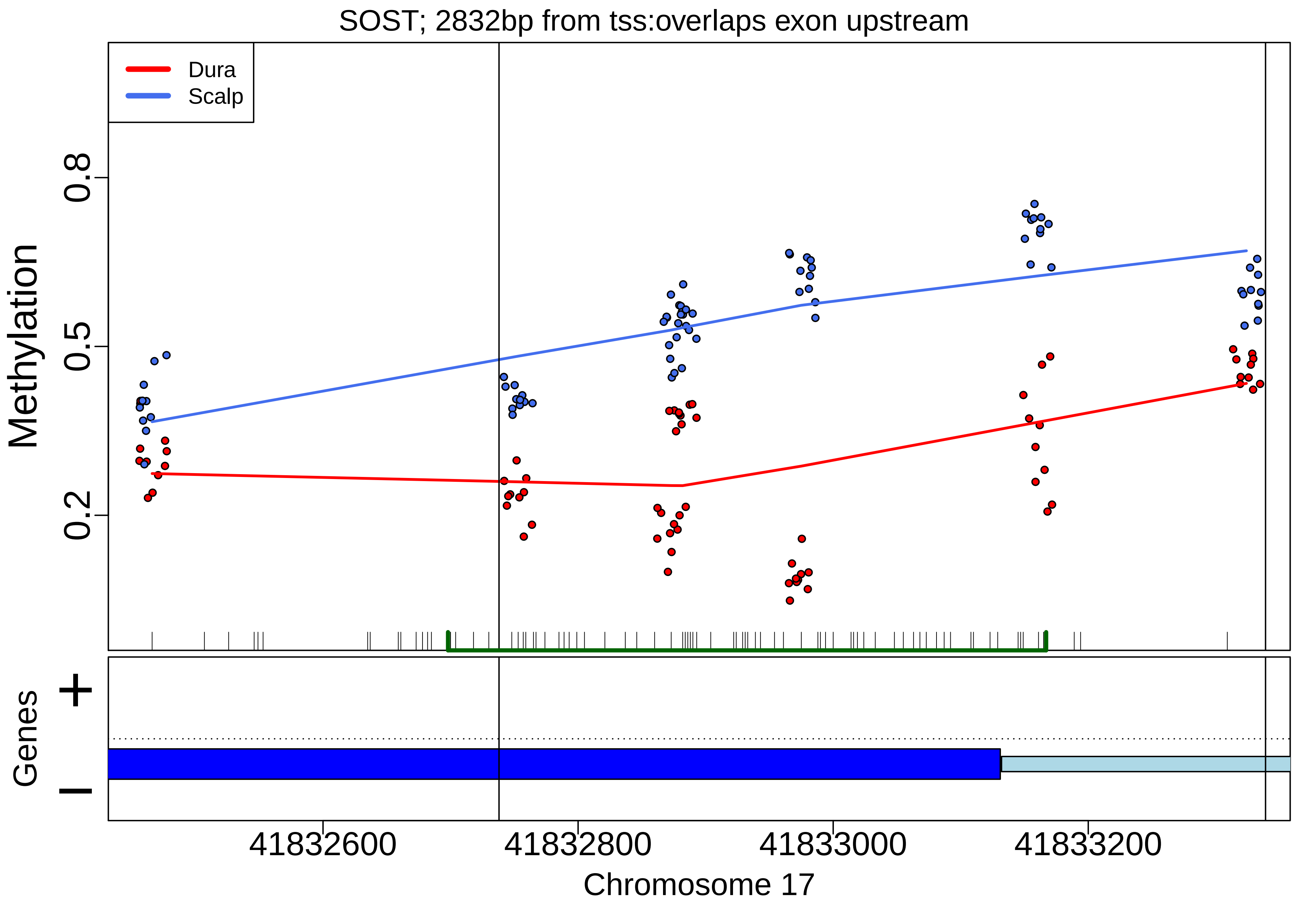
SOST; 2832bp from tss:overlaps exon upstream
Dura
Scalp
Methylation
0.2
0.5
0.8
Genes
+
−
41832600
41832800
41833000
41833200
Chromosome 17

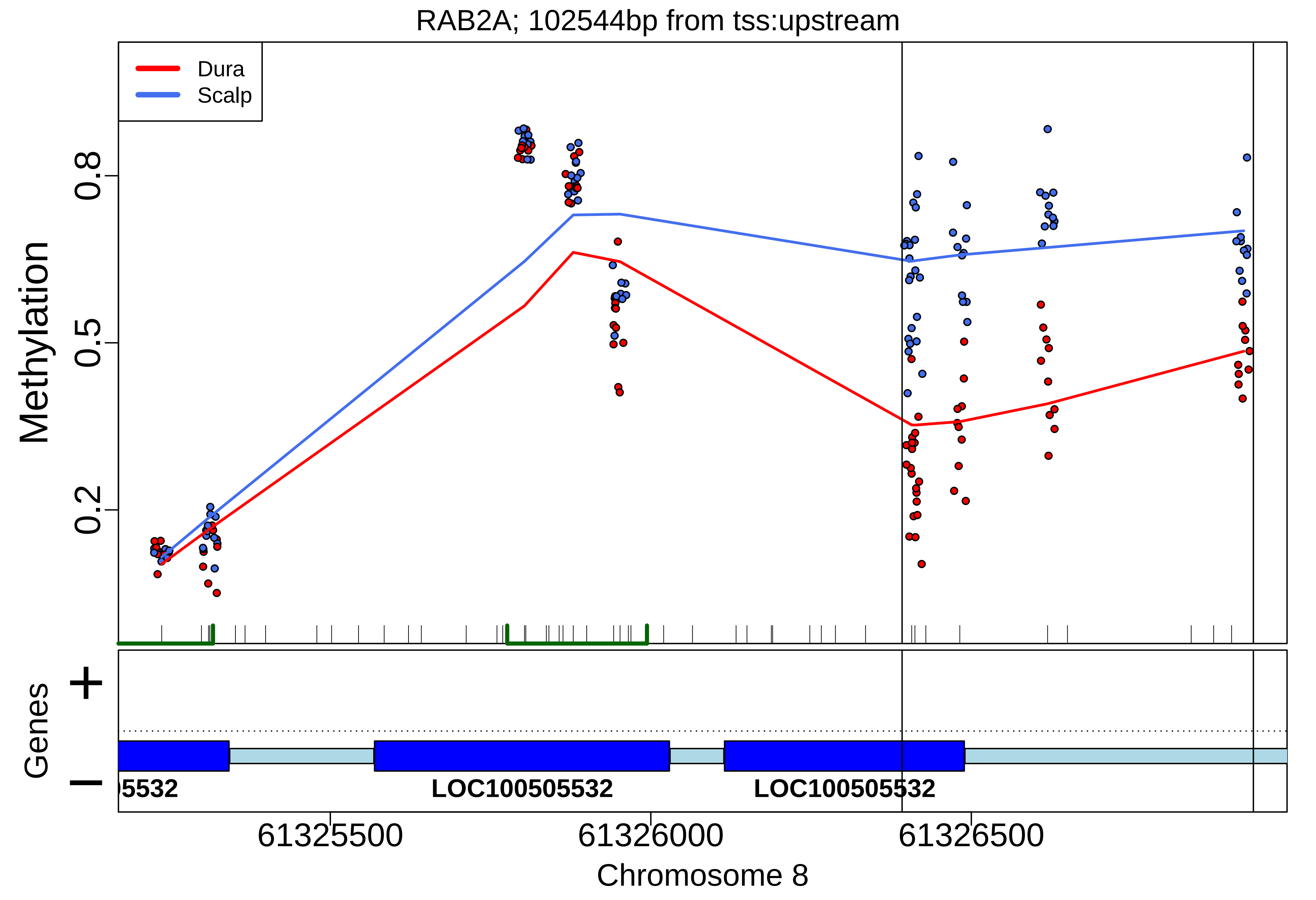
RAB2A; 102544bp from tss:upstream
Dura
Scalp
Methylation
0.2
0.5
0.8
Genes
+
-
)5532
LOC100505532
LOC100505532
61325500
61326000
61326500
Chromosome 8

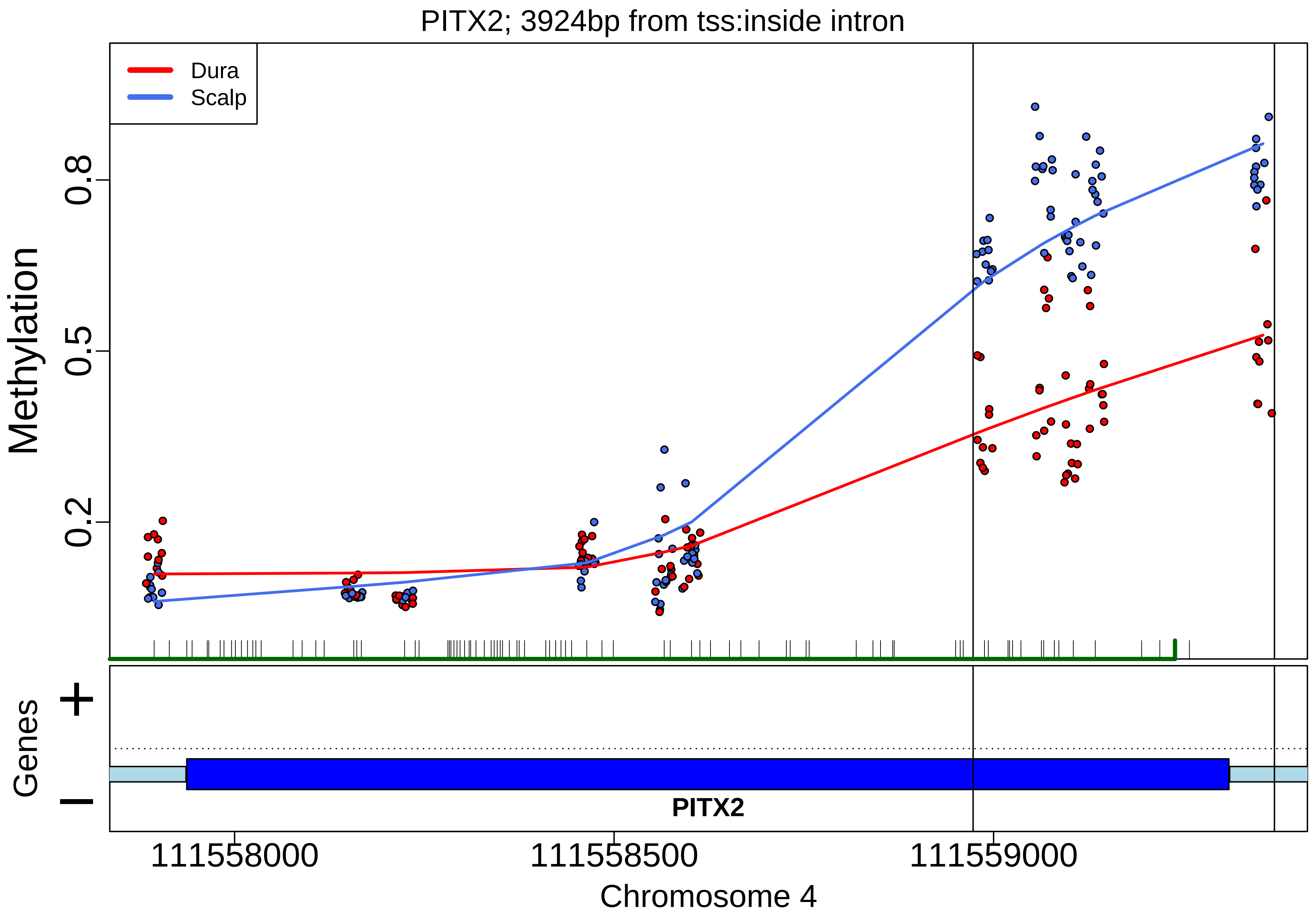
PITX2; 3924bp from tss:inside intron
Dura
Scalp
Methylation
0.2
0.5
0.8
Genes
+
−
PITX2
111558000
111558500
111559000
Chromosome 4

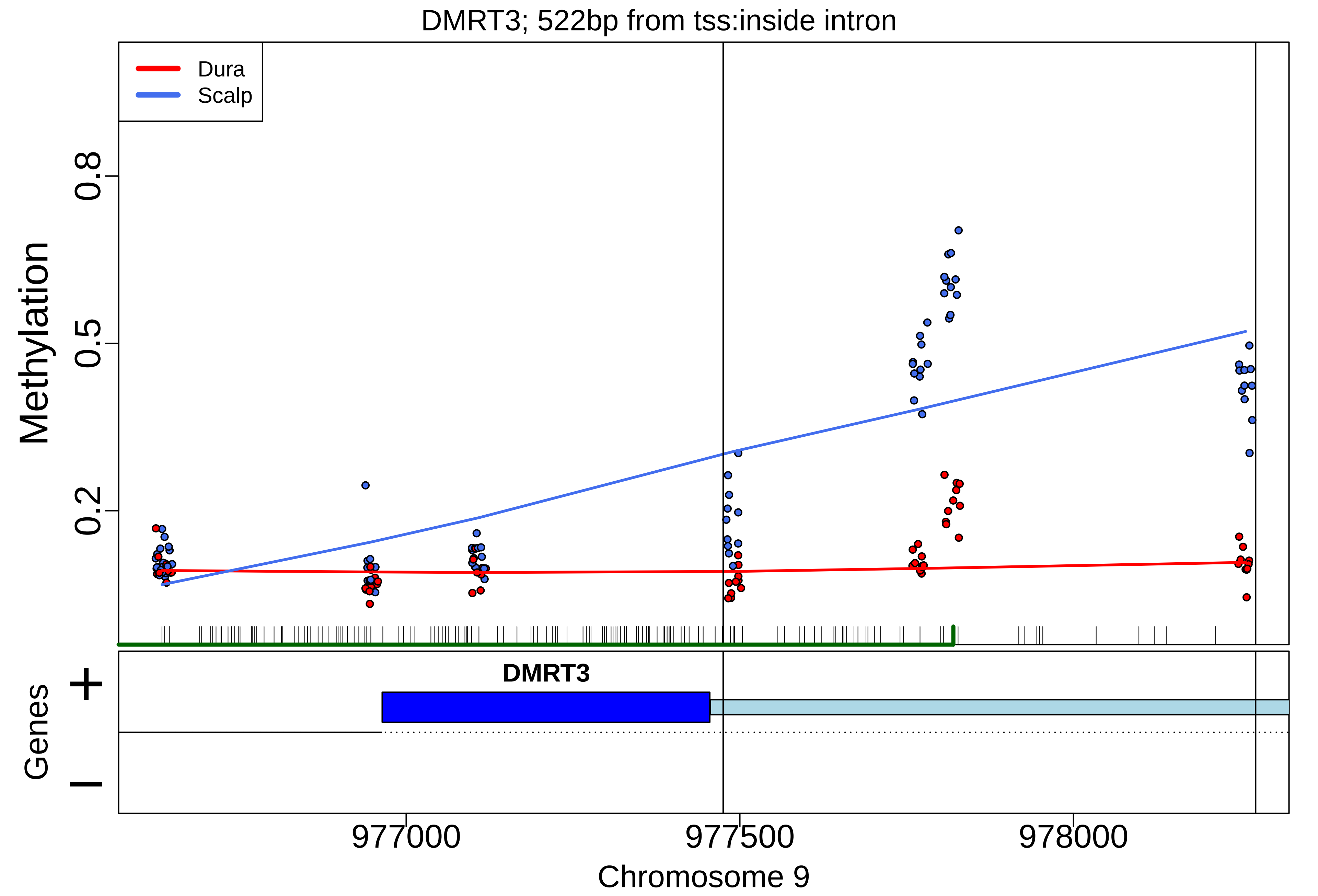

DMRT3; 522bp from tss:inside intron
Dura
Scalp
Methylation
0.2
0.5
0.8
Genes
+
-
DMRT3
977000
977500
978000
Chromosome 9

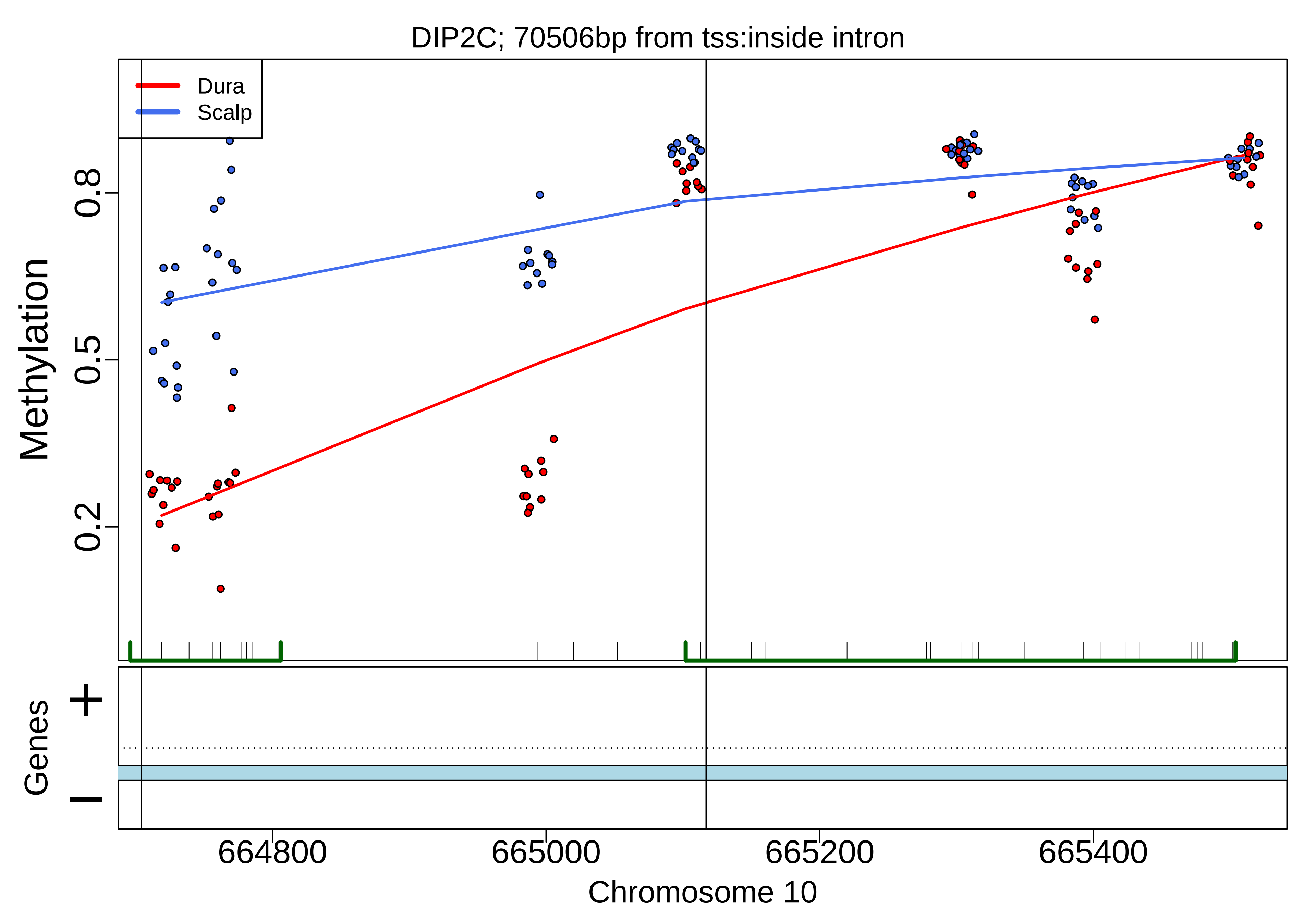
DIP2C; 70506bp from tss:inside intron
Dura
Scalp
Methylation
0.2
0.5
0.8
Genes
+
-
664800
665000
665200
665400
Chromosome 10

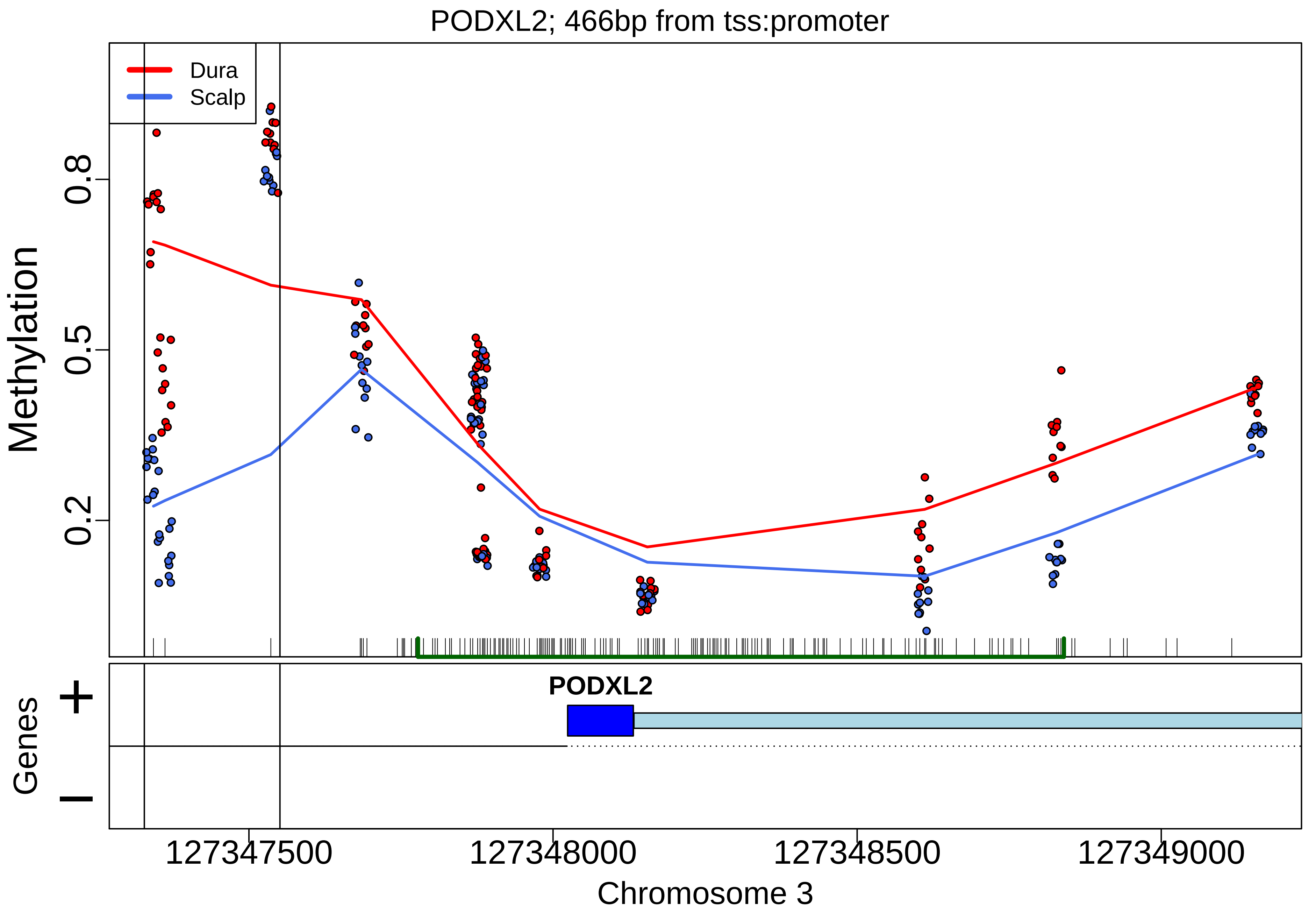
PODXL2; 466bp from tss:promoter
Dura
Scalp
Methylation
0.2
0.5
0.8
Genes
+
-
PODXL2
127347500
127348000
127348500
127349000
Chromosome 3

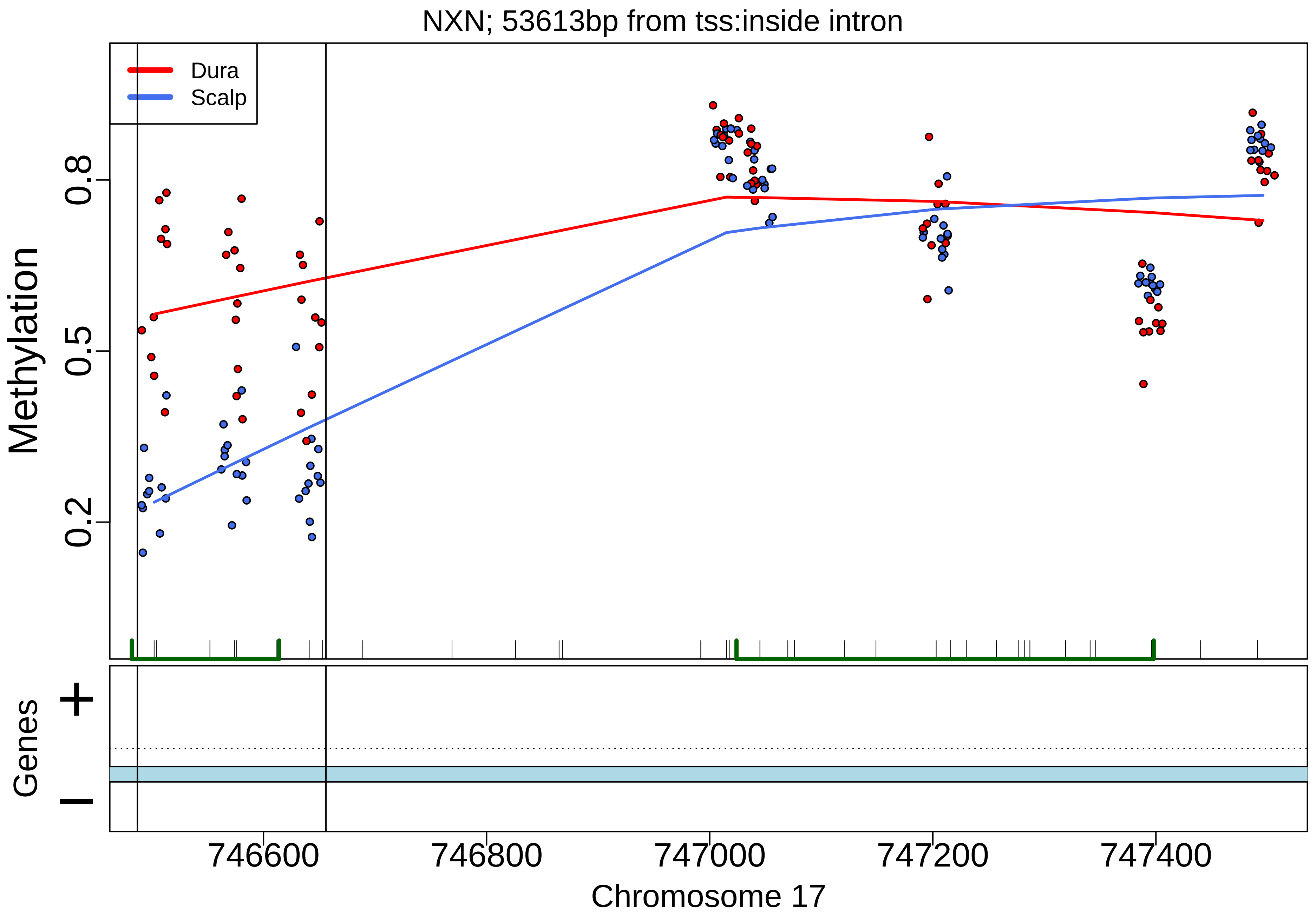

NXN; 53613bp from tss:inside intron
Dura
Scalp
Methylation
0.2
0.5
0.8
Genes
+
−
746600
746800
747000
747200
747400
Chromosome 17

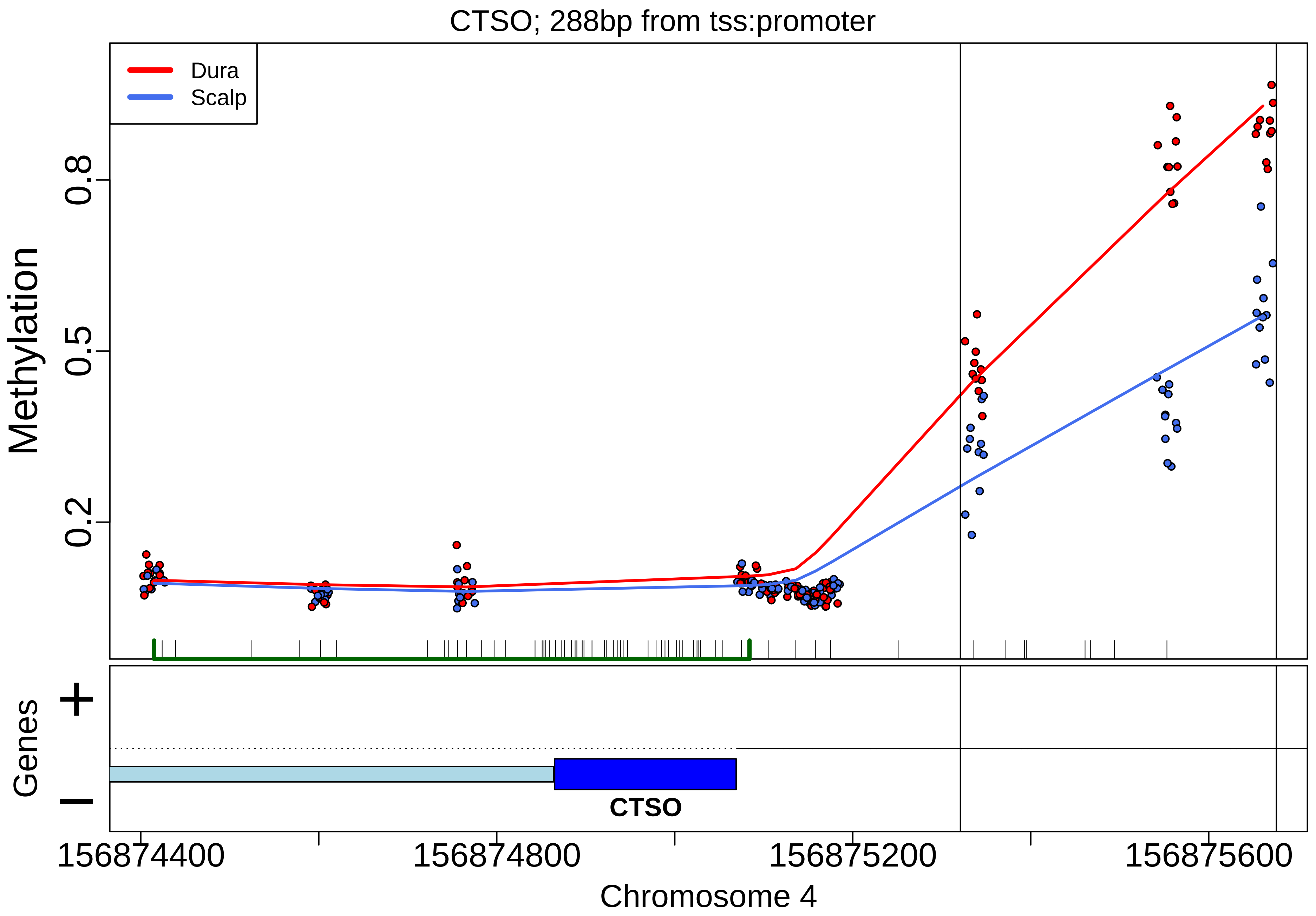
CTSO; 288bp from tss:promoter
Dura
Scalp
Methylation
0.2
0.5
0.8
Genes
+
−
CTSO
156874400
156874800
156875200
156875600
Chromosome 4

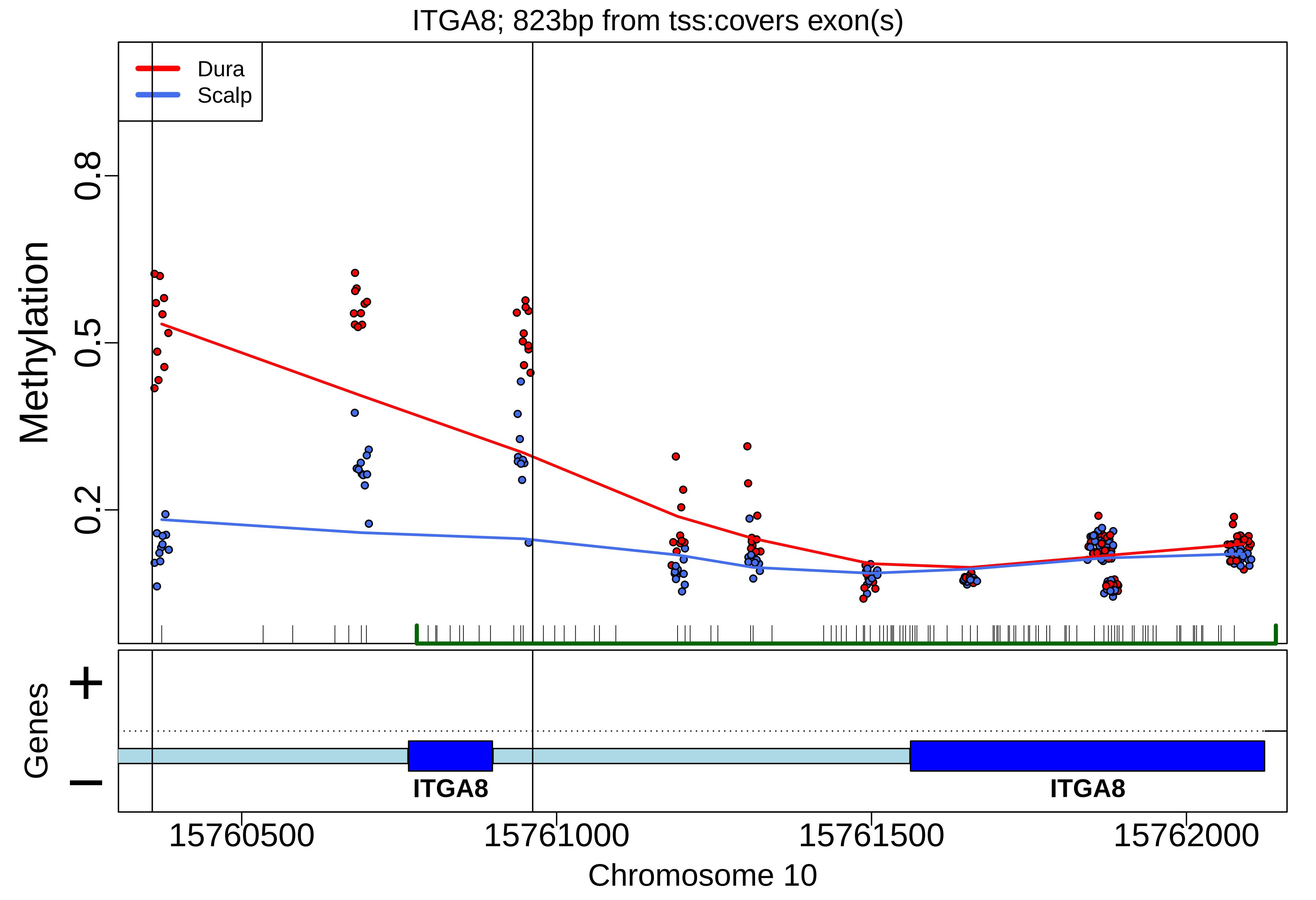

ITGA8; 823bp from tss:covers exon(s)
Dura
Scalp
Methylation
0.8
0.5
0.2
Genes
+
−
ITGA8
ITGA8
15760500
15761000
15761500
15762000
Chromosome 10

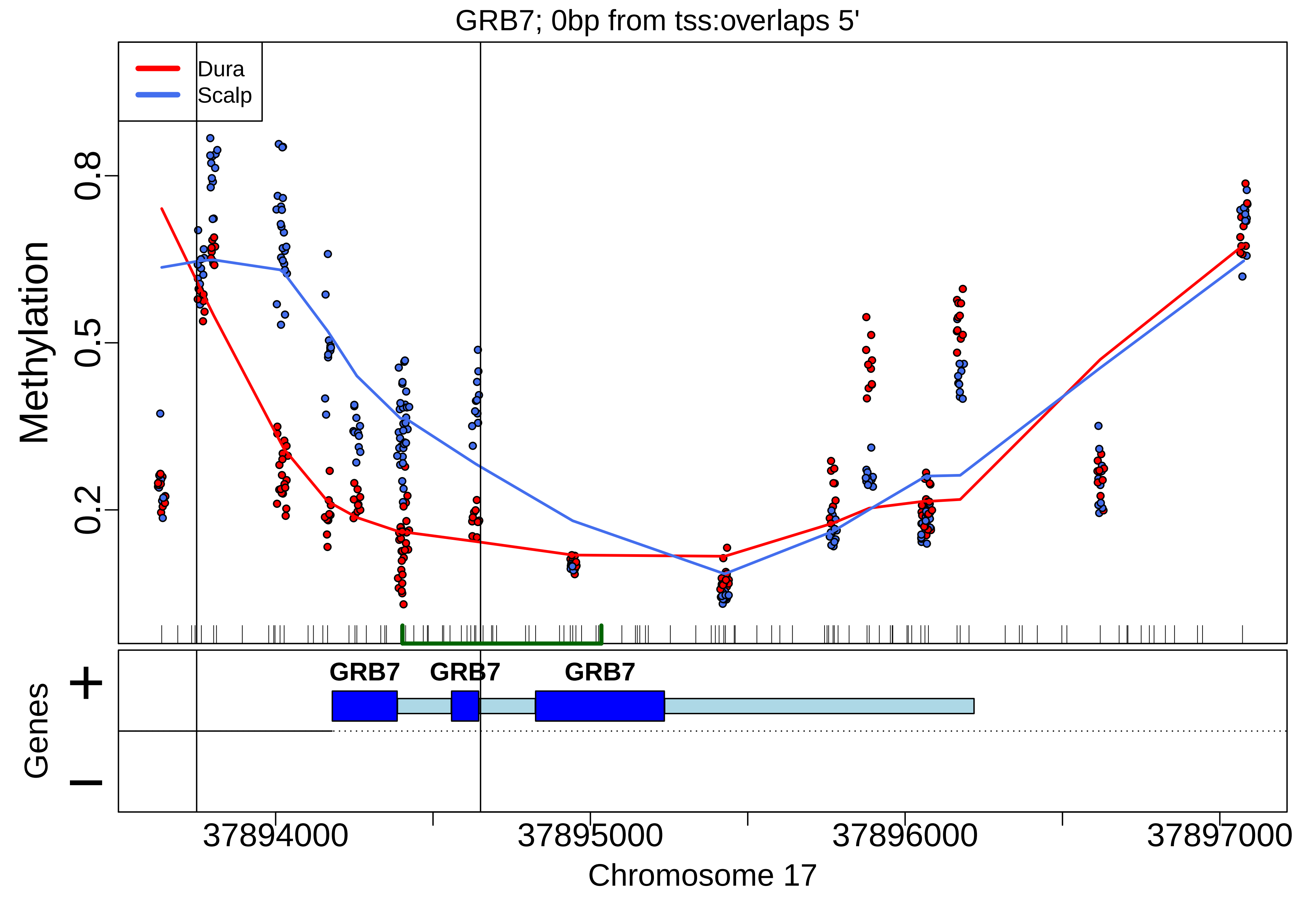
GRB7; 0bp from tss:overlaps 5'
Dura
Scalp
Methylation
0.2
0.5
0.8
Genes
+
-
GRB7
GRB7
GRB7
37894000
37895000
37896000
37897000
Chromosome 17

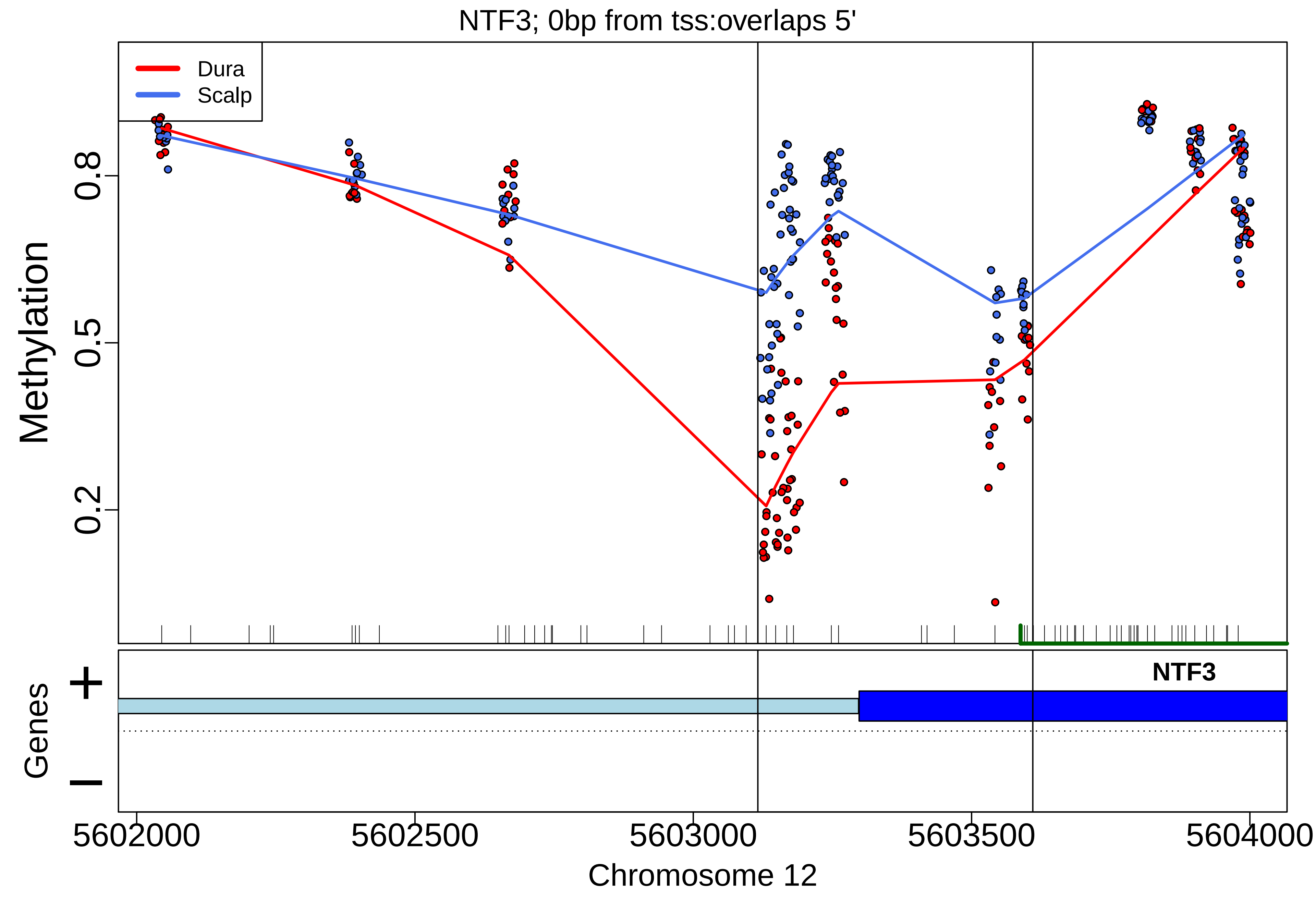

NTF3; 0bp from tss:overlaps 5'
Dura
Scalp
Methylation
0.2
0.5
0.8
Genes
+
-
NTF3
5602000
5602500
5603000
5603500
5604000
Chromosome 12

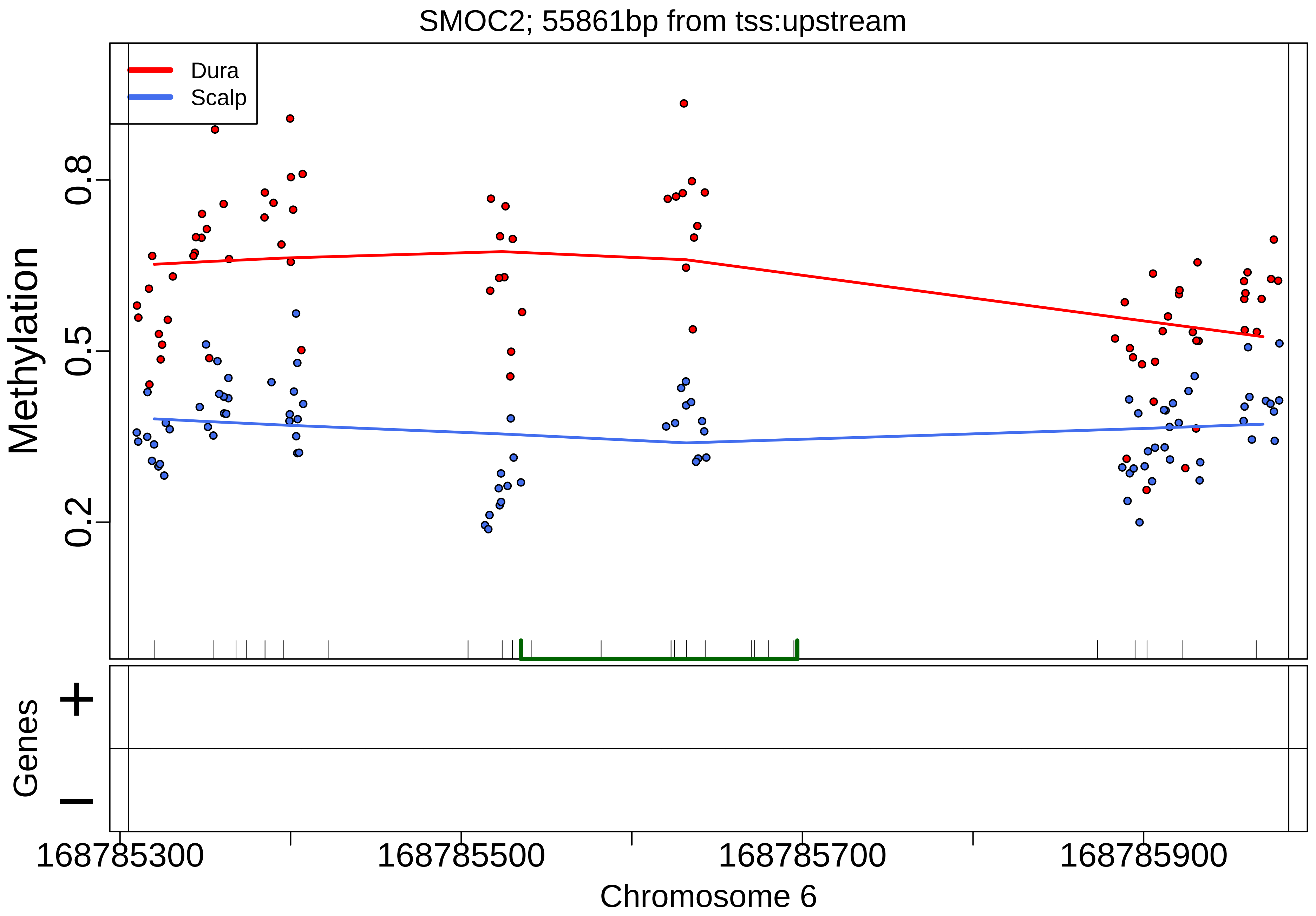
SMOC2; 55861bp from tss:upstream
Dura
Scalp
Methylation
0.2
0.5
0.8
Genes
+
-
168785300
168785500
168785700
168785900
Chromosome 6

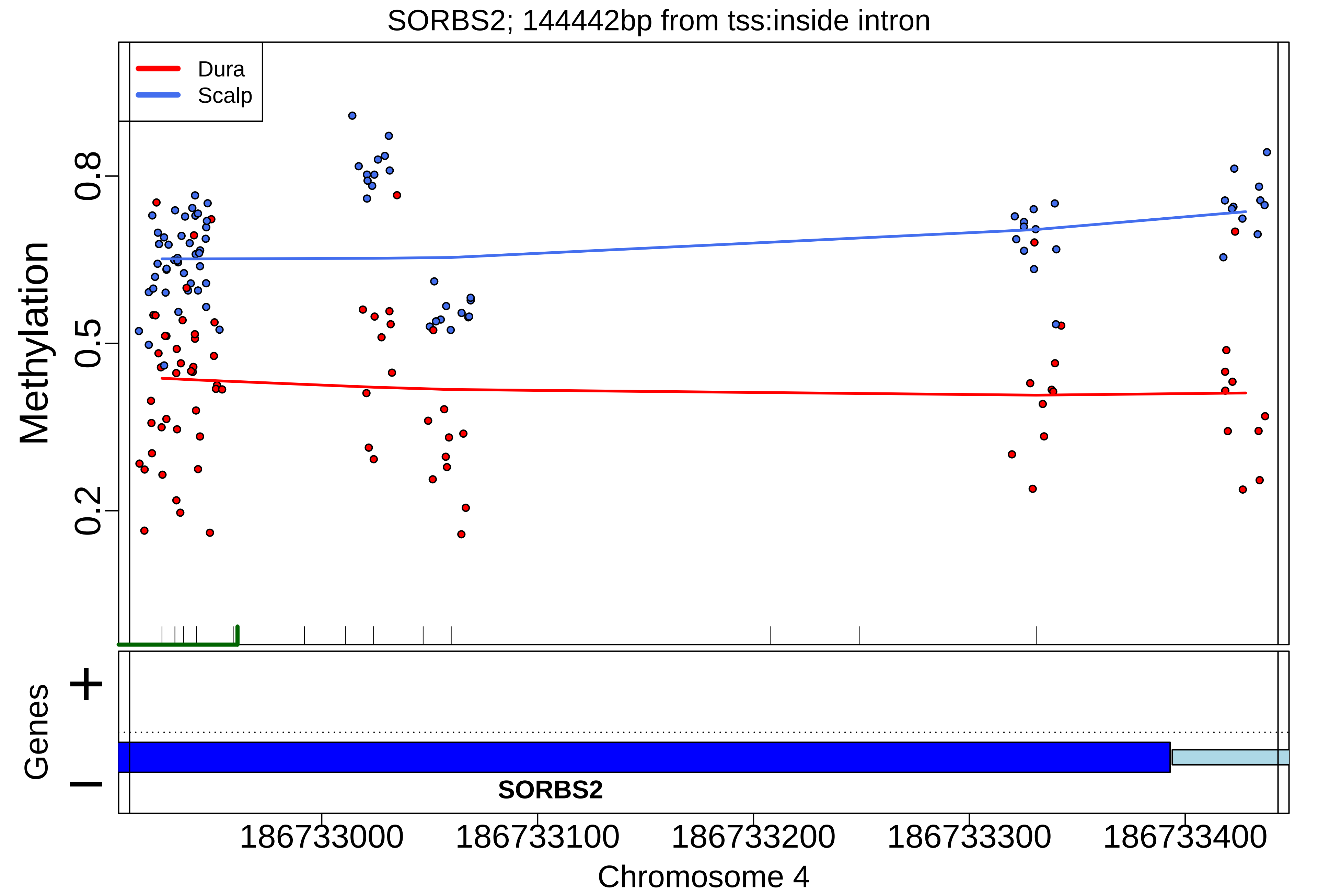
SORBS2; 144442bp from tss:inside intron
Dura
Scalp
Methylation
0.2
0.5
0.8
Genes
+
-
SORBS2
186733000
186733100
186733200
186733300
186733400
Chromosome 4

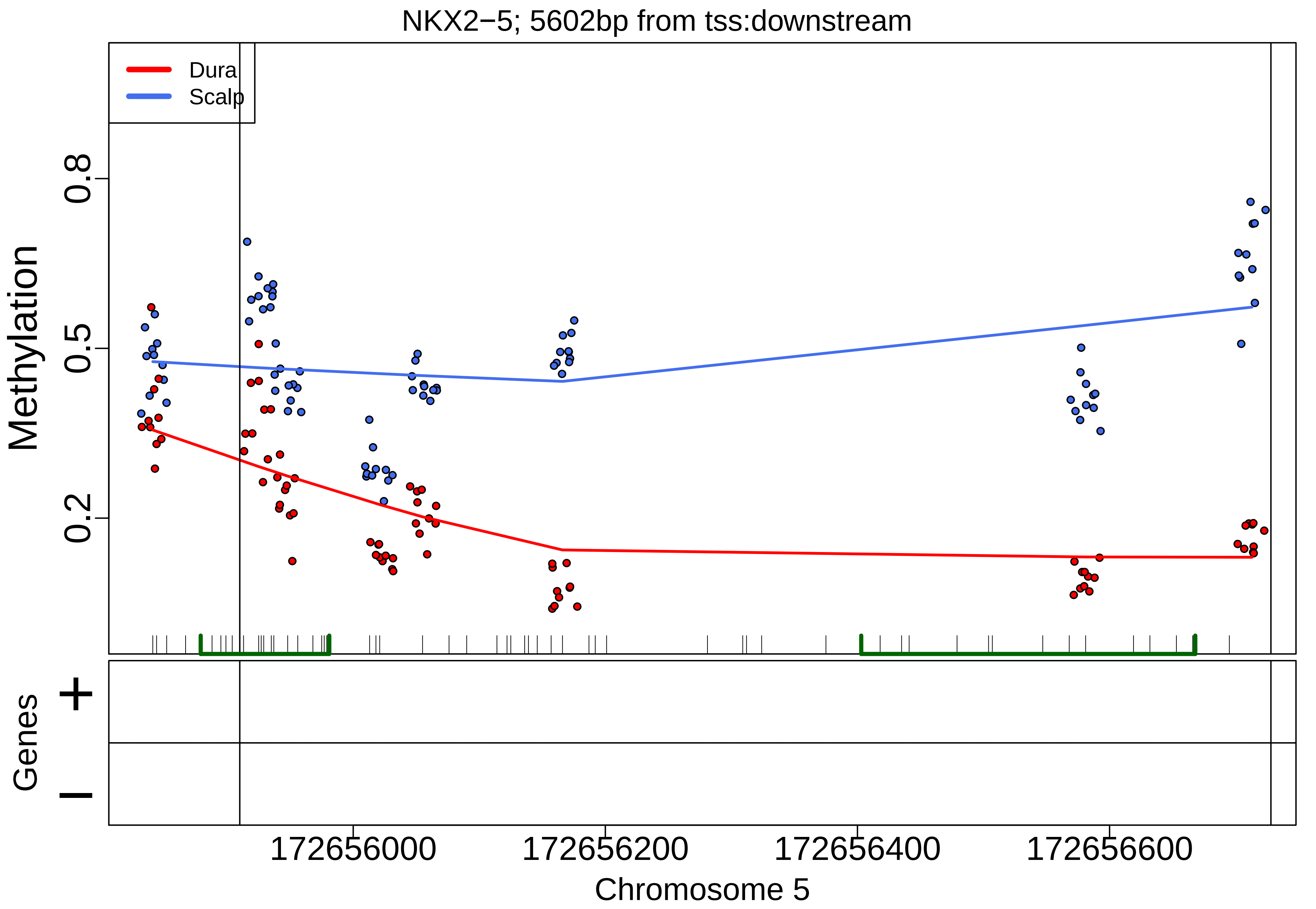

NKX2−5; 5602bp from tss:downstream
Dura
Scalp
Methylation
0.2
0.5
0.8
Genes
+
−
172656000
172656200
172656400
172656600
Chromosome 5

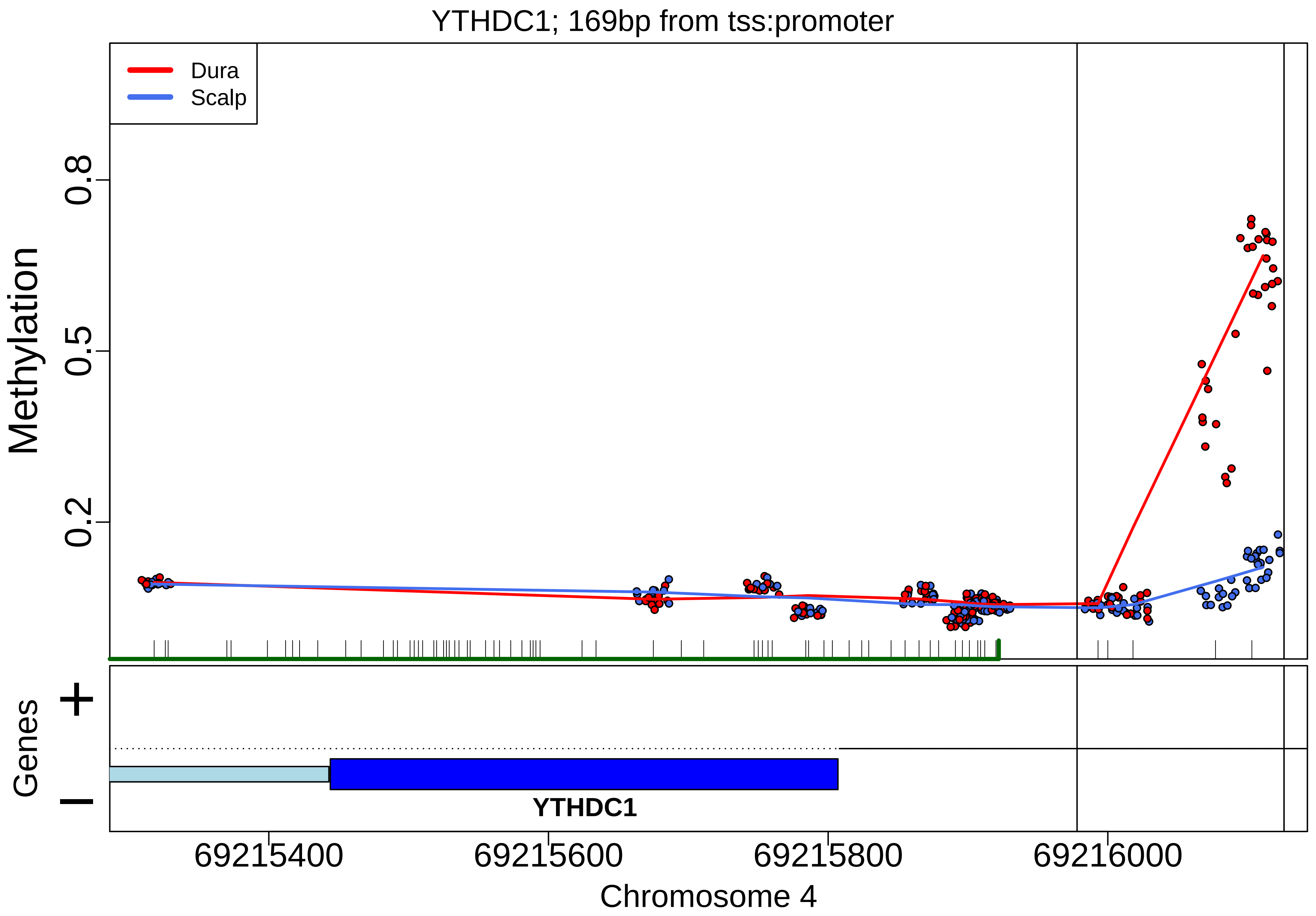
YTHDC1; 169bp from tss:promoter
Dura
Scalp
Methylation
0.2
0.5
0.8
Genes
+
-
YTHDC1
69215400
69215600
69215800
69216000
Chromosome 4

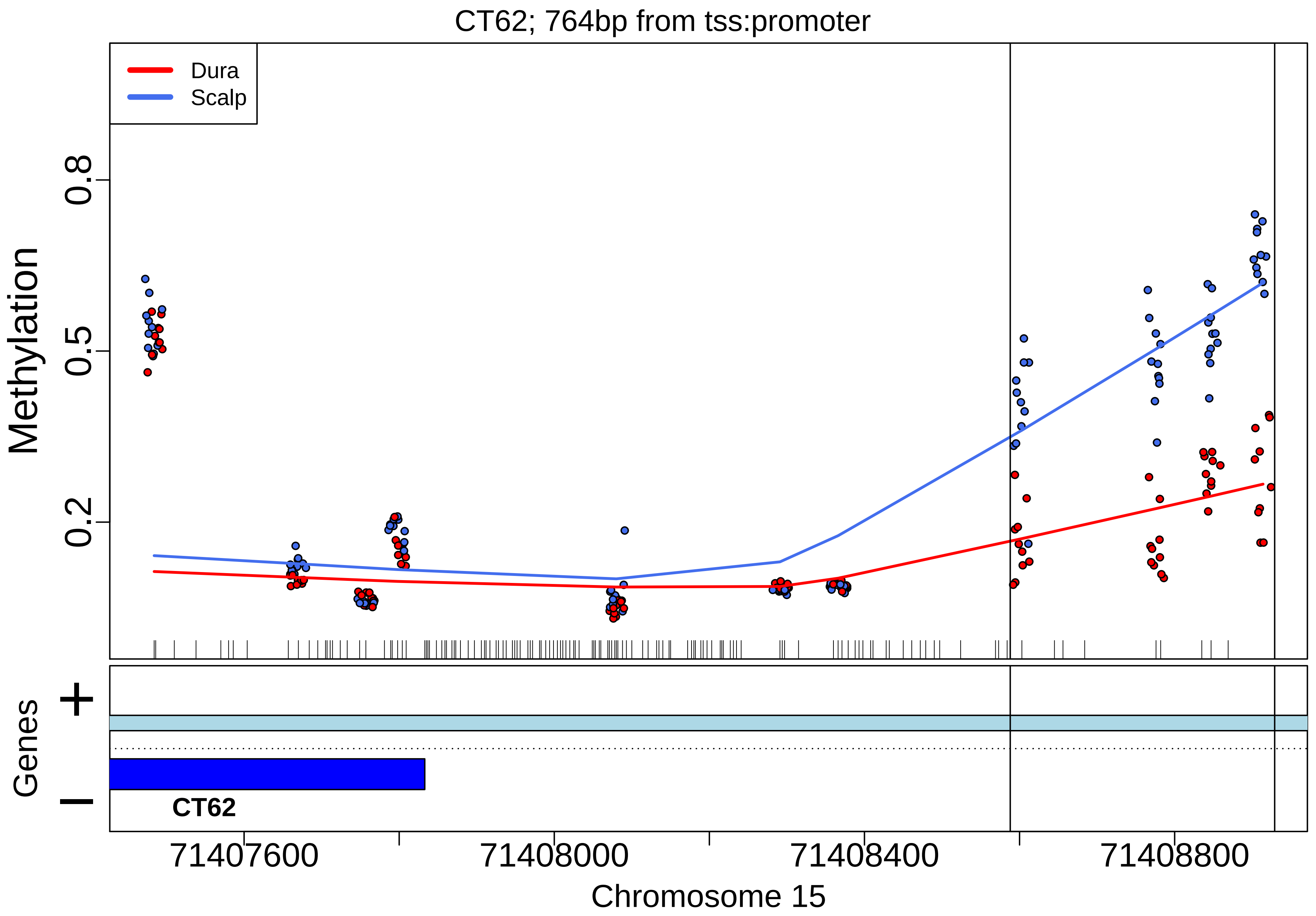

CT62; 764bp from tss:promoter
Dura
Scalp
Methylation
0.2
0.5
0.8
Genes
+
−
CT62
71407600
71408000
71408400
71408800
Chromosome 15

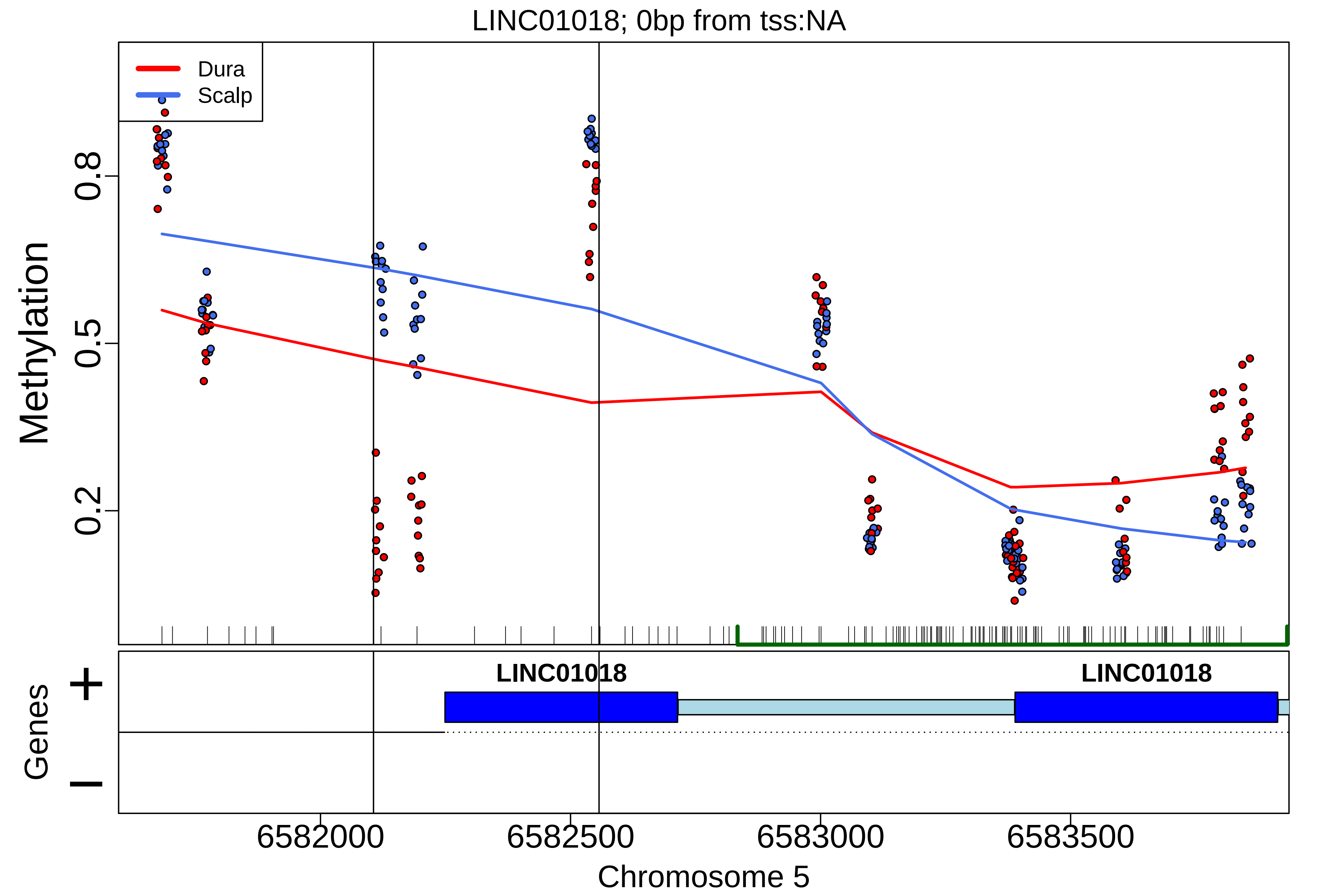

LINC01018; 0bp from tss:NA
Dura
Scalp
Methylation
0.2
0.5
0.8
Genes
+
−
LINC01018
LINC01018
6582000
6582500
6583000
6583500
Chromosome 5

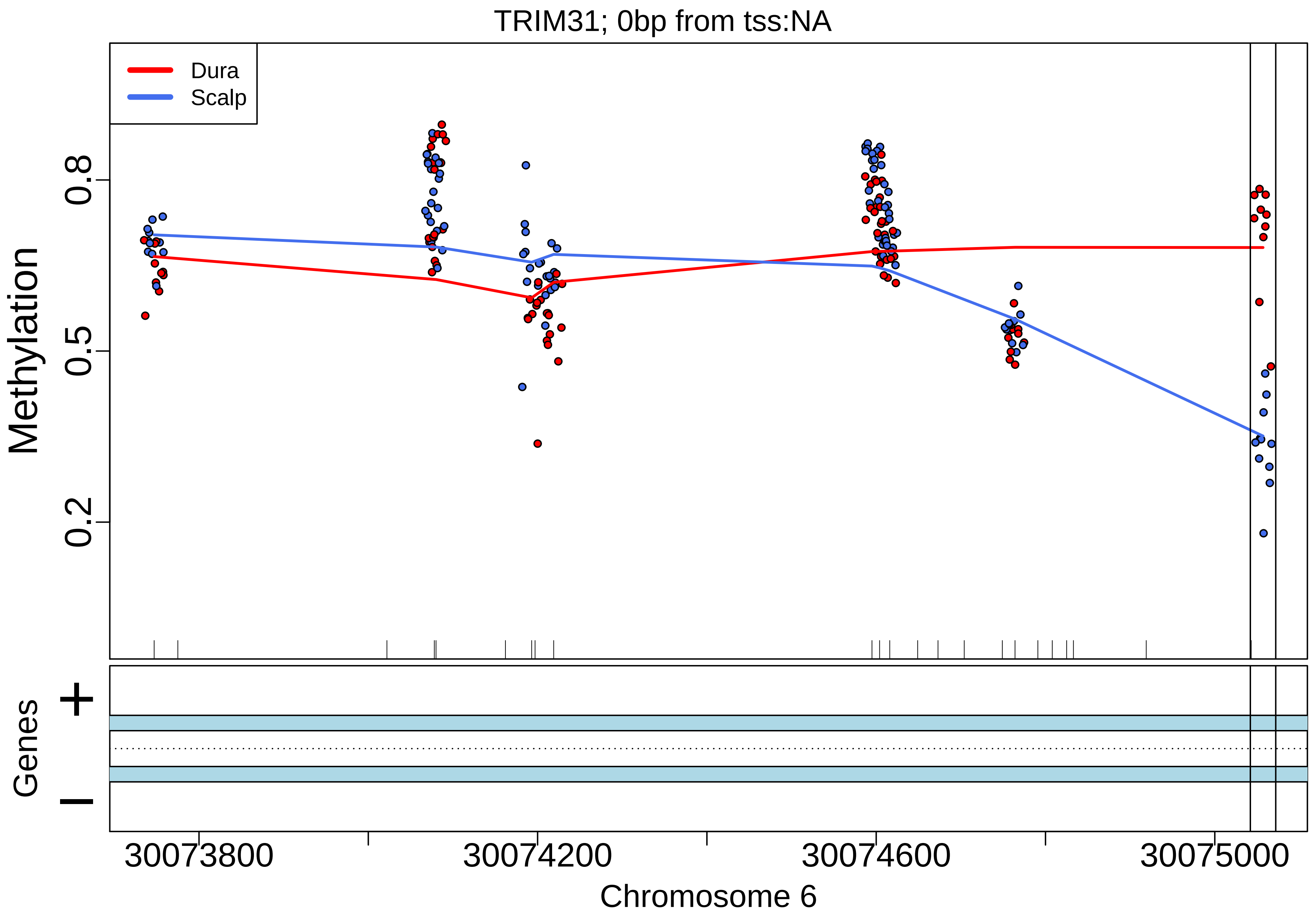

TRIM31; 0bp from tss:NA
Dura
Scalp
Methylation
0.2
0.5
0.8
Genes
+
-
30073800
30074200
30074600
30075000
Chromosome 6

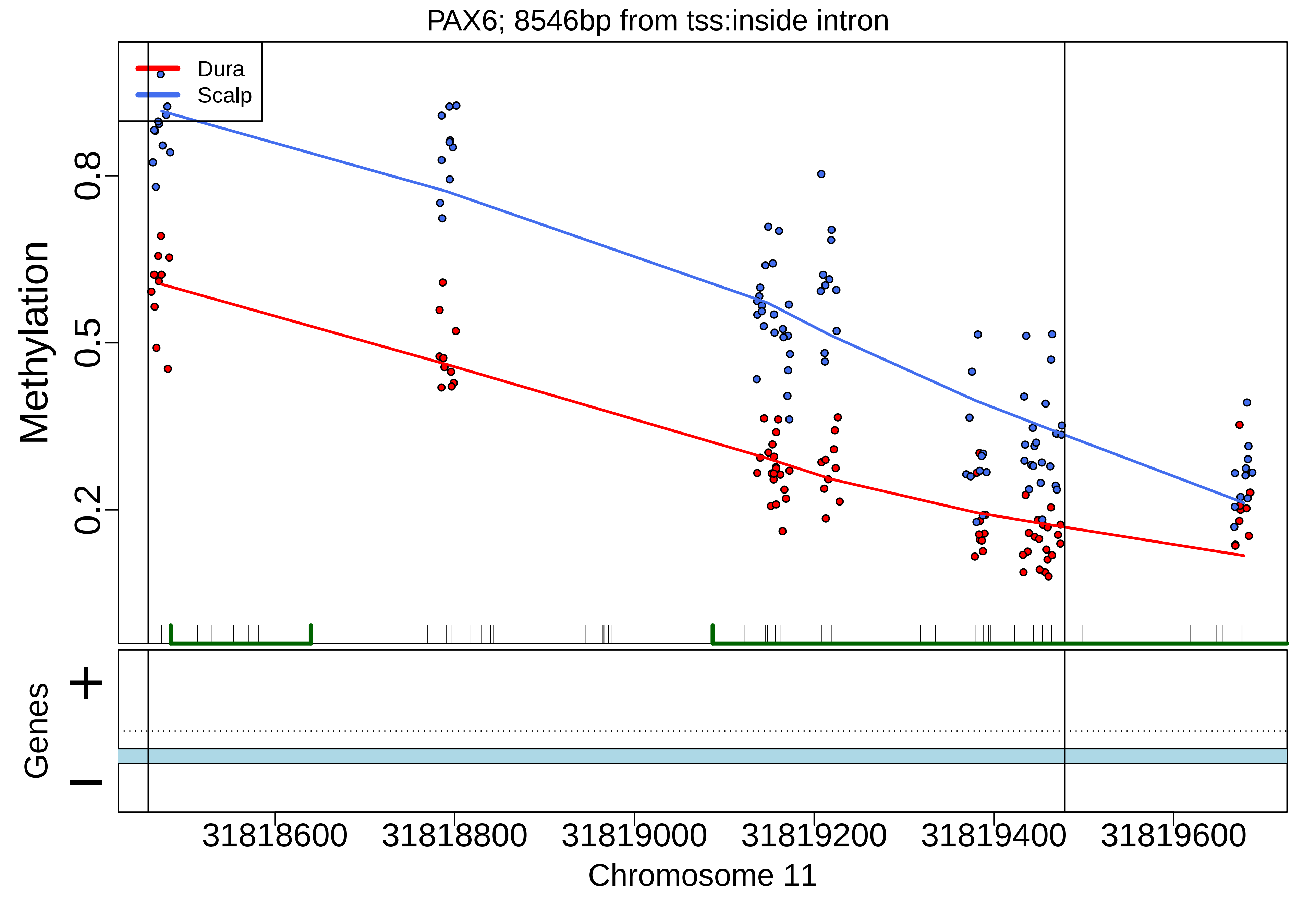
PAX6; 8546bp from tss:inside intron
Dura
Scalp
Methylation
0.2
0.5
0.8
Genes
+
−
31818600
31818800
31819000
31819200
31819400
31819600
Chromosome 11

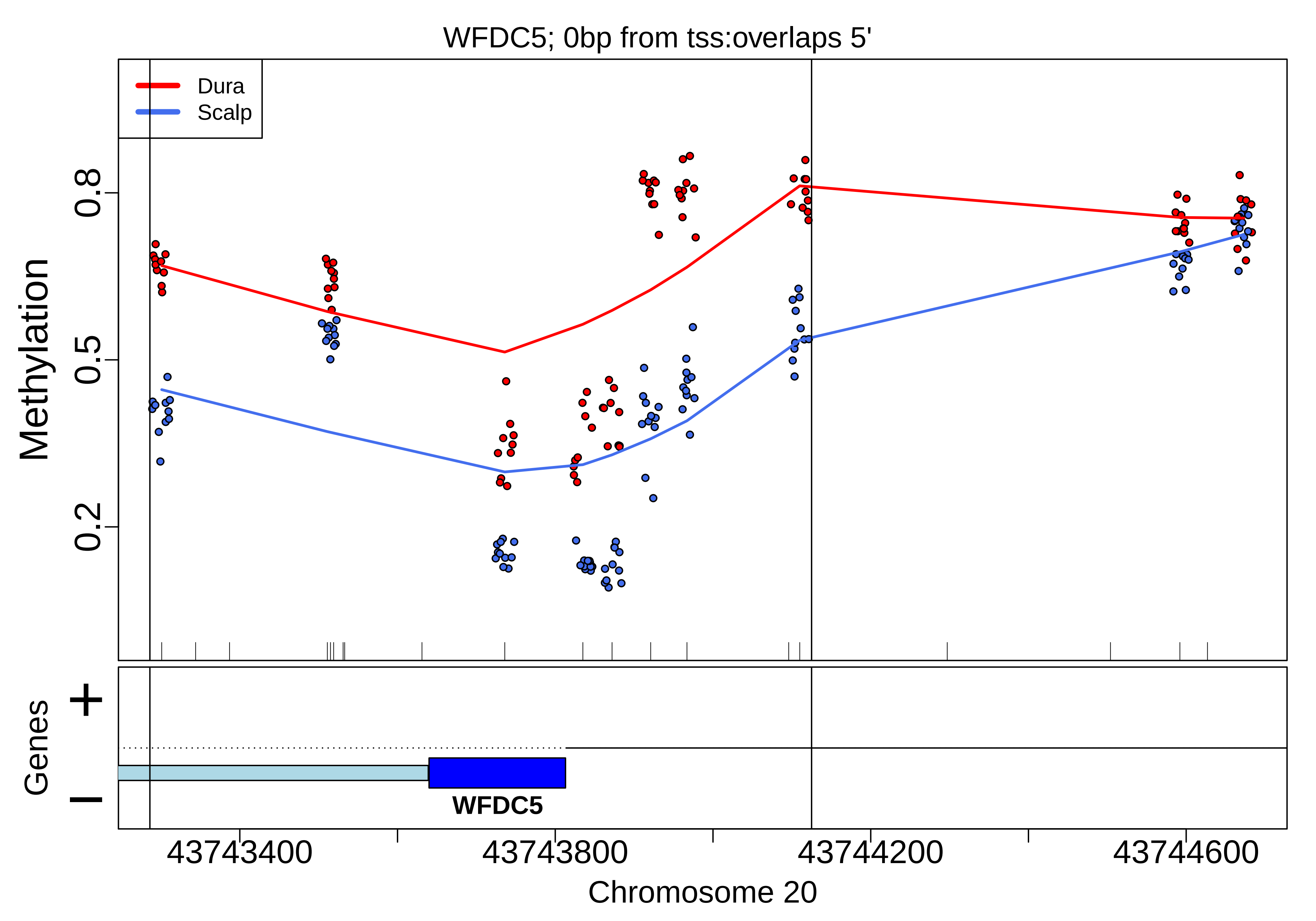

WFDC5; 0bp from tss:overlaps 5'
Dura
Scalp
Methylation
0.2
0.5
0.8
Genes
+
−
WFDC5
43743400
43743800
43744200
43744600
Chromosome 20

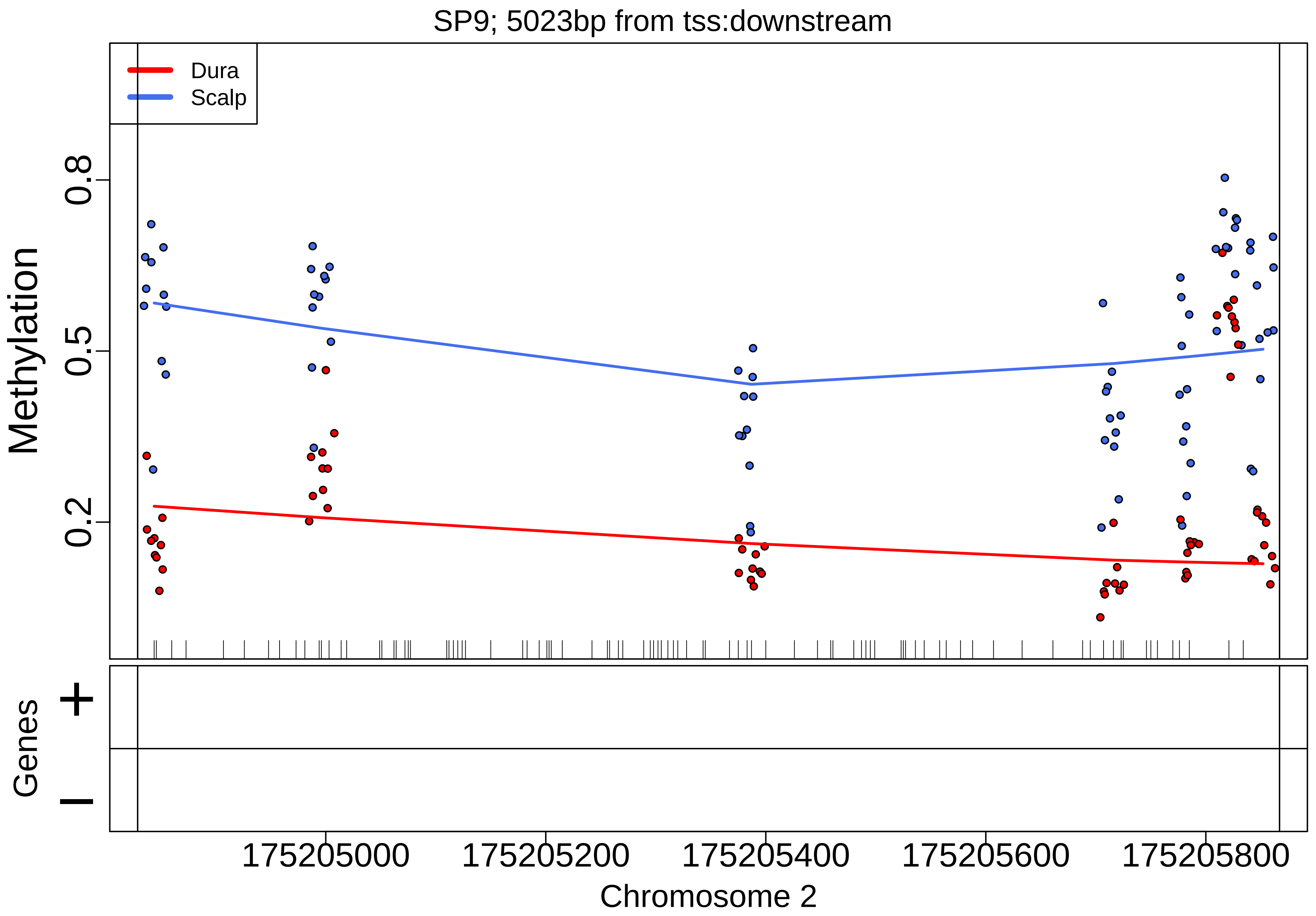

SP9; 5023bp from tss:downstream
Dura
Scalp
Methylation
0.2
0.5
0.8
Genes
+
−
175205000
175205200
175205400
175205600
175205800
Chromosome 2

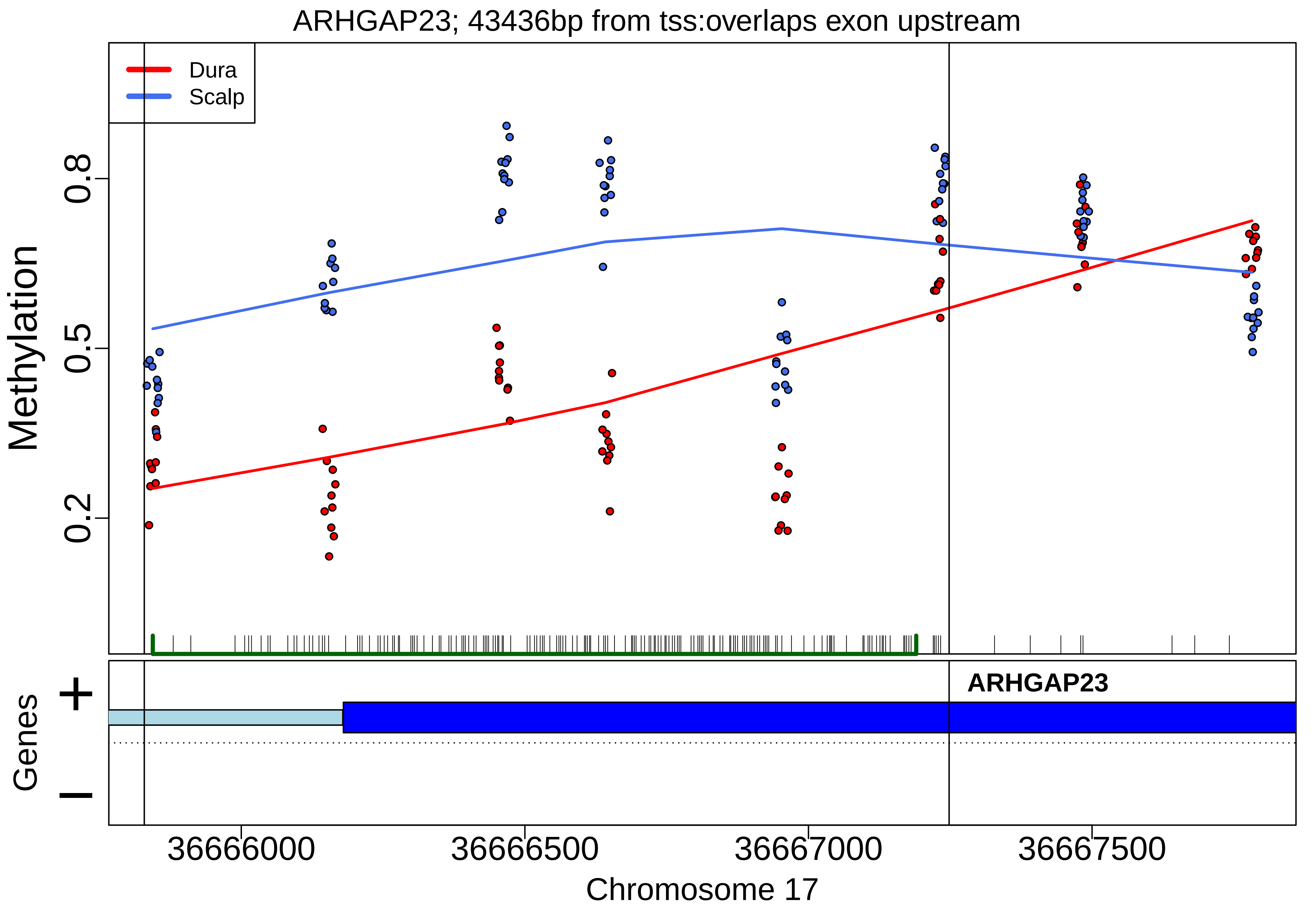
ARHGAP23; 43436bp from tss:overlaps exon upstream
Dura
Scalp
Methylation
0.2
0.5
0.8
Genes
+
-
ARHGAP23
36666000
36666500
36667000
36667500
Chromosome 17

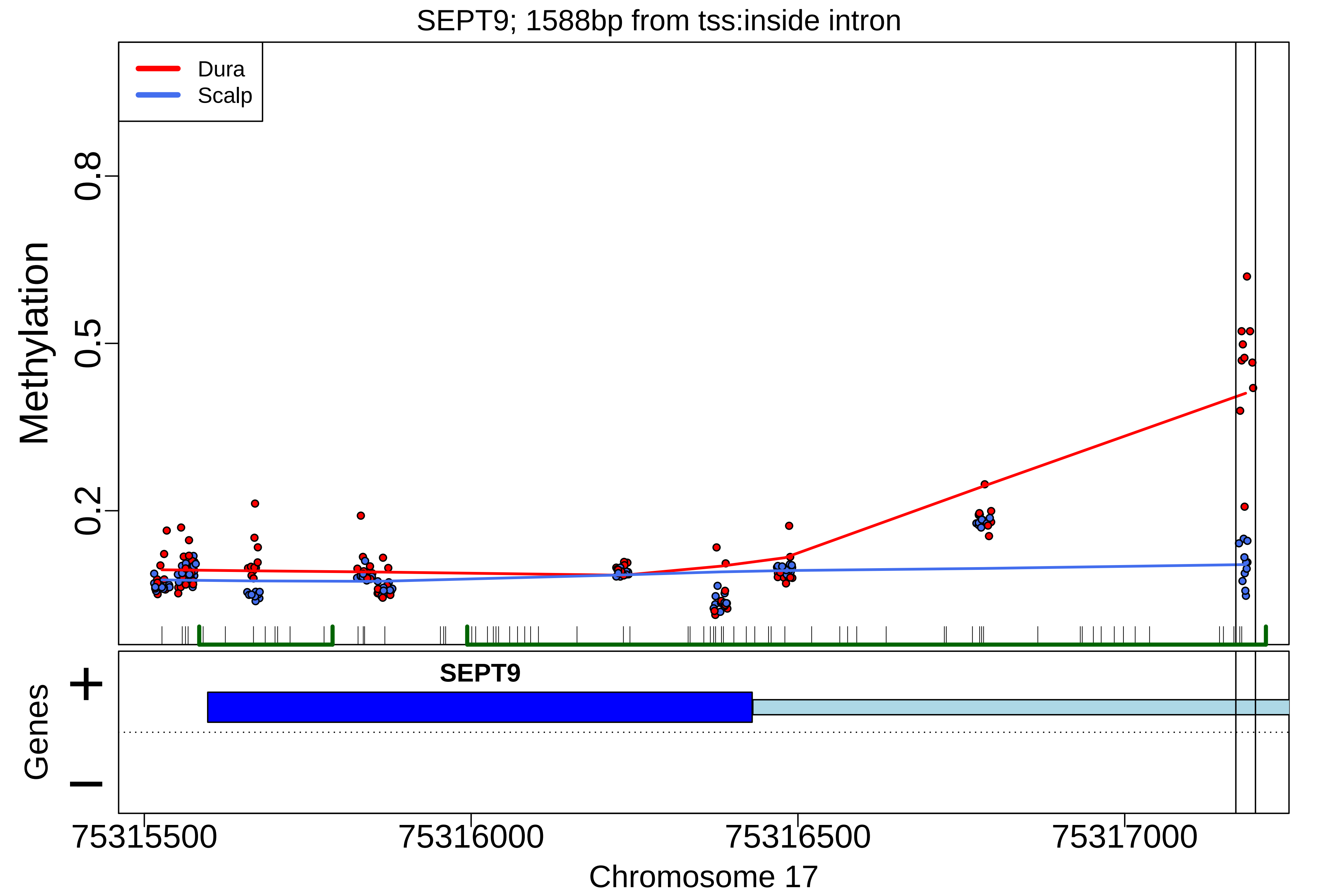
SEPT9; 1588bp from tss:inside intron
Dura
Scalp
Methylation
0.8
0.5
0.2
Genes
+
-
SEPT9
75315500
75316000
75316500
75317000
Chromosome 17

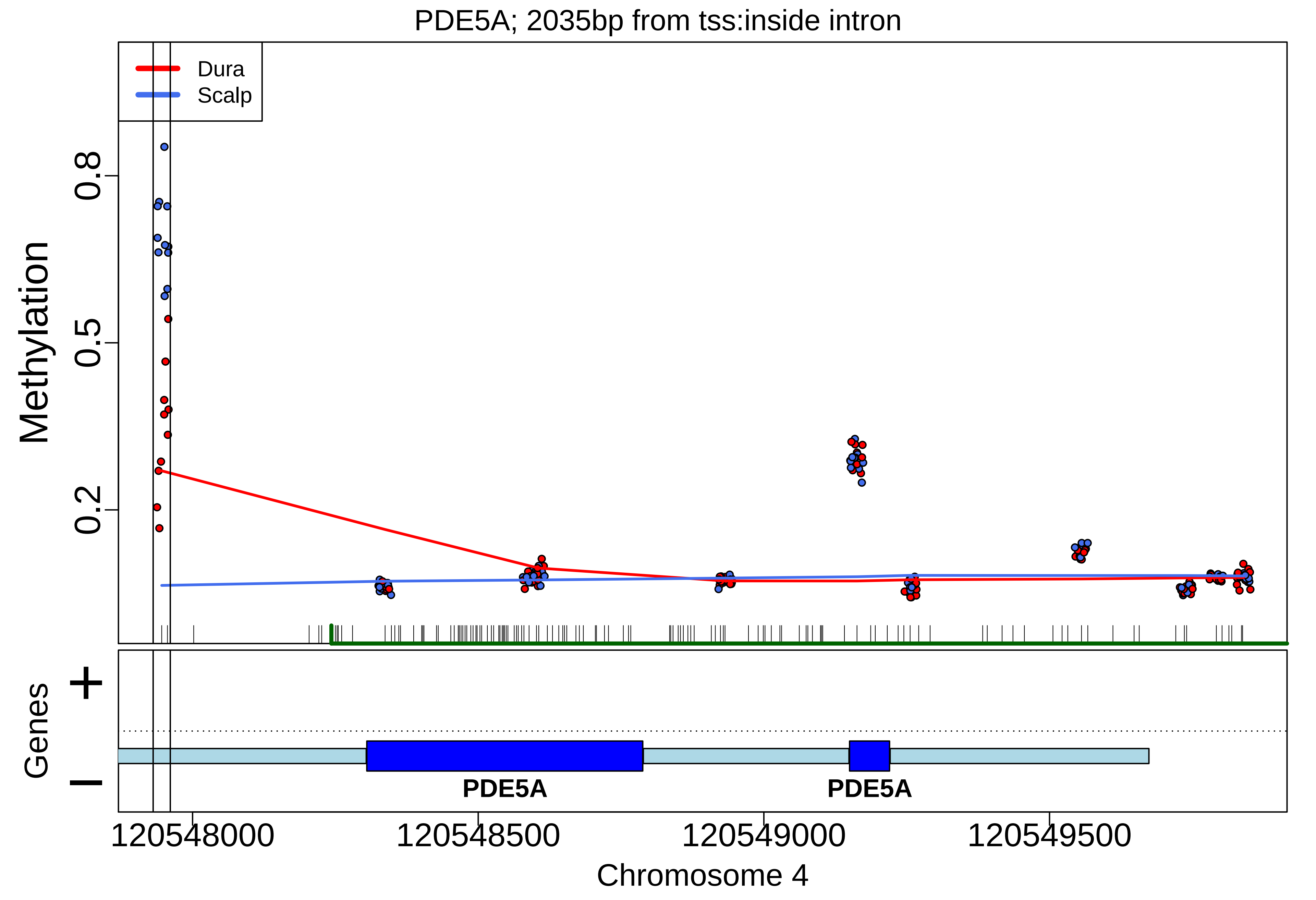

PDE5A; 2035bp from tss:inside intron
Dura
Scalp
Methylation
0.2
0.5
0.8
Genes
+
-
PDE5A
PDE5A
120548000
120548500
120549000
120549500
Chromosome 4

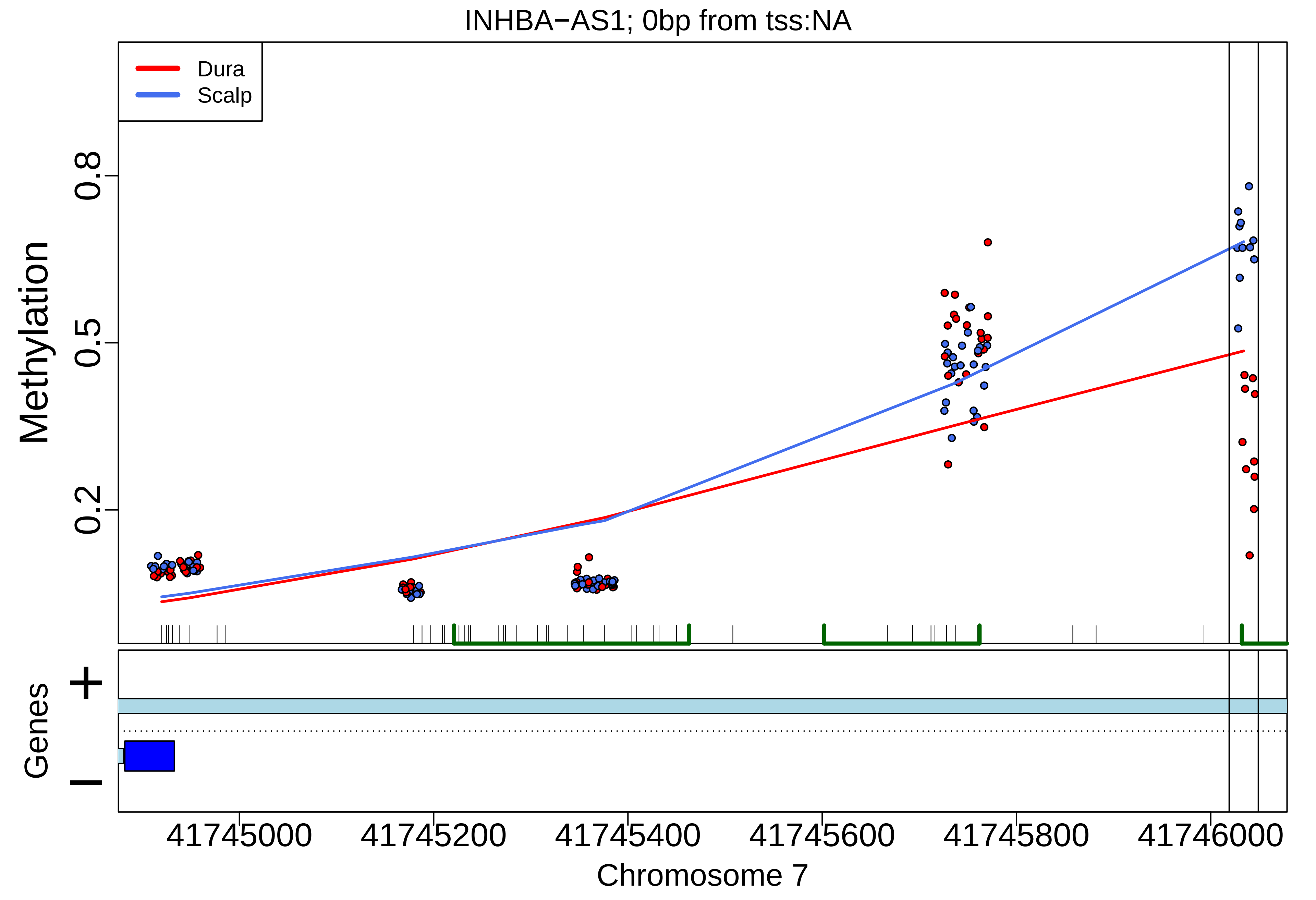

INHBA−AS1; 0bp from tss:NA
Dura
Scalp
Methylation
0.2
0.5
0.8
Genes
+
−
41745000
41745200
41745400
41745600
41745800
41746000
Chromosome 7

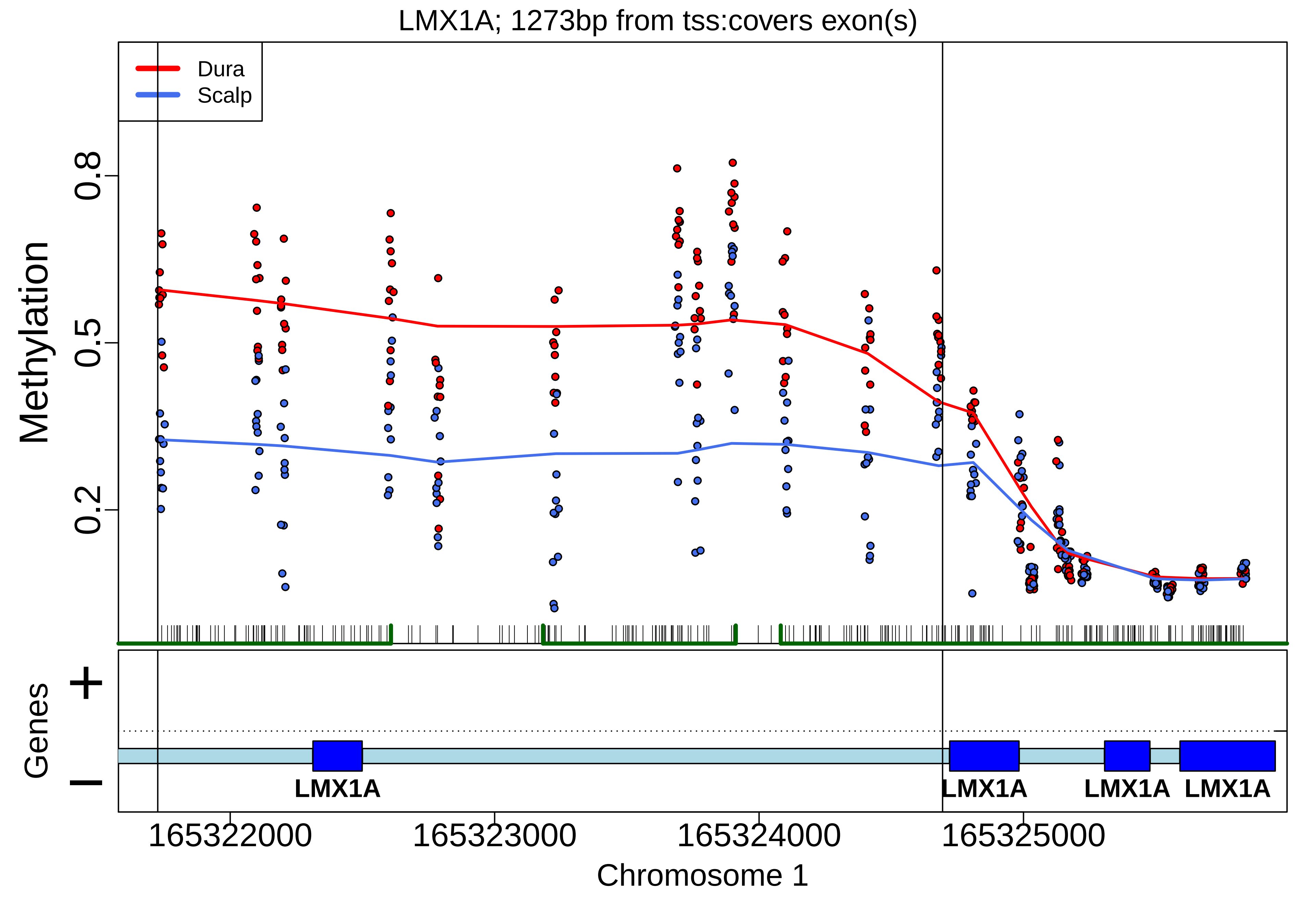
LMX1A; 1273bp from tss:covers exon(s)
Dura
Scalp
Methylation
0.2
0.5
0.8
Genes
+
−
LMX1A
LMX1A
LMX1A
LMX1A
165322000
165323000
165324000
165325000
Chromosome 1

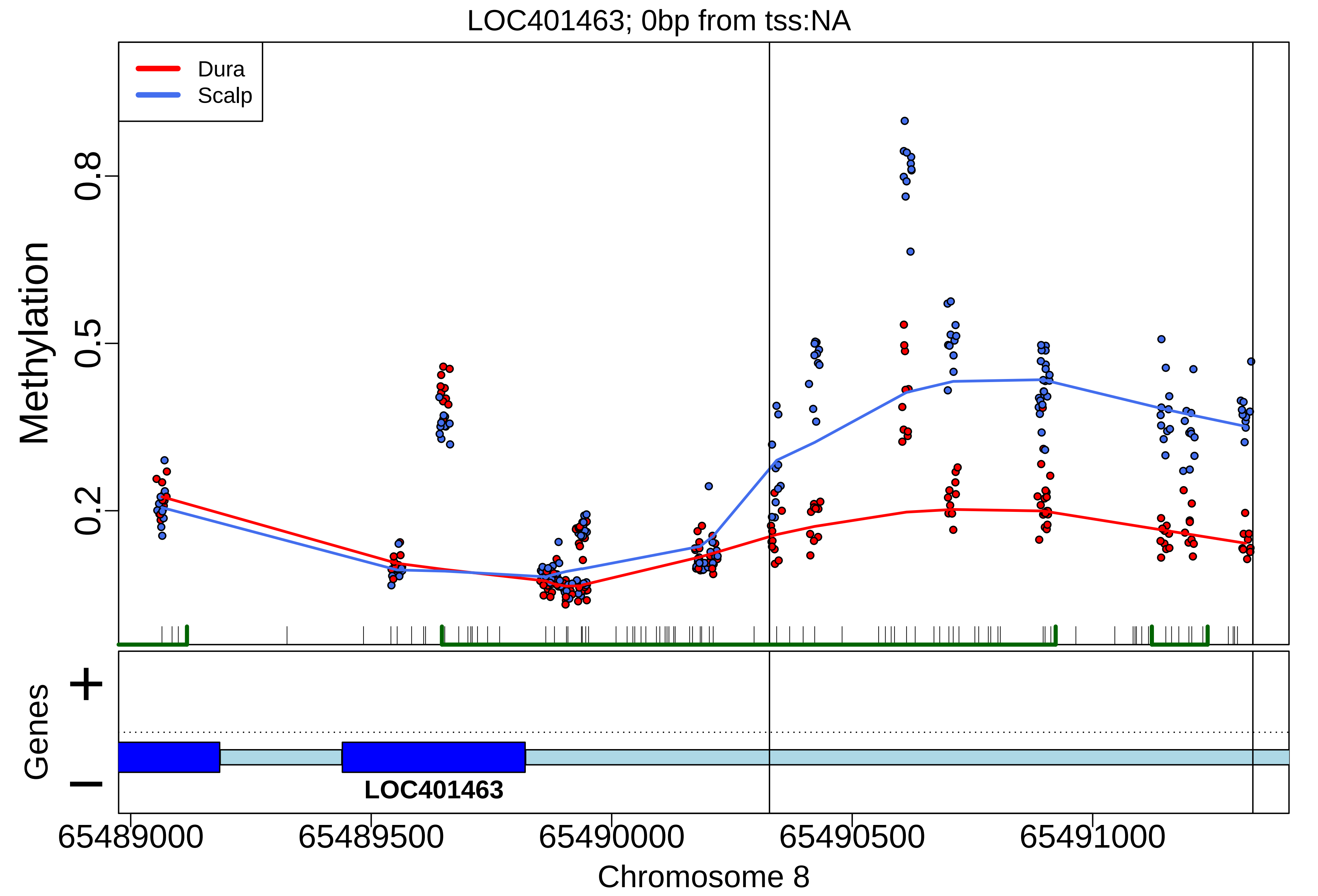

LOC401463; 0bp from tss:NA
Dura
Scalp
Methylation
0.2
0.5
0.8
Genes
+
−
LOC401463
65489000
65489500
65490000
65490500
65491000
Chromosome 8

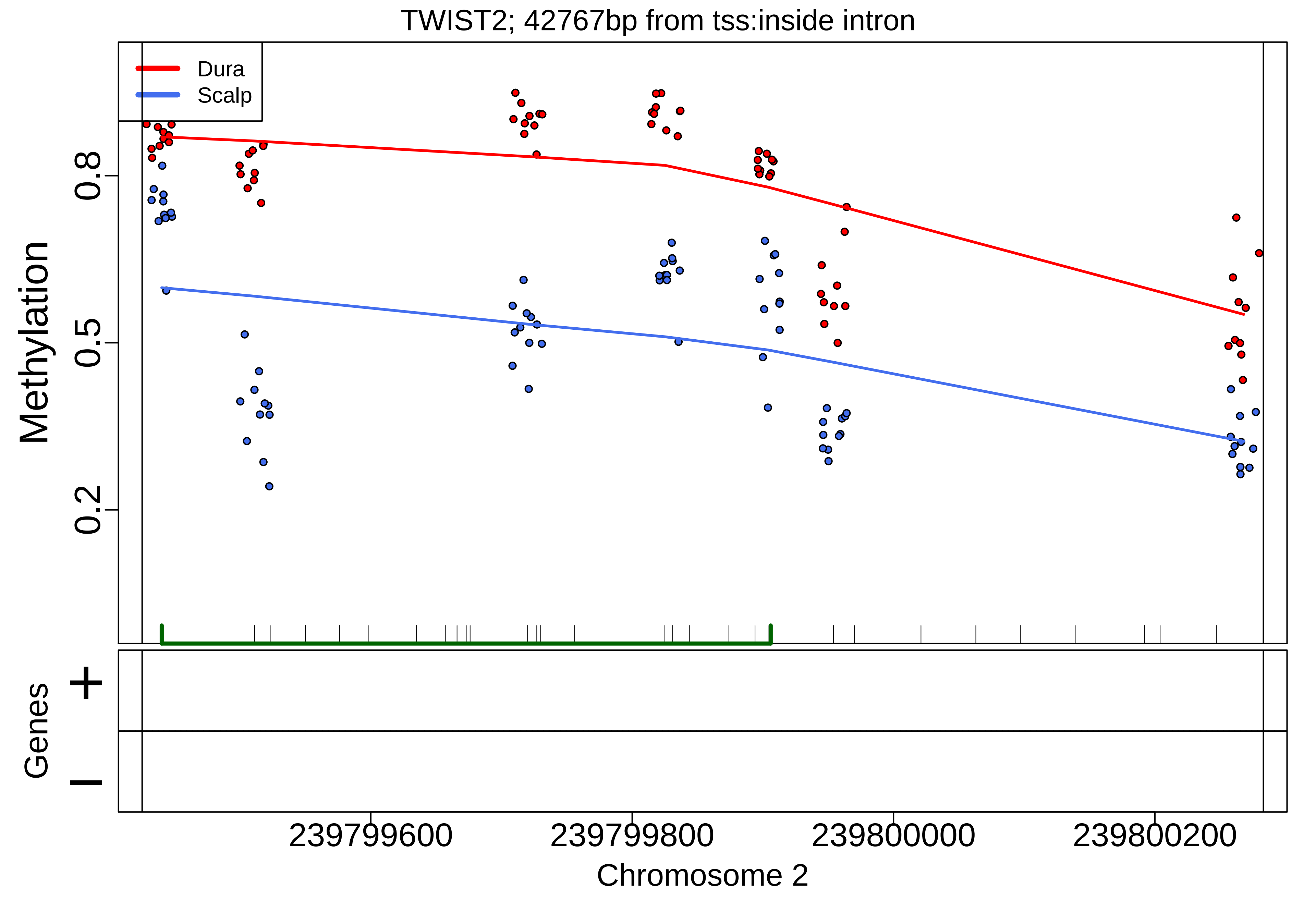

TWIST2; 42767bp from tss:inside intron
Dura
Scalp
Methylation
0.2
0.5
0.8
Genes
+
−
239799600
239799800
239800000
239800200
Chromosome 2

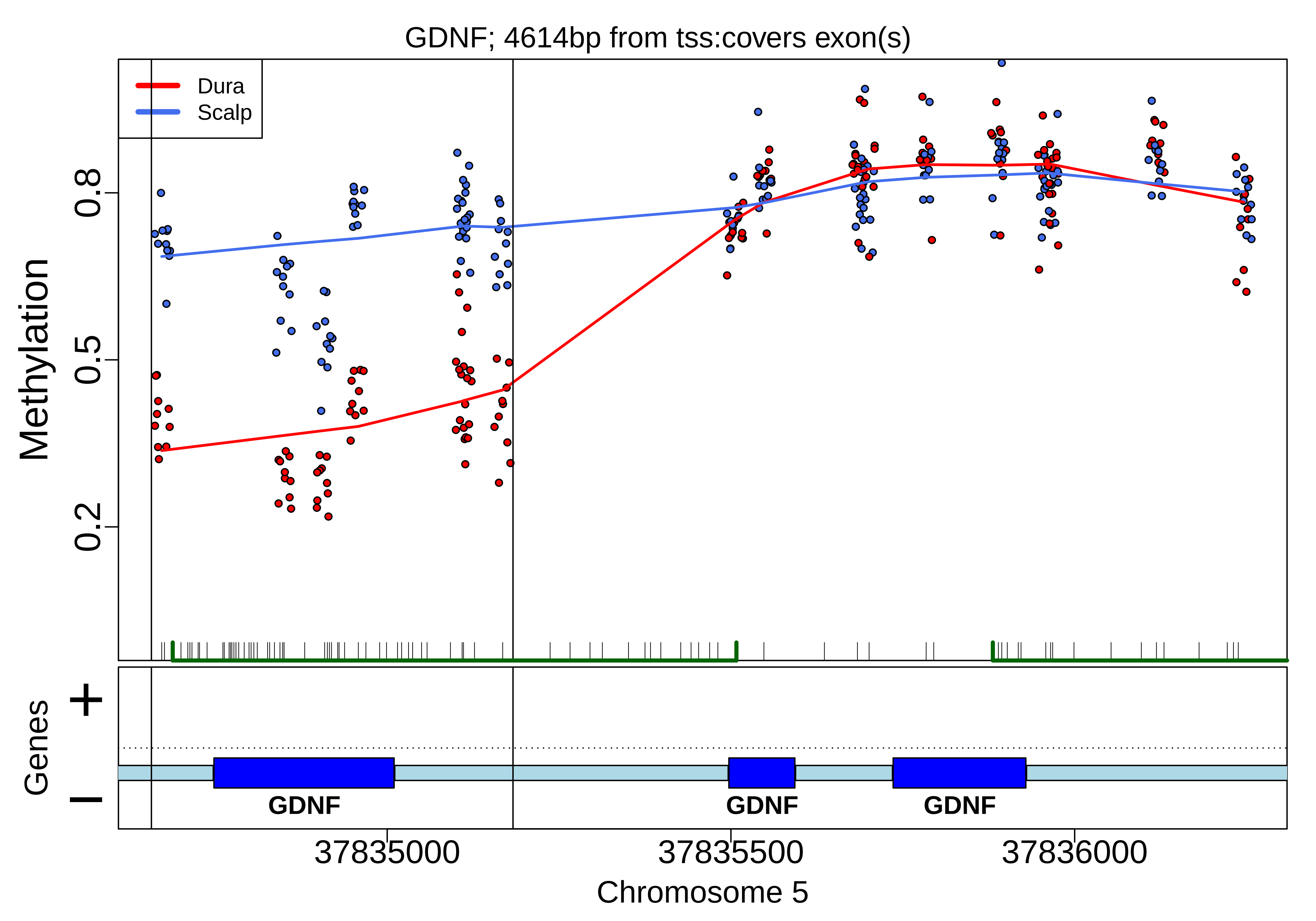

GDNF; 4614bp from tss:covers exon(s)
Dura
Scalp
Methylation
0.2
0.5
0.8
Genes
+
−
GDNF
GDNF
GDNF
37835000
37835500
37836000
Chromosome 5

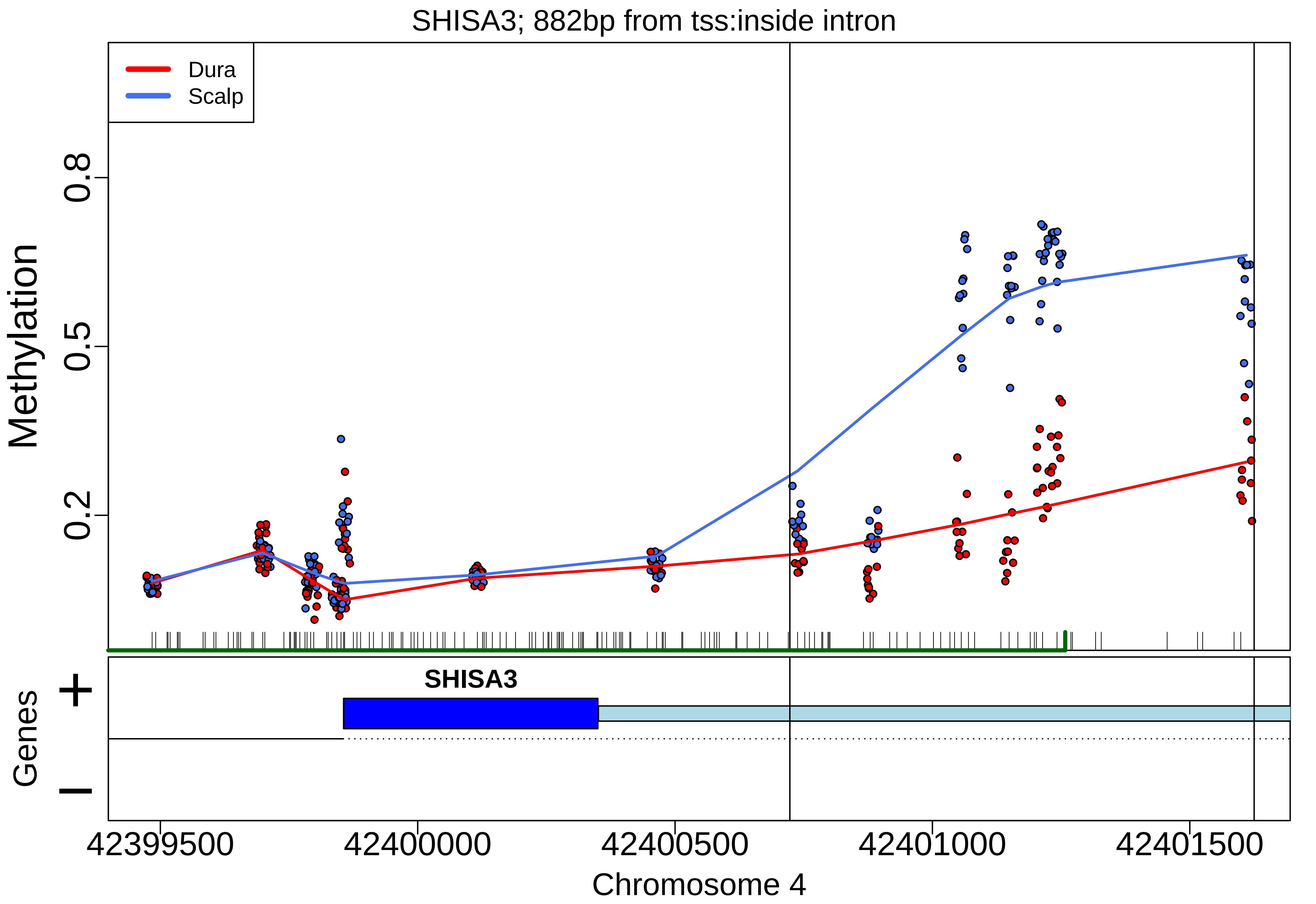

SHISA3; 882bp from tss:inside intron
Dura
Scalp
Methylation
0.2
0.5
0.8
Genes
+
−
SHISA3
42399500
42400000
42400500
42401000
42401500
Chromosome 4

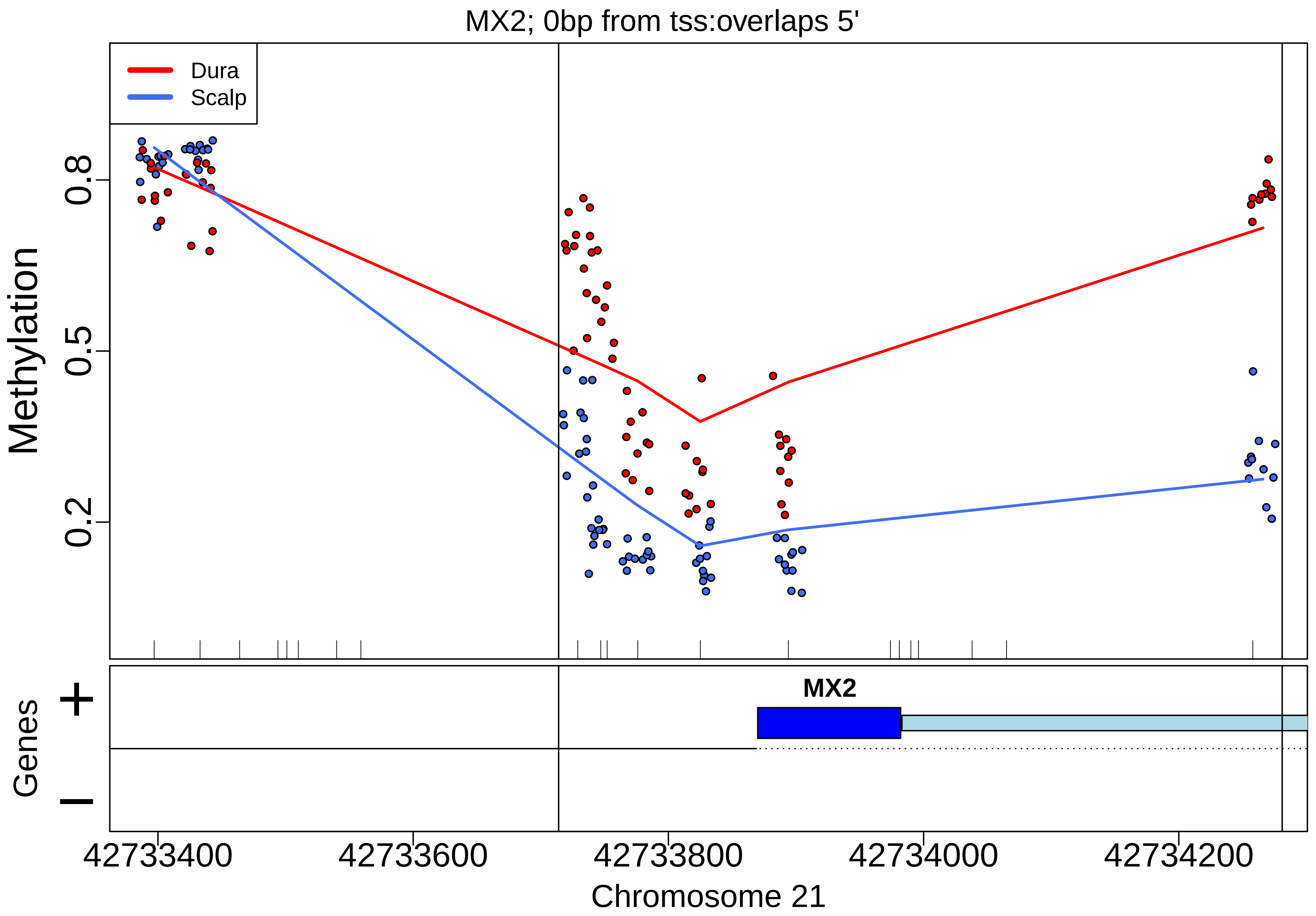

MX2; 0bp from tss:overlaps 5'
Dura
Scalp
Methylation
0.2
0.5
0.8
Genes
+
MX2
42733400
42733600
42733800
42734000
42734200
Chromosome 21

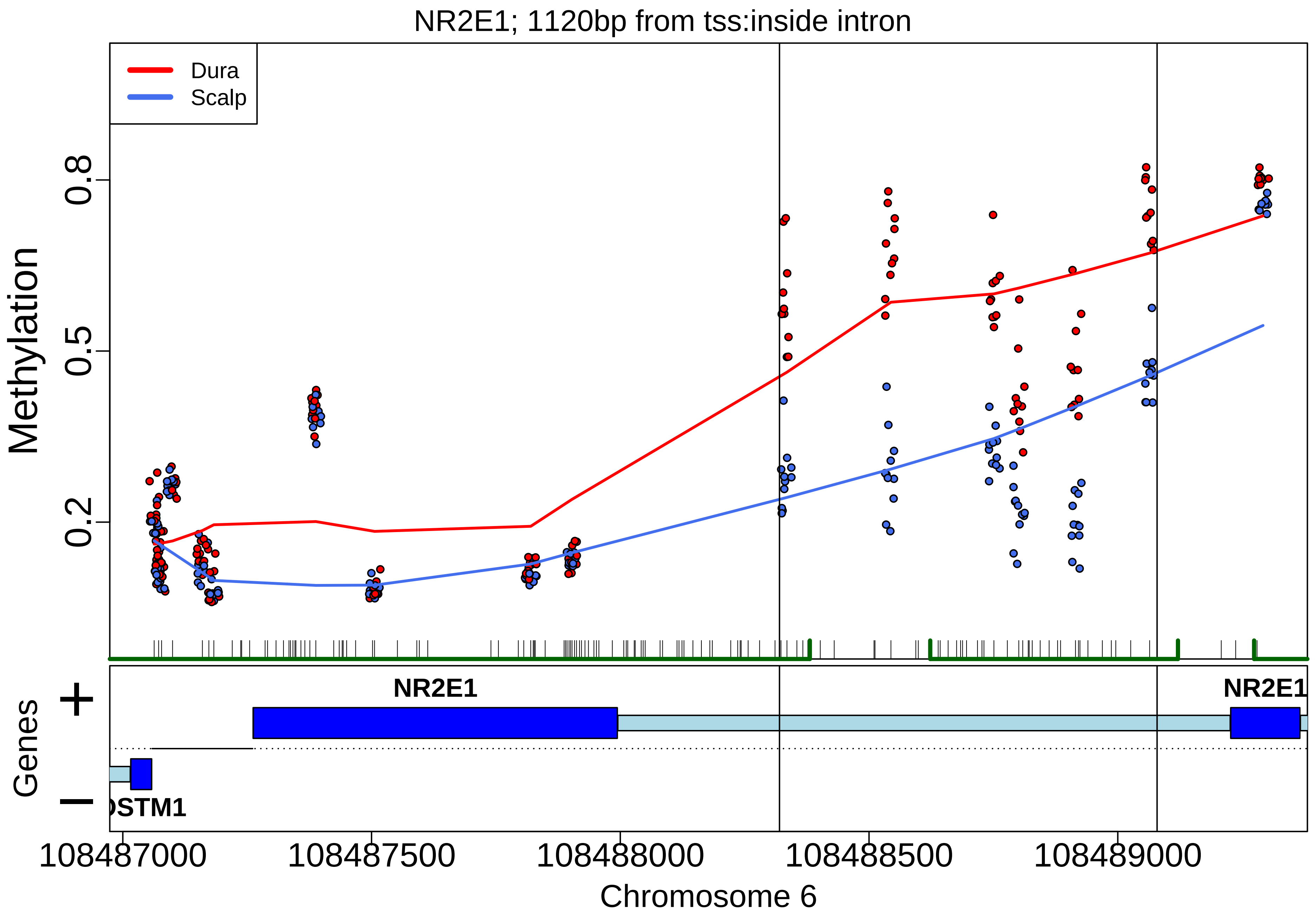

NR2E1; 1120bp from tss:inside intron
Dura
Scalp
Methylation
0.2
0.5
0.8
Genes
+
−
NR2E1
NR2E1
OSTM1
108487000
108487500
108488000
108488500
108489000
Chromosome 6

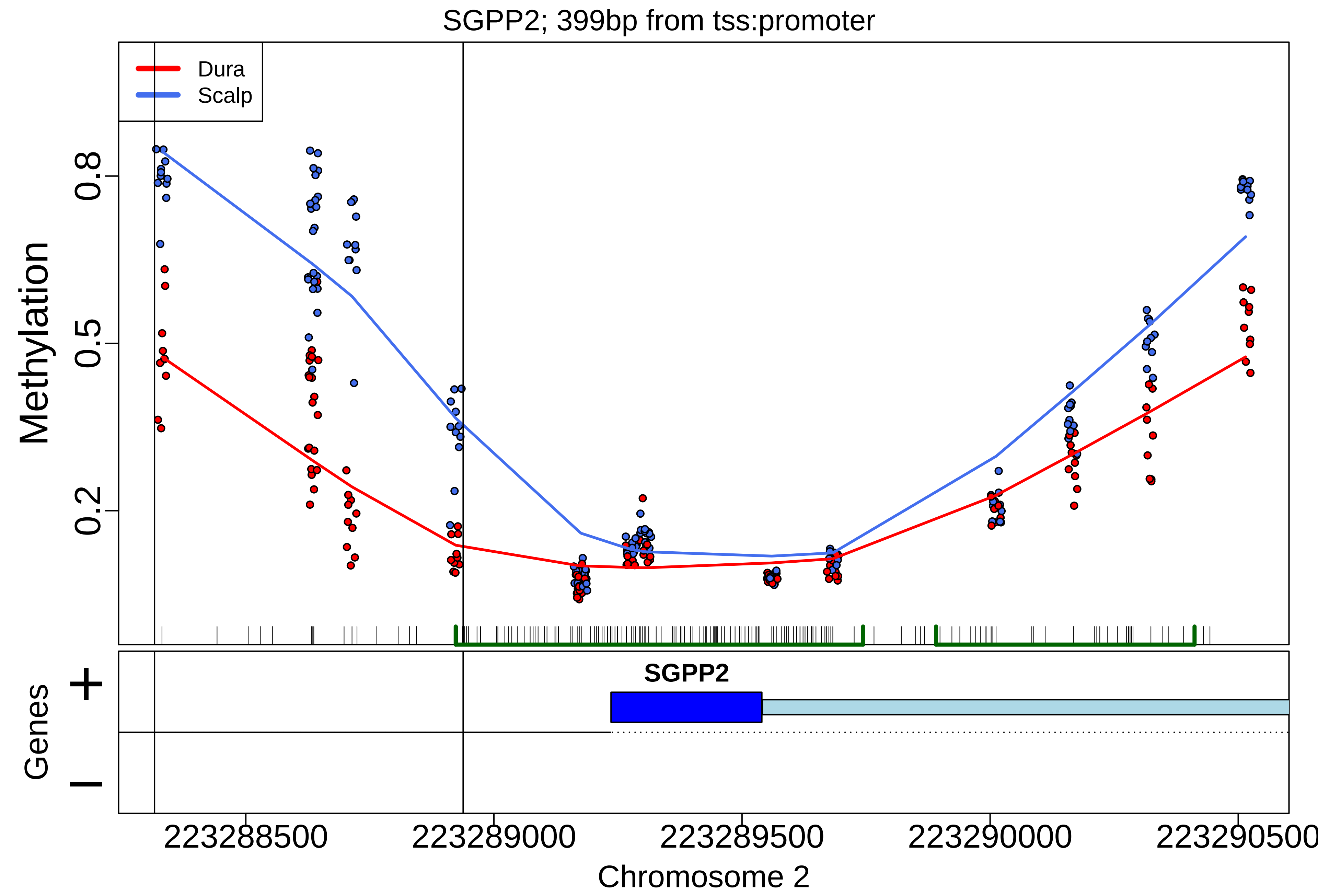
SGPP2; 399bp from tss:promoter
Dura
Scalp
Methylation
0.2
0.5
0.8
Genes
+
−
SGPP2
223288500
223289000
223289500
223290000
223290500
Chromosome 2

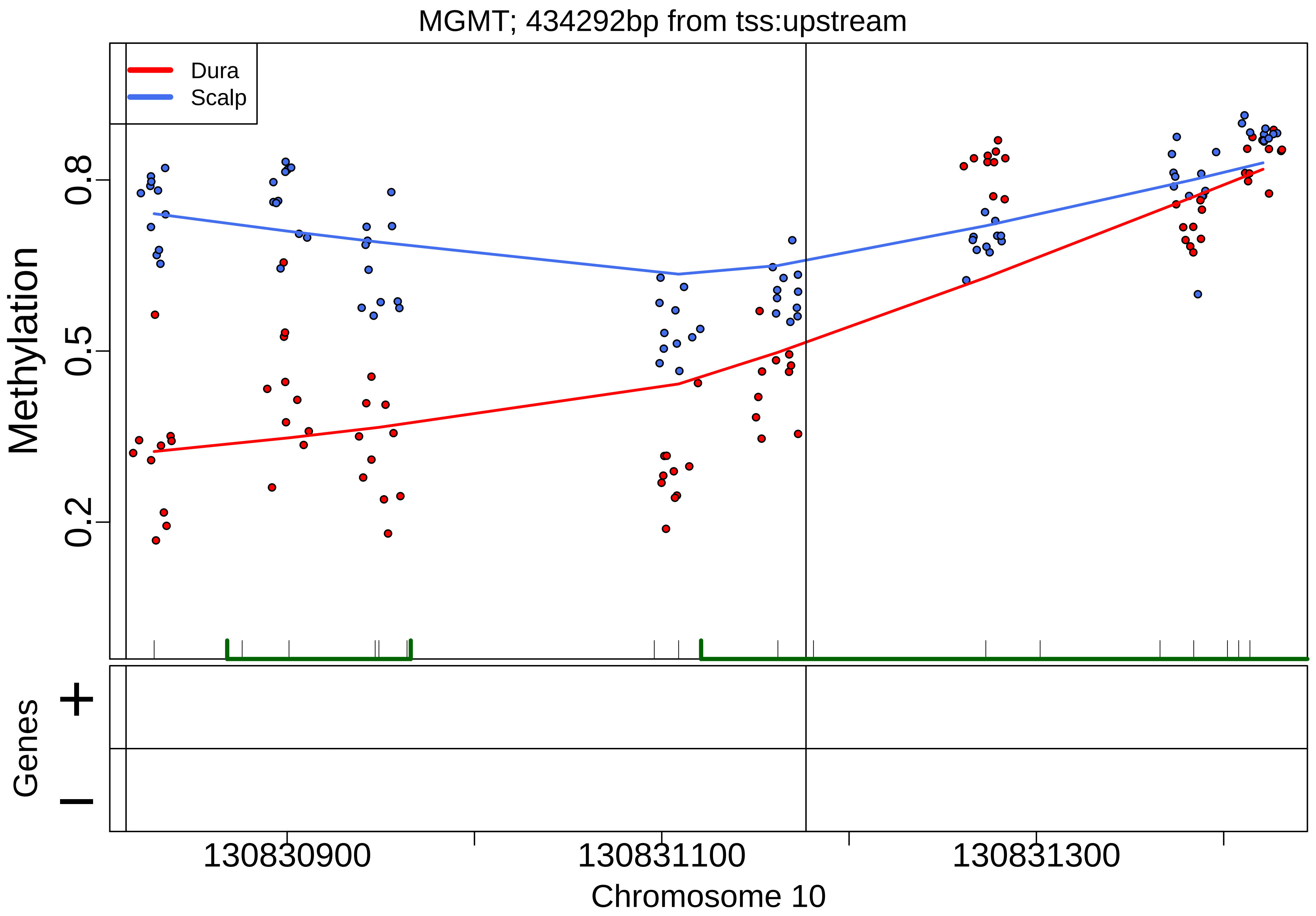
MGMT; 434292bp from tss:upstream
Dura
Scalp
Methylation
0.2
0.5
0.8
Genes
+
−
130830900
130831100
130831300
Chromosome 10

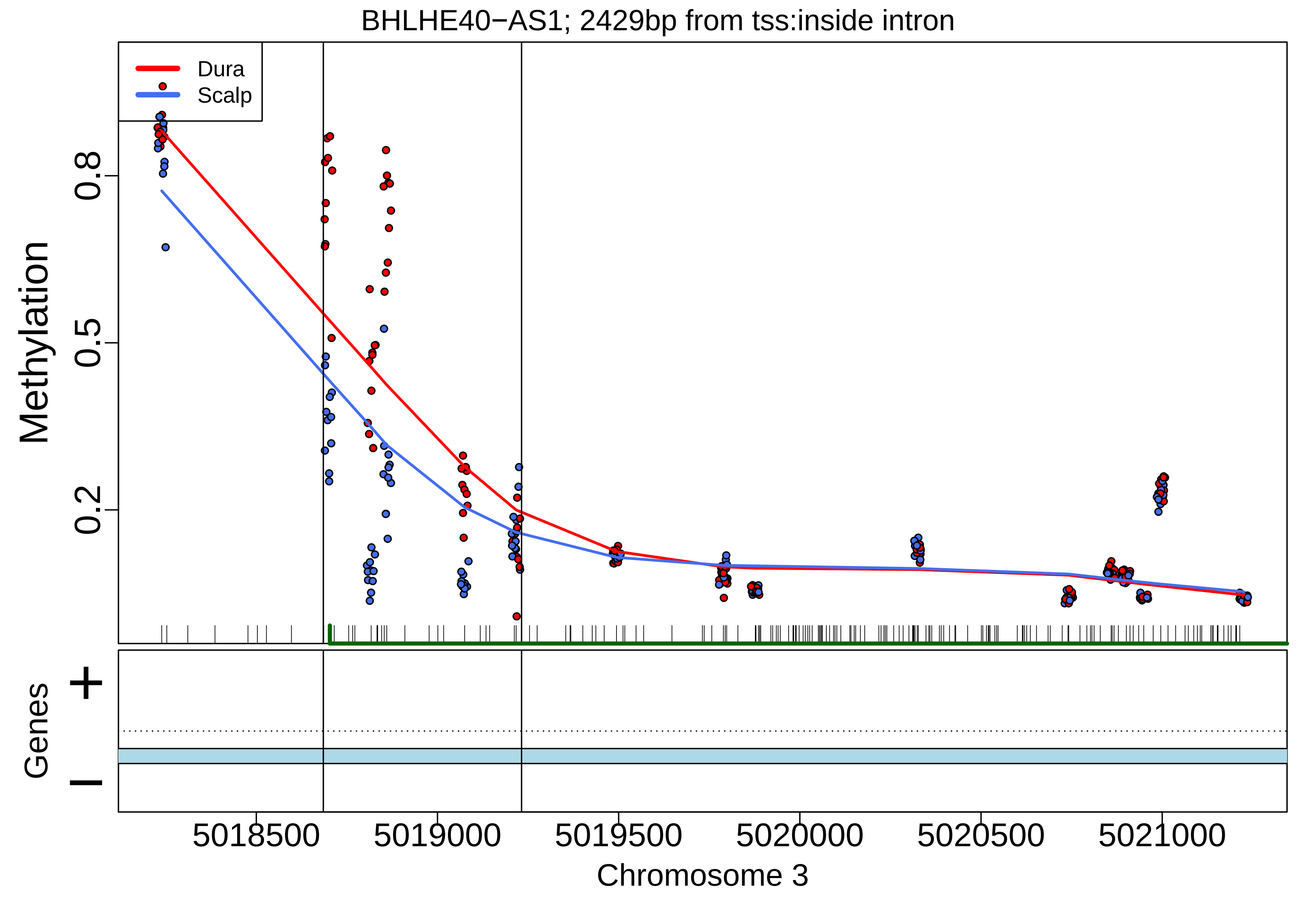

BHLHE40−AS1; 2429bp from tss:inside intron
Dura
Scalp
Methylation
0.2
0.5
0.8
Genes
+
−
5018500
5019000
5019500
5020000
5020500
5021000
Chromosome 3

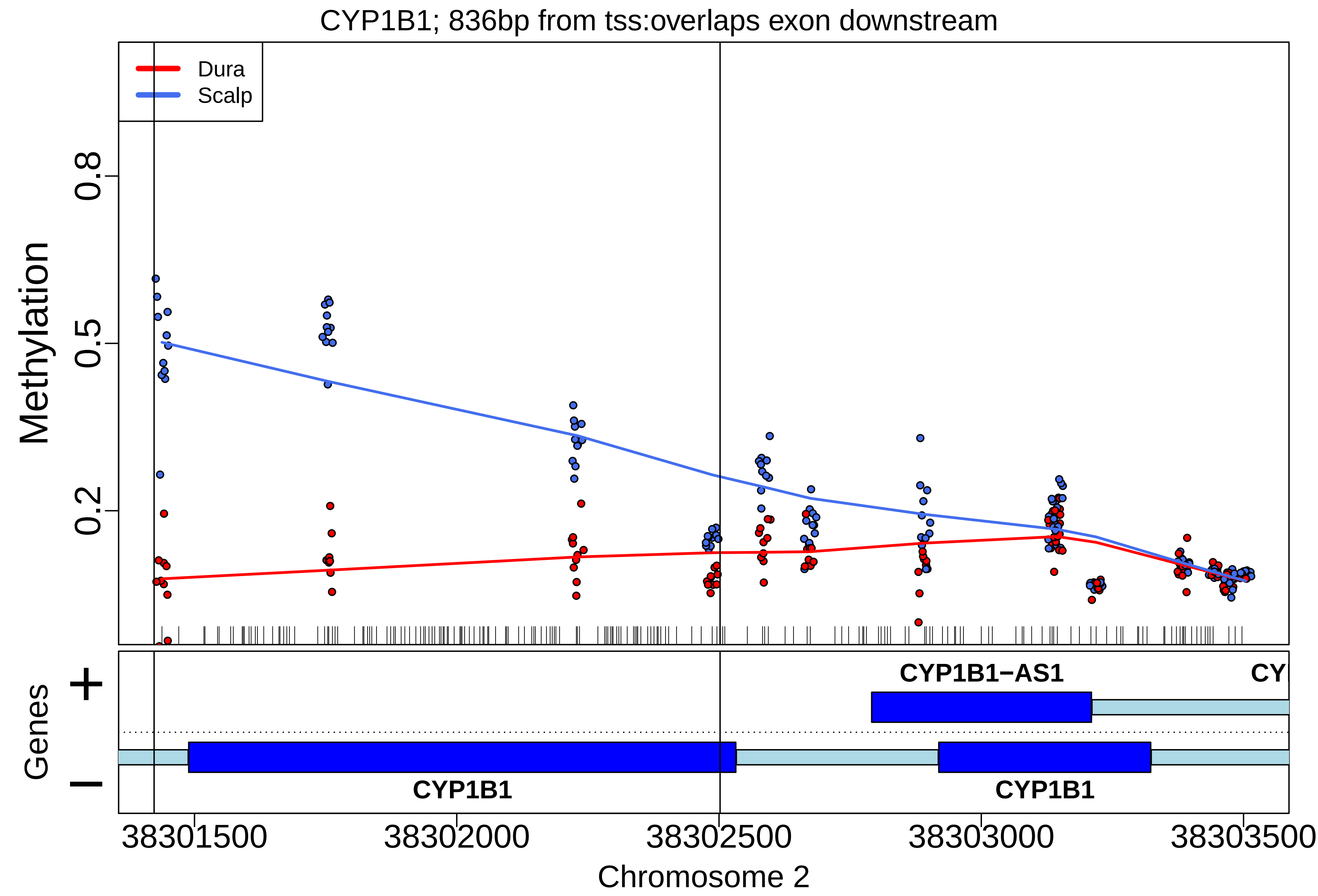
CYP1B1; 836bp from tss:overlaps exon downstream
Dura
Scalp
Methylation
0.2
0.5
0.8
Genes
+
−
CYP1B1−AS1
CY
CYP1B1
CYP1B1
38301500
38302000
38302500
38303000
38303500
Chromosome 2

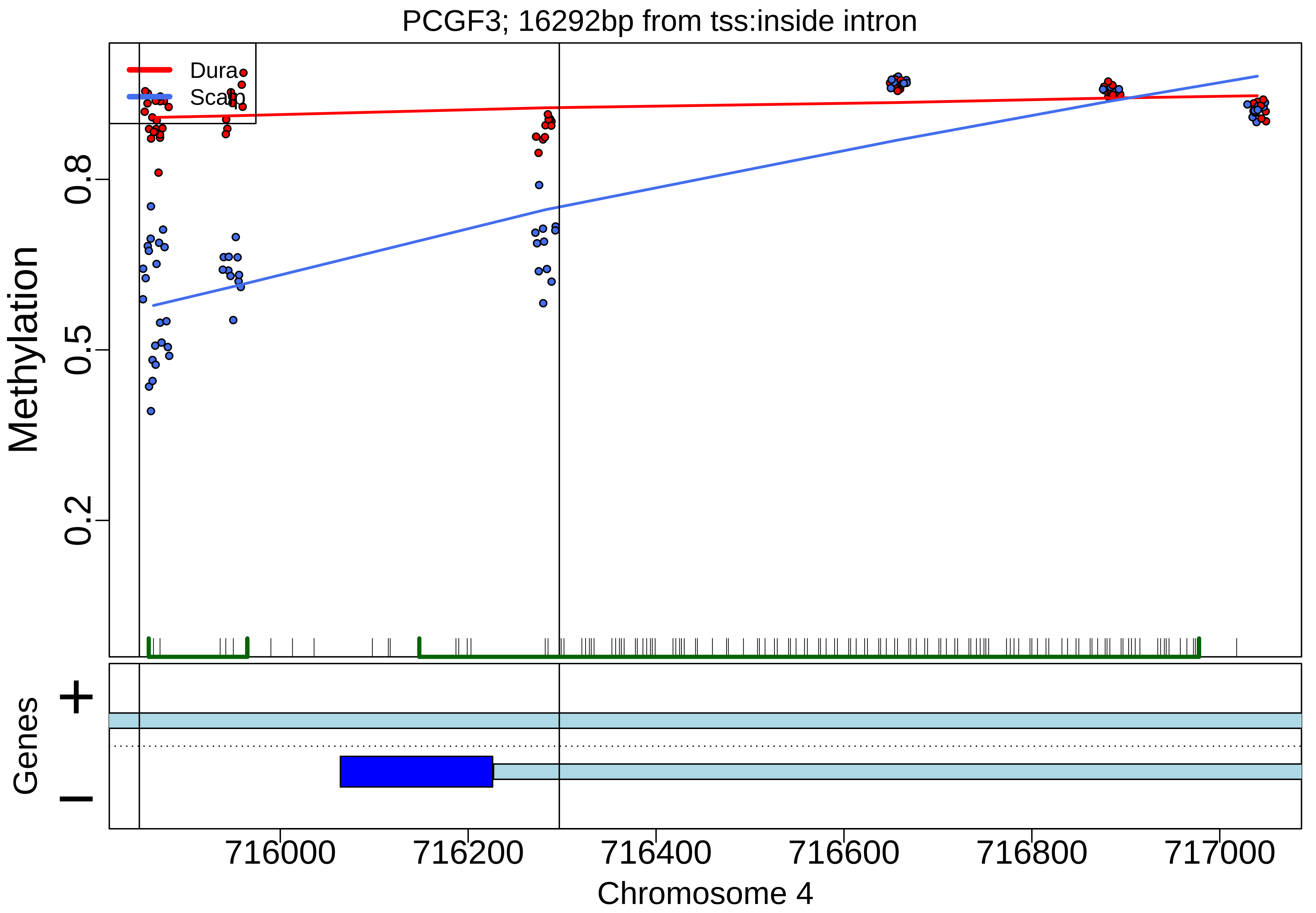
PCGF3; 16292bp from tss:inside intron
Dura
Scalp
Methylation
0.2
0.5
0.8
Genes
+
-
716000
716200
716400
716600
716800
717000
Chromosome 4

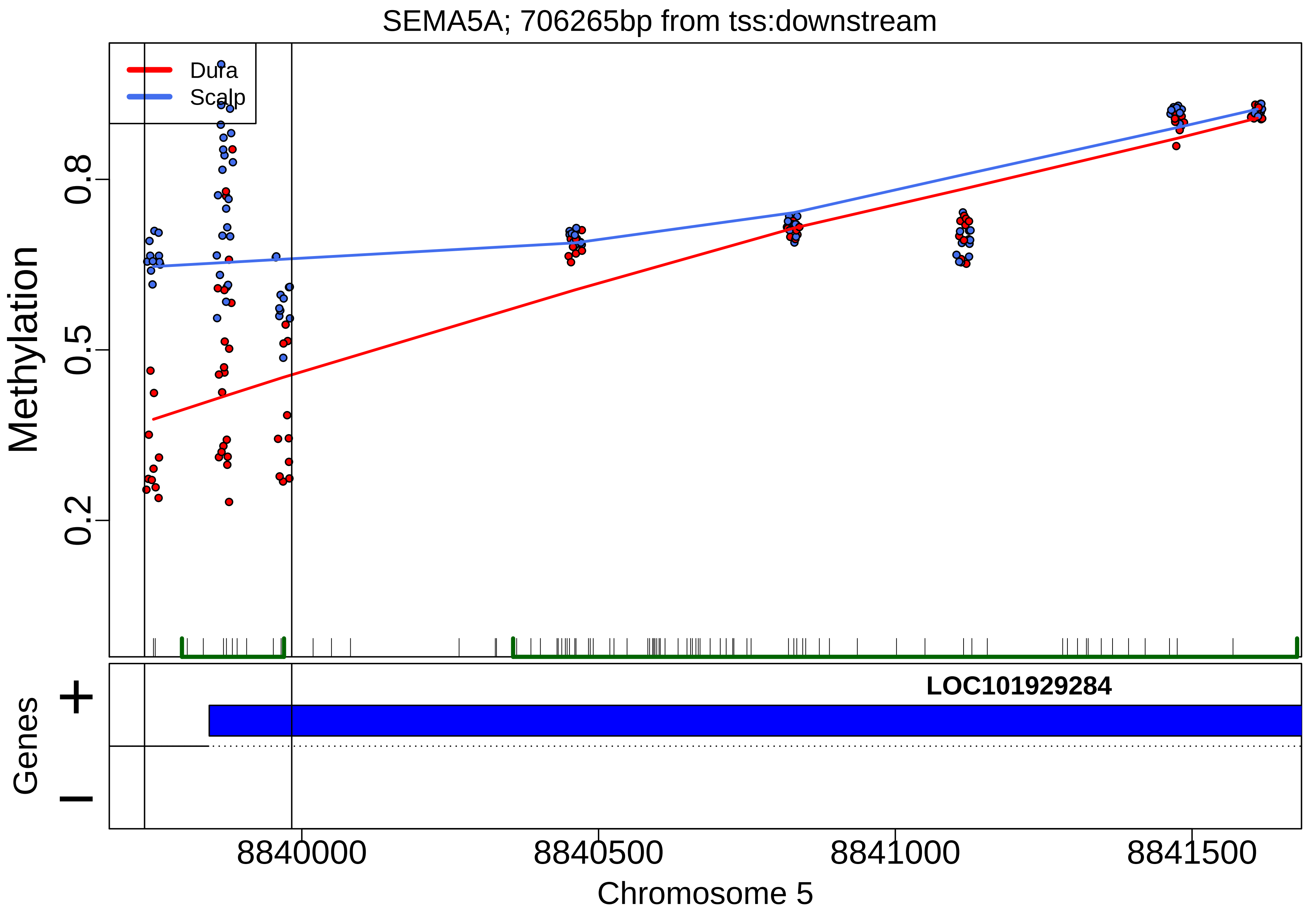

SEMA5A; 706265bp from tss:downstream
Dura
Scalp
Methylation
0.2
0.5
0.8
Genes
+
-
LOC101929284
8840000
8840500
8841000
8841500
Chromosome 5

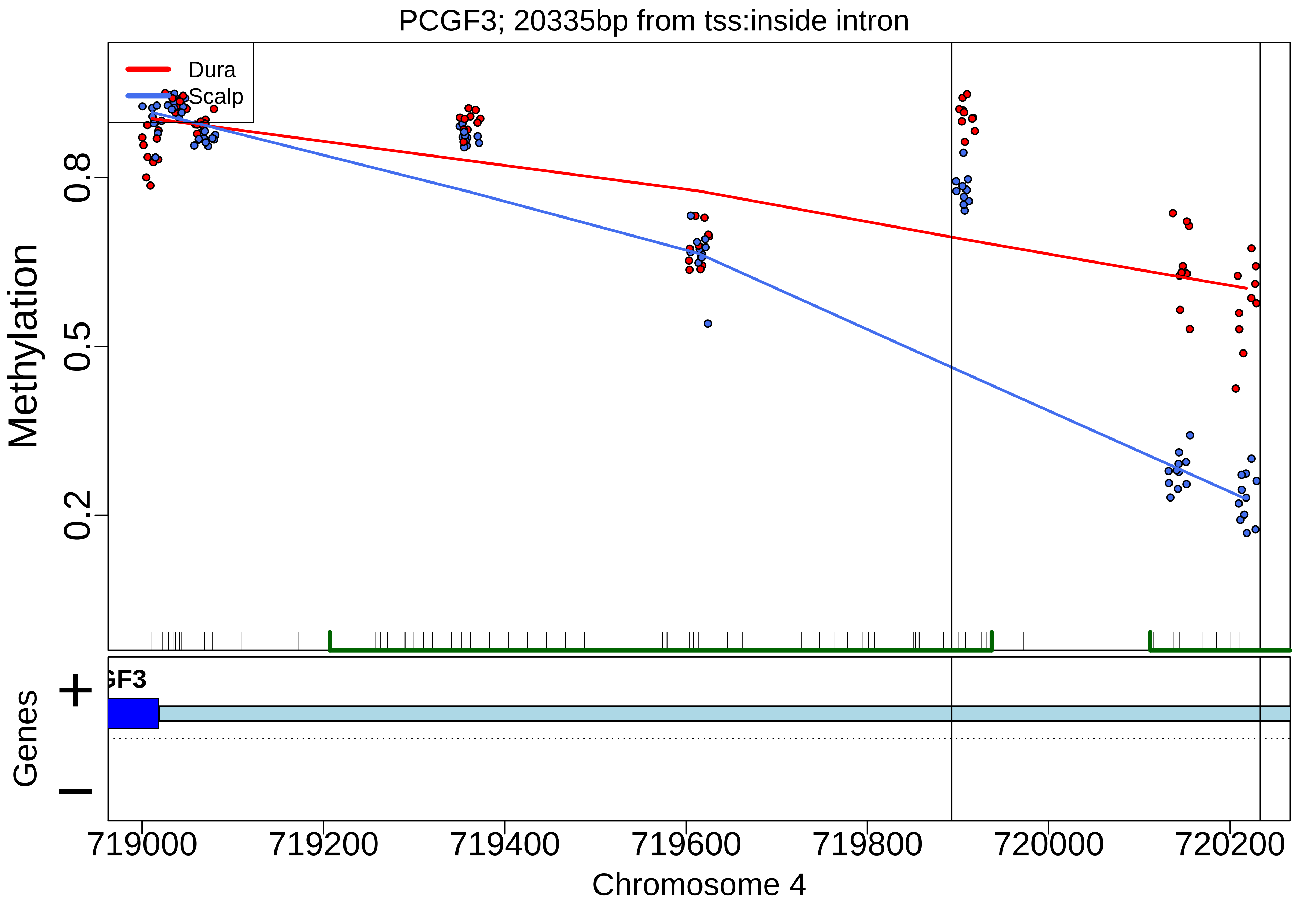

PCGF3; 20335bp from tss:inside intron
Dura
Scalp
Methylation
0.2
0.5
0.8
Genes
+
-
GF3
719000
719200
719400
719600
719800
720000
720200
Chromosome 4

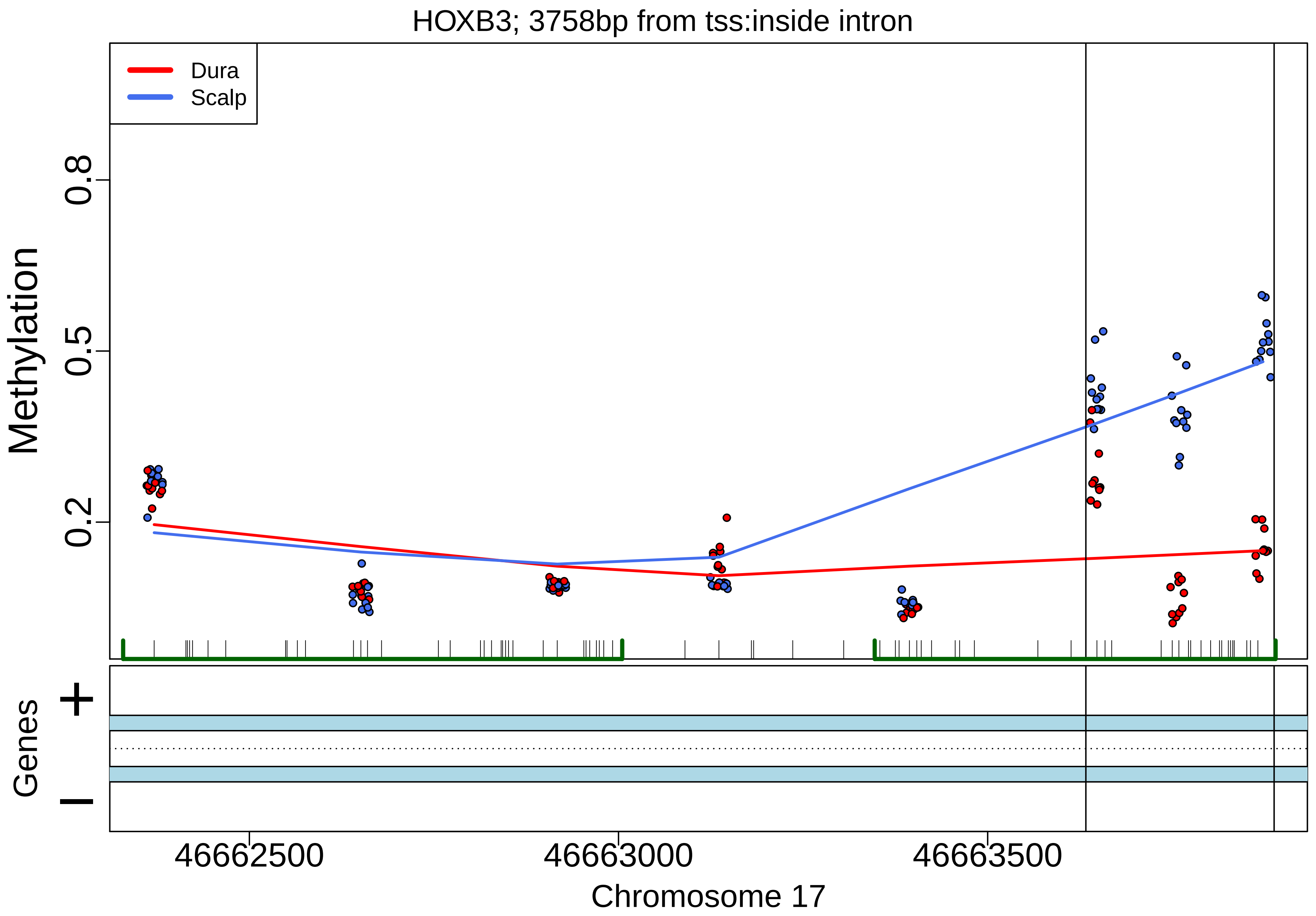
HOXB3; 3758bp from tss:inside intron
Dura
Scalp
Methylation
0.2
0.5
0.8
Genes
+
-
46662500
46663000
46663500
Chromosome 17

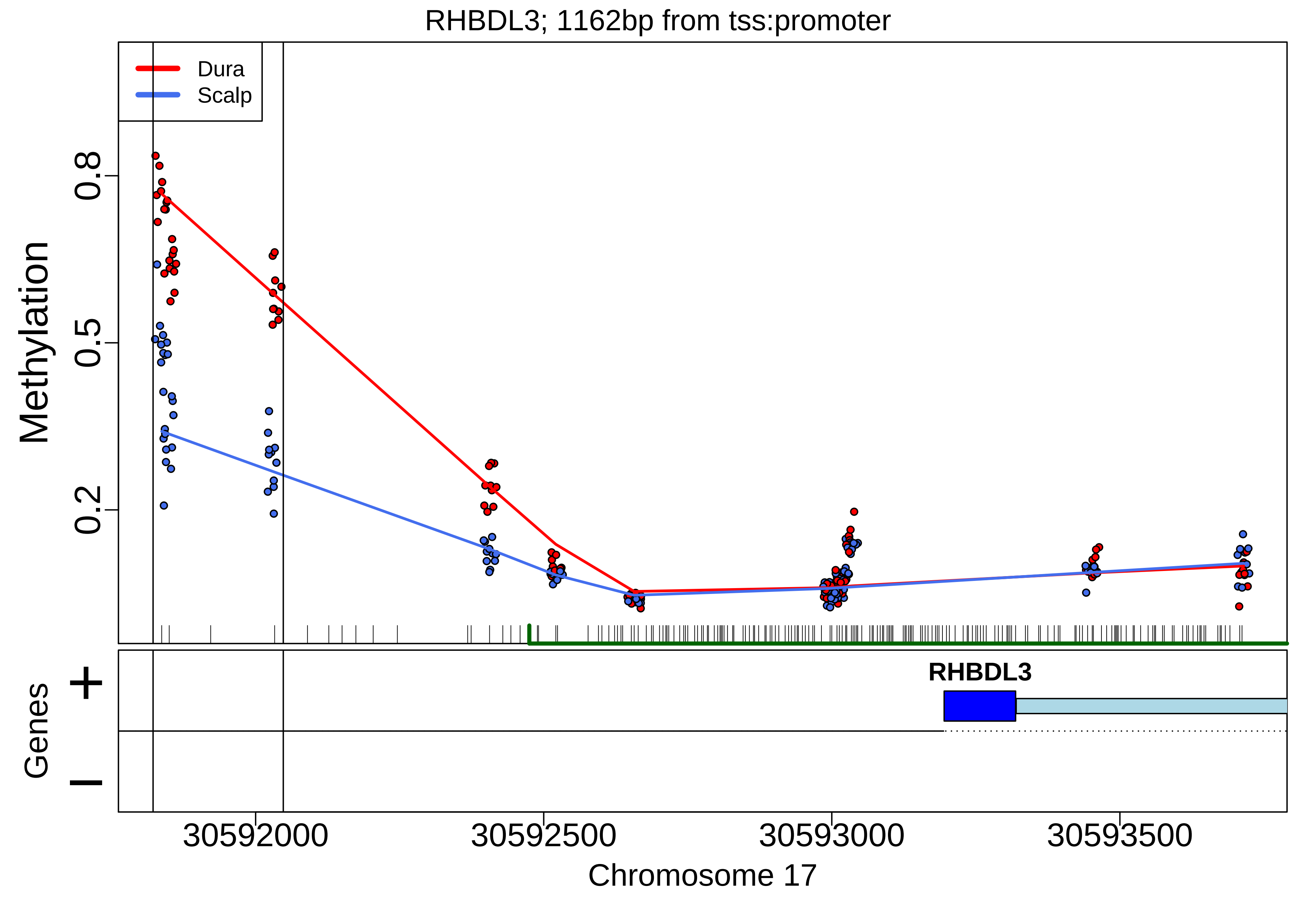
RHBDL3; 1162bp from tss:promoter
Dura
Scalp
Methylation
0.8
0.5
0.2
Genes
+
-
RHBDL3
30592000
30592500
30593000
30593500
Chromosome 17

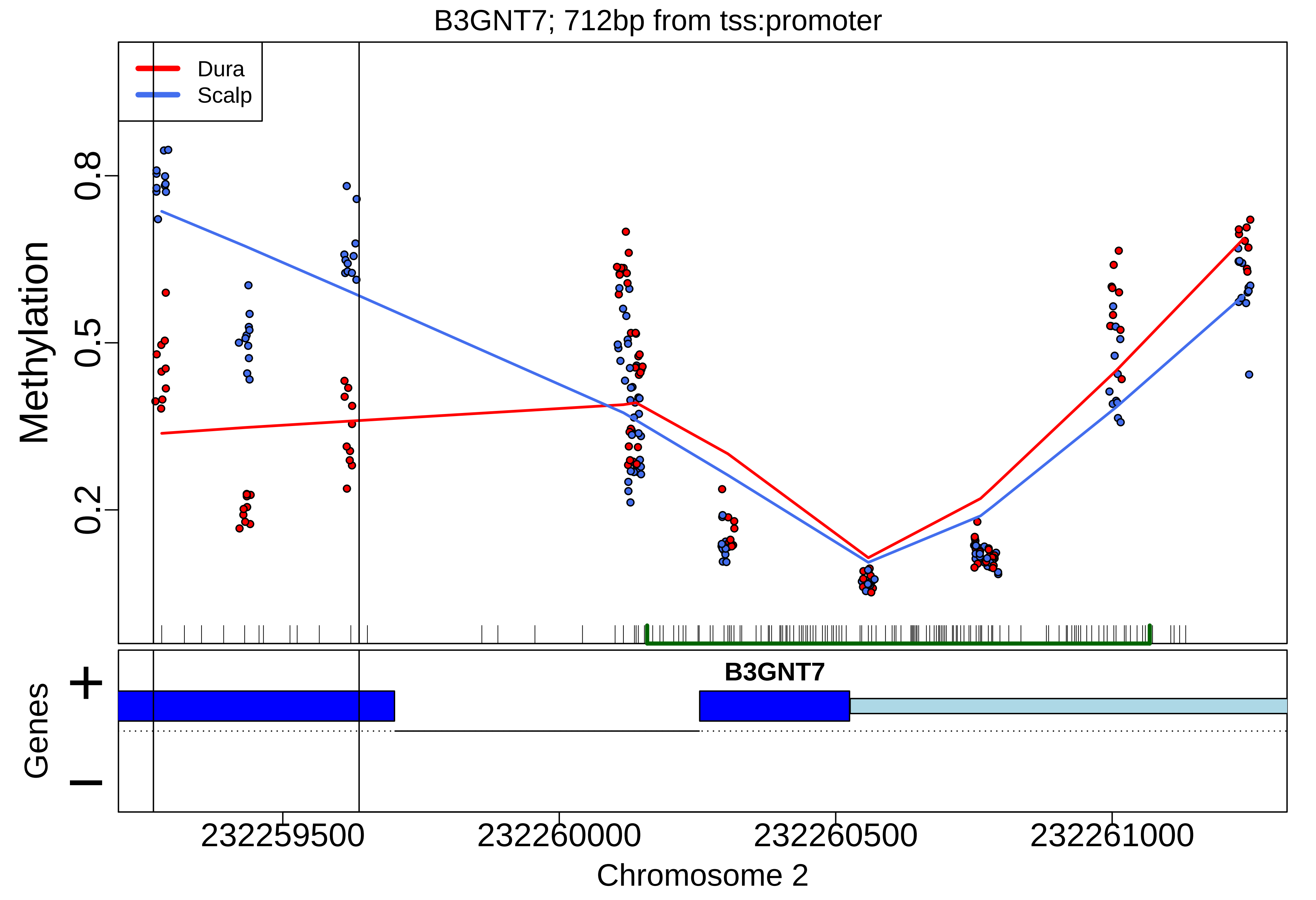
B3GNT7; 712bp from tss:promoter
Dura
Scalp
Methylation
0.2
0.5
0.8
Genes
+
−
B3GNT7
232259500
232260000
232260500
232261000
Chromosome 2

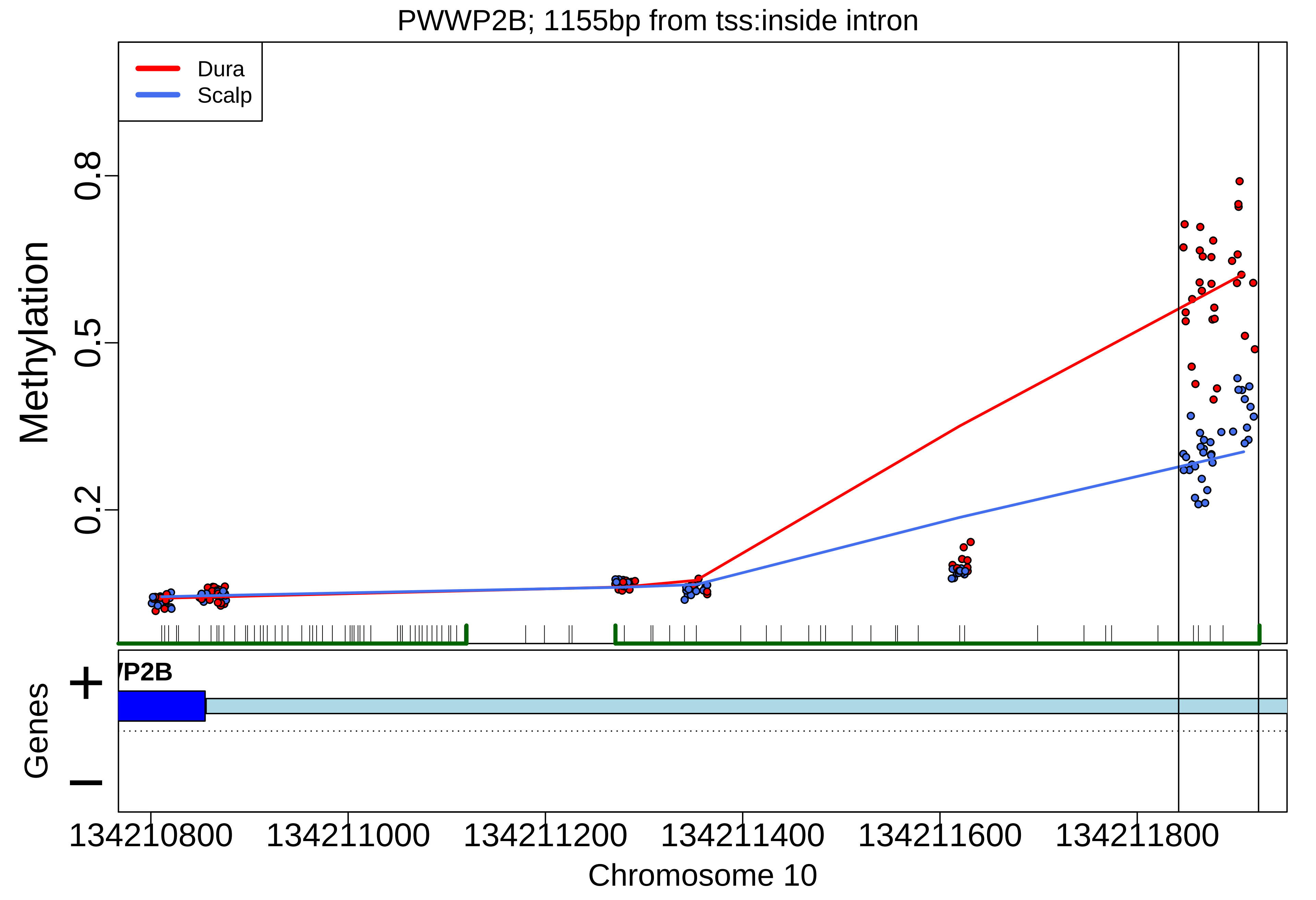

PWWP2B; 1155bp from tss:inside intron
Dura
Scalp
Methylation
0.8
0.5
0.2
Genes
+
−
/P2B
134210800
134211000
134211200
134211400
134211600
134211800
Chromosome 10

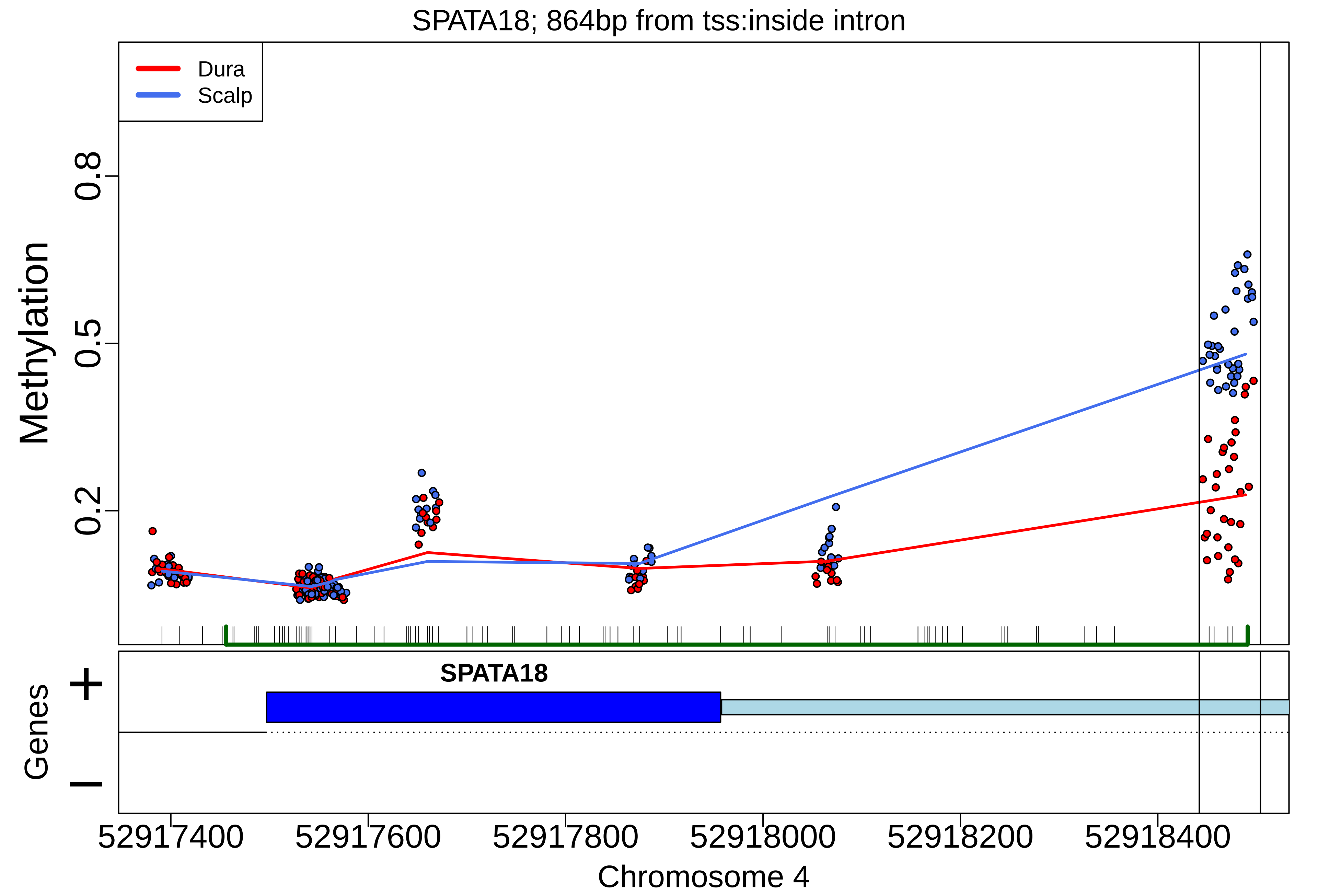

SPATA18; 864bp from tss:inside intron
Dura
Scalp
Methylation
0.2
0.5
0.8
Genes
+
−
SPATA18
52917400
52917600
52917800
52918000
52918200
52918400
Chromosome 4

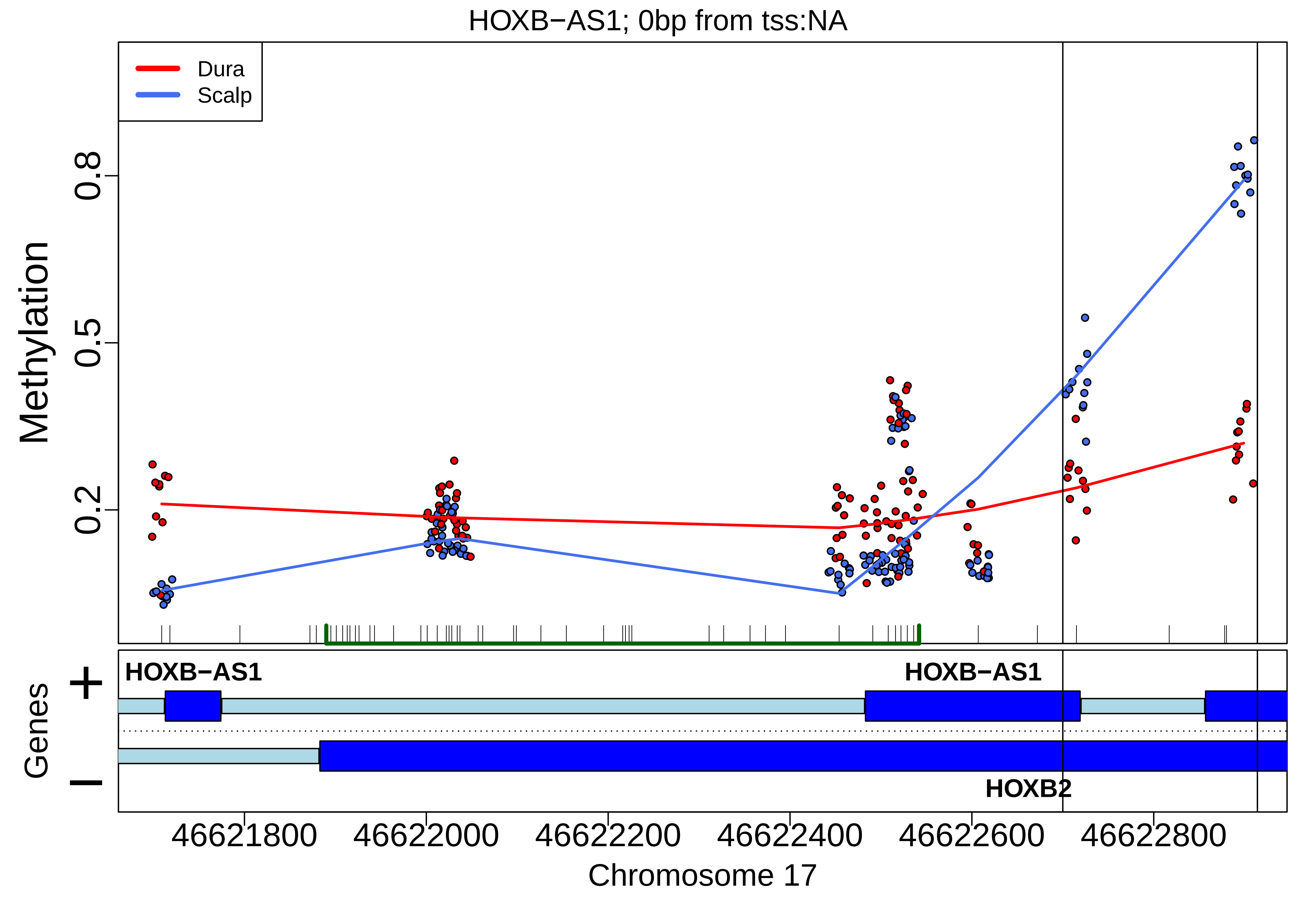

HOXB−AS1; 0bp from tss:NA
Dura
Scalp
Methylation
0.2
0.5
0.8
Genes
+
−
HOXB−AS1
HOXB−AS1
HOXB2
46621800
46622000
46622200
46622400
46622600
46622800
Chromosome 17

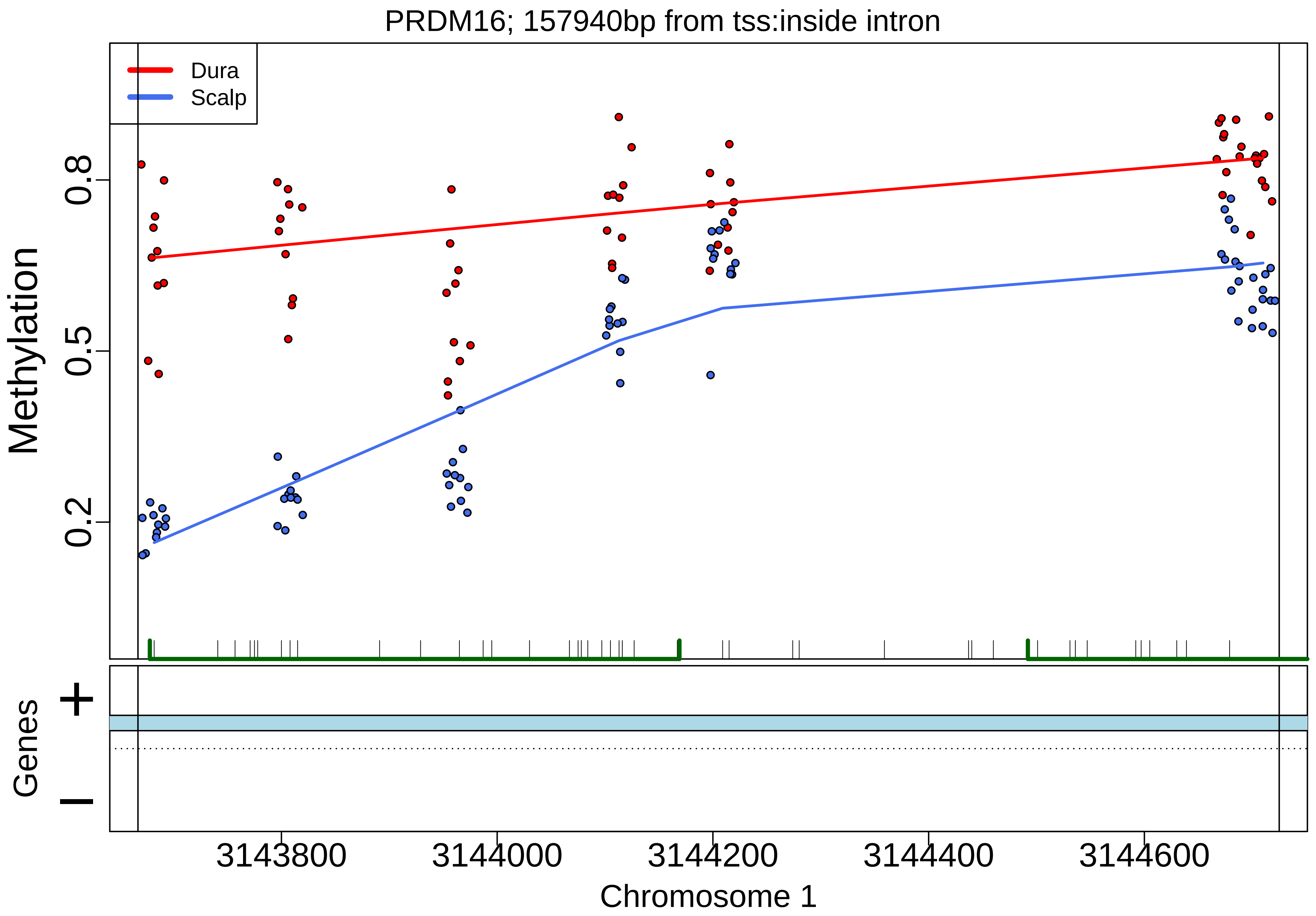

PRDM16; 157940bp from tss:inside intron
Dura
Scalp
Methylation
0.2
0.5
0.8
Genes
+
−
3143800
3144000
3144200
3144400
3144600
Chromosome 1

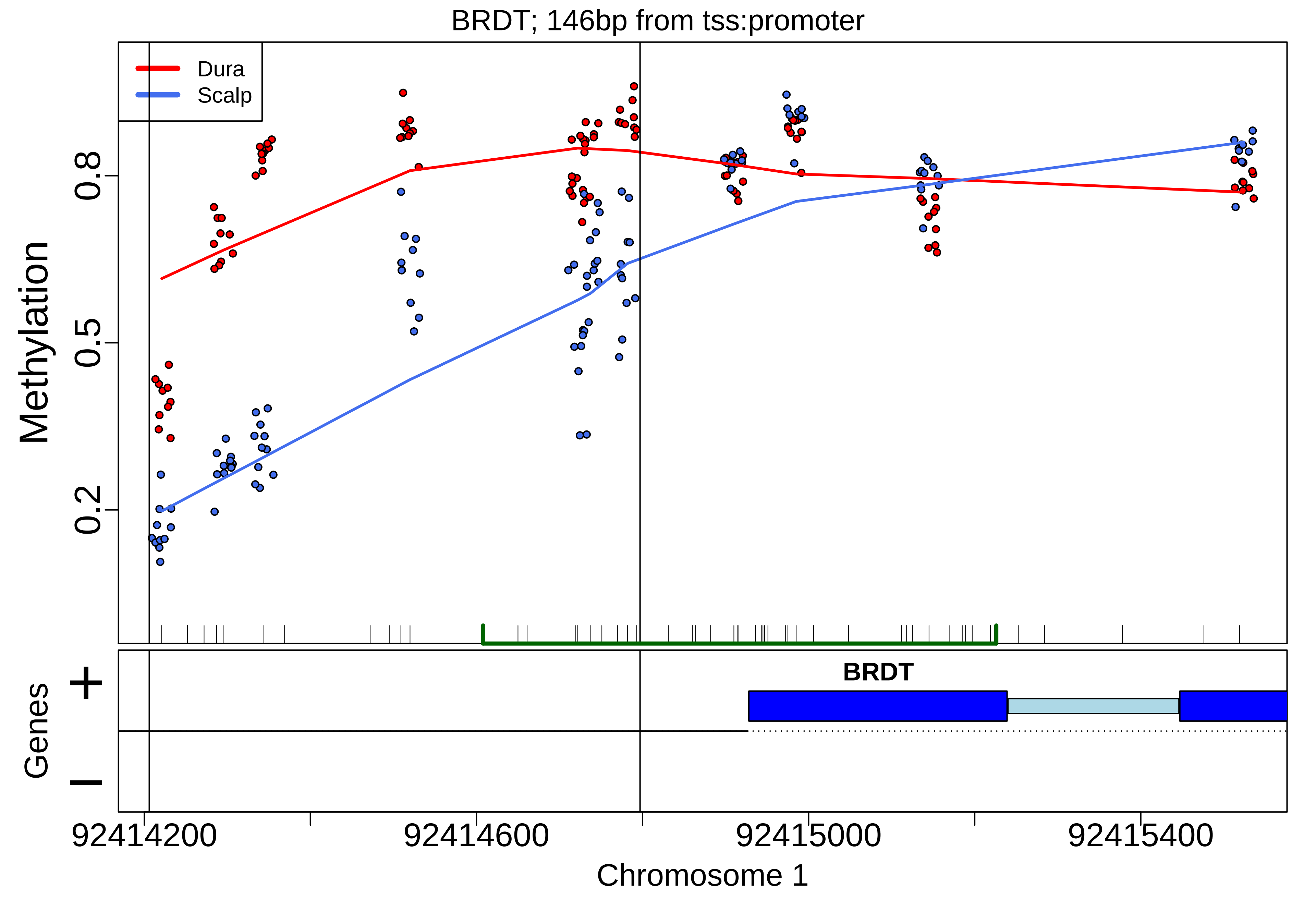

BRDT; 146bp from tss:promoter
Dura
Scalp
Methylation
0.2
0.5
0.8
Genes
+
-
BRDT
92414200
92414600
92415000
92415400
Chromosome 1

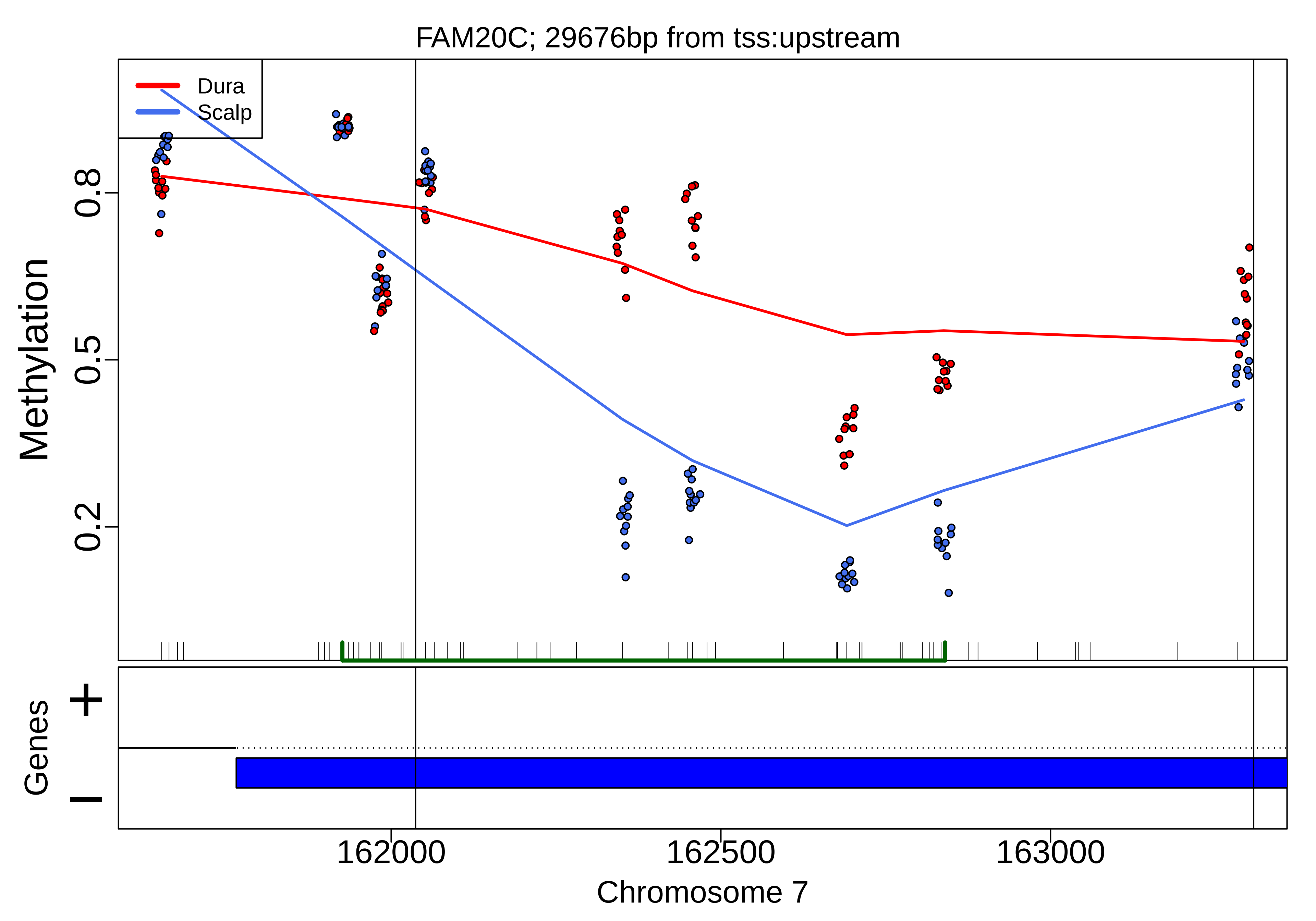
FAM20C; 29676bp from tss:upstream
Dura
Scalp
Methylation
0.2
0.5
0.8
Genes
+
-
162000
162500
163000
Chromosome 7

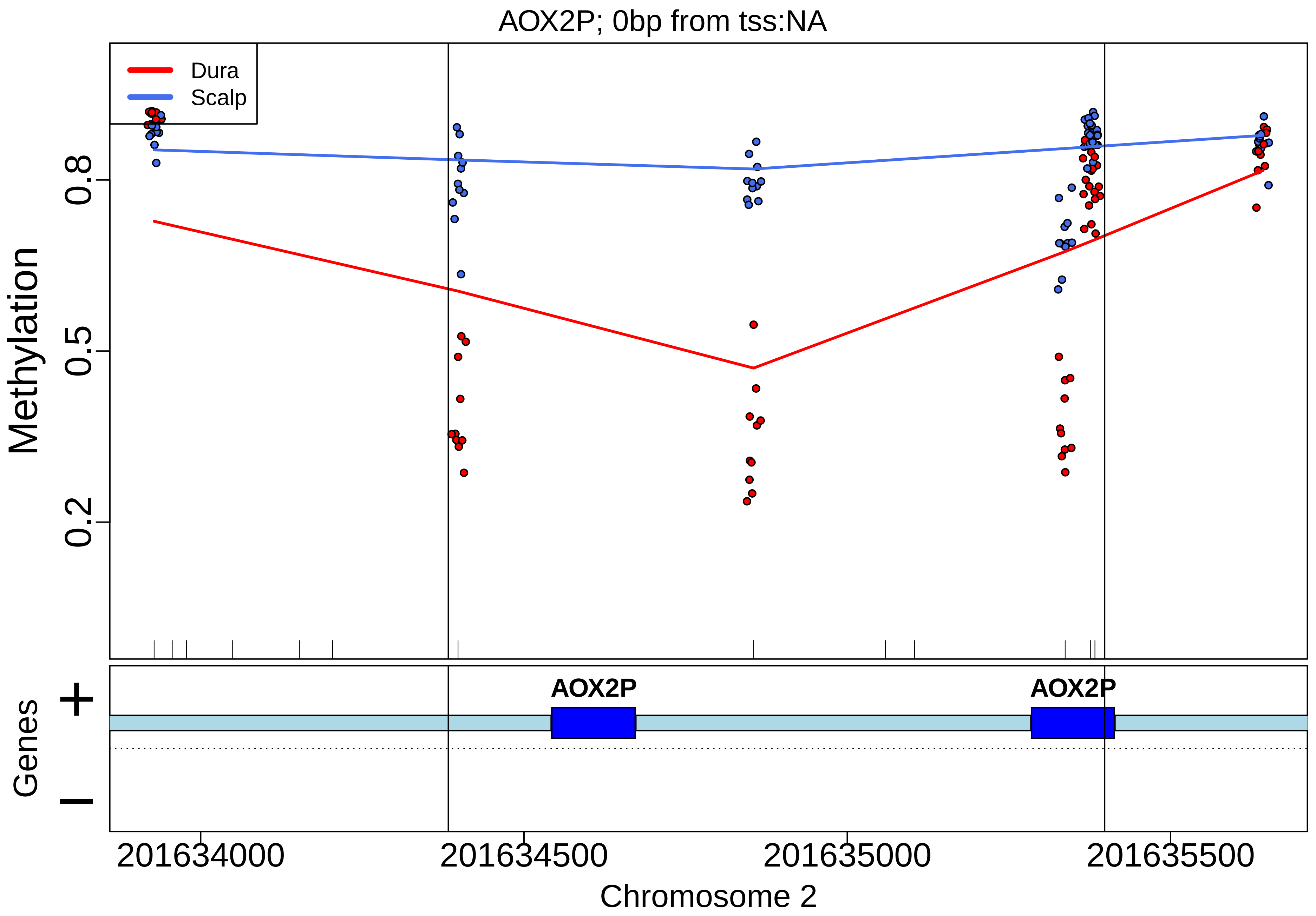
AOX2P; 0bp from tss:NA
Dura
Scalp
Methylation
0.2
0.5
0.8
Genes
+
AOX2P
AOX2P
201634000
201634500
201635000
201635500
Chromosome 2

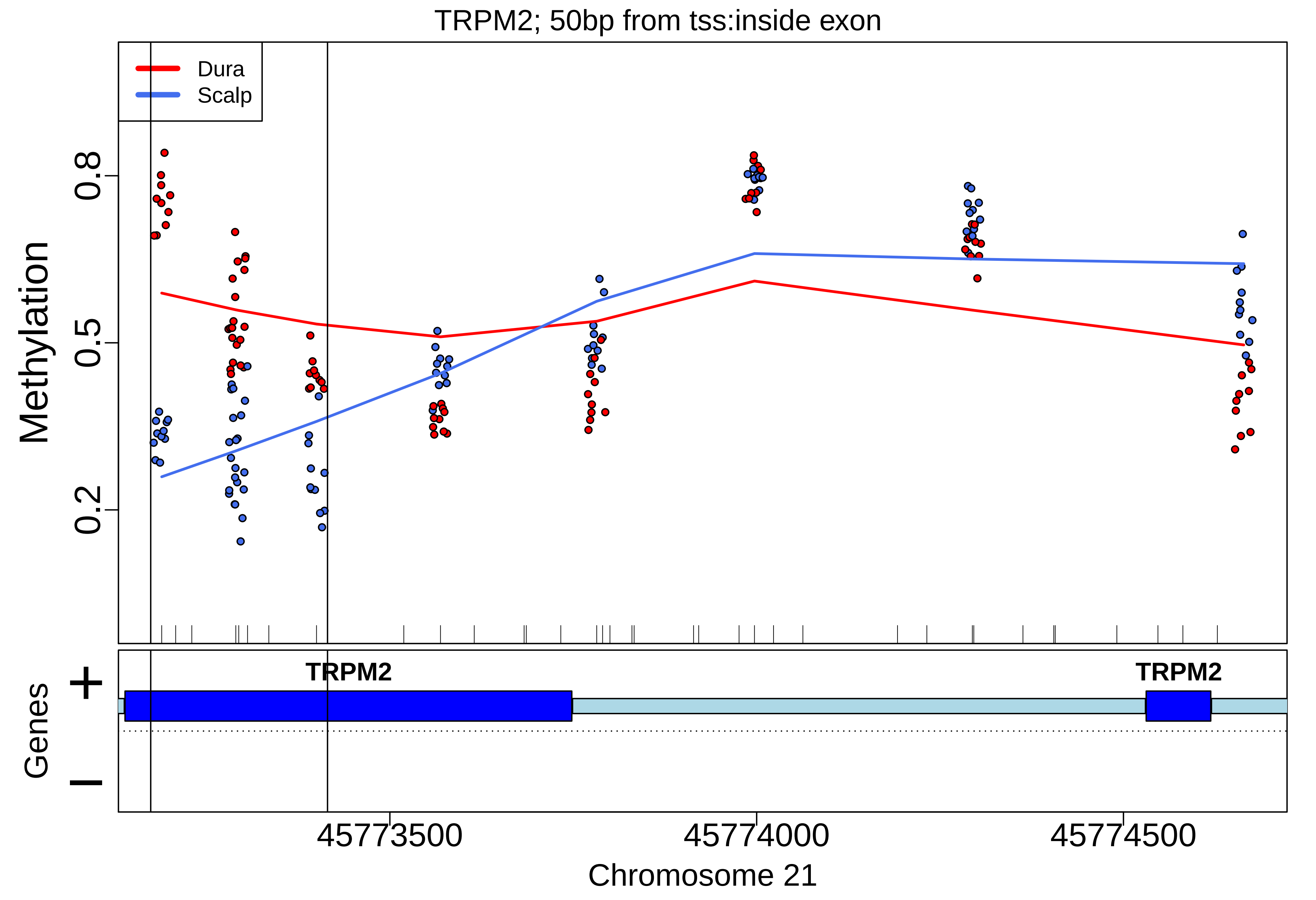

TRPM2; 50bp from tss:inside exon
Dura
Scalp
Methylation
0.2
0.5
0.8
Genes
+
-
TRPM2
TRPM2
45773500
45774000
45774500
Chromosome 21

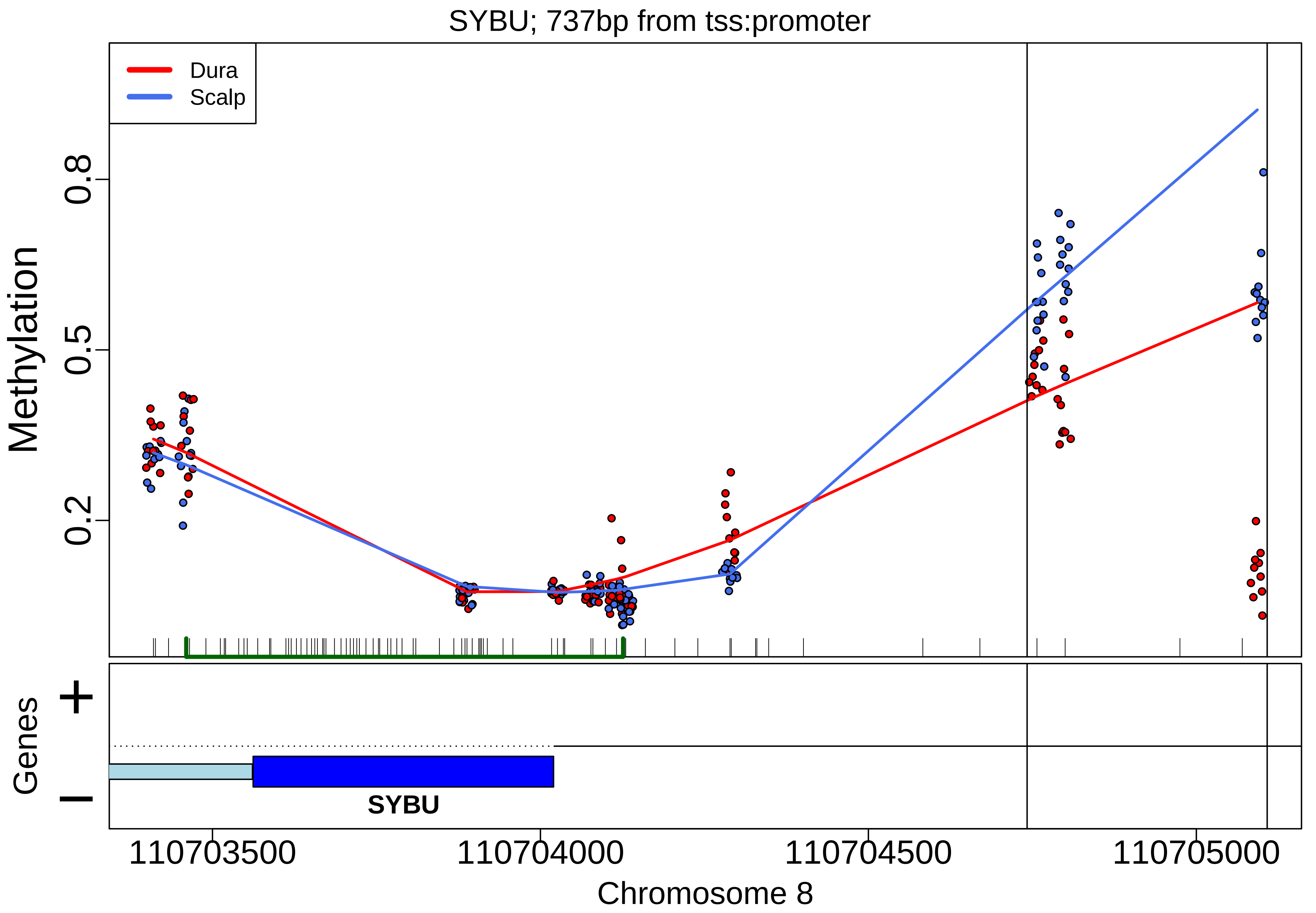
SYBU; 737bp from tss:promoter
Dura
Scalp
Methylation
0.2
0.5
0.8
Genes
+
−
SYBU
110703500
110704000
110704500
110705000
Chromosome 8

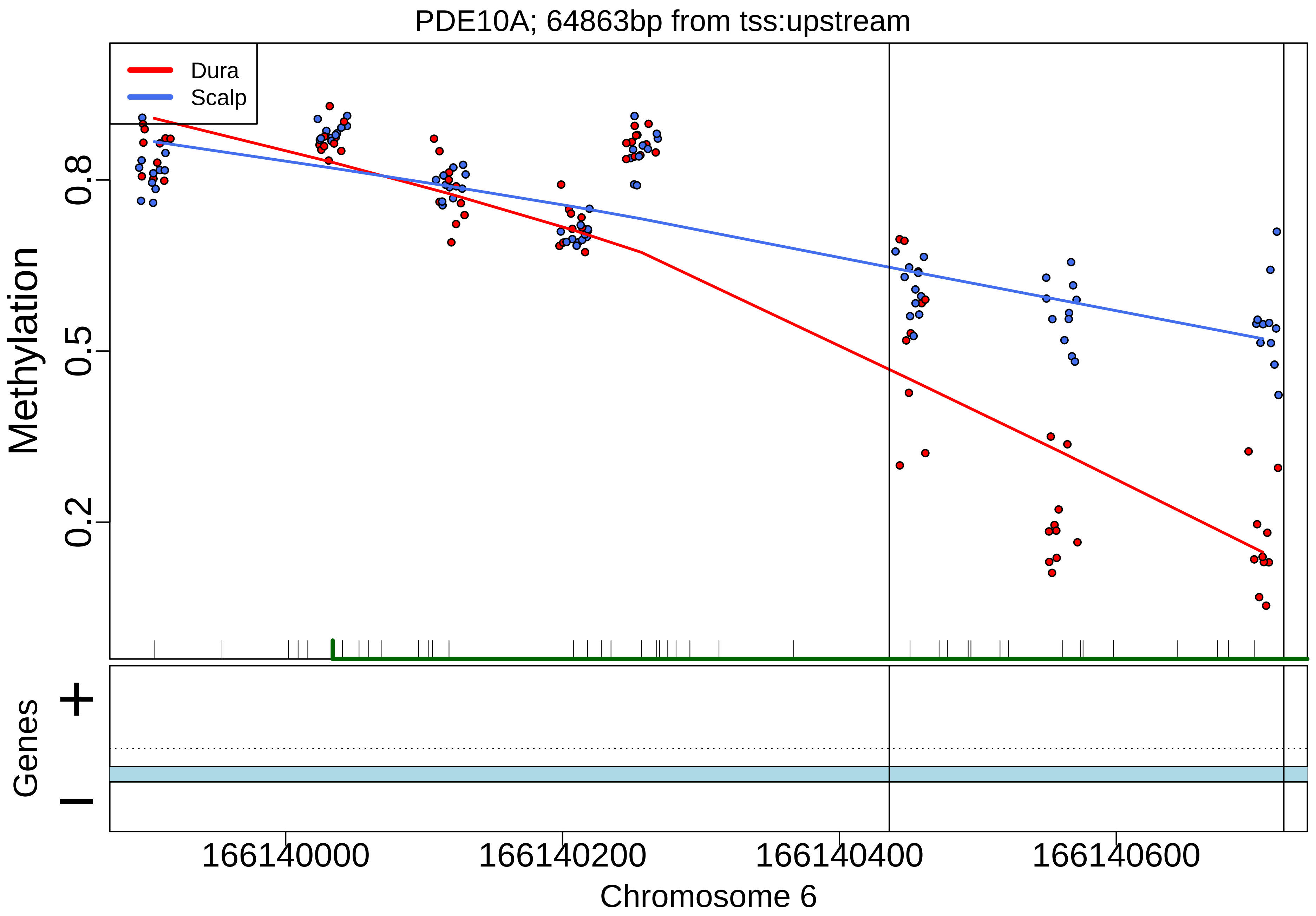

PDE10A; 64863bp from tss:upstream
Dura
Scalp
Methylation
0.2
0.5
0.8
Genes
+
-
166140000
166140200
166140400
166140600
Chromosome 6

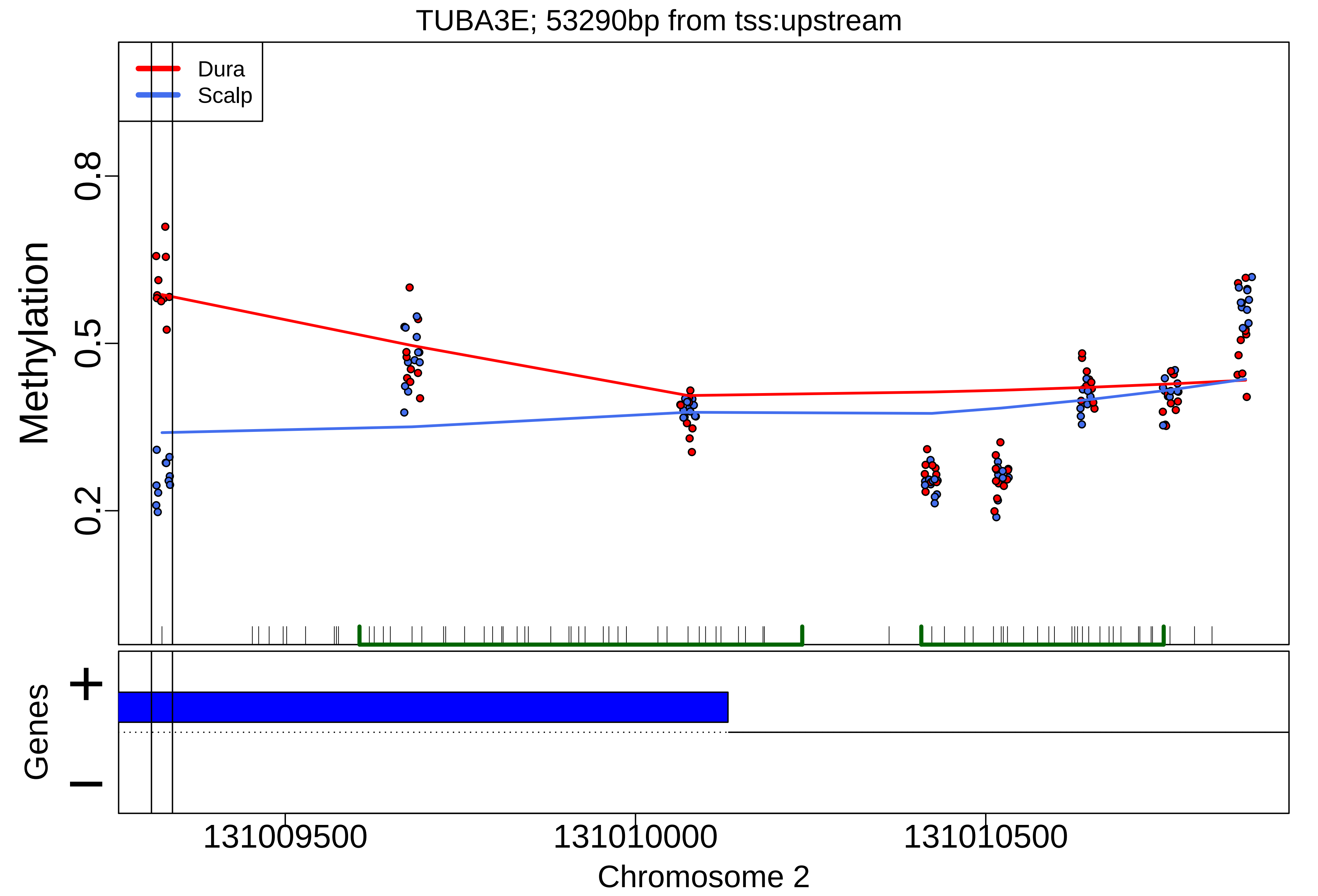

TUBA3E; 53290bp from tss:upstream
Dura
Scalp
Methylation
0.2
0.5
0.8
Genes
+
−
131009500
131010000
131010500
Chromosome 2

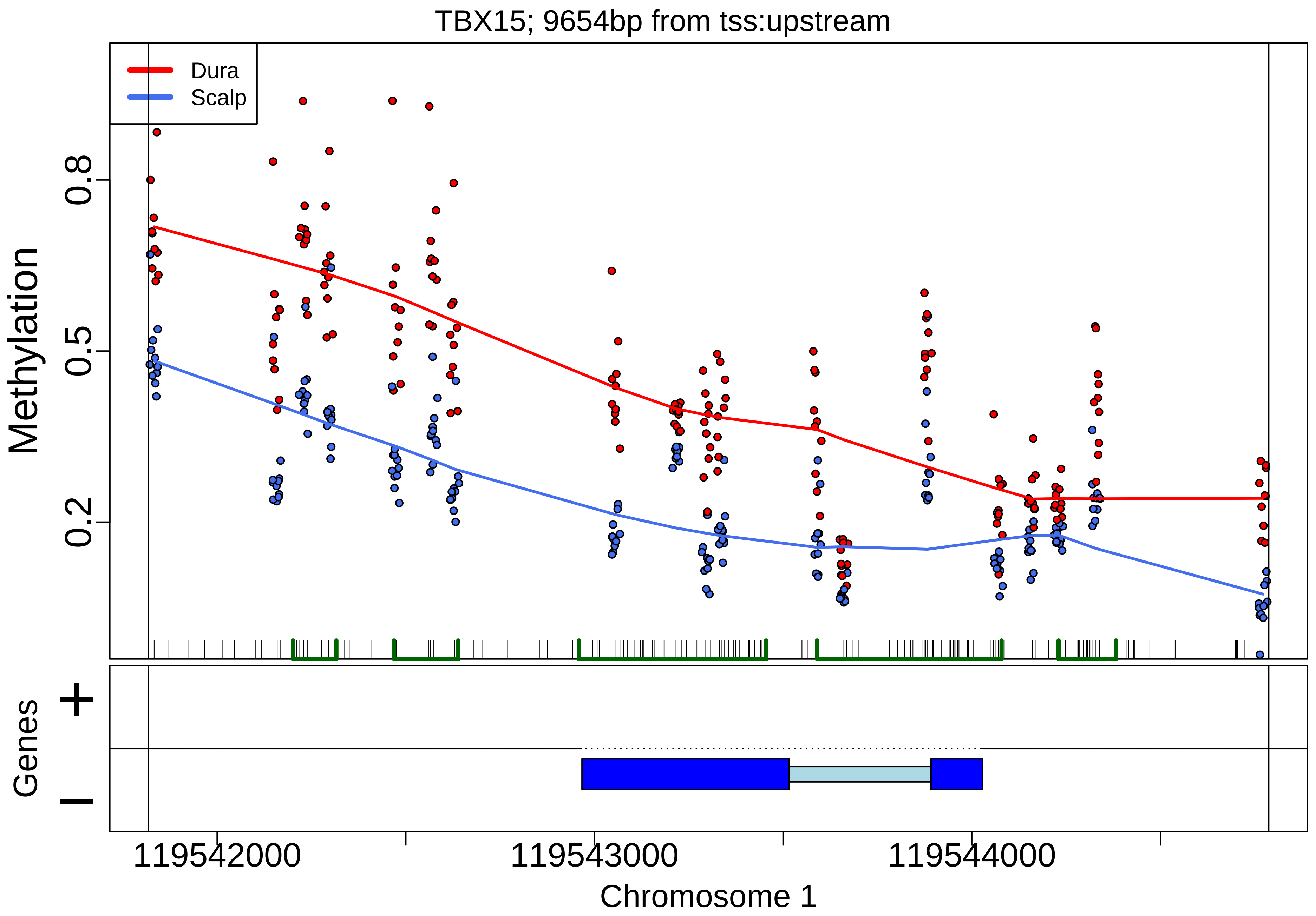
TBX15; 9654bp from tss:upstream
Dura
Scalp
Methylation
0.2
0.5
0.8
Genes
+
−
119542000
119543000
119544000
Chromosome 1

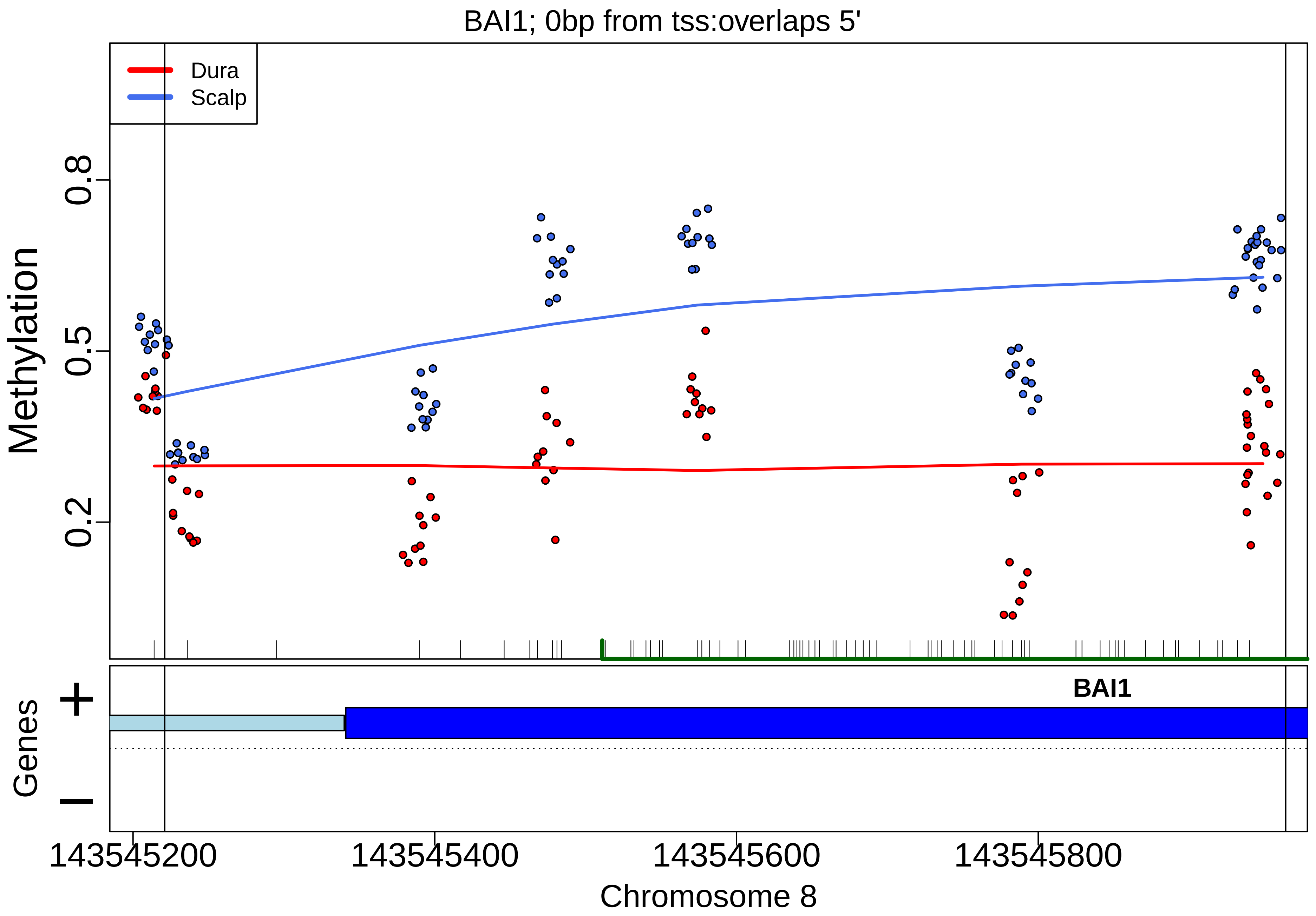

BAI1; 0bp from tss:overlaps 5'
Dura
Scalp
Methylation
0.2
0.5
0.8
Genes
+
−
BAI1
143545200
143545400
143545600
143545800
Chromosome 8

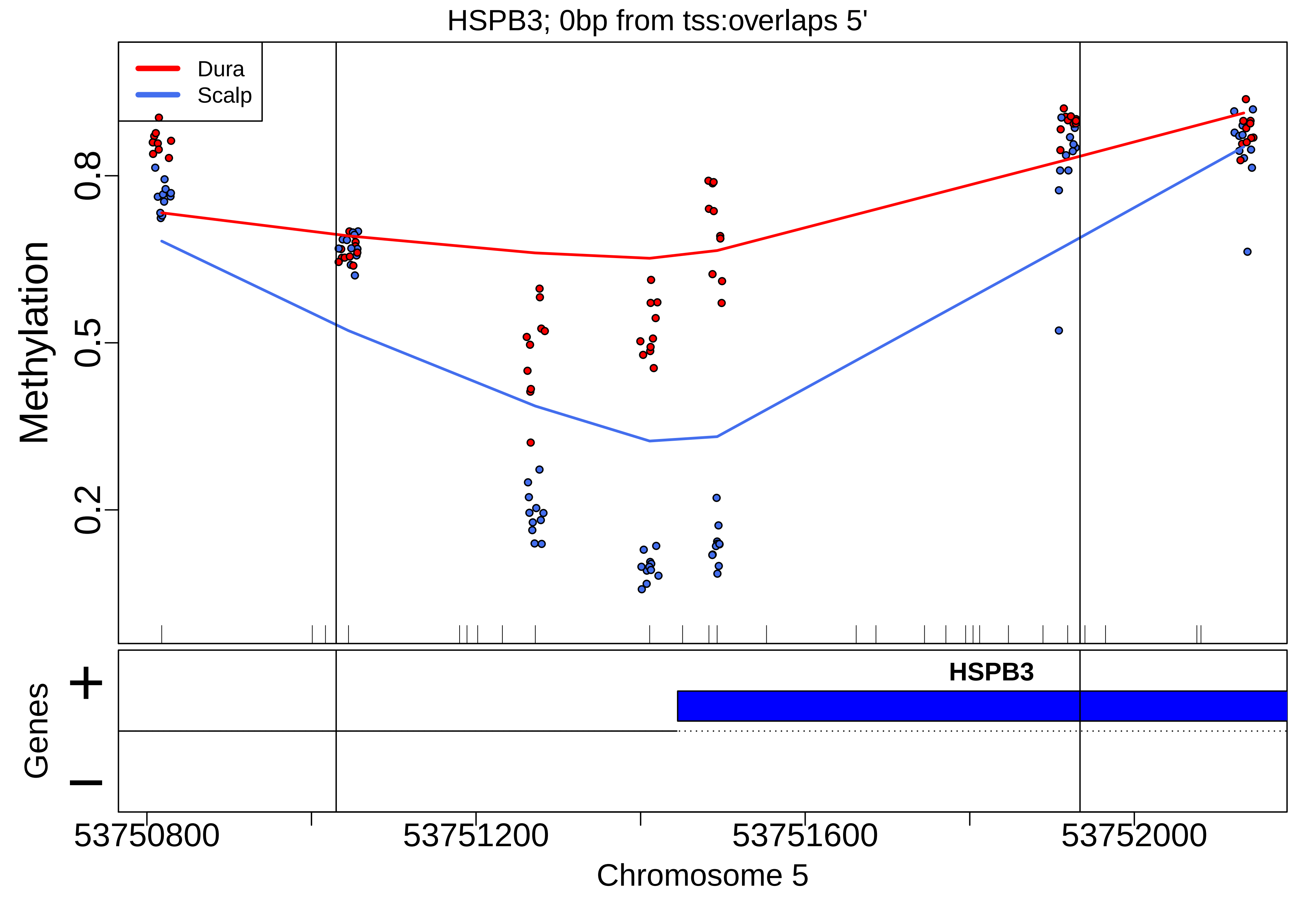

HSPB3; 0bp from tss:overlaps 5'
Dura
Scalp
Methylation
0.2
0.5
0.8
Genes
+
-
HSPB3
53750800
53751200
53751600
53752000
Chromosome 5

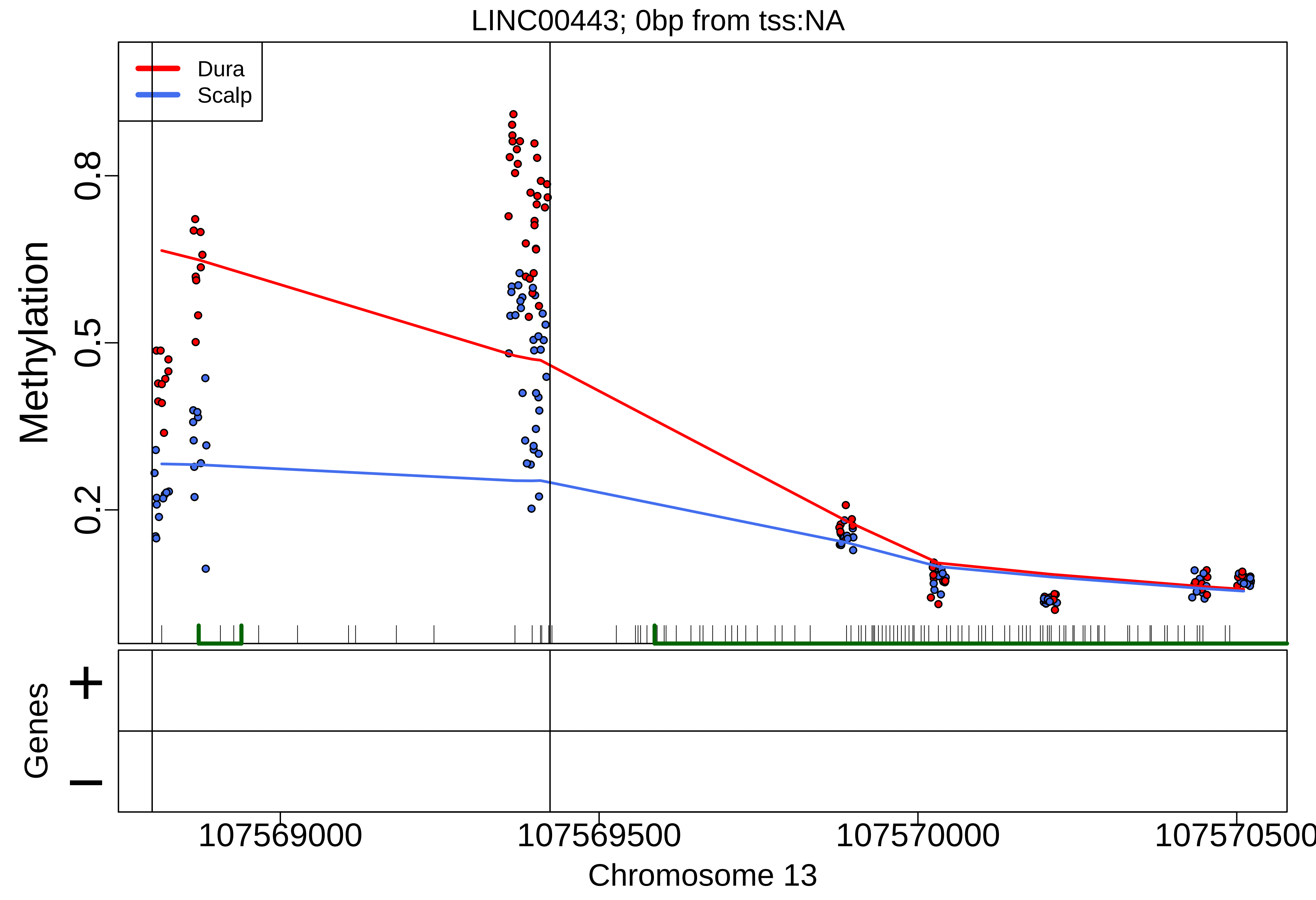

LINC00443; 0bp from tss:NA
Dura
Scalp
Methylation
0.2
0.5
0.8
Genes
+
−
107569000
107569500
107570000
107570500
Chromosome 13

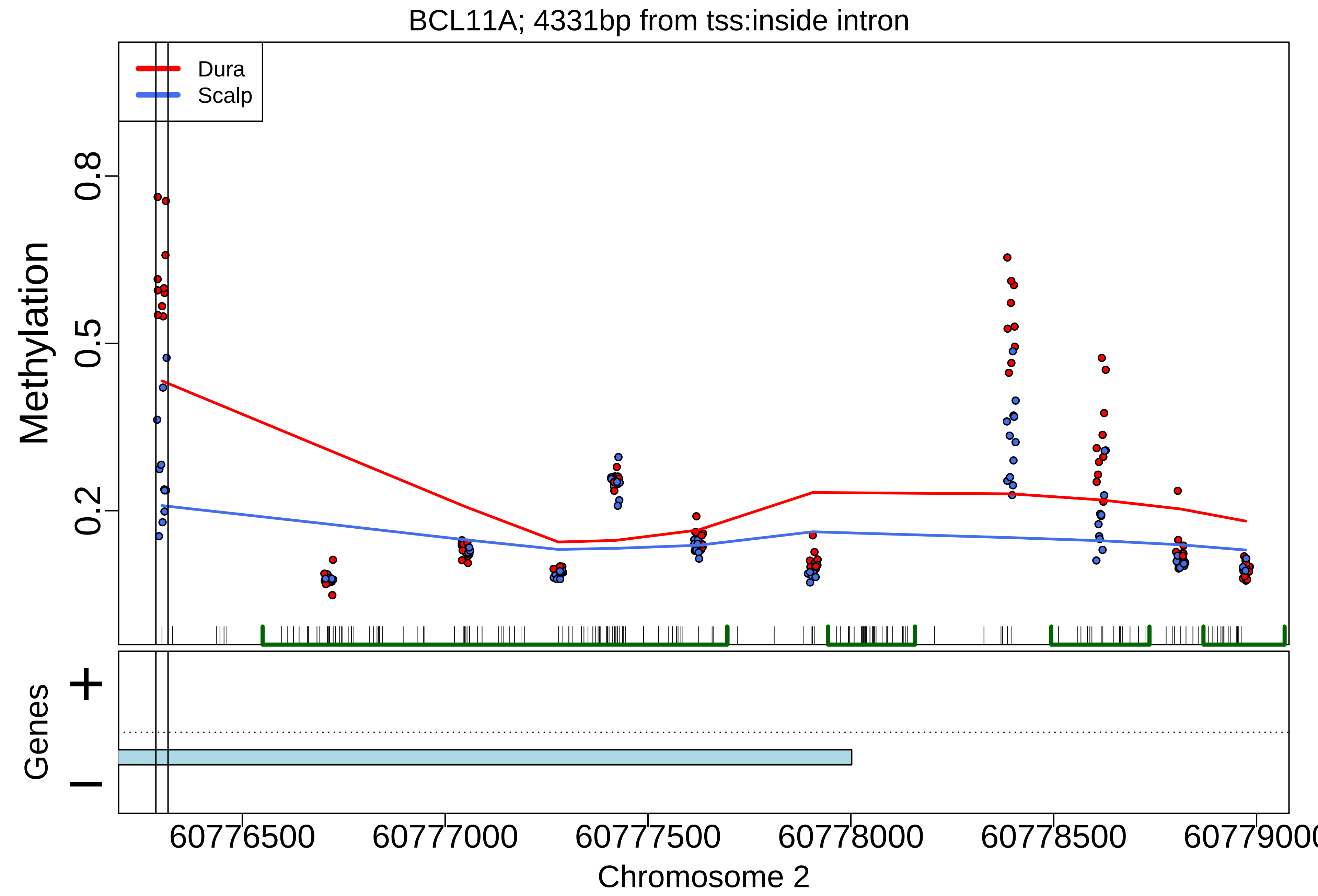

BCL11A; 4331bp from tss:inside intron
Dura
Scalp
Methylation
0.2
0.5
0.8
Genes
+
-
60776500
60777000
60777500
60778000
60778500
60779000
Chromosome 2

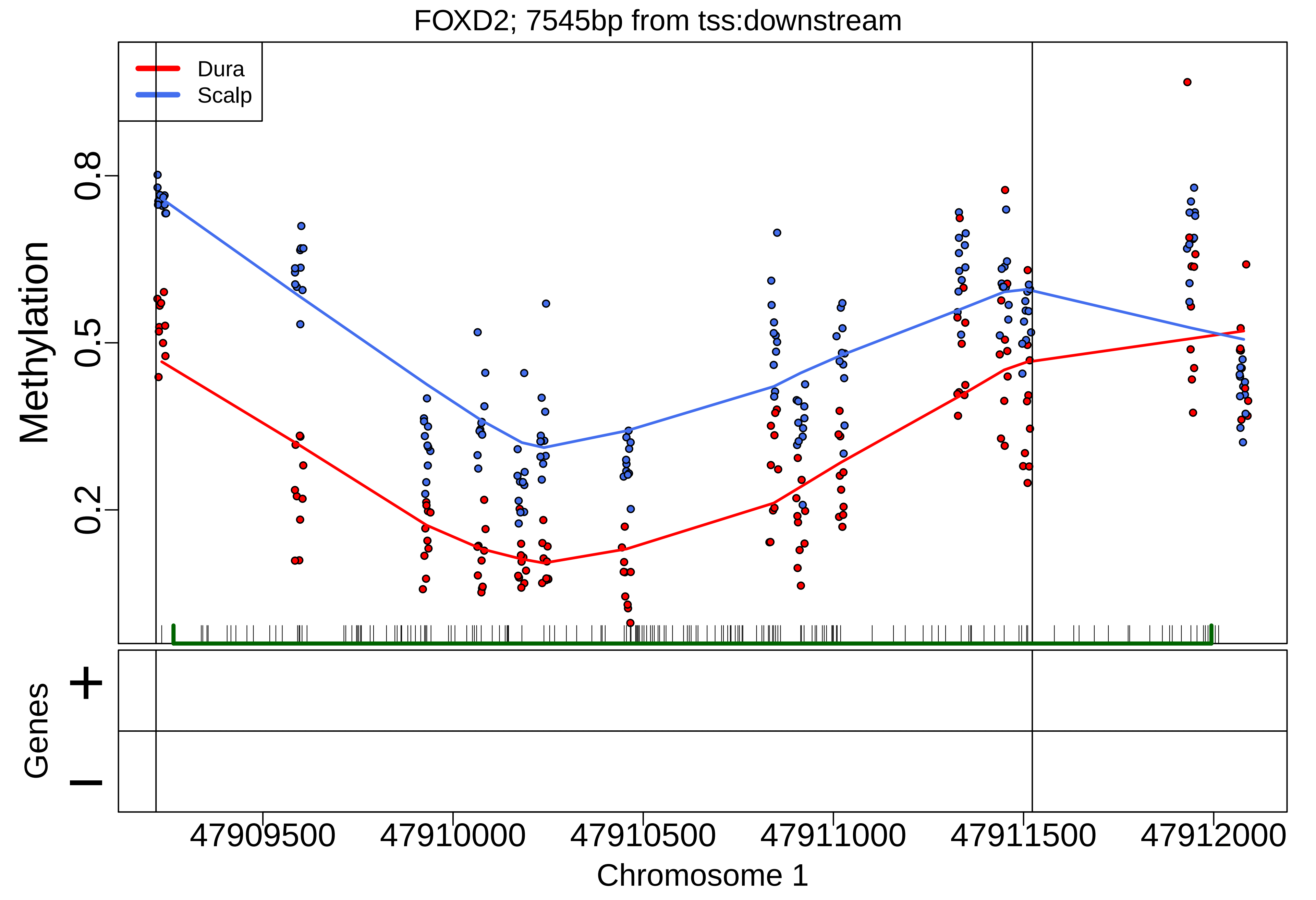
FOXD2; 7545bp from tss:downstream
Dura
Scalp
Methylation
0.2
0.5
0.8
Genes
+
-
47909500
47910000
47910500
47911000
47911500
47912000
Chromosome 1

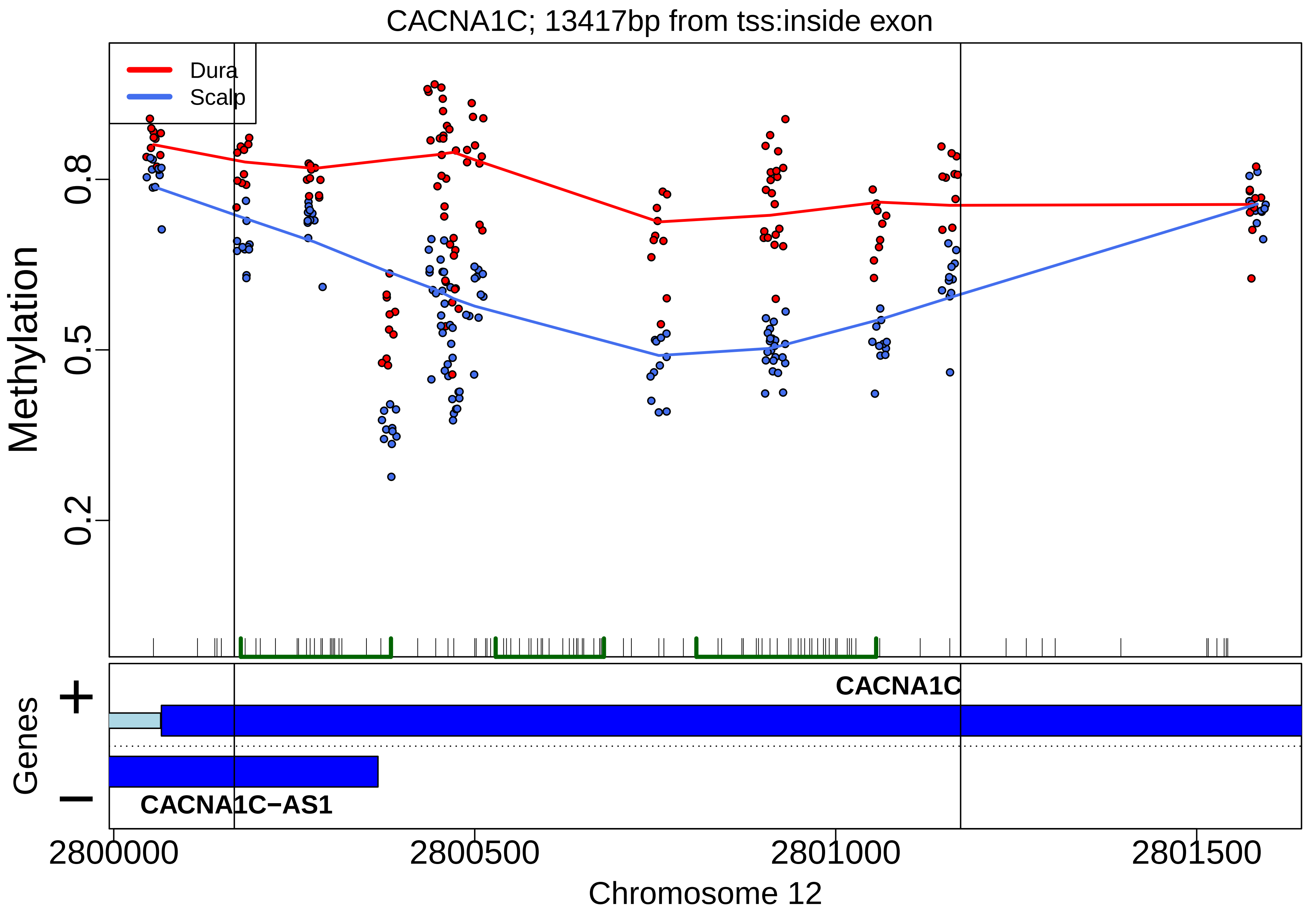
CACNA1C; 13417bp from tss:inside exon
Dura
Scalp
Methylation
0.2
0.5
0.8
Genes
+
−
CACNA1C
CACNA1C−AS1
2800000
2800500
2801000
2801500
Chromosome 12

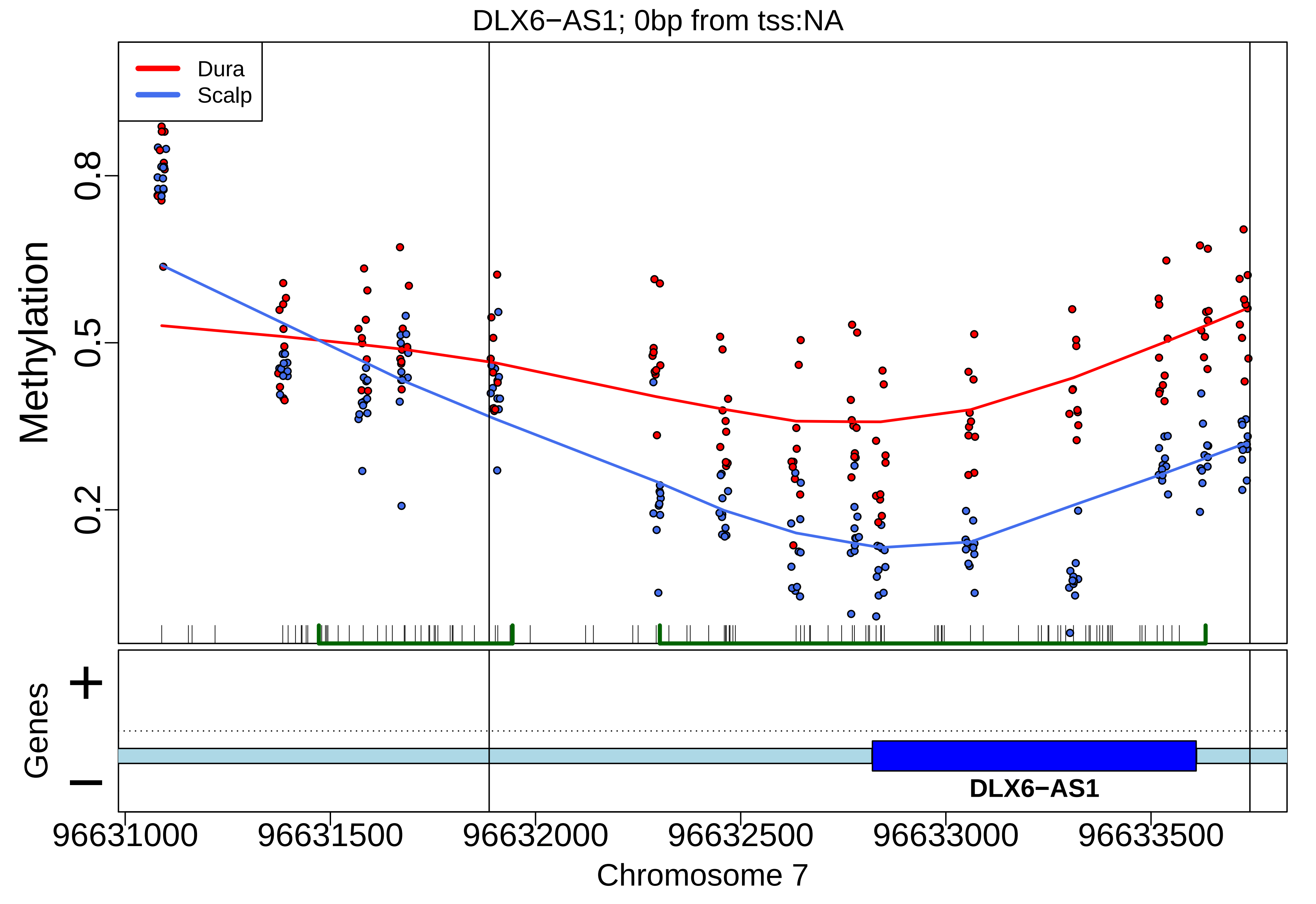

DLX6−AS1; 0bp from tss:NA
Dura
Scalp
Methylation
0.2
0.5
0.8
Genes
+
−
DLX6−AS1
96631000
96631500
96632000
96632500
96633000
96633500
Chromosome 7

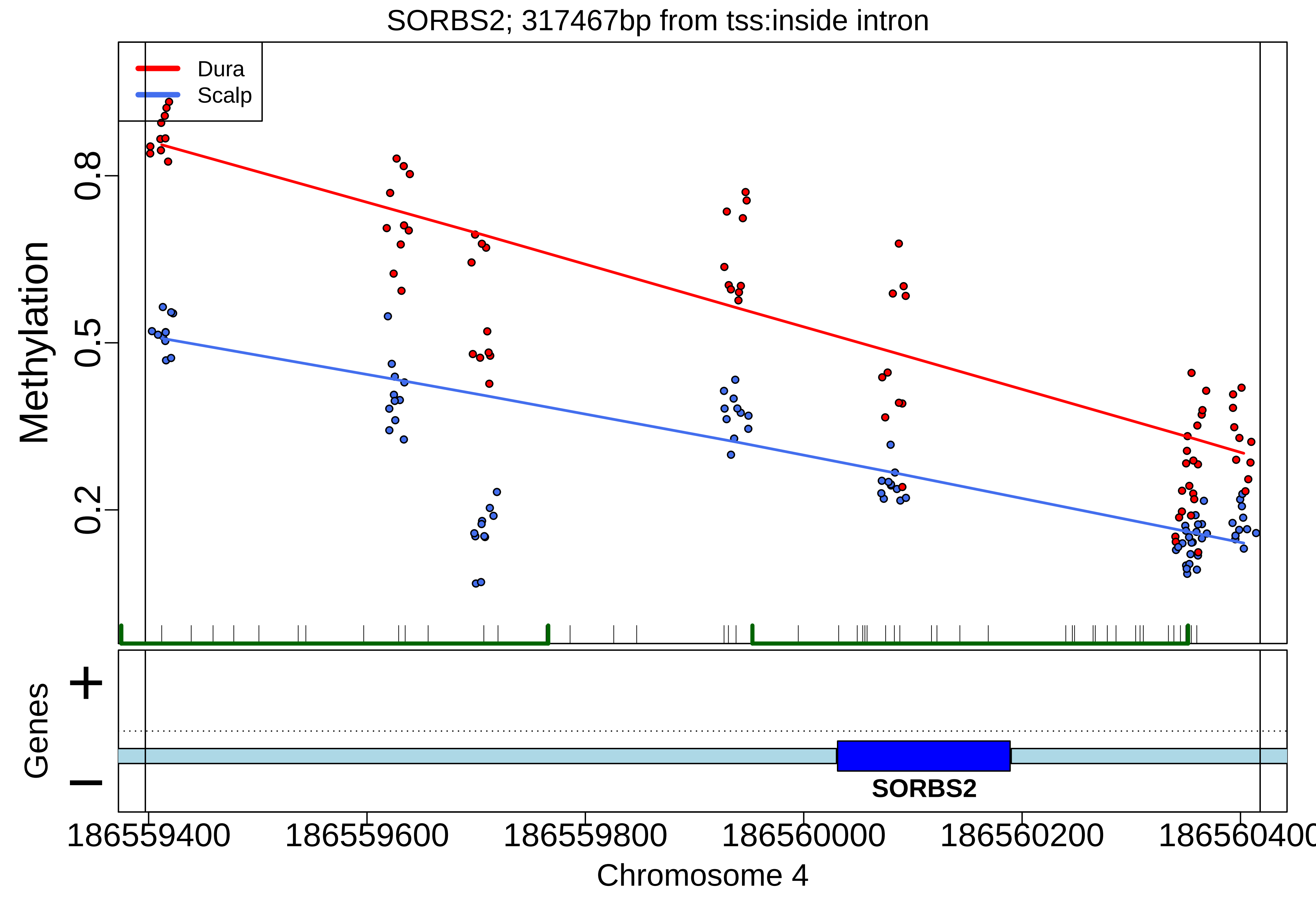
SORBS2; 317467bp from tss:inside intron
Dura
Scalp
Methylation
0.2
0.5
0.8
Genes
+
-
SORBS2
186559400
186559600
186559800
186560000
186560200
186560400
Chromosome 4

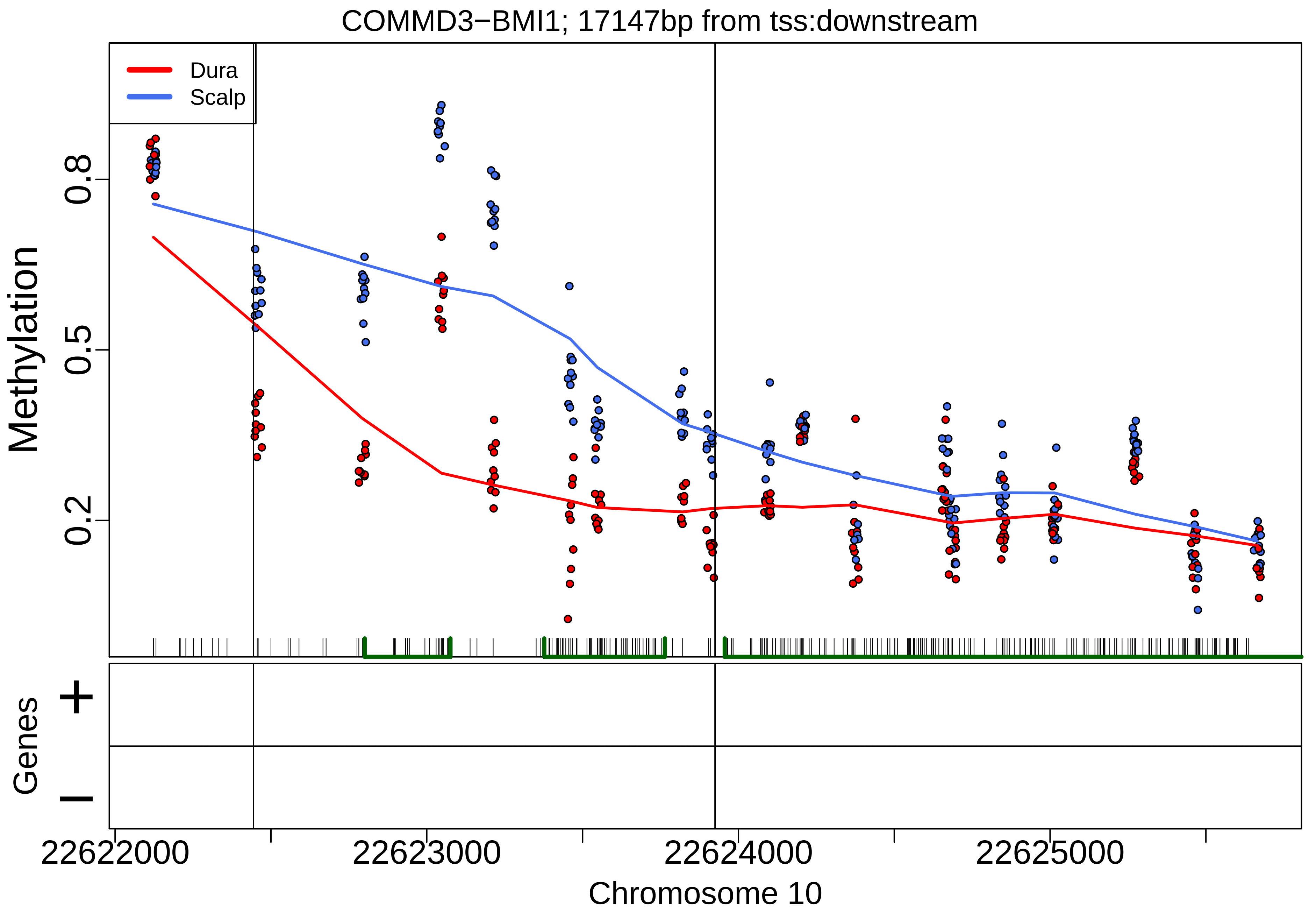
COMMD3−BMI1; 17147bp from tss:downstream
Dura
Scalp
Methylation
0.2
0.5
0.8
Genes
+
−
22622000
22623000
22624000
22625000
Chromosome 10

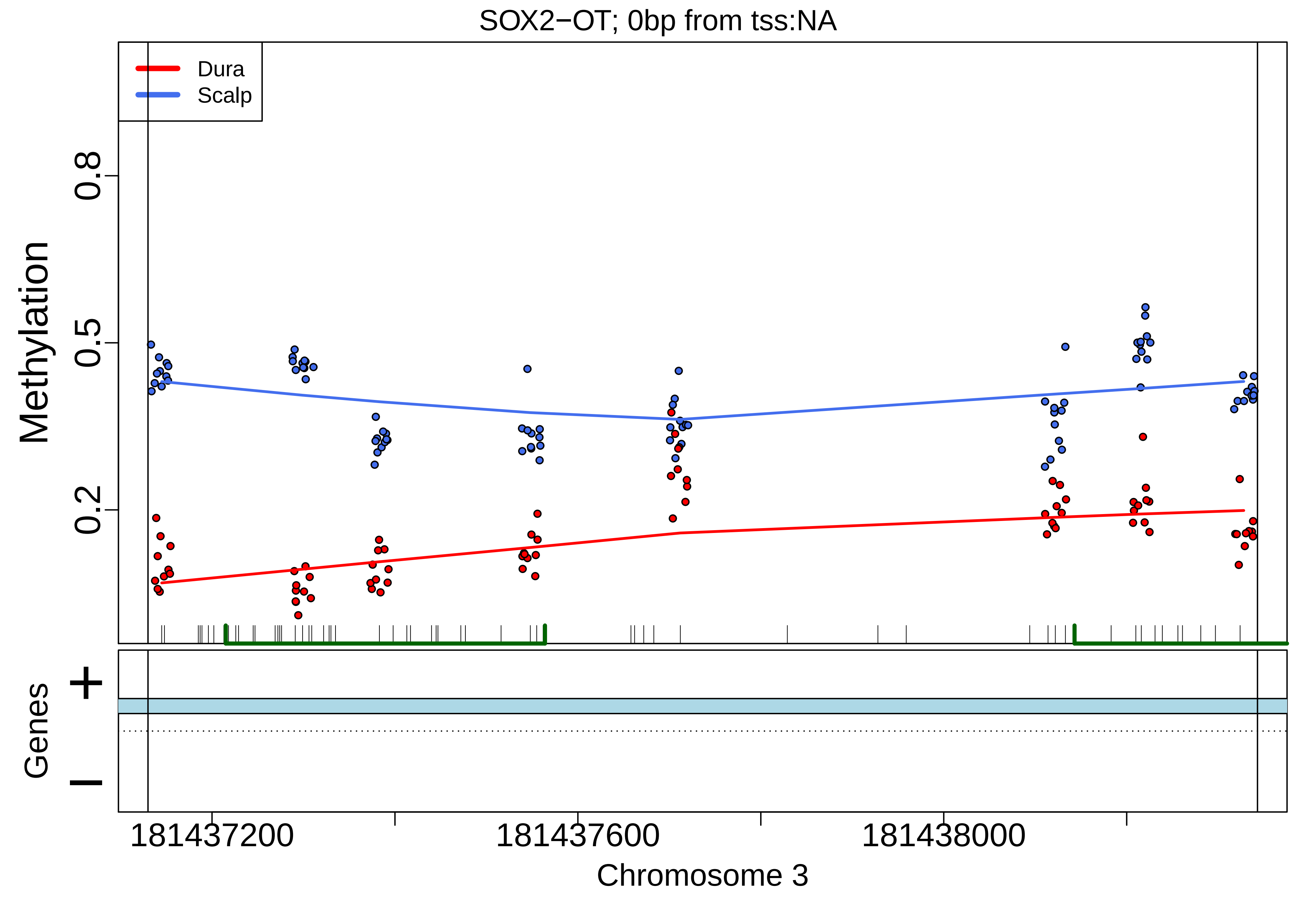

SOX2−OT; 0bp from tss:NA
Dura
Scalp
Methylation
0.2
0.5
0.8
Genes
+
−
181437200
181437600
181438000
Chromosome 3

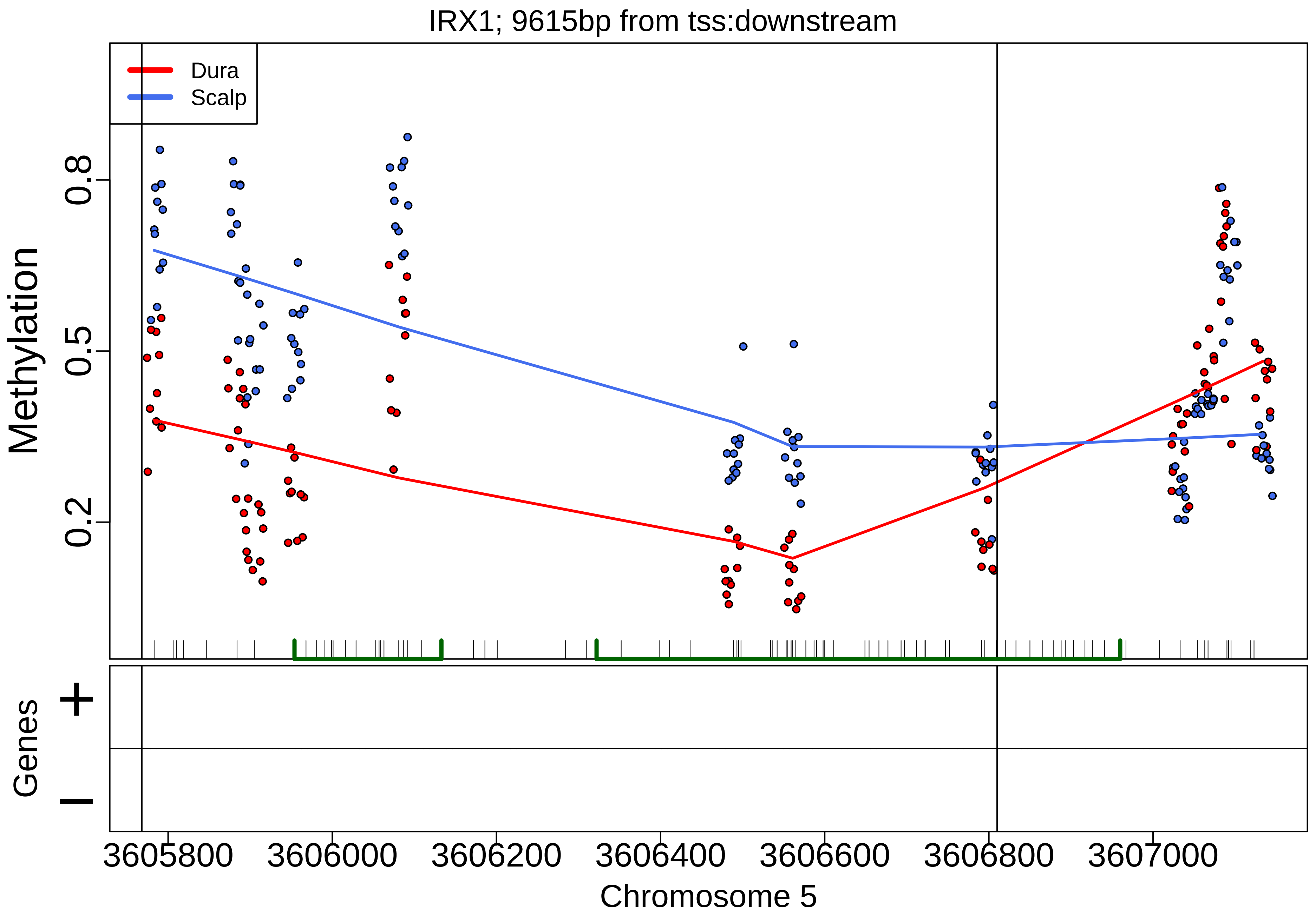
IRX1; 9615bp from tss:downstream
Dura
Scalp
Methylation
0.2
0.5
0.8
Genes
+
-
3605800
3606000
3606200
3606400
3606600
3606800
3607000
Chromosome 5

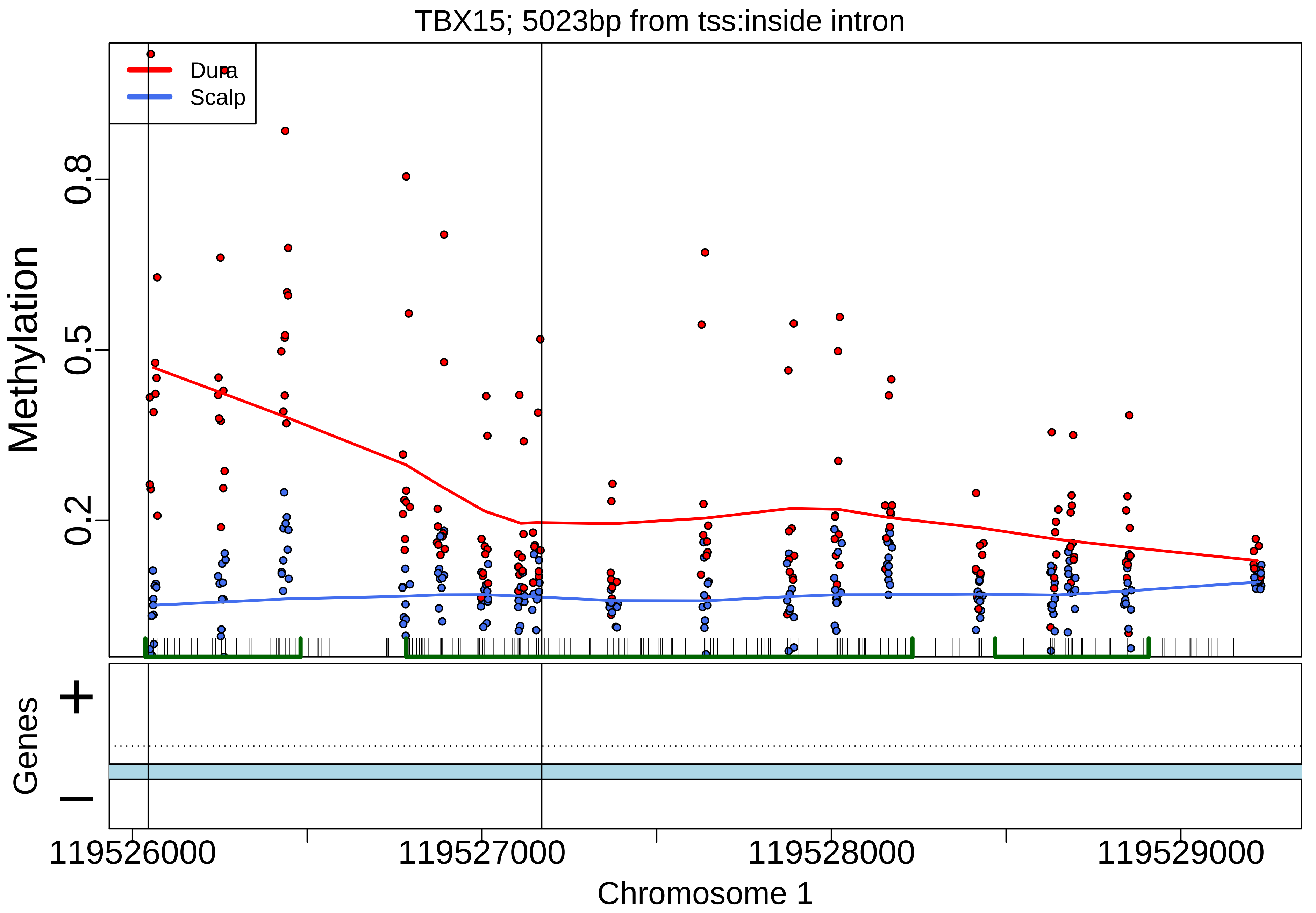
TBX15; 5023bp from tss:inside intron
Dura
Scalp
Methylation
0.2
0.5
0.8
Genes
+
-
119526000
119527000
119528000
119529000
Chromosome 1

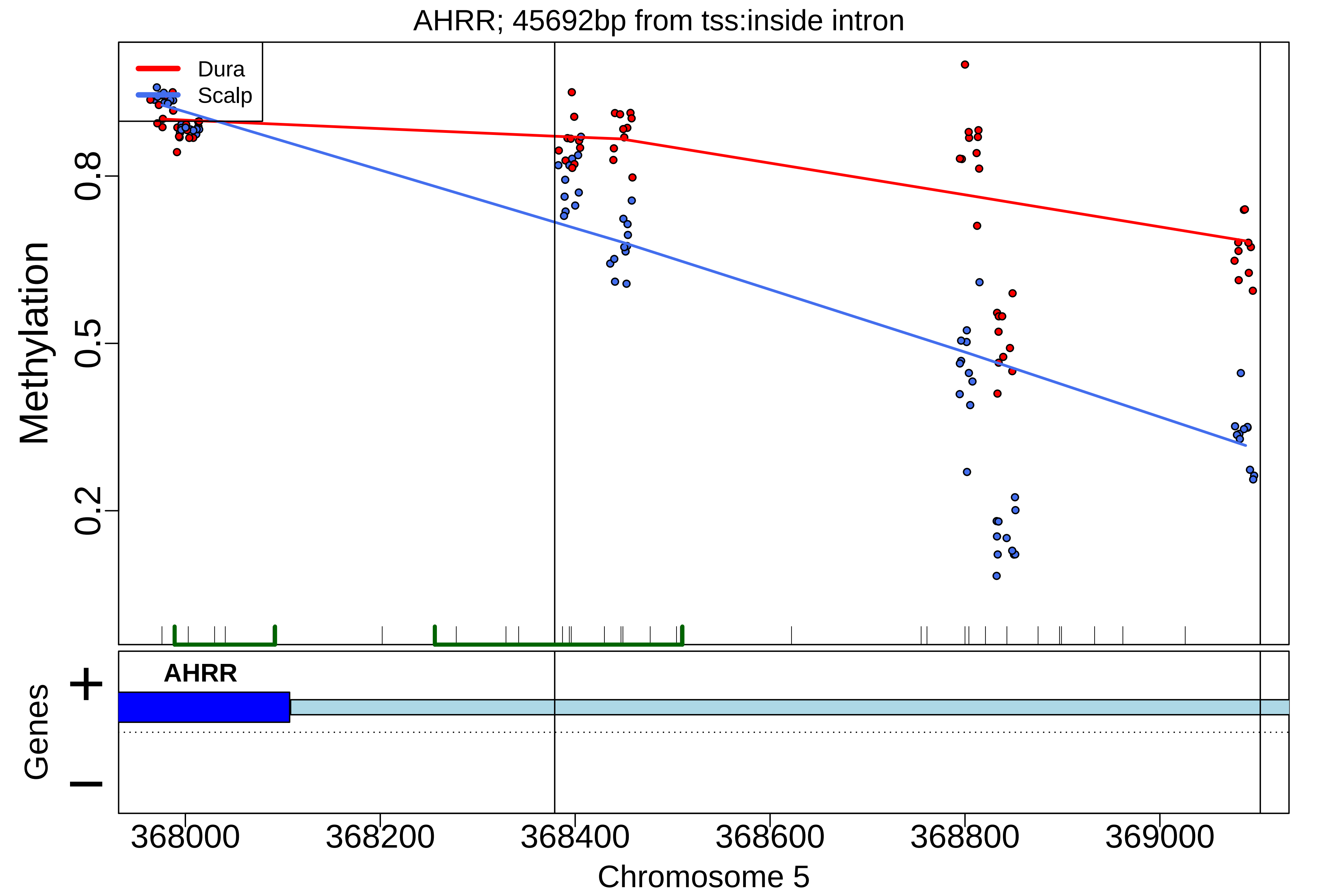

AHRR; 45692bp from tss:inside intron
Dura
Scalp
Methylation
0.2
0.5
0.8
Genes
+
-
AHRR
368000
368200
368400
368600
368800
369000
Chromosome 5

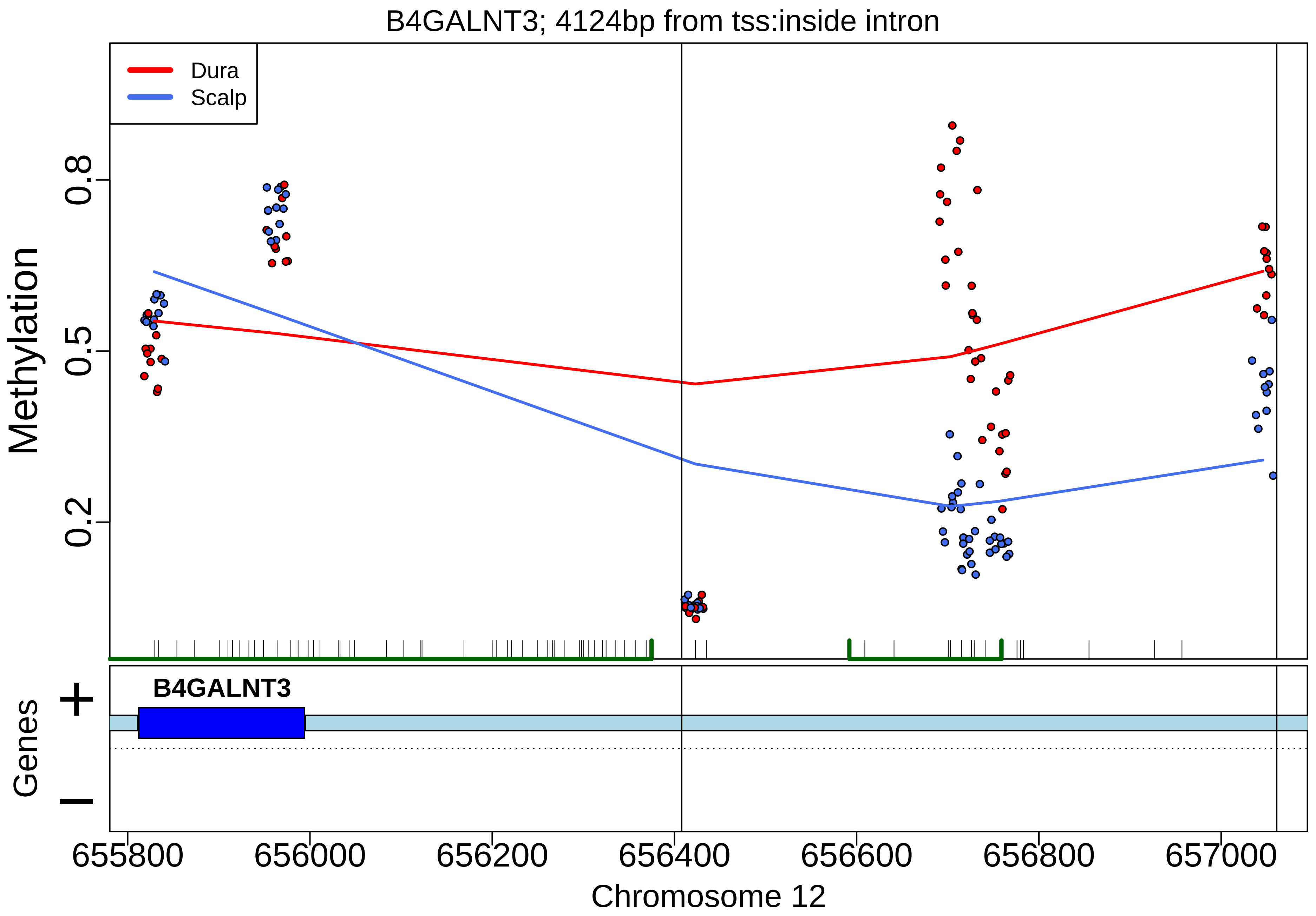
B4GALNT3; 4124bp from tss:inside intron
Dura
Scalp
Methylation
0.2
0.5
0.8
Genes
+
−
B4GALNT3
655800
656000
656200
656400
656600
656800
657000
Chromosome 12

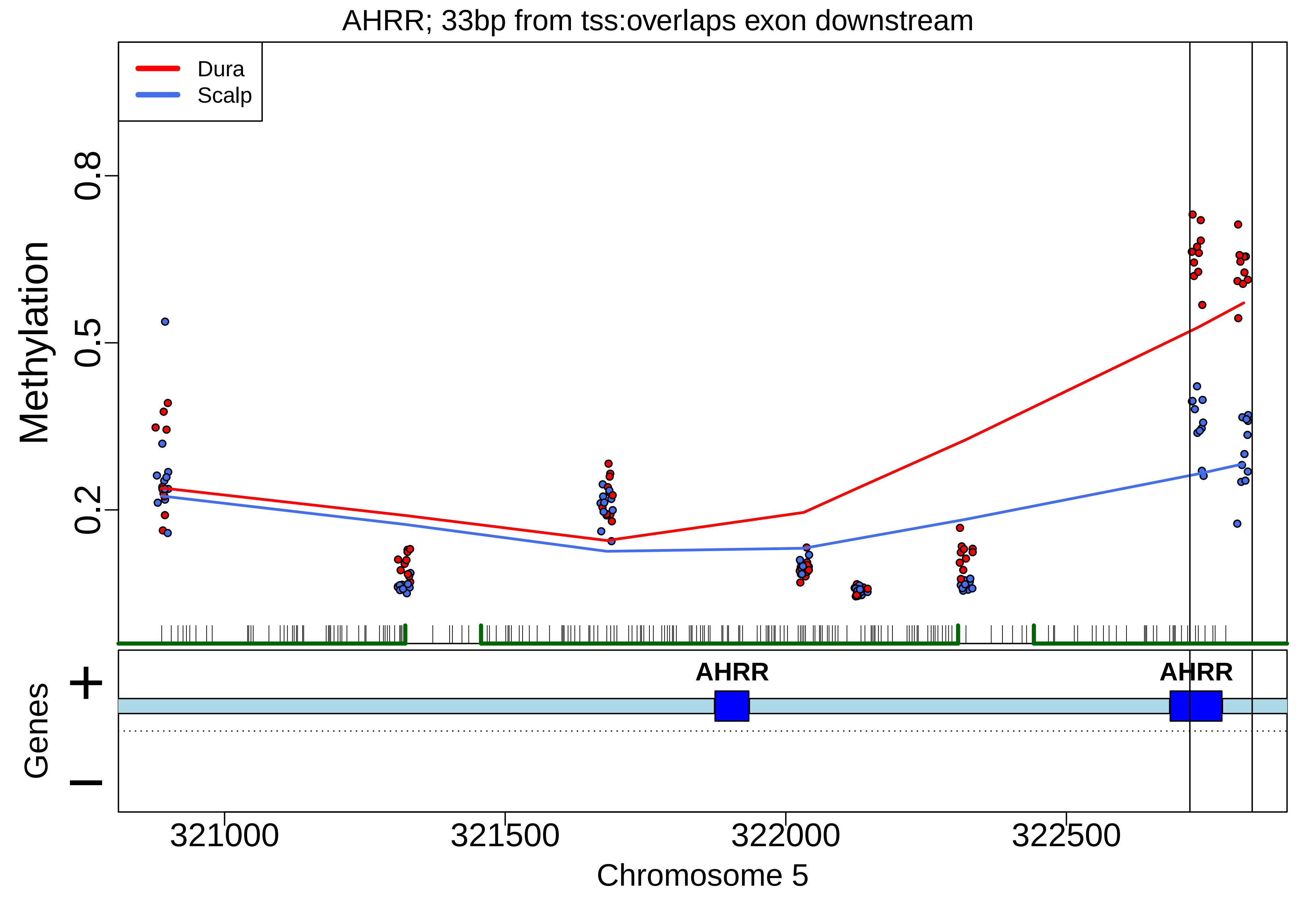

AHRR; 33bp from tss:overlaps exon downstream
Dura
Scalp
Methylation
0.2
0.5
0.8
Genes
+
-
AHRR
AHRR
321000
321500
322000
322500
Chromosome 5

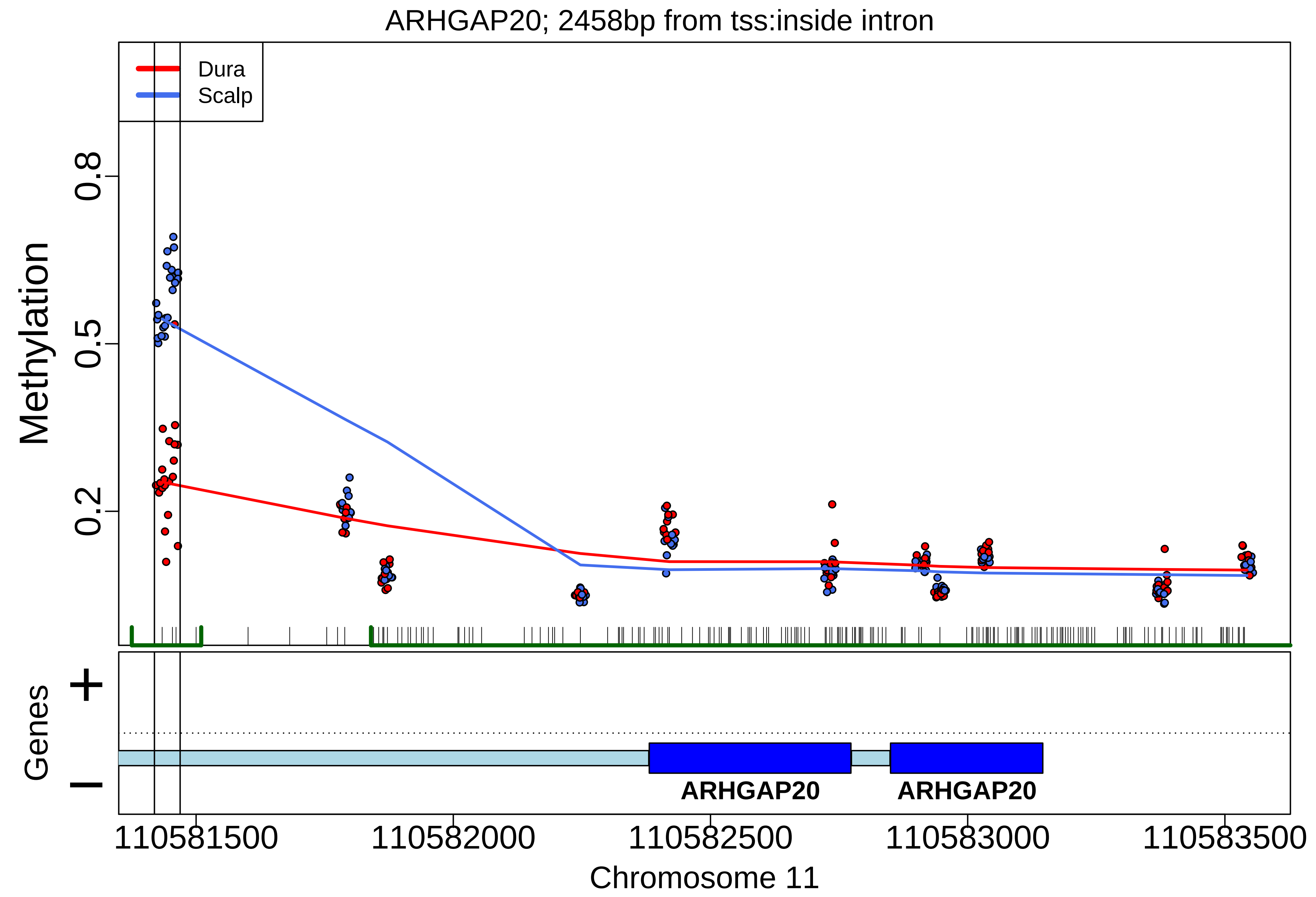

ARHGAP20; 2458bp from tss:inside intron
Dura
Scalp
Methylation
0.2
0.5
0.8
Genes
+
-
ARHGAP20
ARHGAP20
110581500
110582000
110582500
110583000
110583500
Chromosome 11

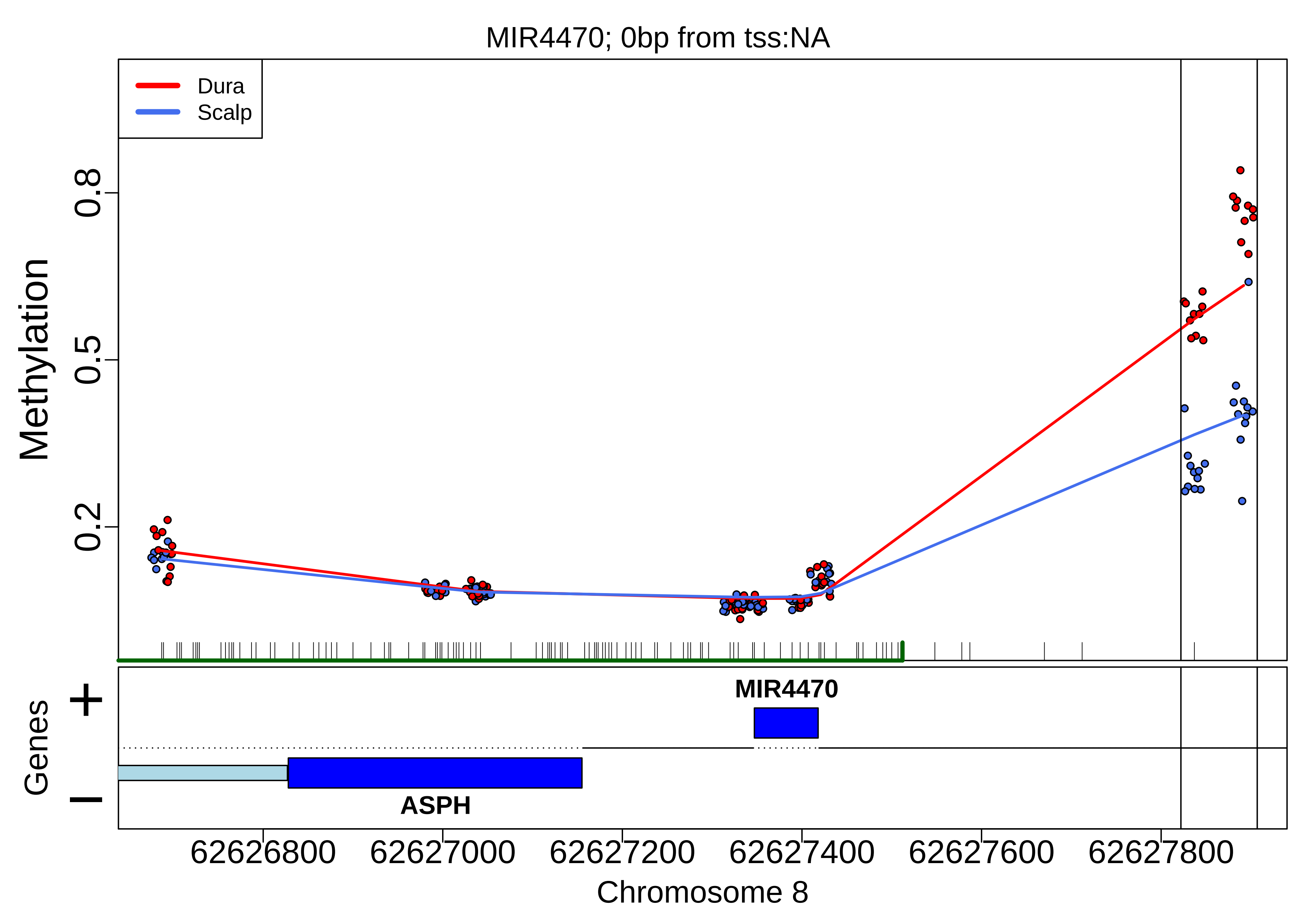
MIR4470; 0bp from tss:NA
Dura
Scalp
Methylation
0.2
0.5
0.8
Genes
+
-
MIR4470
ASPH
62626800
62627000
62627200
62627400
62627600
62627800
Chromosome 8

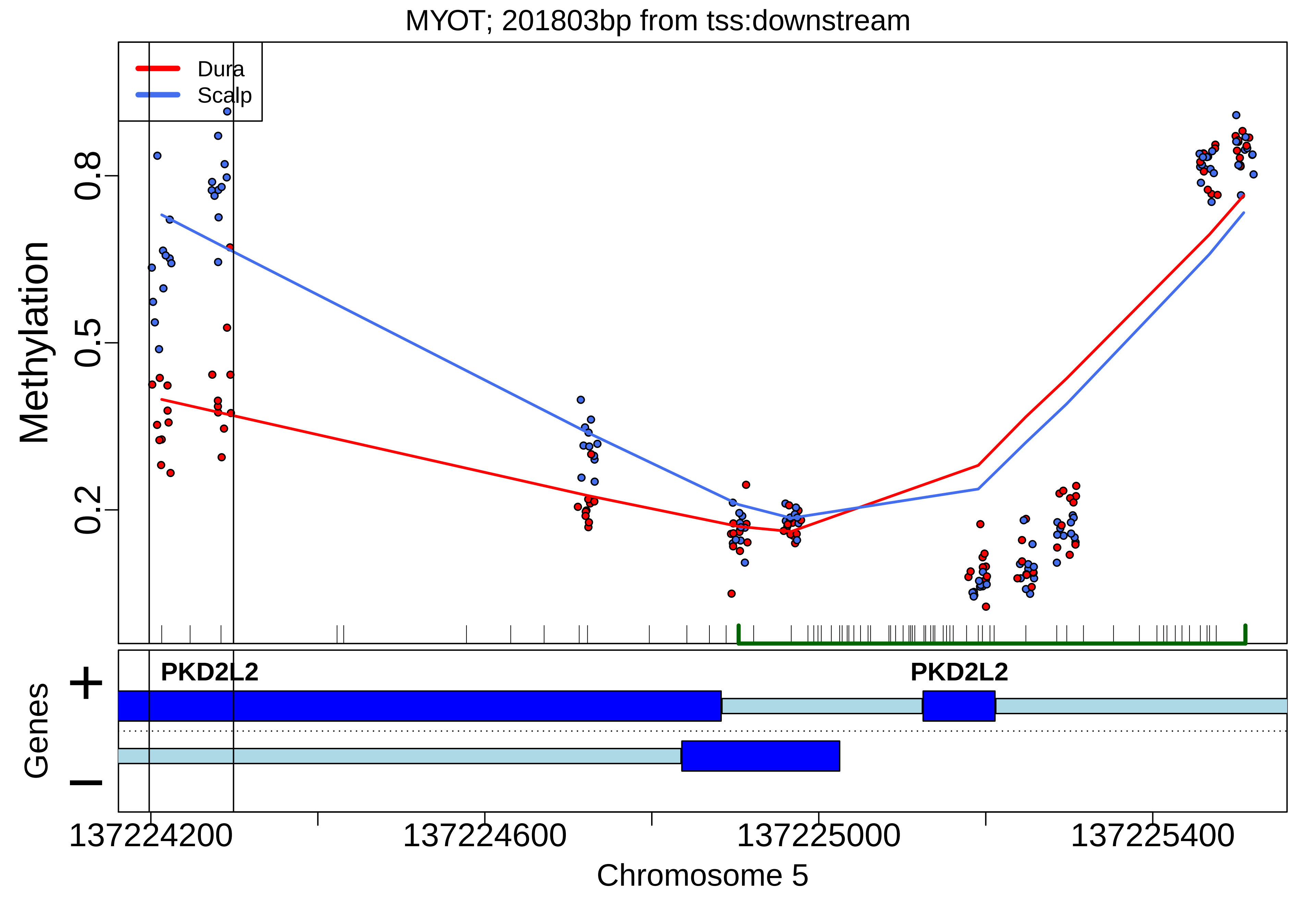

MYOT; 201803bp from tss:downstream
Dura
Scalp
Methylation
0.2
0.5
0.8
Genes
+
-
PKD2L2
PKD2L2
137224200
137224600
137225000
137225400
Chromosome 5

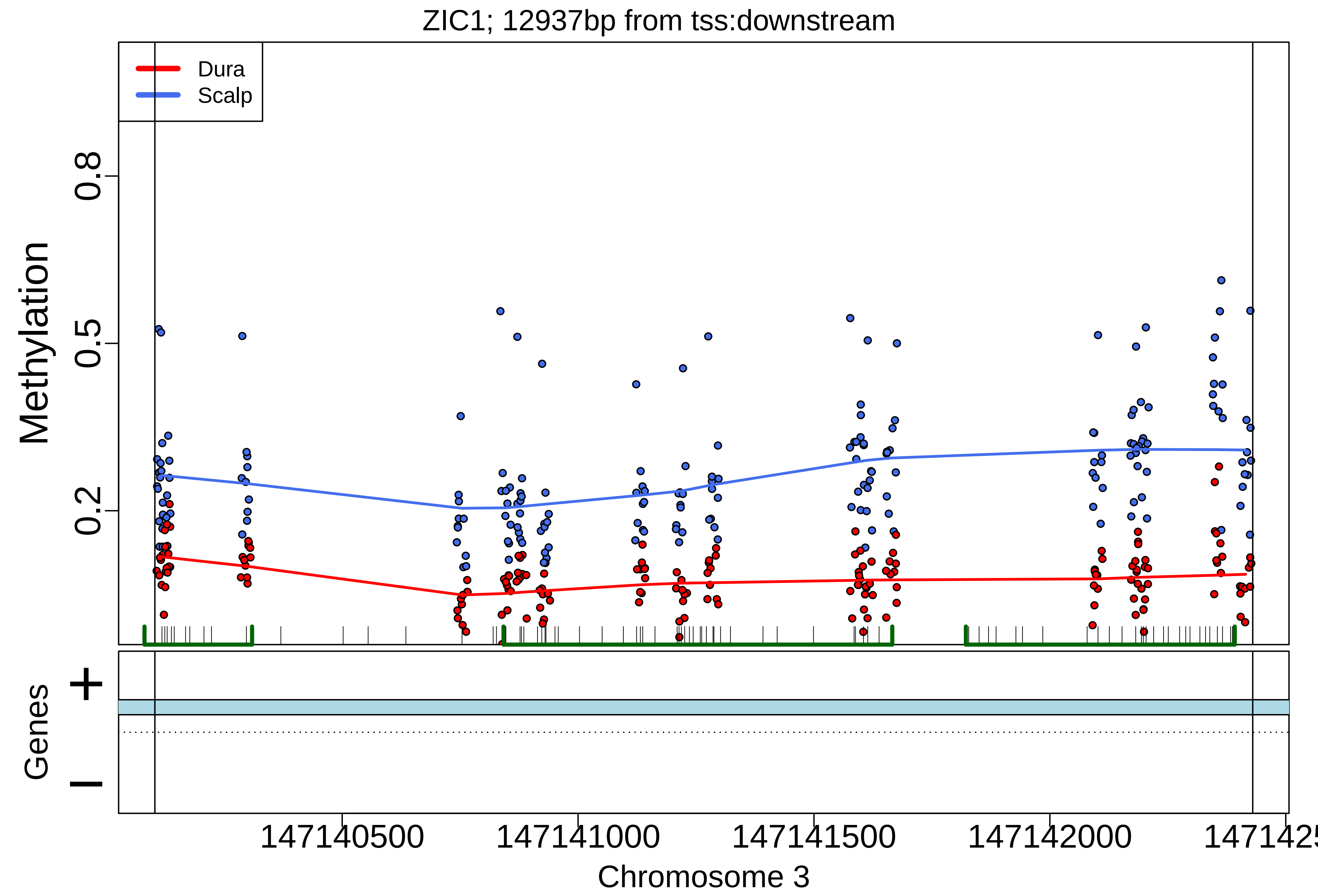

ZIC1; 12937bp from tss:downstream
Dura
Scalp
Methylation
0.2
0.5
0.8
Genes
+
-
147140500
147141000
147141500
147142000
Chromosome 3

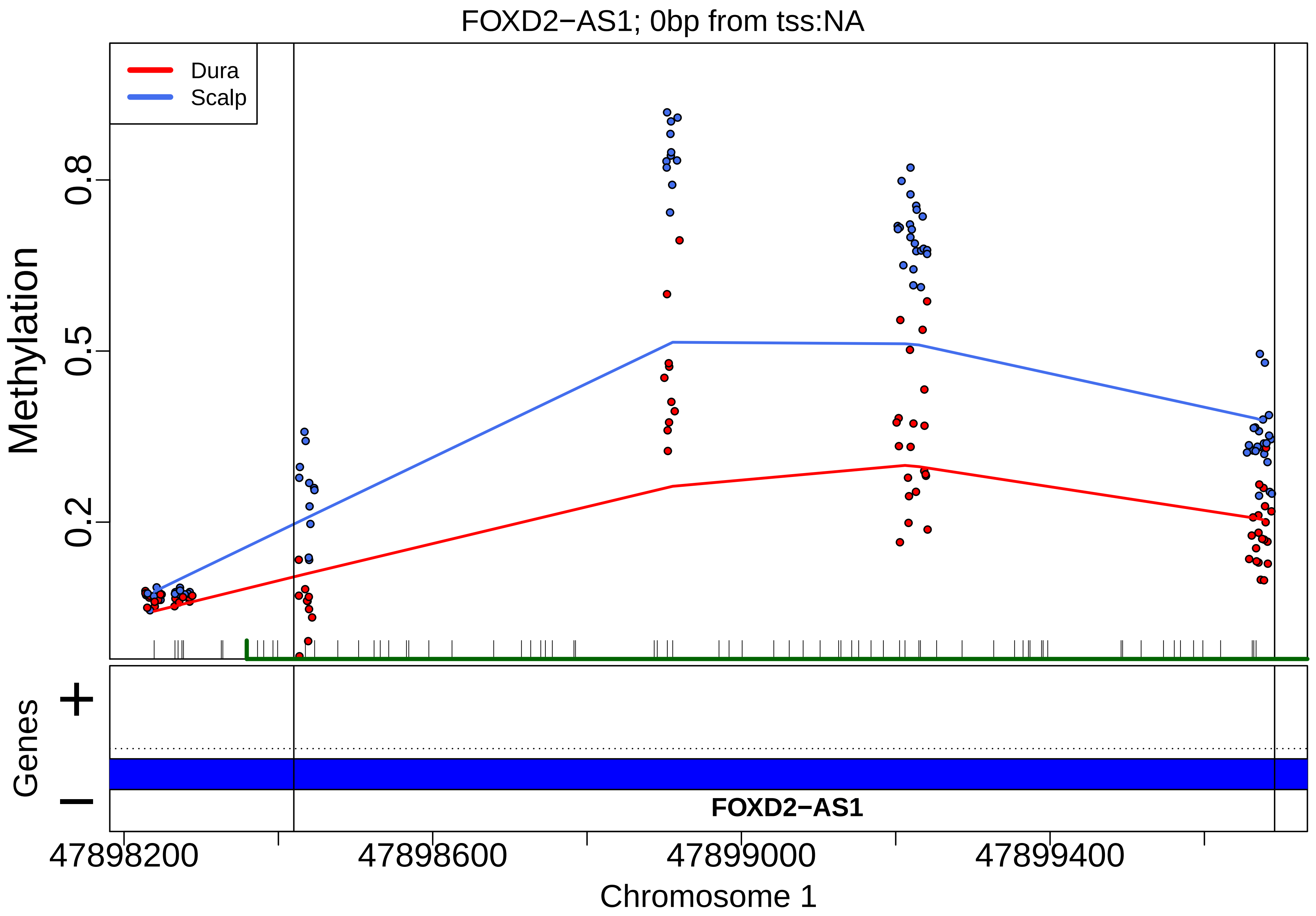

FOXD2−AS1; 0bp from tss:NA
Dura
Scalp
Methylation
0.2
0.5
0.8
Genes
+
−
FOXD2−AS1
47898200
47898600
47899000
47899400
Chromosome 1

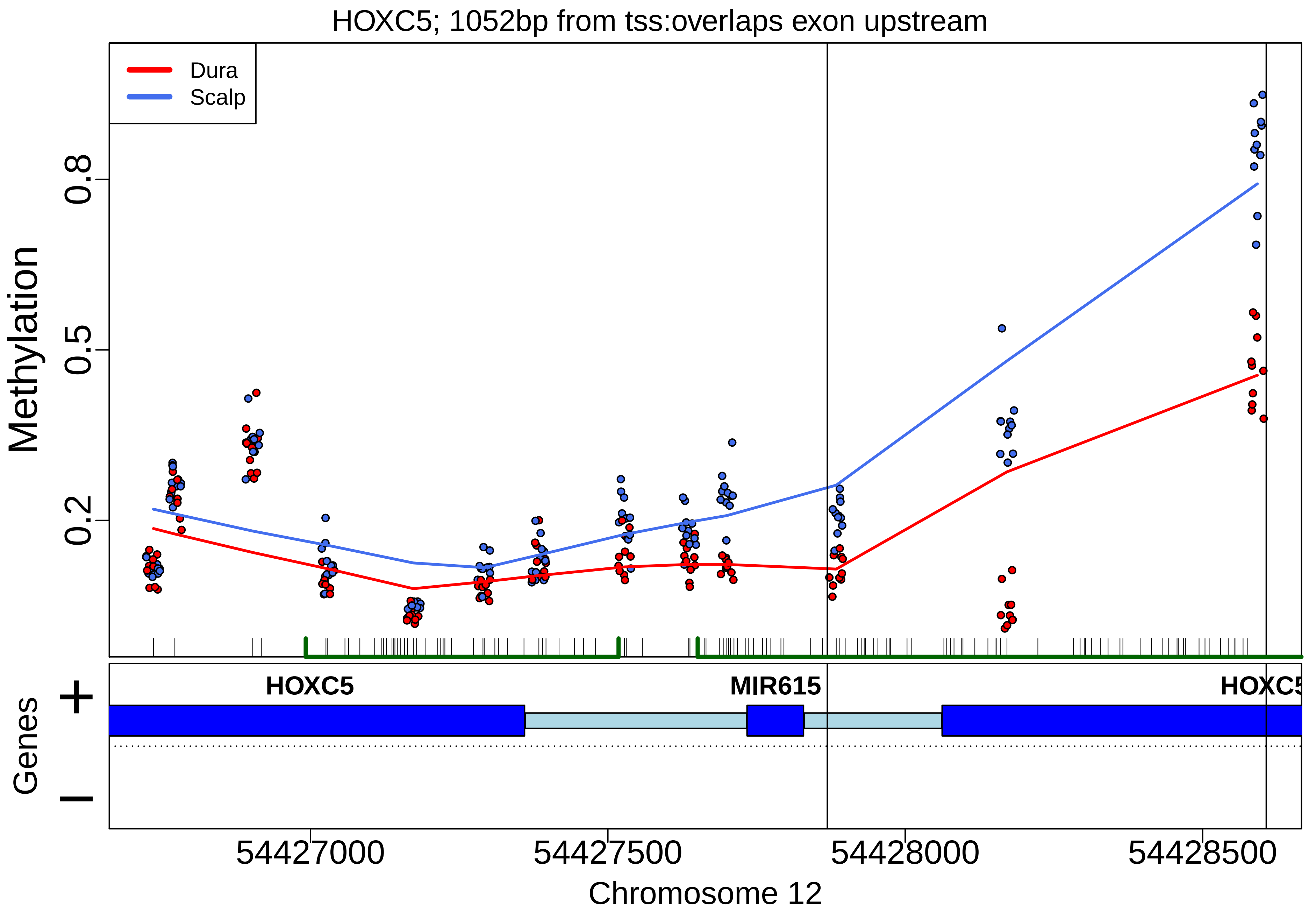
HOXC5; 1052bp from tss:overlaps exon upstream
Dura
Scalp
Methylation
0.2
0.5
0.8
Genes
+
-
HOXC5
MIR615
HOXC5
54427000
54427500
54428000
54428500
Chromosome 12

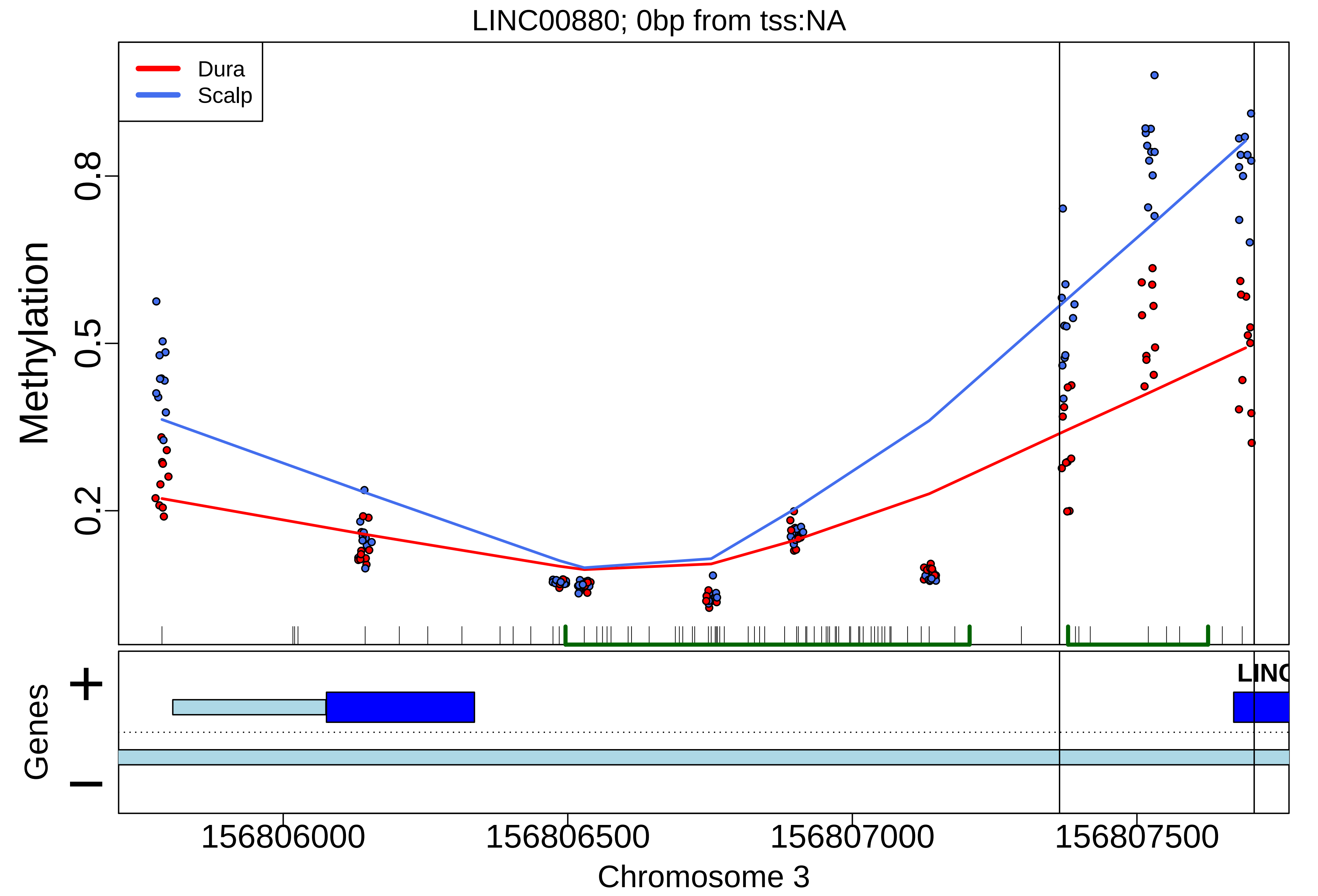

LINC00880; 0bp from tss:NA
Dura
Scalp
Methylation
0.2
0.5
0.8
Genes
+
-
LINC
156806000
156806500
156807000
156807500
Chromosome 3

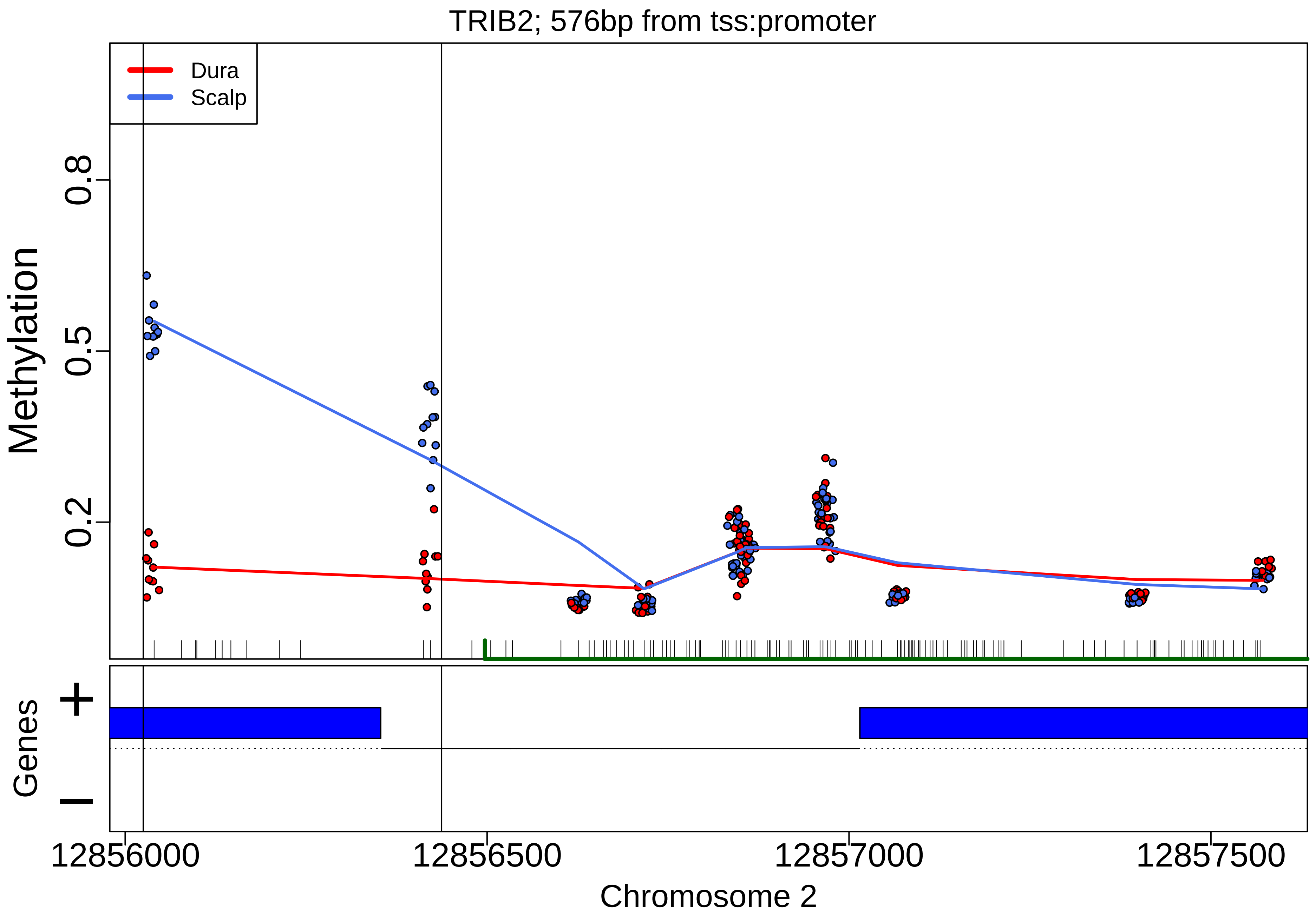
TRIB2; 576bp from tss:promoter
Dura
Scalp
Methylation
0.2
0.5
0.8
Genes
+
-
12856000
12856500
12857000
12857500
Chromosome 2

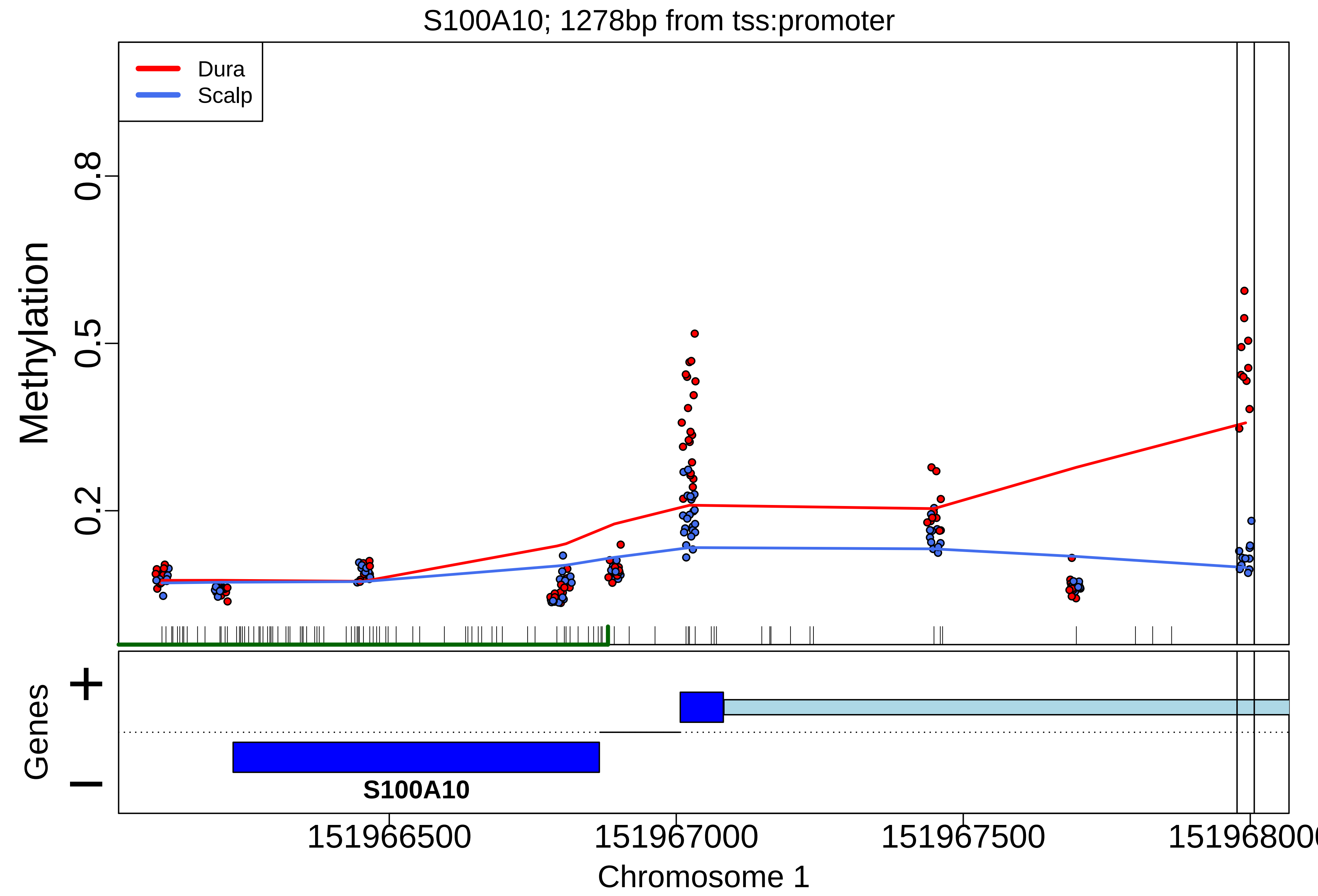

S100A10; 1278bp from tss:promoter
Dura
Scalp
Methylation
0.2
0.5
0.8
Genes
+
-
S100A10
151966500
151967000
151967500
151968000
Chromosome 1

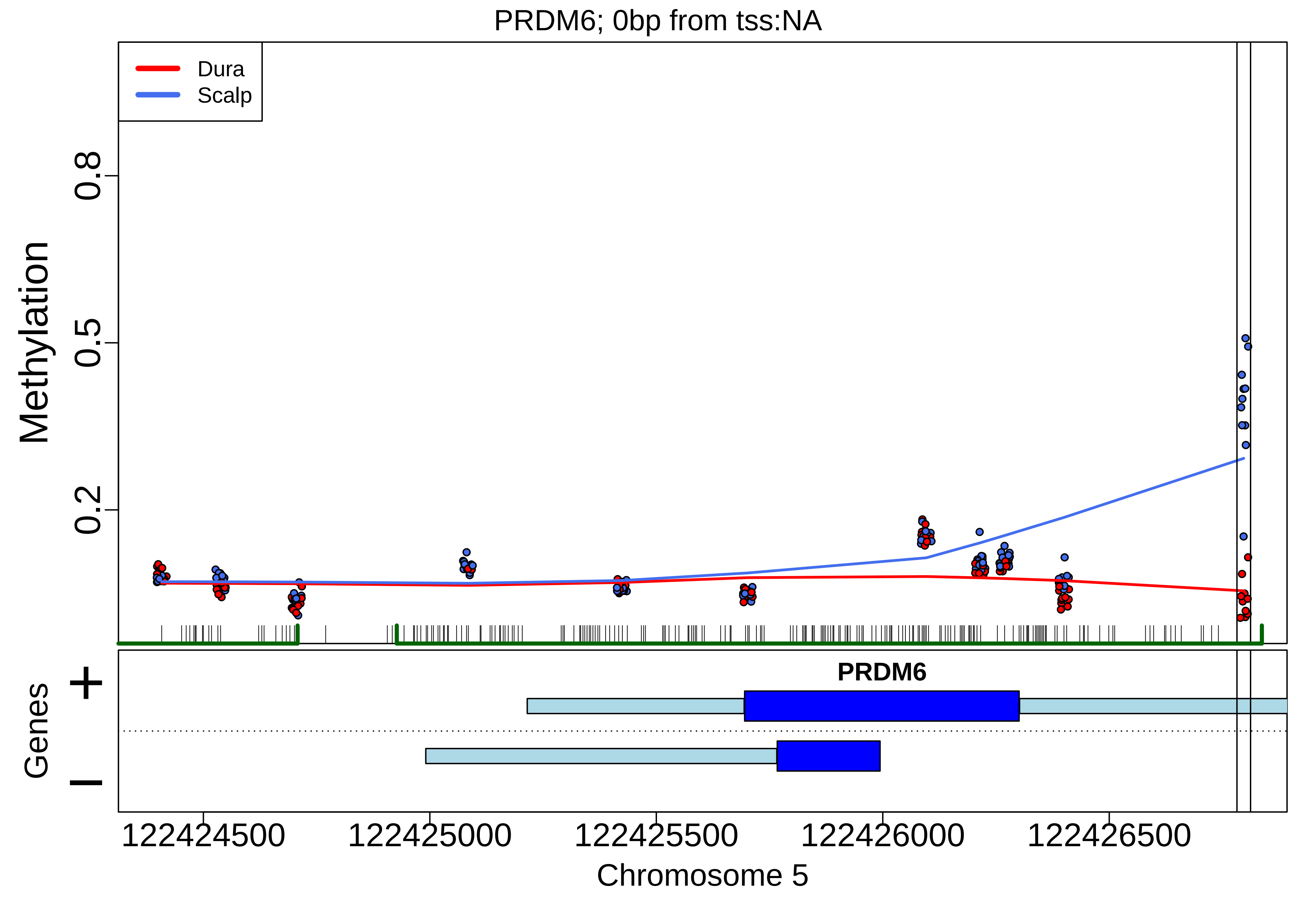

PRDM6; 0bp from tss:NA
Dura
Scalp
Methylation
0.2
0.5
0.8
Genes
+
-
PRDM6
122424500
122425000
122425500
122426000
122426500
Chromosome 5

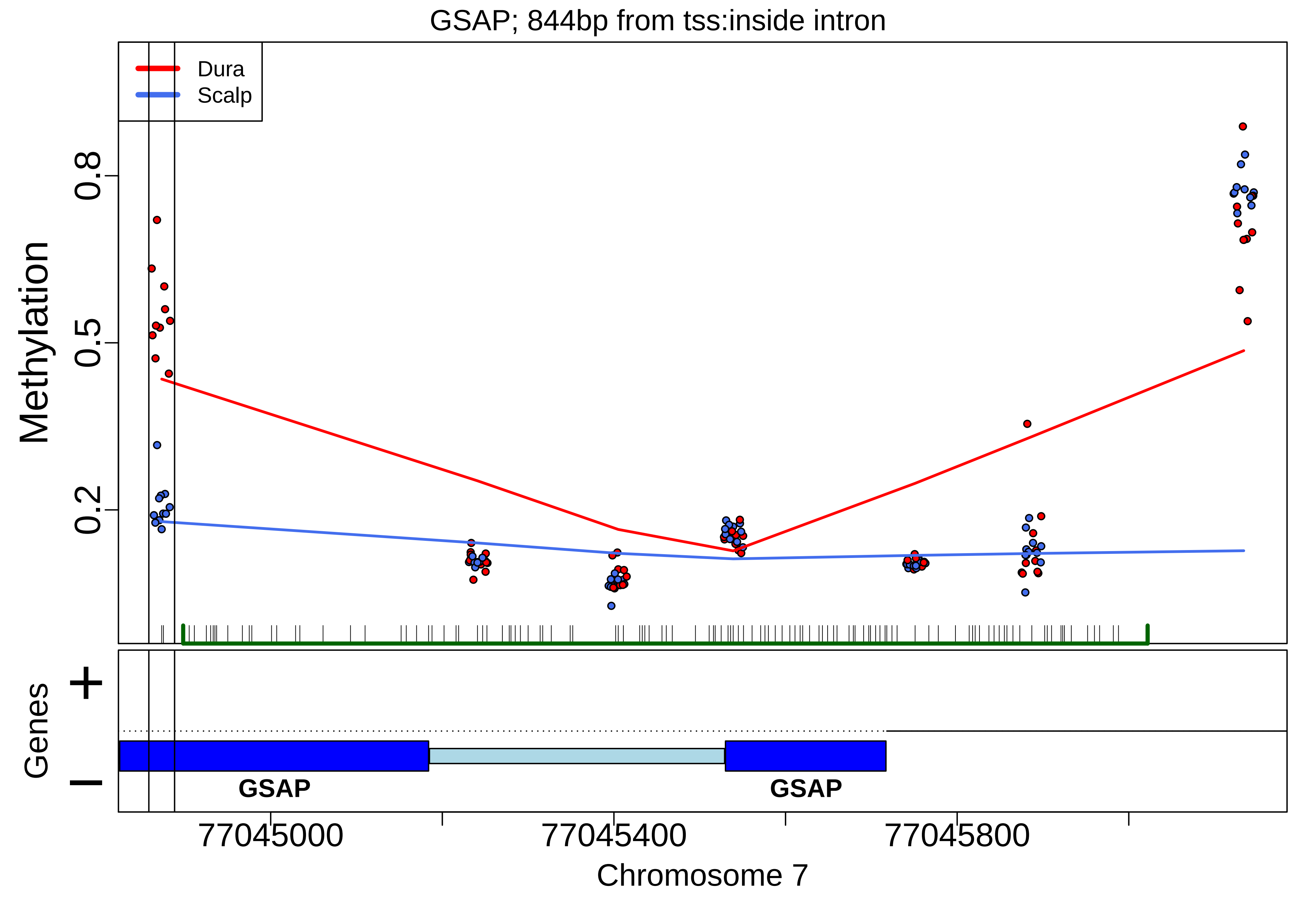

GSAP; 844bp from tss:inside intron
Dura
Scalp
Methylation
0.2
0.5
0.8
Genes
+
-
GSAP
GSAP
77045000
77045400
77045800
Chromosome 7

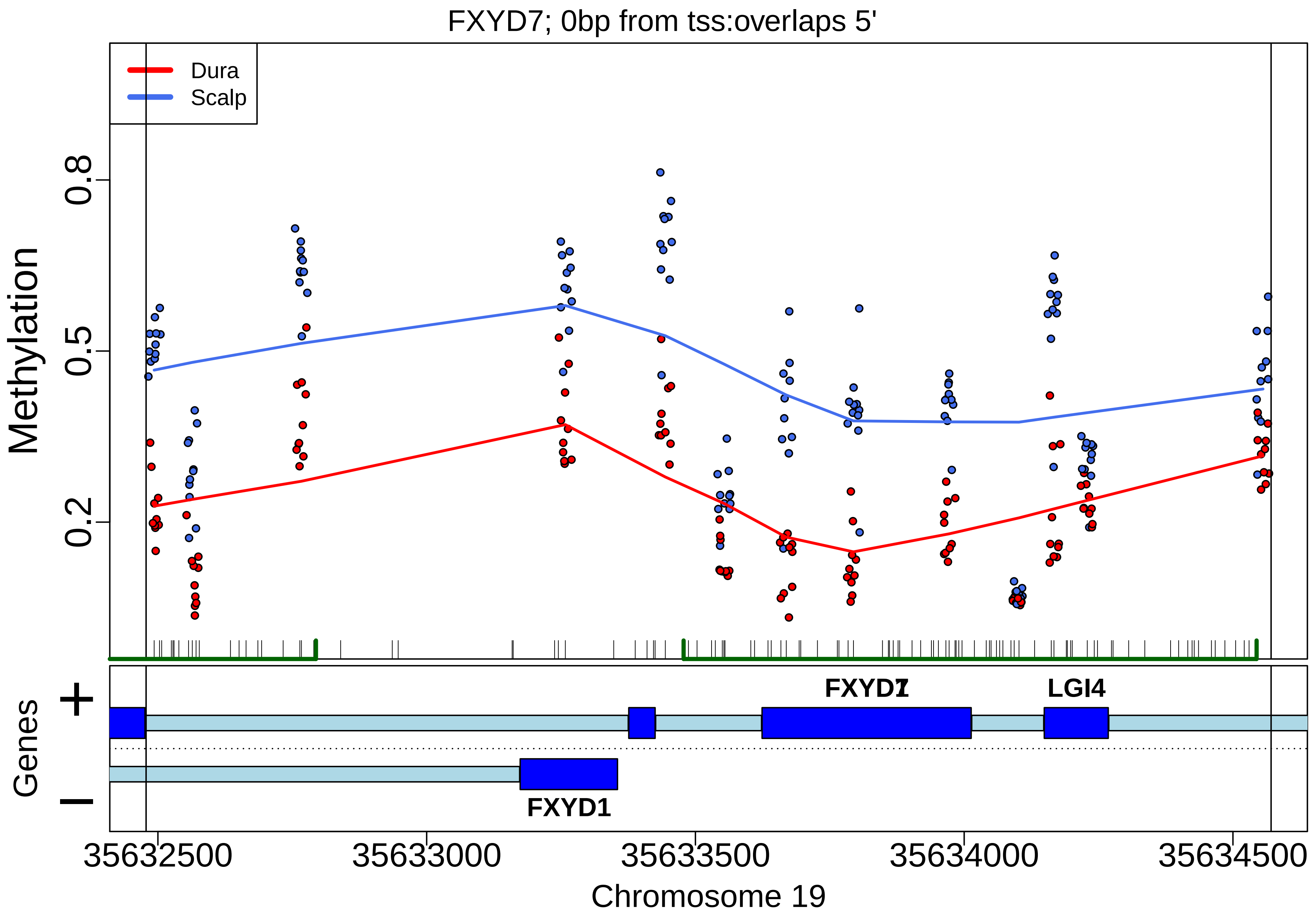

FXYD7; 0bp from tss:overlaps 5'
Dura
Scalp
Methylation
0.2
0.5
0.8
Genes
+
−
FXYD7
LGI4
FXYD1
35632500
35633000
35633500
35634000
35634500
Chromosome 19

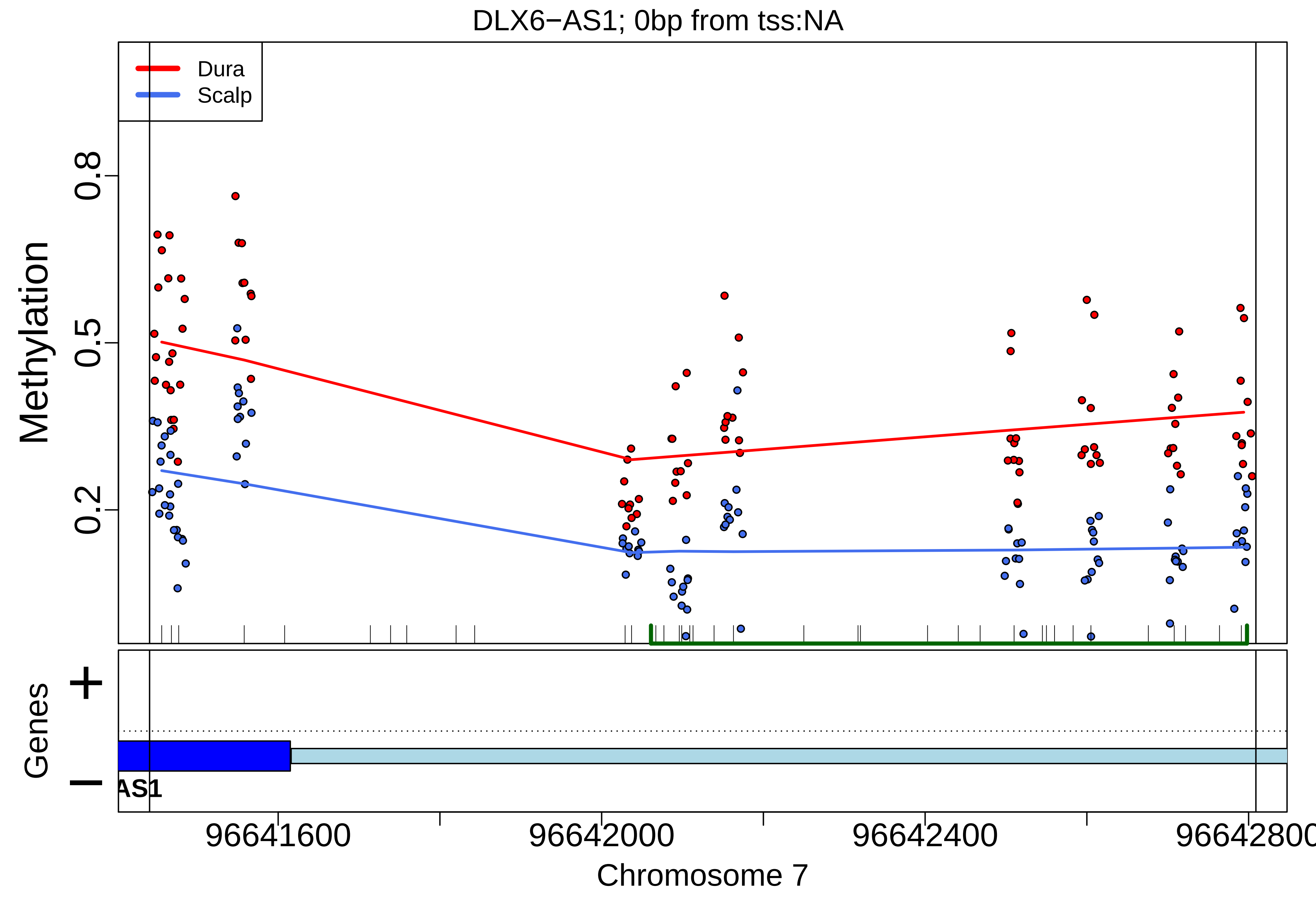

DLX6−AS1; 0bp from tss:NA
Dura
Scalp
Methylation
0.2
0.5
0.8
Genes
+
−
AS1
96641600
96642000
96642400
96642800
Chromosome 7

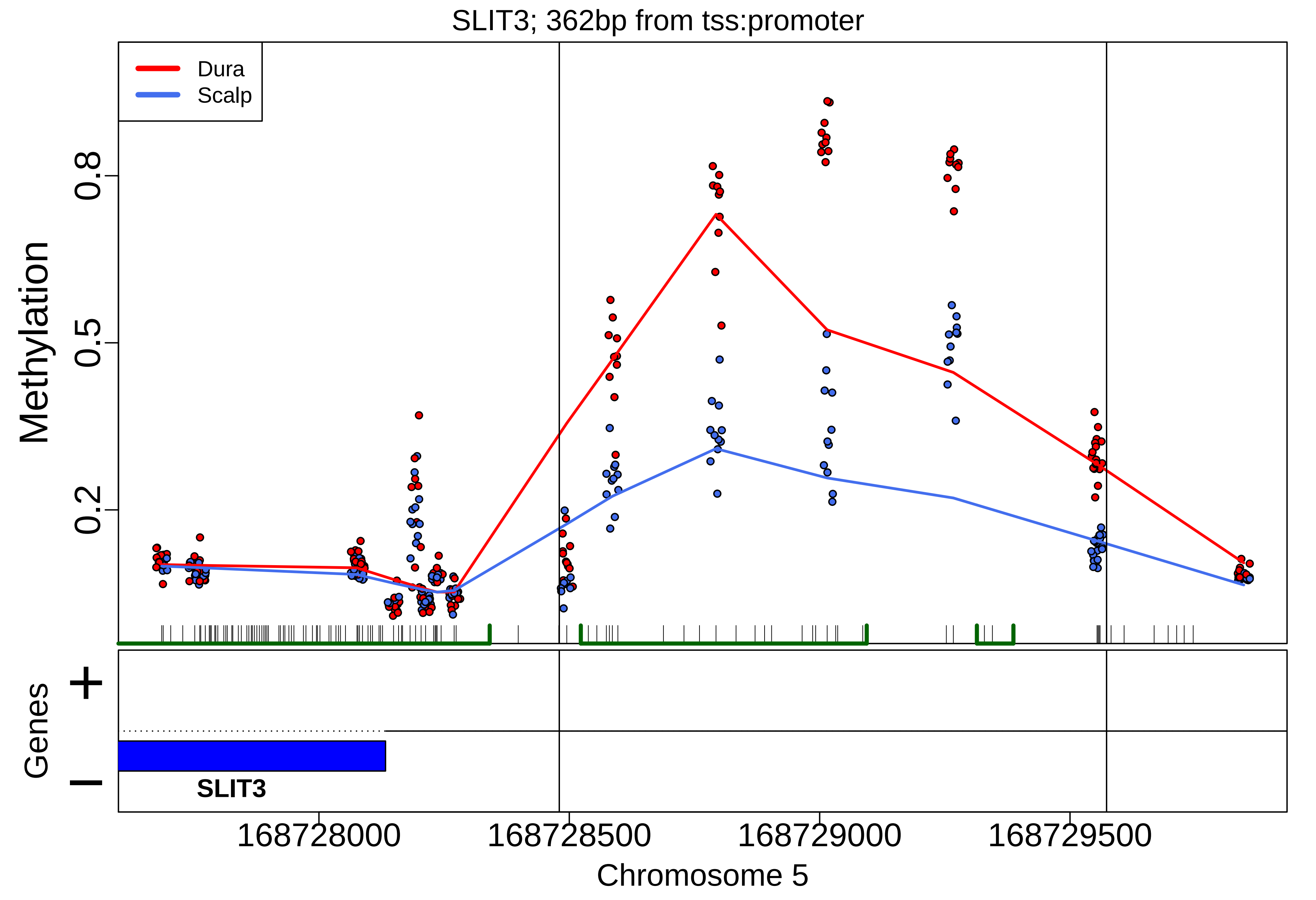
SLIT3; 362bp from tss:promoter
Dura
Scalp
Methylation
0.2
0.5
0.8
Genes
+
−
SLIT3
168728000
168728500
168729000
168729500
Chromosome 5

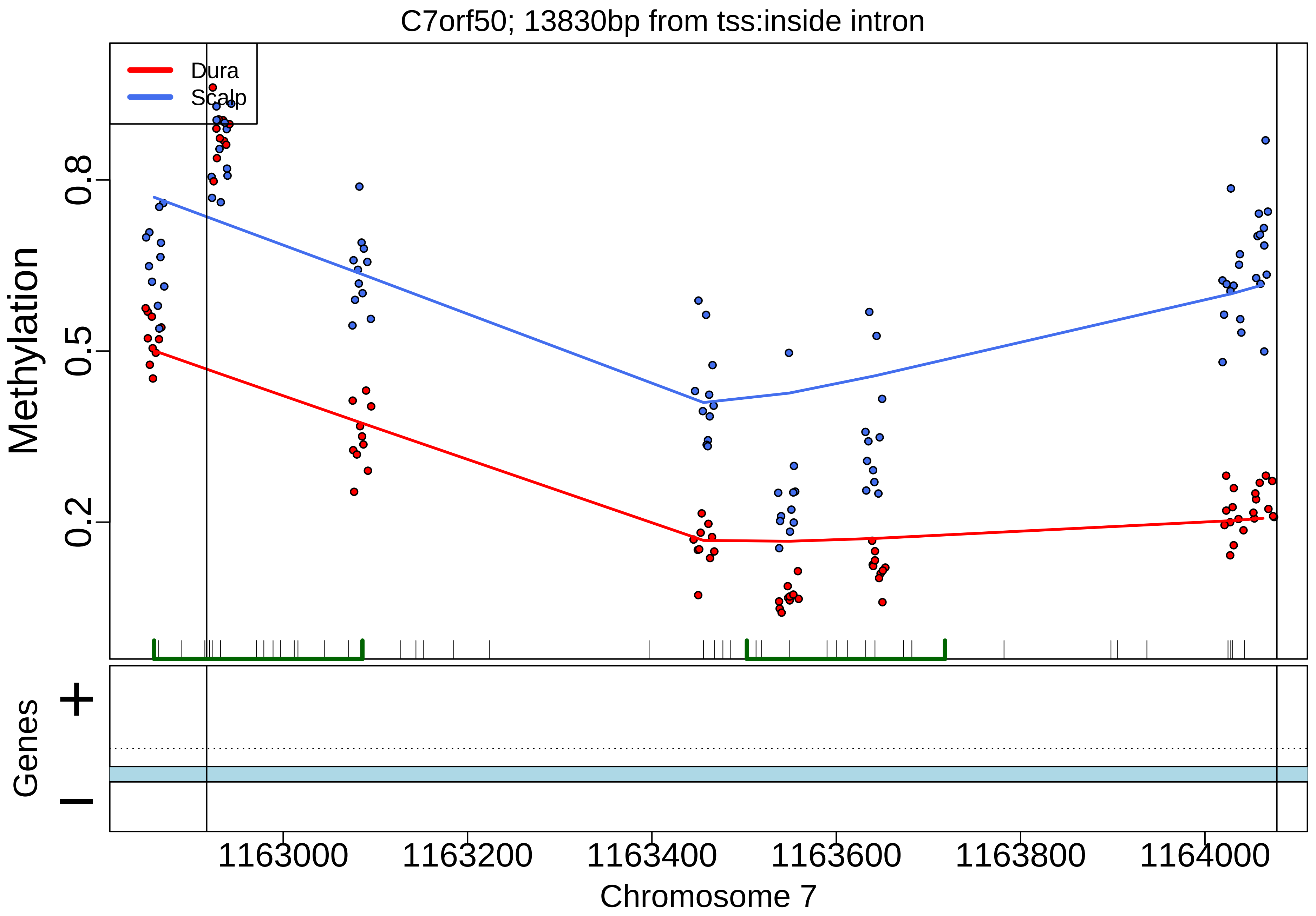

C7orf50; 13830bp from tss:inside intron
Dura
Scalp
Methylation
0.2
0.5
0.8
Genes
+
-
1163000
1163200
1163400
1163600
1163800
1164000
Chromosome 7

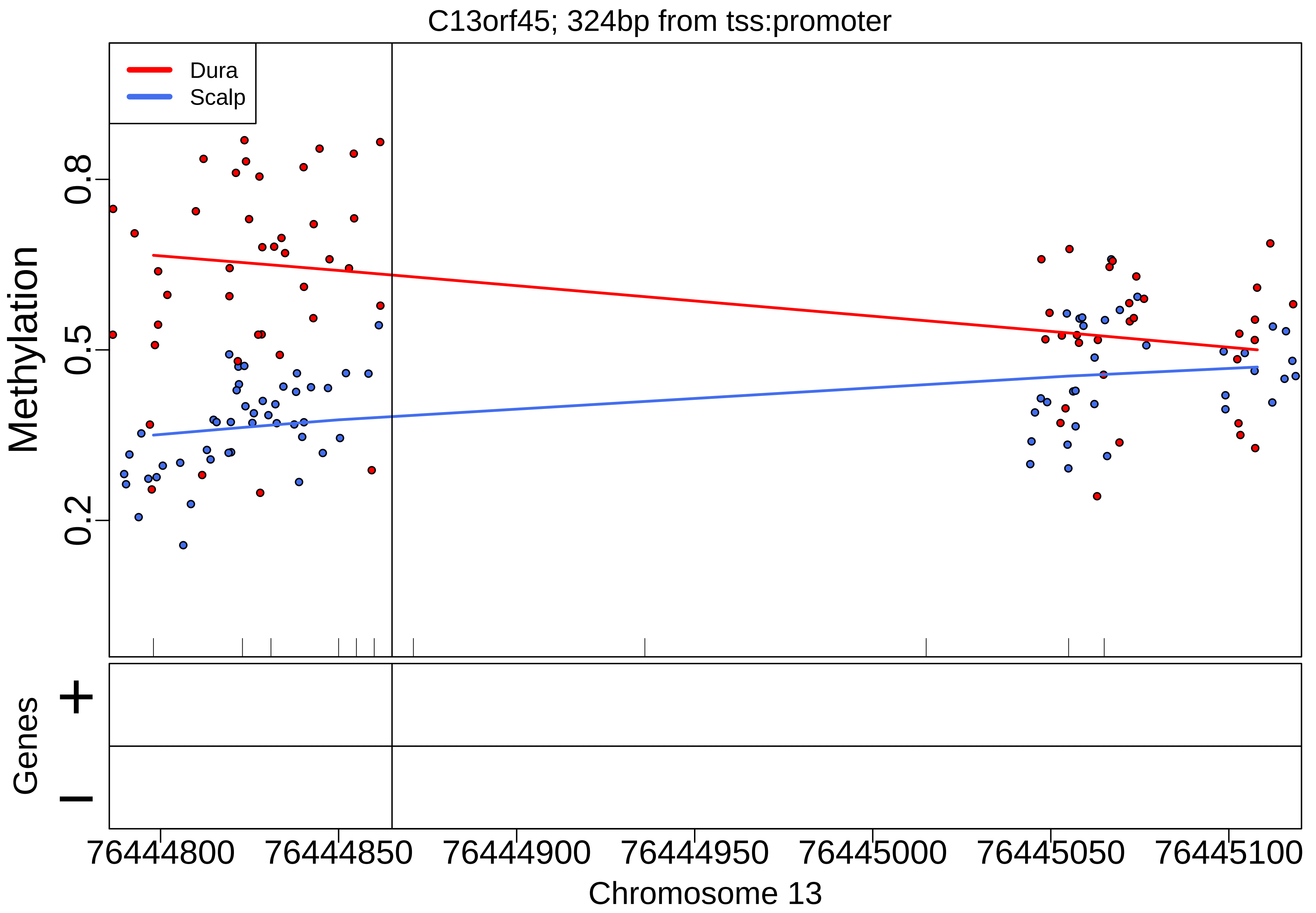

C13orf45; 324bp from tss:promoter
Dura
Scalp
Methylation
0.2
0.5
0.8
Genes
+
−
76444800
76444850
76444900
76444950
76445000
76445050
76445100
Chromosome 13

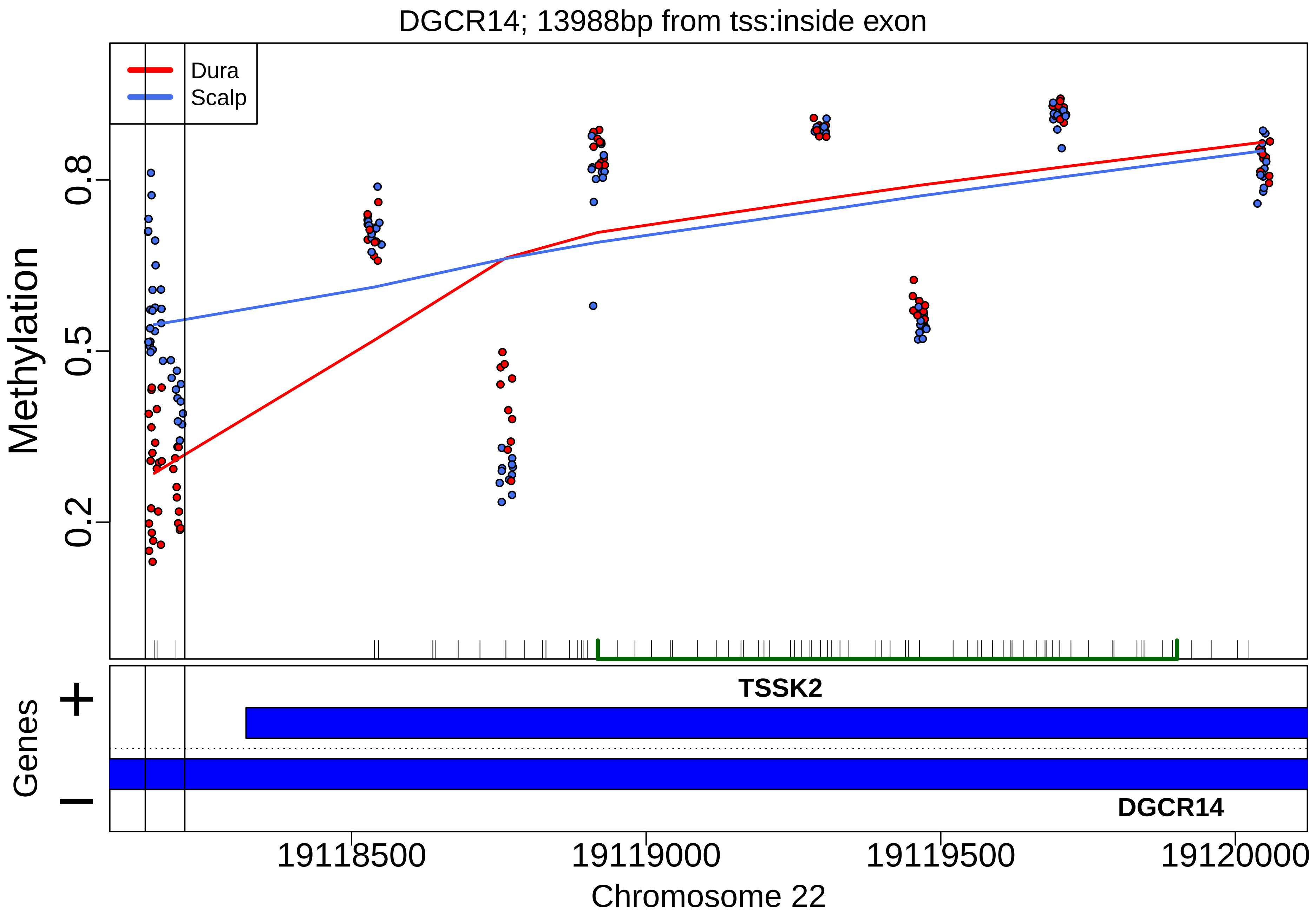
DGCR14; 13988bp from tss:inside exon
Dura
Scalp
Methylation
0.2
0.5
0.8
Genes
+
−
TSSK2
DGCR14
19118500
19119000
19119500
19120000
Chromosome 22

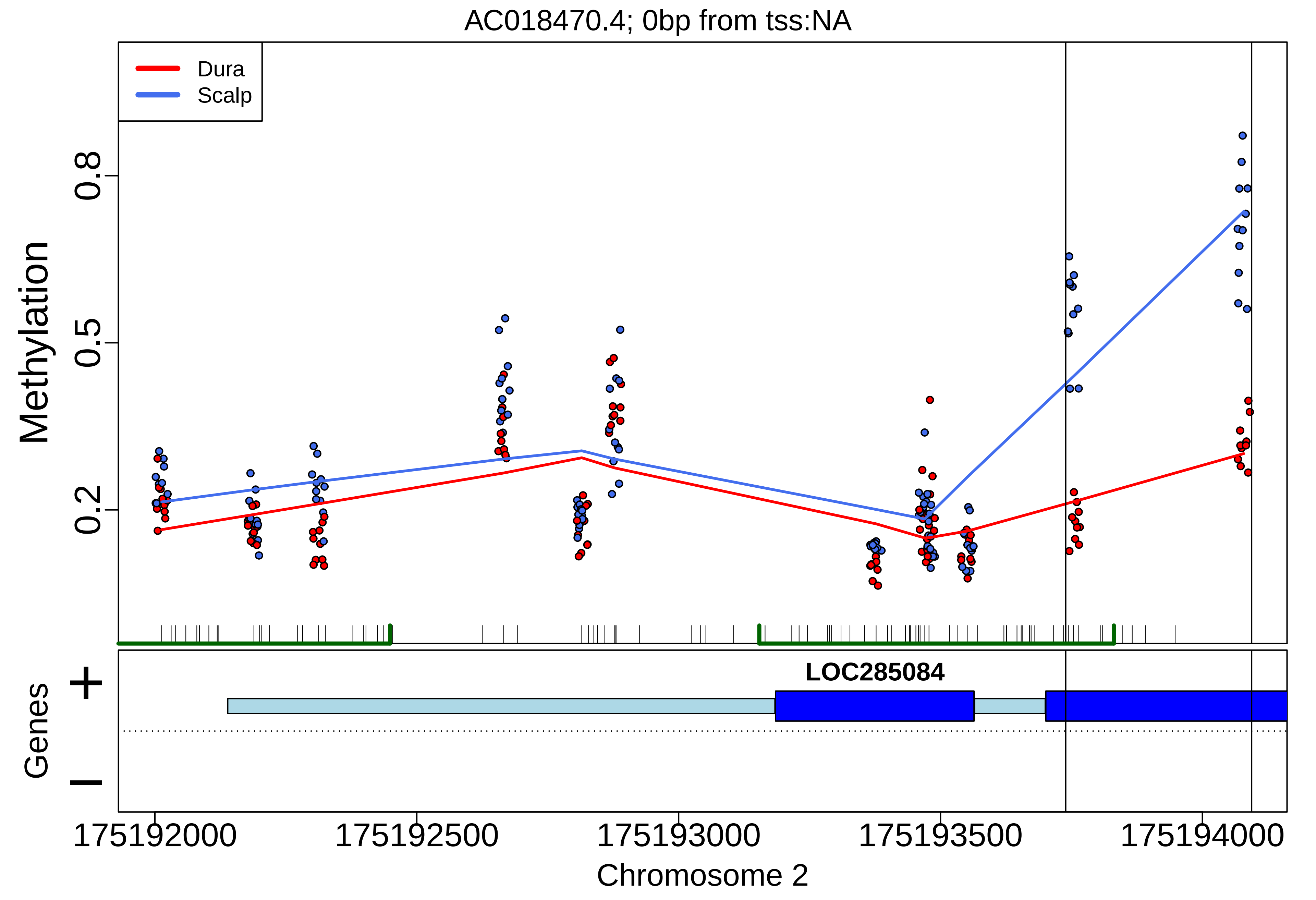

AC018470.4; 0bp from tss:NA
Dura
Scalp
Methylation
0.2
0.5
0.8
Genes
+
−
LOC285084
175192000
175192500
175193000
175193500
175194000
Chromosome 2

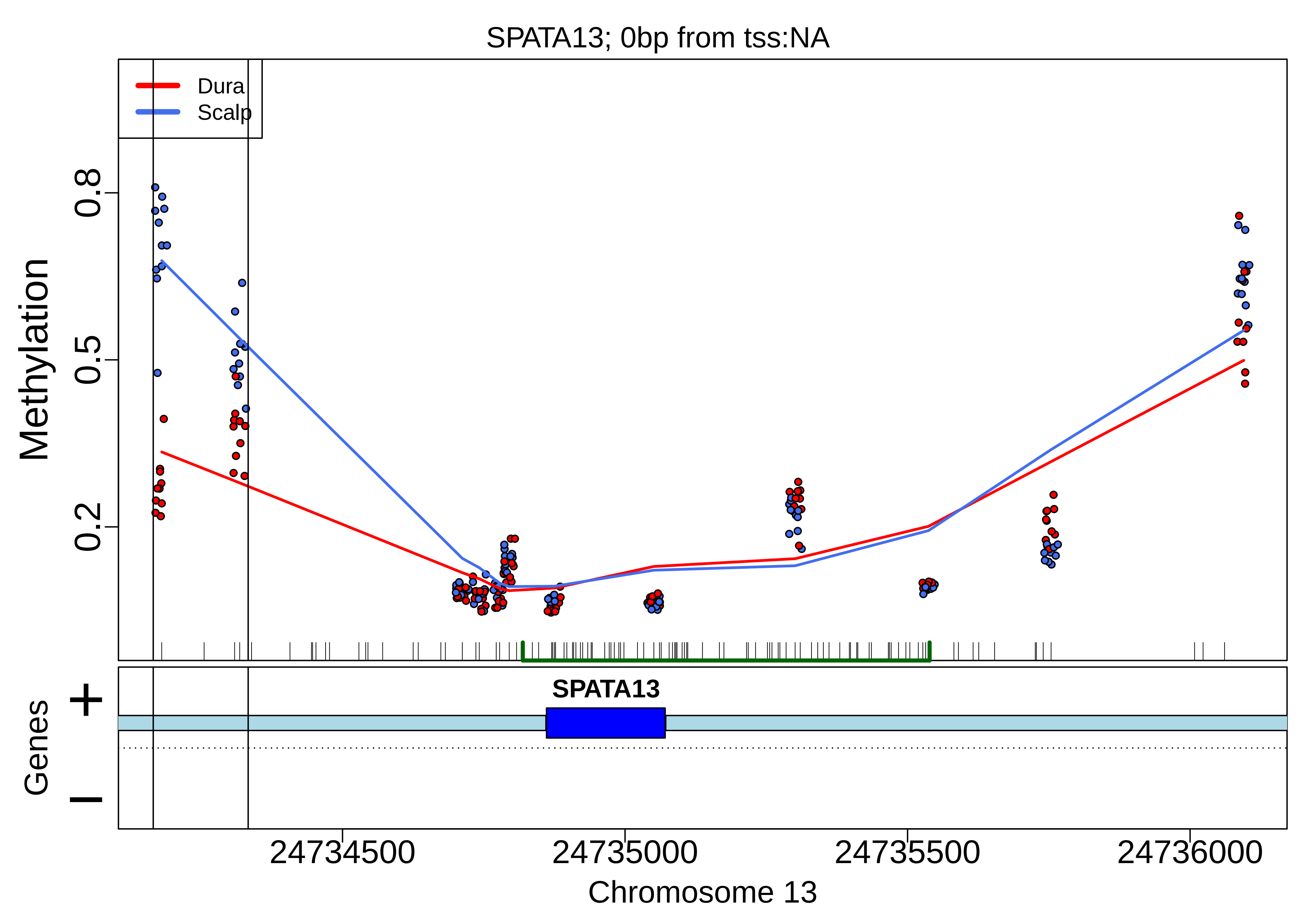
SPATA13; 0bp from tss:NA
Dura
Scalp
Methylation
0.2
0.5
0.8
Genes
+
−
SPATA13
24734500
24735000
24735500
24736000
Chromosome 13

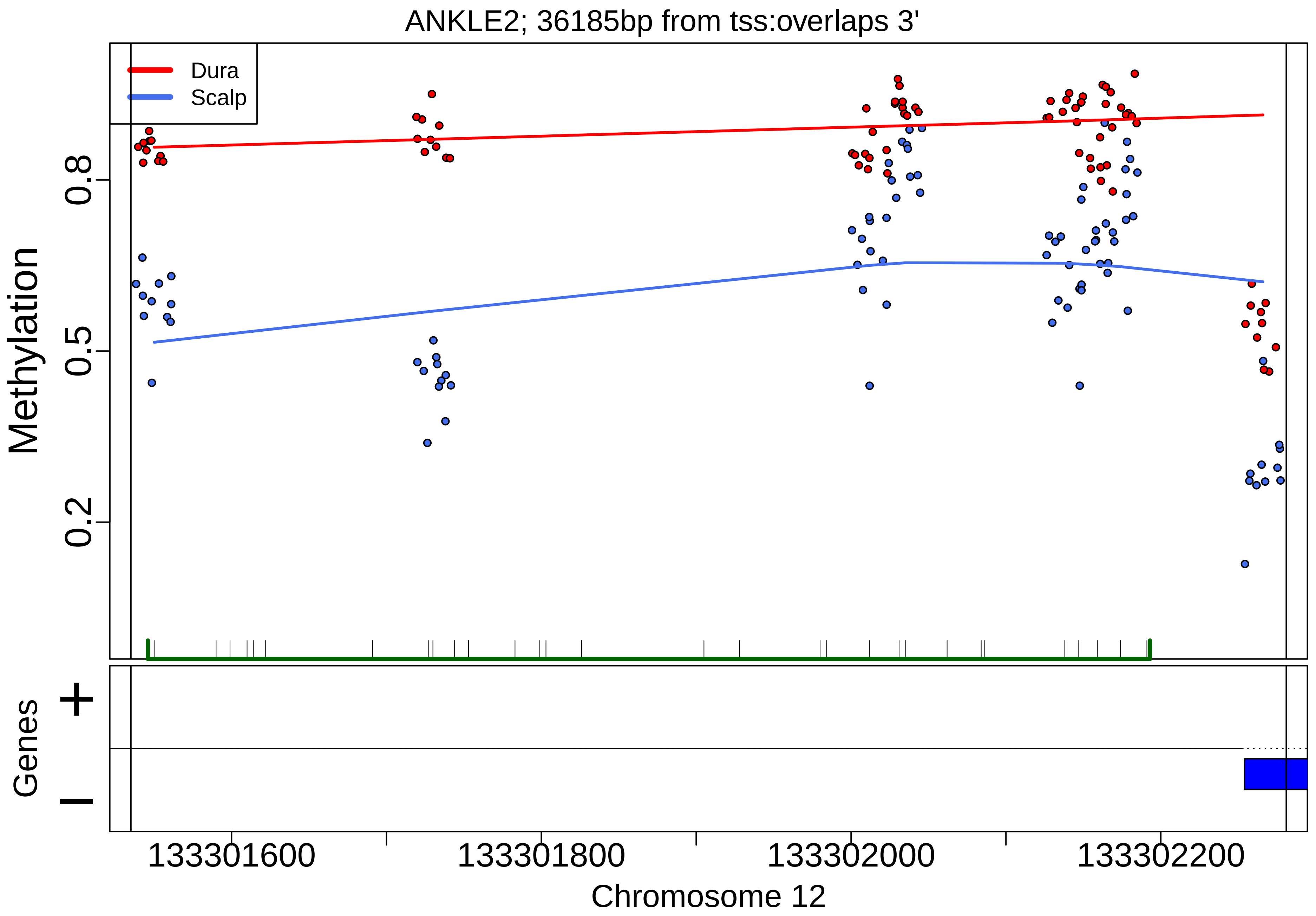

ANKLE2; 36185bp from tss:overlaps 3'
Dura
Scalp
Methylation
0.2
0.5
0.8
Genes
+
-
133301600
133301800
133302000
133302200
Chromosome 12

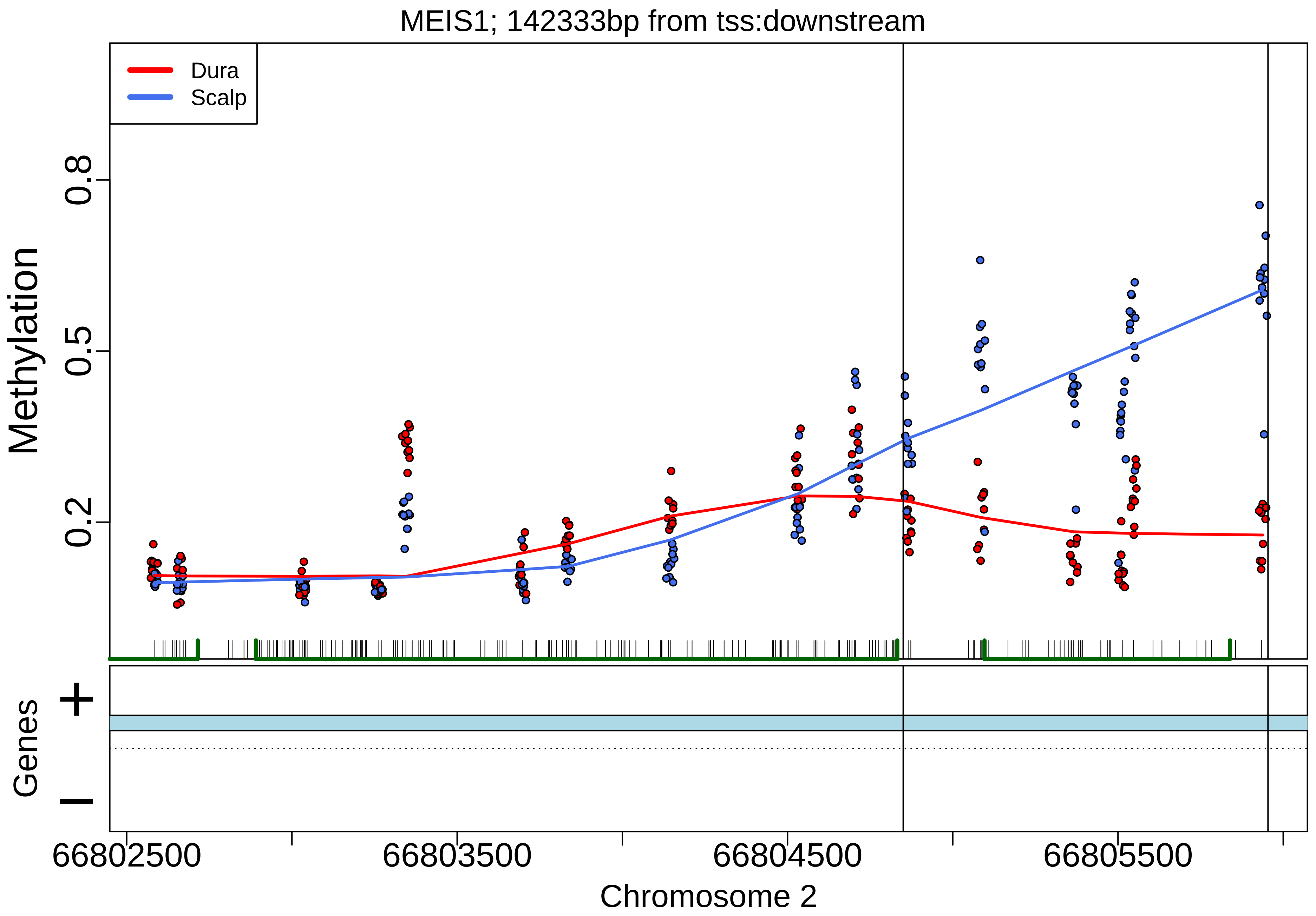
MEIS1; 142333bp from tss:downstream
Dura
Scalp
Methylation
0.2
0.5
0.8
Genes
+
−
66802500
66803500
66804500
66805500
Chromosome 2

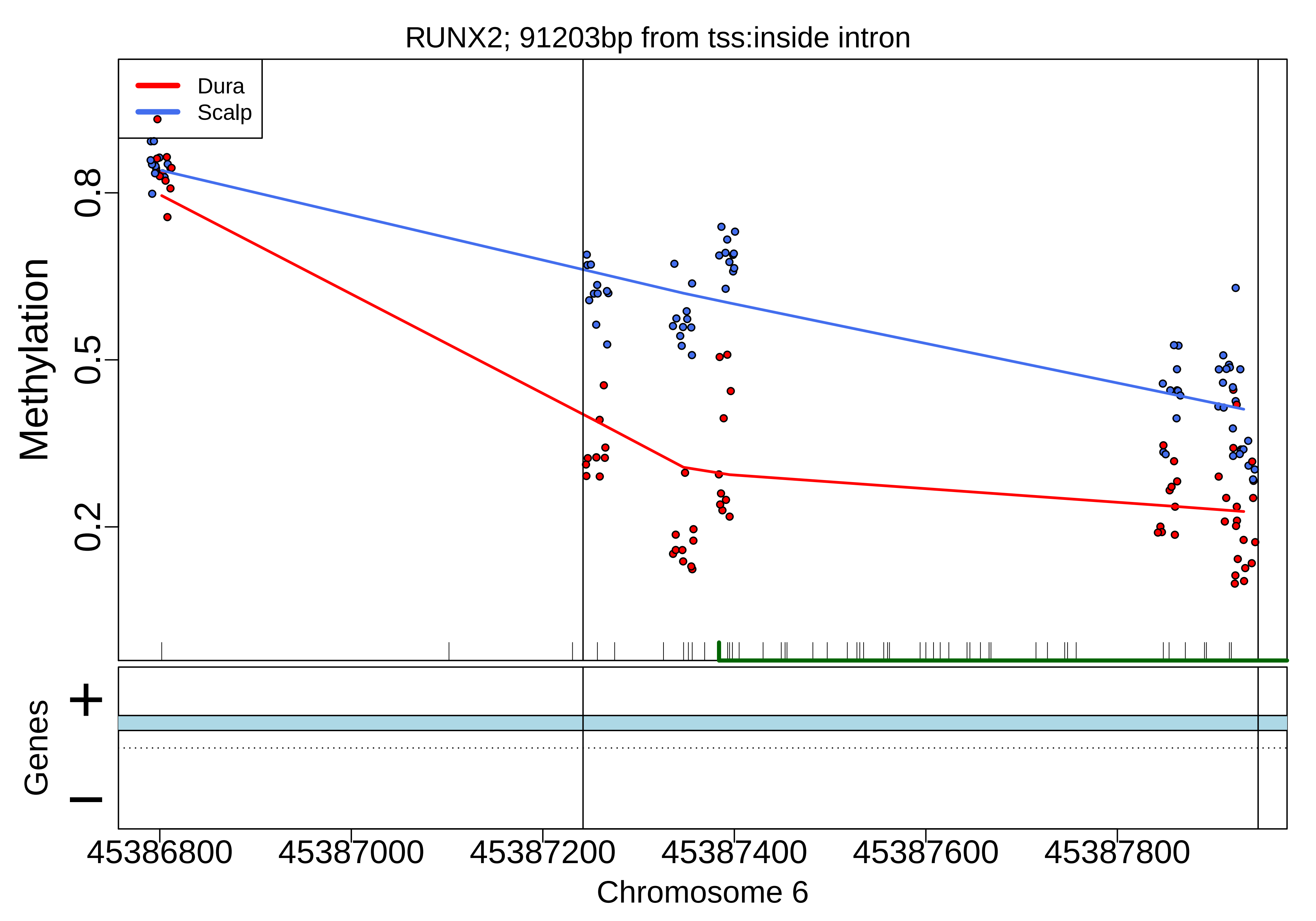

RUNX2; 91203bp from tss:inside intron
Dura
Scalp
Methylation
0.2
0.5
0.8
Genes
+
−
45386800
45387000
45387200
45387400
45387600
45387800
Chromosome 6

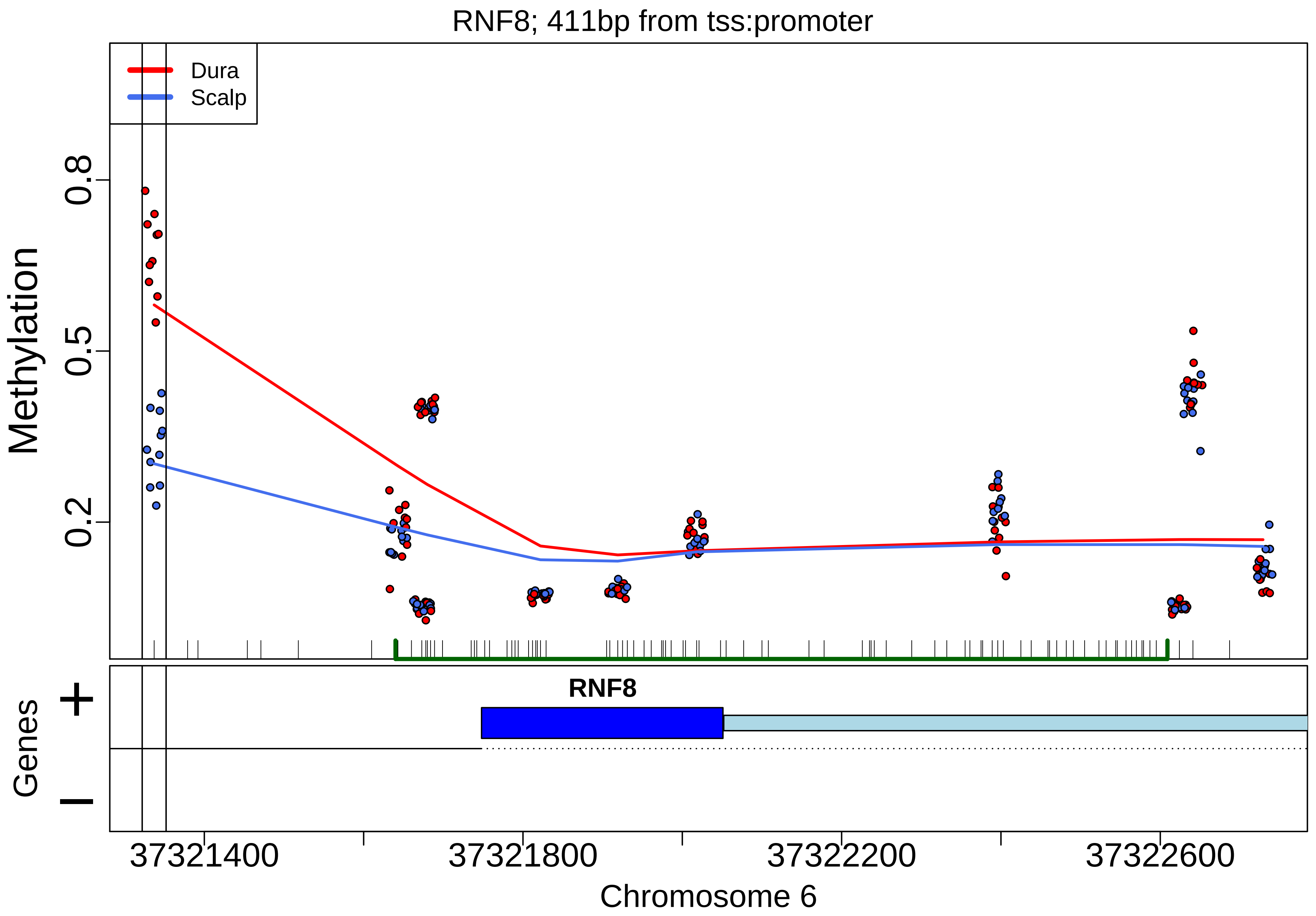
RNF8; 411bp from tss:promoter
Dura
Scalp
Methylation
0.2
0.5
0.8
Genes
+
-
RNF8
37321400
37321800
37322200
37322600
Chromosome 6

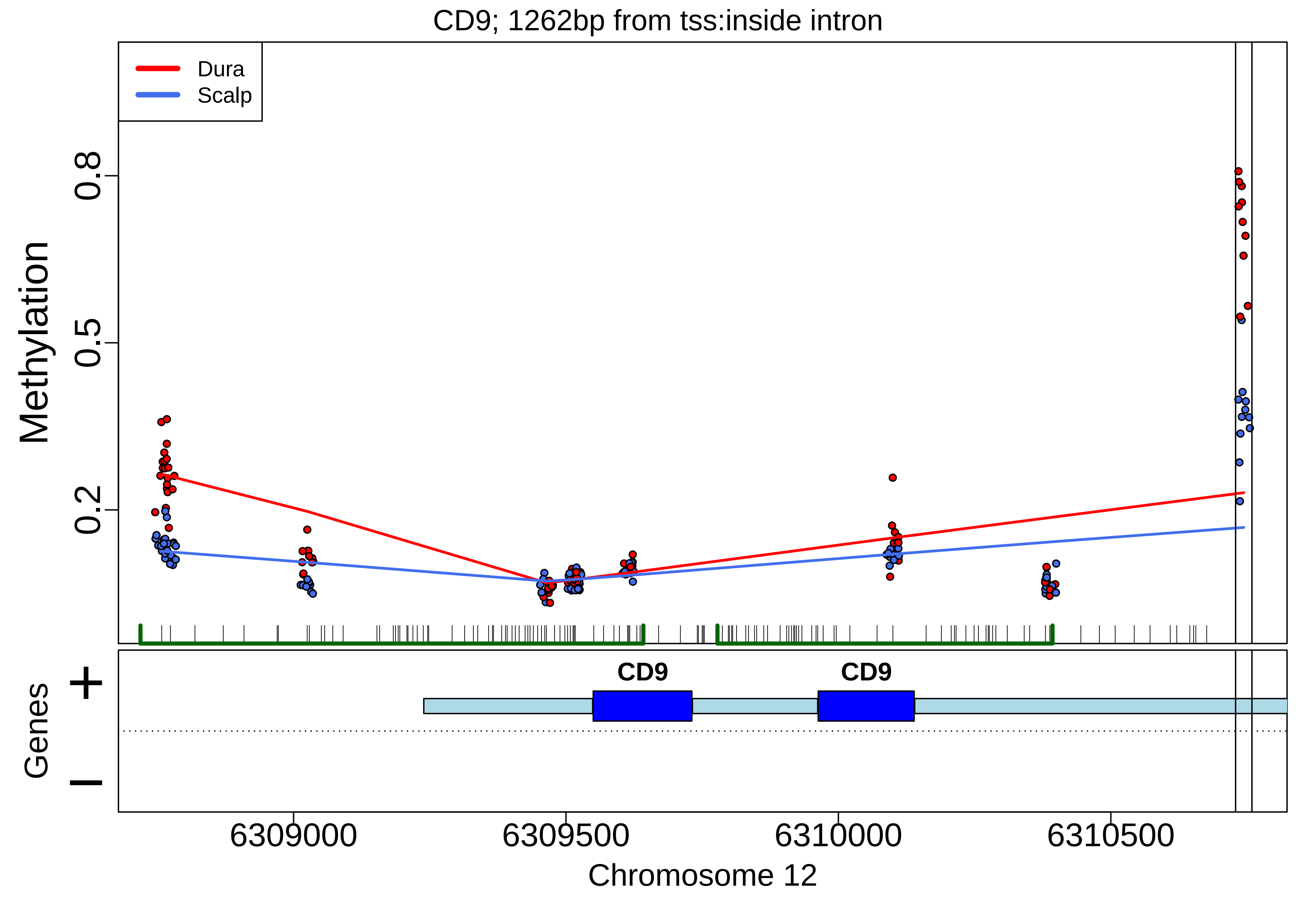
CD9; 1262bp from tss:inside intron
Dura
Scalp
Methylation
0.8
0.5
0.2
Genes
+
−
CD9
CD9
6309000
6309500
6310000
6310500
Chromosome 12

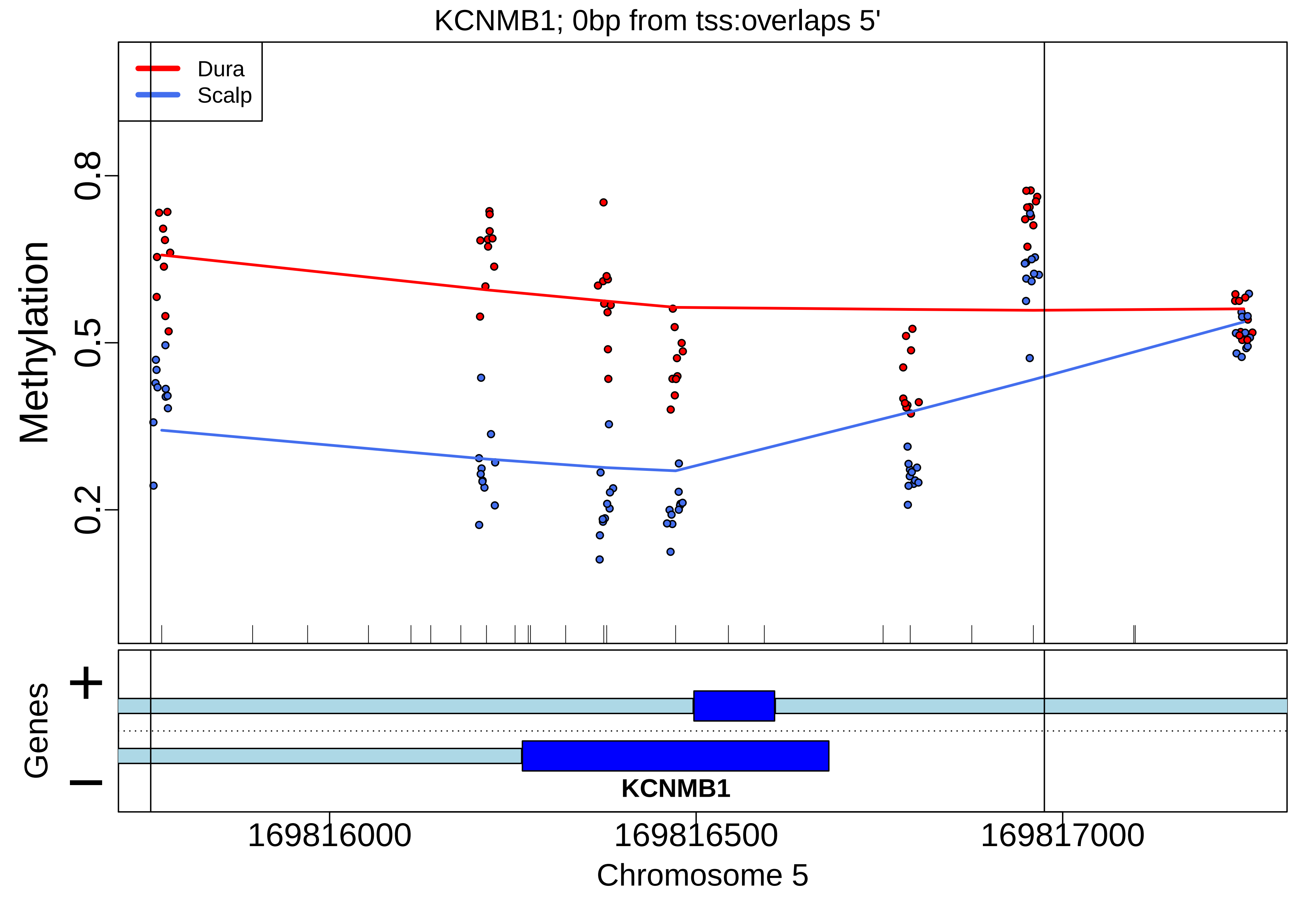

KCNMB1; 0bp from tss:overlaps 5'
Dura
Scalp
Methylation
0.2
0.5
0.8
Genes
+
−
KCNMB1
169816000
169816500
169817000
Chromosome 5

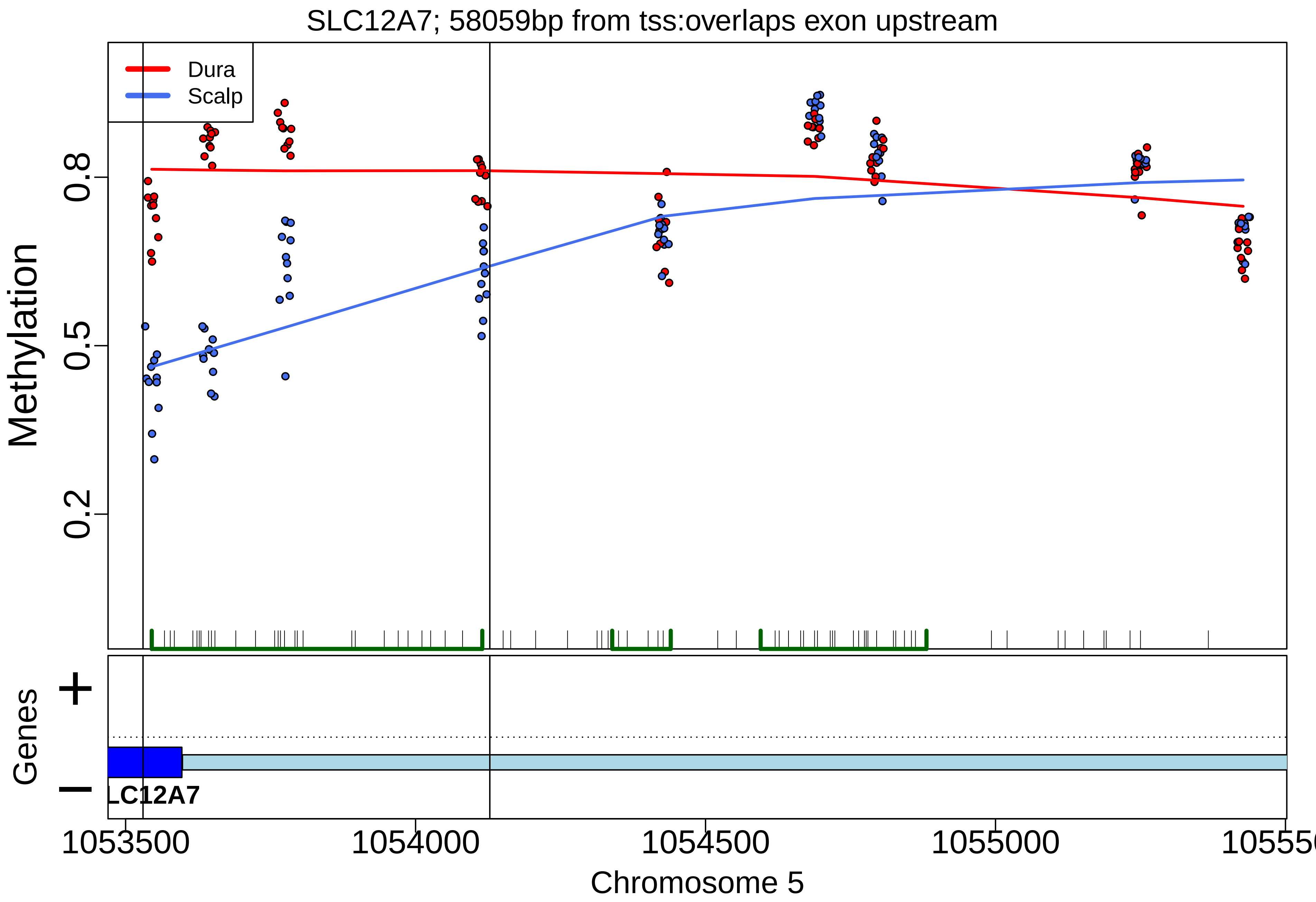

SLC12A7; 58059bp from tss:overlaps exon upstream
Dura
Scalp
Methylation
0.2
0.5
0.8
Genes
+
−
LC12A7
1053500
1054000
1054500
1055000
Chromosome 5

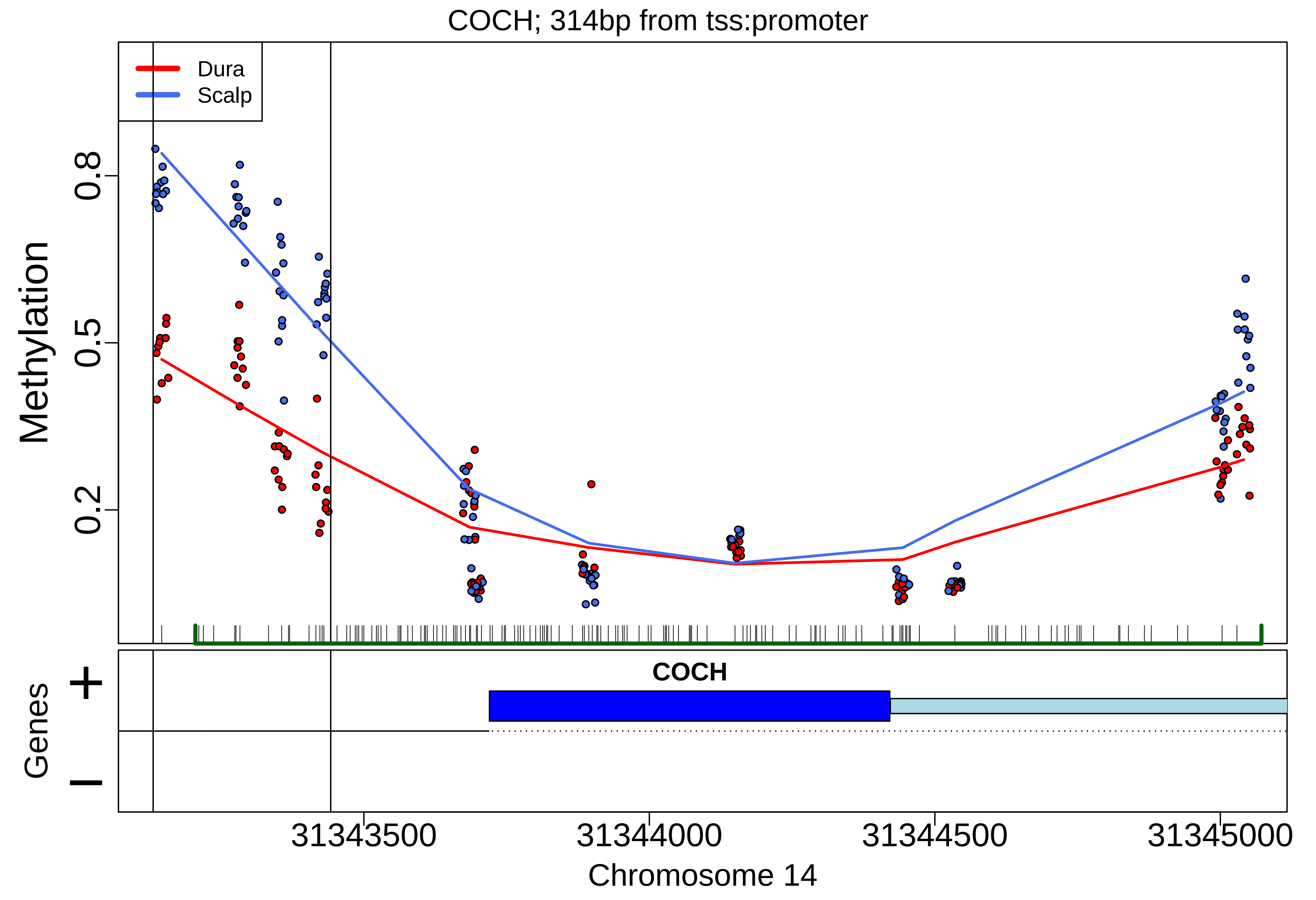

COCH; 314bp from tss:promoter
Dura
Scalp
Methylation
0.2
0.5
0.8
Genes
+
-
COCH
31343500
31344000
31344500
31345000
Chromosome 14

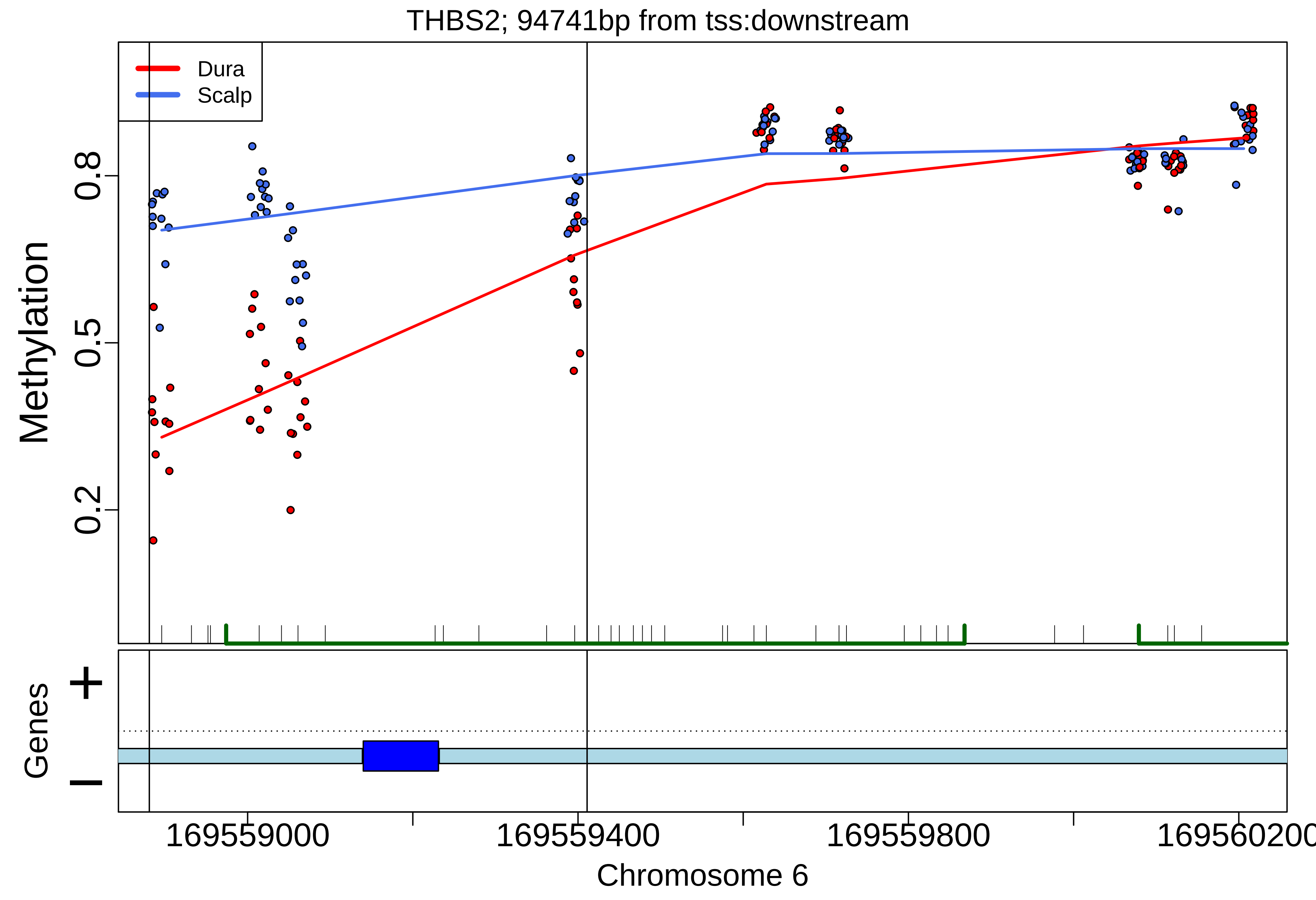

THBS2; 94741bp from tss:downstream
Dura
Scalp
Methylation
0.2
0.5
0.8
Genes
+
-
169559000
169559400
169559800
169560200
Chromosome 6

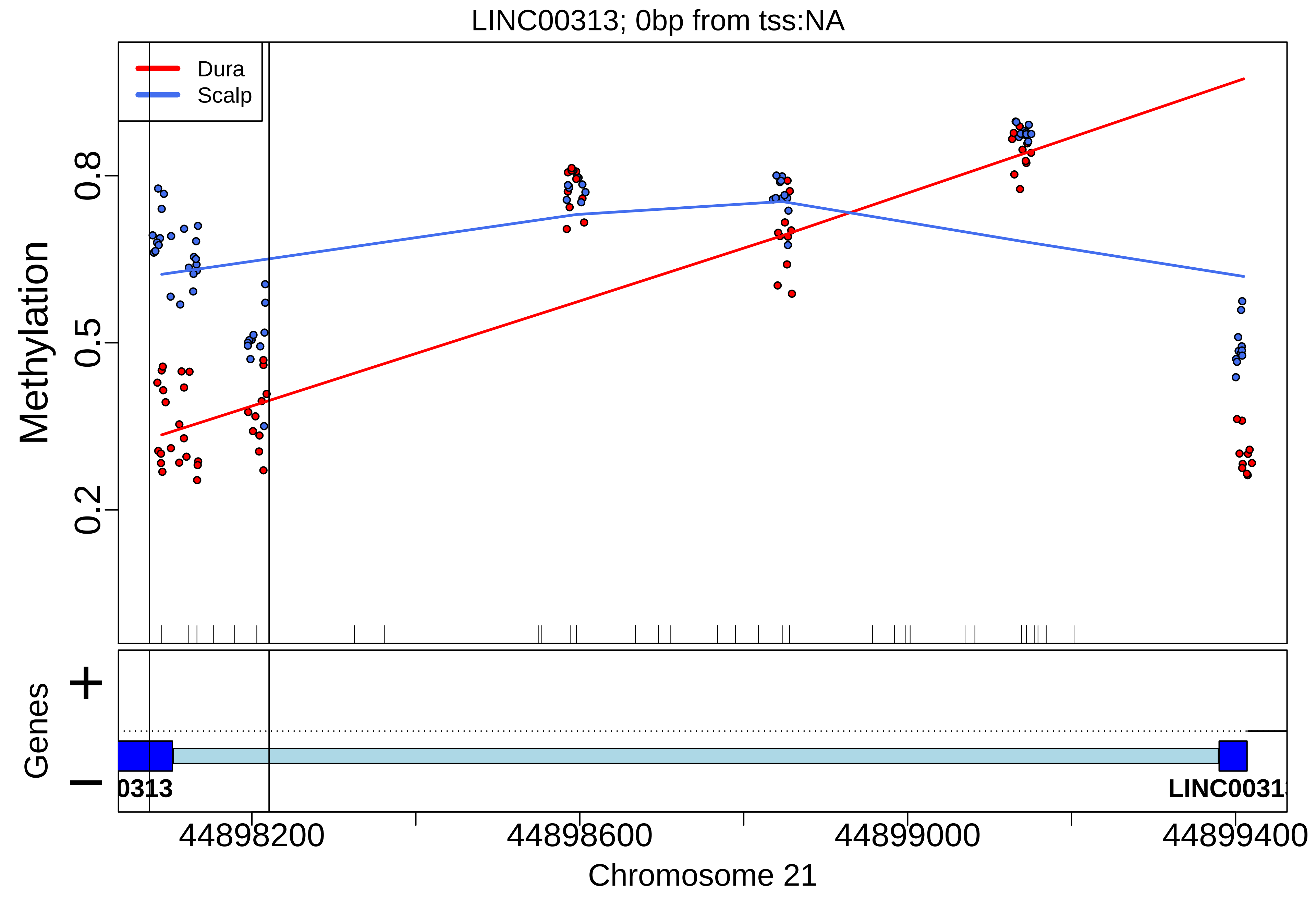

LINC00313; 0bp from tss:NA
Dura
Scalp
Methylation
0.2
0.5
0.8
Genes
+
-
0313
LINC0031
44898200
44898600
44899000
44899400
Chromosome 21

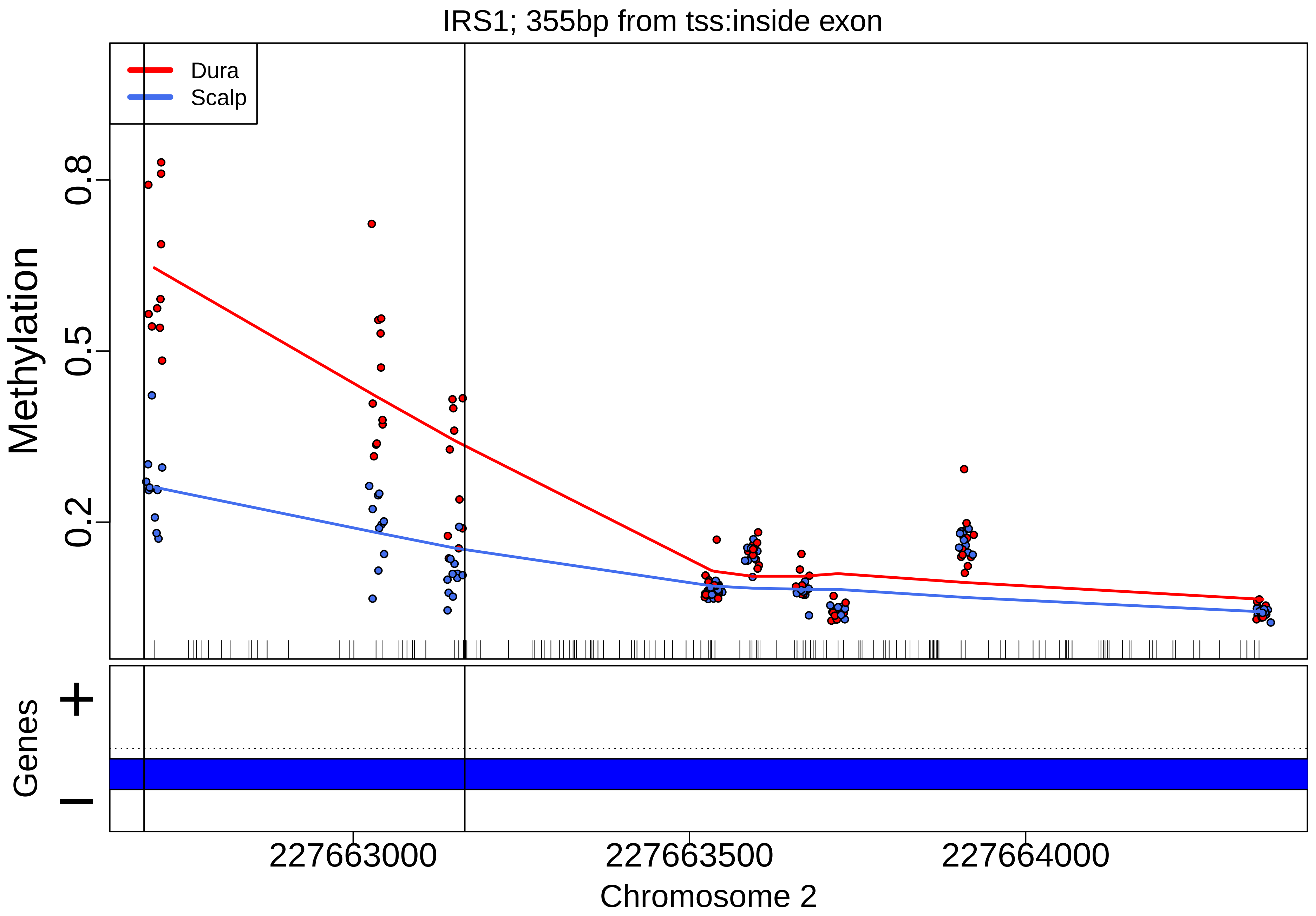

IRS1; 355bp from tss:inside exon
Dura
Scalp
Methylation
0.2
0.5
0.8
Genes
+
−
227663000
227663500
227664000
Chromosome 2

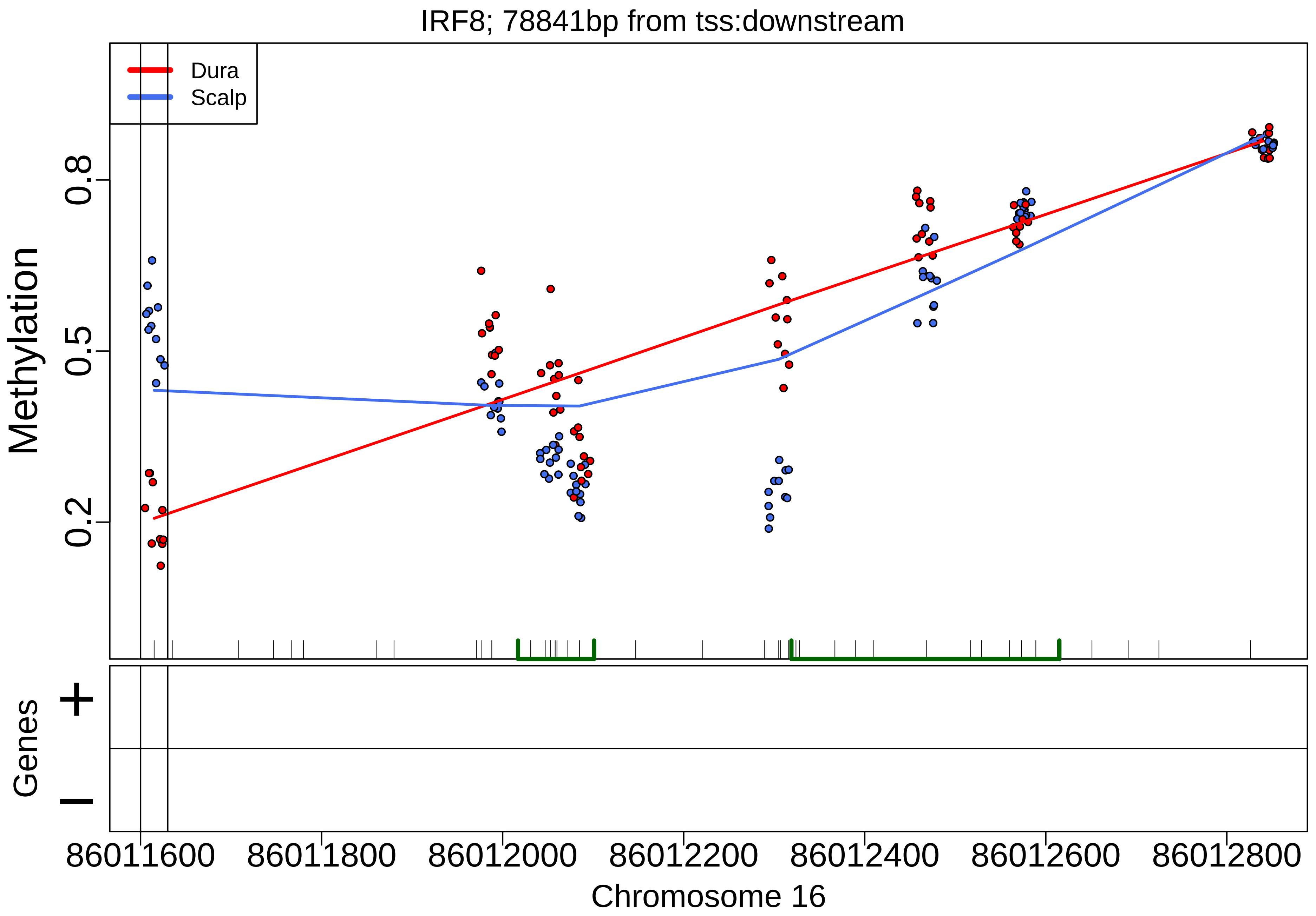
IRF8; 78841bp from tss:downstream
Dura
Scalp
Methylation
0.2
0.5
0.8
Genes
+
−
86011600
86011800
86012000
86012200
86012400
86012600
86012800
Chromosome 16

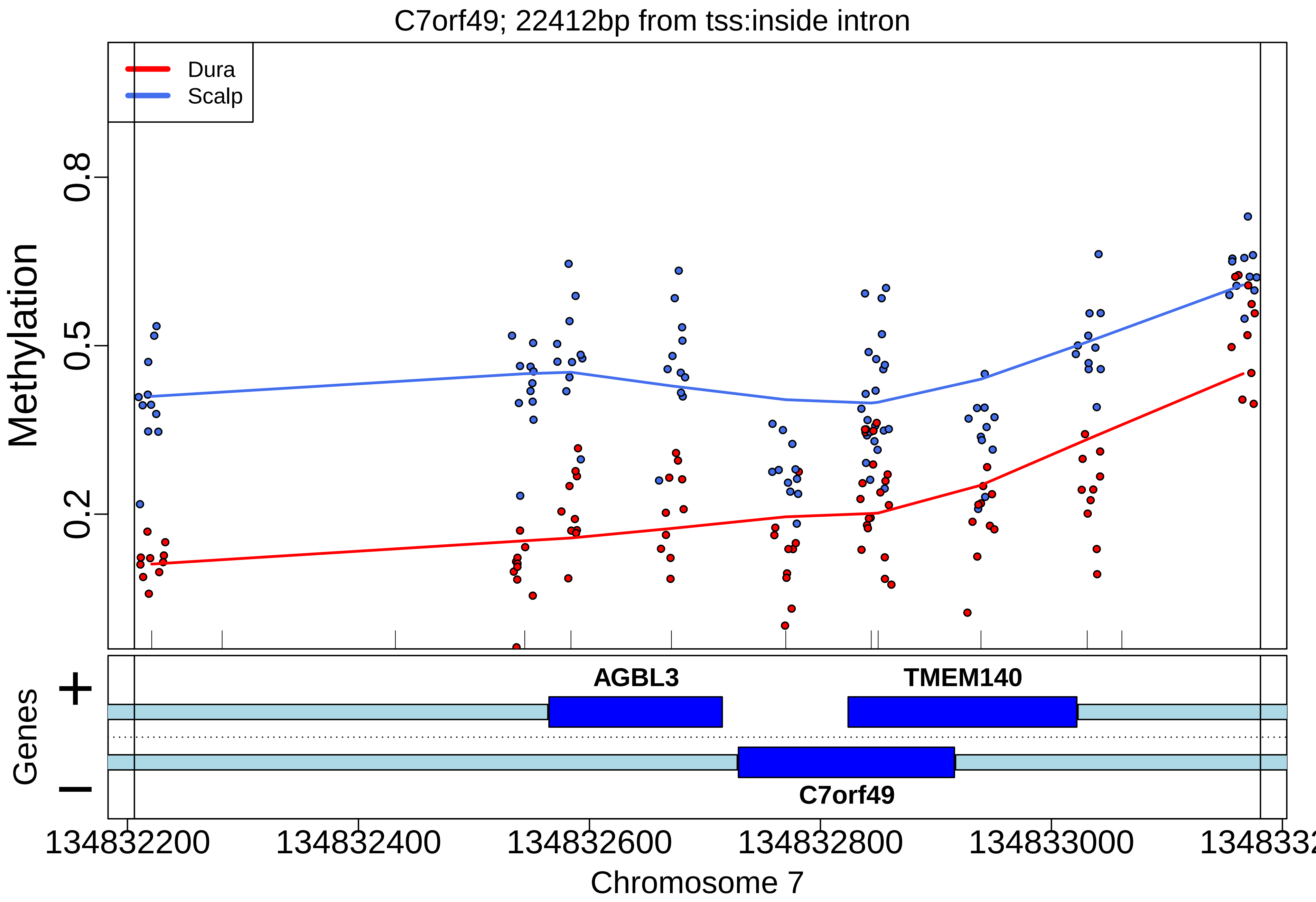

C7orf49; 22412bp from tss:inside intron
Dura
Scalp
Methylation
0.2
0.5
0.8
Genes
+
−
AGBL3
TMEM140
C7orf49
134832200
134832400
134832600
134832800
134833000
Chromosome 7

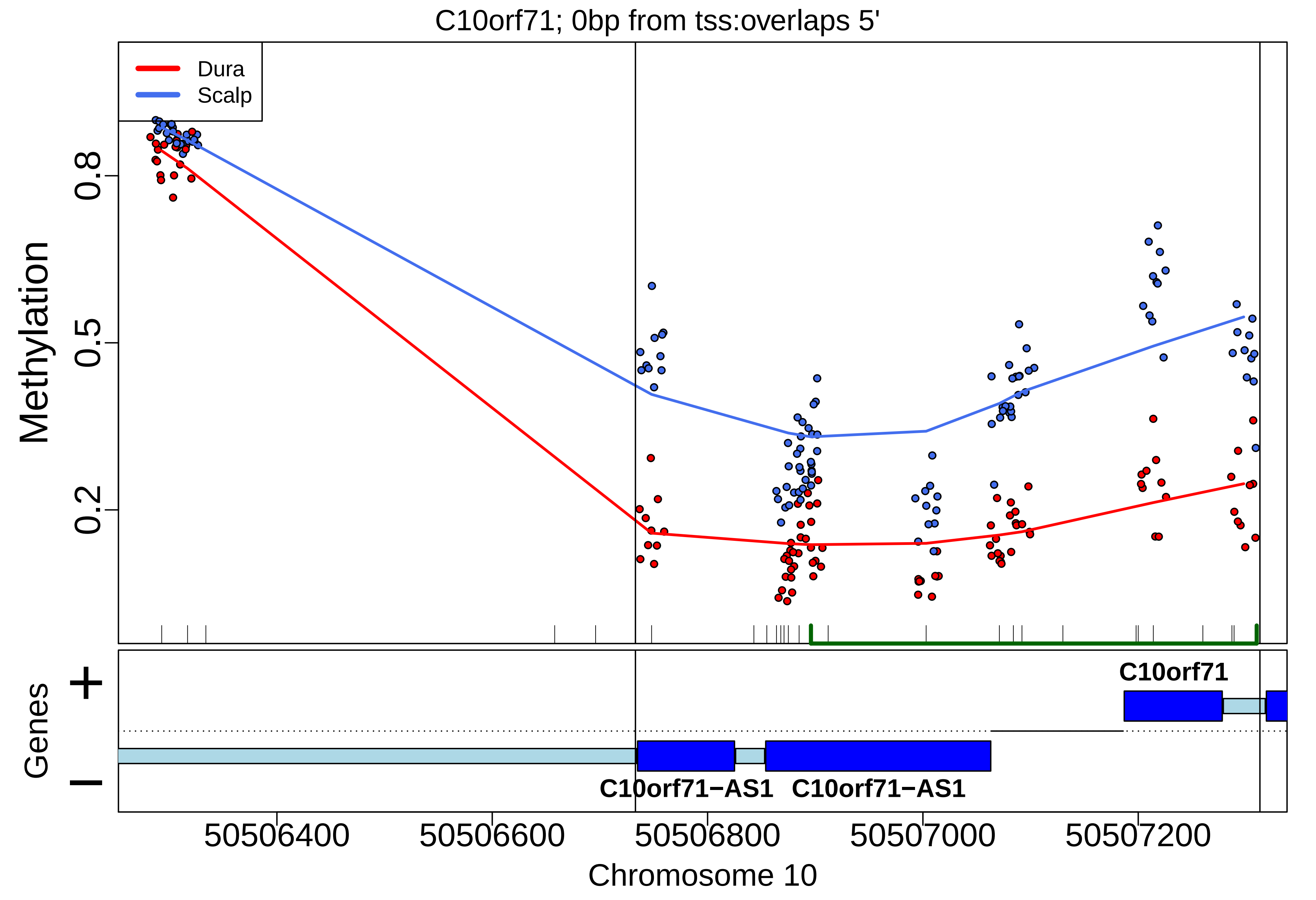
C10orf71; 0bp from tss:overlaps 5'
Dura
Scalp
Methylation
0.2
0.5
0.8
Genes
+
−
C10orf71
C10orf71−AS1
C10orf71−AS1
50506400
50506600
50506800
50507000
50507200
Chromosome 10

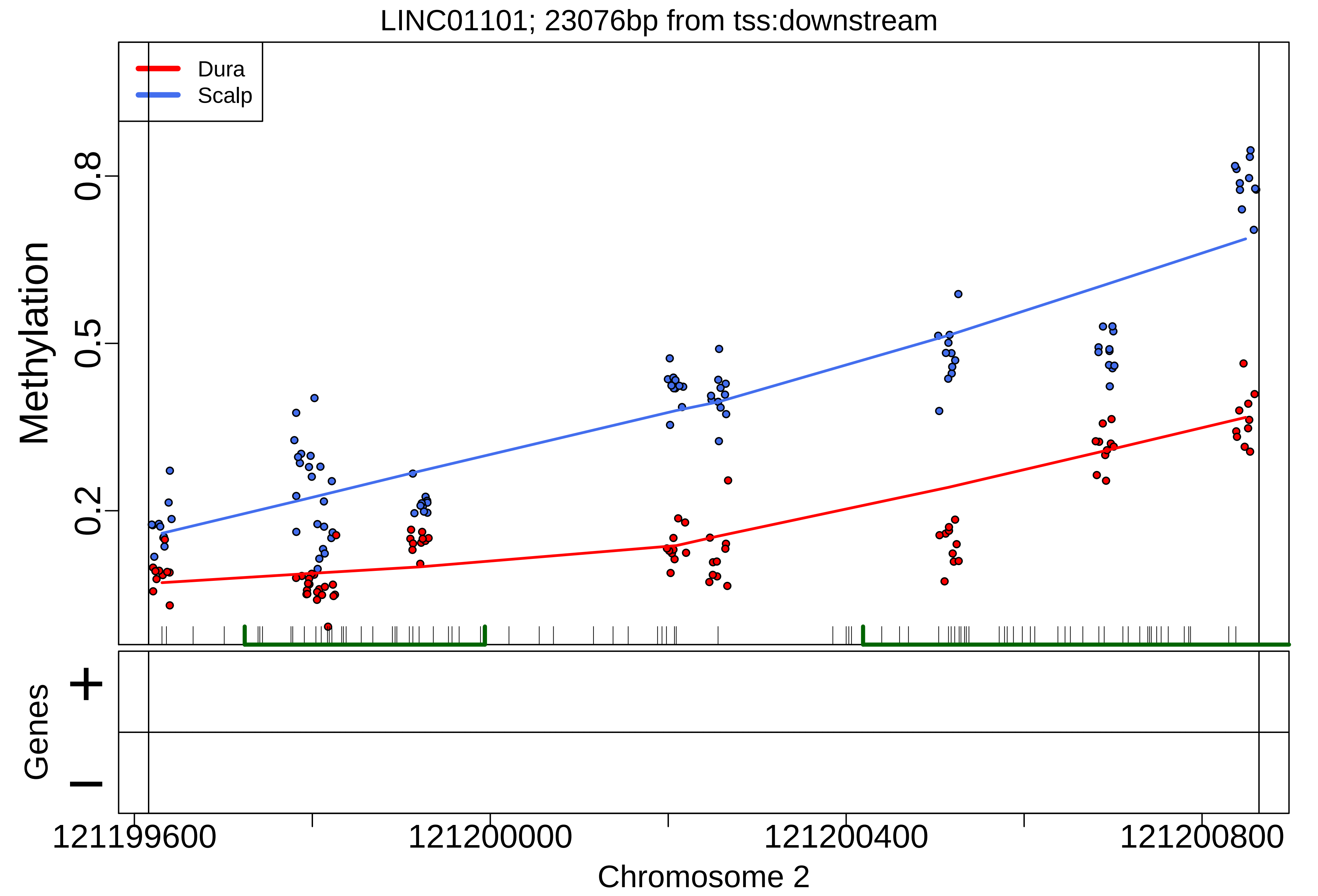

LINC01101; 23076bp from tss:downstream
Dura
Scalp
Methylation
0.2
0.5
0.8
Genes
+
-
121199600
121200000
121200400
121200800
Chromosome 2

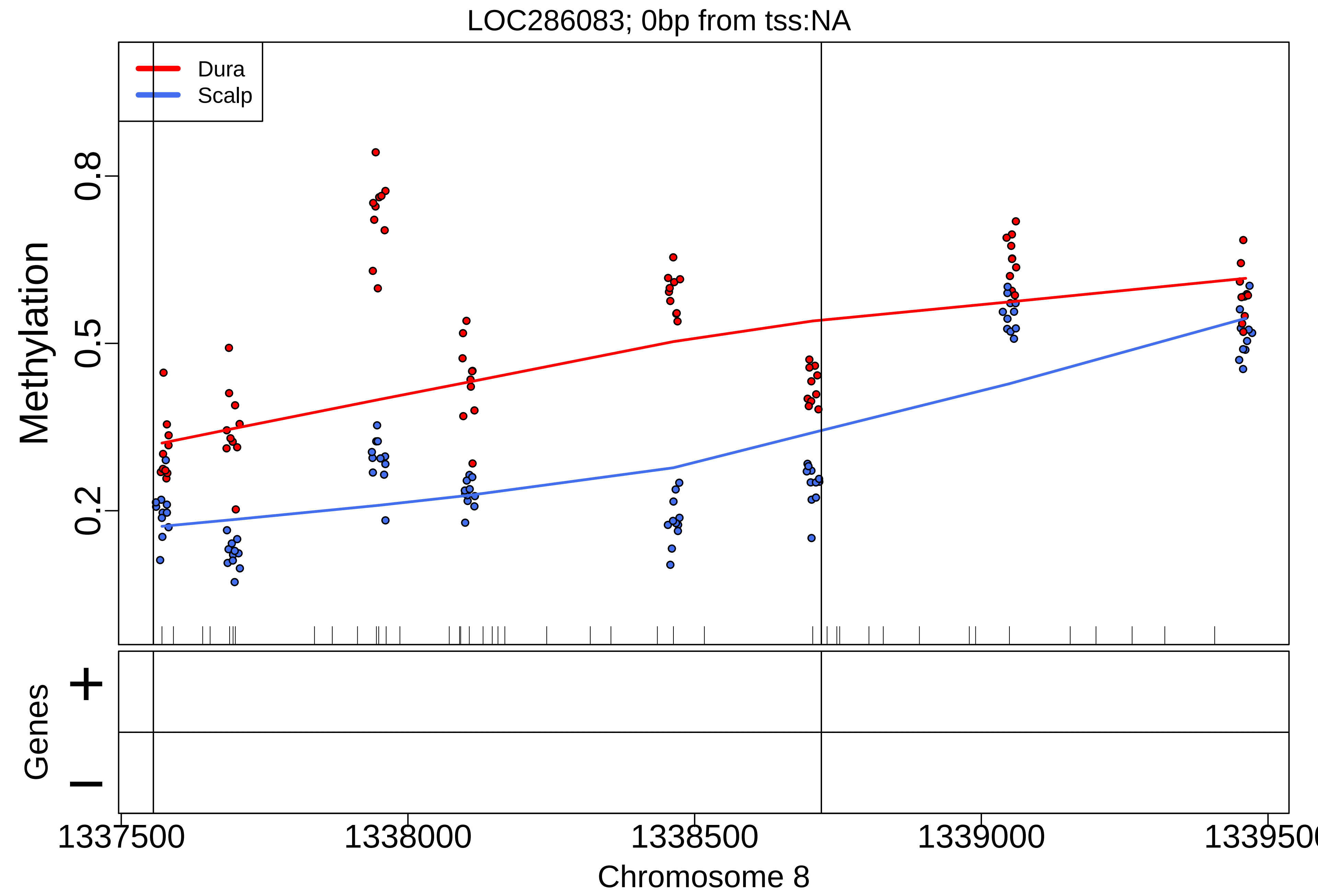
LOC286083; 0bp from tss:NA
Dura
Scalp
Methylation
0.2
0.5
0.8
Genes
+
−
1337500
1338000
1338500
1339000
1339500
Chromosome 8

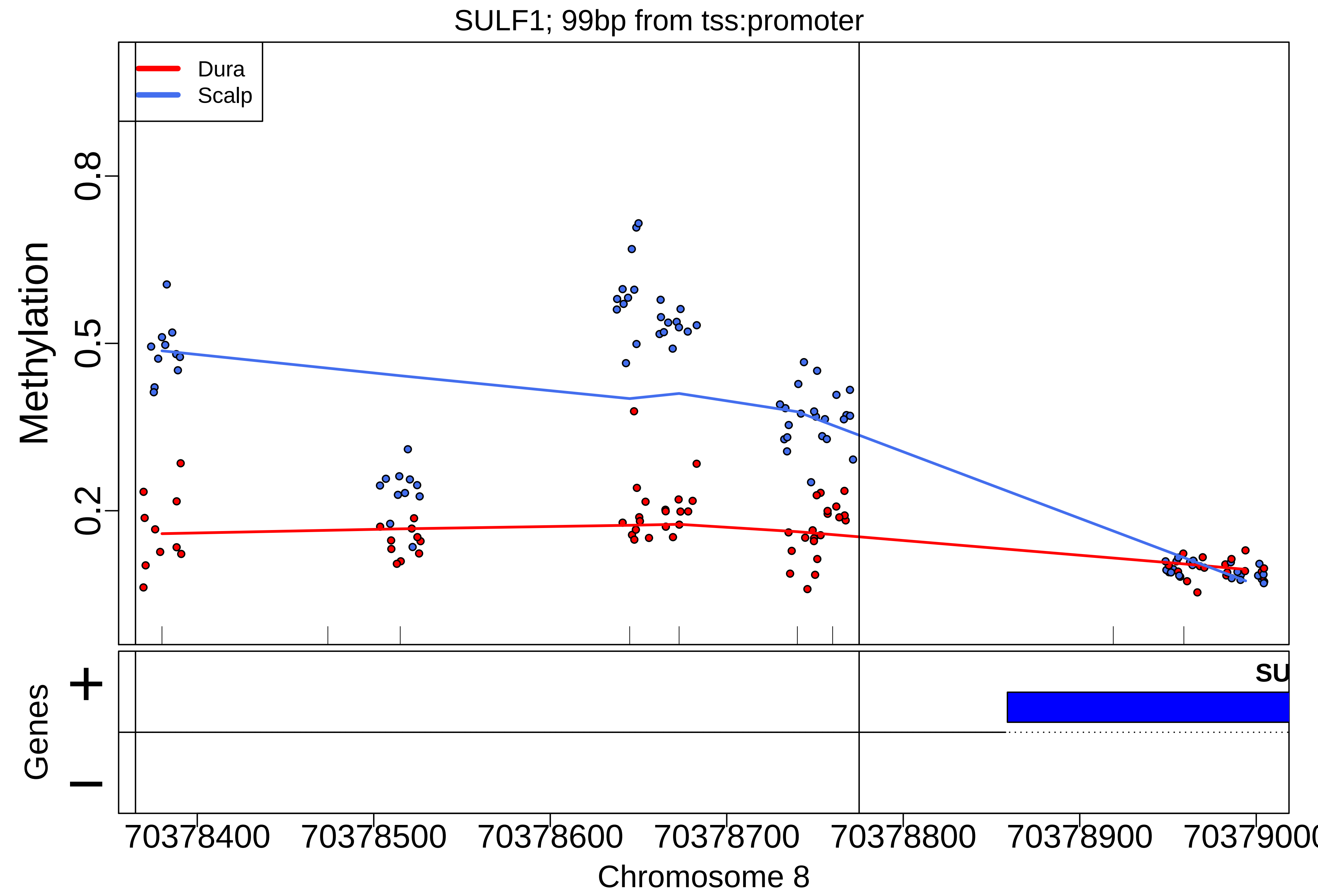

SULF1; 99bp from tss:promoter
Dura
Scalp
Methylation
0.2
0.5
0.8
Genes
+
−
SU
70378400
70378500
70378600
70378700
70378800
70378900
70379000
Chromosome 8

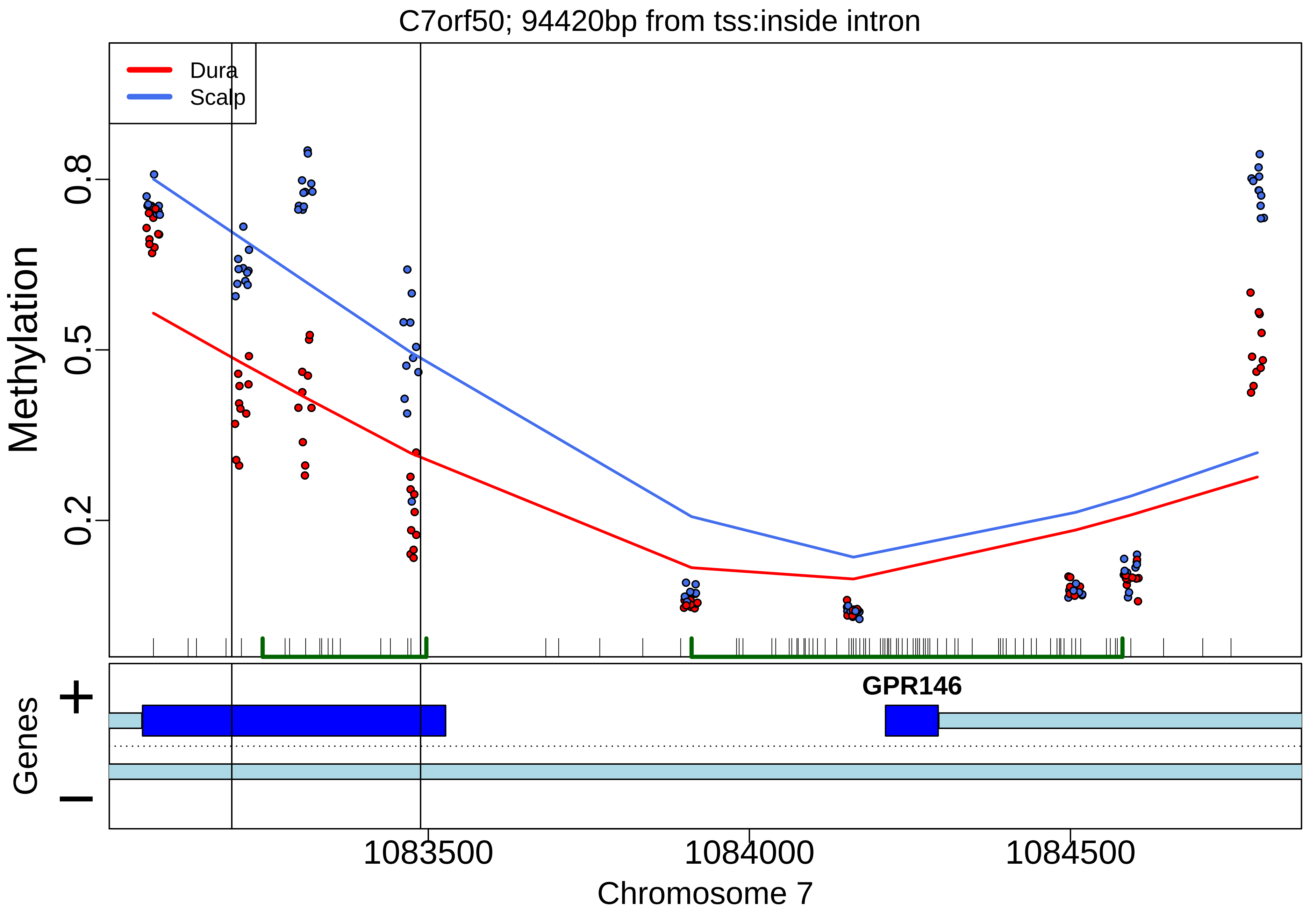
C7orf50; 94420bp from tss:inside intron
Dura
Scalp
Methylation
0.2
0.5
0.8
Genes
+
-
GPR146
1083500
1084000
1084500
Chromosome 7

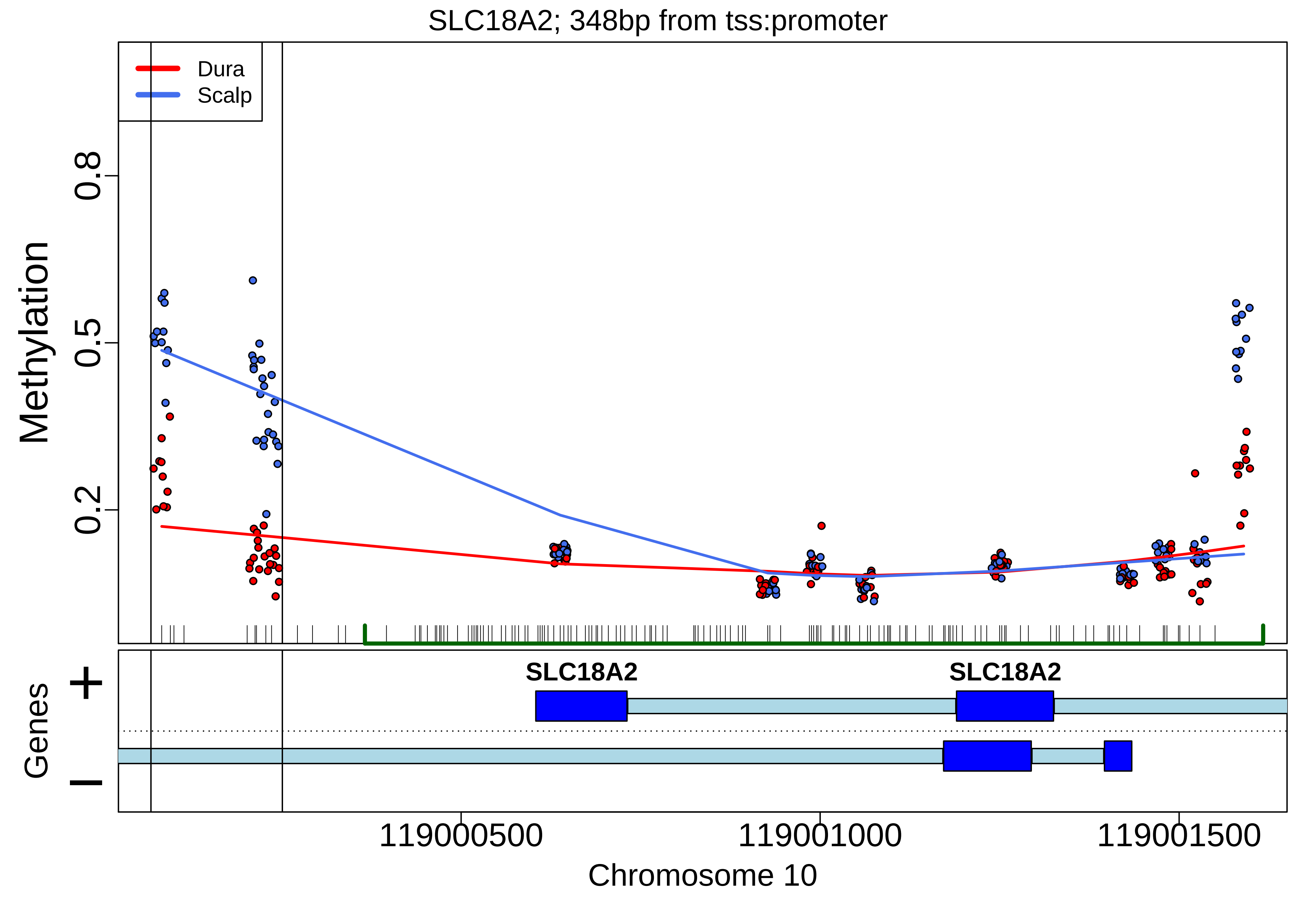
SLC18A2; 348bp from tss:promoter
Dura
Scalp
Methylation
0.2
0.5
0.8
Genes
+
-
SLC18A2
SLC18A2
119000500
119001000
119001500
Chromosome 10

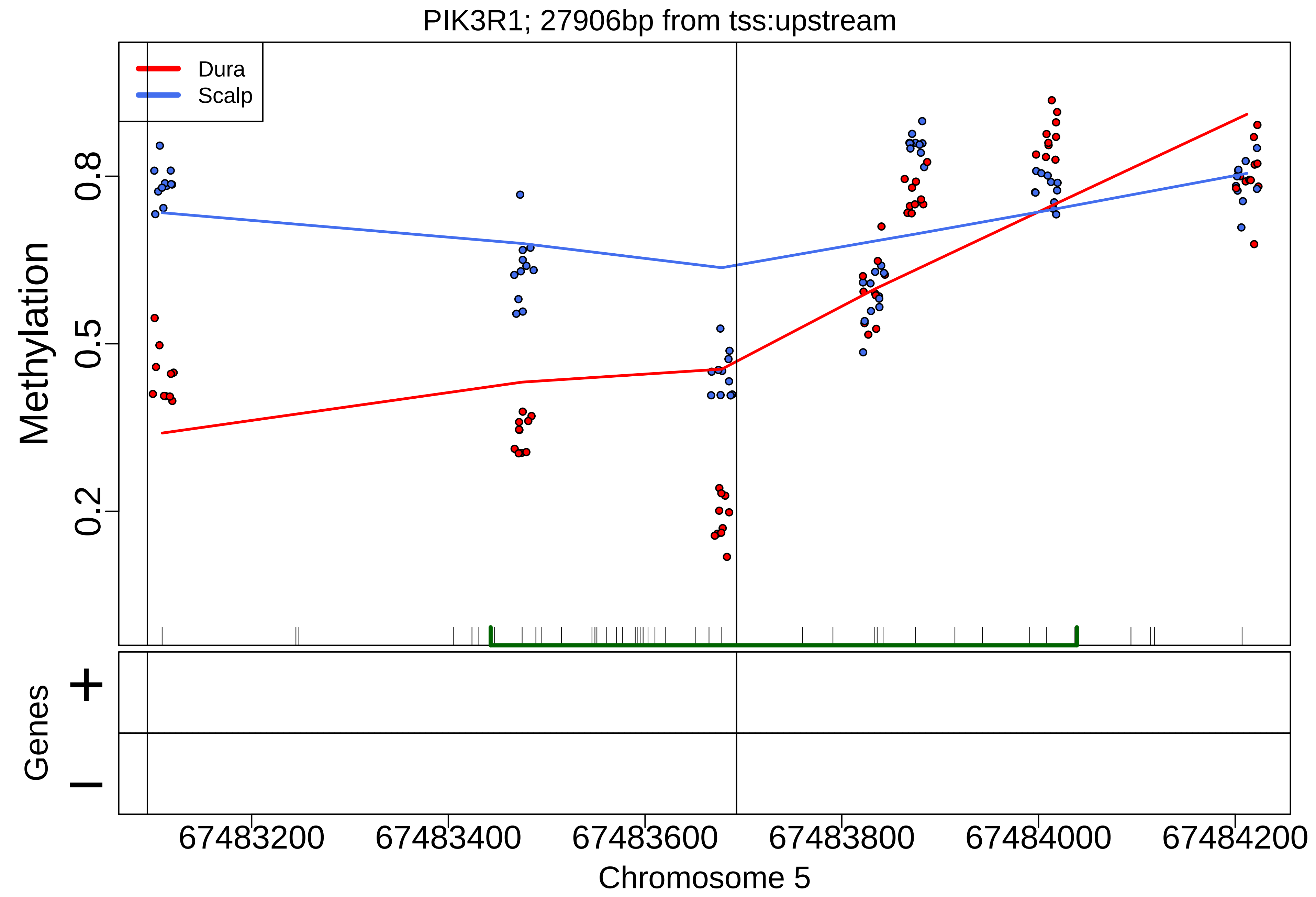

PIK3R1; 27906bp from tss:upstream
Dura
Scalp
Methylation
0.2
0.5
0.8
Genes
+
-
67483200
67483400
67483600
67483800
67484000
67484200
Chromosome 5

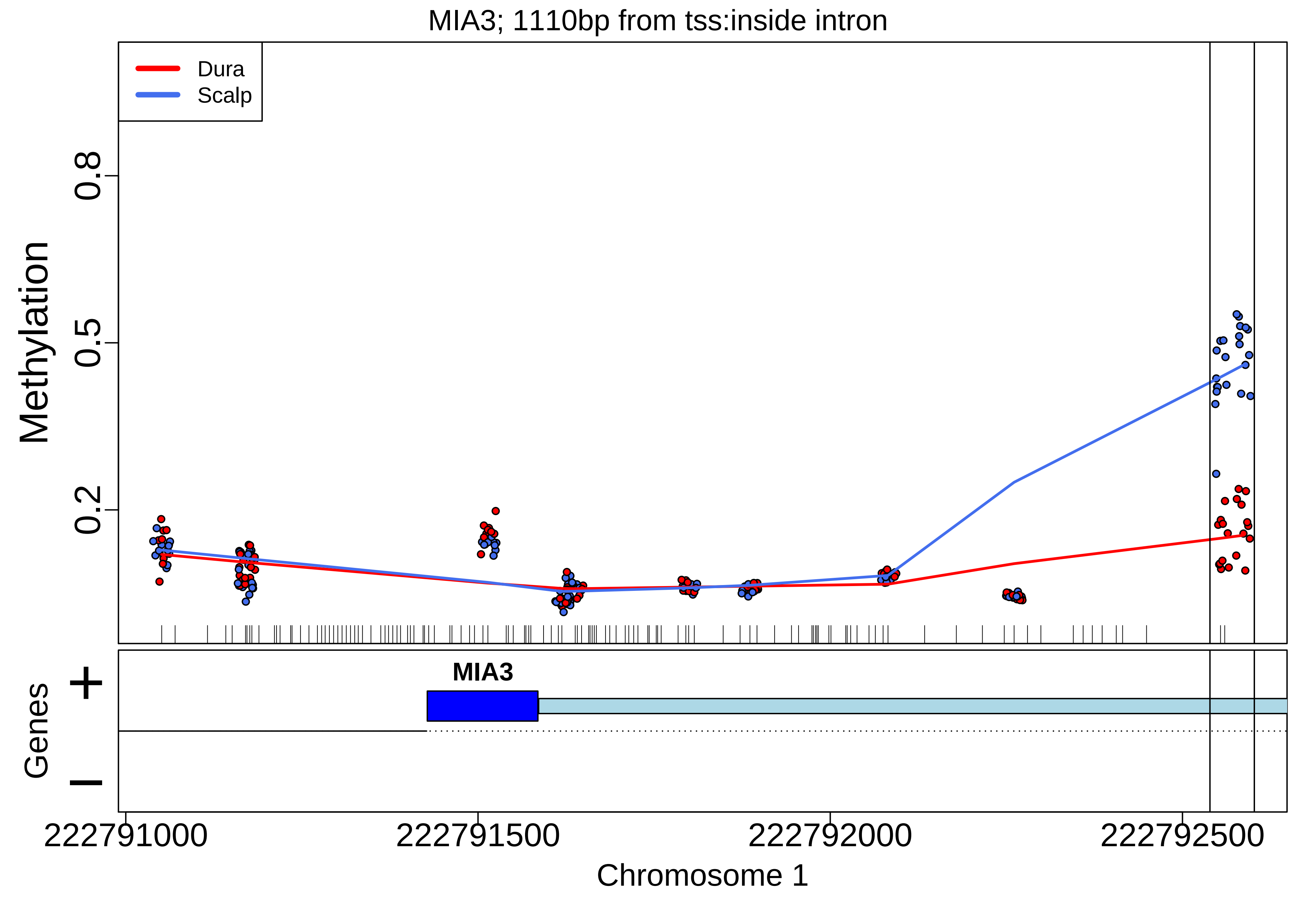

MIA3; 1110bp from tss:inside intron
Dura
Scalp
Methylation
0.2
0.5
0.8
Genes
+
-
MIA3
222791000
222791500
222792000
222792500
Chromosome 1

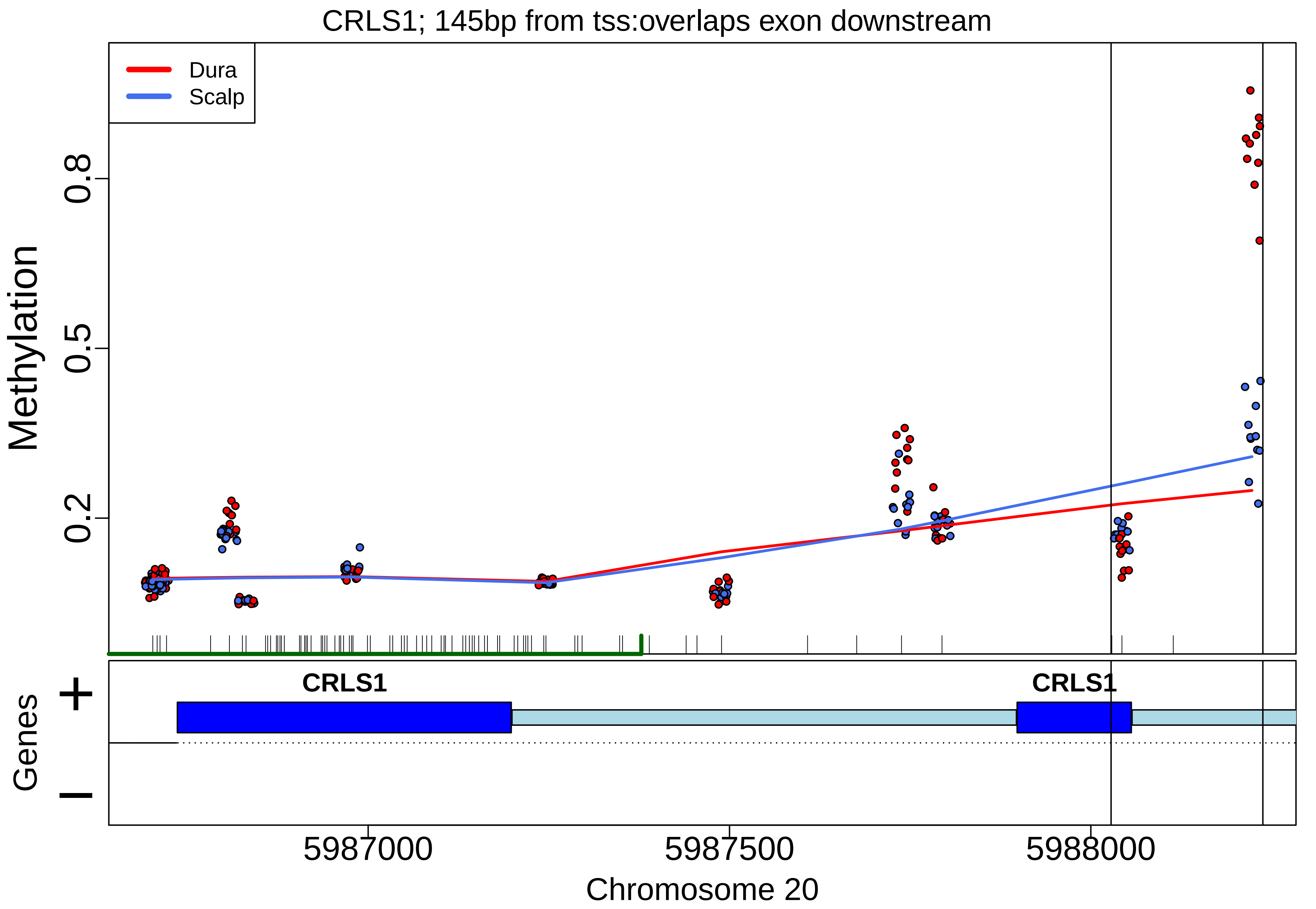

CRLS1; 145bp from tss:overlaps exon downstream
Dura
Scalp
Methylation
0.2
0.5
0.8
Genes
+
-
CRLS1
CRLS1
5987000
5987500
5988000
Chromosome 20

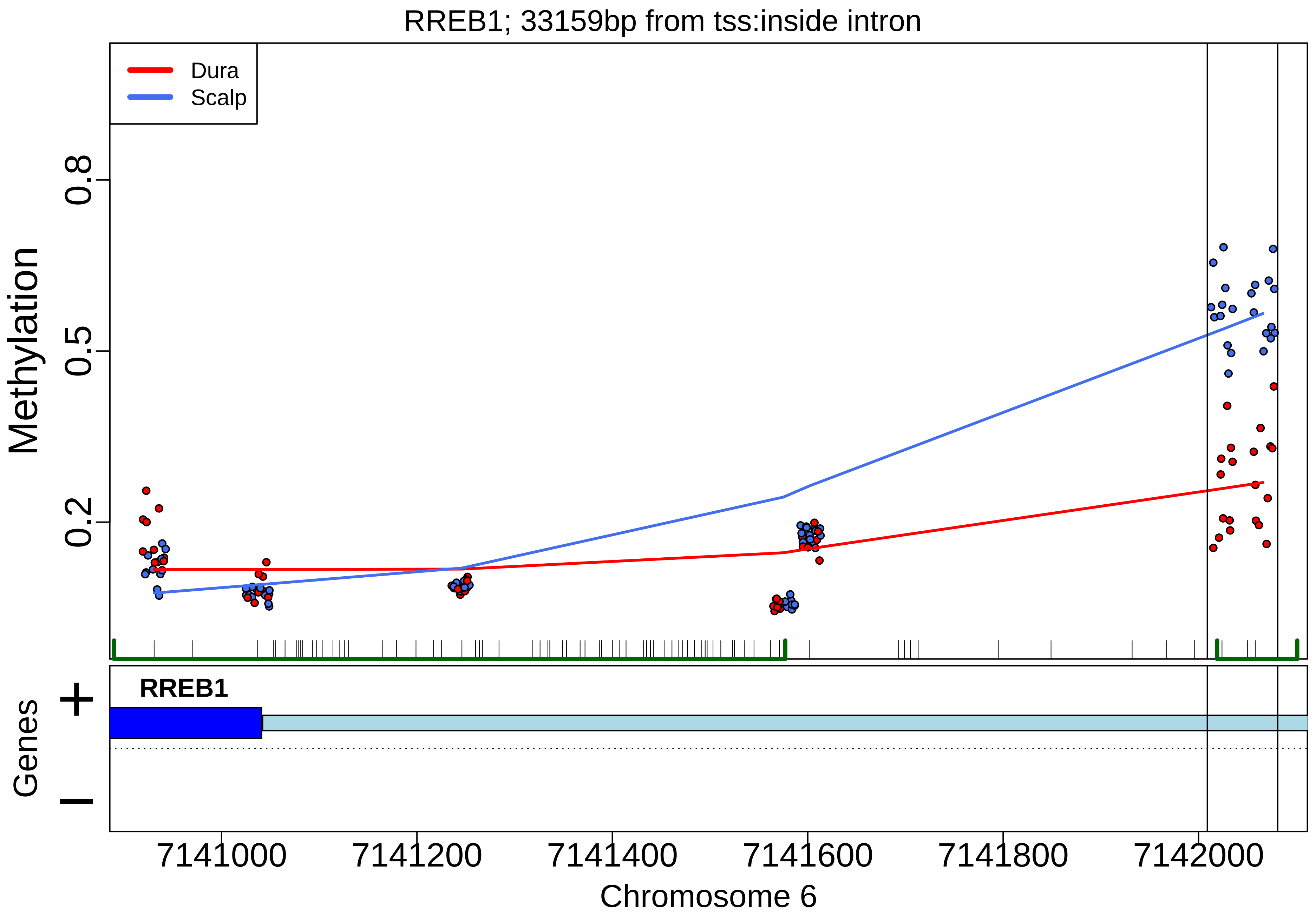

RREB1; 33159bp from tss:inside intron
Dura
Scalp
Methylation
0.2
0.5
0.8
Genes
+
-
RREB1
7141000
7141200
7141400
7141600
7141800
7142000
Chromosome 6

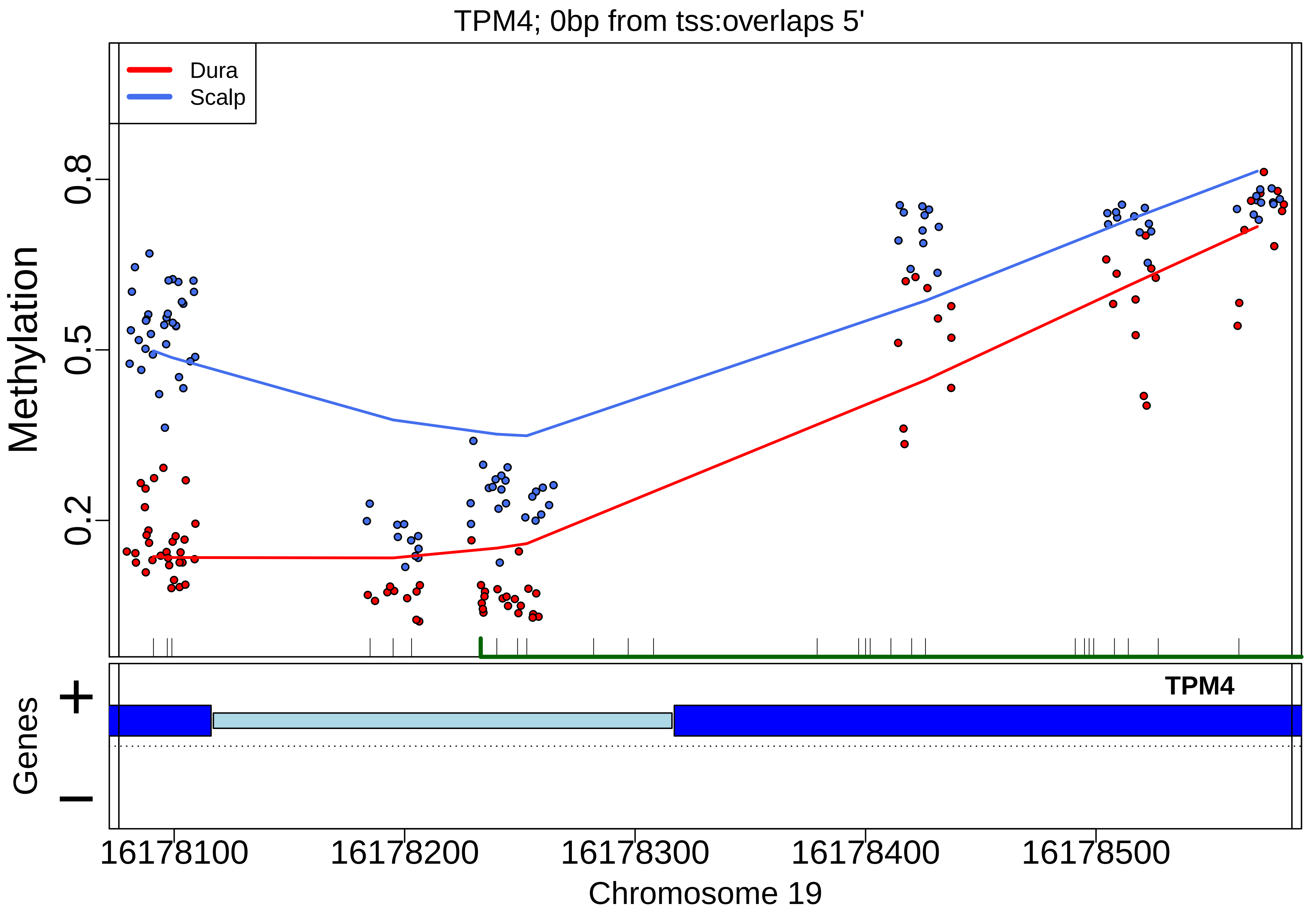

TPM4; 0bp from tss:overlaps 5'
Dura
Scalp
Methylation
0.2
0.5
0.8
Genes
+
−
TPM4
16178100
16178200
16178300
16178400
16178500
Chromosome 19

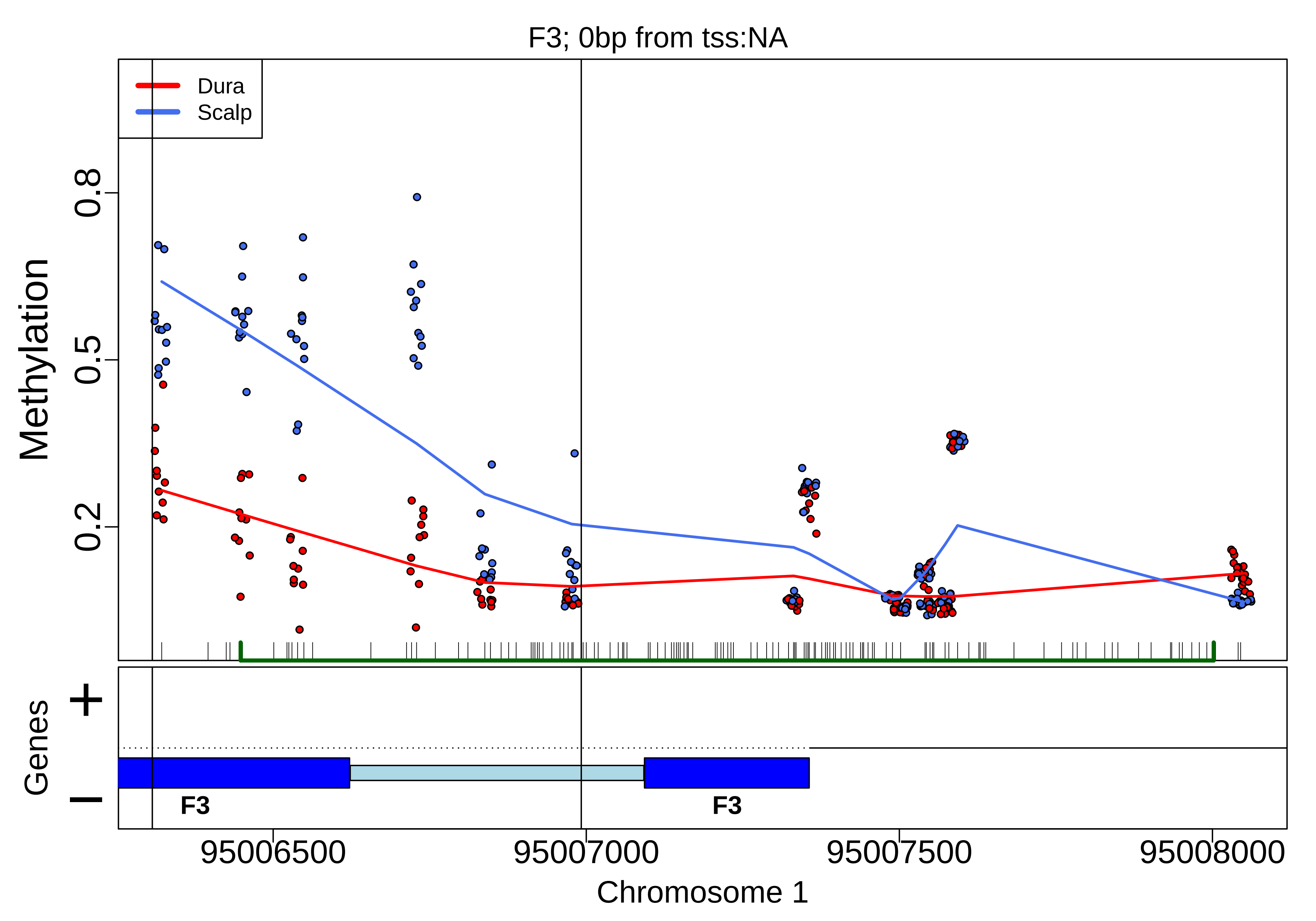
F3; 0bp from tss:NA
Dura
Scalp
Methylation
0.2
0.5
0.8
Genes
+
-
F3
F3
95006500
95007000
95007500
95008000
Chromosome 1

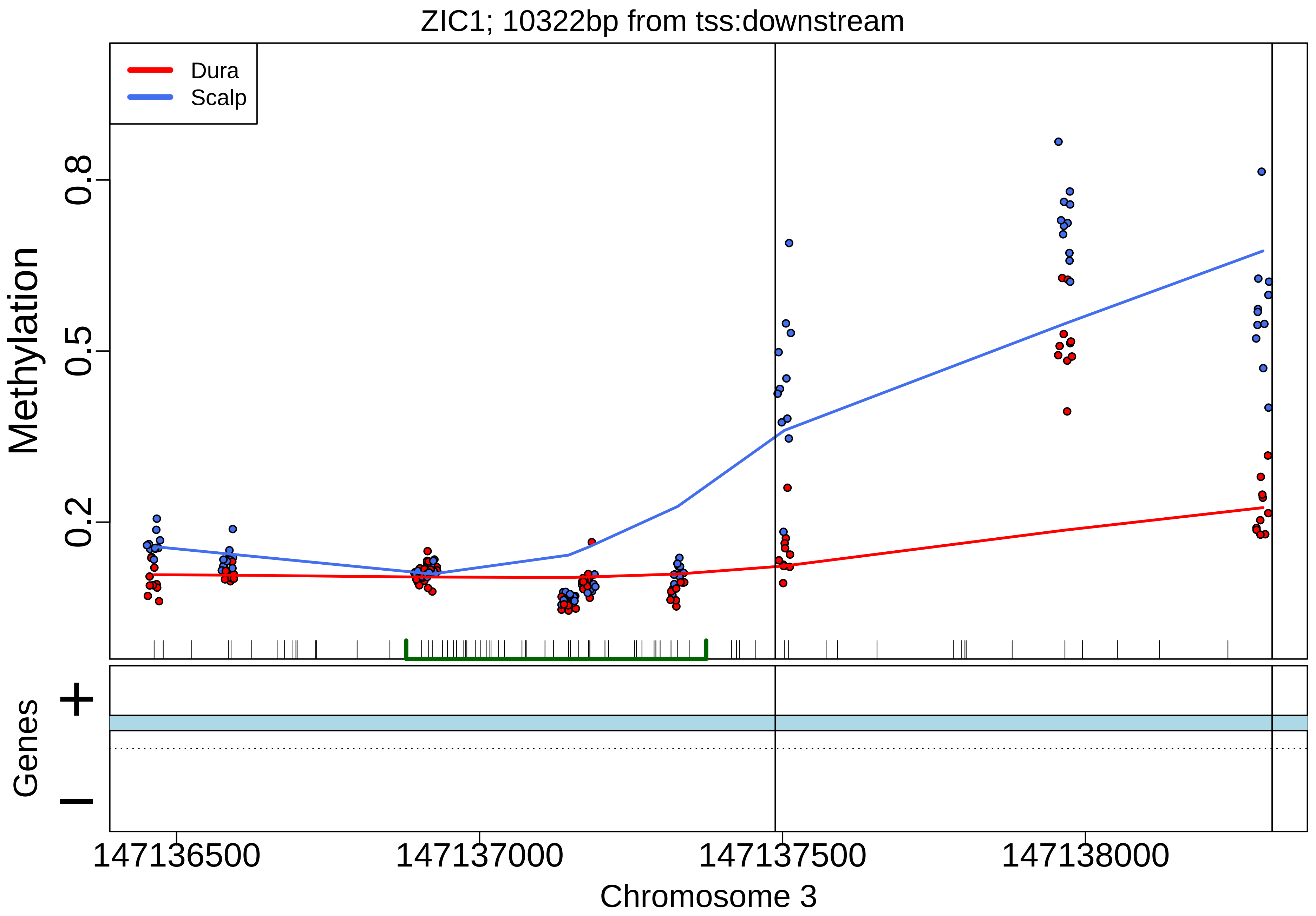
ZIC1; 10322bp from tss:downstream
Dura
Scalp
Methylation
0.2
0.5
0.8
Genes
+
−
147136500
147137000
147137500
147138000
Chromosome 3

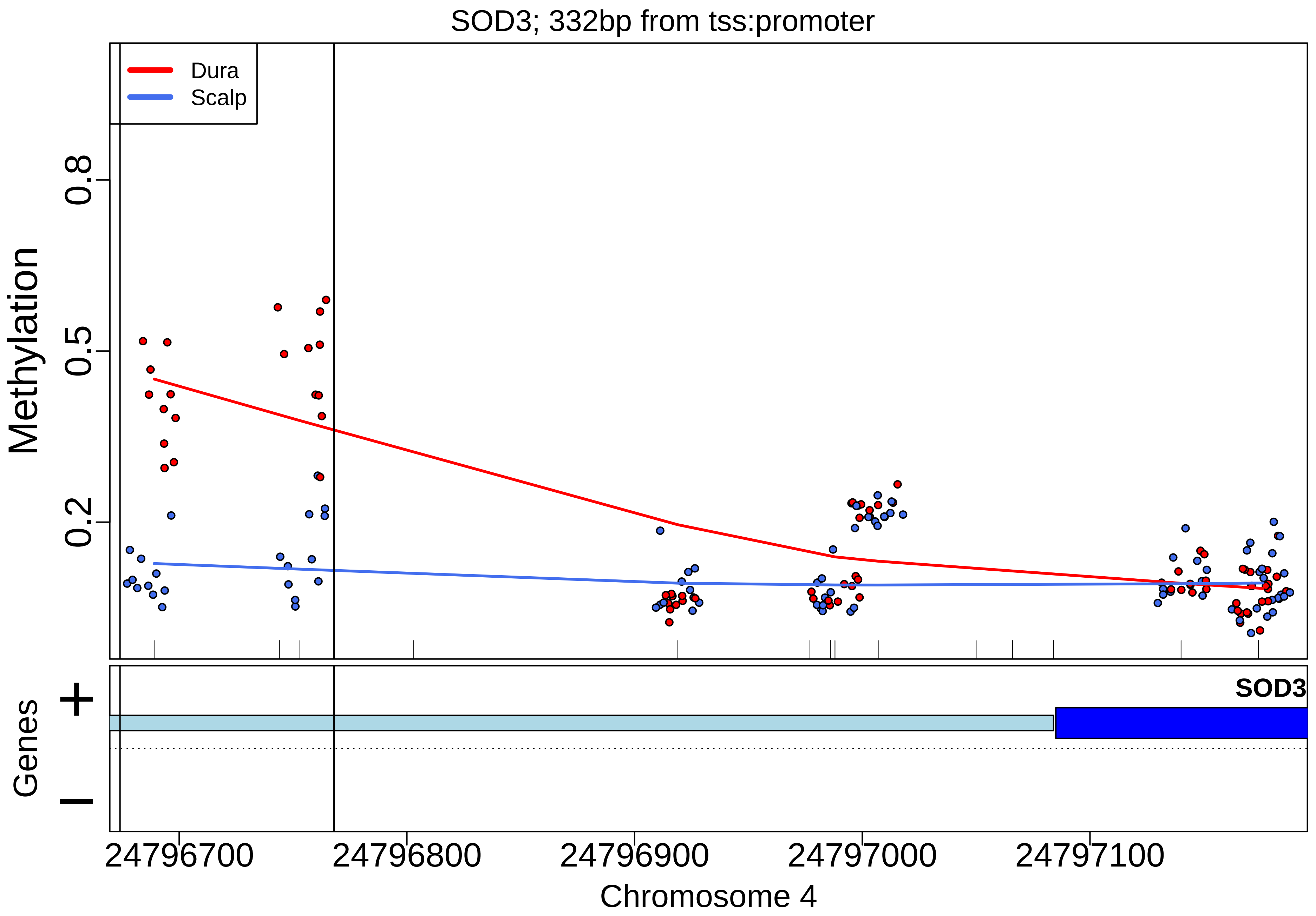

SOD3; 332bp from tss:promoter
Dura
Scalp
Methylation
0.2
0.5
0.8
Genes
+
-
SOD3
24796700
24796800
24796900
24797000
24797100
Chromosome 4

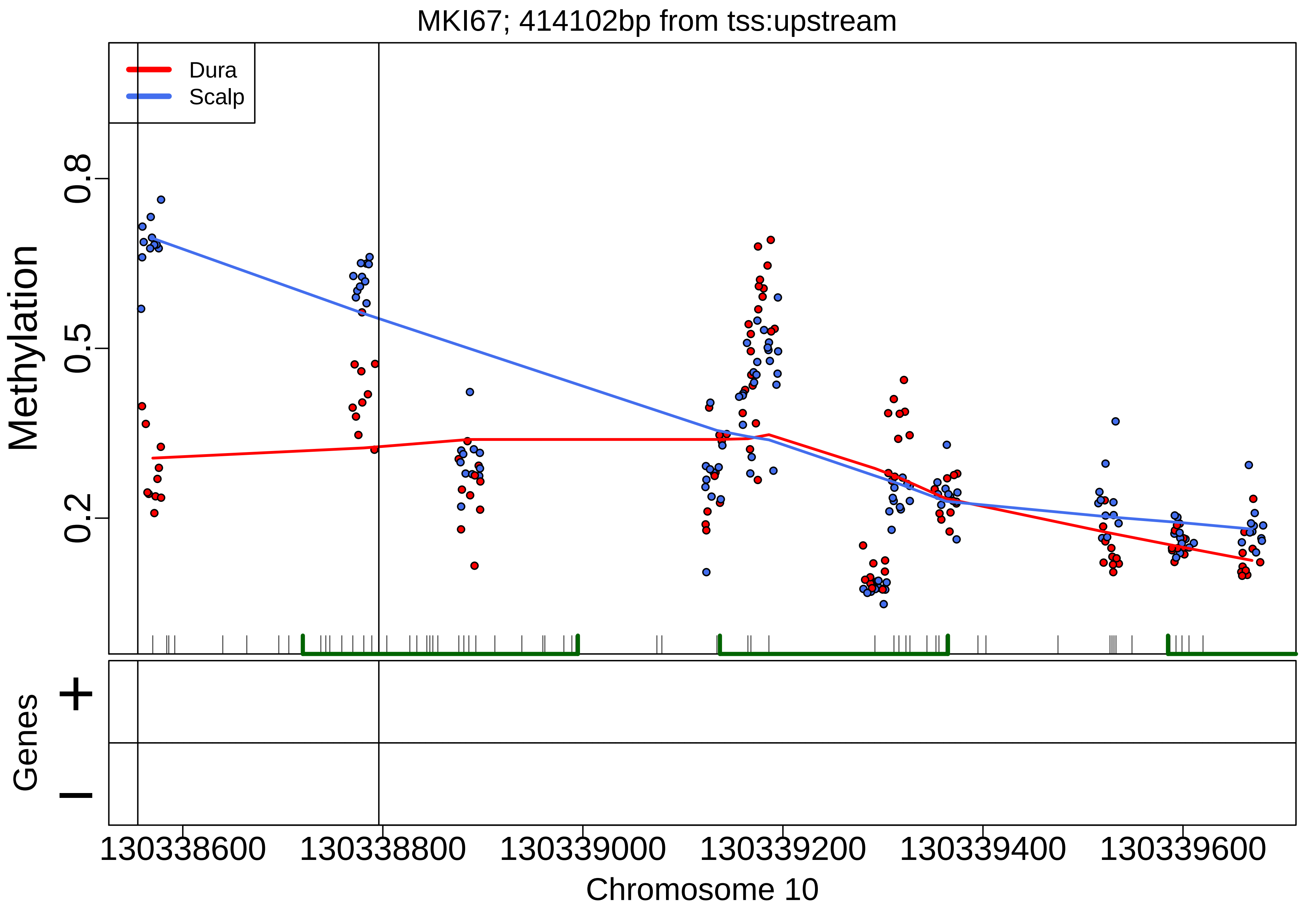
MKI67; 414102bp from tss:upstream
Dura
Scalp
Methylation
0.2
0.5
0.8
Genes
+
-
130338600
130338800
130339000
130339200
130339400
130339600
Chromosome 10

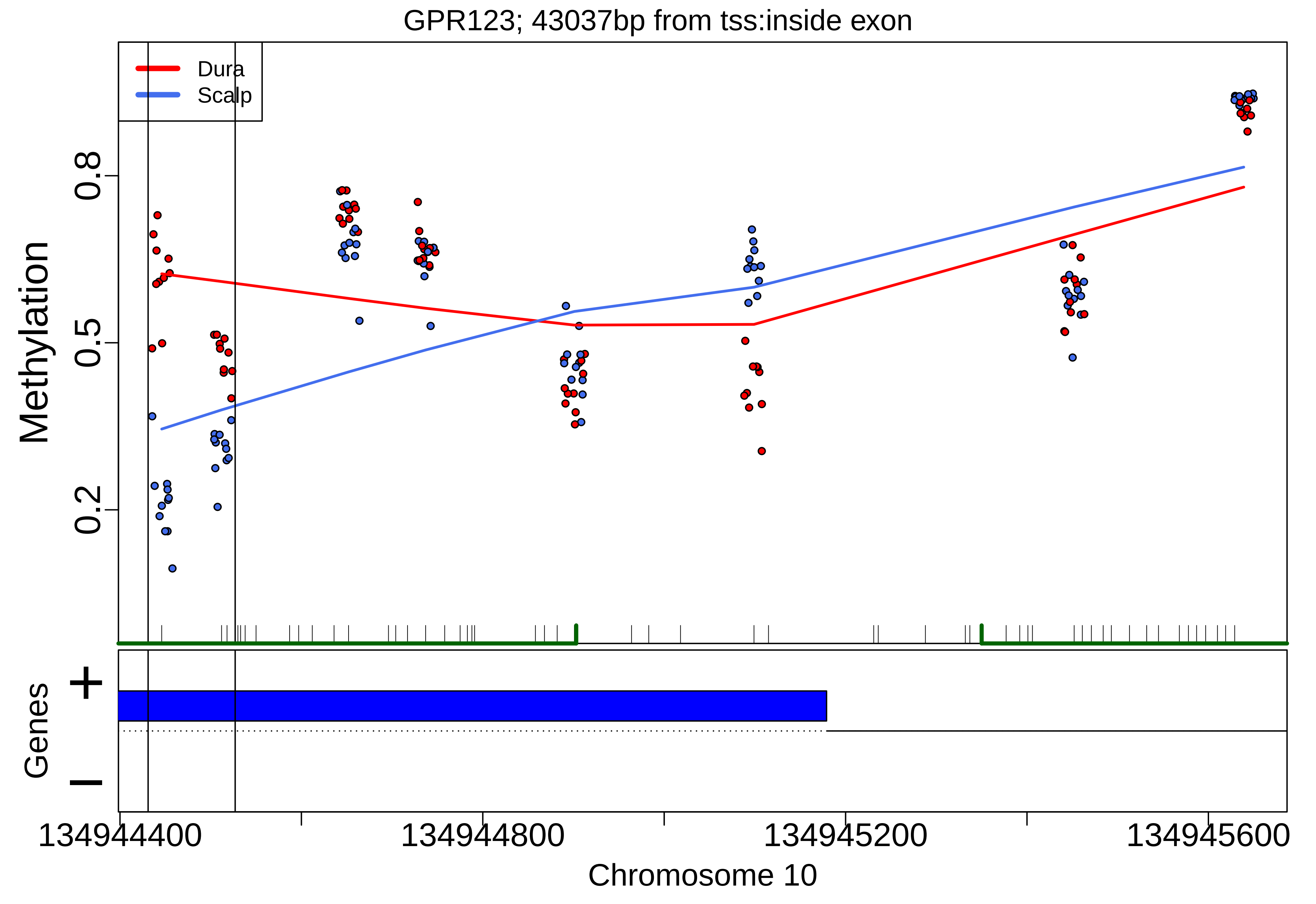

GPR123; 43037bp from tss:inside exon
Dura
Scalp
Methylation
0.2
0.5
0.8
Genes
+
−
134944400
134944800
134945200
134945600
Chromosome 10

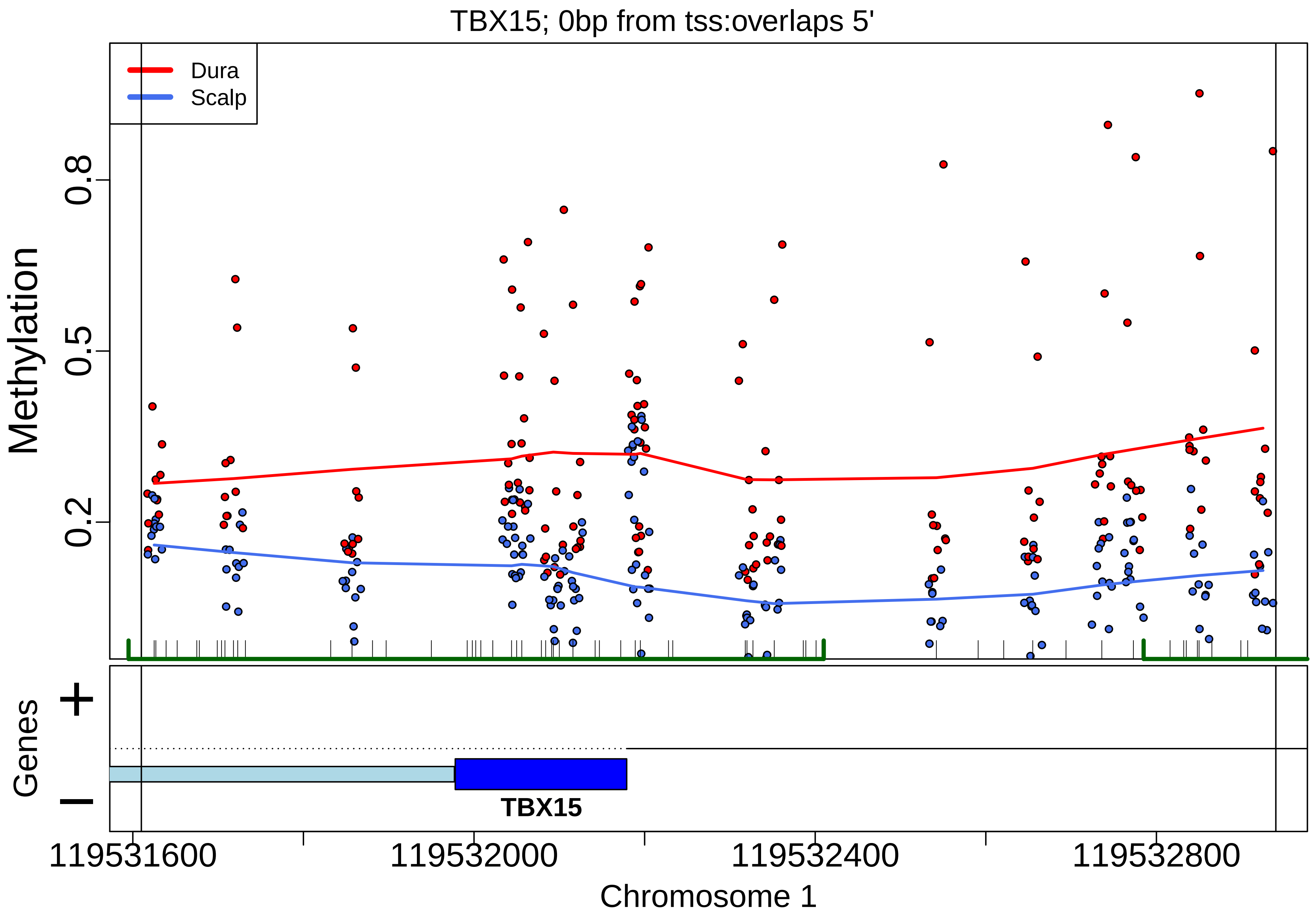
TBX15; 0bp from tss:overlaps 5'
Dura
Scalp
Methylation
0.2
0.5
0.8
Genes
+
−
TBX15
119531600
119532000
119532400
119532800
Chromosome 1

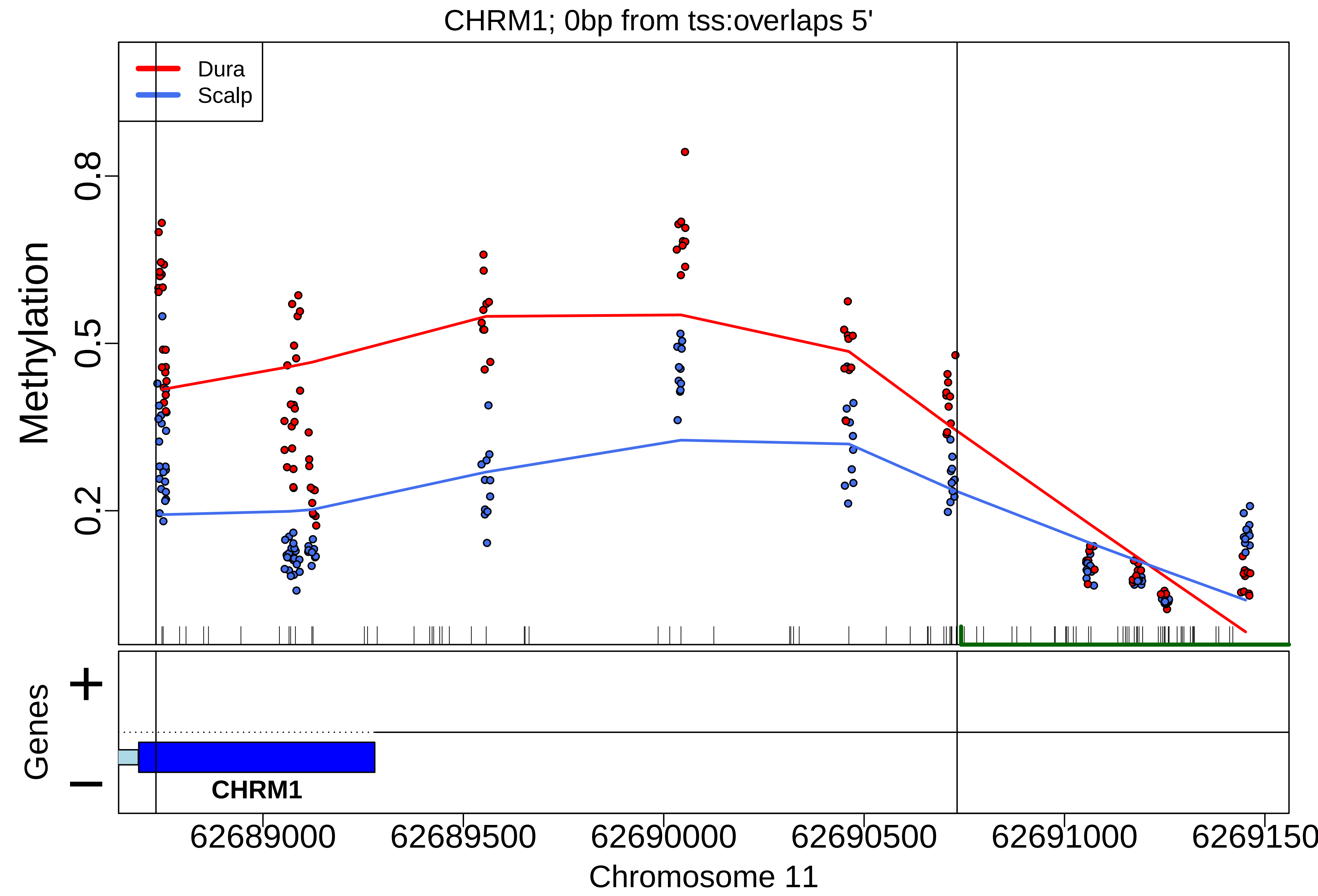

CHRM1; 0bp from tss:overlaps 5'
Dura
Scalp
Methylation
0.2
0.5
0.8
Genes
+
−
CHRM1
62689000
62689500
62690000
62690500
62691000
6269150
Chromosome 11

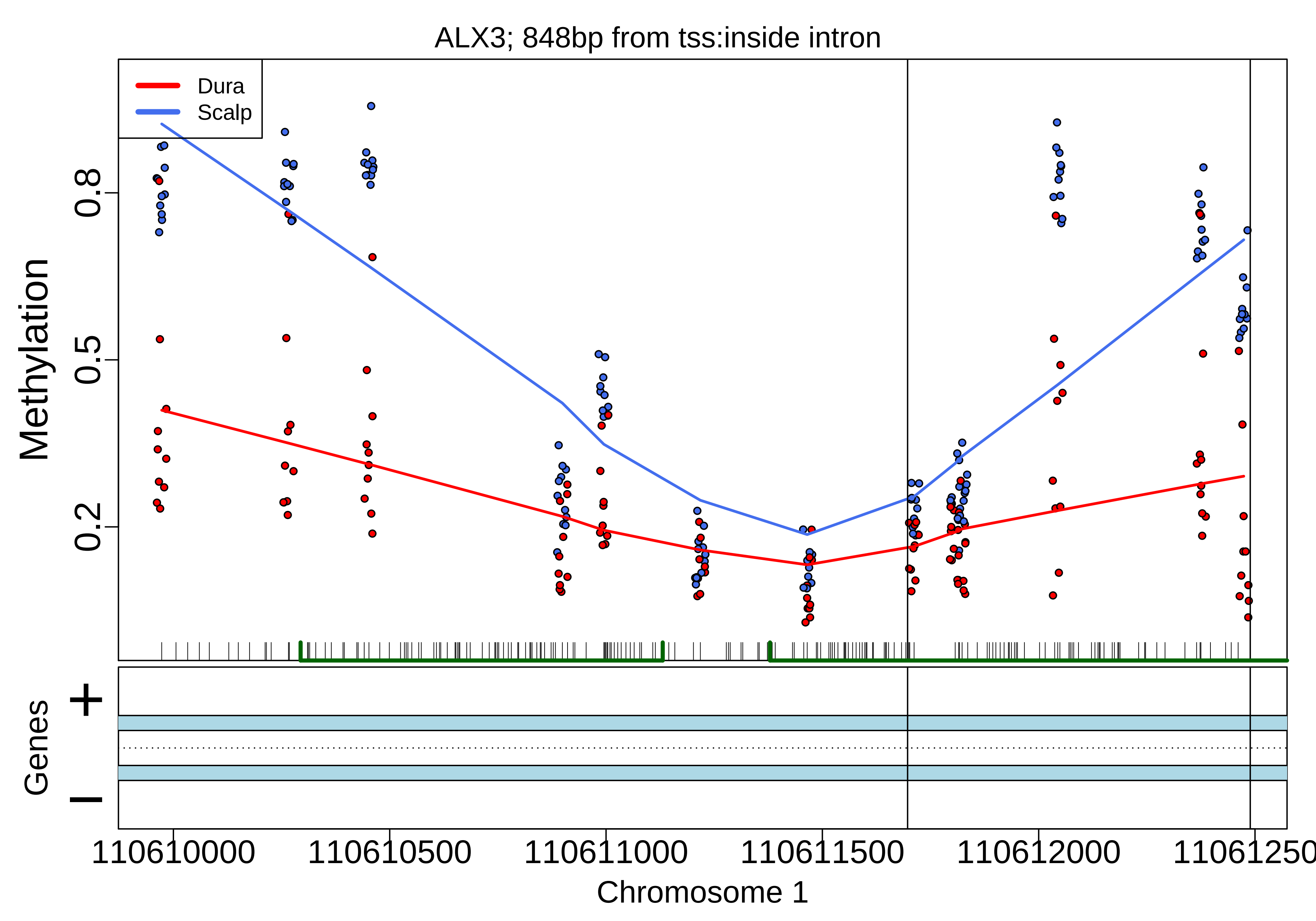

ALX3; 848bp from tss:inside intron
Dura
Scalp
Methylation
0.2
0.5
0.8
Genes
+
−
110610000
110610500
110611000
110611500
110612000
11061250
Chromosome 1

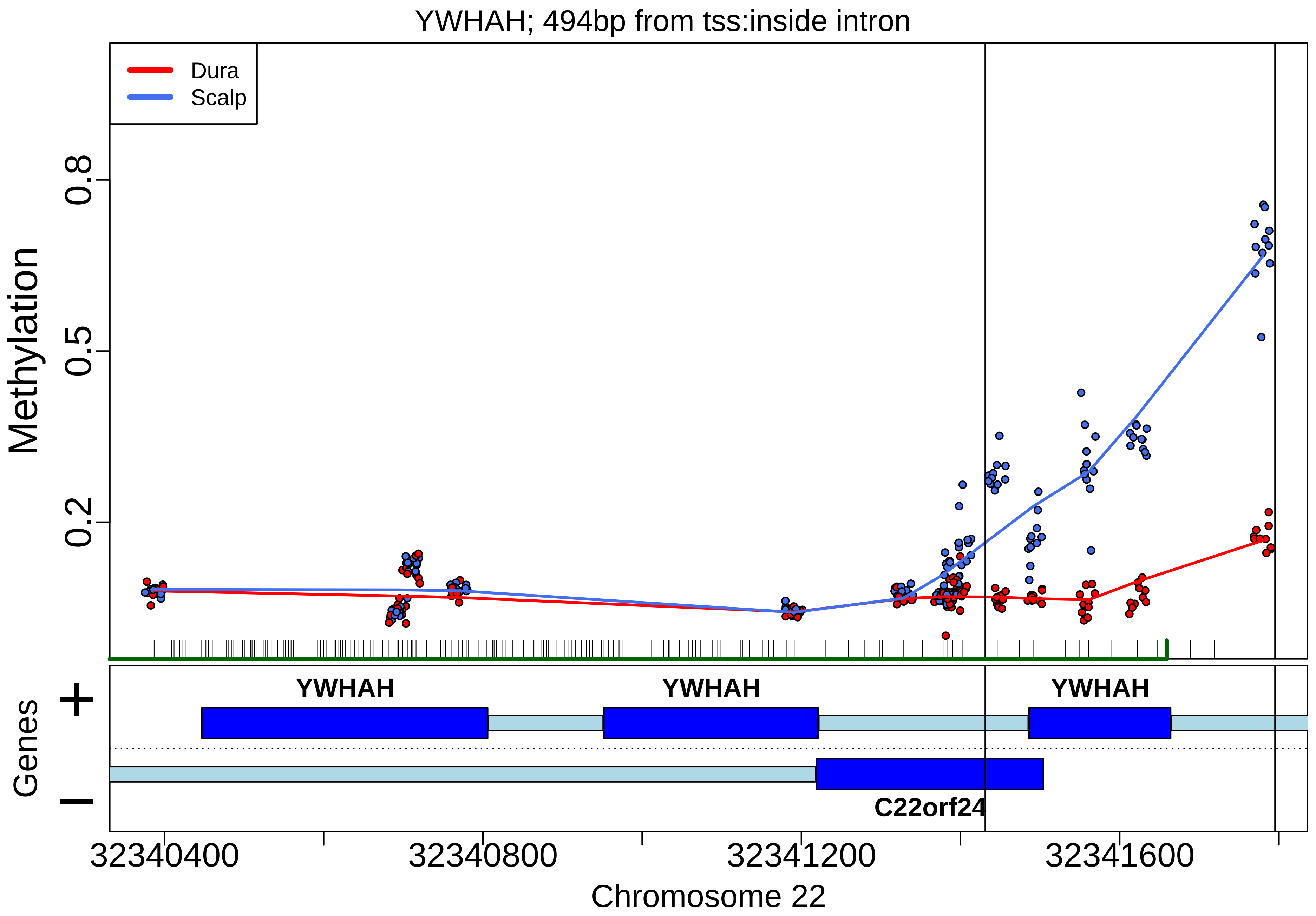

YWHAH; 494bp from tss:inside intron
Dura
Scalp
Methylation
0.2
0.5
0.8
Genes
+
-
YWHAH
YWHAH
YWHAH
C22orf24
32340400
32340800
32341200
32341600
Chromosome 22

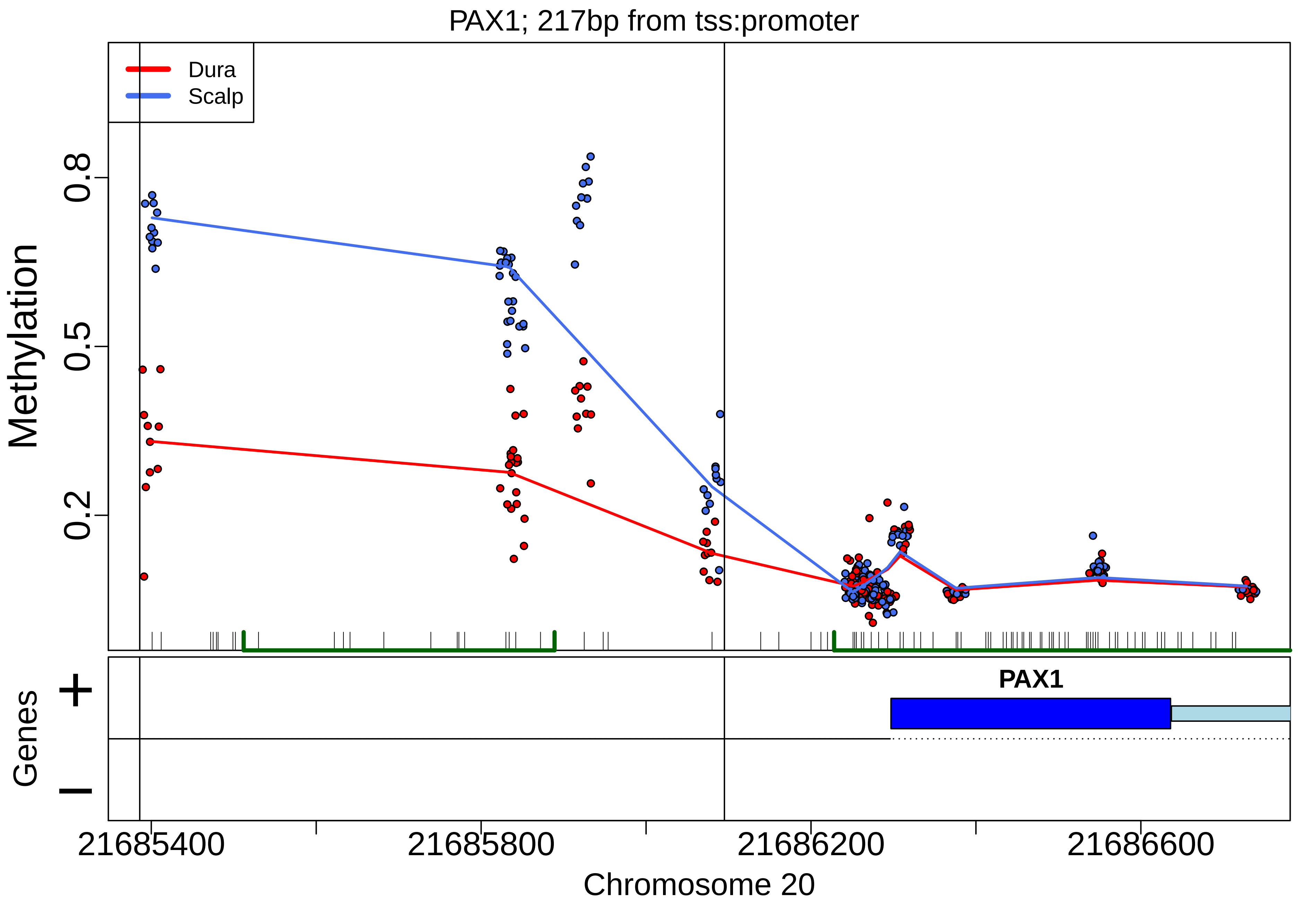
PAX1; 217bp from tss:promoter
Dura
Scalp
Methylation
0.2
0.5
0.8
Genes
+
−
PAX1
21685400
21685800
21686200
21686600
Chromosome 20

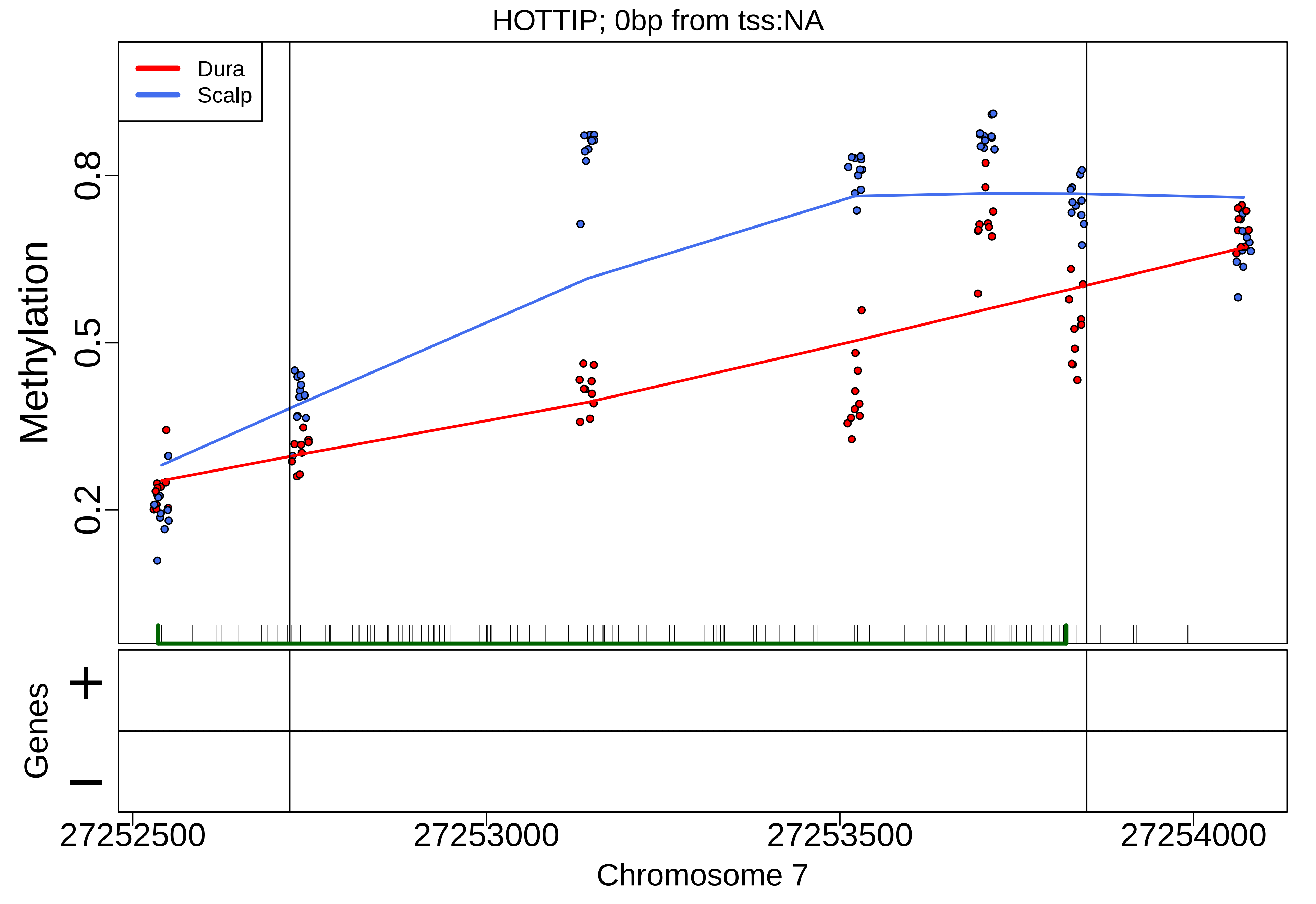

HOTTIP; 0bp from tss:NA
Dura
Scalp
Methylation
0.2
0.5
0.8
Genes
+
−
27252500
27253000
27253500
27254000
Chromosome 7

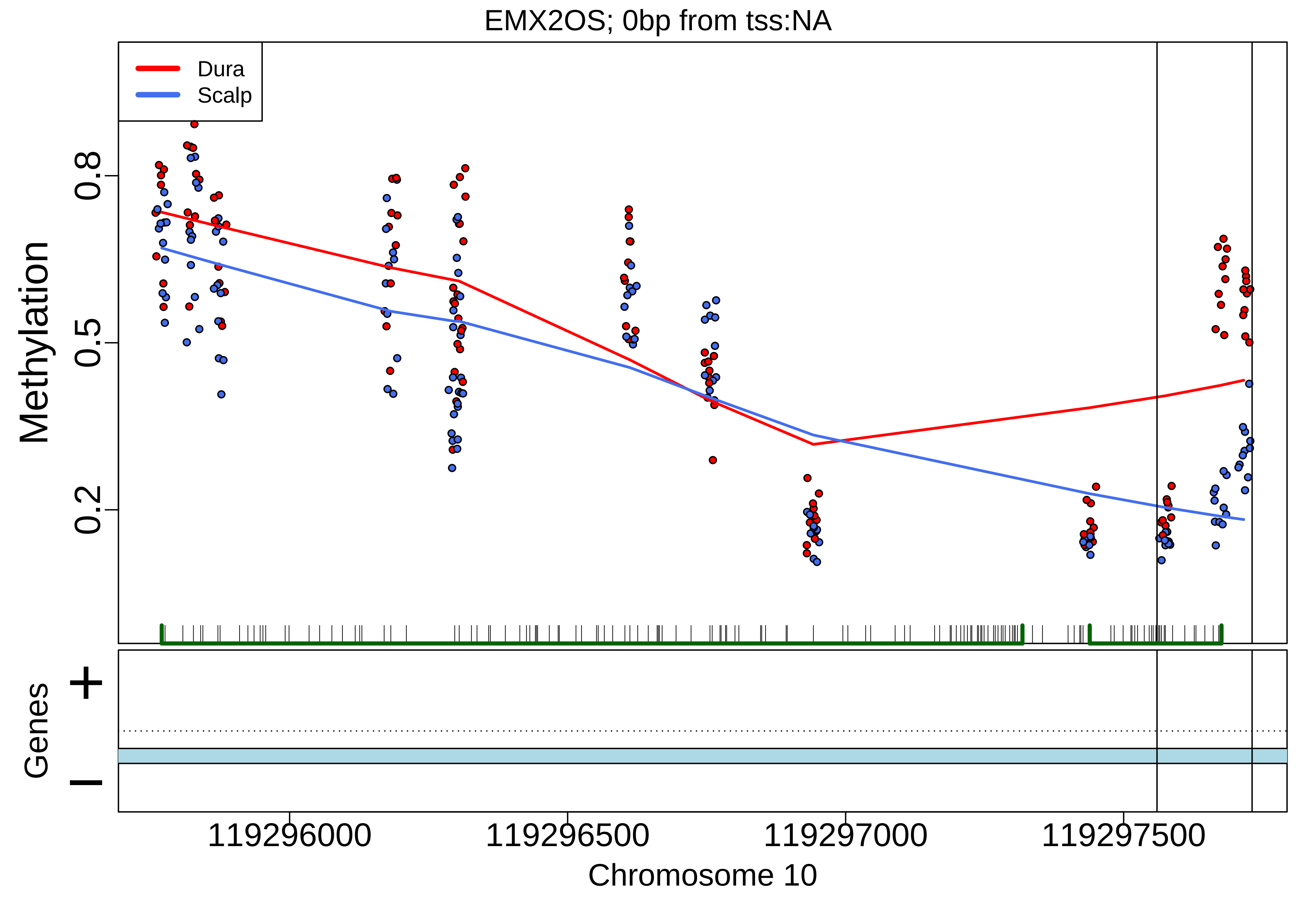
EMX2OS; 0bp from tss:NA
Dura
Scalp
Methylation
0.2
0.5
0.8
Genes
+
-
119296000
119296500
119297000
119297500
Chromosome 10

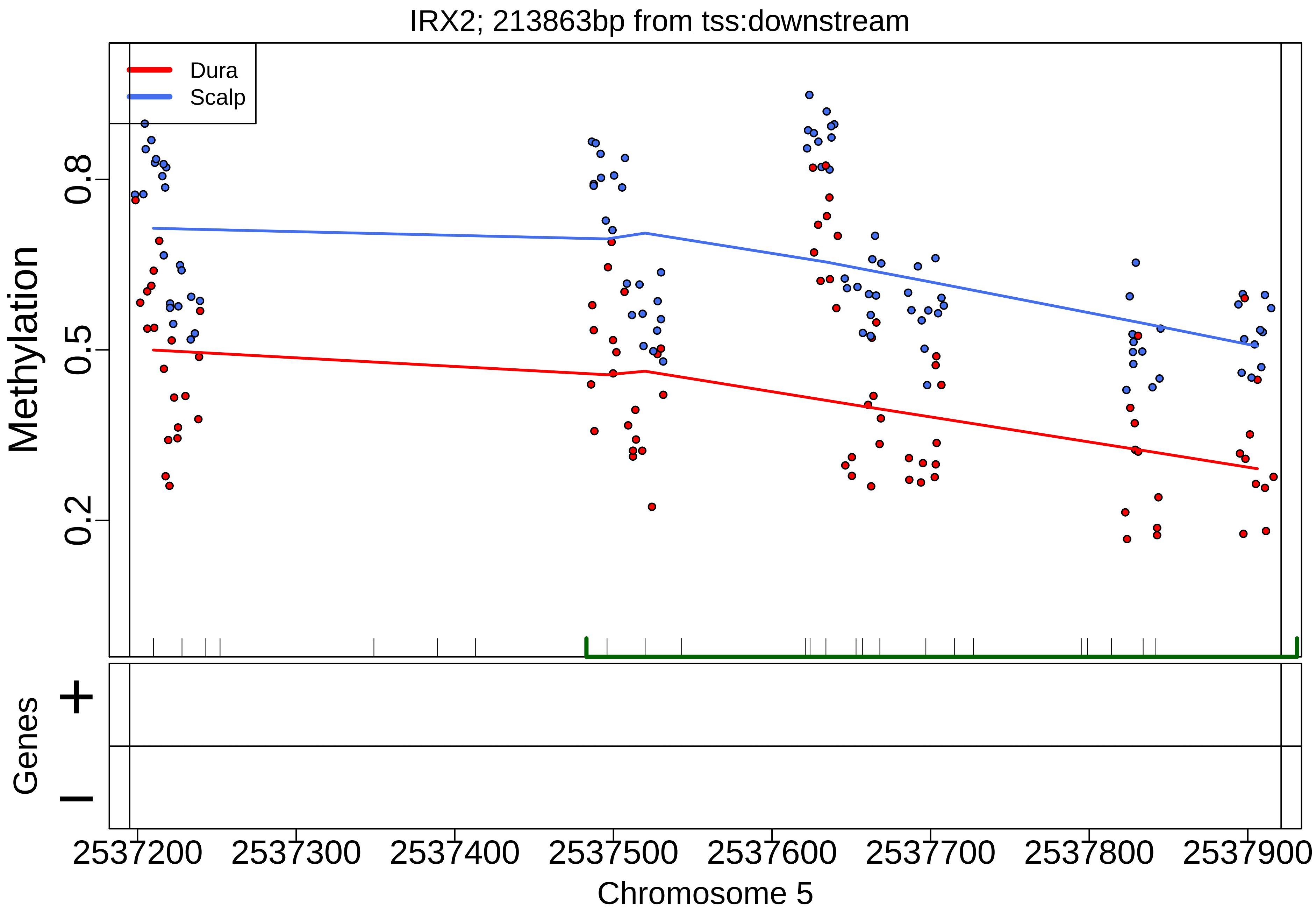

IRX2; 213863bp from tss:downstream
Dura
Scalp
Methylation
0.2
0.5
0.8
Genes
+
-
2537200
2537300
2537400
2537500
2537600
2537700
2537800
2537900
Chromosome 5

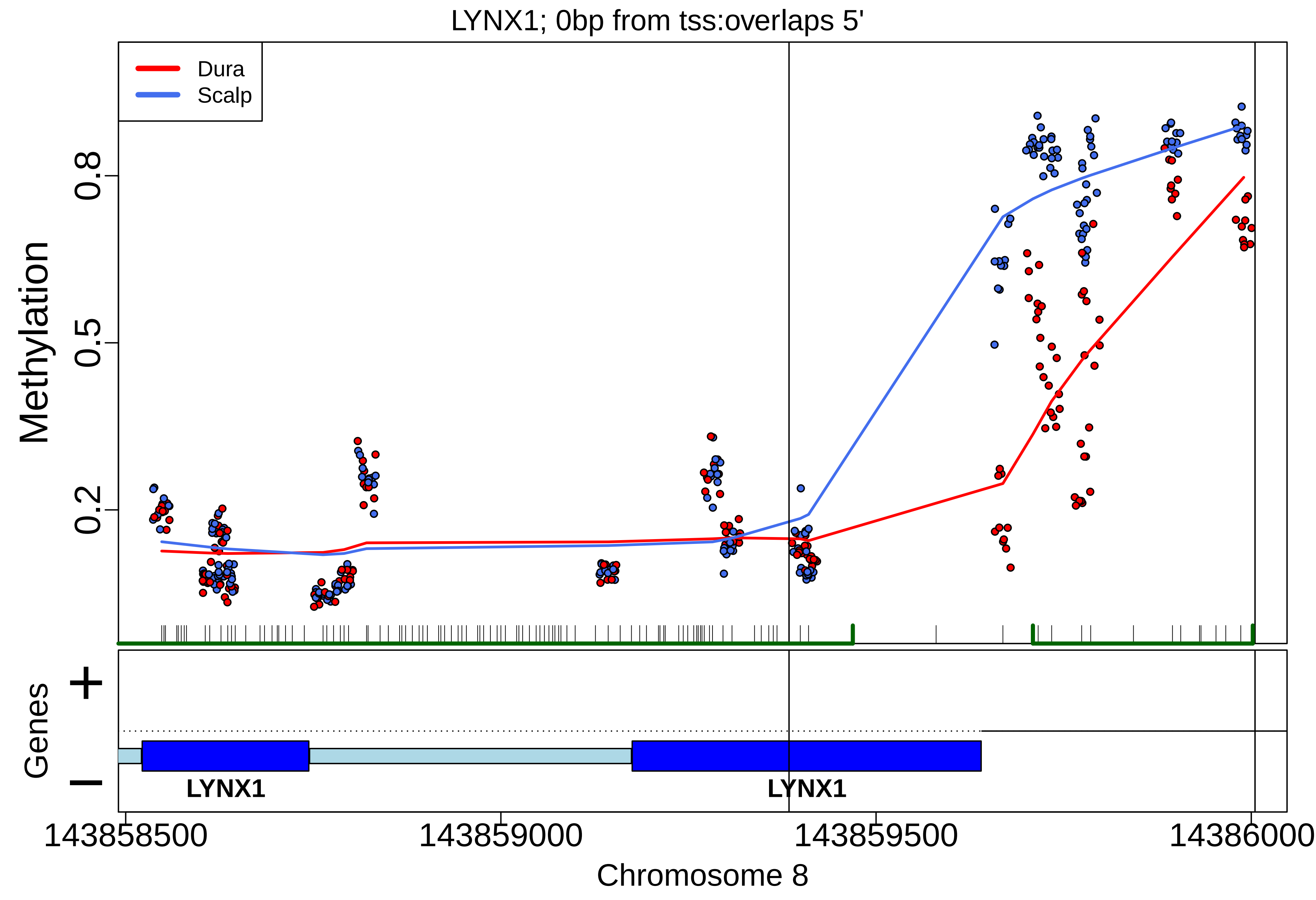

LYNX1; 0bp from tss:overlaps 5'
Dura
Scalp
Methylation
0.2
0.5
0.8
Genes
+
−
LYNX1
LYNX1
143858500
143859000
143859500
14386000
Chromosome 8

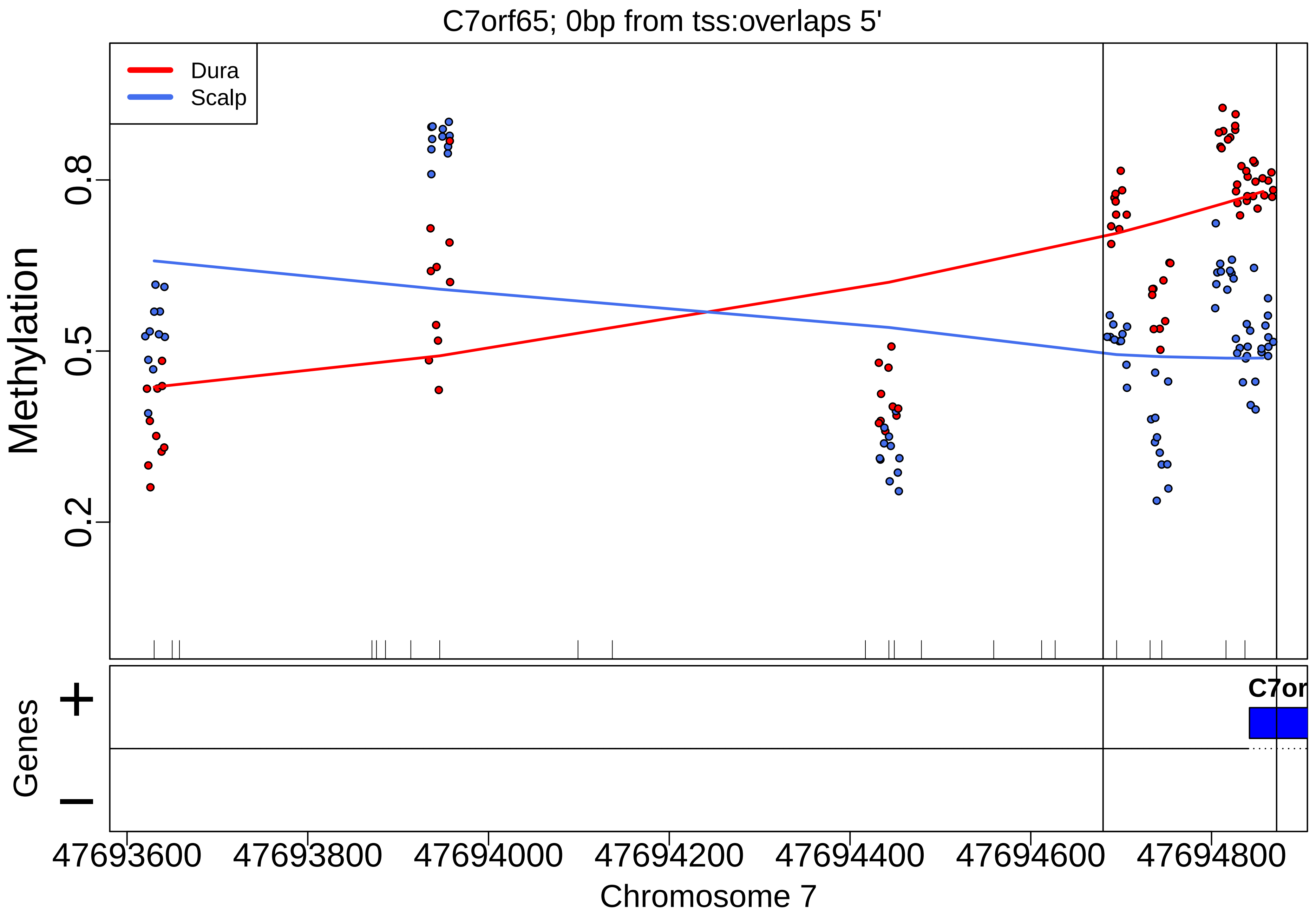

C7orf65; 0bp from tss:overlaps 5'
Dura
Scalp
Methylation
0.2
0.5
0.8
Genes
+
–
C7or
47693600
47693800
47694000
47694200
47694400
47694600
47694800
Chromosome 7

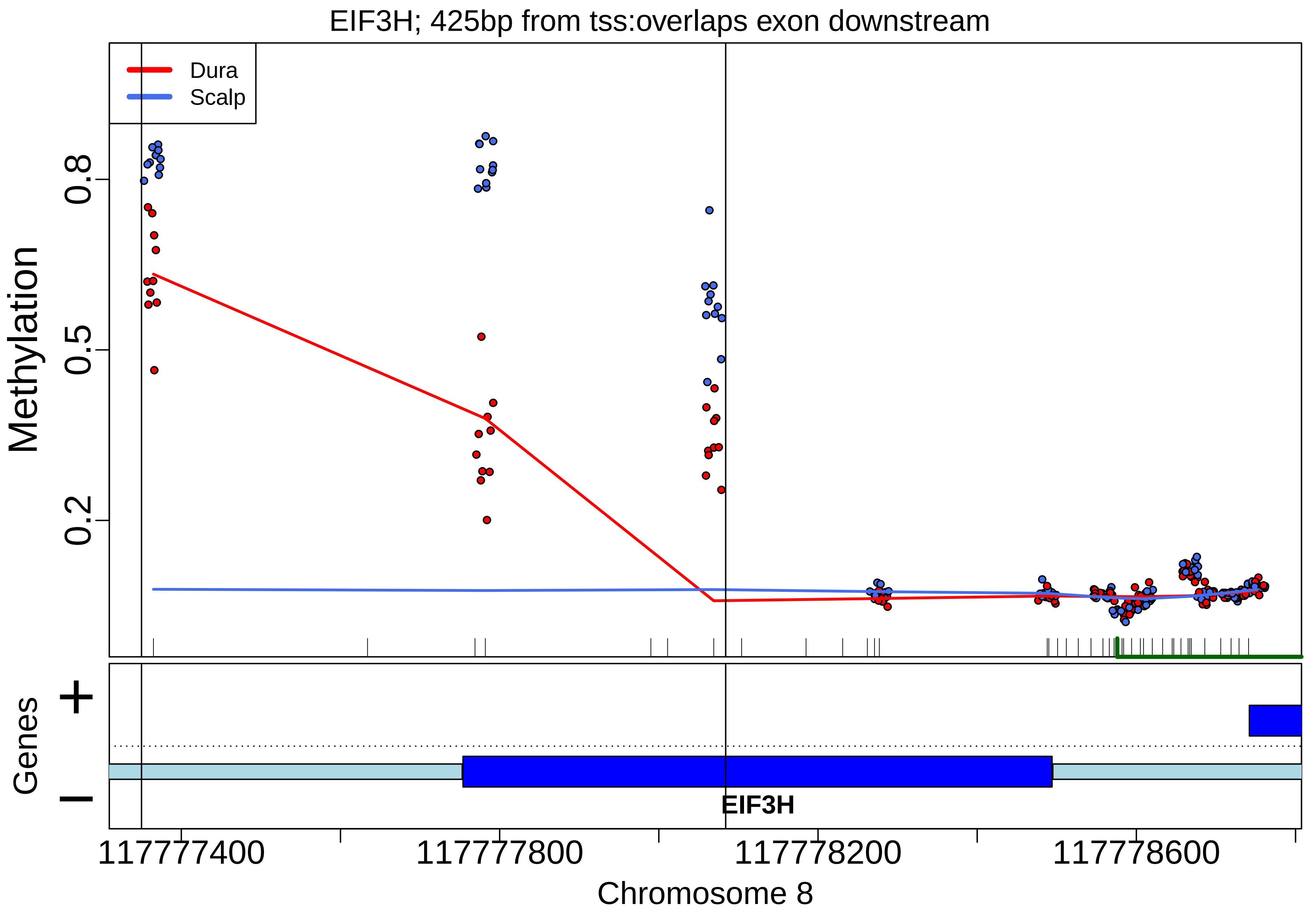
EIF3H; 425bp from tss:overlaps exon downstream
Dura
Scalp
Methylation
0.2
0.5
0.8
Genes
+
−
EIF3H
117777400
117777800
117778200
117778600
Chromosome 8

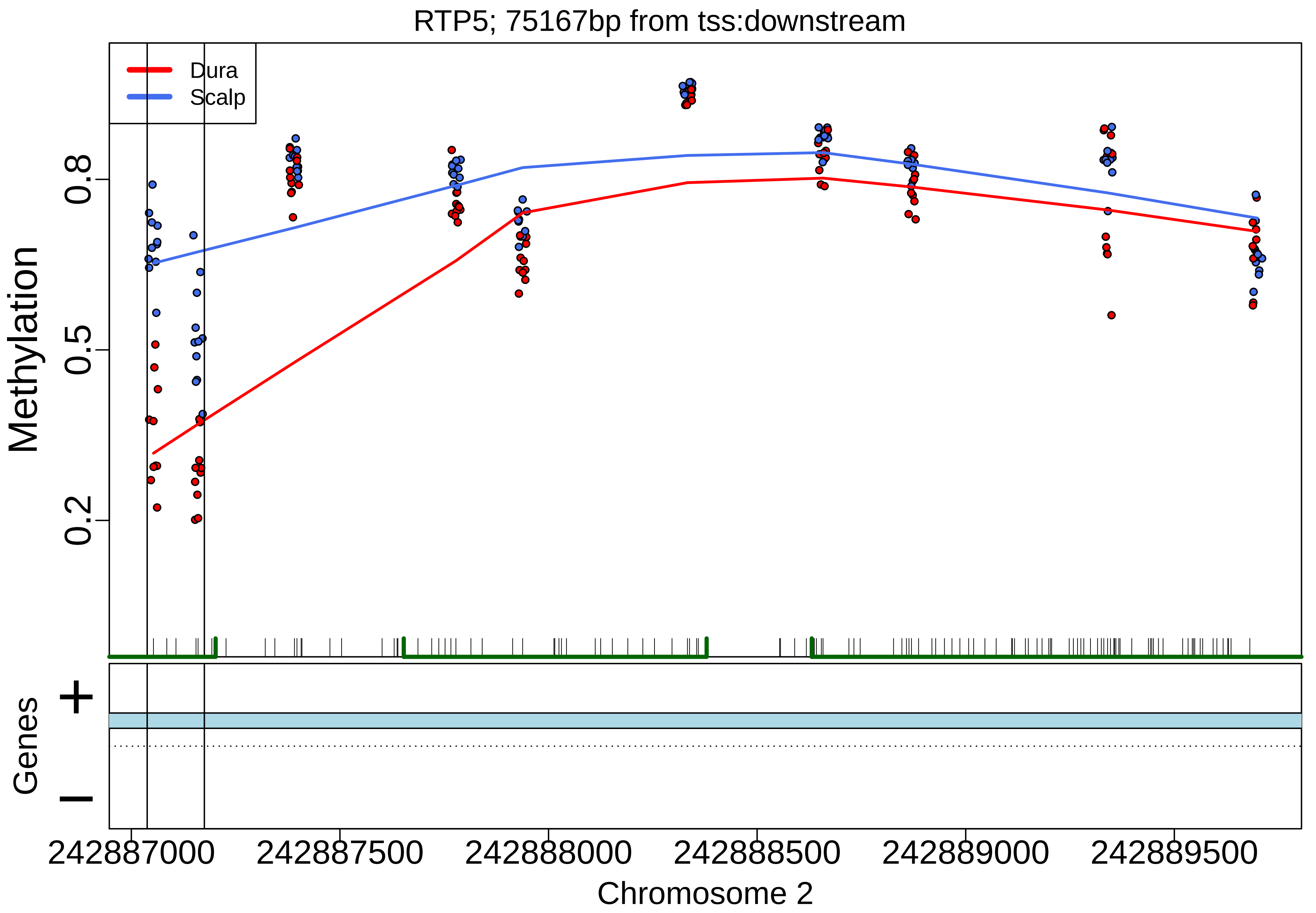

RTP5; 75167bp from tss:downstream
Dura
Scalp
Methylation
0.2
0.5
0.8
Genes
+
−
242887000
242887500
242888000
242888500
242889000
242889500
Chromosome 2

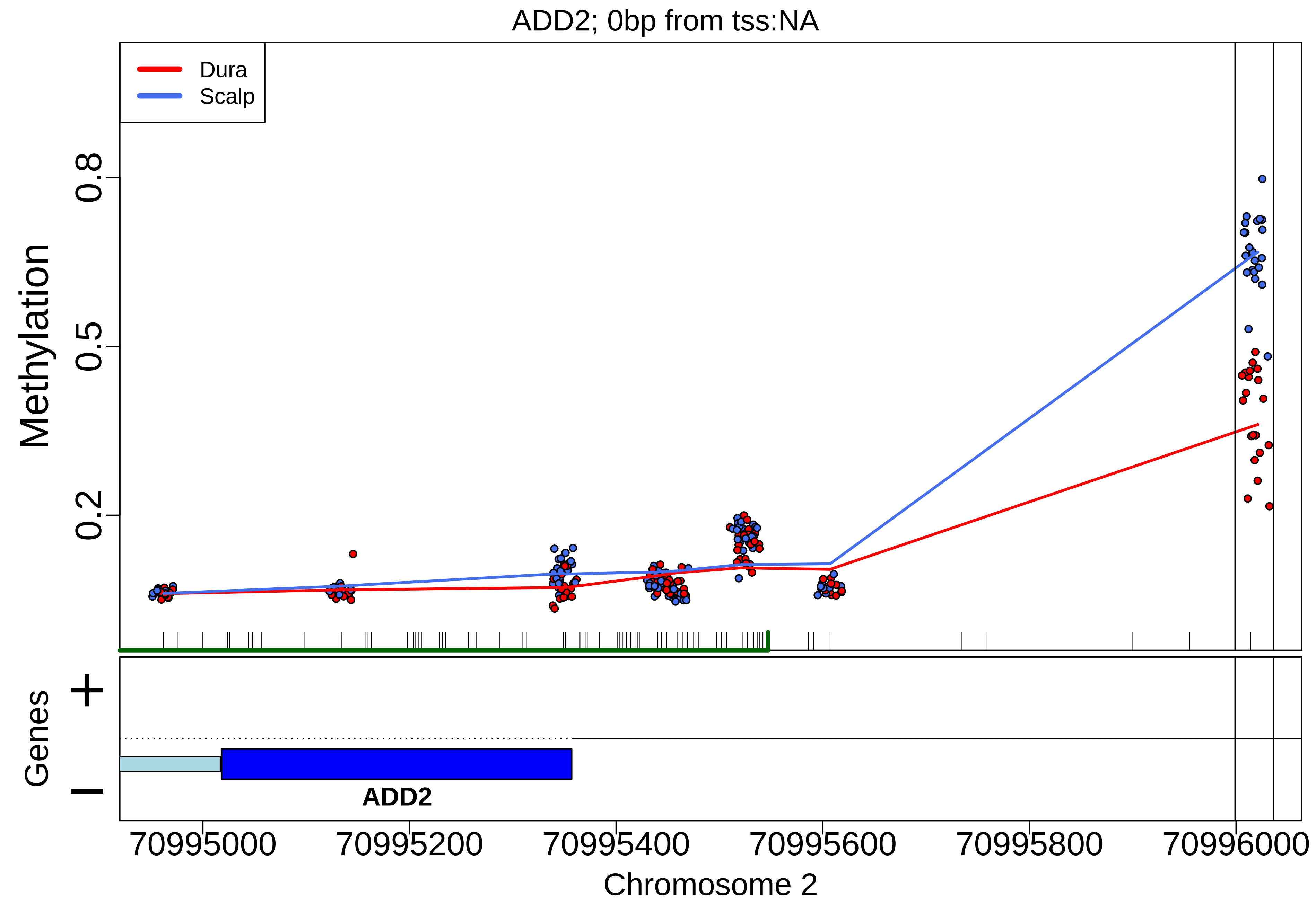
ADD2; 0bp from tss:NA
Dura
Scalp
Methylation
0.2
0.5
0.8
Genes
+
-
ADD2
70995000
70995200
70995400
70995600
70995800
70996000
Chromosome 2

LSMEM1; 0bp from tss:overlaps 5'

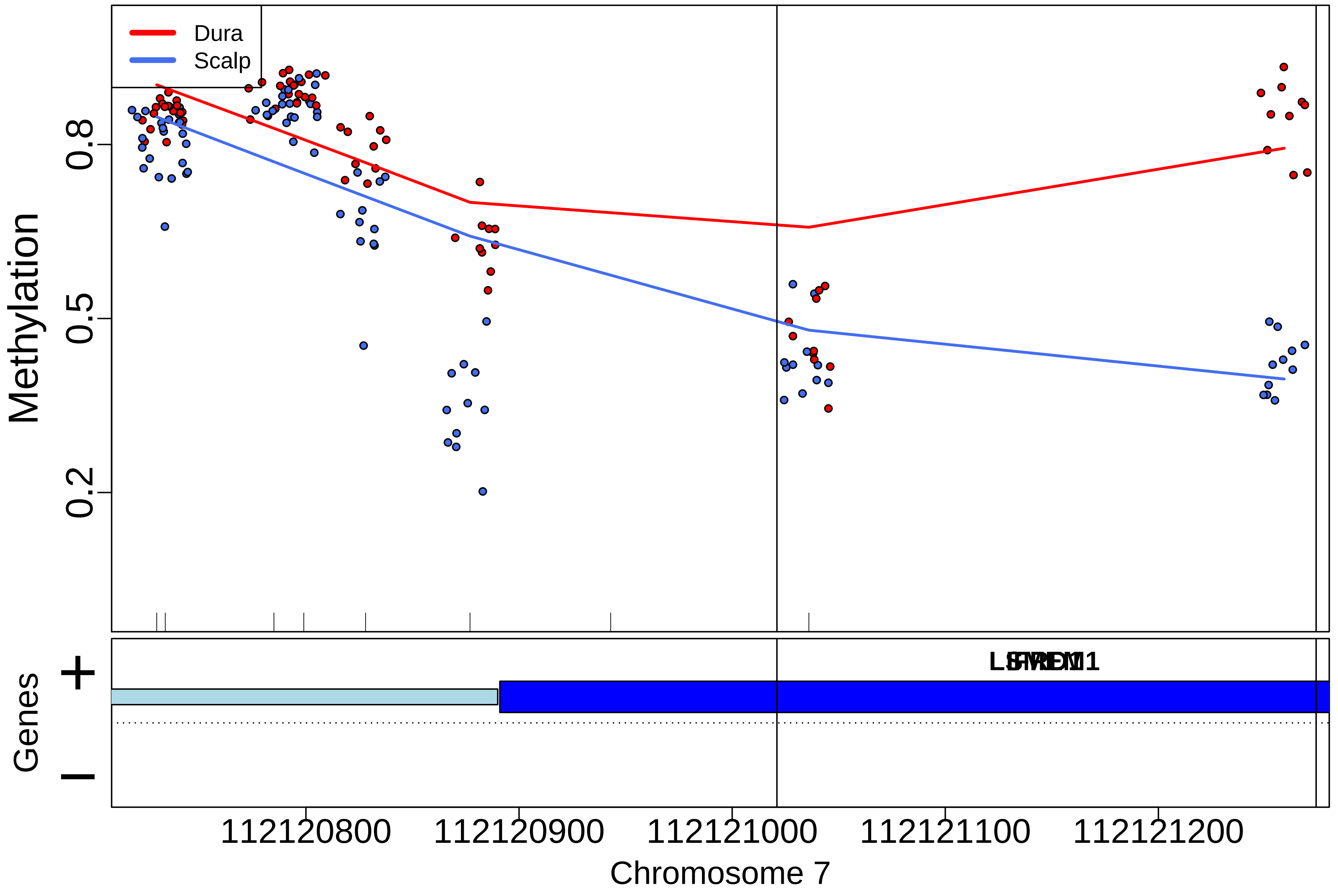

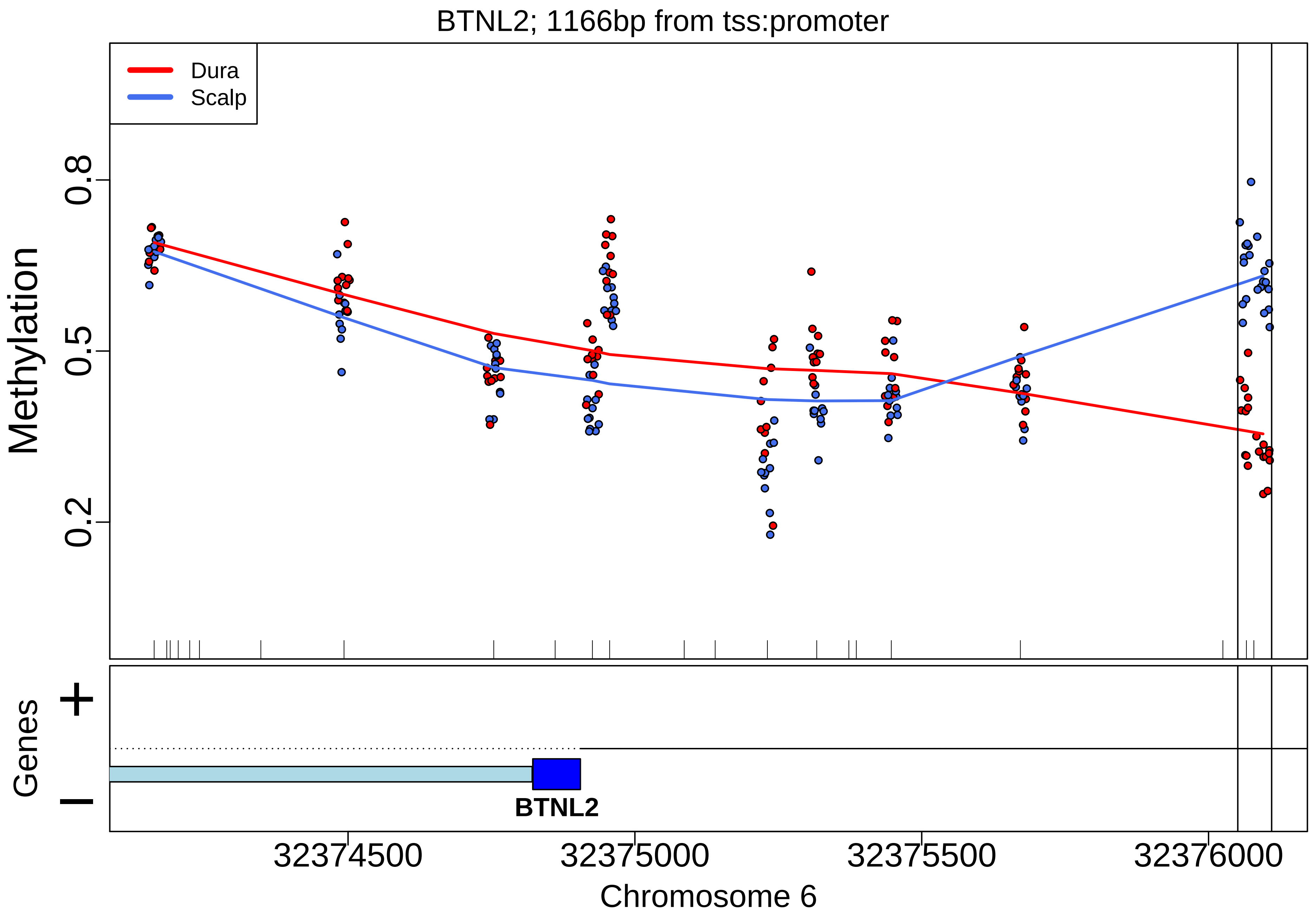

BTNL2; 1166bp from tss:promoter
Dura
Scalp
Methylation
0.2
0.5
0.8
Genes
+
-
BTNL2
32374500
32375000
32375500
32376000
Chromosome 6

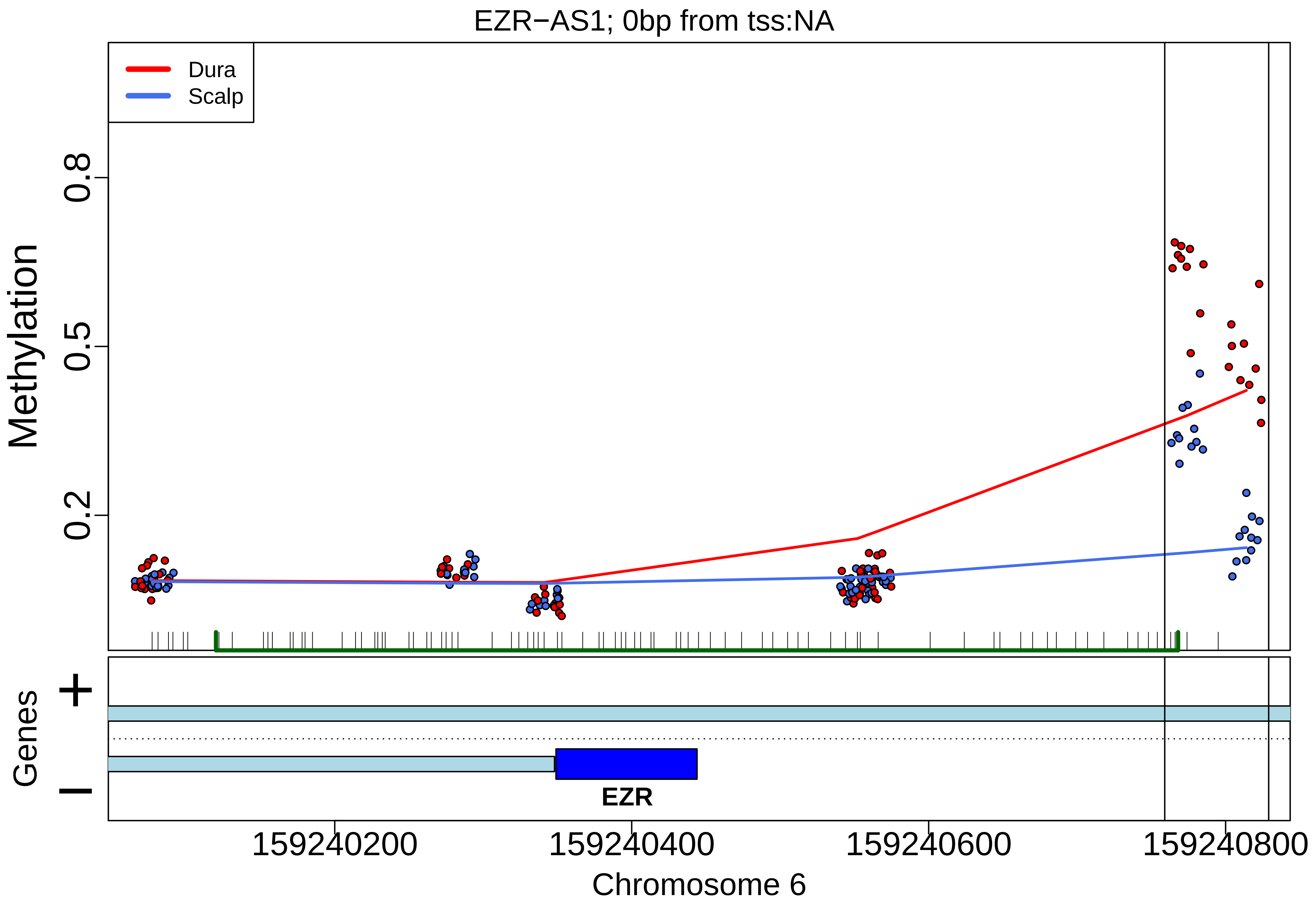

EZR−AS1; 0bp from tss:NA
Dura
Scalp
Methylation
0.2
0.5
0.8
Genes
+
−
EZR
159240200
159240400
159240600
159240800
Chromosome 6

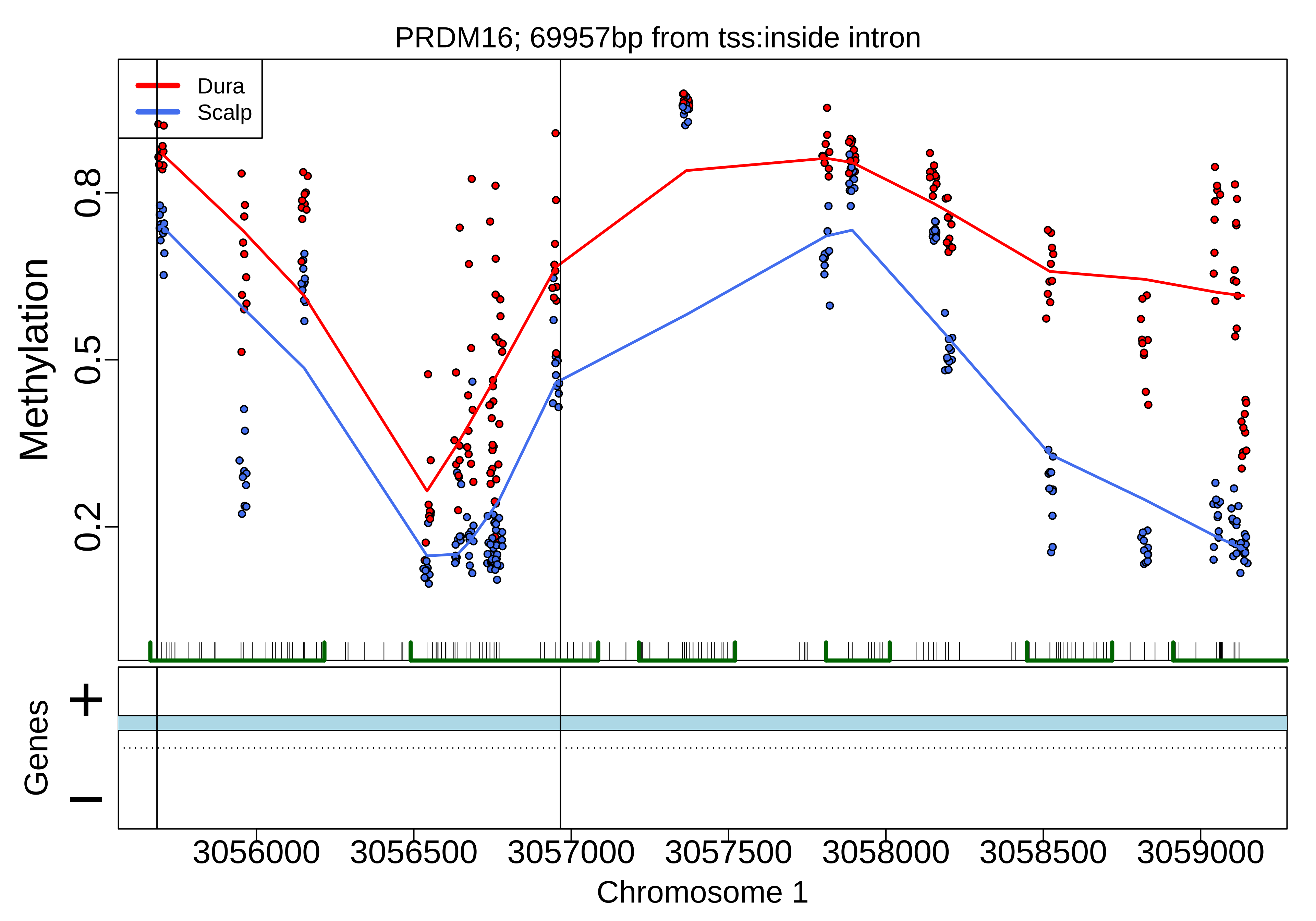

PRDM16; 69957bp from tss:inside intron
Dura
Scalp
Methylation
0.2
0.5
0.8
Genes
+
−
3056000
3056500
3057000
3057500
3058000
3058500
3059000
Chromosome 1

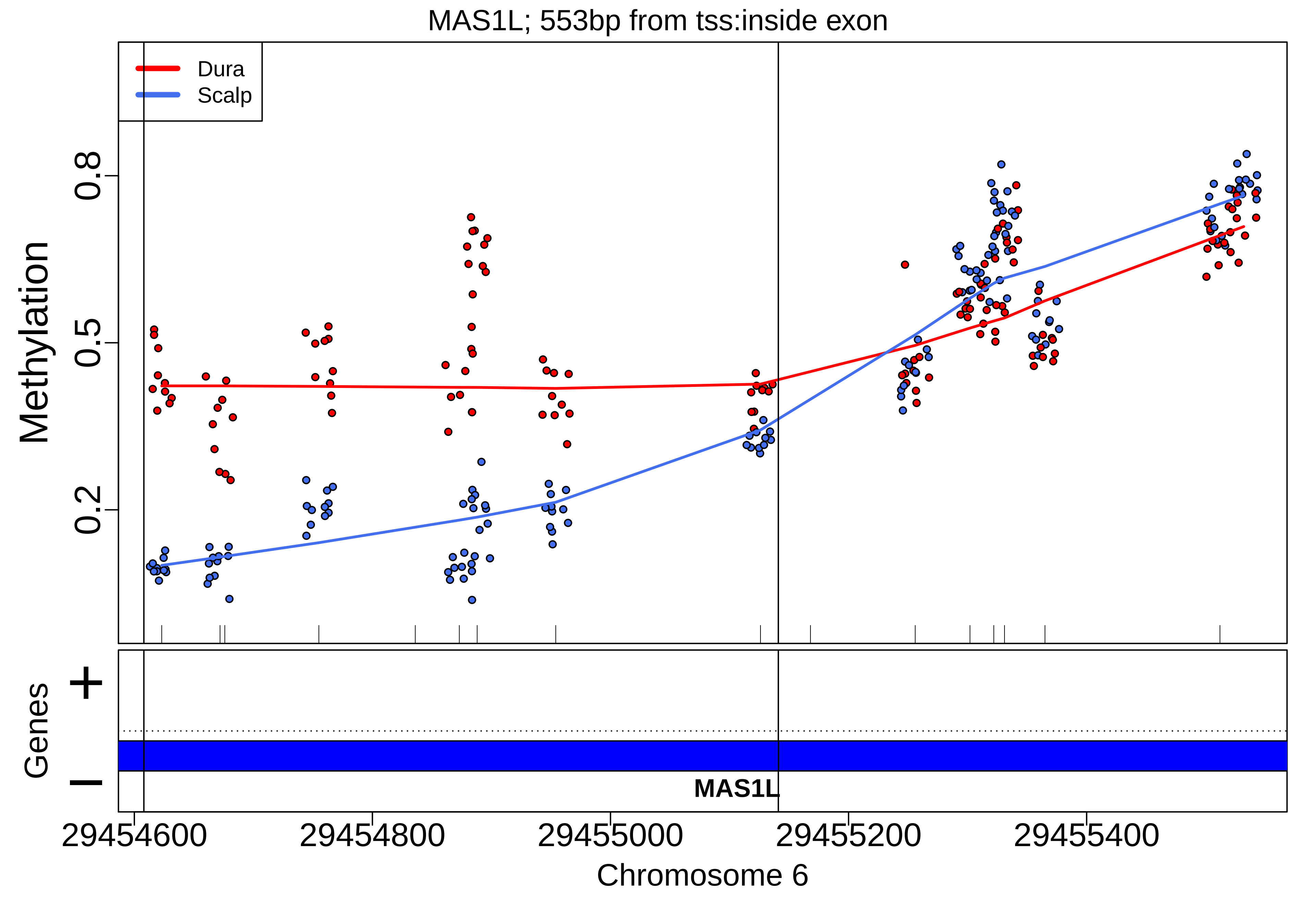
MAS1L; 553bp from tss:inside exon
Dura
Scalp
Methylation
0.2
0.5
0.8
Genes
+
−
MAS1L
29454600
29454800
29455000
29455200
29455400
Chromosome 6

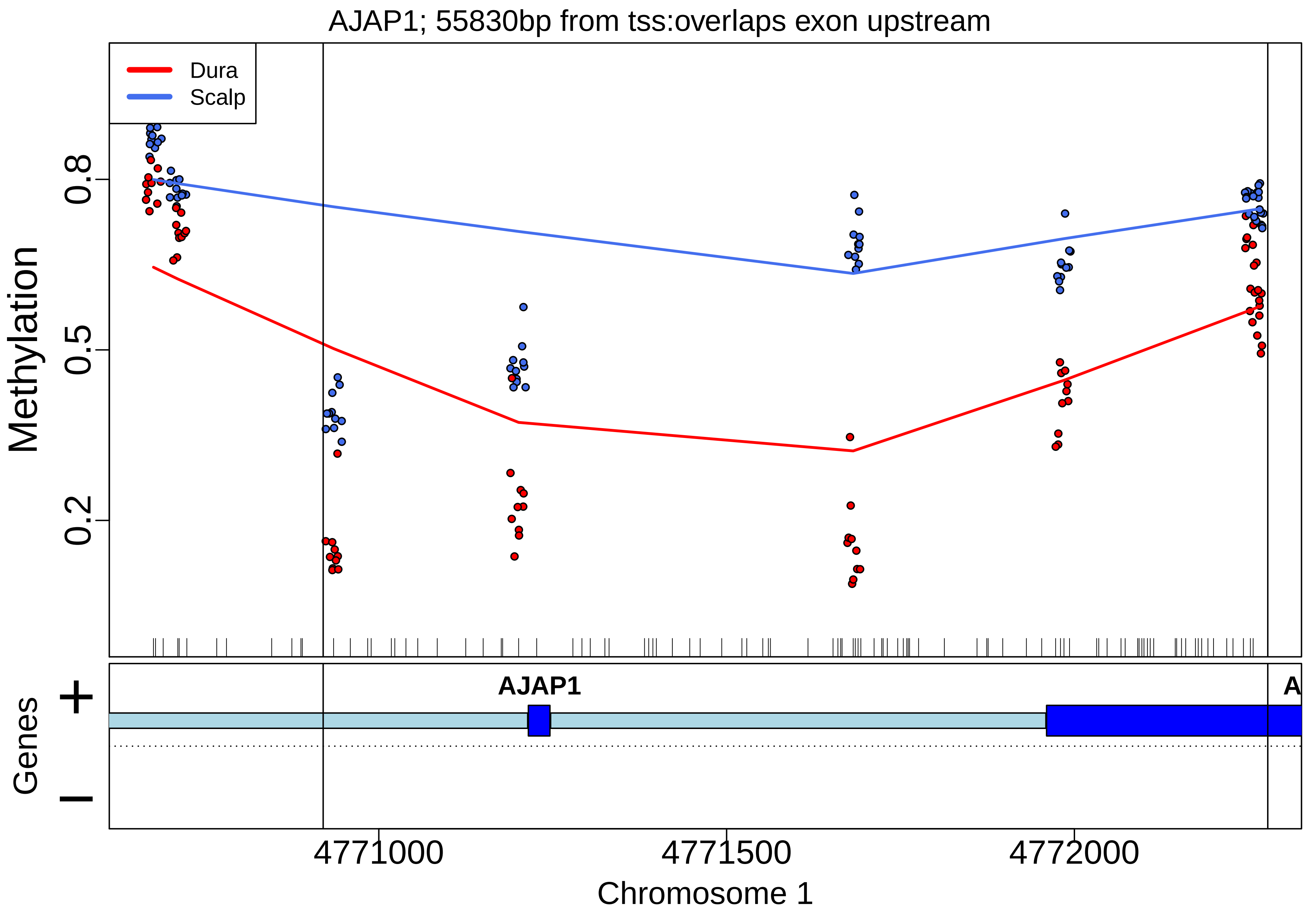
AJAP1; 55830bp from tss:overlaps exon upstream
Dura
Scalp
Methylation
0.2
0.5
0.8
Genes
+
-
AJAP1
A
4771000
4771500
4772000
Chromosome 1

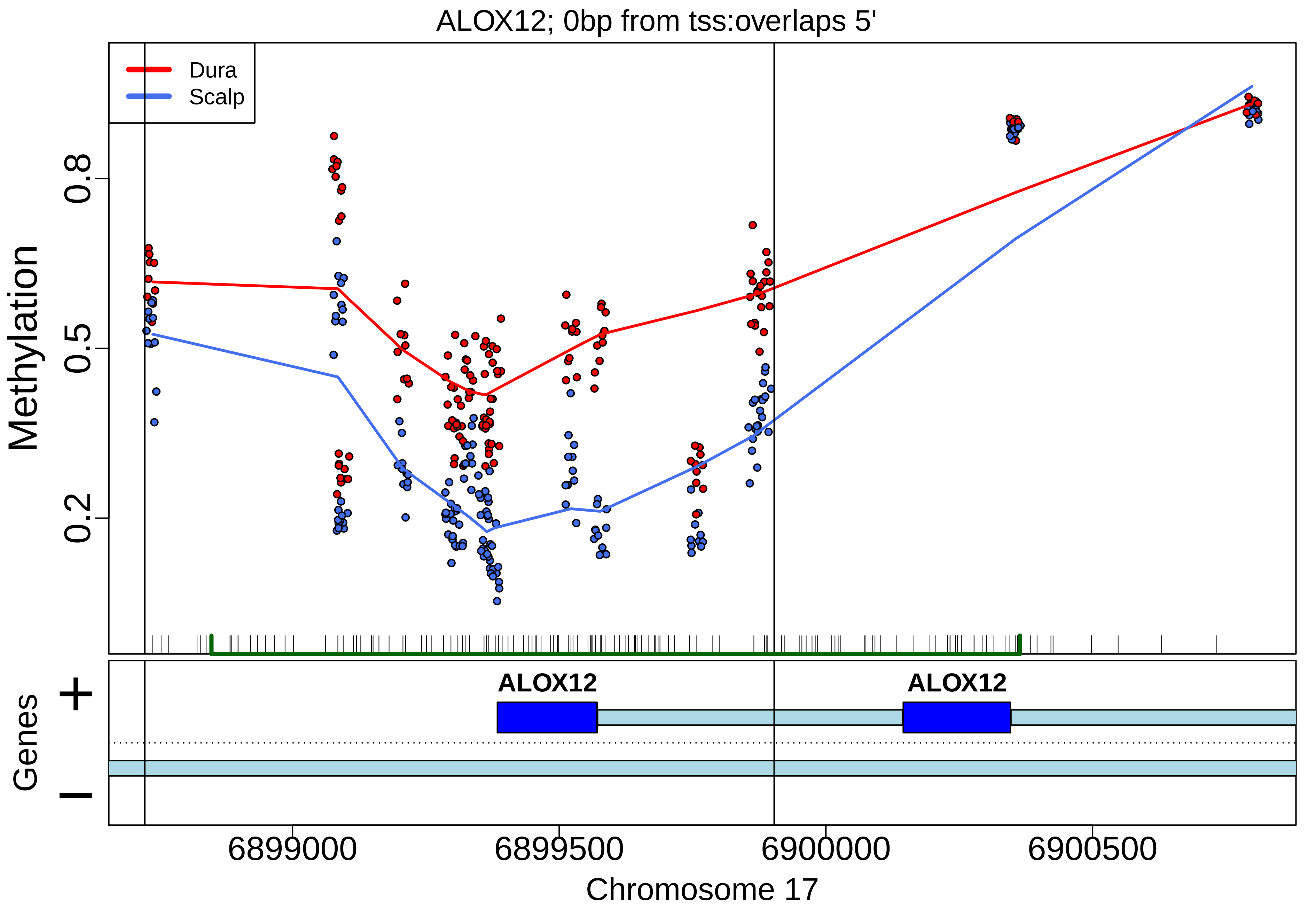

ALOX12; 0bp from tss:overlaps 5'
Dura
Scalp
Methylation
0.2
0.5
0.8
Genes
+
-
ALOX12
ALOX12
6899000
6899500
6900000
6900500
Chromosome 17

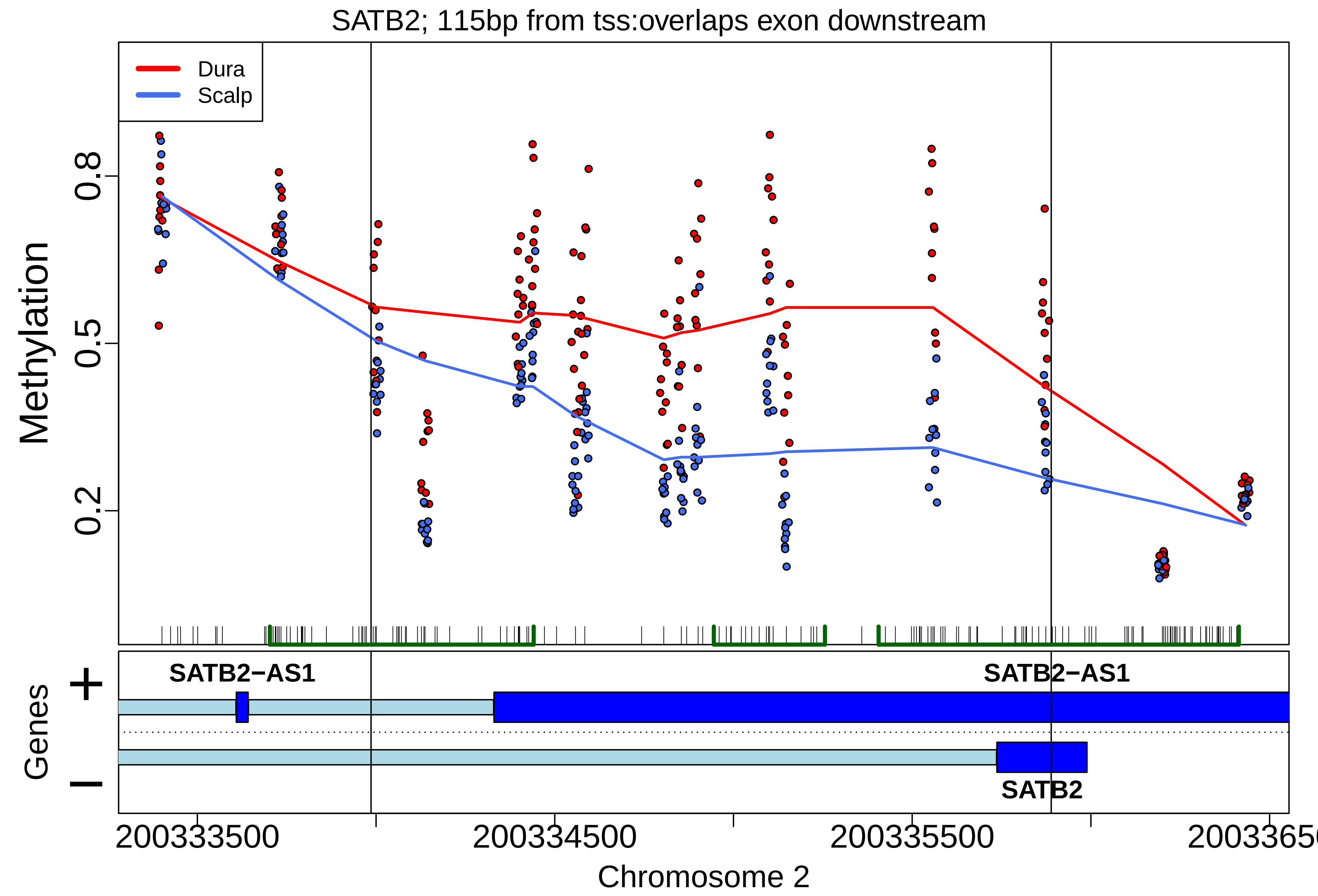
SATB2; 115bp from tss:overlaps exon downstream
Dura
Scalp
Methylation
0.2
0.5
0.8
Genes
+
−
SATB2−AS1
SATB2−AS1
SATB2
200333500
200334500
200335500
Chromosome 2

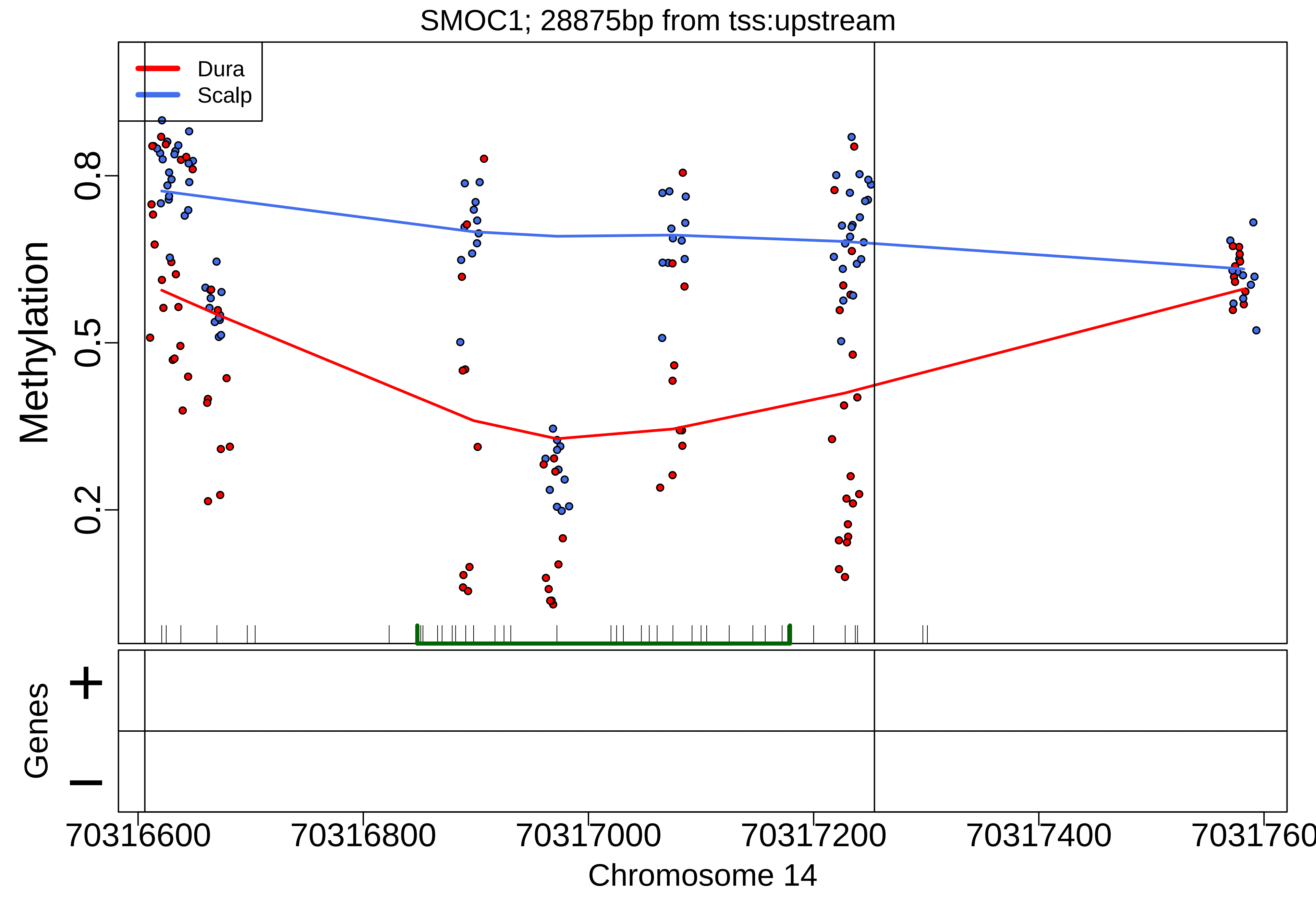

SMOC1; 28875bp from tss:upstream
Dura
Scalp
Methylation
0.2
0.5
0.8
Genes
+
-
70316600
70316800
70317000
70317200
70317400
7031760
Chromosome 14

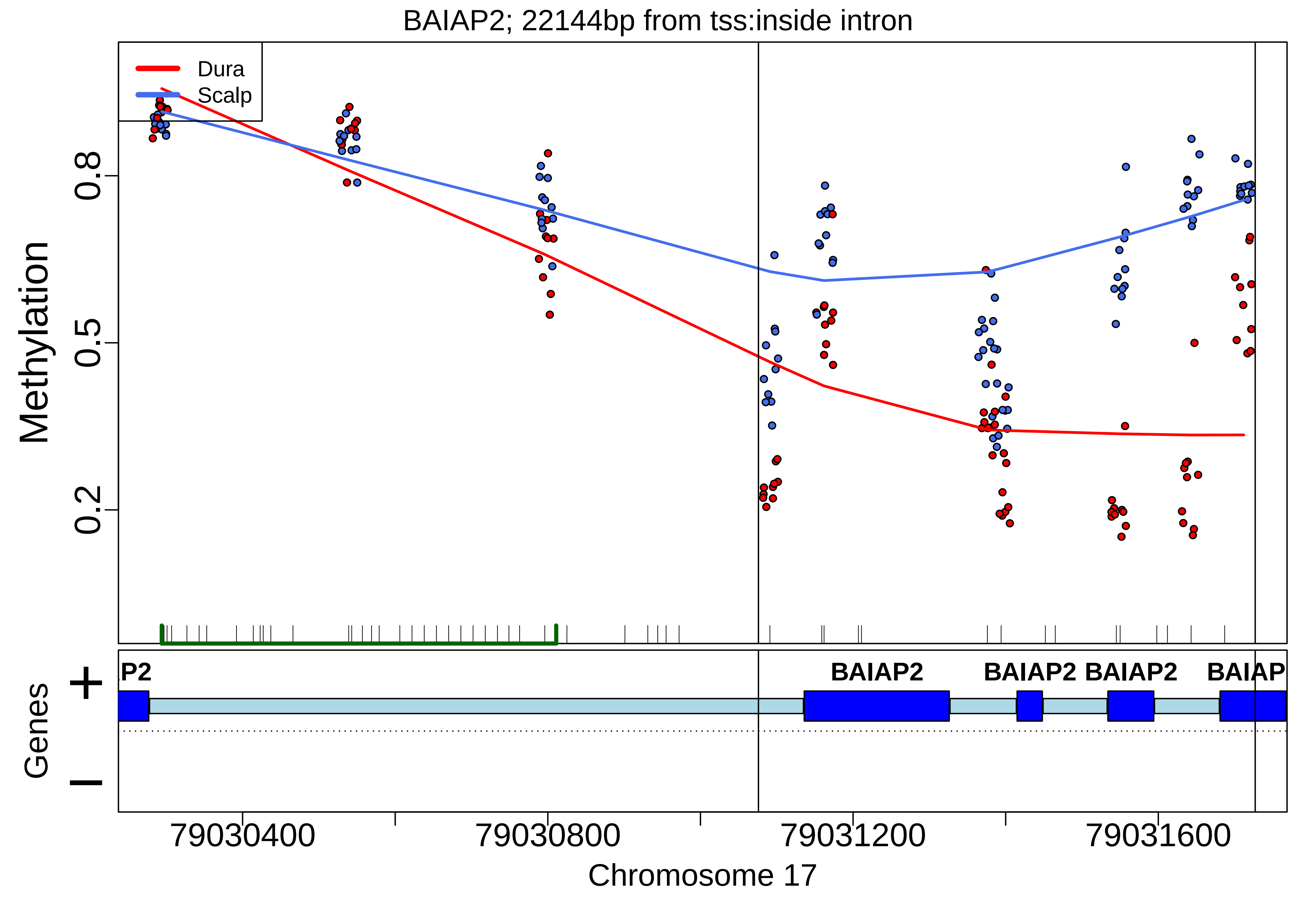

BAIAP2; 22144bp from tss:inside intron
Dura
Scalp
Methylation
0.2
0.5
0.8
Genes
+
-
P2
BAIAP2
BAIAP2
BAIAP2
BAIAP
79030400
79030800
79031200
79031600
Chromosome 17

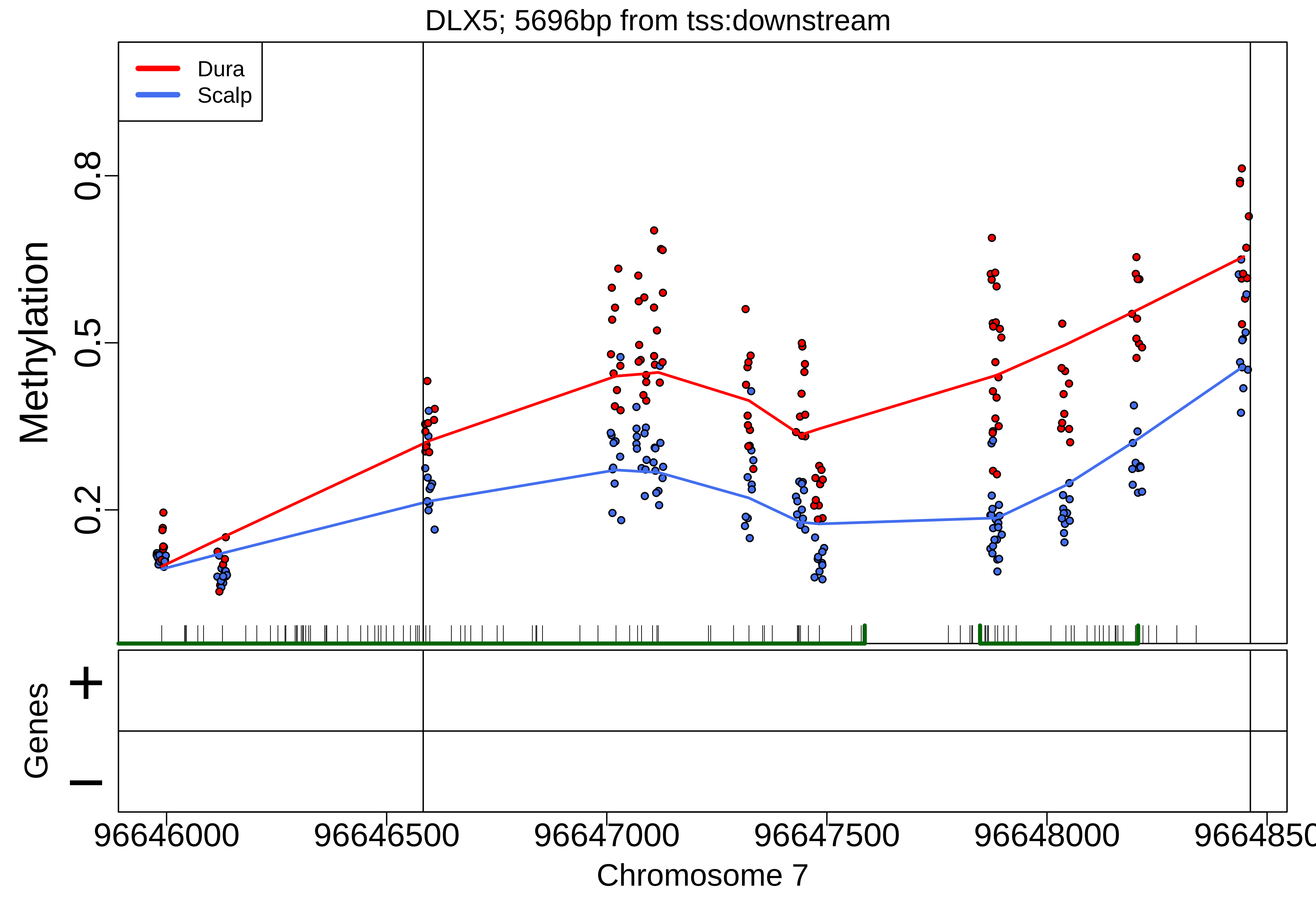

DLX5; 5696bp from tss:downstream
Dura
Scalp
Methylation
0.2
0.5
0.8
Genes
+
-
96646000
96646500
96647000
96647500
96648000
9664850
Chromosome 7

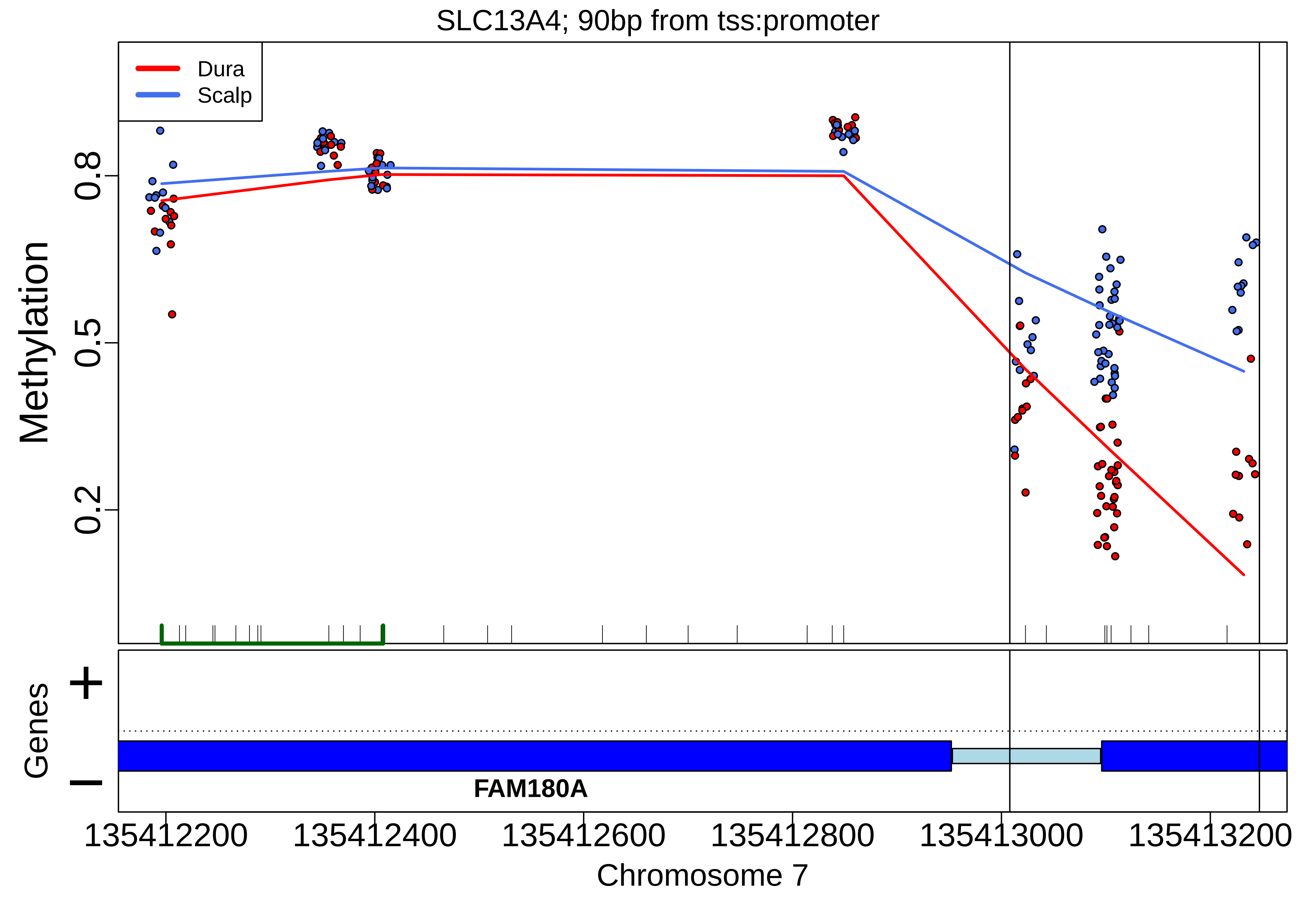
SLC13A4; 90bp from tss:promoter
Dura
Scalp
Methylation
0.8
0.5
0.2
Genes
+
-
FAM180A
135412200
135412400
135412600
135412800
135413000
135413200
Chromosome 7

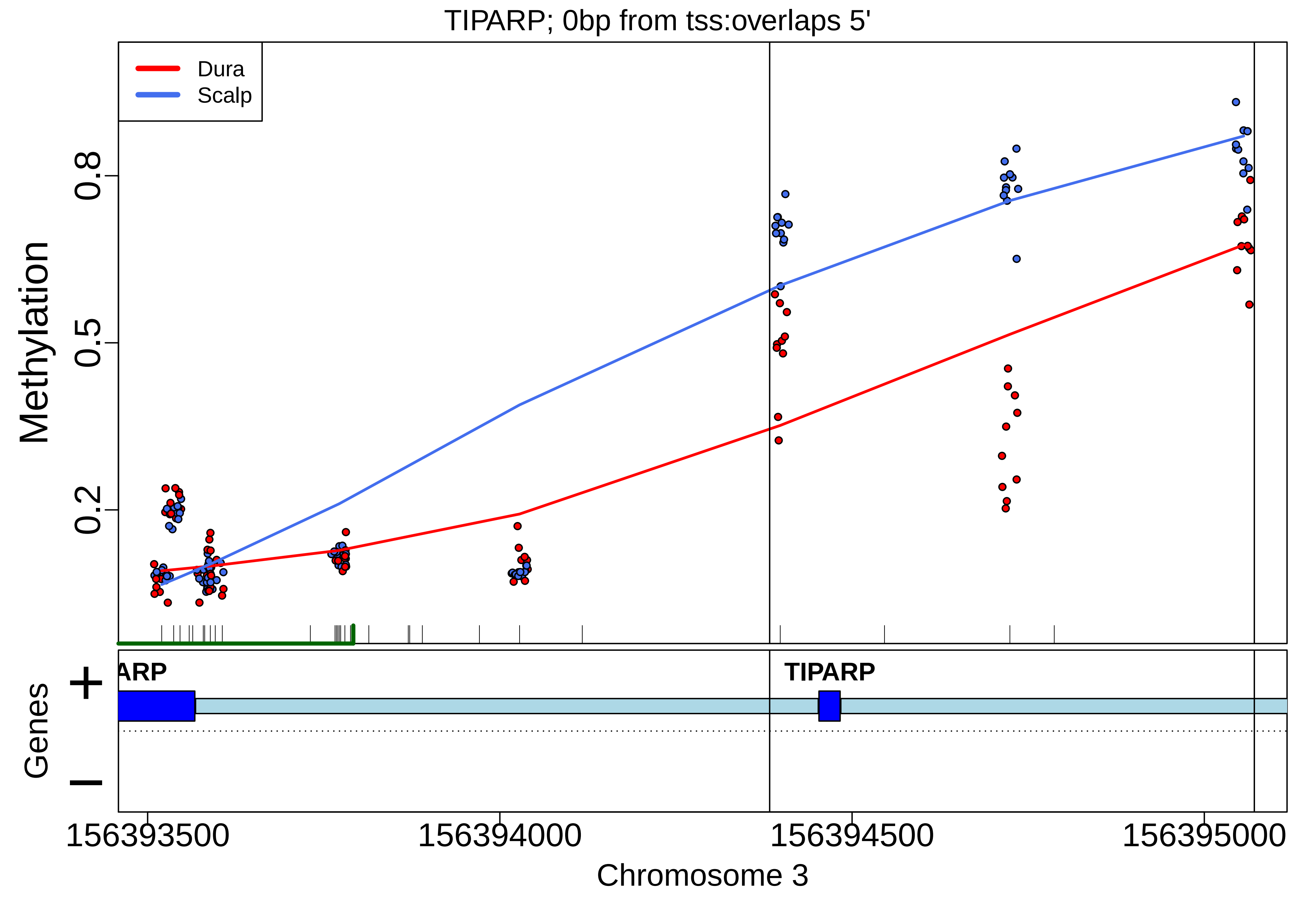
TIPARP; 0bp from tss:overlaps 5'
Dura
Scalp
Methylation
0.2
0.5
0.8
Genes
+
−
ARP
TIPARP
156393500
156394000
156394500
156395000
Chromosome 3

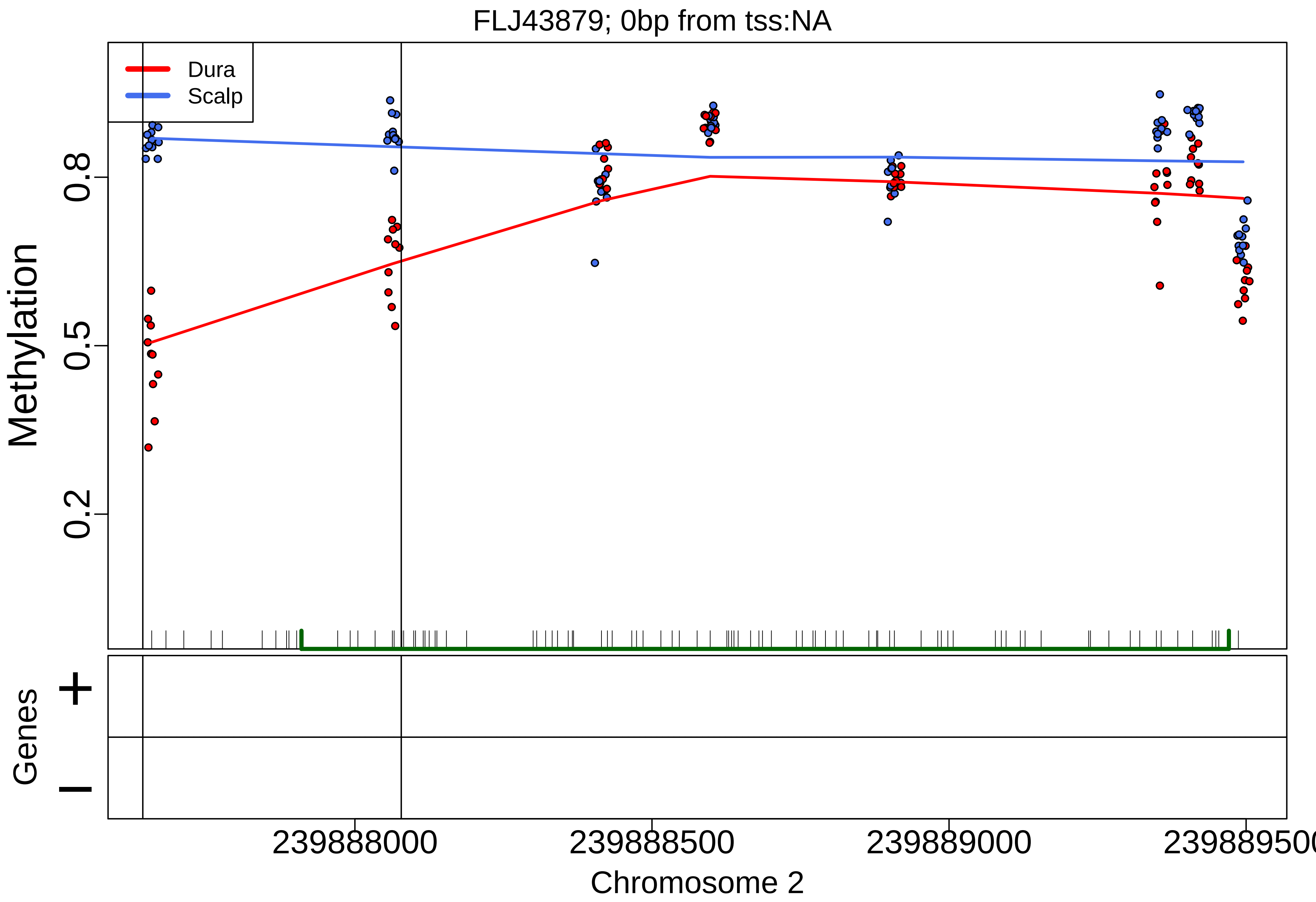
FLJ43879; 0bp from tss:NA
Dura
Scalp
Methylation
0.2
0.5
0.8
Genes
+
-
239888000
239888500
239889000
239889500
Chromosome 2

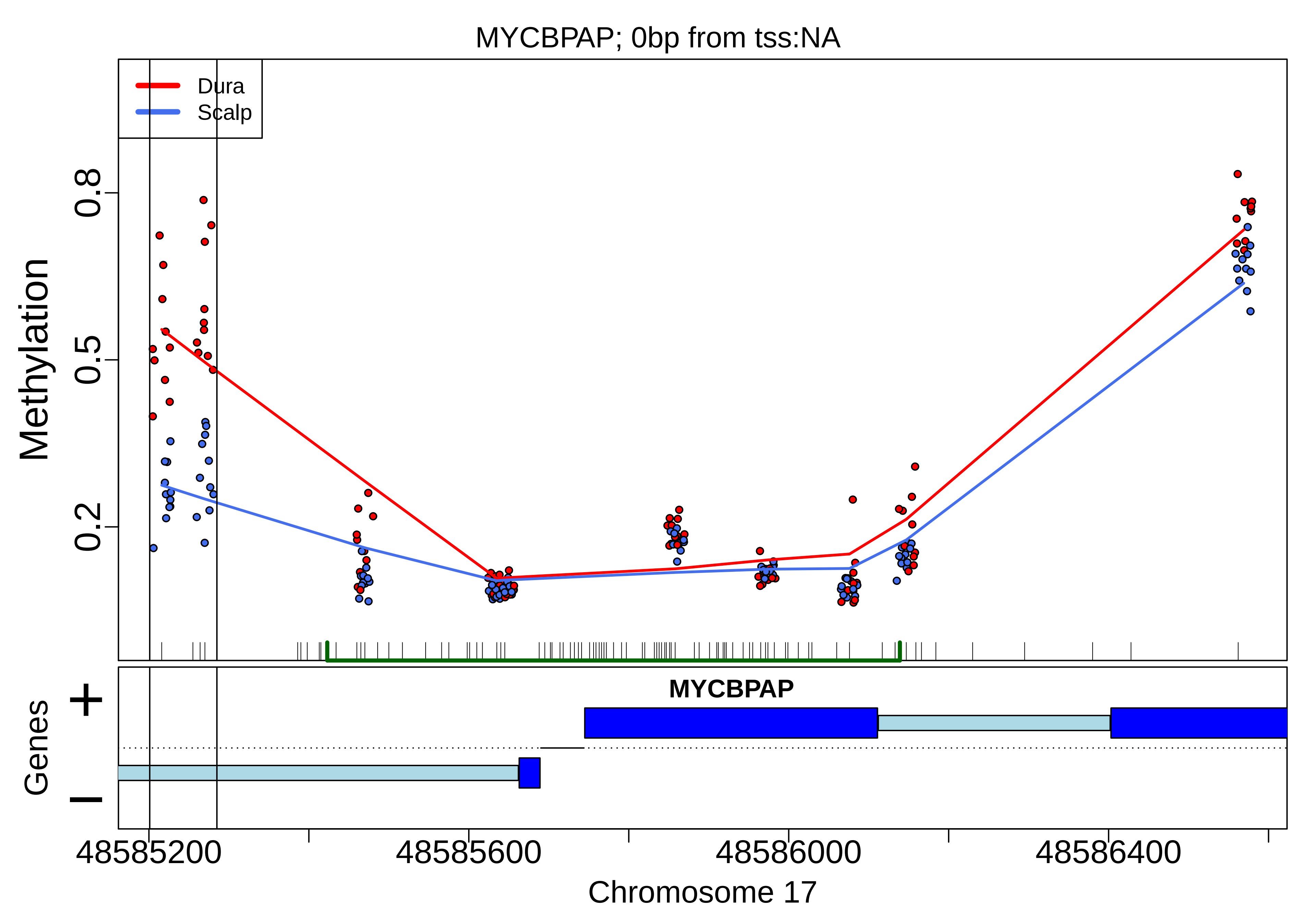

MYCBPAP; 0bp from tss:NA
Dura
Scalp
Methylation
0.2
0.5
0.8
Genes
+
−
MYCBPAP
48585200
48585600
48586000
48586400
Chromosome 17

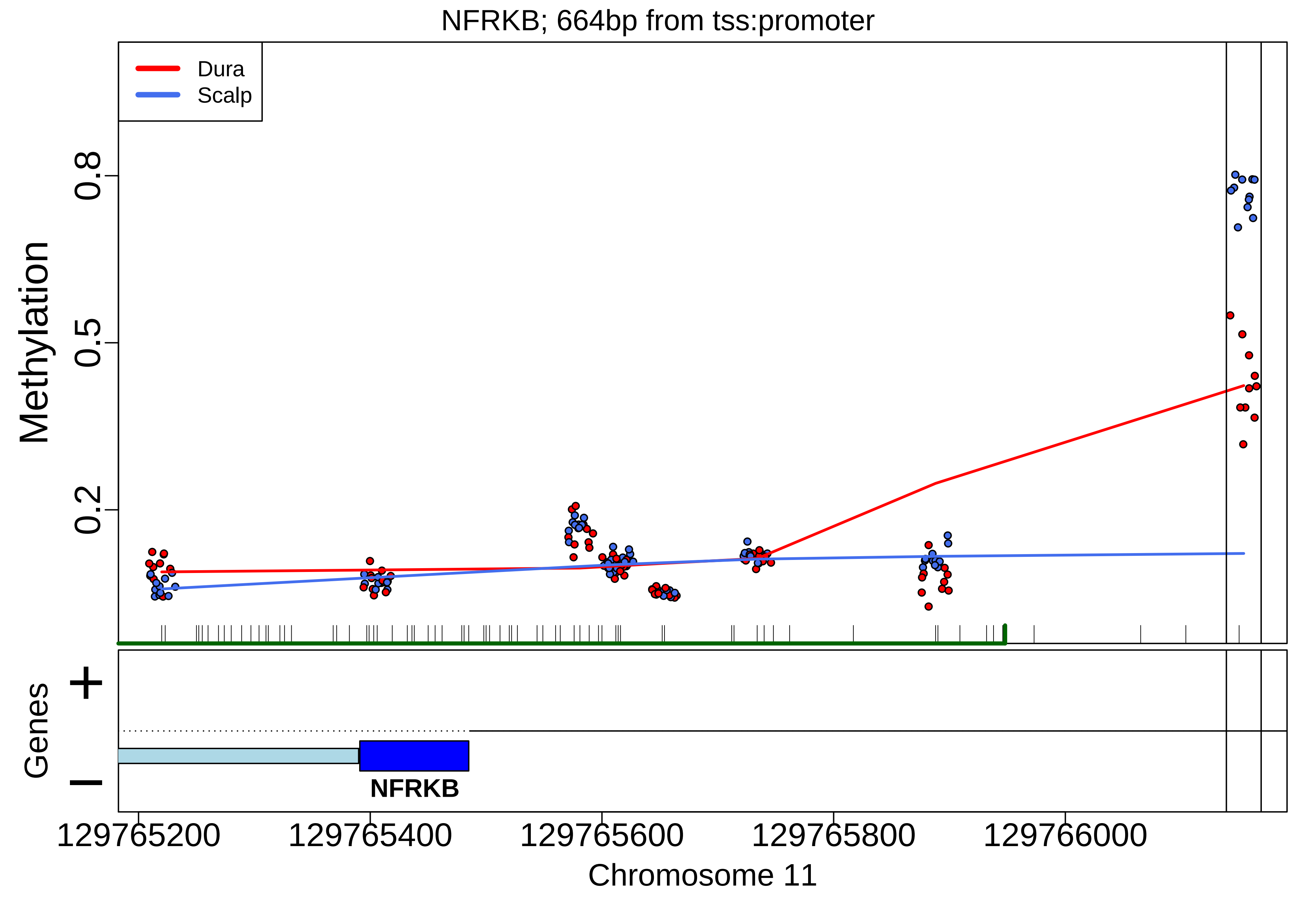
NFRKB; 664bp from tss:promoter
Dura
Scalp
Methylation
0.2
0.5
0.8
Genes
+
−
NFRKB
129765200
129765400
129765600
129765800
129766000
Chromosome 11

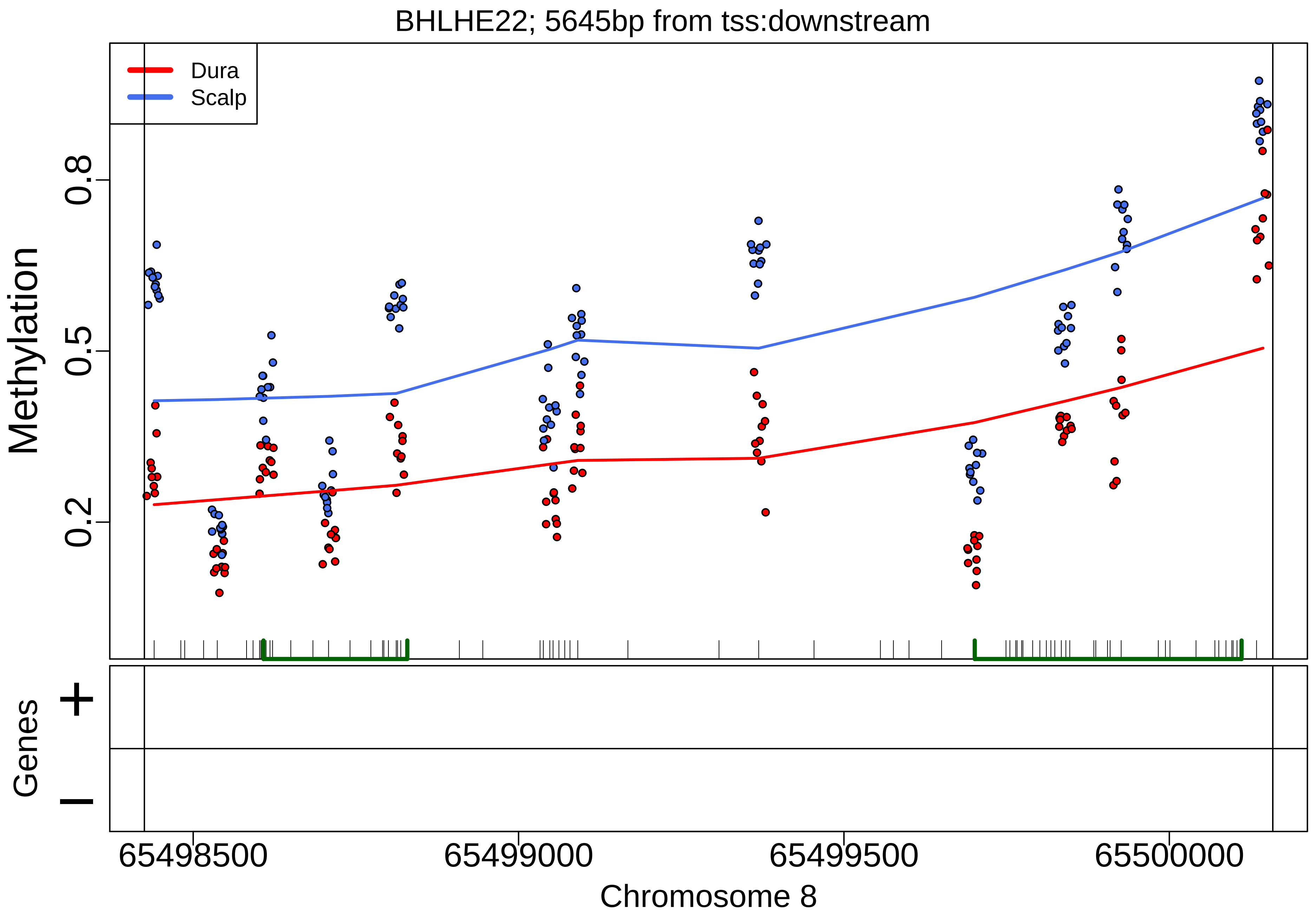

BHLHE22; 5645bp from tss:downstream
Dura
Scalp
Methylation
0.2
0.5
0.8
Genes
+
-
65498500
65499000
65499500
65500000
Chromosome 8

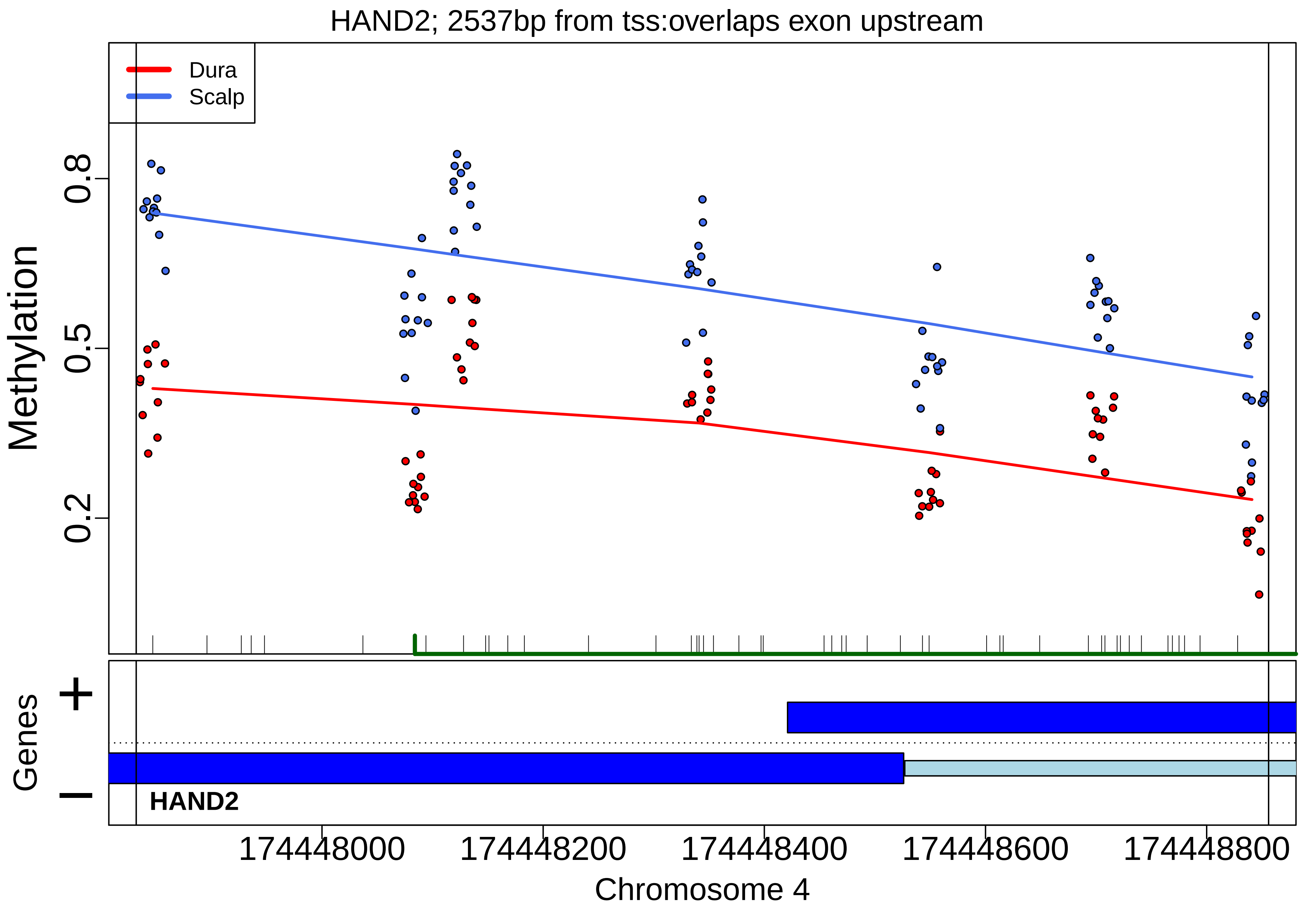

HAND2; 2537bp from tss:overlaps exon upstream
Dura
Scalp
Methylation
0.2
0.5
0.8
Genes
+
−
HAND2
174448000
174448200
174448400
174448600
174448800
Chromosome 4

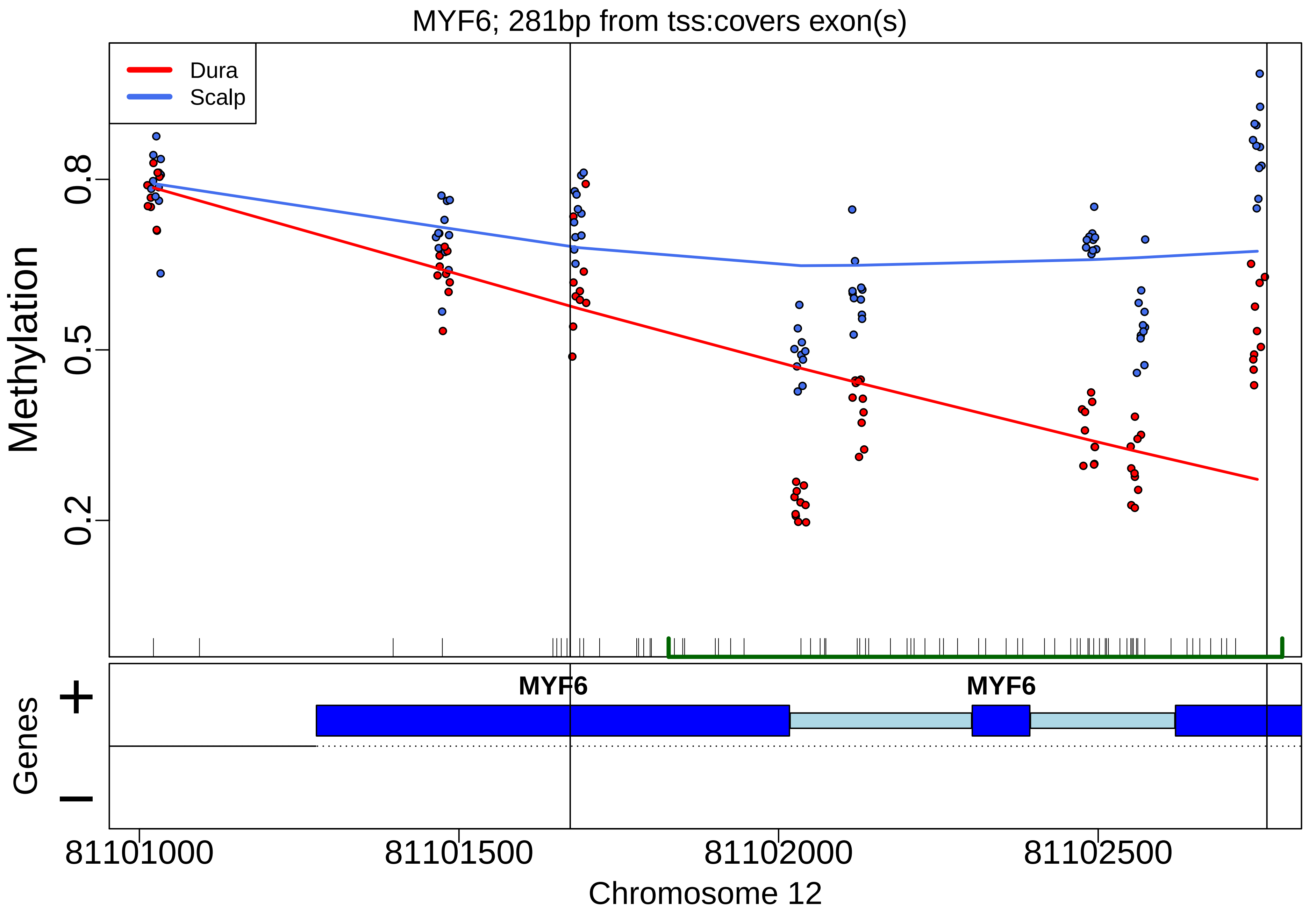

MYF6; 281bp from tss:covers exon(s)
Dura
Scalp
Methylation
0.2
0.5
0.8
Genes
+
-
MYF6
MYF6
81101000
81101500
81102000
81102500
Chromosome 12

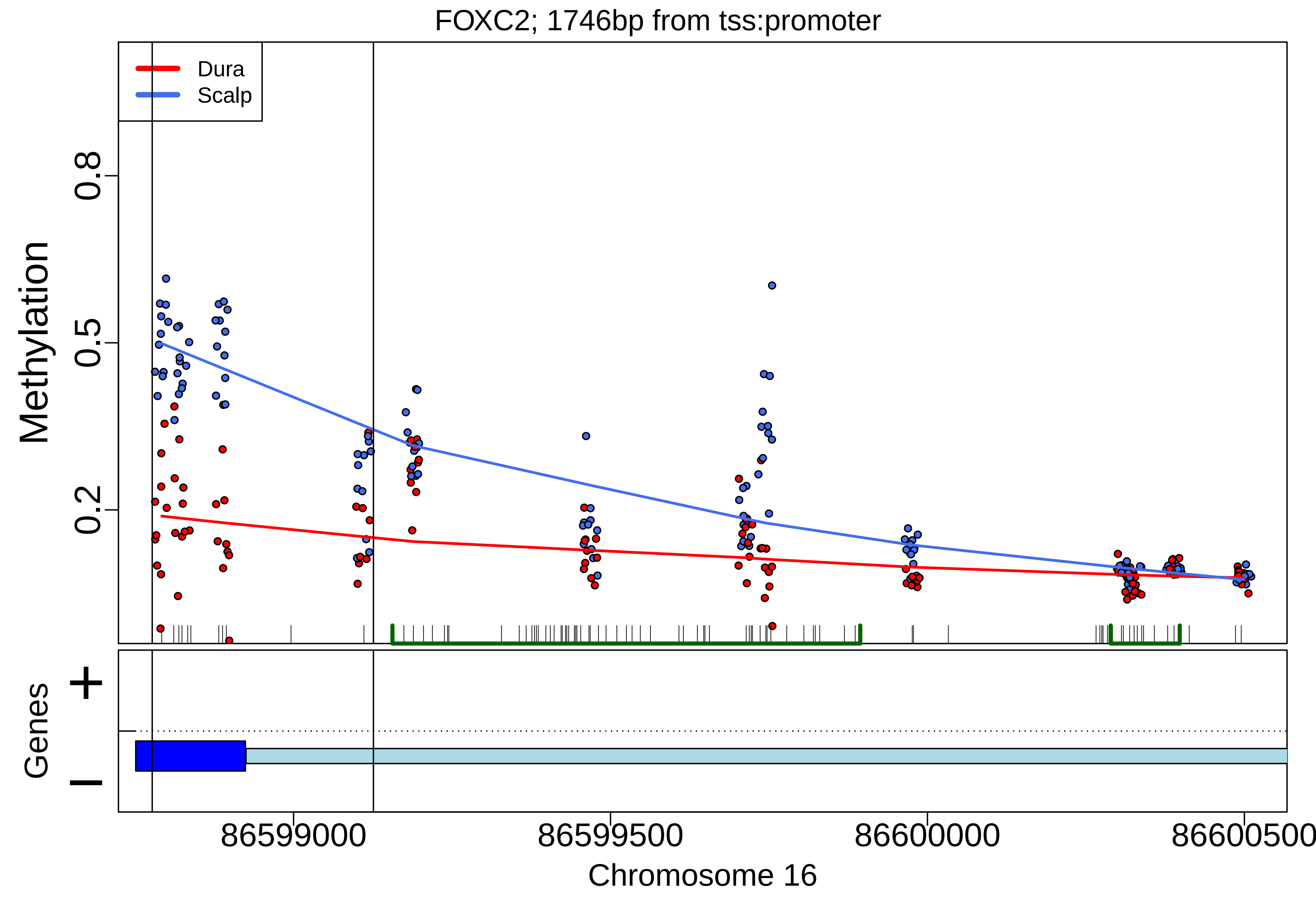

FOXC2; 1746bp from tss:promoter
Dura
Scalp
Methylation
0.2
0.5
0.8
Genes
+
-
86599000
86599500
86600000
86600500
Chromosome 16

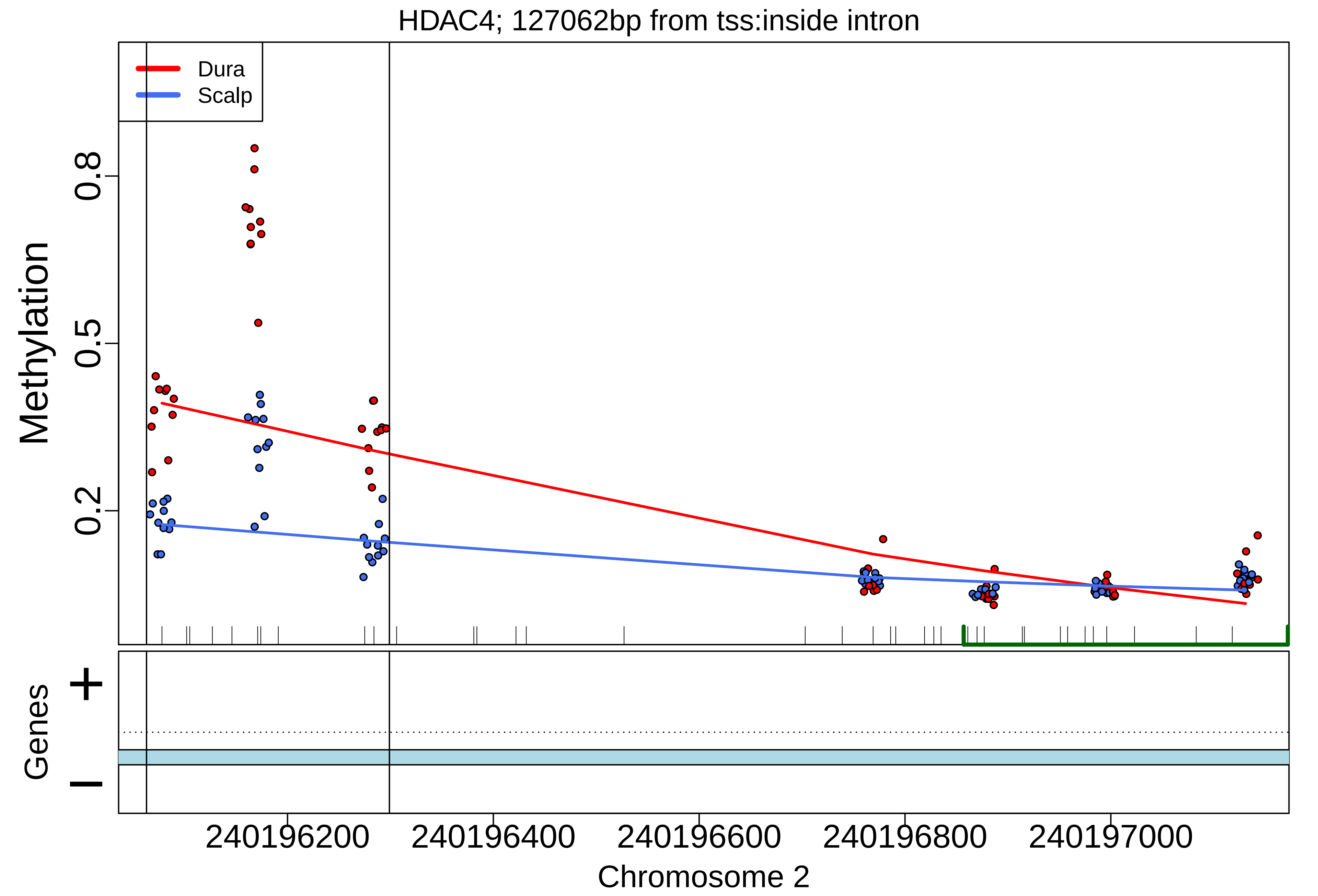
HDAC4; 127062bp from tss:inside intron
Dura
Scalp
Methylation
0.2
0.5
0.8
Genes
+
-
240196200
240196400
240196600
240196800
240197000
Chromosome 2

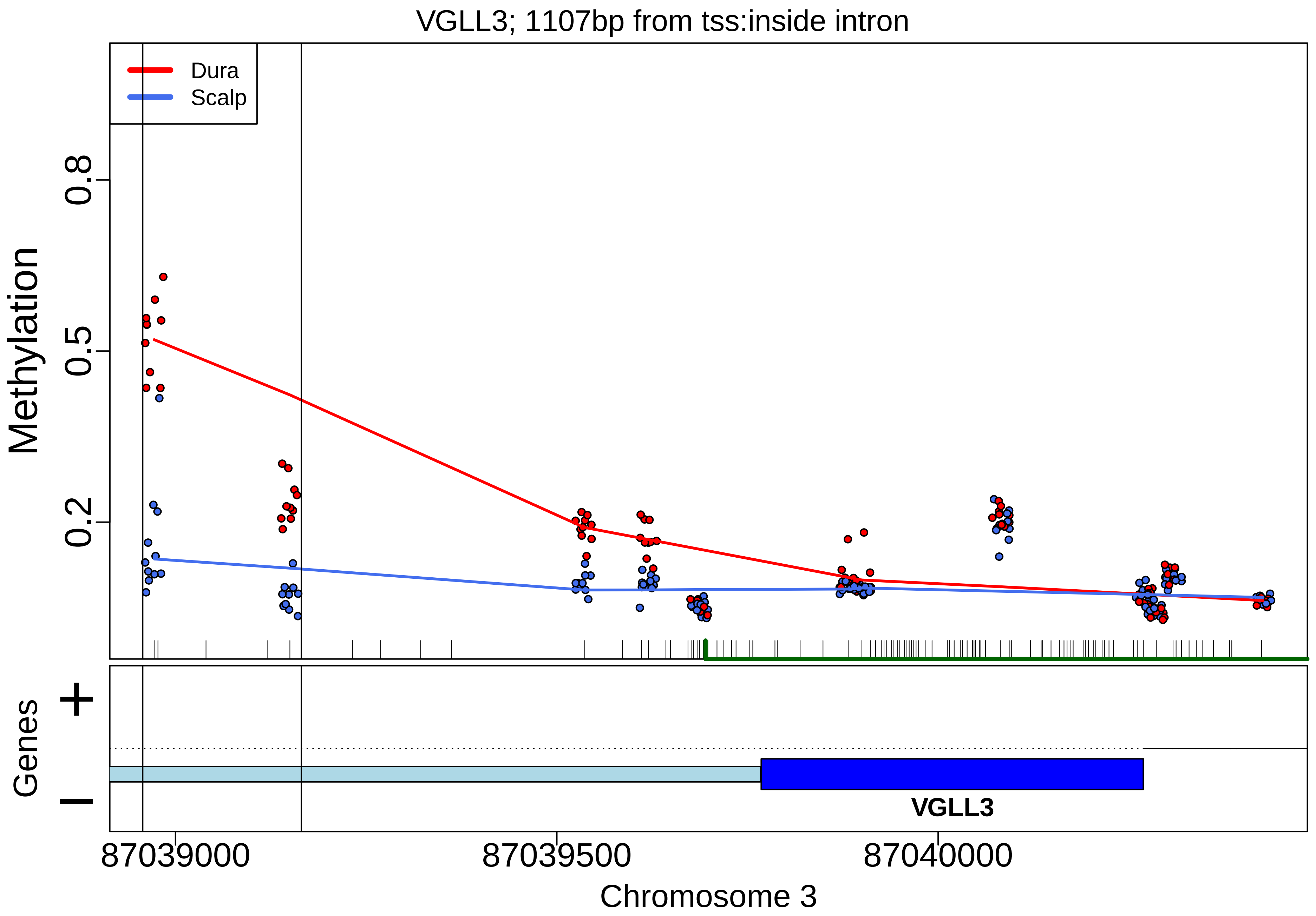

VGLL3; 1107bp from tss:inside intron
Dura
Scalp
Methylation
0.2
0.5
0.8
Genes
+
-
VGLL3
87039000
87039500
87040000
Chromosome 3

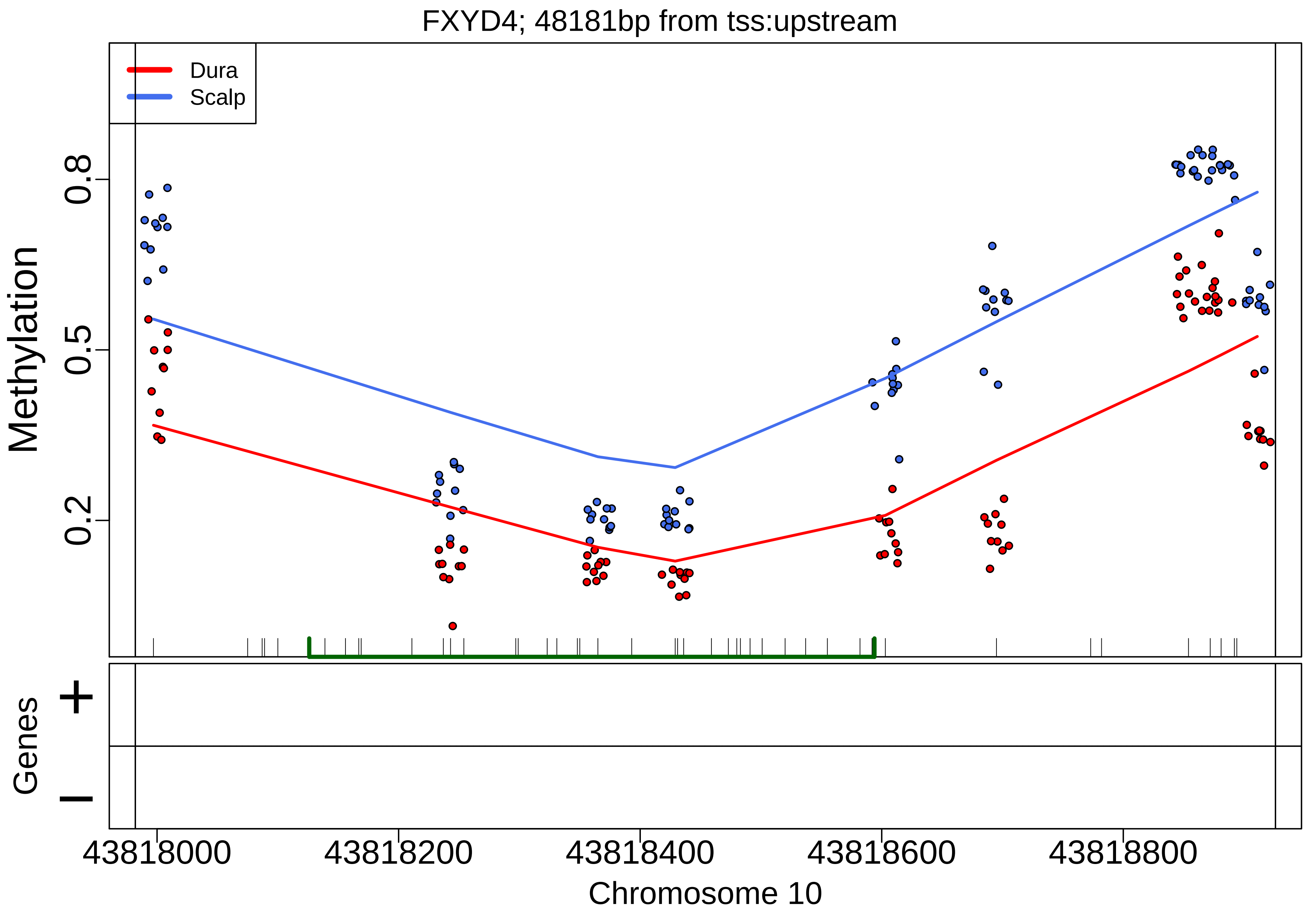

FXYD4; 48181bp from tss:upstream
Dura
Scalp
Methylation
0.2
0.5
0.8
Genes
+
−
43818000
43818200
43818400
43818600
43818800
Chromosome 10

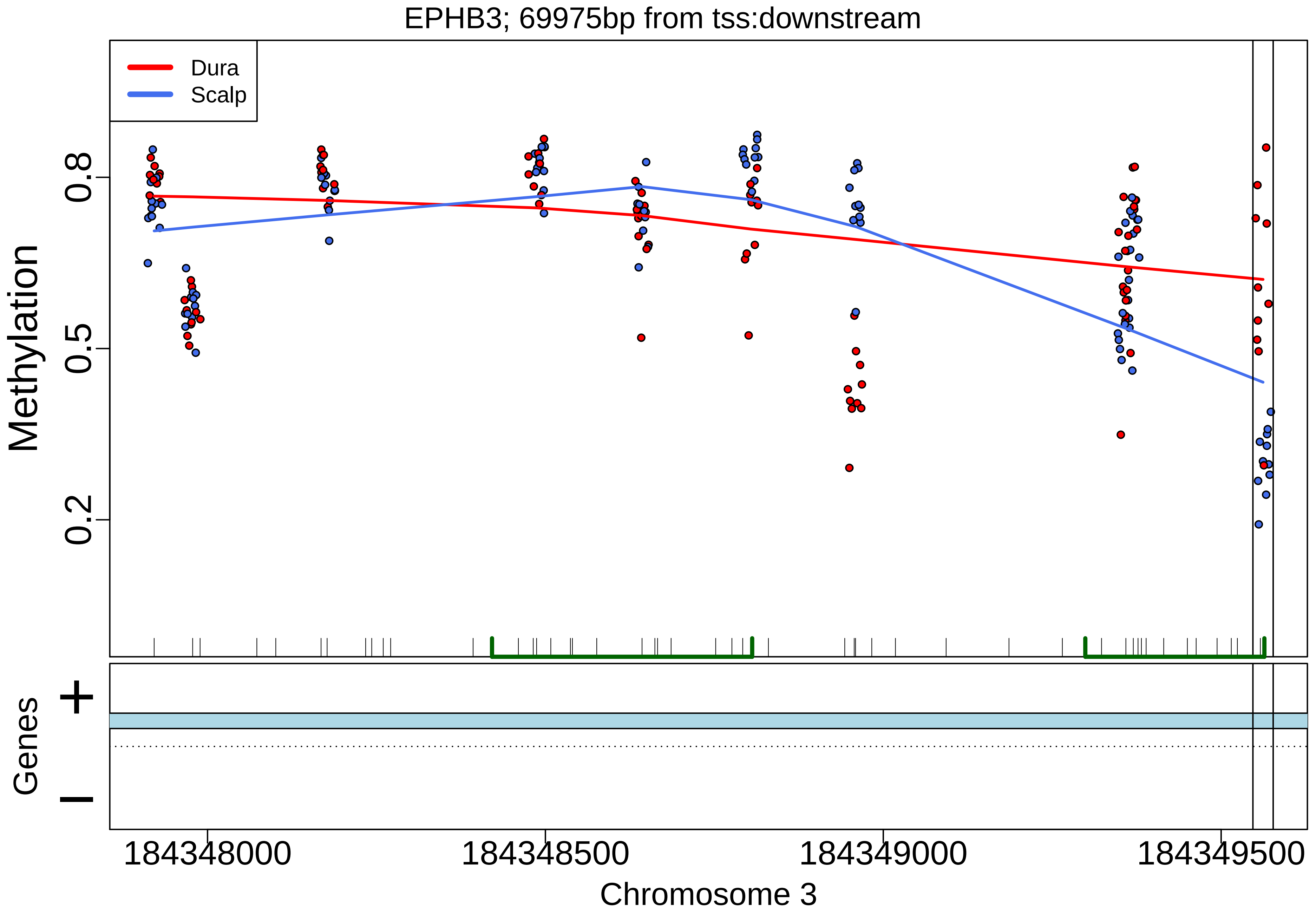

EPHB3; 69975bp from tss:downstream
Dura
Scalp
Methylation
0.2
0.5
0.8
Genes
+
−
184348000
184348500
184349000
184349500
Chromosome 3

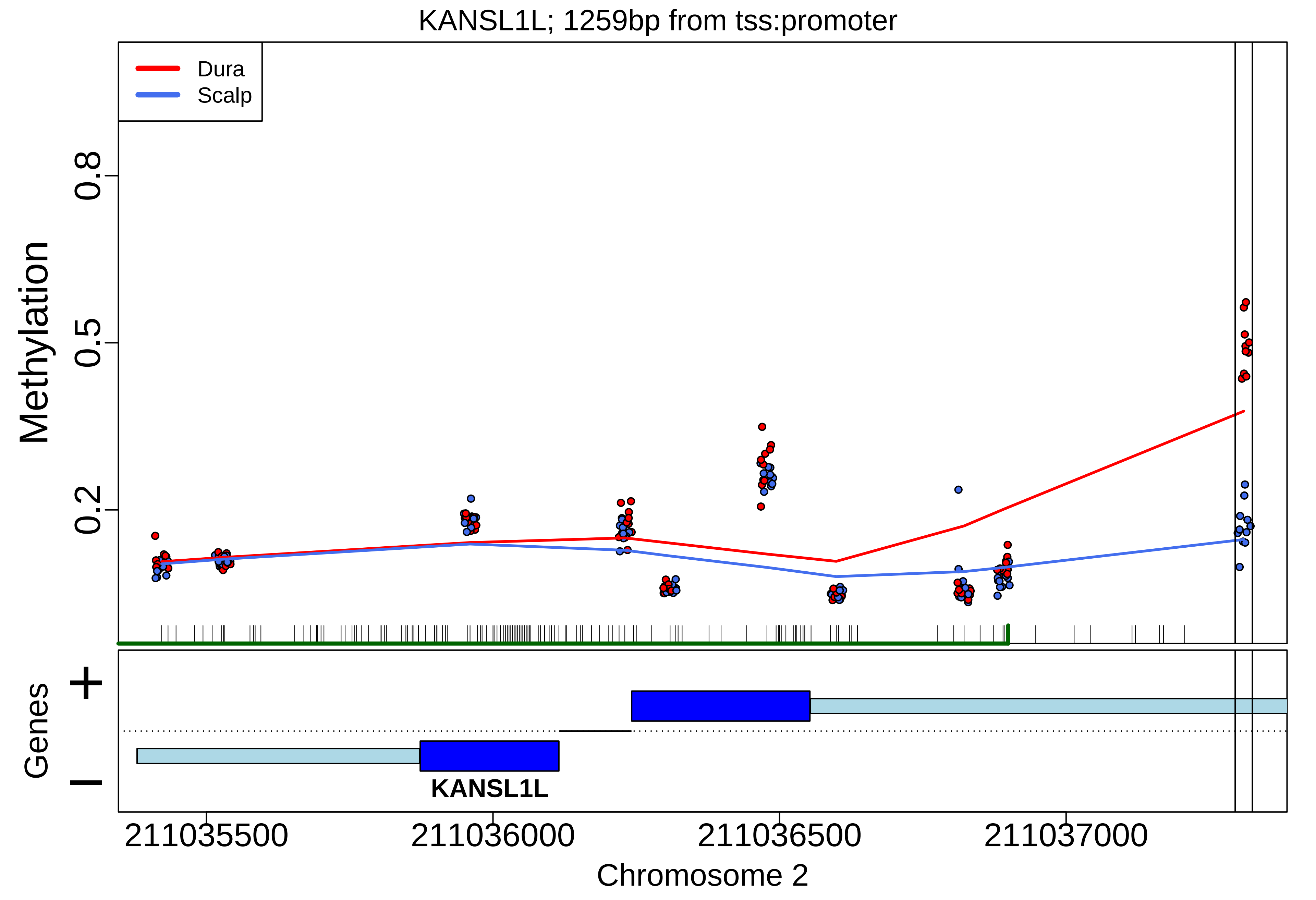

KANSL1L; 1259bp from tss:promoter
Dura
Scalp
Methylation
0.2
0.5
0.8
Genes
+
-
KANSL1L
211035500
211036000
211036500
211037000
Chromosome 2

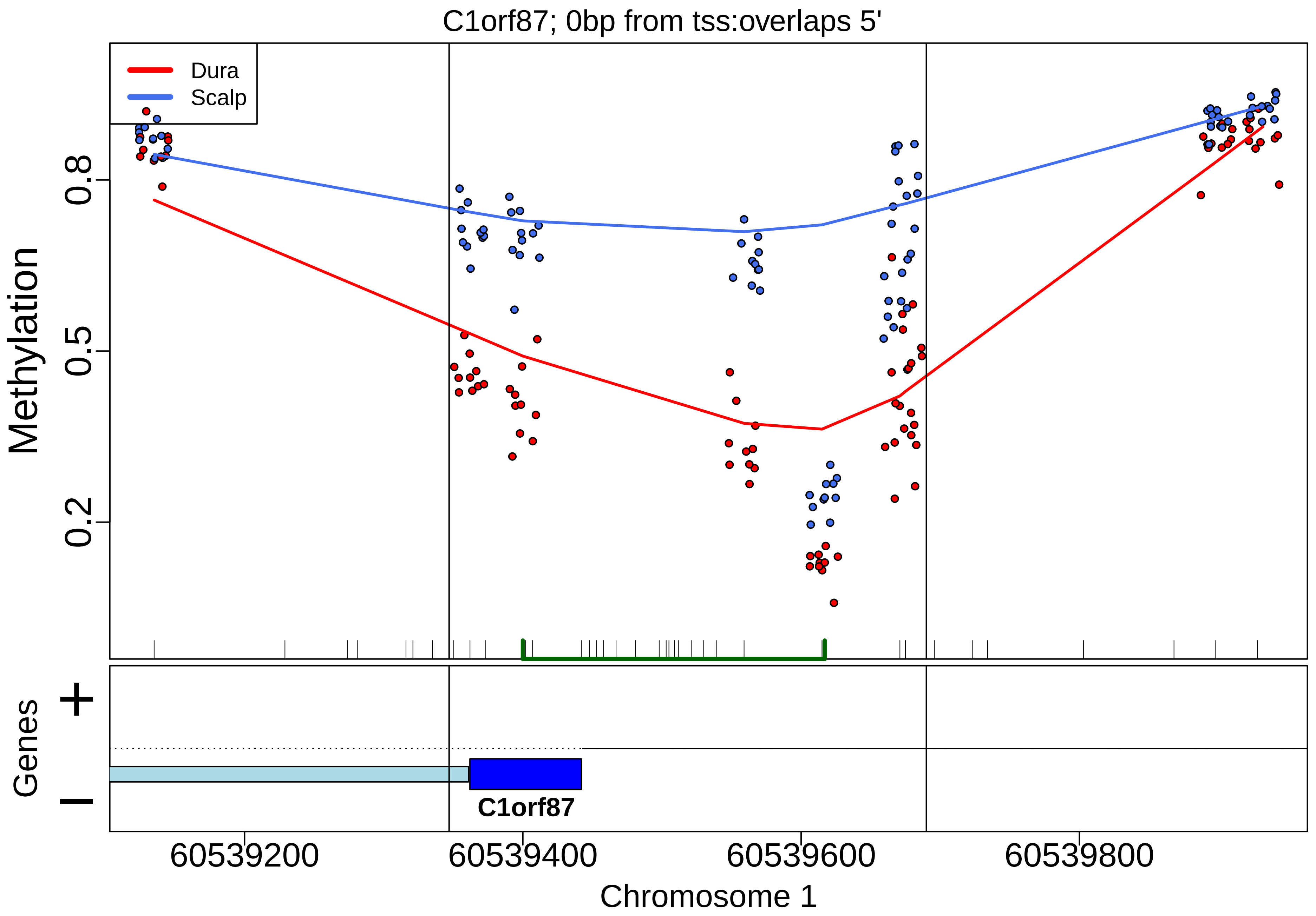
C1orf87; 0bp from tss:overlaps 5'
Dura
Scalp
Methylation
0.2
0.5
0.8
Genes
+
−
C1orf87
60539200
60539400
60539600
60539800
Chromosome 1

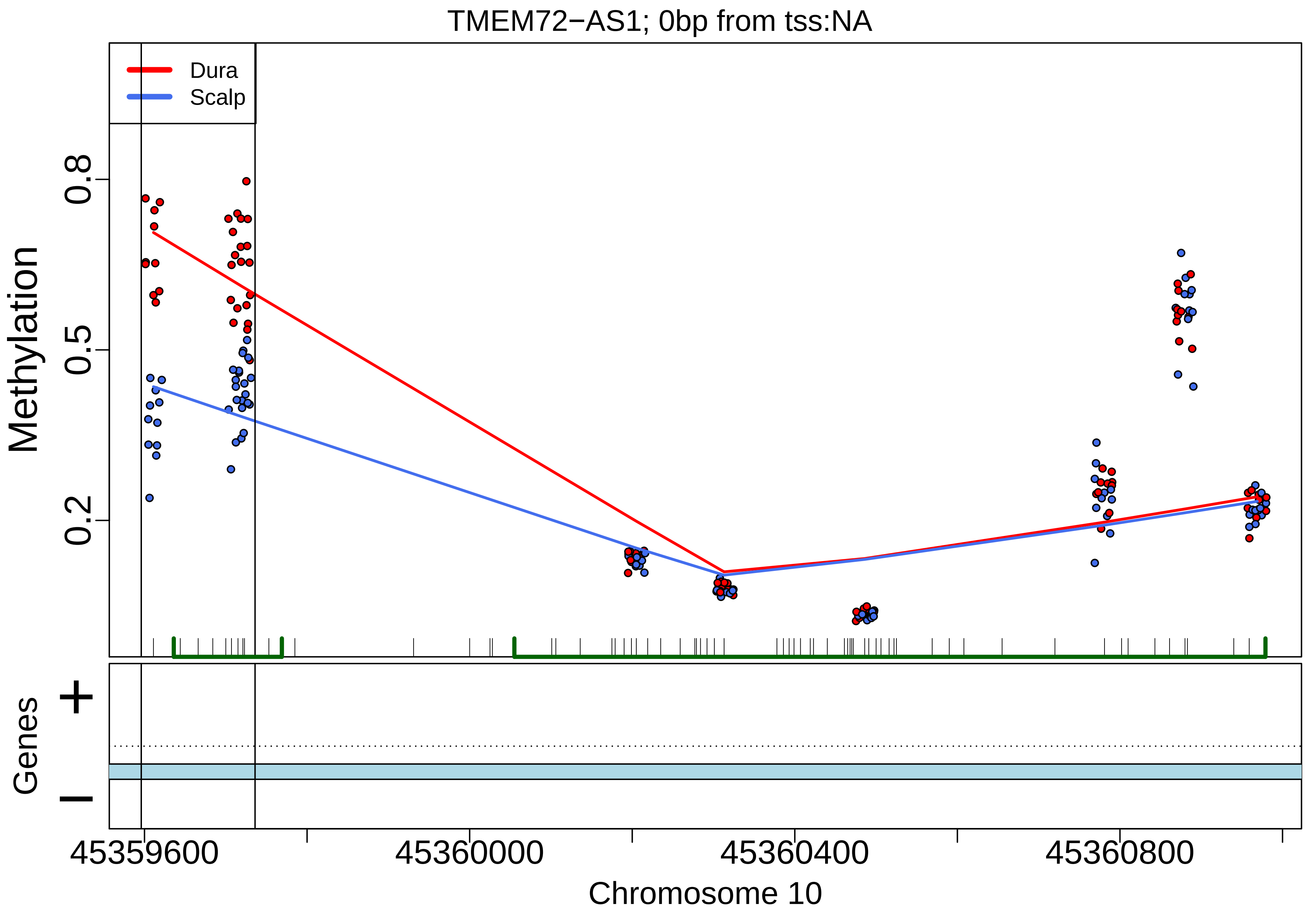

TMEM72−AS1; 0bp from tss:NA
Dura
Scalp
Methylation
0.2
0.5
0.8
Genes
+
−
45359600
45360000
45360400
45360800
Chromosome 10

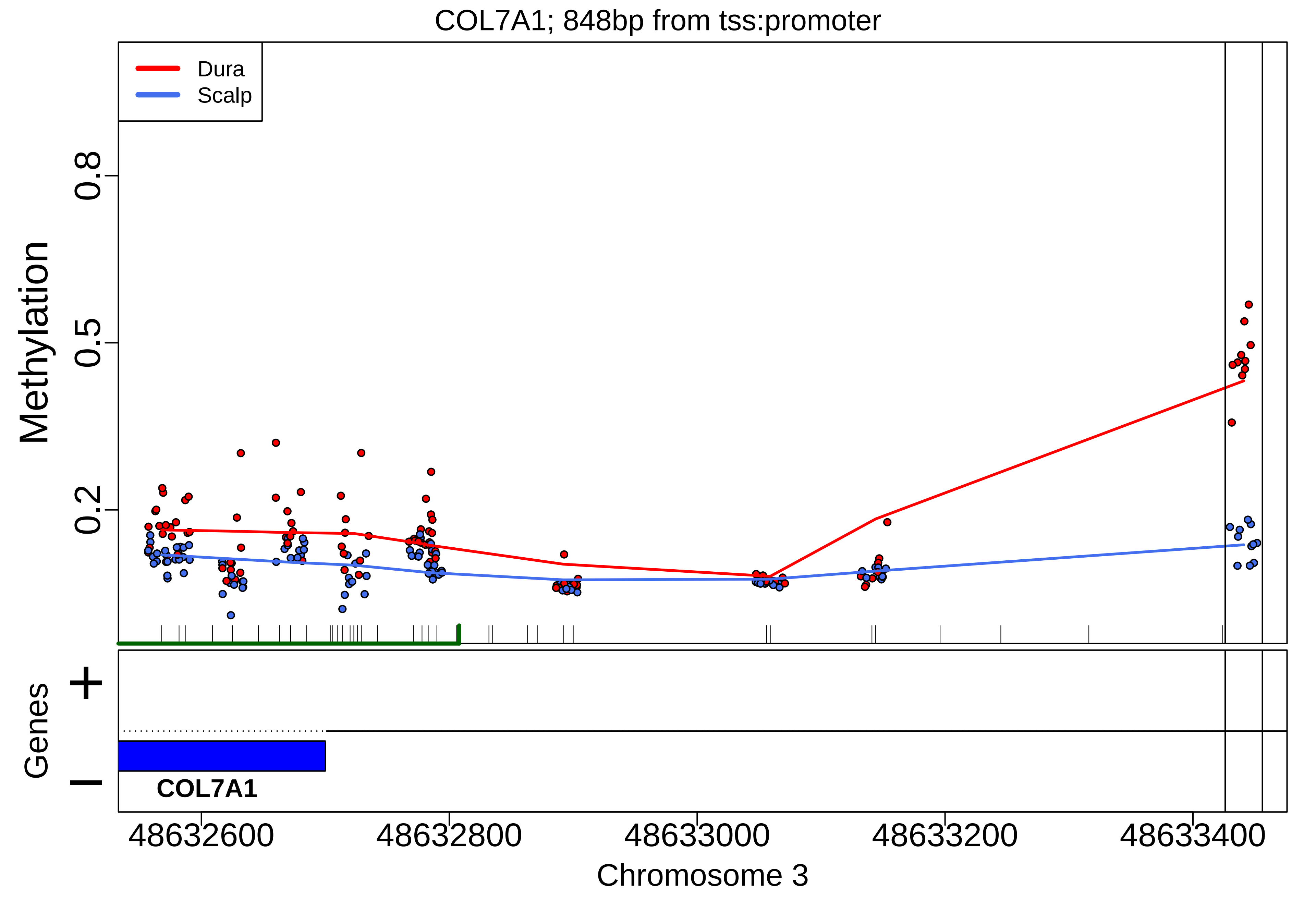
COL7A1; 848bp from tss:promoter
Dura
Scalp
Methylation
0.2
0.5
0.8
Genes
+
−
COL7A1
48632600
48632800
48633000
48633200
48633400
Chromosome 3

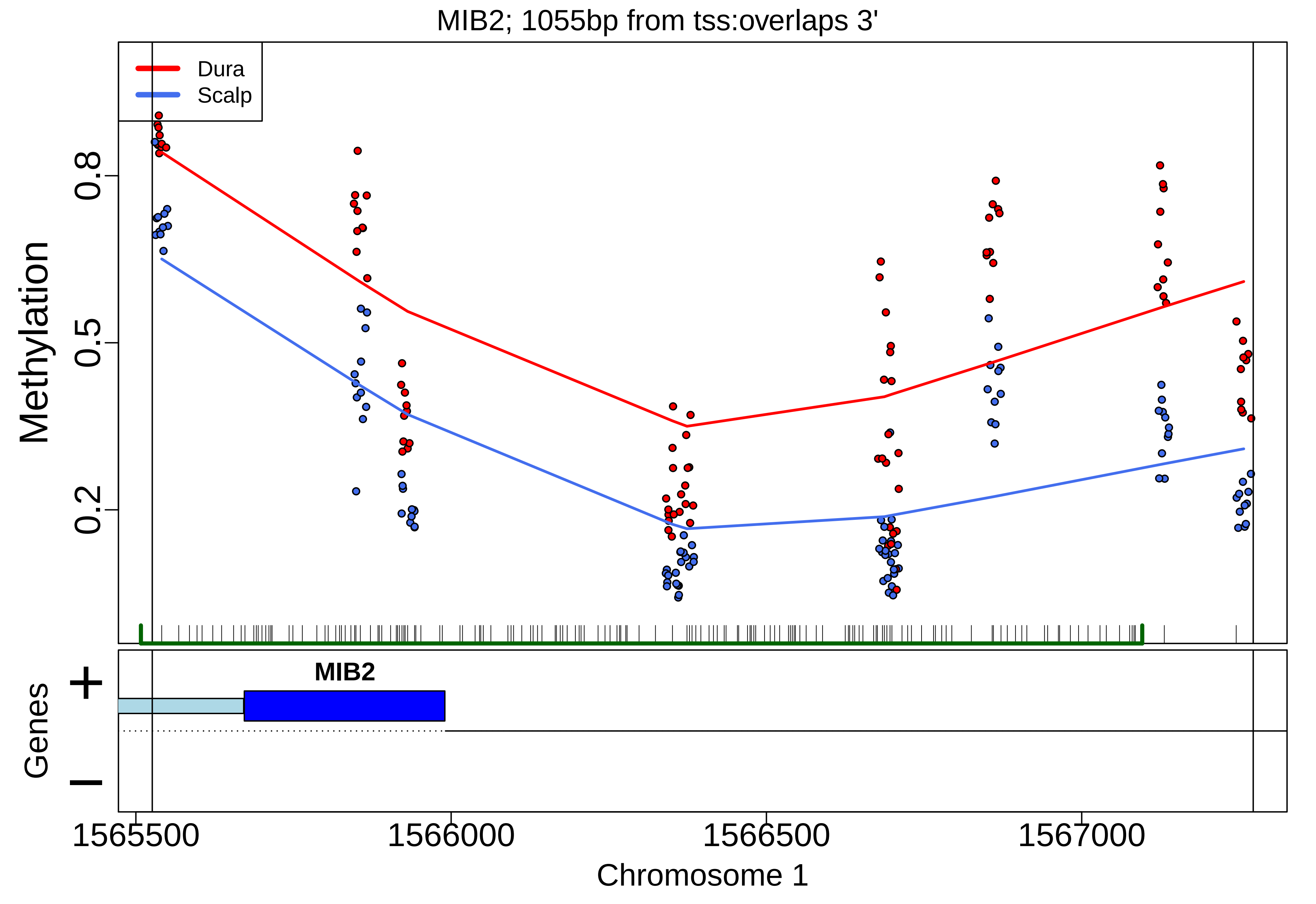
MIB2; 1055bp from tss:overlaps 3'
Dura
Scalp
Methylation
0.2
0.5
0.8
Genes
+
–
MIB2
1565500
1566000
1566500
1567000
Chromosome 1

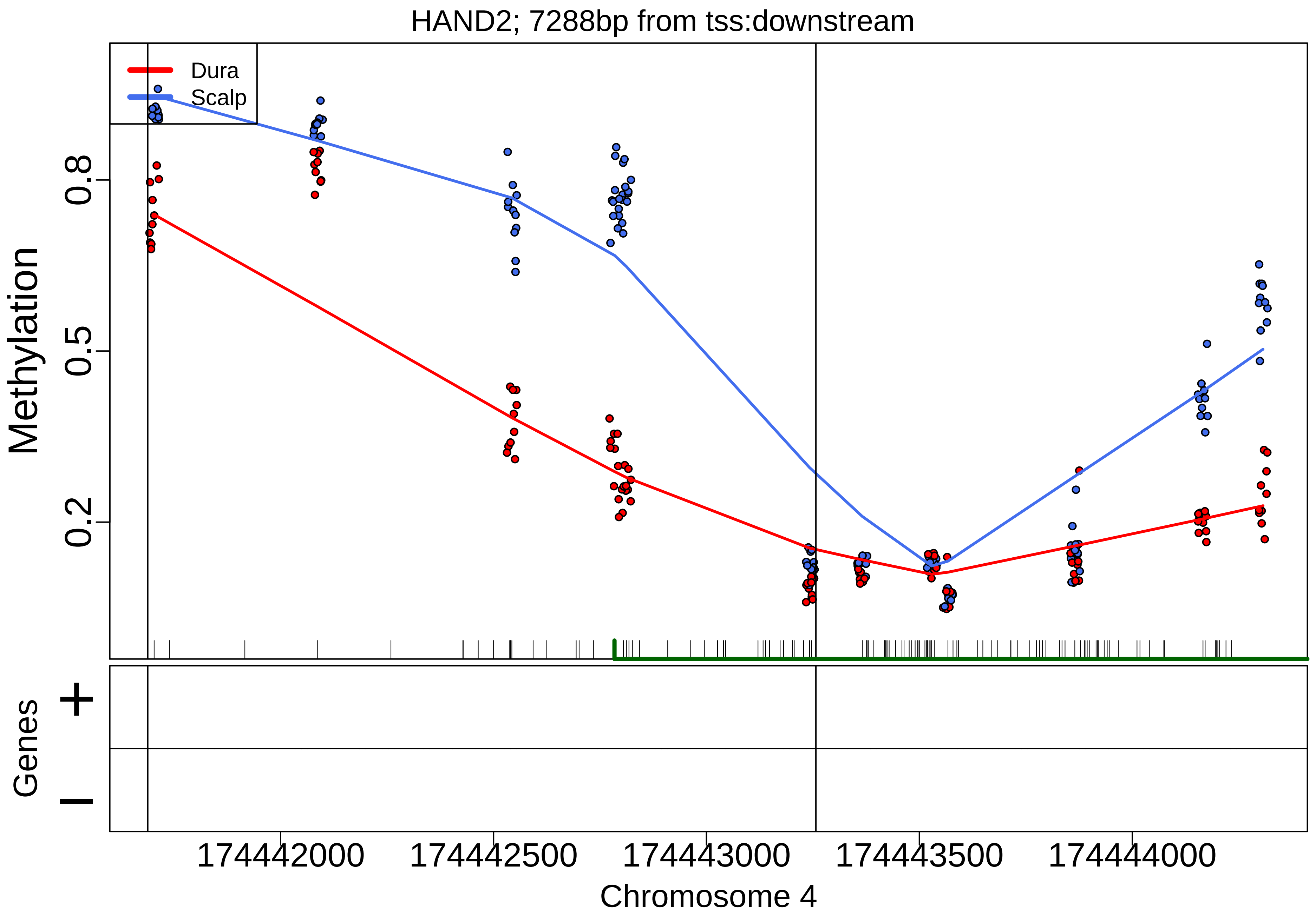
HAND2; 7288bp from tss:downstream
Dura
Scalp
Methylation
0.2
0.5
0.8
Genes
+
–
174442000
174442500
174443000
174443500
174444000
Chromosome 4

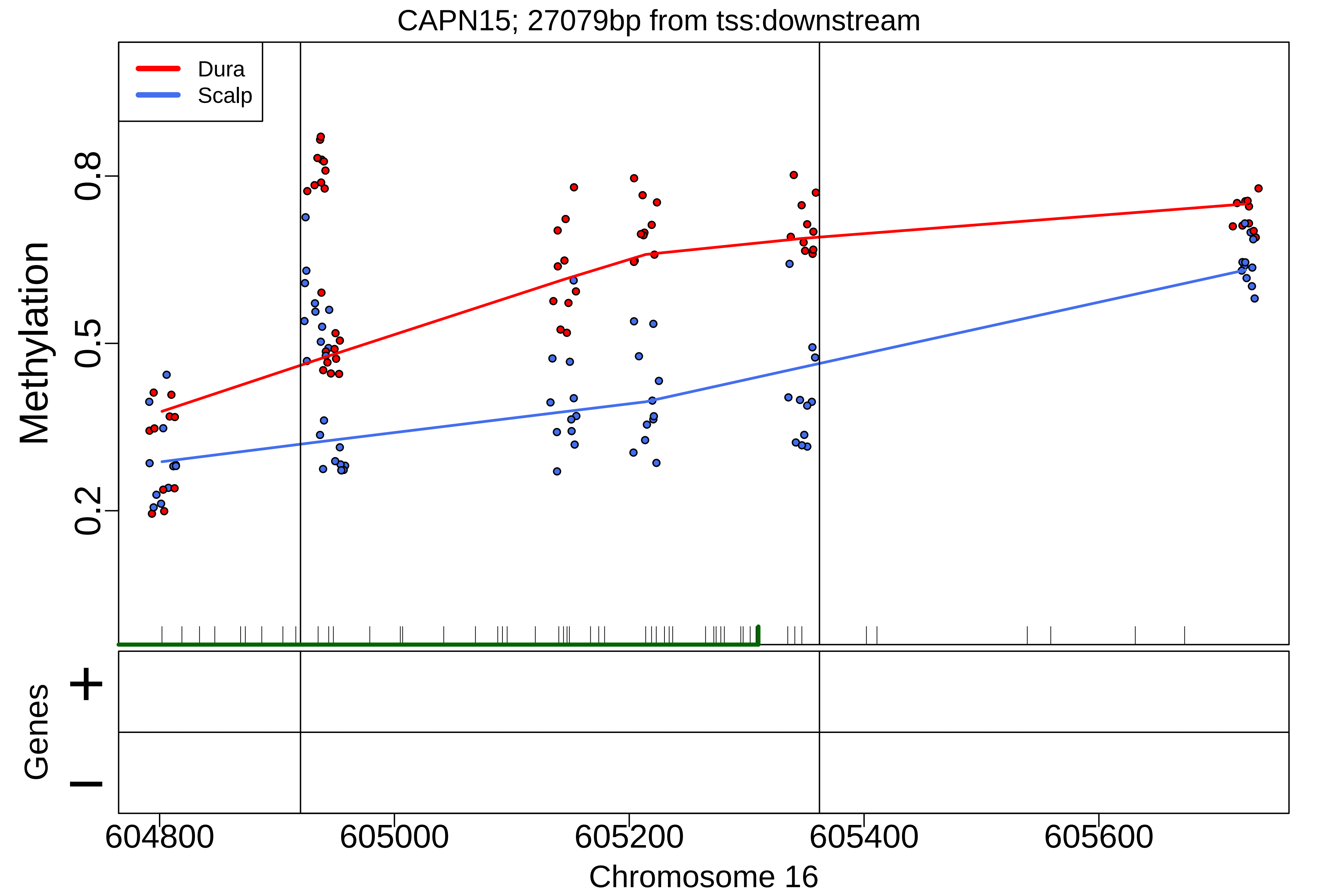
CAPN15; 27079bp from tss:downstream
Dura
Scalp
Methylation
0.2
0.5
0.8
Genes
+
−
604800
605000
605200
605400
605600
Chromosome 16

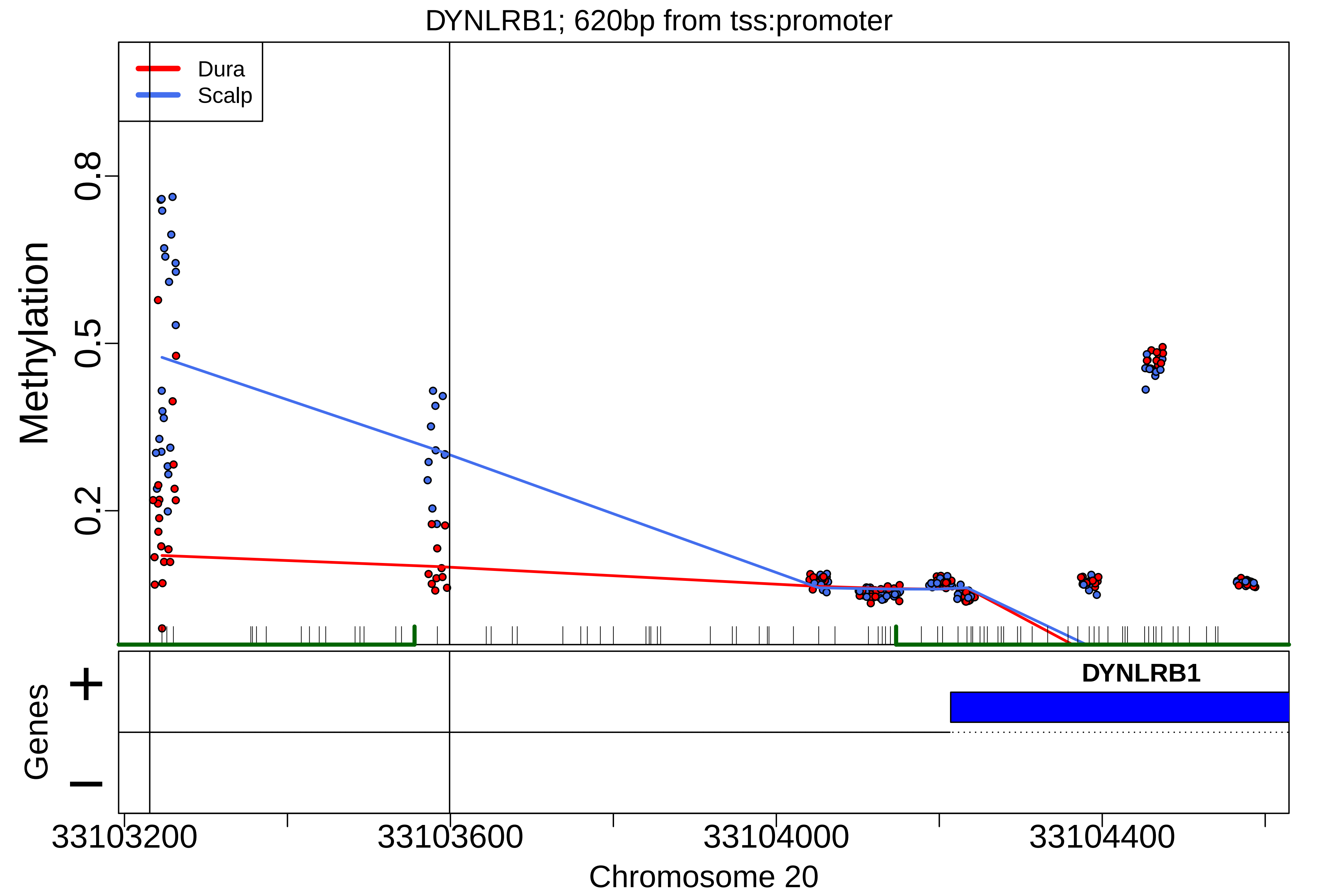

DYNLRB1; 620bp from tss:promoter
Dura
Scalp
Methylation
0.8
0.5
0.2
Genes
+
-
DYNLRB1
33103200
33103600
33104000
33104400
Chromosome 20

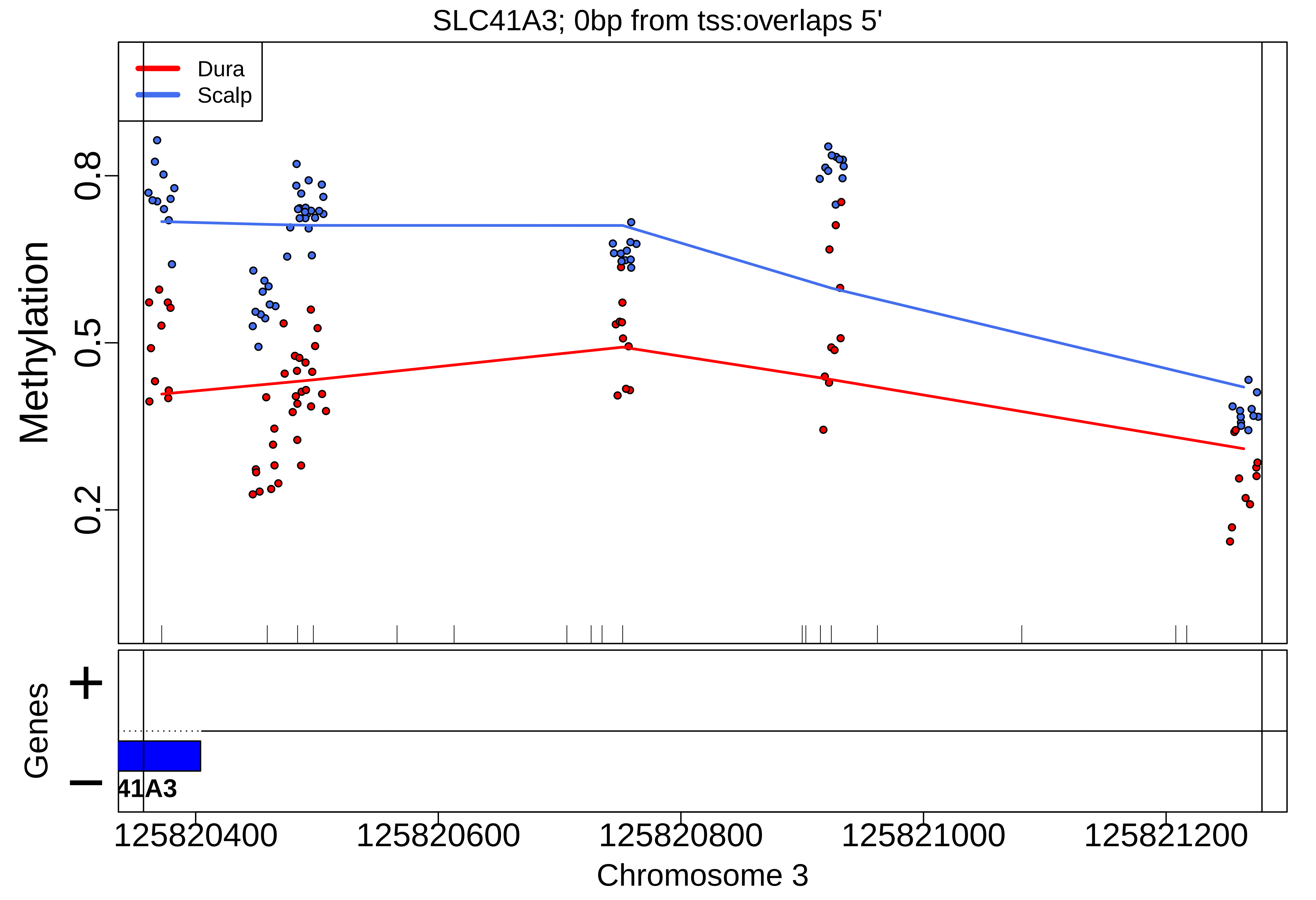
SLC41A3; 0bp from tss:overlaps 5'
Dura
Scalp
Methylation
0.2
0.5
0.8
Genes
+
−
41A3
125820400
125820600
125820800
125821000
125821200
Chromosome 3

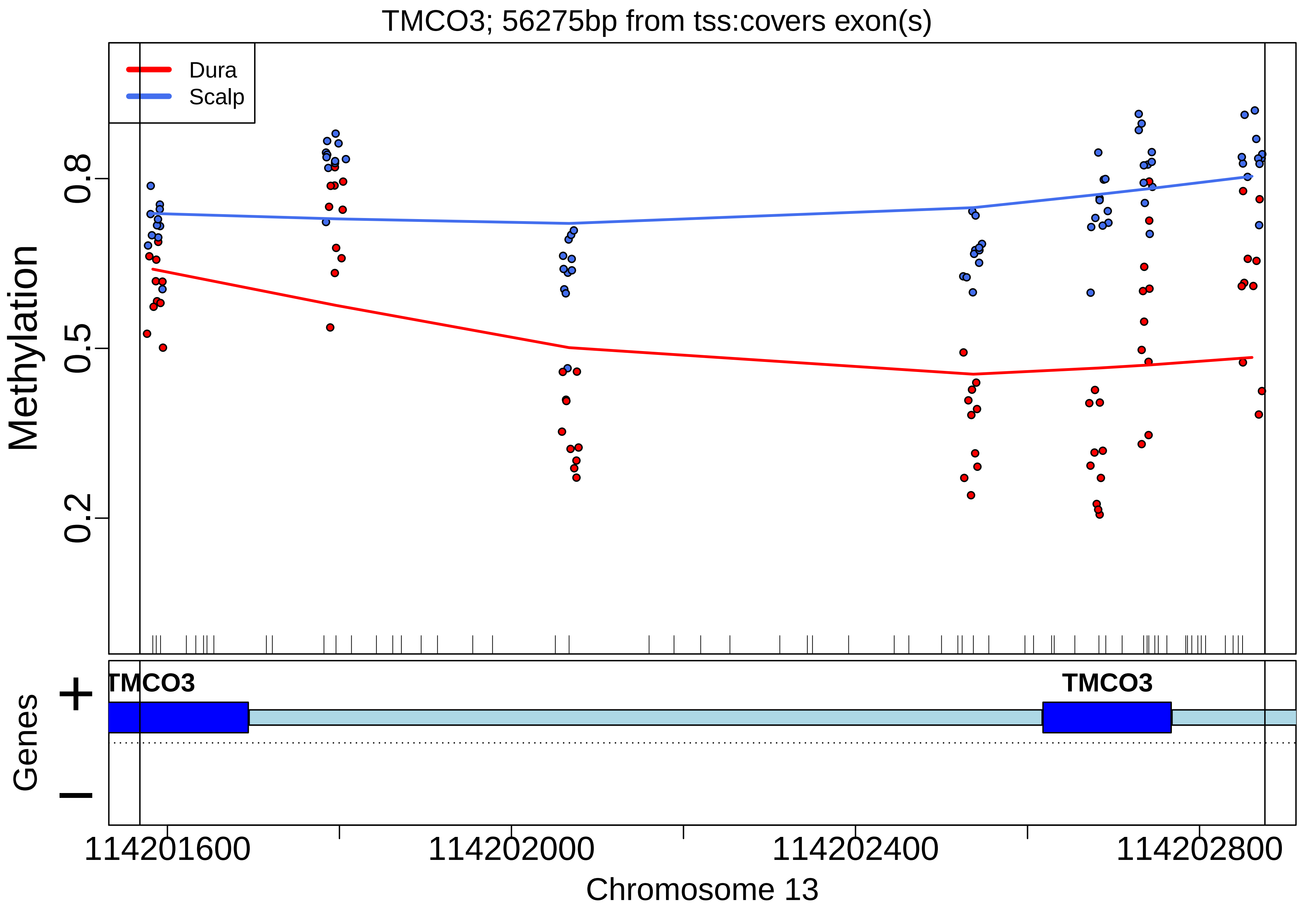
TMCO3; 56275bp from tss:covers exon(s)
Dura
Scalp
Methylation
0.2
0.5
0.8
Genes
+
−
TMCO3
TMCO3
114201600
114202000
114202400
114202800
Chromosome 13

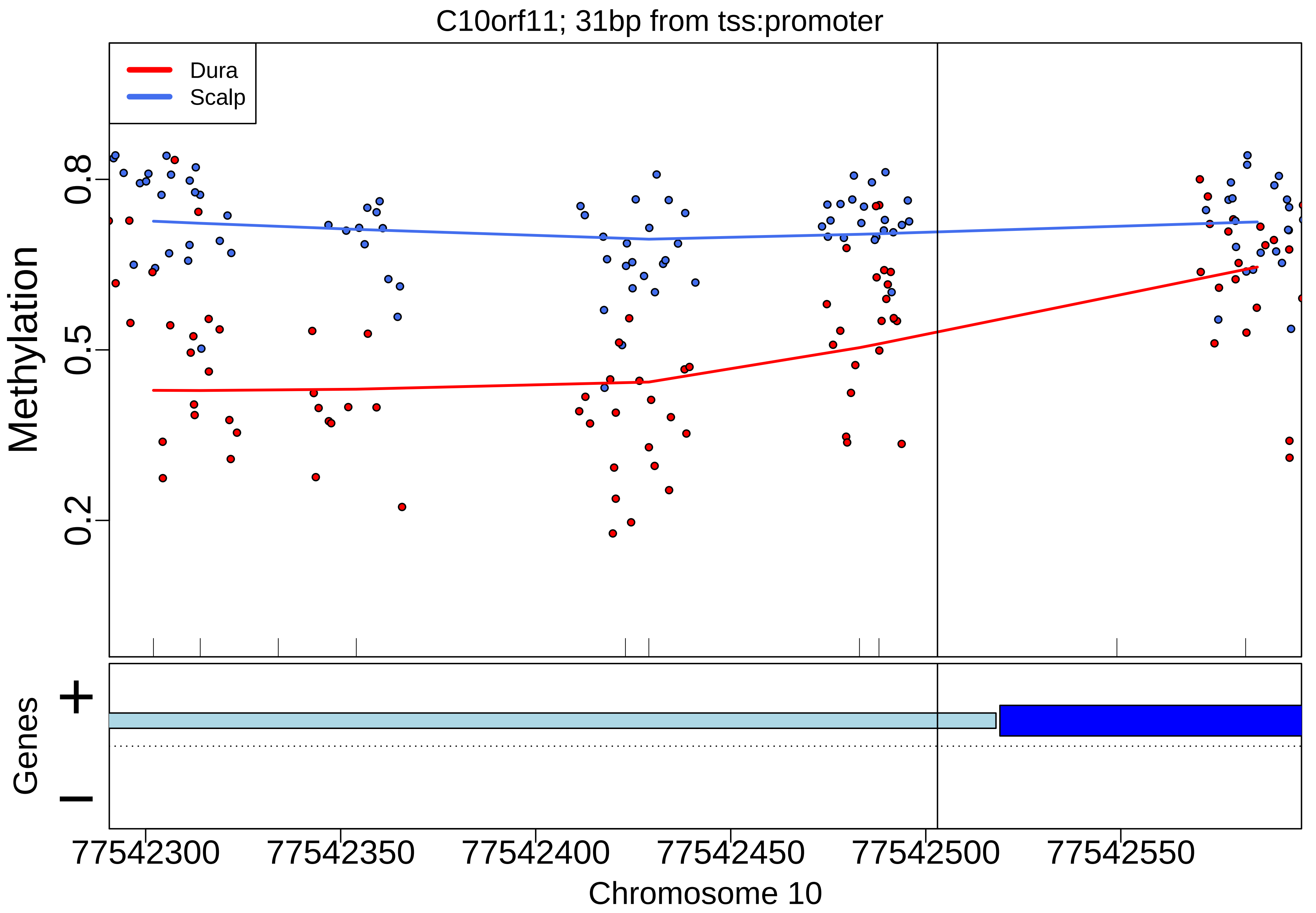

C10orf11; 31bp from tss:promoter
Dura
Scalp
Methylation
0.2
0.5
0.8
Genes
+
−
77542300
77542350
77542400
77542450
77542500
77542550
Chromosome 10

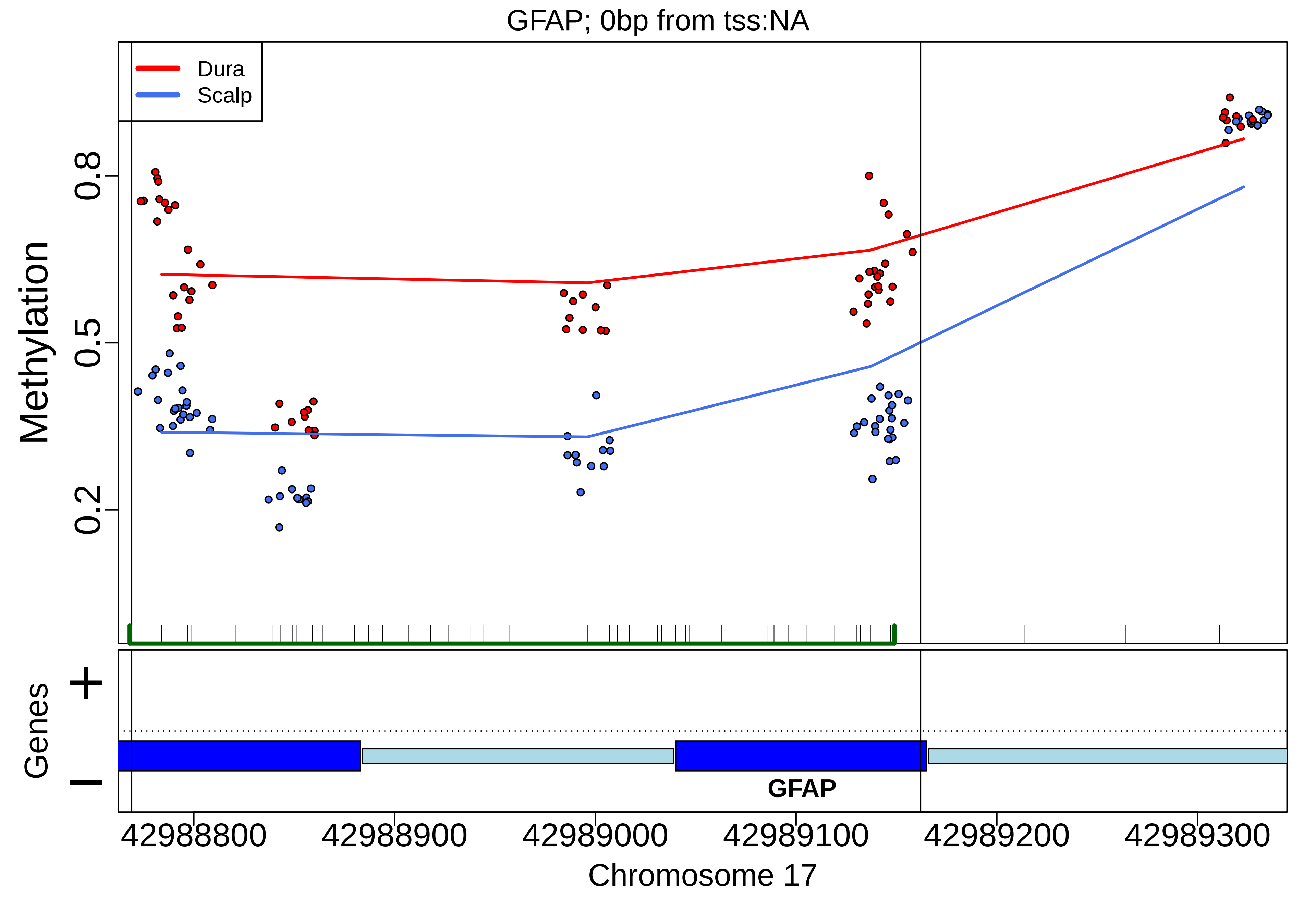

GFAP; 0bp from tss:NA
Dura
Scalp
Methylation
0.2
0.5
0.8
Genes
+
-
GFAP
42988800
42988900
42989000
42989100
42989200
42989300
Chromosome 17

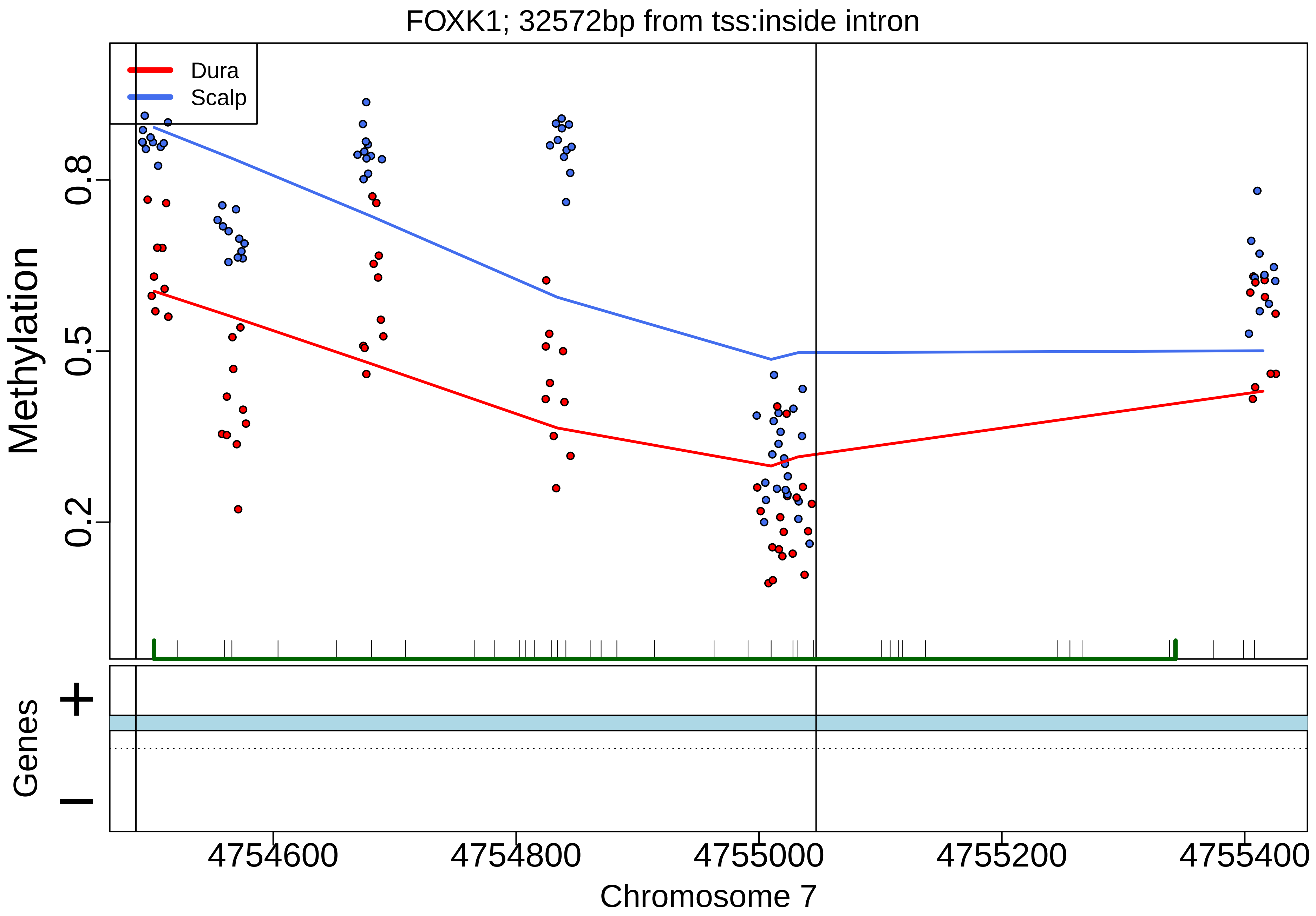

FOXK1; 32572bp from tss:inside intron
Dura
Scalp
Methylation
0.2
0.5
0.8
Genes
+
-
4754600
4754800
4755000
4755200
4755400
Chromosome 7

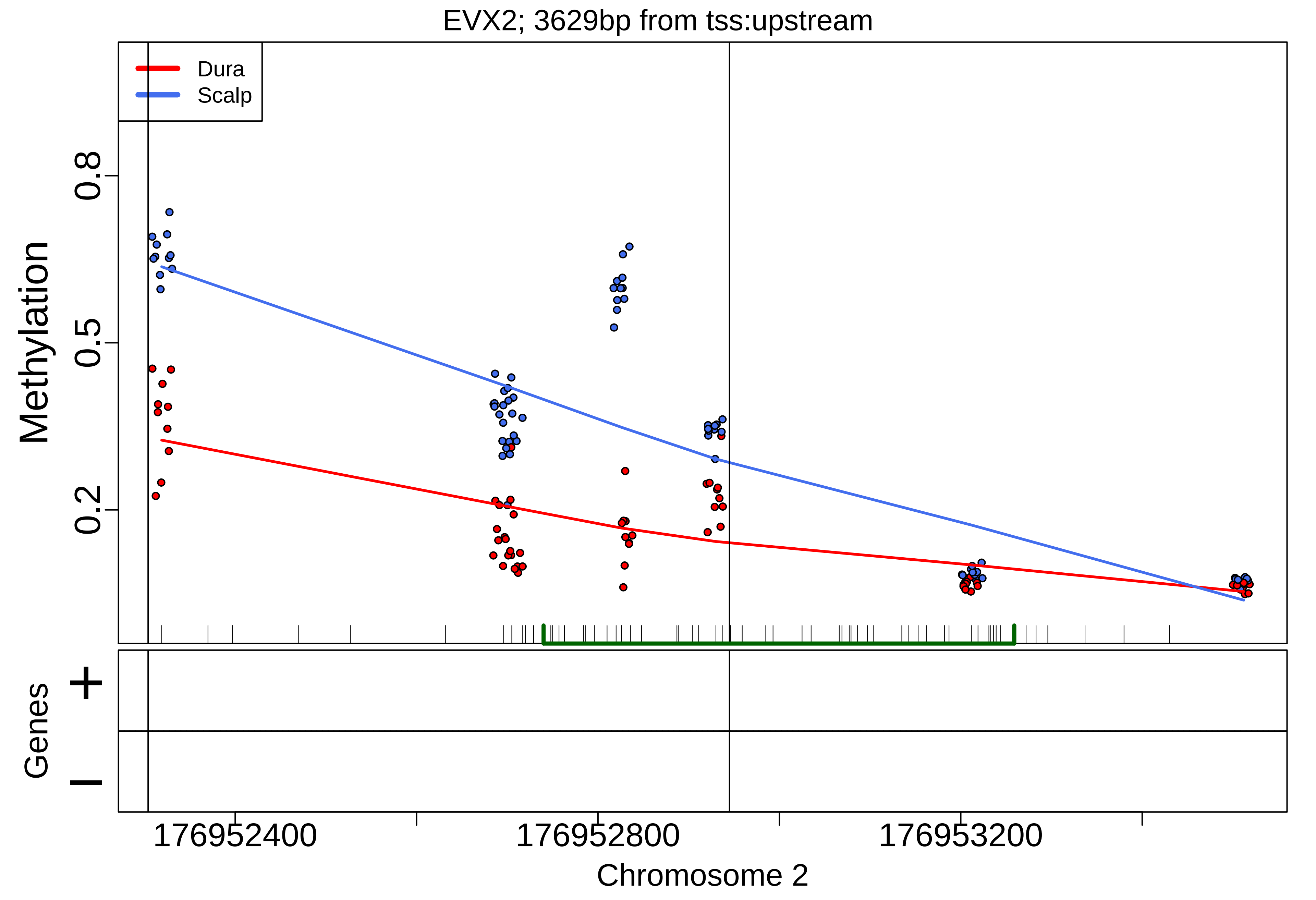
EVX2; 3629bp from tss:upstream
Dura
Scalp
Methylation
0.2
0.5
0.8
Genes
+
−
176952400
176952800
176953200
Chromosome 2

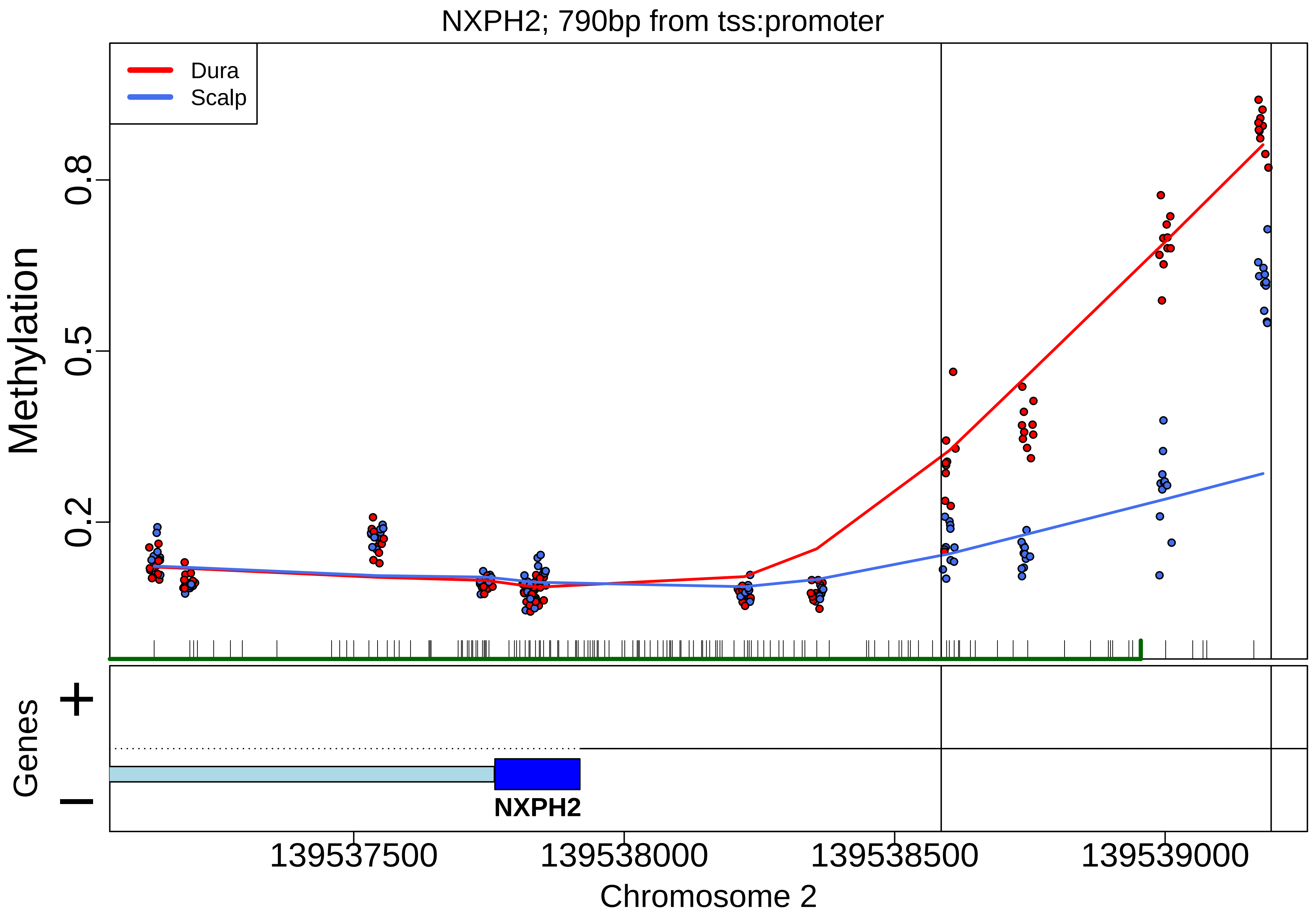

NXPH2; 790bp from tss:promoter
Dura
Scalp
Methylation
0.2
0.5
0.8
Genes
+
−
NXPH2
139537500
139538000
139538500
139539000
Chromosome 2

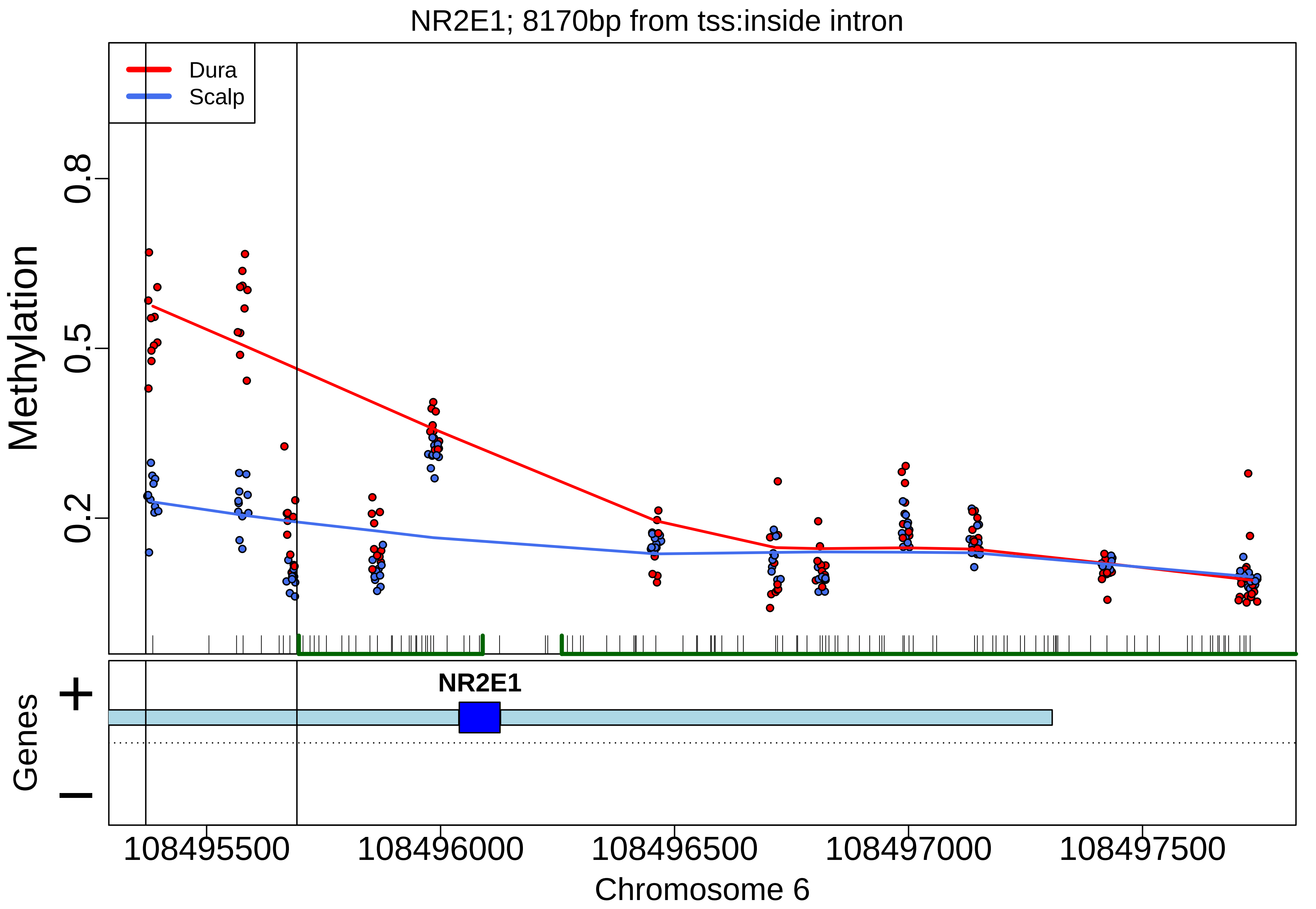

NR2E1; 8170bp from tss:inside intron
Dura
Scalp
Methylation
0.2
0.5
0.8
Genes
+
-
NR2E1
108495500
108496000
108496500
108497000
108497500
Chromosome 6

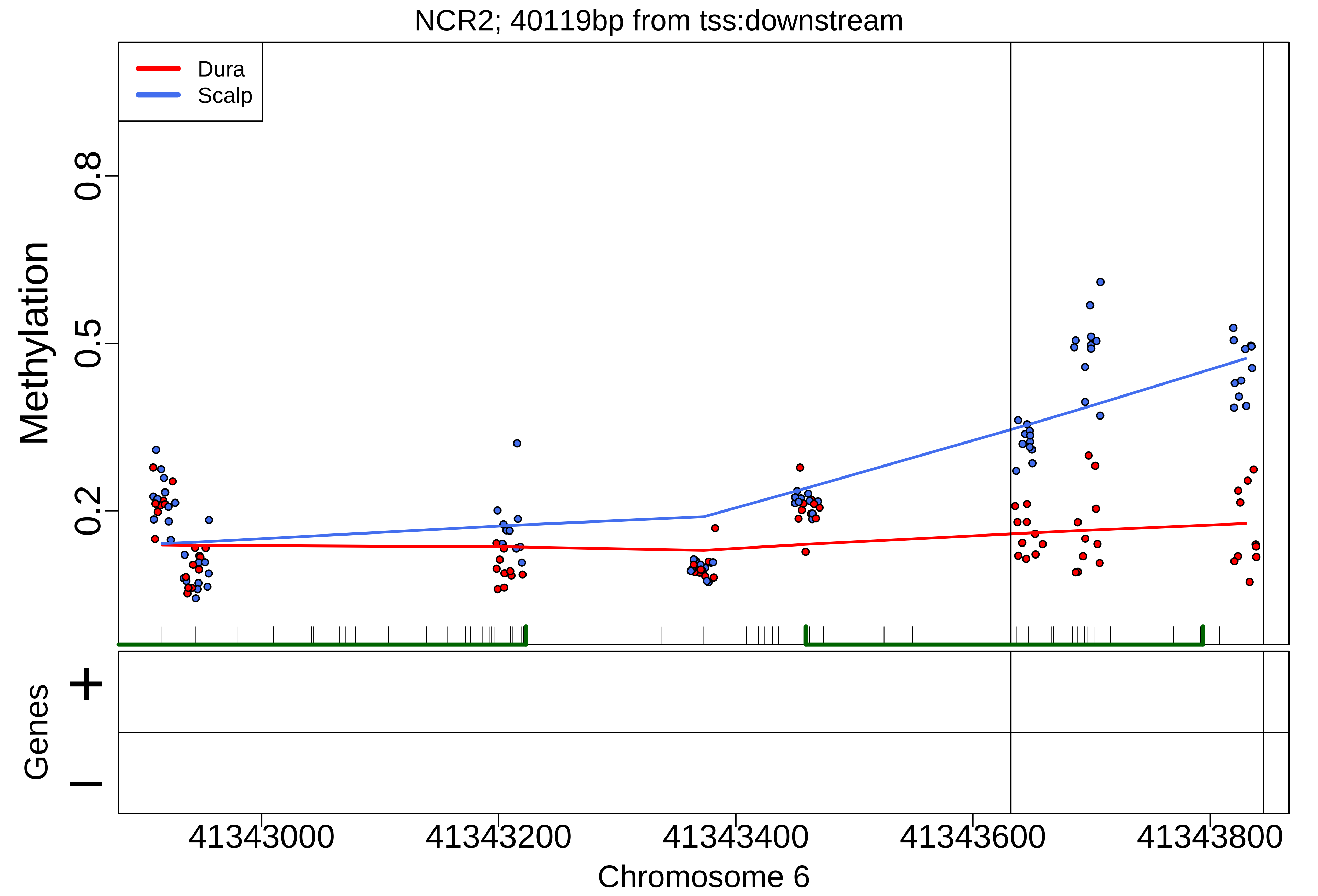
NCR2; 40119bp from tss:downstream
Dura
Scalp
Methylation
0.2
0.5
0.8
Genes
+
−
41343000
41343200
41343400
41343600
41343800
Chromosome 6

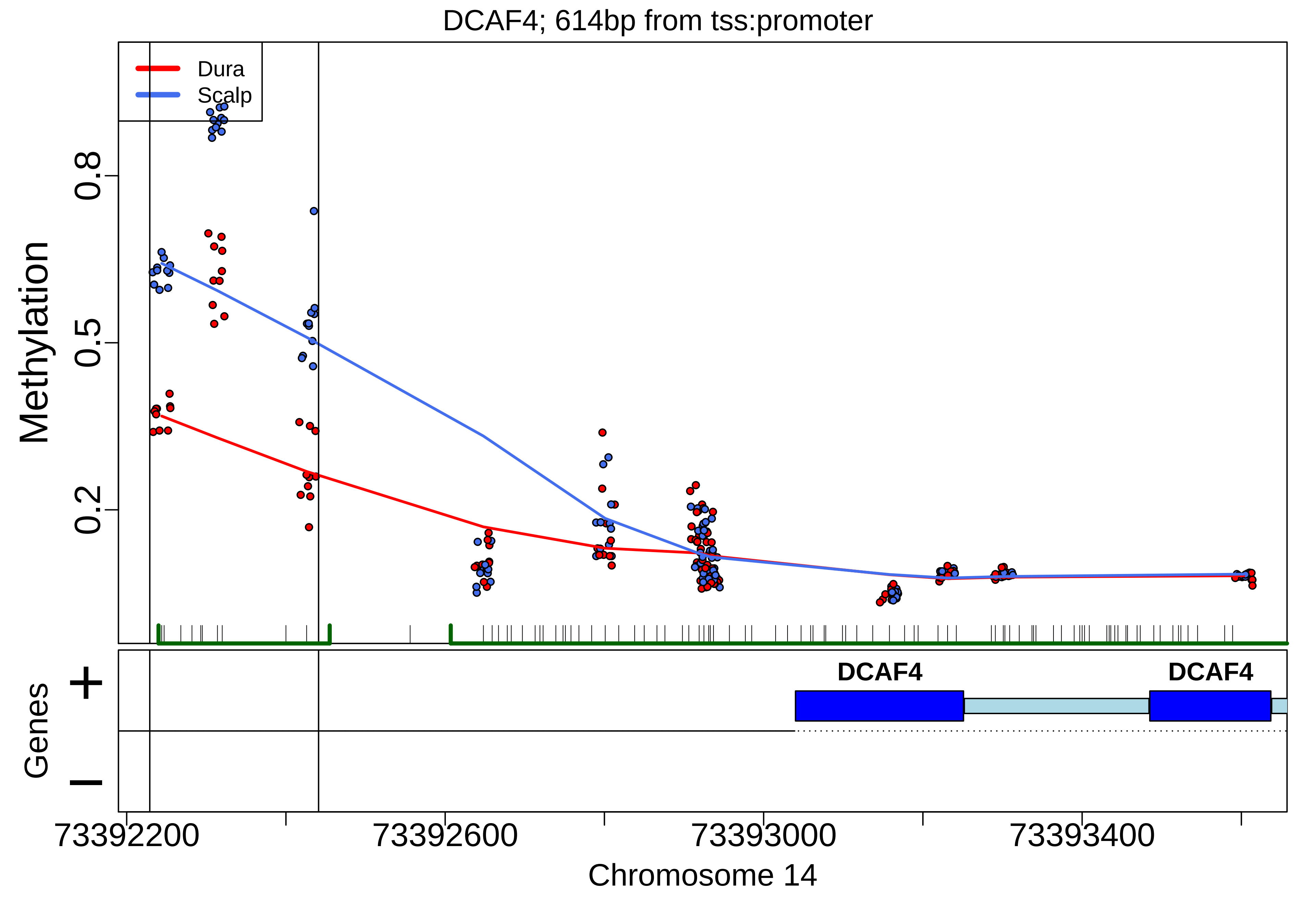
DCAF4; 614bp from tss:promoter
Dura
Scalp
Methylation
0.2
0.5
0.8
Genes
+
-
DCAF4
DCAF4
73392200
73392600
73393000
73393400
Chromosome 14

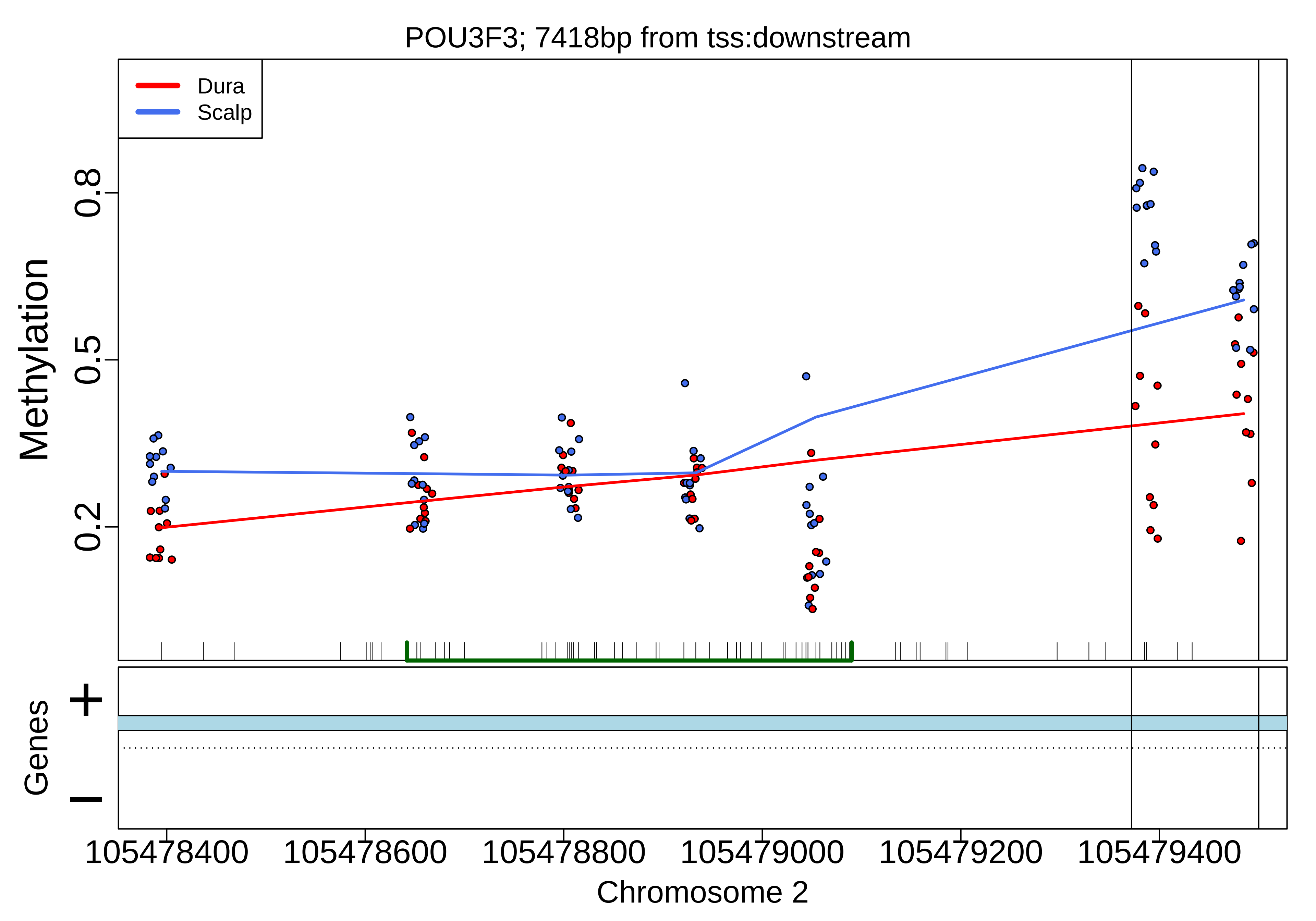

POU3F3; 7418bp from tss:downstream
Dura
Scalp
Methylation
0.2
0.5
0.8
Genes
+
−
105478400
105478600
105478800
105479000
105479200
105479400
Chromosome 2

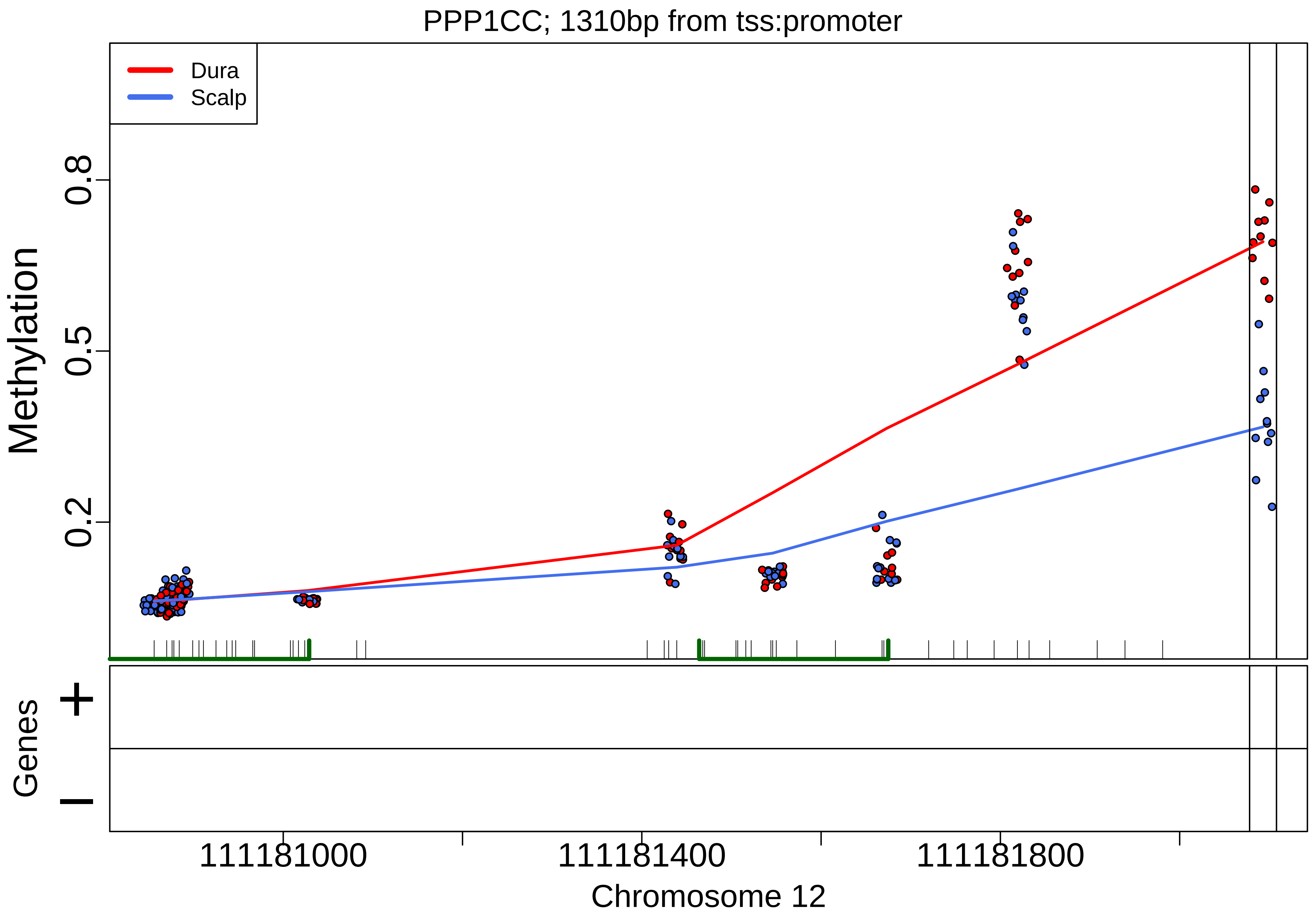
PPP1CC; 1310bp from tss:promoter
Dura
Scalp
Methylation
0.2
0.5
0.8
Genes
+
−
111181000
111181400
111181800
Chromosome 12

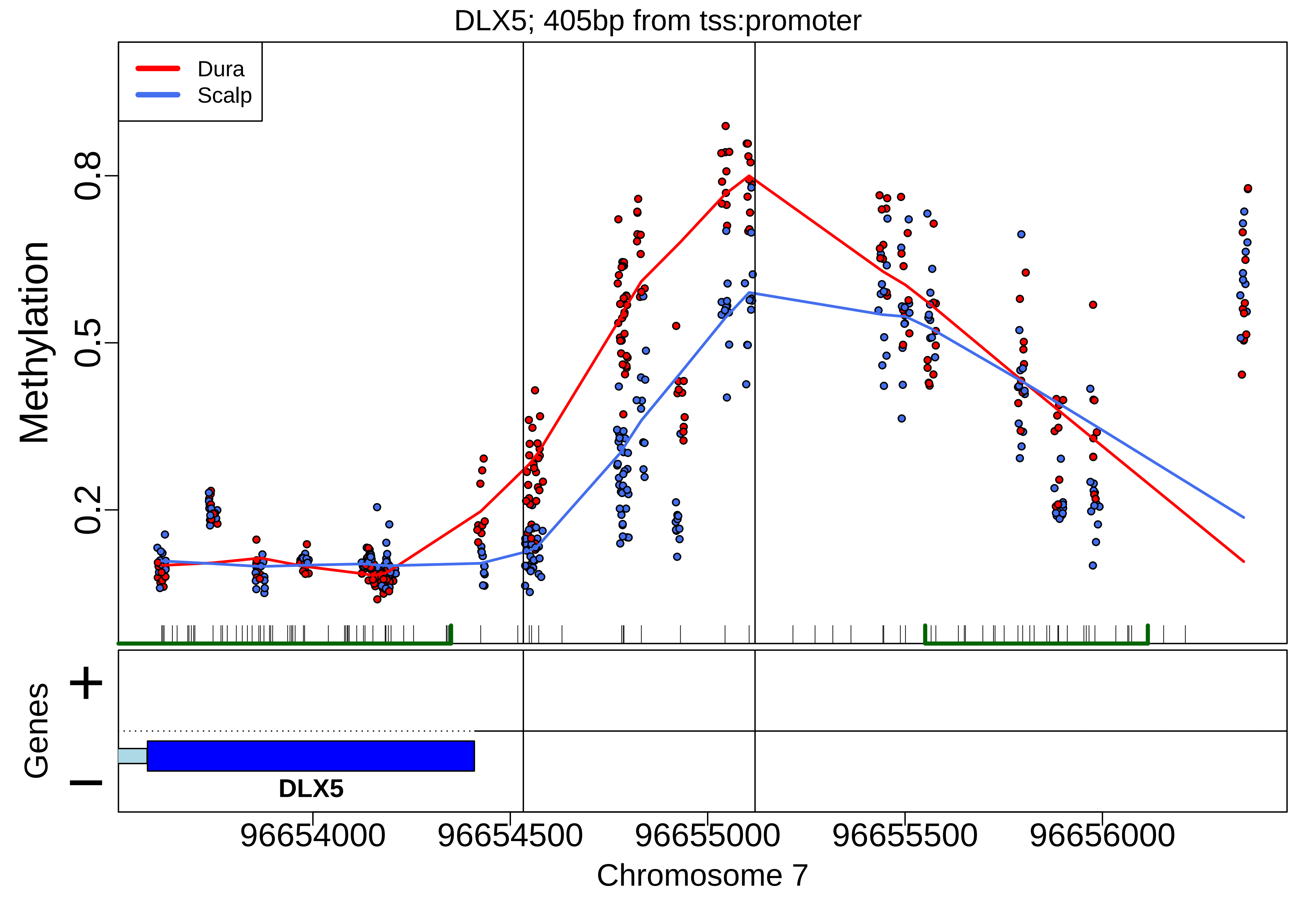
DLX5; 405bp from tss:promoter
Dura
Scalp
Methylation
0.2
0.5
0.8
Genes
+
−
DLX5
96654000
96654500
96655000
96655500
96656000
Chromosome 7

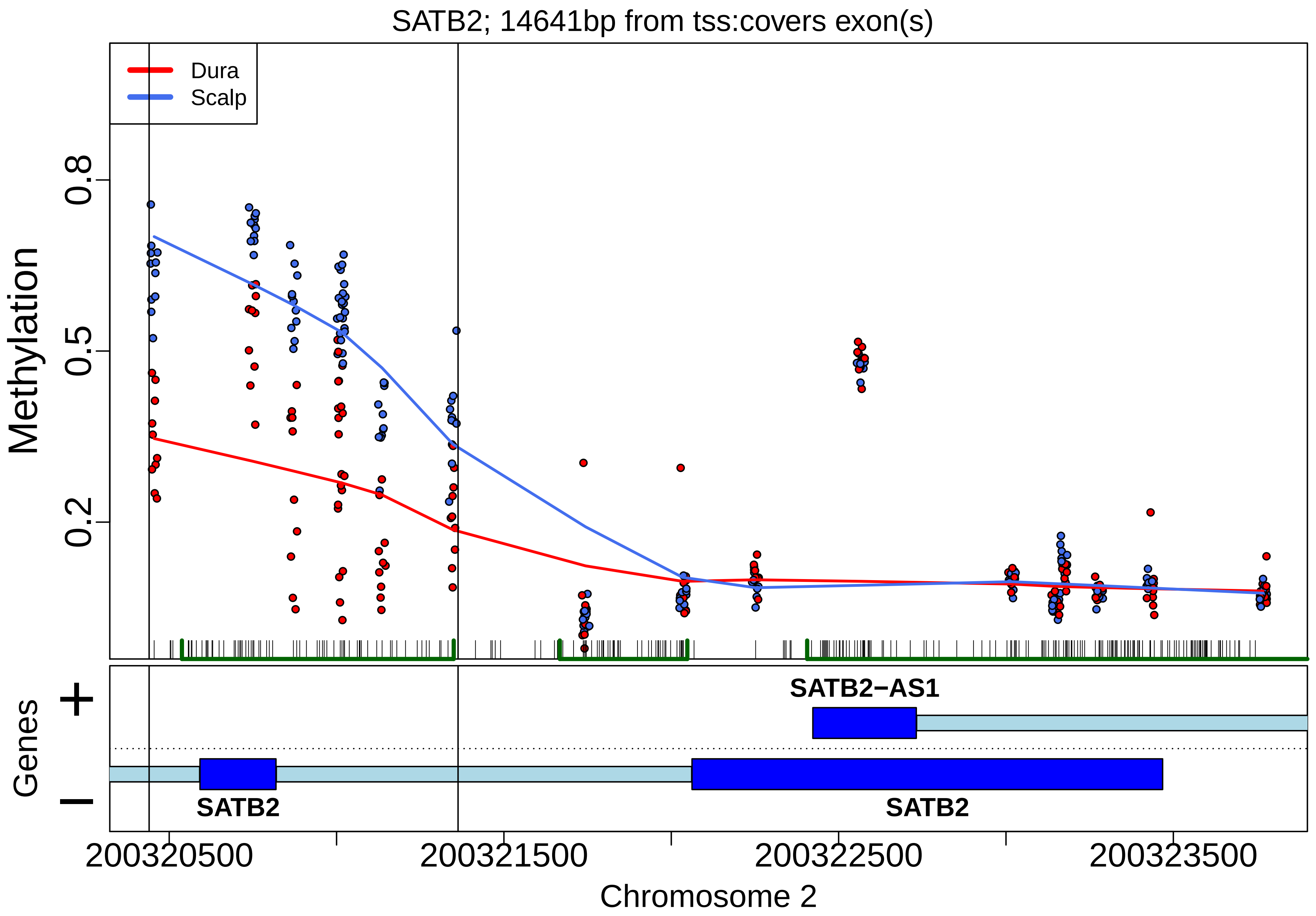

SATB2; 14641bp from tss:covers exon(s)
Dura
Scalp
Methylation
0.8
0.5
0.2
Genes
+
−
SATB2−AS1
SATB2
SATB2
200320500
200321500
200322500
200323500
Chromosome 2

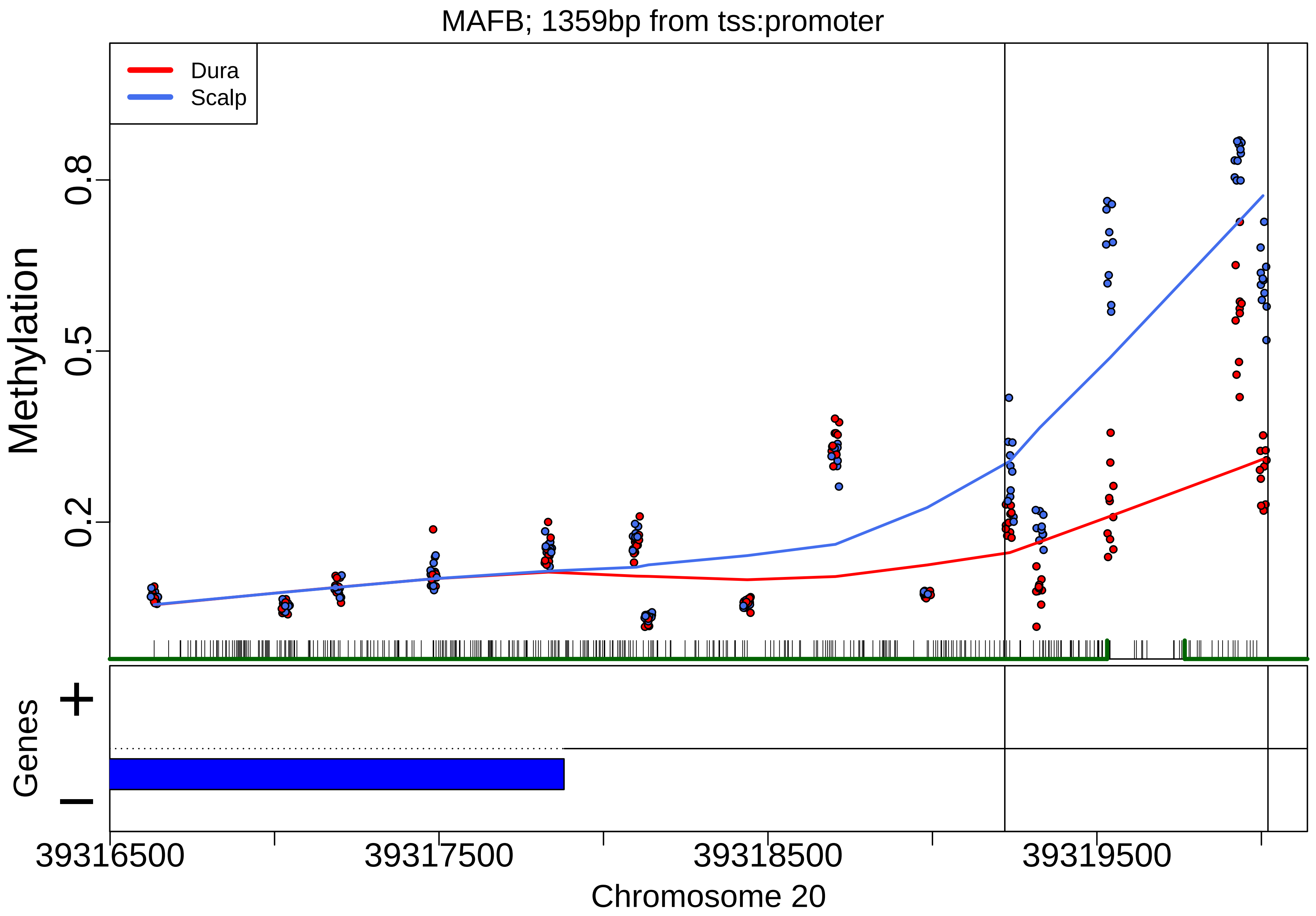
MAFB; 1359bp from tss:promoter
Dura
Scalp
Methylation
0.2
0.5
0.8
Genes
+
−
39316500
39317500
39318500
39319500
Chromosome 20

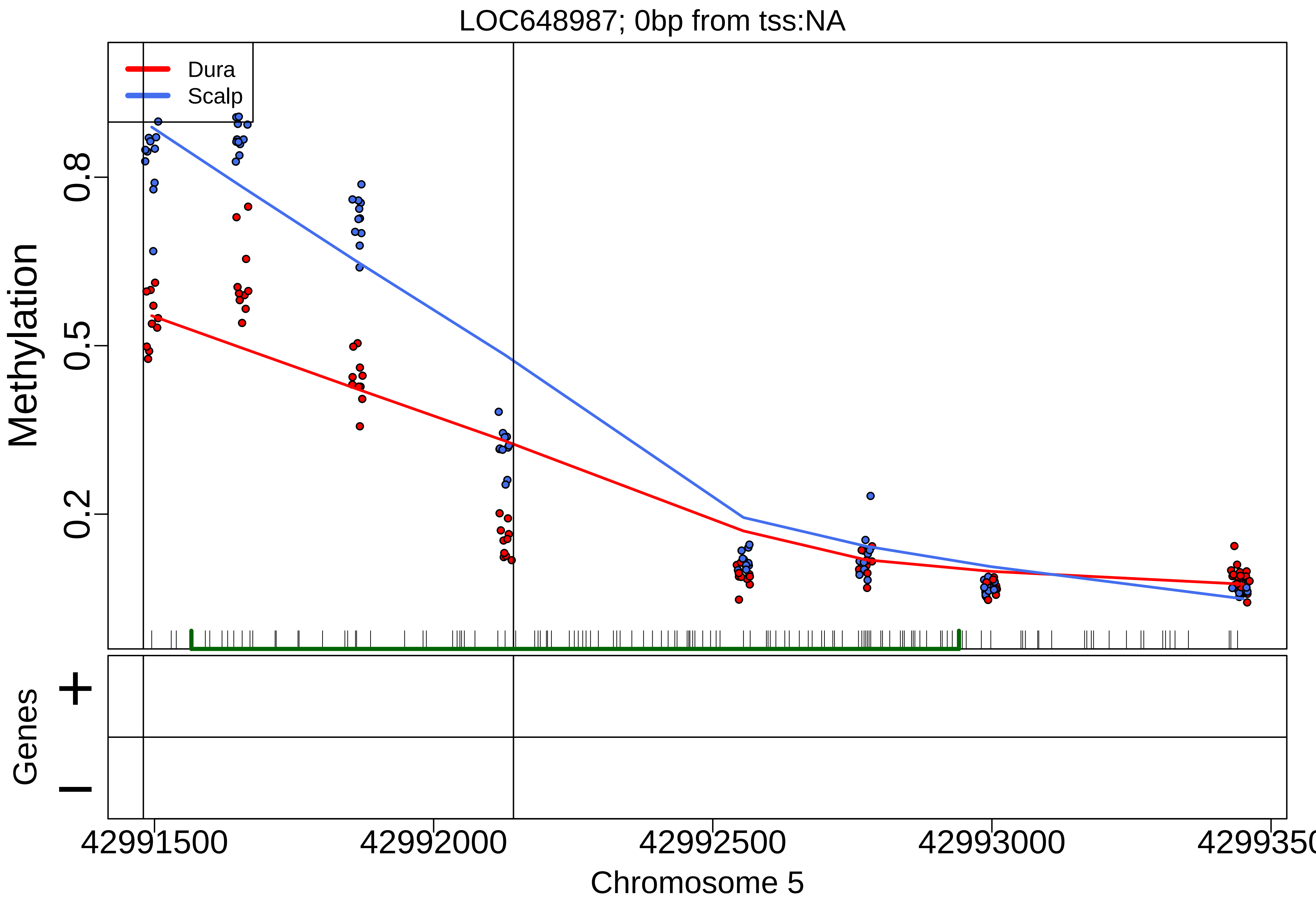

LOC648987; 0bp from tss:NA
Dura
Scalp
Methylation
0.8
0.5
0.2
Genes
+
−
42991500
42992000
42992500
42993000
4299350
Chromosome 5

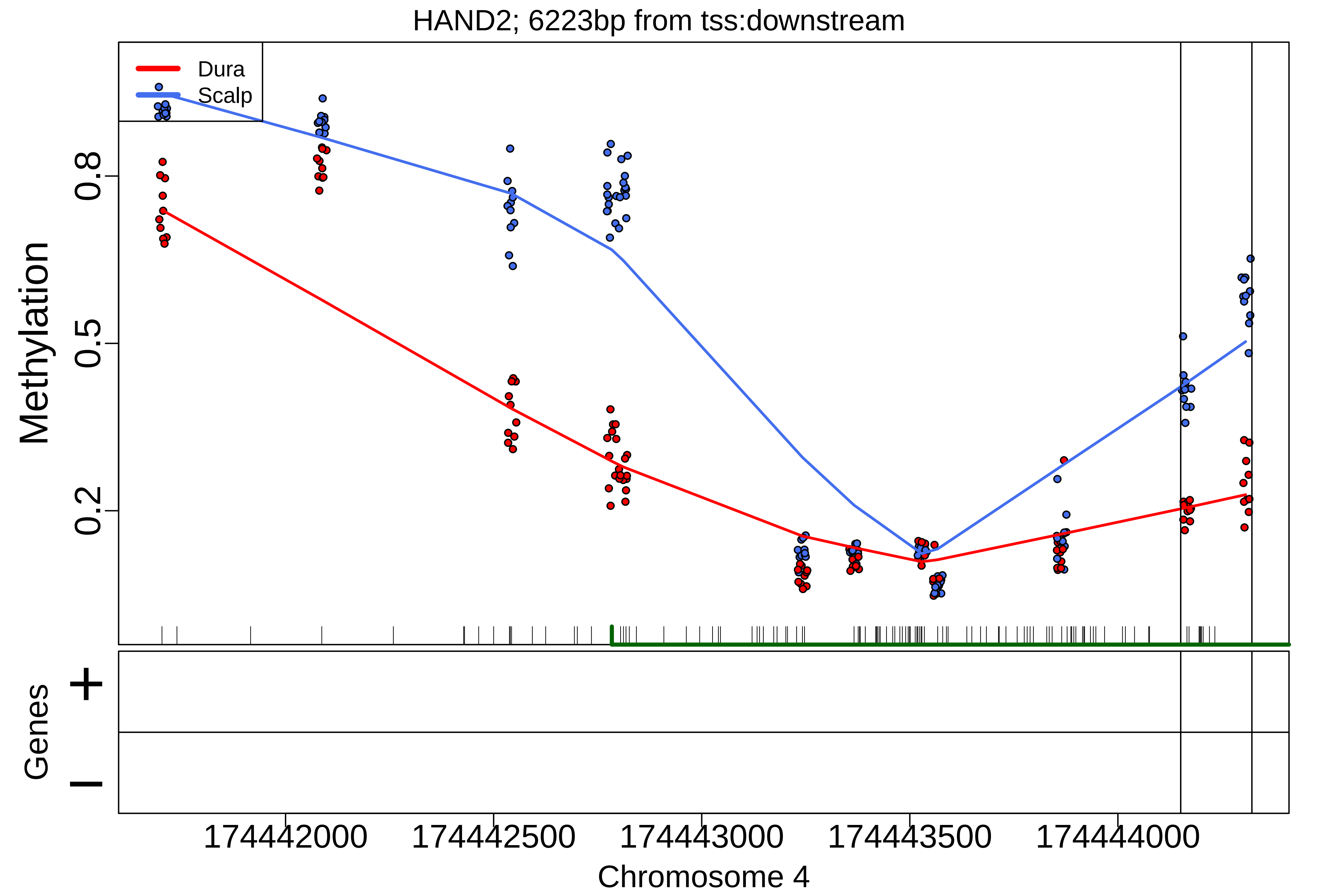

HAND2; 6223bp from tss:downstream
Dura
Scalp
Methylation
0.2
0.5
0.8
Genes
+
−
174442000
174442500
174443000
174443500
174444000
Chromosome 4

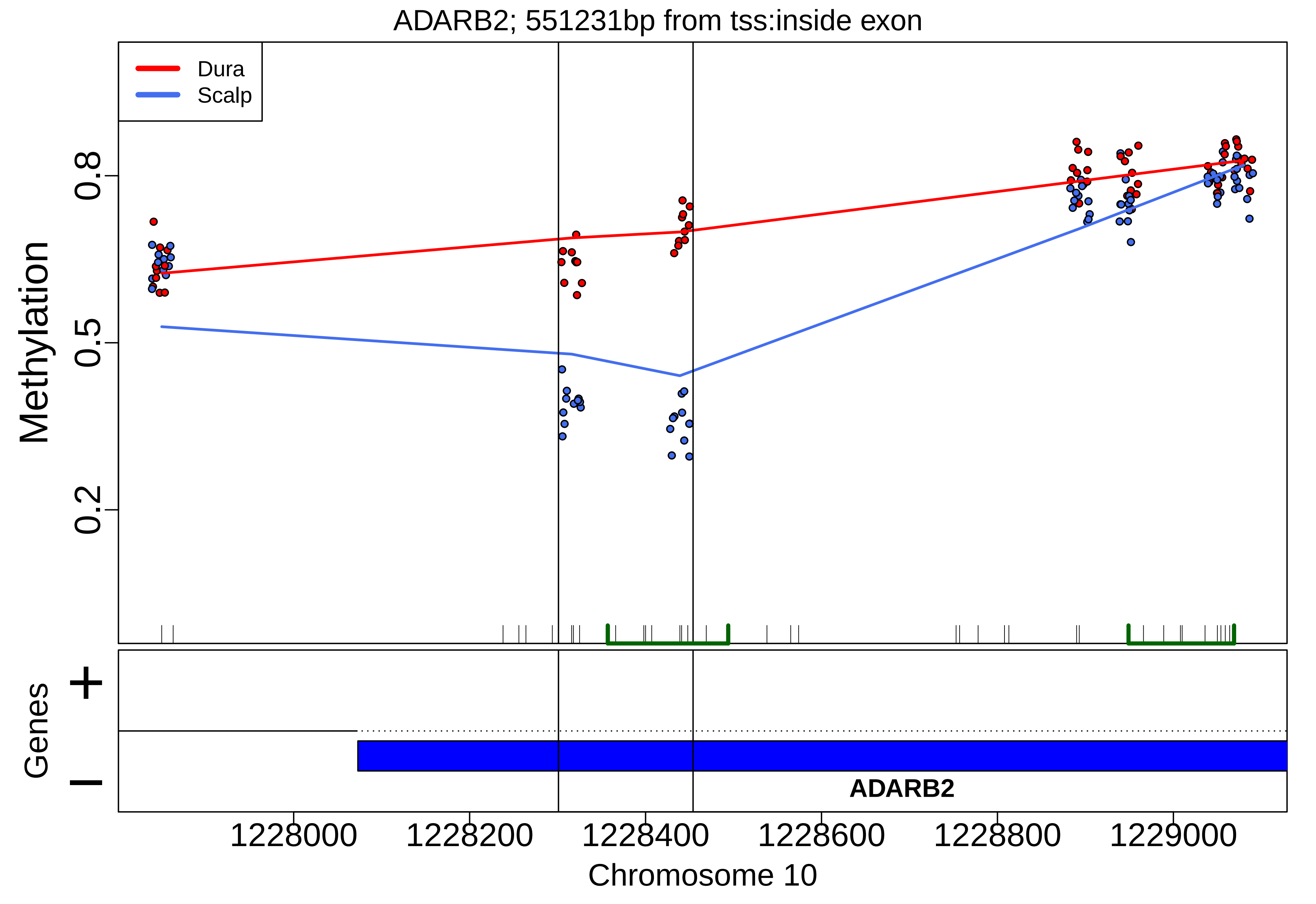
ADARB2; 551231bp from tss:inside exon
Dura
Scalp
Methylation
0.2
0.5
0.8
Genes
+
–
ADARB2
1228000
1228200
1228400
1228600
1228800
1229000
Chromosome 10

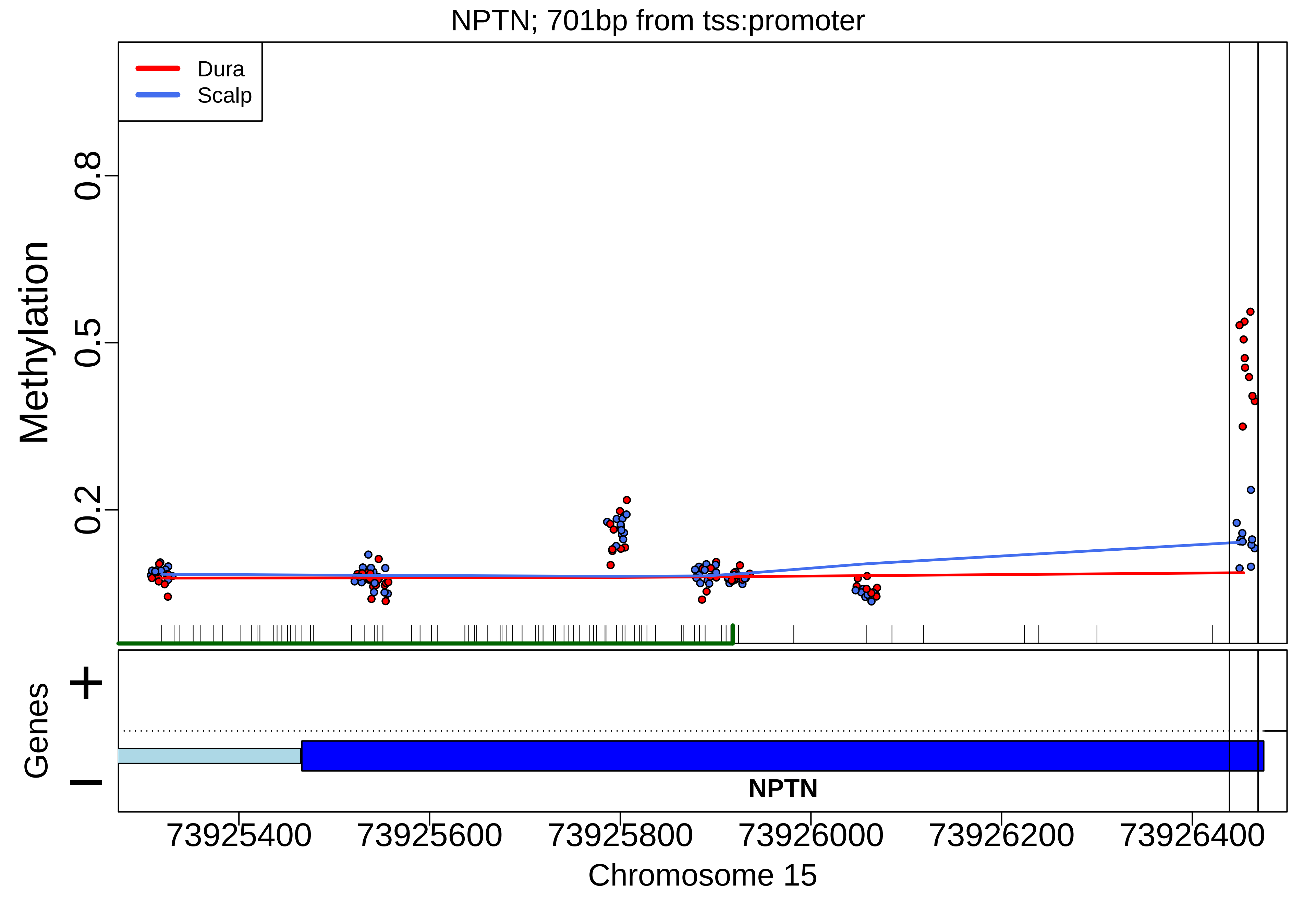

NPTN; 701bp from tss:promoter
Dura
Scalp
Methylation
0.2
0.5
0.8
Genes
+
-
NPTN
73925400
73925600
73925800
73926000
73926200
73926400
Chromosome 15

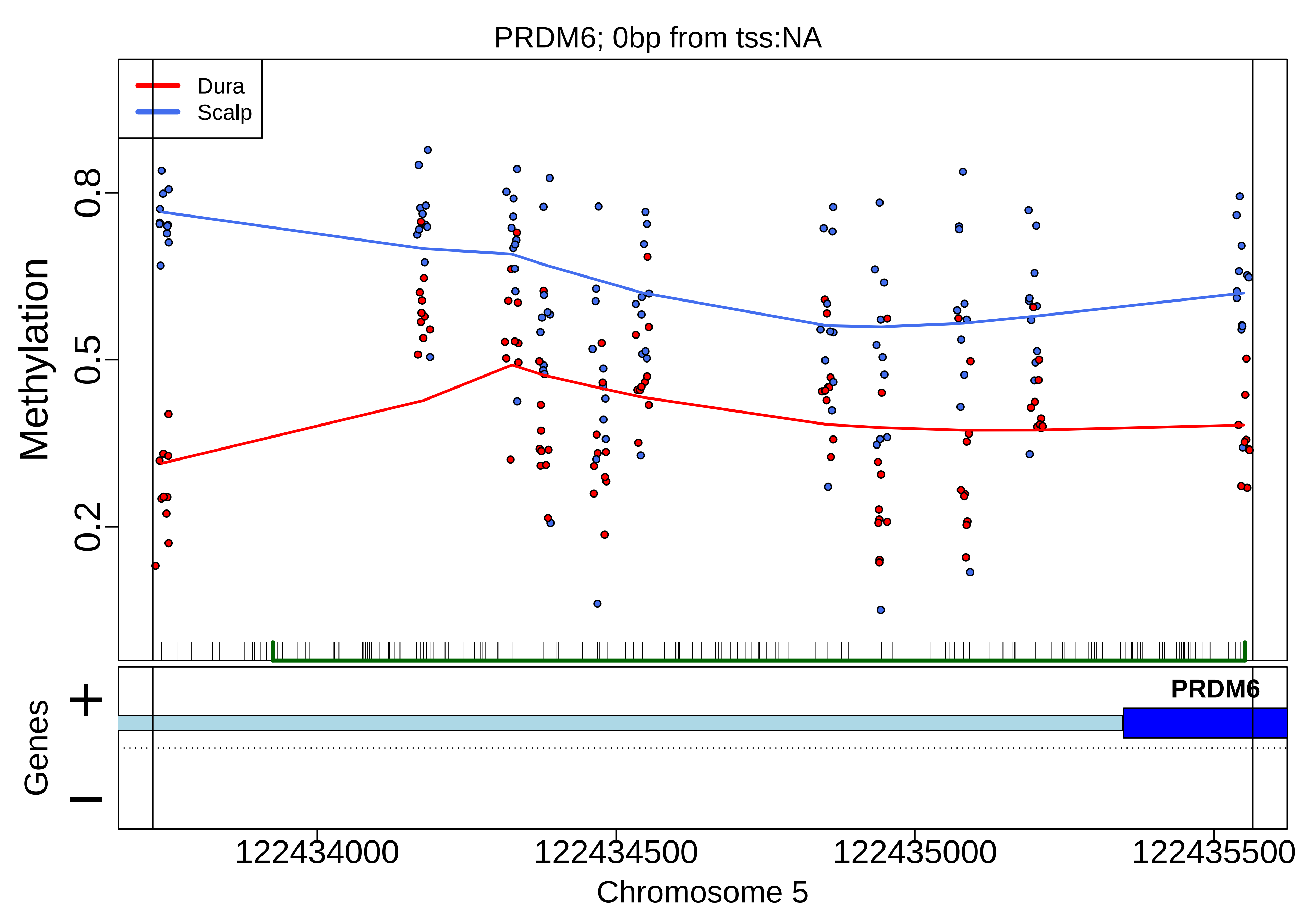
PRDM6; 0bp from tss:NA
Dura
Scalp
Methylation
0.8
0.5
0.2
Genes
+
-
PRDM6
122434000
122434500
122435000
122435500
Chromosome 5

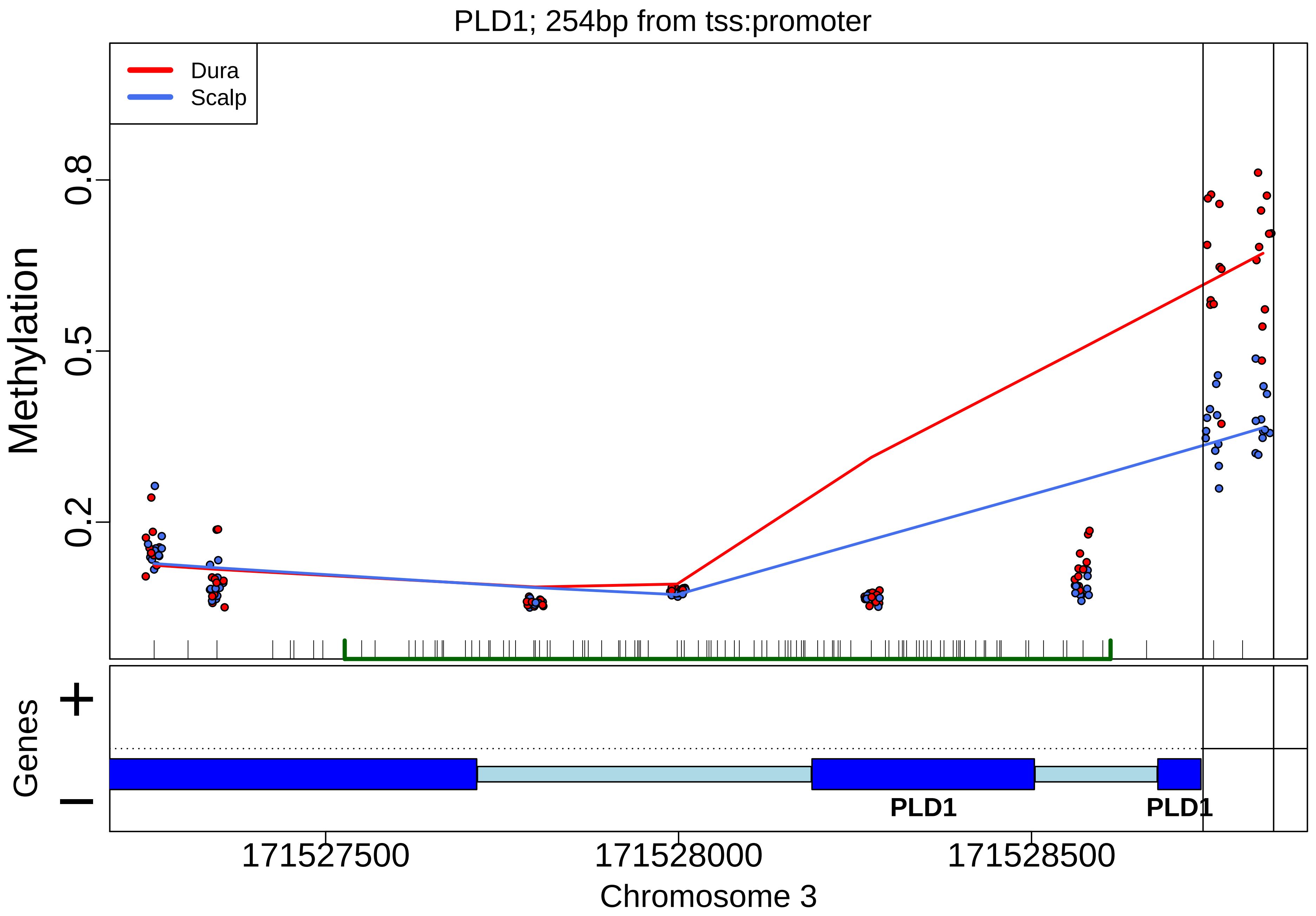

PLD1; 254bp from tss:promoter
Dura
Scalp
Methylation
0.2
0.5
0.8
Genes
+
-
PLD1
PLD1
171527500
171528000
171528500
Chromosome 3

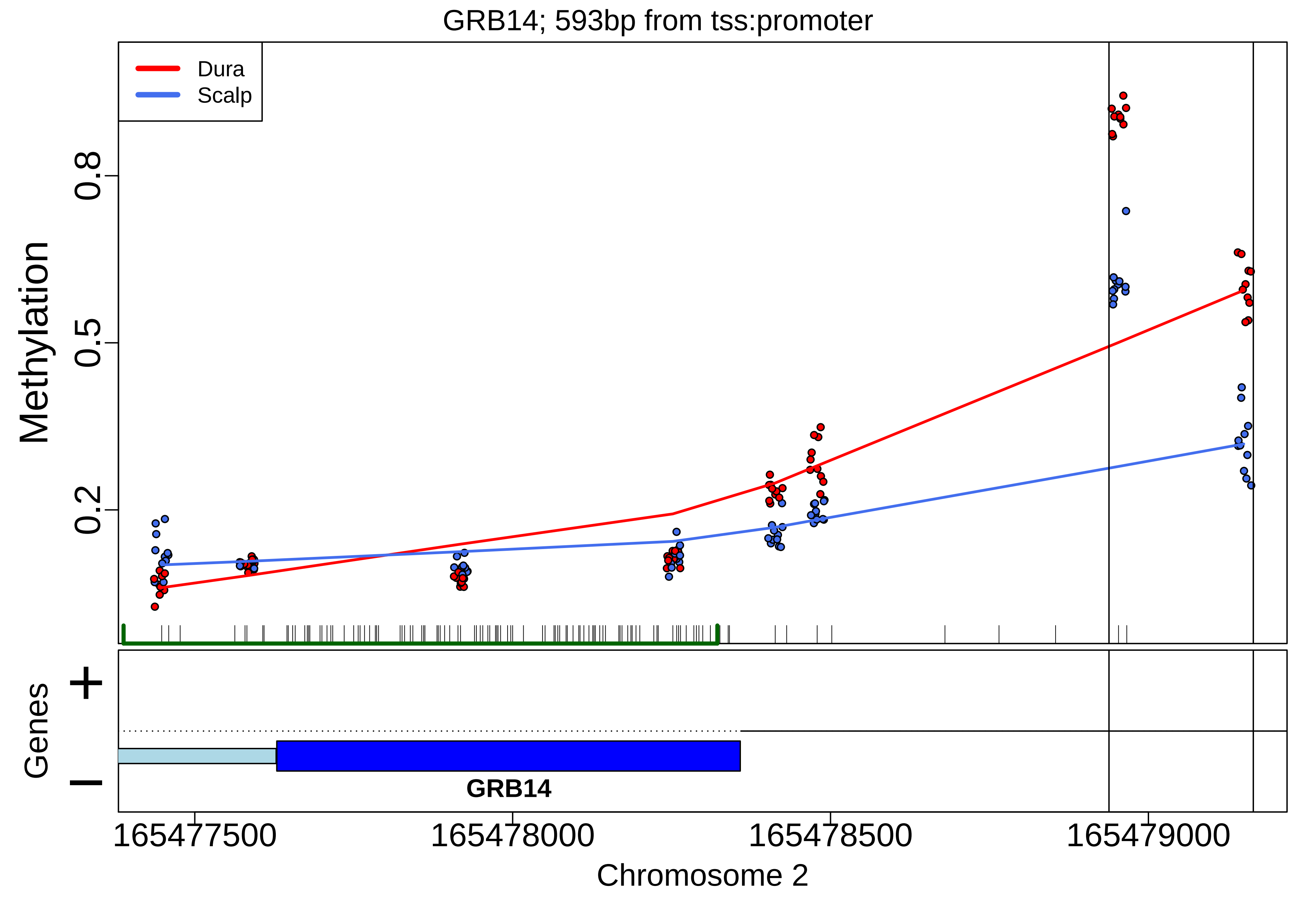
GRB14; 593bp from tss:promoter
Dura
Scalp
Methylation
0.2
0.5
0.8
Genes
+
-
GRB14
165477500
165478000
165478500
165479000
Chromosome 2

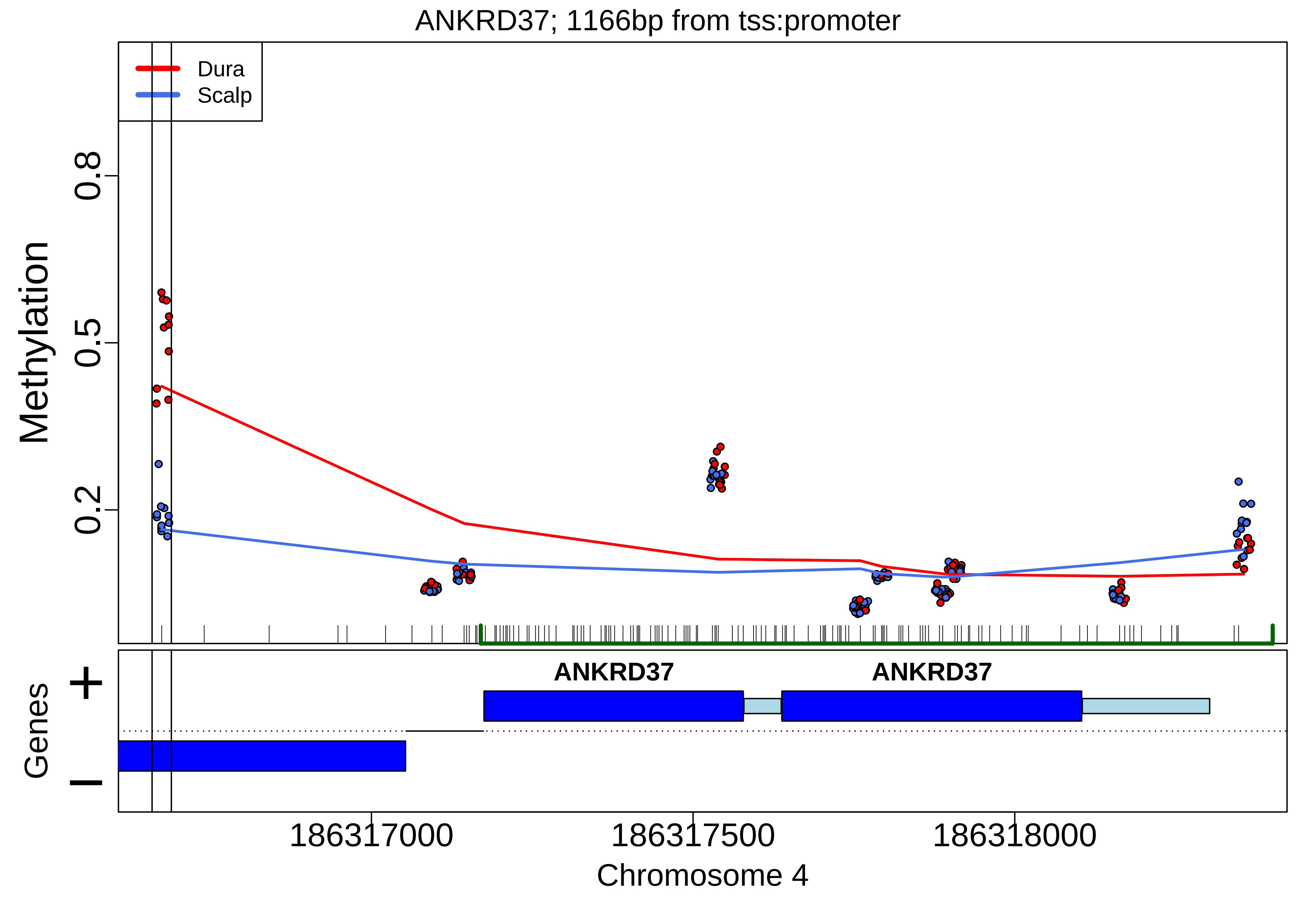

ANKRD37; 1166bp from tss:promoter
Dura
Scalp
Methylation
0.2
0.5
0.8
Genes
+
-
ANKRD37
ANKRD37
186317000
186317500
186318000
Chromosome 4

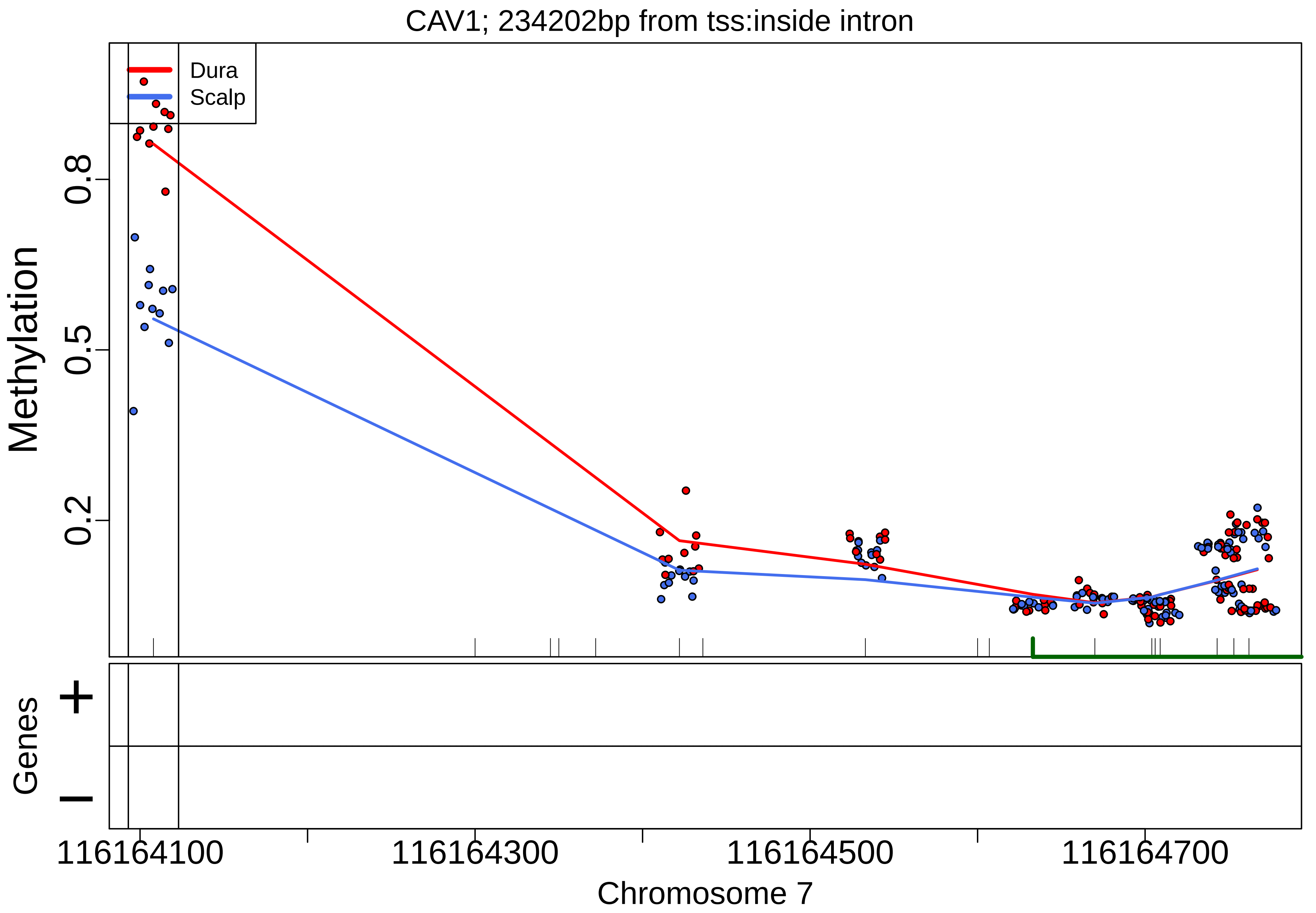

CAV1; 234202bp from tss:inside intron
Dura
Scalp
Methylation
0.8
0.5
0.2
Genes
+
-
116164100
116164300
116164500
116164700
Chromosome 7

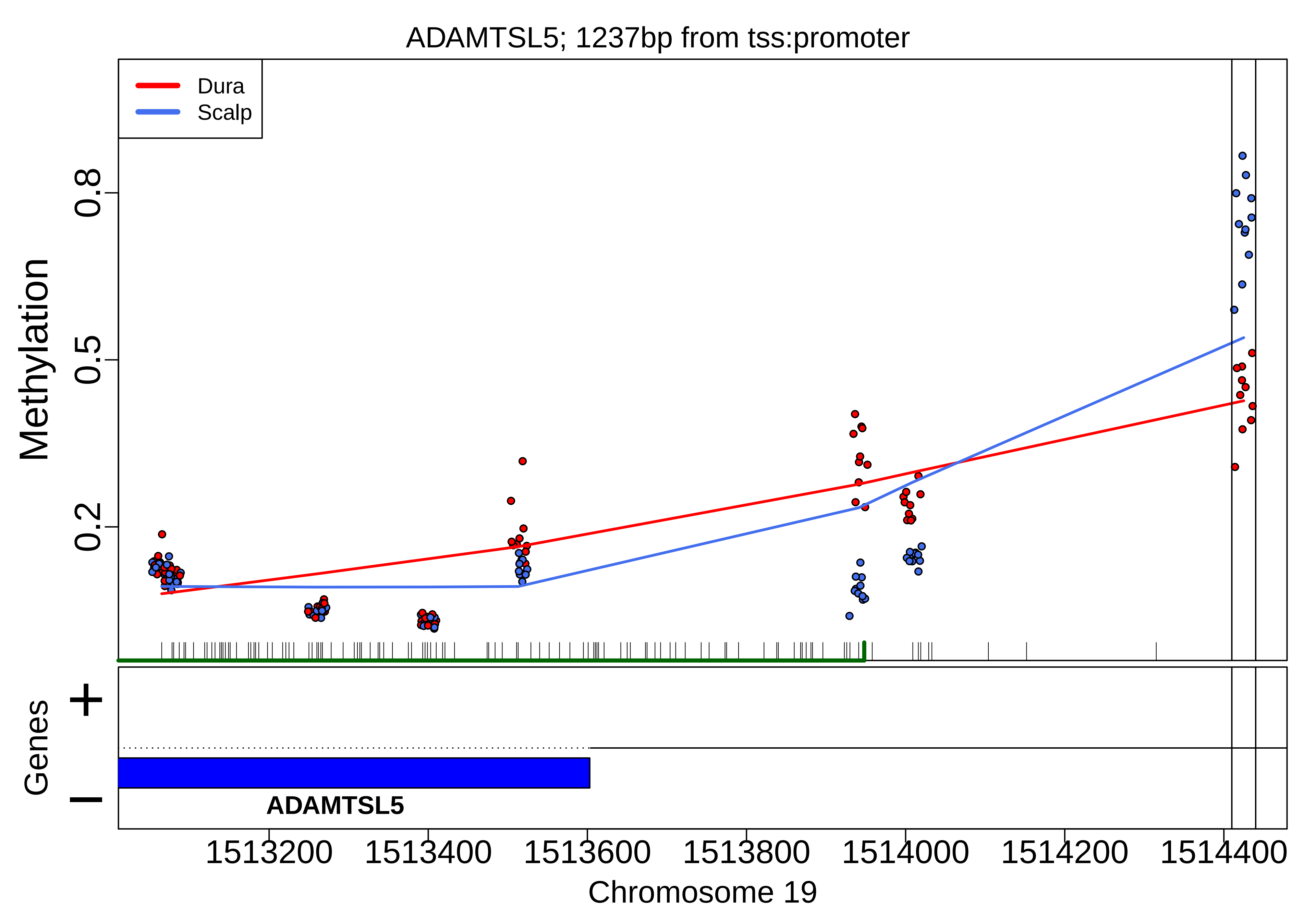

ADAMTSL5; 1237bp from tss:promoter
Dura
Scalp
Methylation
0.2
0.5
0.8
Genes
+
−
ADAMTSL5
1513200
1513400
1513600
1513800
1514000
1514200
1514400
Chromosome 19

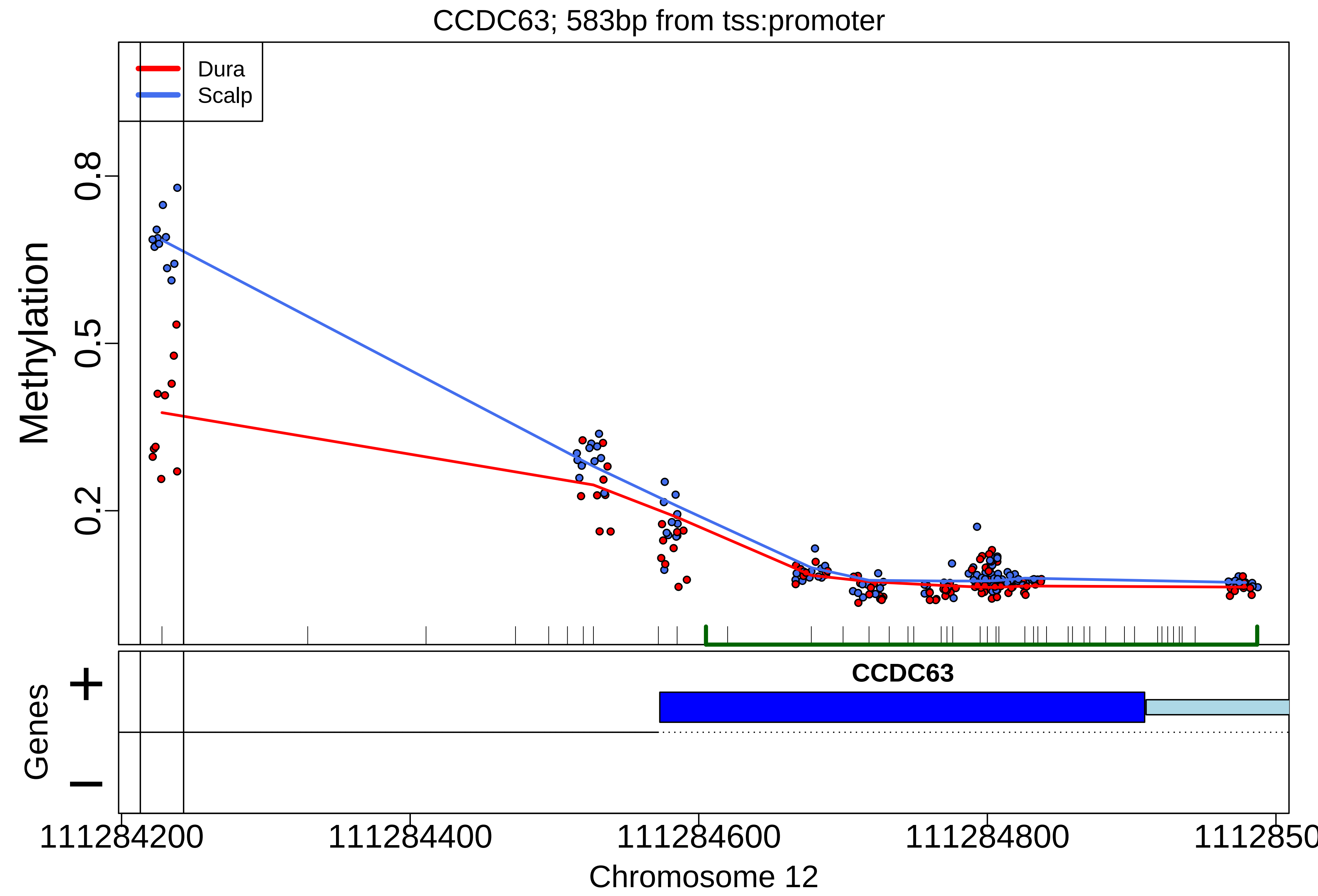
CCDC63; 583bp from tss:promoter
Dura
Scalp
Methylation
0.2
0.5
0.8
Genes
+
−
CCDC63
111284200
111284400
111284600
111284800
1112850
Chromosome 12

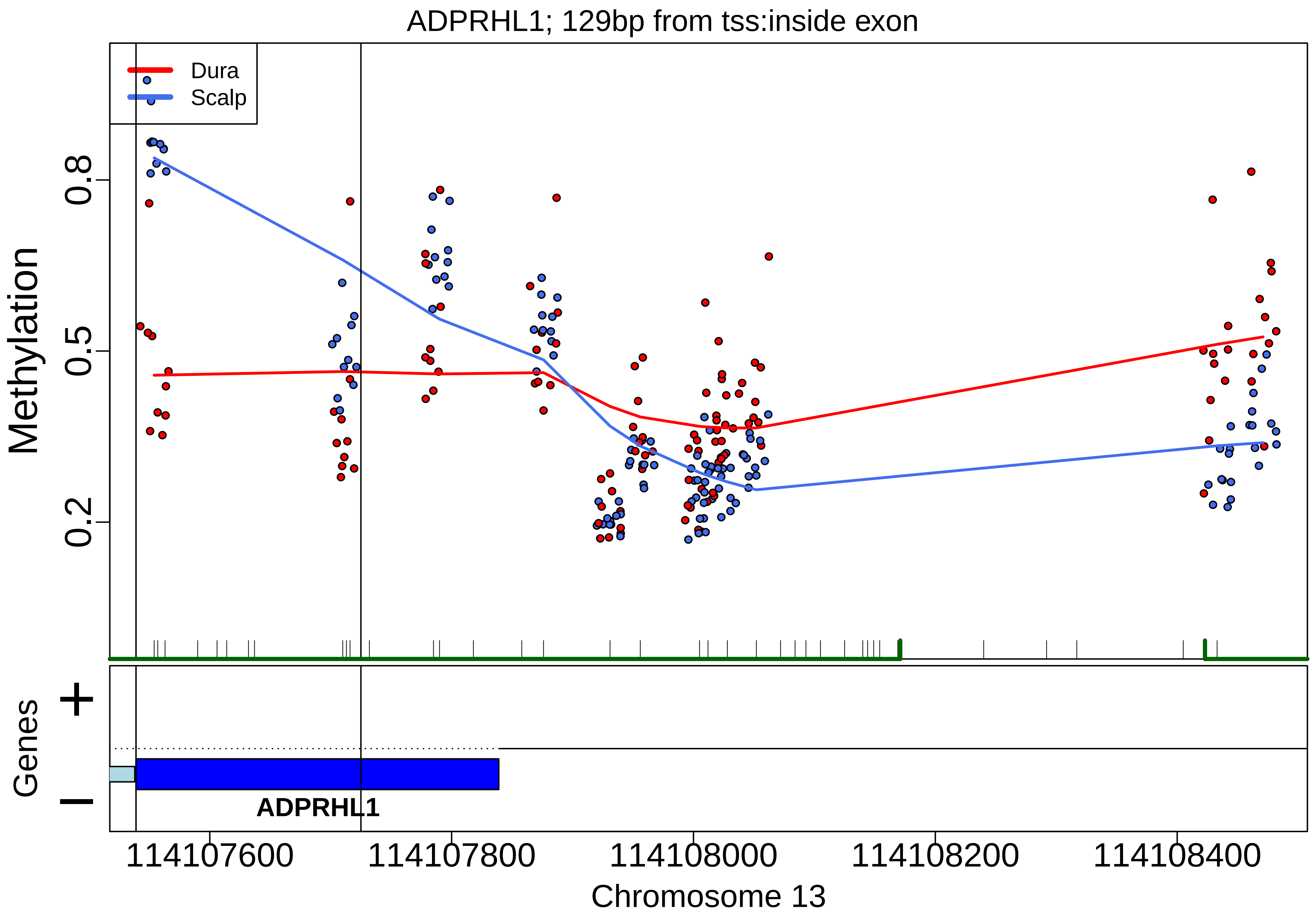

ADPRHL1; 129bp from tss:inside exon
Dura
Scalp
Methylation
0.2
0.5
0.8
Genes
+
−
ADPRHL1
114107600
114107800
114108000
114108200
114108400
Chromosome 13

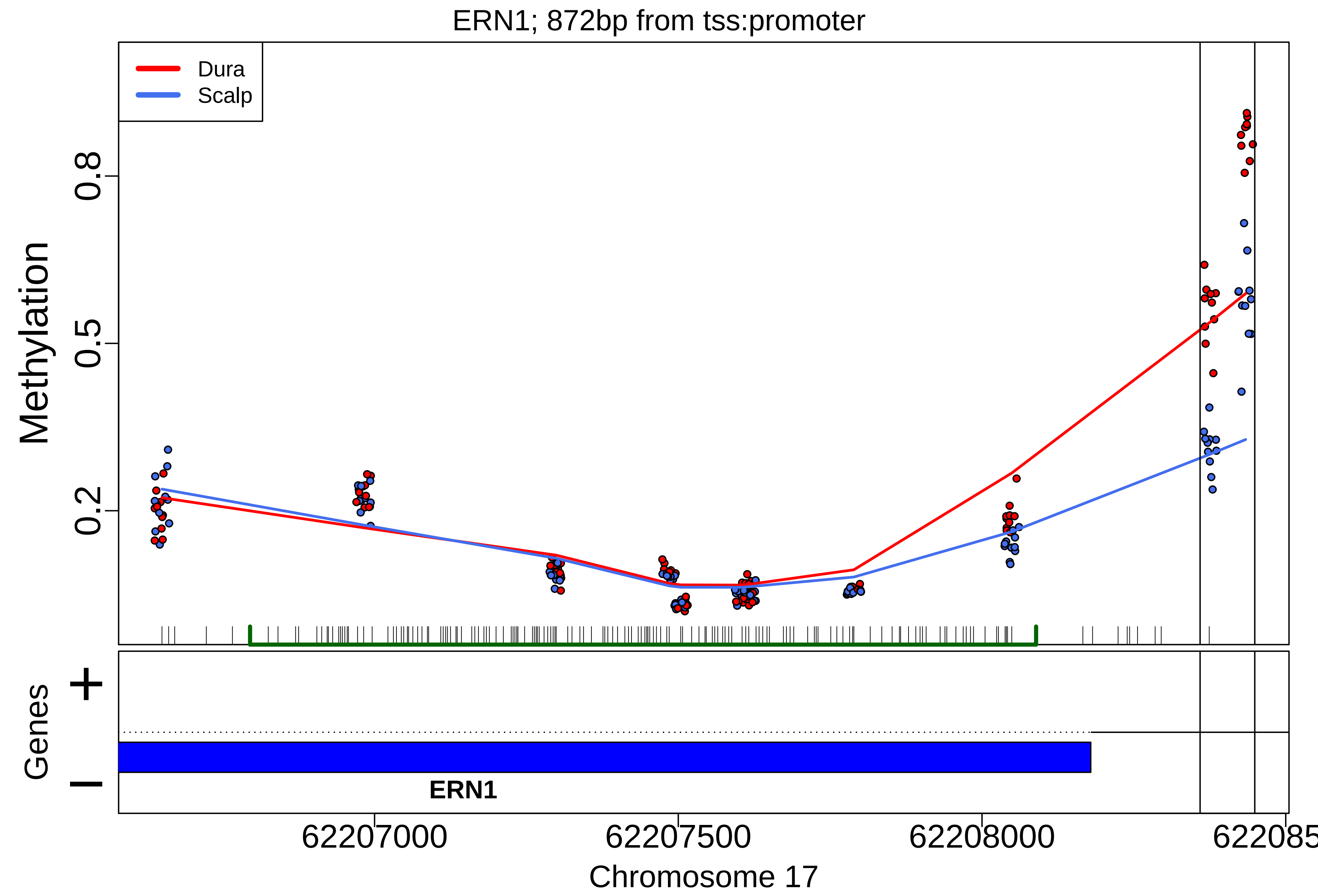

ERN1; 872bp from tss:promoter
Dura
Scalp
Methylation
0.2
0.5
0.8
Genes
+
-
ERN1
62207000
62207500
62208000
622085
Chromosome 17

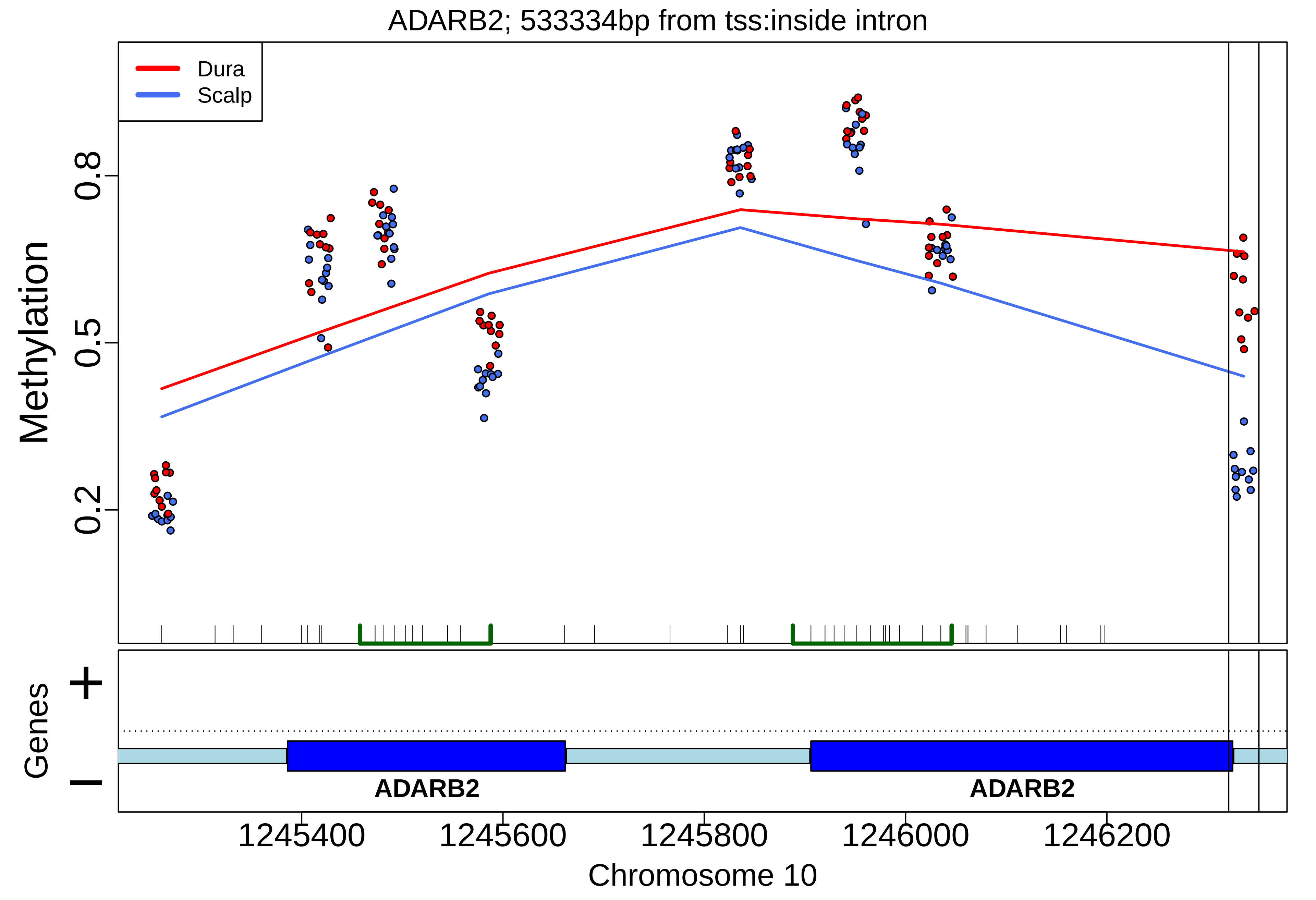
ADARB2; 533334bp from tss:inside intron
Dura
Scalp
Methylation
0.2
0.5
0.8
Genes
+
−
ADARB2
ADARB2
1245400
1245600
1245800
1246000
1246200
Chromosome 10

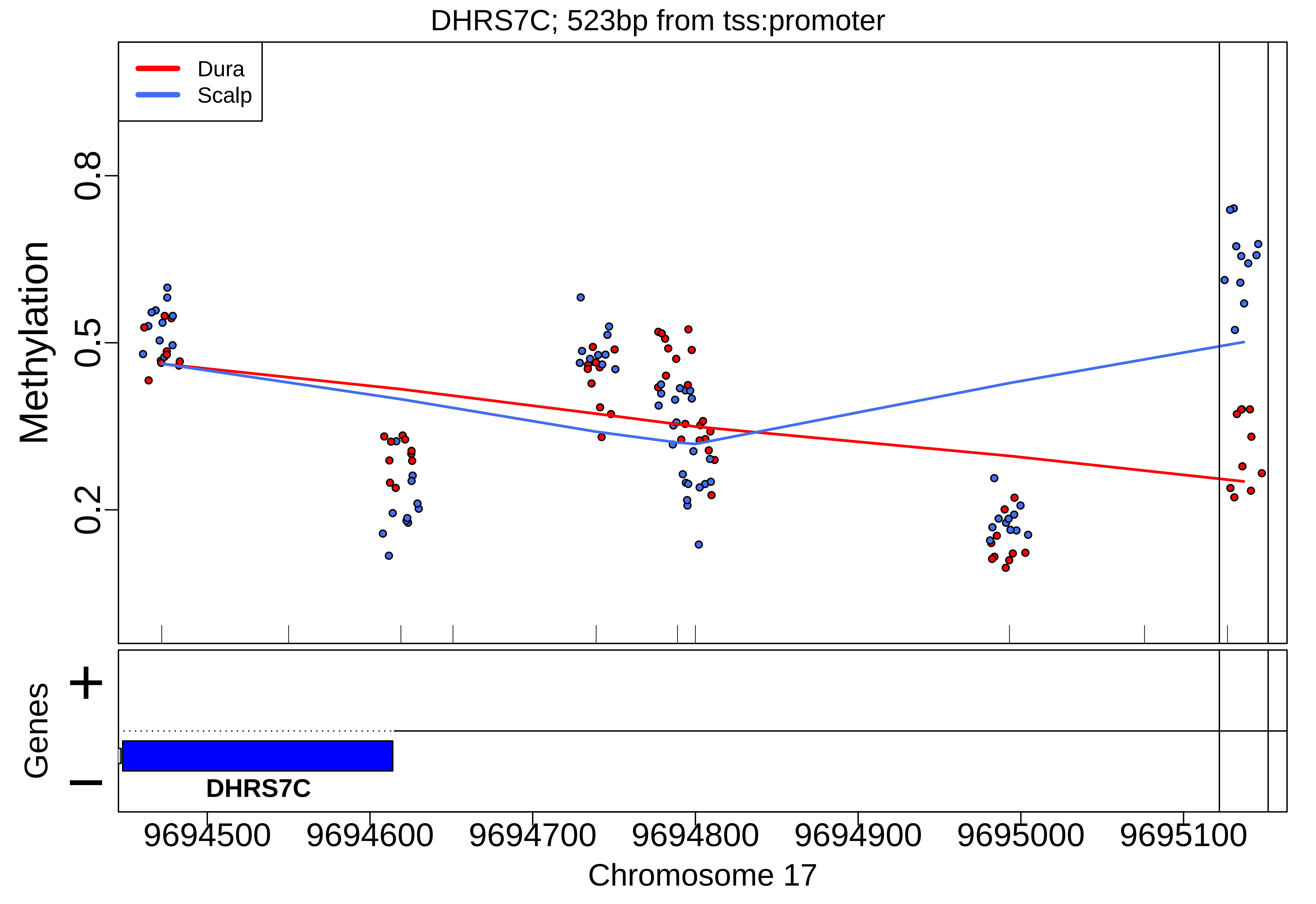

DHRS7C; 523bp from tss:promoter
Dura
Scalp
Methylation
0.2
0.5
0.8
Genes
+
-
DHRS7C
9694500
9694600
9694700
9694800
9694900
9695000
9695100
Chromosome 17

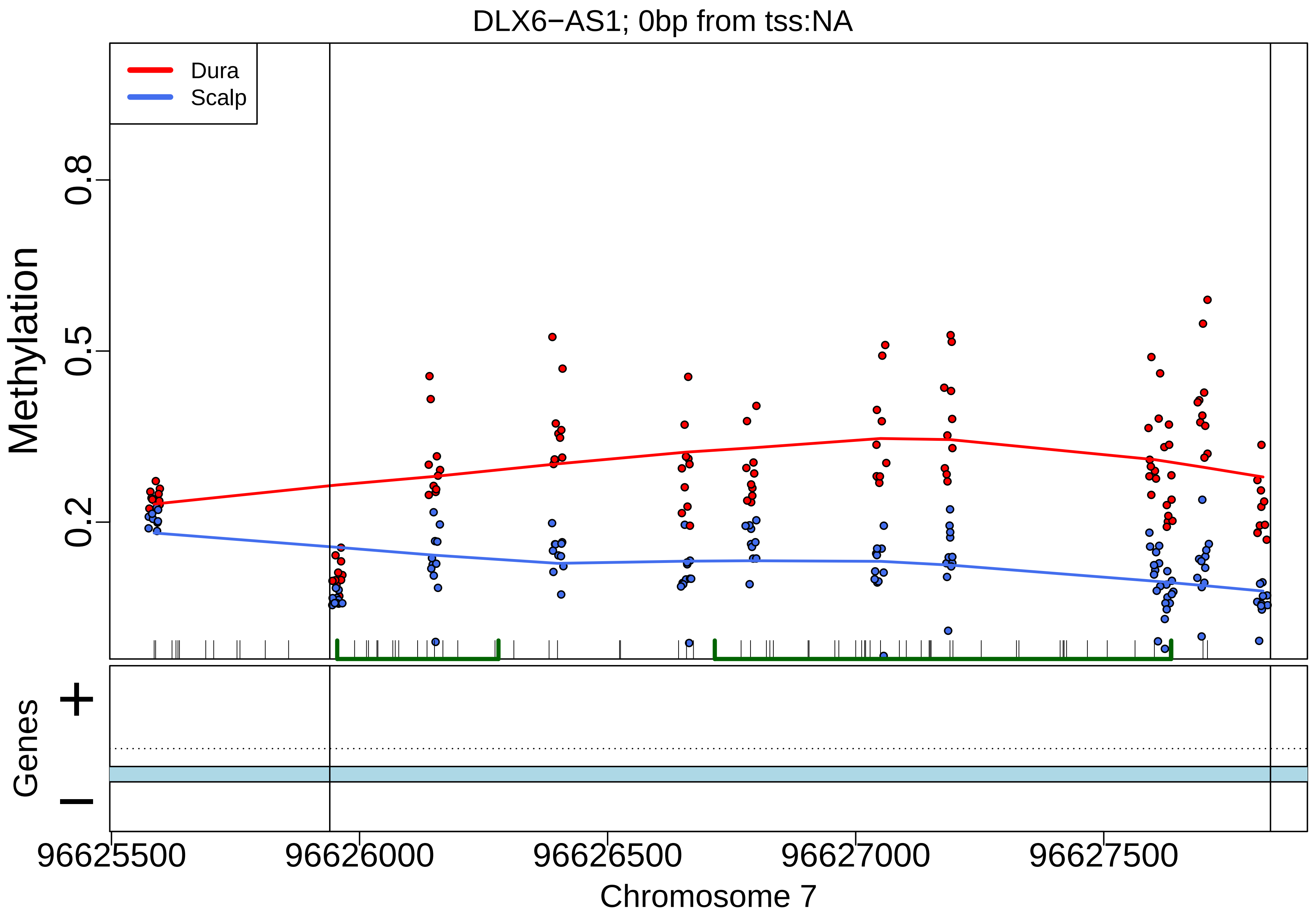
DLX6−AS1; 0bp from tss:NA
Dura
Scalp
Methylation
0.2
0.5
0.8
Genes
+
−
96625500
96626000
96626500
96627000
96627500
Chromosome 7

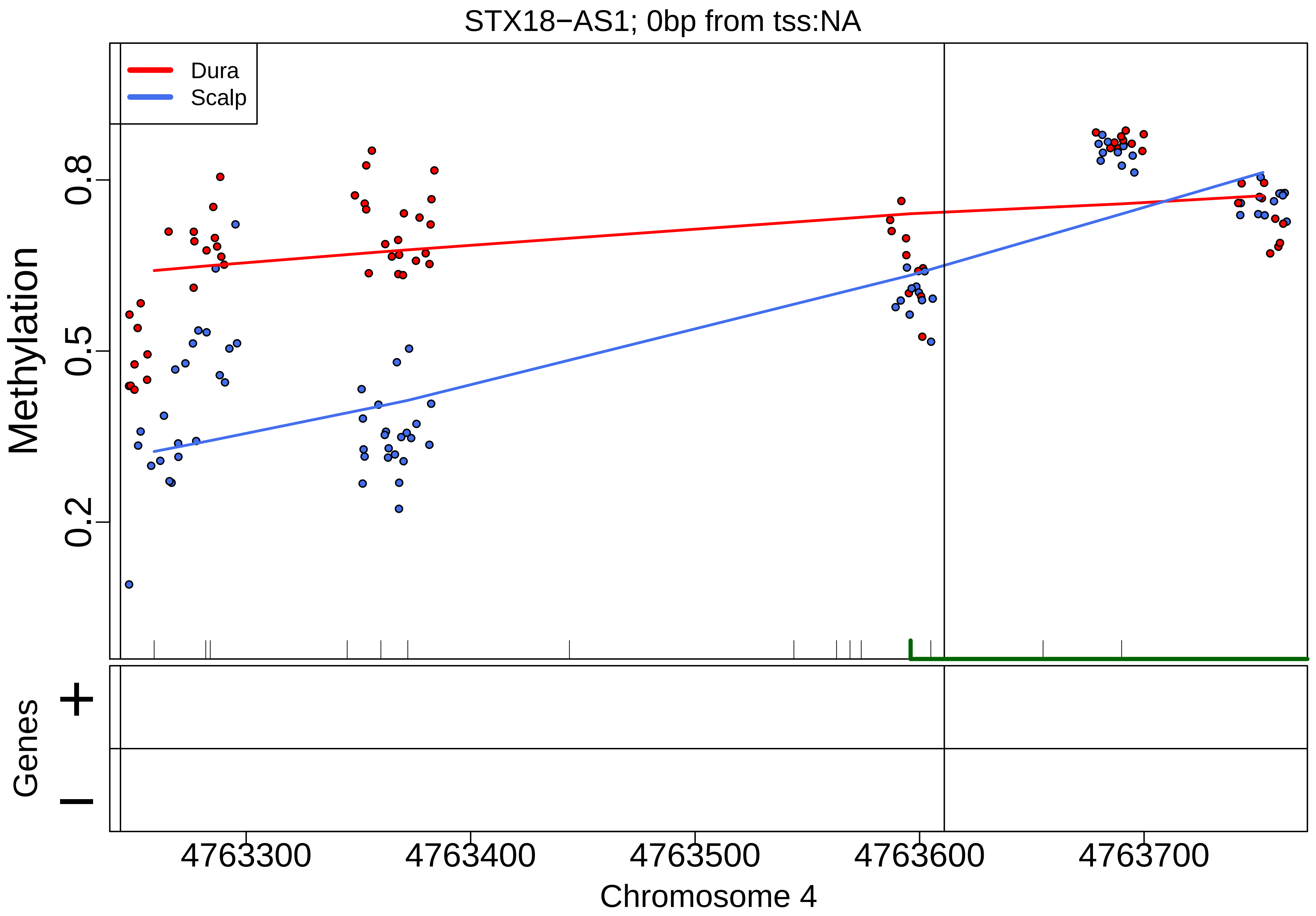
STX18−AS1; 0bp from tss:NA
Dura
Scalp
Methylation
0.2
0.5
0.8
Genes
+
−
4763300
4763400
4763500
4763600
4763700
Chromosome 4

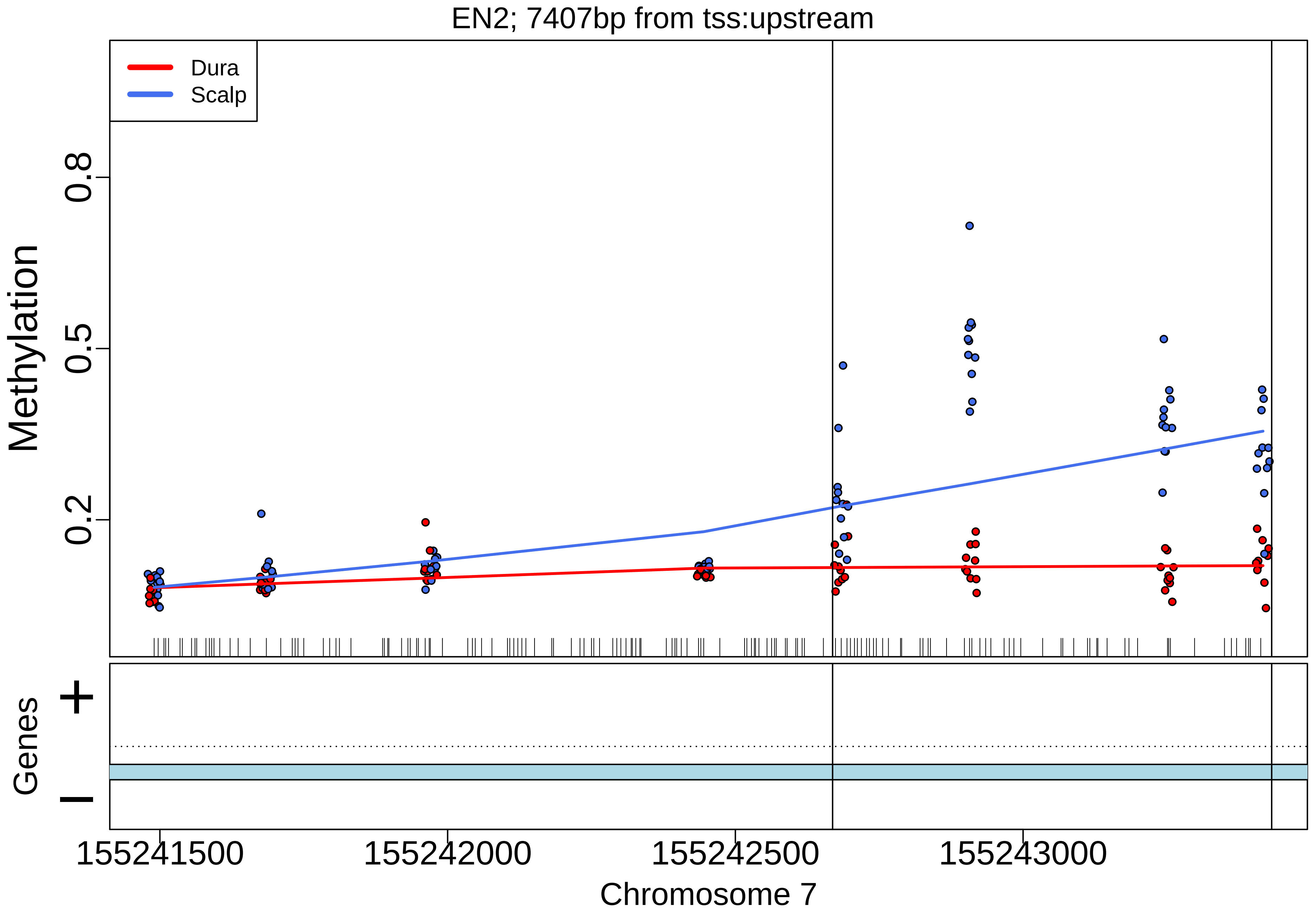
EN2; 7407bp from tss:upstream
Dura
Scalp
Methylation
0.2
0.5
0.8
Genes
+
155241500
155242000
155242500
155243000
Chromosome 7

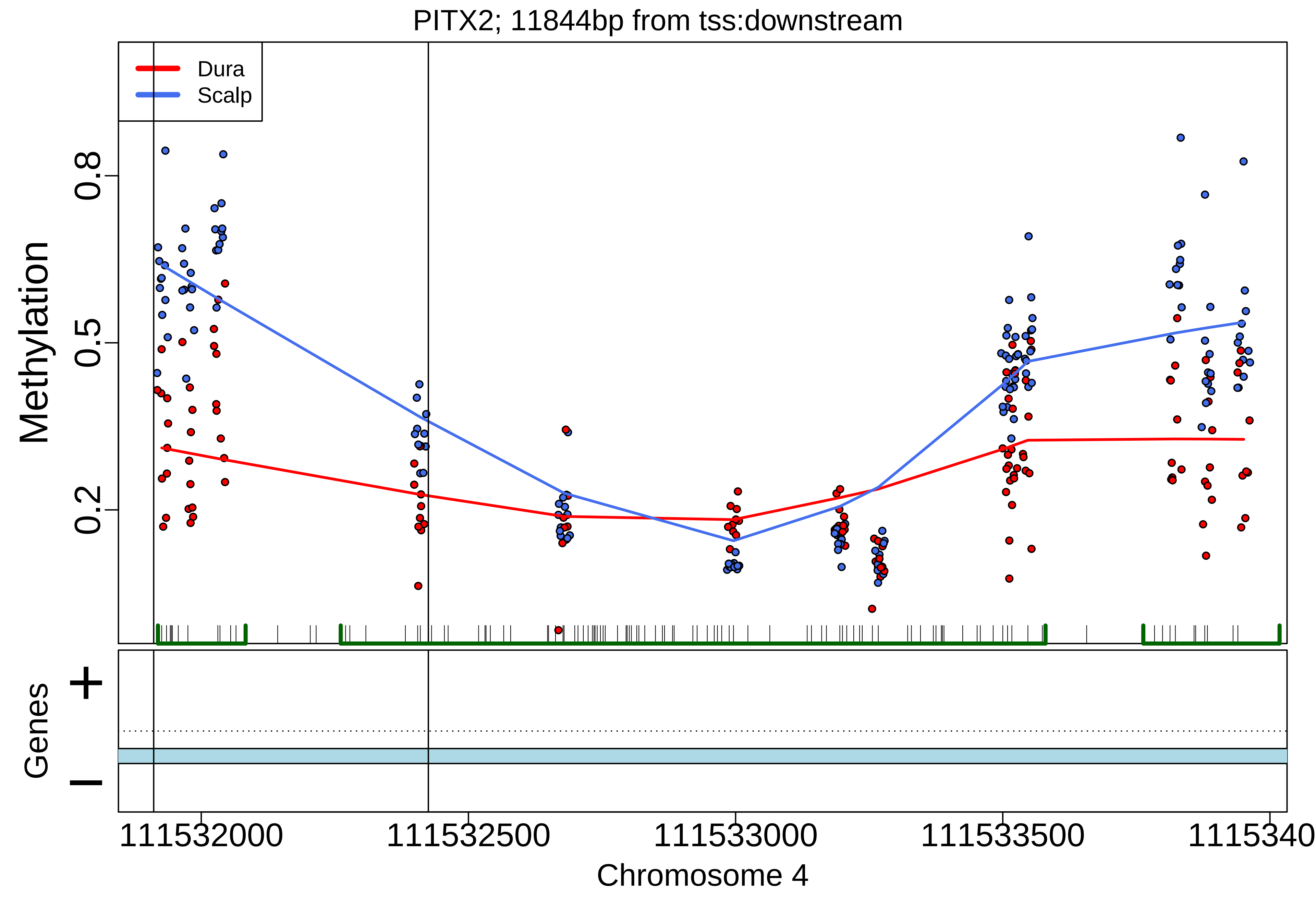
PITX2; 11844bp from tss:downstream
Dura
Scalp
Methylation
0.2
0.5
0.8
Genes
+
−
111532000
111532500
111533000
111533500
1115340
Chromosome 4

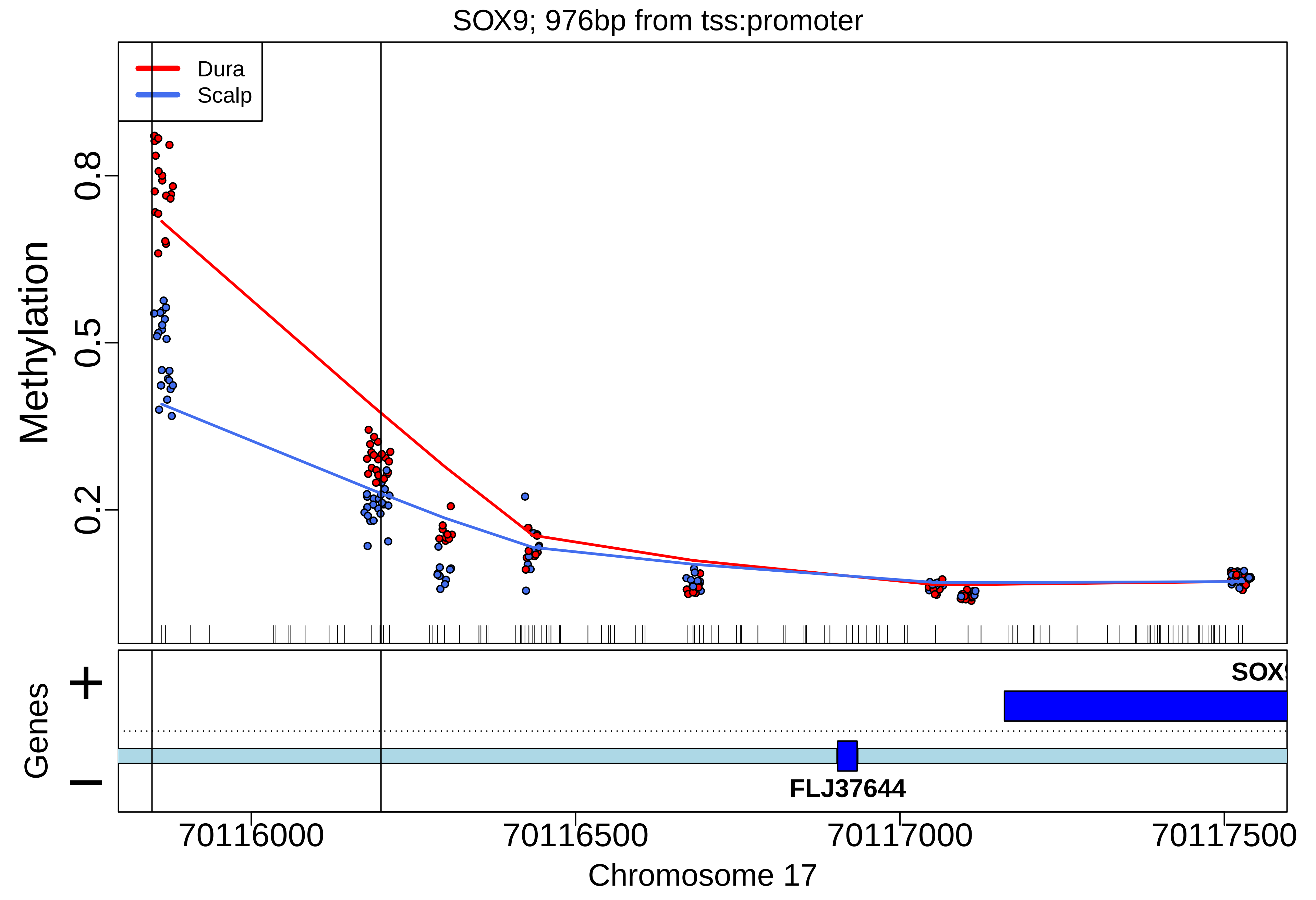

SOX9; 976bp from tss:promoter
Dura
Scalp
Methylation
0.2
0.5
0.8
Genes
+
-
SOX
FLJ37644
70116000
70116500
70117000
70117500
Chromosome 17

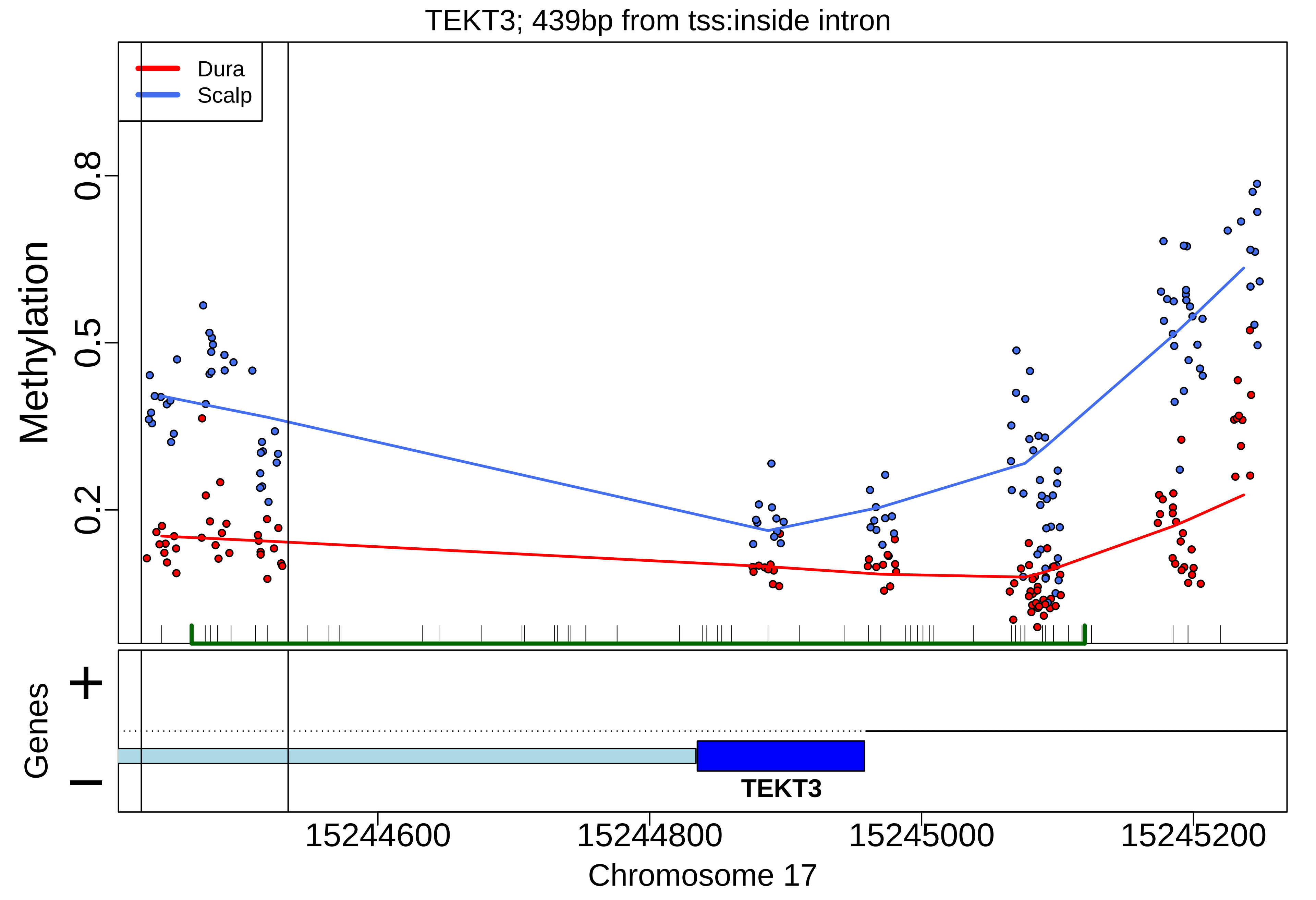

TEKT3; 439bp from tss:inside intron
Dura
Scalp
Methylation
0.2
0.5
0.8
Genes
+
−
TEKT3
15244600
15244800
15245000
15245200
Chromosome 17

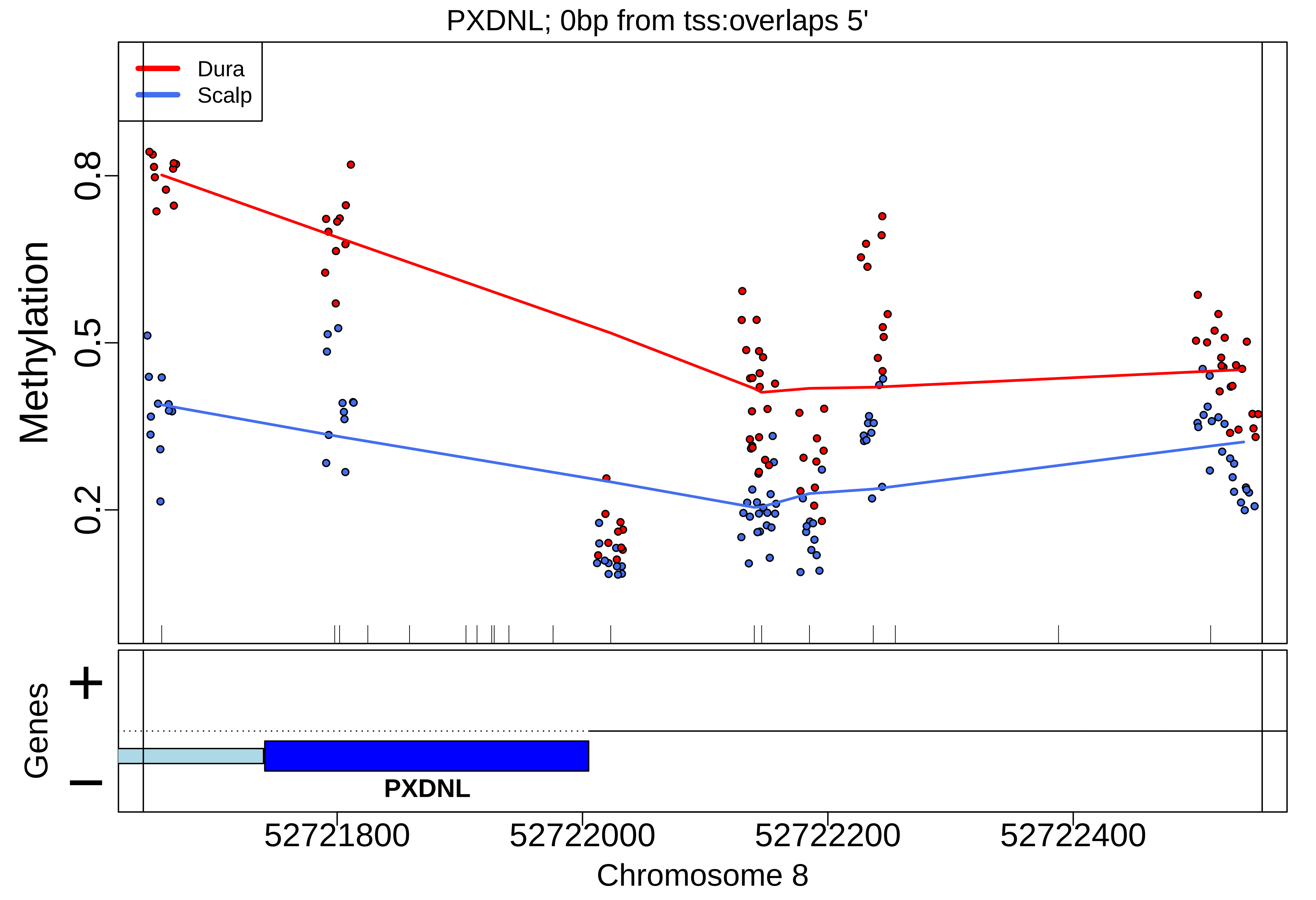

PXDNL; 0bp from tss:overlaps 5'
Dura
Scalp
Methylation
0.2
0.5
0.8
Genes
+
-
PXDNL
52721800
52722000
52722200
52722400
Chromosome 8

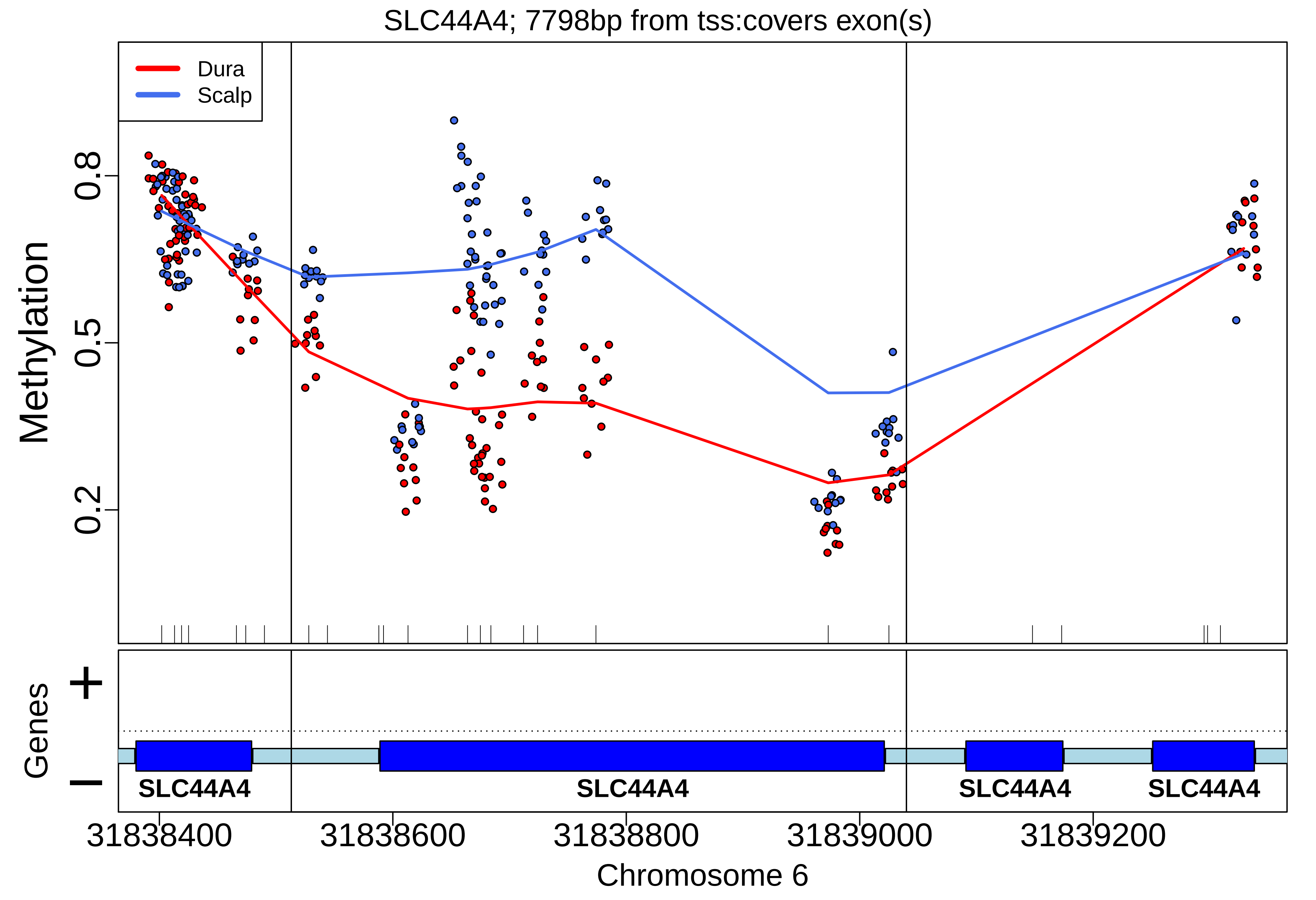

SLC44A4; 7798bp from tss:covers exon(s)
Dura
Scalp
Methylation
0.2
0.5
0.8
Genes
+
-
SLC44A4
SLC44A4
SLC44A4
SLC44A4
31838400
31838600
31838800
31839000
31839200
Chromosome 6

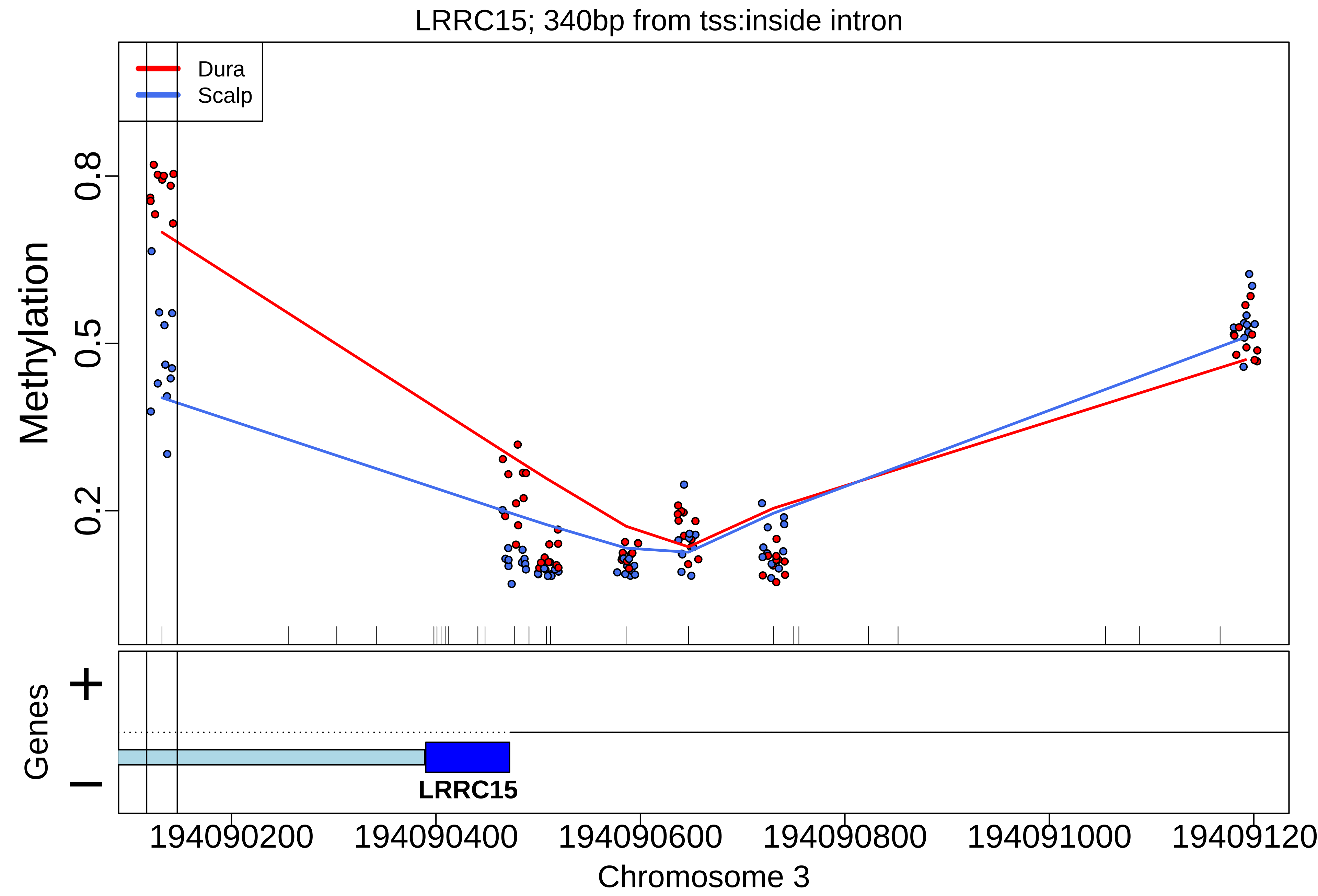

LRRC15; 340bp from tss:inside intron
Dura
Scalp
Methylation
0.2
0.5
0.8
Genes
+
-
LRRC15
194090200
194090400
194090600
194090800
194091000
19409120
Chromosome 3

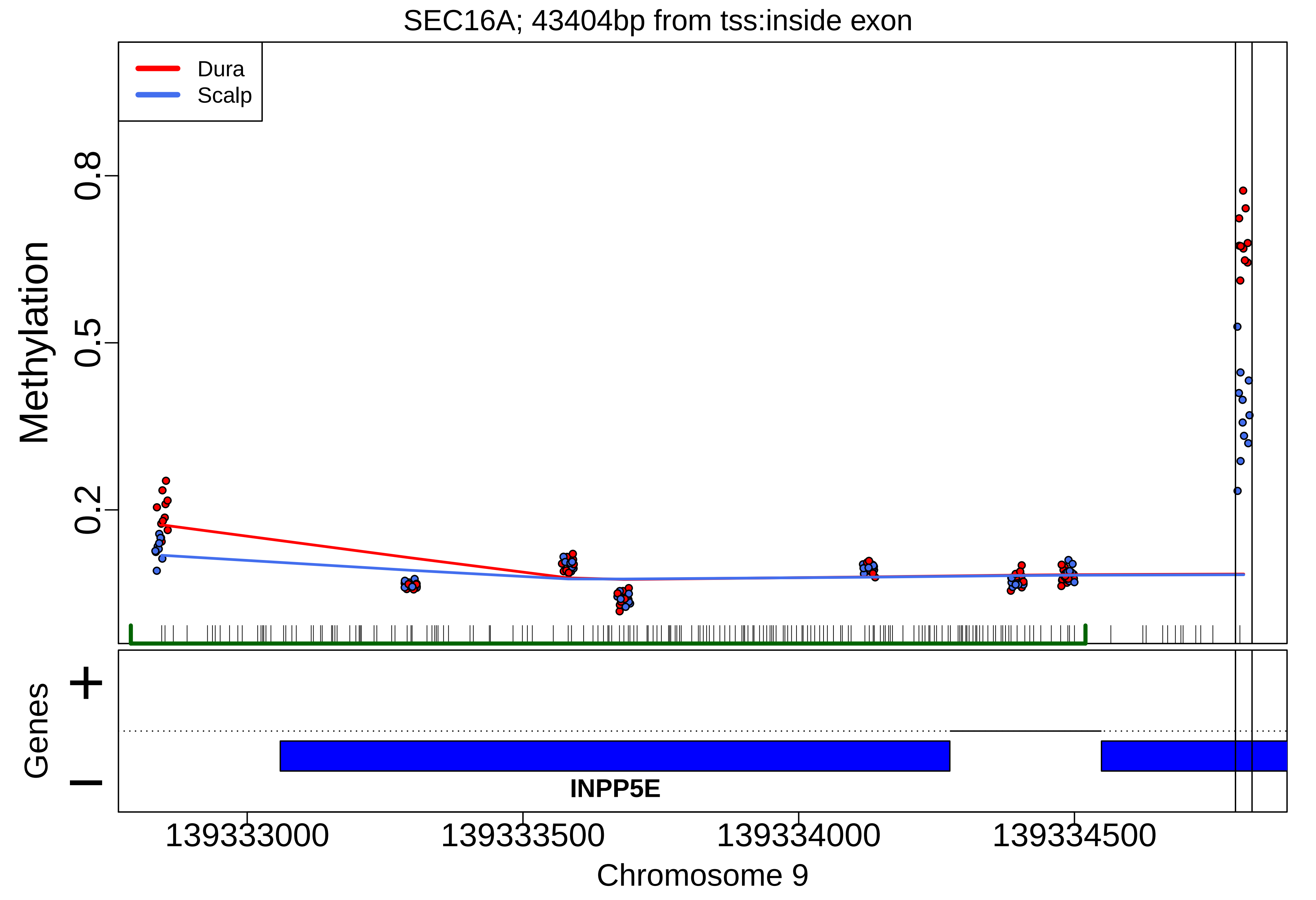
SEC16A; 43404bp from tss:inside exon
Dura
Scalp
Methylation
0.2
0.5
0.8
Genes
+
−
INPP5E
139333000
139333500
139334000
139334500
Chromosome 9

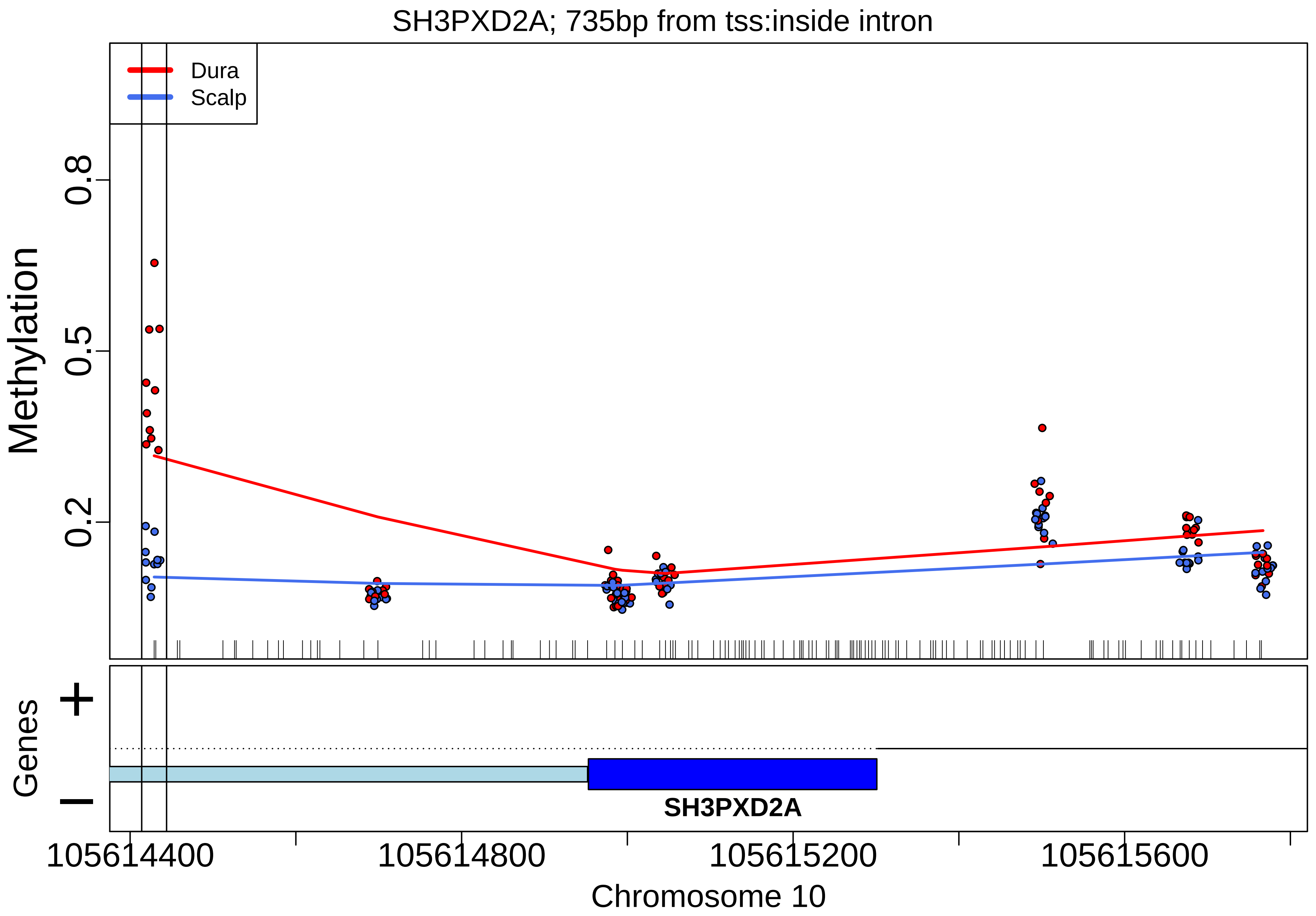
SH3PXD2A; 735bp from tss:inside intron
Dura
Scalp
Methylation
0.8
0.5
0.2
Genes
+
-
SH3PXD2A
105614400
105614800
105615200
105615600
Chromosome 10

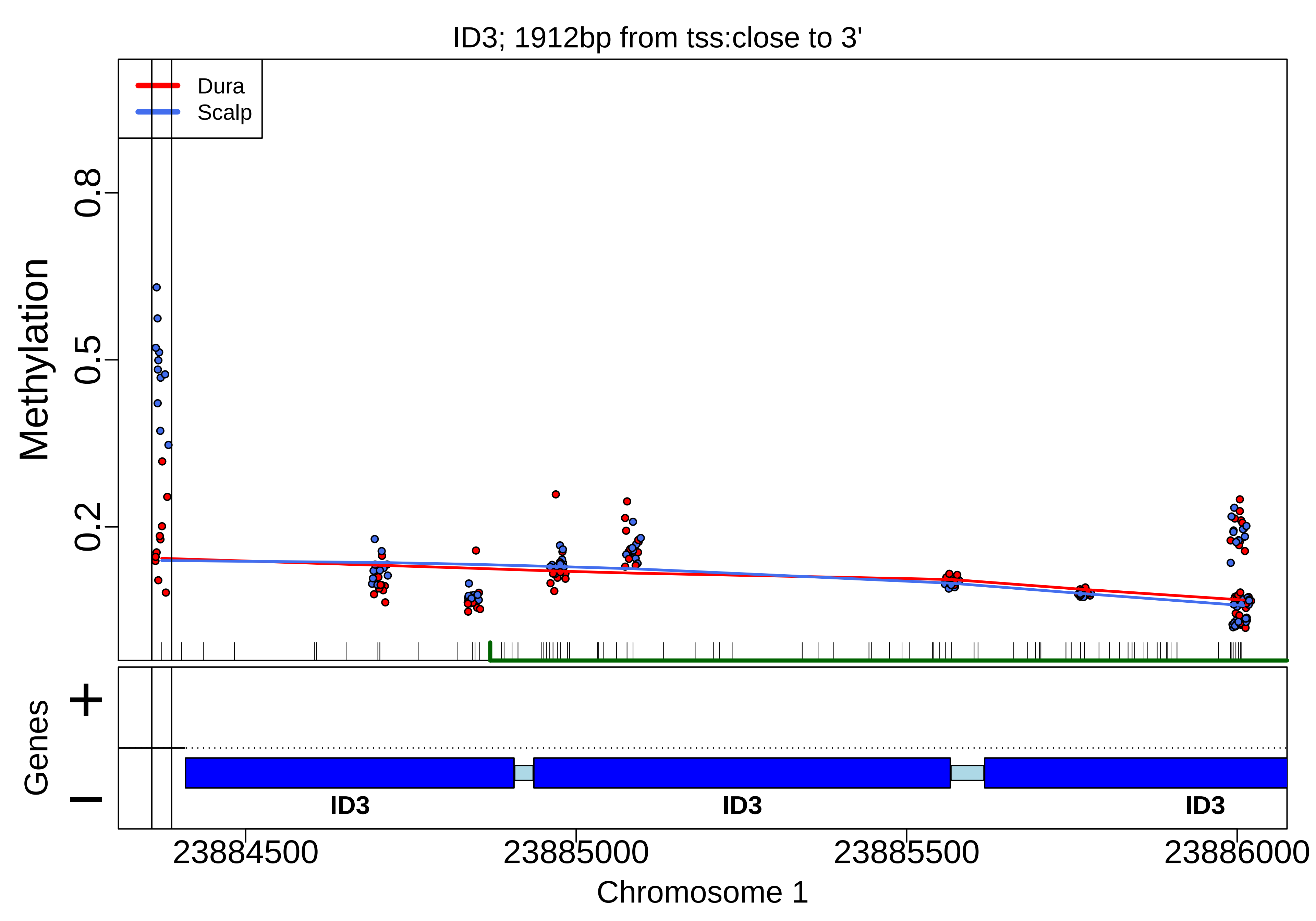

ID3; 1912bp from tss:close to 3'
Dura
Scalp
Methylation
0.2
0.5
0.8
Genes
+
-
ID3
ID3
ID3
23884500
23885000
23885500
23886000
Chromosome 1

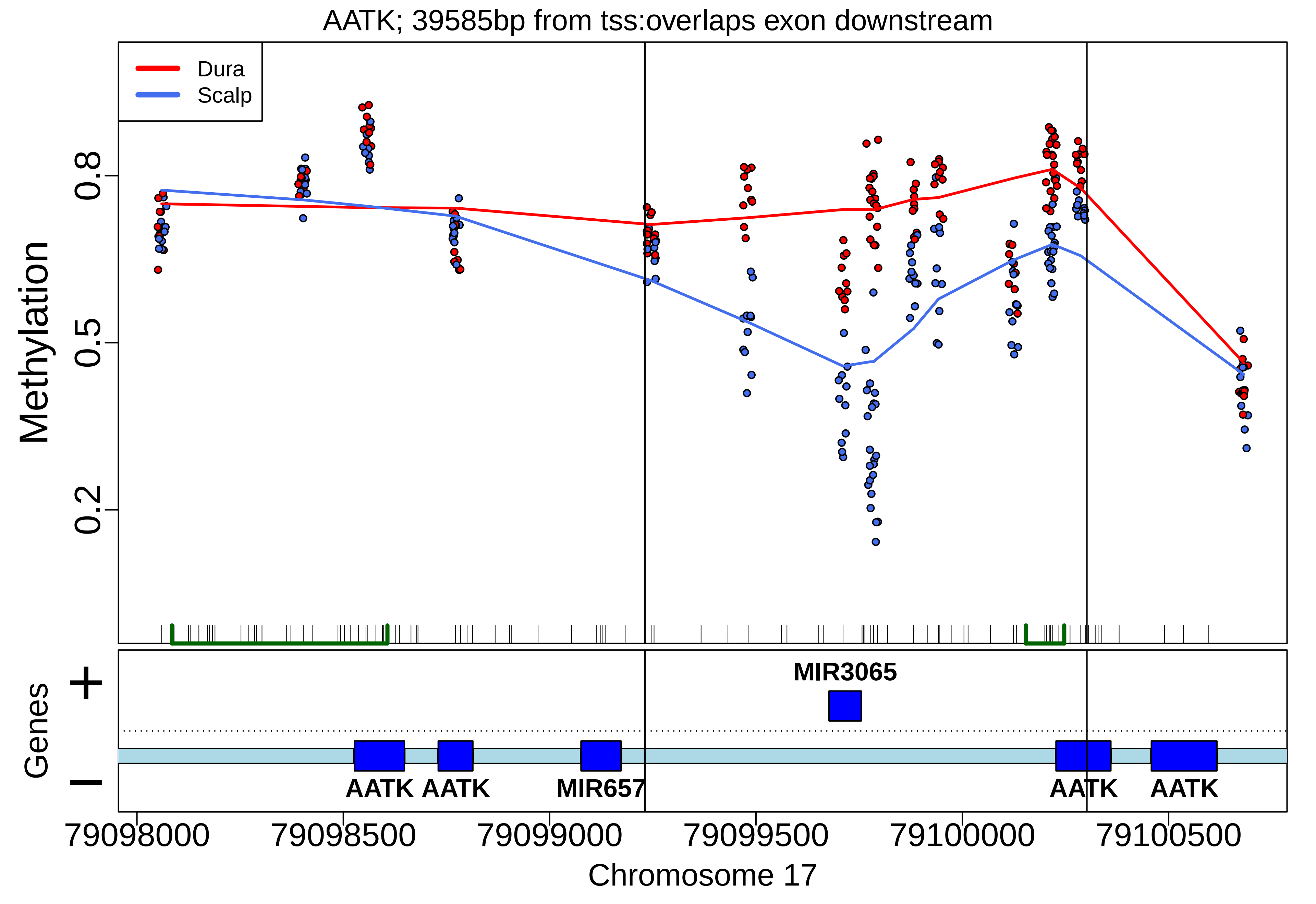

AATK; 39585bp from tss:overlaps exon downstream
Dura
Scalp
Methylation
0.2
0.5
0.8
Genes
+
–
MIR3065
AATK
AATK
MIR657
AATK
AATK
79098000
79098500
79099000
79099500
79100000
79100500
Chromosome 17

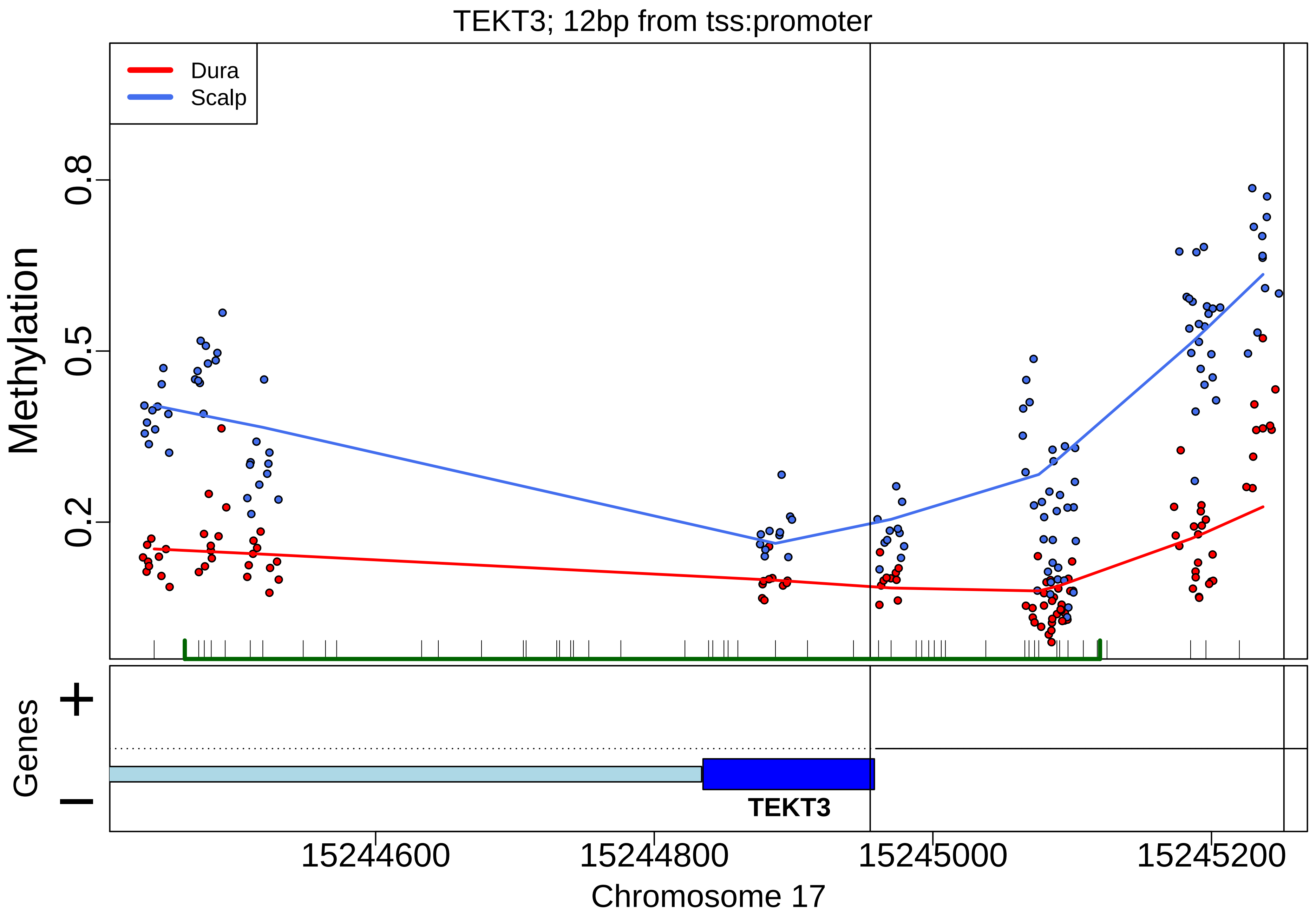

TEKT3; 12bp from tss:promoter
Dura
Scalp
Methylation
0.2
0.5
0.8
Genes
+
-
TEKT3
15244600
15244800
15245000
15245200
Chromosome 17

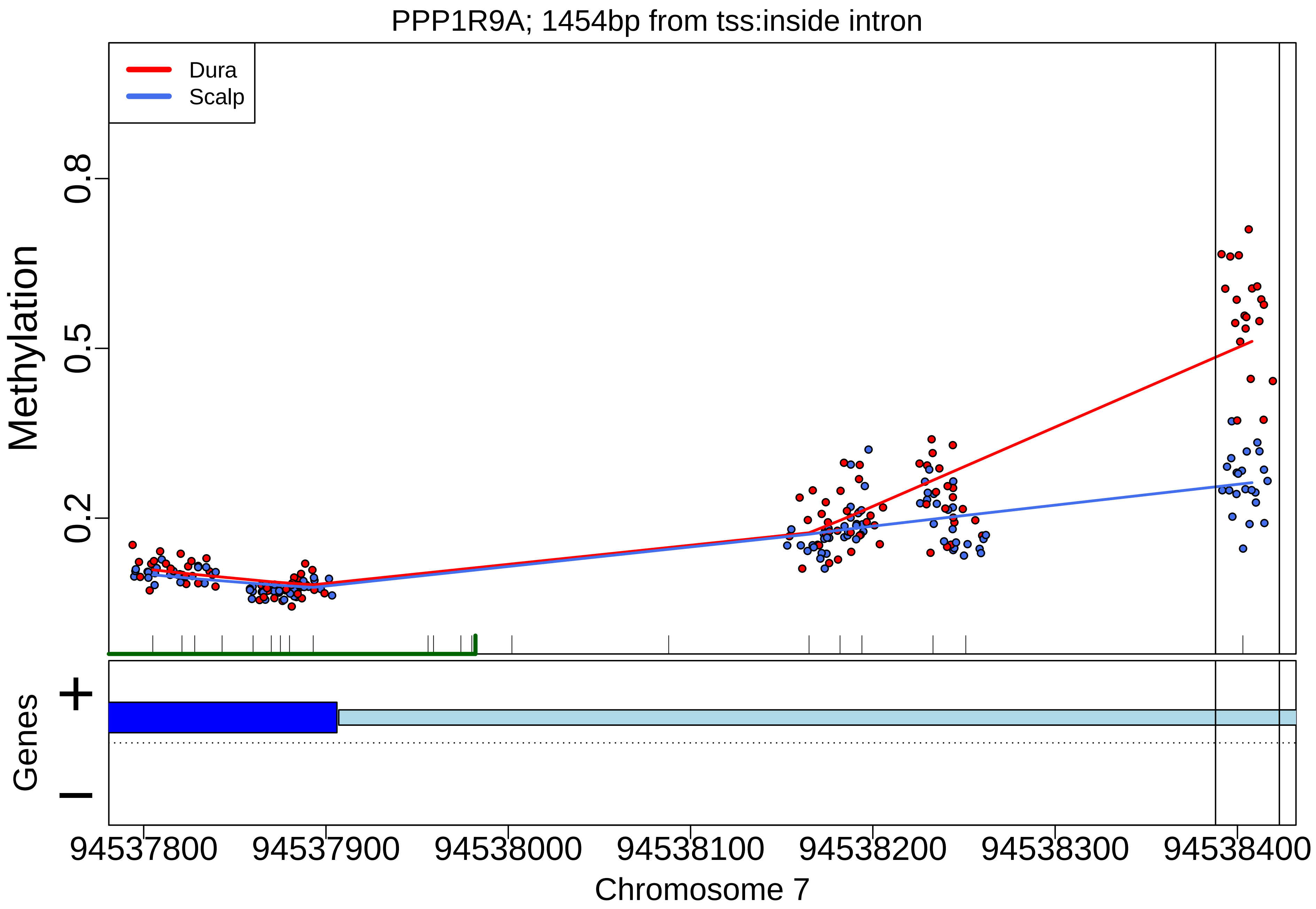

PPP1R9A; 1454bp from tss:inside intron
Dura
Scalp
Methylation
0.2
0.5
0.8
Genes
+
-
94537800
94537900
94538000
94538100
94538200
94538300
94538400
Chromosome 7

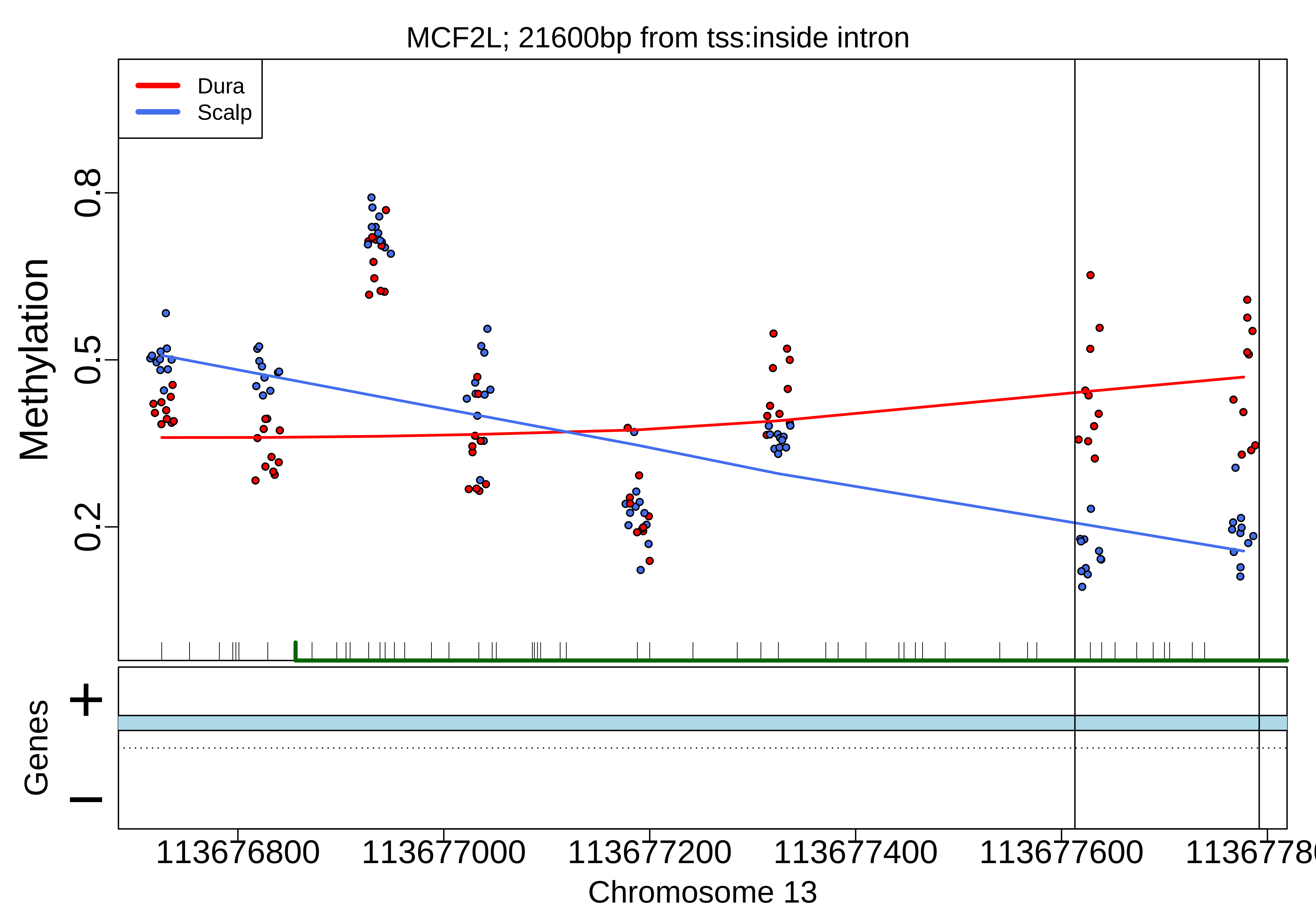

MCF2L; 21600bp from tss:inside intron
Dura
Scalp
Methylation
0.2
0.5
0.8
Genes
+
-
113676800
113677000
113677200
113677400
113677600
Chromosome 13

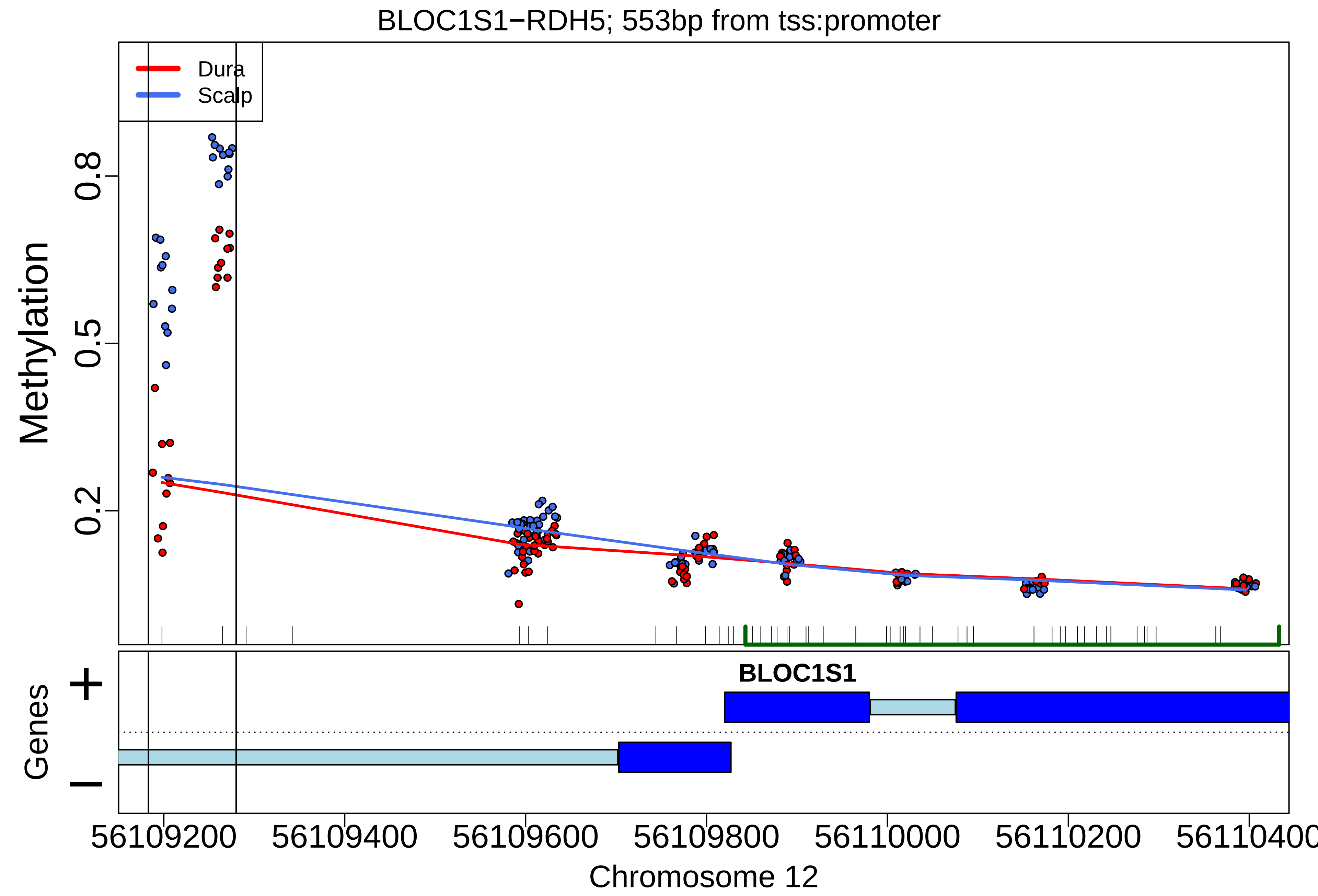

BLOC1S1−RDH5; 553bp from tss:promoter
Dura
Scalp
Methylation
0.2
0.5
0.8
Genes
+
−
BLOC1S1
56109200
56109400
56109600
56109800
56110000
56110200
56110400
Chromosome 12

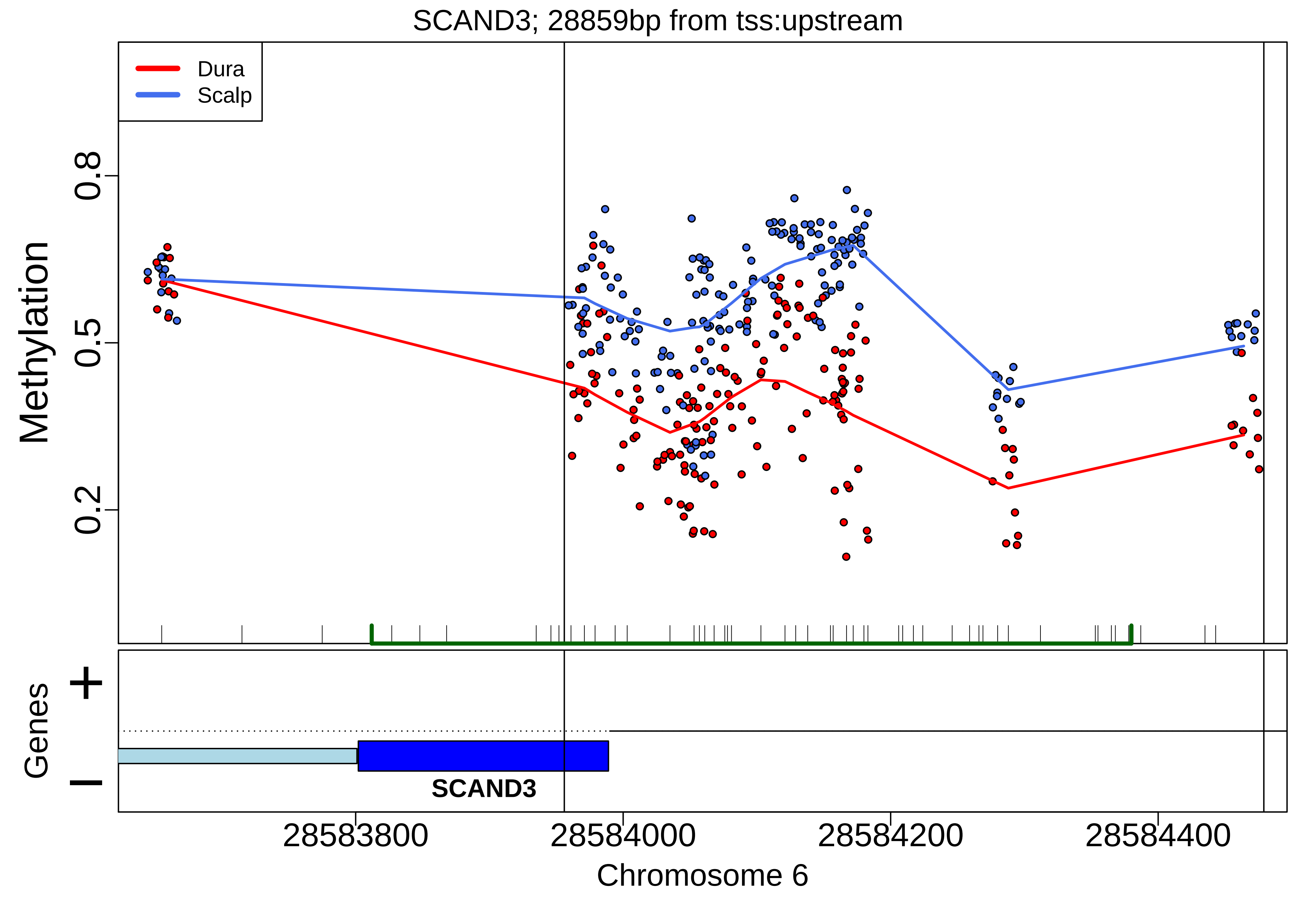
SCAND3; 28859bp from tss:upstream
Dura
Scalp
Methylation
0.2
0.5
0.8
Genes
+
−
SCAND3
28583800
28584000
28584200
28584400
Chromosome 6

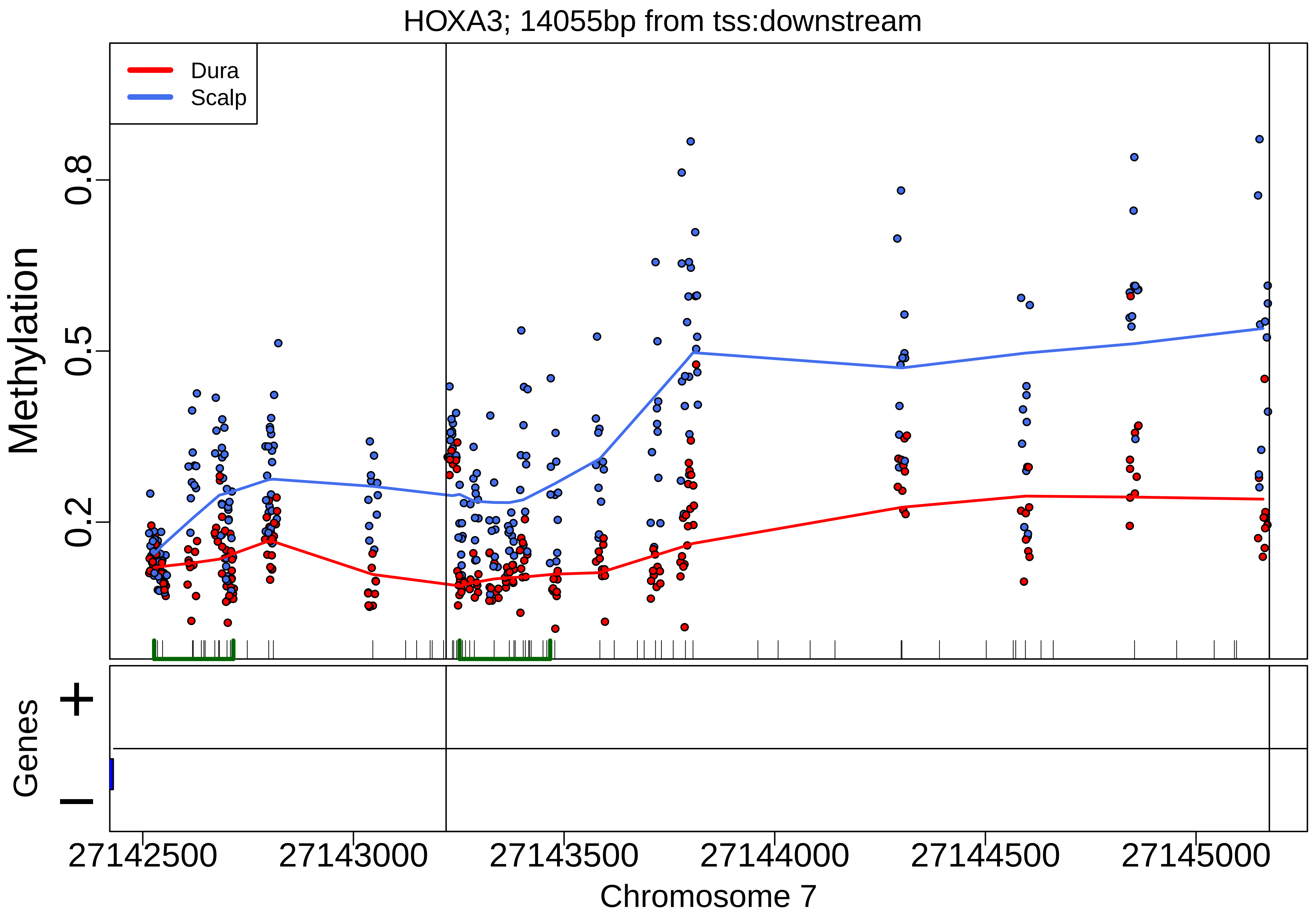

HOXA3; 14055bp from tss:downstream
Dura
Scalp
Methylation
0.2
0.5
0.8
Genes
+
−
27142500
27143000
27143500
27144000
27144500
27145000
Chromosome 7

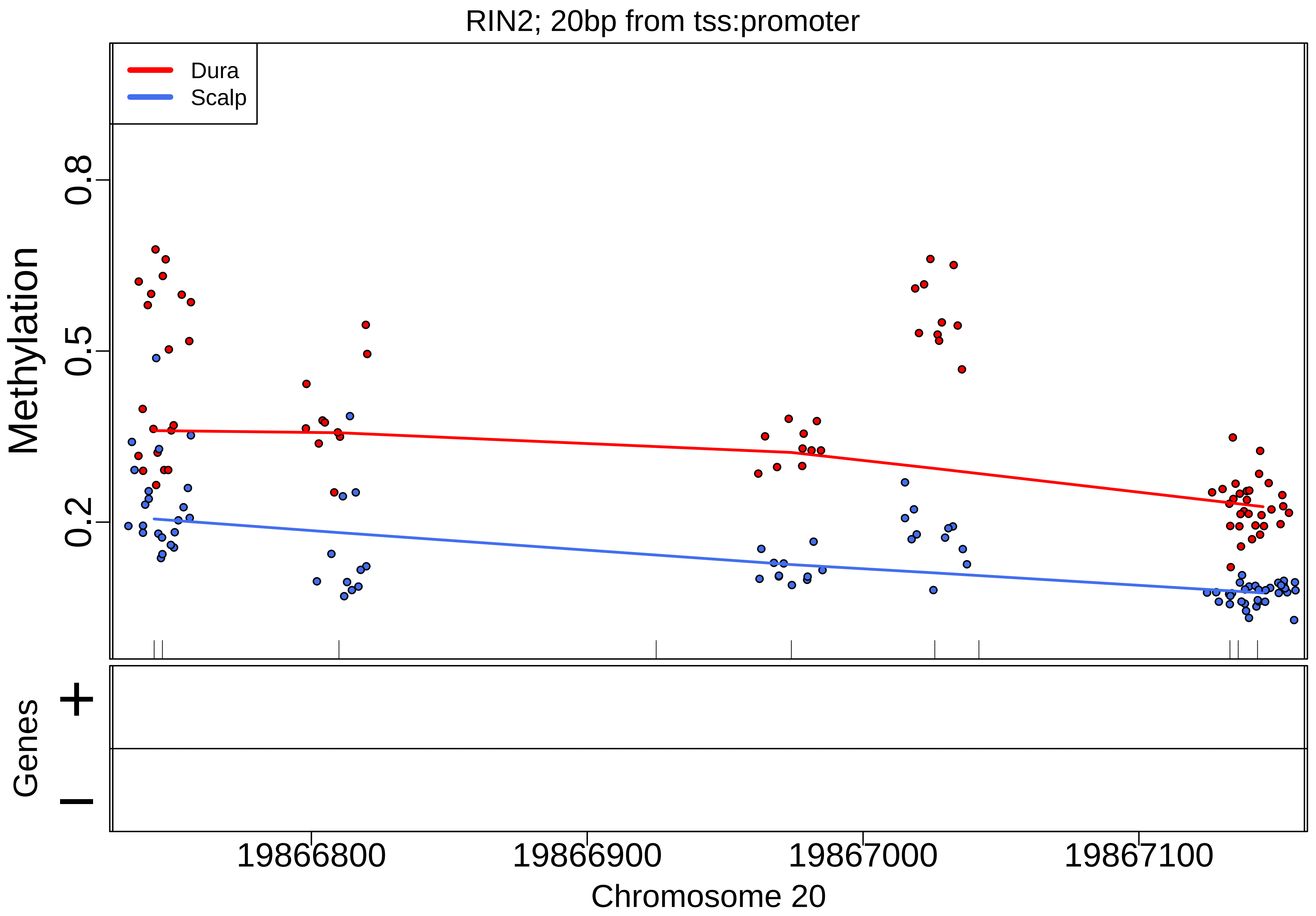
RIN2; 20bp from tss:promoter
Dura
Scalp
Methylation
0.2
0.5
0.8
Genes
+
-
19866800
19866900
19867000
19867100
Chromosome 20

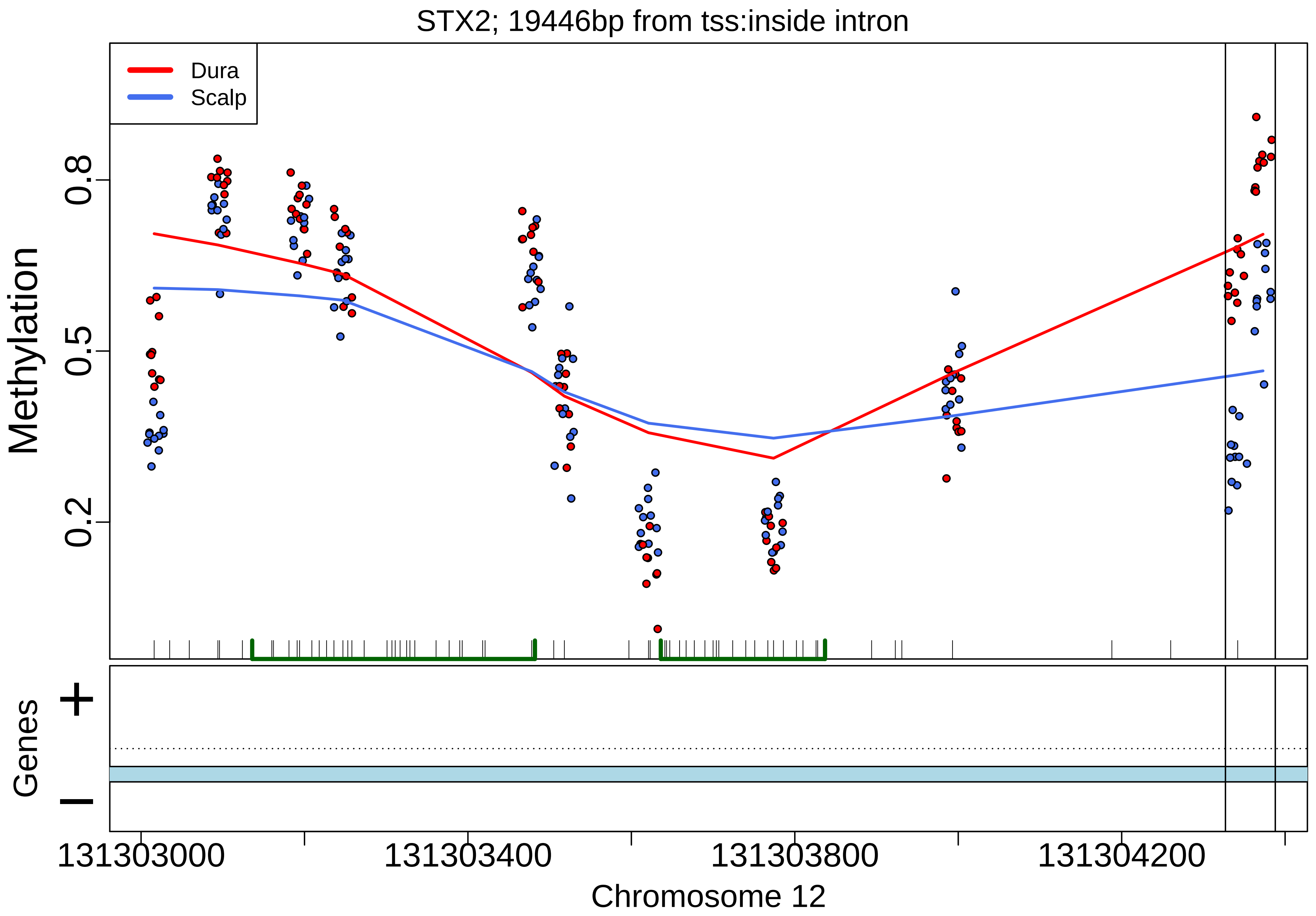

STX2; 19446bp from tss:inside intron
Dura
Scalp
Methylation
0.2
0.5
0.8
Genes
+
−
131303000
131303400
131303800
131304200
Chromosome 12

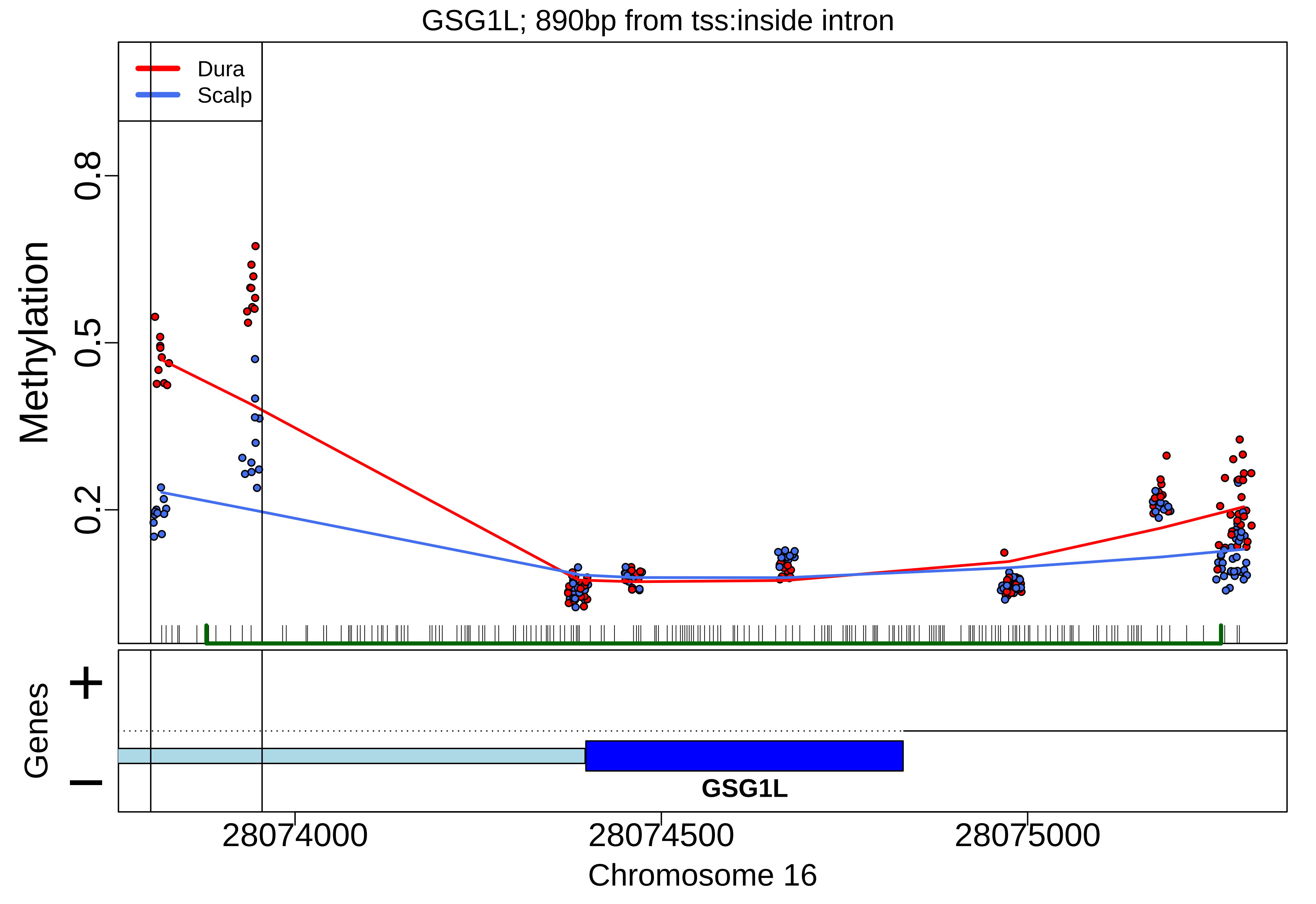
GSG1L; 890bp from tss:inside intron
Dura
Scalp
Methylation
0.2
0.5
0.8
Genes
+
−
GSG1L
28074000
28074500
28075000
Chromosome 16

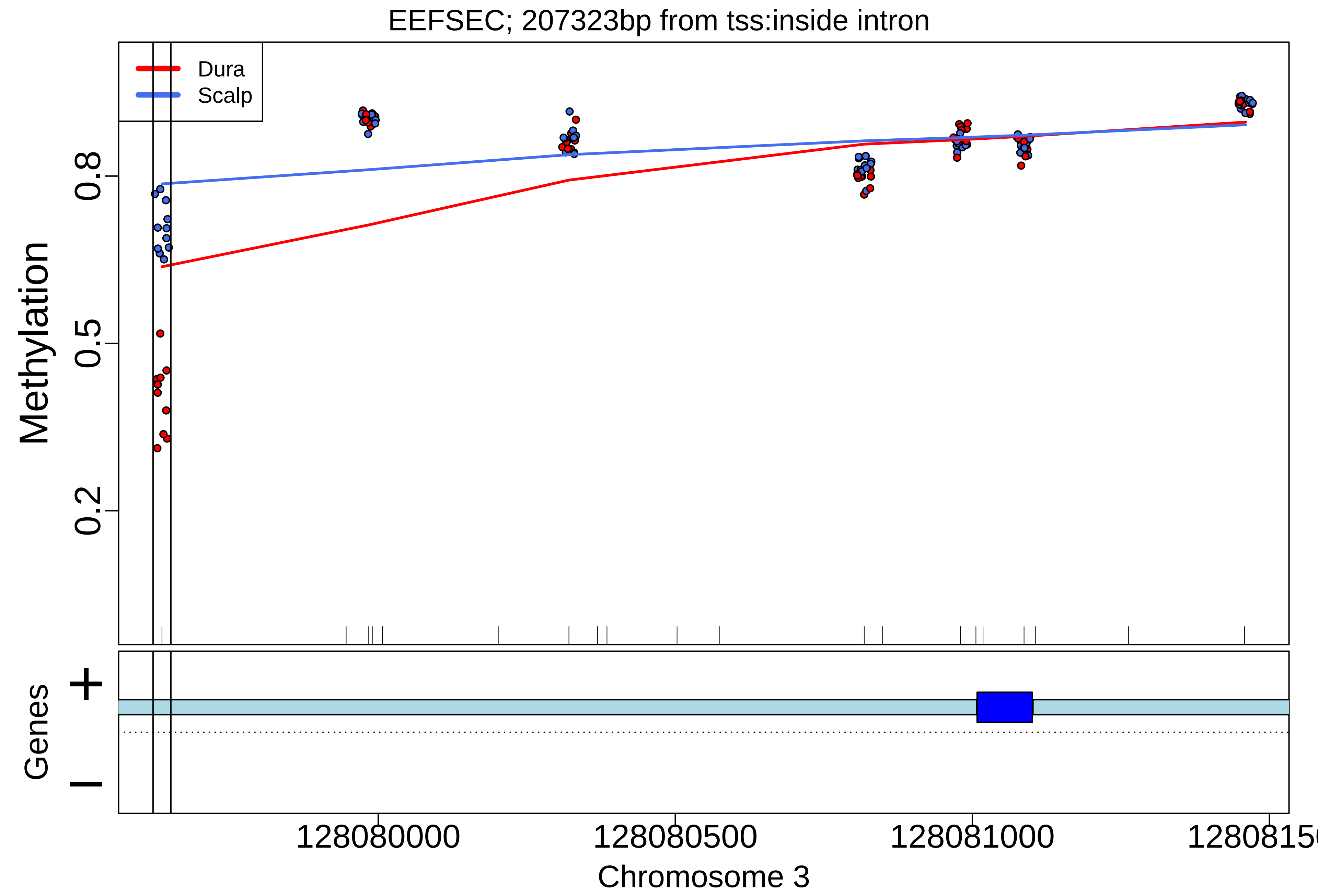

EEFSEC; 207323bp from tss:inside intron
Dura
Scalp
Methylation
0.2
0.5
0.8
Genes
+
-
128080000
128080500
128081000
Chromosome 3

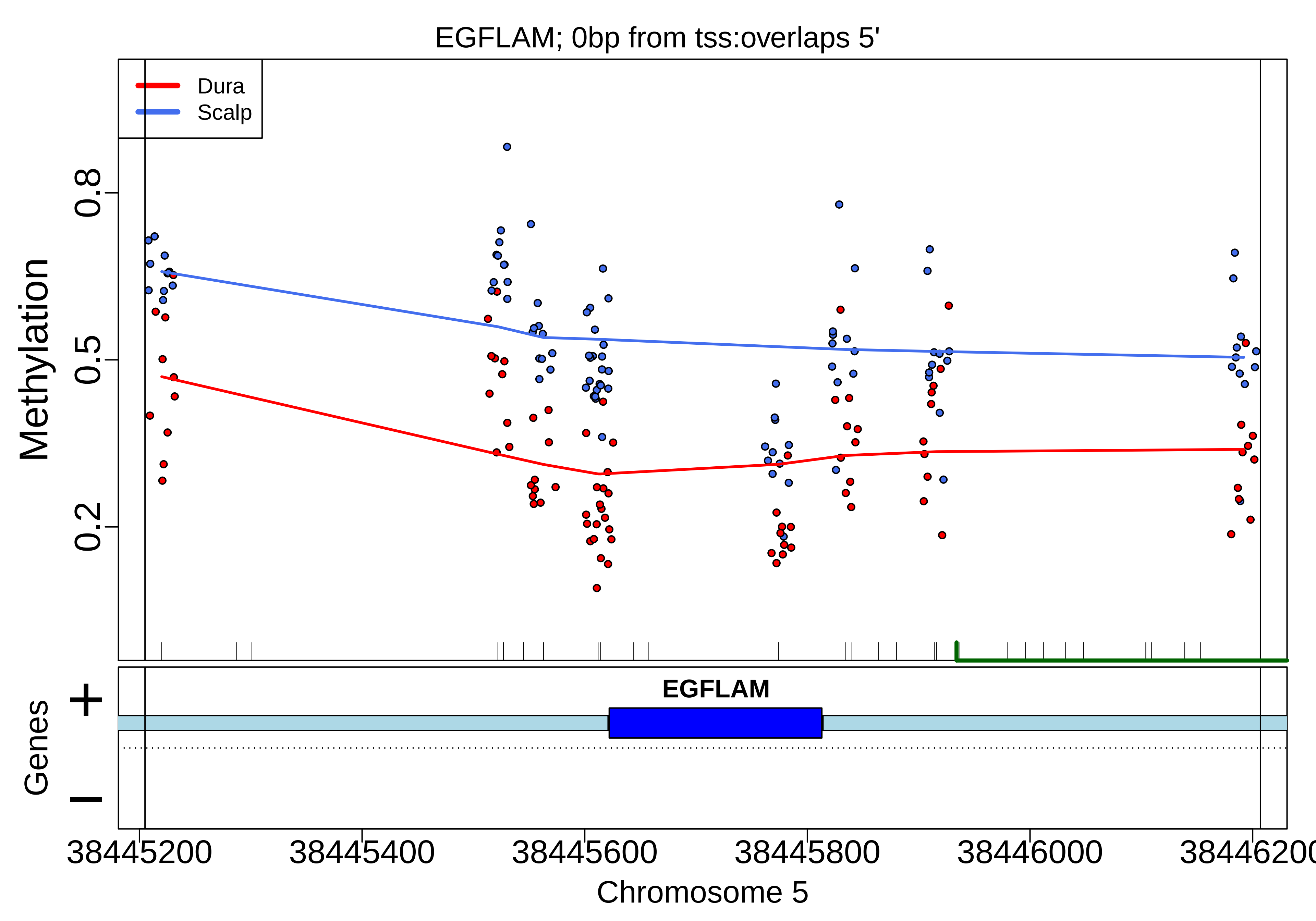

EGFLAM; 0bp from tss:overlaps 5'
Dura
Scalp
Methylation
0.2
0.5
0.8
Genes
+
−
EGFLAM
38445200
38445400
38445600
38445800
38446000
38446200
Chromosome 5

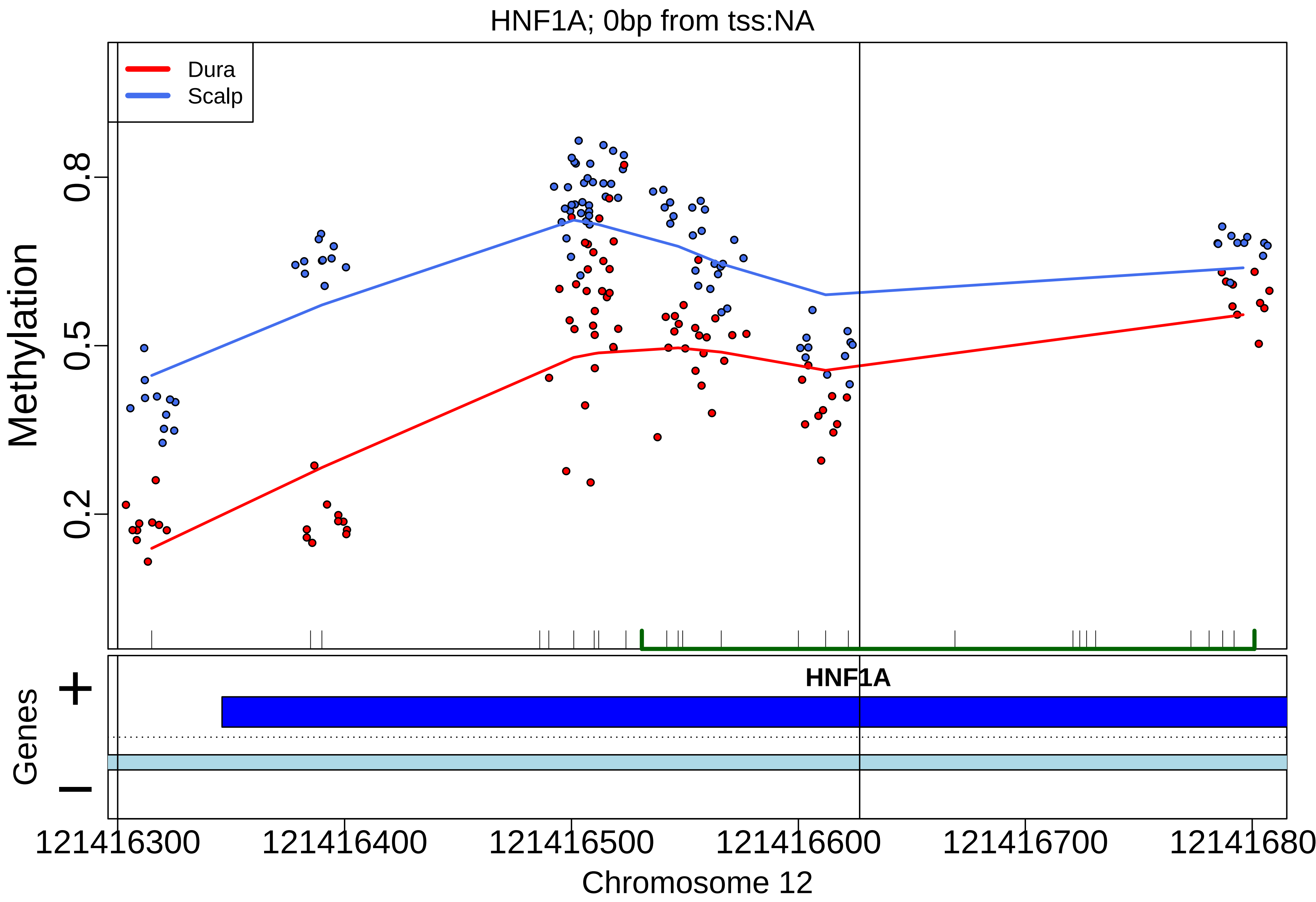

HNF1A; 0bp from tss:NA
Dura
Scalp
Methylation
0.2
0.5
0.8
Genes
+
-
HNF1A
121416300
121416400
121416500
121416600
121416700
12141680
Chromosome 12

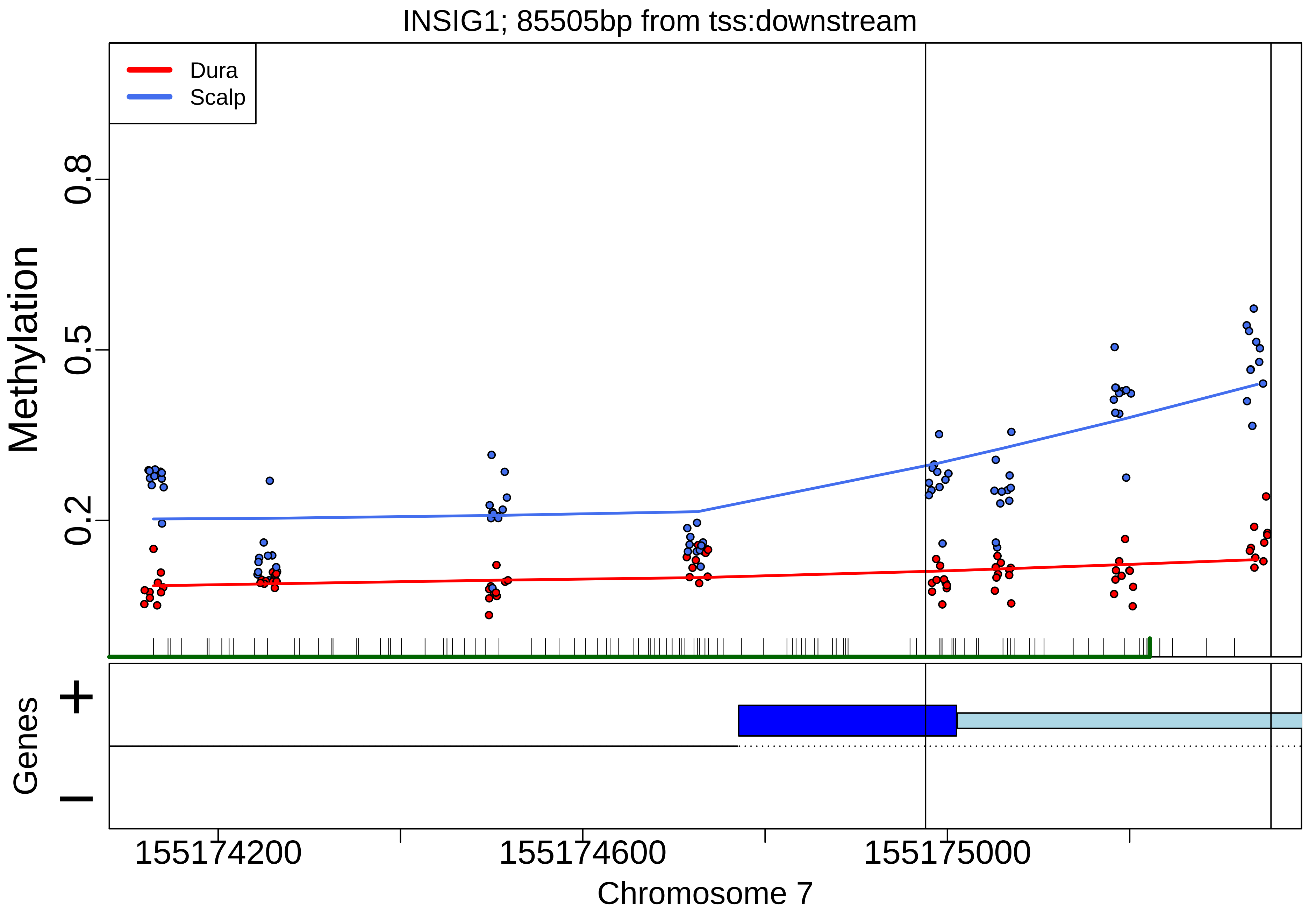

INSIG1; 85505bp from tss:downstream
Dura
Scalp
Methylation
0.2
0.5
0.8
Genes
+
−
155174200
155174600
155175000
Chromosome 7

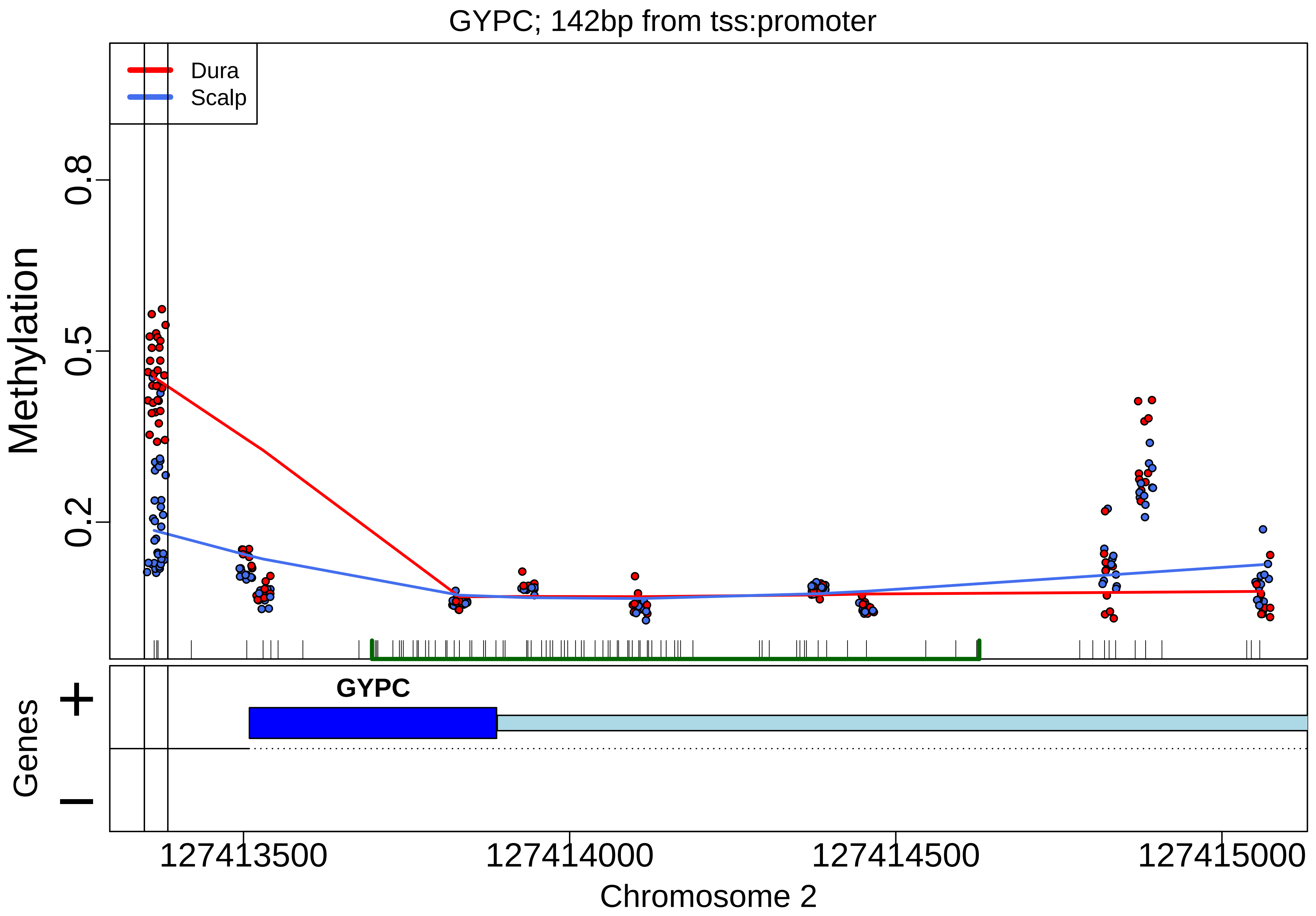

GYPC; 142bp from tss:promoter
Dura
Scalp
Methylation
0.2
0.5
0.8
GYPC
Genes
+
-
127413500
127414000
127414500
127415000
Chromosome 2

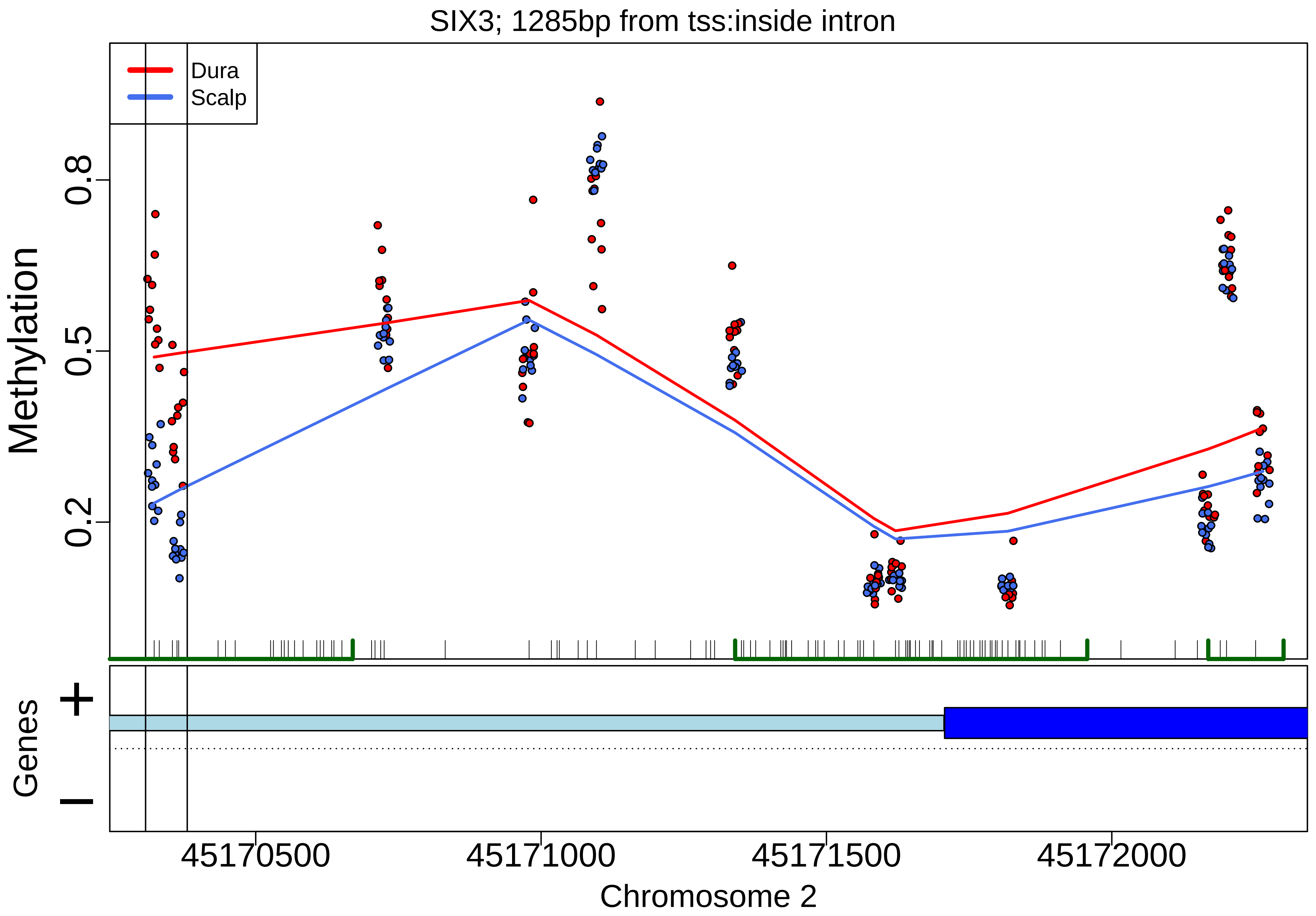

SIX3; 1285bp from tss:inside intron
Dura
Scalp
Methylation
0.2
0.5
0.8
Genes
+
−
45170500
45171000
45171500
45172000
Chromosome 2

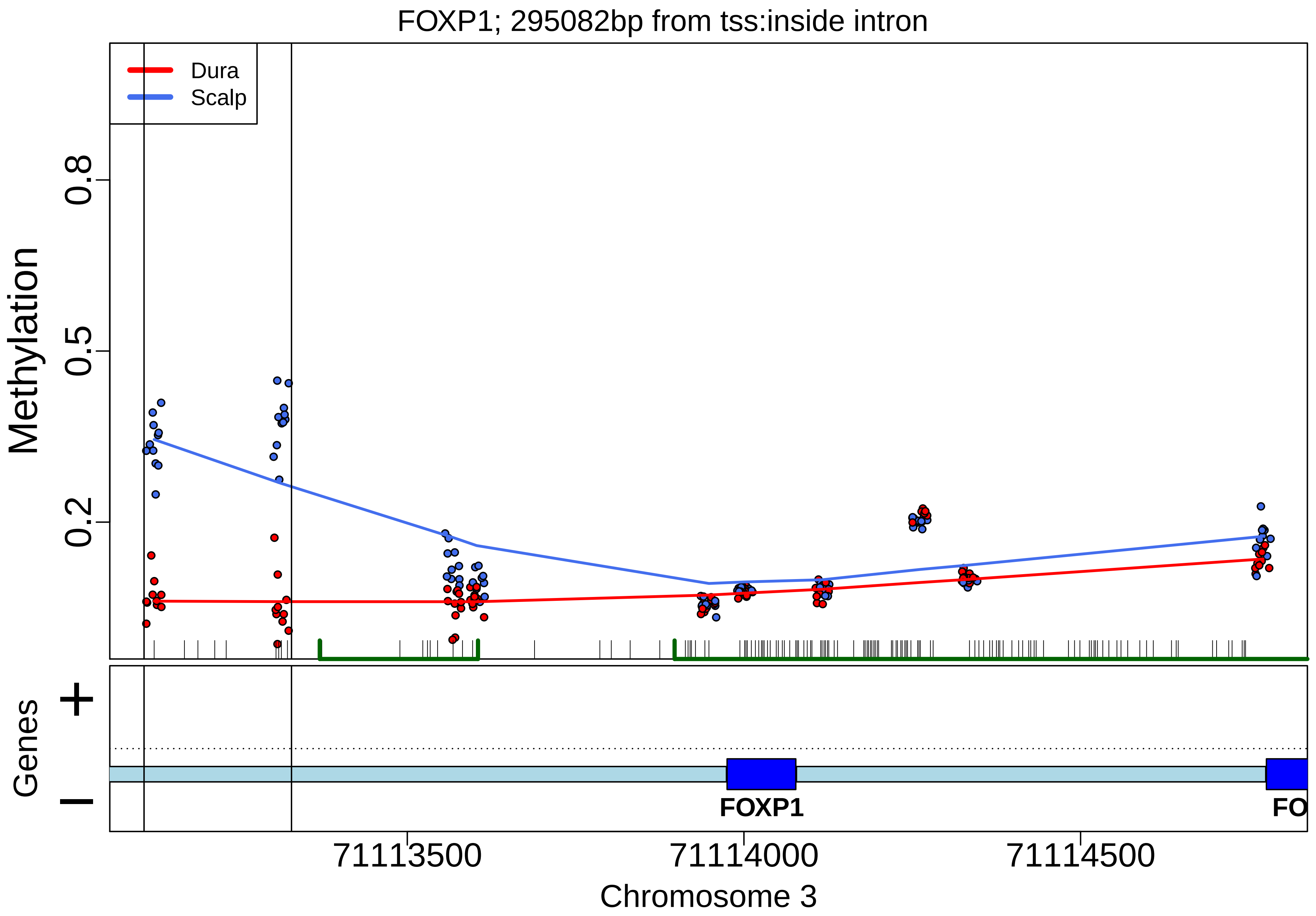

FOXP1; 295082bp from tss:inside intron
Dura
Scalp
Methylation
0.2
0.5
0.8
Genes
+
-
FOXP1
FO
71113500
71114000
71114500
Chromosome 3

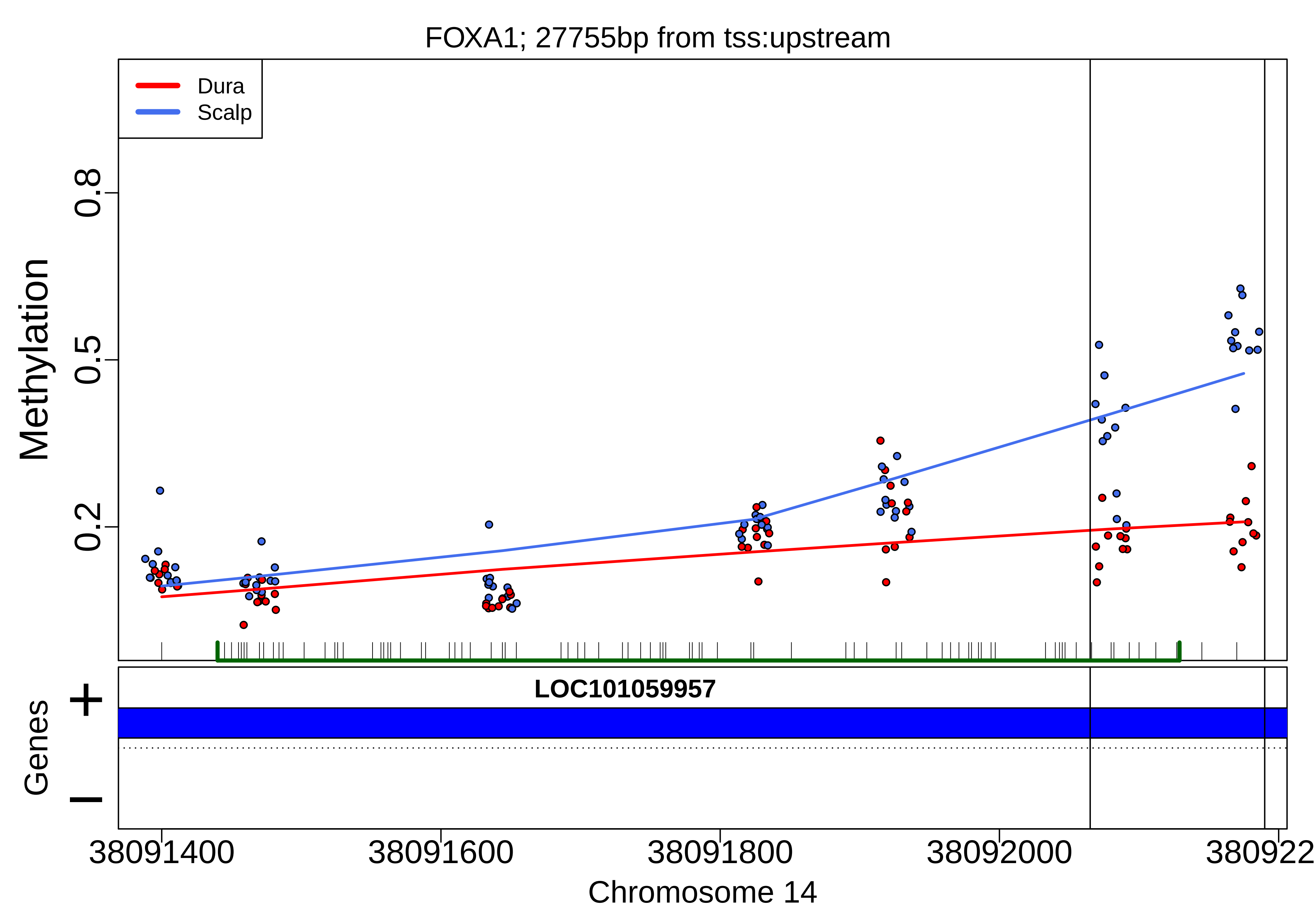

FOXA1; 27755bp from tss:upstream
Dura
Scalp
Methylation
0.2
0.5
0.8
Genes
+
–
LOC101059957
38091400
38091600
38091800
38092000
380922
Chromosome 14

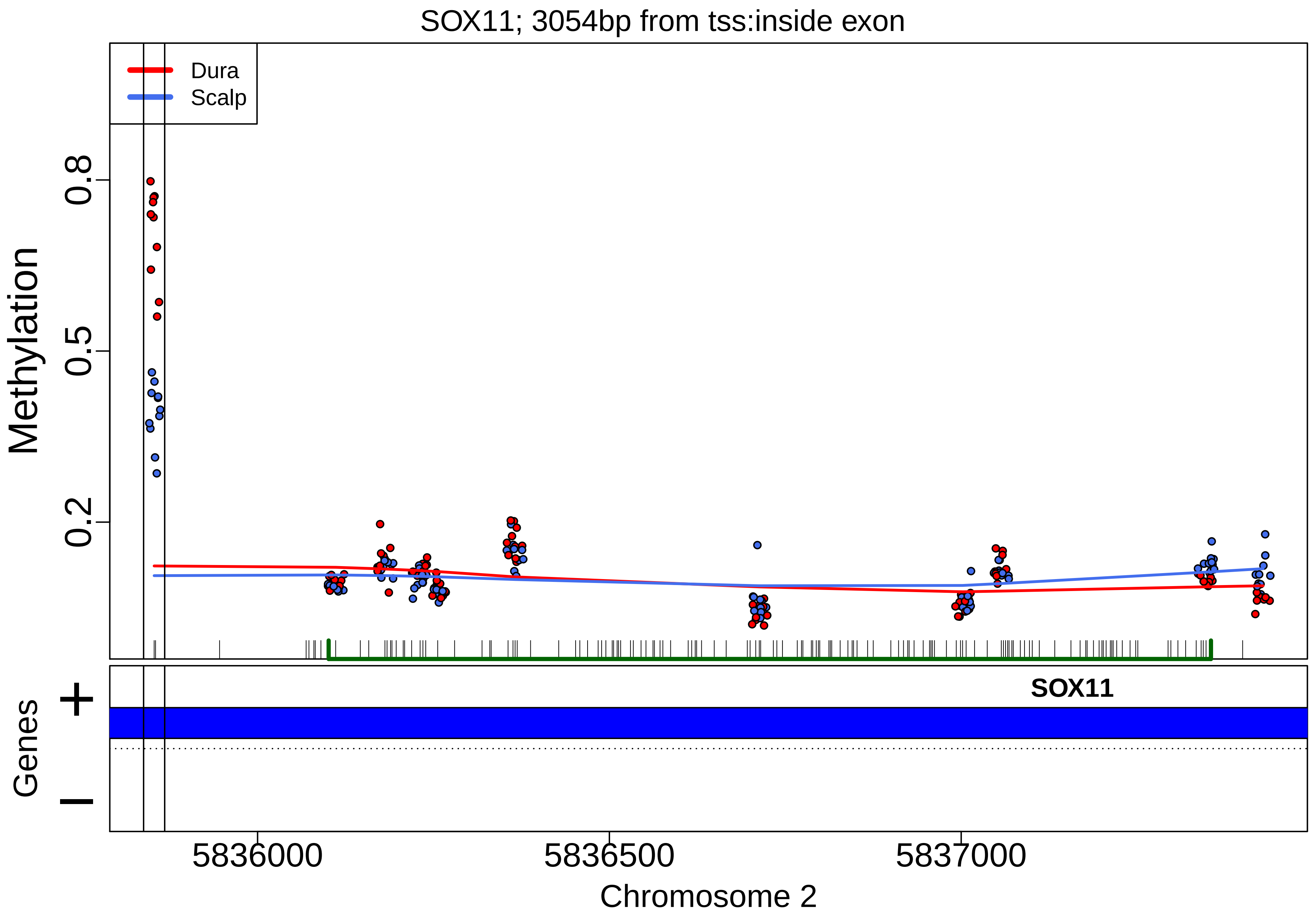

SOX11; 3054bp from tss:inside exon
Dura
Scalp
Methylation
0.8
0.5
0.2
Genes
+
−
SOX11
5836000
5836500
5837000
Chromosome 2

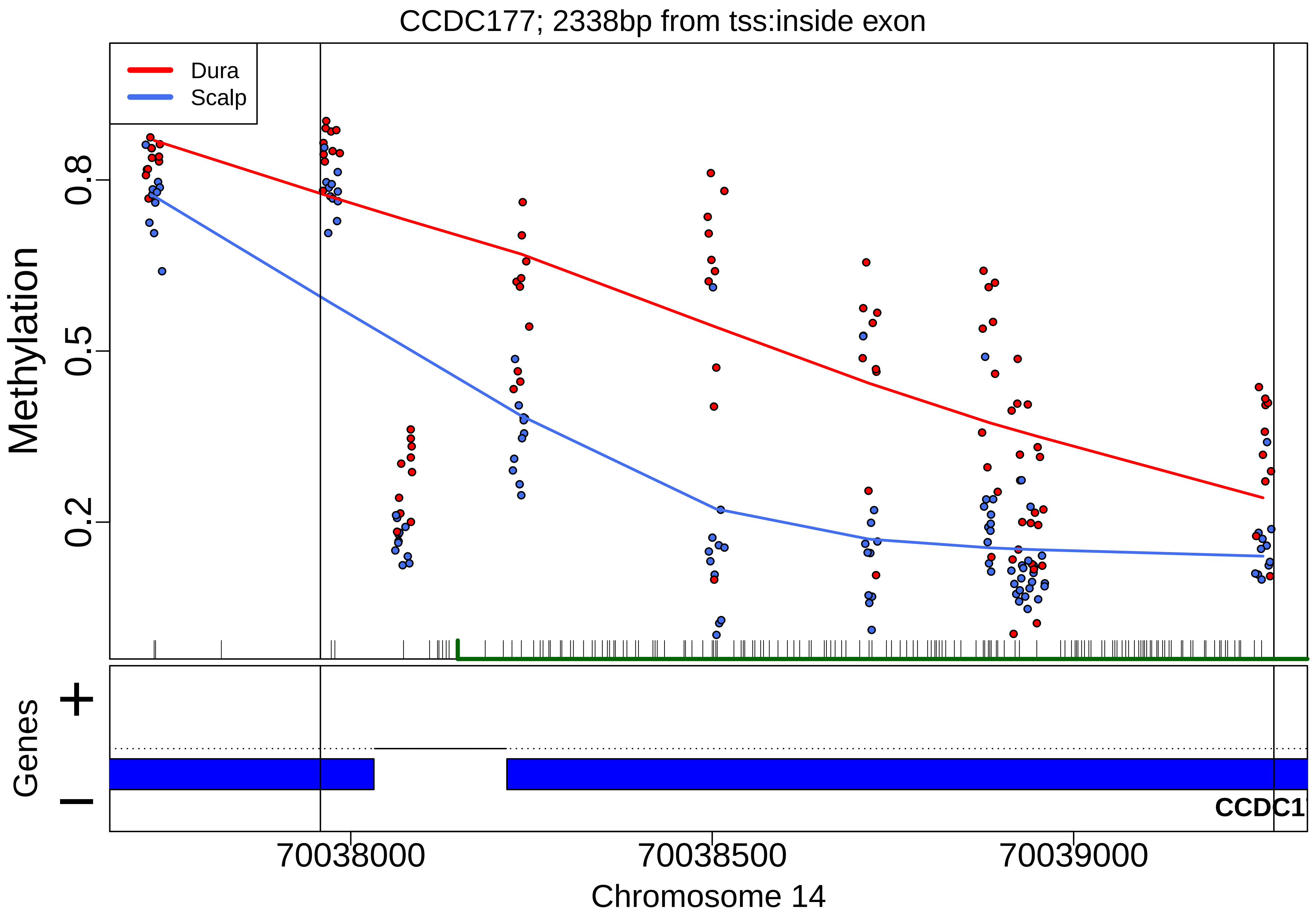

CCDC177; 2338bp from tss:inside exon
Dura
Scalp
Methylation
0.2
0.5
0.8
Genes
+
−
CCDC17
70038000
70038500
70039000
Chromosome 14

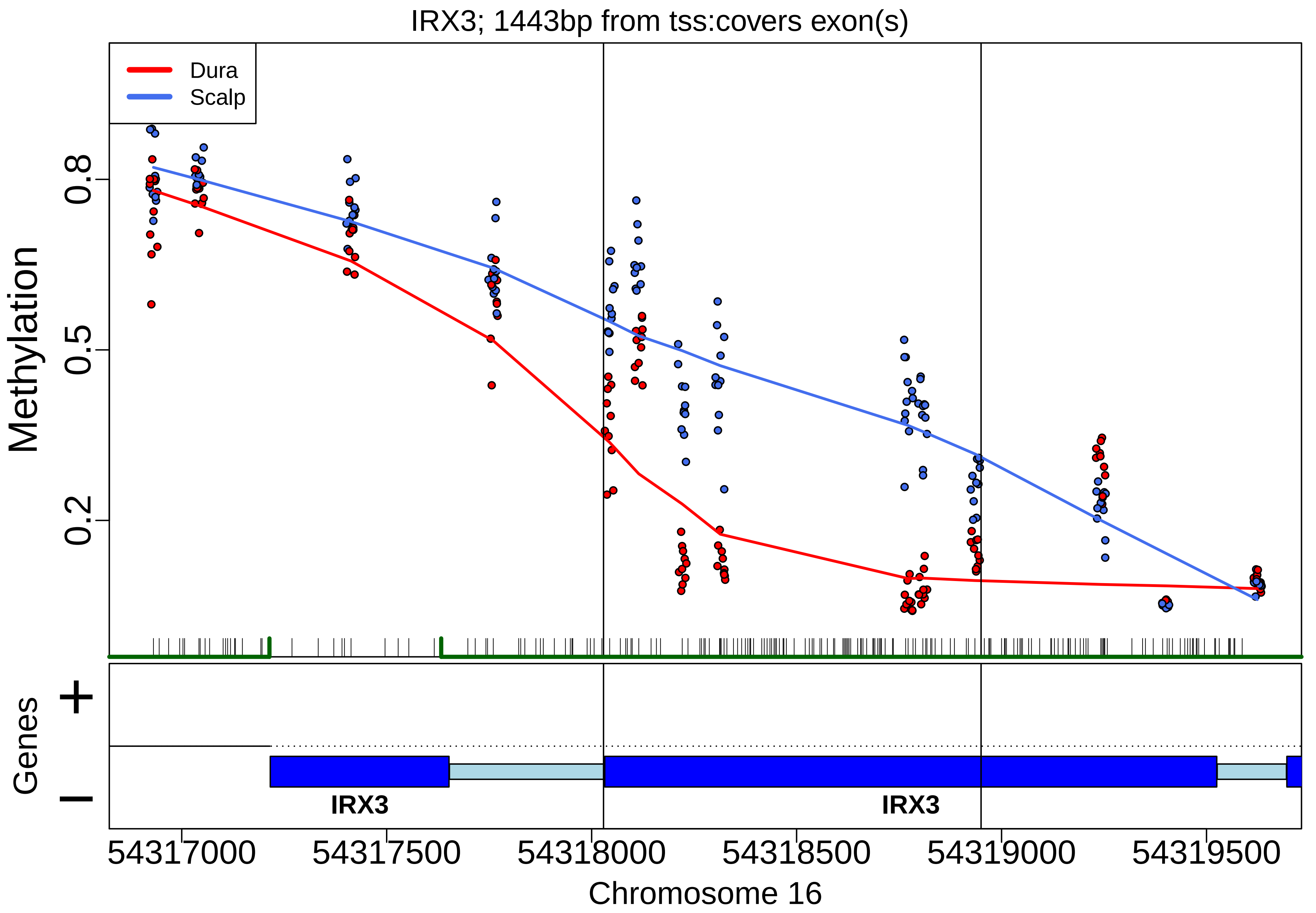

IRX3; 1443bp from tss:covers exon(s)
Dura
Scalp
Methylation
0.2
0.5
0.8
Genes
+
–
IRX3
IRX3
54317000
54317500
54318000
54318500
54319000
54319500
Chromosome 16

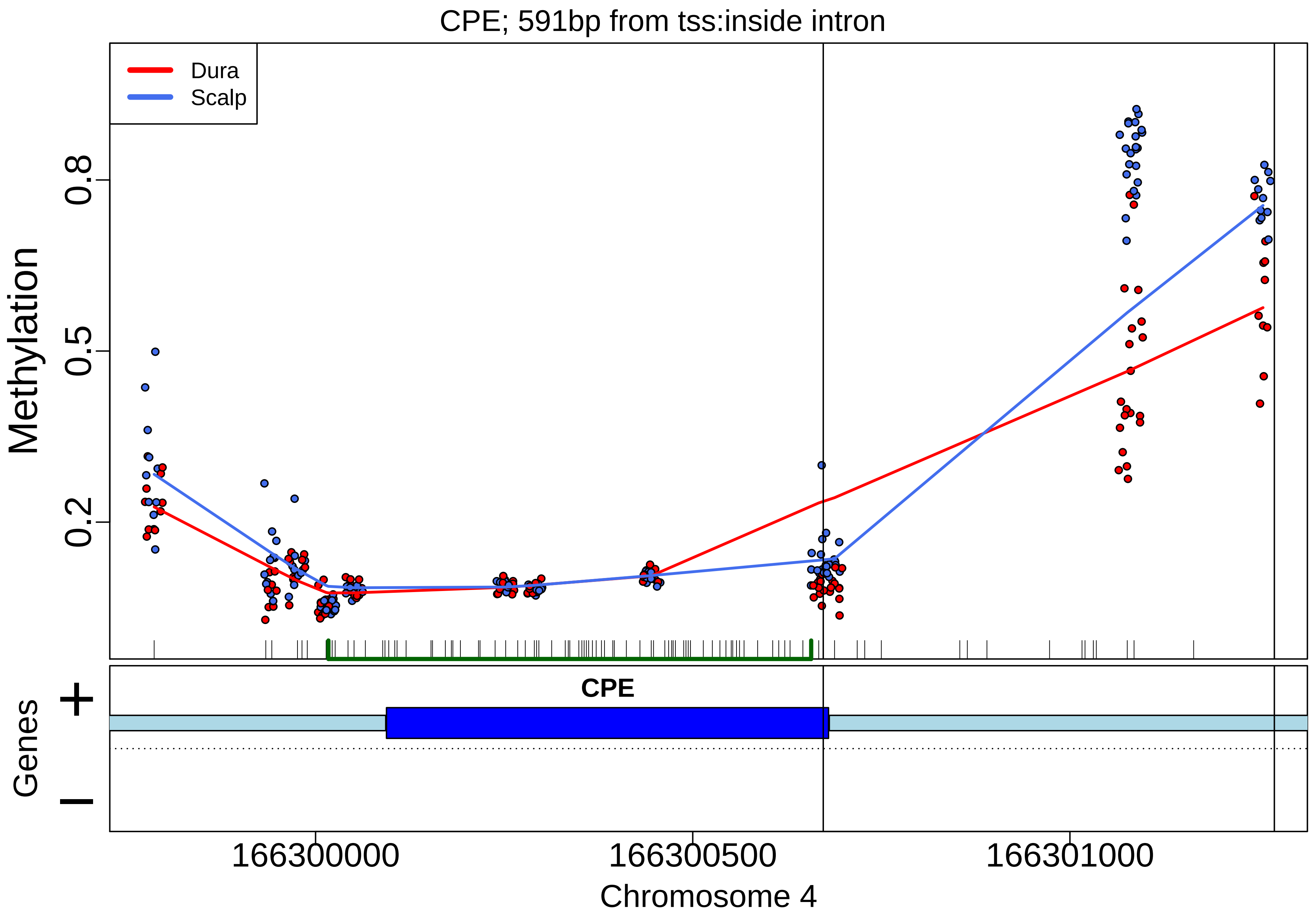

CPE; 591bp from tss:inside intron
Dura
Scalp
Methylation
0.2
0.5
0.8
Genes
+
−
CPE
166300000
166300500
166301000
Chromosome 4

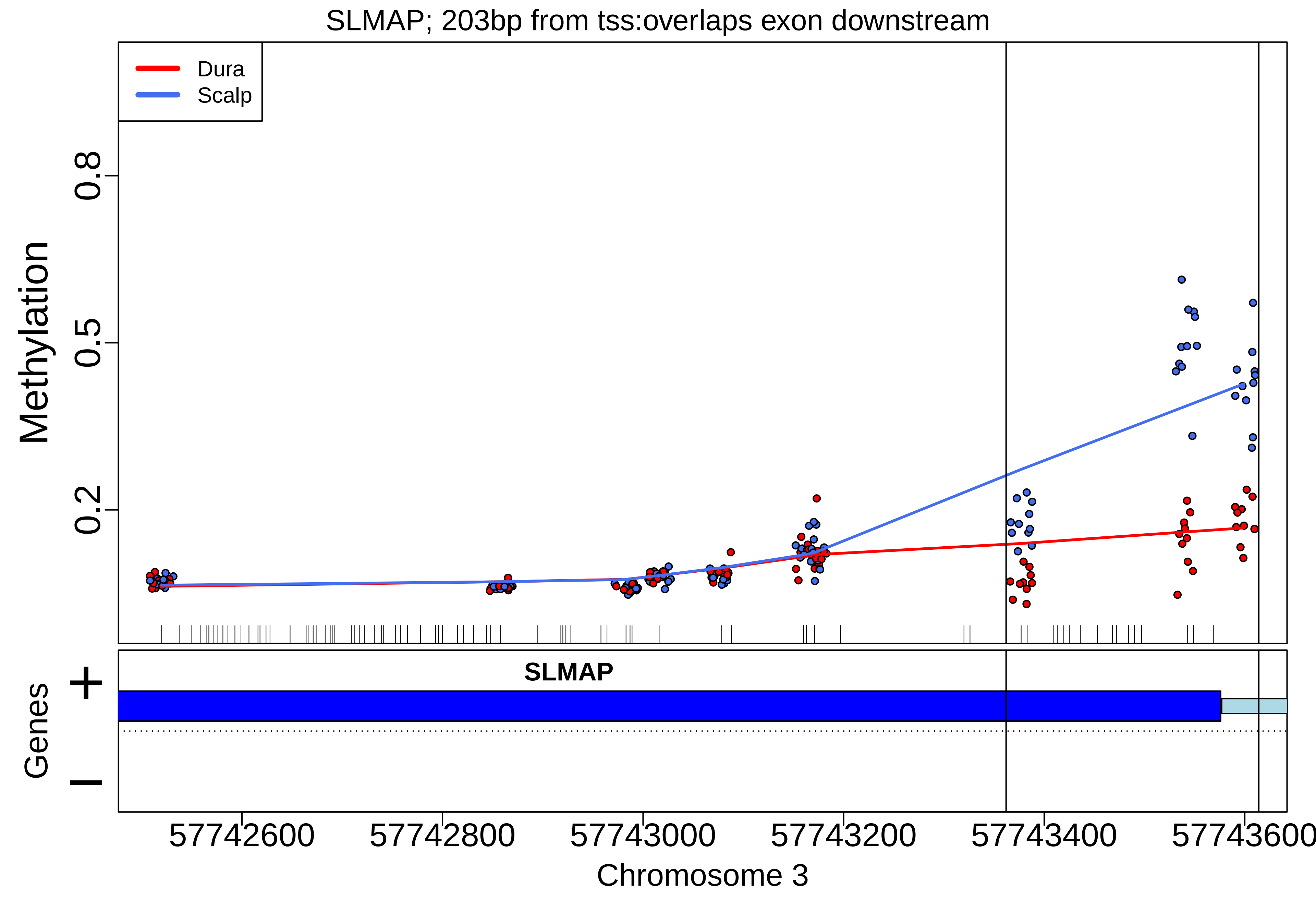
SLMAP; 203bp from tss:overlaps exon downstream
Dura
Scalp
Methylation
0.2
0.5
0.8
Genes
+
-
SLMAP
57742600
57742800
57743000
57743200
57743400
57743600
Chromosome 3

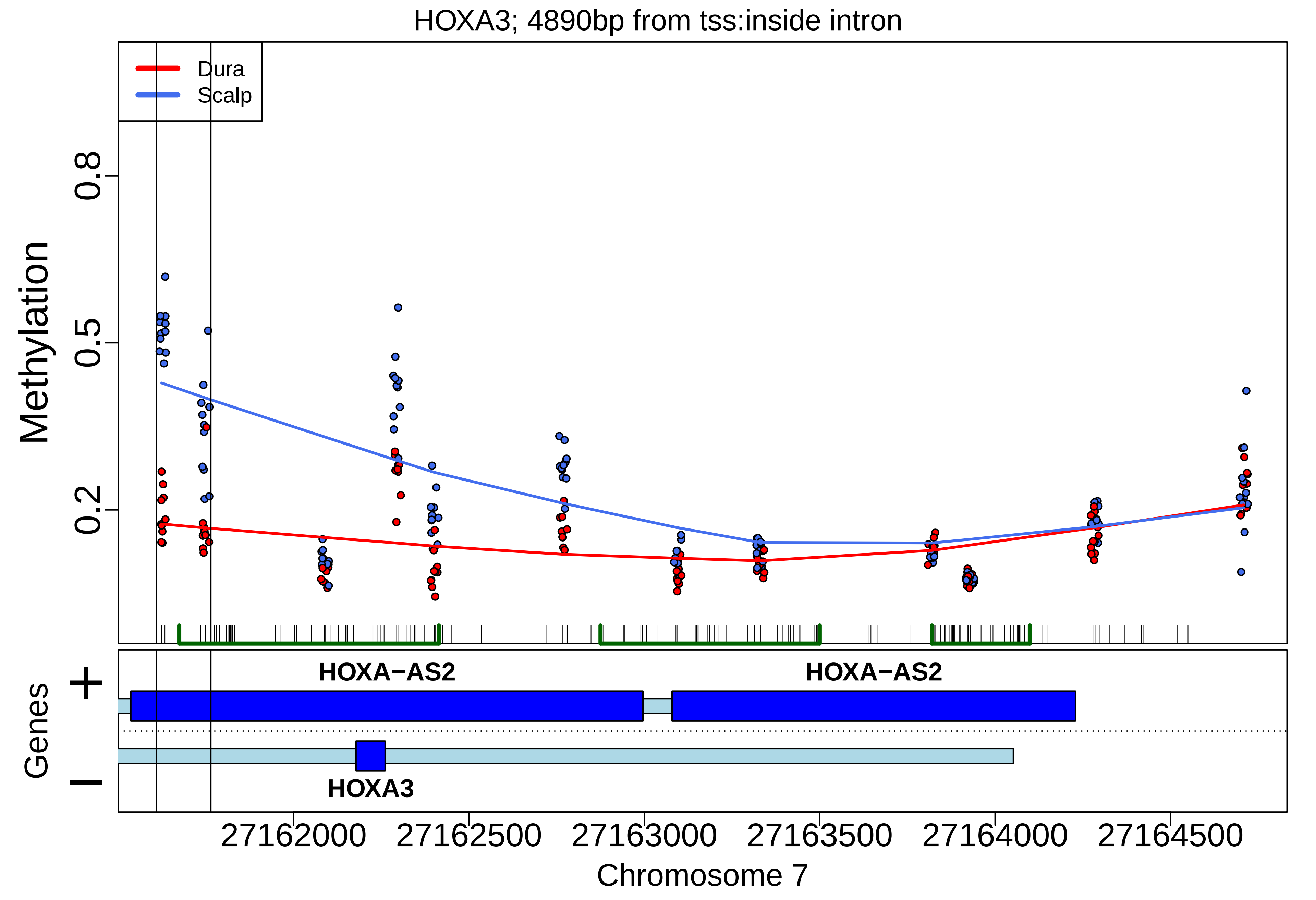

HOXA3; 4890bp from tss:inside intron
Dura
Scalp
Methylation
0.2
0.5
0.8
Genes
+
−
HOXA−AS2
HOXA−AS2
HOXA3
27162000
27162500
27163000
27163500
27164000
27164500
Chromosome 7

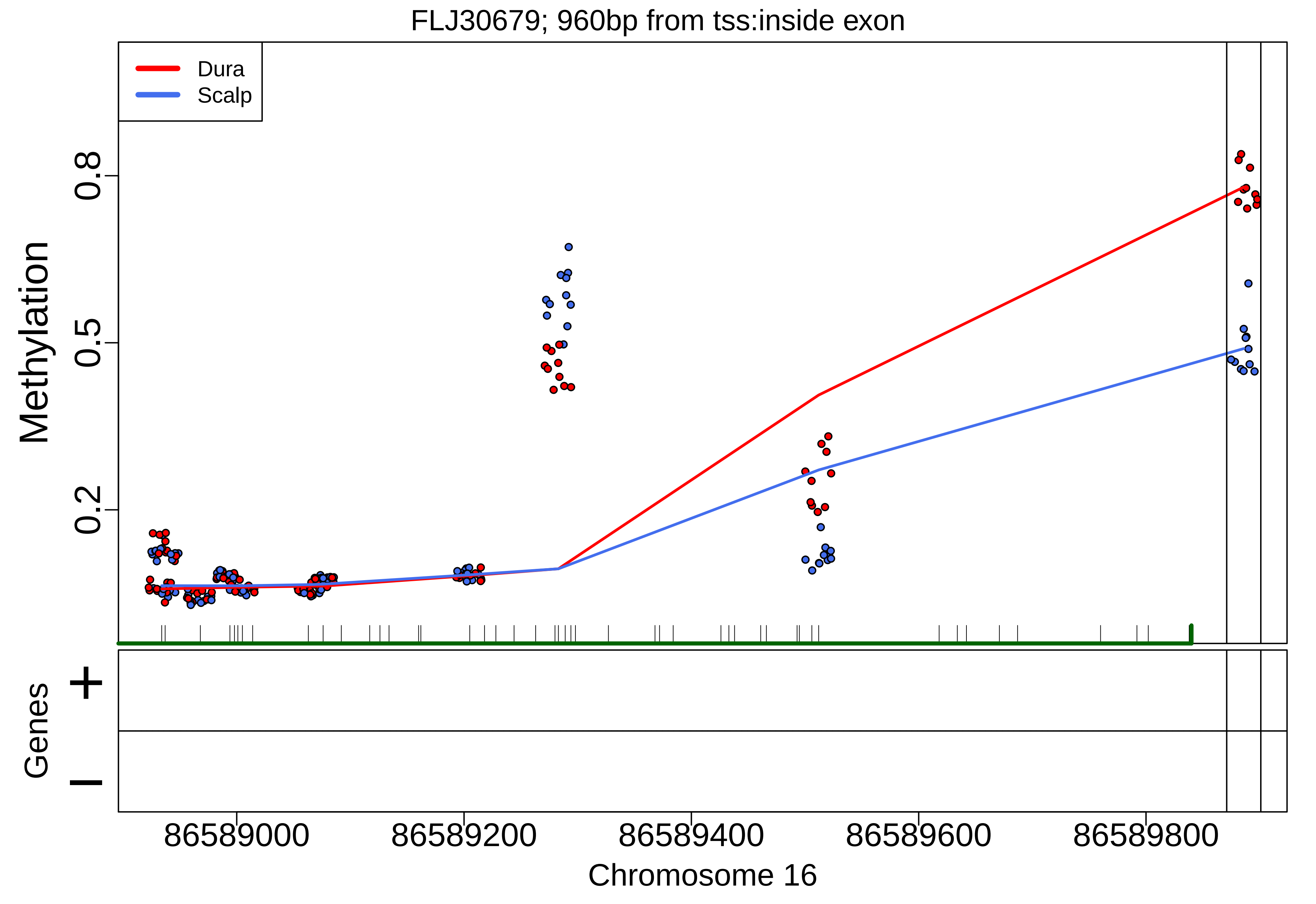
FLJ30679; 960bp from tss:inside exon
Dura
Scalp
Methylation
0.2
0.5
0.8
Genes
+
-
86589000
86589200
86589400
86589600
86589800
Chromosome 16

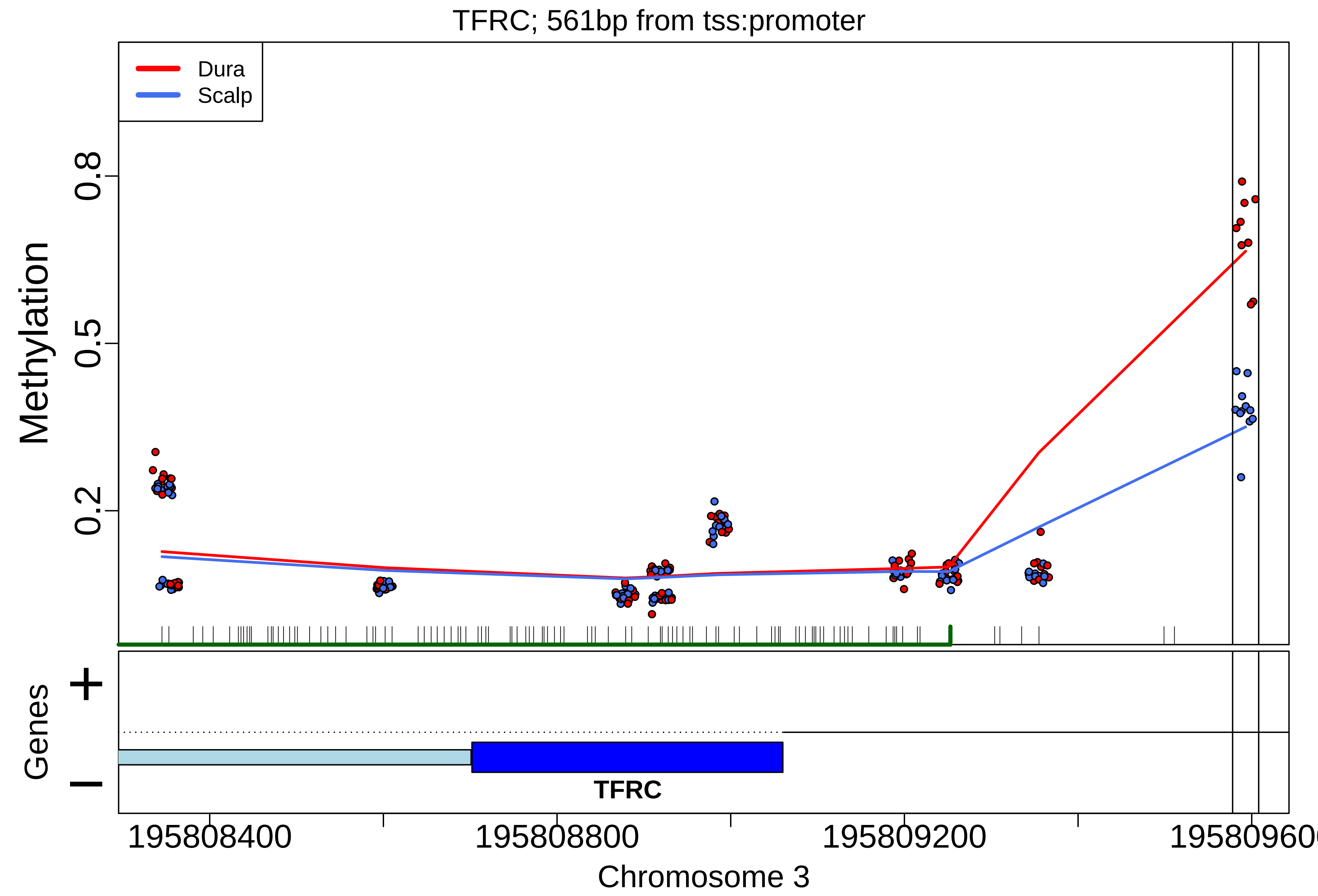

TFRC; 561bp from tss:promoter
Dura
Scalp
Methylation
0.2
0.5
0.8
Genes
+
-
TFRC
195808400
195808800
195809200
19580960
Chromosome 3

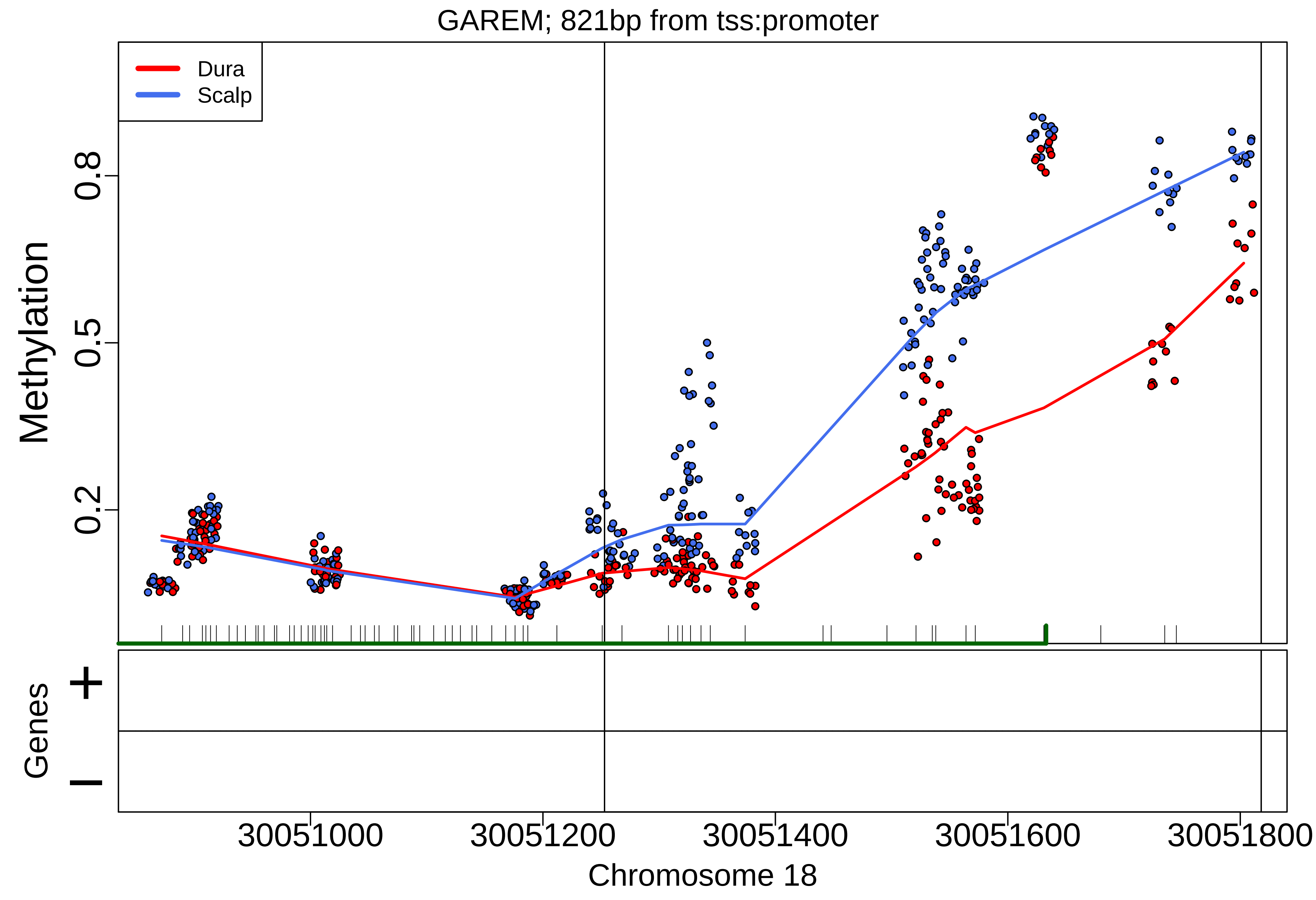

GAREM; 821bp from tss:promoter
Dura
Scalp
Methylation
0.2
0.5
0.8
Genes
+
-
30051000
30051200
30051400
30051600
30051800
Chromosome 18

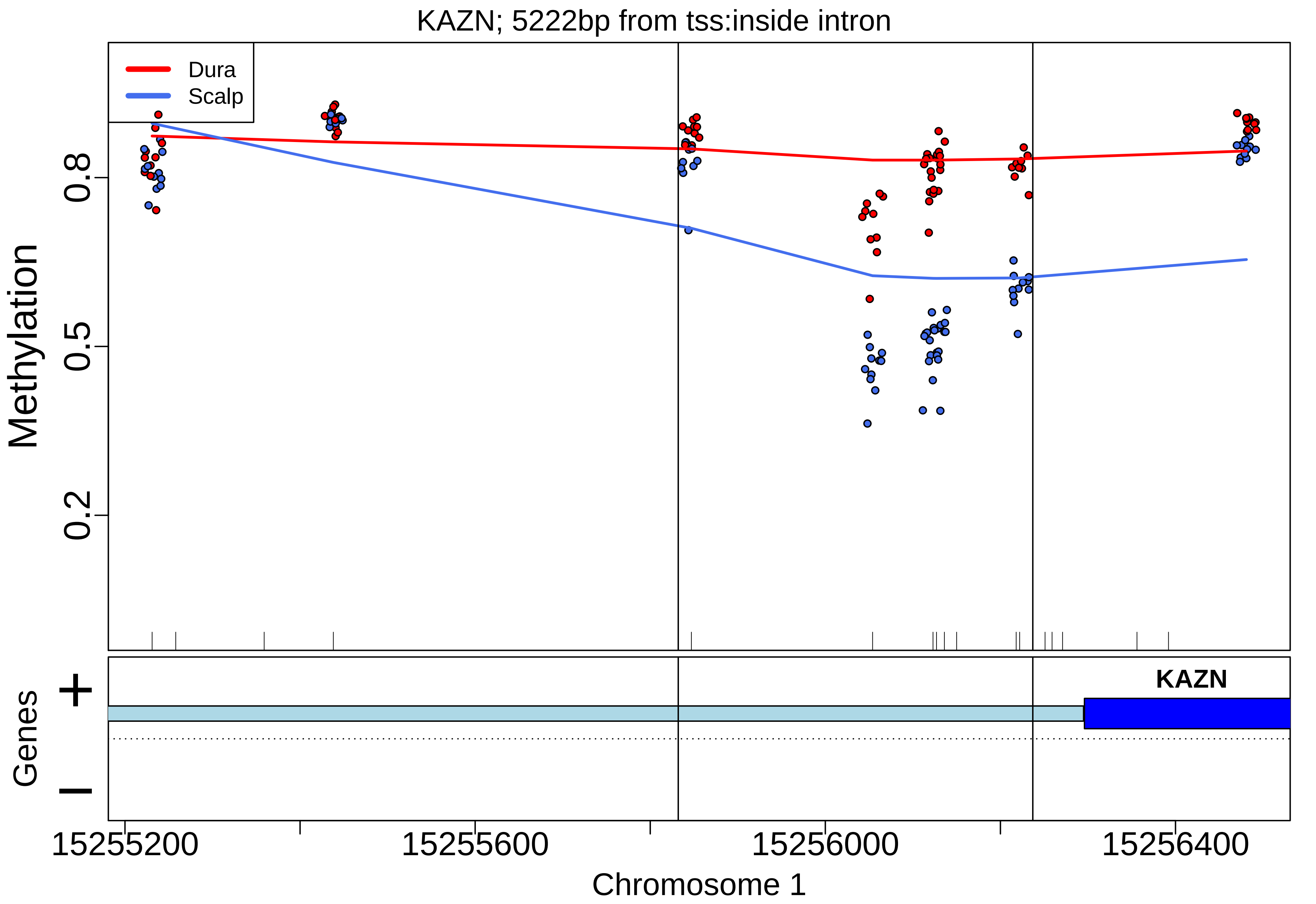

KAZN; 5222bp from tss:inside intron
Dura
Scalp
Methylation
0.8
0.5
0.2
Genes
+
−
KAZN
15255200
15255600
15256000
15256400
Chromosome 1

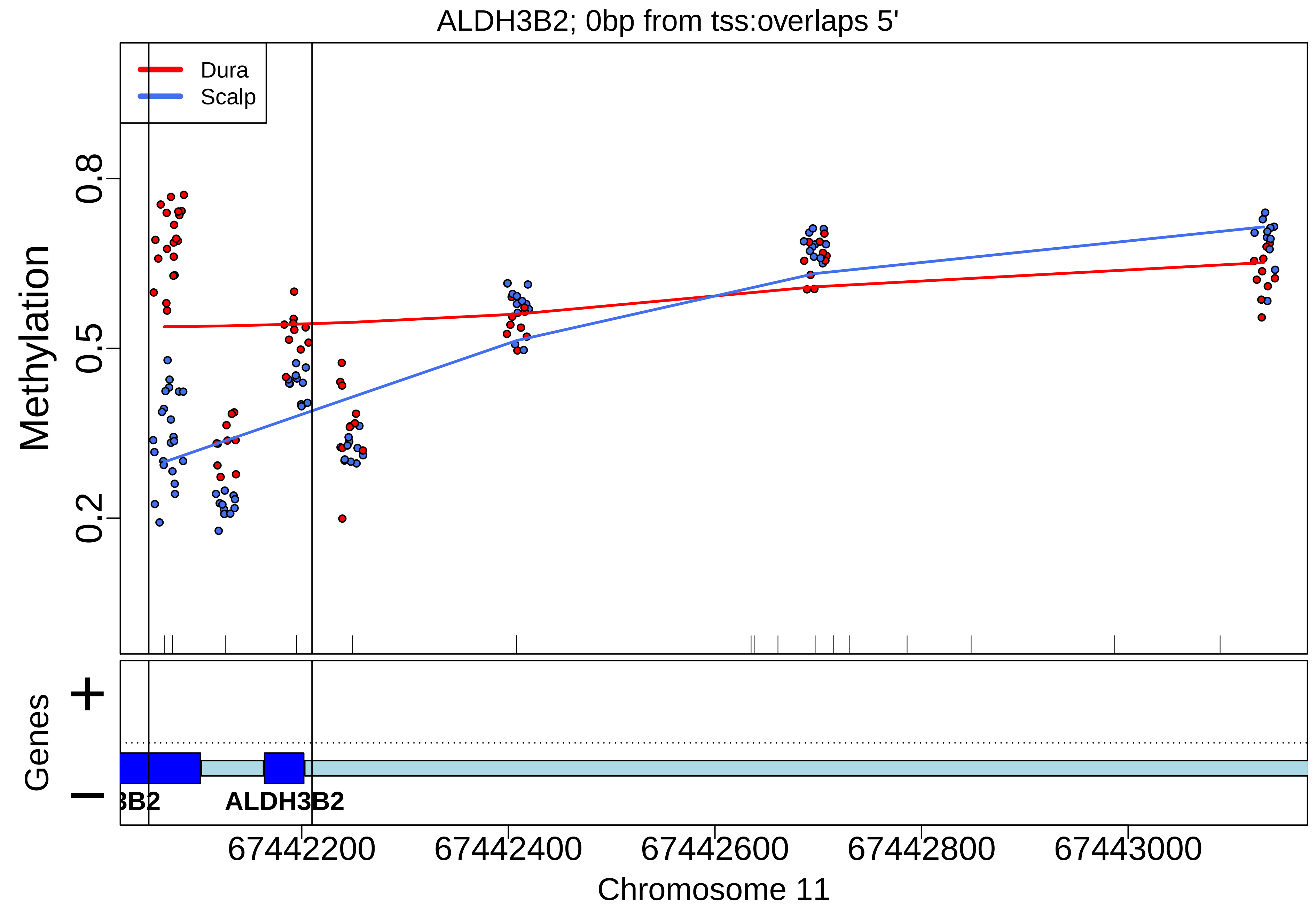

ALDH3B2; 0bp from tss:overlaps 5'
Dura
Scalp
Methylation
0.2
0.5
0.8
Genes
+
-
3B2
ALDH3B2
67442200
67442400
67442600
67442800
67443000
Chromosome 11

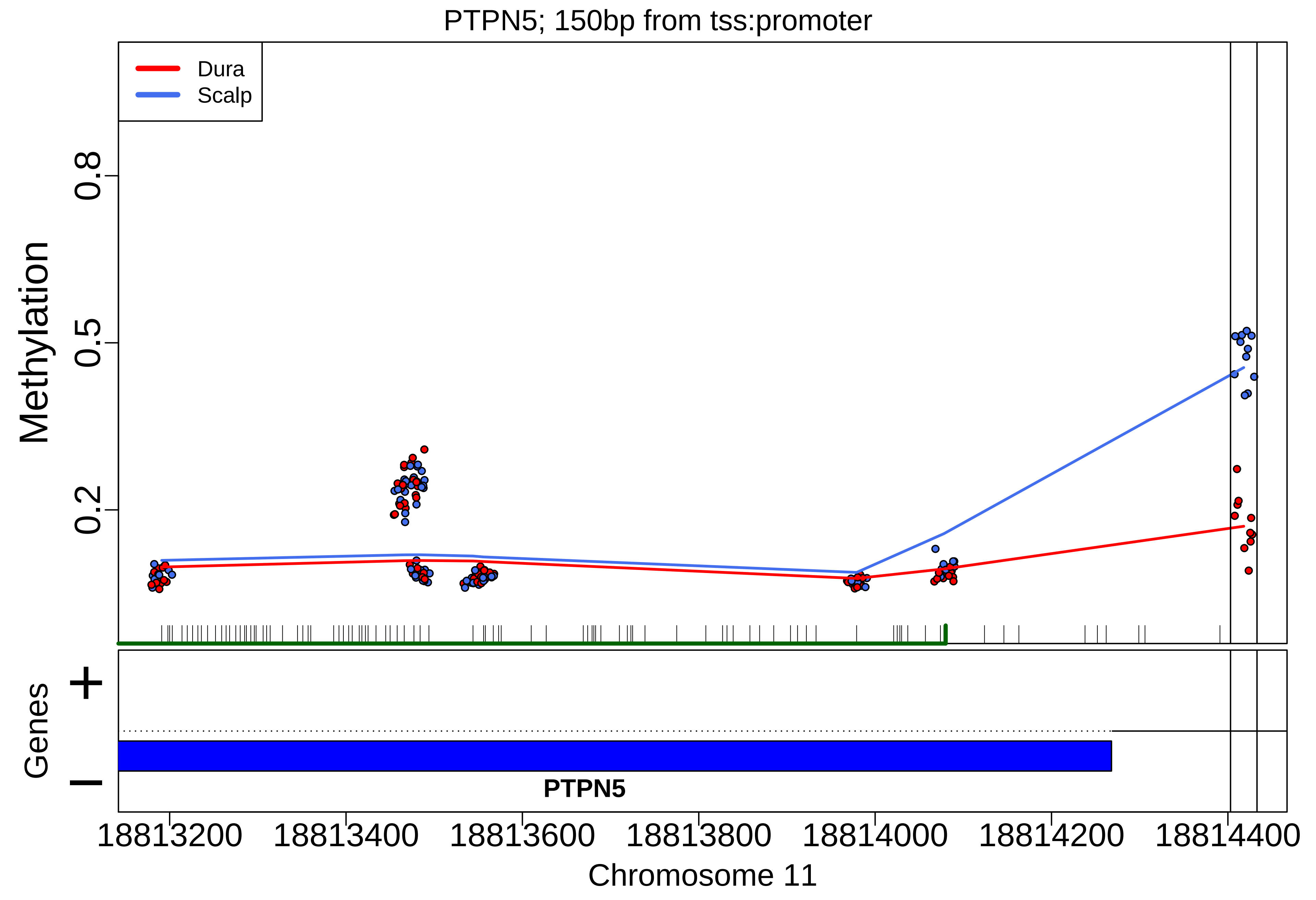
PTPN5; 150bp from tss:promoter
Dura
Scalp
Methylation
0.2
0.5
0.8
Genes
+
-
PTPN5
18813200
18813400
18813600
18813800
18814000
18814200
18814400
Chromosome 11

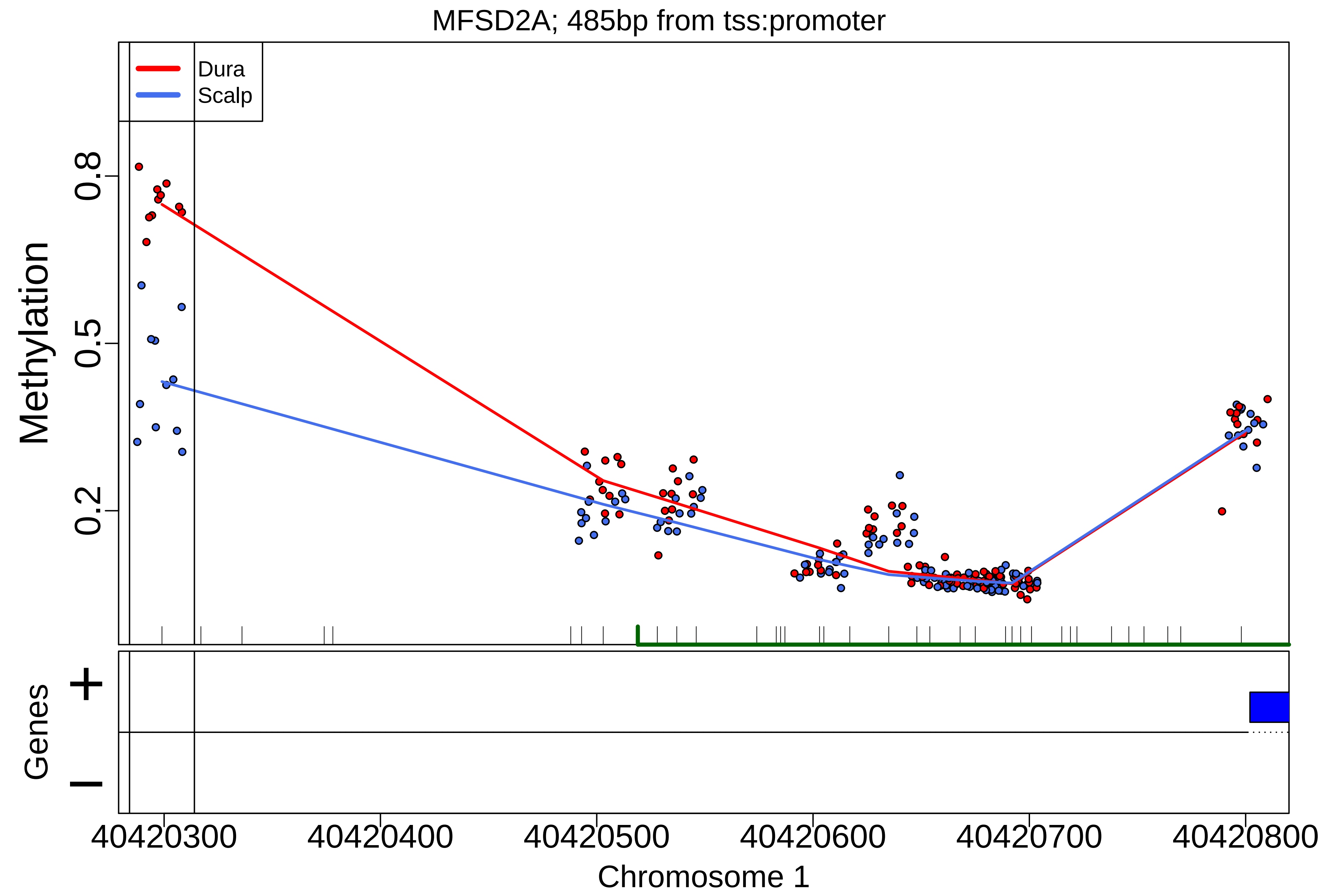
MFSD2A; 485bp from tss:promoter
Dura
Scalp
Methylation
0.2
0.5
0.8
Genes
+
-
40420300
40420400
40420500
40420600
40420700
40420800
Chromosome 1

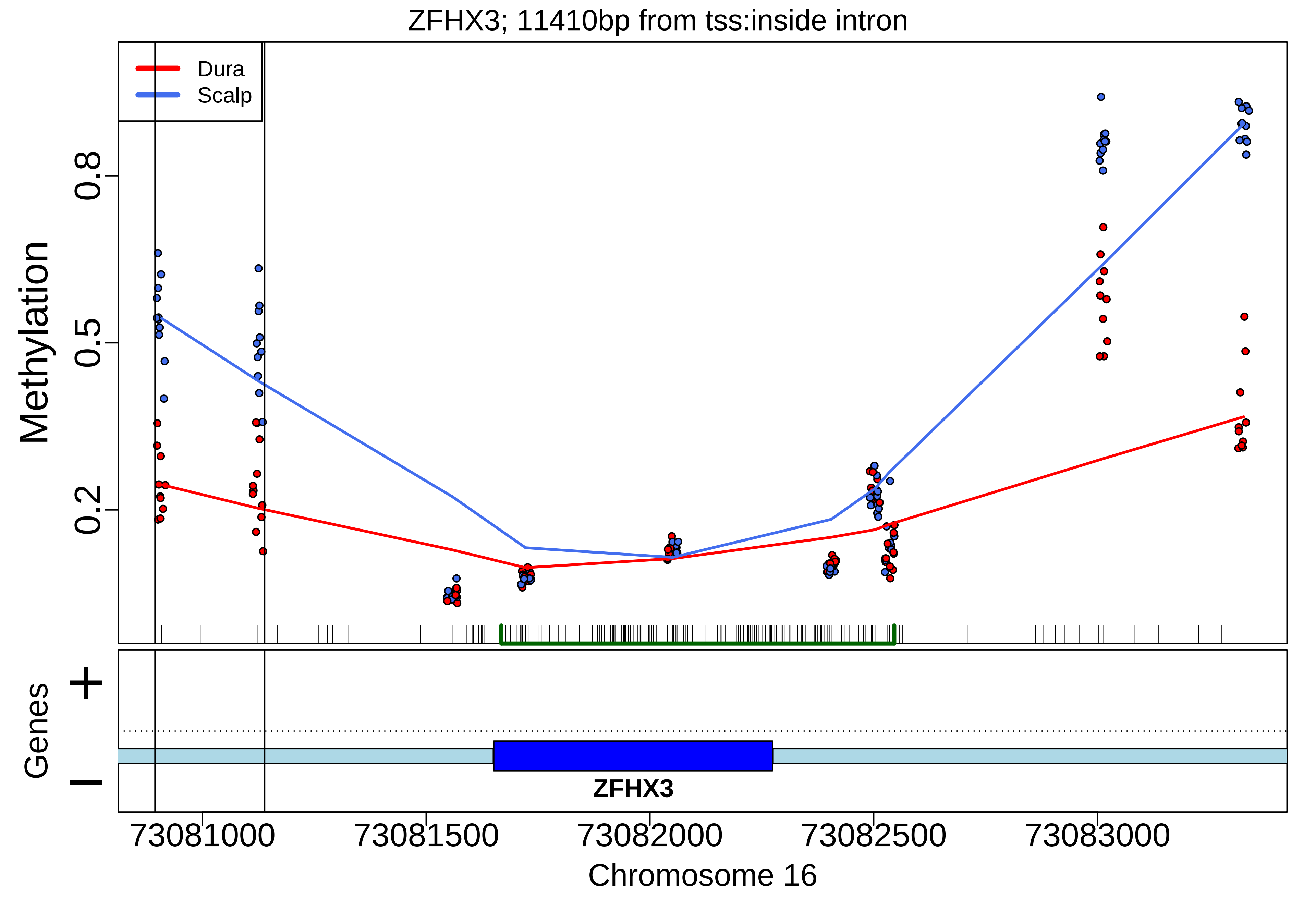

ZFHX3; 11410bp from tss:inside intron
Dura
Scalp
Methylation
0.2
0.5
0.8
Genes
+
−
ZFHX3
73081000
73081500
73082000
73082500
73083000
Chromosome 16

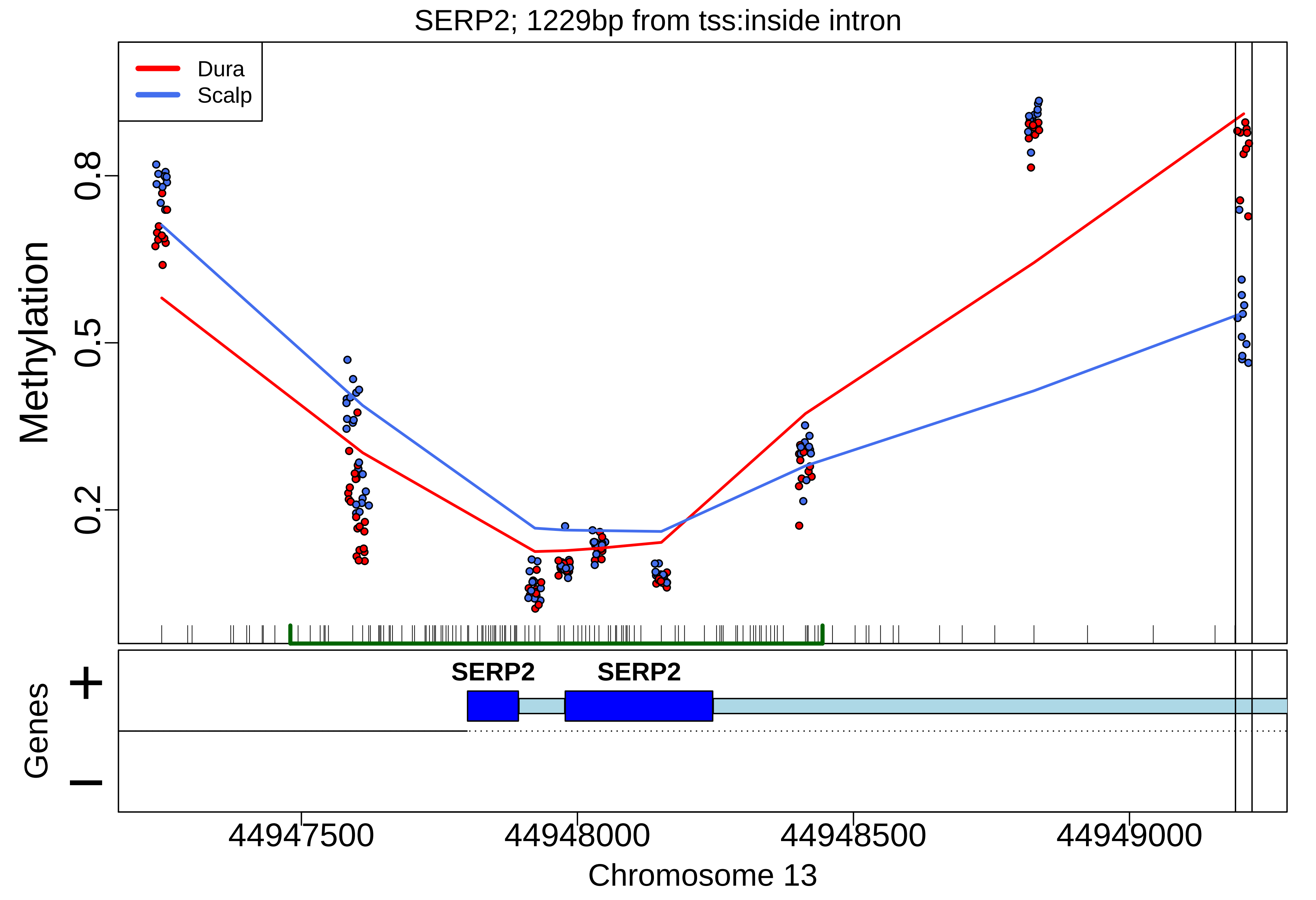
SERP2; 1229bp from tss:inside intron
Dura
Scalp
Methylation
0.2
0.5
0.8
Genes
+
-
SERP2
SERP2
44947500
44948000
44948500
44949000
Chromosome 13

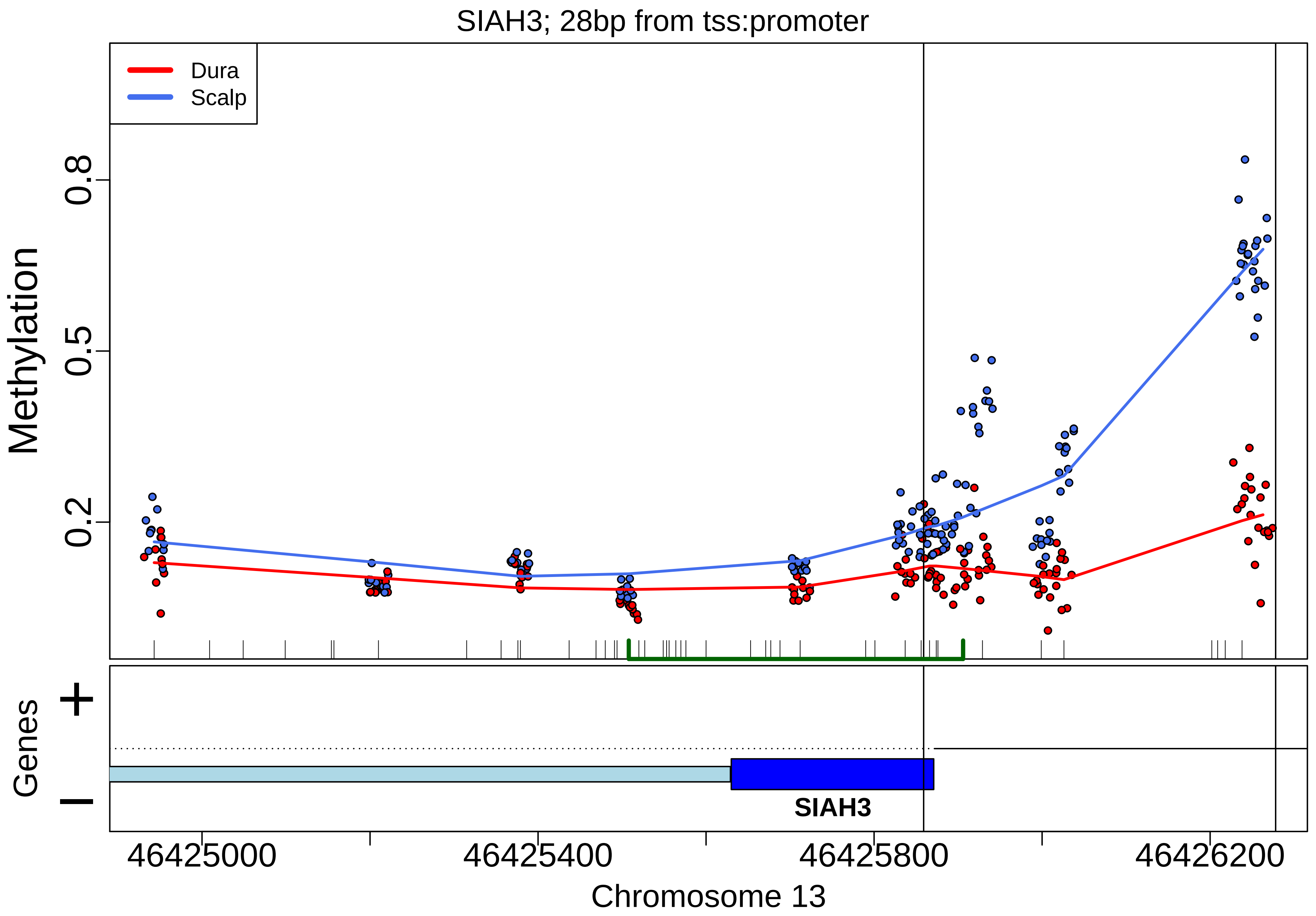
SIAH3; 28bp from tss:promoter
Dura
Scalp
Methylation
0.2
0.5
0.8
Genes
+
−
SIAH3
46425000
46425400
46425800
46426200
Chromosome 13

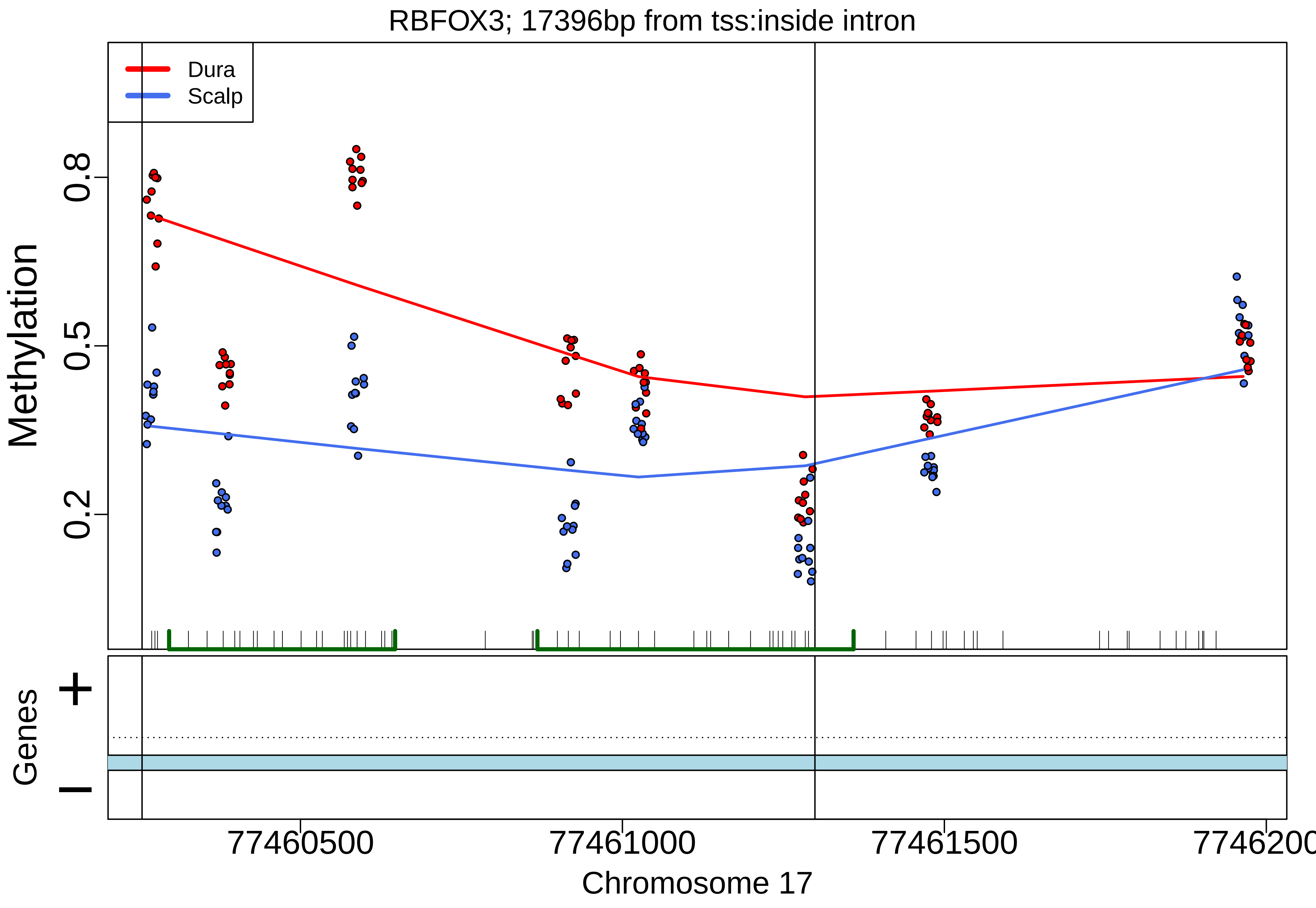

RBFOX3; 17396bp from tss:inside intron
Dura
Scalp
Methylation
0.2
0.5
0.8
Genes
+
-
77460500
77461000
77461500
7746200
Chromosome 17

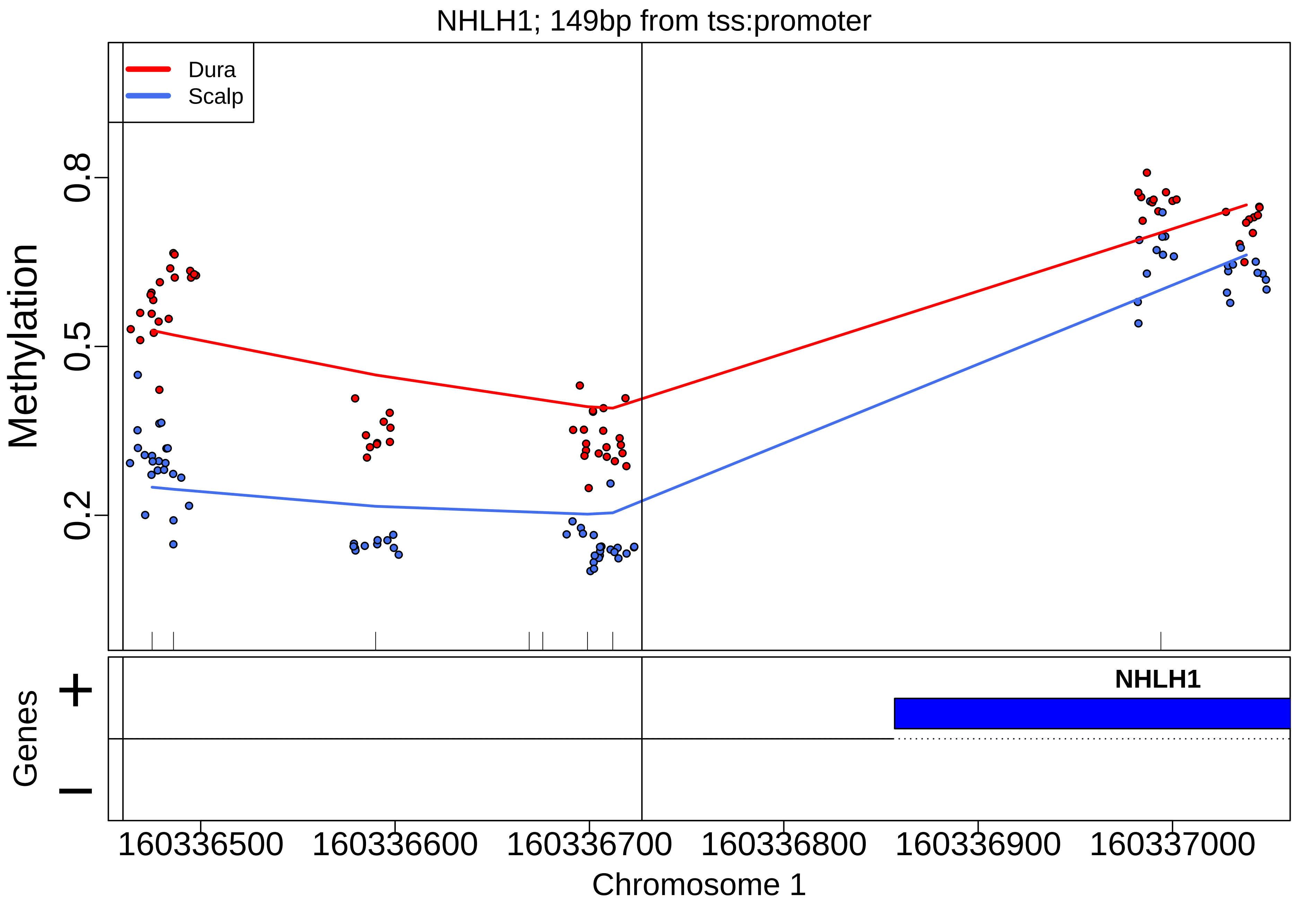
NHLH1; 149bp from tss:promoter
Dura
Scalp
Methylation
0.2
0.5
0.8
Genes
+
−
NHLH1
160336500
160336600
160336700
160336800
160336900
160337000
Chromosome 1

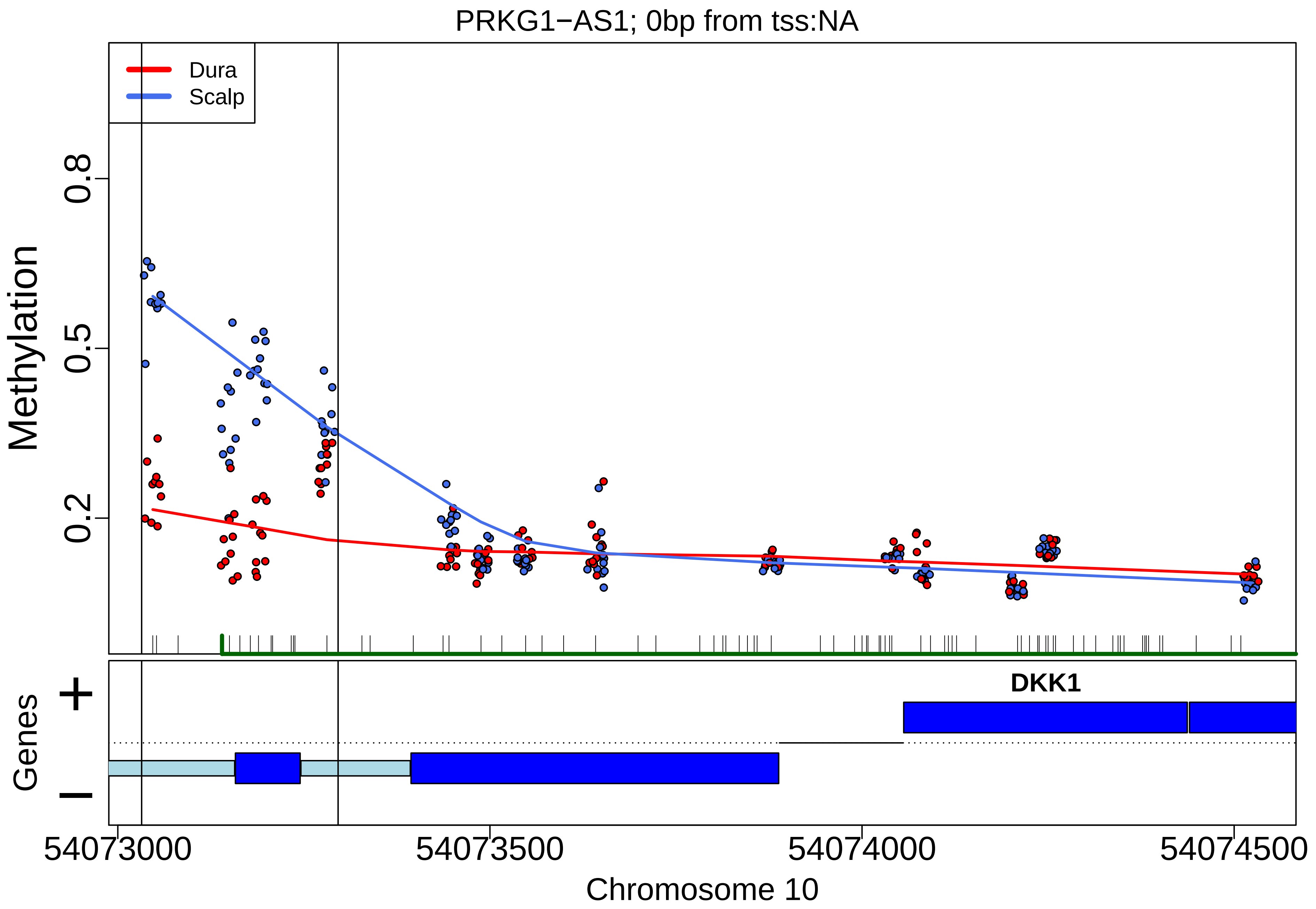
PRKG1−AS1; 0bp from tss:NA
Dura
Scalp
Methylation
0.2
0.5
0.8
Genes
+
−
DKK1
54073000
54073500
54074000
54074500
Chromosome 10

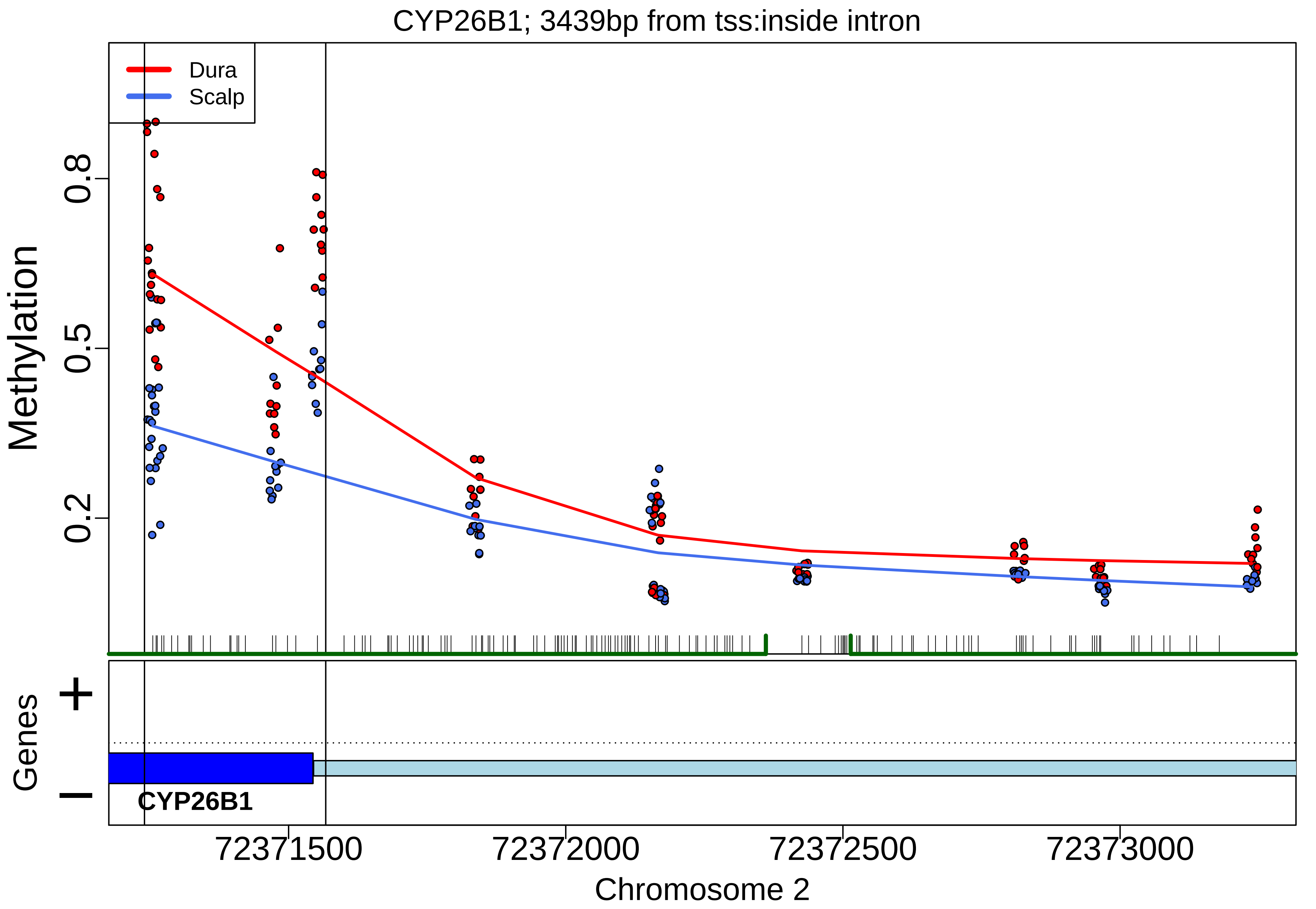

CYP26B1; 3439bp from tss:inside intron
Dura
Scalp
Methylation
0.2
0.5
0.8
Genes
+
-
CYP26B1
72371500
72372000
72372500
72373000
Chromosome 2

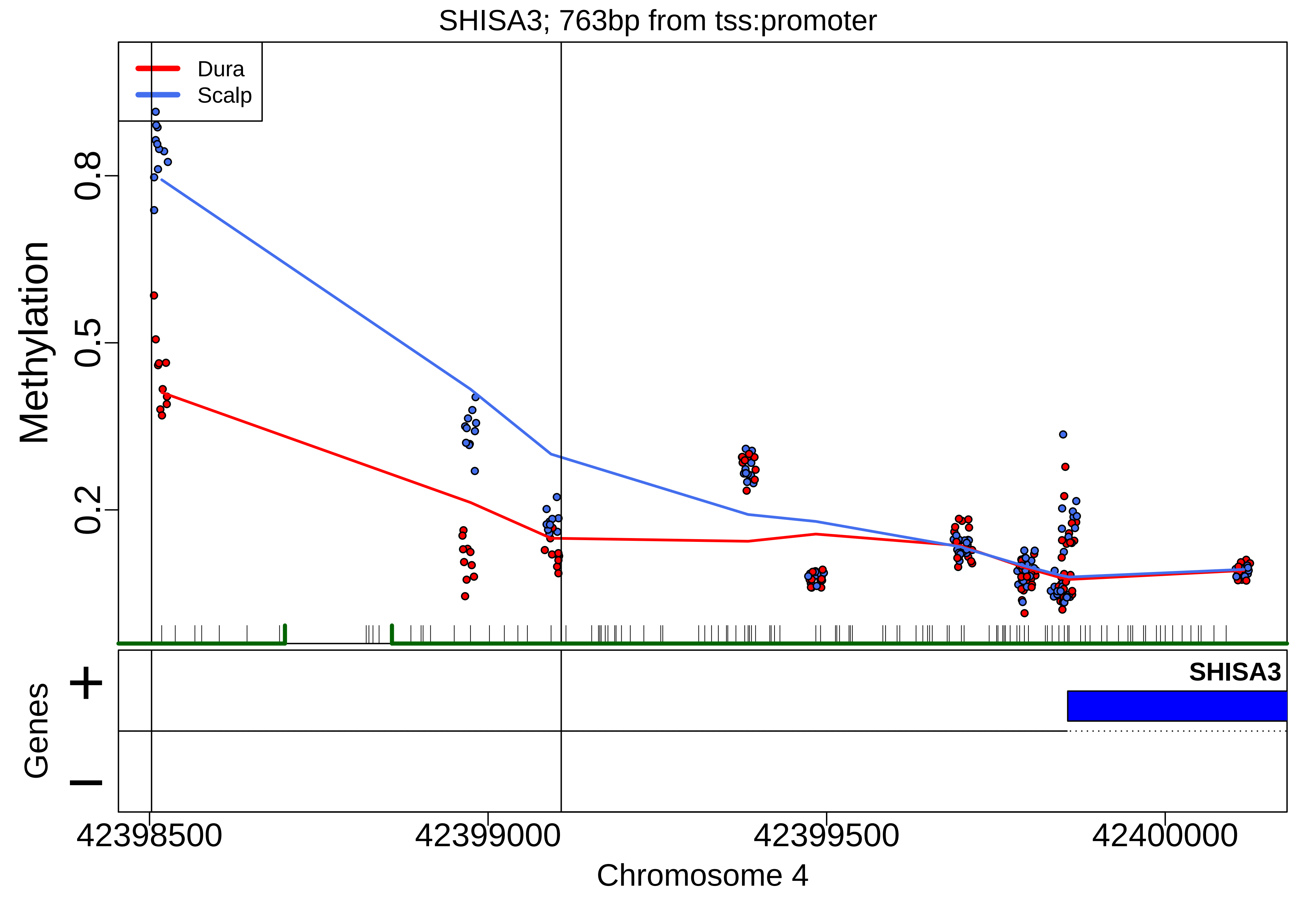
SHISA3; 763bp from tss:promoter
Dura
Scalp
Methylation
0.2
0.5
0.8
Genes
+
-
SHISA3
42398500
42399000
42399500
42400000
Chromosome 4

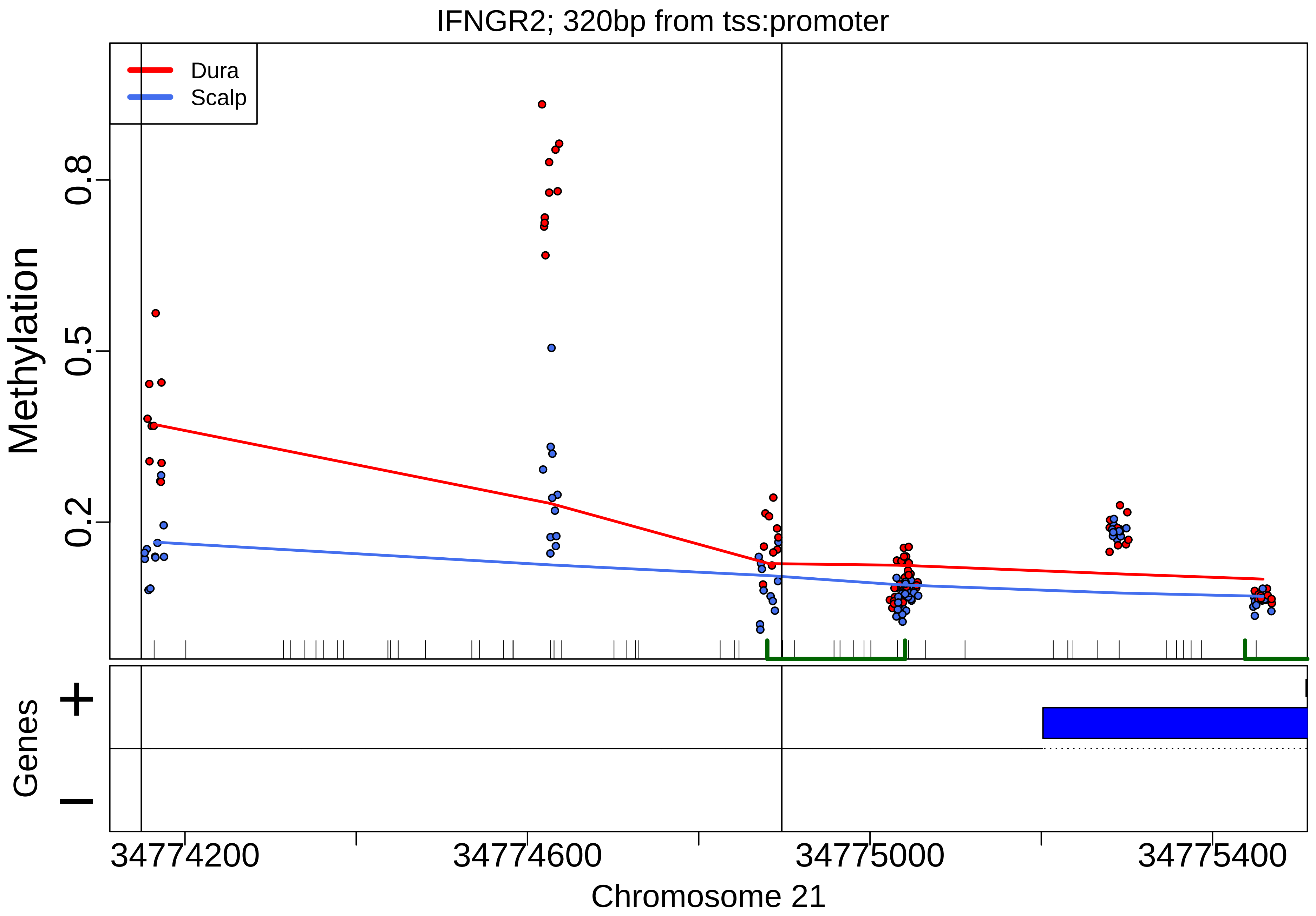

IFNGR2; 320bp from tss:promoter
Dura
Scalp
Methylation
0.2
0.5
0.8
Genes
+
-
34774200
34774600
34775000
34775400
Chromosome 21

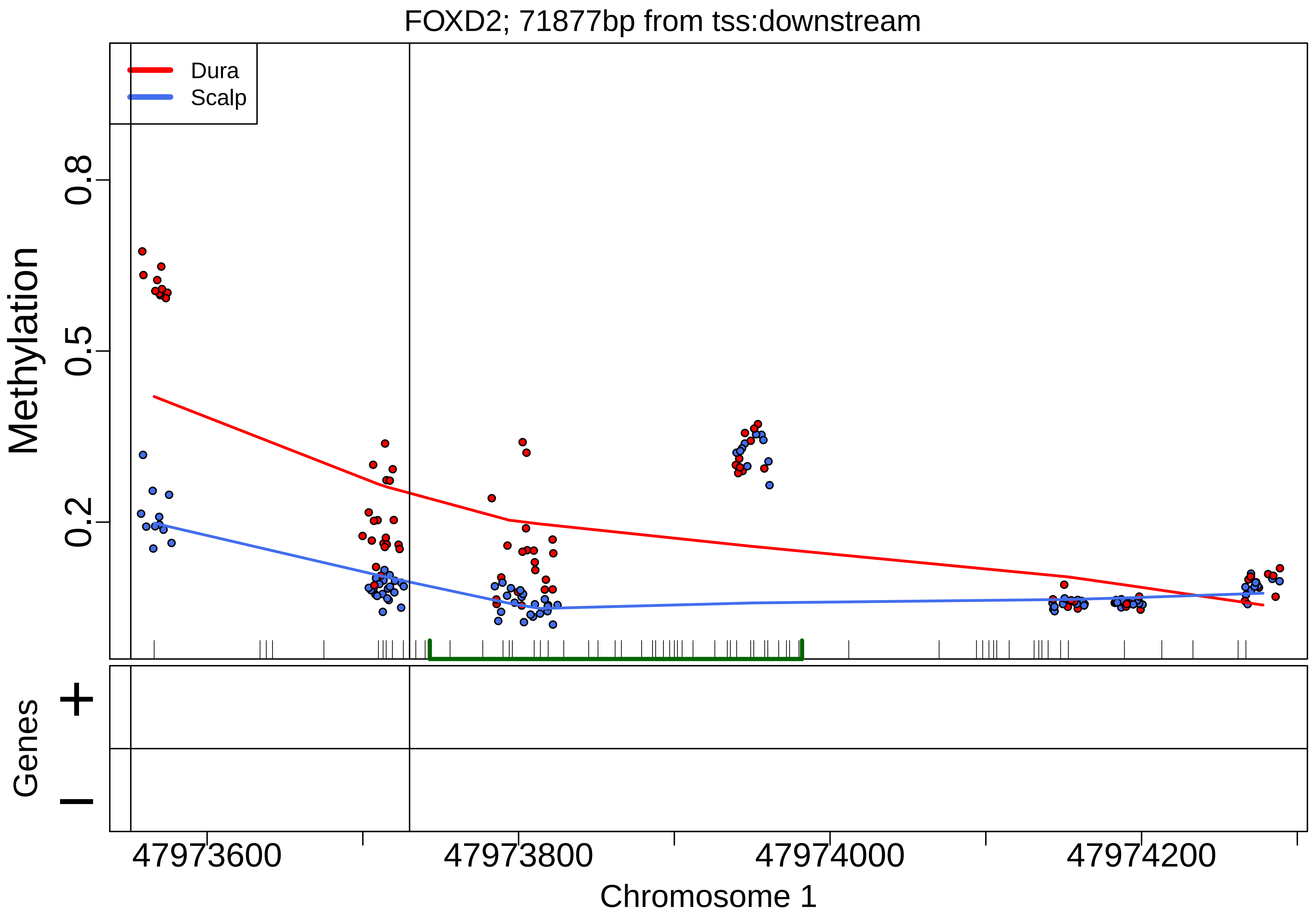
FOXD2; 71877bp from tss:downstream
Dura
Scalp
Methylation
0.2
0.5
0.8
Genes
+
-
47973600
47973800
47974000
47974200
Chromosome 1

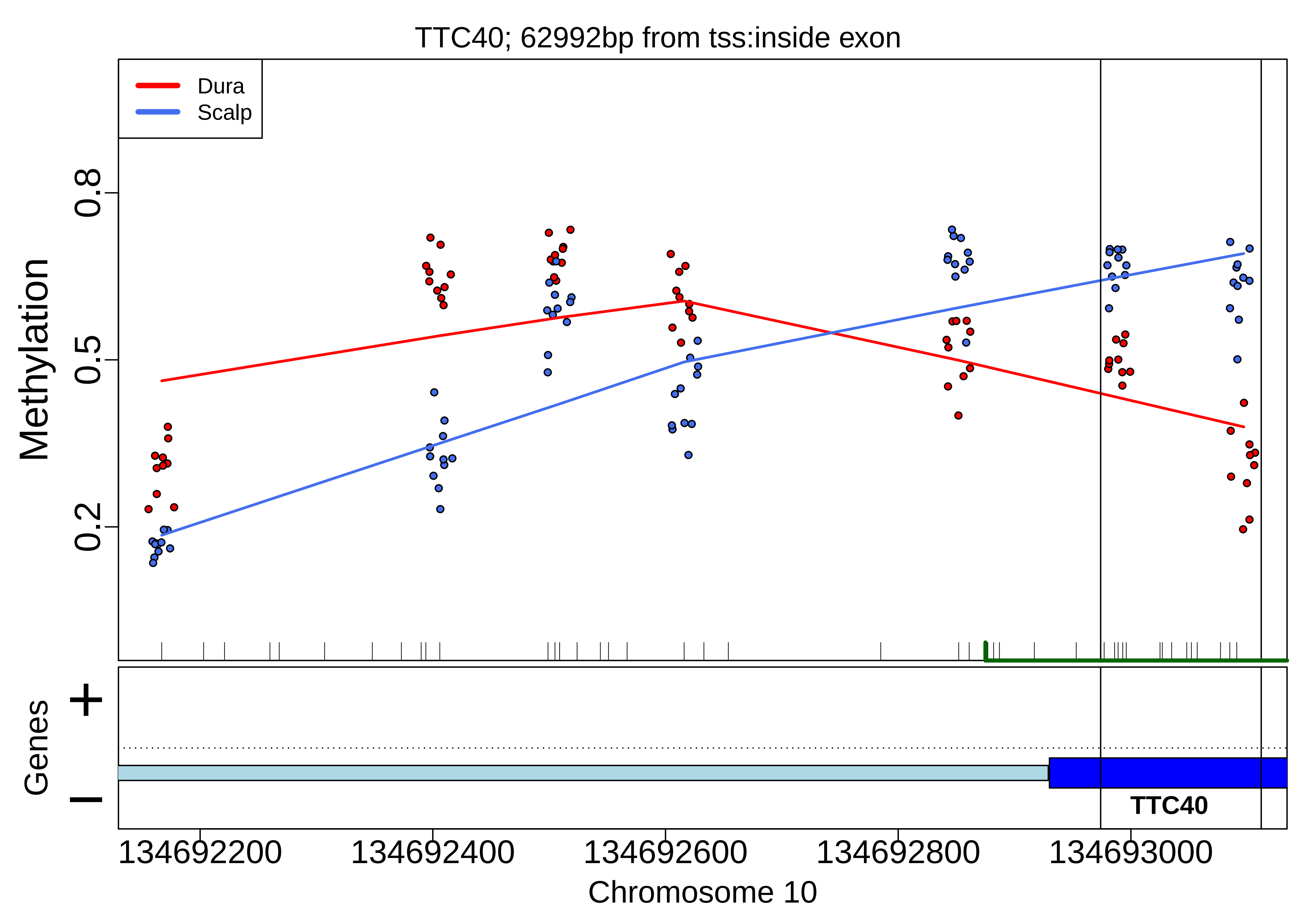

TTC40; 62992bp from tss:inside exon
Dura
Scalp
Methylation
0.2
0.5
0.8
Genes
+
-
TTC40
134692200
134692400
134692600
134692800
134693000
Chromosome 10

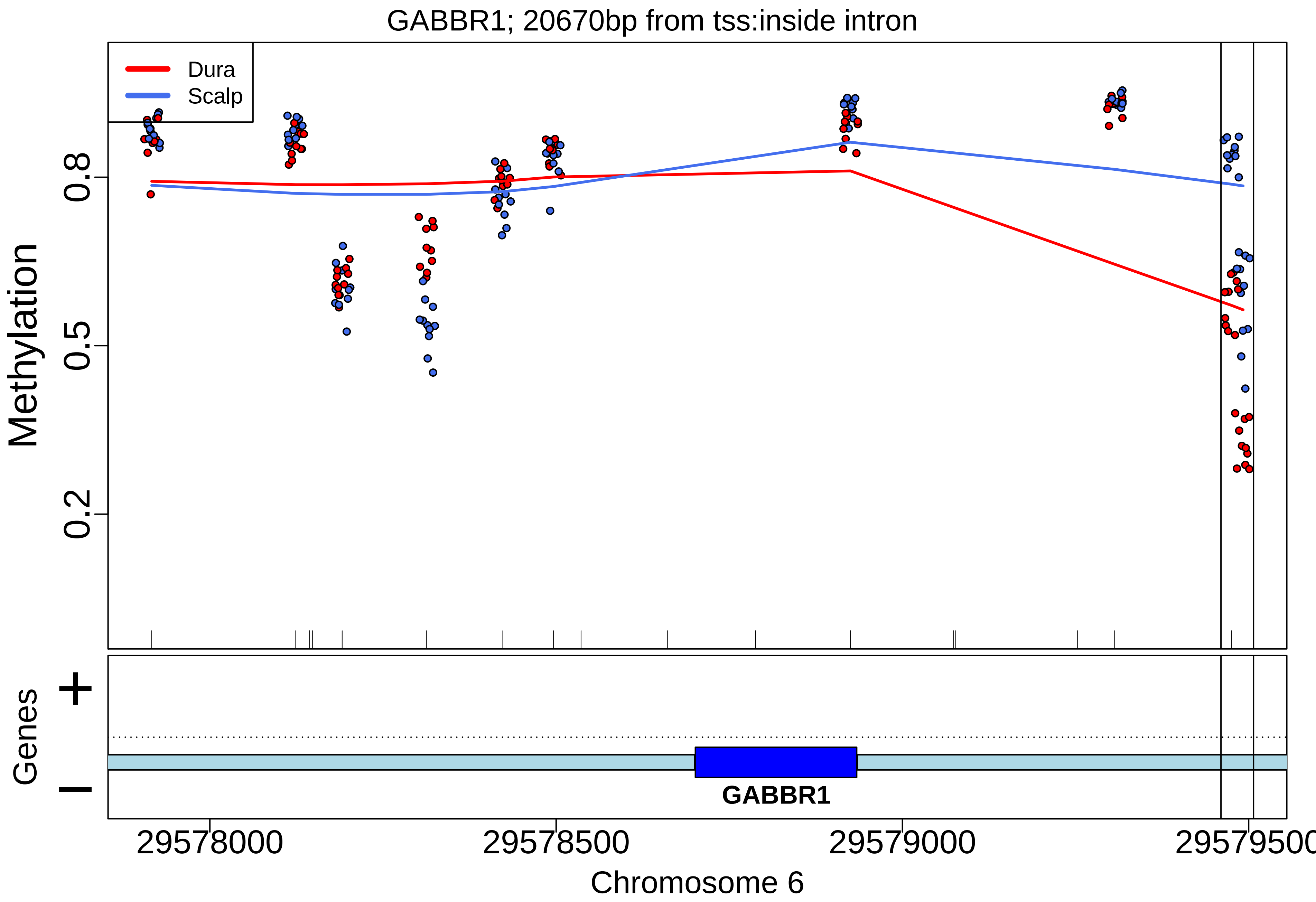
GABBR1; 20670bp from tss:inside intron
Dura
Scalp
Methylation
0.2
0.5
0.8
Genes
+
-
GABBR1
29578000
29578500
29579000
29579500
Chromosome 6

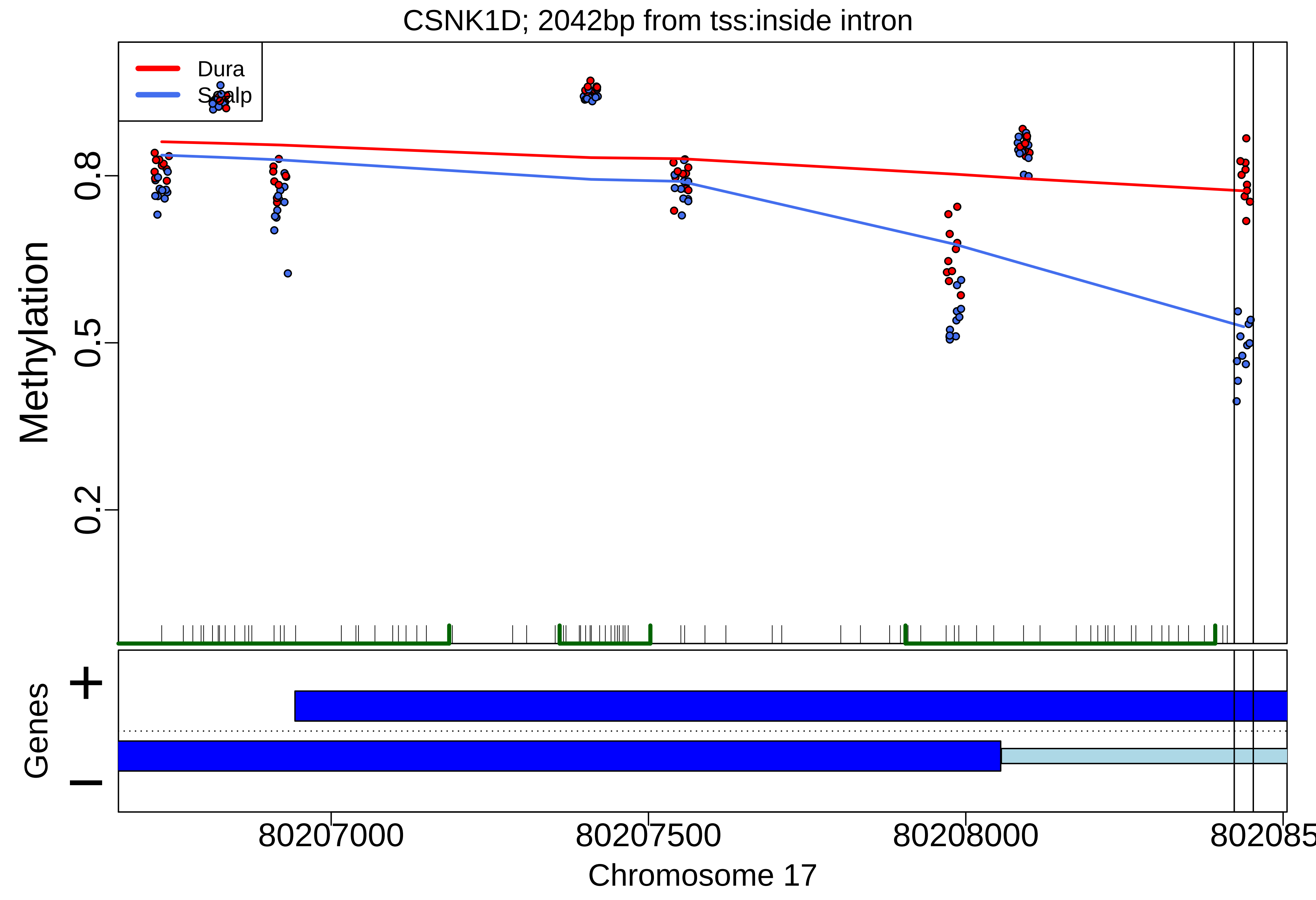

CSNK1D; 2042bp from tss:inside intron
Dura
Scalp
Methylation
0.2
0.5
0.8
Genes
+
-
80207000
80207500
80208000
802085
Chromosome 17

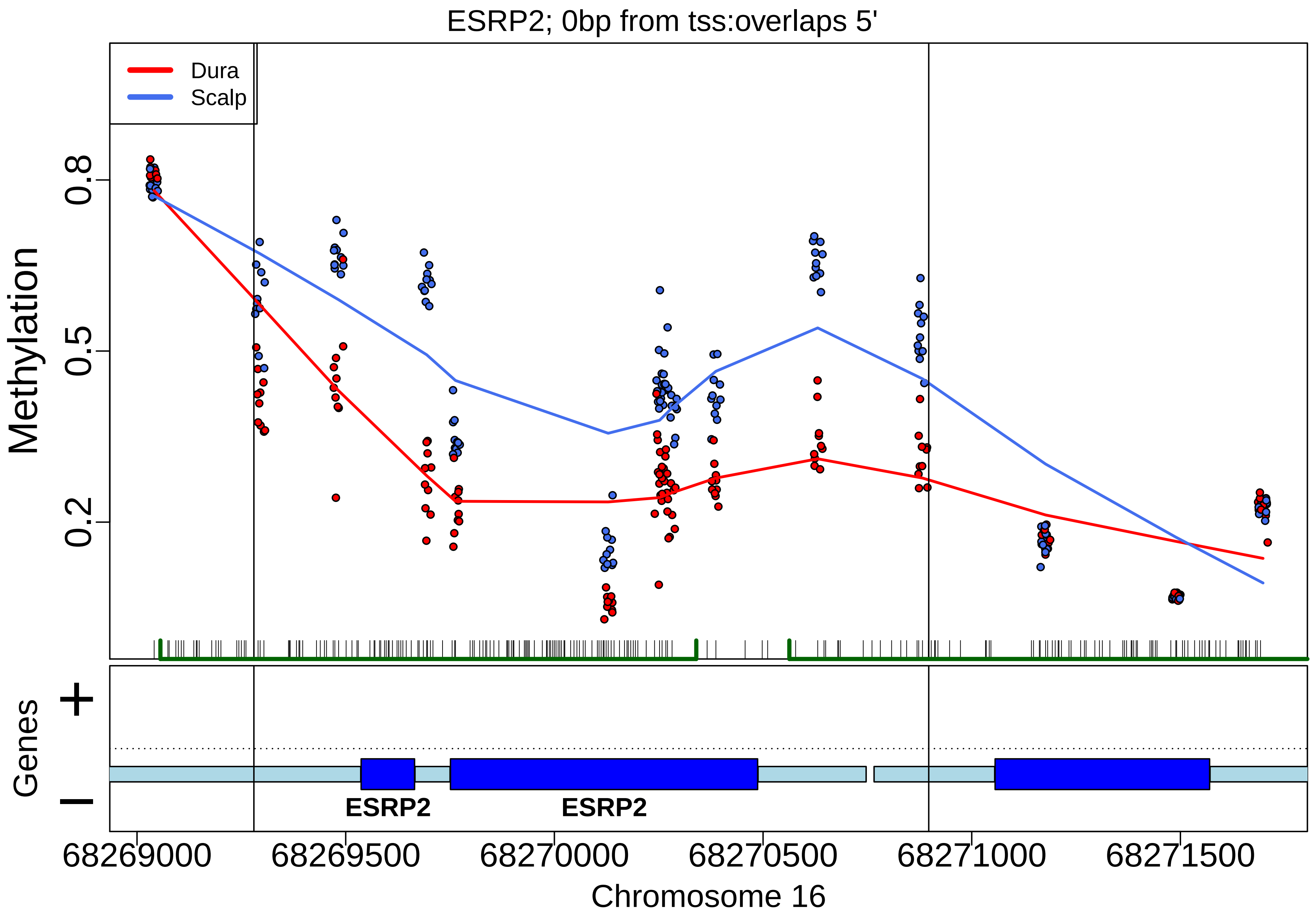

ESRP2; 0bp from tss:overlaps 5'
Dura
Scalp
Methylation
0.2
0.5
0.8
Genes
+
−
ESRP2
ESRP2
68269000
68269500
68270000
68270500
68271000
68271500
Chromosome 16

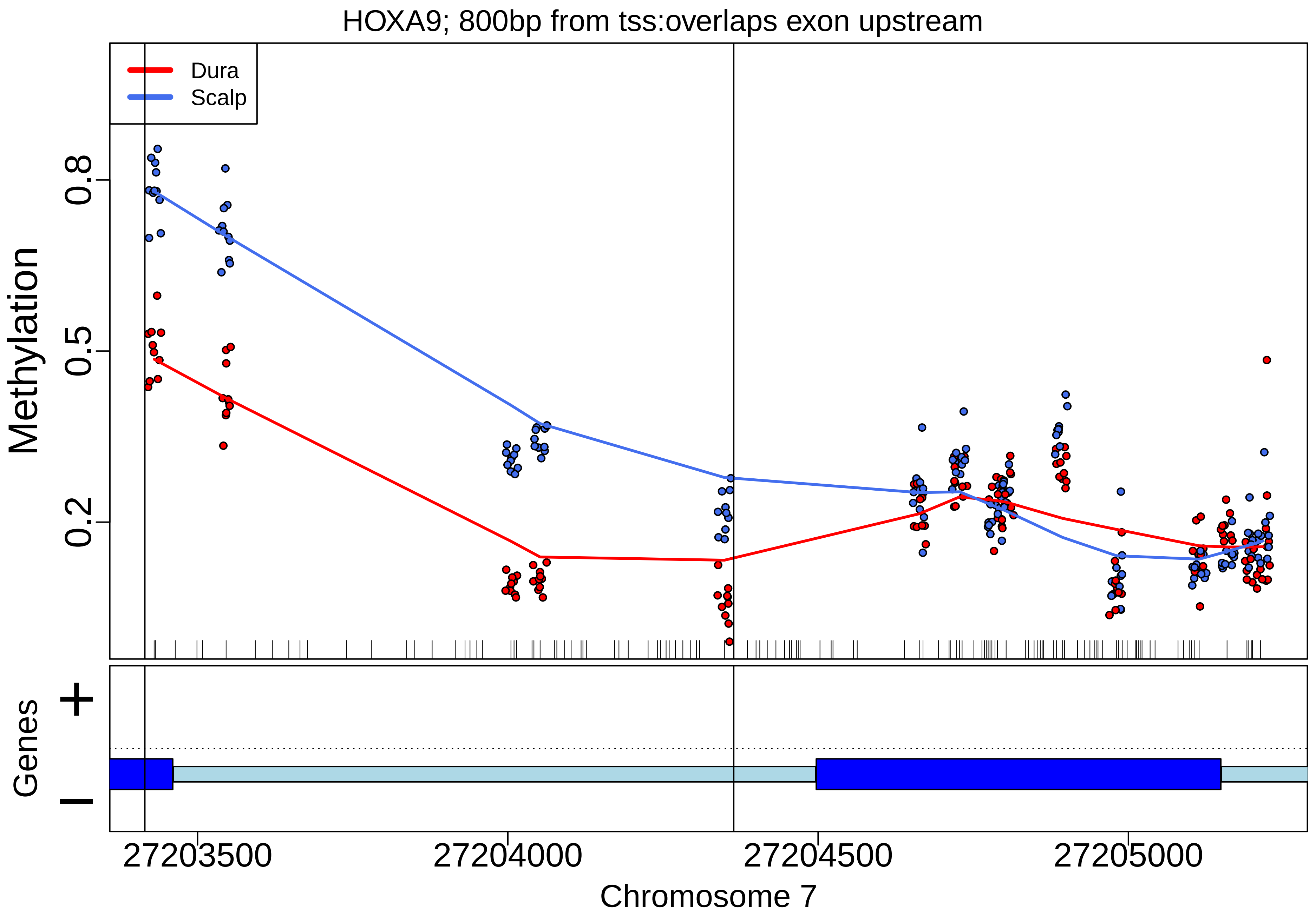
HOXA9; 800bp from tss:overlaps exon upstream
Dura
Scalp
Methylation
0.2
0.5
0.8
Genes
+
-
27203500
27204000
27204500
27205000
Chromosome 7

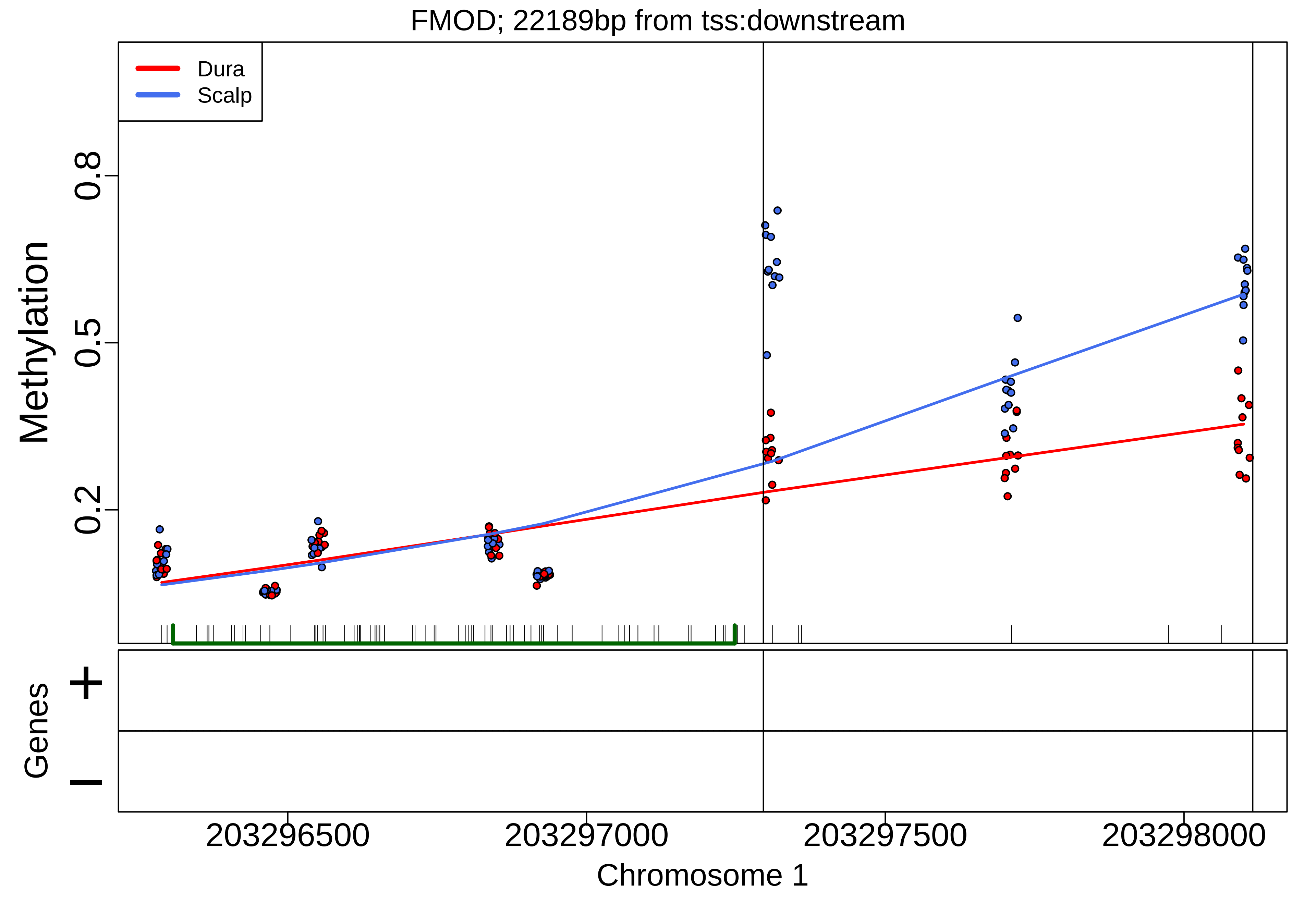

FMOD; 22189bp from tss:downstream
Dura
Scalp
Methylation
0.2
0.5
0.8
Genes
+
−
203296500
203297000
203297500
203298000
Chromosome 1

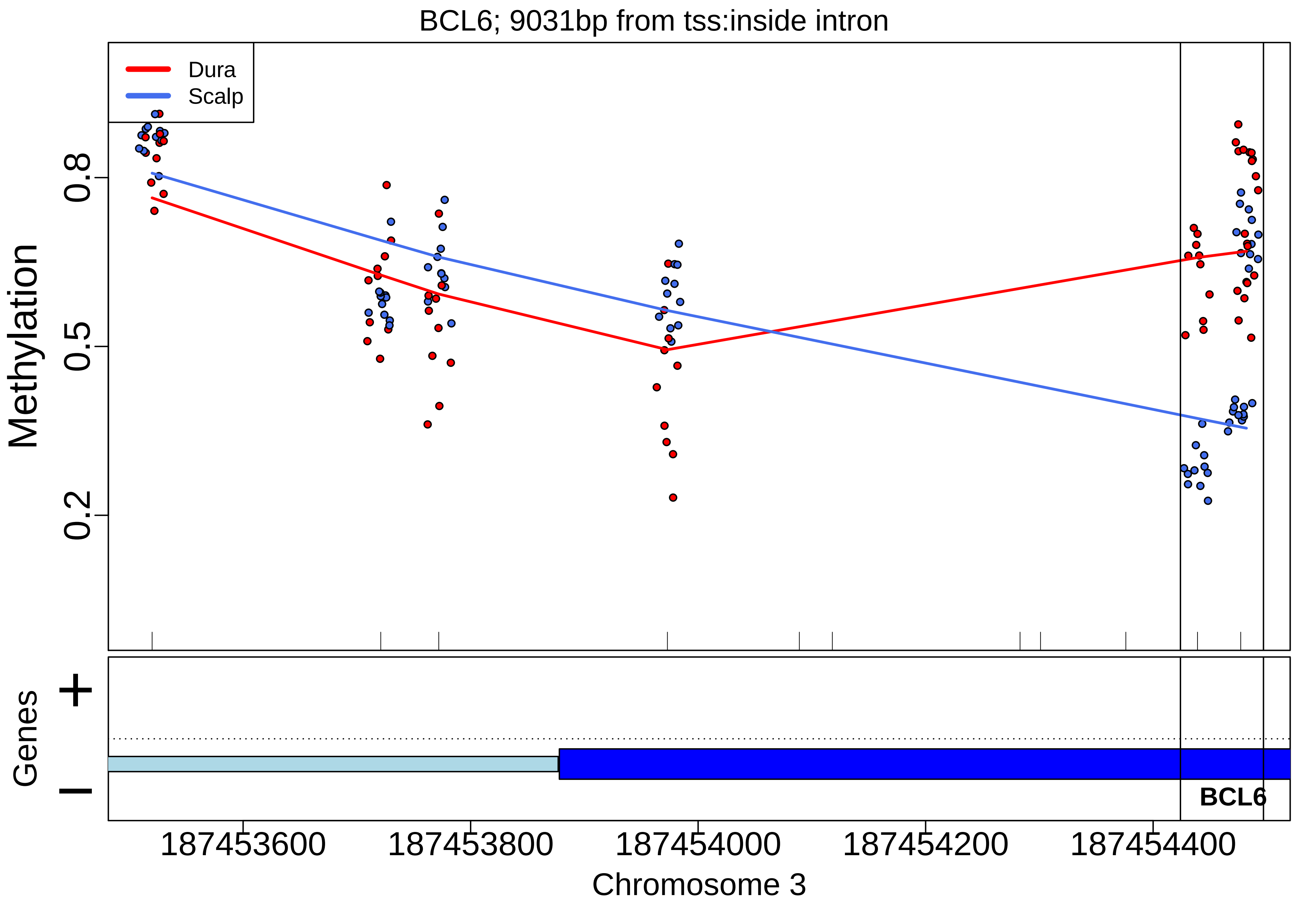

BCL6; 9031bp from tss:inside intron
Dura
Scalp
Methylation
0.2
0.5
0.8
Genes
+
-
BCL6
187453600
187453800
187454000
187454200
187454400
Chromosome 3

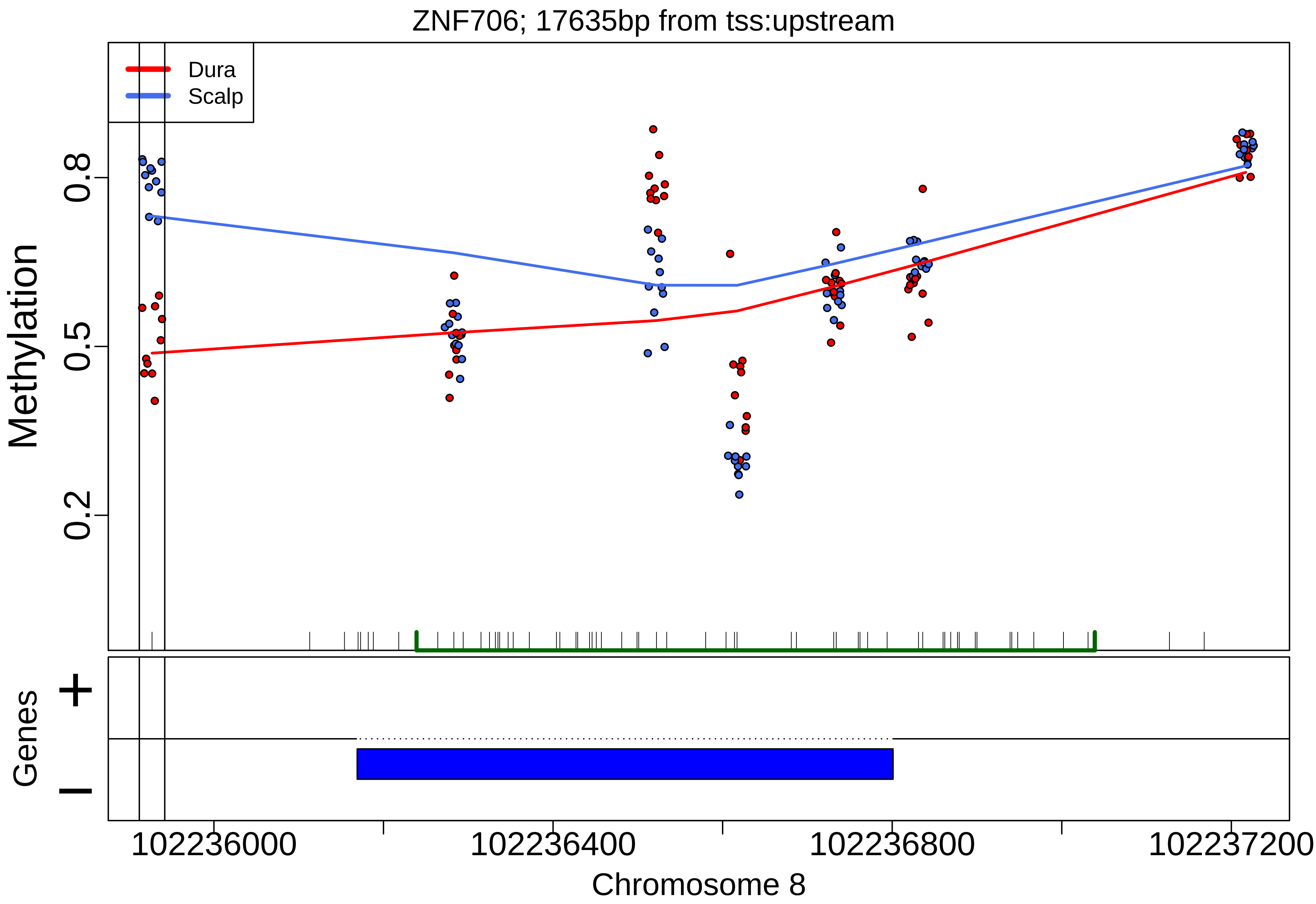
ZNF706; 17635bp from tss:upstream
Dura
Scalp
Methylation
0.2
0.5
0.8
Genes
+
-
102236000
102236400
102236800
102237200
Chromosome 8

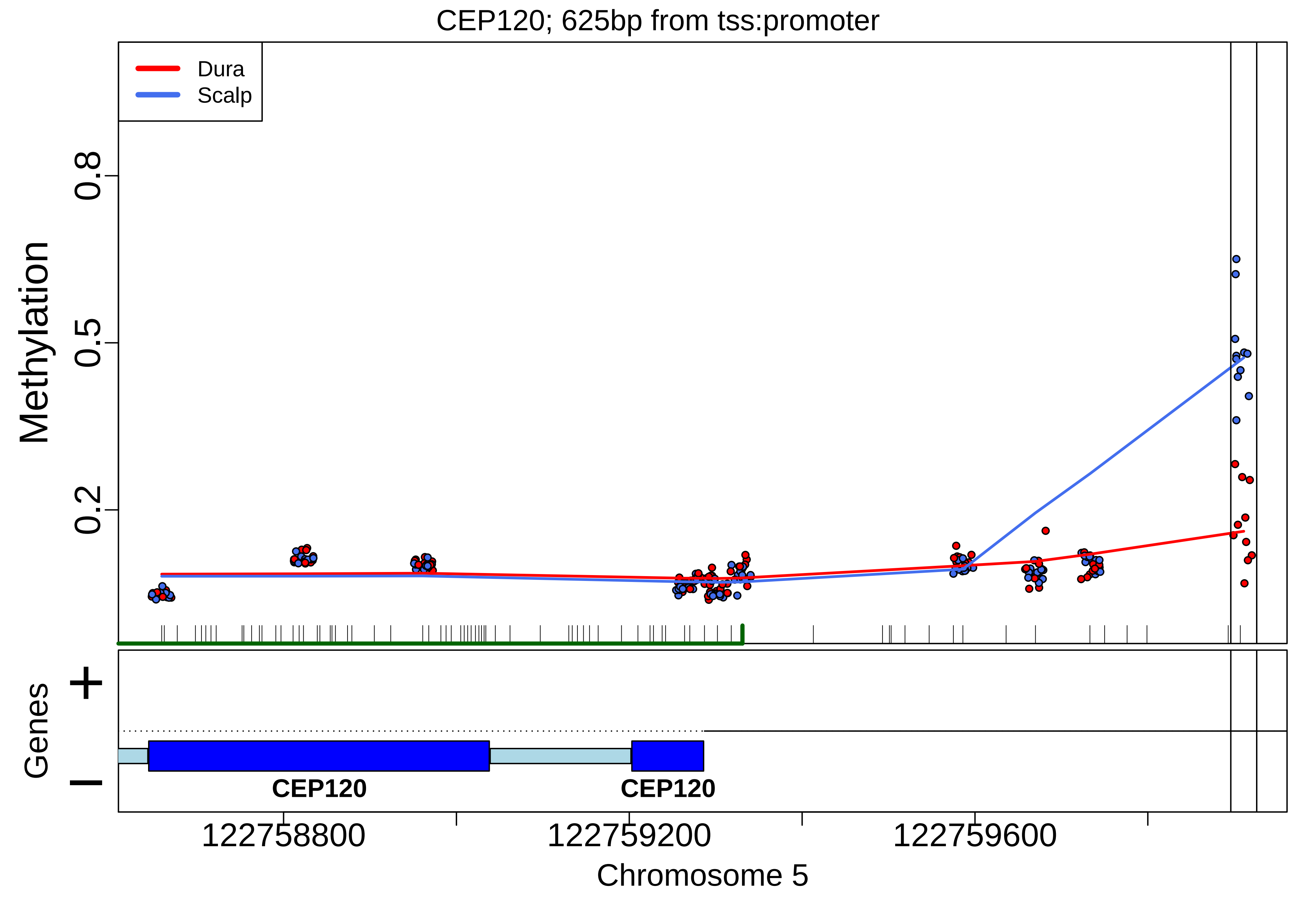

CEP120; 625bp from tss:promoter
Dura
Scalp
Methylation
0.2
0.5
0.8
Genes
+
−
CEP120
CEP120
122758800
122759200
122759600
Chromosome 5

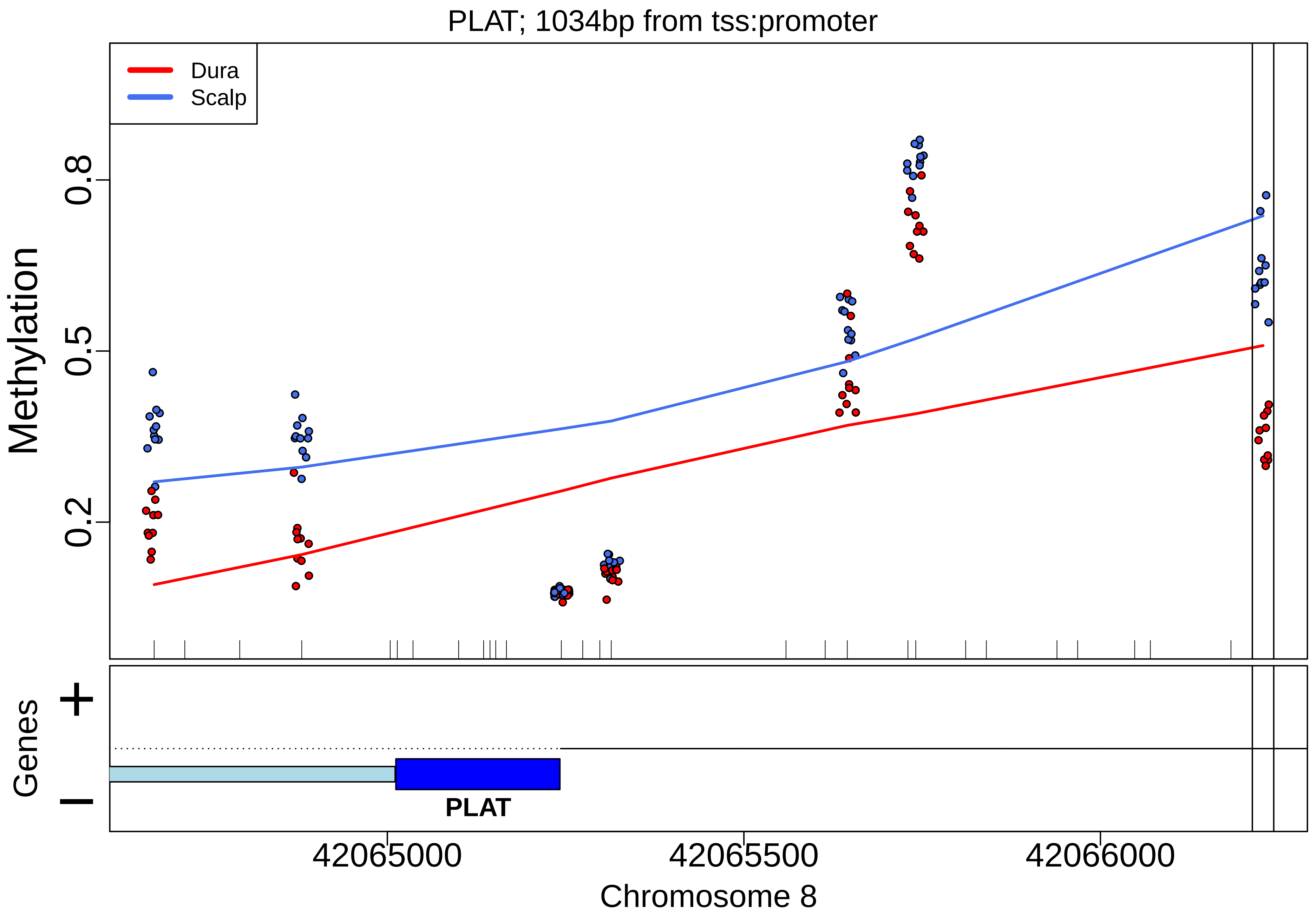

PLAT; 1034bp from tss:promoter
Dura
Scalp
Methylation
0.2
0.5
0.8
Genes
+
−
PLAT
42065000
42065500
42066000
Chromosome 8

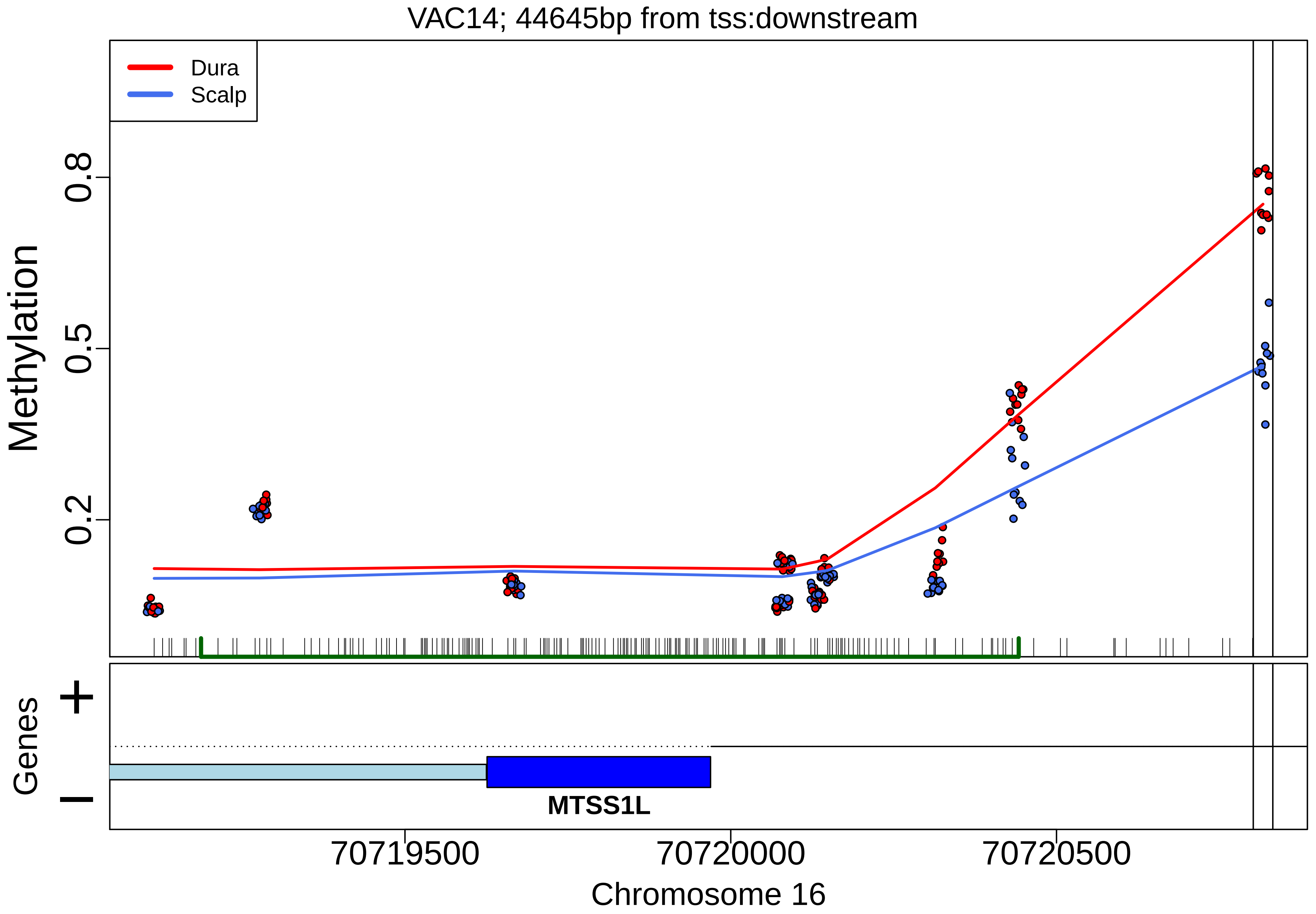

VAC14; 44645bp from tss:downstream
Dura
Scalp
Methylation
0.2
0.5
0.8
Genes
+
−
MTSS1L
70719500
70720000
70720500
Chromosome 16

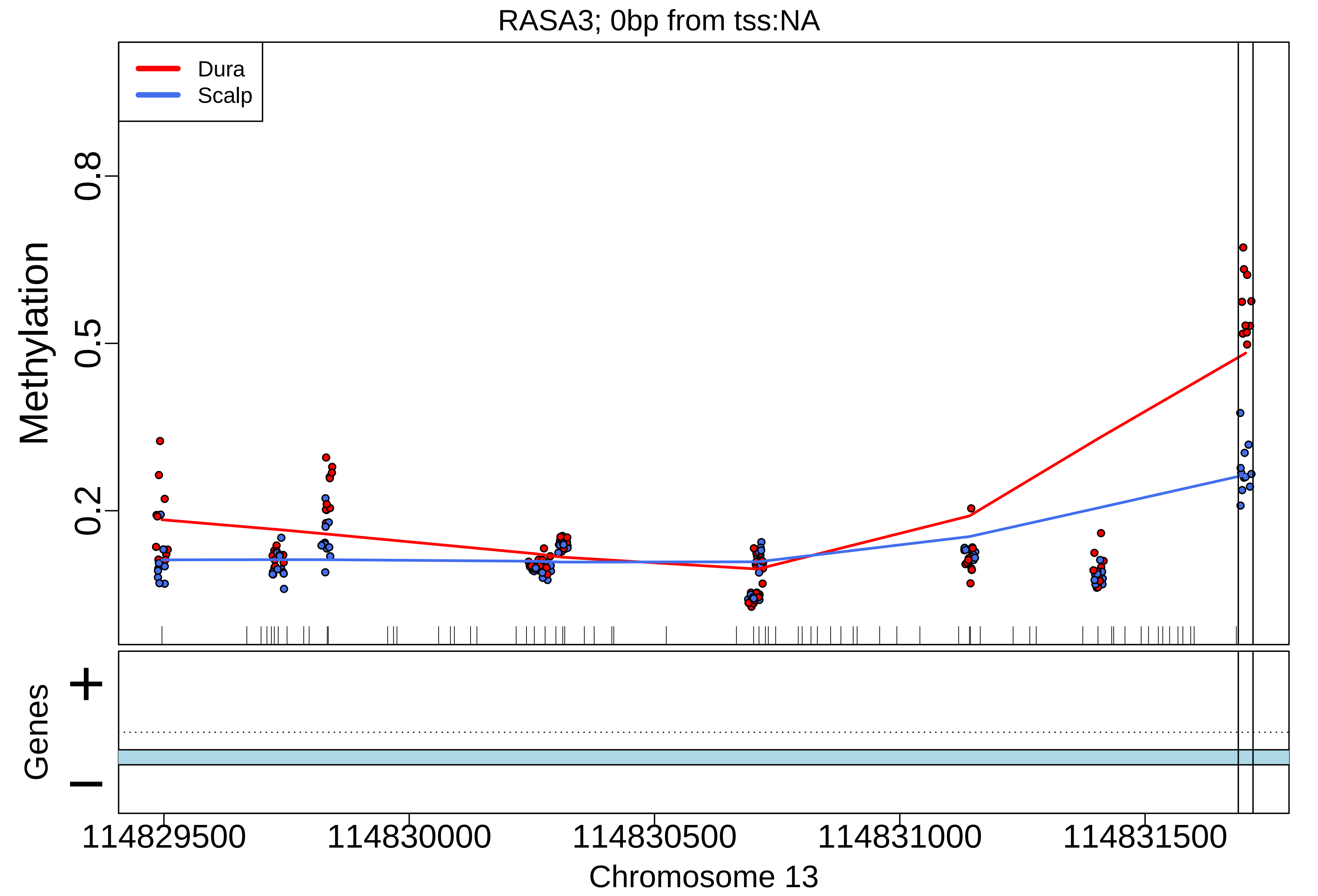

RASA3; 0bp from tss:NA
Dura
Scalp
Methylation
0.2
0.5
0.8
Genes
+
−
114829500
114830000
114830500
114831000
114831500
Chromosome 13

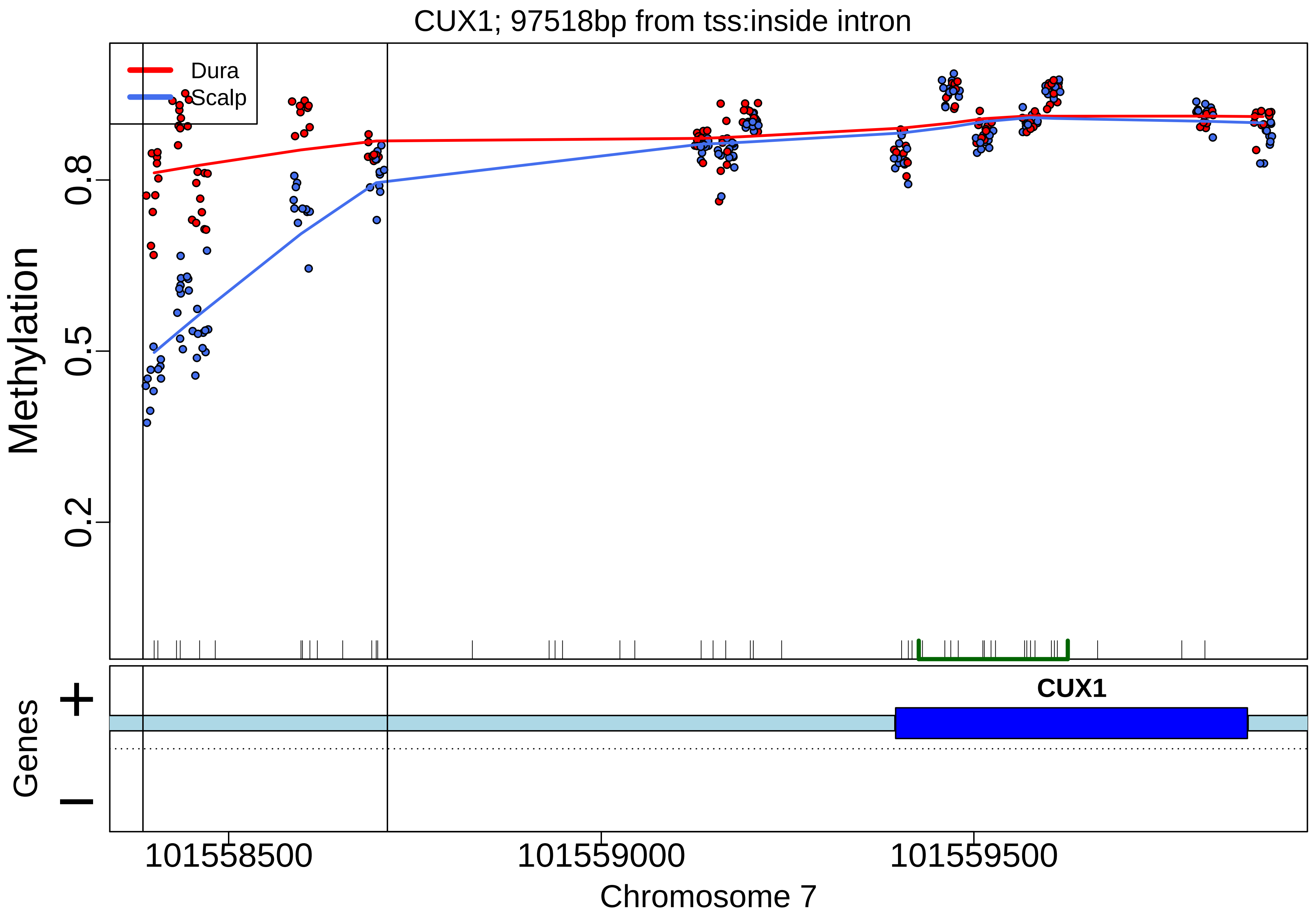

CUX1; 97518bp from tss:inside intron
Dura
Scalp
Methylation
0.2
0.5
0.8
Genes
+
-
CUX1
101558500
101559000
101559500
Chromosome 7

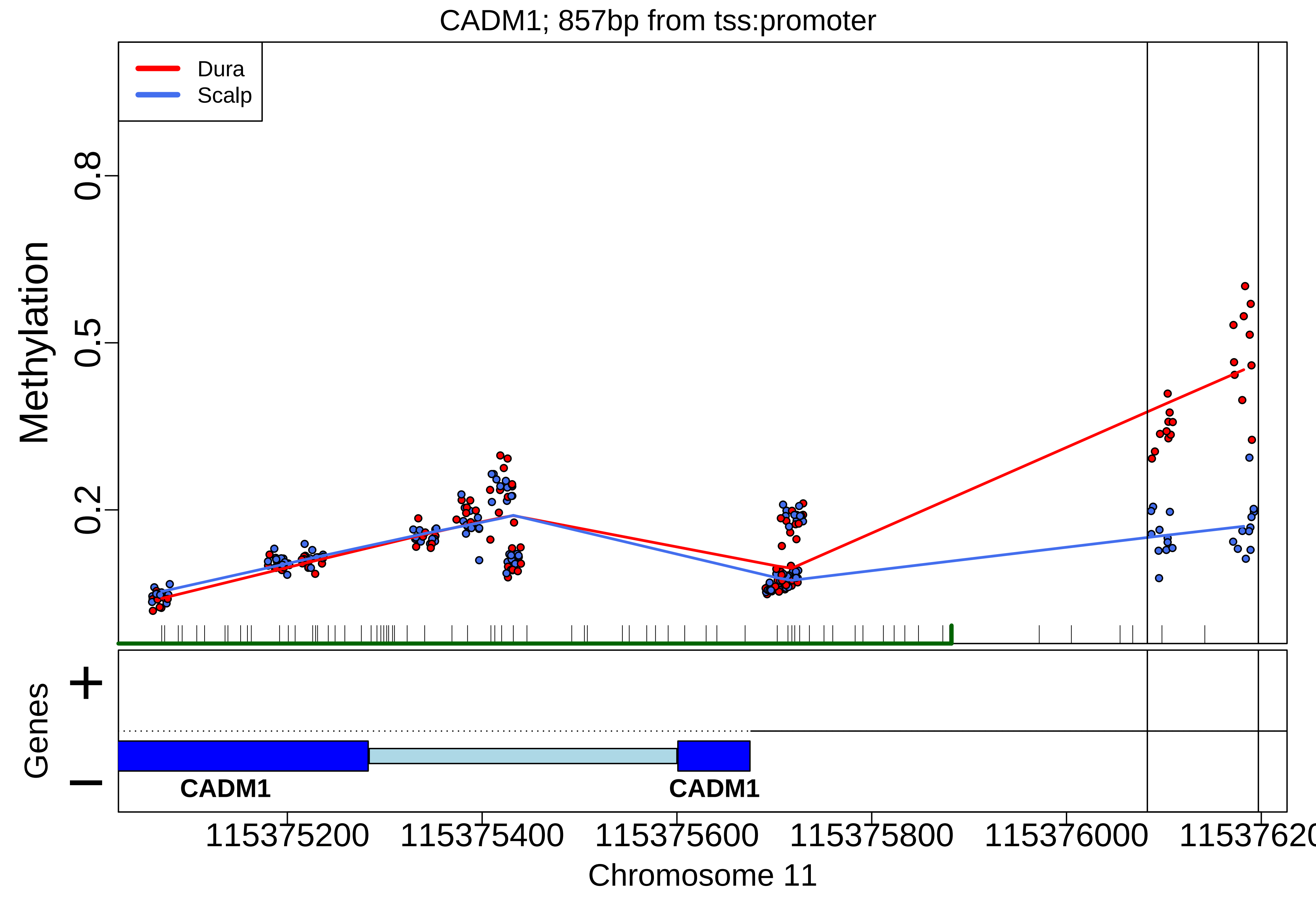
CADM1; 857bp from tss:promoter
Dura
Scalp
Methylation
0.2
0.5
0.8
Genes
+
-
CADM1
CADM1
115375200
115375400
115375600
115375800
115376000
Chromosome 11

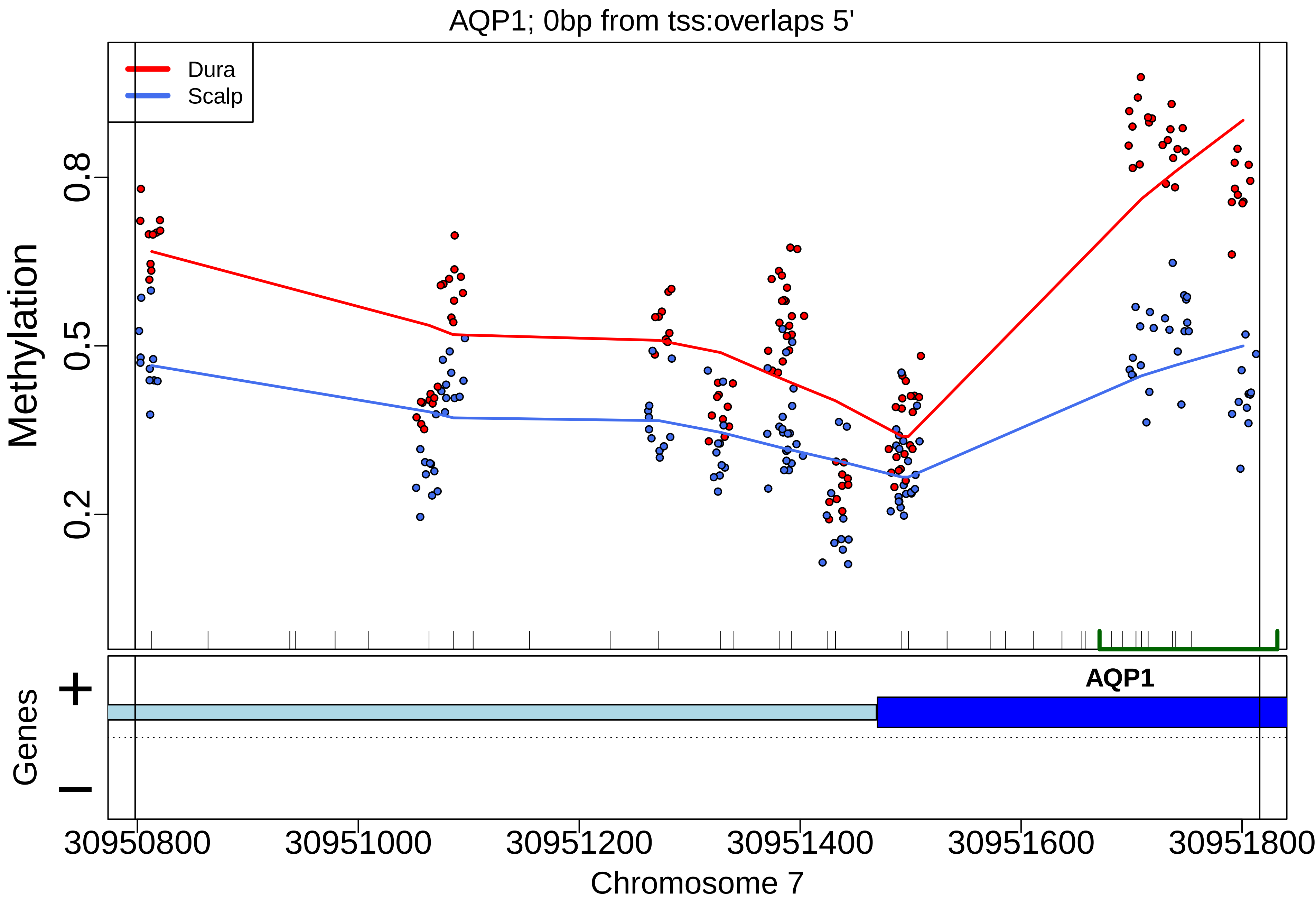

AQP1; 0bp from tss:overlaps 5'
Dura
Scalp
Methylation
0.2
0.5
0.8
Genes
+
–
AQP1
30950800
30951000
30951200
30951400
30951600
30951800
Chromosome 7

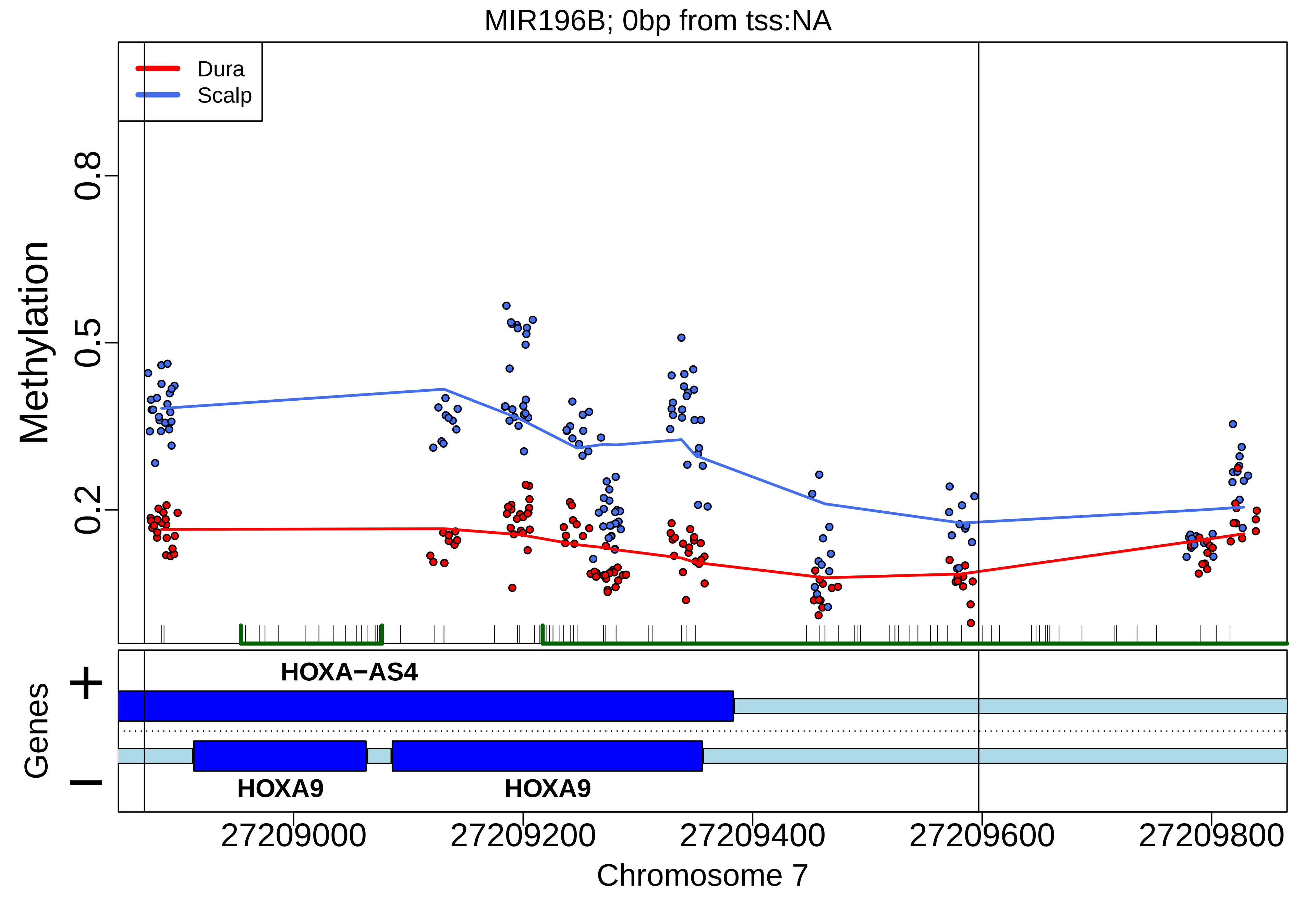

MIR196B; 0bp from tss:NA
Dura
Scalp
Methylation
0.2
0.5
0.8
Genes
+
−
HOXA−AS4
HOXA9
HOXA9
27209000
27209200
27209400
27209600
27209800
Chromosome 7

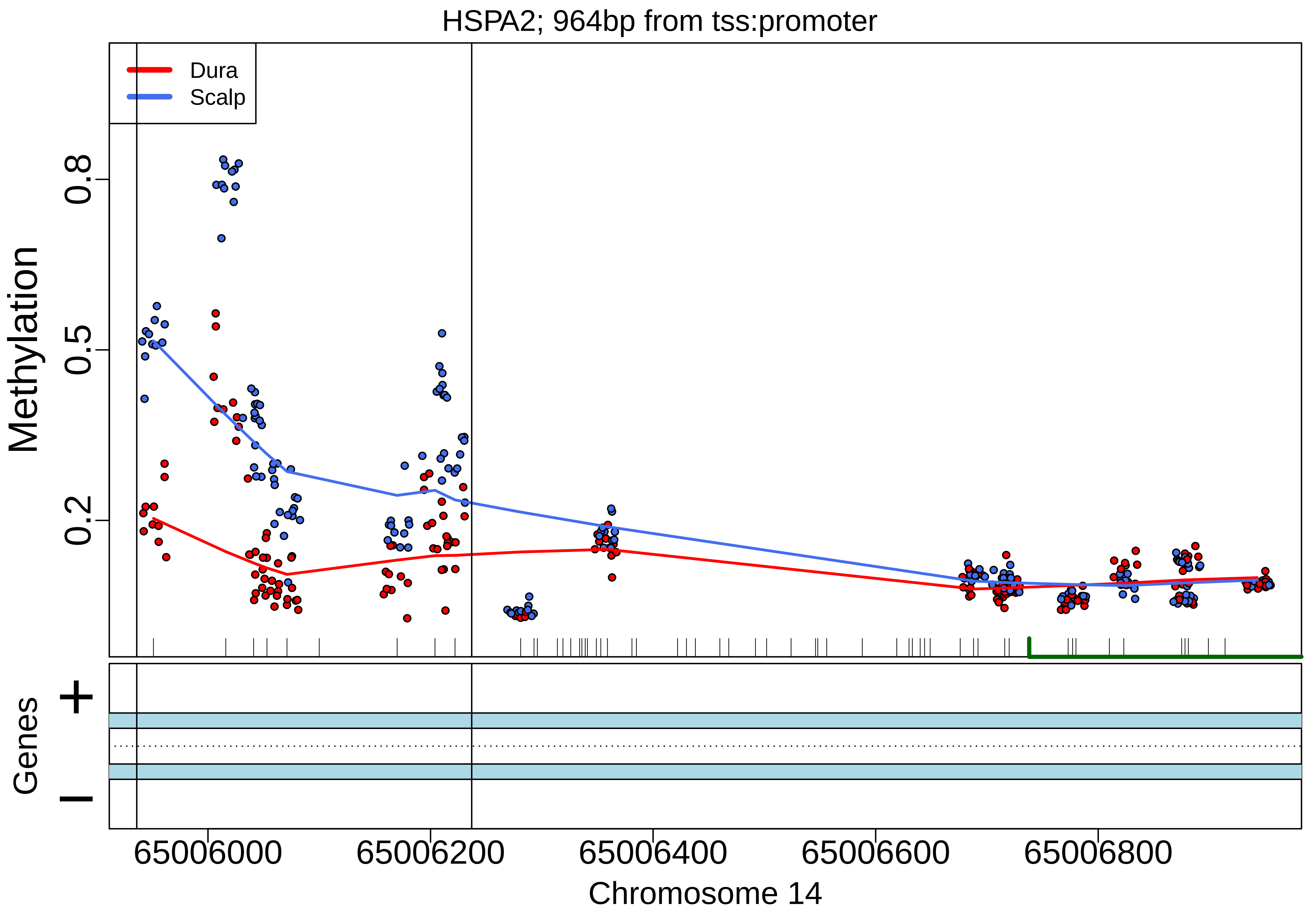
HSPA2; 964bp from tss:promoter
Dura
Scalp
Methylation
0.2
0.5
0.8
Genes
+
−
65006000
65006200
65006400
65006600
65006800
Chromosome 14

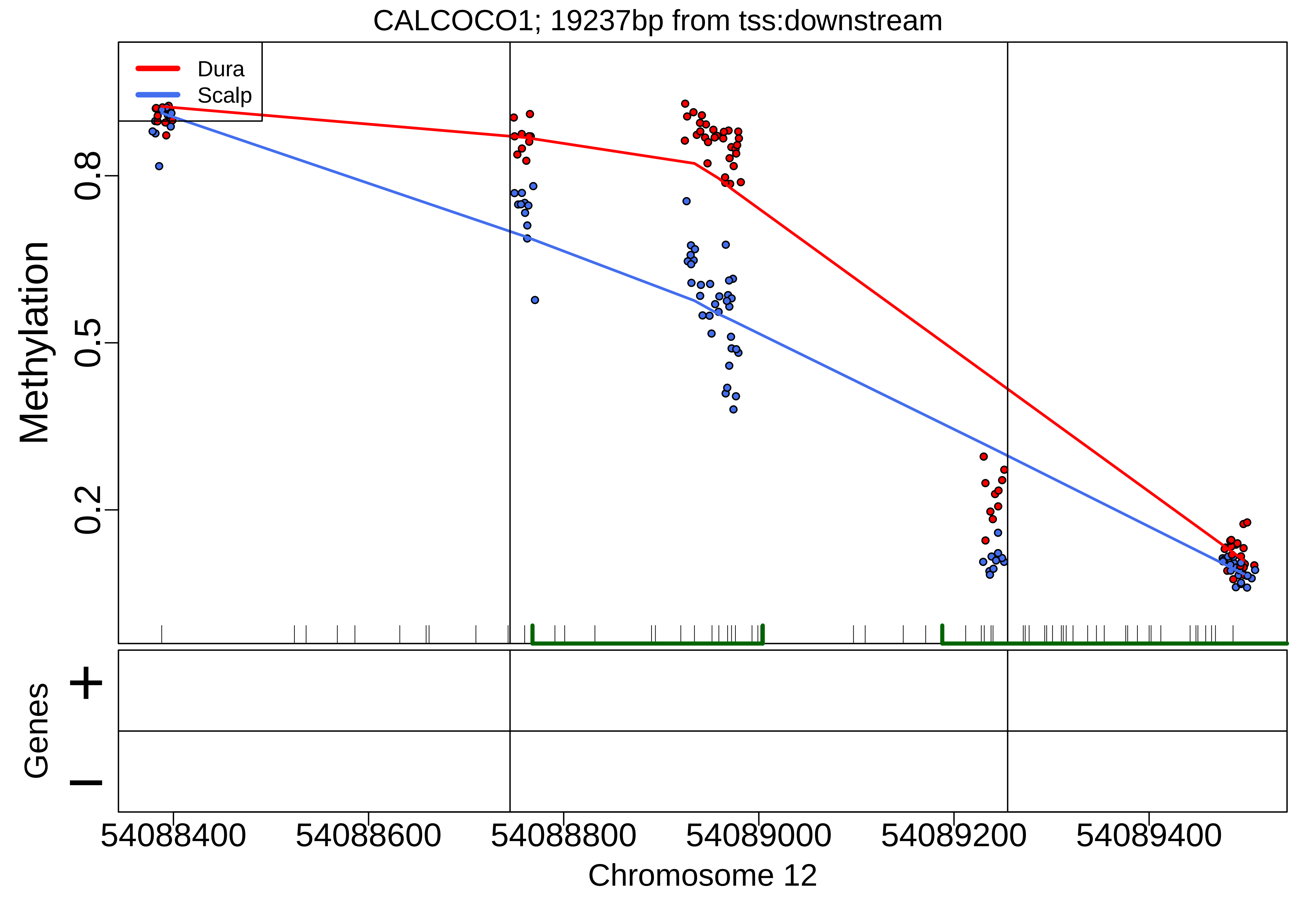

CALCOCO1; 19237bp from tss:downstream
Dura
Scalp
Methylation
0.2
0.5
0.8
Genes
+
−
54088400
54088600
54088800
54089000
54089200
54089400
Chromosome 12

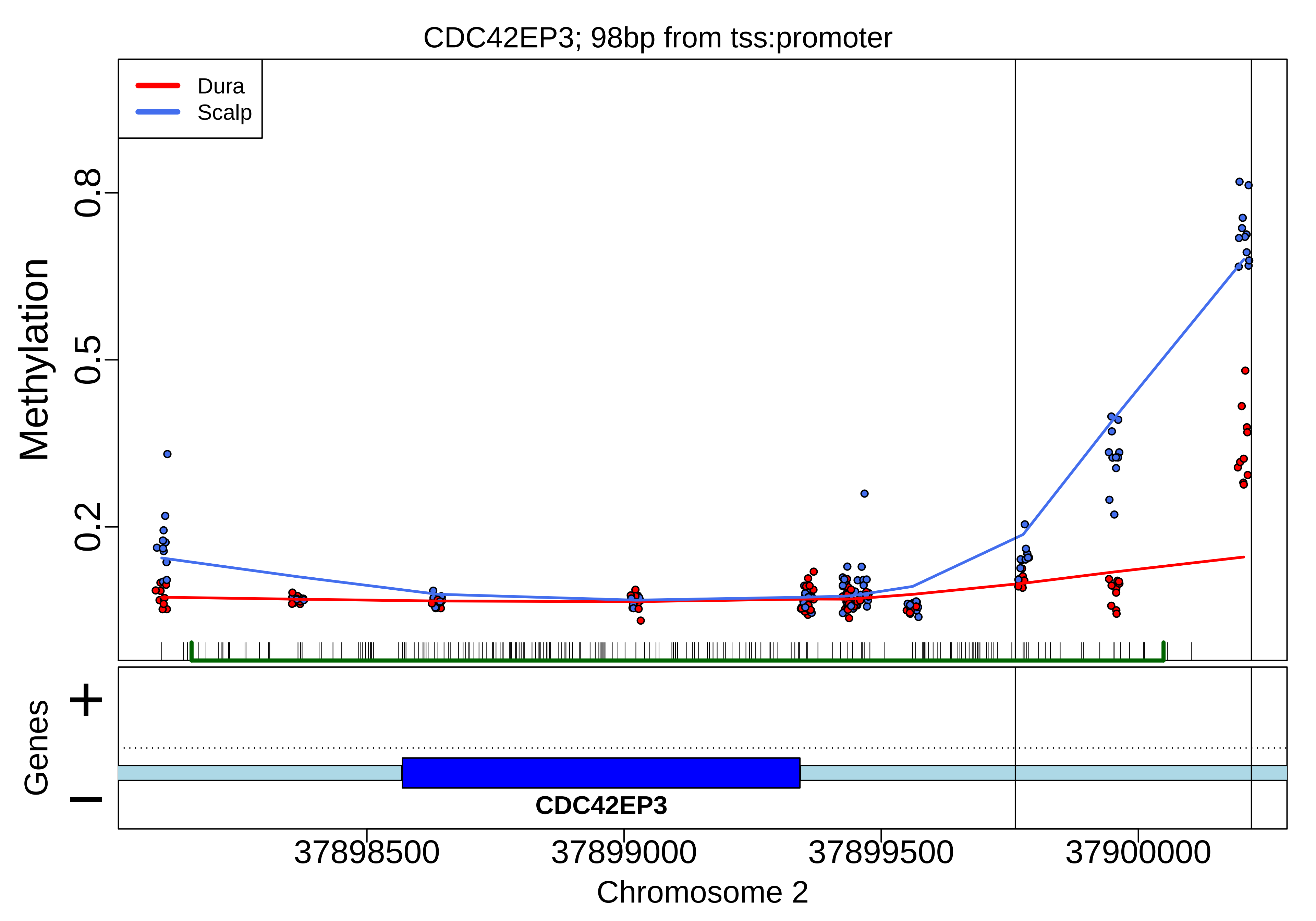
CDC42EP3; 98bp from tss:promoter
Dura
Scalp
Methylation
0.2
0.5
0.8
Genes
+
-
CDC42EP3
37898500
37899000
37899500
37900000
Chromosome 2

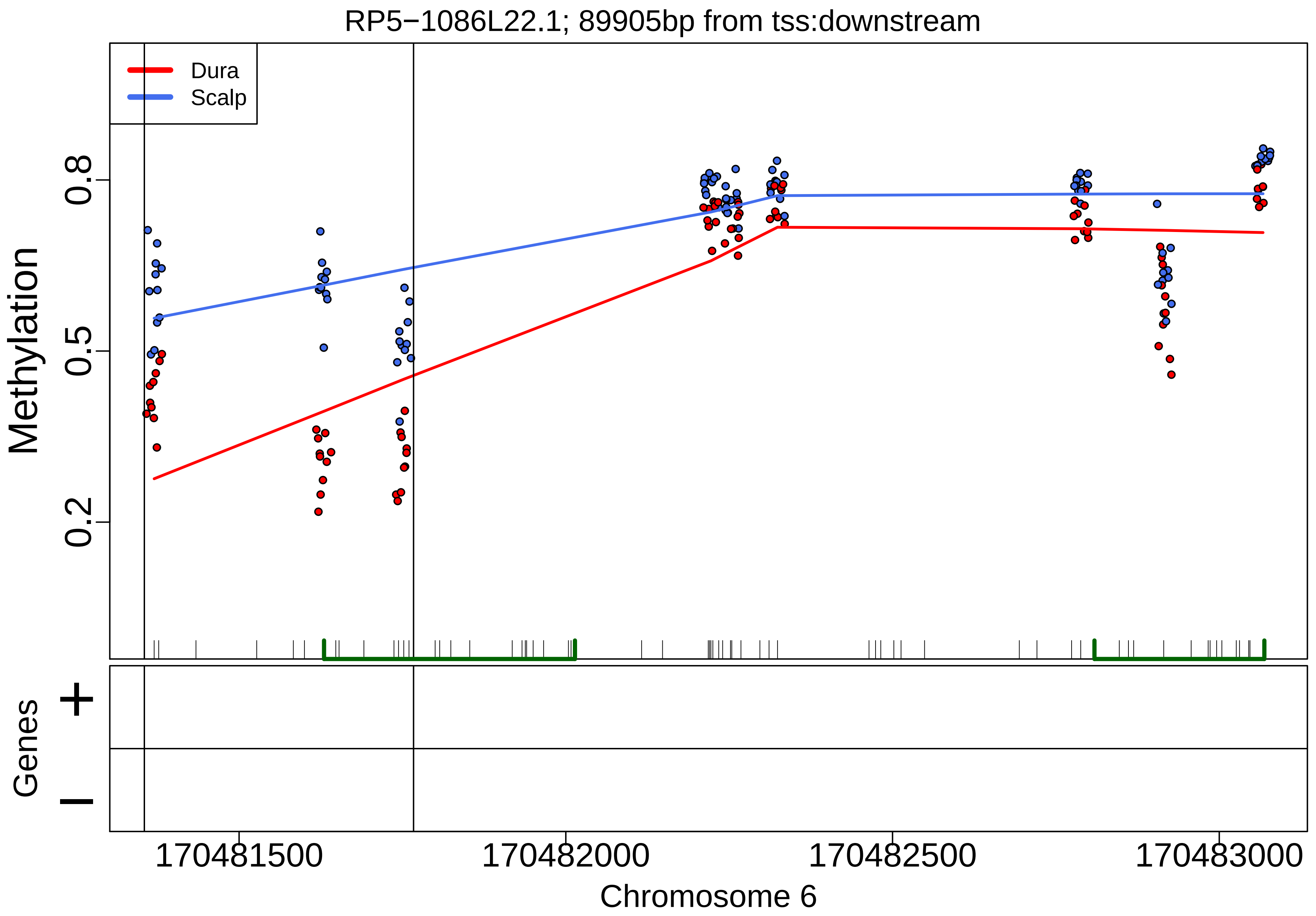

RP5−1086L22.1; 89905bp from tss:downstream
Dura
Scalp
Methylation
0.2
0.5
0.8
Genes
+
−
170481500
170482000
170482500
170483000
Chromosome 6

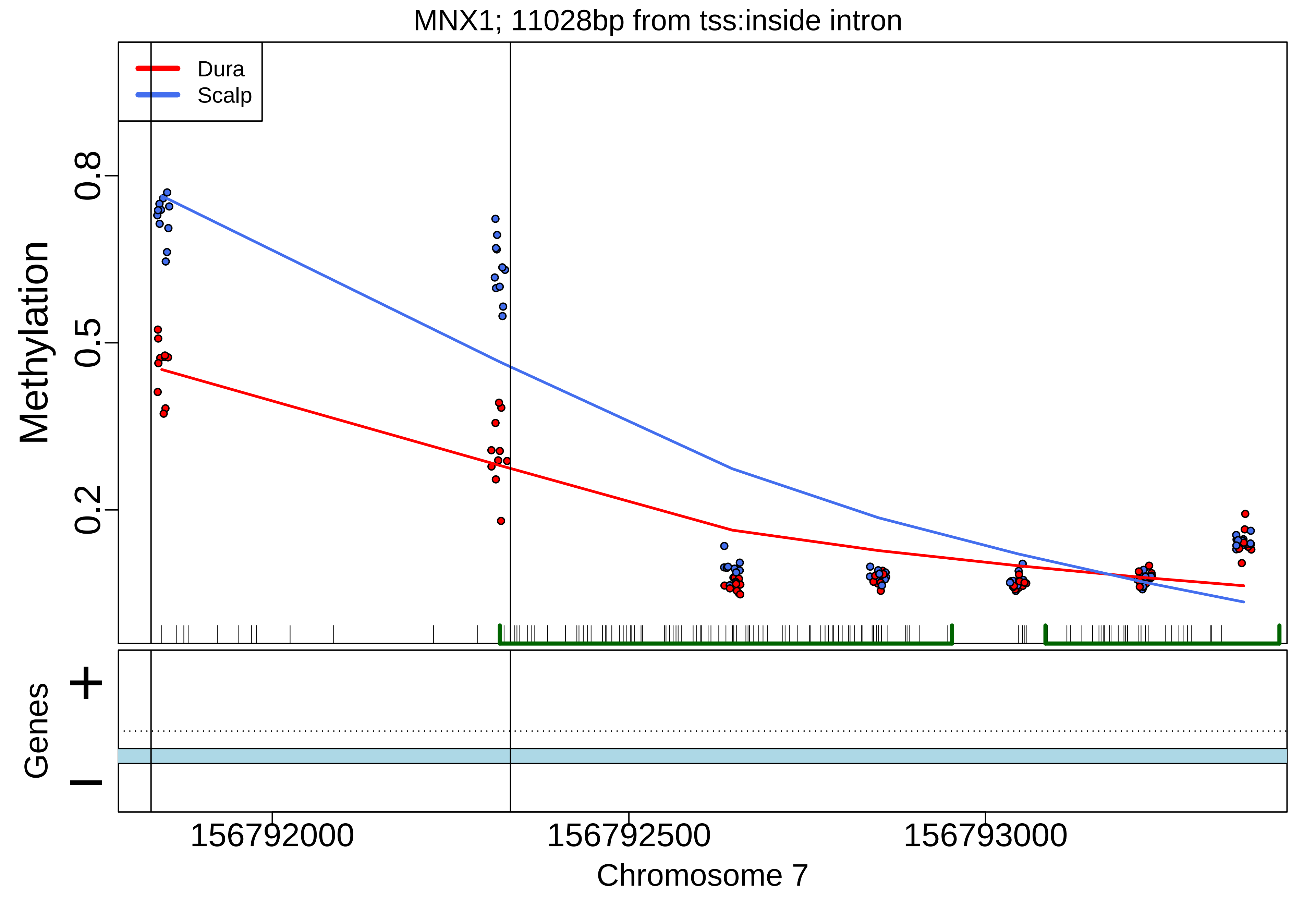

MNX1; 11028bp from tss:inside intron
Dura
Scalp
Methylation
0.2
0.5
0.8
Genes
+
−
156792000
156792500
156793000
Chromosome 7

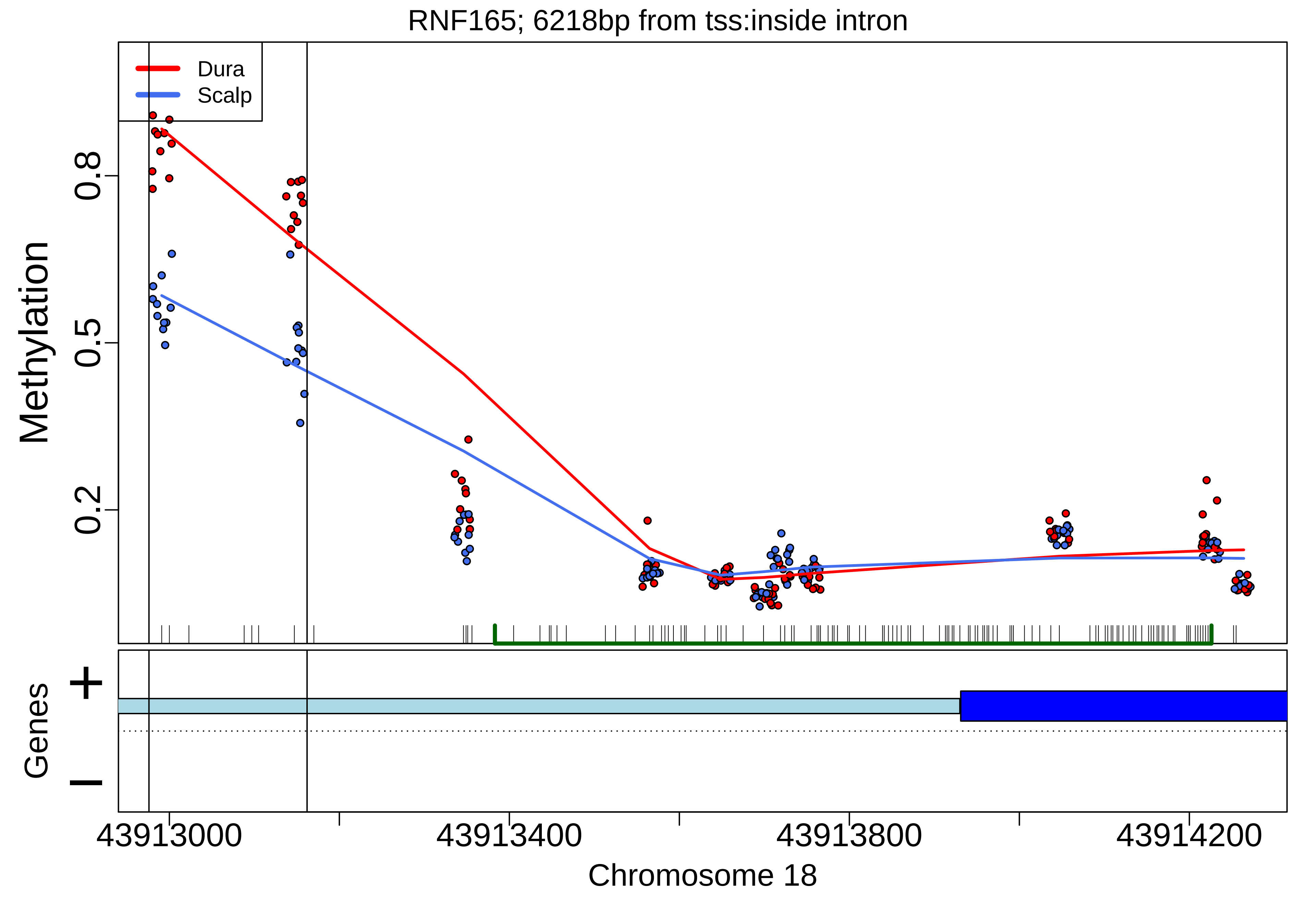

RNF165; 6218bp from tss:inside intron
Dura
Scalp
Methylation
0.2
0.5
0.8
Genes
+
-
43913000
43913400
43913800
43914200
Chromosome 18

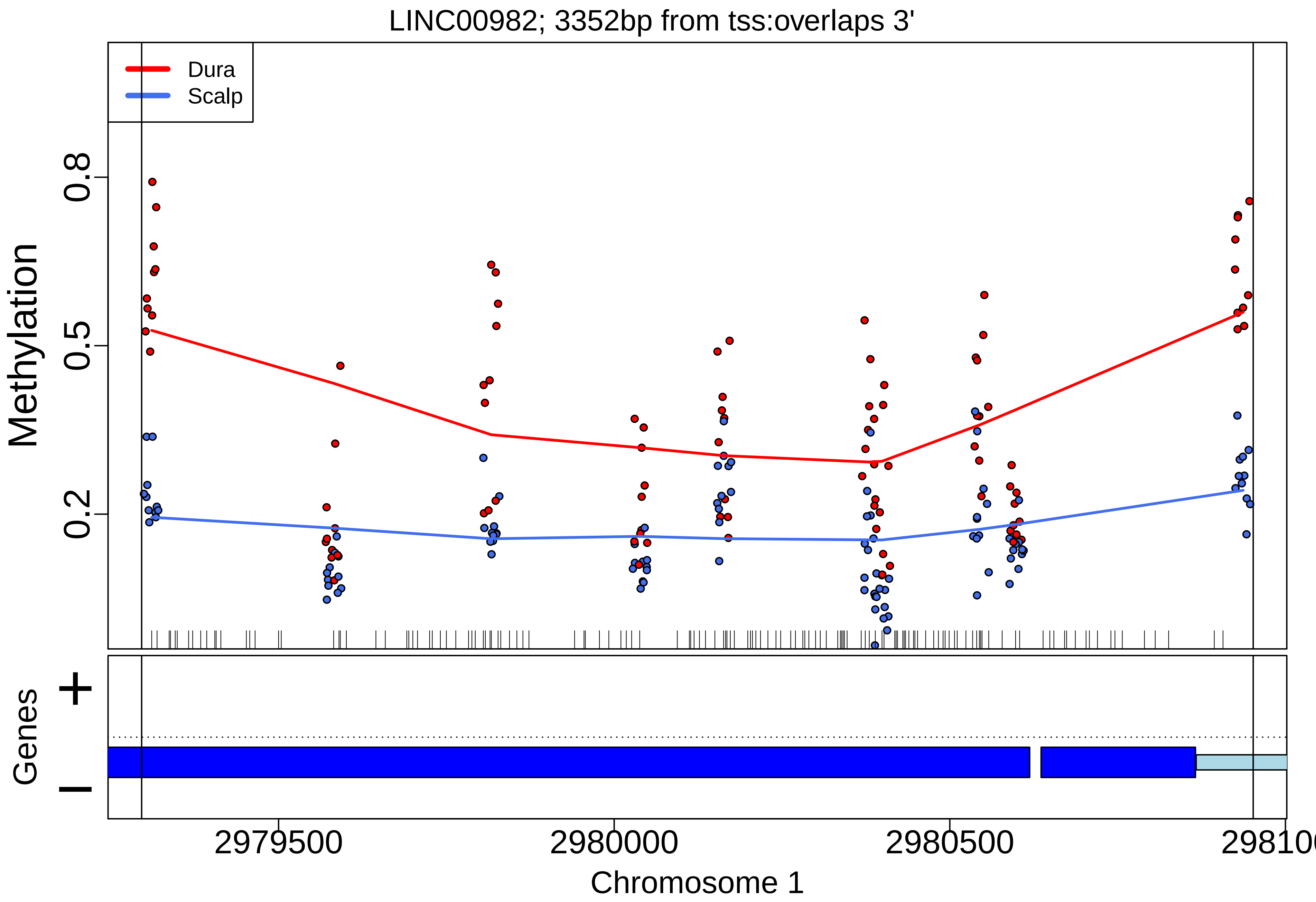

LINC00982; 3352bp from tss:overlaps 3'
Dura
Scalp
Methylation
0.2
0.5
0.8
Genes
+
-
2979500
2980000
2980500
298100
Chromosome 1

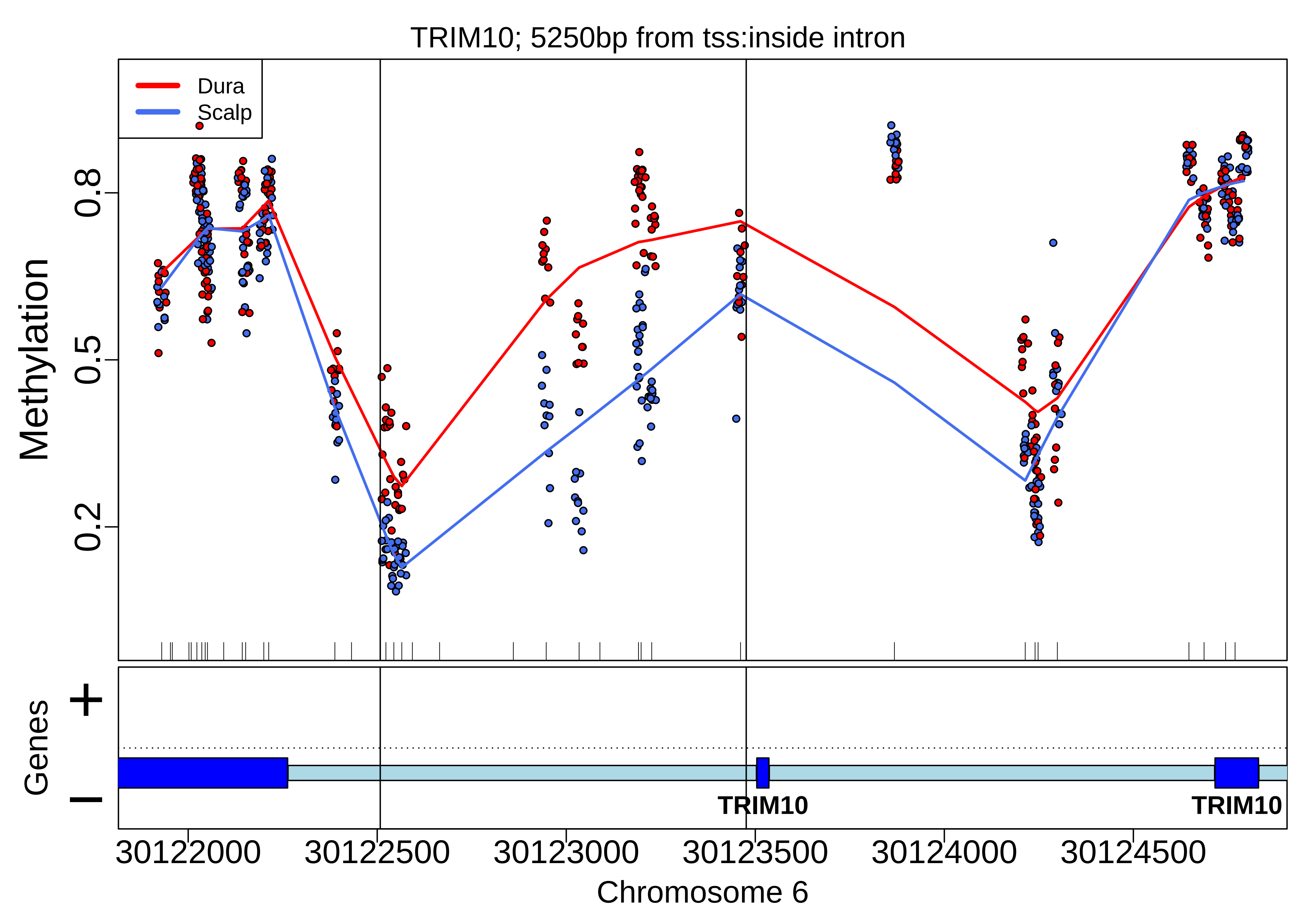

TRIM10; 5250bp from tss:inside intron
Dura
Scalp
Methylation
0.2
0.5
0.8
Genes
+
−
TRIM10
TRIM10
30122000
30122500
30123000
30123500
30124000
30124500
Chromosome 6

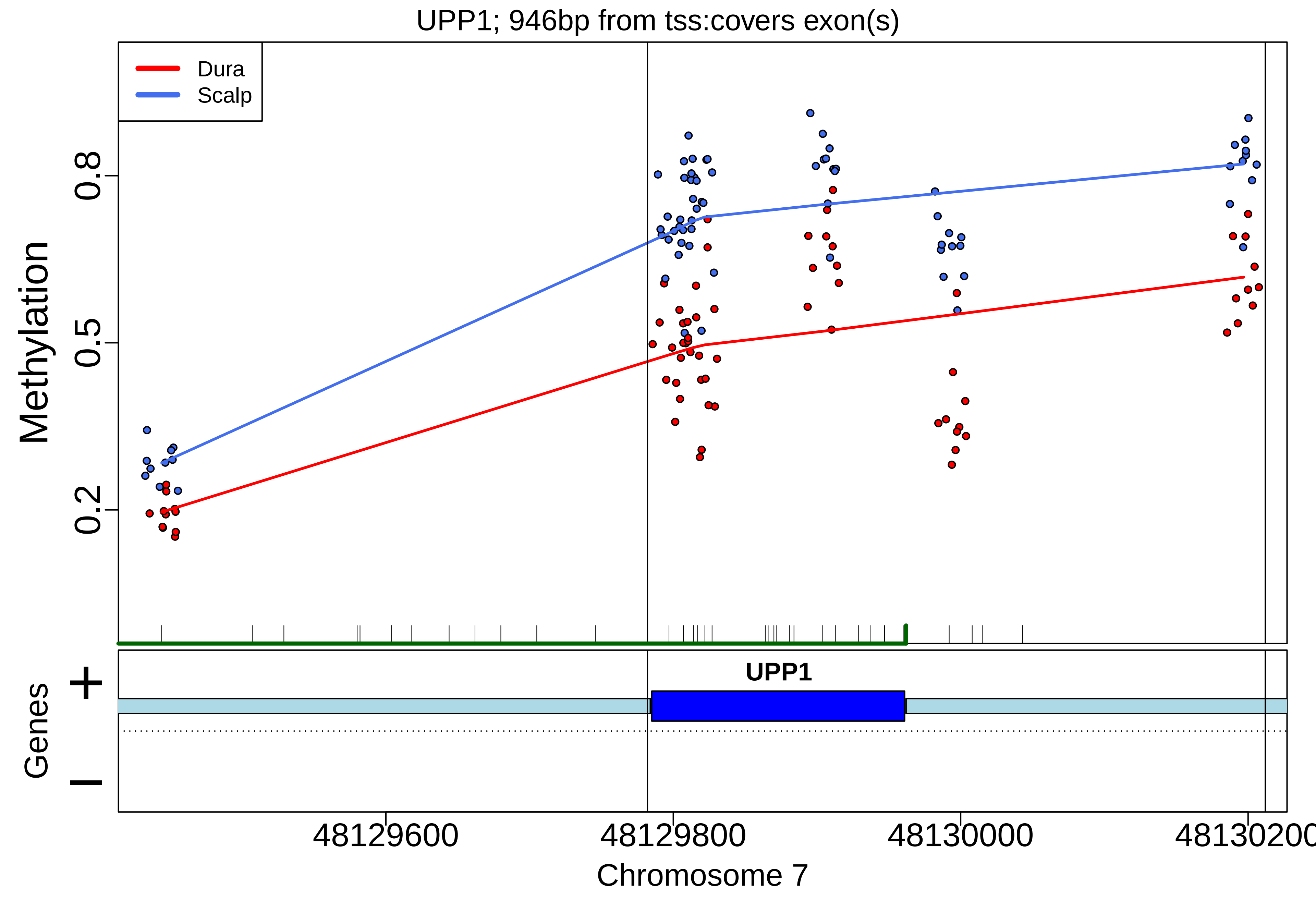

UPP1; 946bp from tss:covers exon(s)
Dura
Scalp
Methylation
0.2
0.5
0.8
Genes
+
UPP1
48129600
48129800
48130000
48130200
Chromosome 7

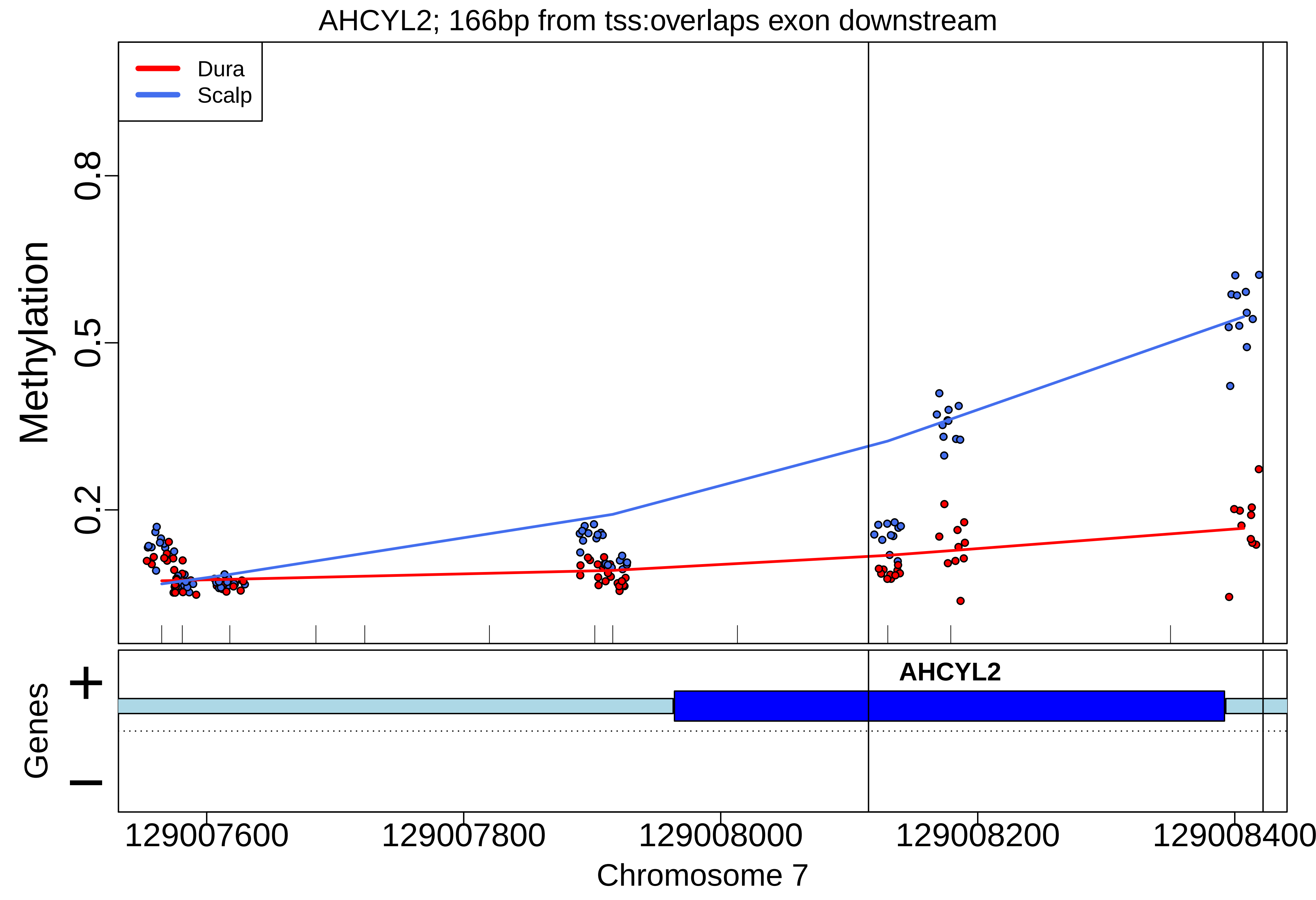
AHCYL2; 166bp from tss:overlaps exon downstream
Dura
Scalp
Methylation
0.2
0.5
0.8
Genes
+
-
AHCYL2
129007600
129007800
129008000
129008200
129008400
Chromosome 7

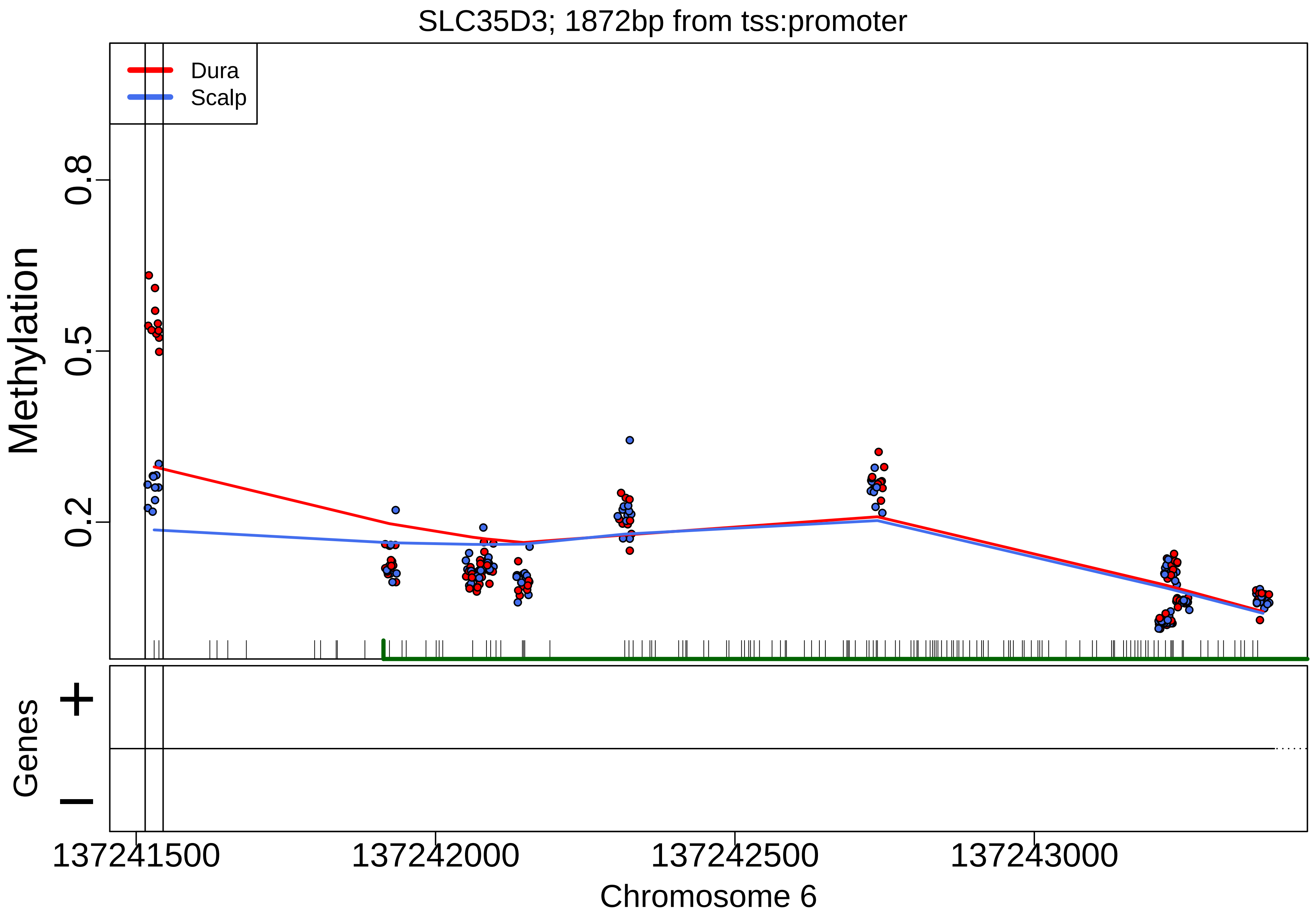

SLC35D3; 1872bp from tss:promoter
Dura
Scalp
Methylation
0.2
0.5
0.8
Genes
+
-
137241500
137242000
137242500
137243000
Chromosome 6

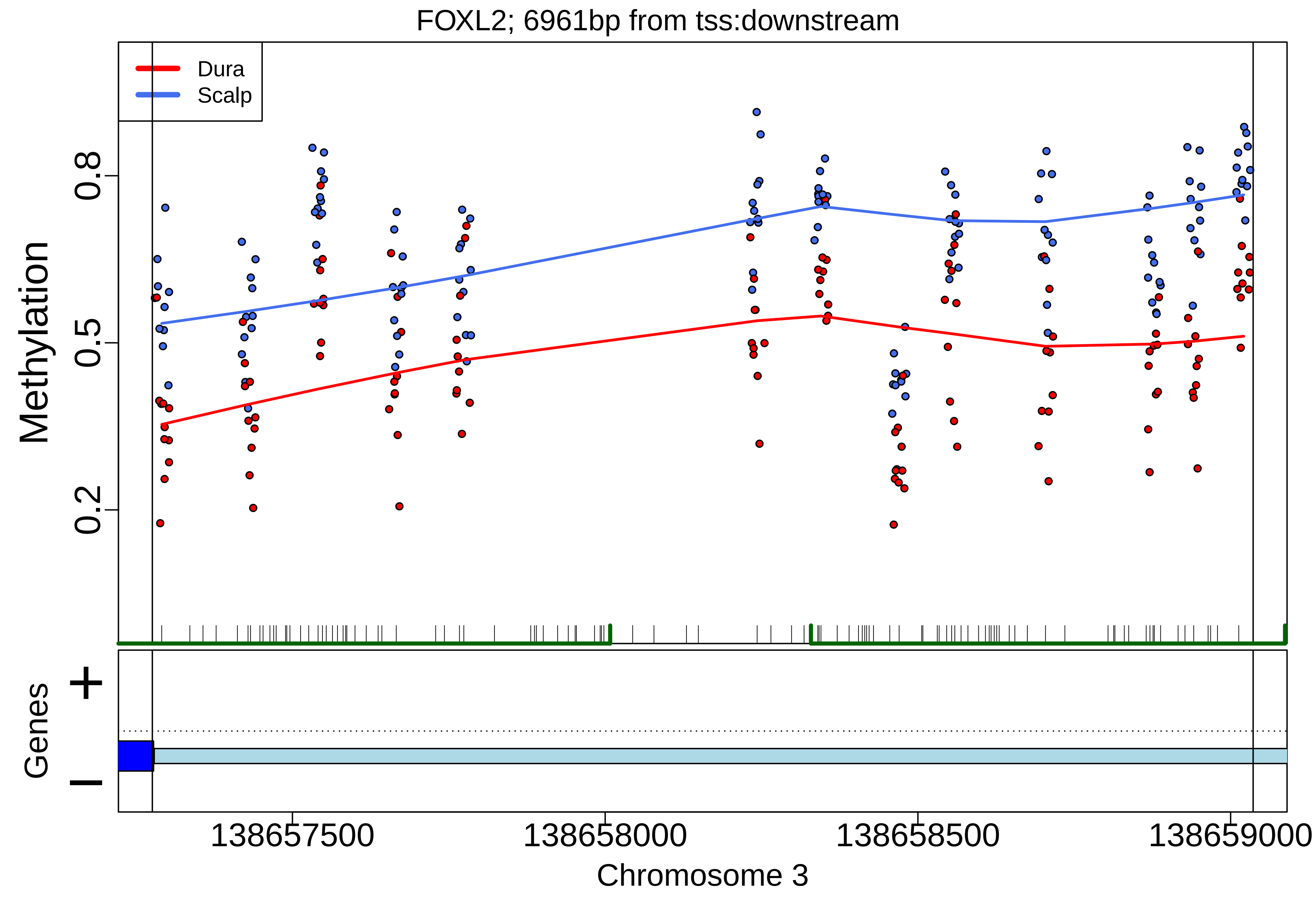

FOXL2; 6961bp from tss:downstream
Dura
Scalp
Methylation
0.2
0.5
0.8
Genes
+
−
138657500
138658000
138658500
138659000
Chromosome 3

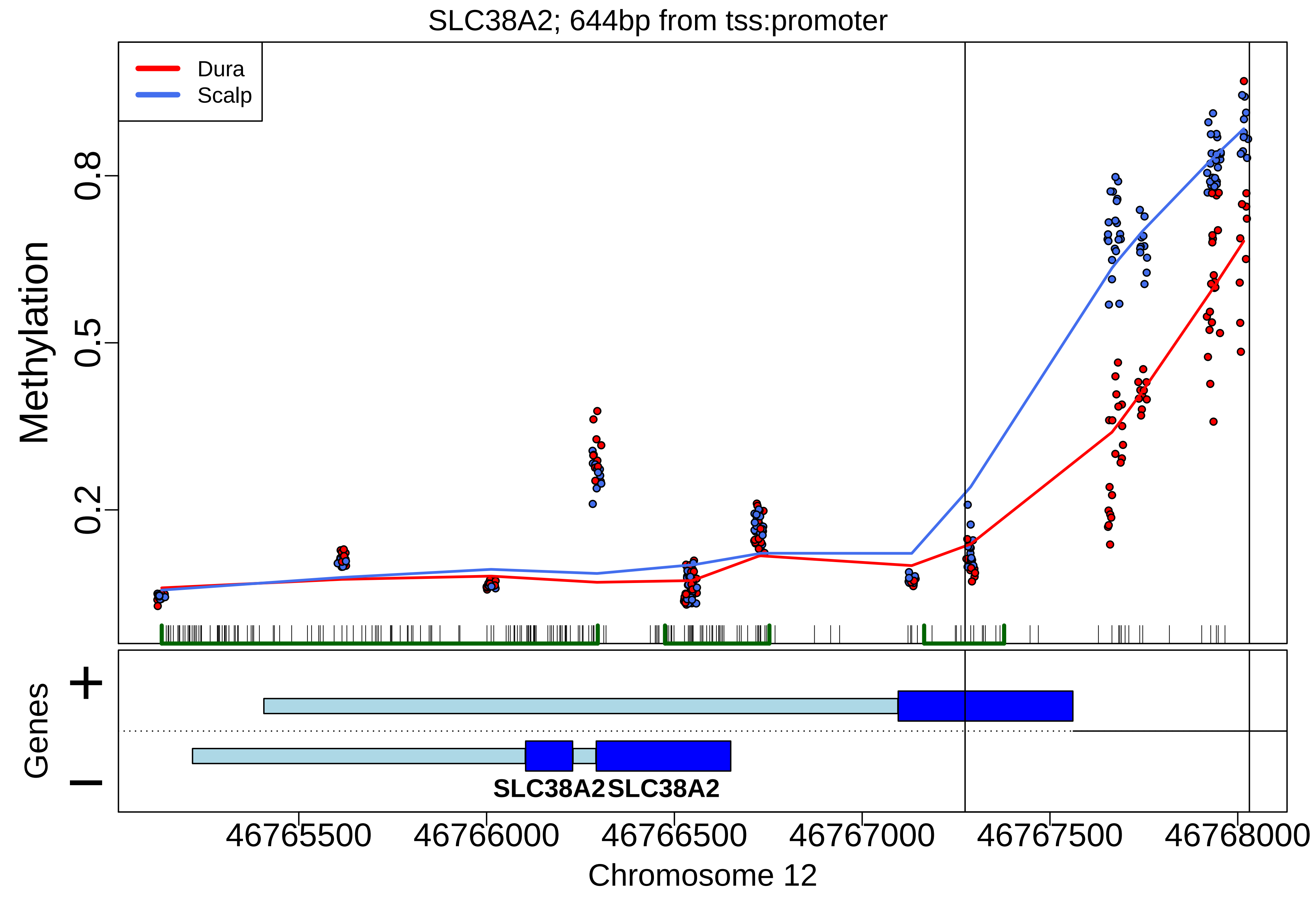

SLC38A2; 644bp from tss:promoter
Dura
Scalp
Methylation
0.2
0.5
0.8
Genes
+
−
SLC38A2
SLC38A2
46765500
46766000
46766500
46767000
46767500
46768000
Chromosome 12

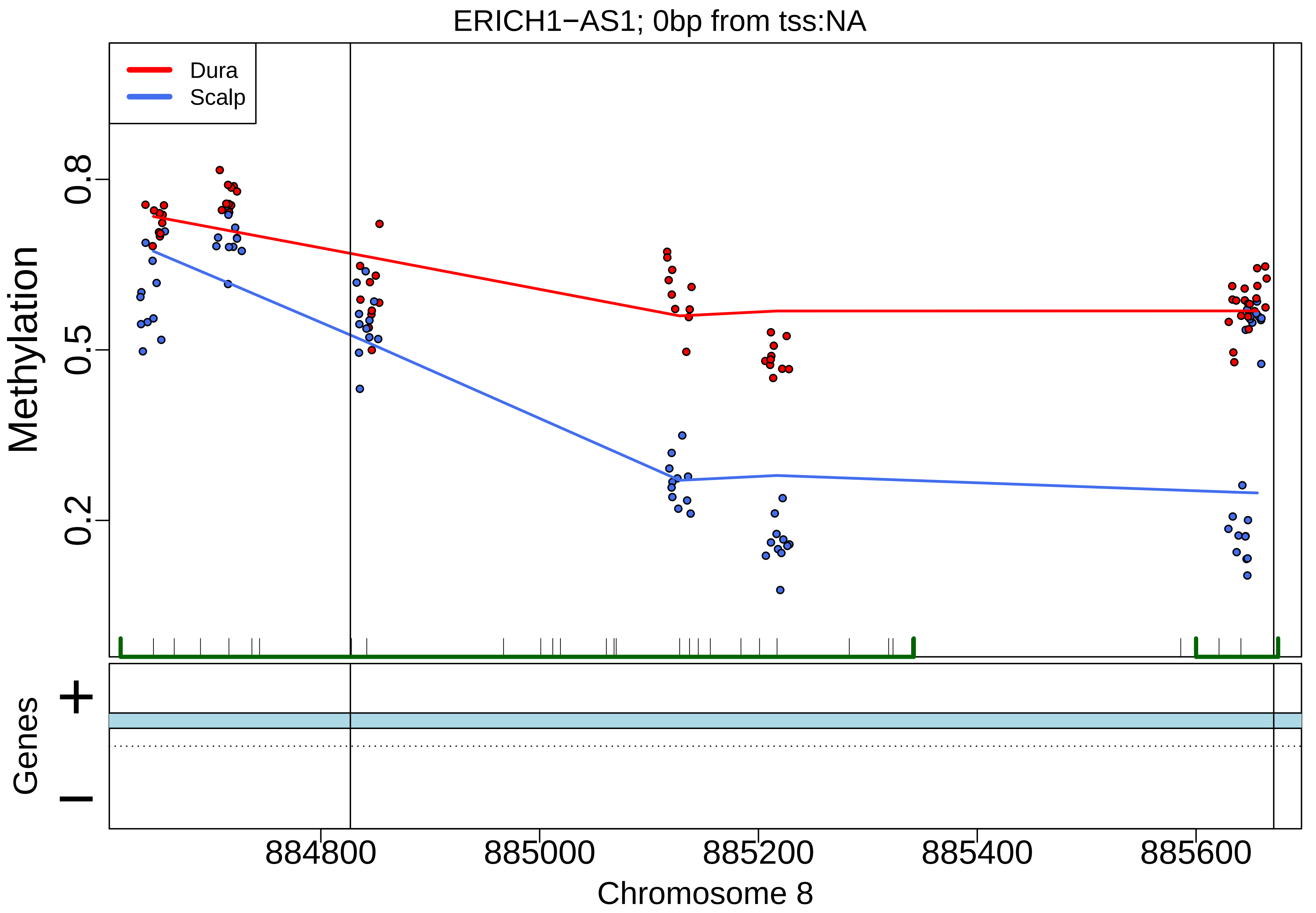
ERICH1−AS1; 0bp from tss:NA
Dura
Scalp
Methylation
0.8
0.5
0.2
Genes
+
−
884800
885000
885200
885400
885600
Chromosome 8

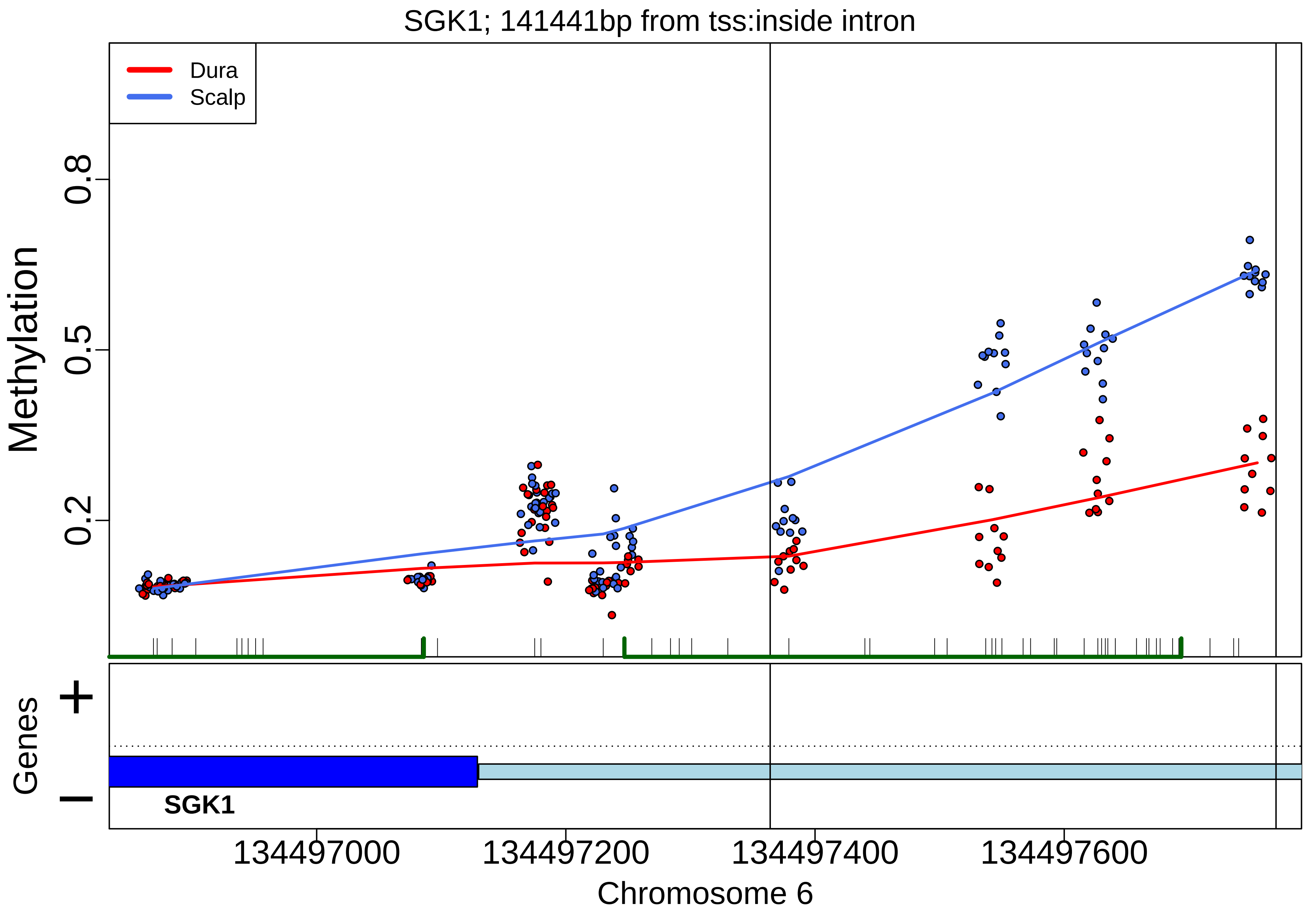

SGK1; 141441bp from tss:inside intron
Dura
Scalp
Methylation
0.2
0.5
0.8
Genes
+
−
SGK1
134497000
134497200
134497400
134497600
Chromosome 6

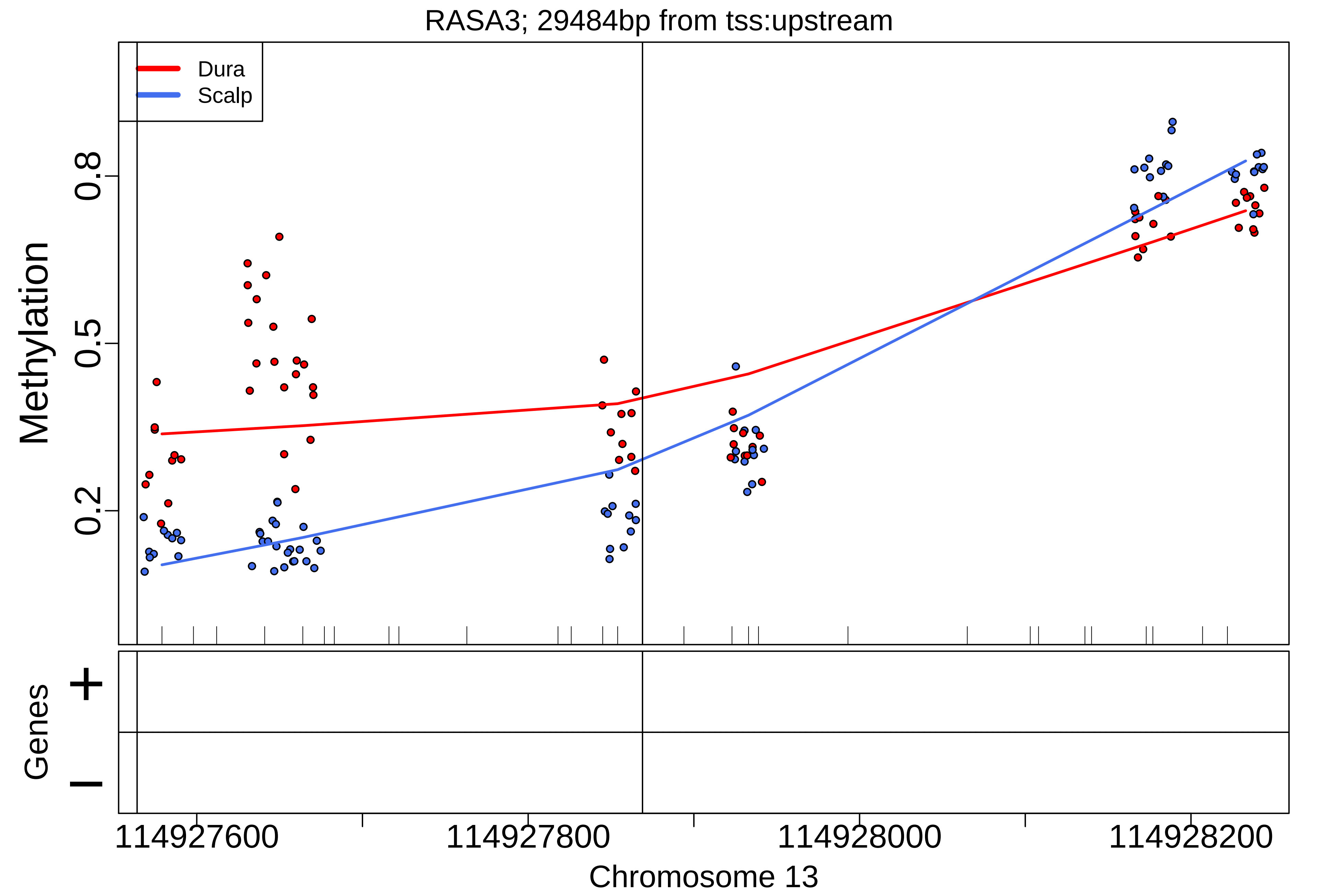
RASA3; 29484bp from tss:upstream
Dura
Scalp
Methylation
0.2
0.5
0.8
Genes
+
−
114927600
114927800
114928000
114928200
Chromosome 13

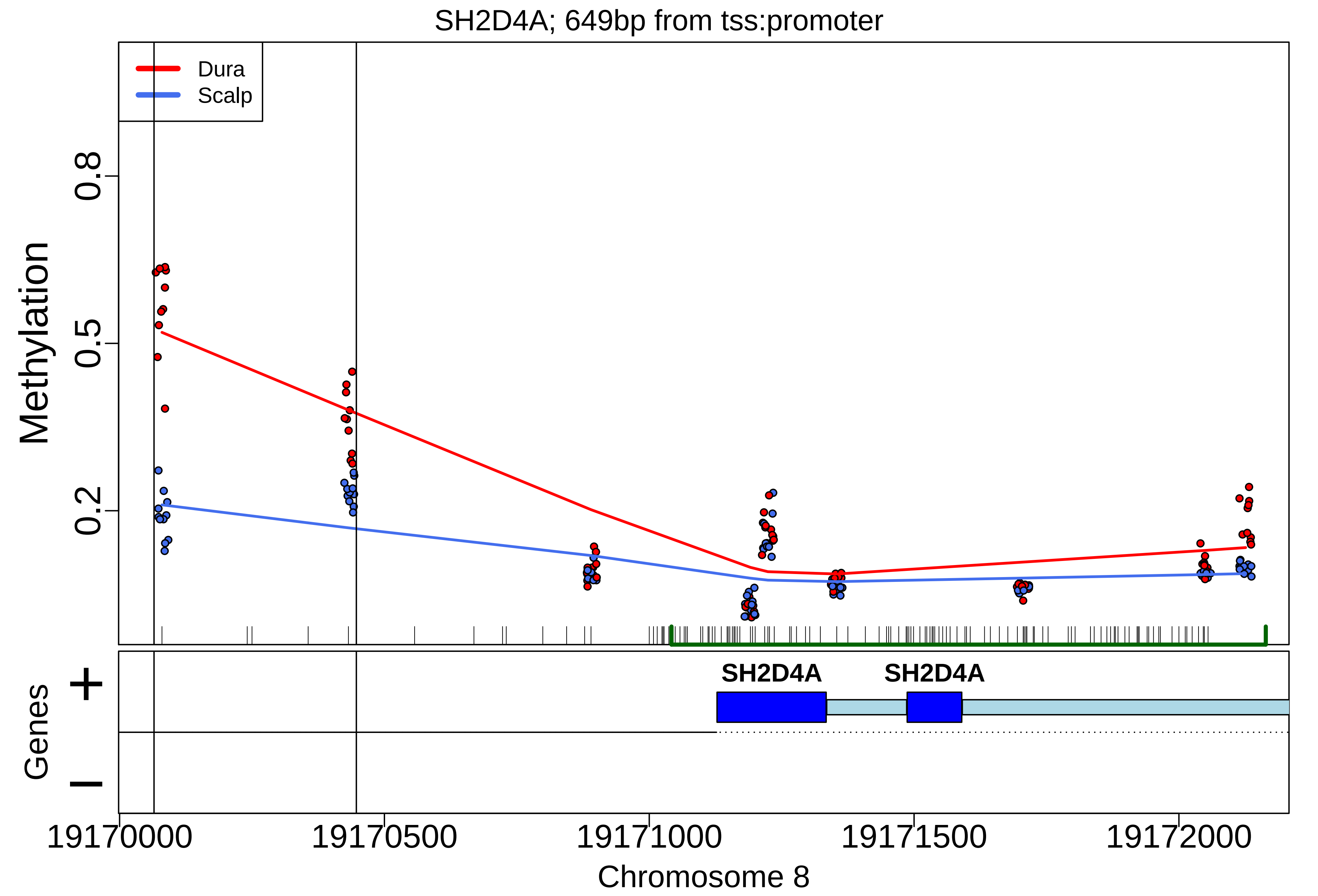

SH2D4A; 649bp from tss:promoter
Dura
Scalp
Methylation
0.2
0.5
0.8
Genes
+
-
SH2D4A
SH2D4A
19170000
19170500
19171000
19171500
19172000
Chromosome 8

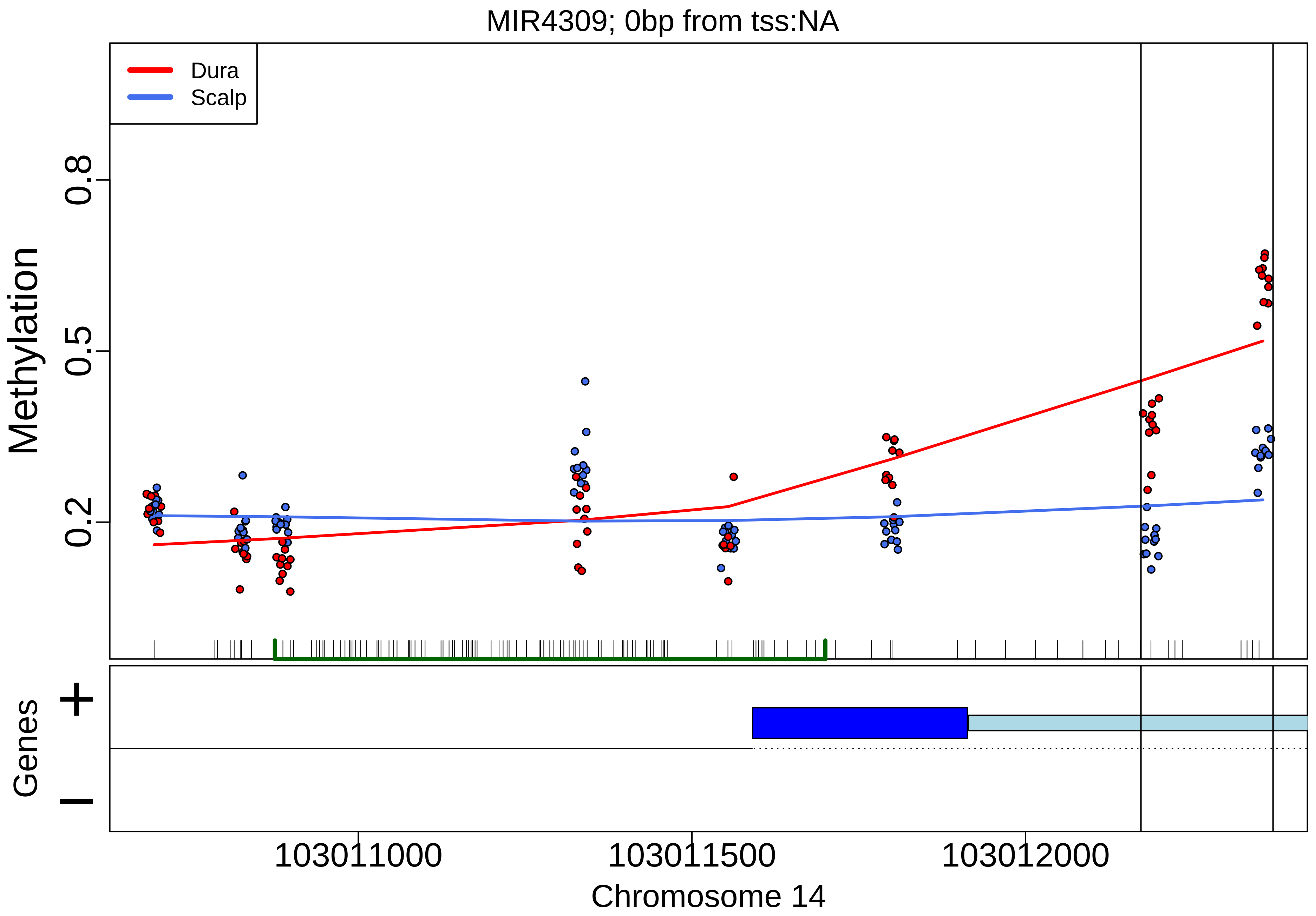

MIR4309; 0bp from tss:NA
Dura
Scalp
Methylation
0.2
0.5
0.8
Genes
+
−
103011000
103011500
103012000
Chromosome 14

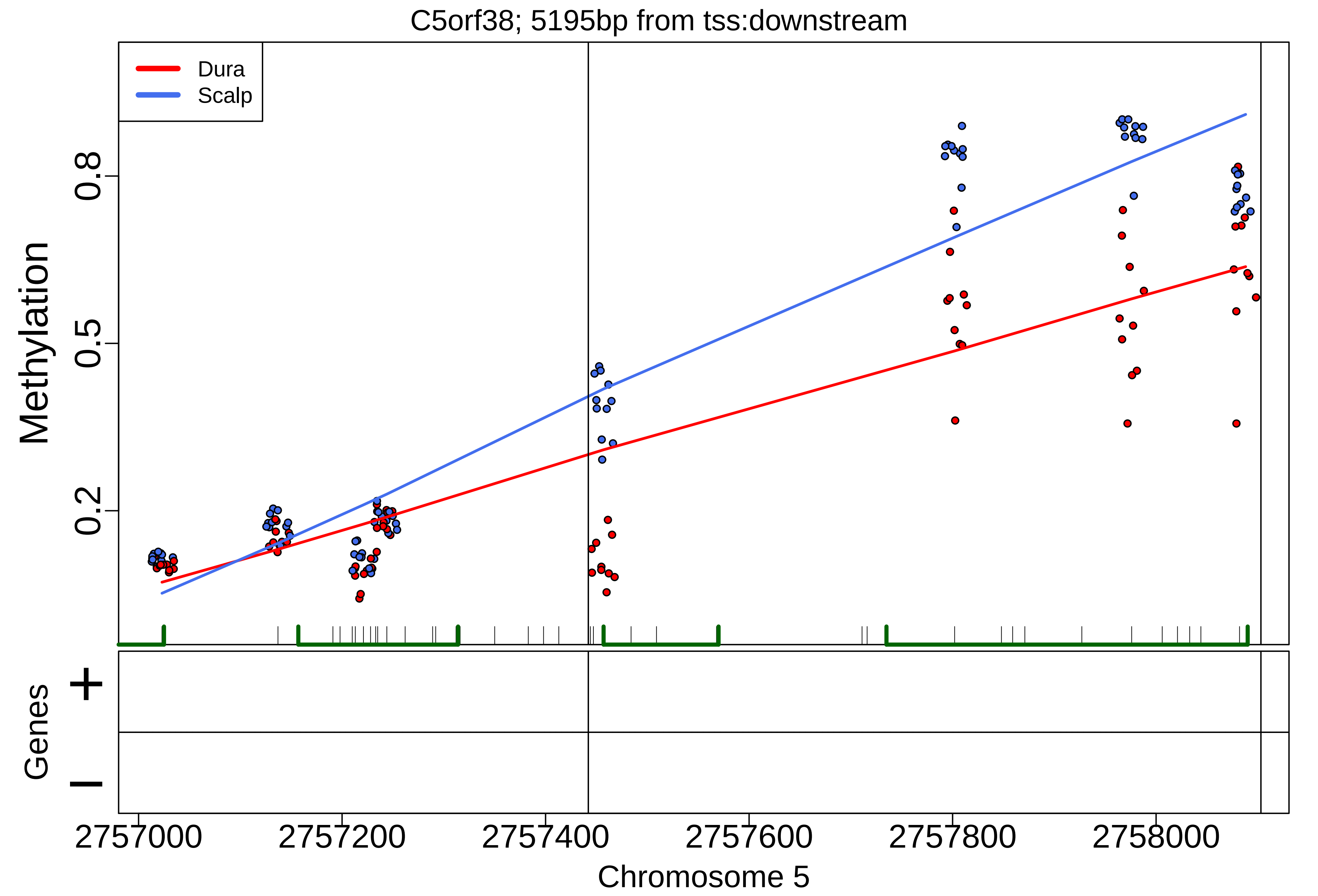
C5orf38; 5195bp from tss:downstream
Dura
Scalp
Methylation
0.2
0.5
0.8
Genes
+
−
2757000
2757200
2757400
2757600
2757800
2758000
Chromosome 5

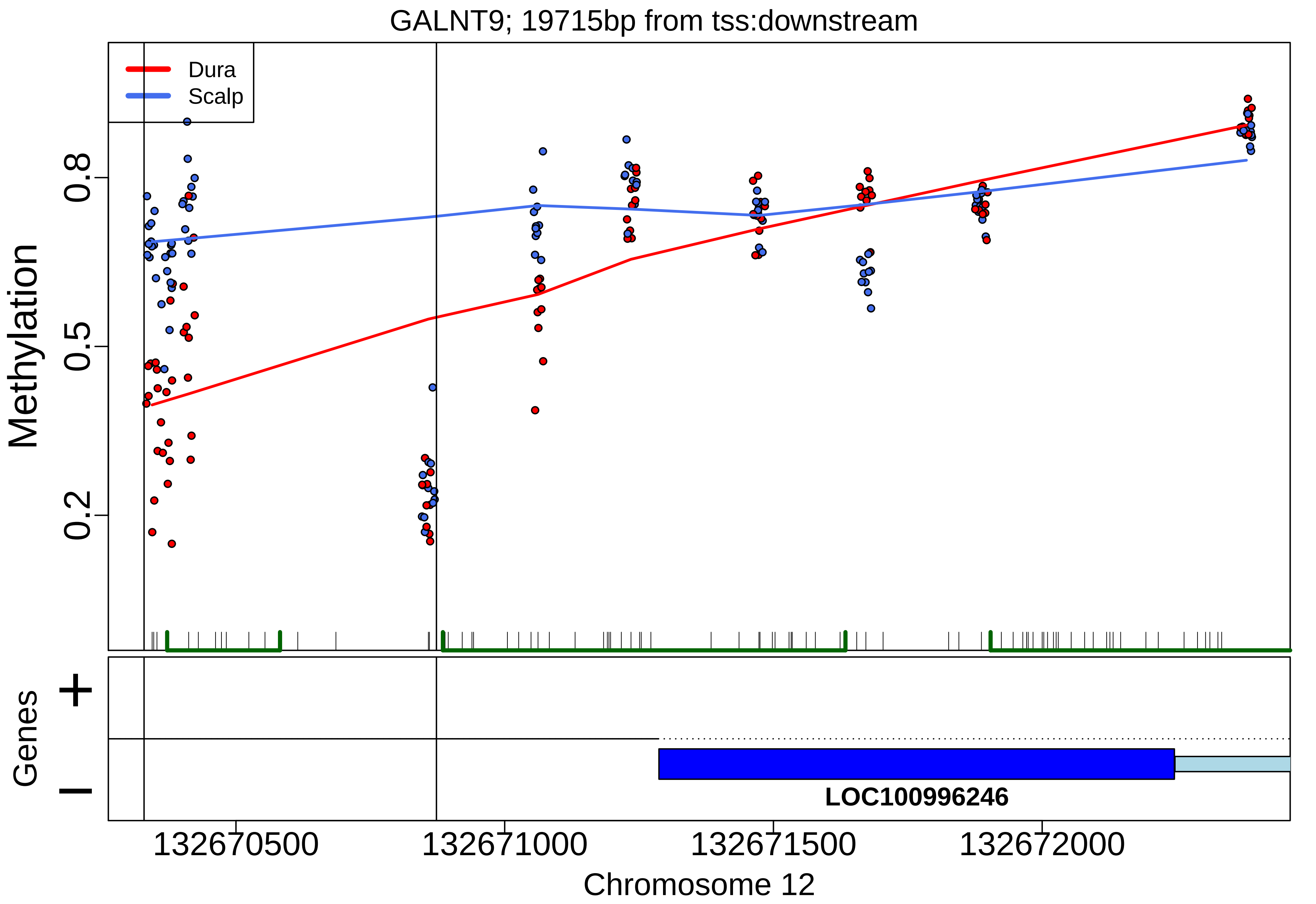
GALNT9; 19715bp from tss:downstream
Dura
Scalp
Methylation
0.2
0.5
0.8
Genes
+
−
LOC100996246
132670500
132671000
132671500
132672000
Chromosome 12

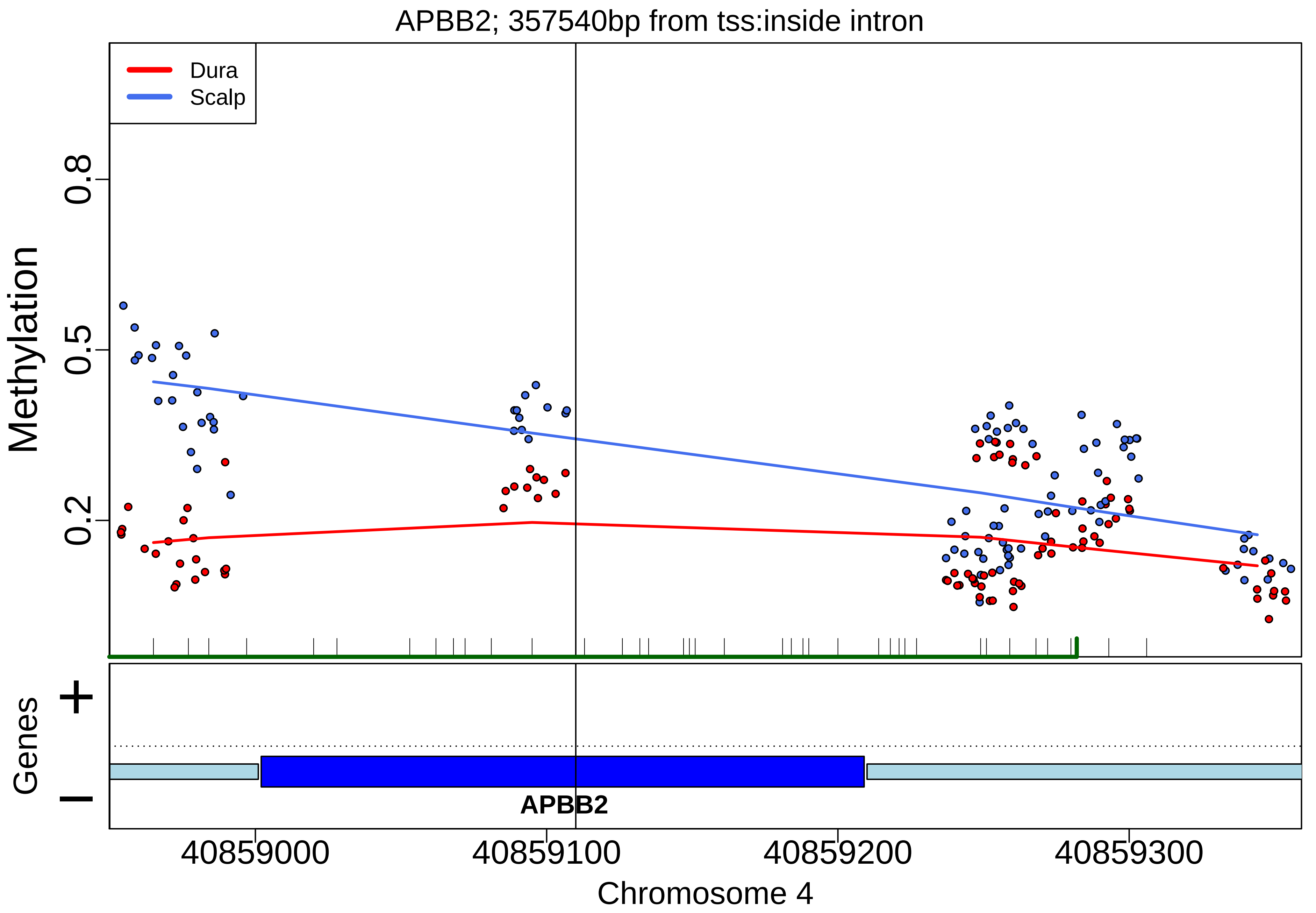

APBB2; 357540bp from tss:inside intron
Dura
Scalp
Methylation
0.2
0.5
0.8
Genes
+
-
APBB2
40859000
40859100
40859200
40859300
Chromosome 4

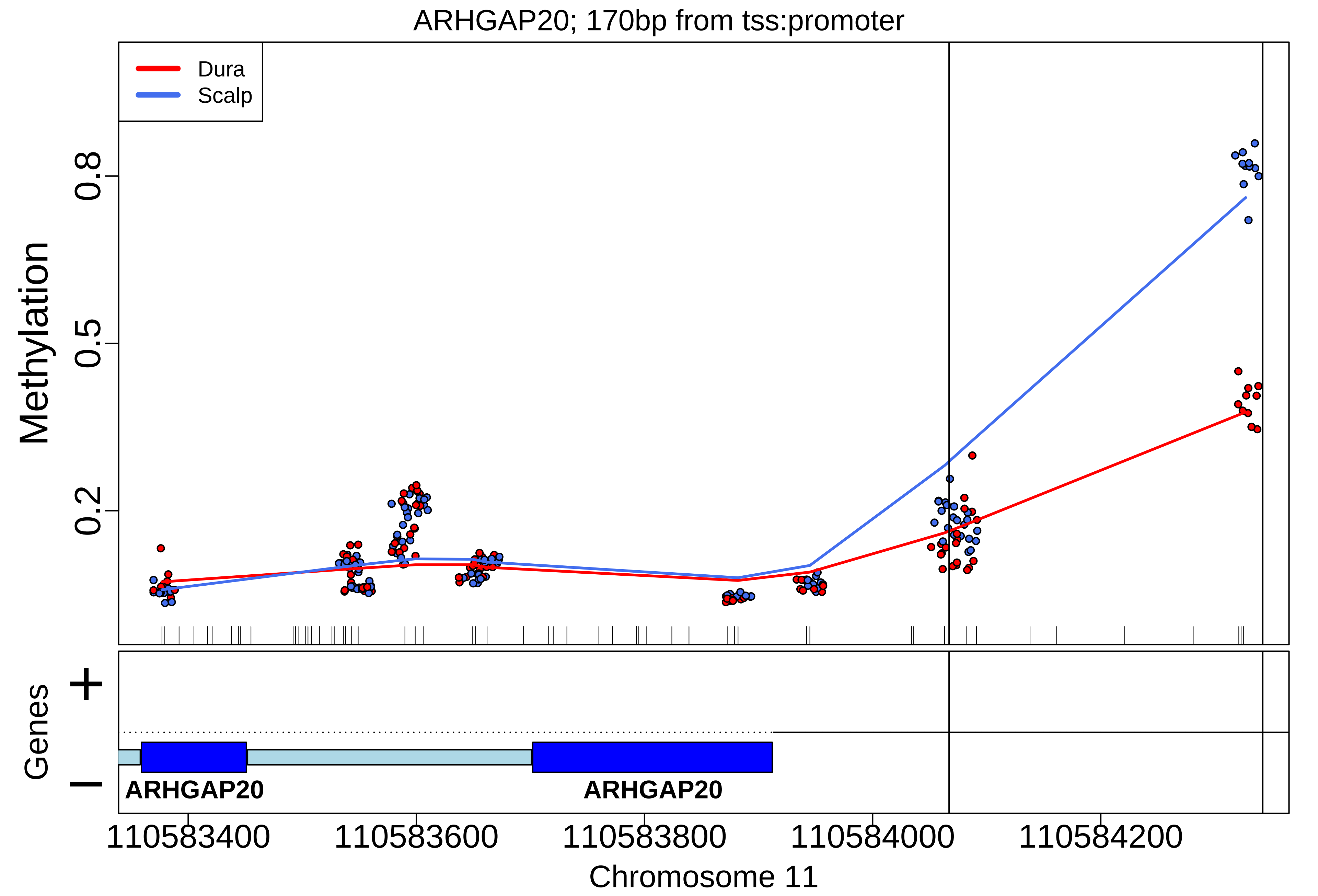
ARHGAP20; 170bp from tss:promoter
Dura
Scalp
Methylation
0.2
0.5
0.8
Genes
+
−
ARHGAP20
ARHGAP20
110583400
110583600
110583800
110584000
110584200
Chromosome 11

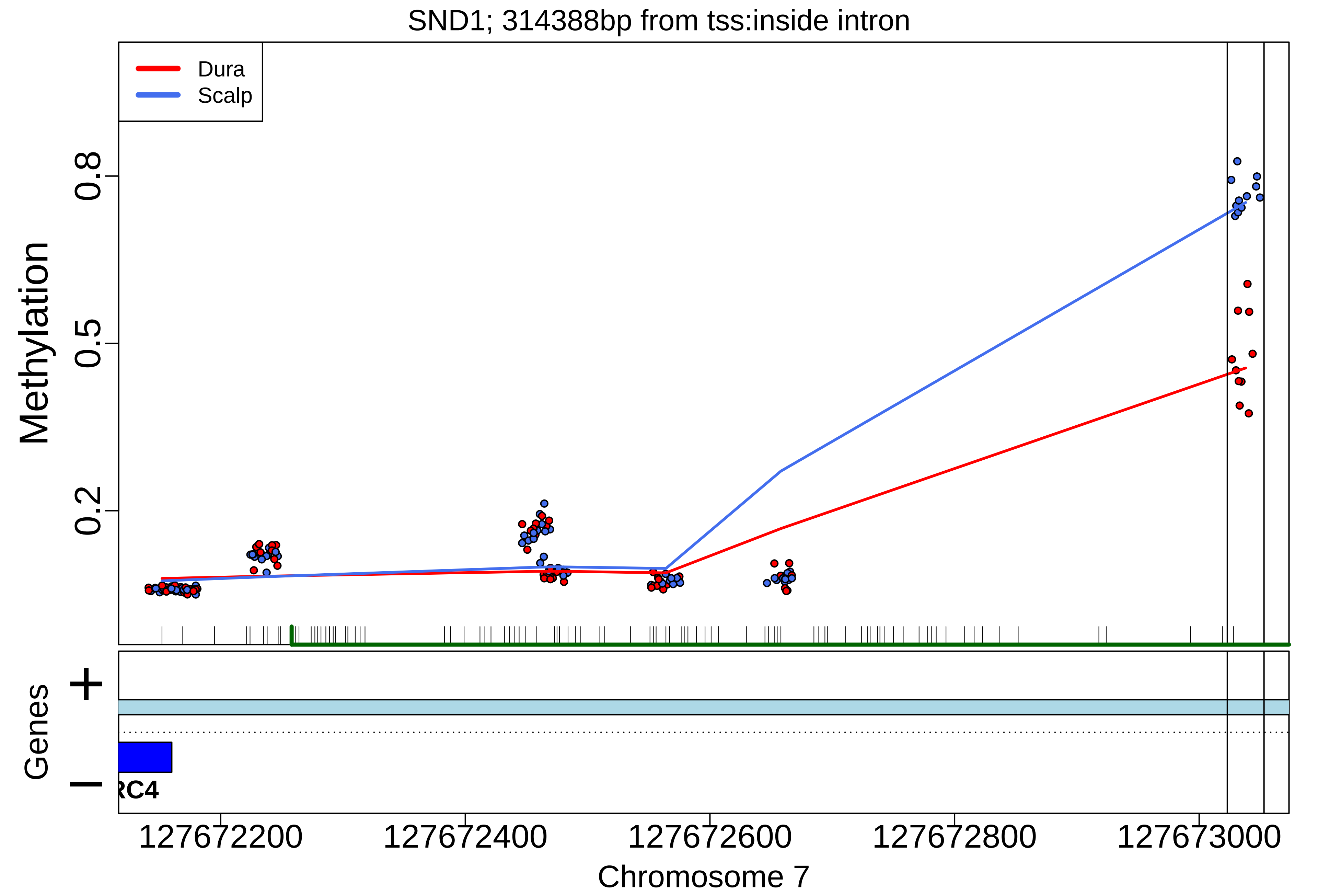

SND1; 314388bp from tss:inside intron
Dura
Scalp
Methylation
0.2
0.5
0.8
Genes
+
–
RC4
127672200
127672400
127672600
127672800
127673000
Chromosome 7

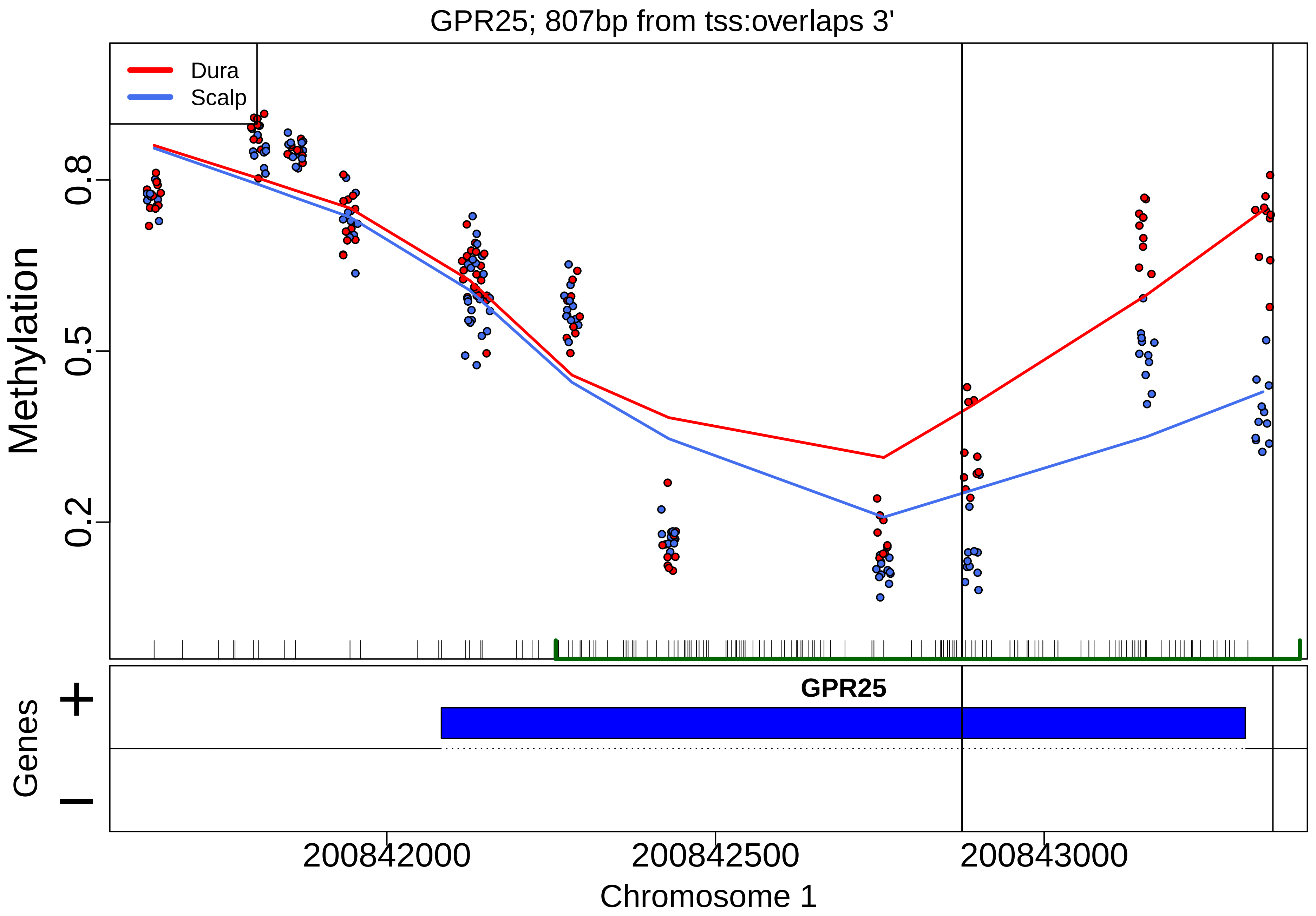
GPR25; 807bp from tss:overlaps 3'
Dura
Scalp
Methylation
0.2
0.5
0.8
Genes
+
-
GPR25
200842000
200842500
200843000
Chromosome 1

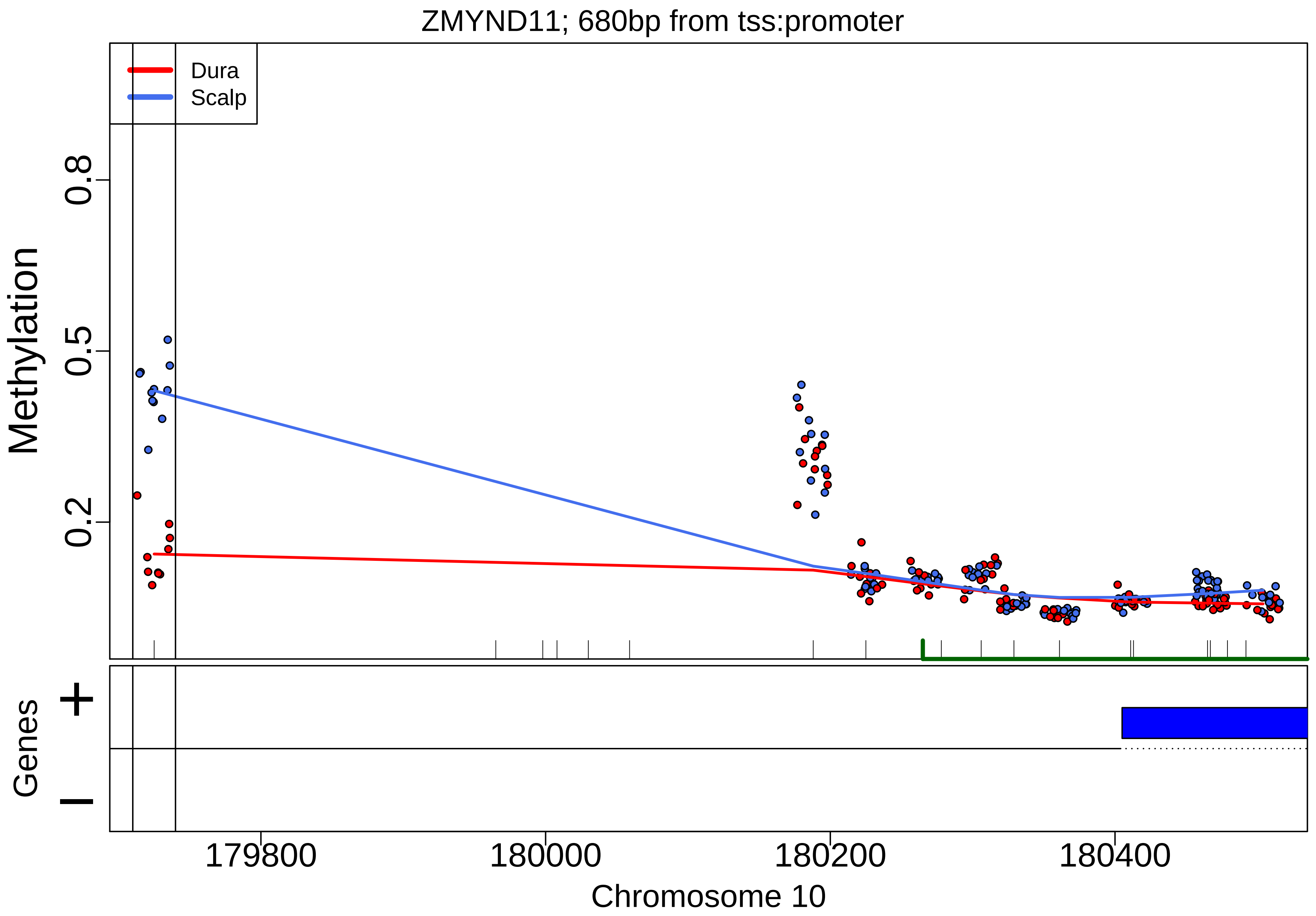
ZMYND11; 680bp from tss:promoter
Dura
Scalp
Methylation
0.2
0.5
0.8
Genes
+
−
179800
180000
180200
180400
Chromosome 10

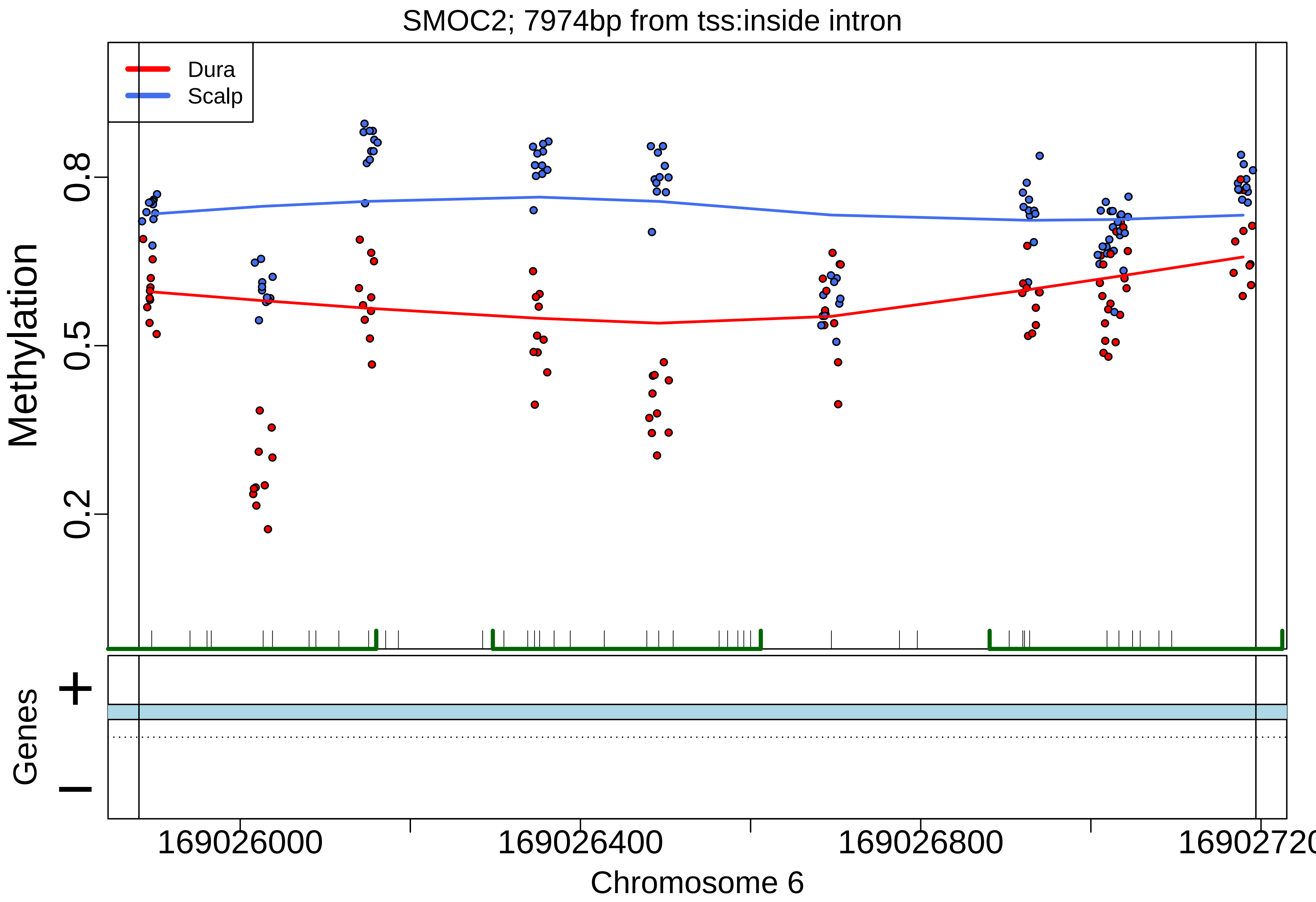

SMOC2; 7974bp from tss:inside intron
Dura
Scalp
Methylation
0.2
0.5
0.8
Genes
+
-
169026000
169026400
169026800
16902720
Chromosome 6

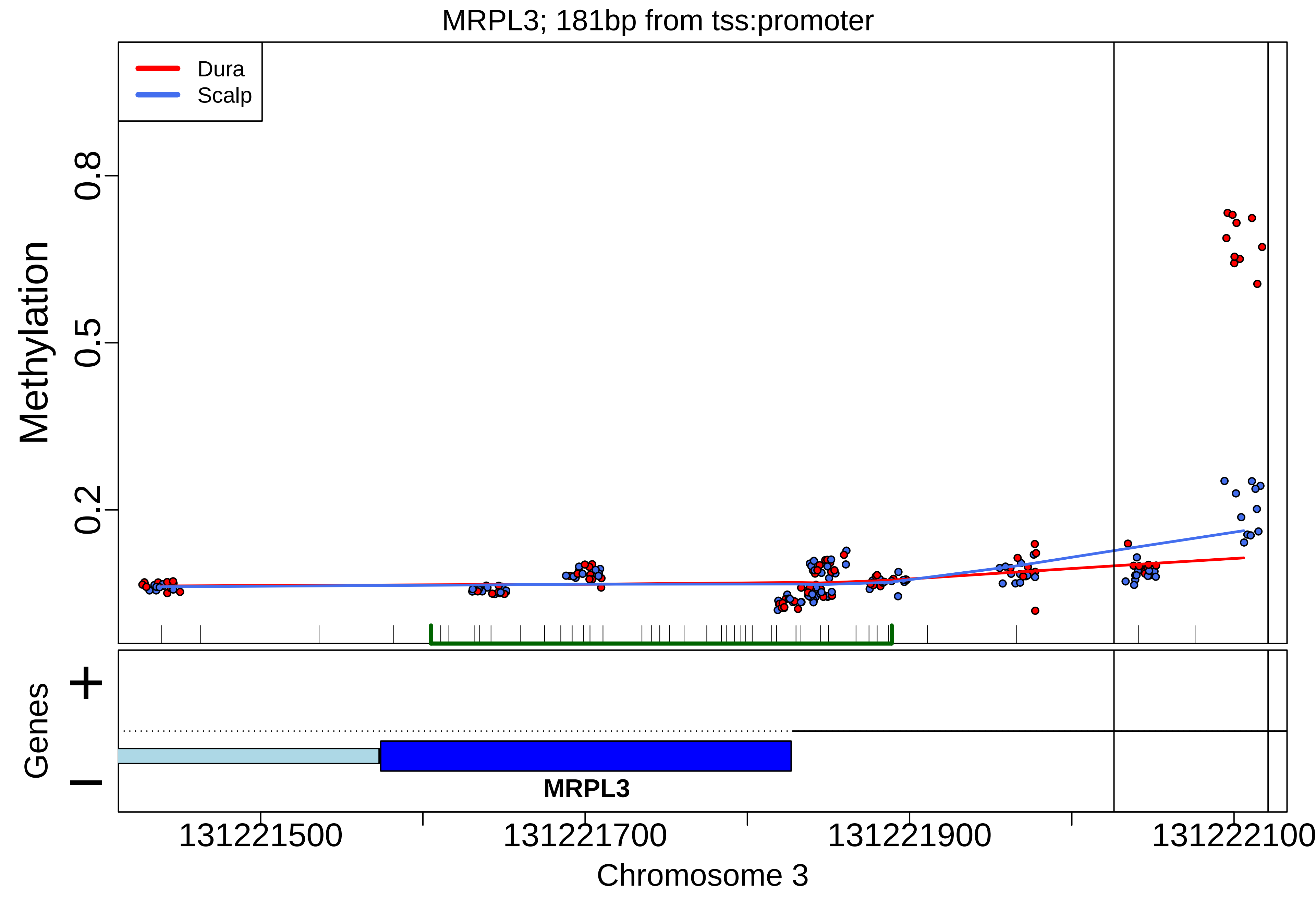

MRPL3; 181bp from tss:promoter
Dura
Scalp
Methylation
0.2
0.5
0.8
Genes
+
-
MRPL3
131221500
131221700
131221900
131222100
Chromosome 3

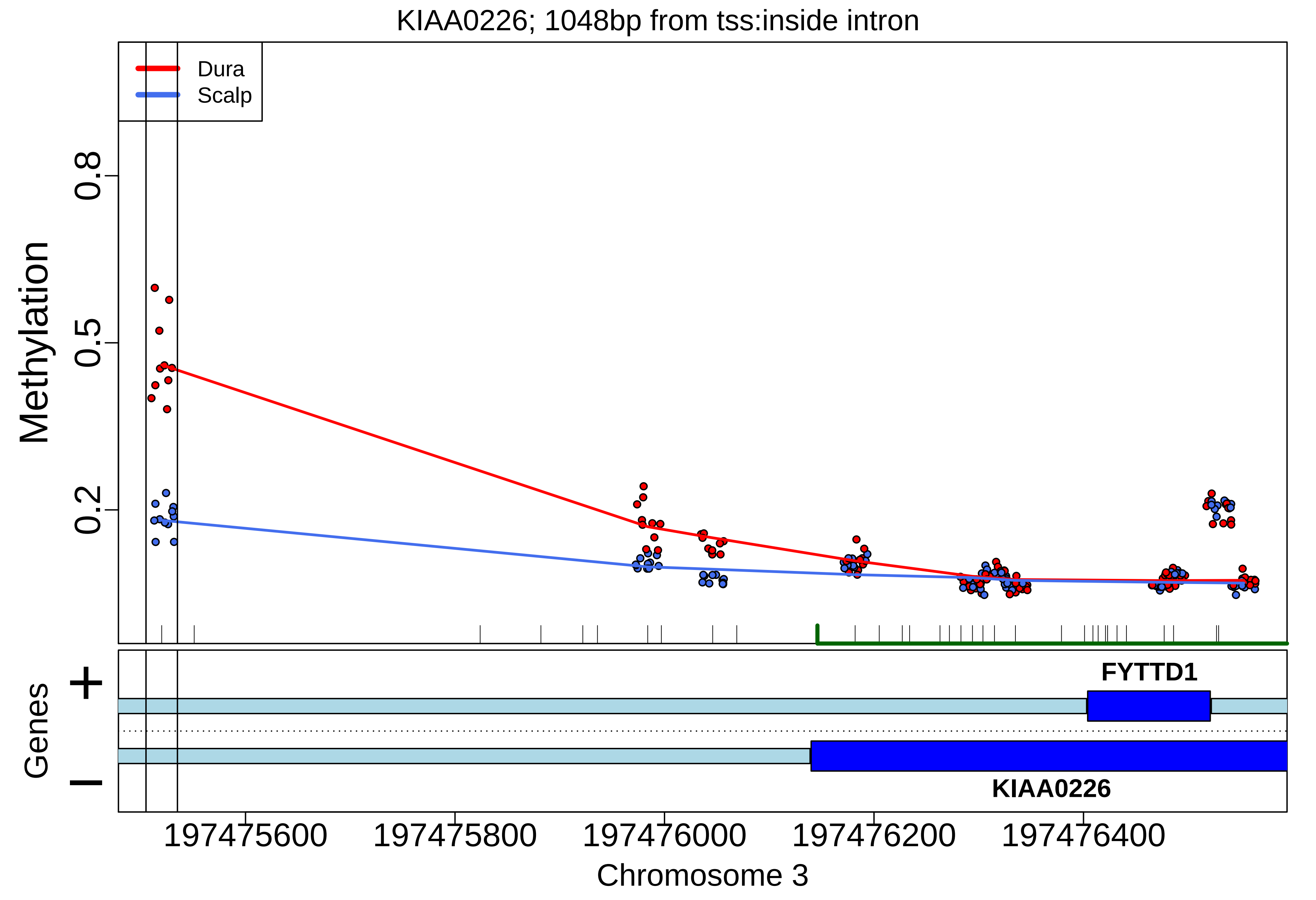
KIAA0226; 1048bp from tss:inside intron
Dura
Scalp
Methylation
0.8
0.5
0.2
Genes
+
−
FYTTD1
KIAA0226
197475600
197475800
197476000
197476200
197476400
Chromosome 3

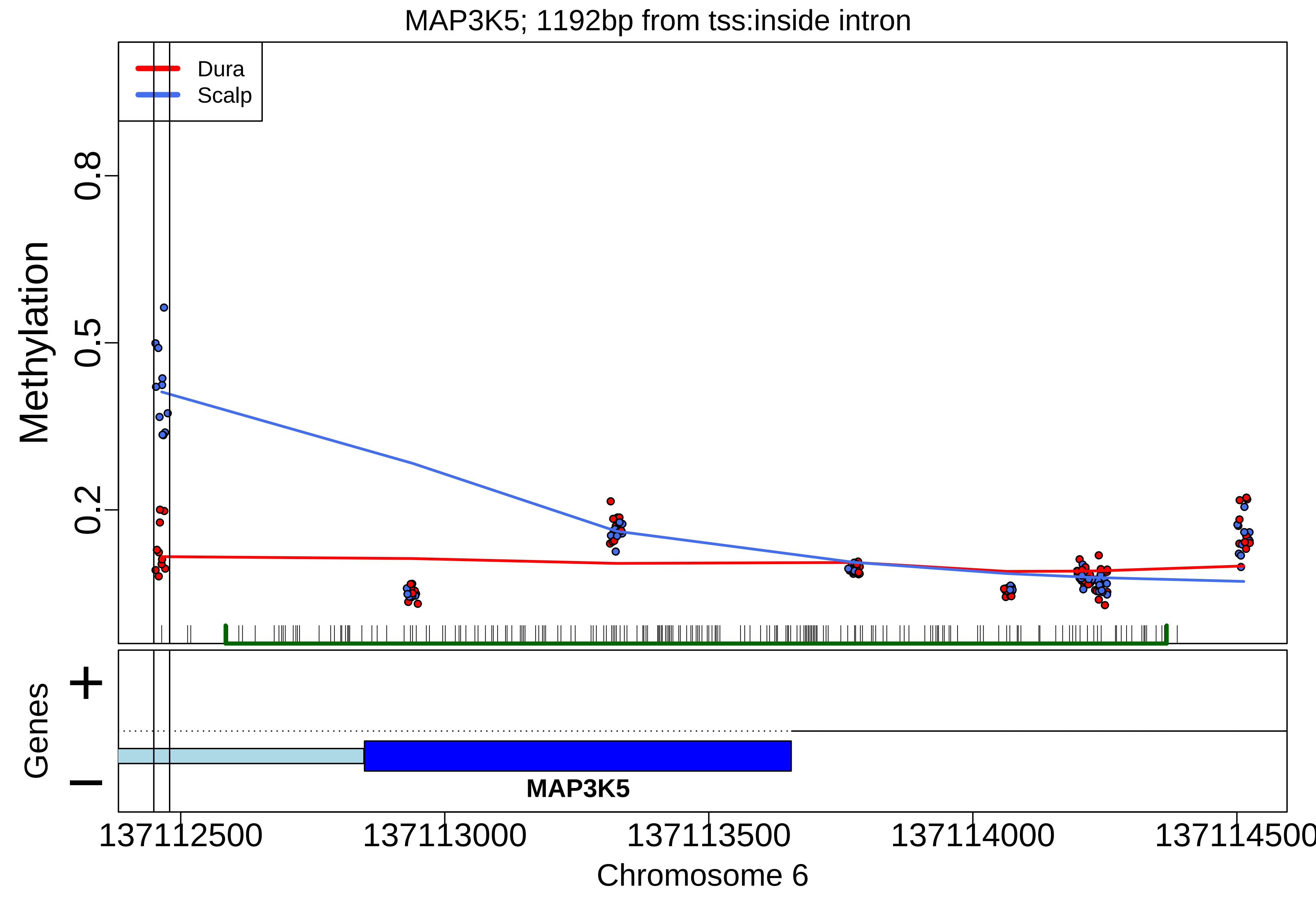

MAP3K5; 1192bp from tss:inside intron
Dura
Scalp
Methylation
0.2
0.5
0.8
Genes
+
−
MAP3K5
137112500
137113000
137113500
137114000
137114500
Chromosome 6

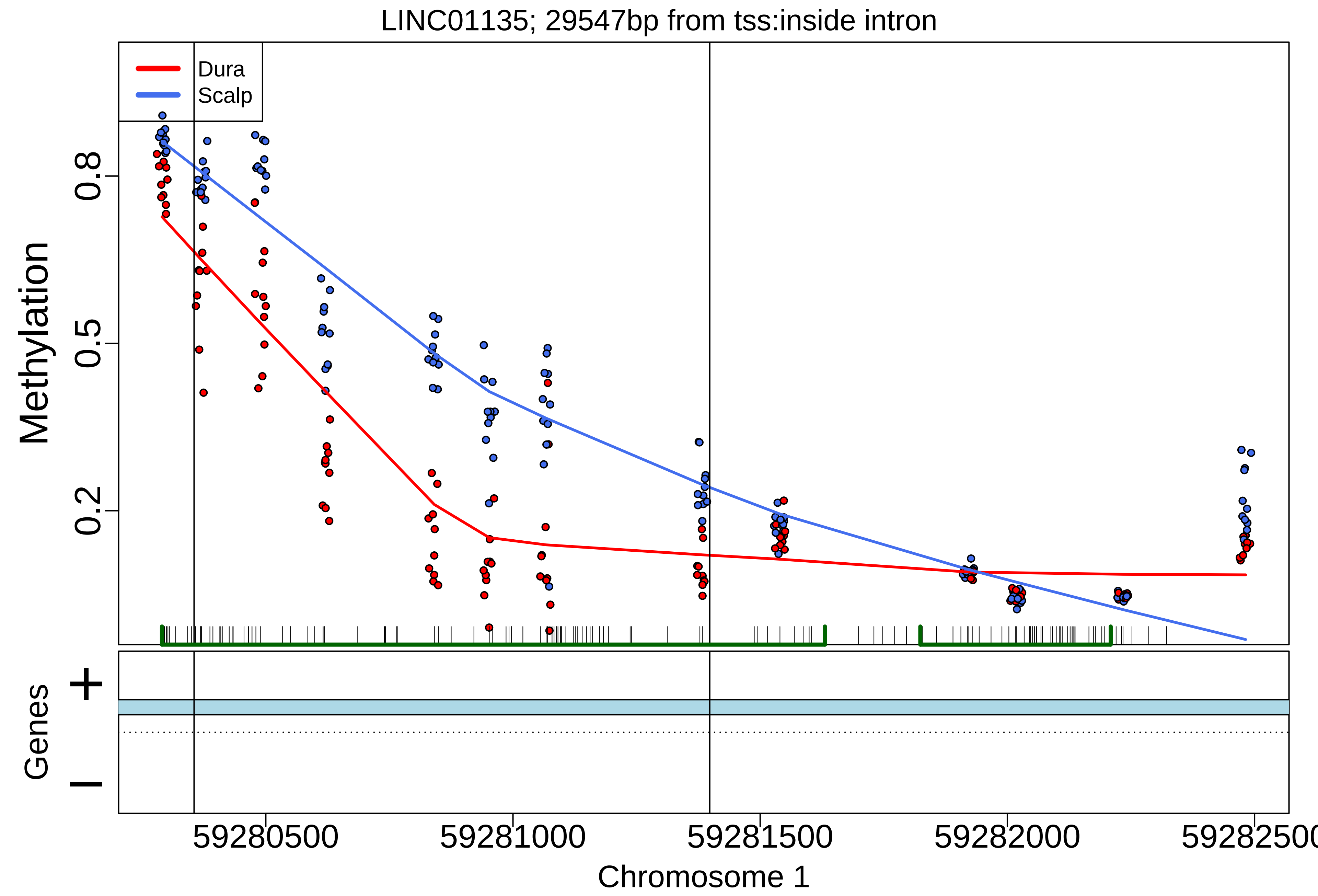

LINC01135; 29547bp from tss:inside intron
Dura
Scalp
Methylation
0.2
0.5
0.8
Genes
+
-
59280500
59281000
59281500
59282000
59282500
Chromosome 1

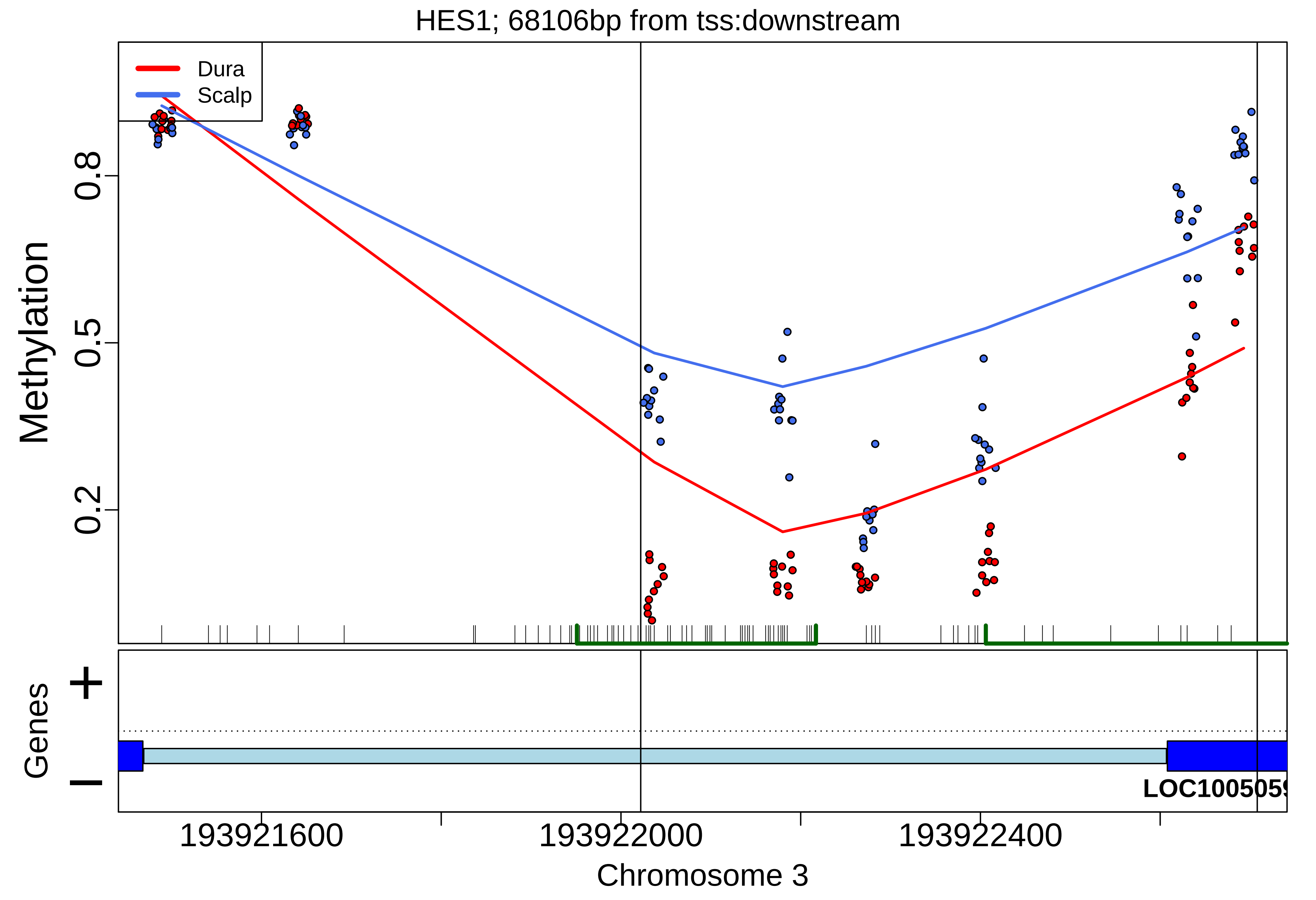
HES1; 68106bp from tss:downstream
Dura
Scalp
Methylation
0.2
0.5
0.8
Genes
+
−
LOC100505
193921600
193922000
193922400
Chromosome 3

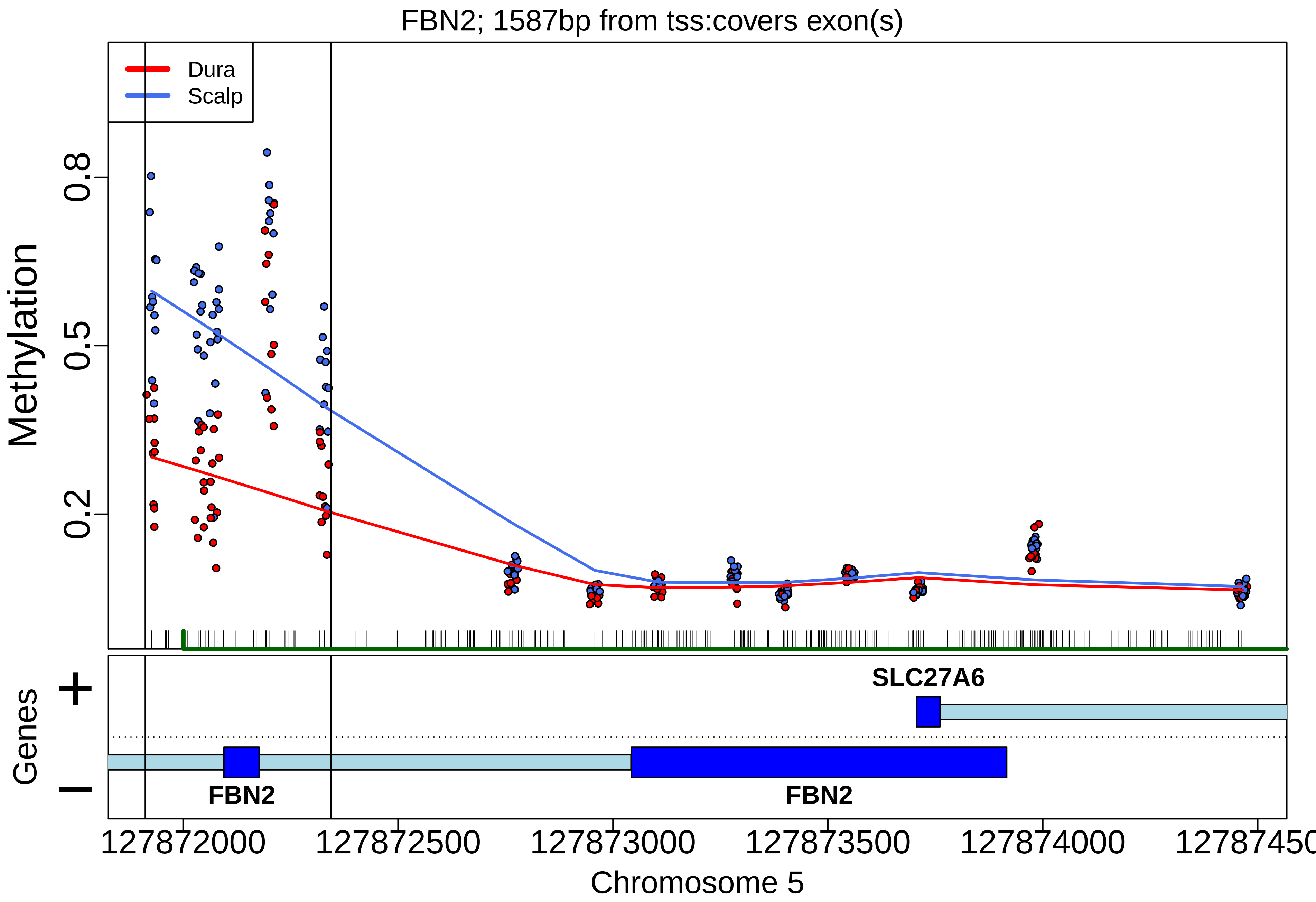
FBN2; 1587bp from tss:covers exon(s)
Dura
Scalp
Methylation
0.2
0.5
0.8
Genes
+
-
SLC27A6
FBN2
FBN2
127872000
127872500
127873000
127873500
127874000
Chromosome 5

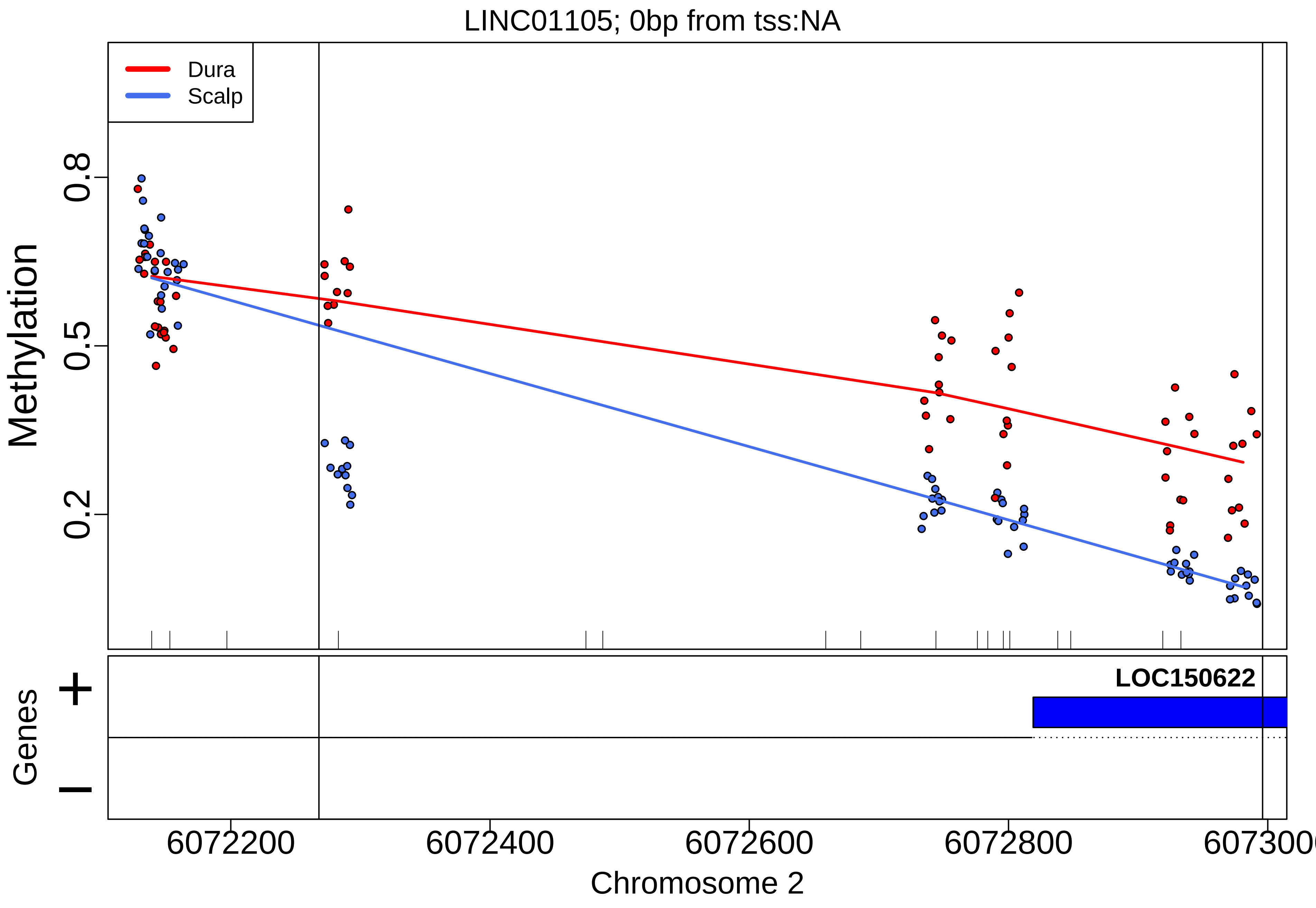
LINC01105; 0bp from tss:NA
Dura
Scalp
Methylation
0.2
0.5
0.8
Genes
+
-
LOC150622
6072200
6072400
6072600
6072800
607300
Chromosome 2

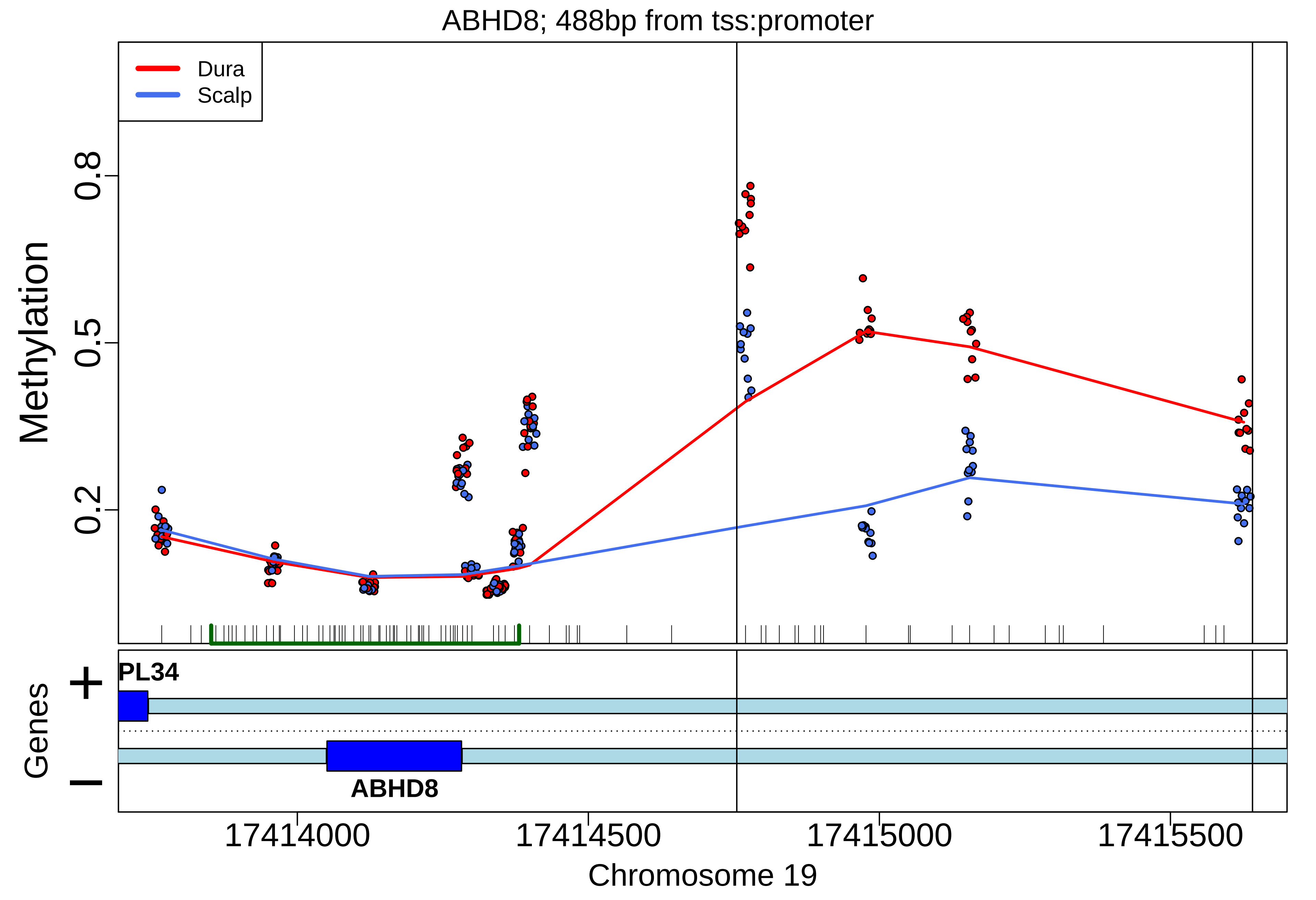
ABHD8; 488bp from tss:promoter
Dura
Scalp
Methylation
0.2
0.5
0.8
Genes
+
-
PL34
ABHD8
17414000
17414500
17415000
17415500
Chromosome 19

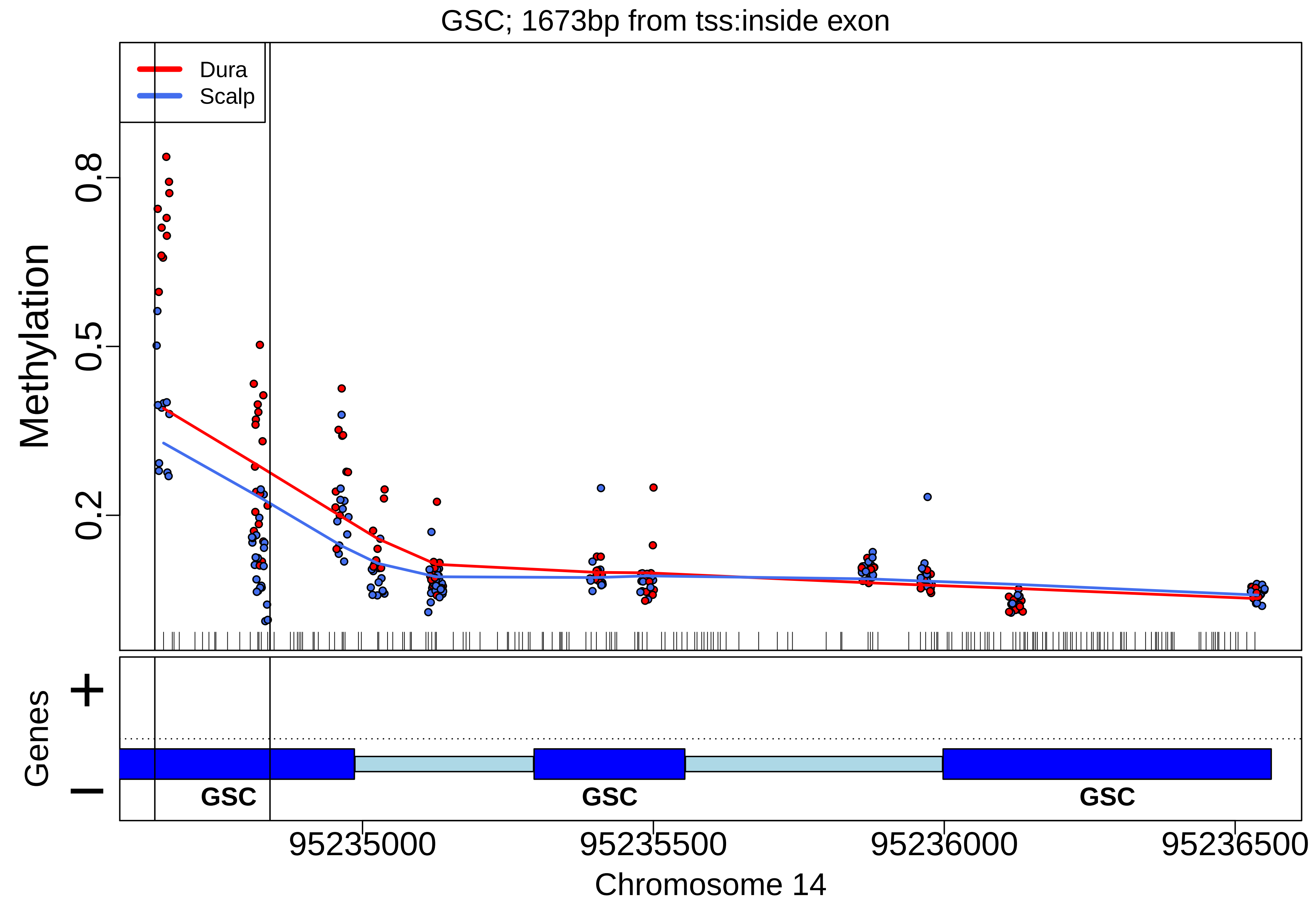

GSC; 1673bp from tss:inside exon
Dura
Scalp
Methylation
0.8
0.5
0.2
Genes
+
−
GSC
GSC
GSC
95235000
95235500
95236000
95236500
Chromosome 14

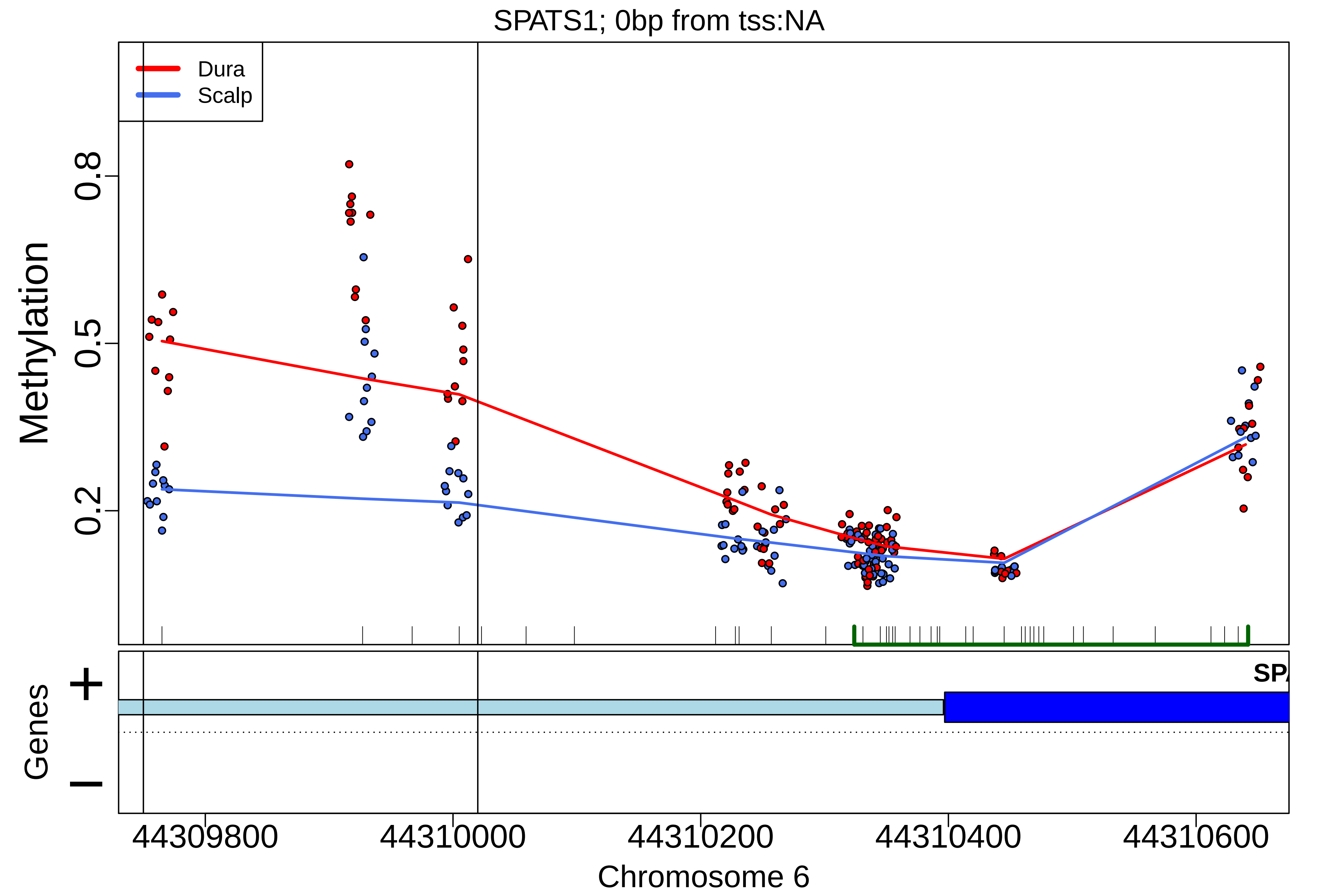

SPATS1; 0bp from tss:NA
Dura
Scalp
Methylation
0.2
0.5
0.8
Genes
+
-
SP
44309800
44310000
44310200
44310400
44310600
Chromosome 6

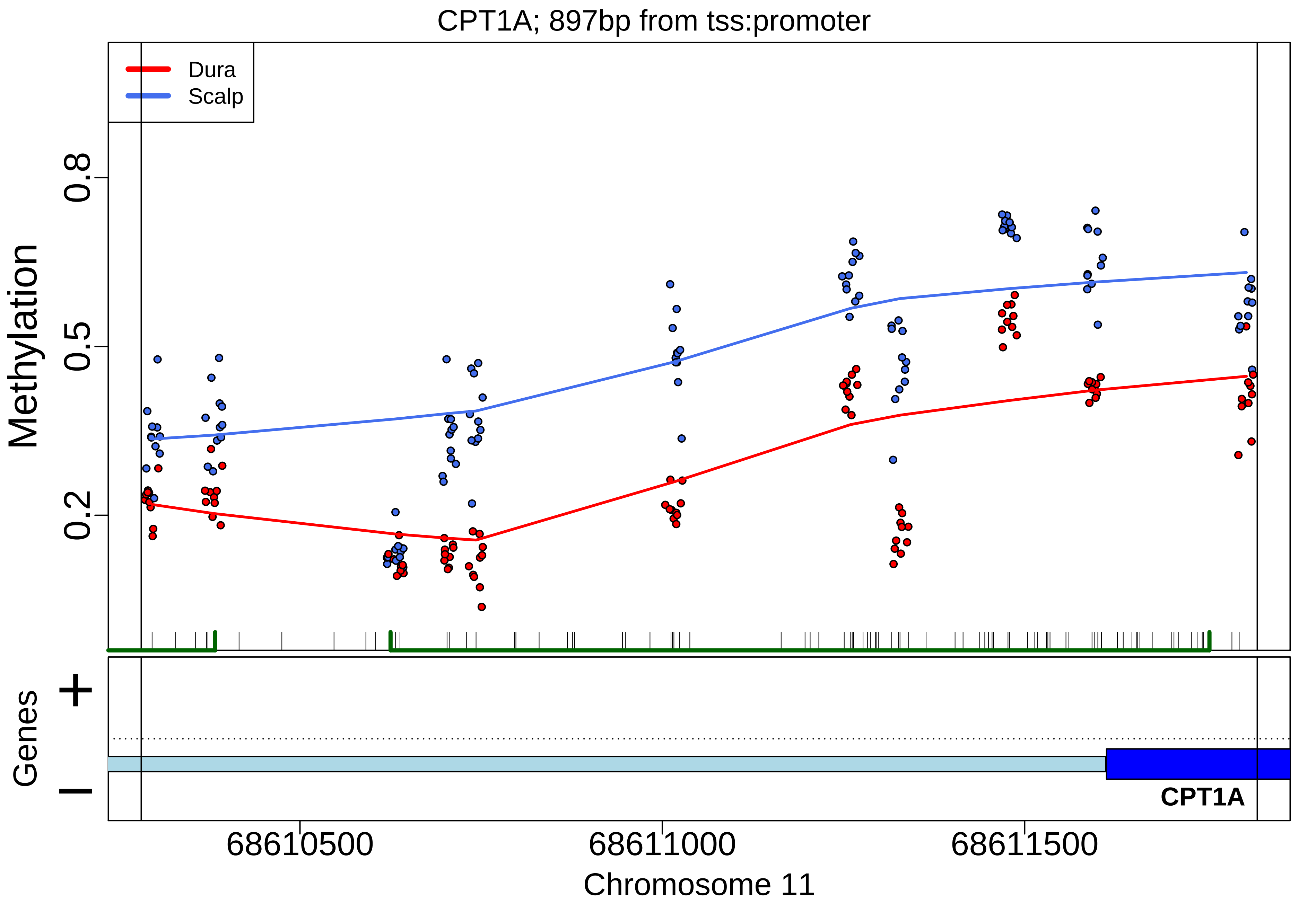

CPT1A; 897bp from tss:promoter
Dura
Scalp
Methylation
0.2
0.5
0.8
Genes
+
−
CPT1A
68610500
68611000
68611500
Chromosome 11

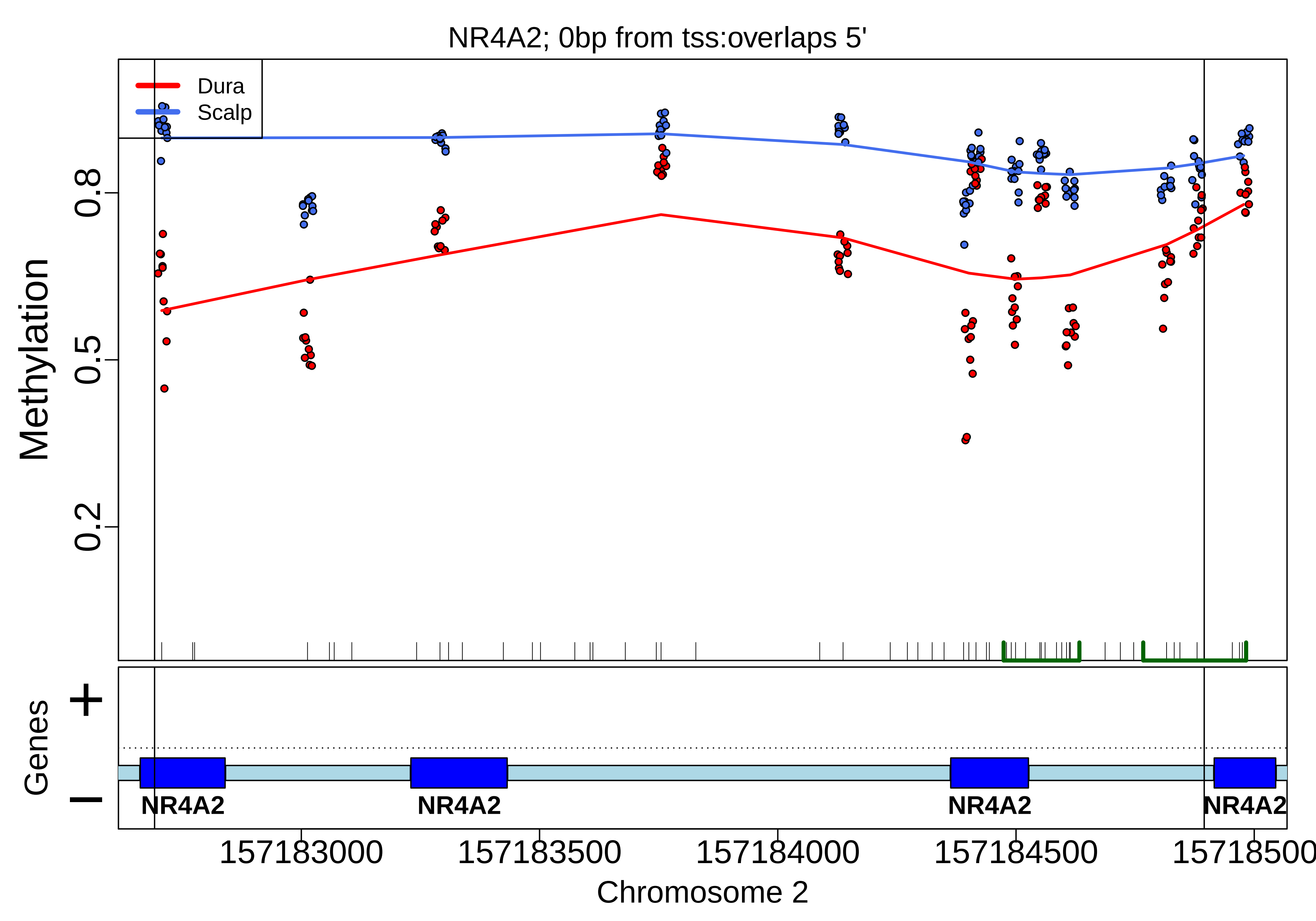

NR4A2; 0bp from tss:overlaps 5'
Dura
Scalp
Methylation
0.2
0.5
0.8
Genes
+
-
NR4A2
NR4A2
NR4A2
NR4A2
157183000
157183500
157184000
157184500
15718500
Chromosome 2

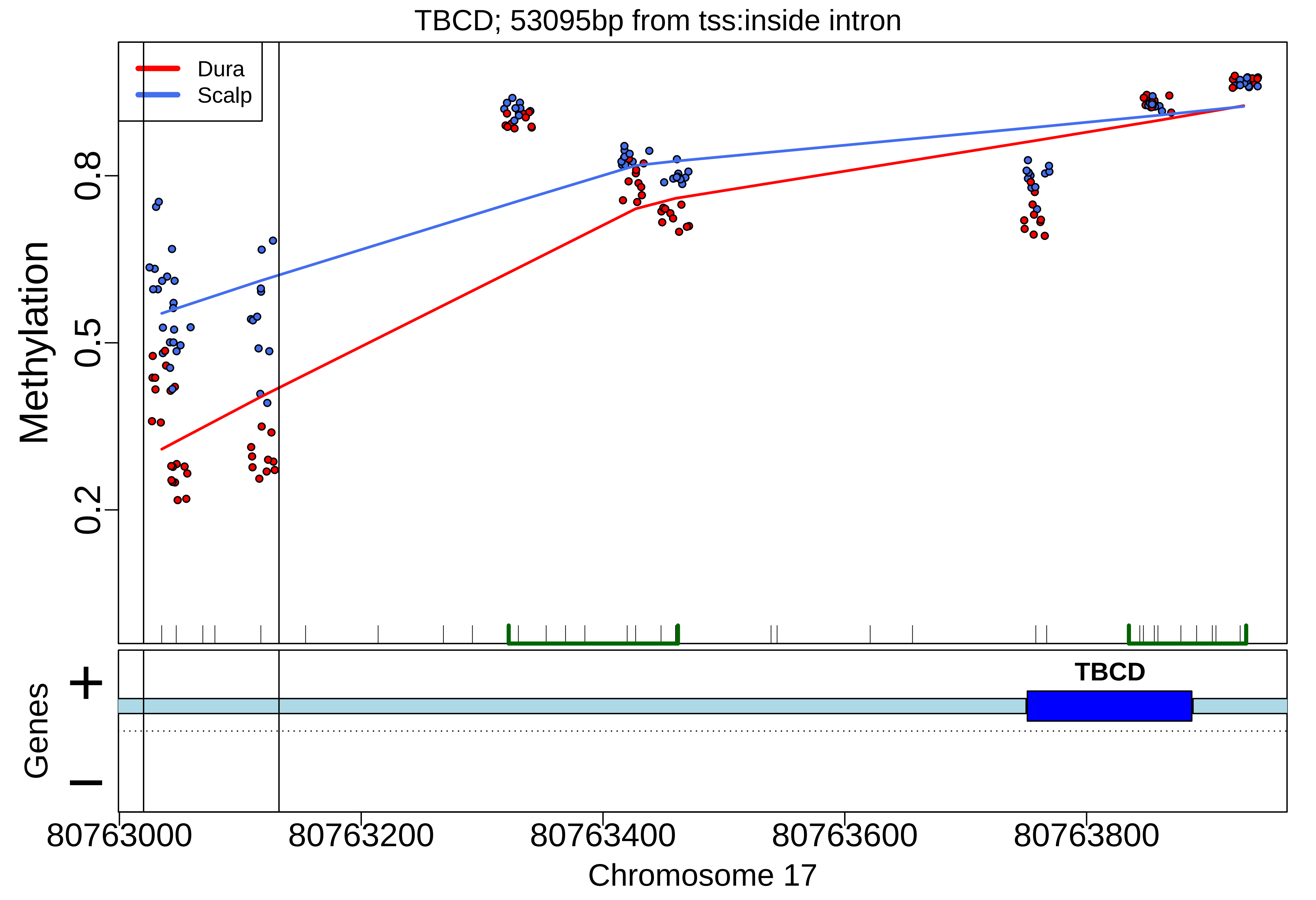

TBCD; 53095bp from tss:inside intron
Dura
Scalp
Methylation
0.2
0.5
0.8
Genes
+
−
TBCD
80763000
80763200
80763400
80763600
80763800
Chromosome 17

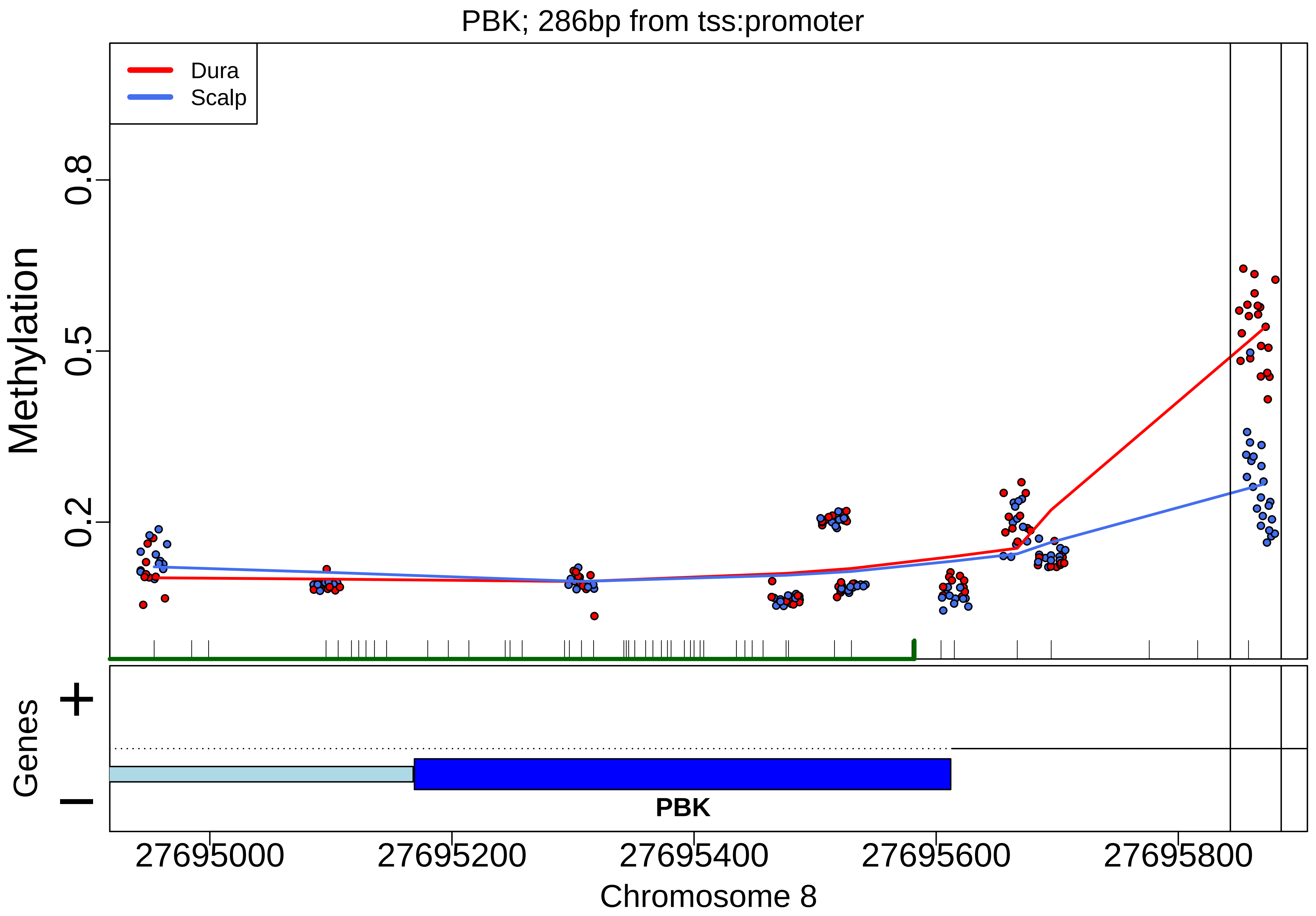

PBK; 286bp from tss:promoter
Dura
Scalp
Methylation
0.2
0.5
0.8
Genes
+
-
PBK
27695000
27695200
27695400
27695600
27695800
Chromosome 8

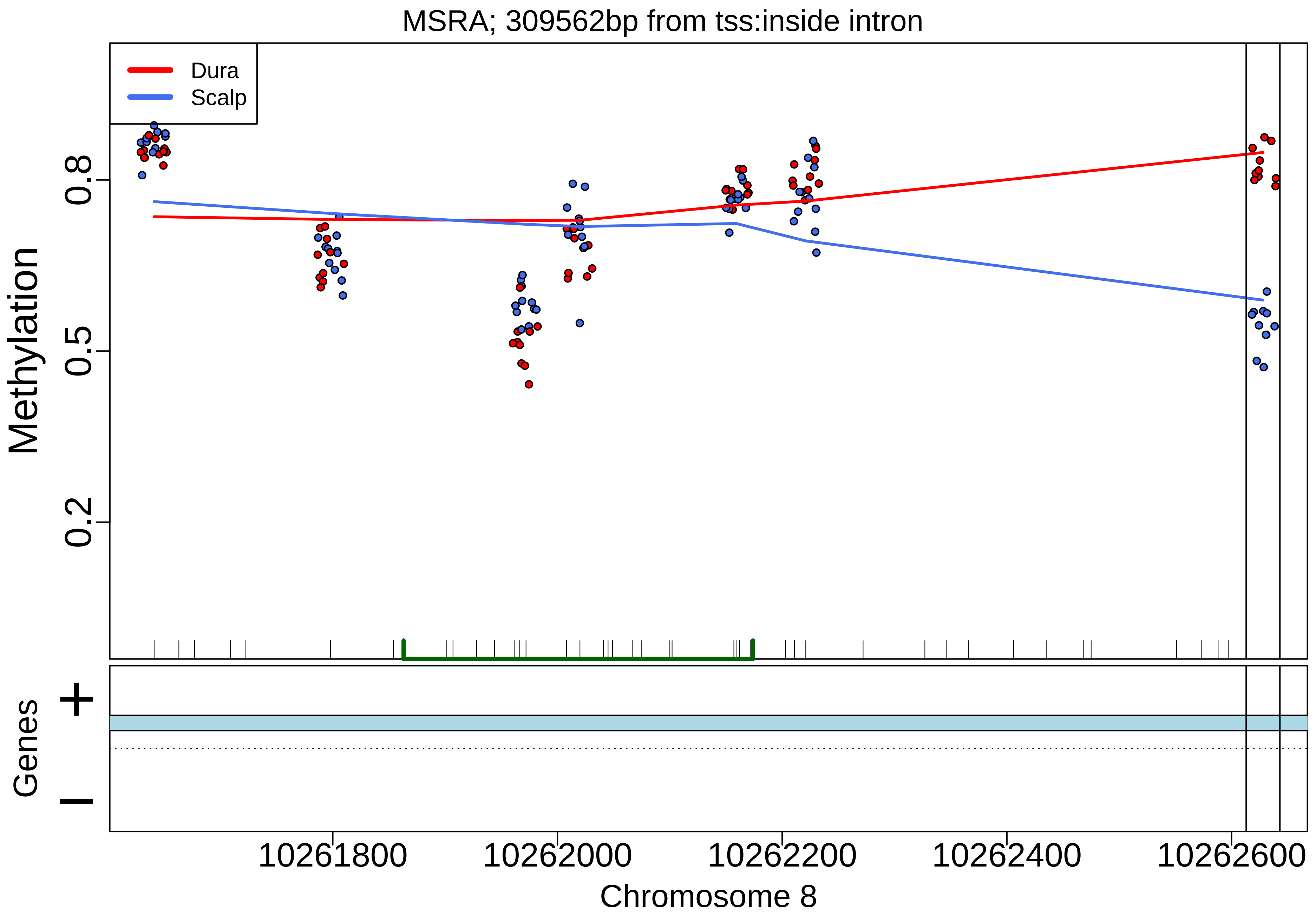

MSRA; 309562bp from tss:inside intron
Dura
Scalp
Methylation
0.2
0.5
0.8
Genes
+
-
10261800
10262000
10262200
10262400
10262600
Chromosome 8

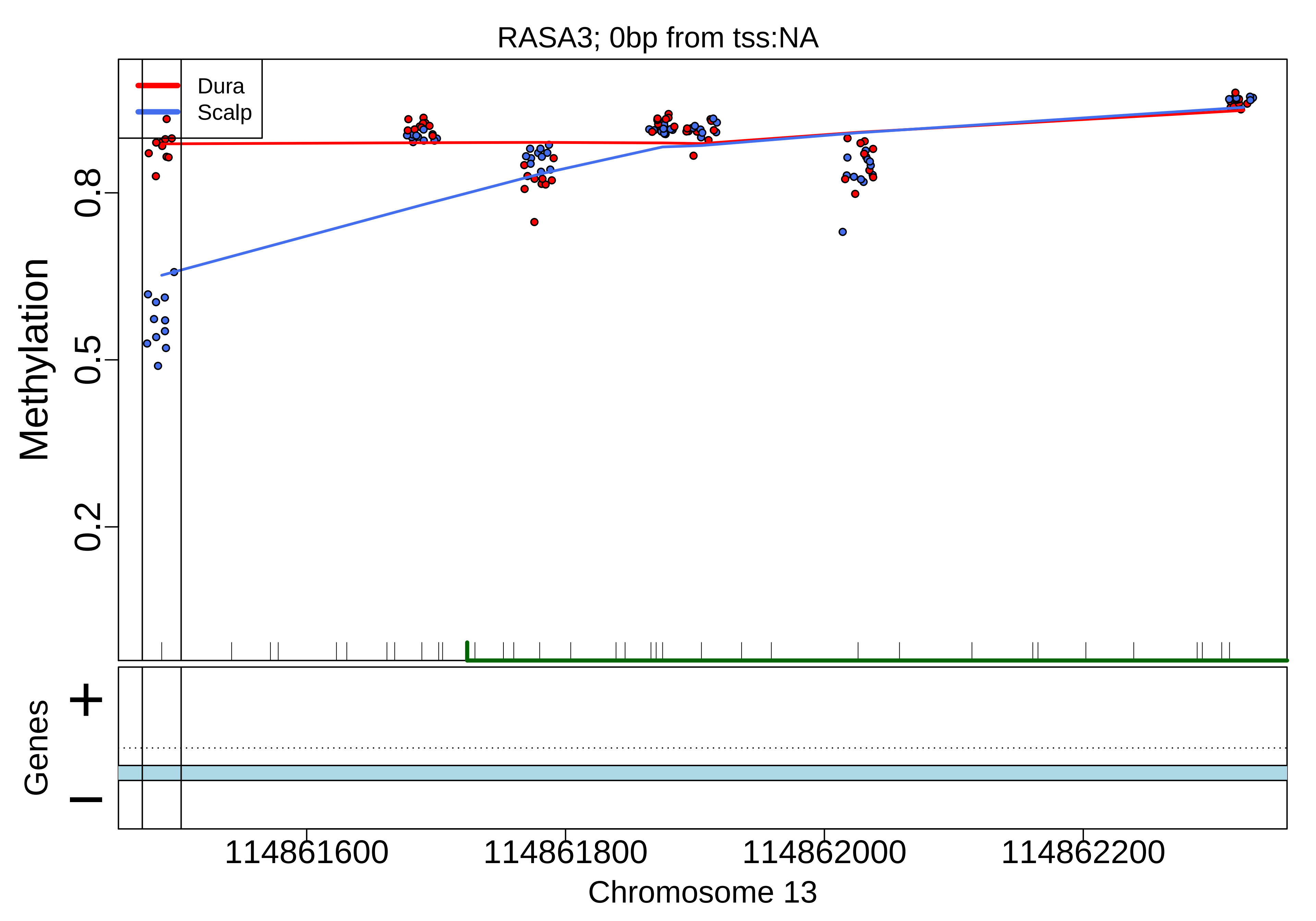

RASA3; 0bp from tss:NA
Dura
Scalp
Methylation
0.2
0.5
0.8
Genes
+
−
114861600
114861800
114862000
114862200
Chromosome 13

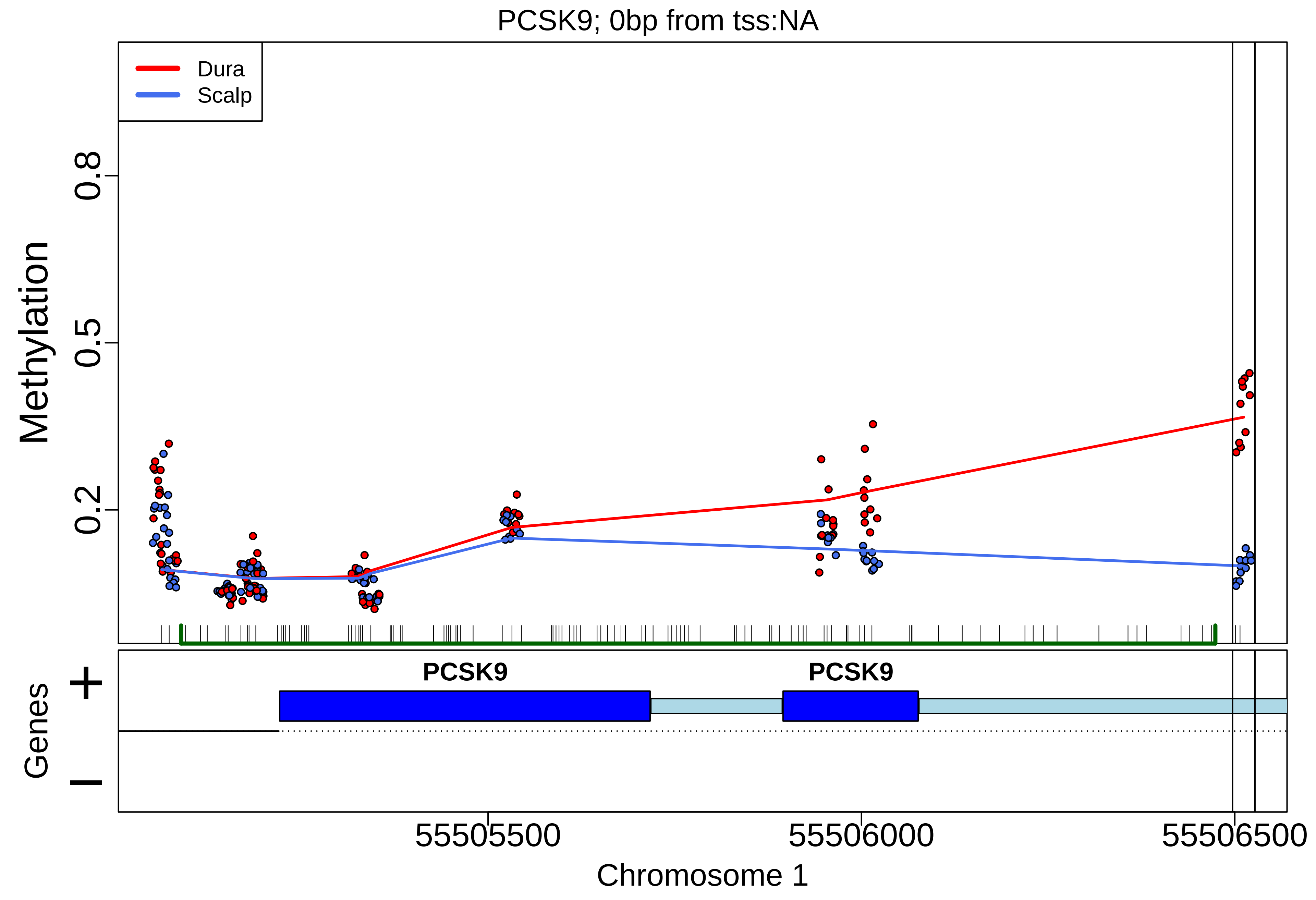

PCSK9; 0bp from tss:NA
Dura
Scalp
Methylation
0.2
0.5
0.8
Genes
+
-
PCSK9
PCSK9
55505500
55506000
55506500
Chromosome 1

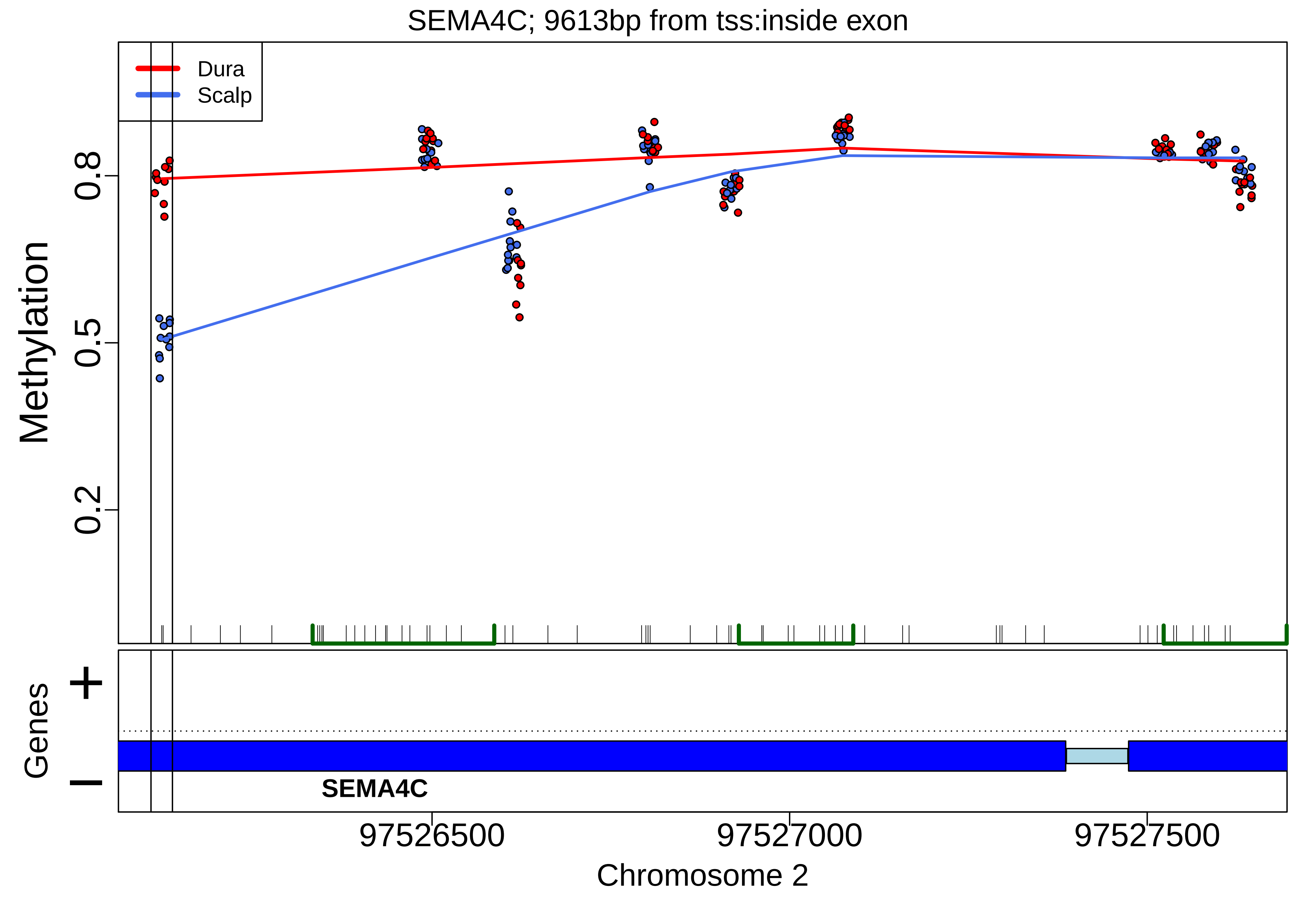
SEMA4C; 9613bp from tss:inside exon
Dura
Scalp
Methylation
0.2
0.5
0.8
Genes
+
−
SEMA4C
97526500
97527000
97527500
Chromosome 2

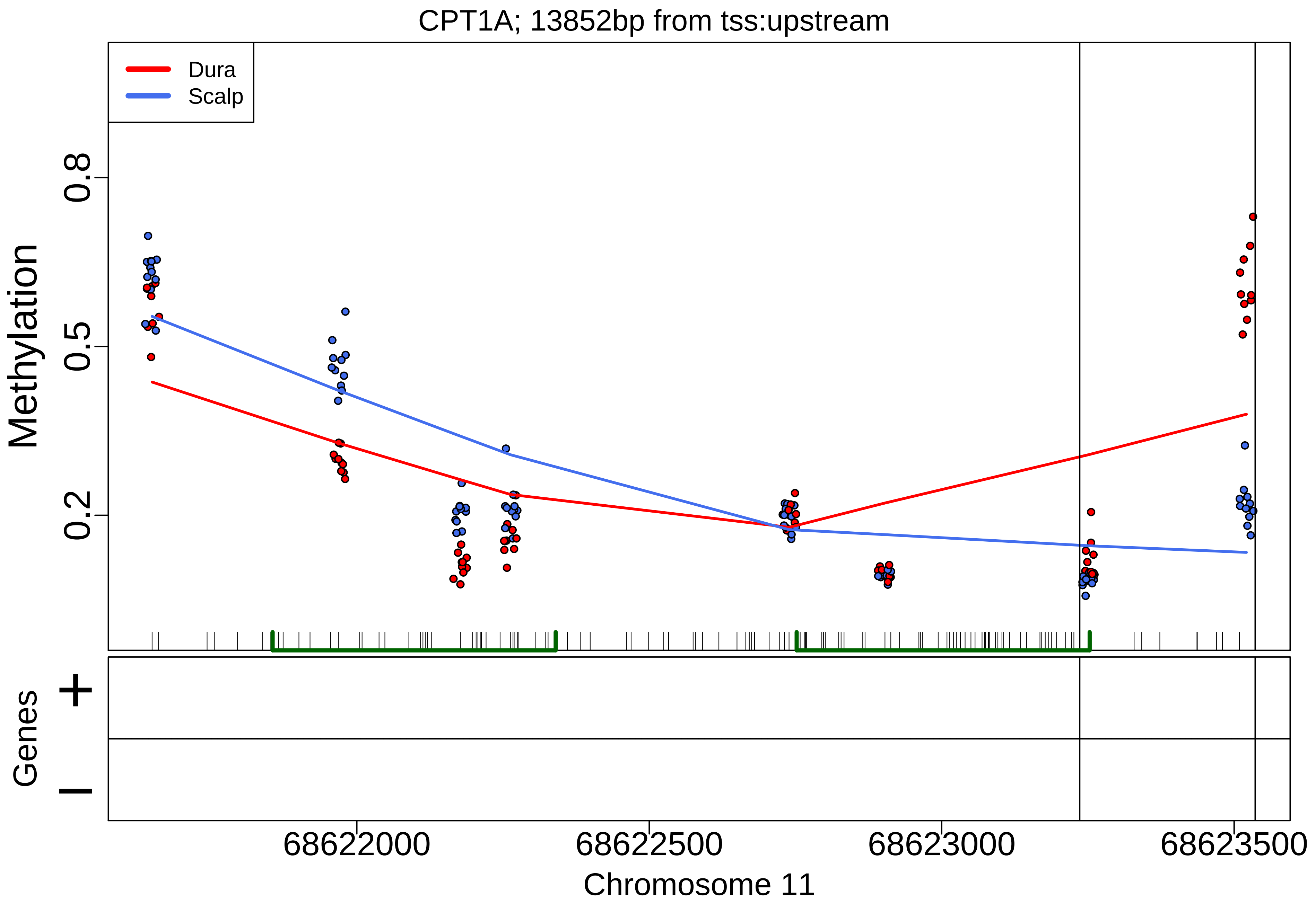
CPT1A; 13852bp from tss:upstream
Dura
Scalp
Methylation
0.2
0.5
0.8
Genes
+
-
68622000
68622500
68623000
68623500
Chromosome 11

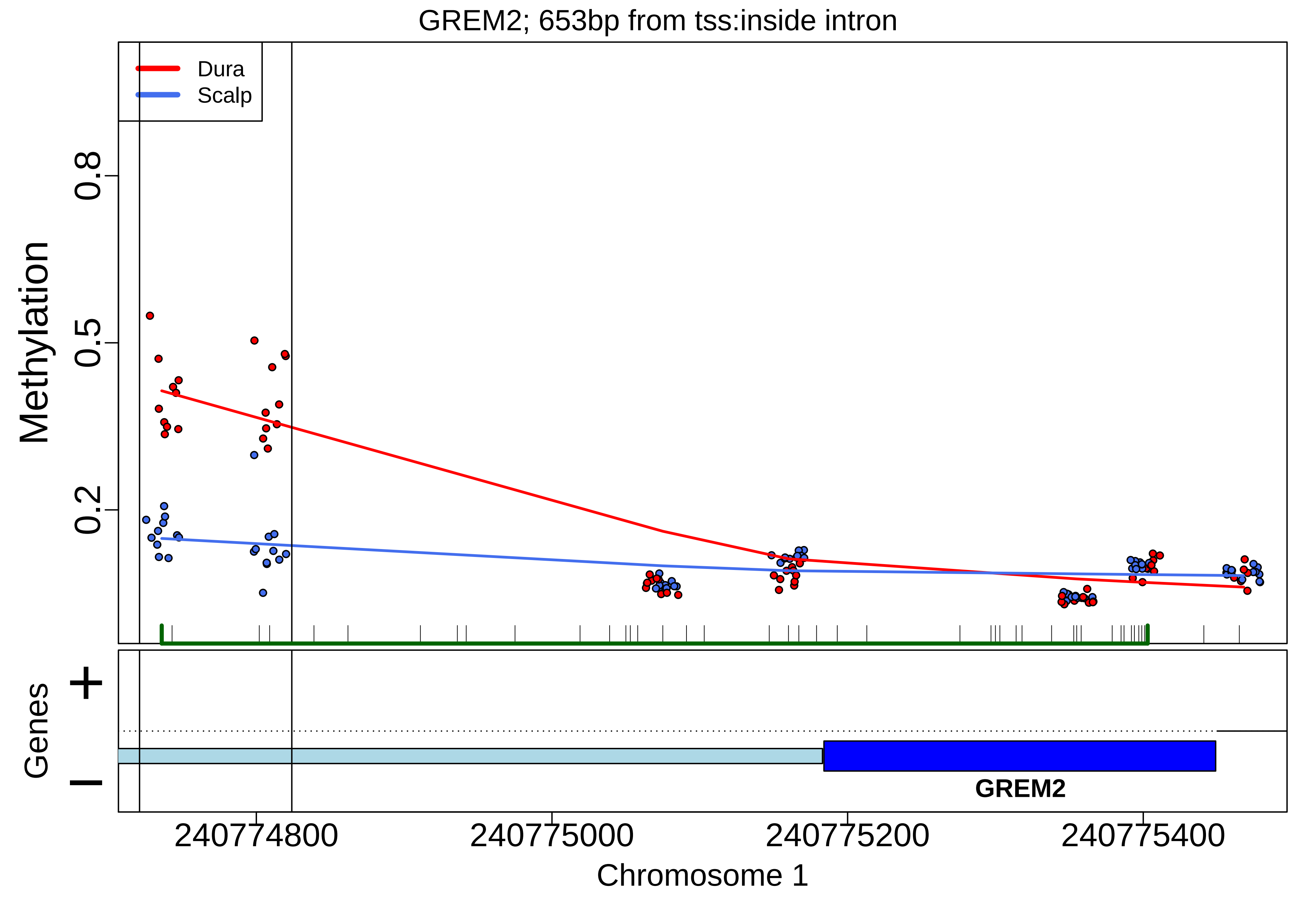
GREM2; 653bp from tss:inside intron
Dura
Scalp
Methylation
0.2
0.5
0.8
Genes
+
-
GREM2
240774800
240775000
240775200
240775400
Chromosome 1

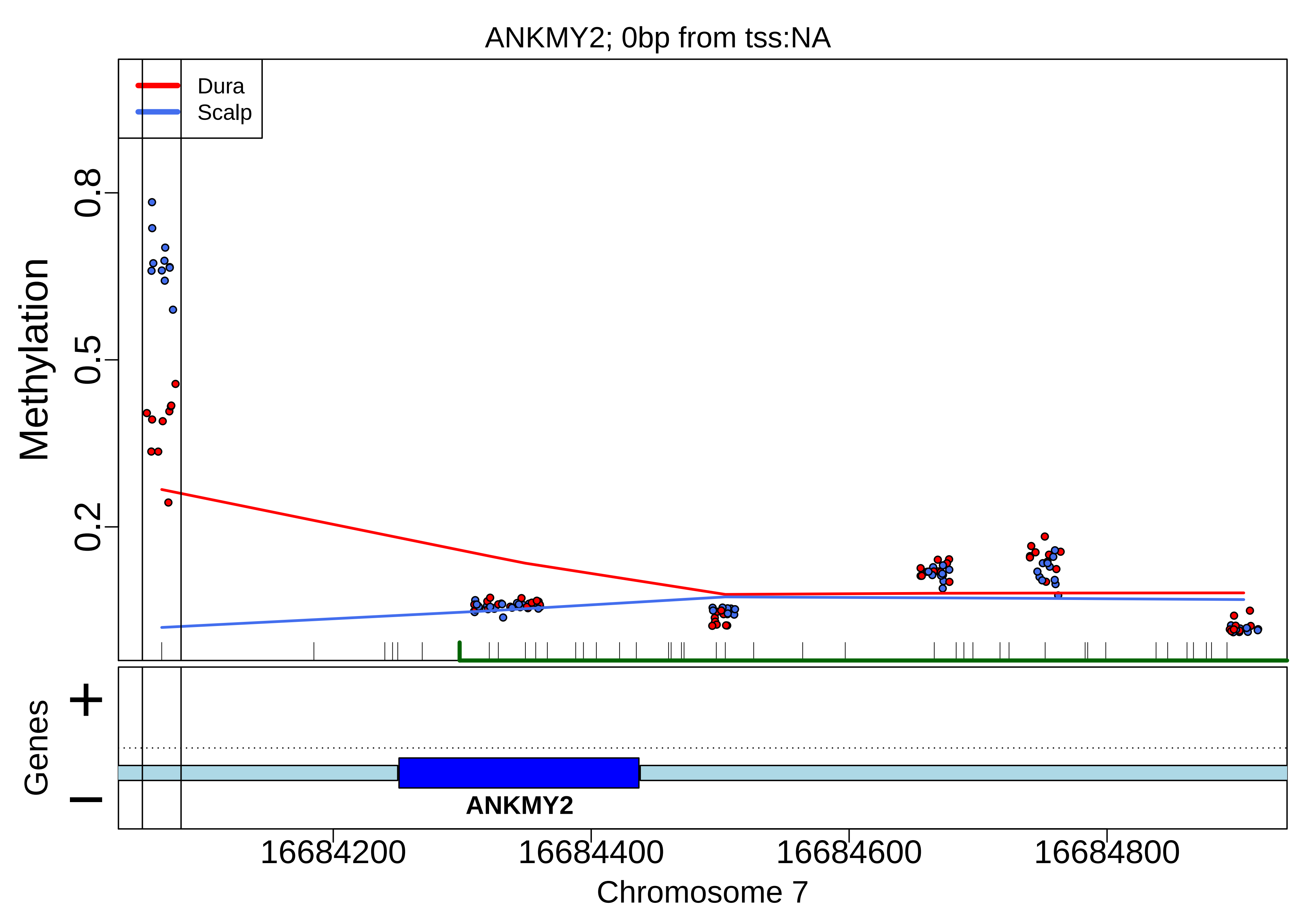
ANKMY2; 0bp from tss:NA
Dura
Scalp
Methylation
0.2
0.5
0.8
Genes
+
−
ANKMY2
16684200
16684400
16684600
16684800
Chromosome 7

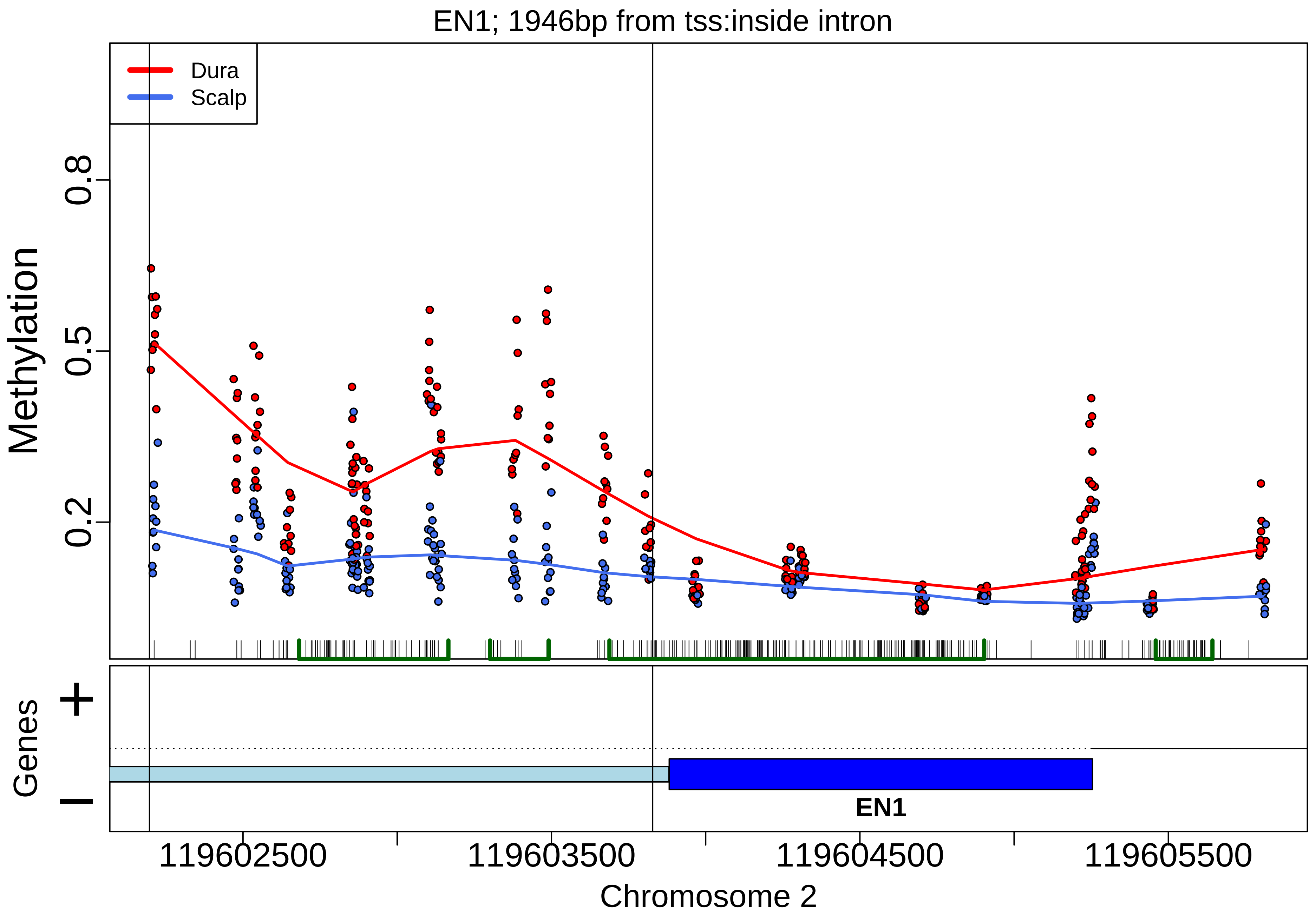

EN1; 1946bp from tss:inside intron
Dura
Scalp
Methylation
0.2
0.5
0.8
Genes
+
-
EN1
119602500
119603500
119604500
119605500
Chromosome 2

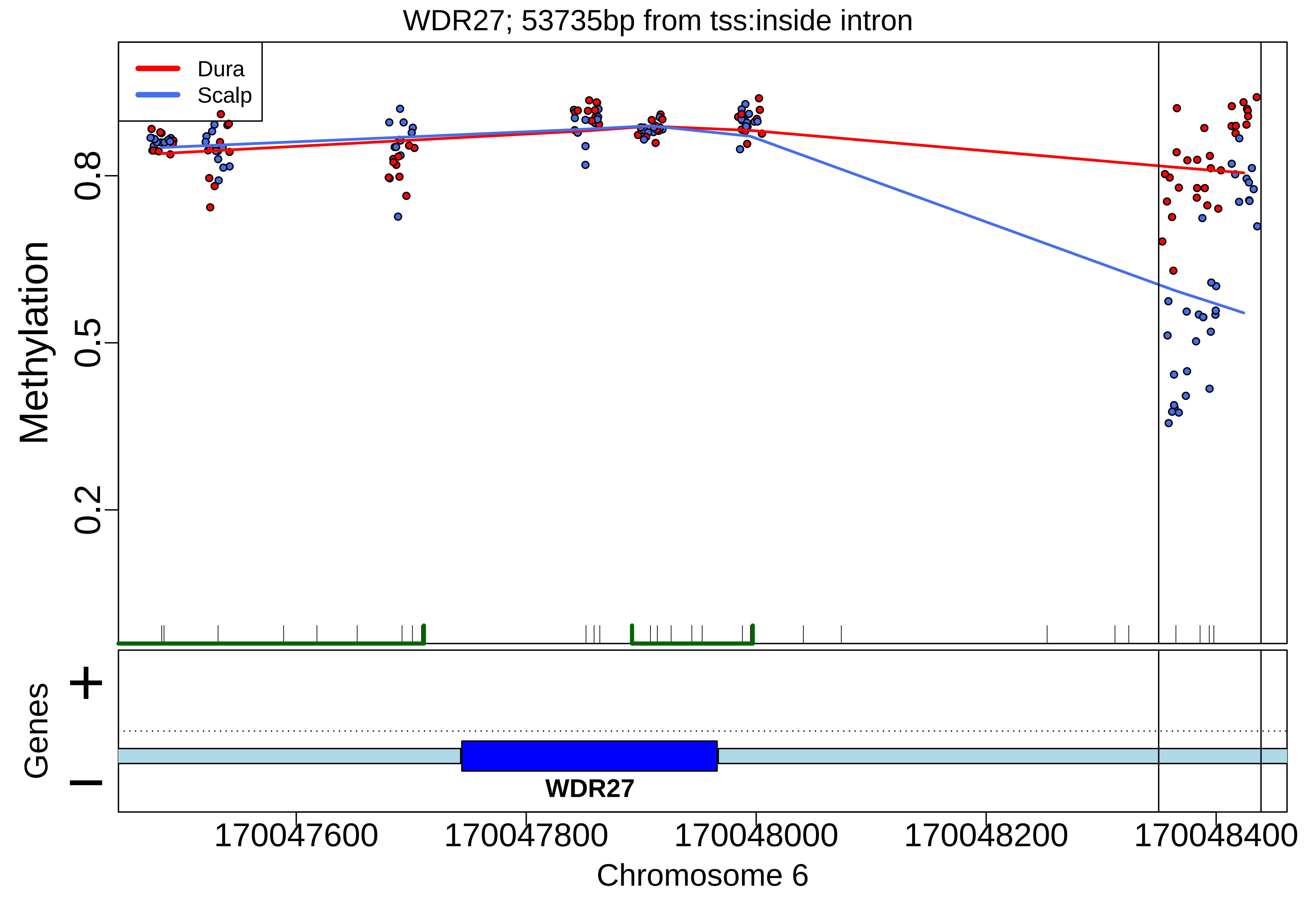
WDR27; 53735bp from tss:inside intron
Dura
Scalp
Methylation
0.2
0.5
0.8
Genes
+
-
WDR27
170047600
170047800
170048000
170048200
170048400
Chromosome 6

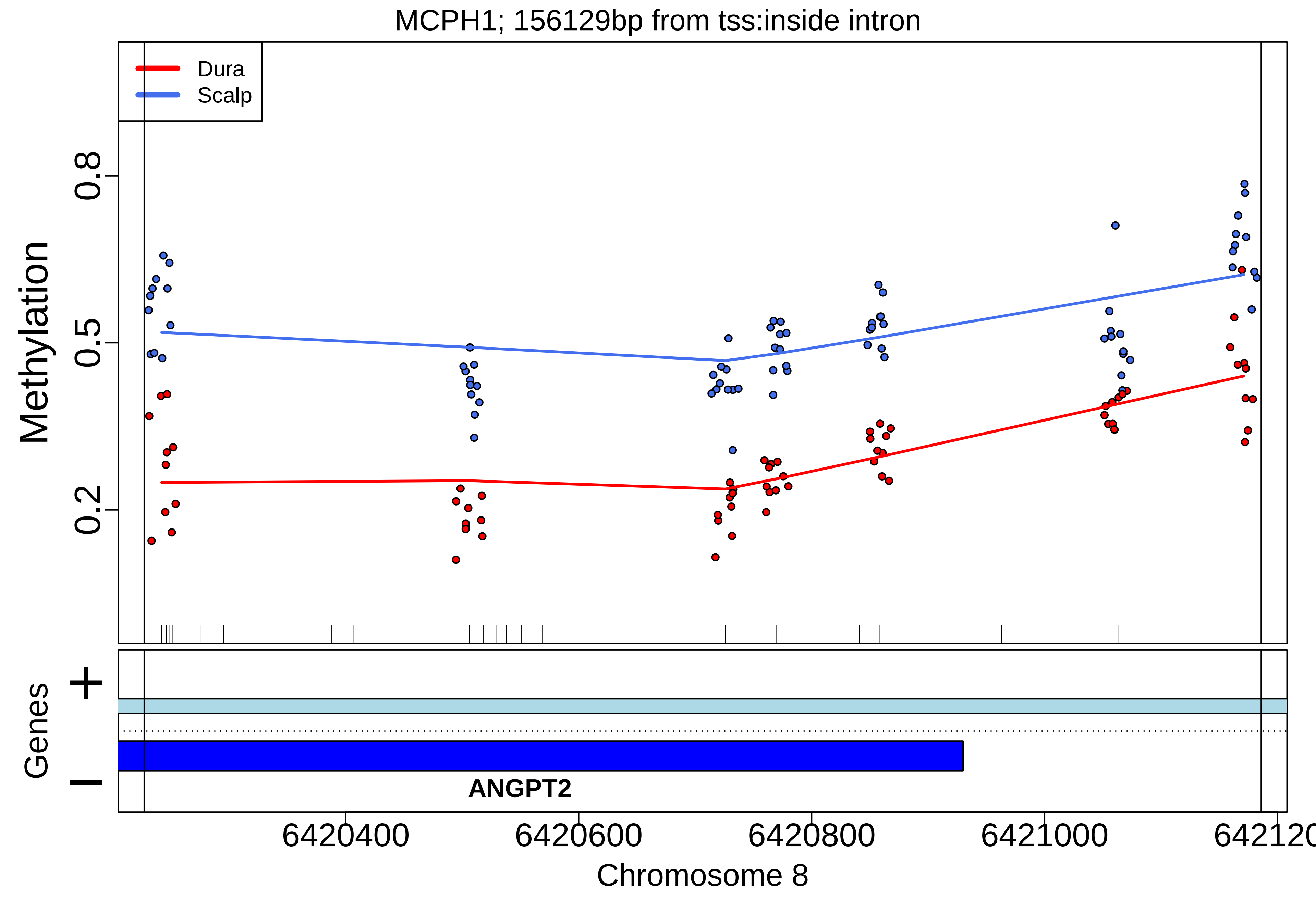

MCPH1; 156129bp from tss:inside intron
Dura
Scalp
Methylation
0.2
0.5
0.8
Genes
+
-
ANGPT2
6420400
6420600
6420800
6421000
Chromosome 8

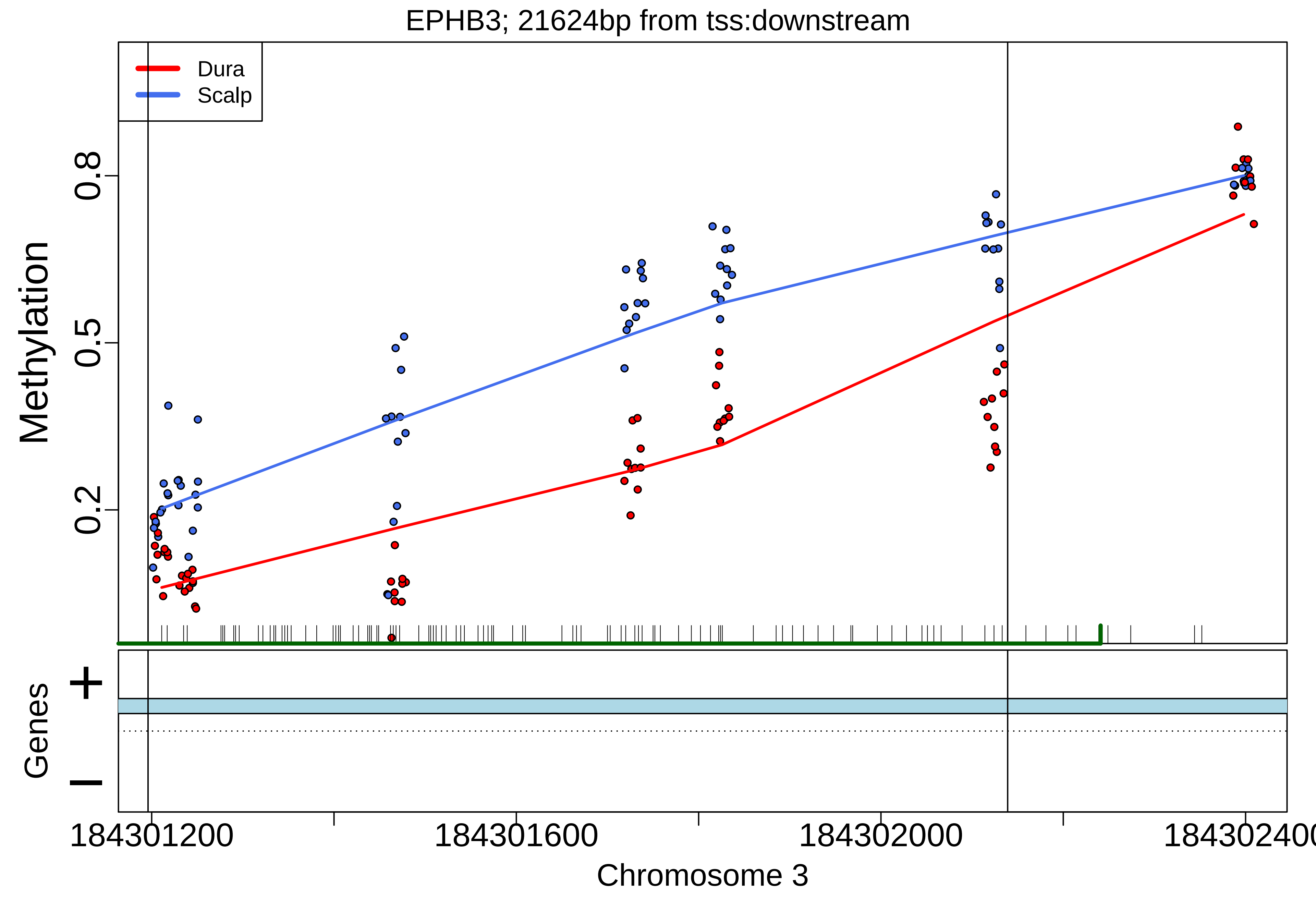
EPHB3; 21624bp from tss:downstream
Dura
Scalp
Methylation
0.2
0.5
0.8
Genes
+
−
184301200
184301600
184302000
184302400
Chromosome 3

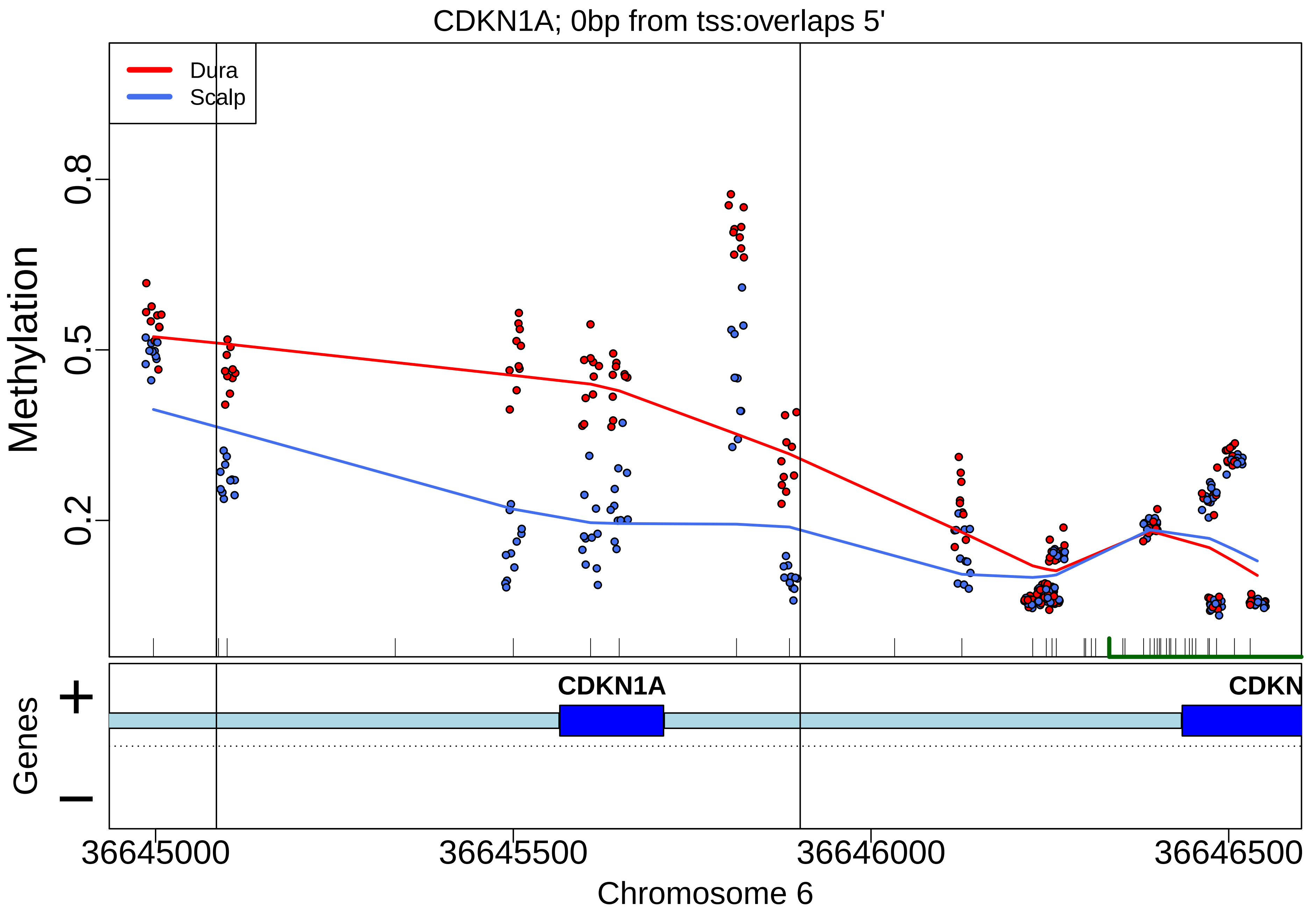

CDKN1A; 0bp from tss:overlaps 5'
Dura
Scalp
Methylation
0.2
0.5
0.8
Genes
+
−
CDKN1A
CDKN
36645000
36645500
36646000
36646500
Chromosome 6

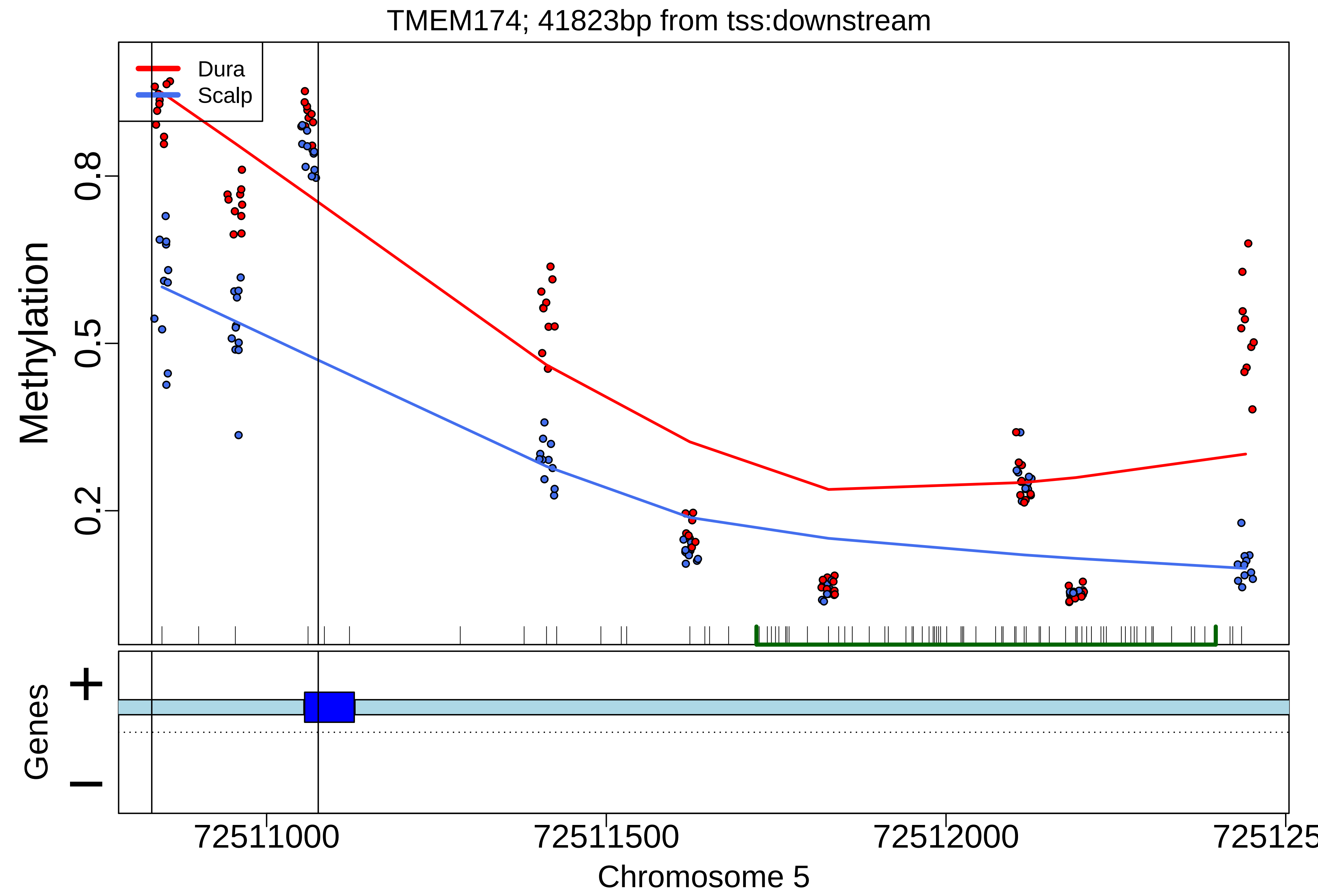

TMEM174; 41823bp from tss:downstream
Dura
Scalp
Methylation
0.2
0.5
0.8
Genes
+
-
72511000
72511500
72512000
725125
Chromosome 5

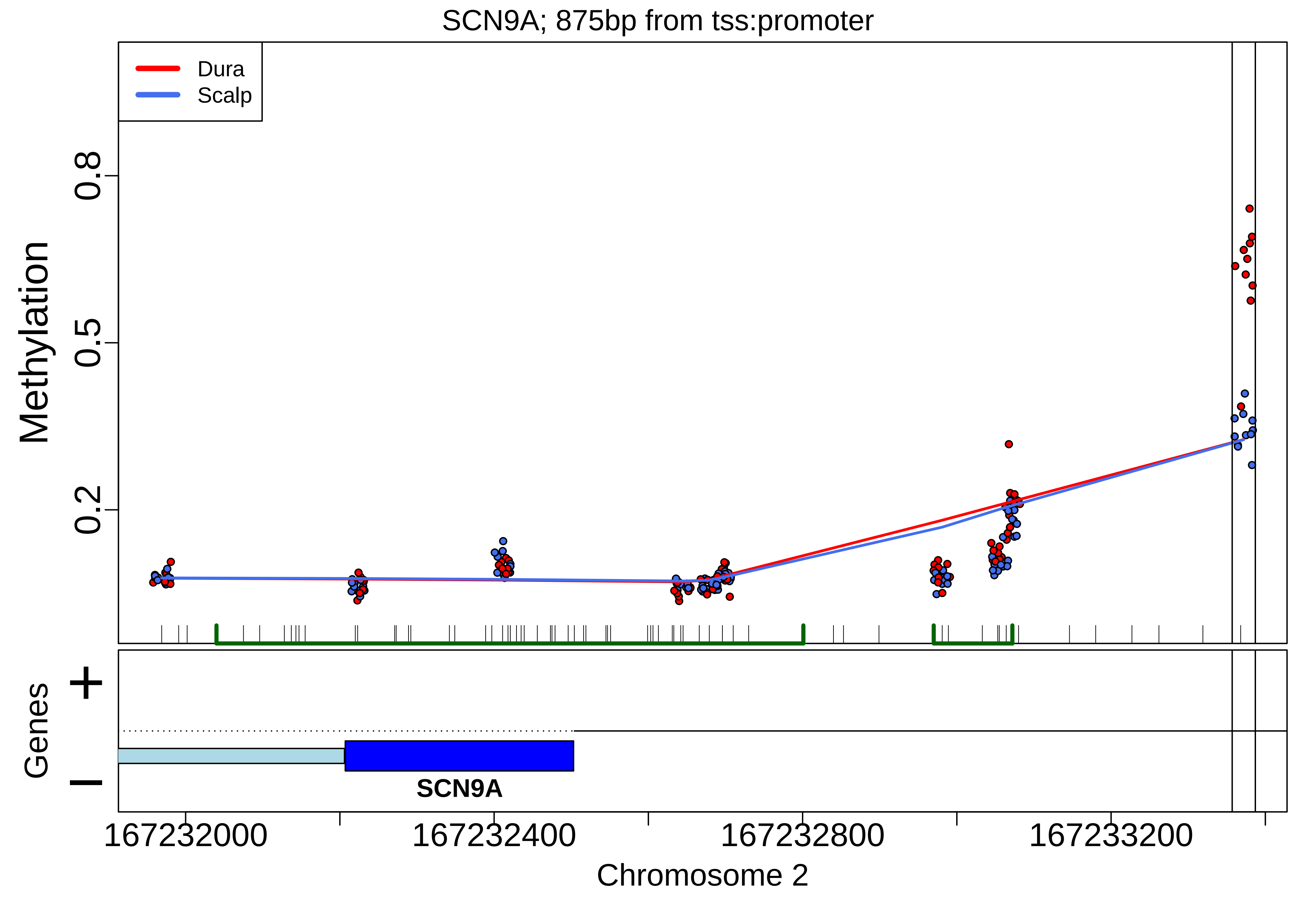

SCN9A; 875bp from tss:promoter
Dura
Scalp
Methylation
0.2
0.5
0.8
Genes
+
−
SCN9A
167232000
167232400
167232800
167233200
Chromosome 2

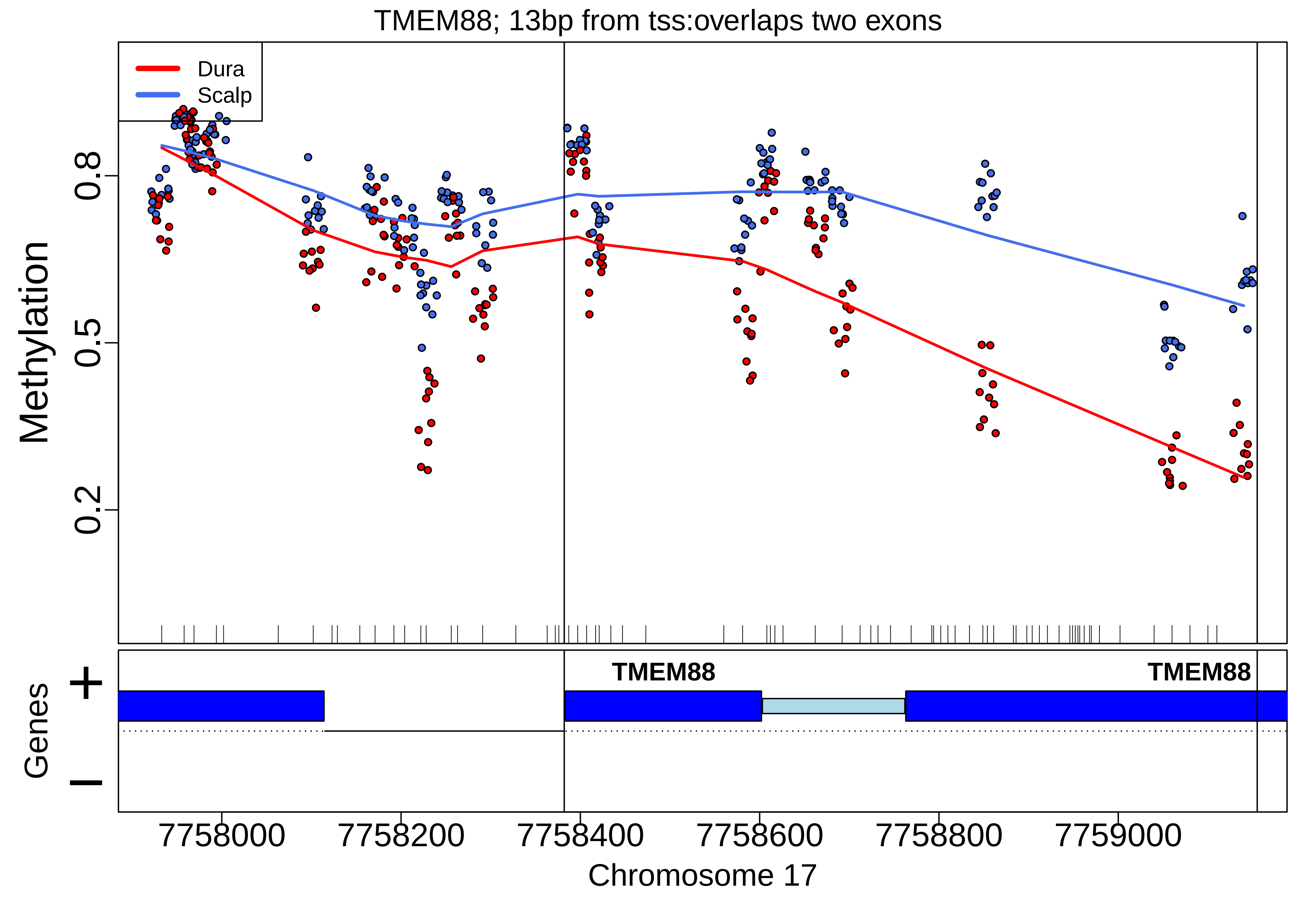
TMEM88; 13bp from tss:overlaps two exons
Dura
Scalp
Methylation
0.2
0.5
0.8
Genes
+
−
TMEM88
TMEM88
7758000
7758200
7758400
7758600
7758800
7759000
Chromosome 17

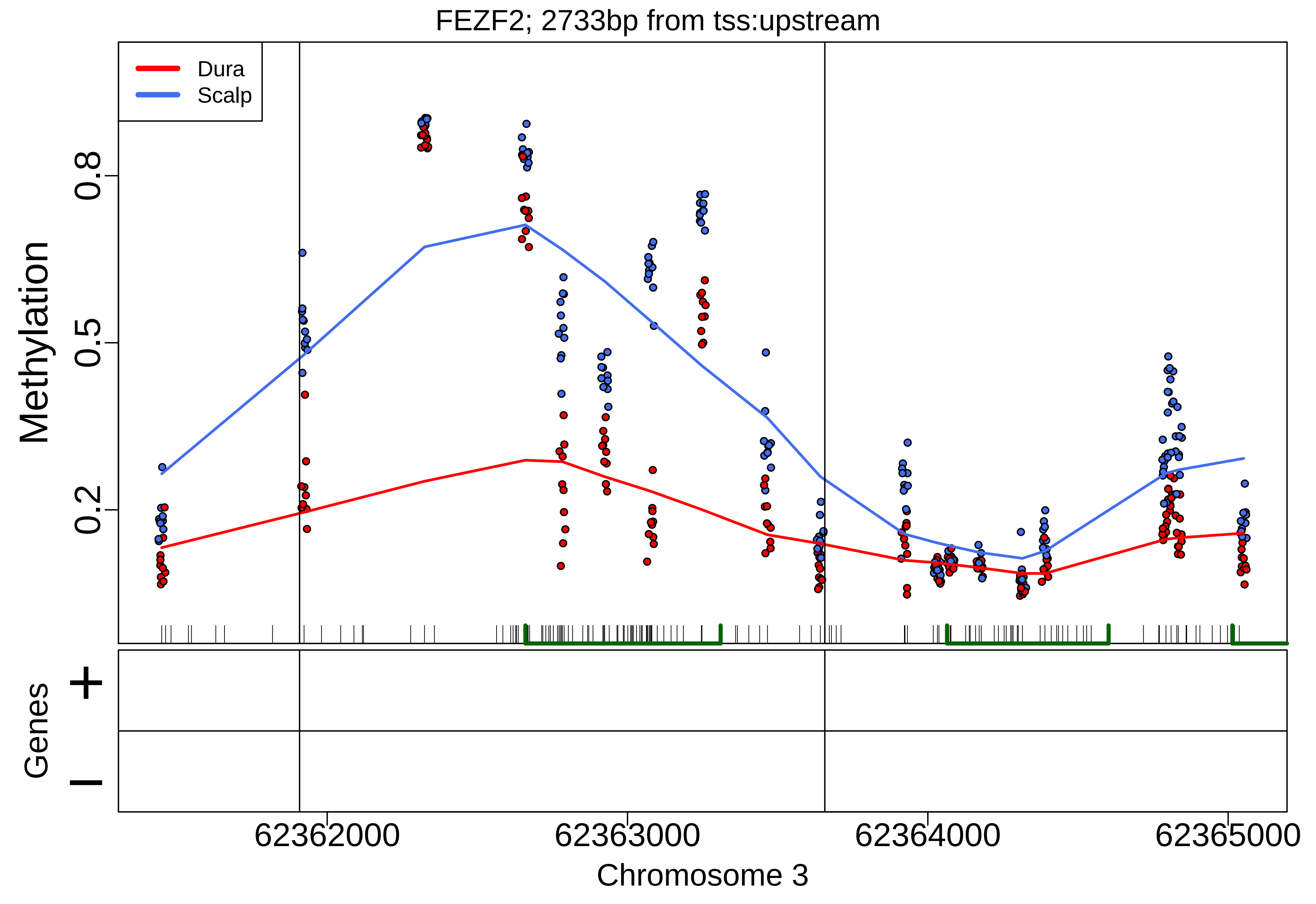

FEZF2; 2733bp from tss:upstream
Dura
Scalp
Methylation
0.2
0.5
0.8
Genes
+
−
62362000
62363000
62364000
62365000
Chromosome 3

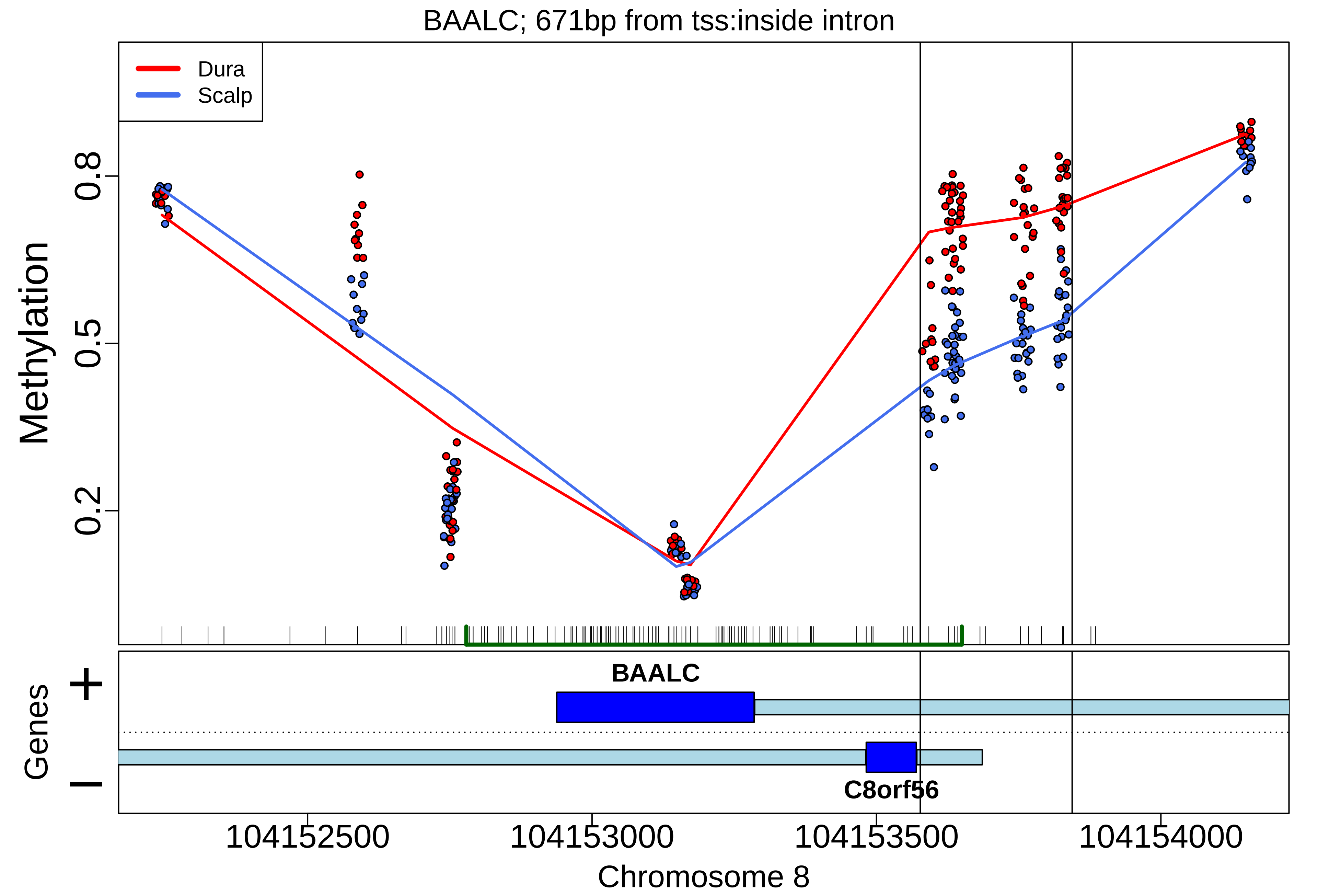
BAALC; 671bp from tss:inside intron
Dura
Scalp
Methylation
0.2
0.5
0.8
Genes
+
-
BAALC
C8orf56
104152500
104153000
104153500
104154000
Chromosome 8

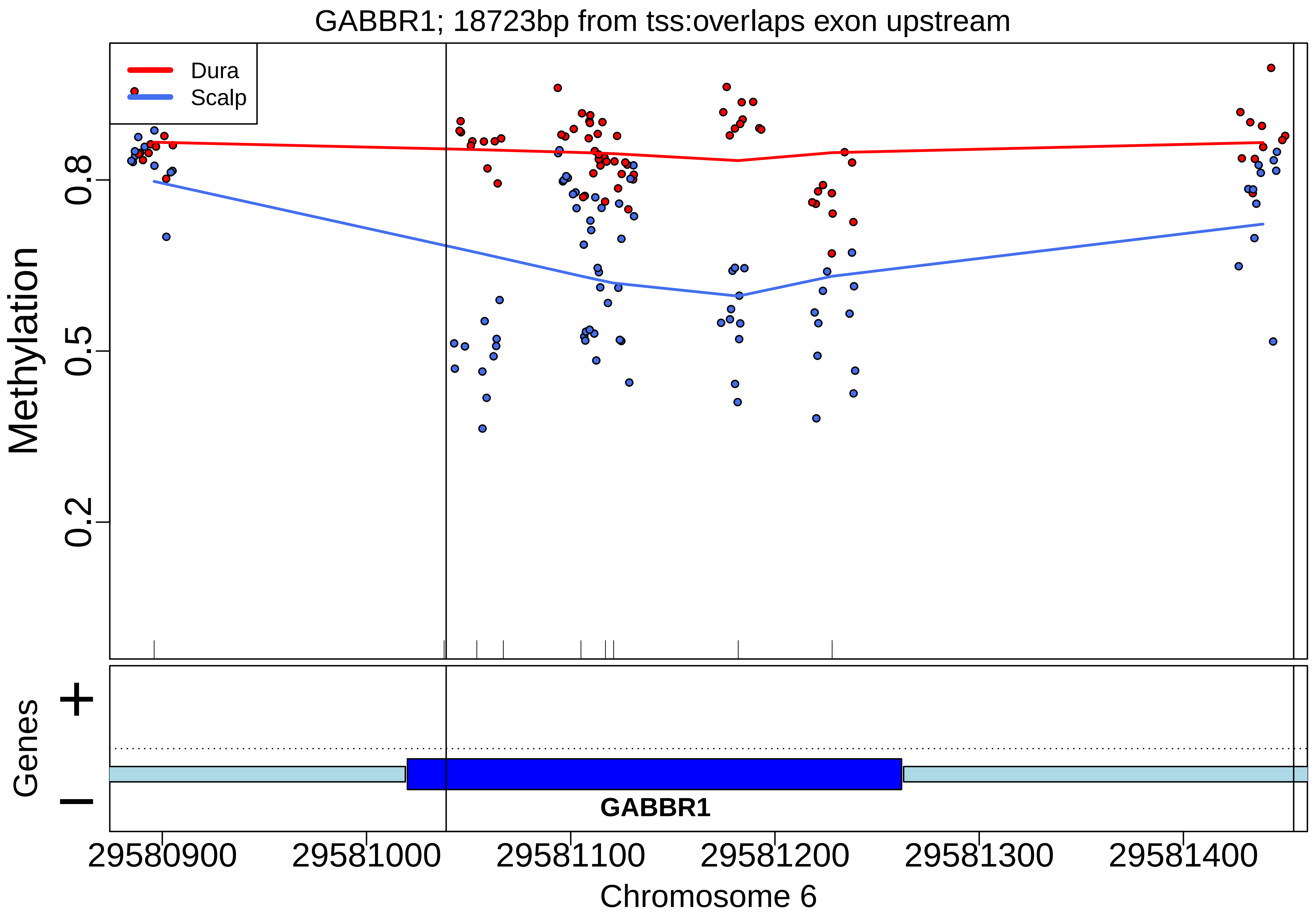
GABBR1; 18723bp from tss:overlaps exon upstream
Dura
Scalp
Methylation
0.2
0.5
0.8
Genes
+
-
GABBR1
29580900
29581000
29581100
29581200
29581300
29581400
Chromosome 6

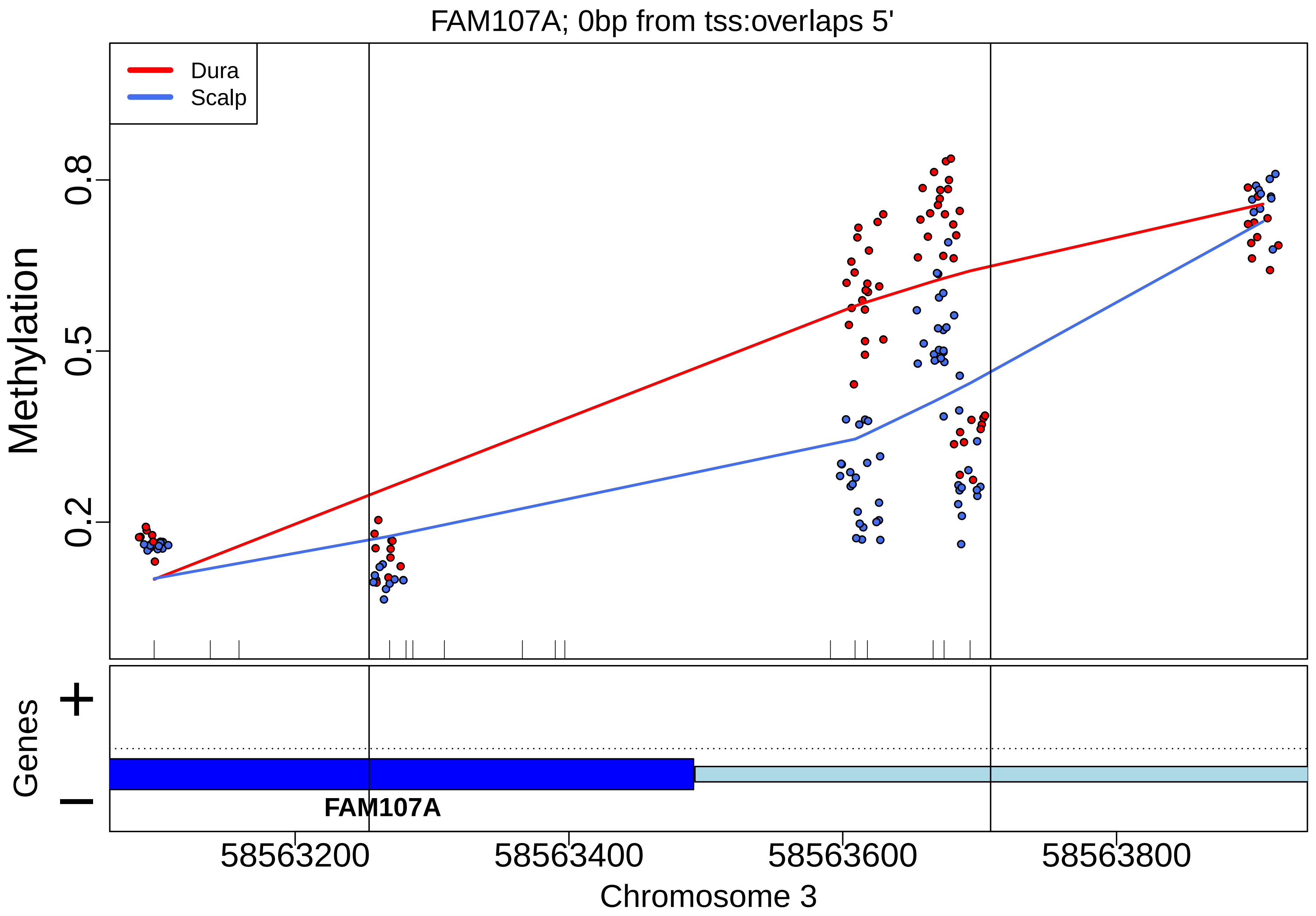

FAM107A; 0bp from tss:overlaps 5'
Dura
Scalp
Methylation
0.2
0.5
0.8
Genes
+
-
FAM107A
58563200
58563400
58563600
58563800
Chromosome 3

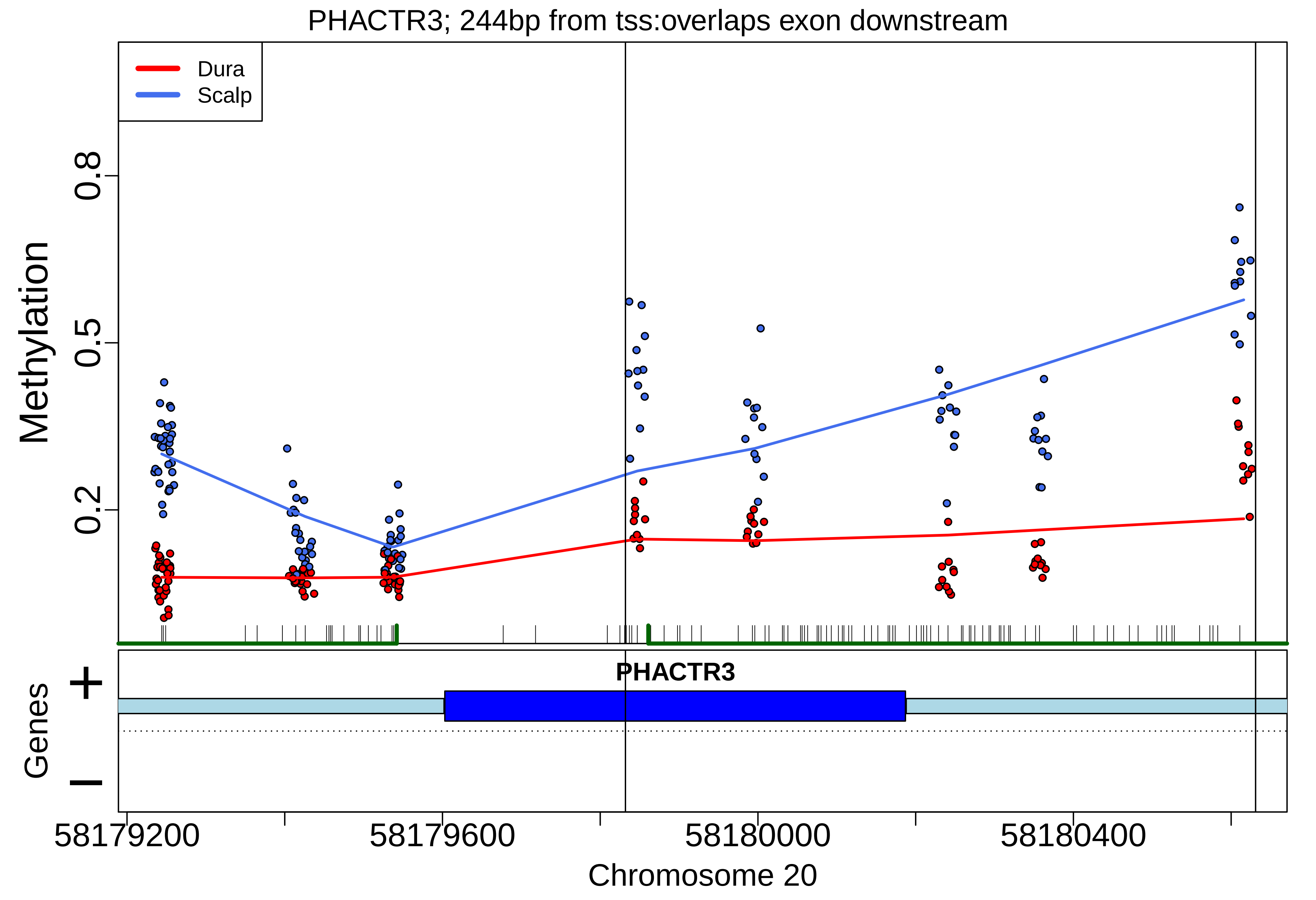

PHACTR3; 244bp from tss:overlaps exon downstream
Dura
Scalp
Methylation
0.2
0.5
0.8
Genes
+
–
PHACTR3
58179200
58179600
58180000
58180400
Chromosome 20

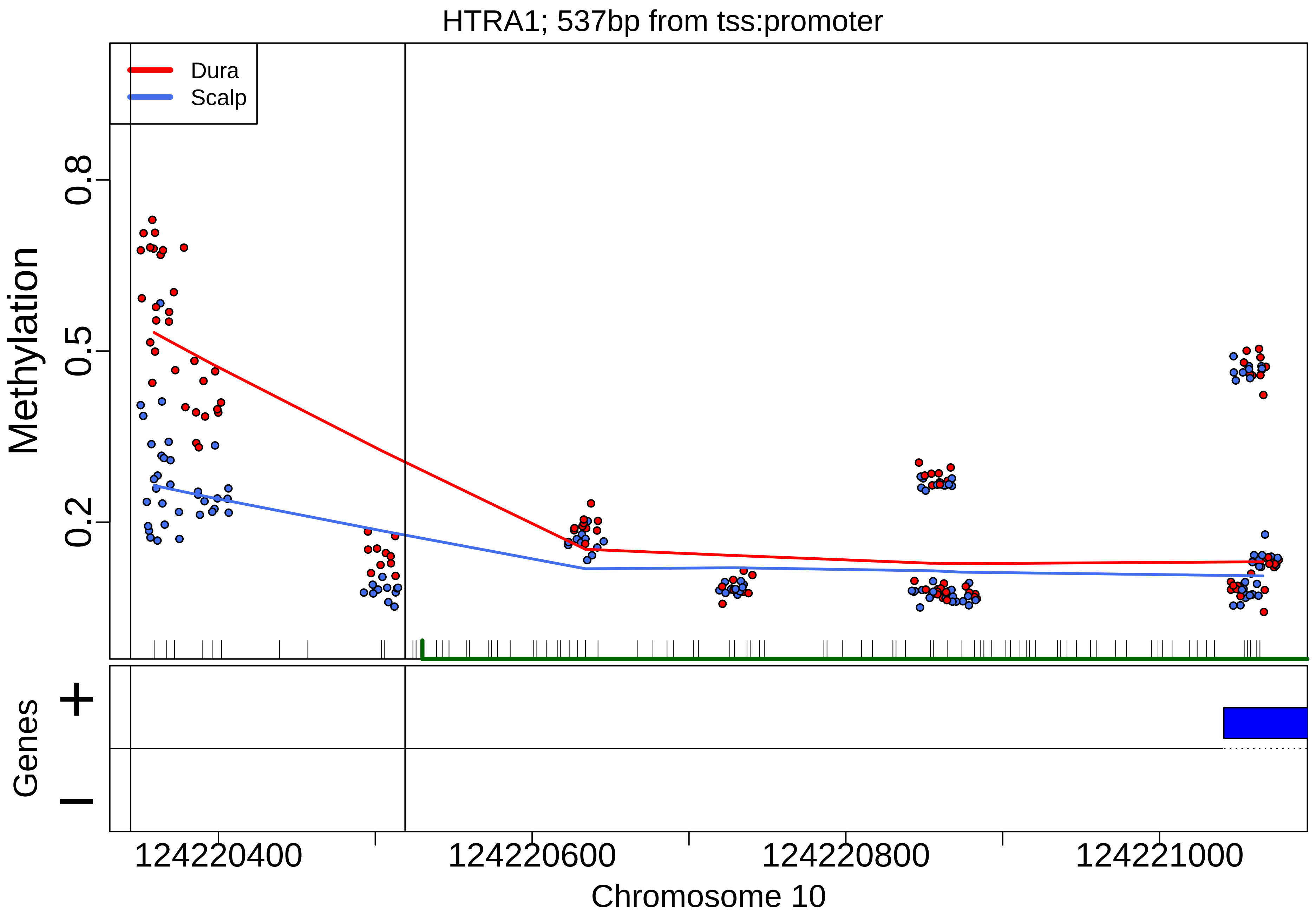

HTRA1; 537bp from tss:promoter
Dura
Scalp
Methylation
0.8
0.5
0.2
Genes
+
-
124220400
124220600
124220800
124221000
Chromosome 10

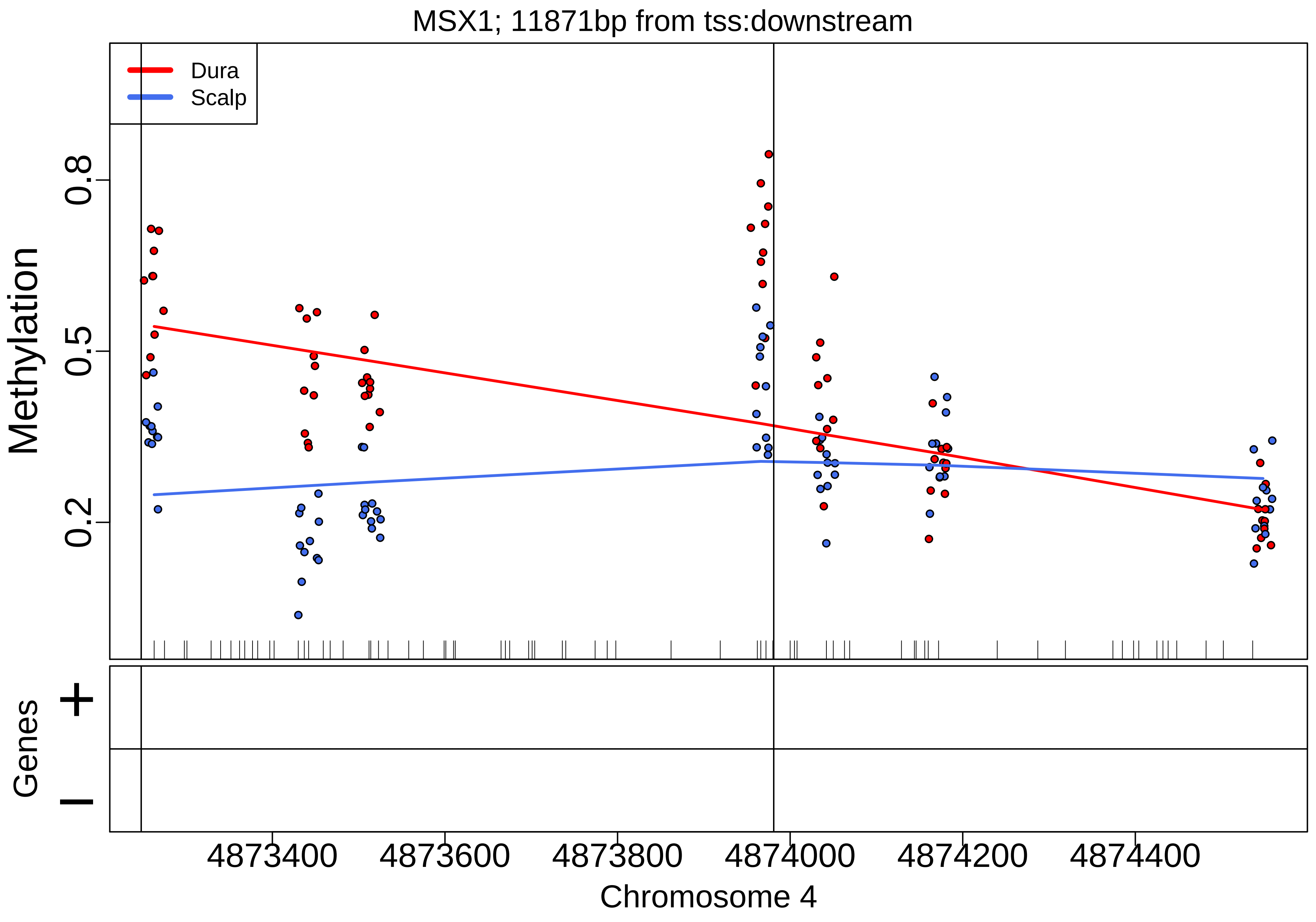

MSX1; 11871bp from tss:downstream
Dura
Scalp
Methylation
0.2
0.5
0.8
Genes
+
−
4873400
4873600
4873800
4874000
4874200
4874400
Chromosome 4

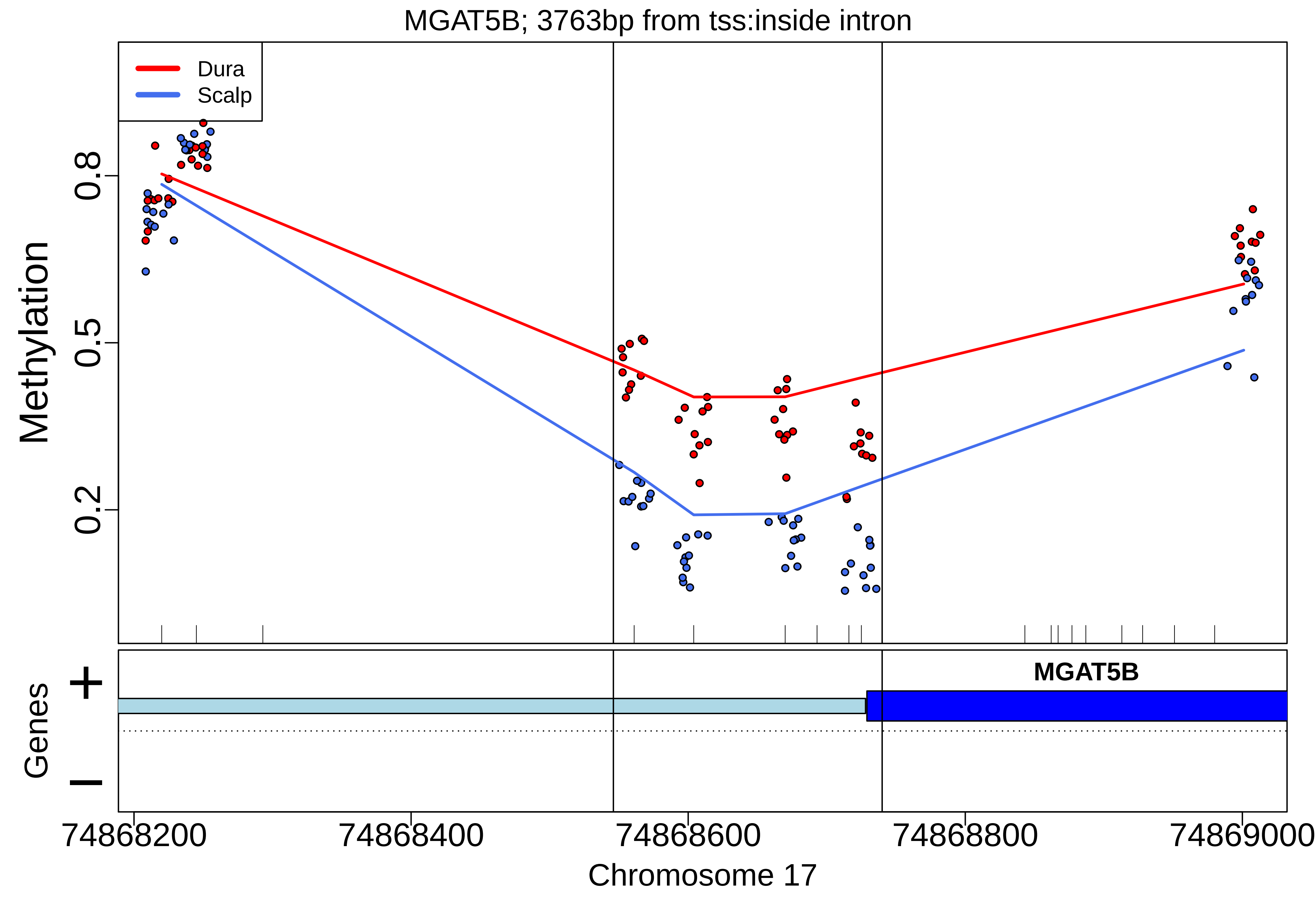
MGAT5B; 3763bp from tss:inside intron
Dura
Scalp
Methylation
0.2
0.5
0.8
Genes
+
−
MGAT5B
74868200
74868400
74868600
74868800
74869000
Chromosome 17

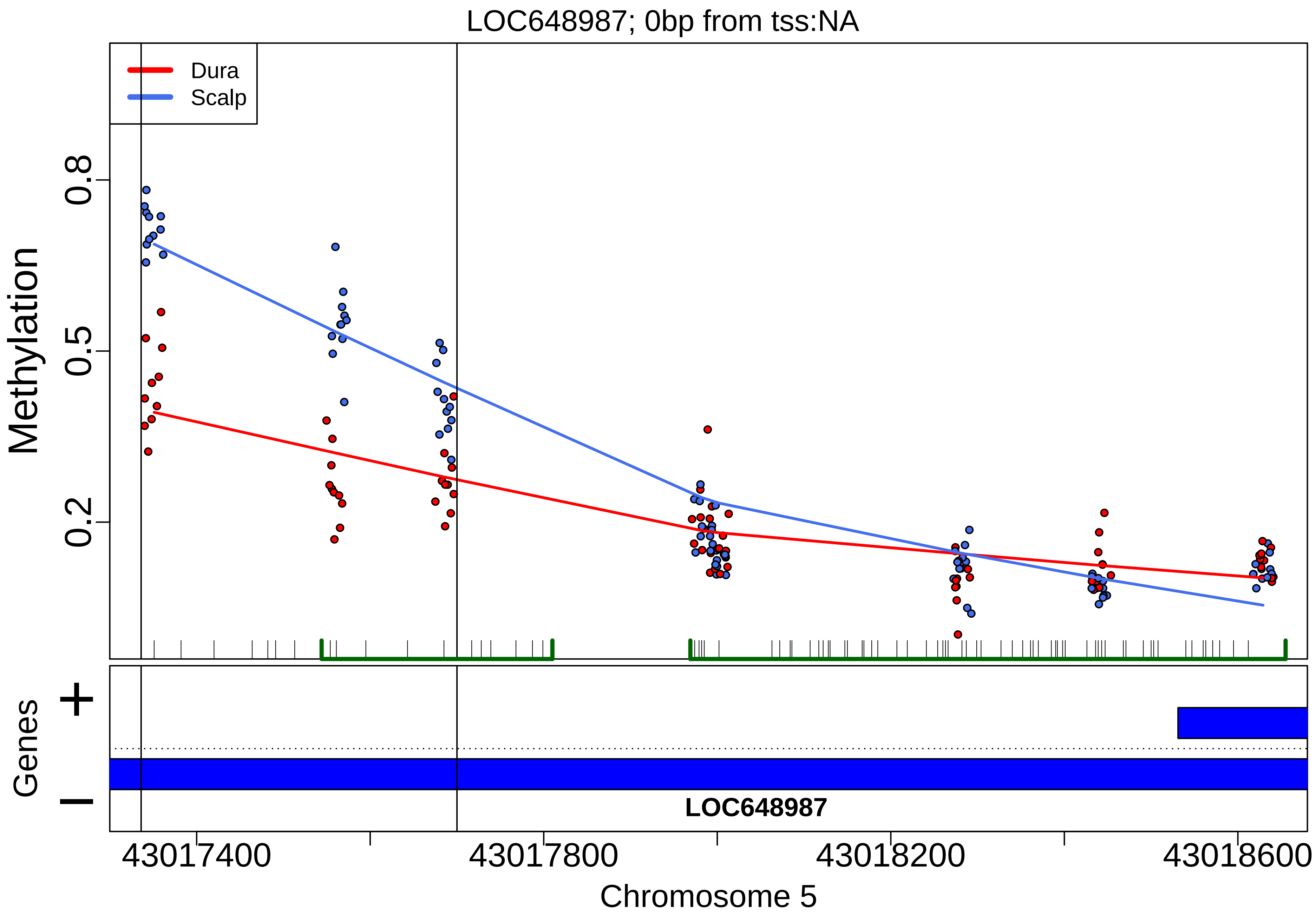

LOC648987; 0bp from tss:NA
Dura
Scalp
Methylation
0.8
0.5
0.2
Genes
+
-
LOC648987
43017400
43017800
43018200
43018600
Chromosome 5

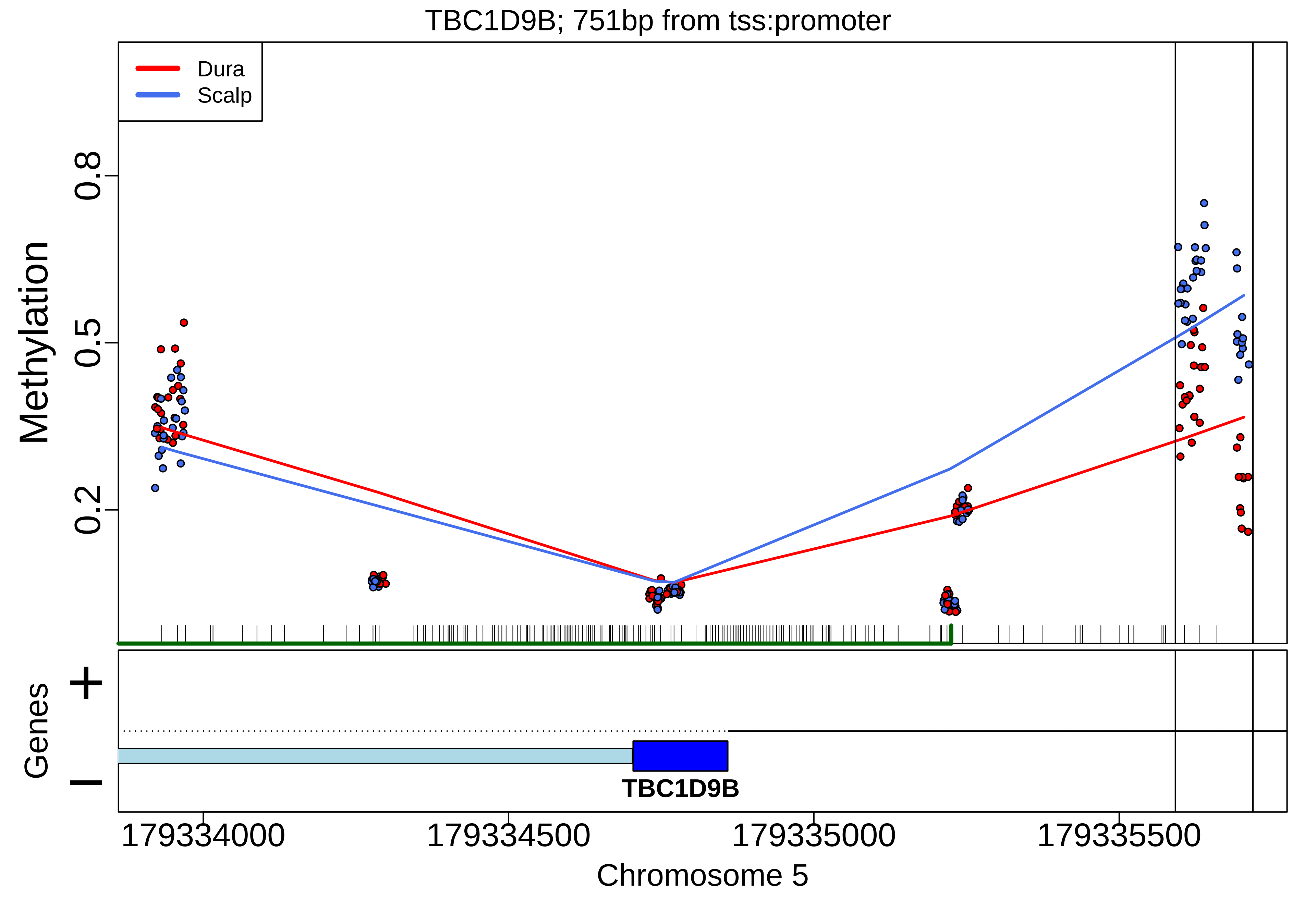
TBC1D9B; 751bp from tss:promoter
Dura
Scalp
Methylation
0.2
0.5
0.8
Genes
+
−
TBC1D9B
179334000
179334500
179335000
179335500
Chromosome 5

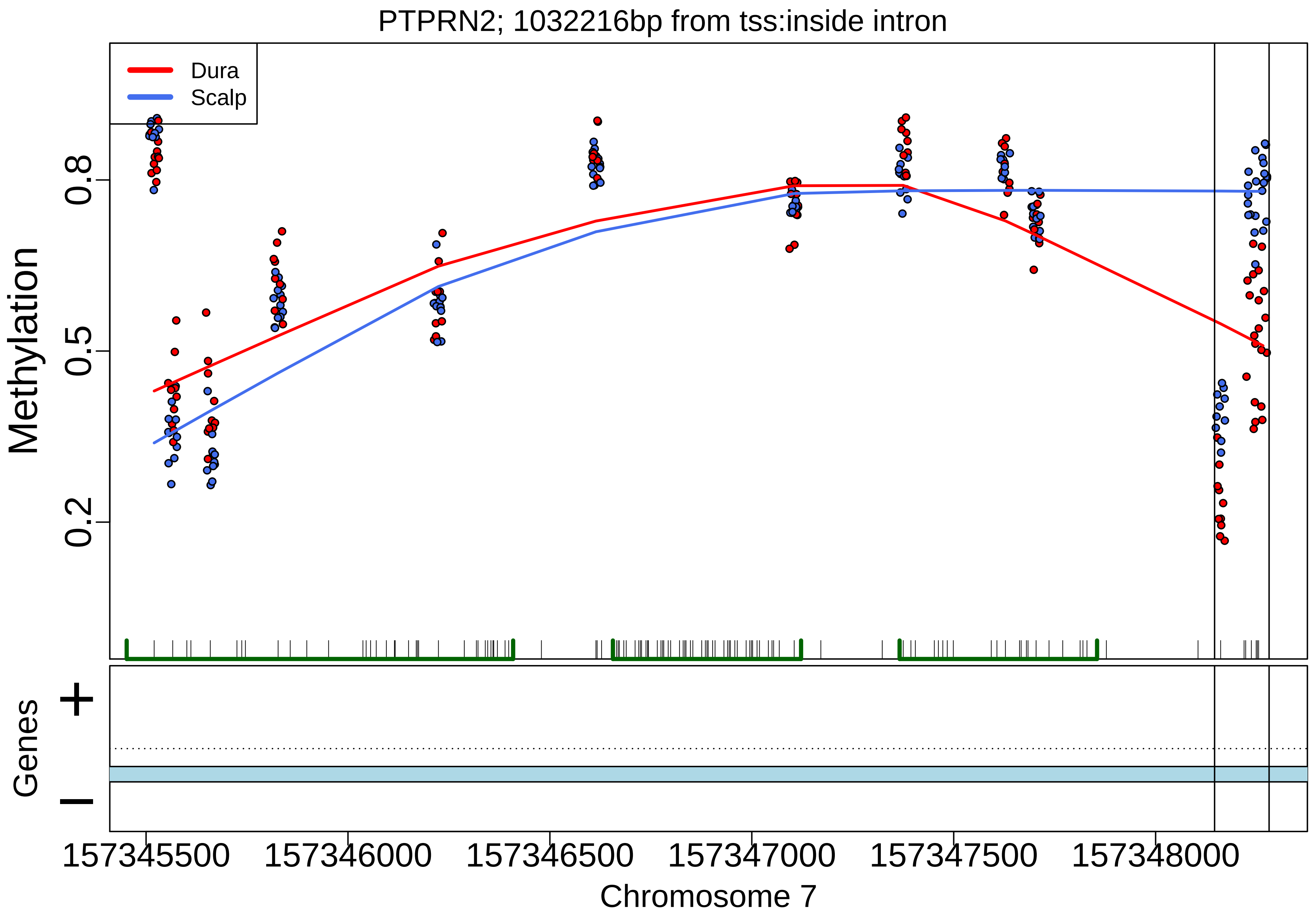

PTPRN2; 1032216bp from tss:inside intron
Dura
Scalp
Methylation
0.2
0.5
0.8
Genes
+
−
157345500
157346000
157346500
157347000
157347500
157348000
Chromosome 7

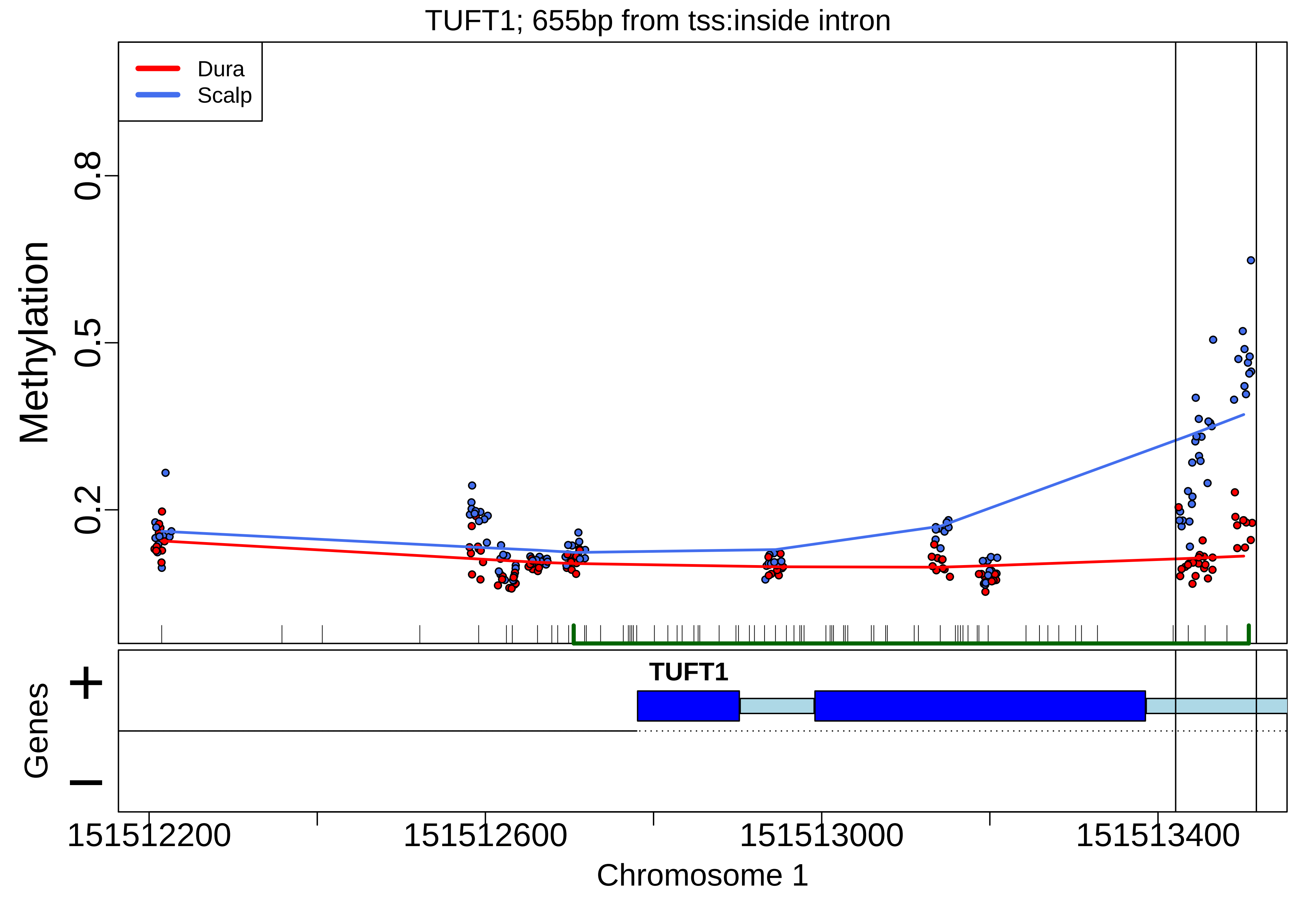
TUFT1; 655bp from tss:inside intron
Dura
Scalp
Methylation
0.2
0.5
0.8
Genes
+
-
TUFT1
151512200
151512600
151513000
151513400
Chromosome 1

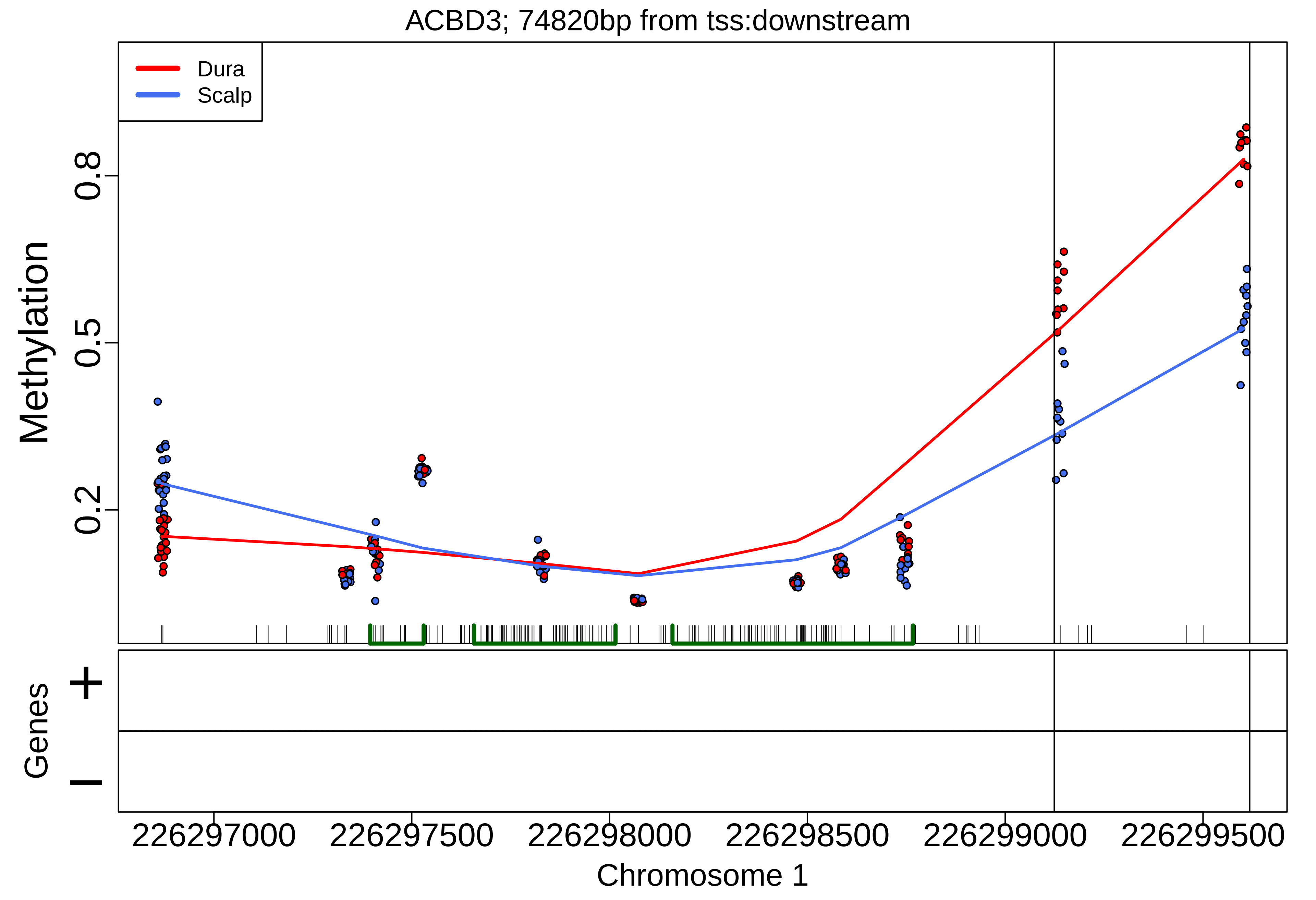

ACBD3; 74820bp from tss:downstream
Dura
Scalp
Methylation
0.2
0.5
0.8
Genes
+
−
226297000
226297500
226298000
226298500
226299000
226299500
Chromosome 1

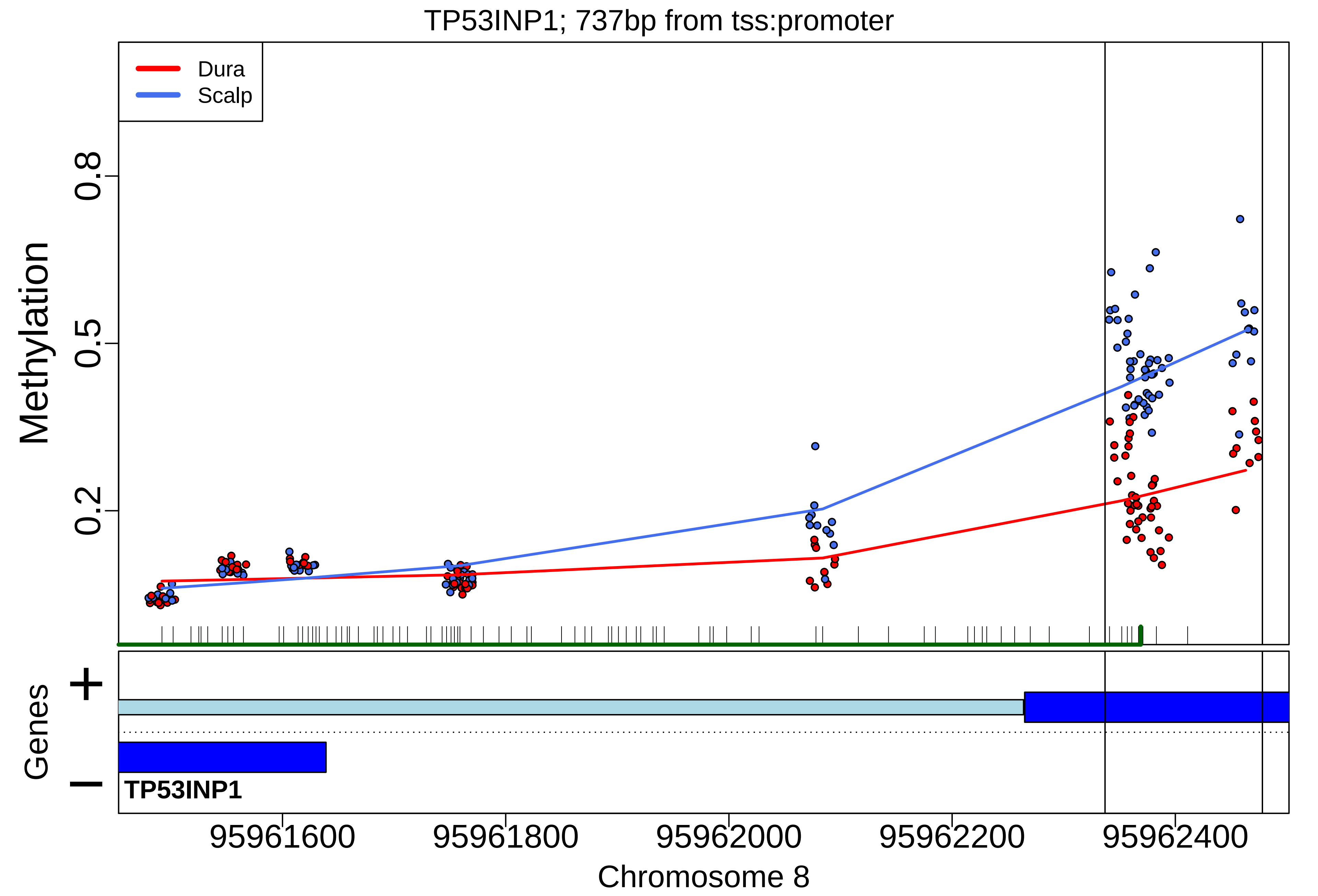

TP53INP1; 737bp from tss:promoter
Dura
Scalp
Methylation
0.2
0.5
0.8
Genes
+
−
TP53INP1
95961600
95961800
95962000
95962200
95962400
Chromosome 8

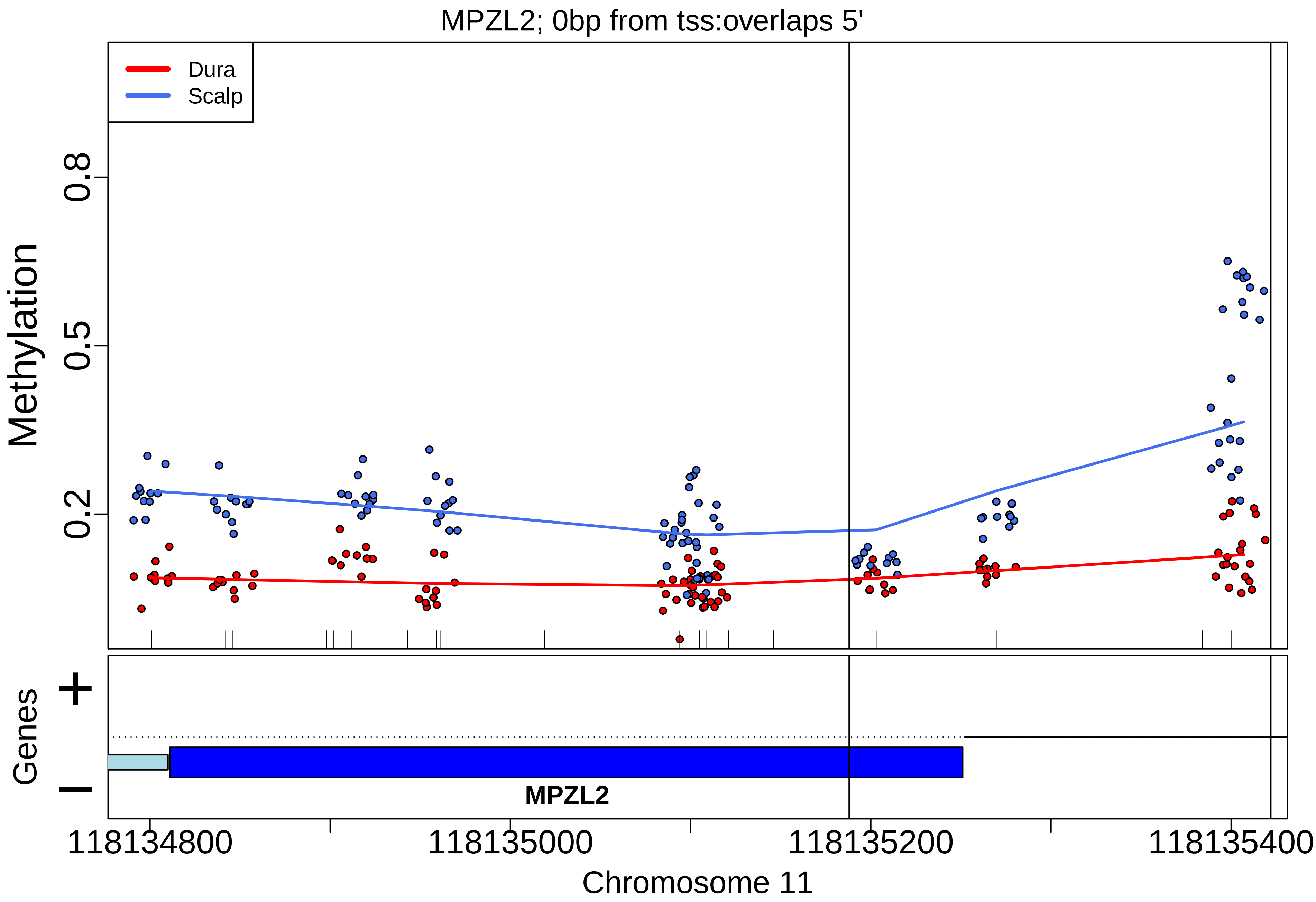
MPZL2; 0bp from tss:overlaps 5'
Dura
Scalp
Methylation
0.2
0.5
0.8
Genes
+
−
MPZL2
118134800
118135000
118135200
118135400
Chromosome 11

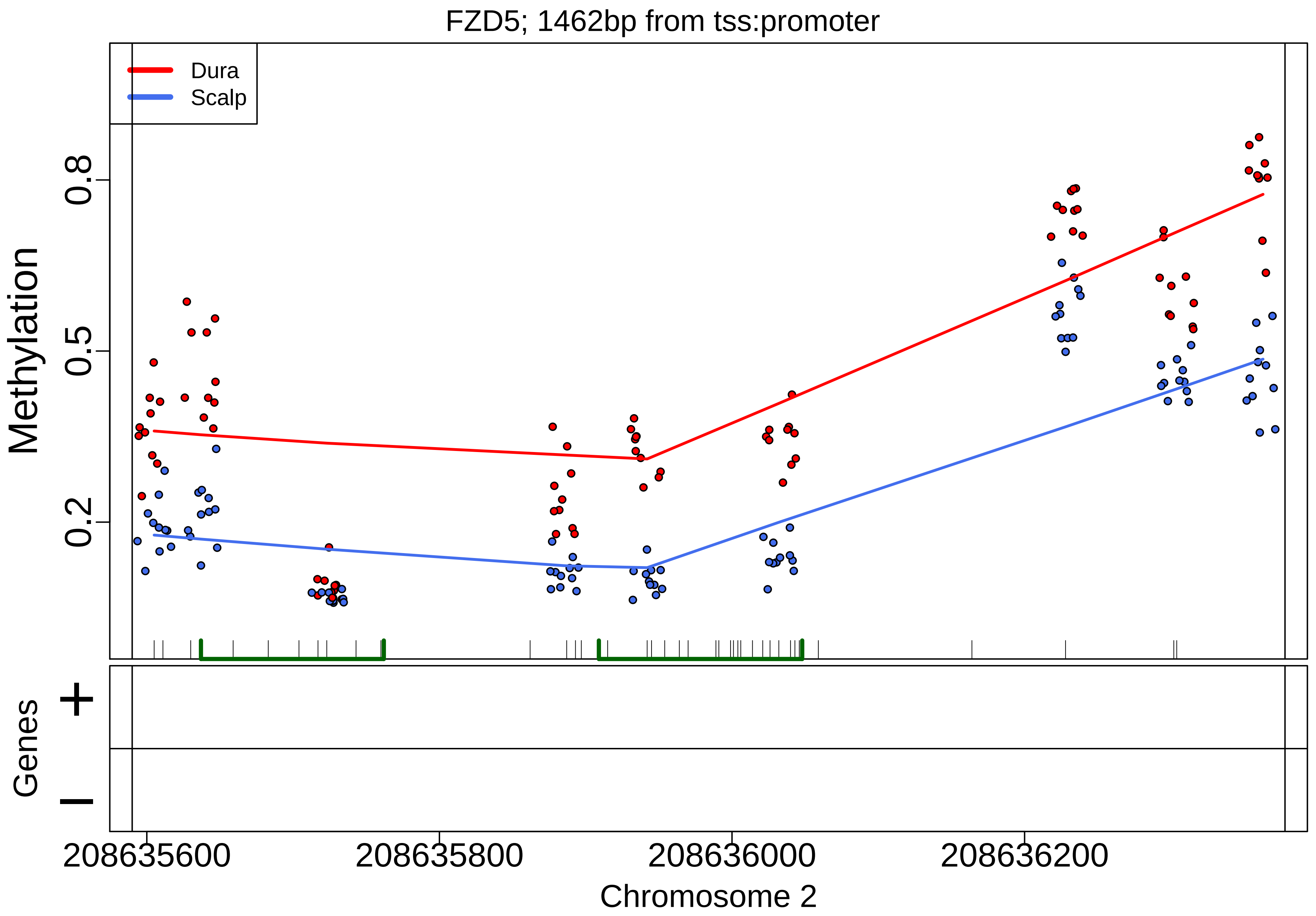

FZD5; 1462bp from tss:promoter
Dura
Scalp
Methylation
0.2
0.5
0.8
Genes
+
−
208635600
208635800
208636000
208636200
Chromosome 2

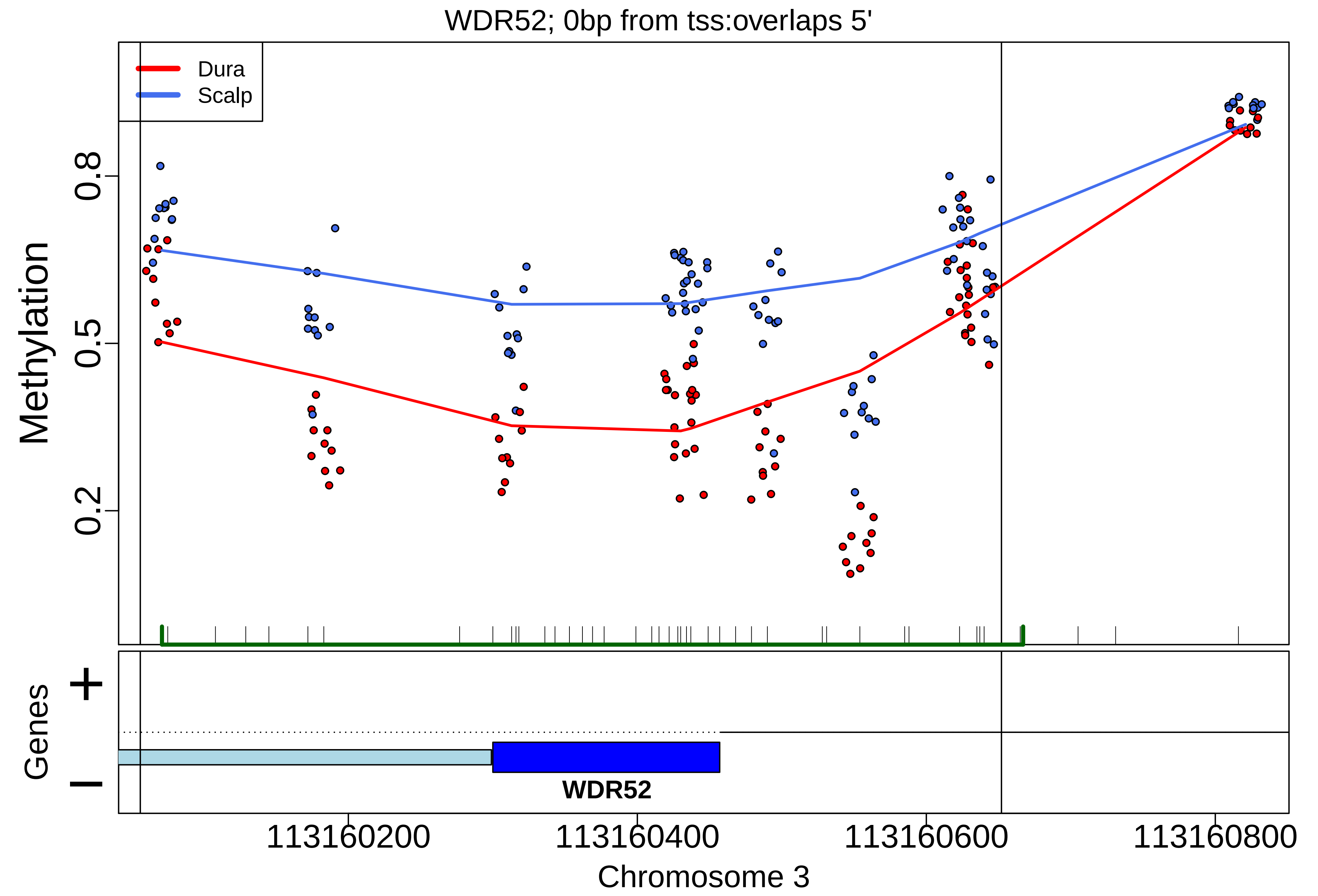

WDR52; 0bp from tss:overlaps 5'
Dura
Scalp
Methylation
0.2
0.5
0.8
Genes
+
-
WDR52
113160200
113160400
113160600
113160800
Chromosome 3

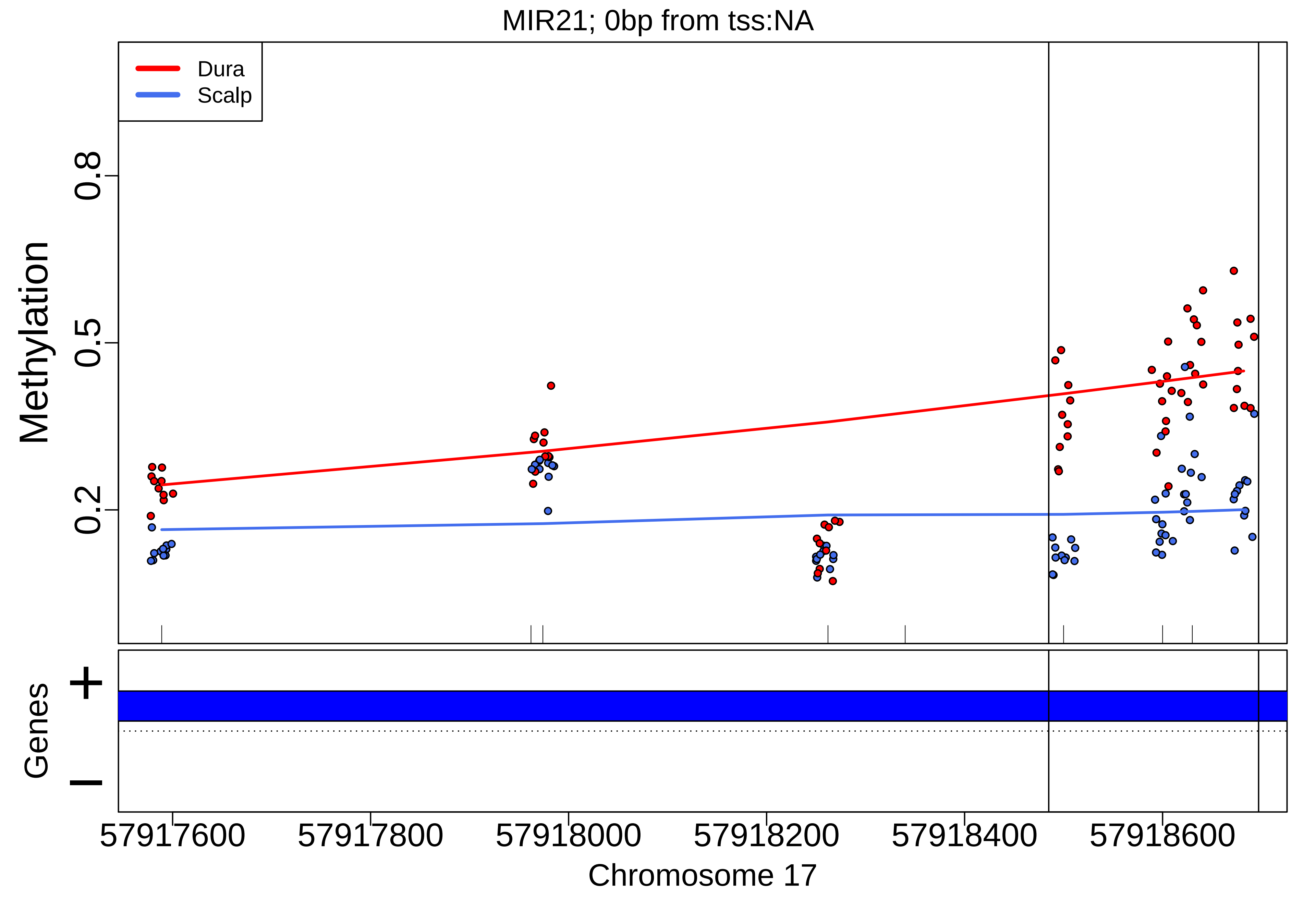

MIR21; 0bp from tss:NA
Dura
Scalp
Methylation
0.2
0.5
0.8
Genes
+
-
57917600
57917800
57918000
57918200
57918400
57918600
Chromosome 17

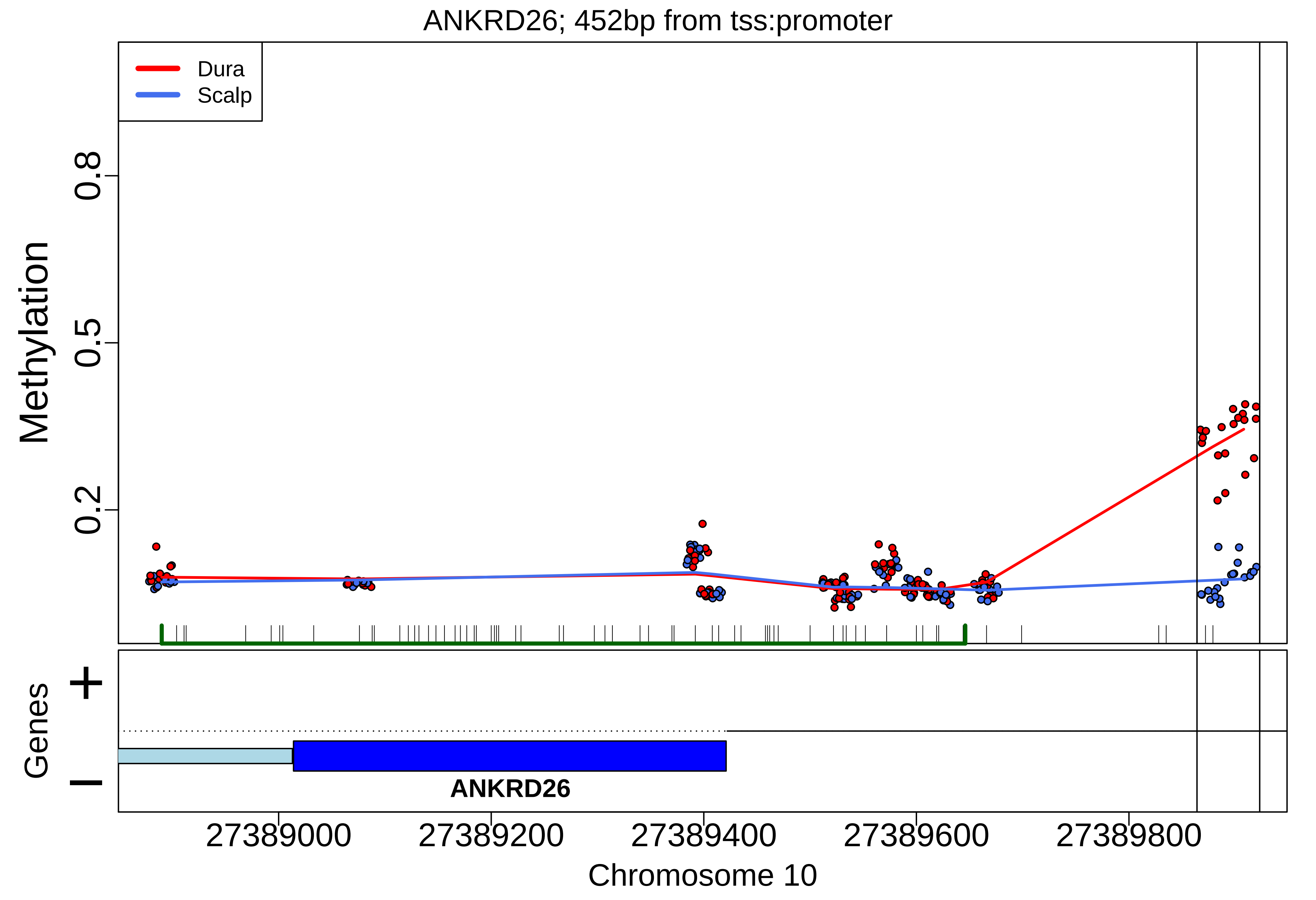

ANKRD26; 452bp from tss:promoter
Dura
Scalp
Methylation
0.2
0.5
0.8
Genes
+
-
ANKRD26
27389000
27389200
27389400
27389600
27389800
Chromosome 10

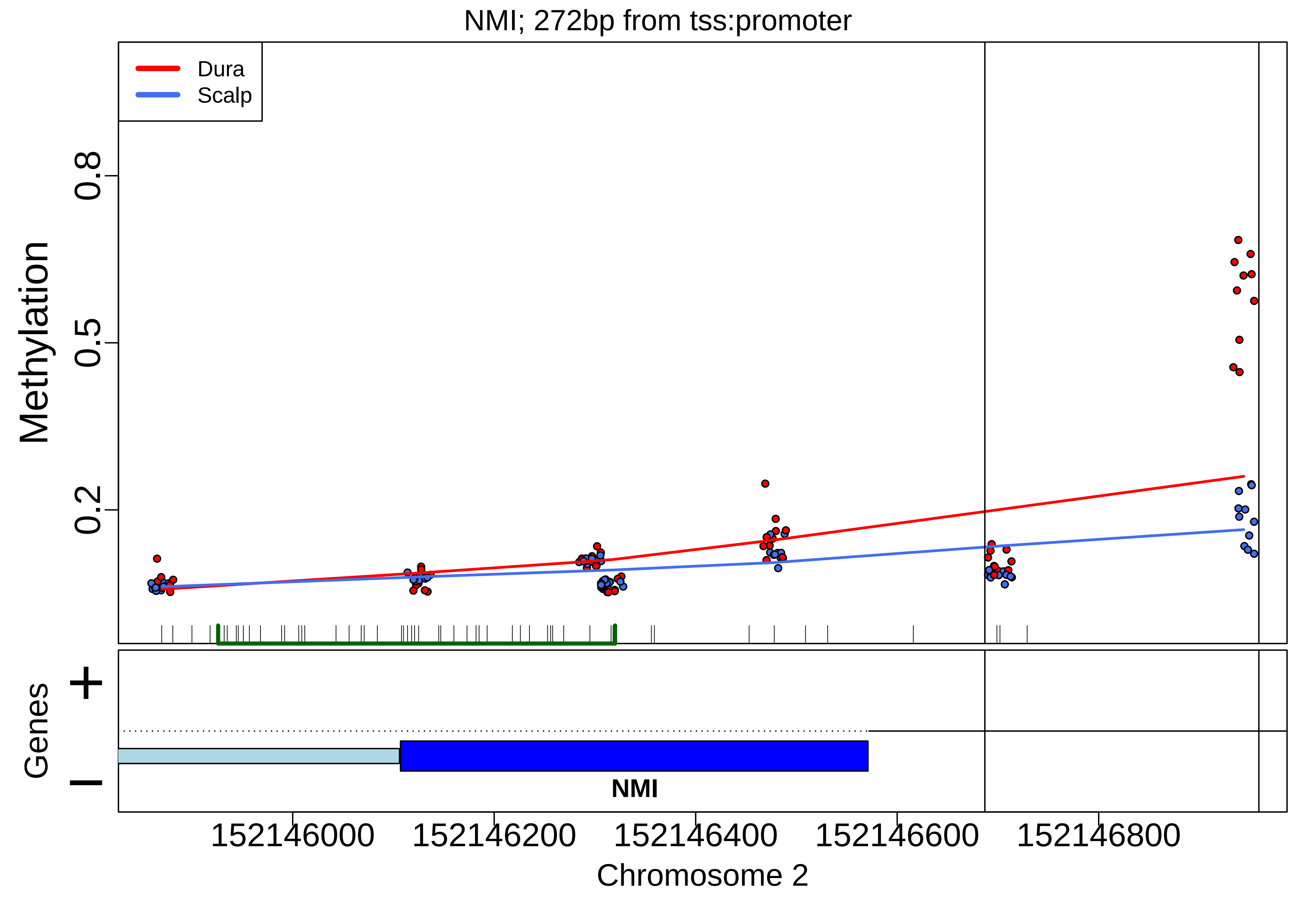

NMI; 272bp from tss:promoter
Dura
Scalp
Methylation
0.2
0.5
0.8
Genes
+
−
NMI
152146000
152146200
152146400
152146600
152146800
Chromosome 2

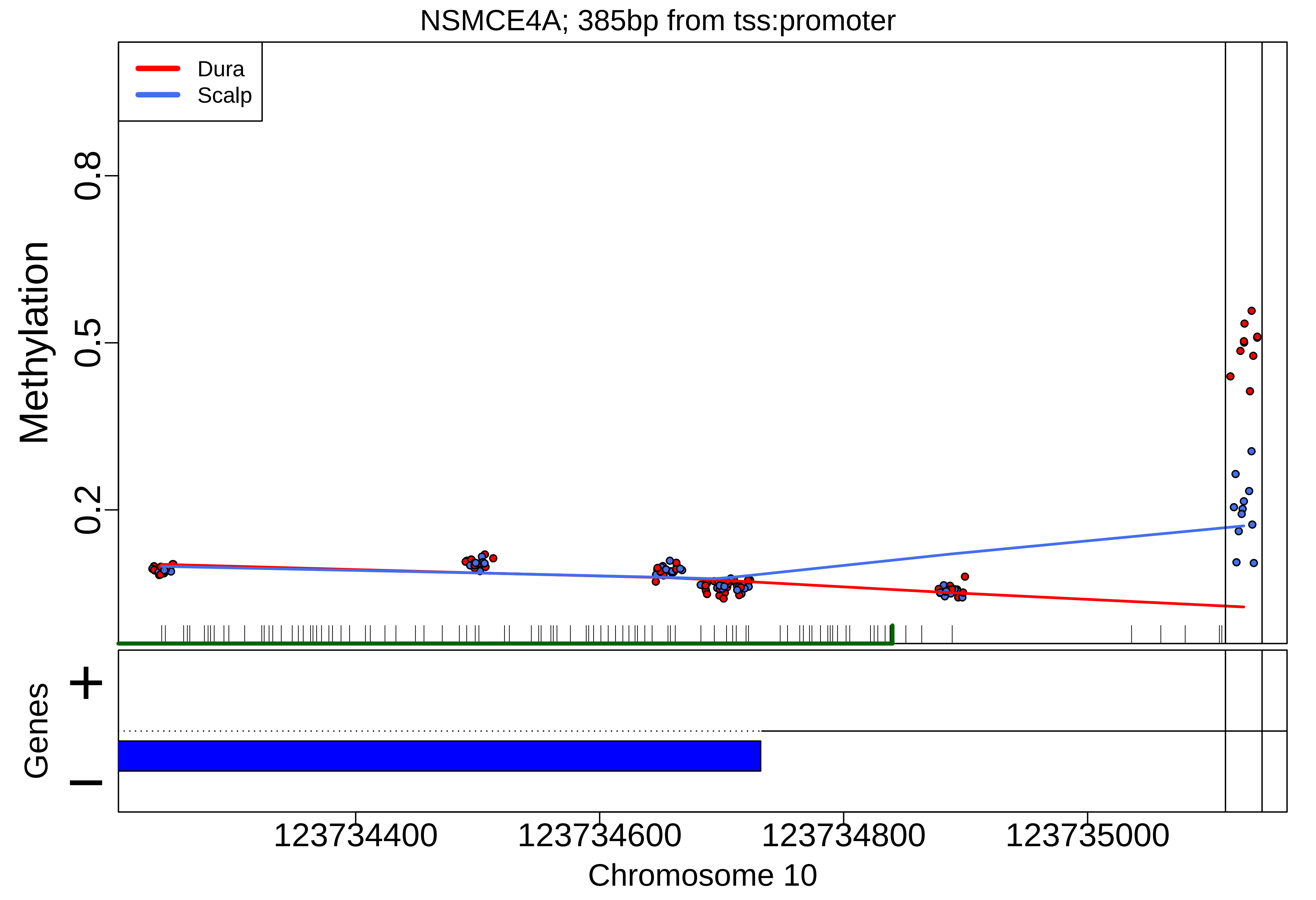

NSMCE4A; 385bp from tss:promoter
Dura
Scalp
Methylation
0.2
0.5
0.8
Genes
+
−
123734400
123734600
123734800
123735000
Chromosome 10

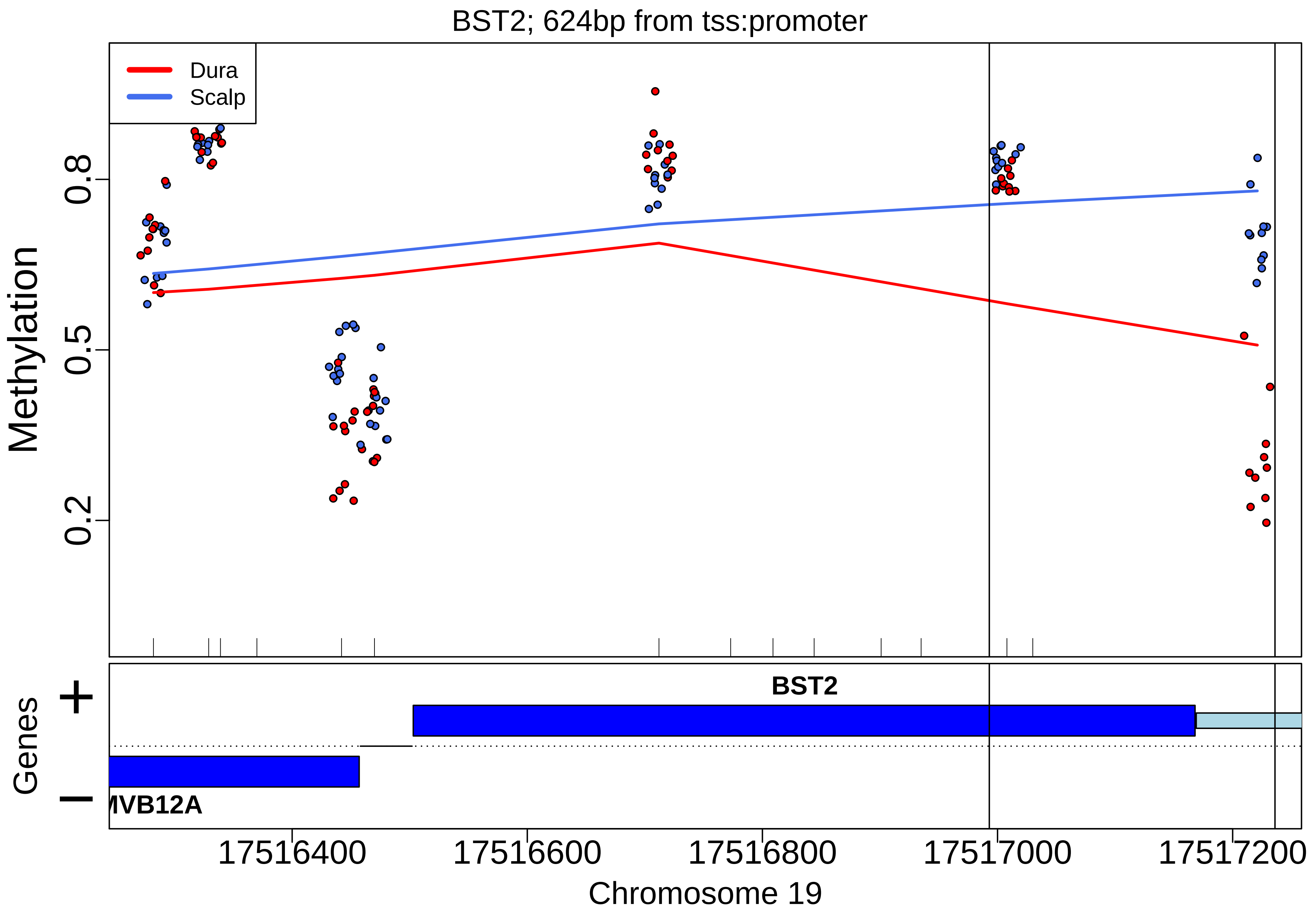
BST2; 624bp from tss:promoter
Dura
Scalp
Methylation
0.2
0.5
0.8
Genes
+
−
BST2
MVB12A
17516400
17516600
17516800
17517000
17517200
Chromosome 19

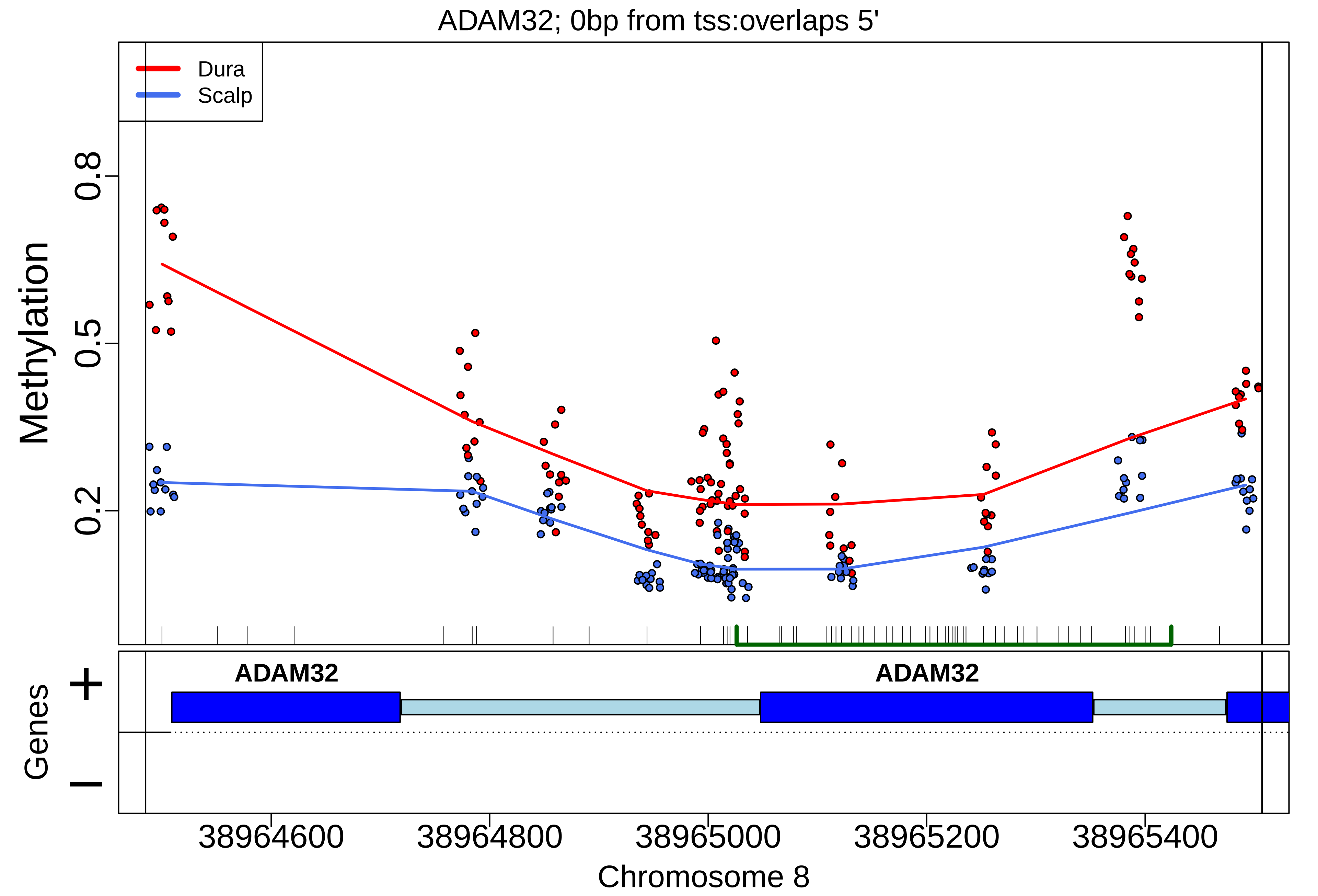
ADAM32; 0bp from tss:overlaps 5'
Dura
Scalp
Methylation
0.2
0.5
0.8
Genes
+
-
ADAM32
ADAM32
38964600
38964800
38965000
38965200
38965400
Chromosome 8

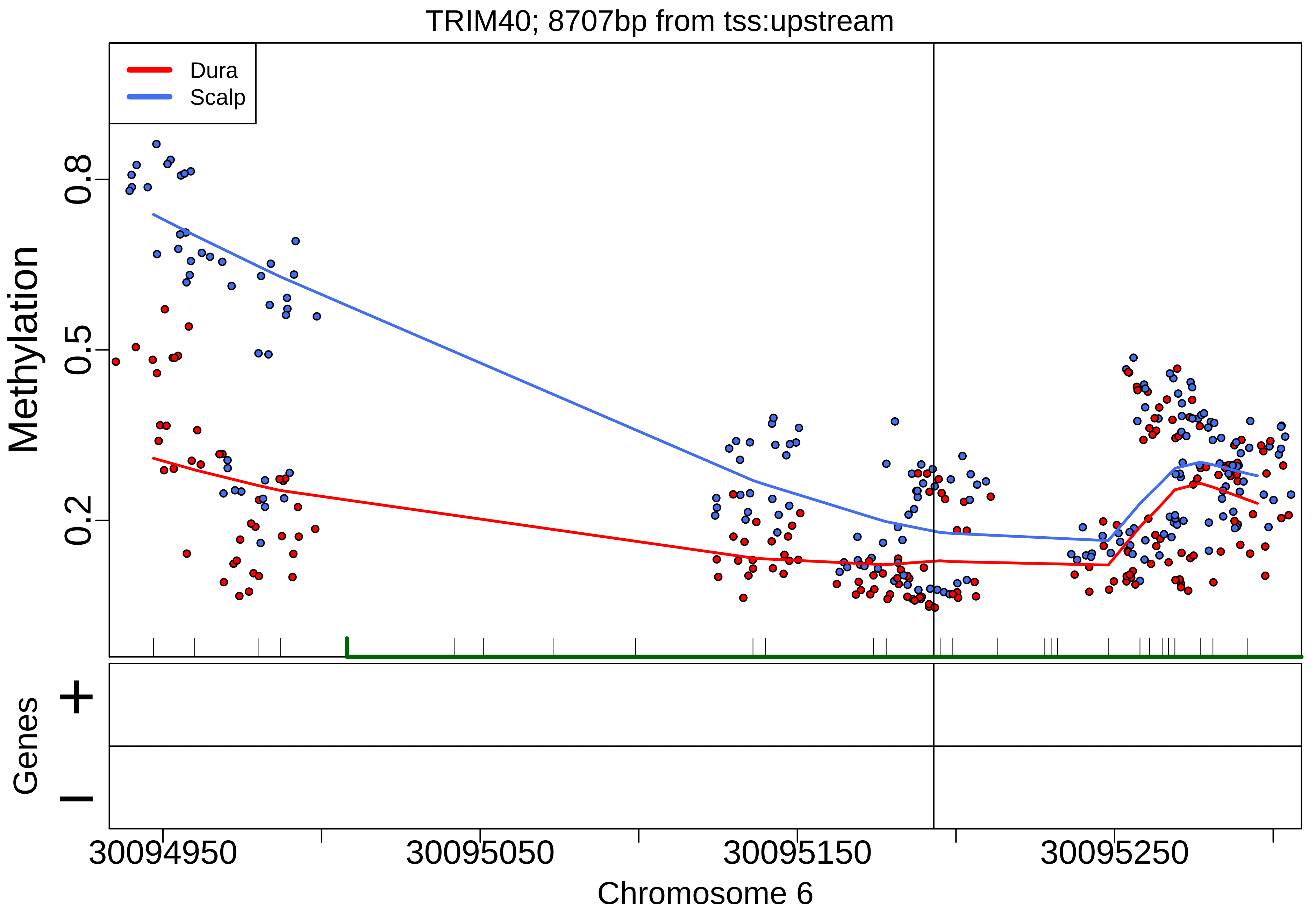
TRIM40; 8707bp from tss:upstream
Dura
Scalp
Methylation
0.2
0.5
0.8
Genes
+
−
30094950
30095050
30095150
30095250
Chromosome 6

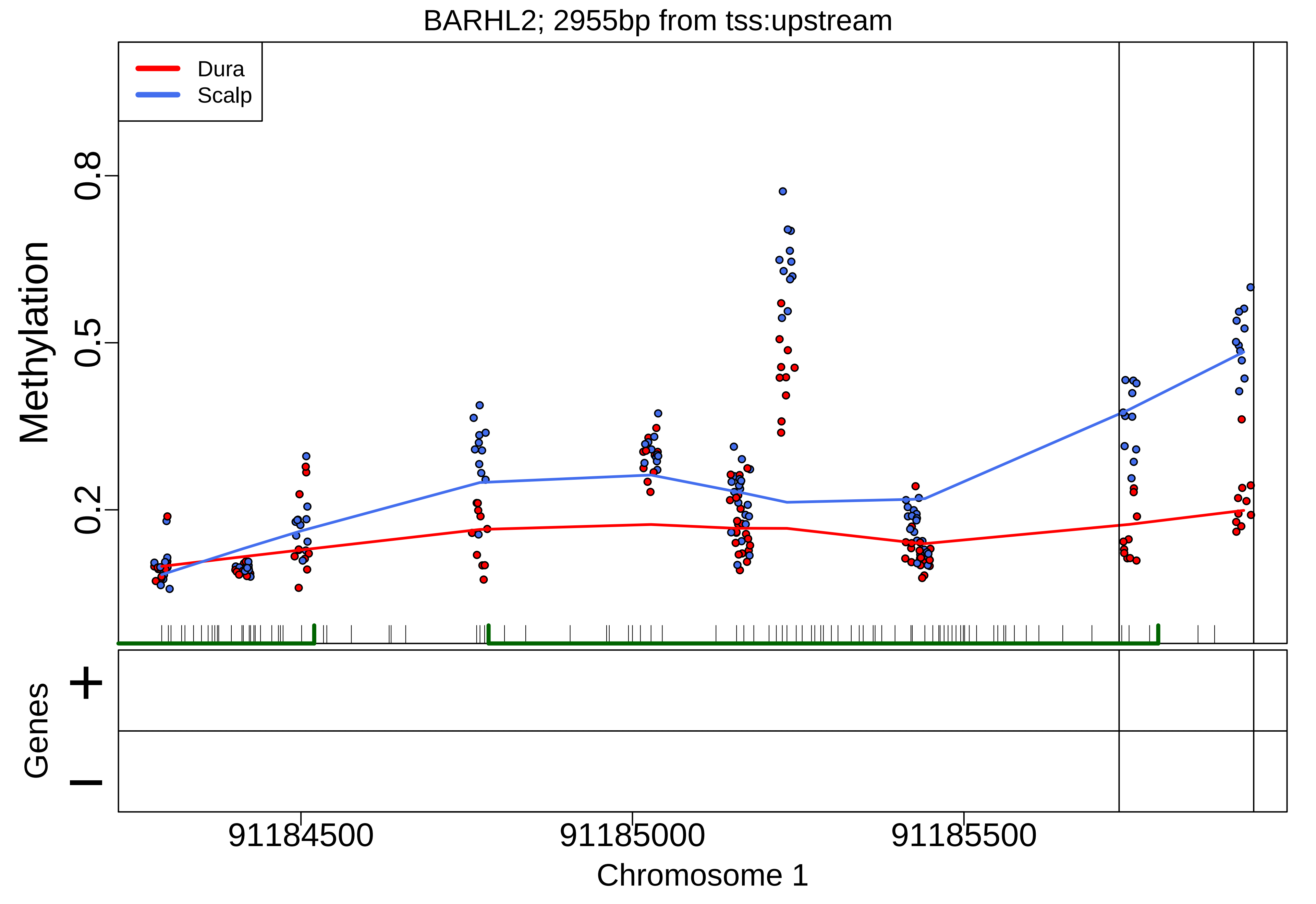
BARHL2; 2955bp from tss:upstream
Dura
Scalp
Methylation
0.2
0.5
0.8
Genes
+
−
91184500
91185000
91185500
Chromosome 1

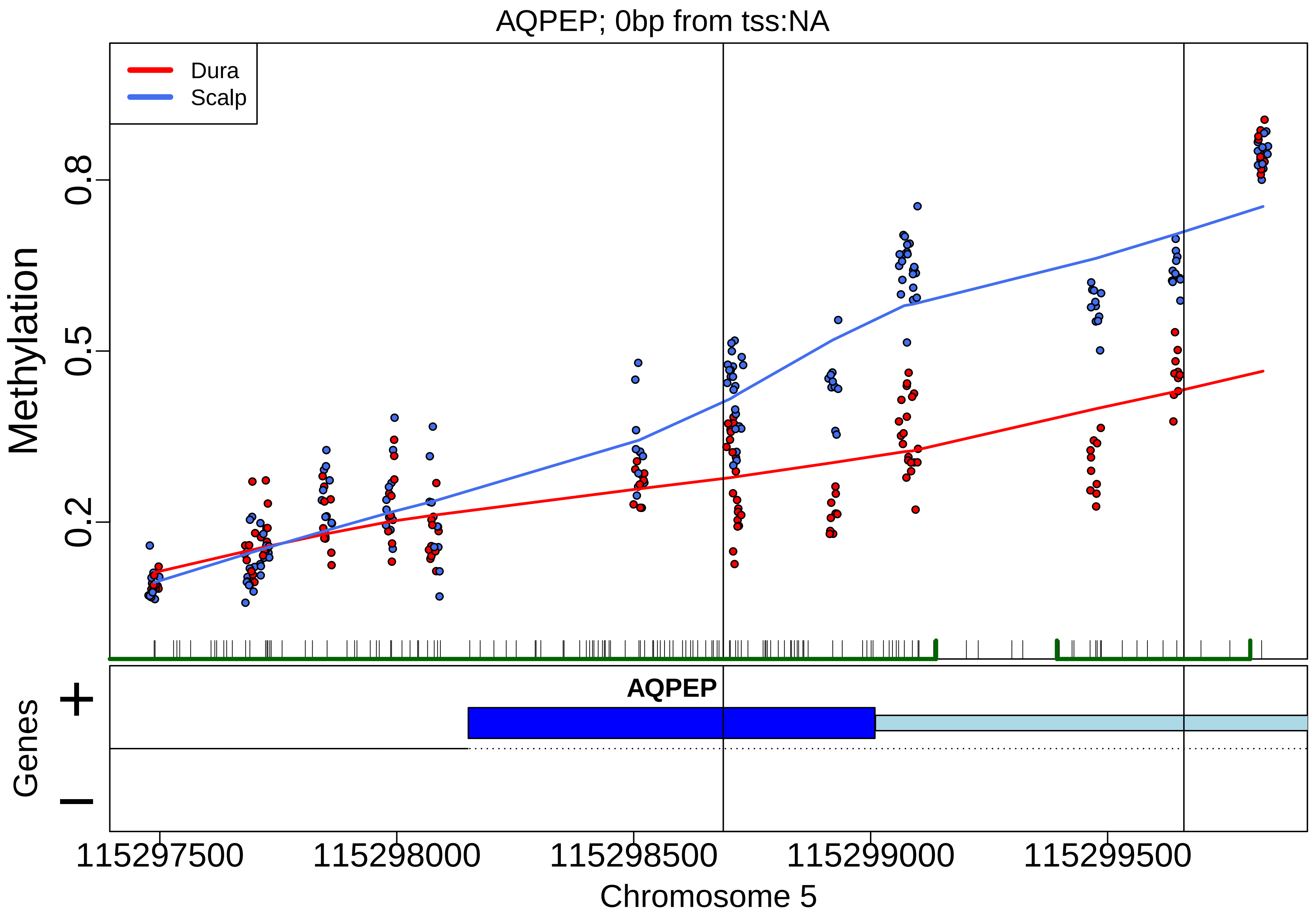
AQPEP; 0bp from tss:NA
Dura
Scalp
Methylation
0.2
0.5
0.8
Genes
+
−
AQPEP
115297500
115298000
115298500
115299000
115299500
Chromosome 5

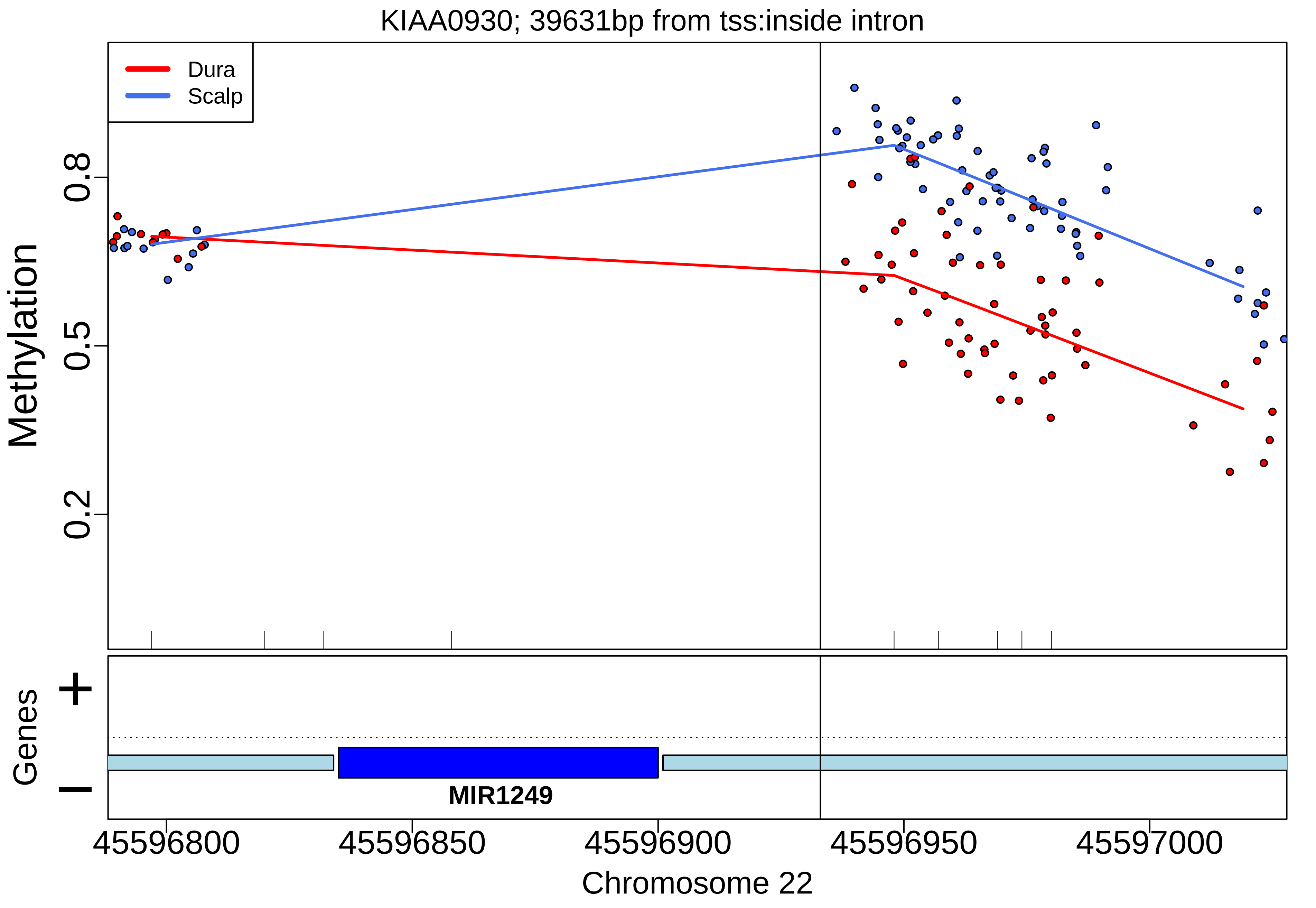
KIAA0930; 39631bp from tss:inside intron
Dura
Scalp
Methylation
0.2
0.5
0.8
Genes
+
-
MIR1249
45596800
45596850
45596900
45596950
45597000
Chromosome 22

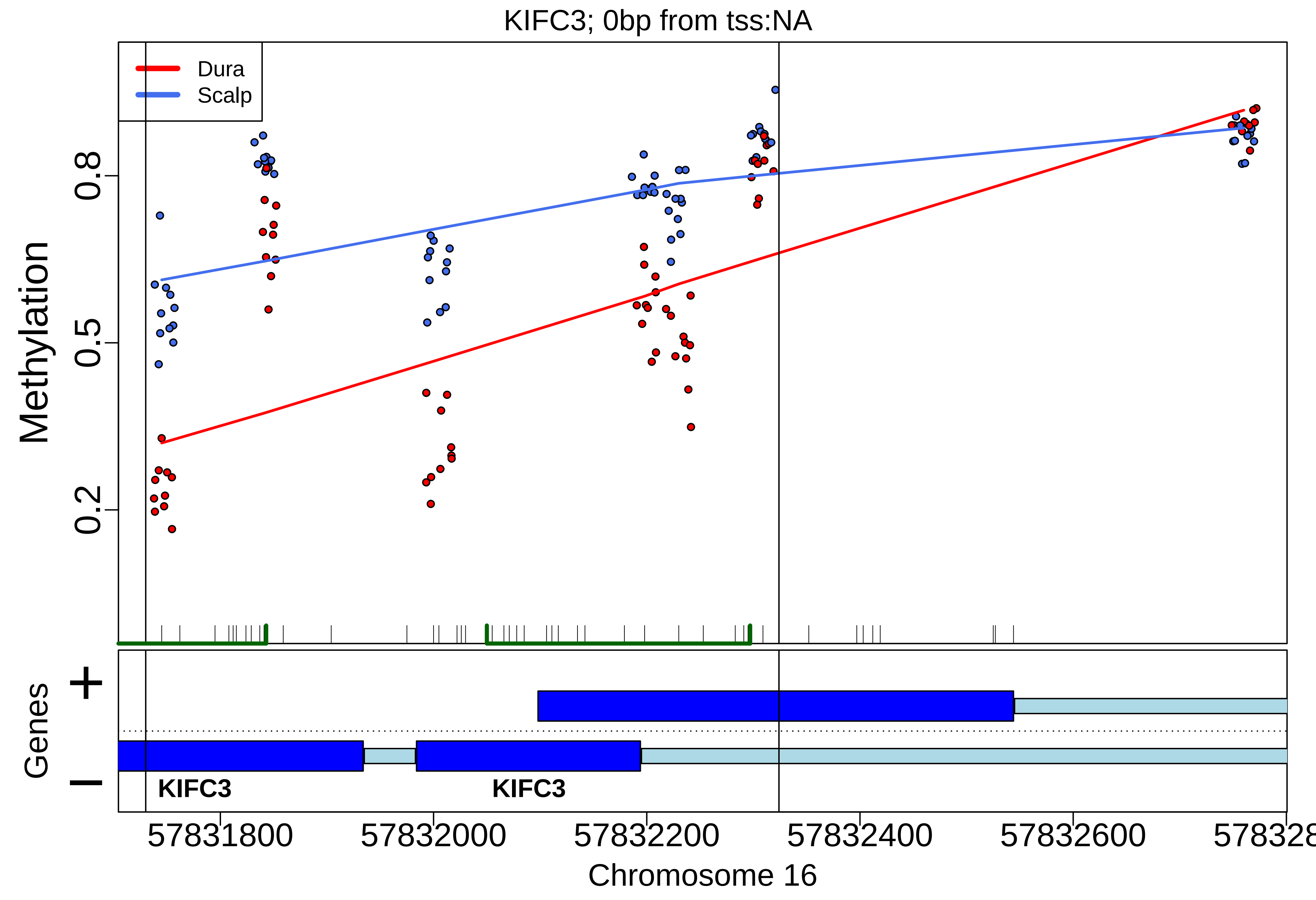

KIFC3; 0bp from tss:NA
Dura
Scalp
Methylation
0.2
0.5
0.8
Genes
+
–
KIFC3
KIFC3
57831800
57832000
57832200
57832400
57832600
Chromosome 16

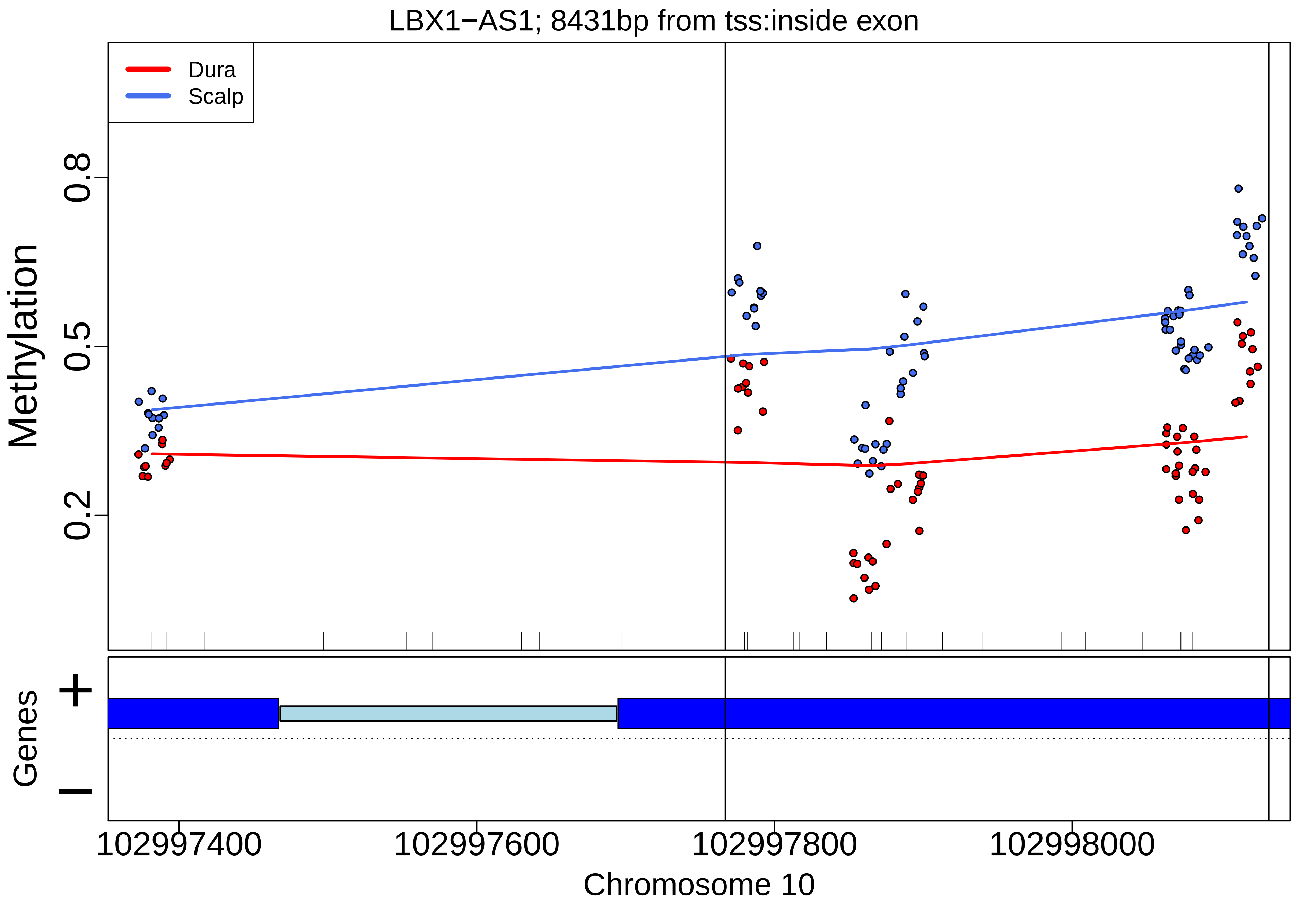

LBX1−AS1; 8431bp from tss:inside exon
Dura
Scalp
Methylation
0.2
0.5
0.8
Genes
+
−
102997400
102997600
102997800
102998000
Chromosome 10

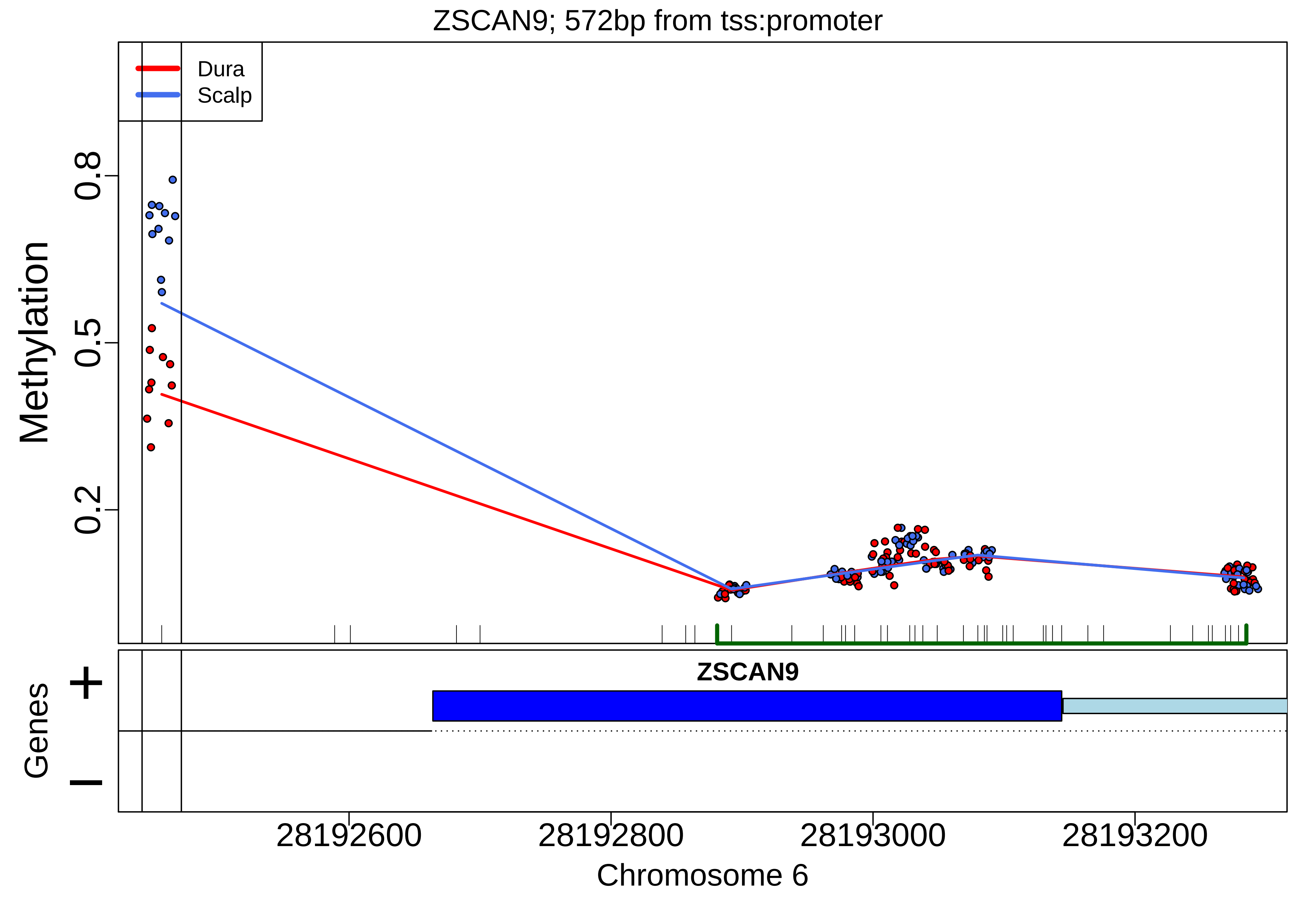

ZSCAN9; 572bp from tss:promoter
Dura
Scalp
Methylation
0.2
0.5
0.8
Genes
+
-
ZSCAN9
28192600
28192800
28193000
28193200
Chromosome 6

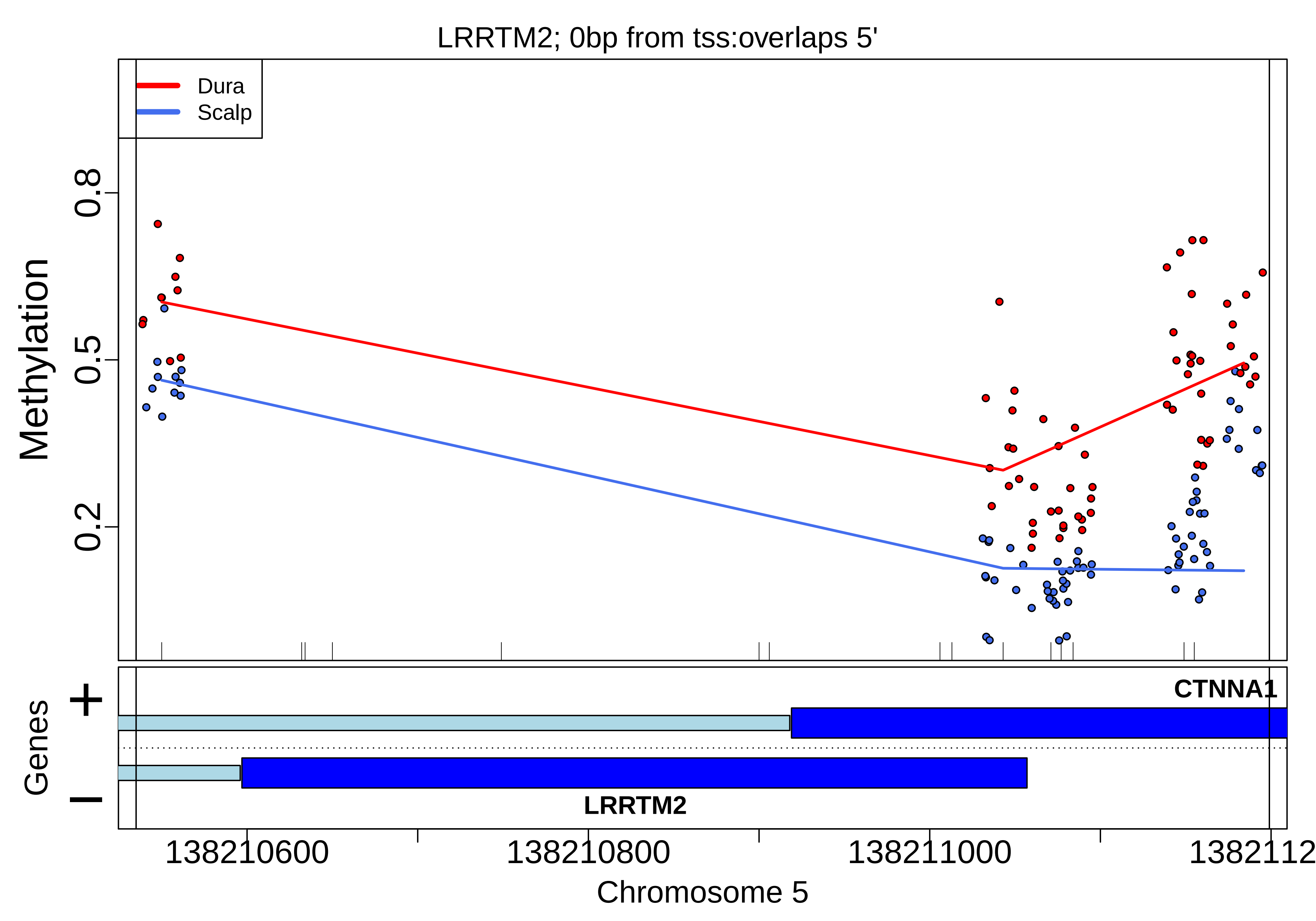

LRRTM2; 0bp from tss:overlaps 5'
Dura
Scalp
Methylation
0.2
0.5
0.8
Genes
+
-
CTNNA1
LRRTM2
138210600
138210800
138211000
1382112
Chromosome 5

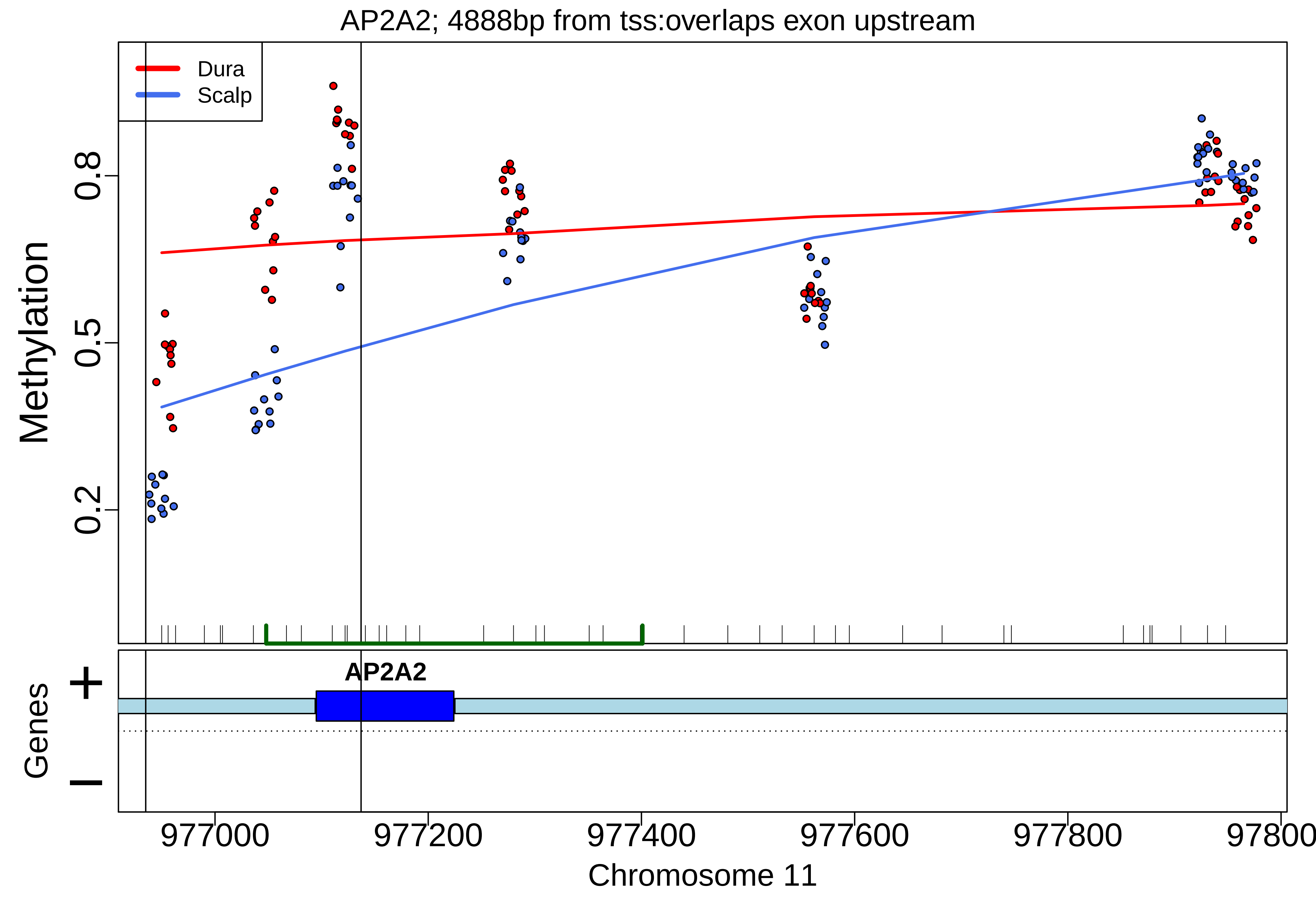

AP2A2; 4888bp from tss:overlaps exon upstream
Dura
Scalp
Methylation
0.2
0.5
0.8
Genes
+
−
AP2A2
977000
977200
977400
977600
977800
97800
Chromosome 11

WBP1L; 0bp from tss:NA

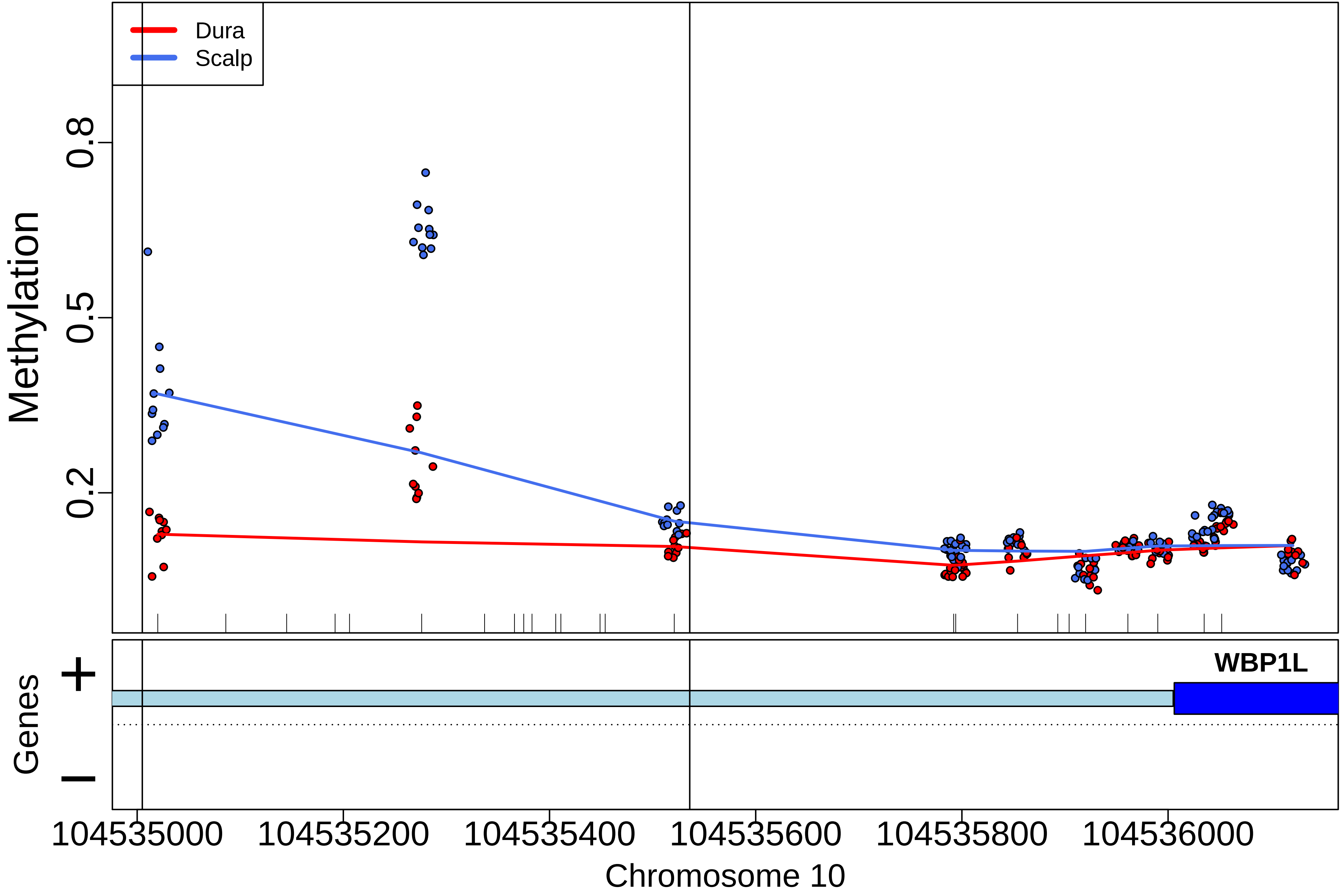

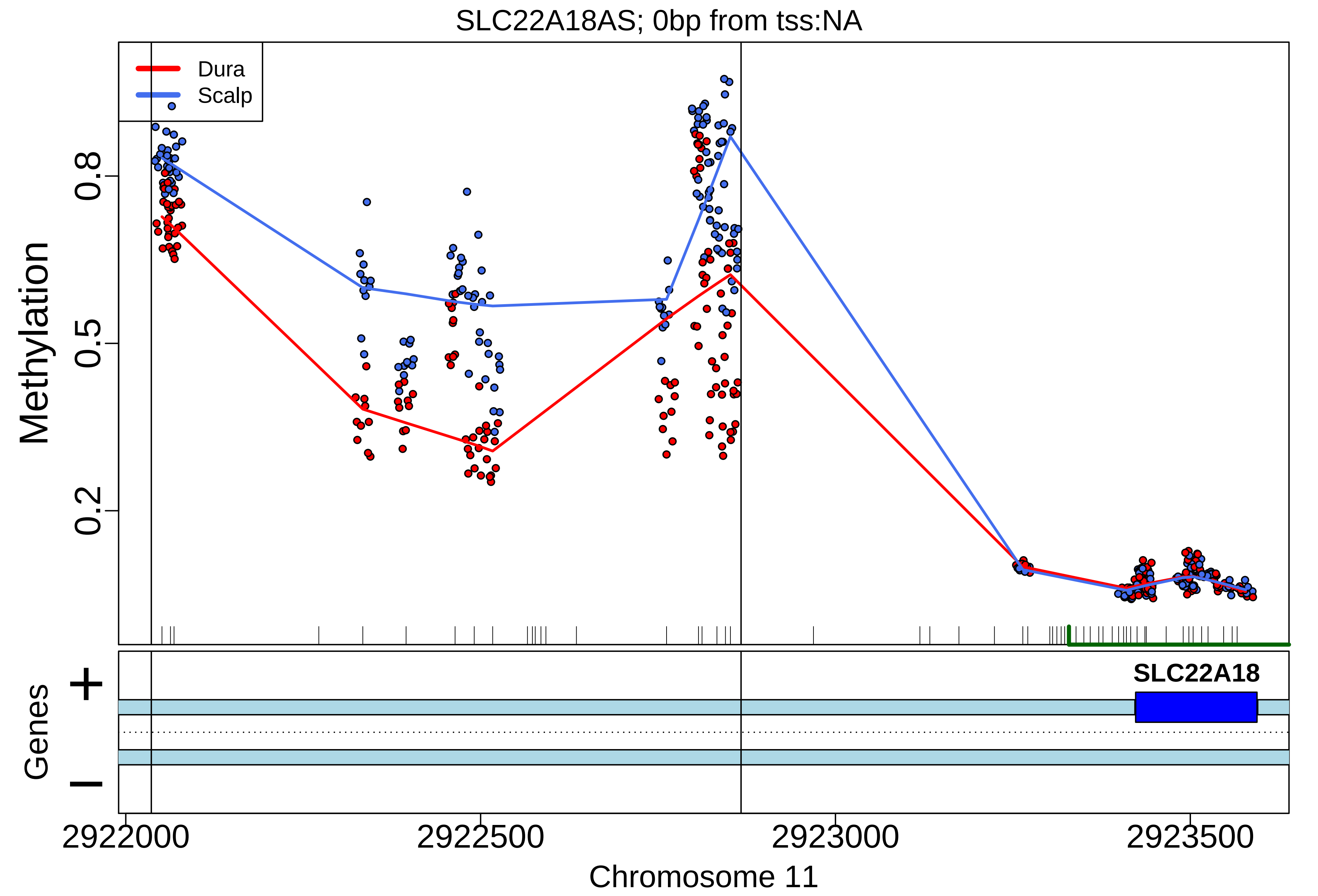
SLC22A18AS; 0bp from tss:NA
Dura
Scalp
Methylation
0.2
0.5
0.8
Genes
+
−
SLC22A18
2922000
2922500
2923000
2923500
Chromosome 11

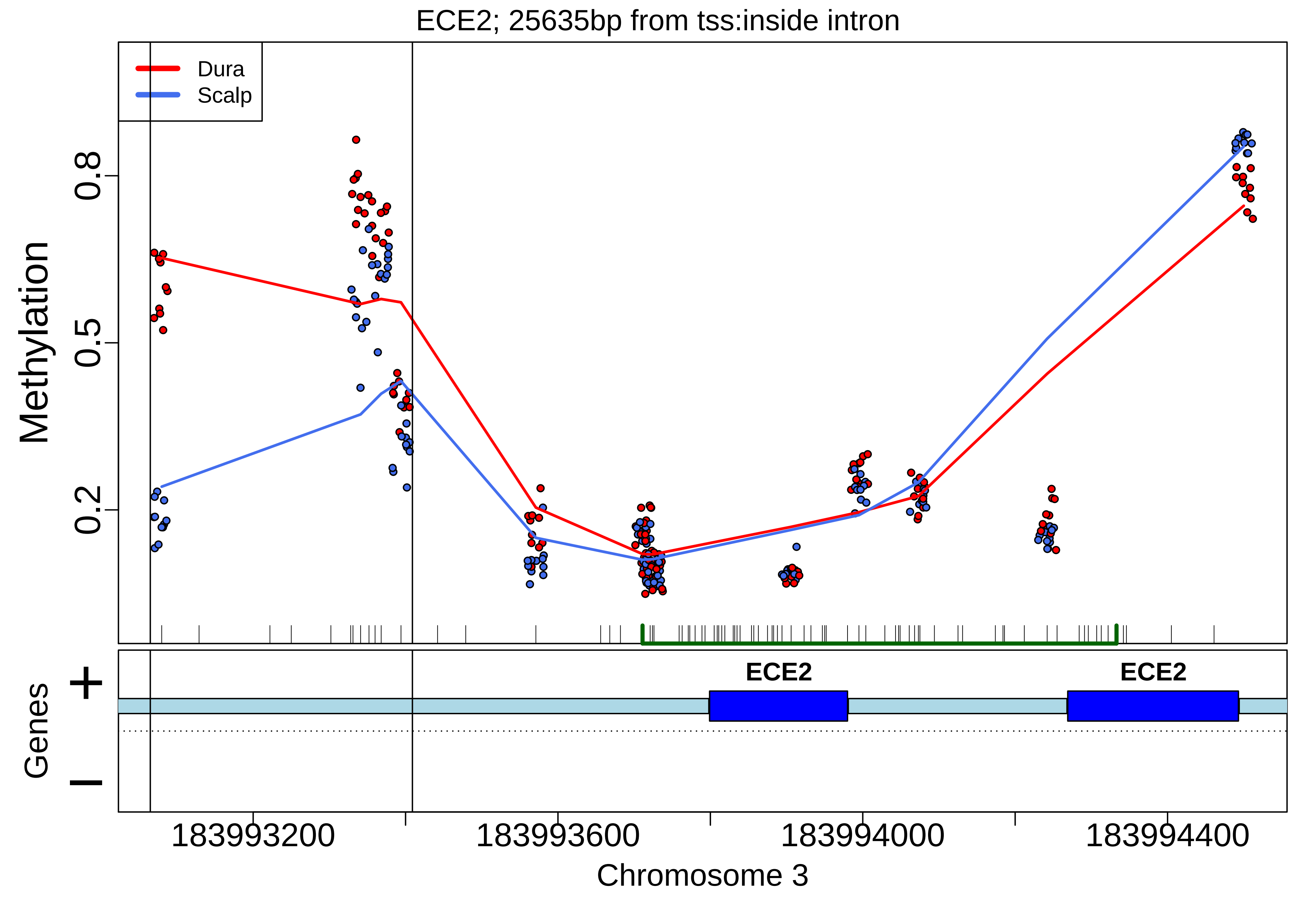
ECE2; 25635bp from tss:inside intron
Dura
Scalp
Methylation
0.2
0.5
0.8
Genes
+
-
ECE2
ECE2
183993200
183993600
183994000
183994400
Chromosome 3

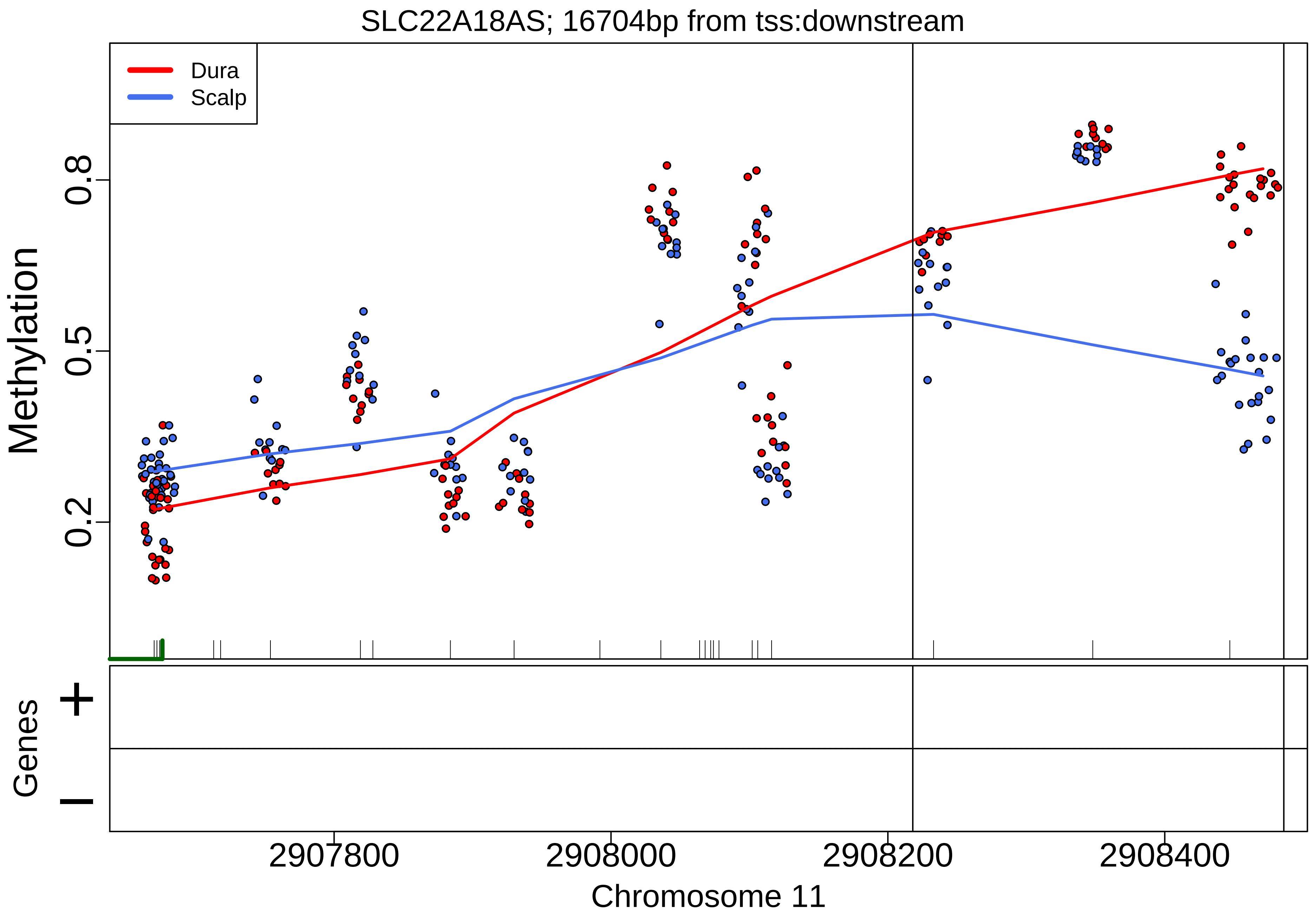

SLC22A18AS; 16704bp from tss:downstream
Dura
Scalp
Methylation
0.2
0.5
0.8
Genes
+
-
2907800
2908000
2908200
2908400
Chromosome 11

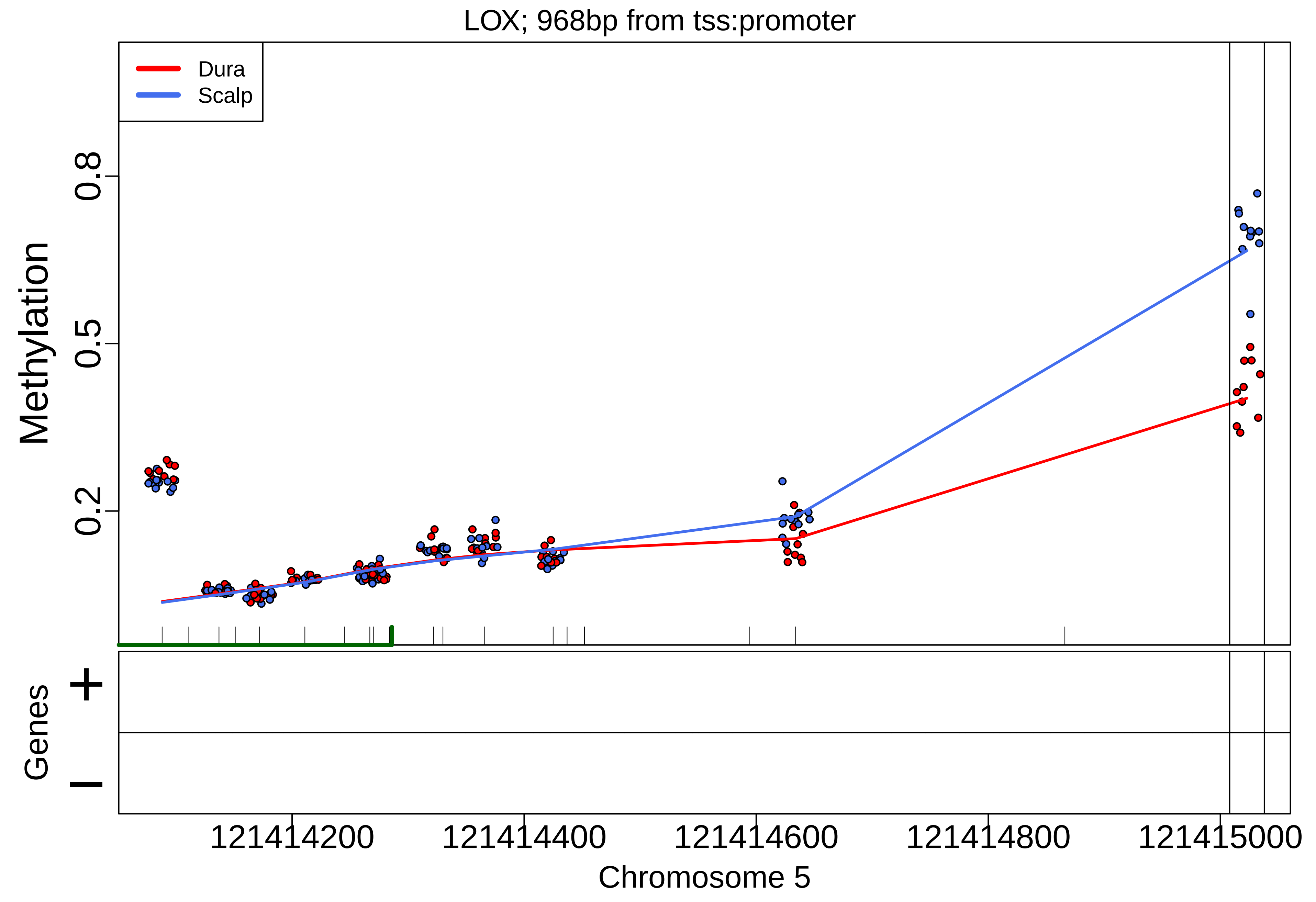

LOX; 968bp from tss:promoter
Dura
Scalp
Methylation
0.2
0.5
0.8
Genes
+
−
121414200
121414400
121414600
121414800
121415000
Chromosome 5

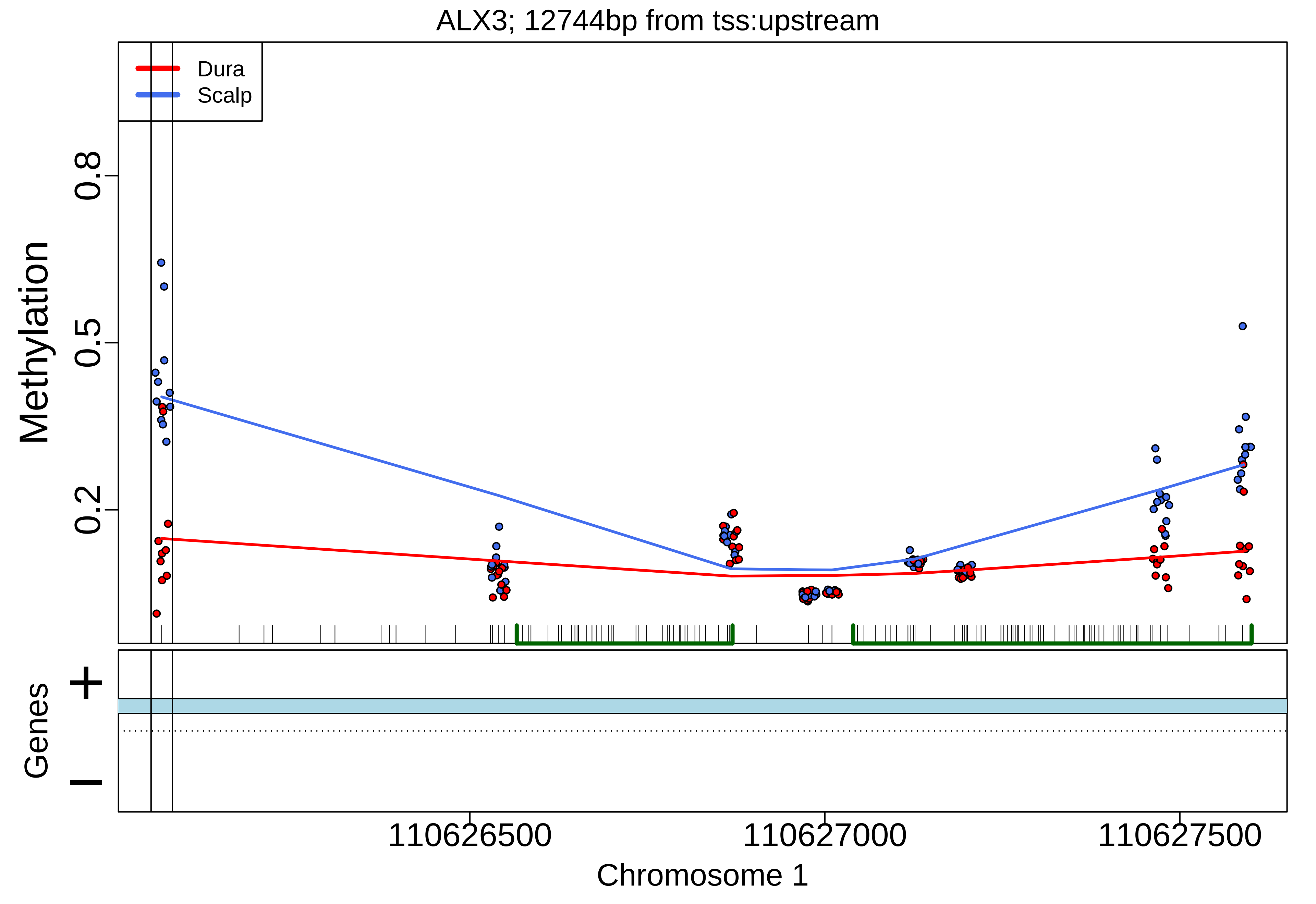

ALX3; 12744bp from tss:upstream
Dura
Scalp
Methylation
0.2
0.5
0.8
Genes
+
-
110626500
110627000
110627500
Chromosome 1

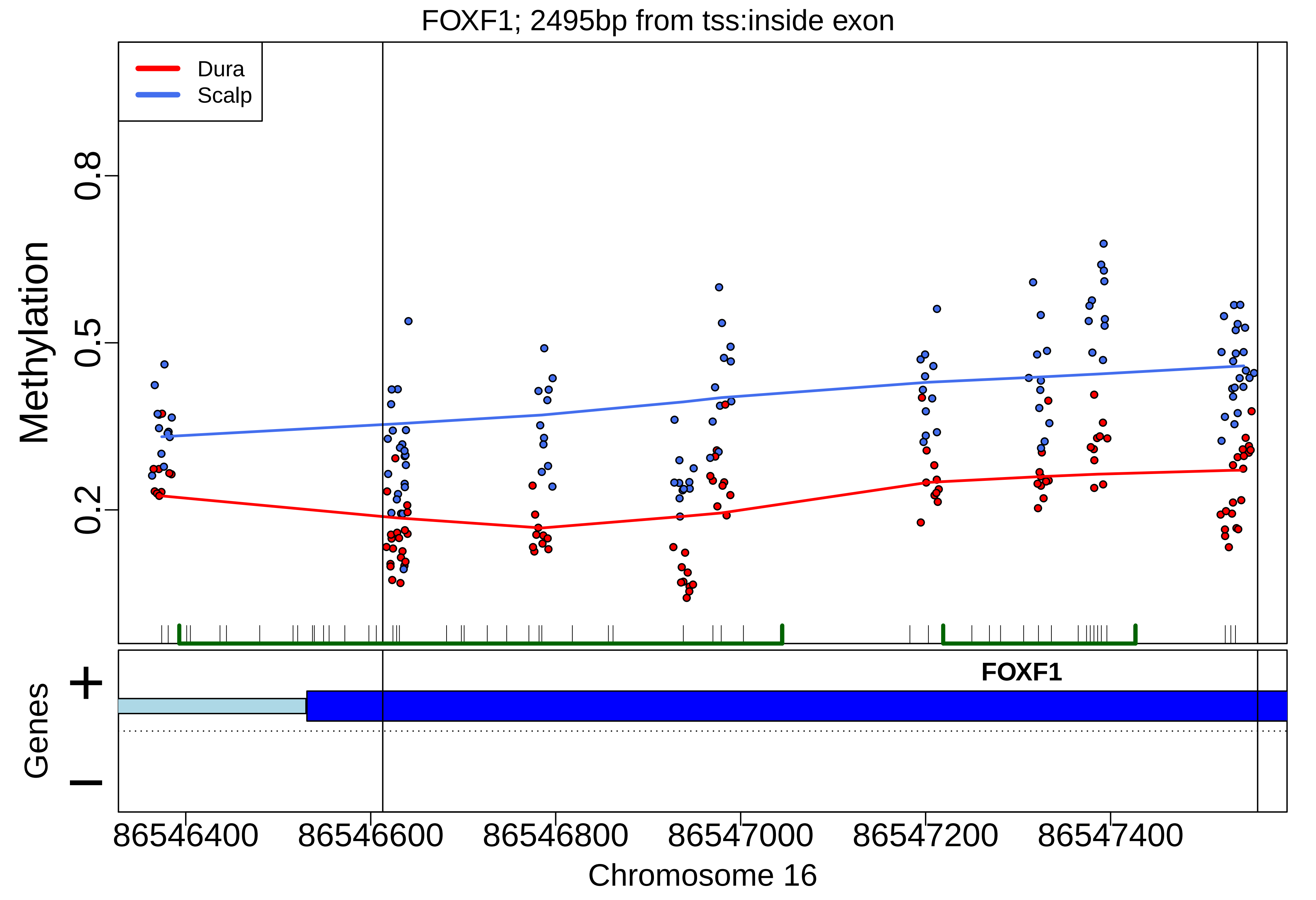

FOXF1; 2495bp from tss:inside exon
Dura
Scalp
Methylation
0.2
0.5
0.8
Genes
+
-
FOXF1
86546400
86546600
86546800
86547000
86547200
86547400
Chromosome 16

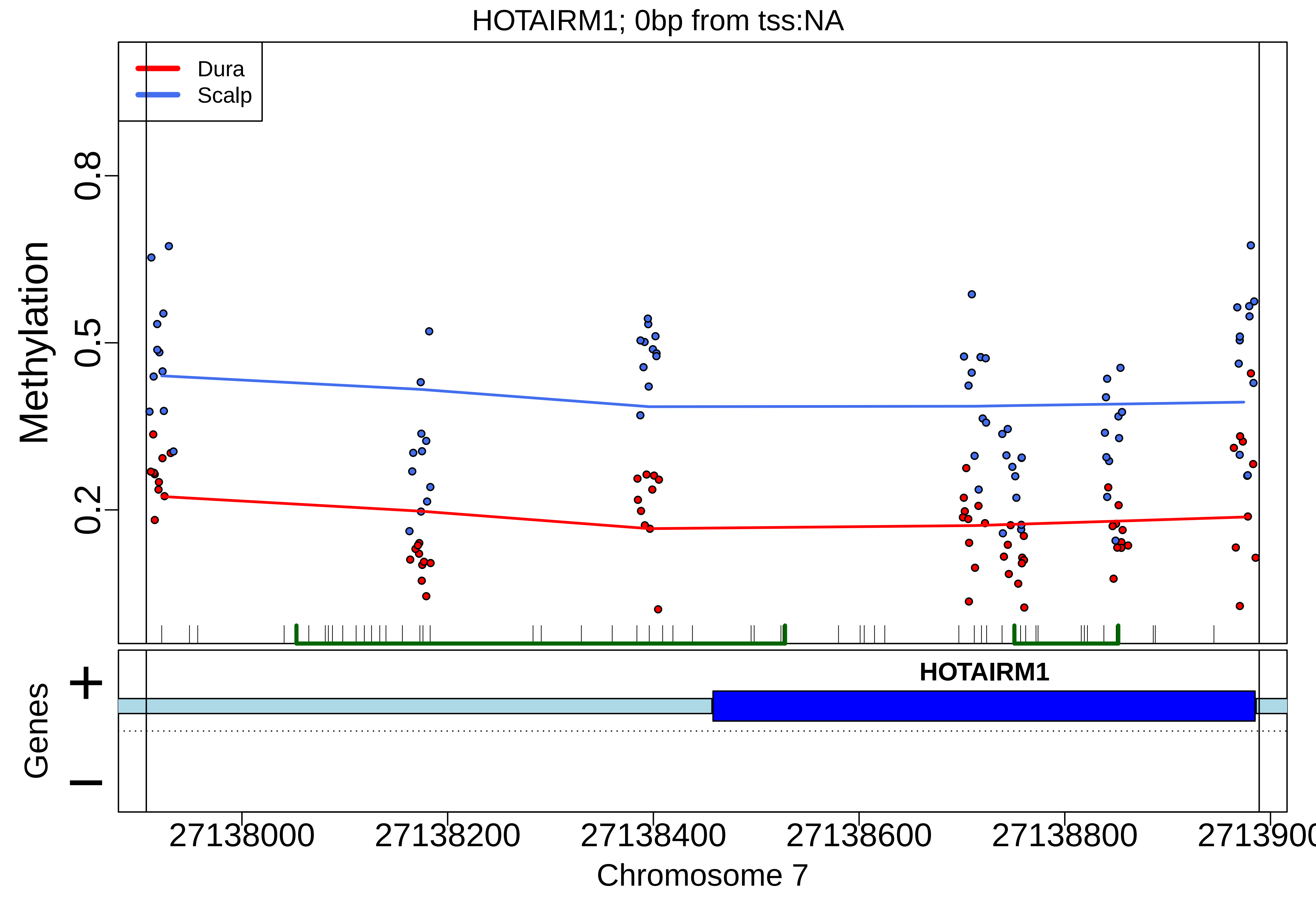

HOTAIRM1; 0bp from tss:NA
Dura
Scalp
Methylation
0.2
0.5
0.8
Genes
+
-
HOTAIRM1
27138000
27138200
27138400
27138600
27138800
2713900
Chromosome 7

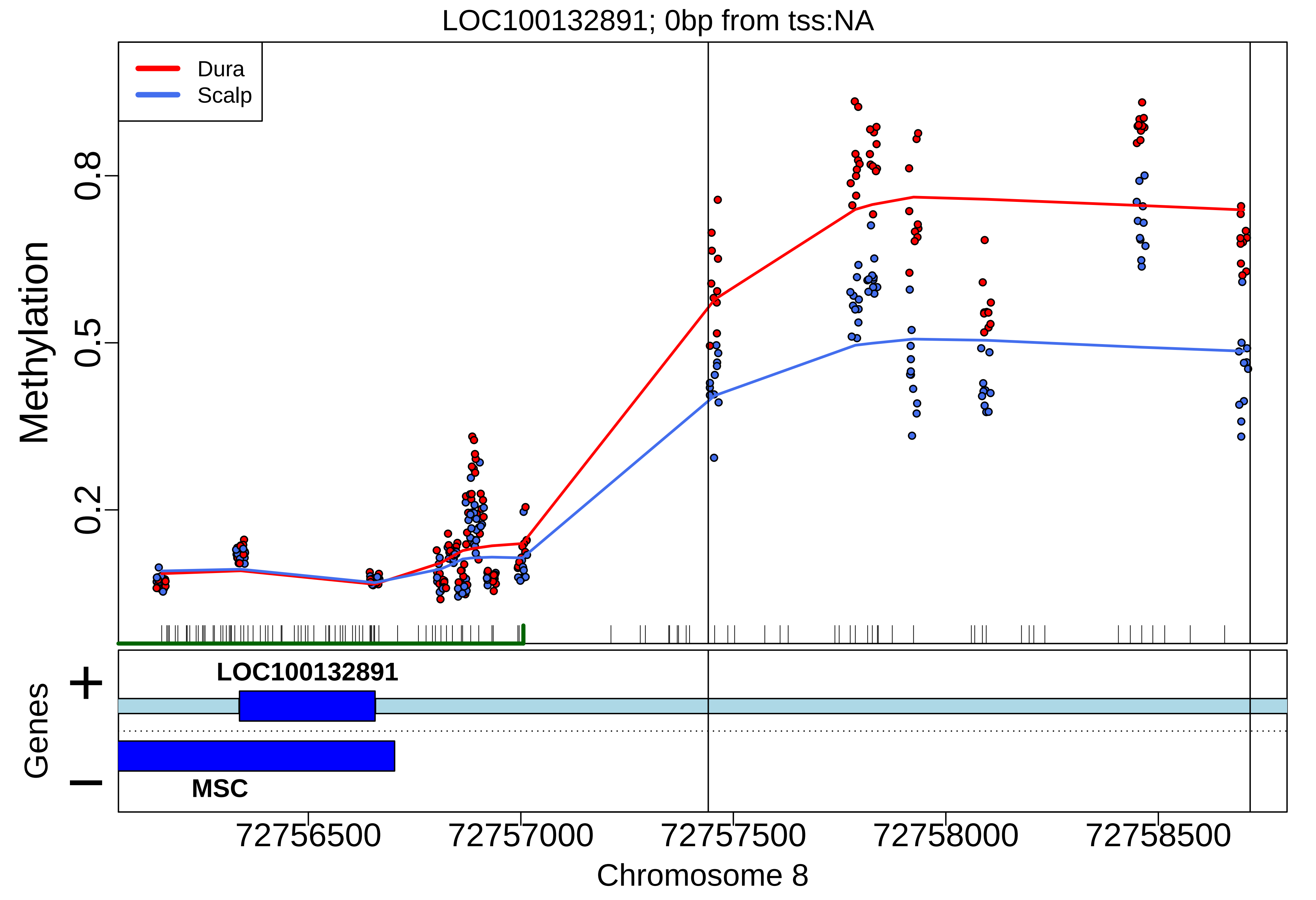

LOC100132891; 0bp from tss:NA
Dura
Scalp
Methylation
0.2
0.5
0.8
Genes
+
−
LOC100132891
MSC
72756500
72757000
72757500
72758000
72758500
Chromosome 8

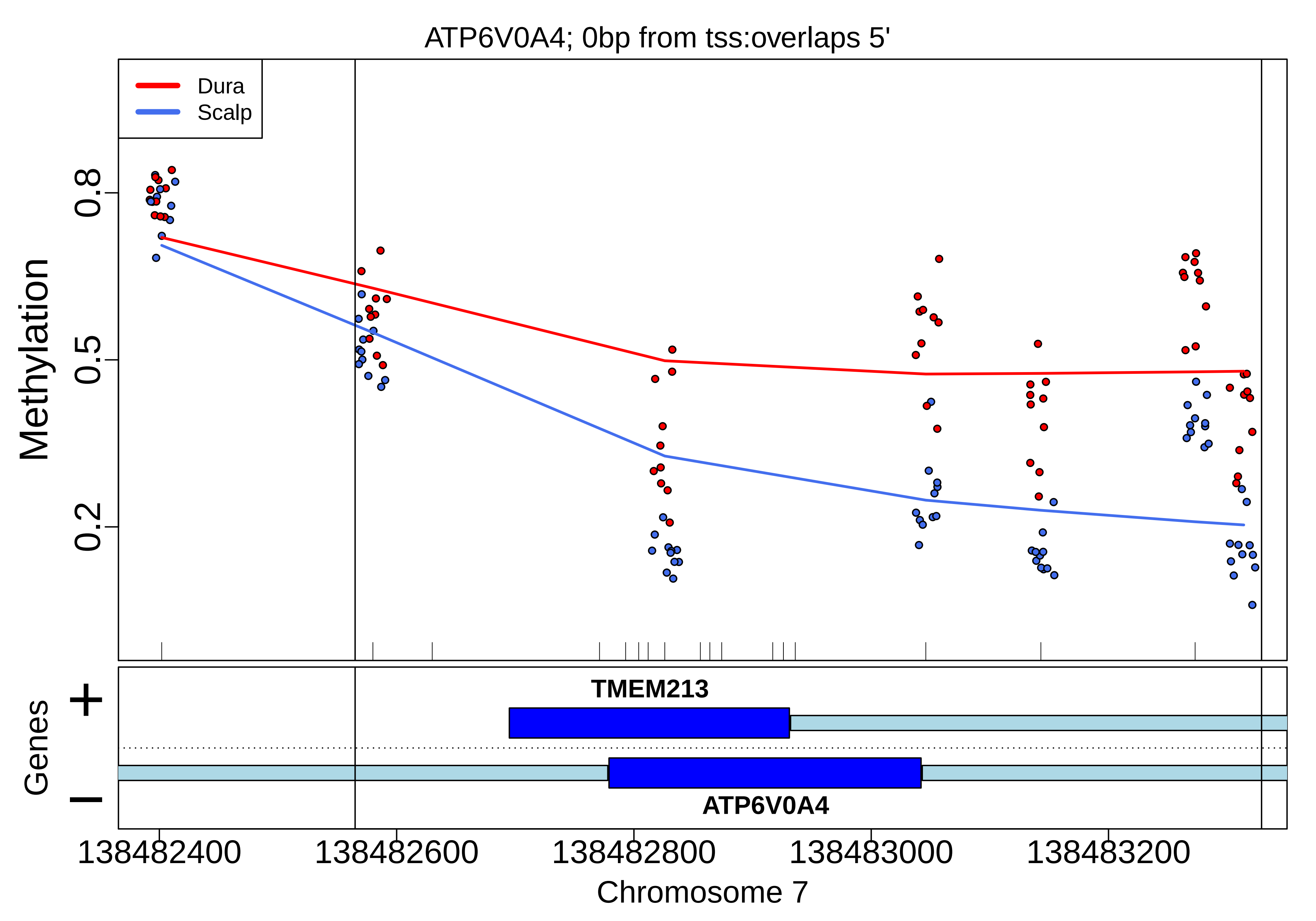

ATP6V0A4; 0bp from tss:overlaps 5'
Dura
Scalp
Methylation
0.2
0.5
0.8
Genes
+
−
TMEM213
ATP6V0A4
138482400
138482600
138482800
138483000
138483200
Chromosome 7

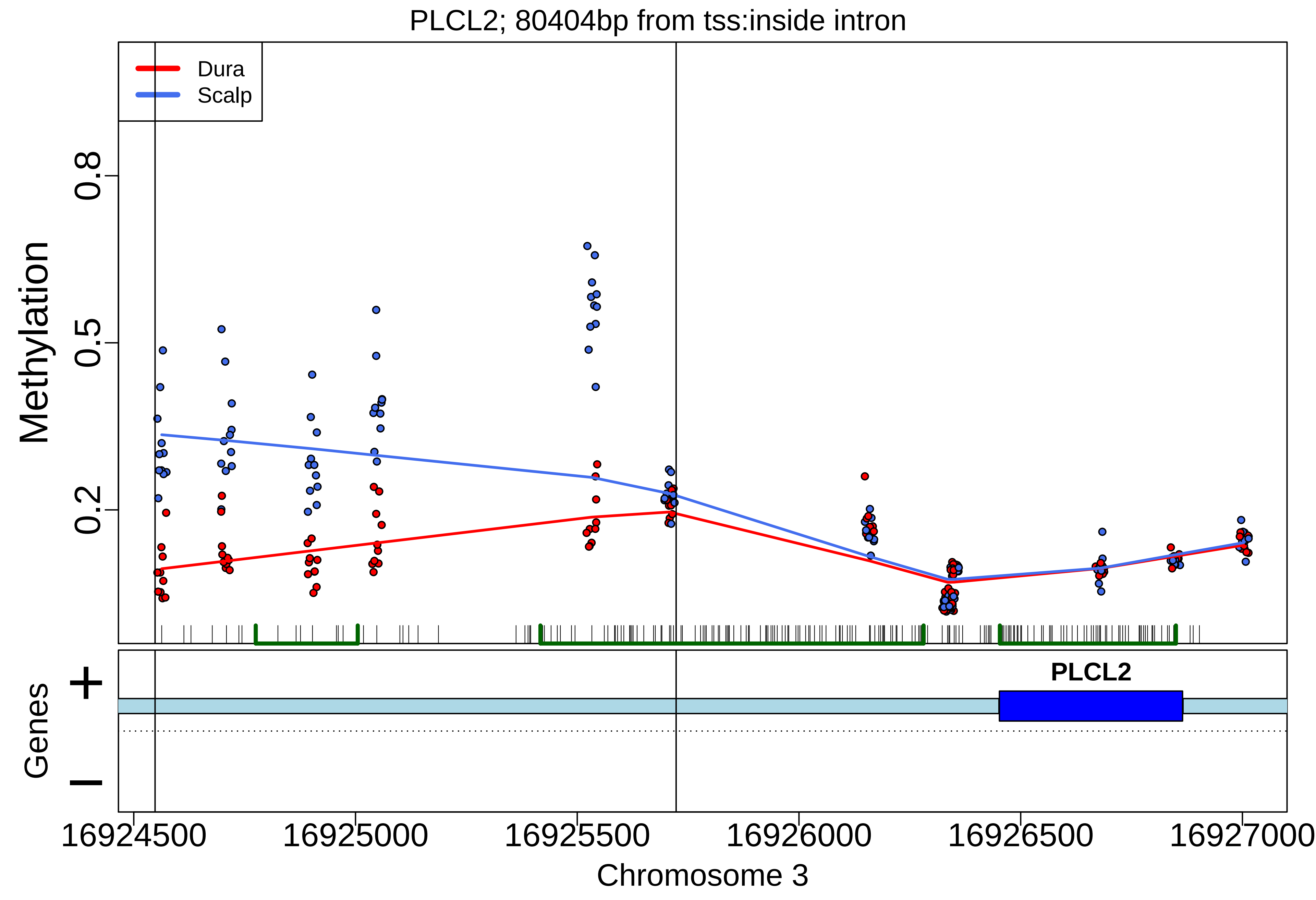
PLCL2; 80404bp from tss:inside intron
Dura
Scalp
Methylation
0.2
0.5
0.8
Genes
+
-
PLCL2
16924500
16925000
16925500
16926000
16926500
16927000
Chromosome 3

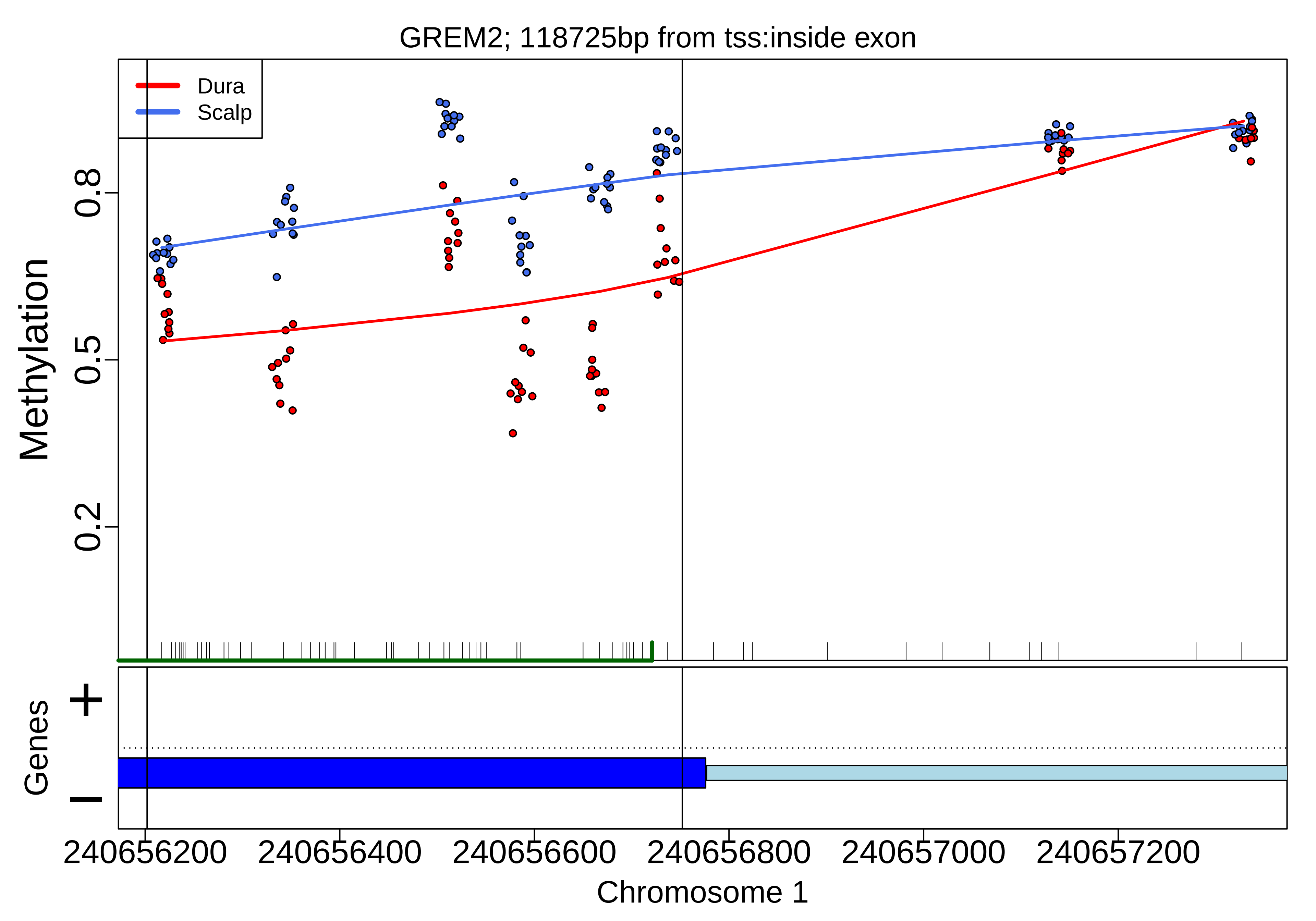
GREM2; 118725bp from tss:inside exon
Dura
Scalp
Methylation
0.2
0.5
0.8
Genes
+
−
240656200
240656400
240656600
240656800
240657000
240657200
Chromosome 1

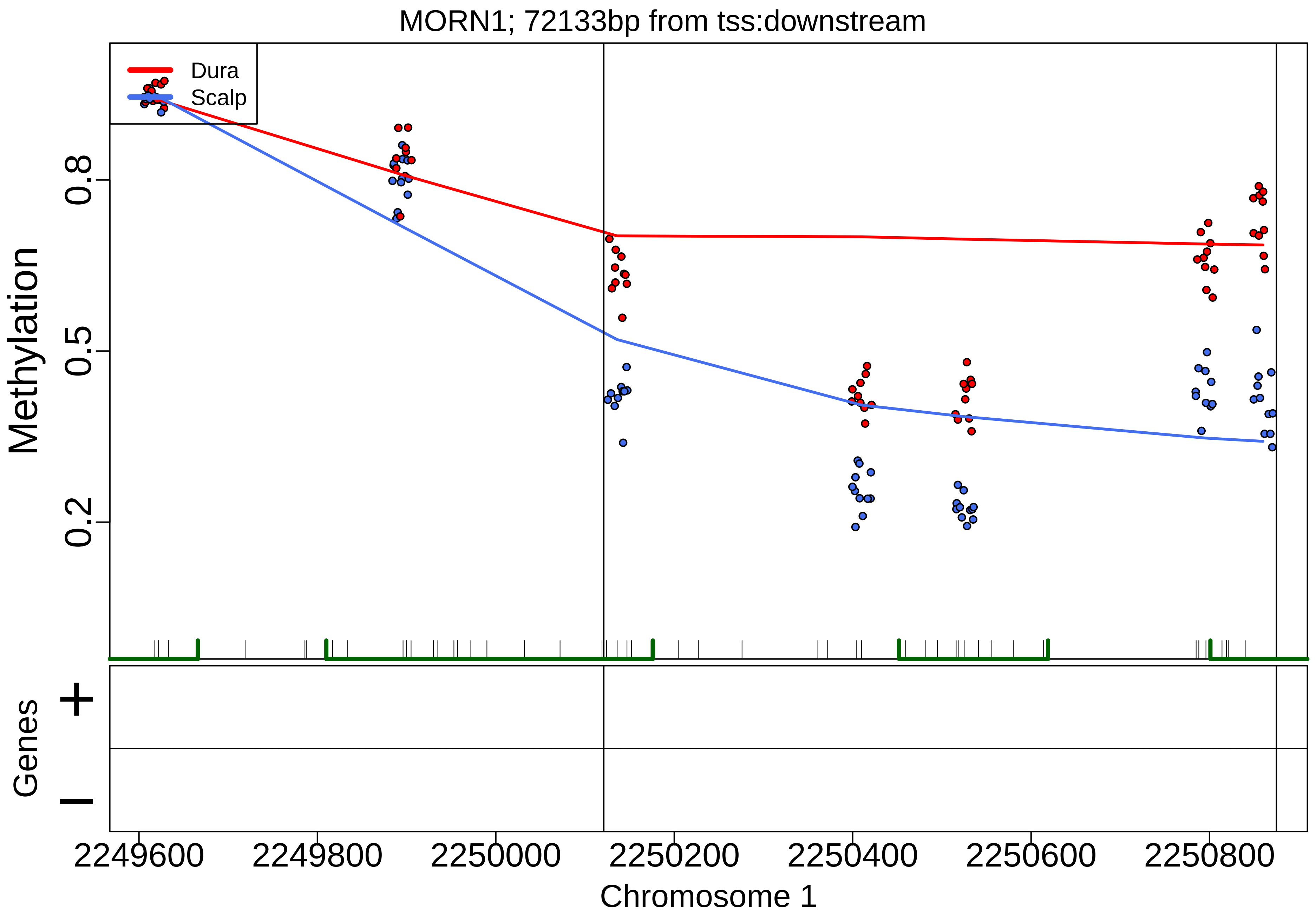
MORN1; 72133bp from tss:downstream
Dura
Scalp
Methylation
0.2
0.5
0.8
Genes
+
-
2249600
2249800
2250000
2250200
2250400
2250600
2250800
Chromosome 1

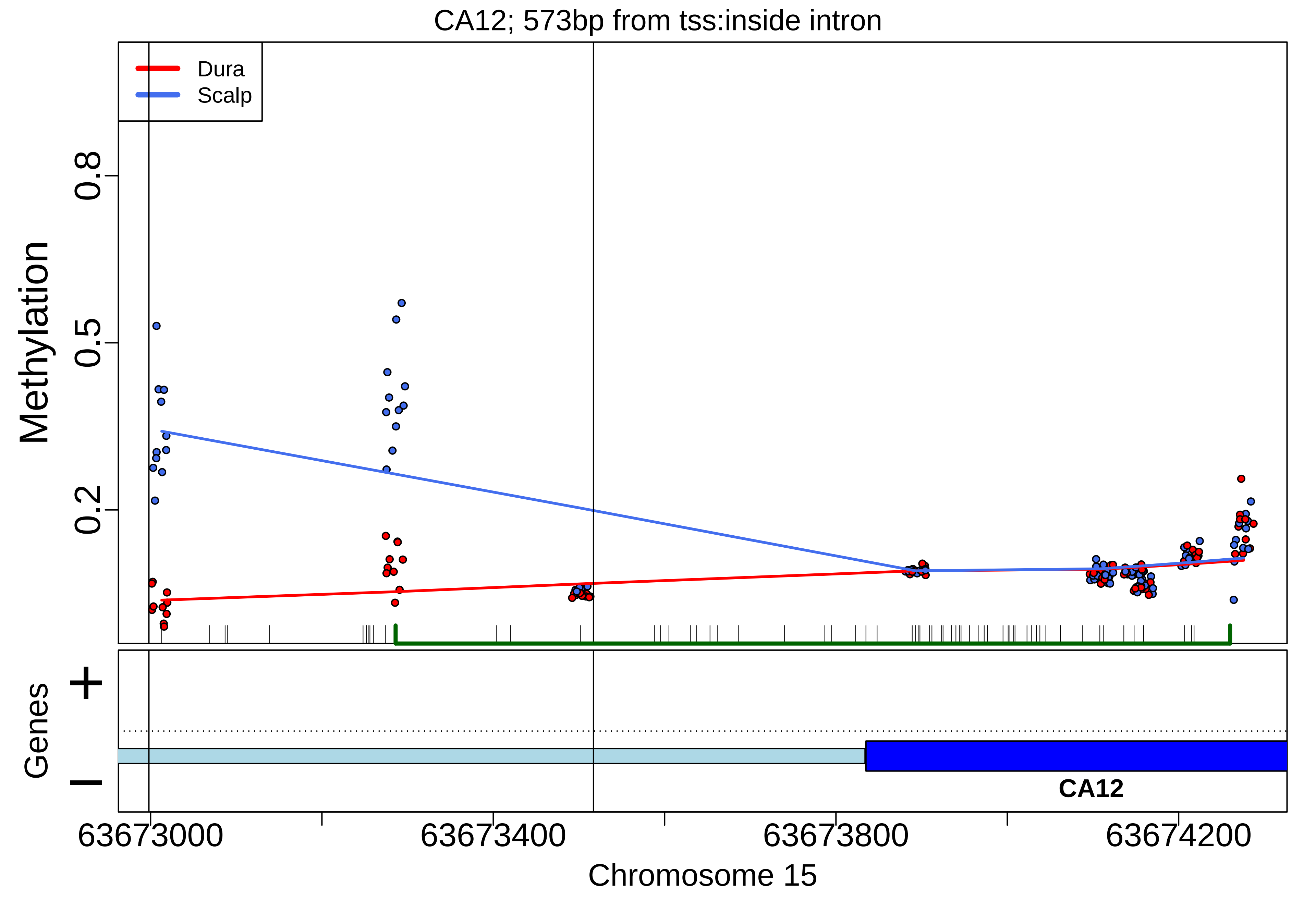
CA12; 573bp from tss:inside intron
Dura
Scalp
Methylation
0.8
0.5
0.2
Genes
+
–
CA12
63673000
63673400
63673800
63674200
Chromosome 15

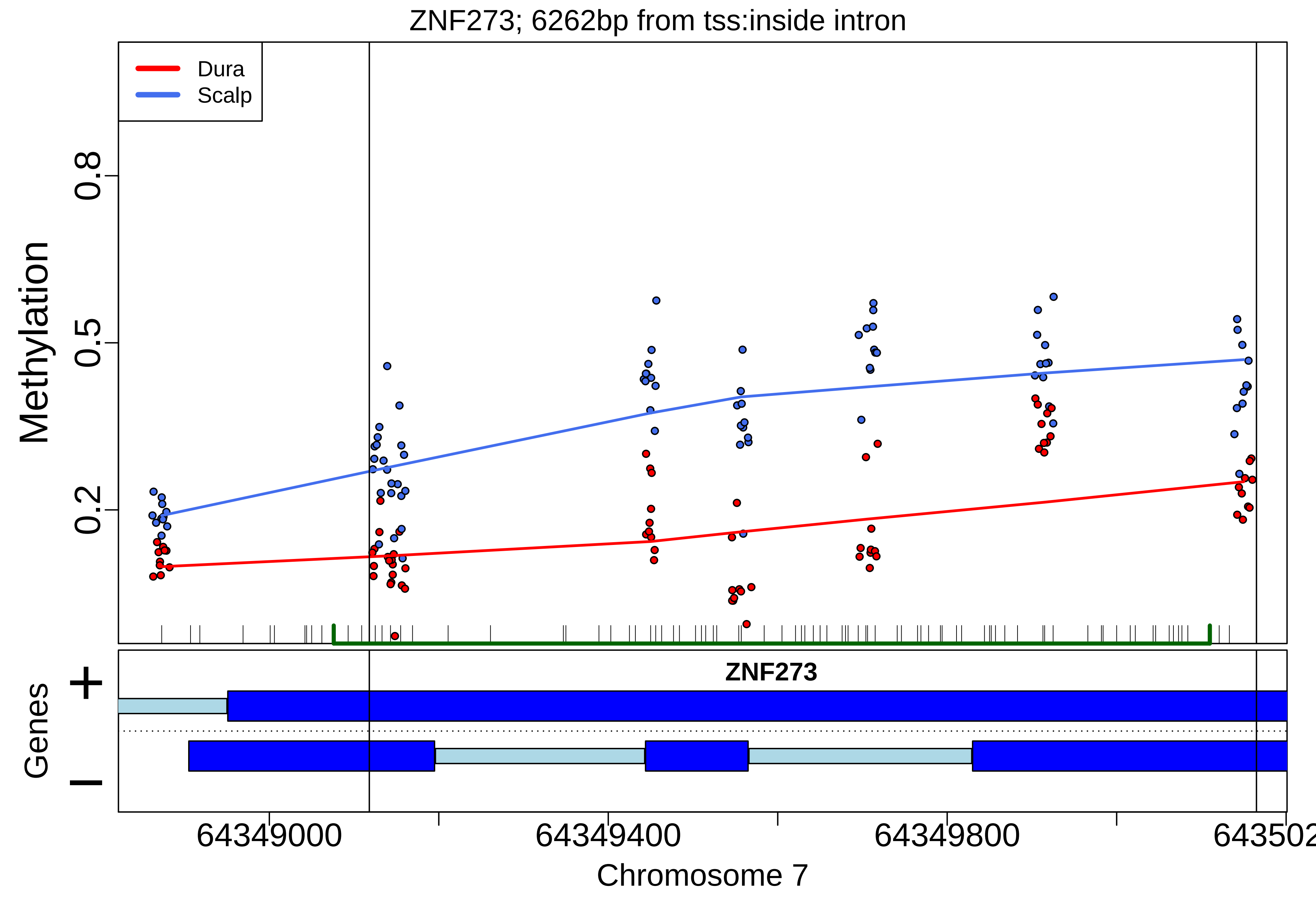

ZNF273; 6262bp from tss:inside intron
Dura
Scalp
Methylation
0.2
0.5
0.8
Genes
+
-
ZNF273
64349000
64349400
64349800
643502
Chromosome 7

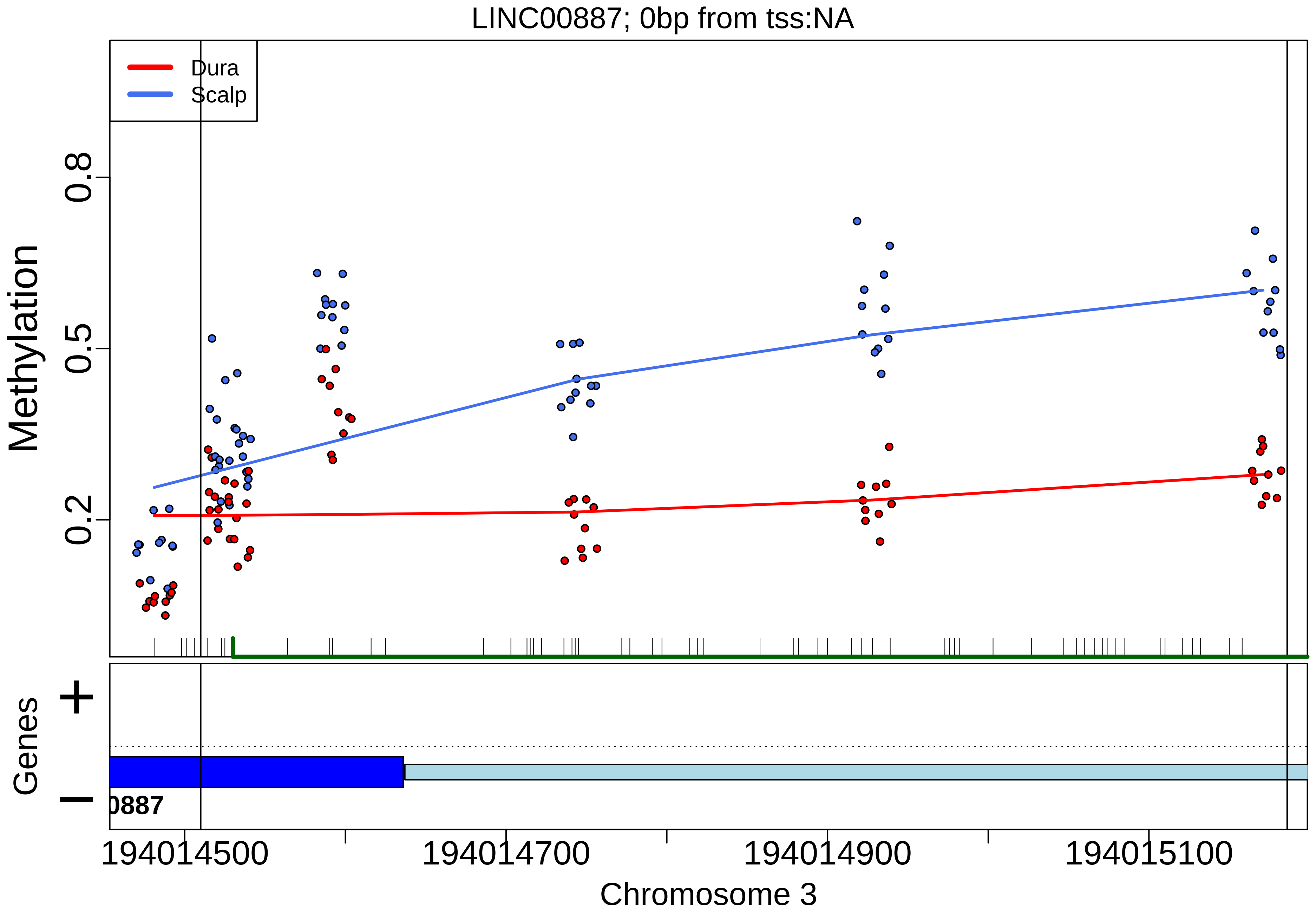

LINC00887; 0bp from tss:NA
Dura
Scalp
Methylation
0.2
0.5
0.8
Genes
+
–
0887
194014500
194014700
194014900
194015100
Chromosome 3

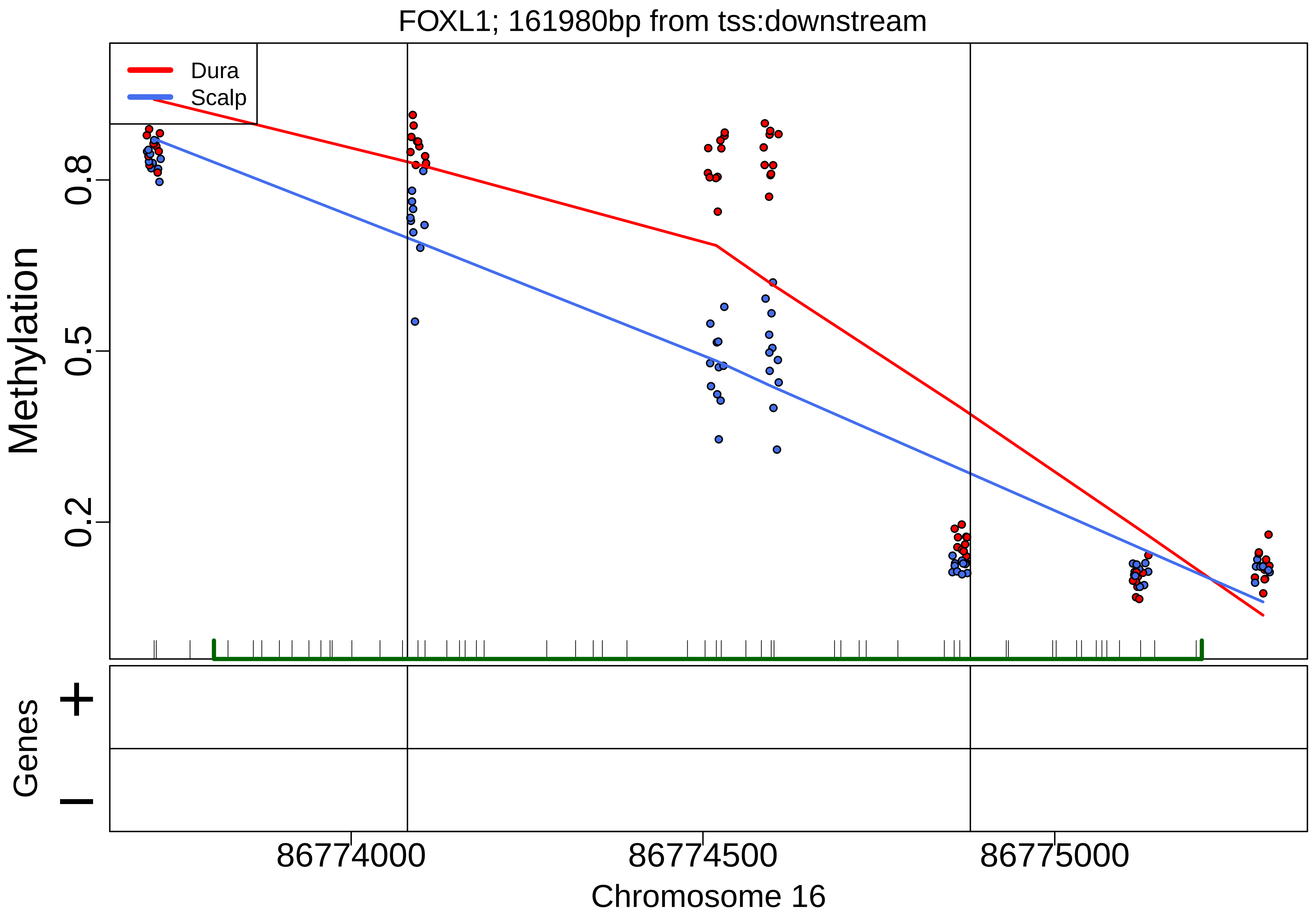
FOXL1; 161980bp from tss:downstream
Dura
Scalp
Methylation
0.2
0.5
0.8
Genes
+
-
86774000
86774500
86775000
Chromosome 16

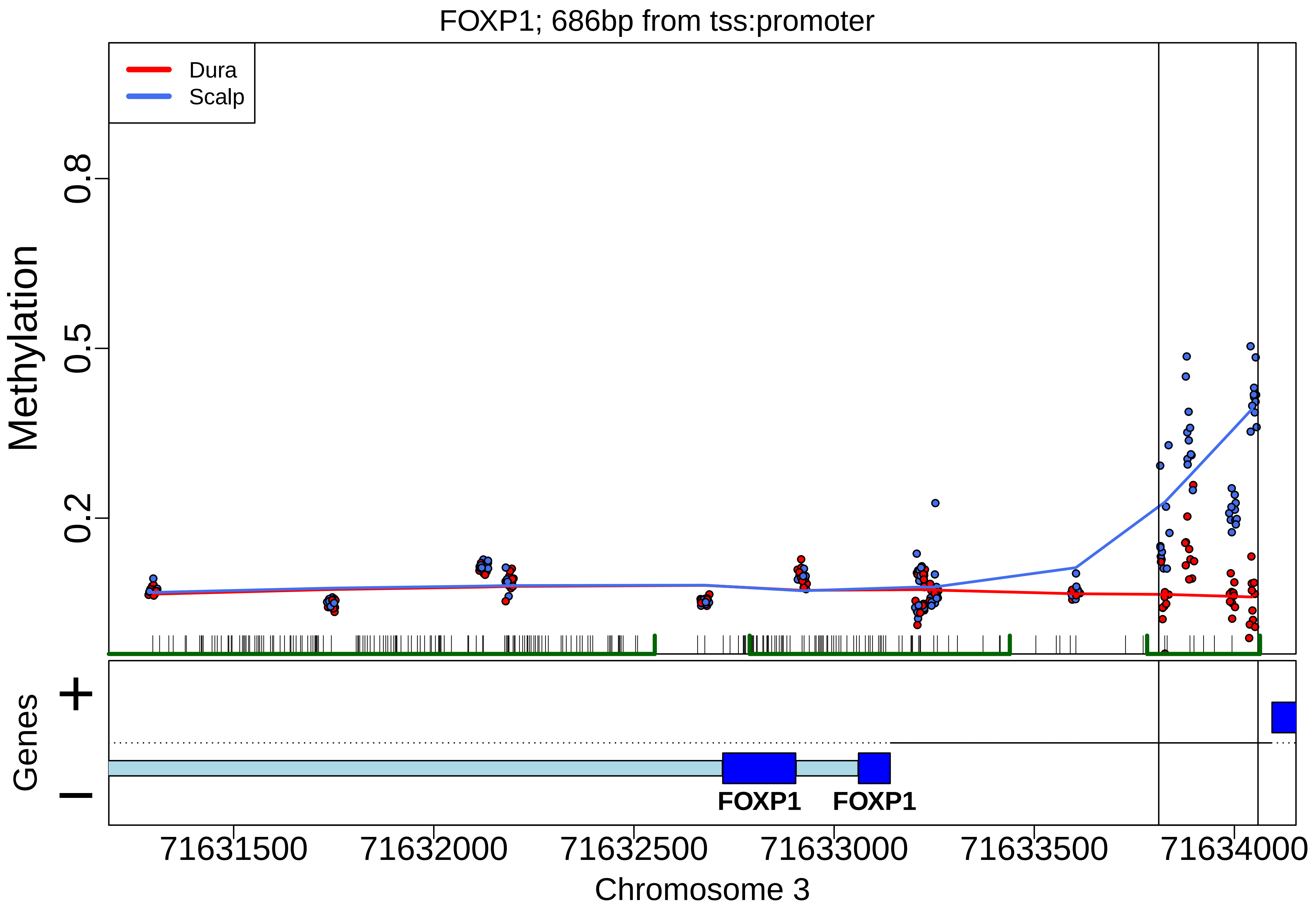
FOXP1; 686bp from tss:promoter
Dura
Scalp
Methylation
0.2
0.5
0.8
Genes
+
−
FOXP1
FOXP1
71631500
71632000
71632500
71633000
71633500
71634000
Chromosome 3

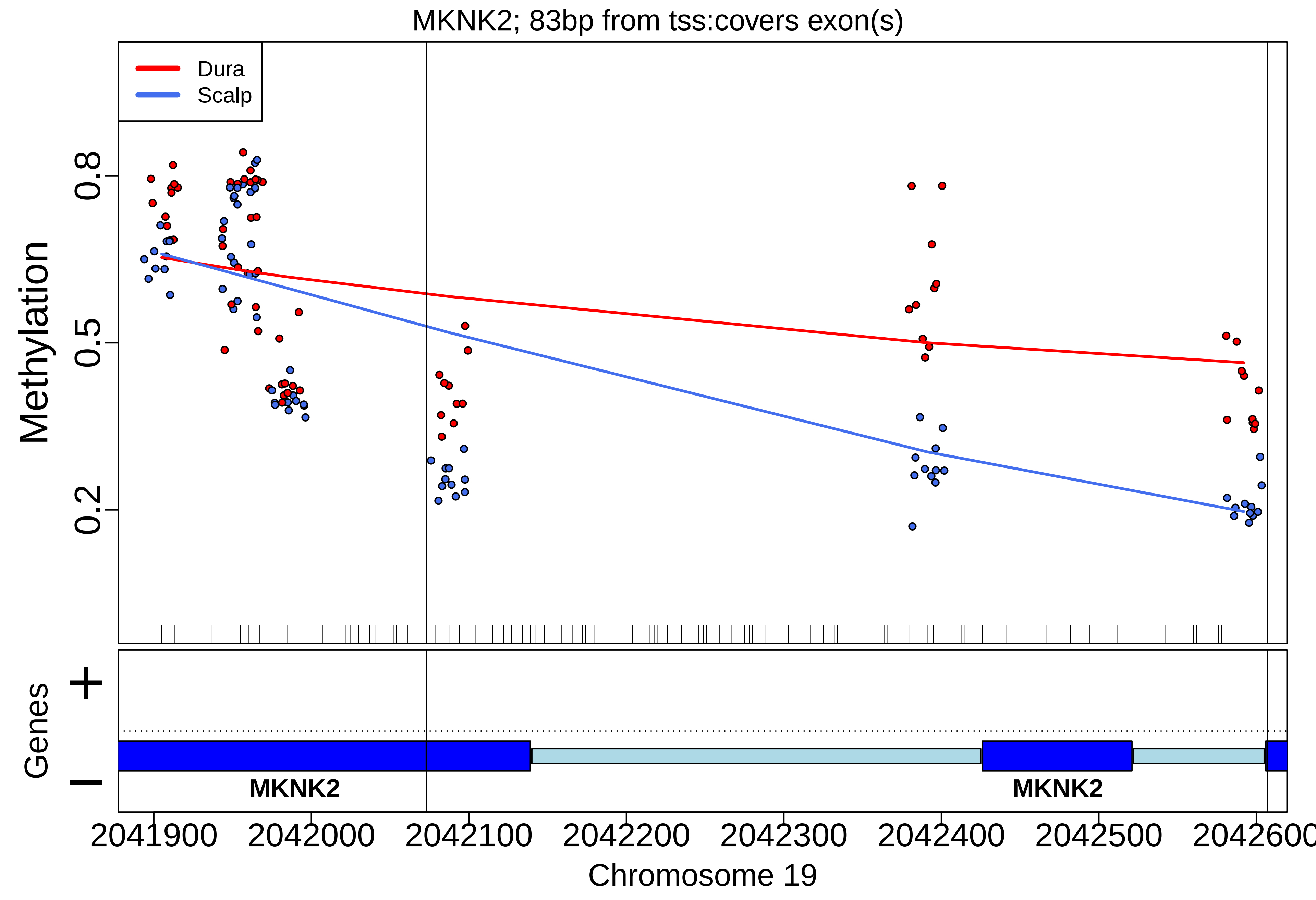

MKNK2; 83bp from tss:covers exon(s)
Dura
Scalp
Methylation
0.2
0.5
0.8
Genes
+
-
MKNK2
MKNK2
2041900
2042000
2042100
2042200
2042300
2042400
2042500
2042600
Chromosome 19

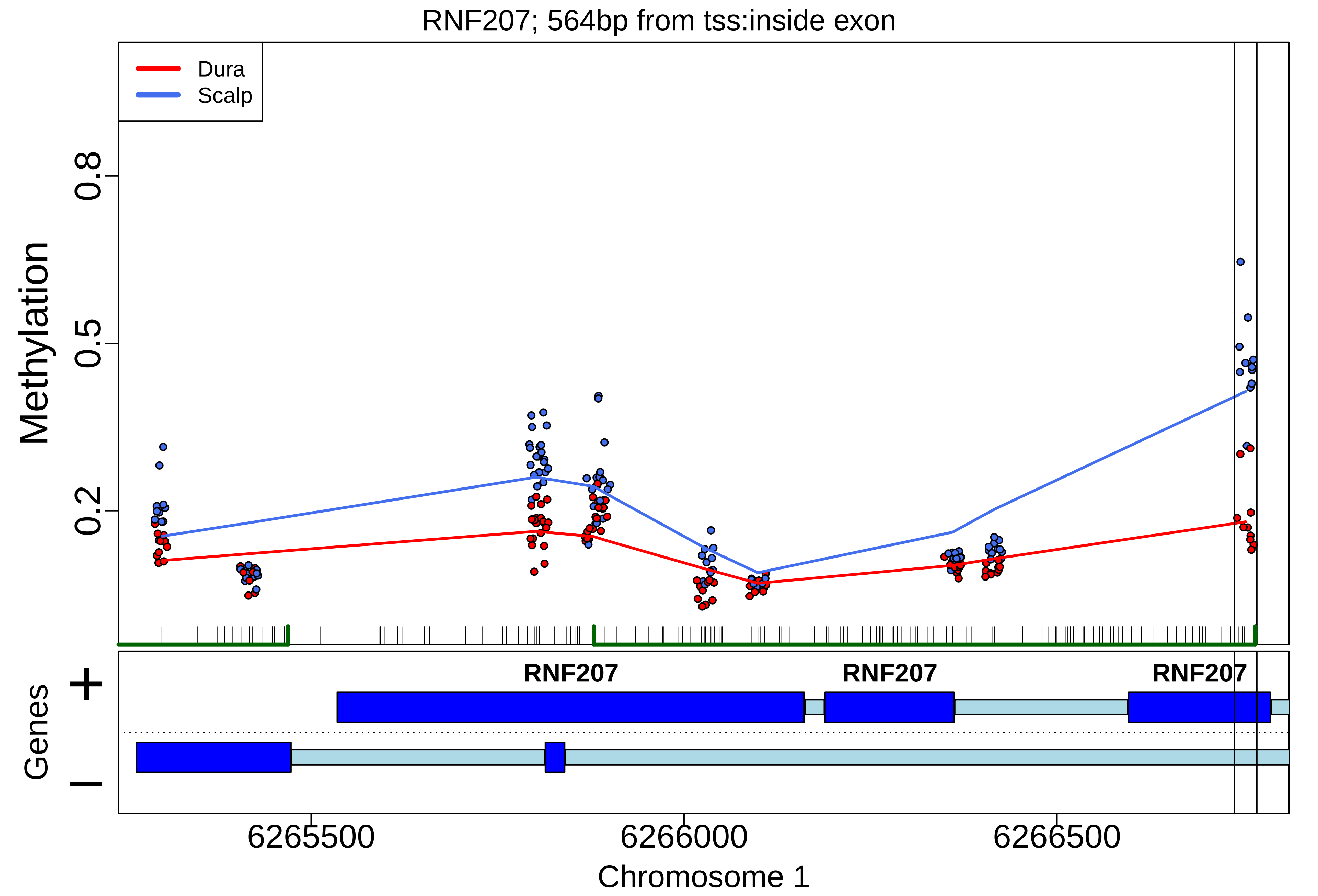
RNF207; 564bp from tss:inside exon
Dura
Scalp
Methylation
0.2
0.5
0.8
Genes
+
-
RNF207
RNF207
RNF207
6265500
6266000
6266500
Chromosome 1

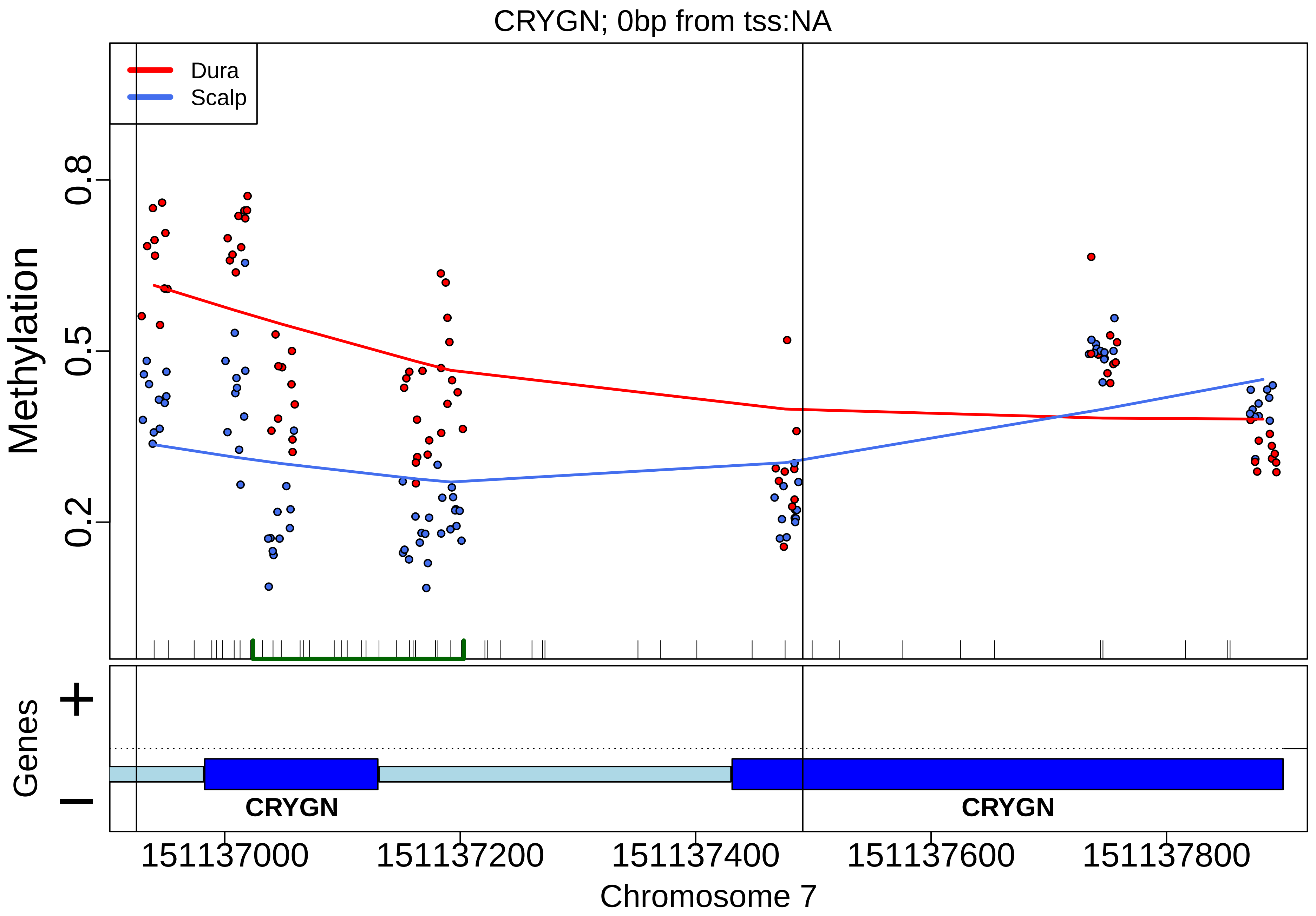
CRYGN; 0bp from tss:NA
Dura
Scalp
Methylation
0.8
0.5
0.2
Genes
+
-
CRYGN
CRYGN
151137000
151137200
151137400
151137600
151137800
Chromosome 7

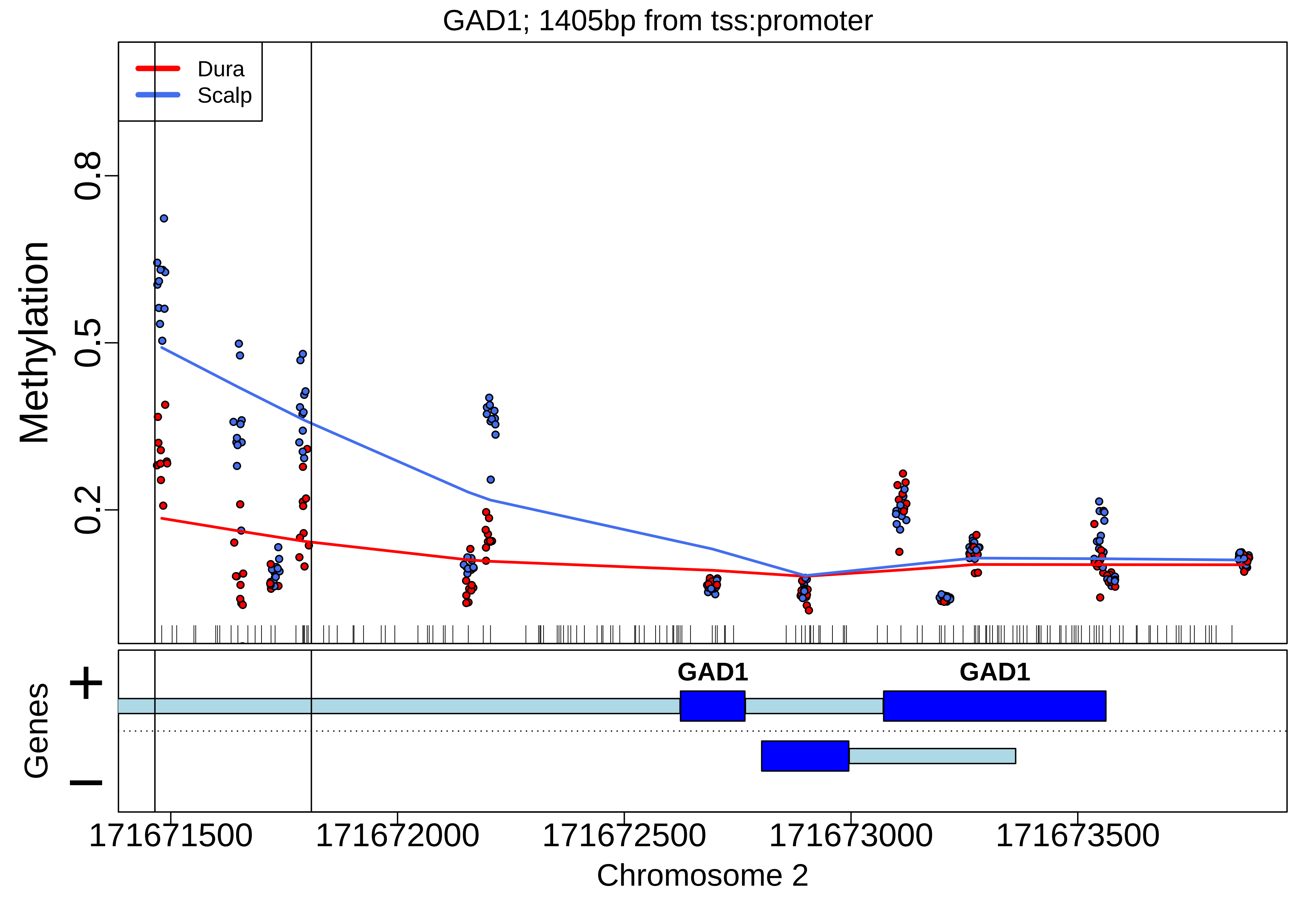
GAD1; 1405bp from tss:promoter
Dura
Scalp
Methylation
0.2
0.5
0.8
Genes
+
–
GAD1
GAD1
171671500
171672000
171672500
171673000
171673500
Chromosome 2

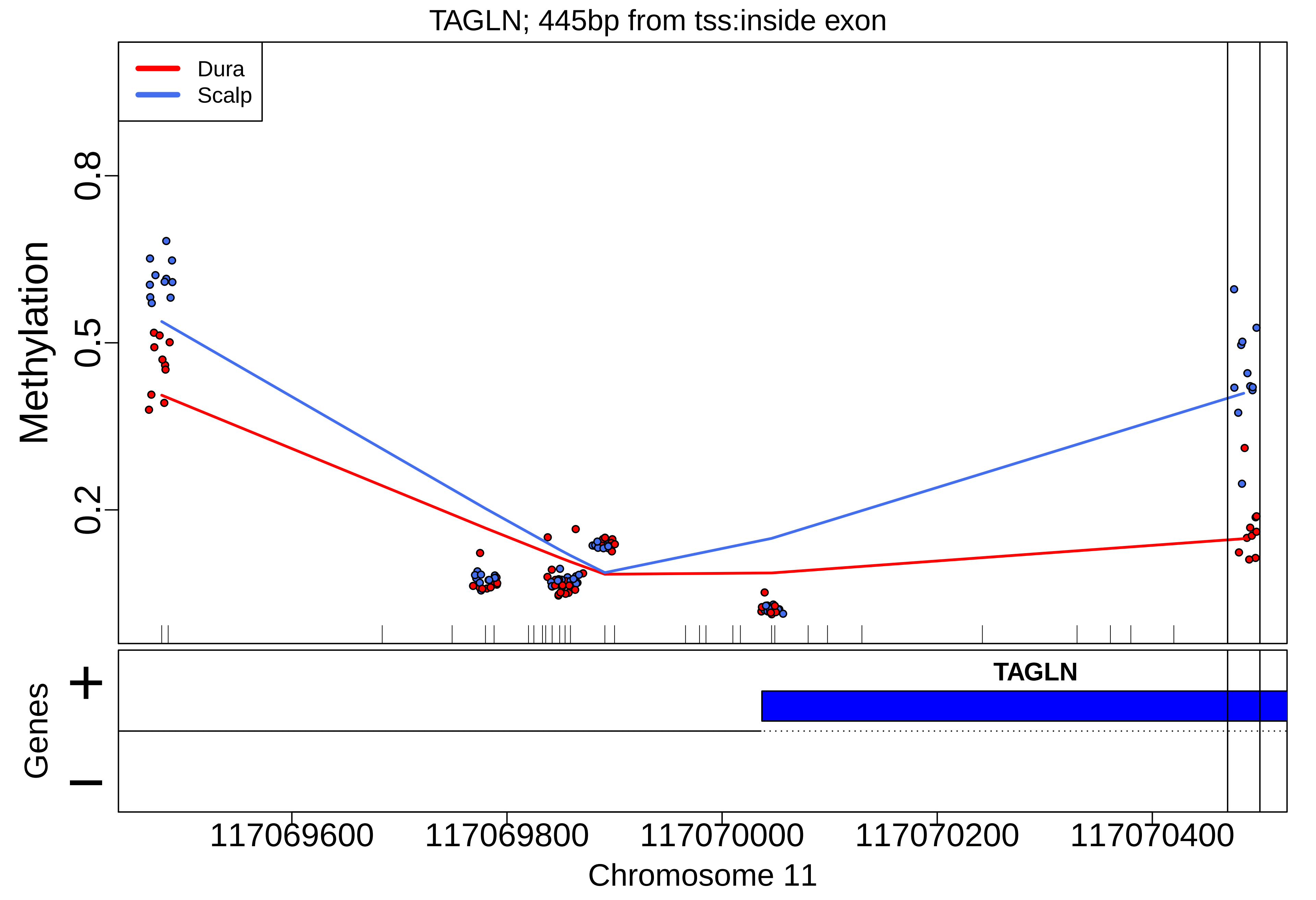
TAGLN; 445bp from tss:inside exon
Dura
Scalp
Methylation
0.2
0.5
0.8
Genes
+
−
TAGLN
117069600
117069800
117070000
117070200
117070400
Chromosome 11

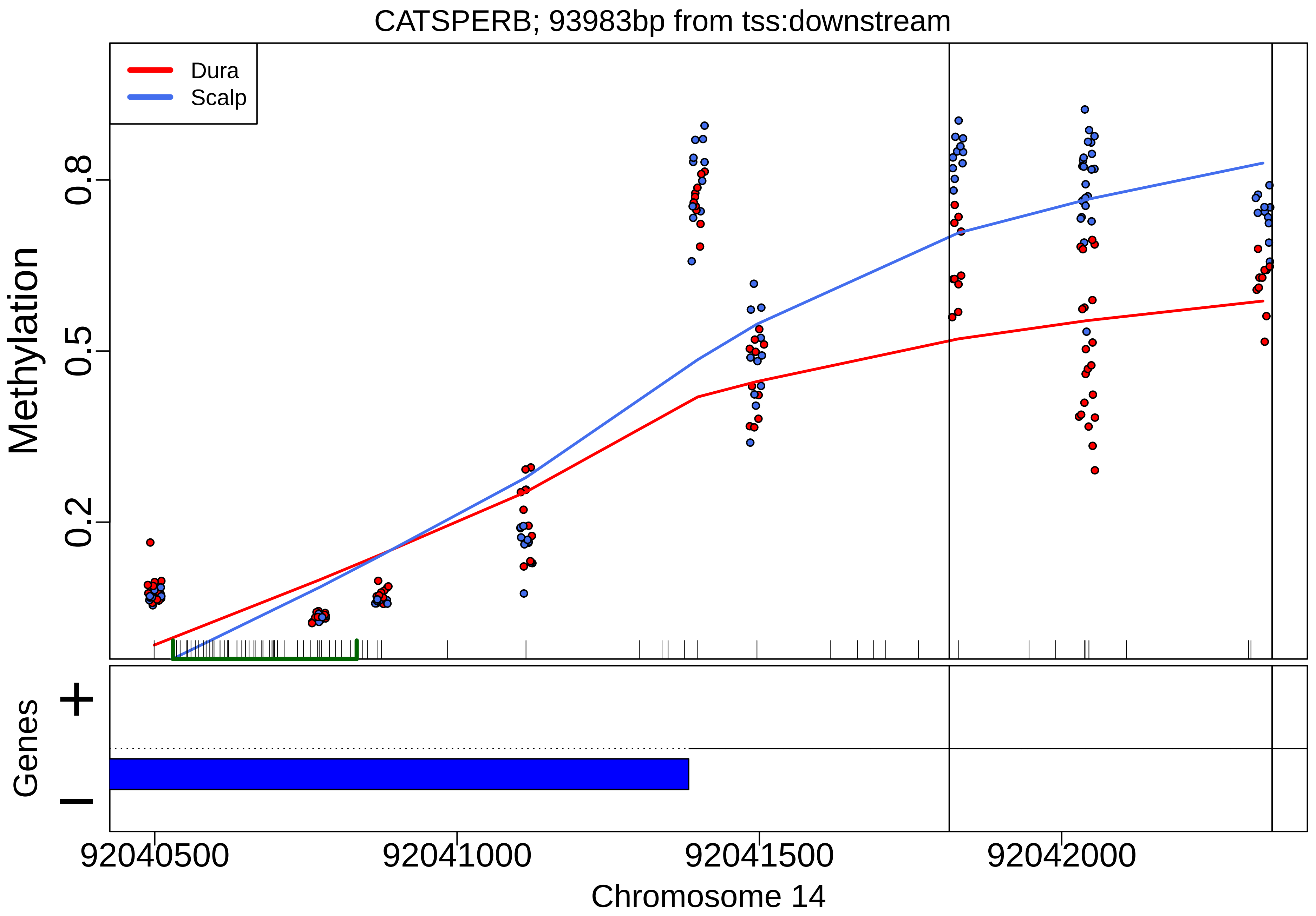

CATSPERB; 93983bp from tss:downstream
Dura
Scalp
Methylation
0.2
0.5
0.8
Genes
+
−
92040500
92041000
92041500
92042000
Chromosome 14

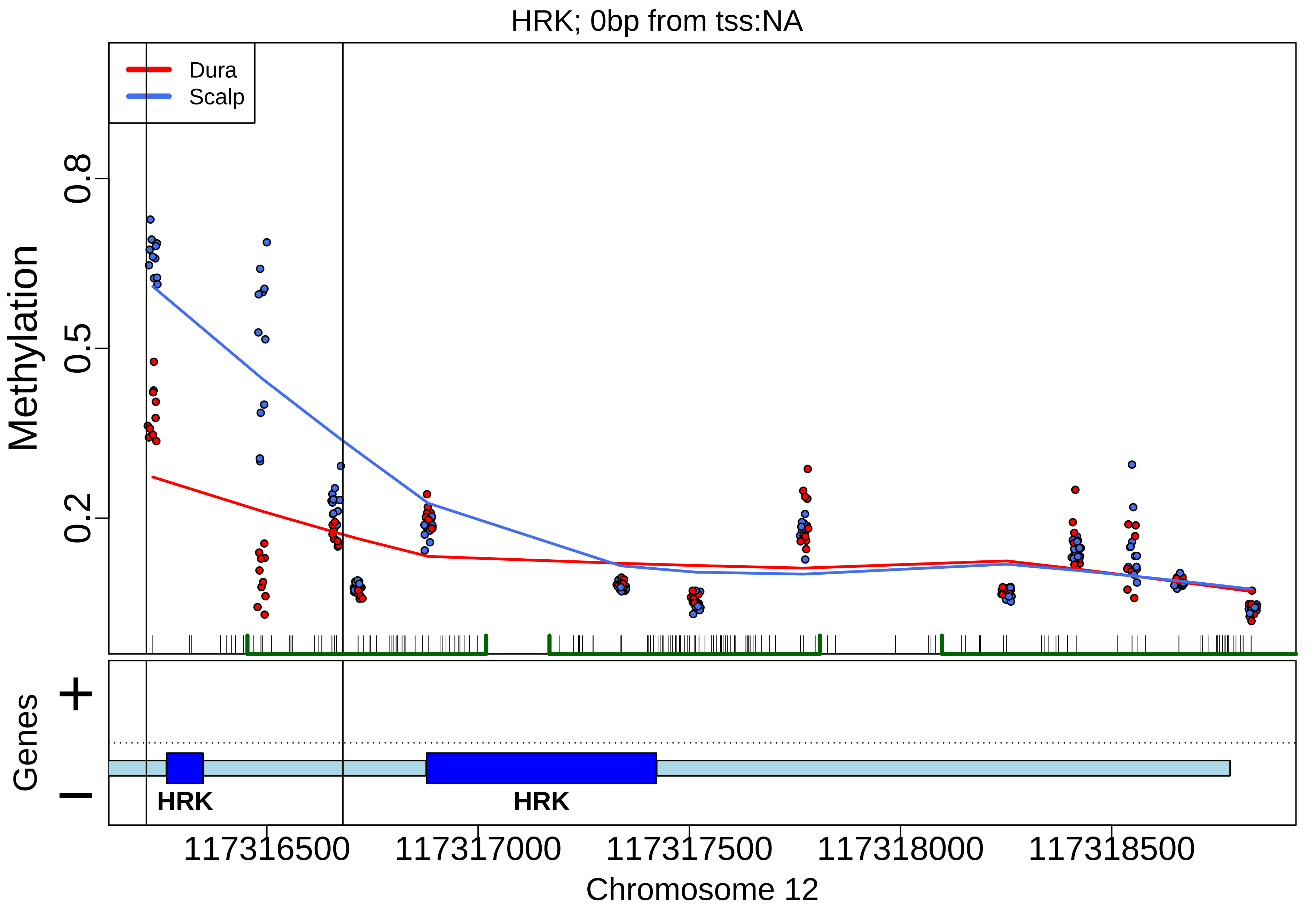
HRK; 0bp from tss:NA
Dura
Scalp
Methylation
0.2
0.5
0.8
Genes
+
-
HRK
HRK
117316500
117317000
117317500
117318000
117318500
Chromosome 12

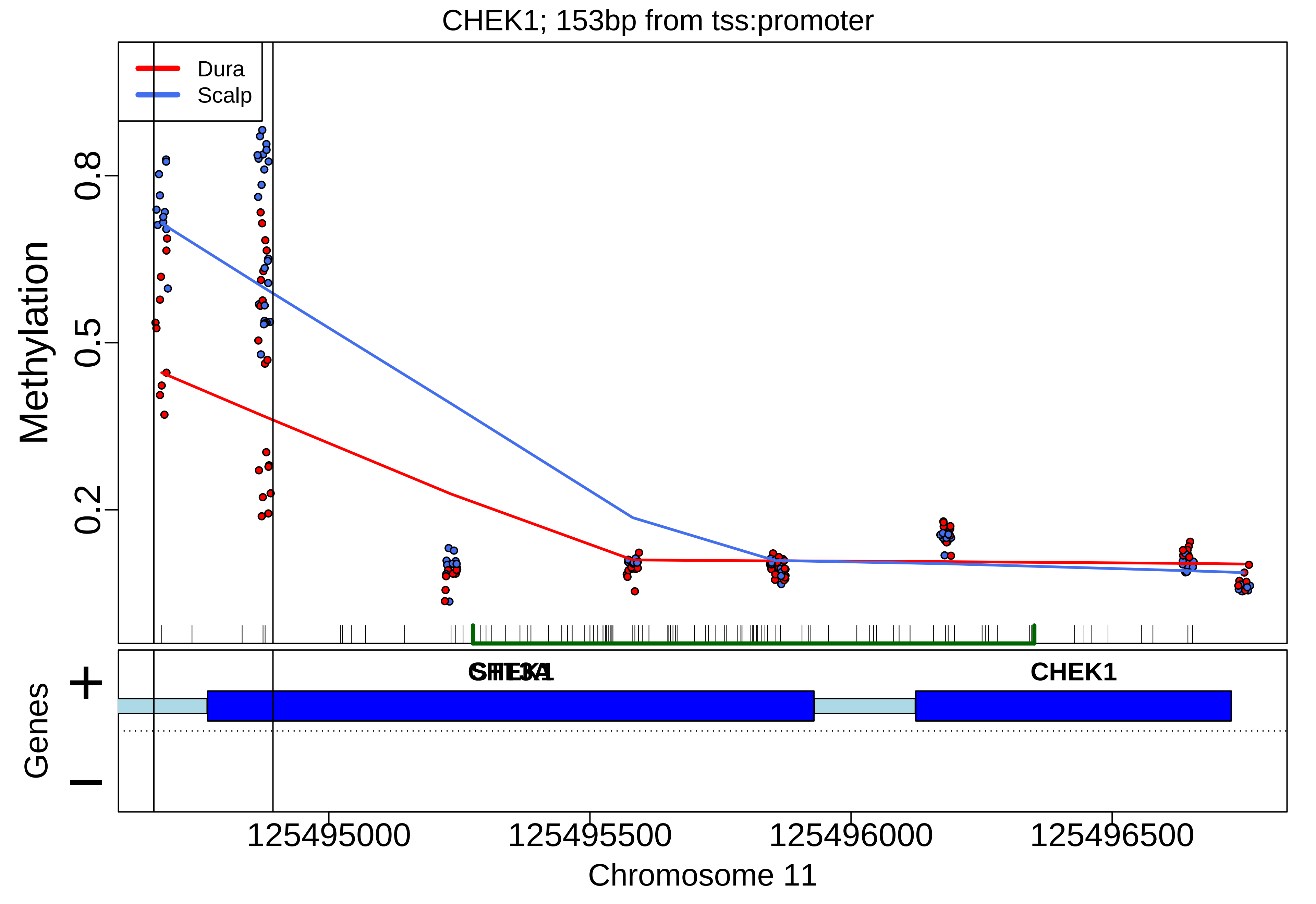

CHEK1; 153bp from tss:promoter
Dura
Scalp
Methylation
0.2
0.5
0.8
Genes
+
-
CHEK1
STT3A
CHEK1
125495000
125495500
125496000
125496500
Chromosome 11

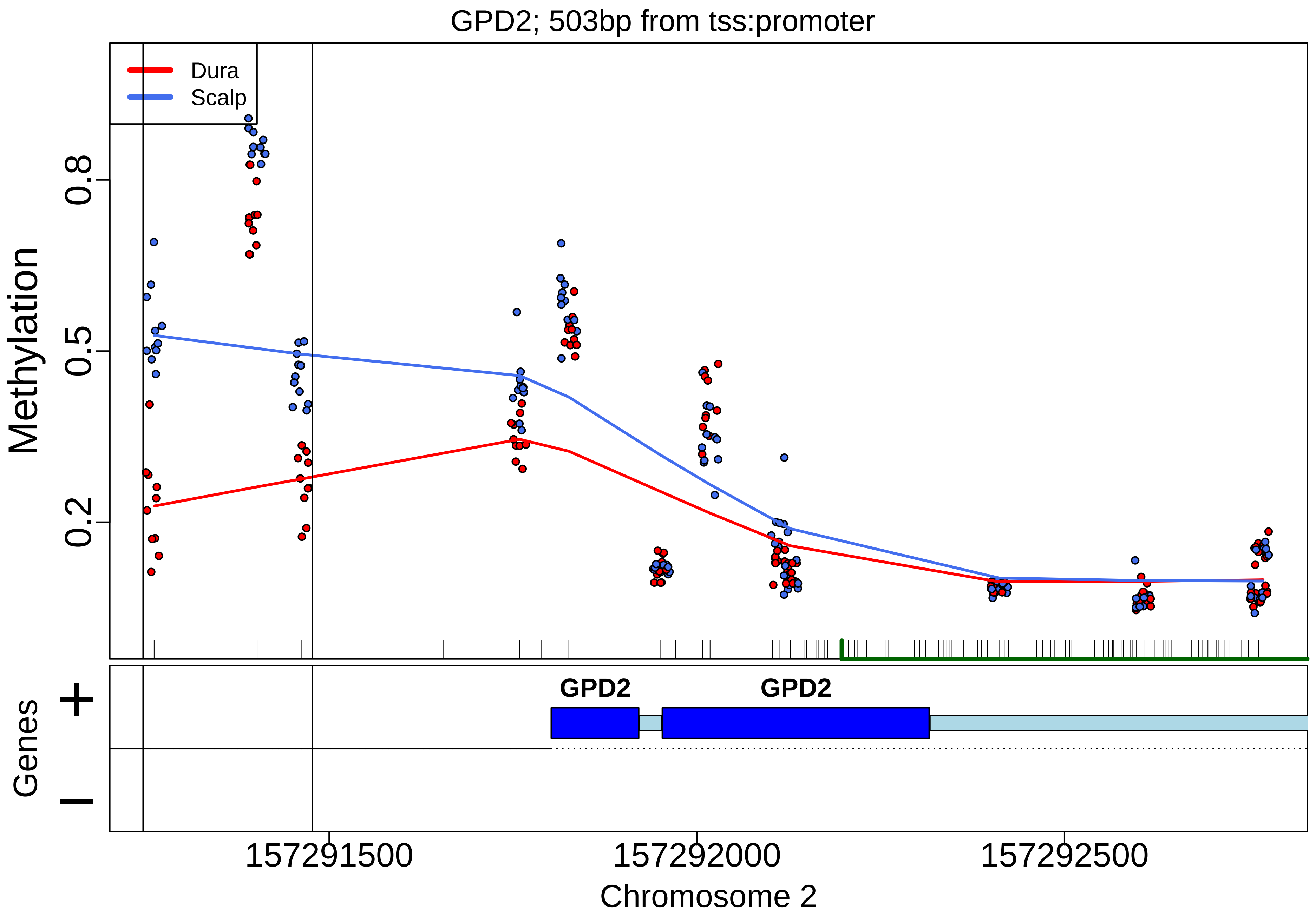
GPD2; 503bp from tss:promoter
Dura
Scalp
Methylation
0.2
0.5
0.8
Genes
+
-
GPD2
GPD2
157291500
157292000
157292500
Chromosome 2

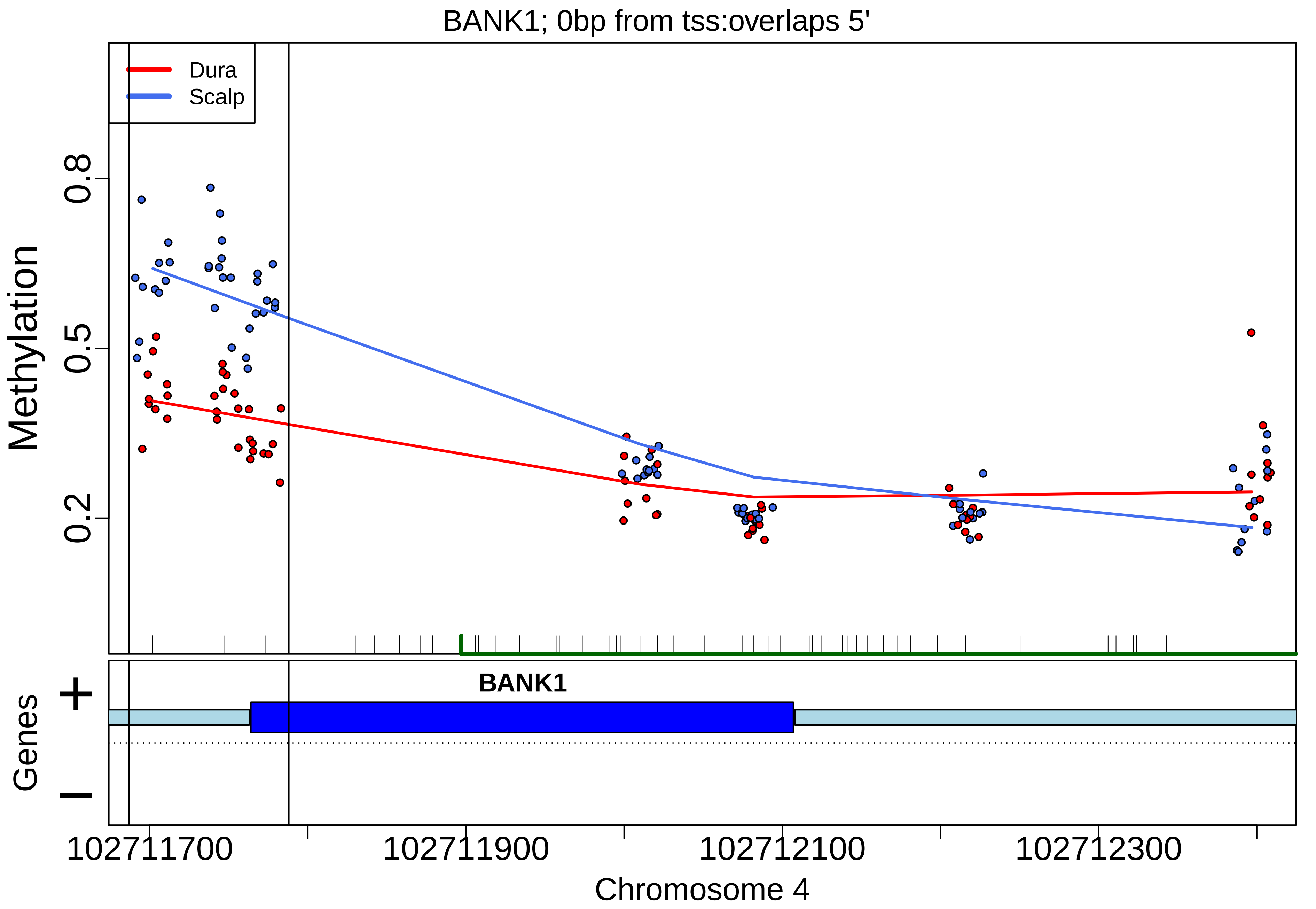
BANK1; 0bp from tss:overlaps 5'
Dura
Scalp
Methylation
0.2
0.5
0.8
Genes
+
-
BANK1
102711700
102711900
102712100
102712300
Chromosome 4

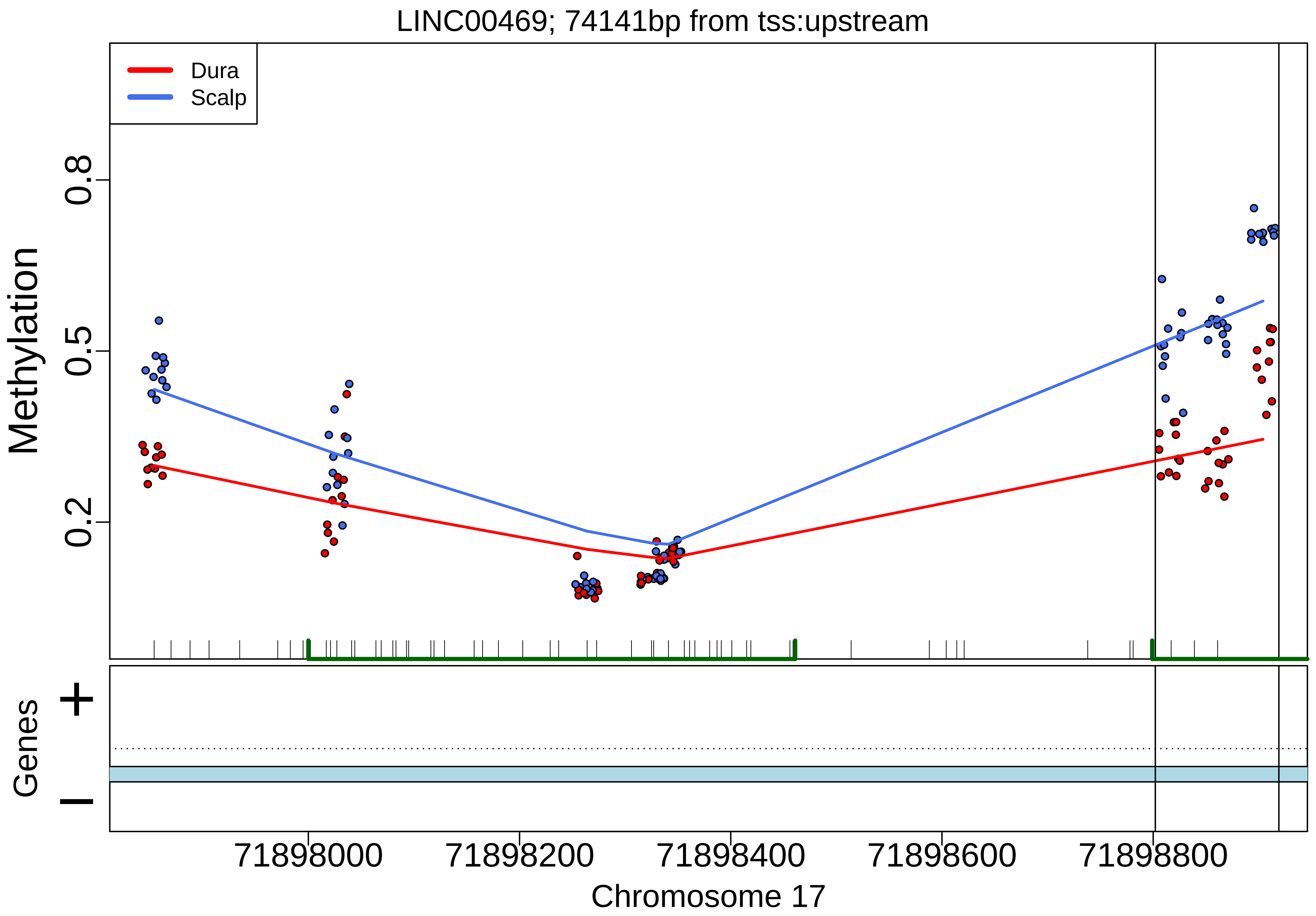

LINC00469; 74141bp from tss:upstream
Dura
Scalp
Methylation
0.2
0.5
0.8
Genes
+
-
71898000
71898200
71898400
71898600
71898800
Chromosome 17

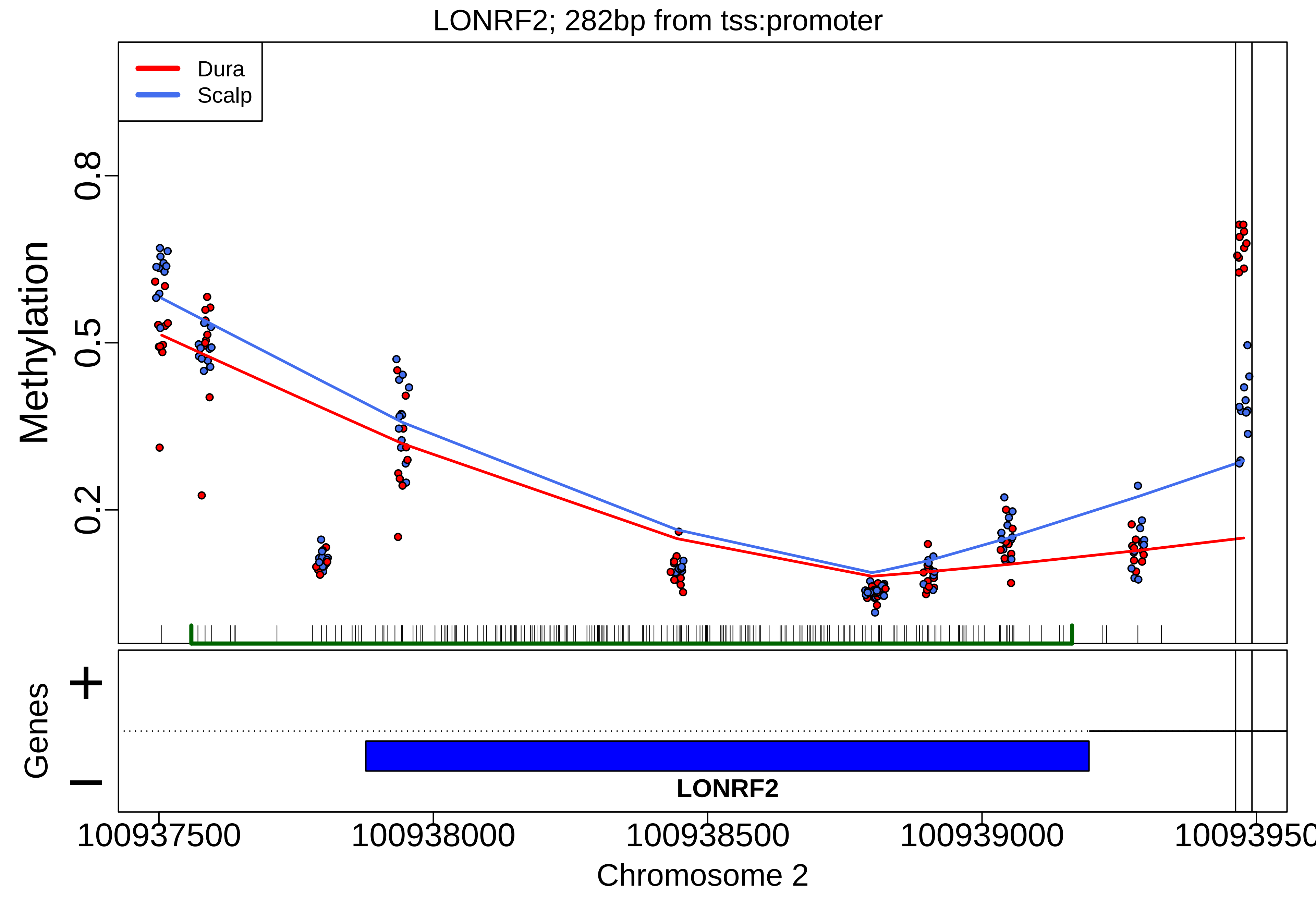
LONRF2; 282bp from tss:promoter
Dura
Scalp
Methylation
0.2
0.5
0.8
Genes
+
-
LONRF2
100937500
100938000
100938500
100939000
10093950
Chromosome 2

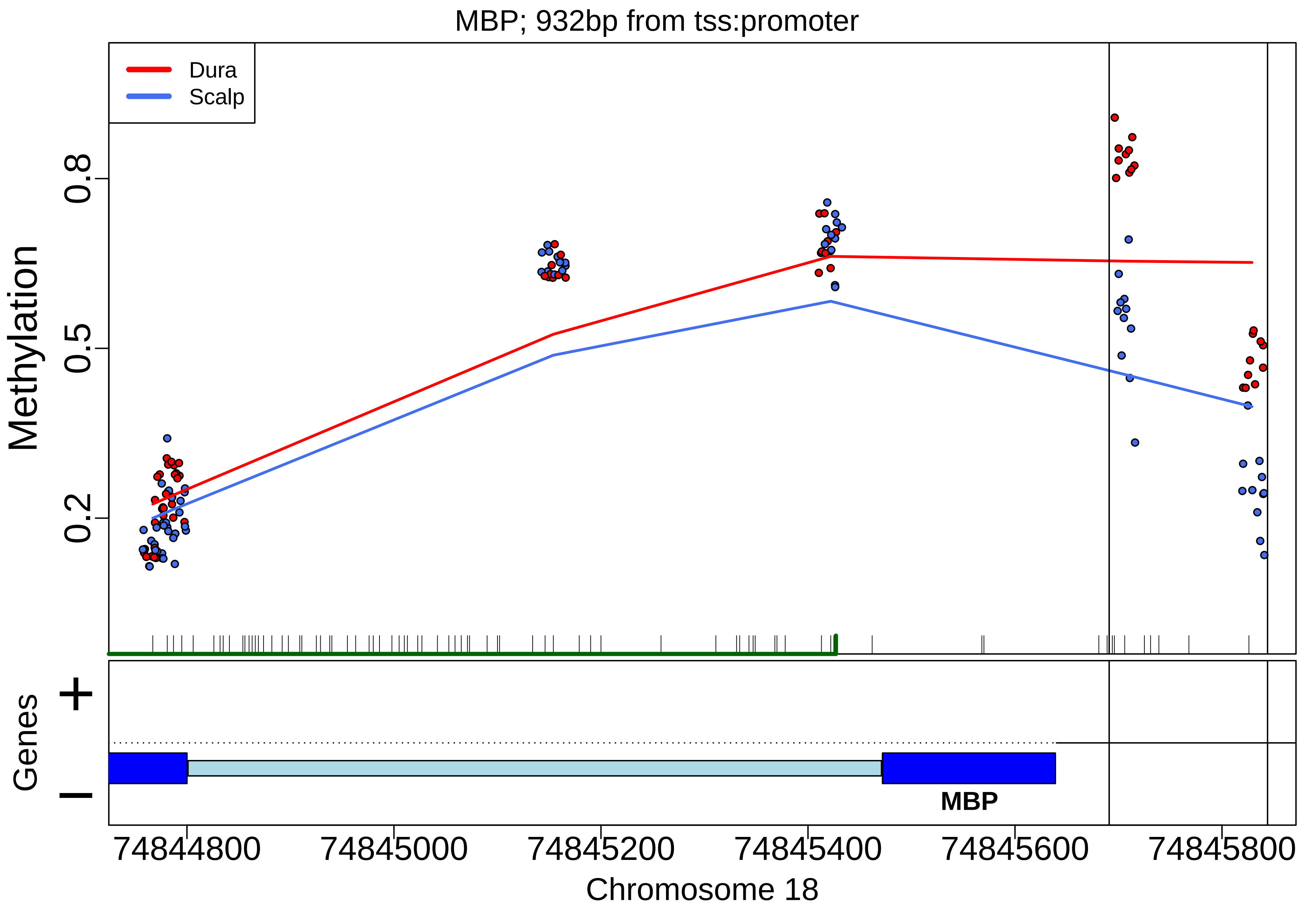

MBP; 932bp from tss:promoter
Dura
Scalp
Methylation
0.2
0.5
0.8
Genes
+
−
MBP
74844800
74845000
74845200
74845400
74845600
74845800
Chromosome 18

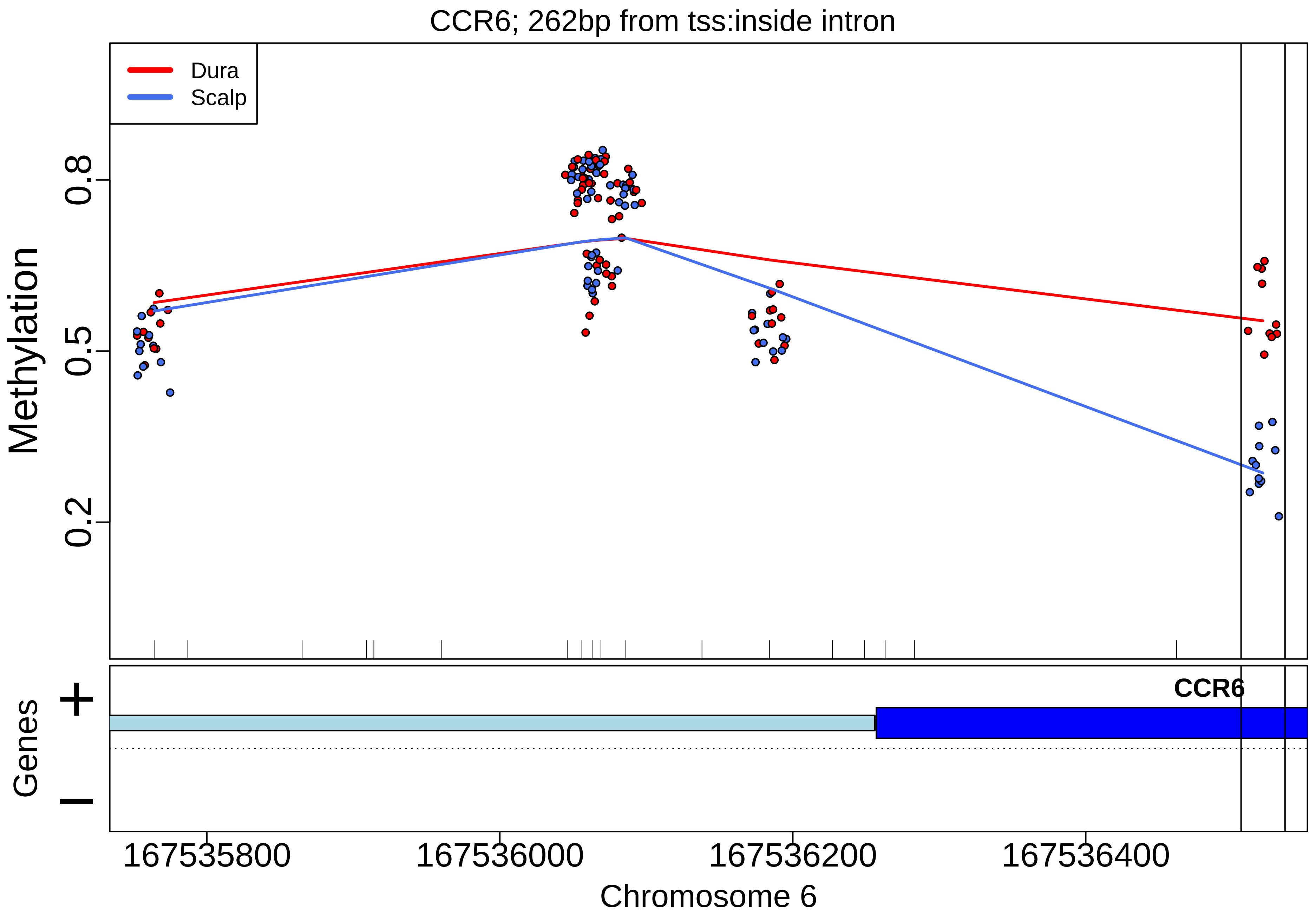
CCR6; 262bp from tss:inside intron
Dura
Scalp
Methylation
0.2
0.5
0.8
Genes
+
–
CCR6
167535800
167536000
167536200
167536400
Chromosome 6

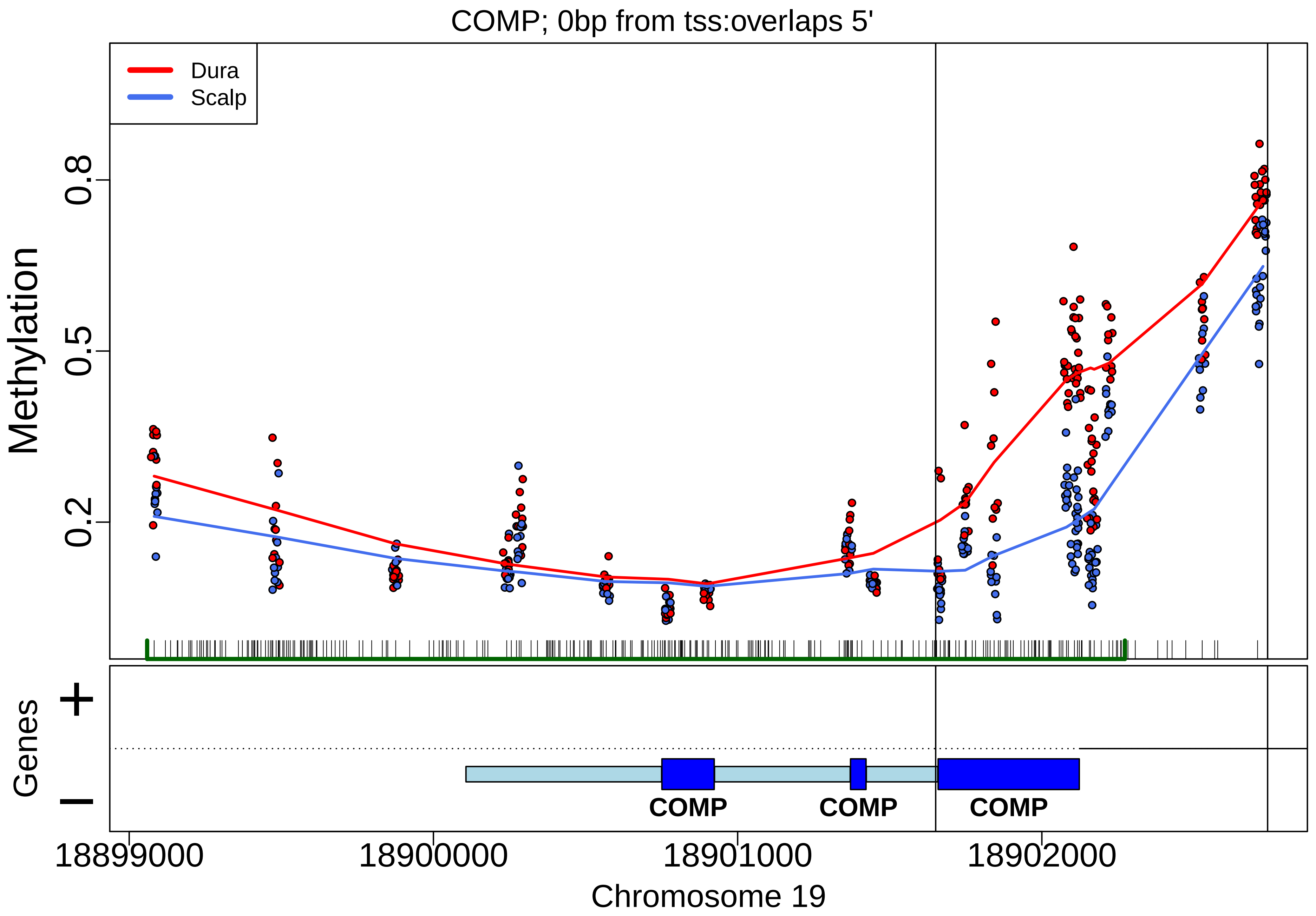
COMP; 0bp from tss:overlaps 5'
Dura
Scalp
Methylation
0.2
0.5
0.8
Genes
+
−
COMP
COMP
COMP
18899000
18900000
18901000
18902000
Chromosome 19

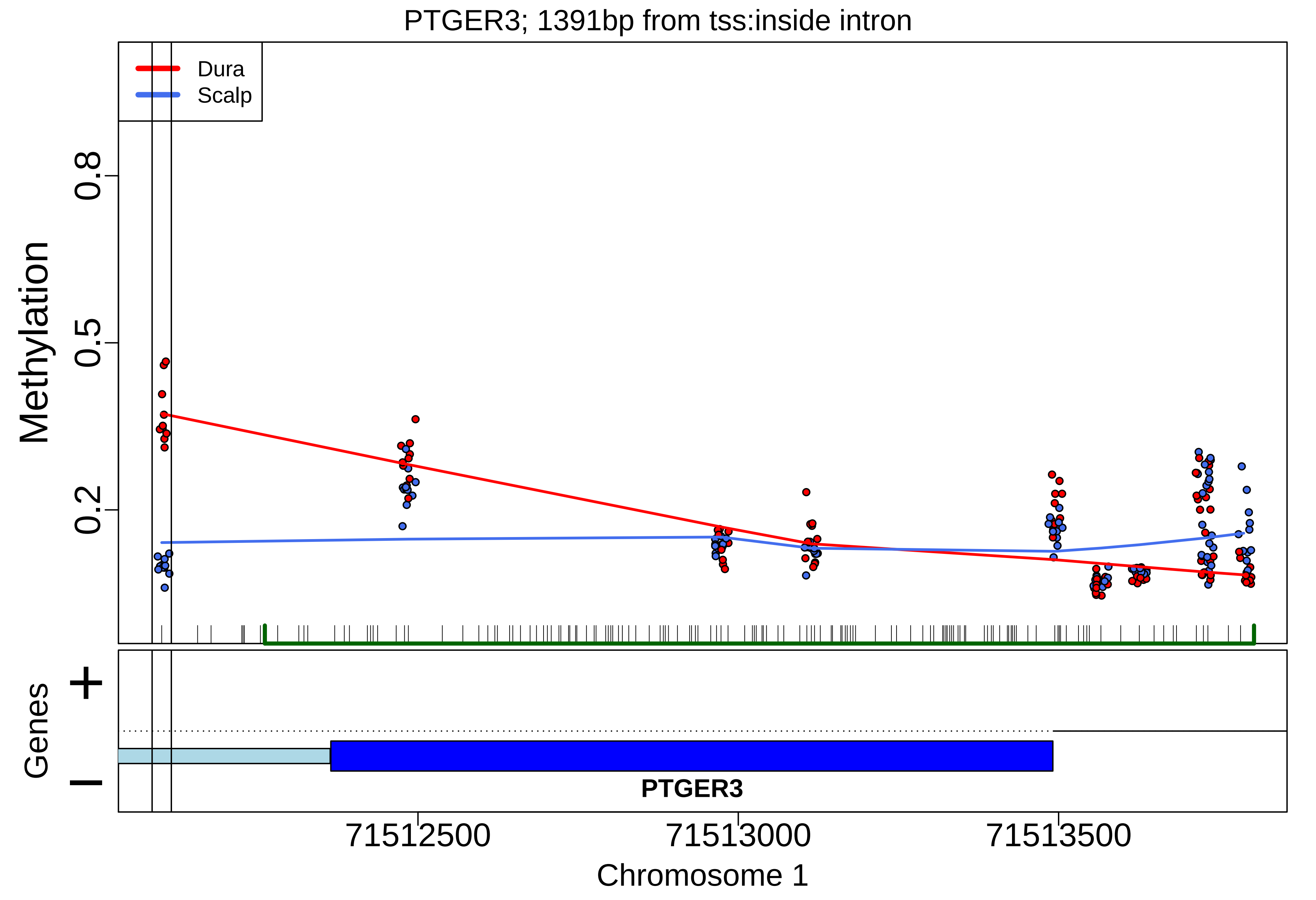

PTGER3; 1391bp from tss:inside intron
Dura
Scalp
Methylation
0.8
0.5
0.2
Genes
+
-
PTGER3
71512500
71513000
71513500
Chromosome 1

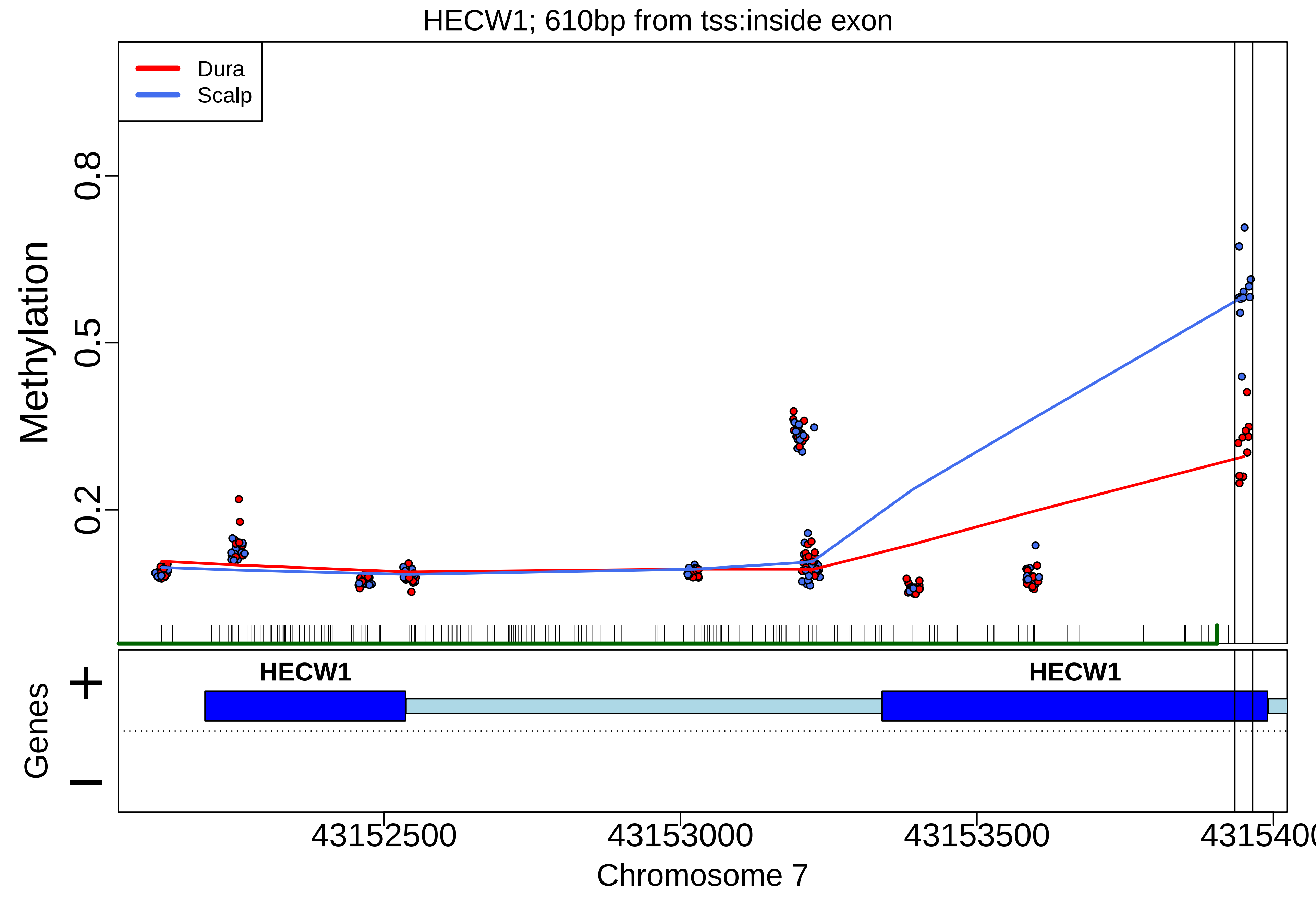

HECW1; 610bp from tss:inside exon
Dura
Scalp
Methylation
0.2
0.5
0.8
Genes
+
−
HECW1
HECW1
43152500
43153000
43153500
4315400
Chromosome 7

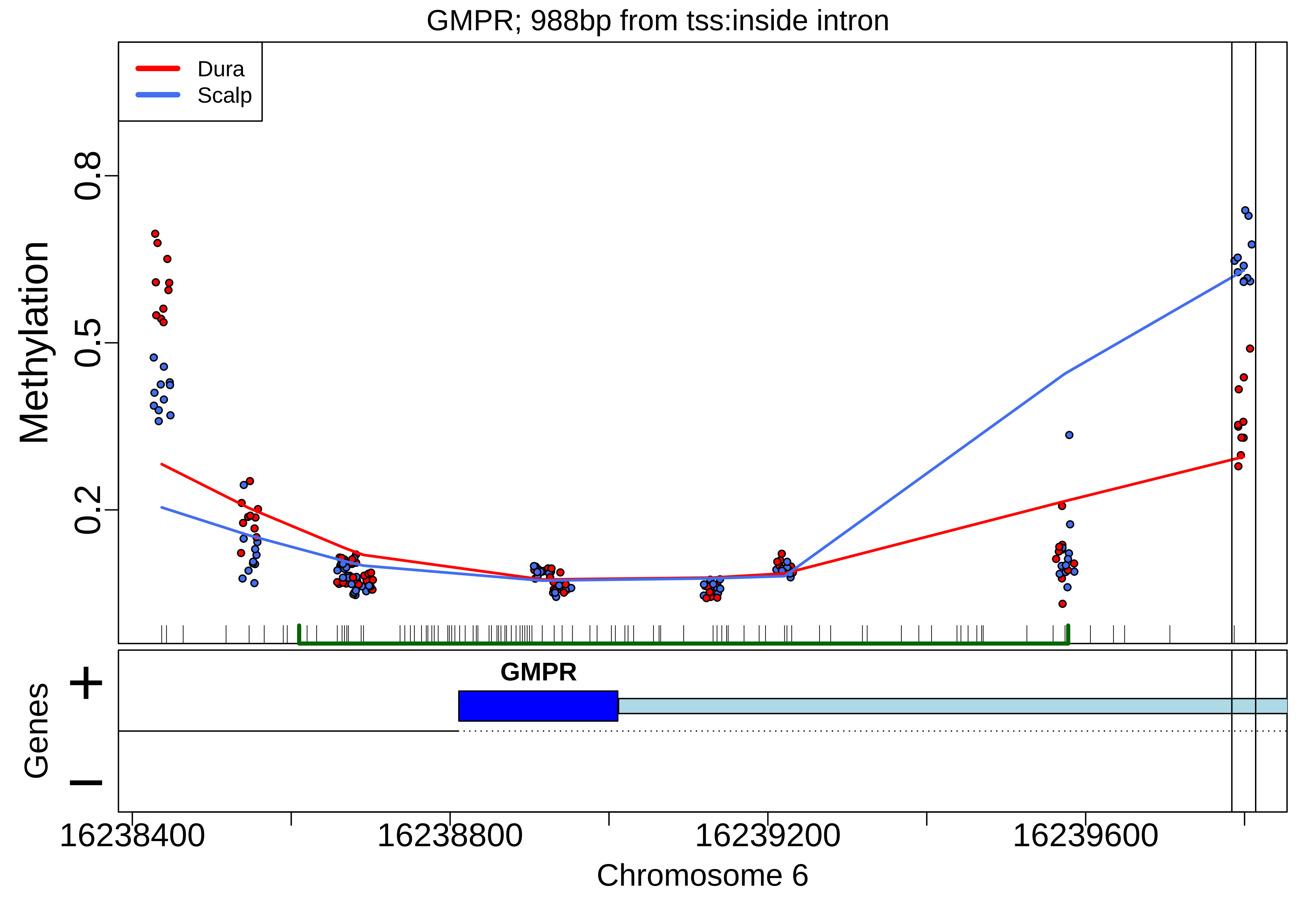

GMPR; 988bp from tss:inside intron
Dura
Scalp
Methylation
0.2
0.5
0.8
Genes
+
–
GMPR
16238400
16238800
16239200
16239600
Chromosome 6

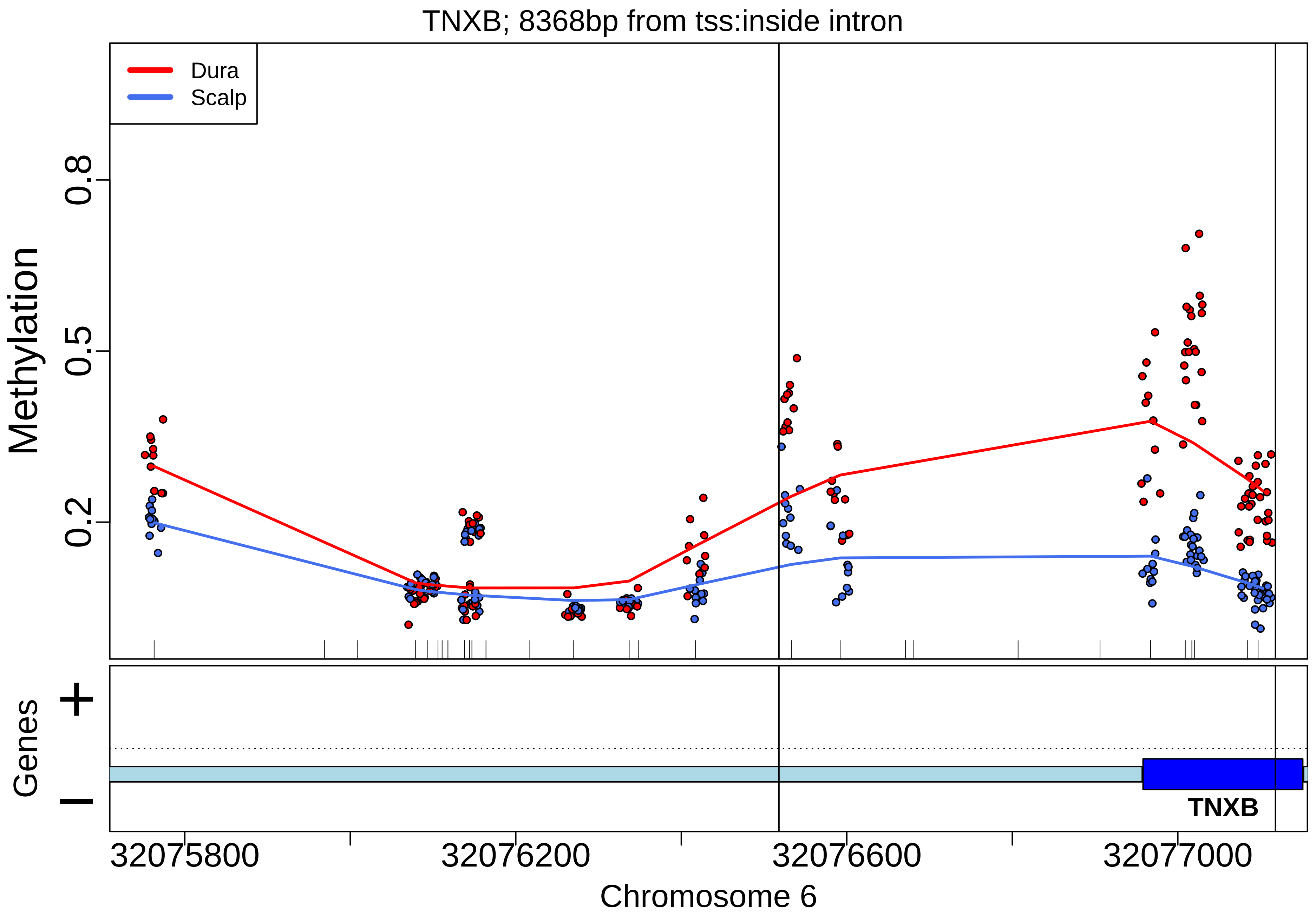
TNXB; 8368bp from tss:inside intron
Dura
Scalp
Methylation
0.2
0.5
0.8
Genes
+
−
TNXB
32075800
32076200
32076600
32077000
Chromosome 6

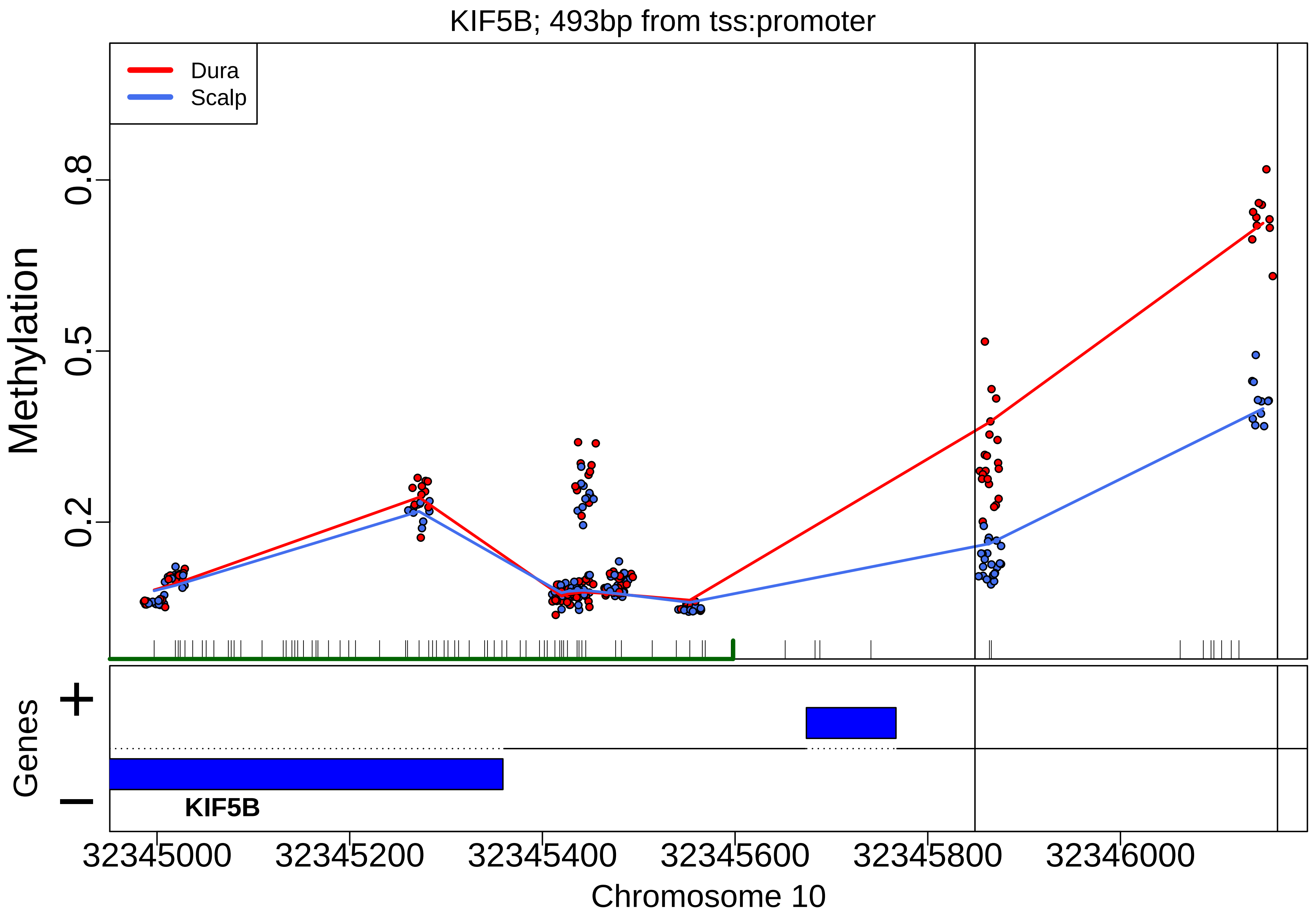

KIF5B; 493bp from tss:promoter
Dura
Scalp
Methylation
0.2
0.5
0.8
Genes
+
−
KIF5B
32345000
32345200
32345400
32345600
32345800
32346000
Chromosome 10

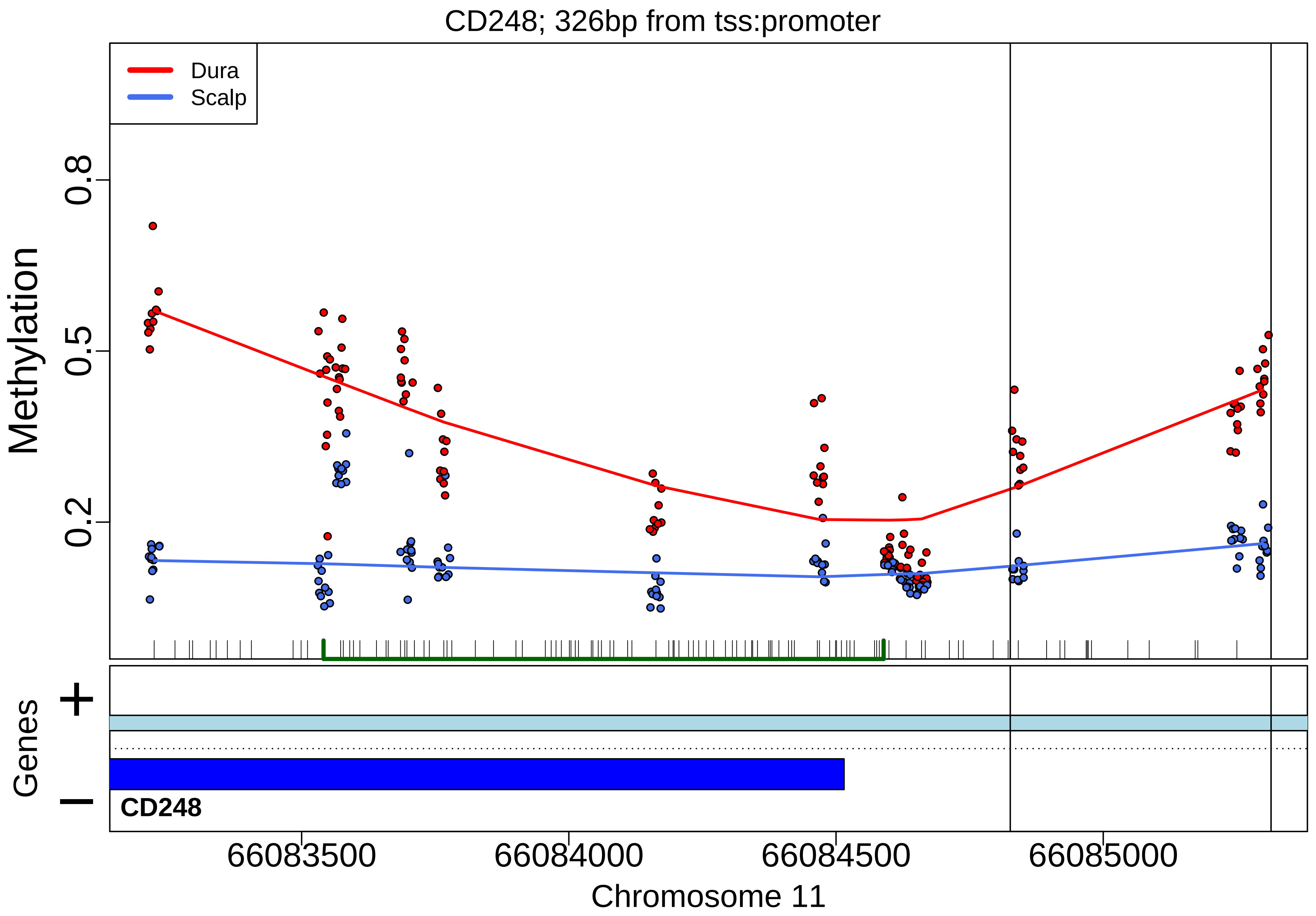

CD248; 326bp from tss:promoter
Dura
Scalp
Methylation
0.2
0.5
0.8
Genes
+
−
CD248
66083500
66084000
66084500
66085000
Chromosome 11

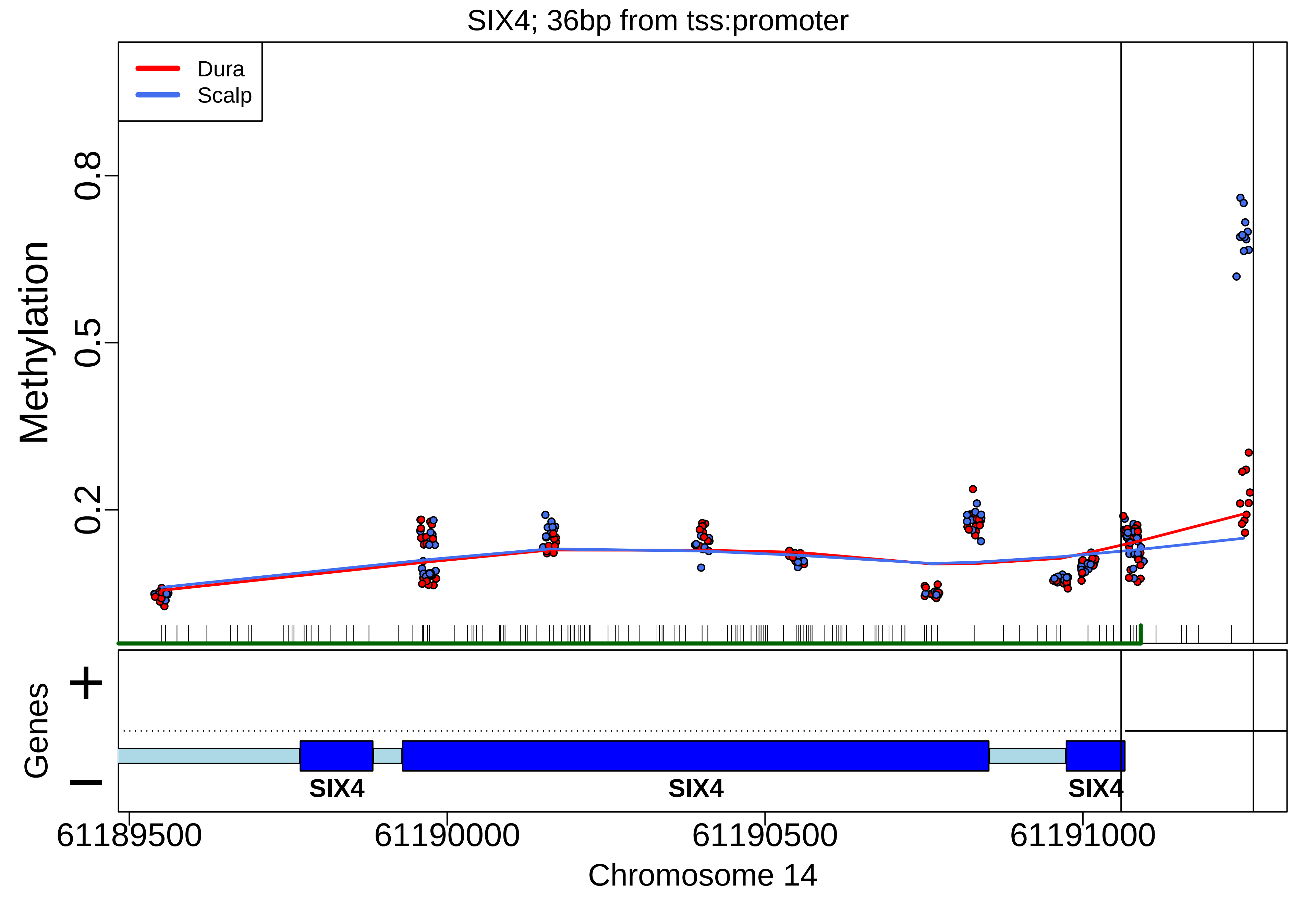
SIX4; 36bp from tss:promoter
Dura
Scalp
Methylation
0.8
0.5
0.2
Genes
+
−
SIX4
SIX4
SIX4
61189500
61190000
61190500
61191000
Chromosome 14

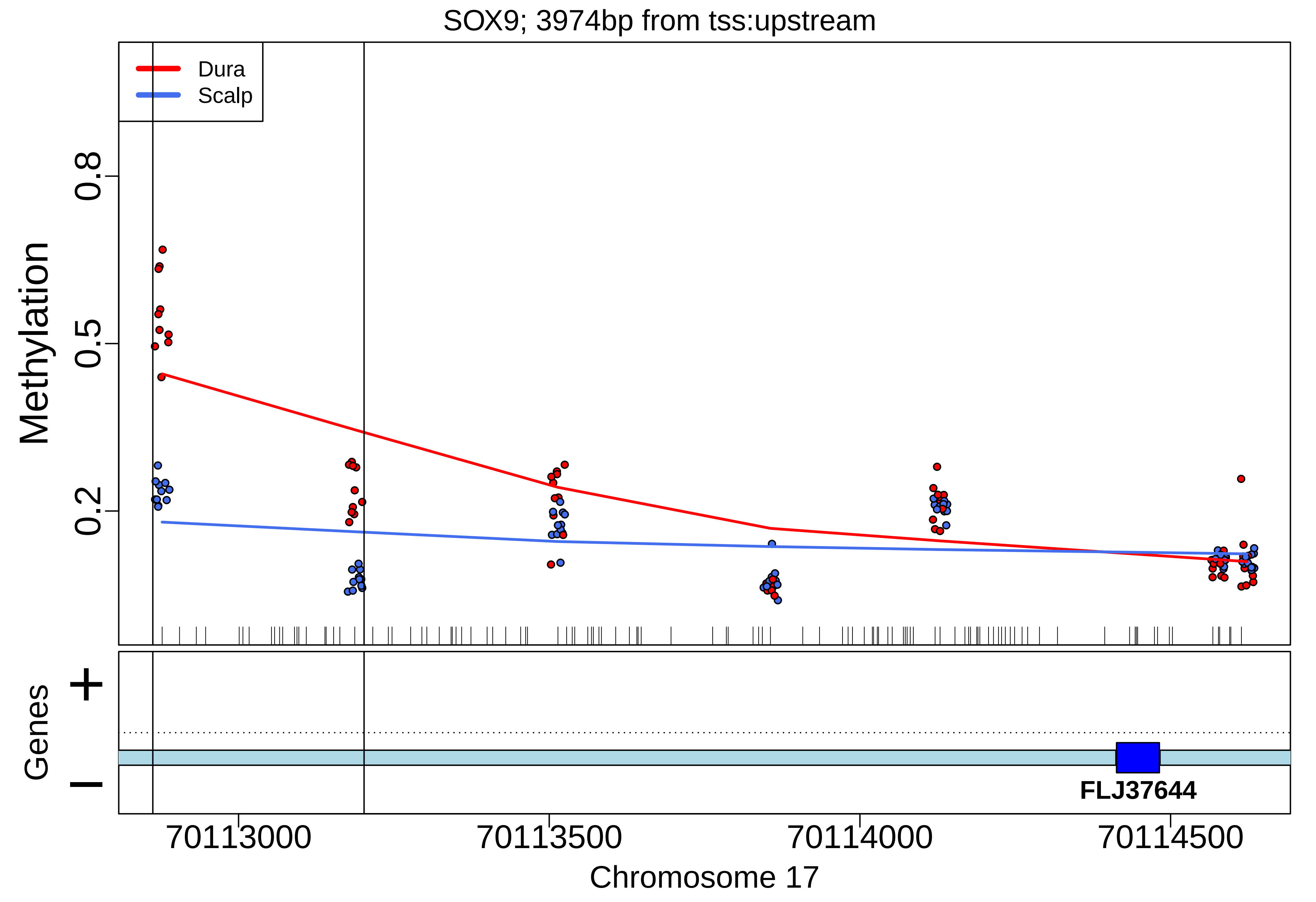

SOX9; 3974bp from tss:upstream
Dura
Scalp
Methylation
0.2
0.5
0.8
Genes
+
−
FLJ37644
70113000
70113500
70114000
70114500
Chromosome 17

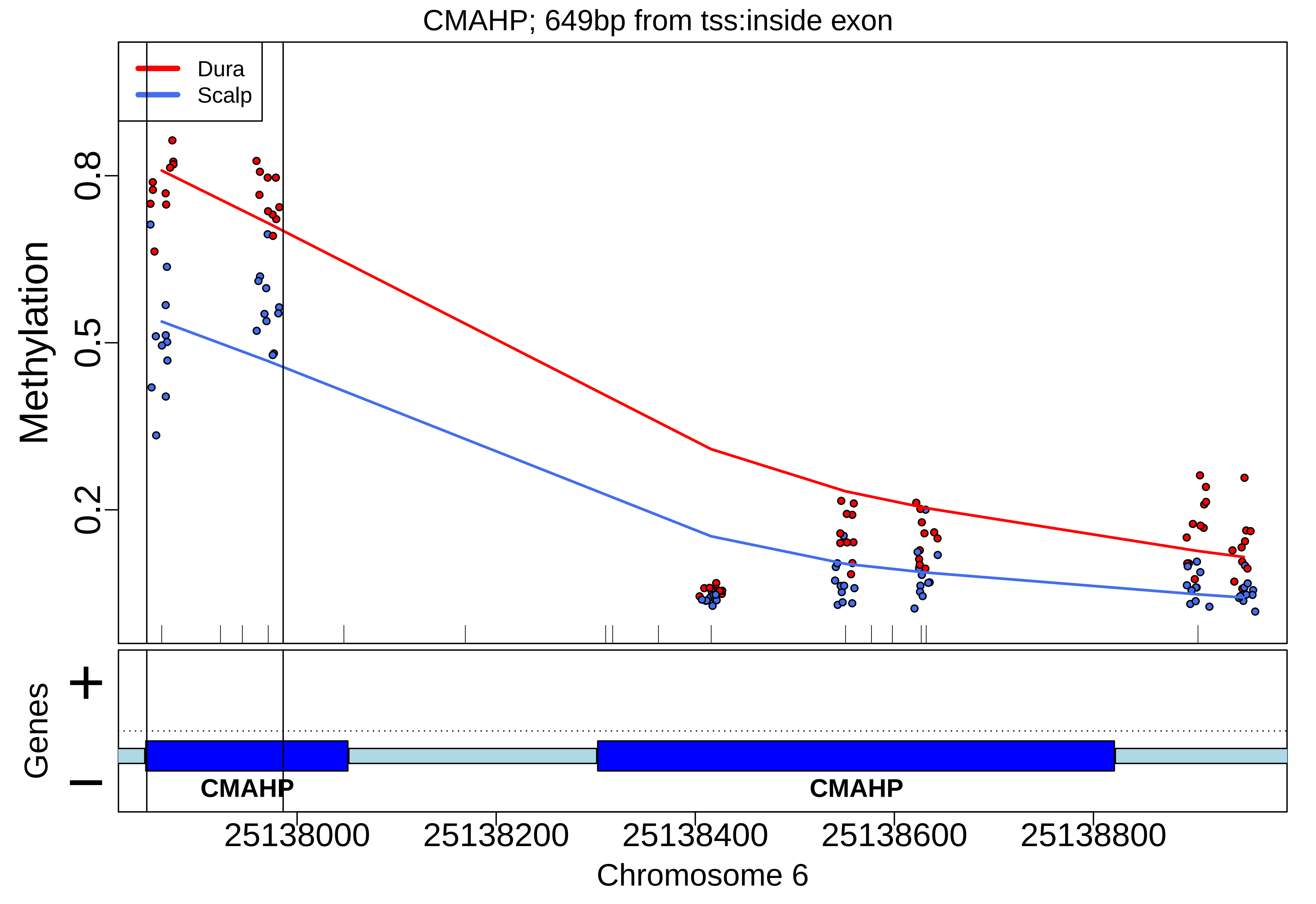
CMAHP; 649bp from tss:inside exon
Dura
Scalp
Methylation
0.2
0.5
0.8
Genes
+
−
CMAHP
CMAHP
25138000
25138200
25138400
25138600
25138800
Chromosome 6

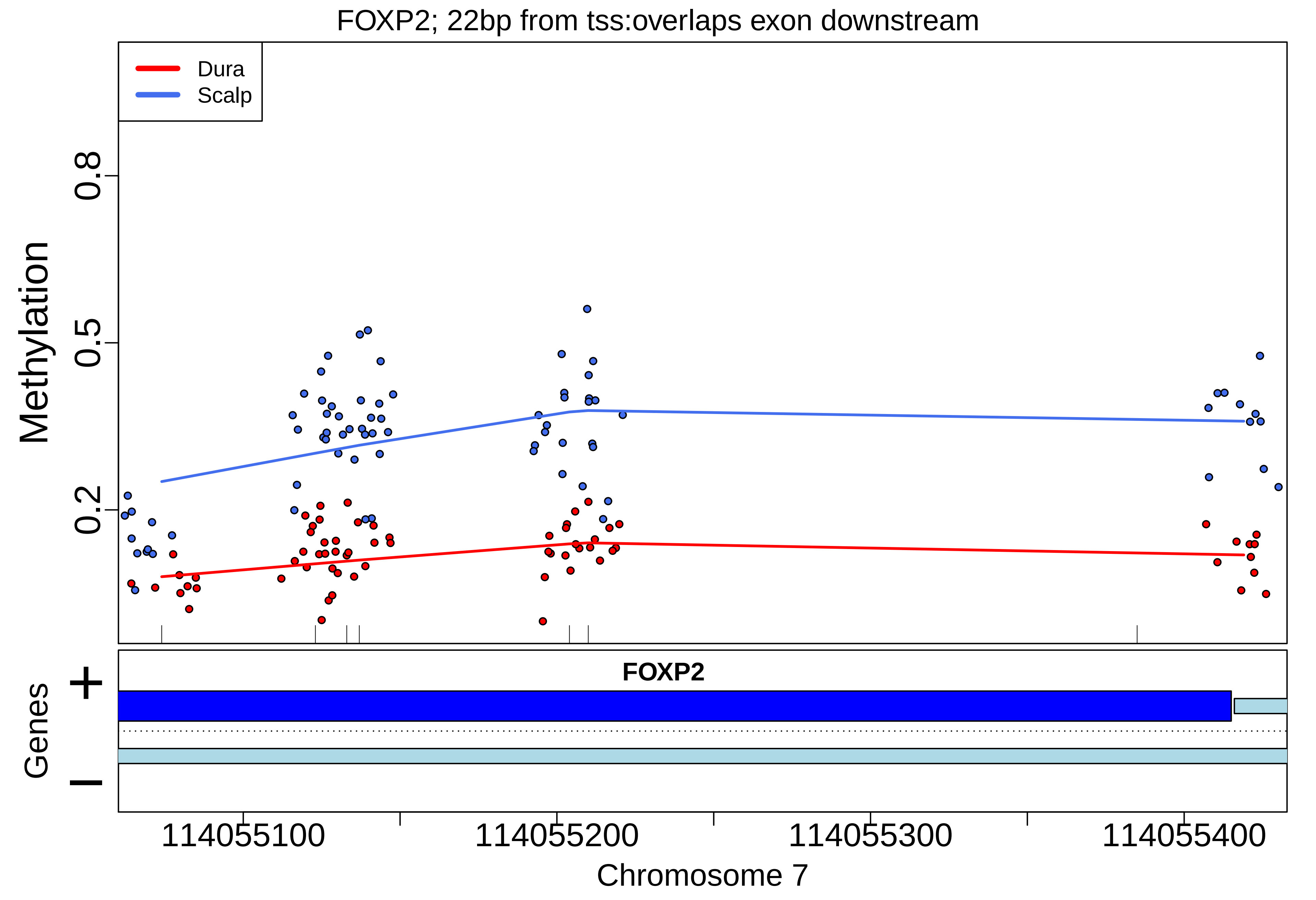

FOXP2; 22bp from tss:overlaps exon downstream
Dura
Scalp
Methylation
0.8
0.5
0.2
Genes
+
-
FOXP2
114055100
114055200
114055300
114055400
Chromosome 7

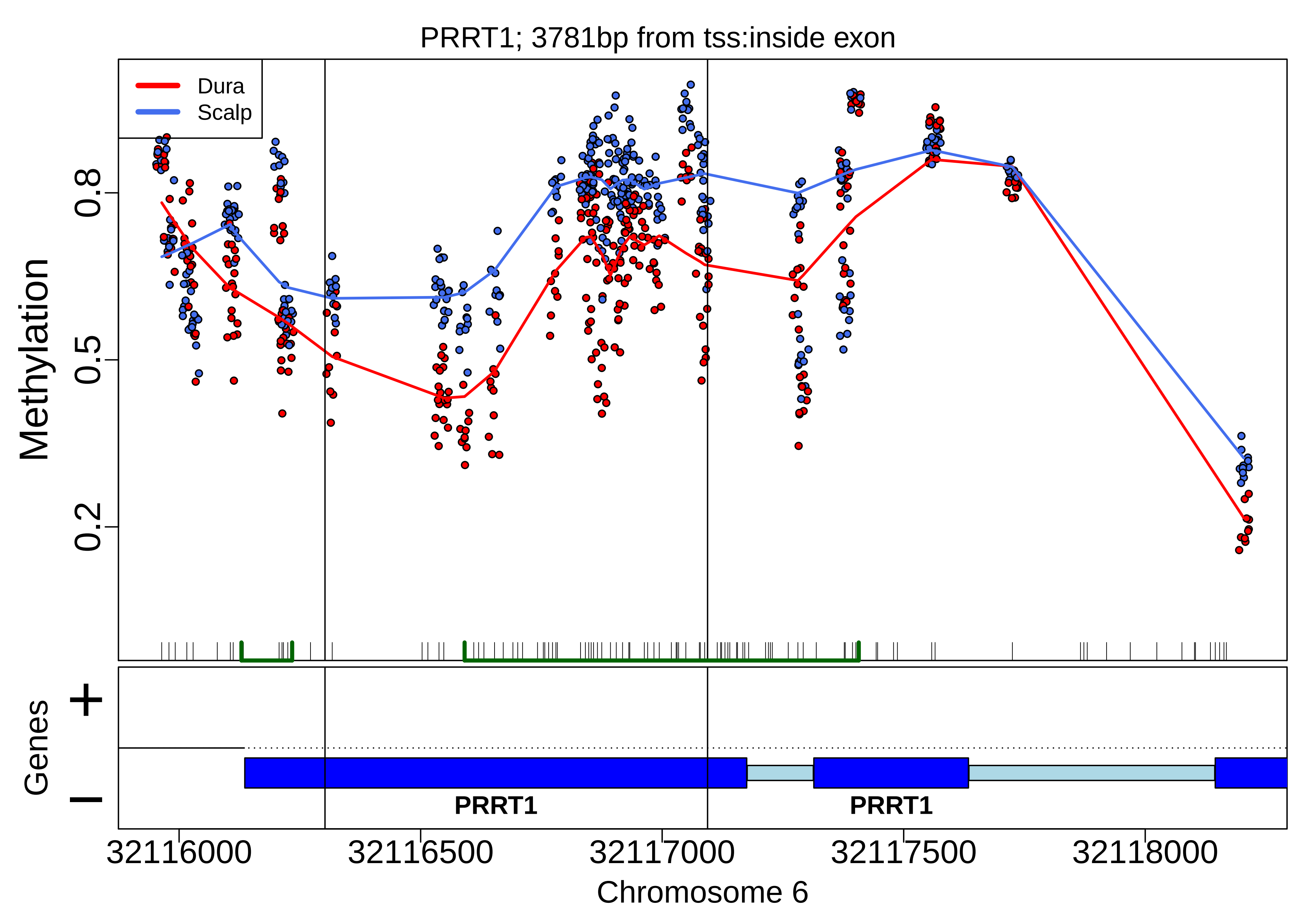

PRRT1; 3781bp from tss:inside exon
Dura
Scalp
Methylation
0.2
0.5
0.8
Genes
+
-
PRRT1
PRRT1
32116000
32116500
32117000
32117500
32118000
Chromosome 6

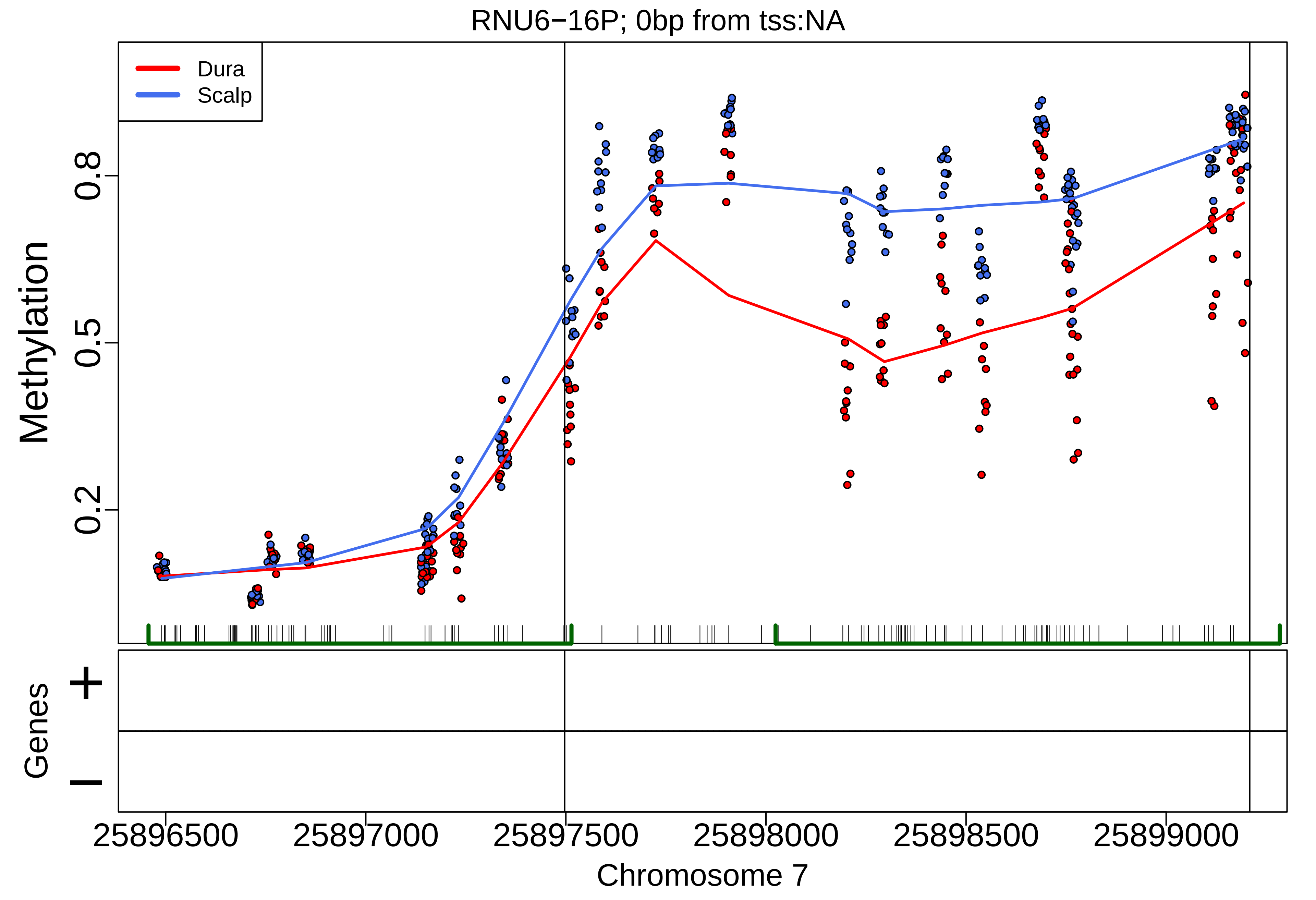

RNU6−16P; 0bp from tss:NA
Dura
Scalp
Methylation
0.2
0.5
0.8
Genes
+
−
25896500
25897000
25897500
25898000
25898500
25899000
Chromosome 7

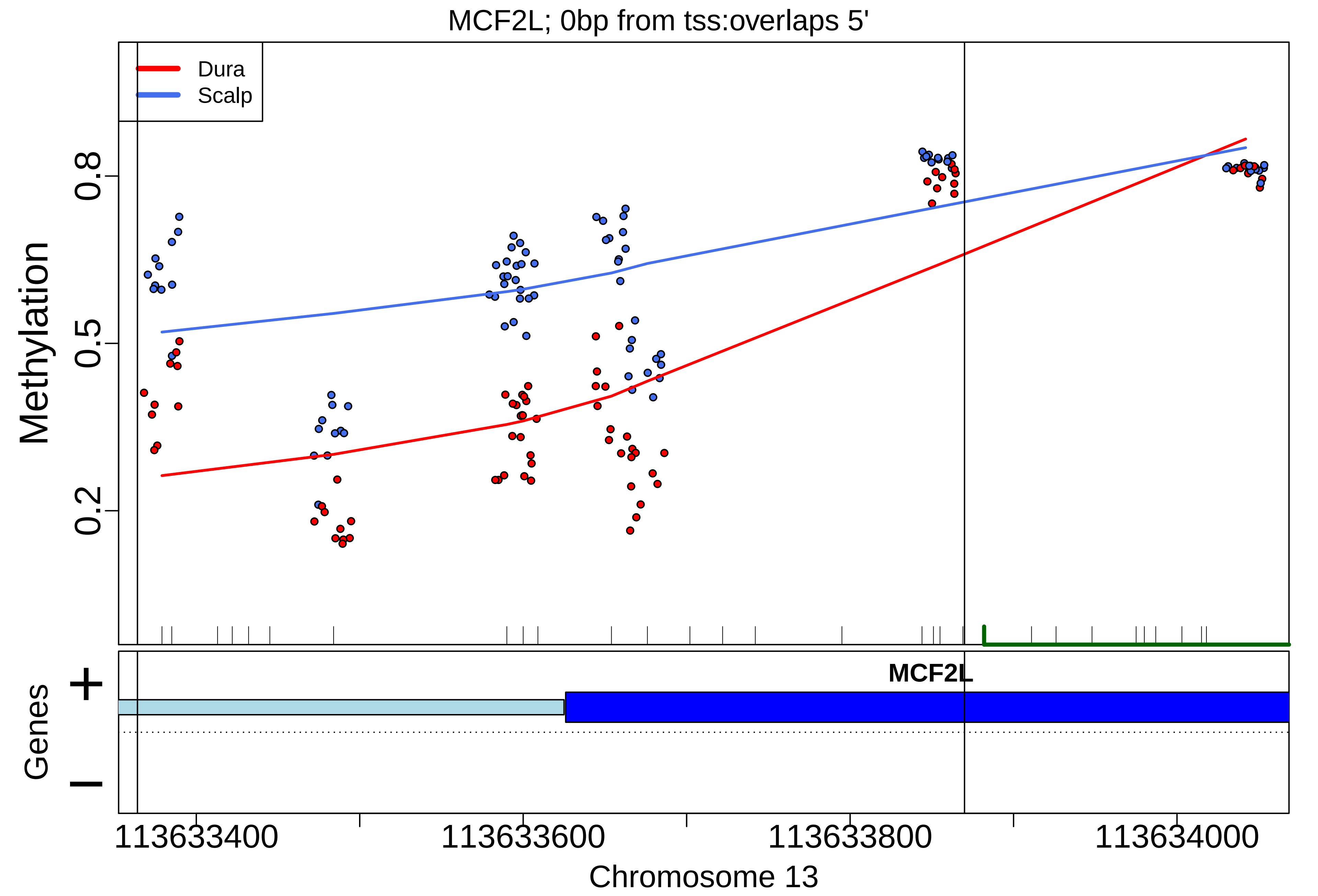

MCF2L; 0bp from tss:overlaps 5'
Dura
Scalp
Methylation
0.2
0.5
0.8
Genes
+
-
MCF2L
113633400
113633600
113633800
113634000
Chromosome 13

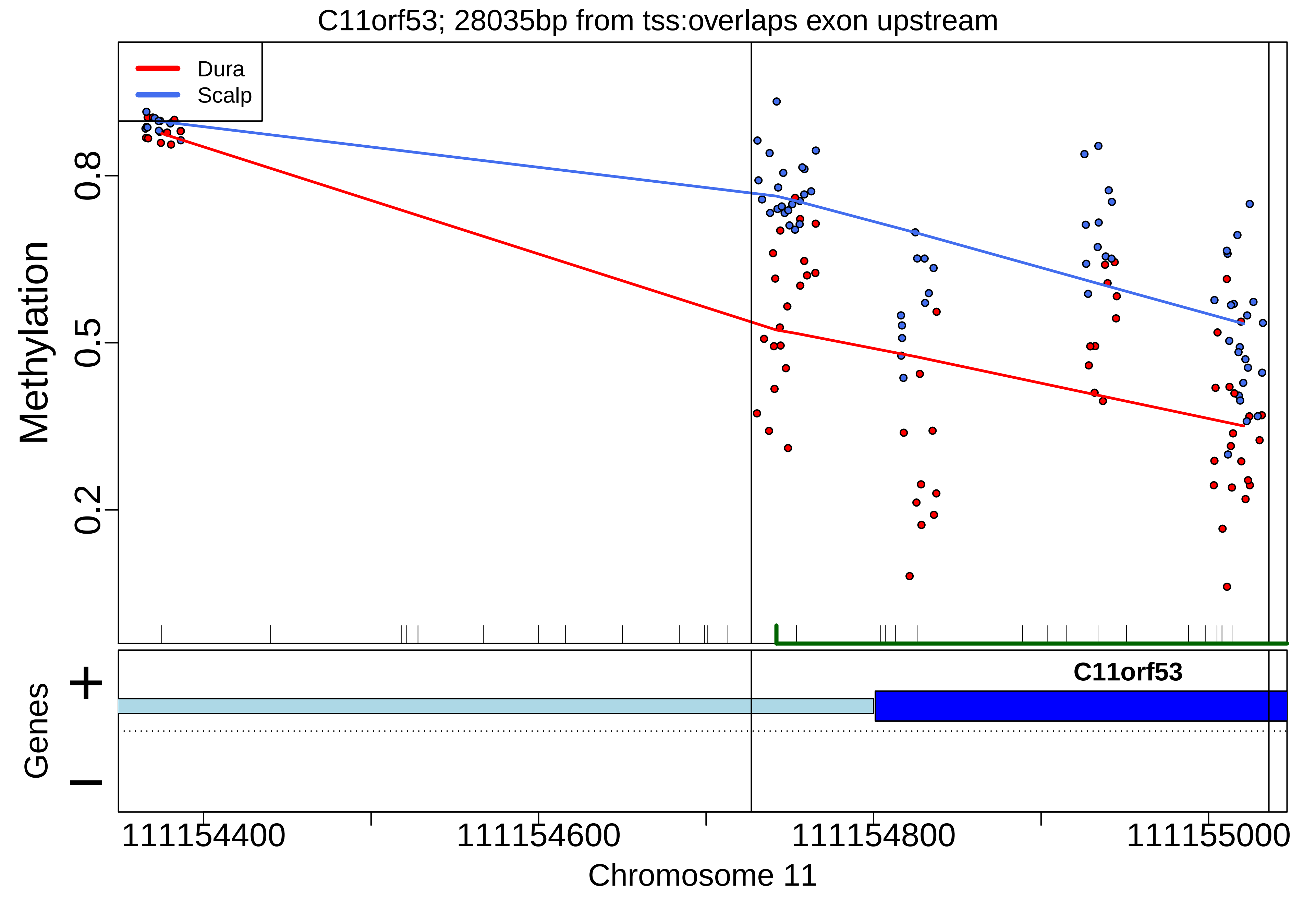
C11orf53; 28035bp from tss:overlaps exon upstream
Dura
Scalp
Methylation
0.2
0.5
0.8
Genes
+
-
C11orf53
111154400
111154600
111154800
111155000
Chromosome 11

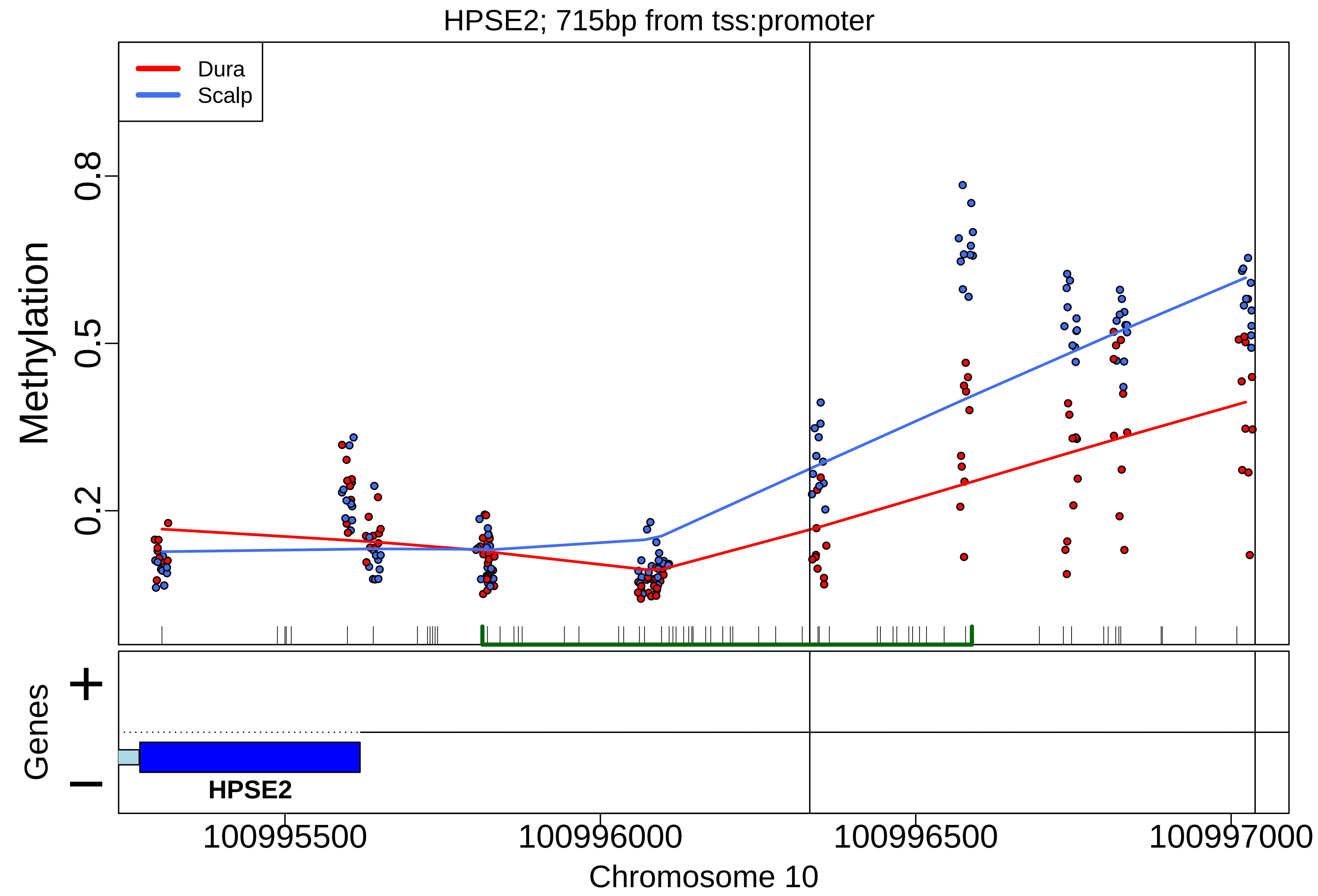
HPSE2; 715bp from tss:promoter
Dura
Scalp
Methylation
0.2
0.5
0.8
Genes
+
-
HPSE2
100995500
100996000
100996500
100997000
Chromosome 10

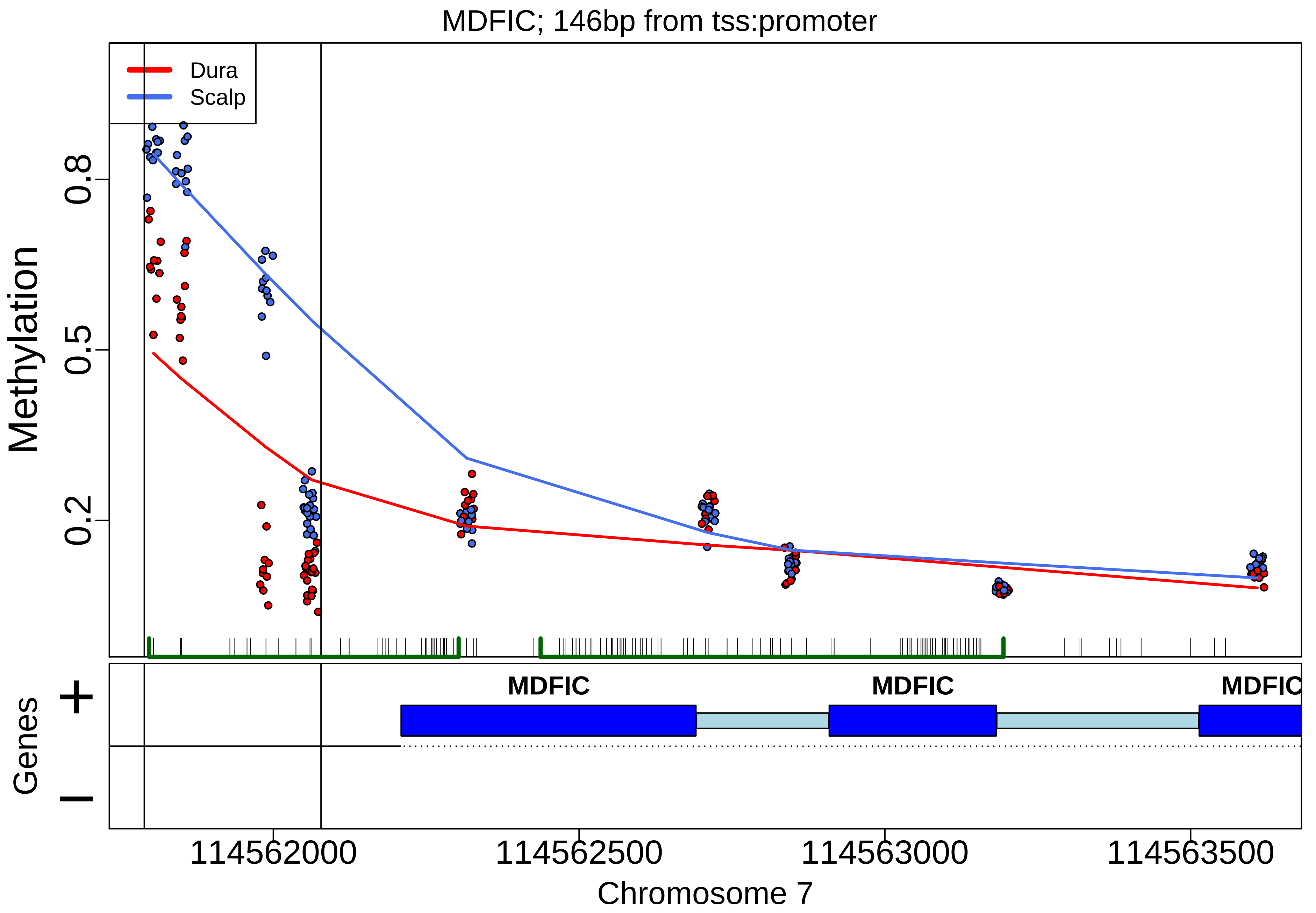

MDFIC; 146bp from tss:promoter
Dura
Scalp
Methylation
0.2
0.5
0.8
Genes
+
–
MDFIC
MDFIC
MDFIC
114562000
114562500
114563000
114563500
Chromosome 7

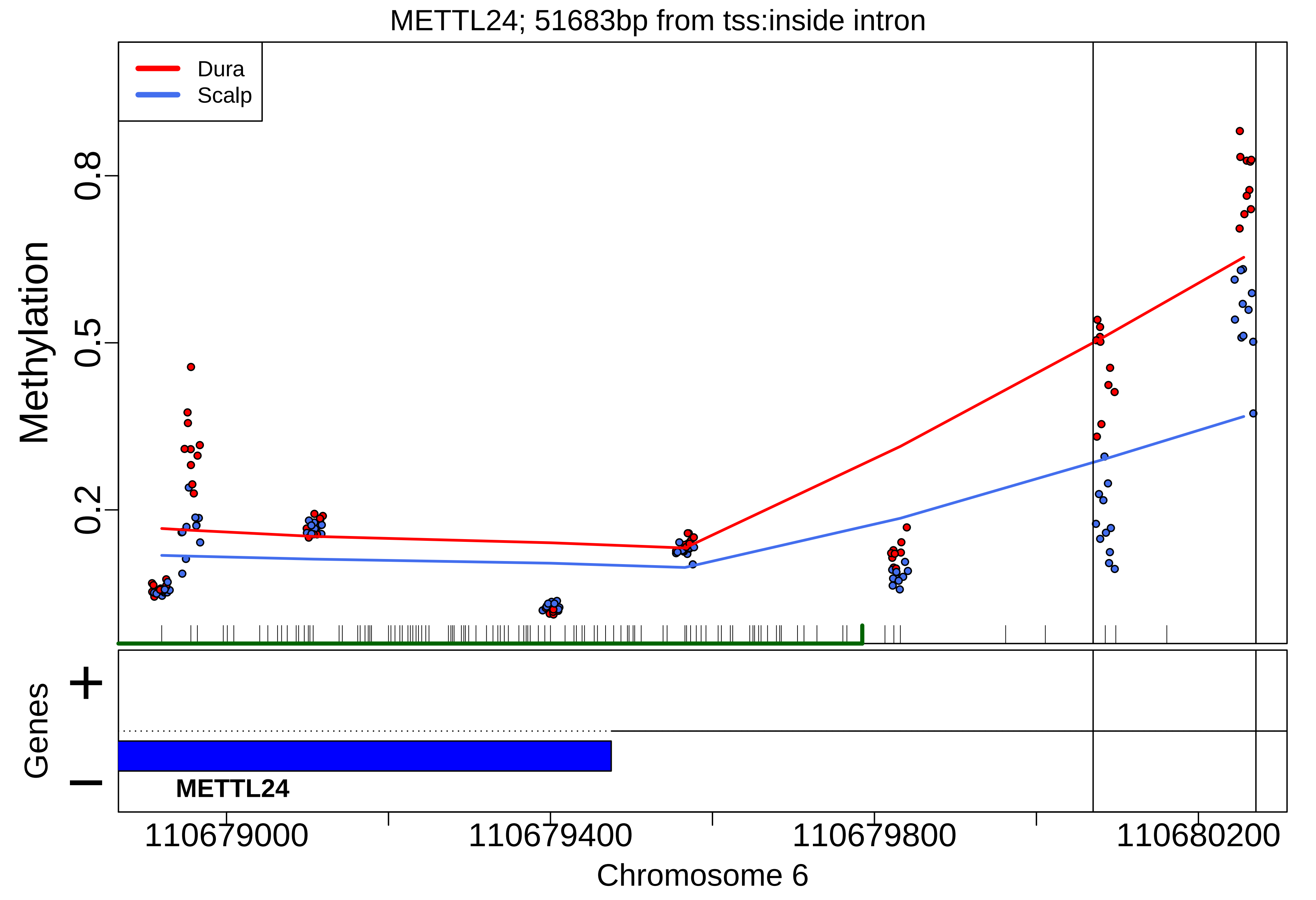

METTL24; 51683bp from tss:inside intron
Dura
Scalp
Methylation
0.2
0.5
0.8
Genes
+
-
METTL24
110679000
110679400
110679800
110680200
Chromosome 6

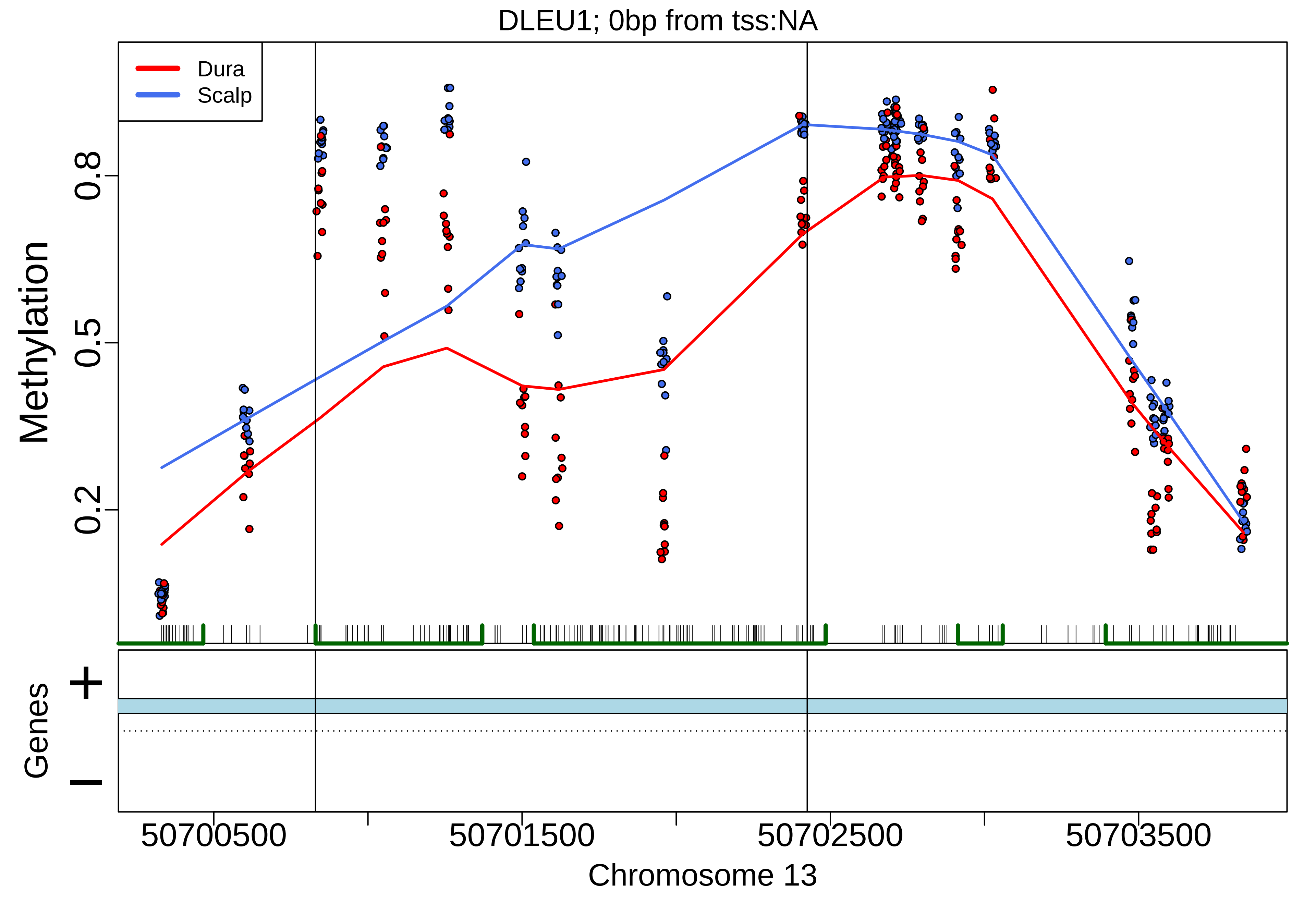

DLEU1; 0bp from tss:NA
Dura
Scalp
Methylation
0.2
0.5
0.8
Genes
+
−
50700500
50701500
50702500
50703500
Chromosome 13

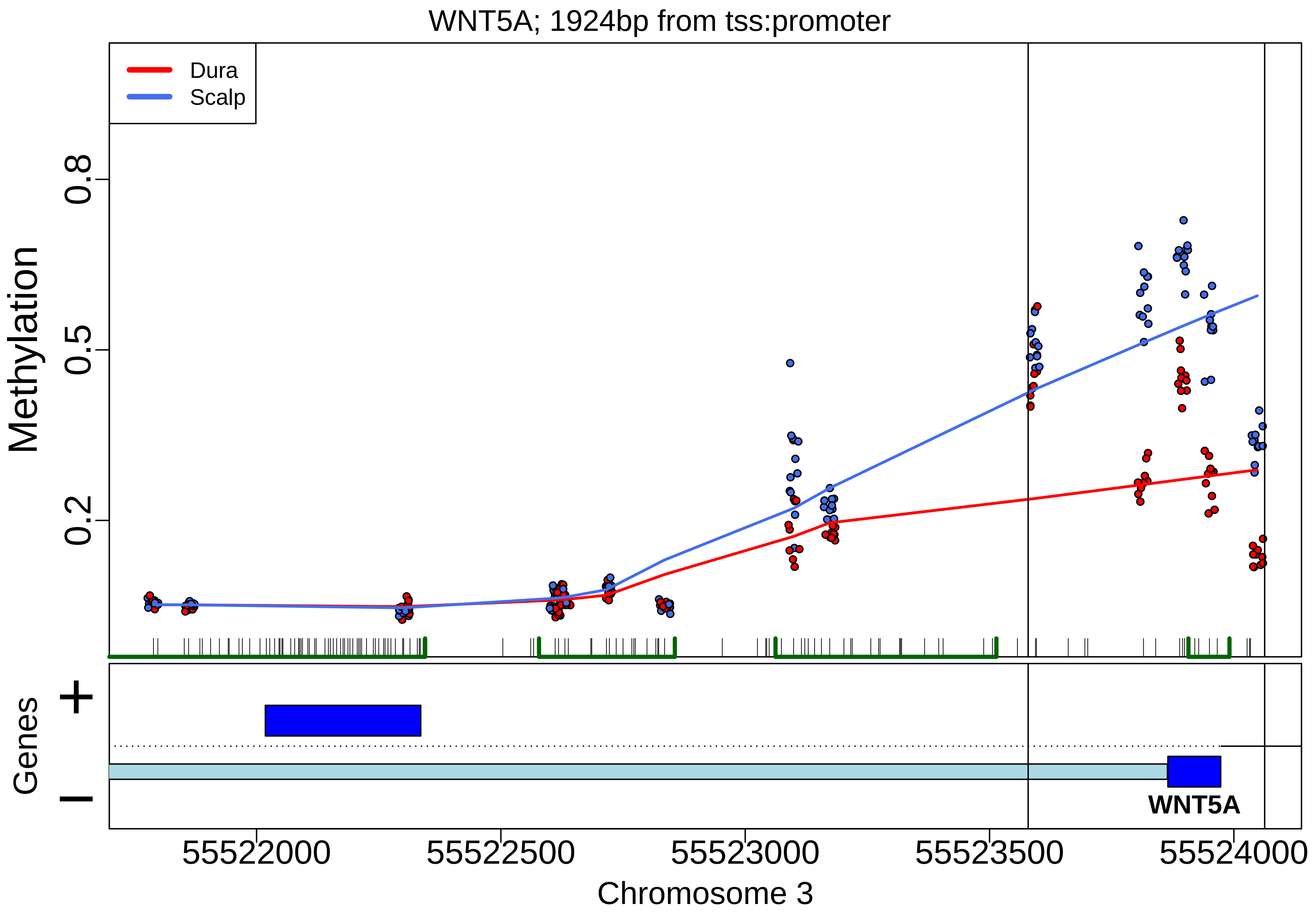

WNT5A; 1924bp from tss:promoter
Dura
Scalp
Methylation
0.2
0.5
0.8
Genes
+
-
WNT5A
55522000
55522500
55523000
55523500
55524000
Chromosome 3

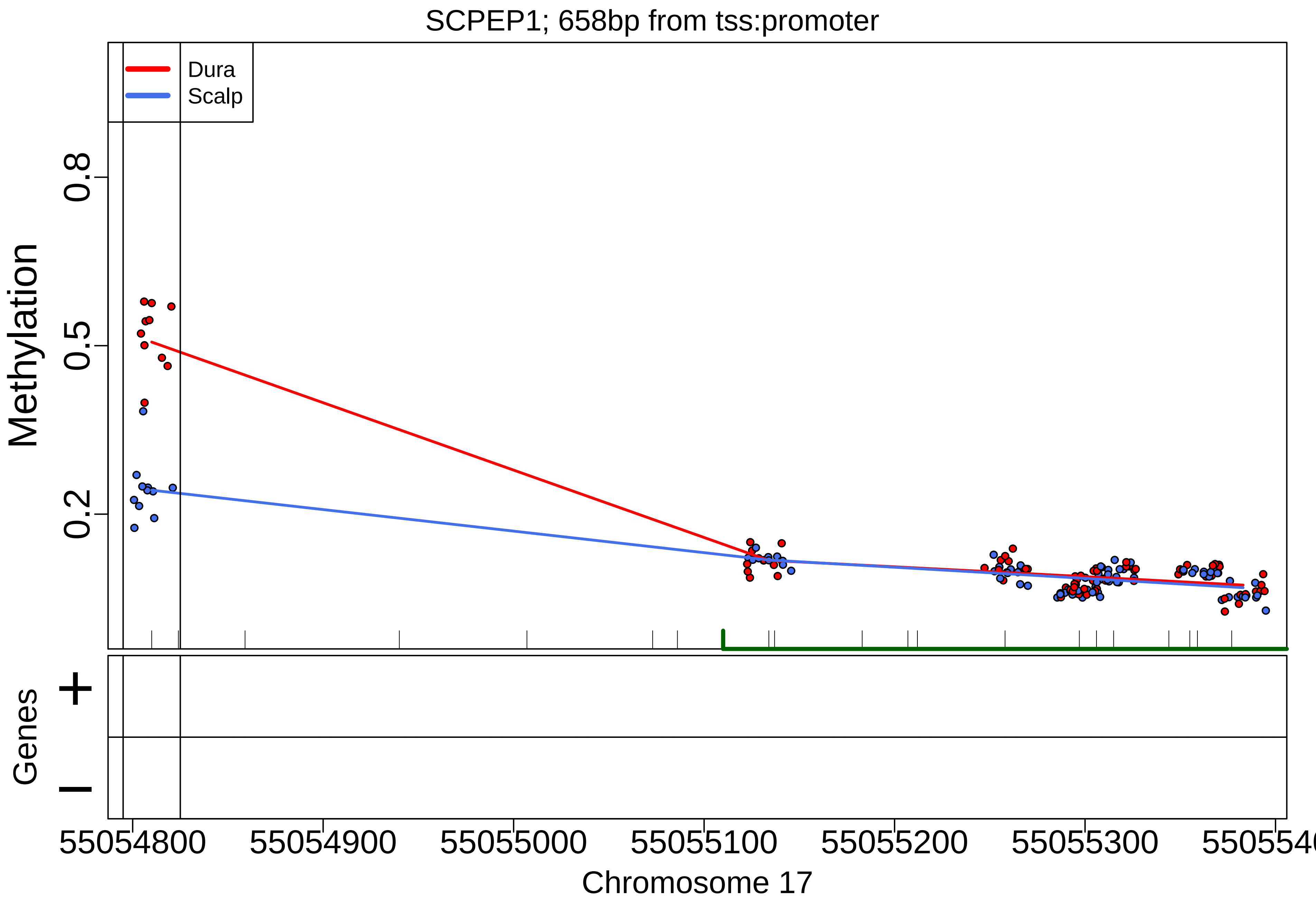

SCPEP1; 658bp from tss:promoter
Dura
Scalp
Methylation
0.2
0.5
0.8
Genes
+
−
55054800
55054900
55055000
55055100
55055200
55055300
Chromosome 17

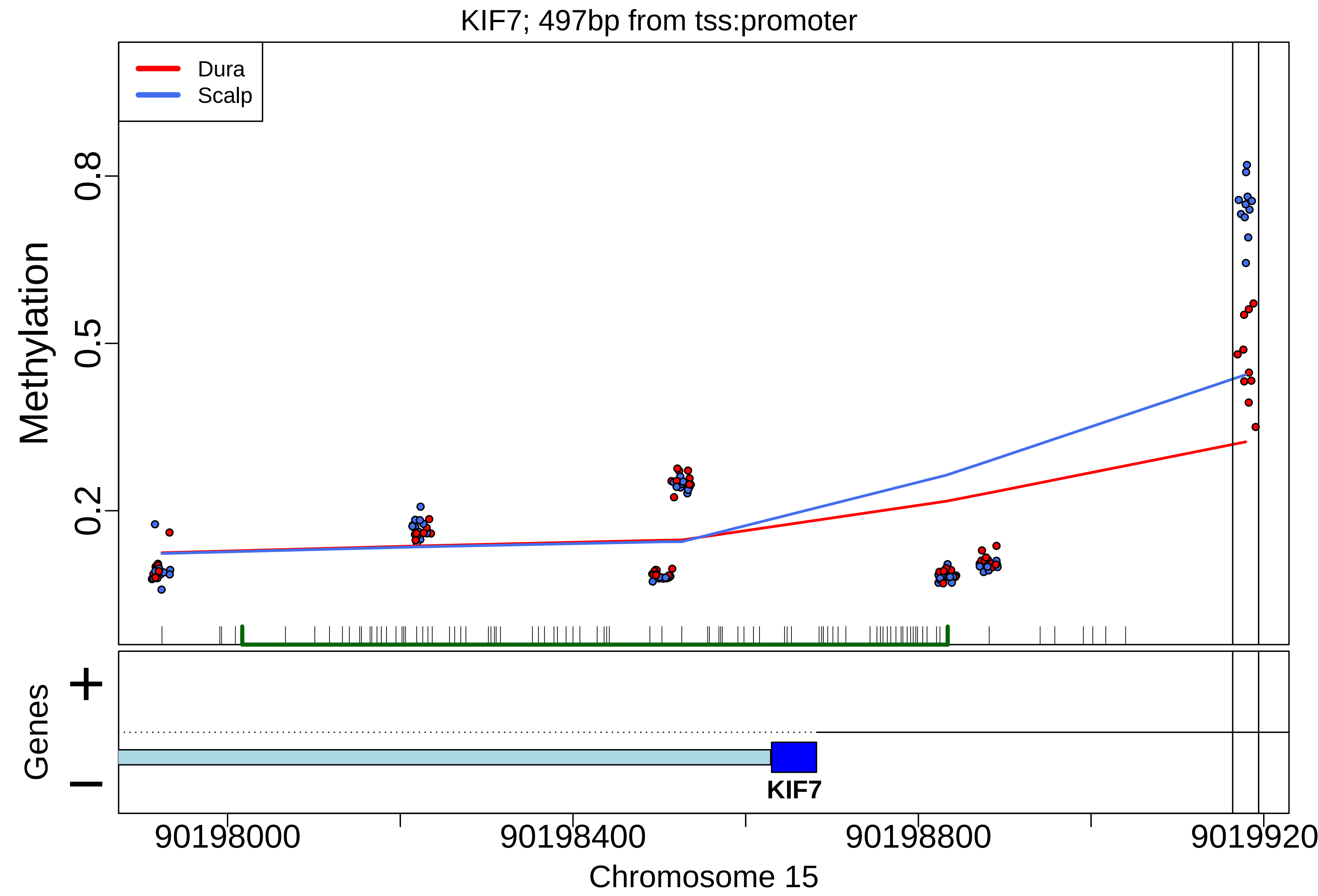

KIF7; 497bp from tss:promoter
Dura
Scalp
Methylation
0.2
0.5
0.8
Genes
+
-
KIF7
90198000
90198400
90198800
9019920
Chromosome 15

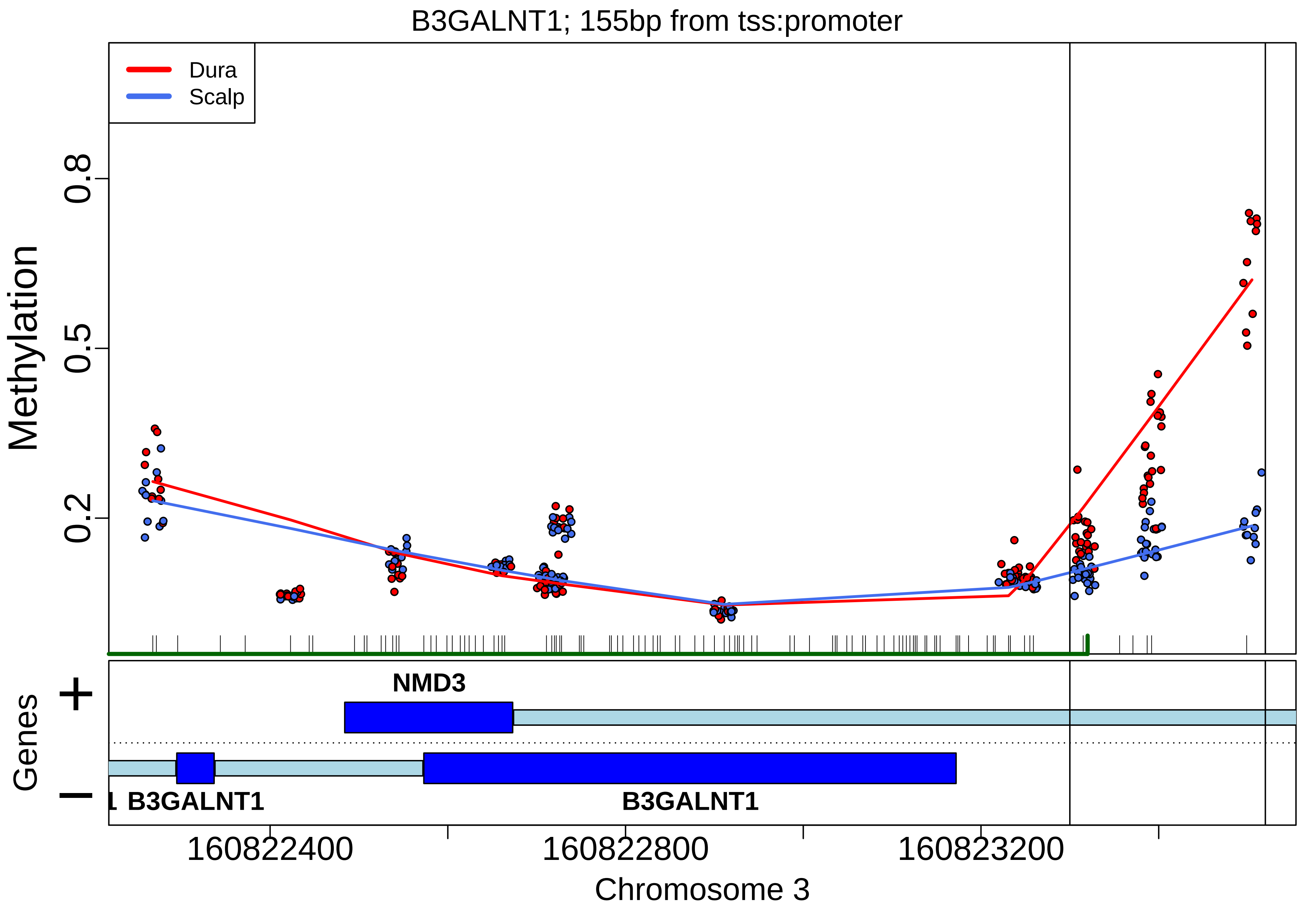

B3GALNT1; 155bp from tss:promoter
Dura
Scalp
Methylation
0.8
0.5
0.2
Genes
+
−
NMD3
B3GALNT1
B3GALNT1
160822400
160822800
160823200
Chromosome 3

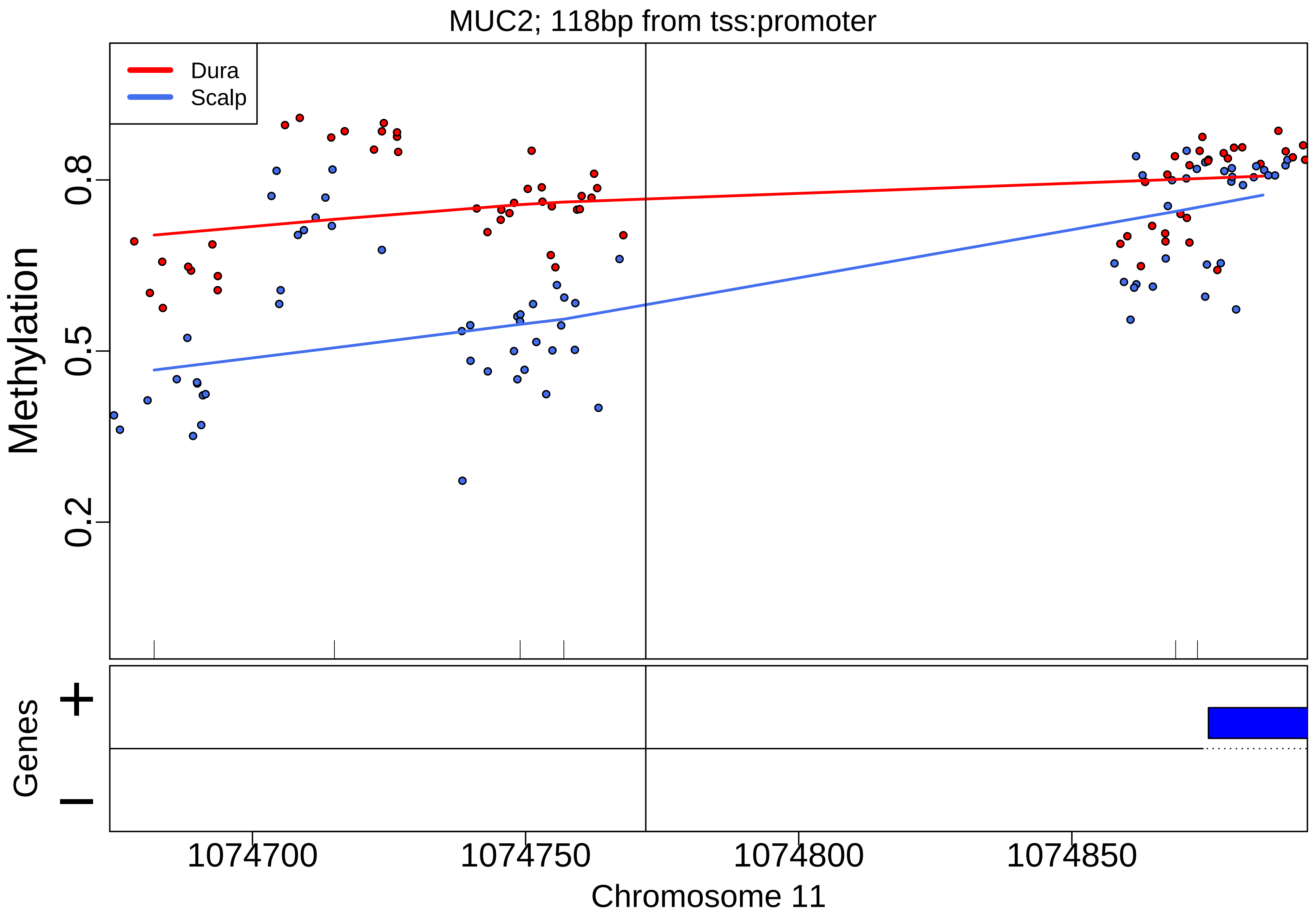
MUC2; 118bp from tss:promoter
Dura
Scalp
Methylation
0.2
0.5
0.8
Genes
+
-
1074700
1074750
1074800
1074850
Chromosome 11

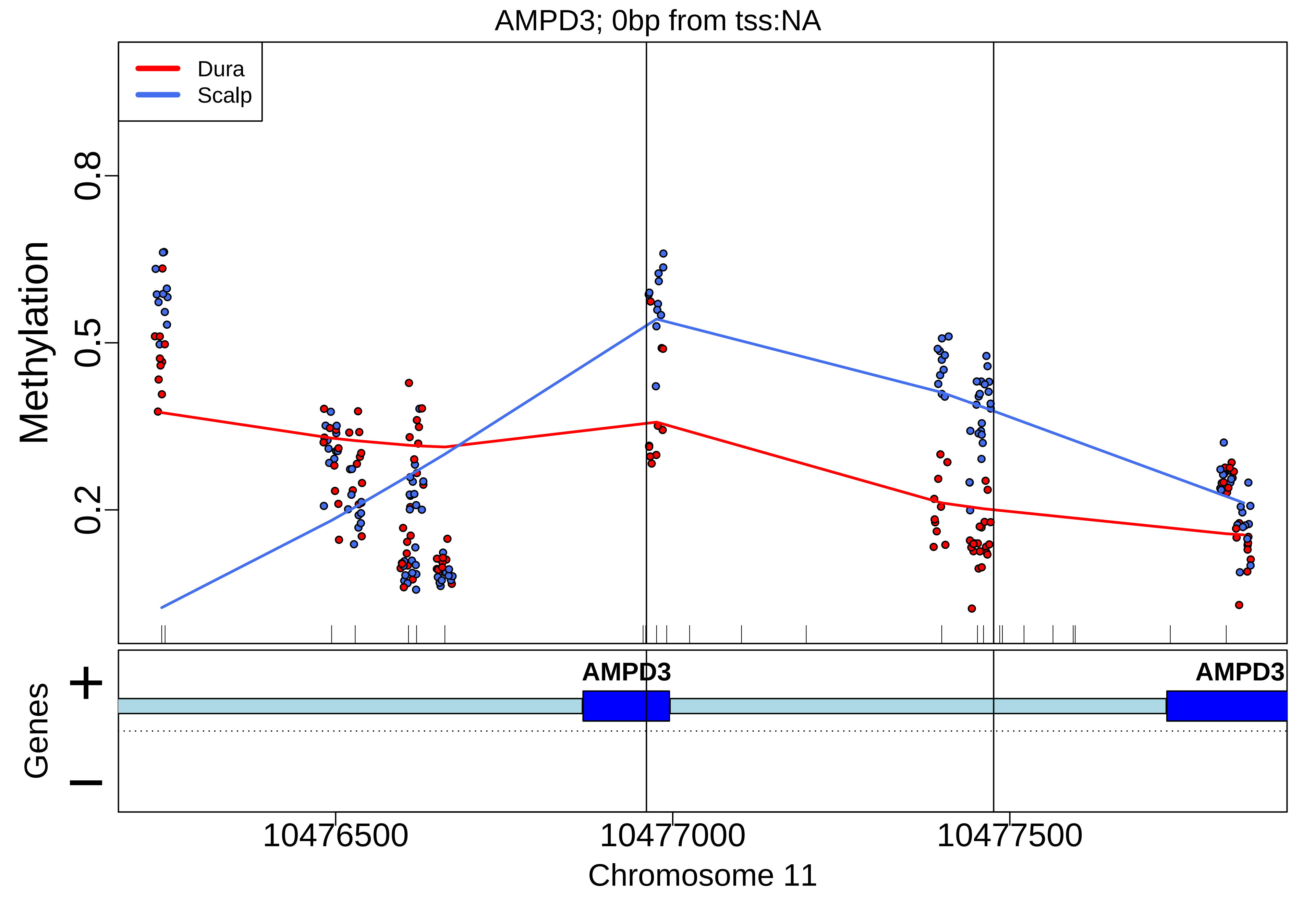
AMPD3; 0bp from tss:NA
Dura
Scalp
Methylation
0.2
0.5
0.8
Genes
+
-
AMPD3
AMPD3
10476500
10477000
10477500
Chromosome 11

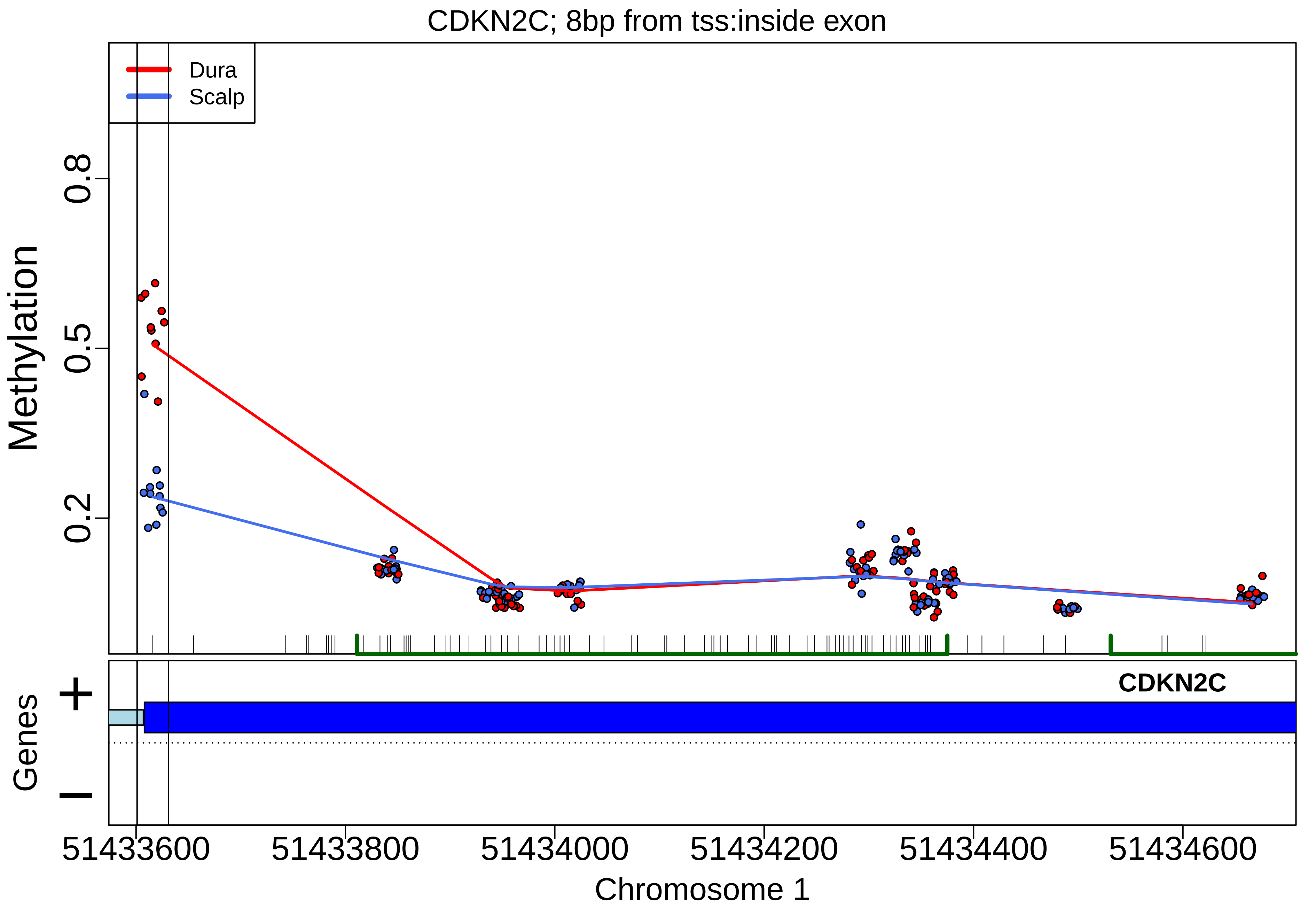
CDKN2C; 8bp from tss:inside exon
Dura
Scalp
Methylation
0.2
0.5
0.8
Genes
+
-
CDKN2C
51433600
51433800
51434000
51434200
51434400
51434600
Chromosome 1

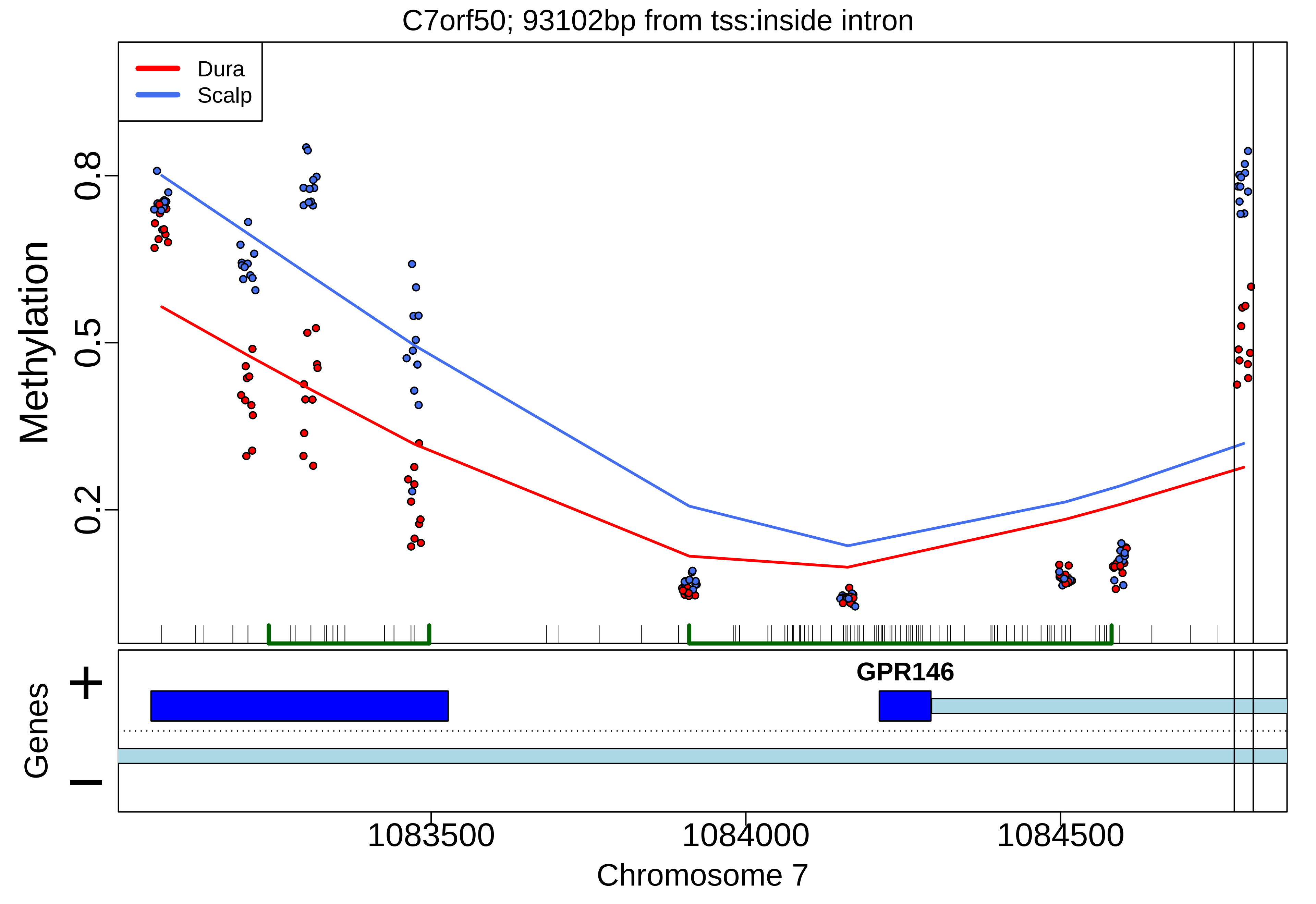
C7orf50; 93102bp from tss:inside intron
Dura
Scalp
Methylation
0.2
0.5
0.8
Genes
+
-
GPR146
1083500
1084000
1084500
Chromosome 7

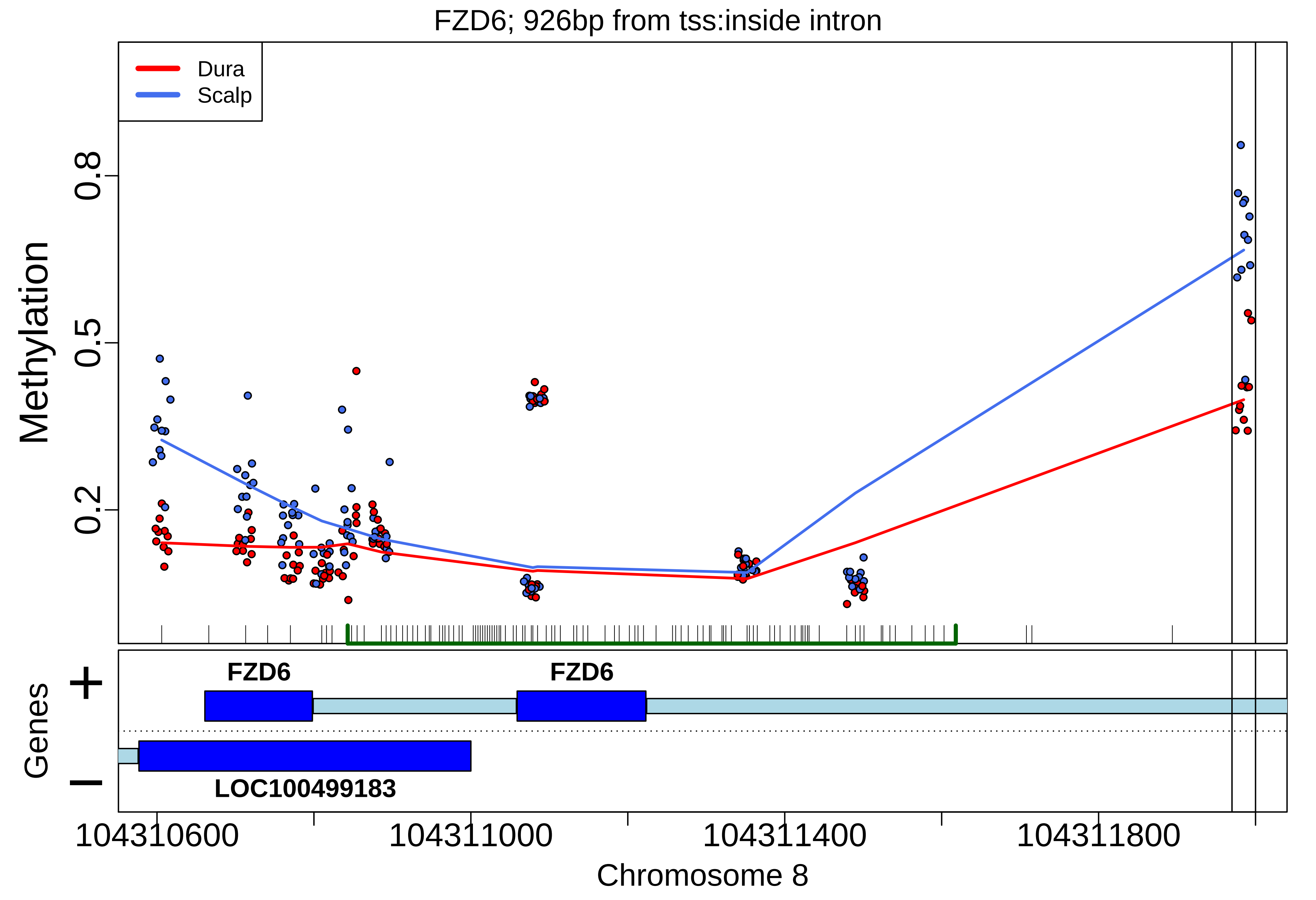

FZD6; 926bp from tss:inside intron
Dura
Scalp
Methylation
0.2
0.5
0.8
Genes
+
−
FZD6
FZD6
LOC100499183
104310600
104311000
104311400
104311800
Chromosome 8

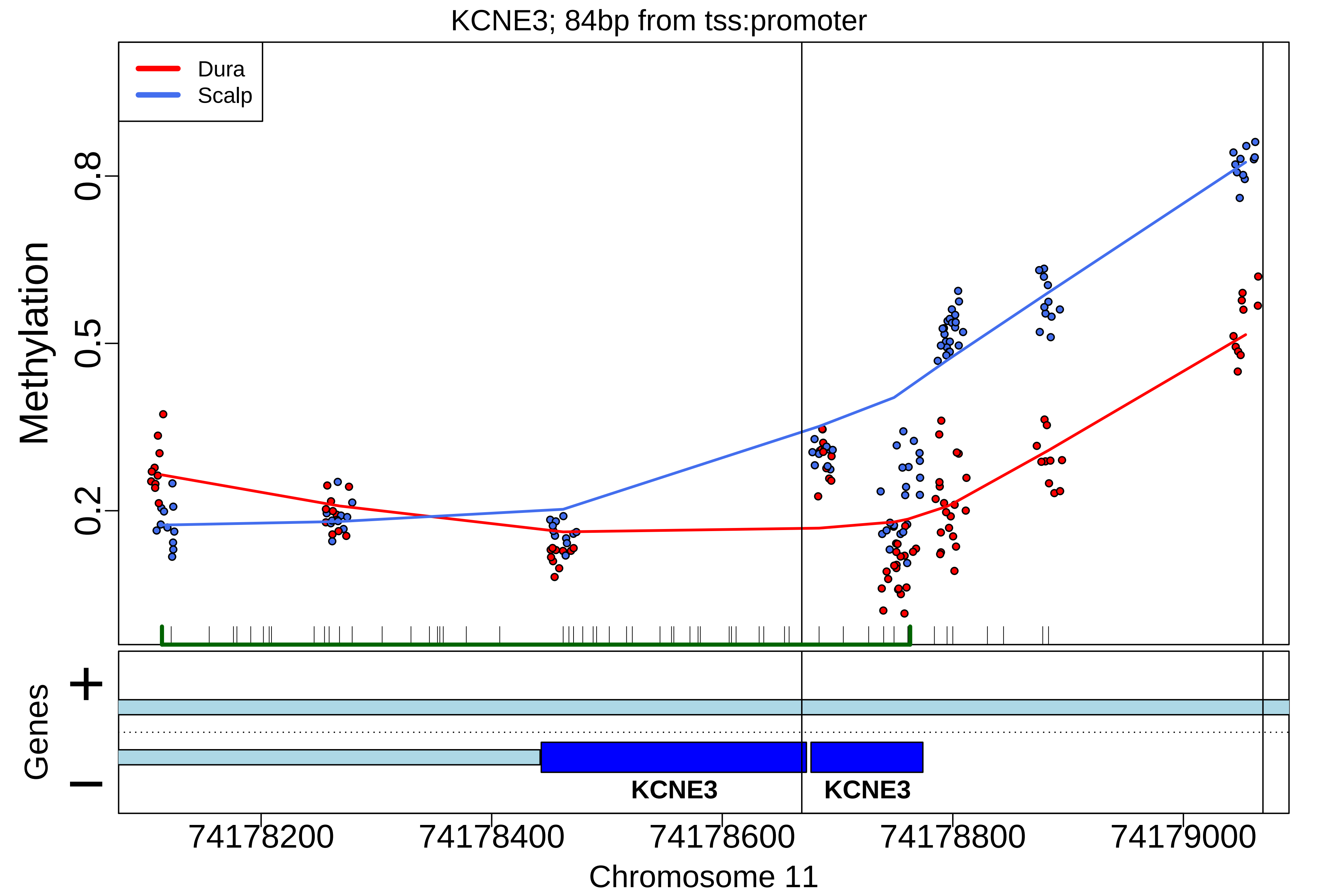
KCNE3; 84bp from tss:promoter
Dura
Scalp
Methylation
0.2
0.5
0.8
Genes
+
-
KCNE3
KCNE3
74178200
74178400
74178600
74178800
74179000
Chromosome 11

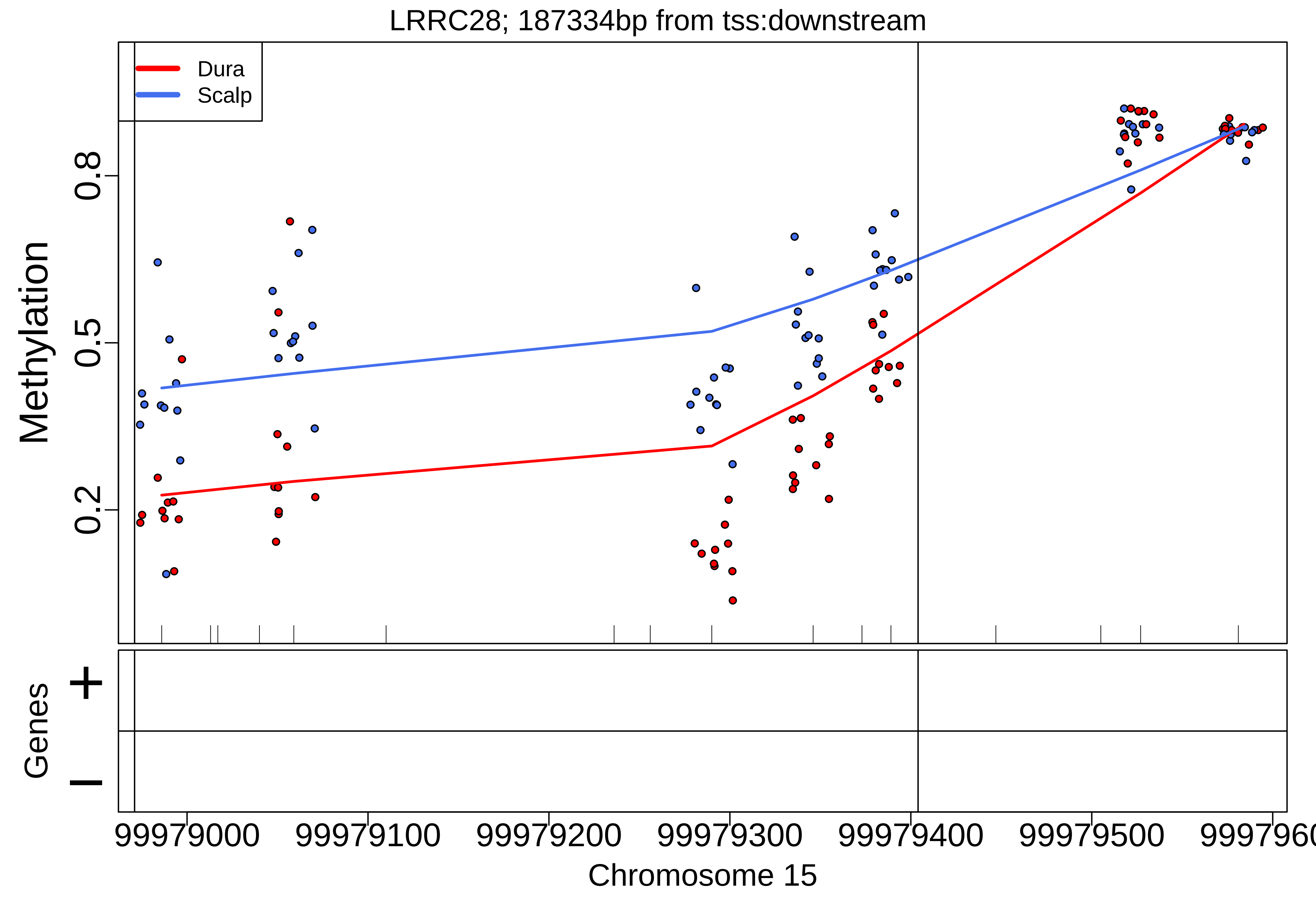
LRRC28; 187334bp from tss:downstream
Dura
Scalp
Methylation
0.2
0.5
0.8
Genes
+
−
99979000
99979100
99979200
99979300
99979400
99979500
Chromosome 15

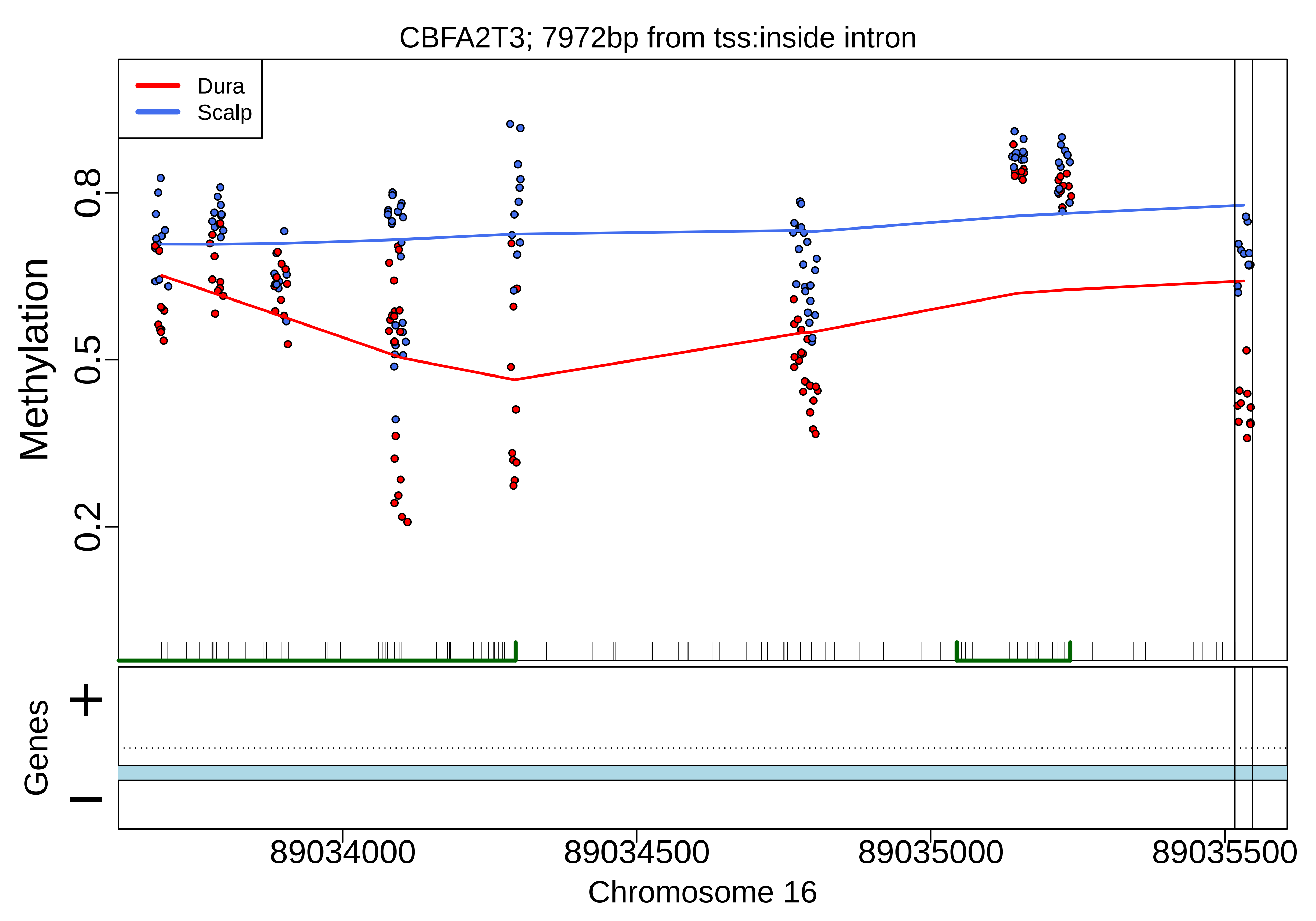
CBFA2T3; 7972bp from tss:inside intron
Dura
Scalp
Methylation
0.2
0.5
0.8
Genes
+
-
89034000
89034500
89035000
89035500
Chromosome 16
