# Supplementary material for: Strong Components of Epigenetic Memory in Cultured Human Fibroblasts Related to Site of Origin and Donor Age
Source: PLoS Genet. 2016 Feb 25;12(2):e1005819. doi: 10.1371/journal.pgen.1005819 (PMC4767228; doi:10.1371/journal.pgen.1005819)
Supplement: S8 Fig — DNAm plotted with respect to age for all 7,265 CpGs significantly associated with diverging DNAm levels across aging (at FDR < 10%). (PDF) [file pgen.1005819.s008.pdf]

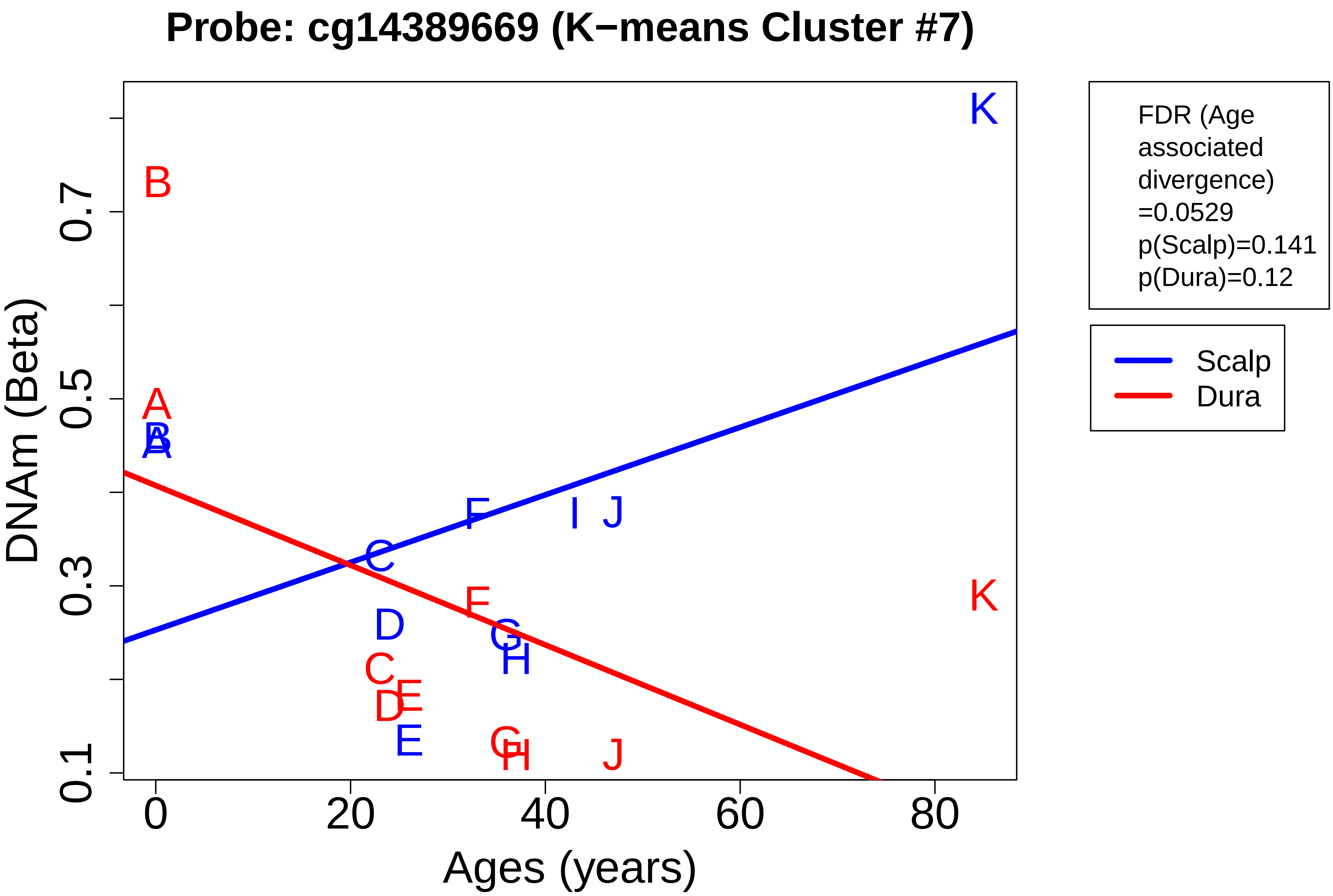

Probe: cg14389669 (K−means Cluster #7)
DNAm (Beta)
0.1
0.3
0.5
0.7
Ages (years)
0
20
40
60
80
FDR (Age associated divergence) =0.0529
p(Scalp)=0.141
p(Dura)=0.12
Scalp
Dura

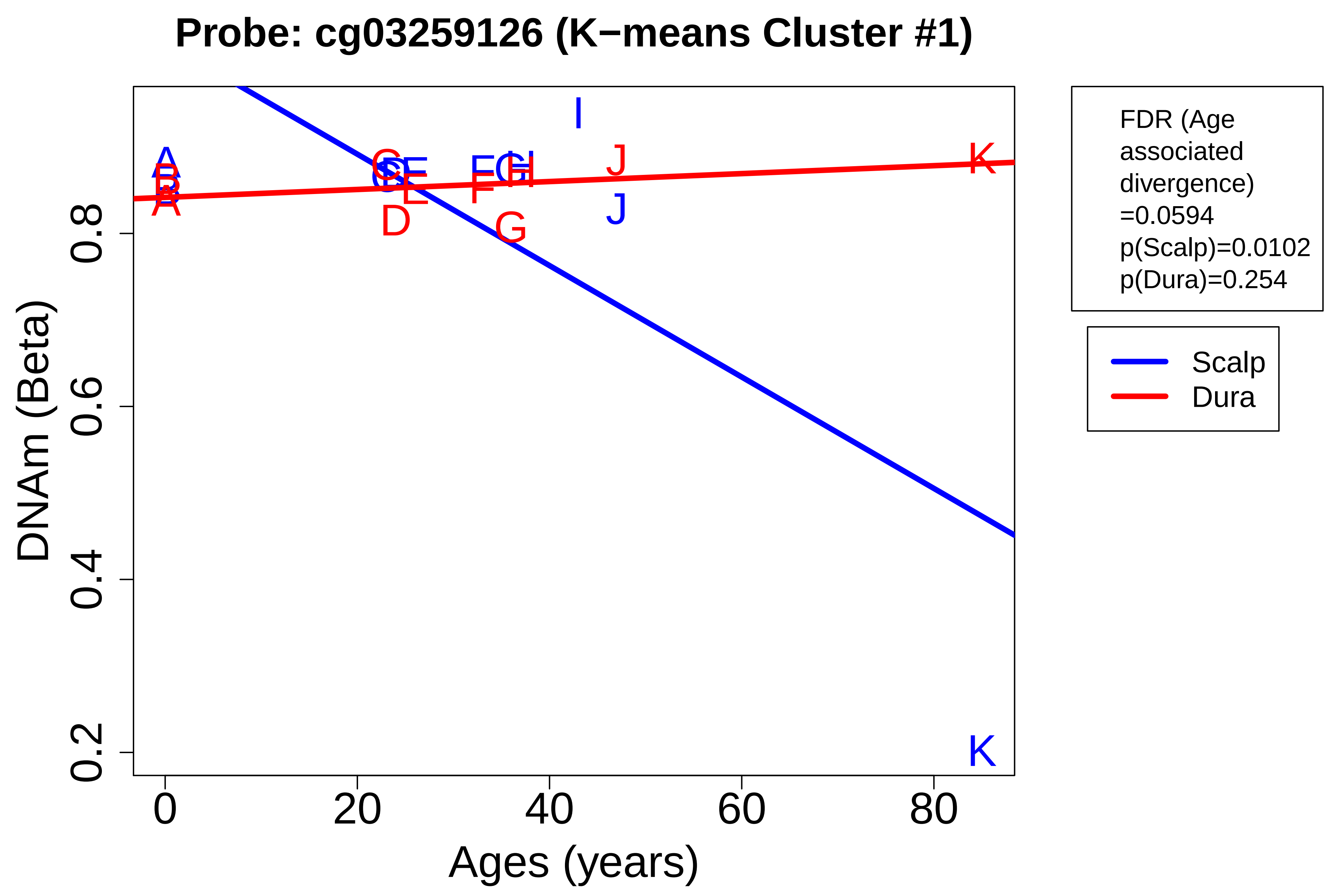

Probe: cg03259126 (K−means Cluster #1)
DNAm (Beta)
0.2
0.4
0.6
0.8
Ages (years)
0
20
40
60
80
FDR (Age associated divergence) =0.0594
p(Scalp)=0.0102
p(Dura)=0.254
Scalp
Dura

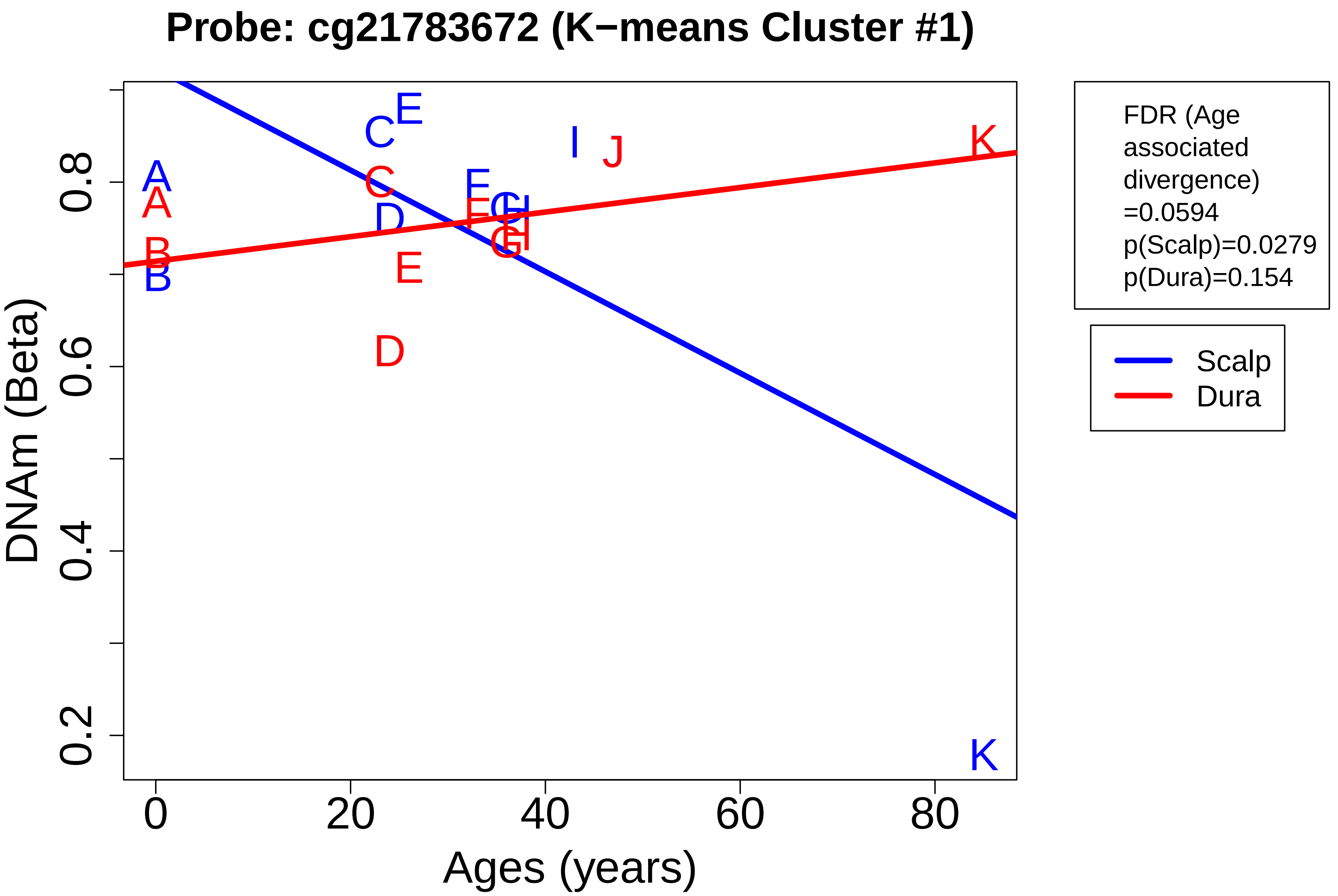

Probe: cg21783672 (K−means Cluster #1)
DNAm (Beta)
0.2
0.4
0.6
0.8
0
20
40
60
80
Ages (years)
FDR (Age associated divergence) =0.0594
p(Scalp)=0.0279
p(Dura)=0.154
Scalp
Dura

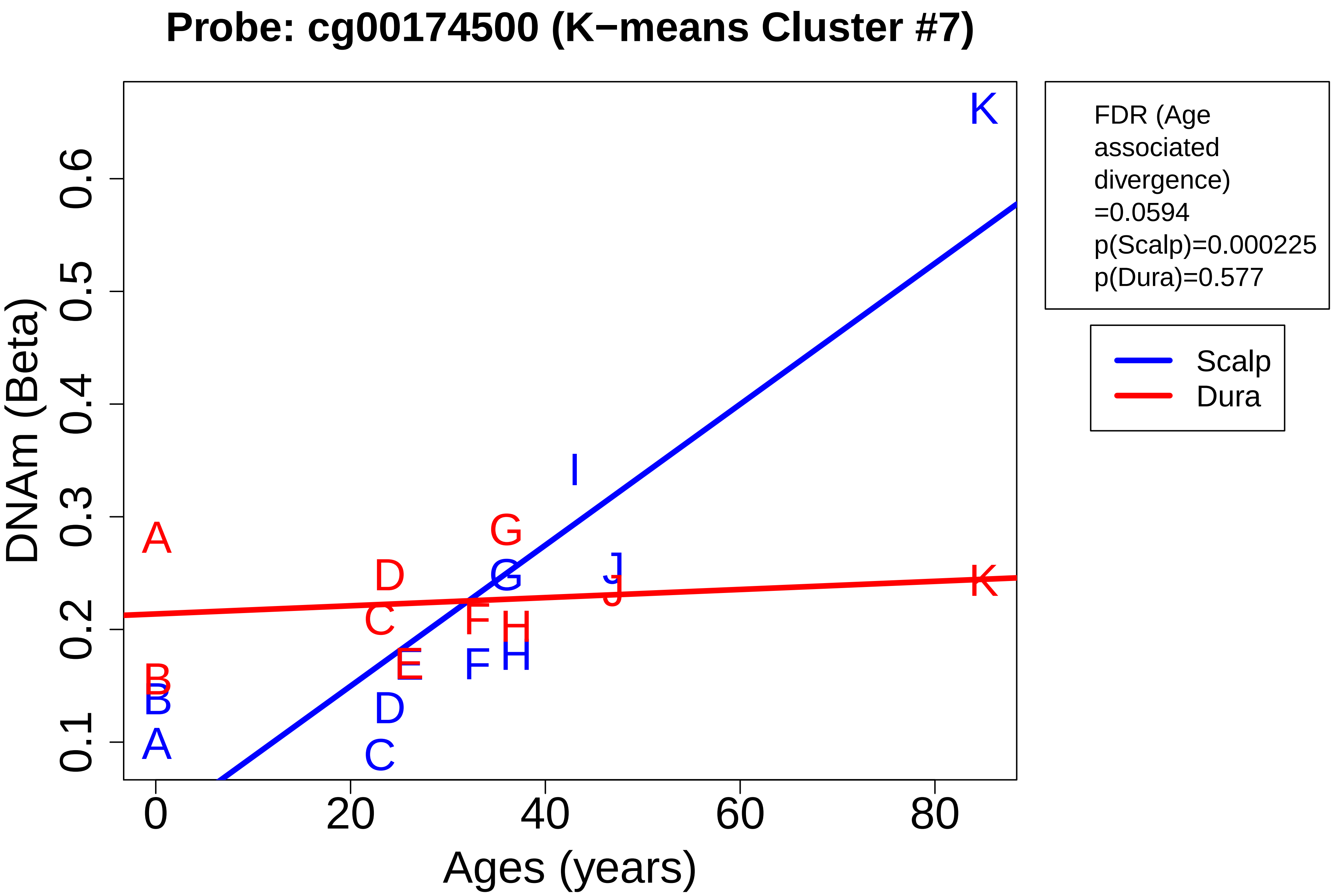

Probe: cg00174500 (K−means Cluster #7)
DNAm (Beta)
0.1
0.2
0.3
0.4
0.5
0.6
Ages (years)
0
20
40
60
80
FDR (Age associated divergence) =0.0594
p(Scalp)=0.000225
p(Dura)=0.577
Scalp
Dura
A
B
C
D
E
F
G
H
I
J
K

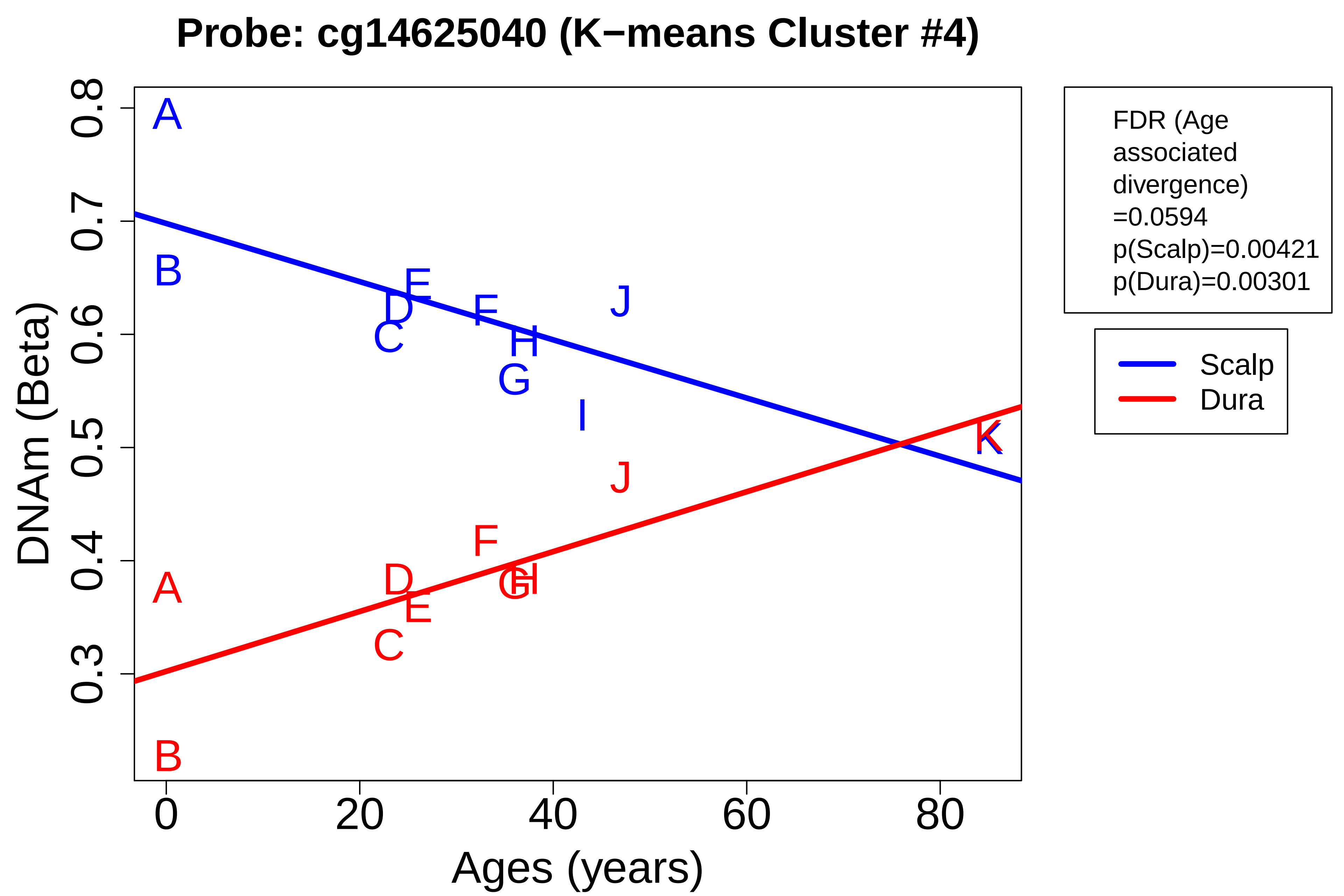

Probe: cg14625040 (K−means Cluster #4)
DNAm (Beta)
0.3
0.4
0.5
0.6
0.7
0.8
0
20
40
60
80
Ages (years)
A
B
C
D
E
F
G
H
I
J
K
FDR (Age associated divergence) =0.0594
p(Scalp)=0.00421
p(Dura)=0.00301
Scalp
Dura

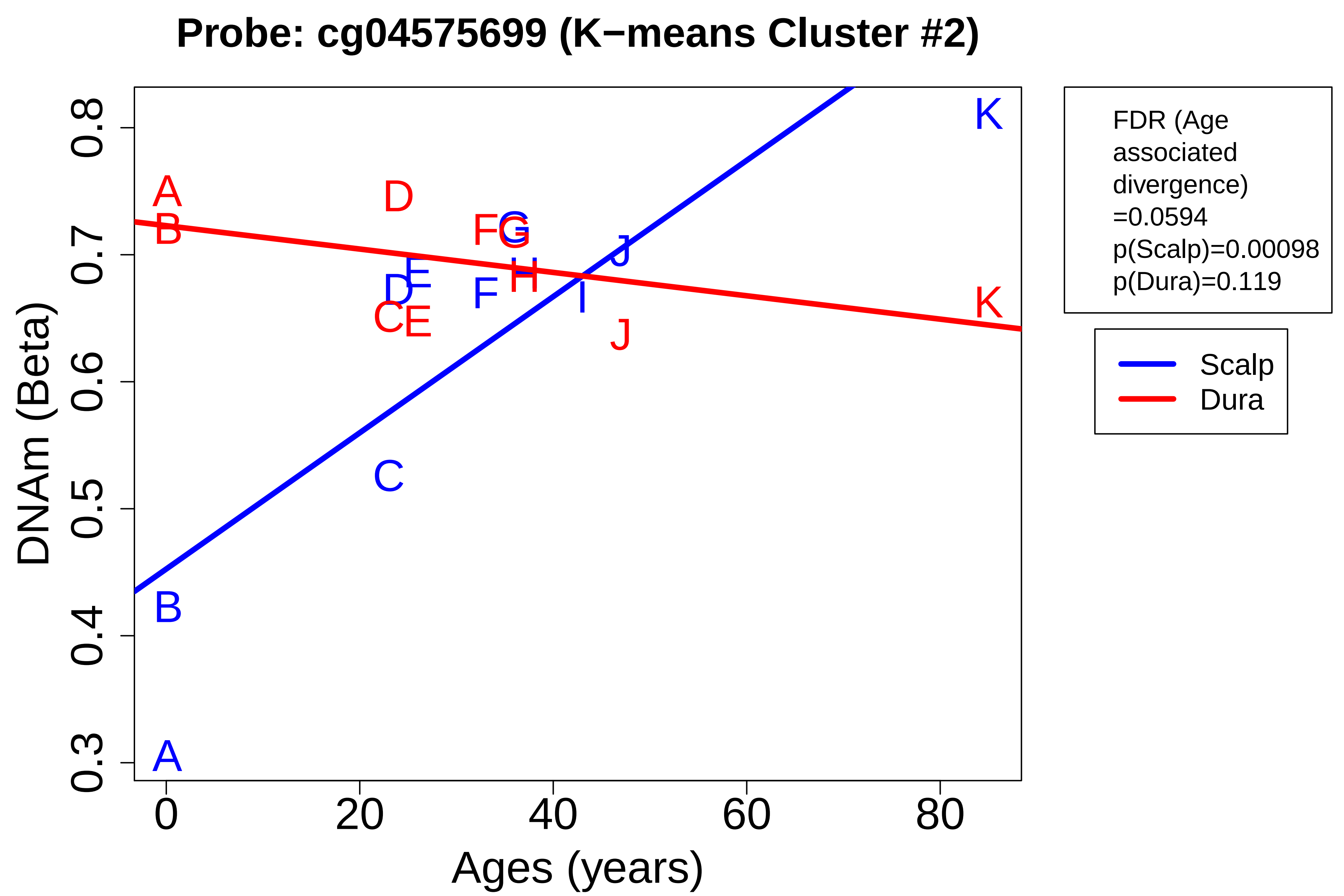
Probe: cg04575699 (K−means Cluster #2)
DNAm (Beta)
Ages (years)
0.3
0.4
0.5
0.6
0.7
0.8
0
20
40
60
80
FDR (Age associated divergence) =0.0594
p(Scalp)=0.00098
p(Dura)=0.119
Scalp
Dura

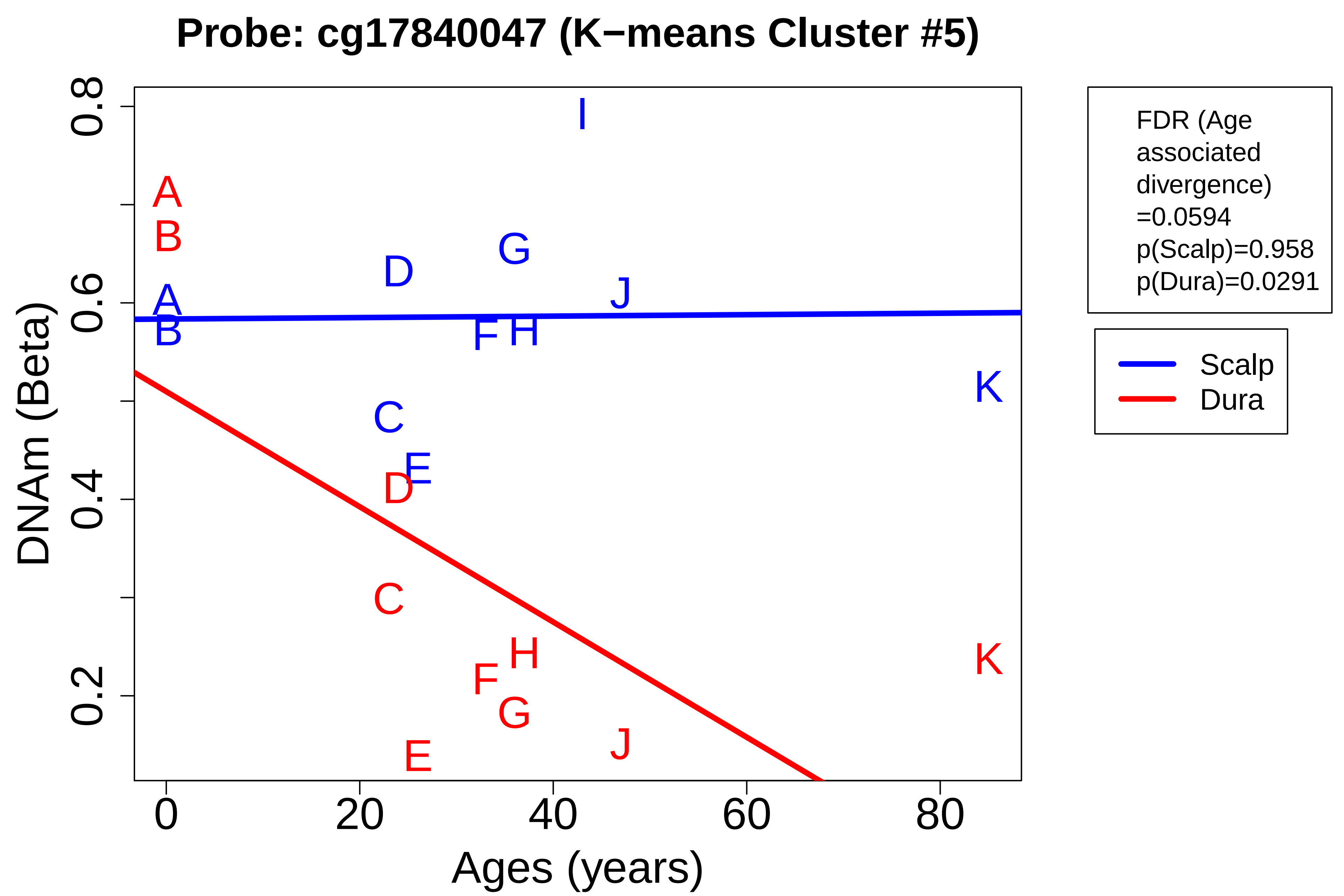
Probe: cg17840047 (K−means Cluster #5)
DNAm (Beta)
Ages (years)
0.2
0.4
0.6
0.8
0
20
40
60
80
FDR (Age associated divergence) =0.0594
p(Scalp)=0.958
p(Dura)=0.0291
Scalp
Dura
A
B
C
D
E
F
G
H
I
J
K

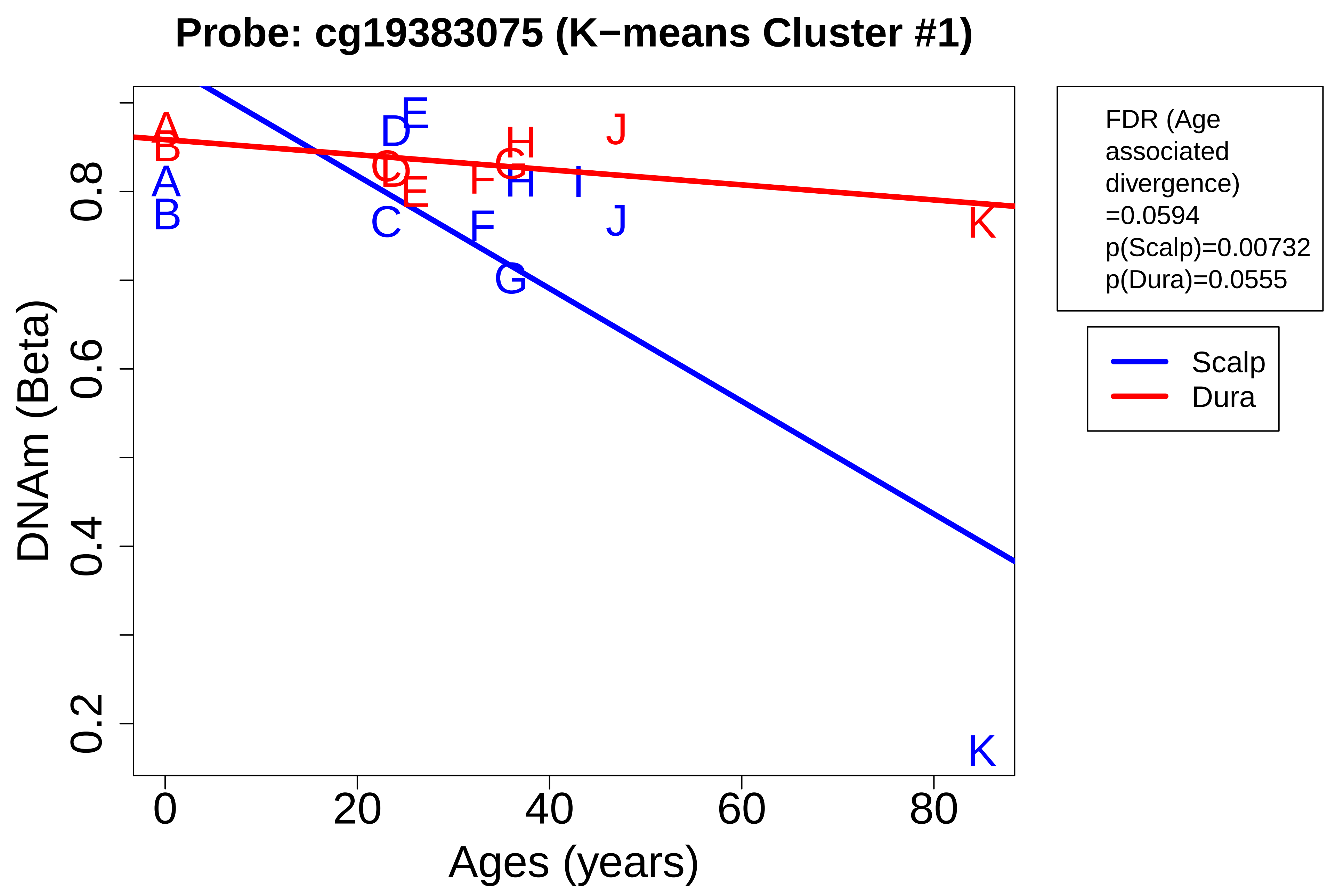

Probe: cg19383075 (K−means Cluster #1)
DNAm (Beta)
0.2
0.4
0.6
0.8
Ages (years)
0
20
40
60
80
FDR (Age associated divergence) =0.0594
p(Scalp)=0.00732
p(Dura)=0.0555
Scalp
Dura
A
B
C
D
E
F
G
H
I
J
K

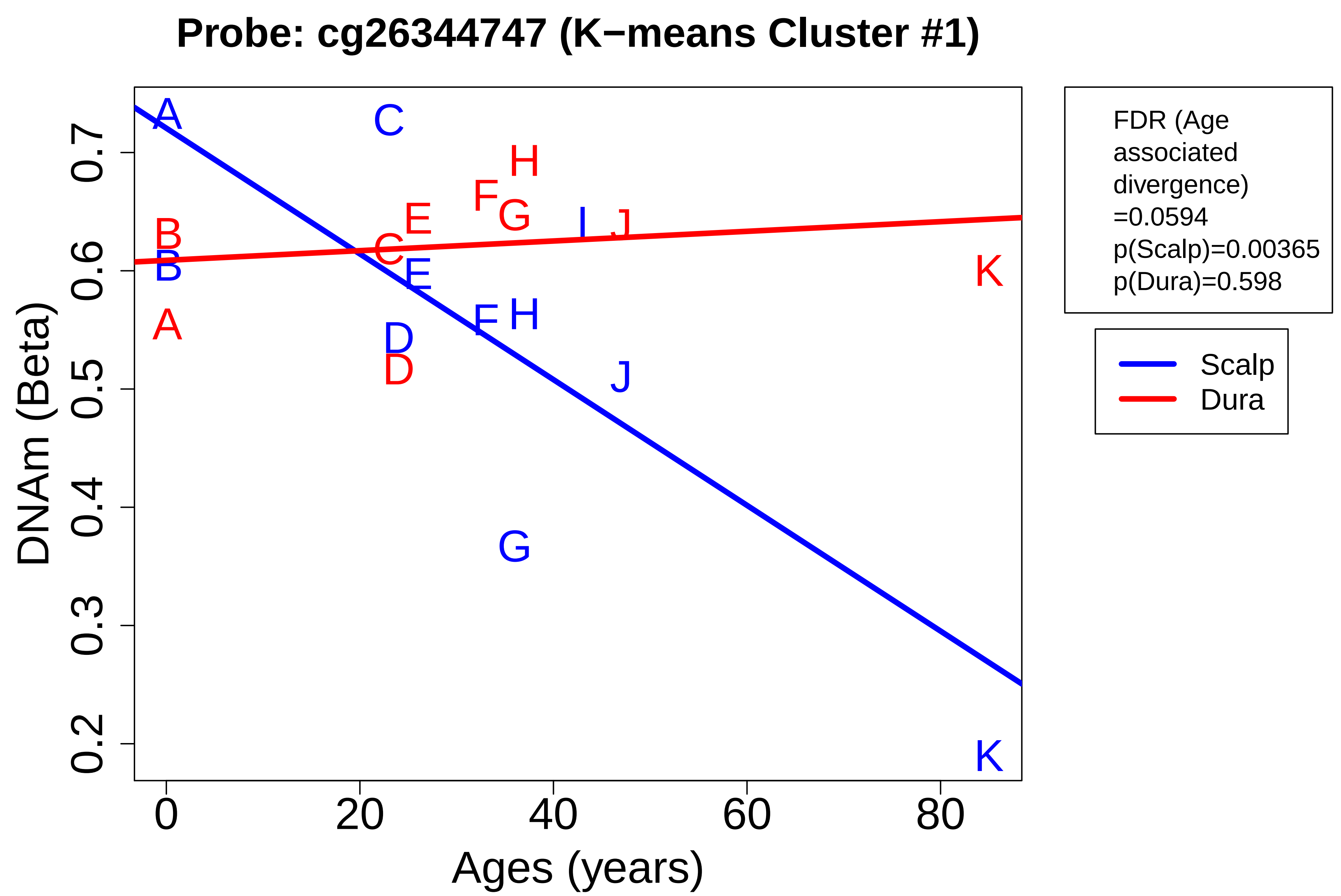
Probe: cg26344747 (K−means Cluster #1)
DNAm (Beta)
Ages (years)
0.2
0.3
0.4
0.5
0.6
0.7
0
20
40
60
80
FDR (Age associated divergence) =0.0594
p(Scalp)=0.00365
p(Dura)=0.598
Scalp
Dura
A
B
C
D
E
F
G
H
I
J
K

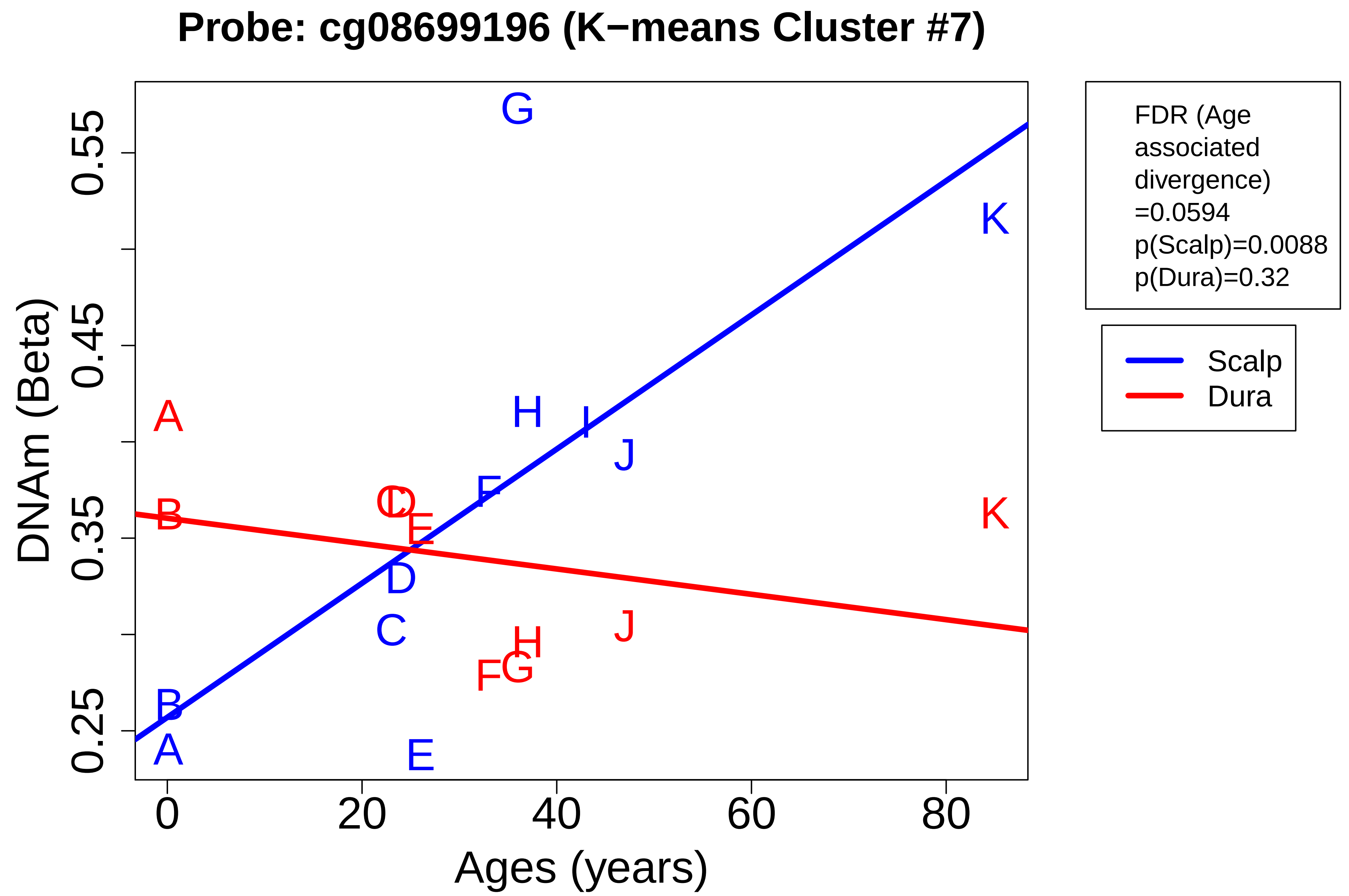

Probe: cg08699196 (K−means Cluster #7)
FDR (Age associated divergence) =0.0594
p(Scalp)=0.0088
p(Dura)=0.32
Scalp
Dura
DNAm (Beta)
0.25
0.35
0.45
0.55
Ages (years)
0
20
40
60
80
A
B
C
D
E
F
G
H
I
J
K

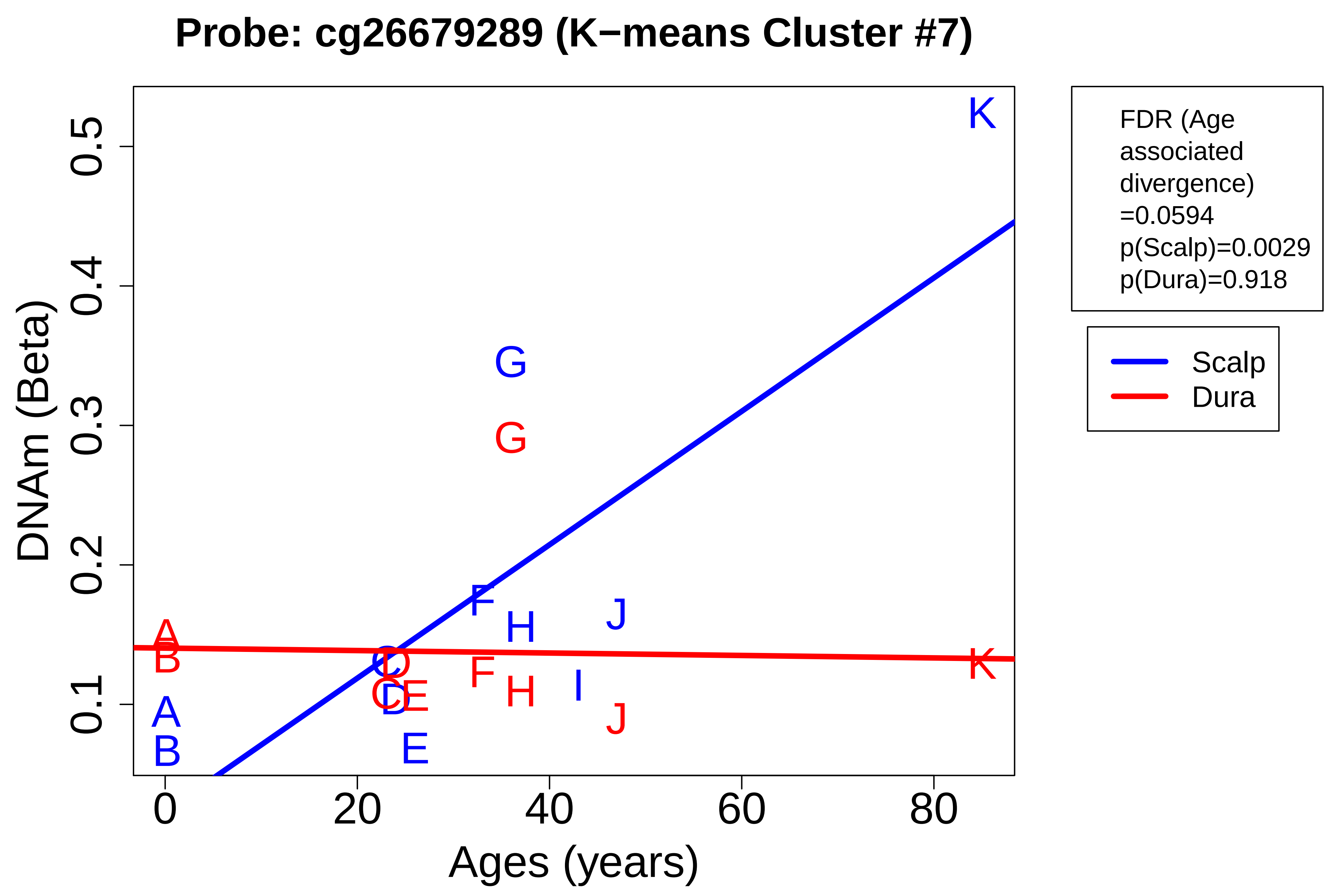

Probe: cg26679289 (K−means Cluster #7)
DNAm (Beta)
0.1
0.2
0.3
0.4
0.5
0
20
40
60
80
Ages (years)
A
B
C
D
E
F
G
H
I
J
K
FDR (Age associated divergence) =0.0594
p(Scalp)=0.0029
p(Dura)=0.918
Scalp
Dura

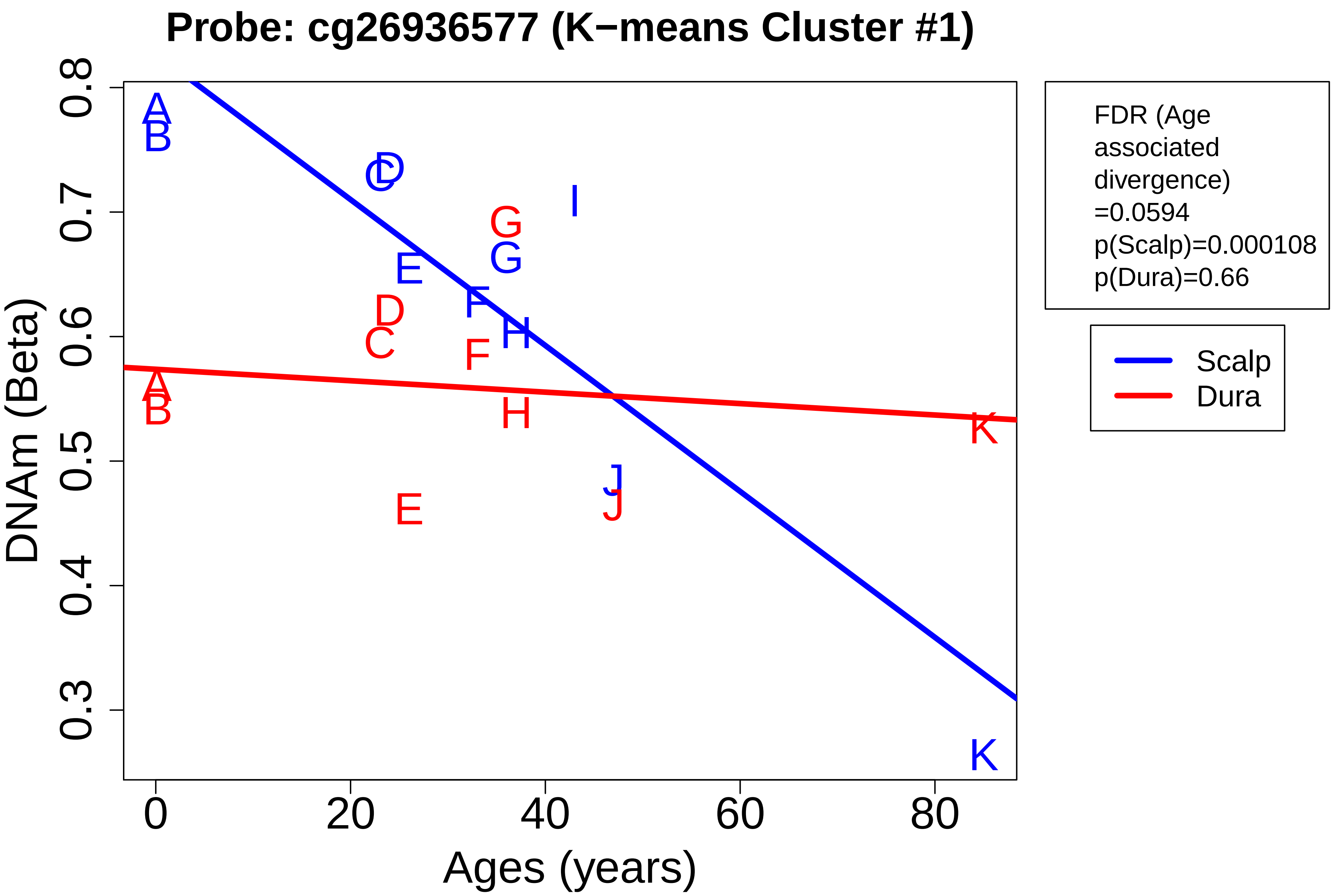

Probe: cg26936577 (K−means Cluster #1)
DNAm (Beta)
0.3
0.4
0.5
0.6
0.7
0.8
Ages (years)
0
20
40
60
80
A
B
C
D
E
F
G
H
I
J
K
FDR (Age associated divergence) =0.0594
p(Scalp)=0.000108
p(Dura)=0.66
Scalp
Dura

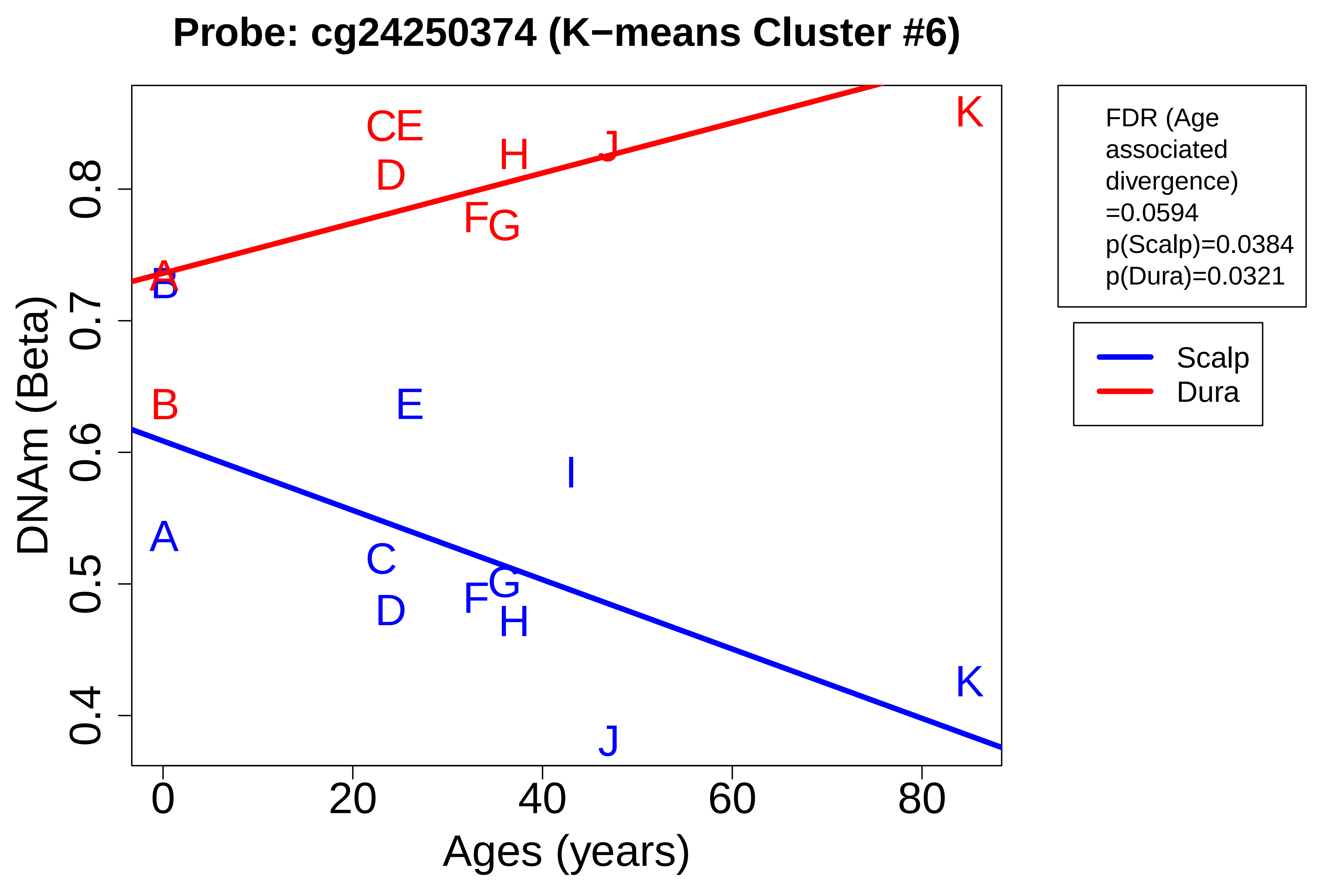

Probe: cg24250374 (K−means Cluster #6)
DNAm (Beta)
Ages (years)
0.4
0.5
0.6
0.7
0.8
0
20
40
60
80
FDR (Age associated divergence) =0.0594
p(Scalp)=0.0384
p(Dura)=0.0321
Scalp
Dura

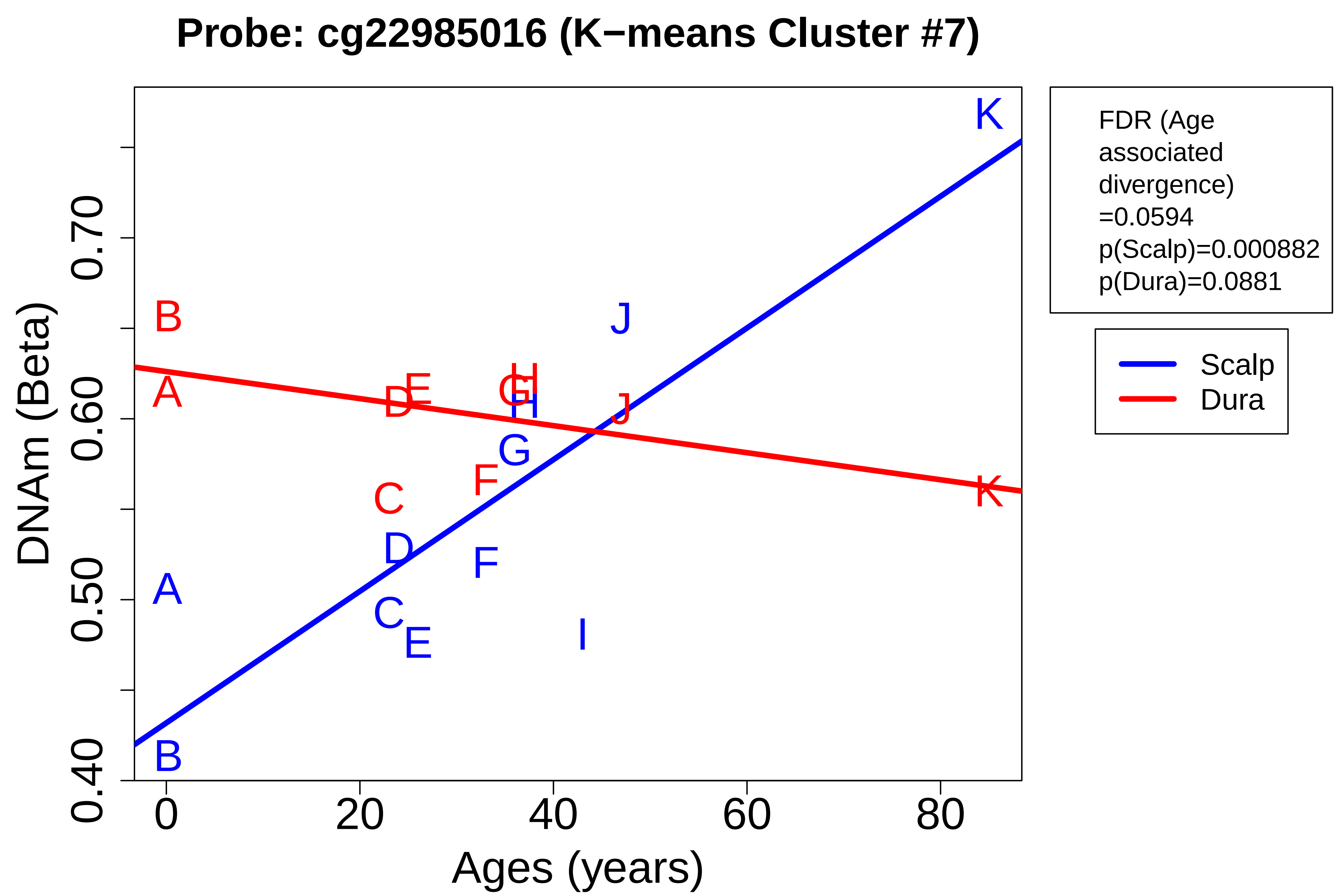

Probe: cg22985016 (K−means Cluster #7)
FDR (Age associated divergence) =0.0594
p(Scalp)=0.000882
p(Dura)=0.0881
Scalp
Dura
DNAm (Beta)
0.40
0.50
0.60
0.70
0
20
40
60
80
Ages (years)
A
B
C
D
E
F
G
H
I
J
K

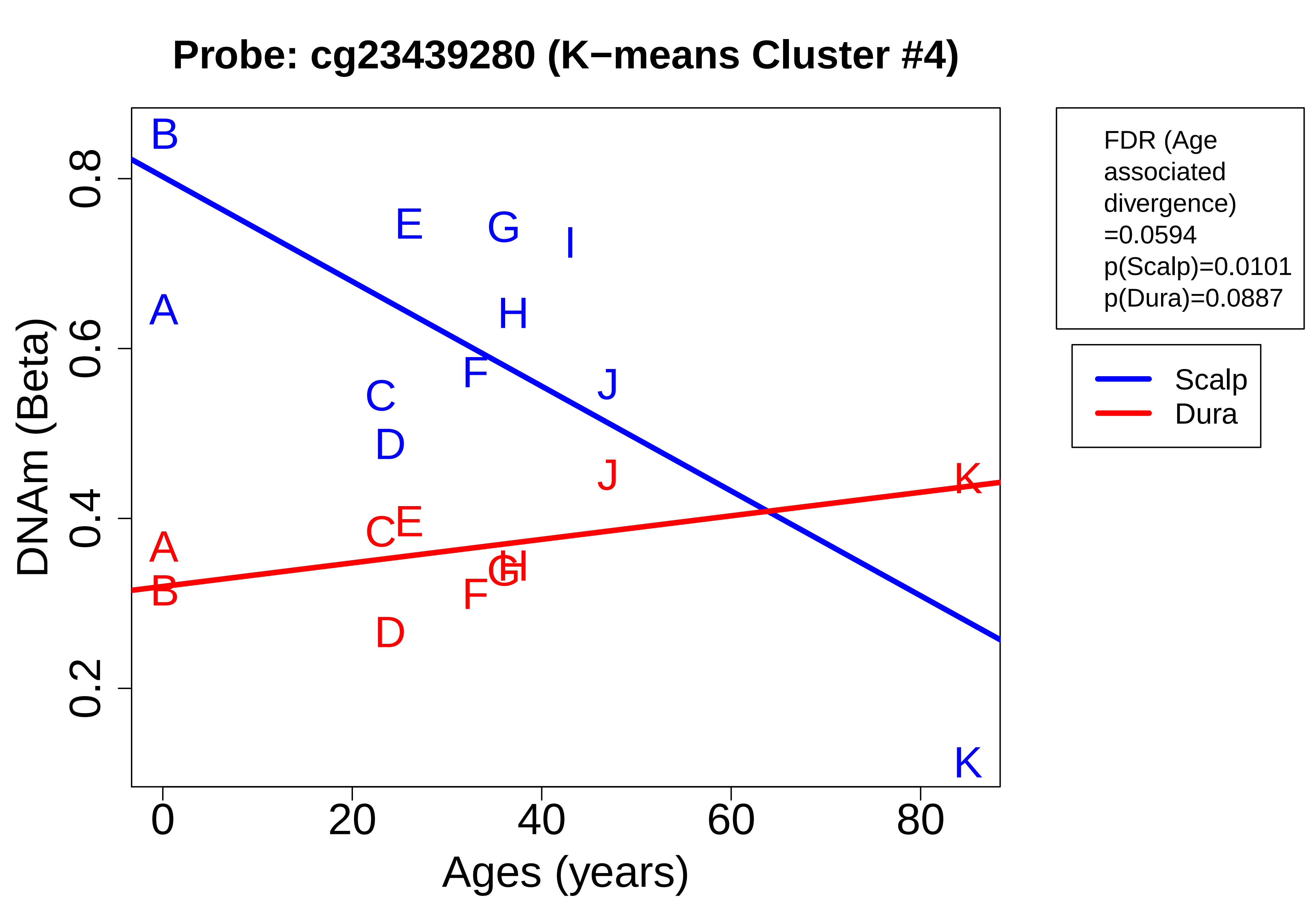

Probe: cg23439280 (K−means Cluster #4)
DNAm (Beta)
0.2
0.4
0.6
0.8
Ages (years)
0
20
40
60
80
A
B
C
D
E
F
G
H
I
J
K
FDR (Age associated divergence) =0.0594
p(Scalp)=0.0101
p(Dura)=0.0887
Scalp
Dura

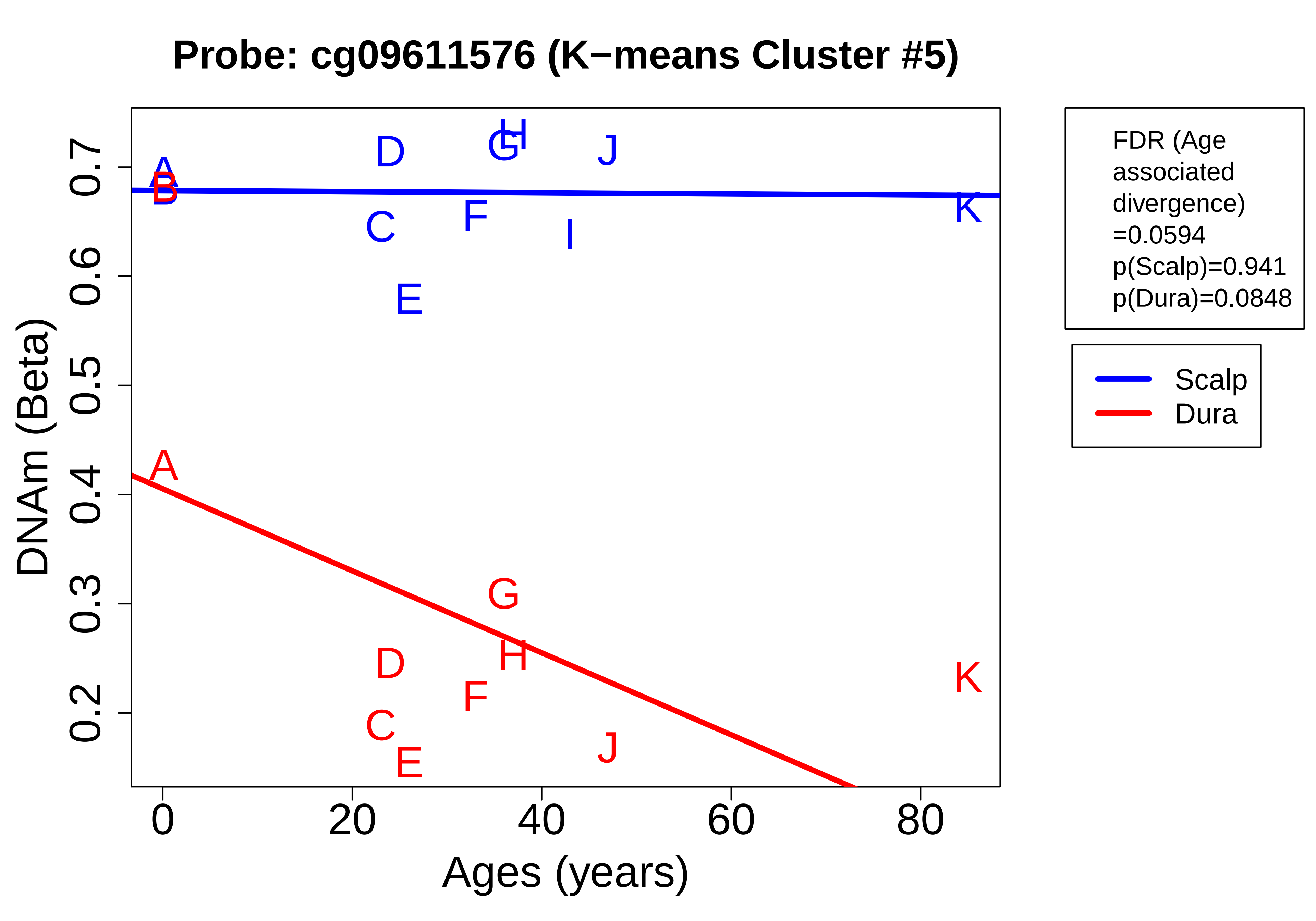
Probe: cg09611576 (K−means Cluster #5)
DNAm (Beta)
0.2
0.3
0.4
0.5
0.6
0.7
Ages (years)
0
20
40
60
80
A
B
C
D
E
F
G
H
I
J
K
FDR (Age associated divergence) =0.0594
p(Scalp)=0.941
p(Dura)=0.0848
Scalp
Dura

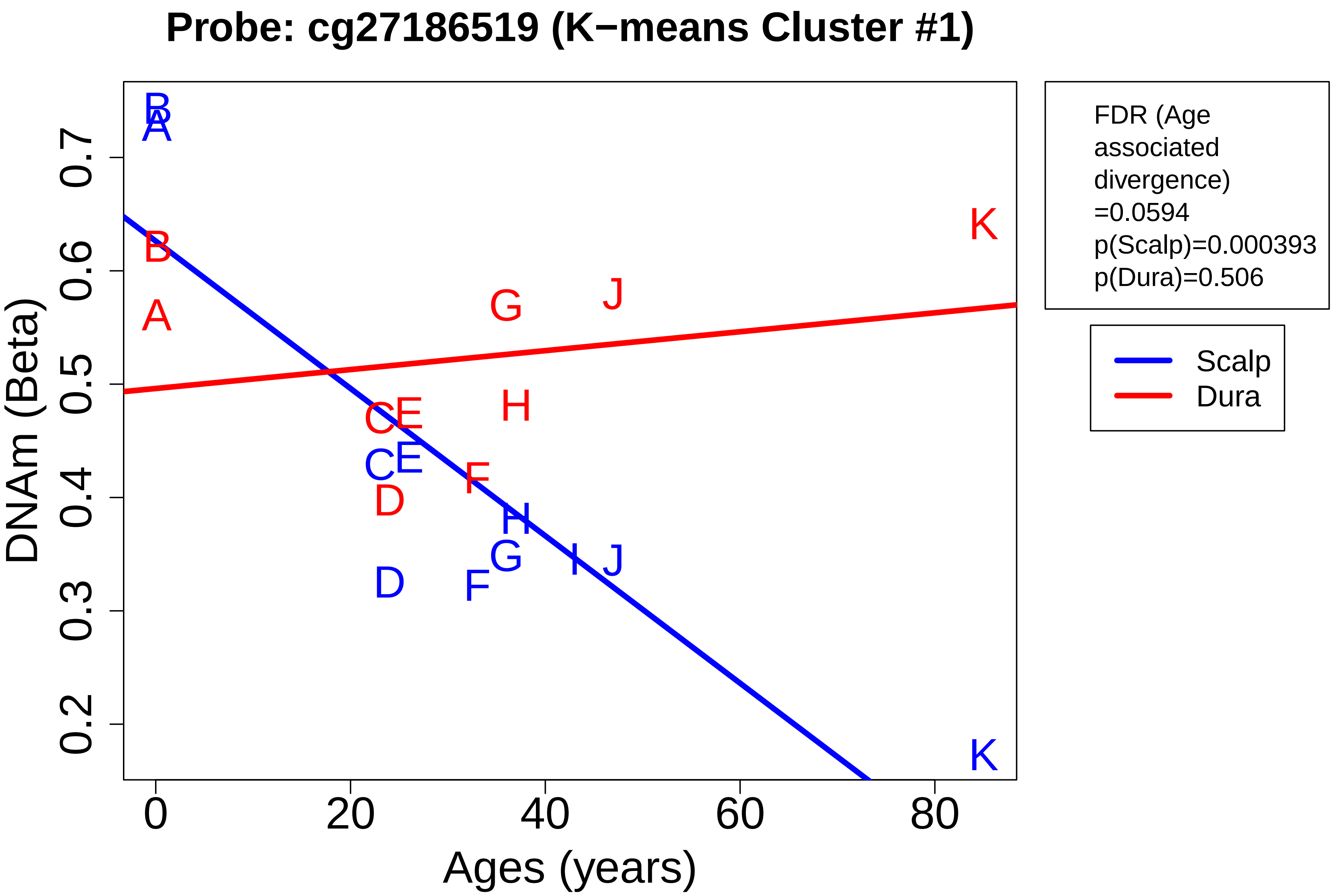
Probe: cg27186519 (K−means Cluster #1)
DNAm (Beta)
Ages (years)
0.2
0.3
0.4
0.5
0.6
0.7
0
20
40
60
80
FDR (Age associated divergence) =0.0594
p(Scalp)=0.000393
p(Dura)=0.506
Scalp
Dura
A
B
C
D
E
F
G
H
I
J
K

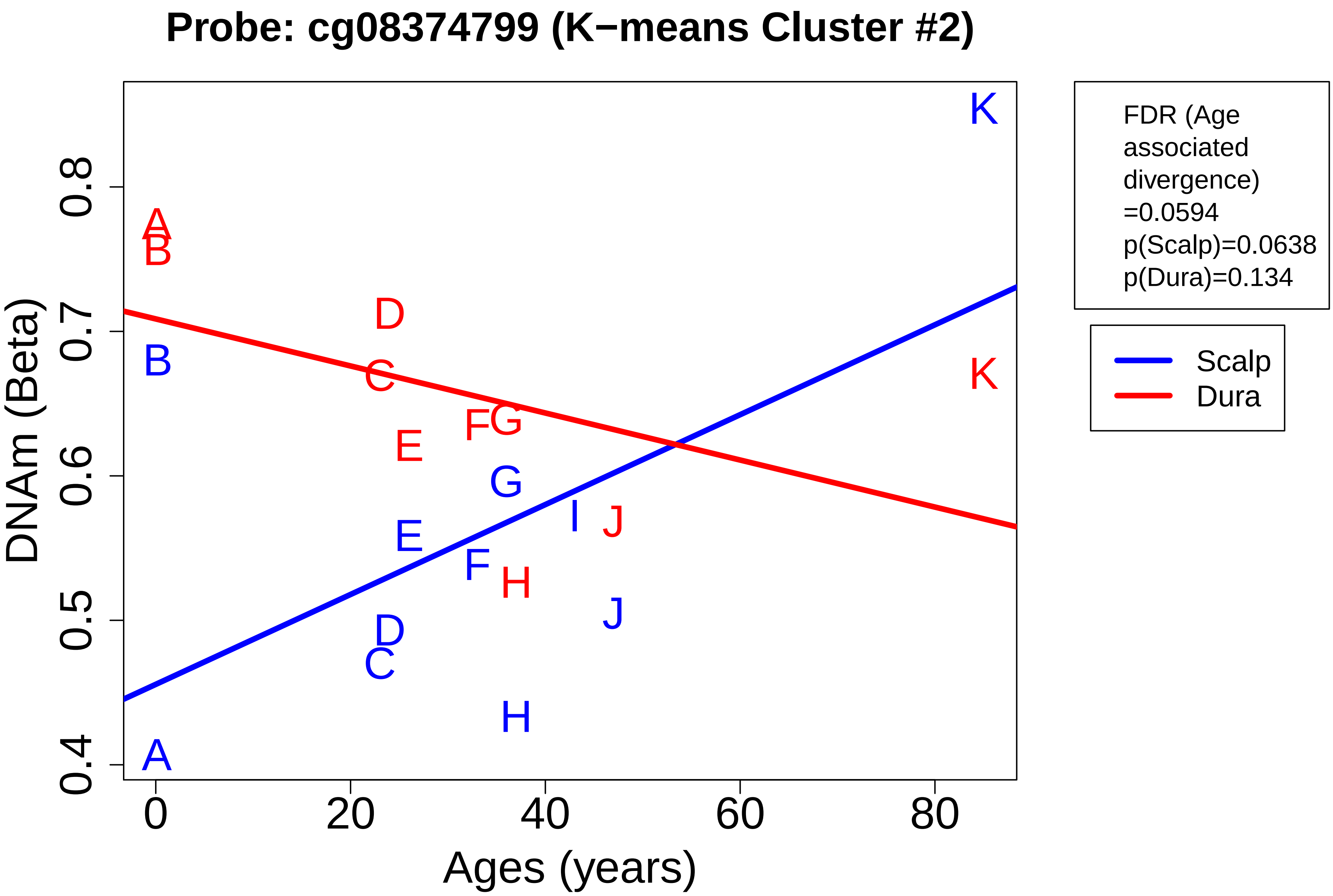

Probe: cg08374799 (K−means Cluster #2)
DNAm (Beta)
0.4
0.5
0.6
0.7
0.8
Ages (years)
0
20
40
60
80
A
B
C
D
E
F
G
H
I
J
K
FDR (Age associated divergence) =0.0594
p(Scalp)=0.0638
p(Dura)=0.134
Scalp
Dura

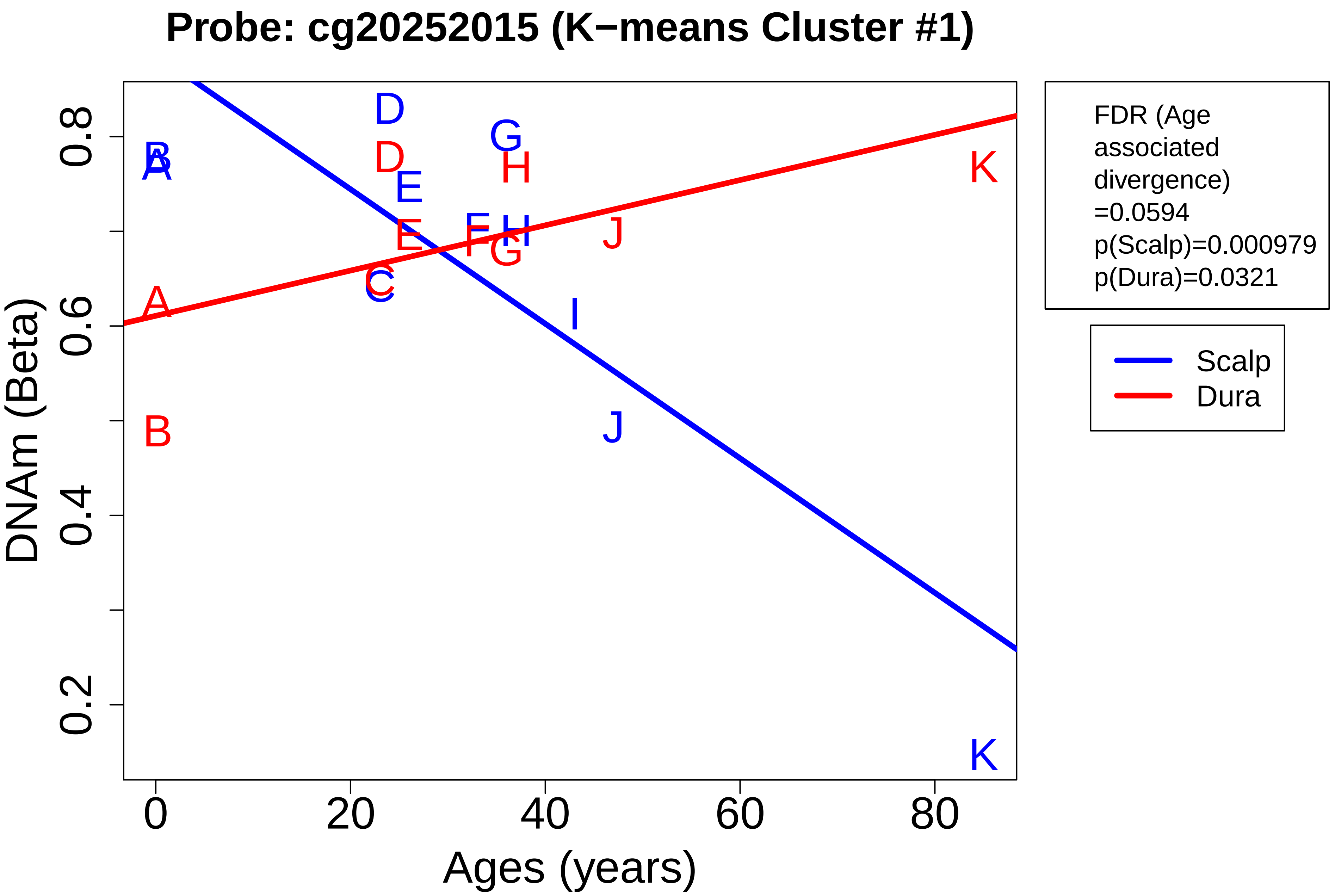
Probe: cg20252015 (K−means Cluster #1)
DNAm (Beta)
0.2
0.4
0.6
0.8
0
20
40
60
80
Ages (years)
FDR (Age associated divergence) =0.0594
p(Scalp)=0.000979
p(Dura)=0.0321
Scalp
Dura

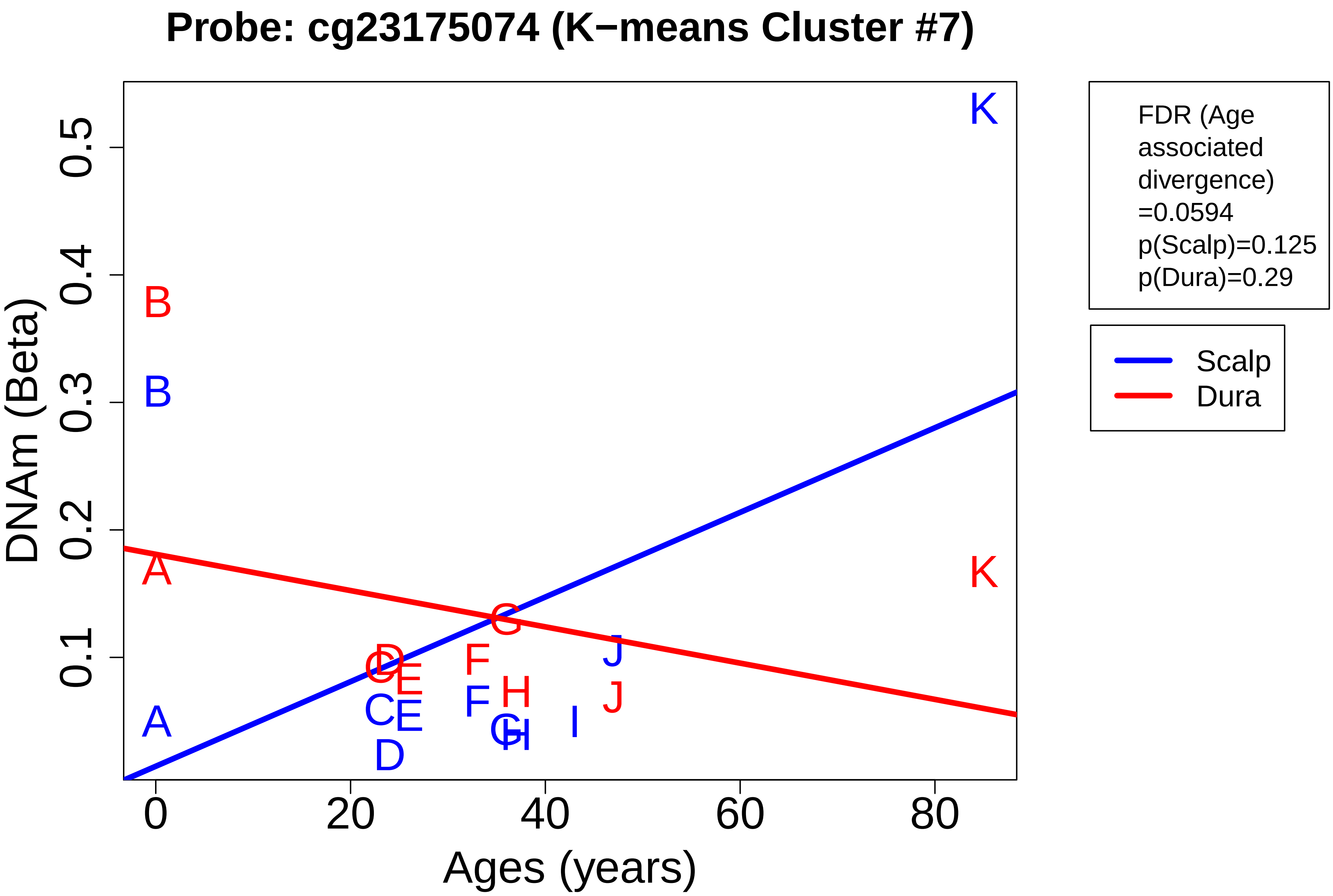

Probe: cg23175074 (K−means Cluster #7)
DNAm (Beta)
0.1
0.2
0.3
0.4
0.5
Ages (years)
0
20
40
60
80
FDR (Age associated divergence) =0.0594
p(Scalp)=0.125
p(Dura)=0.29
Scalp
Dura

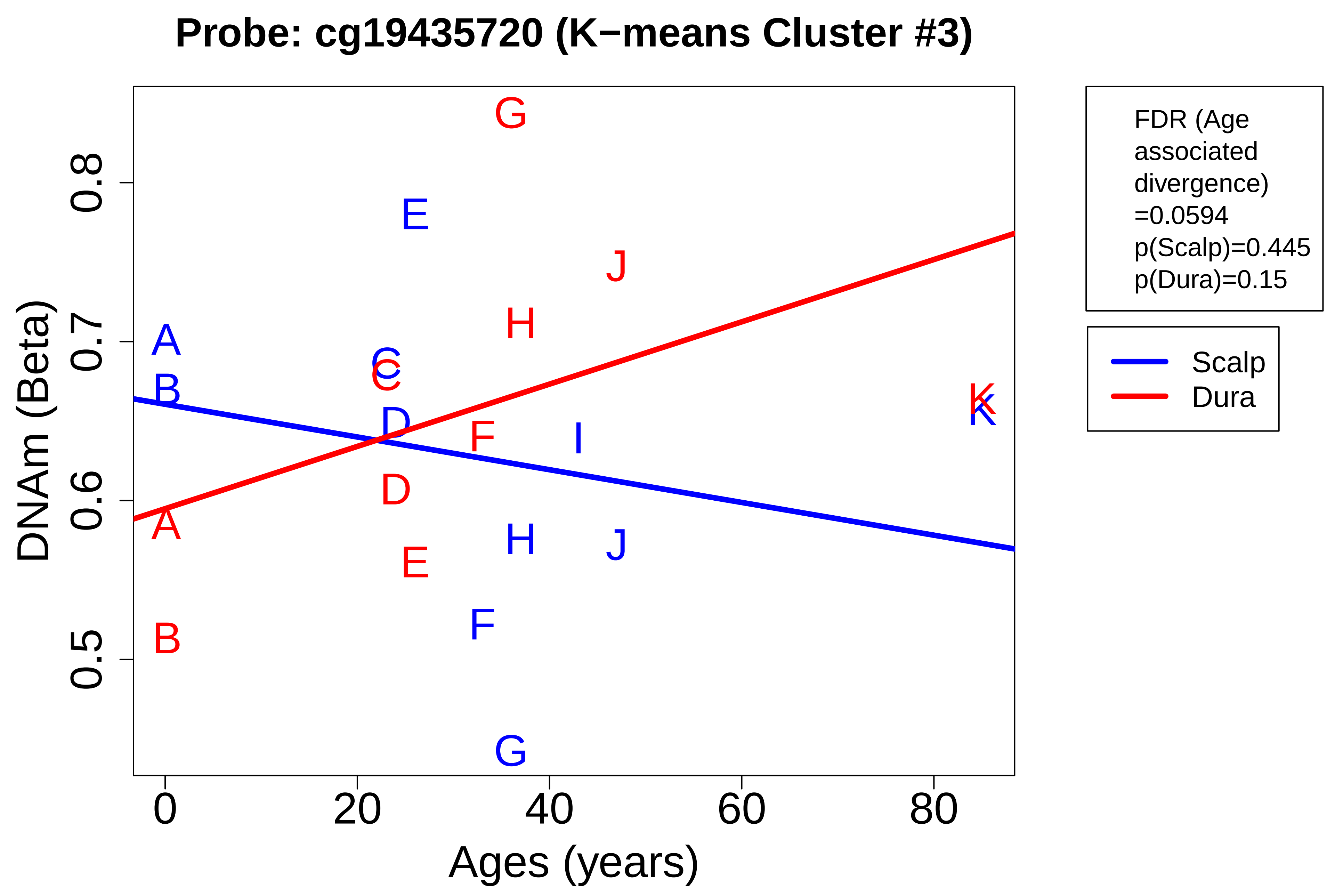
Probe: cg19435720 (K−means Cluster #3)
DNAm (Beta)
0.5
0.6
0.7
0.8
0
20
40
60
80
Ages (years)
A
B
C
D
E
F
G
H
I
J
K
FDR (Age associated divergence) =0.0594
p(Scalp)=0.445
p(Dura)=0.15
Scalp
Dura

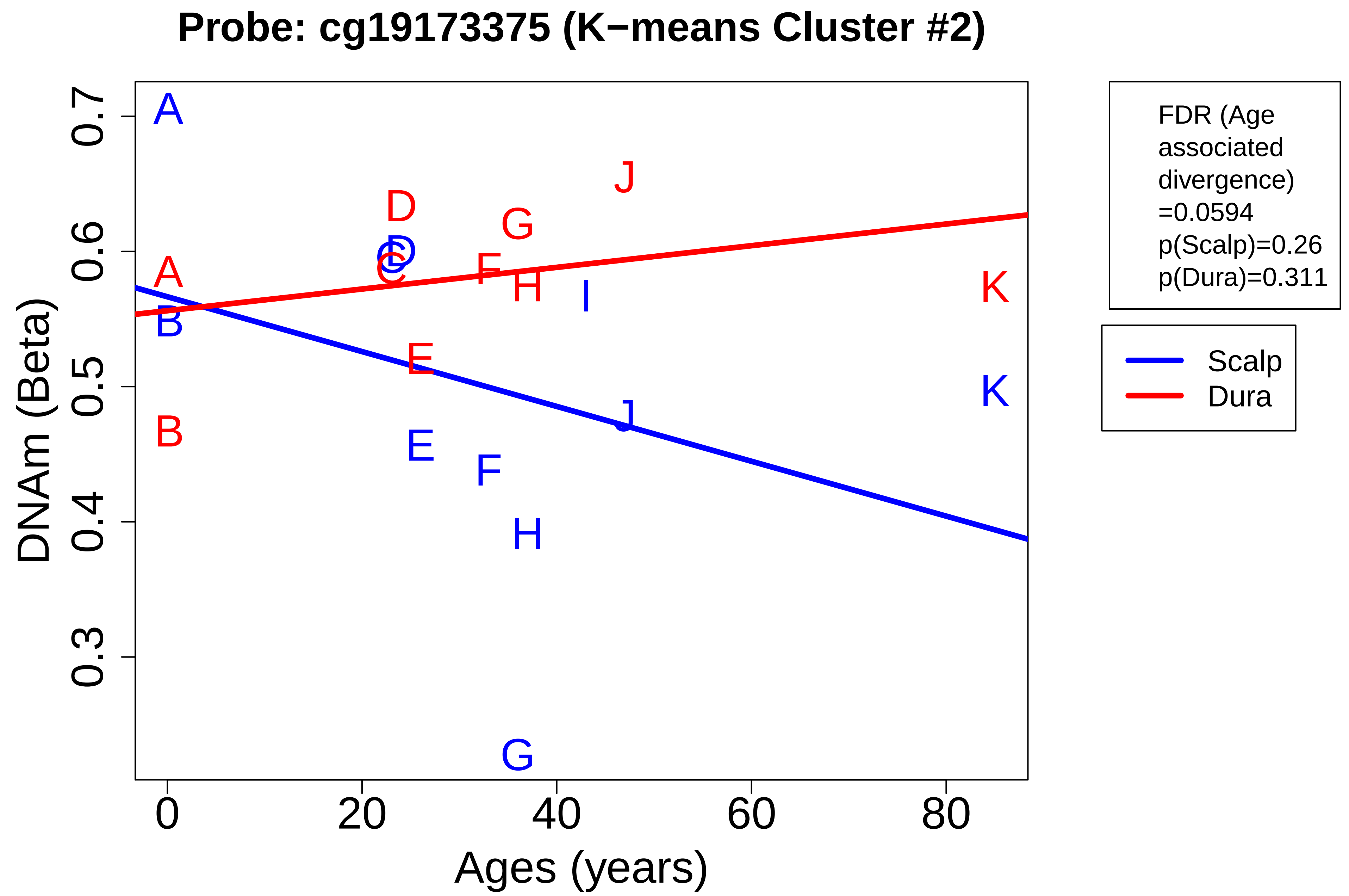

Probe: cg19173375 (K−means Cluster #2)
DNAm (Beta)
0.3
0.4
0.5
0.6
0.7
0
20
40
60
80
Ages (years)
FDR (Age associated divergence) =0.0594
p(Scalp)=0.26
p(Dura)=0.311
Scalp
Dura

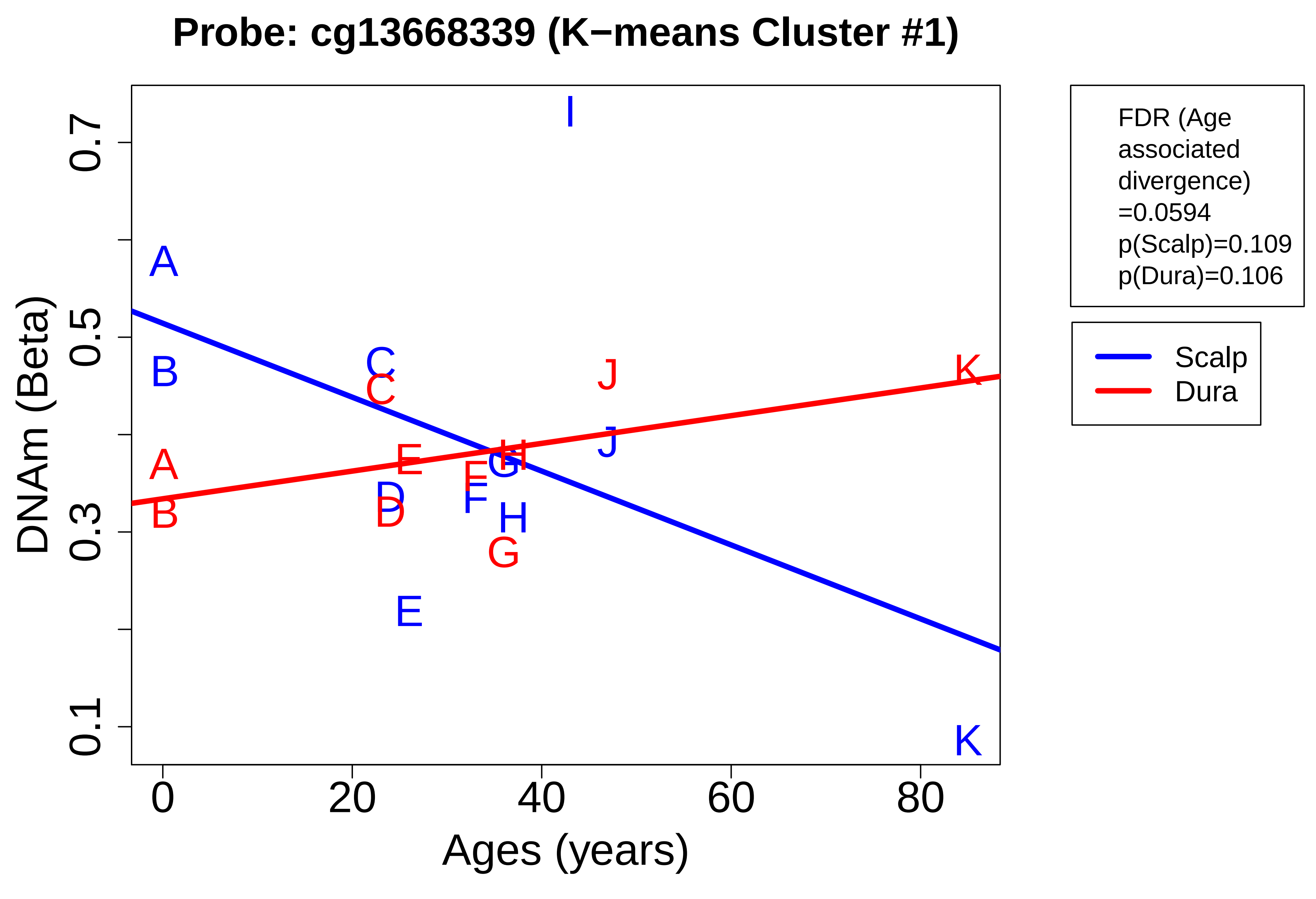

Probe: cg13668339 (K−means Cluster #1)
FDR (Age associated divergence) =0.0594
p(Scalp)=0.109
p(Dura)=0.106
Scalp
Dura
DNAm (Beta)
0.1
0.3
0.5
0.7
0
20
40
60
80
Ages (years)
A
B
C
D
E
F
G
H
I
J
K

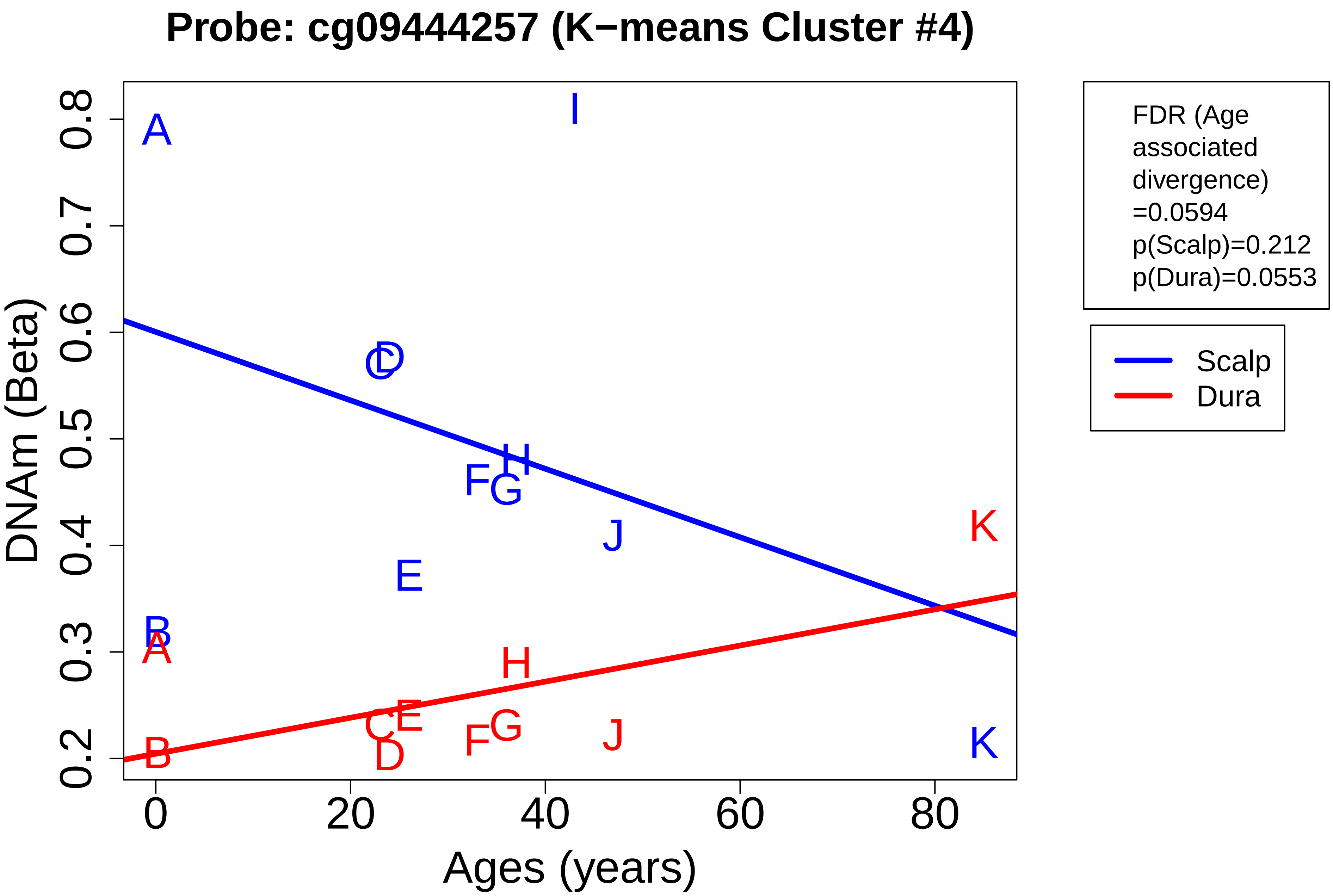

Probe: cg09444257 (K−means Cluster #4)
DNAm (Beta)
0.2
0.3
0.4
0.5
0.6
0.7
0.8
Ages (years)
0
20
40
60
80
FDR (Age associated divergence) =0.0594
p(Scalp)=0.212
p(Dura)=0.0553
Scalp
Dura

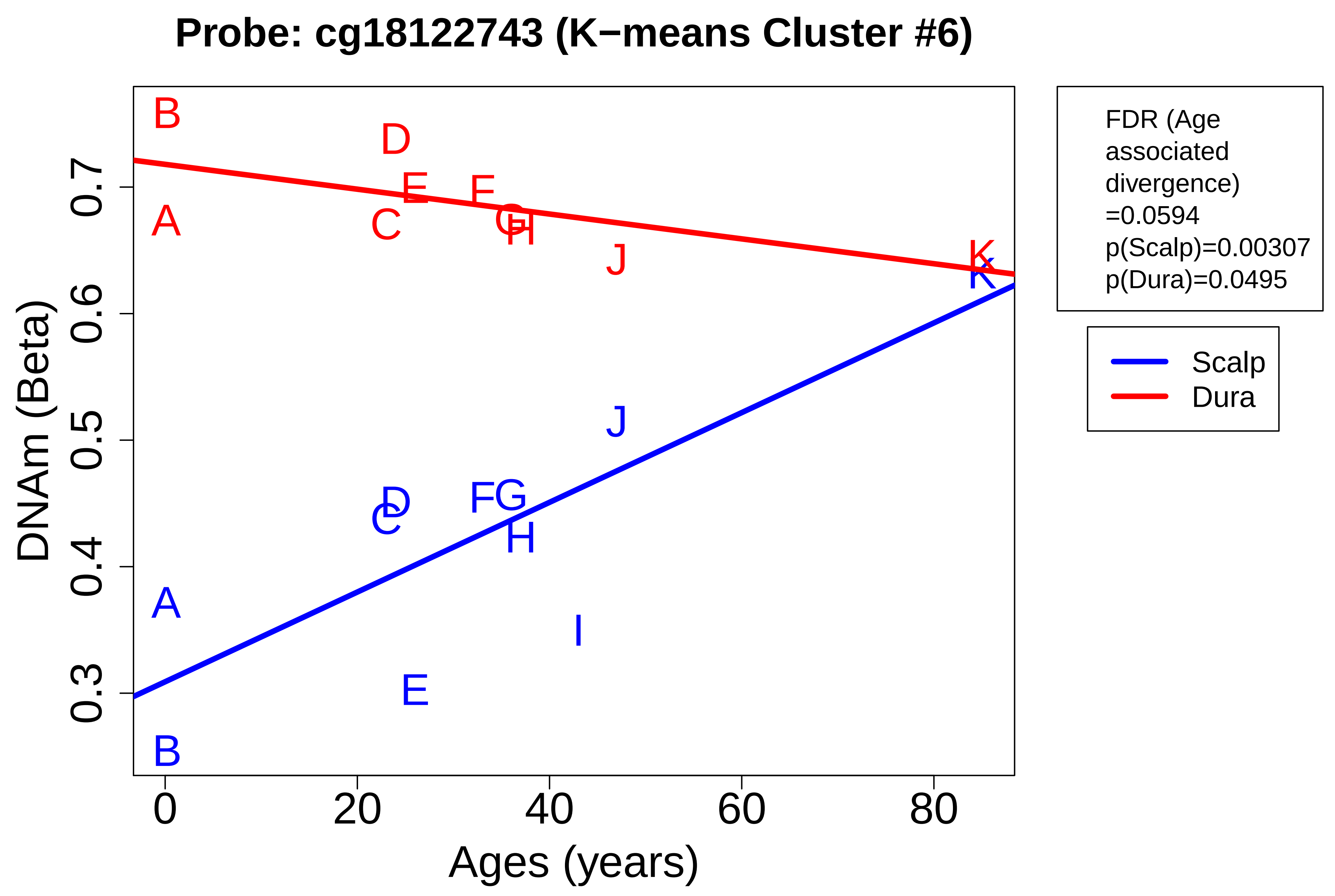

Probe: cg18122743 (K−means Cluster #6)
DNAm (Beta)
0.3
0.4
0.5
0.6
0.7
Ages (years)
0
20
40
60
80
FDR (Age associated divergence) =0.0594
p(Scalp)=0.00307
p(Dura)=0.0495
Scalp
Dura

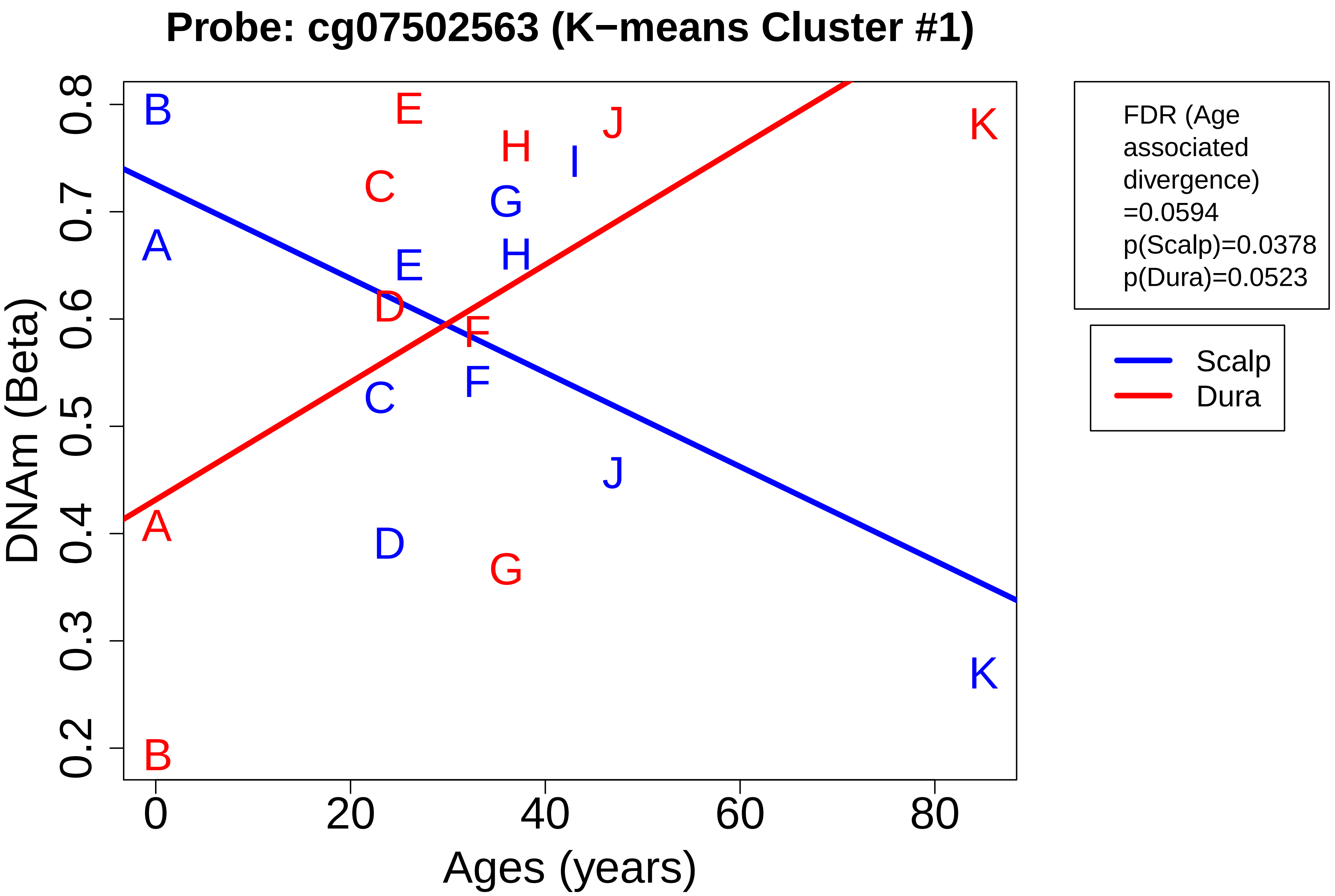

Probe: cg07502563 (K−means Cluster #1)
DNAm (Beta)
0.2
0.3
0.4
0.5
0.6
0.7
0.8
0
20
40
60
80
Ages (years)
A
B
C
D
E
F
G
H
I
J
K
FDR (Age associated divergence) =0.0594
p(Scalp)=0.0378
p(Dura)=0.0523
Scalp
Dura

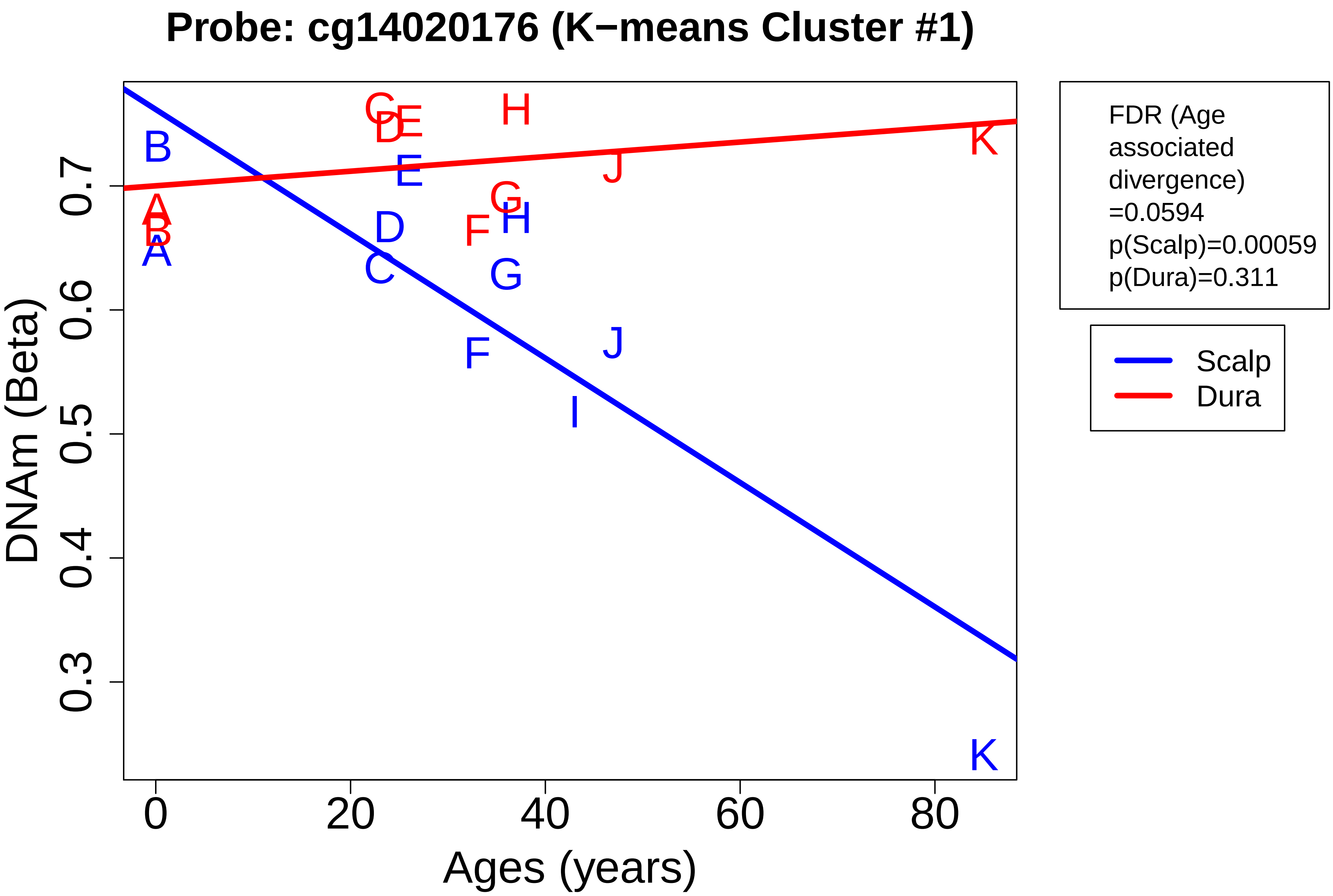
Probe: cg14020176 (K−means Cluster #1)
DNAm (Beta)
0.3
0.4
0.5
0.6
0.7
Ages (years)
0
20
40
60
80
FDR (Age associated divergence) =0.0594
p(Scalp)=0.00059
p(Dura)=0.311
Scalp
Dura

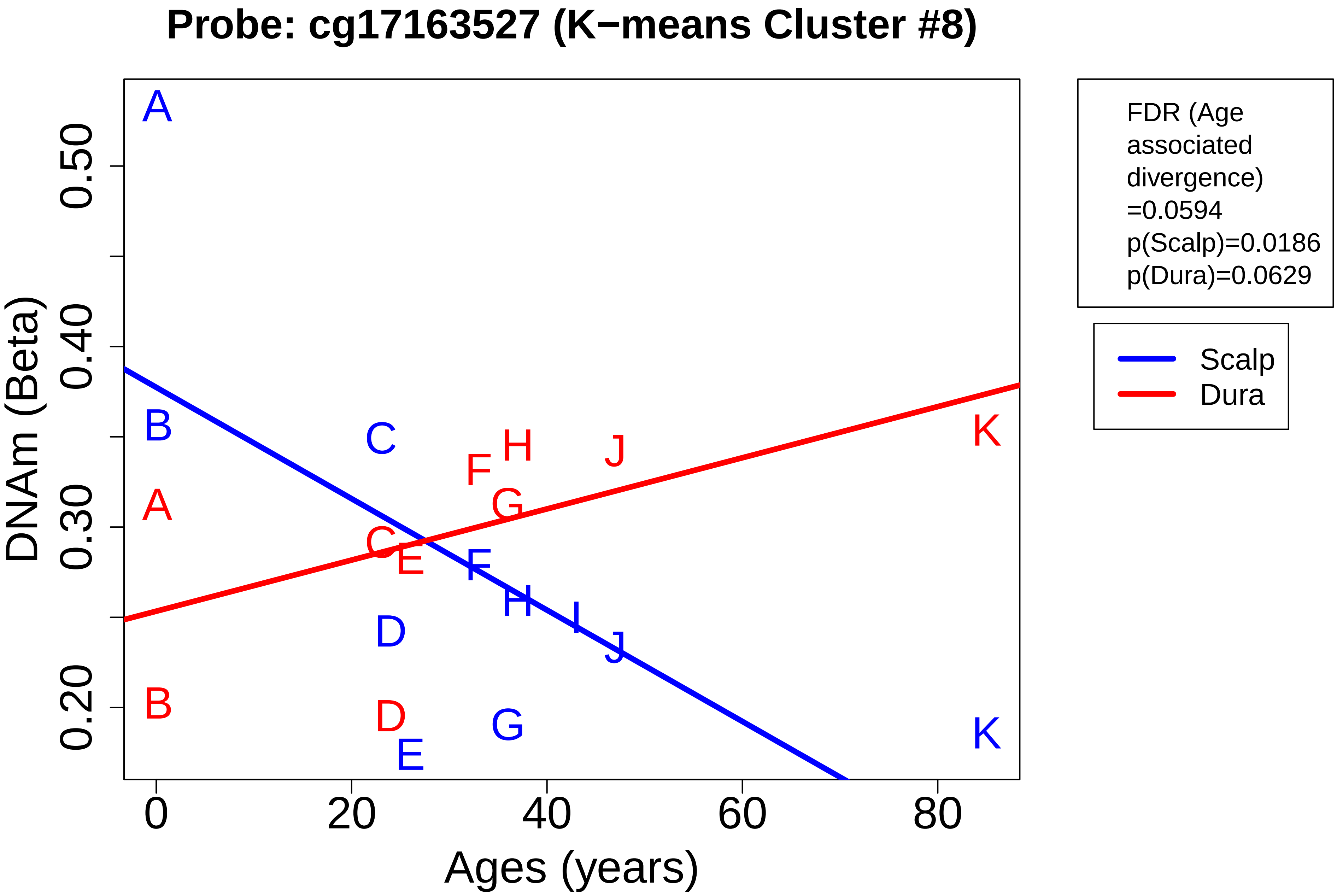

Probe: cg17163527 (K−means Cluster #8)
DNAm (Beta)
0.20
0.30
0.40
0.50
Ages (years)
0
20
40
60
80
A
B
C
D
E
F
G
H
I
J
K
A
B
C
D
E
F
G
H
J
K
FDR (Age associated divergence) =0.0594
p(Scalp)=0.0186
p(Dura)=0.0629
Scalp
Dura

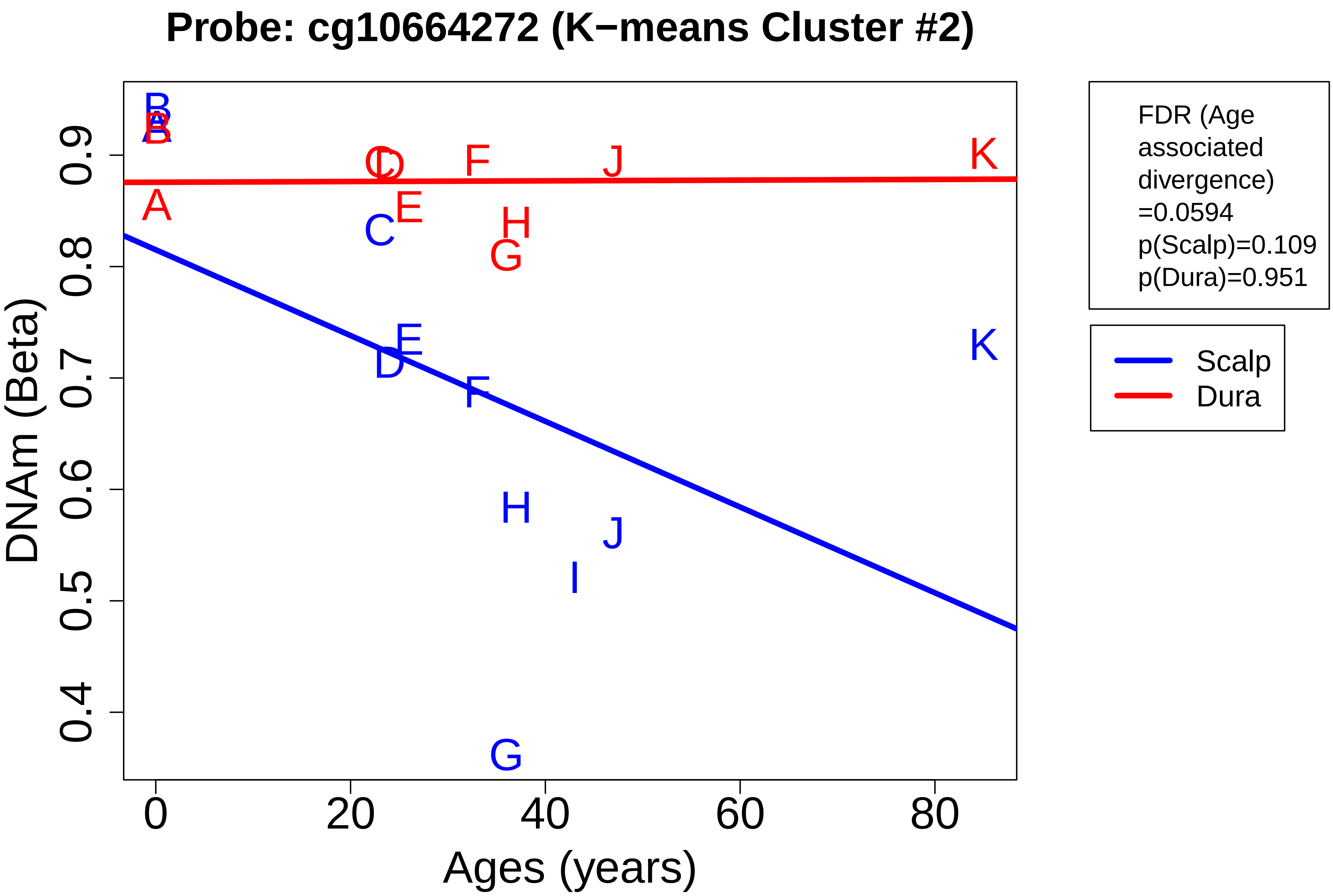
Probe: cg10664272 (K−means Cluster #2)
DNAm (Beta)
Ages (years)
0.4
0.5
0.6
0.7
0.8
0.9
0
20
40
60
80
FDR (Age associated divergence) =0.0594
p(Scalp)=0.109
p(Dura)=0.951
Scalp
Dura

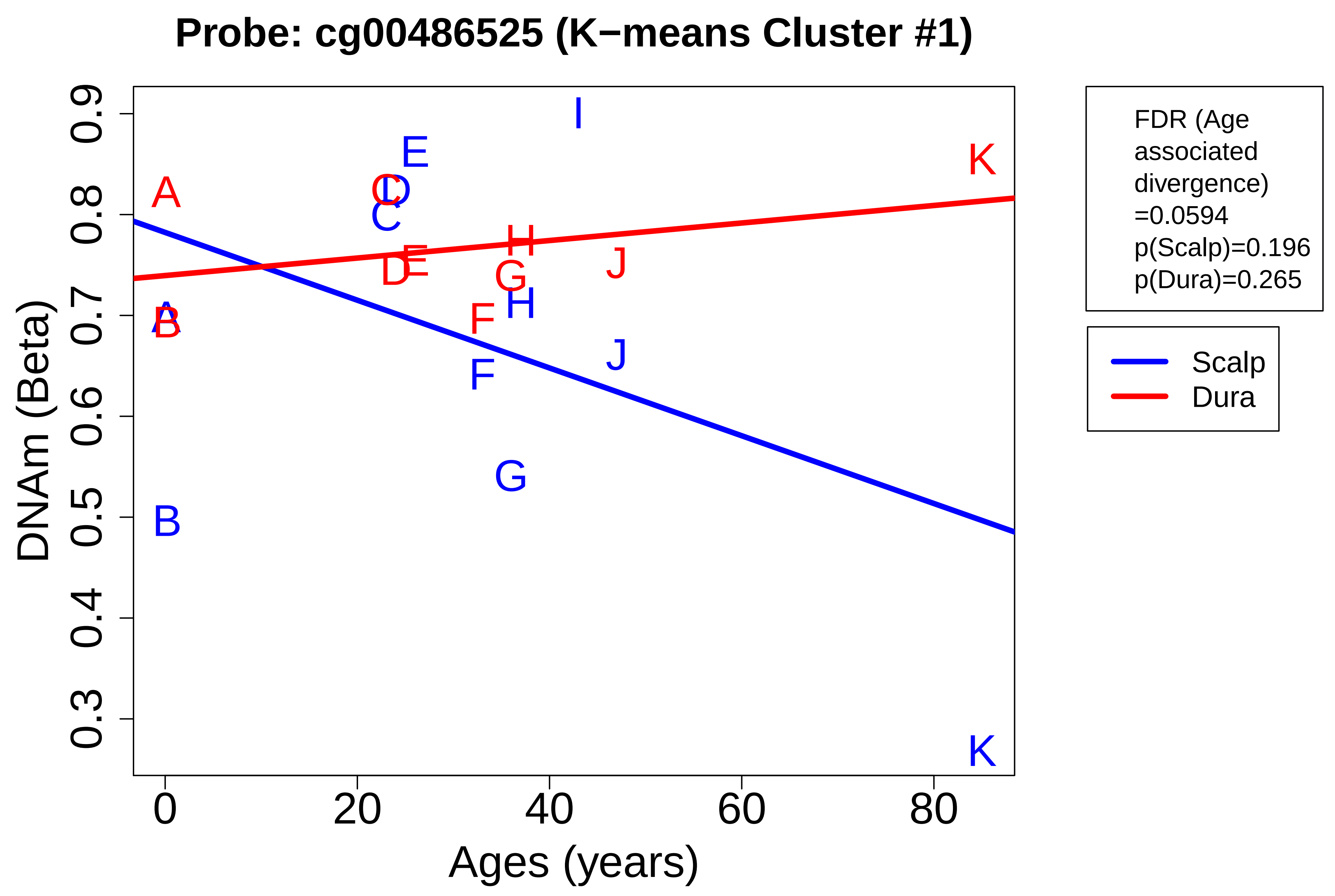
Probe: cg00486525 (K−means Cluster #1)
DNAm (Beta)
0.3
0.4
0.5
0.6
0.7
0.8
0.9
Ages (years)
0
20
40
60
80
FDR (Age associated divergence) =0.0594
p(Scalp)=0.196
p(Dura)=0.265
Scalp
Dura
A
B
C
D
E
F
G
H
I
J
K

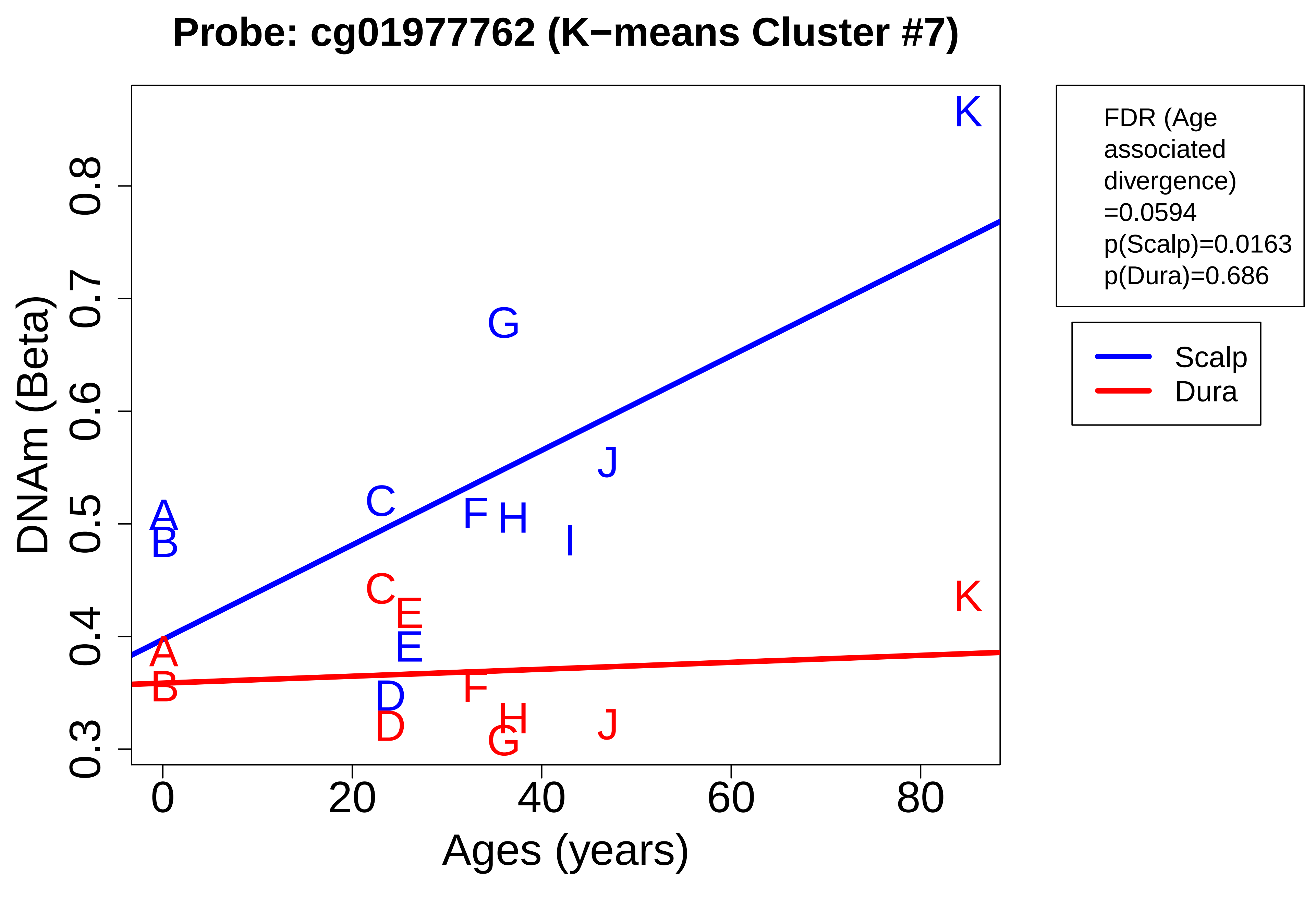

Probe: cg01977762 (K−means Cluster #7)
DNAm (Beta)
0.3
0.4
0.5
0.6
0.7
0.8
0
20
40
60
80
Ages (years)
FDR (Age associated divergence) =0.0594
p(Scalp)=0.0163
p(Dura)=0.686
Scalp
Dura

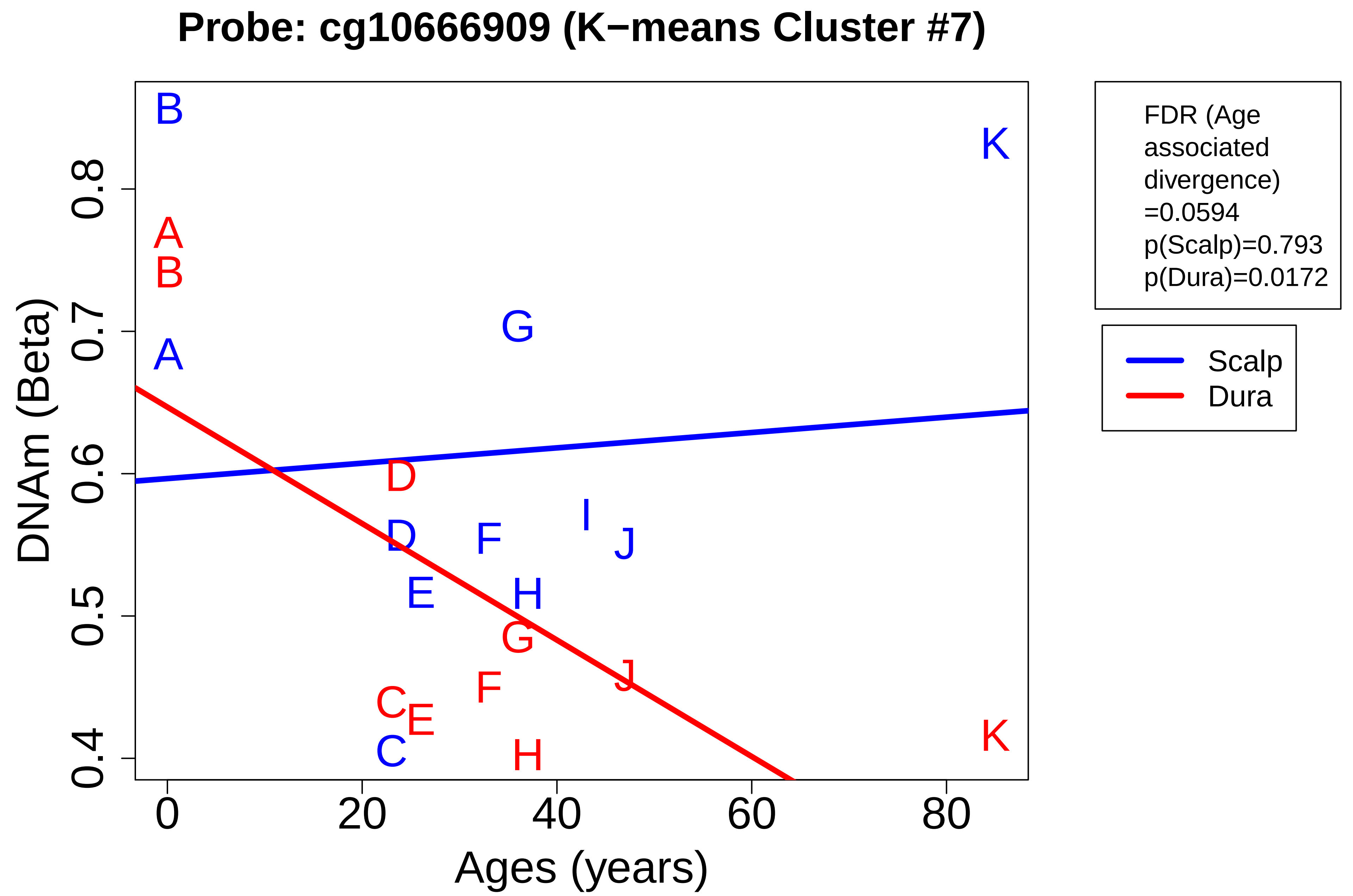
Probe: cg10666909 (K−means Cluster #7)
DNAm (Beta)
0.4
0.5
0.6
0.7
0.8
0
20
40
60
80
Ages (years)
FDR (Age associated divergence) =0.0594
p(Scalp)=0.793
p(Dura)=0.0172
Scalp
Dura

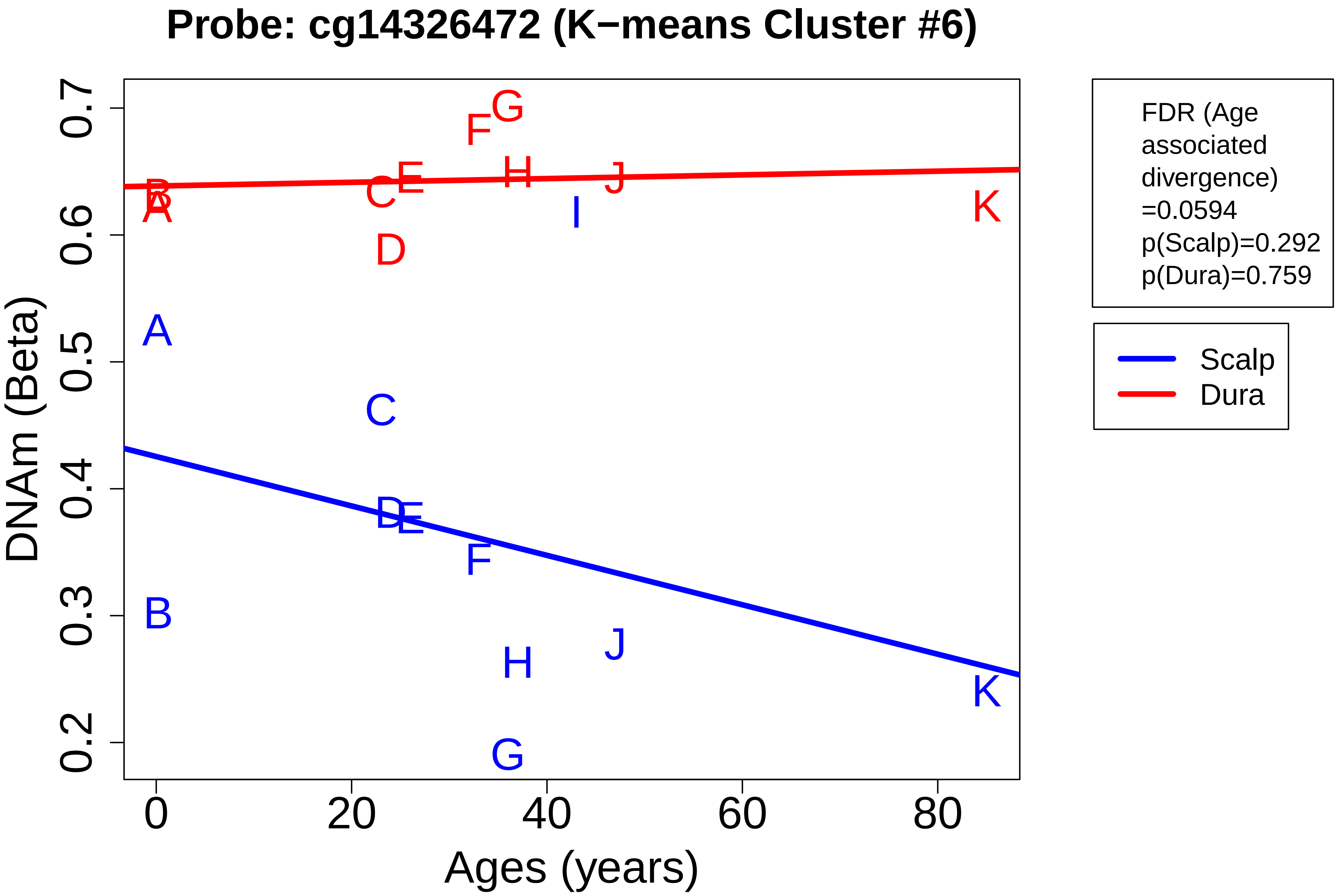

Probe: cg14326472 (K−means Cluster #6)
DNAm (Beta)
0.2
0.3
0.4
0.5
0.6
0.7
Ages (years)
0
20
40
60
80
A
B
C
D
E
F
G
H
I
J
K
FDR (Age associated divergence) =0.0594
p(Scalp)=0.292
p(Dura)=0.759
Scalp
Dura

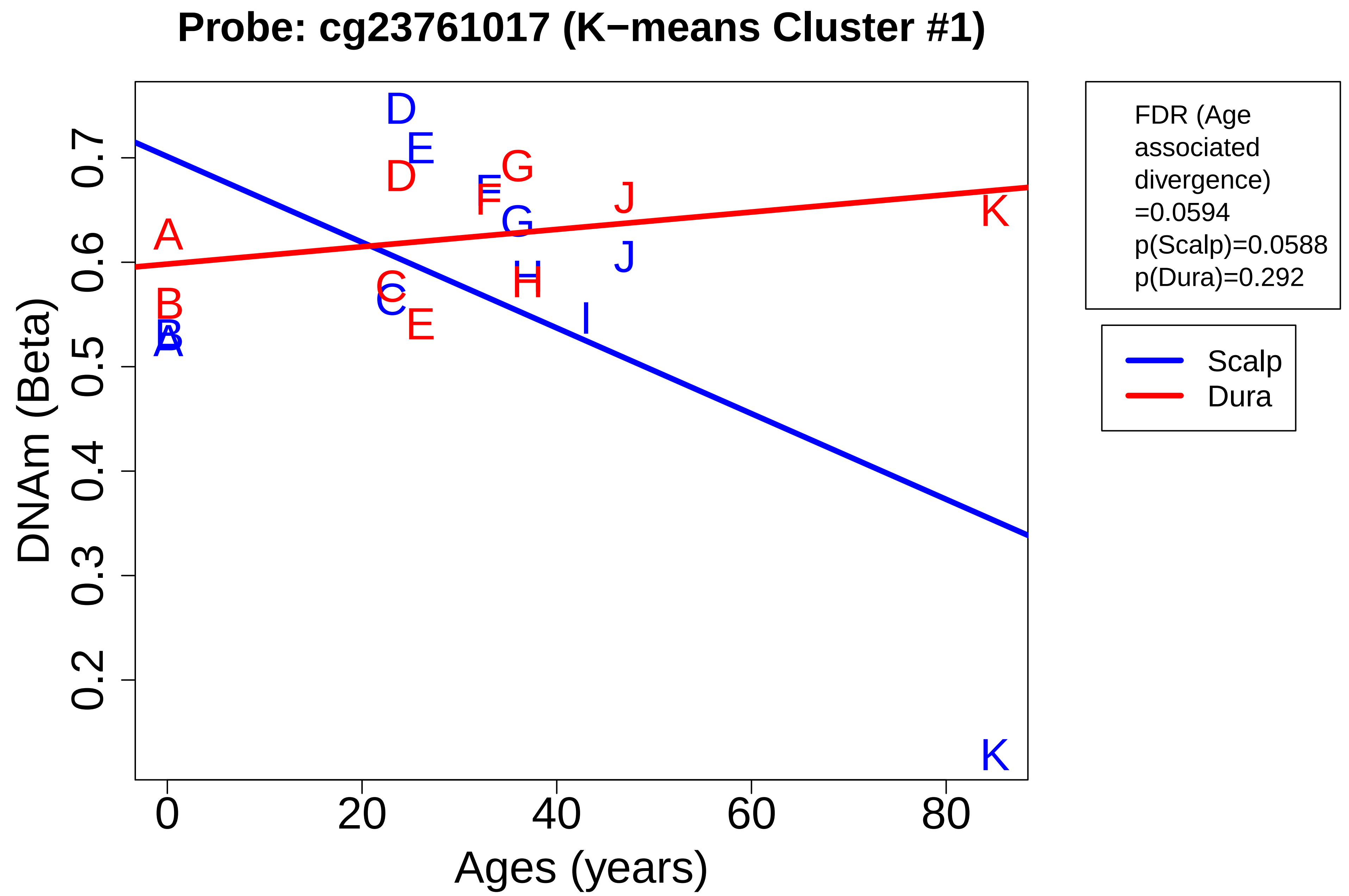
Probe: cg23761017 (K−means Cluster #1)
DNAm (Beta)
Ages (years)
0.2
0.3
0.4
0.5
0.6
0.7
0
20
40
60
80
A
B
C
D
E
F
G
H
I
J
K
FDR (Age associated divergence) =0.0594
p(Scalp)=0.0588
p(Dura)=0.292
Scalp
Dura

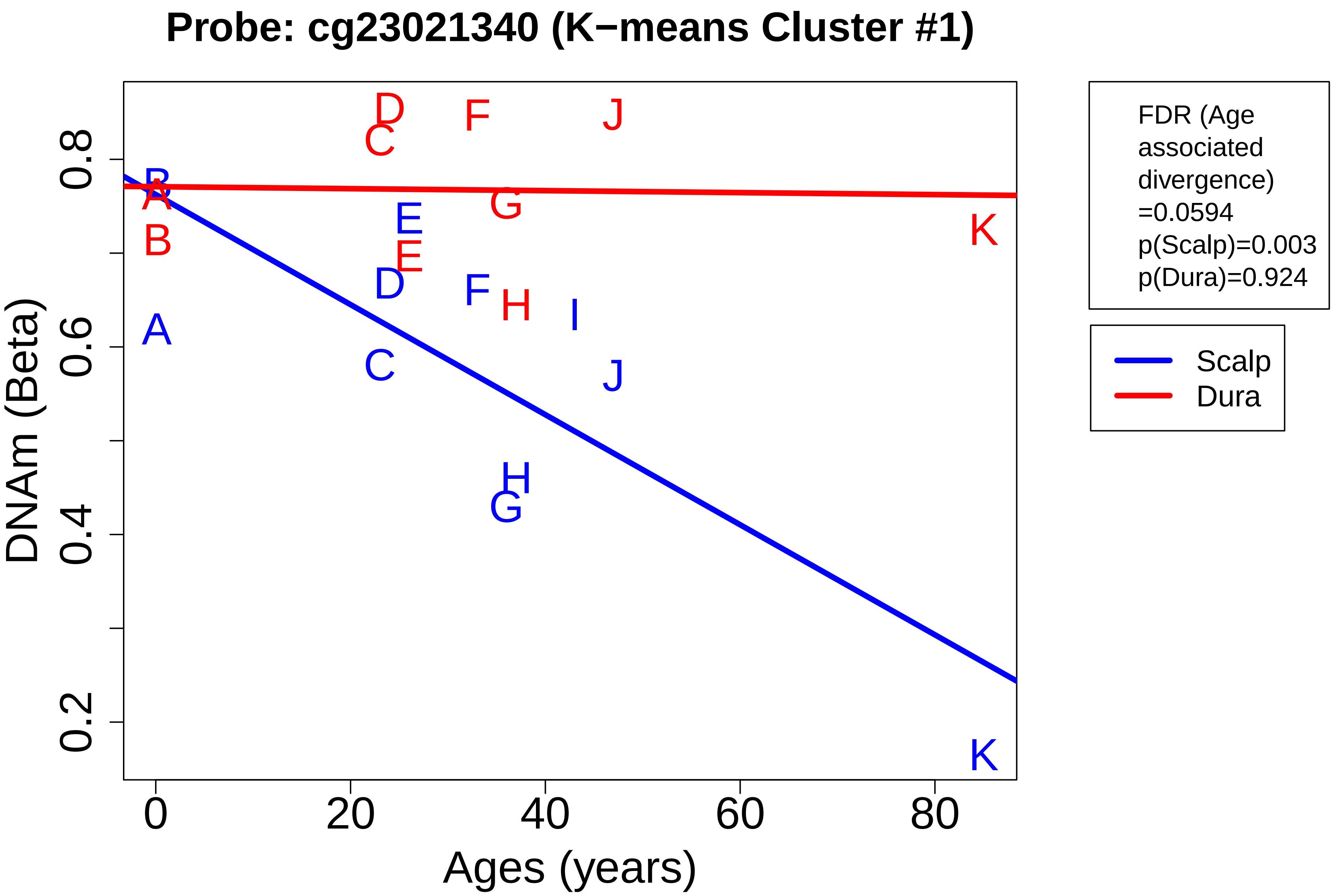

Probe: cg23021340 (K−means Cluster #1)
DNAm (Beta)
0.2
0.4
0.6
0.8
0
20
40
60
80
Ages (years)
A
B
C
D
E
F
G
H
I
J
K
FDR (Age associated divergence) =0.0594
p(Scalp)=0.003
p(Dura)=0.924
Scalp
Dura

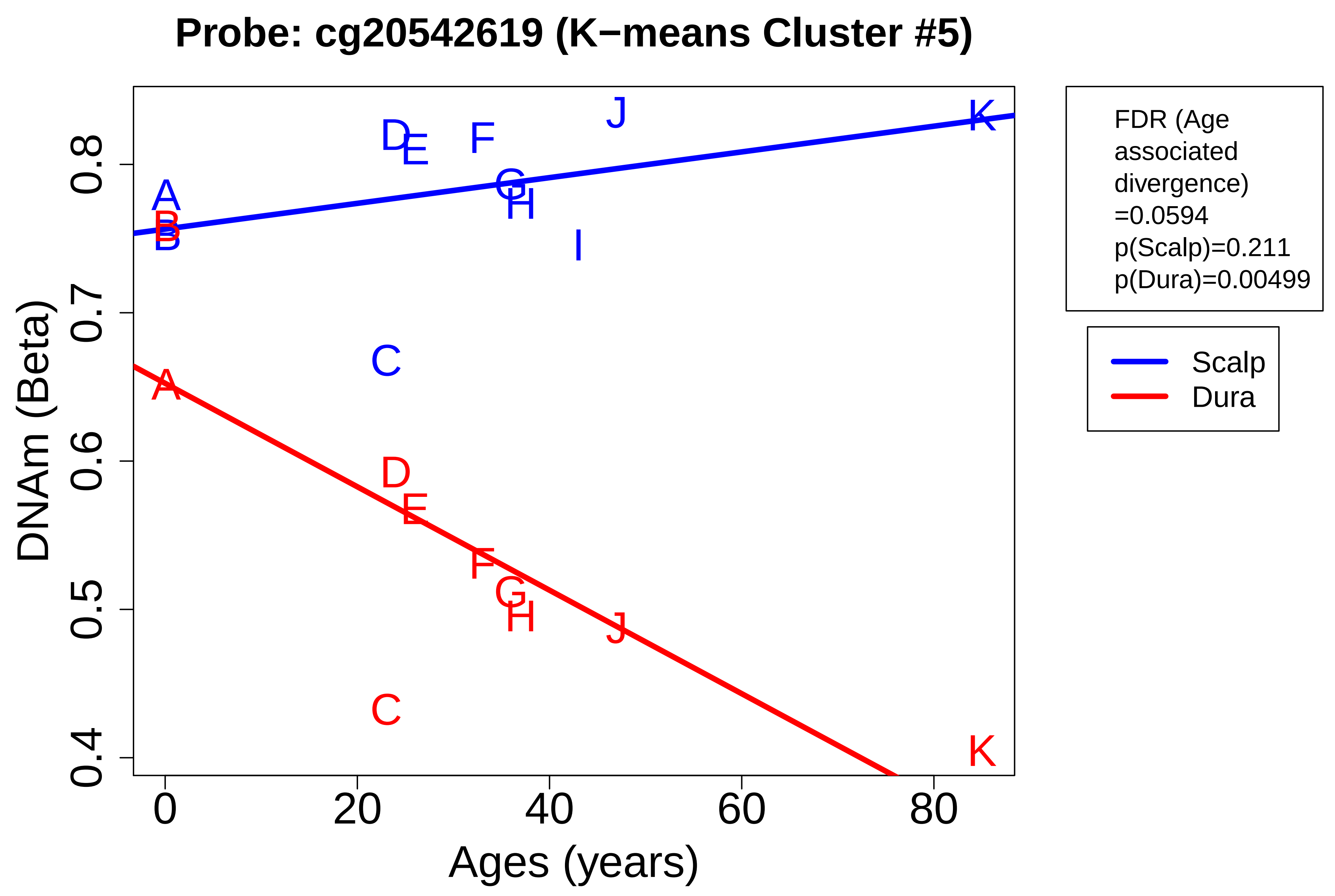

Probe: cg20542619 (K−means Cluster #5)
DNAm (Beta)
0.4
0.5
0.6
0.7
0.8
0
20
40
60
80
Ages (years)
A
B
C
D
E
F
G
H
I
J
K
FDR (Age associated divergence) =0.0594
p(Scalp)=0.211
p(Dura)=0.00499
Scalp
Dura

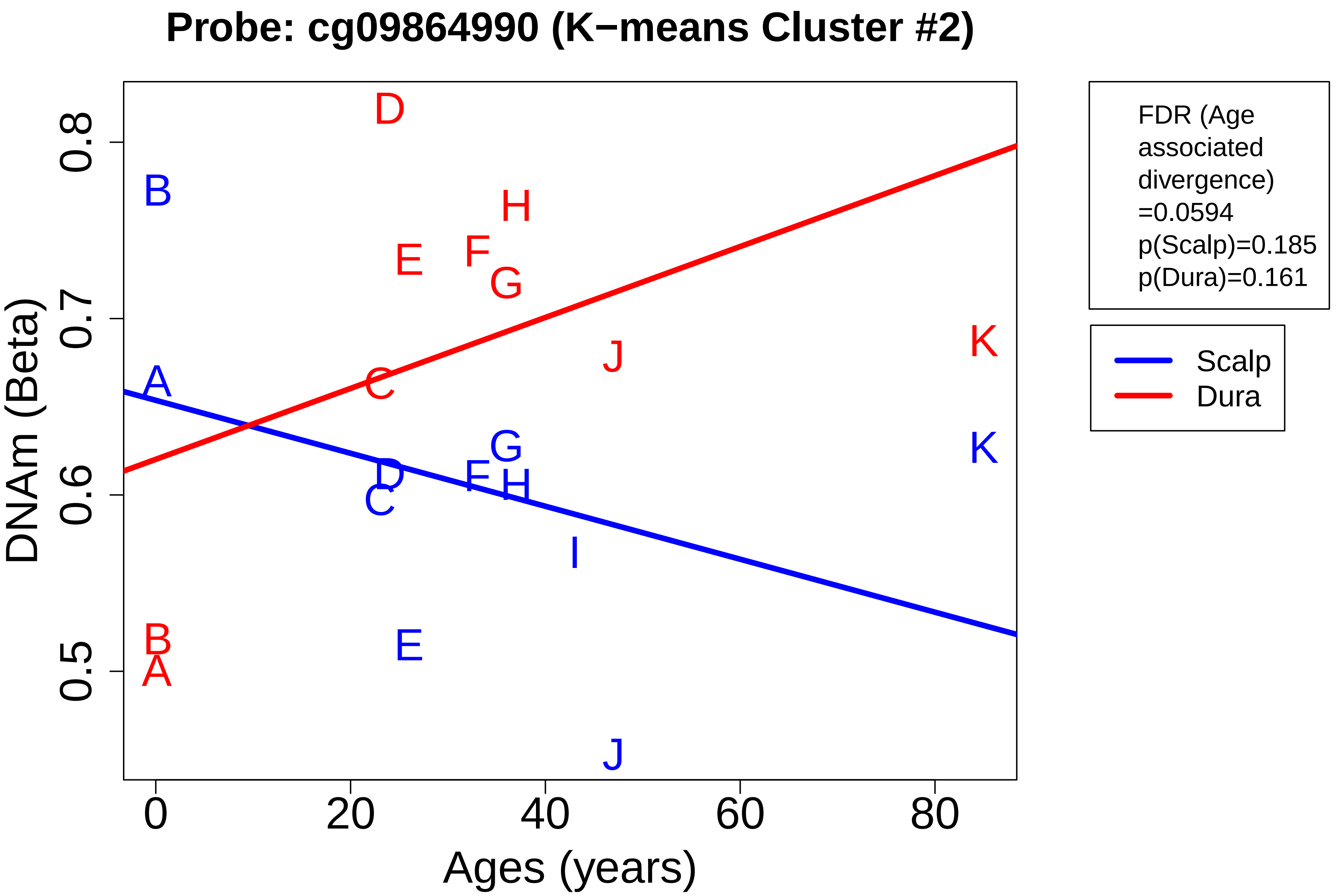

Probe: cg09864990 (K−means Cluster #2)
DNAm (Beta)
0.5
0.6
0.7
0.8
0
20
40
60
80
Ages (years)
FDR (Age associated divergence) =0.0594
p(Scalp)=0.185
p(Dura)=0.161
Scalp
Dura

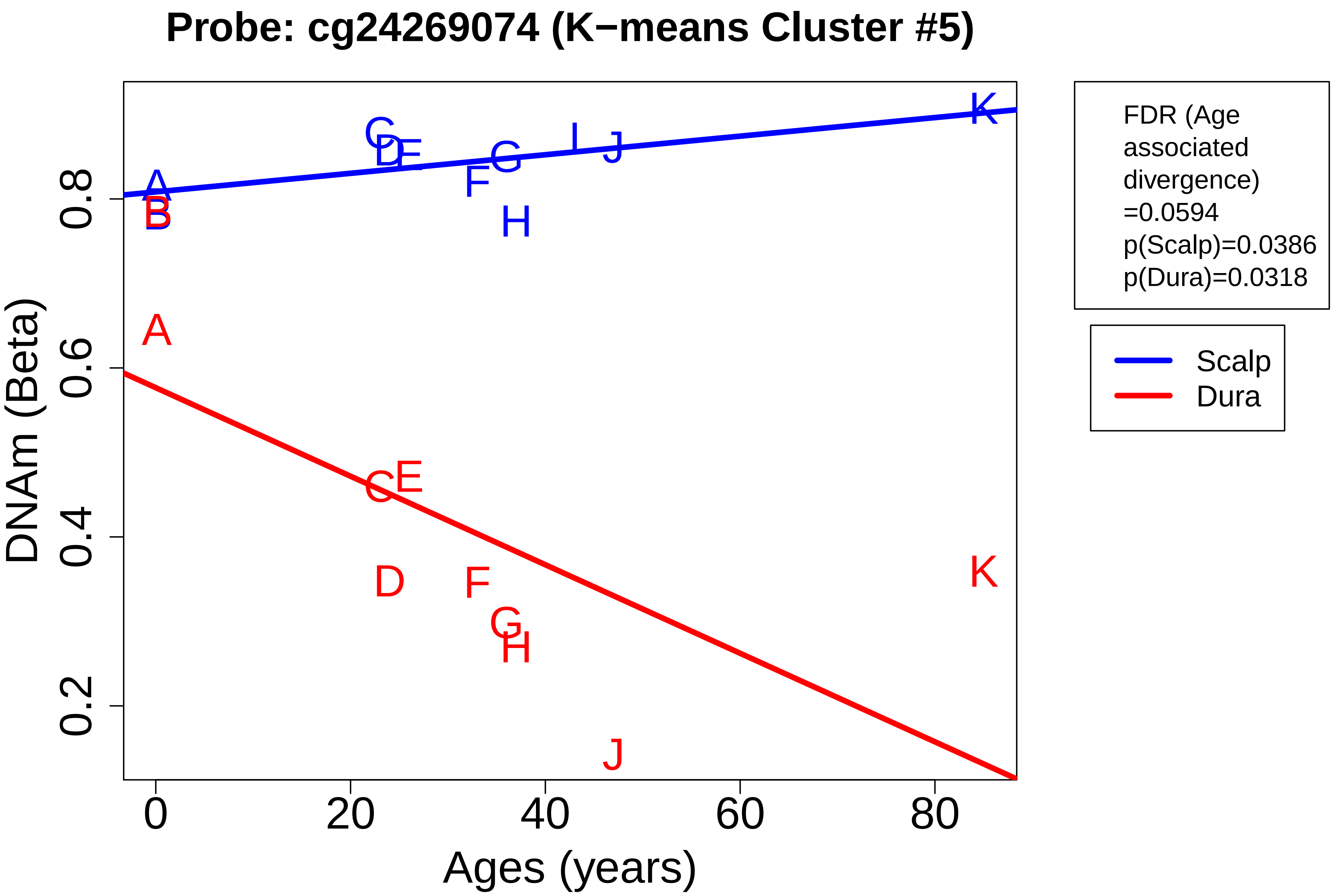

Probe: cg24269074 (K−means Cluster #5)
DNAm (Beta)
0.2
0.4
0.6
0.8
0
20
40
60
80
Ages (years)
FDR (Age associated divergence) =0.0594
p(Scalp)=0.0386
p(Dura)=0.0318
Scalp
Dura

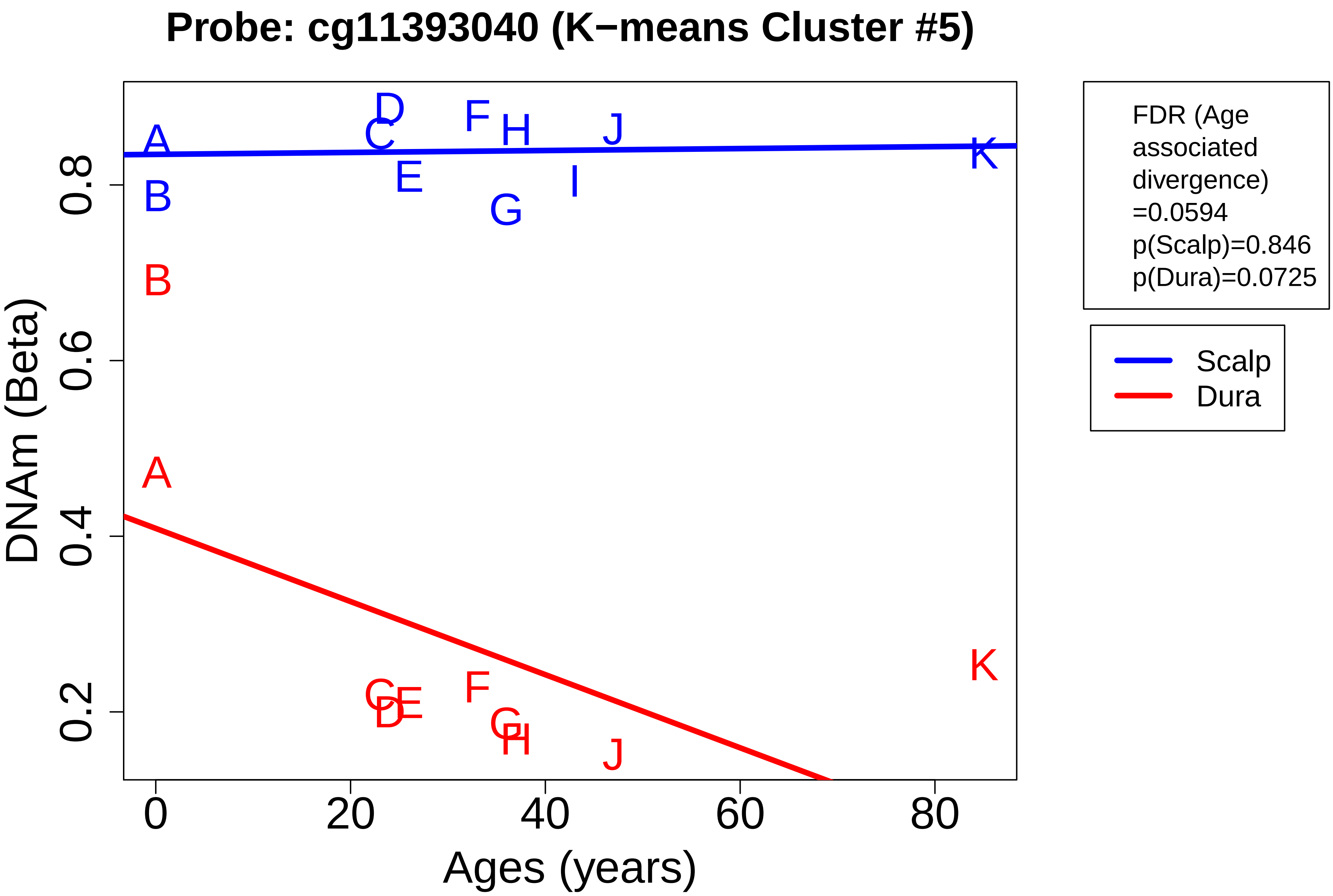

Probe: cg11393040 (K−means Cluster #5)
DNAm (Beta)
0.2
0.4
0.6
0.8
0
20
40
60
80
Ages (years)
FDR (Age associated divergence) =0.0594
p(Scalp)=0.846
p(Dura)=0.0725
Scalp
Dura

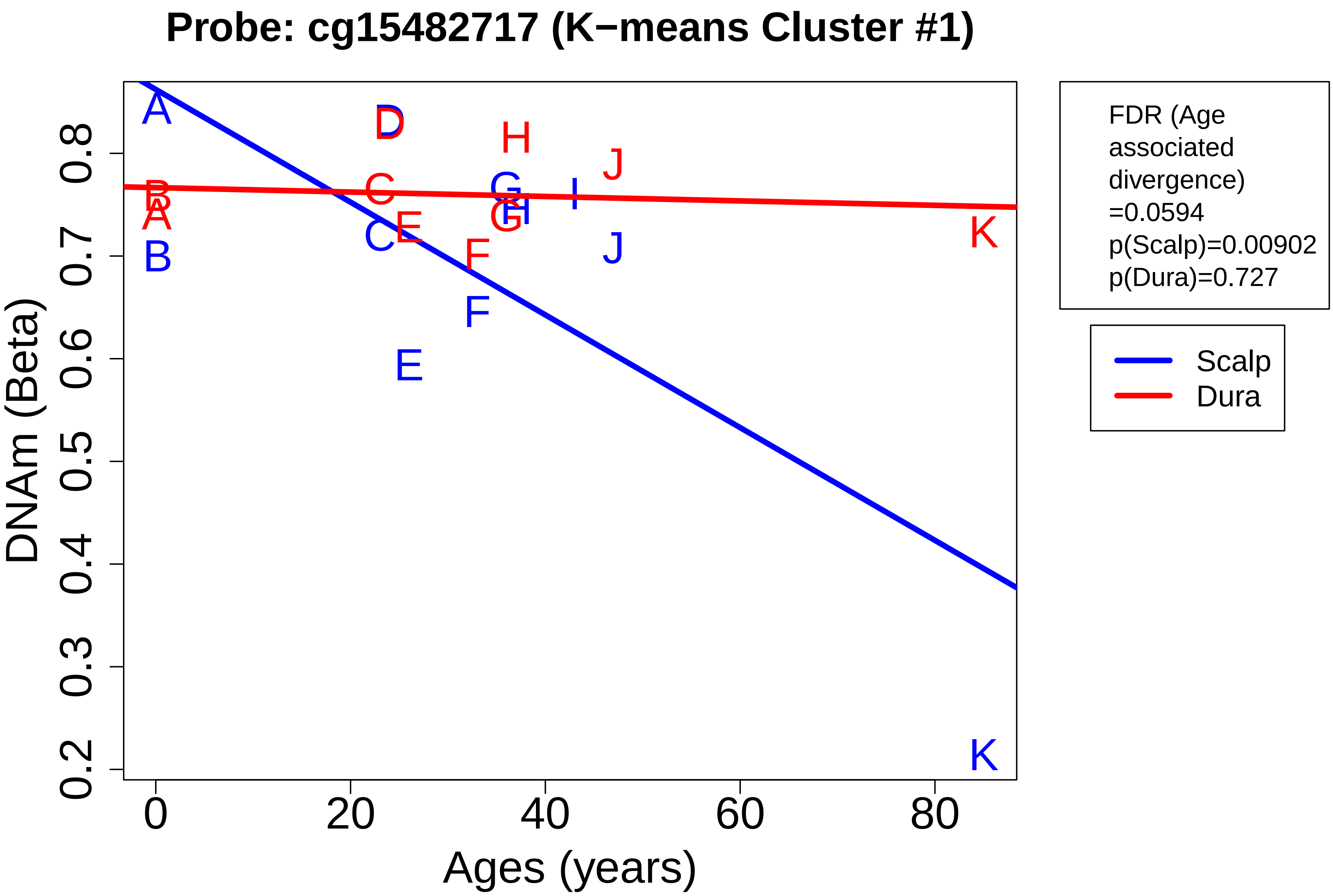

Probe: cg15482717 (K−means Cluster #1)
DNAm (Beta)
0.2
0.3
0.4
0.5
0.6
0.7
0.8
Ages (years)
0
20
40
60
80
A
B
C
D
E
F
G
H
I
J
K
FDR (Age associated divergence) =0.0594
p(Scalp)=0.00902
p(Dura)=0.727
Scalp
Dura

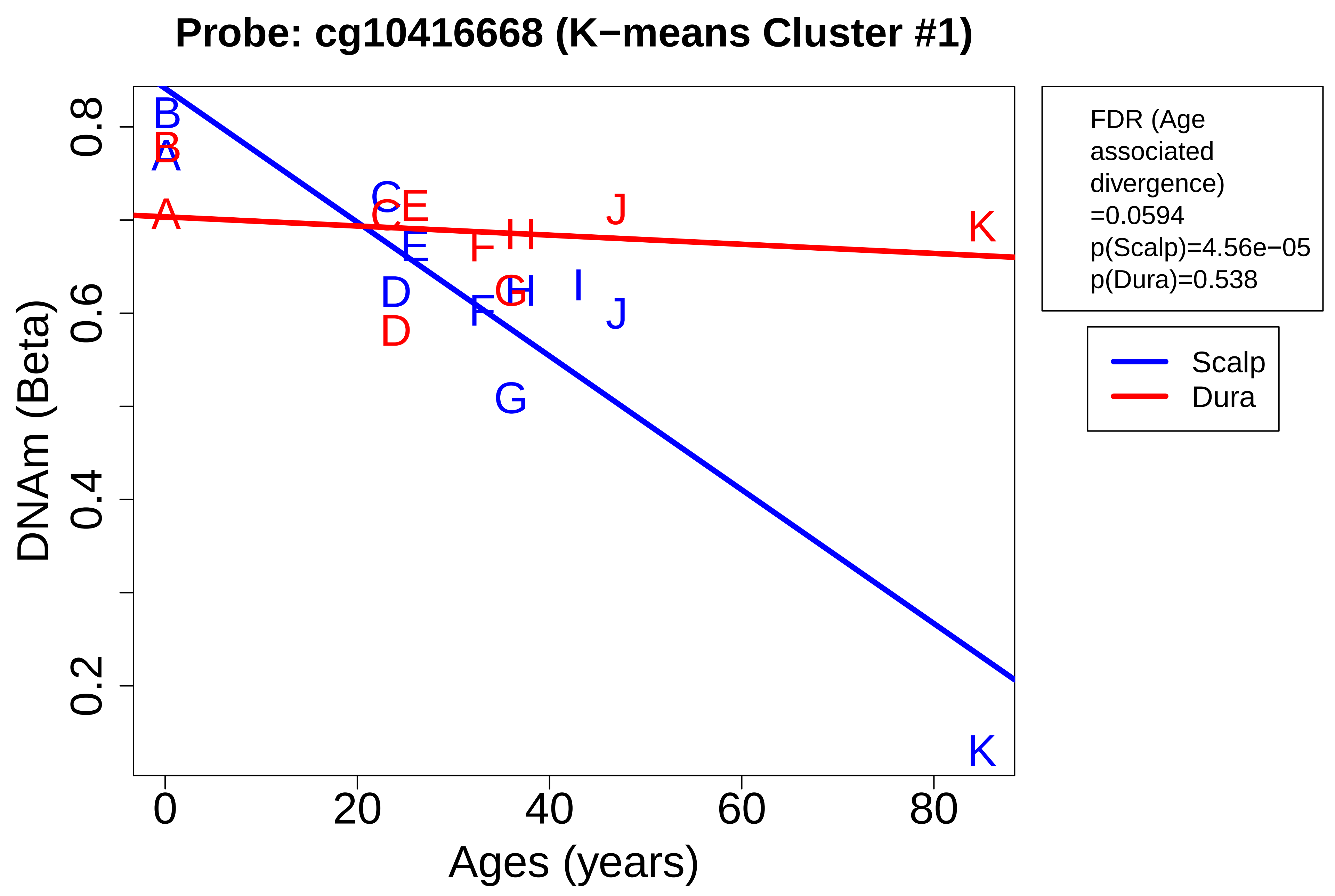

Probe: cg10416668 (K−means Cluster #1)
DNAm (Beta)
0.2
0.4
0.6
0.8
0
20
40
60
80
Ages (years)
A
B
C
D
E
F
G
H
I
J
K
FDR (Age associated divergence) =0.0594
p(Scalp)=4.56e−05
p(Dura)=0.538
Scalp
Dura

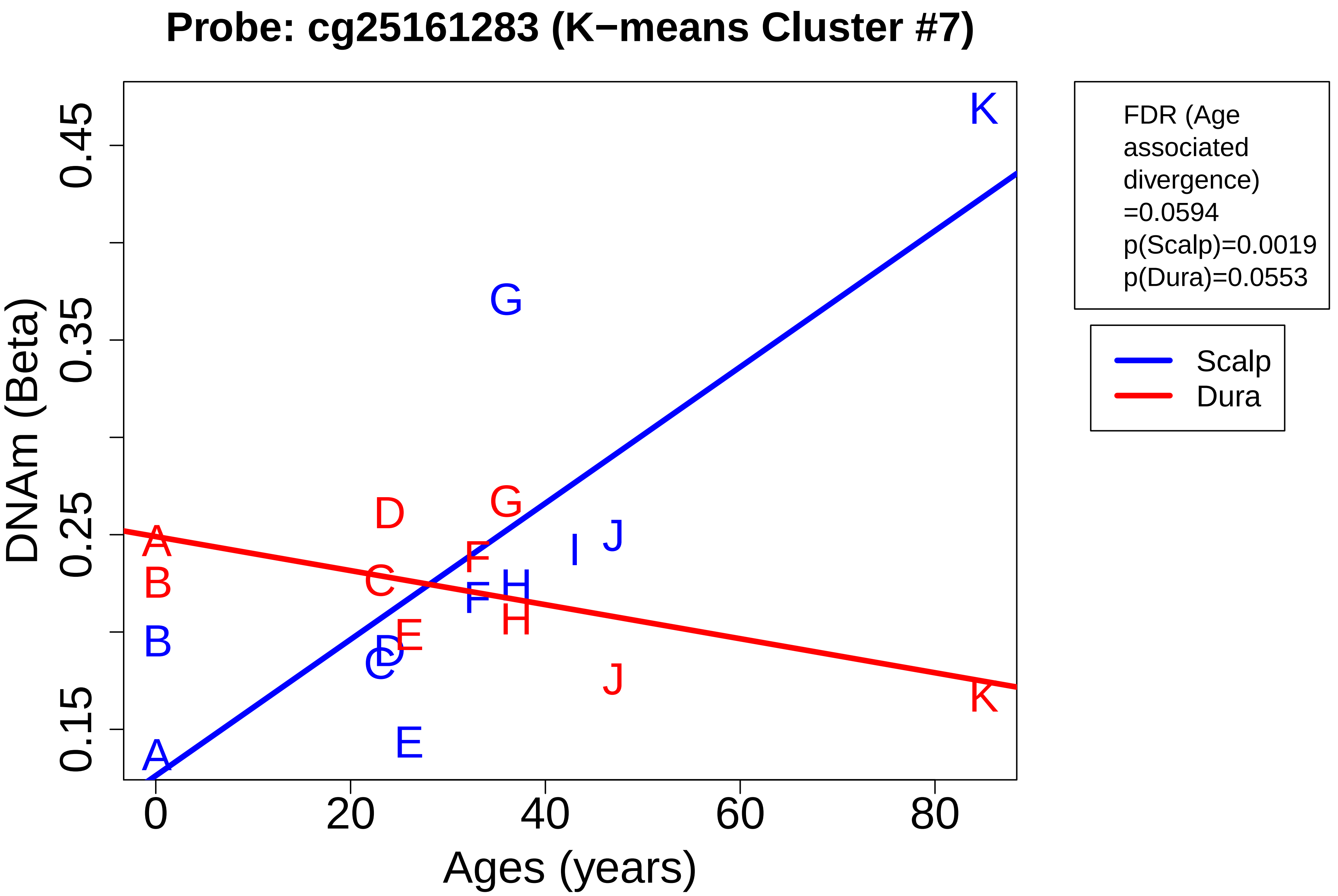
Probe: cg25161283 (K−means Cluster #7)
DNAm (Beta)
0.15
0.25
0.35
0.45
Ages (years)
0
20
40
60
80
FDR (Age associated divergence) =0.0594
p(Scalp)=0.0019
p(Dura)=0.0553
Scalp
Dura

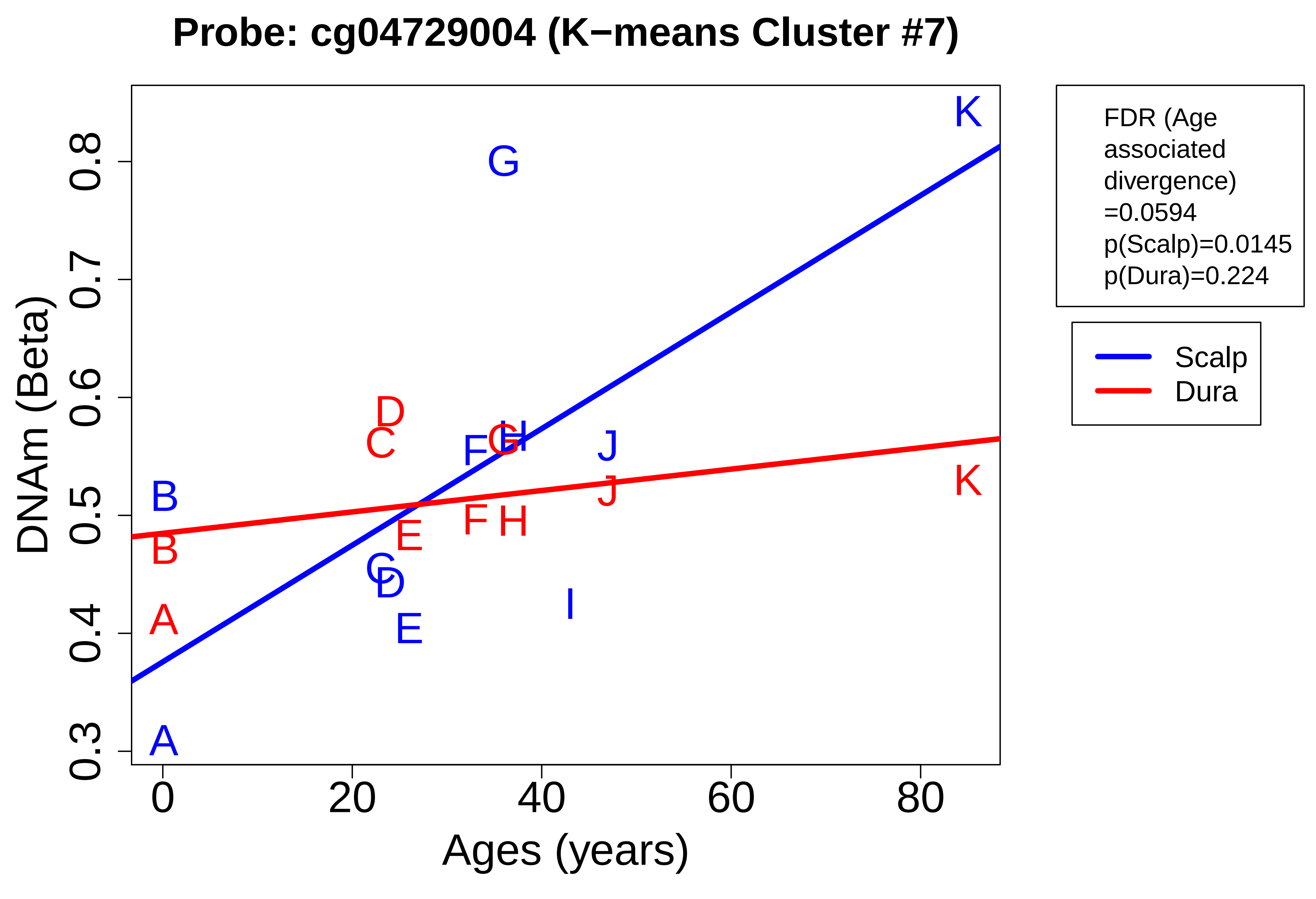

Probe: cg04729004 (K−means Cluster #7)
DNAm (Beta)
Ages (years)
0.3
0.4
0.5
0.6
0.7
0.8
0
20
40
60
80
FDR (Age associated divergence) =0.0594
p(Scalp)=0.0145
p(Dura)=0.224
Scalp
Dura

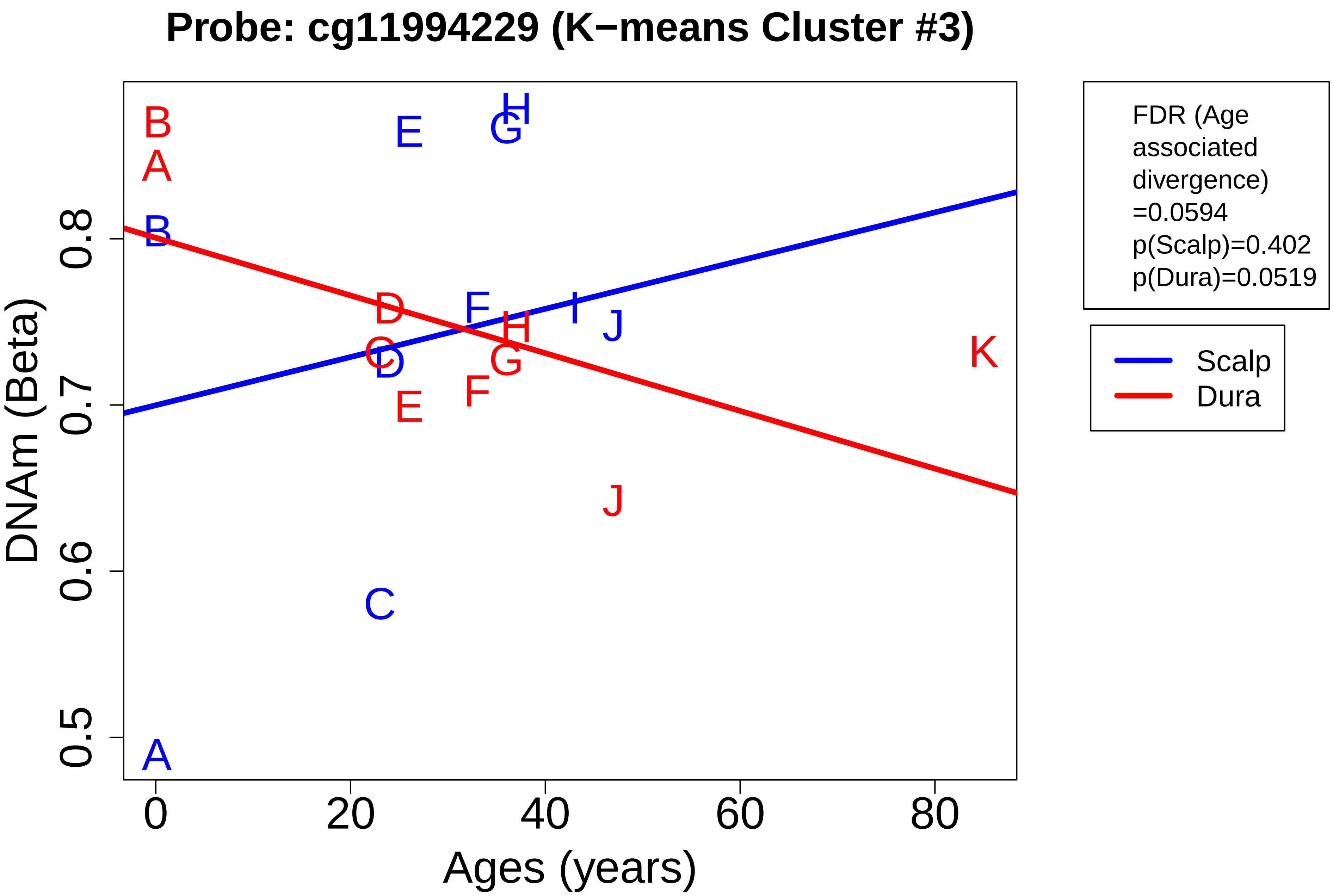
Probe: cg11994229 (K−means Cluster #3)
DNAm (Beta)
0.5
0.6
0.7
0.8
Ages (years)
0
20
40
60
80
FDR (Age associated divergence) =0.0594
p(Scalp)=0.402
p(Dura)=0.0519
Scalp
Dura
A
B
C
D
E
F
G
H
I
J
K

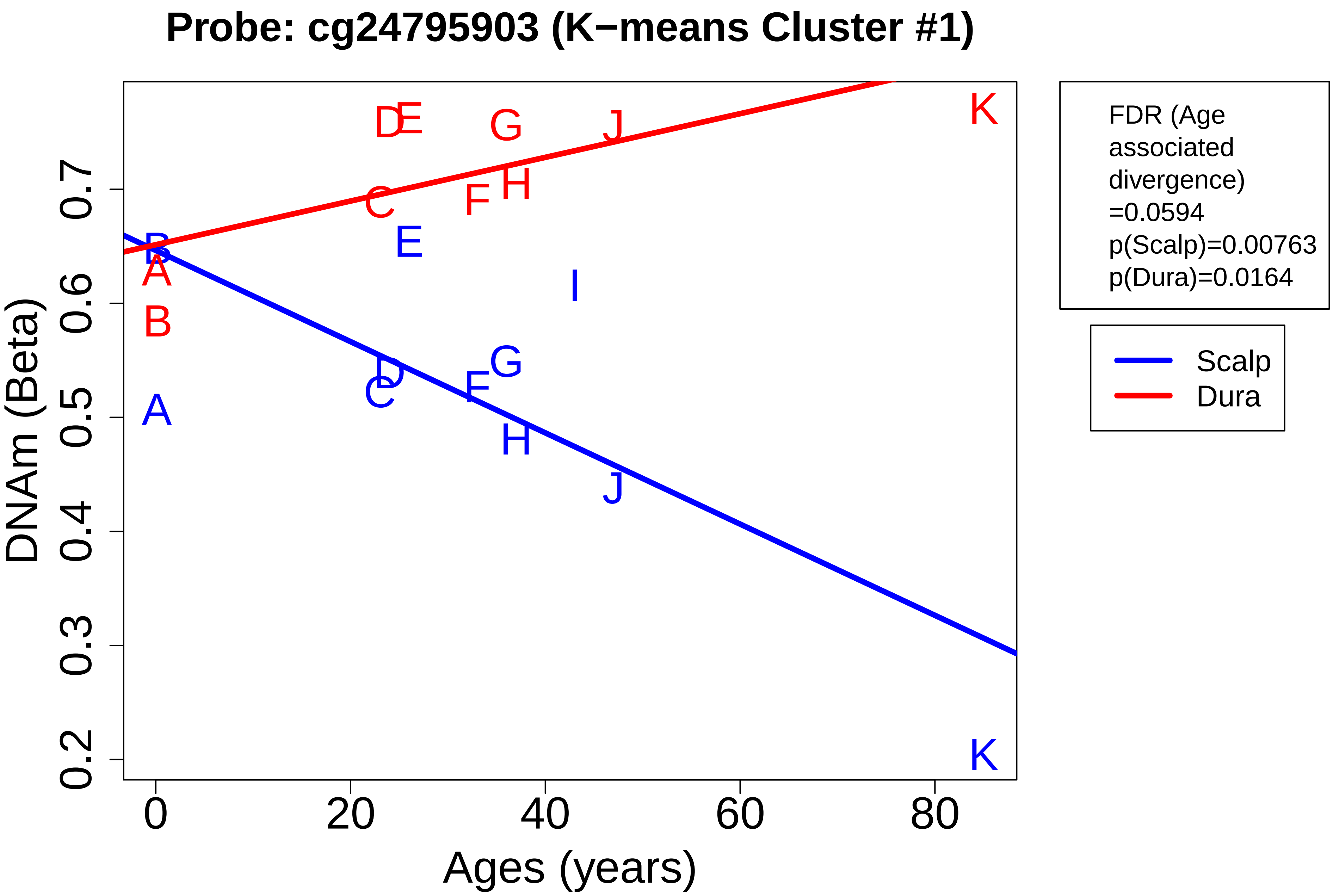
Probe: cg24795903 (K−means Cluster #1)
DNAm (Beta)
Ages (years)
0.2
0.3
0.4
0.5
0.6
0.7
0
20
40
60
80
FDR (Age associated divergence) =0.0594
p(Scalp)=0.00763
p(Dura)=0.0164
Scalp
Dura

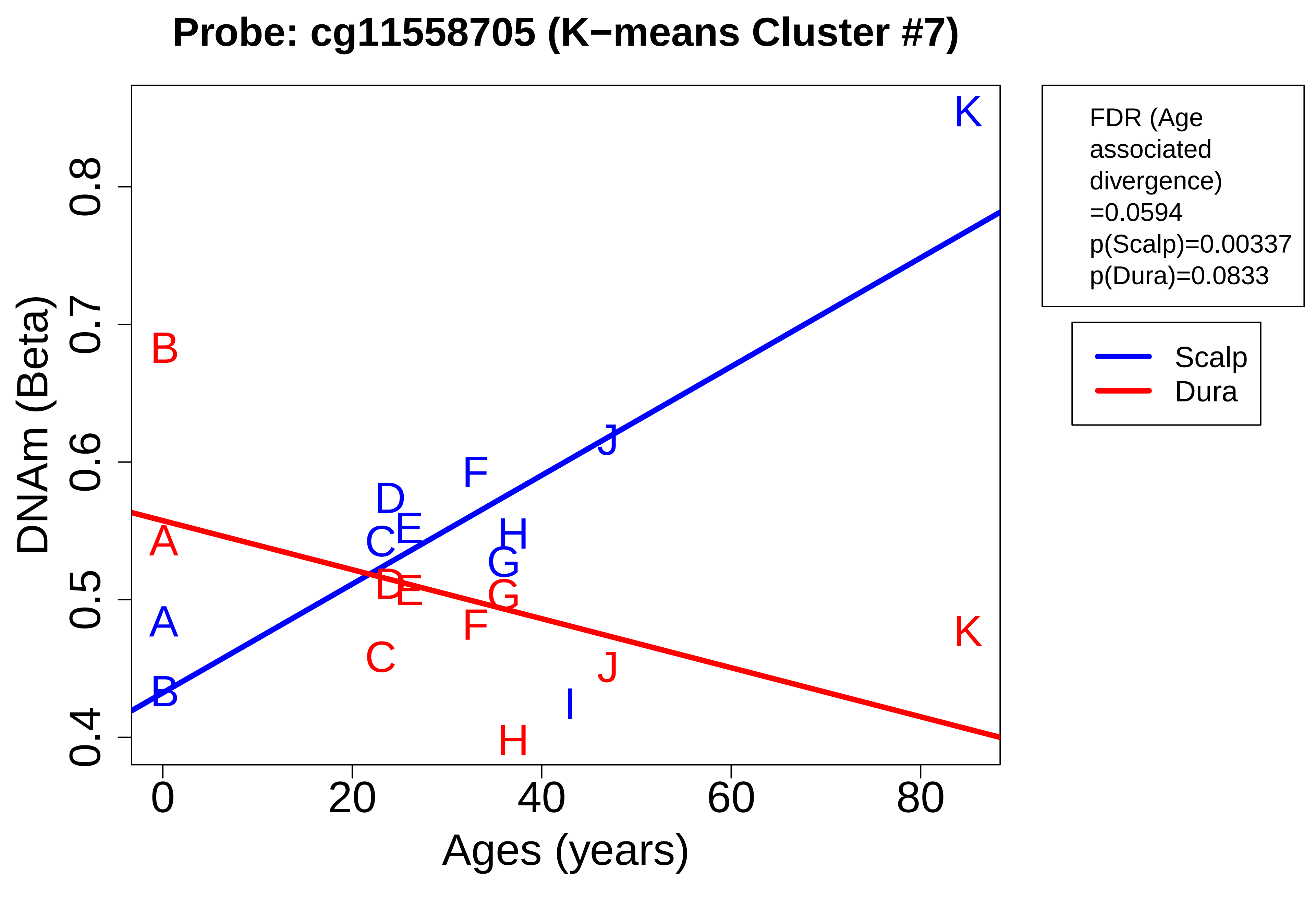
Probe: cg11558705 (K−means Cluster #7)
DNAm (Beta)
0.4
0.5
0.6
0.7
0.8
0
20
40
60
80
Ages (years)
FDR (Age associated divergence) =0.0594
p(Scalp)=0.00337
p(Dura)=0.0833
Scalp
Dura
A
B
C
D
E
F
G
H
I
J
K

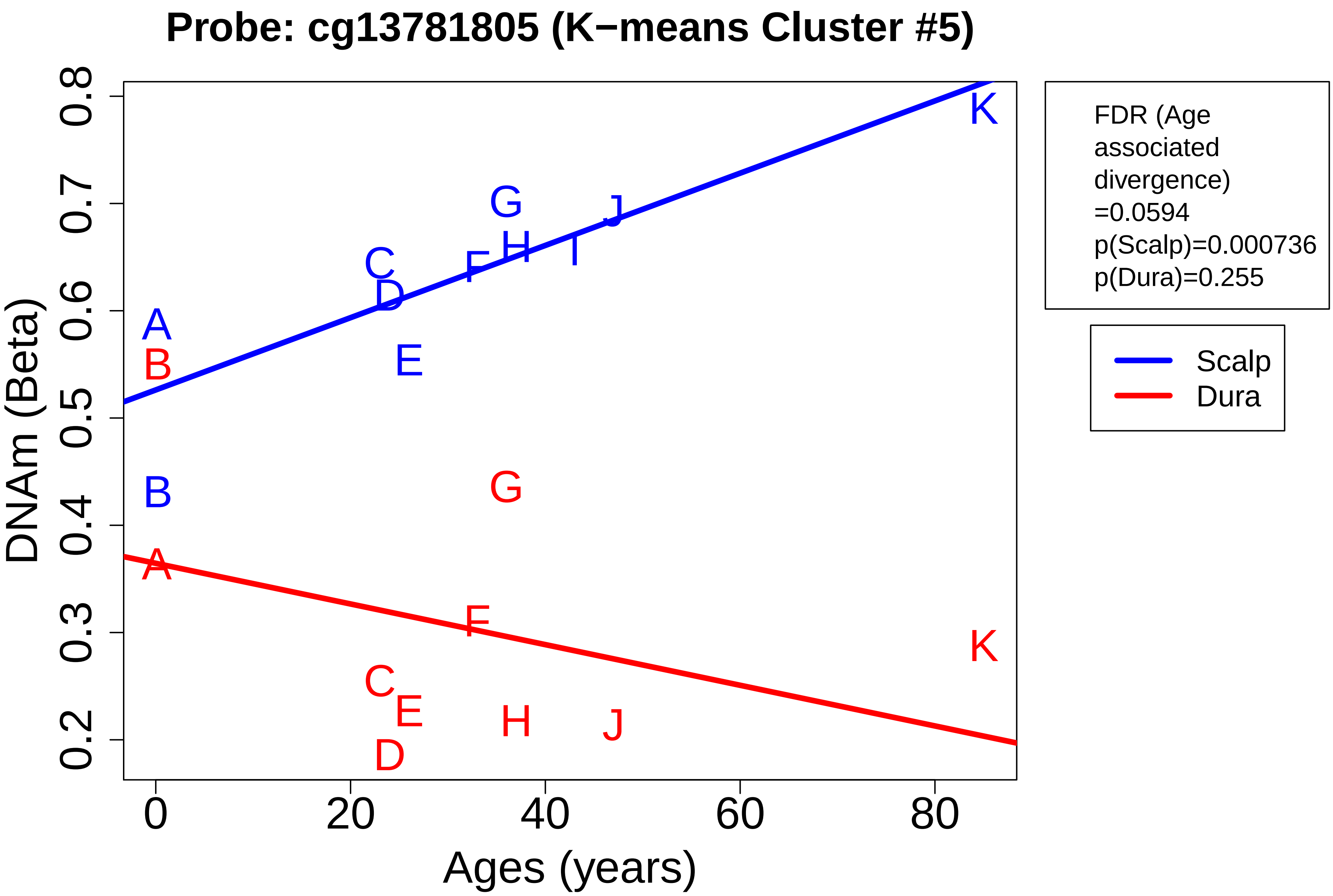

Probe: cg13781805 (K−means Cluster #5)
DNAm (Beta)
Ages (years)
0.2
0.3
0.4
0.5
0.6
0.7
0.8
0
20
40
60
80
A
B
C
D
E
F
G
H
I
J
K
FDR (Age associated divergence) =0.0594
p(Scalp)=0.000736
p(Dura)=0.255
Scalp
Dura

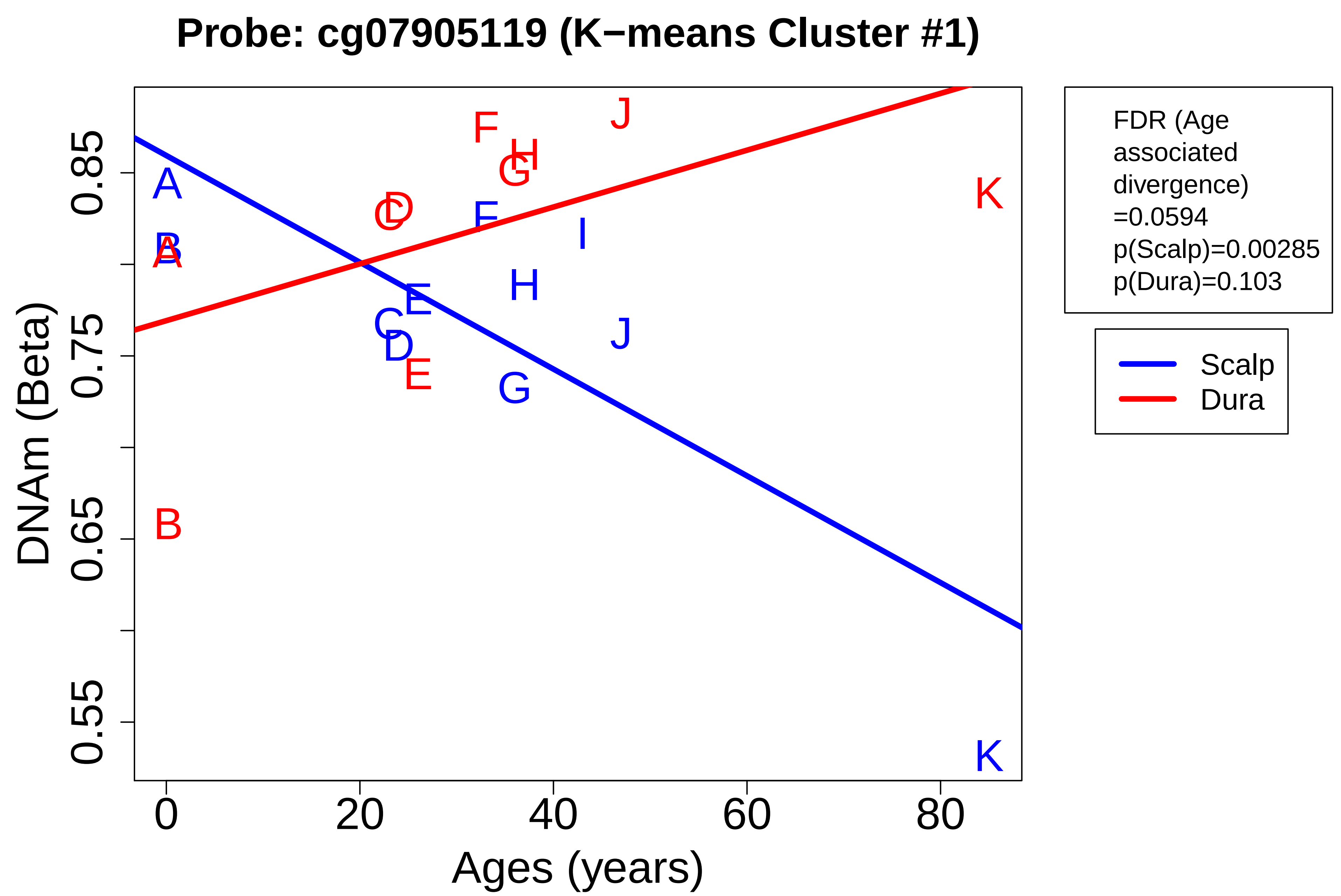

Probe: cg07905119 (K−means Cluster #1)
DNAm (Beta)
0.55
0.65
0.75
0.85
Ages (years)
0
20
40
60
80
FDR (Age associated divergence) =0.0594
p(Scalp)=0.00285
p(Dura)=0.103
Scalp
Dura

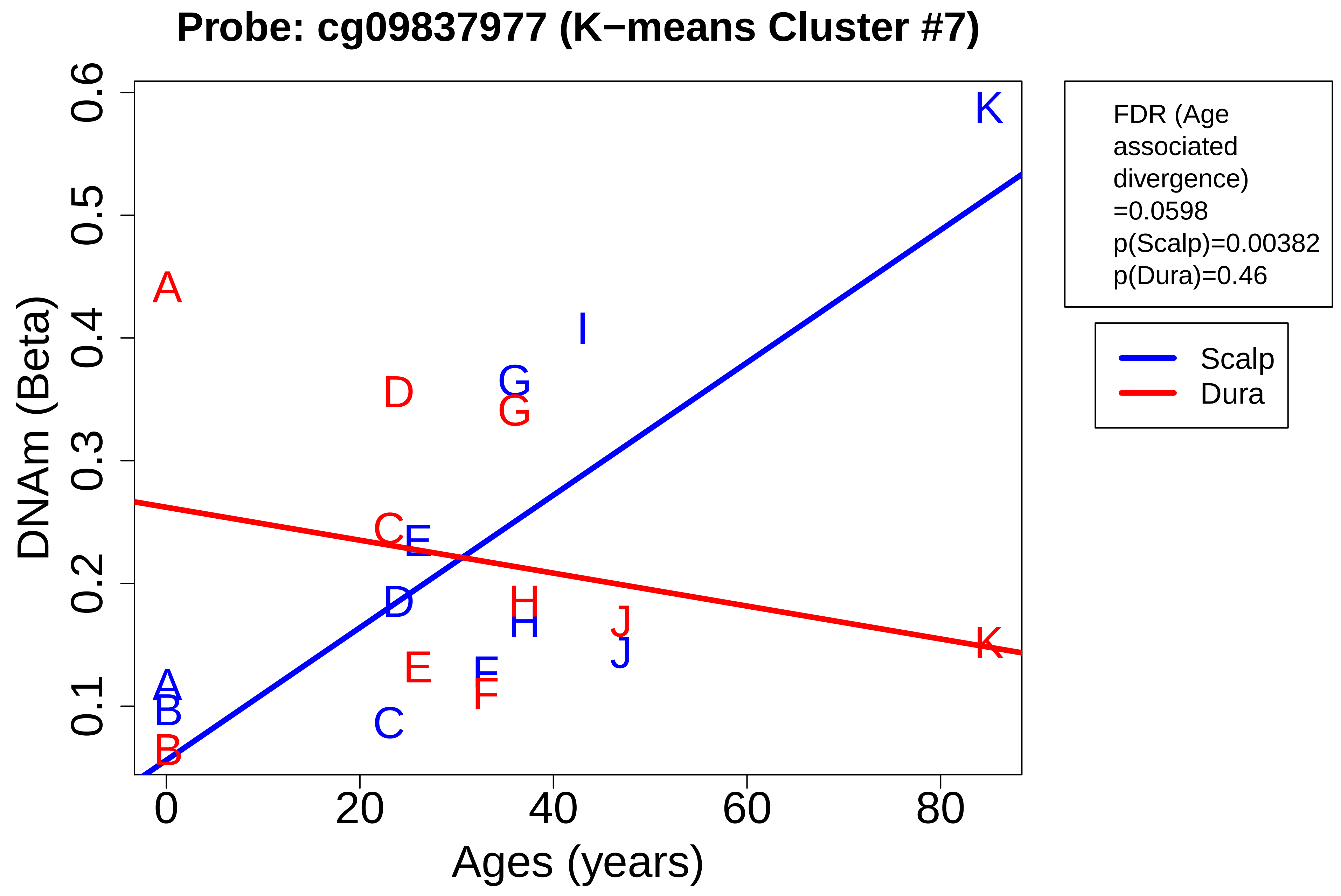

Probe: cg09837977 (K−means Cluster #7)
DNAm (Beta)
0.1
0.2
0.3
0.4
0.5
0.6
Ages (years)
0
20
40
60
80
A
B
C
D
E
F
G
H
I
J
K
FDR (Age associated divergence) =0.0598
p(Scalp)=0.00382
p(Dura)=0.46
Scalp
Dura

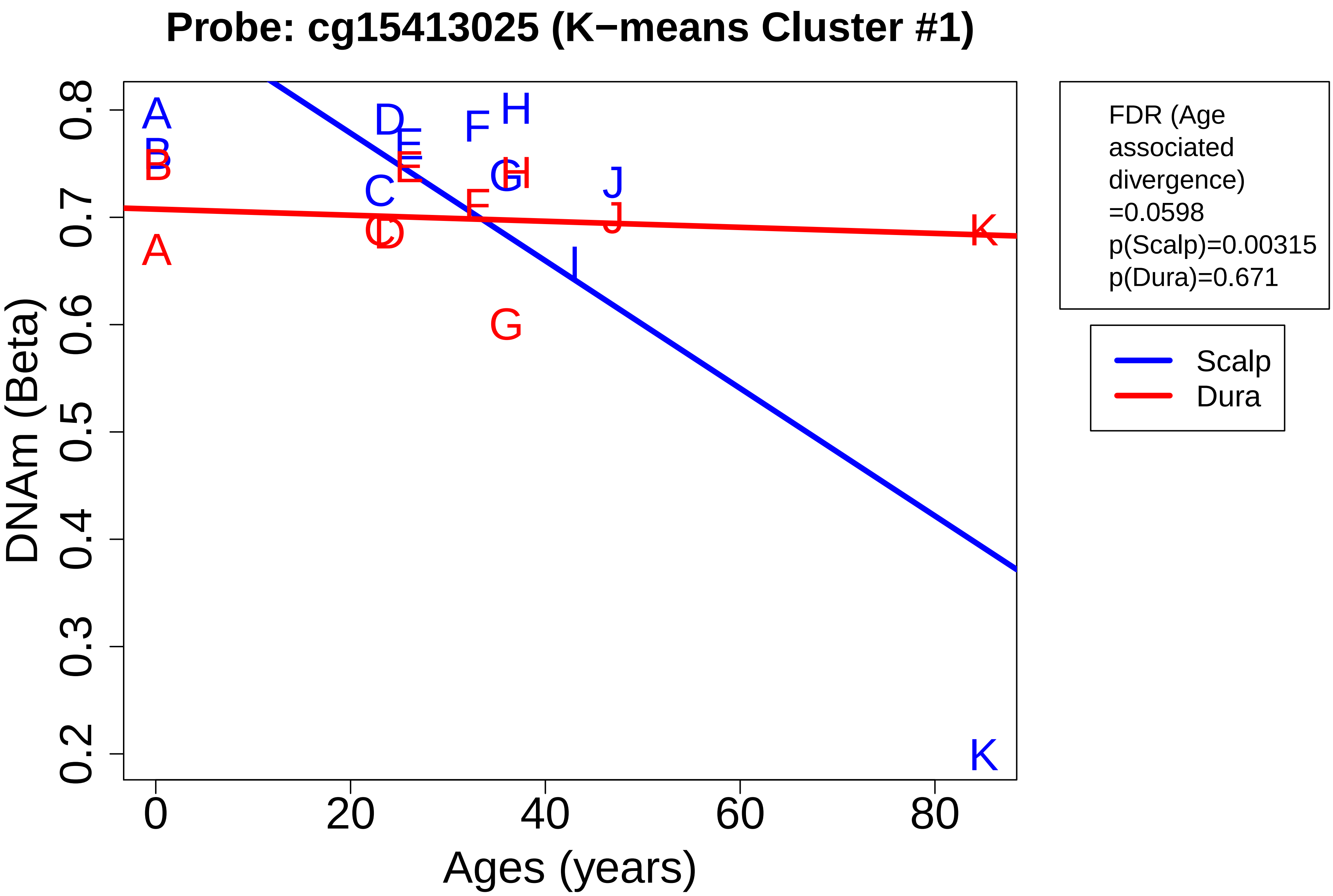

Probe: cg15413025 (K−means Cluster #1)
DNAm (Beta)
0.2
0.3
0.4
0.5
0.6
0.7
0.8
Ages (years)
0
20
40
60
80
A
B
C
D
E
F
G
H
I
J
K
FDR (Age associated divergence) =0.0598
p(Scalp)=0.00315
p(Dura)=0.671
Scalp
Dura

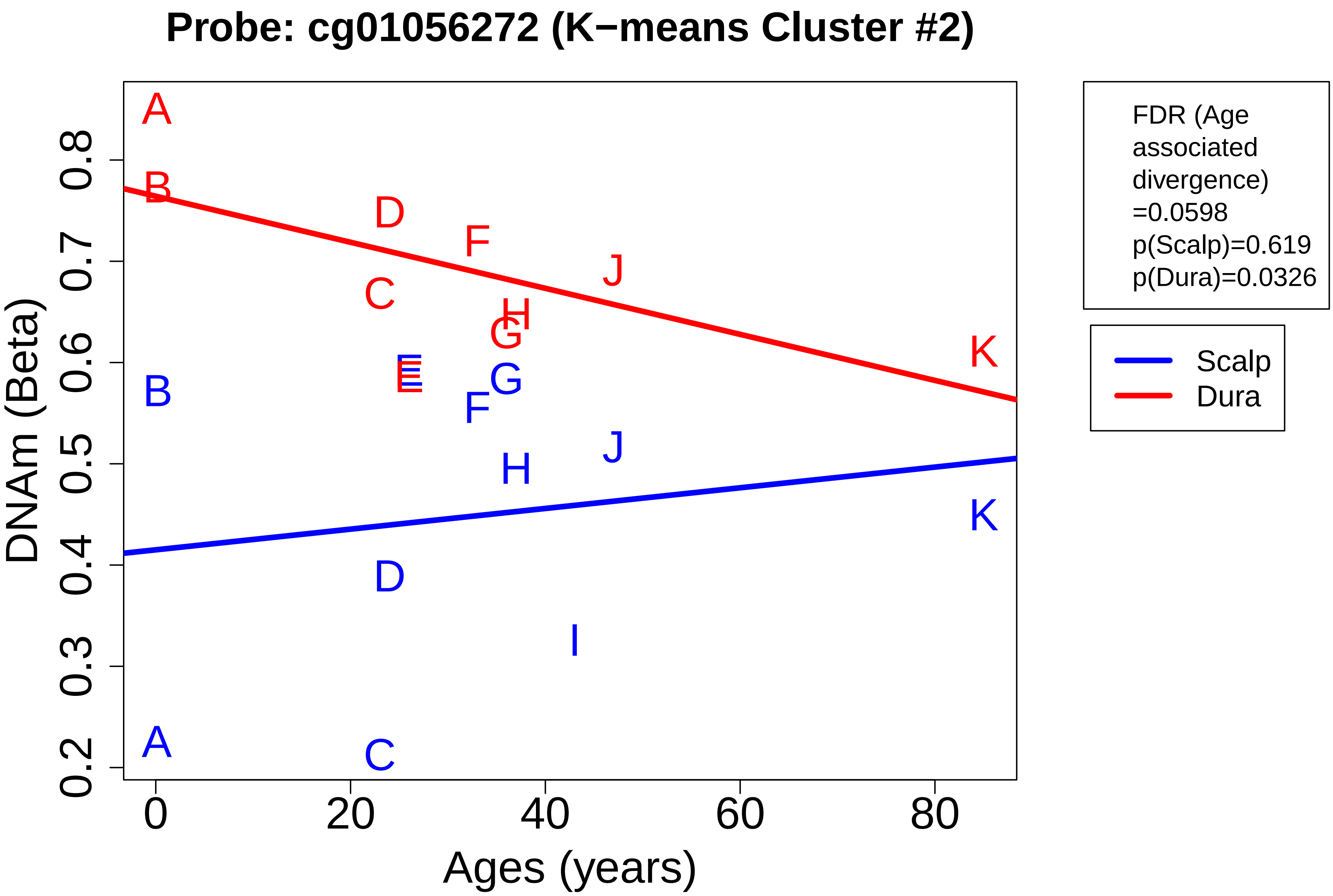
Probe: cg01056272 (K−means Cluster #2)
DNAm (Beta)
Ages (years)
0.2
0.3
0.4
0.5
0.6
0.7
0.8
0
20
40
60
80
A
B
C
D
E
F
G
H
I
J
K
FDR (Age associated divergence) =0.0598
p(Scalp)=0.619
p(Dura)=0.0326
Scalp
Dura

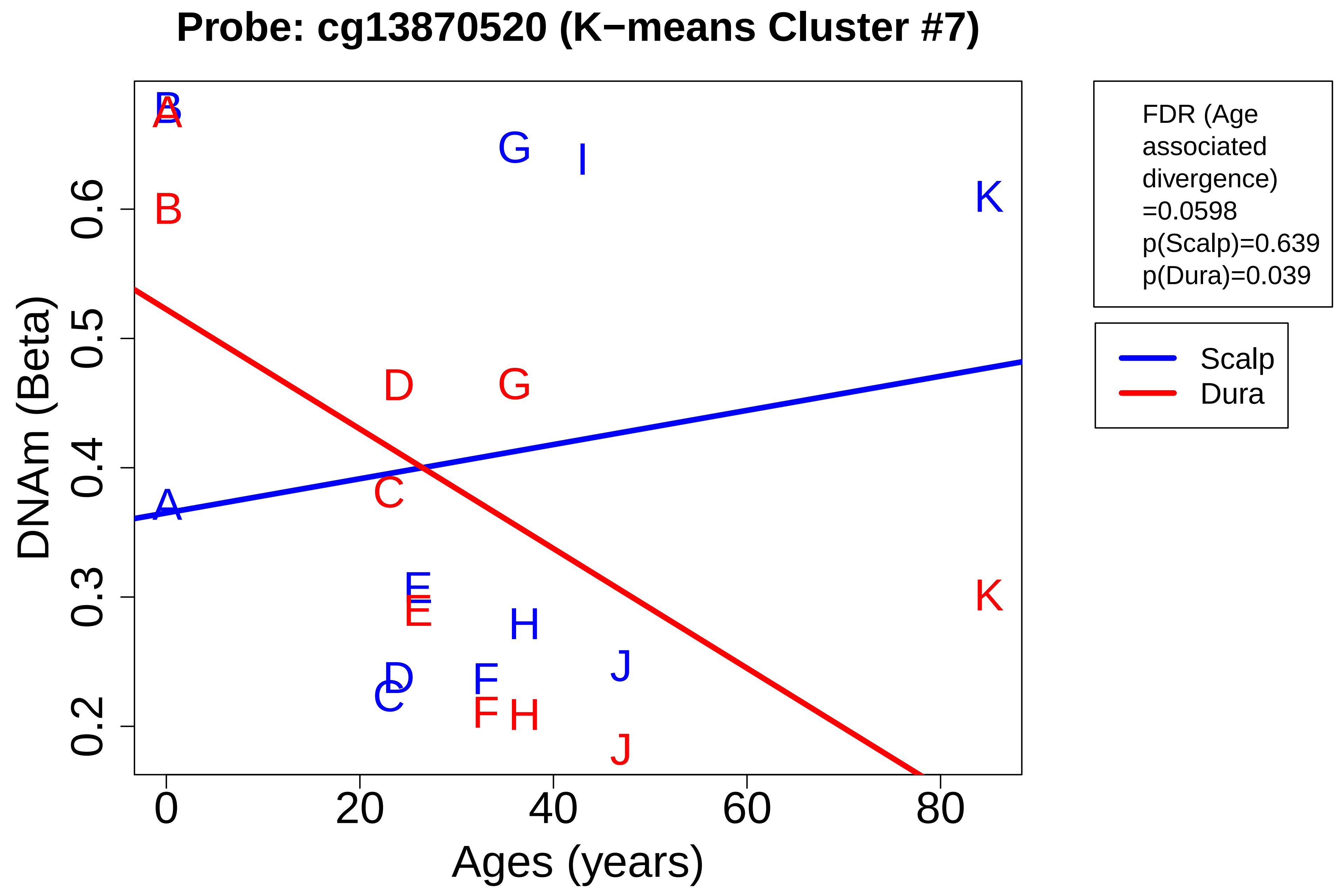
Probe: cg13870520 (K−means Cluster #7)
DNAm (Beta)
0.2
0.3
0.4
0.5
0.6
0
20
40
60
80
Ages (years)
B
A
G
I
K
B
D
G
C
A
E
E
H
K
J
D
C
F
F
H
J
FDR (Age associated divergence) =0.0598
p(Scalp)=0.639
p(Dura)=0.039
Scalp
Dura

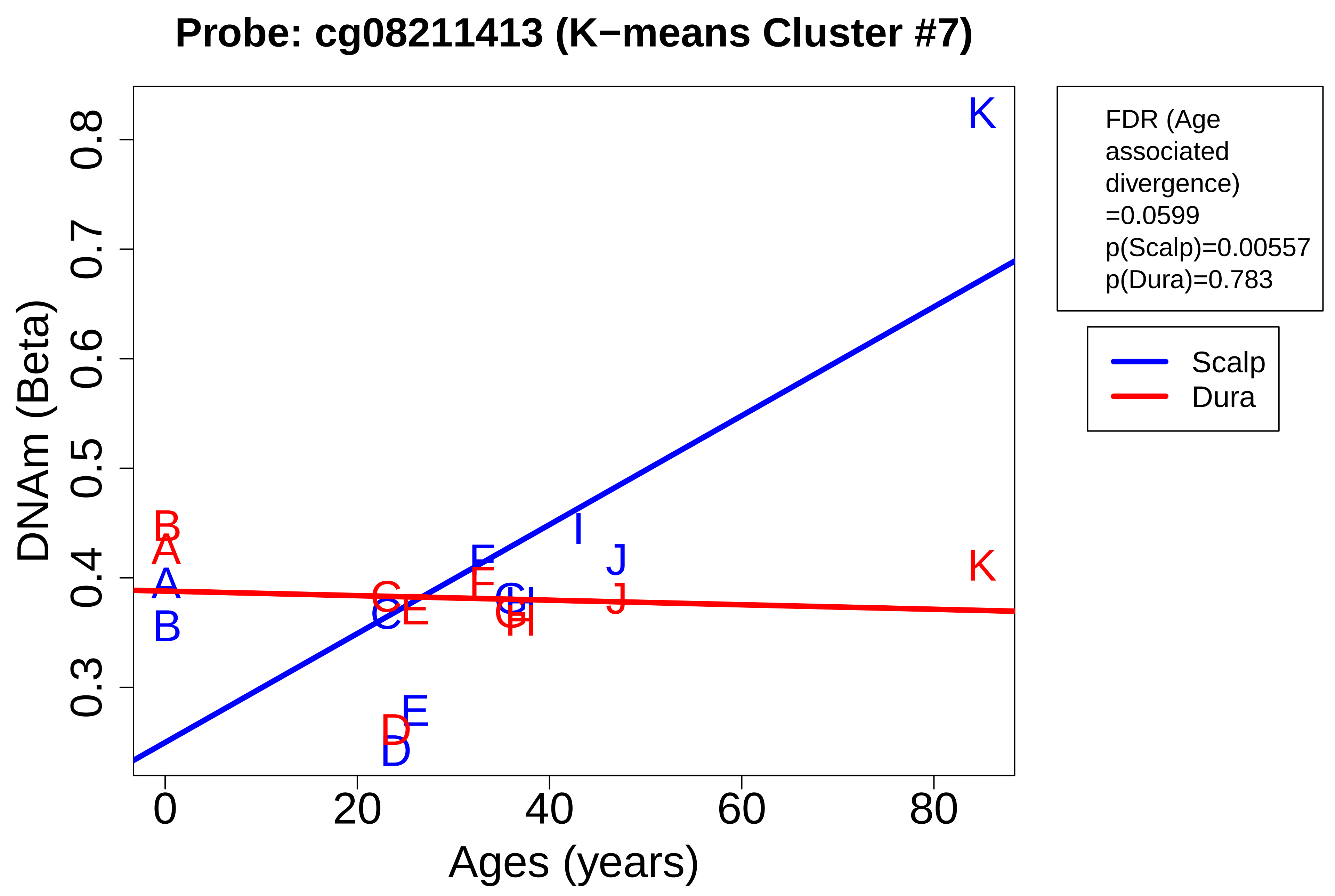
Probe: cg08211413 (K−means Cluster #7)
DNAm (Beta)
0.3
0.4
0.5
0.6
0.7
0.8
0
20
40
60
80
Ages (years)
FDR (Age associated divergence) =0.0599
p(Scalp)=0.00557
p(Dura)=0.783
Scalp
Dura

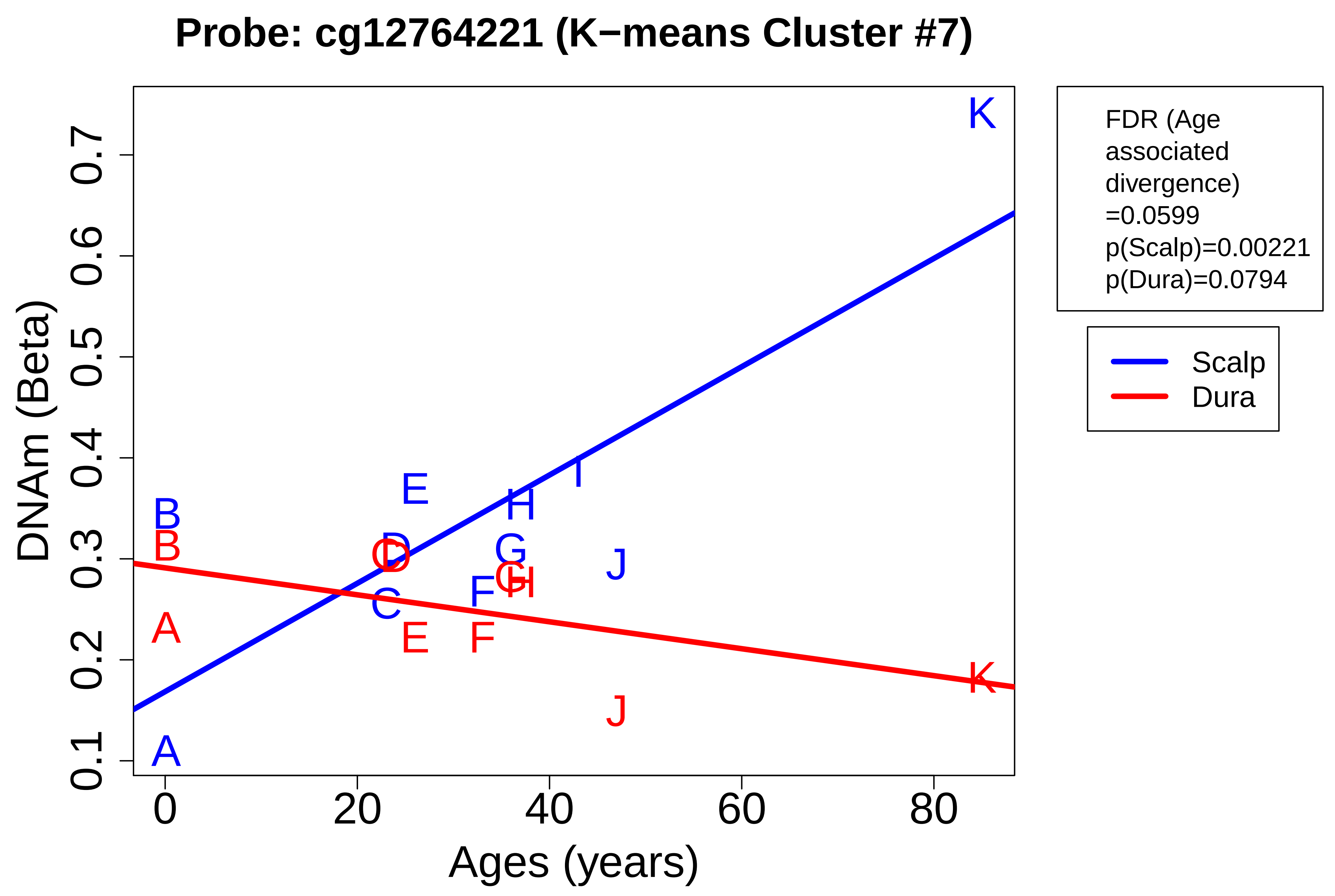
Probe: cg12764221 (K−means Cluster #7)
DNAm (Beta)
0.1
0.2
0.3
0.4
0.5
0.6
0.7
Ages (years)
0
20
40
60
80
FDR (Age associated divergence) =0.0599
p(Scalp)=0.00221
p(Dura)=0.0794
Scalp
Dura

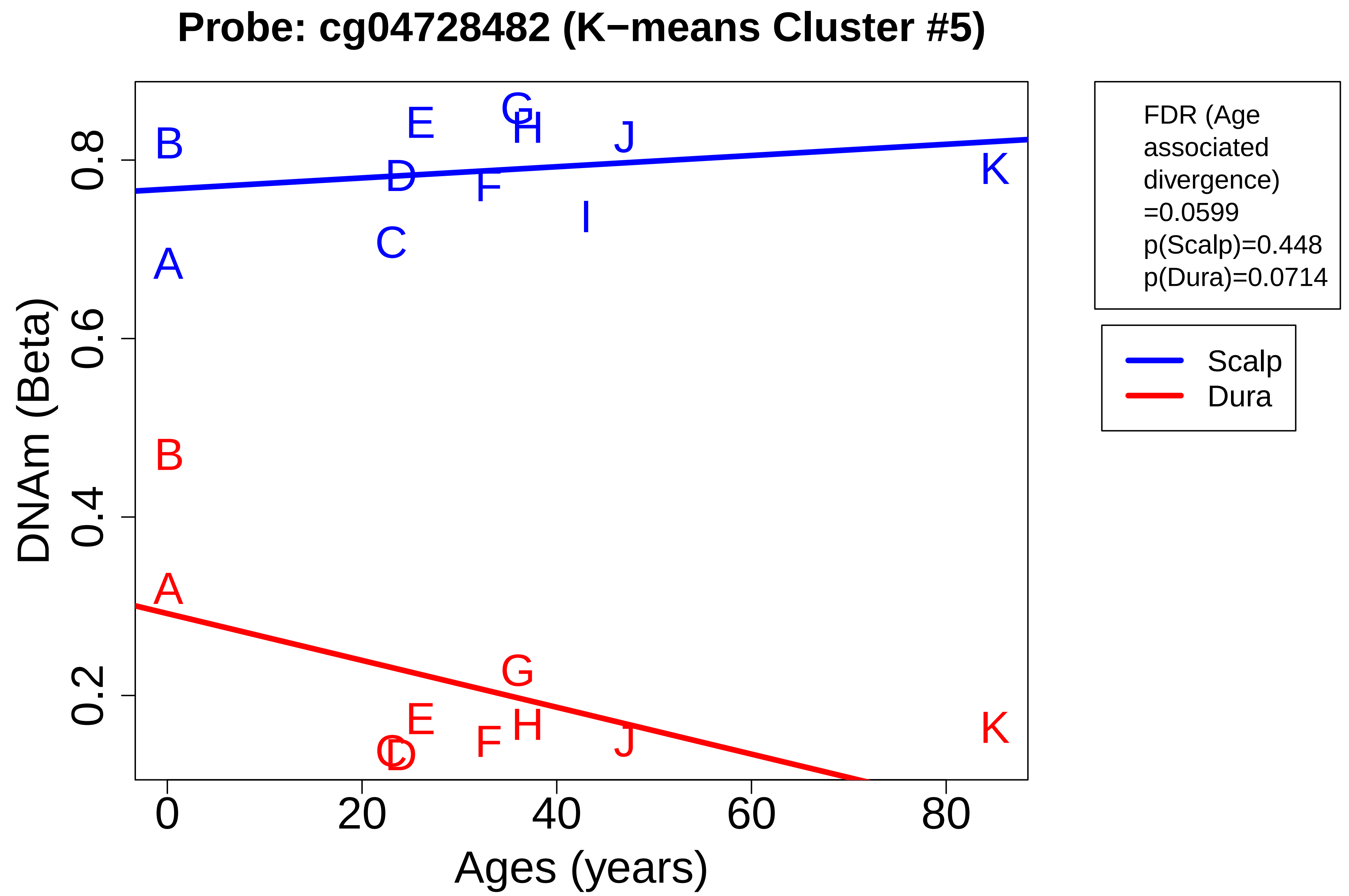

Probe: cg04728482 (K−means Cluster #5)
DNAm (Beta)
0.2
0.4
0.6
0.8
Ages (years)
0
20
40
60
80
A
B
C
D
E
F
G
H
I
J
K
FDR (Age associated divergence) =0.0599
p(Scalp)=0.448
p(Dura)=0.0714
Scalp
Dura

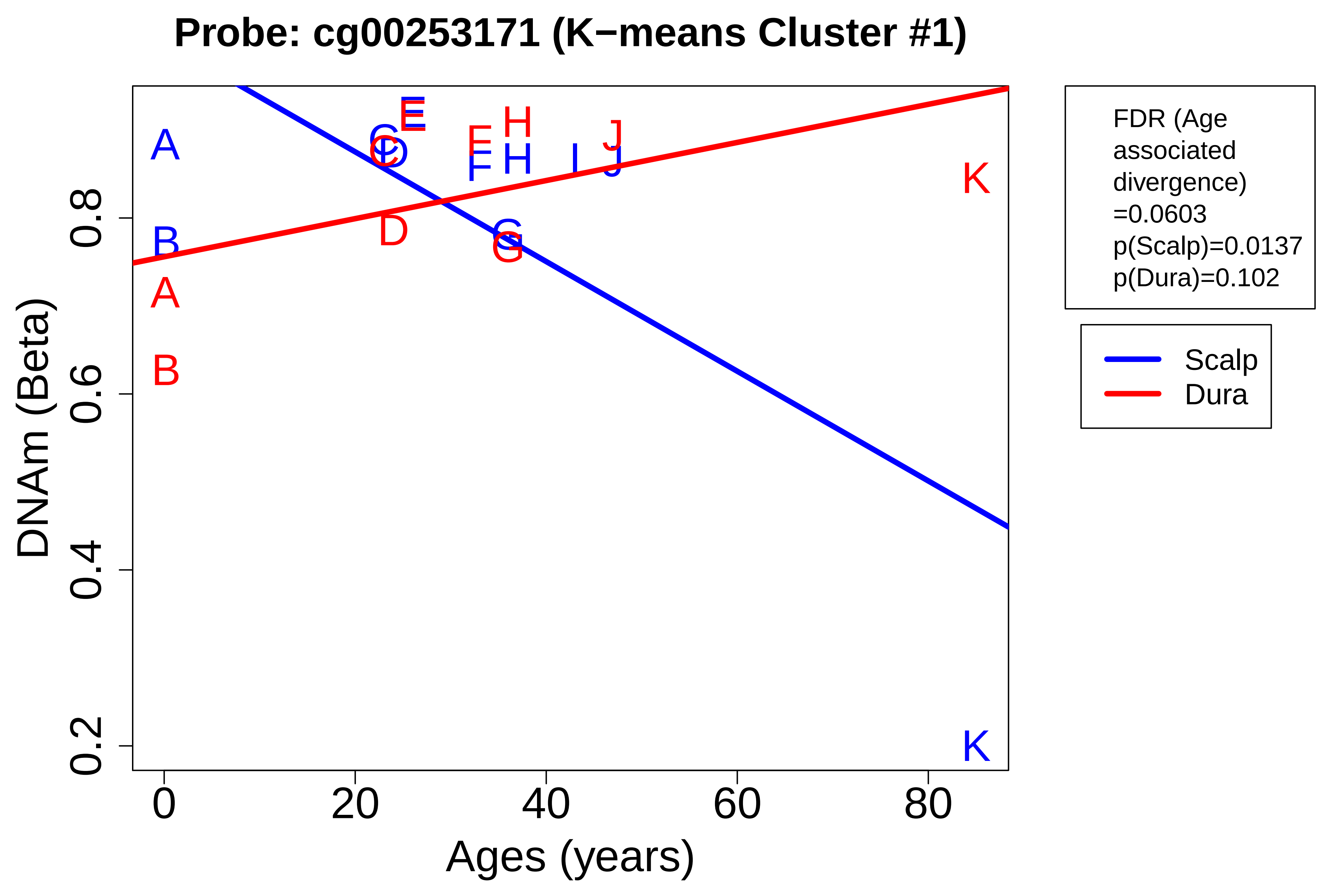
Probe: cg00253171 (K−means Cluster #1)
DNAm (Beta)
0.2
0.4
0.6
0.8
0
20
40
60
80
Ages (years)
A
B
C
D
E
F
G
H
I
J
K
FDR (Age associated divergence) =0.0603
p(Scalp)=0.0137
p(Dura)=0.102
Scalp
Dura

Probe: cg13446366 (K−means Cluster #1)

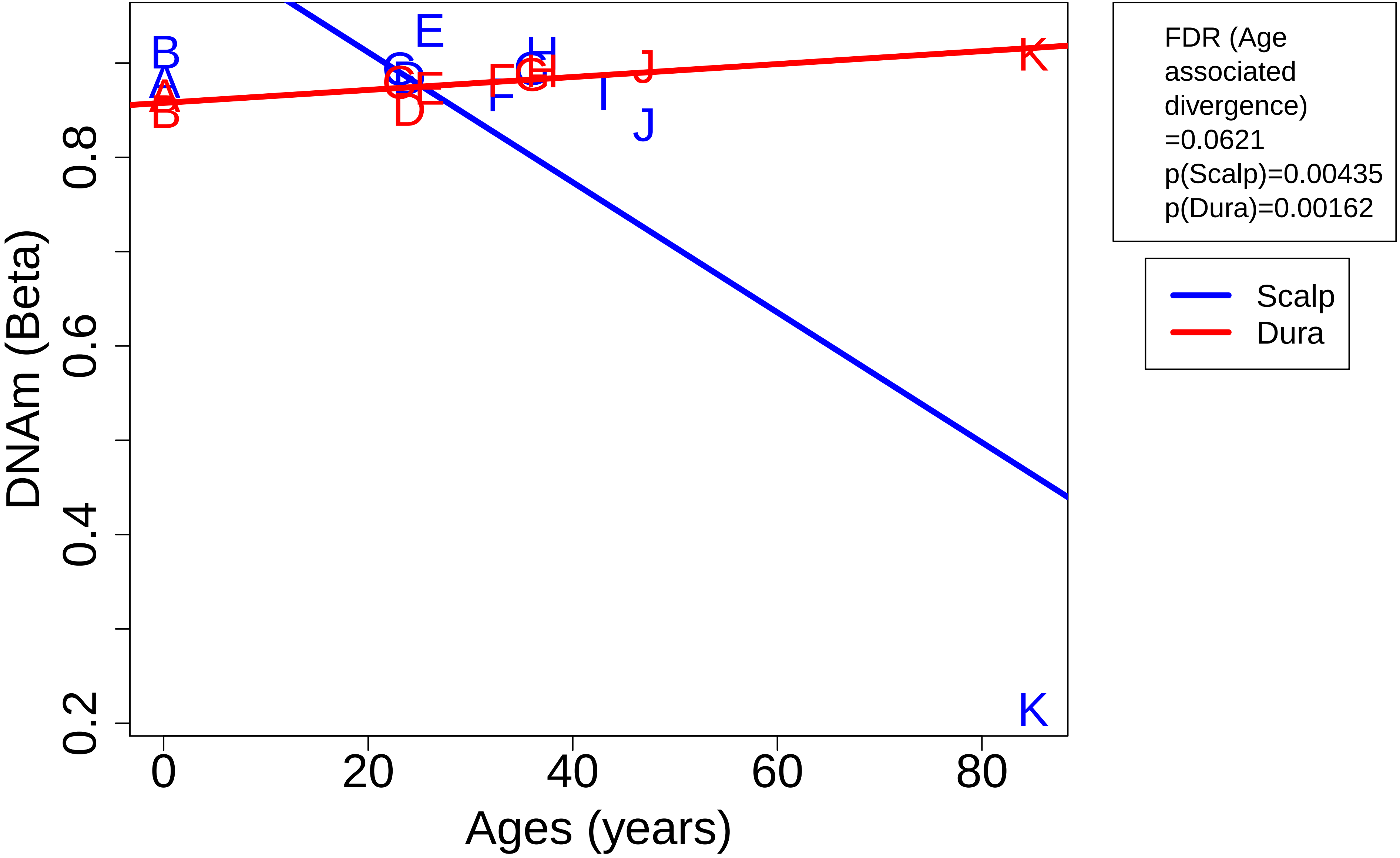

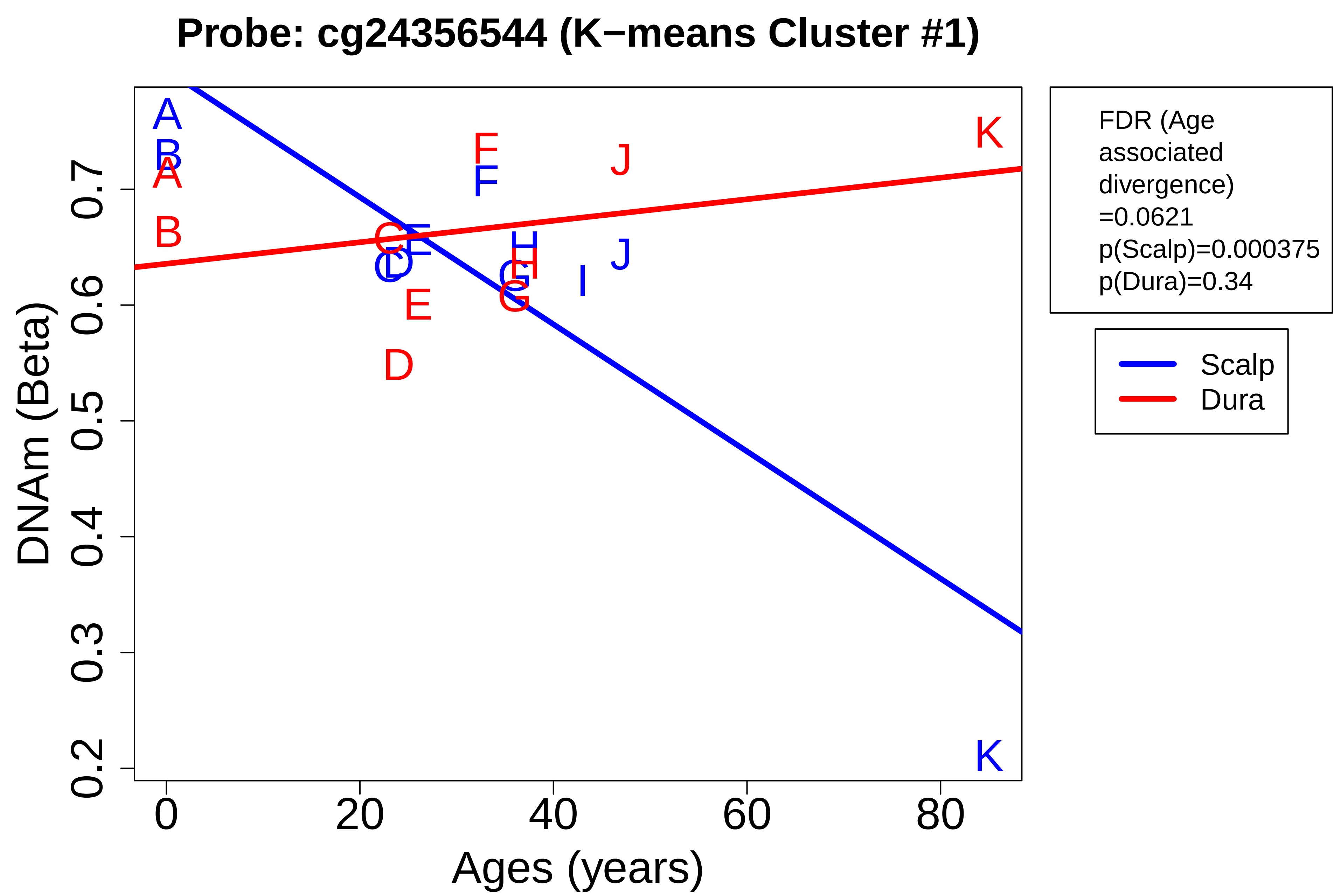

Probe: cg24356544 (K−means Cluster #1)
DNAm (Beta)
0.2
0.3
0.4
0.5
0.6
0.7
0
20
40
60
80
Ages (years)
A
B
C
D
E
F
G
H
I
J
K
FDR (Age associated divergence) =0.0621
p(Scalp)=0.000375
p(Dura)=0.34
Scalp
Dura

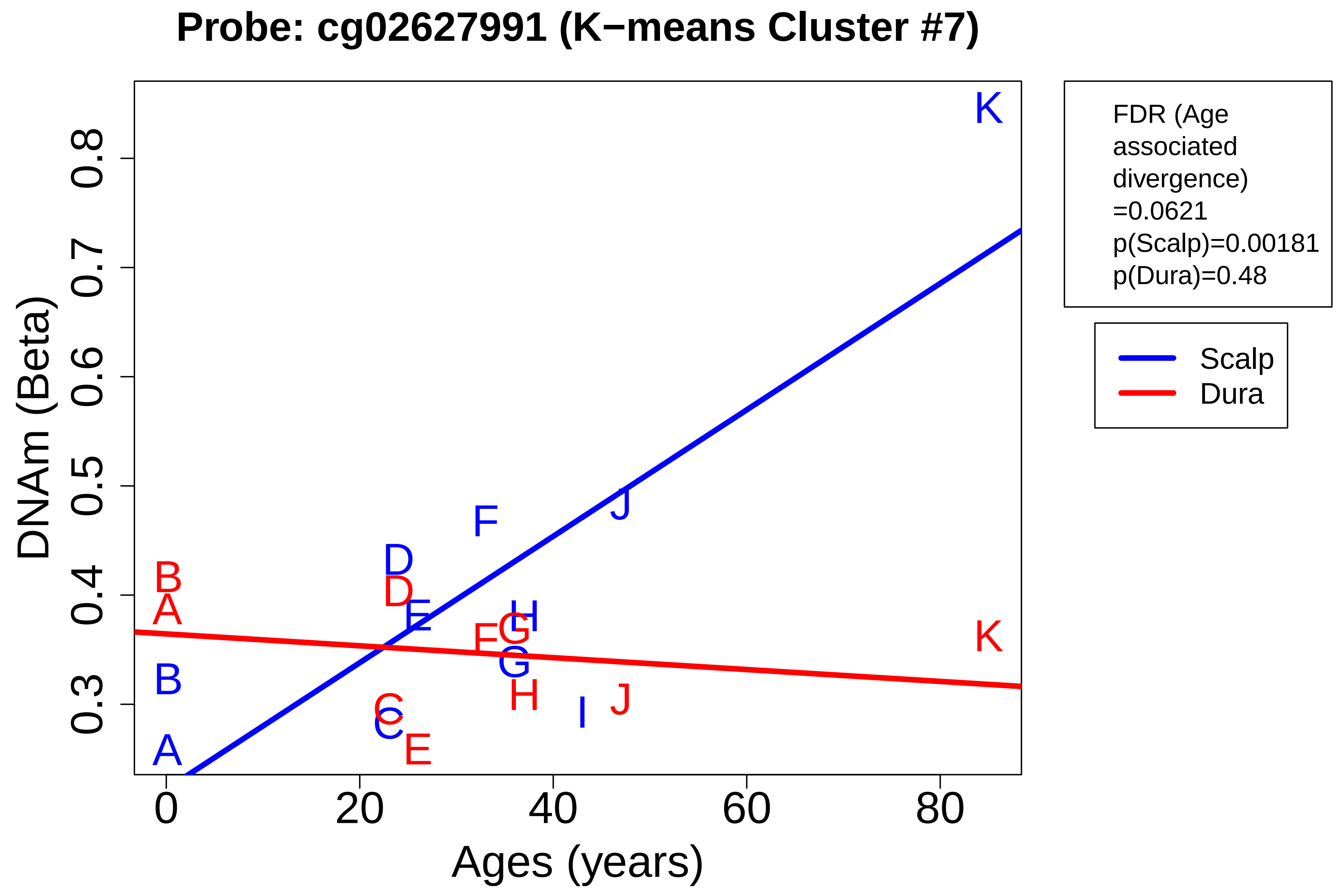

Probe: cg02627991 (K−means Cluster #7)
DNAm (Beta)
Ages (years)
0.3
0.4
0.5
0.6
0.7
0.8
0
20
40
60
80
FDR (Age associated divergence) =0.0621
p(Scalp)=0.00181
p(Dura)=0.48
Scalp
Dura

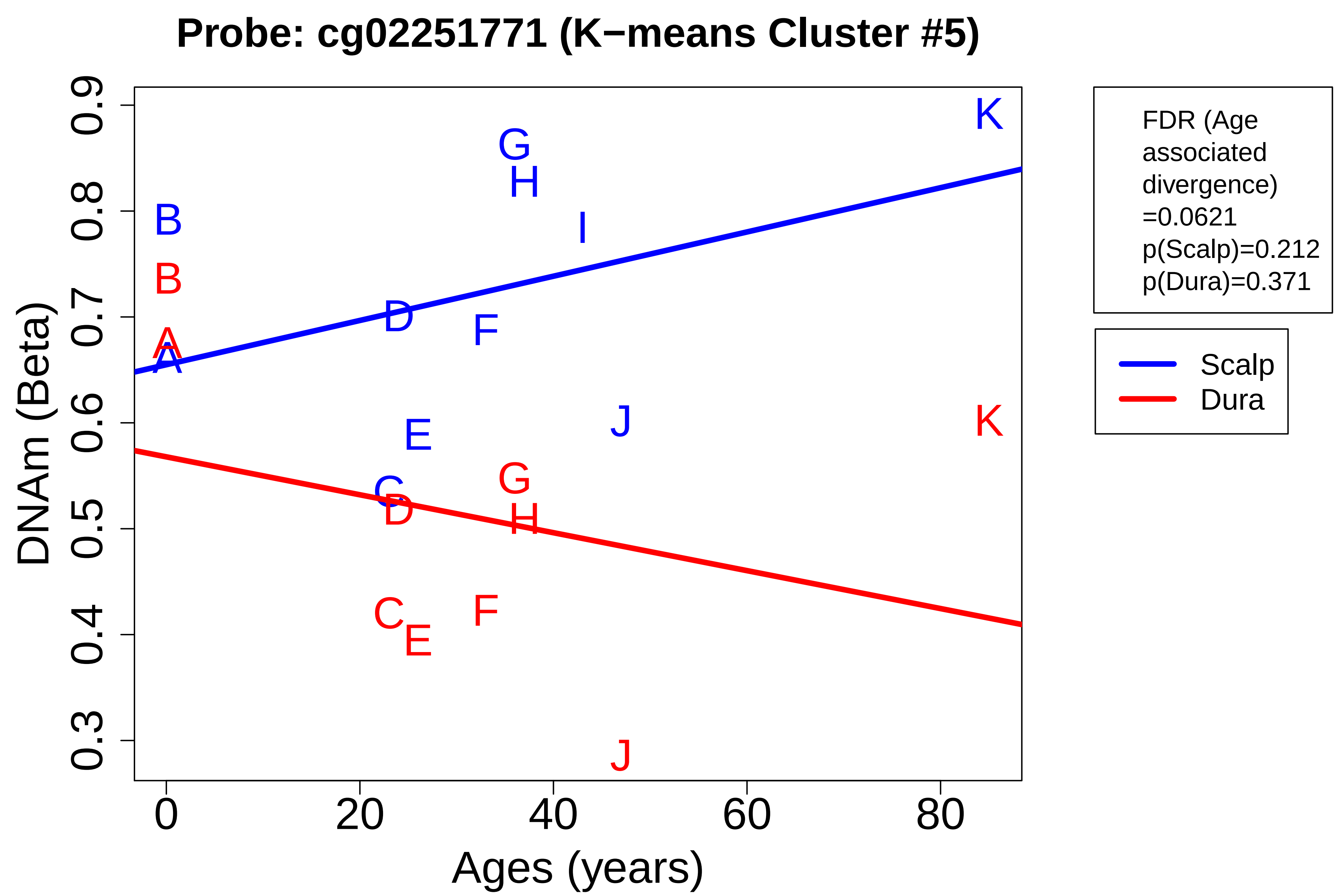

Probe: cg02251771 (K−means Cluster #5)
DNAm (Beta)
0.3
0.4
0.5
0.6
0.7
0.8
0.9
0
20
40
60
80
Ages (years)
FDR (Age associated divergence) =0.0621
p(Scalp)=0.212
p(Dura)=0.371
Scalp
Dura
A
B
C
D
E
F
G
H
I
J
K

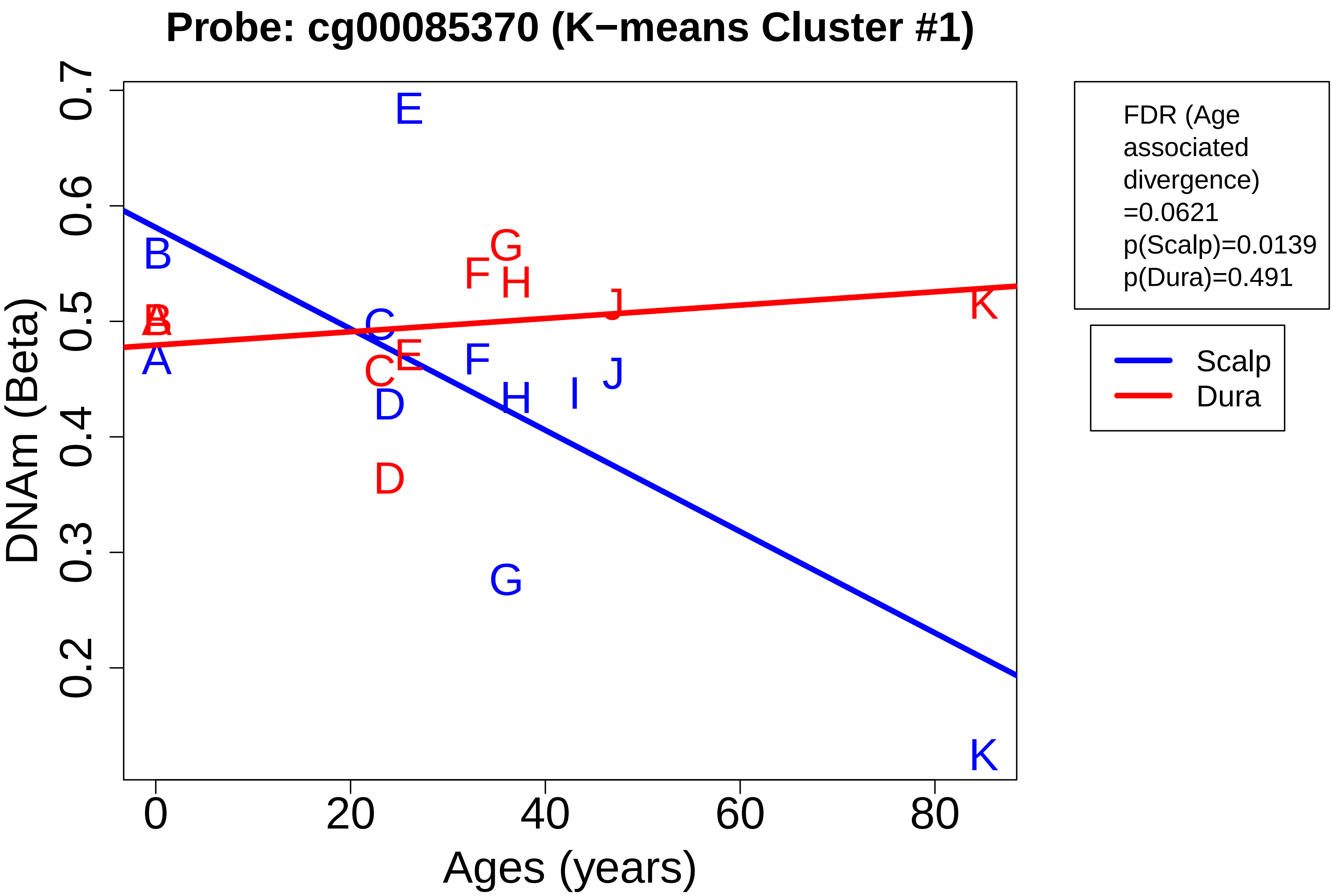
Probe: cg00085370 (K−means Cluster #1)
DNAm (Beta)
Ages (years)
0.2
0.3
0.4
0.5
0.6
0.7
0
20
40
60
80
FDR (Age associated divergence) =0.0621
p(Scalp)=0.0139
p(Dura)=0.491
Scalp
Dura

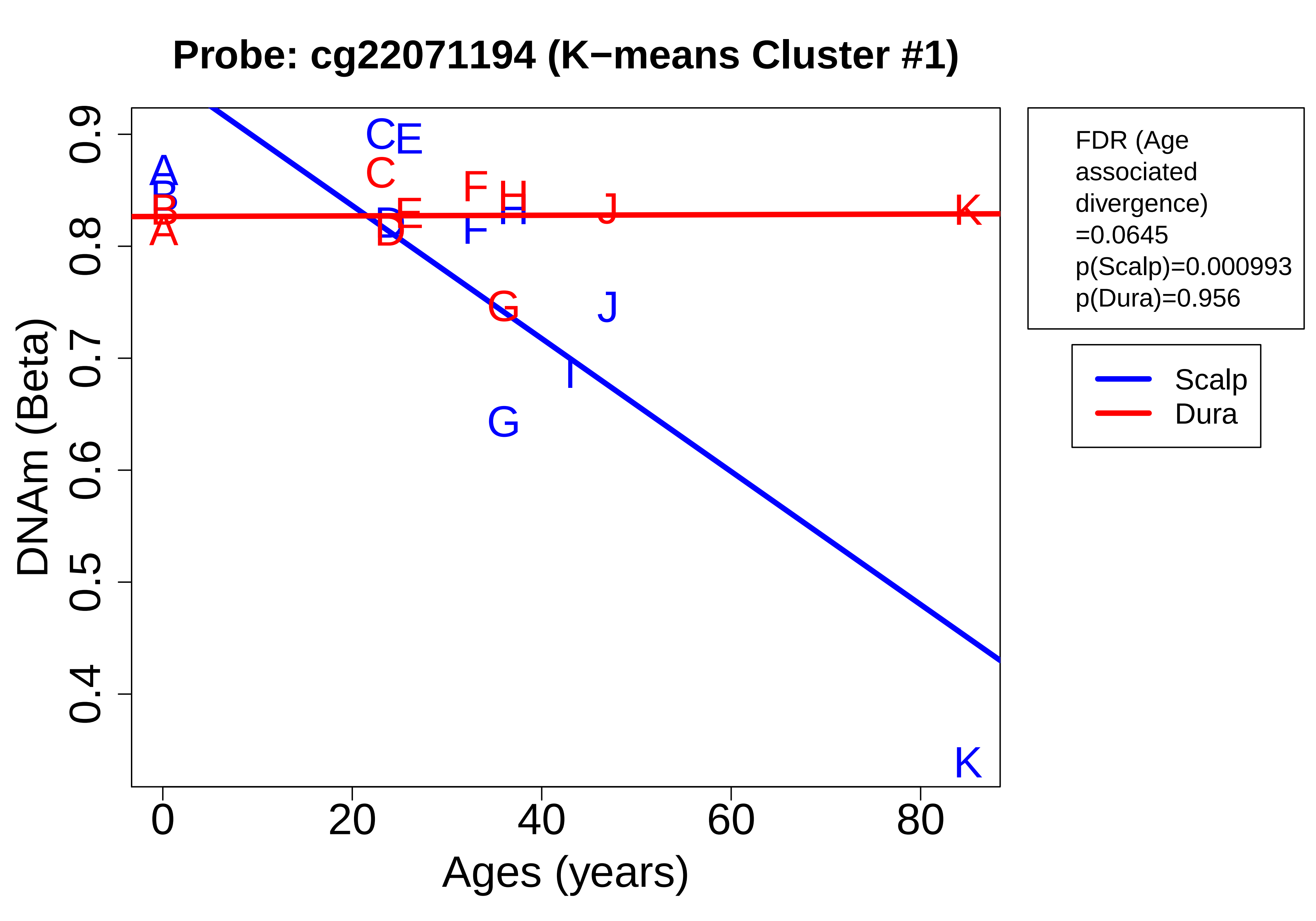

Probe: cg22071194 (K−means Cluster #1)
DNAm (Beta)
0.4
0.5
0.6
0.7
0.8
0.9
0
20
40
60
80
Ages (years)
FDR (Age associated divergence) =0.0645
p(Scalp)=0.000993
p(Dura)=0.956
Scalp
Dura

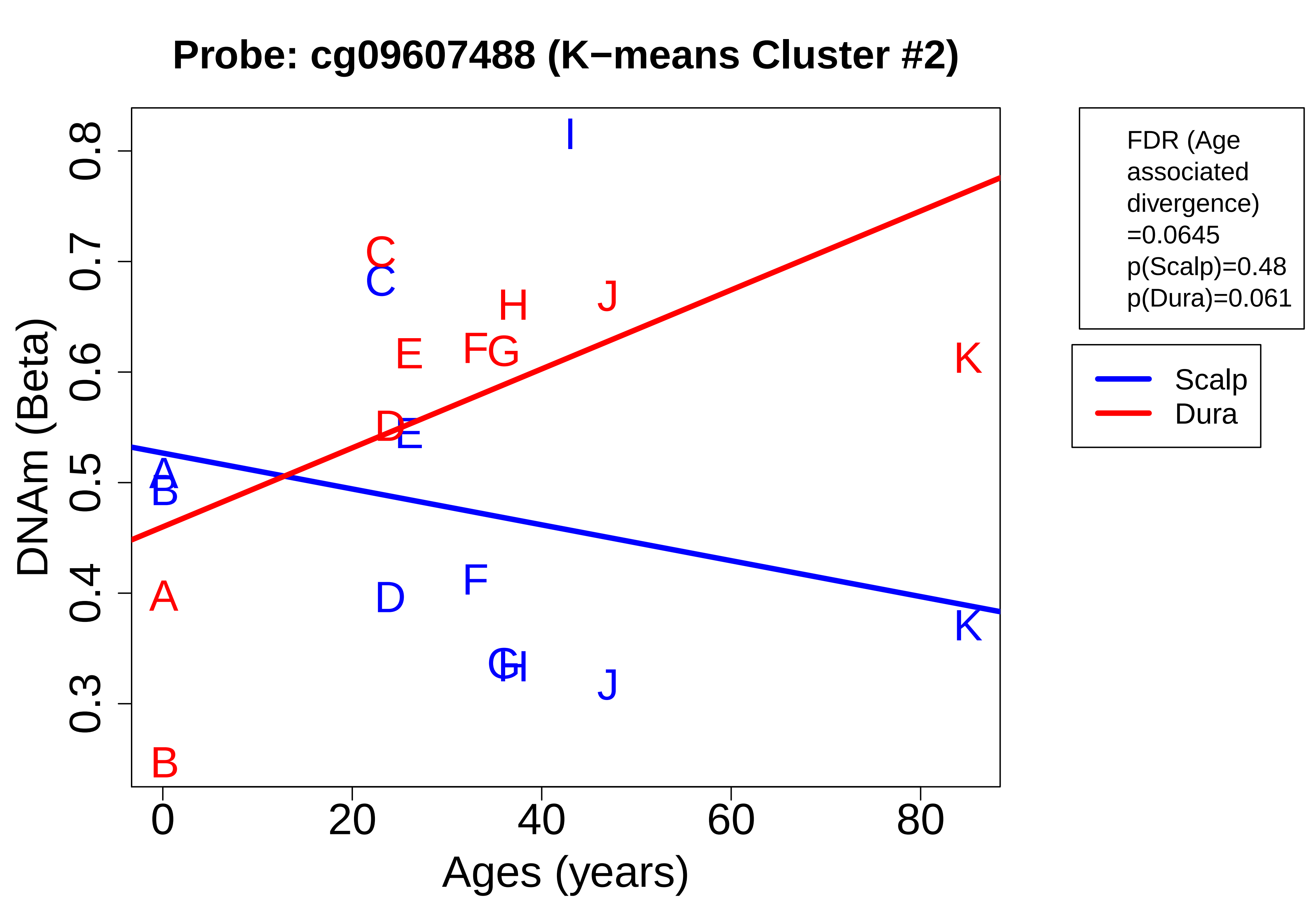

Probe: cg09607488 (K−means Cluster #2)
DNAm (Beta)
0.3
0.4
0.5
0.6
0.7
0.8
0
20
40
60
80
Ages (years)
FDR (Age associated divergence) =0.0645
p(Scalp)=0.48
p(Dura)=0.061
Scalp
Dura

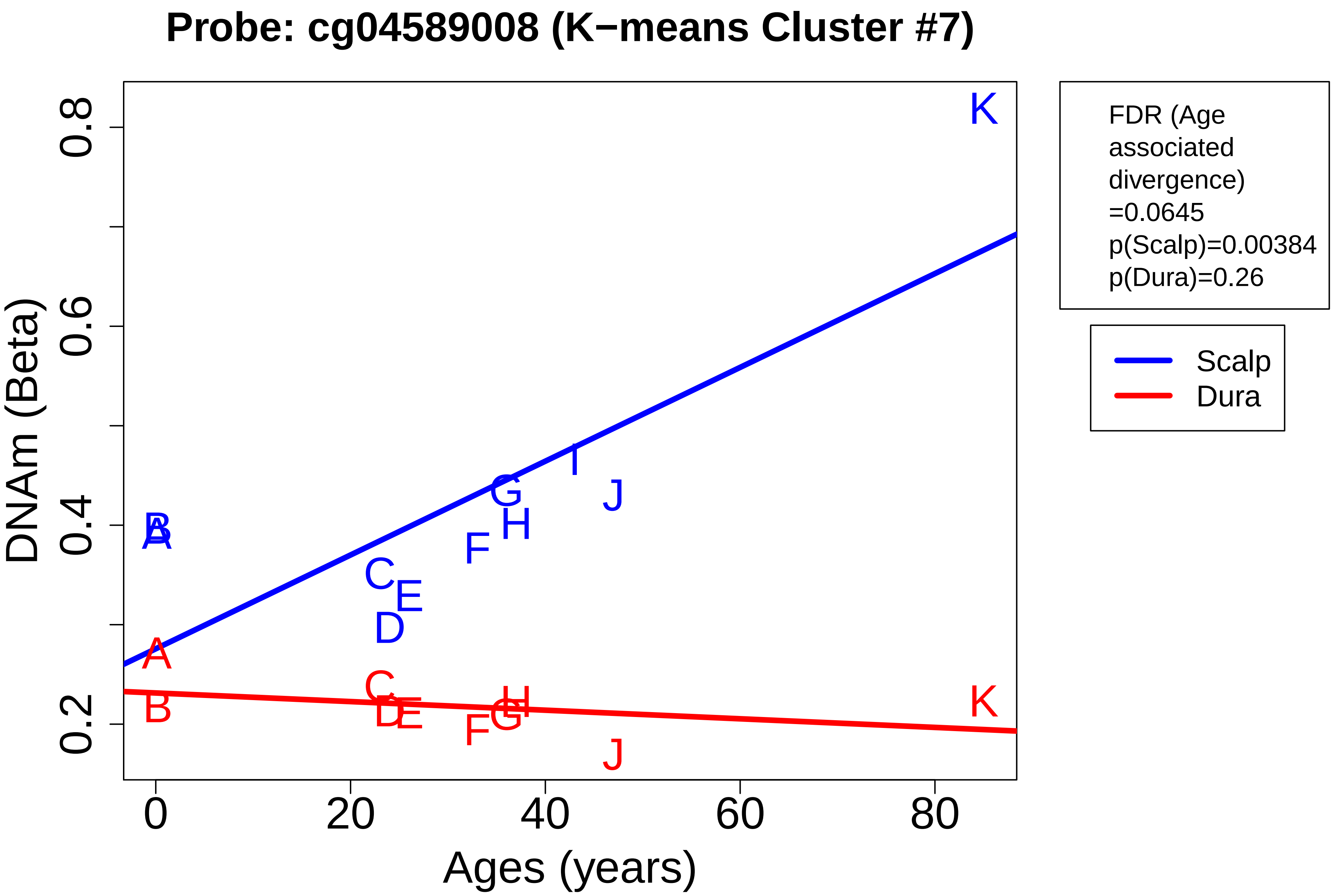
Probe: cg04589008 (K−means Cluster #7)
FDR (Age associated divergence) =0.0645
p(Scalp)=0.00384
p(Dura)=0.26
Scalp
Dura
DNAm (Beta)
0.2
0.4
0.6
0.8
0
20
40
60
80
Ages (years)
A
B
C
D
E
F
G
H
I
J
K

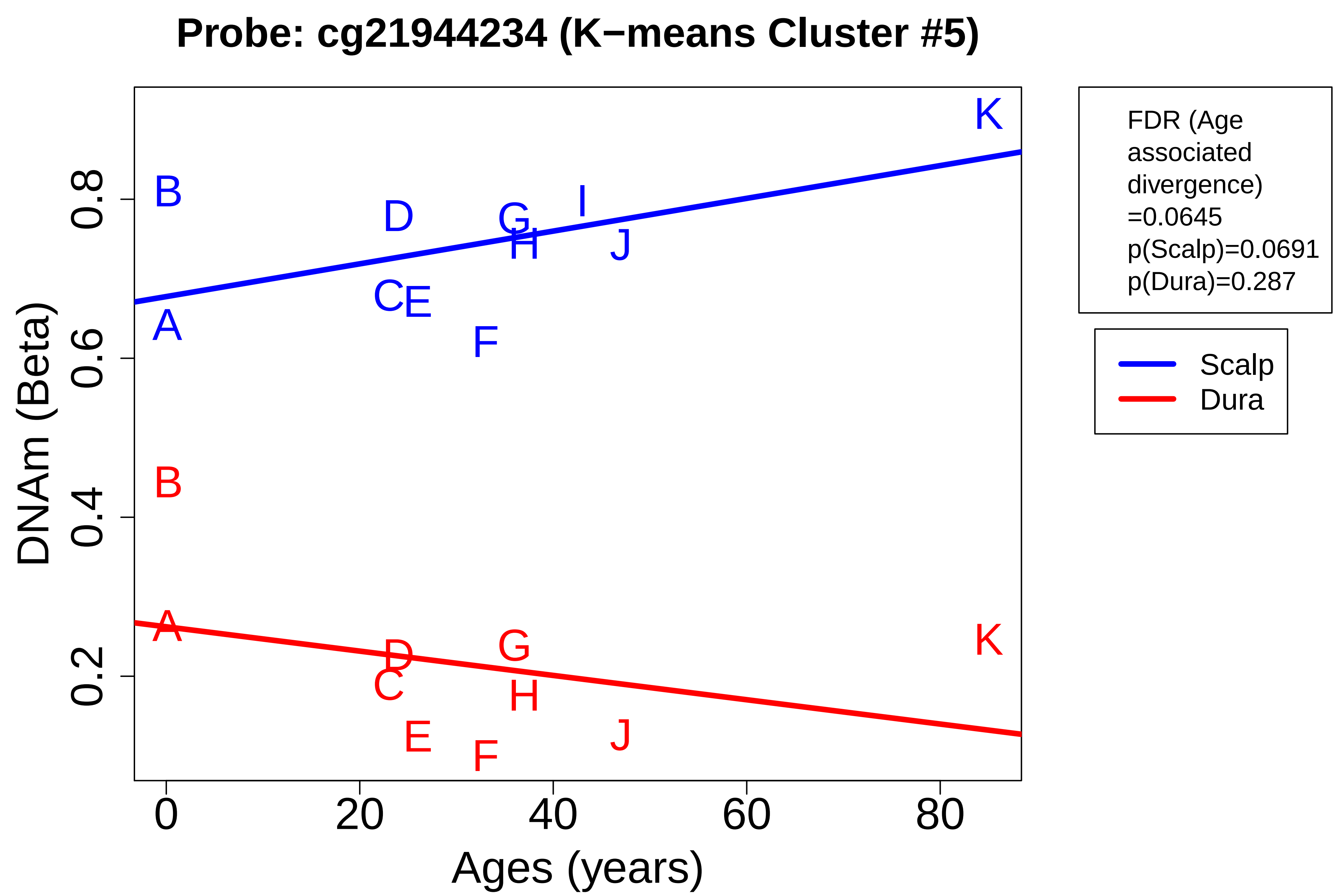
Probe: cg21944234 (K−means Cluster #5)
DNAm (Beta)
0.2
0.4
0.6
0.8
0
20
40
60
80
Ages (years)
A
B
C
D
E
F
G
H
I
J
K
FDR (Age associated divergence) =0.0645
p(Scalp)=0.0691
p(Dura)=0.287
Scalp
Dura

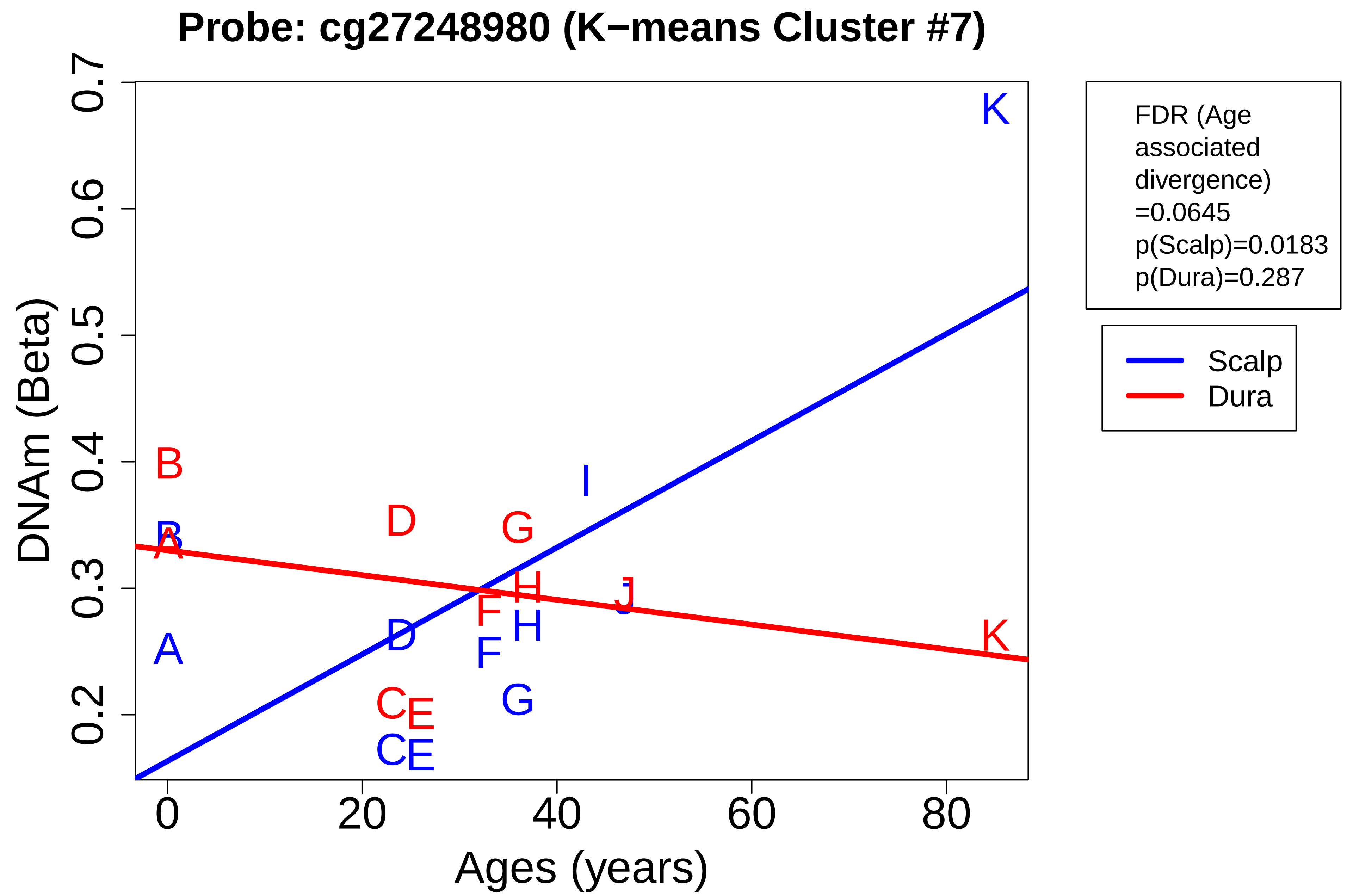
Probe: cg27248980 (K−means Cluster #7)
DNAm (Beta)
Ages (years)
0.2
0.3
0.4
0.5
0.6
0.7
0
20
40
60
80
FDR (Age associated divergence) =0.0645
p(Scalp)=0.0183
p(Dura)=0.287
Scalp
Dura
A
B
C
D
E
F
G
H
I
J
K

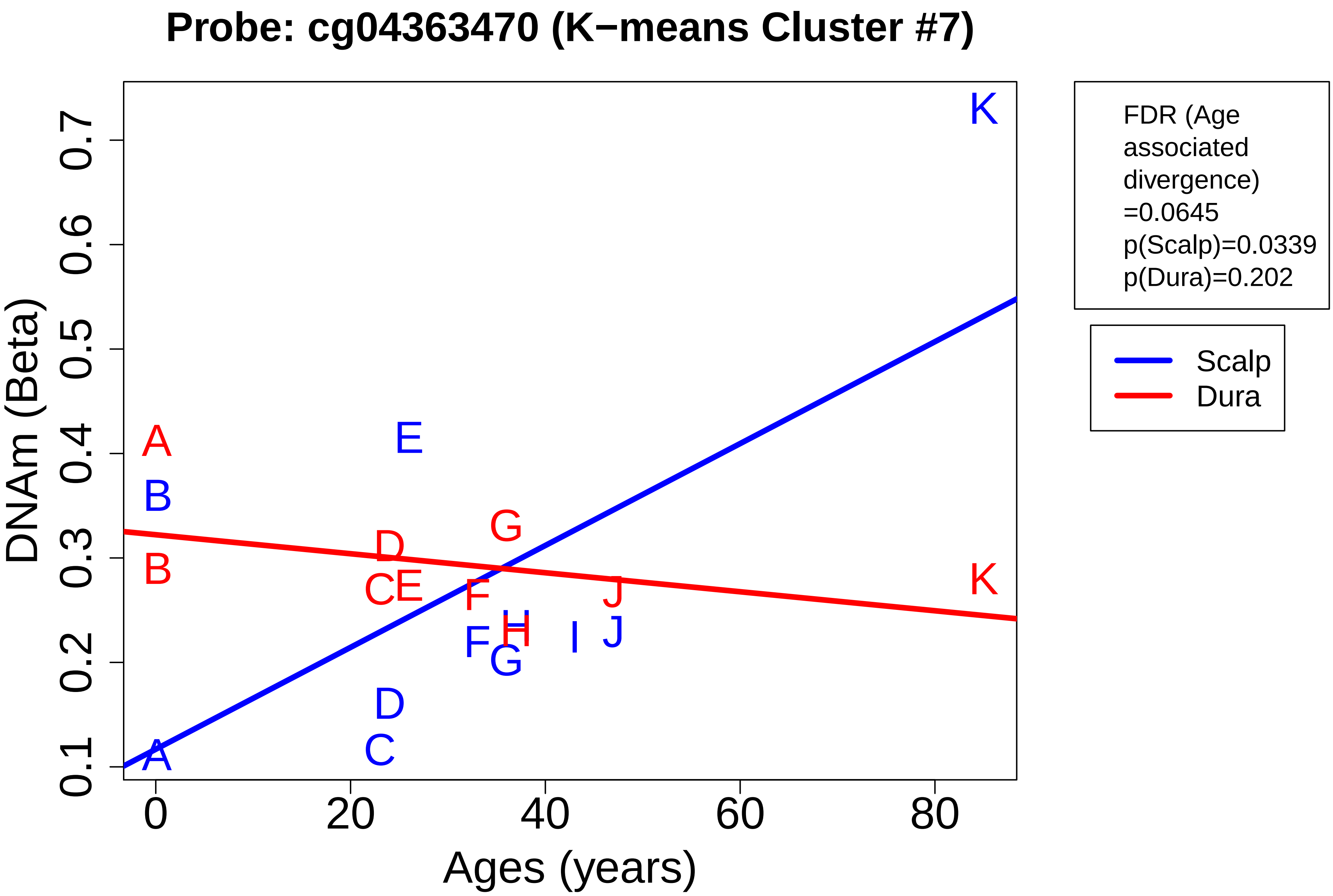

Probe: cg04363470 (K−means Cluster #7)
DNAm (Beta)
Ages (years)
0.1
0.2
0.3
0.4
0.5
0.6
0.7
0
20
40
60
80
A
B
C
D
E
F
G
H
I
J
K
FDR (Age associated divergence) =0.0645
p(Scalp)=0.0339
p(Dura)=0.202
Scalp
Dura

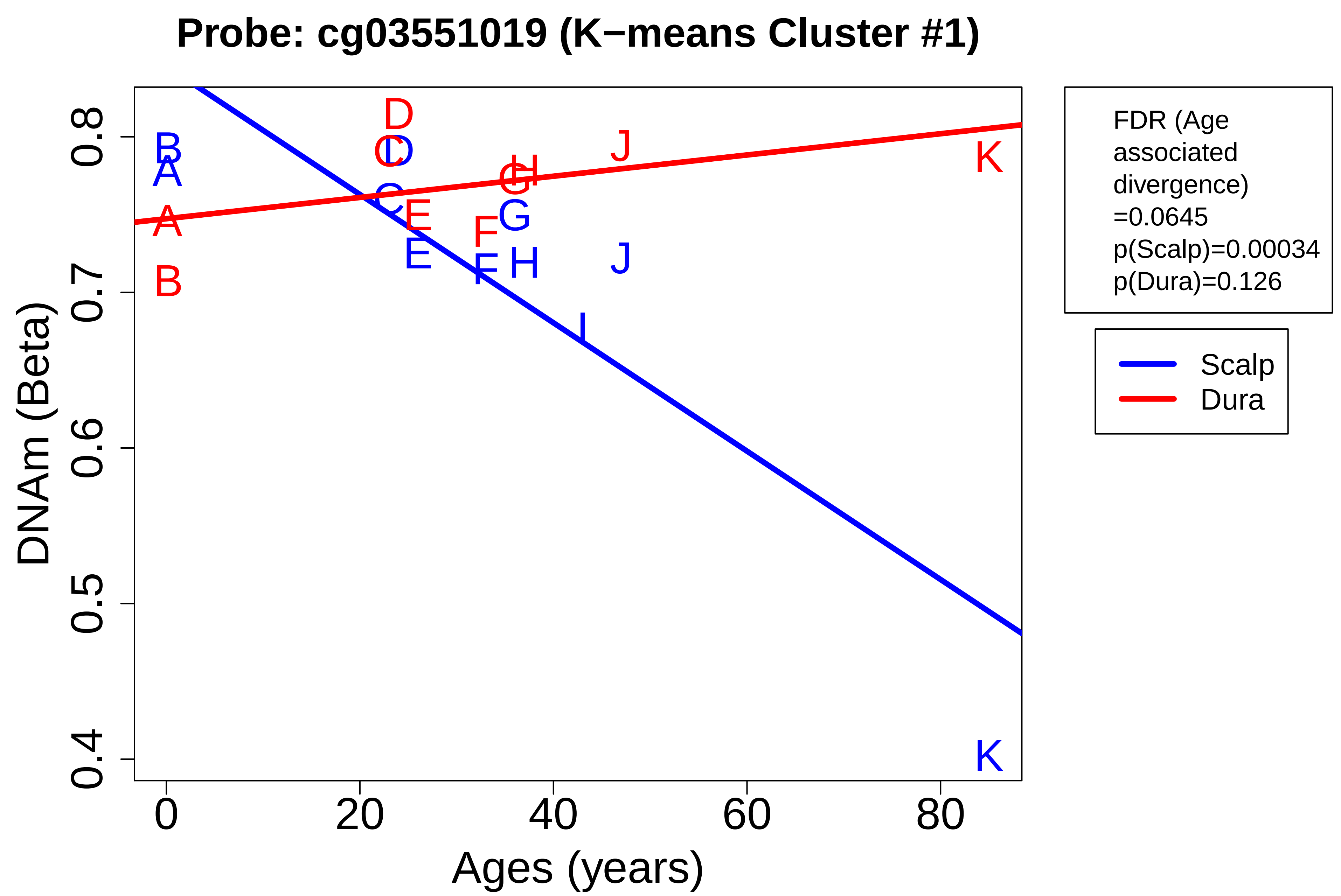

Probe: cg03551019 (K−means Cluster #1)
DNAm (Beta)
0.4
0.5
0.6
0.7
0.8
0
20
40
60
80
Ages (years)
FDR (Age associated divergence) =0.0645
p(Scalp)=0.00034
p(Dura)=0.126
Scalp
Dura

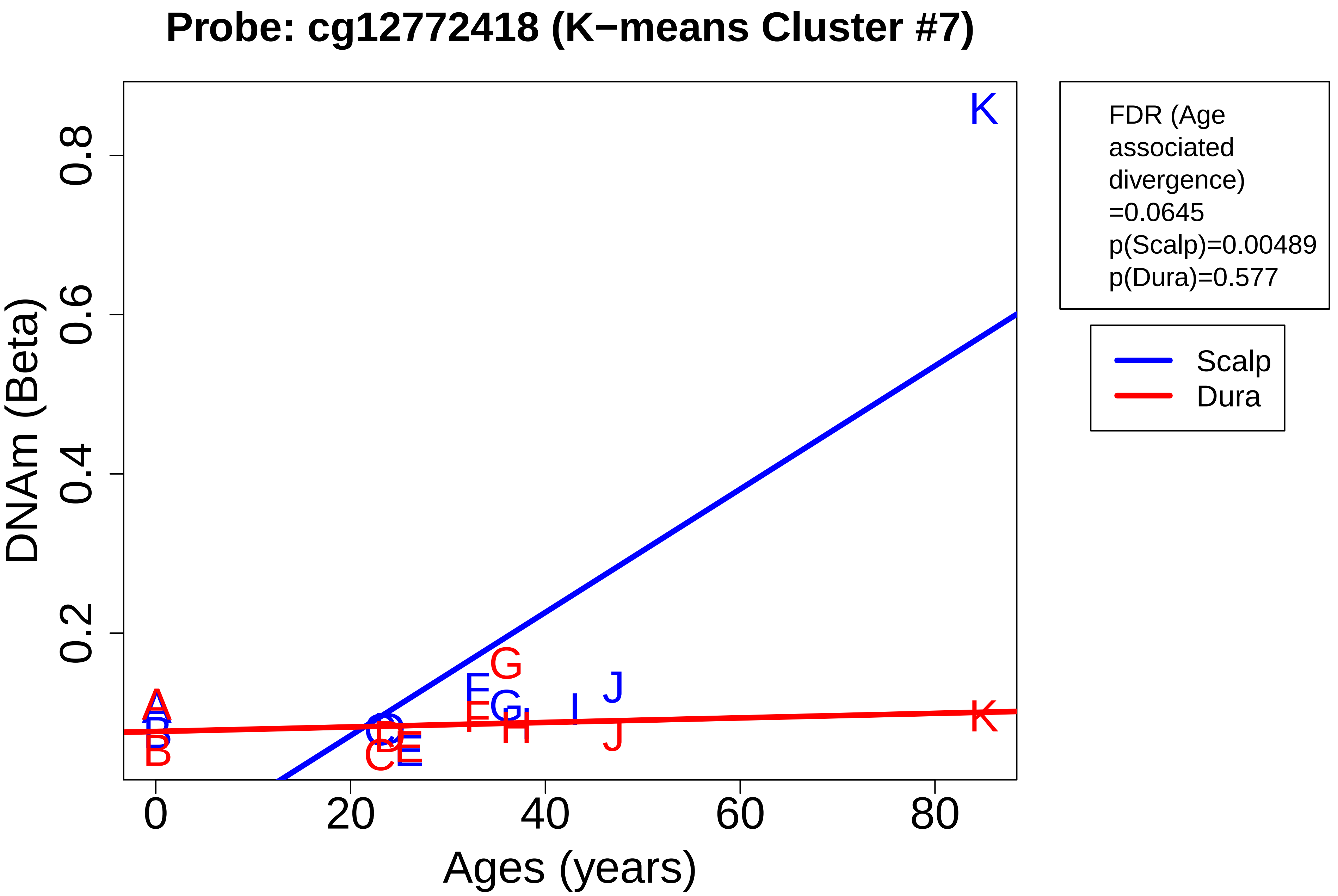

Probe: cg12772418 (K−means Cluster #7)
DNAm (Beta)
0.2
0.4
0.6
0.8
0
20
40
60
80
Ages (years)
FDR (Age associated divergence) =0.0645
p(Scalp)=0.00489
p(Dura)=0.577
Scalp
Dura

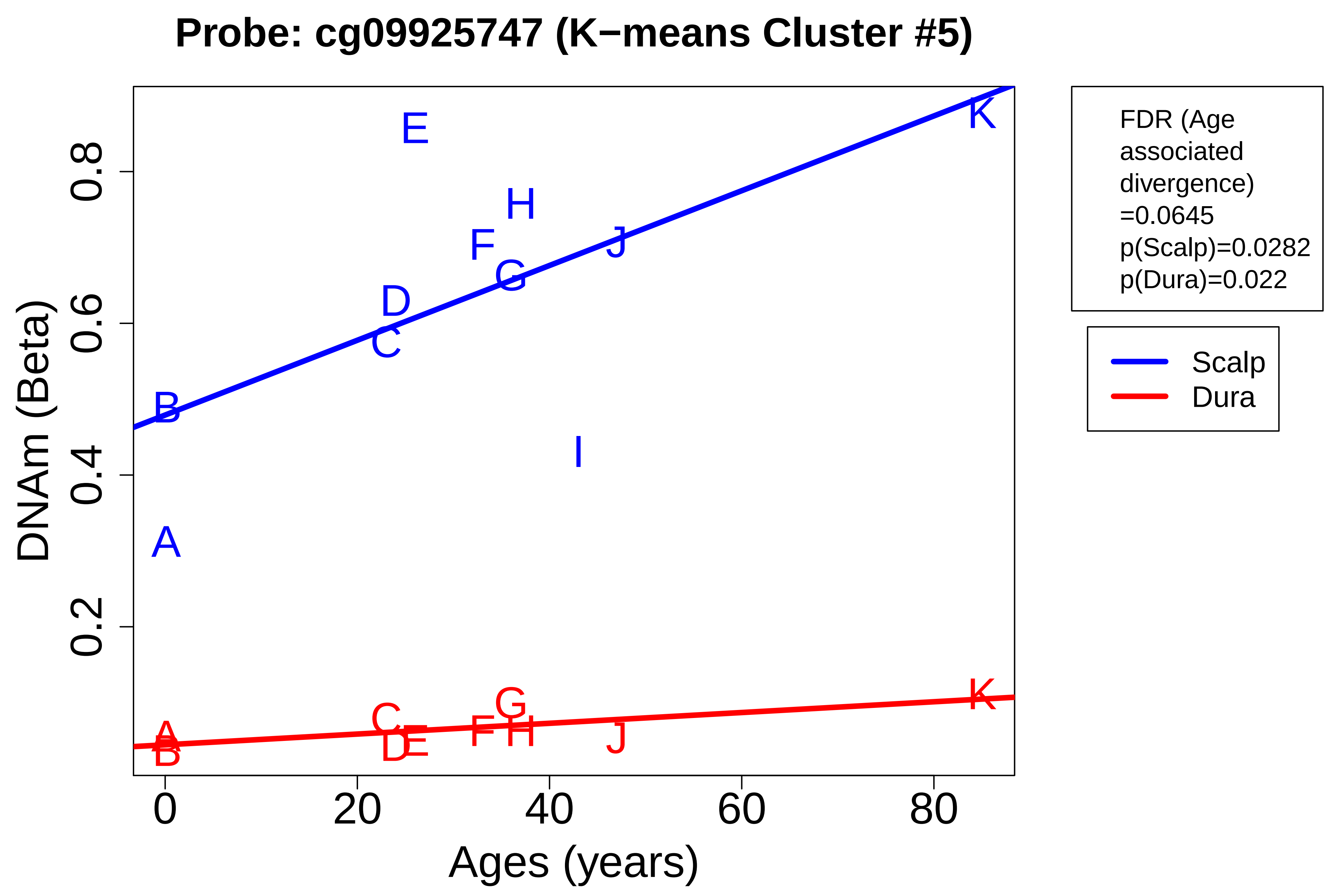

Probe: cg09925747 (K−means Cluster #5)
DNAm (Beta)
0.2
0.4
0.6
0.8
Ages (years)
0
20
40
60
80
A
B
C
D
E
F
G
H
I
J
K
FDR (Age associated divergence) =0.0645
p(Scalp)=0.0282
p(Dura)=0.022
Scalp
Dura

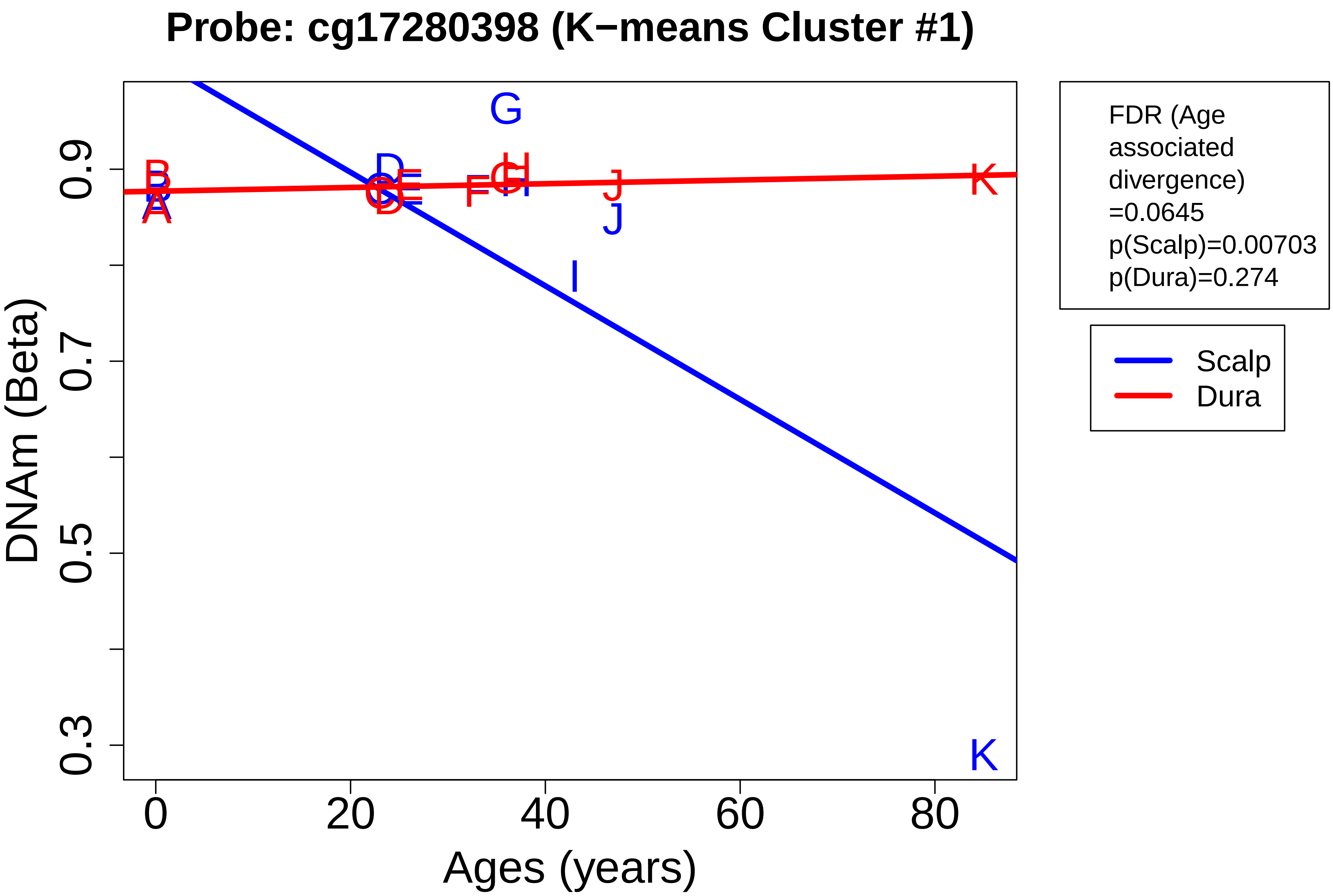
Probe: cg17280398 (K−means Cluster #1)
DNAm (Beta)
0.3
0.5
0.7
0.9
0
20
40
60
80
Ages (years)
A
B
C
D
E
F
G
H
I
J
K
FDR (Age associated divergence) =0.0645
p(Scalp)=0.00703
p(Dura)=0.274
Scalp
Dura

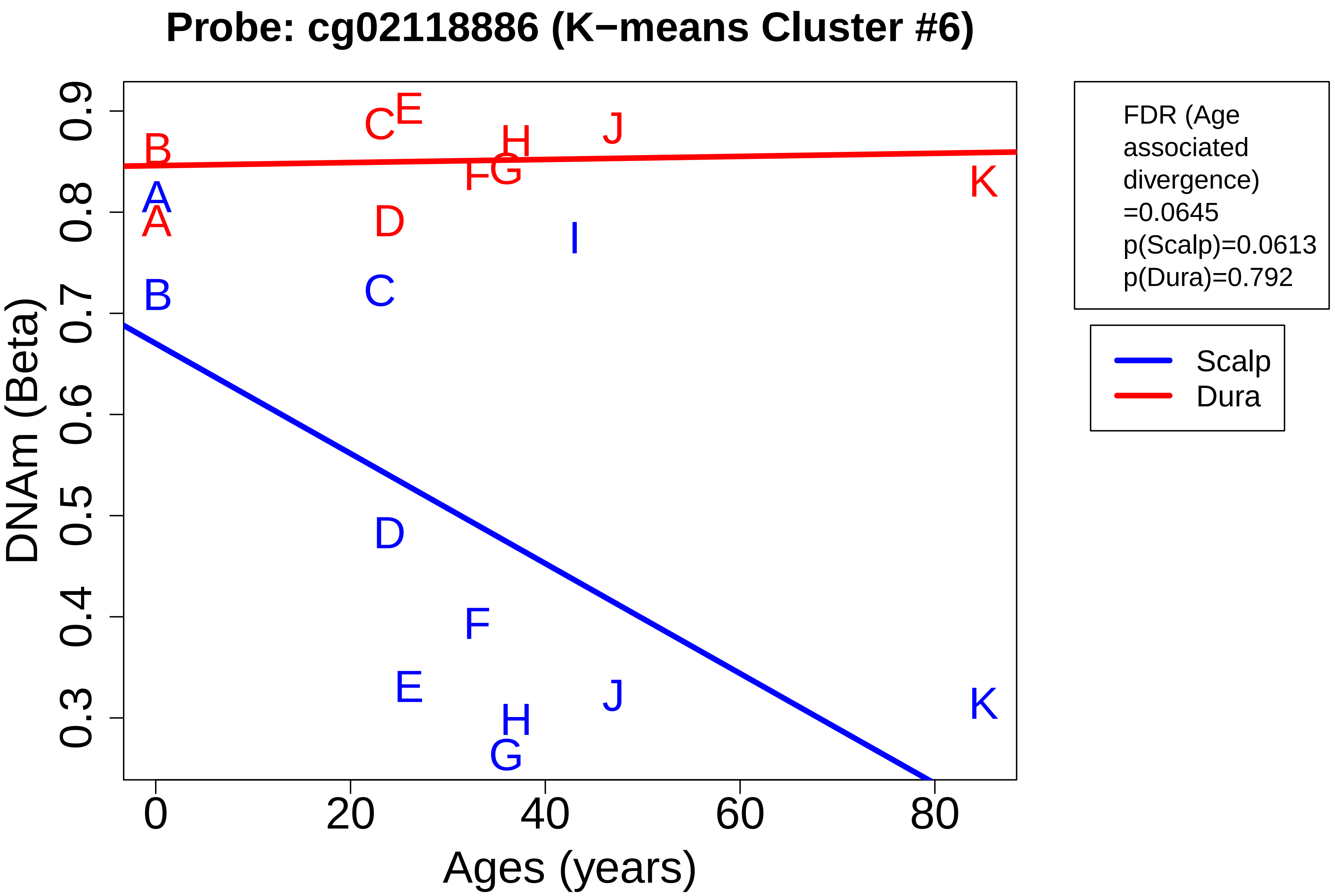

Probe: cg02118886 (K−means Cluster #6)
DNAm (Beta)
0.3
0.4
0.5
0.6
0.7
0.8
0.9
0
20
40
60
80
Ages (years)
FDR (Age associated divergence) =0.0645
p(Scalp)=0.0613
p(Dura)=0.792
Scalp
Dura

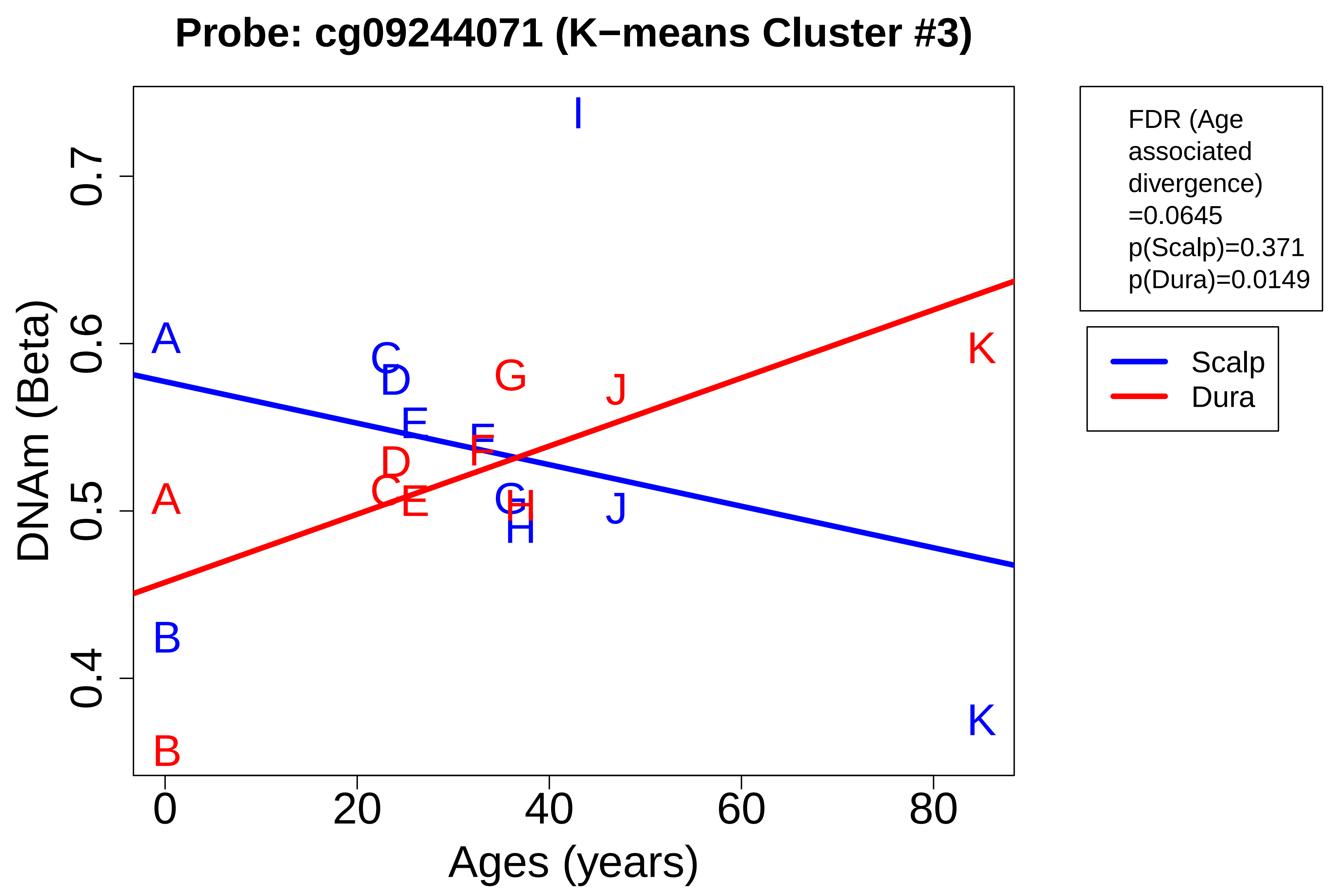
Probe: cg09244071 (K−means Cluster #3)
DNAm (Beta)
0.4
0.5
0.6
0.7
0
20
40
60
80
Ages (years)
FDR (Age associated divergence) =0.0645
p(Scalp)=0.371
p(Dura)=0.0149
Scalp
Dura
A
B
C
D
E
F
G
H
I
J
K

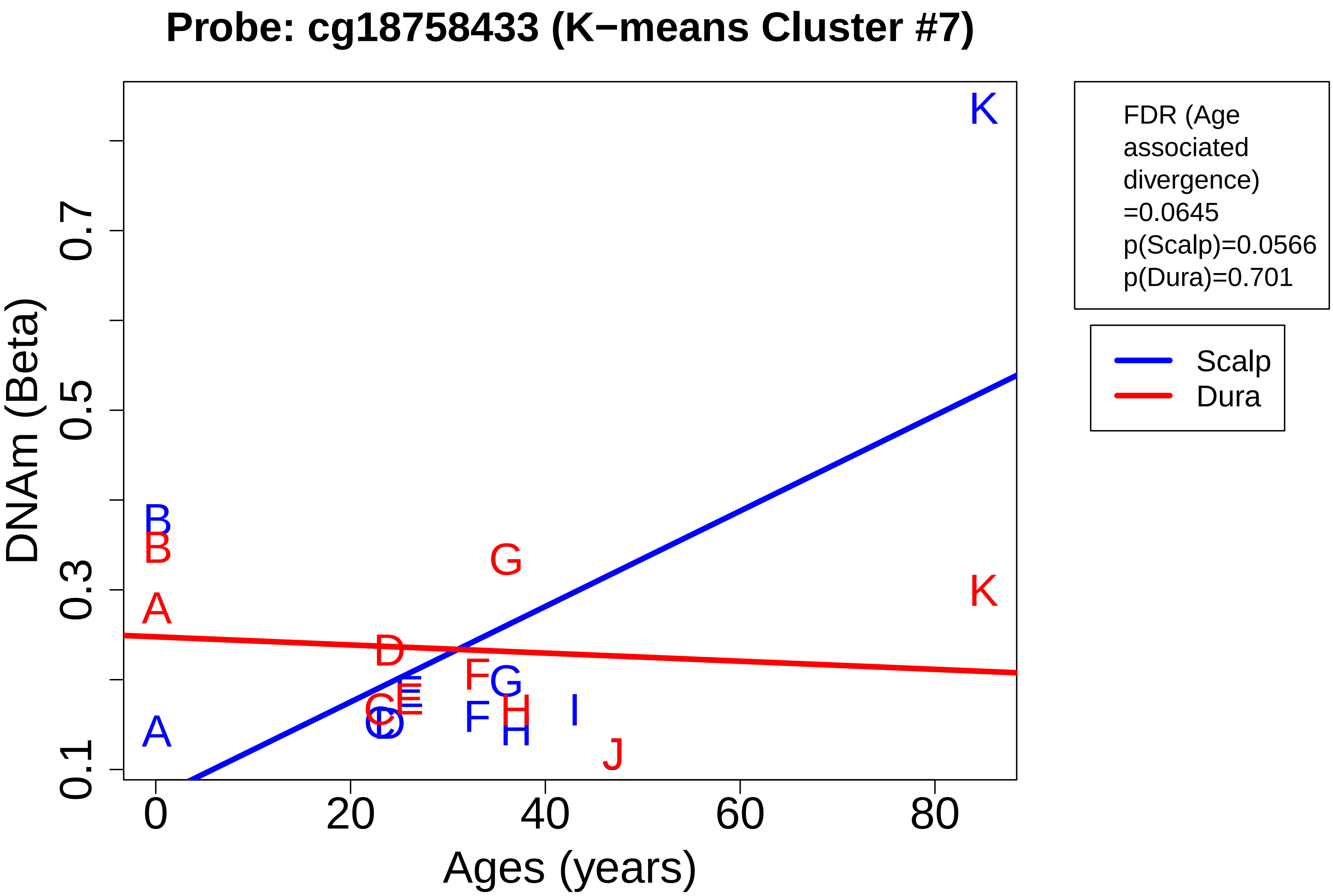

Probe: cg18758433 (K−means Cluster #7)
DNAm (Beta)
0.1
0.3
0.5
0.7
0
20
40
60
80
Ages (years)
FDR (Age associated divergence) =0.0645
p(Scalp)=0.0566
p(Dura)=0.701
Scalp
Dura
A
B
C
D
E
F
G
H
I
J
K

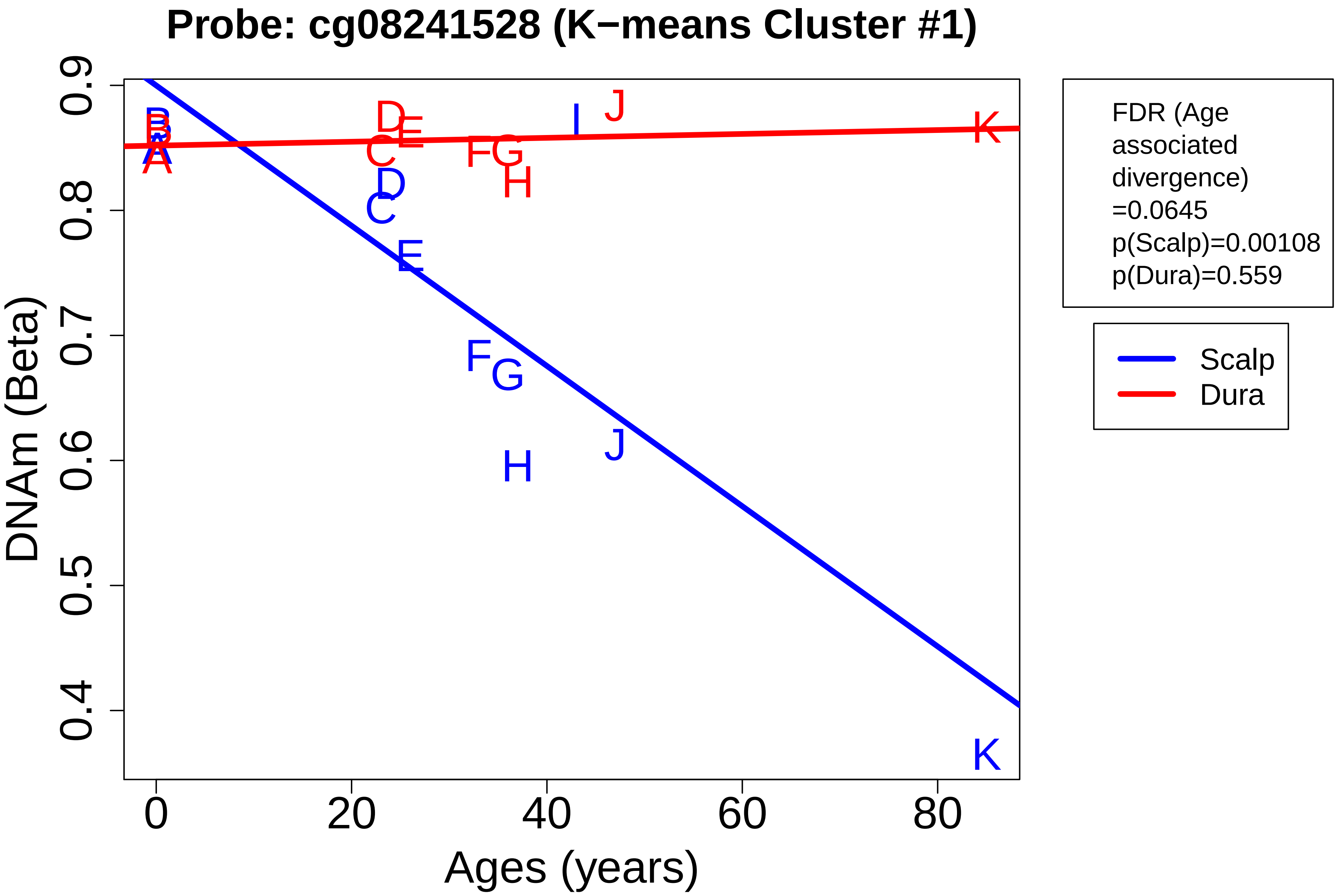
Probe: cg08241528 (K−means Cluster #1)
DNAm (Beta)
0.4
0.5
0.6
0.7
0.8
0.9
0
20
40
60
80
Ages (years)
FDR (Age associated divergence) =0.0645
p(Scalp)=0.00108
p(Dura)=0.559
Scalp
Dura

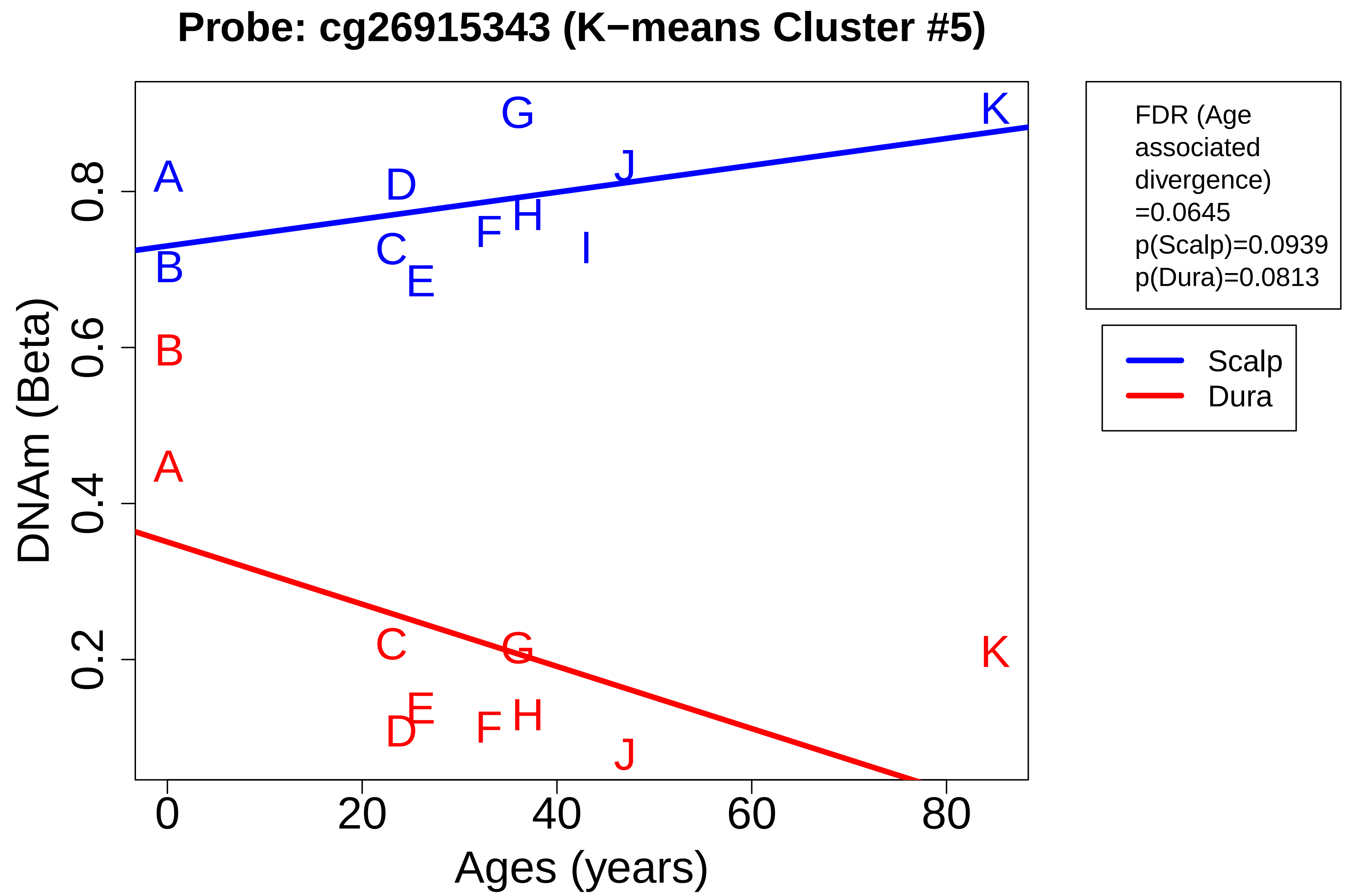

Probe: cg26915343 (K−means Cluster #5)
DNAm (Beta)
0.2
0.4
0.6
0.8
0
20
40
60
80
Ages (years)
A
B
C
D
E
F
G
H
I
J
K
FDR (Age associated divergence) =0.0645
p(Scalp)=0.0939
p(Dura)=0.0813
Scalp
Dura

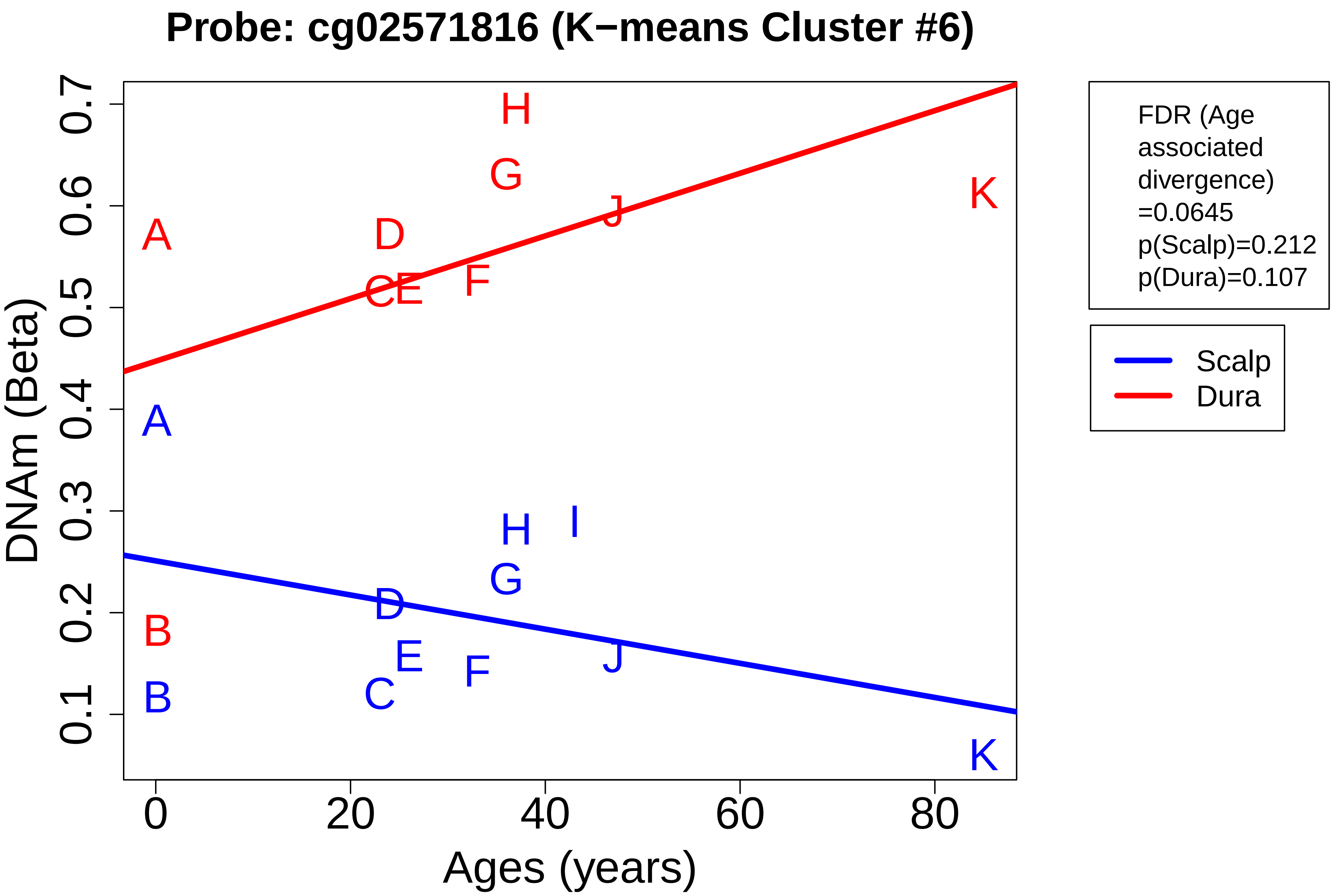
Probe: cg02571816 (K−means Cluster #6)
DNAm (Beta)
0.1
0.2
0.3
0.4
0.5
0.6
0.7
Ages (years)
0
20
40
60
80
A
B
CE
D
F
G
H
J
K
A
B
C
D
E
F
G
H
I
J
K
FDR (Age associated divergence) =0.0645
p(Scalp)=0.212
p(Dura)=0.107
Scalp
Dura

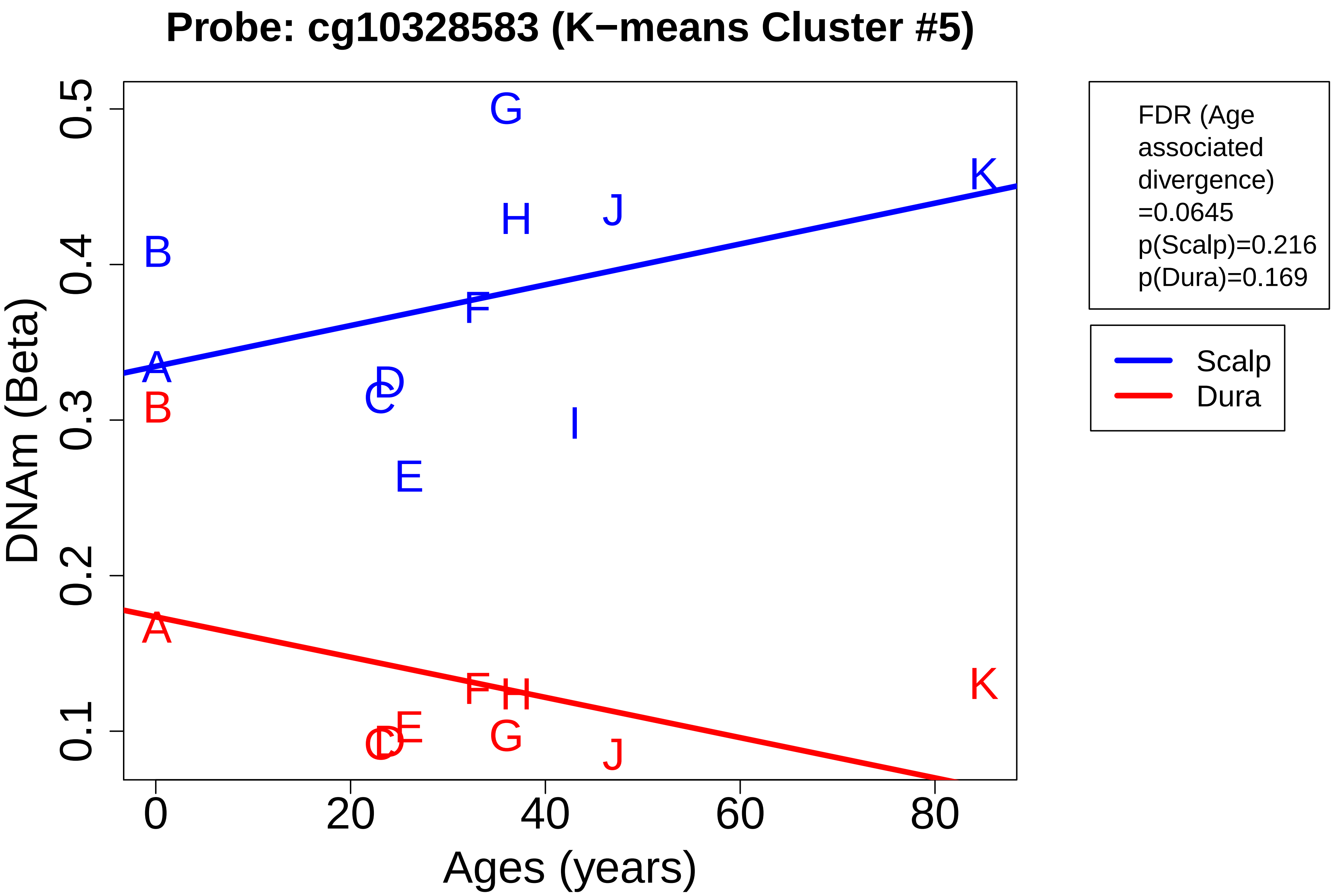

Probe: cg10328583 (K−means Cluster #5)
DNAm (Beta)
0.1
0.2
0.3
0.4
0.5
Ages (years)
0
20
40
60
80
FDR (Age associated divergence) =0.0645
p(Scalp)=0.216
p(Dura)=0.169
Scalp
Dura

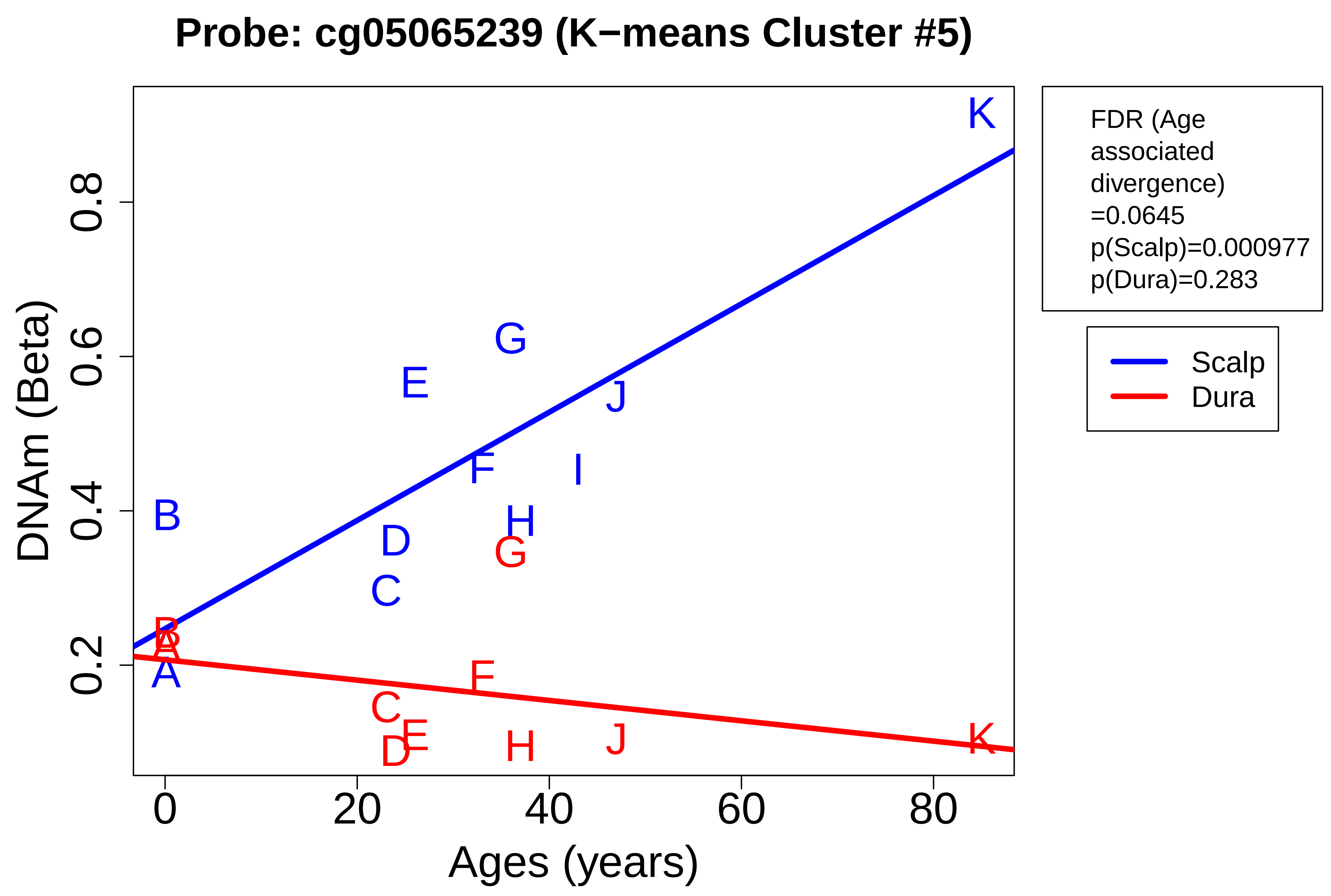
Probe: cg05065239 (K−means Cluster #5)
DNAm (Beta)
0.2
0.4
0.6
0.8
Ages (years)
0
20
40
60
80
FDR (Age associated divergence) =0.0645
p(Scalp)=0.000977
p(Dura)=0.283
Scalp
Dura
A B C D E F G H I J K

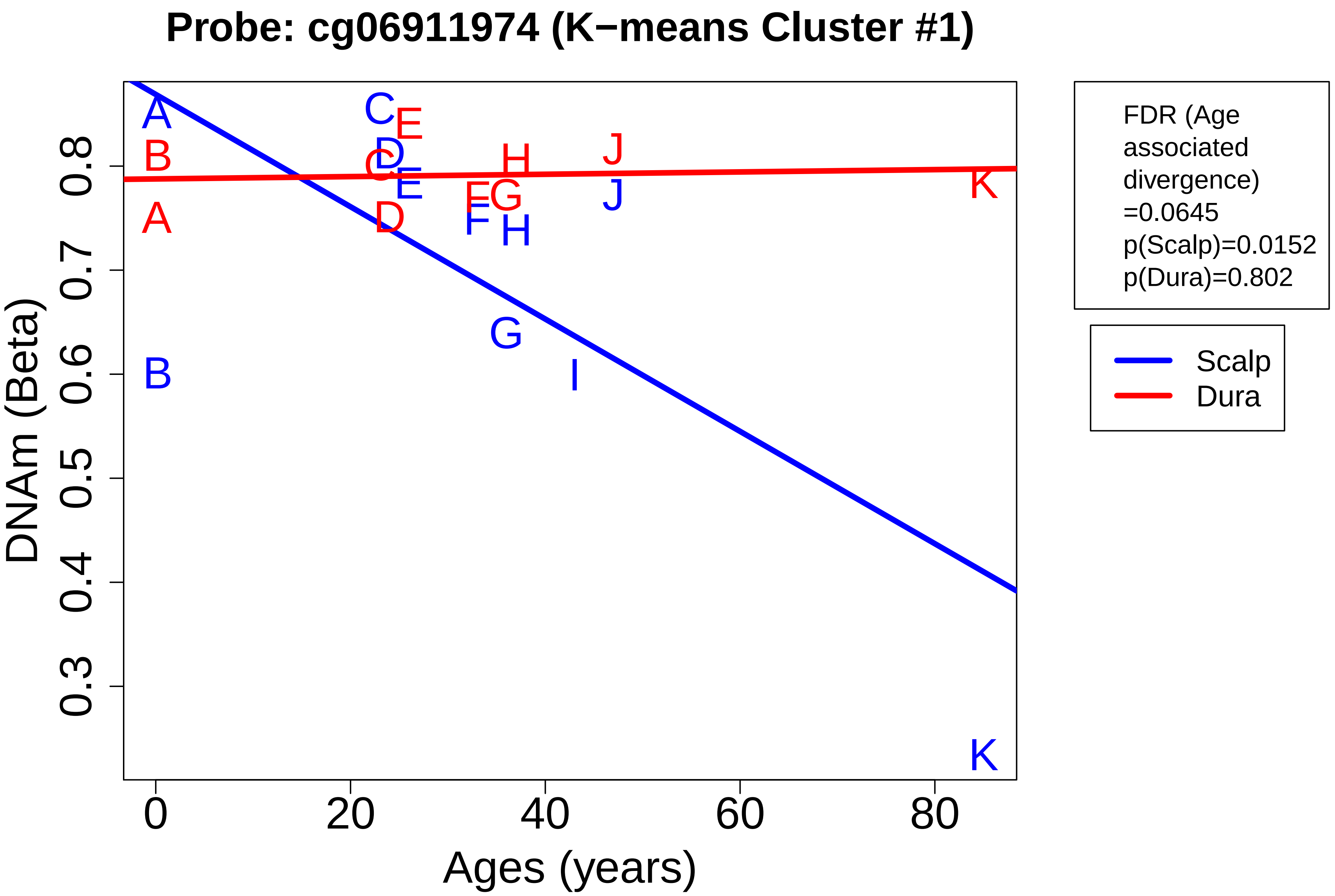
Probe: cg06911974 (K−means Cluster #1)
FDR (Age associated divergence) =0.0645
p(Scalp)=0.0152
p(Dura)=0.802
Scalp
Dura
DNAm (Beta)
Ages (years)
0.3
0.4
0.5
0.6
0.7
0.8
0
20
40
60
80

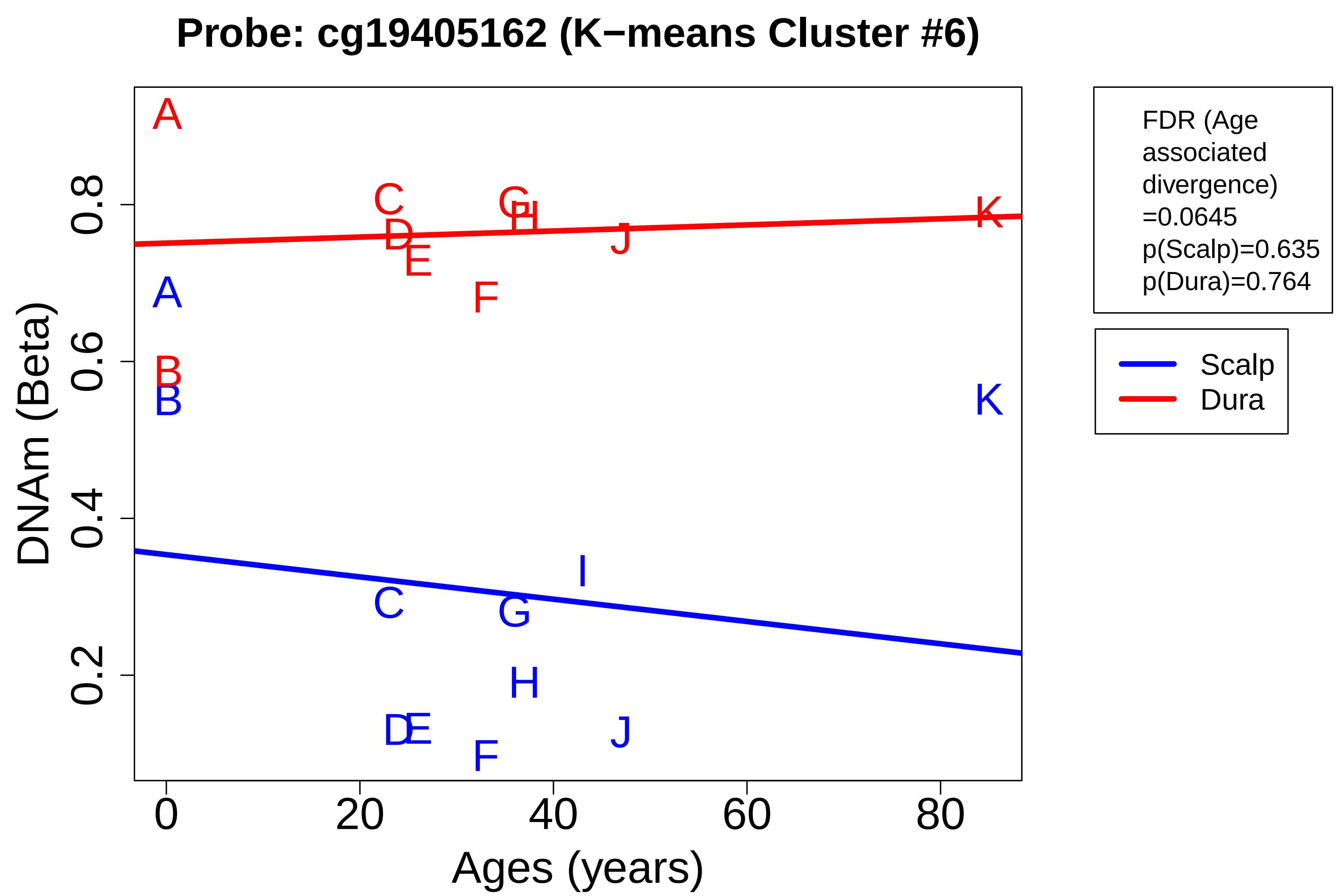
Probe: cg19405162 (K−means Cluster #6)
DNAm (Beta)
0.2
0.4
0.6
0.8
0
20
40
60
80
Ages (years)
A
B
C
D
E
F
G
H
I
J
K
FDR (Age associated divergence) =0.0645
p(Scalp)=0.635
p(Dura)=0.764
Scalp
Dura

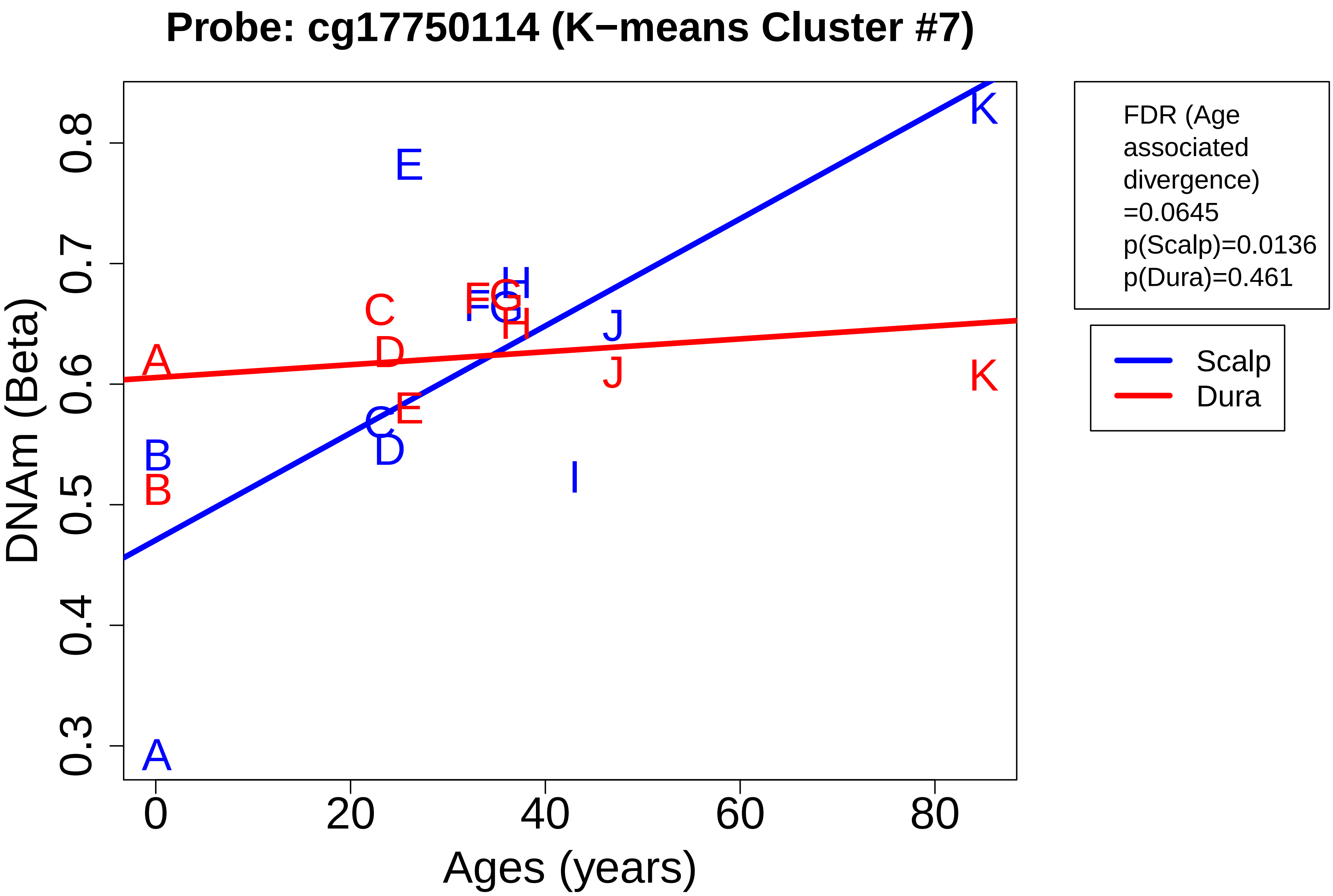

Probe: cg17750114 (K−means Cluster #7)
DNAm (Beta)
0.3
0.4
0.5
0.6
0.7
0.8
Ages (years)
0
20
40
60
80
A
B
C
D
E
F
G
H
I
J
K
FDR (Age associated divergence) =0.0645
p(Scalp)=0.0136
p(Dura)=0.461
Scalp
Dura

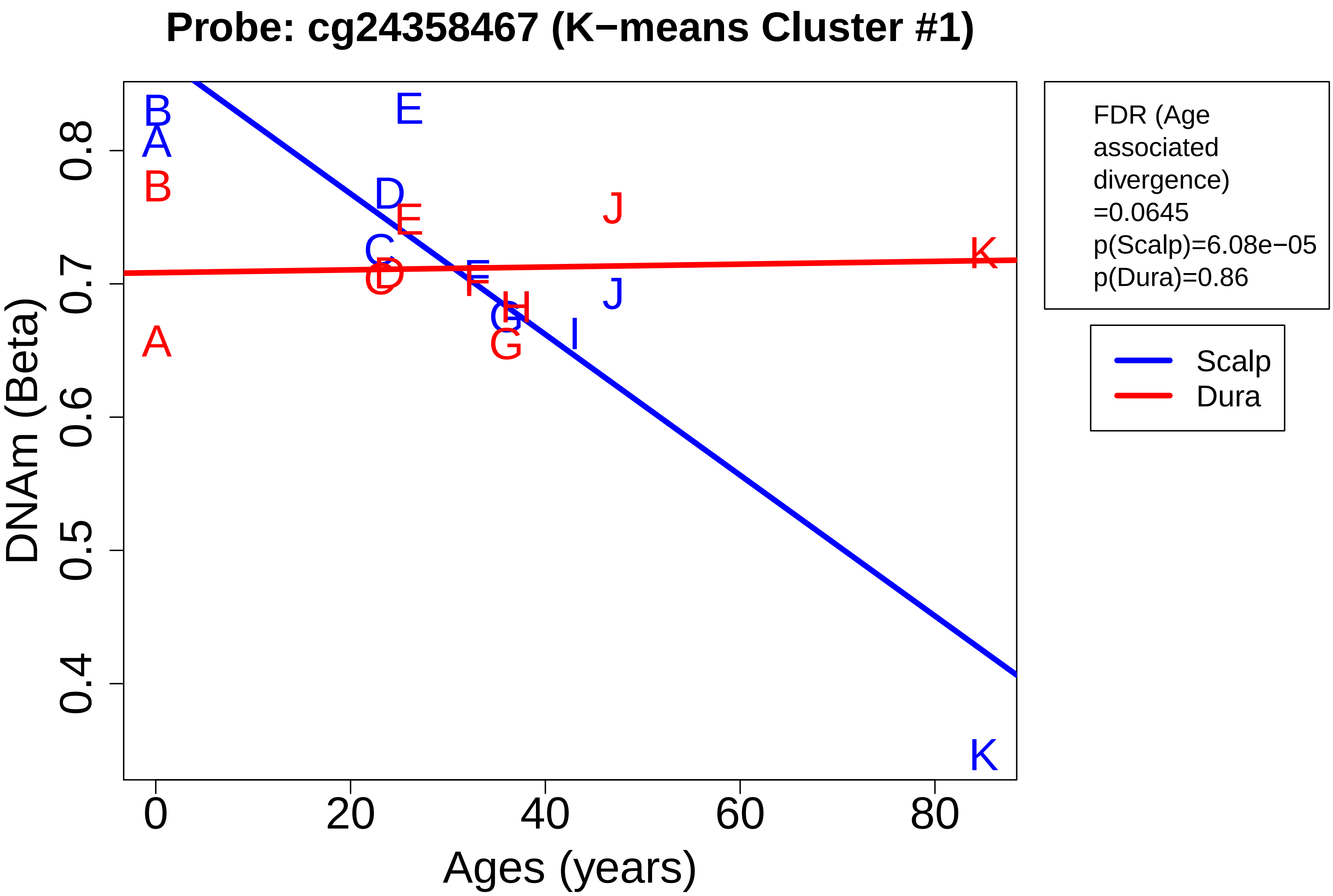
Probe: cg24358467 (K−means Cluster #1)
DNAm (Beta)
0.4
0.5
0.6
0.7
0.8
0
20
40
60
80
Ages (years)
FDR (Age associated divergence) =0.0645
p(Scalp)=6.08e−05
p(Dura)=0.86
Scalp
Dura

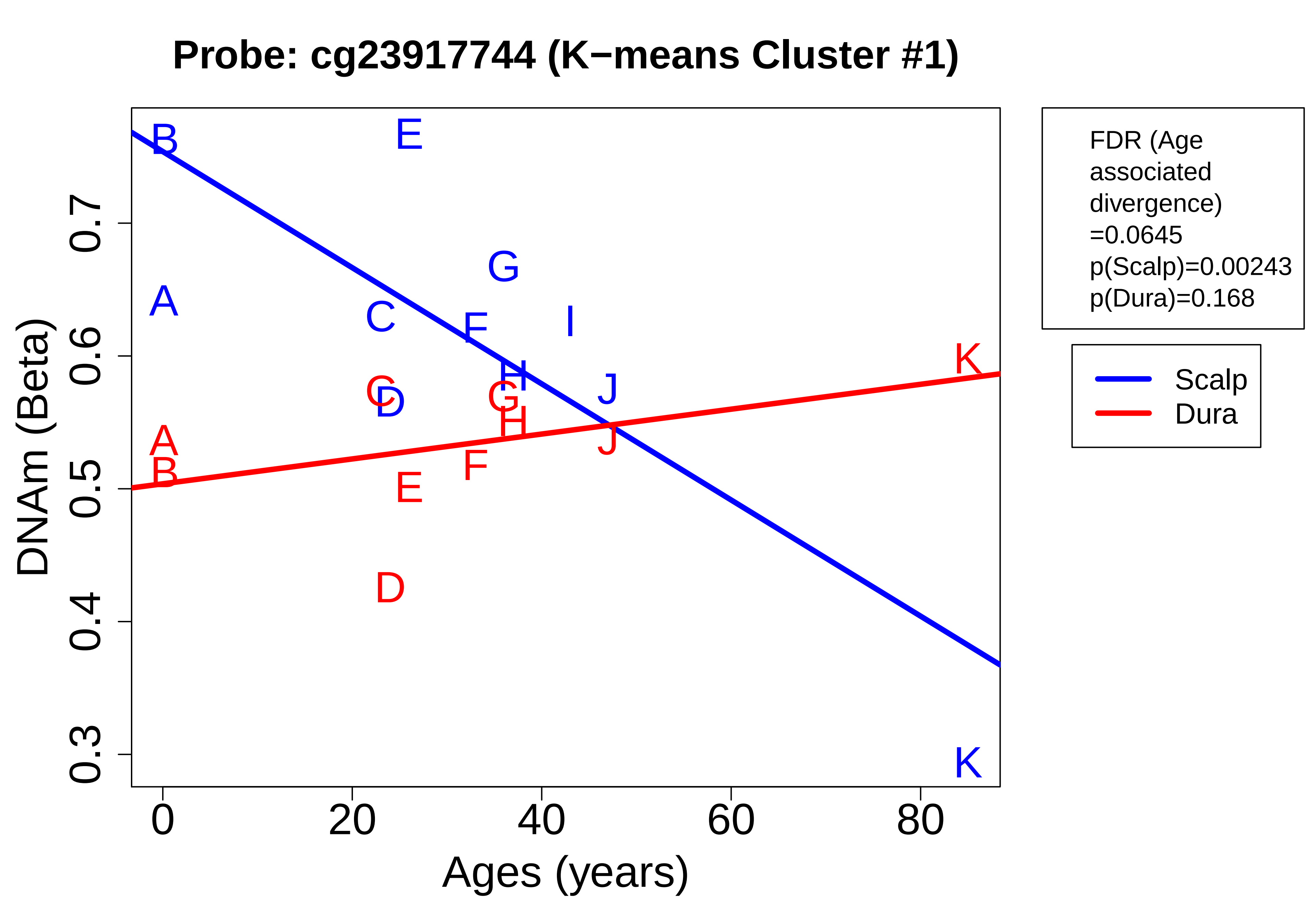

Probe: cg23917744 (K−means Cluster #1)
FDR (Age associated divergence) =0.0645
p(Scalp)=0.00243
p(Dura)=0.168
Scalp
Dura
DNAm (Beta)
0.3
0.4
0.5
0.6
0.7
Ages (years)
0
20
40
60
80
A
B
C
D
E
F
G
H
I
J
K

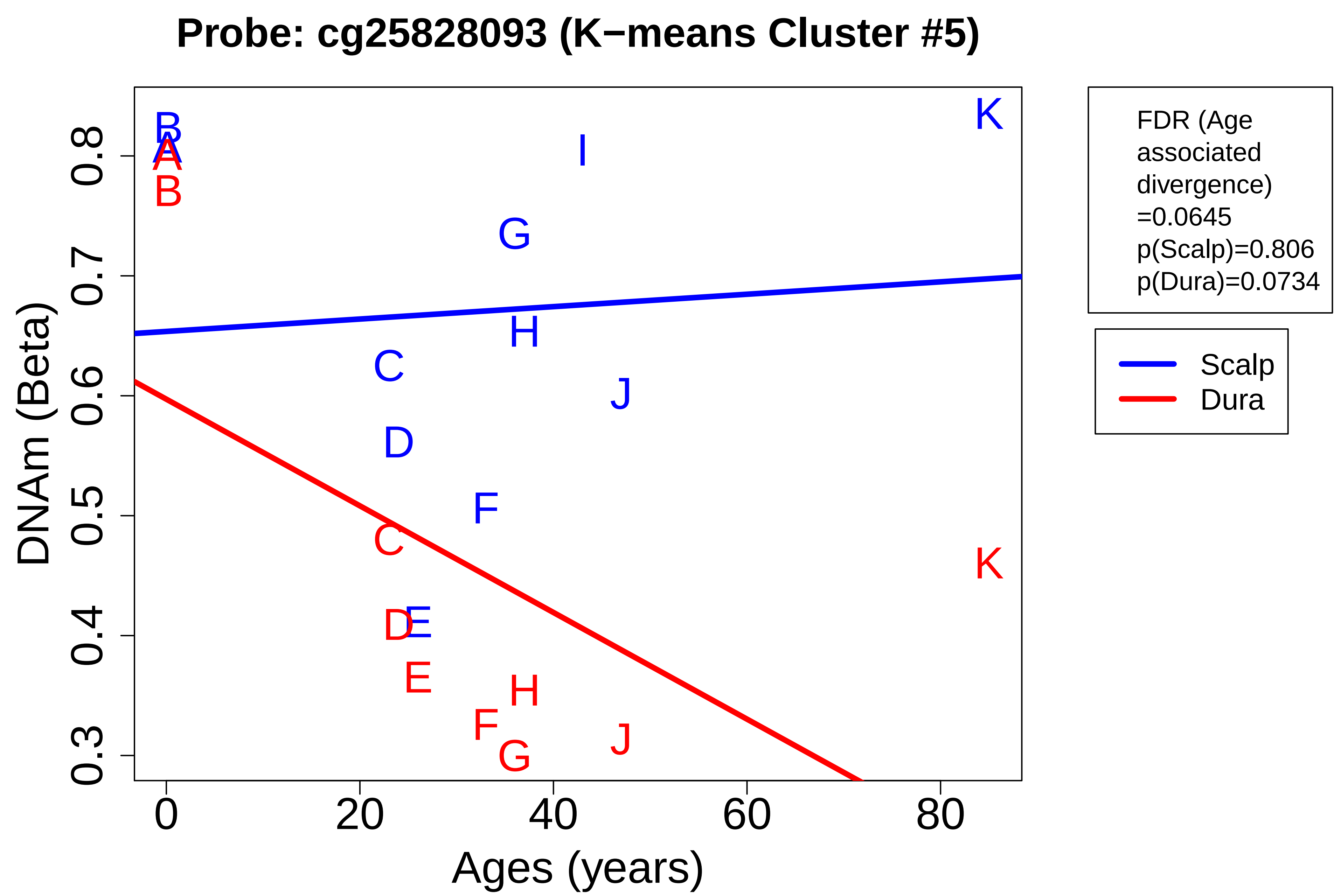
Probe: cg25828093 (K−means Cluster #5)
DNAm (Beta)
0.3
0.4
0.5
0.6
0.7
0.8
Ages (years)
0
20
40
60
80
FDR (Age associated divergence) =0.0645
p(Scalp)=0.806
p(Dura)=0.0734
Scalp
Dura

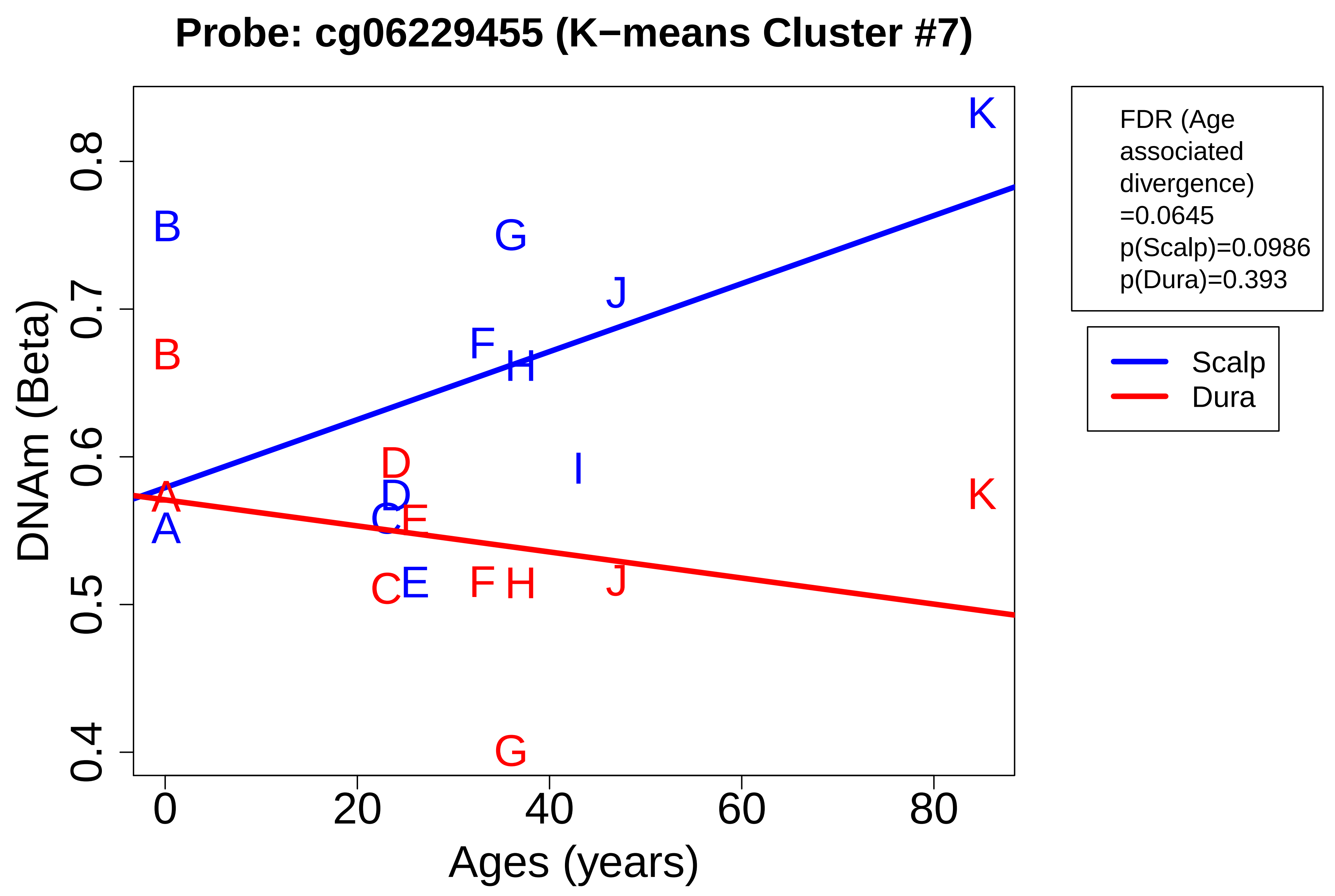

Probe: cg06229455 (K−means Cluster #7)
DNAm (Beta)
Ages (years)
0.4
0.5
0.6
0.7
0.8
0
20
40
60
80
FDR (Age associated divergence) =0.0645
p(Scalp)=0.0986
p(Dura)=0.393
Scalp
Dura
A B C D E F G H I J K
A B C D E F G H J K

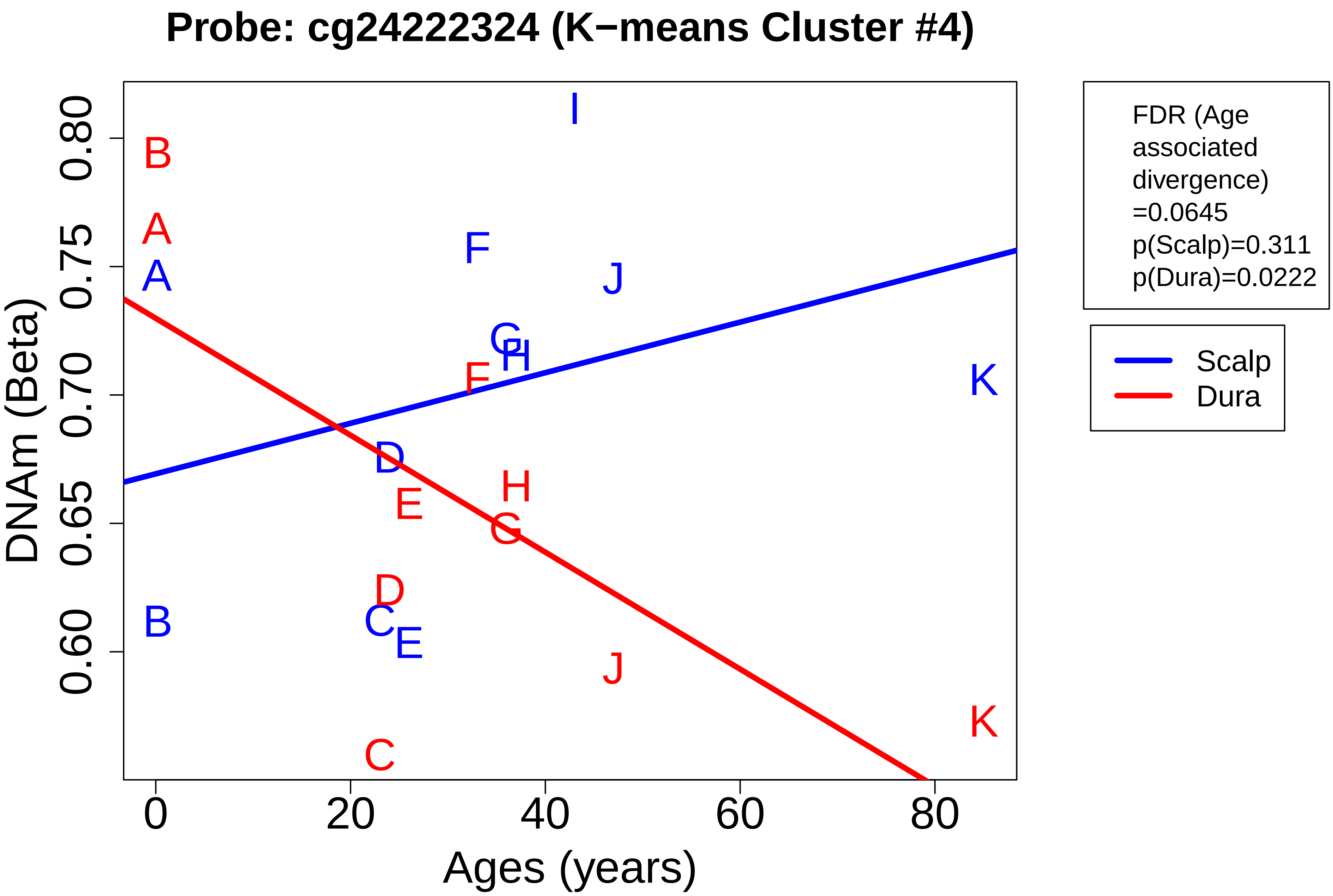

Probe: cg24222324 (K−means Cluster #4)
FDR (Age associated divergence) =0.0645
p(Scalp)=0.311
p(Dura)=0.0222
Scalp
Dura
DNAm (Beta)
0.60
0.65
0.70
0.75
0.80
0
20
40
60
80
Ages (years)
A
B
C
D
E
F
G
H
I
J
K

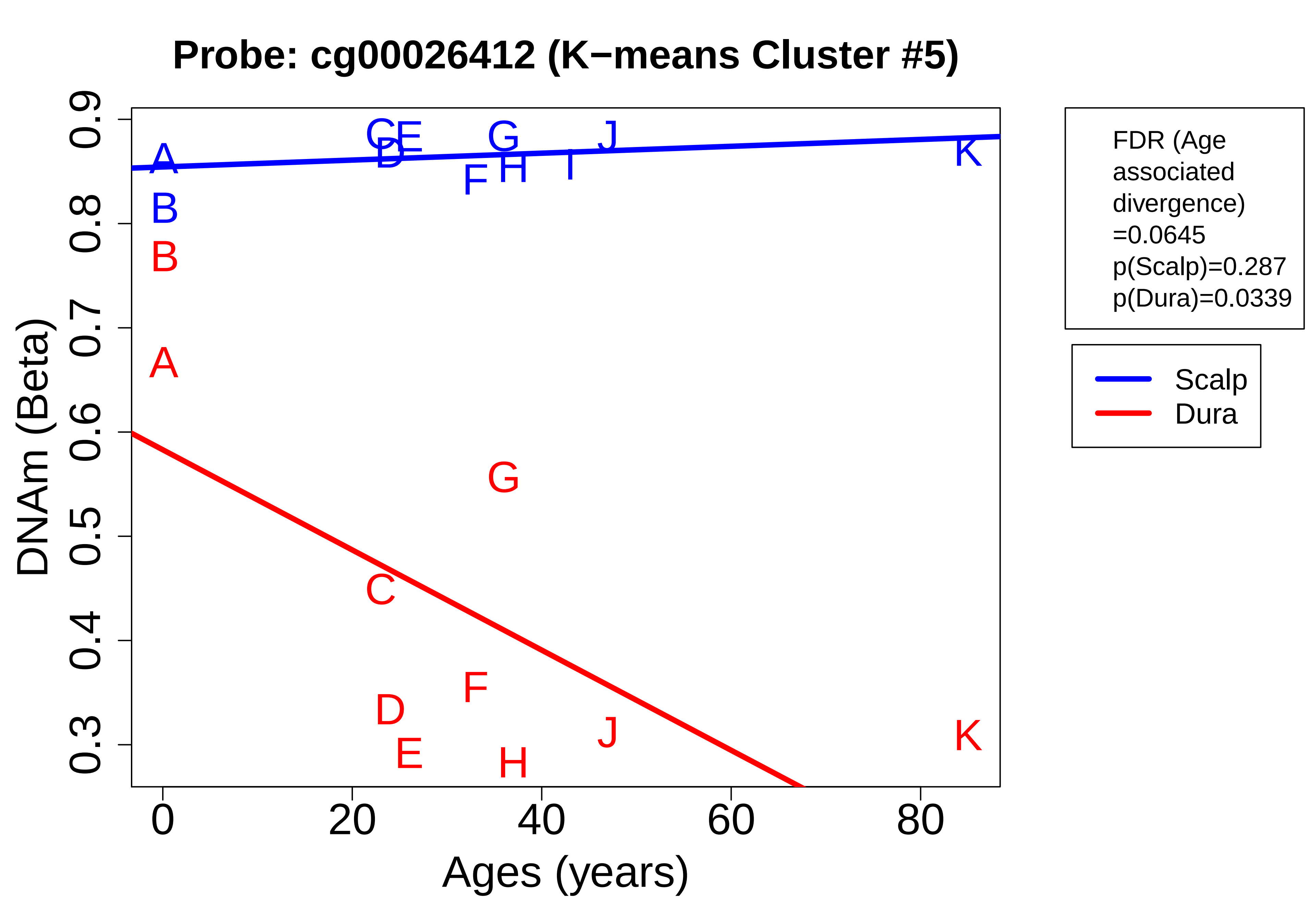
Probe: cg00026412 (K−means Cluster #5)
DNAm (Beta)
Ages (years)
0.3
0.4
0.5
0.6
0.7
0.8
0.9
0
20
40
60
80
FDR (Age associated divergence) =0.0645
p(Scalp)=0.287
p(Dura)=0.0339
Scalp
Dura
A
B
C
D
E
F
G
H
I
J
K

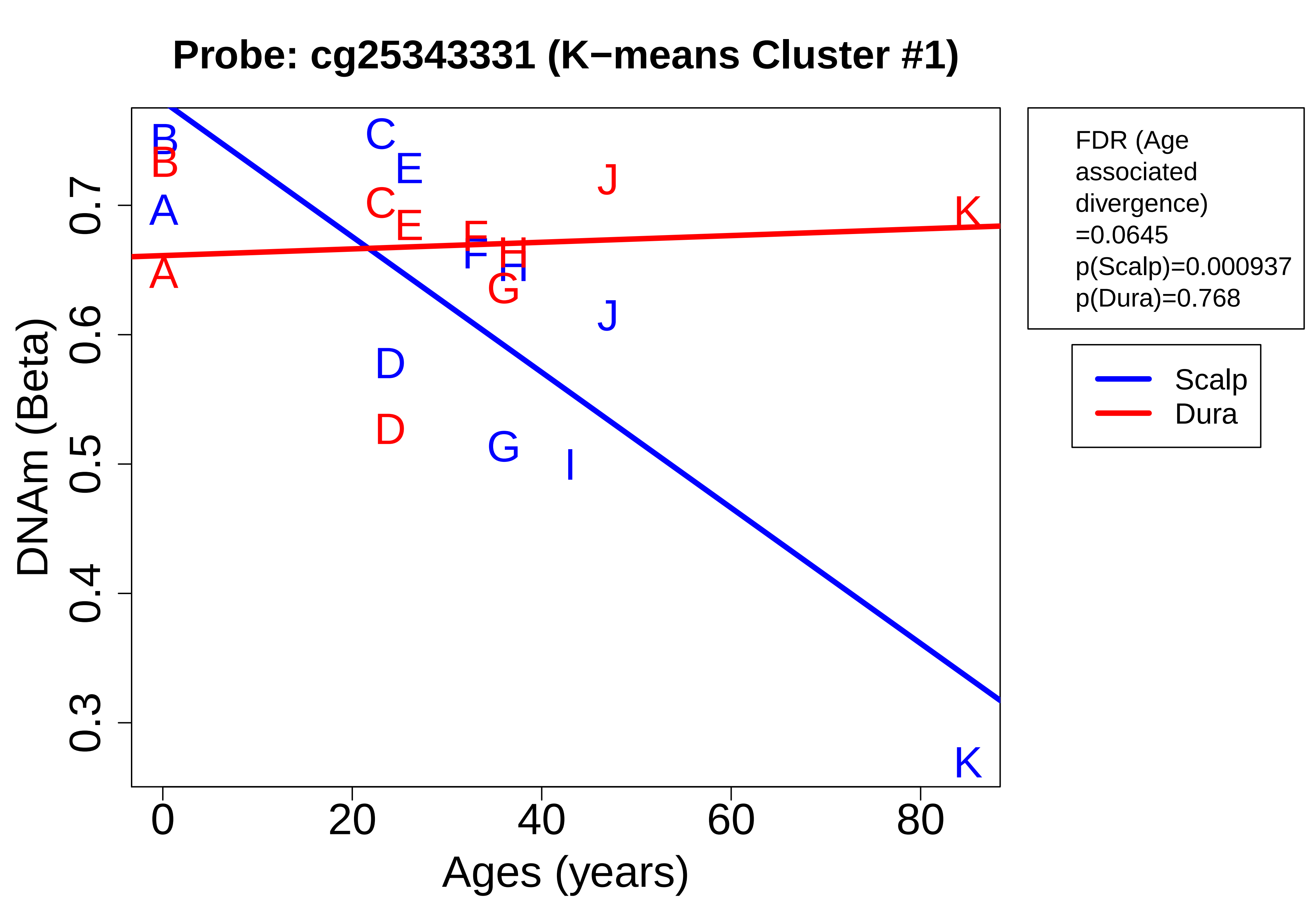

Probe: cg25343331 (K−means Cluster #1)
DNAm (Beta)
0.3
0.4
0.5
0.6
0.7
0
20
40
60
80
Ages (years)
A
B
C
D
E
F
G
H
I
J
K
FDR (Age associated divergence) =0.0645
p(Scalp)=0.000937
p(Dura)=0.768
Scalp
Dura

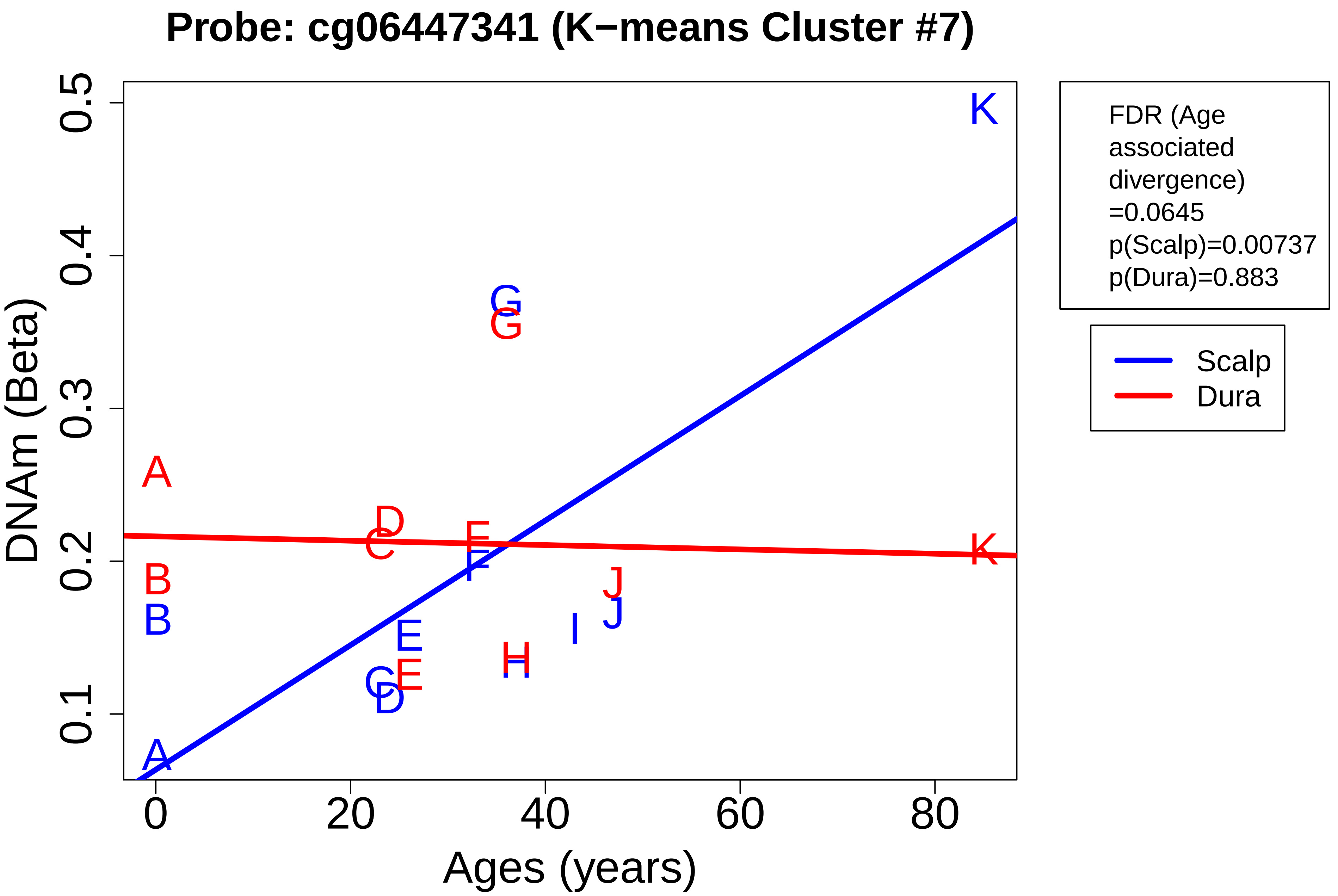

Probe: cg06447341 (K−means Cluster #7)
DNAm (Beta)
0.1
0.2
0.3
0.4
0.5
Ages (years)
0
20
40
60
80
A
B
C
D
E
F
G
H
I
J
K
FDR (Age associated divergence) =0.0645
p(Scalp)=0.00737
p(Dura)=0.883
Scalp
Dura

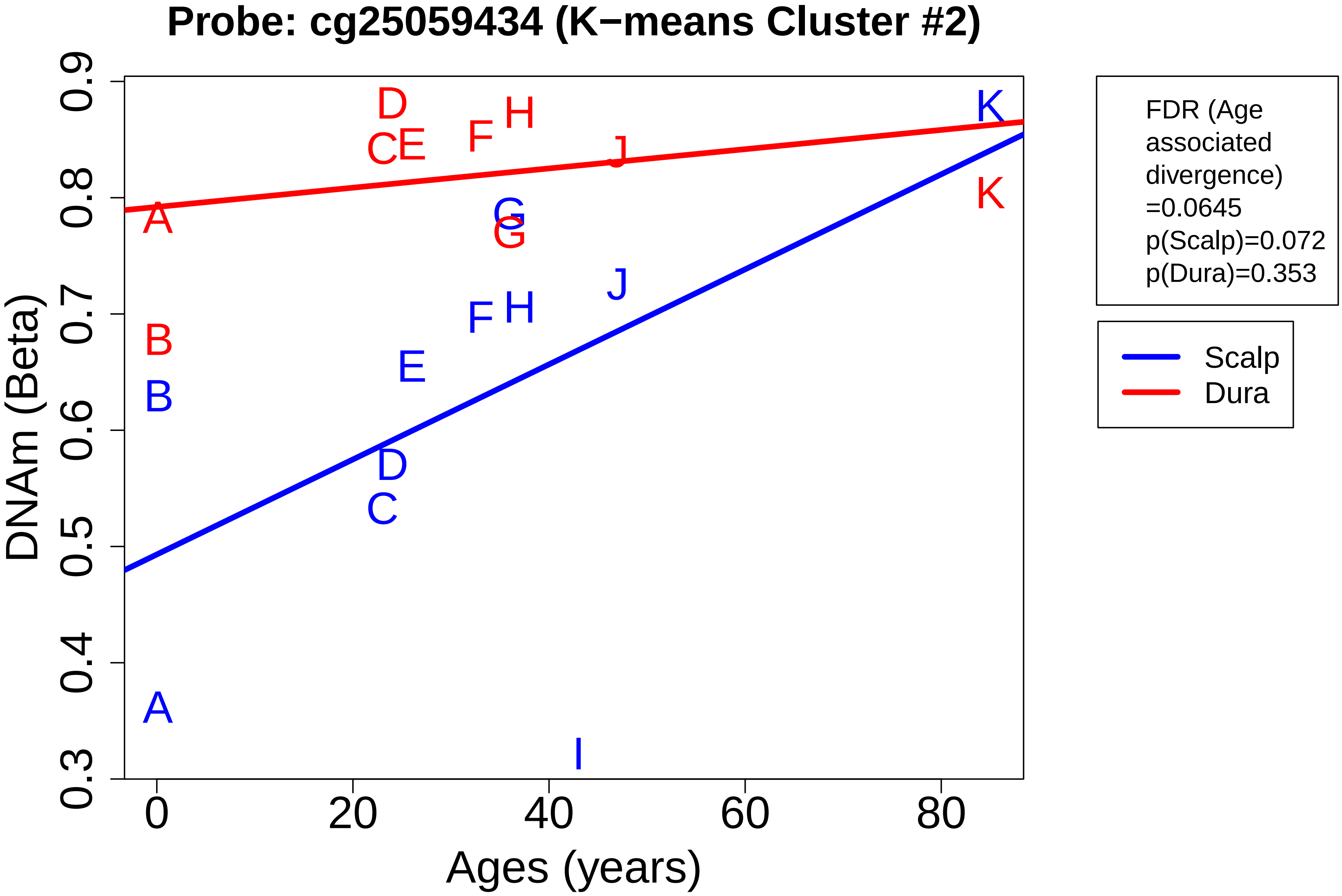

Probe: cg25059434 (K−means Cluster #2)
DNAm (Beta)
0.3
0.4
0.5
0.6
0.7
0.8
0.9
0
20
40
60
80
Ages (years)
A
B
C
D
E
F
G
H
I
J
K
A
B
CE
D
F
G
H
J
K
FDR (Age associated divergence) =0.0645
p(Scalp)=0.072
p(Dura)=0.353
Scalp
Dura

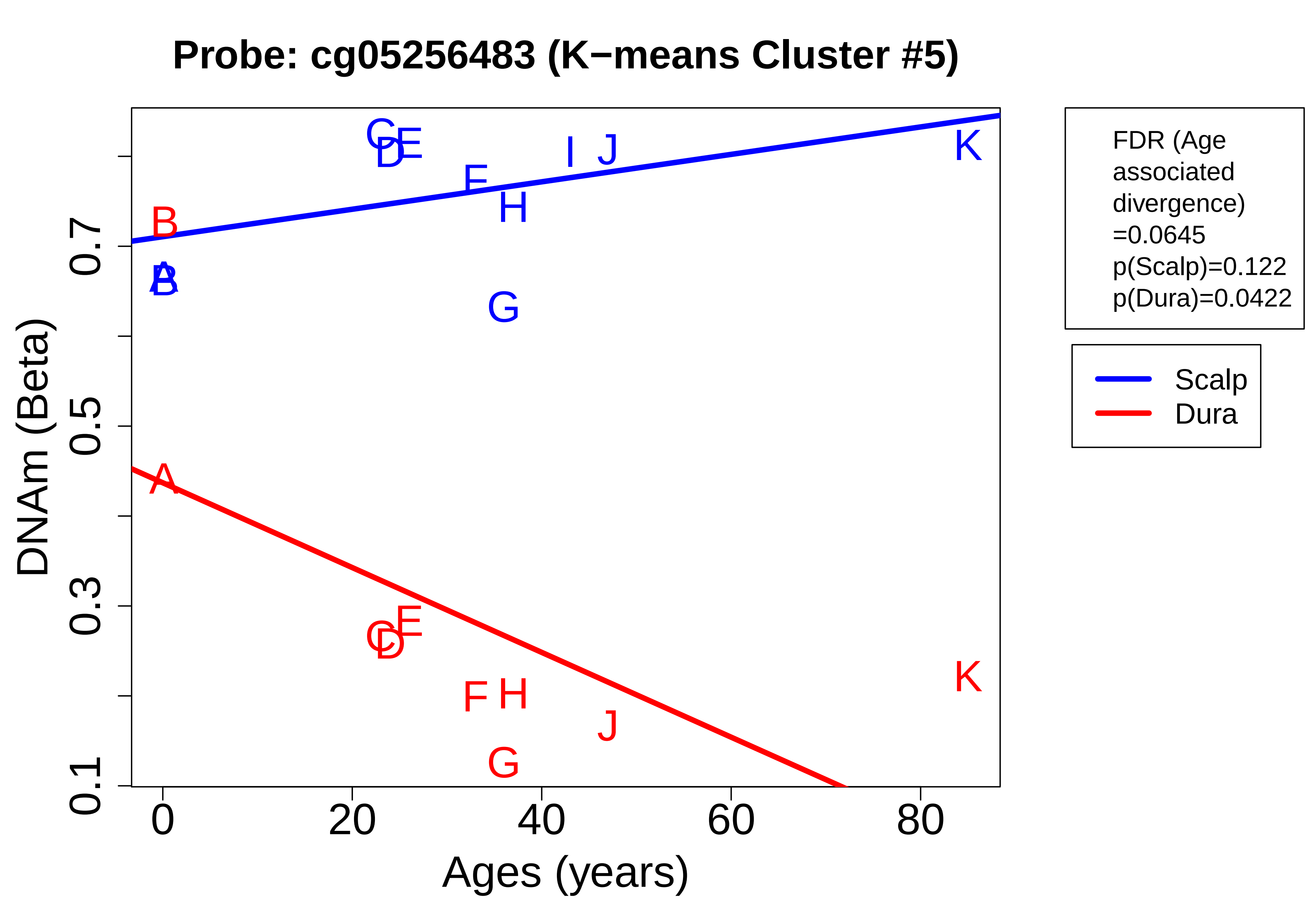
Probe: cg05256483 (K−means Cluster #5)
DNAm (Beta)
0.1
0.3
0.5
0.7
Ages (years)
0
20
40
60
80
FDR (Age associated divergence) =0.0645
p(Scalp)=0.122
p(Dura)=0.0422
Scalp
Dura
A
B
C
D
E
F
G
H
I
J
K

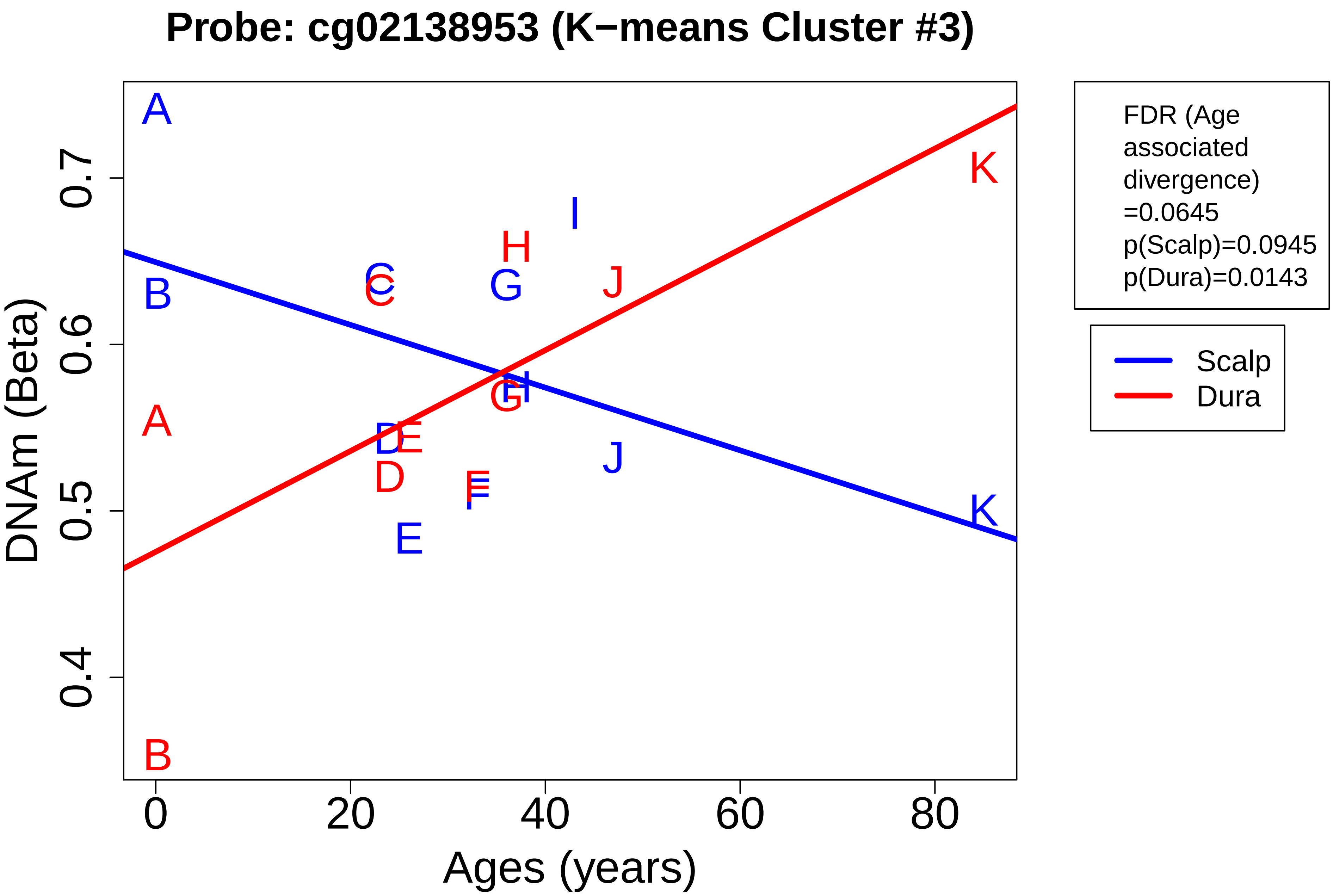
Probe: cg02138953 (K−means Cluster #3)
DNAm (Beta)
Ages (years)
0.4
0.5
0.6
0.7
0
20
40
60
80
FDR (Age associated divergence) =0.0645
p(Scalp)=0.0945
p(Dura)=0.0143
Scalp
Dura

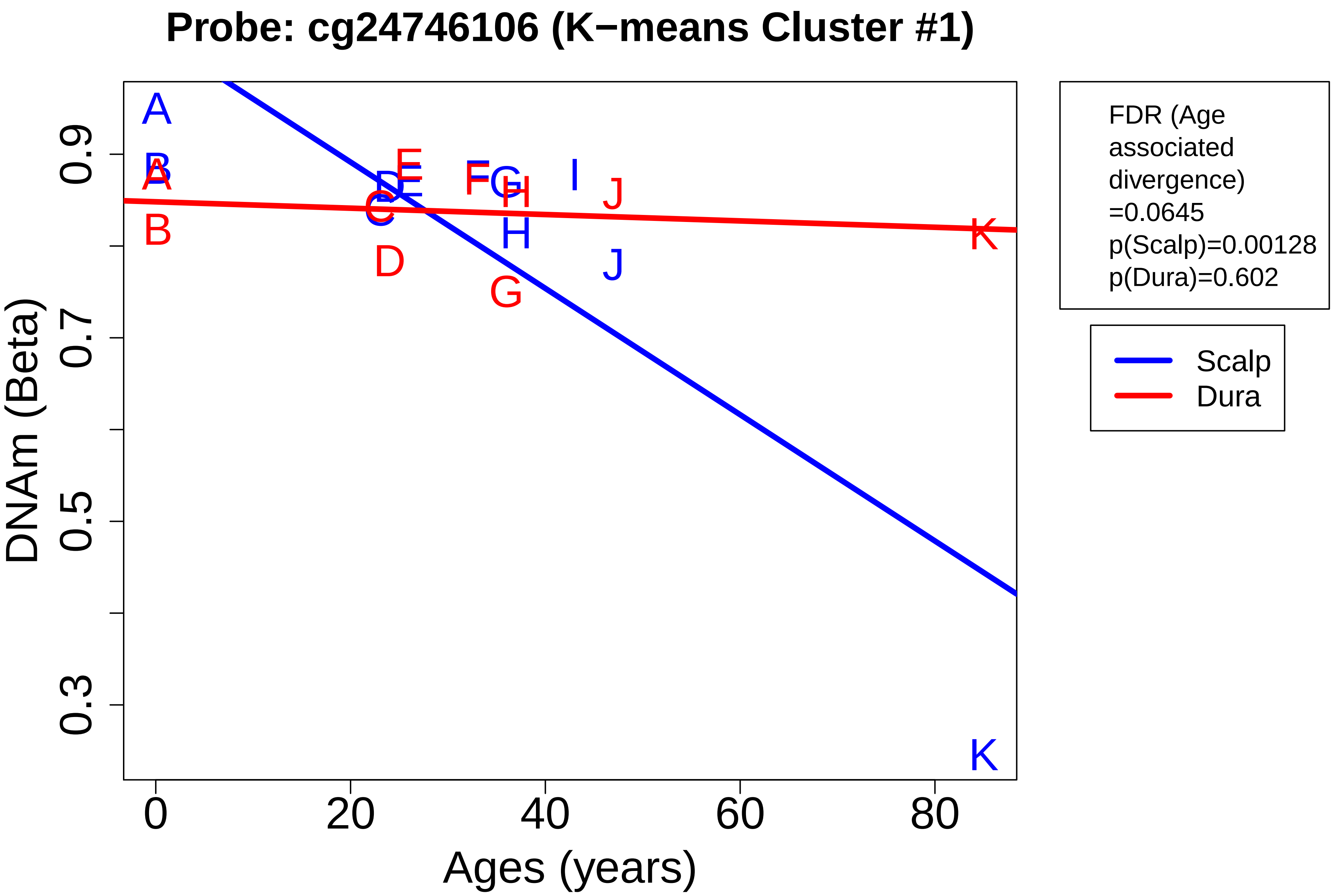

Probe: cg24746106 (K−means Cluster #1)
FDR (Age associated divergence) =0.0645
p(Scalp)=0.00128
p(Dura)=0.602
Scalp
Dura
DNAm (Beta)
0.3
0.5
0.7
0.9
0
20
40
60
80
Ages (years)

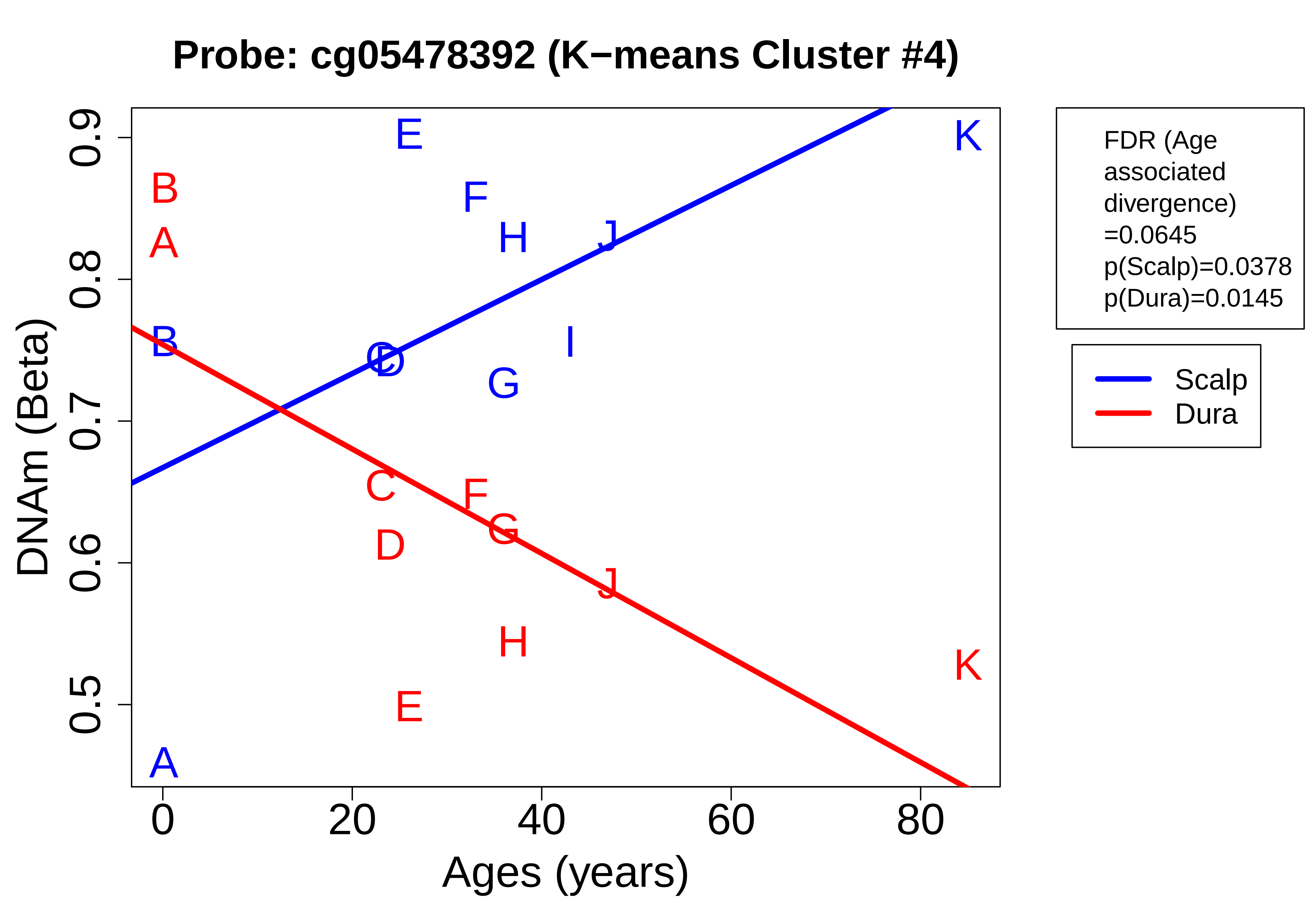
Probe: cg05478392 (K−means Cluster #4)
DNAm (Beta)
Ages (years)
0.5
0.6
0.7
0.8
0.9
0
20
40
60
80
FDR (Age associated divergence) =0.0645
p(Scalp)=0.0378
p(Dura)=0.0145
Scalp
Dura

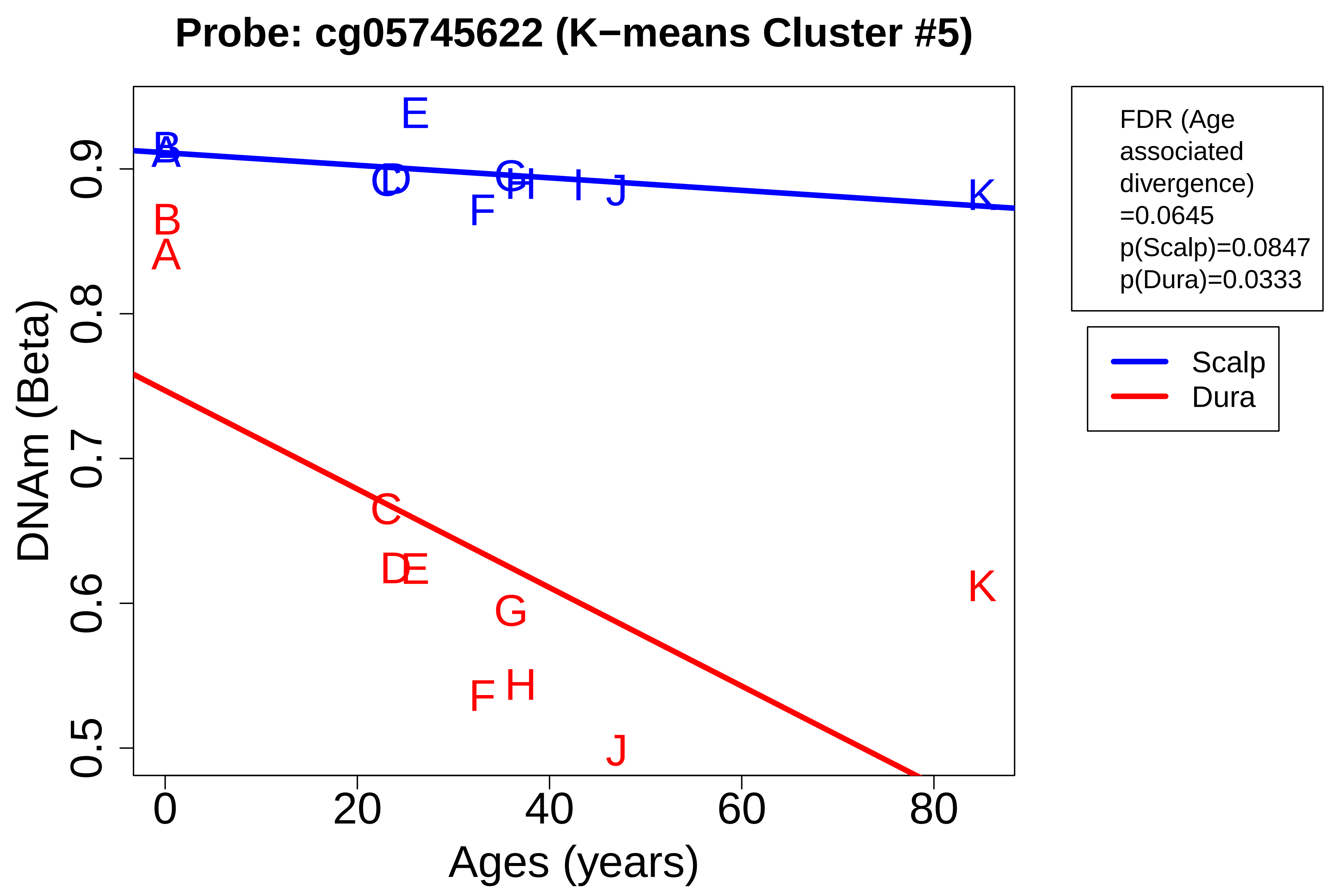

Probe: cg05745622 (K−means Cluster #5)
DNAm (Beta)
0.5
0.6
0.7
0.8
0.9
Ages (years)
0
20
40
60
80
A
B
C
D
E
F
G
H
I
J
K
FDR (Age associated divergence) =0.0645
p(Scalp)=0.0847
p(Dura)=0.0333
Scalp
Dura

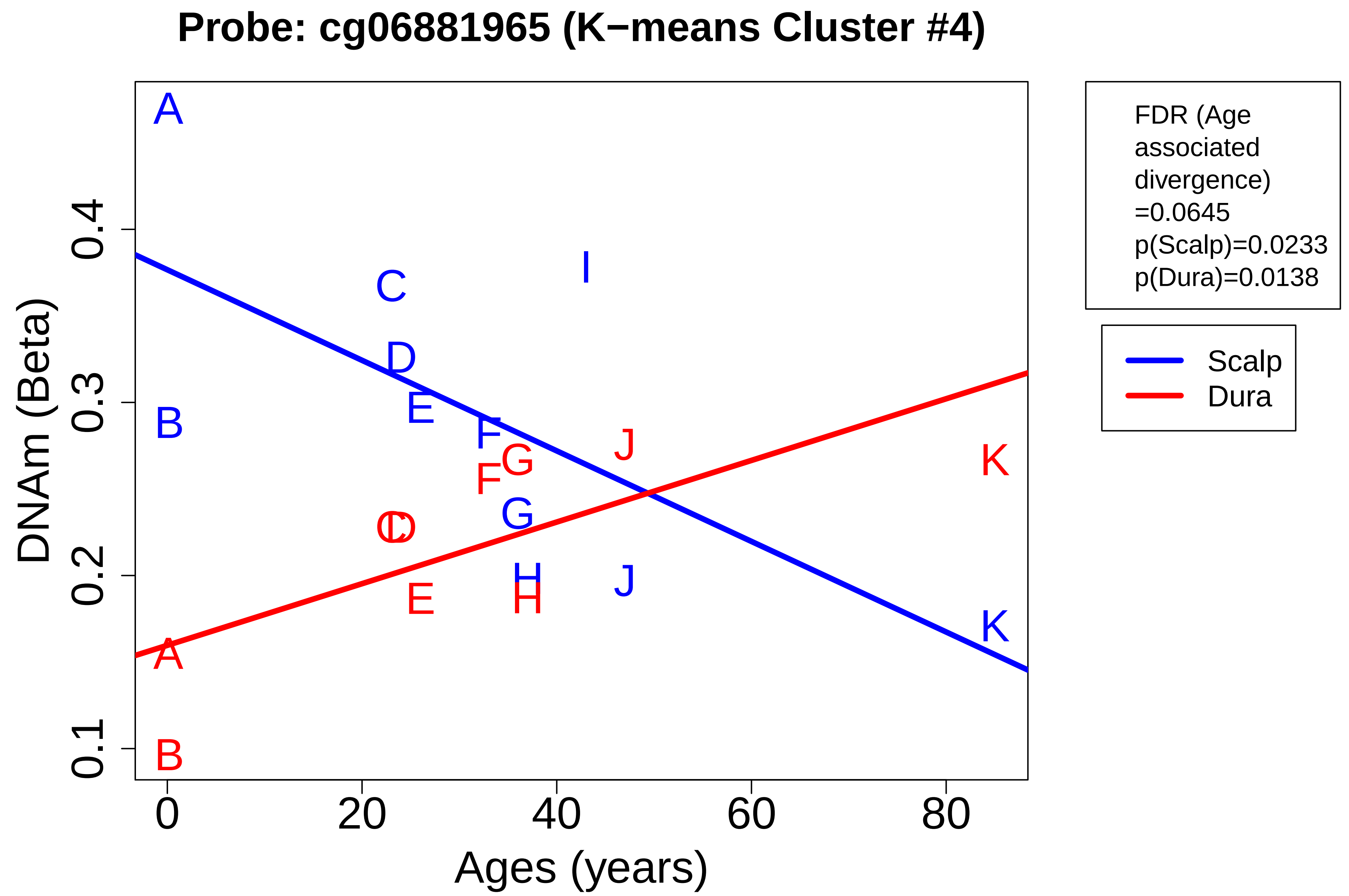

Probe: cg06881965 (K−means Cluster #4)
FDR (Age associated divergence) =0.0645
p(Scalp)=0.0233
p(Dura)=0.0138
Scalp
Dura
DNAm (Beta)
0.1
0.2
0.3
0.4
0
20
40
60
80
Ages (years)
A
B
C
D
E
F
G
H
I
J
K

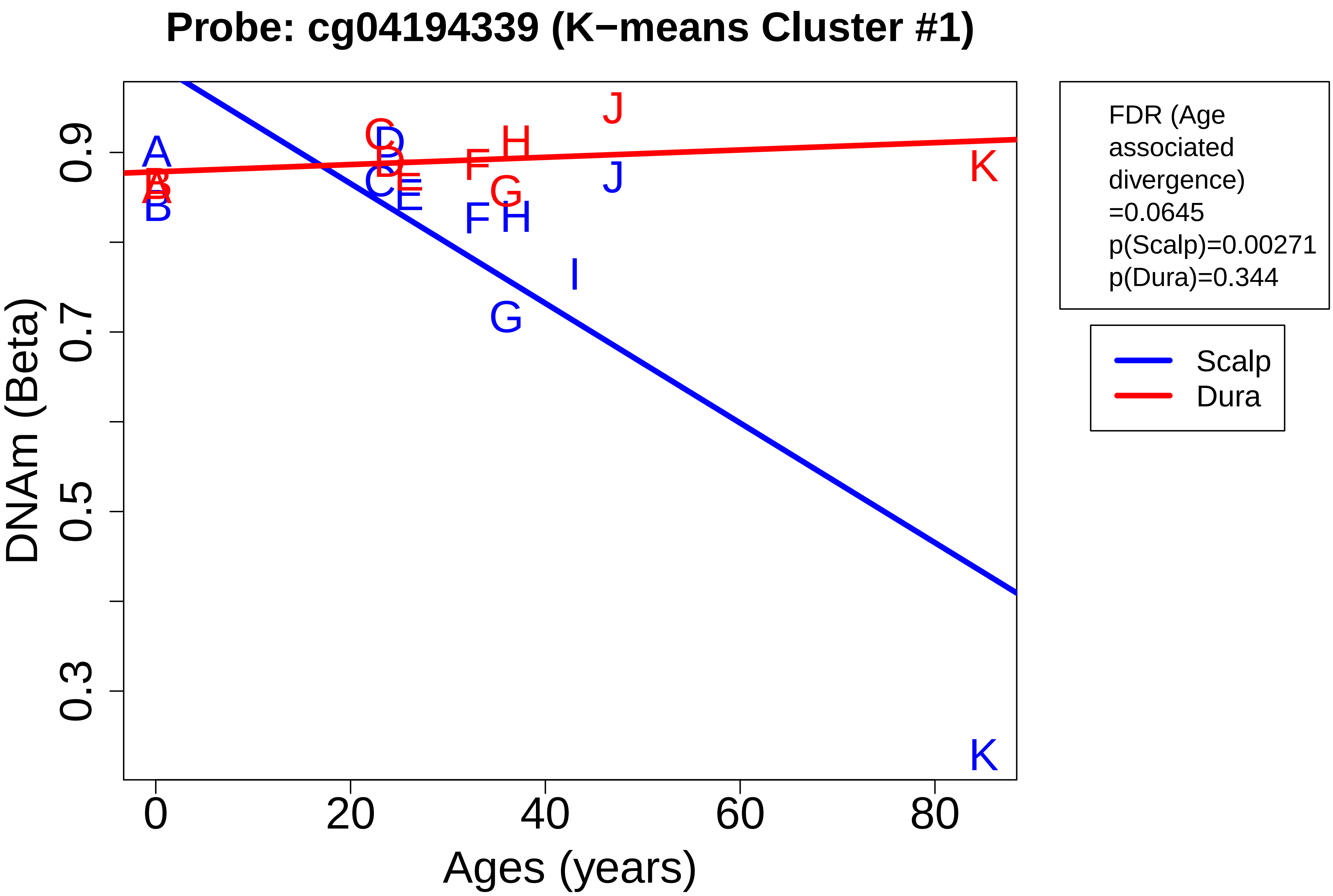
Probe: cg04194339 (K−means Cluster #1)
DNAm (Beta)
0.3
0.5
0.7
0.9
0
20
40
60
80
Ages (years)
FDR (Age associated divergence) =0.0645
p(Scalp)=0.00271
p(Dura)=0.344
Scalp
Dura

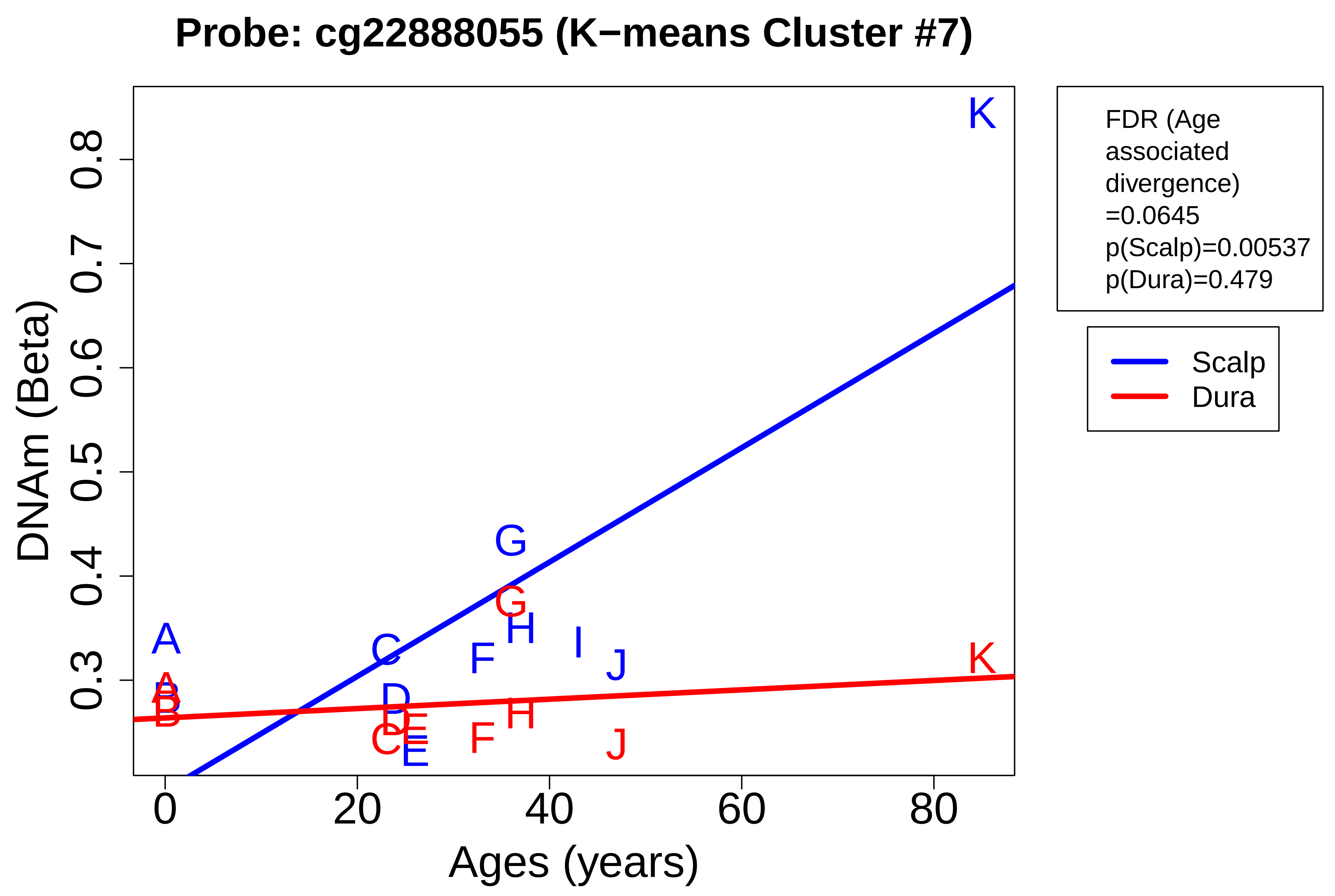
Probe: cg22888055 (K−means Cluster #7)
DNAm (Beta)
0.3
0.4
0.5
0.6
0.7
0.8
Ages (years)
0
20
40
60
80
FDR (Age associated divergence) =0.0645
p(Scalp)=0.00537
p(Dura)=0.479
Scalp
Dura
A
B
C
D
E
F
G
H
I
J
K

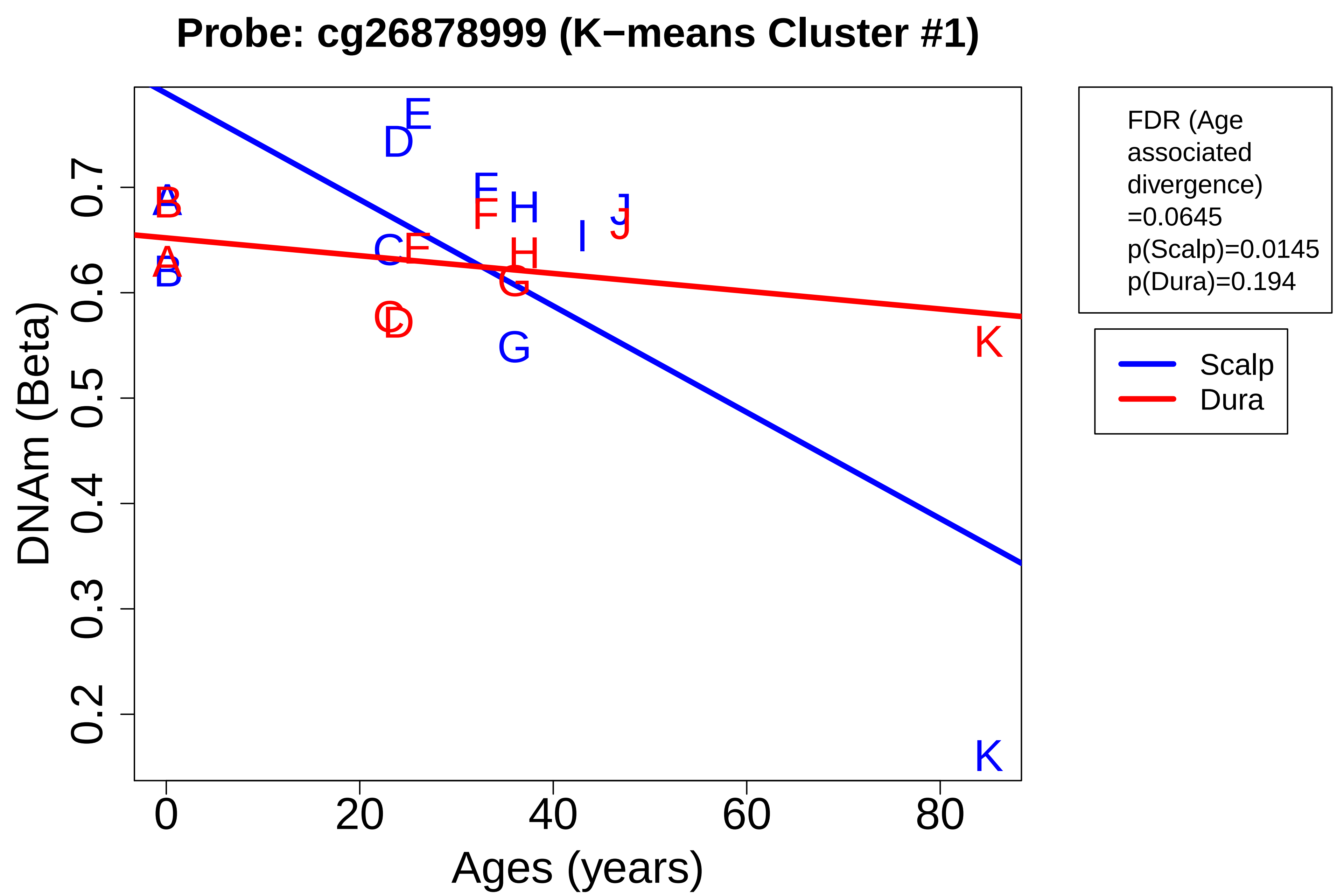

Probe: cg26878999 (K−means Cluster #1)
DNAm (Beta)
0.2
0.3
0.4
0.5
0.6
0.7
Ages (years)
0
20
40
60
80
FDR (Age associated divergence) =0.0645
p(Scalp)=0.0145
p(Dura)=0.194
Scalp
Dura
A B C D E F G H I J K

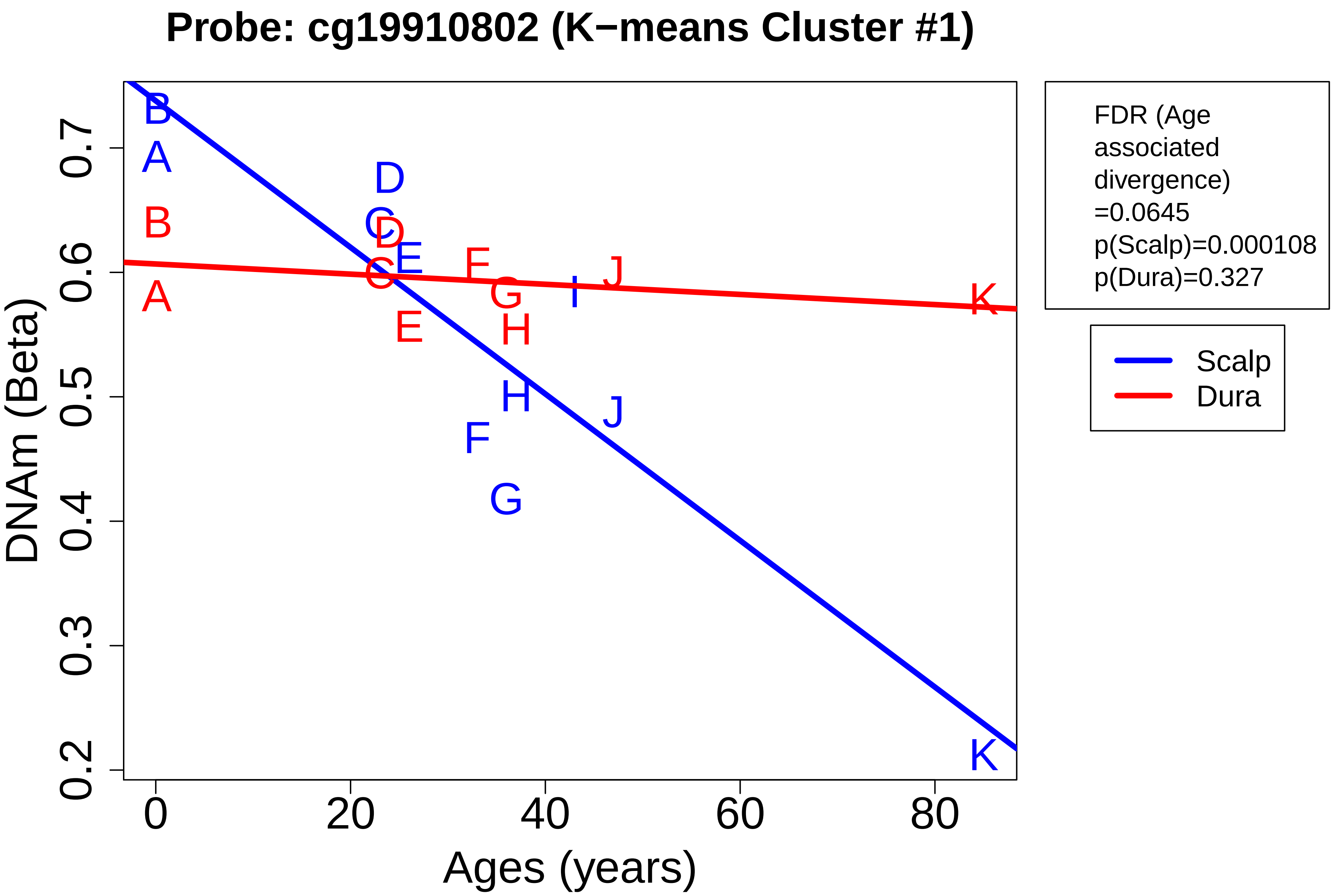

Probe: cg19910802 (K−means Cluster #1)
DNAm (Beta)
0.2
0.3
0.4
0.5
0.6
0.7
0
20
40
60
80
Ages (years)
A
B
C
D
E
F
G
H
I
J
K
FDR (Age associated divergence) =0.0645
p(Scalp)=0.000108
p(Dura)=0.327
Scalp
Dura

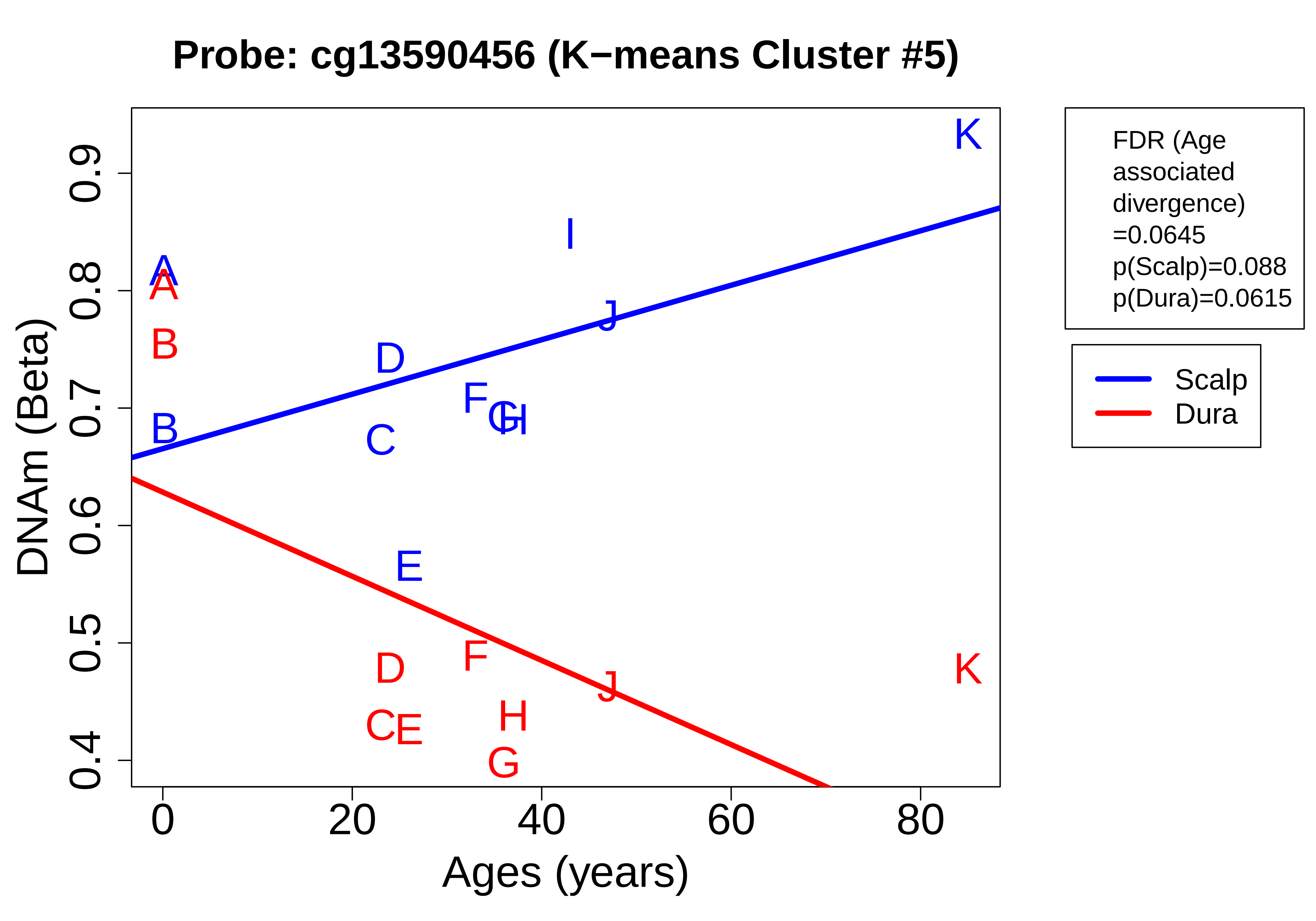

Probe: cg13590456 (K−means Cluster #5)
DNAm (Beta)
0.4
0.5
0.6
0.7
0.8
0.9
Ages (years)
0
20
40
60
80
FDR (Age associated divergence) =0.0645
p(Scalp)=0.088
p(Dura)=0.0615
Scalp
Dura

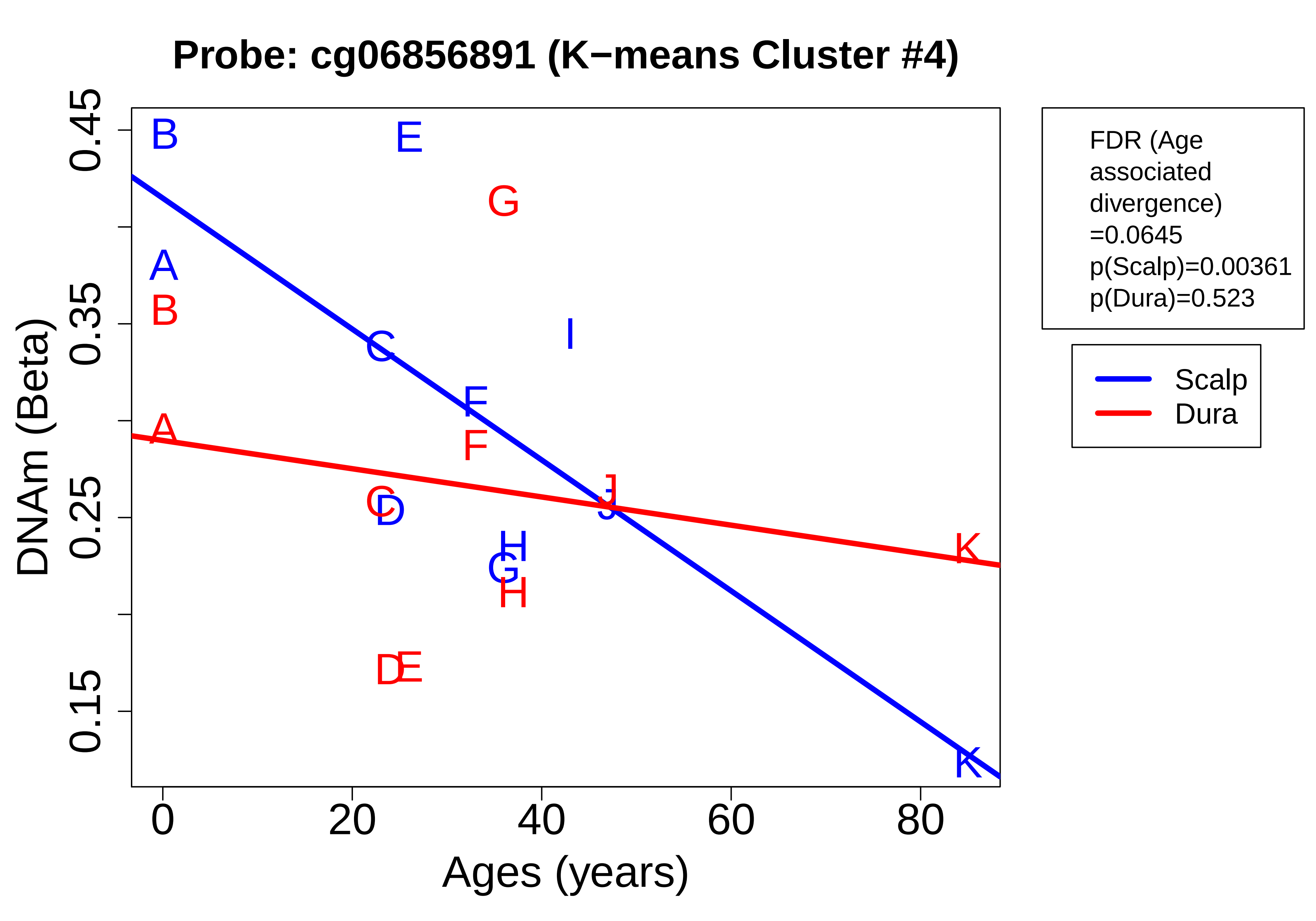

Probe: cg06856891 (K−means Cluster #4)
DNAm (Beta)
Ages (years)
0.15
0.25
0.35
0.45
0
20
40
60
80
B
E
G
A
B
C
I
F
A
F
J
C
D
H
K
G
H
D
E
K
FDR (Age associated divergence) =0.0645
p(Scalp)=0.00361
p(Dura)=0.523
Scalp
Dura

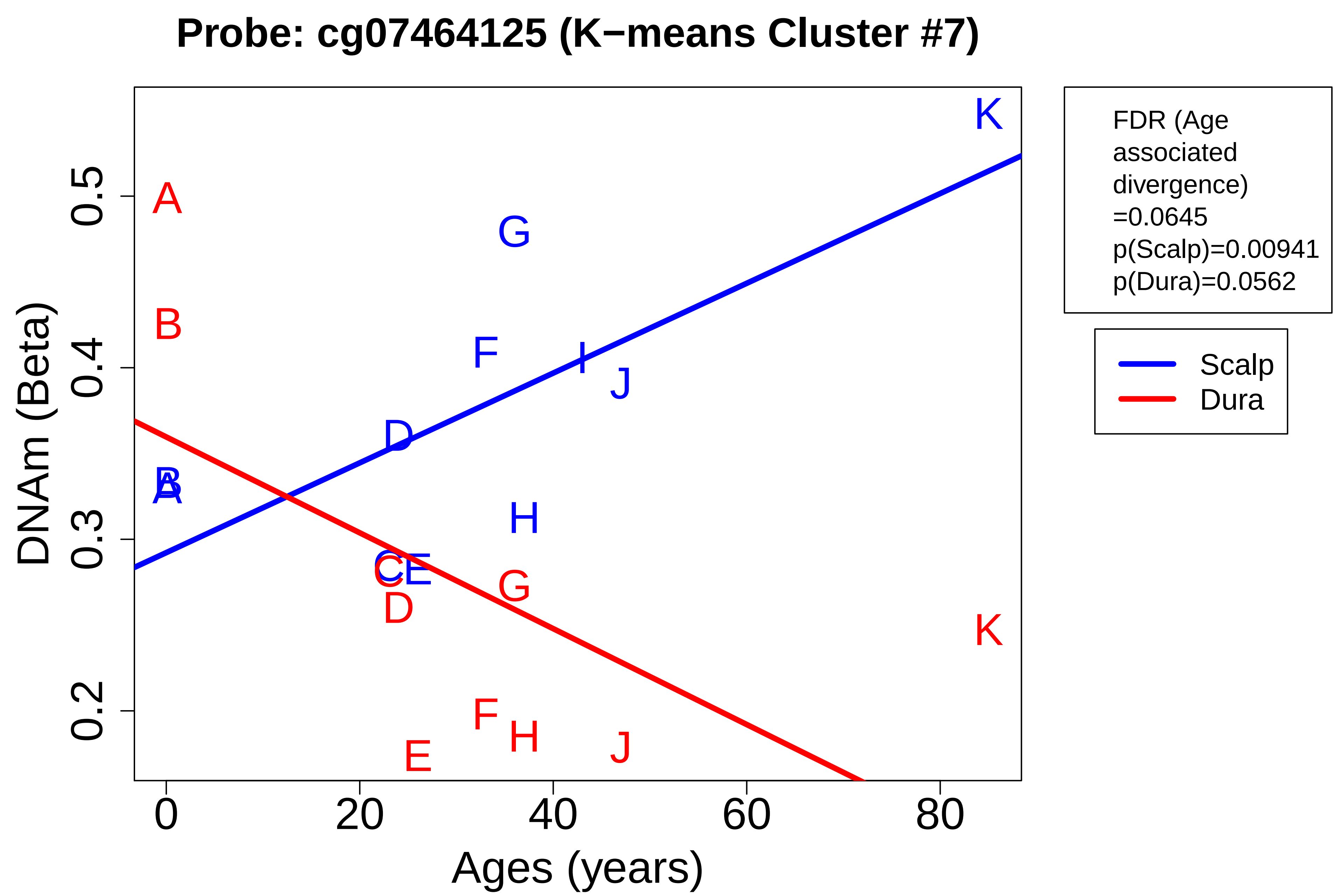
Probe: cg07464125 (K−means Cluster #7)
DNAm (Beta)
0.2
0.3
0.4
0.5
0
20
40
60
80
Ages (years)
FDR (Age associated divergence) =0.0645
p(Scalp)=0.00941
p(Dura)=0.0562
Scalp
Dura
A
B
C
D
E
F
G
H
I
J
K

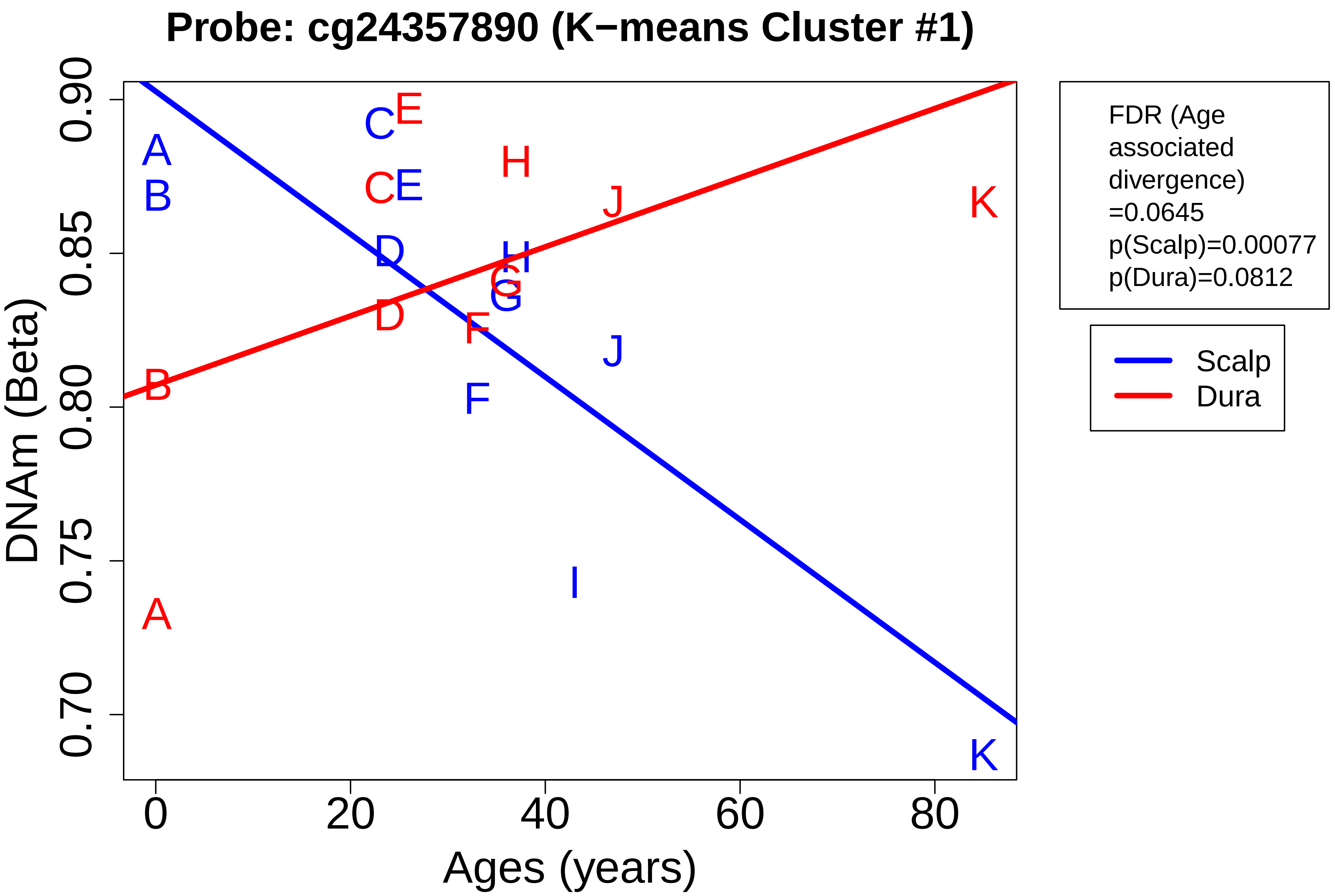
Probe: cg24357890 (K−means Cluster #1)
DNAm (Beta)
0.70
0.75
0.80
0.85
0.90
Ages (years)
0
20
40
60
80
A
B
C
D
E
F
G
H
I
J
K
FDR (Age associated divergence) =0.0645
p(Scalp)=0.00077
p(Dura)=0.0812
Scalp
Dura

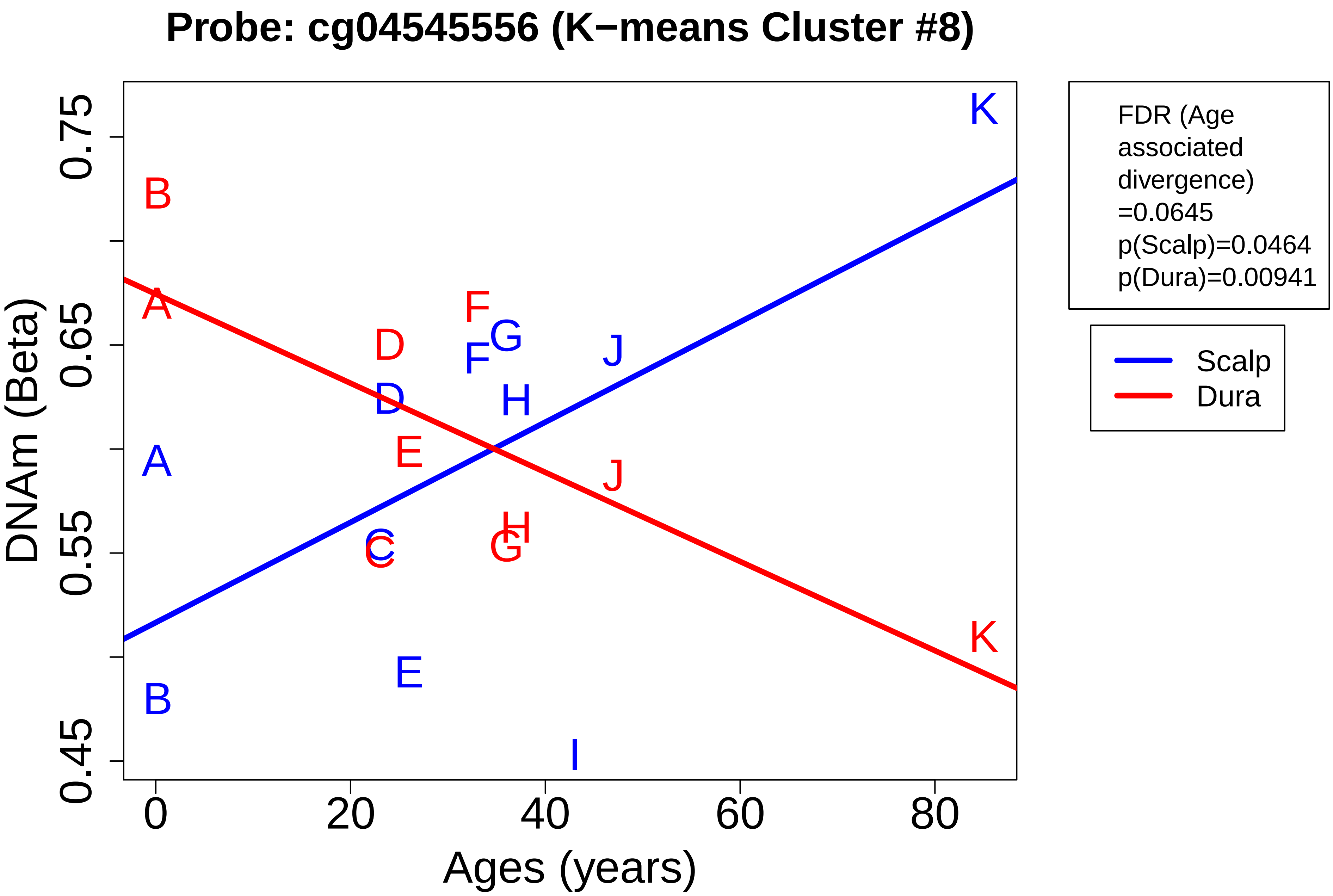

Probe: cg04545556 (K−means Cluster #8)
DNAm (Beta)
0.45
0.55
0.65
0.75
0
20
40
60
80
Ages (years)
FDR (Age associated divergence) =0.0645
p(Scalp)=0.0464
p(Dura)=0.00941
Scalp
Dura

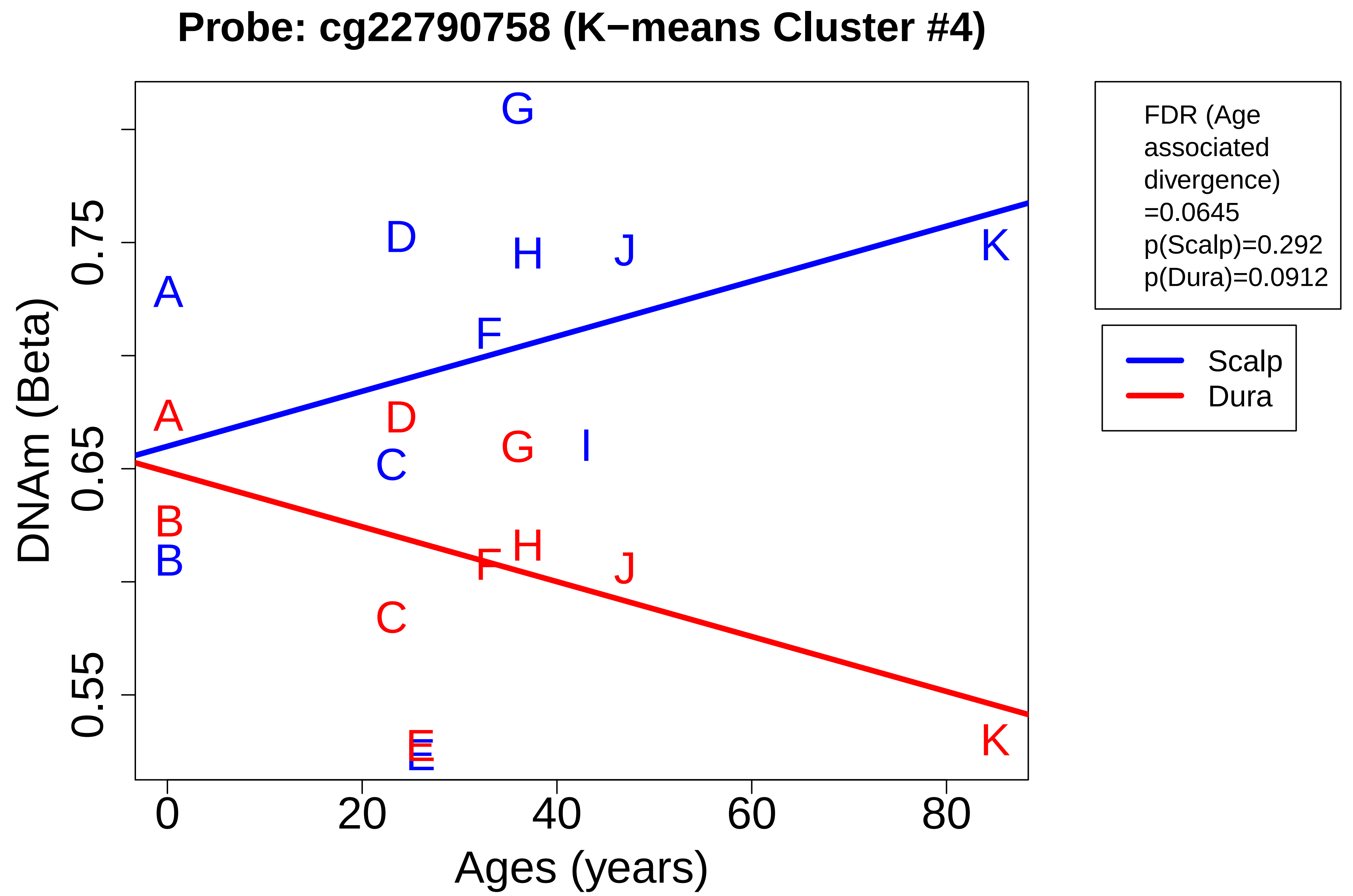
Probe: cg22790758 (K−means Cluster #4)
DNAm (Beta)
0.55
0.65
0.75
Ages (years)
0
20
40
60
80
FDR (Age associated divergence) =0.0645
p(Scalp)=0.292
p(Dura)=0.0912
Scalp
Dura
A
B
C
D
E
F
G
H
I
J
K

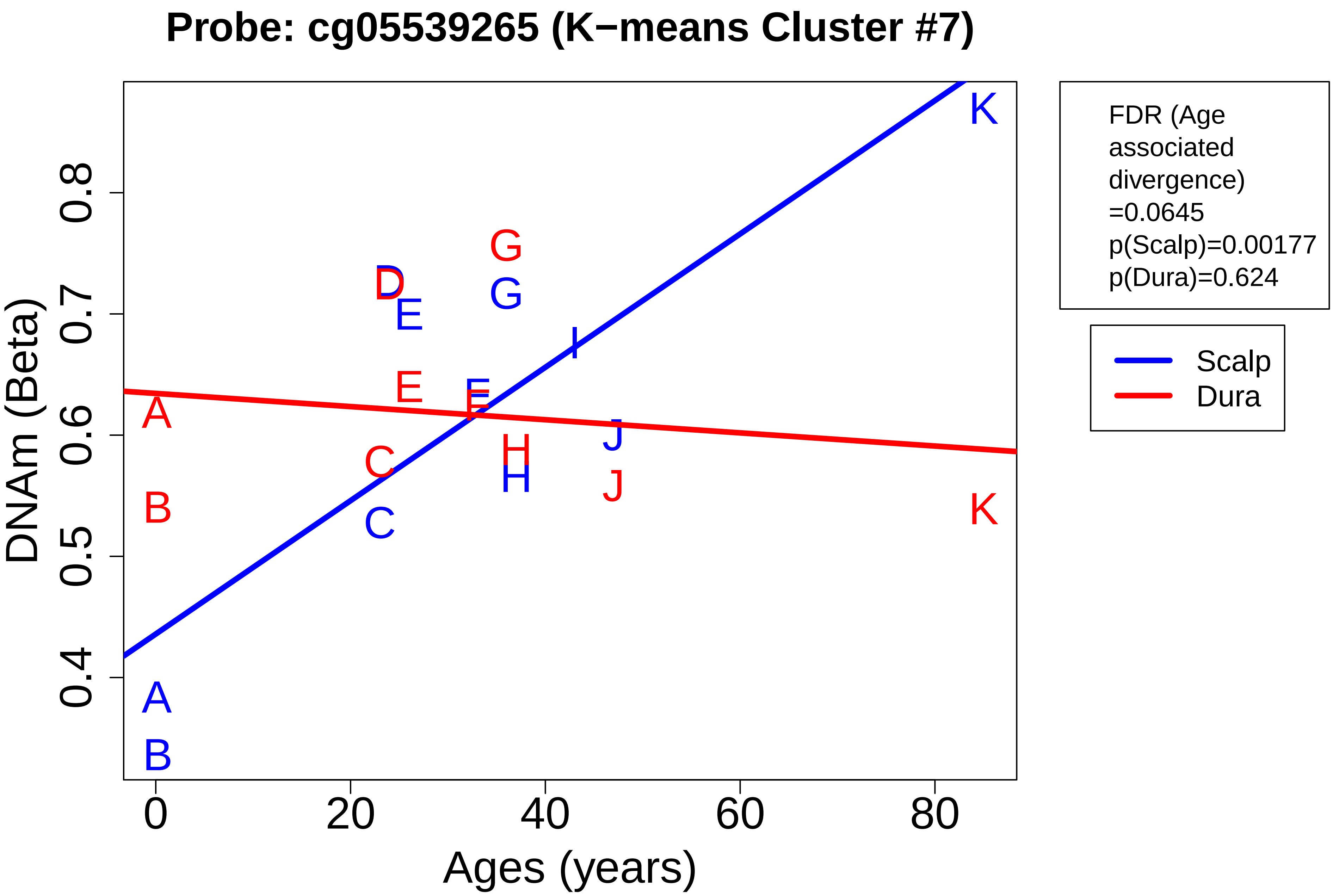
Probe: cg05539265 (K−means Cluster #7)
DNAm (Beta)
0.4
0.5
0.6
0.7
0.8
0
20
40
60
80
Ages (years)
A
B
C
D
E
F
G
H
I
J
K
FDR (Age associated divergence) =0.0645
p(Scalp)=0.00177
p(Dura)=0.624
Scalp
Dura

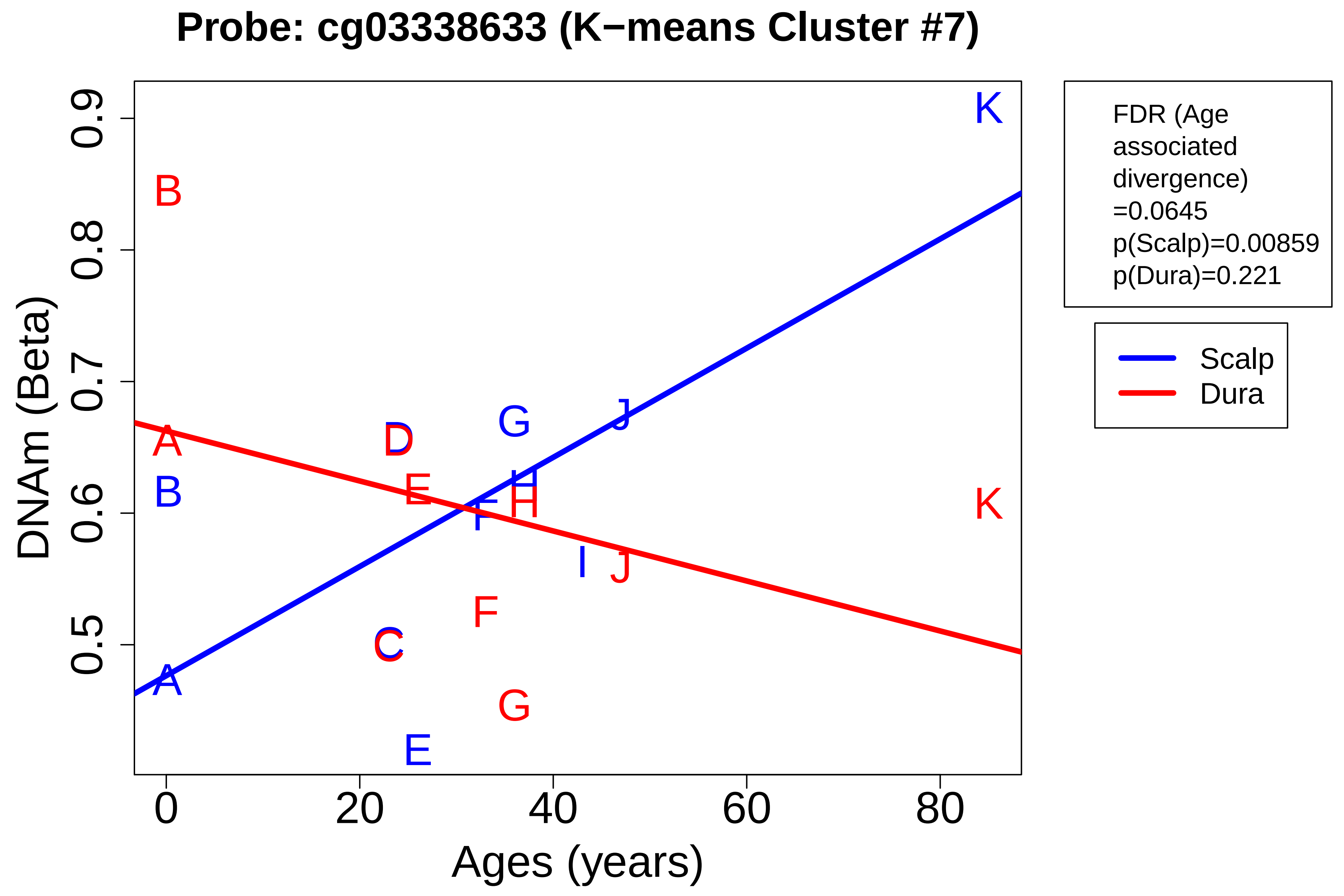

Probe: cg03338633 (K−means Cluster #7)
DNAm (Beta)
0.5
0.6
0.7
0.8
0.9
Ages (years)
0
20
40
60
80
A
B
C
D
E
F
G
H
I
J
K
FDR (Age associated divergence) =0.0645
p(Scalp)=0.00859
p(Dura)=0.221
Scalp
Dura

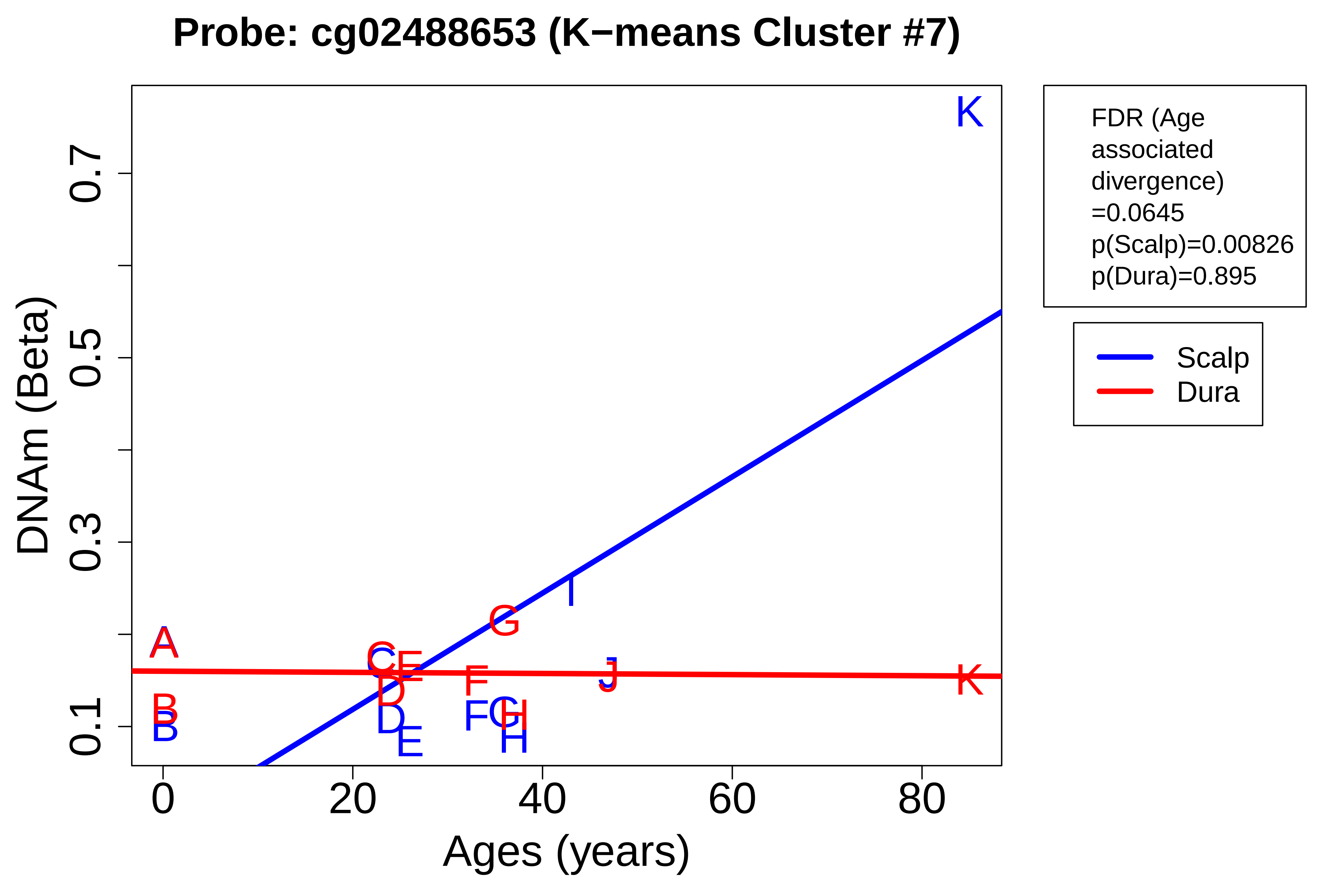

Probe: cg02488653 (K−means Cluster #7)
FDR (Age associated divergence) =0.0645
p(Scalp)=0.00826
p(Dura)=0.895
Scalp
Dura
DNAm (Beta)
0.1
0.3
0.5
0.7
Ages (years)
0
20
40
60
80
A
B
C
D
E
F
G
H
I
J
K

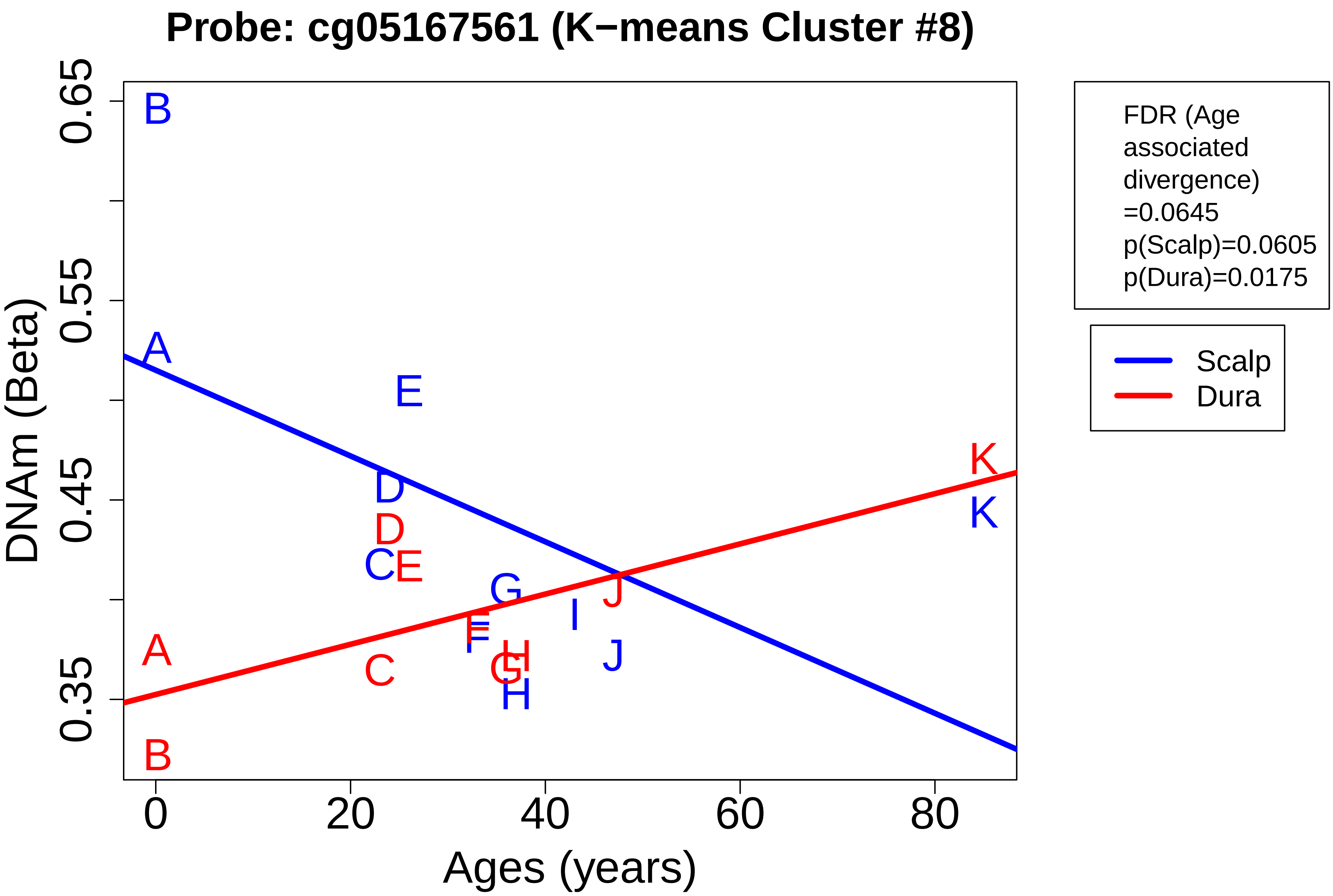

Probe: cg05167561 (K−means Cluster #8)
DNAm (Beta)
0.65
0.55
0.45
0.35
0
20
40
60
80
Ages (years)
FDR (Age associated divergence) =0.0645
p(Scalp)=0.0605
p(Dura)=0.0175
Scalp
Dura

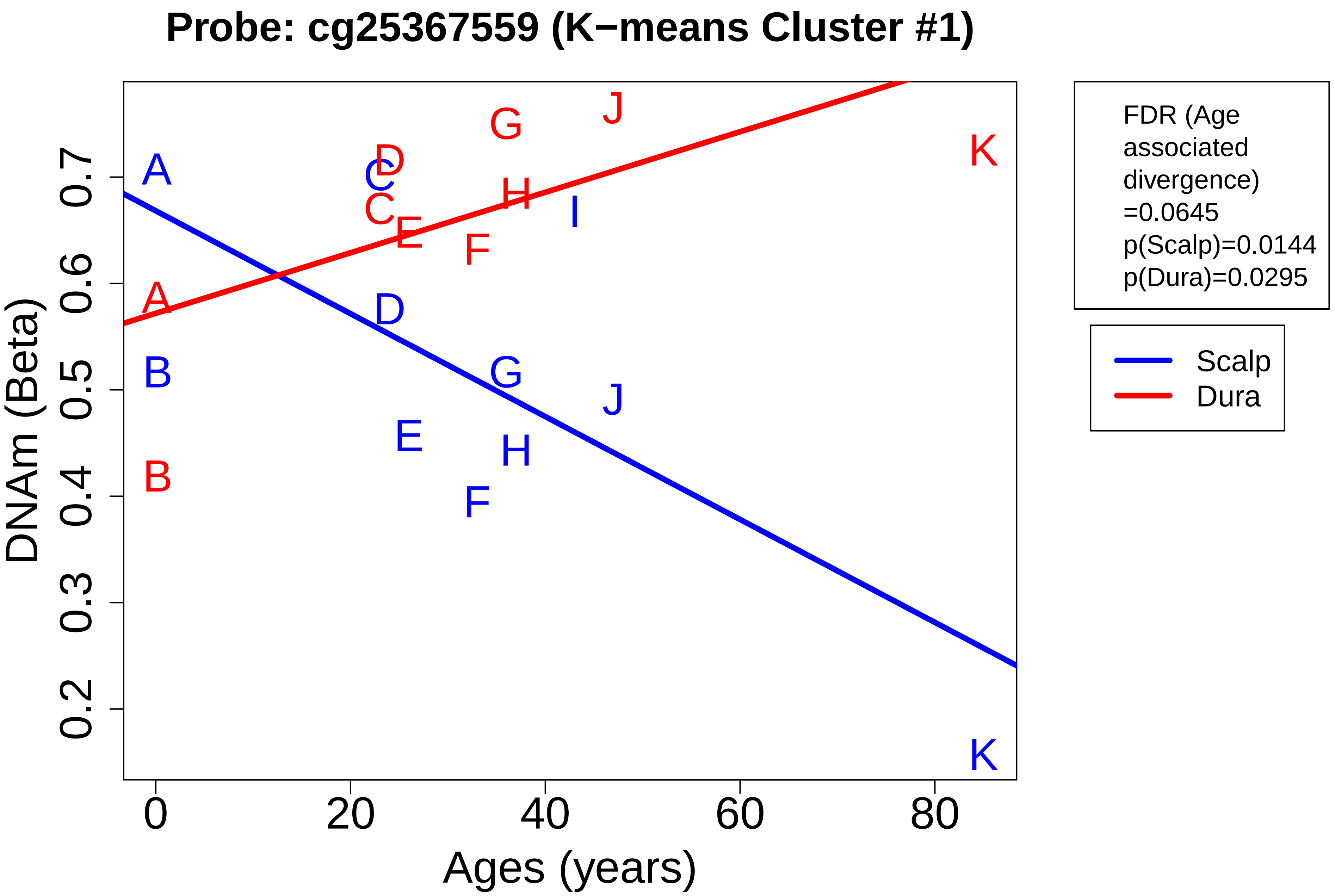
Probe: cg25367559 (K−means Cluster #1)
DNAm (Beta)
0.2
0.3
0.4
0.5
0.6
0.7
0
20
40
60
80
Ages (years)
FDR (Age associated divergence) =0.0645
p(Scalp)=0.0144
p(Dura)=0.0295
Scalp
Dura
A
B
C
D
E
F
G
H
I
J
K

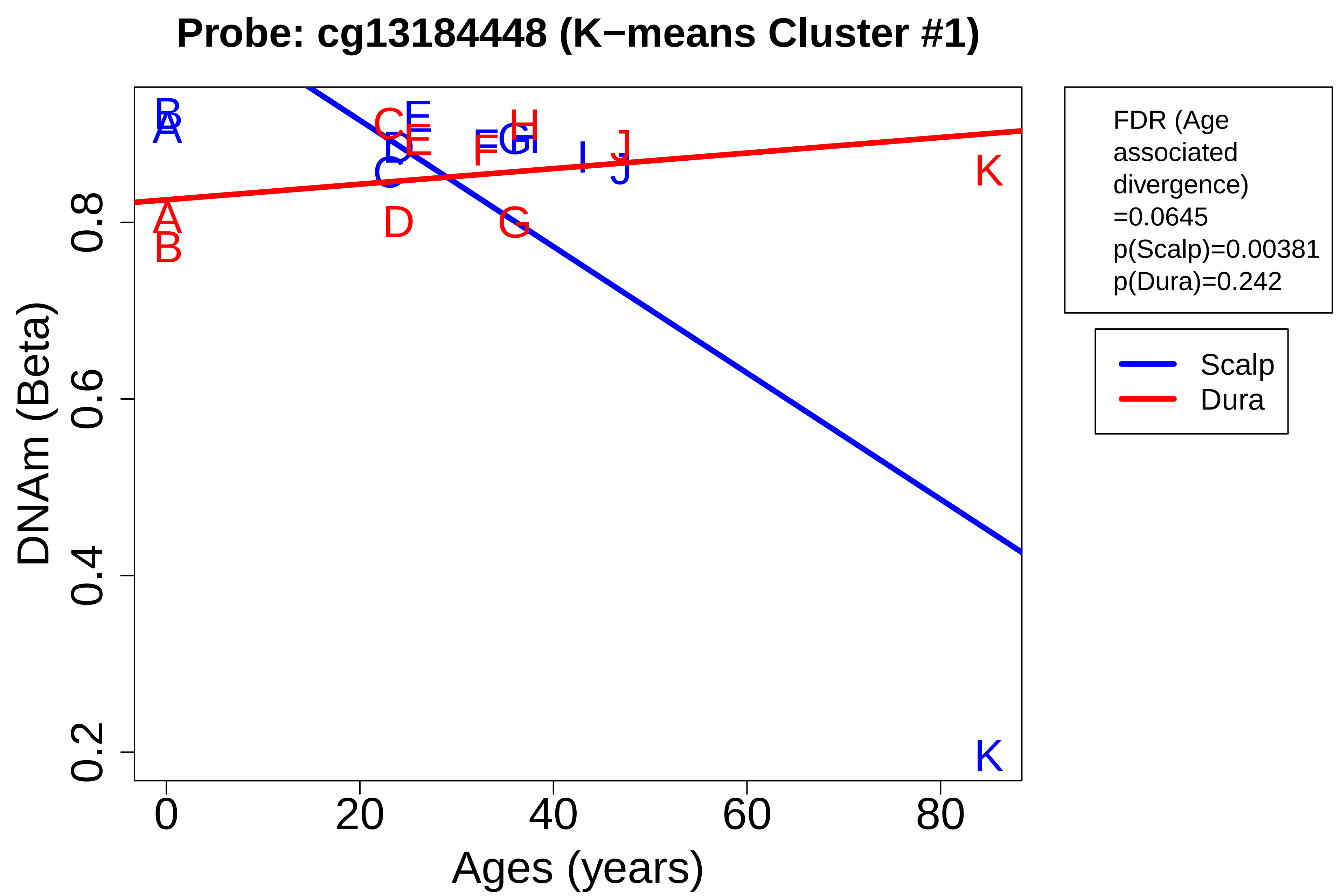

Probe: cg13184448 (K−means Cluster #1)
DNAm (Beta)
0.2
0.4
0.6
0.8
Ages (years)
0
20
40
60
80
FDR (Age associated divergence) =0.0645
p(Scalp)=0.00381
p(Dura)=0.242
Scalp
Dura
A
B
C
D
E
F
G
H
I
J
K

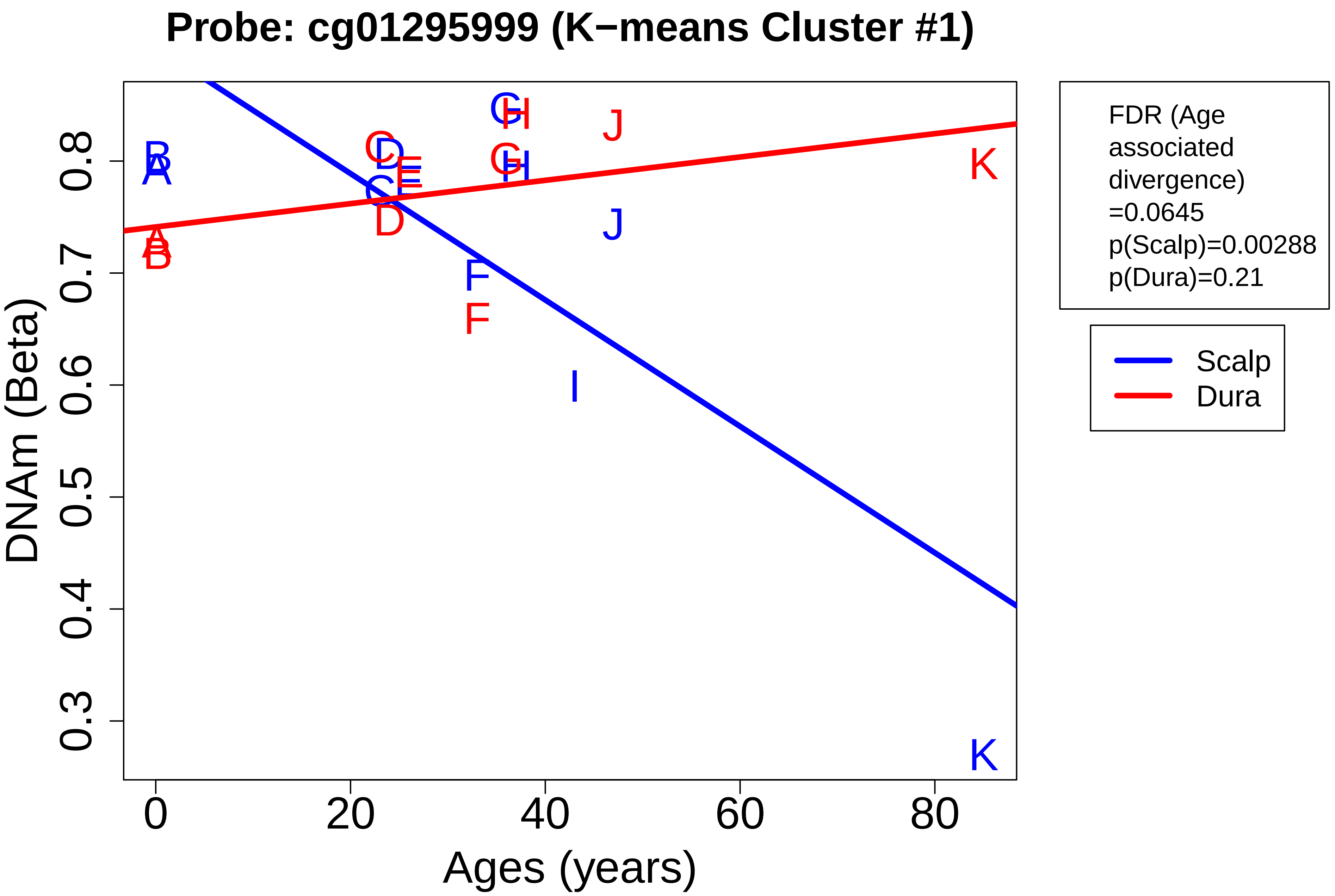

Probe: cg01295999 (K−means Cluster #1)
DNAm (Beta)
0.3
0.4
0.5
0.6
0.7
0.8
0
20
40
60
80
Ages (years)
FDR (Age associated divergence) =0.0645
p(Scalp)=0.00288
p(Dura)=0.21
Scalp
Dura

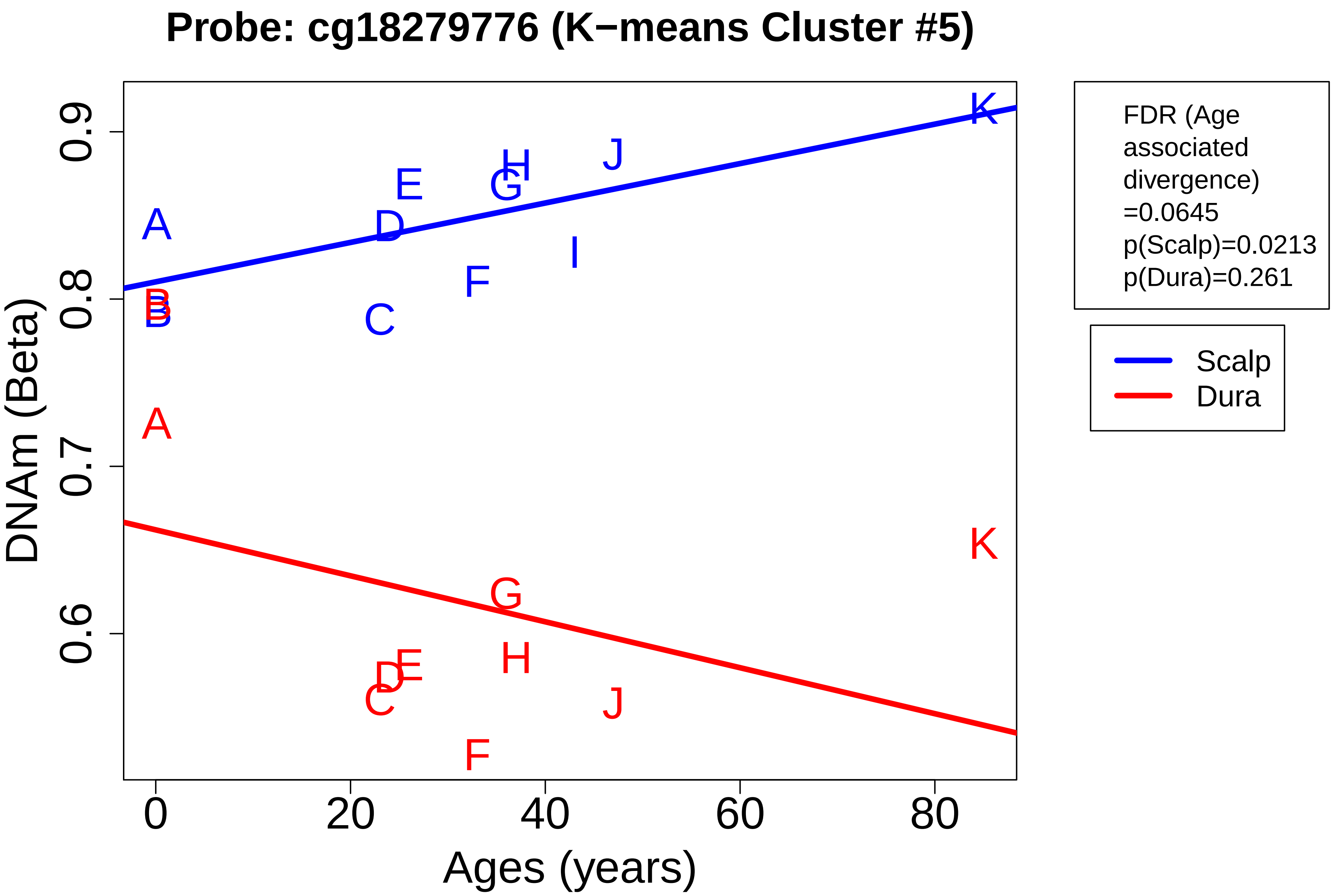

Probe: cg18279776 (K−means Cluster #5)
DNAm (Beta)
0.6
0.7
0.8
0.9
Ages (years)
0
20
40
60
80
A
B
C
D
E
F
G
H
I
J
K
FDR (Age associated divergence) =0.0645
p(Scalp)=0.0213
p(Dura)=0.261
Scalp
Dura

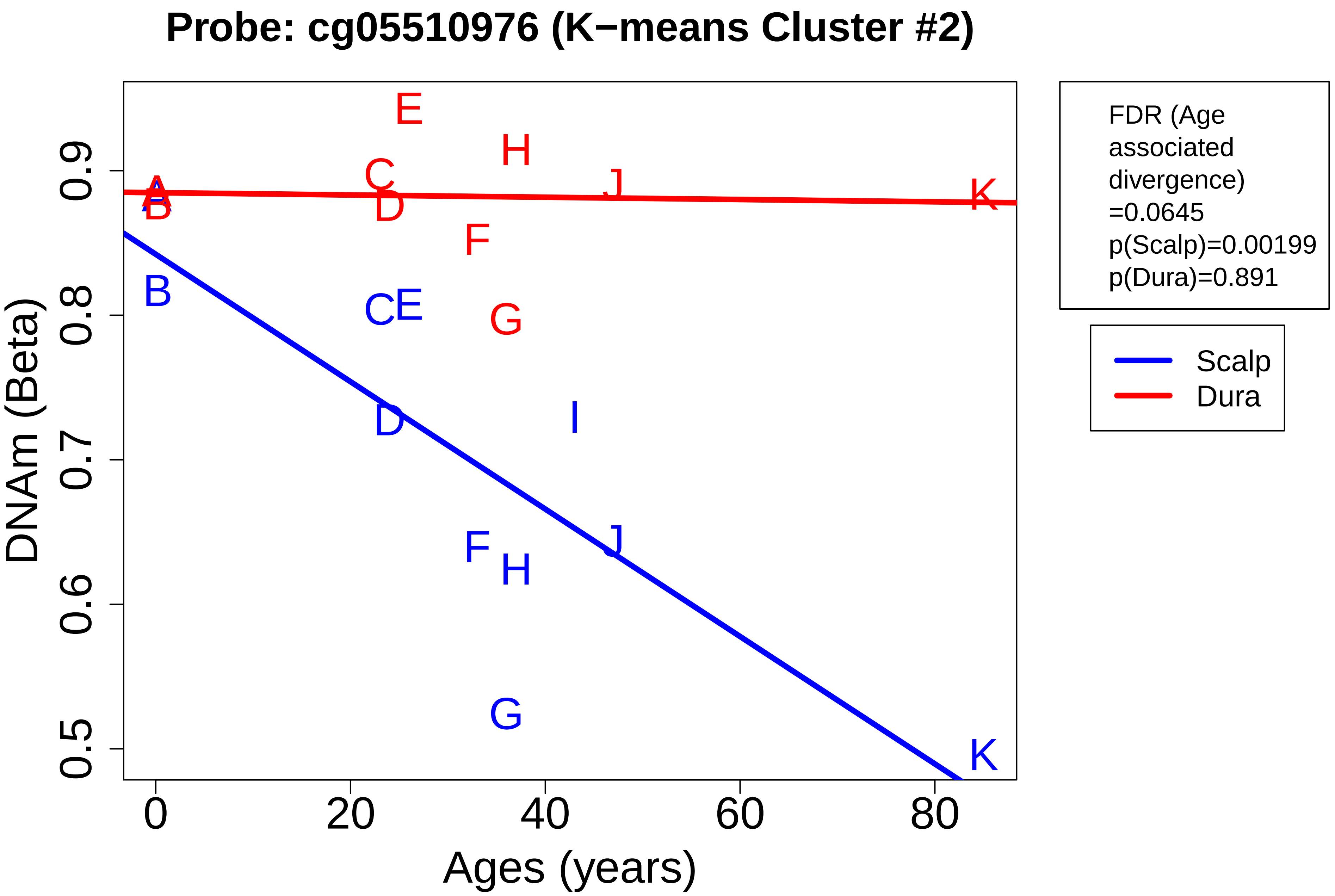
Probe: cg05510976 (K−means Cluster #2)
DNAm (Beta)
Ages (years)
0.5
0.6
0.7
0.8
0.9
0
20
40
60
80
FDR (Age associated divergence) =0.0645
p(Scalp)=0.00199
p(Dura)=0.891
Scalp
Dura
A
B
C
D
E
F
G
H
I
J
K

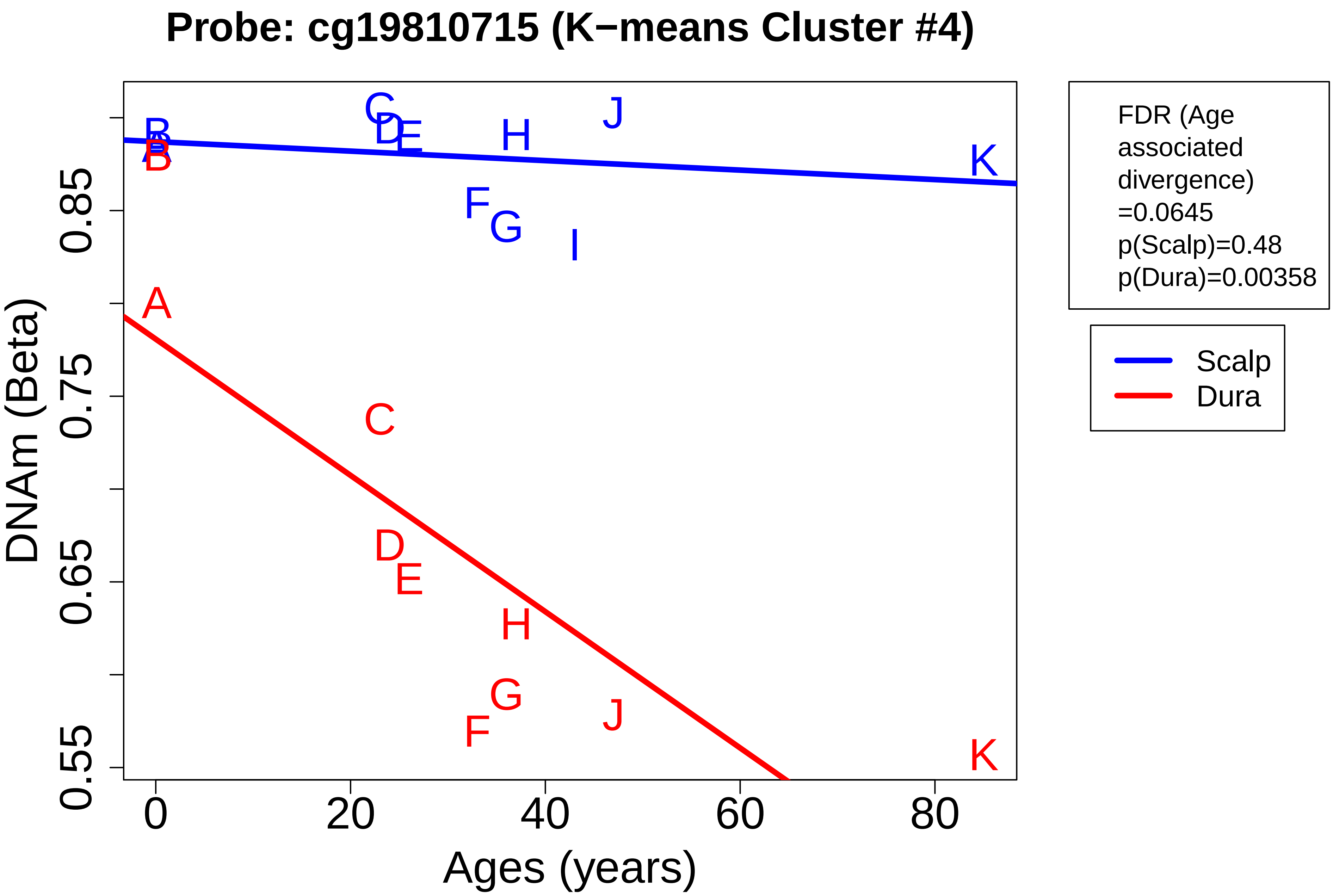
Probe: cg19810715 (K−means Cluster #4)
DNAm (Beta)
0.55
0.65
0.75
0.85
Ages (years)
0
20
40
60
80
FDR (Age associated divergence) =0.0645
p(Scalp)=0.48
p(Dura)=0.00358
Scalp
Dura
A
B
C
D
E
F
G
H
I
J
K

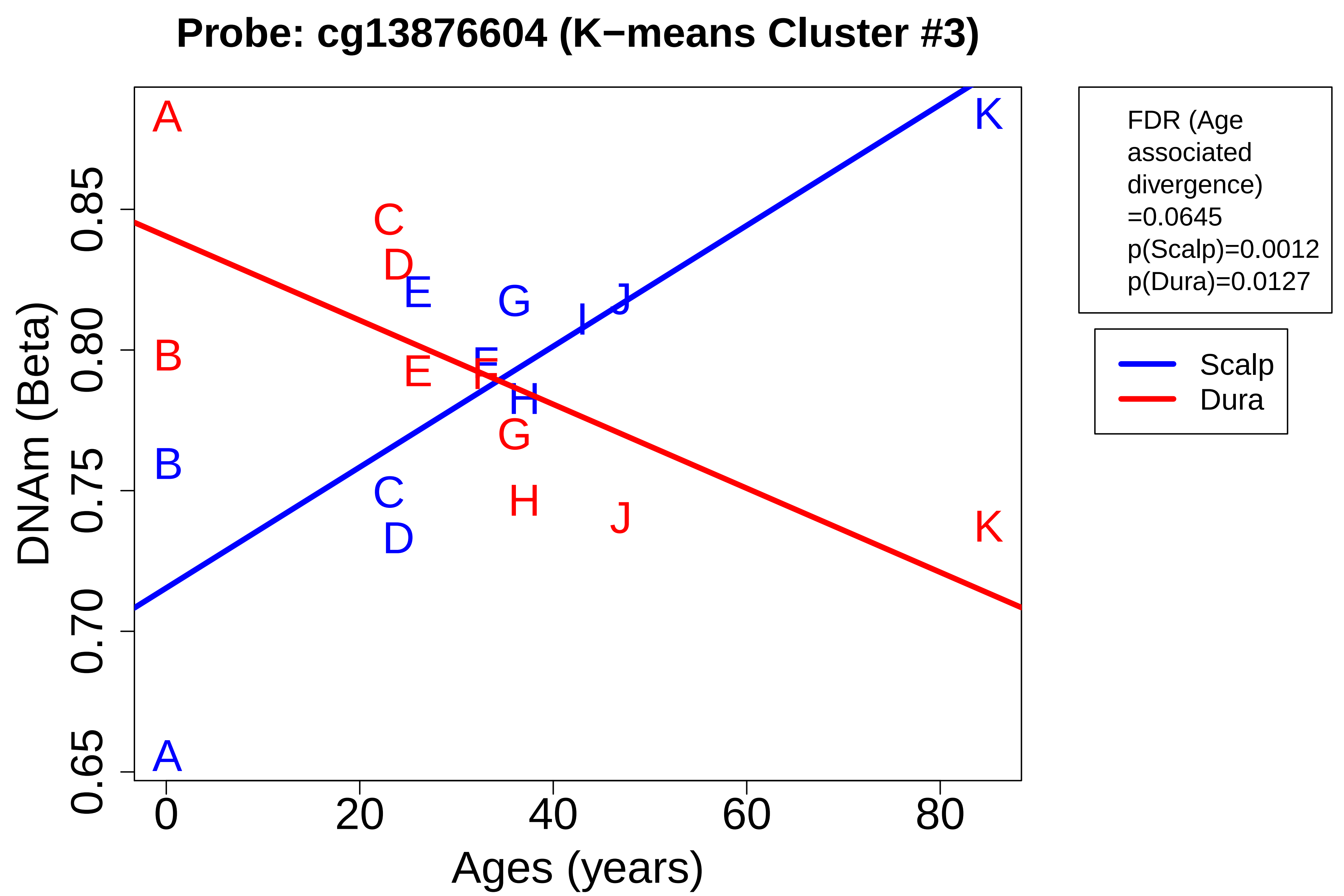

Probe: cg13876604 (K−means Cluster #3)
DNAm (Beta)
0.65
0.70
0.75
0.80
0.85
0
20
40
60
80
Ages (years)
A
B
C
D
E
F
G
H
I
J
K
FDR (Age associated divergence) =0.0645
p(Scalp)=0.0012
p(Dura)=0.0127
Scalp
Dura

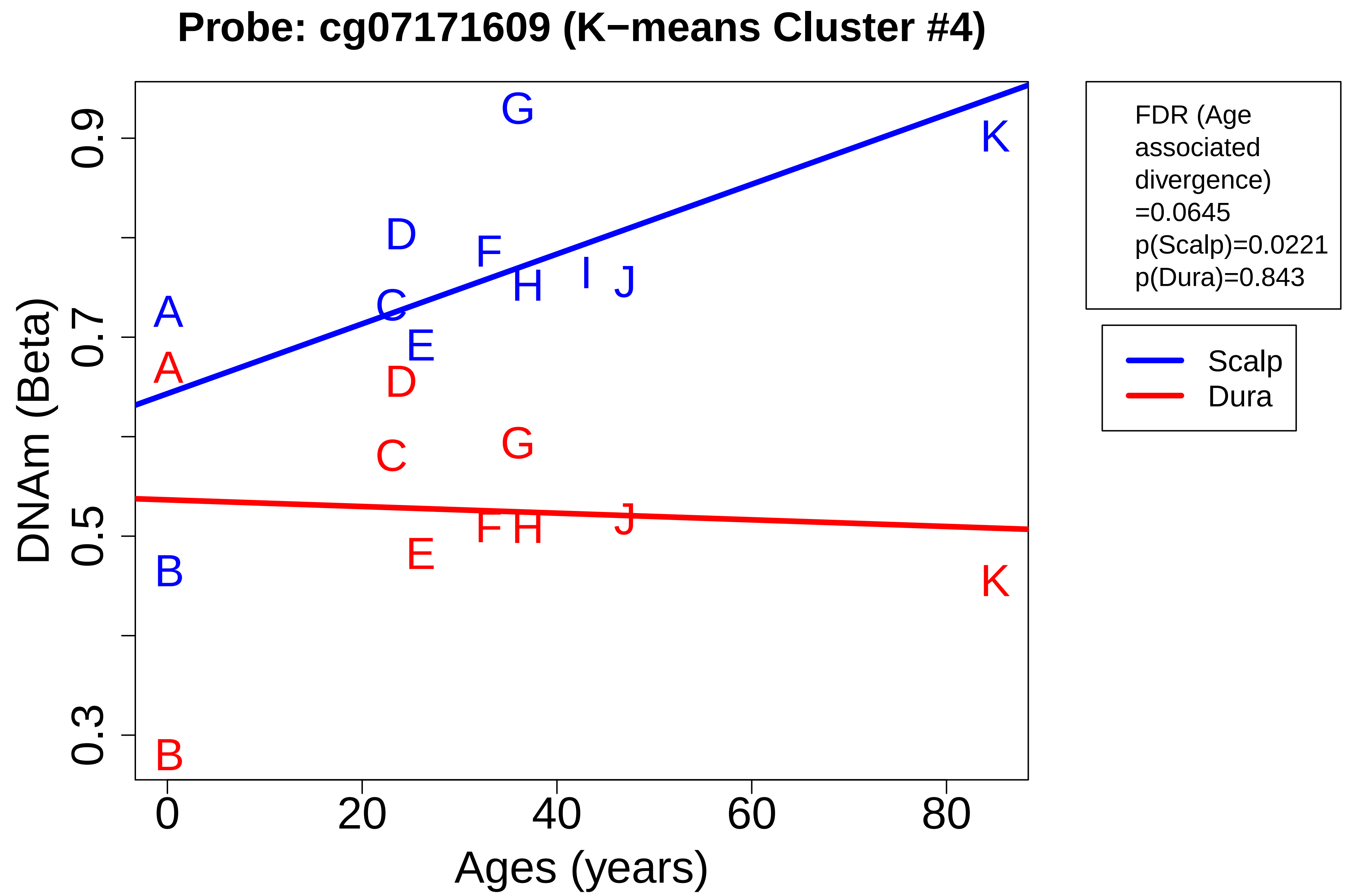

Probe: cg07171609 (K−means Cluster #4)
DNAm (Beta)
0.3
0.5
0.7
0.9
0
20
40
60
80
Ages (years)
FDR (Age associated divergence) =0.0645
p(Scalp)=0.0221
p(Dura)=0.843
Scalp
Dura
A
B
C
D
E
F
G
H
I
J
K

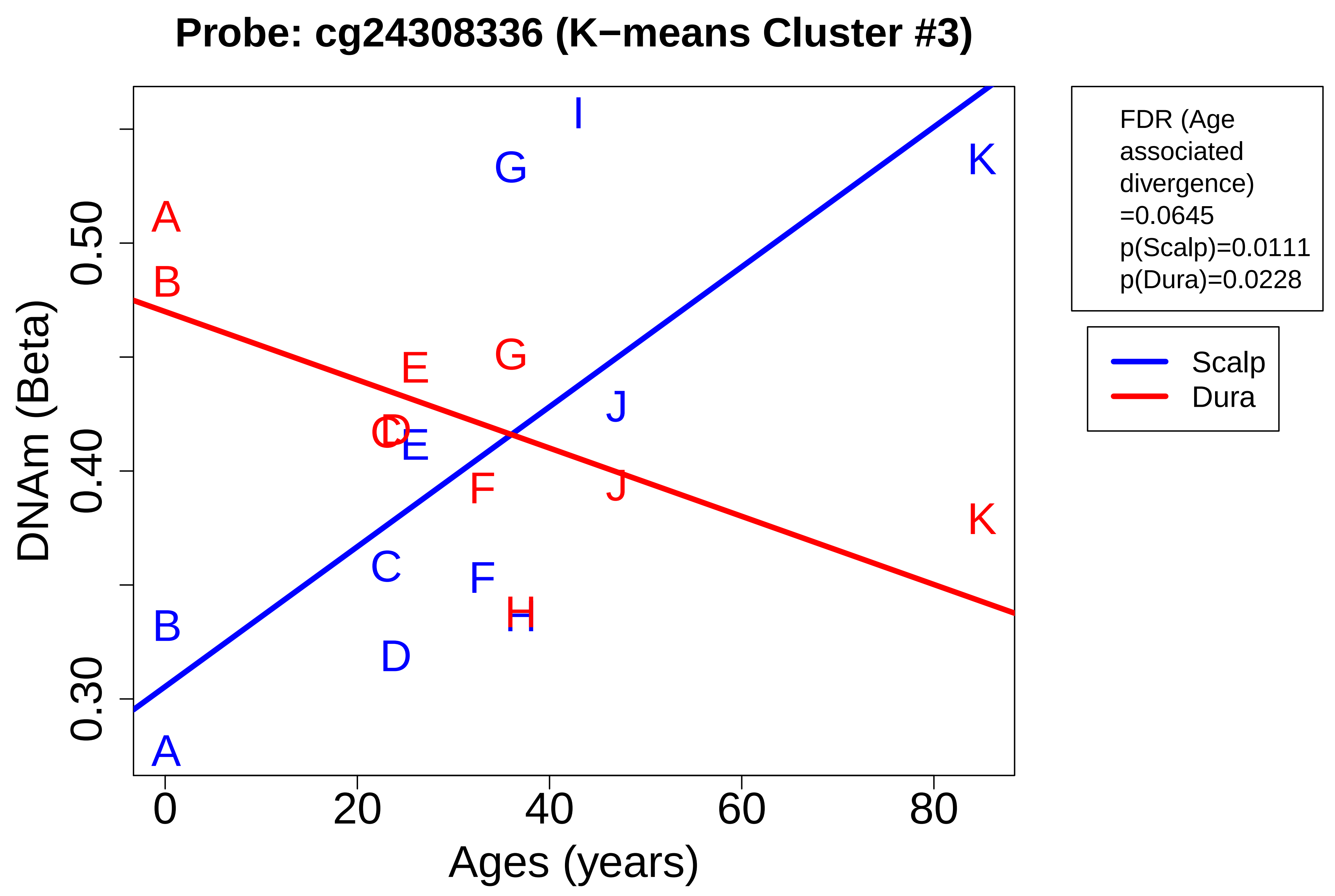

Probe: cg24308336 (K−means Cluster #3)
FDR (Age associated divergence) =0.0645
p(Scalp)=0.0111
p(Dura)=0.0228
Scalp
Dura
DNAm (Beta)
0.30
0.40
0.50
0
20
40
60
80
Ages (years)
A
B
C
D
E
F
G
H
I
J
K

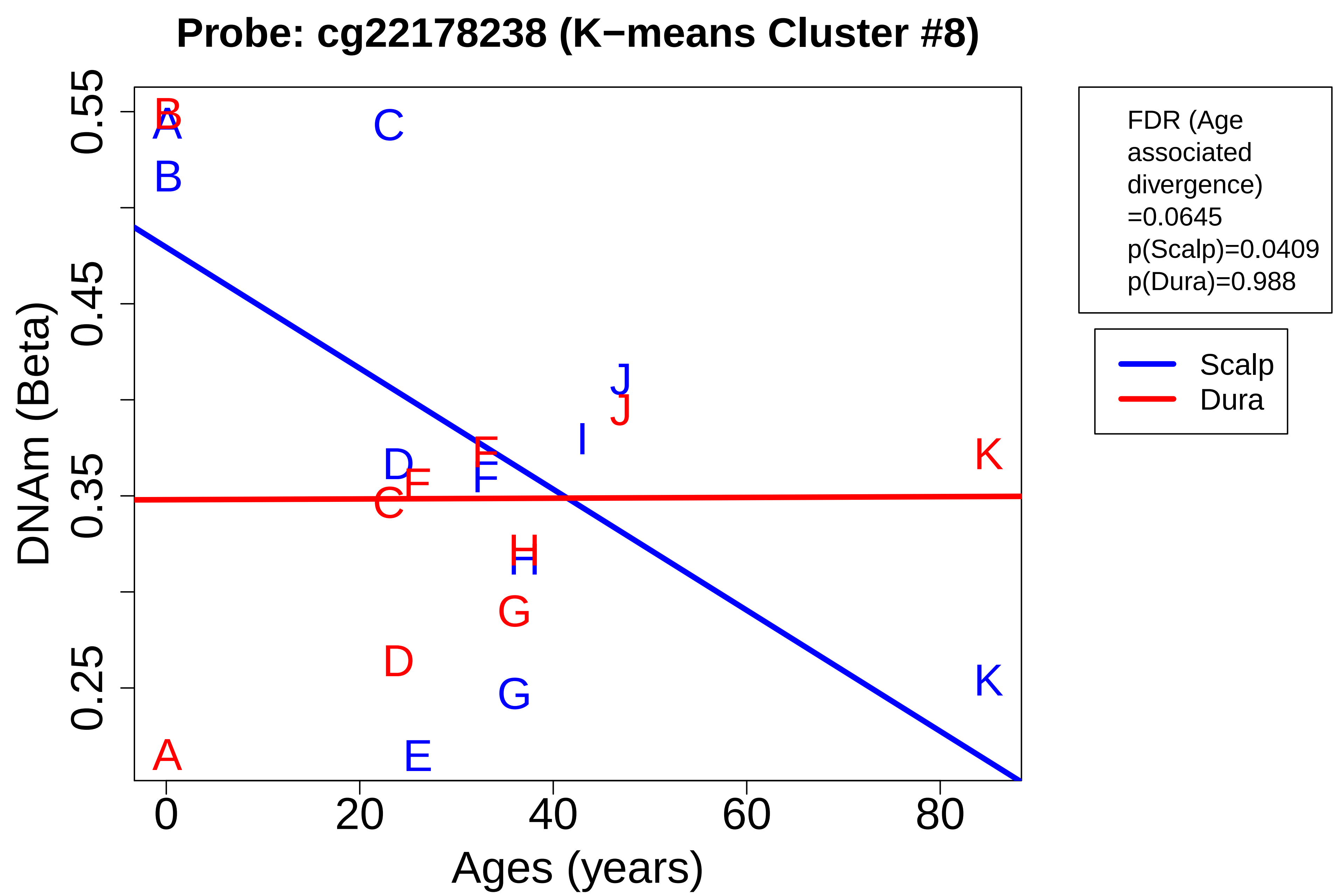

Probe: cg22178238 (K−means Cluster #8)
DNAm (Beta)
0.25
0.35
0.45
0.55
Ages (years)
0
20
40
60
80
FDR (Age associated divergence) =0.0645
p(Scalp)=0.0409
p(Dura)=0.988
Scalp
Dura
A
B
C
D
E
F
G
H
I
J
K

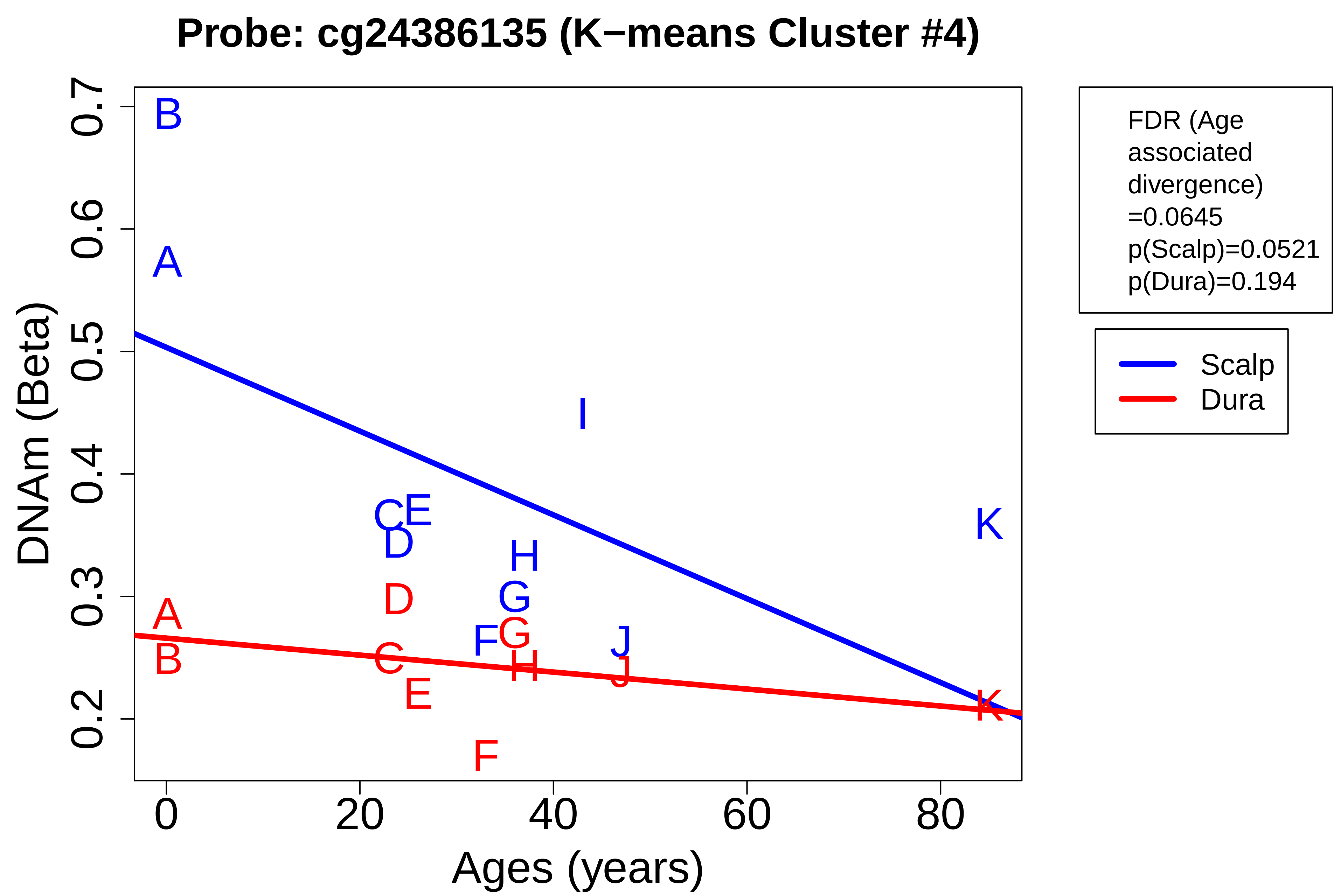
Probe: cg24386135 (K−means Cluster #4)
DNAm (Beta)
0.2
0.3
0.4
0.5
0.6
0.7
Ages (years)
0
20
40
60
80
FDR (Age associated divergence) =0.0645
p(Scalp)=0.0521
p(Dura)=0.194
Scalp
Dura
A B C D E F G H I J K

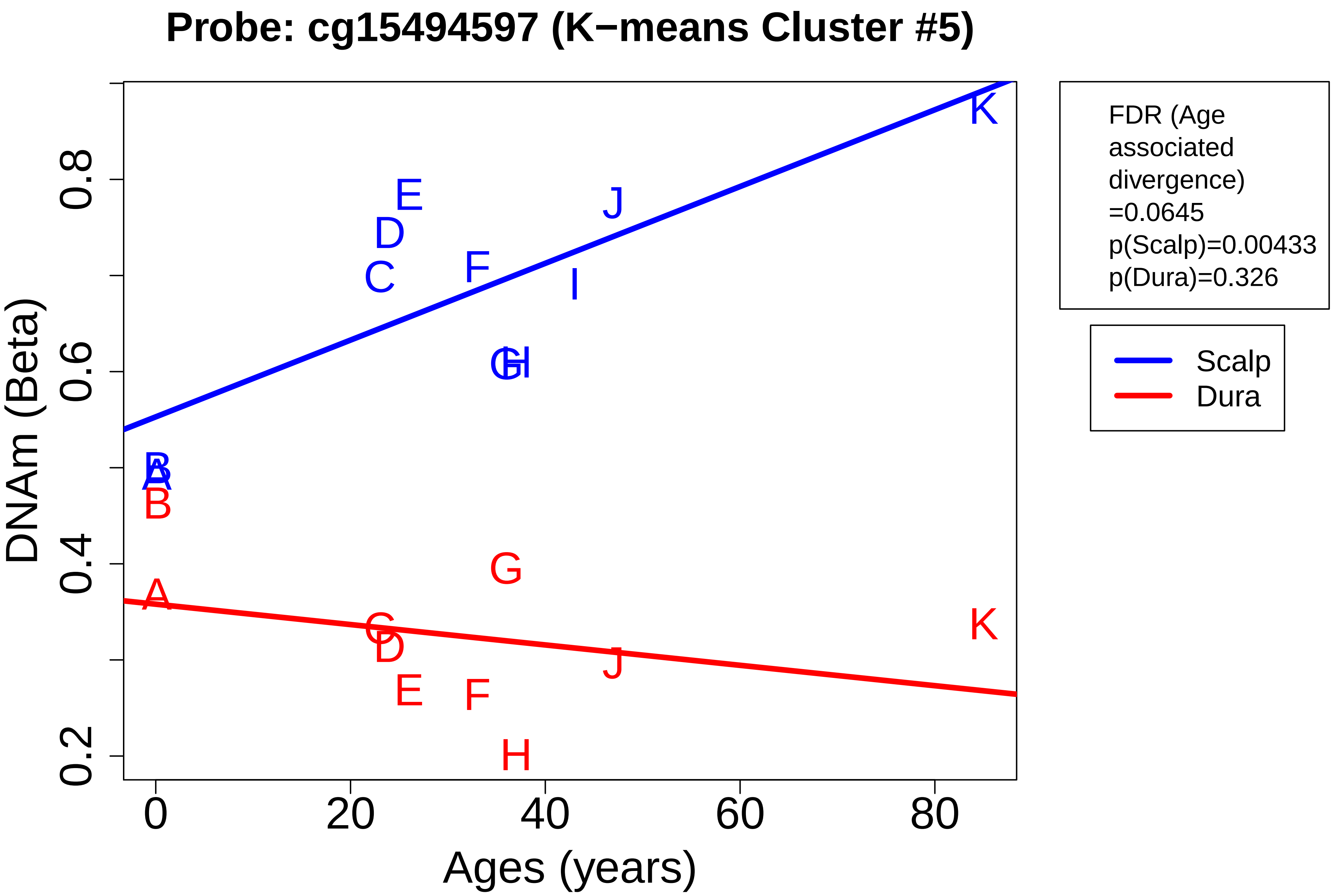

Probe: cg15494597 (K−means Cluster #5)
DNAm (Beta)
0.2
0.4
0.6
0.8
0
20
40
60
80
Ages (years)
FDR (Age associated divergence) =0.0645
p(Scalp)=0.00433
p(Dura)=0.326
Scalp
Dura
A
B
C
D
E
F
G
H
I
J
K

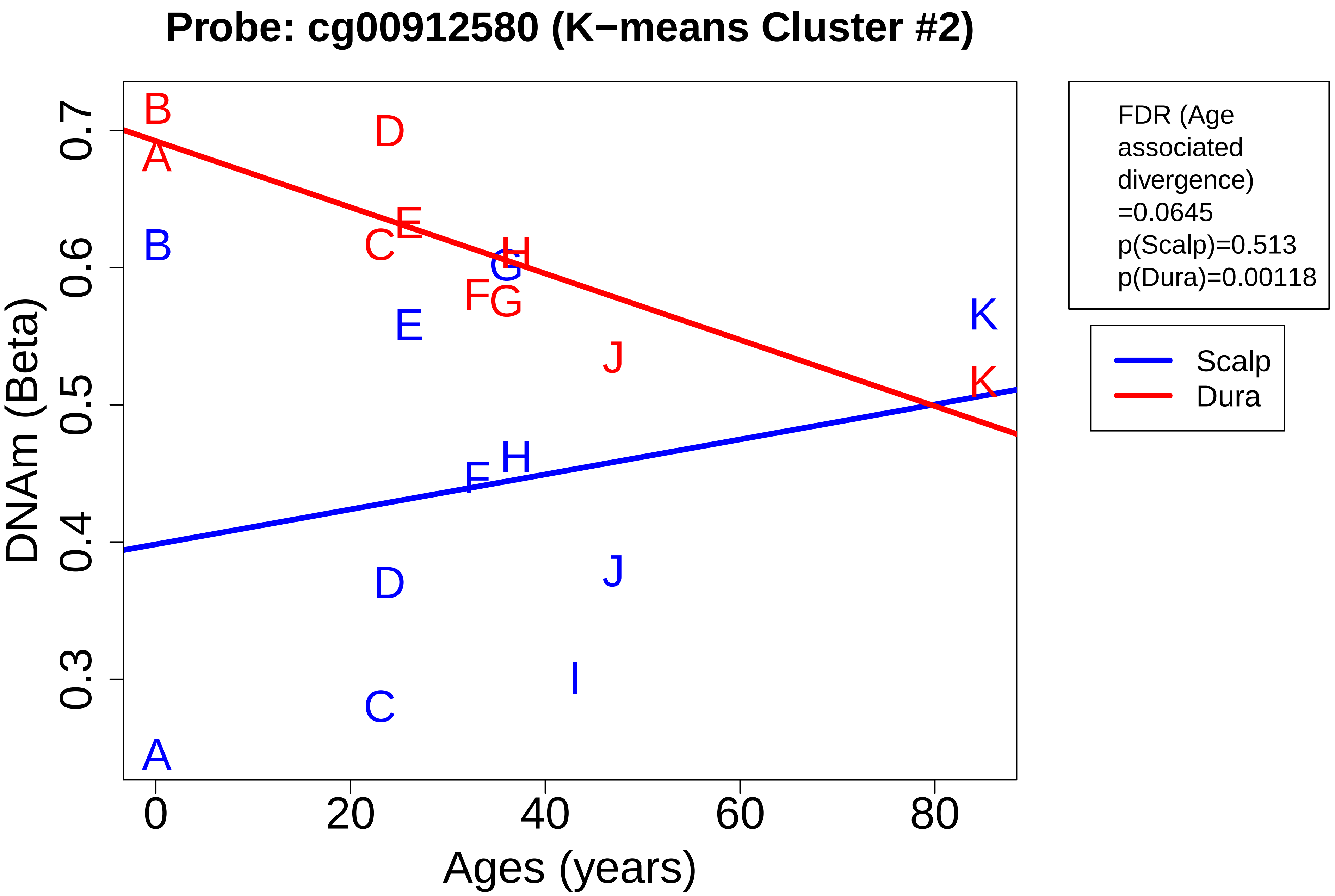
Probe: cg00912580 (K−means Cluster #2)
DNAm (Beta)
Ages (years)
0.3
0.4
0.5
0.6
0.7
0
20
40
60
80
FDR (Age associated divergence) =0.0645
p(Scalp)=0.513
p(Dura)=0.00118
Scalp
Dura

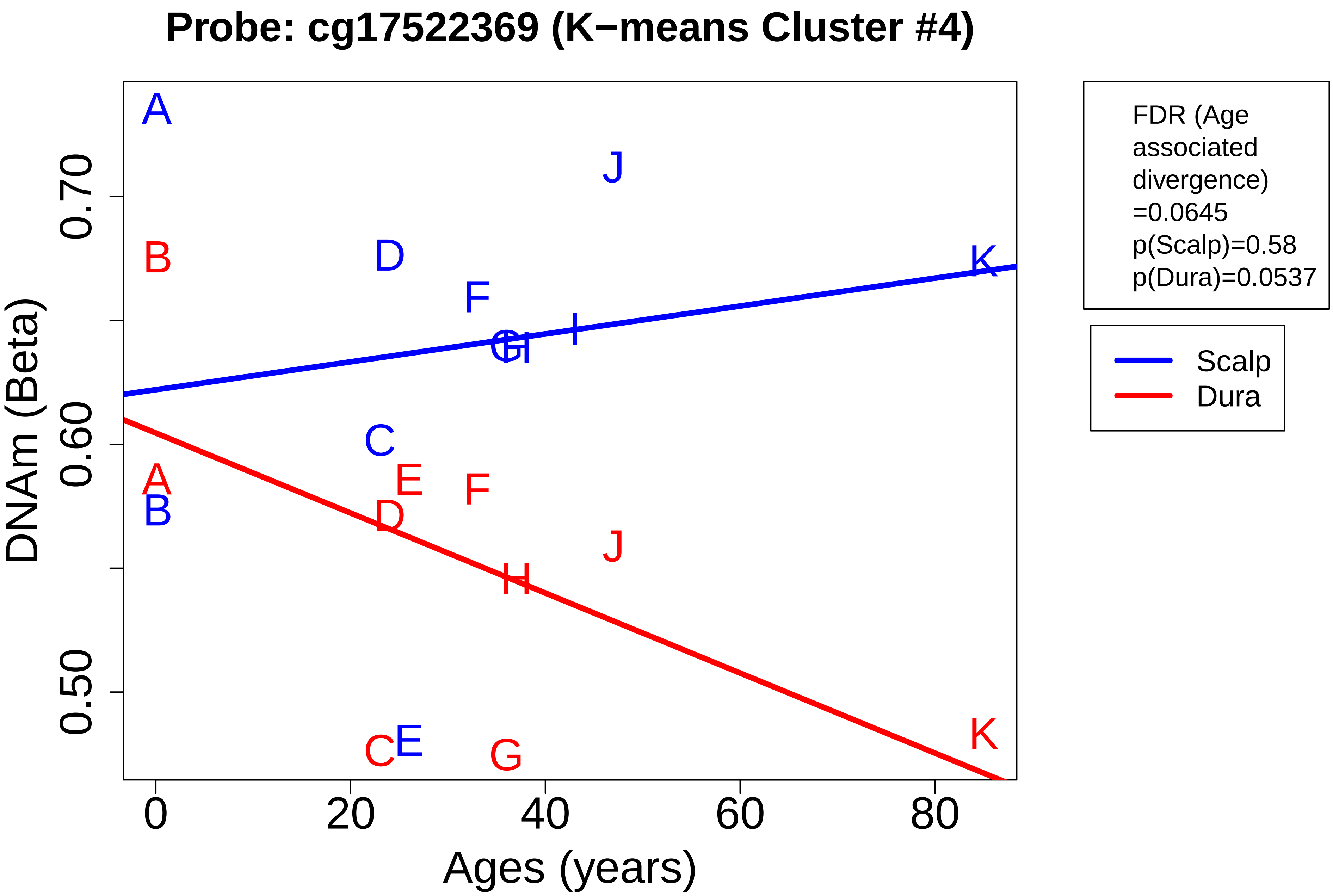

Probe: cg17522369 (K−means Cluster #4)
DNAm (Beta)
0.50
0.60
0.70
Ages (years)
0
20
40
60
80
A
B
C
D
E
F
G
H
I
J
K
FDR (Age associated divergence) =0.0645
p(Scalp)=0.58
p(Dura)=0.0537
Scalp
Dura

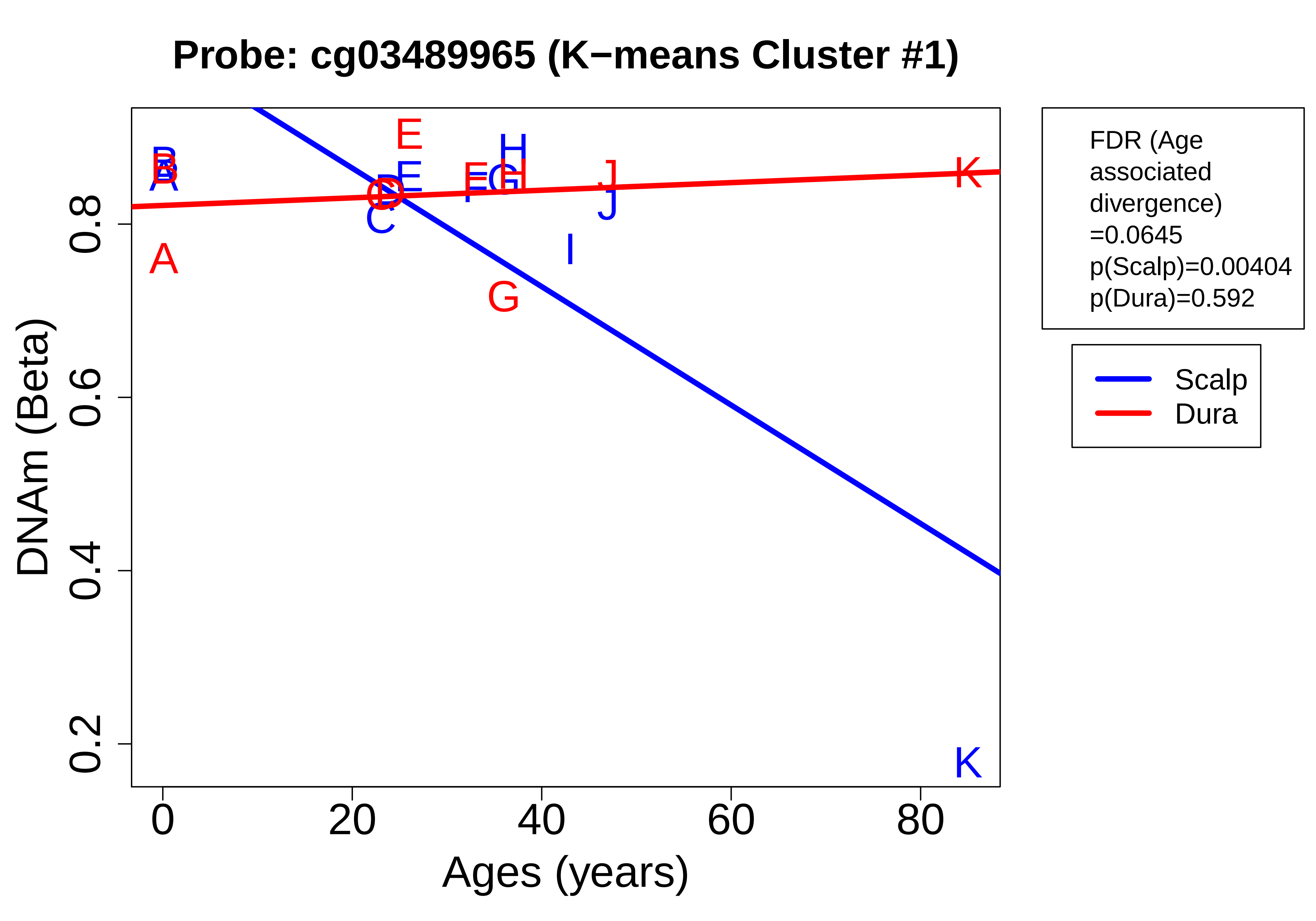

Probe: cg03489965 (K−means Cluster #1)
FDR (Age associated divergence) =0.0645
p(Scalp)=0.00404
p(Dura)=0.592
Scalp
Dura
DNAm (Beta)
0.2
0.4
0.6
0.8
0
20
40
60
80
Ages (years)

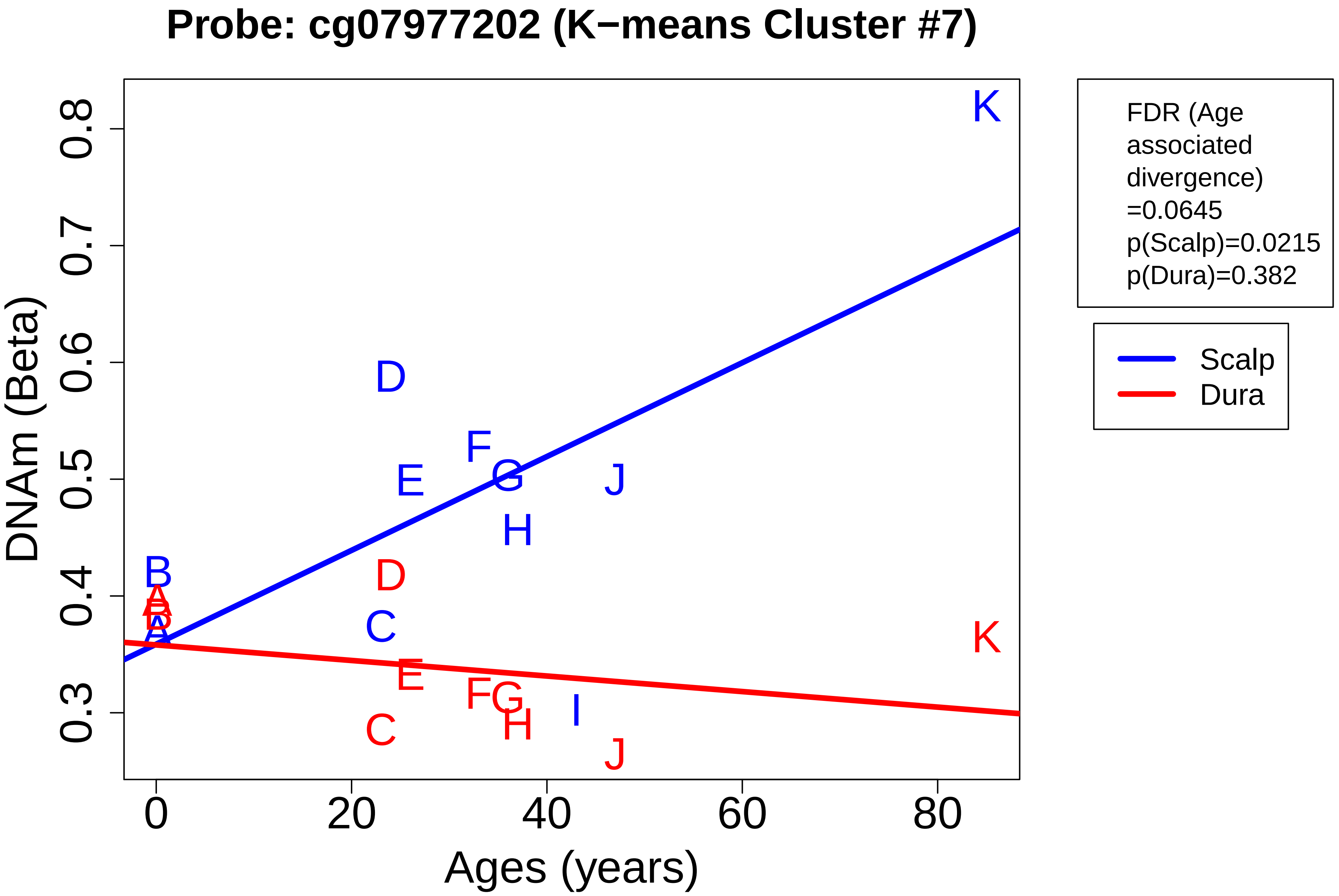

Probe: cg07977202 (K−means Cluster #7)
DNAm (Beta)
0.3
0.4
0.5
0.6
0.7
0.8
Ages (years)
0
20
40
60
80
FDR (Age associated divergence) =0.0645
p(Scalp)=0.0215
p(Dura)=0.382
Scalp
Dura
A
B
C
D
E
F
G
H
I
J
K

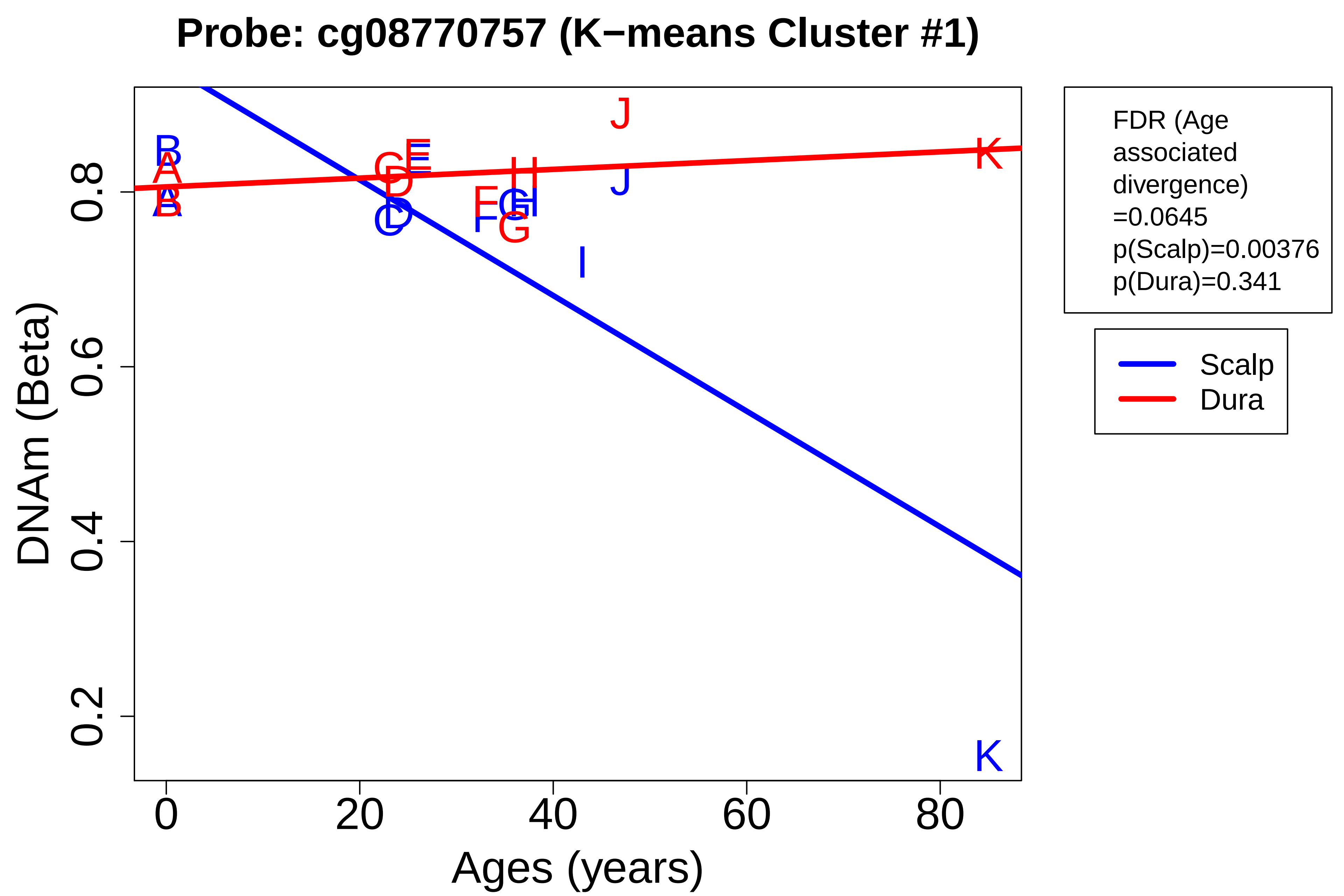
Probe: cg08770757 (K−means Cluster #1)
FDR (Age associated divergence) =0.0645
p(Scalp)=0.00376
p(Dura)=0.341
Scalp
Dura
DNAm (Beta)
Ages (years)
0.2
0.4
0.6
0.8
0
20
40
60
80

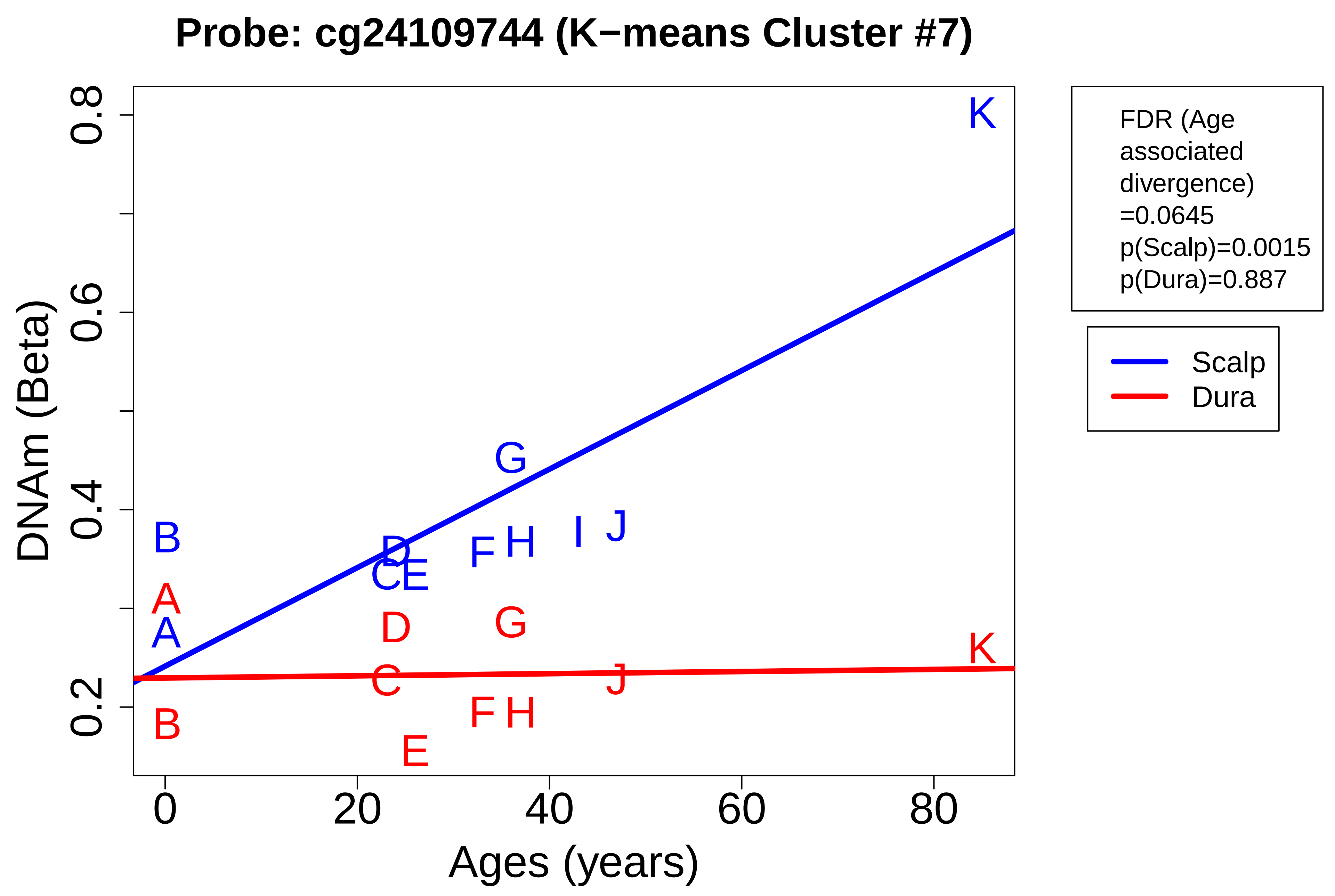

Probe: cg24109744 (K−means Cluster #7)
DNAm (Beta)
0.2
0.4
0.6
0.8
Ages (years)
0
20
40
60
80
FDR (Age associated divergence) =0.0645
p(Scalp)=0.0015
p(Dura)=0.887
Scalp
Dura
A
B
C
D
E
F
G
H
I
J
K

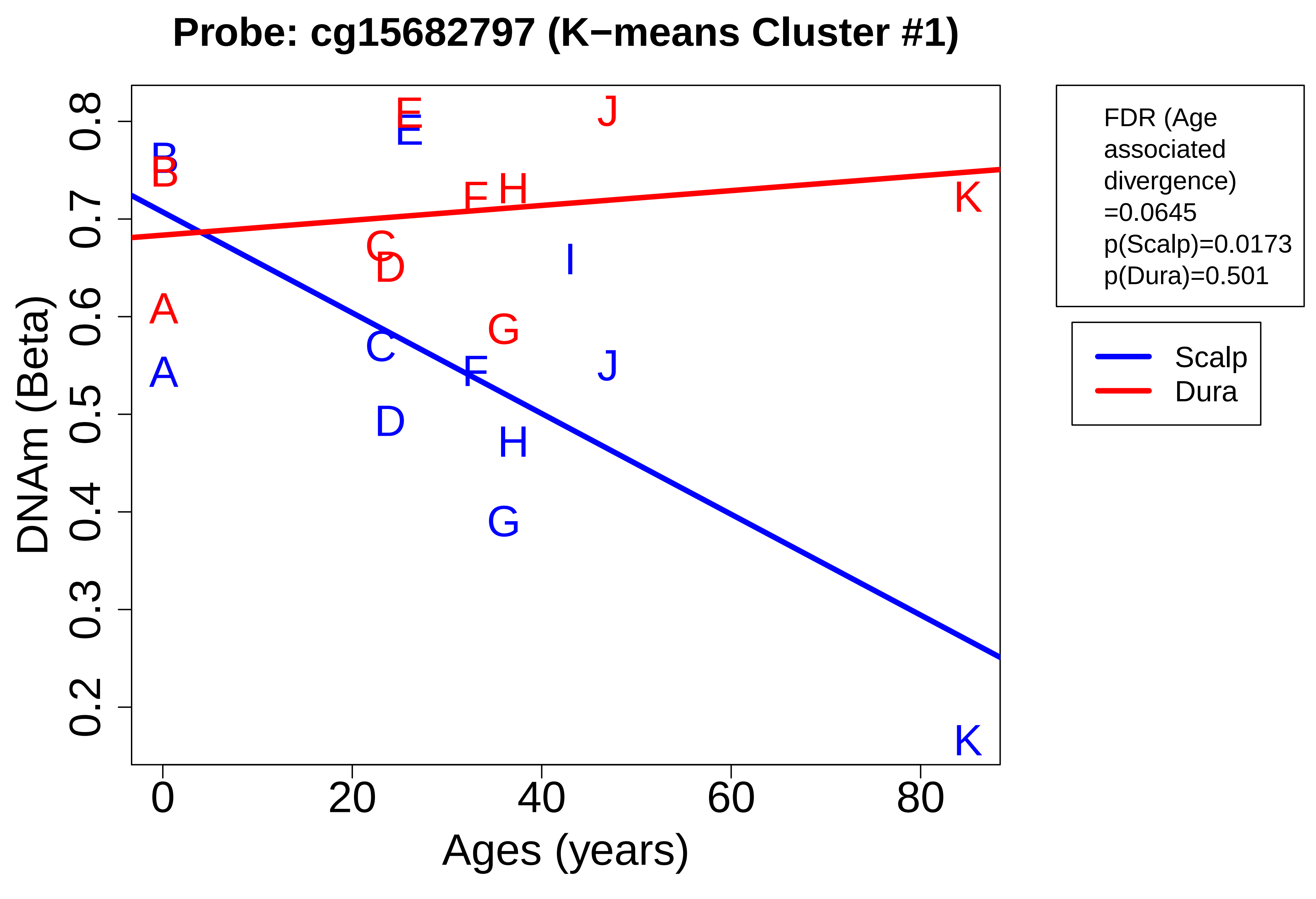

Probe: cg15682797 (K−means Cluster #1)
DNAm (Beta)
0.2
0.3
0.4
0.5
0.6
0.7
0.8
Ages (years)
0
20
40
60
80
FDR (Age associated divergence) =0.0645
p(Scalp)=0.0173
p(Dura)=0.501
Scalp
Dura
A
B
C
D
E
F
G
H
I
J
K

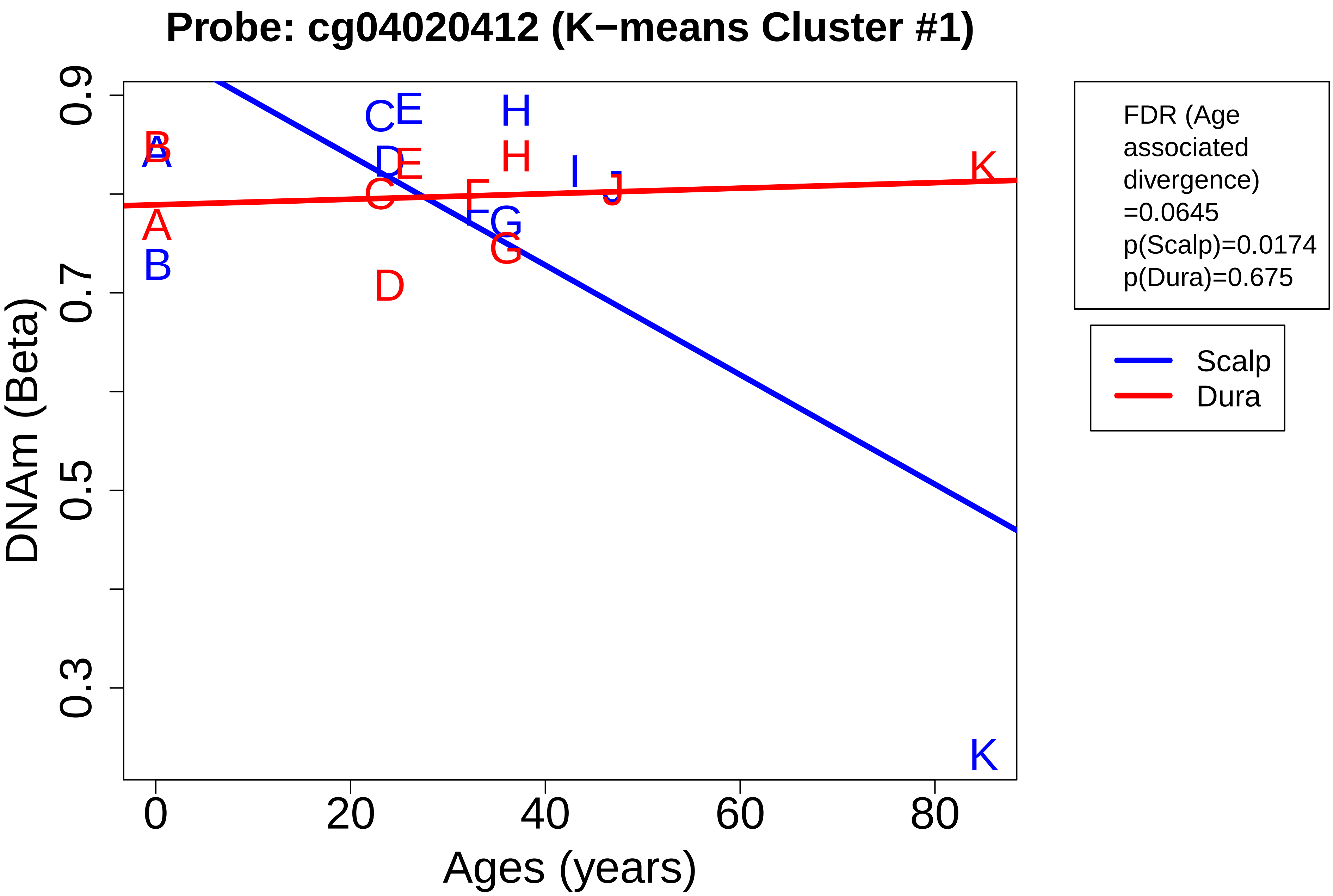

Probe: cg04020412 (K−means Cluster #1)
DNAm (Beta)
0.9
0.7
0.5
0.3
0
20
40
60
80
Ages (years)
FDR (Age associated divergence) =0.0645
p(Scalp)=0.0174
p(Dura)=0.675
Scalp
Dura
A
B
C
D
E
F
G
H
I
J
K

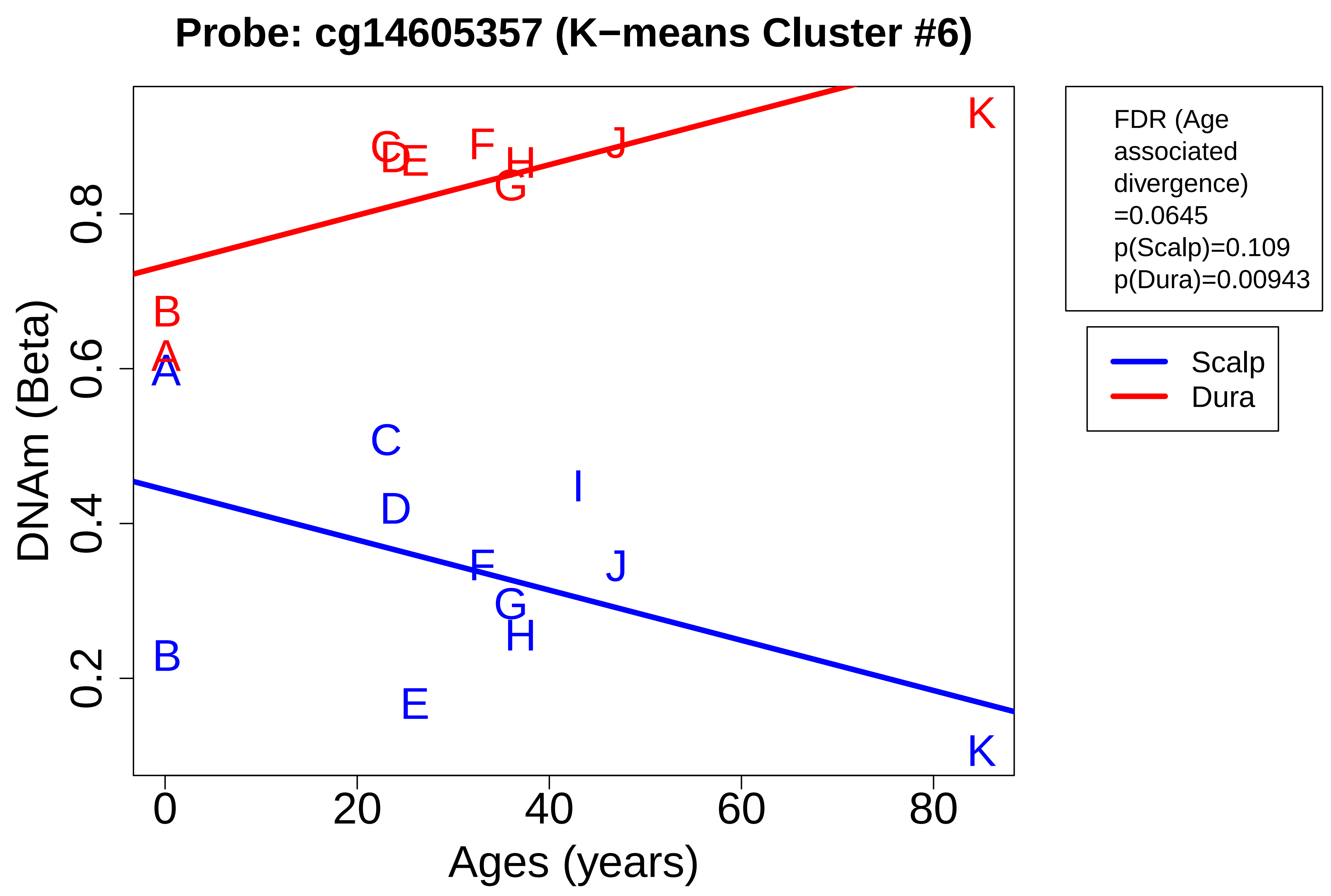
Probe: cg14605357 (K−means Cluster #6)
DNAm (Beta)
0.2
0.4
0.6
0.8
0
20
40
60
80
Ages (years)
FDR (Age associated divergence) =0.0645
p(Scalp)=0.109
p(Dura)=0.00943
Scalp
Dura
A
B
C
D
E
F
G
H
I
J
K

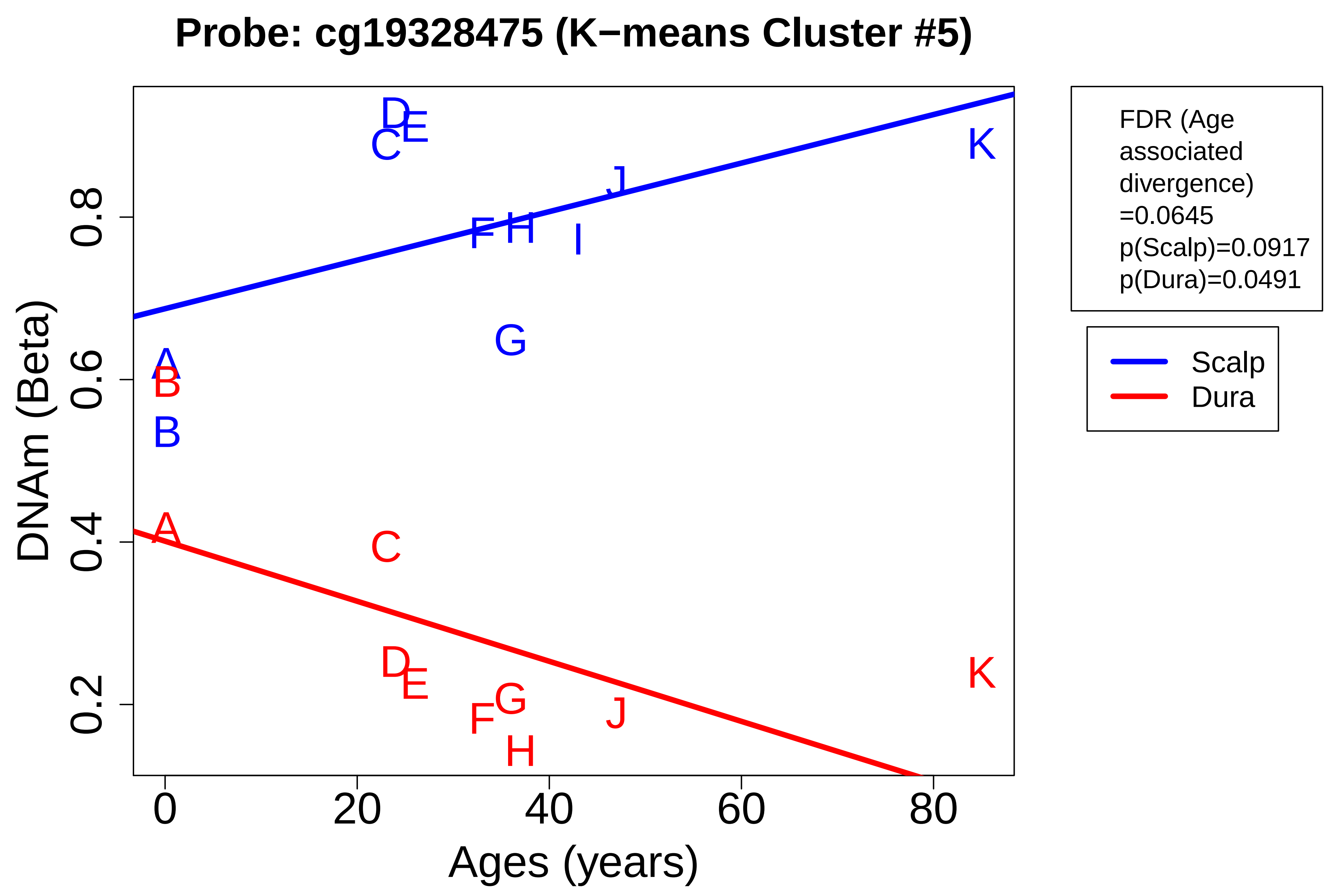

Probe: cg19328475 (K−means Cluster #5)
DNAm (Beta)
0.2
0.4
0.6
0.8
0
20
40
60
80
Ages (years)
FDR (Age associated divergence) =0.0645
p(Scalp)=0.0917
p(Dura)=0.0491
Scalp
Dura

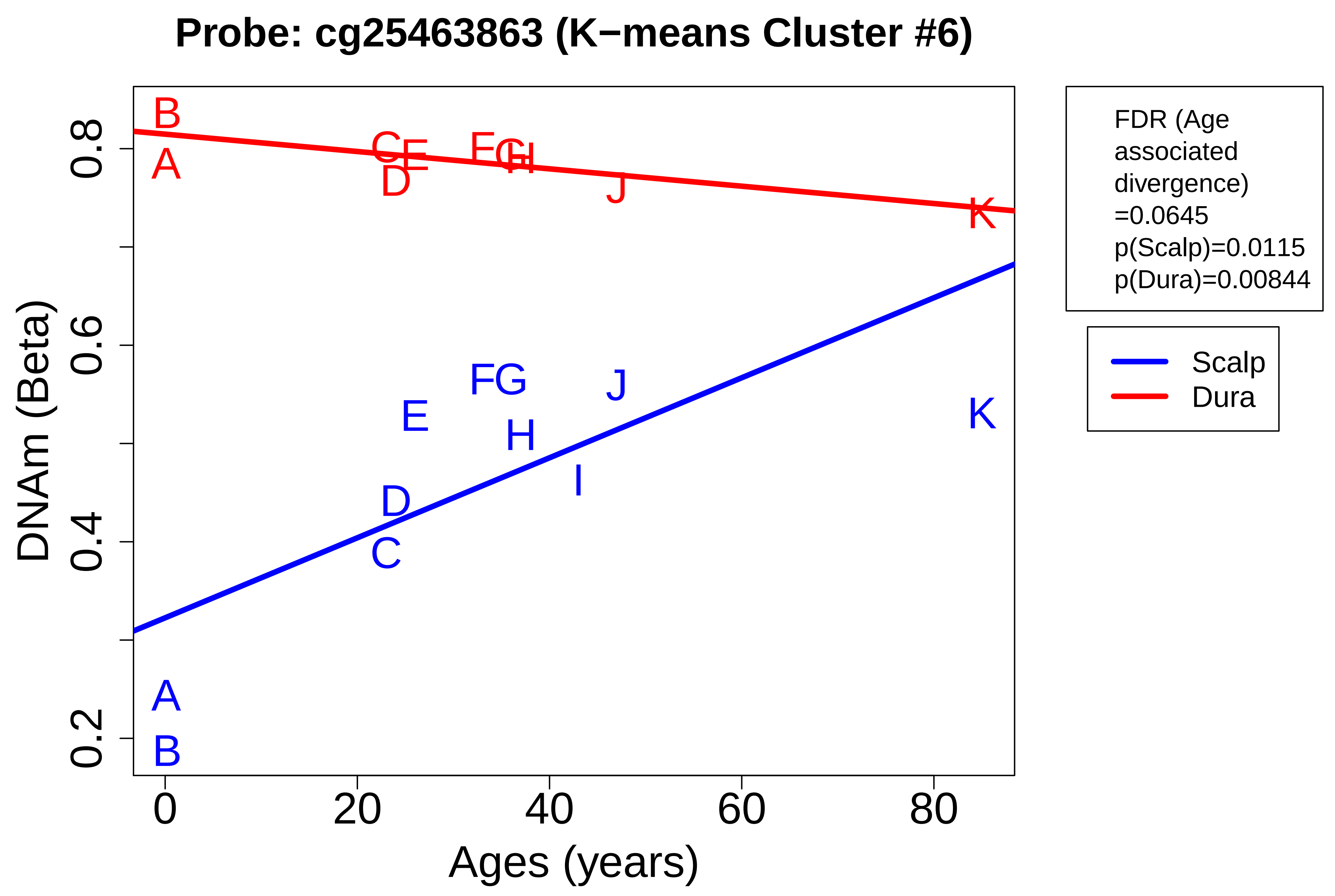

Probe: cg25463863 (K−means Cluster #6)
FDR (Age associated divergence) =0.0645
p(Scalp)=0.0115
p(Dura)=0.00844
Scalp
Dura
DNAm (Beta)
0.2
0.4
0.6
0.8
0
20
40
60
80
Ages (years)

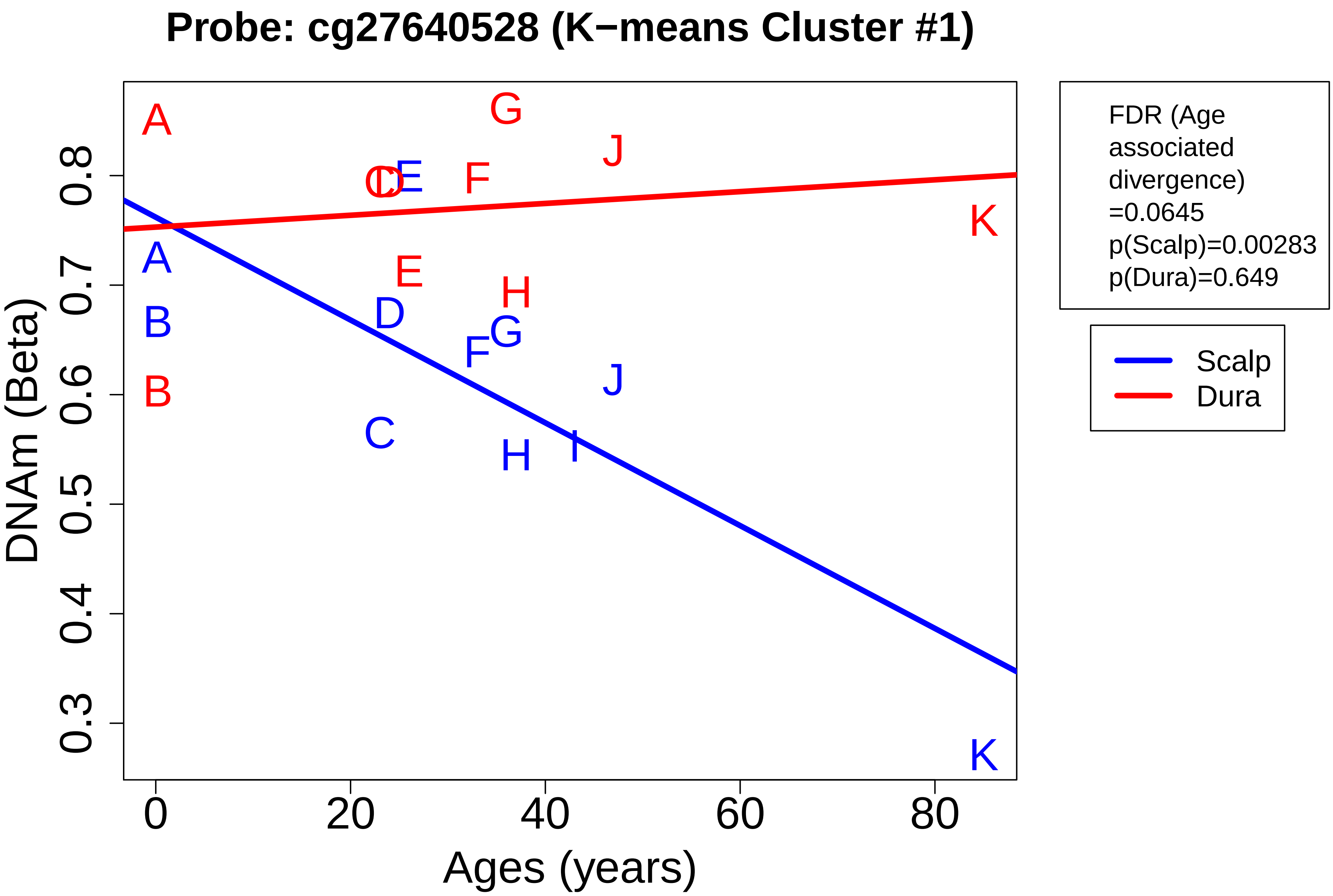

Probe: cg27640528 (K−means Cluster #1)
DNAm (Beta)
0.3
0.4
0.5
0.6
0.7
0.8
0
20
40
60
80
Ages (years)
A
B
C
D
E
F
G
H
I
J
K
FDR (Age associated divergence) =0.0645
p(Scalp)=0.00283
p(Dura)=0.649
Scalp
Dura

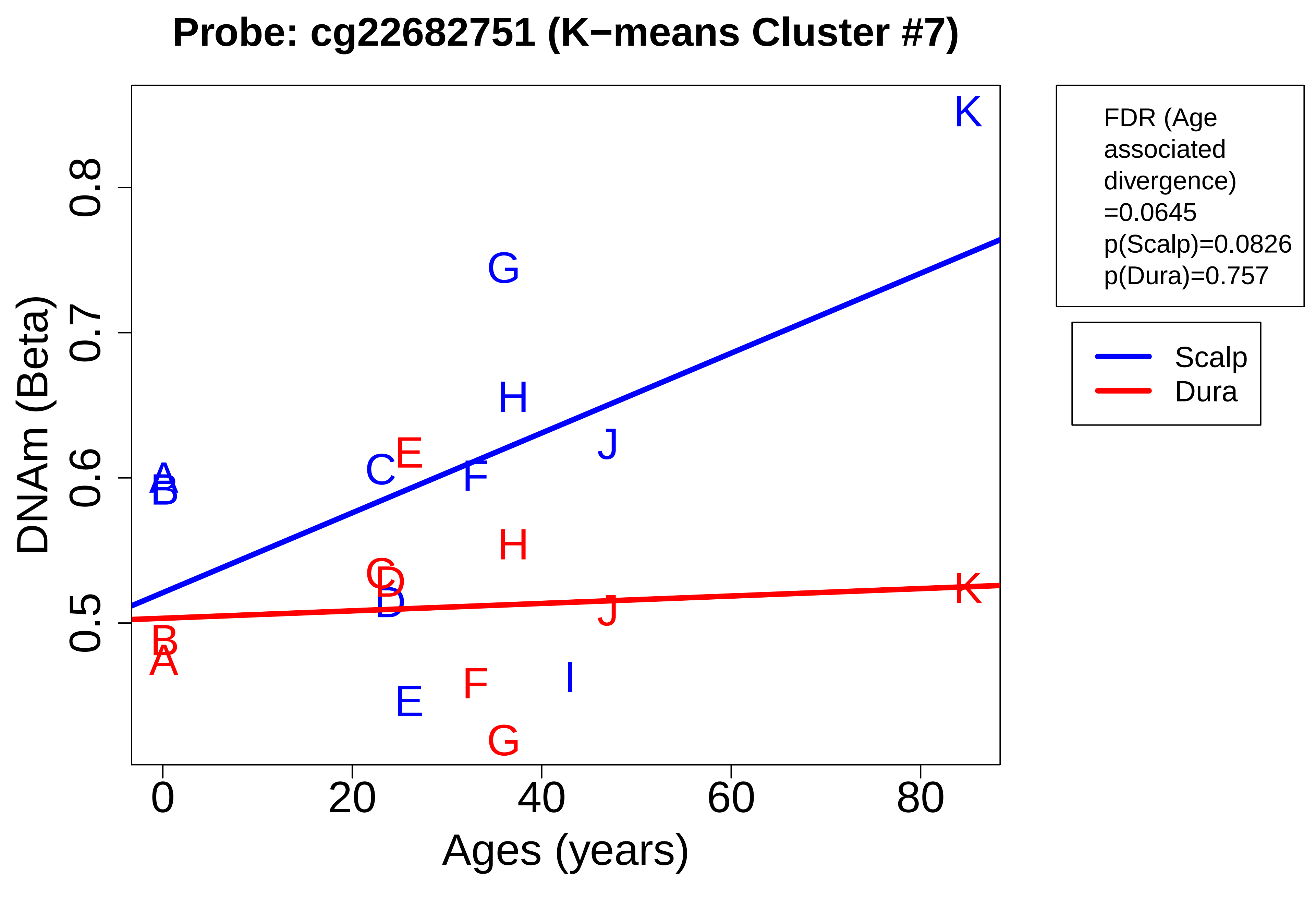
Probe: cg22682751 (K−means Cluster #7)
DNAm (Beta)
0.5
0.6
0.7
0.8
0
20
40
60
80
Ages (years)
FDR (Age associated divergence) =0.0645
p(Scalp)=0.0826
p(Dura)=0.757
Scalp
Dura
A
B
C
D
E
F
G
H
I
J
K
A
B
C
D
E
F
G
H
J
K

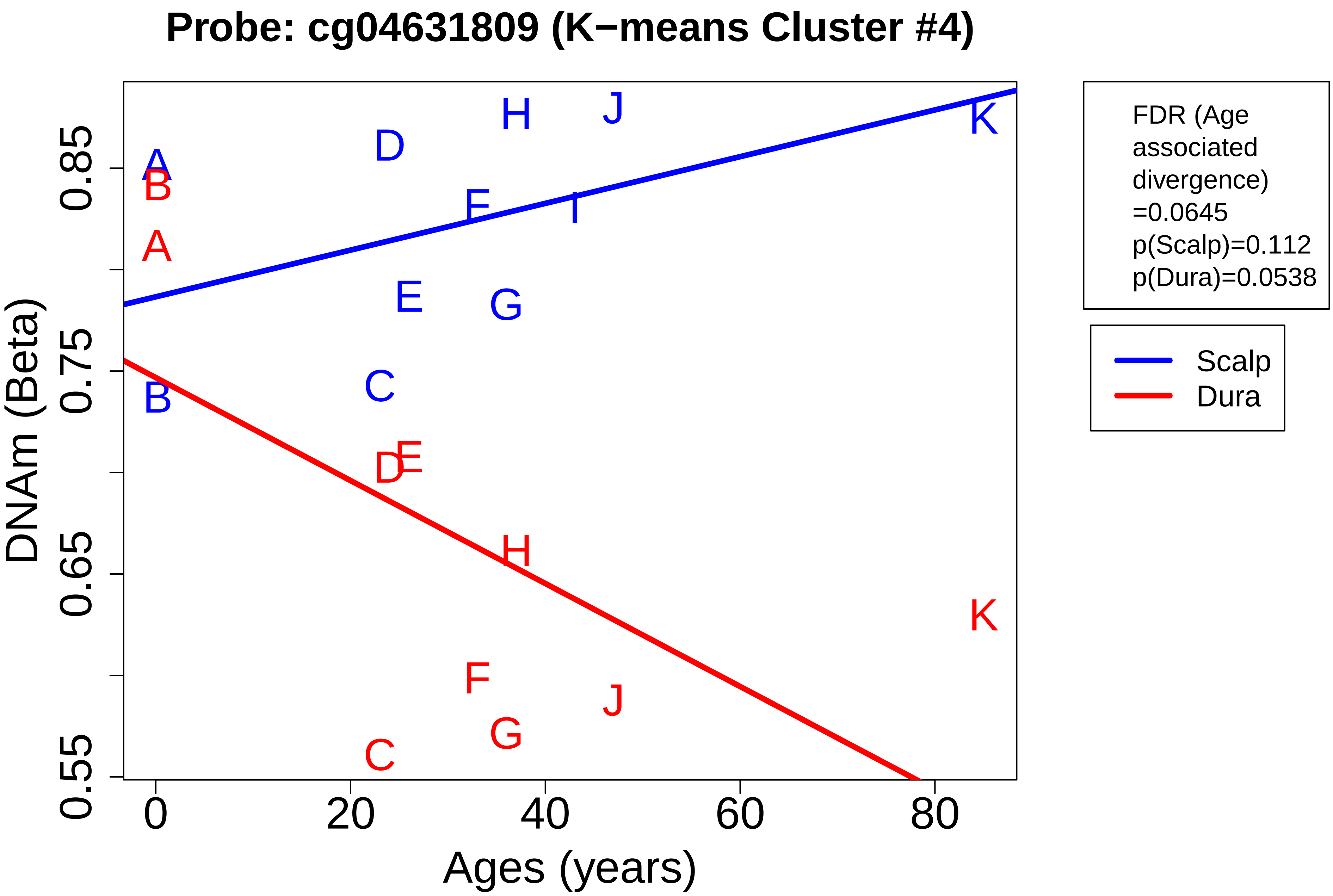
Probe: cg04631809 (K−means Cluster #4)
DNAm (Beta)
0.55
0.65
0.75
0.85
0
20
40
60
80
Ages (years)
FDR (Age associated divergence) =0.0645
p(Scalp)=0.112
p(Dura)=0.0538
Scalp
Dura

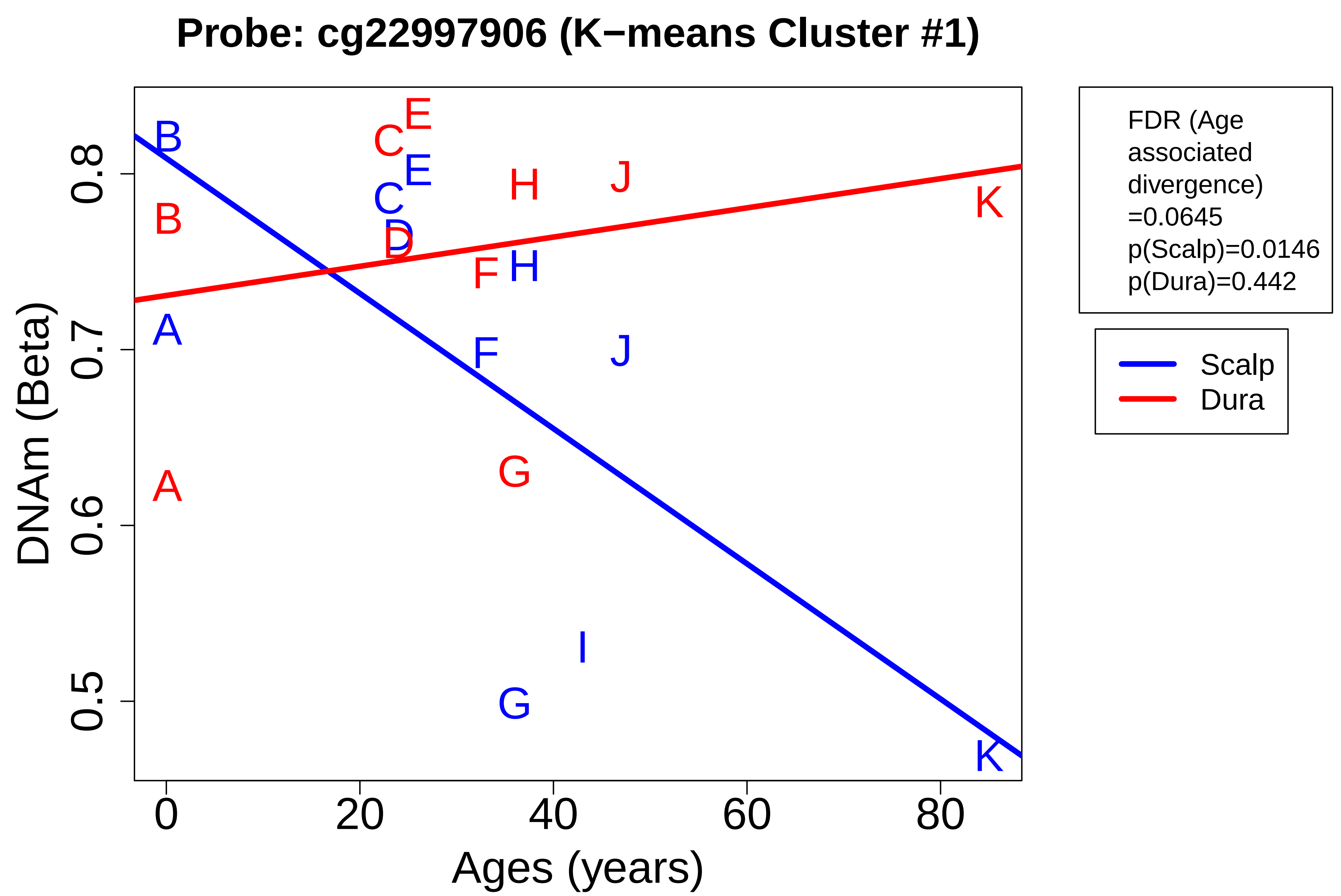

Probe: cg22997906 (K−means Cluster #1)
DNAm (Beta)
0.5
0.6
0.7
0.8
Ages (years)
0
20
40
60
80
FDR (Age associated divergence) =0.0645
p(Scalp)=0.0146
p(Dura)=0.442
Scalp
Dura

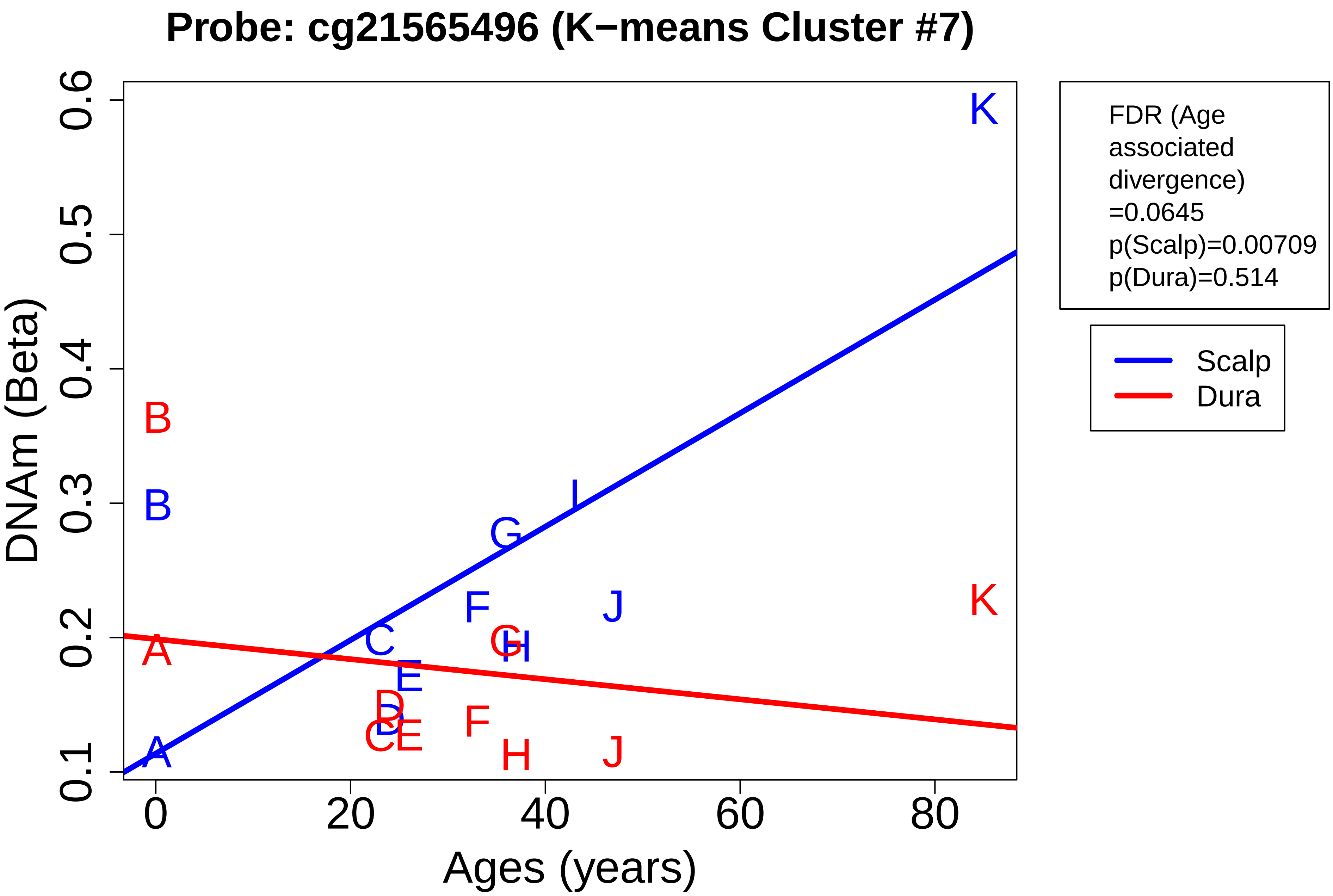

Probe: cg21565496 (K−means Cluster #7)
DNAm (Beta)
0.1
0.2
0.3
0.4
0.5
0.6
Ages (years)
0
20
40
60
80
FDR (Age associated divergence) =0.0645
p(Scalp)=0.00709
p(Dura)=0.514
Scalp
Dura

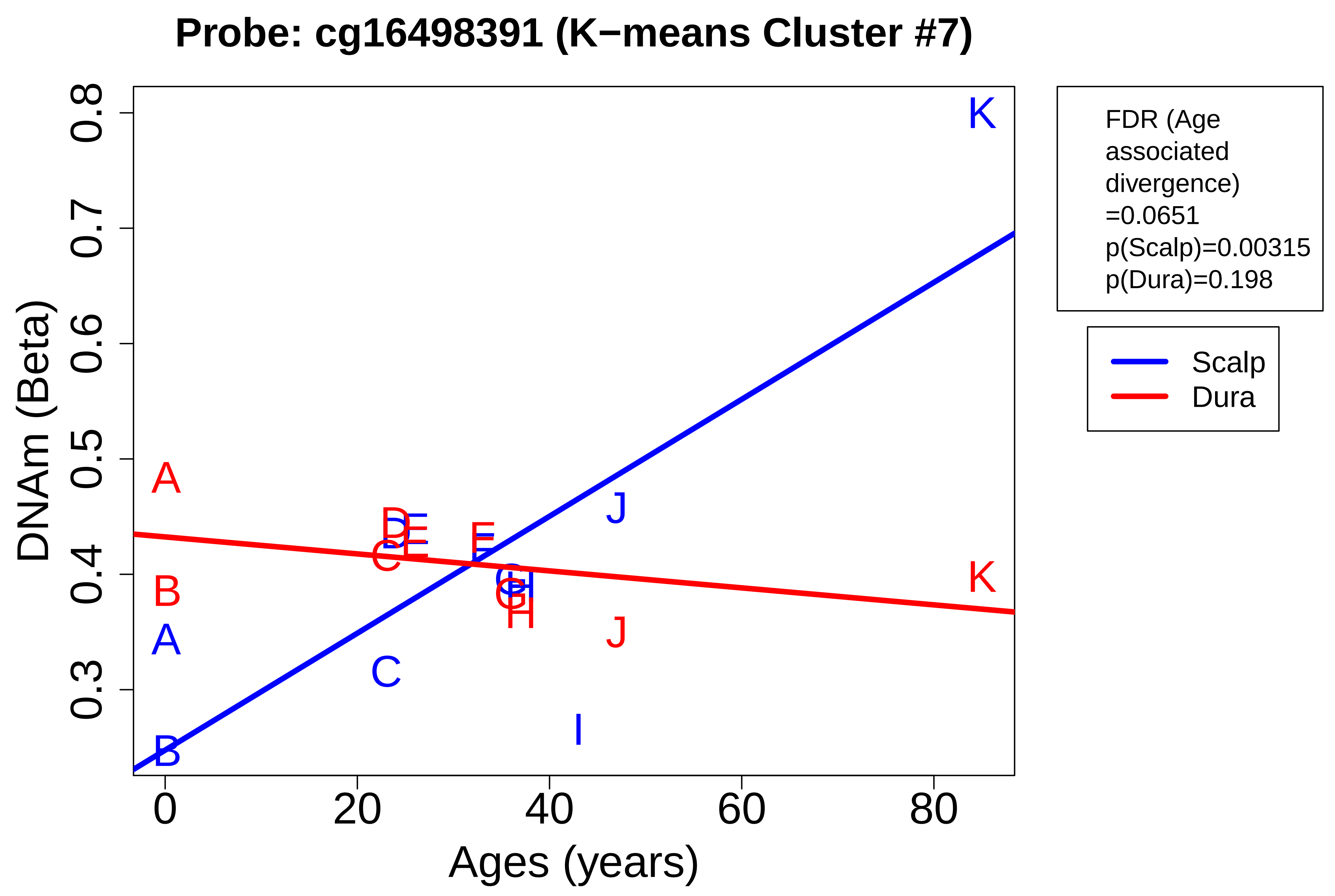

Probe: cg16498391 (K−means Cluster #7)
DNAm (Beta)
Ages (years)
0.3
0.4
0.5
0.6
0.7
0.8
0
20
40
60
80
FDR (Age associated divergence) =0.0651
p(Scalp)=0.00315
p(Dura)=0.198
Scalp
Dura

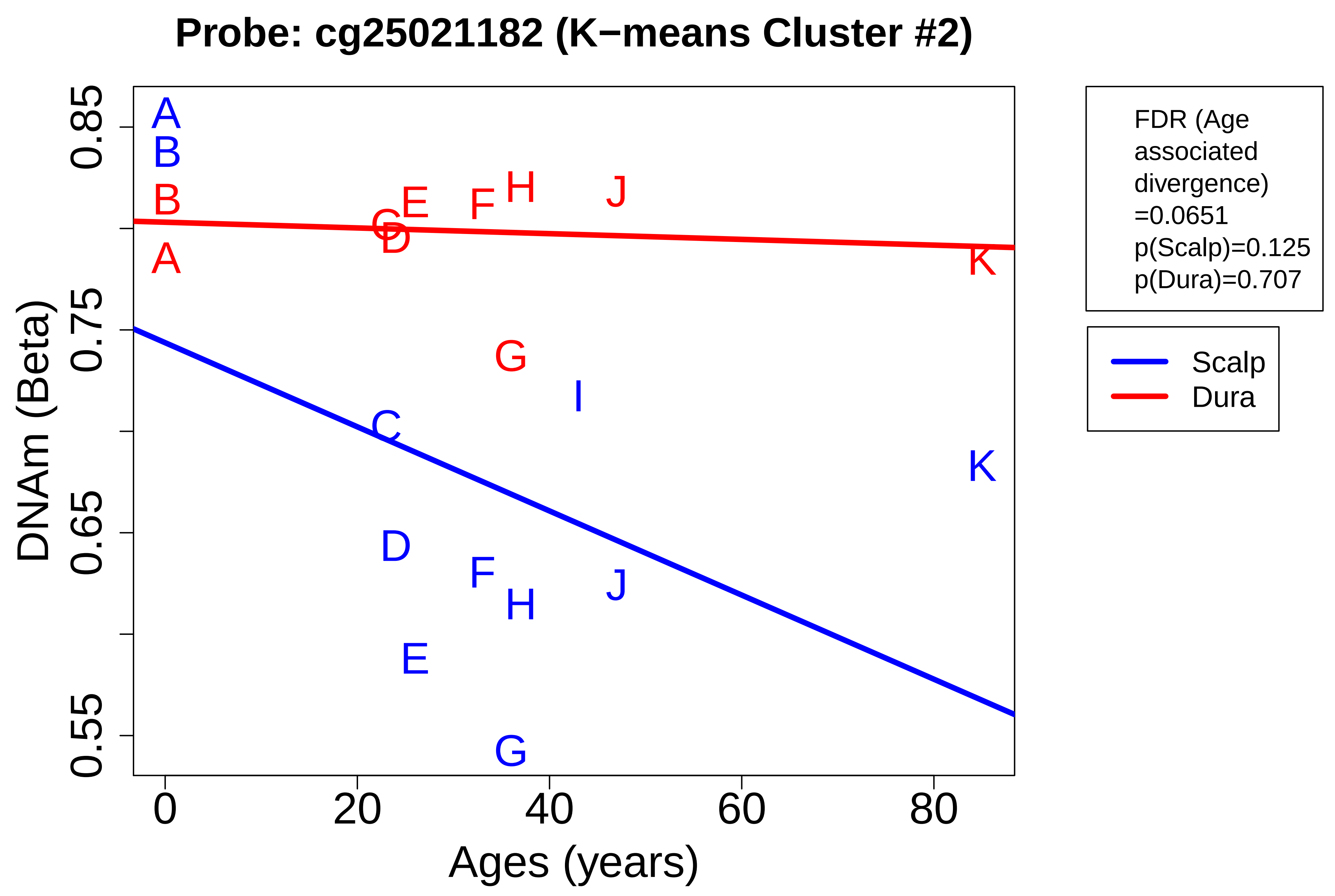

Probe: cg25021182 (K−means Cluster #2)
DNAm (Beta)
0.55
0.65
0.75
0.85
Ages (years)
0
20
40
60
80
A
B
B
E
F
H
J
G
D
A
K
G
I
C
K
D
F
J
H
E
G
FDR (Age associated divergence) =0.0651
p(Scalp)=0.125
p(Dura)=0.707
Scalp
Dura

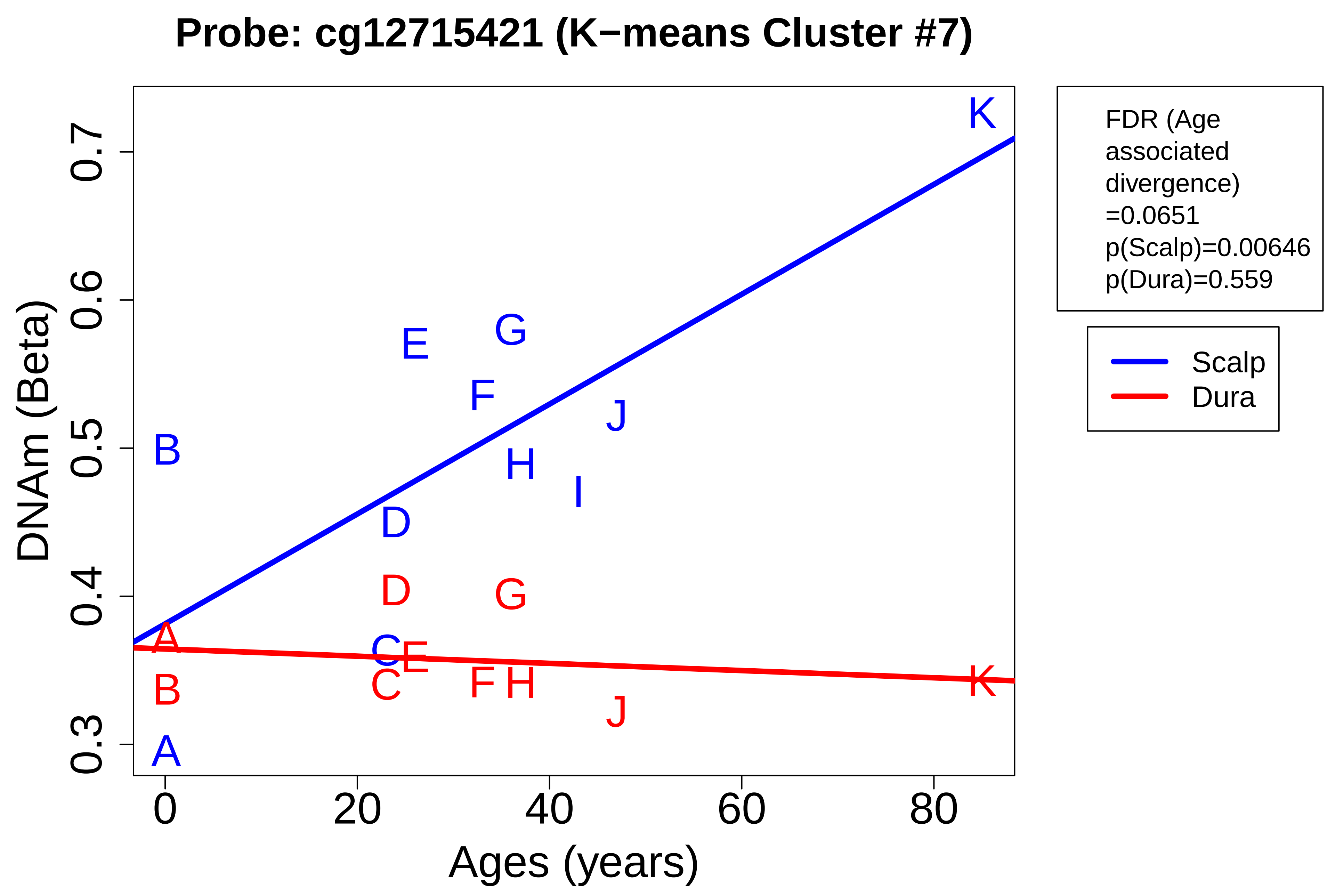
Probe: cg12715421 (K−means Cluster #7)
DNAm (Beta)
0.3
0.4
0.5
0.6
0.7
Ages (years)
0
20
40
60
80
FDR (Age associated divergence) =0.0651
p(Scalp)=0.00646
p(Dura)=0.559
Scalp
Dura
A
B
C
D
E
F
G
H
I
J
K

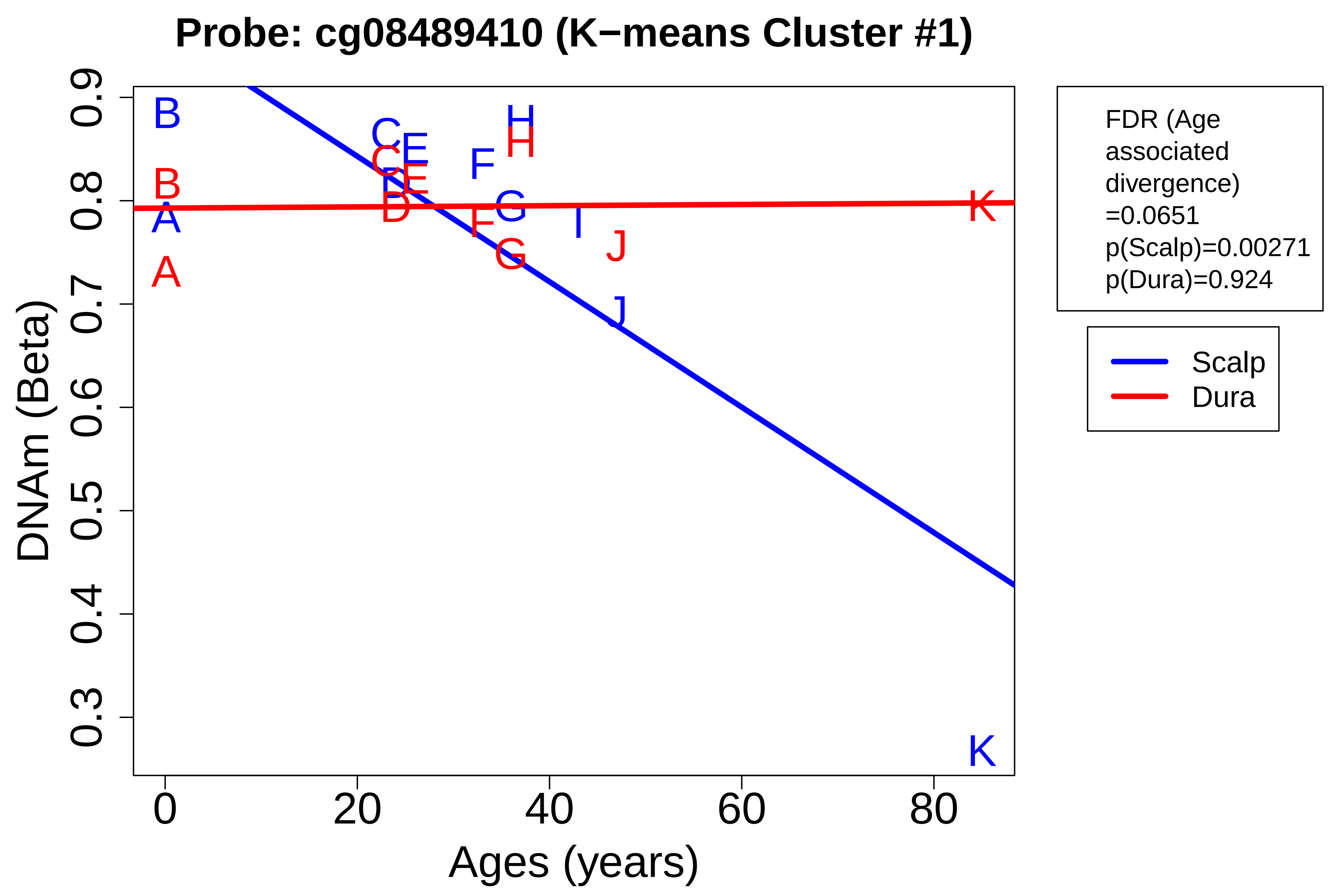
Probe: cg08489410 (K−means Cluster #1)
DNAm (Beta)
0.3
0.4
0.5
0.6
0.7
0.8
0.9
Ages (years)
0
20
40
60
80
A
B
C
D
E
F
G
H
I
J
K
FDR (Age
associated
divergence)
=0.0651
p(Scalp)=0.00271
p(Dura)=0.924
Scalp
Dura

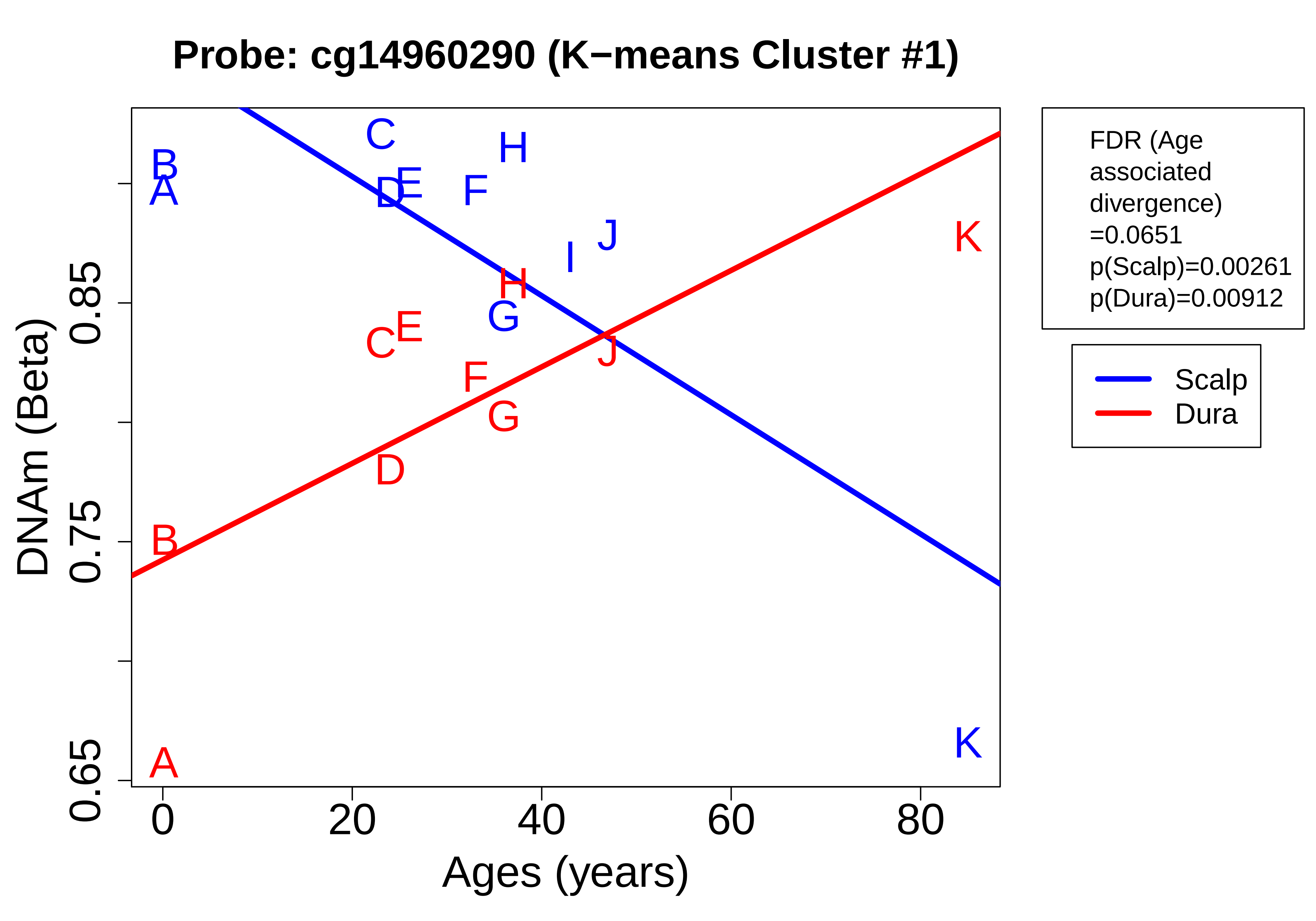

Probe: cg14960290 (K−means Cluster #1)
FDR (Age associated divergence) =0.0651
p(Scalp)=0.00261
p(Dura)=0.00912
Scalp
Dura
DNAm (Beta)
0.65
0.75
0.85
0
20
40
60
80
Ages (years)
A
B
C
D
E
F
G
H
I
J
K

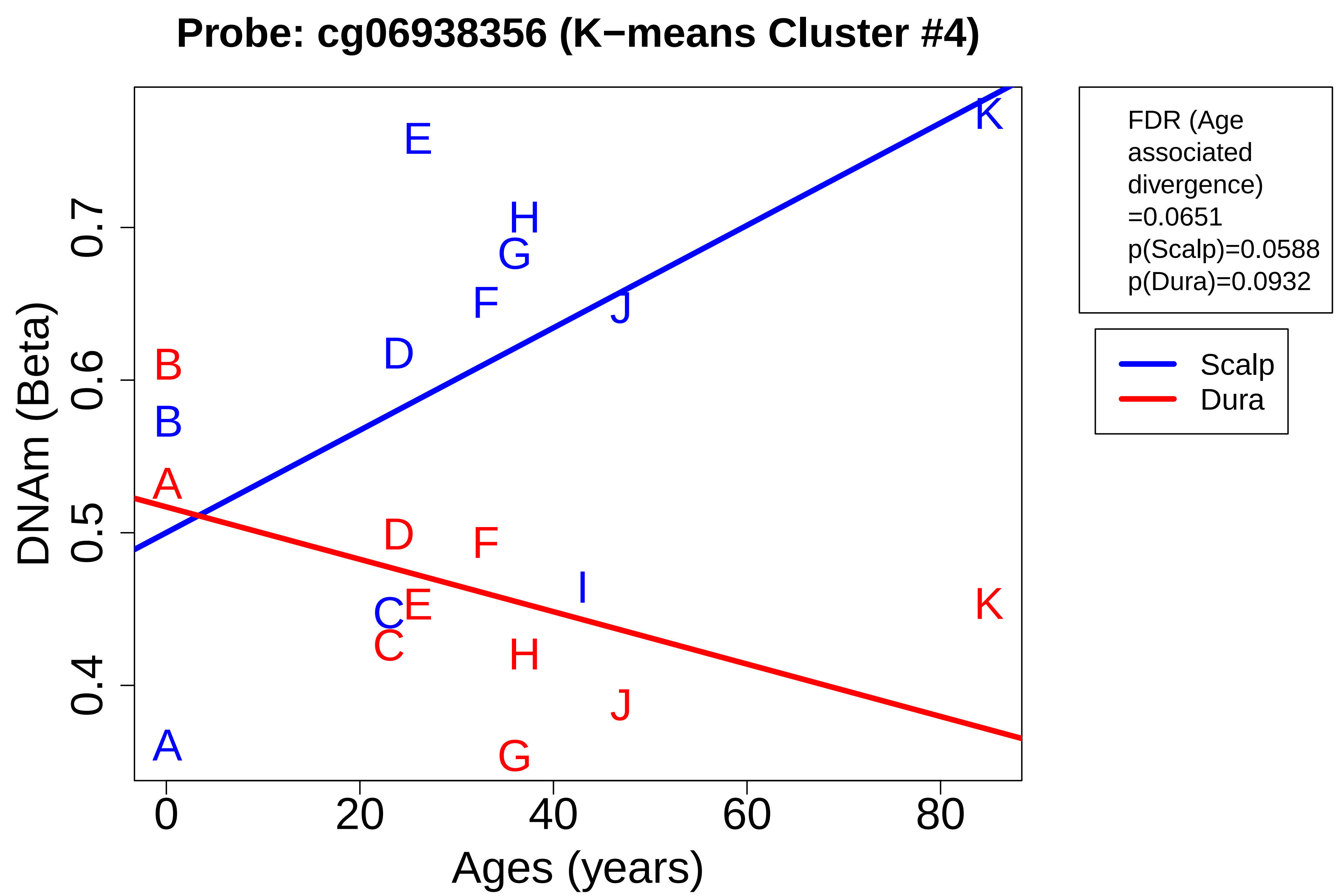

Probe: cg06938356 (K−means Cluster #4)
DNAm (Beta)
0.4
0.5
0.6
0.7
0
20
40
60
80
Ages (years)
A
B
C
D
E
F
G
H
I
J
K
A
B
C
D
E
F
G
H
J
K
FDR (Age associated divergence) =0.0651
p(Scalp)=0.0588
p(Dura)=0.0932
Scalp
Dura

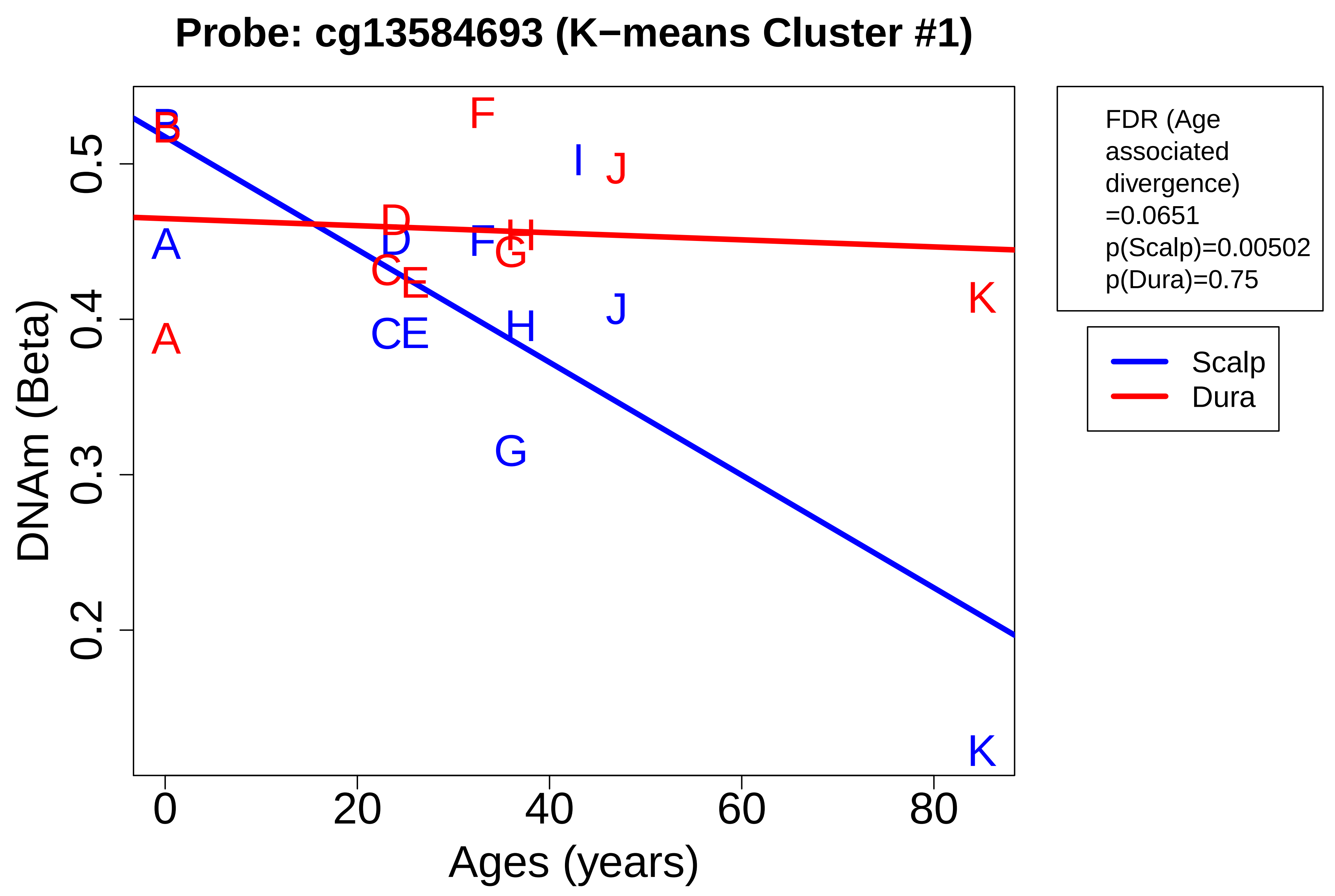

Probe: cg13584693 (K−means Cluster #1)
DNAm (Beta)
0.2
0.3
0.4
0.5
0
20
40
60
80
Ages (years)
A
B
C
D
E
F
G
H
I
J
K
FDR (Age associated divergence) =0.0651
p(Scalp)=0.00502
p(Dura)=0.75
Scalp
Dura

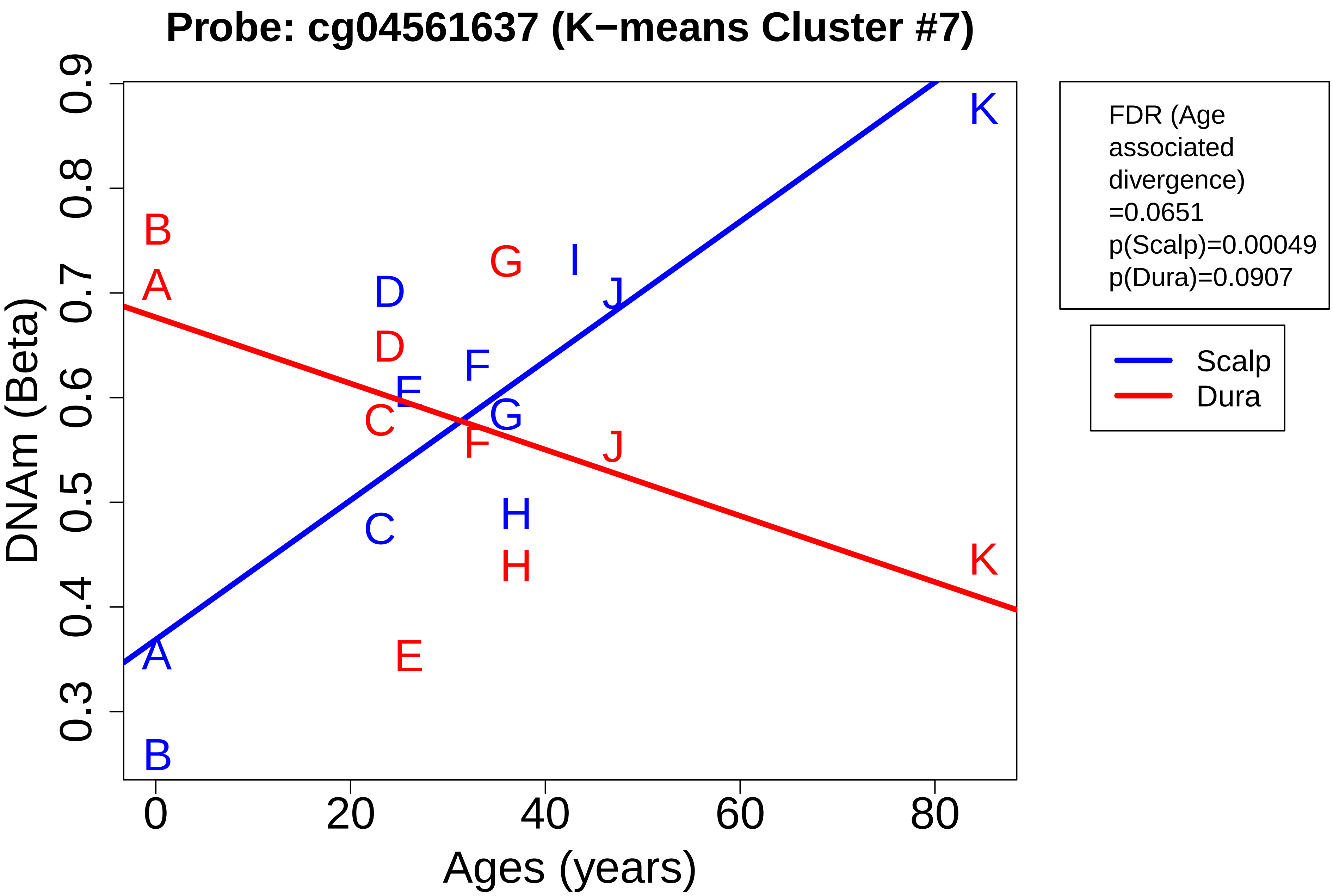

Probe: cg04561637 (K−means Cluster #7)
DNAm (Beta)
0.3
0.4
0.5
0.6
0.7
0.8
0.9
Ages (years)
0
20
40
60
80
A
B
C
D
E
F
G
H
I
J
K
A
B
C
D
E
F
G
H
J
K
FDR (Age associated divergence) =0.0651
p(Scalp)=0.00049
p(Dura)=0.0907
Scalp
Dura

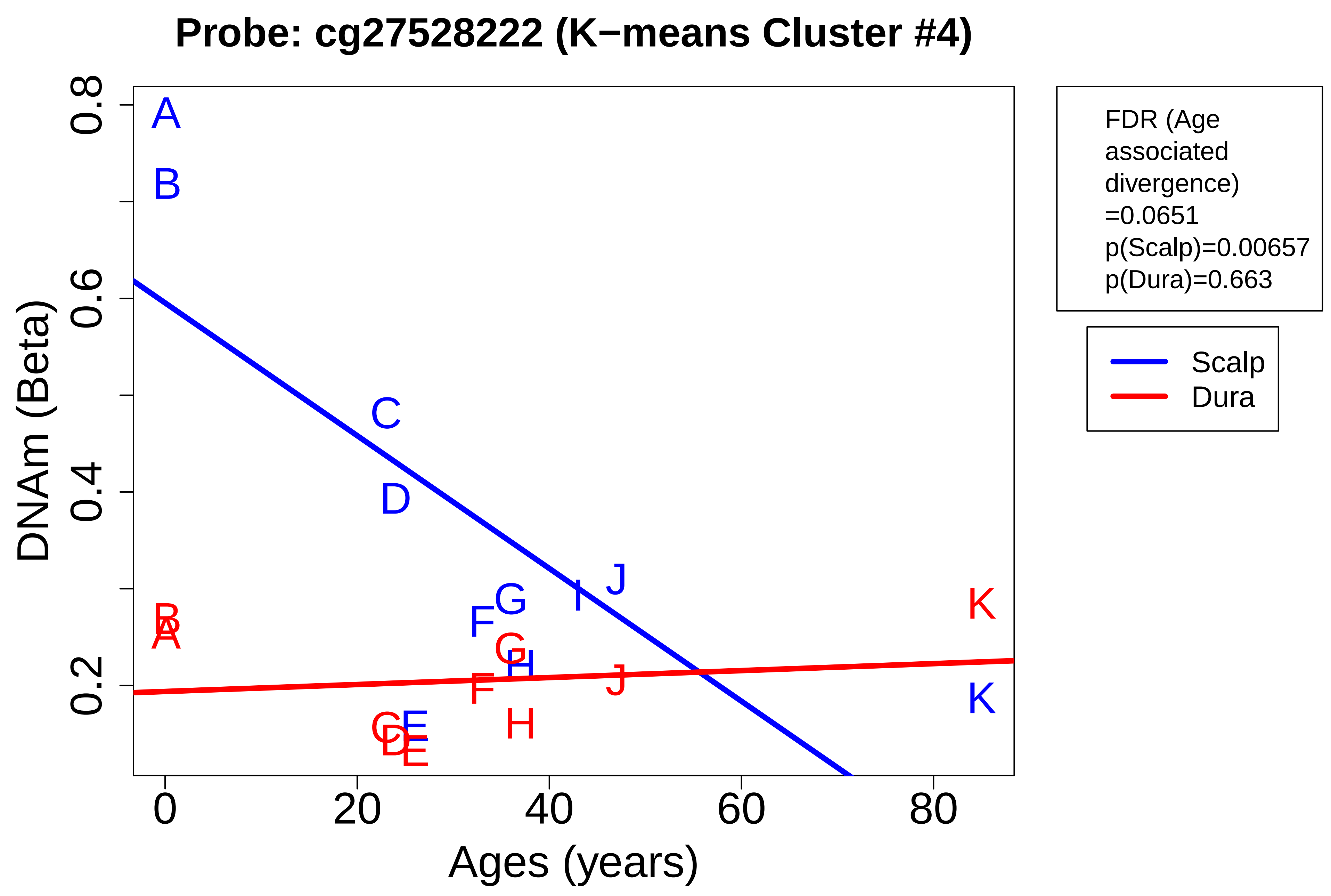

Probe: cg27528222 (K−means Cluster #4)
DNAm (Beta)
0.2
0.4
0.6
0.8
0
20
40
60
80
Ages (years)
FDR (Age associated divergence) =0.0651
p(Scalp)=0.00657
p(Dura)=0.663
Scalp
Dura
A
B
C
D
E
F
G
H
I
J
K

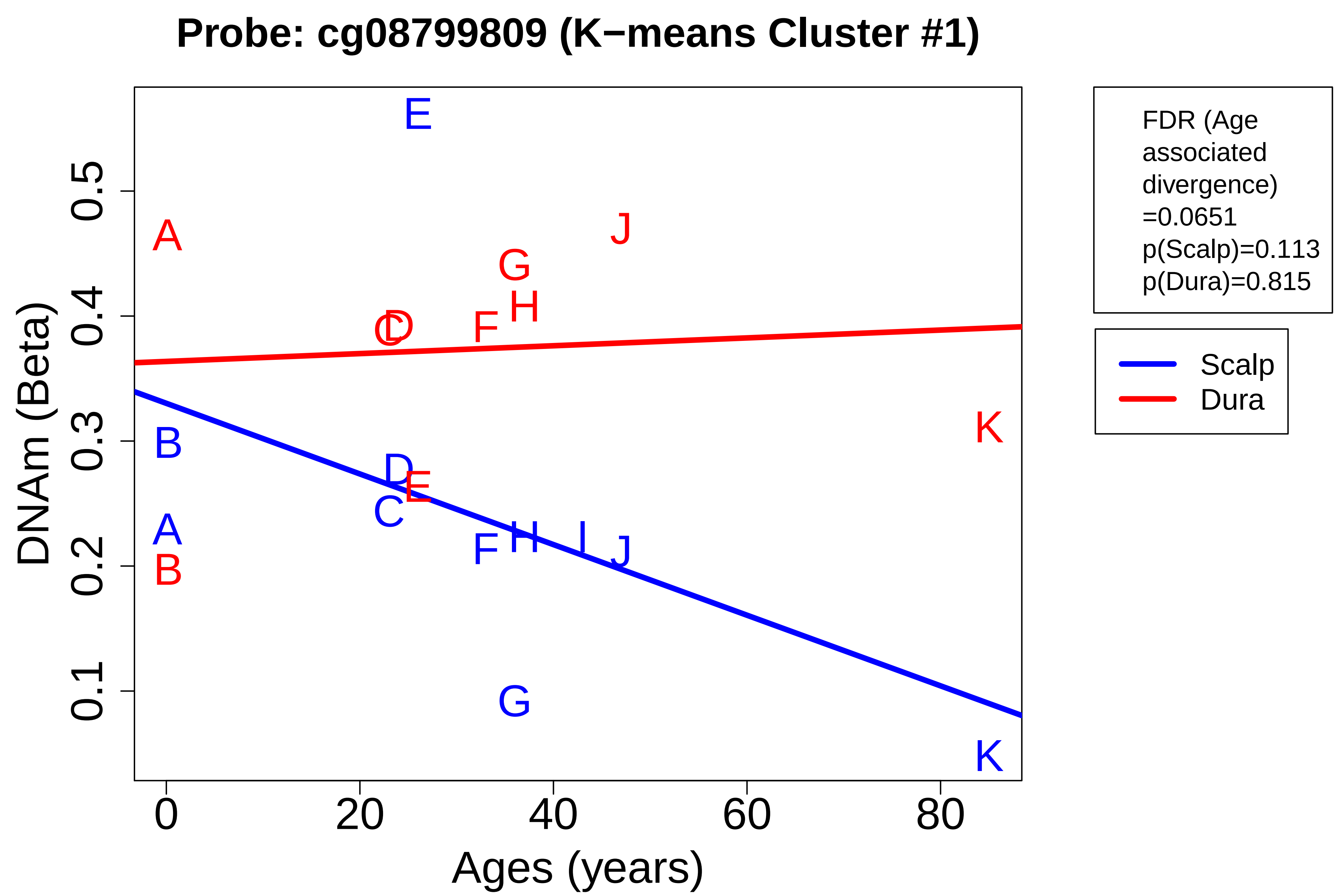
Probe: cg08799809 (K−means Cluster #1)
DNAm (Beta)
Ages (years)
0.1
0.2
0.3
0.4
0.5
0
20
40
60
80
FDR (Age associated divergence) =0.0651
p(Scalp)=0.113
p(Dura)=0.815
Scalp
Dura
A
B
C
D
E
F
G
H
I
J
K

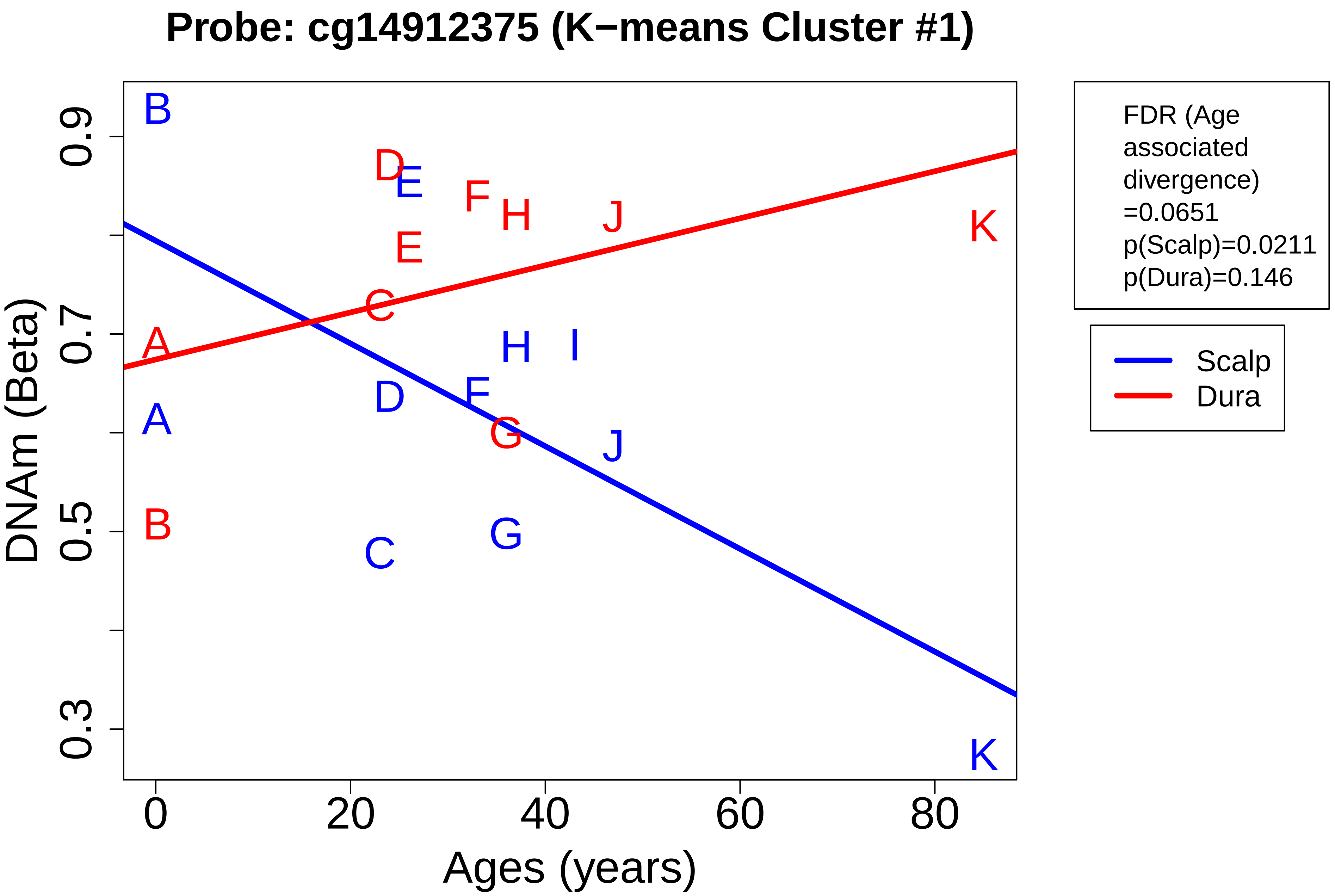
Probe: cg14912375 (K−means Cluster #1)
DNAm (Beta)
0.3
0.5
0.7
0.9
Ages (years)
0
20
40
60
80
FDR (Age associated divergence) =0.0651
p(Scalp)=0.0211
p(Dura)=0.146
Scalp
Dura

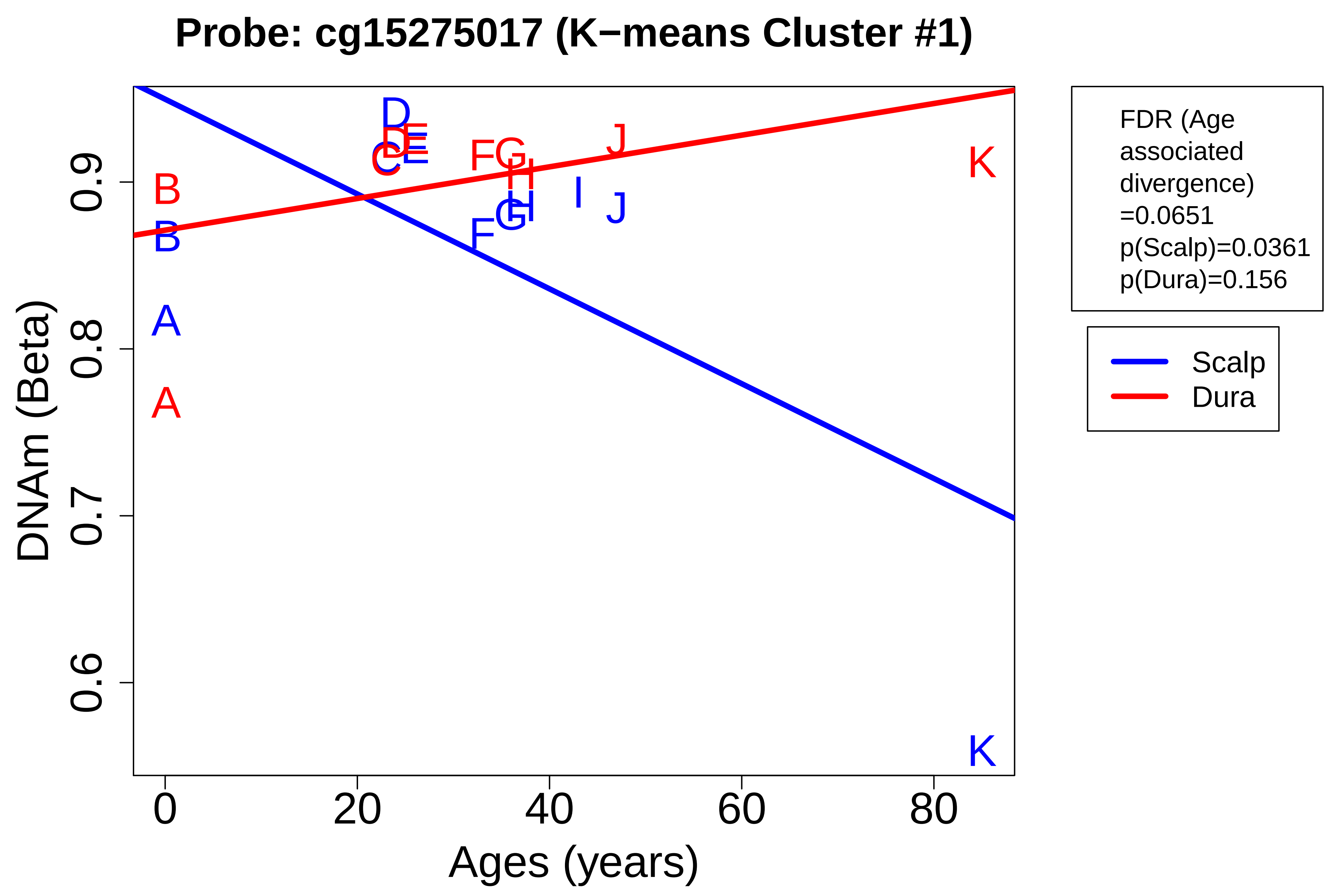

Probe: cg15275017 (K−means Cluster #1)
DNAm (Beta)
0.6
0.7
0.8
0.9
0
20
40
60
80
Ages (years)
FDR (Age associated divergence) =0.0651
p(Scalp)=0.0361
p(Dura)=0.156
Scalp
Dura

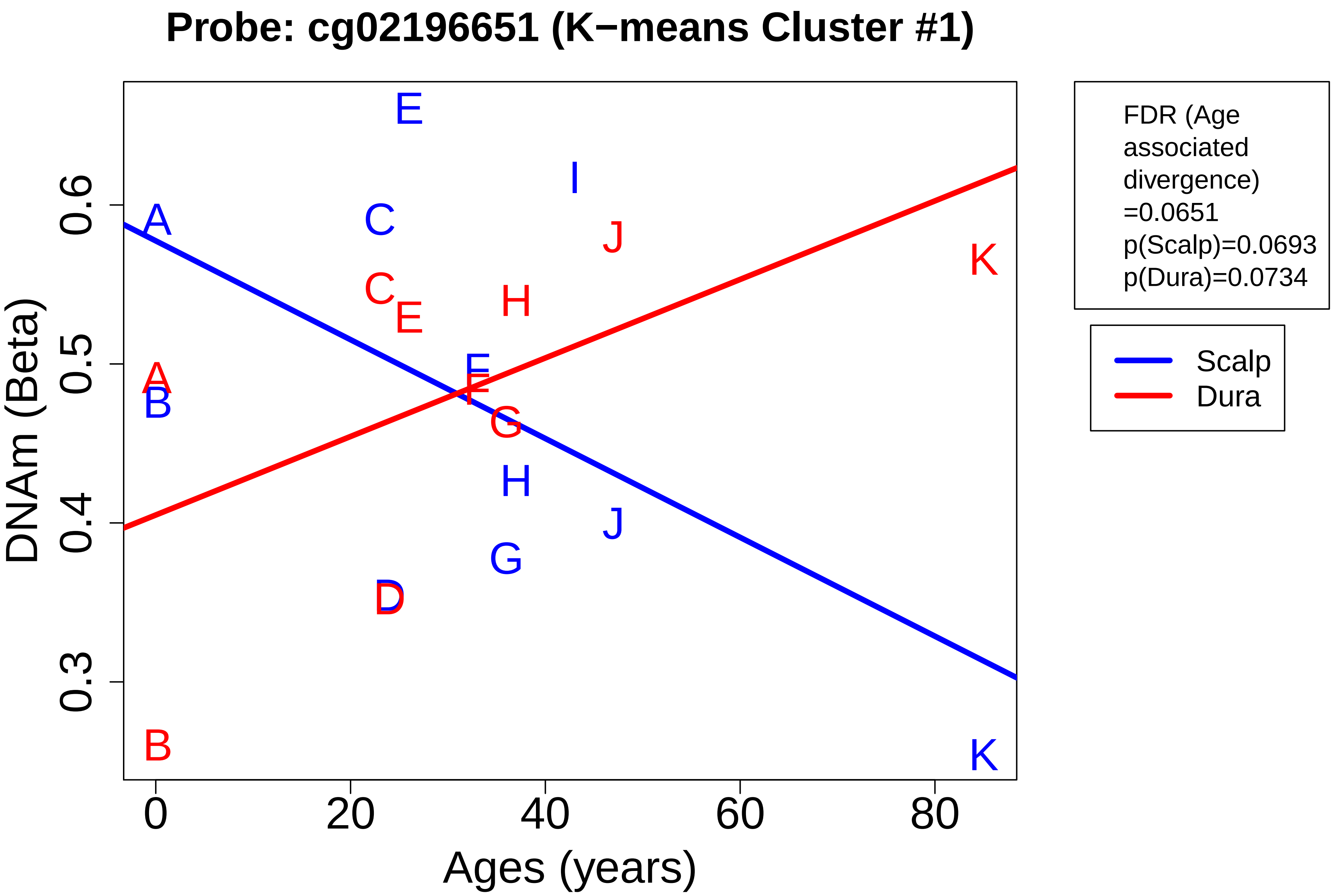

Probe: cg02196651 (K−means Cluster #1)
DNAm (Beta)
0.3
0.4
0.5
0.6
Ages (years)
0
20
40
60
80
A
B
C
D
E
F
G
H
I
J
K
FDR (Age associated divergence) =0.0651
p(Scalp)=0.0693
p(Dura)=0.0734
Scalp
Dura

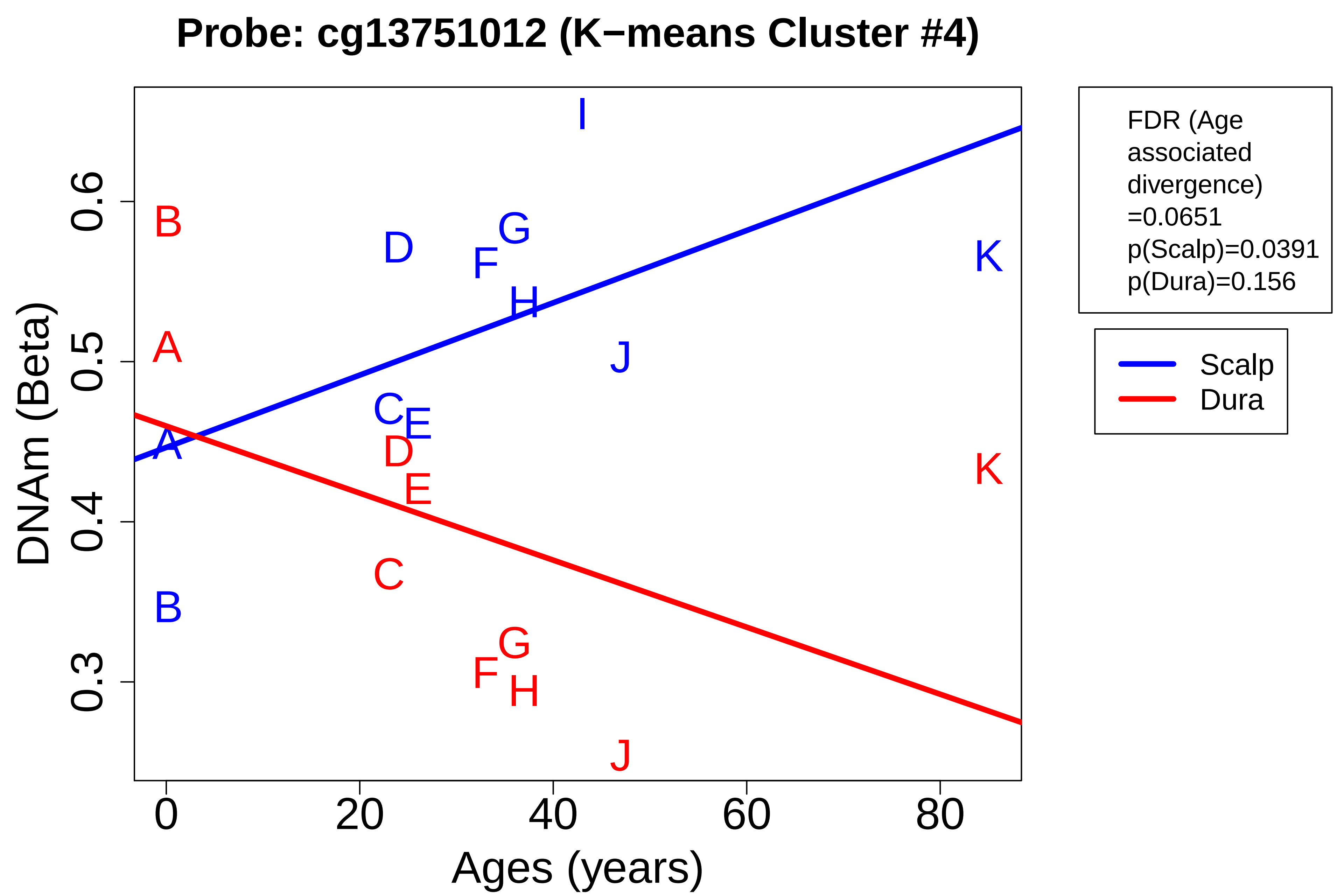
Probe: cg13751012 (K−means Cluster #4)
DNAm (Beta)
0.3
0.4
0.5
0.6
0
20
40
60
80
Ages (years)
A
B
C
D
E
F
G
H
I
J
K
FDR (Age associated divergence) =0.0651
p(Scalp)=0.0391
p(Dura)=0.156
Scalp
Dura

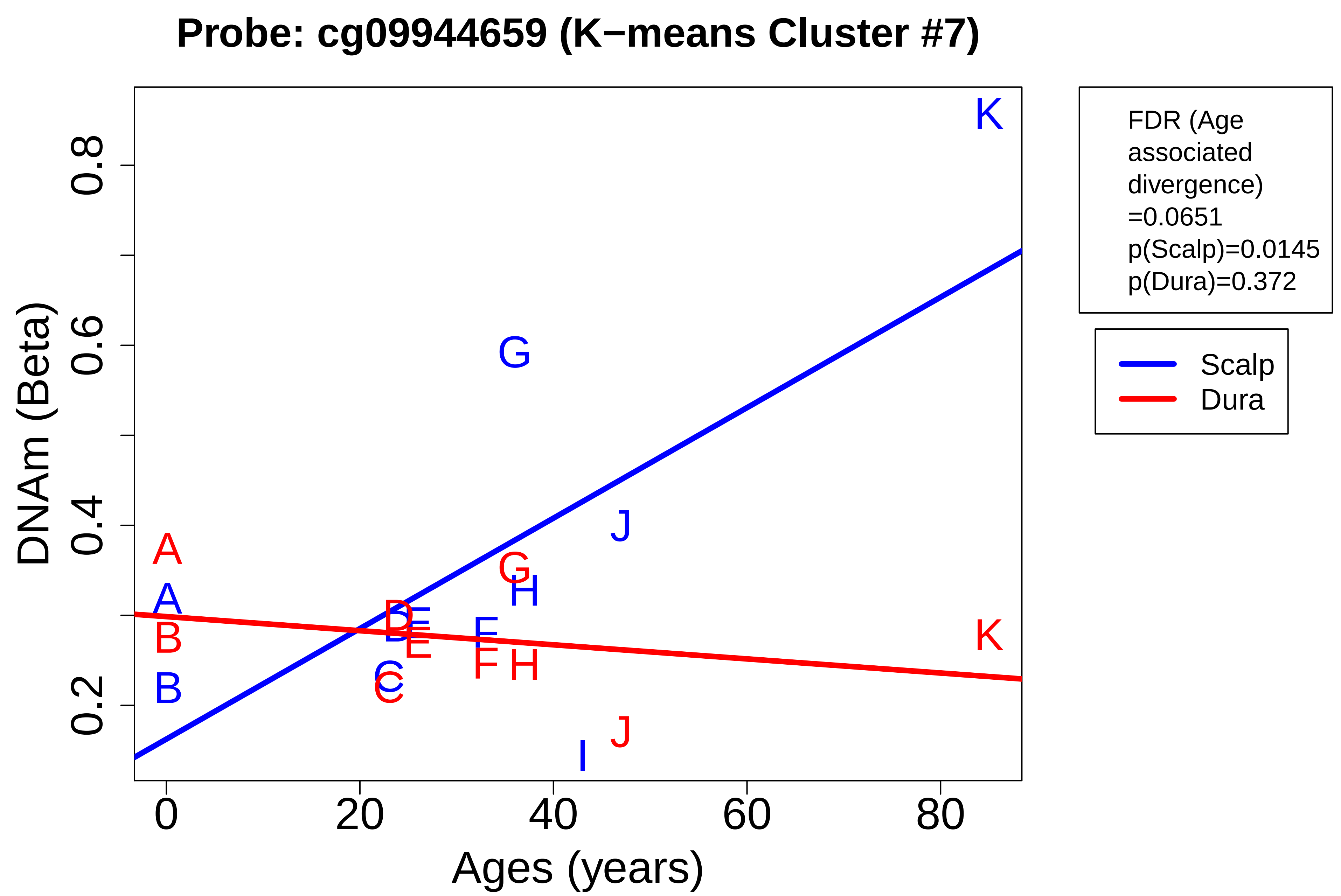

Probe: cg09944659 (K−means Cluster #7)
DNAm (Beta)
0.2
0.4
0.6
0.8
0
20
40
60
80
Ages (years)
FDR (Age associated divergence) =0.0651
p(Scalp)=0.0145
p(Dura)=0.372
Scalp
Dura

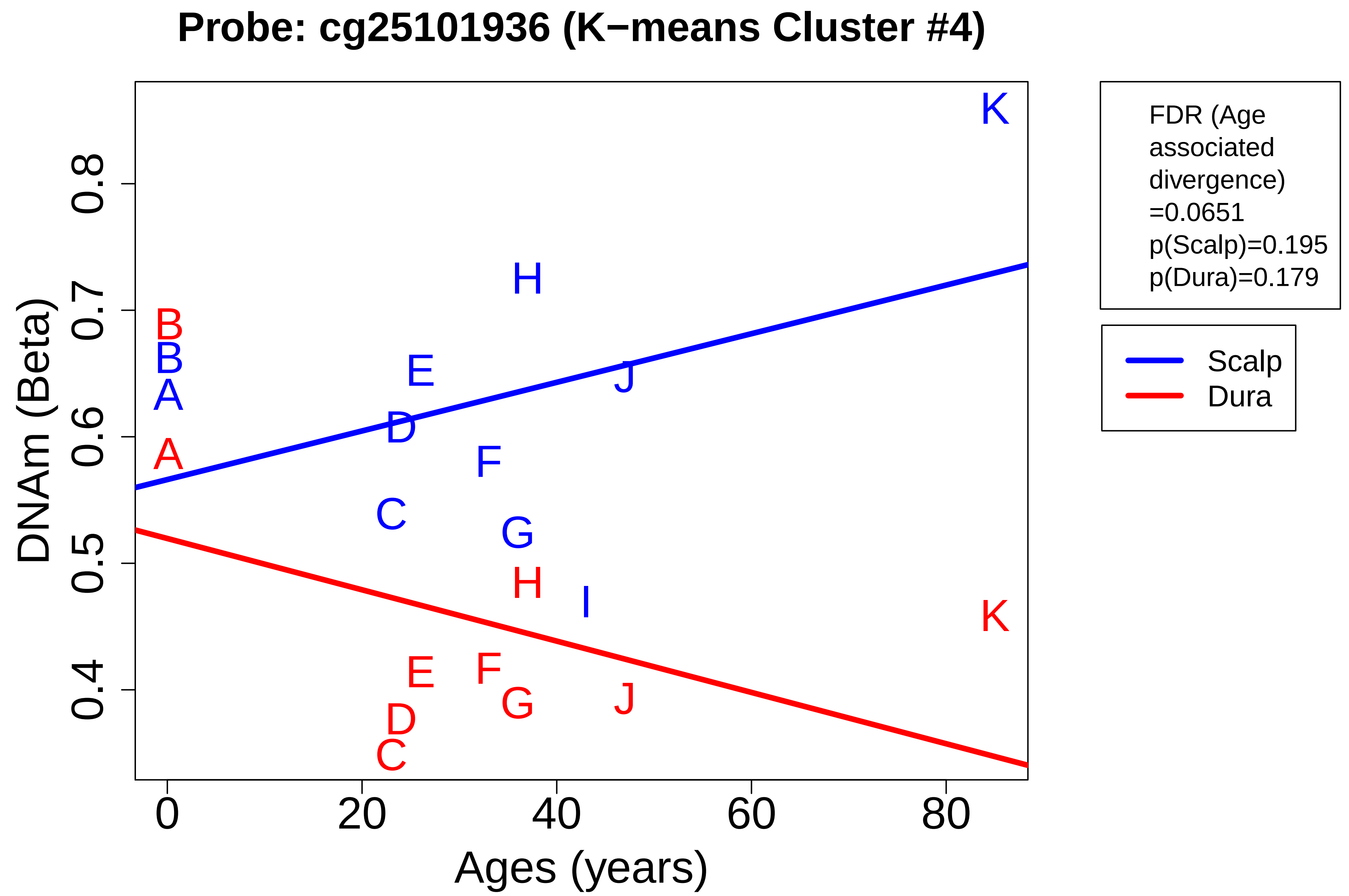

Probe: cg25101936 (K−means Cluster #4)
FDR (Age associated divergence) =0.0651
p(Scalp)=0.195
p(Dura)=0.179
Scalp
Dura
DNAm (Beta)
0.4
0.5
0.6
0.7
0.8
0
20
40
60
80
Ages (years)
A
B
C
D
E
F
G
H
I
J
K

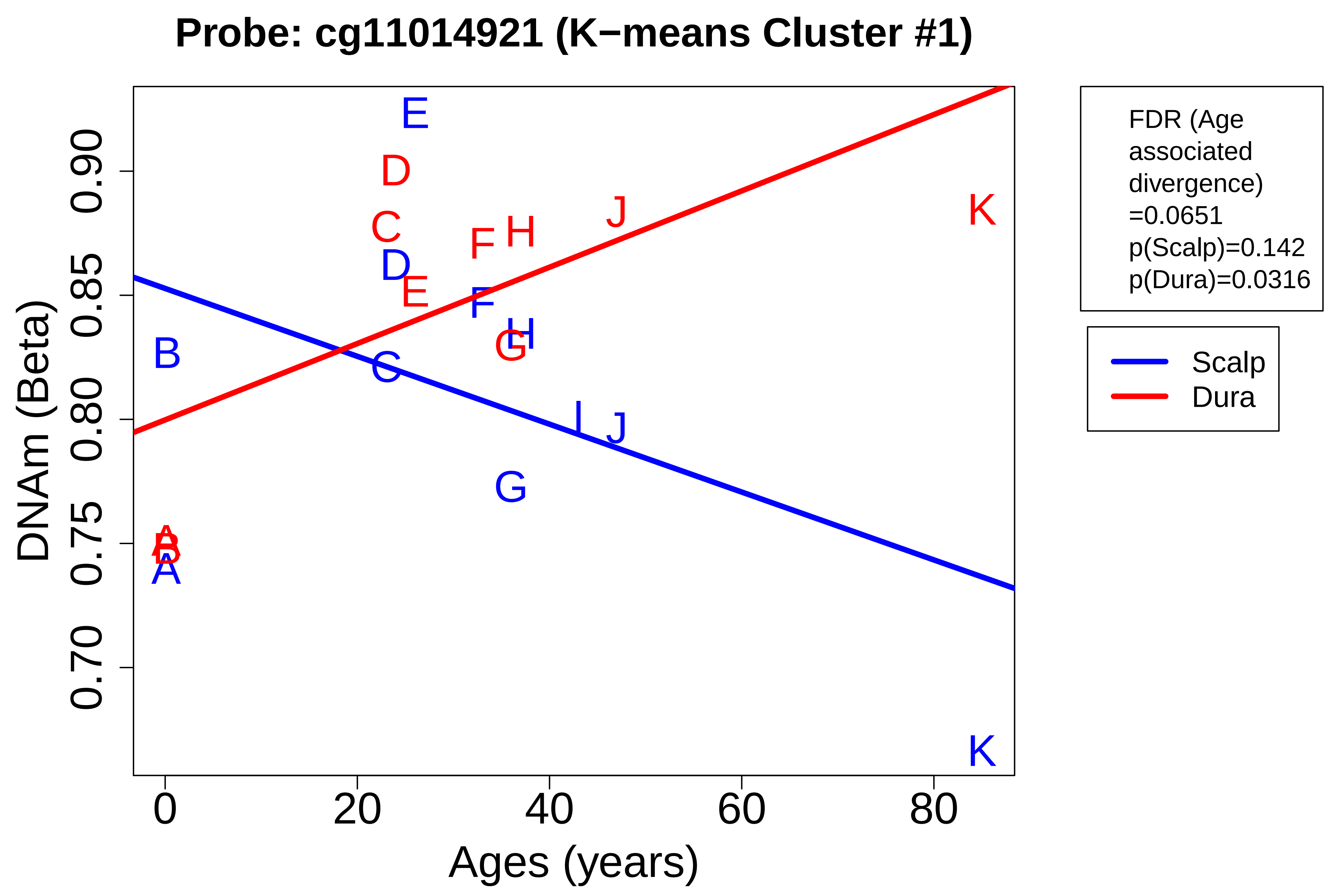

Probe: cg11014921 (K−means Cluster #1)
DNAm (Beta)
0.70
0.75
0.80
0.85
0.90
Ages (years)
0
20
40
60
80
FDR (Age associated divergence) =0.0651
p(Scalp)=0.142
p(Dura)=0.0316
Scalp
Dura

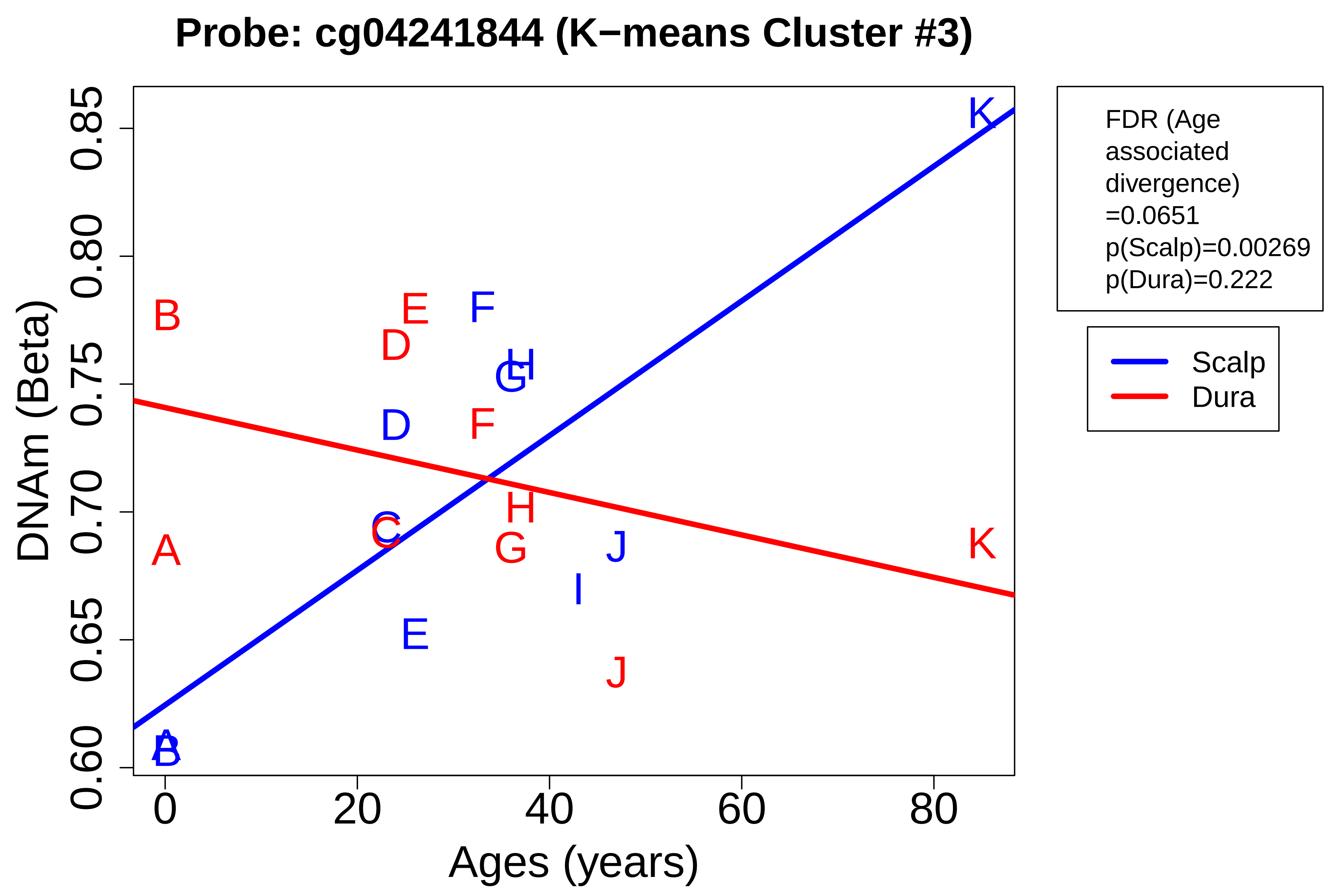
Probe: cg04241844 (K−means Cluster #3)
FDR (Age associated divergence) =0.0651
p(Scalp)=0.00269
p(Dura)=0.222
Scalp
Dura
DNAm (Beta)
0.60
0.65
0.70
0.75
0.80
0.85
0
20
40
60
80
Ages (years)
A
B
C
D
E
F
G
H
I
J
K

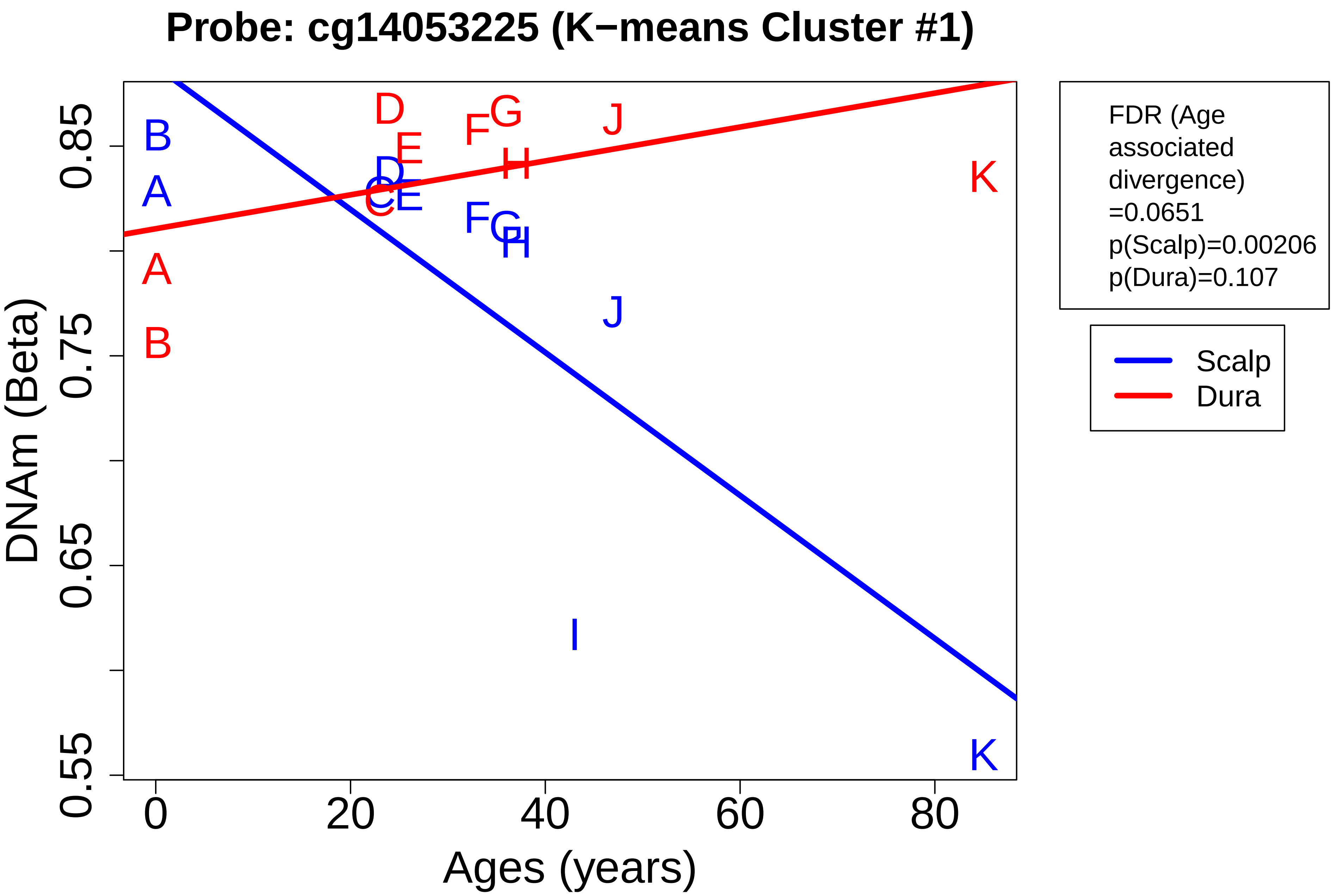
Probe: cg14053225 (K−means Cluster #1)
DNAm (Beta)
0.55
0.65
0.75
0.85
0
20
40
60
80
Ages (years)
FDR (Age associated divergence) =0.0651
p(Scalp)=0.00206
p(Dura)=0.107
Scalp
Dura

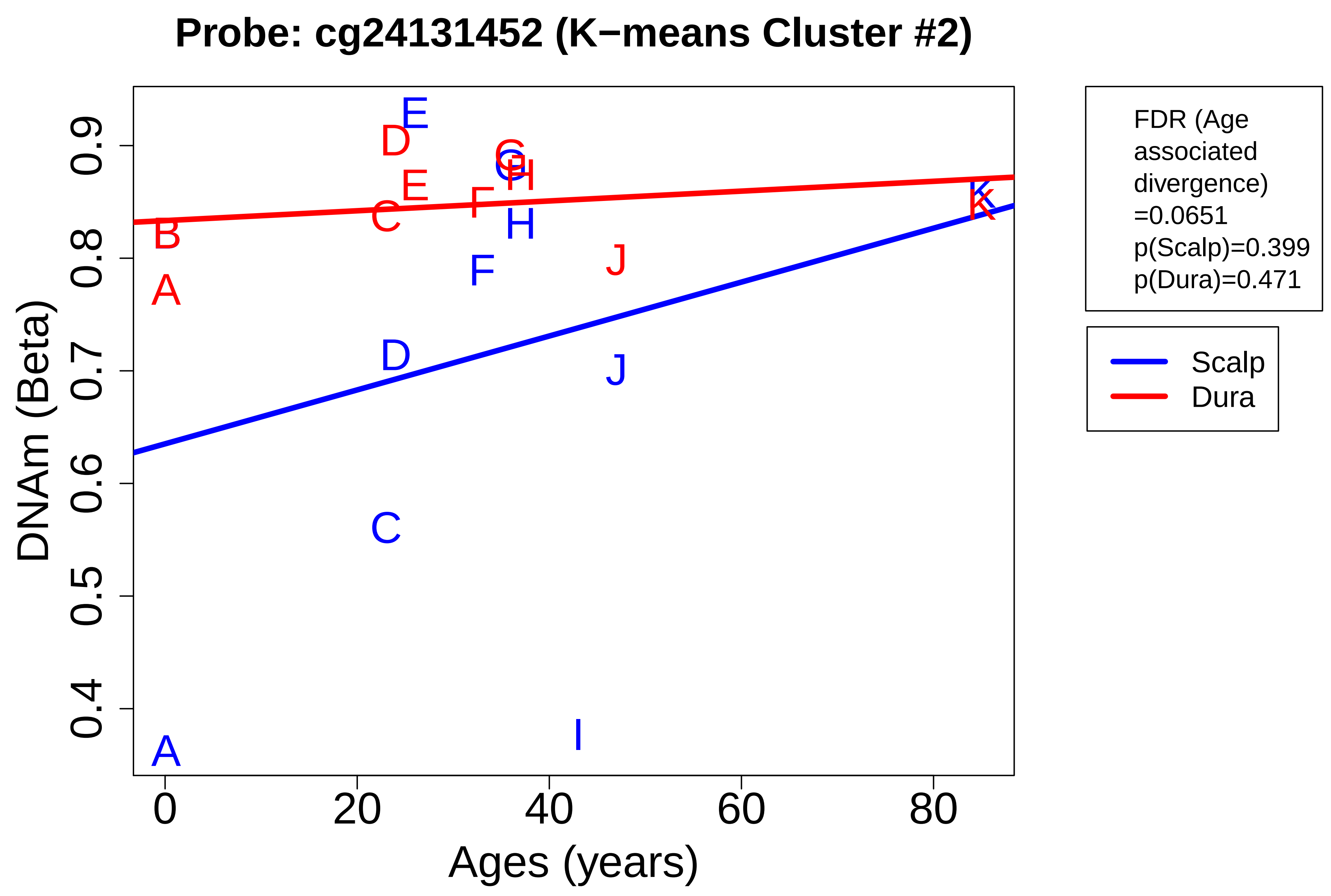

Probe: cg24131452 (K−means Cluster #2)
FDR (Age associated divergence) =0.0651
p(Scalp)=0.399
p(Dura)=0.471
Scalp
Dura
DNAm (Beta)
0.4
0.5
0.6
0.7
0.8
0.9
0
20
40
60
80
Ages (years)

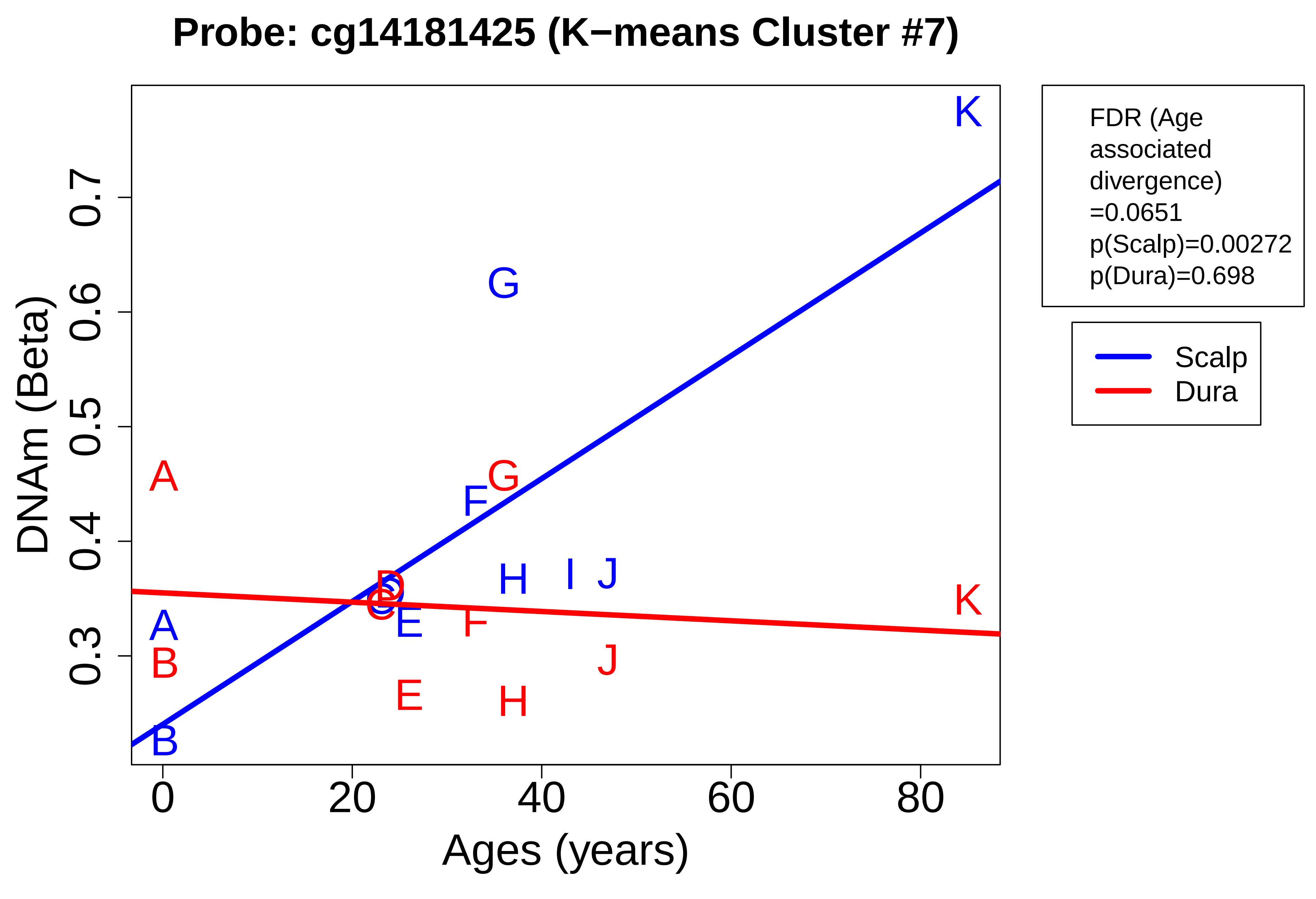

Probe: cg14181425 (K−means Cluster #7)
DNAm (Beta)
0.3
0.4
0.5
0.6
0.7
0
20
40
60
80
Ages (years)
A
B
C
D
E
F
G
H
I
J
K
A
B
C
D
E
F
G
H
J
K
FDR (Age associated divergence) =0.0651
p(Scalp)=0.00272
p(Dura)=0.698
Scalp
Dura

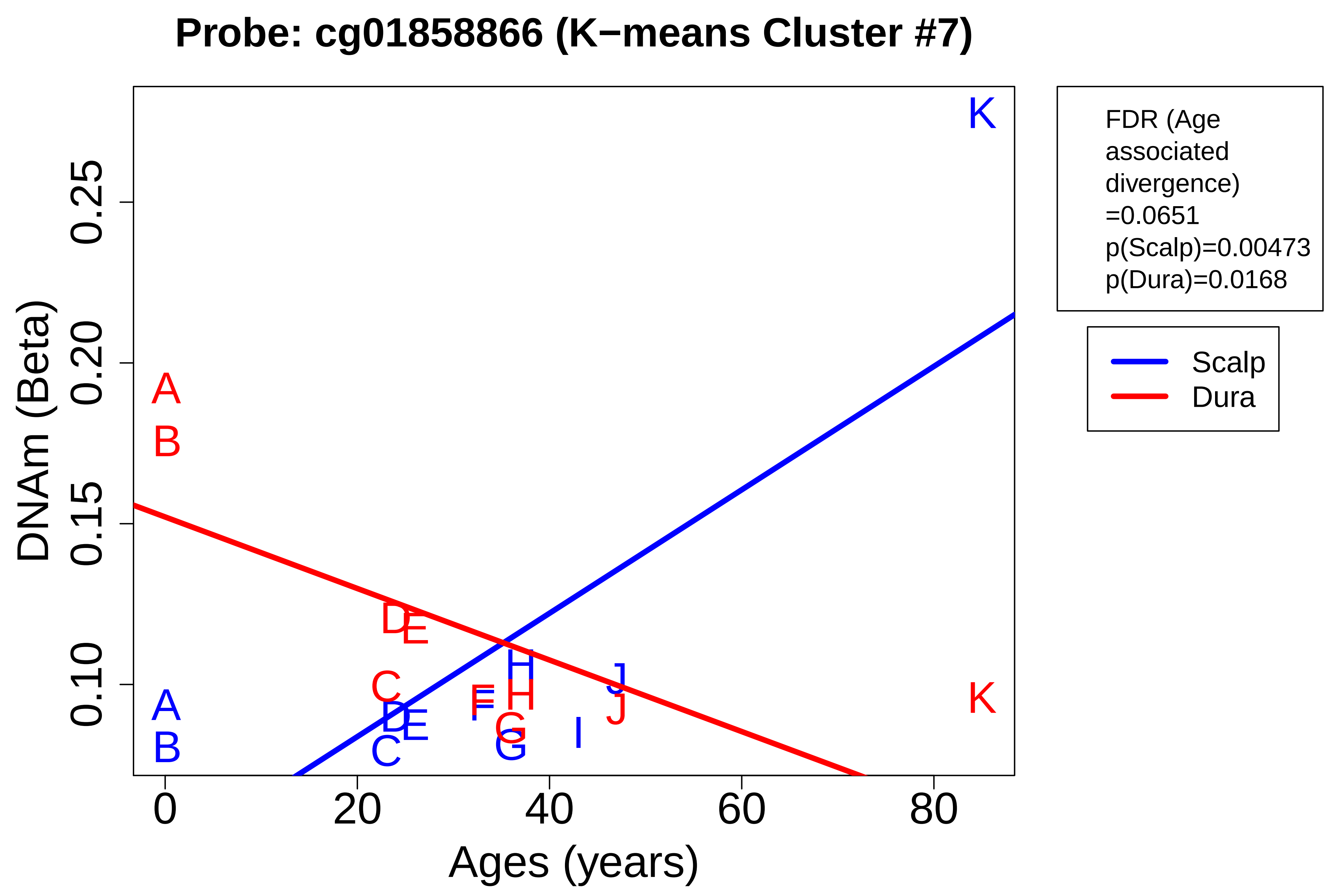

Probe: cg01858866 (K−means Cluster #7)
DNAm (Beta)
0.10
0.15
0.20
0.25
Ages (years)
0
20
40
60
80
FDR (Age associated divergence) =0.0651
p(Scalp)=0.00473
p(Dura)=0.0168
Scalp
Dura
A
B
C
D
E
F
G
H
I
J
K

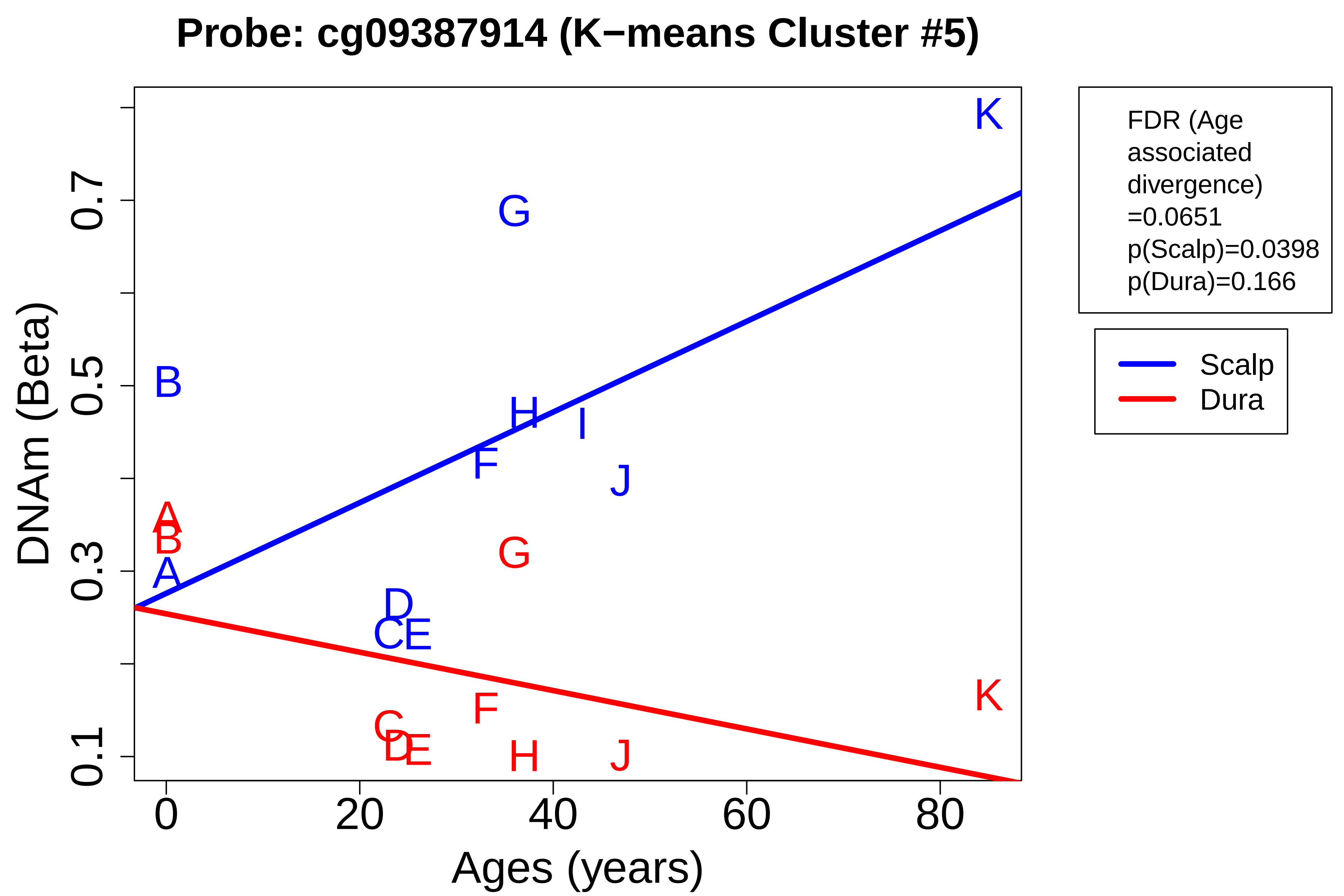
Probe: cg09387914 (K−means Cluster #5)
DNAm (Beta)
0.1
0.3
0.5
0.7
0
20
40
60
80
Ages (years)
FDR (Age associated divergence) =0.0651
p(Scalp)=0.0398
p(Dura)=0.166
Scalp
Dura
A
B
C
D
E
F
G
H
I
J
K

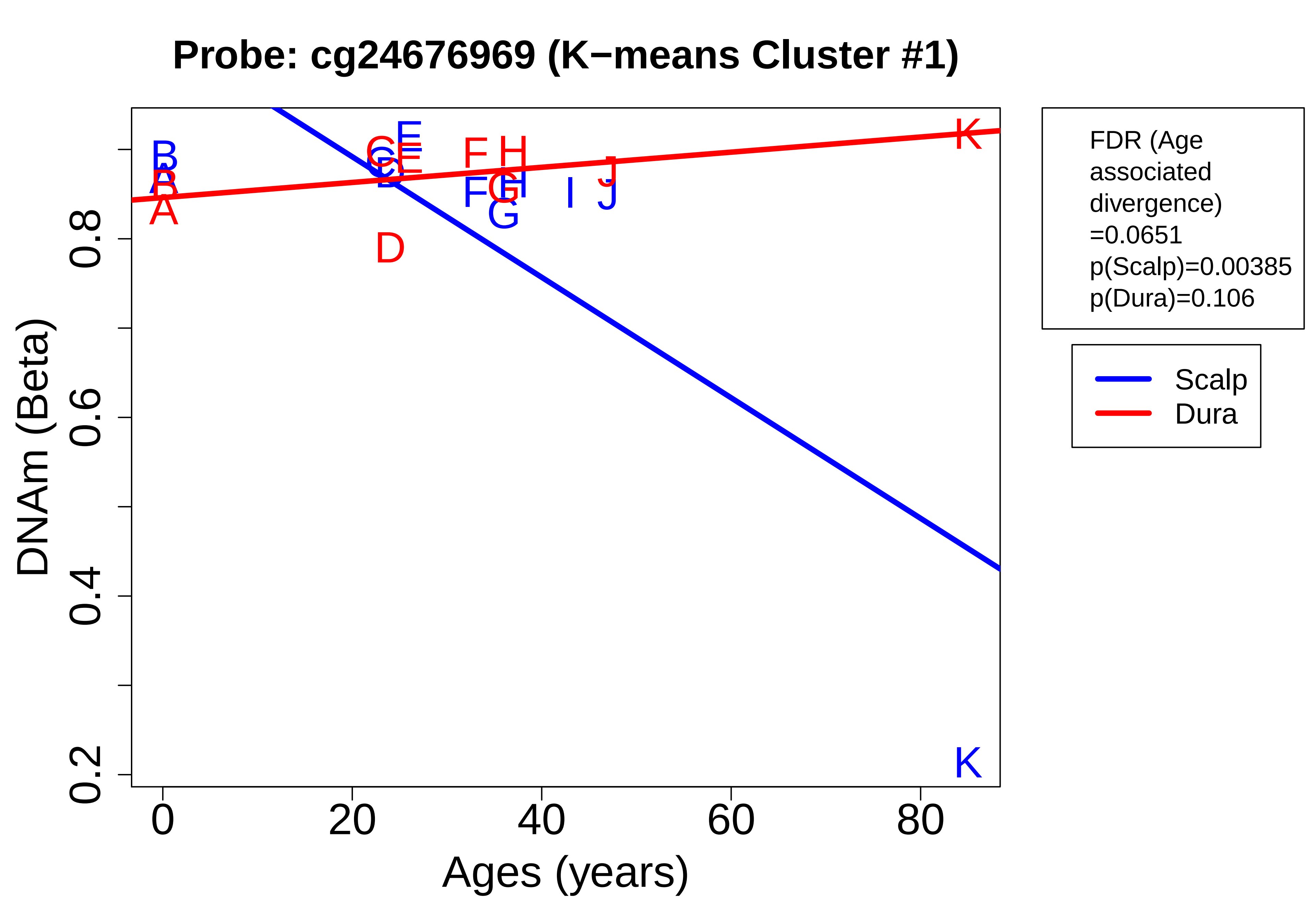
Probe: cg24676969 (K−means Cluster #1)
DNAm (Beta)
0.2
0.4
0.6
0.8
0
20
40
60
80
Ages (years)
FDR (Age associated divergence) =0.0651
p(Scalp)=0.00385
p(Dura)=0.106
Scalp
Dura
A
B
C
D
E
F
G
H
I
J
K

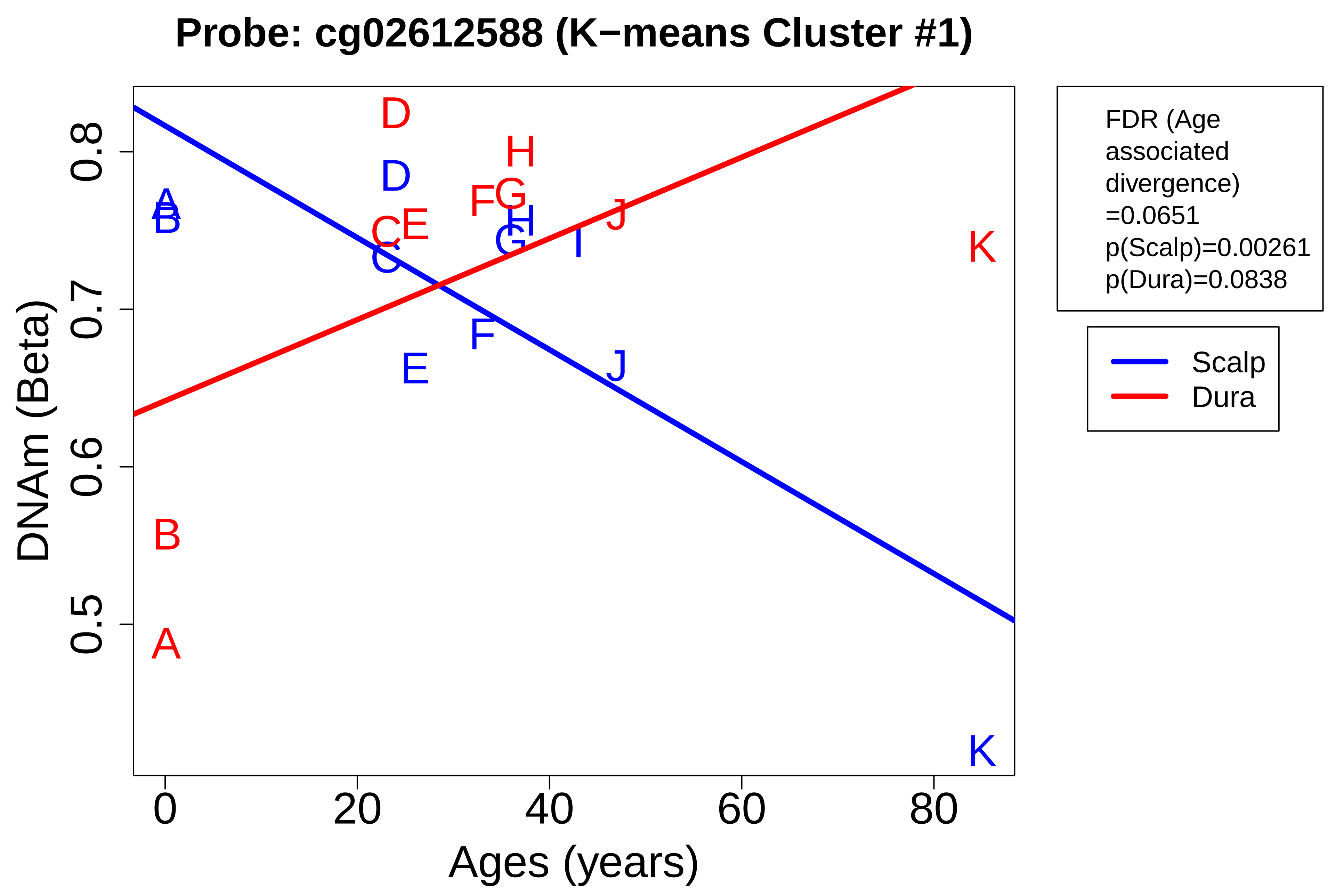

Probe: cg02612588 (K−means Cluster #1)
FDR (Age associated divergence) =0.0651
p(Scalp)=0.00261
p(Dura)=0.0838
Scalp
Dura
DNAm (Beta)
0.5
0.6
0.7
0.8
0
20
40
60
80
Ages (years)
A
B
C
D
E
F
G
H
I
J
K

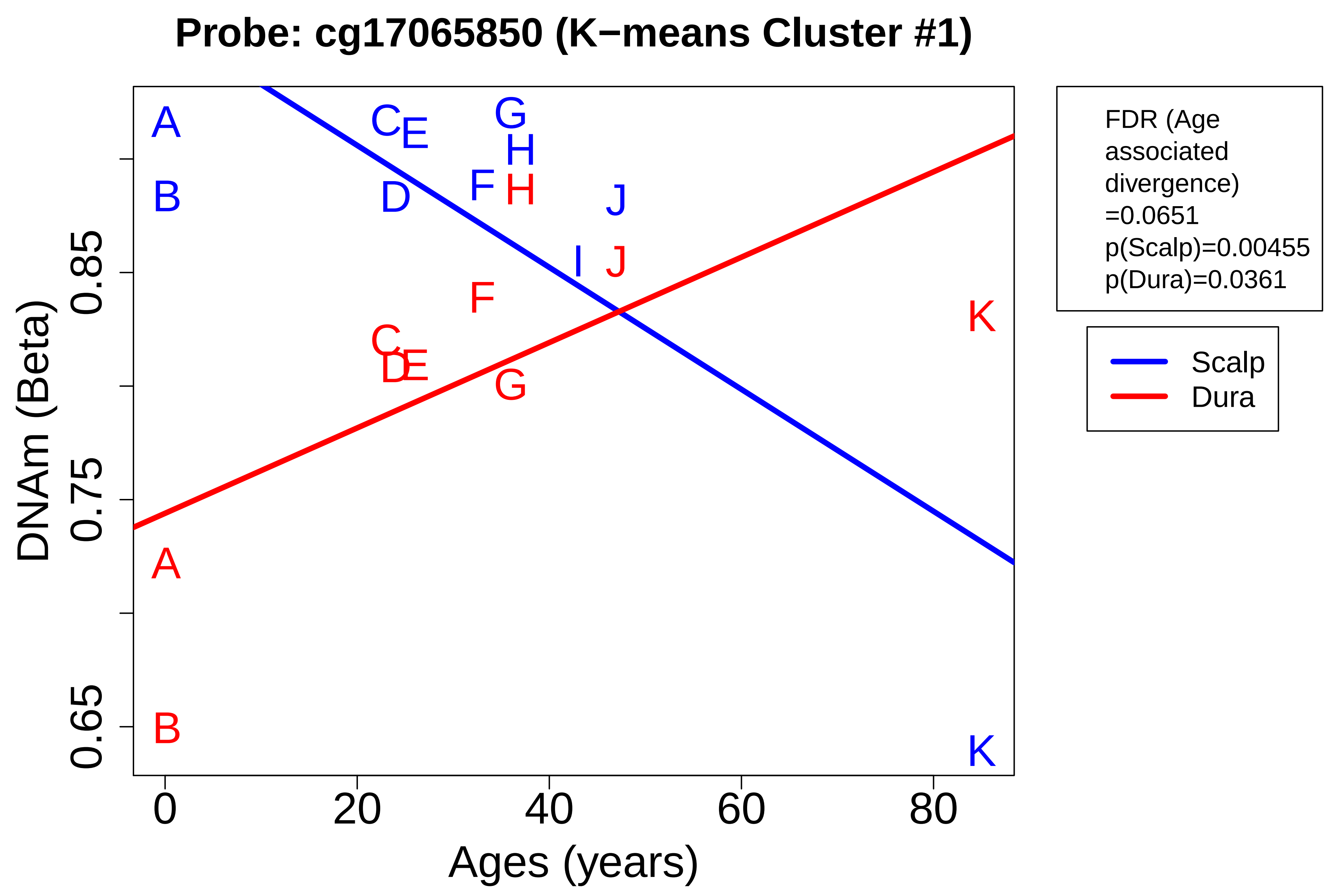
Probe: cg17065850 (K−means Cluster #1)
DNAm (Beta)
0.65
0.75
0.85
0
20
40
60
80
Ages (years)
A
B
C
D
E
F
G
H
I
J
K
FDR (Age associated divergence) =0.0651
p(Scalp)=0.00455
p(Dura)=0.0361
Scalp
Dura

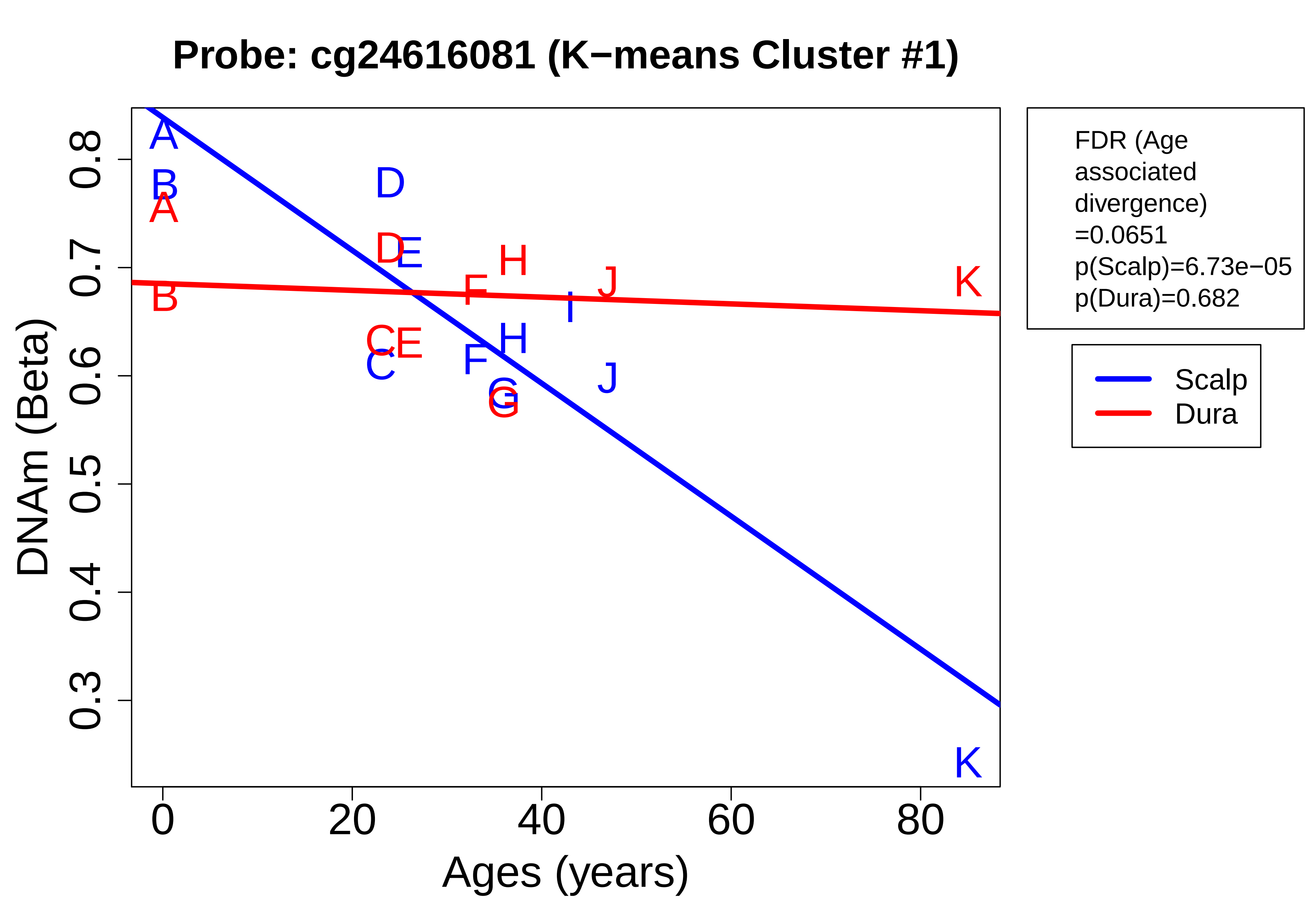

Probe: cg24616081 (K−means Cluster #1)
DNAm (Beta)
0.3
0.4
0.5
0.6
0.7
0.8
0
20
40
60
80
Ages (years)
A
B
C
D
E
F
G
H
I
J
K
FDR (Age associated divergence) =0.0651
p(Scalp)=6.73e−05
p(Dura)=0.682
Scalp
Dura

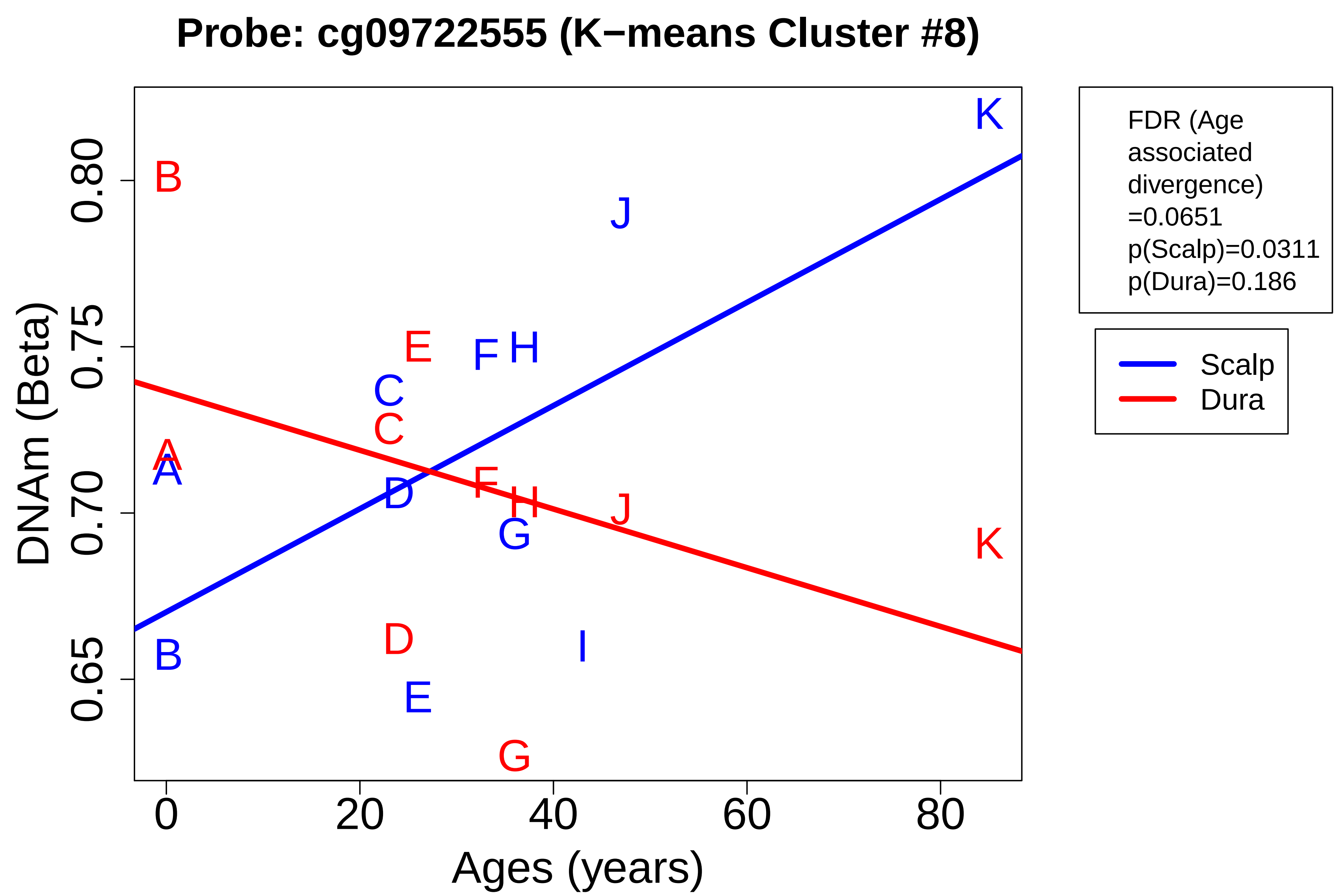
Probe: cg09722555 (K−means Cluster #8)
DNAm (Beta)
0.65
0.70
0.75
0.80
0
20
40
60
80
Ages (years)
A
B
C
D
E
F
G
H
I
J
K
FDR (Age associated divergence) =0.0651
p(Scalp)=0.0311
p(Dura)=0.186
Scalp
Dura

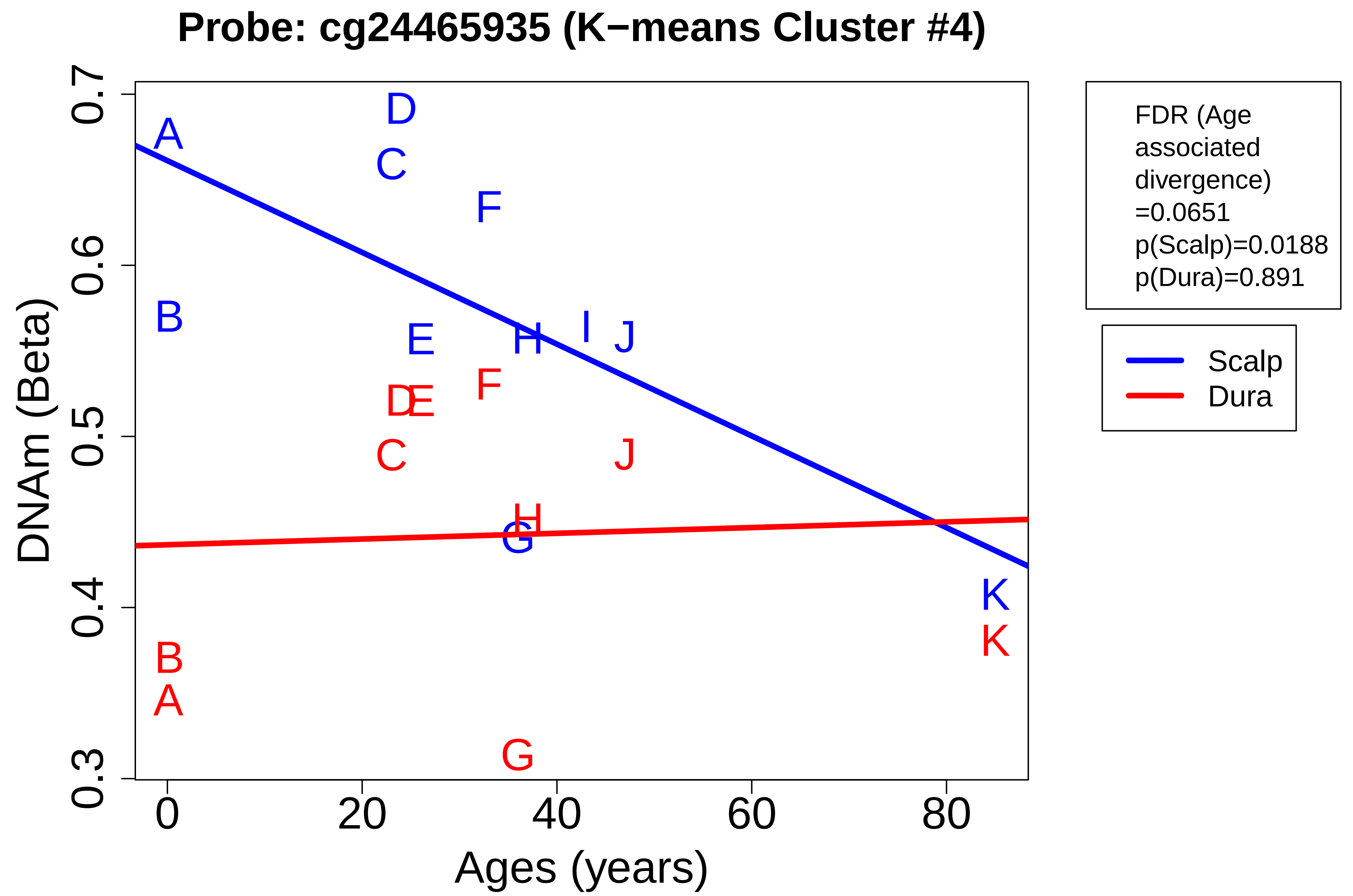

Probe: cg24465935 (K−means Cluster #4)
FDR (Age associated divergence) =0.0651
p(Scalp)=0.0188
p(Dura)=0.891
Scalp
Dura
DNAm (Beta)
Ages (years)
0.3
0.4
0.5
0.6
0.7
0
20
40
60
80

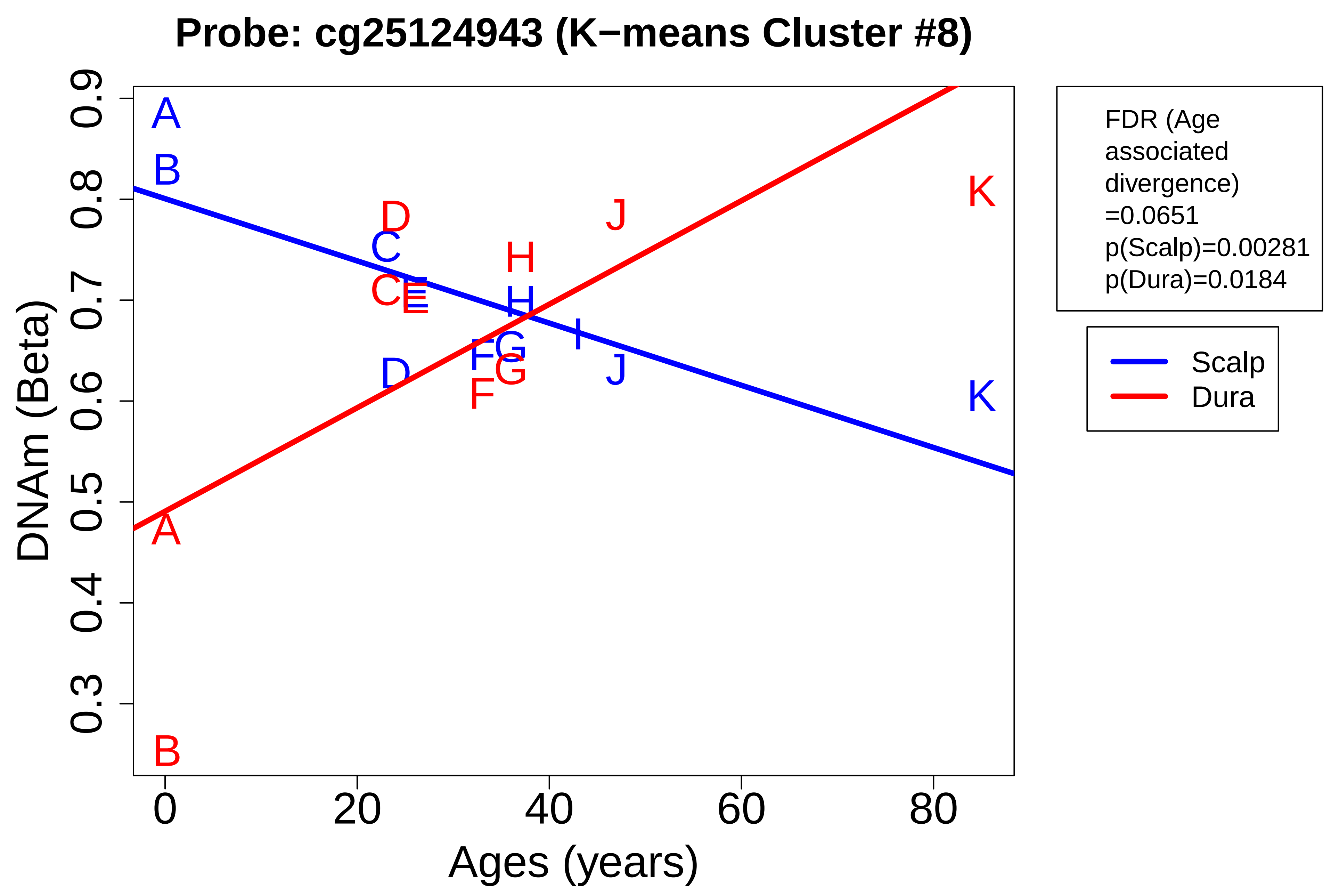

Probe: cg25124943 (K−means Cluster #8)
DNAm (Beta)
0.3
0.4
0.5
0.6
0.7
0.8
0.9
Ages (years)
0
20
40
60
80
A
B
C
D
E
F
G
H
I
J
K
FDR (Age associated divergence) =0.0651
p(Scalp)=0.00281
p(Dura)=0.0184
Scalp
Dura

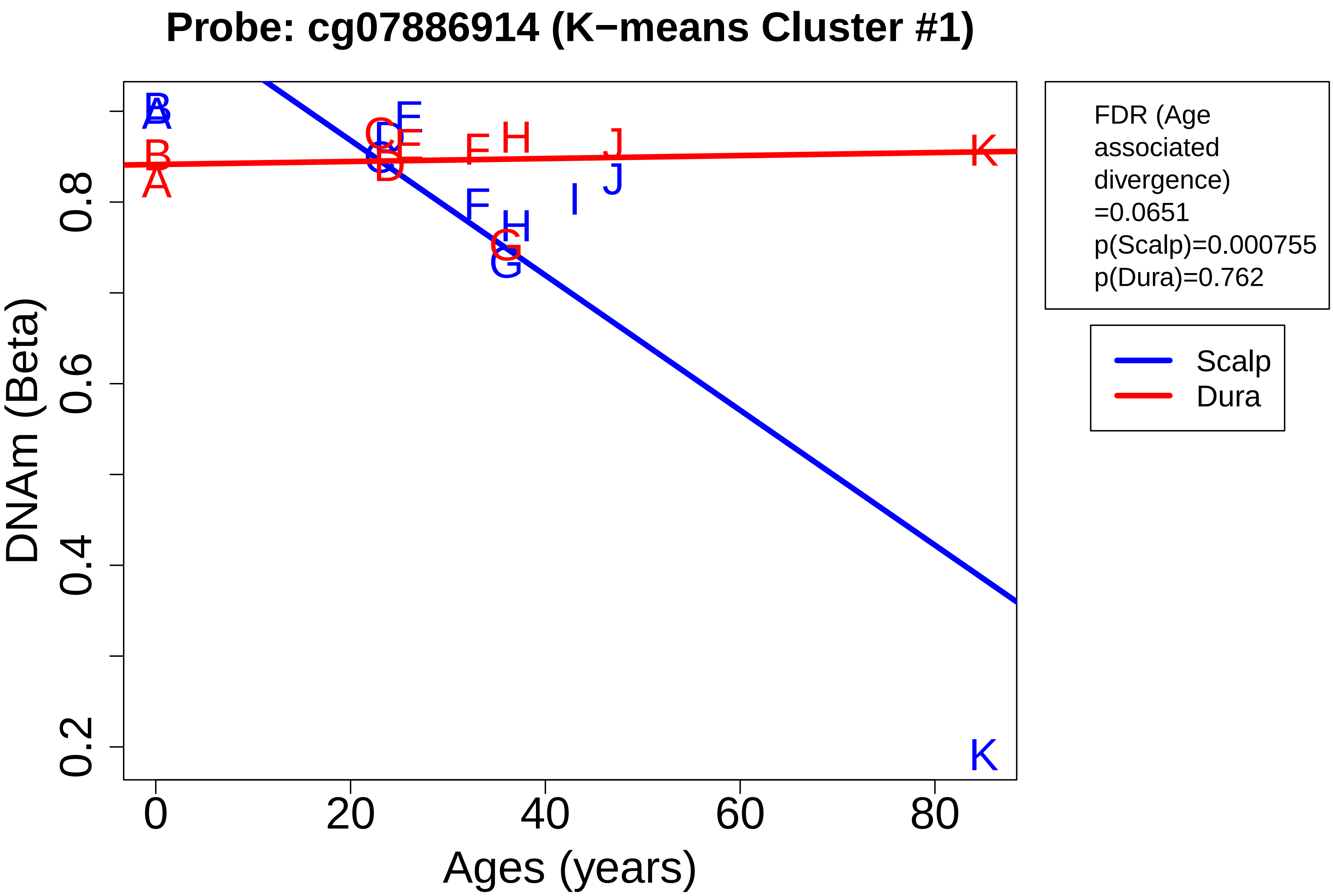

Probe: cg07886914 (K−means Cluster #1)
DNAm (Beta)
0.2
0.4
0.6
0.8
0
20
40
60
80
Ages (years)
FDR (Age associated divergence) =0.0651
p(Scalp)=0.000755
p(Dura)=0.762
Scalp
Dura

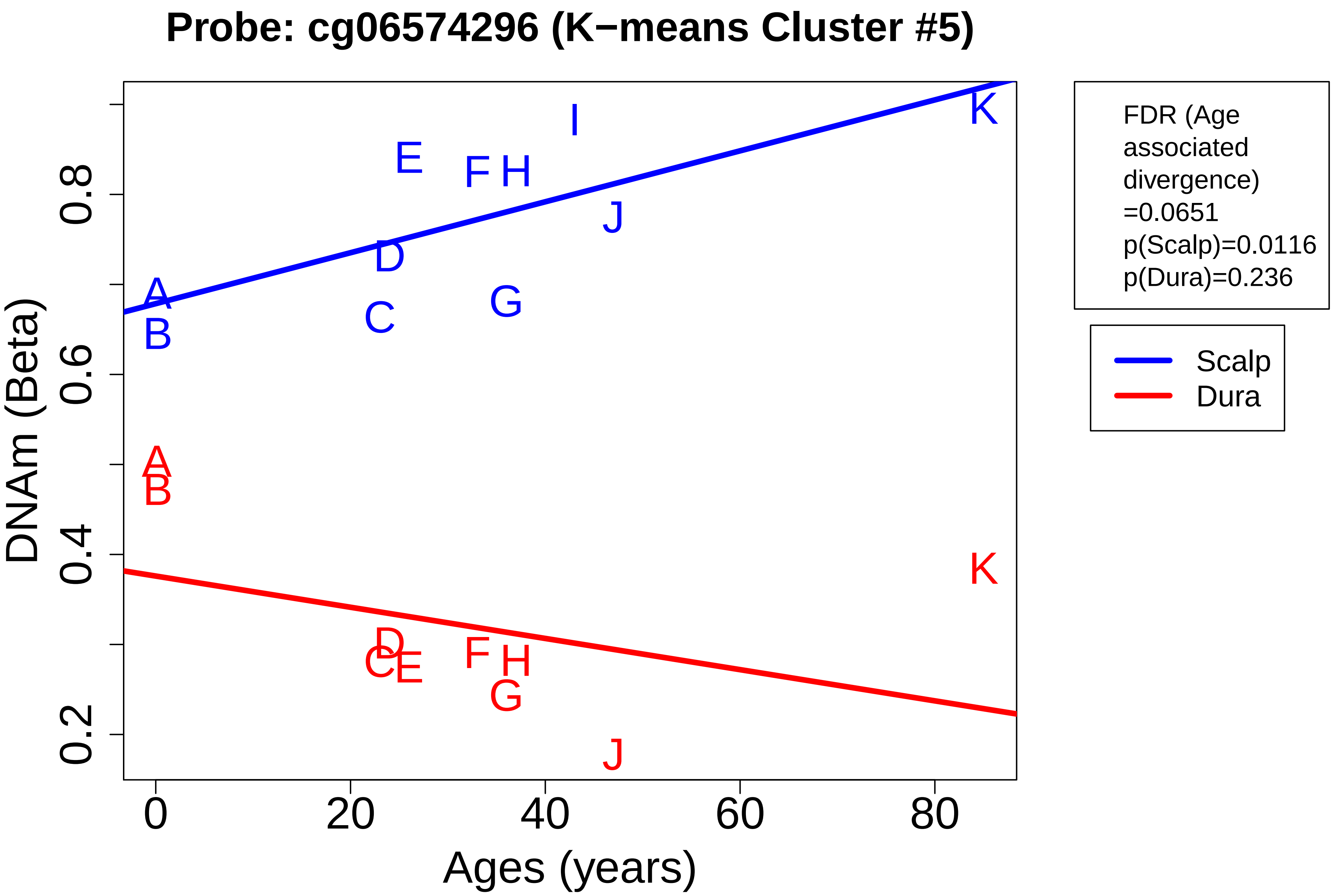

Probe: cg06574296 (K−means Cluster #5)
DNAm (Beta)
0.2
0.4
0.6
0.8
Ages (years)
0
20
40
60
80
A
B
C
D
E
F
G
H
I
J
K
A
B
C
D
E
F
G
H
J
K
FDR (Age associated divergence) =0.0651
p(Scalp)=0.0116
p(Dura)=0.236
Scalp
Dura

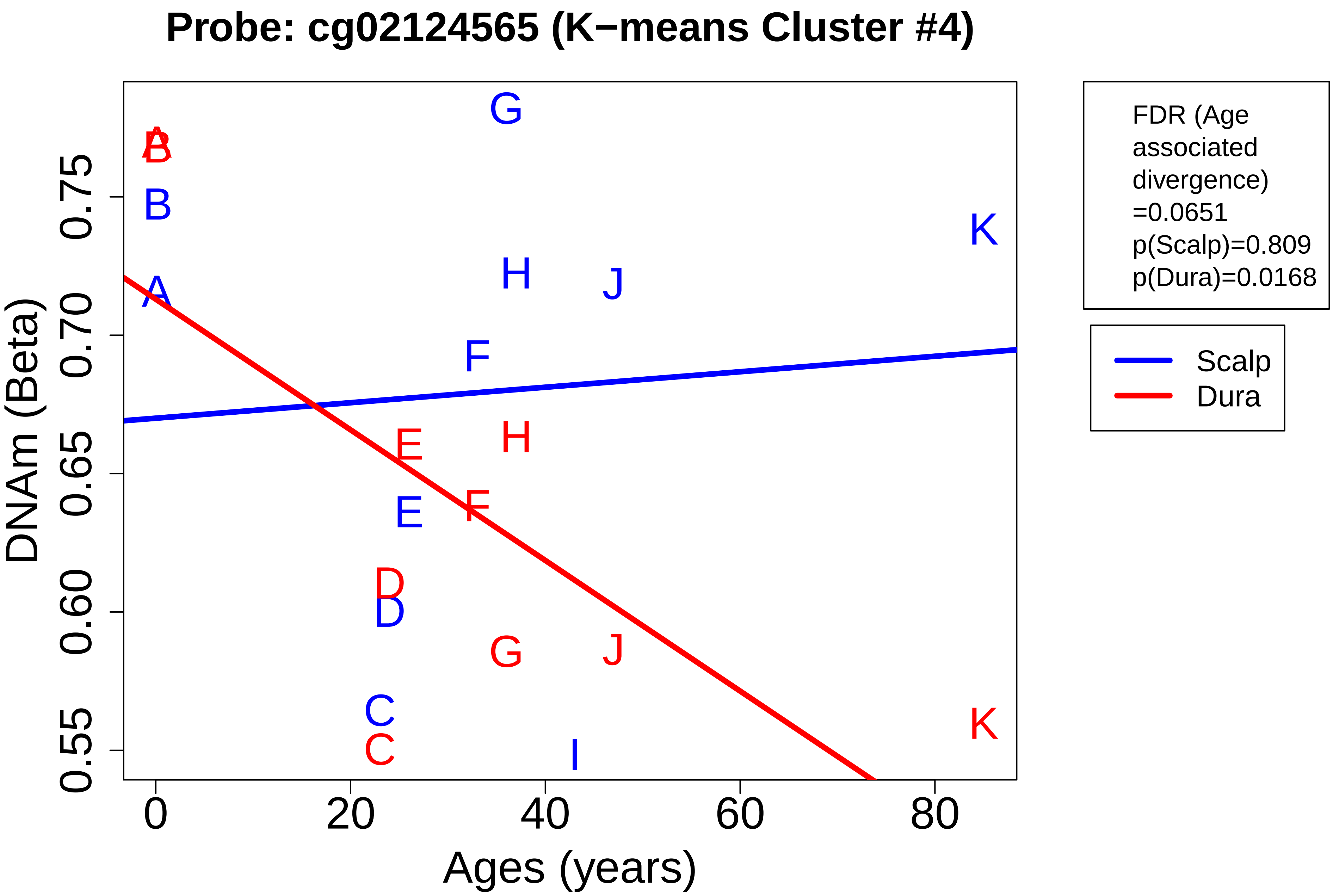

Probe: cg02124565 (K−means Cluster #4)
DNAm (Beta)
0.55
0.60
0.65
0.70
0.75
0
20
40
60
80
Ages (years)
FDR (Age associated divergence) =0.0651
p(Scalp)=0.809
p(Dura)=0.0168
Scalp
Dura

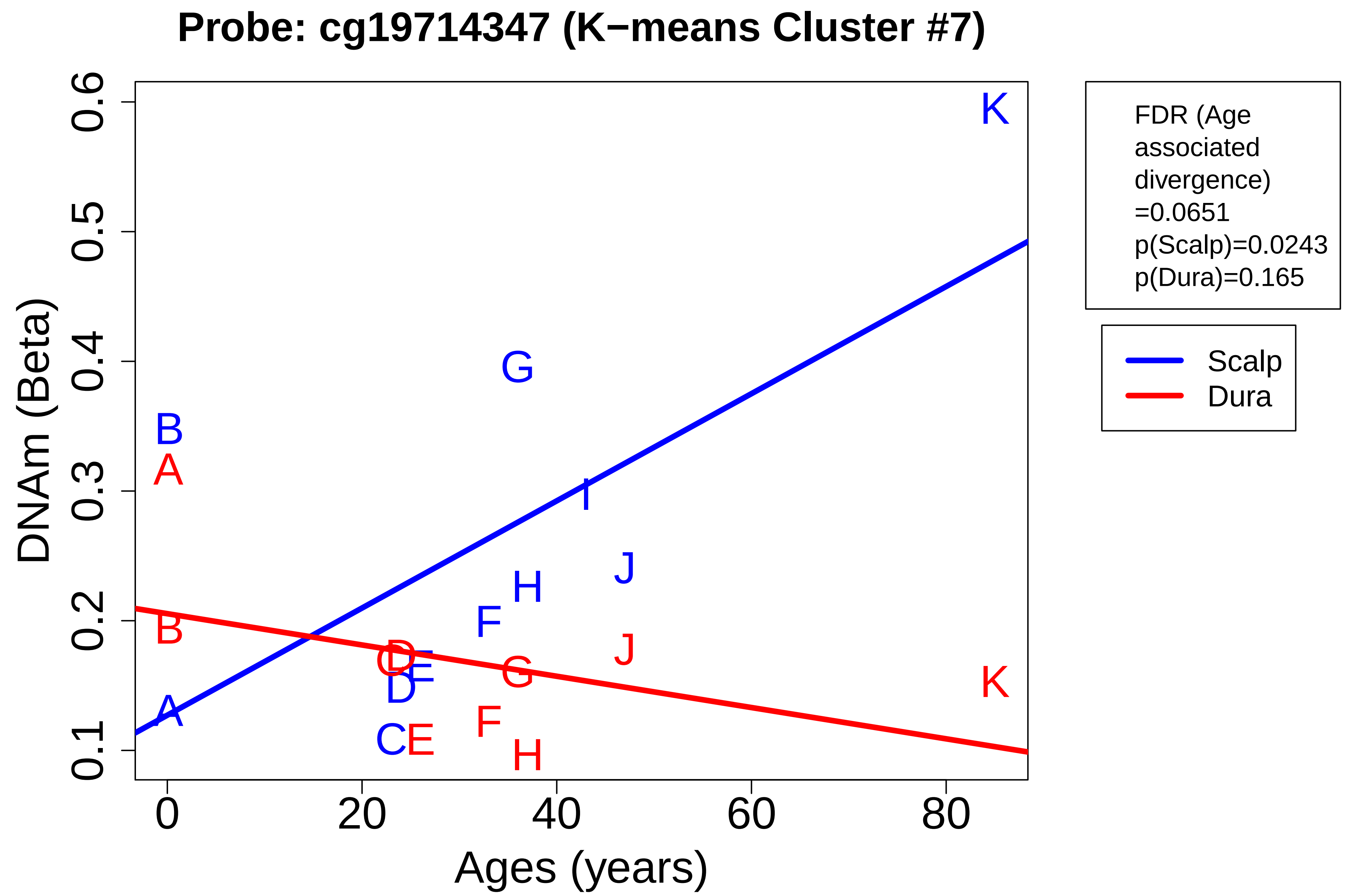

Probe: cg19714347 (K−means Cluster #7)
DNAm (Beta)
0.1
0.2
0.3
0.4
0.5
0.6
0
20
40
60
80
Ages (years)
FDR (Age associated divergence) =0.0651
p(Scalp)=0.0243
p(Dura)=0.165
Scalp
Dura
A
B
C
D
E
F
G
H
I
J
K

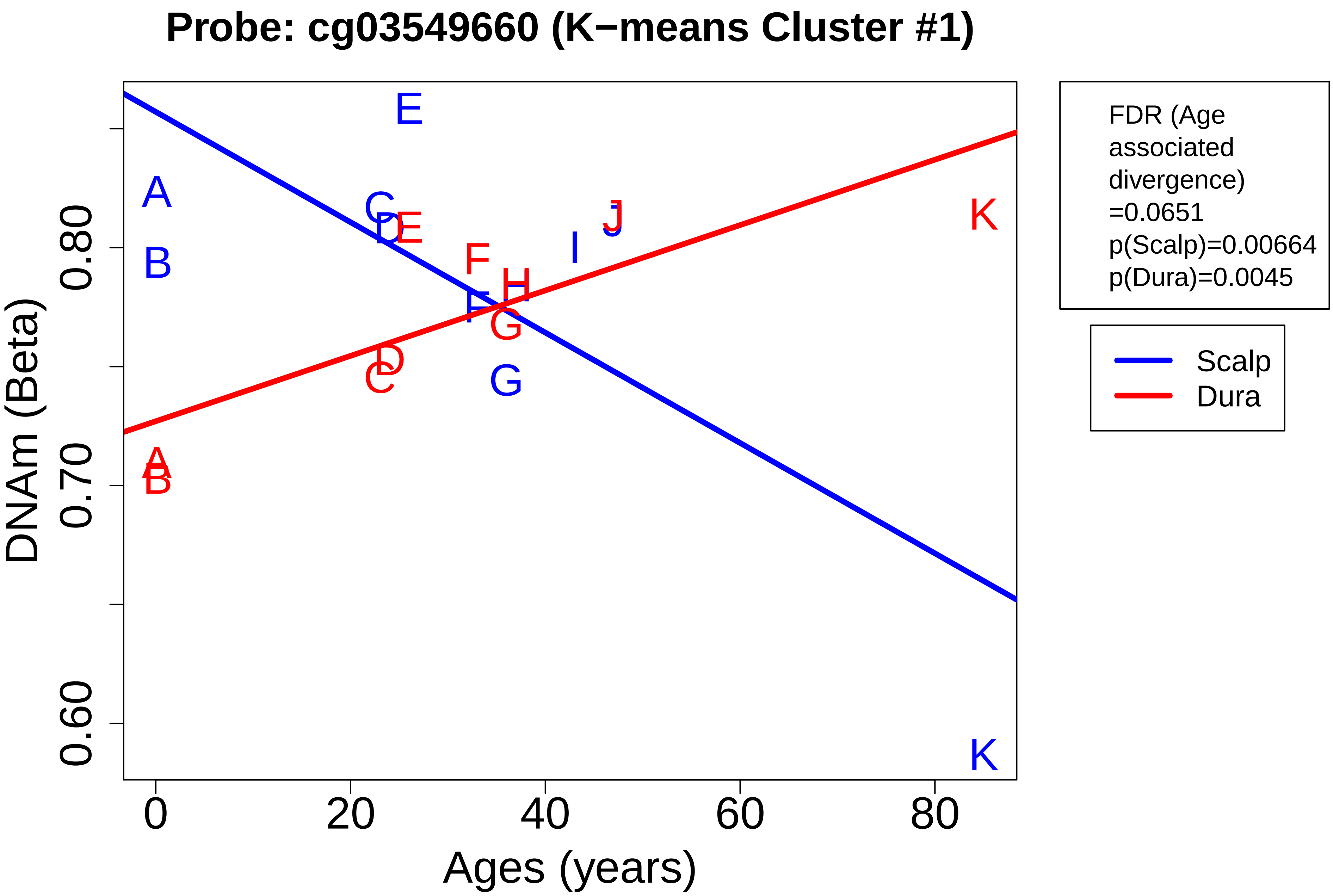

Probe: cg03549660 (K−means Cluster #1)
DNAm (Beta)
0.60
0.70
0.80
Ages (years)
0
20
40
60
80
A
B
C
D
E
F
G
H
I
J
K
FDR (Age associated divergence) =0.0651
p(Scalp)=0.00664
p(Dura)=0.0045
Scalp
Dura

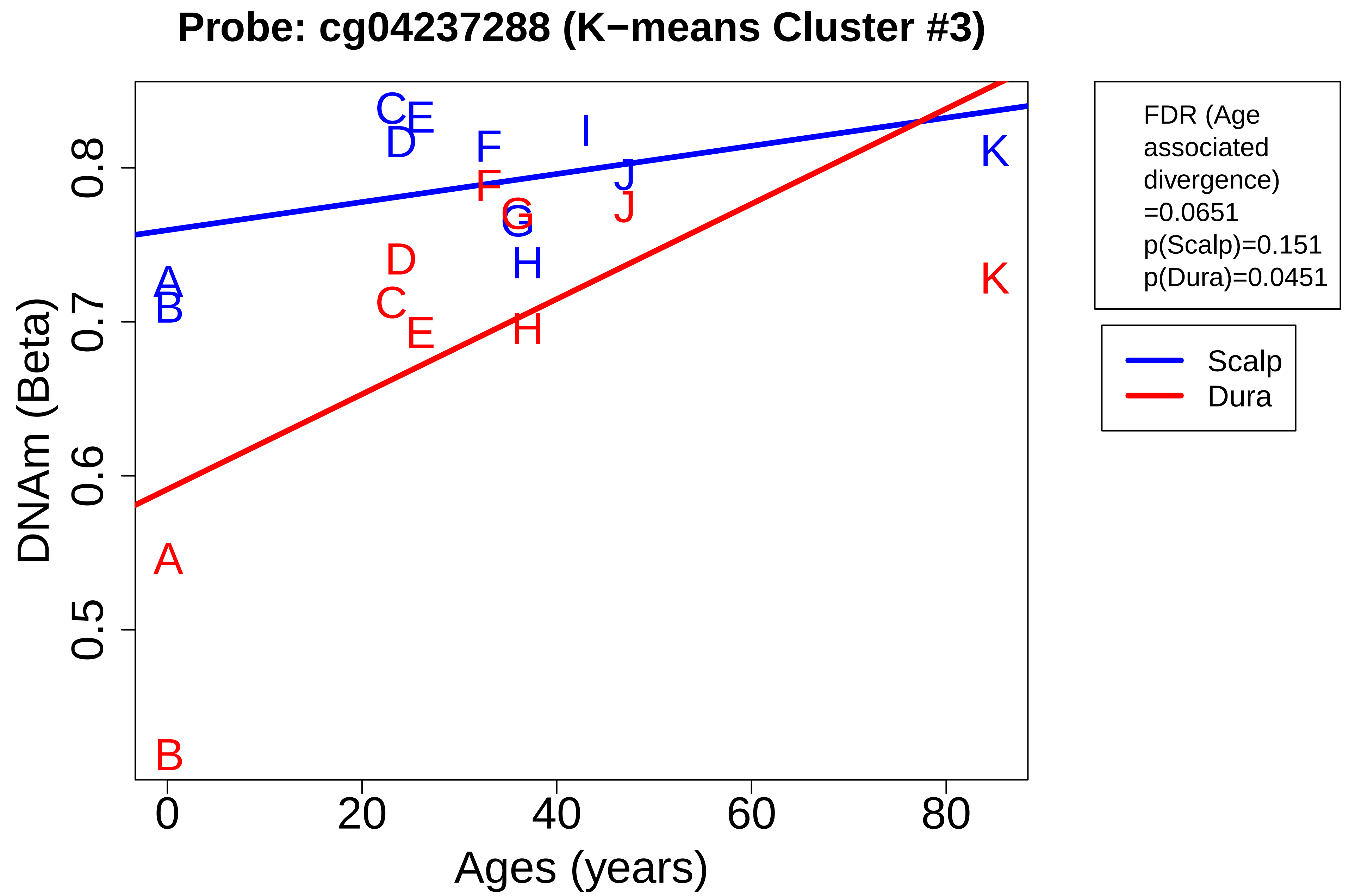

Probe: cg04237288 (K−means Cluster #3)
DNAm (Beta)
0.5
0.6
0.7
0.8
Ages (years)
0
20
40
60
80
FDR (Age associated divergence) =0.0651
p(Scalp)=0.151
p(Dura)=0.0451
Scalp
Dura

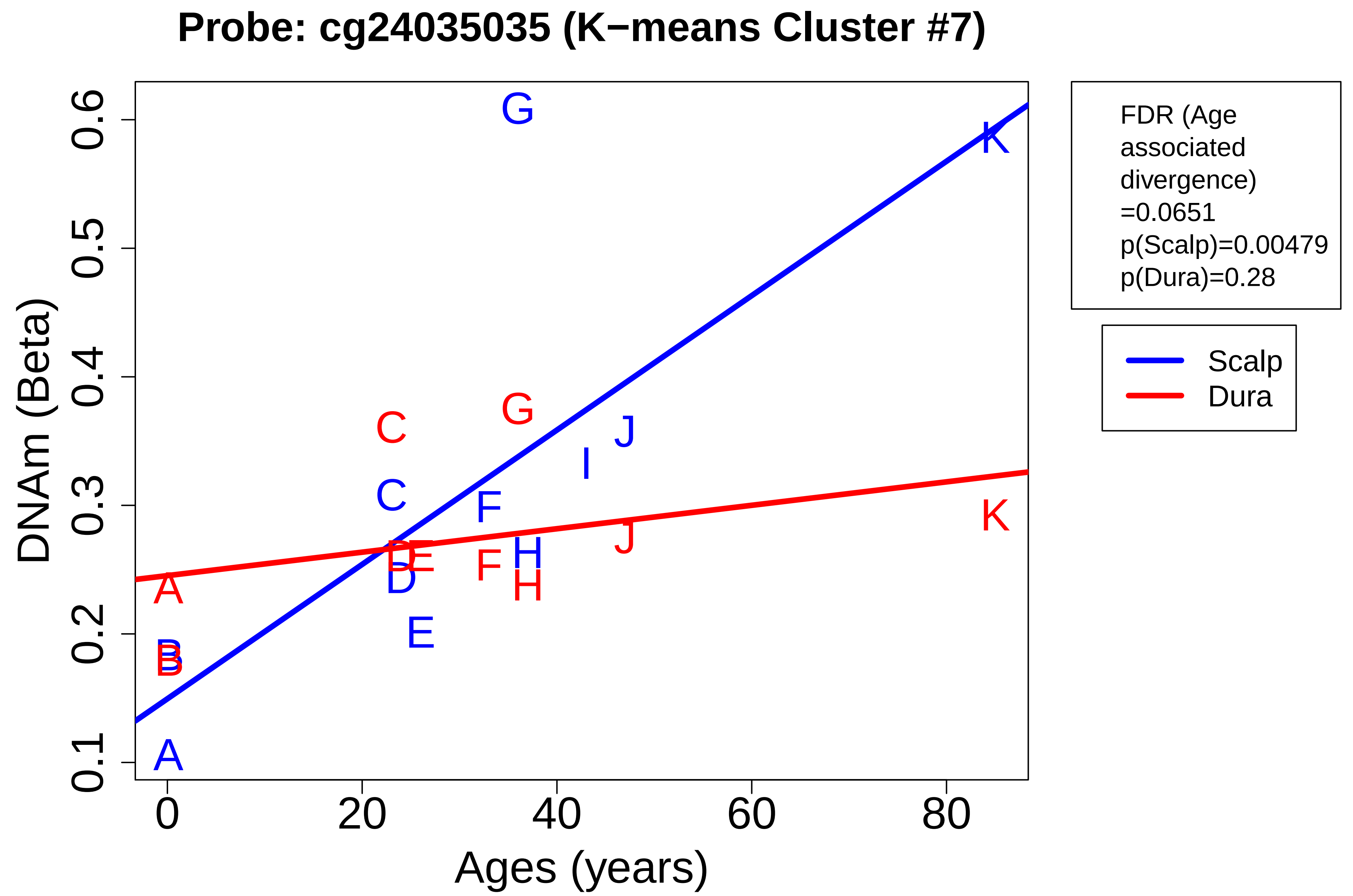

Probe: cg24035035 (K−means Cluster #7)
FDR (Age associated divergence) =0.0651
p(Scalp)=0.00479
p(Dura)=0.28
Scalp
Dura
DNAm (Beta)
Ages (years)
0.1
0.2
0.3
0.4
0.5
0.6
0
20
40
60
80

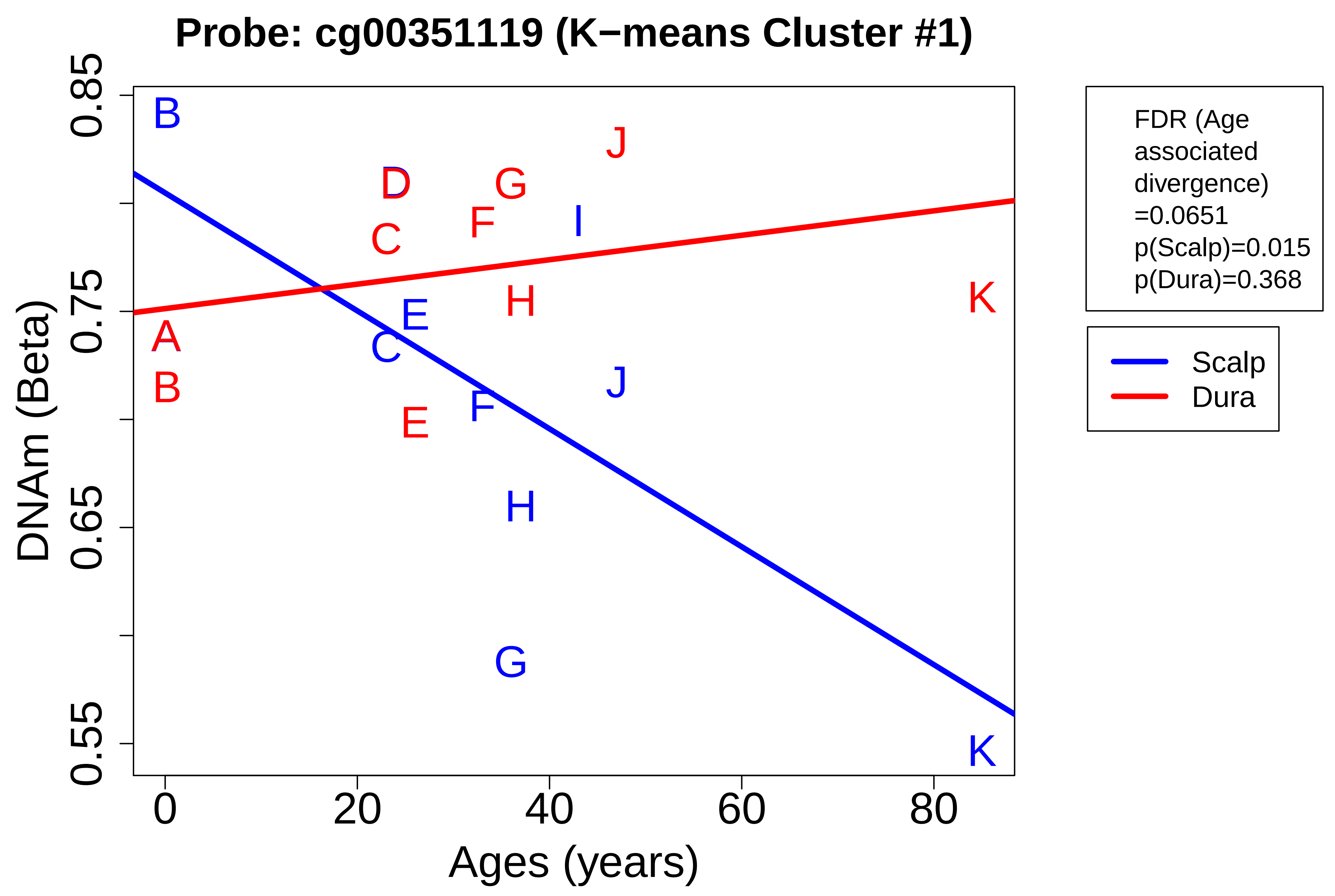

Probe: cg00351119 (K−means Cluster #1)
DNAm (Beta)
0.55
0.65
0.75
0.85
Ages (years)
0
20
40
60
80
B
J
D
G
F
I
C
H
K
E
A
C
B
J
F
E
H
G
K
FDR (Age associated divergence) =0.0651
p(Scalp)=0.015
p(Dura)=0.368
Scalp
Dura

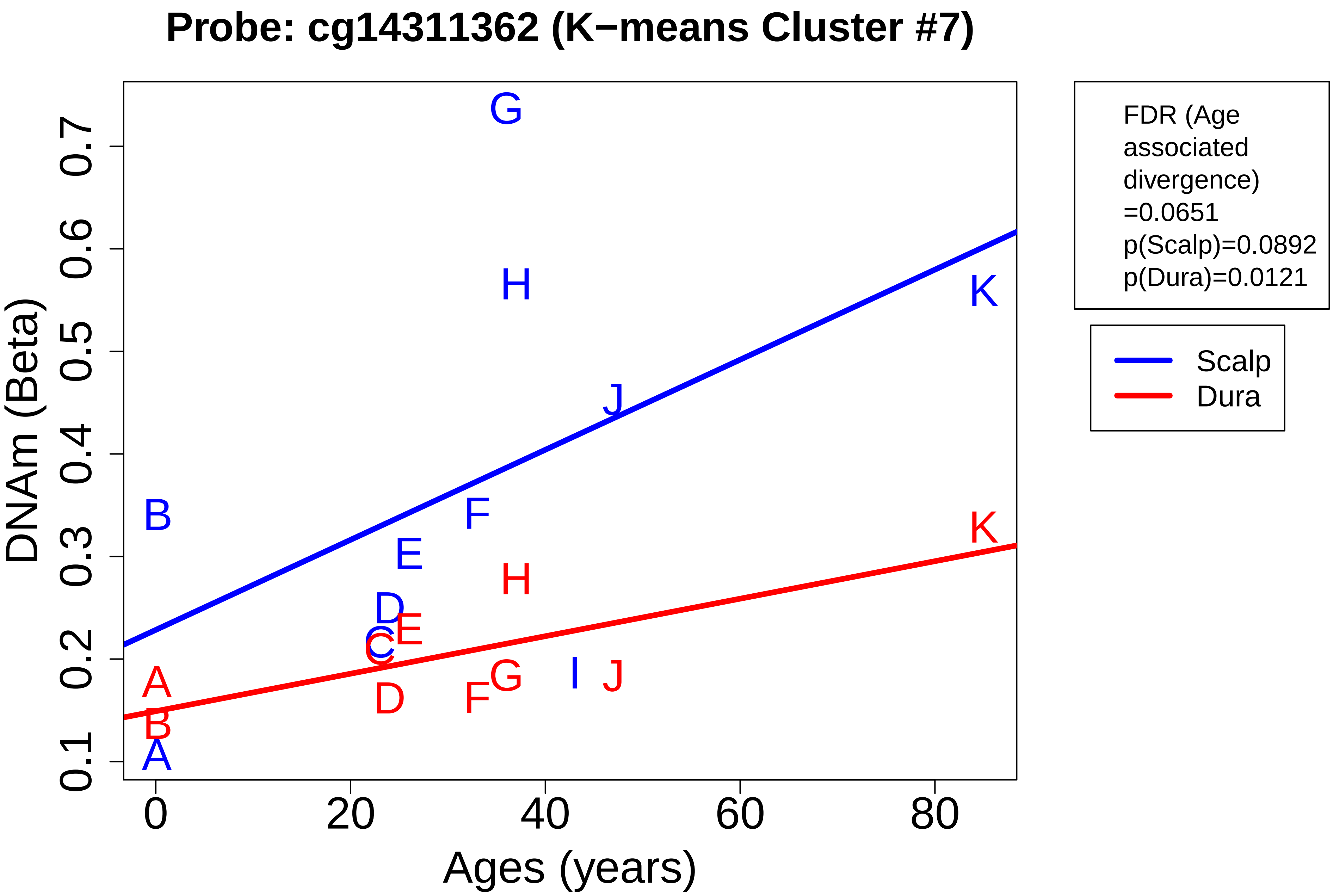
Probe: cg14311362 (K−means Cluster #7)
DNAm (Beta)
0.1
0.2
0.3
0.4
0.5
0.6
0.7
0
20
40
60
80
Ages (years)
A
B
C
D
E
F
G
H
I
J
K
FDR (Age associated divergence) =0.0651
p(Scalp)=0.0892
p(Dura)=0.0121
Scalp
Dura

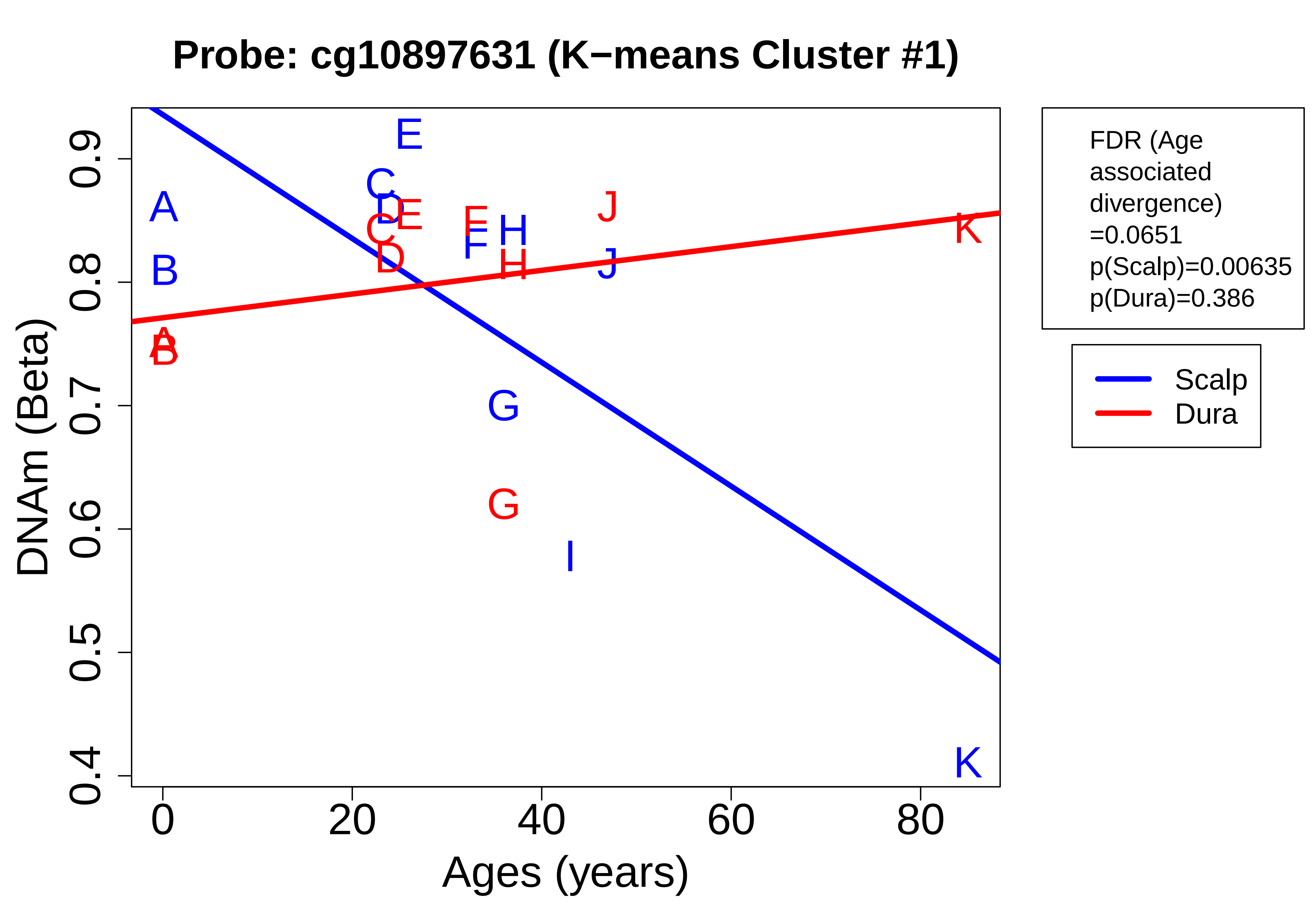
Probe: cg10897631 (K−means Cluster #1)
DNAm (Beta)
0.4
0.5
0.6
0.7
0.8
0.9
0
20
40
60
80
Ages (years)
FDR (Age associated divergence) =0.0651
p(Scalp)=0.00635
p(Dura)=0.386
Scalp
Dura

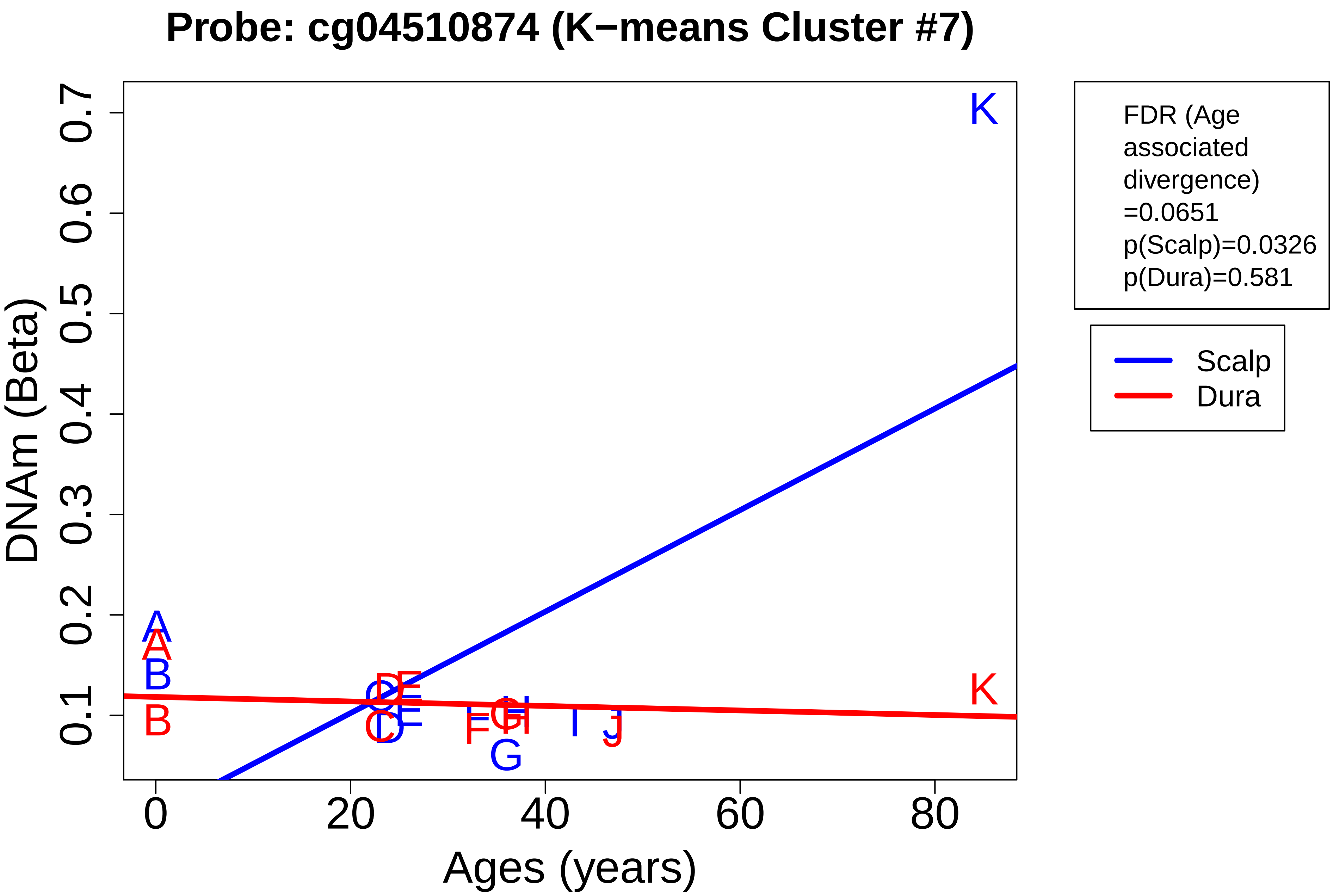

Probe: cg04510874 (K−means Cluster #7)
DNAm (Beta)
0.1
0.2
0.3
0.4
0.5
0.6
0.7
0
20
40
60
80
Ages (years)
FDR (Age associated divergence) =0.0651
p(Scalp)=0.0326
p(Dura)=0.581
Scalp
Dura

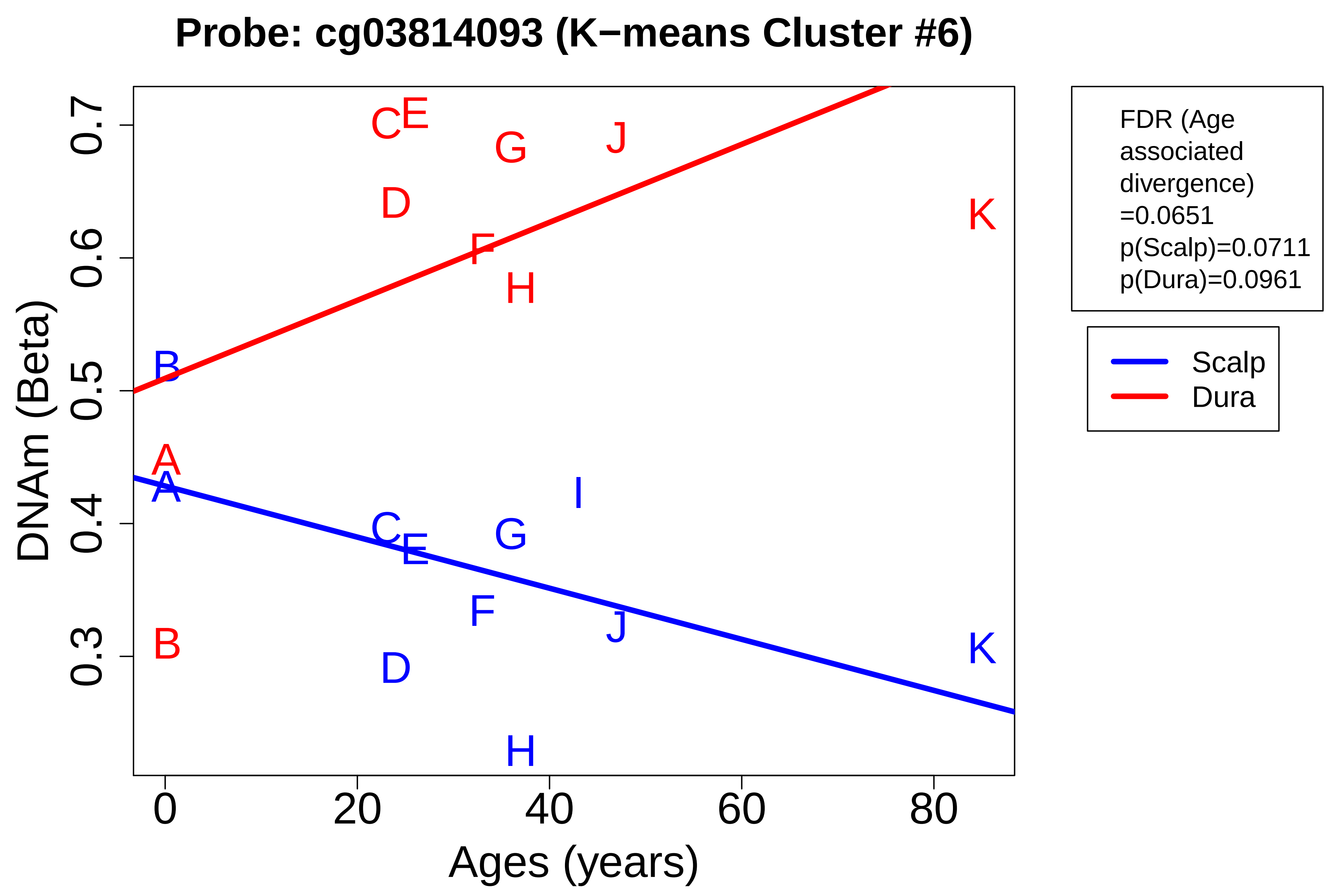

Probe: cg03814093 (K−means Cluster #6)
FDR (Age associated divergence) =0.0651
p(Scalp)=0.0711
p(Dura)=0.0961
Scalp
Dura
DNAm (Beta)
Ages (years)
0.3
0.4
0.5
0.6
0.7
0
20
40
60
80

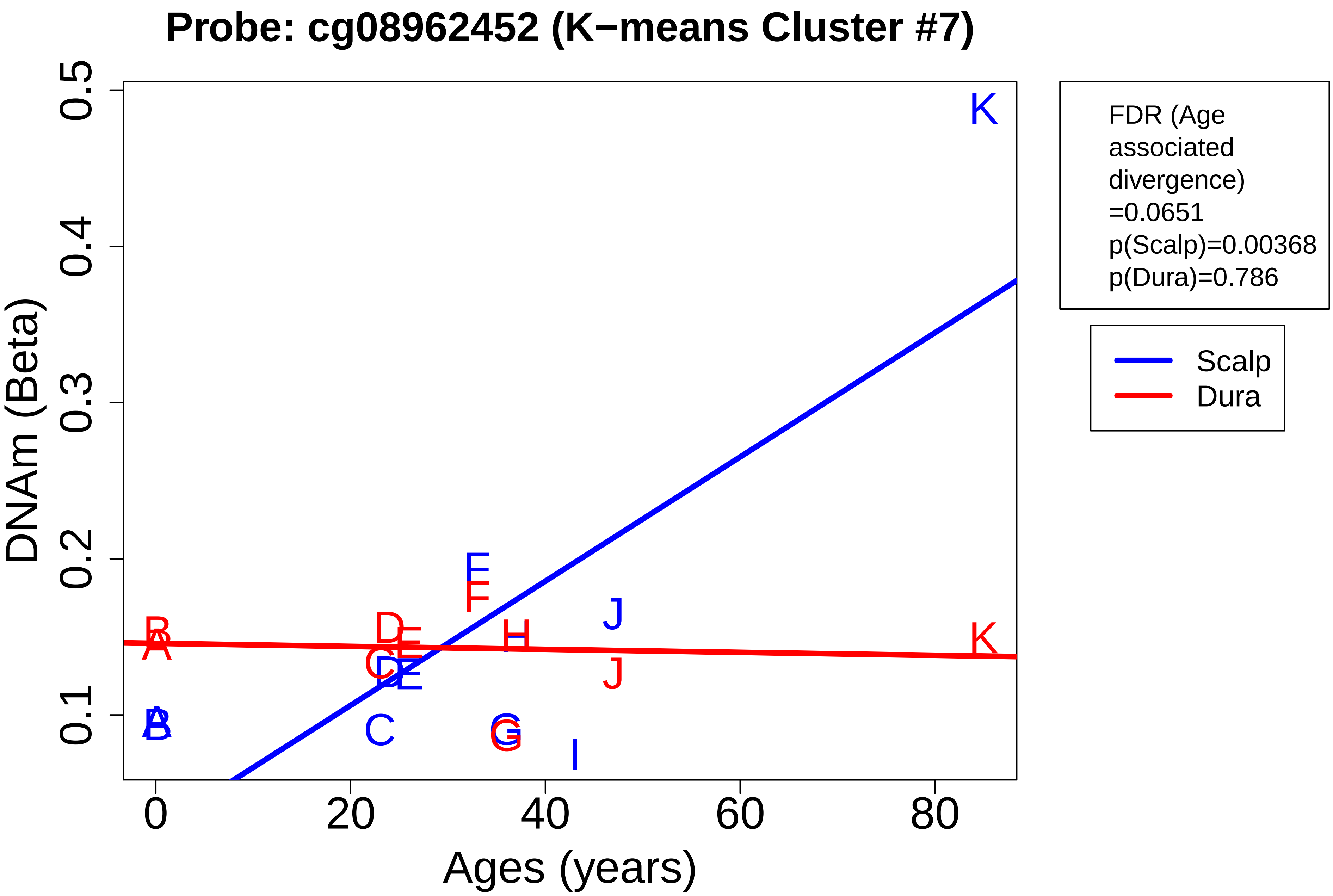

Probe: cg08962452 (K−means Cluster #7)
FDR (Age associated divergence) =0.0651
p(Scalp)=0.00368
p(Dura)=0.786
Scalp
Dura
DNAm (Beta)
Ages (years)
0.1
0.2
0.3
0.4
0.5
0
20
40
60
80

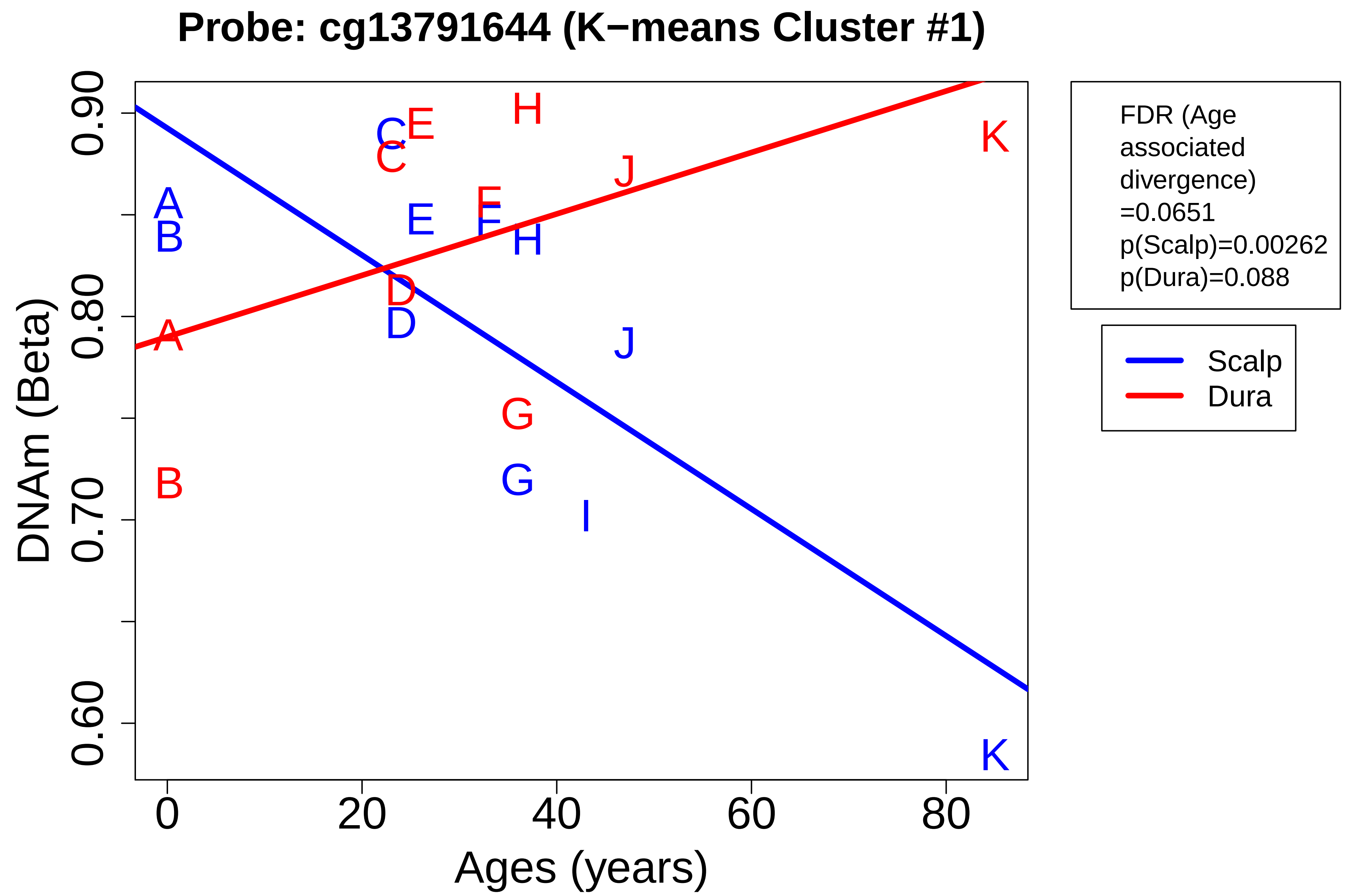

Probe: cg13791644 (K−means Cluster #1)
DNAm (Beta)
0.60
0.70
0.80
0.90
0
20
40
60
80
Ages (years)
FDR (Age associated divergence) =0.0651
p(Scalp)=0.00262
p(Dura)=0.088
Scalp
Dura

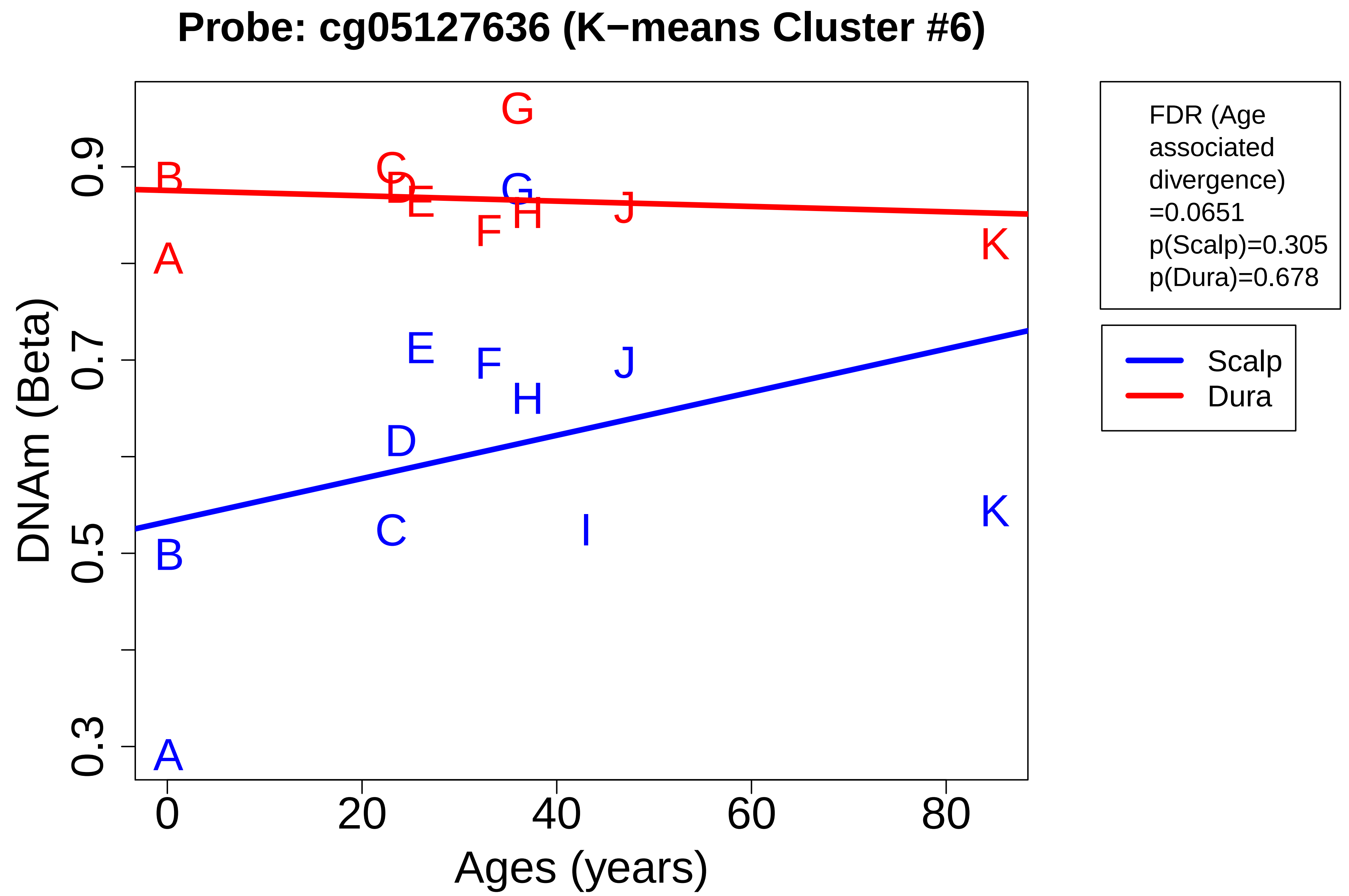

Probe: cg05127636 (K−means Cluster #6)
DNAm (Beta)
Ages (years)
0.3
0.5
0.7
0.9
0
20
40
60
80
A
B
C
D
E
F
G
H
I
J
K
FDR (Age associated divergence) =0.0651
p(Scalp)=0.305
p(Dura)=0.678
Scalp
Dura

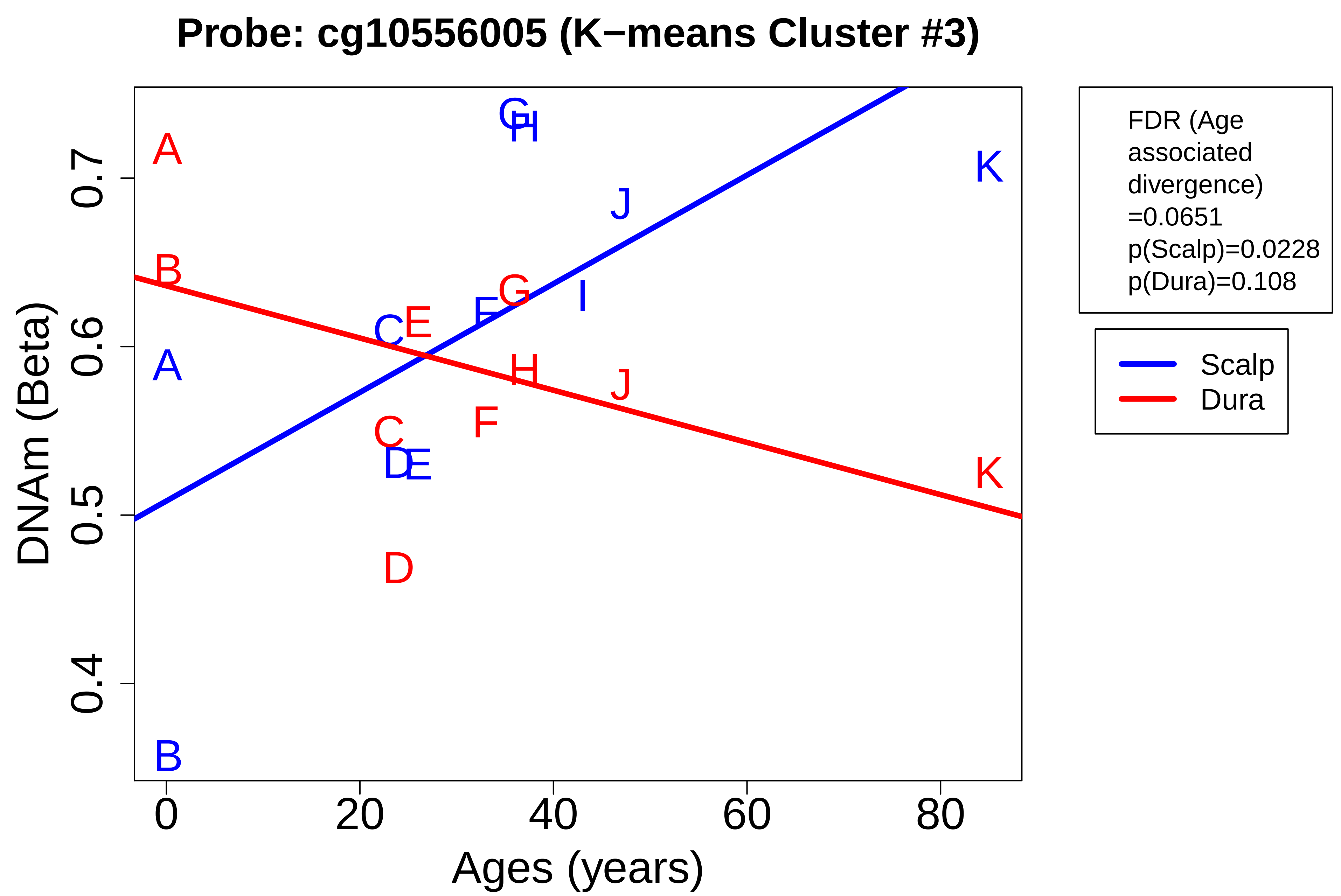
Probe: cg10556005 (K−means Cluster #3)
DNAm (Beta)
0.4
0.5
0.6
0.7
0
20
40
60
80
Ages (years)
FDR (Age associated divergence) =0.0651
p(Scalp)=0.0228
p(Dura)=0.108
Scalp
Dura

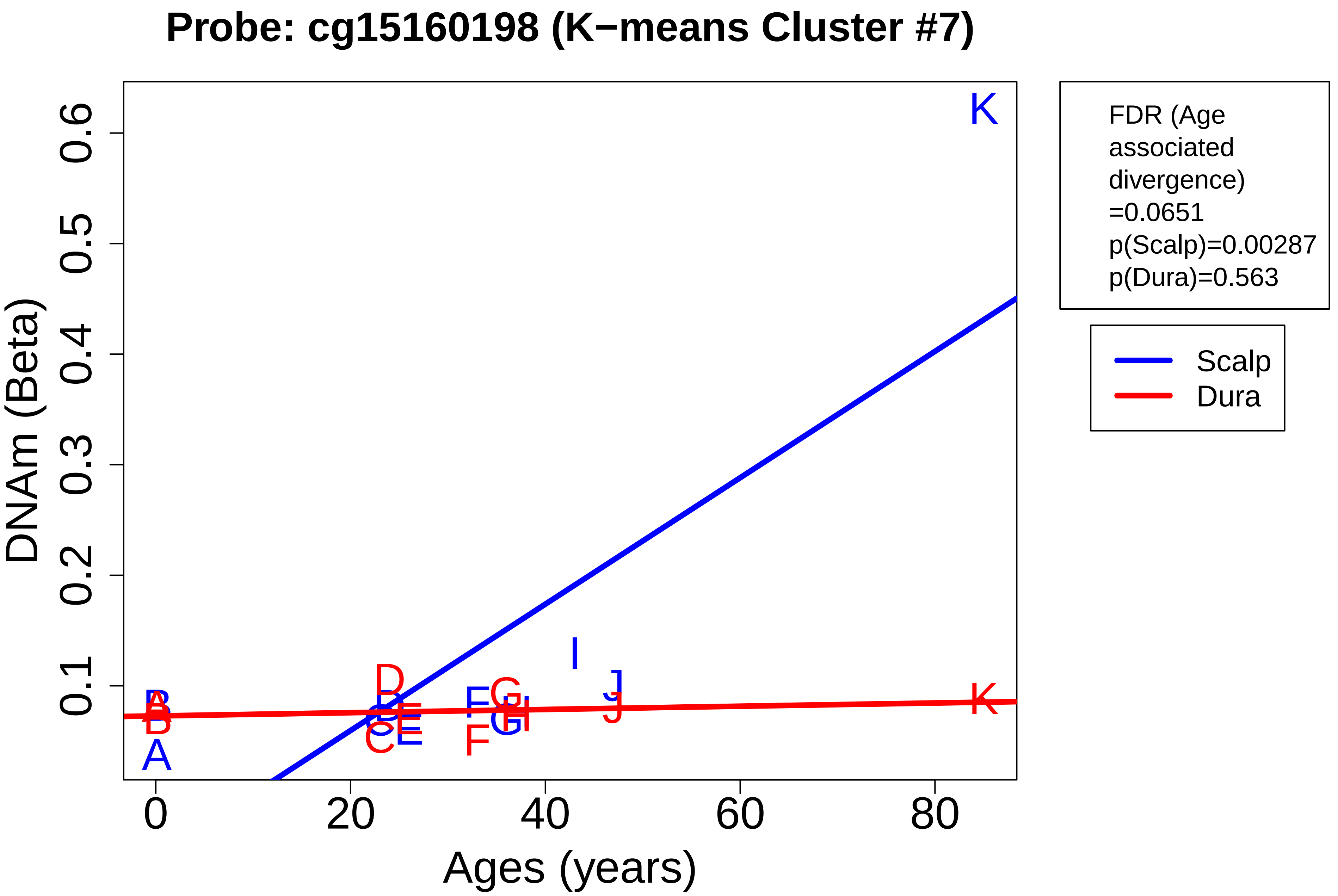

Probe: cg15160198 (K−means Cluster #7)
DNAm (Beta)
0.1
0.2
0.3
0.4
0.5
0.6
0
20
40
60
80
Ages (years)
FDR (Age associated divergence) =0.0651
p(Scalp)=0.00287
p(Dura)=0.563
Scalp
Dura

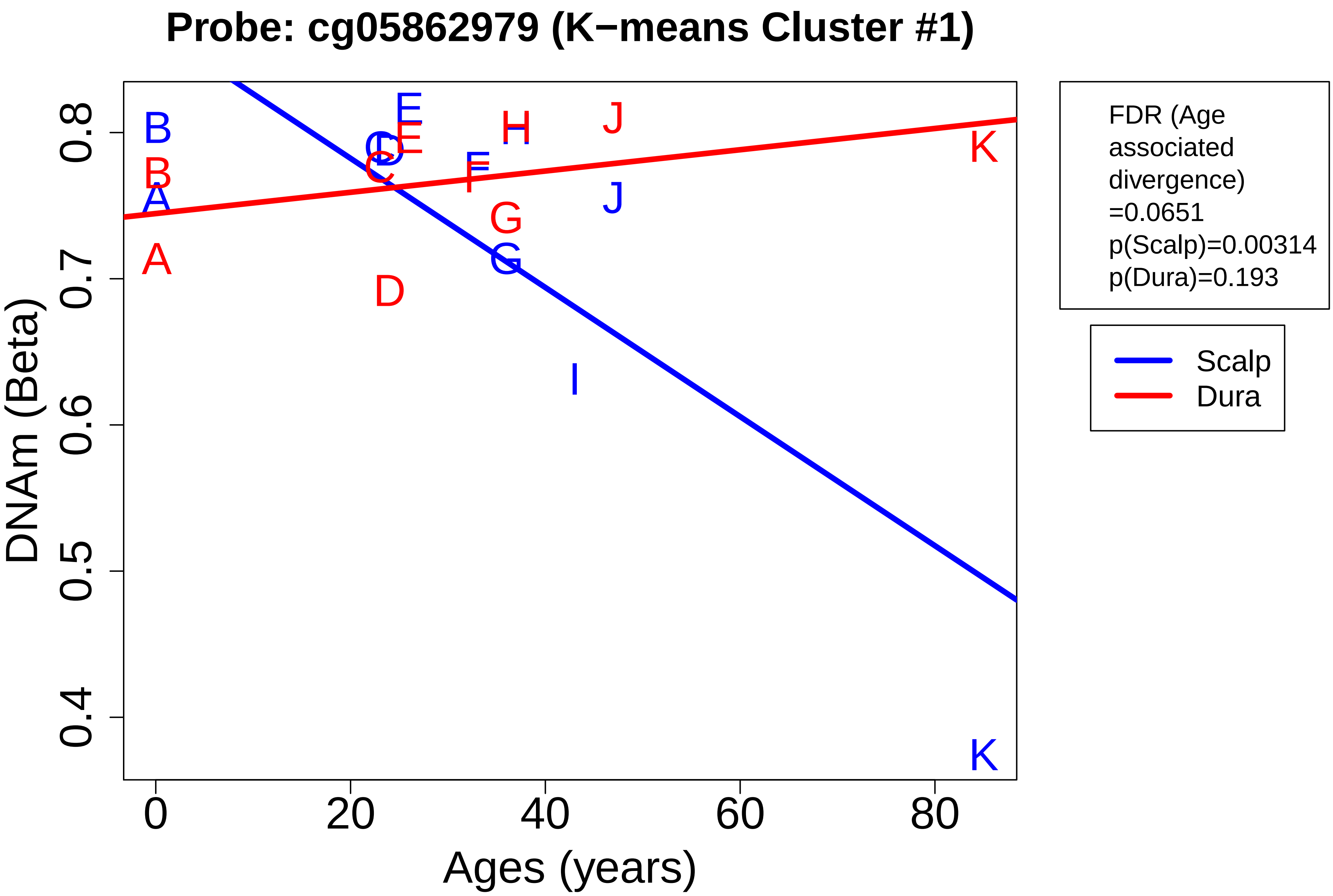

Probe: cg05862979 (K−means Cluster #1)
DNAm (Beta)
0.4
0.5
0.6
0.7
0.8
Ages (years)
0
20
40
60
80
FDR (Age associated divergence) =0.0651
p(Scalp)=0.00314
p(Dura)=0.193
Scalp
Dura

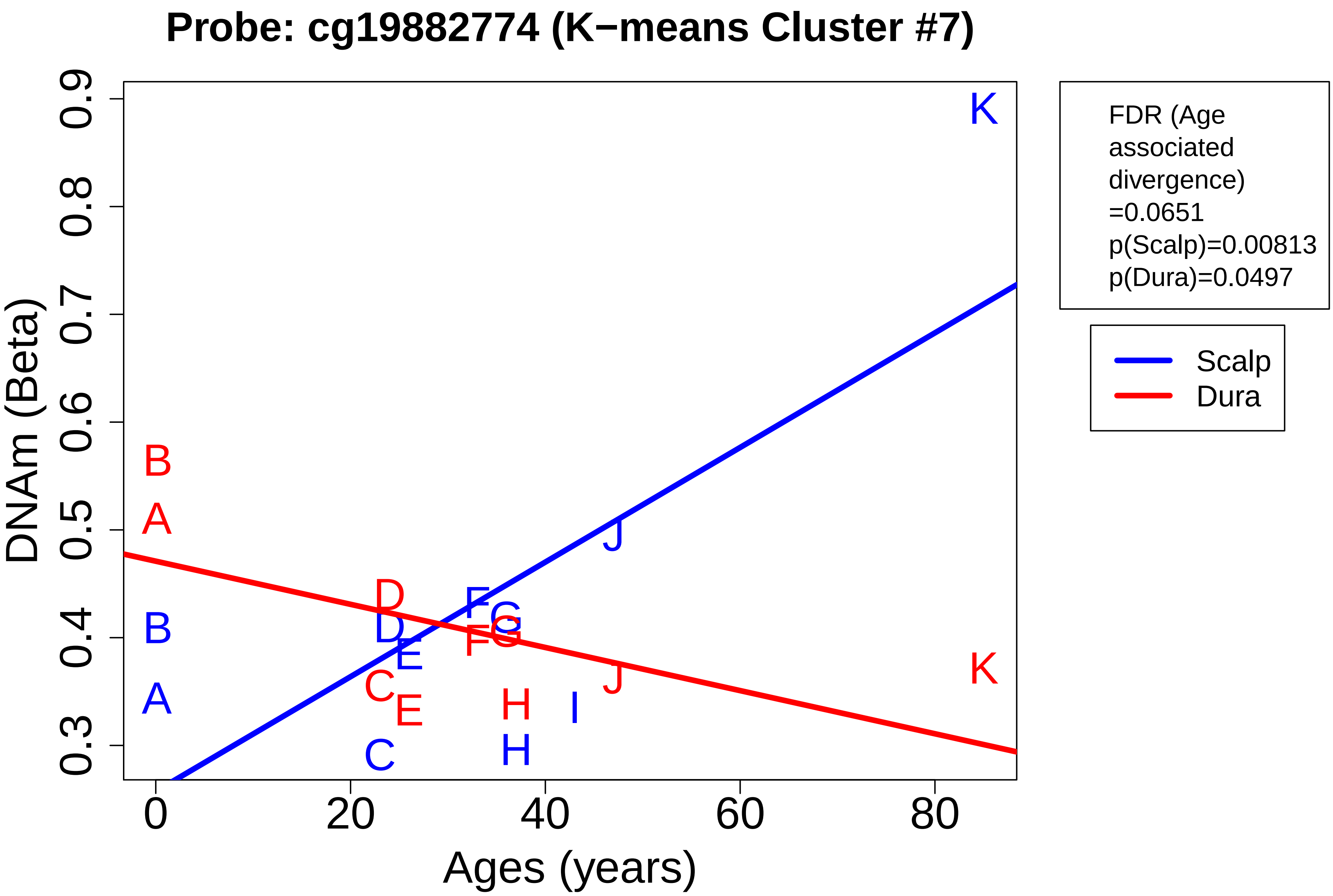

Probe: cg19882774 (K−means Cluster #7)
DNAm (Beta)
Ages (years)
FDR (Age associated divergence) =0.0651
p(Scalp)=0.00813
p(Dura)=0.0497
Scalp
Dura

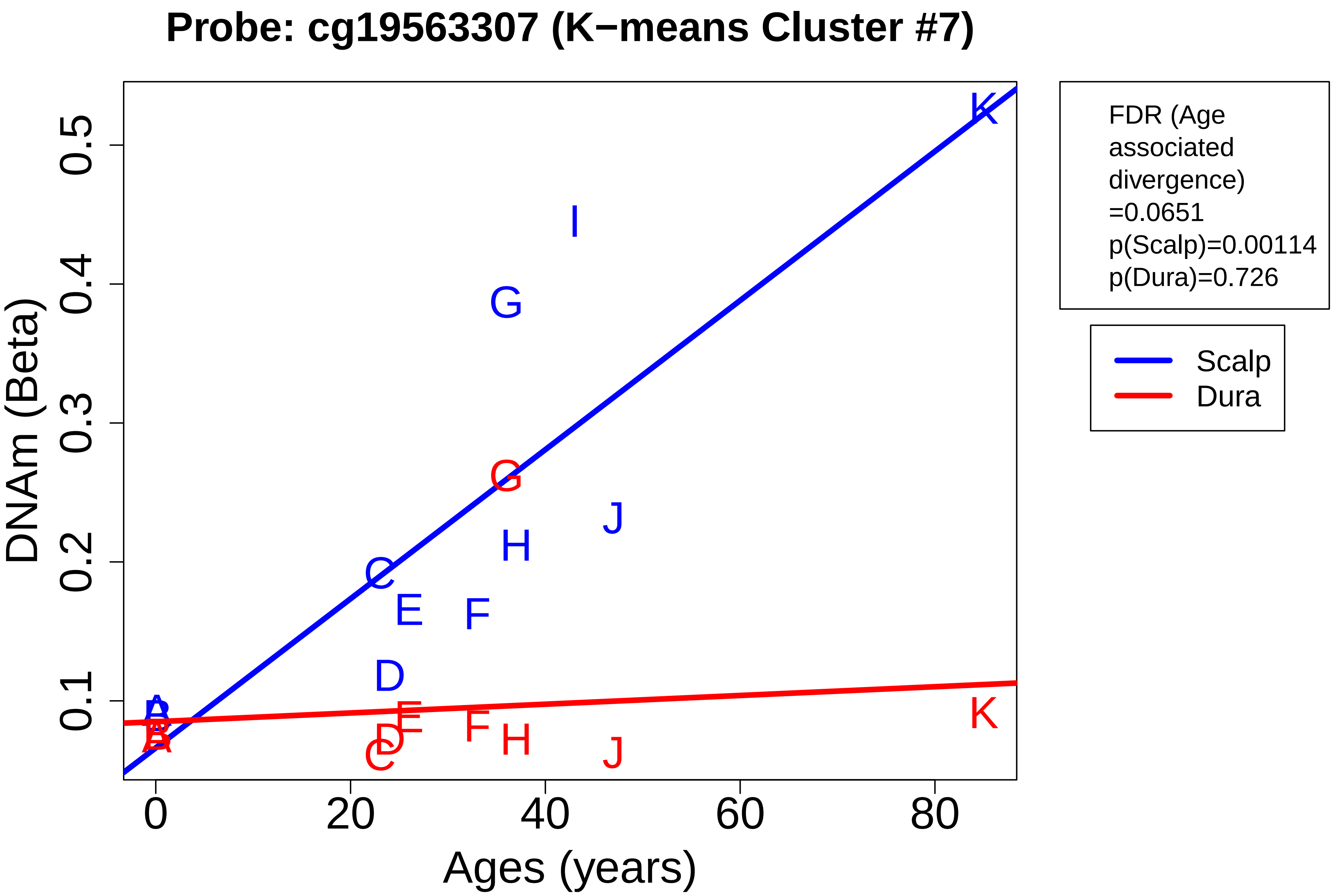

Probe: cg19563307 (K−means Cluster #7)
DNAm (Beta)
0.1
0.2
0.3
0.4
0.5
0
20
40
60
80
Ages (years)
FDR (Age associated divergence) =0.0651
p(Scalp)=0.00114
p(Dura)=0.726
Scalp
Dura

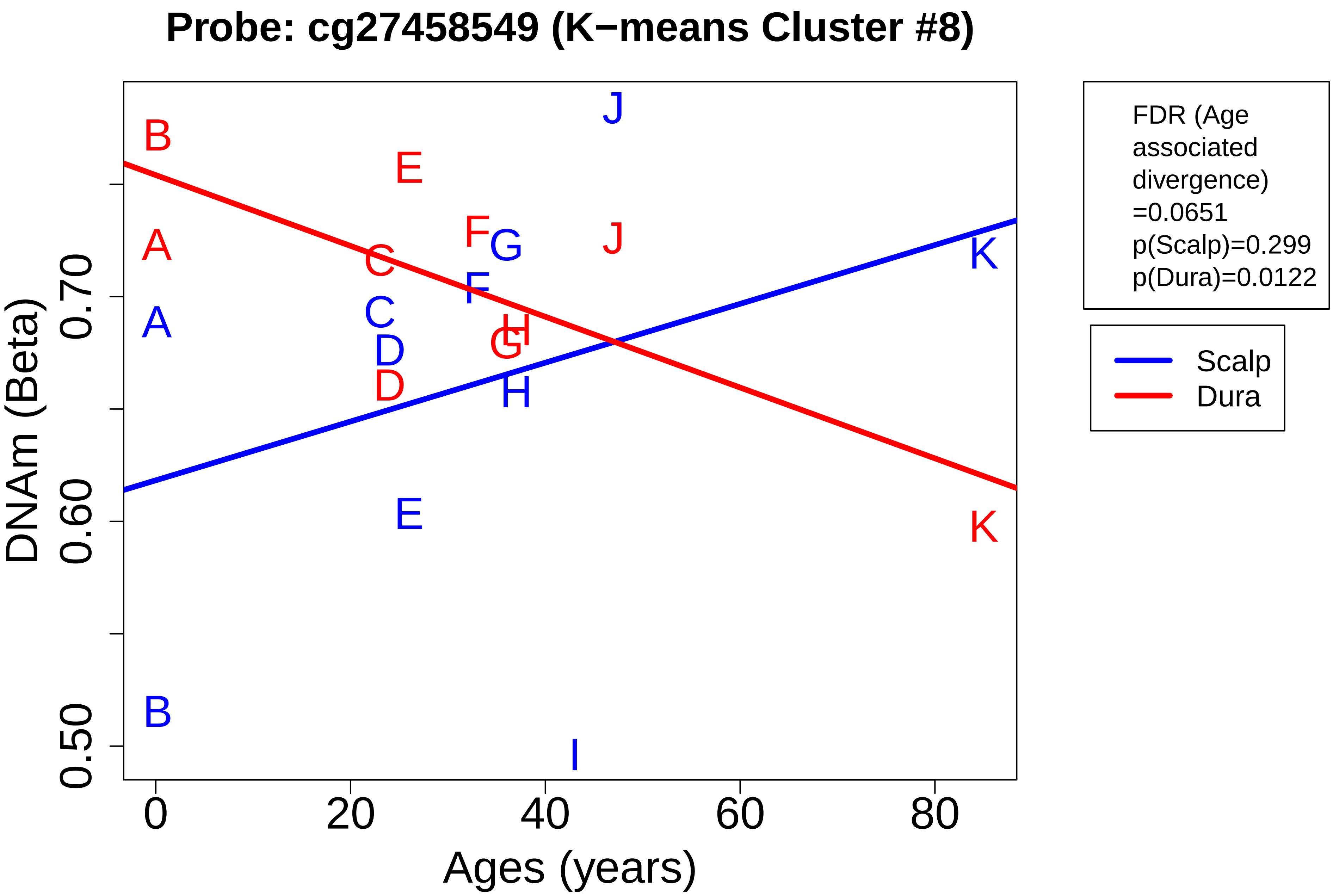

Probe: cg27458549 (K−means Cluster #8)
FDR (Age associated divergence) =0.0651
p(Scalp)=0.299
p(Dura)=0.0122
Scalp
Dura
DNAm (Beta)
0.50
0.60
0.70
0
20
40
60
80
Ages (years)

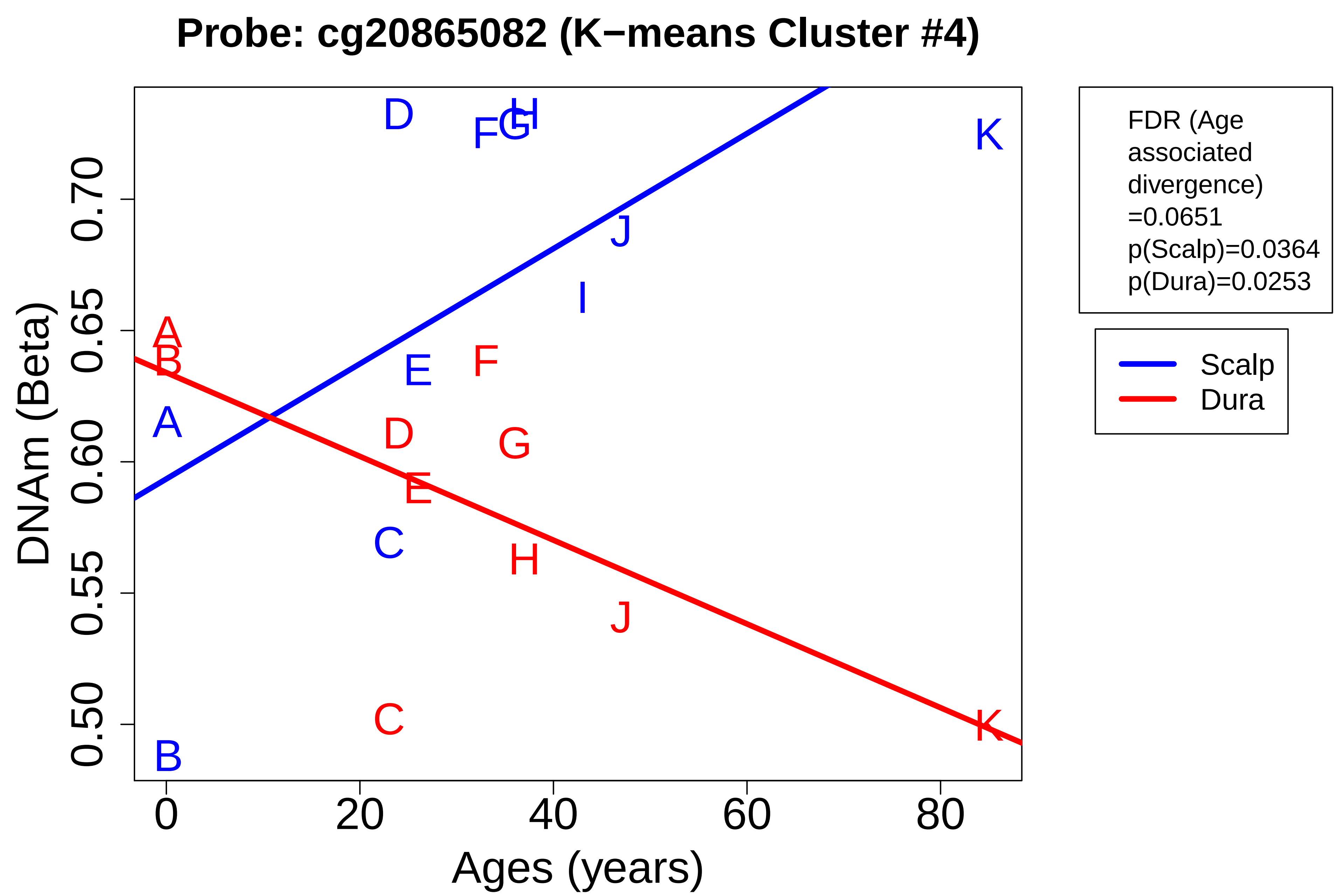

Probe: cg20865082 (K−means Cluster #4)
DNAm (Beta)
0.50
0.55
0.60
0.65
0.70
Ages (years)
0
20
40
60
80
FDR (Age associated divergence) =0.0651
p(Scalp)=0.0364
p(Dura)=0.0253
Scalp
Dura
A
B
C
D
E
F
G
H
I
J
K

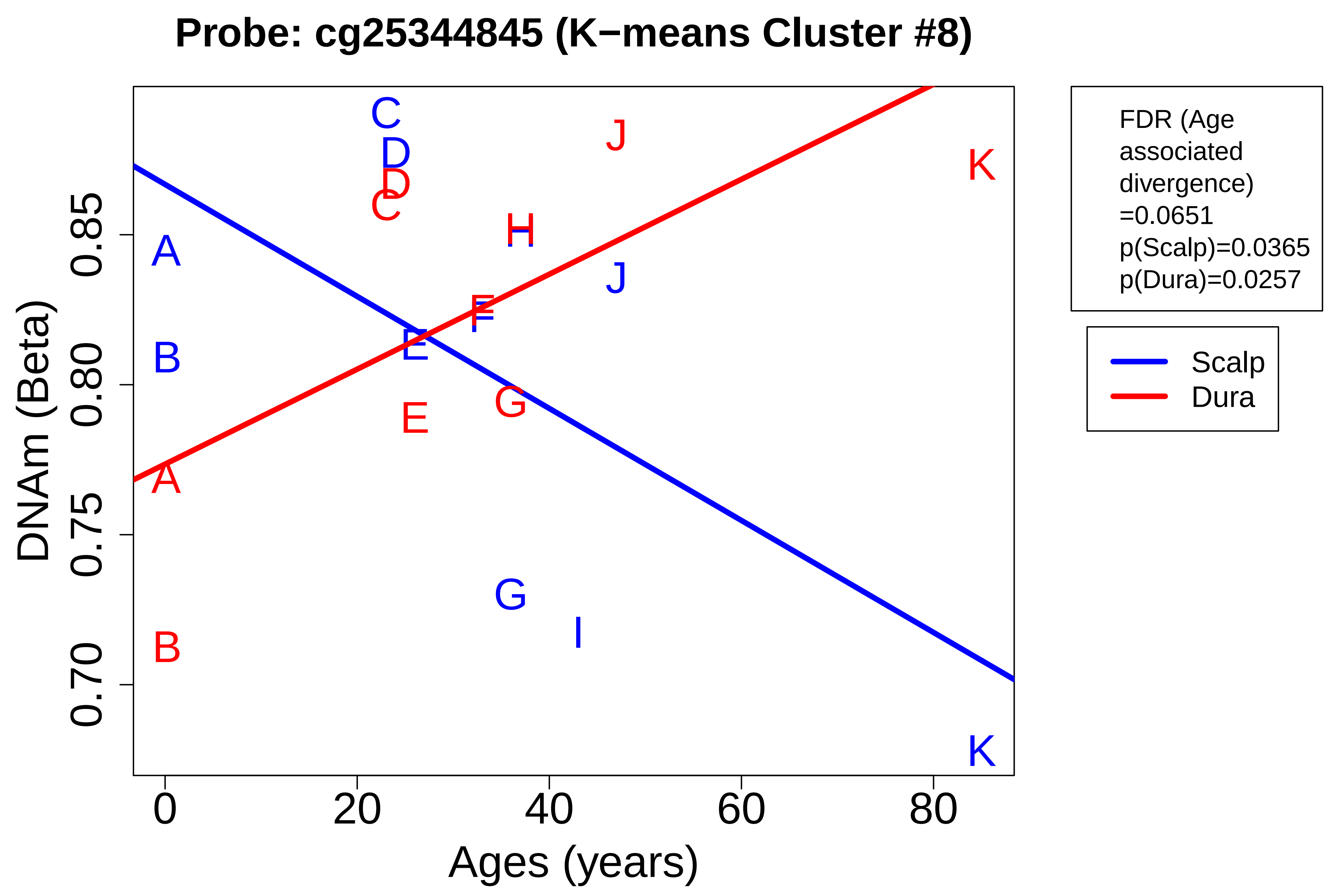

Probe: cg25344845 (K−means Cluster #8)
DNAm (Beta)
0.70
0.75
0.80
0.85
0
20
40
60
80
Ages (years)
A
B
C
D
E
F
G
H
I
J
K
FDR (Age associated divergence) =0.0651
p(Scalp)=0.0365
p(Dura)=0.0257
Scalp
Dura

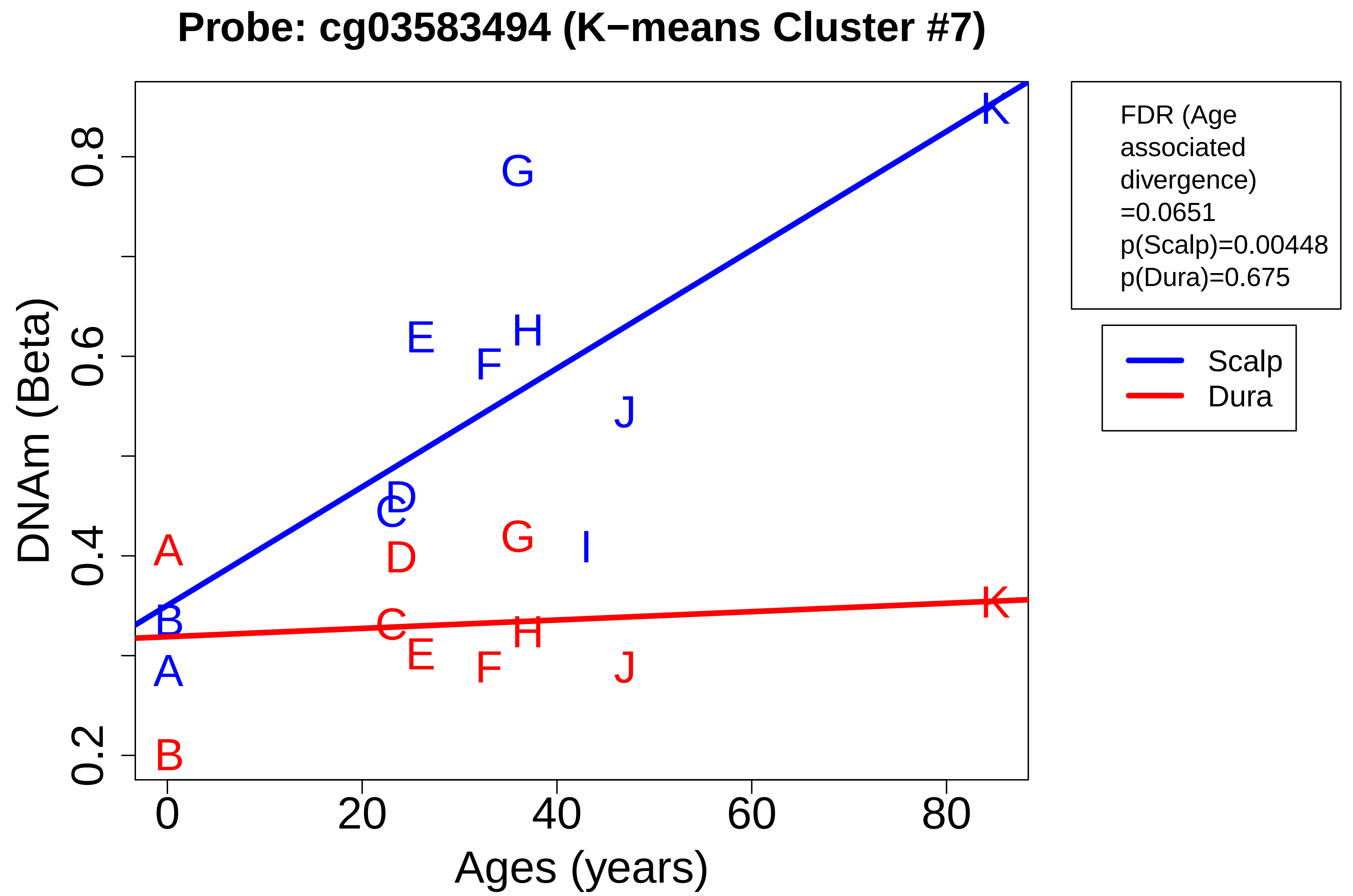
Probe: cg03583494 (K−means Cluster #7)
DNAm (Beta)
Ages (years)
0.2
0.4
0.6
0.8
0
20
40
60
80
FDR (Age associated divergence) =0.0651
p(Scalp)=0.00448
p(Dura)=0.675
Scalp
Dura
A
B
C
D
E
F
G
H
I
J
K

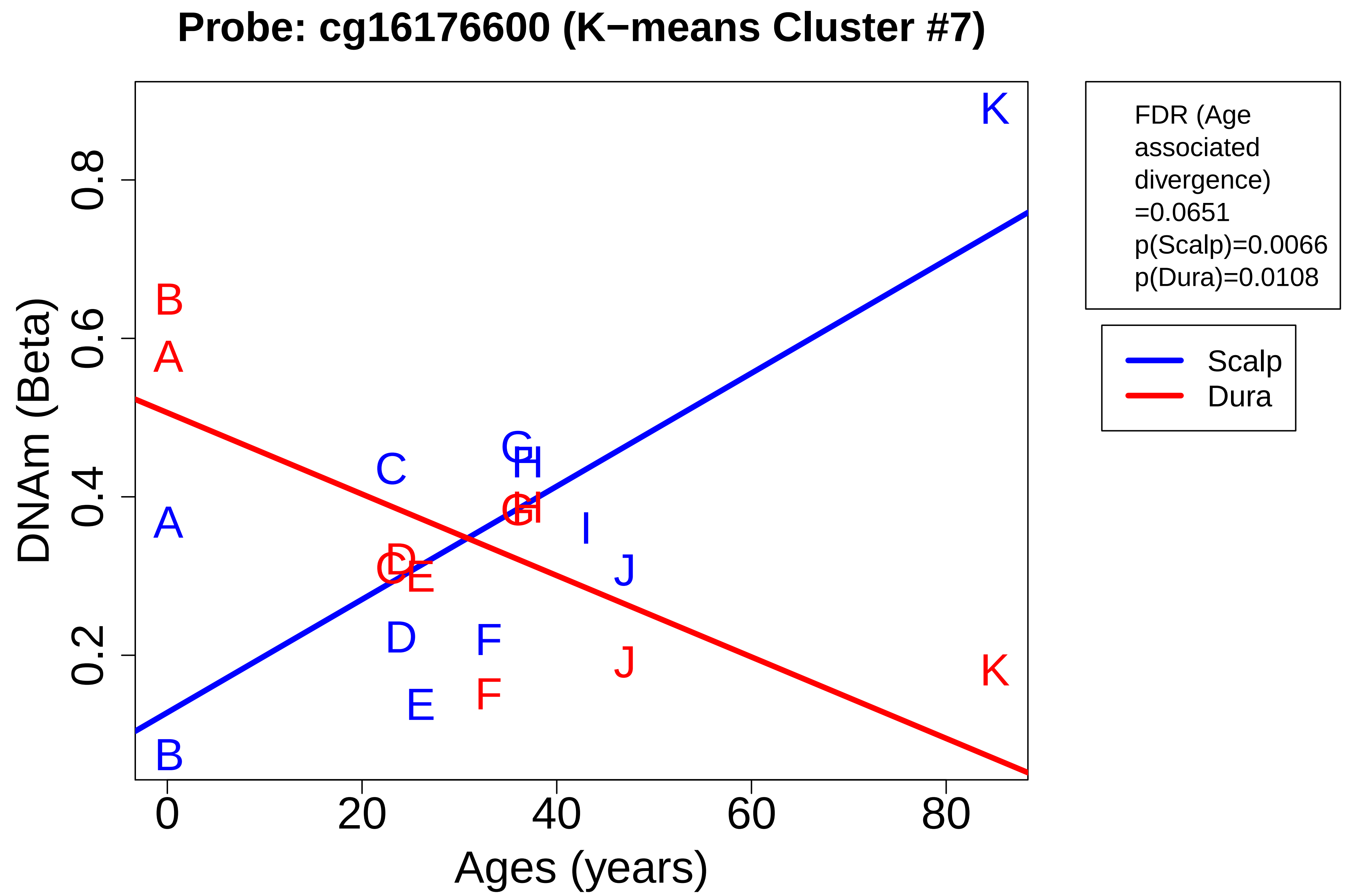

Probe: cg16176600 (K−means Cluster #7)
DNAm (Beta)
0.2
0.4
0.6
0.8
0
20
40
60
80
Ages (years)
FDR (Age associated divergence) =0.0651
p(Scalp)=0.0066
p(Dura)=0.0108
Scalp
Dura

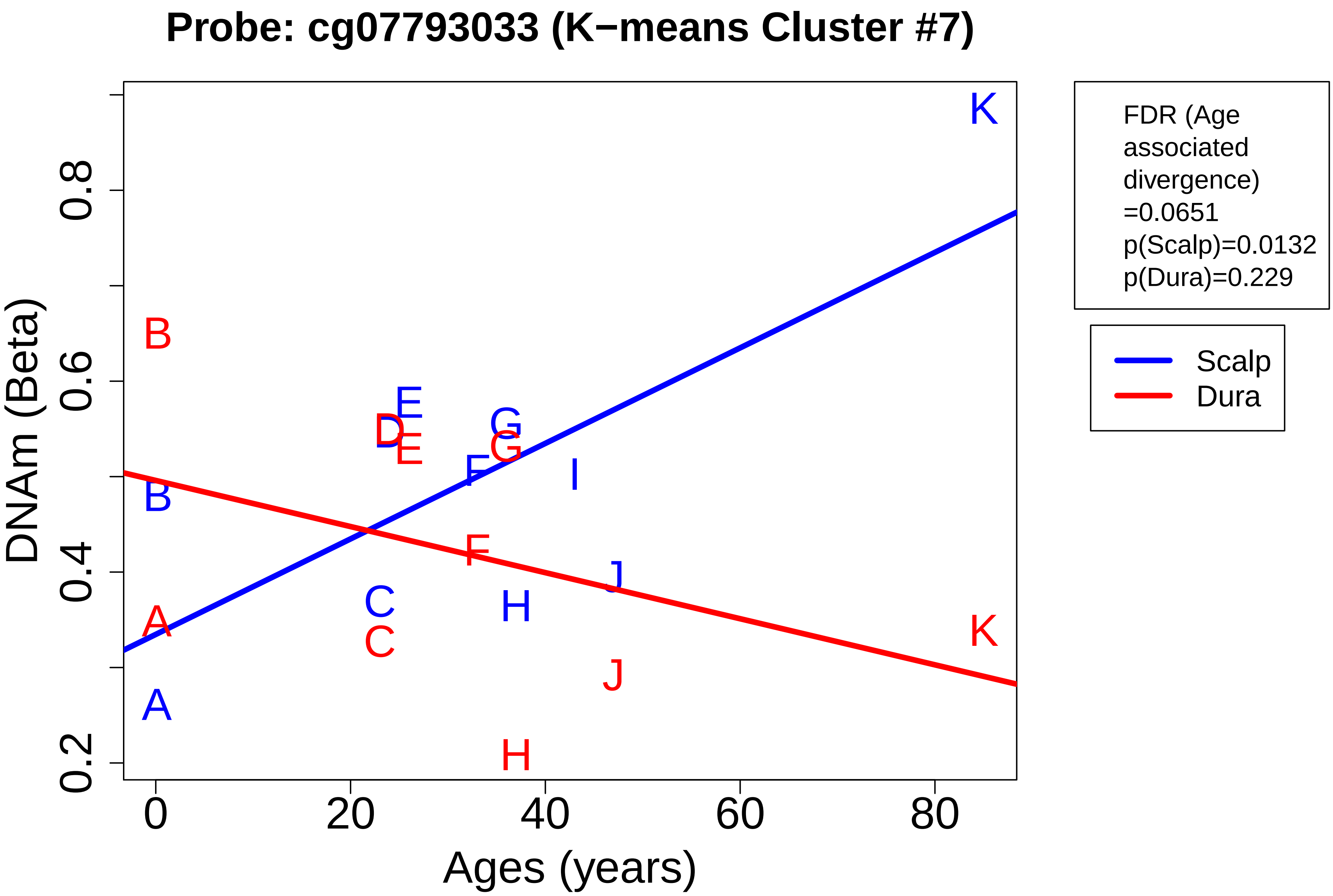
Probe: cg07793033 (K−means Cluster #7)
DNAm (Beta)
0.2
0.4
0.6
0.8
0
20
40
60
80
Ages (years)
FDR (Age associated divergence) =0.0651
p(Scalp)=0.0132
p(Dura)=0.229
Scalp
Dura
A
B
C
D
E
F
G
H
I
J
K

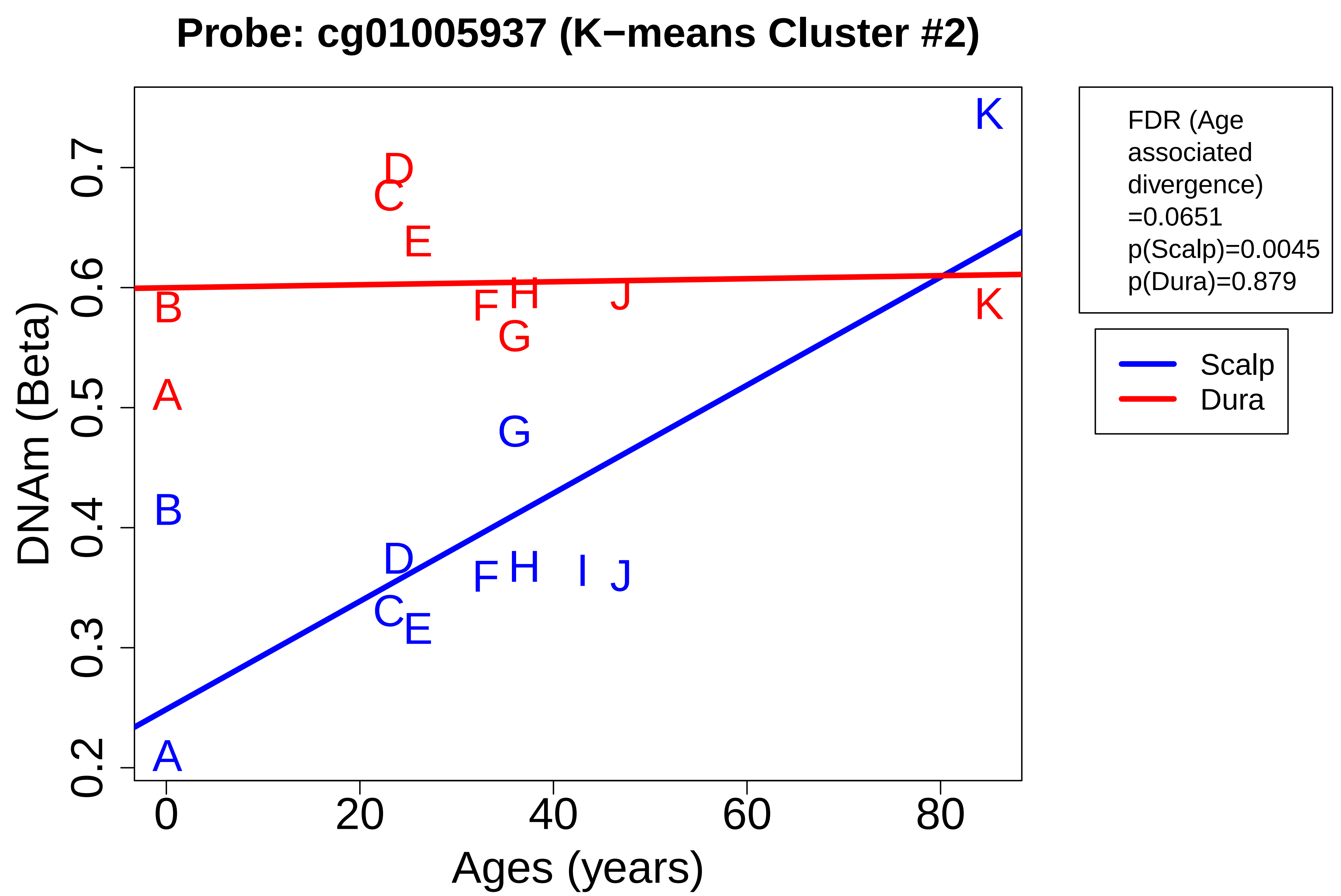

Probe: cg01005937 (K−means Cluster #2)
DNAm (Beta)
Ages (years)
0.2
0.3
0.4
0.5
0.6
0.7
0
20
40
60
80
FDR (Age associated divergence) =0.0651
p(Scalp)=0.0045
p(Dura)=0.879
Scalp
Dura

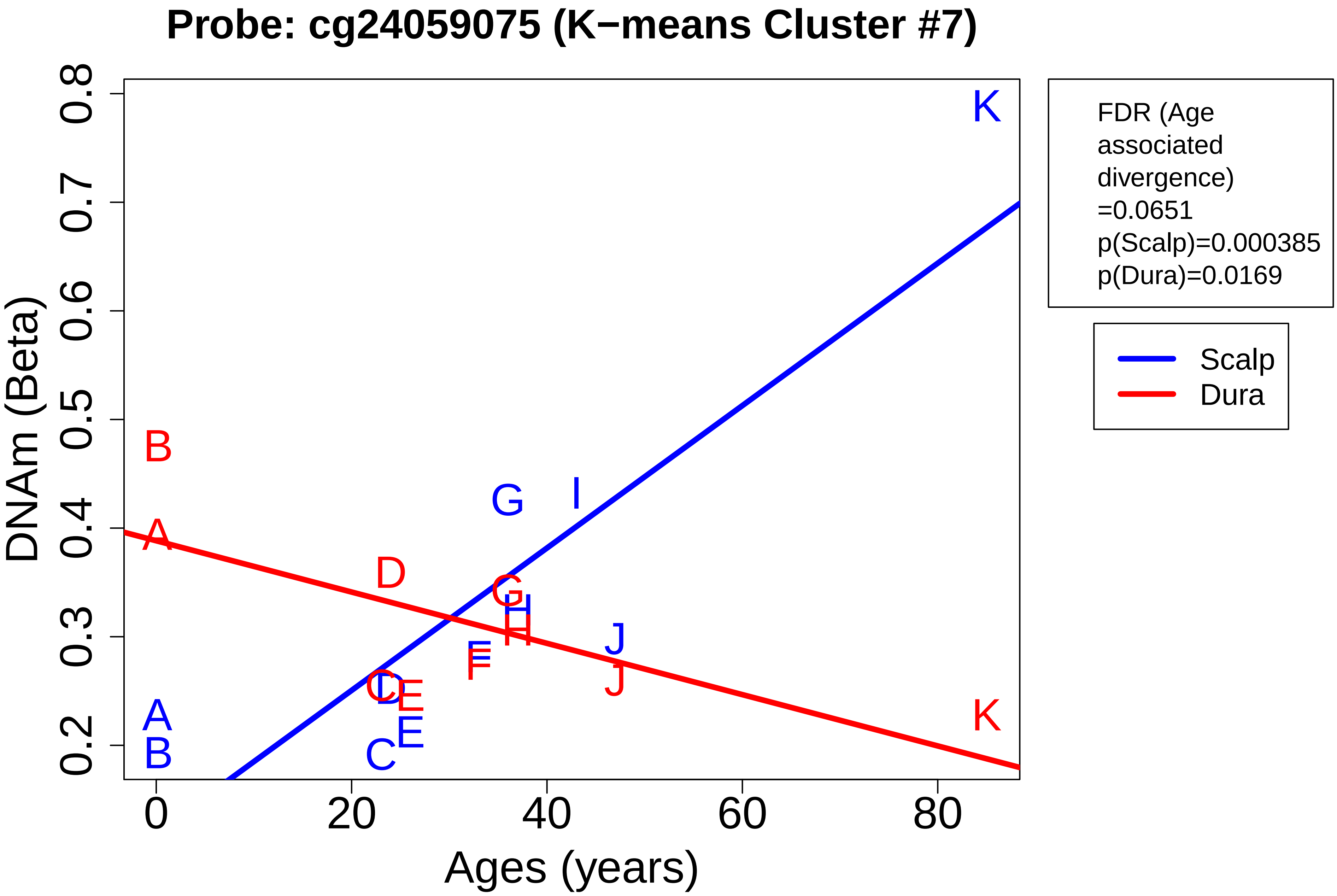

Probe: cg24059075 (K−means Cluster #7)
DNAm (Beta)
0.2
0.3
0.4
0.5
0.6
0.7
0.8
Ages (years)
0
20
40
60
80
A
B
C
D
E
F
G
H
I
J
K
FDR (Age associated divergence) =0.0651
p(Scalp)=0.000385
p(Dura)=0.0169
Scalp
Dura

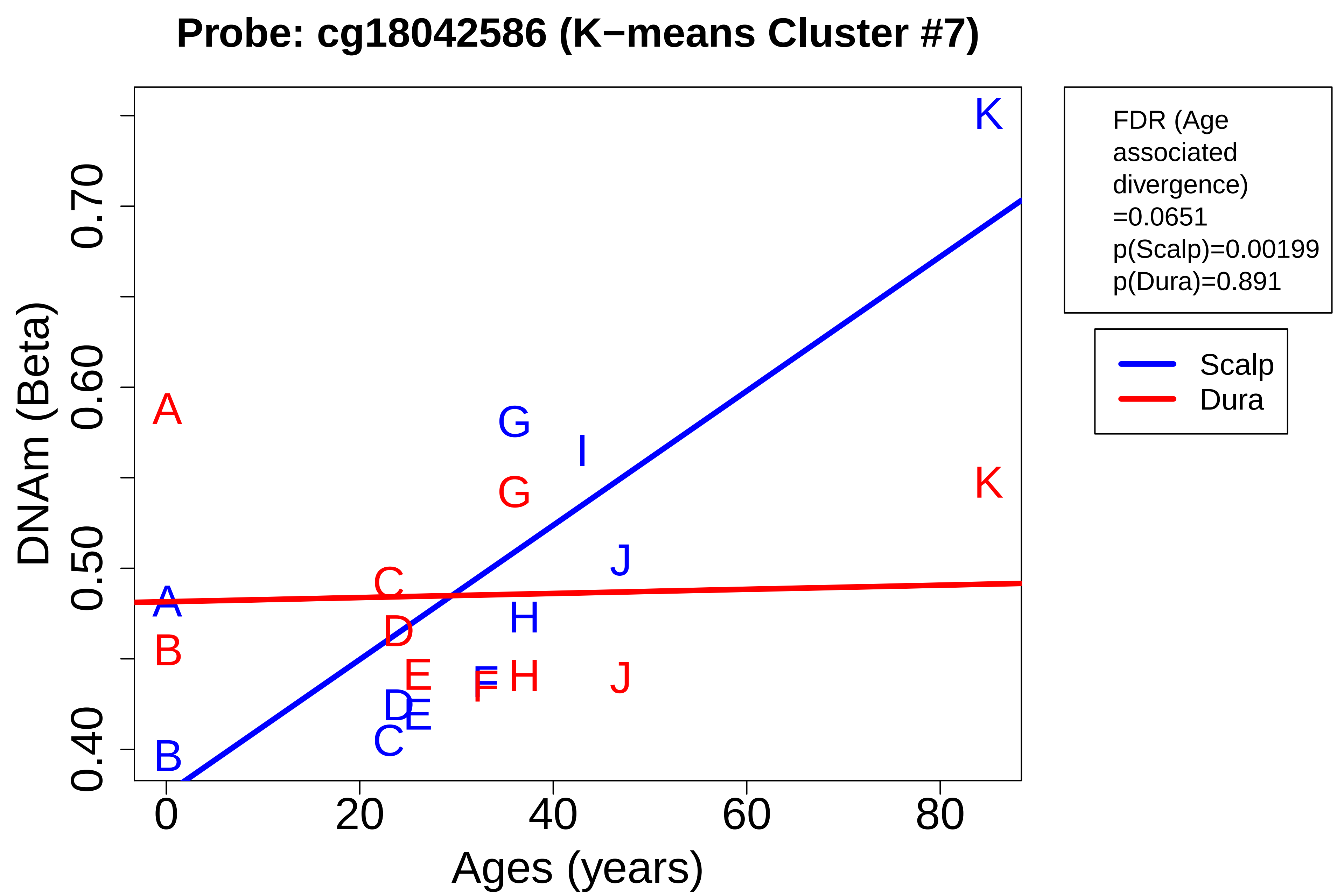

Probe: cg18042586 (K−means Cluster #7)
DNAm (Beta)
0.40
0.50
0.60
0.70
Ages (years)
0
20
40
60
80
A
B
C
D
E
F
G
H
I
J
K
FDR (Age associated divergence) =0.0651
p(Scalp)=0.00199
p(Dura)=0.891
Scalp
Dura

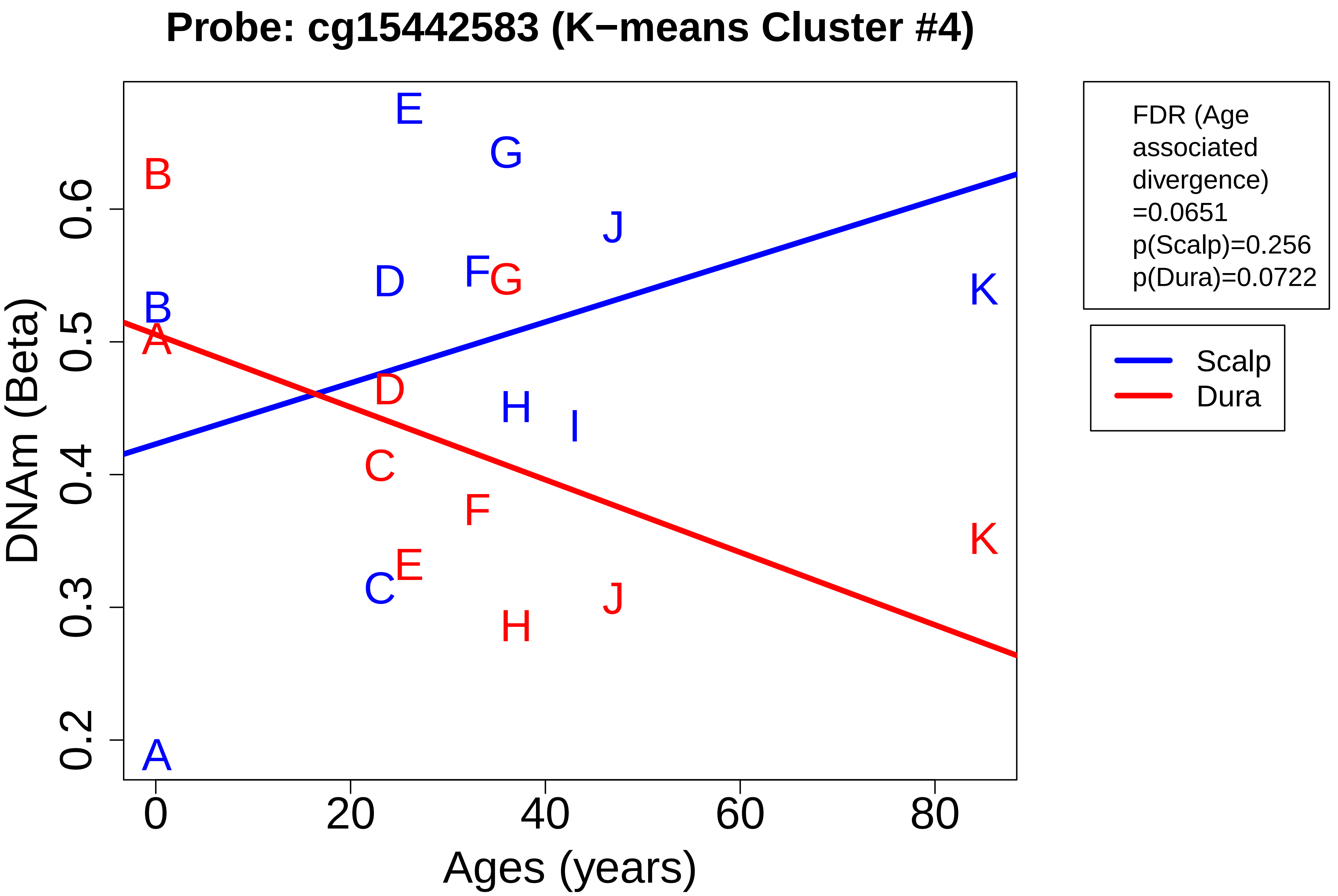

Probe: cg15442583 (K−means Cluster #4)
DNAm (Beta)
0.2
0.3
0.4
0.5
0.6
Ages (years)
0
20
40
60
80
FDR (Age associated divergence) =0.0651
p(Scalp)=0.256
p(Dura)=0.0722
Scalp
Dura

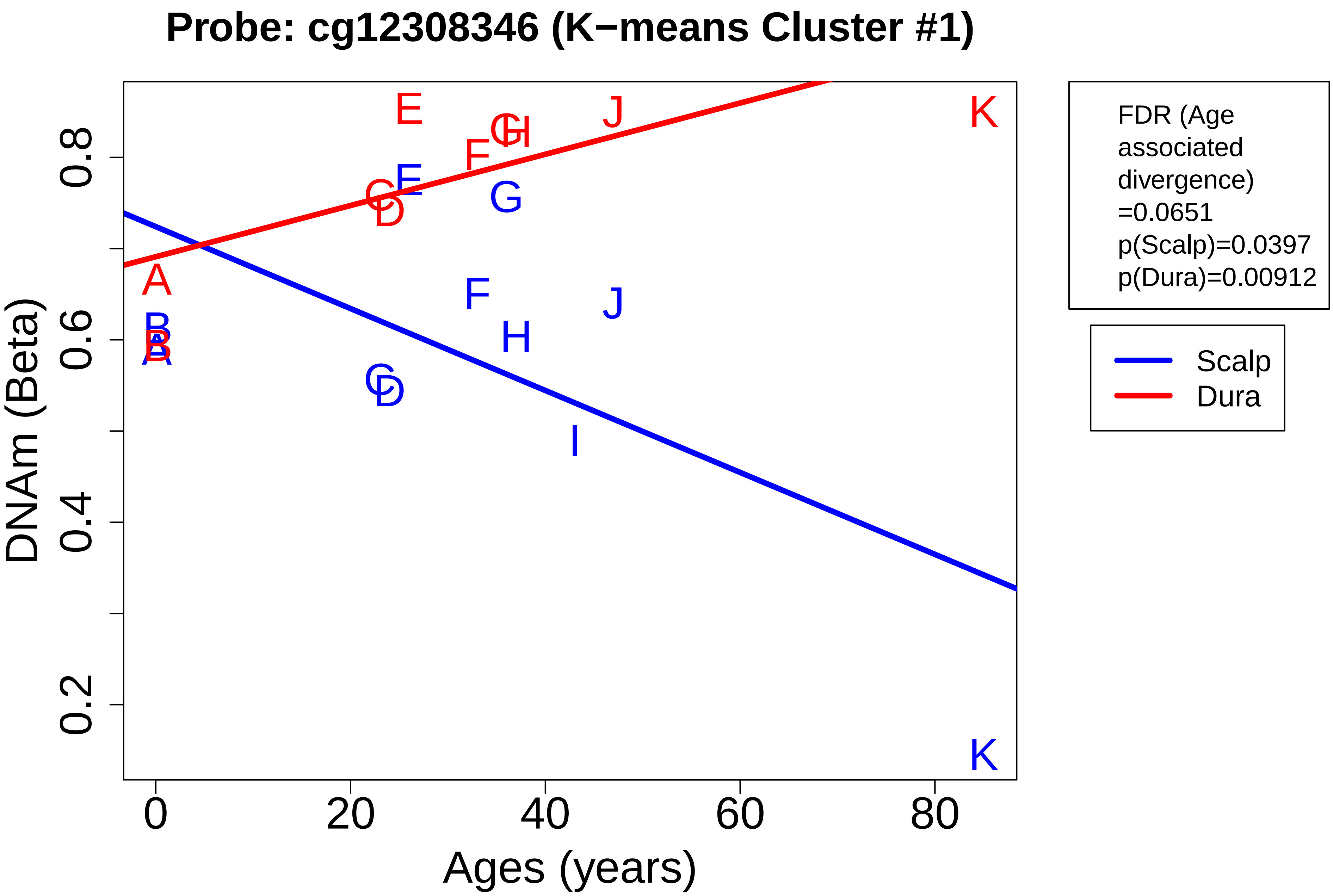

Probe: cg12308346 (K−means Cluster #1)
DNAm (Beta)
0.2
0.4
0.6
0.8
0
20
40
60
80
Ages (years)
FDR (Age associated divergence) =0.0651
p(Scalp)=0.0397
p(Dura)=0.00912
Scalp
Dura

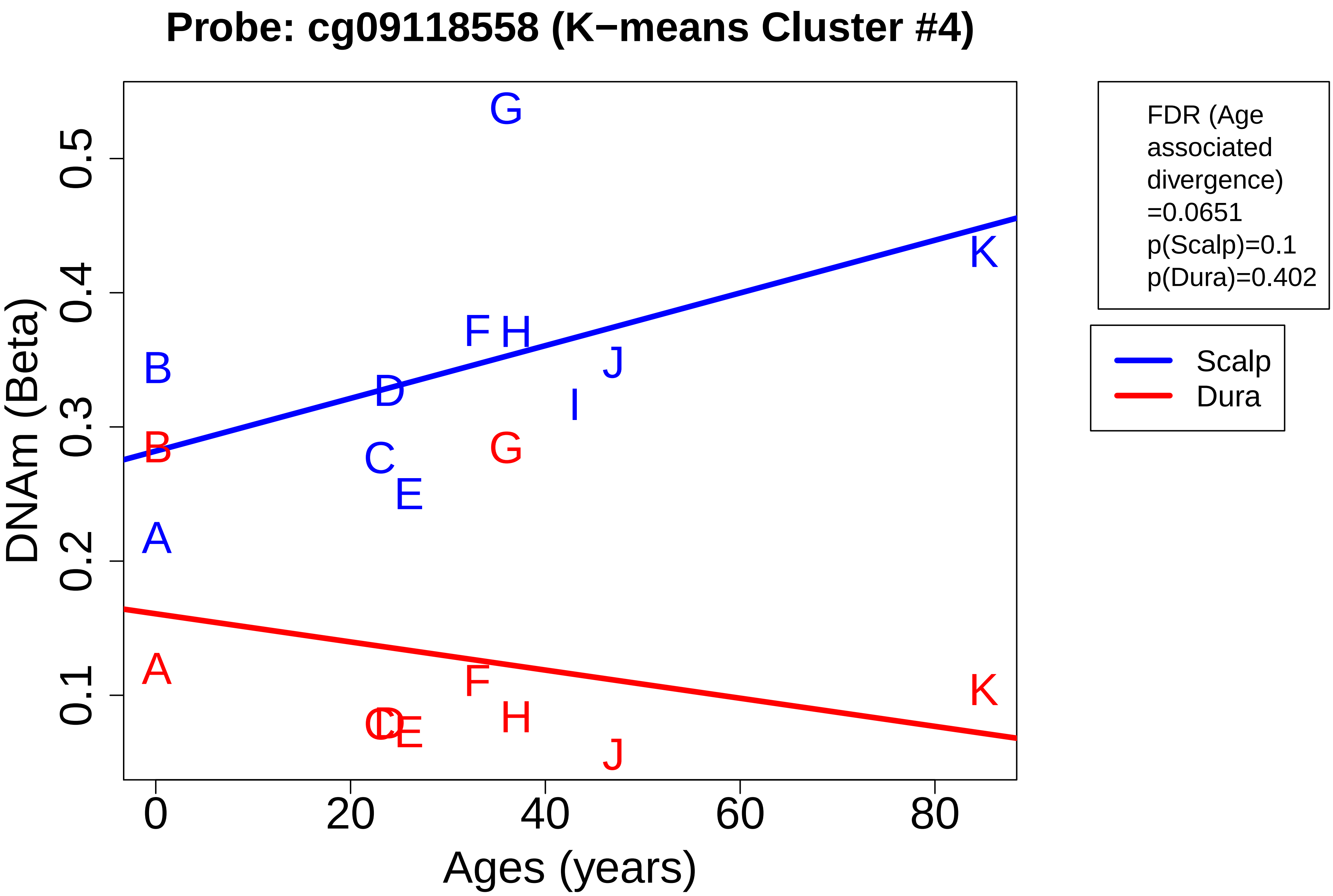

Probe: cg09118558 (K−means Cluster #4)
DNAm (Beta)
Ages (years)
0.1
0.2
0.3
0.4
0.5
0
20
40
60
80
FDR (Age associated divergence) =0.0651
p(Scalp)=0.1
p(Dura)=0.402
Scalp
Dura

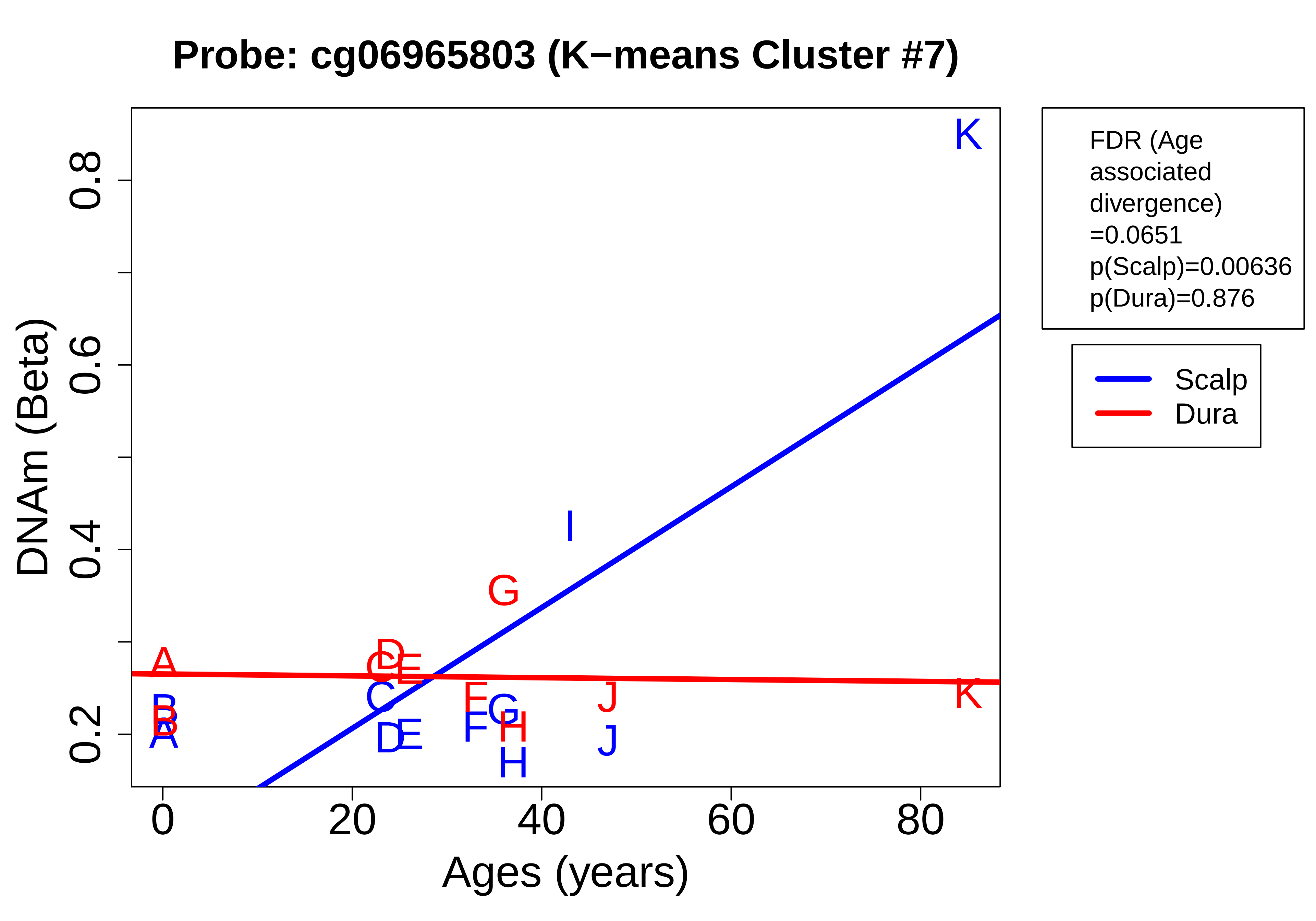
Probe: cg06965803 (K−means Cluster #7)
DNAm (Beta)
0.2
0.4
0.6
0.8
Ages (years)
0
20
40
60
80
FDR (Age associated divergence) =0.0651
p(Scalp)=0.00636
p(Dura)=0.876
Scalp
Dura
A
B
C
D
E
F
G
H
I
J
K

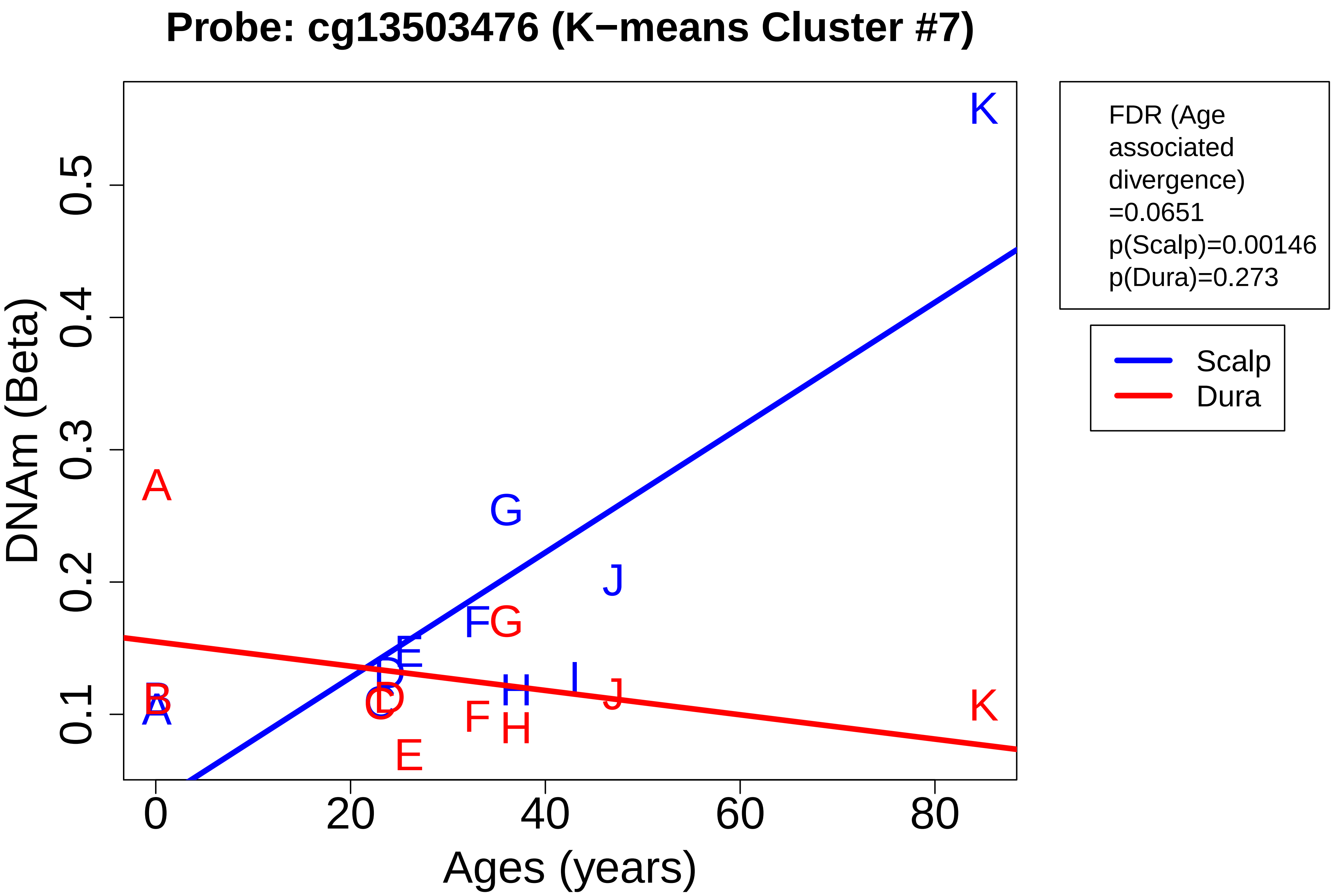

Probe: cg13503476 (K−means Cluster #7)
DNAm (Beta)
0.1
0.2
0.3
0.4
0.5
Ages (years)
0
20
40
60
80
FDR (Age associated divergence) =0.0651
p(Scalp)=0.00146
p(Dura)=0.273
Scalp
Dura
A
B
C
D
E
F
G
H
I
J
K

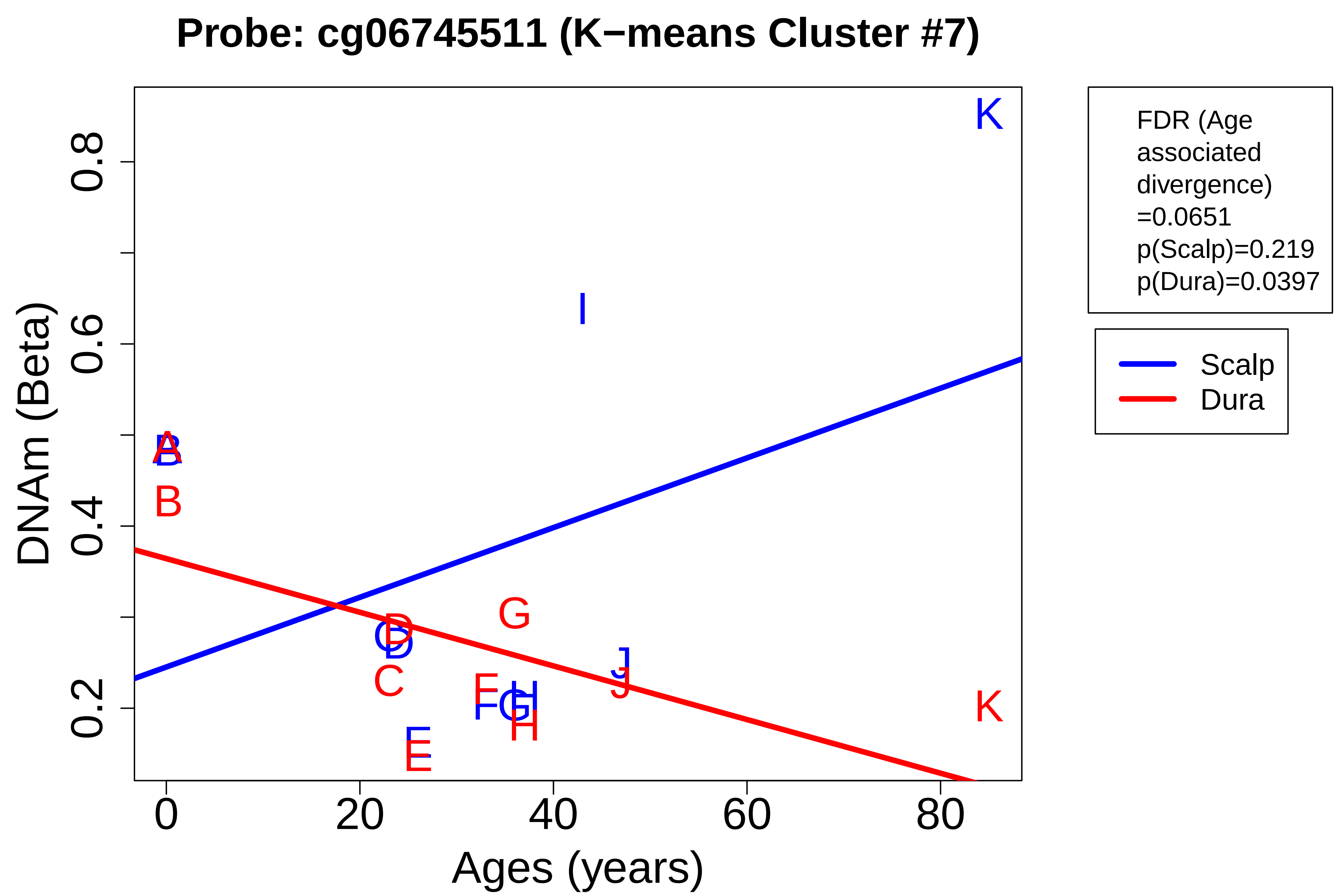

Probe: cg06745511 (K−means Cluster #7)
DNAm (Beta)
0.2
0.4
0.6
0.8
0
20
40
60
80
Ages (years)
FDR (Age associated divergence) =0.0651
p(Scalp)=0.219
p(Dura)=0.0397
Scalp
Dura

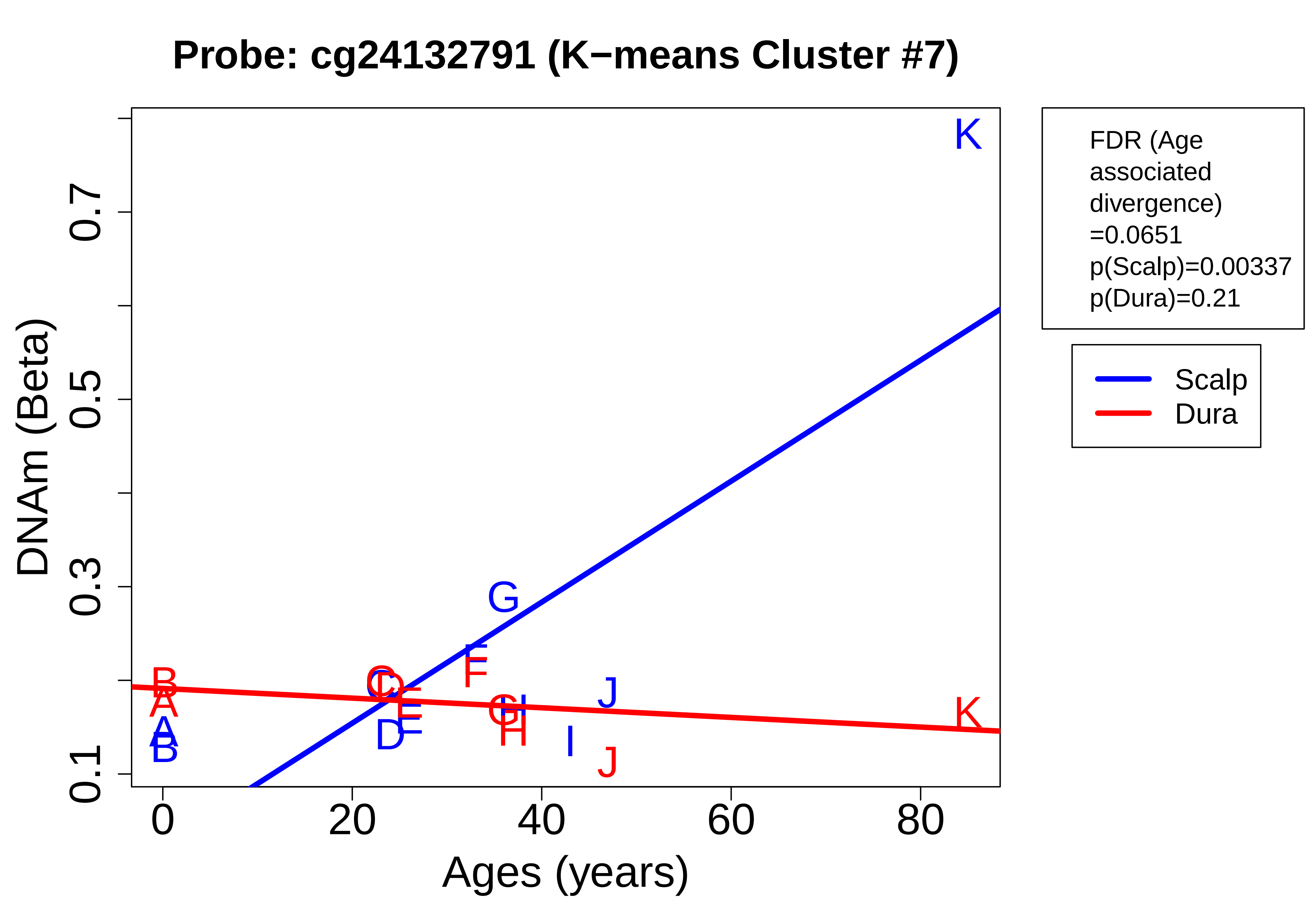

Probe: cg24132791 (K−means Cluster #7)
DNAm (Beta)
0.1
0.3
0.5
0.7
Ages (years)
0
20
40
60
80
FDR (Age associated divergence) =0.0651
p(Scalp)=0.00337
p(Dura)=0.21
Scalp
Dura

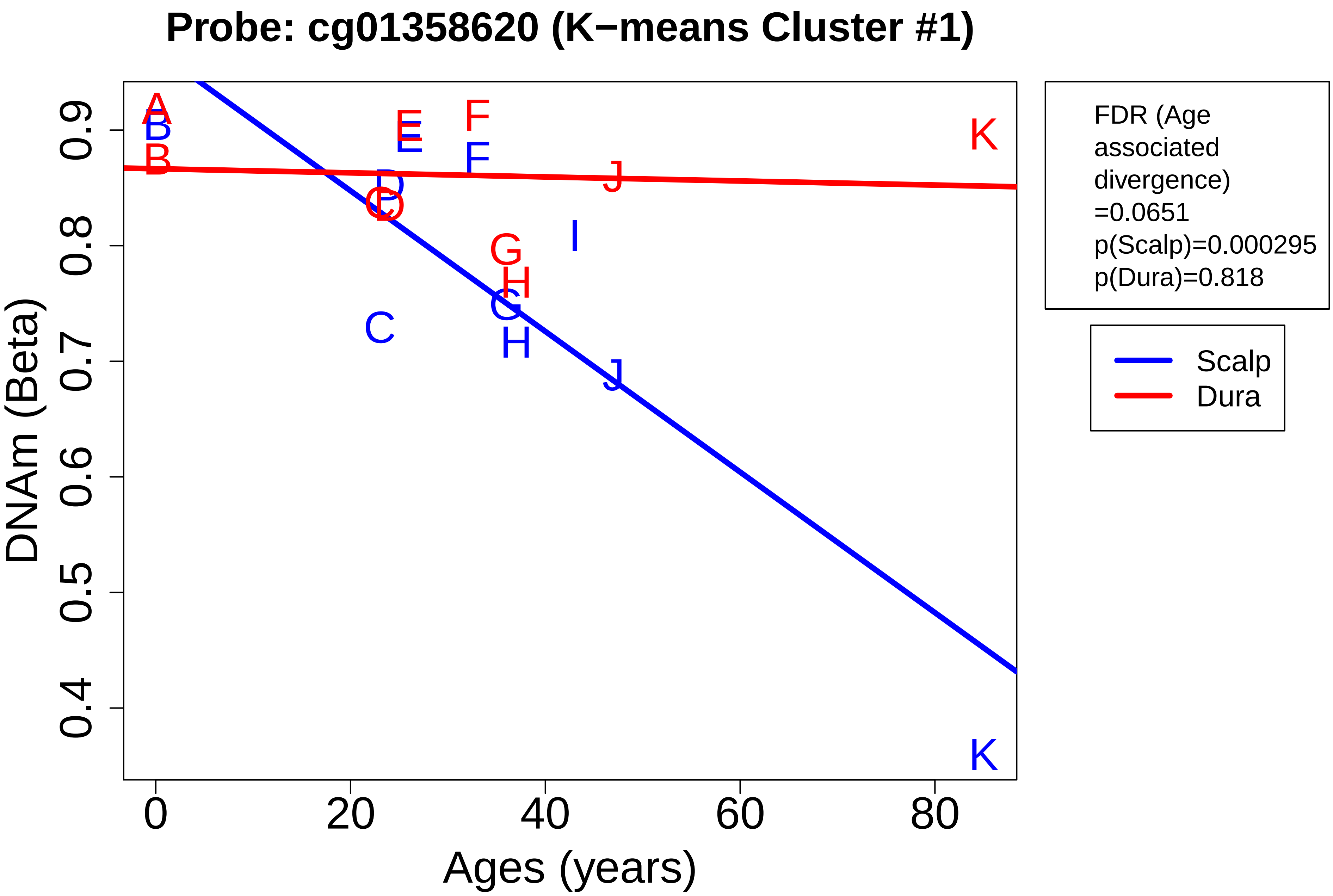

Probe: cg01358620 (K−means Cluster #1)
DNAm (Beta)
0.4
0.5
0.6
0.7
0.8
0.9
0
20
40
60
80
Ages (years)
FDR (Age associated divergence) =0.0651
p(Scalp)=0.000295
p(Dura)=0.818
Scalp
Dura

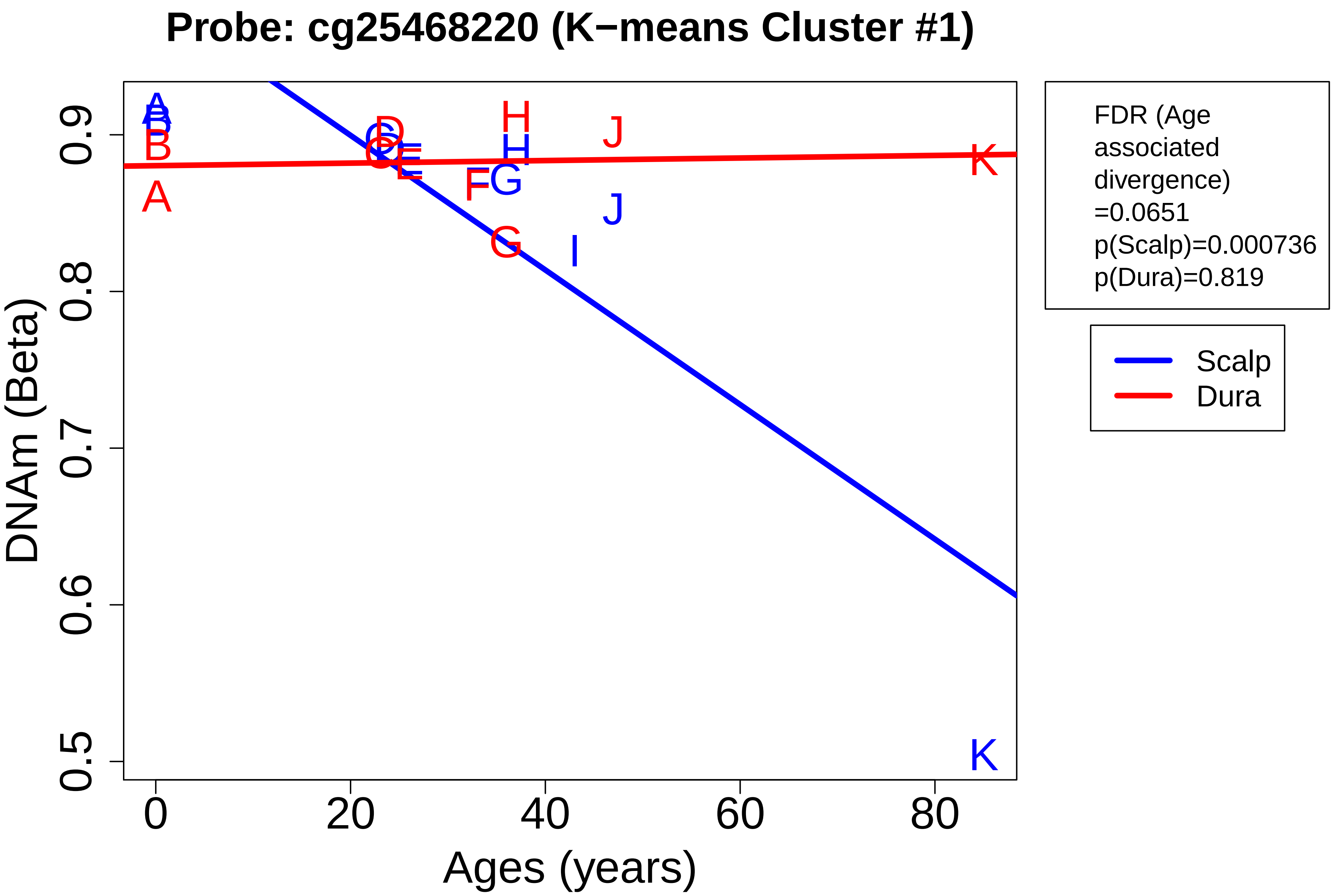

Probe: cg25468220 (K−means Cluster #1)
DNAm (Beta)
0.5
0.6
0.7
0.8
0.9
0
20
40
60
80
Ages (years)
A
B
C
D
E
F
G
H
I
J
K
FDR (Age associated divergence) =0.0651
p(Scalp)=0.000736
p(Dura)=0.819
Scalp
Dura

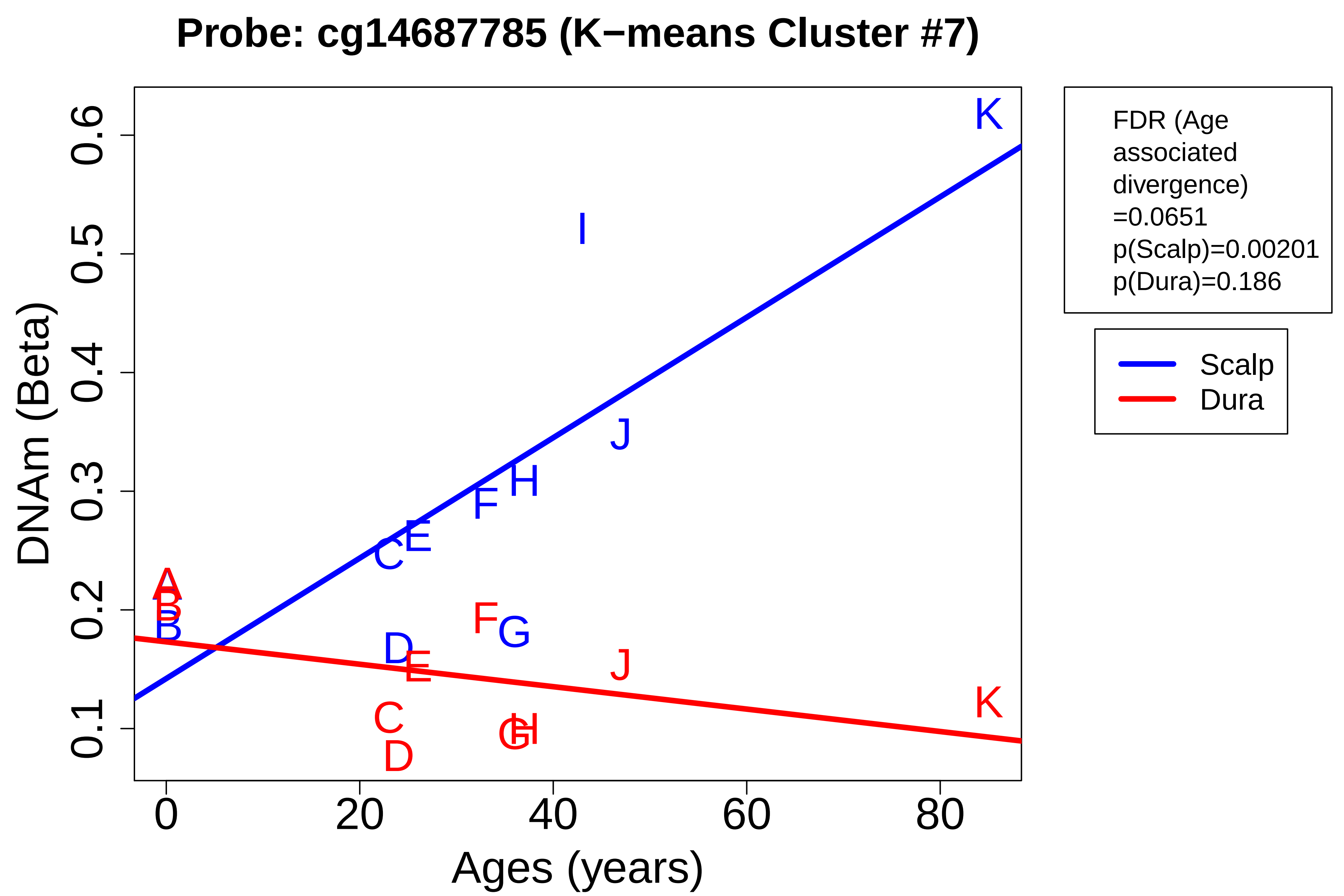
Probe: cg14687785 (K−means Cluster #7)
DNAm (Beta)
Ages (years)
0.1
0.2
0.3
0.4
0.5
0.6
0
20
40
60
80
FDR (Age associated divergence) =0.0651
p(Scalp)=0.00201
p(Dura)=0.186
Scalp
Dura

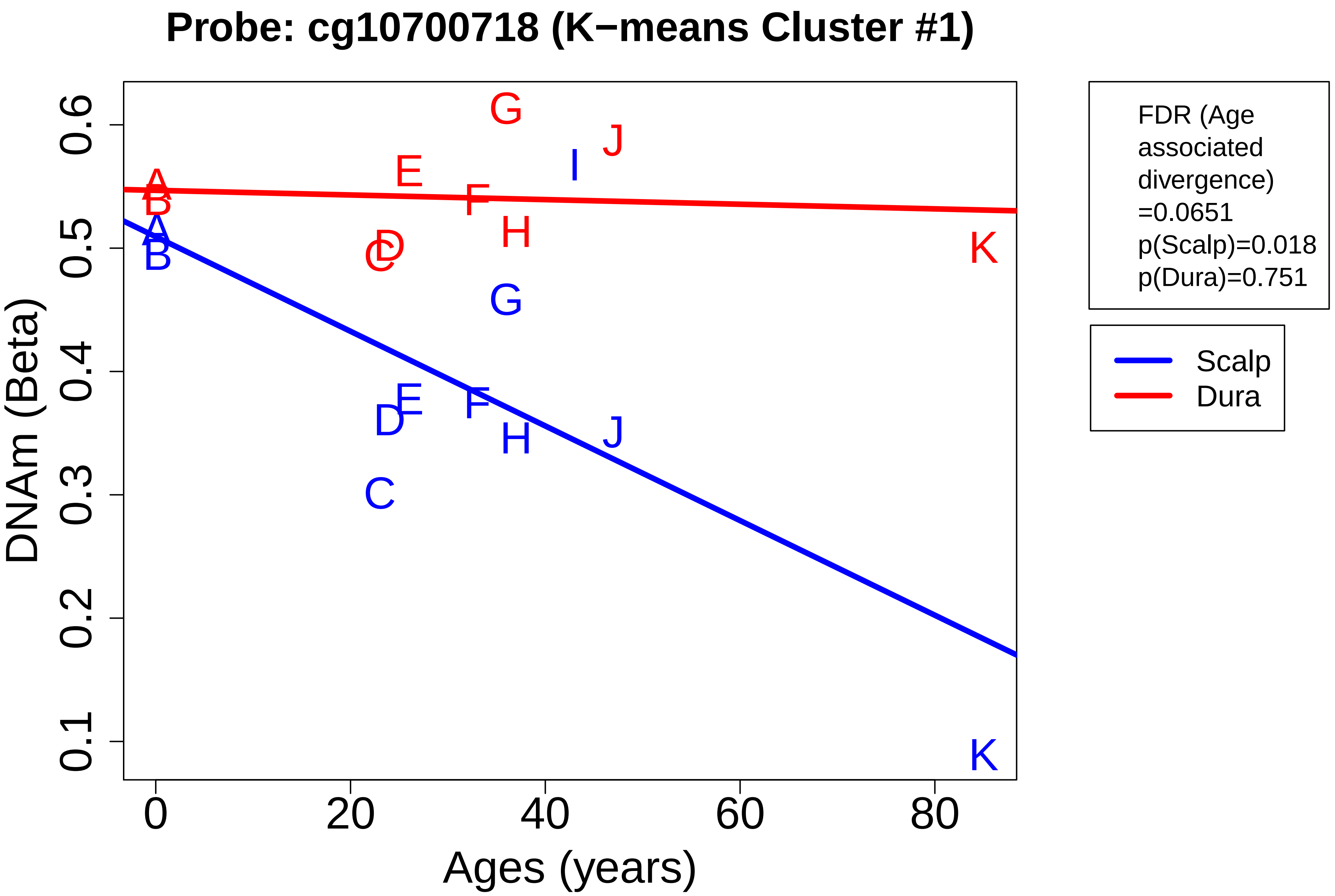

Probe: cg10700718 (K−means Cluster #1)
DNAm (Beta)
Ages (years)
0.1
0.2
0.3
0.4
0.5
0.6
0
20
40
60
80
A
B
C
D
E
F
G
H
I
J
K
FDR (Age associated divergence) =0.0651
p(Scalp)=0.018
p(Dura)=0.751
Scalp
Dura

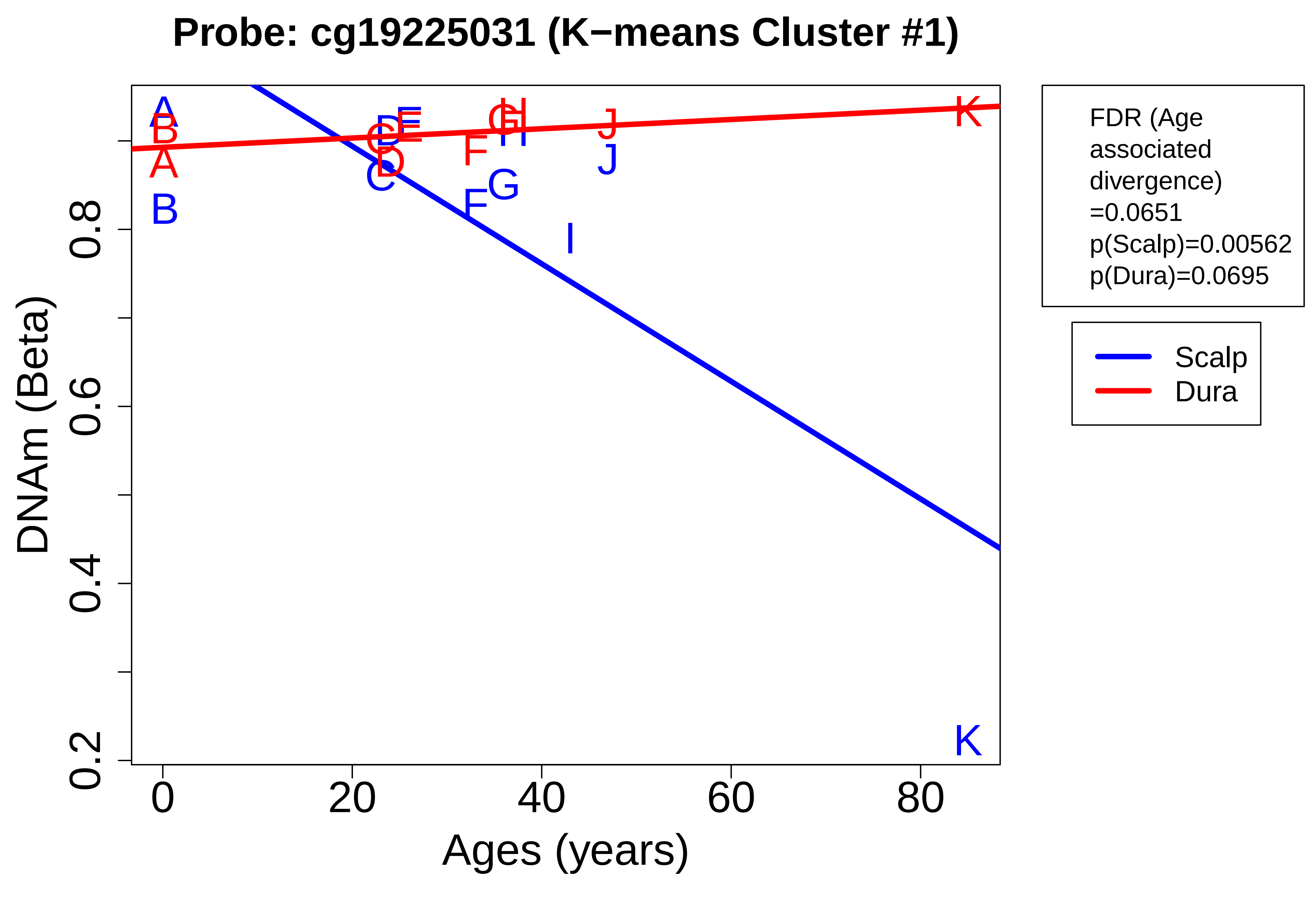
Probe: cg19225031 (K−means Cluster #1)
DNAm (Beta)
0.2
0.4
0.6
0.8
Ages (years)
0
20
40
60
80
A
B
C
D
E
F
G
H
I
J
K
FDR (Age associated divergence) =0.0651
p(Scalp)=0.00562
p(Dura)=0.0695
Scalp
Dura

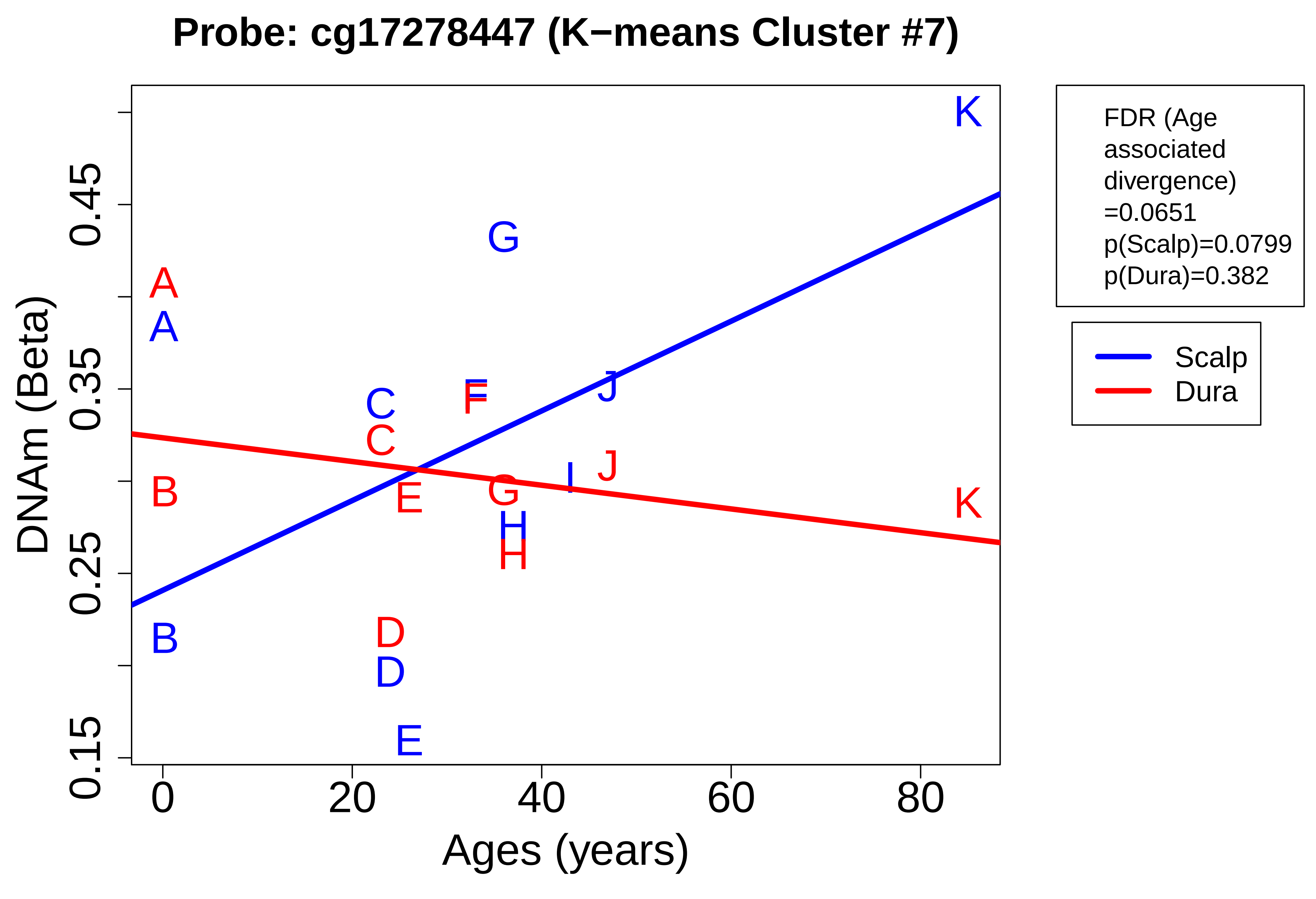

Probe: cg17278447 (K−means Cluster #7)
DNAm (Beta)
0.15
0.25
0.35
0.45
Ages (years)
0
20
40
60
80
FDR (Age associated divergence) =0.0651
p(Scalp)=0.0799
p(Dura)=0.382
Scalp
Dura
A
B
C
D
E
F
G
H
I
J
K

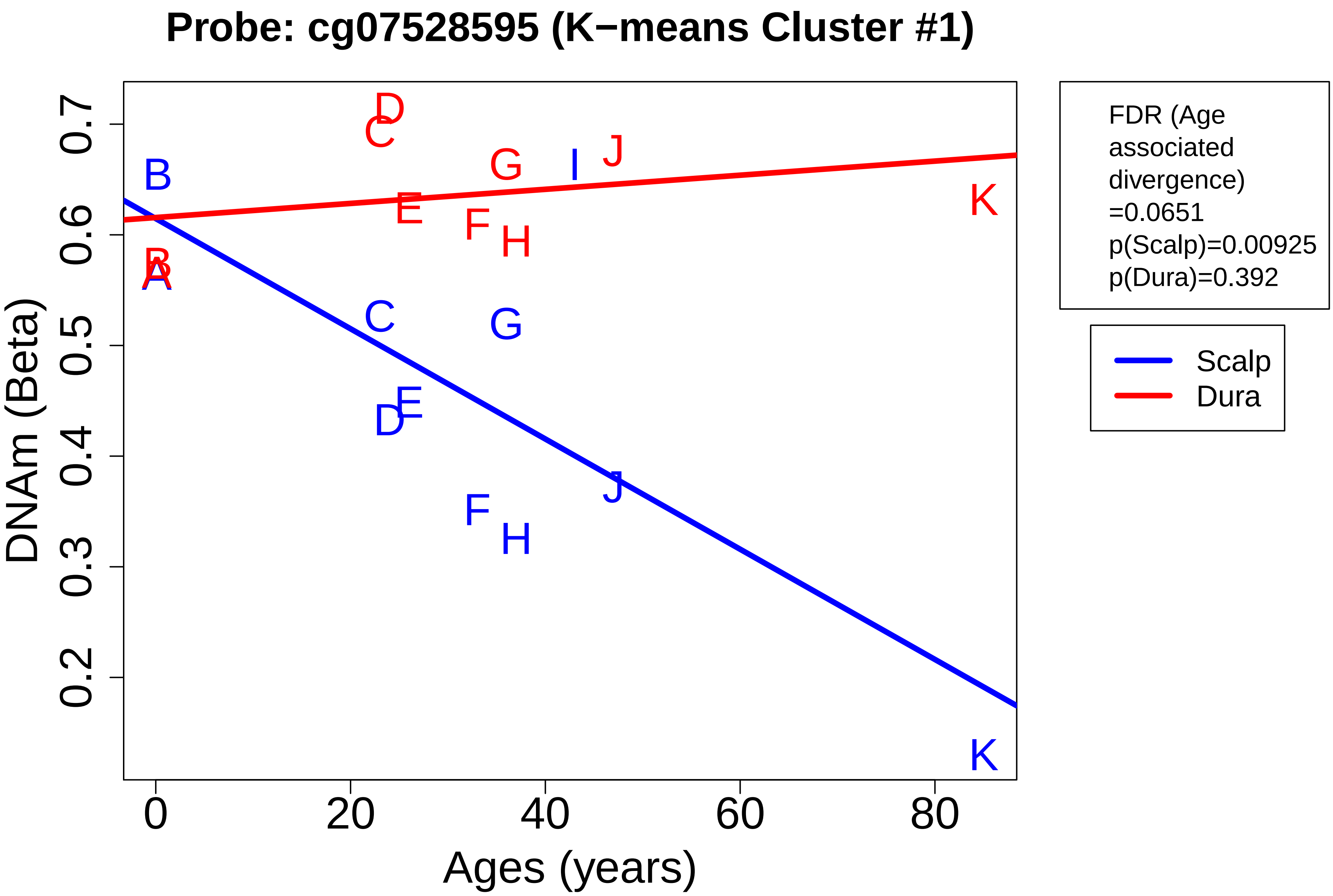

Probe: cg07528595 (K−means Cluster #1)
DNAm (Beta)
0.2
0.3
0.4
0.5
0.6
0.7
Ages (years)
0
20
40
60
80
FDR (Age associated divergence) =0.0651
p(Scalp)=0.00925
p(Dura)=0.392
Scalp
Dura
A
B
C
D
E
F
G
H
I
J
K

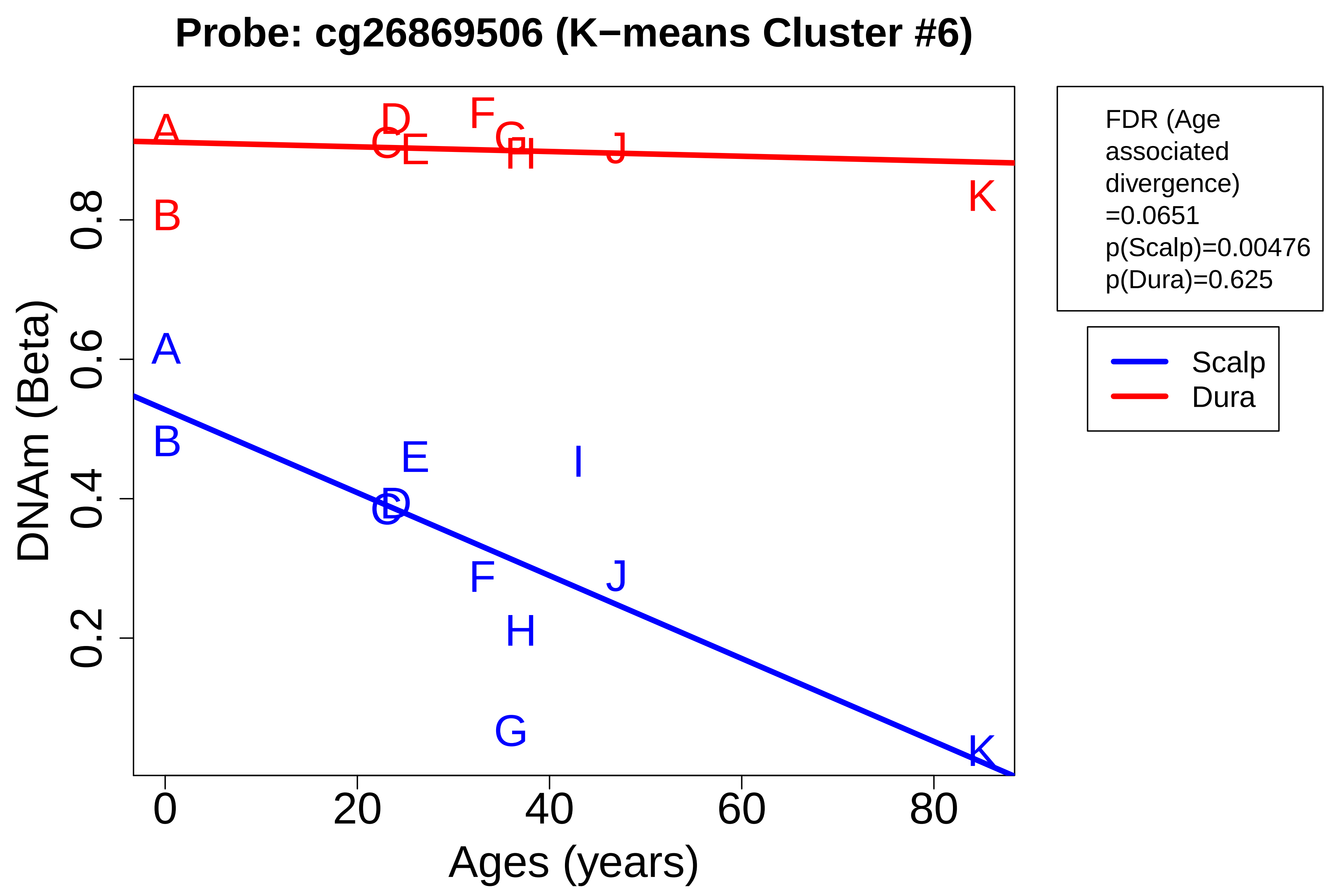

Probe: cg26869506 (K−means Cluster #6)
DNAm (Beta)
0.2
0.4
0.6
0.8
0
20
40
60
80
Ages (years)
FDR (Age associated divergence) =0.0651
p(Scalp)=0.00476
p(Dura)=0.625
Scalp
Dura

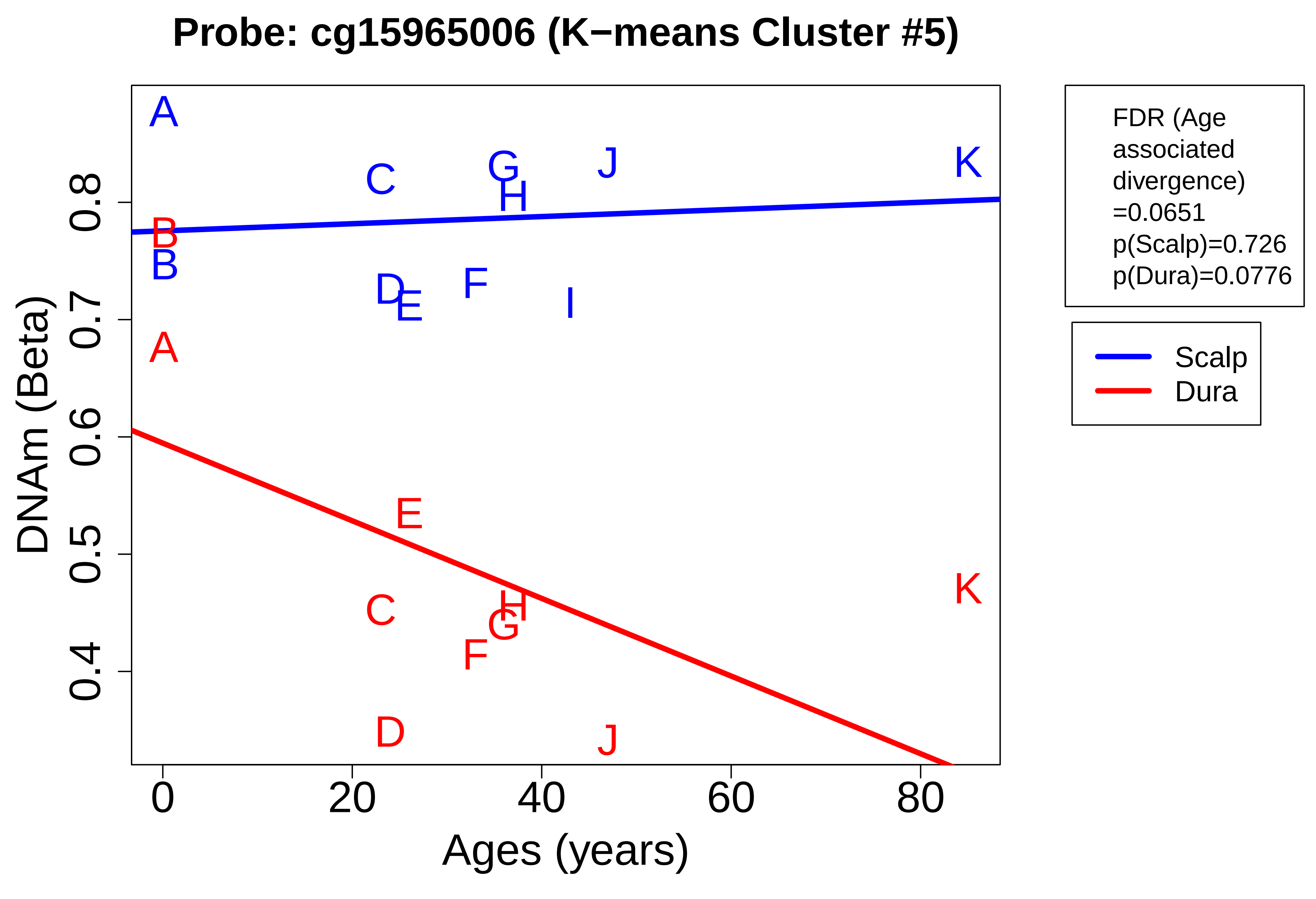
Probe: cg15965006 (K−means Cluster #5)
DNAm (Beta)
0.4
0.5
0.6
0.7
0.8
0
20
40
60
80
Ages (years)
A
B
C
D
E
F
G
H
I
J
K
FDR (Age associated divergence) =0.0651
p(Scalp)=0.726
p(Dura)=0.0776
Scalp
Dura

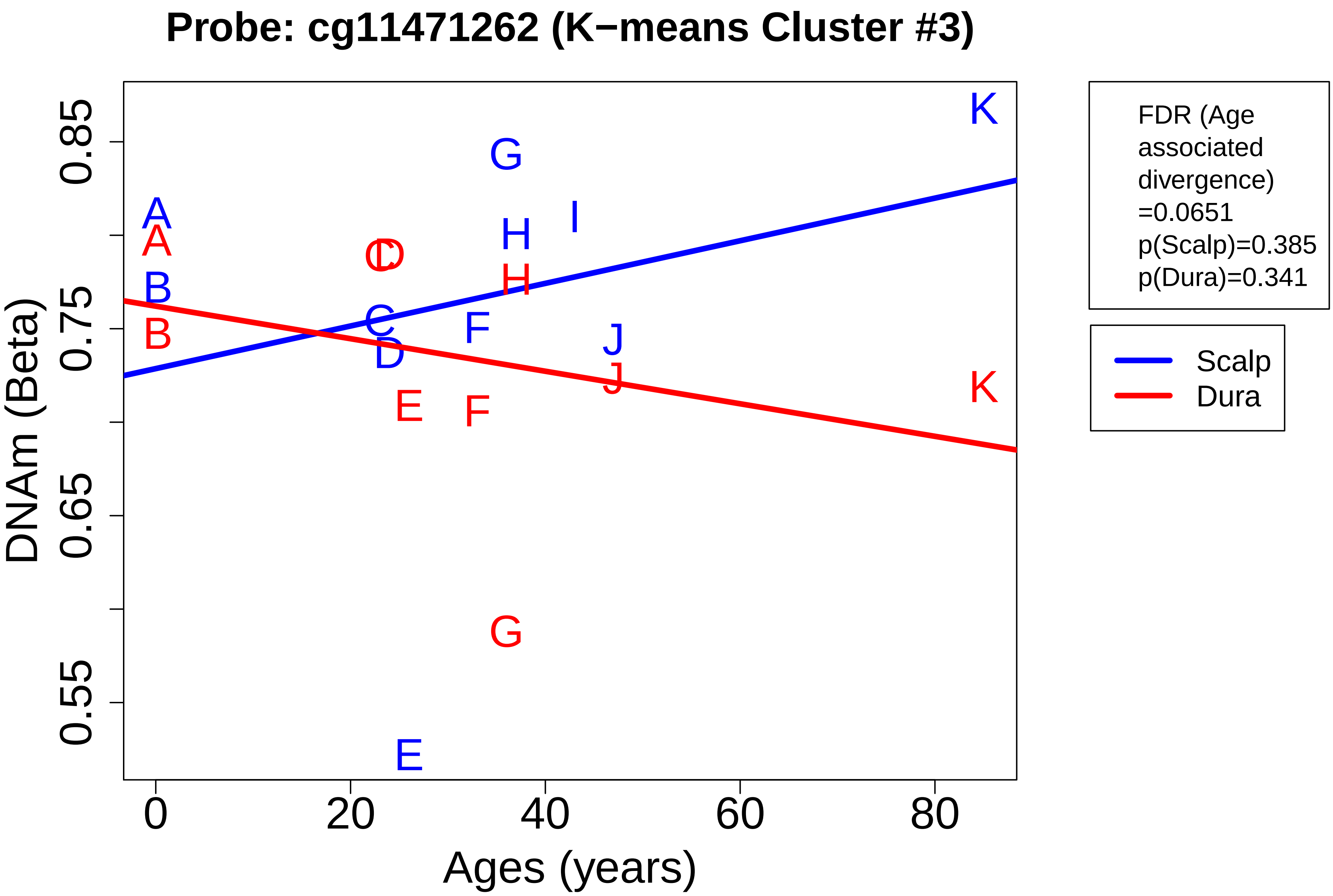
Probe: cg11471262 (K−means Cluster #3)
DNAm (Beta)
0.55
0.65
0.75
0.85
Ages (years)
0
20
40
60
80
A
B
C
D
E
F
G
H
I
J
K
FDR (Age associated divergence) =0.0651
p(Scalp)=0.385
p(Dura)=0.341
Scalp
Dura

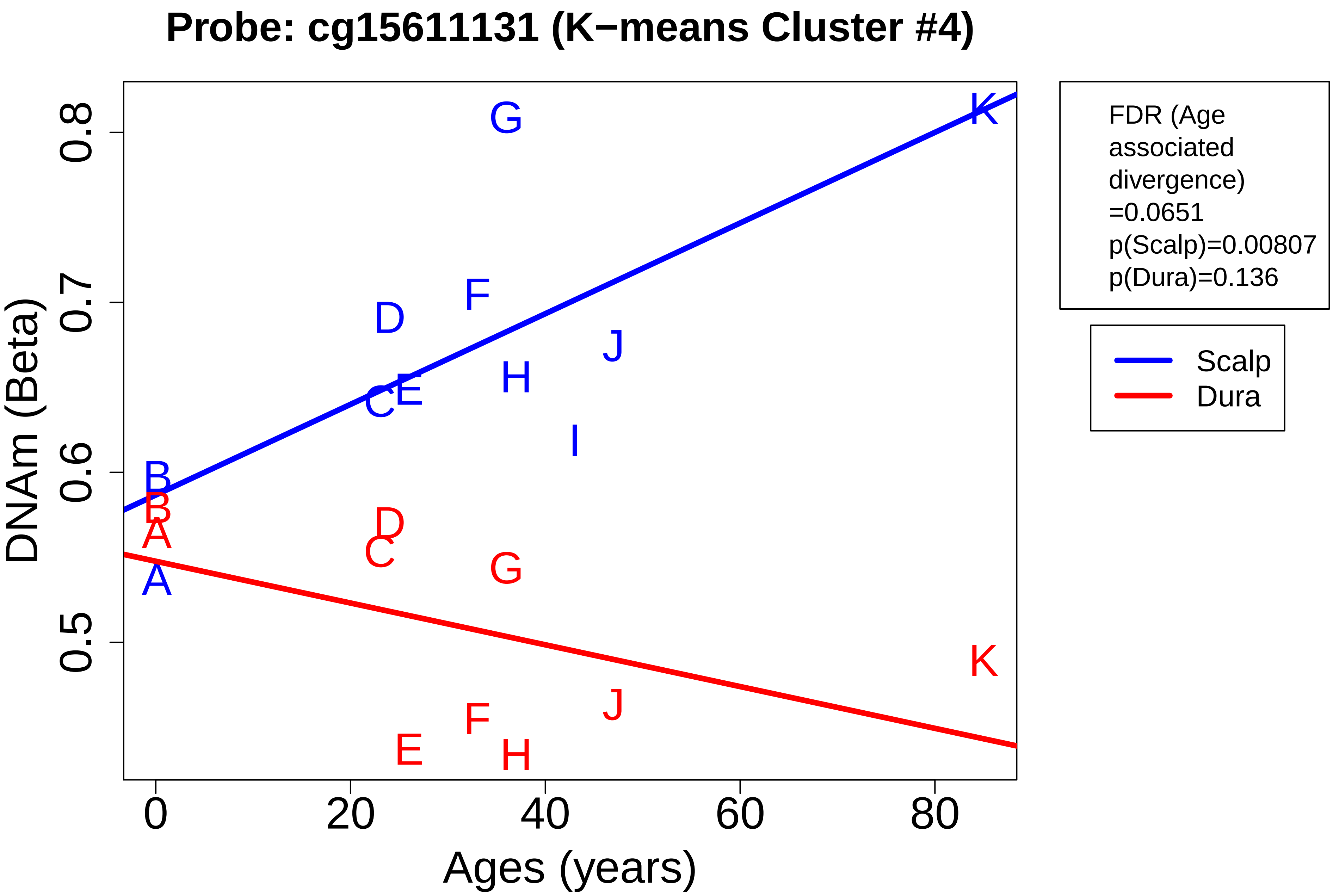
Probe: cg15611131 (K−means Cluster #4)
DNAm (Beta)
0.5
0.6
0.7
0.8
Ages (years)
0
20
40
60
80
FDR (Age associated divergence) =0.0651
p(Scalp)=0.00807
p(Dura)=0.136
Scalp
Dura
A
B
C
D
E
F
G
H
I
J
K

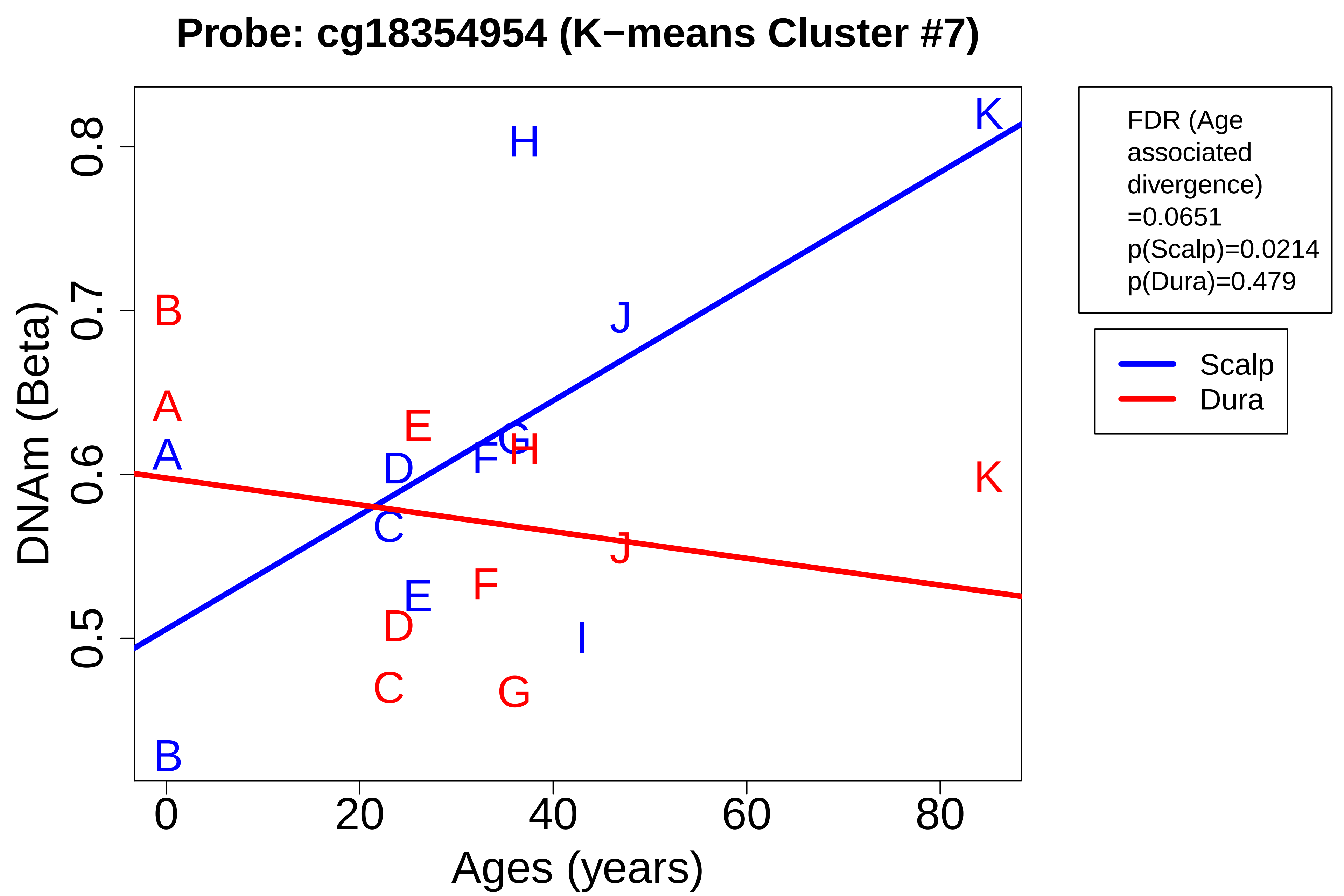

Probe: cg18354954 (K−means Cluster #7)
DNAm (Beta)
0.5
0.6
0.7
0.8
0
20
40
60
80
Ages (years)
FDR (Age associated divergence) =0.0651
p(Scalp)=0.0214
p(Dura)=0.479
Scalp
Dura
A
B
C
D
E
F
G
H
I
J
K

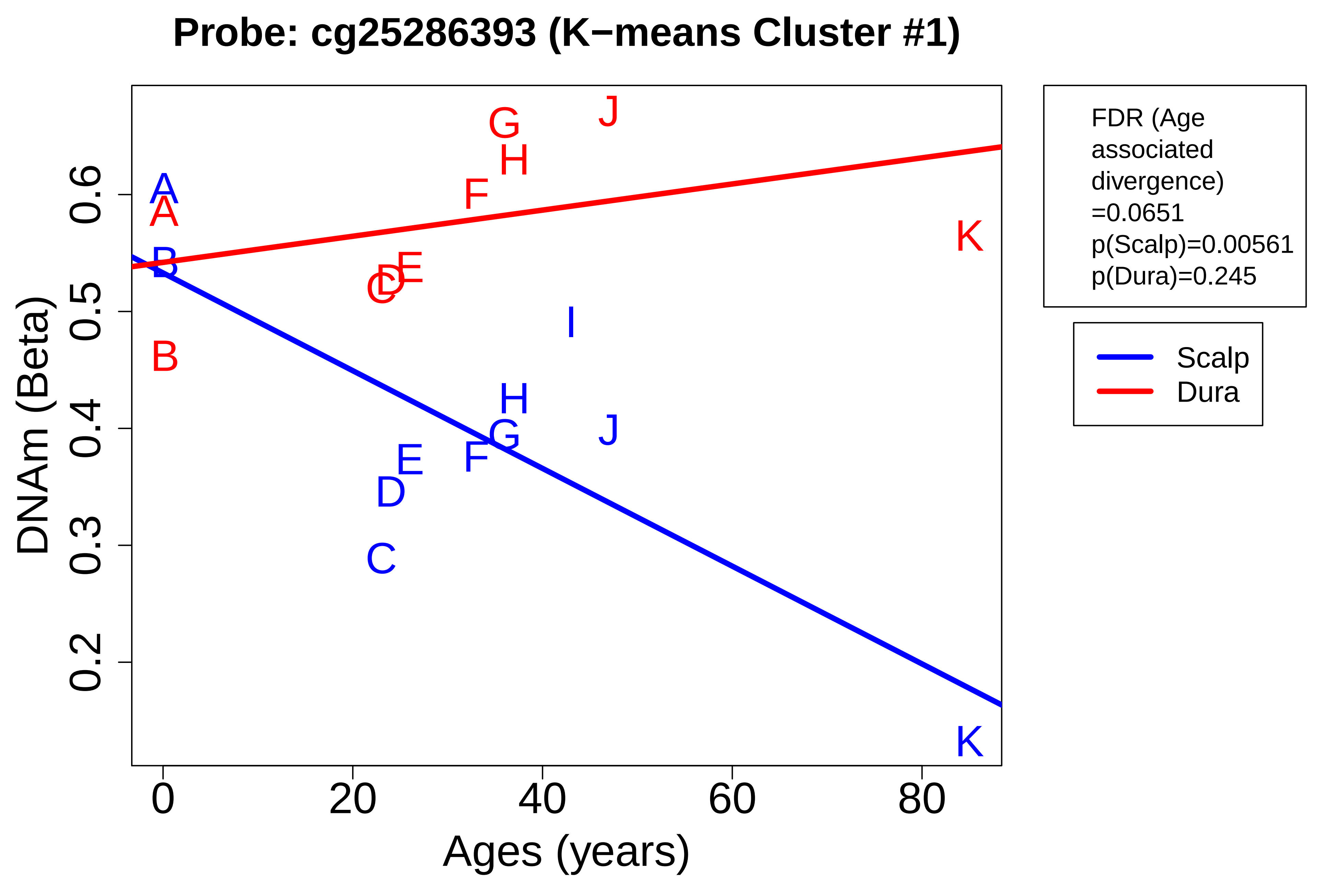

Probe: cg25286393 (K−means Cluster #1)
DNAm (Beta)
0.2
0.3
0.4
0.5
0.6
Ages (years)
0
20
40
60
80
A
B
C
D
E
F
G
H
I
J
K
FDR (Age associated divergence) =0.0651
p(Scalp)=0.00561
p(Dura)=0.245
Scalp
Dura

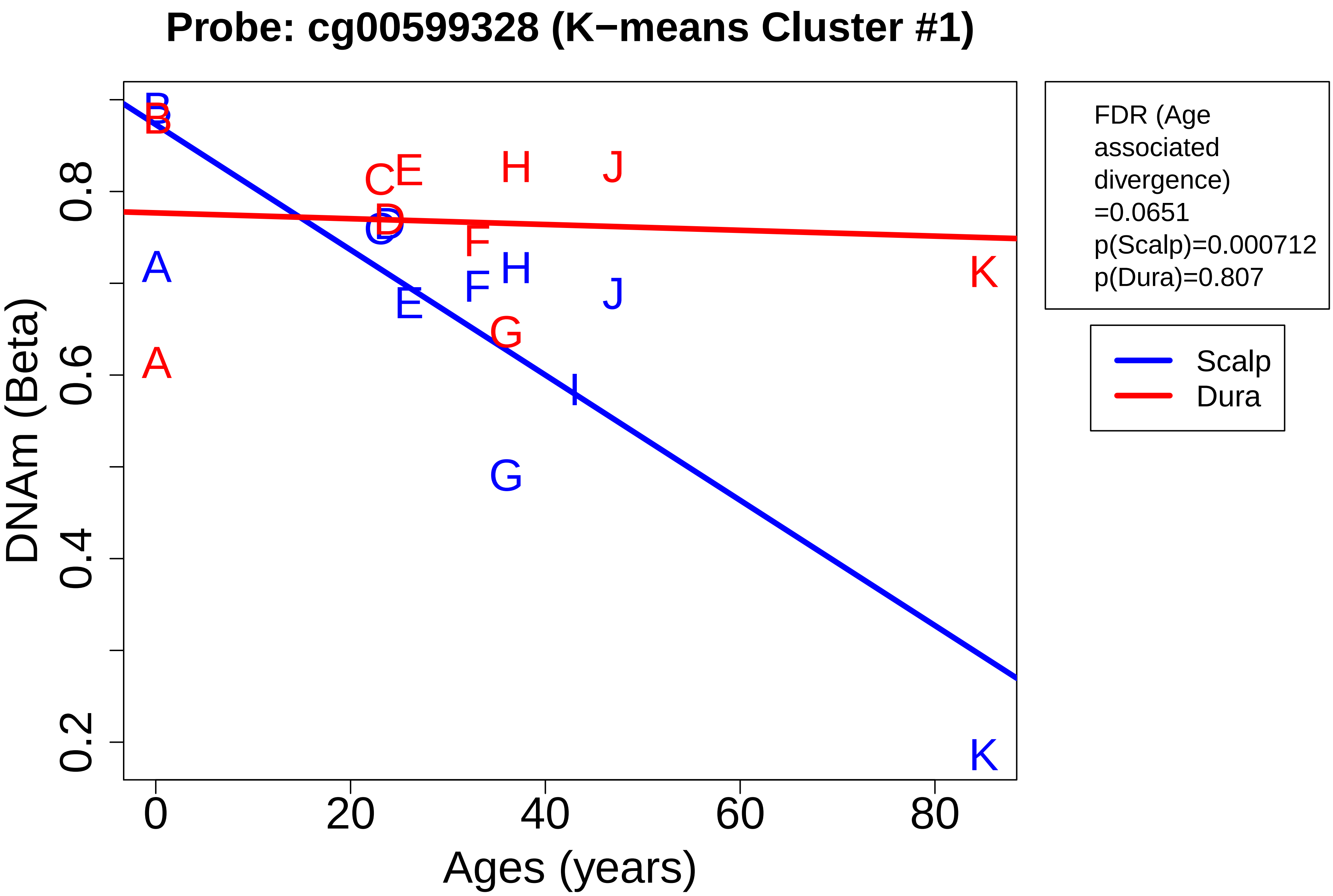

Probe: cg00599328 (K−means Cluster #1)
DNAm (Beta)
0.2
0.4
0.6
0.8
Ages (years)
0
20
40
60
80
FDR (Age associated divergence) =0.0651
p(Scalp)=0.000712
p(Dura)=0.807
Scalp
Dura

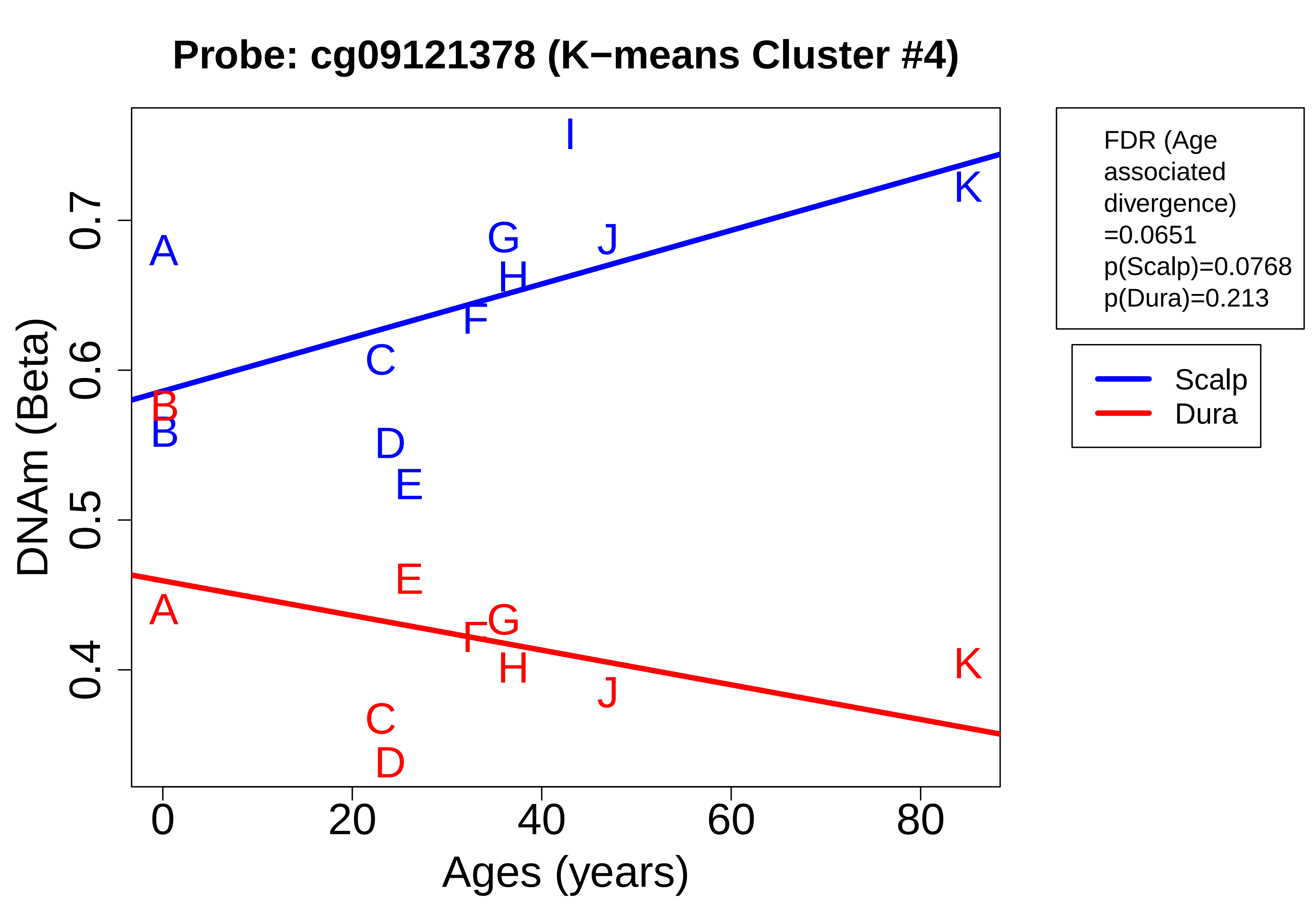

Probe: cg09121378 (K−means Cluster #4)
DNAm (Beta)
0.4
0.5
0.6
0.7
Ages (years)
0
20
40
60
80
FDR (Age associated divergence) =0.0651
p(Scalp)=0.0768
p(Dura)=0.213
Scalp
Dura
A
B
C
D
E
F
G
H
I
J
K

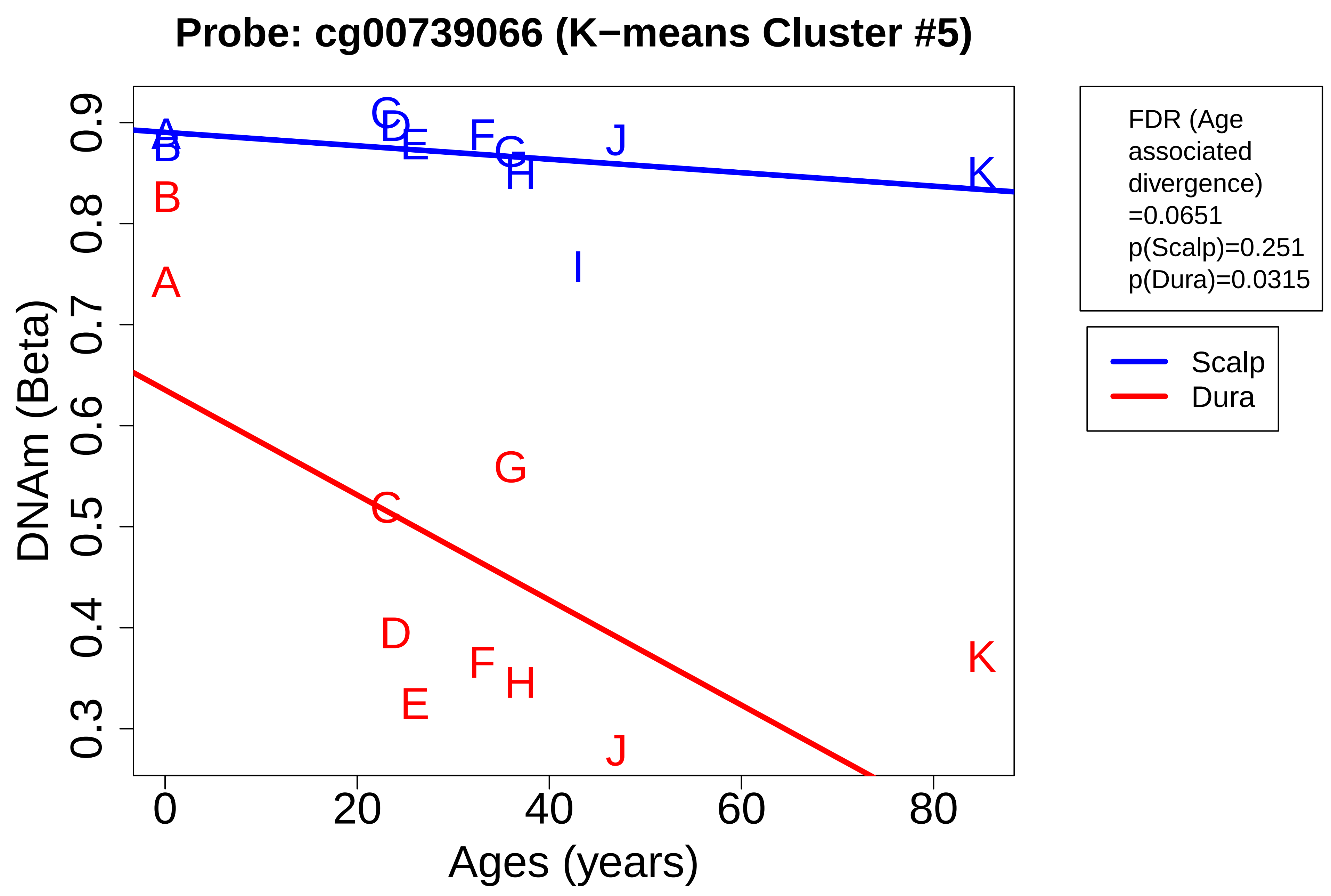

Probe: cg00739066 (K−means Cluster #5)
DNAm (Beta)
0.3
0.4
0.5
0.6
0.7
0.8
0.9
0
20
40
60
80
Ages (years)
A
B
C
D
E
F
G
H
I
J
K
FDR (Age associated divergence) =0.0651
p(Scalp)=0.251
p(Dura)=0.0315
Scalp
Dura

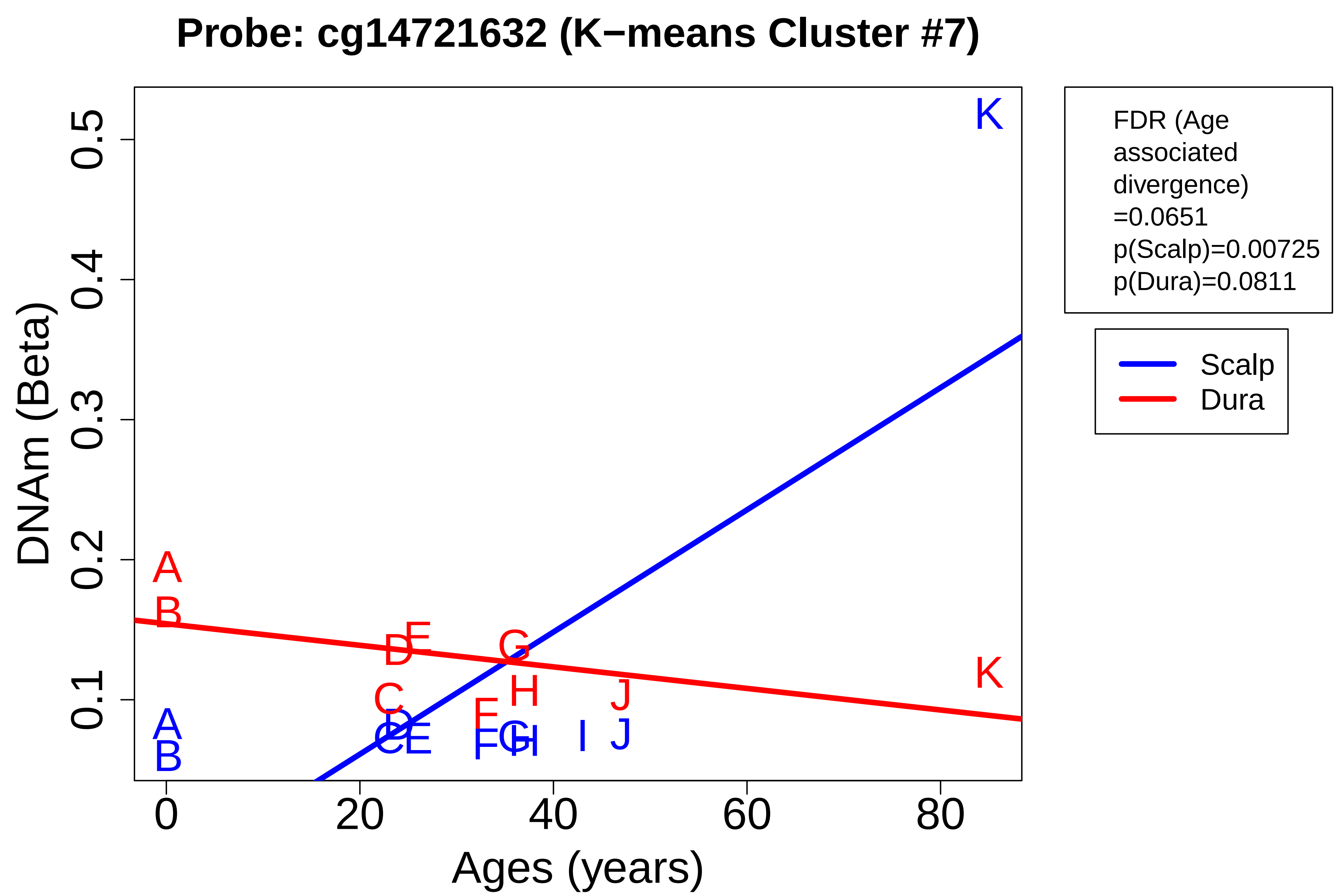

Probe: cg14721632 (K−means Cluster #7)
FDR (Age associated divergence) =0.0651
p(Scalp)=0.00725
p(Dura)=0.0811
Scalp
Dura
DNAm (Beta)
0.1
0.2
0.3
0.4
0.5
0
20
40
60
80
Ages (years)
A
B
C
D
E
F
G
H
I
J
K

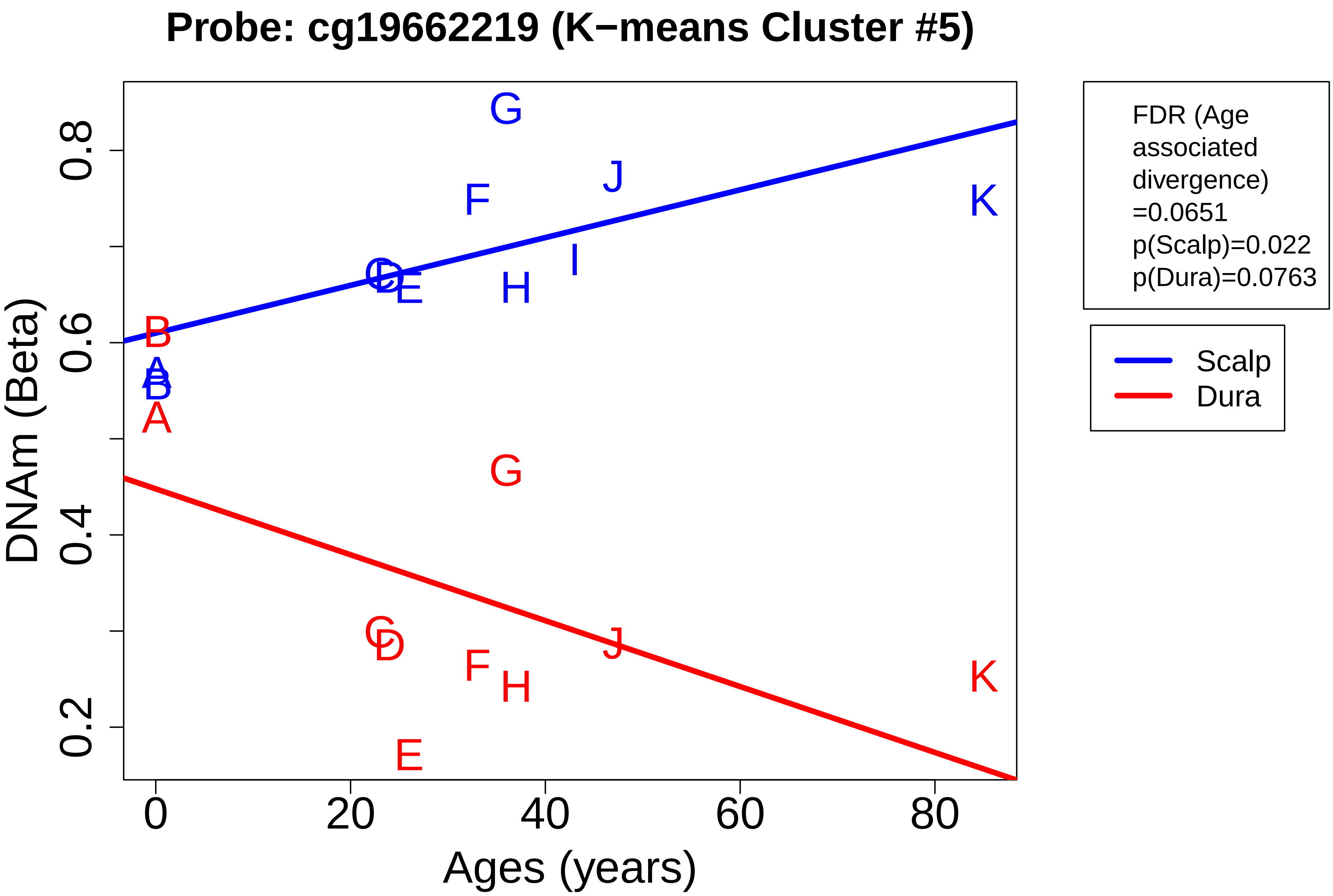

Probe: cg19662219 (K−means Cluster #5)
DNAm (Beta)
0.2
0.4
0.6
0.8
Ages (years)
0
20
40
60
80
FDR (Age associated divergence) =0.0651
p(Scalp)=0.022
p(Dura)=0.0763
Scalp
Dura

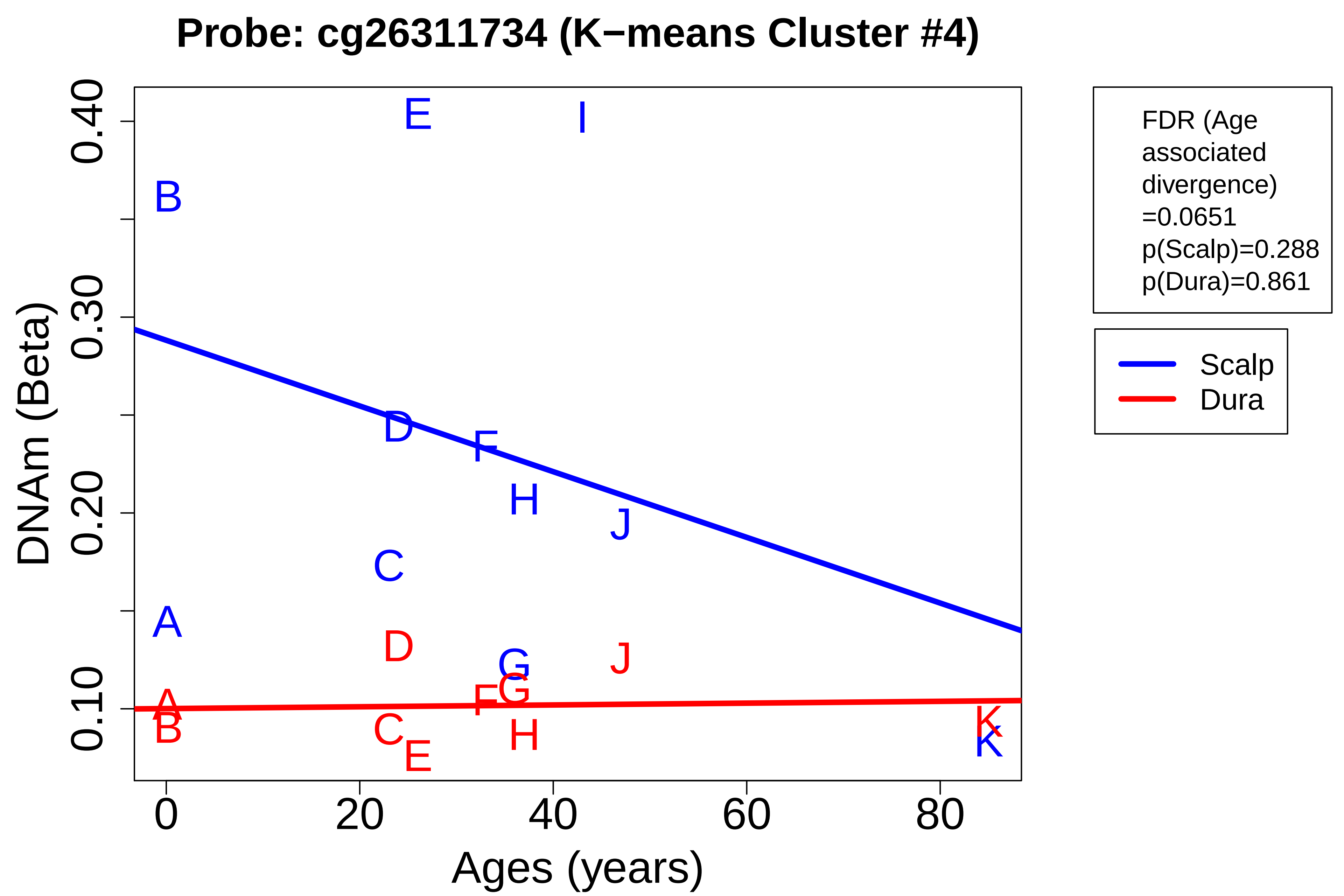

Probe: cg26311734 (K−means Cluster #4)
DNAm (Beta)
0.40
0.30
0.20
0.10
0
20
40
60
80
Ages (years)
FDR (Age associated divergence) =0.0651
p(Scalp)=0.288
p(Dura)=0.861
Scalp
Dura

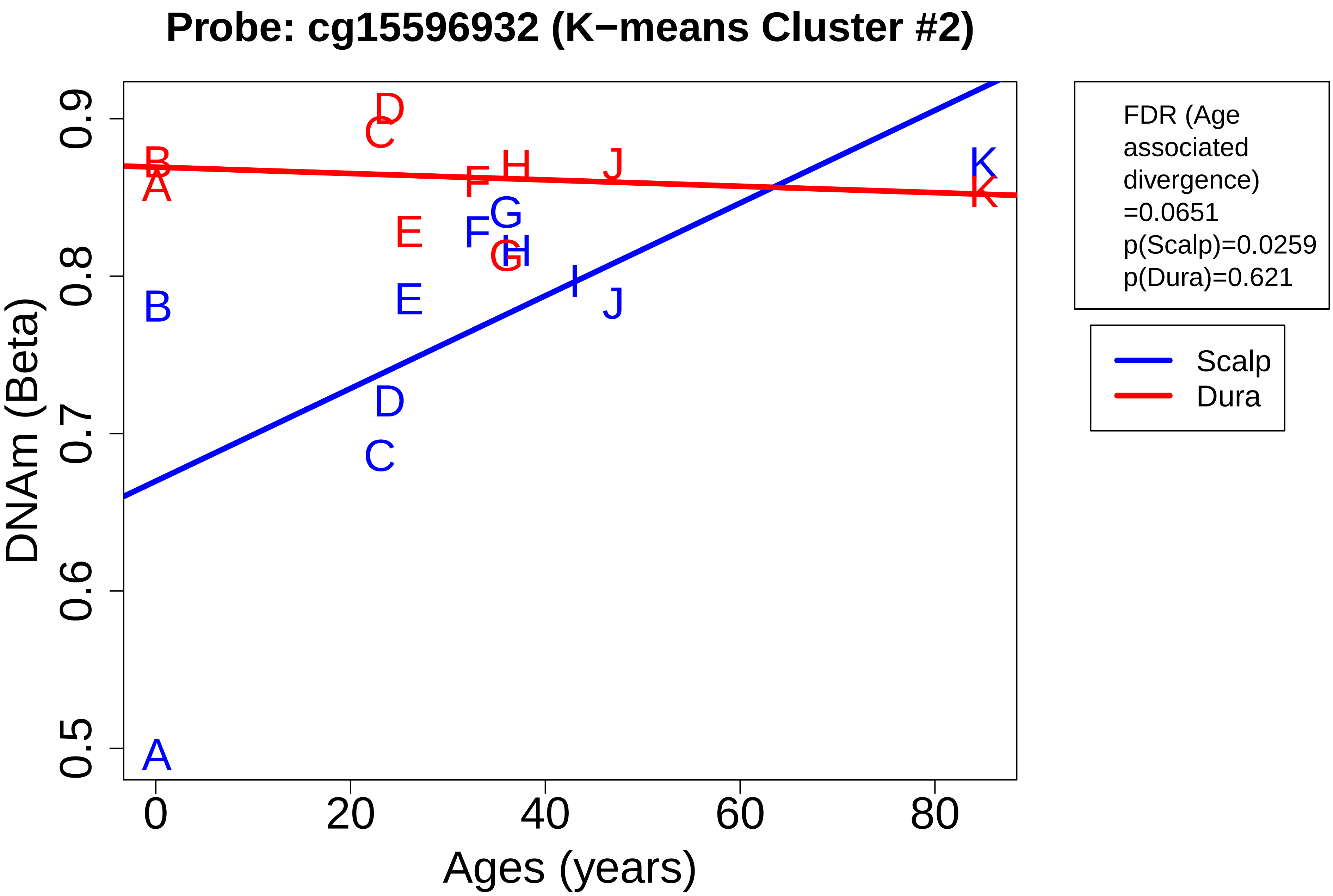
Probe: cg15596932 (K−means Cluster #2)
DNAm (Beta)
0.5
0.6
0.7
0.8
0.9
Ages (years)
0
20
40
60
80
FDR (Age associated divergence) =0.0651
p(Scalp)=0.0259
p(Dura)=0.621
Scalp
Dura

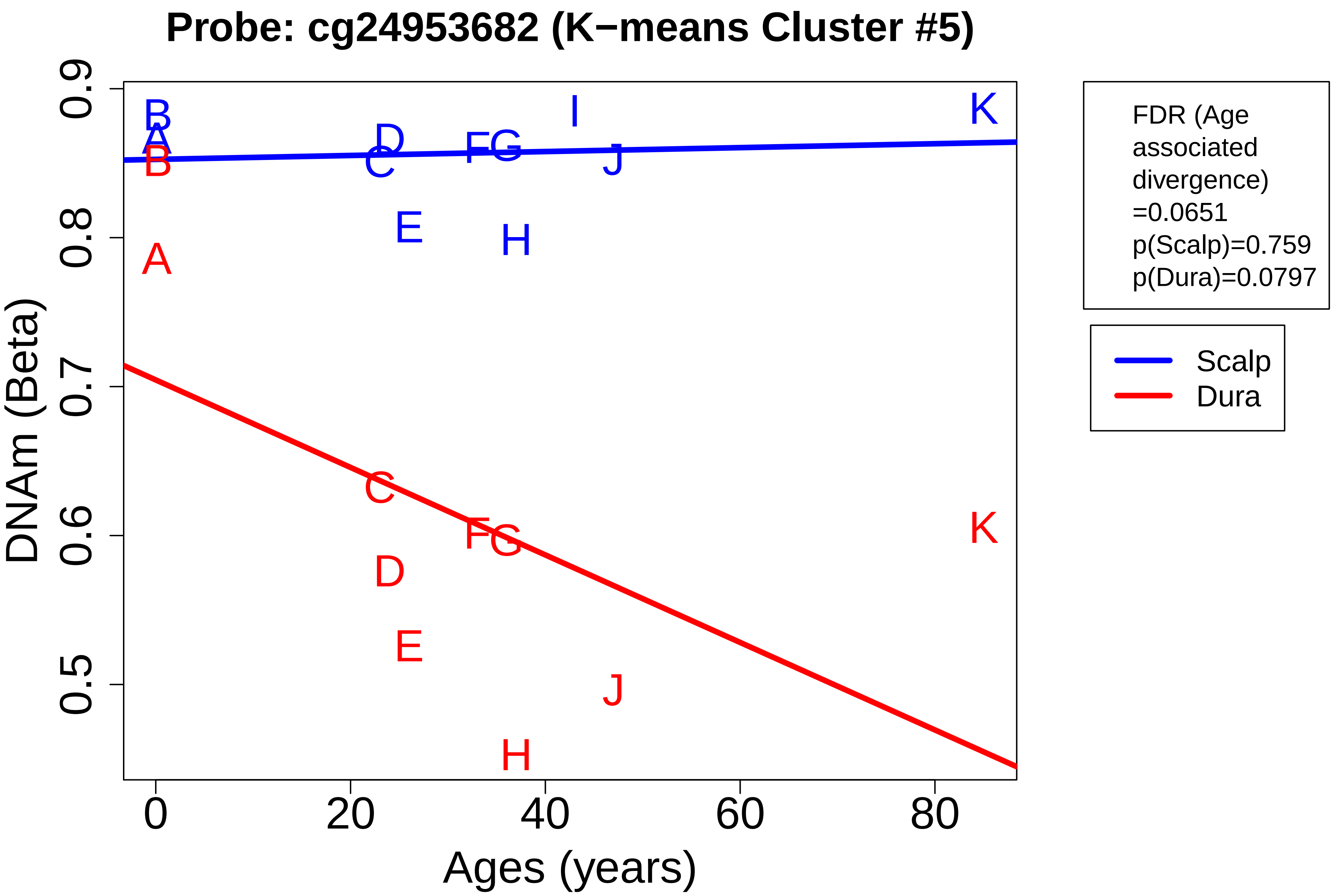

Probe: cg24953682 (K−means Cluster #5)
DNAm (Beta)
0.5
0.6
0.7
0.8
0.9
0
20
40
60
80
Ages (years)
A B C D E F G H I J K
FDR (Age associated divergence) =0.0651
p(Scalp)=0.759
p(Dura)=0.0797
Scalp
Dura

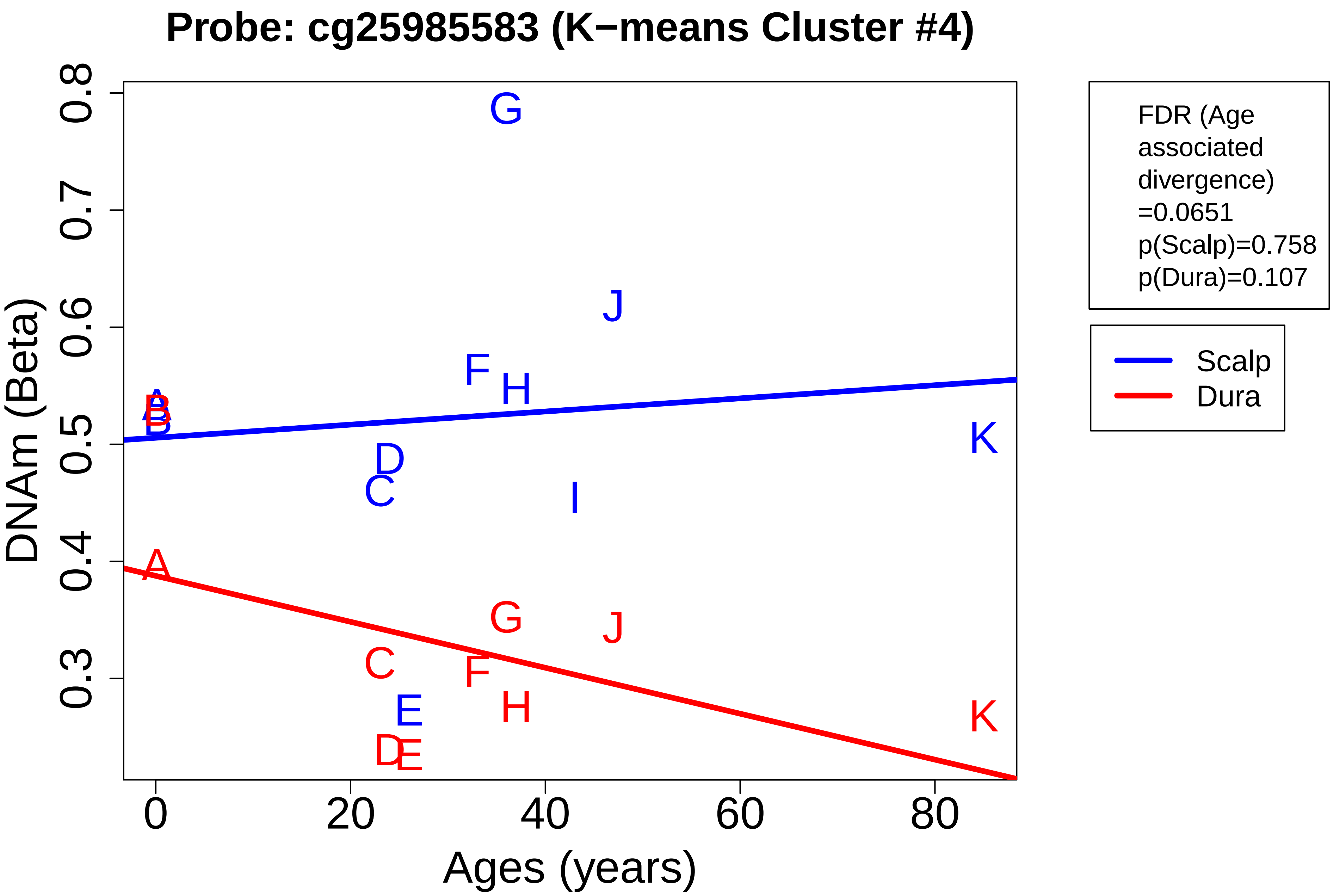

Probe: cg25985583 (K−means Cluster #4)
DNAm (Beta)
0.3
0.4
0.5
0.6
0.7
0.8
0
20
40
60
80
Ages (years)
FDR (Age associated divergence) =0.0651
p(Scalp)=0.758
p(Dura)=0.107
Scalp
Dura

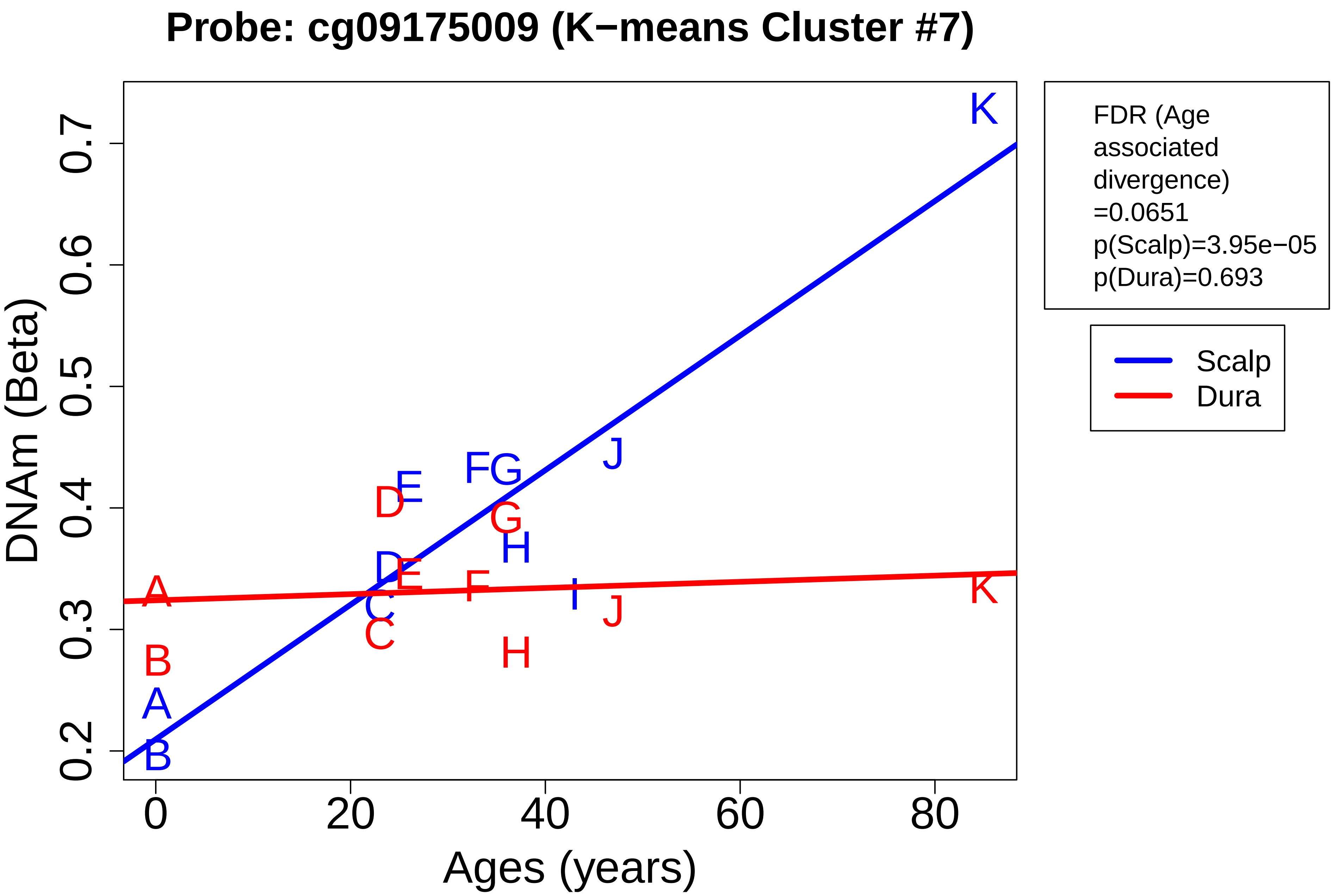

Probe: cg09175009 (K−means Cluster #7)
DNAm (Beta)
0.2
0.3
0.4
0.5
0.6
0.7
Ages (years)
0
20
40
60
80
FDR (Age associated divergence) =0.0651
p(Scalp)=3.95e−05
p(Dura)=0.693
Scalp
Dura
A
B
C
D
E
F
G
H
I
J
K

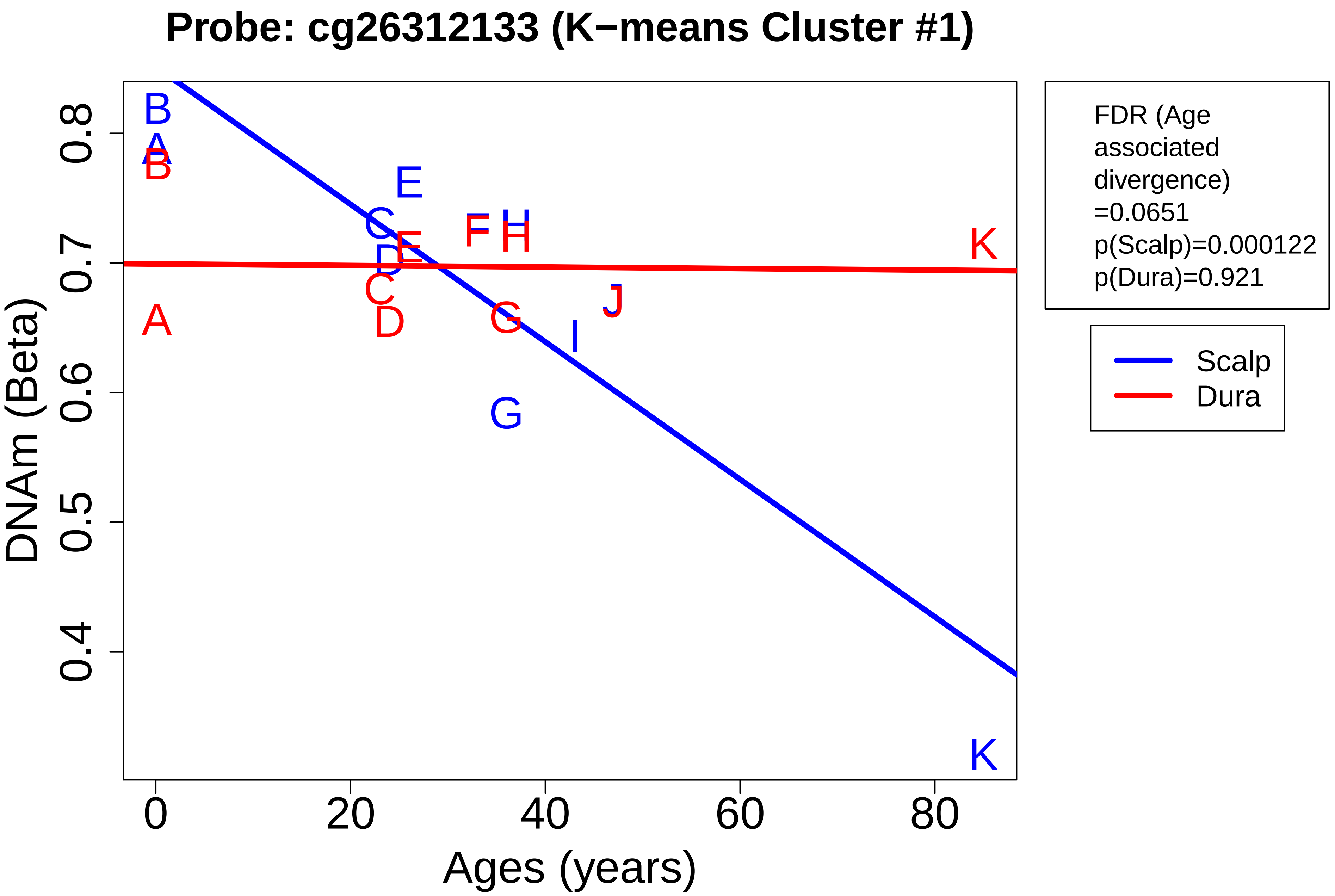
Probe: cg26312133 (K−means Cluster #1)
DNAm (Beta)
0.4
0.5
0.6
0.7
0.8
Ages (years)
0
20
40
60
80
FDR (Age associated divergence) =0.0651
p(Scalp)=0.000122
p(Dura)=0.921
Scalp
Dura

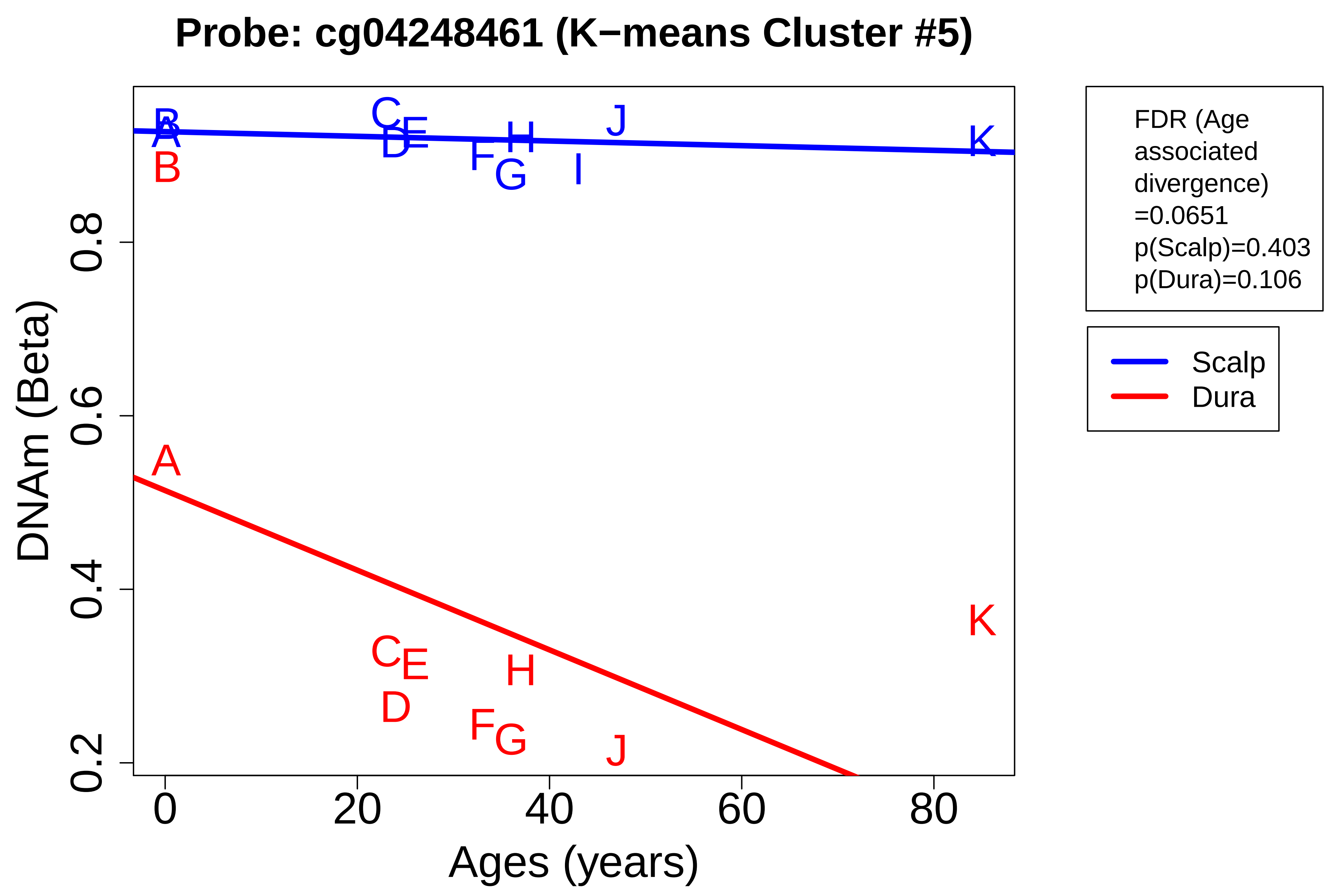

Probe: cg04248461 (K−means Cluster #5)
DNAm (Beta)
0.2
0.4
0.6
0.8
0
20
40
60
80
Ages (years)
B
A
B
C
E
D
F
H
G
J
I
K
A
C
E
H
D
F
G
J
K
FDR (Age associated divergence) =0.0651
p(Scalp)=0.403
p(Dura)=0.106
Scalp
Dura

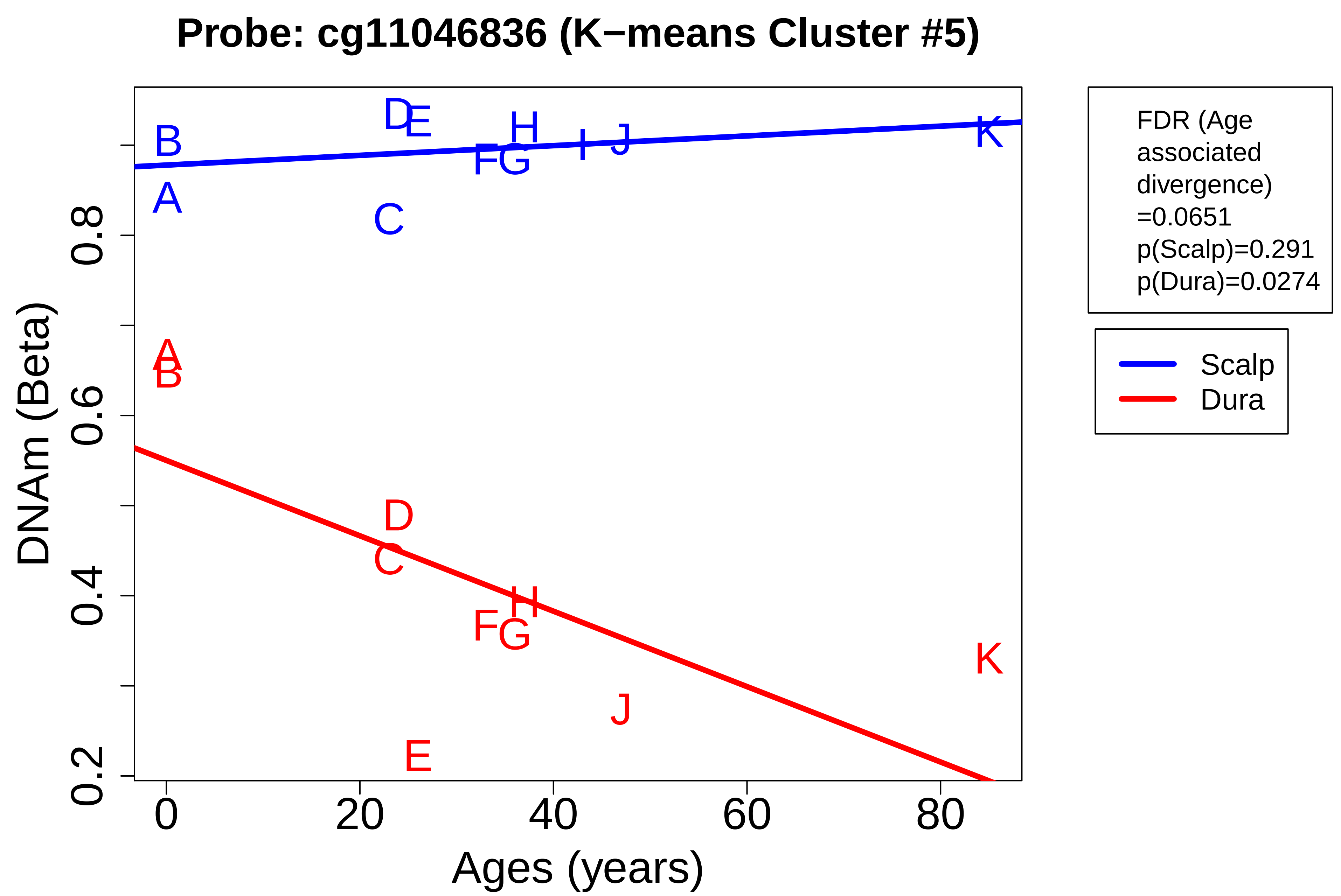
Probe: cg11046836 (K−means Cluster #5)
DNAm (Beta)
0.2
0.4
0.6
0.8
Ages (years)
0
20
40
60
80
A
B
C
D
E
F
G
H
I
J
K
FDR (Age associated divergence) =0.0651
p(Scalp)=0.291
p(Dura)=0.0274
Scalp
Dura

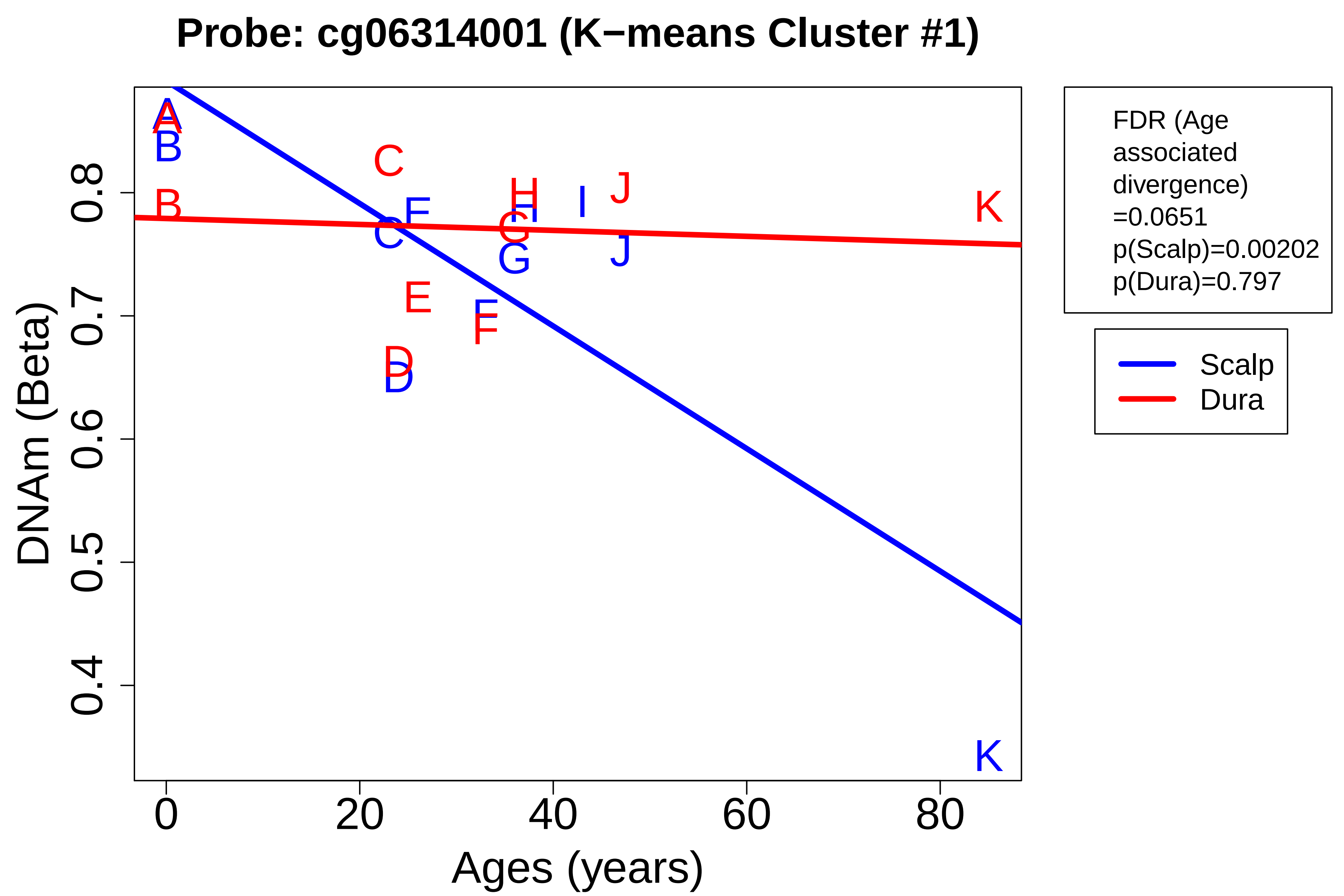
Probe: cg06314001 (K−means Cluster #1)
DNAm (Beta)
0.4
0.5
0.6
0.7
0.8
0
20
40
60
80
Ages (years)
FDR (Age associated divergence) =0.0651
p(Scalp)=0.00202
p(Dura)=0.797
Scalp
Dura

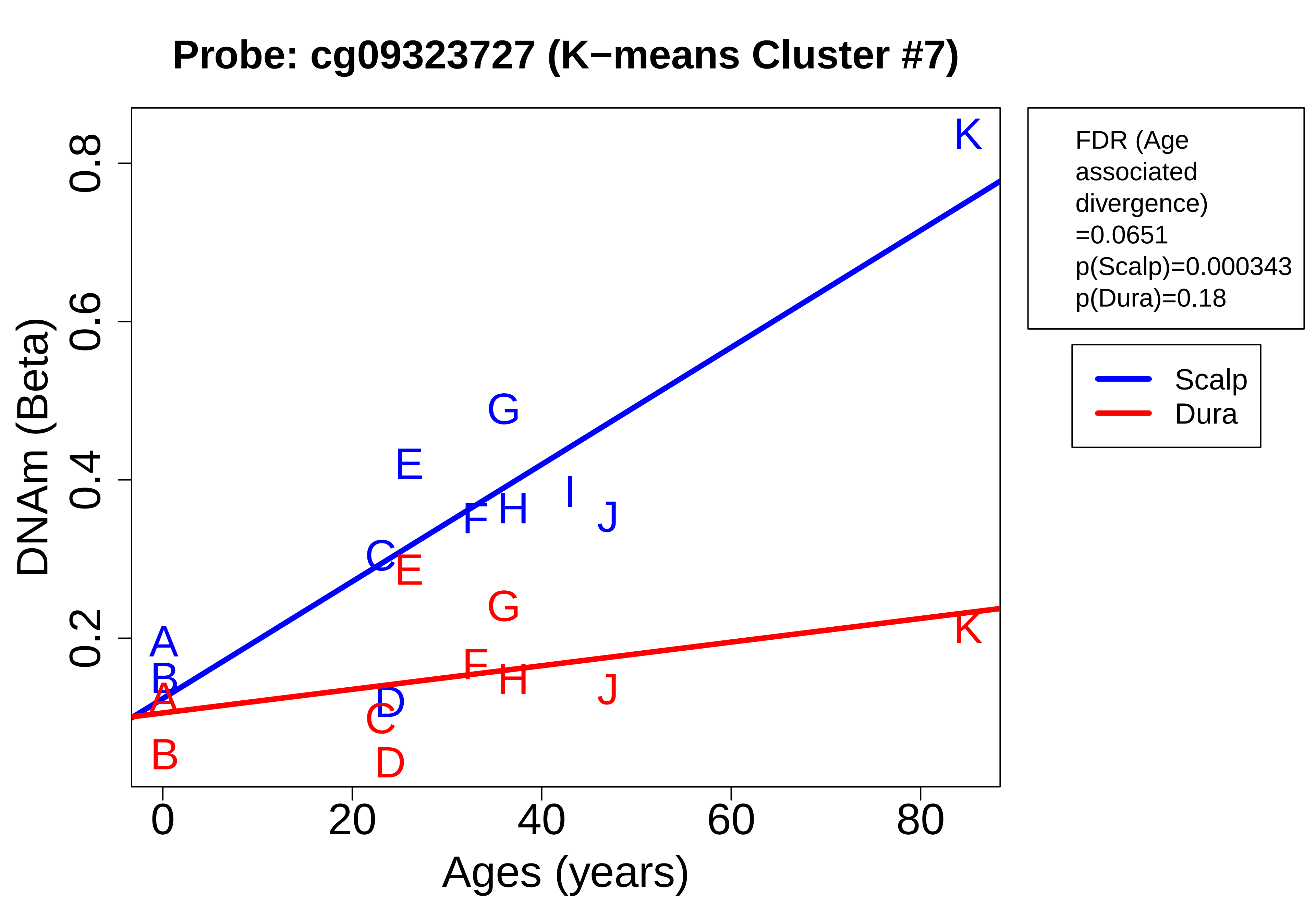

Probe: cg09323727 (K−means Cluster #7)
DNAm (Beta)
0.2
0.4
0.6
0.8
0
20
40
60
80
Ages (years)
A
B
C
D
E
F
G
H
I
J
K
A
B
C
D
E
F
G
H
J
K
FDR (Age associated divergence) =0.0651
p(Scalp)=0.000343
p(Dura)=0.18
Scalp
Dura

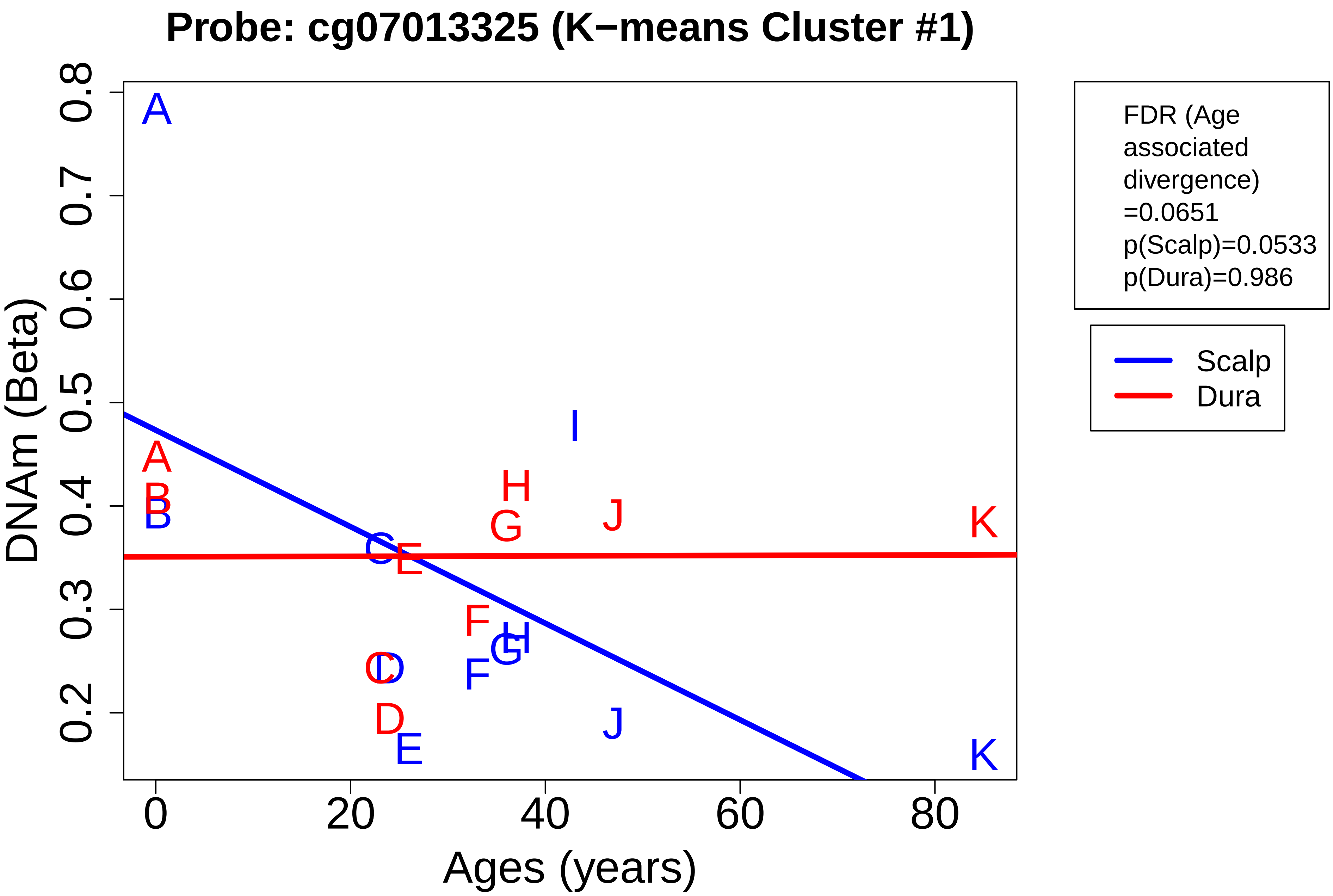

Probe: cg07013325 (K−means Cluster #1)
DNAm (Beta)
Ages (years)
0.2
0.3
0.4
0.5
0.6
0.7
0.8
0
20
40
60
80
FDR (Age associated divergence) =0.0651
p(Scalp)=0.0533
p(Dura)=0.986
Scalp
Dura
A
B
C
D
E
F
G
H
I
J
K

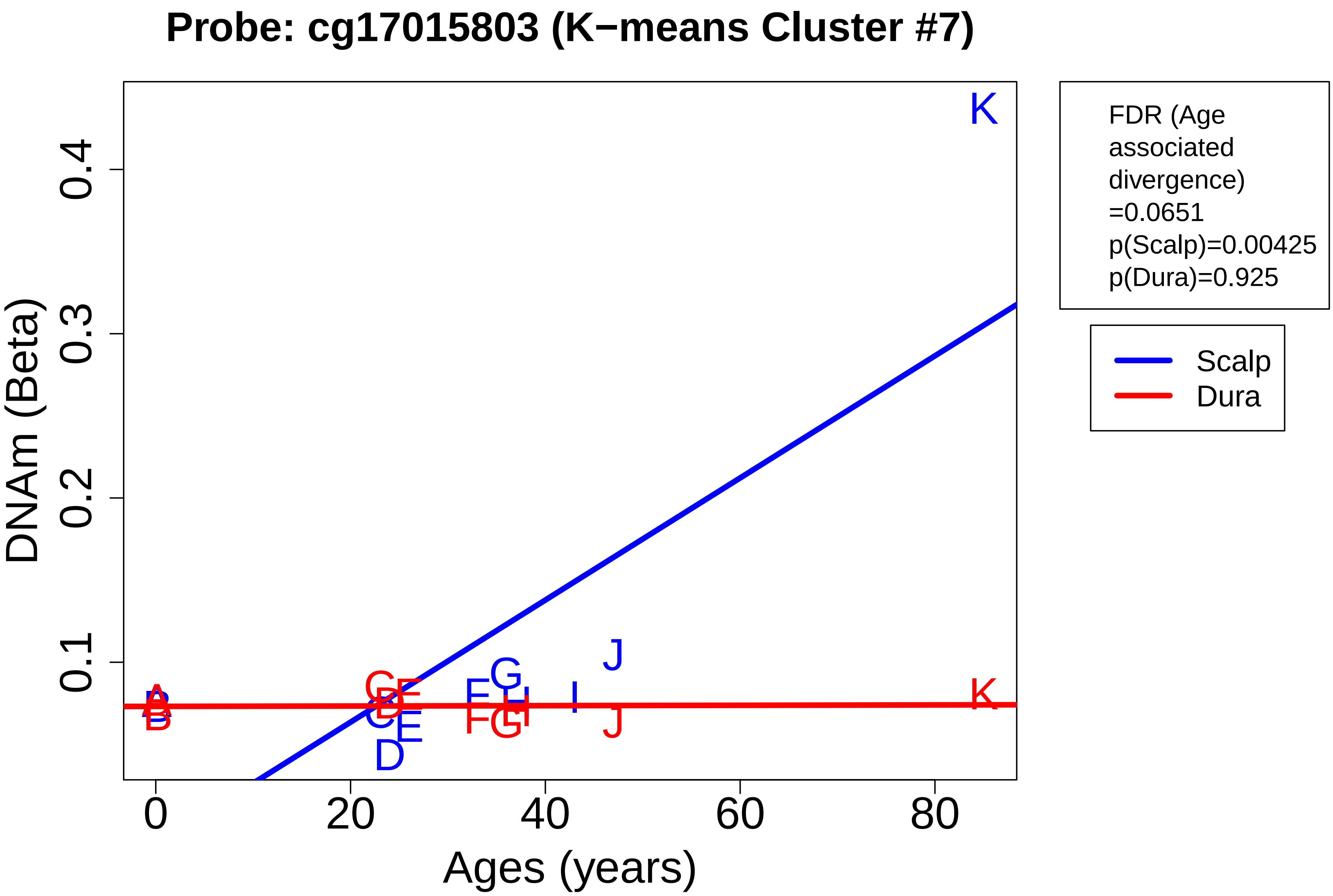
Probe: cg17015803 (K−means Cluster #7)
DNAm (Beta)
0.1
0.2
0.3
0.4
Ages (years)
0
20
40
60
80
FDR (Age associated divergence) =0.0651
p(Scalp)=0.00425
p(Dura)=0.925
Scalp
Dura

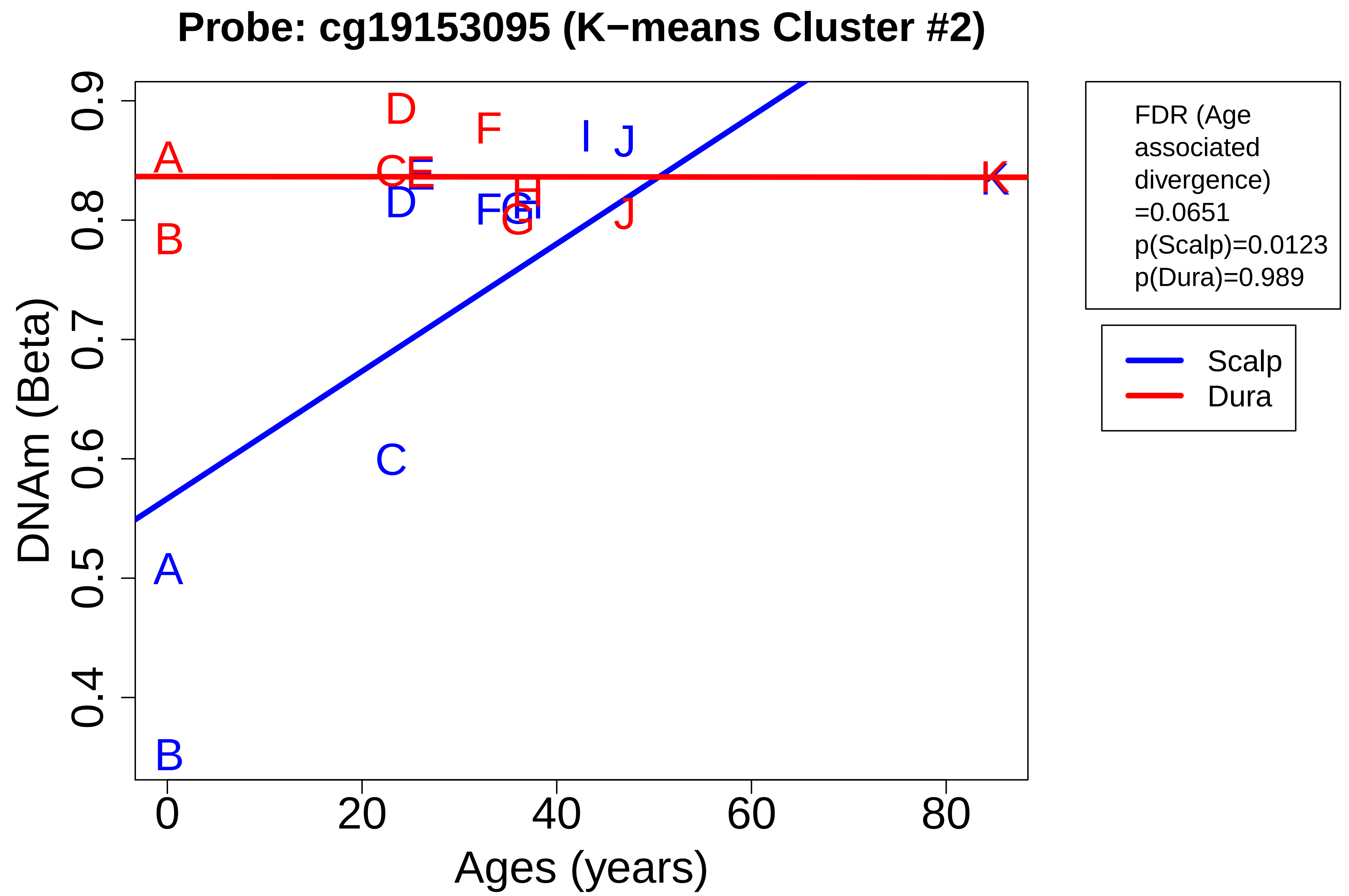
Probe: cg19153095 (K−means Cluster #2)
DNAm (Beta)
0.4
0.5
0.6
0.7
0.8
0.9
0
20
40
60
80
Ages (years)
FDR (Age associated divergence) =0.0651
p(Scalp)=0.0123
p(Dura)=0.989
Scalp
Dura

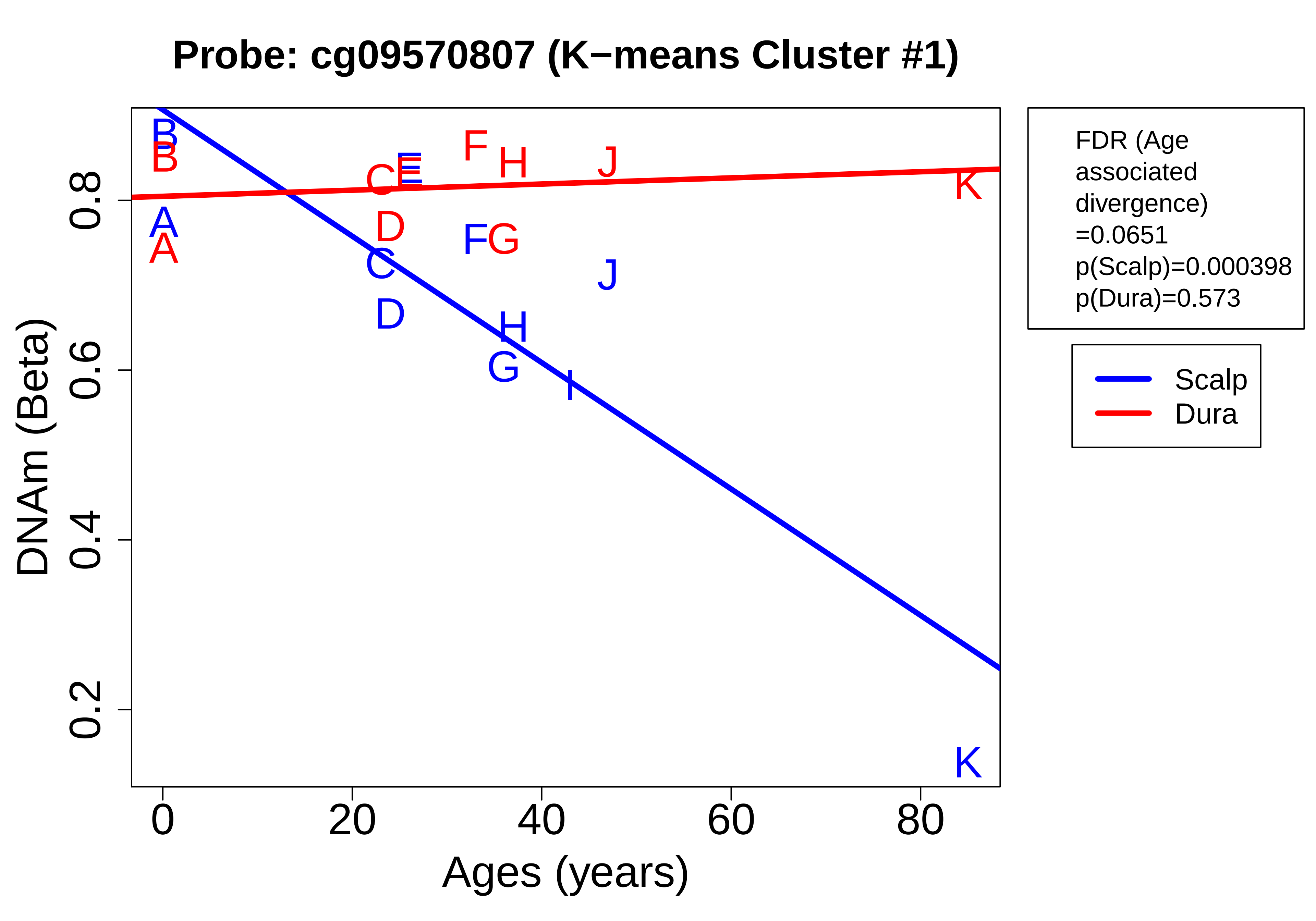

Probe: cg09570807 (K−means Cluster #1)
FDR (Age associated divergence) =0.0651
p(Scalp)=0.000398
p(Dura)=0.573
Scalp
Dura
DNAm (Beta)
0.2
0.4
0.6
0.8
Ages (years)
0
20
40
60
80

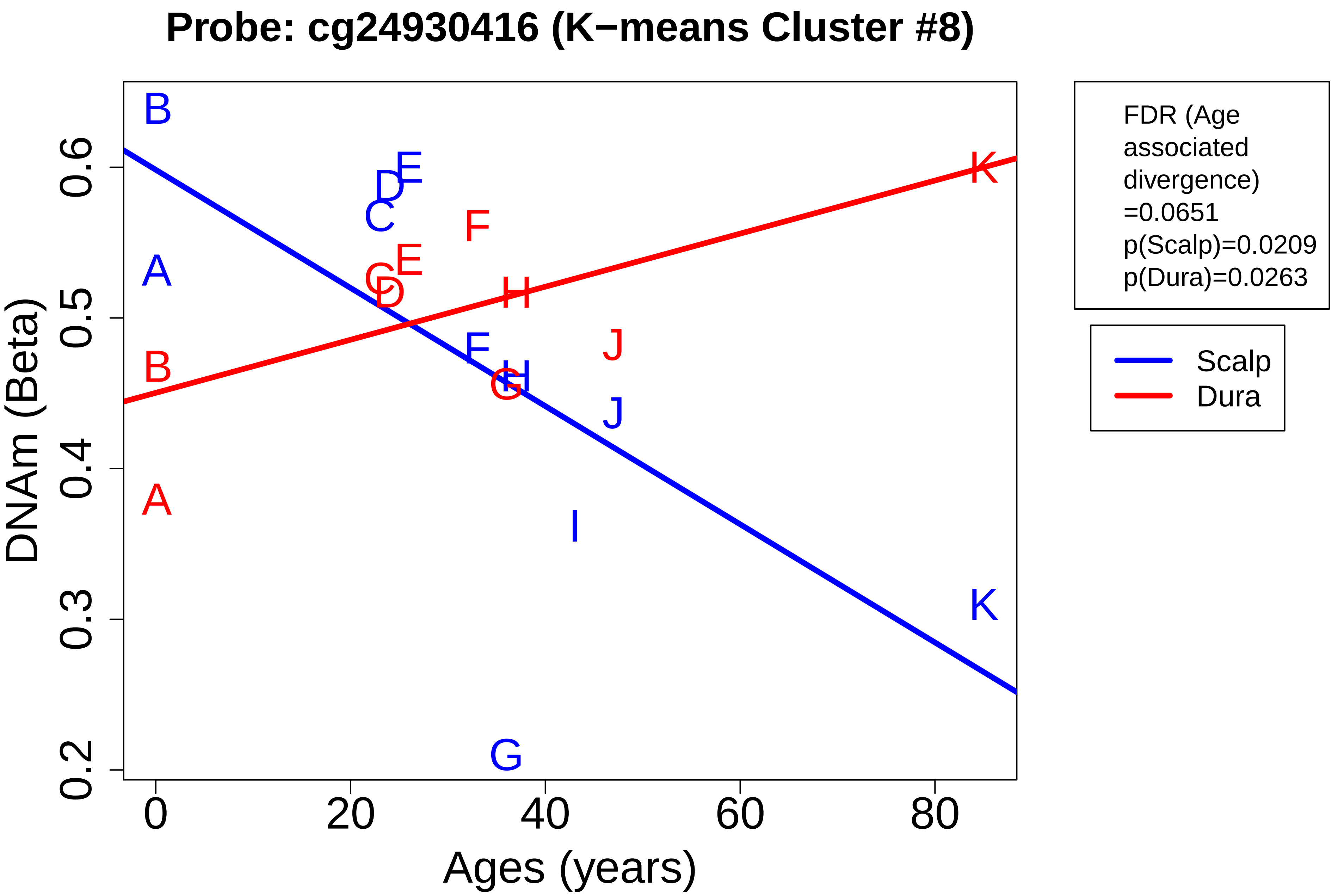

Probe: cg24930416 (K−means Cluster #8)
DNAm (Beta)
0.2
0.3
0.4
0.5
0.6
0
20
40
60
80
Ages (years)
FDR (Age associated divergence) =0.0651
p(Scalp)=0.0209
p(Dura)=0.0263
Scalp
Dura
A
B
C
D
E
F
G
H
I
J
K

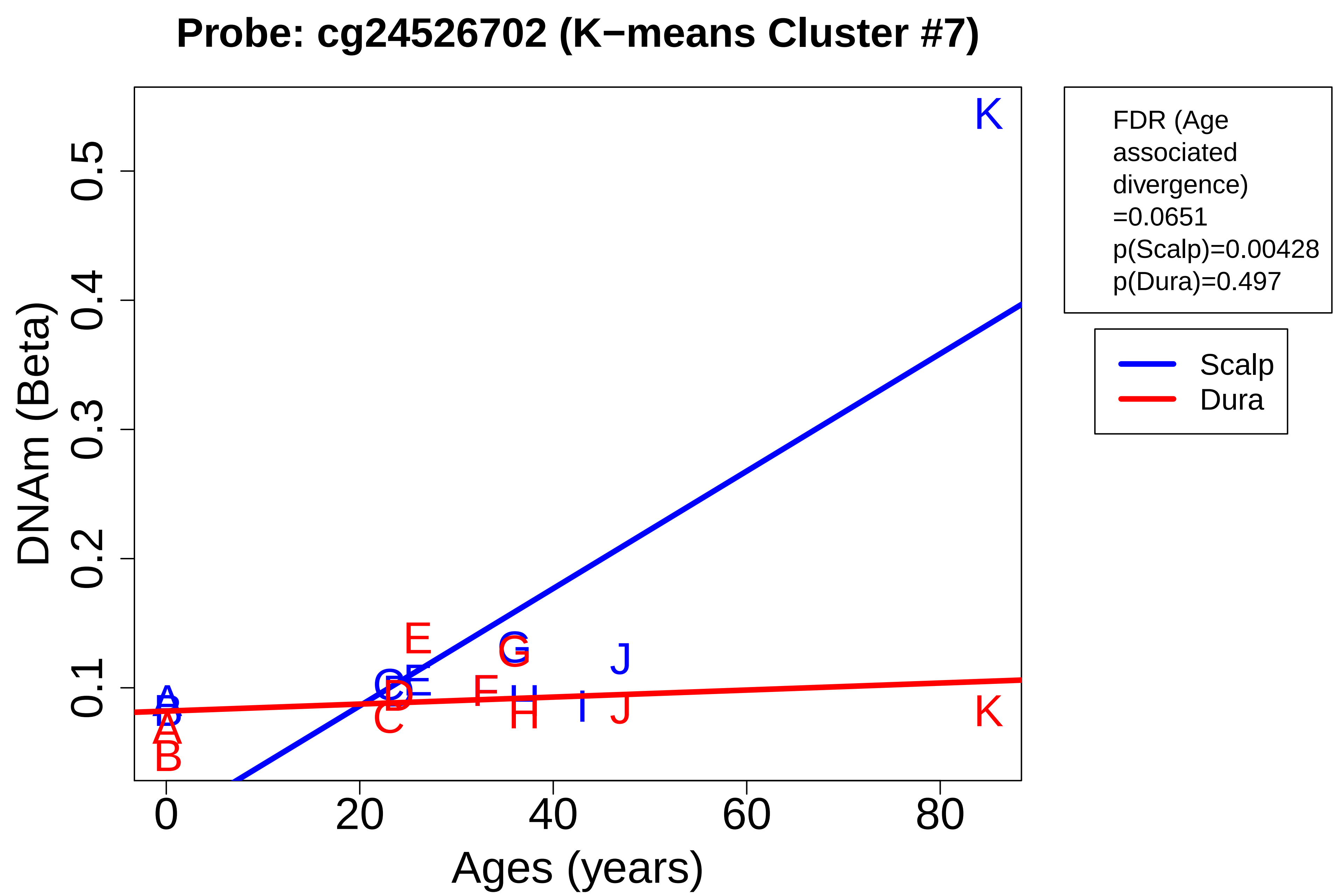
Probe: cg24526702 (K−means Cluster #7)
DNAm (Beta)
0.1
0.2
0.3
0.4
0.5
0
20
40
60
80
Ages (years)
FDR (Age associated divergence) =0.0651
p(Scalp)=0.00428
p(Dura)=0.497
Scalp
Dura
A
B
C
D
E
F
G
H
I
J
K

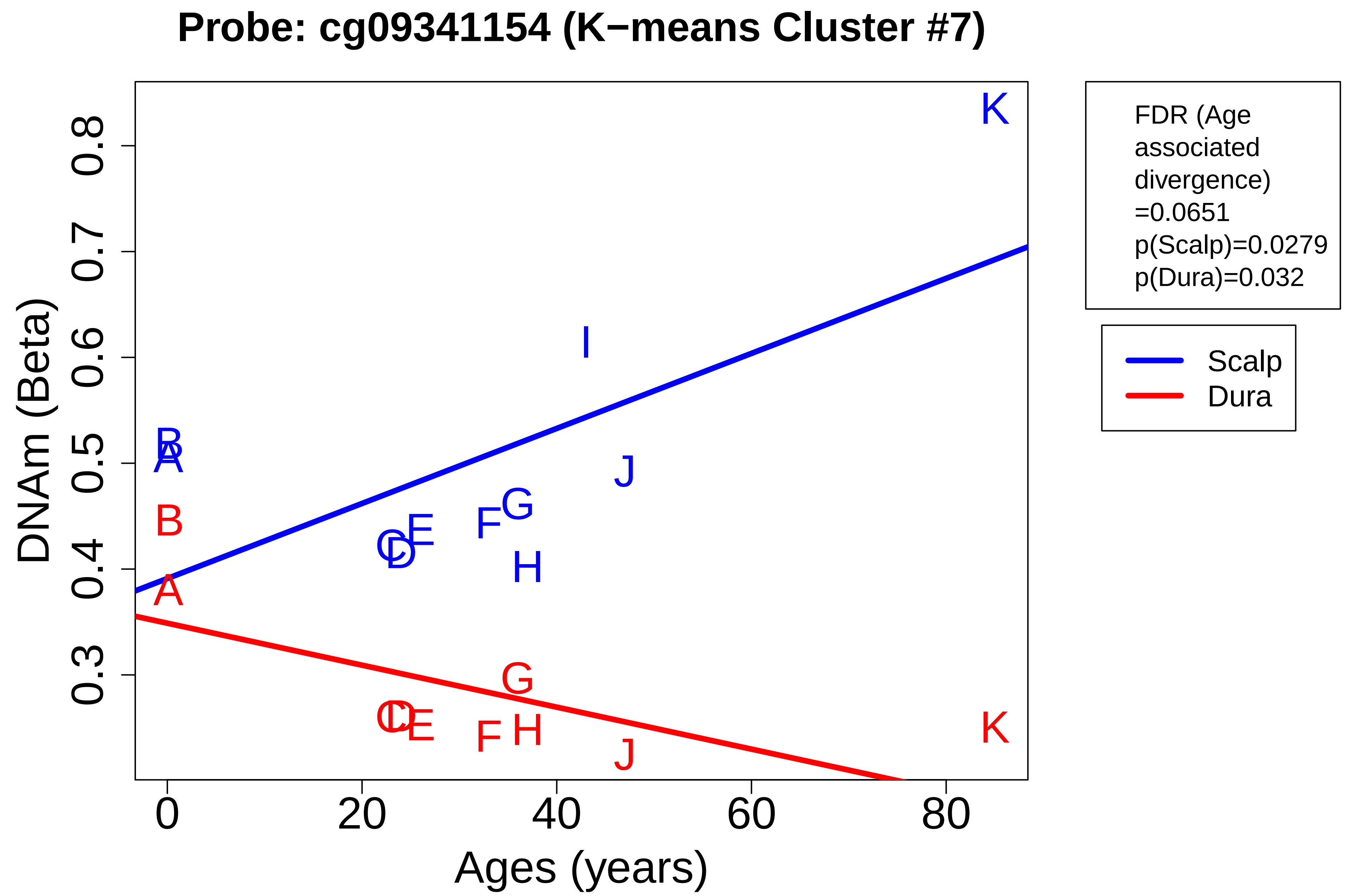

Probe: cg09341154 (K−means Cluster #7)
DNAm (Beta)
0.3
0.4
0.5
0.6
0.7
0.8
0
20
40
60
80
Ages (years)
FDR (Age associated divergence) =0.0651
p(Scalp)=0.0279
p(Dura)=0.032
Scalp
Dura

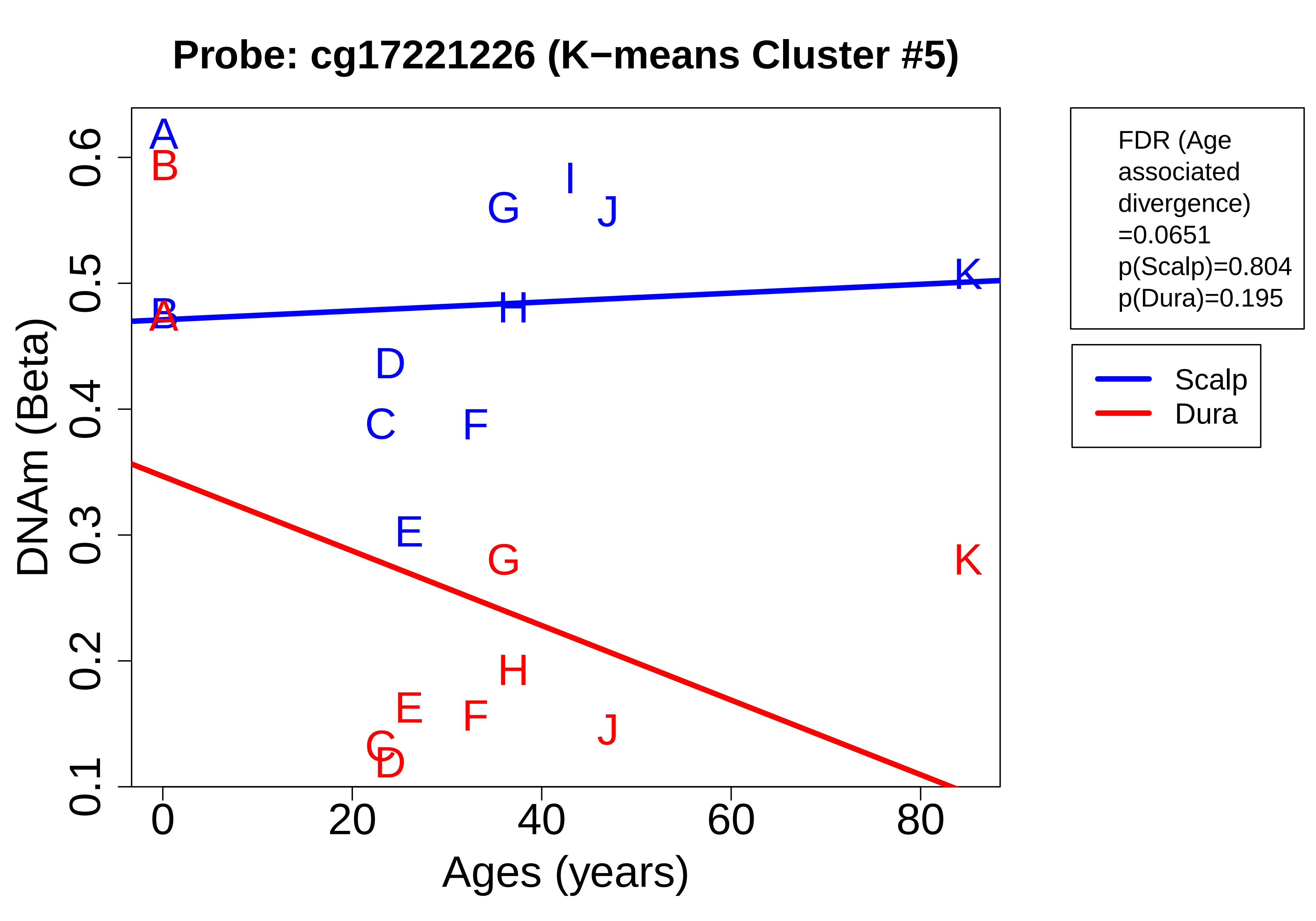

Probe: cg17221226 (K−means Cluster #5)
DNAm (Beta)
Ages (years)
0.1
0.2
0.3
0.4
0.5
0.6
0
20
40
60
80
A
B
C
D
E
F
G
H
I
J
K
FDR (Age associated divergence) =0.0651
p(Scalp)=0.804
p(Dura)=0.195
Scalp
Dura

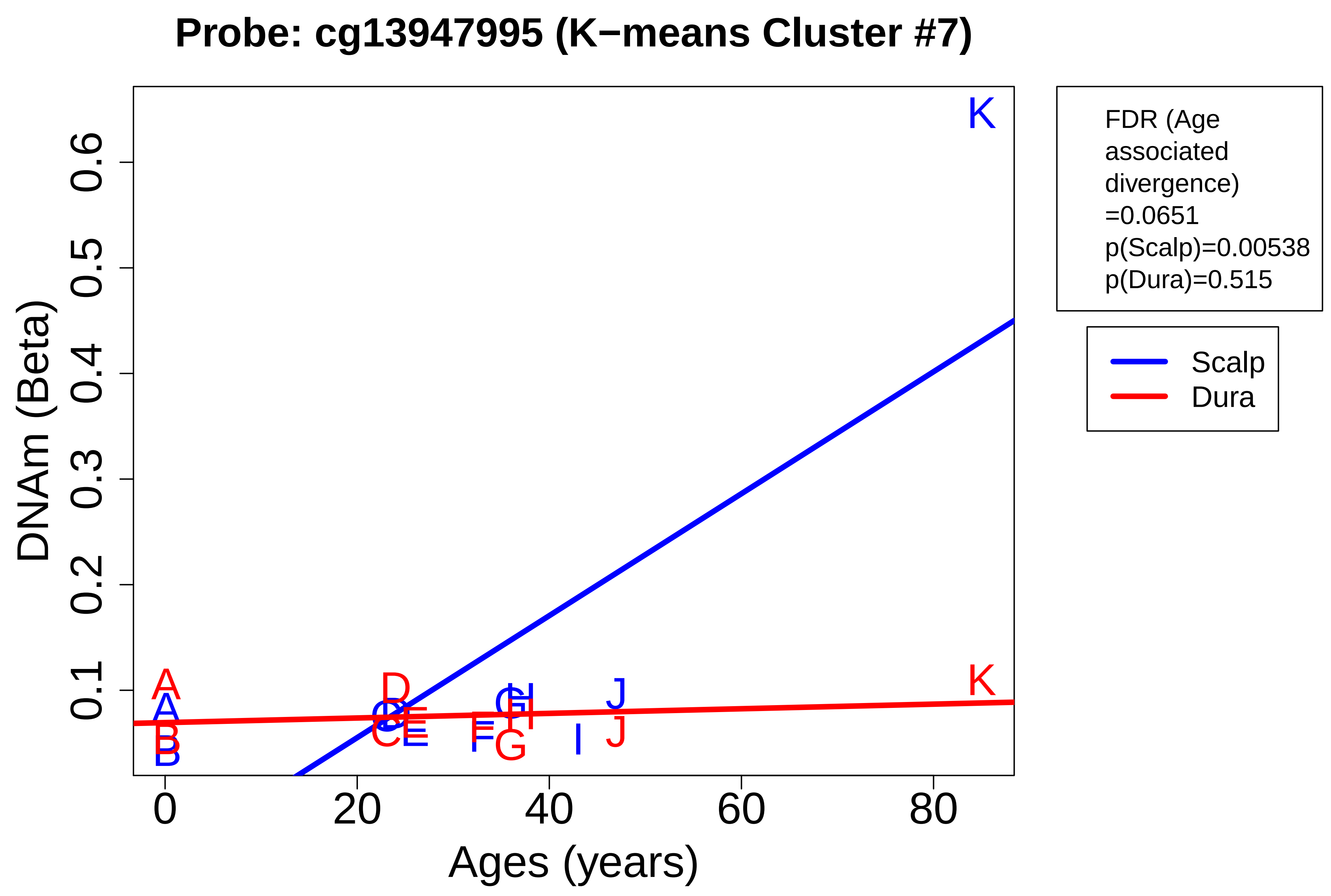
Probe: cg13947995 (K−means Cluster #7)
DNAm (Beta)
0.1
0.2
0.3
0.4
0.5
0.6
0
20
40
60
80
Ages (years)
A
B
C
D
E
F
G
H
I
J
K
FDR (Age associated divergence) =0.0651
p(Scalp)=0.00538
p(Dura)=0.515
Scalp
Dura

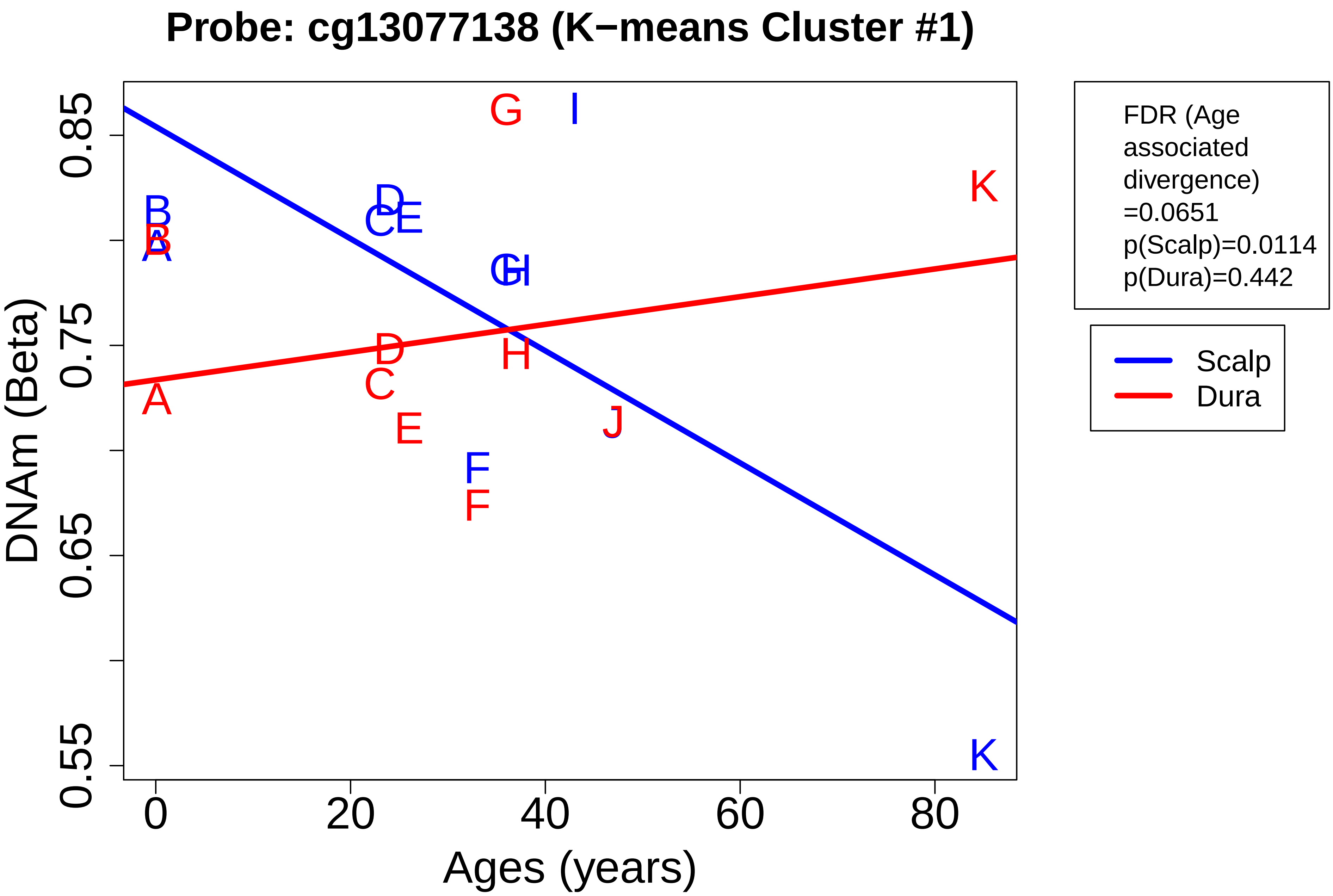
Probe: cg13077138 (K−means Cluster #1)
DNAm (Beta)
0.55
0.65
0.75
0.85
Ages (years)
0
20
40
60
80
FDR (Age associated divergence) =0.0651
p(Scalp)=0.0114
p(Dura)=0.442
Scalp
Dura
A
B
C
D
E
F
G
H
I
J
K

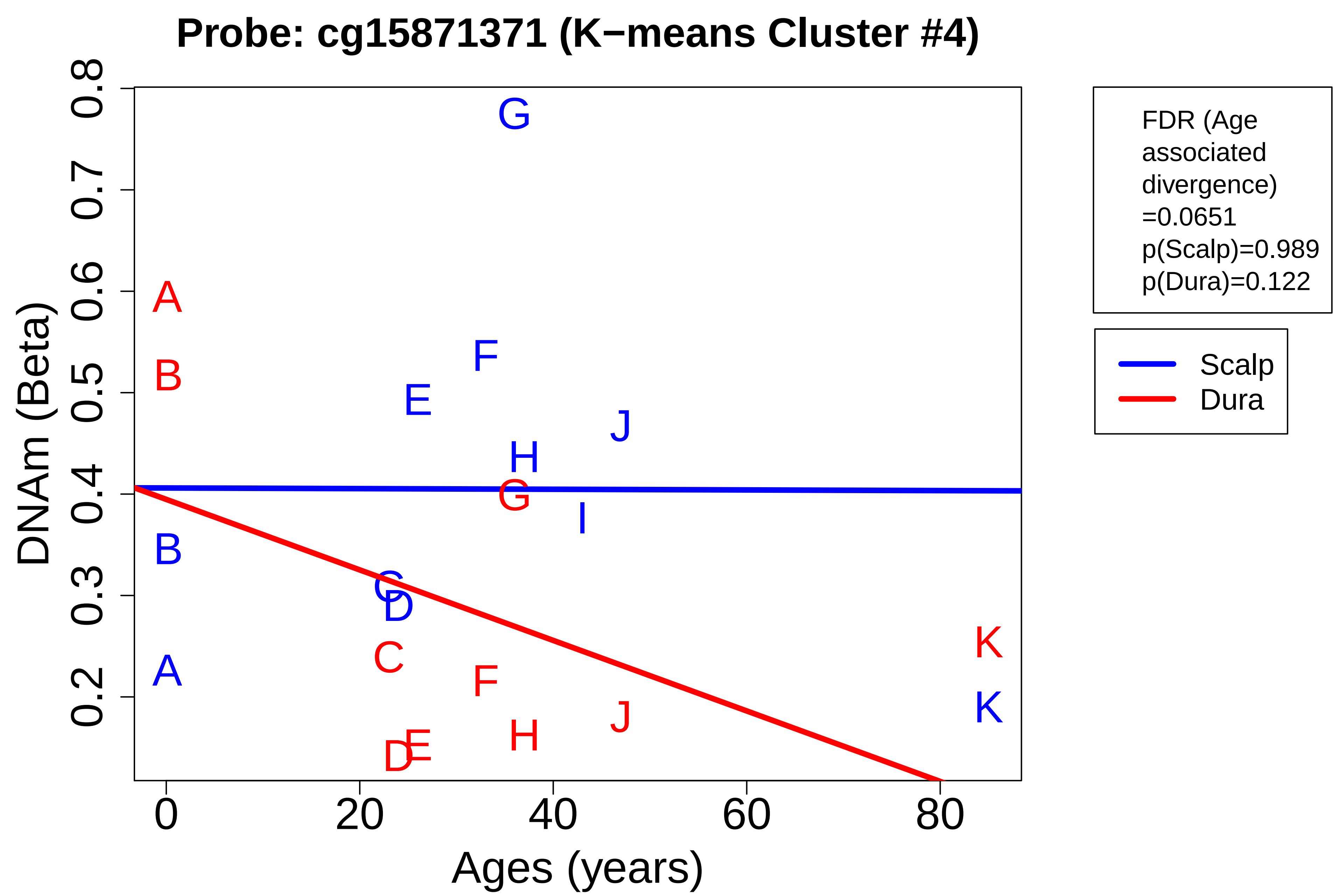
Probe: cg15871371 (K−means Cluster #4)
DNAm (Beta)
0.2
0.3
0.4
0.5
0.6
0.7
0.8
Ages (years)
0
20
40
60
80
FDR (Age associated divergence) =0.0651
p(Scalp)=0.989
p(Dura)=0.122
Scalp
Dura

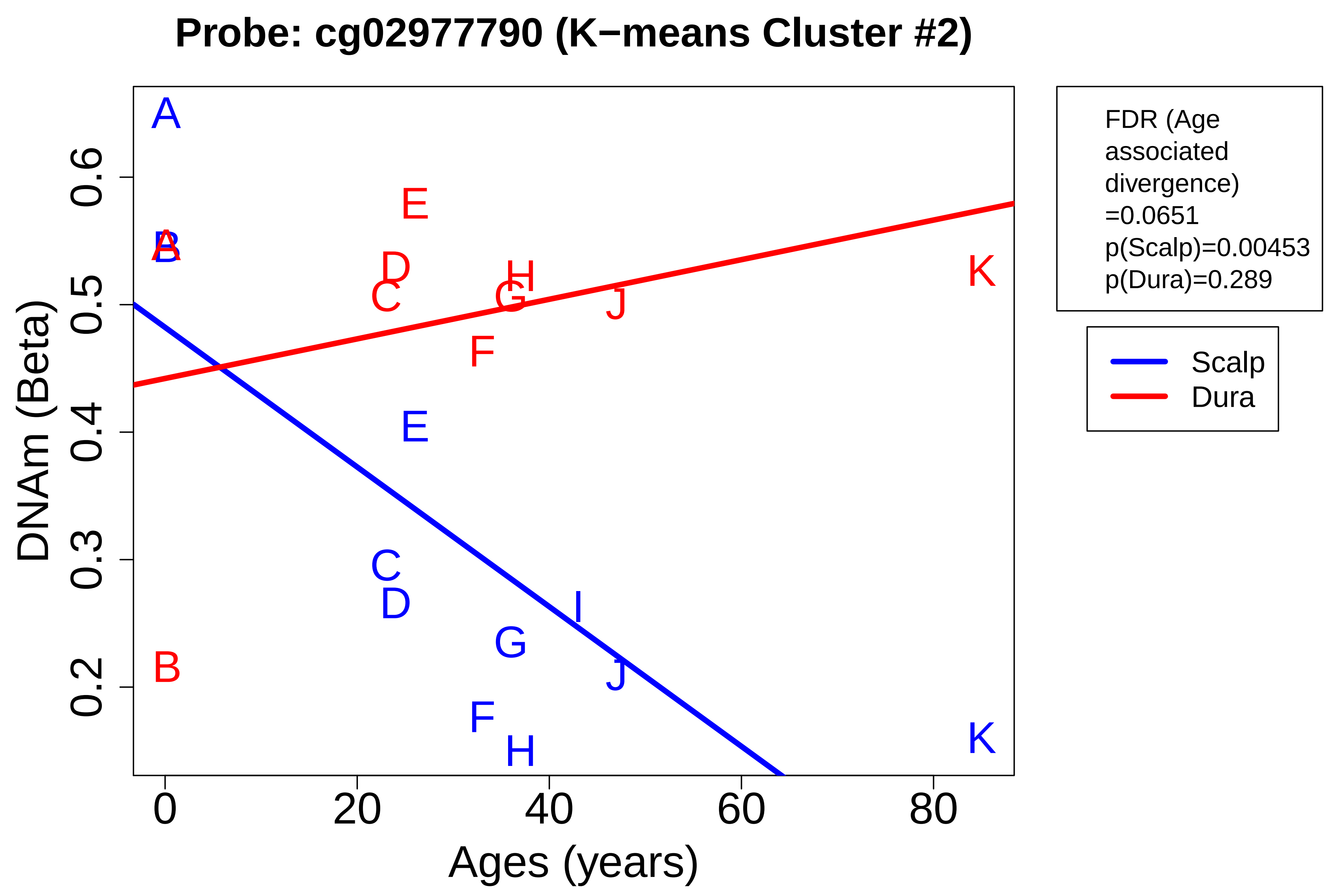

Probe: cg02977790 (K−means Cluster #2)
DNAm (Beta)
0.2
0.3
0.4
0.5
0.6
0
20
40
60
80
Ages (years)
A
B
A
E
D
C
H
G
J
K
F
E
C
D
I
G
B
J
F
H
K
FDR (Age associated divergence) =0.0651
p(Scalp)=0.00453
p(Dura)=0.289
Scalp
Dura

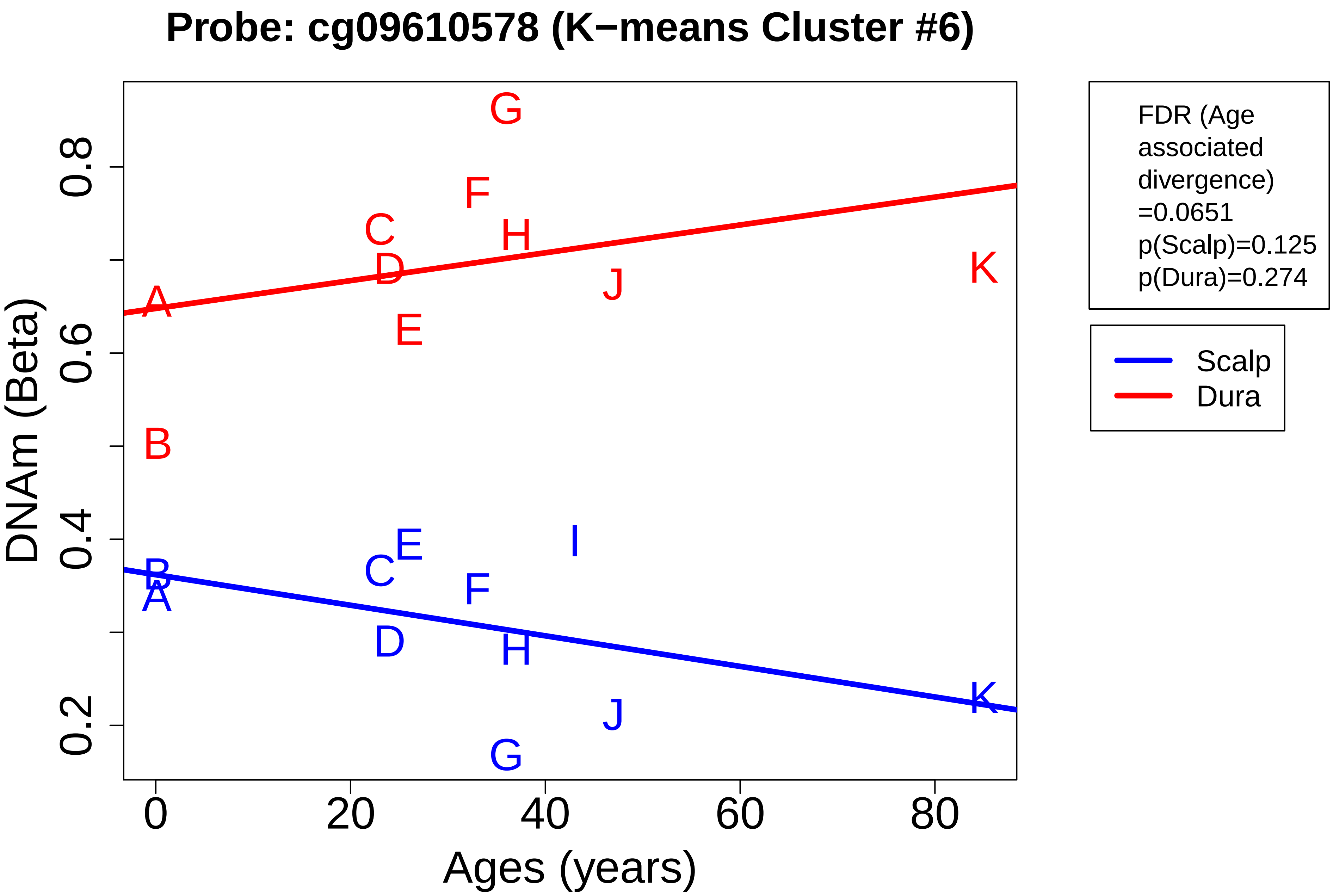
Probe: cg09610578 (K−means Cluster #6)
DNAm (Beta)
0.2
0.4
0.6
0.8
0
20
40
60
80
Ages (years)
FDR (Age associated divergence) =0.0651
p(Scalp)=0.125
p(Dura)=0.274
Scalp
Dura

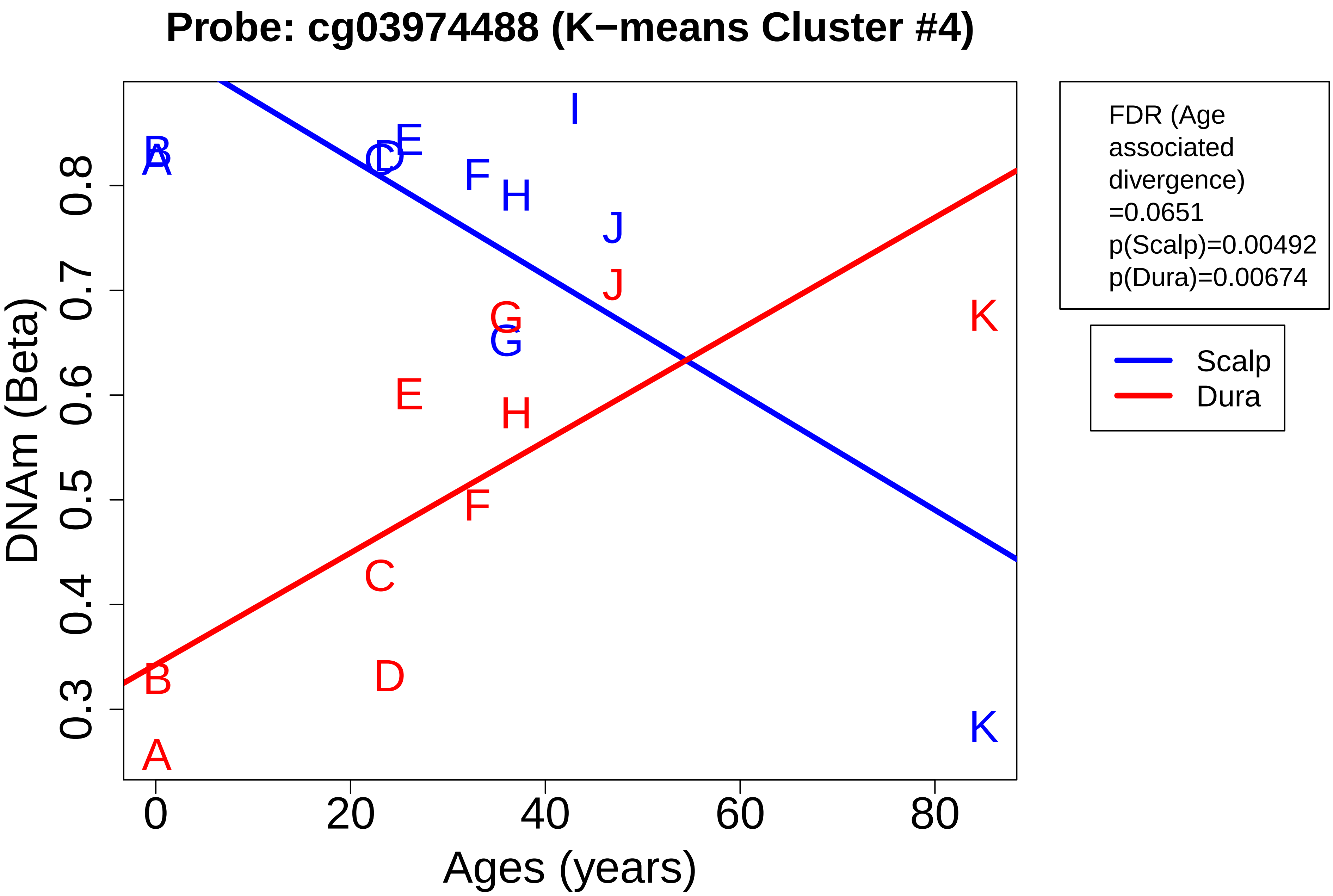
Probe: cg03974488 (K−means Cluster #4)
DNAm (Beta)
0.3
0.4
0.5
0.6
0.7
0.8
Ages (years)
0
20
40
60
80
A
B
C
D
E
F
G
H
I
J
K
FDR (Age associated divergence) =0.0651
p(Scalp)=0.00492
p(Dura)=0.00674
Scalp
Dura

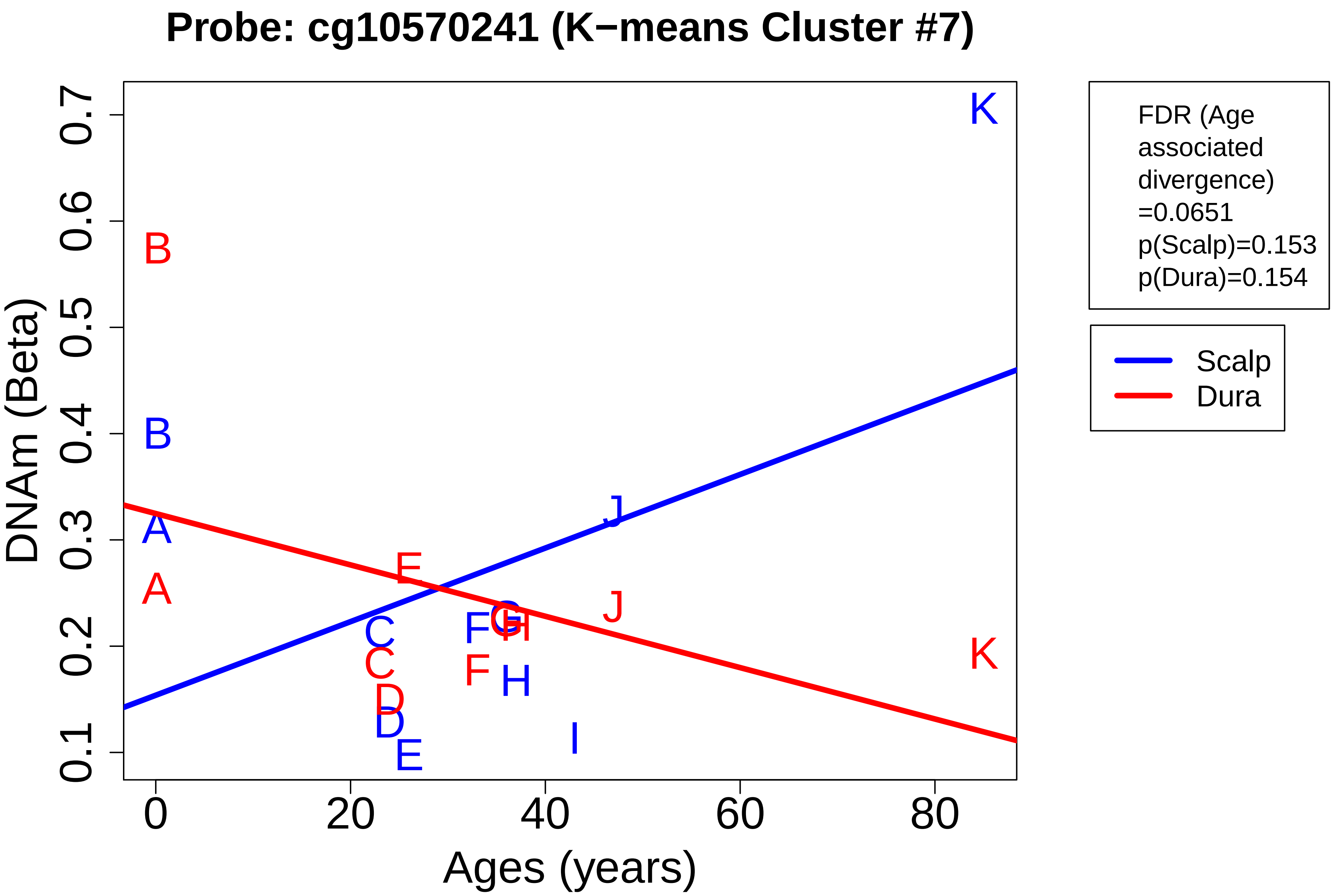

Probe: cg10570241 (K−means Cluster #7)
DNAm (Beta)
Ages (years)
0.1
0.2
0.3
0.4
0.5
0.6
0.7
0
20
40
60
80
FDR (Age associated divergence) =0.0651
p(Scalp)=0.153
p(Dura)=0.154
Scalp
Dura
A
B
C
D
E
F
G
H
I
J
K

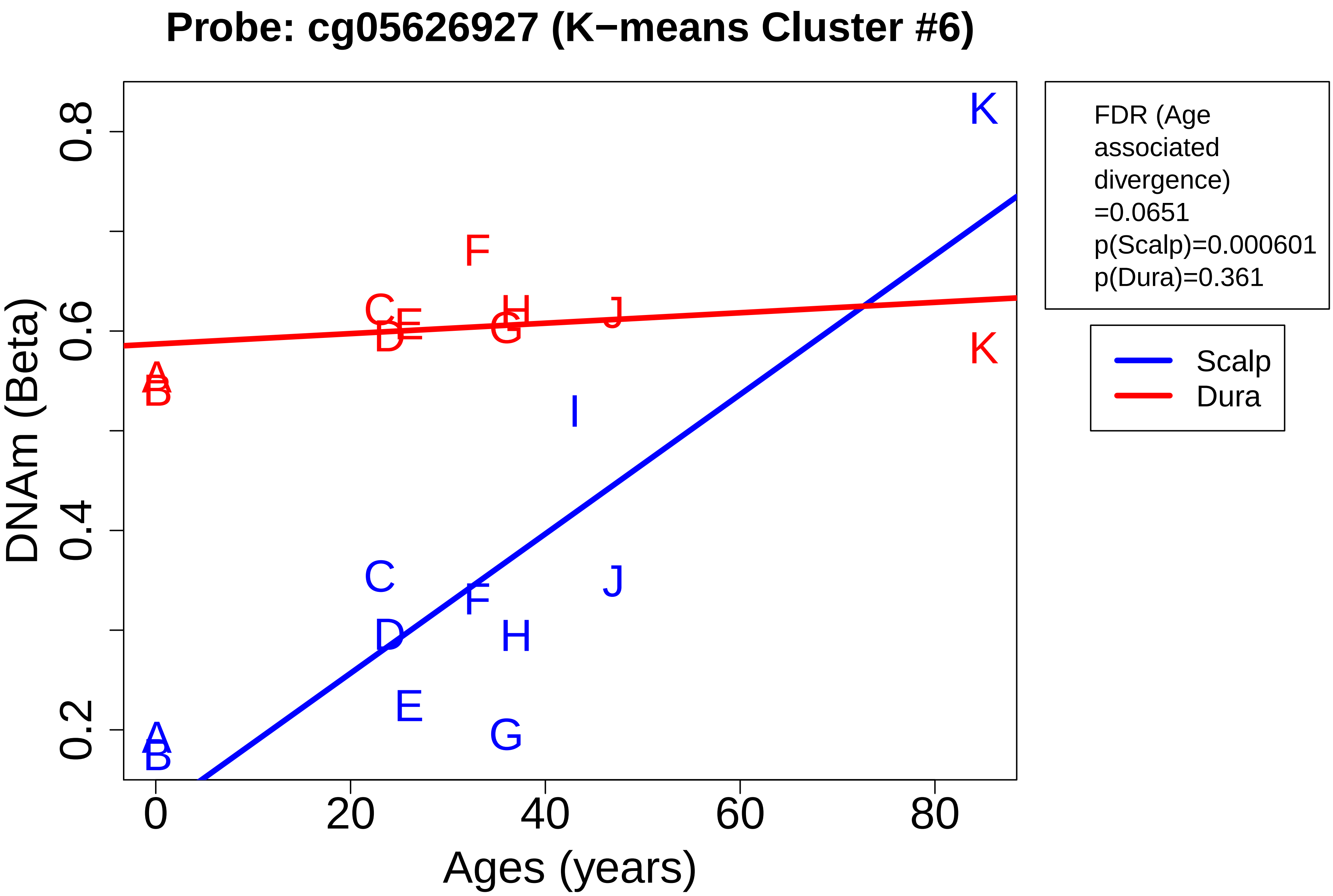

Probe: cg05626927 (K−means Cluster #6)
DNAm (Beta)
0.2
0.4
0.6
0.8
Ages (years)
0
20
40
60
80
FDR (Age associated divergence) =0.0651
p(Scalp)=0.000601
p(Dura)=0.361
Scalp
Dura
A
B
C
D
E
F
G
H
I
J
K

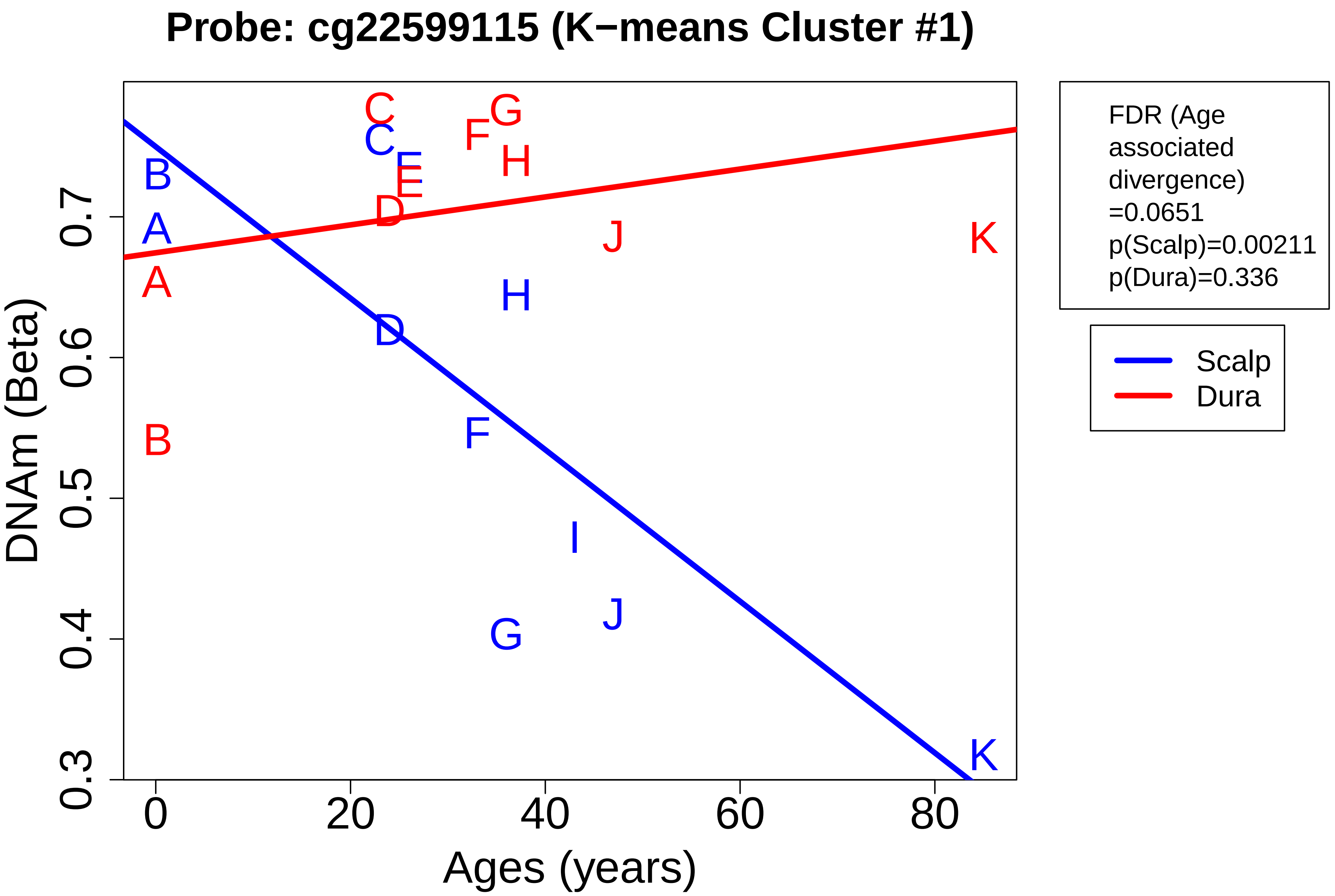

Probe: cg22599115 (K−means Cluster #1)
DNAm (Beta)
Ages (years)
0.3
0.4
0.5
0.6
0.7
0
20
40
60
80
FDR (Age associated divergence) =0.0651
p(Scalp)=0.00211
p(Dura)=0.336
Scalp
Dura

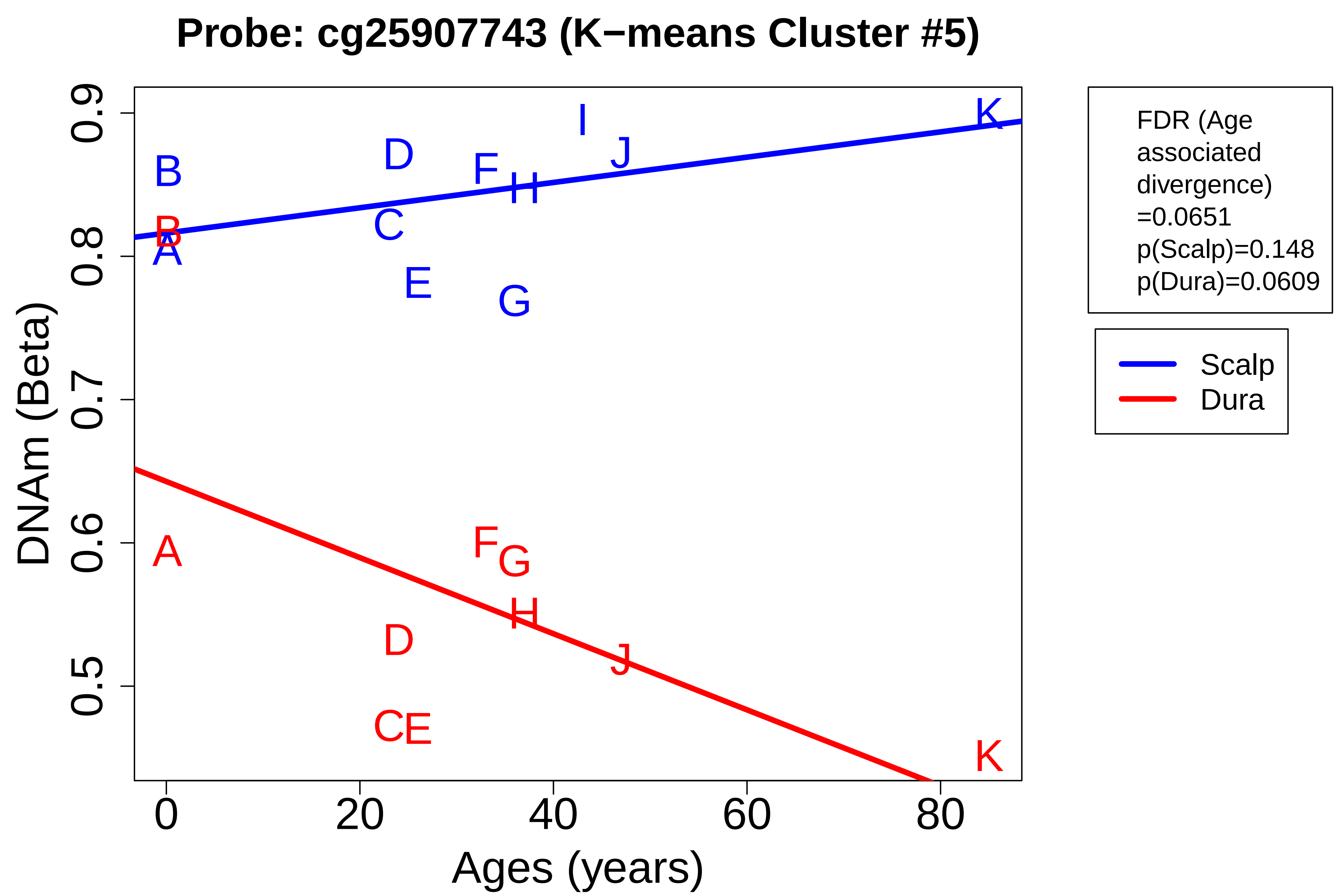

Probe: cg25907743 (K−means Cluster #5)
DNAm (Beta)
0.5
0.6
0.7
0.8
0.9
0
20
40
60
80
Ages (years)
FDR (Age associated divergence) =0.0651
p(Scalp)=0.148
p(Dura)=0.0609
Scalp
Dura
A
B
C
D
E
F
G
H
I
J
K

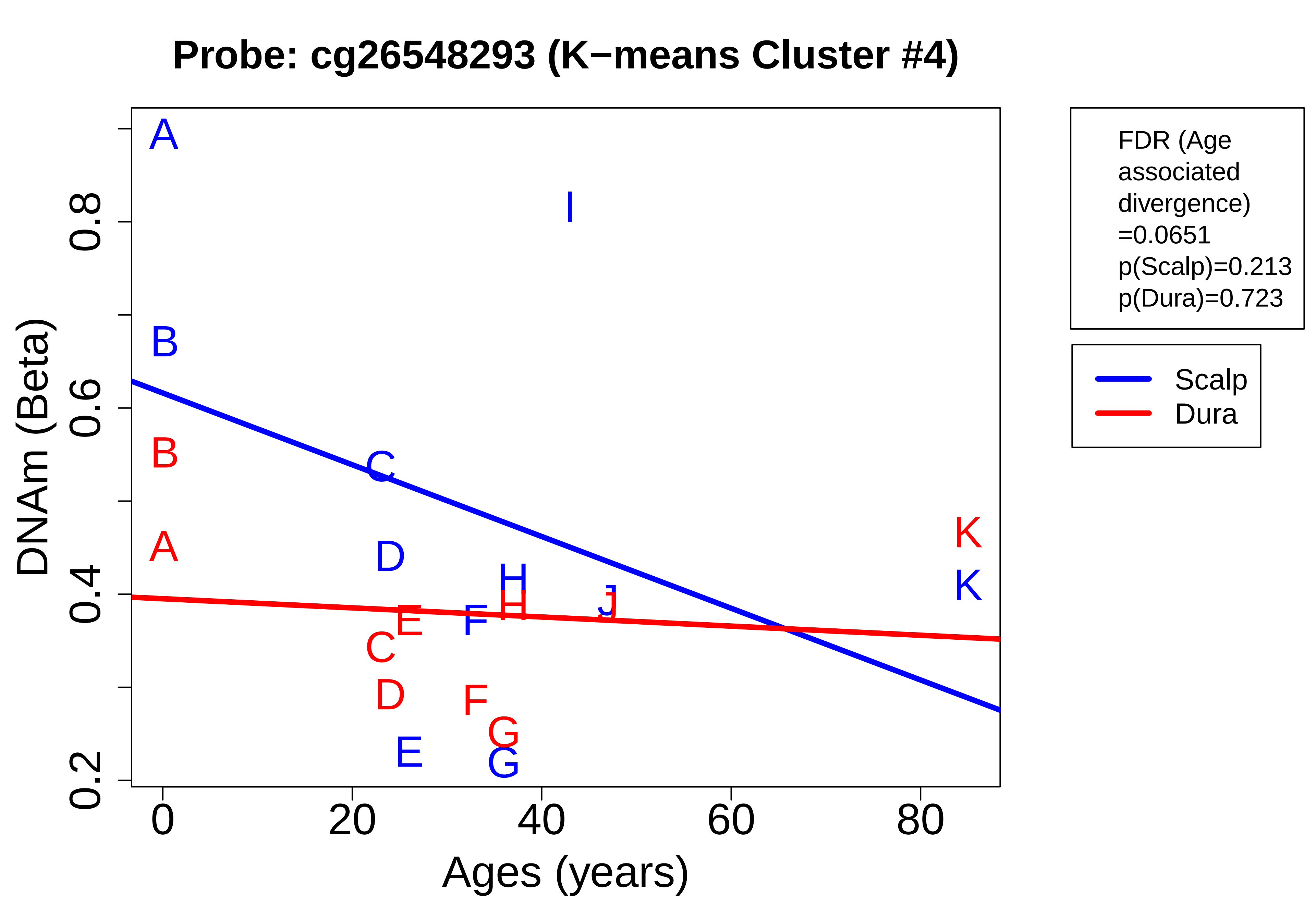

Probe: cg26548293 (K−means Cluster #4)
DNAm (Beta)
0.2
0.4
0.6
0.8
0
20
40
60
80
Ages (years)
FDR (Age associated divergence) =0.0651
p(Scalp)=0.213
p(Dura)=0.723
Scalp
Dura

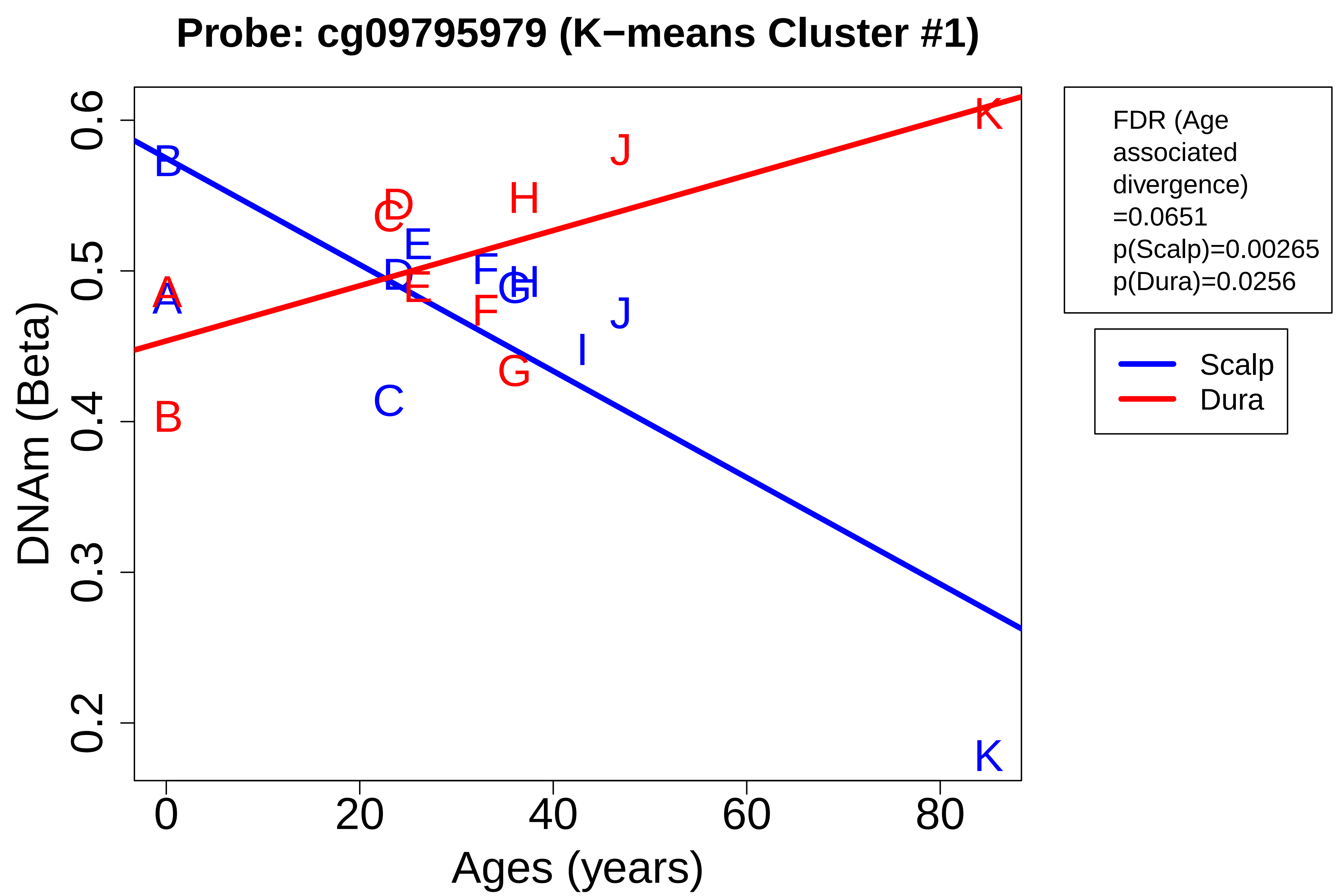

Probe: cg09795979 (K−means Cluster #1)
DNAm (Beta)
0.2
0.3
0.4
0.5
0.6
Ages (years)
0
20
40
60
80
FDR (Age associated divergence) =0.0651
p(Scalp)=0.00265
p(Dura)=0.0256
Scalp
Dura

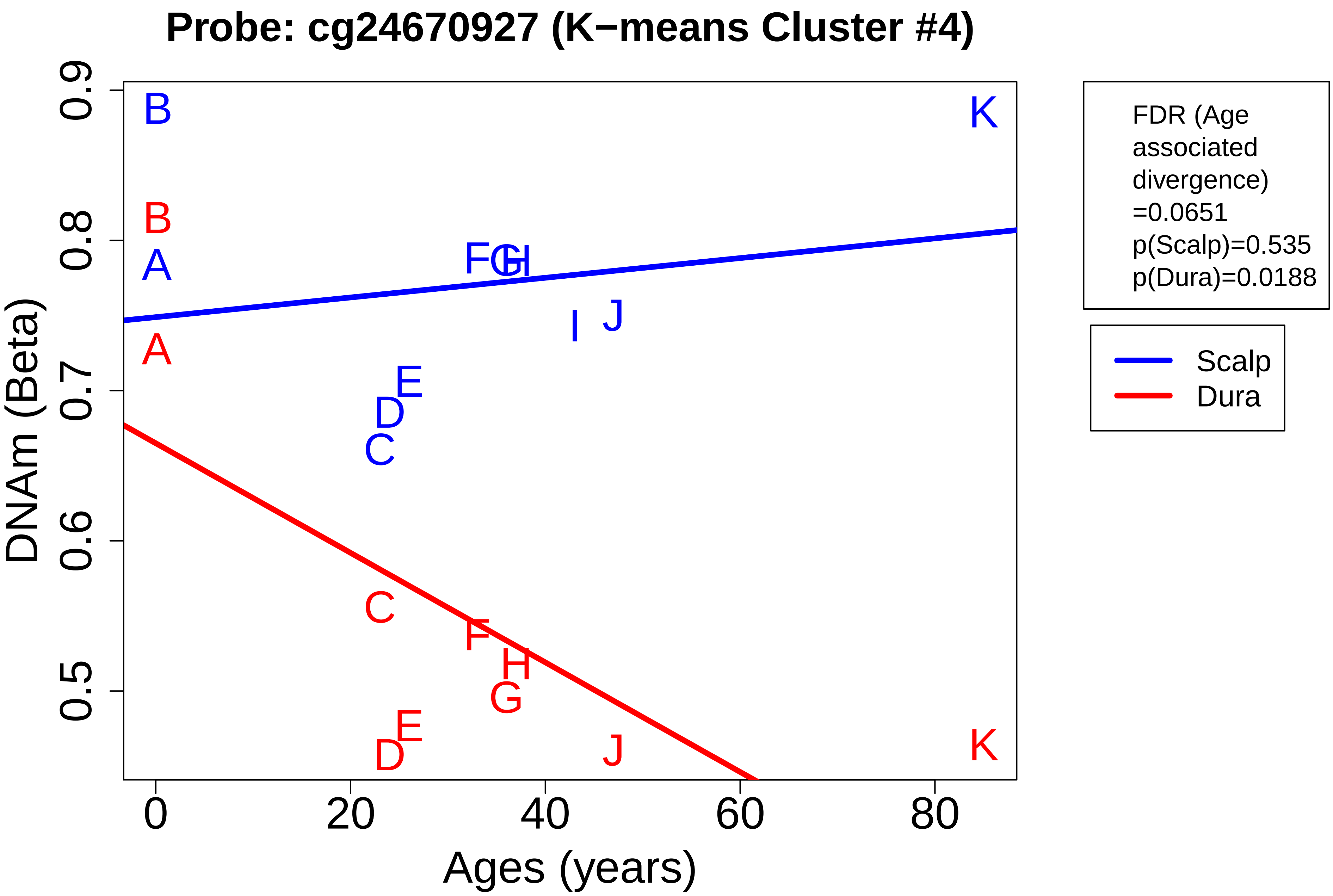
Probe: cg24670927 (K−means Cluster #4)
DNAm (Beta)
Ages (years)
0.5
0.6
0.7
0.8
0.9
0
20
40
60
80
FDR (Age associated divergence) =0.0651
p(Scalp)=0.535
p(Dura)=0.0188
Scalp
Dura

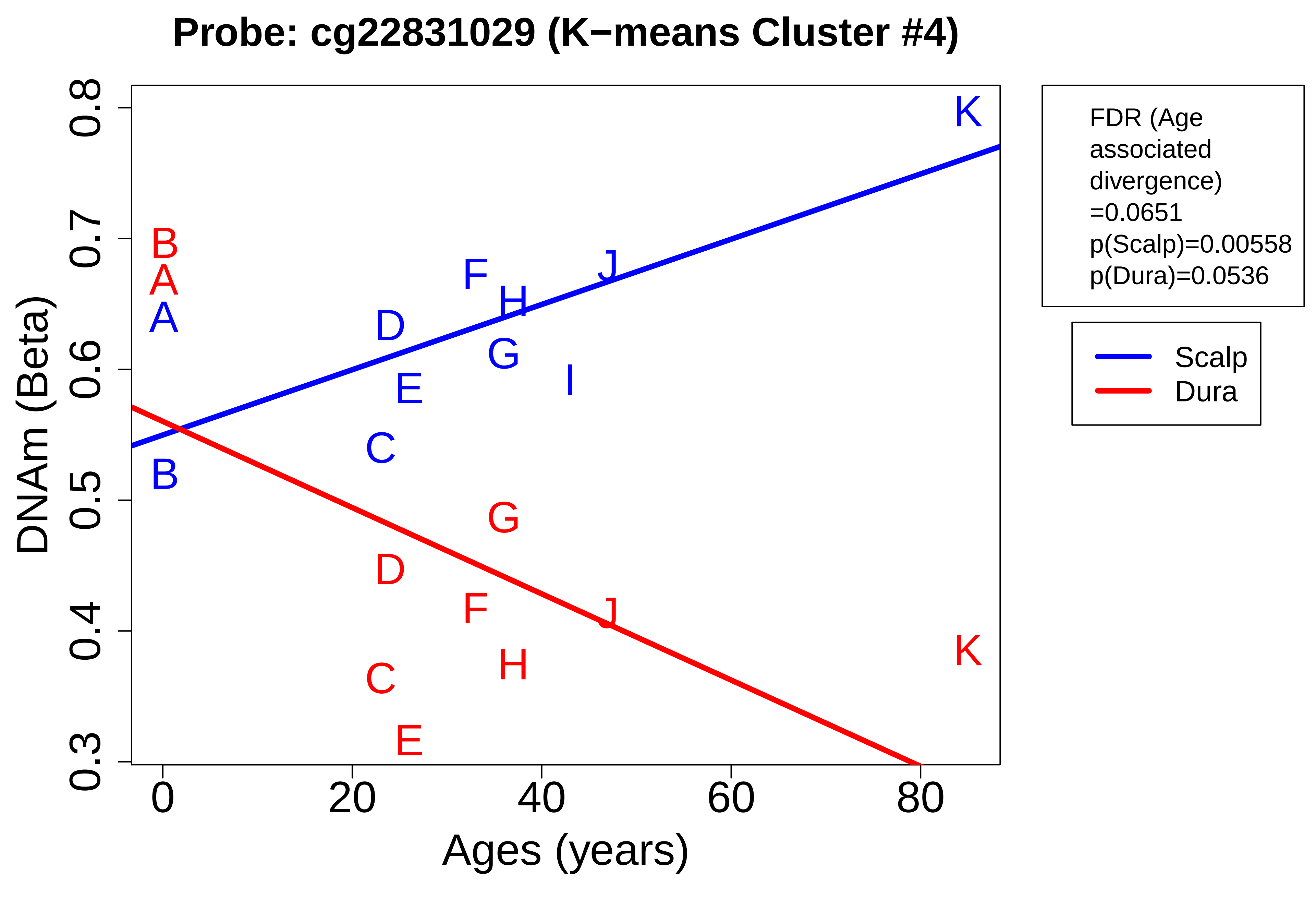
Probe: cg22831029 (K−means Cluster #4)
DNAm (Beta)
0.3
0.4
0.5
0.6
0.7
0.8
0
20
40
60
80
Ages (years)
A
B
C
D
E
F
G
H
I
J
K
FDR (Age associated divergence) =0.0651
p(Scalp)=0.00558
p(Dura)=0.0536
Scalp
Dura

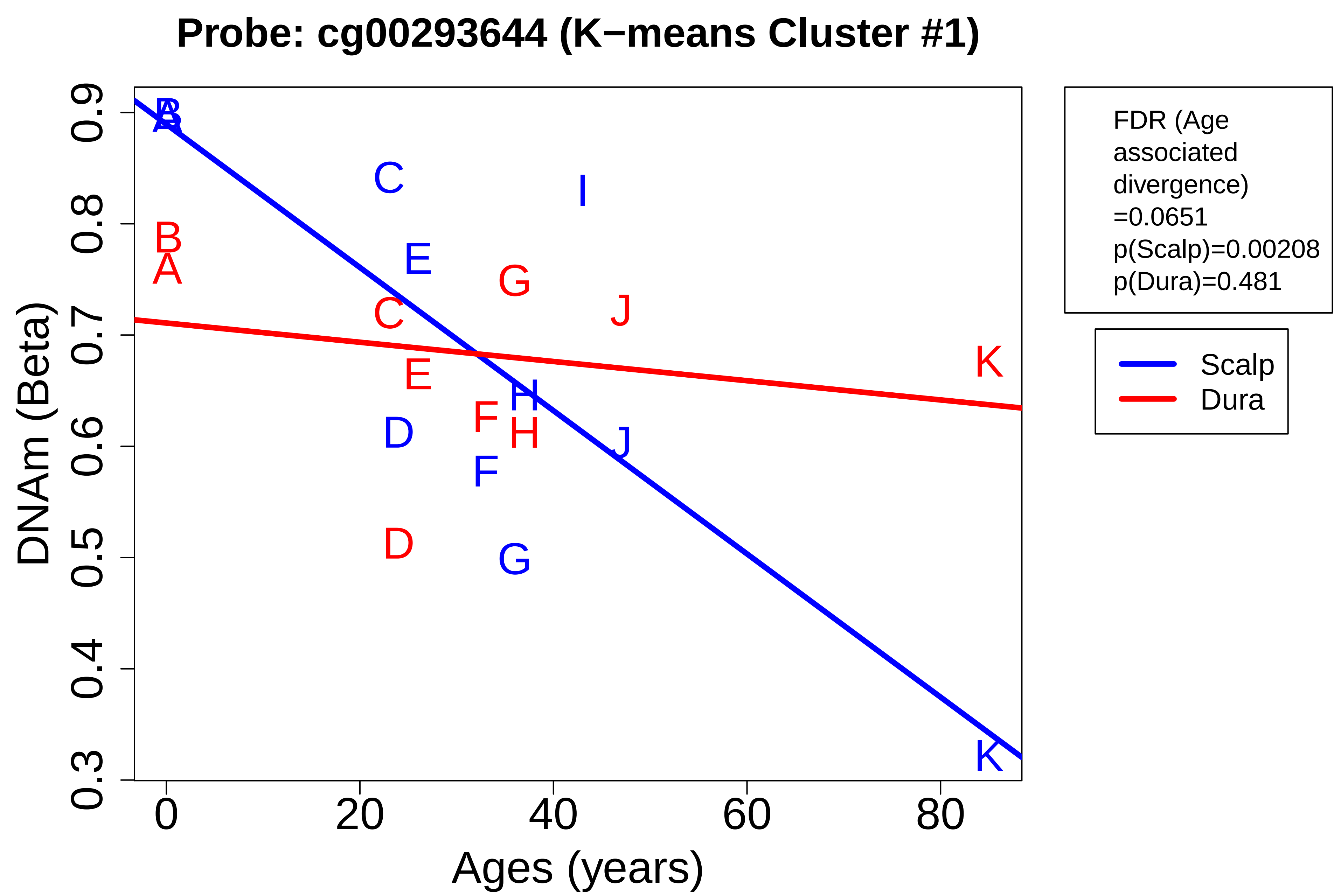

Probe: cg00293644 (K−means Cluster #1)
DNAm (Beta)
0.3
0.4
0.5
0.6
0.7
0.8
0.9
0
20
40
60
80
Ages (years)
FDR (Age associated divergence) =0.0651
p(Scalp)=0.00208
p(Dura)=0.481
Scalp
Dura

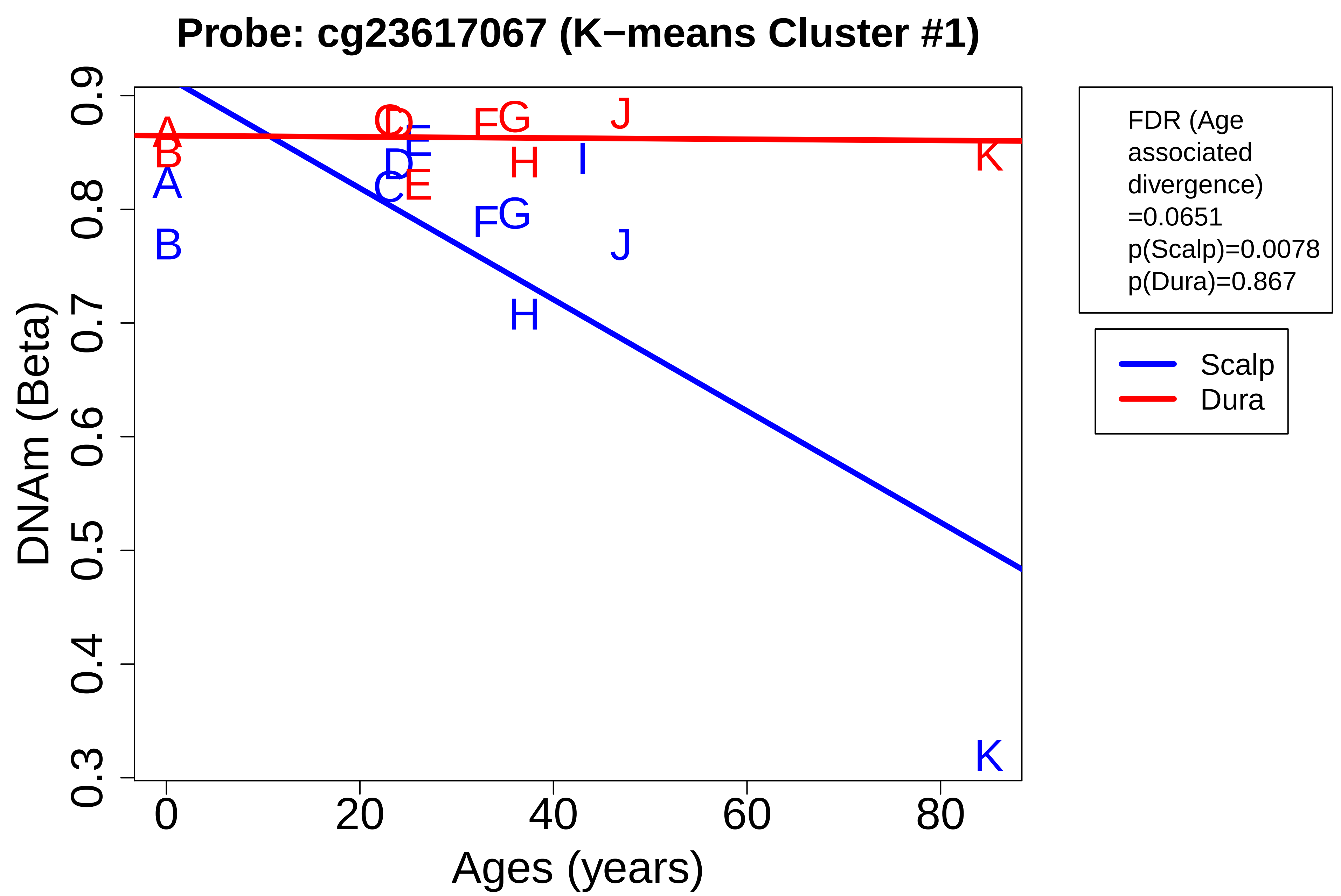

Probe: cg23617067 (K−means Cluster #1)
DNAm (Beta)
Ages (years)
0.3
0.4
0.5
0.6
0.7
0.8
0.9
0
20
40
60
80
FDR (Age associated divergence) =0.0651
p(Scalp)=0.0078
p(Dura)=0.867
Scalp
Dura

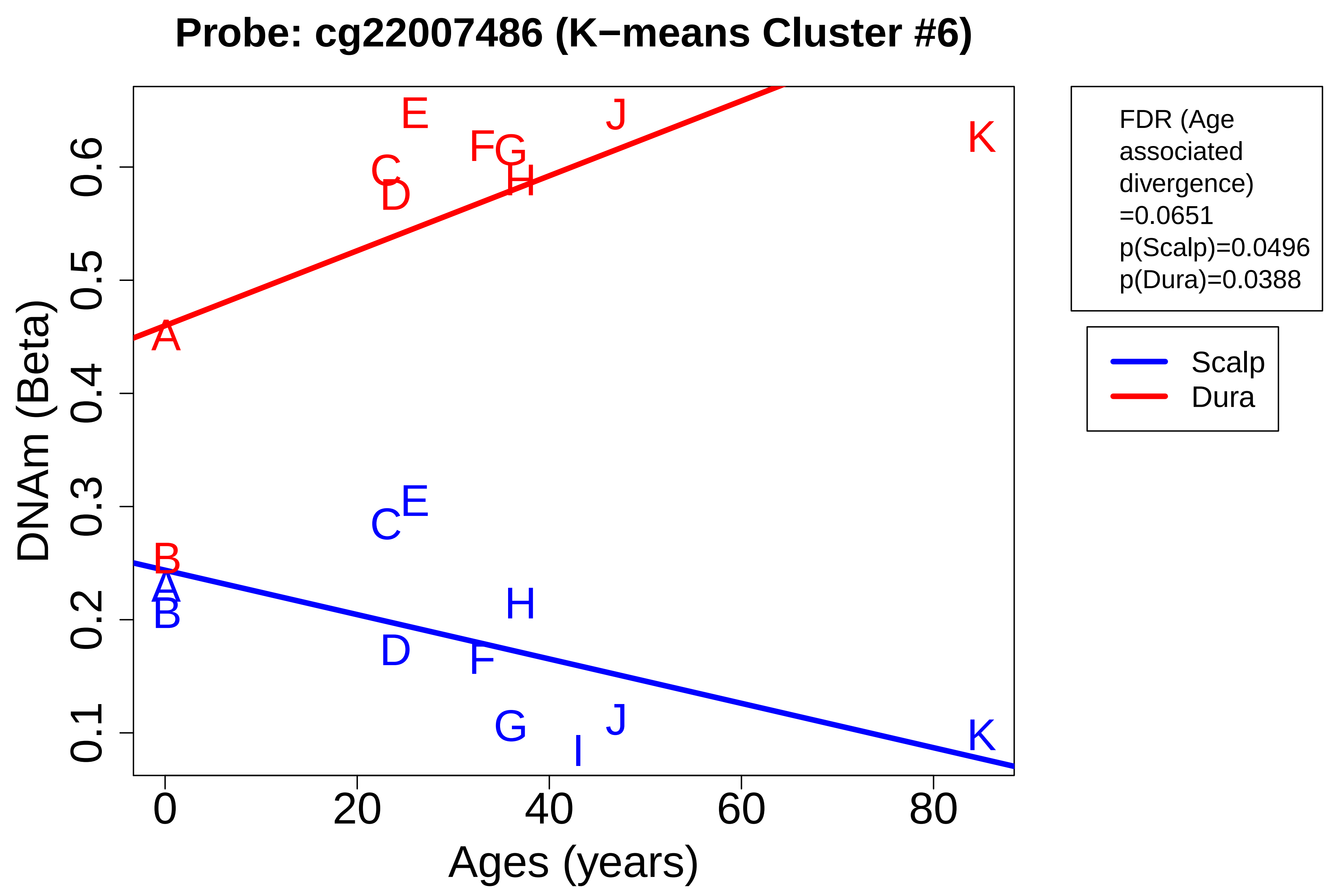

Probe: cg22007486 (K−means Cluster #6)
DNAm (Beta)
0.1
0.2
0.3
0.4
0.5
0.6
0
20
40
60
80
Ages (years)
A
B
C
D
E
F
G
H
I
J
K
FDR (Age associated divergence) =0.0651
p(Scalp)=0.0496
p(Dura)=0.0388
Scalp
Dura

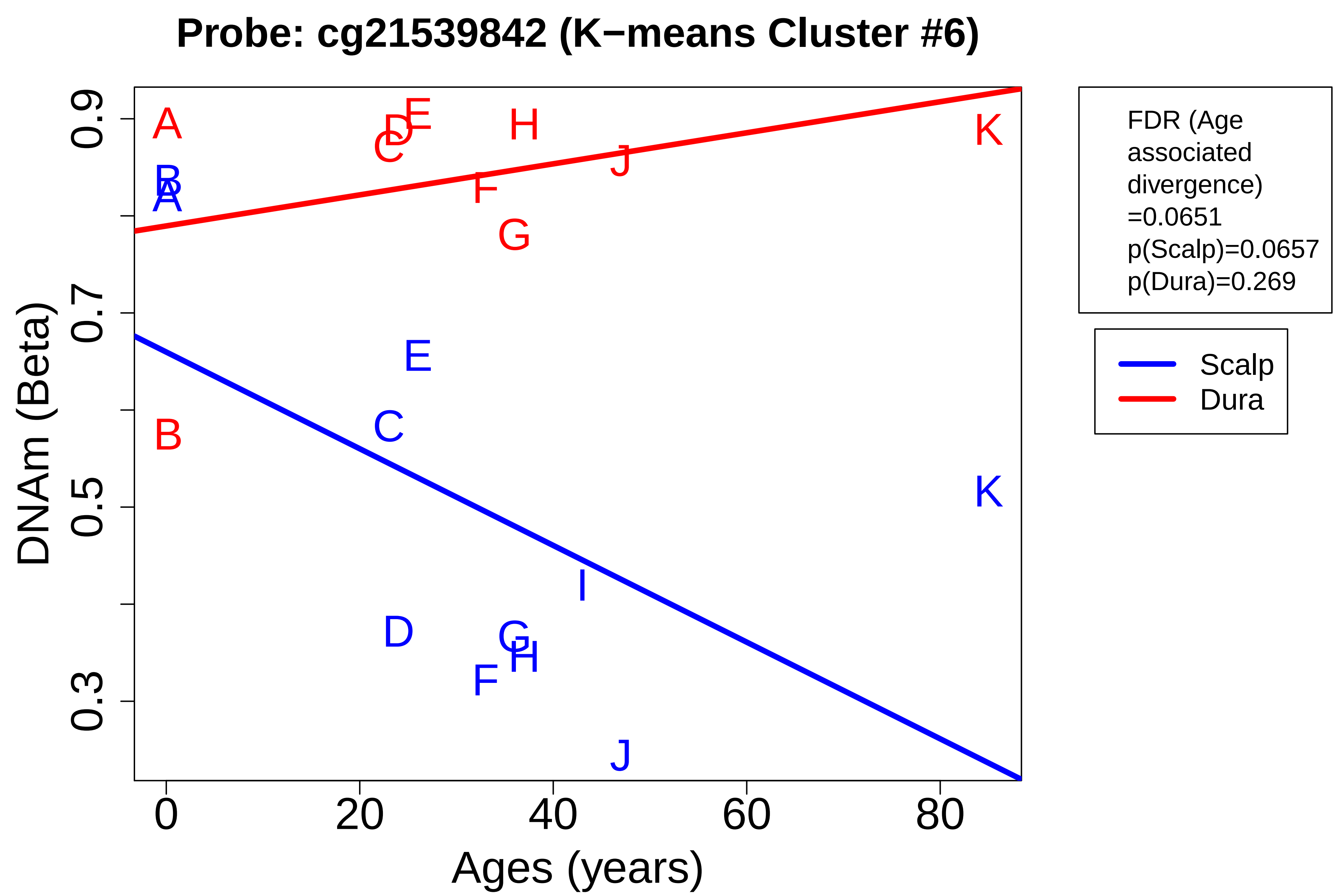
Probe: cg21539842 (K−means Cluster #6)
DNAm (Beta)
0.3
0.5
0.7
0.9
Ages (years)
0
20
40
60
80
A
B
C
D
E
F
G
H
I
J
K
FDR (Age associated divergence) =0.0651
p(Scalp)=0.0657
p(Dura)=0.269
Scalp
Dura

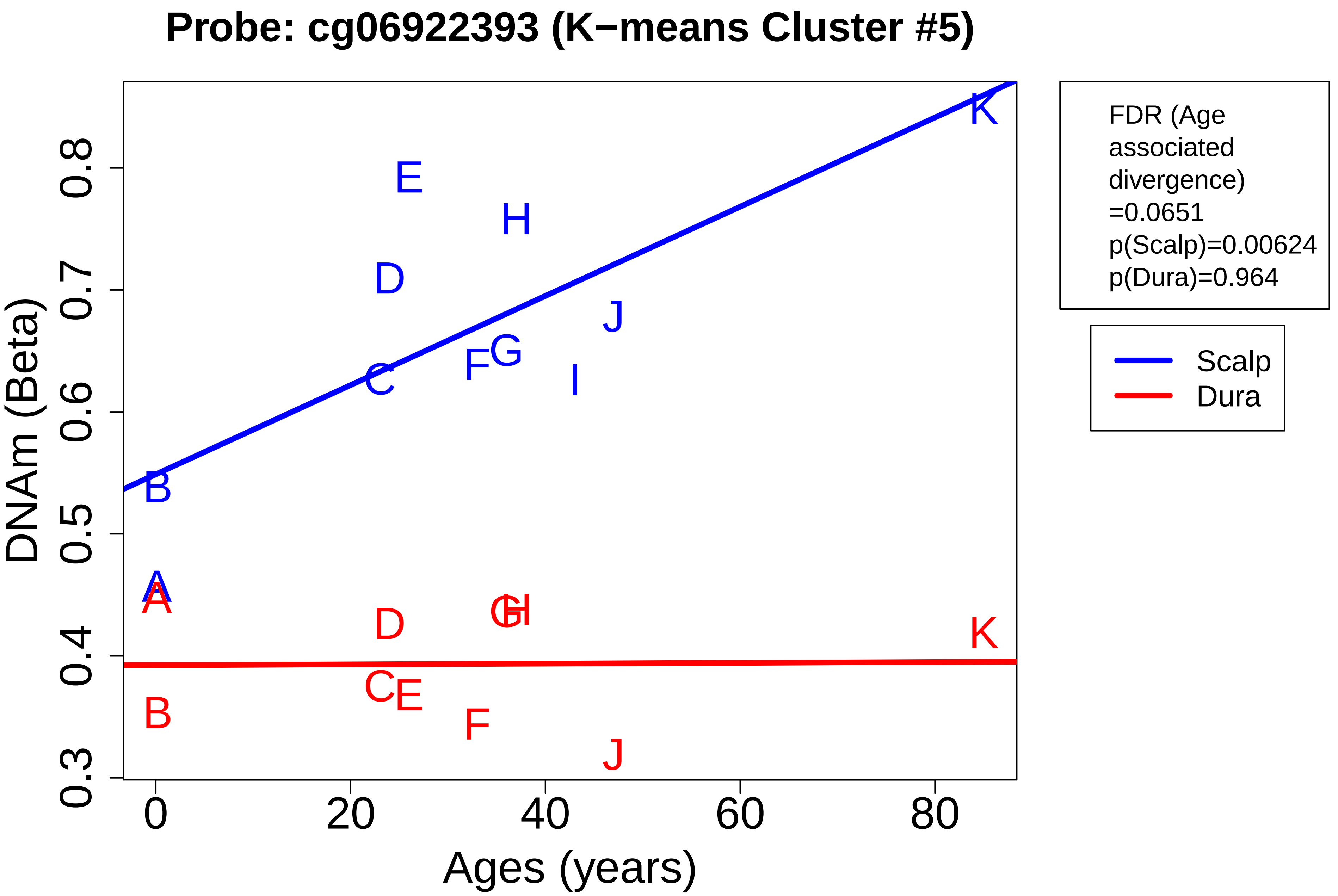

Probe: cg06922393 (K−means Cluster #5)
DNAm (Beta)
0.3
0.4
0.5
0.6
0.7
0.8
0
20
40
60
80
Ages (years)
FDR (Age associated divergence) =0.0651
p(Scalp)=0.00624
p(Dura)=0.964
Scalp
Dura
A
B
C
D
E
F
G
H
I
J
K

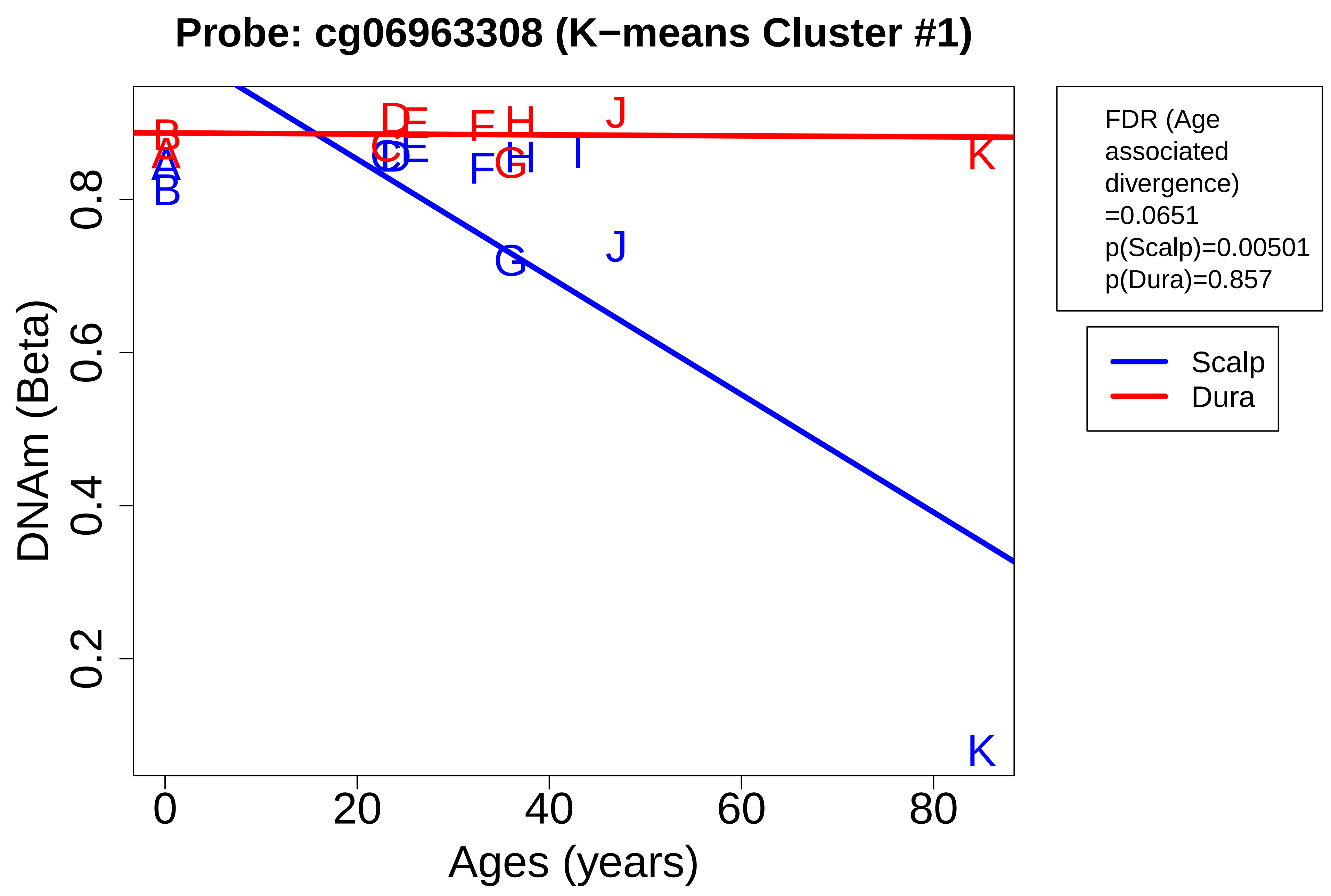

Probe: cg06963308 (K−means Cluster #1)
DNAm (Beta)
0.2
0.4
0.6
0.8
Ages (years)
0
20
40
60
80
FDR (Age associated divergence) =0.0651
p(Scalp)=0.00501
p(Dura)=0.857
Scalp
Dura

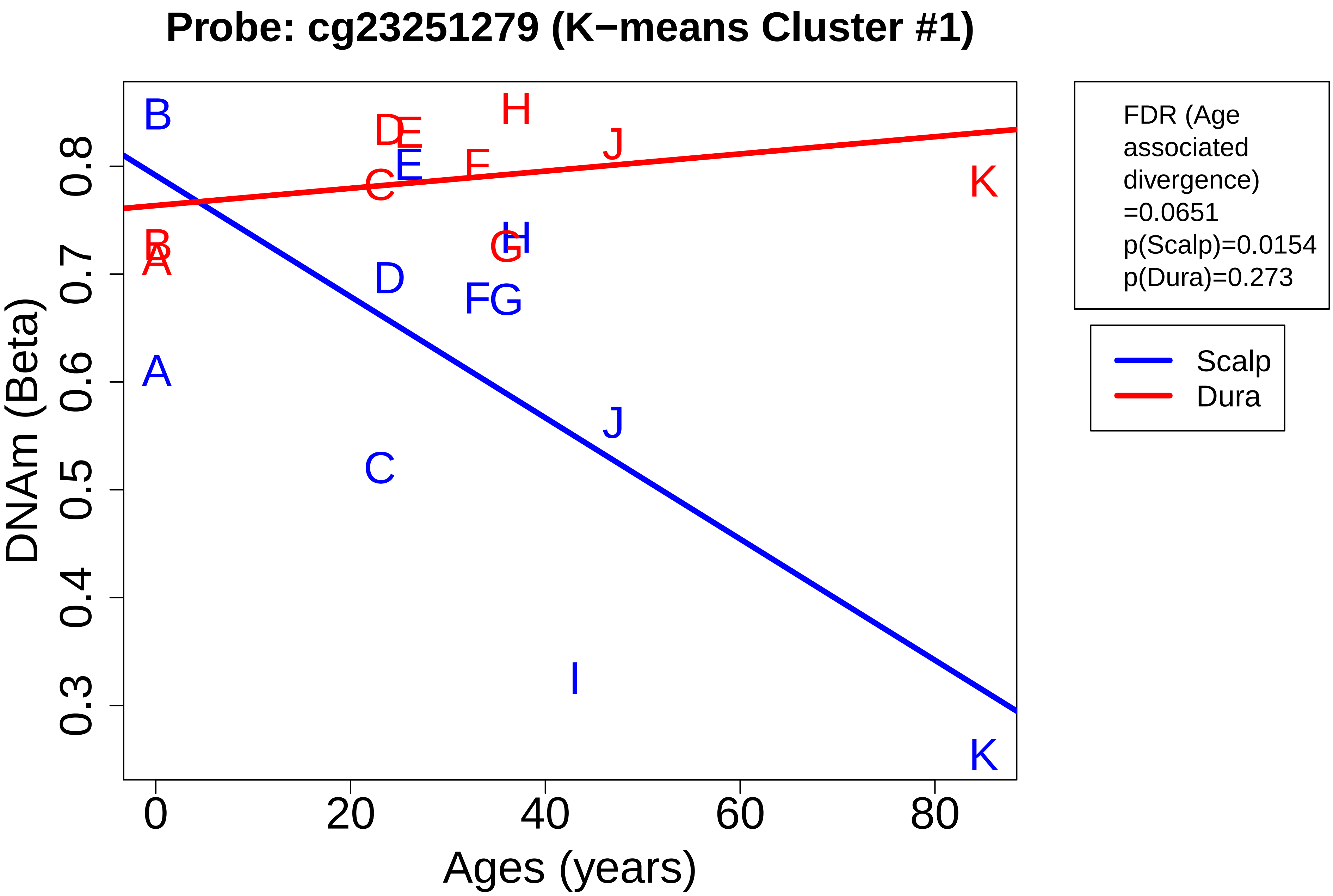

Probe: cg23251279 (K−means Cluster #1)
DNAm (Beta)
0.3
0.4
0.5
0.6
0.7
0.8
0
20
40
60
80
Ages (years)
FDR (Age associated divergence) =0.0651
p(Scalp)=0.0154
p(Dura)=0.273
Scalp
Dura

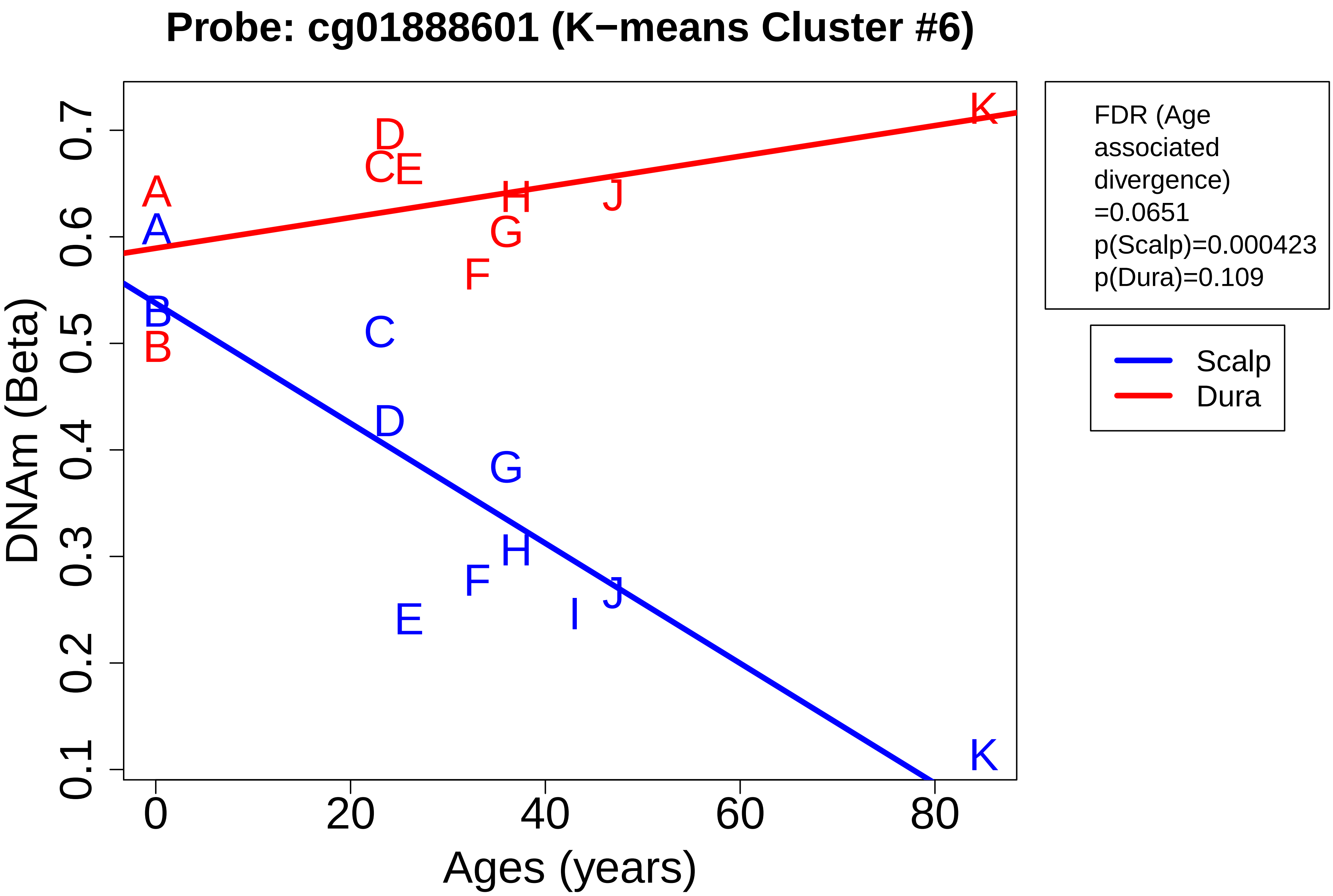

Probe: cg01888601 (K−means Cluster #6)
DNAm (Beta)
0.1
0.2
0.3
0.4
0.5
0.6
0.7
0
20
40
60
80
Ages (years)
A
B
C
D
E
F
G
H
I
J
K
A
B
CE
D
F
G
H
J
K
FDR (Age associated divergence) =0.0651
p(Scalp)=0.000423
p(Dura)=0.109
Scalp
Dura

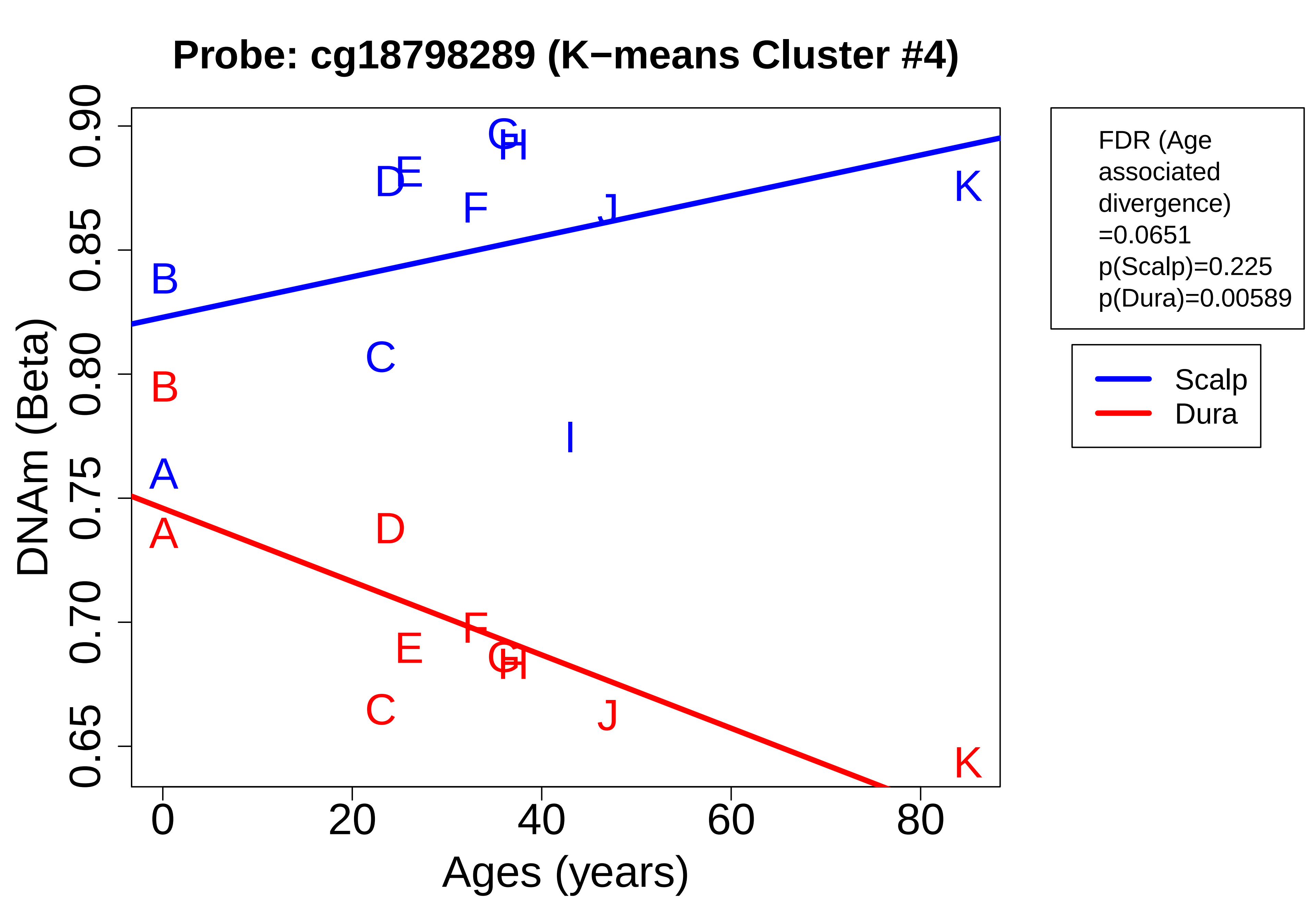

Probe: cg18798289 (K−means Cluster #4)
DNAm (Beta)
0.65
0.70
0.75
0.80
0.85
0.90
0
20
40
60
80
Ages (years)
FDR (Age associated divergence) =0.0651
p(Scalp)=0.225
p(Dura)=0.00589
Scalp
Dura

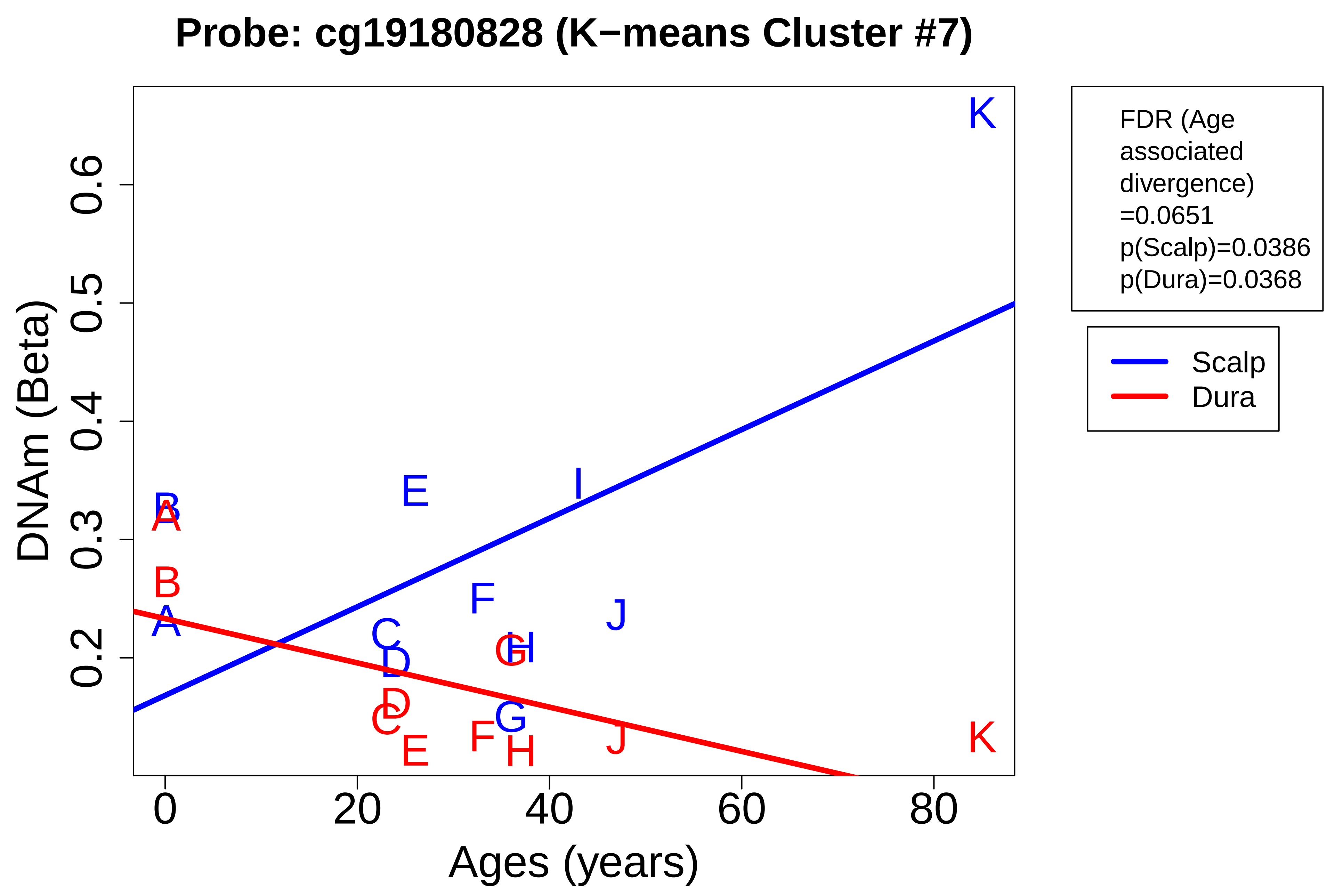

Probe: cg19180828 (K−means Cluster #7)
DNAm (Beta)
0.2
0.3
0.4
0.5
0.6
Ages (years)
0
20
40
60
80
FDR (Age associated divergence) =0.0651
p(Scalp)=0.0386
p(Dura)=0.0368
Scalp
Dura
A
B
C
D
E
F
G
H
I
J
K

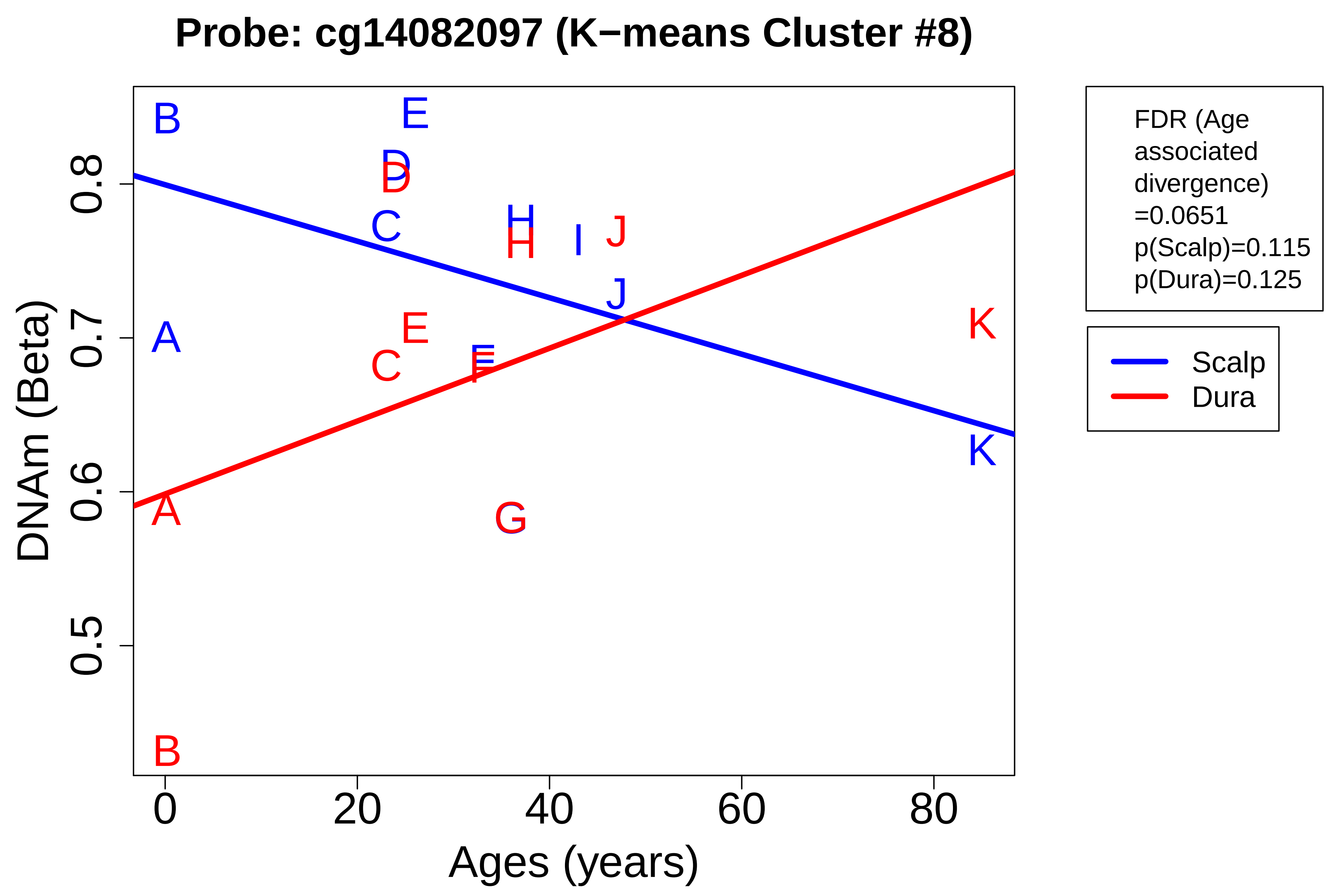

Probe: cg14082097 (K−means Cluster #8)
DNAm (Beta)
0.5
0.6
0.7
0.8
Ages (years)
0
20
40
60
80
A
B
C
D
E
F
G
H
I
J
K
FDR (Age associated divergence) =0.0651
p(Scalp)=0.115
p(Dura)=0.125
Scalp
Dura

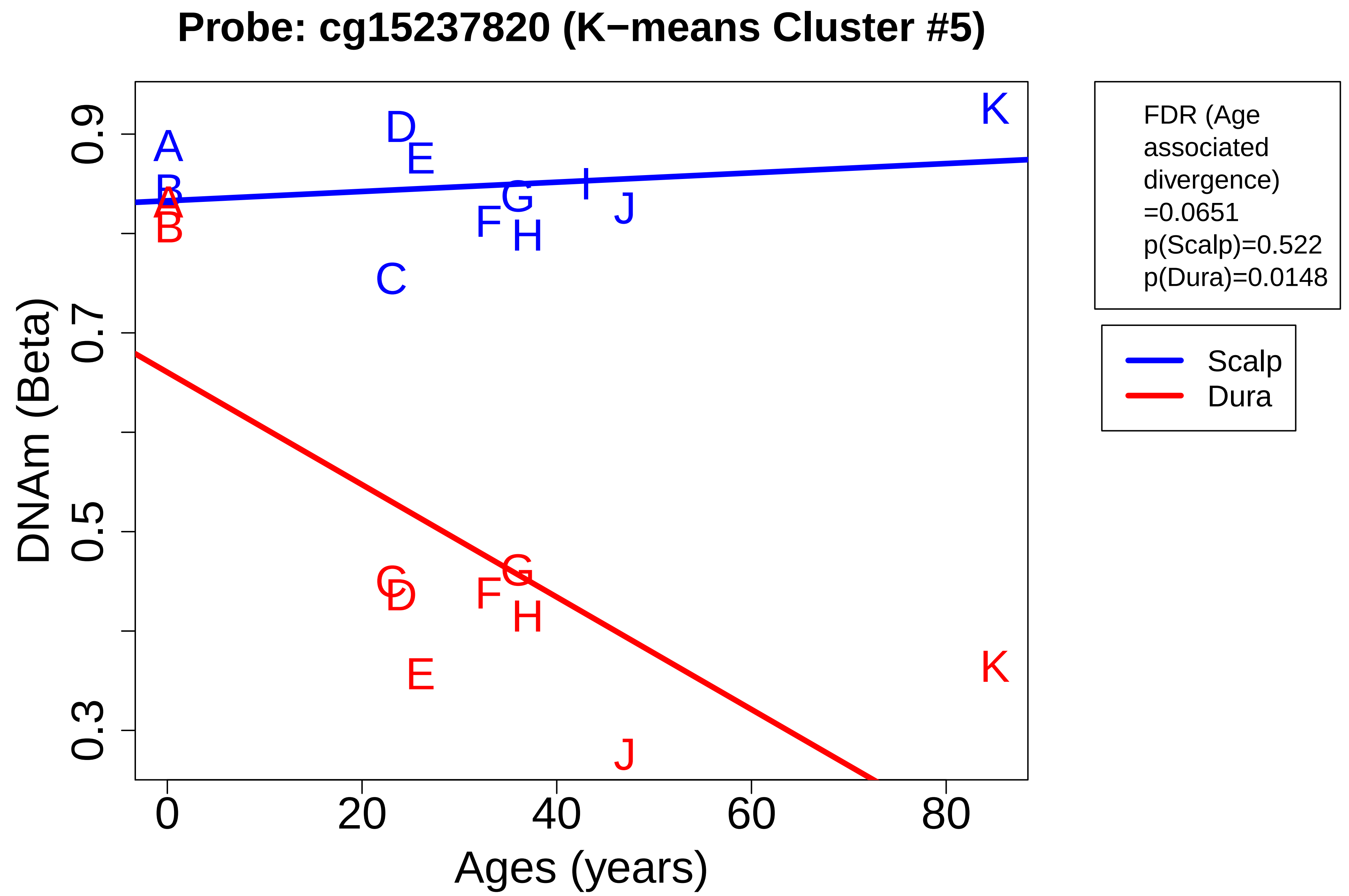

Probe: cg15237820 (K−means Cluster #5)
DNAm (Beta)
0.3
0.5
0.7
0.9
0
20
40
60
80
Ages (years)
A
B
C
D
E
F
G
H
I
J
K
FDR (Age associated divergence) =0.0651
p(Scalp)=0.522
p(Dura)=0.0148
Scalp
Dura

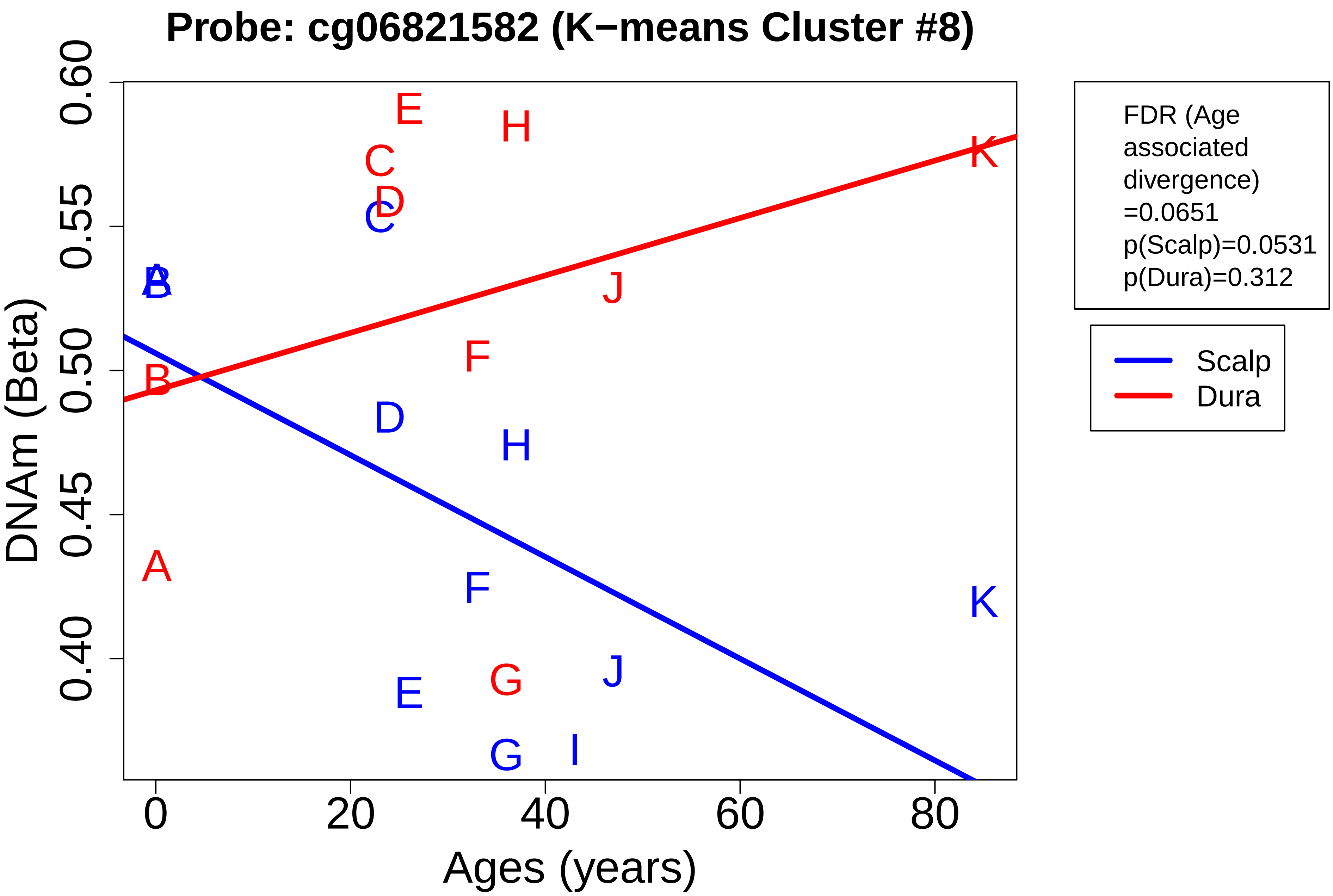

Probe: cg06821582 (K−means Cluster #8)
DNAm (Beta)
0.40
0.45
0.50
0.55
0.60
0
20
40
60
80
Ages (years)
FDR (Age associated divergence) =0.0651
p(Scalp)=0.0531
p(Dura)=0.312
Scalp
Dura

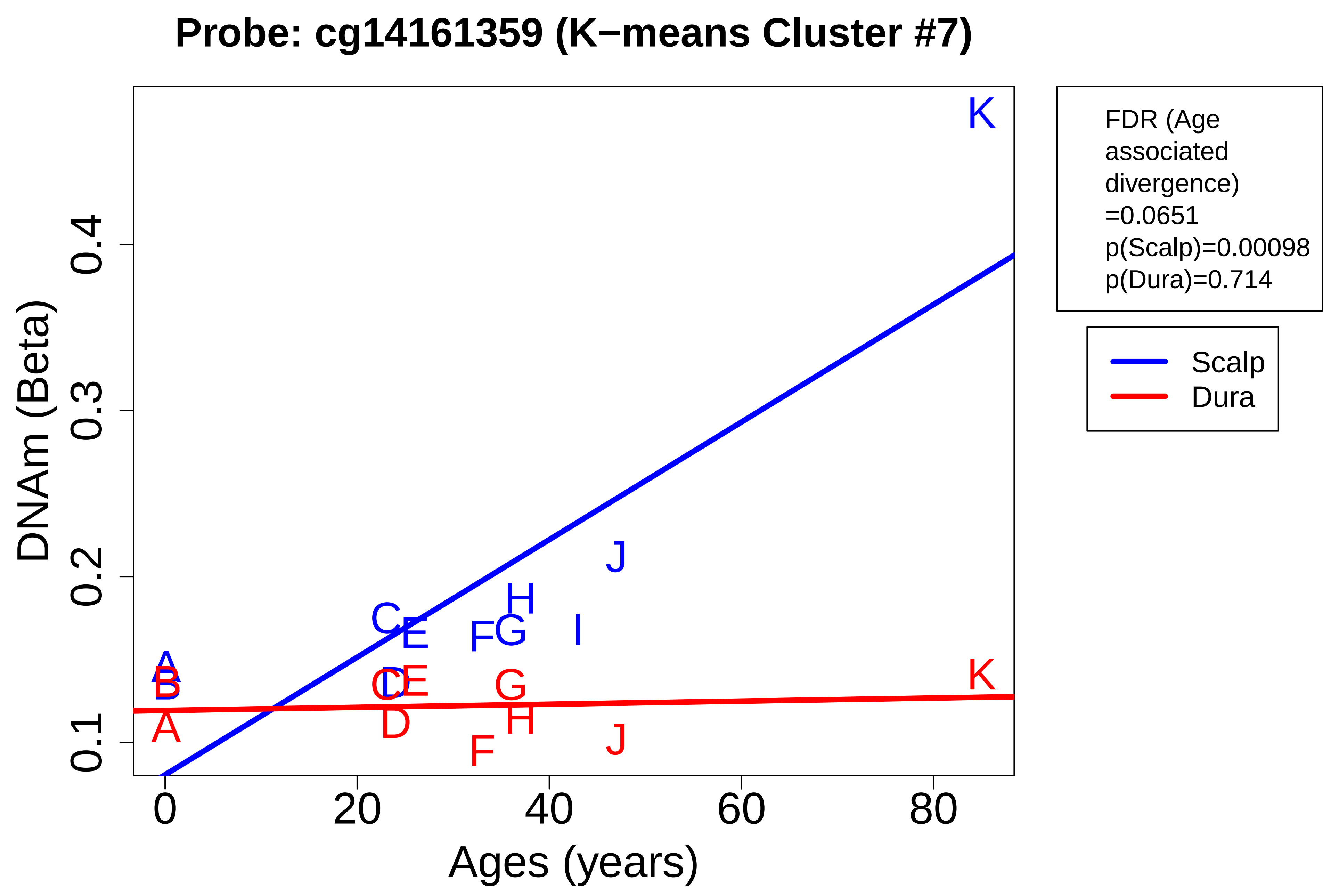

Probe: cg14161359 (K−means Cluster #7)
DNAm (Beta)
0.1
0.2
0.3
0.4
Ages (years)
0
20
40
60
80
A
B
C
D
E
F
G
H
I
J
K
FDR (Age associated divergence) =0.0651
p(Scalp)=0.00098
p(Dura)=0.714
Scalp
Dura

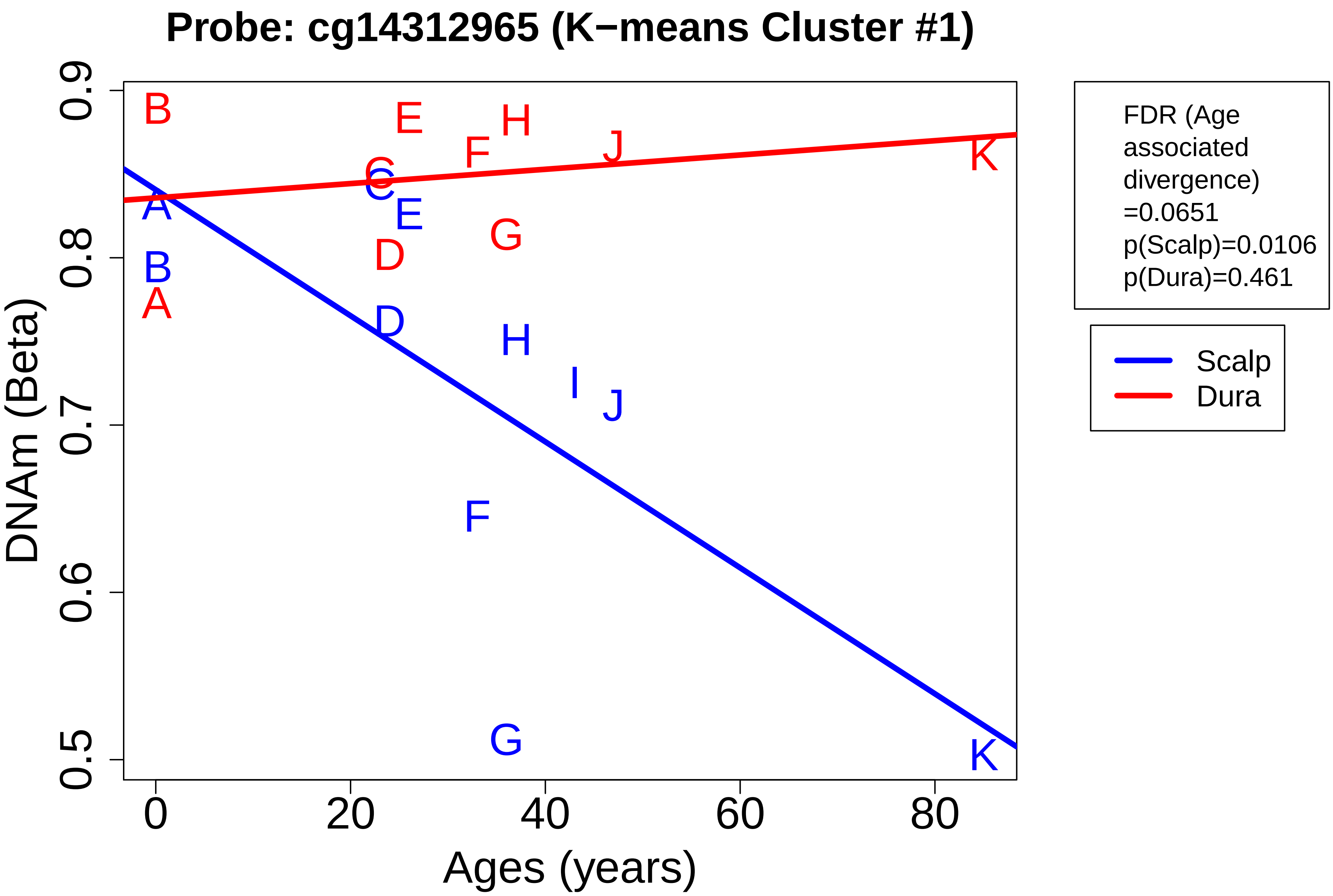

Probe: cg14312965 (K−means Cluster #1)
DNAm (Beta)
0.5
0.6
0.7
0.8
0.9
0
20
40
60
80
Ages (years)
A
B
C
D
E
F
G
H
I
J
K
FDR (Age associated divergence) =0.0651
p(Scalp)=0.0106
p(Dura)=0.461
Scalp
Dura

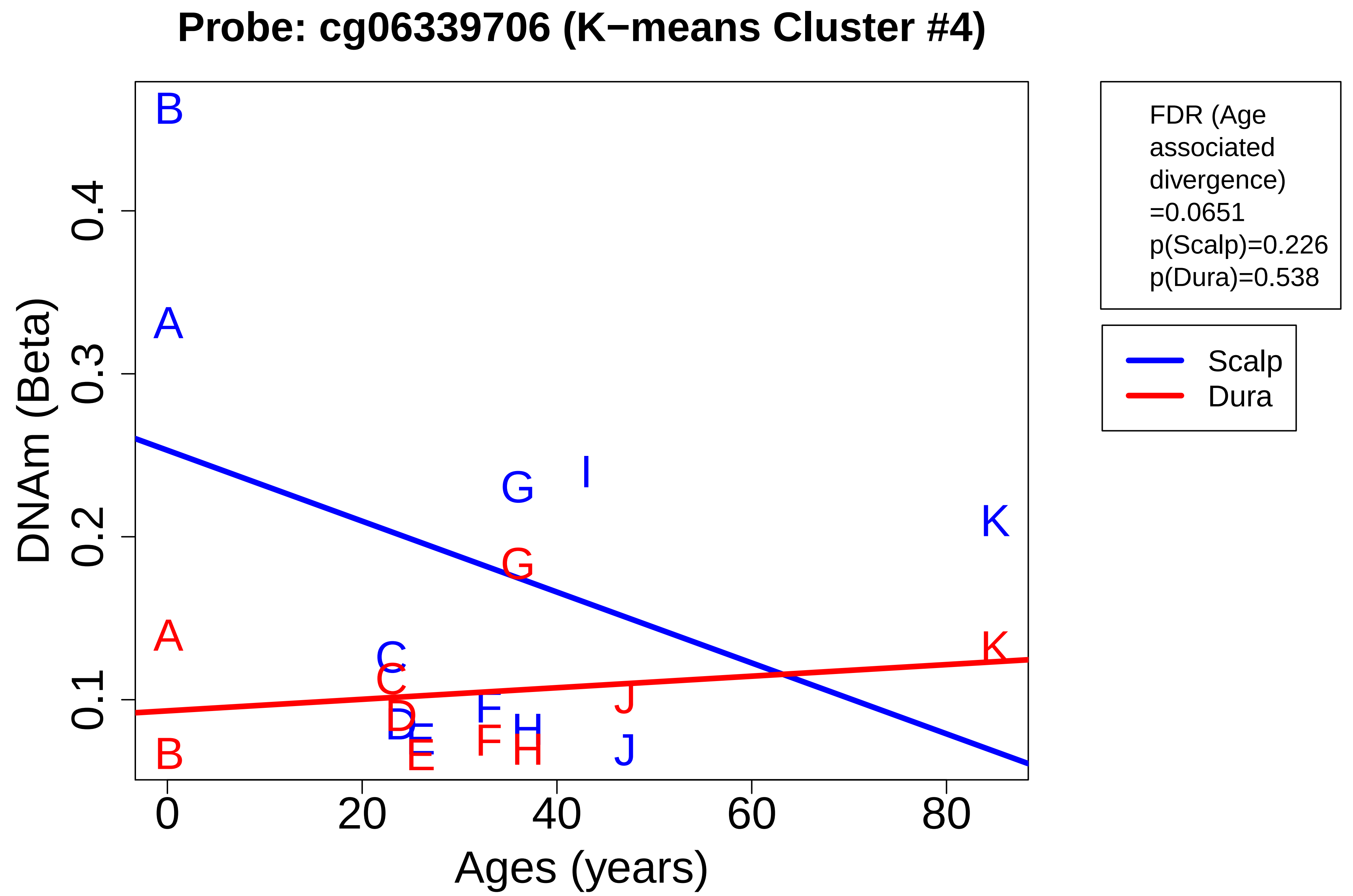
Probe: cg06339706 (K−means Cluster #4)
DNAm (Beta)
0.1
0.2
0.3
0.4
0
20
40
60
80
Ages (years)
FDR (Age associated divergence) =0.0651
p(Scalp)=0.226
p(Dura)=0.538
Scalp
Dura

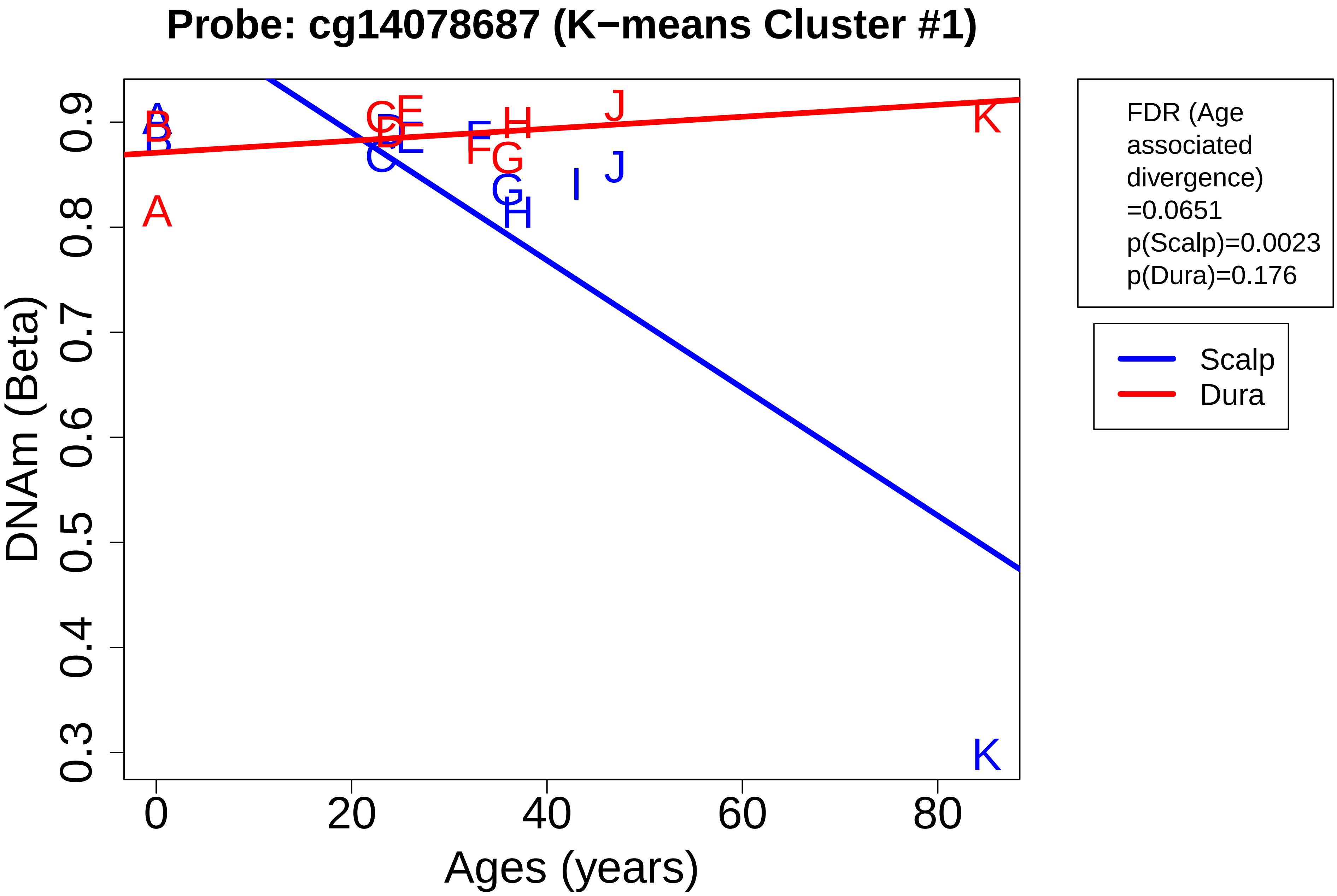

Probe: cg14078687 (K−means Cluster #1)
DNAm (Beta)
0.3
0.4
0.5
0.6
0.7
0.8
0.9
0
20
40
60
80
Ages (years)
FDR (Age associated divergence) =0.0651
p(Scalp)=0.0023
p(Dura)=0.176
Scalp
Dura

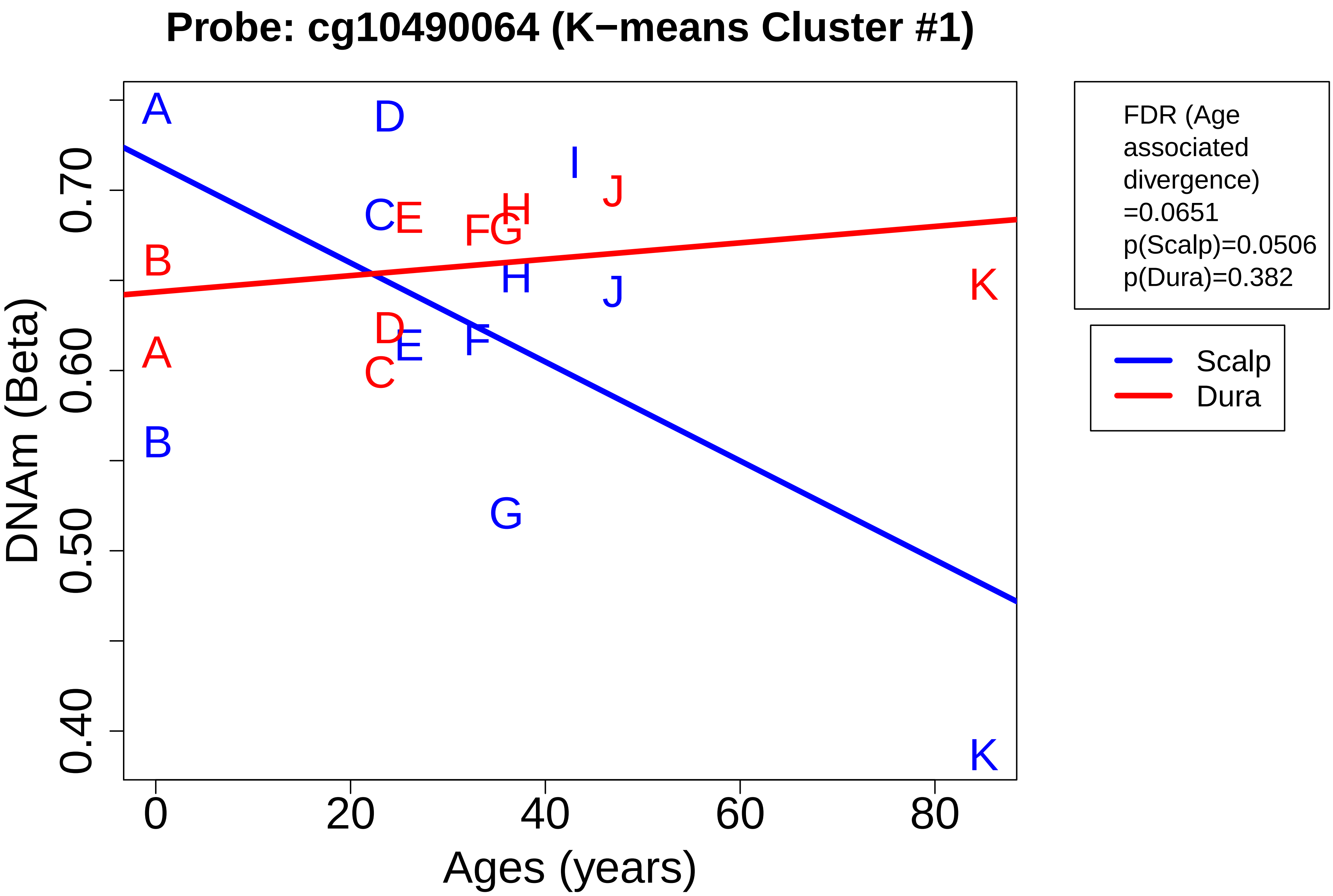
Probe: cg10490064 (K−means Cluster #1)
DNAm (Beta)
0.40
0.50
0.60
0.70
0
20
40
60
80
Ages (years)
A
B
C
D
E
F
G
H
I
J
K
FDR (Age associated divergence) =0.0651
p(Scalp)=0.0506
p(Dura)=0.382
Scalp
Dura

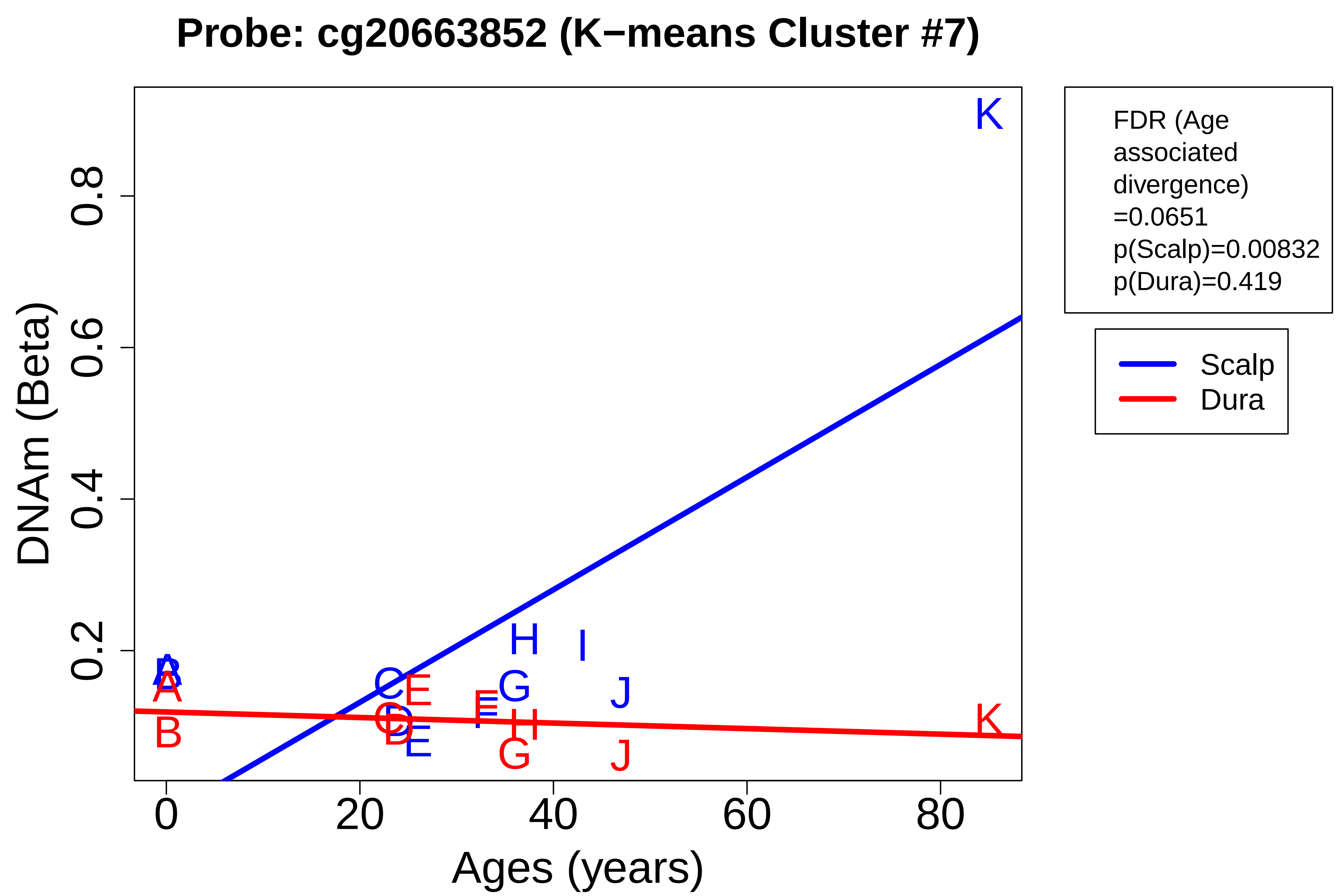

Probe: cg20663852 (K−means Cluster #7)
DNAm (Beta)
0.2
0.4
0.6
0.8
0
20
40
60
80
Ages (years)
FDR (Age associated divergence) =0.0651
p(Scalp)=0.00832
p(Dura)=0.419
Scalp
Dura
A
B
C
D
E
F
G
H
I
J
K

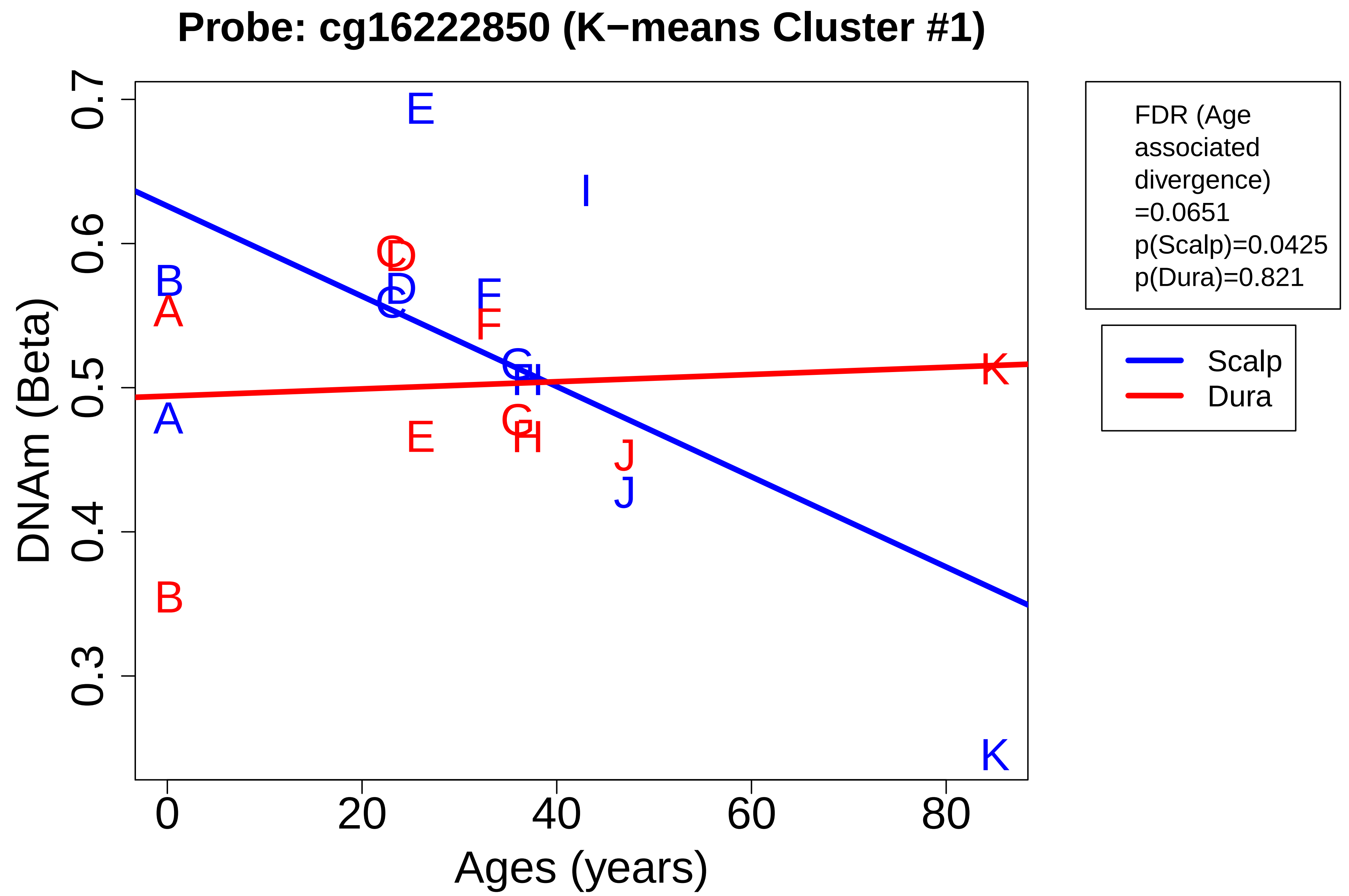

Probe: cg16222850 (K−means Cluster #1)
DNAm (Beta)
0.3
0.4
0.5
0.6
0.7
0
20
40
60
80
Ages (years)
FDR (Age associated divergence) =0.0651
p(Scalp)=0.0425
p(Dura)=0.821
Scalp
Dura
A
B
C
D
E
F
G
H
I
J
K

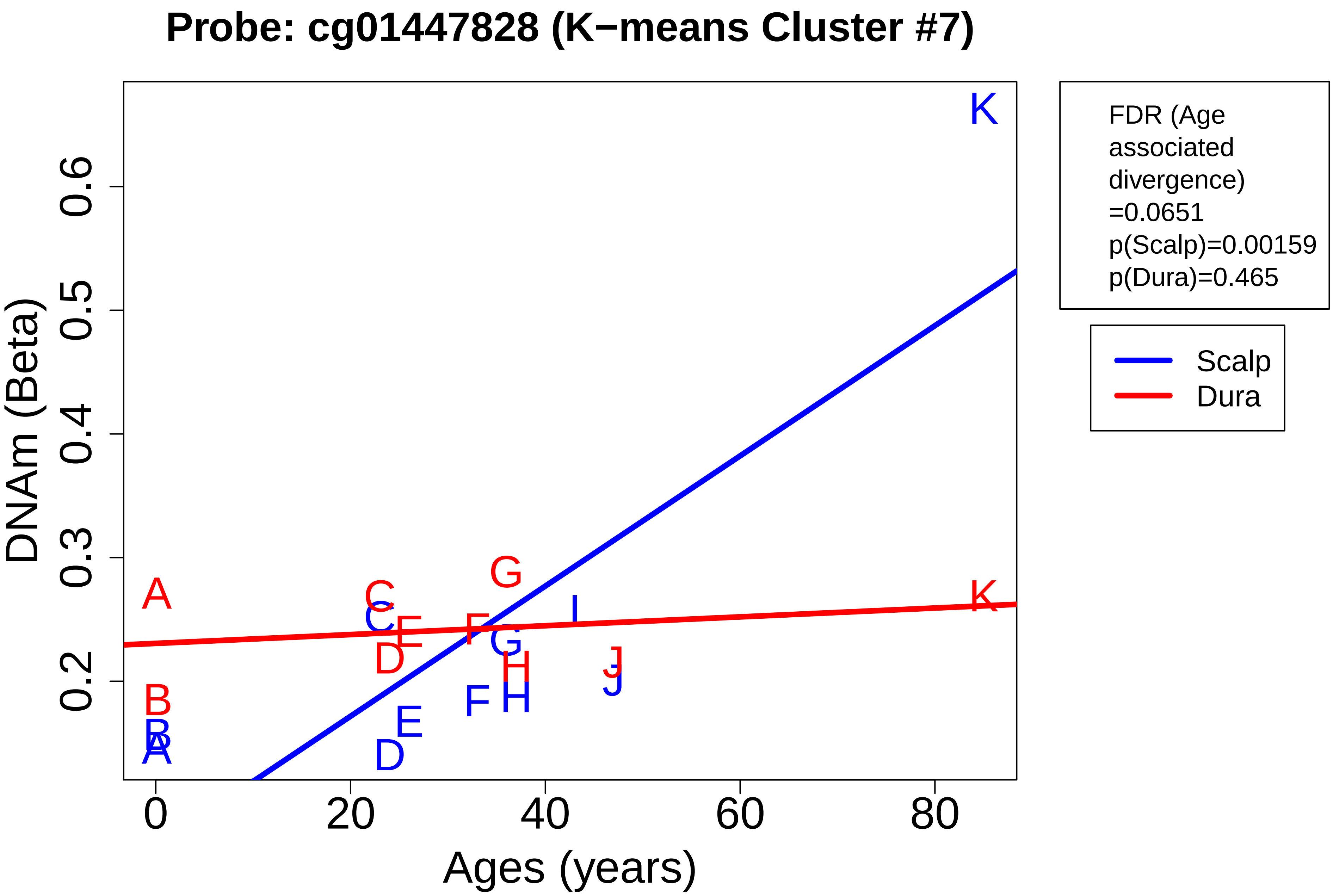

Probe: cg01447828 (K−means Cluster #7)
DNAm (Beta)
0.2
0.3
0.4
0.5
0.6
Ages (years)
0
20
40
60
80
FDR (Age associated divergence) =0.0651
p(Scalp)=0.00159
p(Dura)=0.465
Scalp
Dura

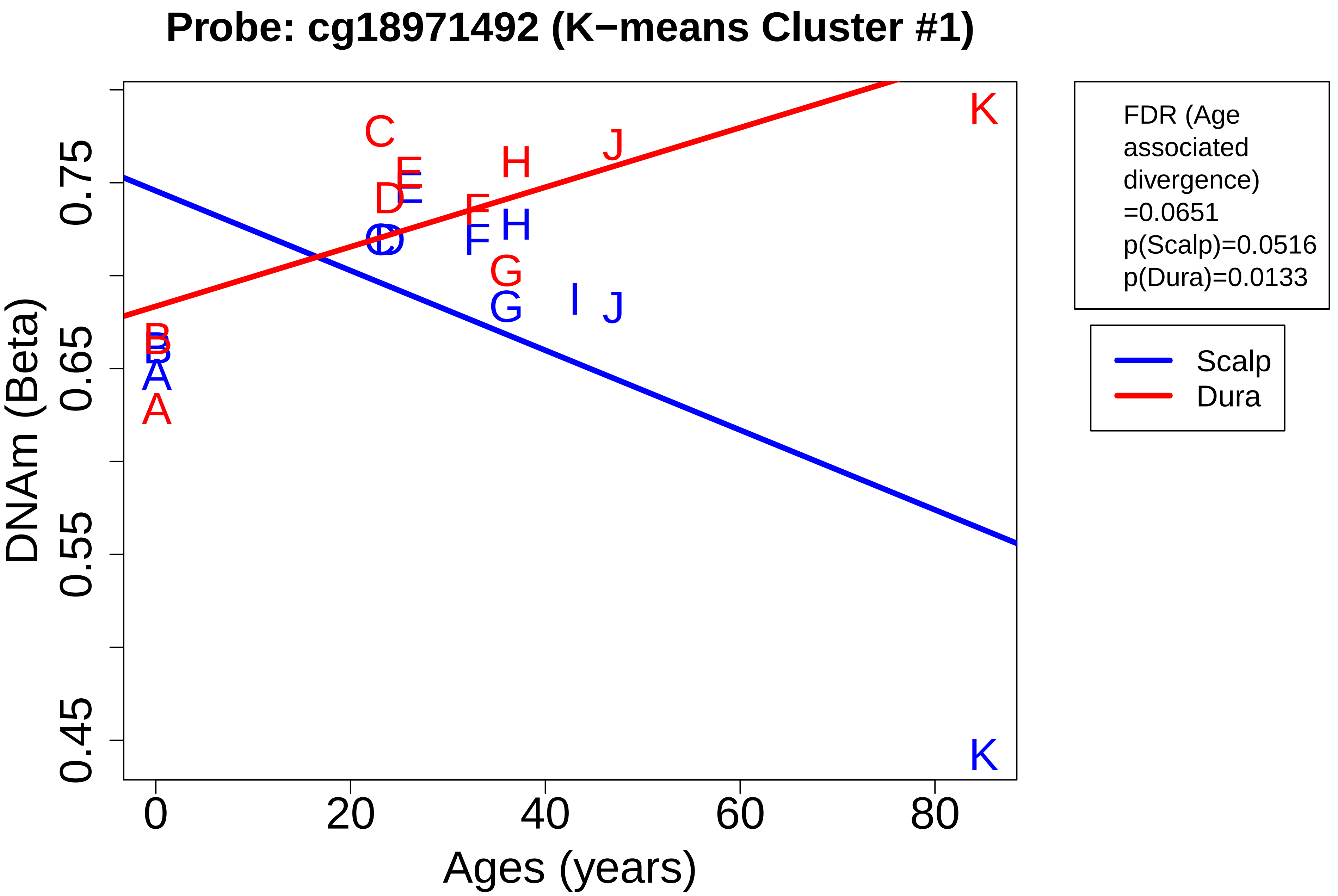

Probe: cg18971492 (K−means Cluster #1)
DNAm (Beta)
0.45
0.55
0.65
0.75
Ages (years)
0
20
40
60
80
A B C D E F G H I J K
FDR (Age associated divergence) =0.0651
p(Scalp)=0.0516
p(Dura)=0.0133
Scalp
Dura

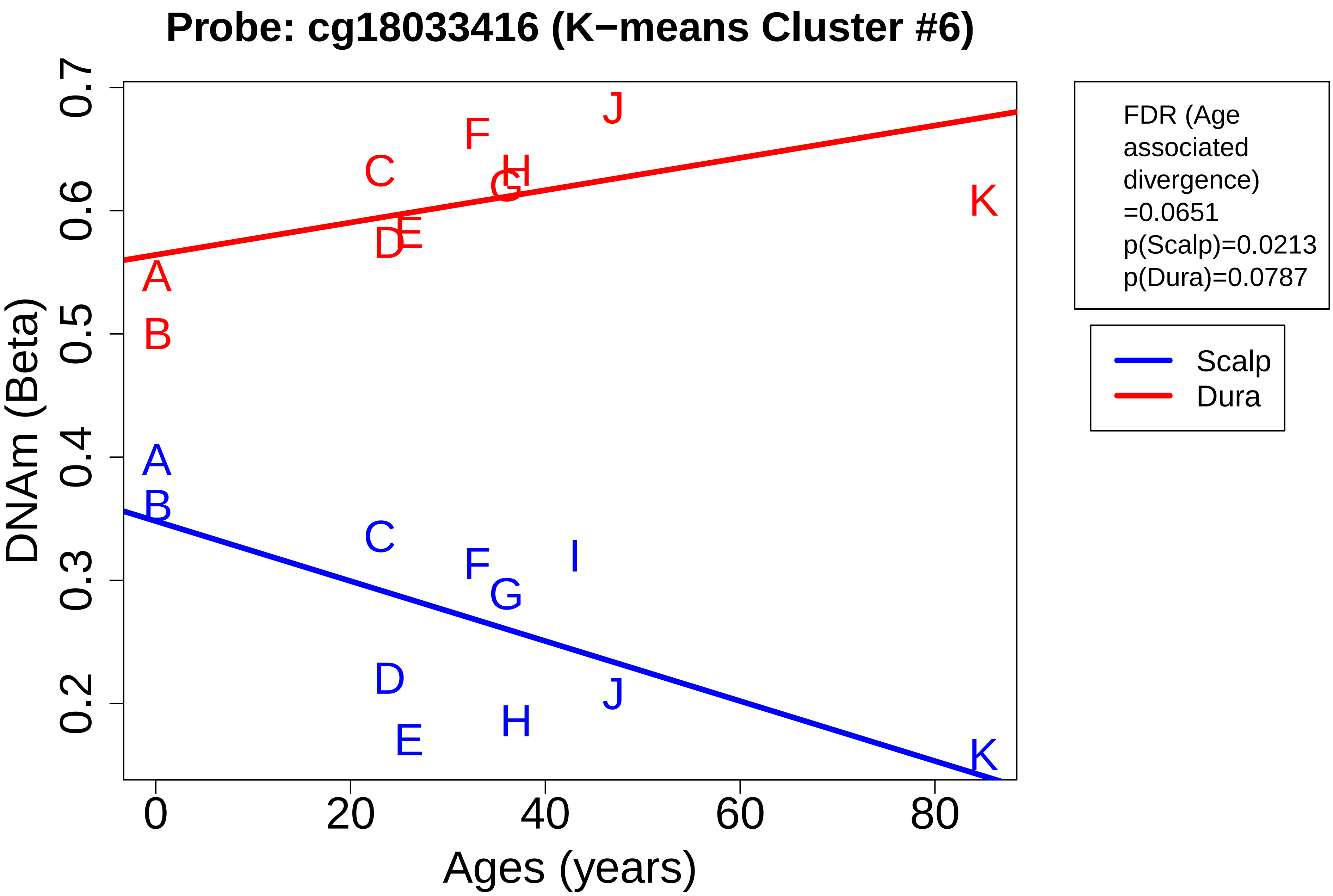

Probe: cg18033416 (K−means Cluster #6)
DNAm (Beta)
0.2
0.3
0.4
0.5
0.6
0.7
0
20
40
60
80
Ages (years)
A
B
C
D
E
F
G
H
J
K
A
B
C
D
E
F
G
H
I
J
K
FDR (Age associated divergence) =0.0651
p(Scalp)=0.0213
p(Dura)=0.0787
Scalp
Dura

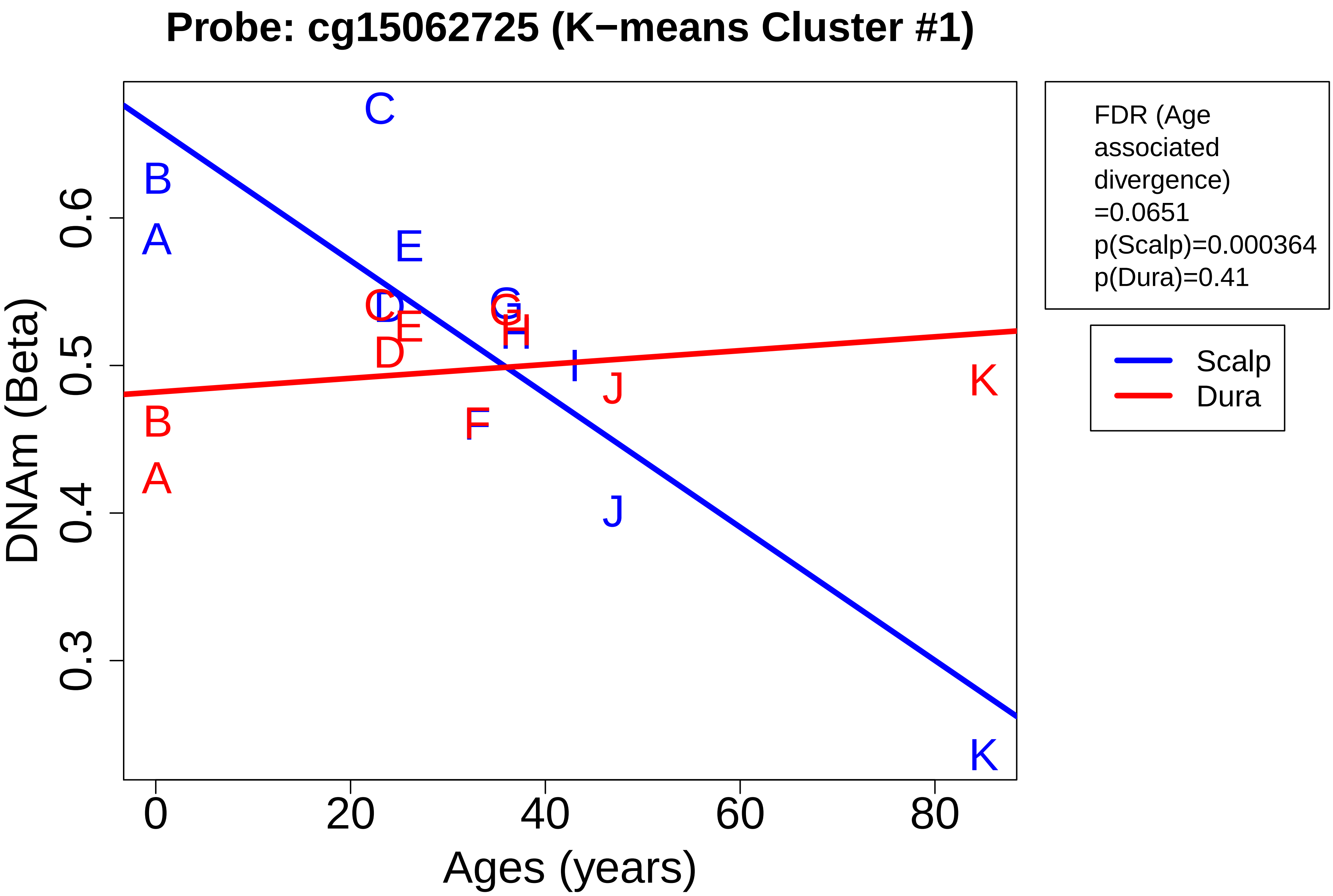

Probe: cg15062725 (K−means Cluster #1)
FDR (Age associated divergence) =0.0651
p(Scalp)=0.000364
p(Dura)=0.41
Scalp
Dura
DNAm (Beta)
0.3
0.4
0.5
0.6
0
20
40
60
80
Ages (years)

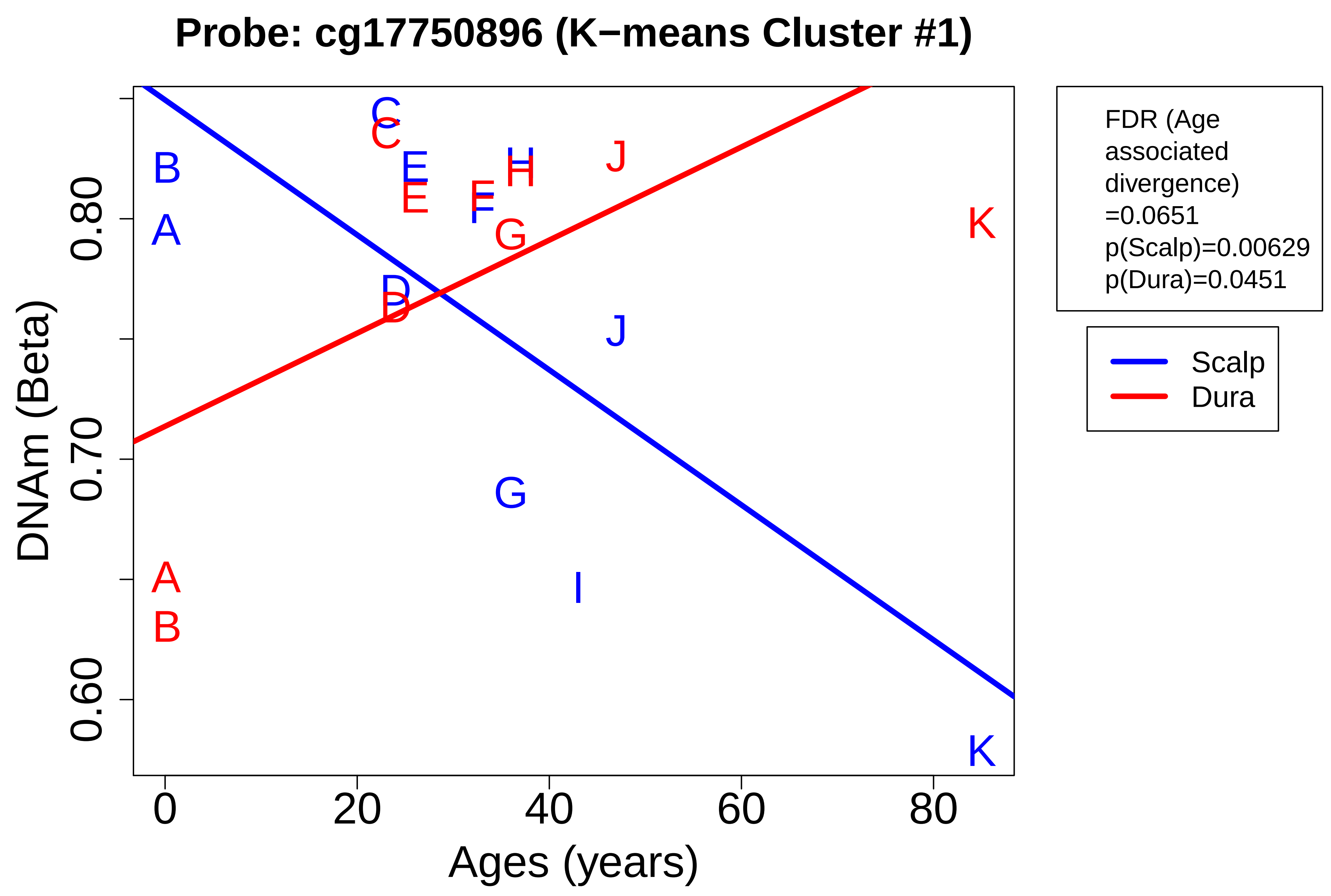

Probe: cg17750896 (K−means Cluster #1)
DNAm (Beta)
0.60
0.70
0.80
Ages (years)
0
20
40
60
80
FDR (Age associated divergence) =0.0651
p(Scalp)=0.00629
p(Dura)=0.0451
Scalp
Dura
A
B
C
D
E
F
G
H
I
J
K

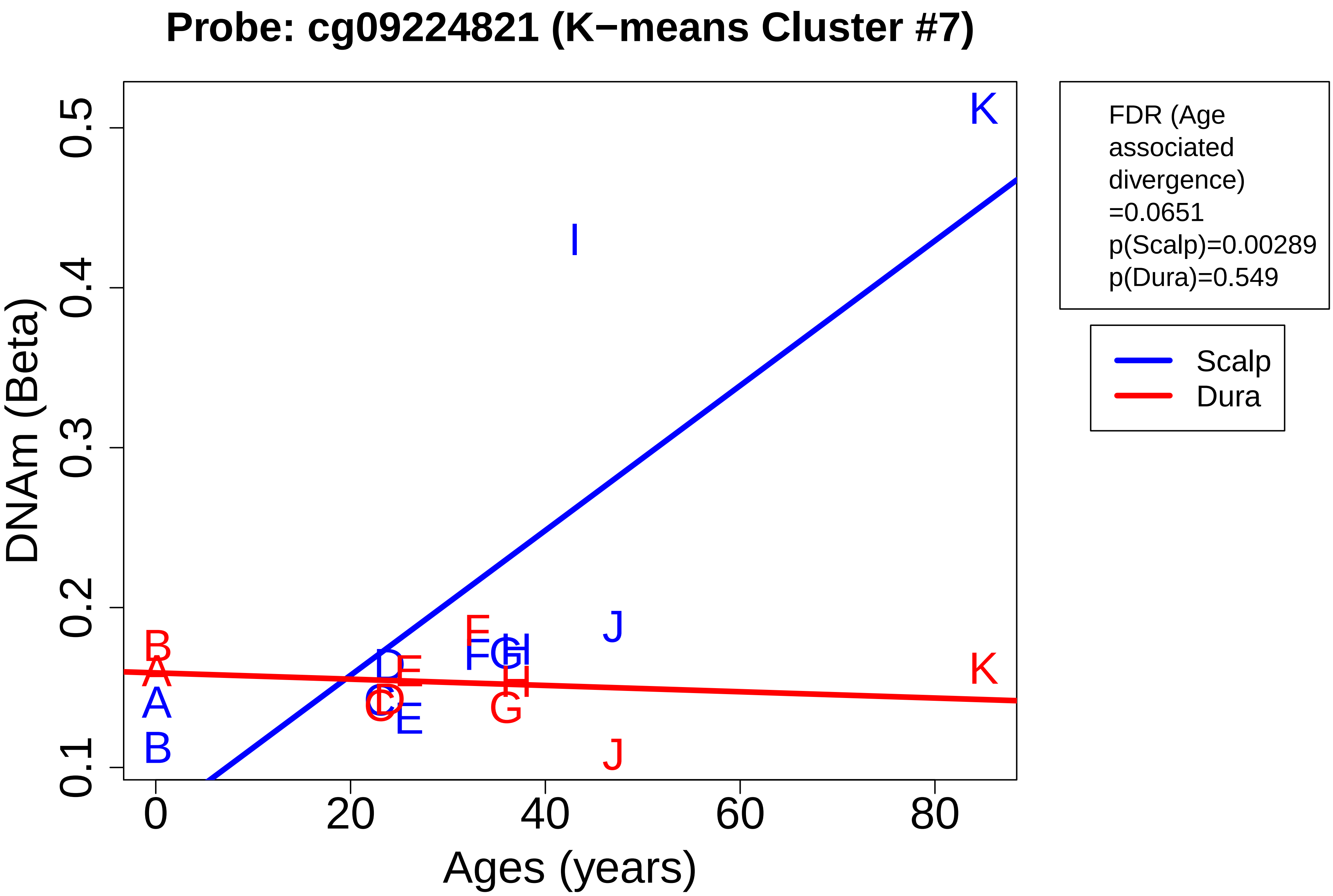
Probe: cg09224821 (K−means Cluster #7)
DNAm (Beta)
Ages (years)
0.1
0.2
0.3
0.4
0.5
0
20
40
60
80
FDR (Age associated divergence) =0.0651
p(Scalp)=0.00289
p(Dura)=0.549
Scalp
Dura

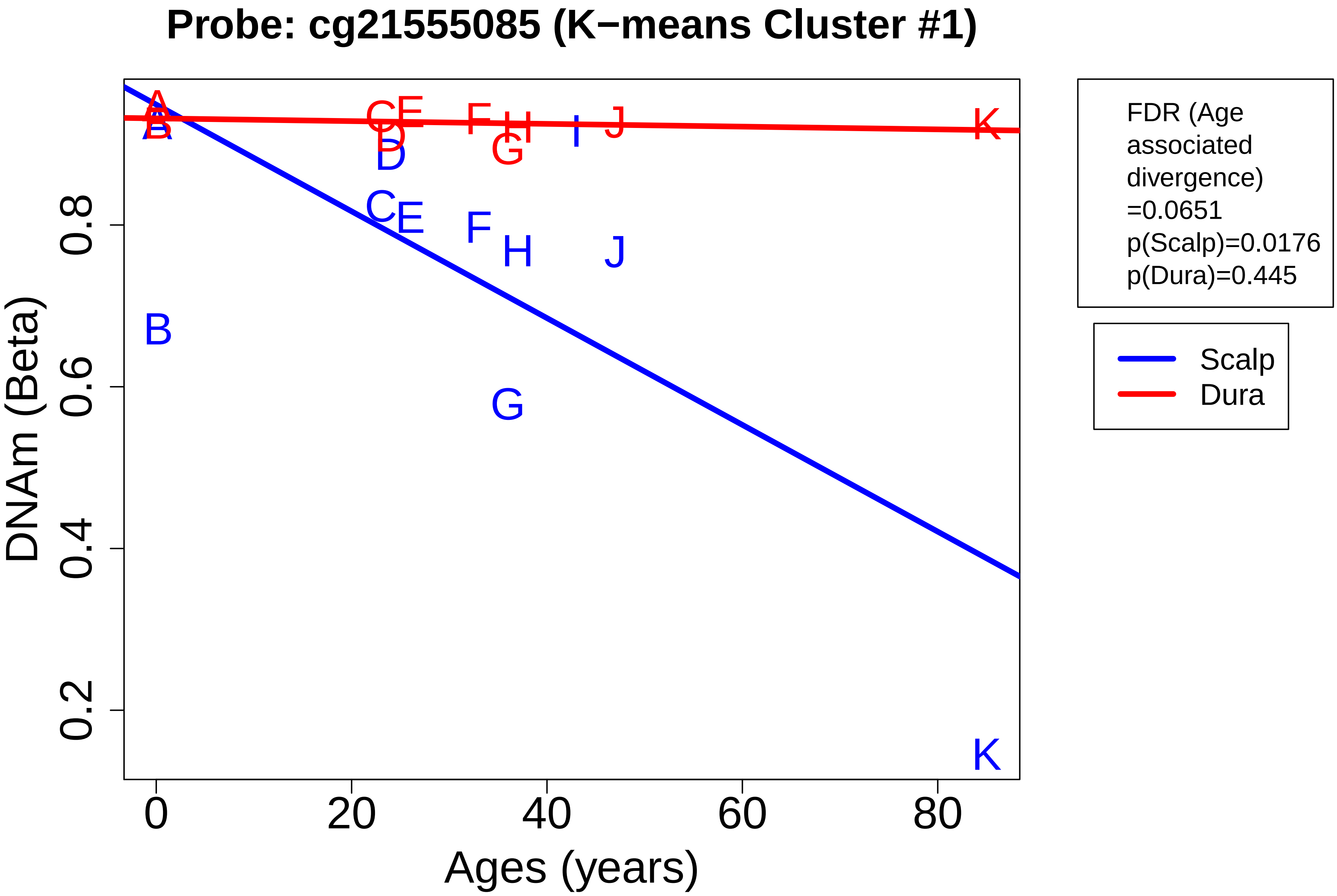

Probe: cg21555085 (K−means Cluster #1)
DNAm (Beta)
0.2
0.4
0.6
0.8
0
20
40
60
80
Ages (years)
A
B
C
D
E
F
G
H
I
J
K
FDR (Age associated divergence) =0.0651
p(Scalp)=0.0176
p(Dura)=0.445
Scalp
Dura

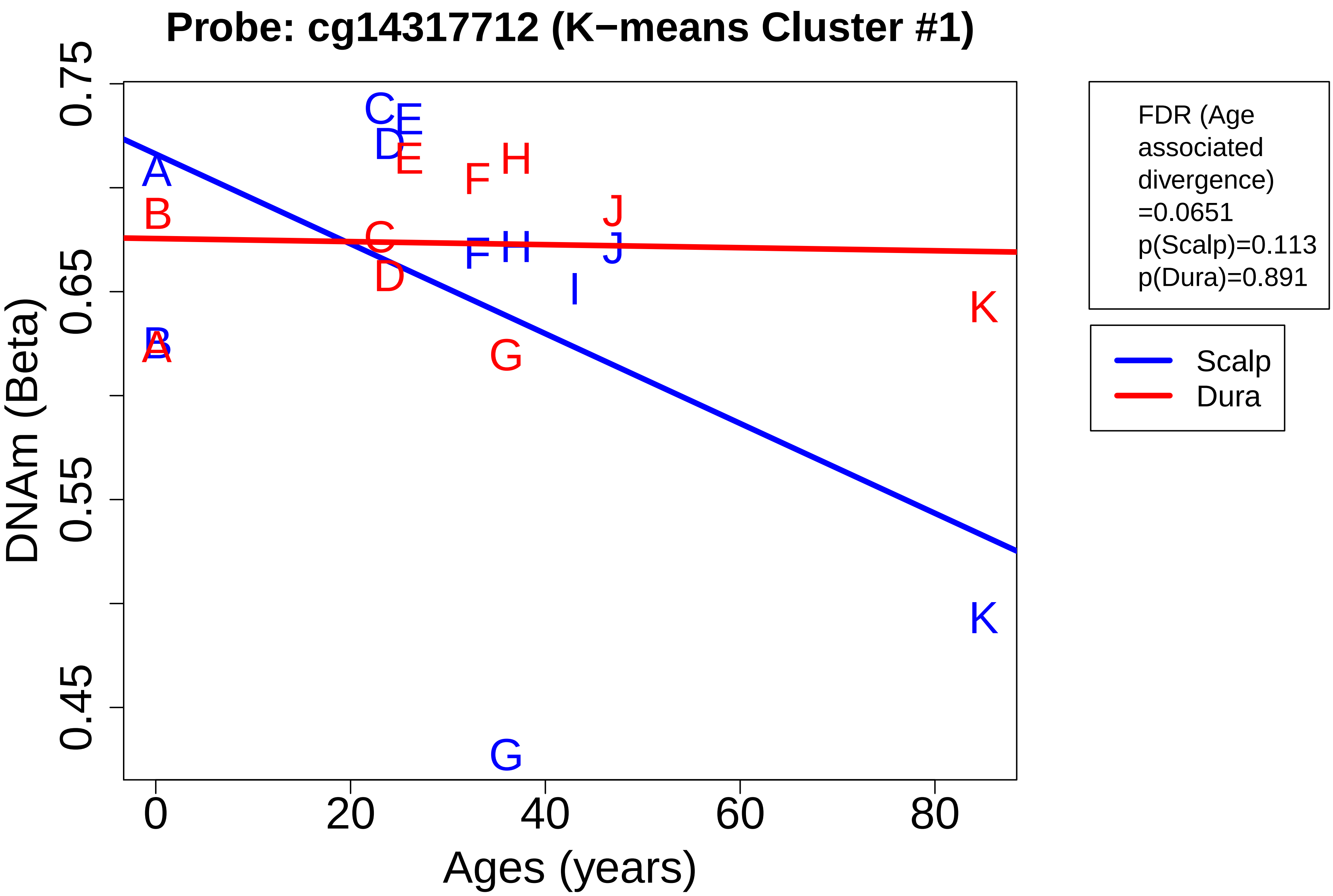

Probe: cg14317712 (K−means Cluster #1)
DNAm (Beta)
0.45
0.55
0.65
0.75
Ages (years)
0
20
40
60
80
A
B
C
D
E
F
G
H
I
J
K
FDR (Age associated divergence) =0.0651
p(Scalp)=0.113
p(Dura)=0.891
Scalp
Dura

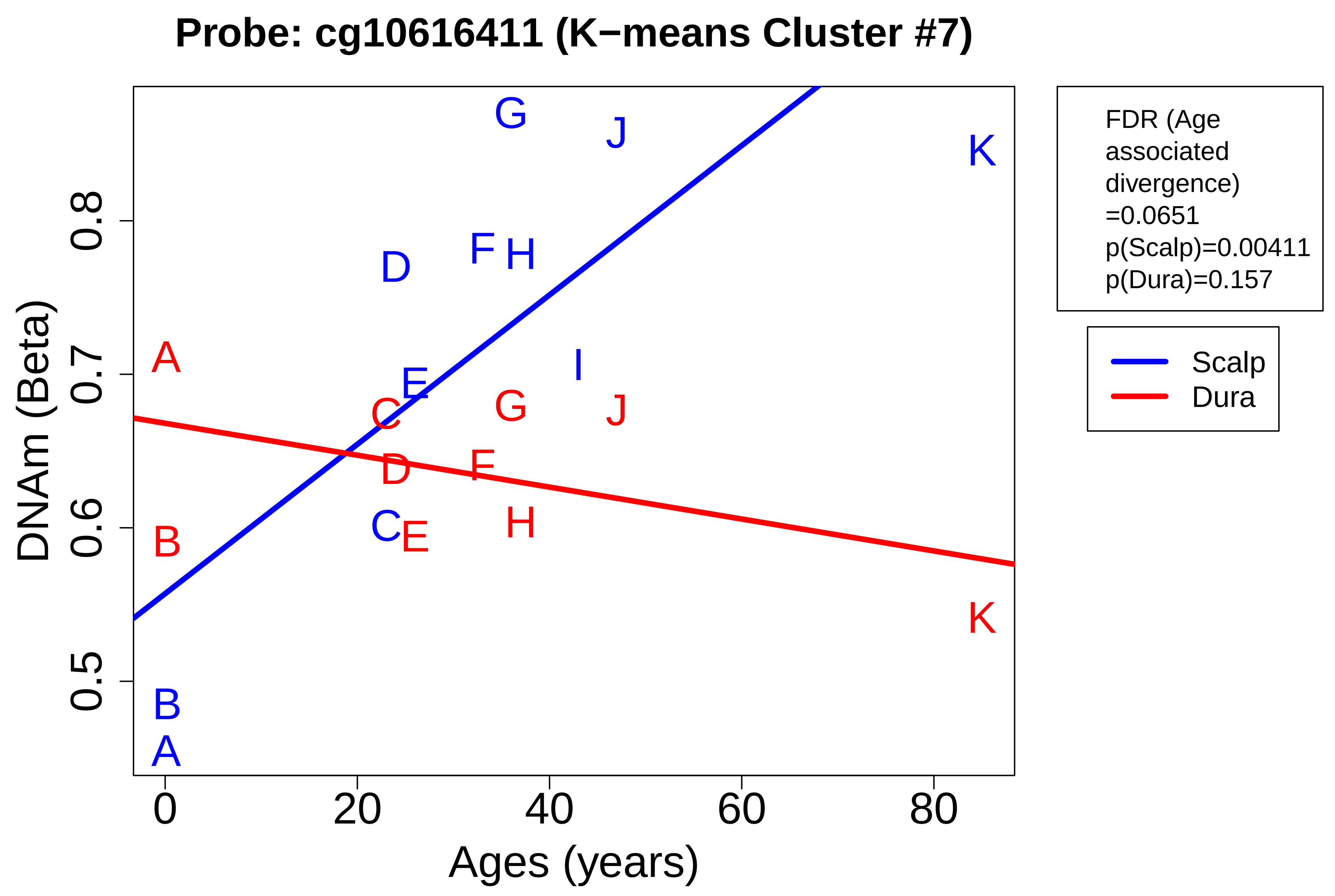
Probe: cg10616411 (K−means Cluster #7)
DNAm (Beta)
0.5
0.6
0.7
0.8
Ages (years)
0
20
40
60
80
FDR (Age associated divergence) =0.0651
p(Scalp)=0.00411
p(Dura)=0.157
Scalp
Dura
A
B
C
D
E
F
G
H
I
J
K

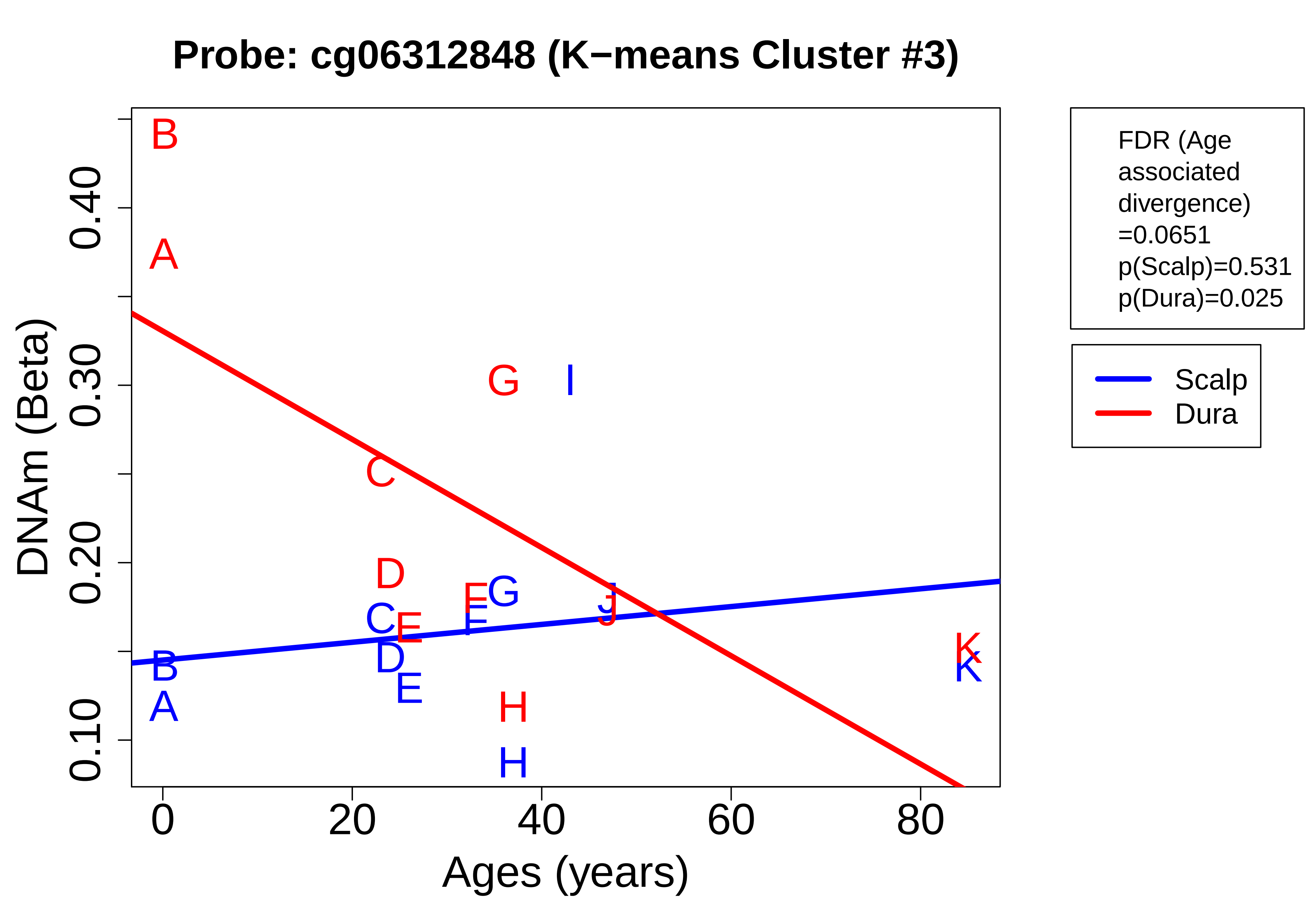

Probe: cg06312848 (K−means Cluster #3)
DNAm (Beta)
0.10
0.20
0.30
0.40
0
20
40
60
80
Ages (years)
FDR (Age associated divergence) =0.0651
p(Scalp)=0.531
p(Dura)=0.025
Scalp
Dura

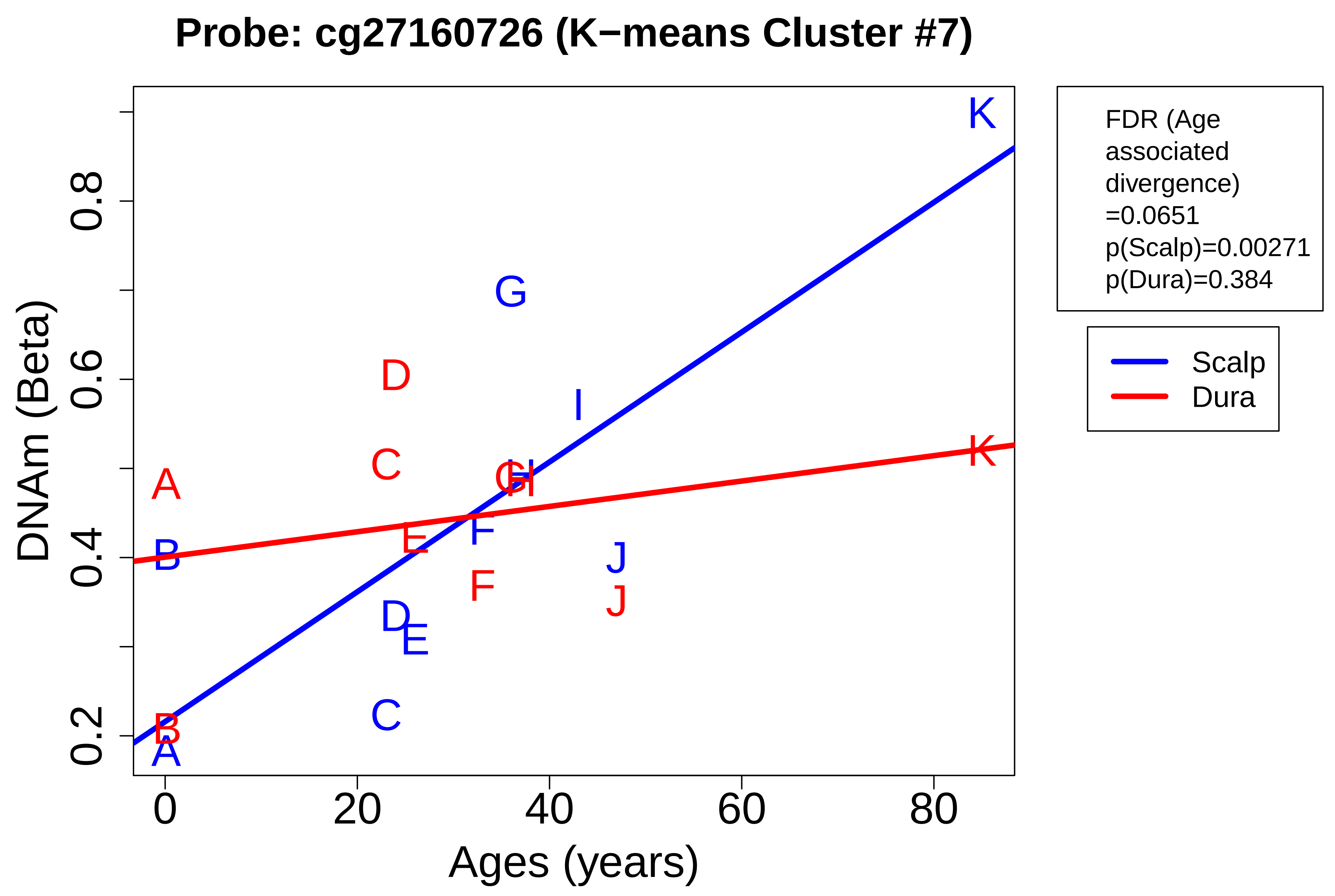
Probe: cg27160726 (K−means Cluster #7)
FDR (Age associated divergence) =0.0651
p(Scalp)=0.00271
p(Dura)=0.384
Scalp
Dura
DNAm (Beta)
0.2
0.4
0.6
0.8
0
20
40
60
80
Ages (years)
A
B
C
D
E
F
G
H
I
J
K

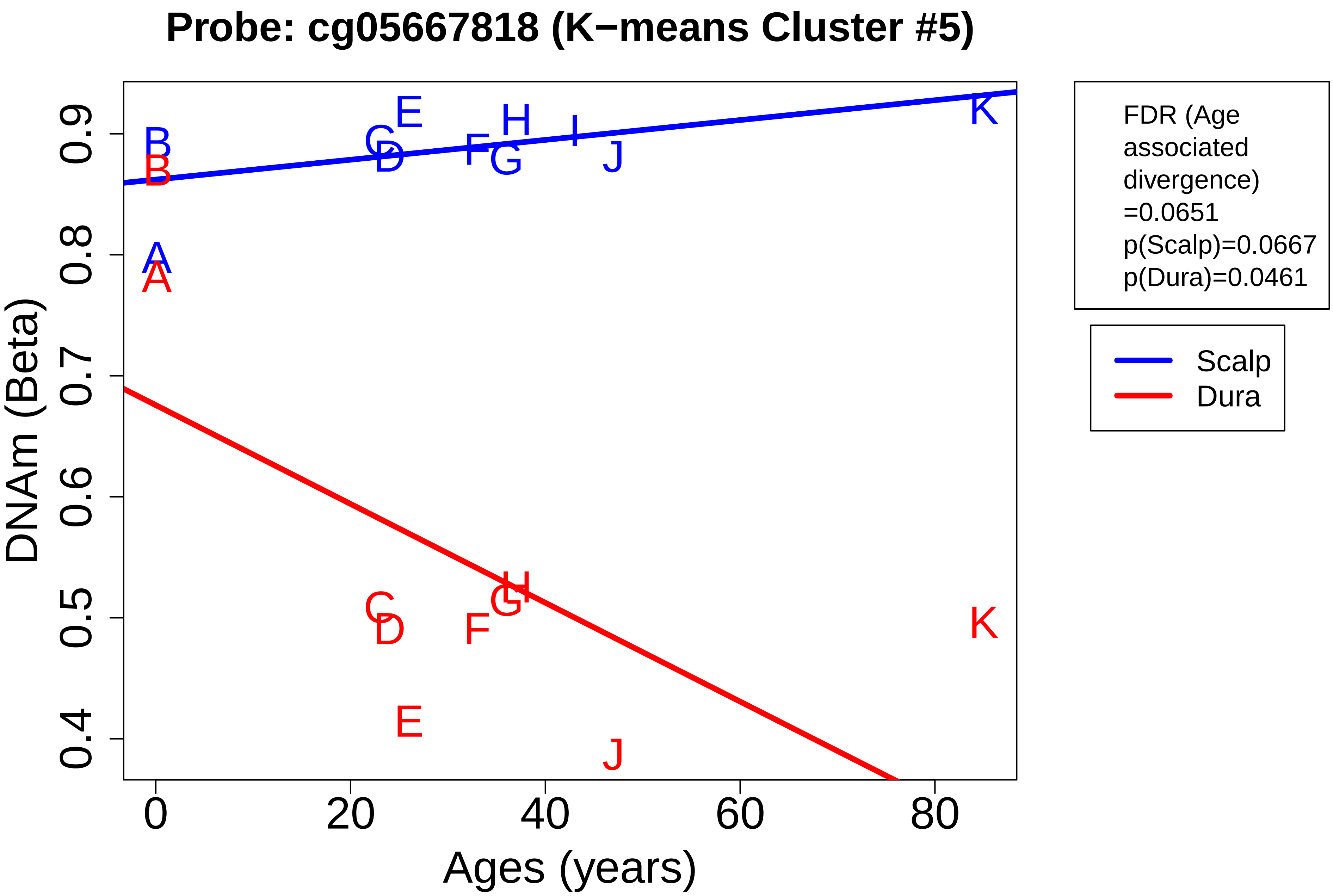

Probe: cg05667818 (K−means Cluster #5)
DNAm (Beta)
0.4
0.5
0.6
0.7
0.8
0.9
0
20
40
60
80
Ages (years)
A
B
C
D
E
F
G
H
I
J
K
FDR (Age associated divergence) =0.0651
p(Scalp)=0.0667
p(Dura)=0.0461
Scalp
Dura

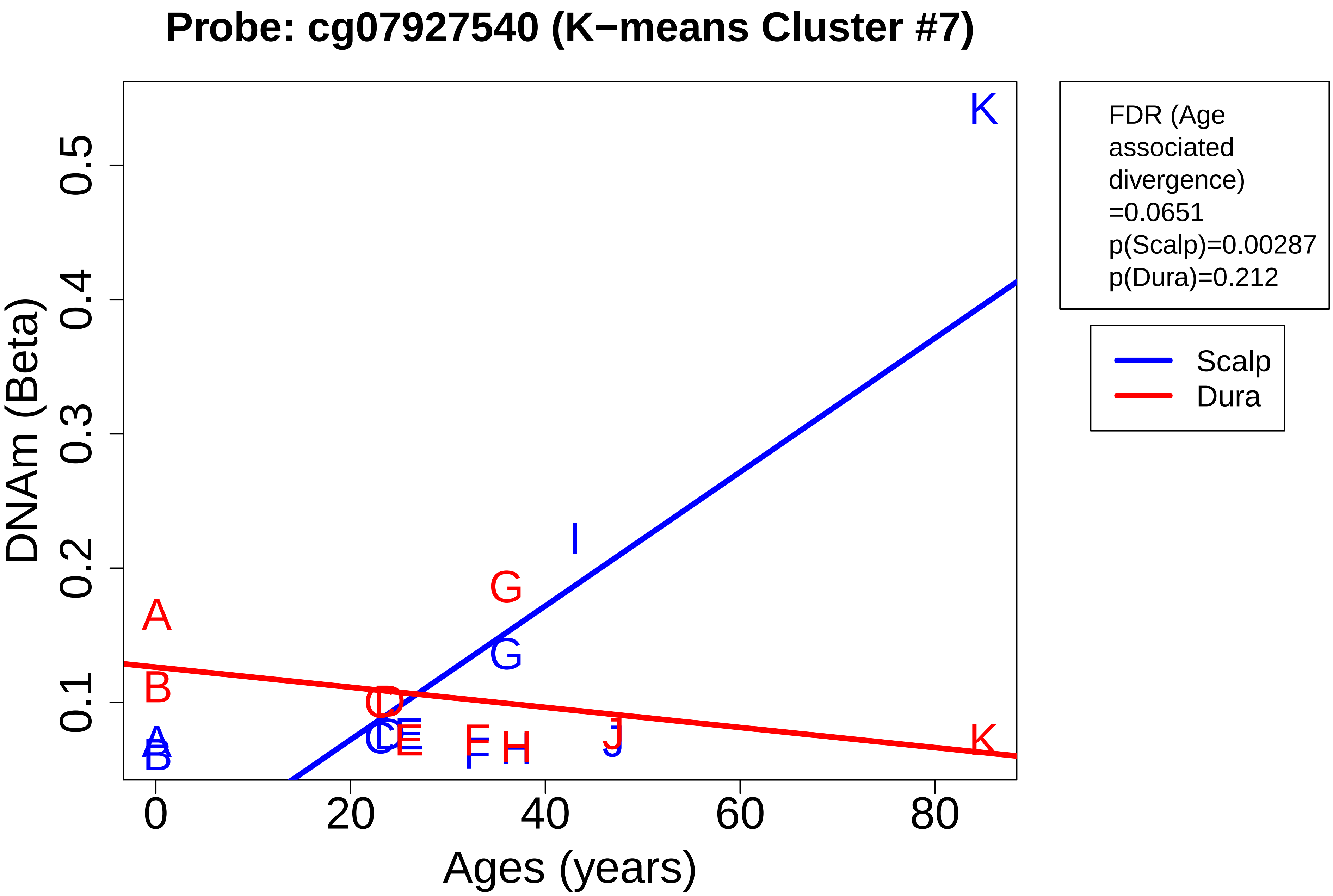

Probe: cg07927540 (K−means Cluster #7)
FDR (Age associated divergence) =0.0651
p(Scalp)=0.00287
p(Dura)=0.212
Scalp
Dura
DNAm (Beta)
Ages (years)
0.1
0.2
0.3
0.4
0.5
0
20
40
60
80
A
B
C
D
E
F
G
H
I
J
K

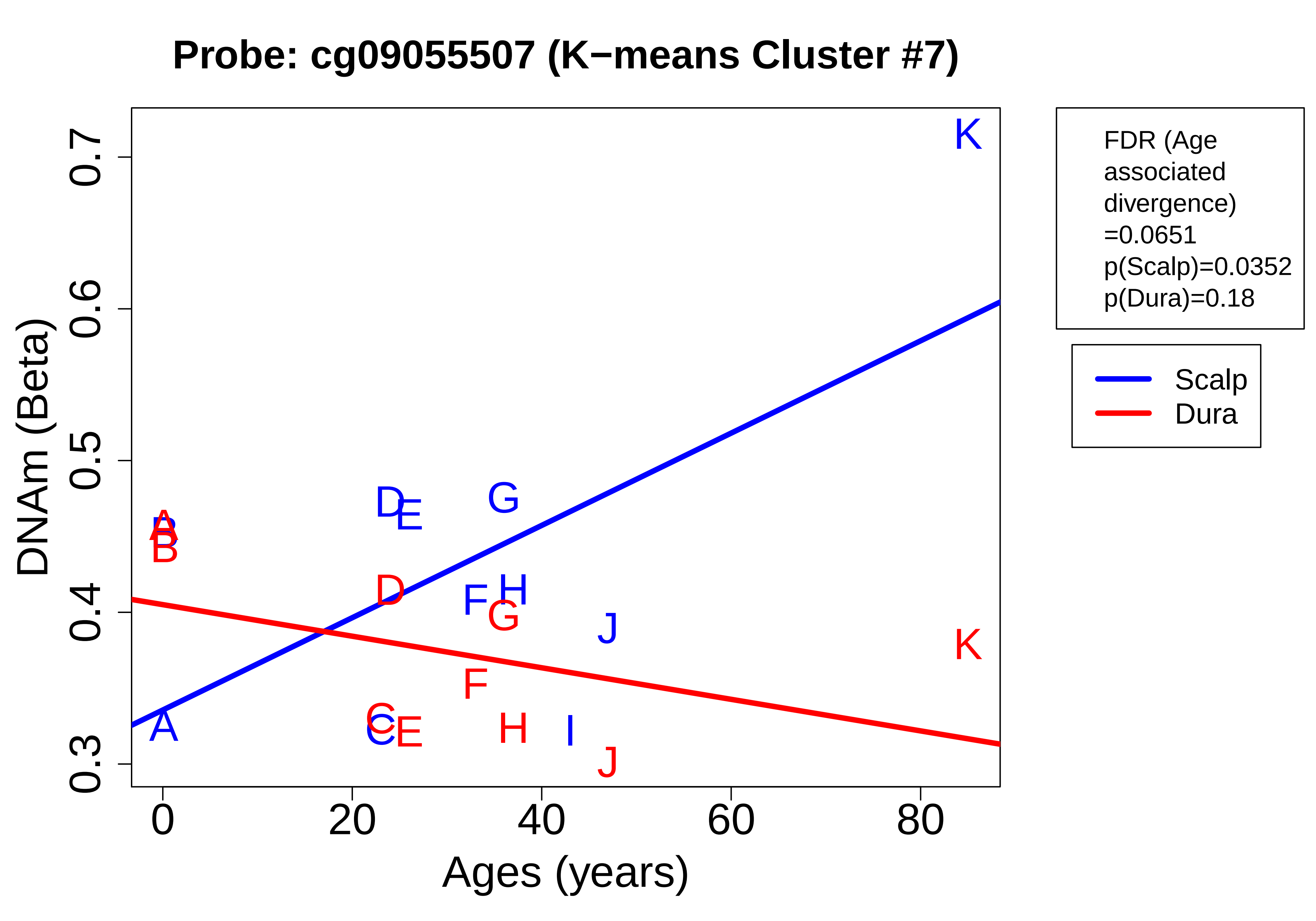

Probe: cg09055507 (K−means Cluster #7)
DNAm (Beta)
0.3
0.4
0.5
0.6
0.7
0
20
40
60
80
Ages (years)
FDR (Age associated divergence) =0.0651
p(Scalp)=0.0352
p(Dura)=0.18
Scalp
Dura
A
B
C
D
E
F
G
H
I
J
K

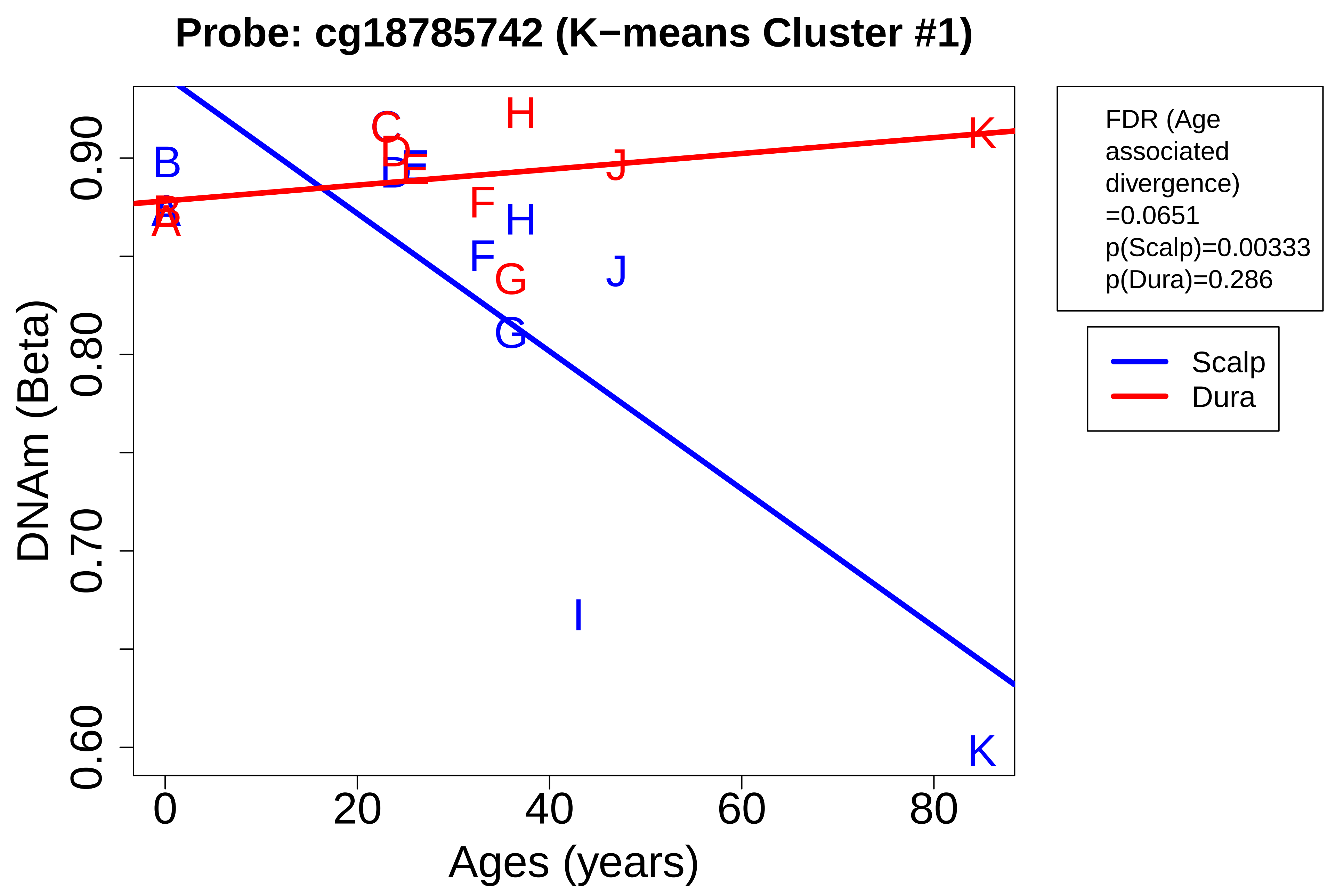

Probe: cg18785742 (K−means Cluster #1)
DNAm (Beta)
0.60
0.70
0.80
0.90
0
20
40
60
80
Ages (years)
FDR (Age associated divergence) =0.0651
p(Scalp)=0.00333
p(Dura)=0.286
Scalp
Dura

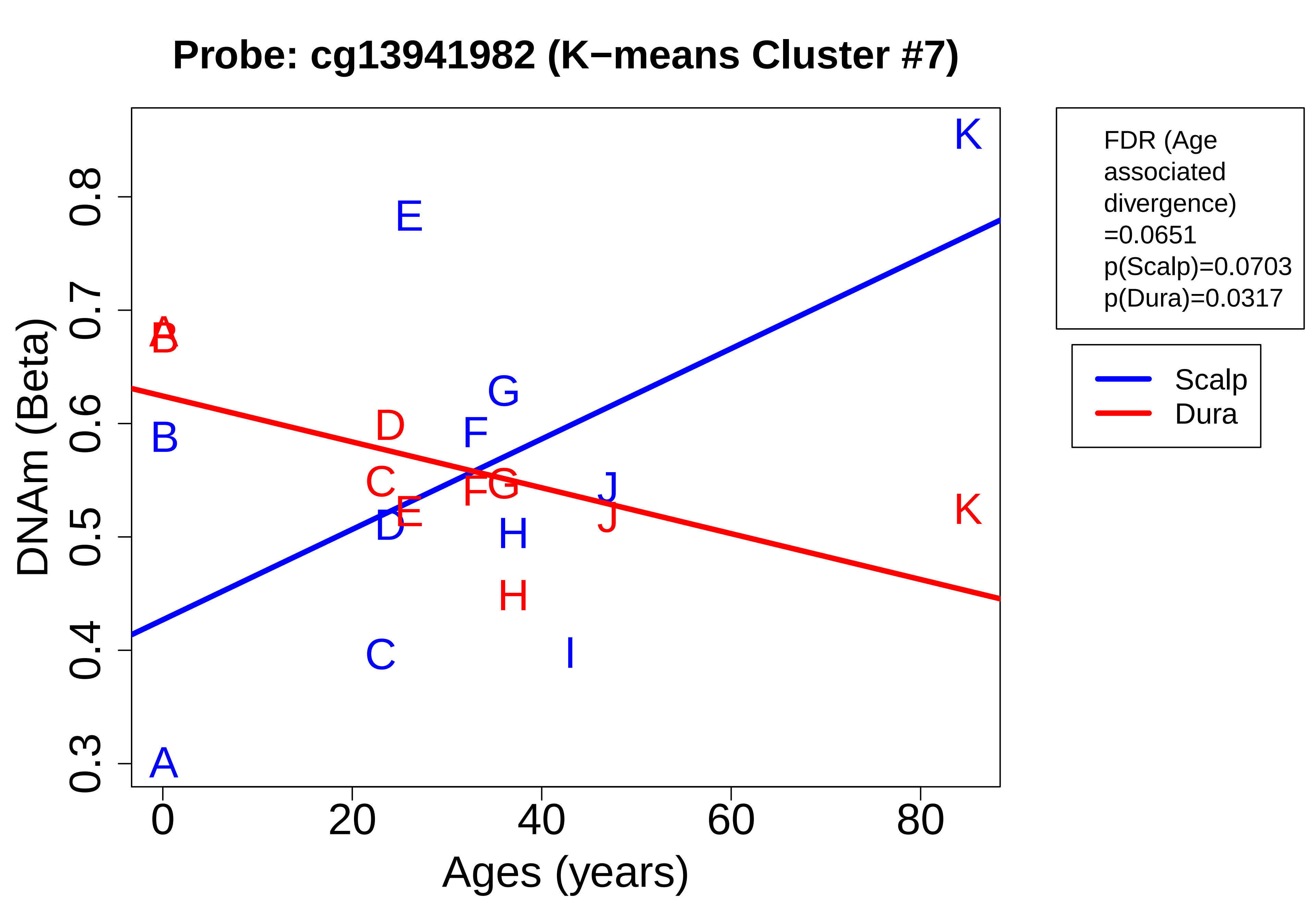

Probe: cg13941982 (K−means Cluster #7)
DNAm (Beta)
0.3
0.4
0.5
0.6
0.7
0.8
0
20
40
60
80
Ages (years)
A
B
C
D
E
F
G
H
I
J
K
A
B
C
D
E
F
G
H
J
K
FDR (Age associated divergence) =0.0651
p(Scalp)=0.0703
p(Dura)=0.0317
Scalp
Dura

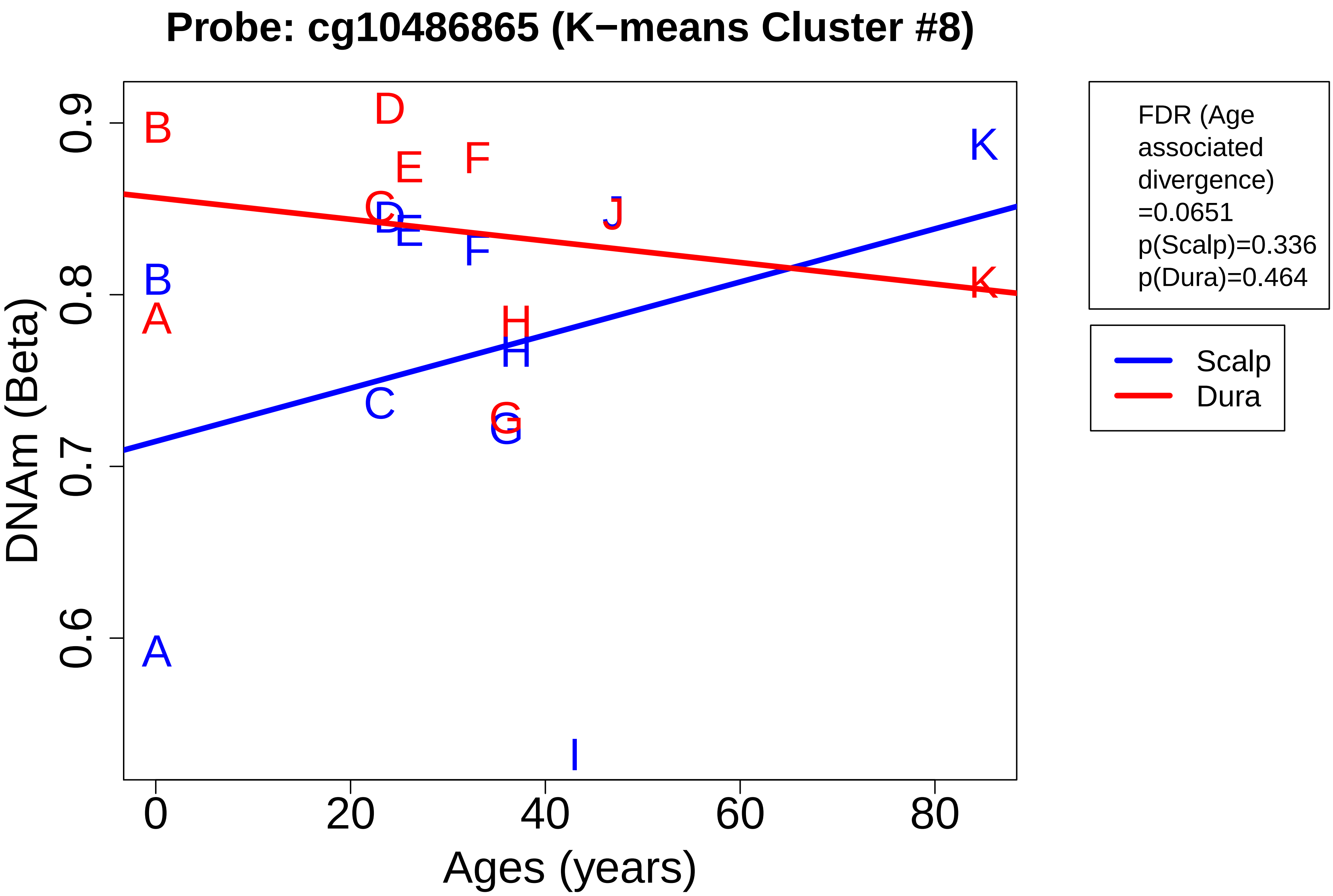

Probe: cg10486865 (K−means Cluster #8)
DNAm (Beta)
Ages (years)
0.6
0.7
0.8
0.9
0
20
40
60
80
FDR (Age associated divergence) =0.0651
p(Scalp)=0.336
p(Dura)=0.464
Scalp
Dura

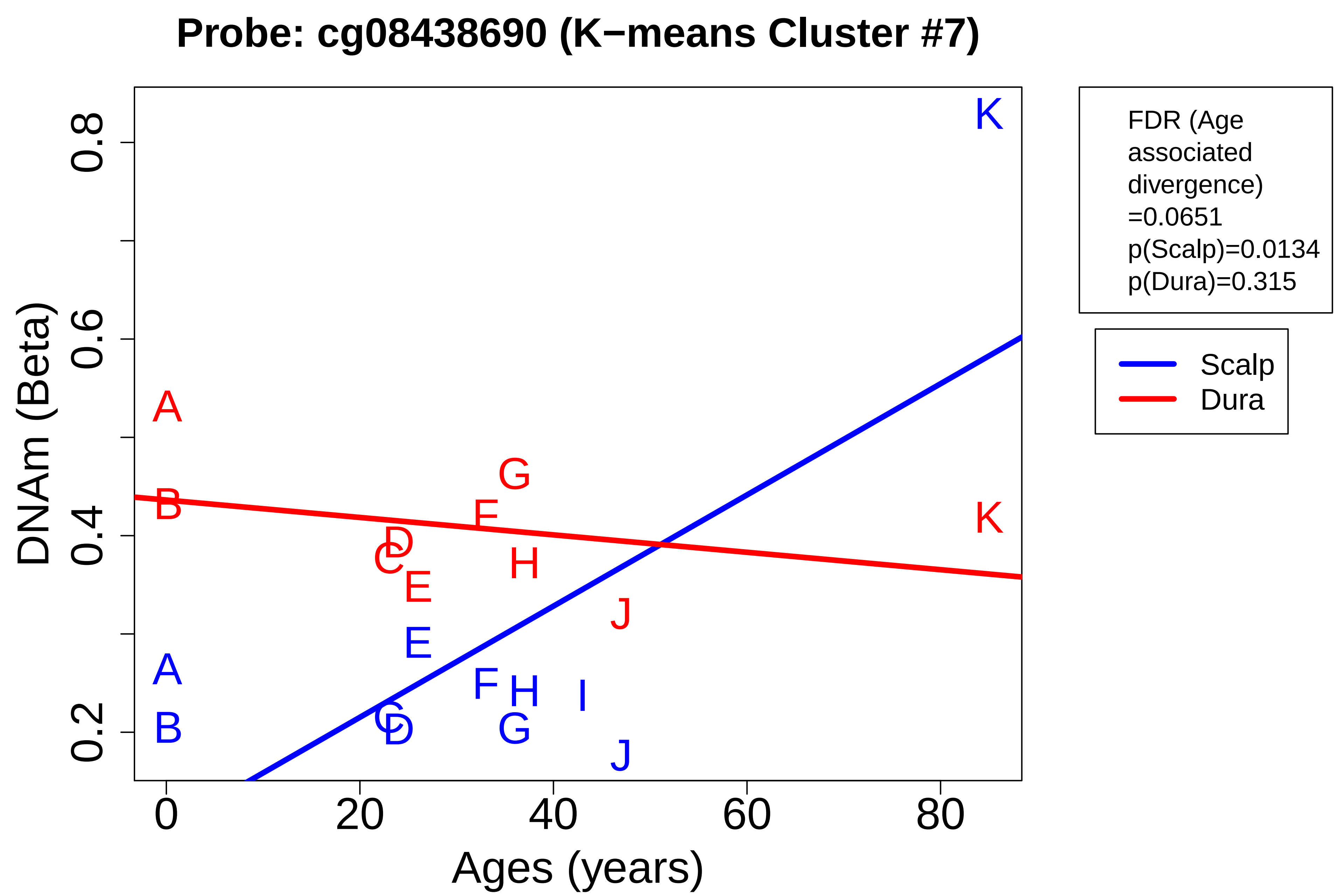

Probe: cg08438690 (K−means Cluster #7)
FDR (Age associated divergence) =0.0651
p(Scalp)=0.0134
p(Dura)=0.315
Scalp
Dura
DNAm (Beta)
0.2
0.4
0.6
0.8
0
20
40
60
80
Ages (years)
A
B
C
D
E
F
G
H
I
J
K

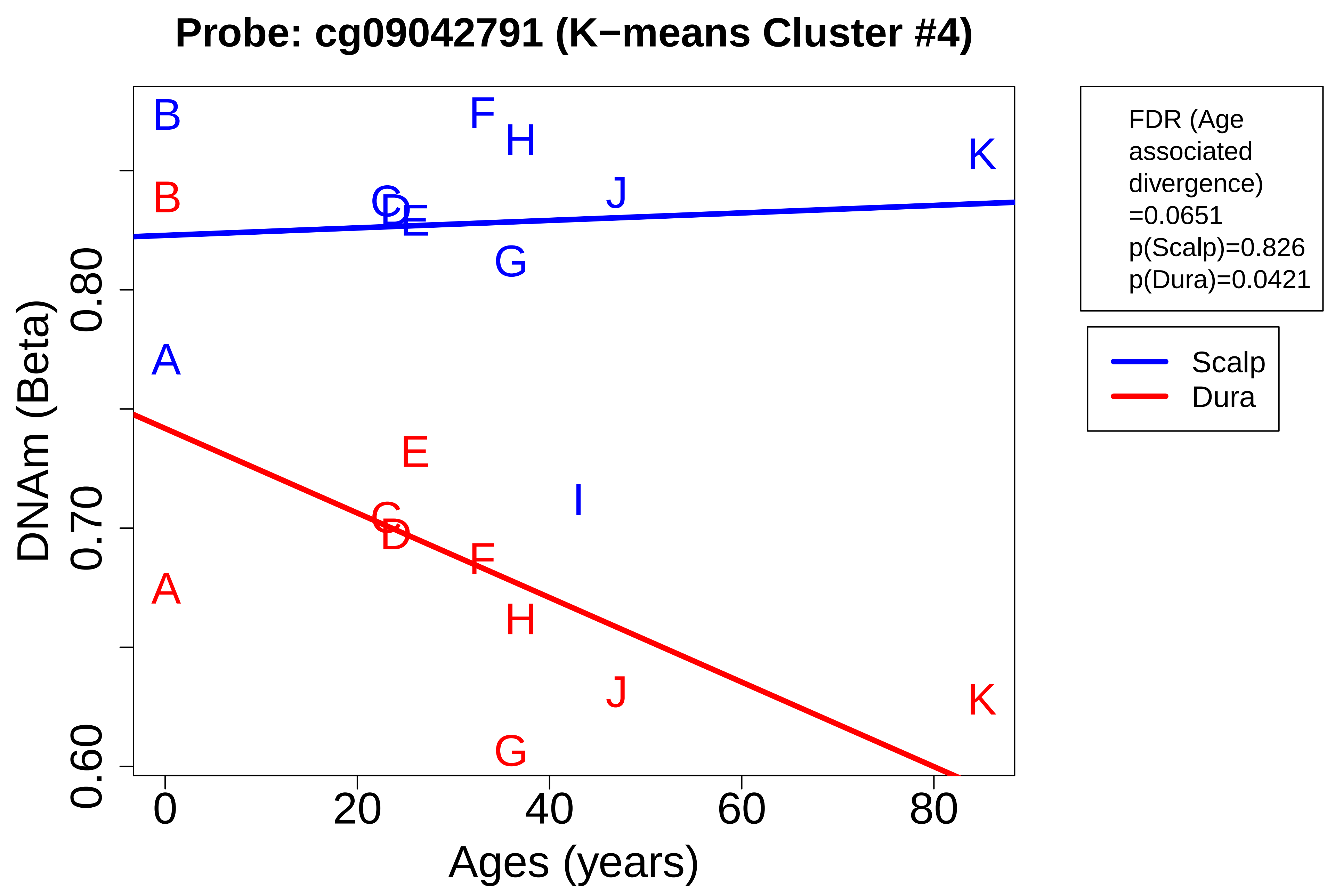

Probe: cg09042791 (K−means Cluster #4)
DNAm (Beta)
0.60
0.70
0.80
0
20
40
60
80
Ages (years)
FDR (Age associated divergence) =0.0651
p(Scalp)=0.826
p(Dura)=0.0421
Scalp
Dura

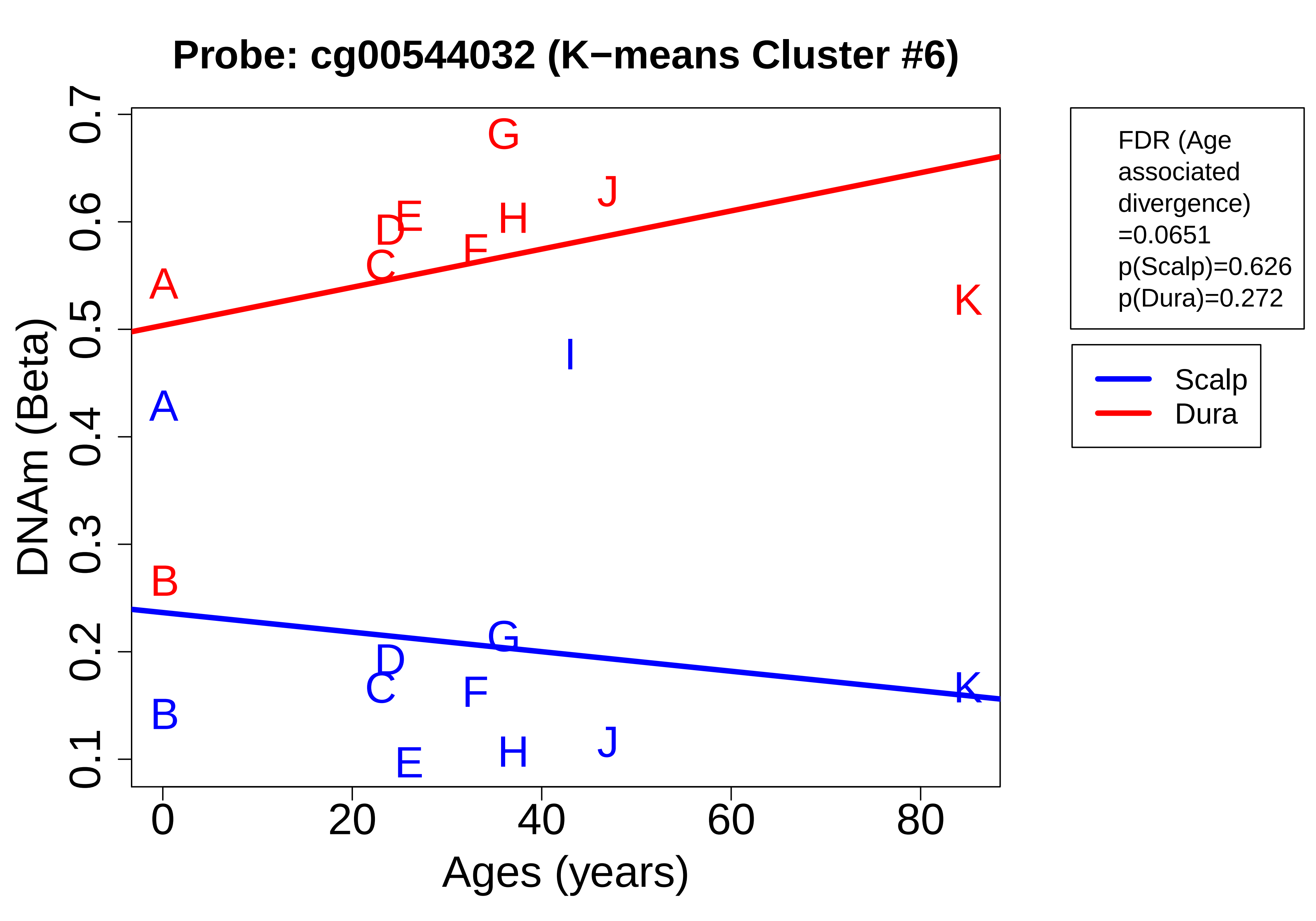
Probe: cg00544032 (K−means Cluster #6)
DNAm (Beta)
Ages (years)
0.1
0.2
0.3
0.4
0.5
0.6
0.7
0
20
40
60
80
FDR (Age associated divergence) =0.0651
p(Scalp)=0.626
p(Dura)=0.272
Scalp
Dura

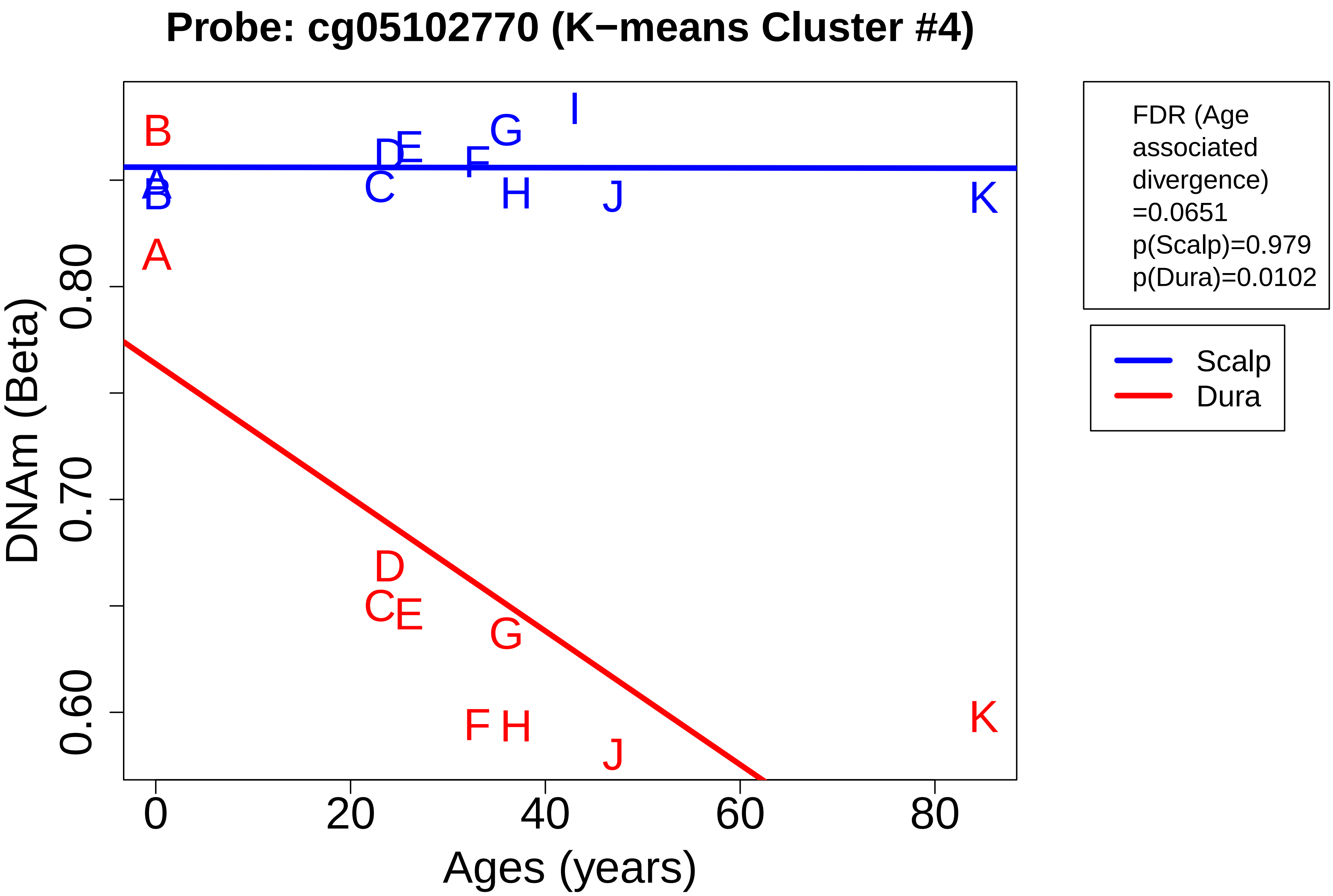

Probe: cg05102770 (K−means Cluster #4)
DNAm (Beta)
0.60
0.70
0.80
Ages (years)
0
20
40
60
80
FDR (Age associated divergence) =0.0651
p(Scalp)=0.979
p(Dura)=0.0102
Scalp
Dura

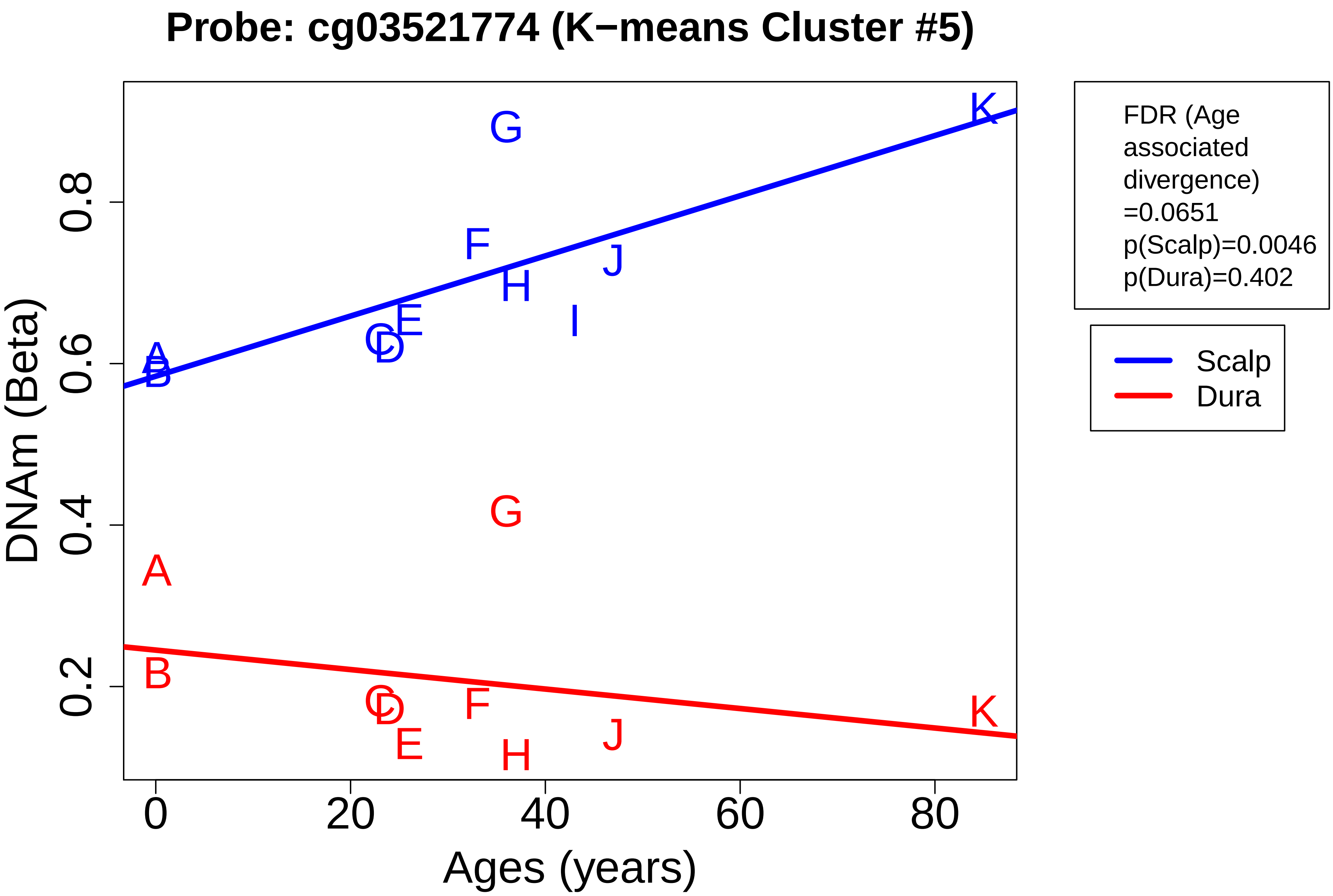

Probe: cg03521774 (K−means Cluster #5)
DNAm (Beta)
0.2
0.4
0.6
0.8
0
20
40
60
80
Ages (years)
FDR (Age associated divergence) =0.0651
p(Scalp)=0.0046
p(Dura)=0.402
Scalp
Dura

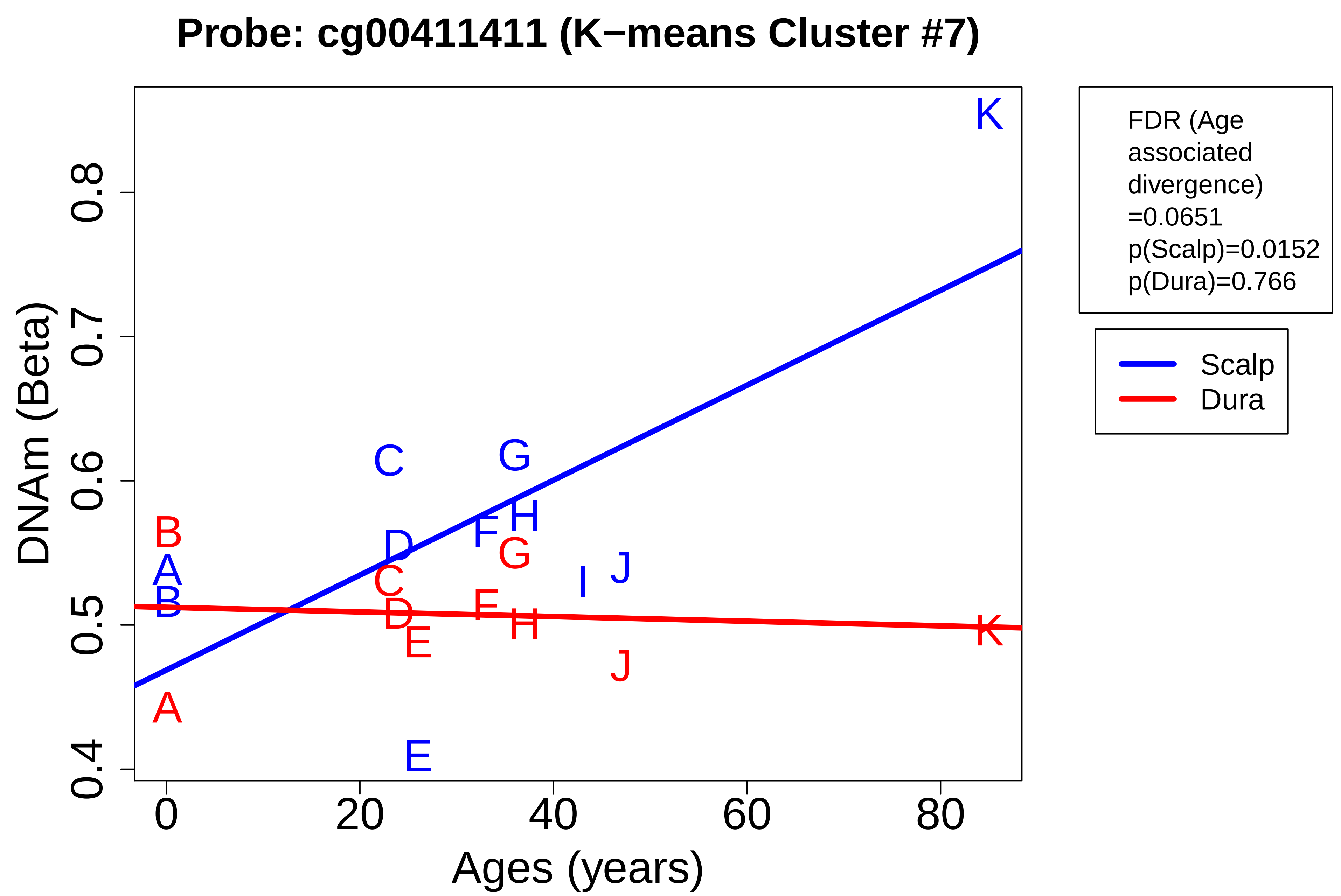

Probe: cg00411411 (K−means Cluster #7)
FDR (Age associated divergence) =0.0651
p(Scalp)=0.0152
p(Dura)=0.766
Scalp
Dura
DNAm (Beta)
0.4
0.5
0.6
0.7
0.8
0
20
40
60
80
Ages (years)

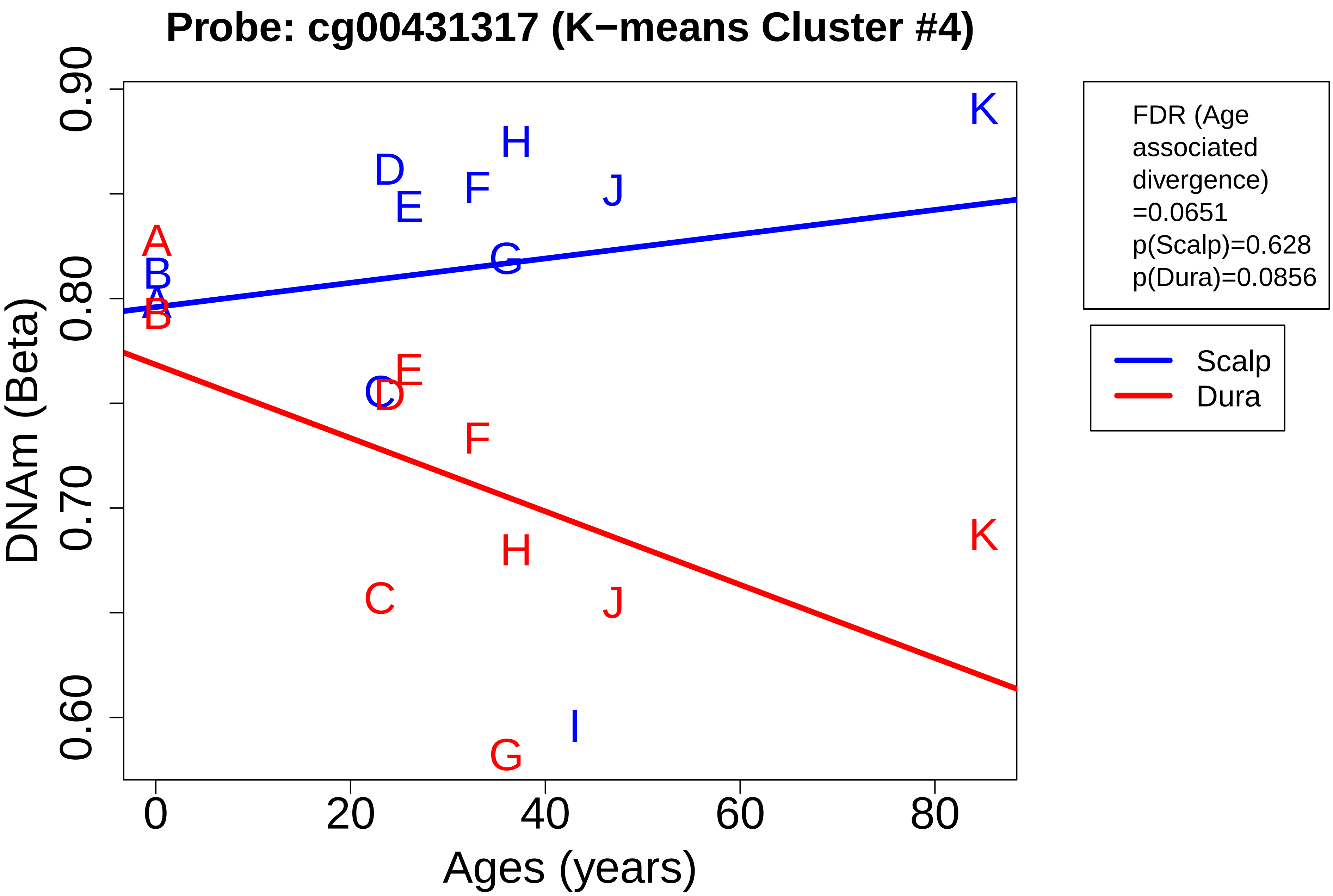
Probe: cg00431317 (K−means Cluster #4)
DNAm (Beta)
0.60
0.70
0.80
0.90
0
20
40
60
80
Ages (years)
FDR (Age associated divergence) =0.0651
p(Scalp)=0.628
p(Dura)=0.0856
Scalp
Dura
A
B
C
D
E
F
G
H
I
J
K

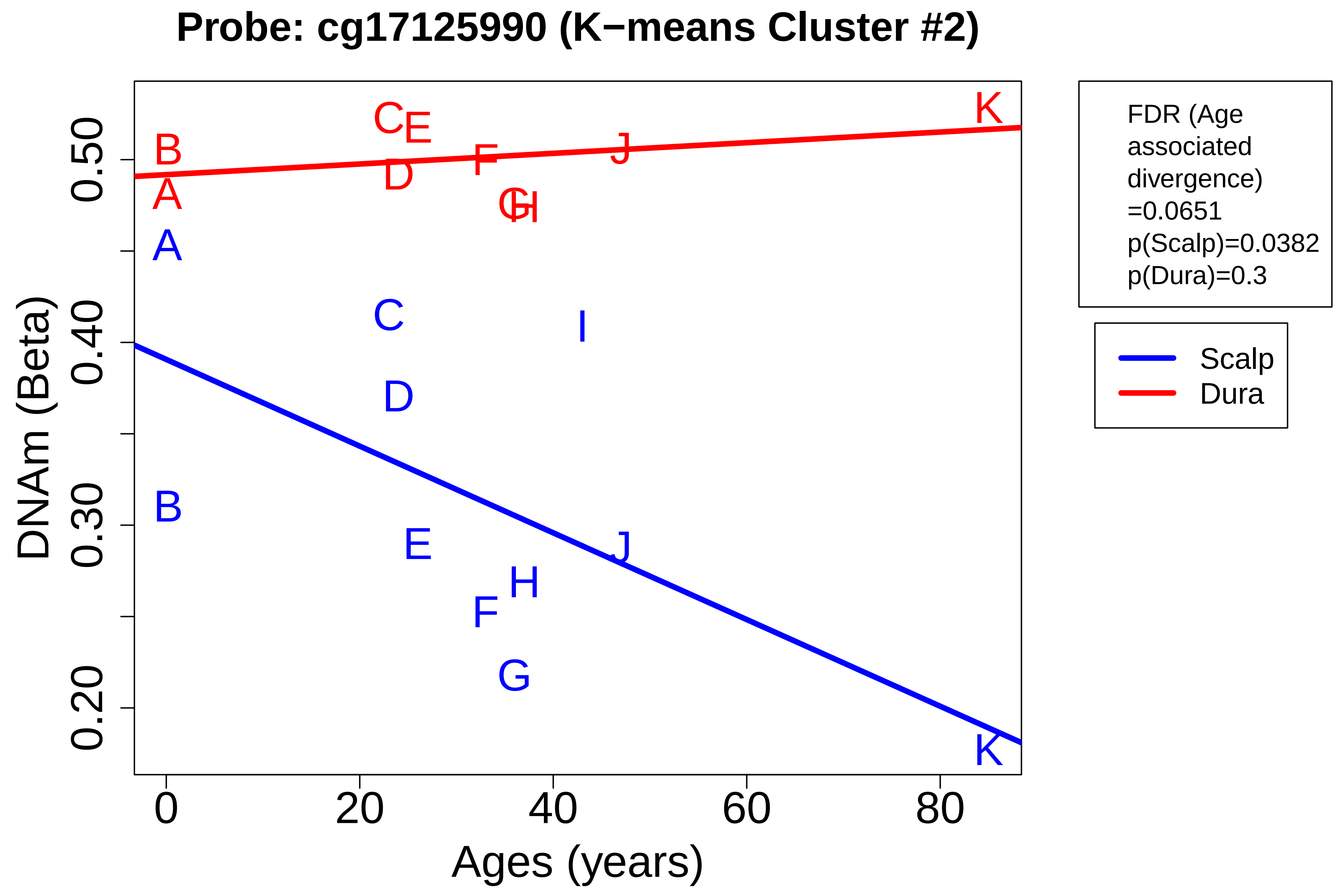
Probe: cg17125990 (K−means Cluster #2)
DNAm (Beta)
0.20
0.30
0.40
0.50
0
20
40
60
80
Ages (years)
A
B
C
D
E
F
G
H
I
J
K
FDR (Age associated divergence) =0.0651
p(Scalp)=0.0382
p(Dura)=0.3
Scalp
Dura

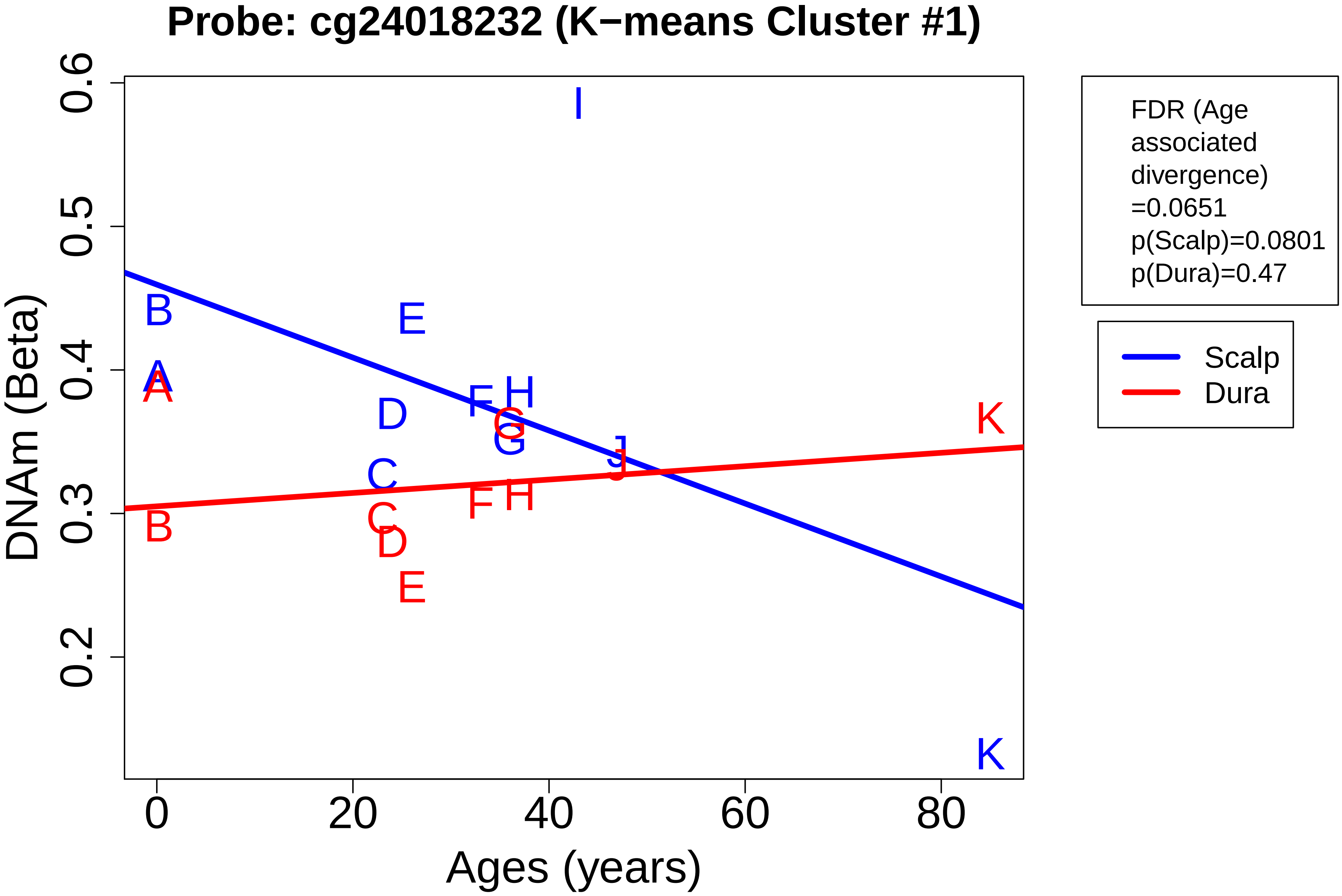
Probe: cg24018232 (K−means Cluster #1)
DNAm (Beta)
0.2
0.3
0.4
0.5
0.6
Ages (years)
0
20
40
60
80
A
B
C
D
E
F
G
H
I
J
K
FDR (Age associated divergence) =0.0651
p(Scalp)=0.0801
p(Dura)=0.47
Scalp
Dura

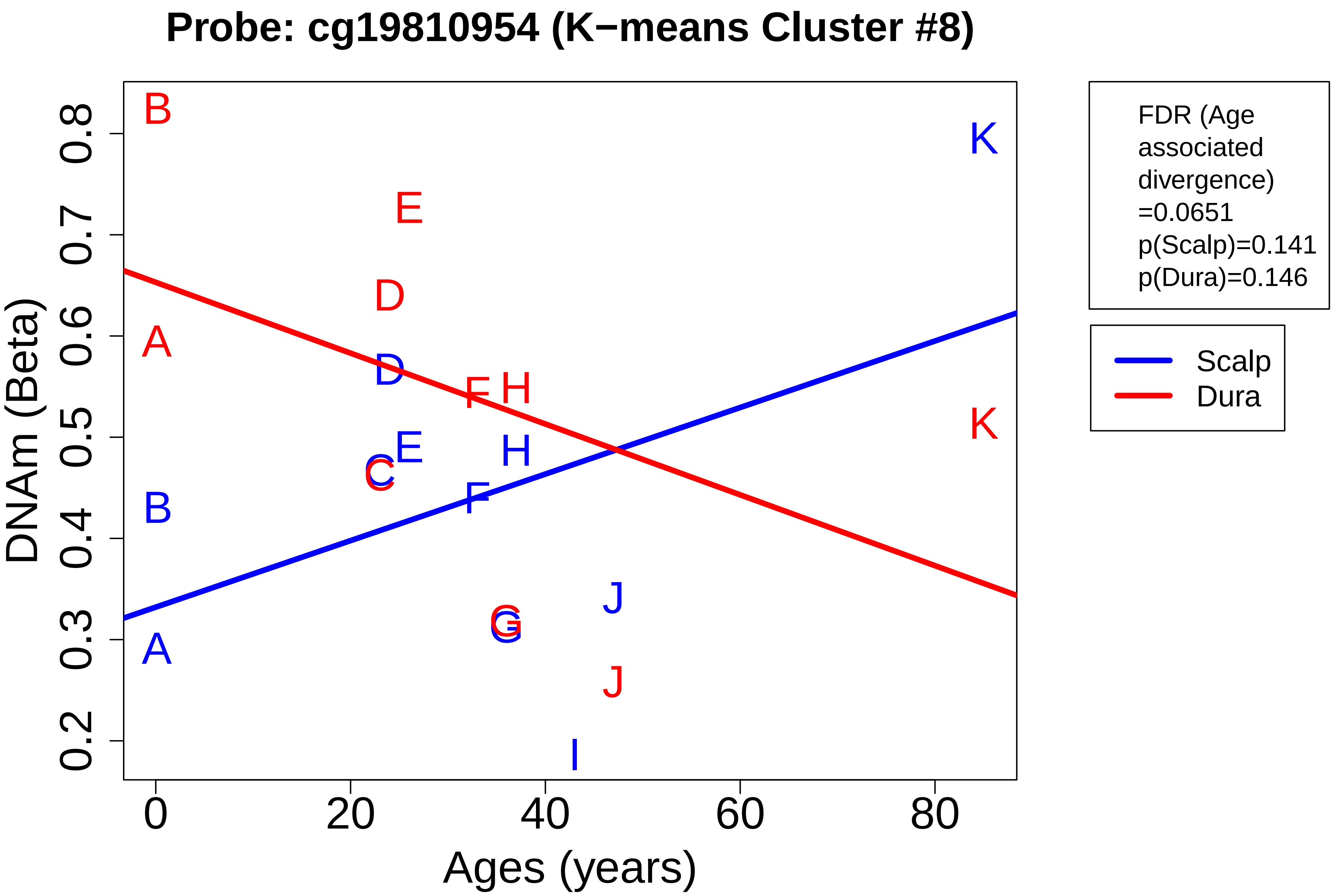
Probe: cg19810954 (K−means Cluster #8)
DNAm (Beta)
0.2
0.3
0.4
0.5
0.6
0.7
0.8
0
20
40
60
80
Ages (years)
FDR (Age associated divergence) =0.0651
p(Scalp)=0.141
p(Dura)=0.146
Scalp
Dura
A
B
C
D
E
F
G
H
I
J
K

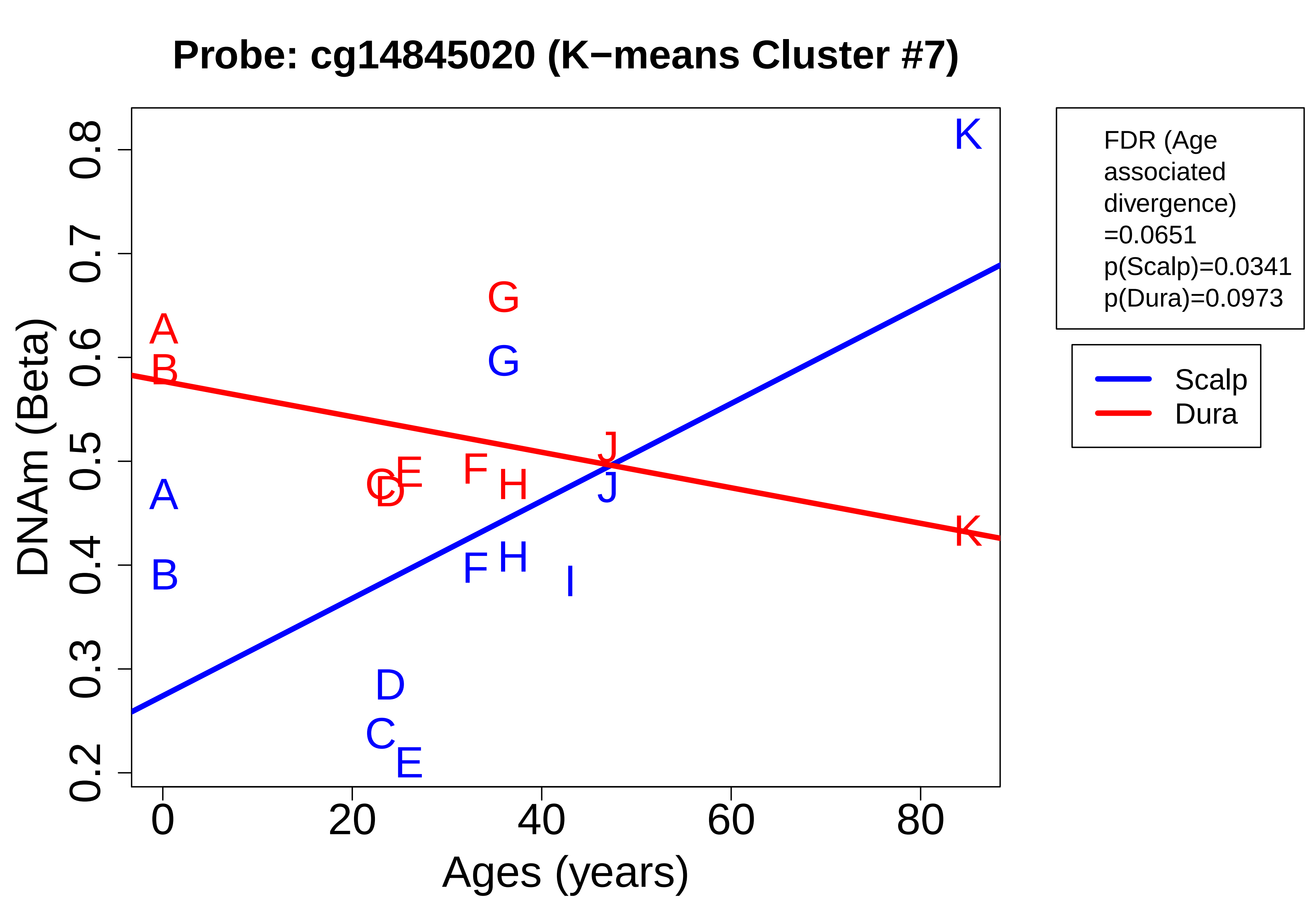

Probe: cg14845020 (K−means Cluster #7)
DNAm (Beta)
0.2
0.3
0.4
0.5
0.6
0.7
0.8
Ages (years)
0
20
40
60
80
FDR (Age associated divergence) =0.0651
p(Scalp)=0.0341
p(Dura)=0.0973
Scalp
Dura
A
B
C
D
E
F
G
H
I
J
K

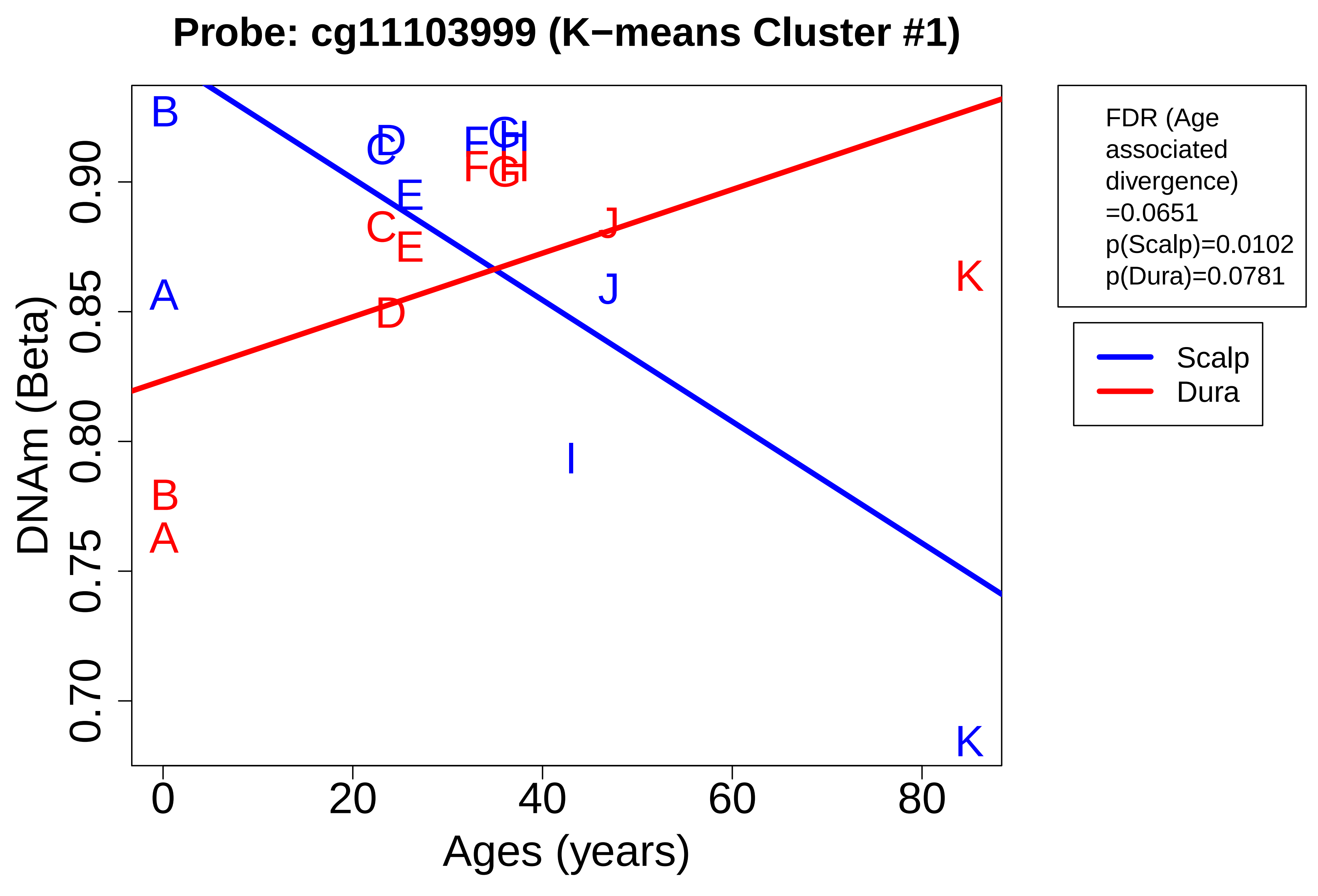

Probe: cg11103999 (K−means Cluster #1)
DNAm (Beta)
0.70
0.75
0.80
0.85
0.90
Ages (years)
0
20
40
60
80
FDR (Age associated divergence) =0.0651
p(Scalp)=0.0102
p(Dura)=0.0781
Scalp
Dura

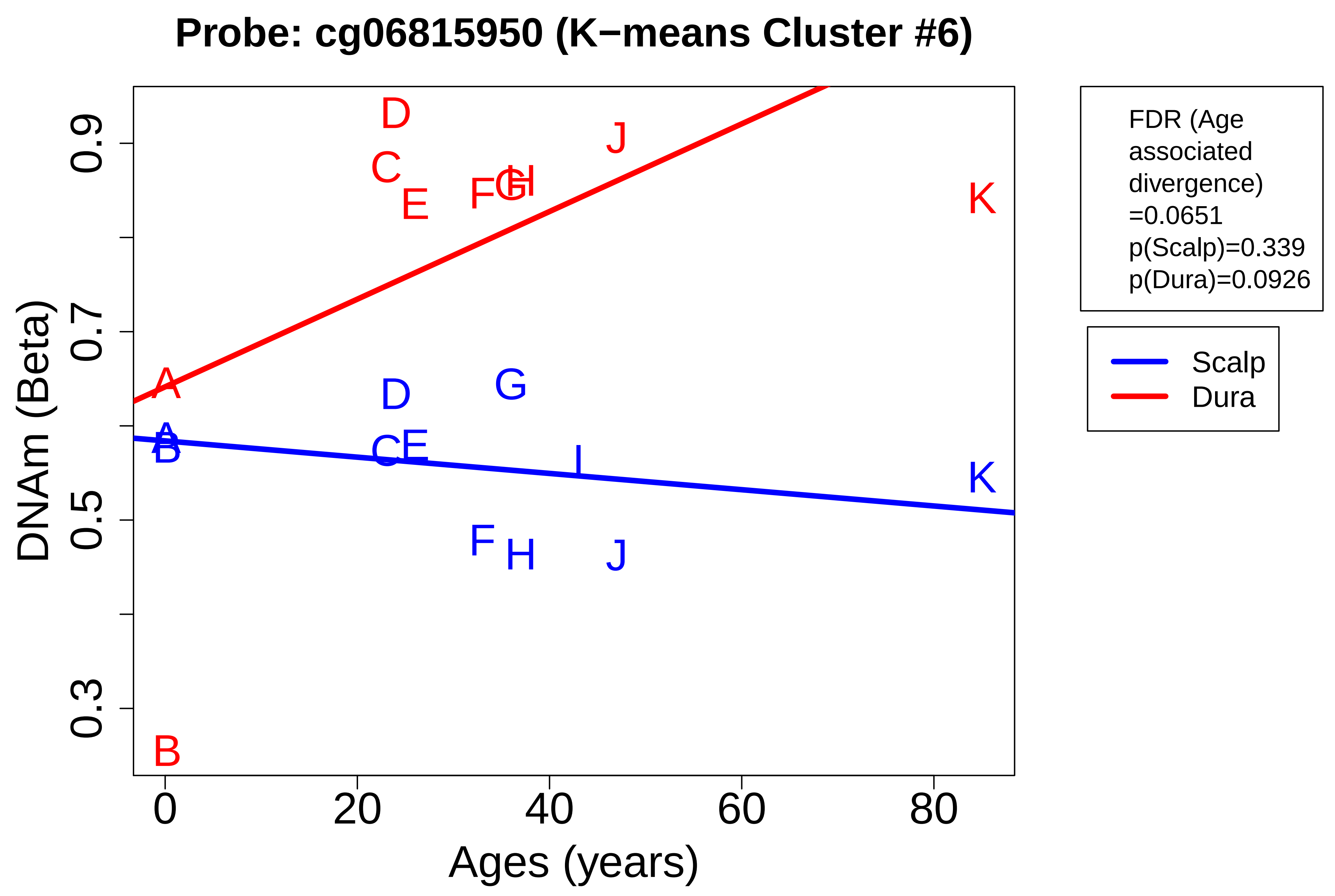
Probe: cg06815950 (K−means Cluster #6)
FDR (Age associated divergence) =0.0651
p(Scalp)=0.339
p(Dura)=0.0926
Scalp
Dura
DNAm (Beta)
Ages (years)
0.3
0.5
0.7
0.9
0
20
40
60
80

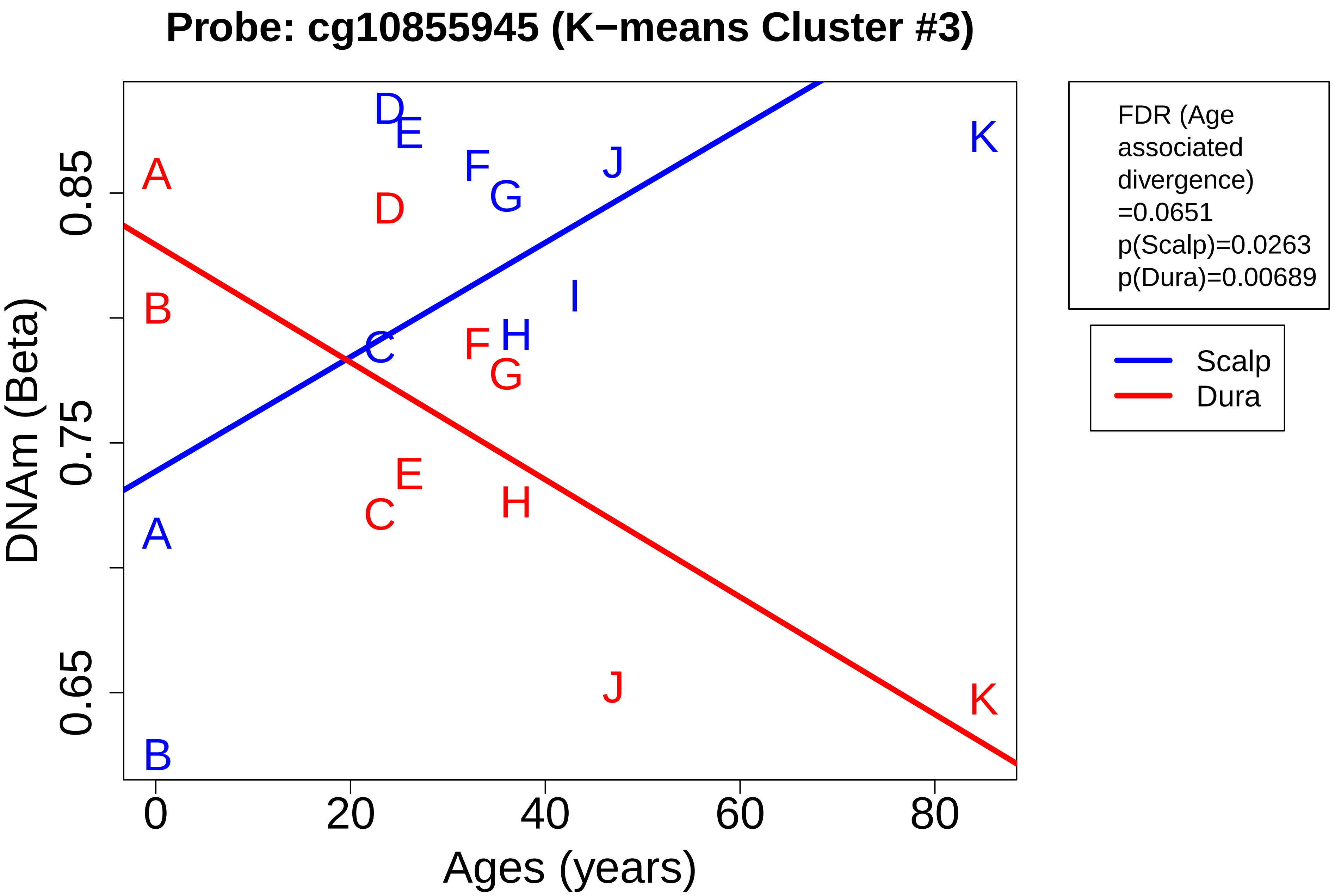
Probe: cg10855945 (K−means Cluster #3)
DNAm (Beta)
0.65
0.75
0.85
Ages (years)
0
20
40
60
80
FDR (Age associated divergence) =0.0651
p(Scalp)=0.0263
p(Dura)=0.00689
Scalp
Dura
A
B
C
D
E
F
G
H
I
J
K

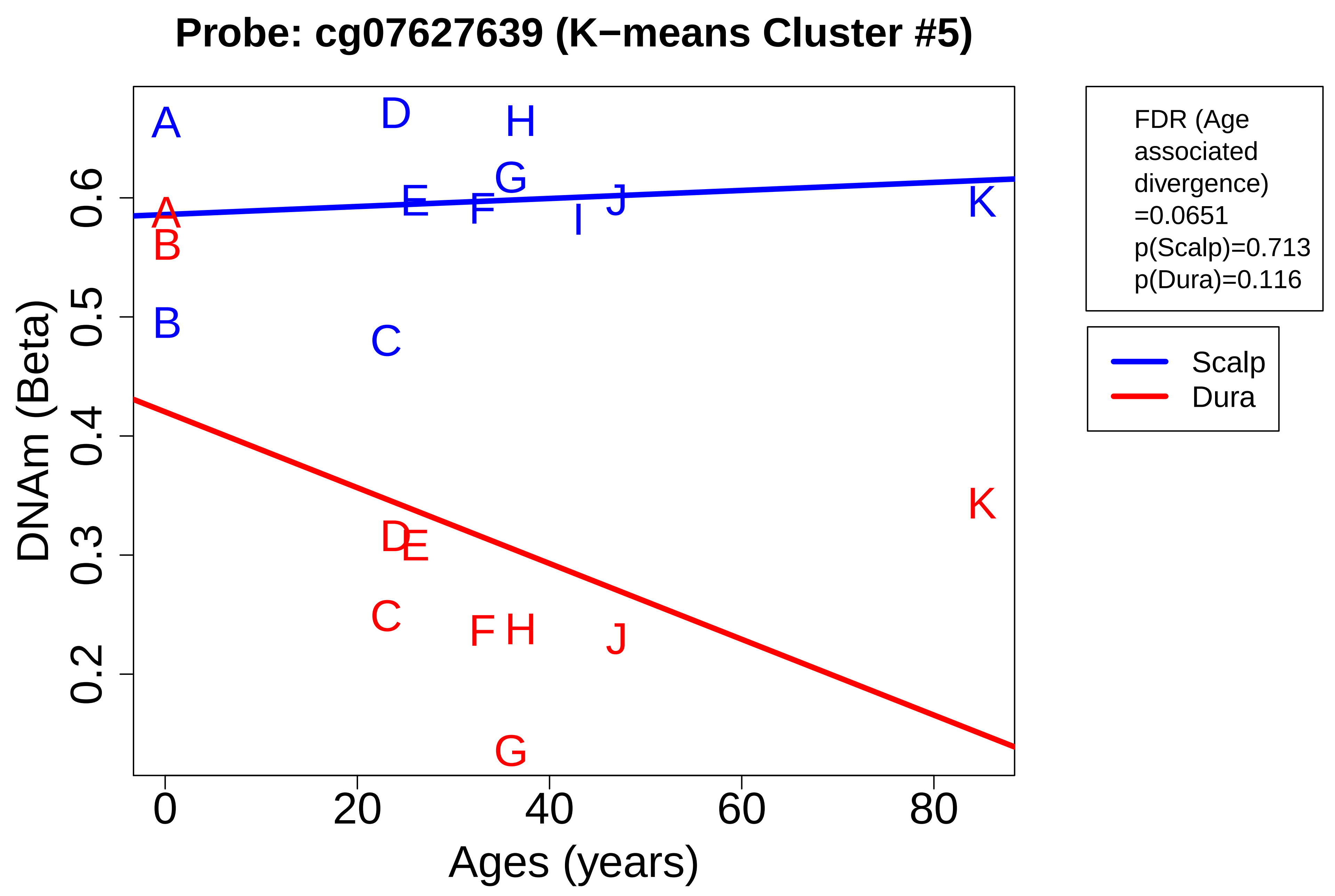
Probe: cg07627639 (K−means Cluster #5)
DNAm (Beta)
0.2
0.3
0.4
0.5
0.6
0
20
40
60
80
Ages (years)
A
B
C
D
E
F
G
H
I
J
K
FDR (Age
associated
divergence)
=0.0651
p(Scalp)=0.713
p(Dura)=0.116
Scalp
Dura

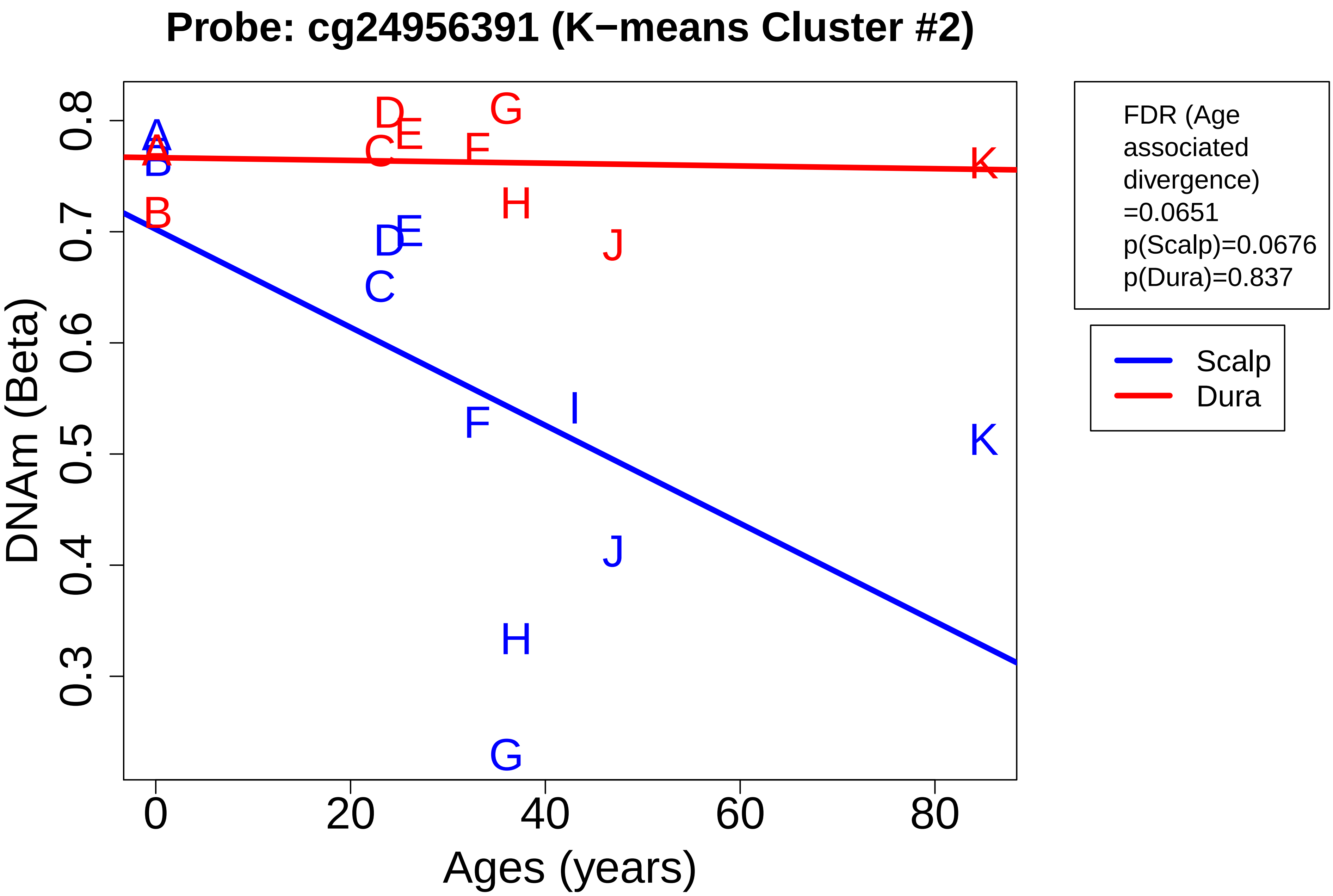

Probe: cg24956391 (K−means Cluster #2)
DNAm (Beta)
0.3
0.4
0.5
0.6
0.7
0.8
0
20
40
60
80
Ages (years)
FDR (Age associated divergence) =0.0651
p(Scalp)=0.0676
p(Dura)=0.837
Scalp
Dura

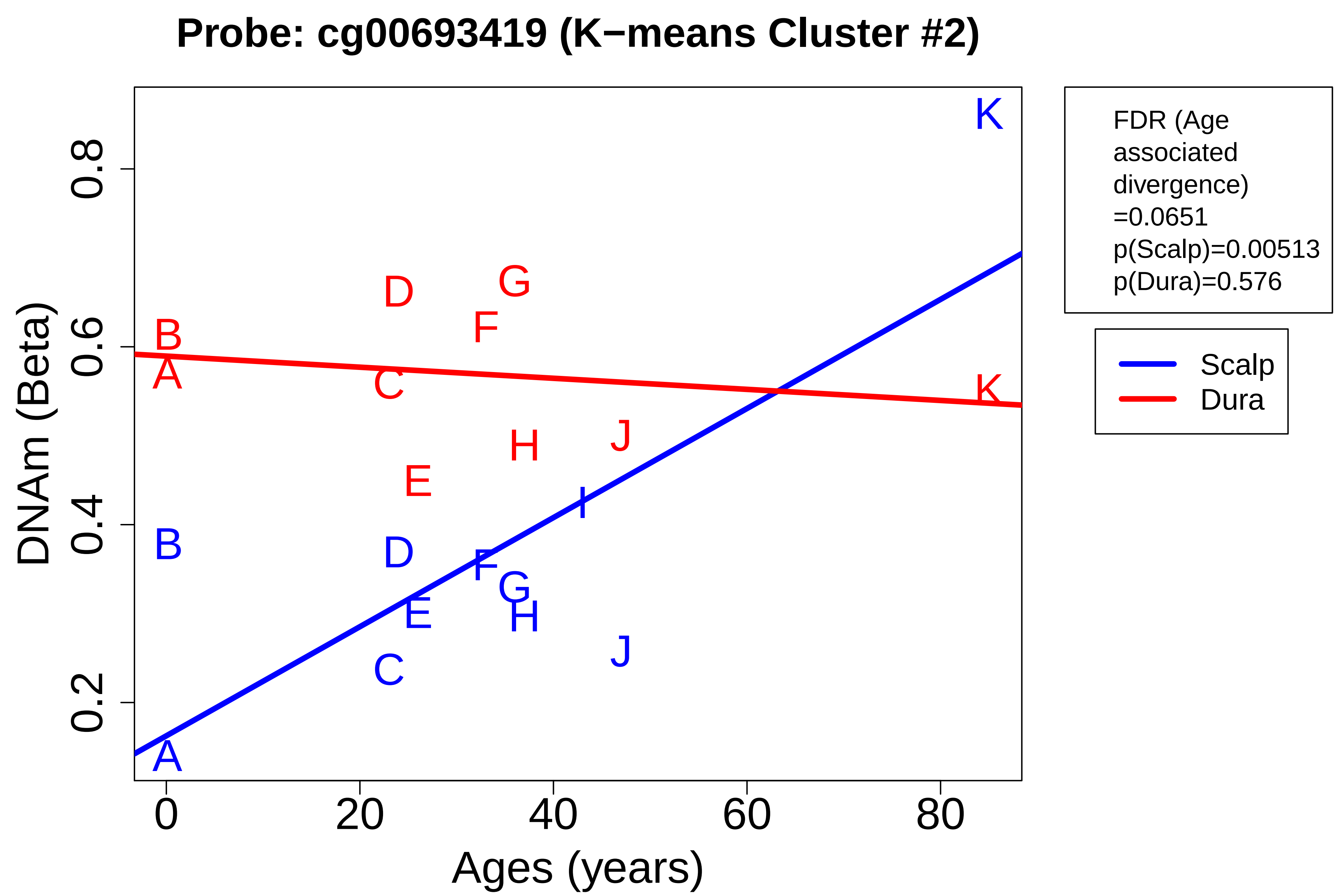

Probe: cg00693419 (K−means Cluster #2)
DNAm (Beta)
0.2
0.4
0.6
0.8
Ages (years)
0
20
40
60
80
A
B
C
D
E
F
G
H
I
J
K
FDR (Age associated divergence) =0.0651
p(Scalp)=0.00513
p(Dura)=0.576
Scalp
Dura

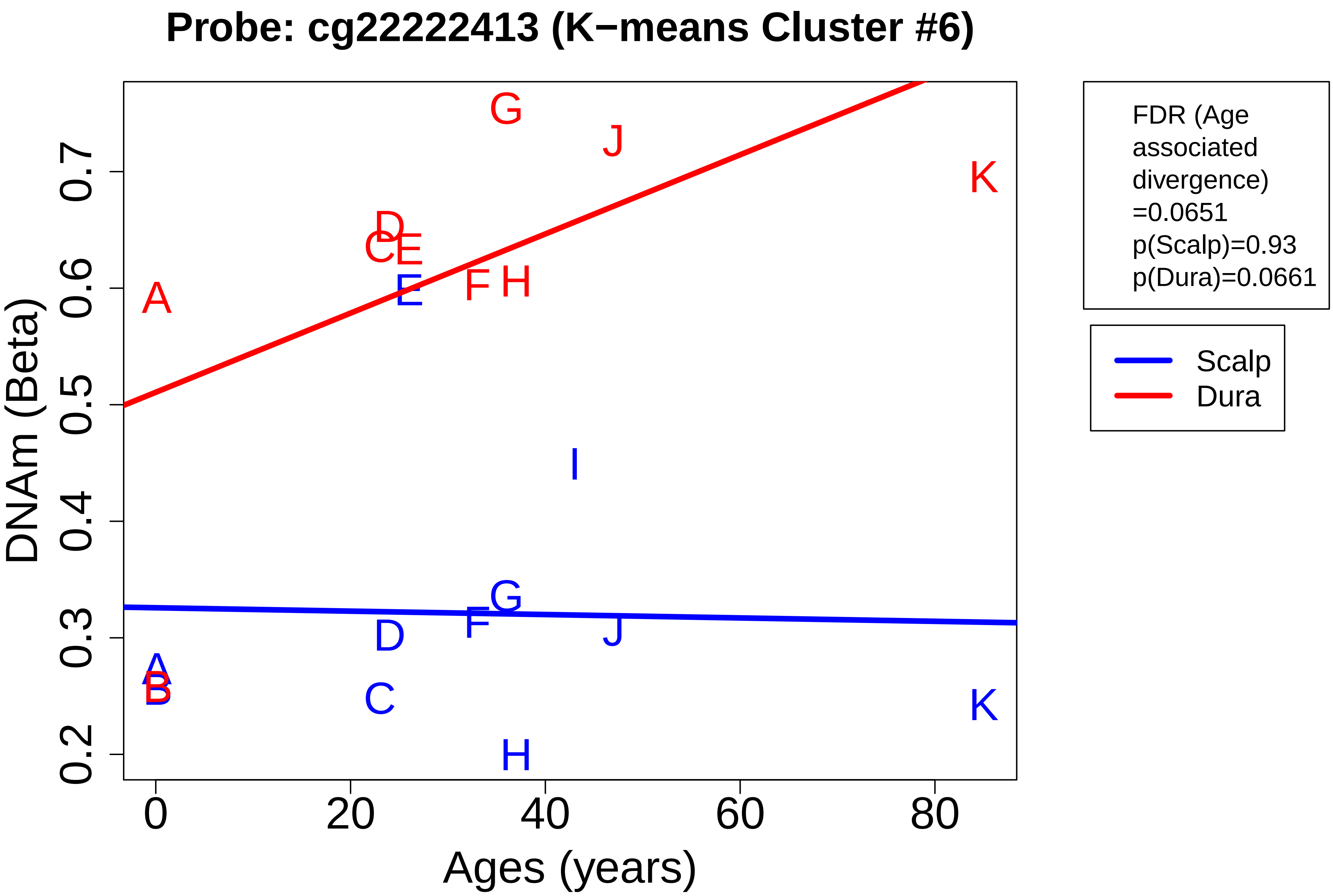

Probe: cg22222413 (K−means Cluster #6)
DNAm (Beta)
Ages (years)
0.2
0.3
0.4
0.5
0.6
0.7
0
20
40
60
80
FDR (Age associated divergence) =0.0651
p(Scalp)=0.93
p(Dura)=0.0661
Scalp
Dura

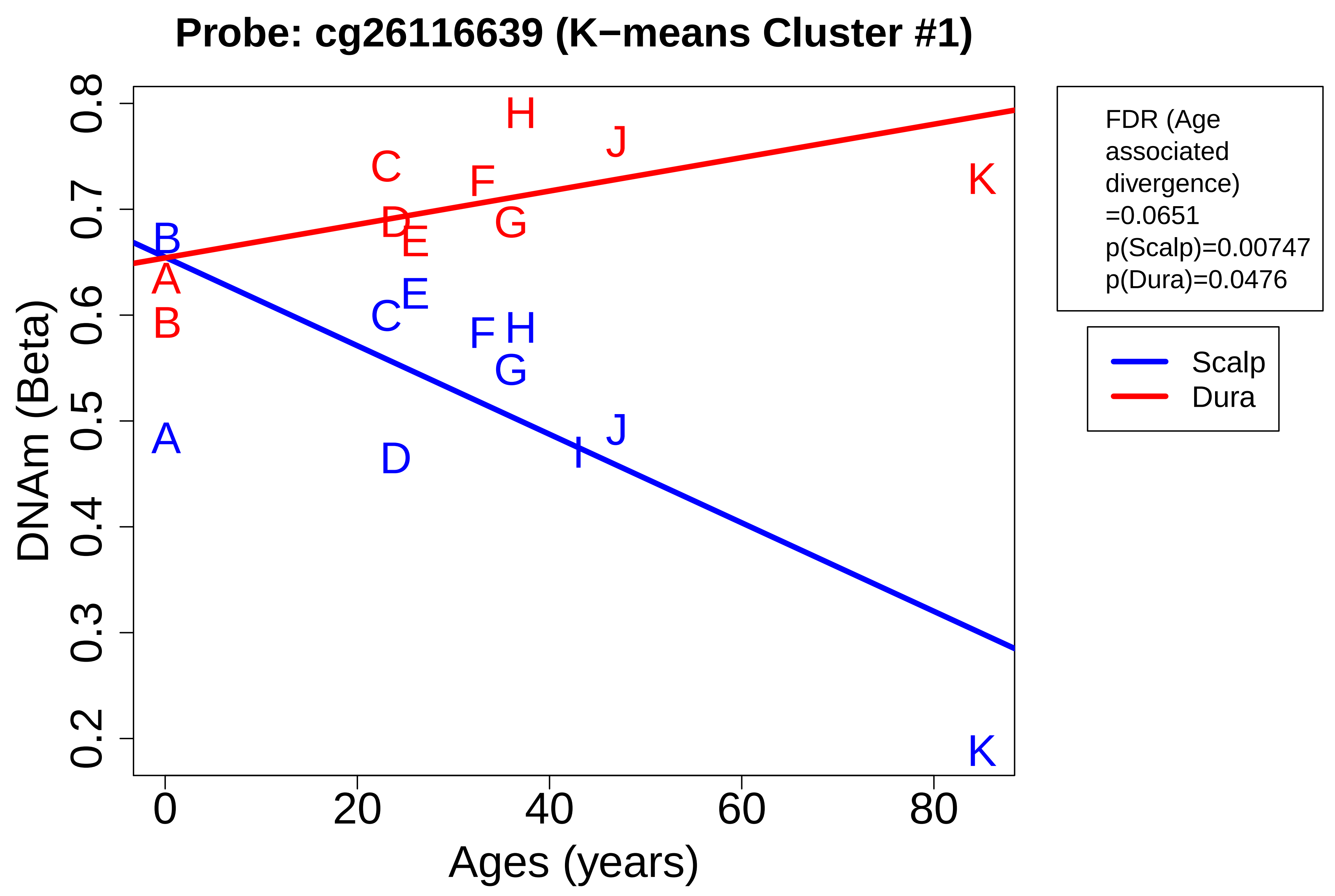
Probe: cg26116639 (K−means Cluster #1)
DNAm (Beta)
0.2
0.3
0.4
0.5
0.6
0.7
0.8
Ages (years)
0
20
40
60
80
A
B
C
D
E
F
G
H
I
J
K
FDR (Age associated divergence) =0.0651
p(Scalp)=0.00747
p(Dura)=0.0476
Scalp
Dura

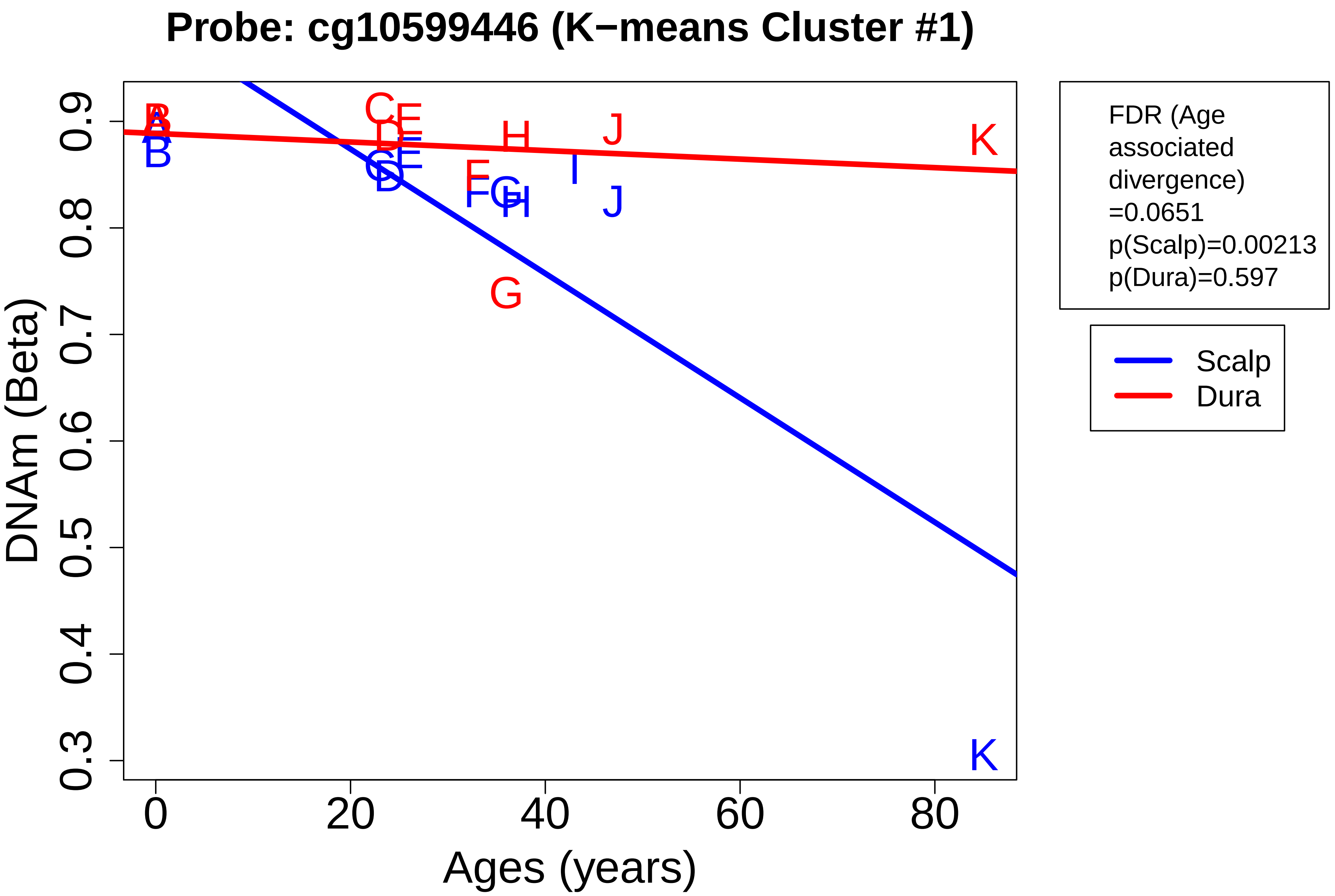

Probe: cg10599446 (K−means Cluster #1)
FDR (Age associated divergence) =0.0651
p(Scalp)=0.00213
p(Dura)=0.597
Scalp
Dura
DNAm (Beta)
Ages (years)
0.3
0.4
0.5
0.6
0.7
0.8
0.9
0
20
40
60
80

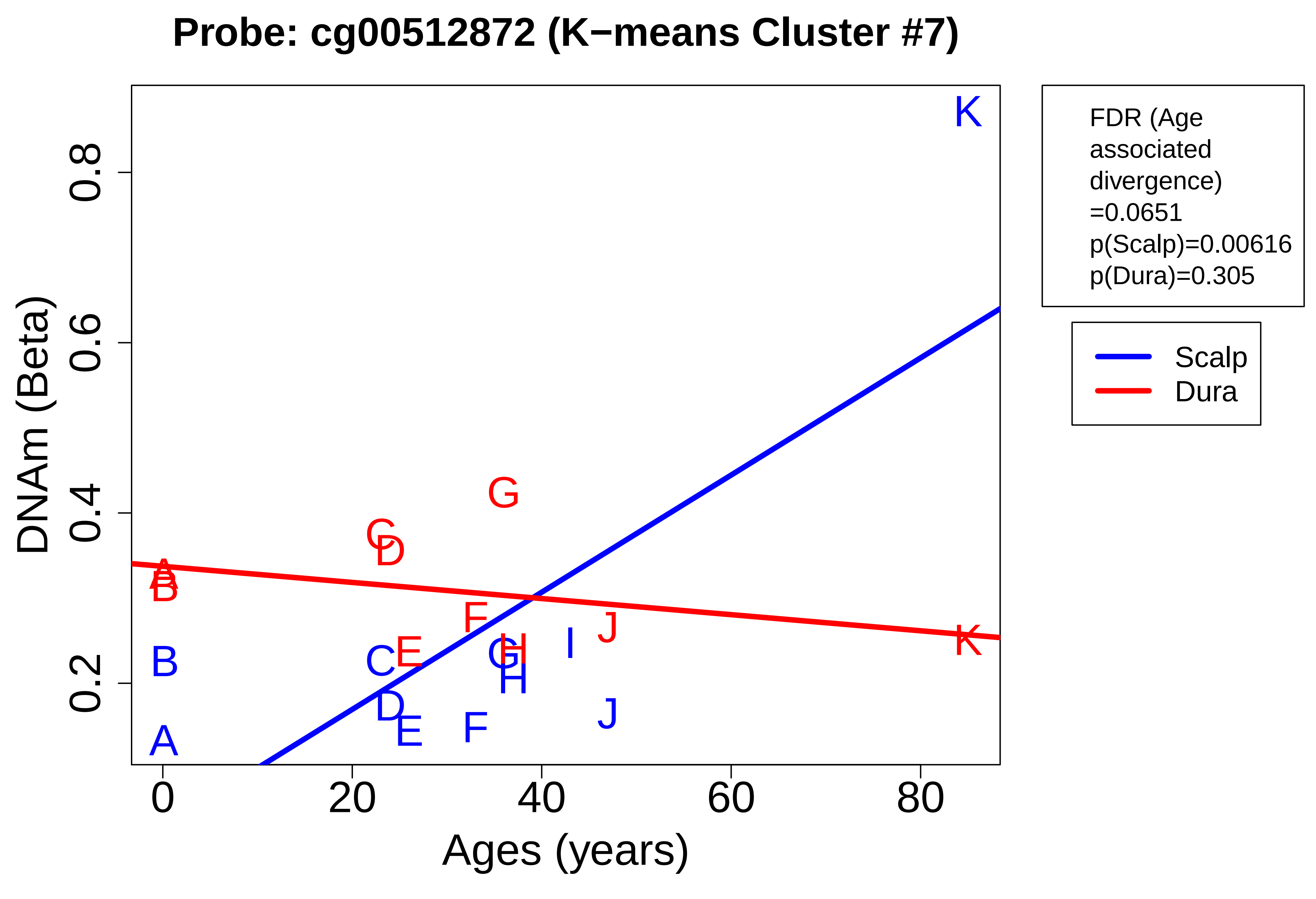
Probe: cg00512872 (K−means Cluster #7)
DNAm (Beta)
0.2
0.4
0.6
0.8
0
20
40
60
80
Ages (years)
FDR (Age associated divergence) =0.0651
p(Scalp)=0.00616
p(Dura)=0.305
Scalp
Dura
A
B
C
D
E
F
G
H
I
J
K

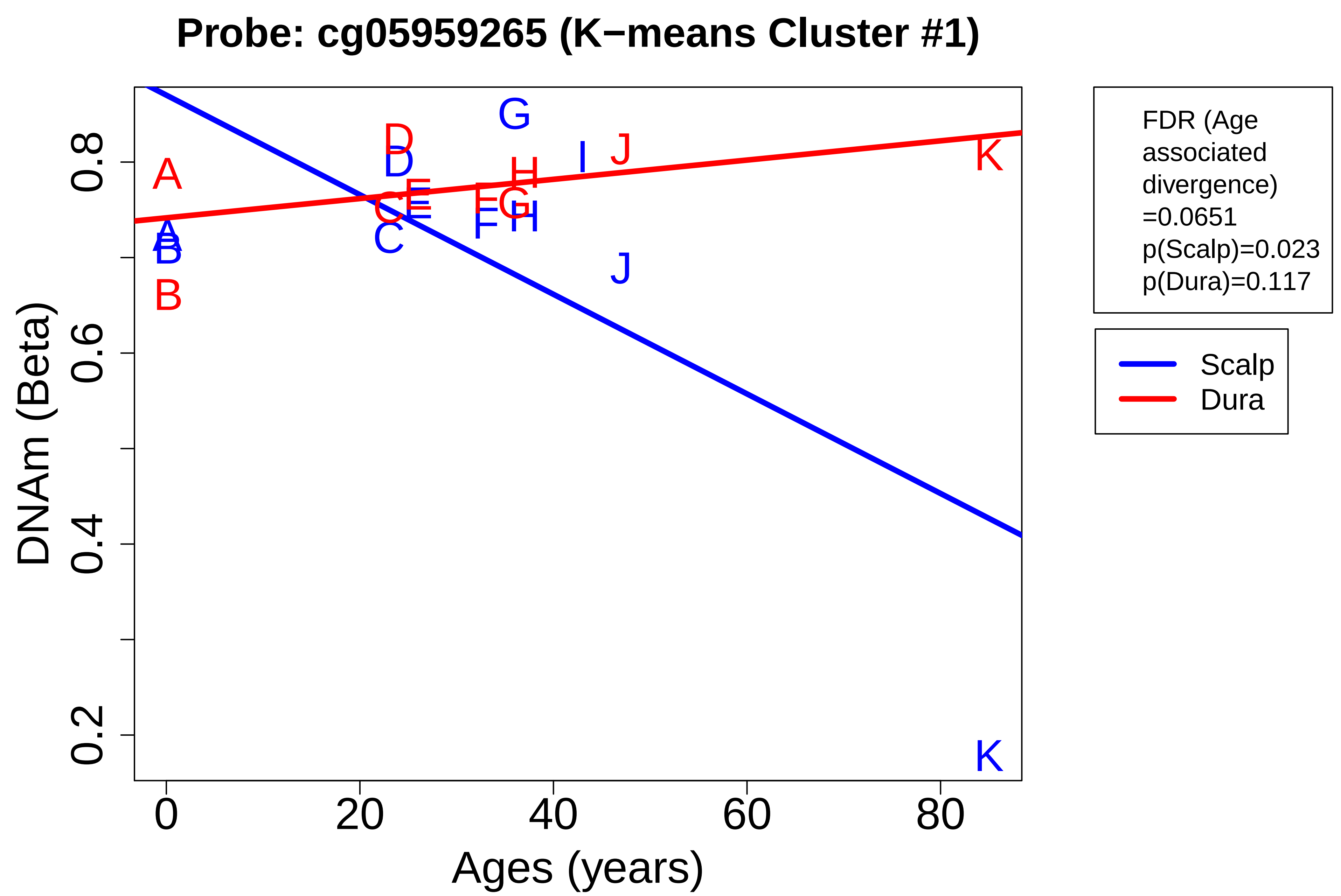

Probe: cg05959265 (K−means Cluster #1)
DNAm (Beta)
0.2
0.4
0.6
0.8
0
20
40
60
80
Ages (years)
A
B
C
D
E
F
G
H
I
J
K
FDR (Age associated divergence) =0.0651
p(Scalp)=0.023
p(Dura)=0.117
Scalp
Dura

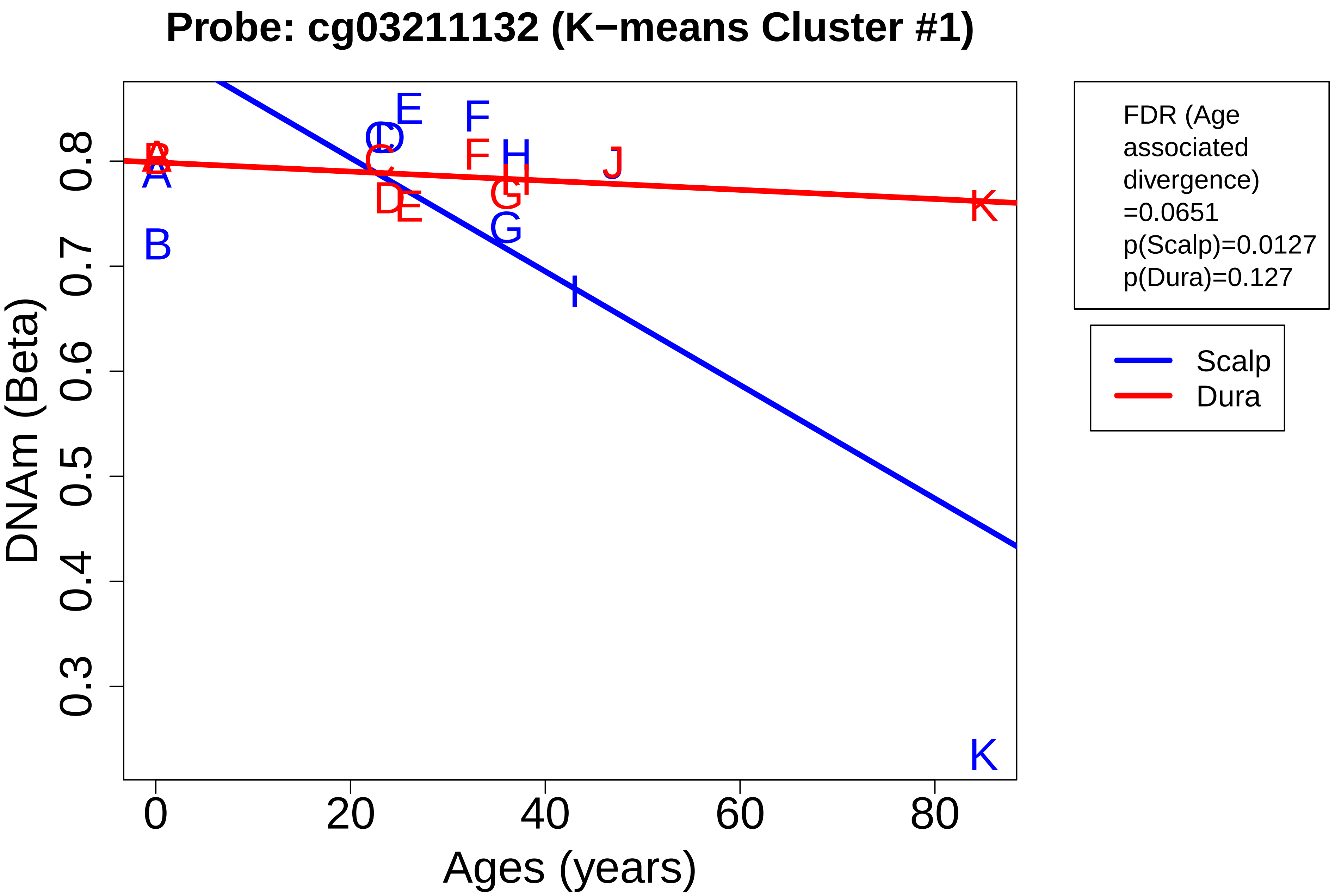

Probe: cg03211132 (K−means Cluster #1)
DNAm (Beta)
0.3
0.4
0.5
0.6
0.7
0.8
Ages (years)
0
20
40
60
80
A
B
C
D
E
F
G
H
I
J
K
FDR (Age associated divergence) =0.0651
p(Scalp)=0.0127
p(Dura)=0.127
Scalp
Dura

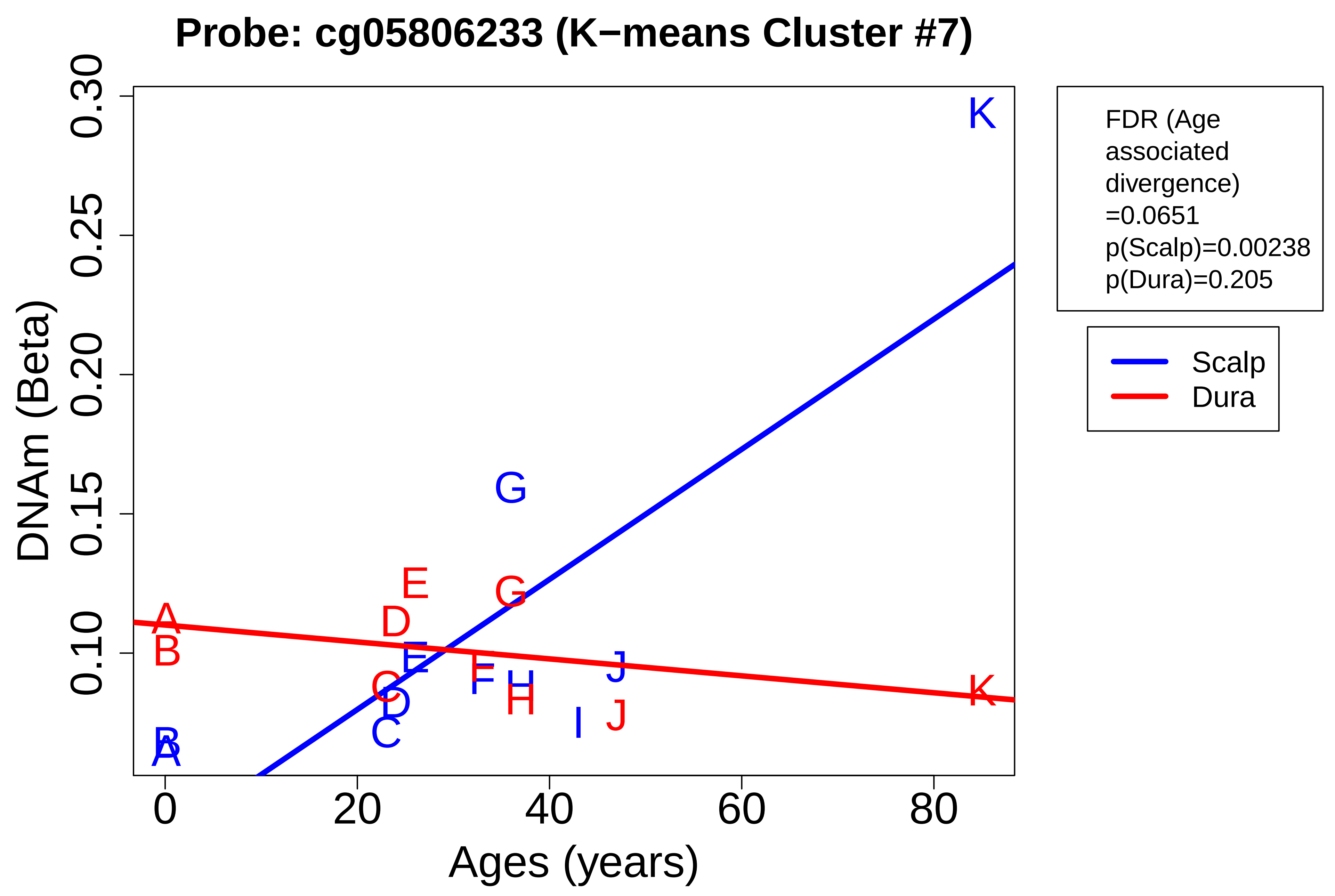
Probe: cg05806233 (K−means Cluster #7)
DNAm (Beta)
0.10
0.15
0.20
0.25
0.30
Ages (years)
0
20
40
60
80
FDR (Age associated divergence) =0.0651
p(Scalp)=0.00238
p(Dura)=0.205
Scalp
Dura
A
B
C
D
E
F
G
H
I
J
K

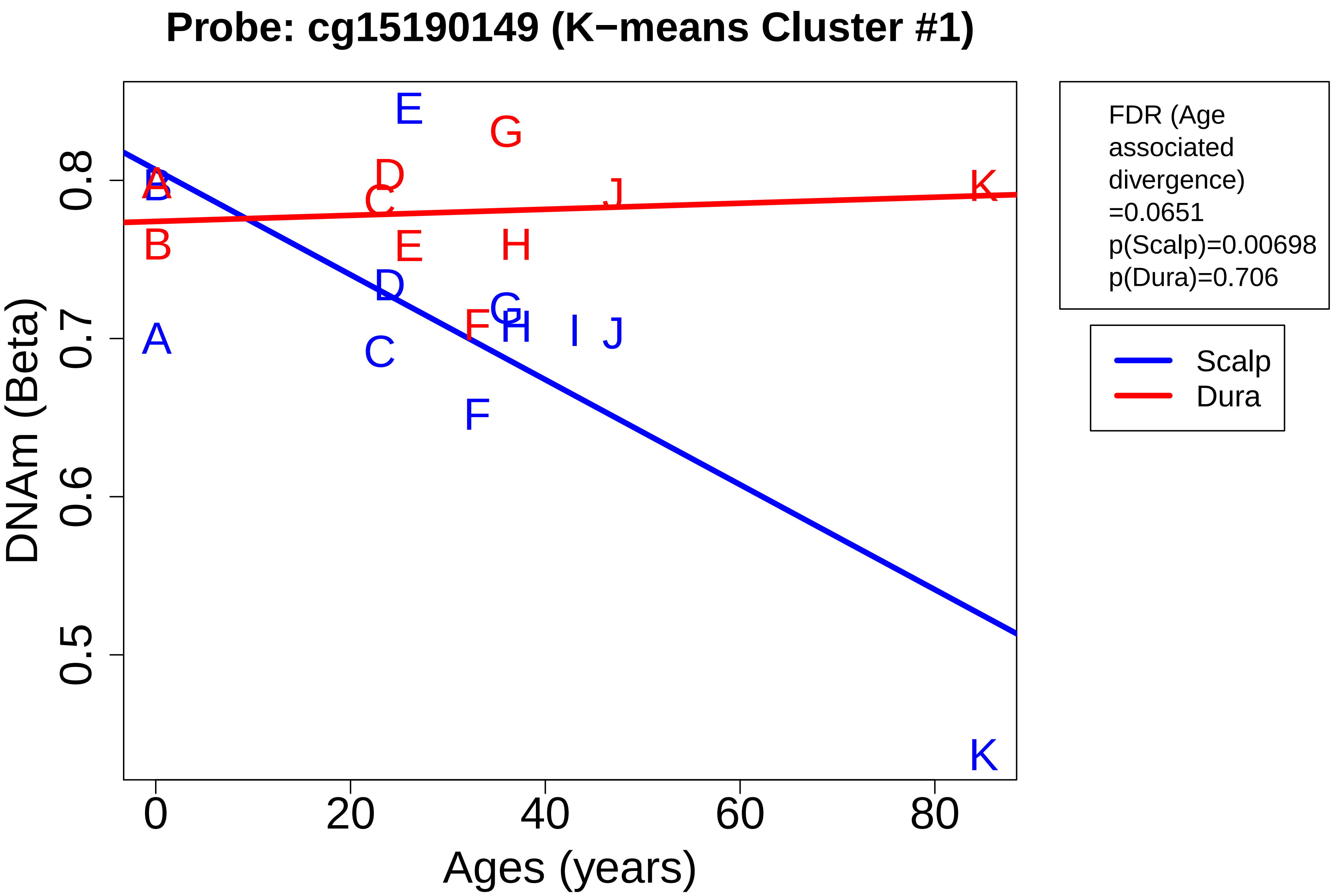

Probe: cg15190149 (K−means Cluster #1)
DNAm (Beta)
0.5
0.6
0.7
0.8
0
20
40
60
80
Ages (years)
A
B
C
D
E
F
G
H
I
J
K
FDR (Age associated divergence) =0.0651
p(Scalp)=0.00698
p(Dura)=0.706
Scalp
Dura

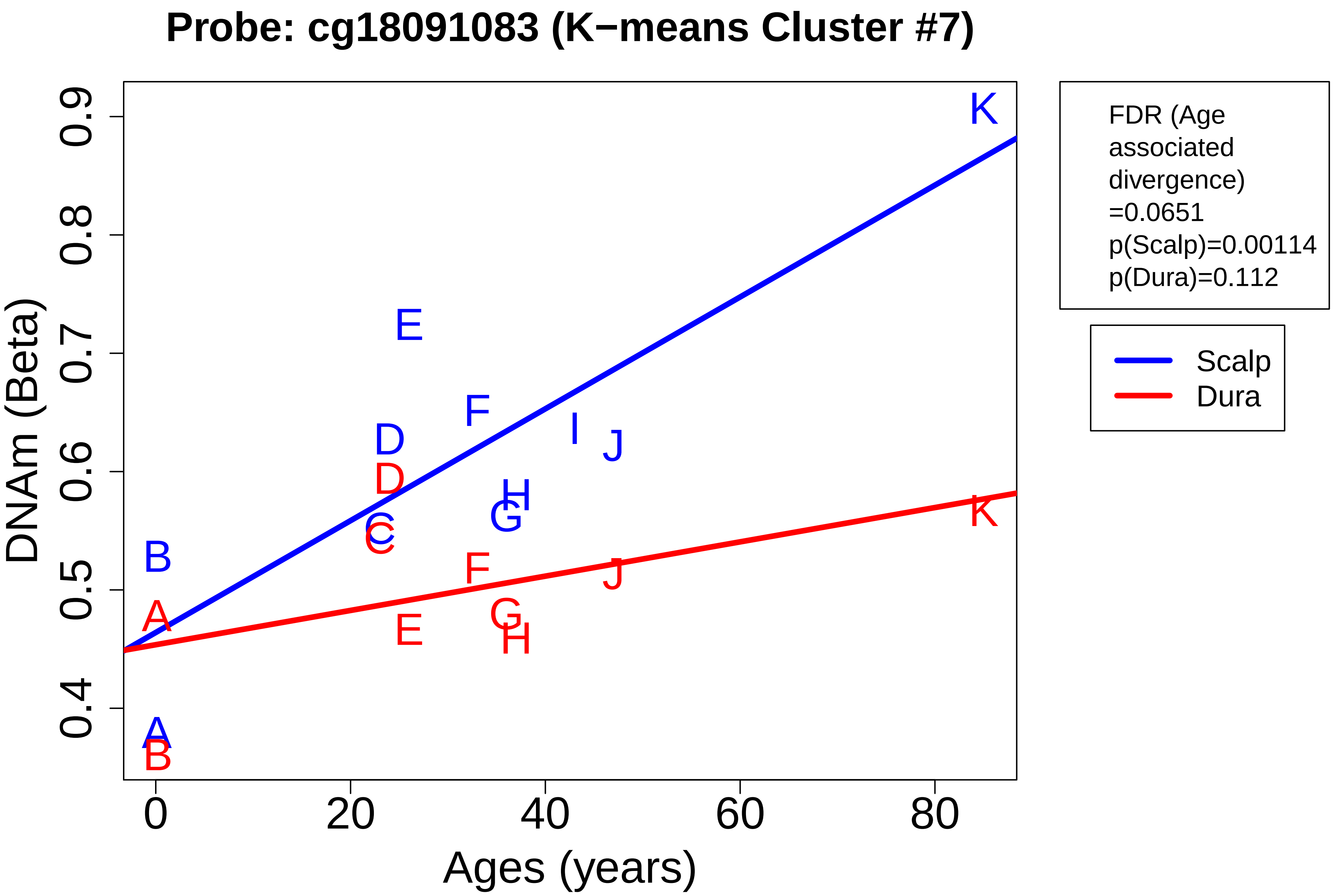
Probe: cg18091083 (K−means Cluster #7)
DNAm (Beta)
0.4
0.5
0.6
0.7
0.8
0.9
Ages (years)
0
20
40
60
80
A
B
C
D
E
F
G
H
I
J
K
FDR (Age associated divergence) =0.0651
p(Scalp)=0.00114
p(Dura)=0.112
Scalp
Dura

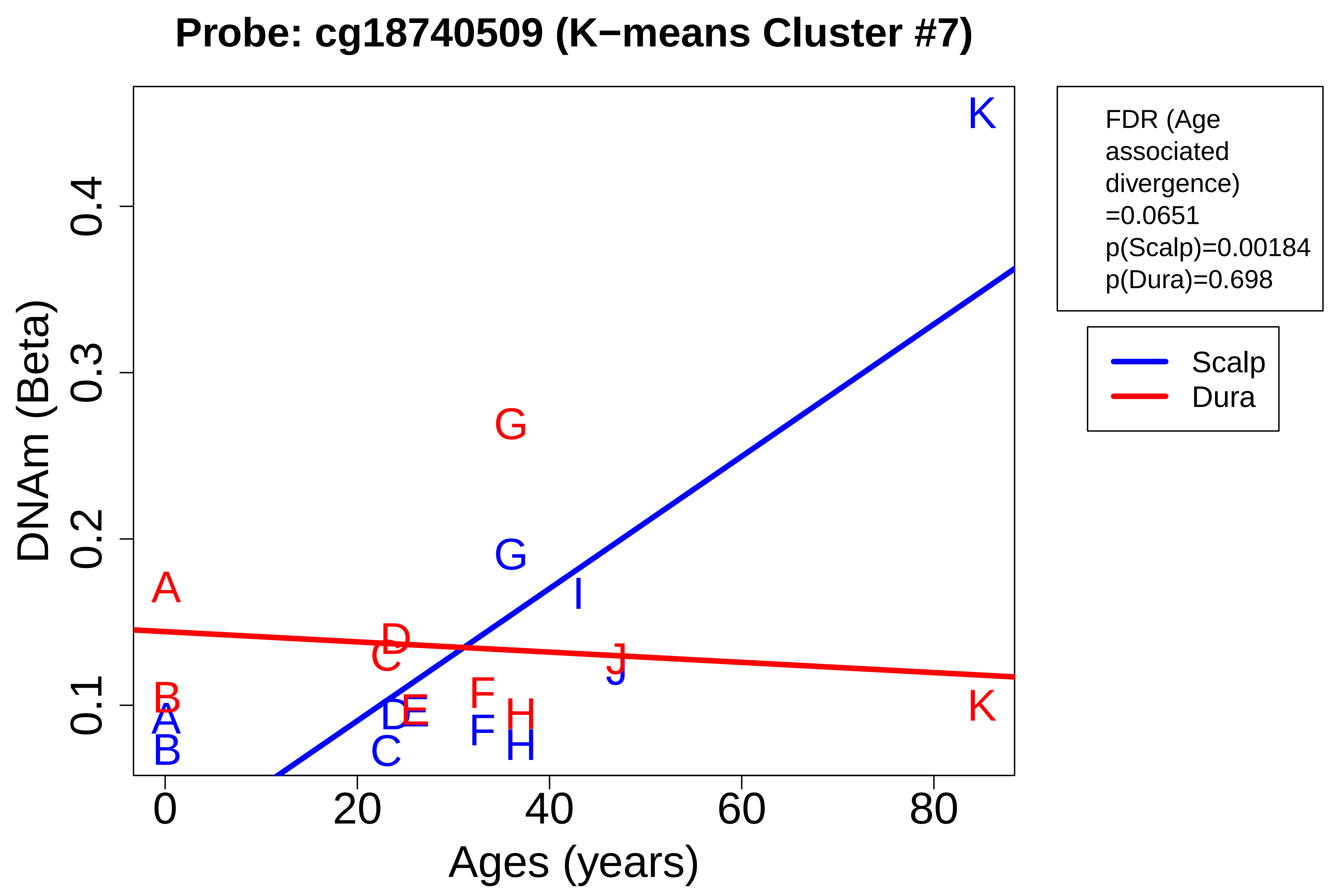

Probe: cg18740509 (K−means Cluster #7)
FDR (Age associated divergence) =0.0651
p(Scalp)=0.00184
p(Dura)=0.698
Scalp
Dura
DNAm (Beta)
0.1
0.2
0.3
0.4
0
20
40
60
80
Ages (years)
A
B
C
D
E
F
G
H
I
J
K

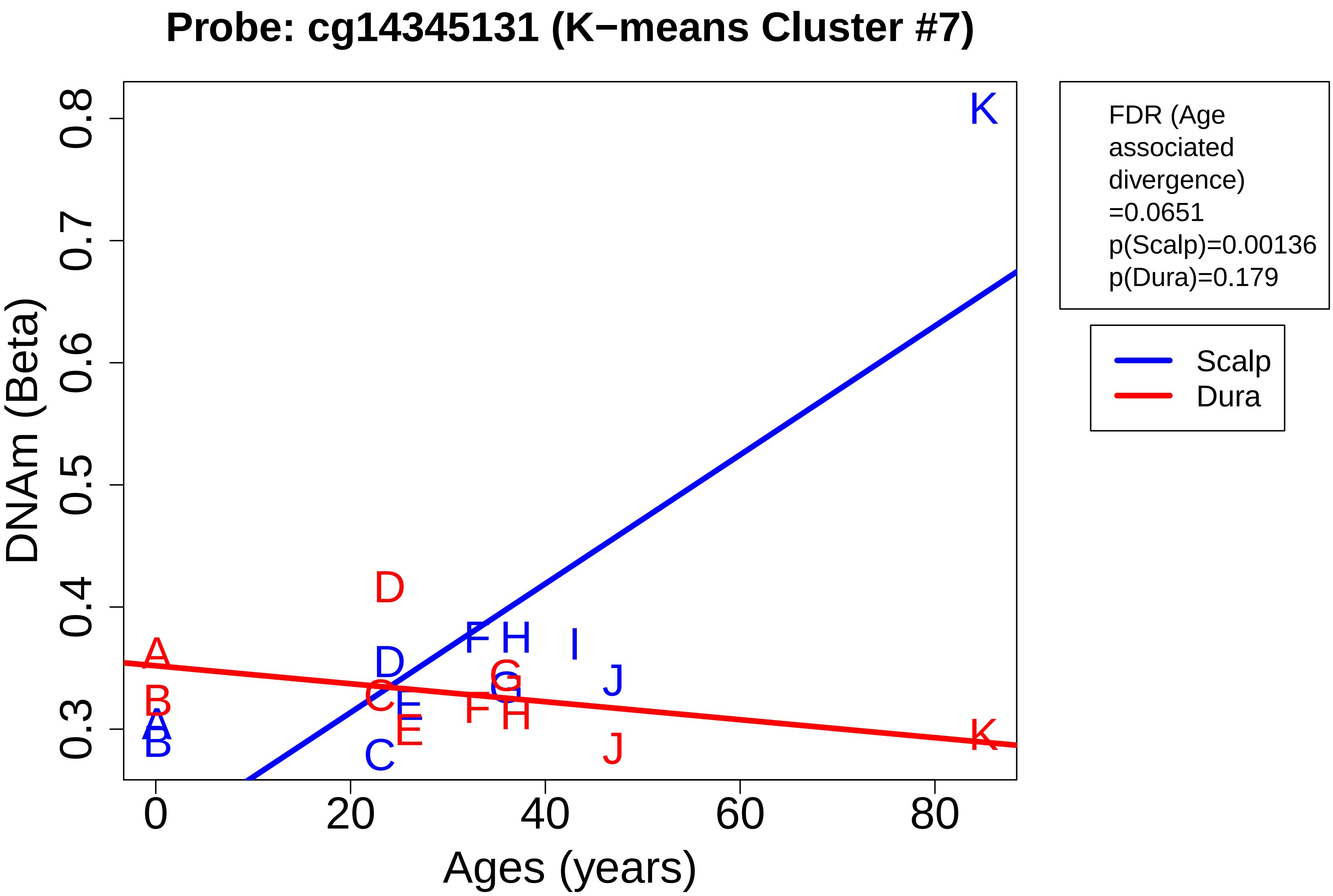
Probe: cg14345131 (K−means Cluster #7)
DNAm (Beta)
0.3
0.4
0.5
0.6
0.7
0.8
Ages (years)
0
20
40
60
80
A
B
C
D
E
F
G
H
I
J
K
FDR (Age associated divergence) =0.0651
p(Scalp)=0.00136
p(Dura)=0.179
Scalp
Dura

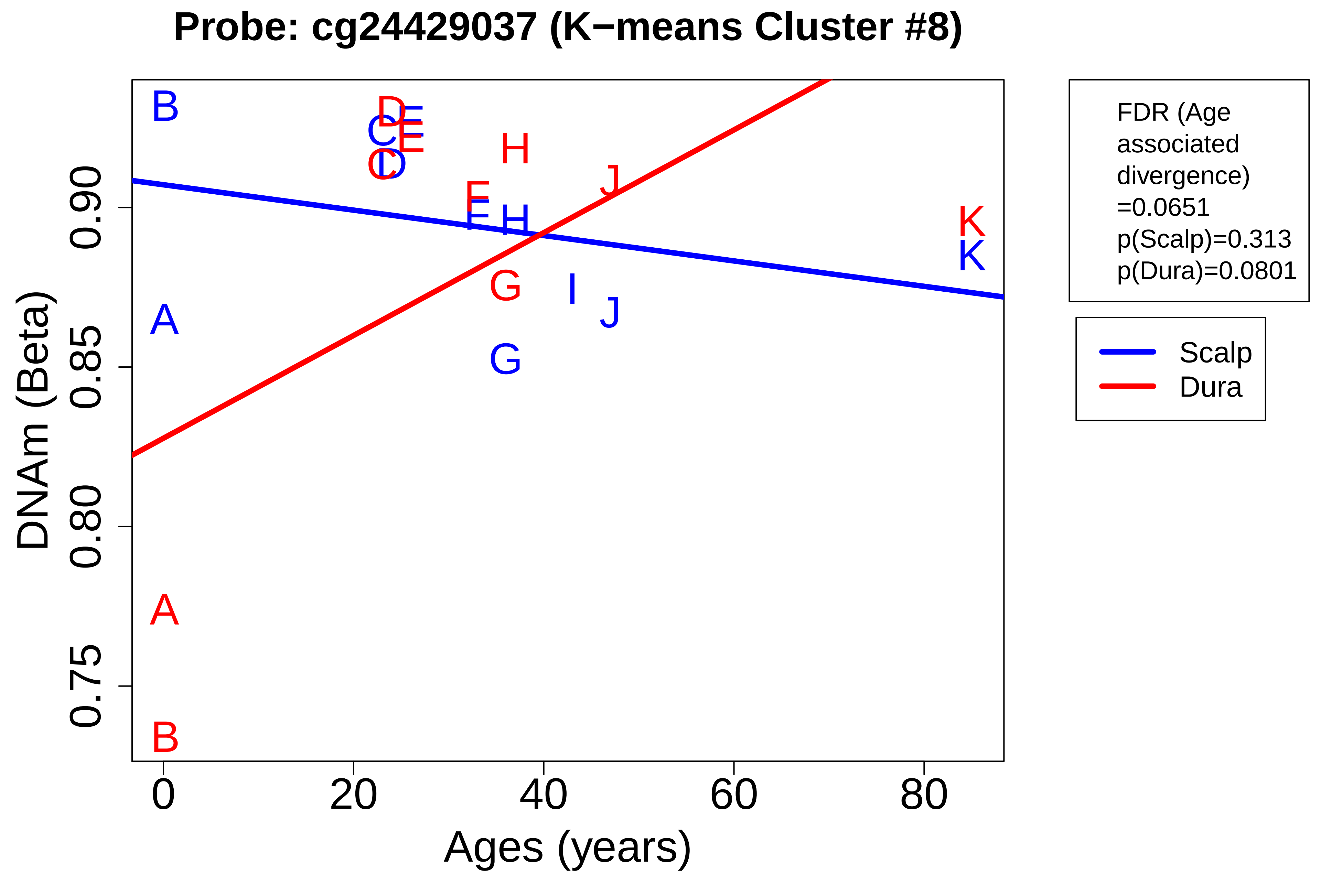

Probe: cg24429037 (K−means Cluster #8)
DNAm (Beta)
0.75
0.80
0.85
0.90
0
20
40
60
80
Ages (years)
A
B
C
D
E
F
G
H
I
J
K
FDR (Age associated divergence) =0.0651
p(Scalp)=0.313
p(Dura)=0.0801
Scalp
Dura

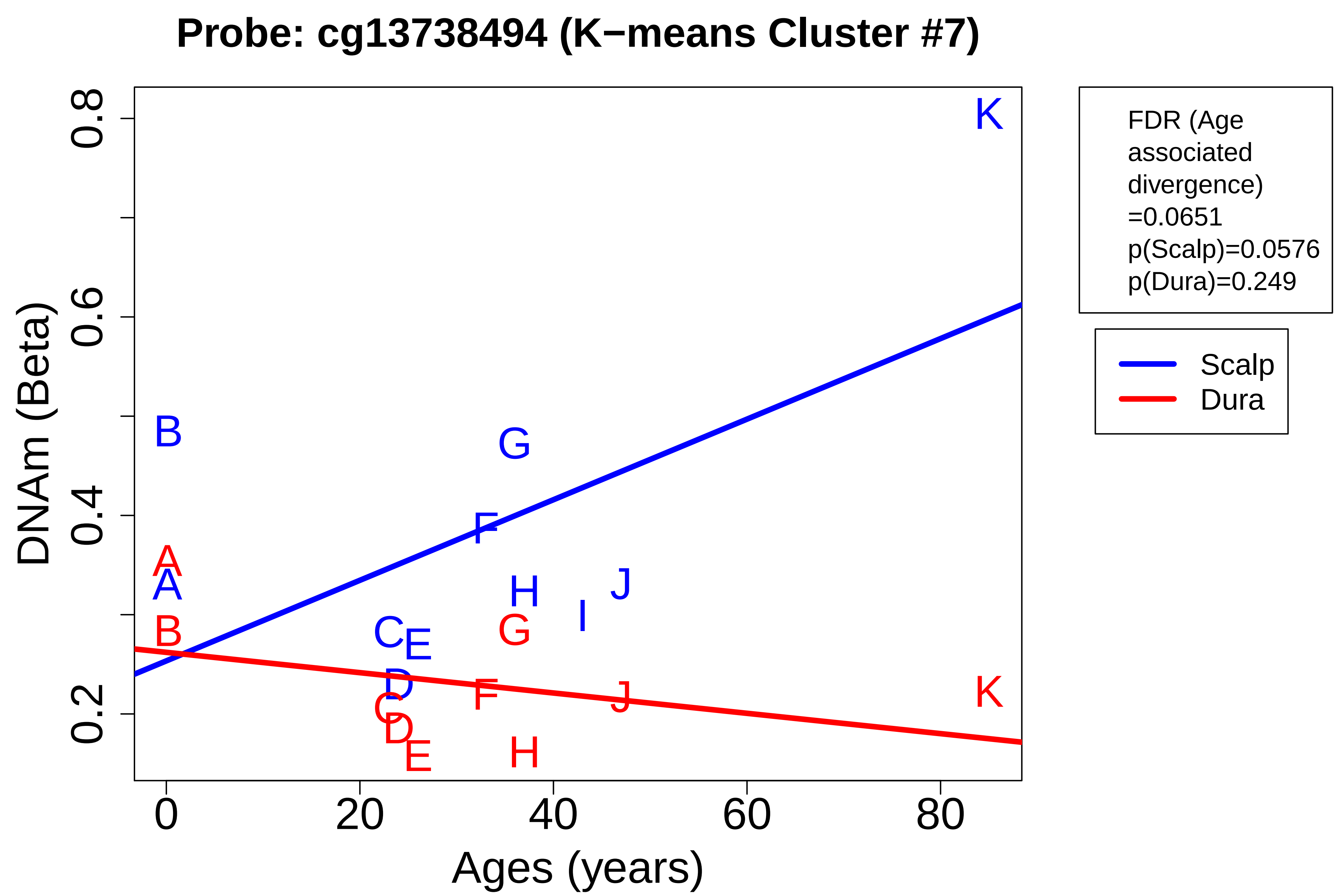

Probe: cg13738494 (K−means Cluster #7)
DNAm (Beta)
0.2
0.4
0.6
0.8
Ages (years)
0
20
40
60
80
FDR (Age associated divergence) =0.0651
p(Scalp)=0.0576
p(Dura)=0.249
Scalp
Dura
A
B
C
D
E
F
G
H
I
J
K

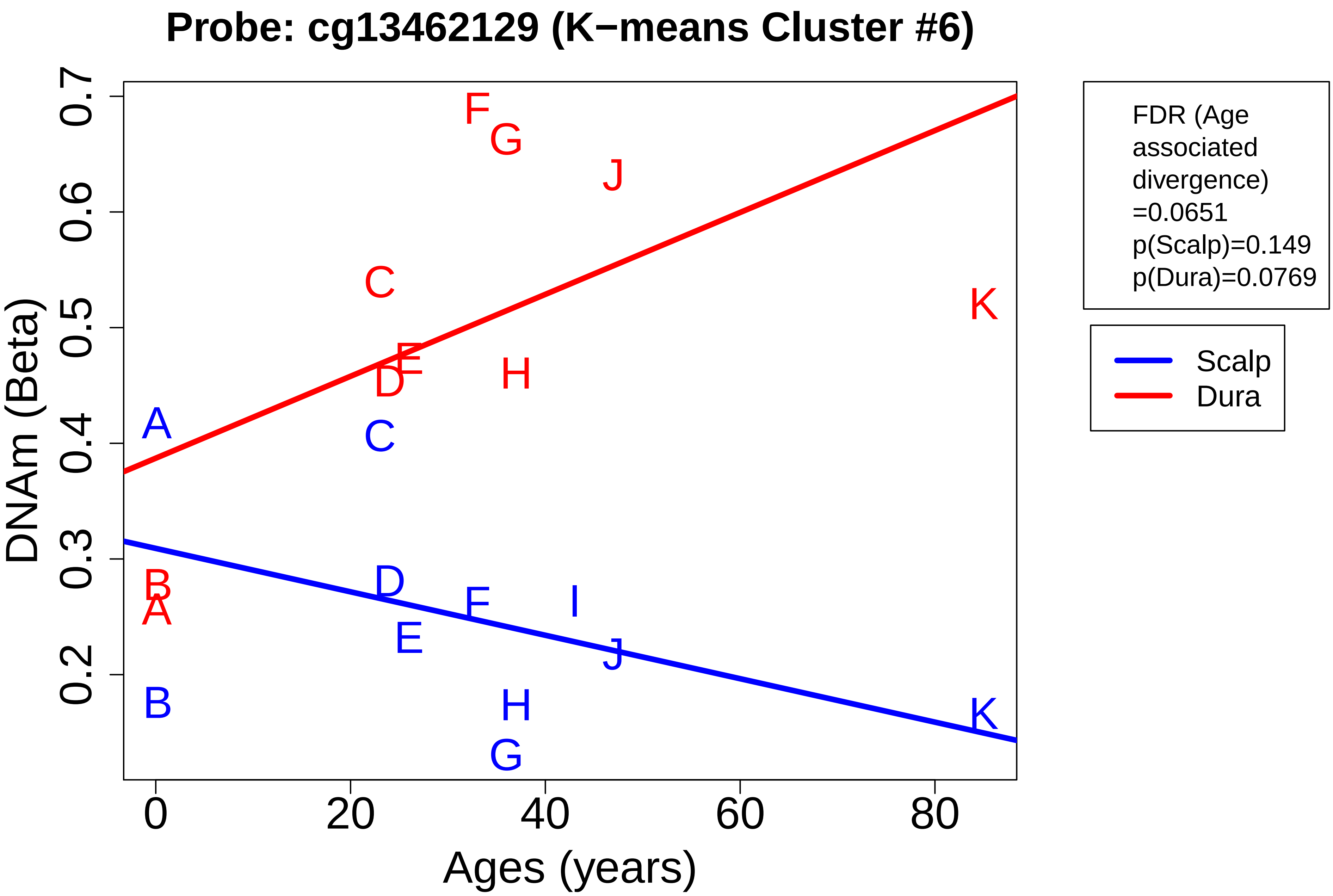
Probe: cg13462129 (K−means Cluster #6)
DNAm (Beta)
Ages (years)
0.2
0.3
0.4
0.5
0.6
0.7
0
20
40
60
80
FDR (Age associated divergence) =0.0651
p(Scalp)=0.149
p(Dura)=0.0769
Scalp
Dura

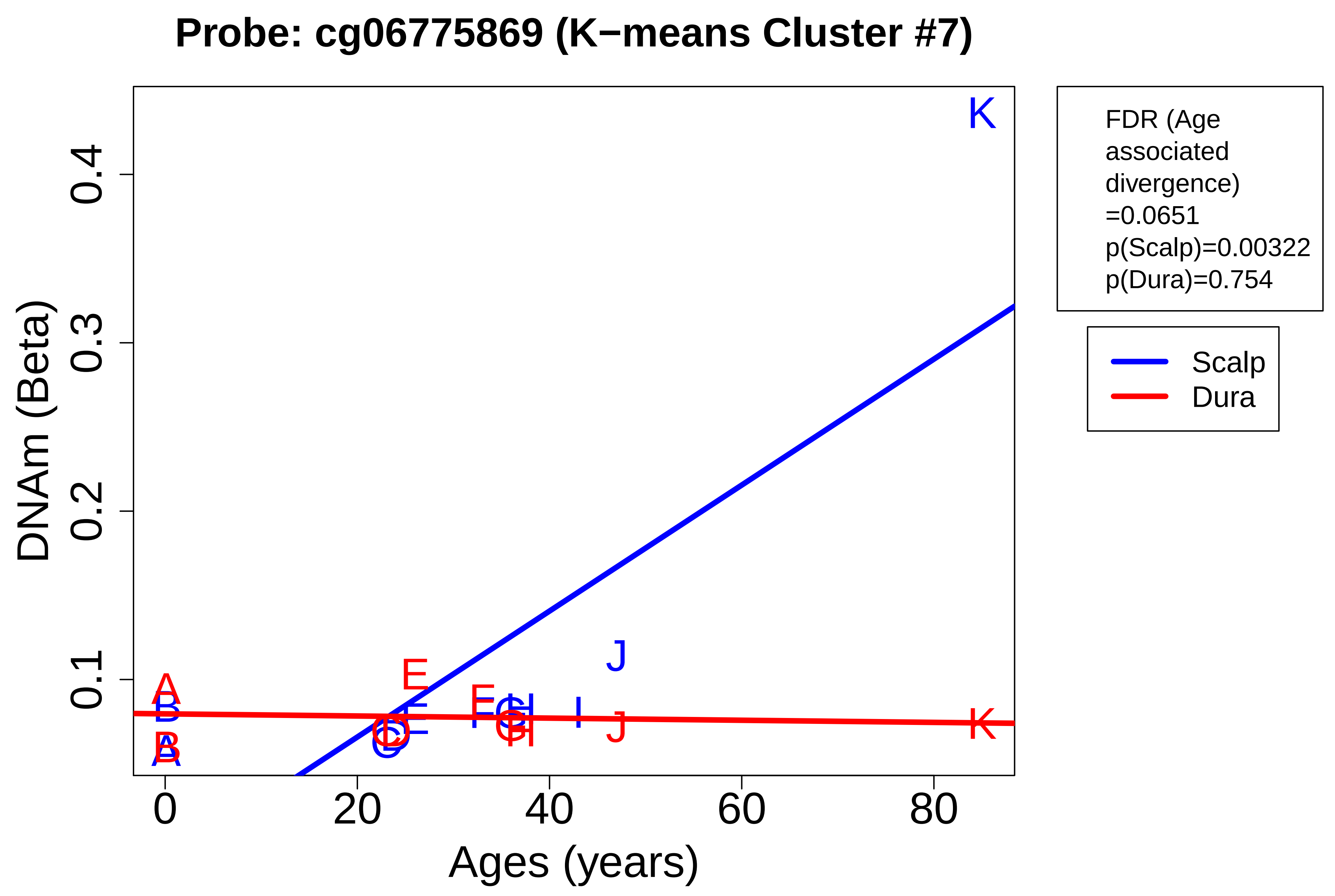
Probe: cg06775869 (K−means Cluster #7)
DNAm (Beta)
0.1
0.2
0.3
0.4
0
20
40
60
80
Ages (years)
FDR (Age associated divergence) =0.0651
p(Scalp)=0.00322
p(Dura)=0.754
Scalp
Dura
A
B
C
D
E
F
G
H
I
J
K

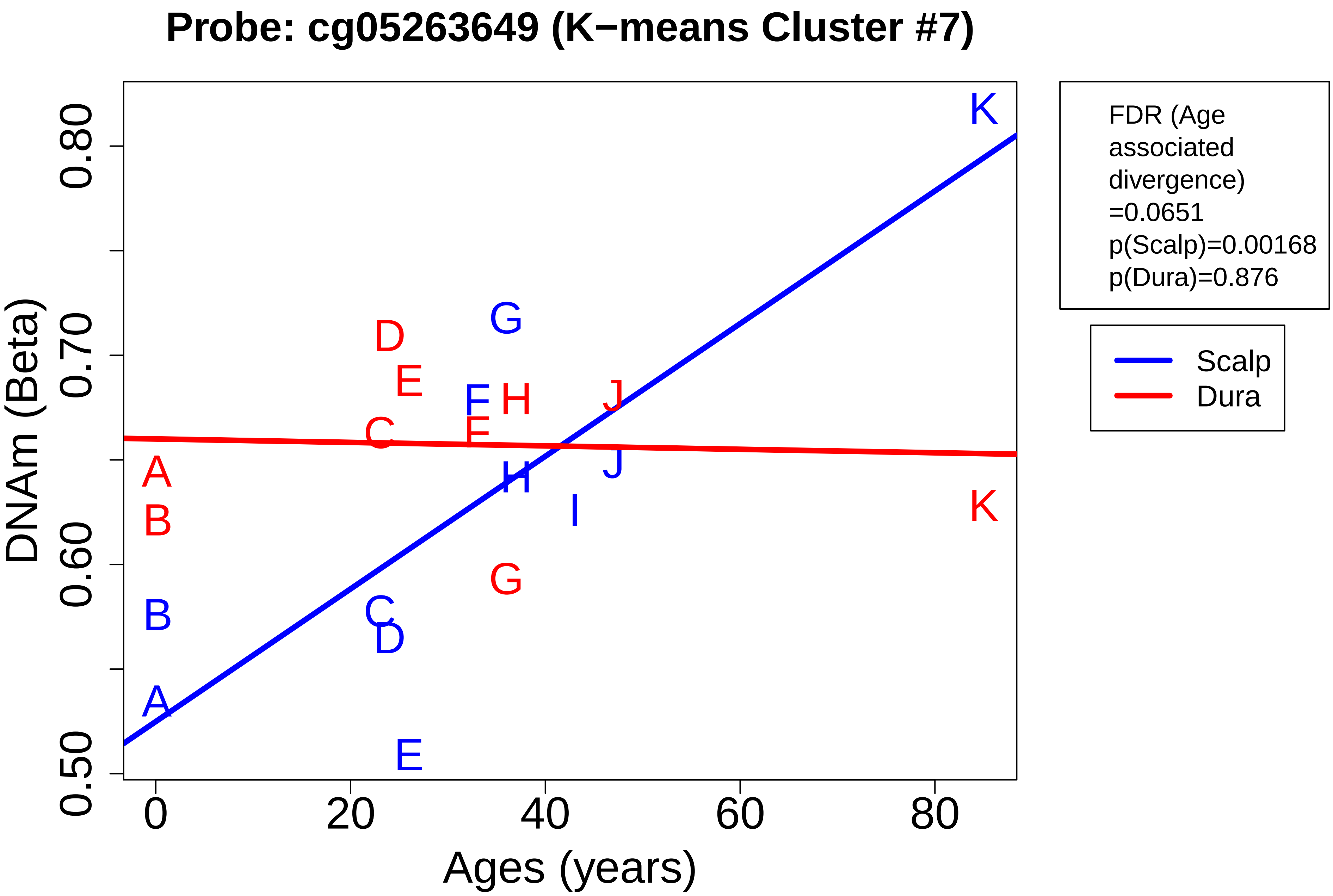
Probe: cg05263649 (K−means Cluster #7)
DNAm (Beta)
0.50
0.60
0.70
0.80
0
20
40
60
80
Ages (years)
FDR (Age associated divergence) =0.0651
p(Scalp)=0.00168
p(Dura)=0.876
Scalp
Dura

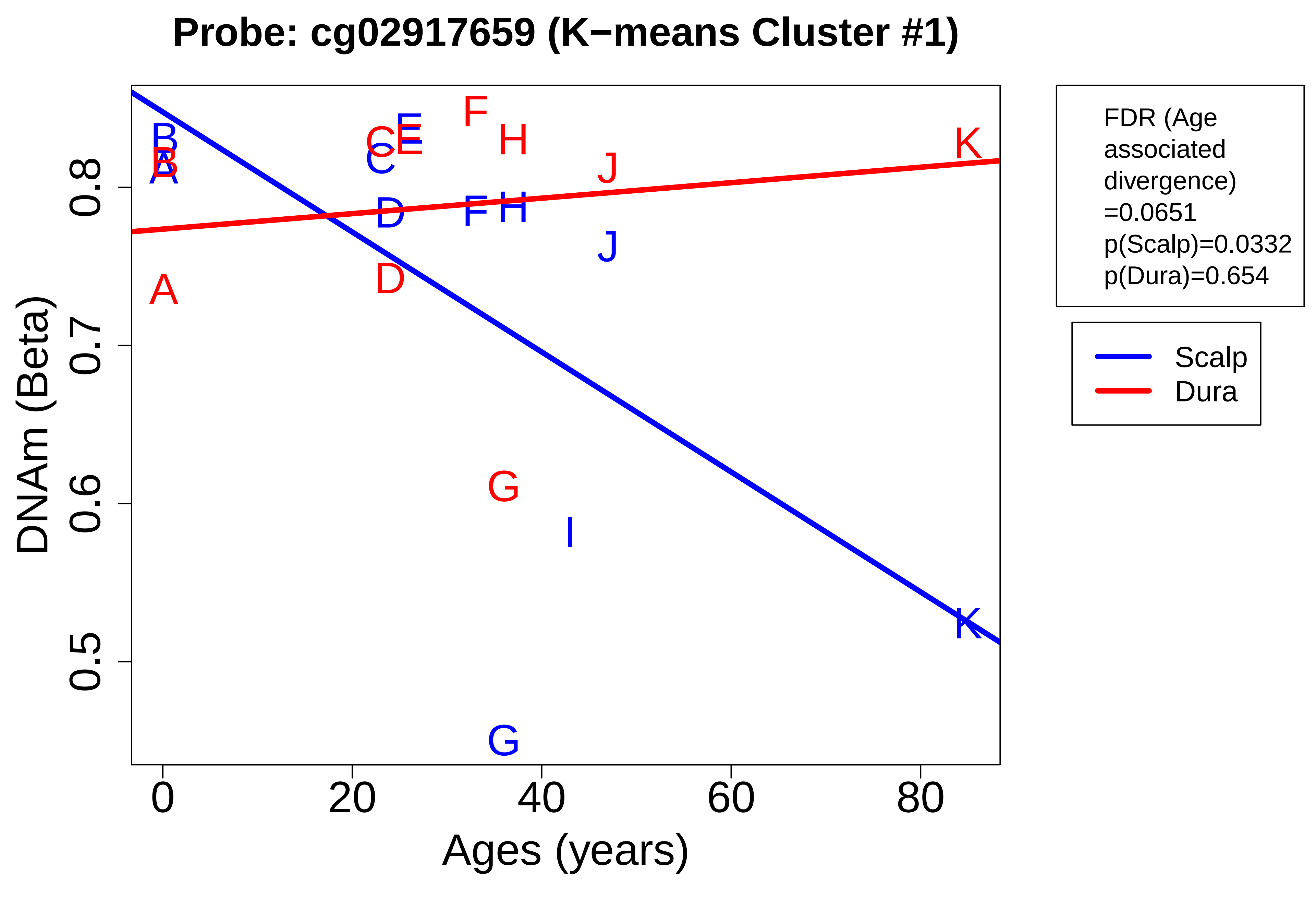

Probe: cg02917659 (K−means Cluster #1)
DNAm (Beta)
0.5
0.6
0.7
0.8
Ages (years)
0
20
40
60
80
A
B
C
D
E
F
G
H
I
J
K
FDR (Age associated divergence) =0.0651
p(Scalp)=0.0332
p(Dura)=0.654
Scalp
Dura

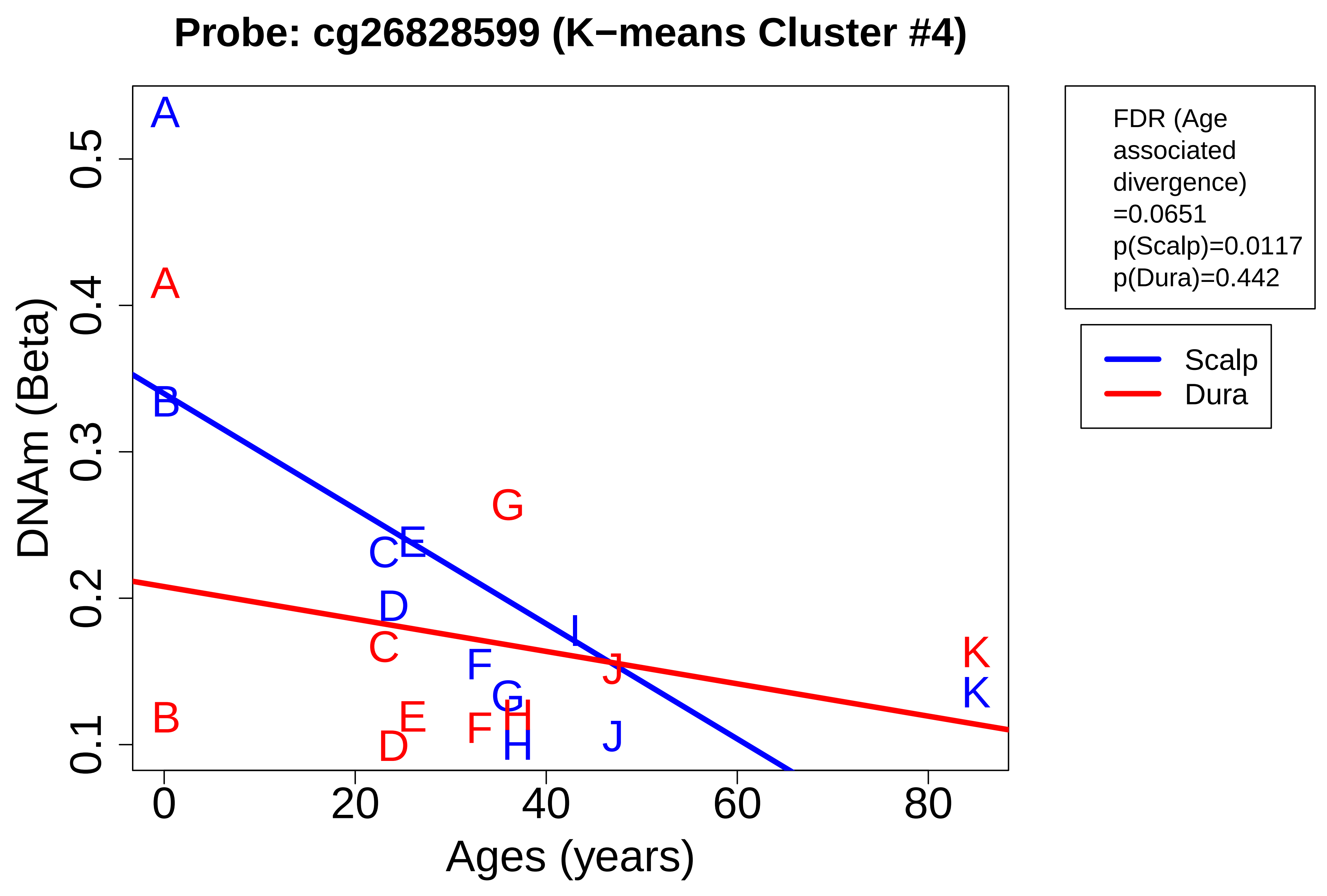
Probe: cg26828599 (K−means Cluster #4)
DNAm (Beta)
0.1
0.2
0.3
0.4
0.5
Ages (years)
0
20
40
60
80
A
B
C
D
E
F
G
H
I
J
K
FDR (Age associated divergence) =0.0651
p(Scalp)=0.0117
p(Dura)=0.442
Scalp
Dura

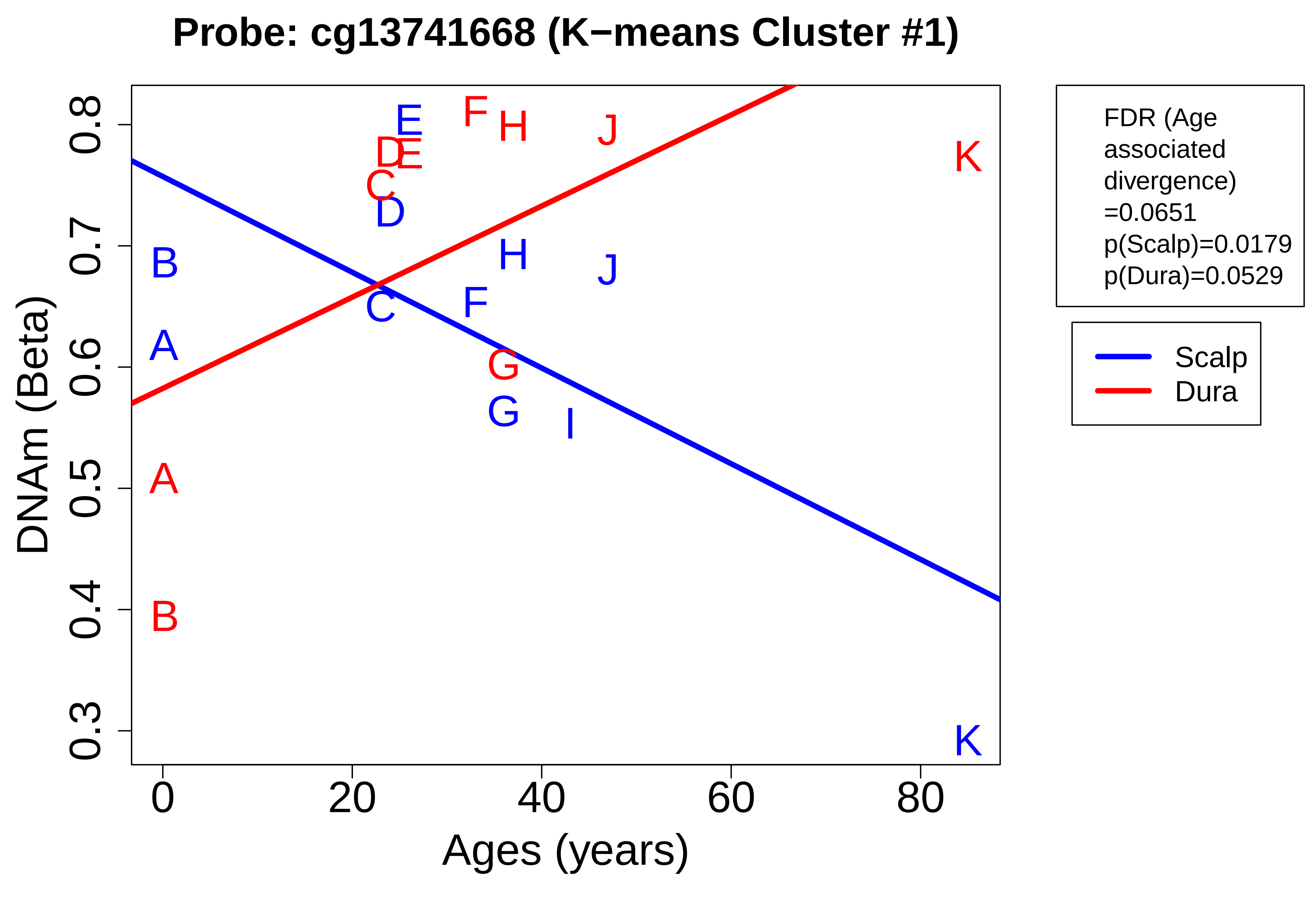
Probe: cg13741668 (K−means Cluster #1)
DNAm (Beta)
0.3
0.4
0.5
0.6
0.7
0.8
0
20
40
60
80
Ages (years)
FDR (Age associated divergence) =0.0651
p(Scalp)=0.0179
p(Dura)=0.0529
Scalp
Dura

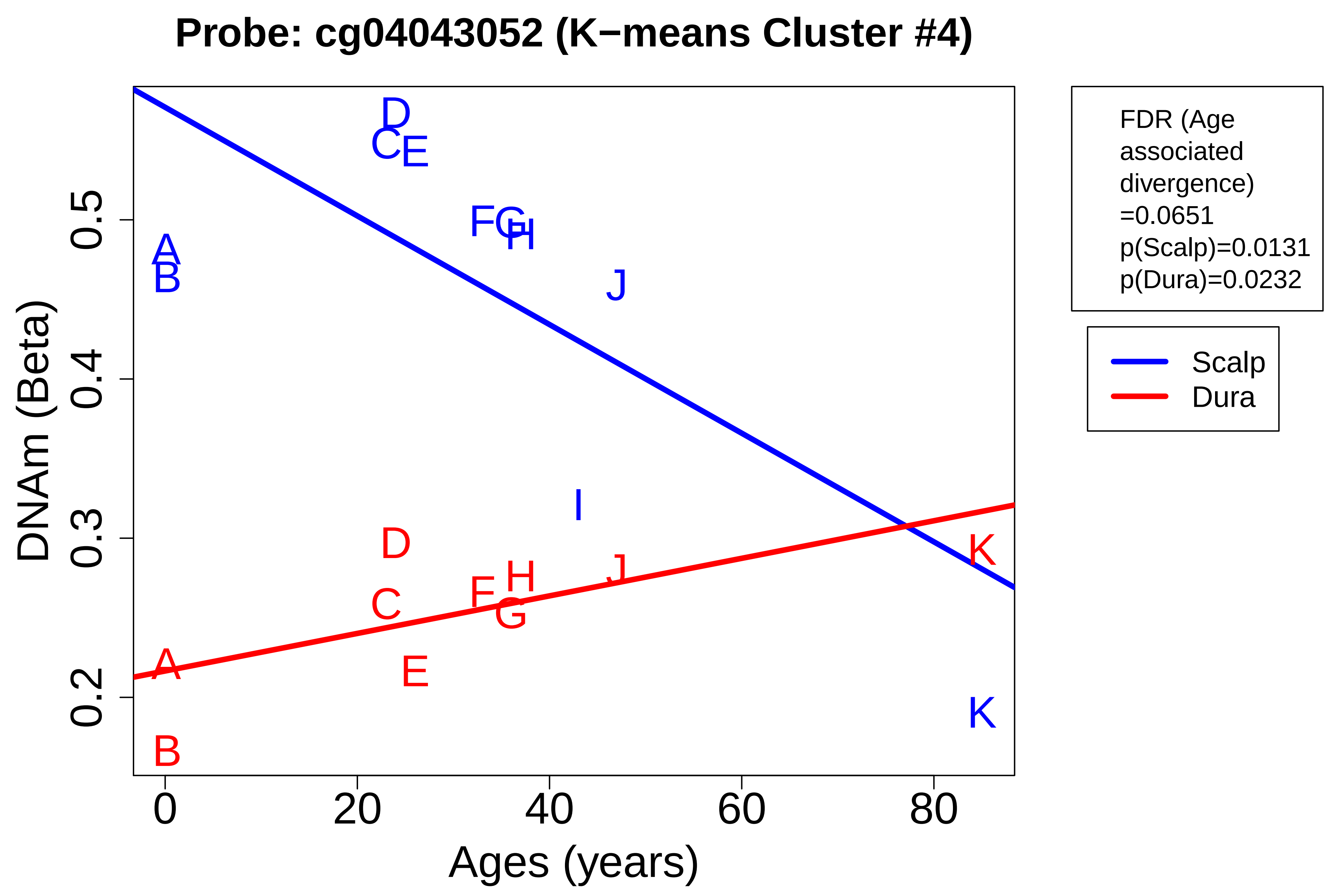

Probe: cg04043052 (K−means Cluster #4)
DNAm (Beta)
0.2
0.3
0.4
0.5
0
20
40
60
80
Ages (years)
A
B
C
D
E
F
G
H
I
J
K
FDR (Age associated divergence) =0.0651
p(Scalp)=0.0131
p(Dura)=0.0232
Scalp
Dura

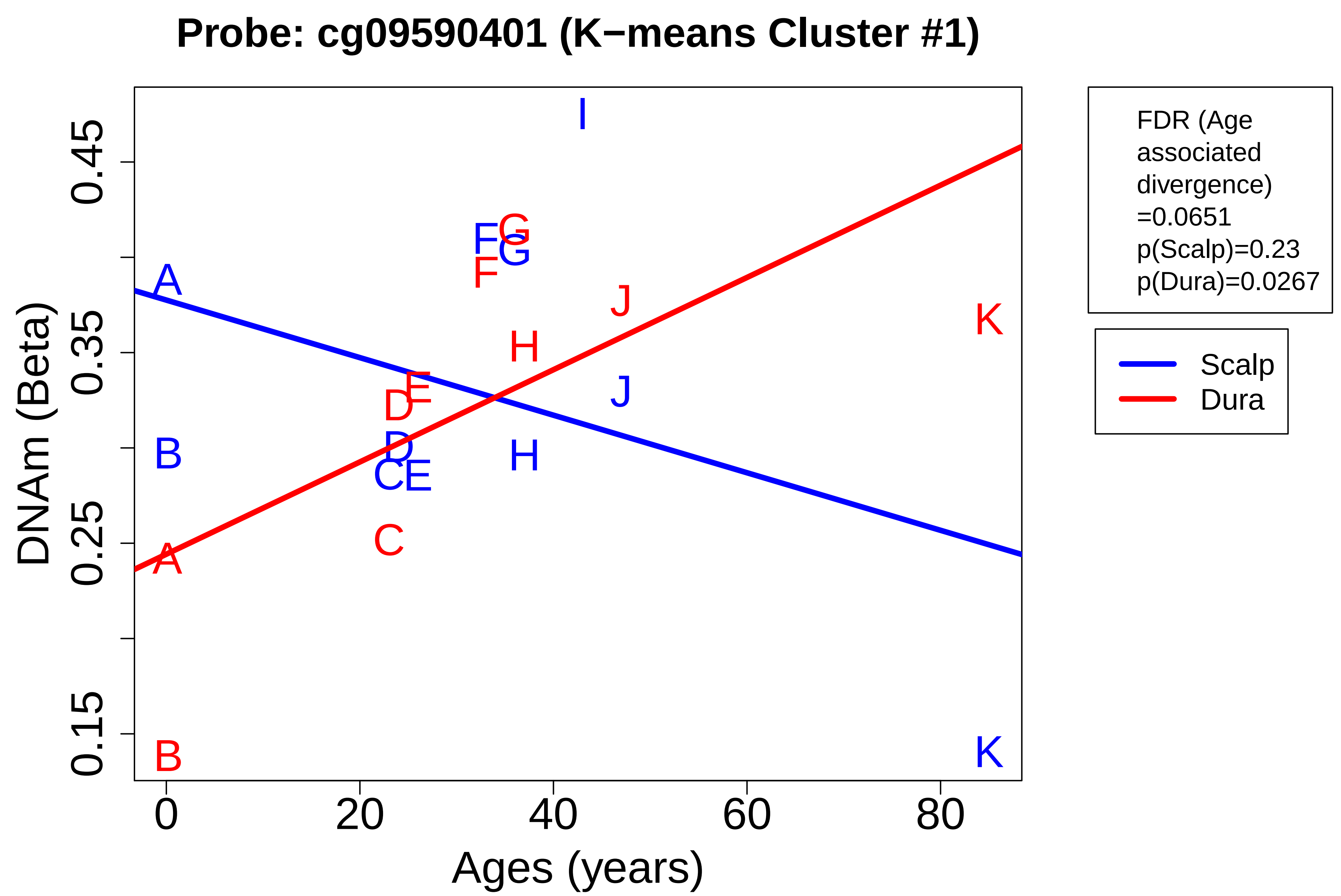

Probe: cg09590401 (K−means Cluster #1)
DNAm (Beta)
0.15
0.25
0.35
0.45
Ages (years)
0
20
40
60
80
FDR (Age associated divergence) =0.0651
p(Scalp)=0.23
p(Dura)=0.0267
Scalp
Dura

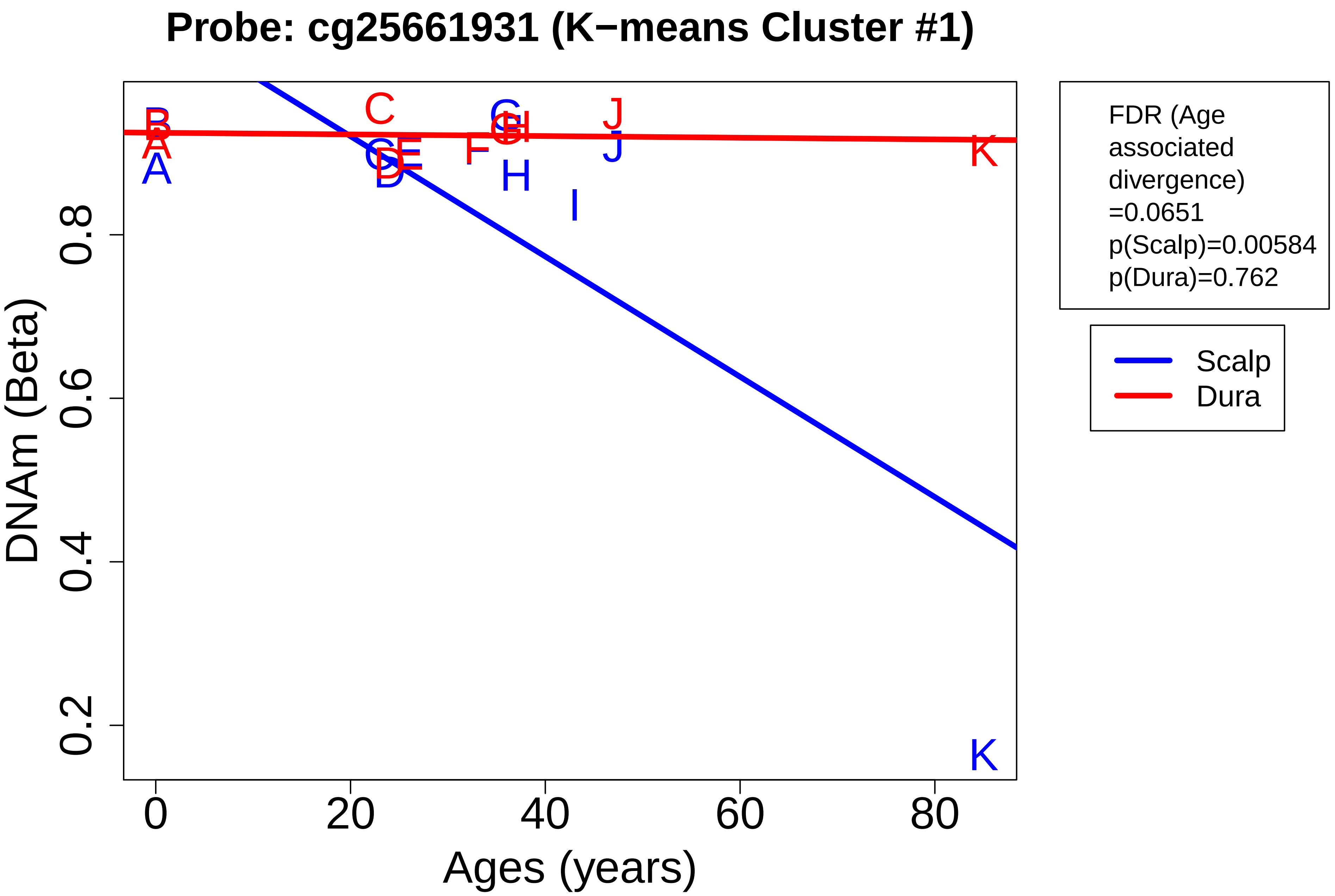

Probe: cg25661931 (K−means Cluster #1)
DNAm (Beta)
0.2
0.4
0.6
0.8
0
20
40
60
80
Ages (years)
FDR (Age associated divergence) =0.0651
p(Scalp)=0.00584
p(Dura)=0.762
Scalp
Dura
A
B
C
D
E
F
G
H
I
J
K

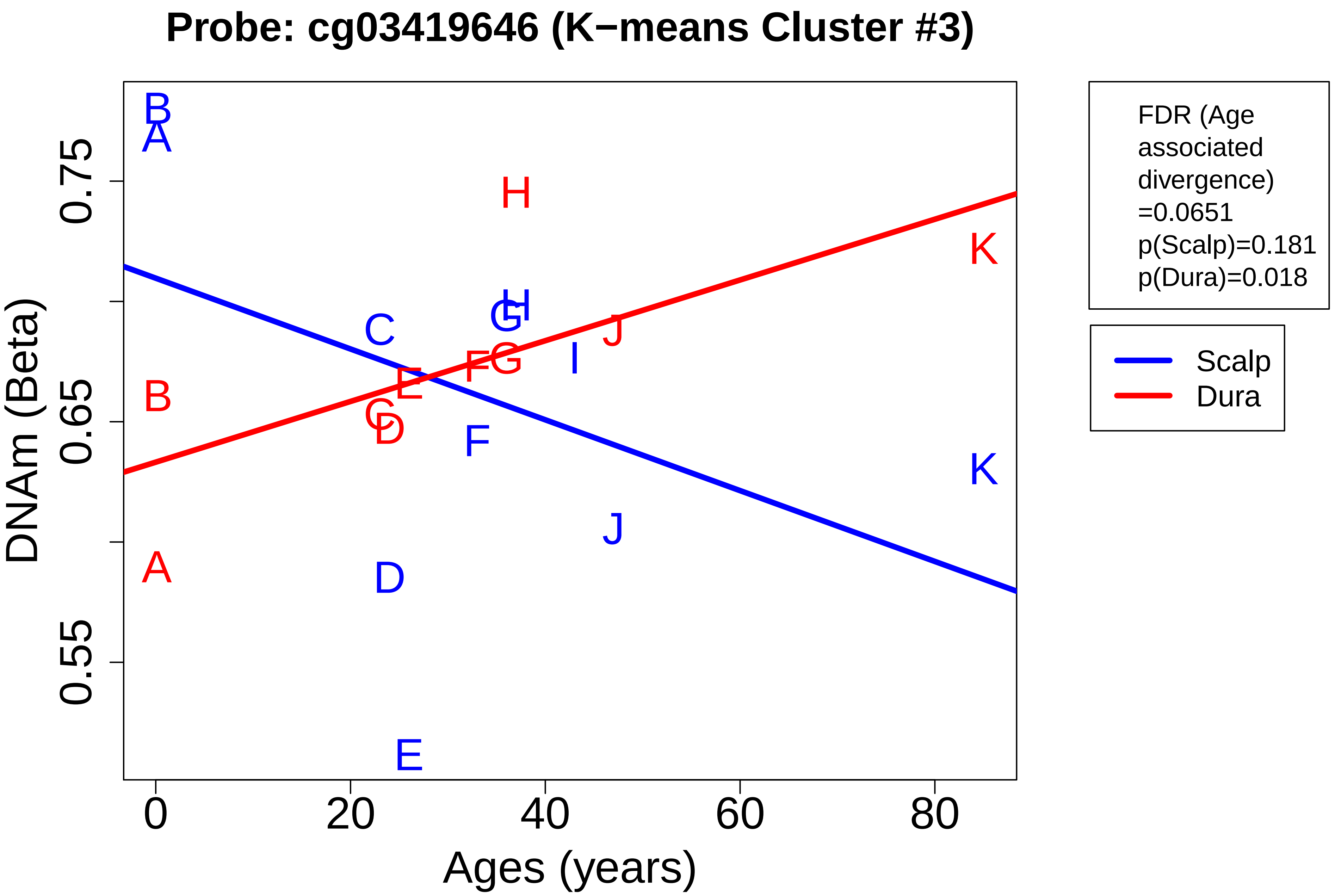

Probe: cg03419646 (K−means Cluster #3)
DNAm (Beta)
0.55
0.65
0.75
Ages (years)
0
20
40
60
80
FDR (Age associated divergence) =0.0651
p(Scalp)=0.181
p(Dura)=0.018
Scalp
Dura

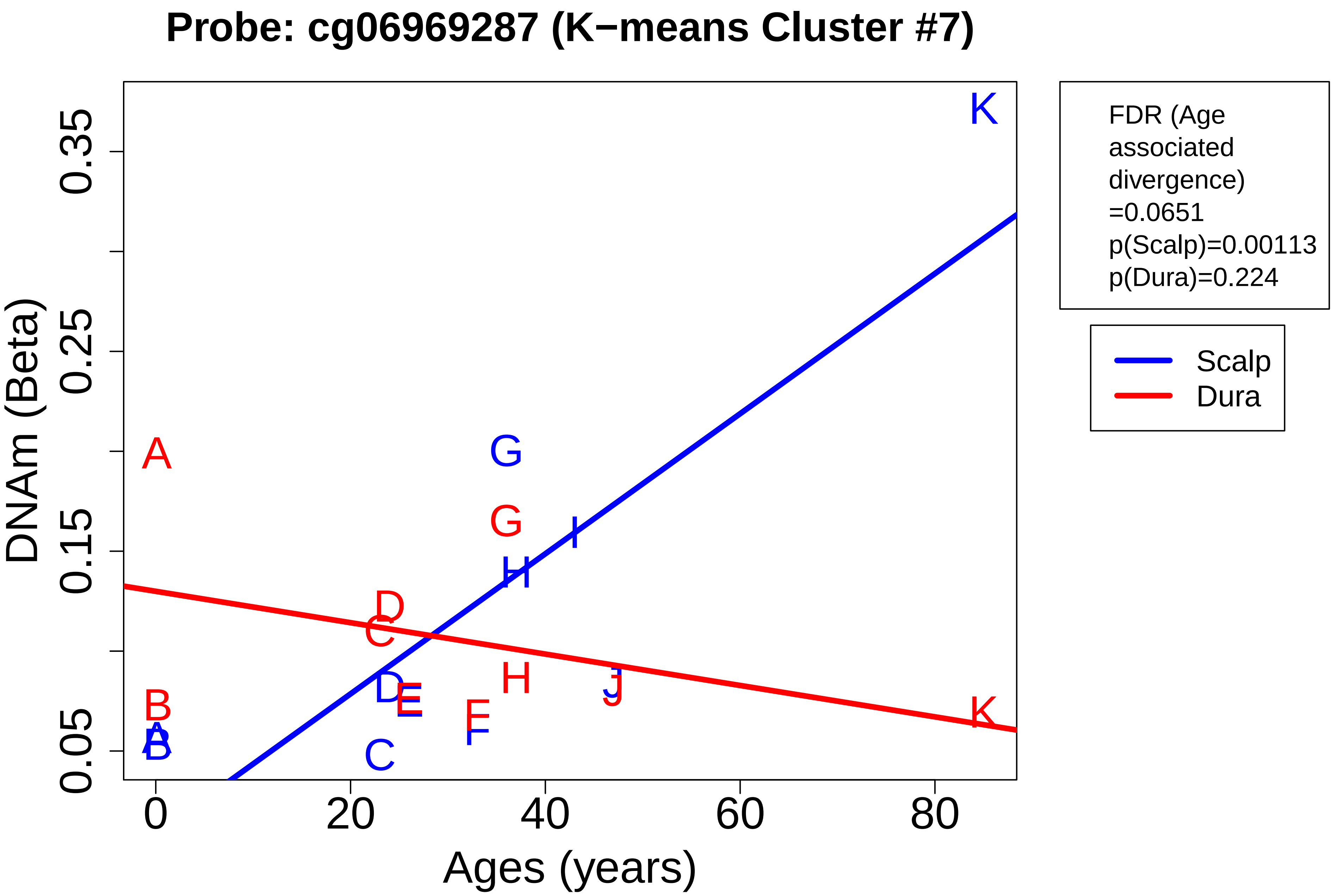
Probe: cg06969287 (K−means Cluster #7)
FDR (Age associated divergence) =0.0651
p(Scalp)=0.00113
p(Dura)=0.224
Scalp
Dura
DNAm (Beta)
Ages (years)
0.05
0.15
0.25
0.35
0
20
40
60
80
A
B
C
D
E
F
G
H
I
J
K

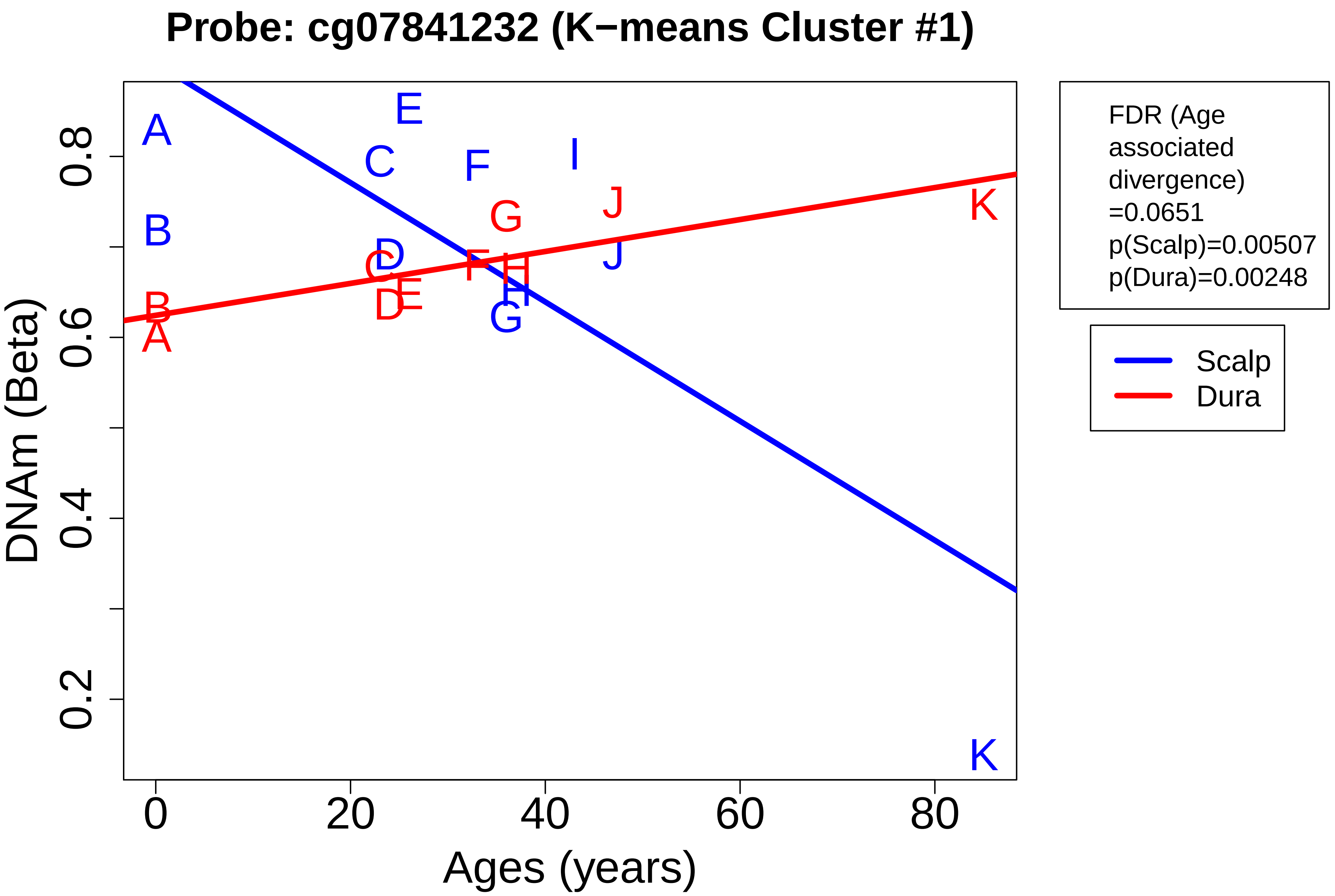

Probe: cg07841232 (K−means Cluster #1)
DNAm (Beta)
0.2
0.4
0.6
0.8
Ages (years)
0
20
40
60
80
A
B
C
D
E
F
G
H
I
J
K
FDR (Age associated divergence) =0.0651
p(Scalp)=0.00507
p(Dura)=0.00248
Scalp
Dura

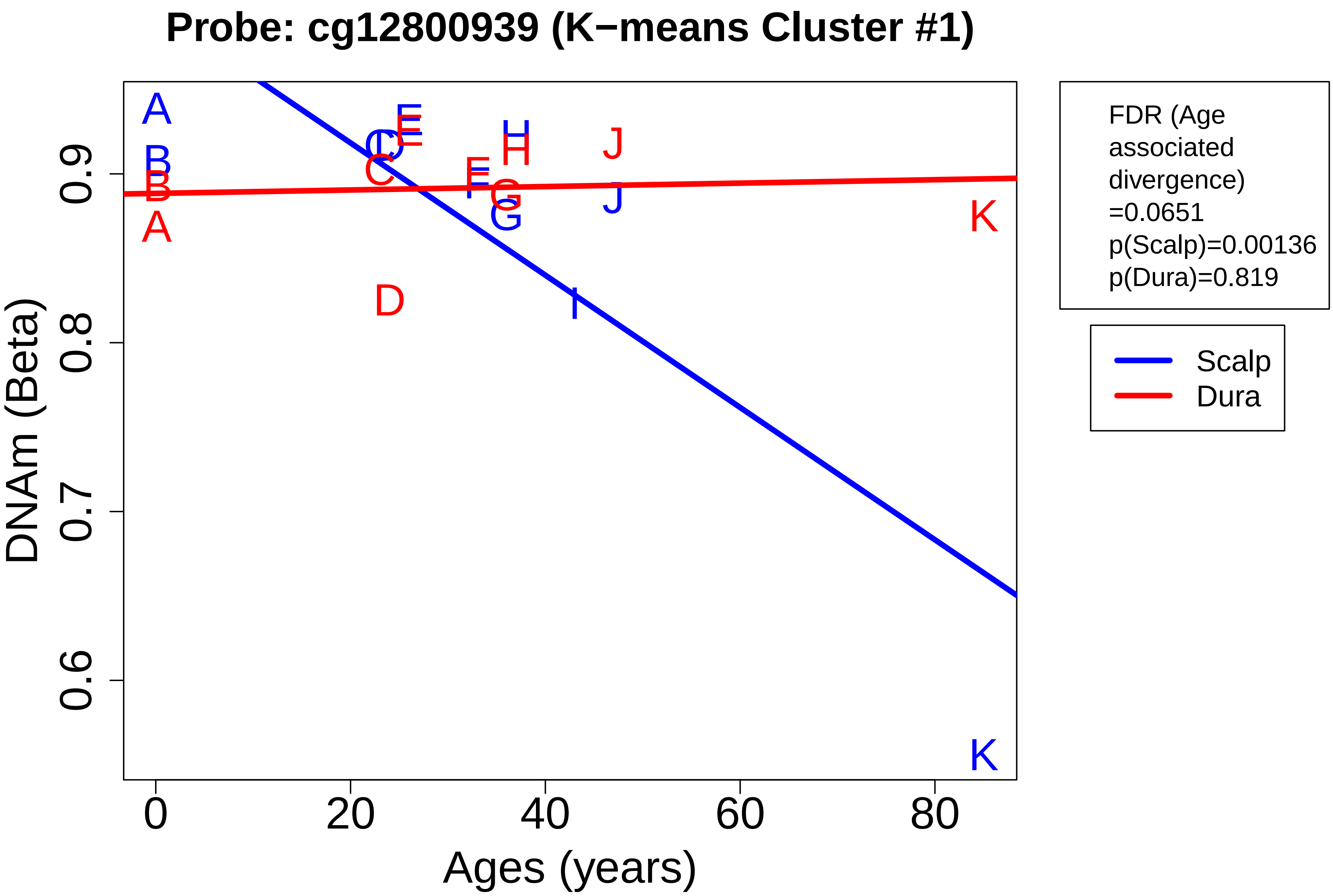

Probe: cg12800939 (K−means Cluster #1)
FDR (Age associated divergence) =0.0651
p(Scalp)=0.00136
p(Dura)=0.819
Scalp
Dura
DNAm (Beta)
0.6
0.7
0.8
0.9
Ages (years)
0
20
40
60
80
A
B
C
D
E
F
G
H
I
J
K

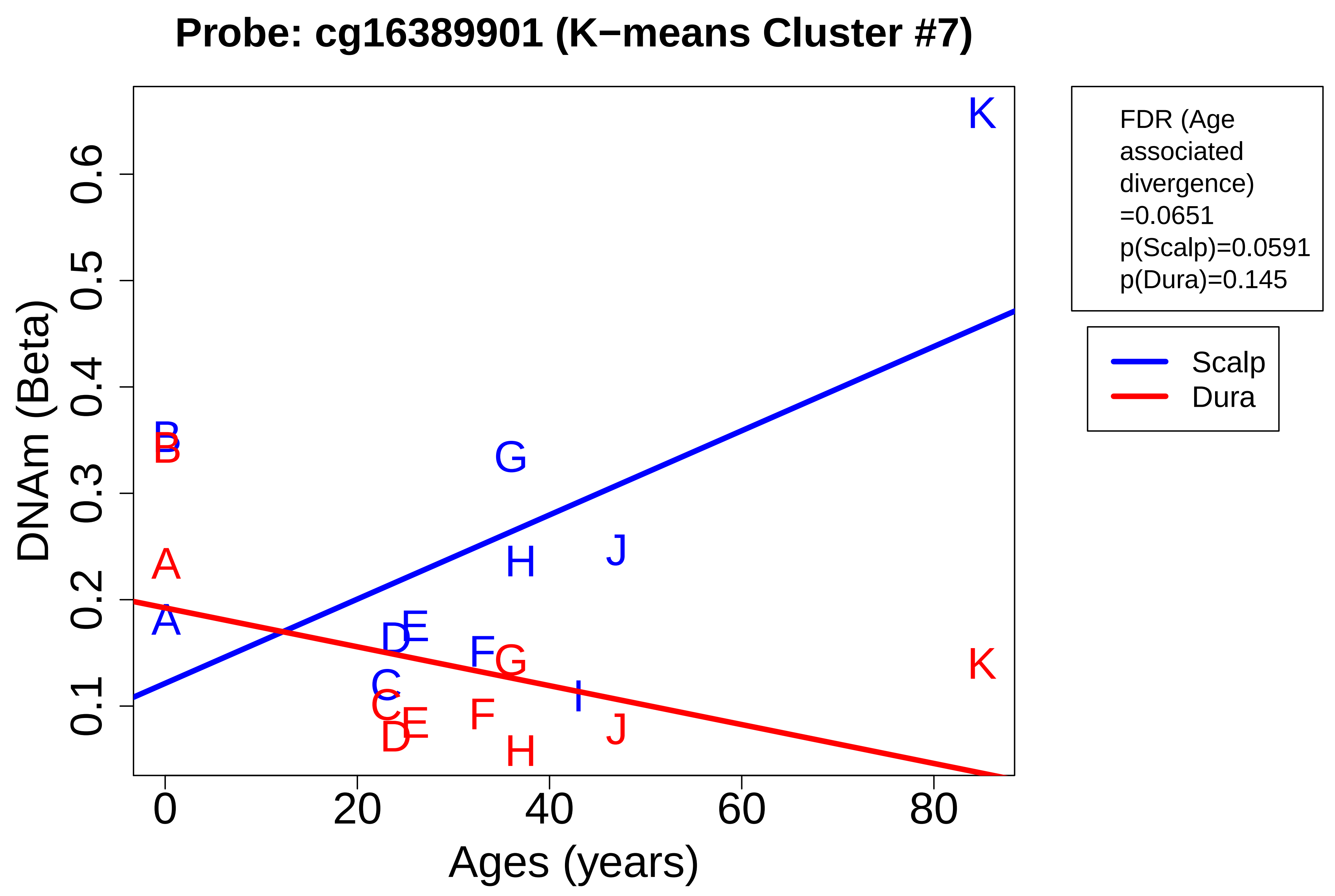

Probe: cg16389901 (K−means Cluster #7)
DNAm (Beta)
0.1
0.2
0.3
0.4
0.5
0.6
0
20
40
60
80
Ages (years)
FDR (Age associated divergence) =0.0651
p(Scalp)=0.0591
p(Dura)=0.145
Scalp
Dura
A
B
C
D
E
F
G
H
I
J
K

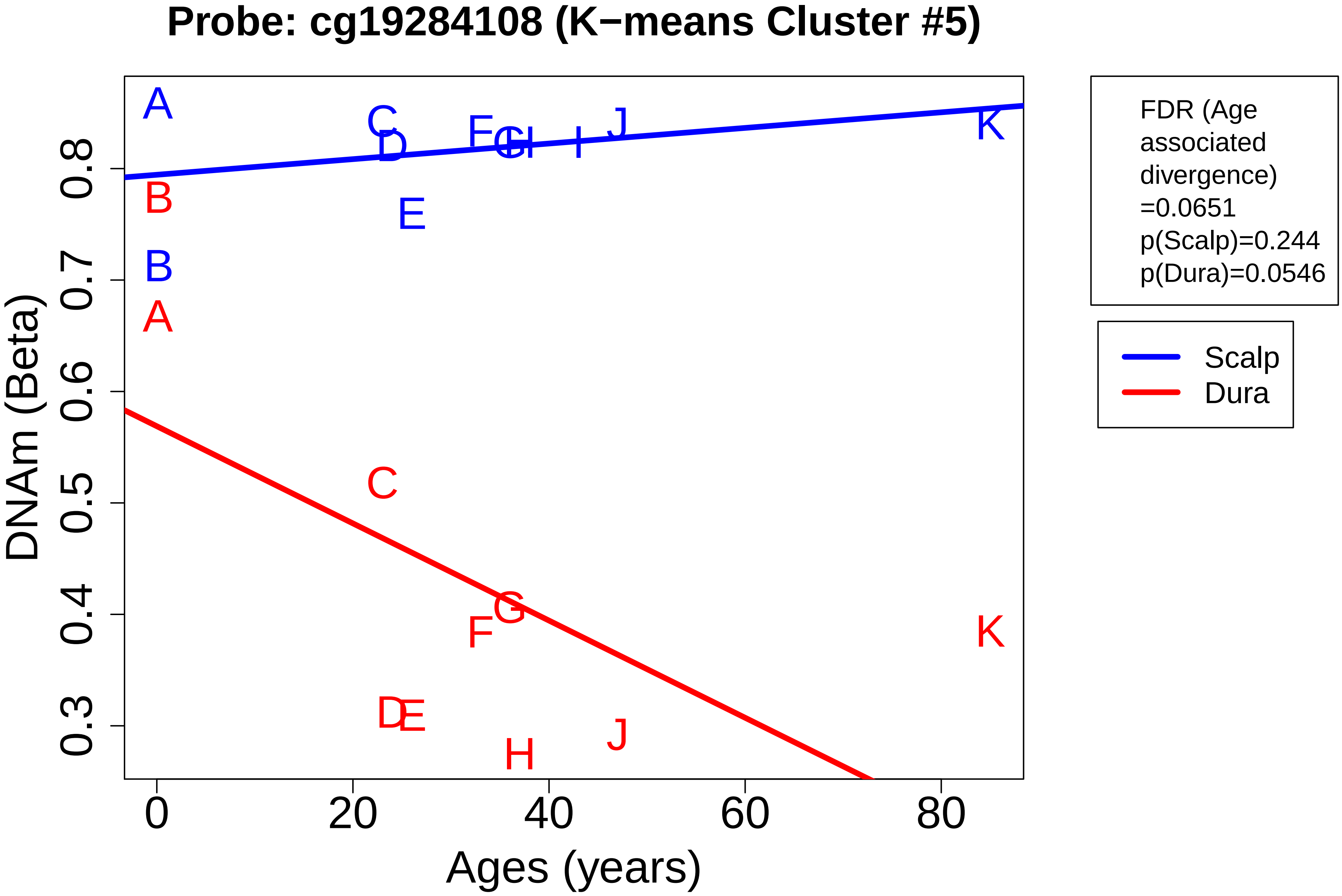

Probe: cg19284108 (K−means Cluster #5)
DNAm (Beta)
0.3
0.4
0.5
0.6
0.7
0.8
0
20
40
60
80
Ages (years)
A
B
C
D
E
F
G
H
I
J
K
FDR (Age associated divergence) =0.0651
p(Scalp)=0.244
p(Dura)=0.0546
Scalp
Dura

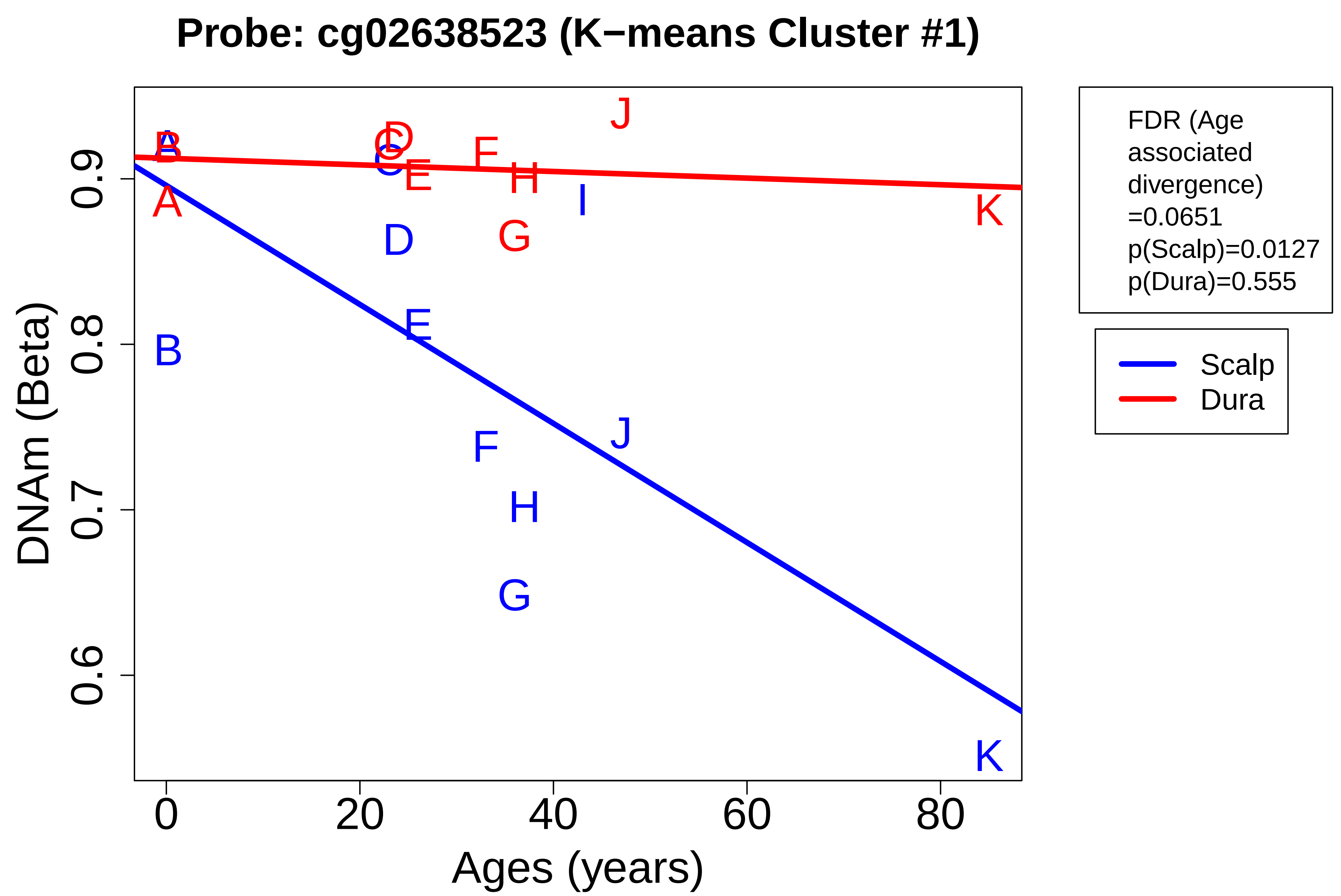
Probe: cg02638523 (K−means Cluster #1)
DNAm (Beta)
0.6
0.7
0.8
0.9
0
20
40
60
80
Ages (years)
A
B
C
D
E
F
G
H
I
J
K
FDR (Age associated divergence) =0.0651
p(Scalp)=0.0127
p(Dura)=0.555
Scalp
Dura

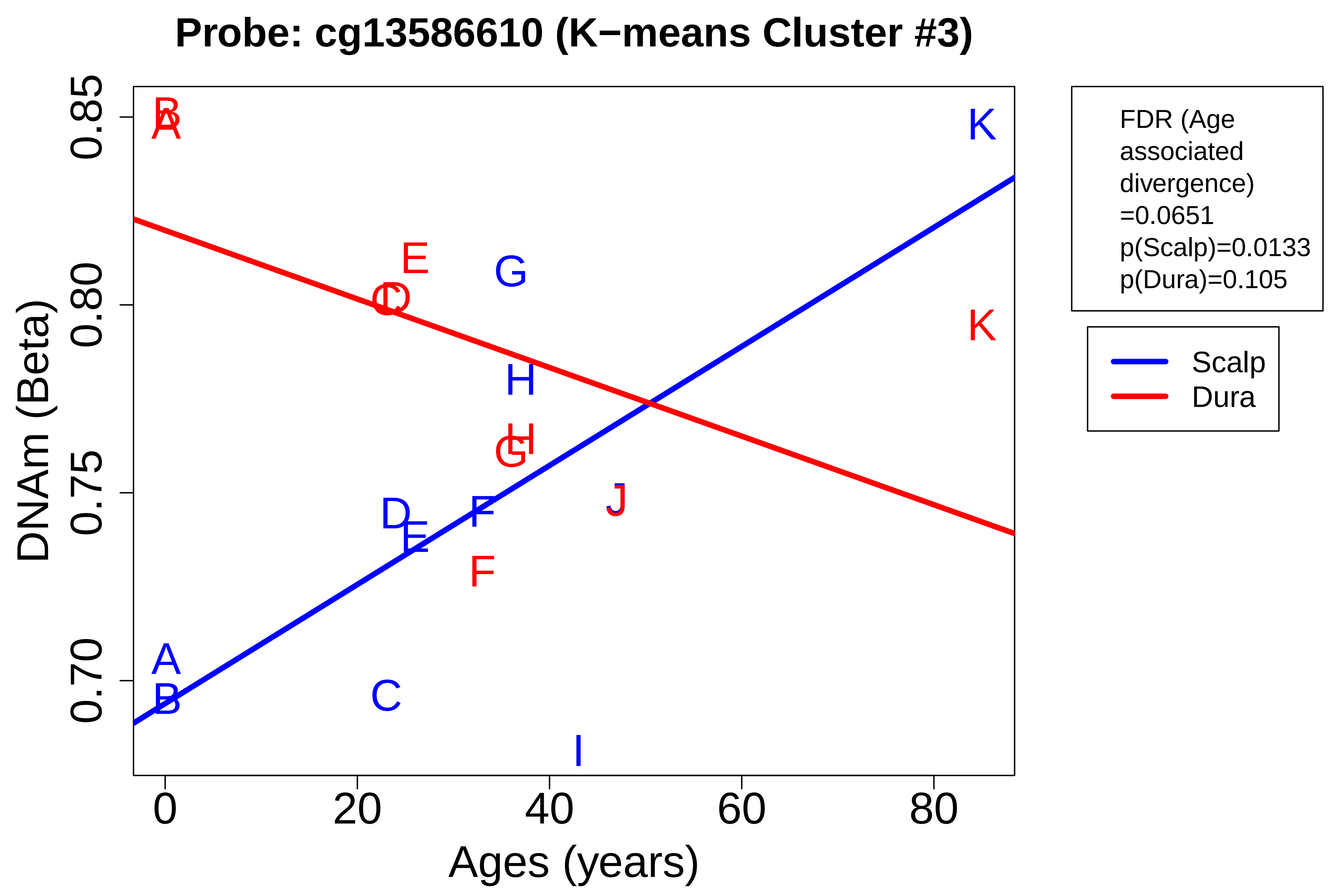

Probe: cg13586610 (K−means Cluster #3)
DNAm (Beta)
0.70
0.75
0.80
0.85
Ages (years)
0
20
40
60
80
A
B
C
D
E
F
G
H
I
J
K
FDR (Age associated divergence) =0.0651
p(Scalp)=0.0133
p(Dura)=0.105
Scalp
Dura

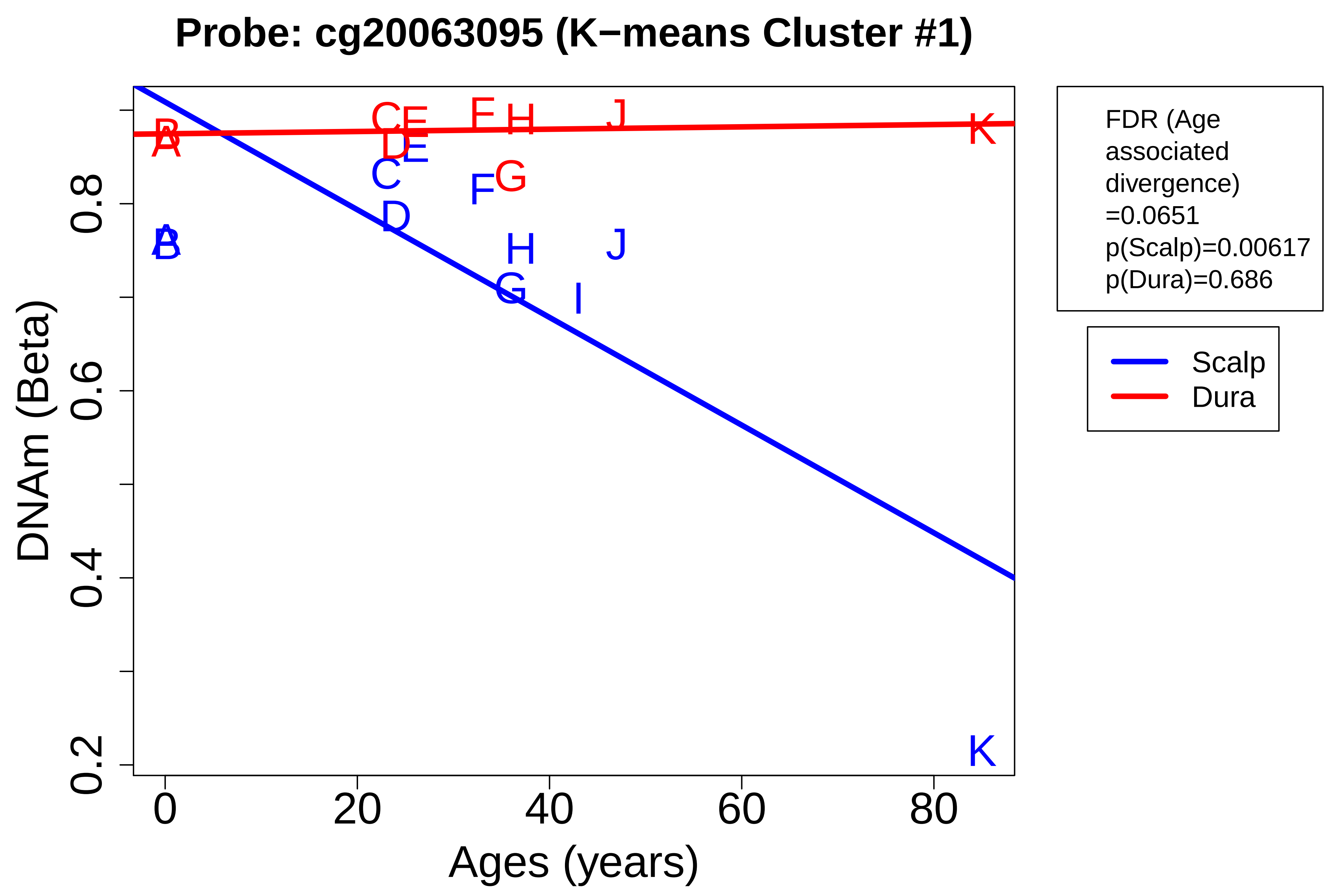

Probe: cg20063095 (K−means Cluster #1)
DNAm (Beta)
0.2
0.4
0.6
0.8
0
20
40
60
80
Ages (years)
FDR (Age associated divergence) =0.0651
p(Scalp)=0.00617
p(Dura)=0.686
Scalp
Dura
A
B
C
D
E
F
G
H
I
J
K

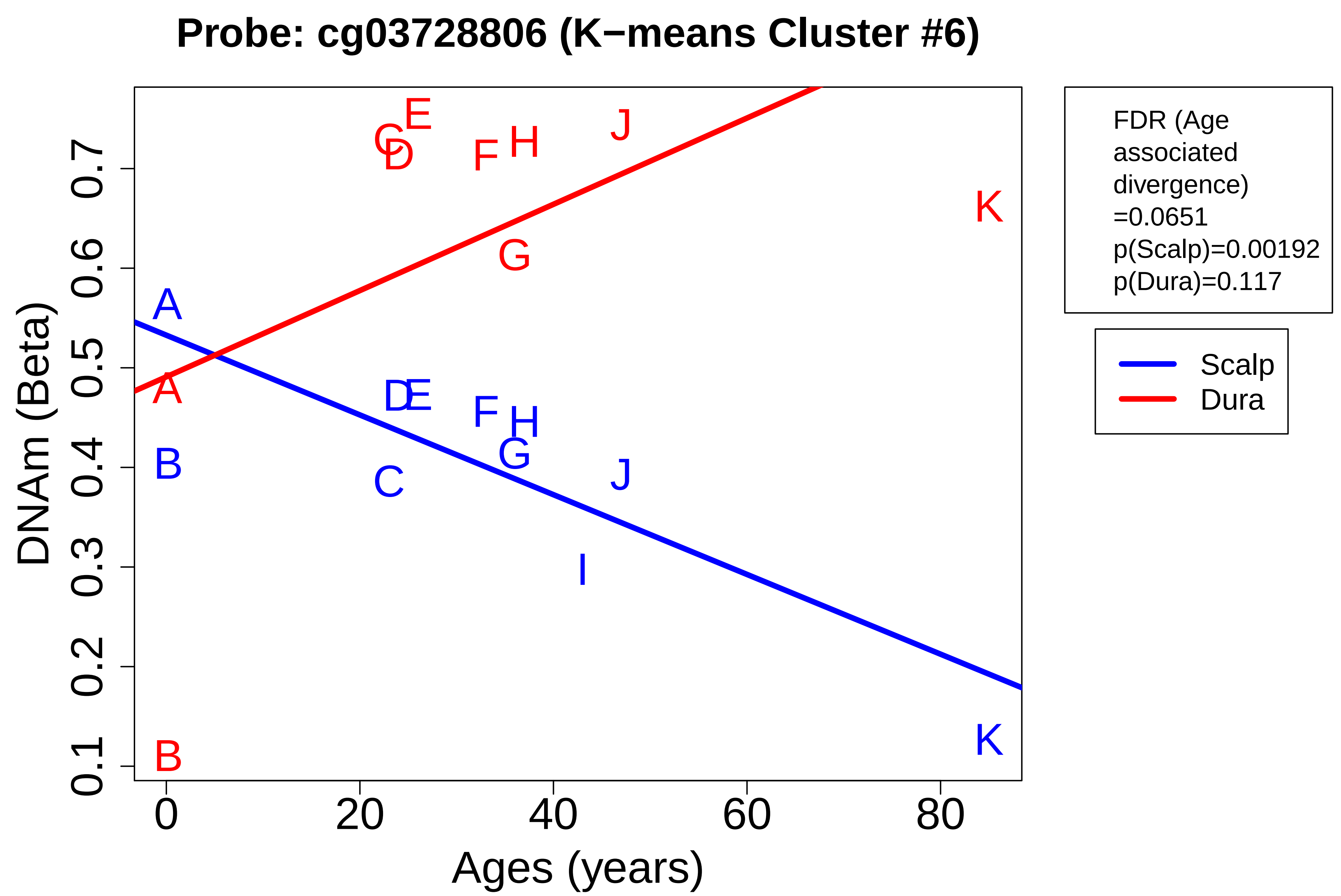
Probe: cg03728806 (K−means Cluster #6)
DNAm (Beta)
0.1
0.2
0.3
0.4
0.5
0.6
0.7
0
20
40
60
80
Ages (years)
A
B
C
D
E
F
G
H
I
J
K
FDR (Age associated divergence) =0.0651
p(Scalp)=0.00192
p(Dura)=0.117
Scalp
Dura

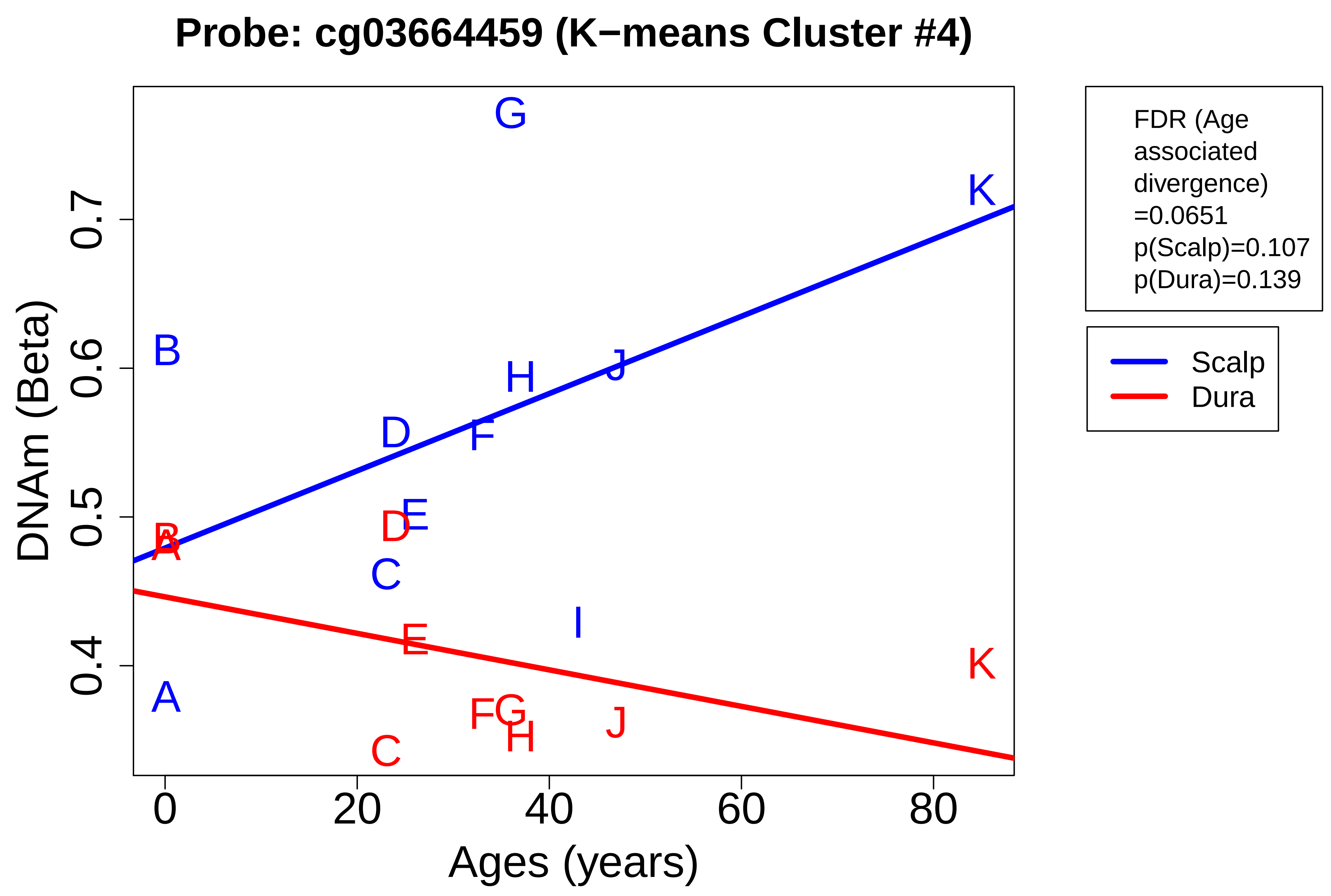

Probe: cg03664459 (K−means Cluster #4)
FDR (Age associated divergence) =0.0651
p(Scalp)=0.107
p(Dura)=0.139
Scalp
Dura
DNAm (Beta)
0.4
0.5
0.6
0.7
0
20
40
60
80
Ages (years)

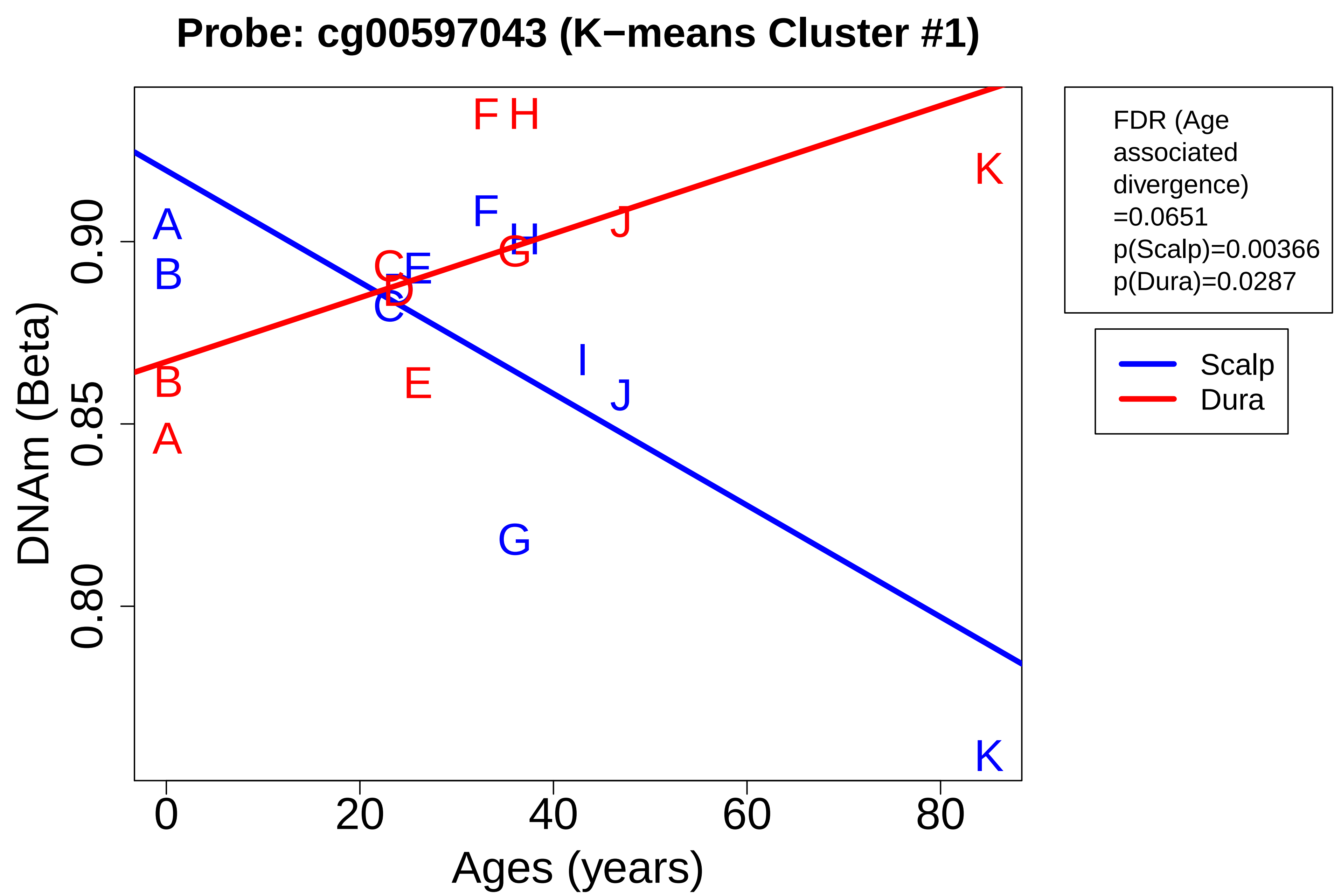
Probe: cg00597043 (K−means Cluster #1)
FDR (Age associated divergence) =0.0651
p(Scalp)=0.00366
p(Dura)=0.0287
Scalp
Dura
DNAm (Beta)
0.80
0.85
0.90
0
20
40
60
80
Ages (years)
A
B
C
D
E
F
G
H
I
J
K

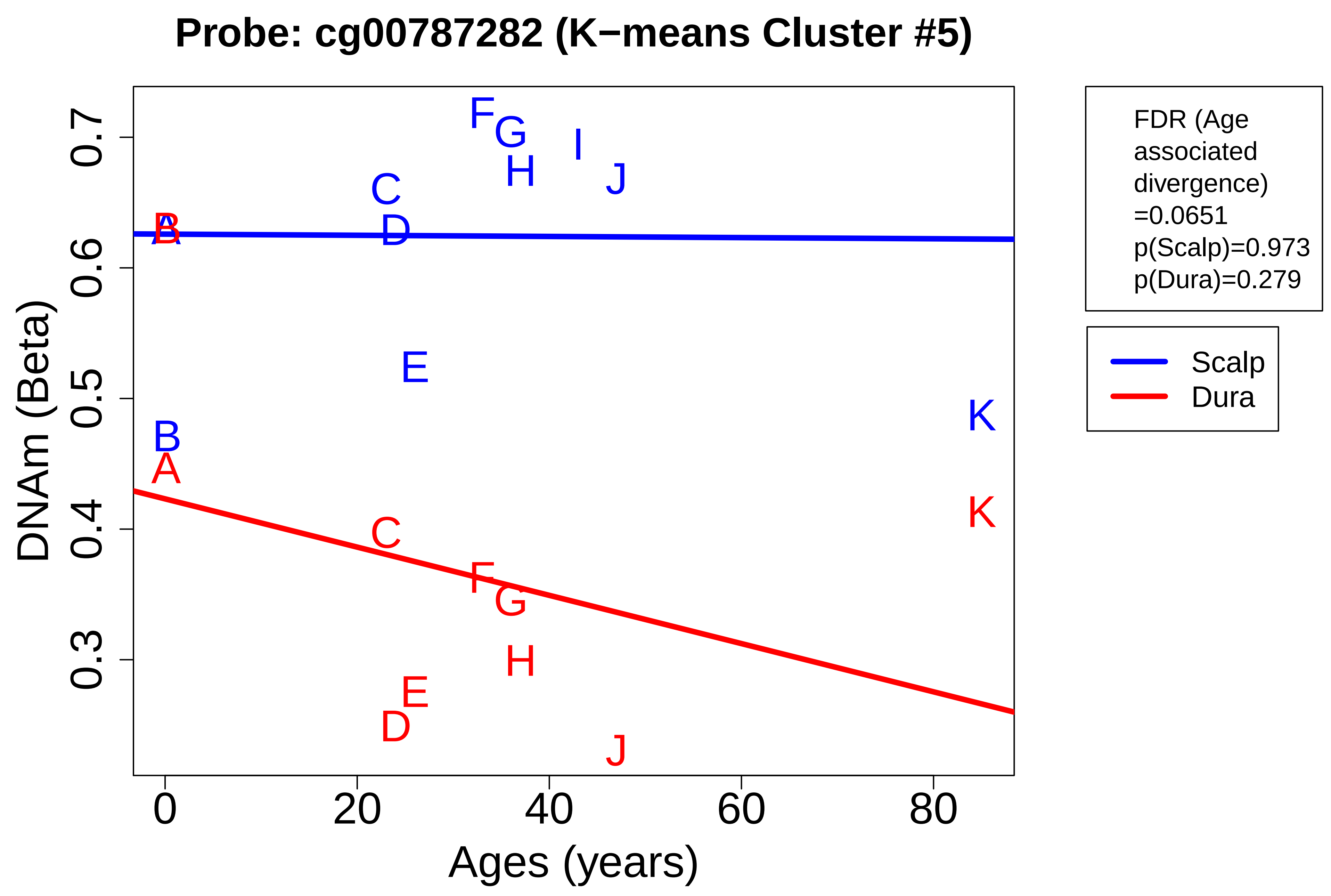
Probe: cg00787282 (K−means Cluster #5)
DNAm (Beta)
0.3
0.4
0.5
0.6
0.7
0
20
40
60
80
Ages (years)
FDR (Age associated divergence) =0.0651
p(Scalp)=0.973
p(Dura)=0.279
Scalp
Dura

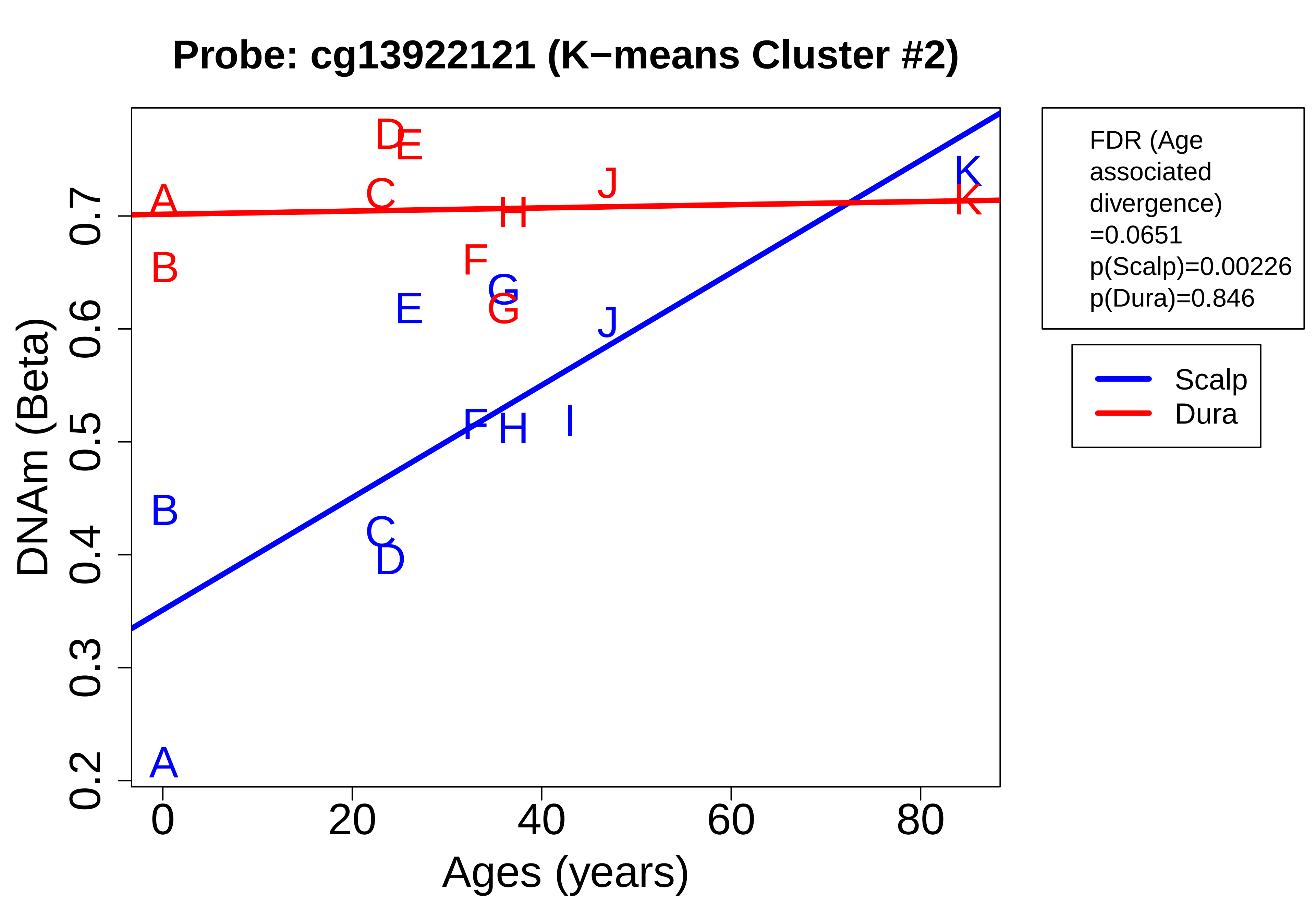

Probe: cg13922121 (K−means Cluster #2)
DNAm (Beta)
0.2
0.3
0.4
0.5
0.6
0.7
0
20
40
60
80
Ages (years)
FDR (Age associated divergence) =0.0651
p(Scalp)=0.00226
p(Dura)=0.846
Scalp
Dura
A
B
C
D
E
F
G
H
I
J
K

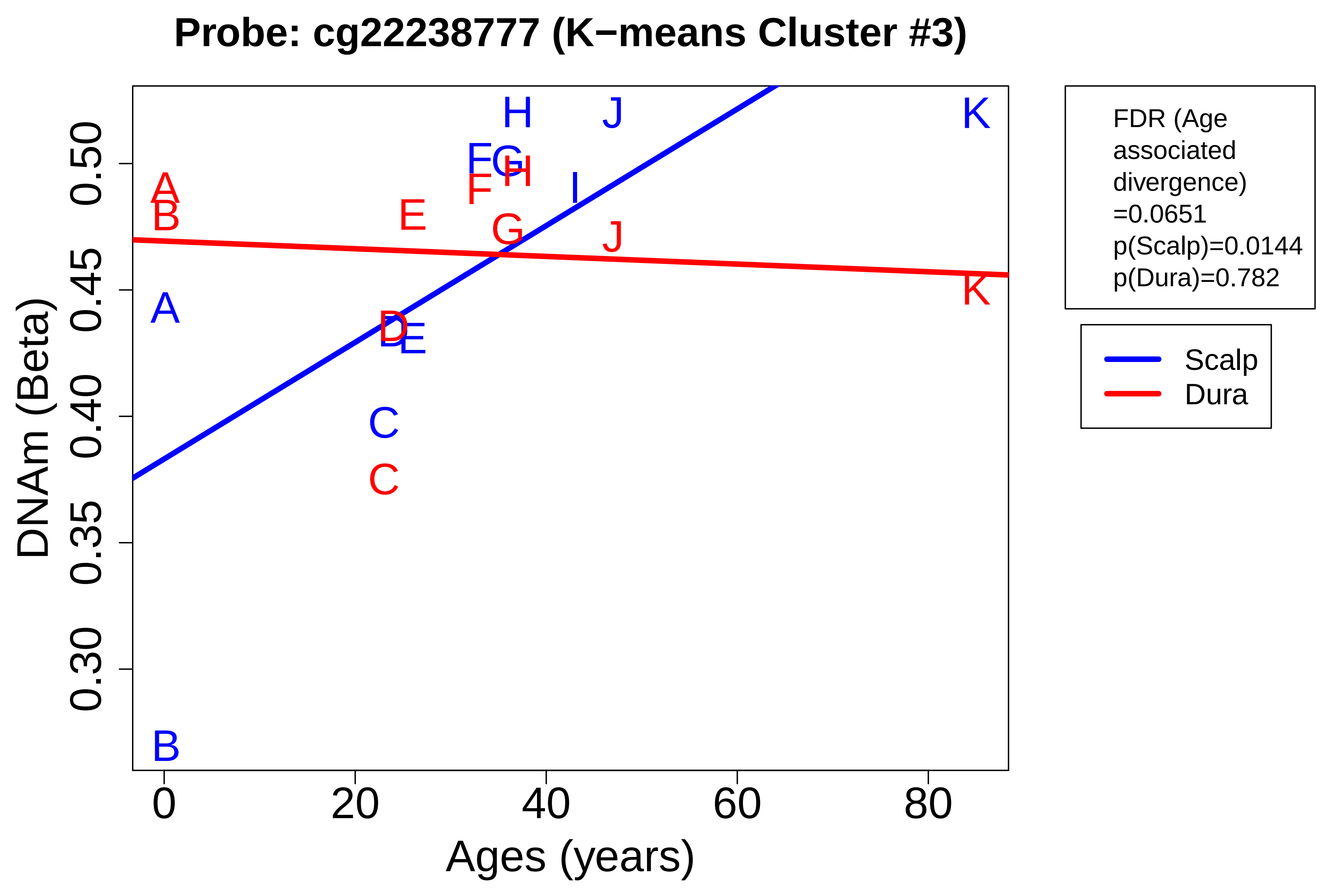

Probe: cg22238777 (K−means Cluster #3)
DNAm (Beta)
0.30
0.35
0.40
0.45
0.50
0
20
40
60
80
Ages (years)
FDR (Age associated divergence) =0.0651
p(Scalp)=0.0144
p(Dura)=0.782
Scalp
Dura

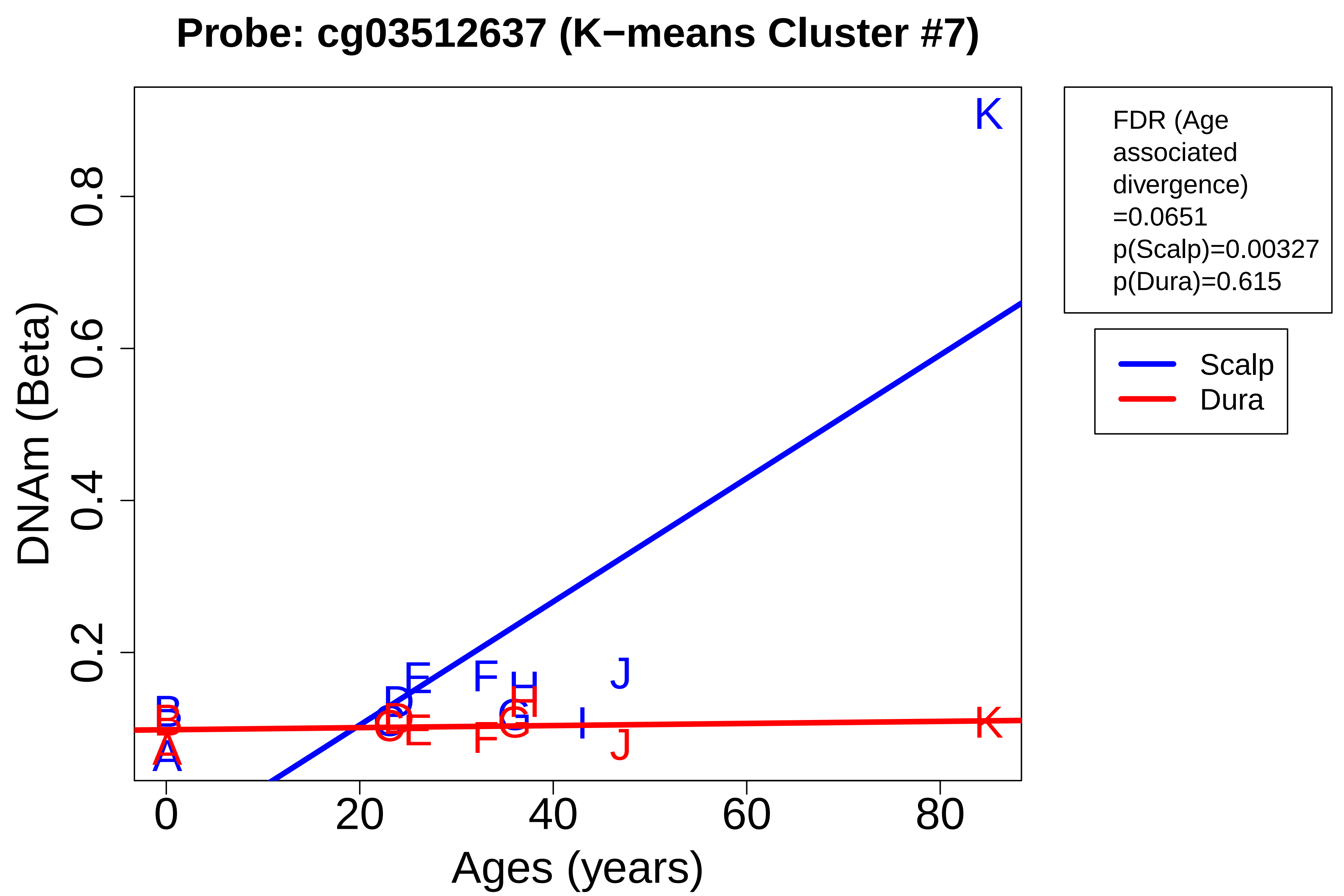

Probe: cg03512637 (K−means Cluster #7)
DNAm (Beta)
0.2
0.4
0.6
0.8
0
20
40
60
80
Ages (years)
FDR (Age associated divergence) =0.0651
p(Scalp)=0.00327
p(Dura)=0.615
Scalp
Dura

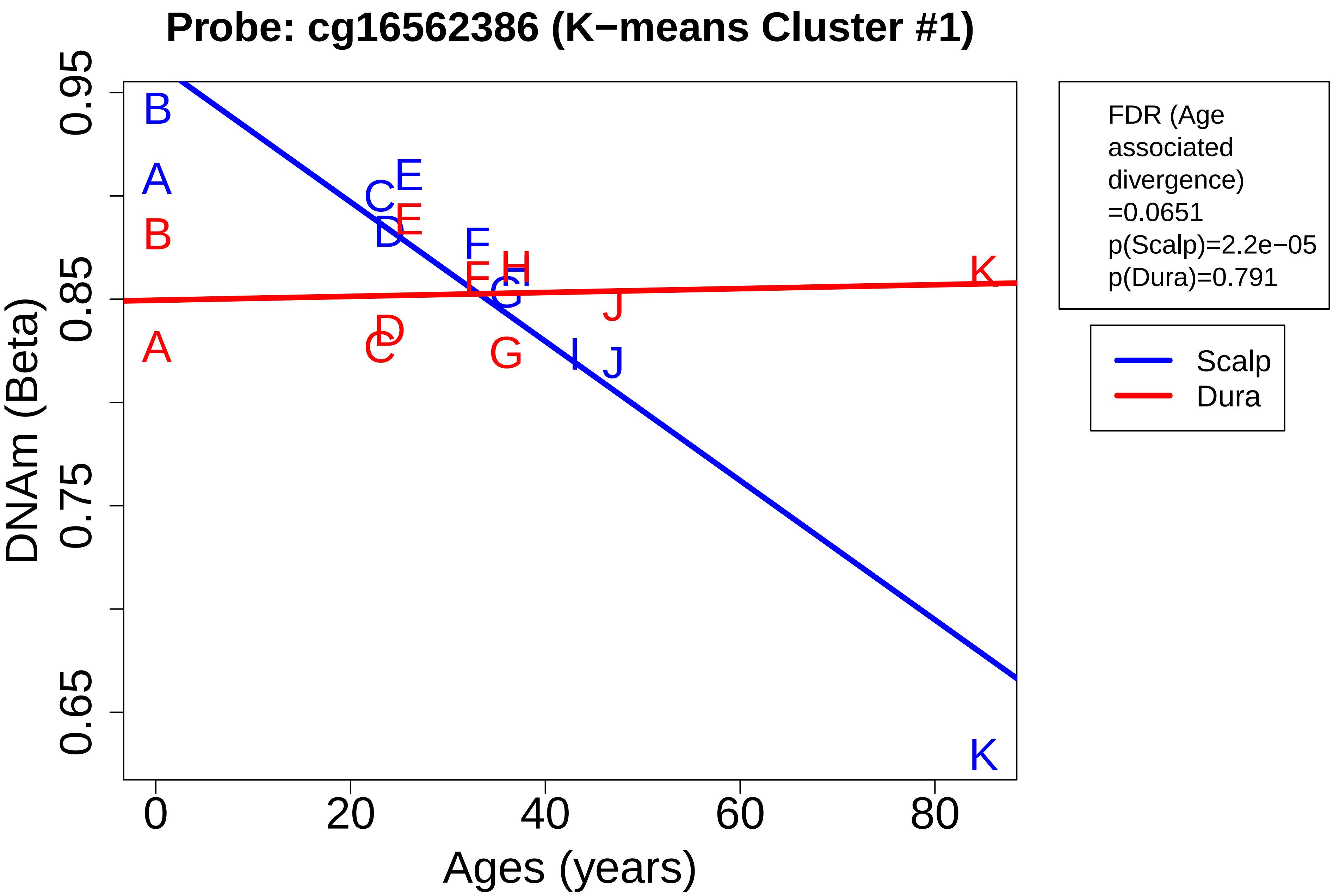

Probe: cg16562386 (K−means Cluster #1)
DNAm (Beta)
0.95
0.85
0.75
0.65
0
20
40
60
80
Ages (years)
FDR (Age associated divergence) =0.0651
p(Scalp)=2.2e−05
p(Dura)=0.791
Scalp
Dura

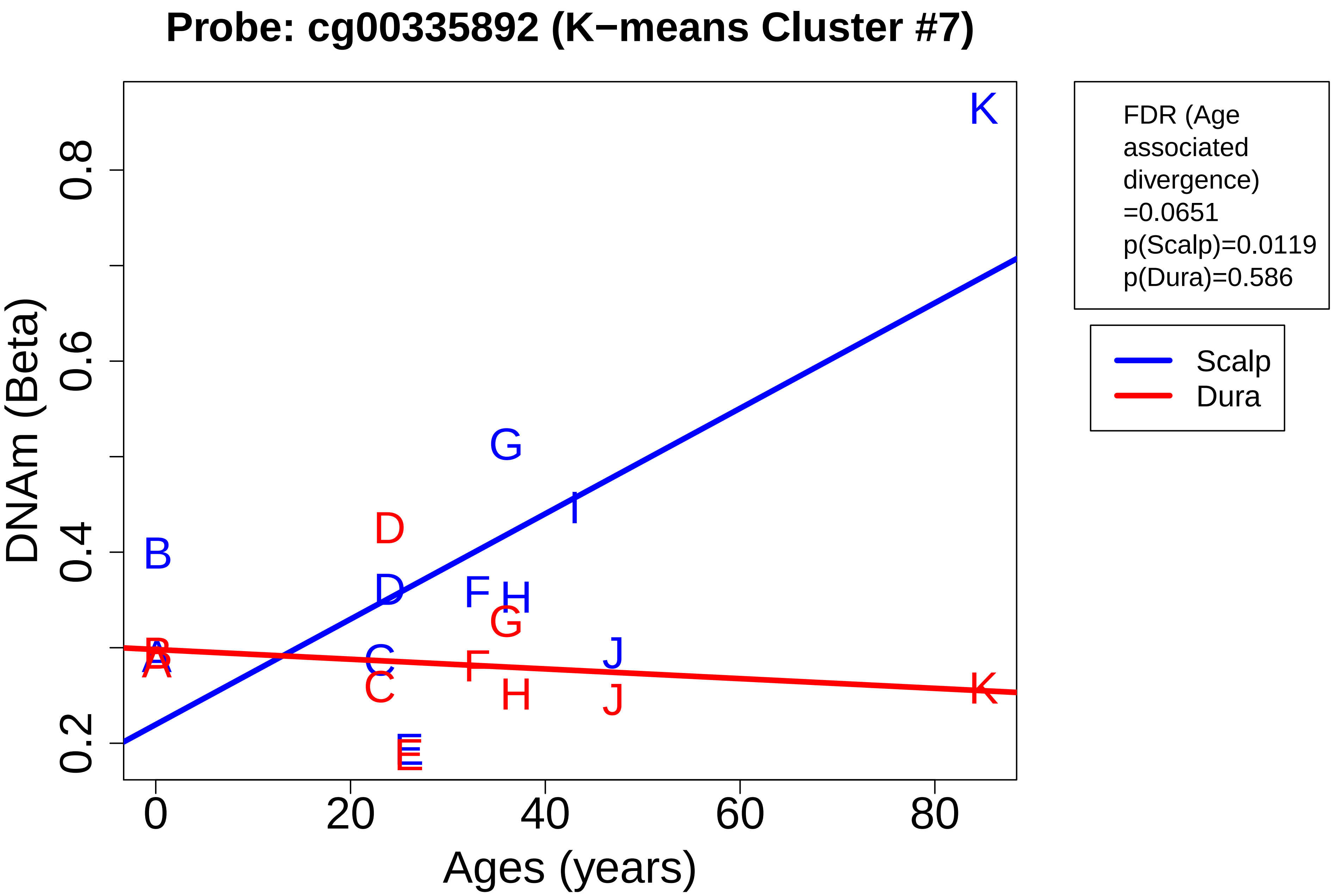

Probe: cg00335892 (K−means Cluster #7)
DNAm (Beta)
0.2
0.4
0.6
0.8
0
20
40
60
80
Ages (years)
FDR (Age associated divergence) =0.0651
p(Scalp)=0.0119
p(Dura)=0.586
Scalp
Dura
A
B
C
D
E
F
G
H
I
J
K

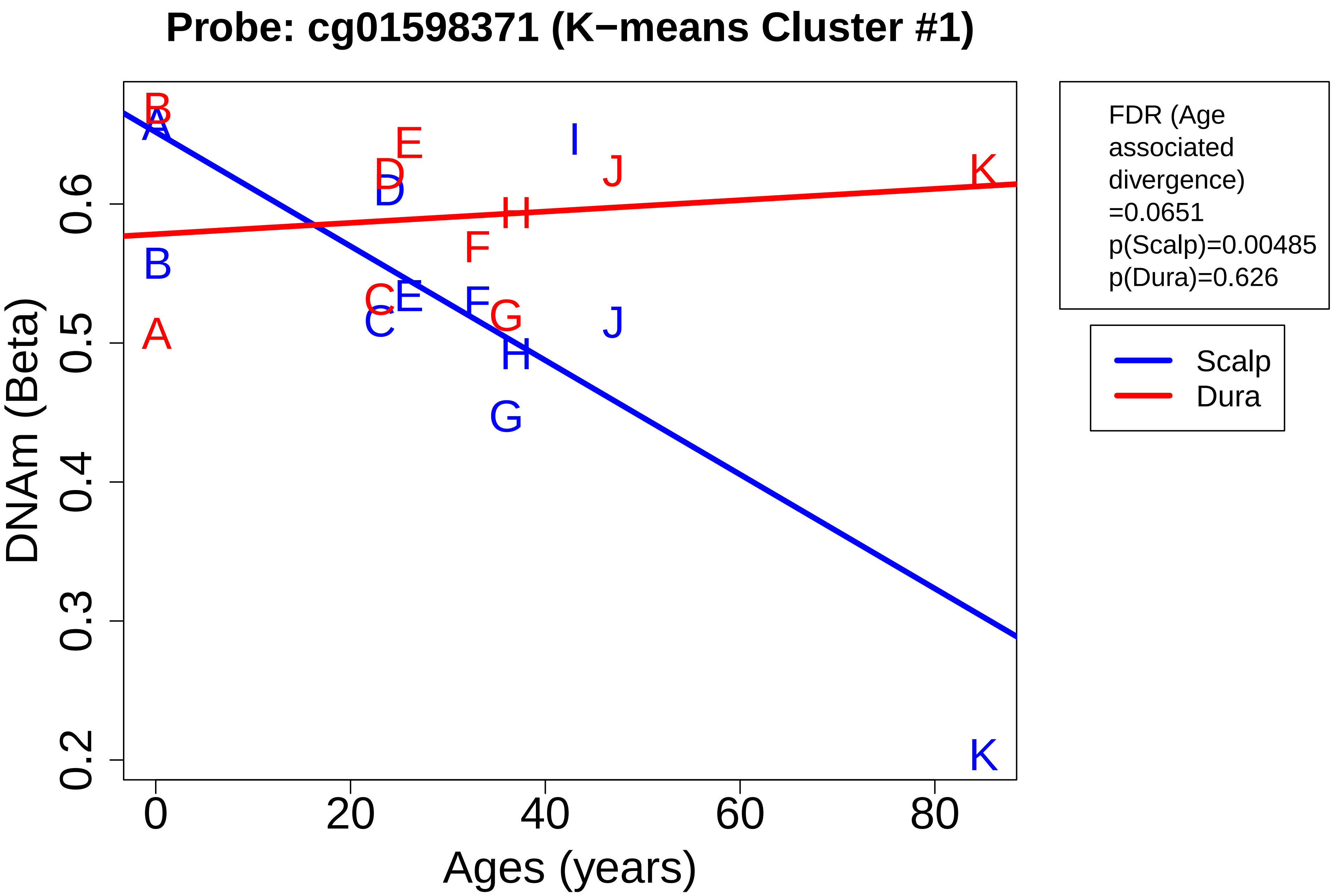

Probe: cg01598371 (K−means Cluster #1)
DNAm (Beta)
0.2
0.3
0.4
0.5
0.6
Ages (years)
0
20
40
60
80
A B C D E F G H I J K
FDR (Age associated divergence) =0.0651
p(Scalp)=0.00485
p(Dura)=0.626
Scalp
Dura

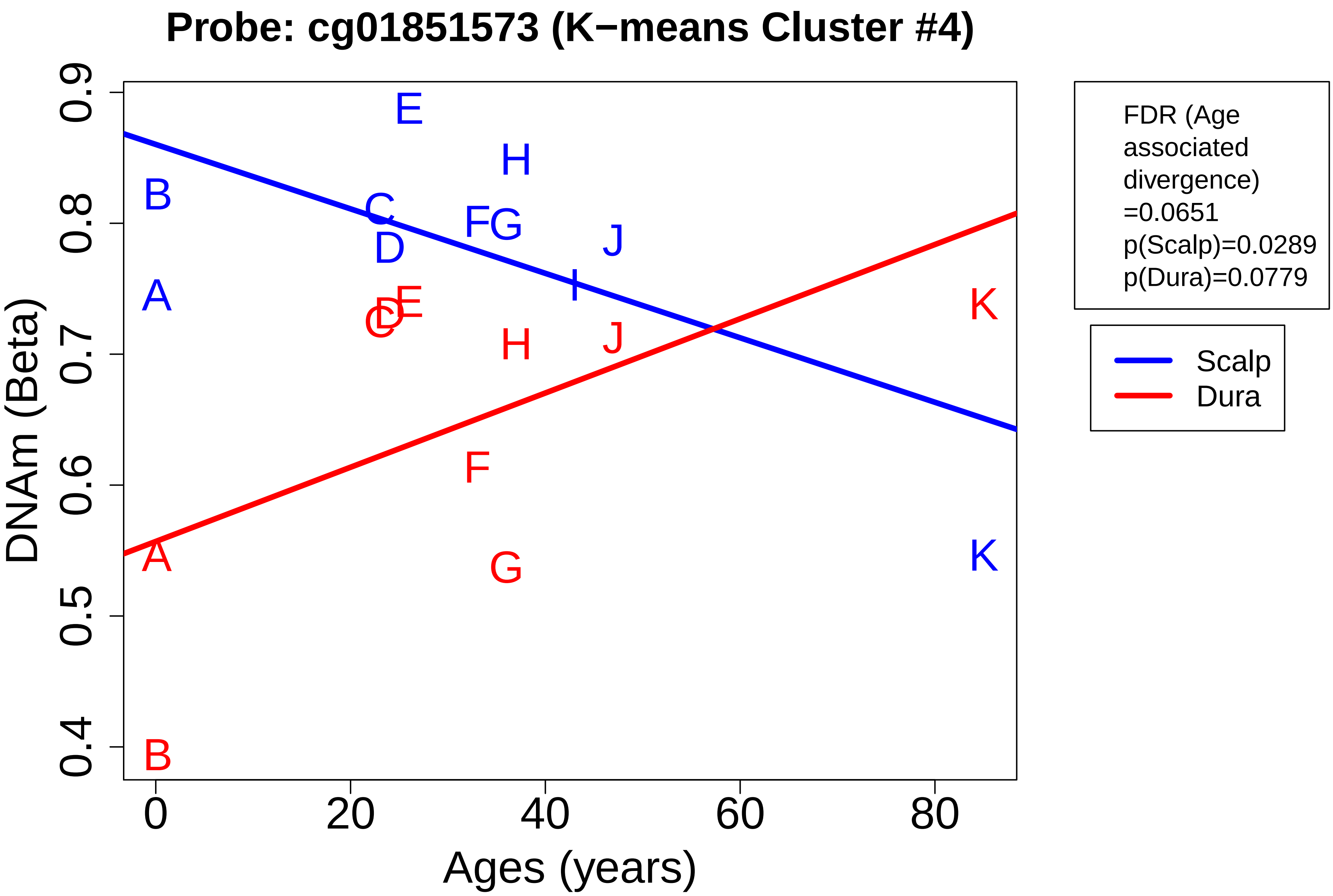

Probe: cg01851573 (K−means Cluster #4)
DNAm (Beta)
0.4
0.5
0.6
0.7
0.8
0.9
0
20
40
60
80
Ages (years)
FDR (Age associated divergence) =0.0651
p(Scalp)=0.0289
p(Dura)=0.0779
Scalp
Dura

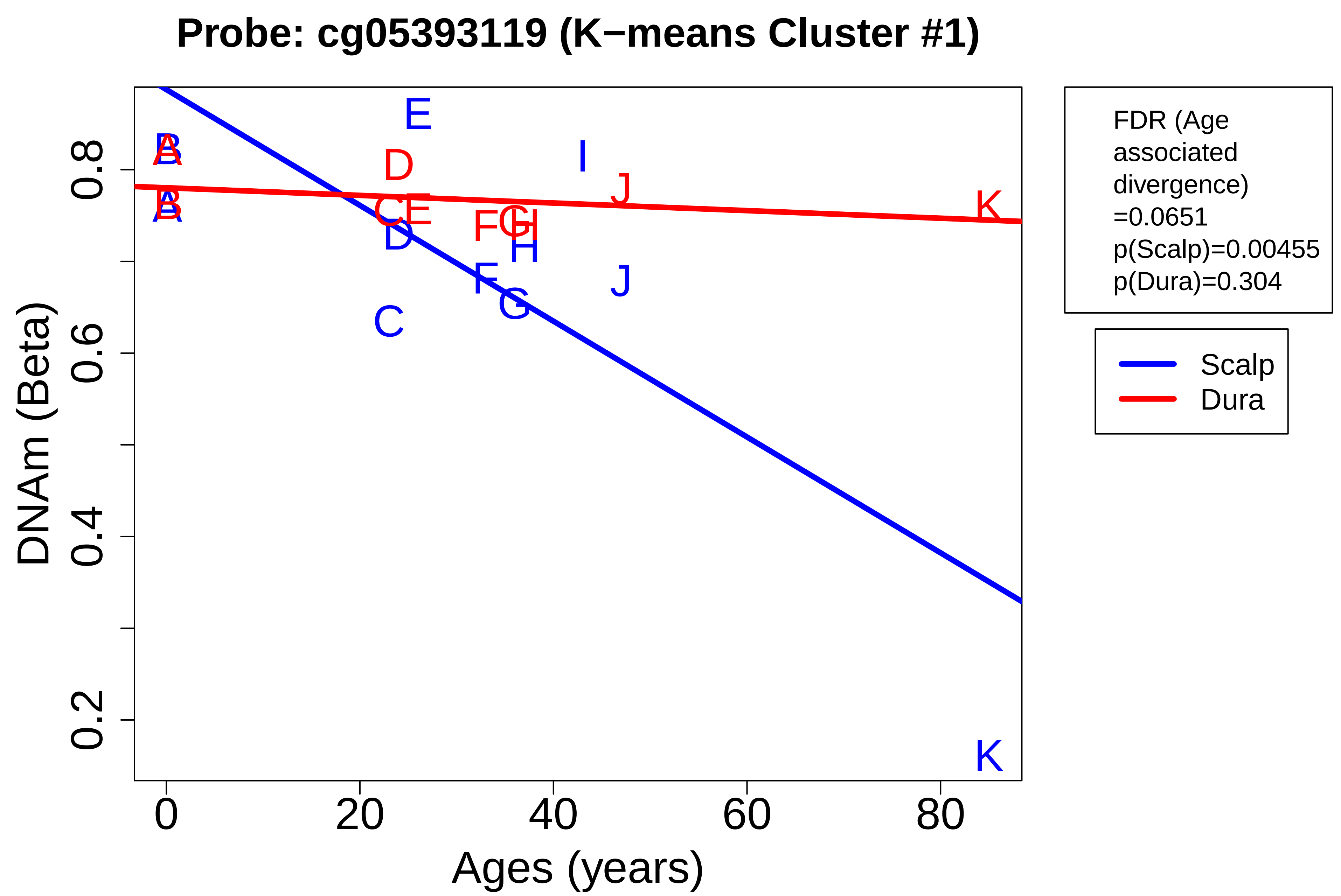

Probe: cg05393119 (K−means Cluster #1)
DNAm (Beta)
0.2
0.4
0.6
0.8
0
20
40
60
80
Ages (years)
A
B
C
D
E
F
G
H
I
J
K
FDR (Age associated divergence) =0.0651
p(Scalp)=0.00455
p(Dura)=0.304
Scalp
Dura

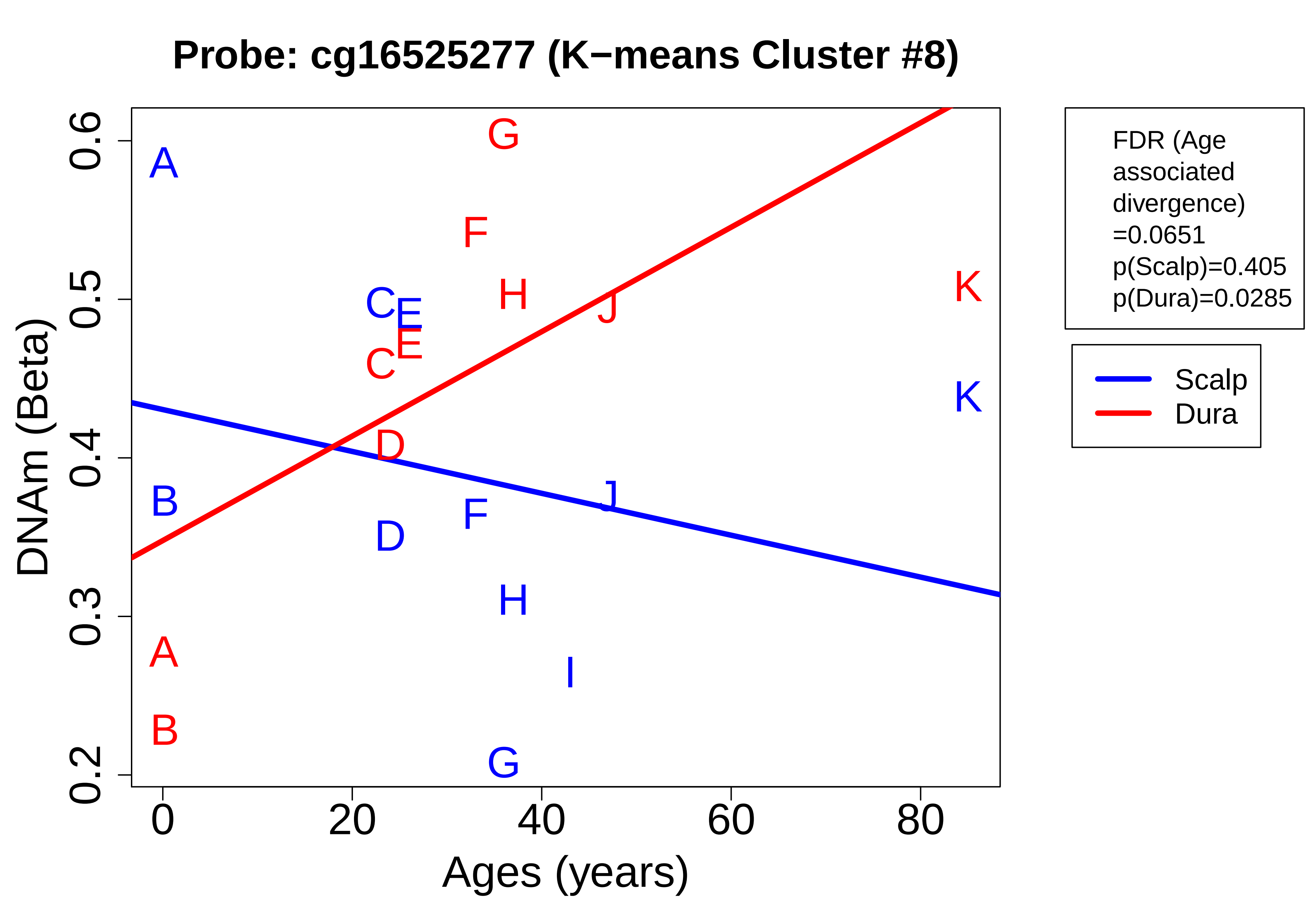

Probe: cg16525277 (K−means Cluster #8)
DNAm (Beta)
0.2
0.3
0.4
0.5
0.6
Ages (years)
0
20
40
60
80
A
B
C
D
E
F
G
H
I
J
K
FDR (Age associated divergence) =0.0651
p(Scalp)=0.405
p(Dura)=0.0285
Scalp
Dura

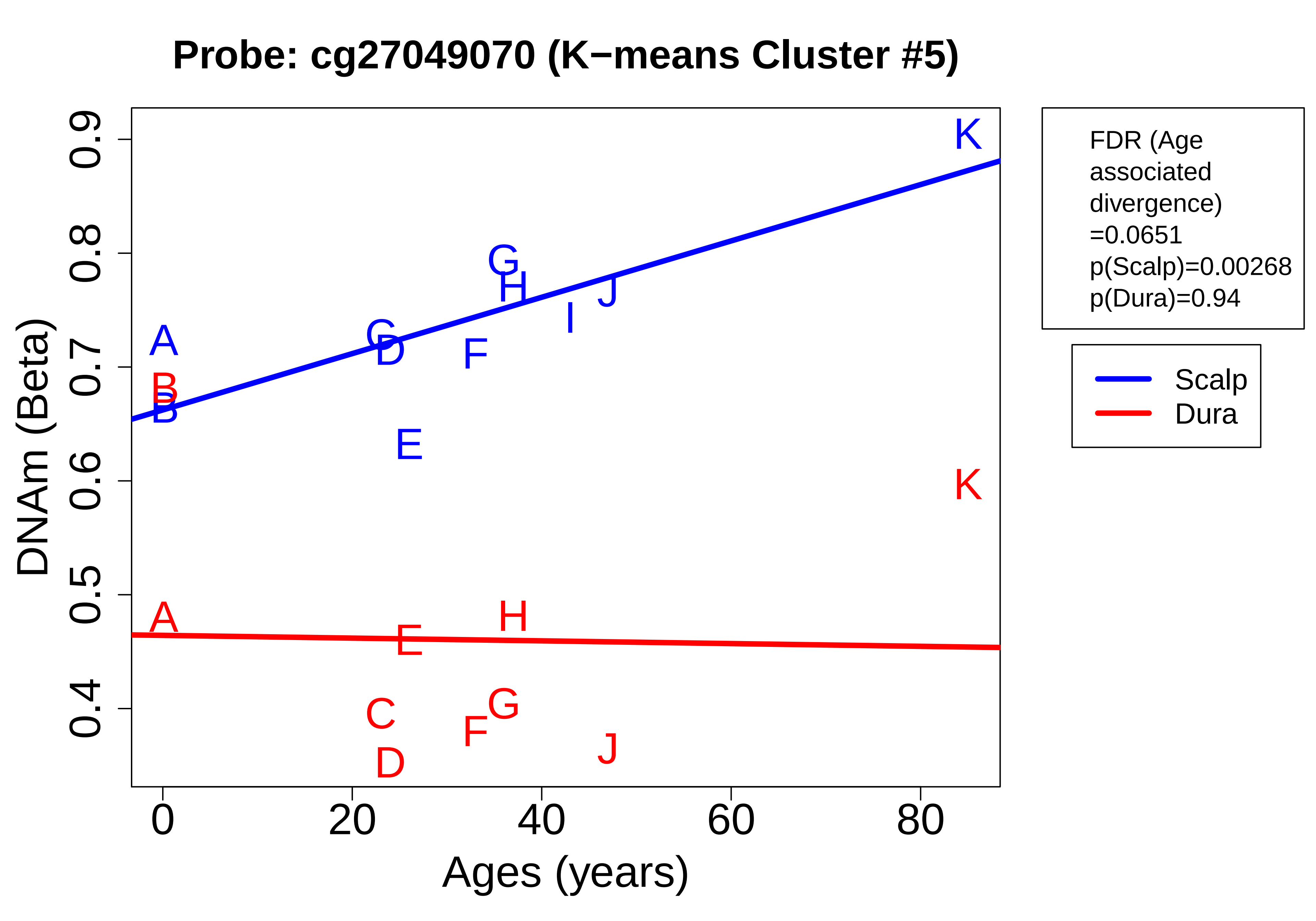

Probe: cg27049070 (K−means Cluster #5)
DNAm (Beta)
0.4
0.5
0.6
0.7
0.8
0.9
Ages (years)
0
20
40
60
80
FDR (Age associated divergence) =0.0651
p(Scalp)=0.00268
p(Dura)=0.94
Scalp
Dura
A
B
C
D
E
F
G
H
I
J
K

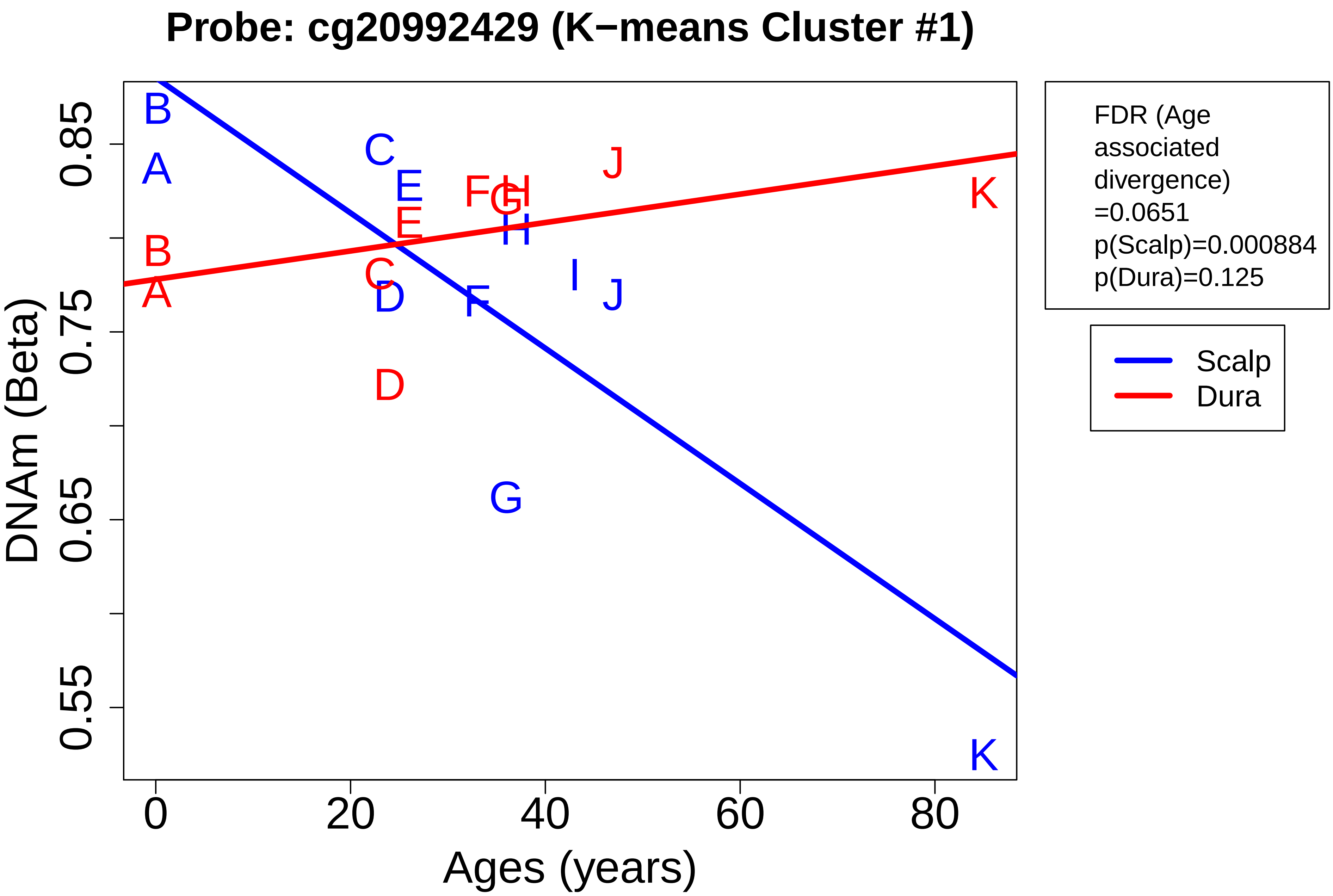

Probe: cg20992429 (K−means Cluster #1)
DNAm (Beta)
0.55
0.65
0.75
0.85
0
20
40
60
80
Ages (years)
FDR (Age associated divergence) =0.0651
p(Scalp)=0.000884
p(Dura)=0.125
Scalp
Dura

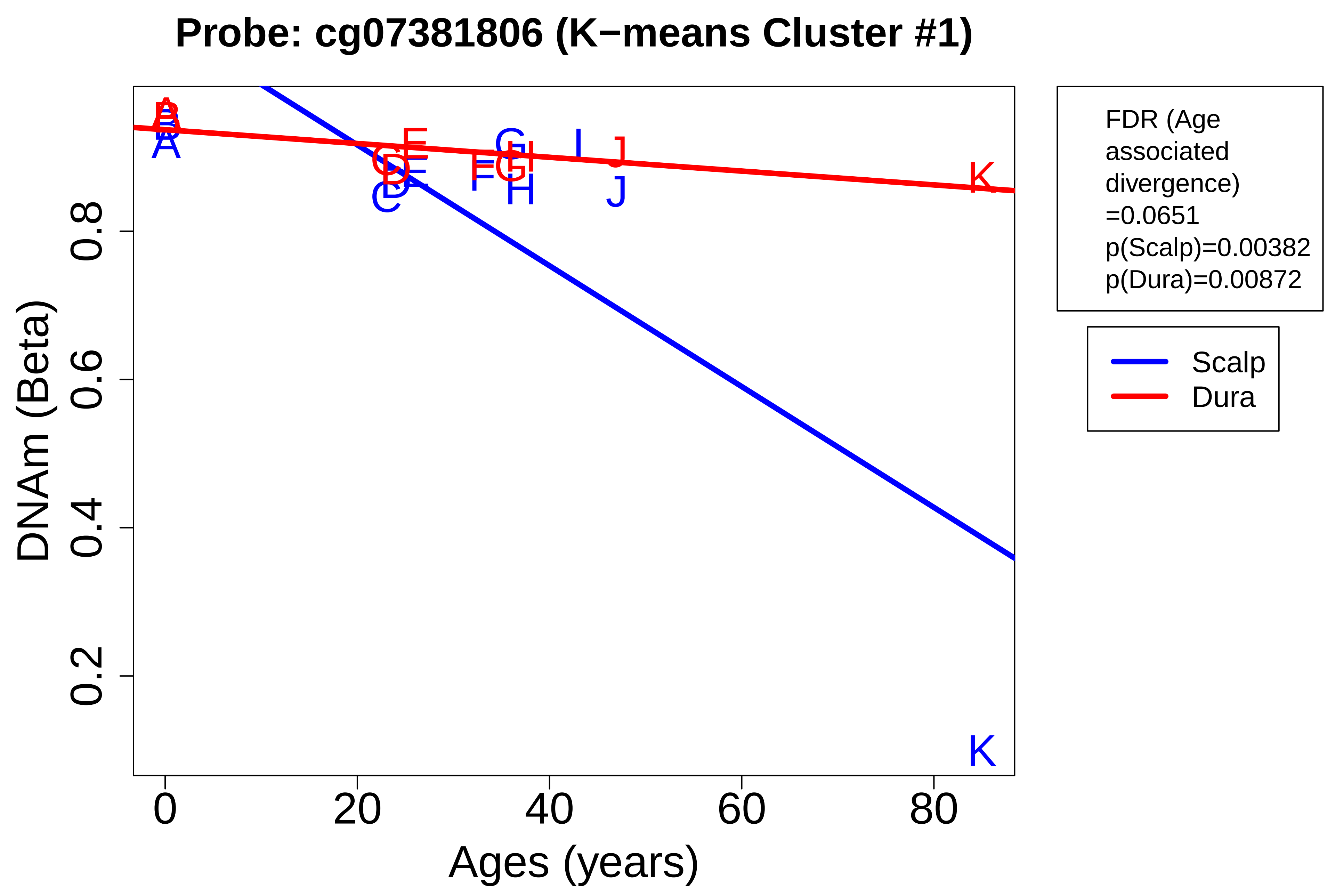

Probe: cg07381806 (K−means Cluster #1)
DNAm (Beta)
0.2
0.4
0.6
0.8
Ages (years)
0
20
40
60
80
FDR (Age associated divergence) =0.0651
p(Scalp)=0.00382
p(Dura)=0.00872
Scalp
Dura
A
B
C
D
E
F
G
H
I
J
K

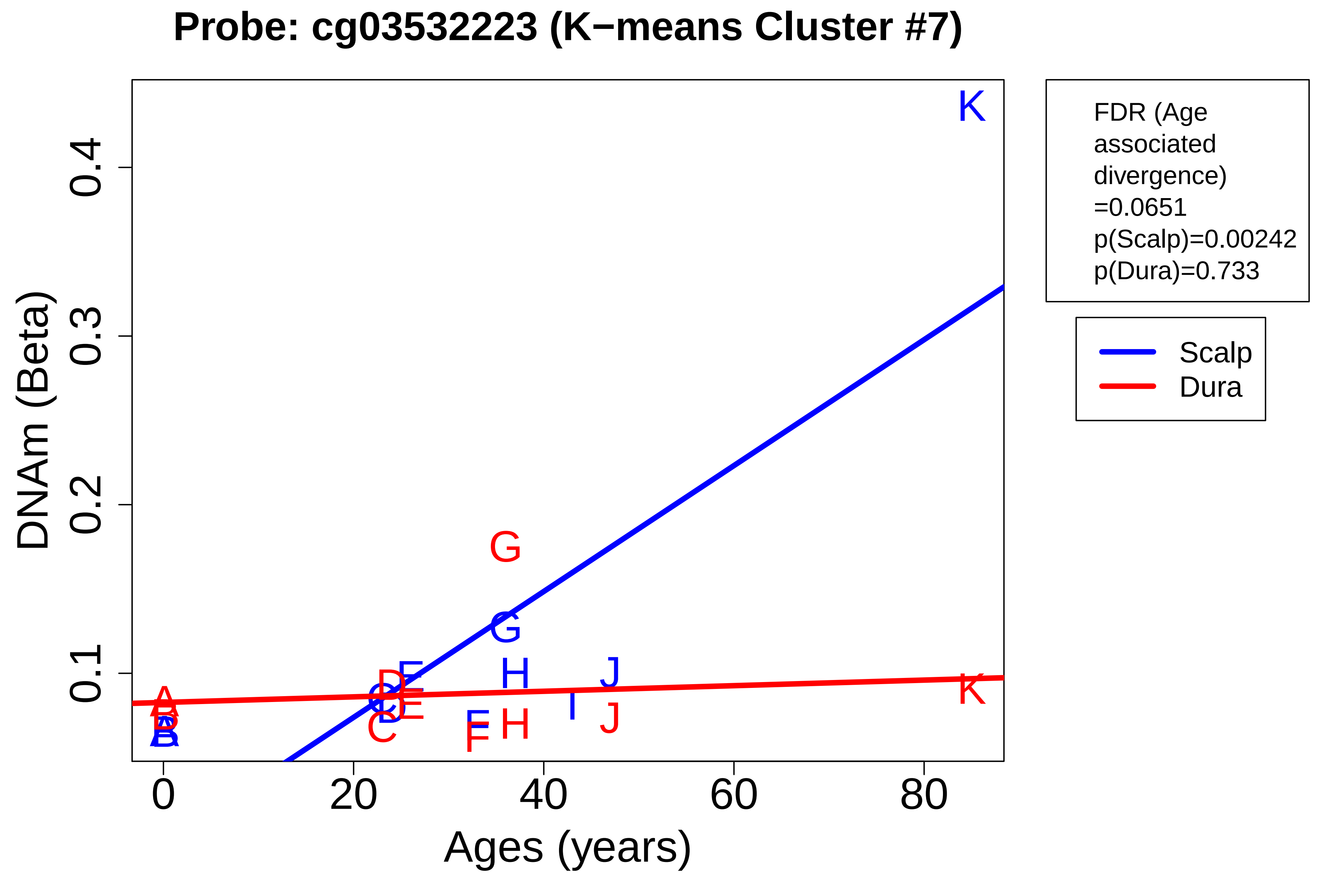

Probe: cg03532223 (K−means Cluster #7)
DNAm (Beta)
0.1
0.2
0.3
0.4
Ages (years)
0
20
40
60
80
A
B
C
D
E
F
G
H
I
J
K
FDR (Age associated divergence) =0.0651
p(Scalp)=0.00242
p(Dura)=0.733
Scalp
Dura

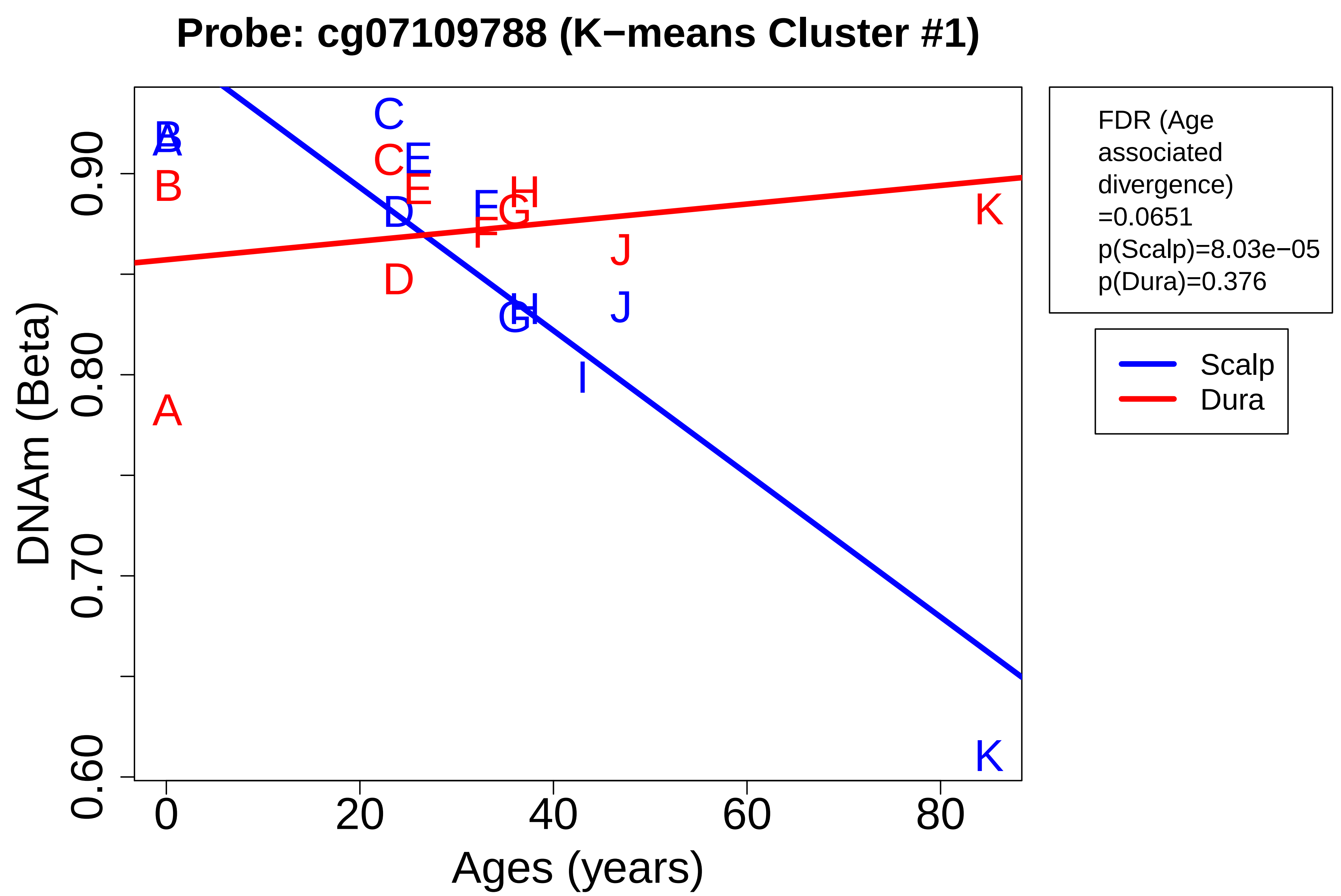
Probe: cg07109788 (K−means Cluster #1)
DNAm (Beta)
0.60
0.70
0.80
0.90
Ages (years)
0
20
40
60
80
A B C D E F G H I J K
FDR (Age associated divergence) =0.0651
p(Scalp)=8.03e−05
p(Dura)=0.376
Scalp
Dura

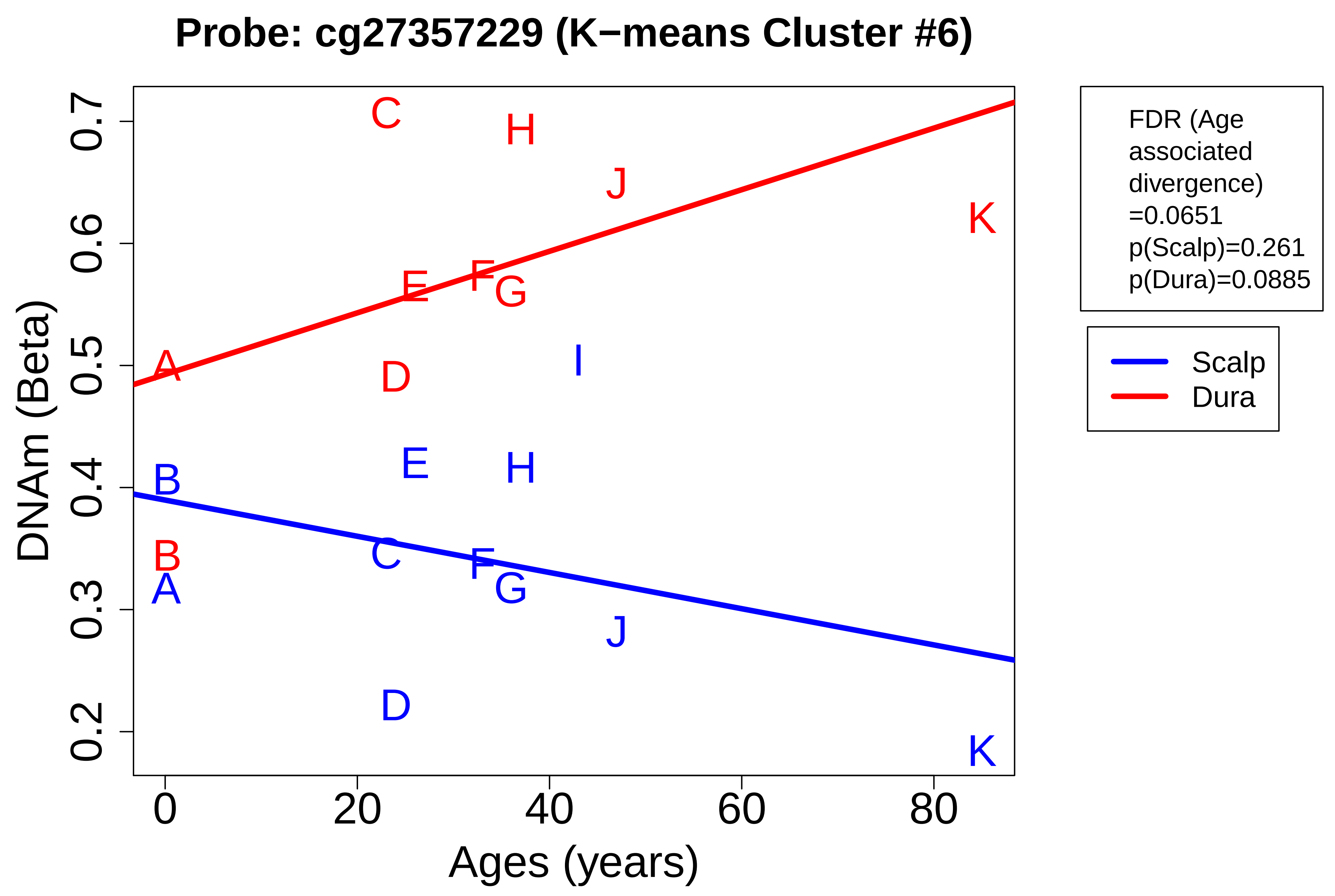
Probe: cg27357229 (K−means Cluster #6)
DNAm (Beta)
Ages (years)
0.2
0.3
0.4
0.5
0.6
0.7
0
20
40
60
80
FDR (Age associated divergence) =0.0651
p(Scalp)=0.261
p(Dura)=0.0885
Scalp
Dura
A
B
C
D
E
F
G
H
I
J
K

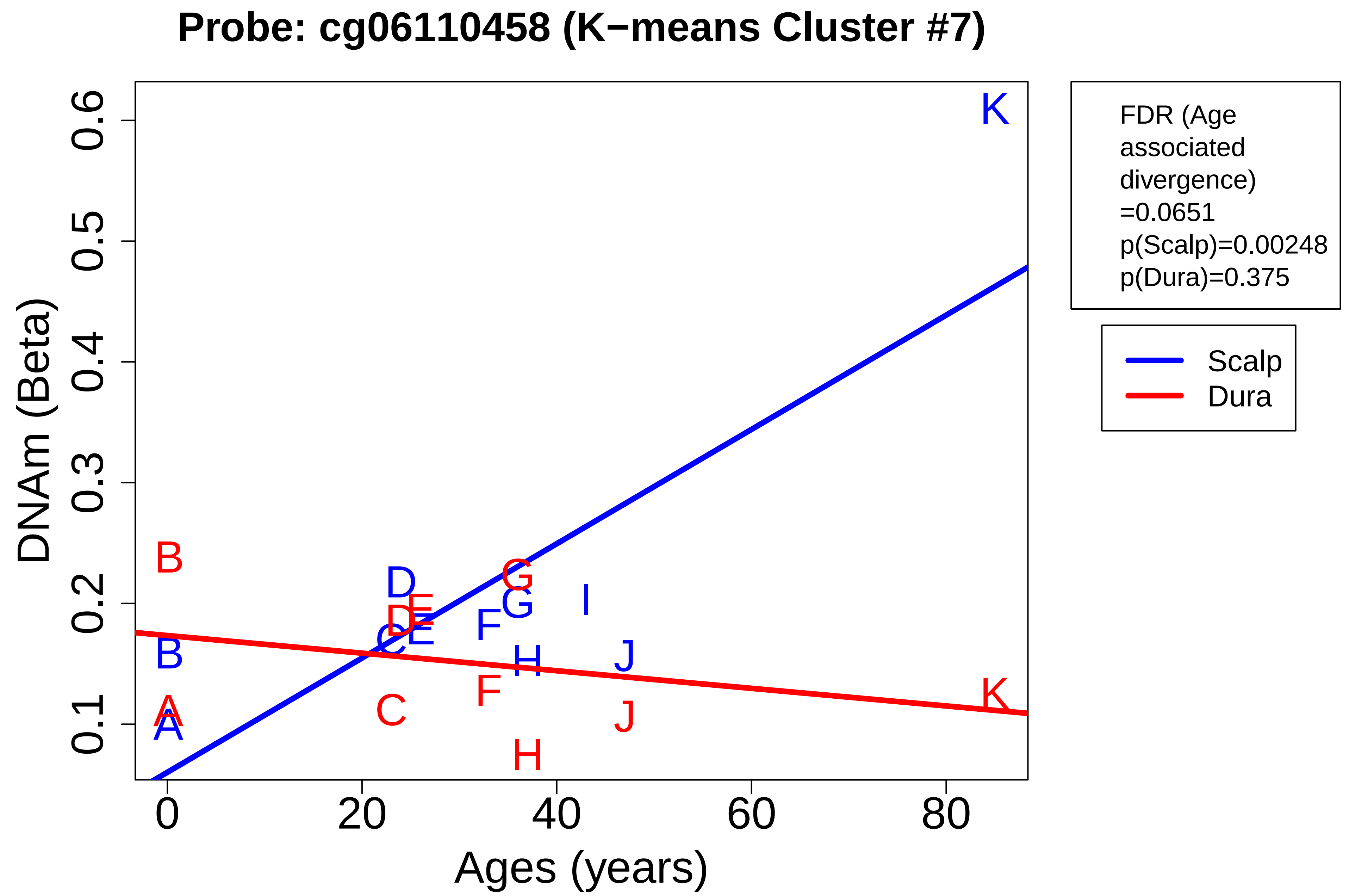
Probe: cg06110458 (K−means Cluster #7)
DNAm (Beta)
0.1
0.2
0.3
0.4
0.5
0.6
Ages (years)
0
20
40
60
80
FDR (Age associated divergence) =0.0651
p(Scalp)=0.00248
p(Dura)=0.375
Scalp
Dura
A
B
C
D
E
F
G
H
I
J
K

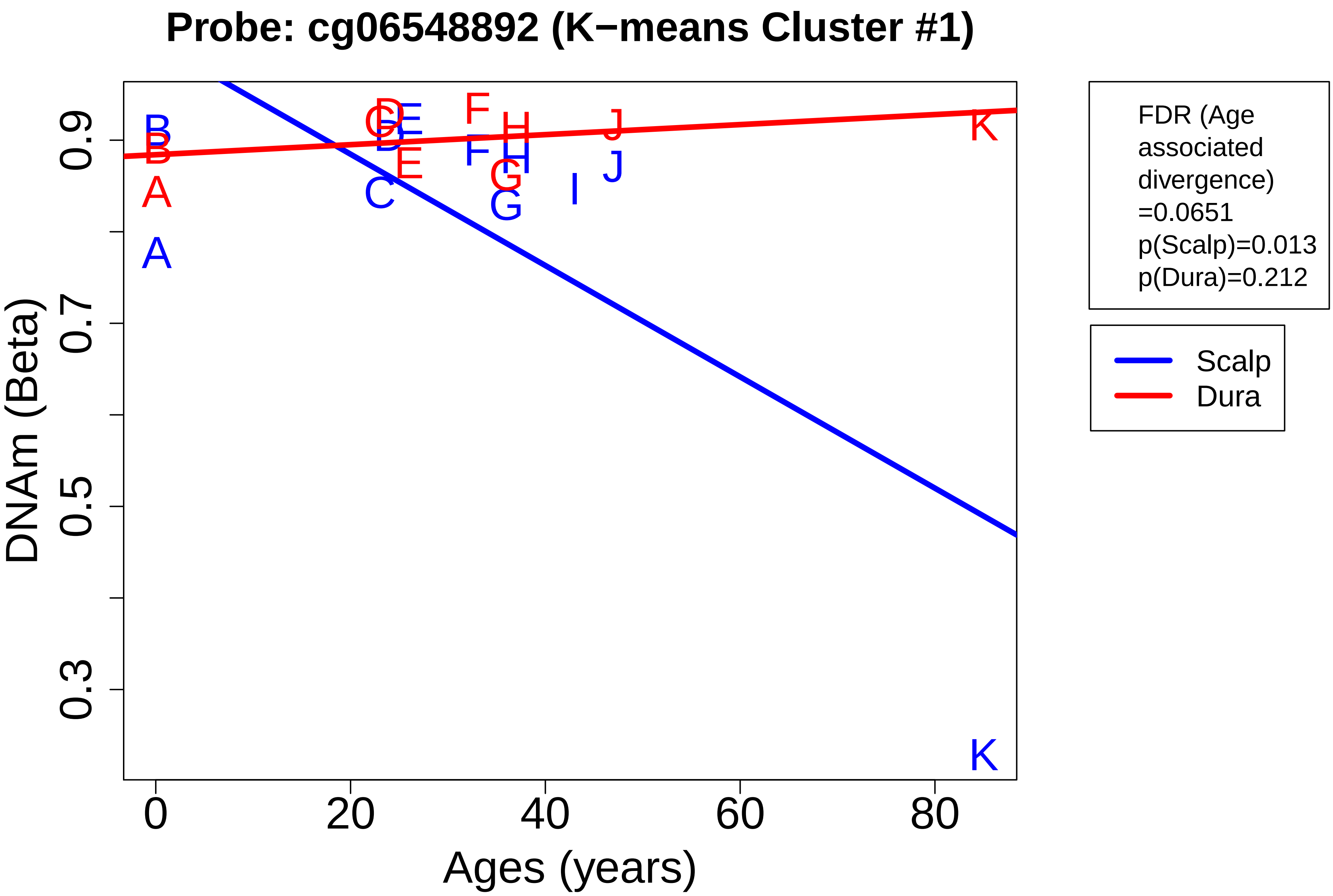
Probe: cg06548892 (K−means Cluster #1)
DNAm (Beta)
0.3
0.5
0.7
0.9
Ages (years)
0
20
40
60
80
A
B
C
D
E
F
G
H
I
J
K
FDR (Age associated divergence) =0.0651
p(Scalp)=0.013
p(Dura)=0.212
Scalp
Dura

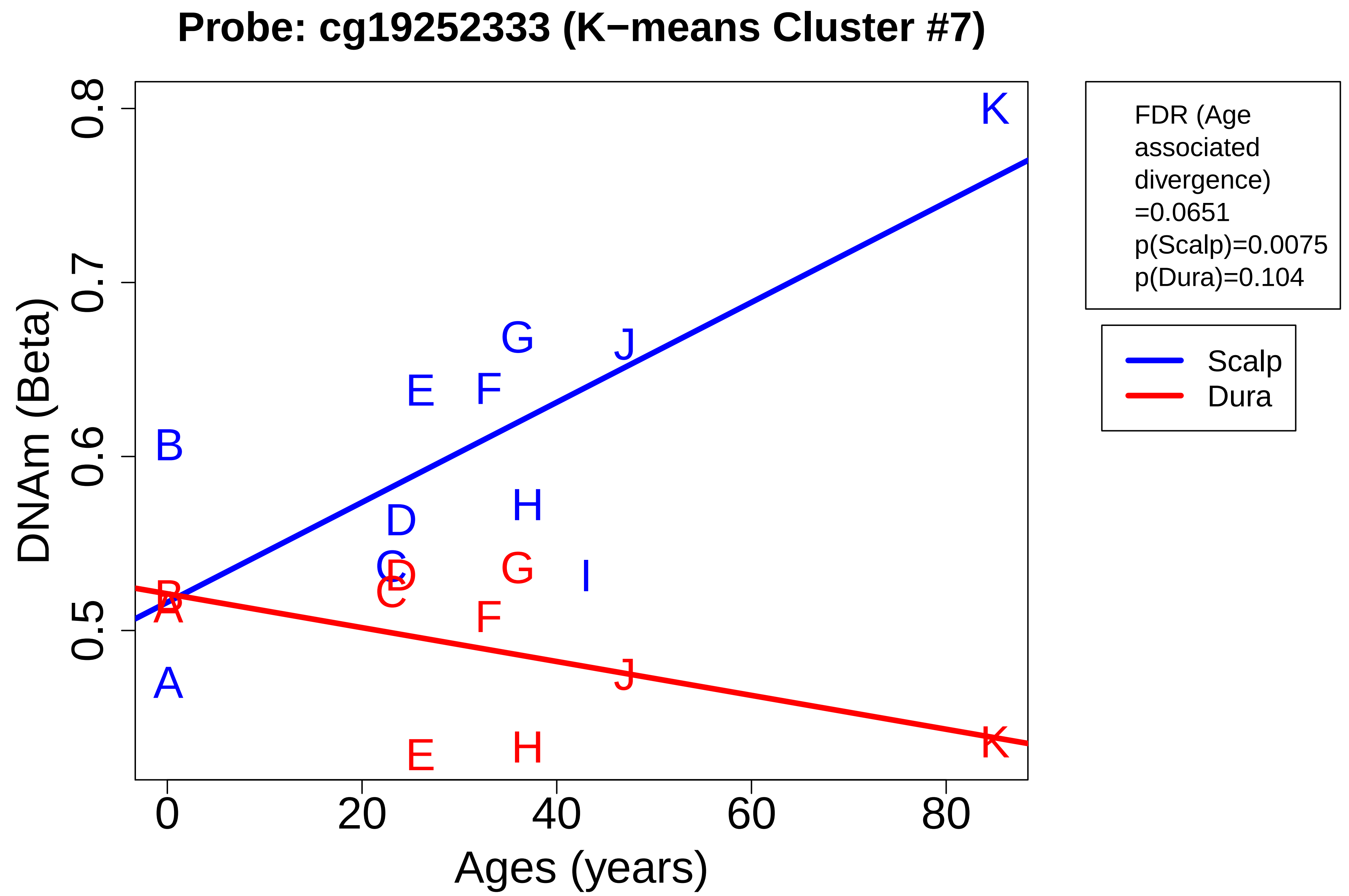

Probe: cg19252333 (K−means Cluster #7)
DNAm (Beta)
0.5
0.6
0.7
0.8
0
20
40
60
80
Ages (years)
FDR (Age associated divergence) =0.0651
p(Scalp)=0.0075
p(Dura)=0.104
Scalp
Dura

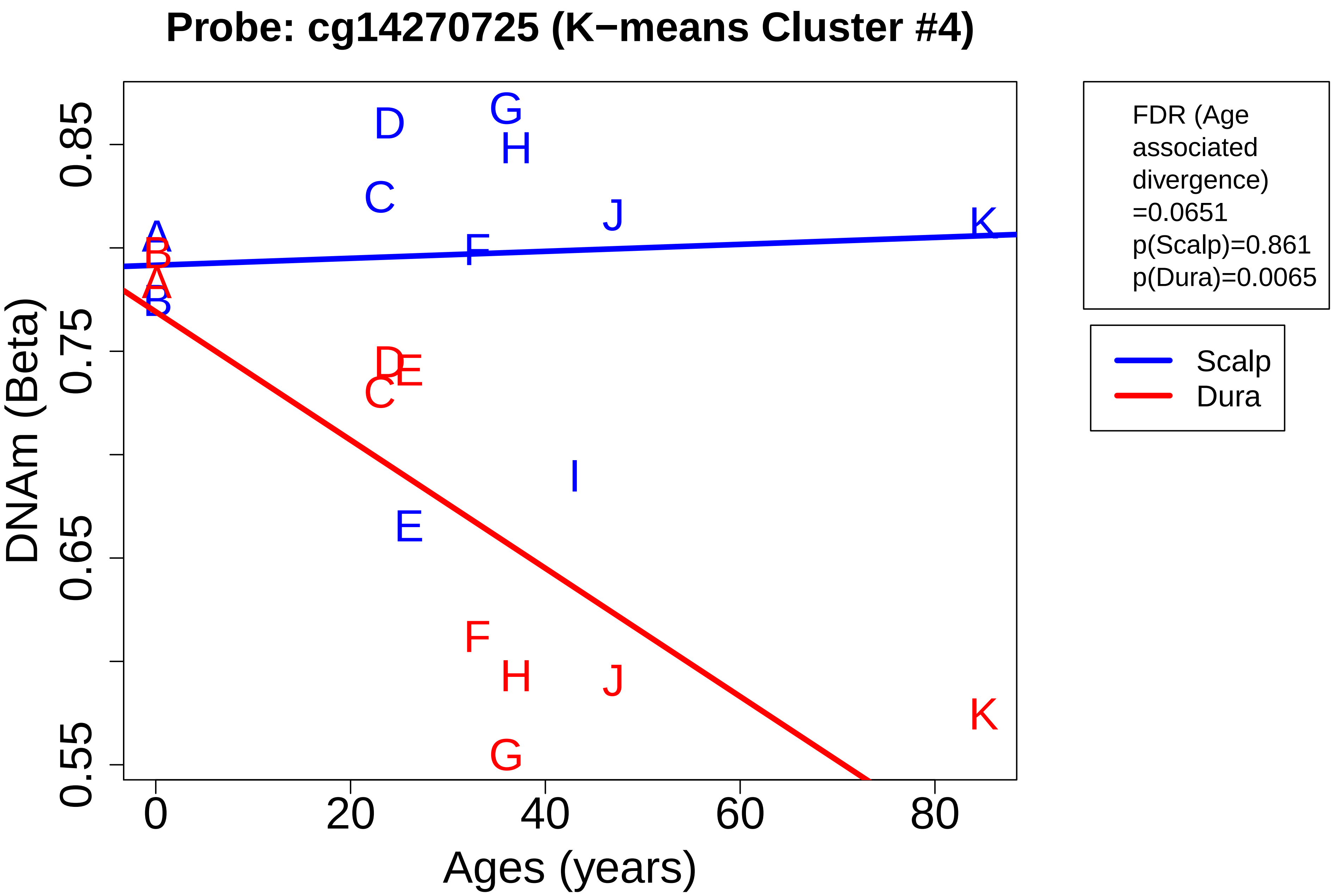

Probe: cg14270725 (K−means Cluster #4)
DNAm (Beta)
0.55
0.65
0.75
0.85
0
20
40
60
80
Ages (years)
FDR (Age associated divergence) =0.0651
p(Scalp)=0.861
p(Dura)=0.0065
Scalp
Dura
A
B
C
D
E
F
G
H
I
J
K

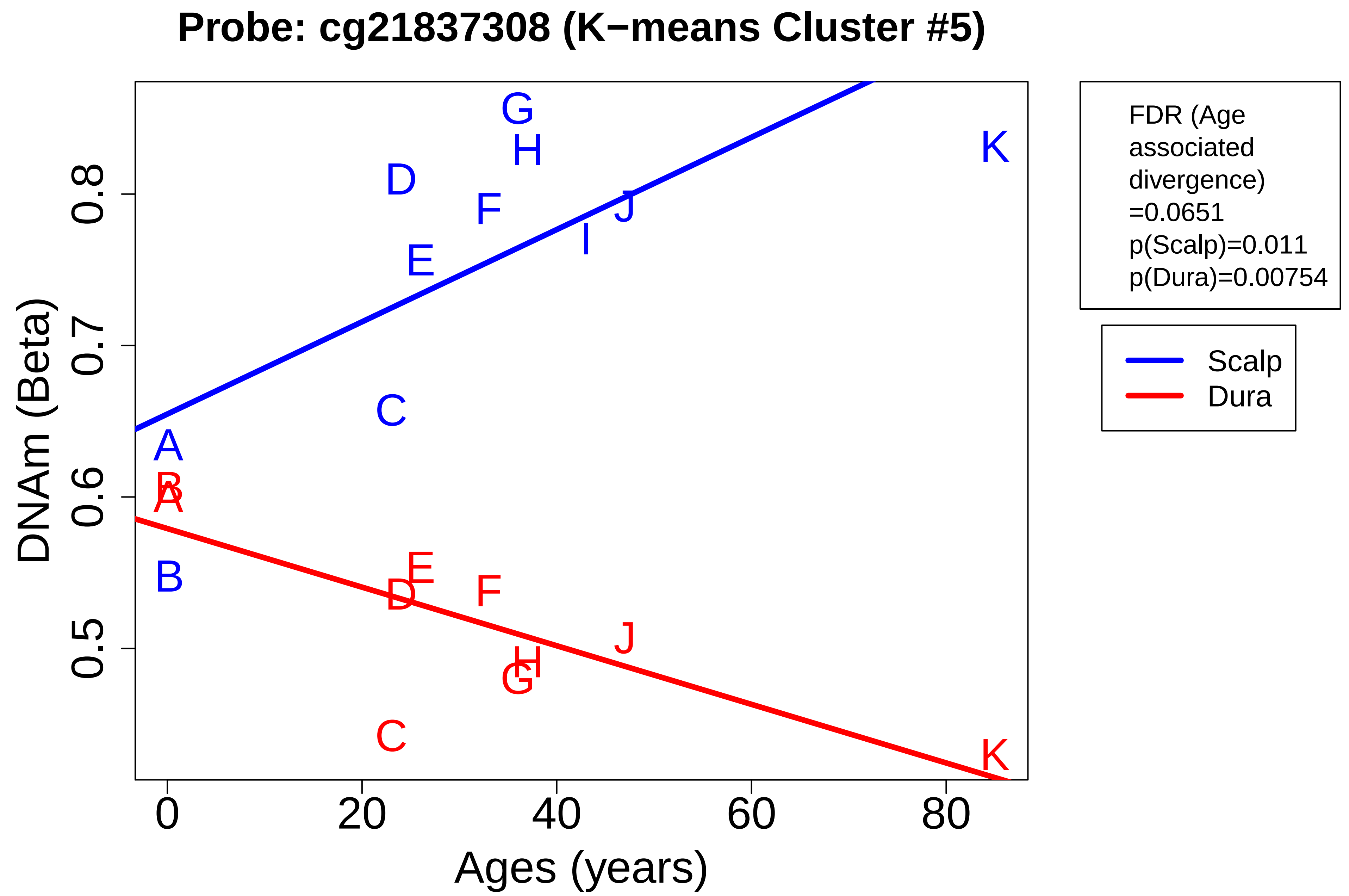
Probe: cg21837308 (K−means Cluster #5)
DNAm (Beta)
Ages (years)
FDR (Age associated divergence) =0.0651
p(Scalp)=0.011
p(Dura)=0.00754
Scalp
Dura

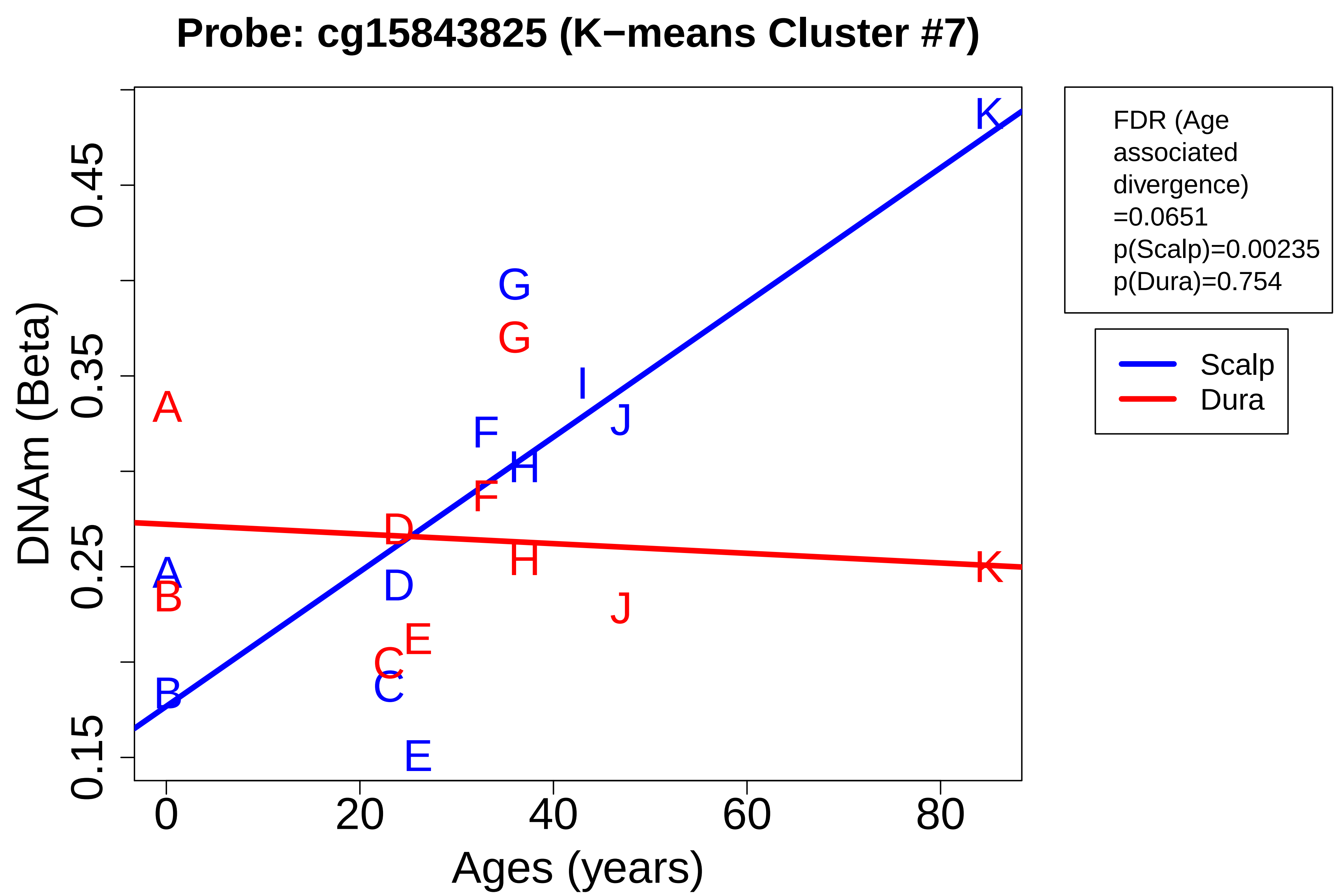
Probe: cg15843825 (K−means Cluster #7)
FDR (Age associated divergence) =0.0651
p(Scalp)=0.00235
p(Dura)=0.754
Scalp
Dura
DNAm (Beta)
0.15
0.25
0.35
0.45
0
20
40
60
80
Ages (years)
A
B
C
D
E
F
G
H
I
J
K

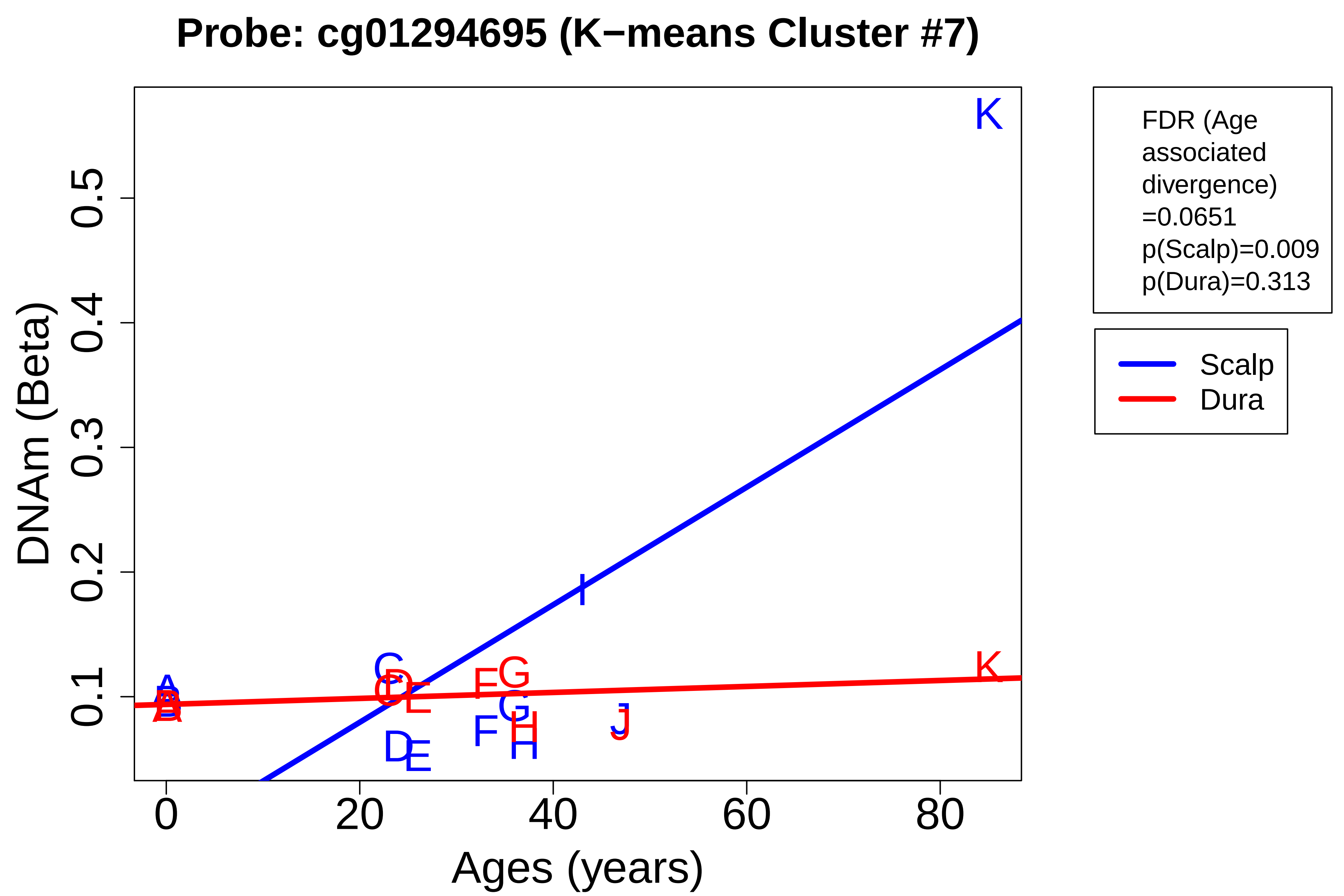

Probe: cg01294695 (K−means Cluster #7)
DNAm (Beta)
0.1
0.2
0.3
0.4
0.5
Ages (years)
0
20
40
60
80
FDR (Age associated divergence) =0.0651
p(Scalp)=0.009
p(Dura)=0.313
Scalp
Dura
A
B
C
D
E
F
G
H
I
J
K

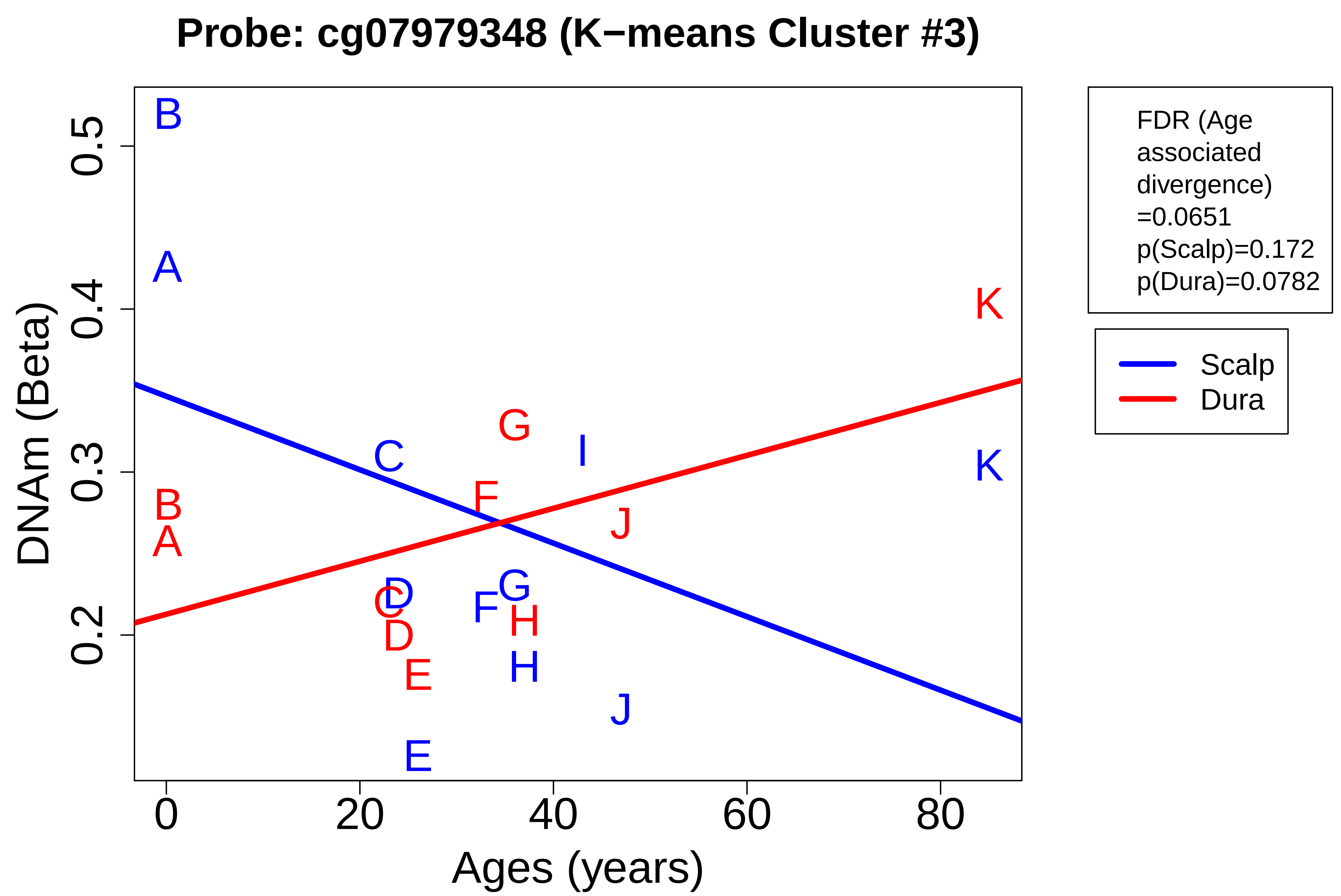
Probe: cg07979348 (K−means Cluster #3)
FDR (Age associated divergence) =0.0651
p(Scalp)=0.172
p(Dura)=0.0782
Scalp
Dura
DNAm (Beta)
0.2
0.3
0.4
0.5
0
20
40
60
80
Ages (years)
A
B
C
D
E
F
G
H
I
J
K

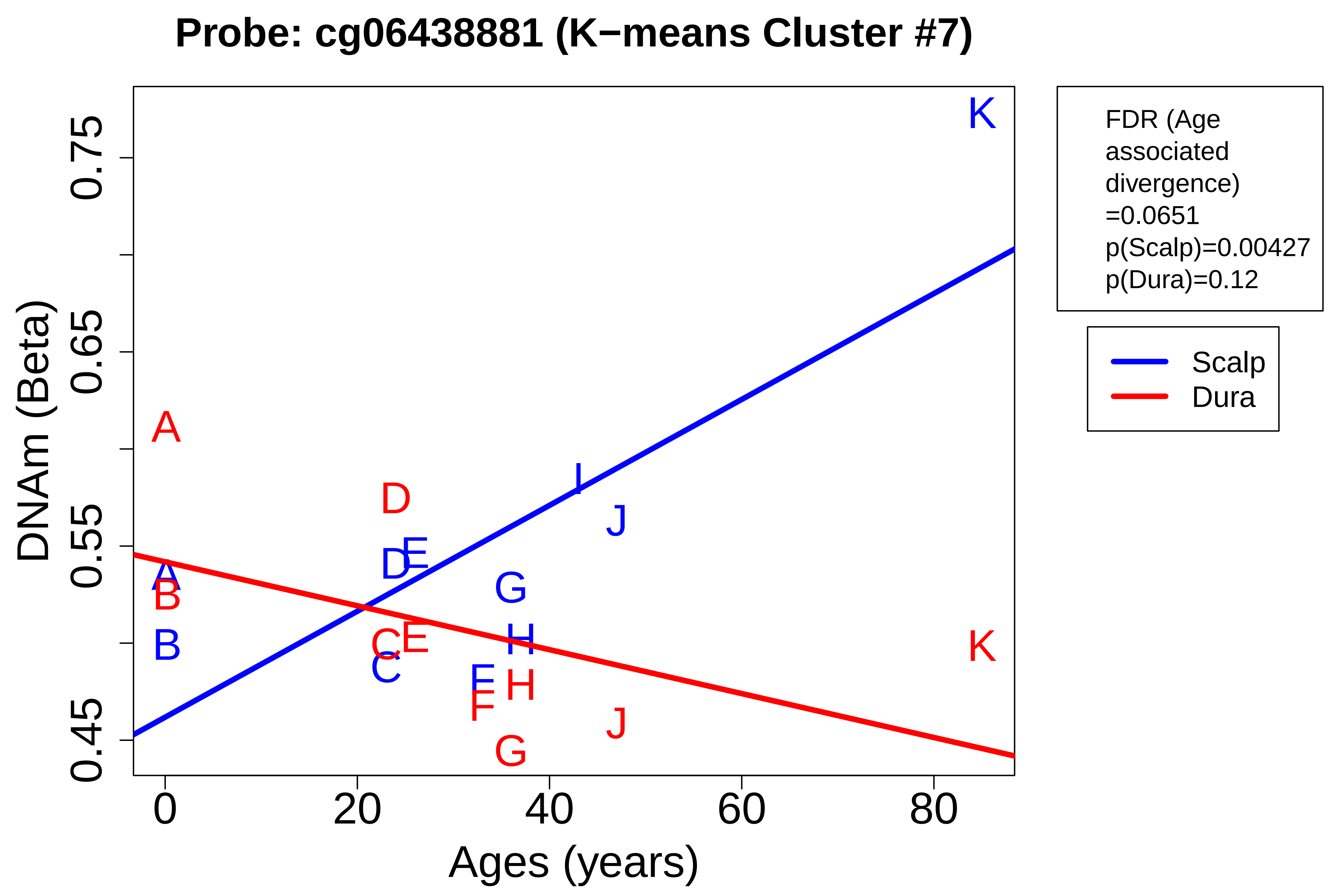

Probe: cg06438881 (K−means Cluster #7)
FDR (Age associated divergence) =0.0651
p(Scalp)=0.00427
p(Dura)=0.12
Scalp
Dura
DNAm (Beta)
0.45
0.55
0.65
0.75
0
20
40
60
80
Ages (years)
A
B
C
D
E
F
G
H
I
J
K

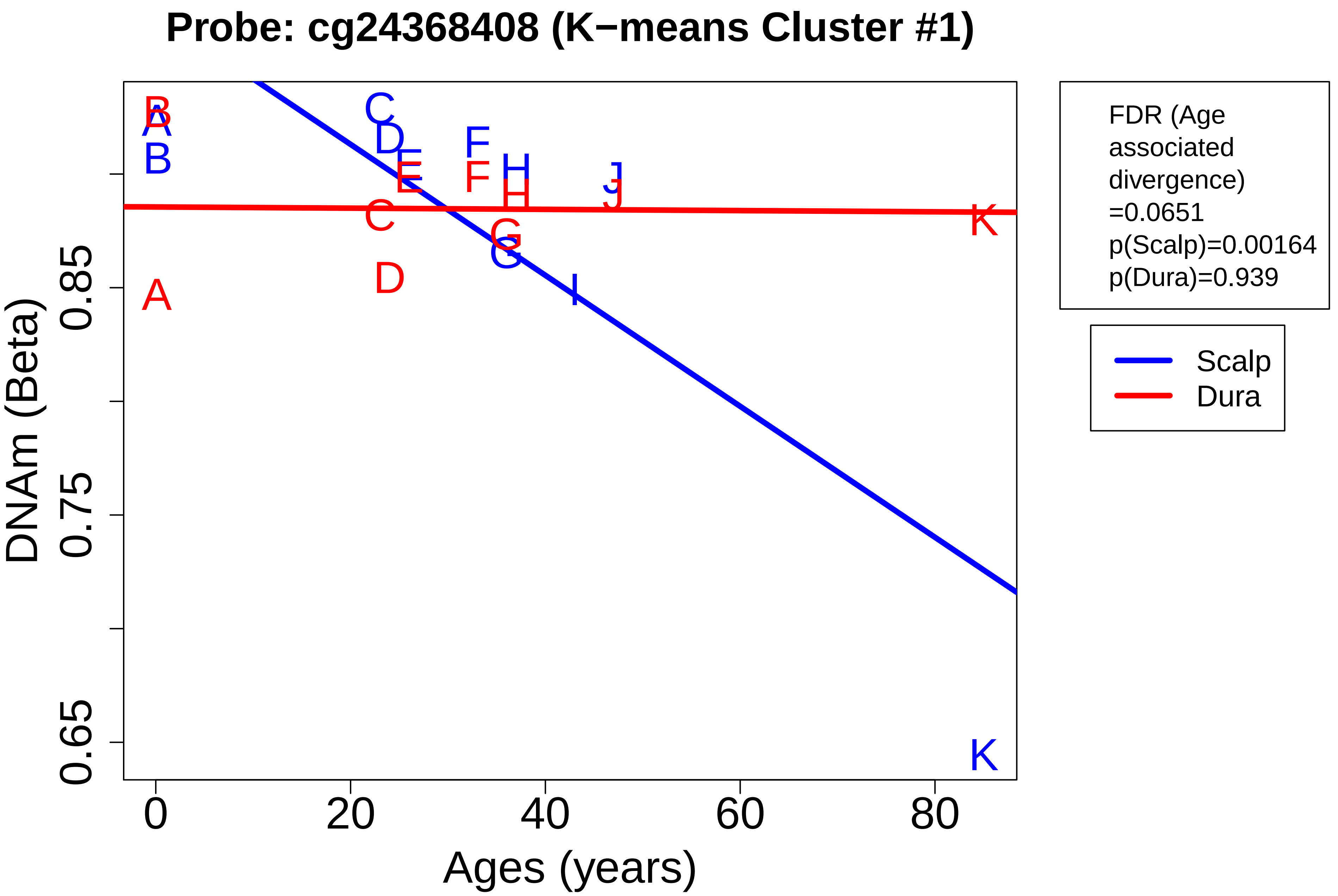

Probe: cg24368408 (K−means Cluster #1)
DNAm (Beta)
0.65
0.75
0.85
Ages (years)
0
20
40
60
80
FDR (Age associated divergence) =0.0651
p(Scalp)=0.00164
p(Dura)=0.939
Scalp
Dura
A
B
C
D
E
F
G
H
I
J
K

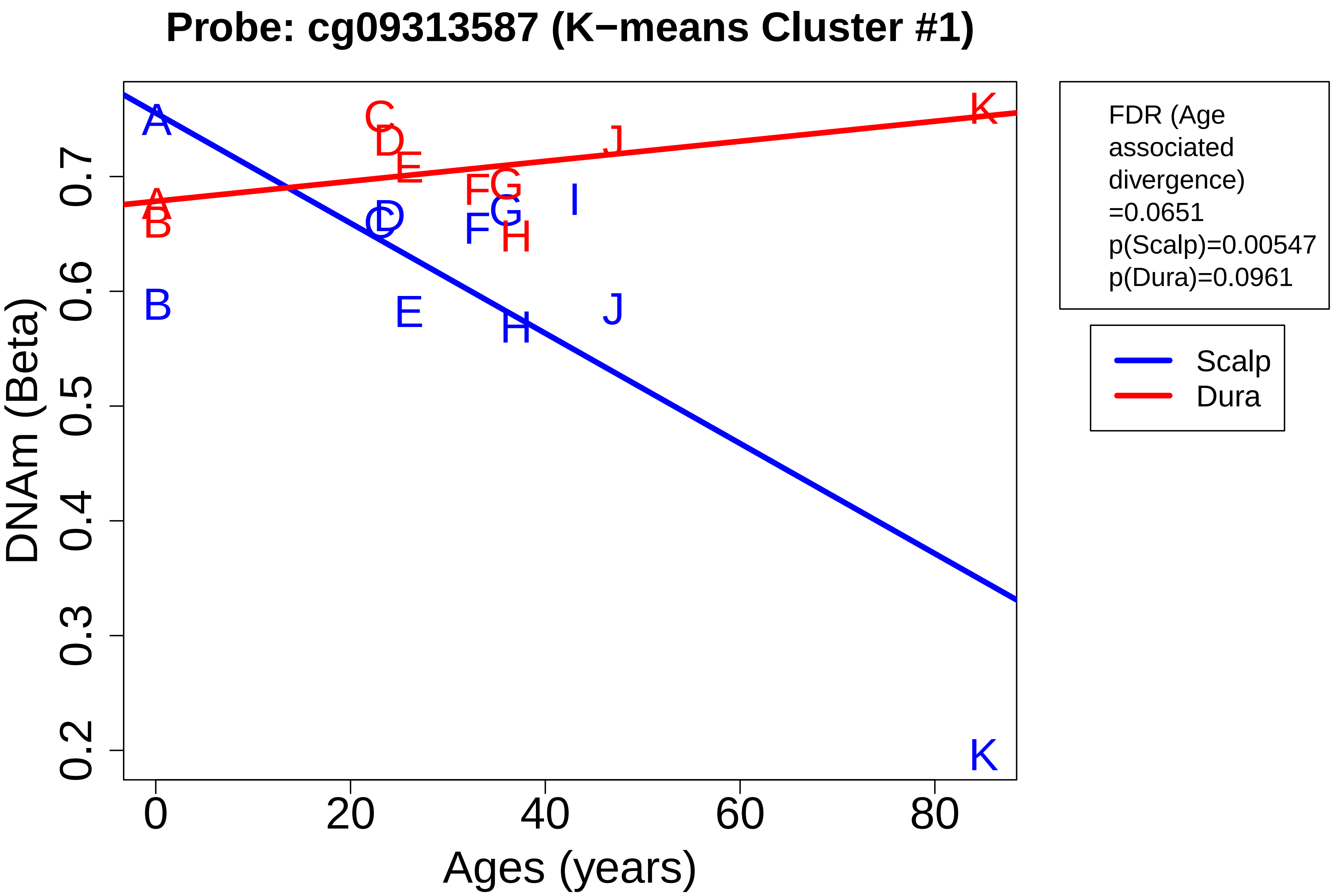

Probe: cg09313587 (K−means Cluster #1)
DNAm (Beta)
0.2
0.3
0.4
0.5
0.6
0.7
Ages (years)
0
20
40
60
80
FDR (Age associated divergence) =0.0651
p(Scalp)=0.00547
p(Dura)=0.0961
Scalp
Dura

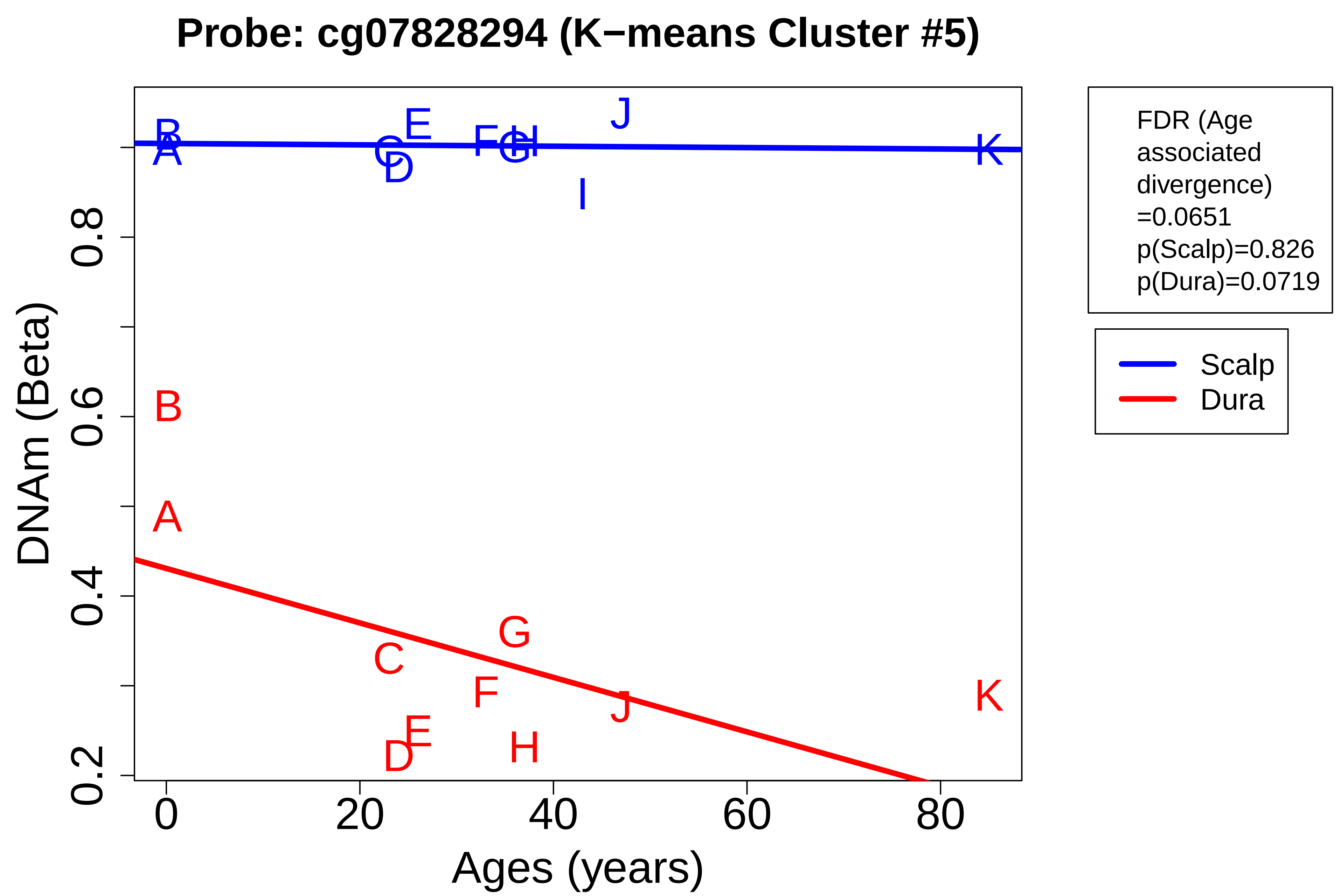

Probe: cg07828294 (K−means Cluster #5)
DNAm (Beta)
0.2
0.4
0.6
0.8
0
20
40
60
80
Ages (years)
FDR (Age associated divergence) =0.0651
p(Scalp)=0.826
p(Dura)=0.0719
Scalp
Dura

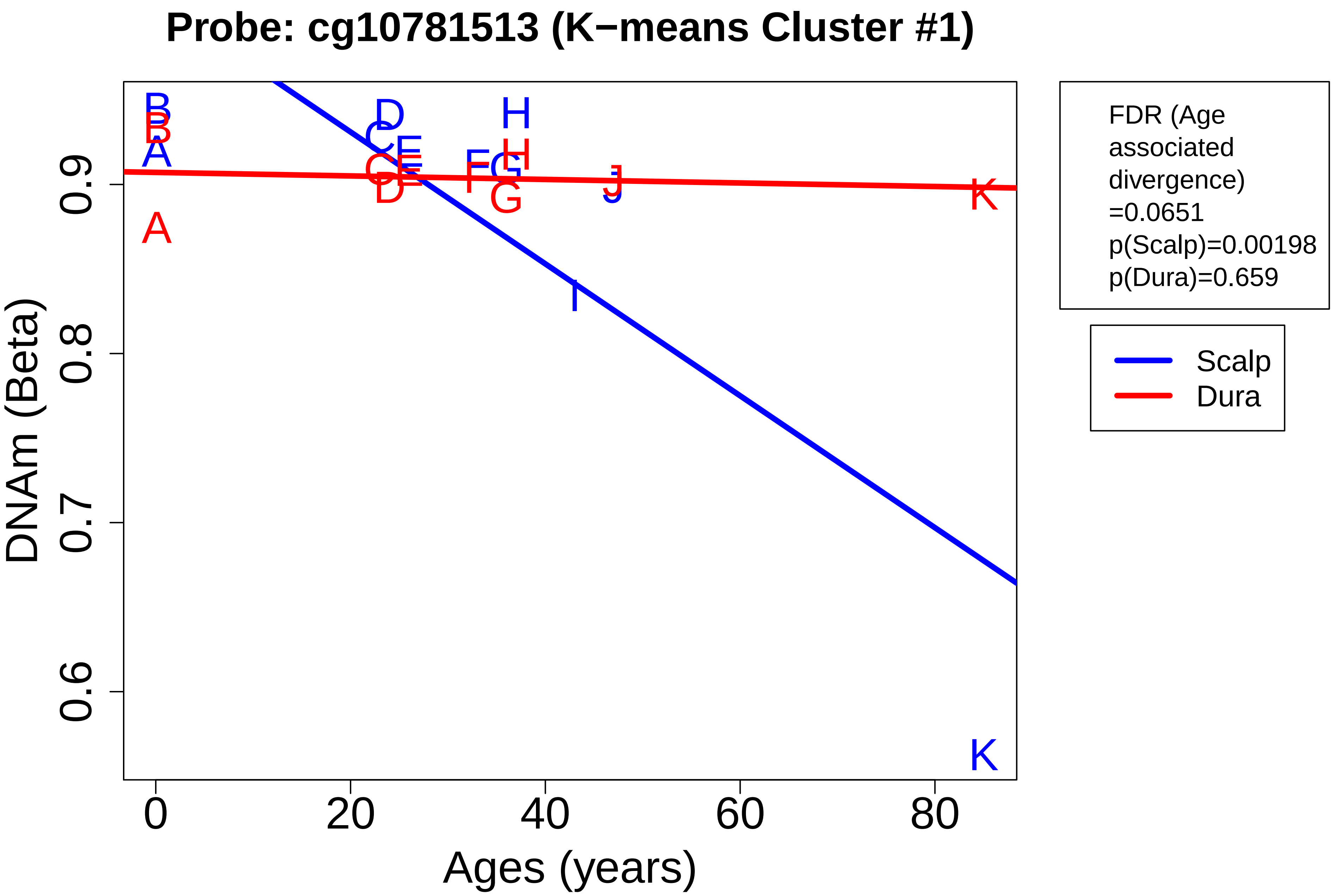

Probe: cg10781513 (K−means Cluster #1)
DNAm (Beta)
Ages (years)
0.6
0.7
0.8
0.9
0
20
40
60
80
FDR (Age associated divergence) =0.0651
p(Scalp)=0.00198
p(Dura)=0.659
Scalp
Dura

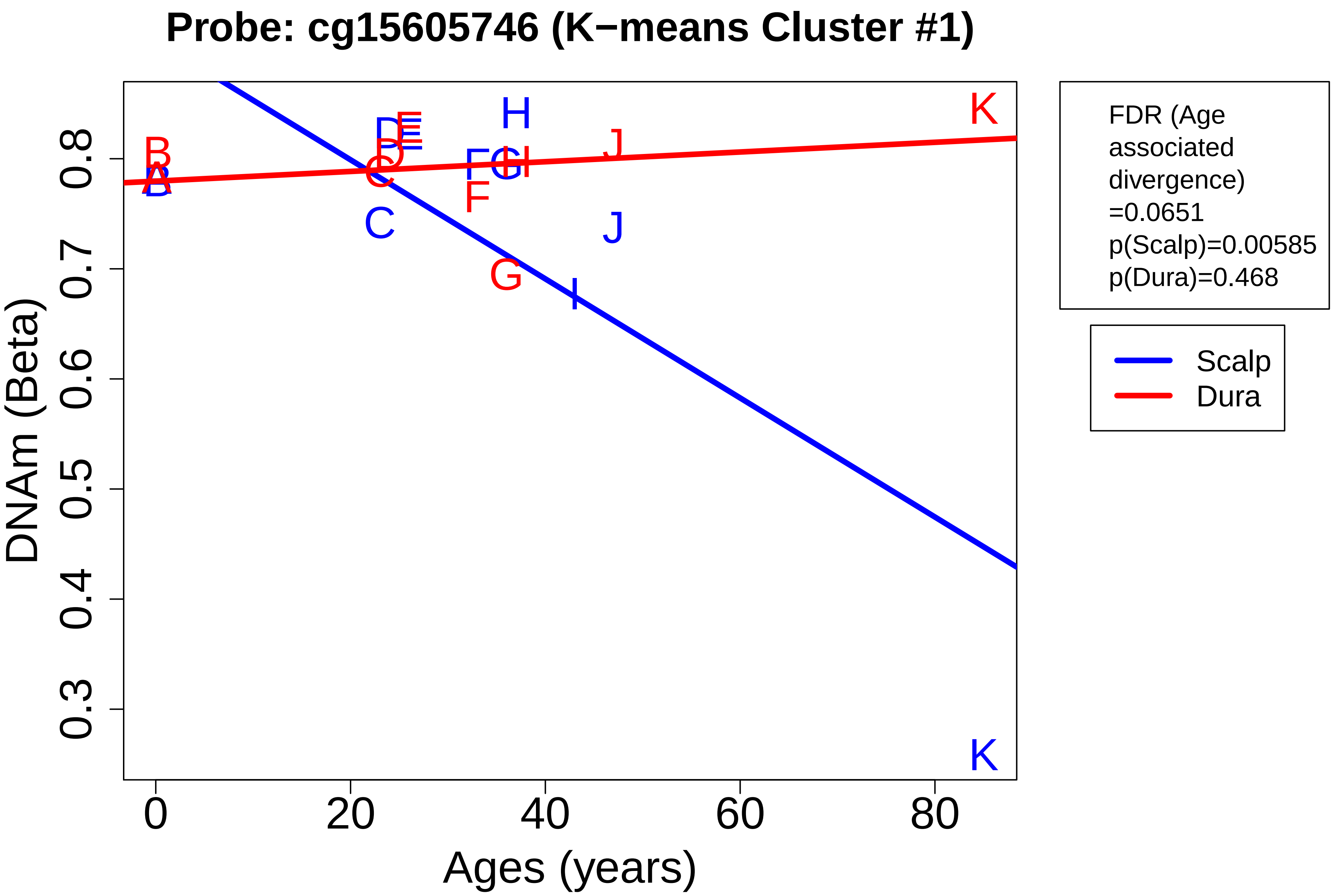

Probe: cg15605746 (K−means Cluster #1)
DNAm (Beta)
0.3
0.4
0.5
0.6
0.7
0.8
0
20
40
60
80
Ages (years)
FDR (Age associated divergence) =0.0651
p(Scalp)=0.00585
p(Dura)=0.468
Scalp
Dura

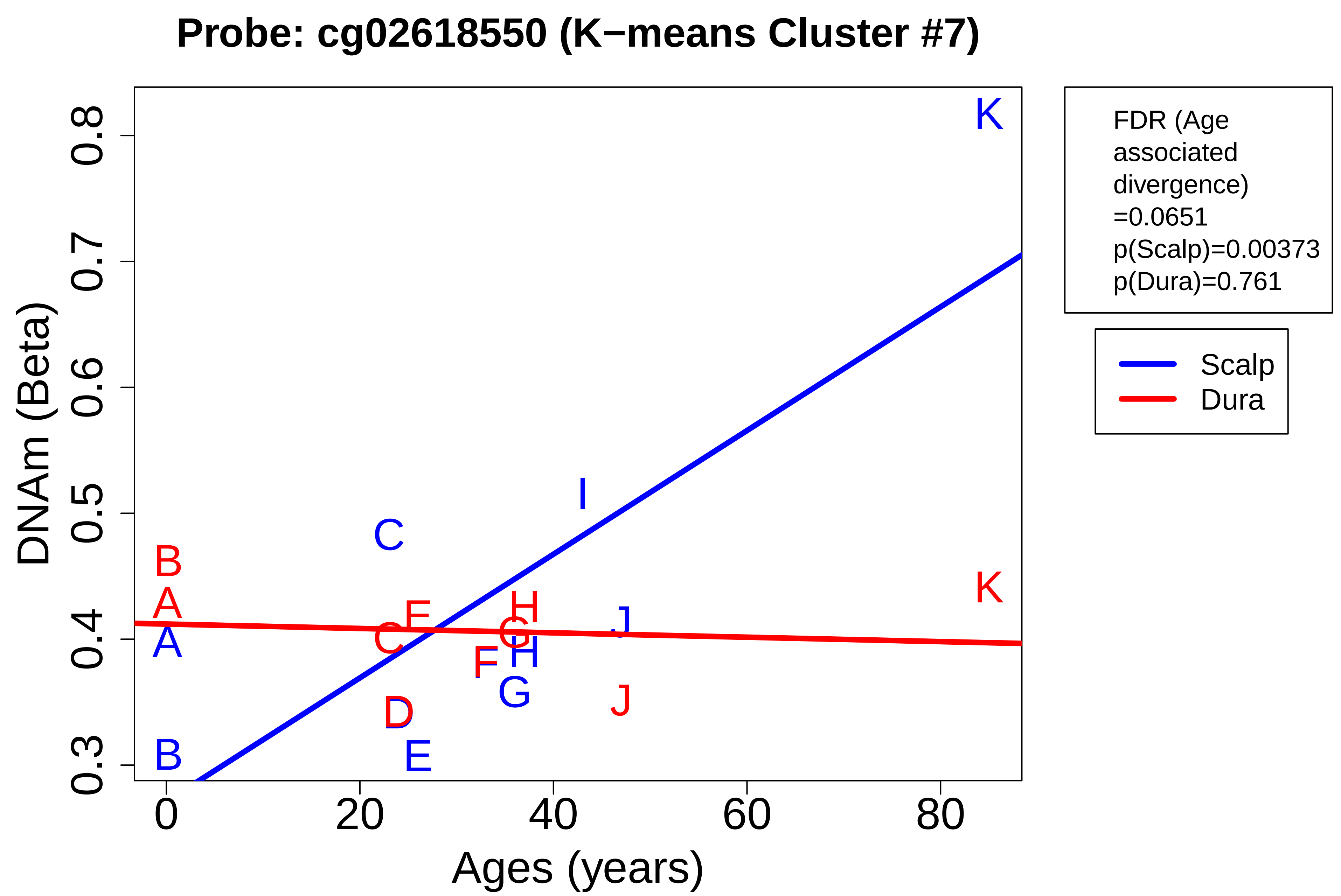
Probe: cg02618550 (K−means Cluster #7)
DNAm (Beta)
0.3
0.4
0.5
0.6
0.7
0.8
Ages (years)
0
20
40
60
80
FDR (Age associated divergence) =0.0651
p(Scalp)=0.00373
p(Dura)=0.761
Scalp
Dura
A
B
C
D
E
F
G
H
I
J
K

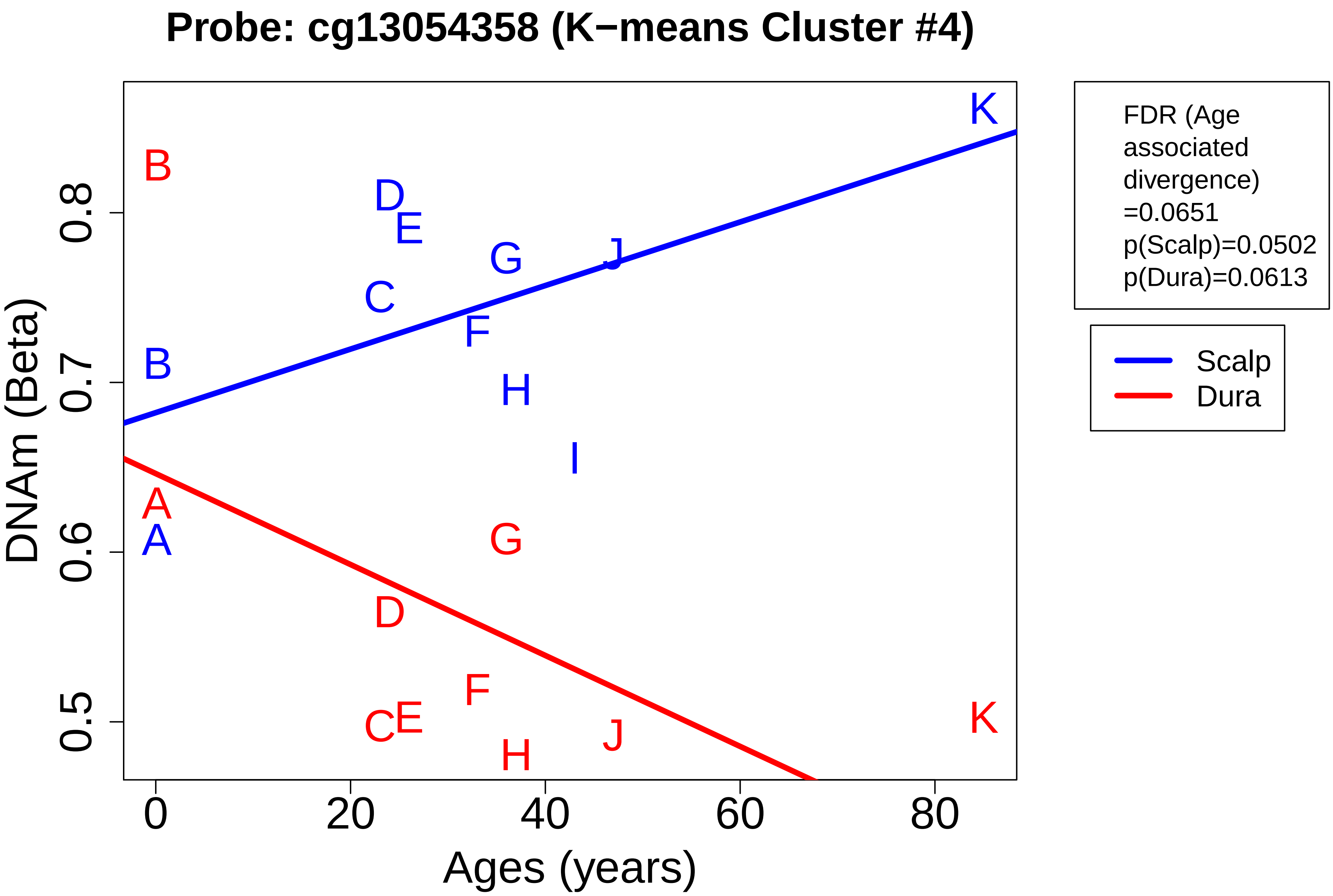

Probe: cg13054358 (K−means Cluster #4)
DNAm (Beta)
0.5
0.6
0.7
0.8
Ages (years)
0
20
40
60
80
A
B
C
D
E
F
G
H
I
J
K
FDR (Age associated divergence) =0.0651
p(Scalp)=0.0502
p(Dura)=0.0613
Scalp
Dura

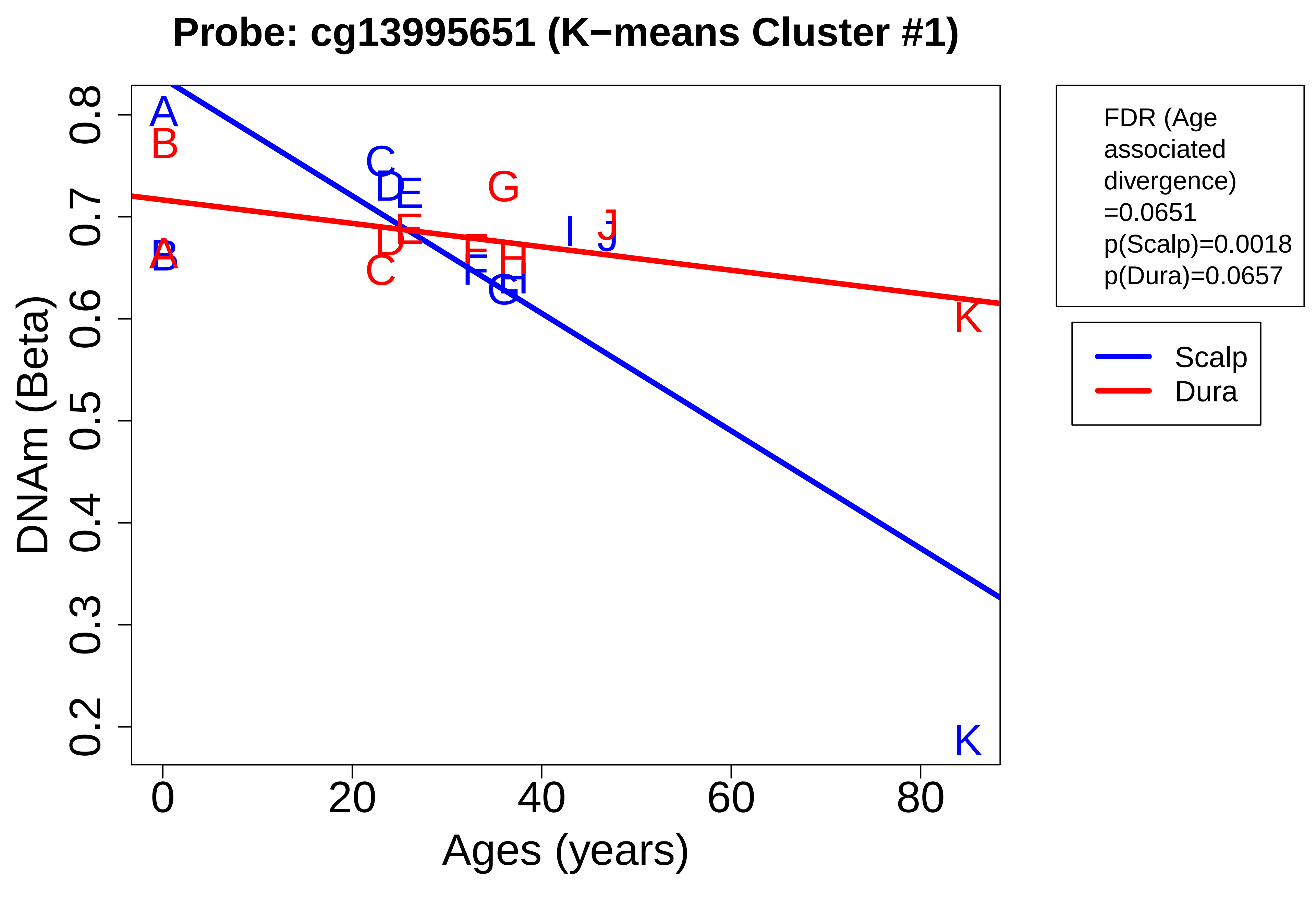

Probe: cg13995651 (K−means Cluster #1)
DNAm (Beta)
Ages (years)
0.2
0.3
0.4
0.5
0.6
0.7
0.8
0
20
40
60
80
FDR (Age associated divergence) =0.0651
p(Scalp)=0.0018
p(Dura)=0.0657
Scalp
Dura

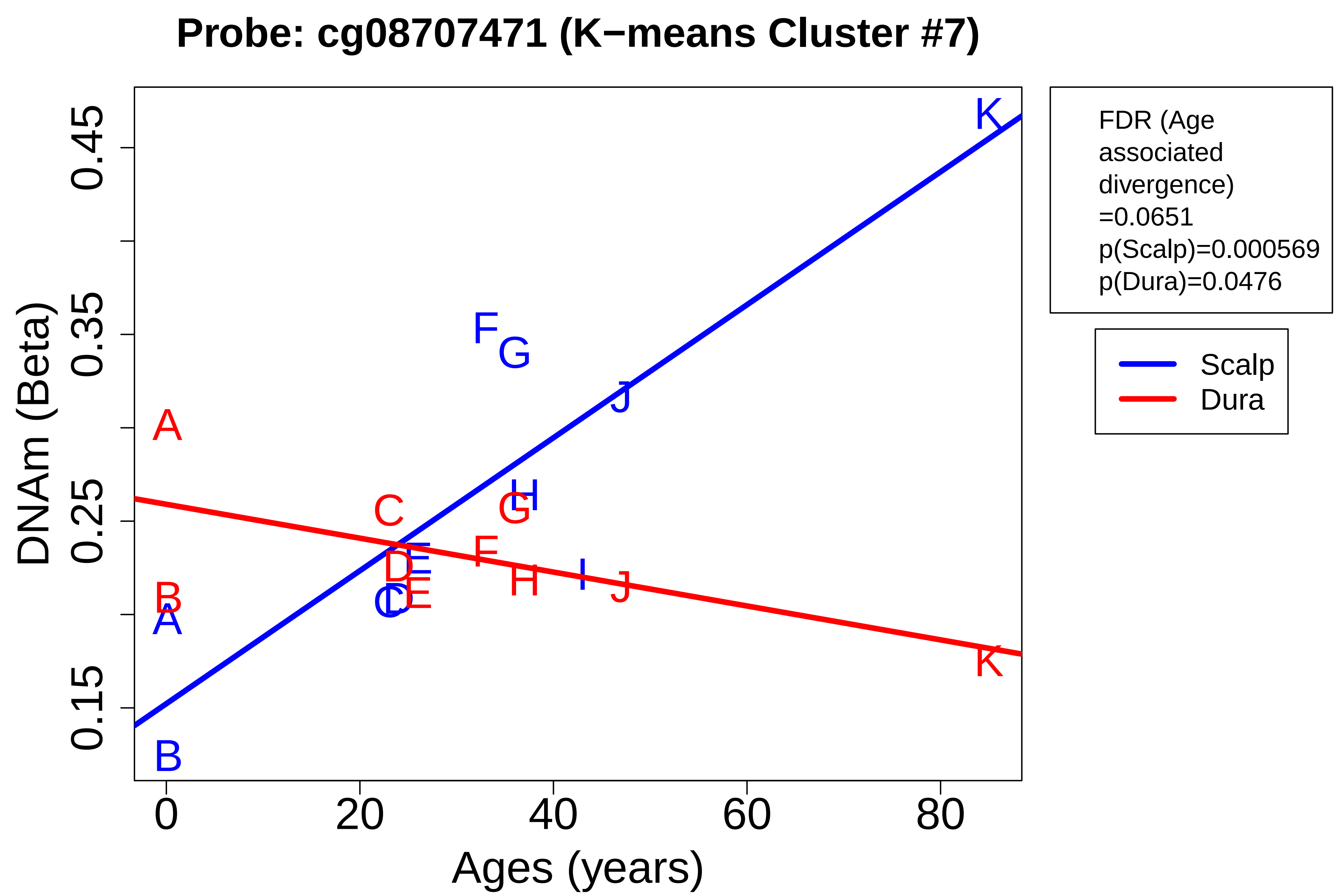

Probe: cg08707471 (K−means Cluster #7)
DNAm (Beta)
0.15
0.25
0.35
0.45
0
20
40
60
80
Ages (years)
FDR (Age associated divergence) =0.0651
p(Scalp)=0.000569
p(Dura)=0.0476
Scalp
Dura
A
B
C
D
E
F
G
H
I
J
K

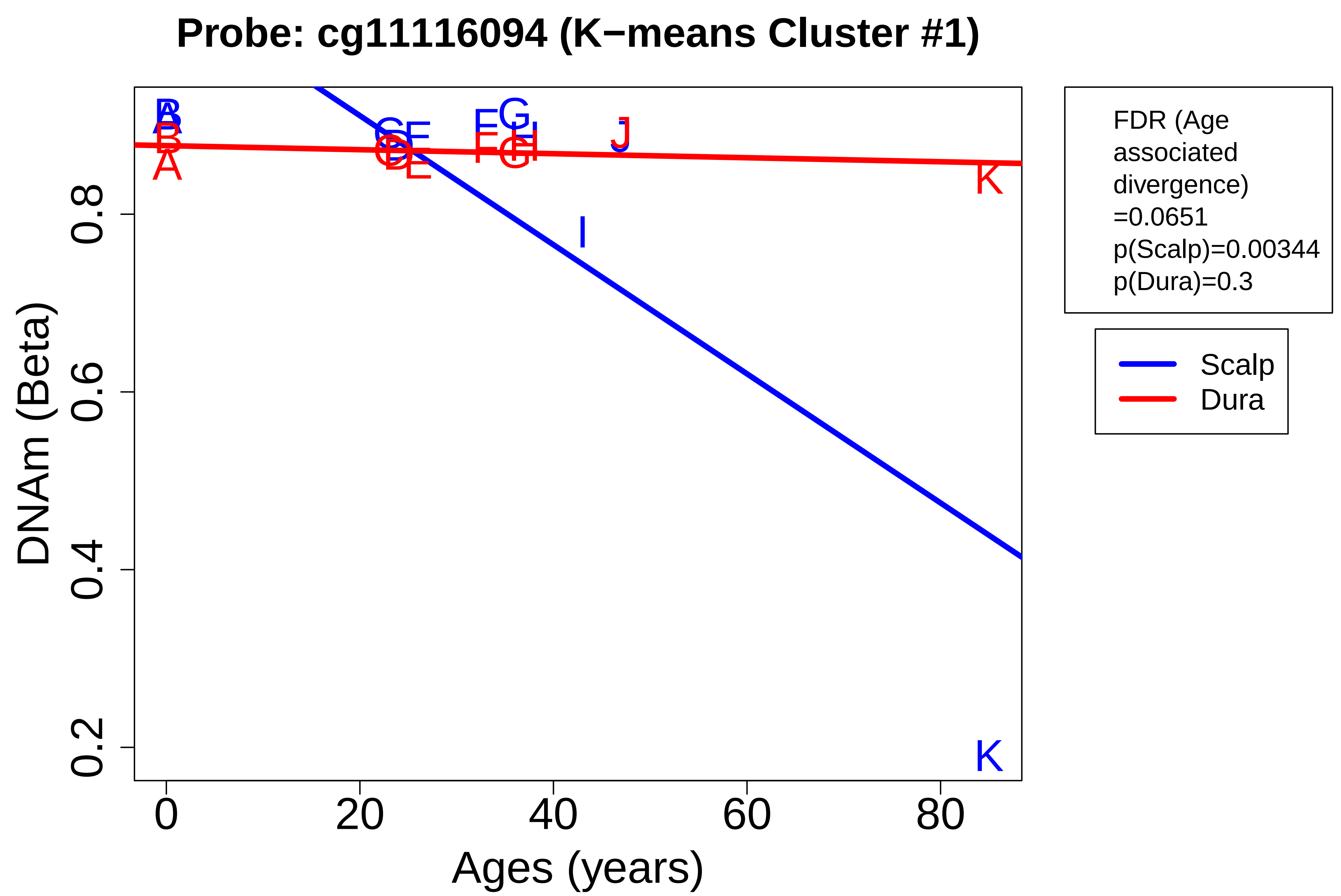

Probe: cg11116094 (K−means Cluster #1)
DNAm (Beta)
0.2
0.4
0.6
0.8
Ages (years)
0
20
40
60
80
FDR (Age associated divergence) =0.0651
p(Scalp)=0.00344
p(Dura)=0.3
Scalp
Dura

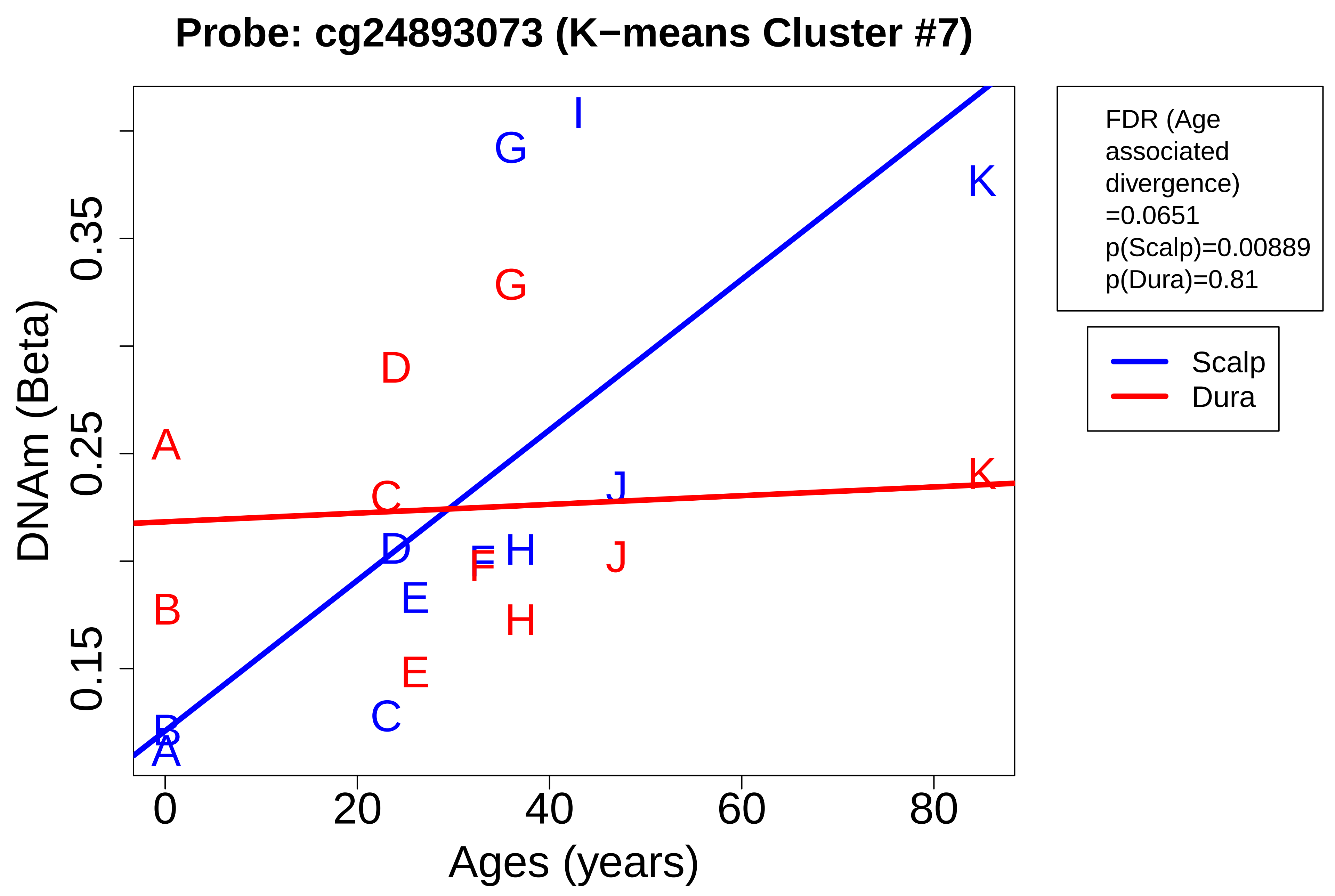

Probe: cg24893073 (K−means Cluster #7)
DNAm (Beta)
0.15
0.25
0.35
Ages (years)
0
20
40
60
80
FDR (Age associated divergence) =0.0651
p(Scalp)=0.00889
p(Dura)=0.81
Scalp
Dura
A
B
C
D
E
F
G
H
I
J
K

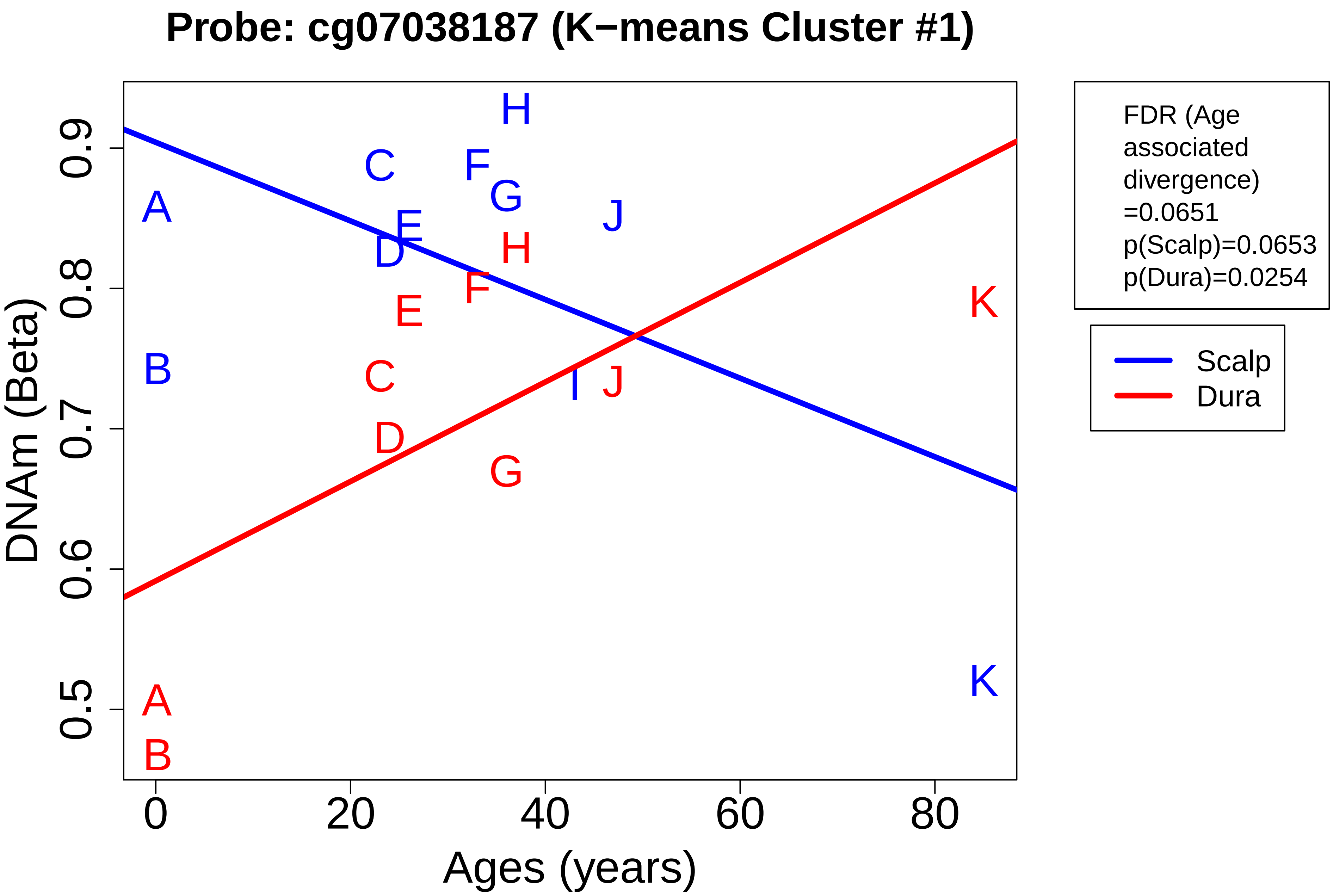

Probe: cg07038187 (K−means Cluster #1)
DNAm (Beta)
0.5
0.6
0.7
0.8
0.9
0
20
40
60
80
Ages (years)
A
B
C
D
E
F
G
H
I
J
K
A
B
C
D
E
F
G
H
J
K
FDR (Age associated divergence) =0.0651
p(Scalp)=0.0653
p(Dura)=0.0254
Scalp
Dura

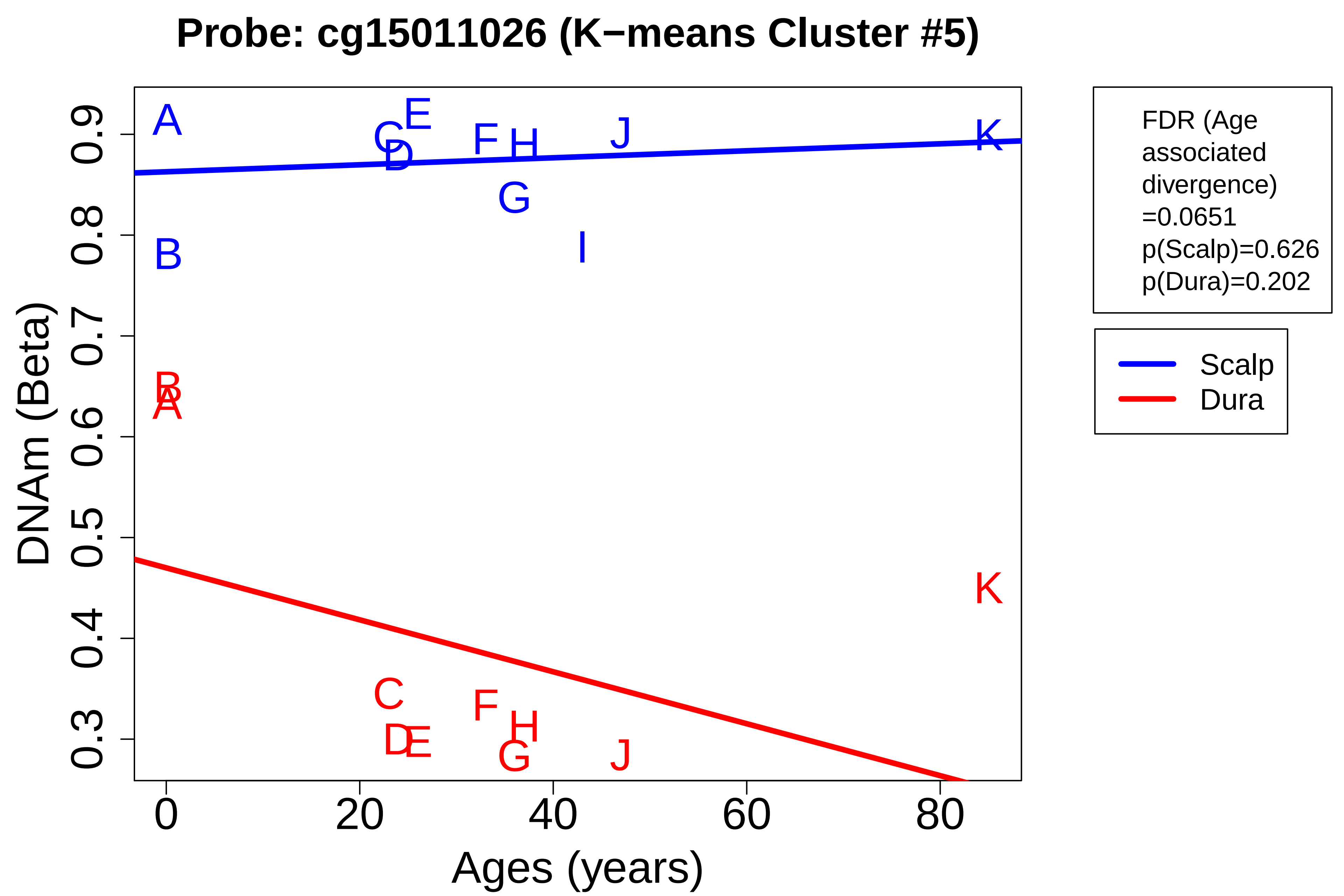

Probe: cg15011026 (K−means Cluster #5)
DNAm (Beta)
0.3
0.4
0.5
0.6
0.7
0.8
0.9
Ages (years)
0
20
40
60
80
A
B
C
D
E
F
G
H
I
J
K
FDR (Age associated divergence) =0.0651
p(Scalp)=0.626
p(Dura)=0.202
Scalp
Dura

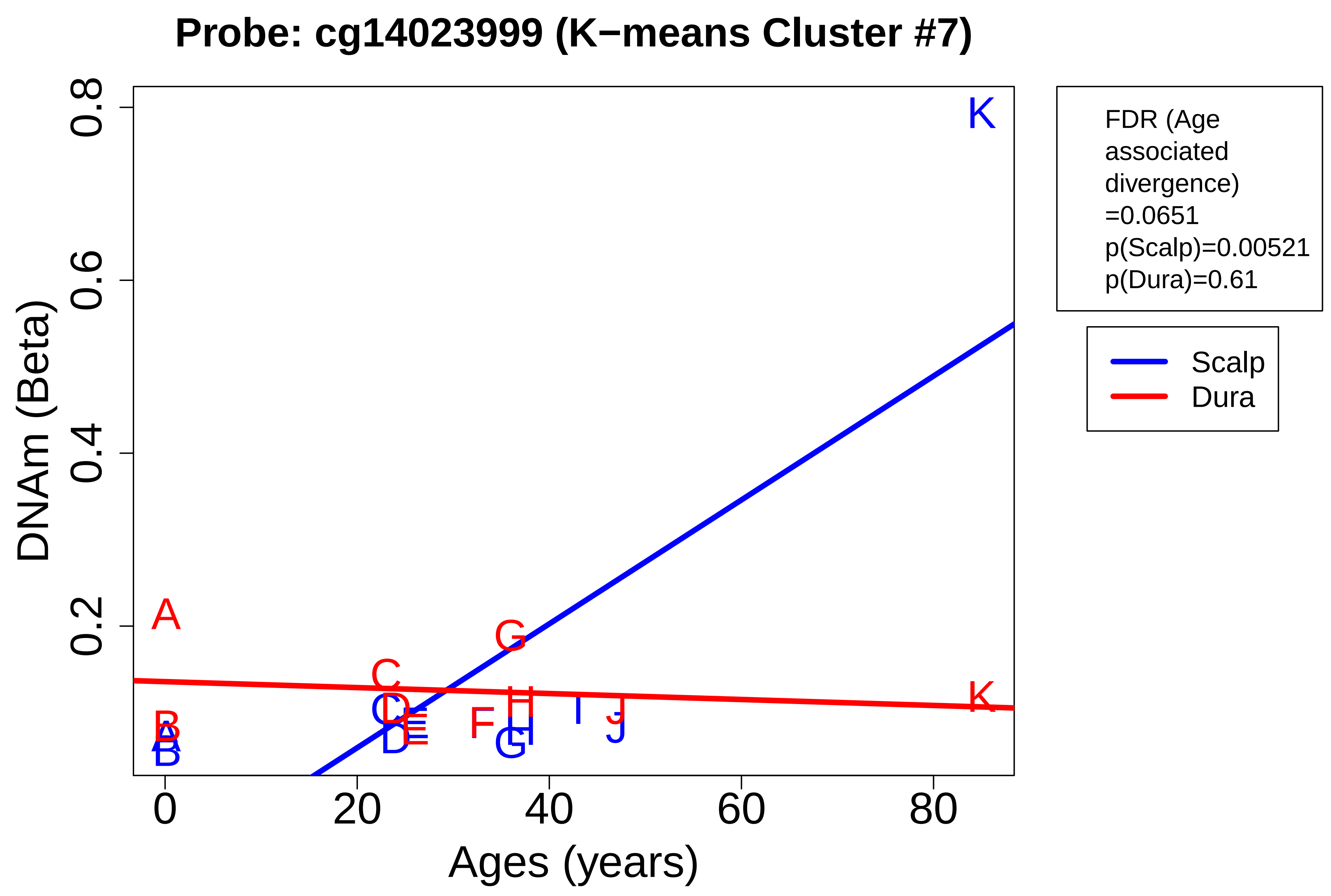

Probe: cg14023999 (K−means Cluster #7)
DNAm (Beta)
0.2
0.4
0.6
0.8
0
20
40
60
80
Ages (years)
A
B
C
D
E
F
G
H
I
J
K
FDR (Age associated divergence) =0.0651
p(Scalp)=0.00521
p(Dura)=0.61
Scalp
Dura

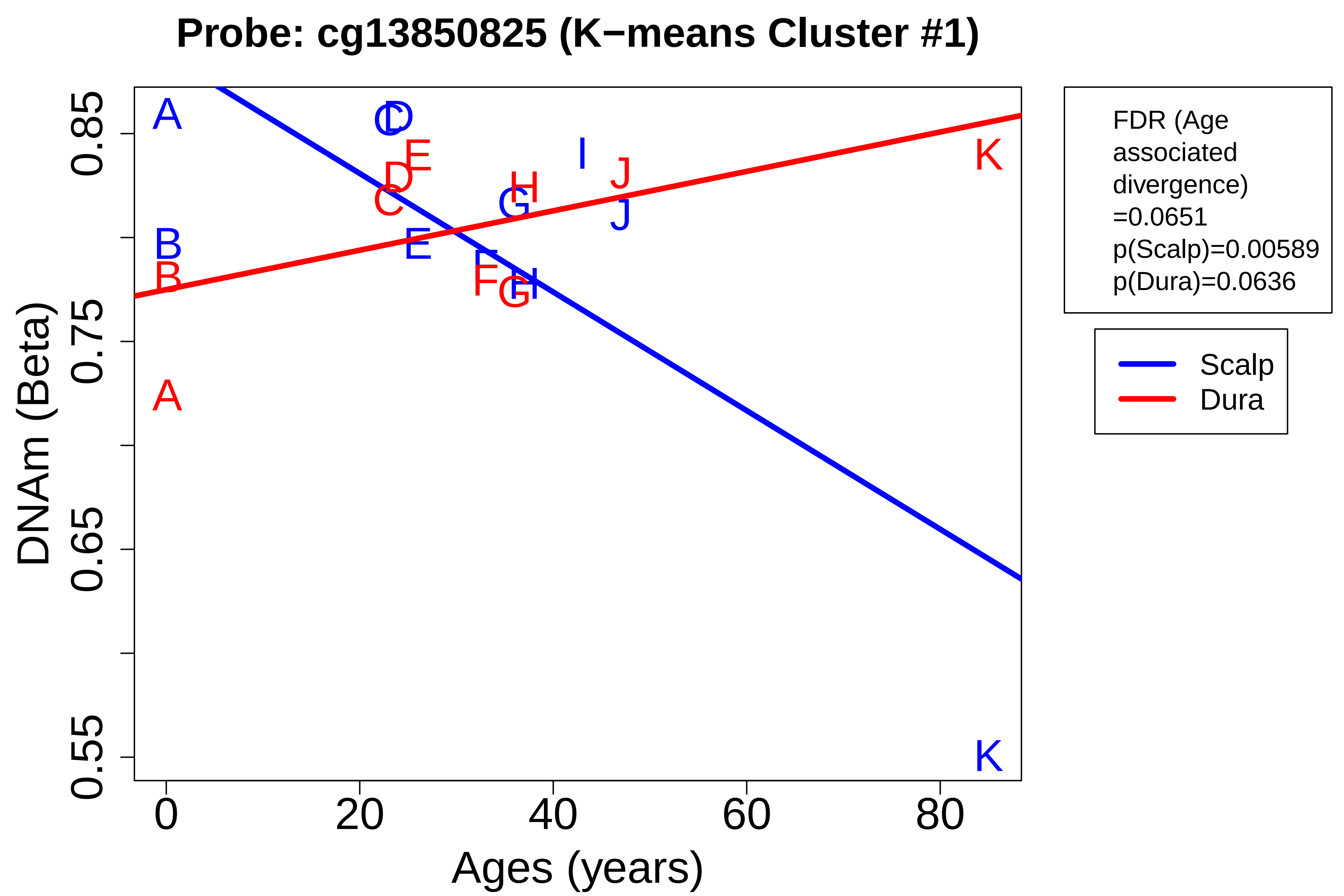
Probe: cg13850825 (K−means Cluster #1)
DNAm (Beta)
0.55
0.65
0.75
0.85
Ages (years)
0
20
40
60
80
FDR (Age associated divergence) =0.0651
p(Scalp)=0.00589
p(Dura)=0.0636
Scalp
Dura

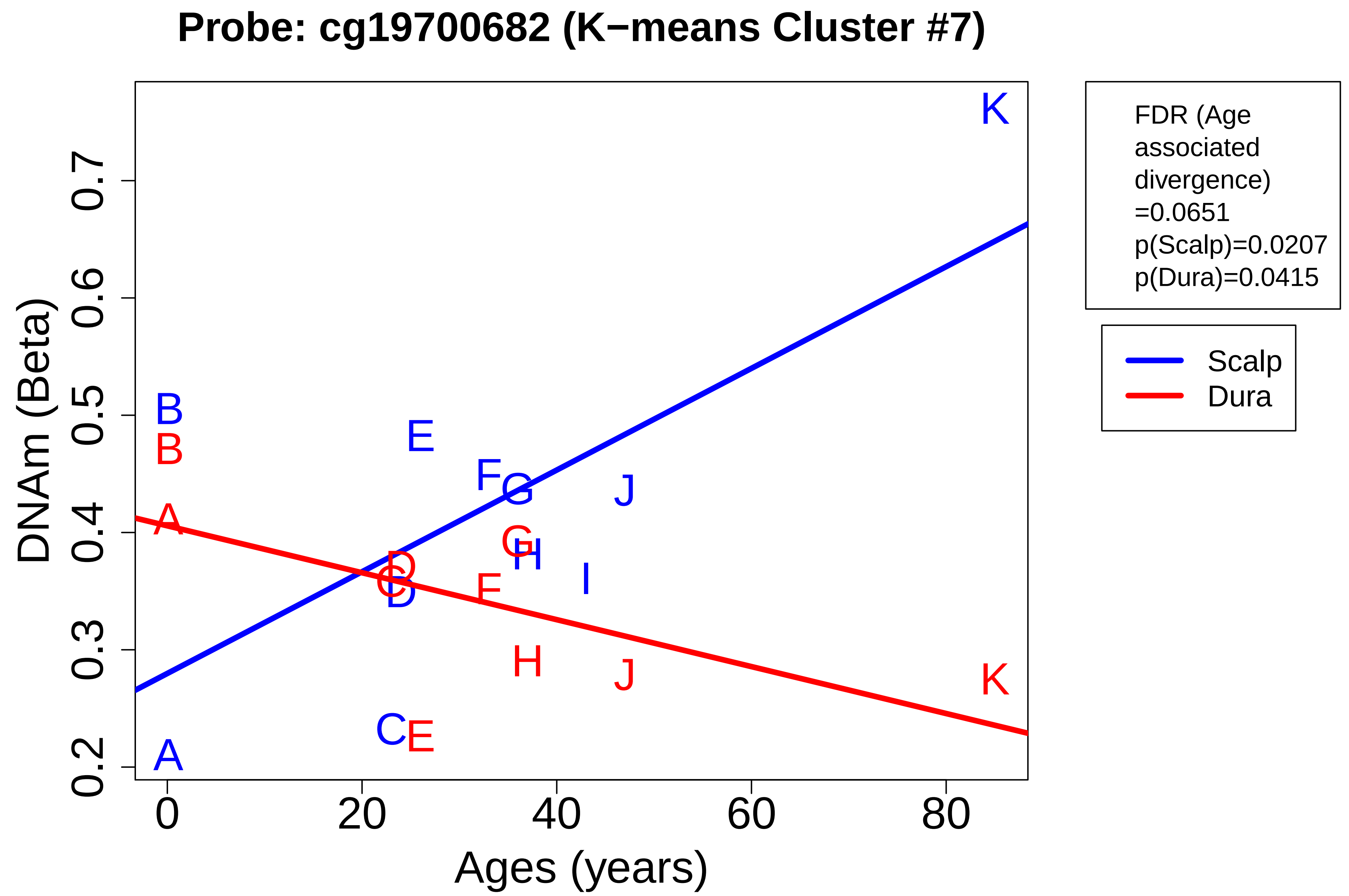

Probe: cg19700682 (K−means Cluster #7)
FDR (Age associated divergence) =0.0651
p(Scalp)=0.0207
p(Dura)=0.0415
Scalp
Dura
DNAm (Beta)
Ages (years)
0.2
0.3
0.4
0.5
0.6
0.7
0
20
40
60
80
A
B
C
D
E
F
G
H
I
J
K

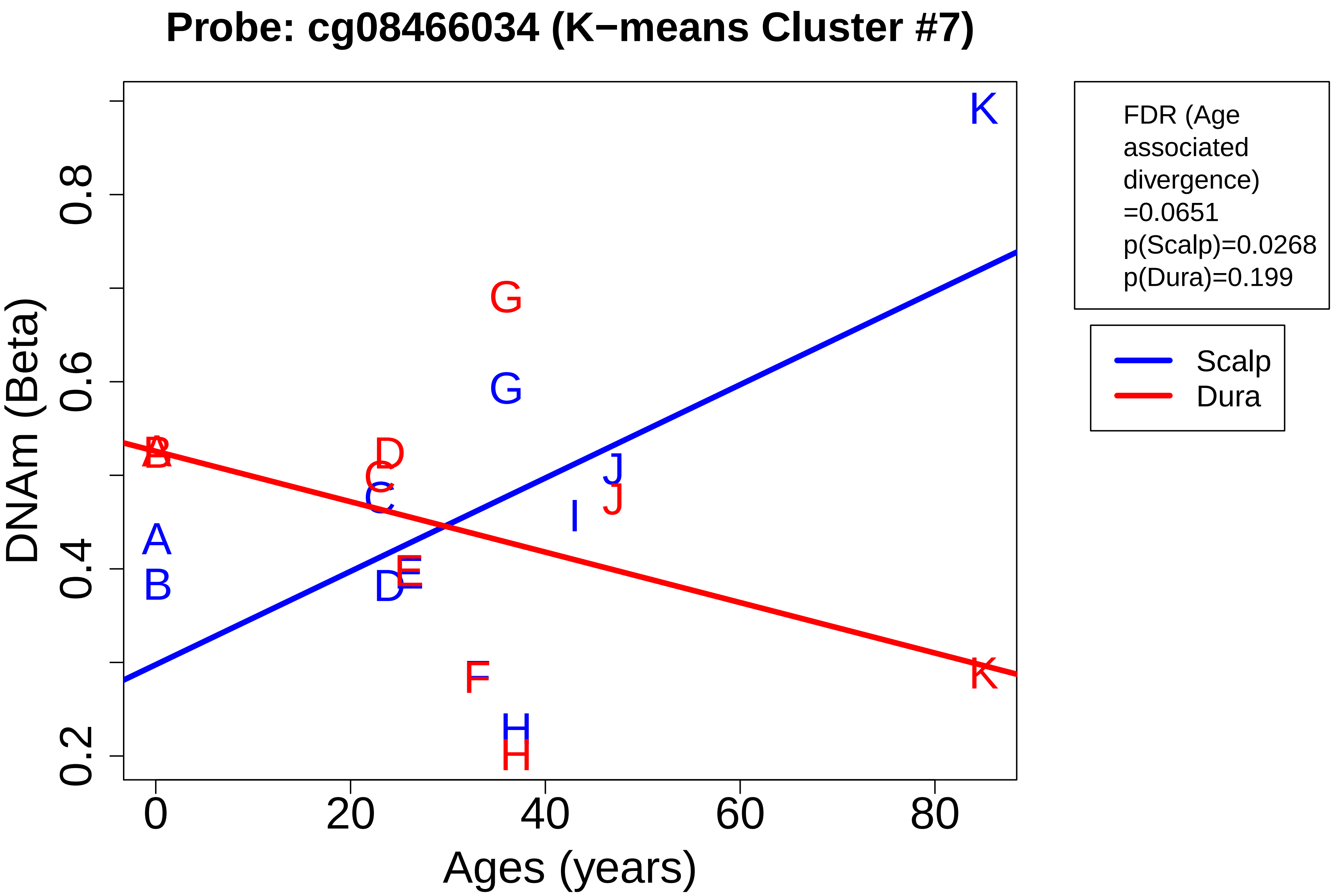

Probe: cg08466034 (K−means Cluster #7)
DNAm (Beta)
0.2
0.4
0.6
0.8
0
20
40
60
80
Ages (years)
FDR (Age associated divergence) =0.0651
p(Scalp)=0.0268
p(Dura)=0.199
Scalp
Dura

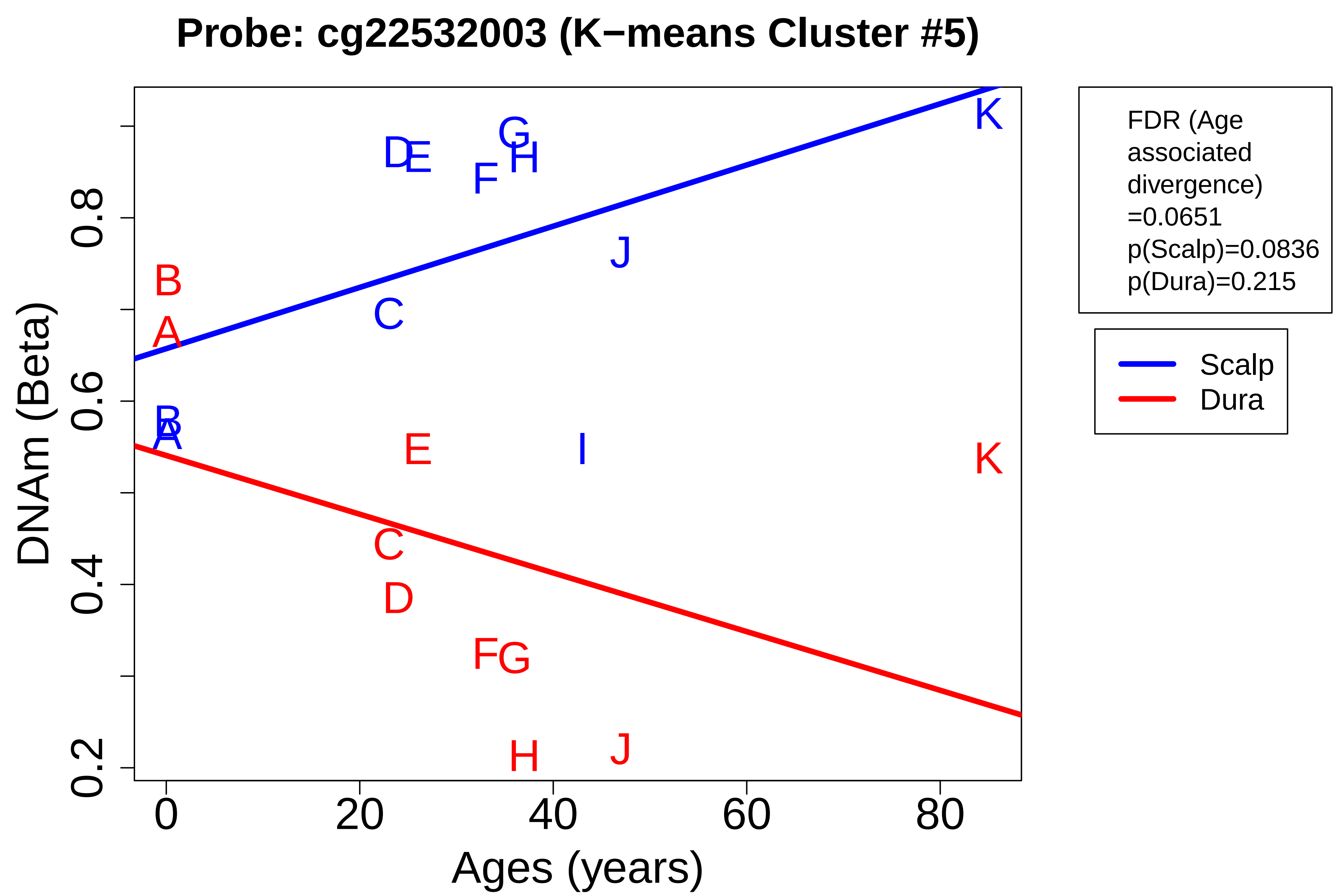

Probe: cg22532003 (K−means Cluster #5)
DNAm (Beta)
0.2
0.4
0.6
0.8
0
20
40
60
80
Ages (years)
FDR (Age associated divergence) =0.0651
p(Scalp)=0.0836
p(Dura)=0.215
Scalp
Dura

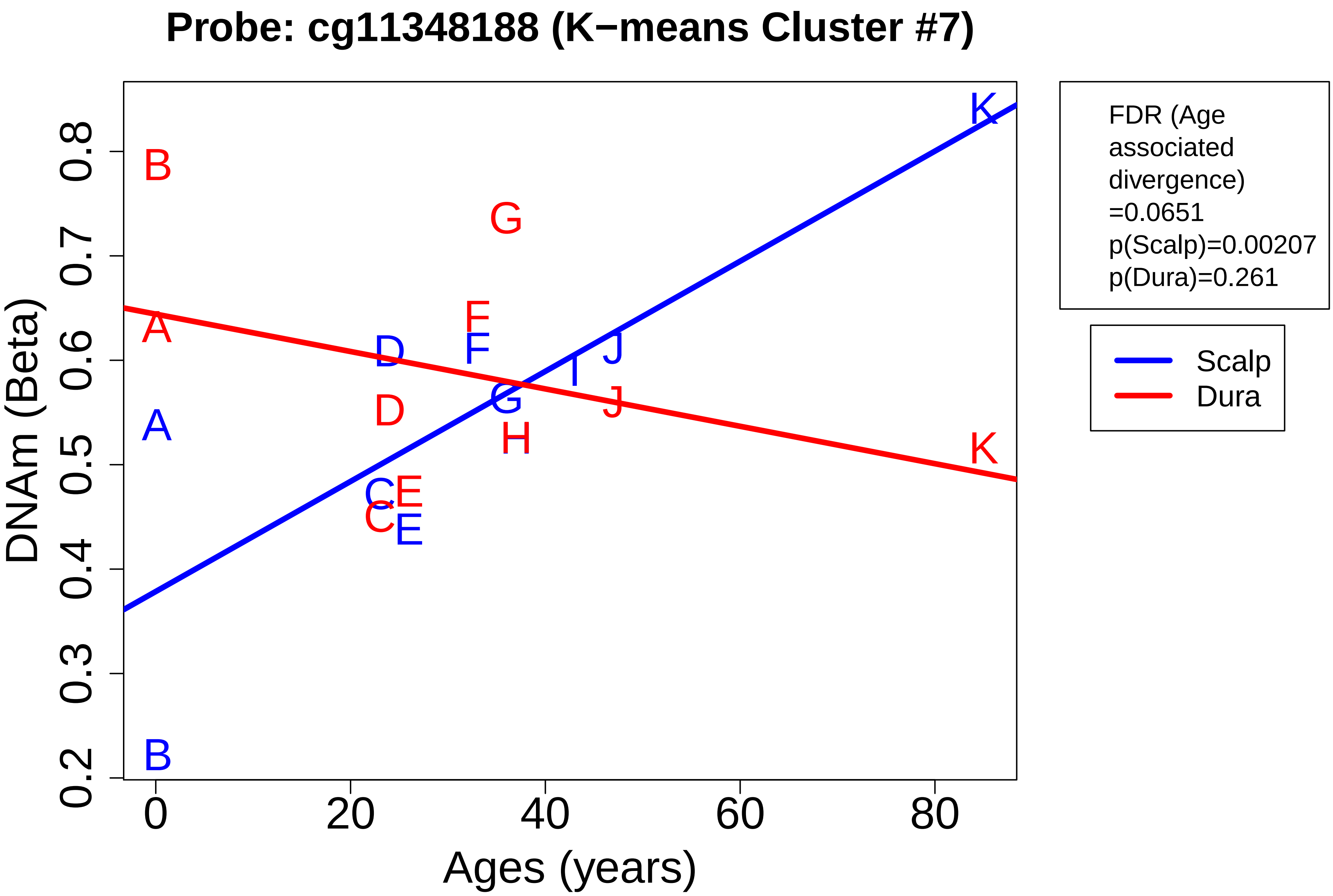

Probe: cg11348188 (K−means Cluster #7)
DNAm (Beta)
Ages (years)
0.2
0.3
0.4
0.5
0.6
0.7
0.8
0
20
40
60
80
FDR (Age associated divergence) =0.0651
p(Scalp)=0.00207
p(Dura)=0.261
Scalp
Dura

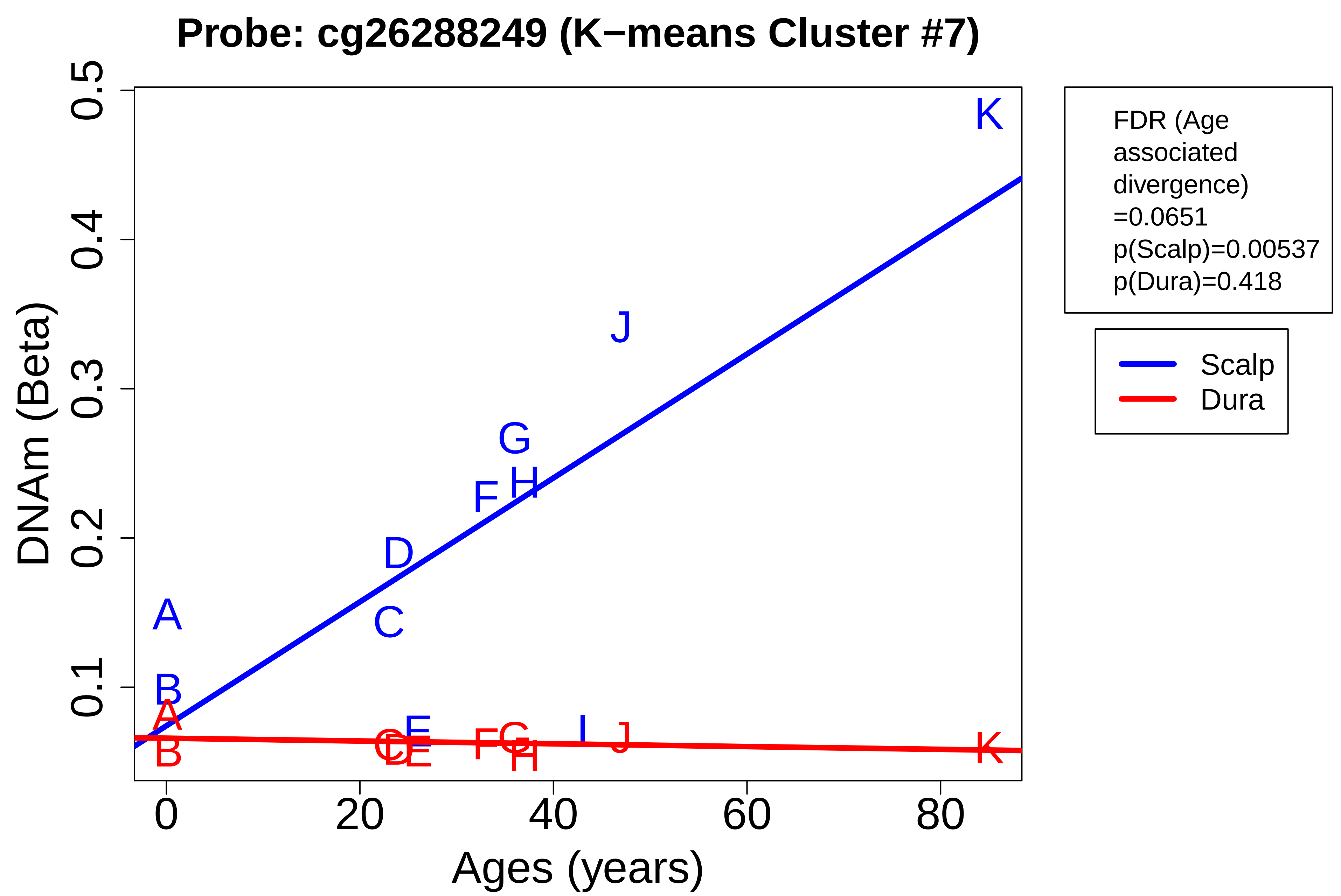

Probe: cg26288249 (K−means Cluster #7)
DNAm (Beta)
Ages (years)
FDR (Age associated divergence) =0.0651
p(Scalp)=0.00537
p(Dura)=0.418
Scalp
Dura

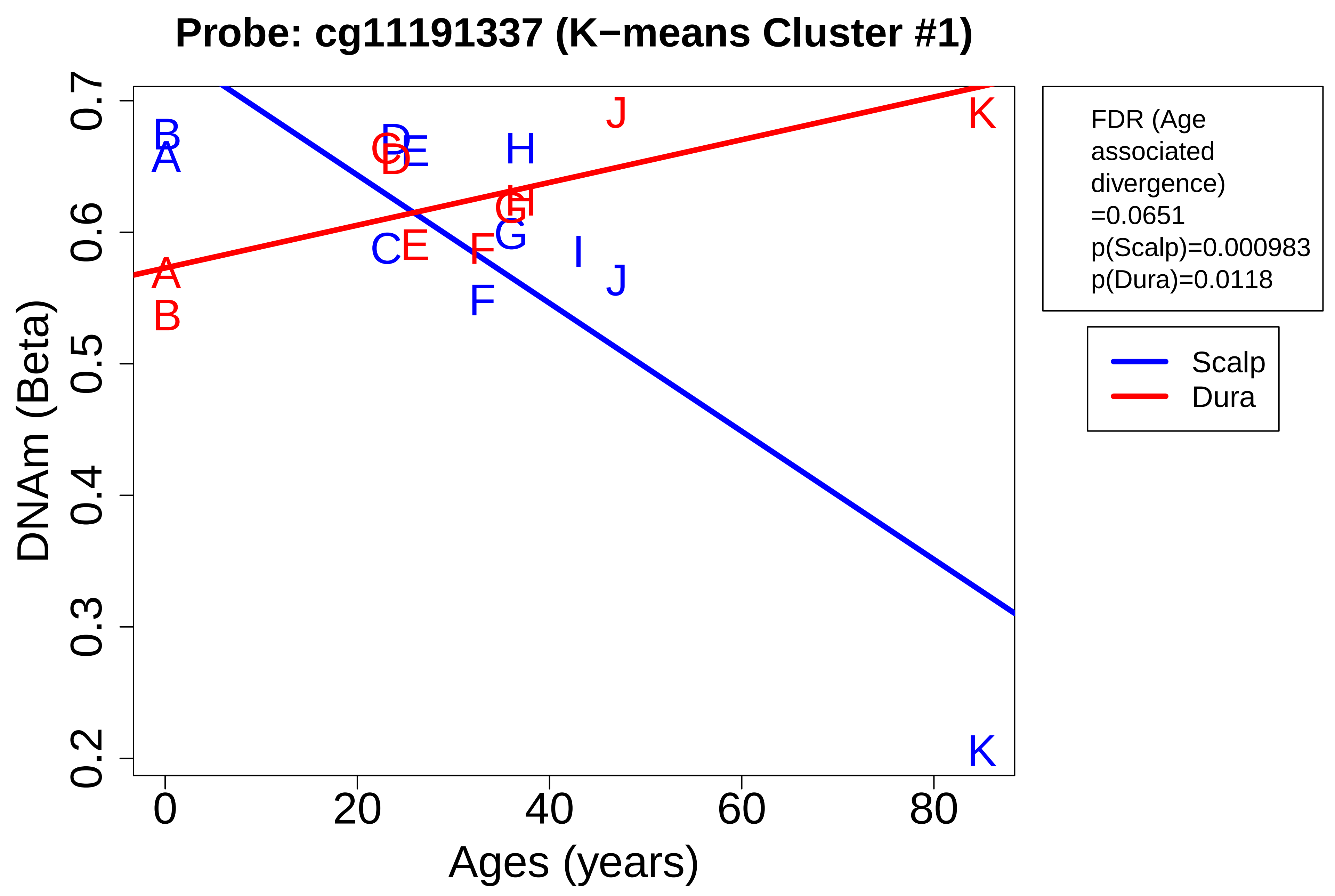
Probe: cg11191337 (K−means Cluster #1)
DNAm (Beta)
0.2
0.3
0.4
0.5
0.6
0.7
0
20
40
60
80
Ages (years)
FDR (Age associated divergence) =0.0651
p(Scalp)=0.000983
p(Dura)=0.0118
Scalp
Dura
A
B
C
D
E
F
G
H
I
J
K

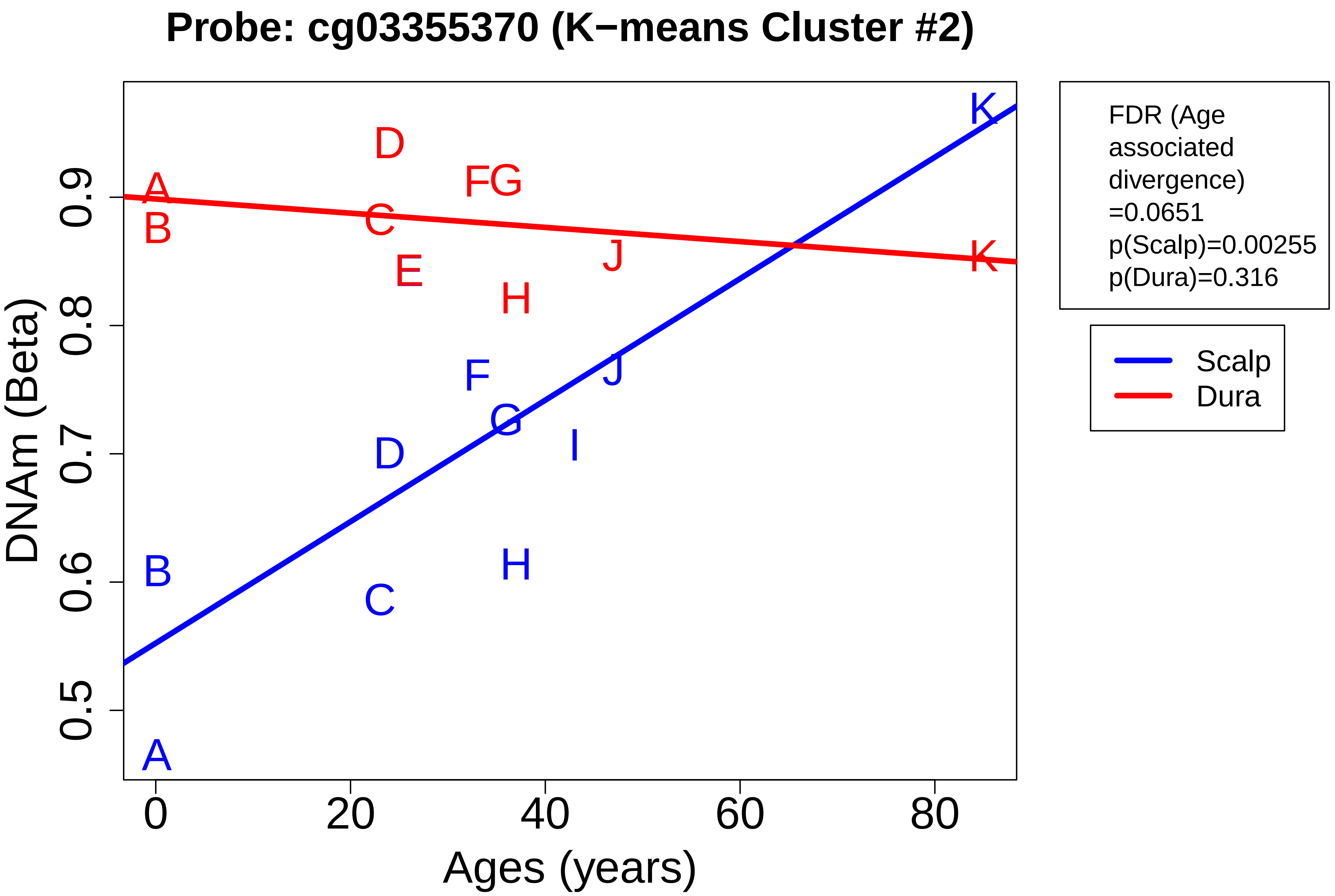

Probe: cg03355370 (K−means Cluster #2)
FDR (Age associated divergence) =0.0651
p(Scalp)=0.00255
p(Dura)=0.316
Scalp
Dura
DNAm (Beta)
0.5
0.6
0.7
0.8
0.9
Ages (years)
0
20
40
60
80
A
B
C
D
E
FG
H
J
K
A
B
C
D
F
G
H
I
J
K

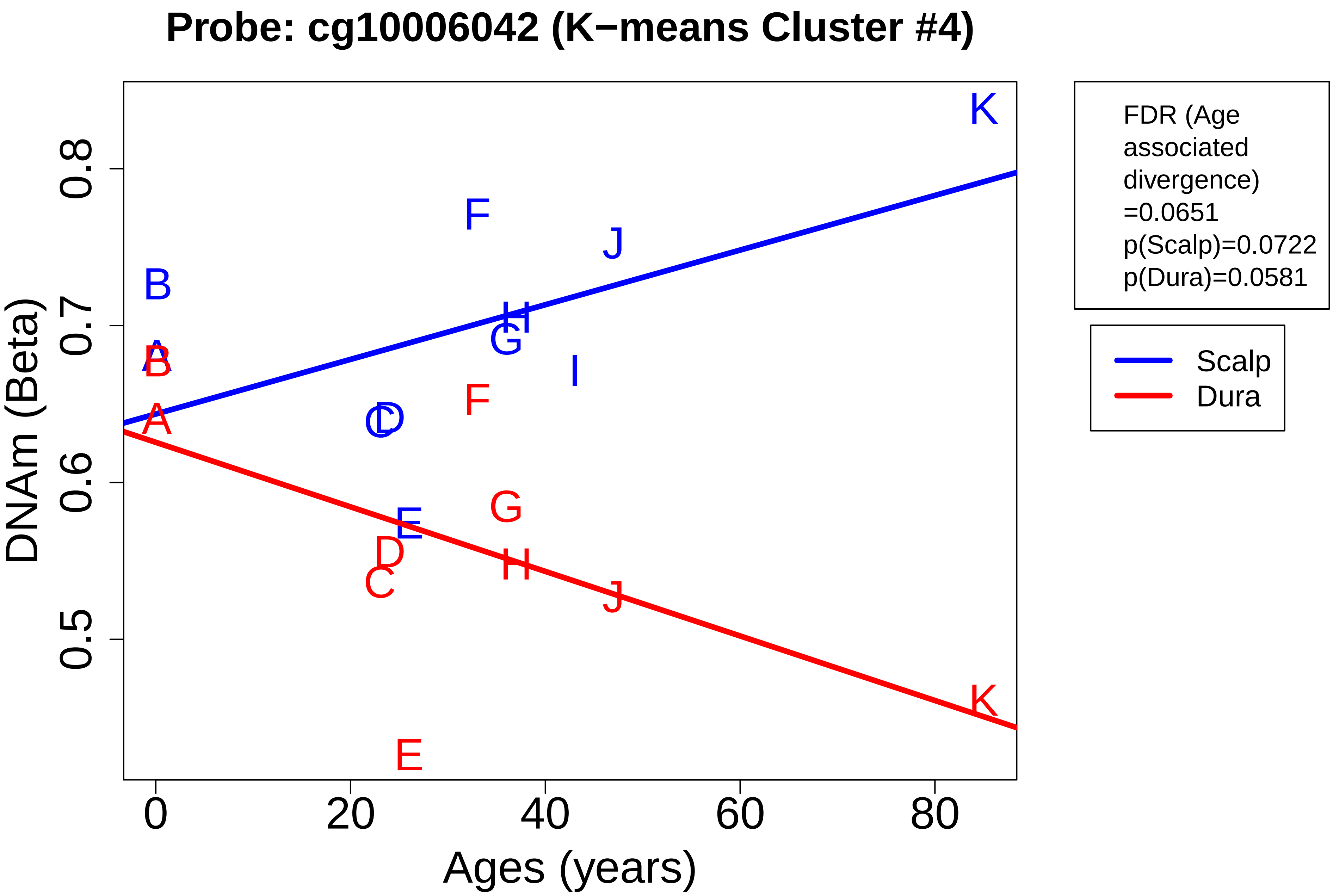
Probe: cg10006042 (K−means Cluster #4)
DNAm (Beta)
0.5
0.6
0.7
0.8
0
20
40
60
80
Ages (years)
FDR (Age associated divergence) =0.0651
p(Scalp)=0.0722
p(Dura)=0.0581
Scalp
Dura

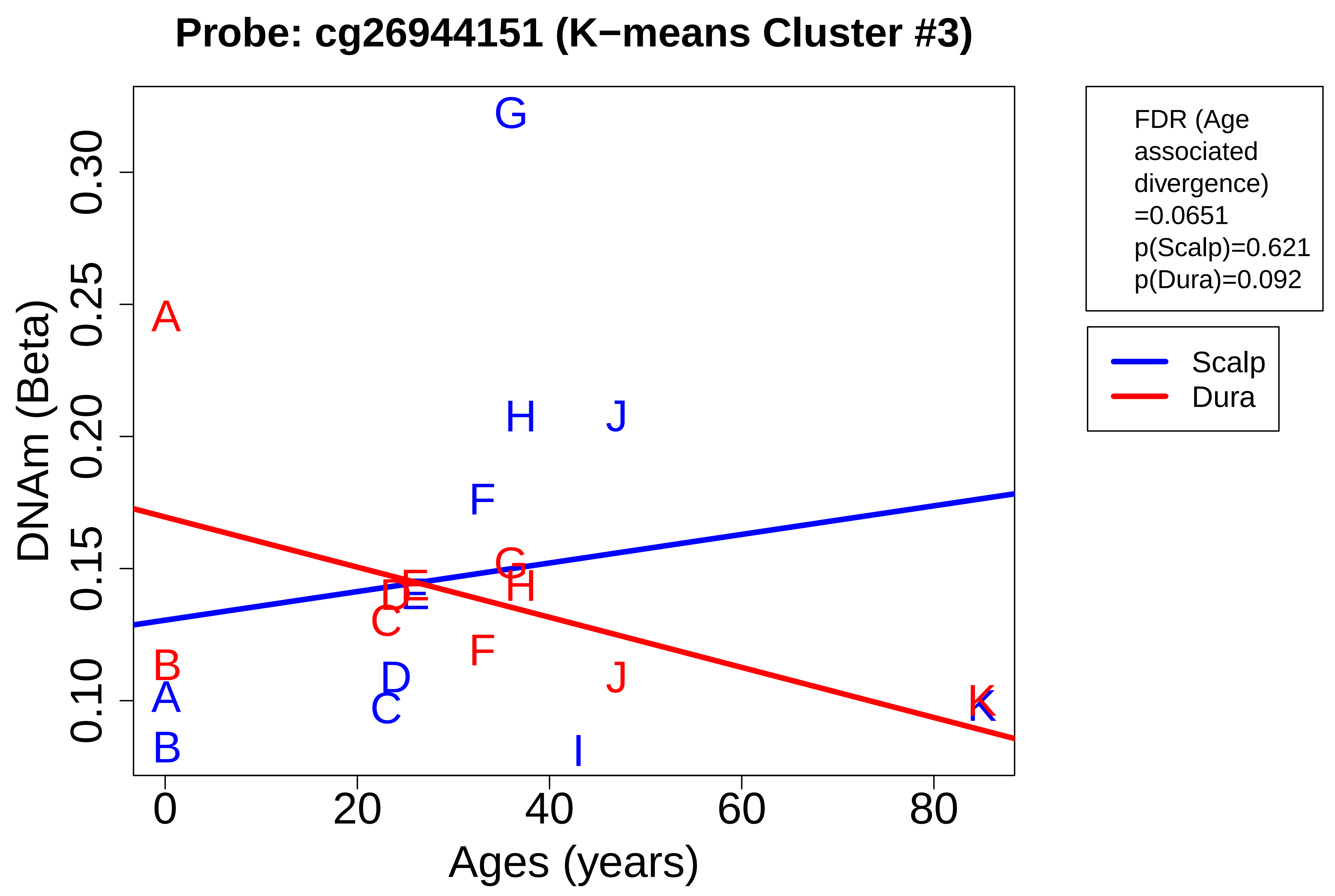

Probe: cg26944151 (K−means Cluster #3)
DNAm (Beta)
0.10
0.15
0.20
0.25
0.30
0
20
40
60
80
Ages (years)
FDR (Age associated divergence) =0.0651
p(Scalp)=0.621
p(Dura)=0.092
Scalp
Dura

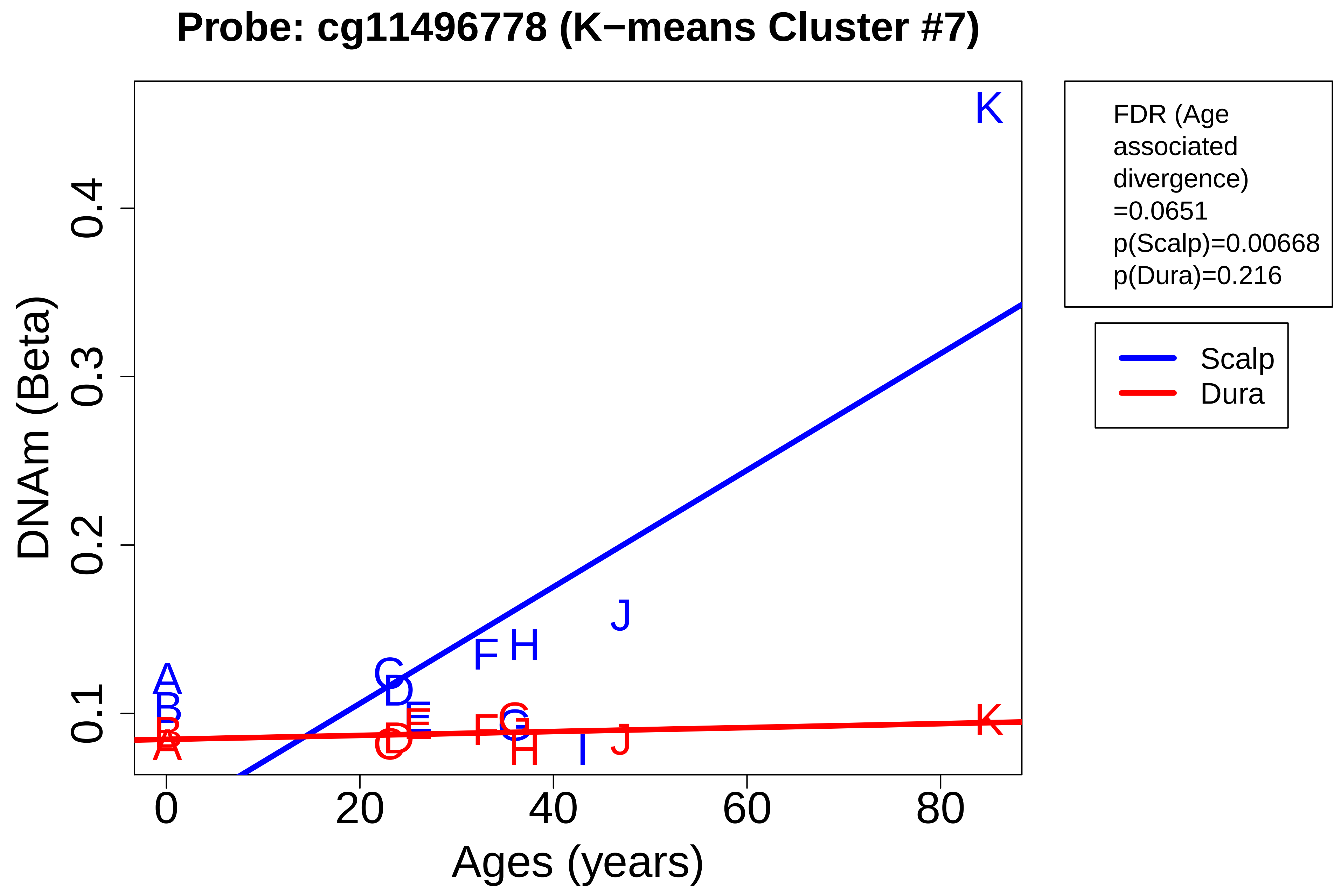
Probe: cg11496778 (K−means Cluster #7)
DNAm (Beta)
0.1
0.2
0.3
0.4
0
20
40
60
80
Ages (years)
A
B
C
D
E
F
G
H
I
J
K
FDR (Age associated divergence) =0.0651
p(Scalp)=0.00668
p(Dura)=0.216
Scalp
Dura

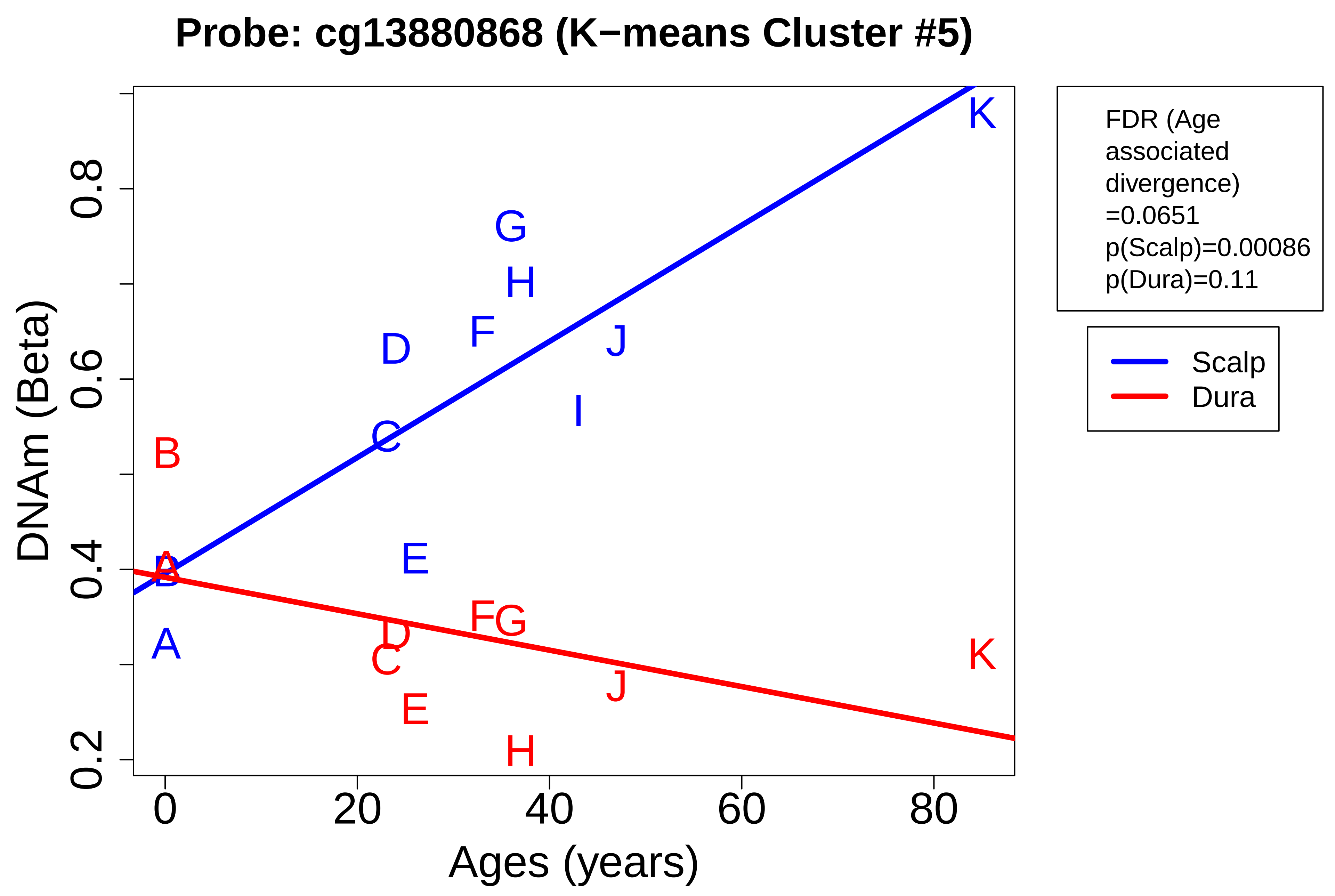

Probe: cg13880868 (K−means Cluster #5)
DNAm (Beta)
0.2
0.4
0.6
0.8
0
20
40
60
80
Ages (years)
FDR (Age associated divergence) =0.0651
p(Scalp)=0.00086
p(Dura)=0.11
Scalp
Dura
A
B
C
D
E
F
G
H
I
J
K
A
B
C
D
E
FG
H
J
K

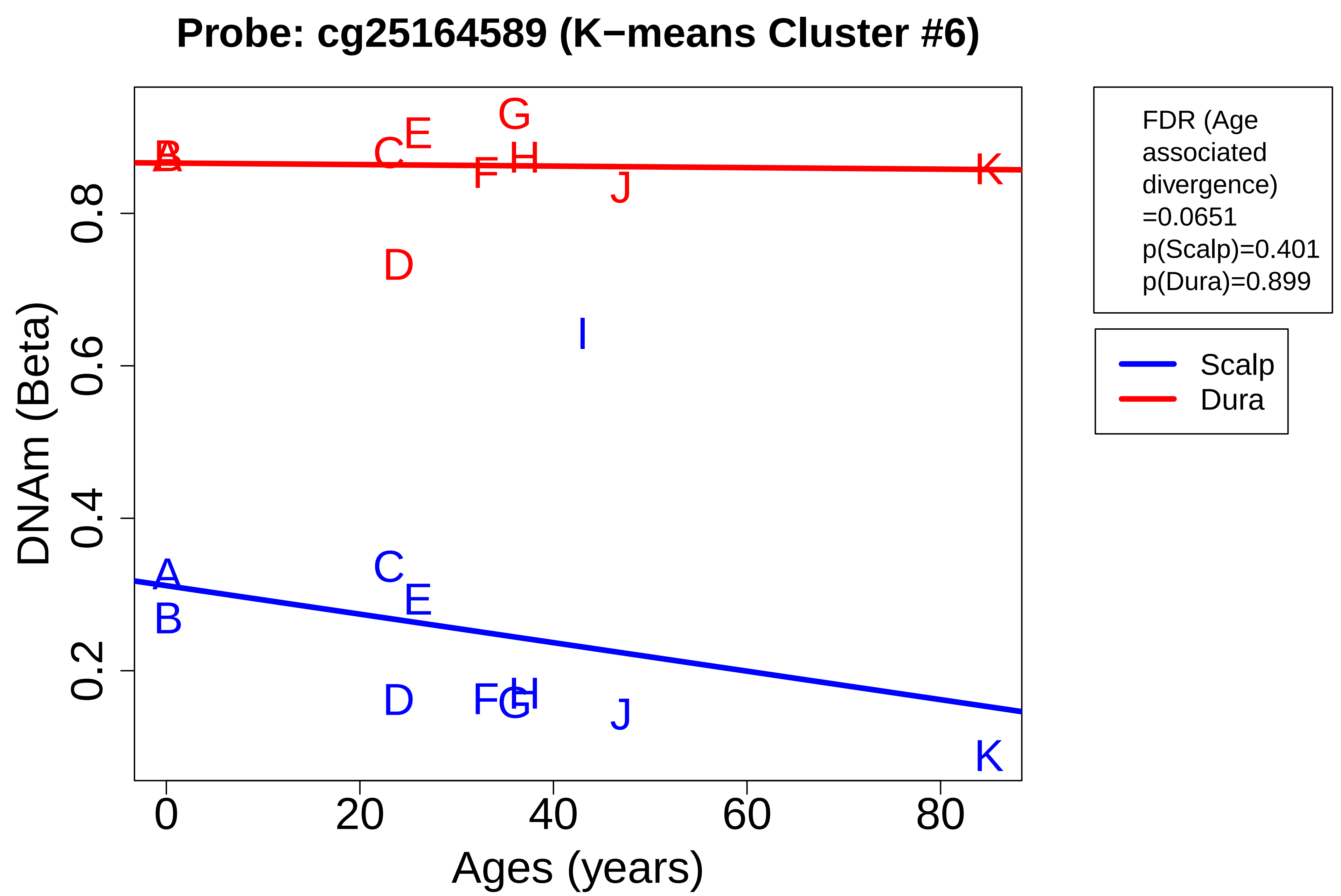

Probe: cg25164589 (K−means Cluster #6)
DNAm (Beta)
0.2
0.4
0.6
0.8
0
20
40
60
80
Ages (years)
A
B
C
D
E
F
G
H
I
J
K
FDR (Age associated divergence) =0.0651
p(Scalp)=0.401
p(Dura)=0.899
Scalp
Dura

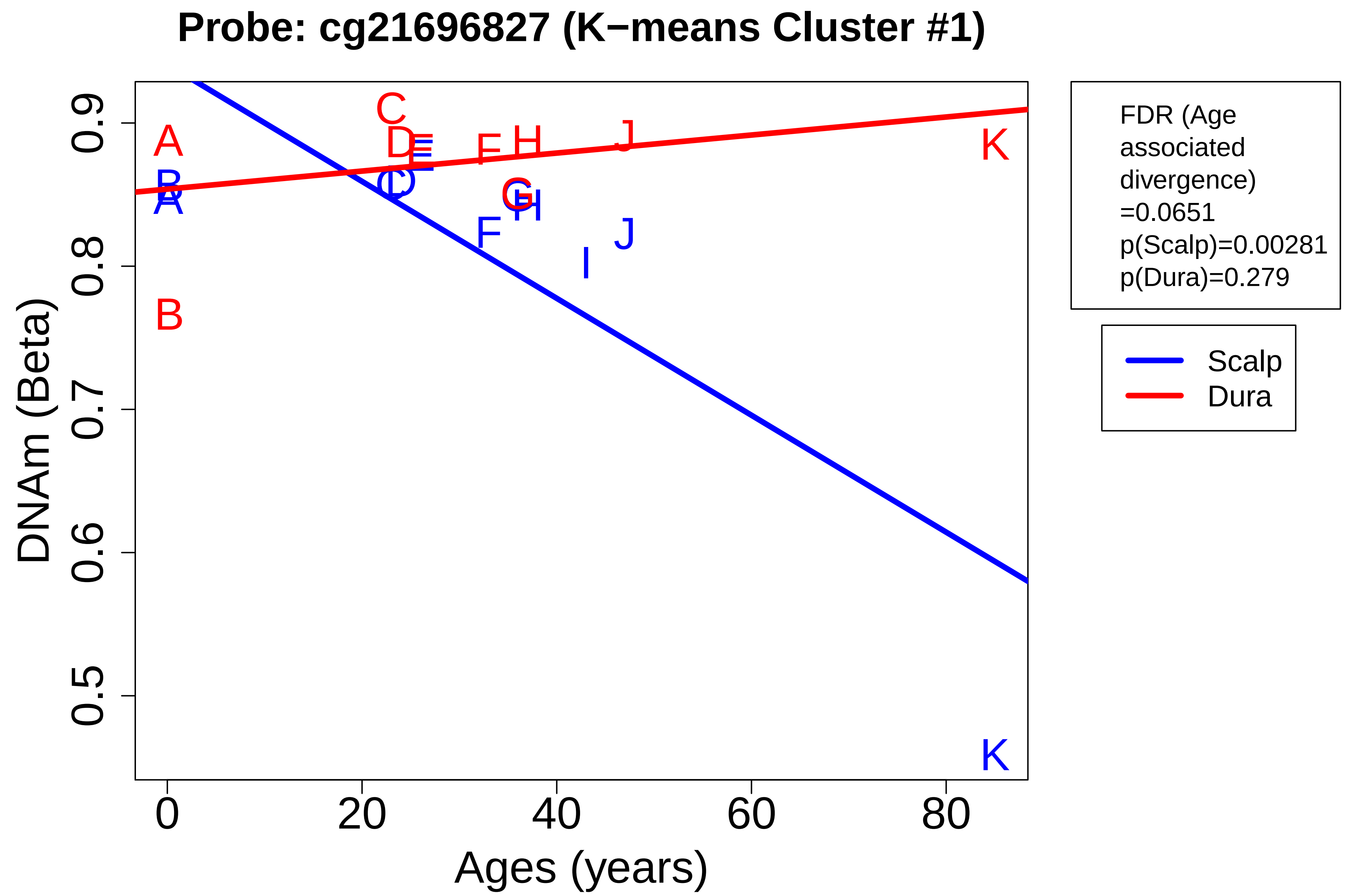
Probe: cg21696827 (K−means Cluster #1)
DNAm (Beta)
Ages (years)
0.5
0.6
0.7
0.8
0.9
0
20
40
60
80
A
B
C
D
E
F
G
H
I
J
K
FDR (Age associated divergence) =0.0651
p(Scalp)=0.00281
p(Dura)=0.279
Scalp
Dura

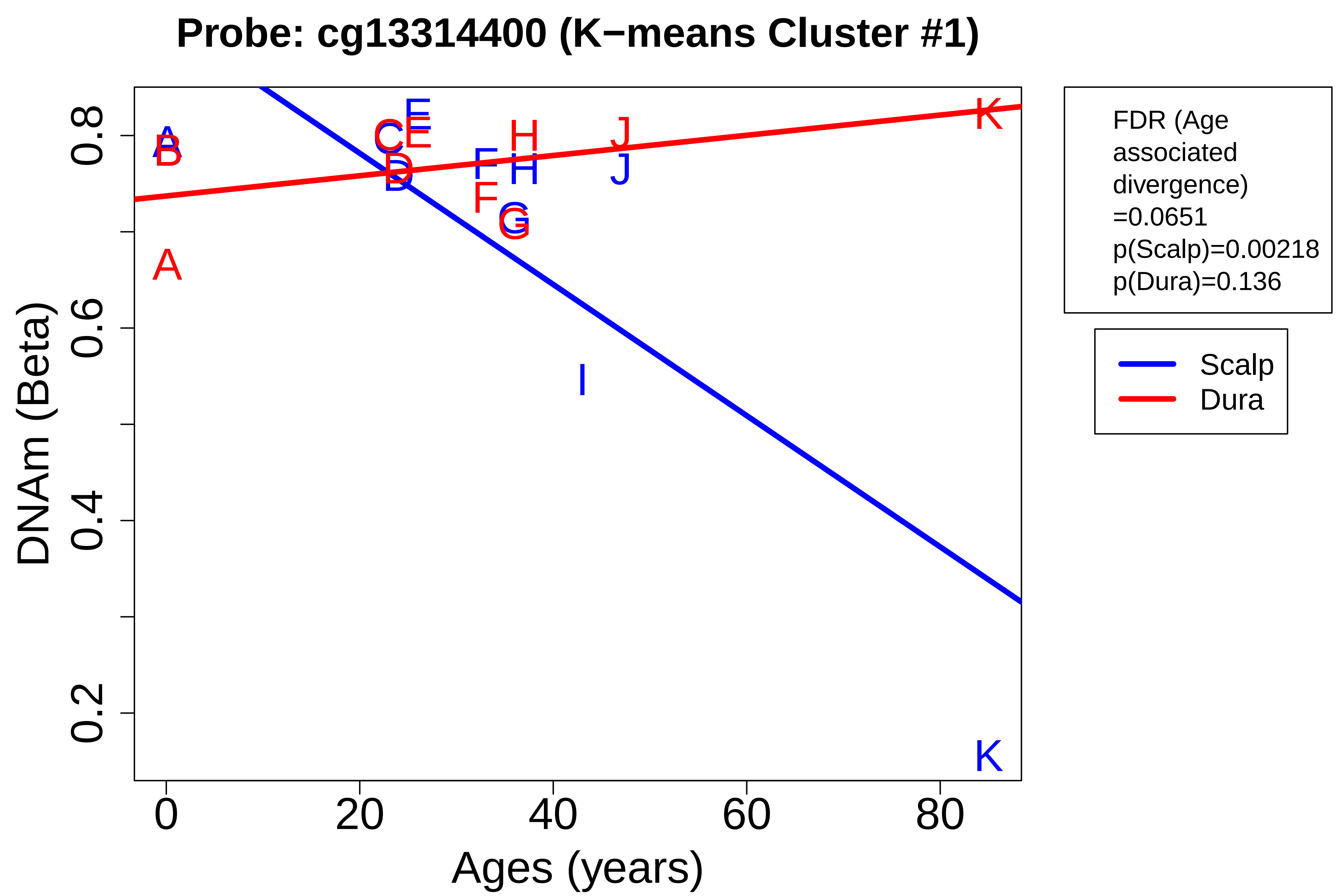

Probe: cg13314400 (K−means Cluster #1)
DNAm (Beta)
0.2
0.4
0.6
0.8
Ages (years)
0
20
40
60
80
A
B
C
D
E
F
G
H
I
J
K
FDR (Age associated divergence) =0.0651
p(Scalp)=0.00218
p(Dura)=0.136
Scalp
Dura

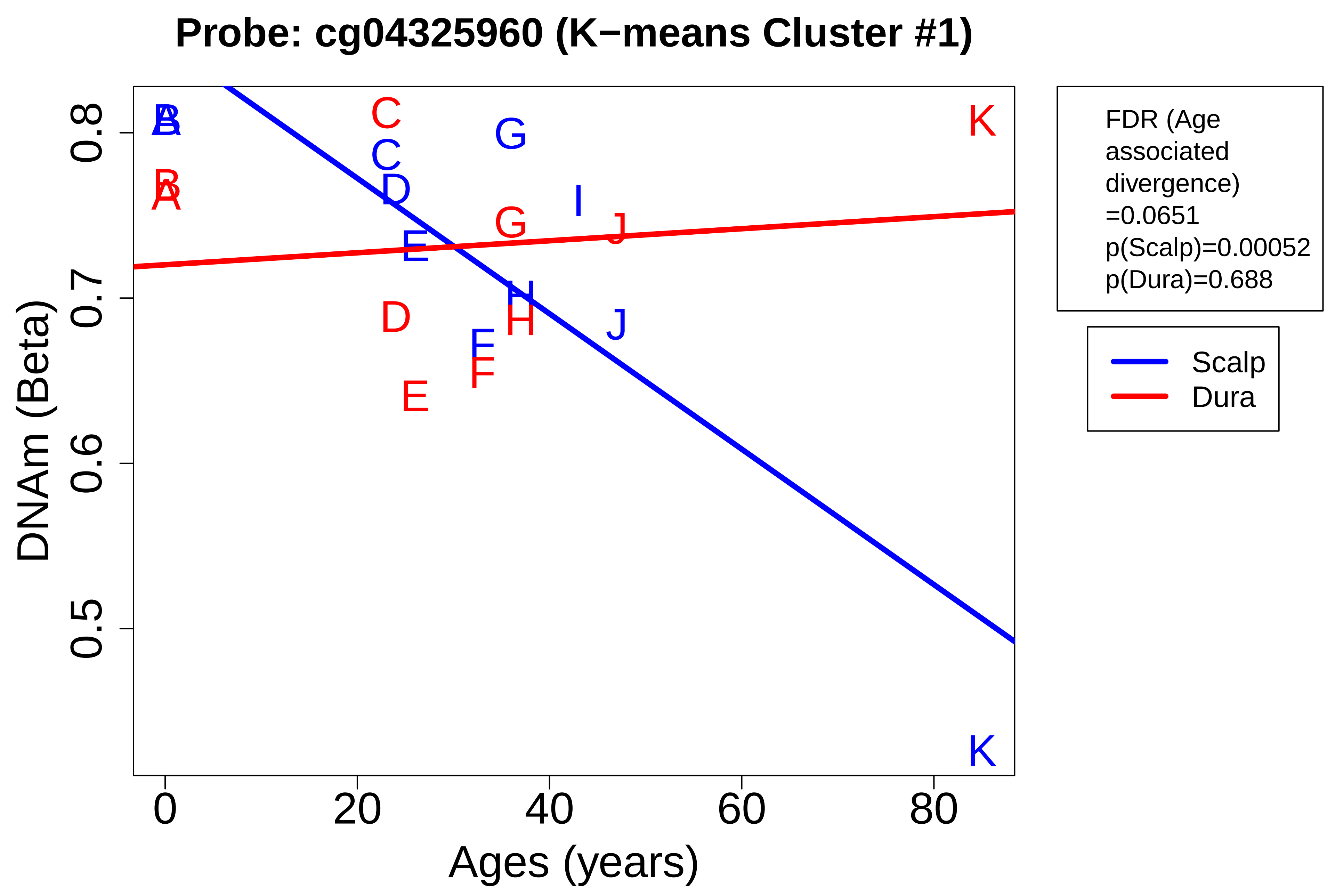

Probe: cg04325960 (K−means Cluster #1)
DNAm (Beta)
Ages (years)
0.5
0.6
0.7
0.8
0
20
40
60
80
FDR (Age associated divergence) =0.0651
p(Scalp)=0.00052
p(Dura)=0.688
Scalp
Dura

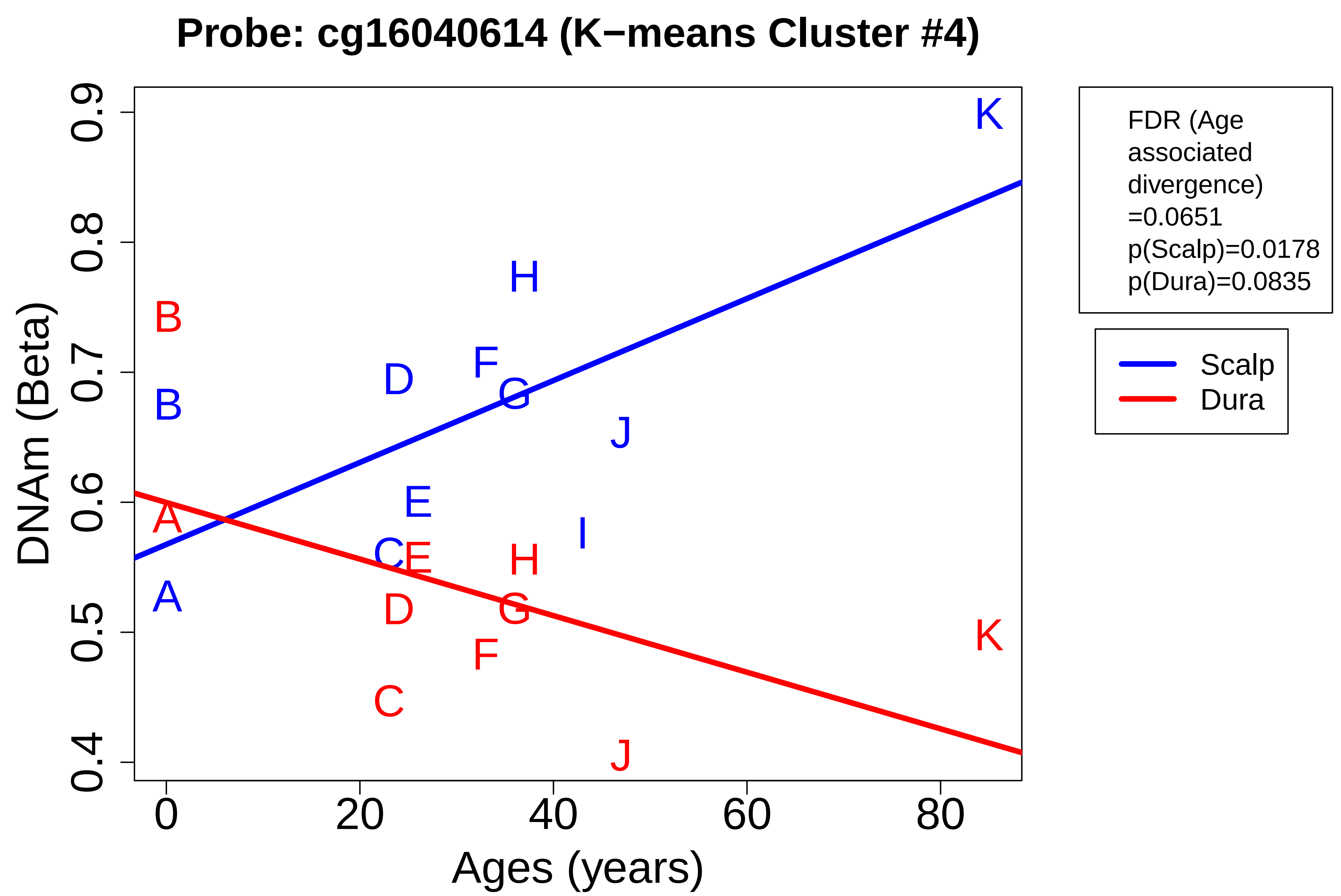
Probe: cg16040614 (K−means Cluster #4)
DNAm (Beta)
0.4
0.5
0.6
0.7
0.8
0.9
0
20
40
60
80
Ages (years)
A
B
C
D
E
F
G
H
I
J
K
A
B
C
D
E
F
G
H
J
K
FDR (Age associated divergence) =0.0651
p(Scalp)=0.0178
p(Dura)=0.0835
Scalp
Dura

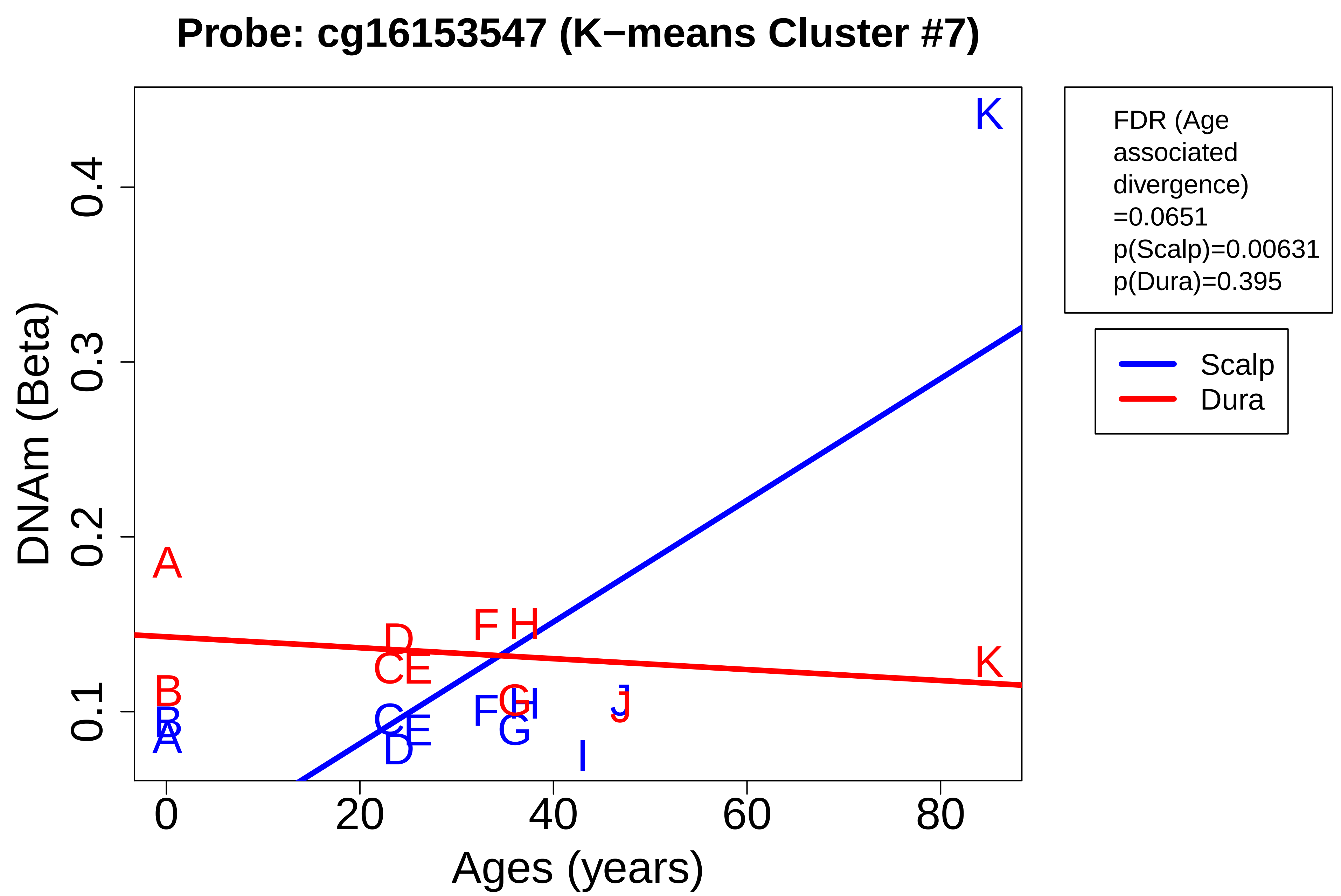
Probe: cg16153547 (K−means Cluster #7)
DNAm (Beta)
0.1
0.2
0.3
0.4
0
20
40
60
80
Ages (years)
A
B
C
D
E
F
G
H
I
J
K
FDR (Age associated divergence) =0.0651
p(Scalp)=0.00631
p(Dura)=0.395
Scalp
Dura

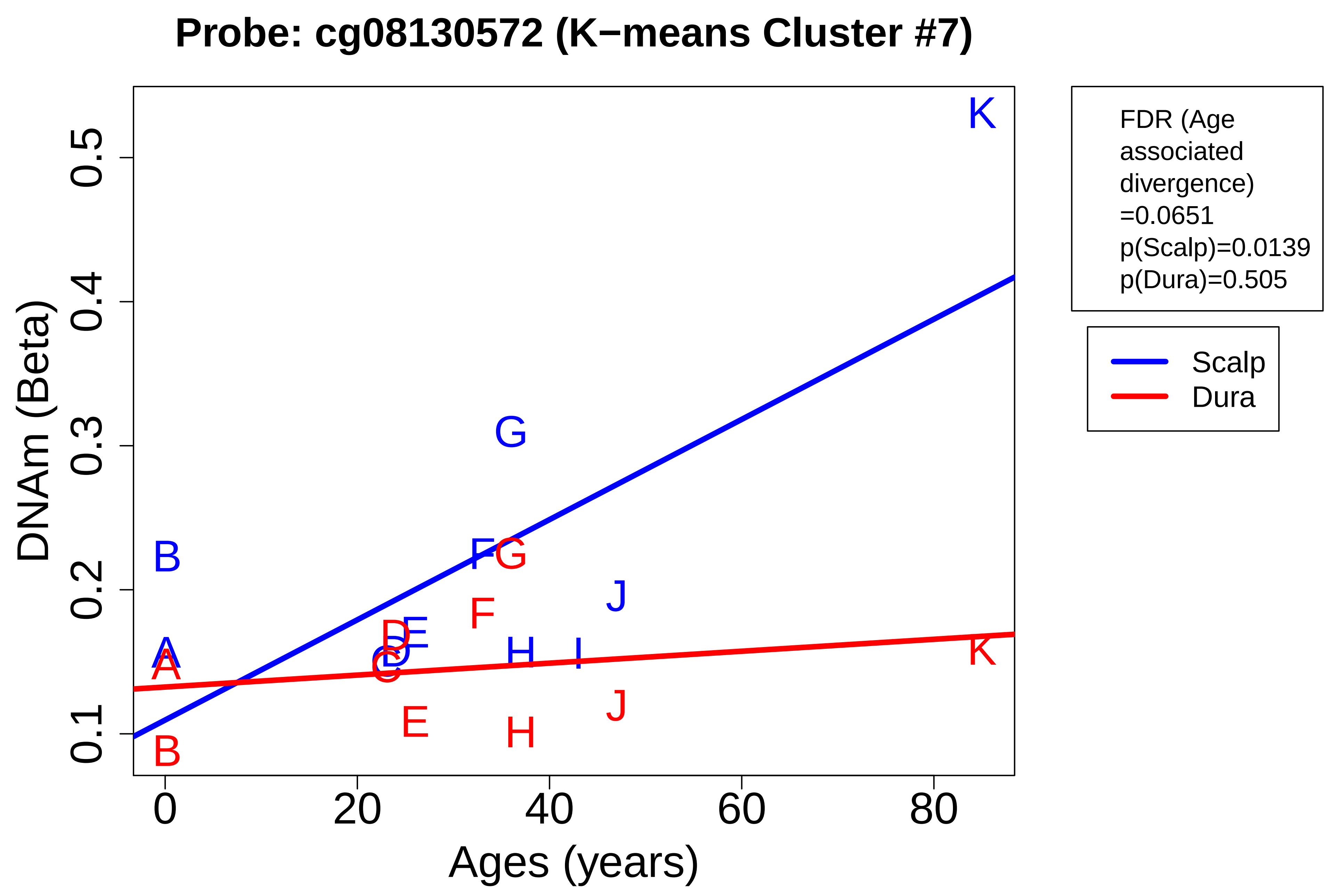

Probe: cg08130572 (K−means Cluster #7)
DNAm (Beta)
0.1
0.2
0.3
0.4
0.5
Ages (years)
0
20
40
60
80
FDR (Age associated divergence) =0.0651
p(Scalp)=0.0139
p(Dura)=0.505
Scalp
Dura

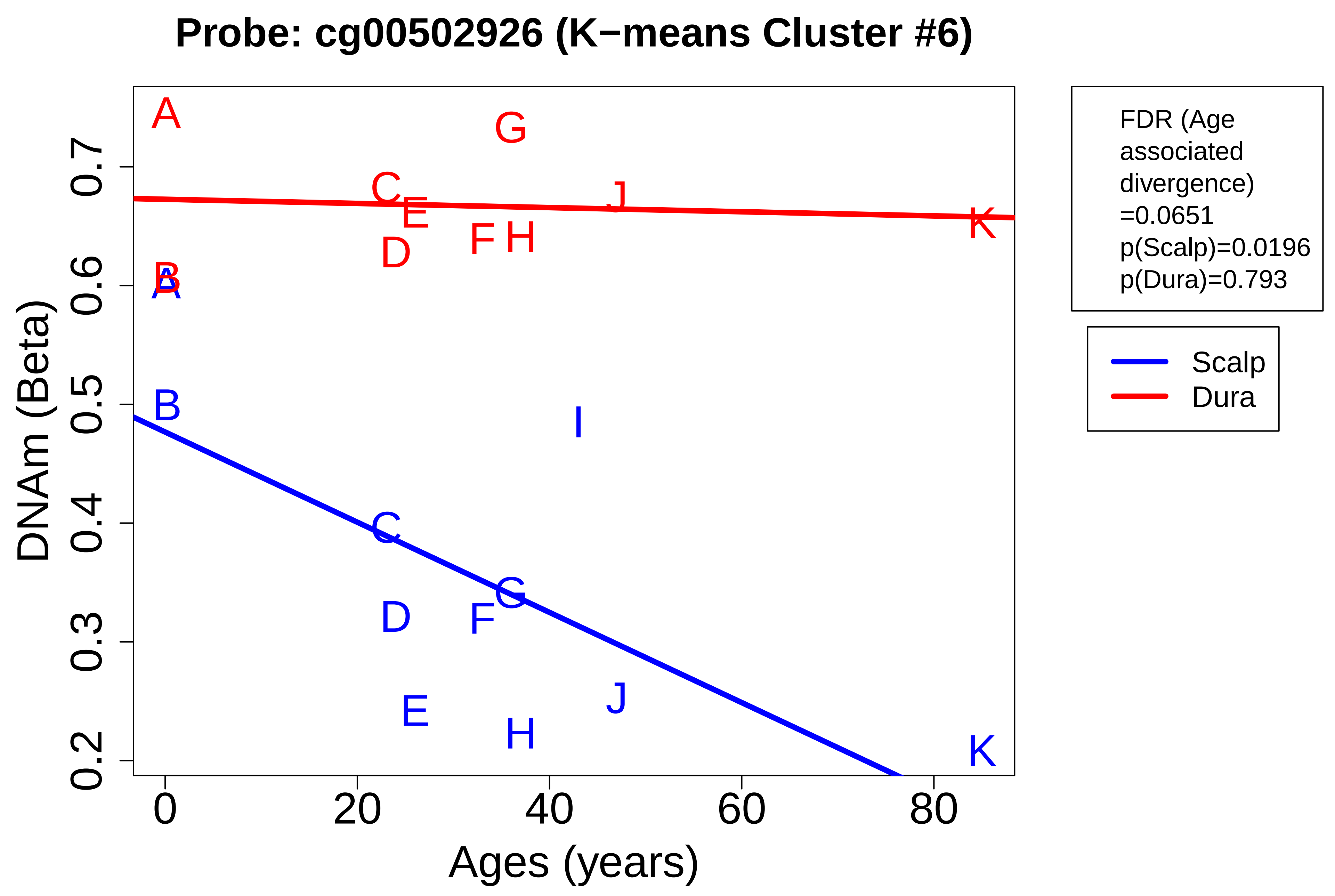

Probe: cg00502926 (K−means Cluster #6)
DNAm (Beta)
Ages (years)
0.2
0.3
0.4
0.5
0.6
0.7
0
20
40
60
80
FDR (Age associated divergence) =0.0651
p(Scalp)=0.0196
p(Dura)=0.793
Scalp
Dura

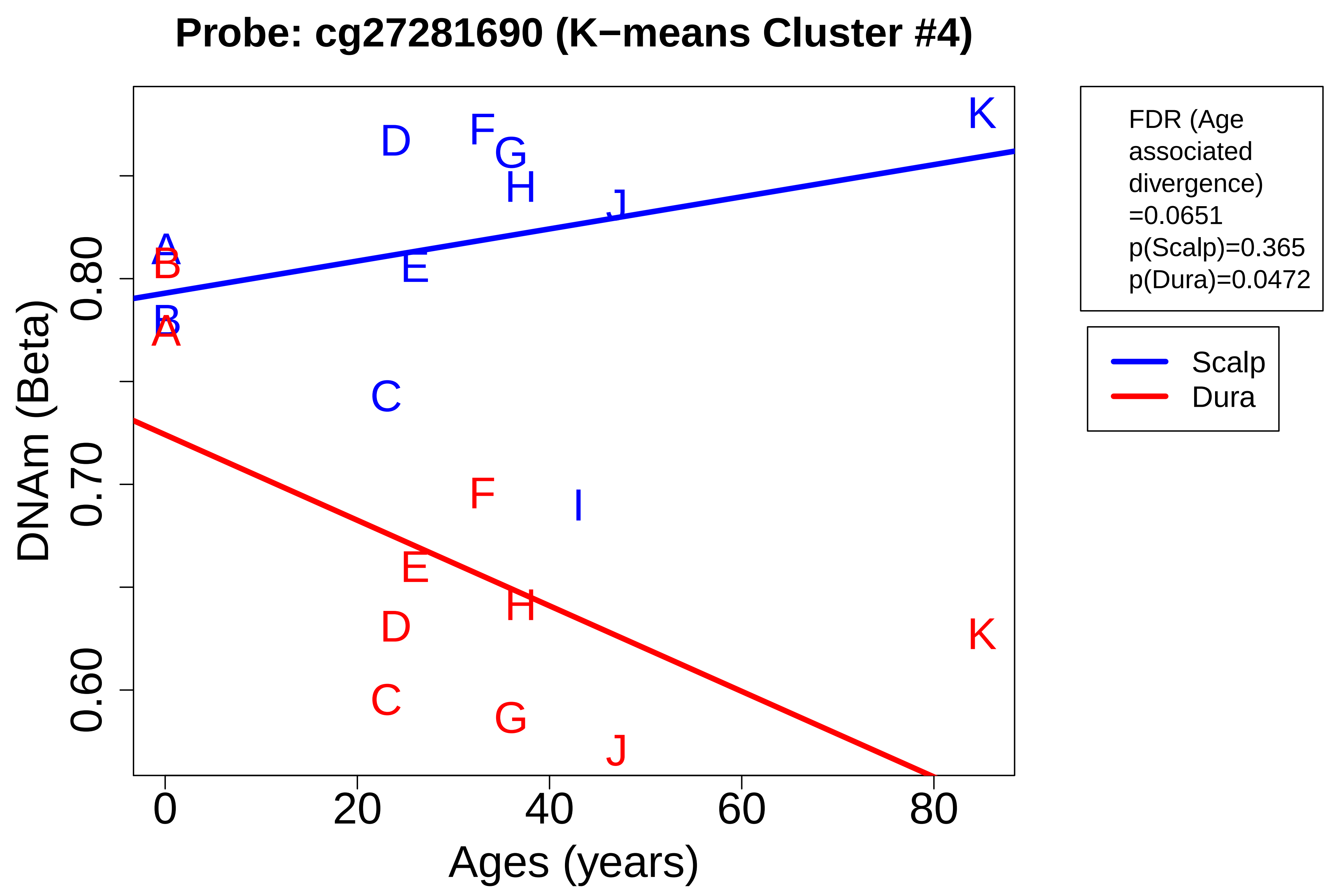

Probe: cg27281690 (K−means Cluster #4)
FDR (Age associated divergence) =0.0651
p(Scalp)=0.365
p(Dura)=0.0472
Scalp
Dura
DNAm (Beta)
0.60
0.70
0.80
0
20
40
60
80
Ages (years)

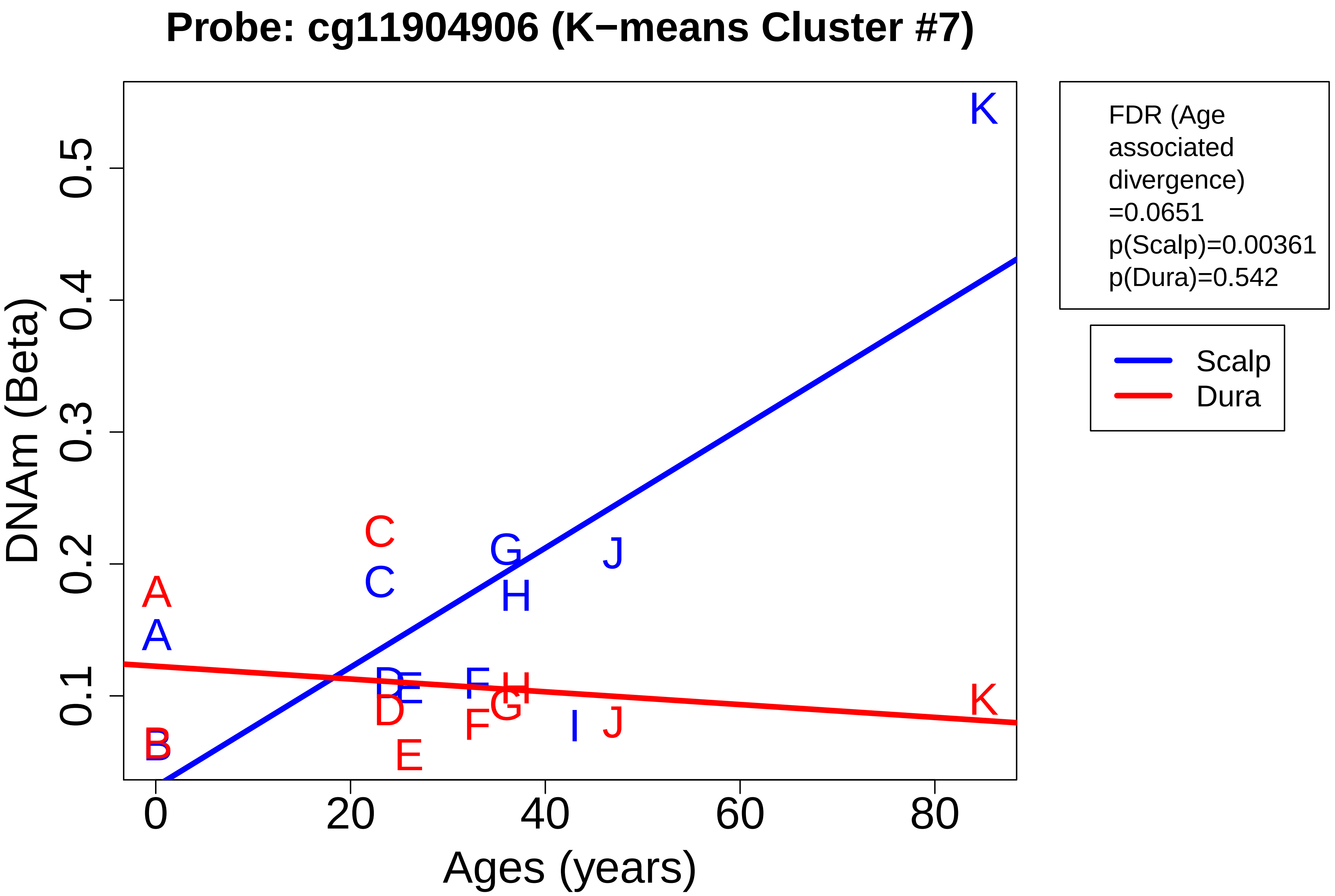

Probe: cg11904906 (K−means Cluster #7)
DNAm (Beta)
0.1
0.2
0.3
0.4
0.5
0
20
40
60
80
Ages (years)
FDR (Age associated divergence) =0.0651
p(Scalp)=0.00361
p(Dura)=0.542
Scalp
Dura

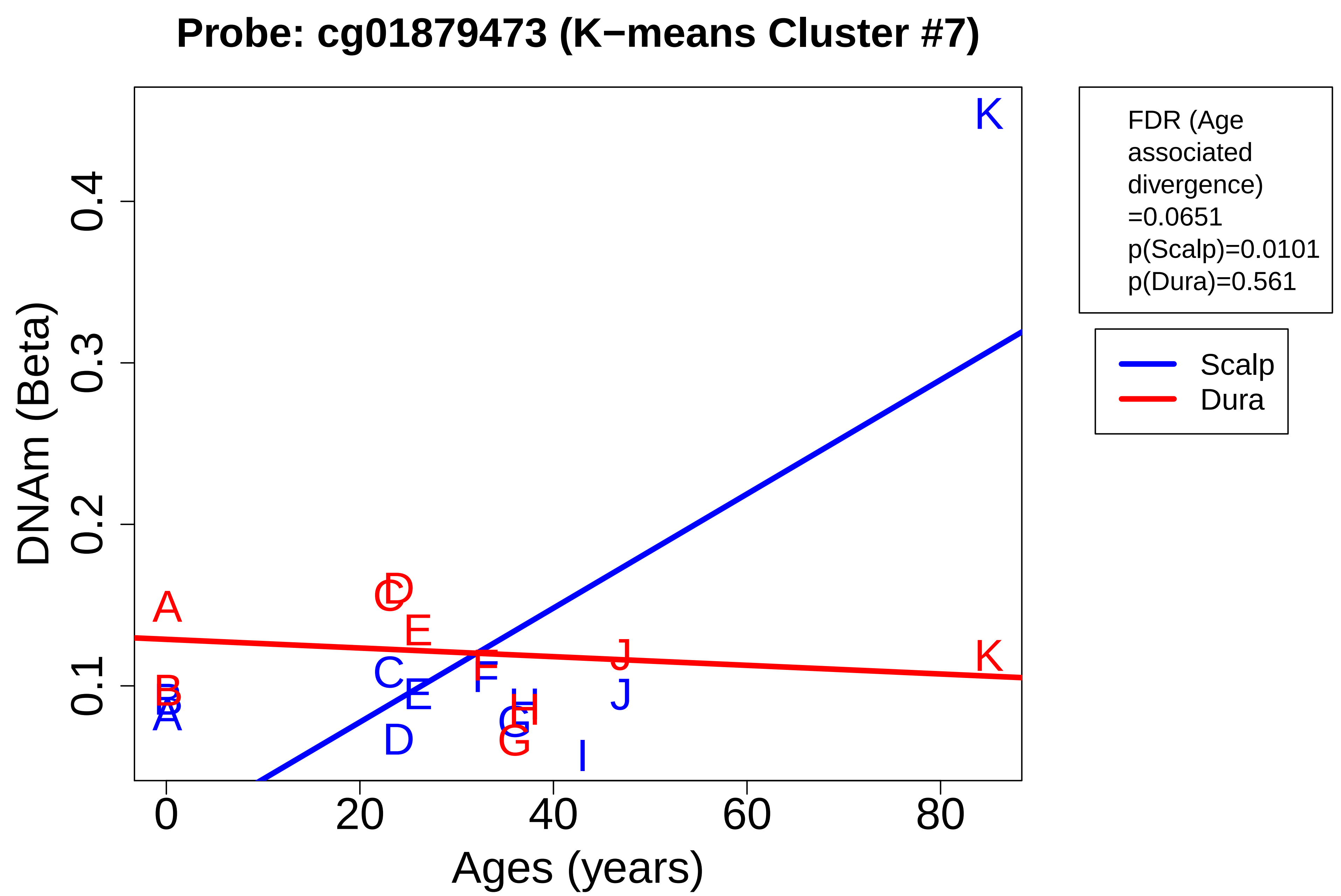
Probe: cg01879473 (K−means Cluster #7)
FDR (Age associated divergence) =0.0651
p(Scalp)=0.0101
p(Dura)=0.561
Scalp
Dura
DNAm (Beta)
0.1
0.2
0.3
0.4
0
20
40
60
80
Ages (years)
A
B
C
D
E
F
G
H
I
J
K

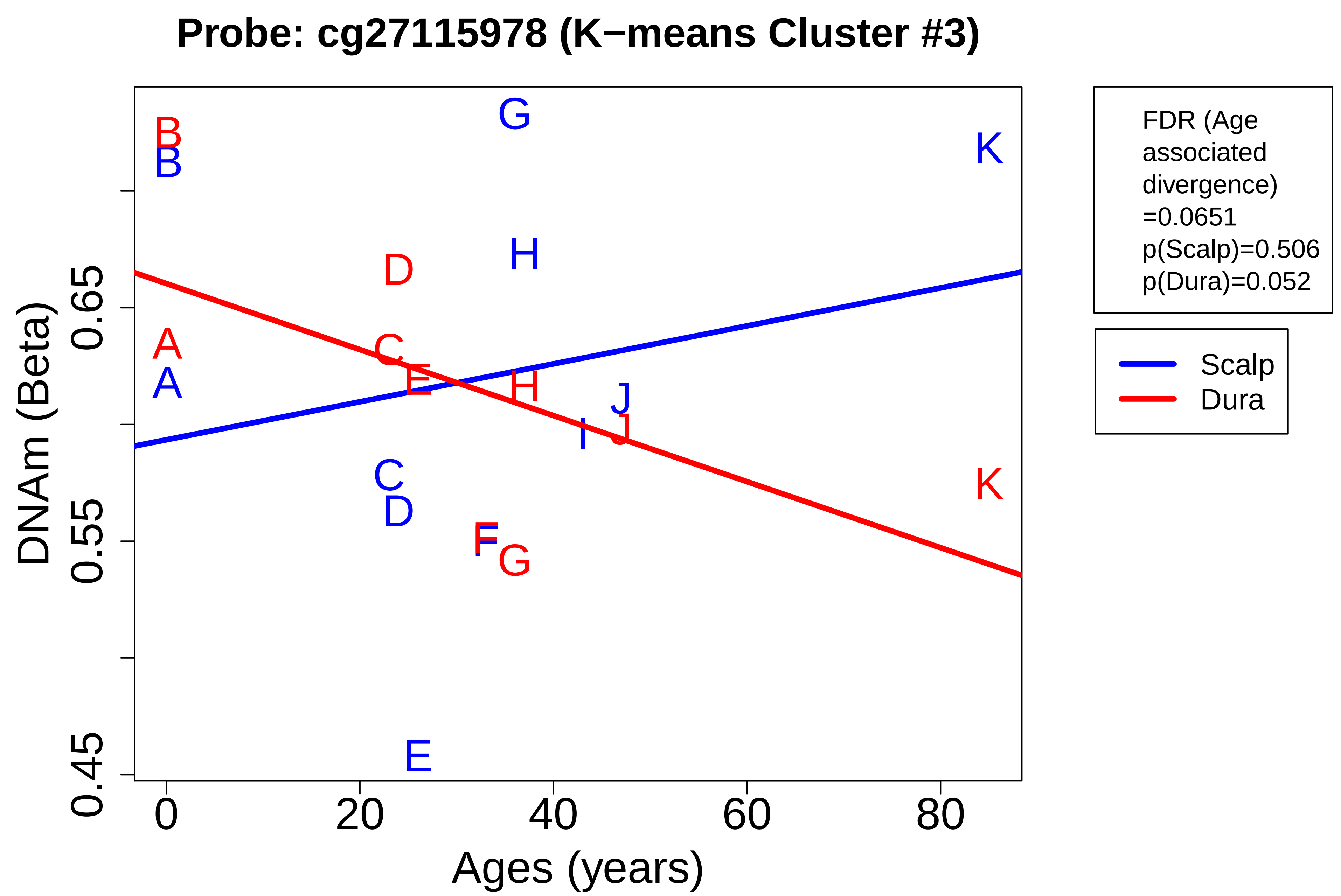

Probe: cg27115978 (K−means Cluster #3)
DNAm (Beta)
0.45
0.55
0.65
0
20
40
60
80
Ages (years)
FDR (Age associated divergence) =0.0651
p(Scalp)=0.506
p(Dura)=0.052
Scalp
Dura
A
B
C
D
E
F
G
H
I
J
K

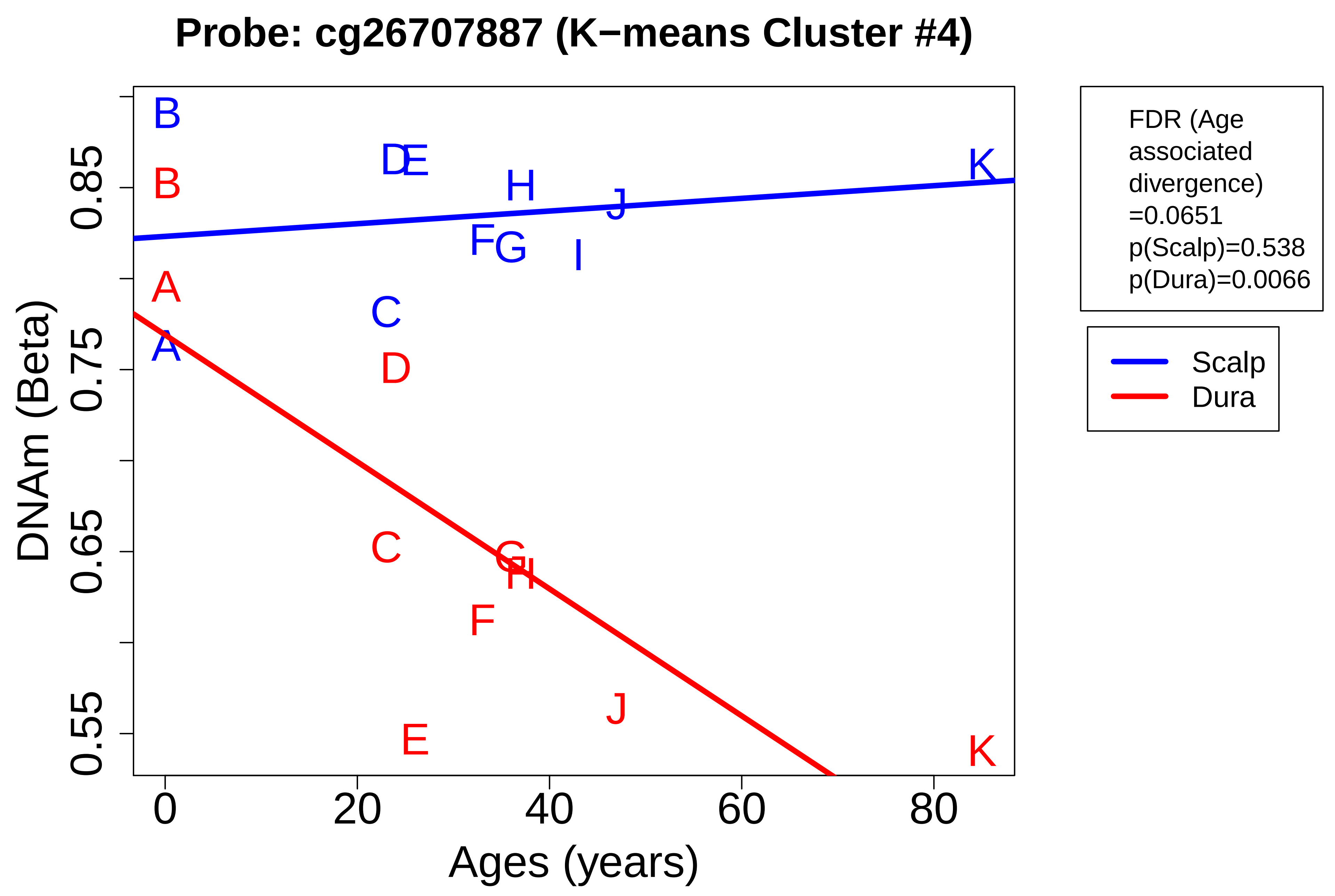

Probe: cg26707887 (K−means Cluster #4)
DNAm (Beta)
0.55
0.65
0.75
0.85
Ages (years)
0
20
40
60
80
A
B
C
D
E
F
G
H
I
J
K
FDR (Age associated divergence) =0.0651
p(Scalp)=0.538
p(Dura)=0.0066
Scalp
Dura

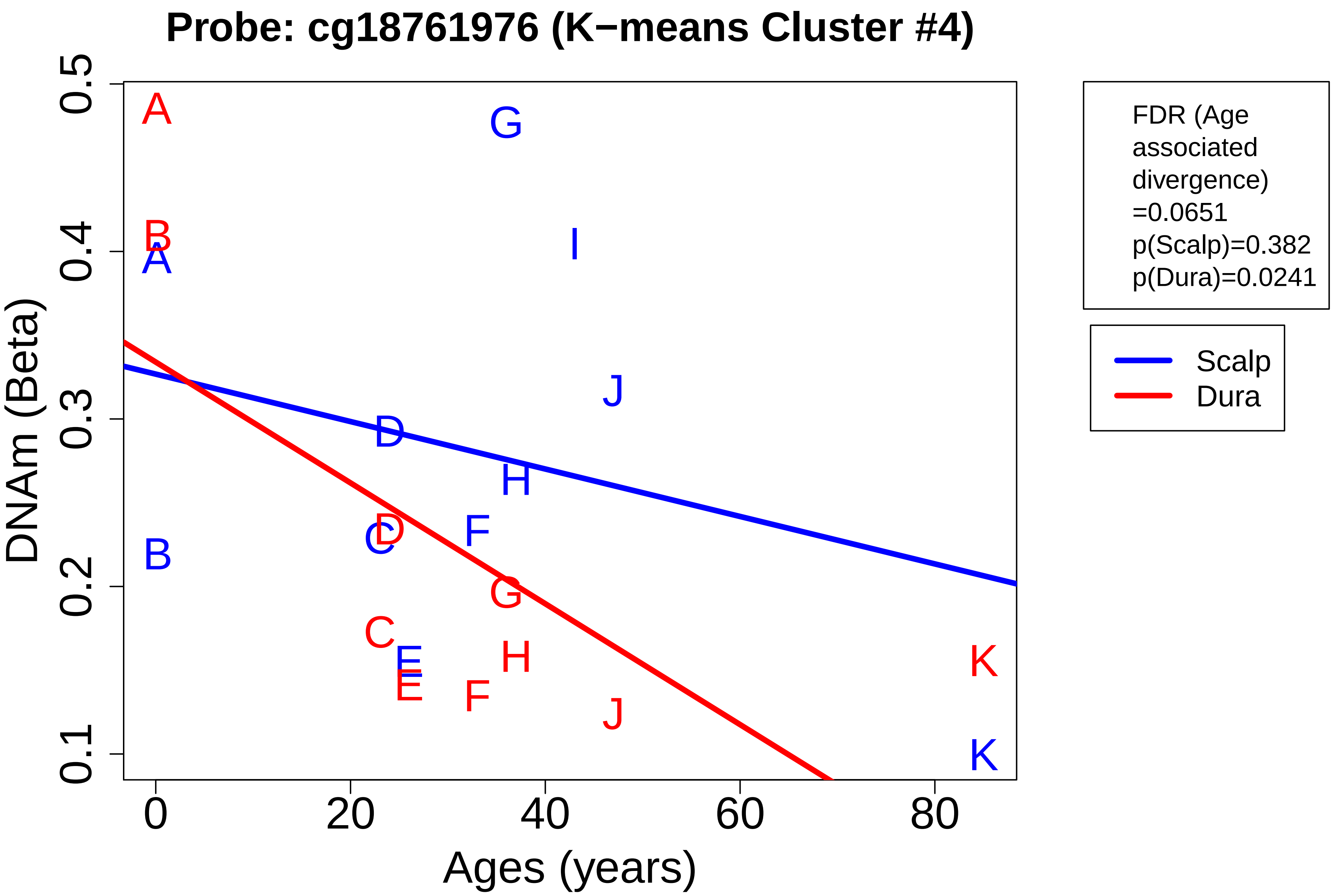

Probe: cg18761976 (K−means Cluster #4)
DNAm (Beta)
0.1
0.2
0.3
0.4
0.5
0
20
40
60
80
Ages (years)
FDR (Age associated divergence) =0.0651
p(Scalp)=0.382
p(Dura)=0.0241
Scalp
Dura
A
B
C
D
E
F
G
H
I
J
K

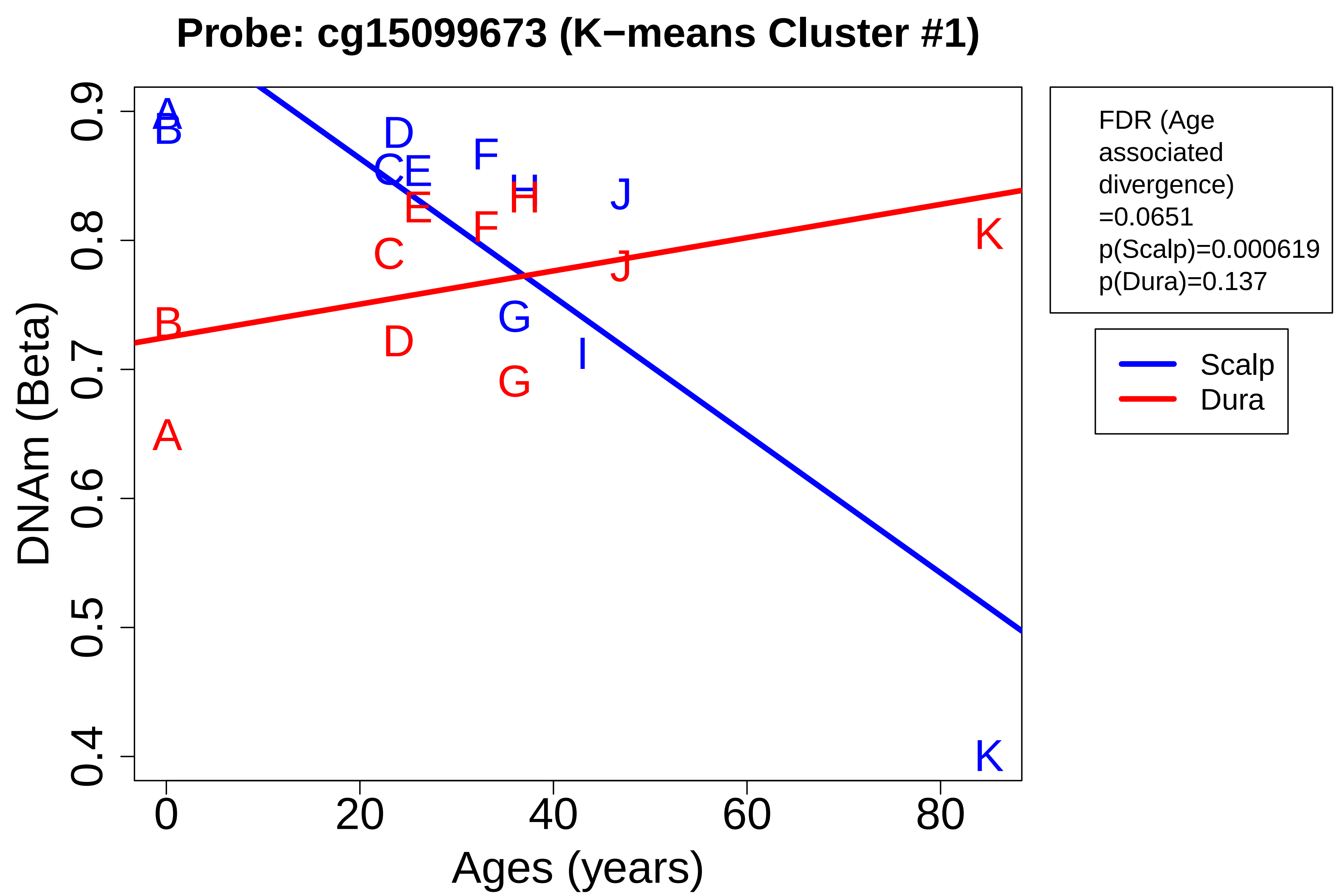

Probe: cg15099673 (K−means Cluster #1)
DNAm (Beta)
0.4
0.5
0.6
0.7
0.8
0.9
0
20
40
60
80
Ages (years)
A
B
C
D
E
F
G
H
I
J
K
FDR (Age associated divergence) =0.0651
p(Scalp)=0.000619
p(Dura)=0.137
Scalp
Dura

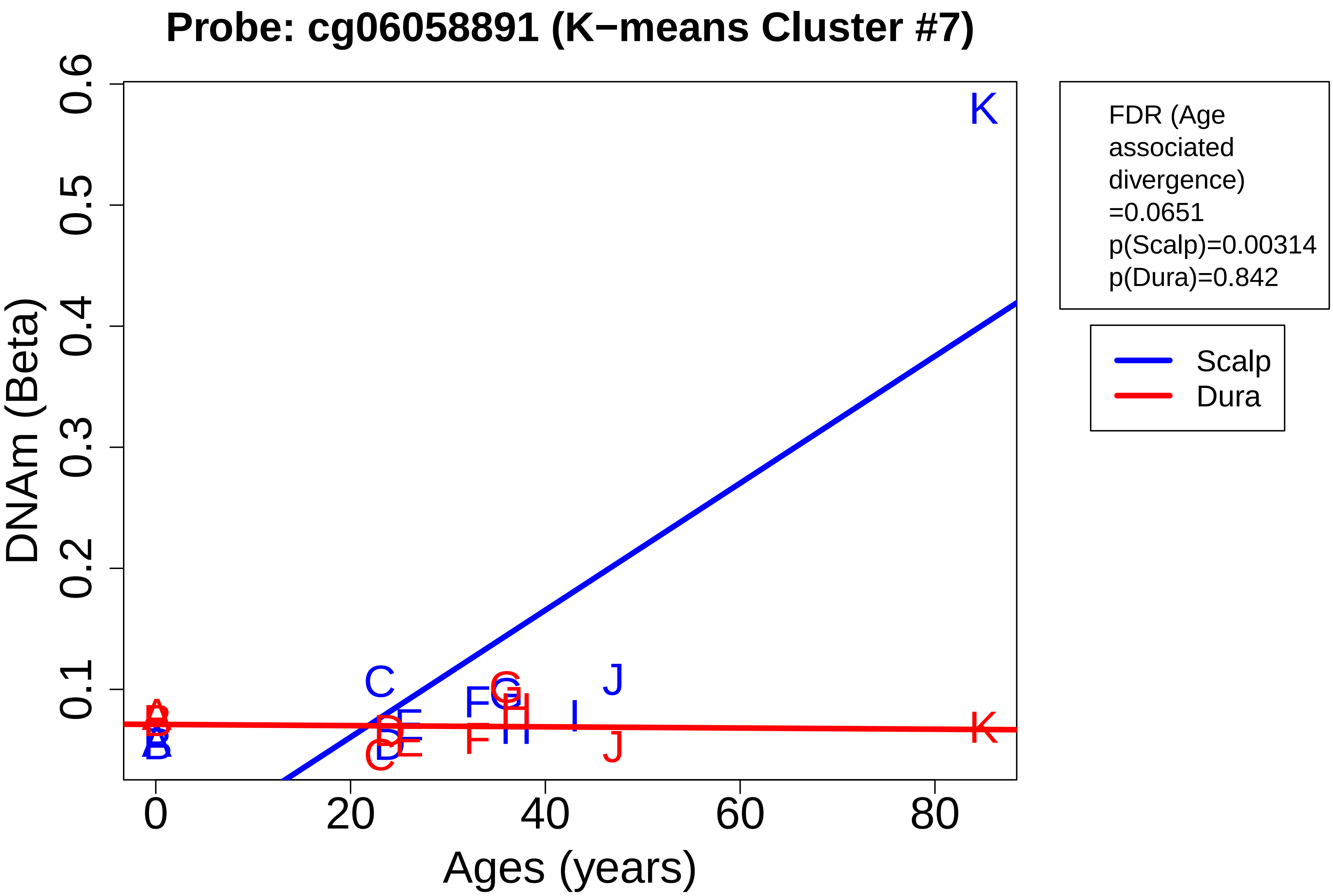

Probe: cg06058891 (K−means Cluster #7)
DNAm (Beta)
0.1
0.2
0.3
0.4
0.5
0.6
0
20
40
60
80
Ages (years)
FDR (Age associated divergence) =0.0651
p(Scalp)=0.00314
p(Dura)=0.842
Scalp
Dura

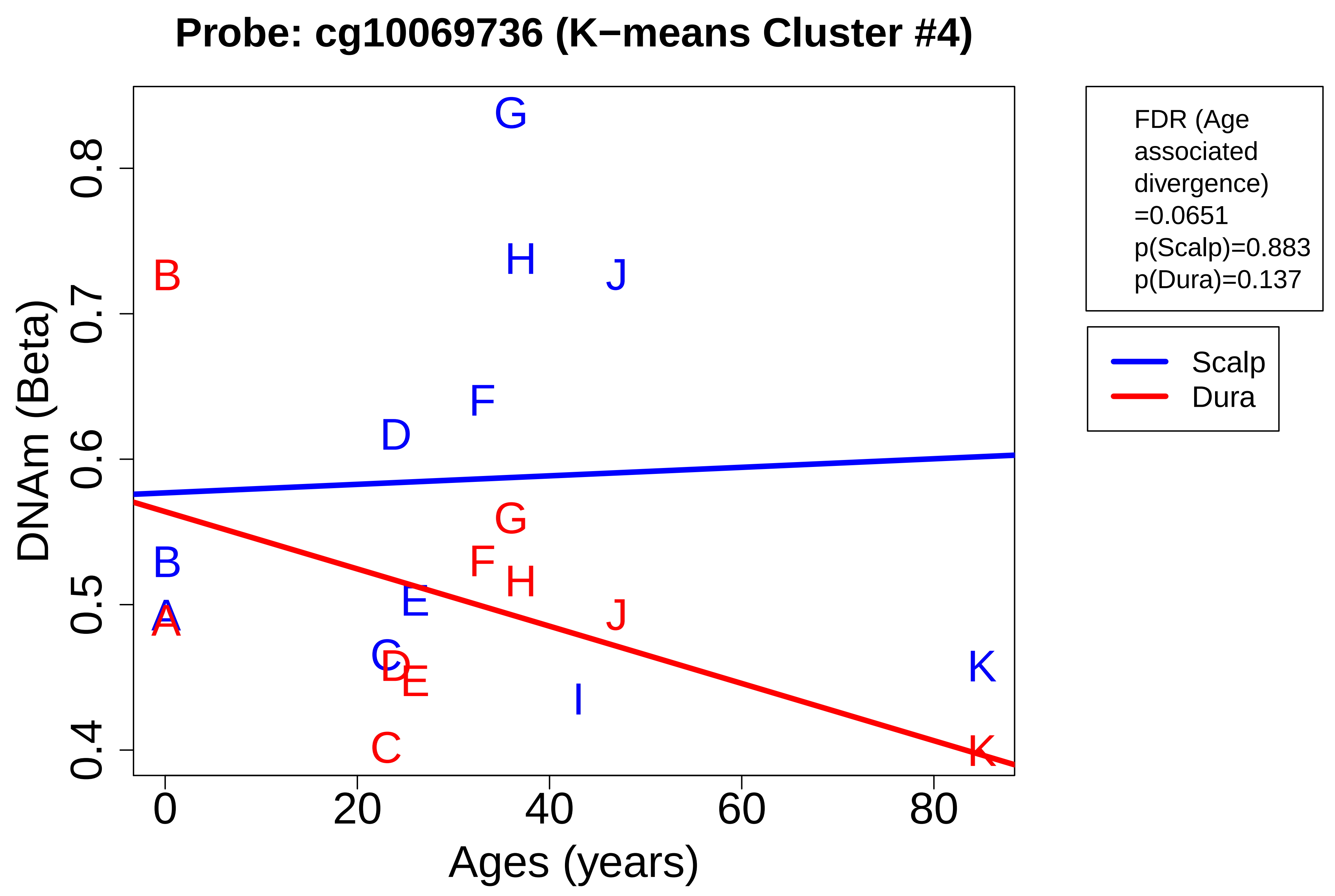
Probe: cg10069736 (K−means Cluster #4)
FDR (Age associated divergence) =0.0651
p(Scalp)=0.883
p(Dura)=0.137
Scalp
Dura
DNAm (Beta)
0.4
0.5
0.6
0.7
0.8
Ages (years)
0
20
40
60
80
A
B
C
D
E
F
G
H
I
J
K

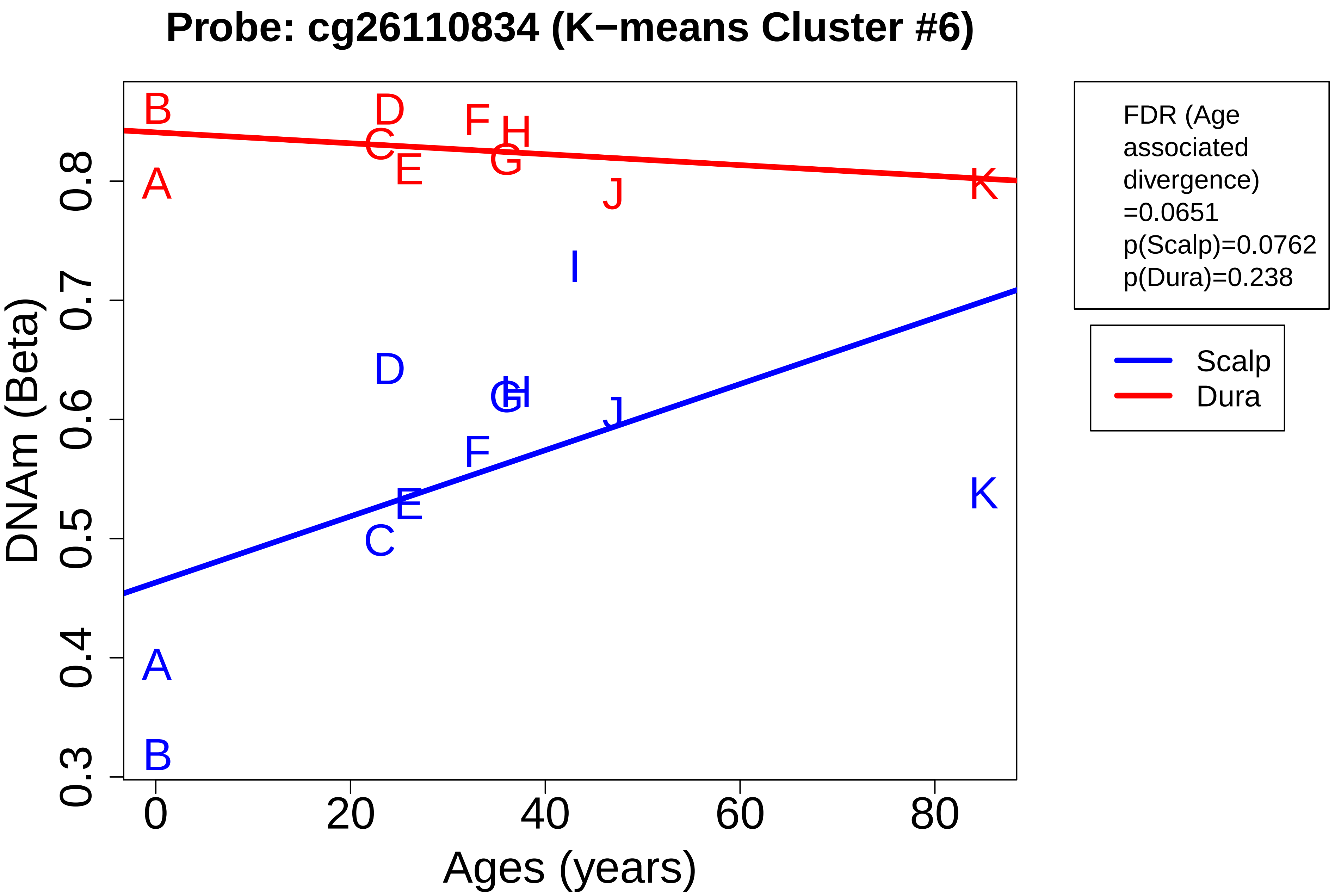
Probe: cg26110834 (K−means Cluster #6)
DNAm (Beta)
0.3
0.4
0.5
0.6
0.7
0.8
0
20
40
60
80
Ages (years)
A
B
C
D
E
F
G
H
I
J
K
FDR (Age associated divergence) =0.0651
p(Scalp)=0.0762
p(Dura)=0.238
Scalp
Dura

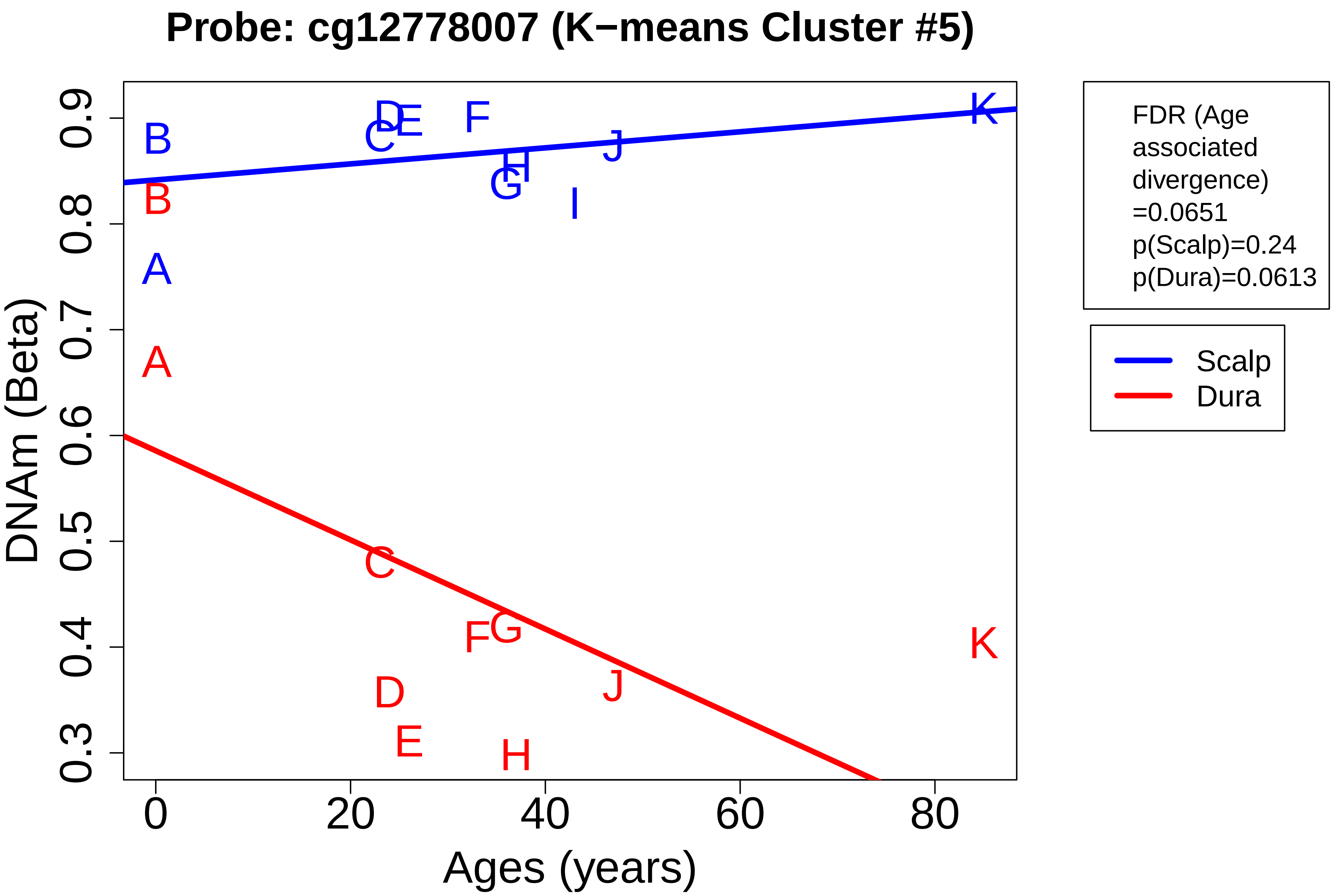

Probe: cg12778007 (K−means Cluster #5)
DNAm (Beta)
Ages (years)
0.3
0.4
0.5
0.6
0.7
0.8
0.9
0
20
40
60
80
A
B
C
D
E
F
G
H
I
J
K
FDR (Age associated divergence) =0.0651
p(Scalp)=0.24
p(Dura)=0.0613
Scalp
Dura

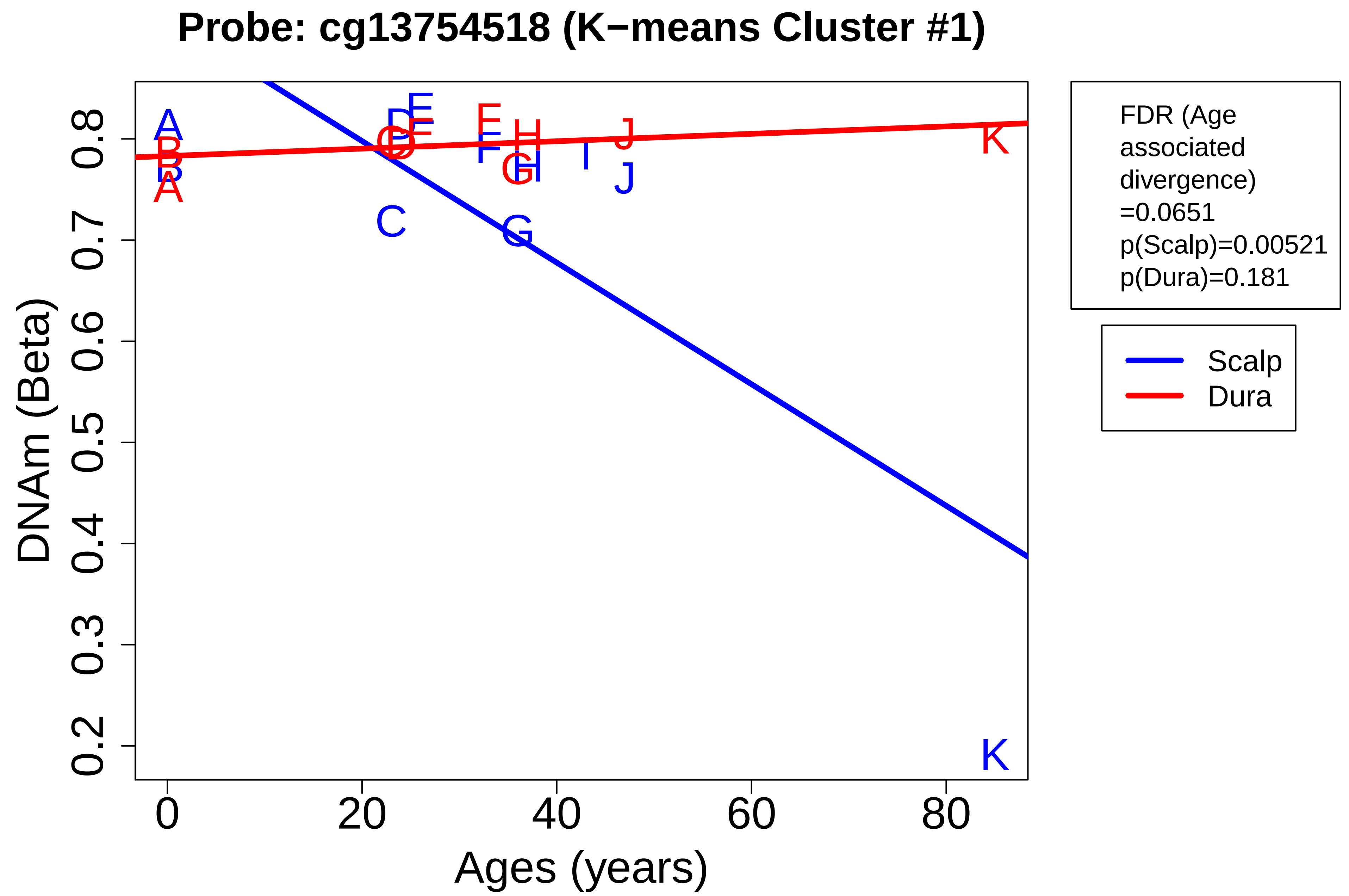
Probe: cg13754518 (K−means Cluster #1)
DNAm (Beta)
0.2
0.3
0.4
0.5
0.6
0.7
0.8
0
20
40
60
80
Ages (years)
FDR (Age associated divergence) =0.0651
p(Scalp)=0.00521
p(Dura)=0.181
Scalp
Dura

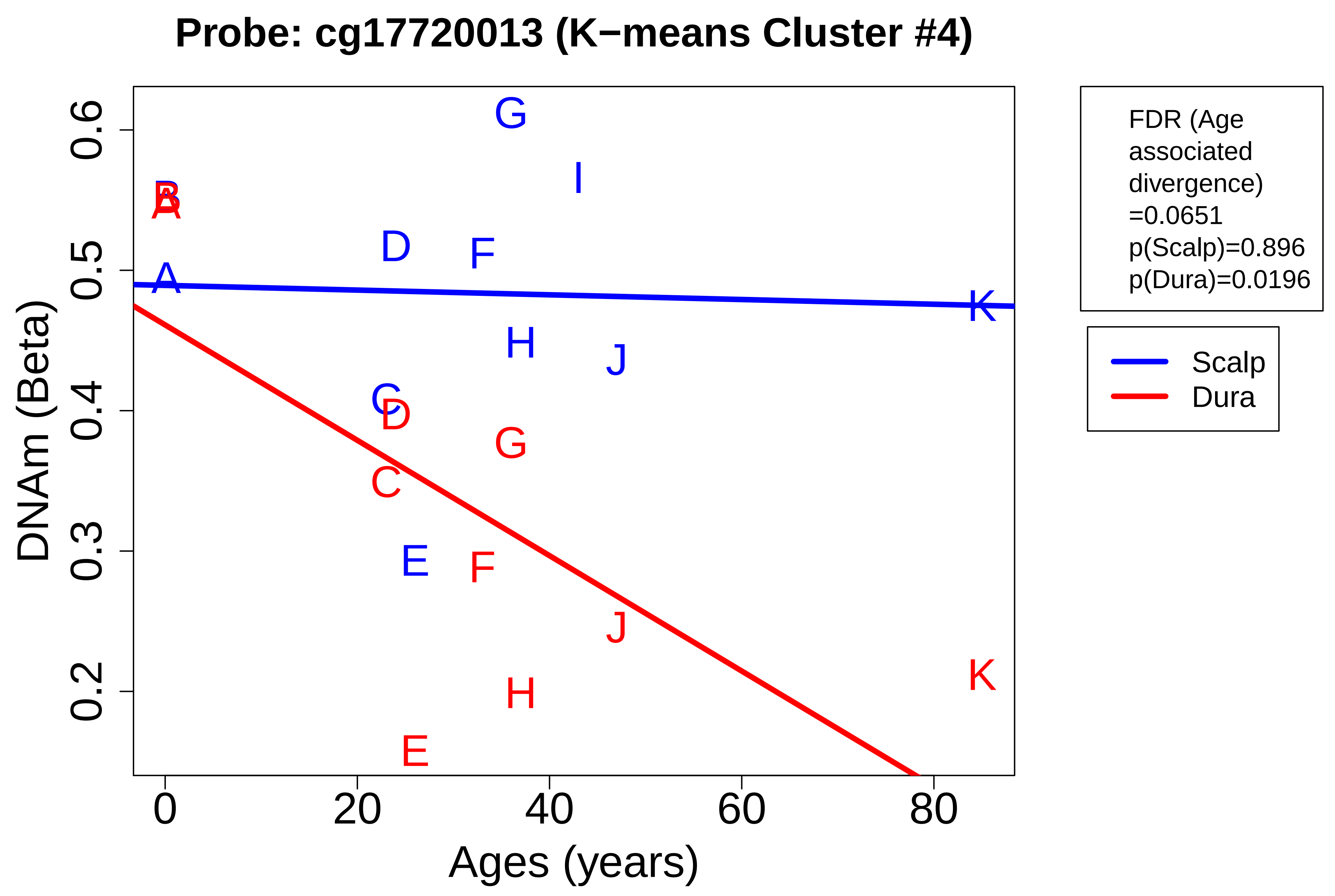

Probe: cg17720013 (K−means Cluster #4)
DNAm (Beta)
Ages (years)
0.2
0.3
0.4
0.5
0.6
0
20
40
60
80
FDR (Age associated divergence) =0.0651
p(Scalp)=0.896
p(Dura)=0.0196
Scalp
Dura
A
B
C
D
E
F
G
H
I
J
K

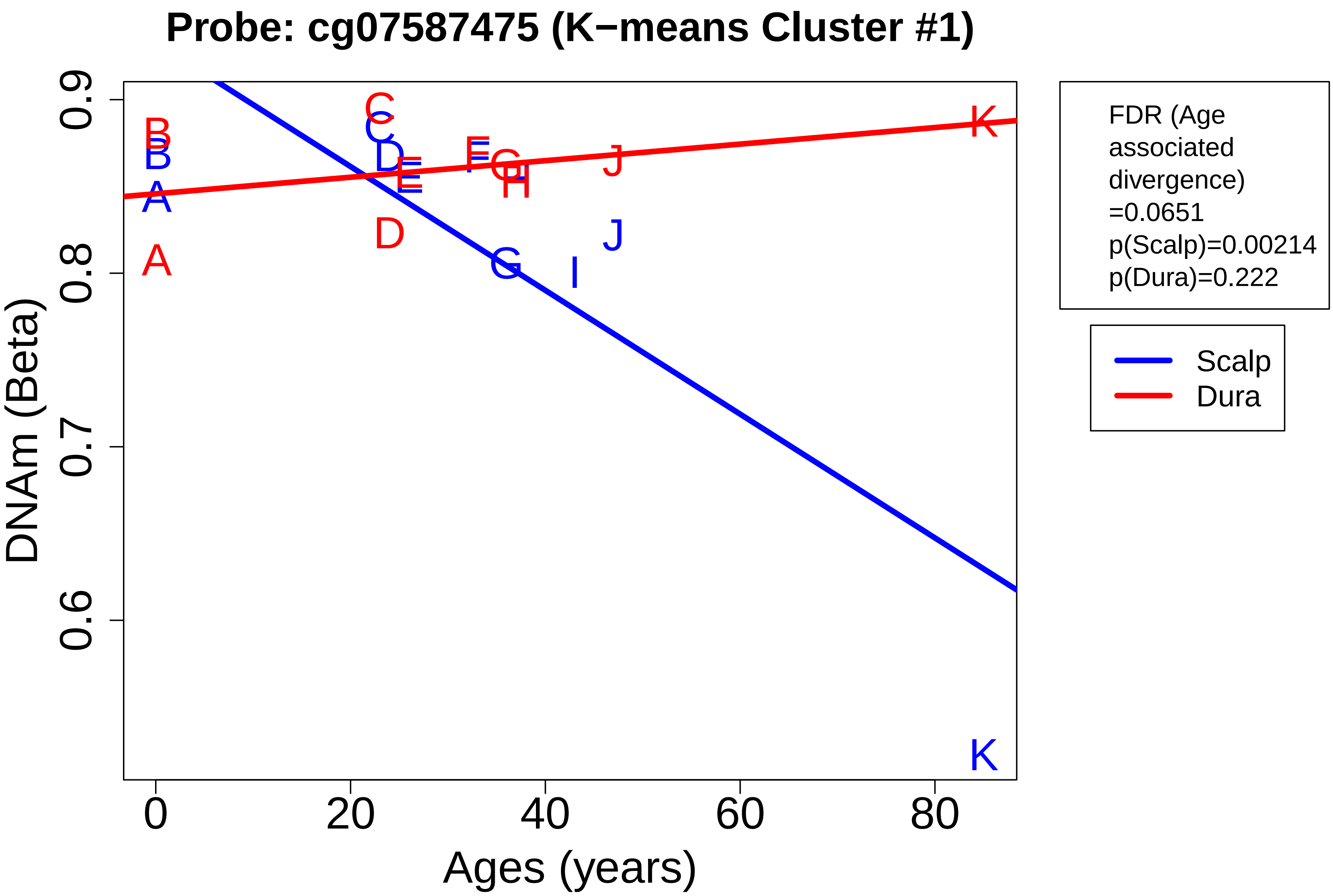

Probe: cg07587475 (K−means Cluster #1)
DNAm (Beta)
0.6
0.7
0.8
0.9
0
20
40
60
80
Ages (years)
FDR (Age associated divergence) =0.0651
p(Scalp)=0.00214
p(Dura)=0.222
Scalp
Dura

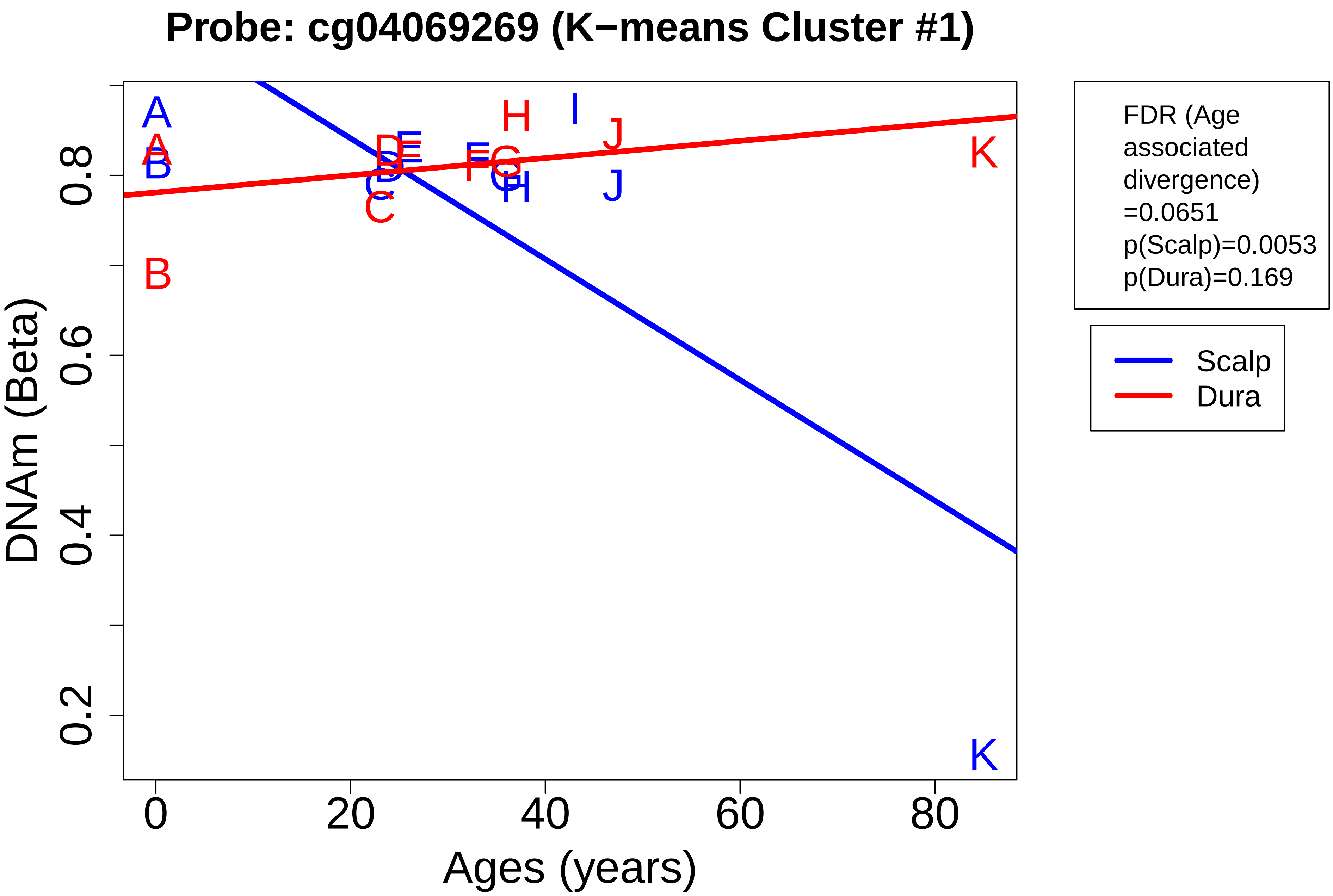

Probe: cg04069269 (K−means Cluster #1)
DNAm (Beta)
0.2
0.4
0.6
0.8
0
20
40
60
80
Ages (years)
FDR (Age associated divergence) =0.0651
p(Scalp)=0.0053
p(Dura)=0.169
Scalp
Dura

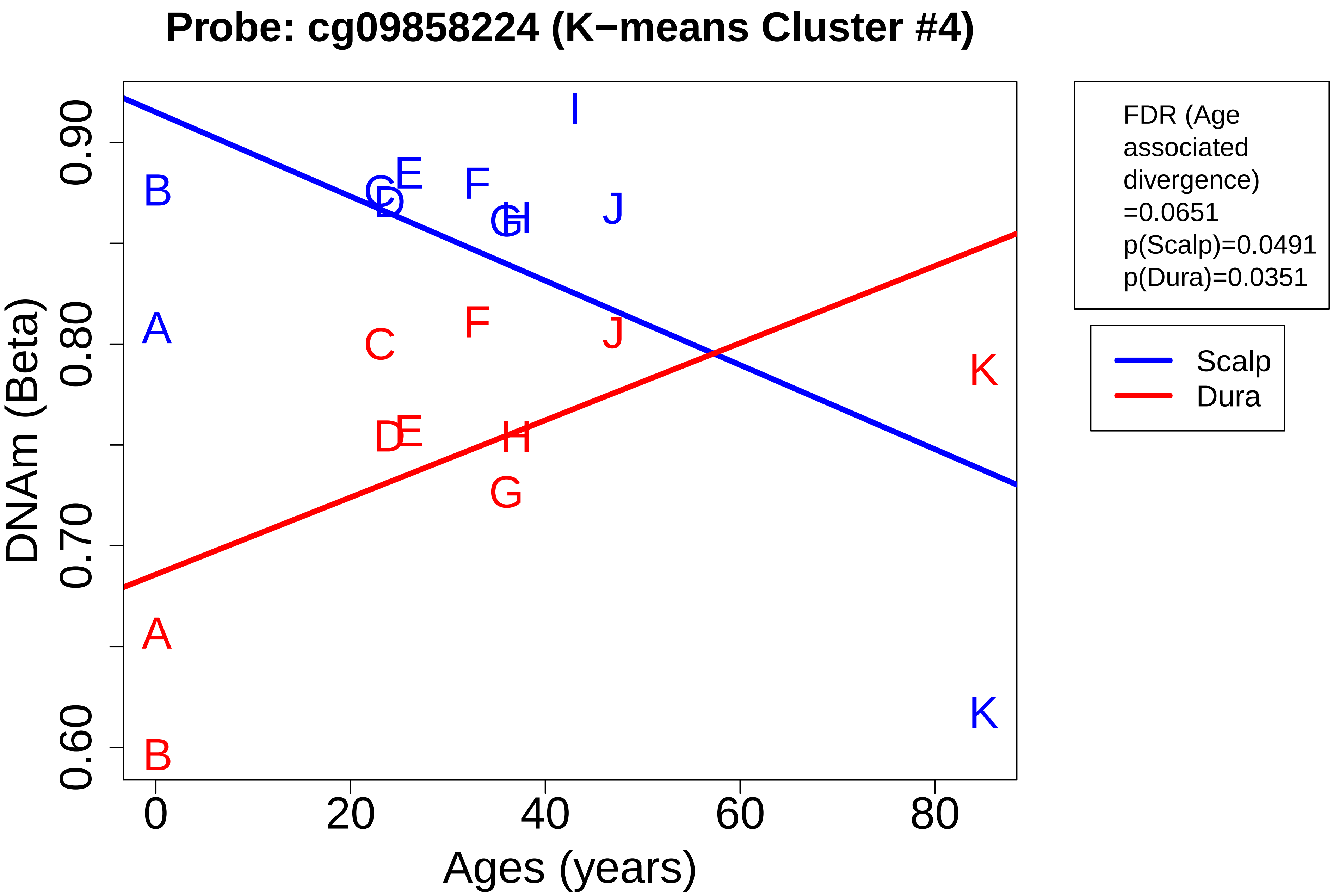
Probe: cg09858224 (K−means Cluster #4)
DNAm (Beta)
0.60
0.70
0.80
0.90
Ages (years)
0
20
40
60
80
FDR (Age associated divergence) =0.0651
p(Scalp)=0.0491
p(Dura)=0.0351
Scalp
Dura
A
B
C
D
E
F
G
H
I
J
K

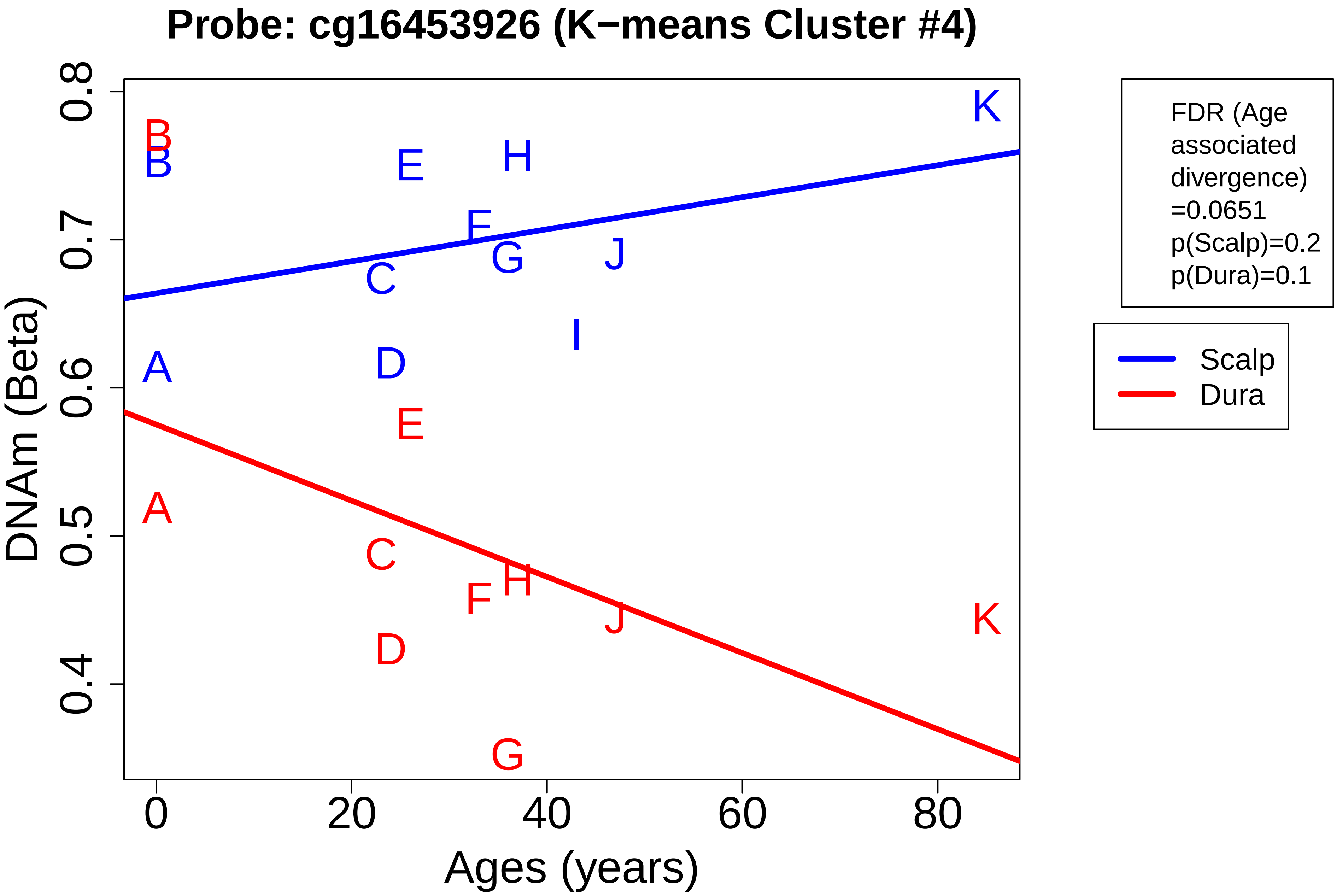

Probe: cg16453926 (K−means Cluster #4)
DNAm (Beta)
0.4
0.5
0.6
0.7
0.8
Ages (years)
0
20
40
60
80
A
B
C
D
E
F
G
H
I
J
K
FDR (Age associated divergence) =0.0651
p(Scalp)=0.2
p(Dura)=0.1
Scalp
Dura

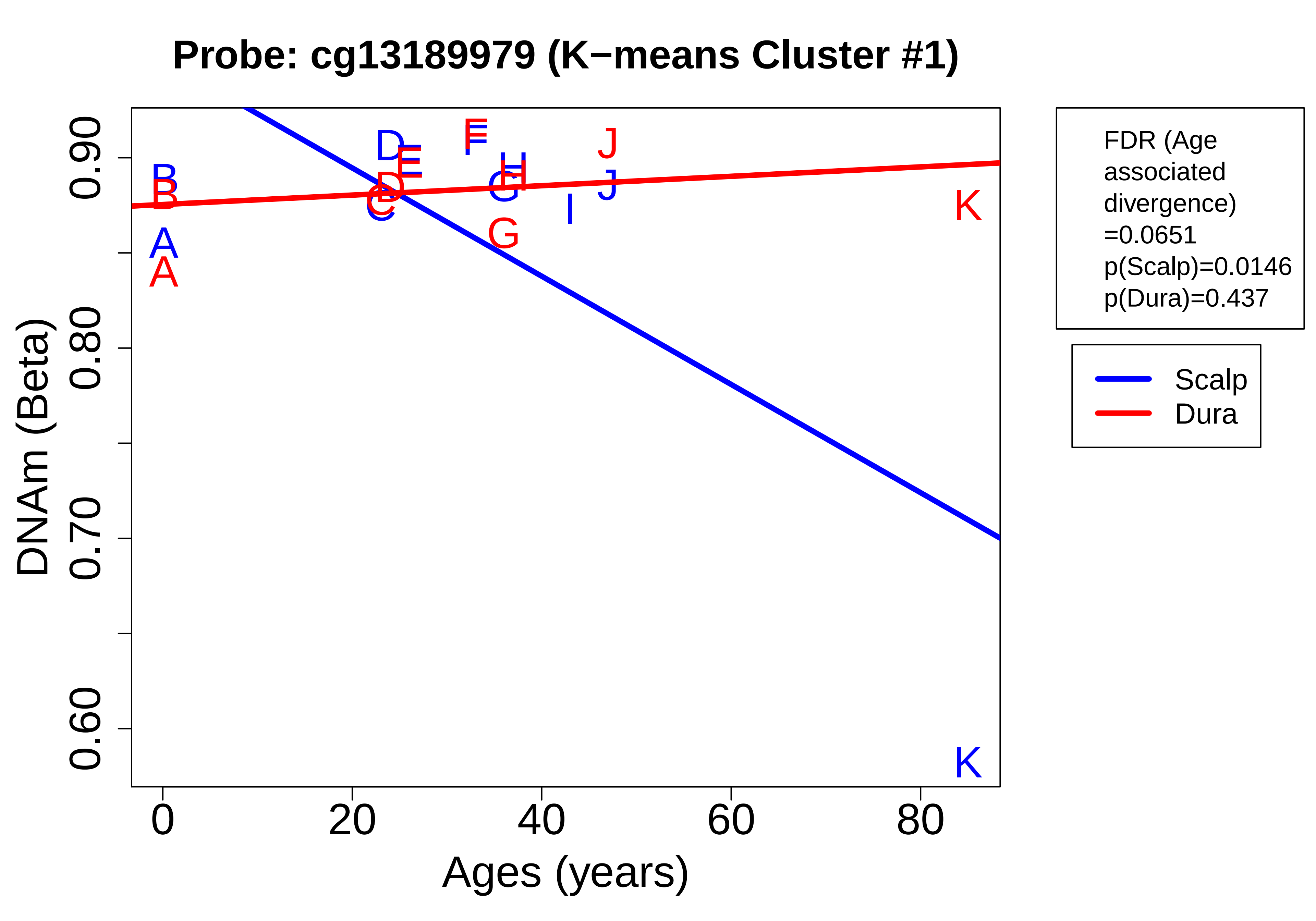

Probe: cg13189979 (K−means Cluster #1)
DNAm (Beta)
0.60
0.70
0.80
0.90
0
20
40
60
80
Ages (years)
A
B
C
D
E
F
G
H
I
J
K
FDR (Age associated divergence) =0.0651
p(Scalp)=0.0146
p(Dura)=0.437
Scalp
Dura

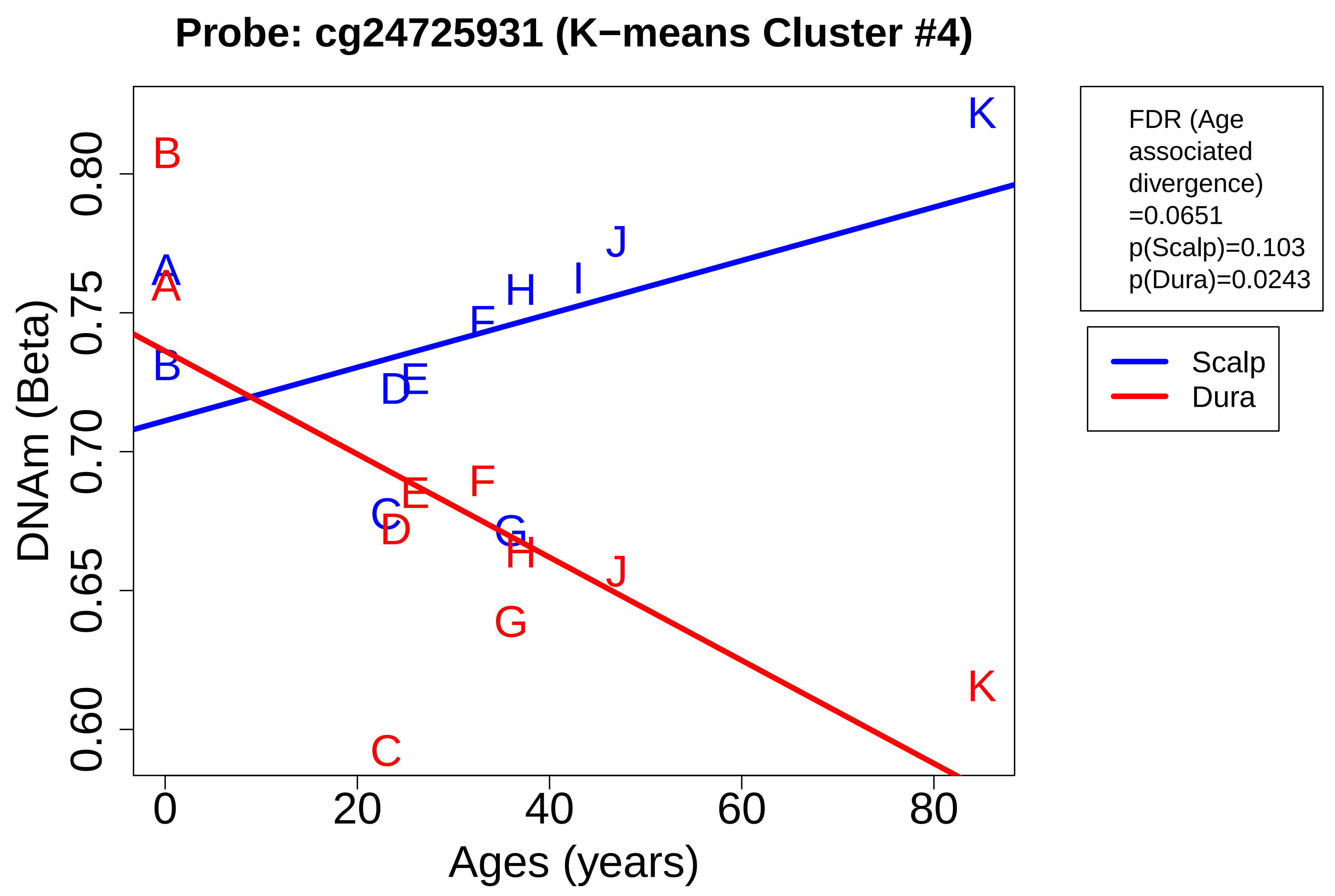
Probe: cg24725931 (K−means Cluster #4)
DNAm (Beta)
0.60
0.65
0.70
0.75
0.80
0
20
40
60
80
Ages (years)
FDR (Age associated divergence) =0.0651
p(Scalp)=0.103
p(Dura)=0.0243
Scalp
Dura
A
B
C
D
E
F
G
H
I
J
K

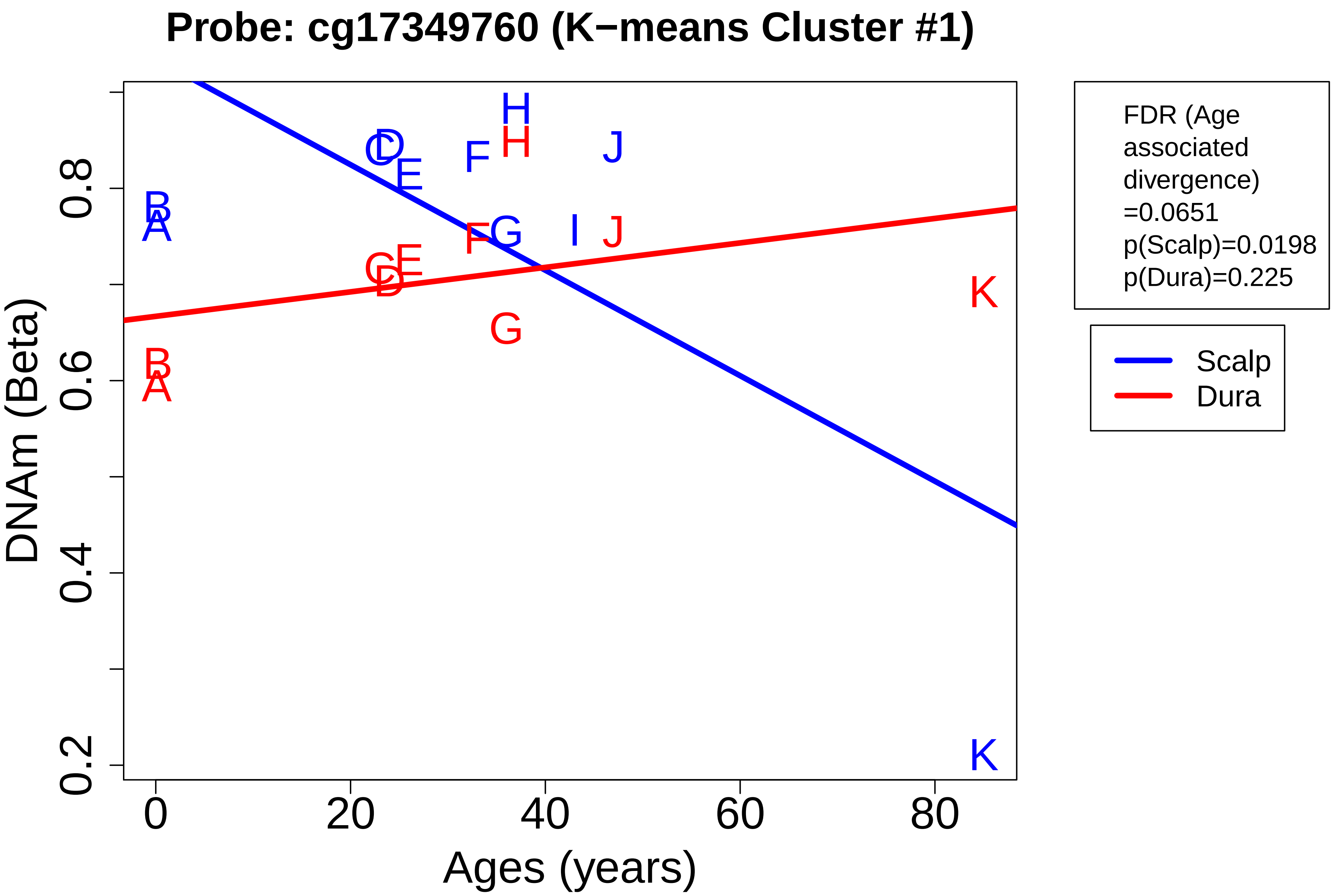

Probe: cg17349760 (K−means Cluster #1)
DNAm (Beta)
0.2
0.4
0.6
0.8
0
20
40
60
80
Ages (years)
FDR (Age associated divergence) =0.0651
p(Scalp)=0.0198
p(Dura)=0.225
Scalp
Dura

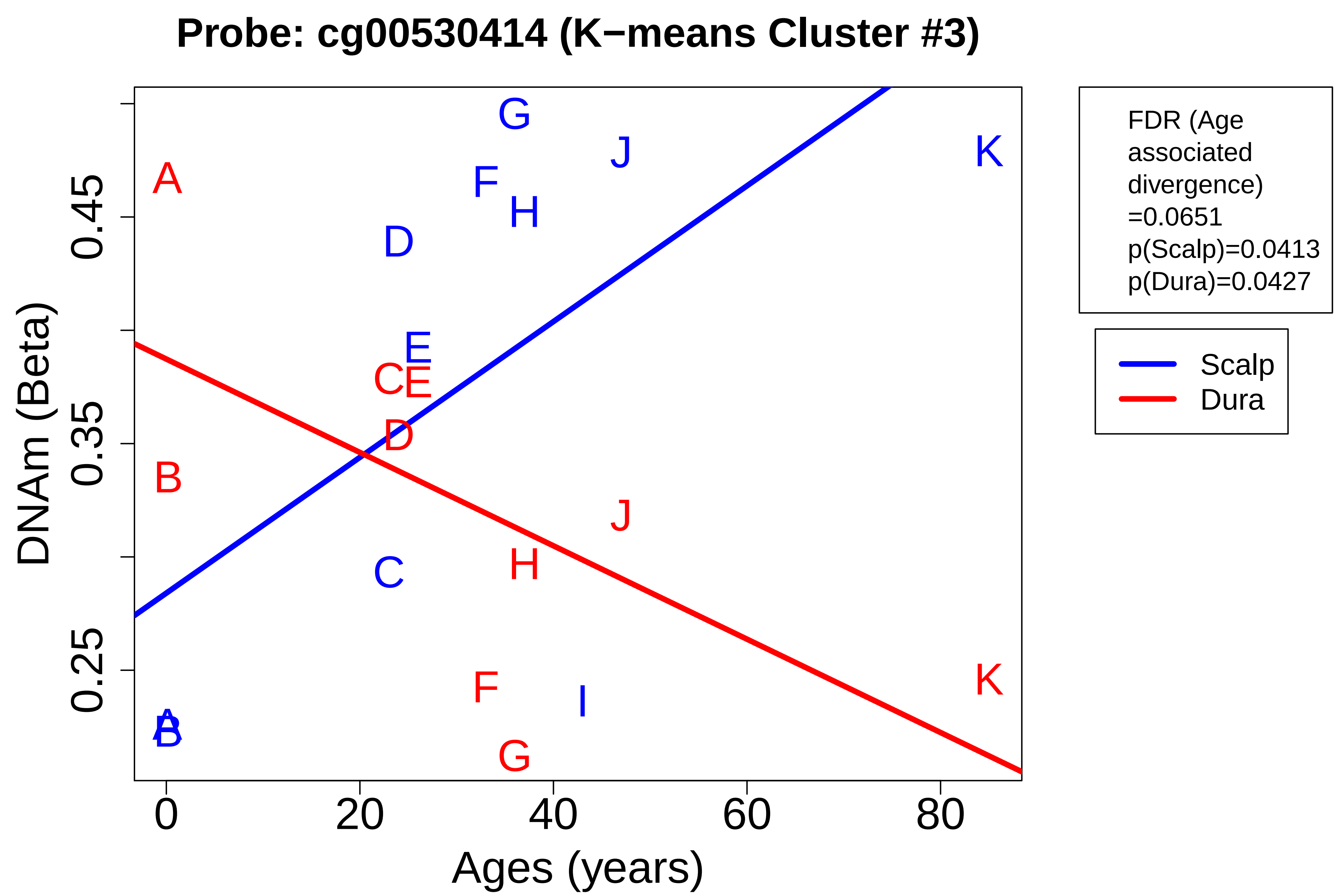

Probe: cg00530414 (K−means Cluster #3)
DNAm (Beta)
0.25
0.35
0.45
Ages (years)
0
20
40
60
80
FDR (Age associated divergence) =0.0651
p(Scalp)=0.0413
p(Dura)=0.0427
Scalp
Dura

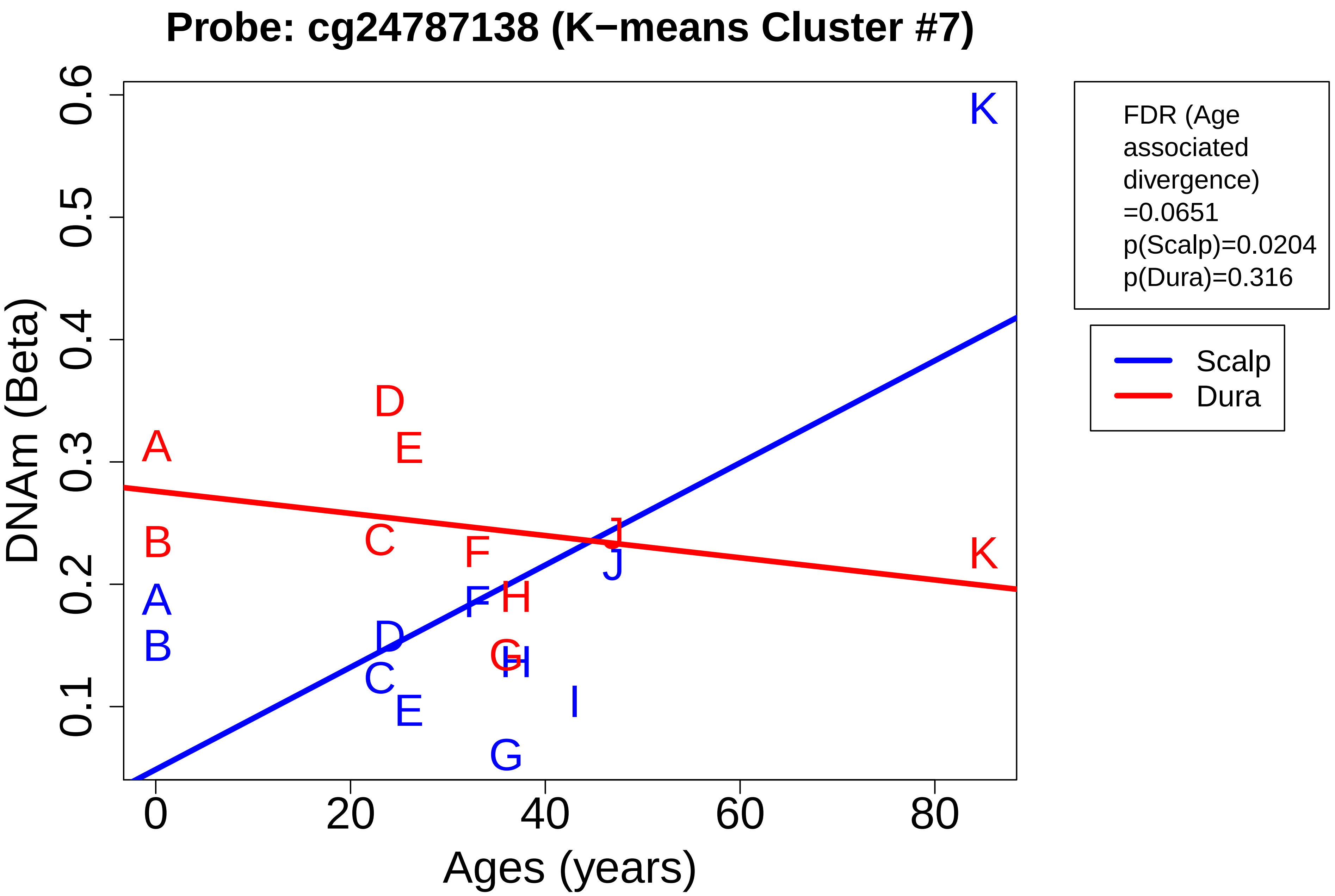

Probe: cg24787138 (K−means Cluster #7)
DNAm (Beta)
0.1
0.2
0.3
0.4
0.5
0.6
0
20
40
60
80
Ages (years)
FDR (Age associated divergence) =0.0651
p(Scalp)=0.0204
p(Dura)=0.316
Scalp
Dura

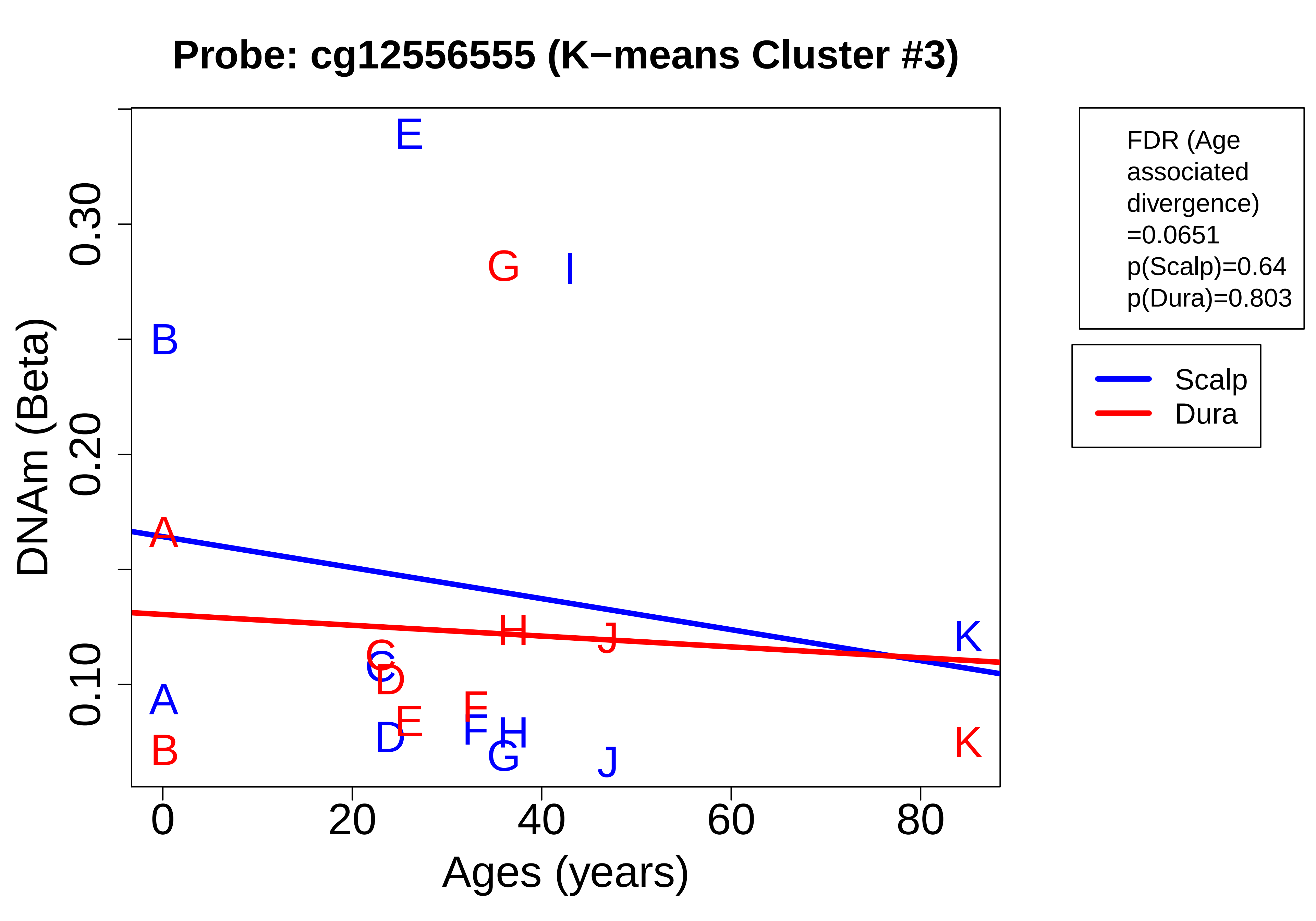

Probe: cg12556555 (K−means Cluster #3)
DNAm (Beta)
0.10
0.20
0.30
0
20
40
60
80
Ages (years)
FDR (Age associated divergence) =0.0651
p(Scalp)=0.64
p(Dura)=0.803
Scalp
Dura
A
B
C
D
E
F
G
H
I
J
K

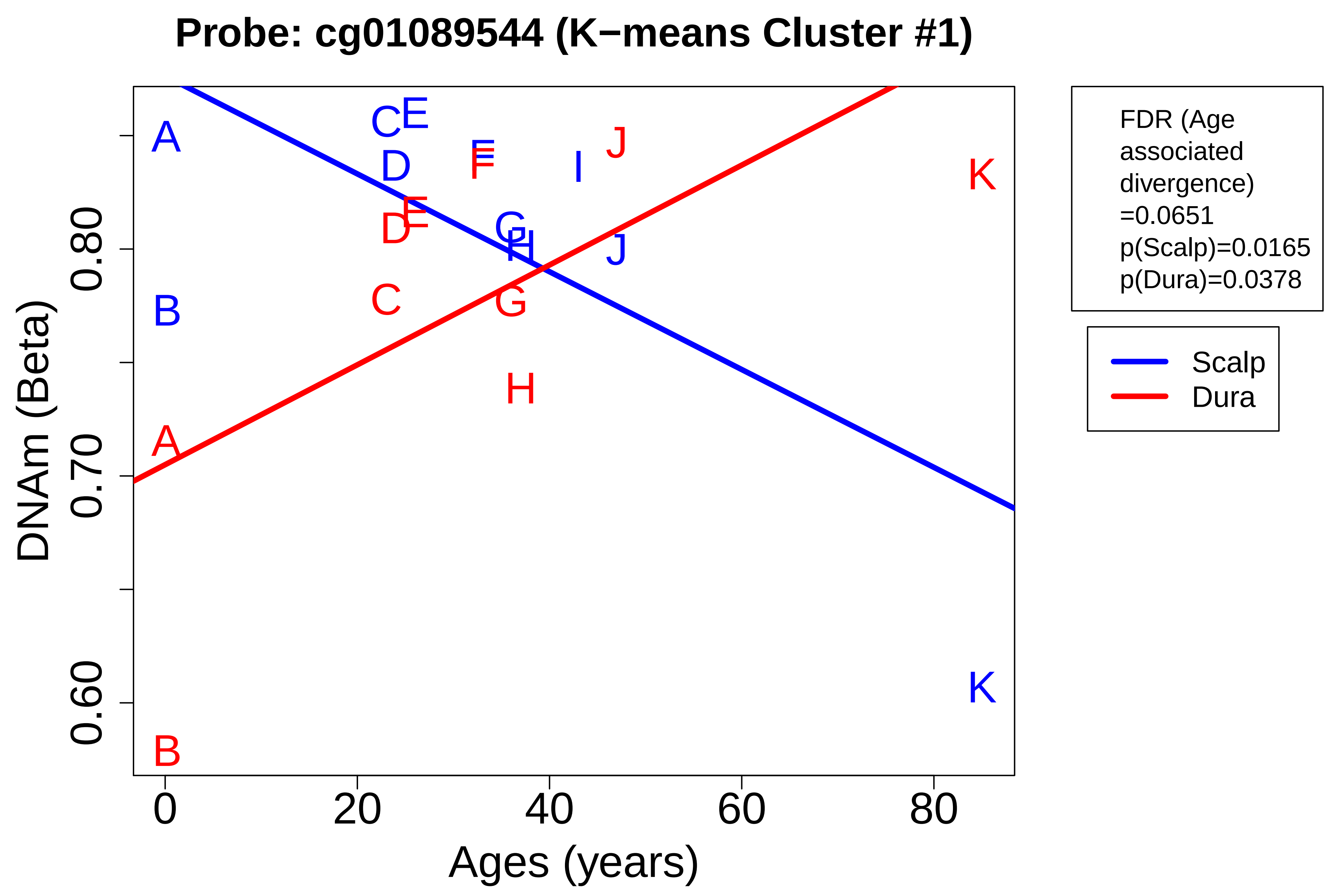

Probe: cg01089544 (K−means Cluster #1)
DNAm (Beta)
0.60
0.70
0.80
Ages (years)
0
20
40
60
80
A
B
C
D
E
F
G
H
I
J
K
FDR (Age associated divergence) =0.0651
p(Scalp)=0.0165
p(Dura)=0.0378
Scalp
Dura

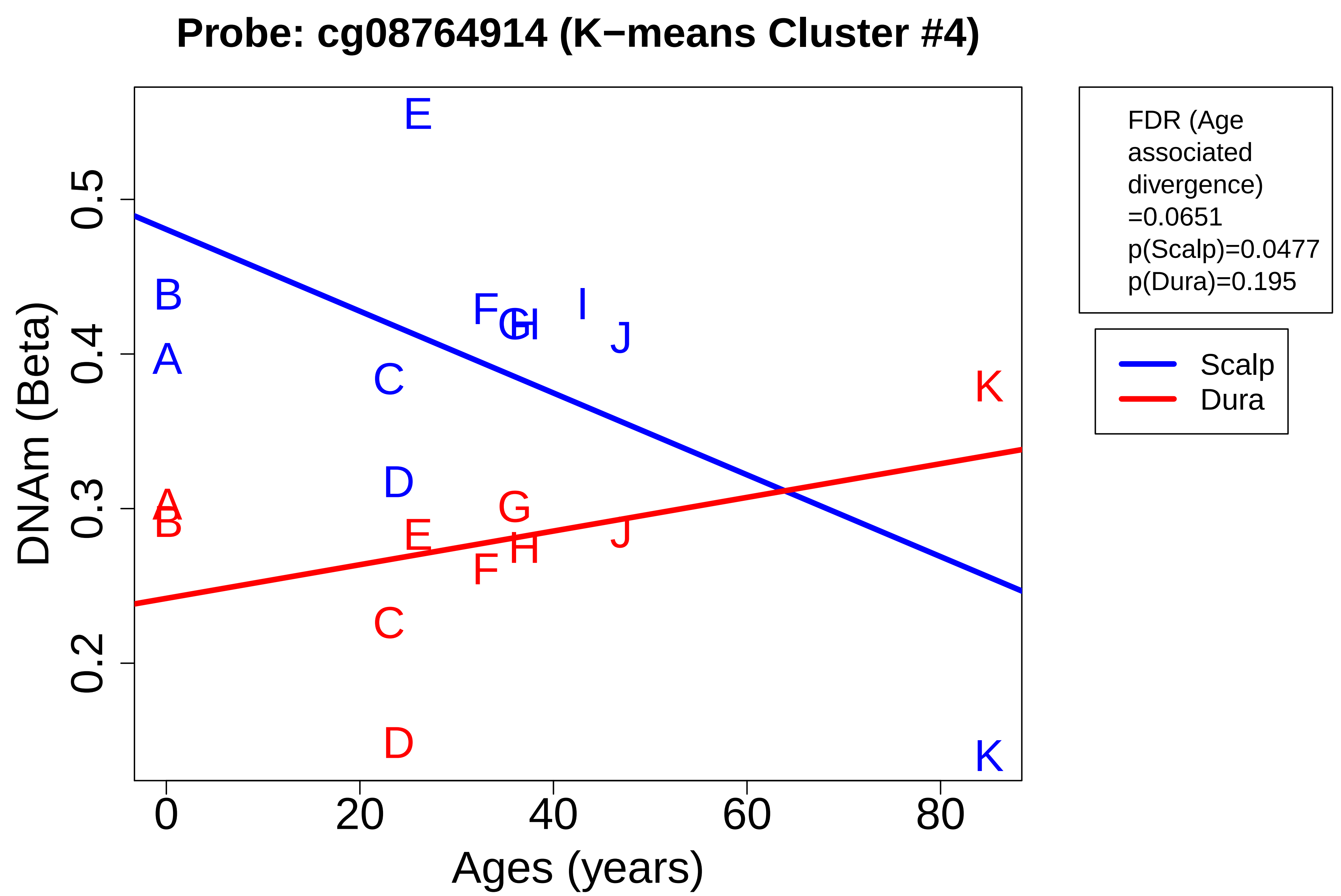

Probe: cg08764914 (K−means Cluster #4)
FDR (Age associated divergence) =0.0651
p(Scalp)=0.0477
p(Dura)=0.195
Scalp
Dura
DNAm (Beta)
0.2
0.3
0.4
0.5
Ages (years)
0
20
40
60
80
A
B
C
D
E
F
G
H
I
J
K

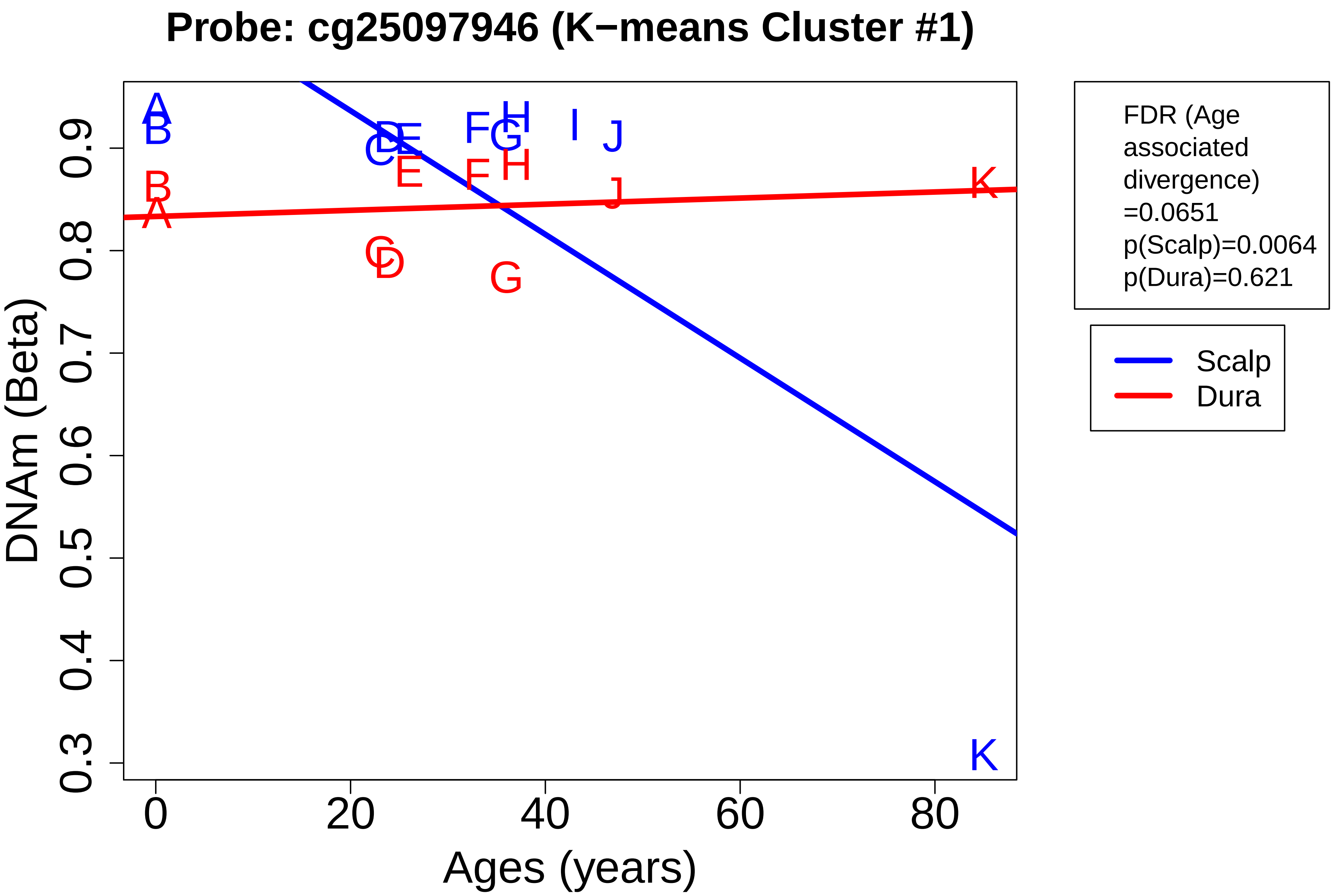

Probe: cg25097946 (K−means Cluster #1)
DNAm (Beta)
0.3
0.4
0.5
0.6
0.7
0.8
0.9
0
20
40
60
80
Ages (years)
FDR (Age associated divergence) =0.0651
p(Scalp)=0.0064
p(Dura)=0.621
Scalp
Dura

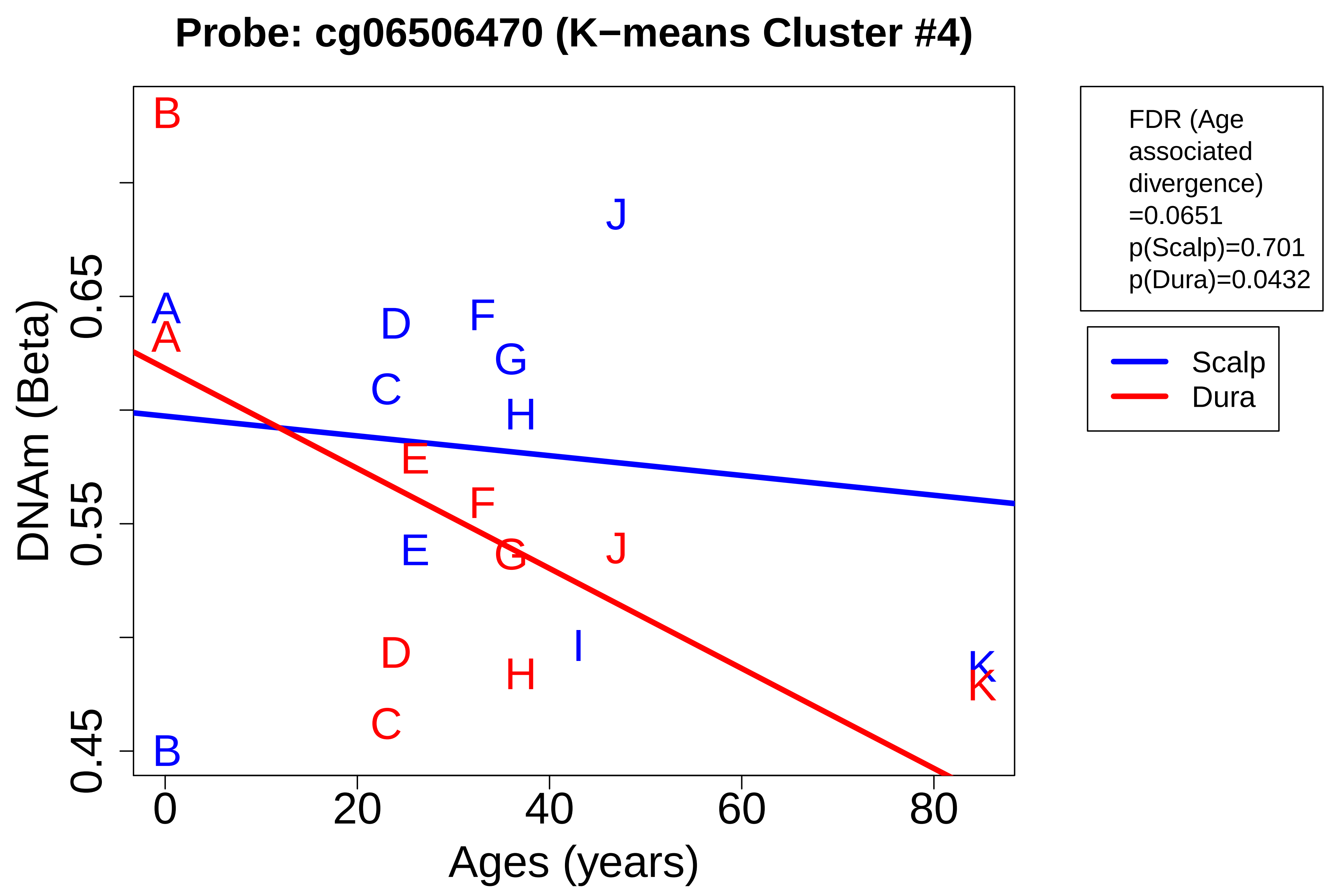

Probe: cg06506470 (K−means Cluster #4)
DNAm (Beta)
0.45
0.55
0.65
Ages (years)
0
20
40
60
80
FDR (Age associated divergence) =0.0651
p(Scalp)=0.701
p(Dura)=0.0432
Scalp
Dura

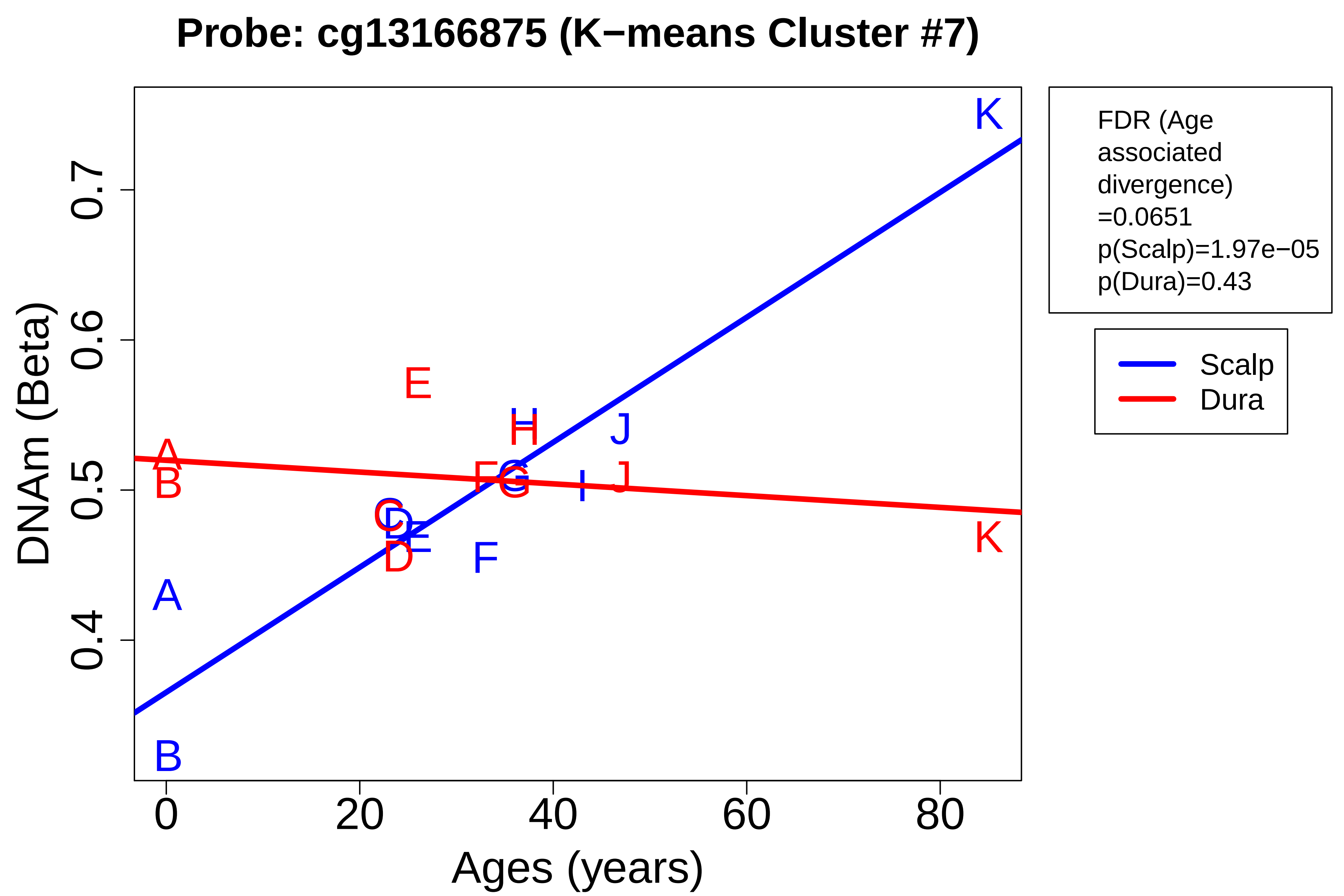

Probe: cg13166875 (K−means Cluster #7)
DNAm (Beta)
Ages (years)
0.4
0.5
0.6
0.7
0
20
40
60
80
FDR (Age associated divergence) =0.0651
p(Scalp)=1.97e−05
p(Dura)=0.43
Scalp
Dura

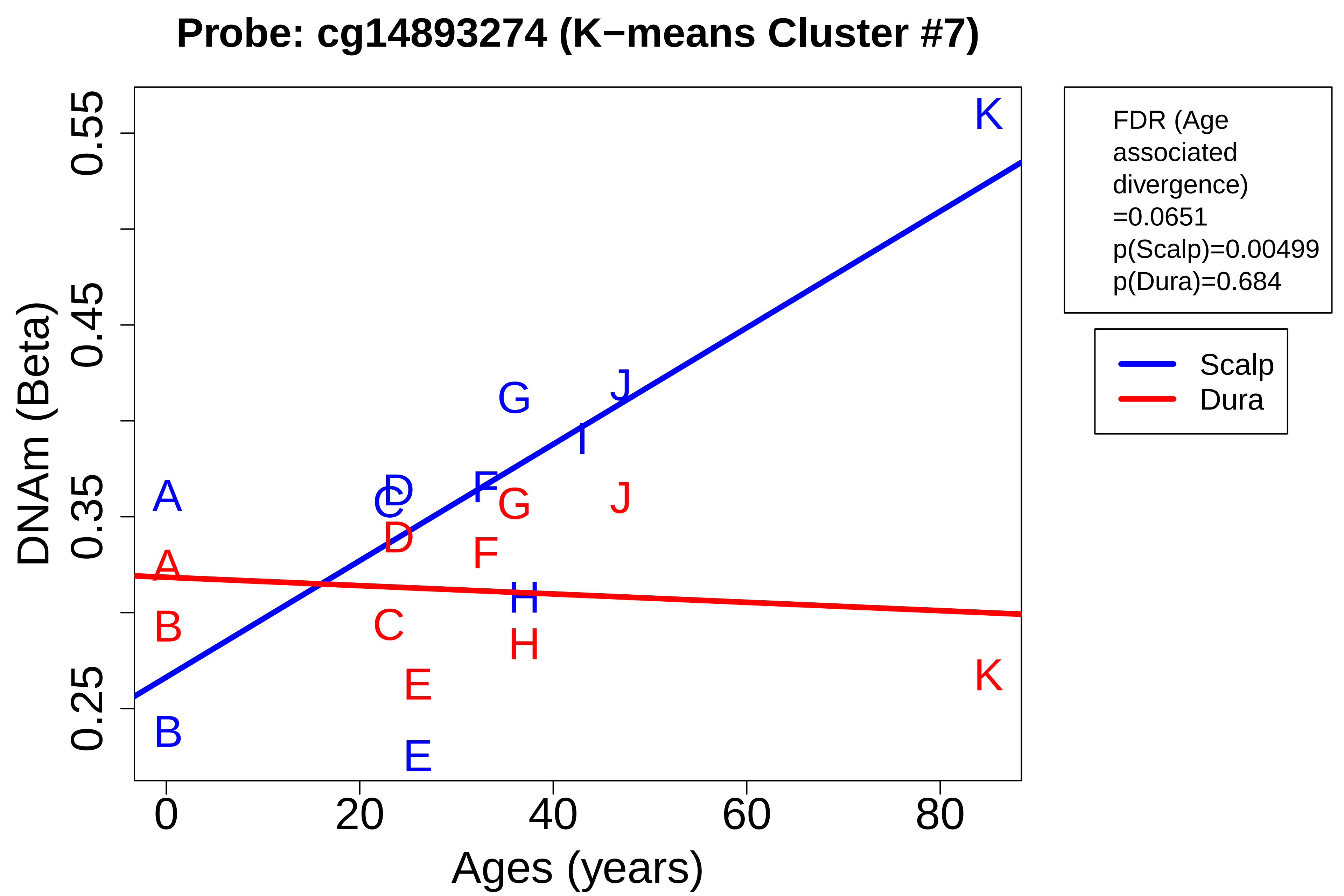
Probe: cg14893274 (K−means Cluster #7)
DNAm (Beta)
0.25
0.35
0.45
0.55
Ages (years)
0
20
40
60
80
A
B
C
D
E
F
G
H
I
J
K
FDR (Age associated divergence) =0.0651
p(Scalp)=0.00499
p(Dura)=0.684
Scalp
Dura

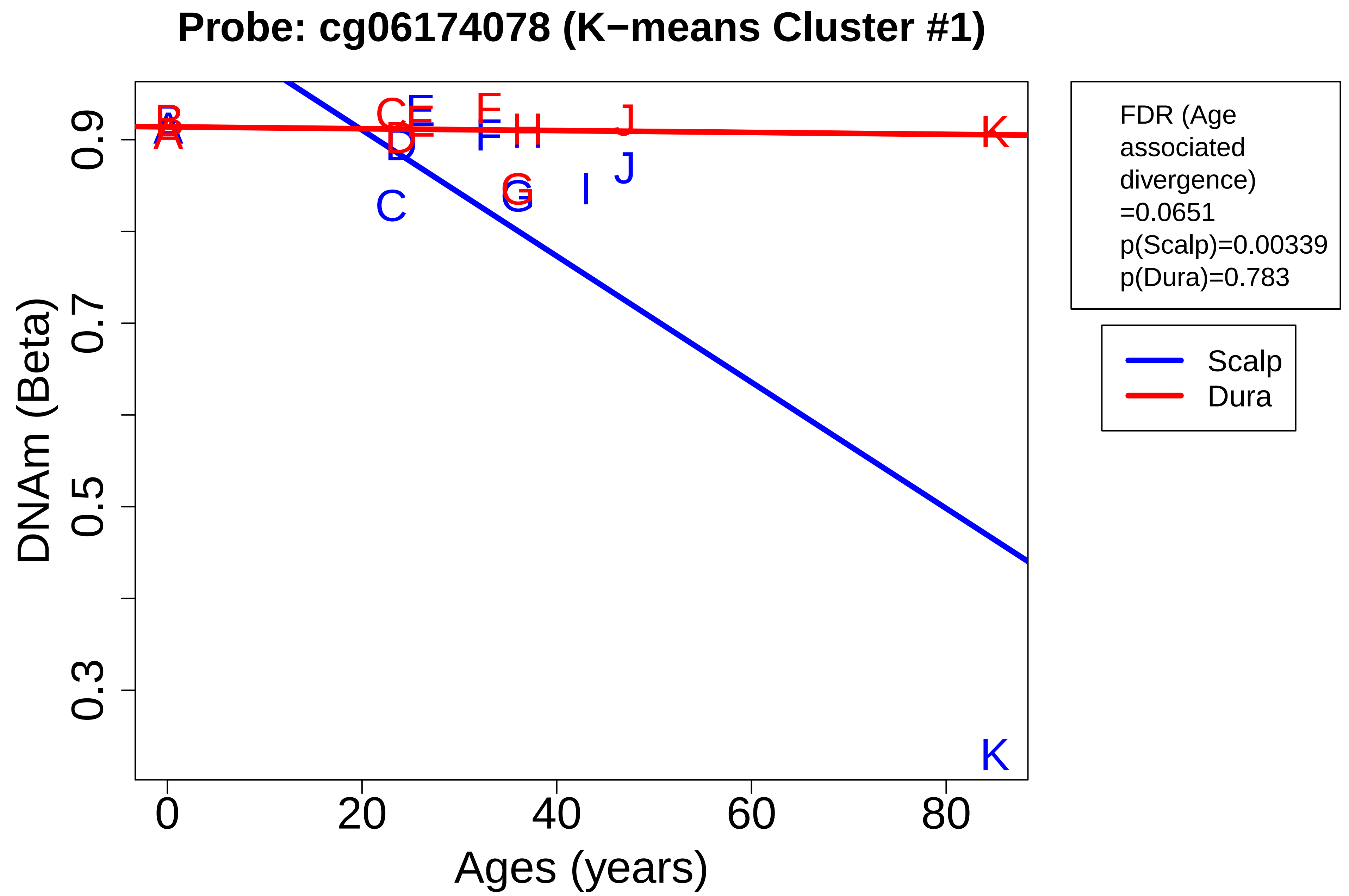

Probe: cg06174078 (K−means Cluster #1)
FDR (Age associated divergence) =0.0651
p(Scalp)=0.00339
p(Dura)=0.783
Scalp
Dura
DNAm (Beta)
0.3
0.5
0.7
0.9
Ages (years)
0
20
40
60
80
A
B
C
D
E
F
G
H
I
J
K

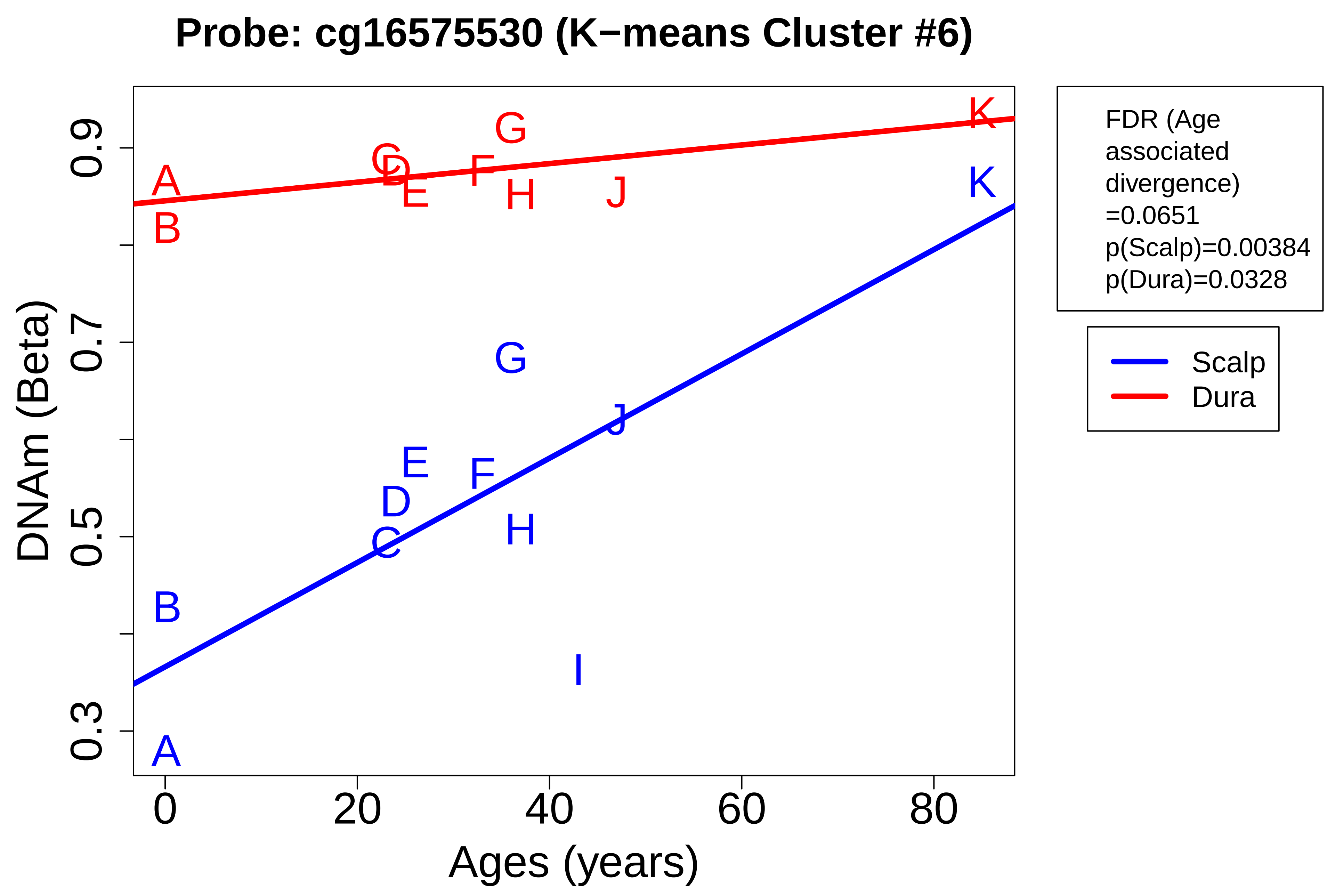

Probe: cg16575530 (K−means Cluster #6)
DNAm (Beta)
0.3
0.5
0.7
0.9
0
20
40
60
80
Ages (years)
A
B
C
D
E
F
G
H
I
J
K
FDR (Age associated divergence) =0.0651
p(Scalp)=0.00384
p(Dura)=0.0328
Scalp
Dura

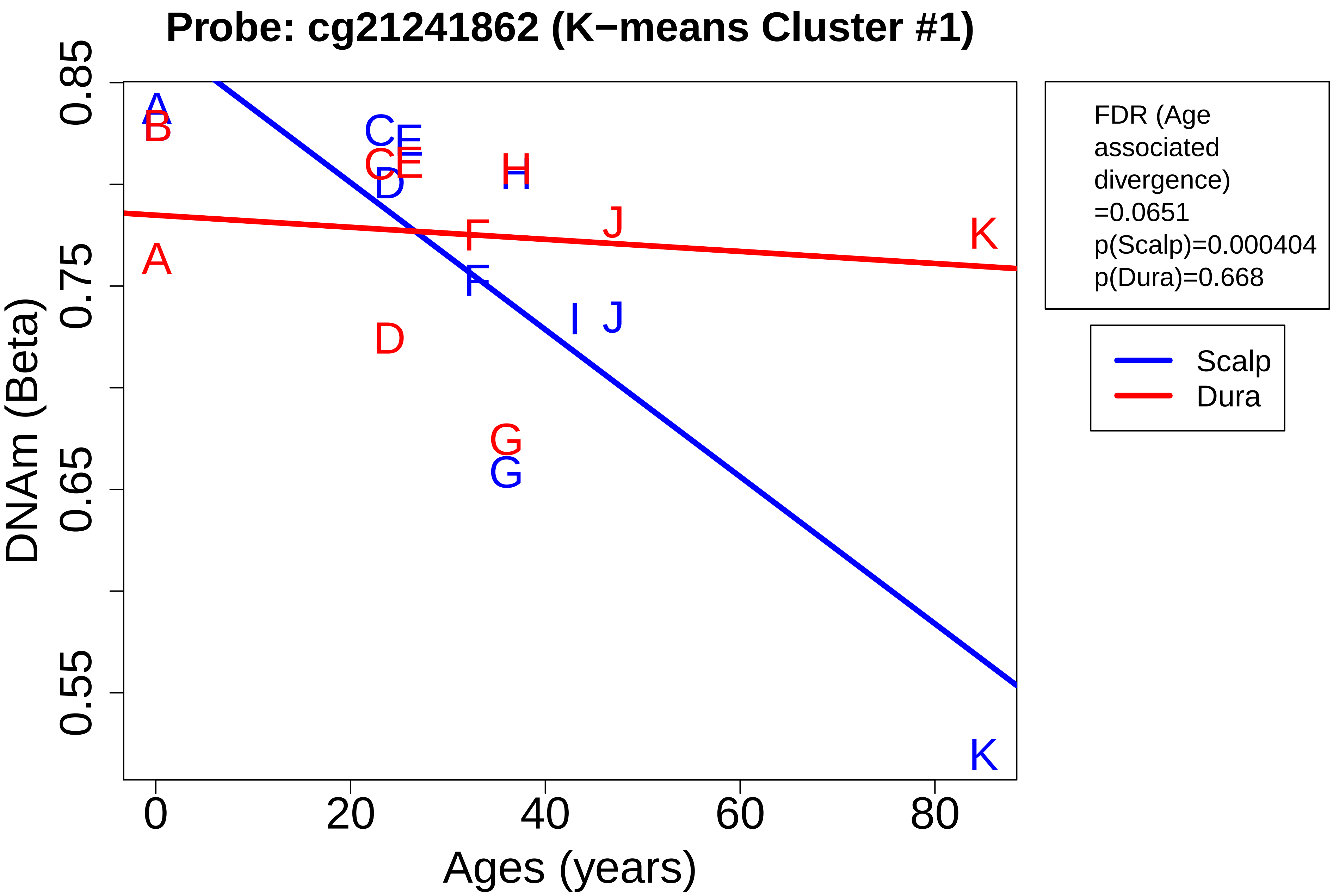

Probe: cg21241862 (K−means Cluster #1)
DNAm (Beta)
0.55
0.65
0.75
0.85
Ages (years)
0
20
40
60
80
FDR (Age associated divergence) =0.0651
p(Scalp)=0.000404
p(Dura)=0.668
Scalp
Dura

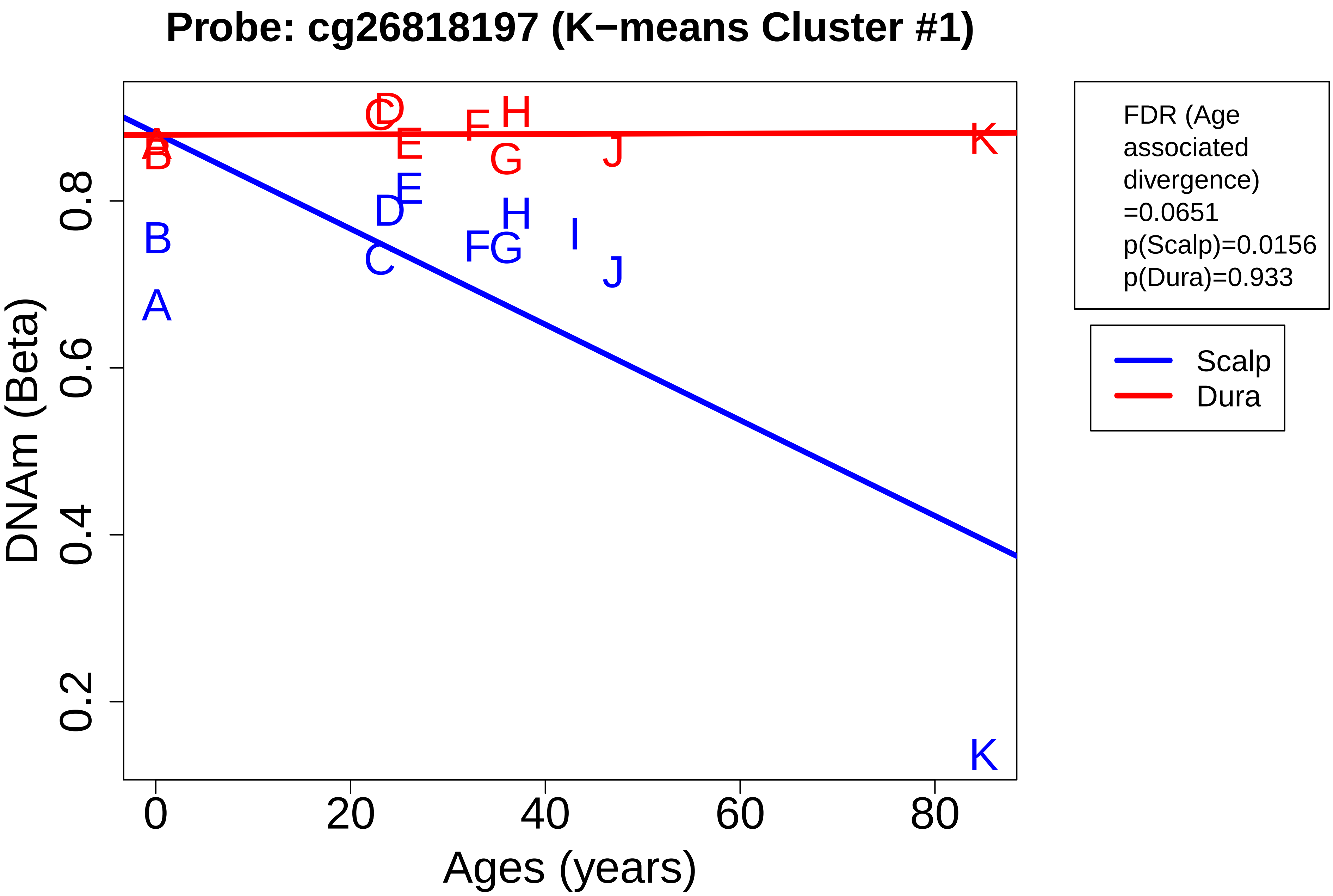

Probe: cg26818197 (K−means Cluster #1)
DNAm (Beta)
0.2
0.4
0.6
0.8
Ages (years)
0
20
40
60
80
FDR (Age associated divergence) =0.0651
p(Scalp)=0.0156
p(Dura)=0.933
Scalp
Dura

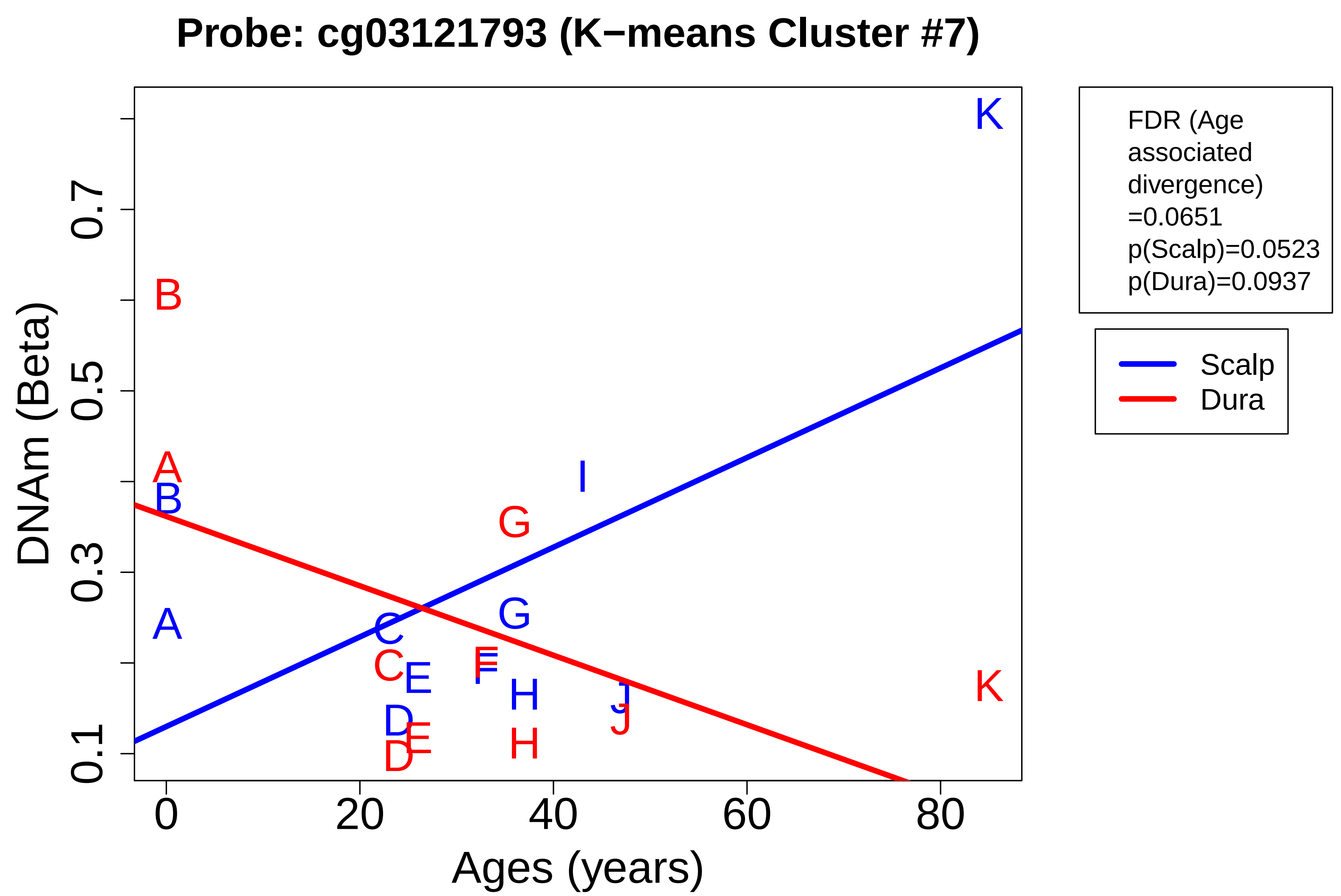
Probe: cg03121793 (K−means Cluster #7)
FDR (Age associated divergence) =0.0651
p(Scalp)=0.0523
p(Dura)=0.0937
Scalp
Dura
DNAm (Beta)
0.1
0.3
0.5
0.7
0
20
40
60
80
Ages (years)
A
B
C
D
E
F
G
H
I
J
K

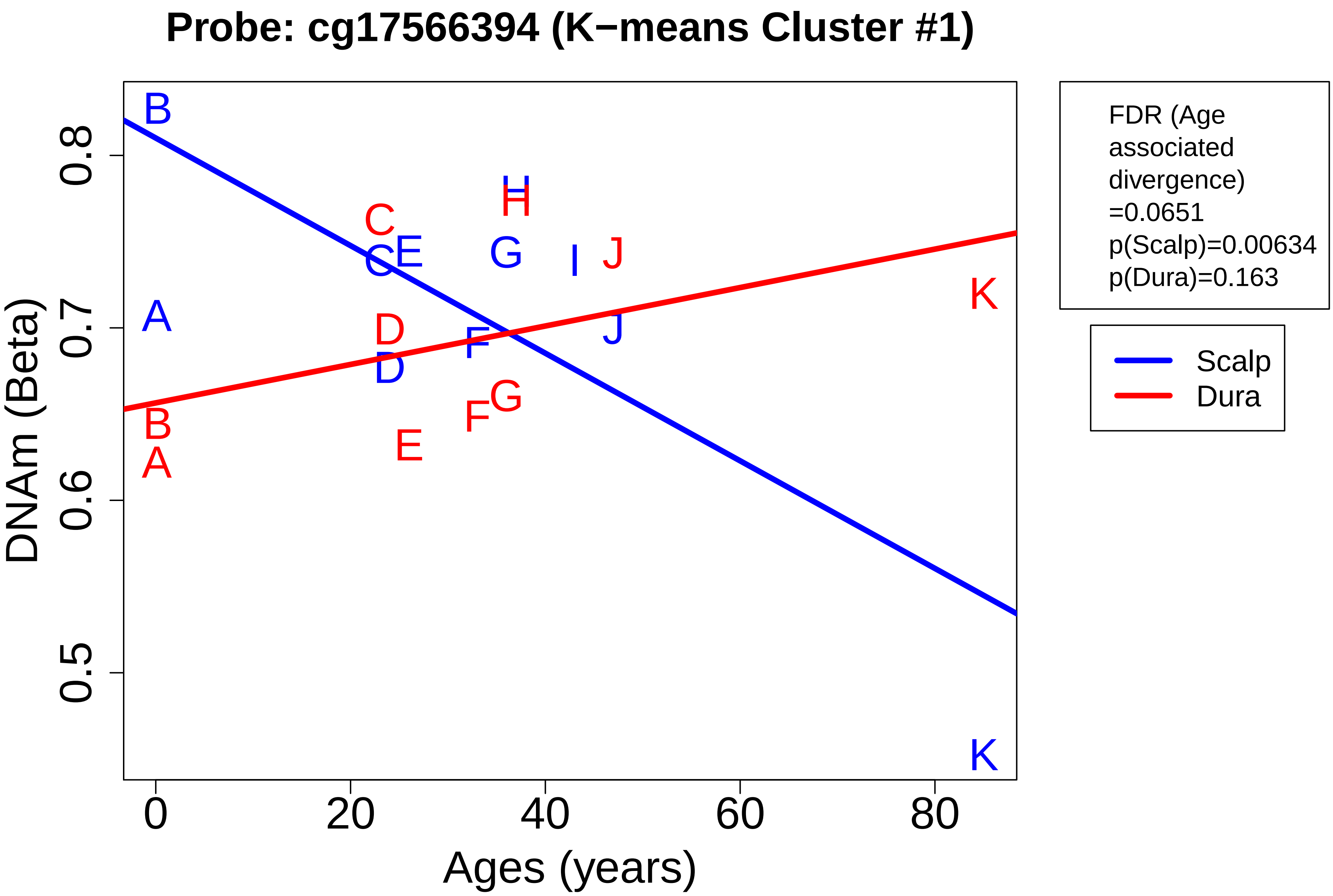
Probe: cg17566394 (K−means Cluster #1)
DNAm (Beta)
0.5
0.6
0.7
0.8
0
20
40
60
80
Ages (years)
A
B
C
D
E
F
G
H
I
J
K
FDR (Age associated divergence) =0.0651
p(Scalp)=0.00634
p(Dura)=0.163
Scalp
Dura

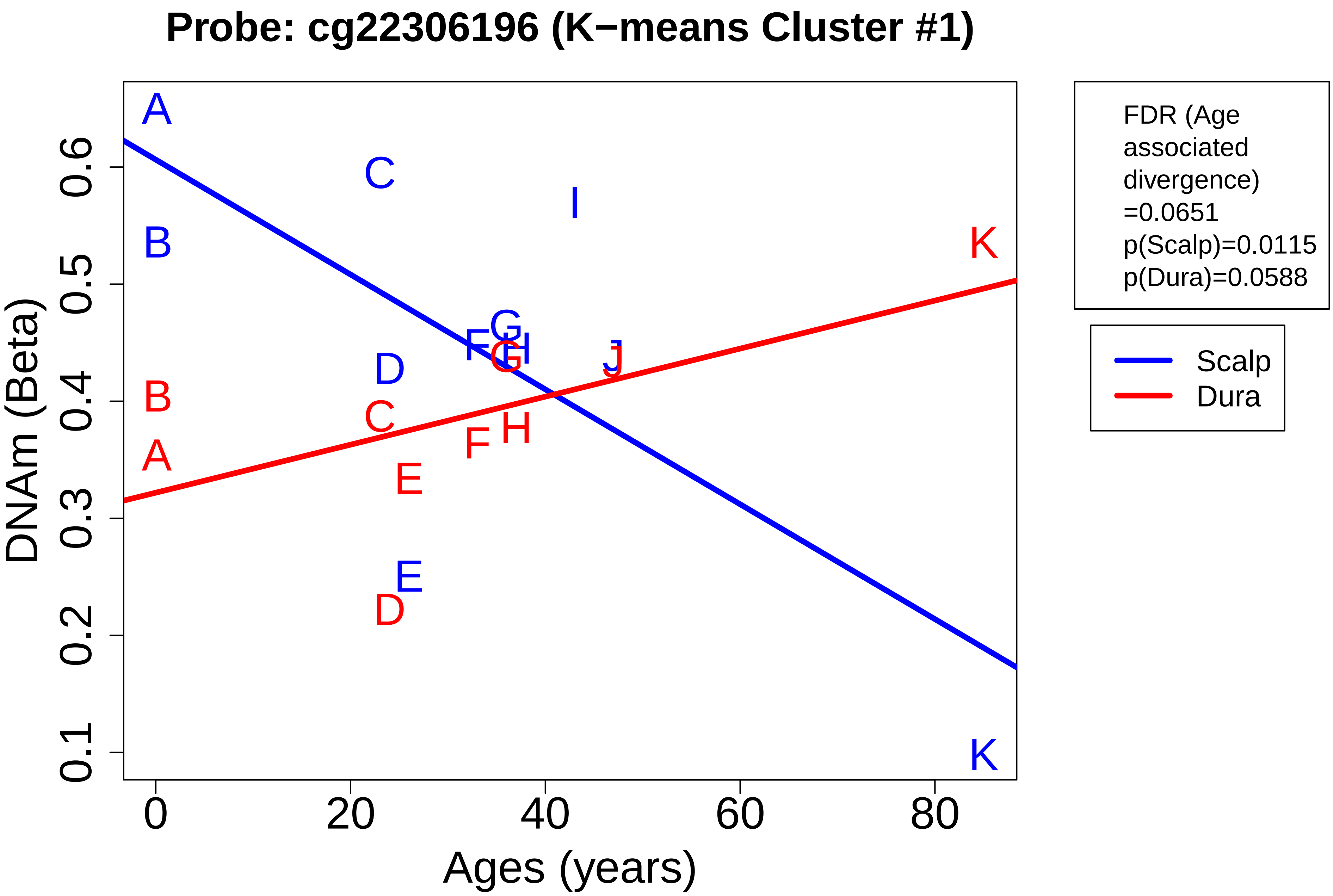

Probe: cg22306196 (K−means Cluster #1)
DNAm (Beta)
0.1
0.2
0.3
0.4
0.5
0.6
0
20
40
60
80
Ages (years)
FDR (Age associated divergence) =0.0651
p(Scalp)=0.0115
p(Dura)=0.0588
Scalp
Dura
A
B
C
D
E
F
G
H
I
J
K

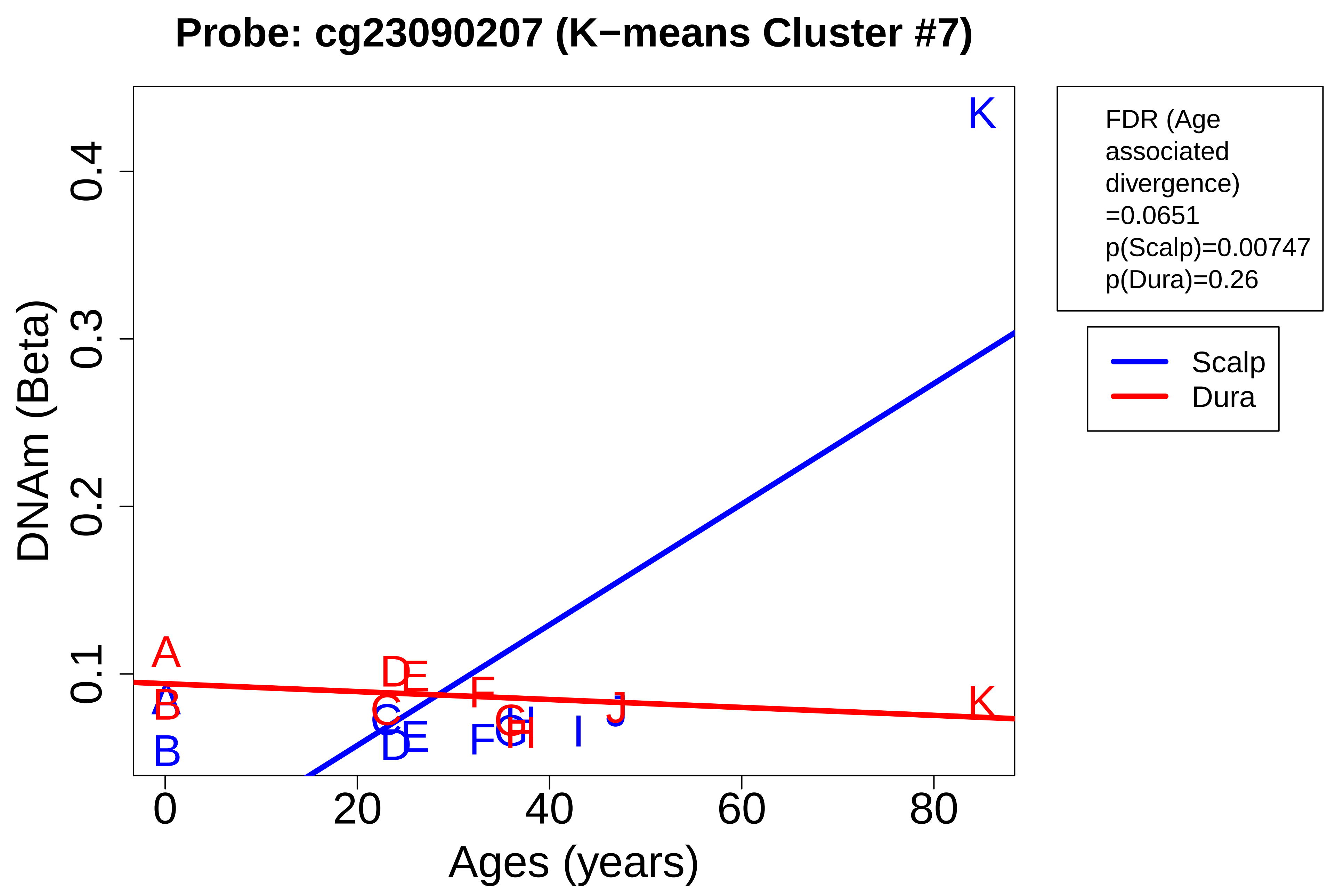

Probe: cg23090207 (K−means Cluster #7)
FDR (Age associated divergence) =0.0651
p(Scalp)=0.00747
p(Dura)=0.26
Scalp
Dura
DNAm (Beta)
0.1
0.2
0.3
0.4
0
20
40
60
80
Ages (years)

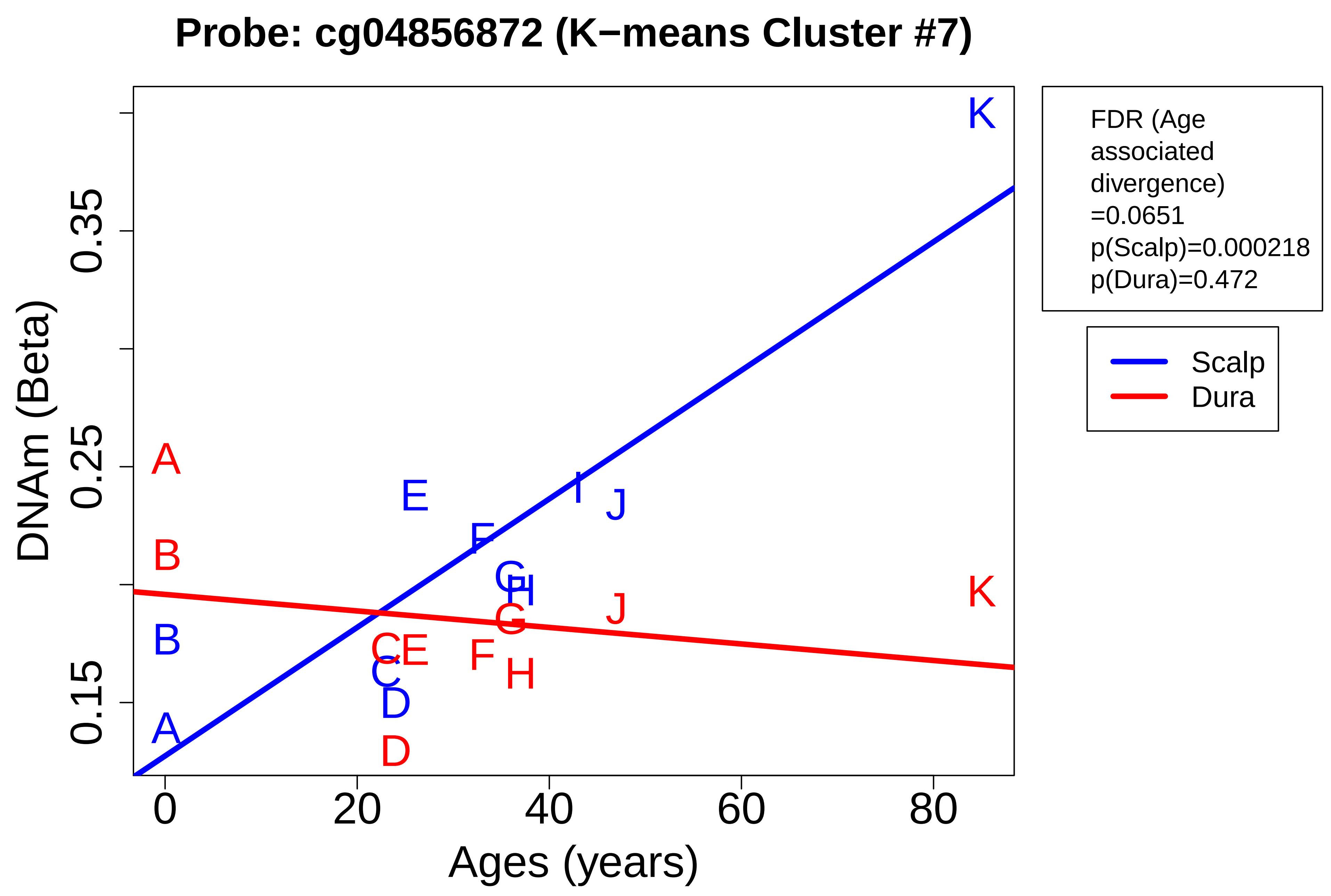
Probe: cg04856872 (K−means Cluster #7)
DNAm (Beta)
0.15
0.25
0.35
Ages (years)
0
20
40
60
80
FDR (Age associated divergence) =0.0651
p(Scalp)=0.000218
p(Dura)=0.472
Scalp
Dura
A
B
C
D
E
F
G
H
I
J
K

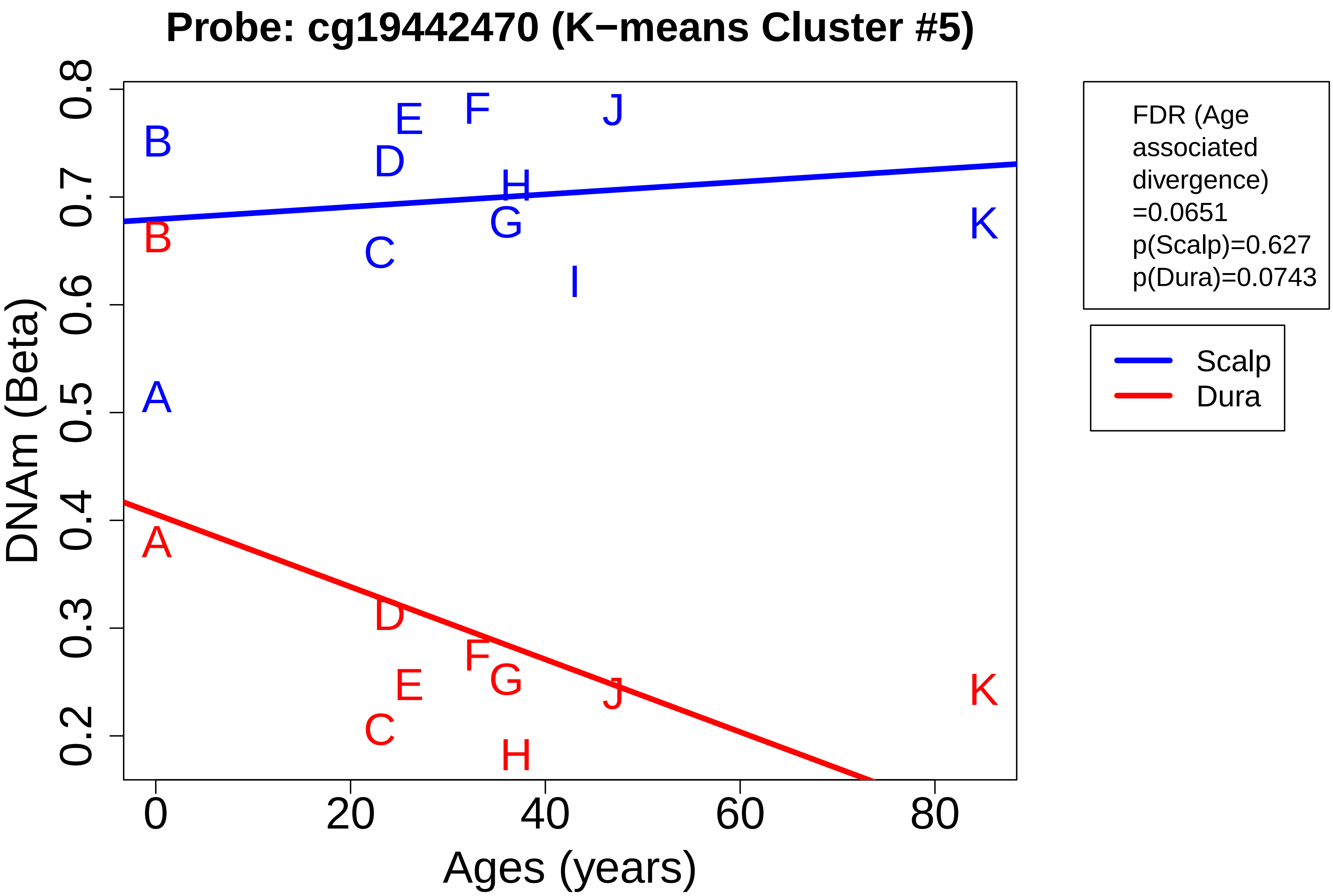

Probe: cg19442470 (K−means Cluster #5)
DNAm (Beta)
0.2
0.3
0.4
0.5
0.6
0.7
0.8
Ages (years)
0
20
40
60
80
FDR (Age associated divergence) =0.0651
p(Scalp)=0.627
p(Dura)=0.0743
Scalp
Dura

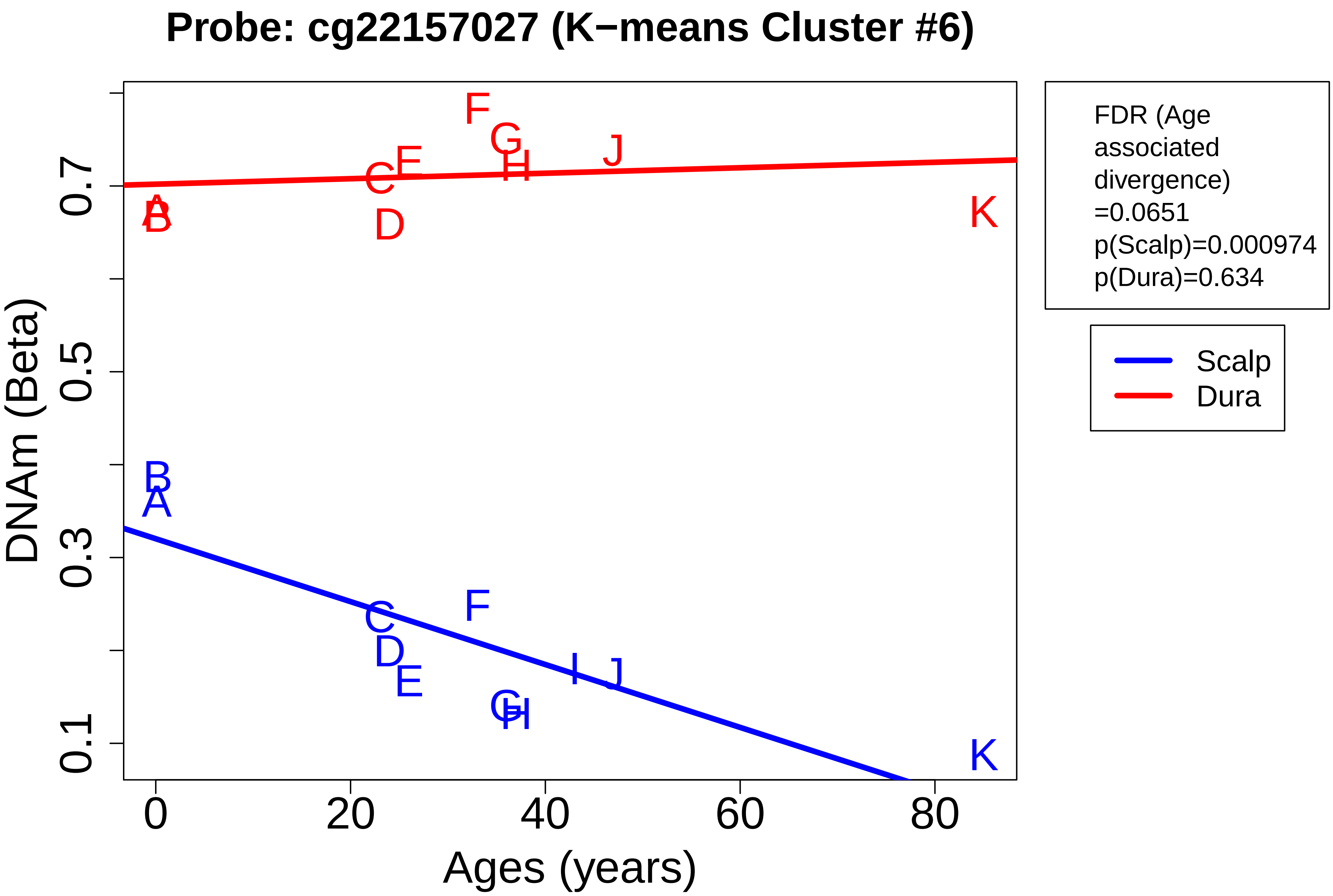

Probe: cg22157027 (K−means Cluster #6)
DNAm (Beta)
0.1
0.3
0.5
0.7
0
20
40
60
80
Ages (years)
FDR (Age associated divergence) =0.0651
p(Scalp)=0.000974
p(Dura)=0.634
Scalp
Dura

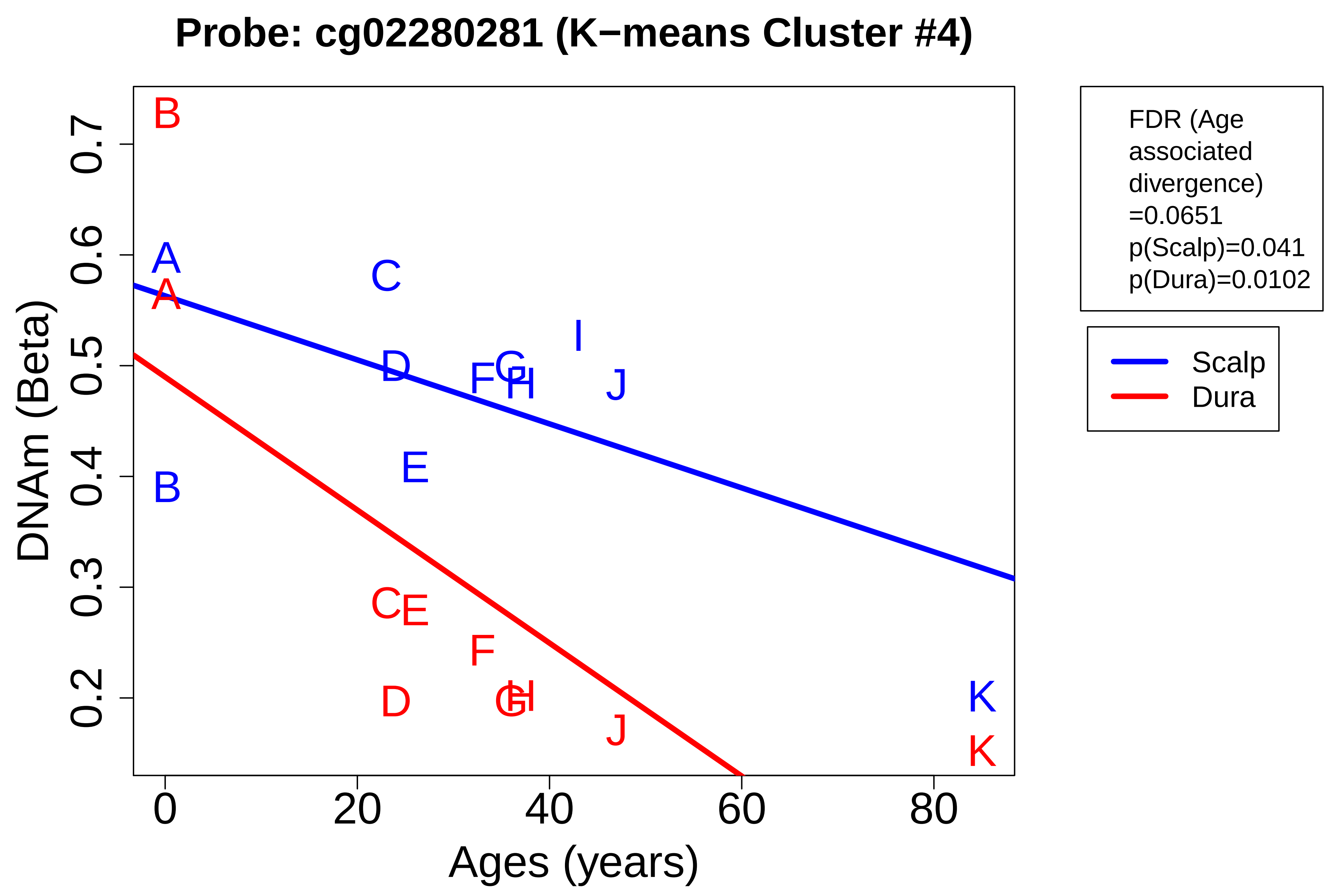
Probe: cg02280281 (K−means Cluster #4)
DNAm (Beta)
Ages (years)
0.2
0.3
0.4
0.5
0.6
0.7
0
20
40
60
80
FDR (Age associated divergence) =0.0651
p(Scalp)=0.041
p(Dura)=0.0102
Scalp
Dura

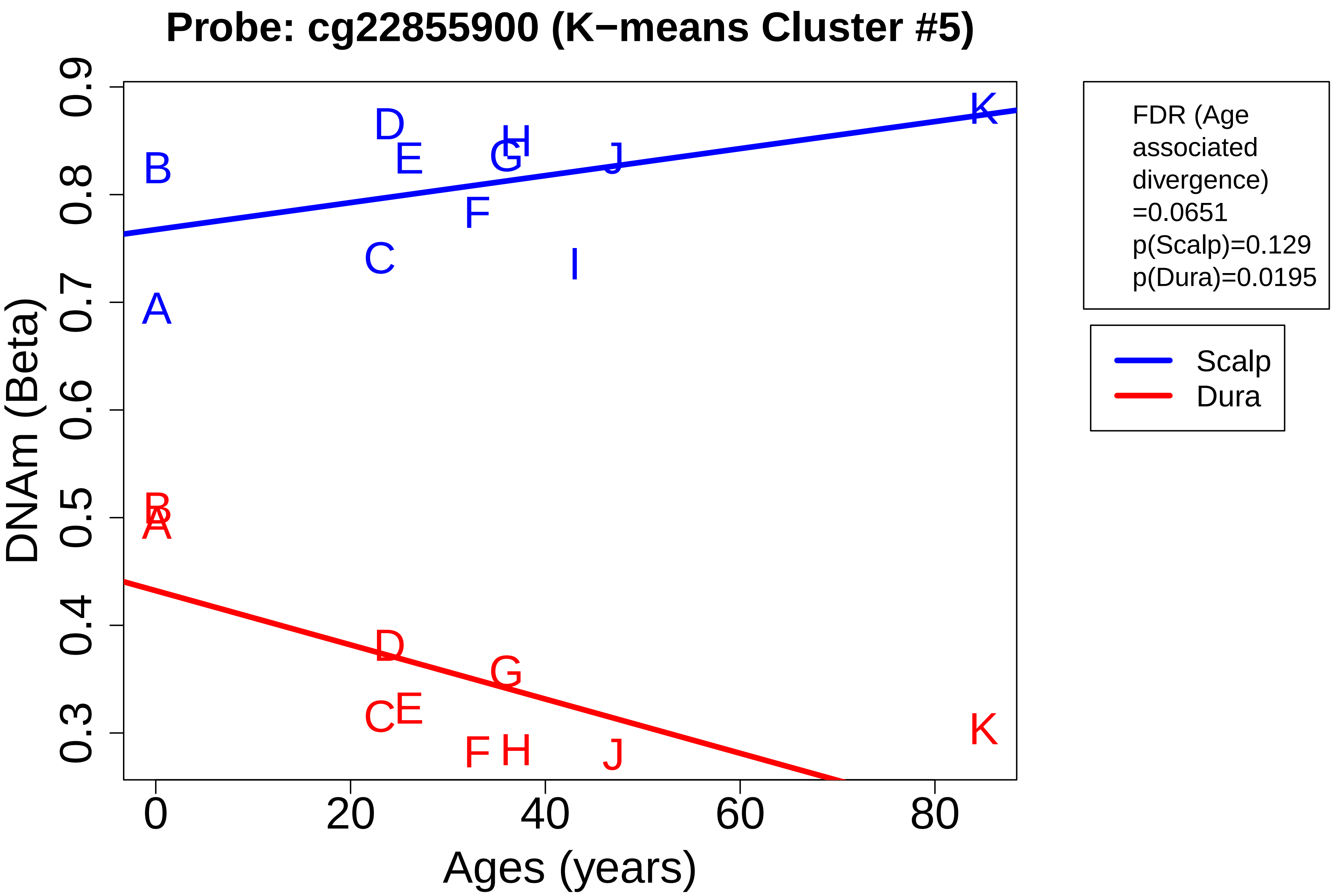

Probe: cg22855900 (K−means Cluster #5)
DNAm (Beta)
0.3
0.4
0.5
0.6
0.7
0.8
0.9
0
20
40
60
80
Ages (years)
A
B
C
D
E
F
G
H
I
J
K
FDR (Age associated divergence) =0.0651
p(Scalp)=0.129
p(Dura)=0.0195
Scalp
Dura

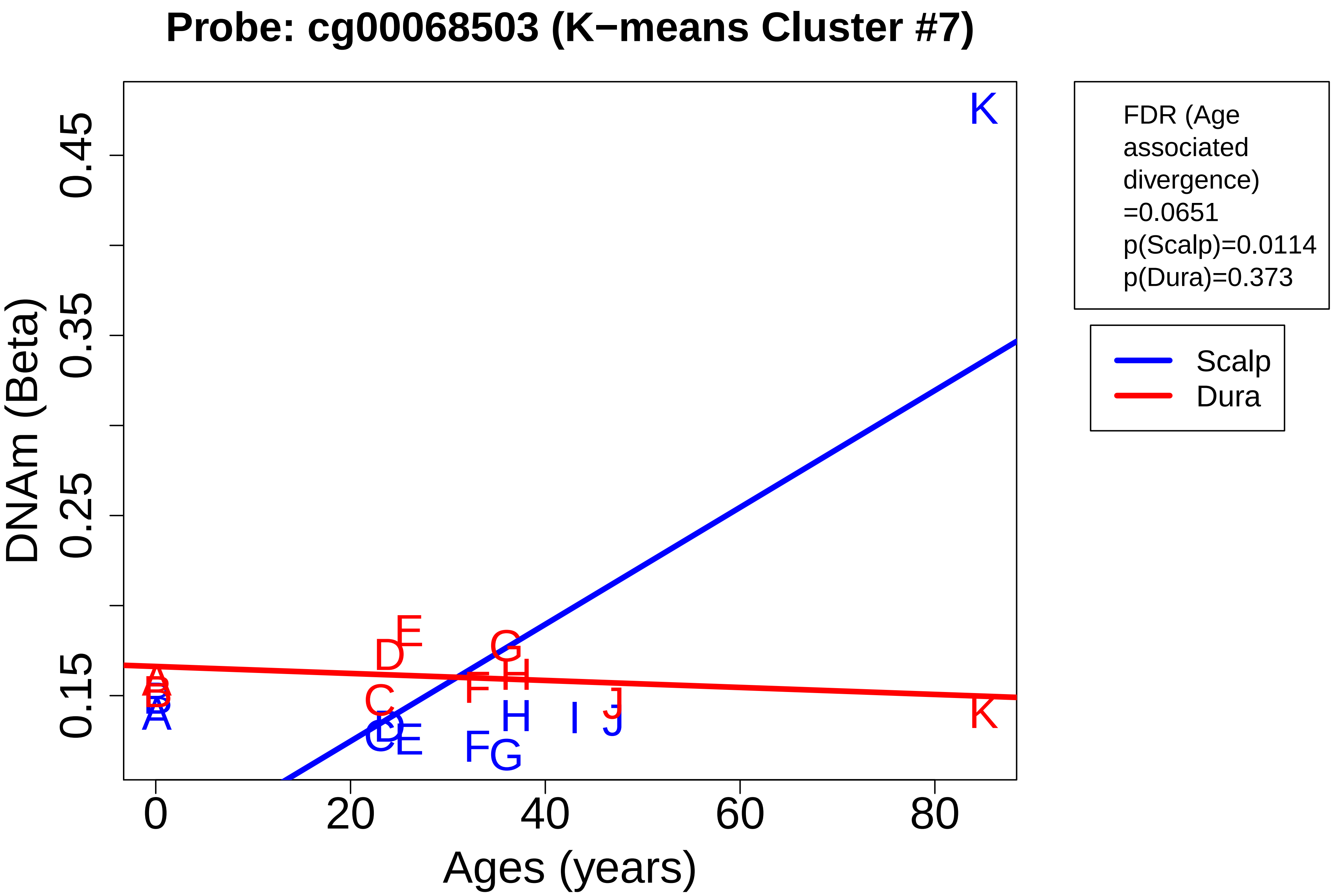

Probe: cg00068503 (K−means Cluster #7)
FDR (Age associated divergence) =0.0651
p(Scalp)=0.0114
p(Dura)=0.373
Scalp
Dura
DNAm (Beta)
0.15
0.25
0.35
0.45
0
20
40
60
80
Ages (years)

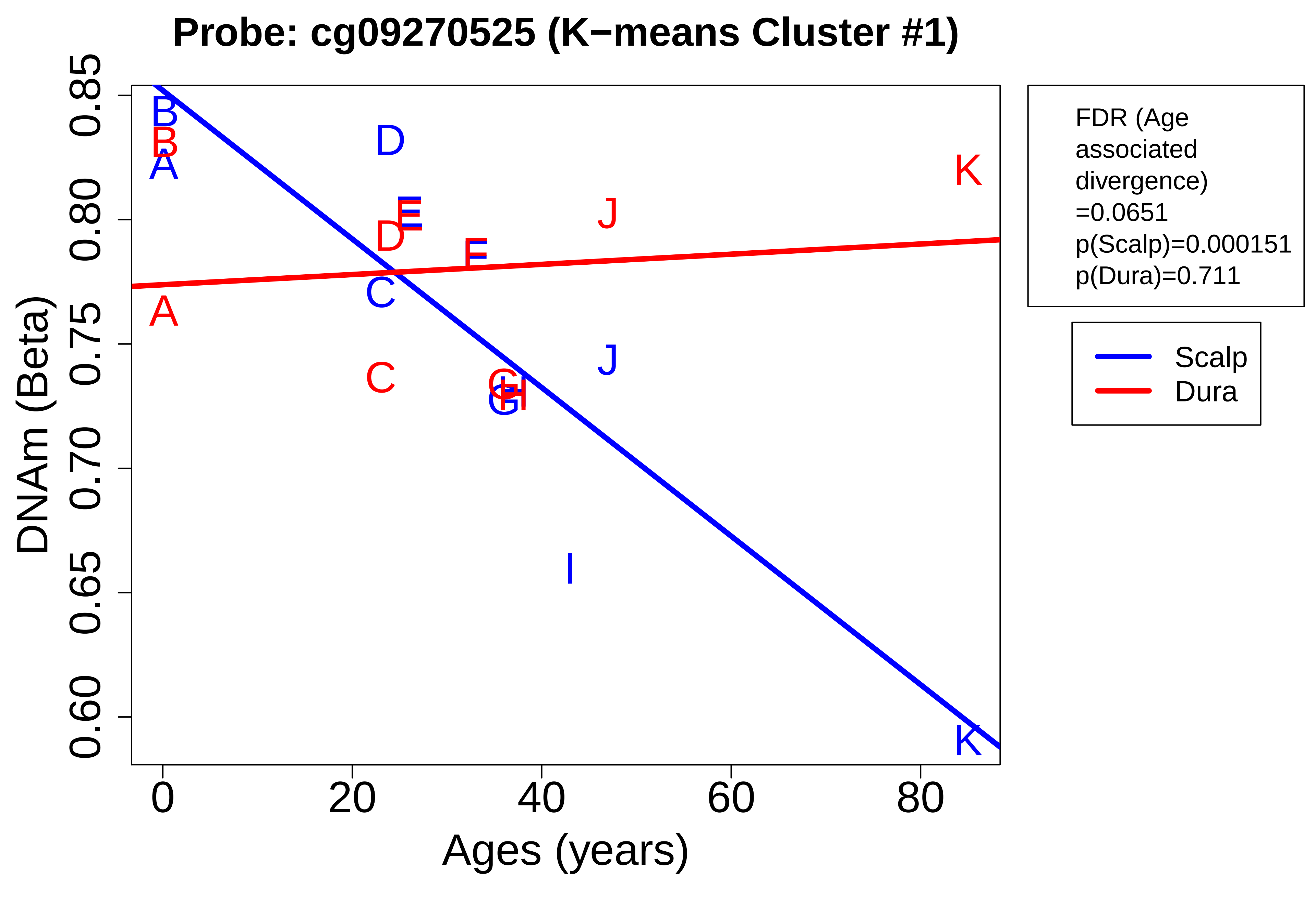

Probe: cg09270525 (K−means Cluster #1)
DNAm (Beta)
0.60
0.65
0.70
0.75
0.80
0.85
Ages (years)
0
20
40
60
80
FDR (Age associated divergence) =0.0651
p(Scalp)=0.000151
p(Dura)=0.711
Scalp
Dura

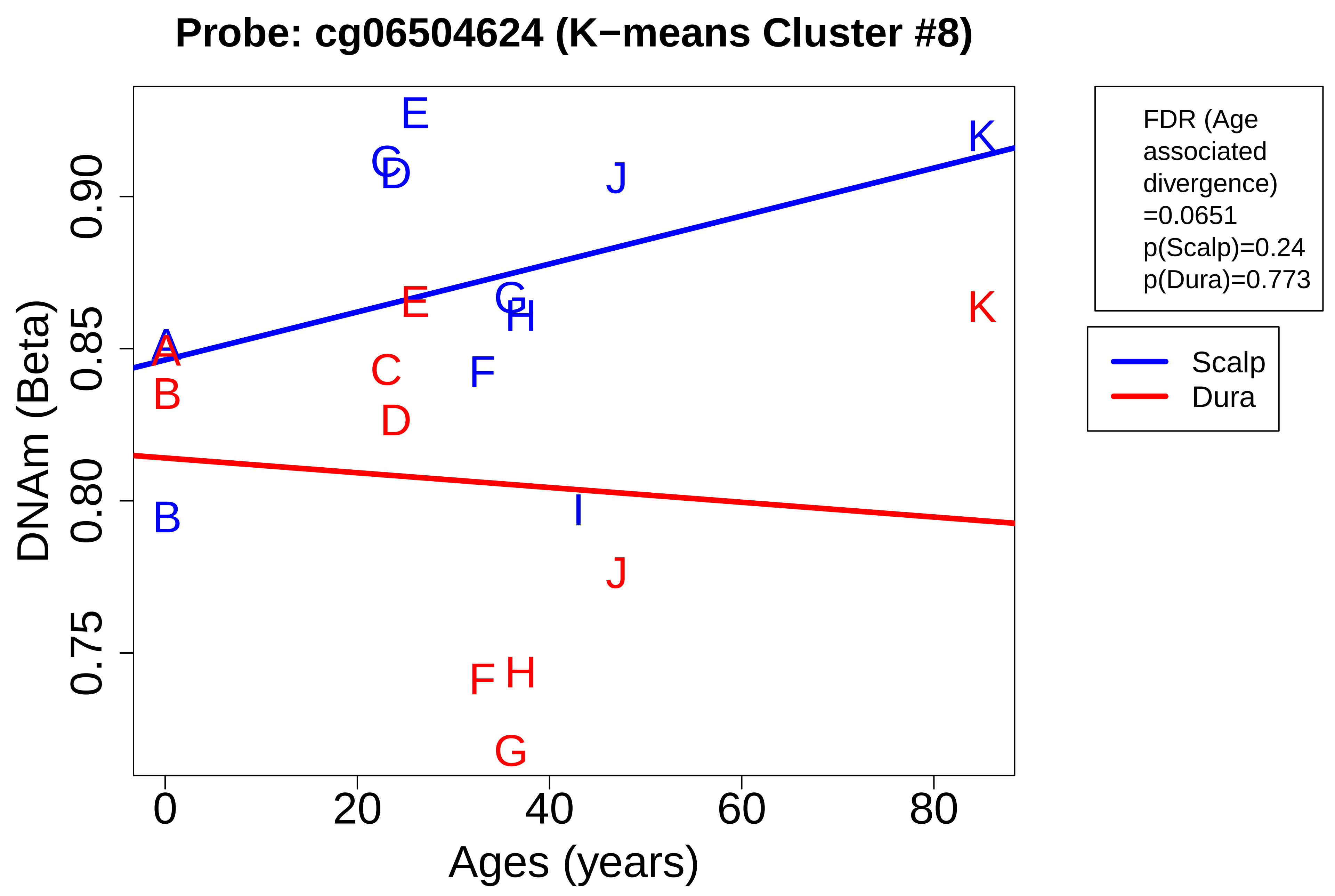

Probe: cg06504624 (K−means Cluster #8)
DNAm (Beta)
0.75
0.80
0.85
0.90
0
20
40
60
80
Ages (years)
FDR (Age associated divergence) =0.0651
p(Scalp)=0.24
p(Dura)=0.773
Scalp
Dura

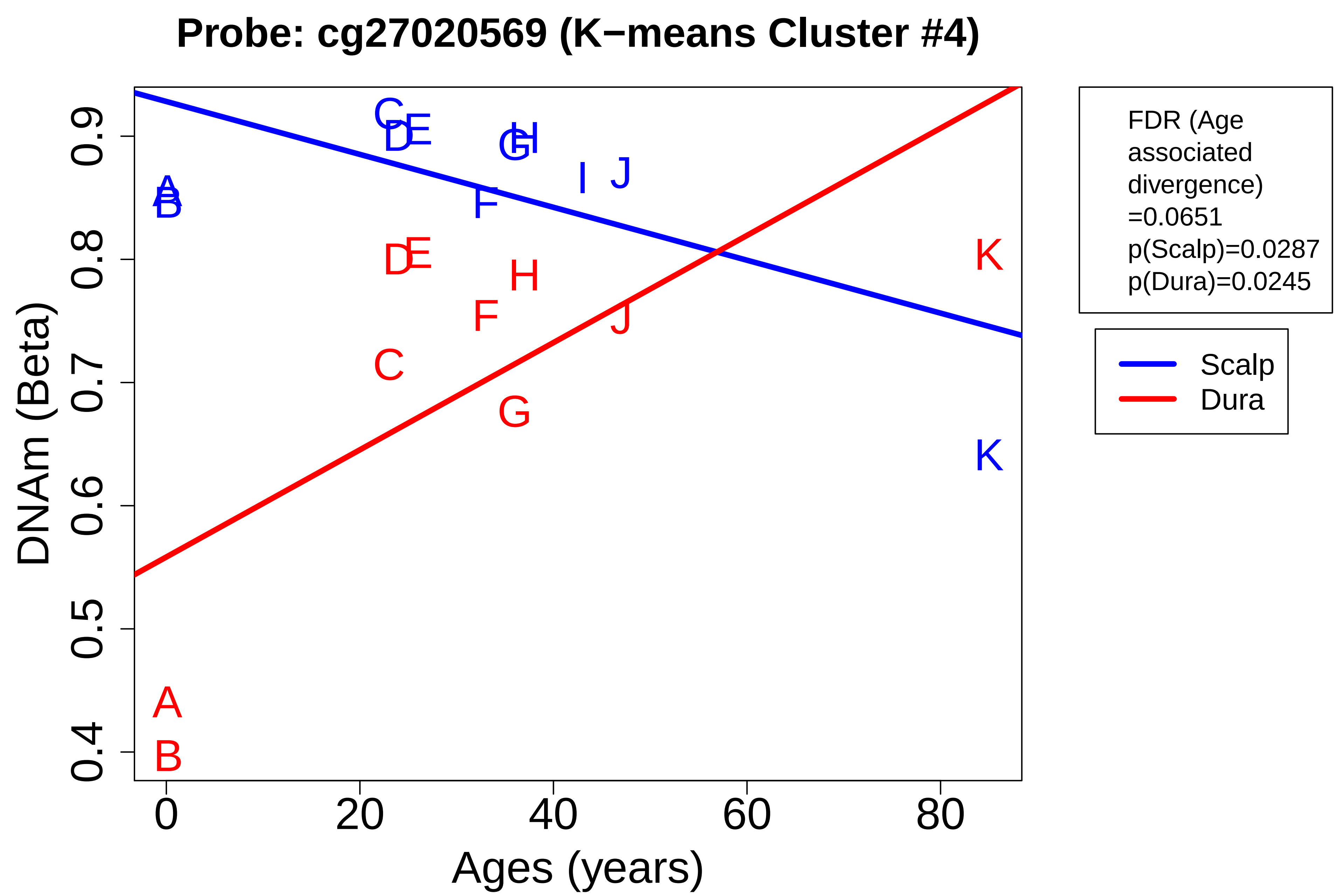

Probe: cg27020569 (K−means Cluster #4)
DNAm (Beta)
0.4
0.5
0.6
0.7
0.8
0.9
0
20
40
60
80
Ages (years)
A
B
C
D
E
F
G
H
I
J
K
FDR (Age associated divergence) =0.0651
p(Scalp)=0.0287
p(Dura)=0.0245
Scalp
Dura

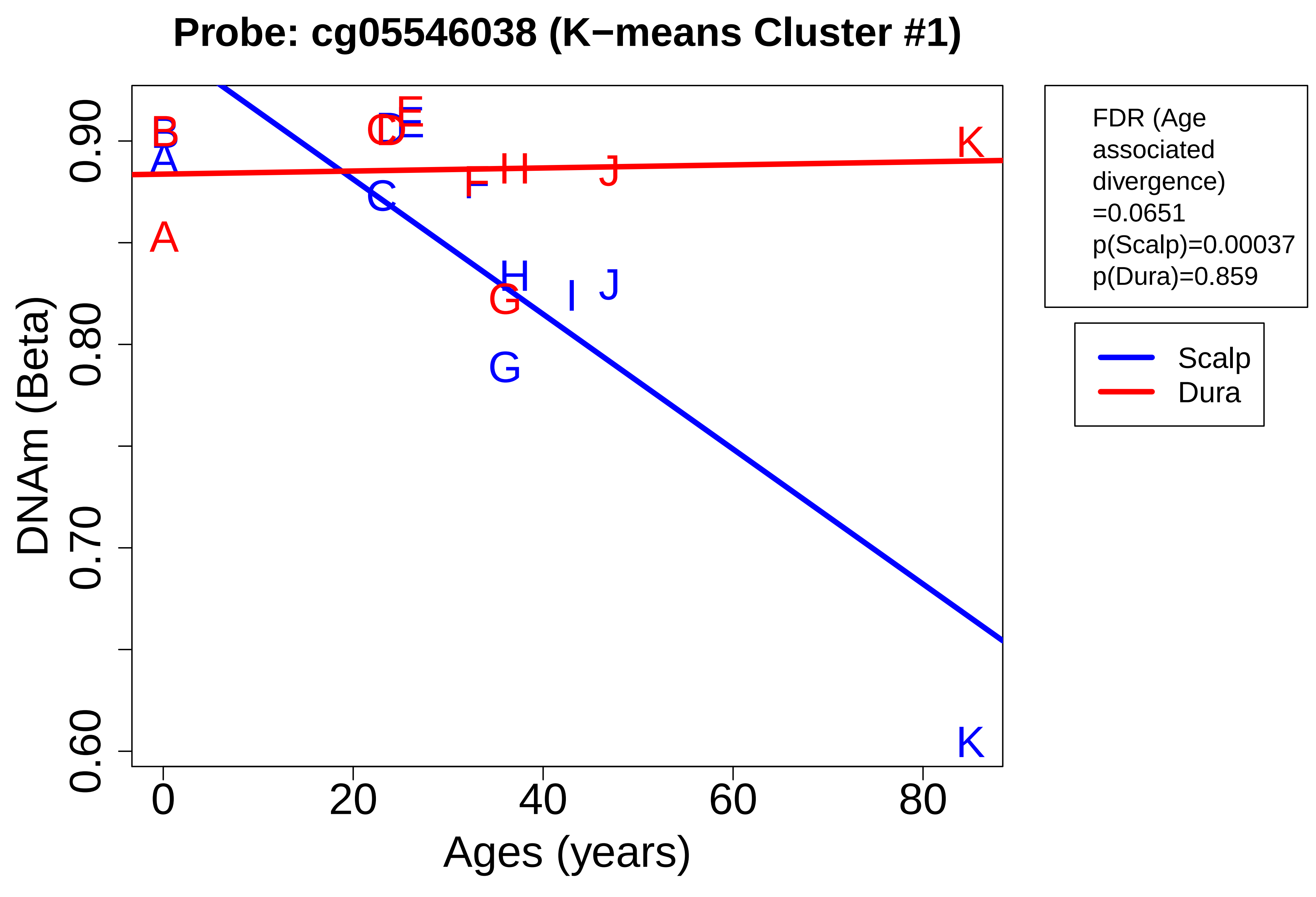

Probe: cg05546038 (K−means Cluster #1)
DNAm (Beta)
0.60
0.70
0.80
0.90
0
20
40
60
80
Ages (years)
A
B
C
D
E
F
G
H
I
J
K
FDR (Age associated divergence) =0.0651
p(Scalp)=0.00037
p(Dura)=0.859
Scalp
Dura

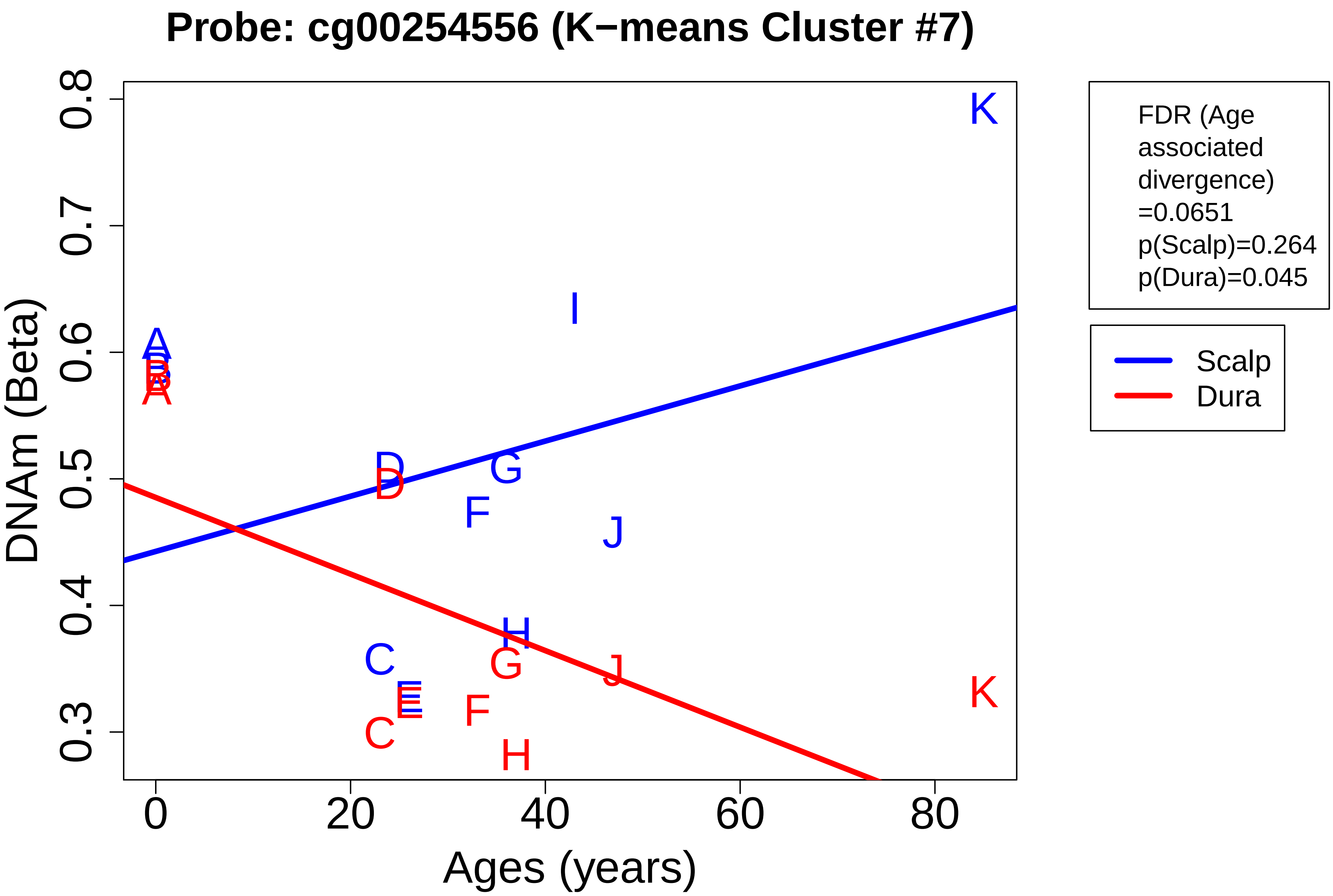
Probe: cg00254556 (K−means Cluster #7)
DNAm (Beta)
0.3
0.4
0.5
0.6
0.7
0.8
0
20
40
60
80
Ages (years)
FDR (Age associated divergence) =0.0651
p(Scalp)=0.264
p(Dura)=0.045
Scalp
Dura

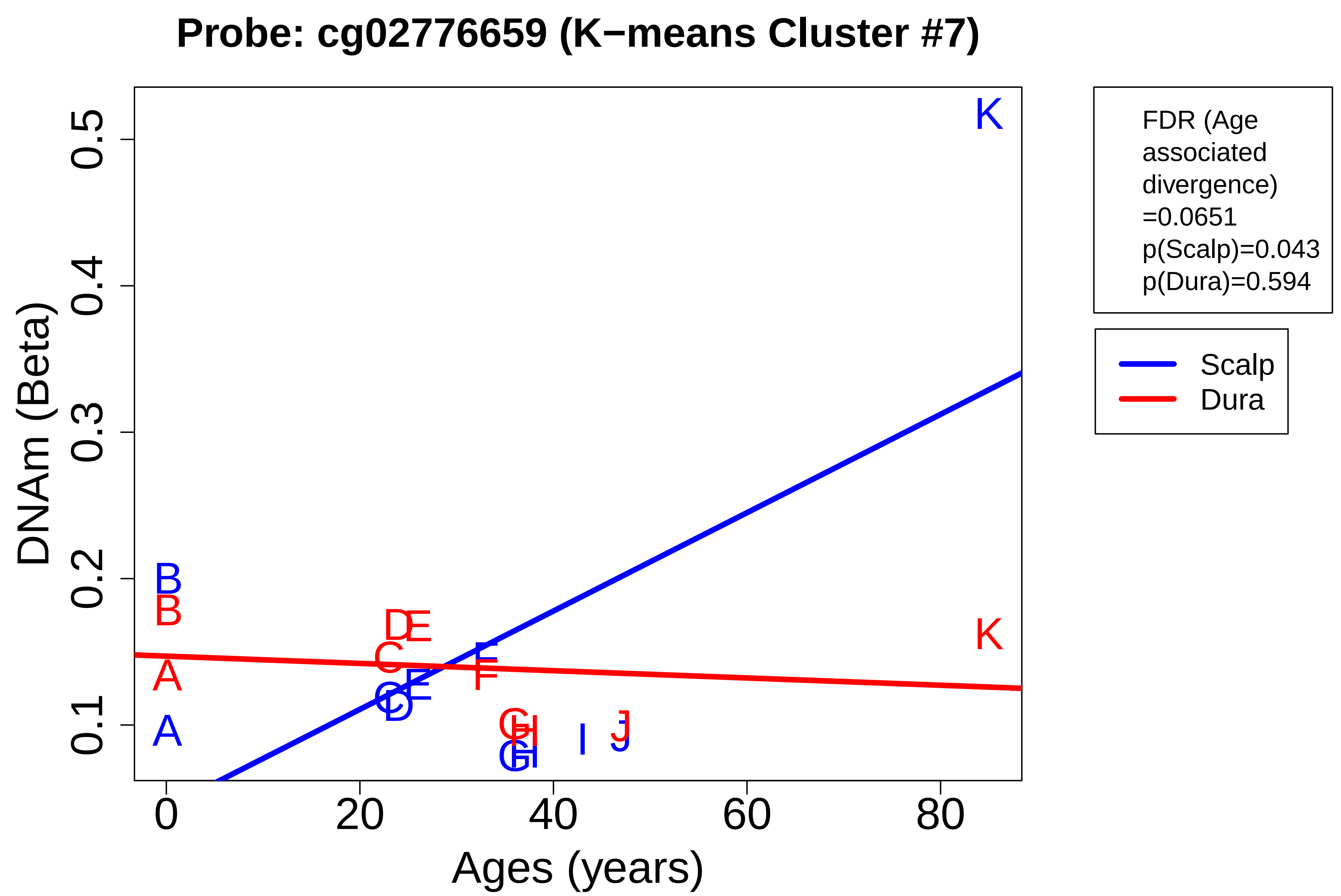
Probe: cg02776659 (K−means Cluster #7)
DNAm (Beta)
Ages (years)
0.1
0.2
0.3
0.4
0.5
0
20
40
60
80
FDR (Age associated divergence) =0.0651
p(Scalp)=0.043
p(Dura)=0.594
Scalp
Dura
A
B
C
D
E
F
G
H
I
J
K

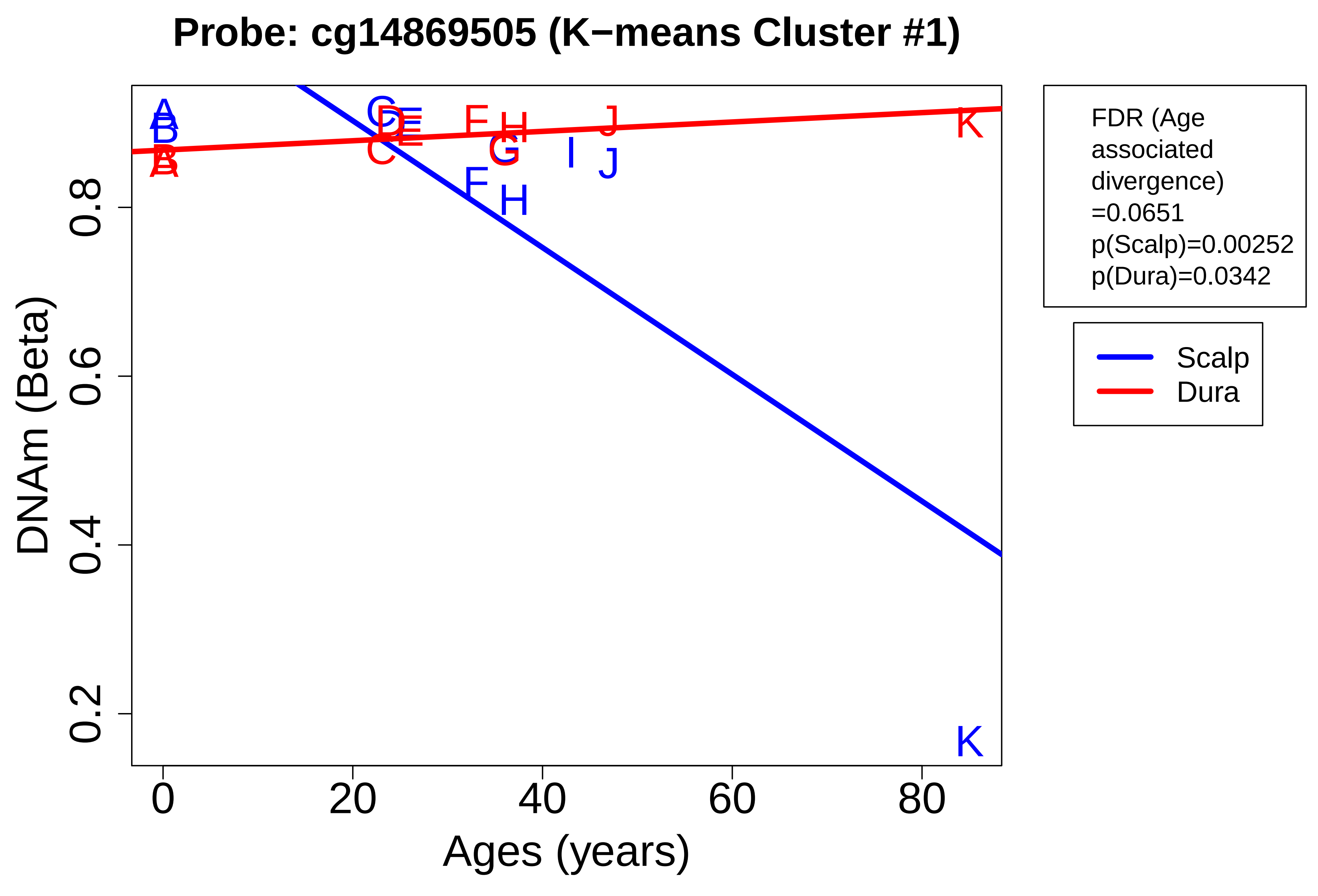
Probe: cg14869505 (K−means Cluster #1)
DNAm (Beta)
0.2
0.4
0.6
0.8
Ages (years)
0
20
40
60
80
A
B
C
D
E
F
G
H
I
J
K
FDR (Age associated divergence) =0.0651
p(Scalp)=0.00252
p(Dura)=0.0342
Scalp
Dura

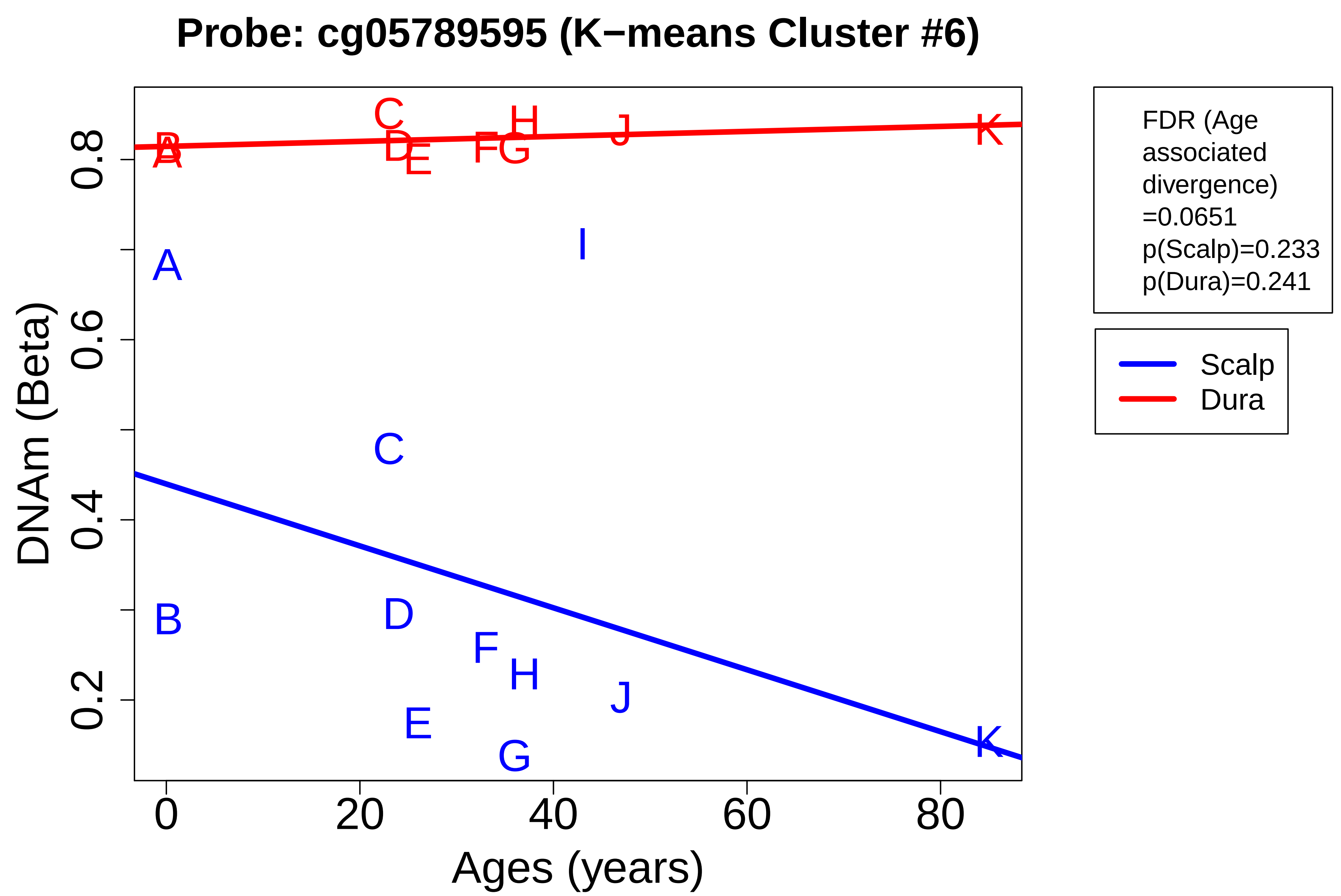

Probe: cg05789595 (K−means Cluster #6)
FDR (Age associated divergence) =0.0651
p(Scalp)=0.233
p(Dura)=0.241
Scalp
Dura
DNAm (Beta)
0.2
0.4
0.6
0.8
0
20
40
60
80
Ages (years)

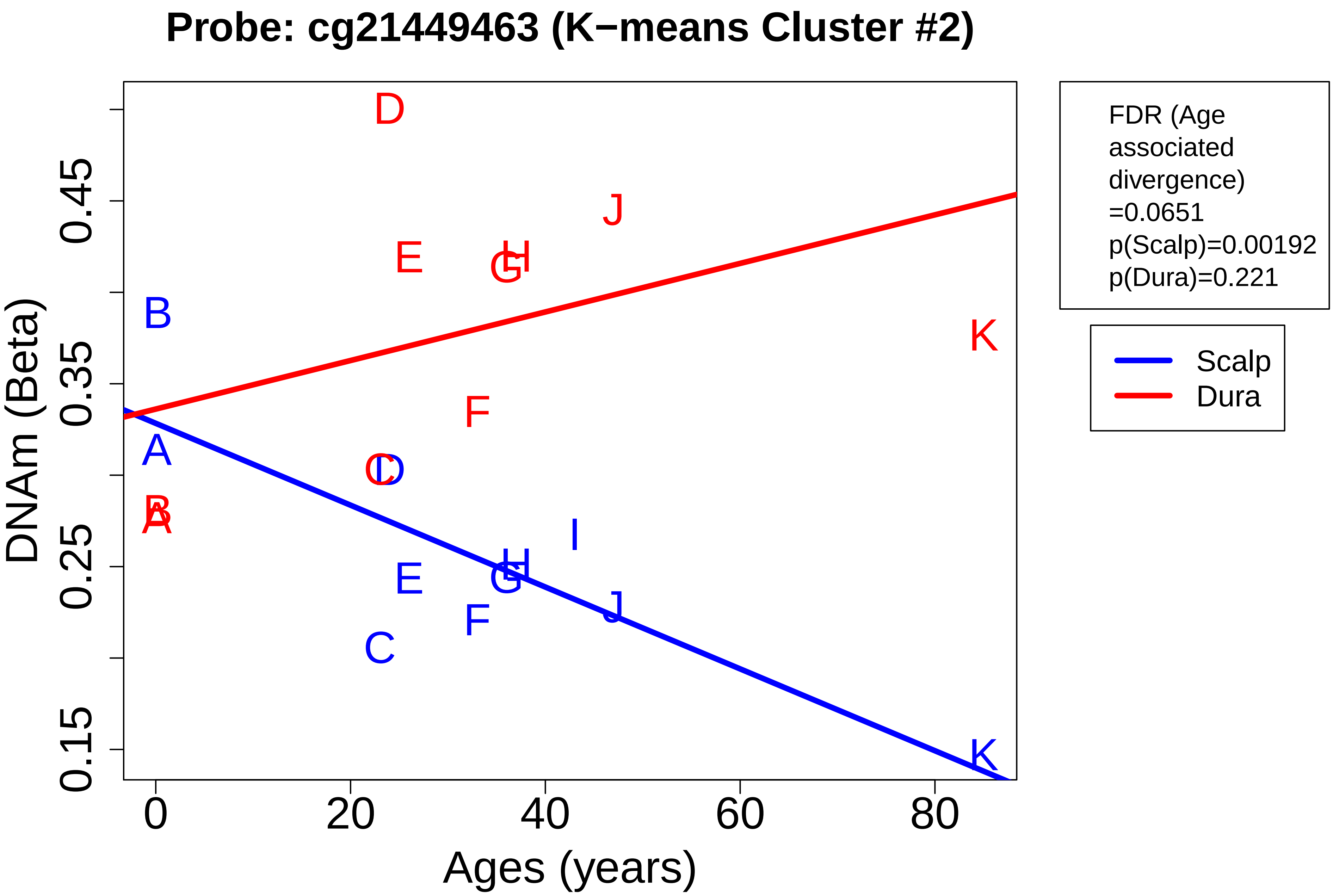

Probe: cg21449463 (K−means Cluster #2)
DNAm (Beta)
0.15
0.25
0.35
0.45
0
20
40
60
80
Ages (years)
FDR (Age associated divergence) =0.0651
p(Scalp)=0.00192
p(Dura)=0.221
Scalp
Dura

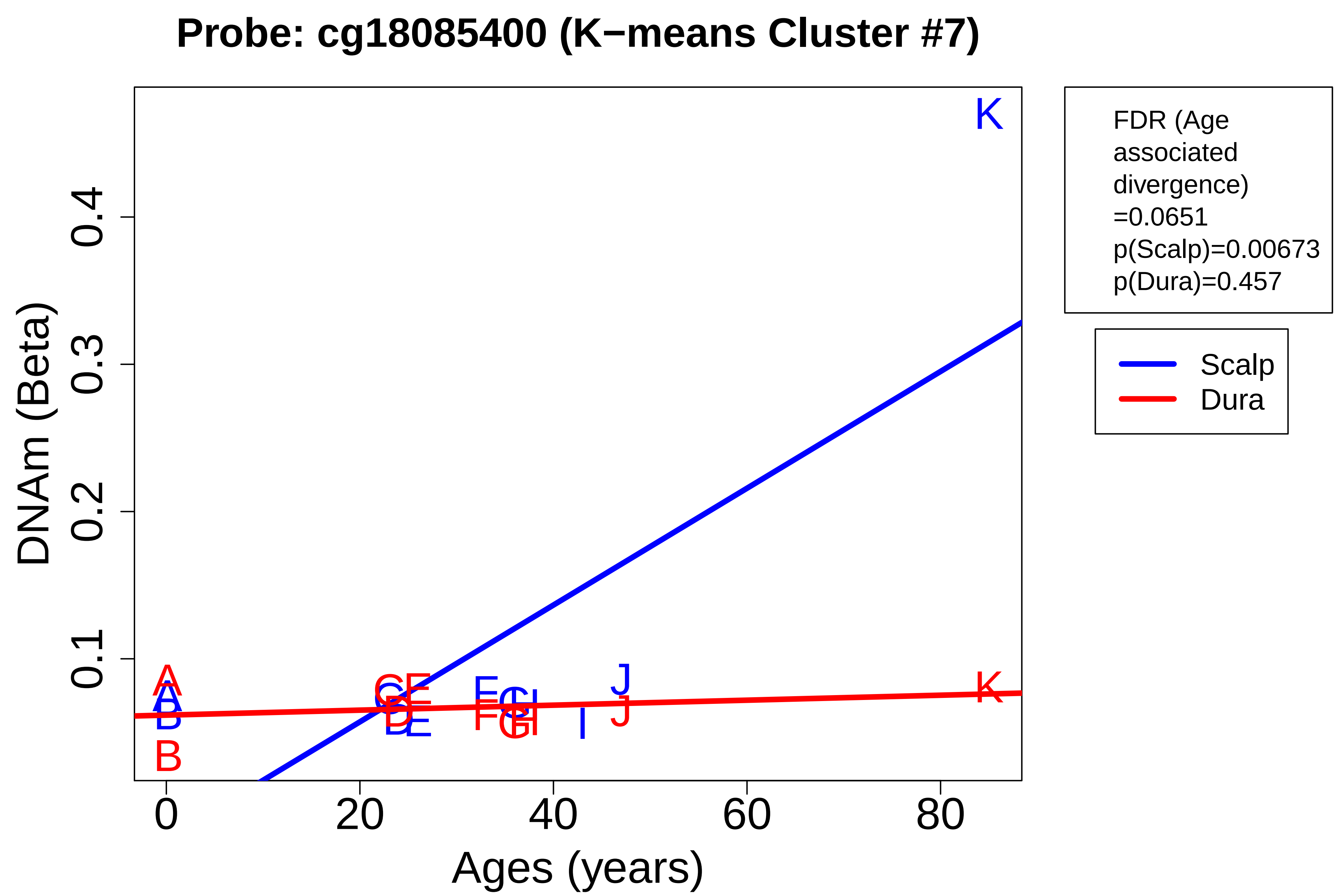
Probe: cg18085400 (K−means Cluster #7)
DNAm (Beta)
0.1
0.2
0.3
0.4
Ages (years)
0
20
40
60
80
FDR (Age associated divergence) =0.0651
p(Scalp)=0.00673
p(Dura)=0.457
Scalp
Dura

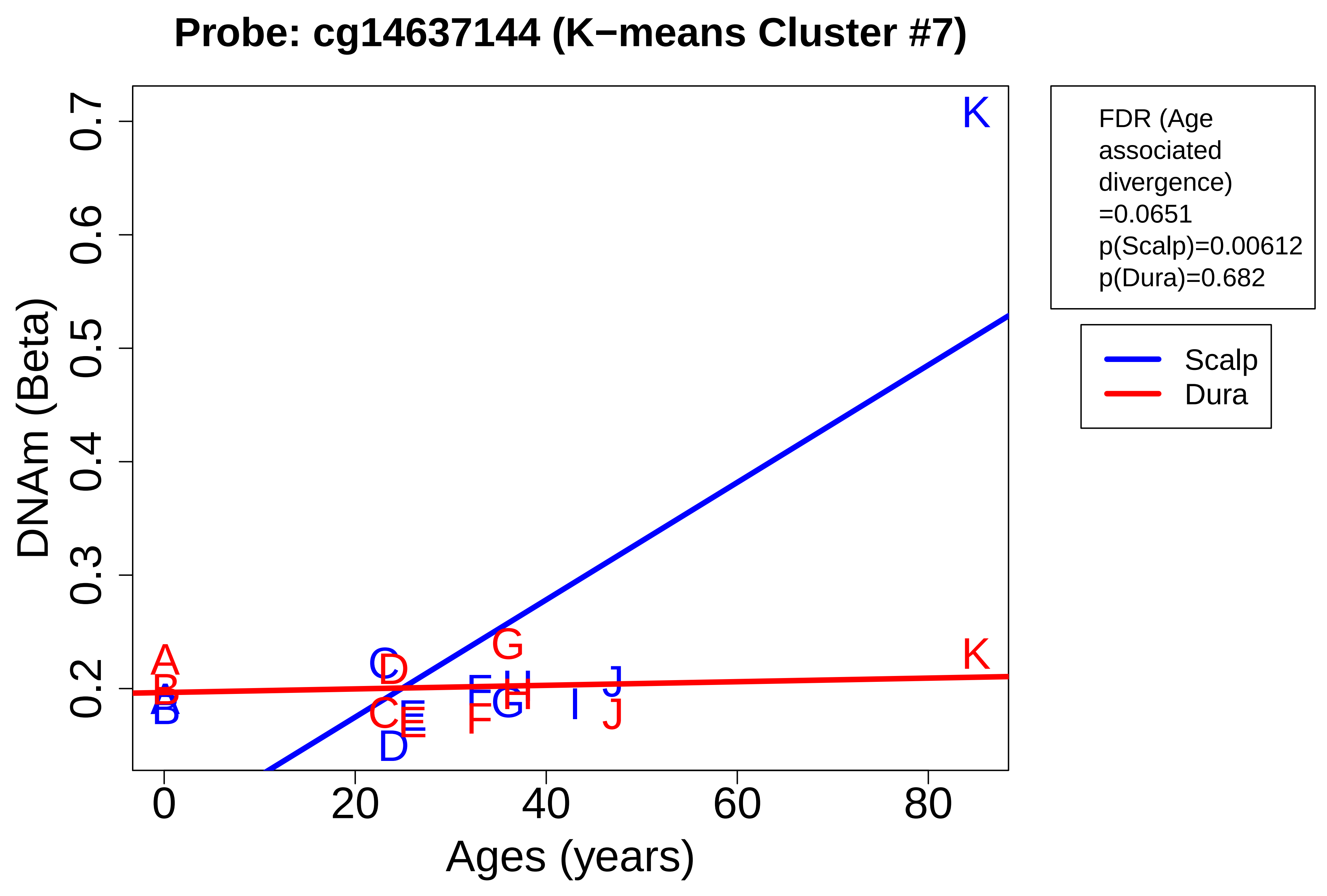
Probe: cg14637144 (K−means Cluster #7)
DNAm (Beta)
0.2
0.3
0.4
0.5
0.6
0.7
0
20
40
60
80
Ages (years)
FDR (Age associated divergence) =0.0651
p(Scalp)=0.00612
p(Dura)=0.682
Scalp
Dura
A
B
C
D
E
F
G
H
I
J
K

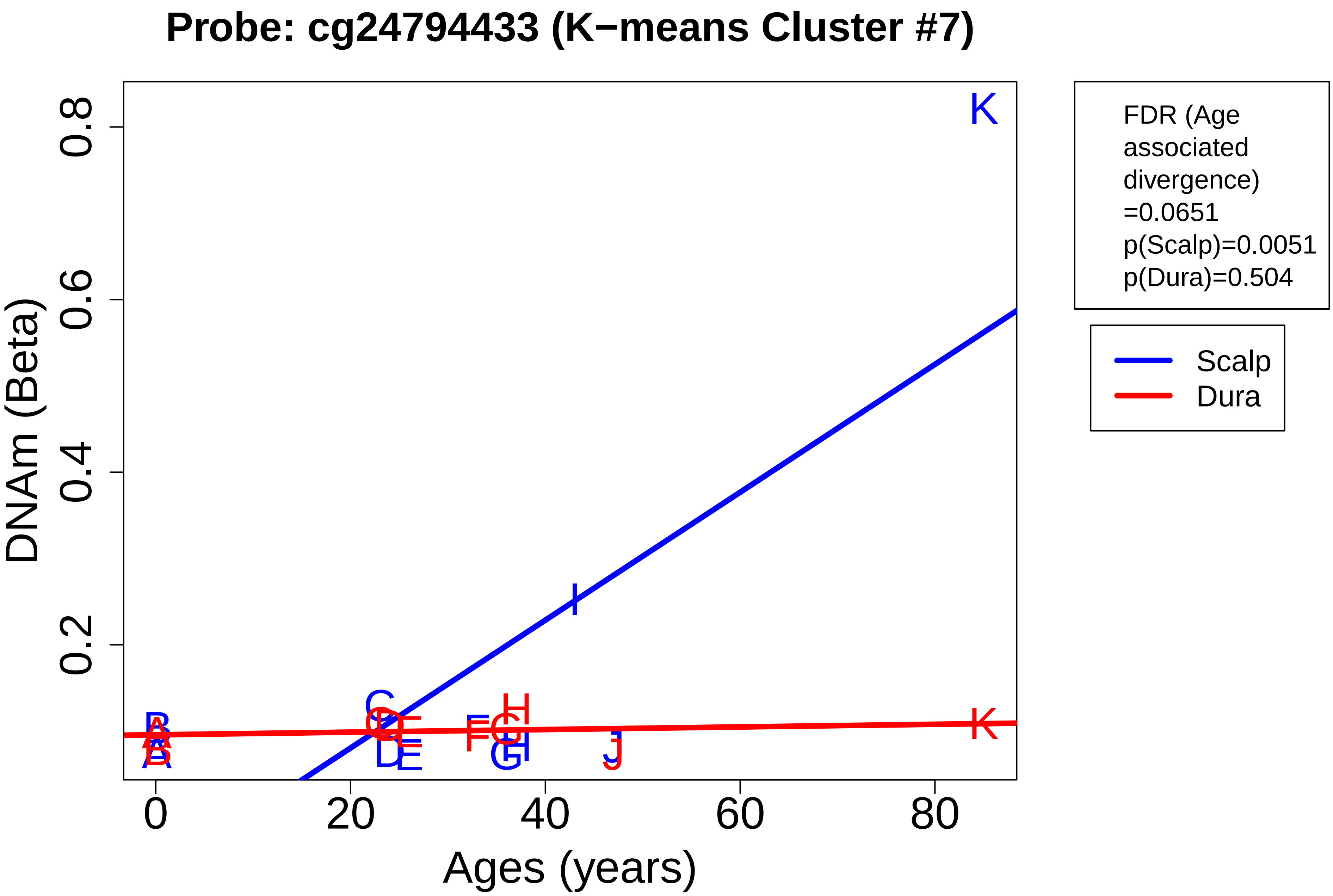
Probe: cg24794433 (K−means Cluster #7)
DNAm (Beta)
0.2
0.4
0.6
0.8
0
20
40
60
80
Ages (years)
FDR (Age associated divergence) =0.0651
p(Scalp)=0.0051
p(Dura)=0.504
Scalp
Dura

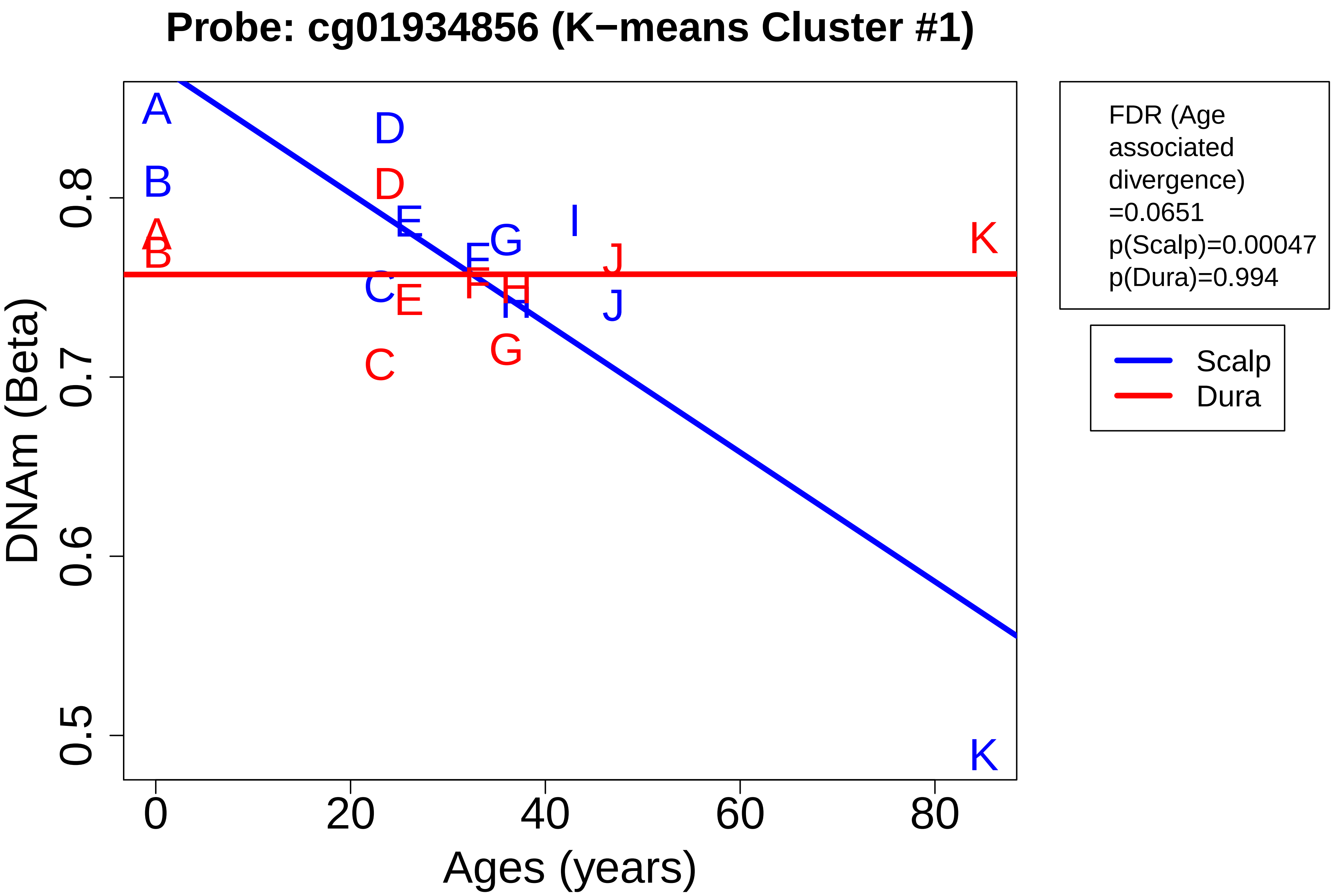

Probe: cg01934856 (K−means Cluster #1)
DNAm (Beta)
0.5
0.6
0.7
0.8
0
20
40
60
80
Ages (years)
FDR (Age associated divergence) =0.0651
p(Scalp)=0.00047
p(Dura)=0.994
Scalp
Dura

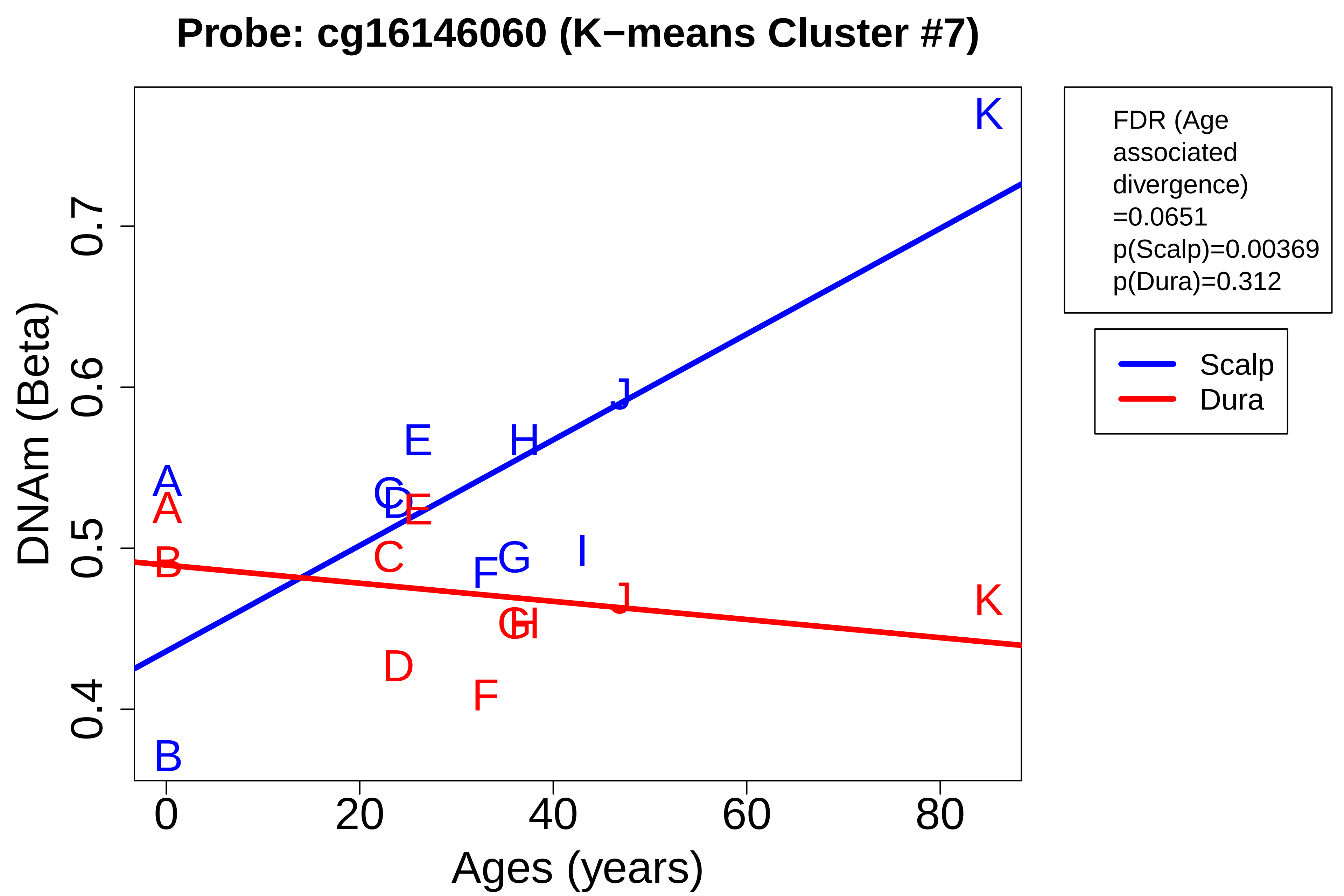
Probe: cg16146060 (K−means Cluster #7)
DNAm (Beta)
0.4
0.5
0.6
0.7
0
20
40
60
80
Ages (years)
A
B
C
D
E
F
G
H
I
J
K
FDR (Age associated divergence) =0.0651
p(Scalp)=0.00369
p(Dura)=0.312
Scalp
Dura

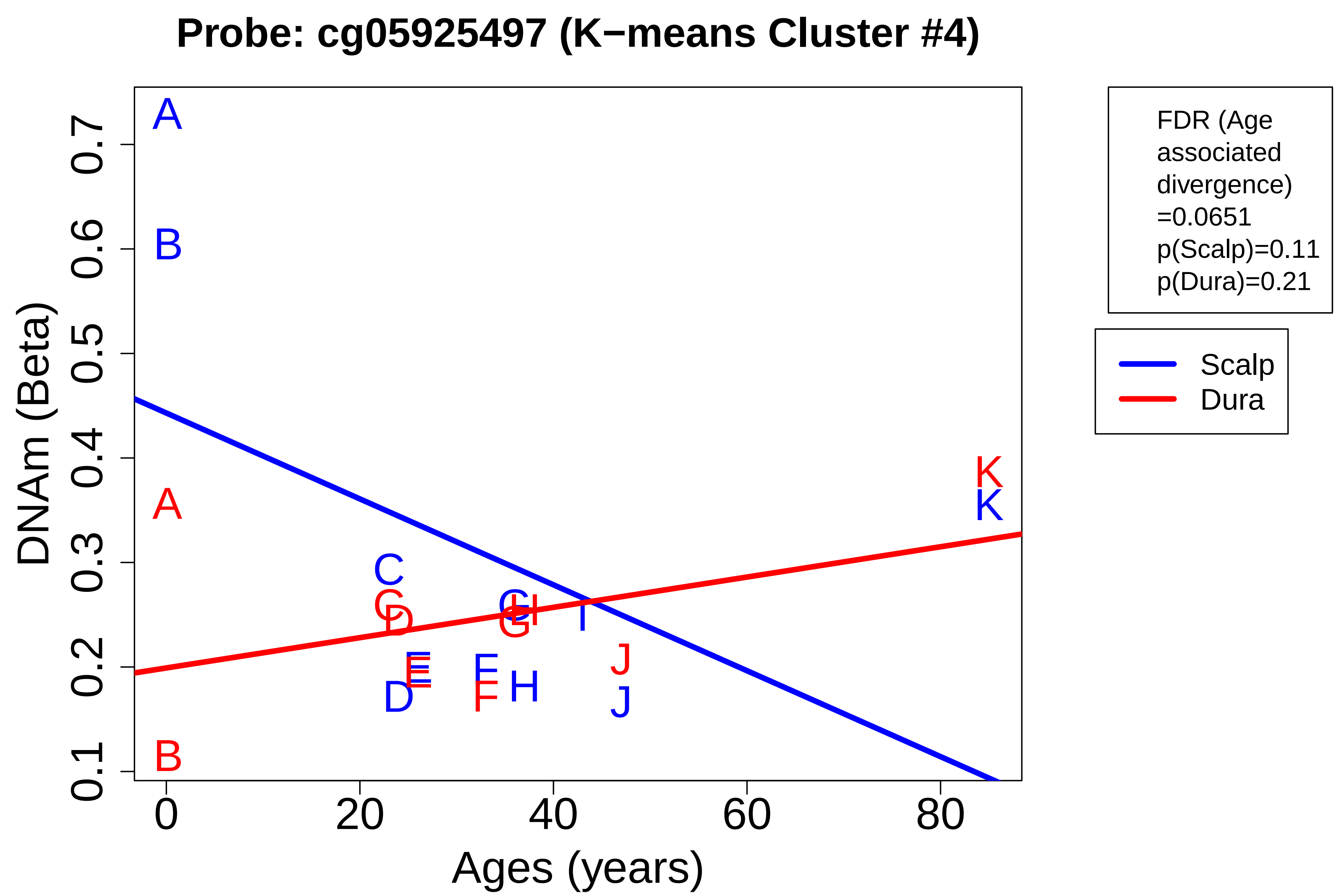
Probe: cg05925497 (K−means Cluster #4)
DNAm (Beta)
Ages (years)
0.1
0.2
0.3
0.4
0.5
0.6
0.7
0
20
40
60
80
FDR (Age associated divergence) =0.0651
p(Scalp)=0.11
p(Dura)=0.21
Scalp
Dura

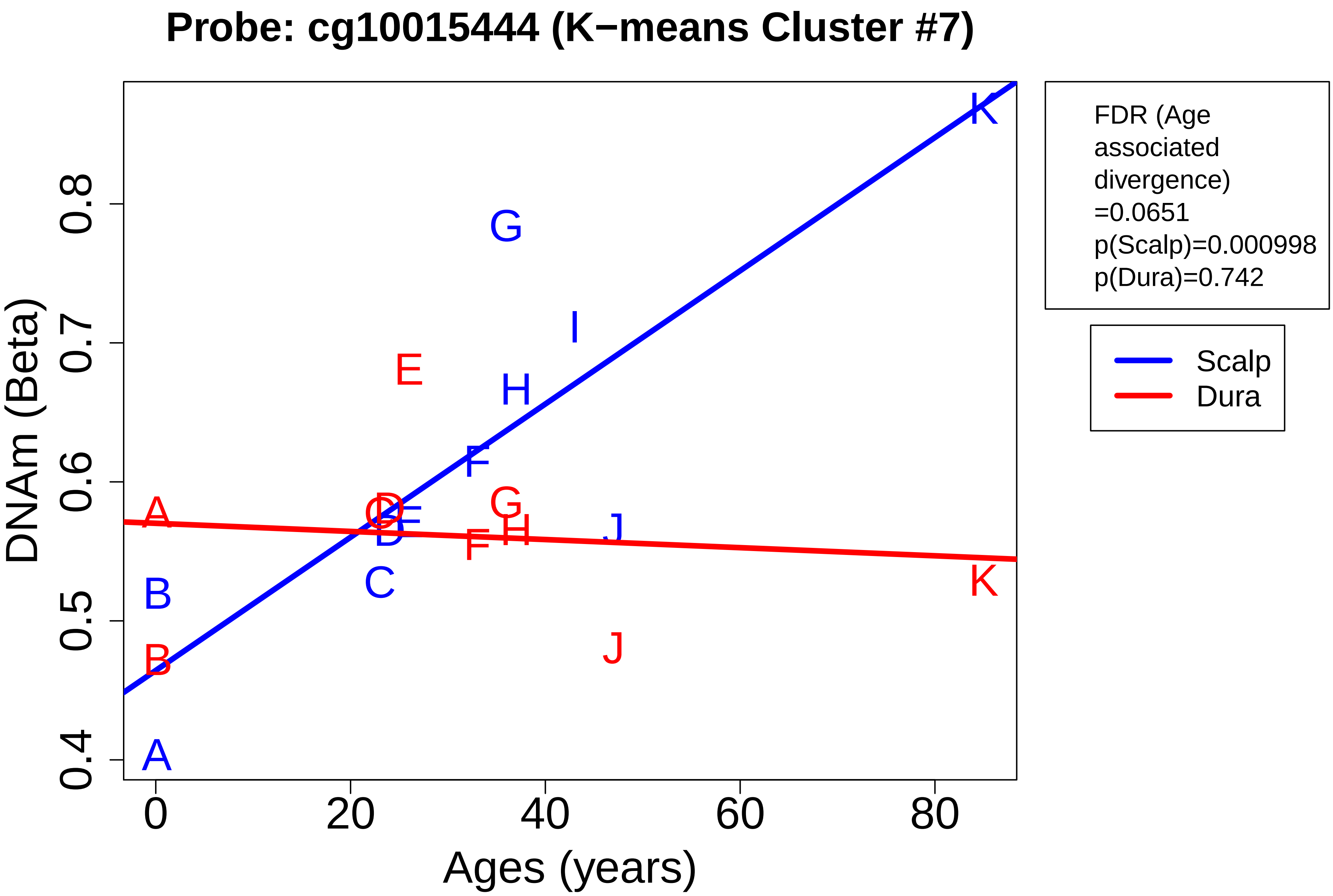
Probe: cg10015444 (K−means Cluster #7)
DNAm (Beta)
0.4
0.5
0.6
0.7
0.8
0
20
40
60
80
Ages (years)
FDR (Age associated divergence) =0.0651
p(Scalp)=0.000998
p(Dura)=0.742
Scalp
Dura
A
B
C
D
E
F
G
H
I
J
K

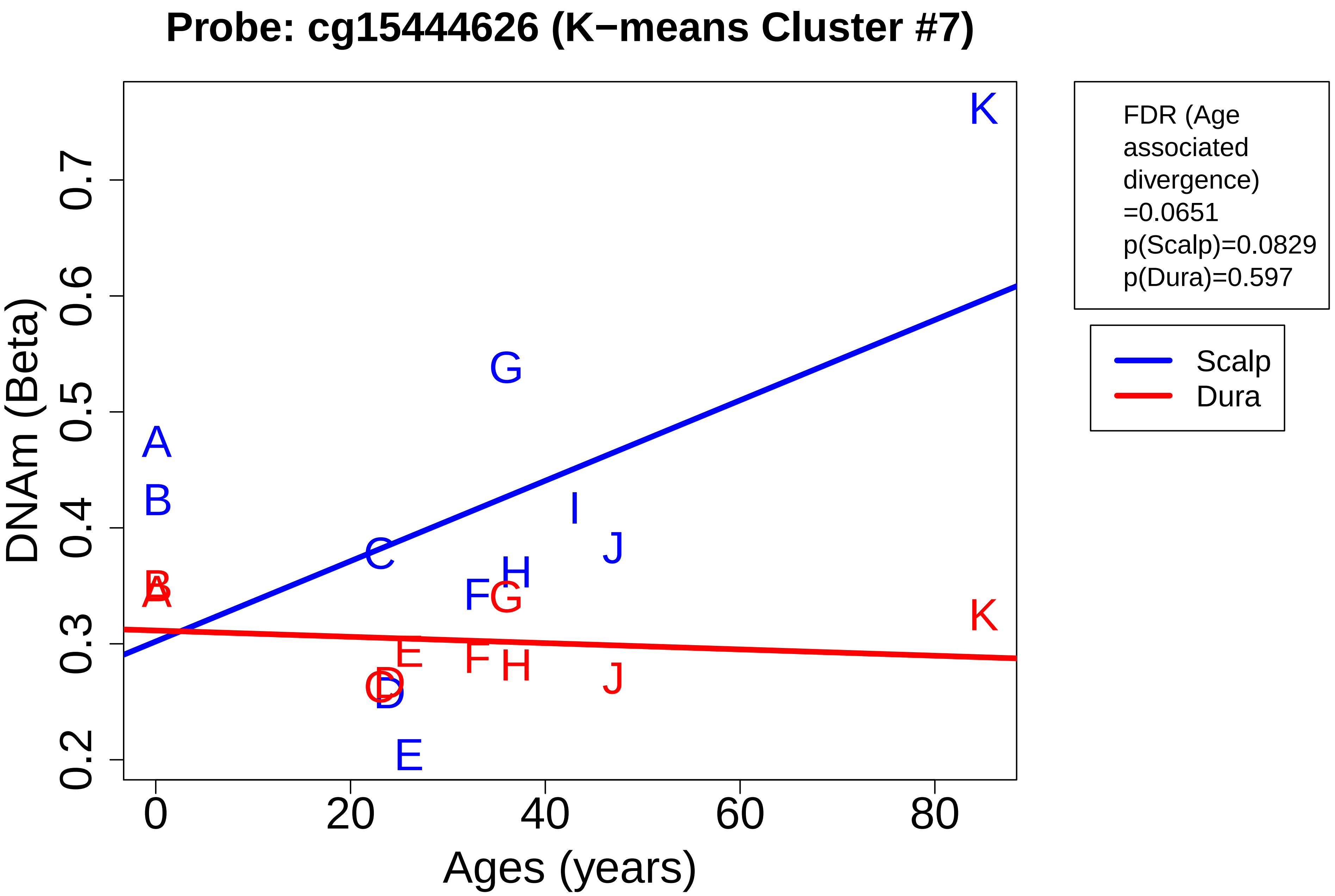
Probe: cg15444626 (K−means Cluster #7)
DNAm (Beta)
Ages (years)
0.2
0.3
0.4
0.5
0.6
0.7
0
20
40
60
80
FDR (Age associated divergence) =0.0651
p(Scalp)=0.0829
p(Dura)=0.597
Scalp
Dura
A
B
C
D
E
F
G
H
I
J
K

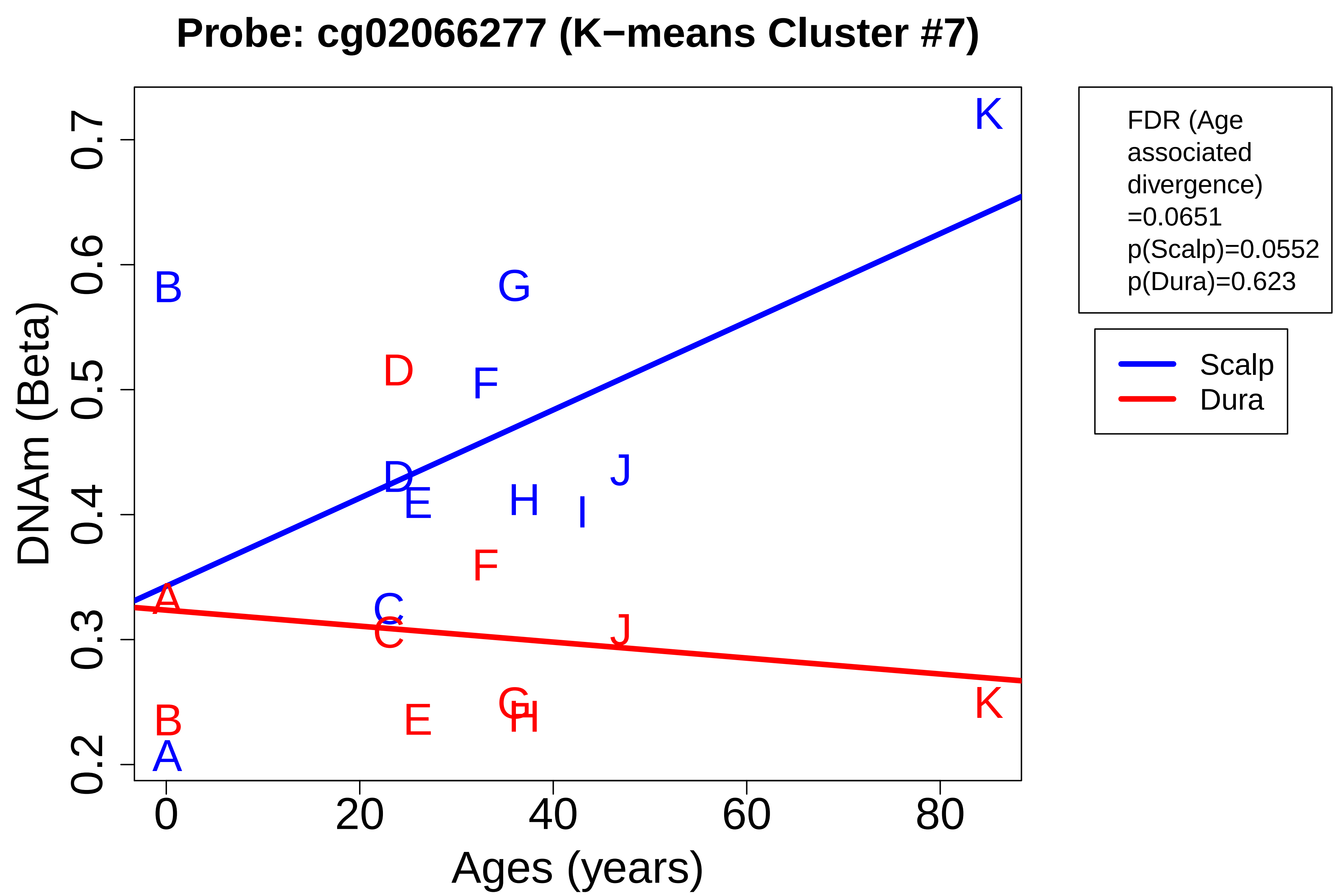
Probe: cg02066277 (K−means Cluster #7)
DNAm (Beta)
Ages (years)
0.2
0.3
0.4
0.5
0.6
0.7
0
20
40
60
80
A
B
C
D
E
F
G
H
I
J
K
FDR (Age associated divergence) =0.0651
p(Scalp)=0.0552
p(Dura)=0.623
Scalp
Dura

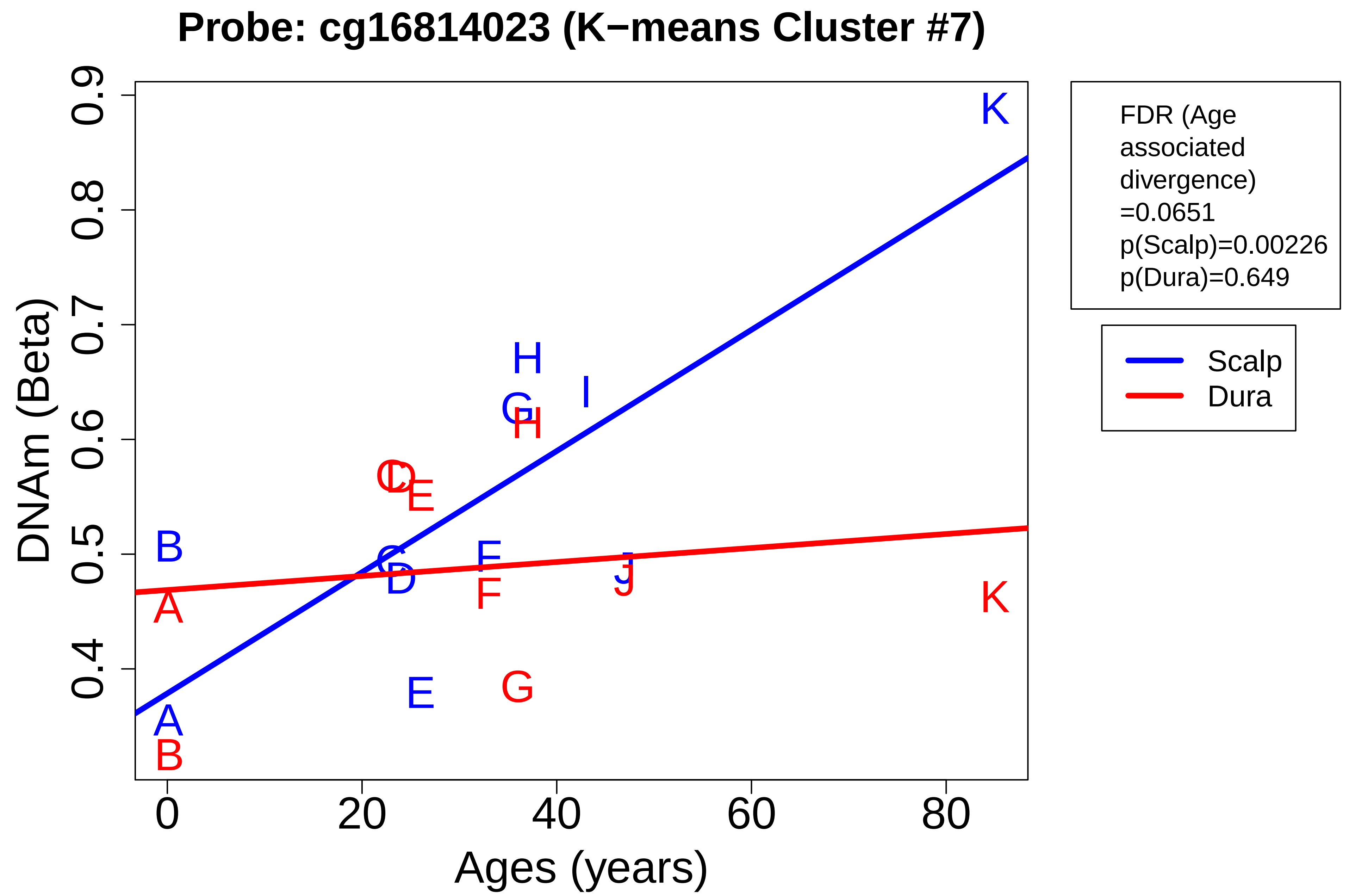

Probe: cg16814023 (K−means Cluster #7)
DNAm (Beta)
0.4
0.5
0.6
0.7
0.8
0.9
0
20
40
60
80
Ages (years)
A
B
C
D
E
F
G
H
I
J
K
FDR (Age associated divergence) =0.0651
p(Scalp)=0.00226
p(Dura)=0.649
Scalp
Dura

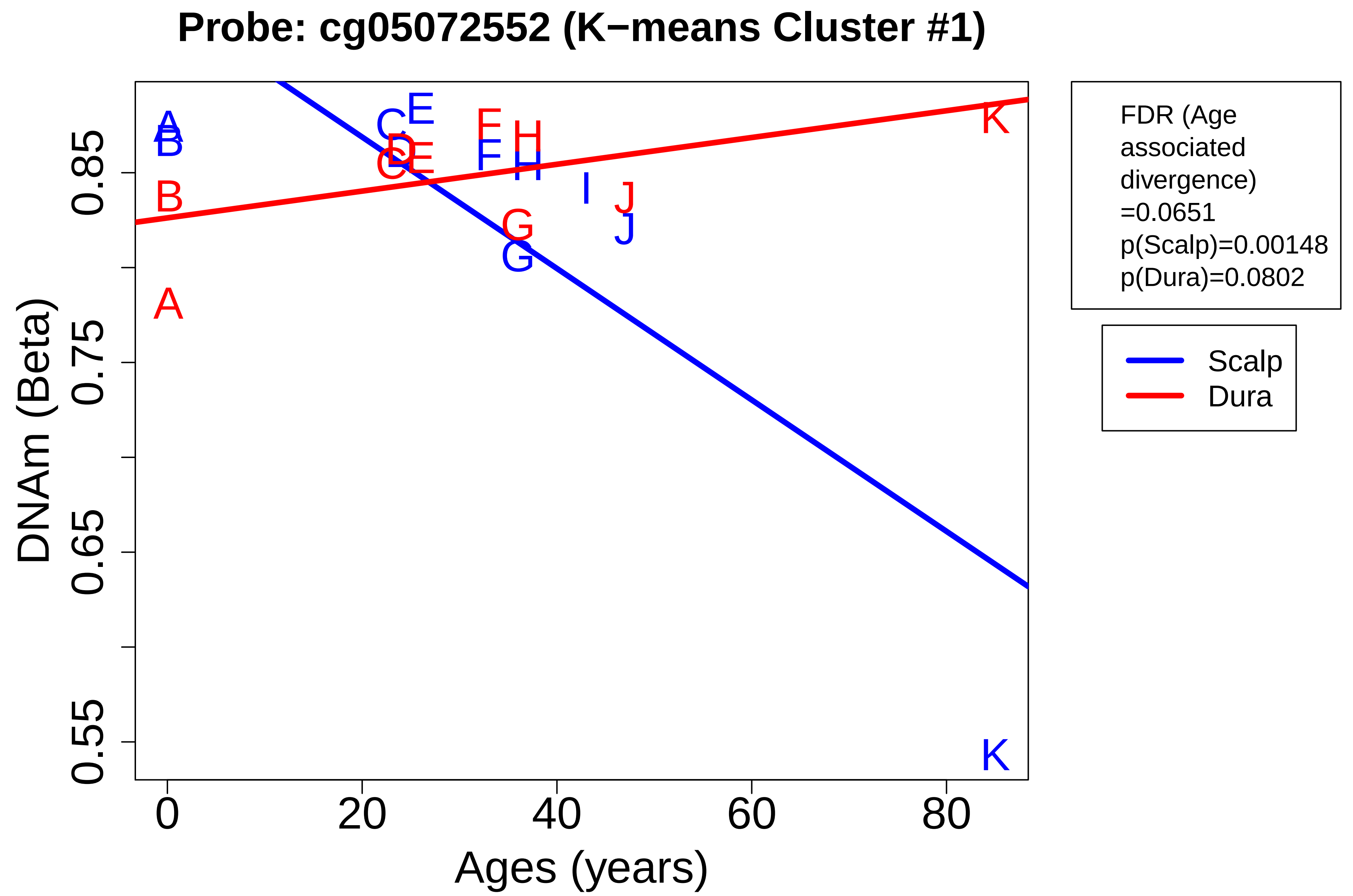

Probe: cg05072552 (K−means Cluster #1)
DNAm (Beta)
0.55
0.65
0.75
0.85
0
20
40
60
80
Ages (years)
A
B
C
D
E
F
G
H
I
J
K
FDR (Age associated divergence) =0.0651
p(Scalp)=0.00148
p(Dura)=0.0802
Scalp
Dura

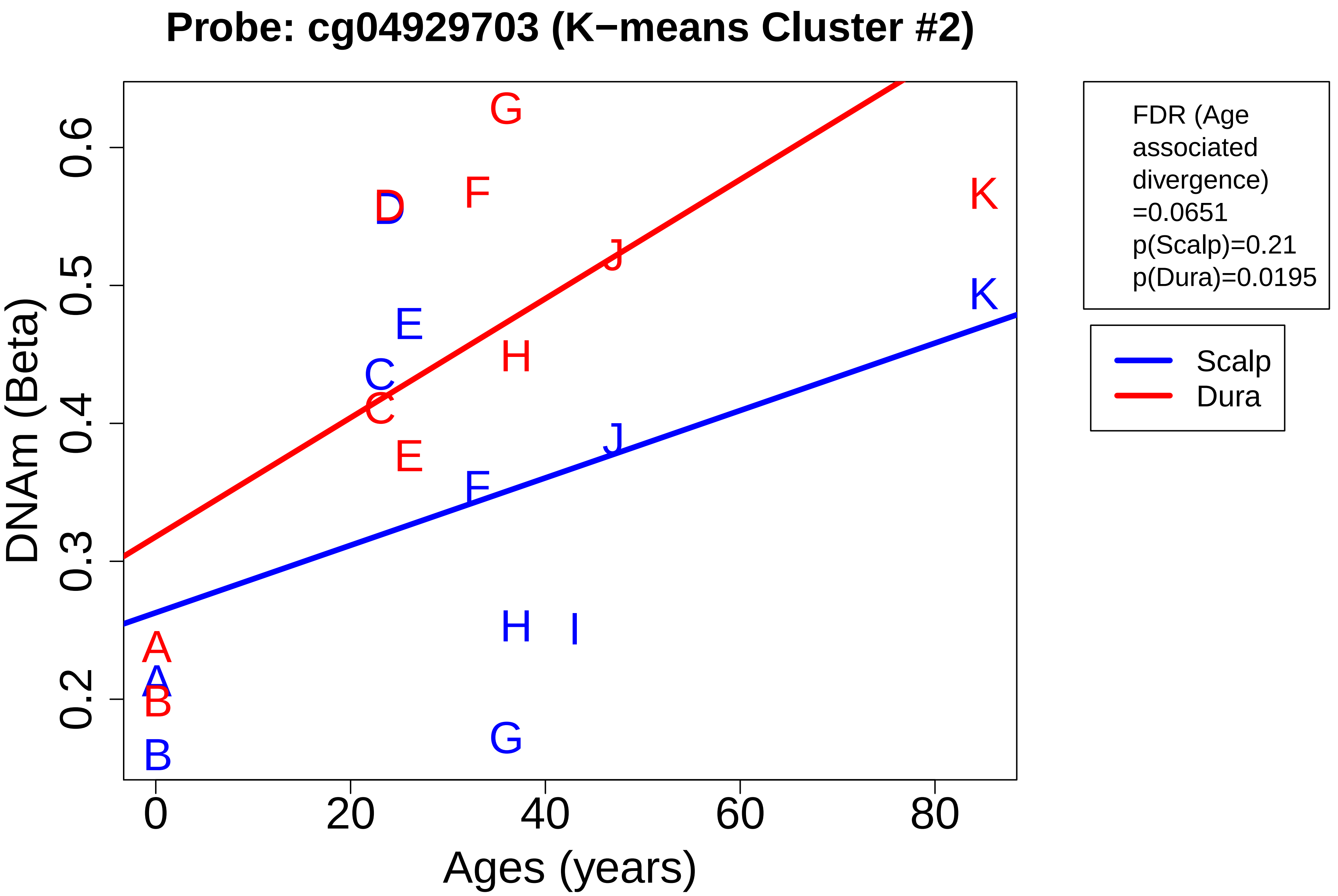

Probe: cg04929703 (K−means Cluster #2)
DNAm (Beta)
0.2
0.3
0.4
0.5
0.6
0
20
40
60
80
Ages (years)
FDR (Age associated divergence) =0.0651
p(Scalp)=0.21
p(Dura)=0.0195
Scalp
Dura
A
B
C
D
E
F
G
H
I
J
K

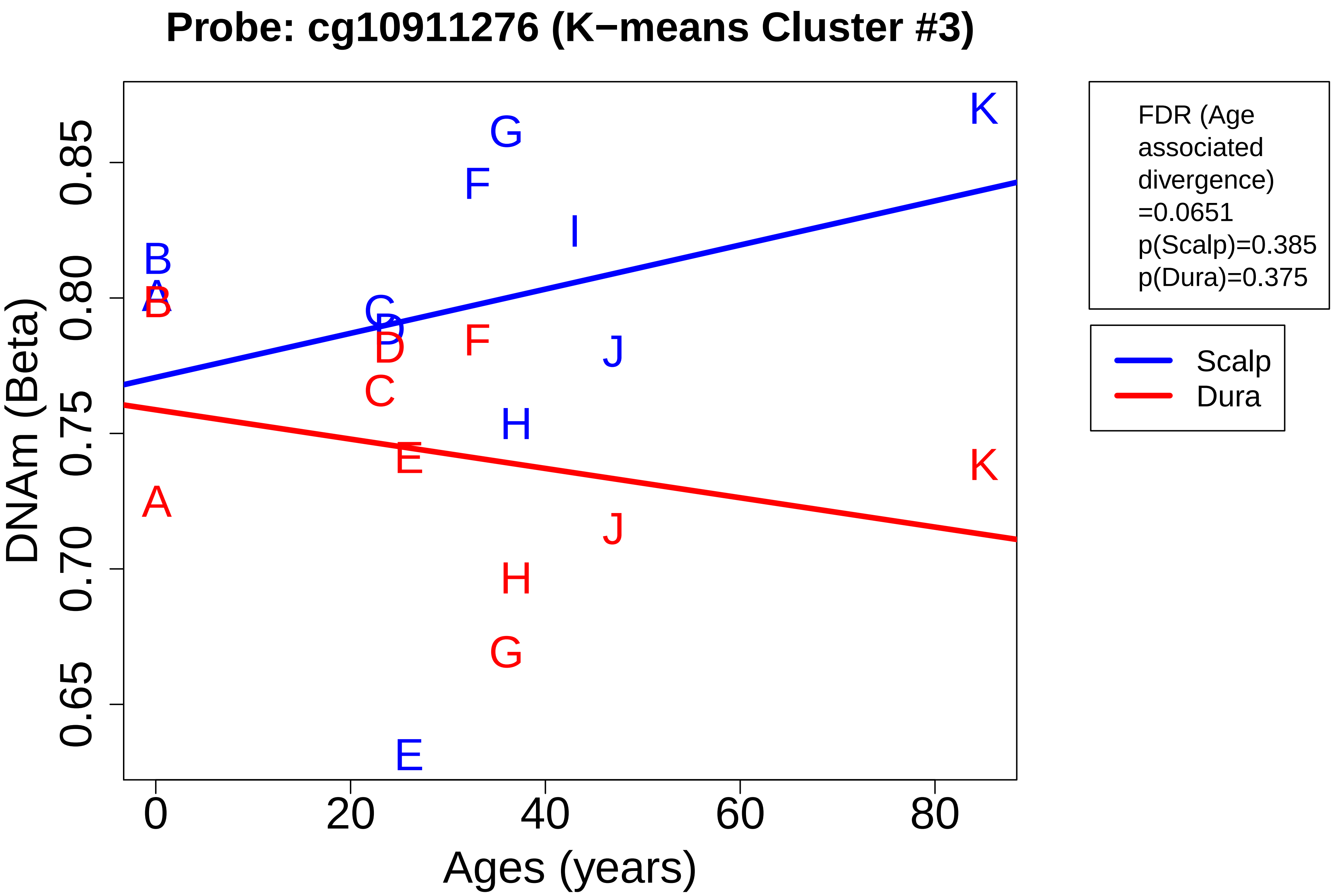

Probe: cg10911276 (K−means Cluster #3)
DNAm (Beta)
0.65
0.70
0.75
0.80
0.85
Ages (years)
0
20
40
60
80
A
B
C
D
E
F
G
H
I
J
K
FDR (Age associated divergence) =0.0651
p(Scalp)=0.385
p(Dura)=0.375
Scalp
Dura

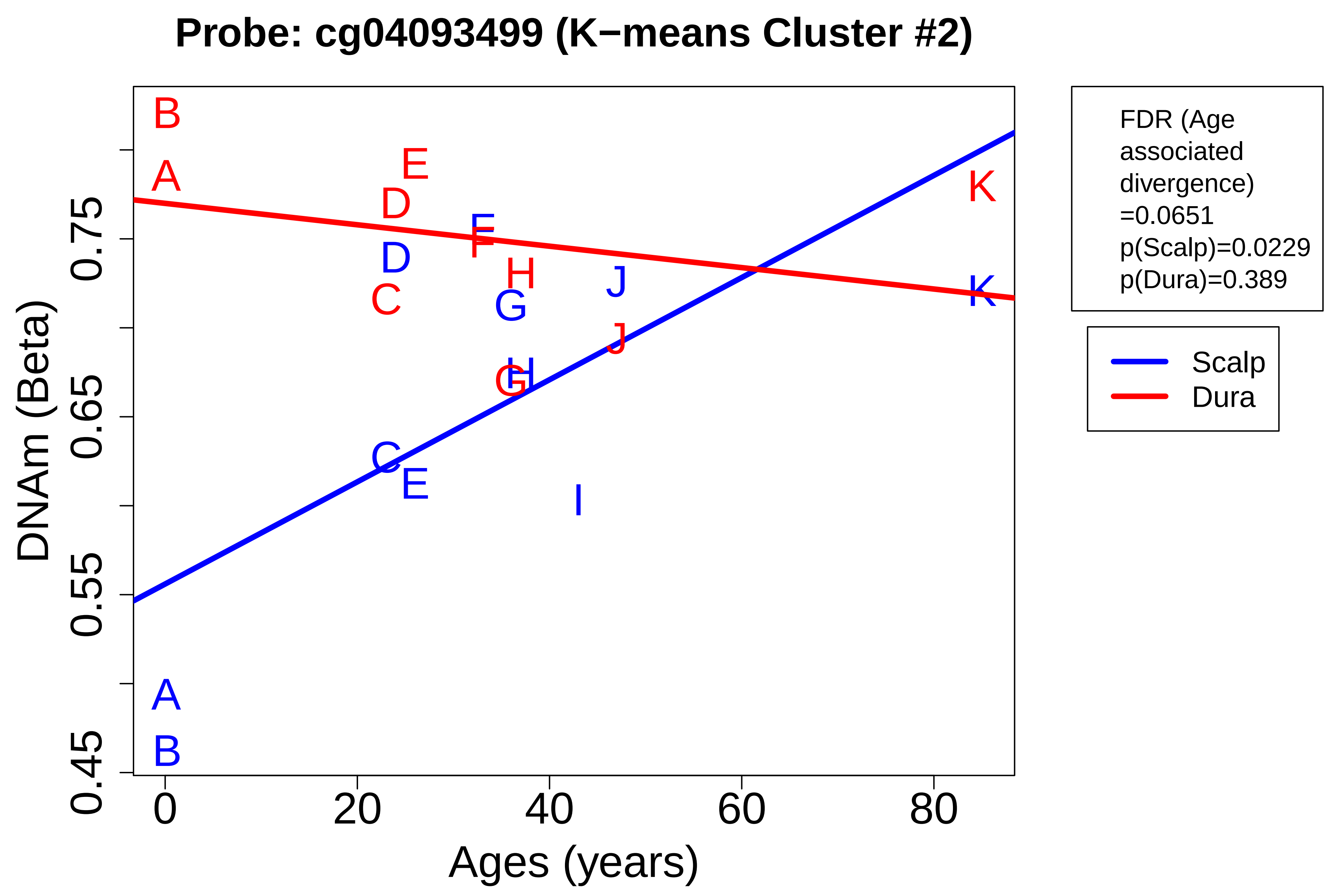

Probe: cg04093499 (K−means Cluster #2)
DNAm (Beta)
0.45
0.55
0.65
0.75
0
20
40
60
80
Ages (years)
FDR (Age associated divergence) =0.0651
p(Scalp)=0.0229
p(Dura)=0.389
Scalp
Dura

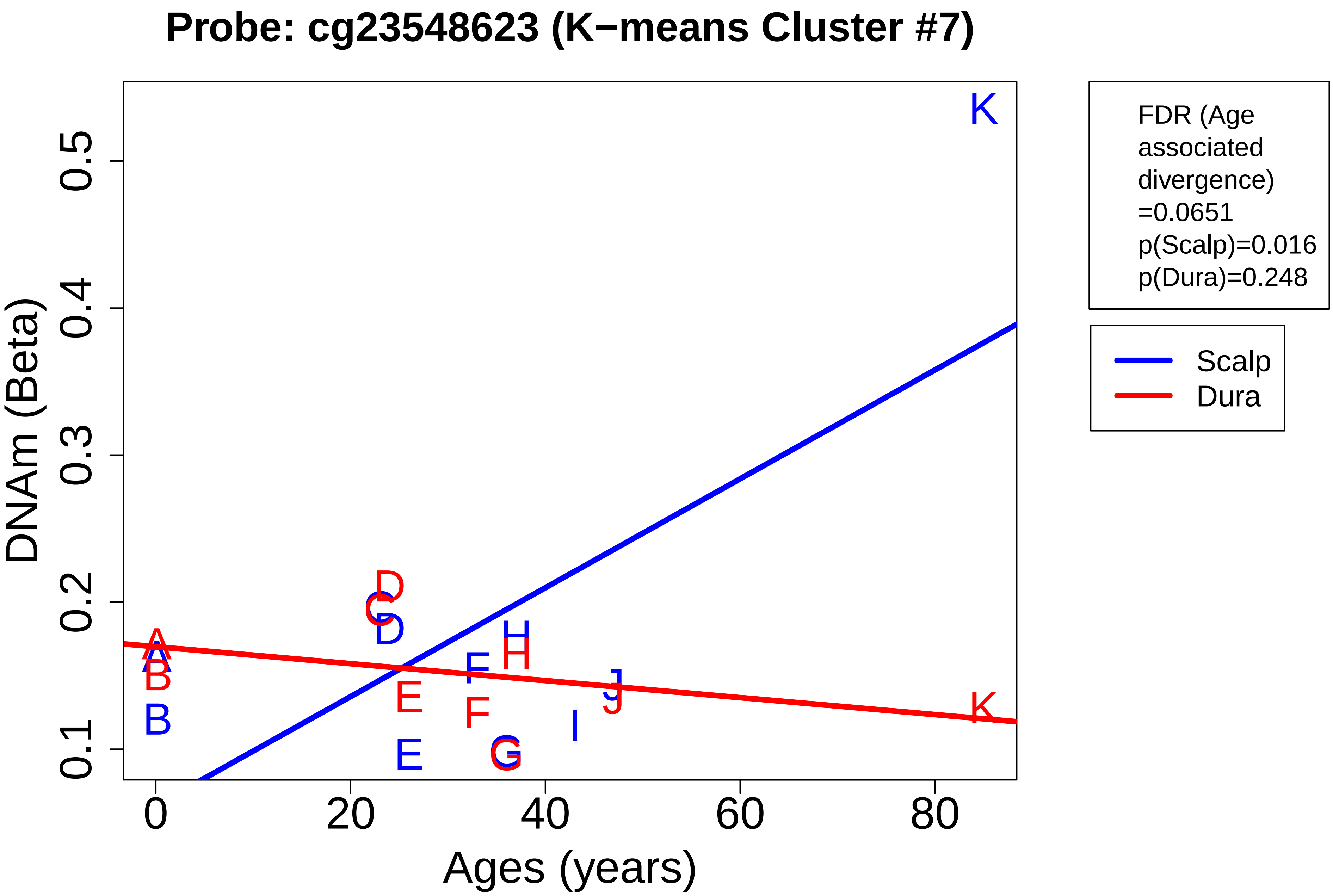

Probe: cg23548623 (K−means Cluster #7)
DNAm (Beta)
0.1
0.2
0.3
0.4
0.5
0
20
40
60
80
Ages (years)
FDR (Age associated divergence) =0.0651
p(Scalp)=0.016
p(Dura)=0.248
Scalp
Dura
A
B
C
D
E
F
G
H
I
J
K

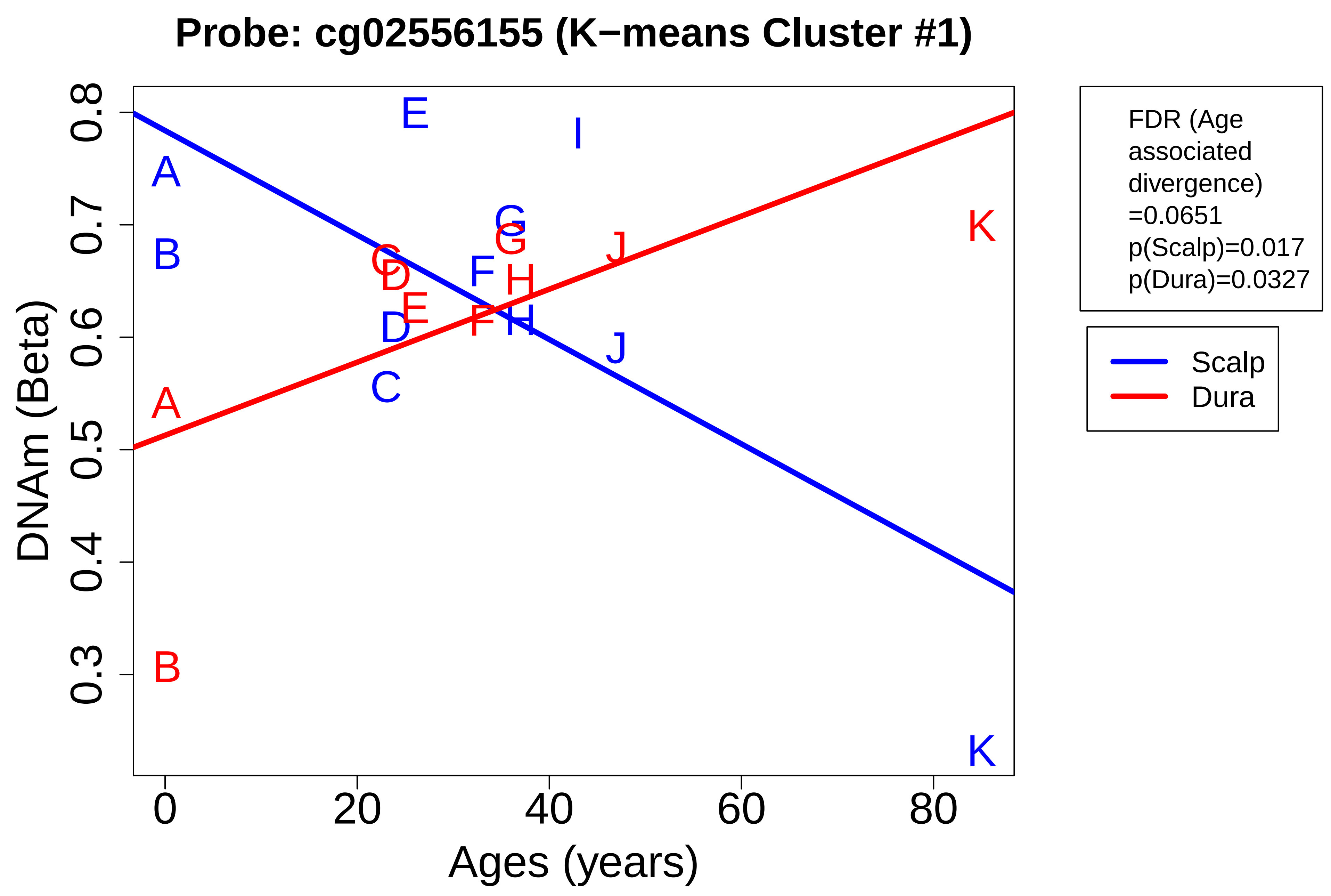

Probe: cg02556155 (K−means Cluster #1)
DNAm (Beta)
0.3
0.4
0.5
0.6
0.7
0.8
0
20
40
60
80
Ages (years)
FDR (Age associated divergence) =0.0651
p(Scalp)=0.017
p(Dura)=0.0327
Scalp
Dura
A
B
C
D
E
F
G
H
I
J
K

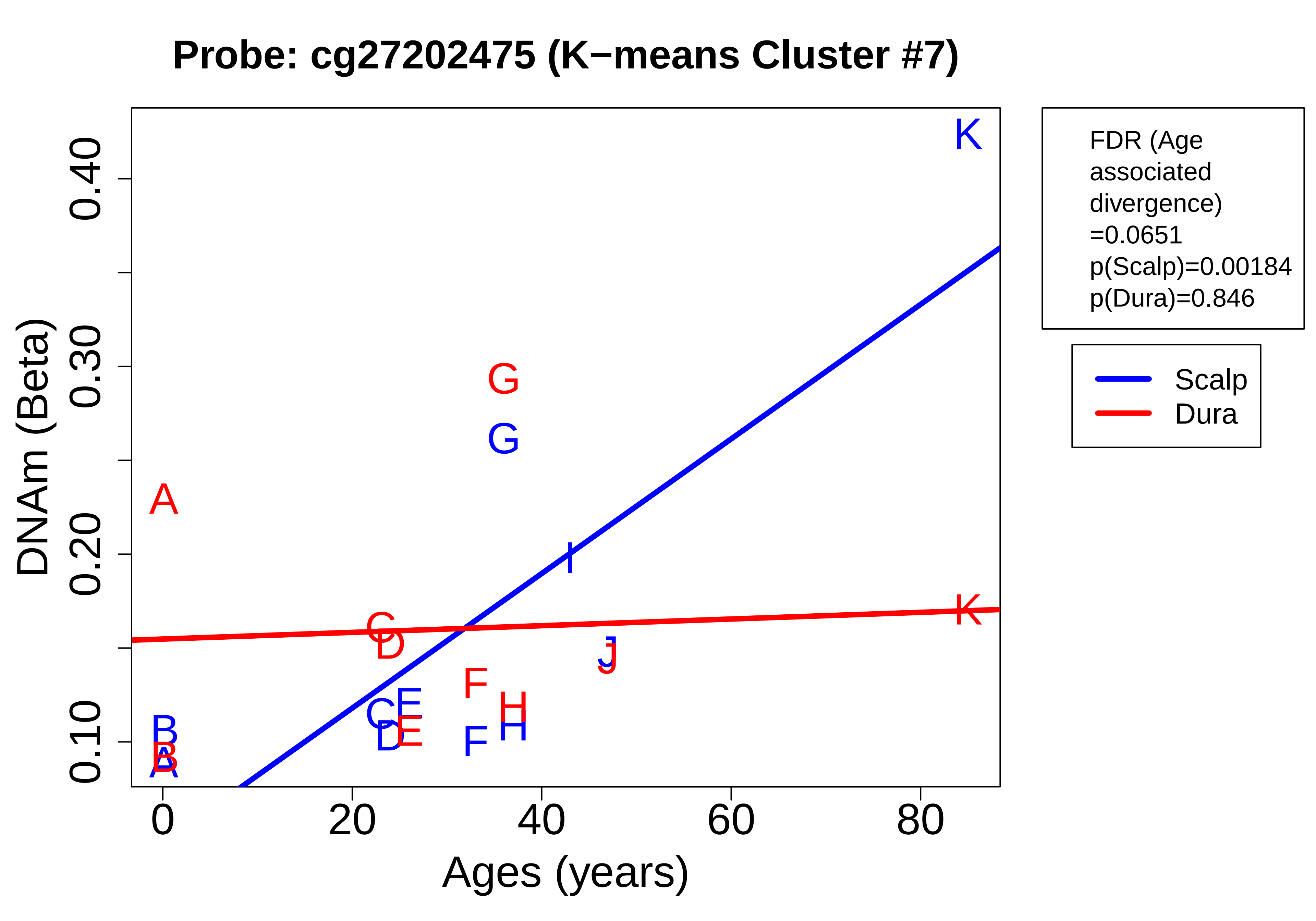

Probe: cg27202475 (K−means Cluster #7)
FDR (Age associated divergence) =0.0651
p(Scalp)=0.00184
p(Dura)=0.846
Scalp
Dura
DNAm (Beta)
0.10
0.20
0.30
0.40
0
20
40
60
80
Ages (years)
A
B
C
D
E
F
G
H
I
J
K

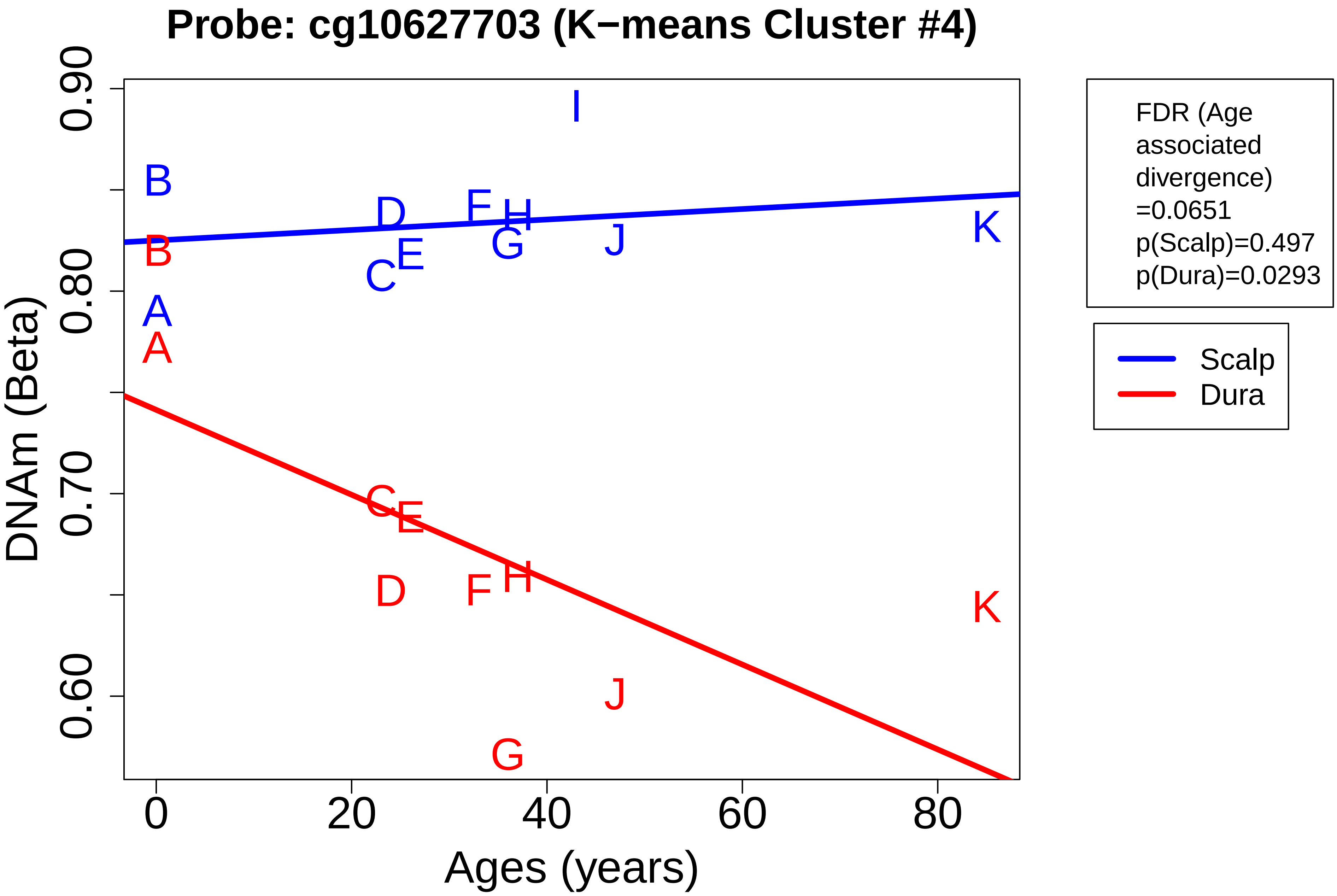
Probe: cg10627703 (K−means Cluster #4)
DNAm (Beta)
0.60
0.70
0.80
0.90
Ages (years)
0
20
40
60
80
FDR (Age associated divergence) =0.0651
p(Scalp)=0.497
p(Dura)=0.0293
Scalp
Dura
A
B
C
D
E
F
G
H
I
J
K

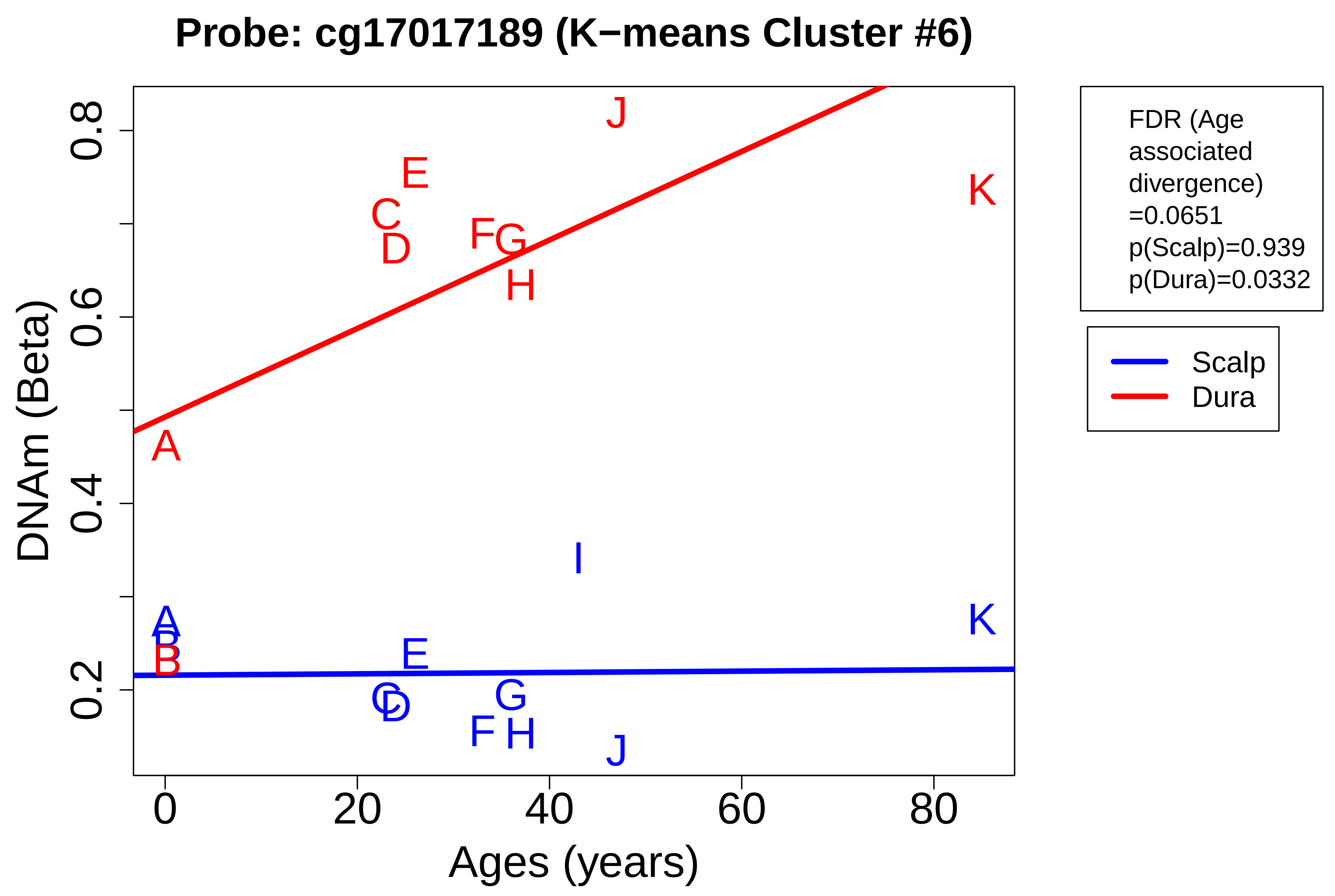
Probe: cg17017189 (K−means Cluster #6)
FDR (Age associated divergence) =0.0651
p(Scalp)=0.939
p(Dura)=0.0332
Scalp
Dura
DNAm (Beta)
Ages (years)
0.2
0.4
0.6
0.8
0
20
40
60
80

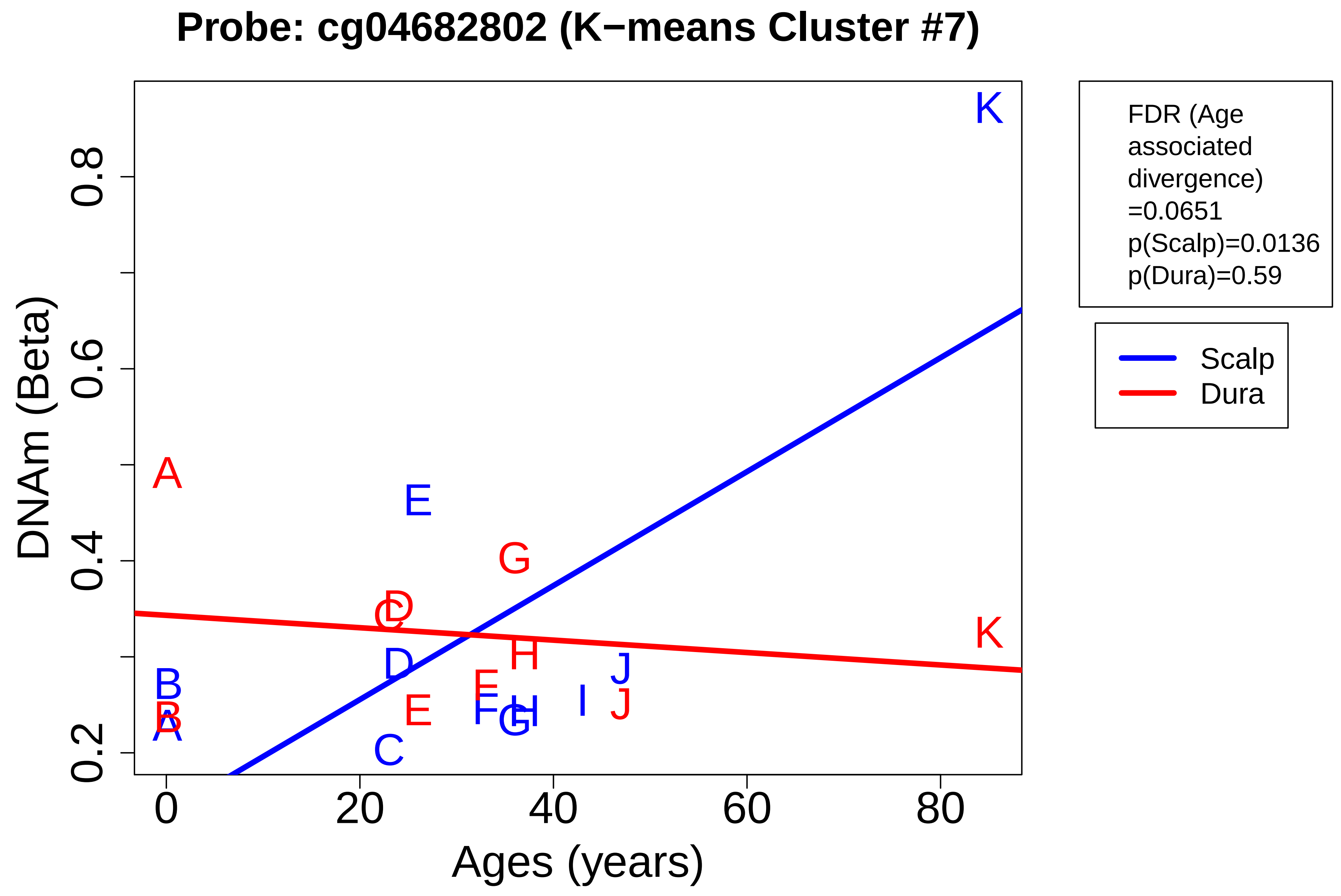
Probe: cg04682802 (K−means Cluster #7)
DNAm (Beta)
0.2
0.4
0.6
0.8
0
20
40
60
80
Ages (years)
A
B
C
D
E
F
G
H
I
J
K
FDR (Age associated divergence) =0.0651
p(Scalp)=0.0136
p(Dura)=0.59
Scalp
Dura

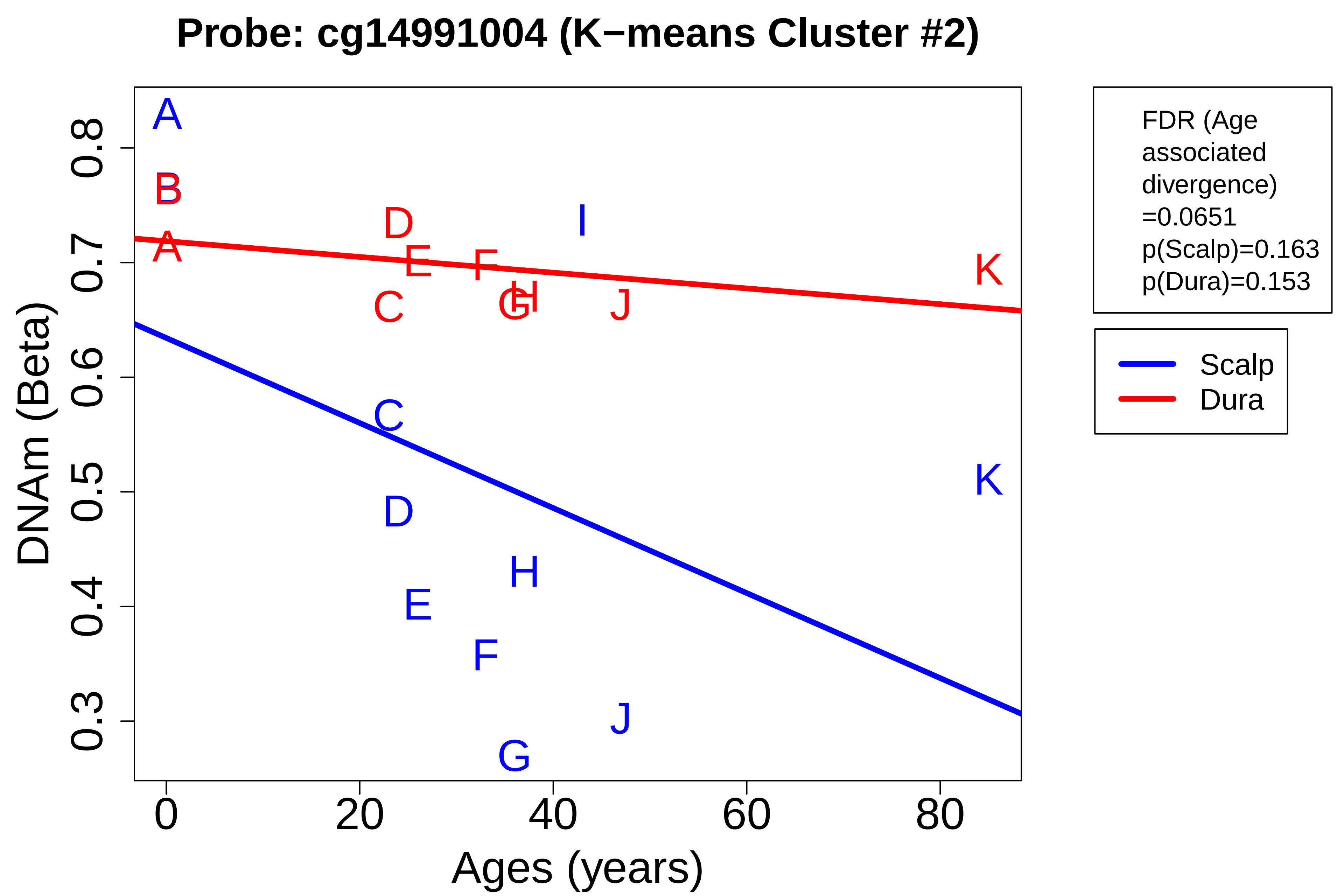

Probe: cg14991004 (K−means Cluster #2)
DNAm (Beta)
0.3
0.4
0.5
0.6
0.7
0.8
0
20
40
60
80
Ages (years)
A
B
C
D
E
F
G
H
I
J
K
FDR (Age associated divergence) =0.0651
p(Scalp)=0.163
p(Dura)=0.153
Scalp
Dura

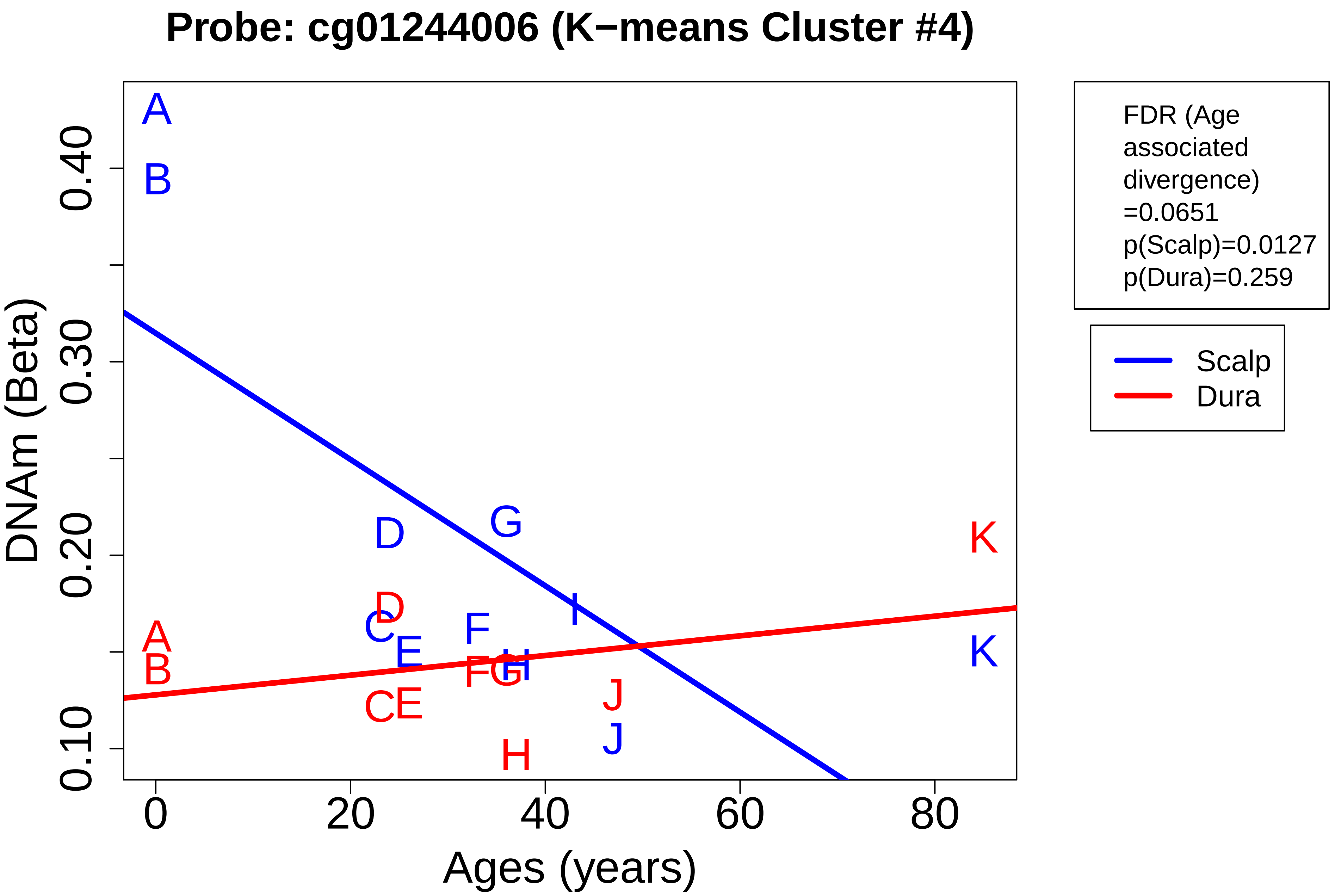

Probe: cg01244006 (K−means Cluster #4)
FDR (Age associated divergence) =0.0651
p(Scalp)=0.0127
p(Dura)=0.259
Scalp
Dura
DNAm (Beta)
0.10
0.20
0.30
0.40
0
20
40
60
80
Ages (years)

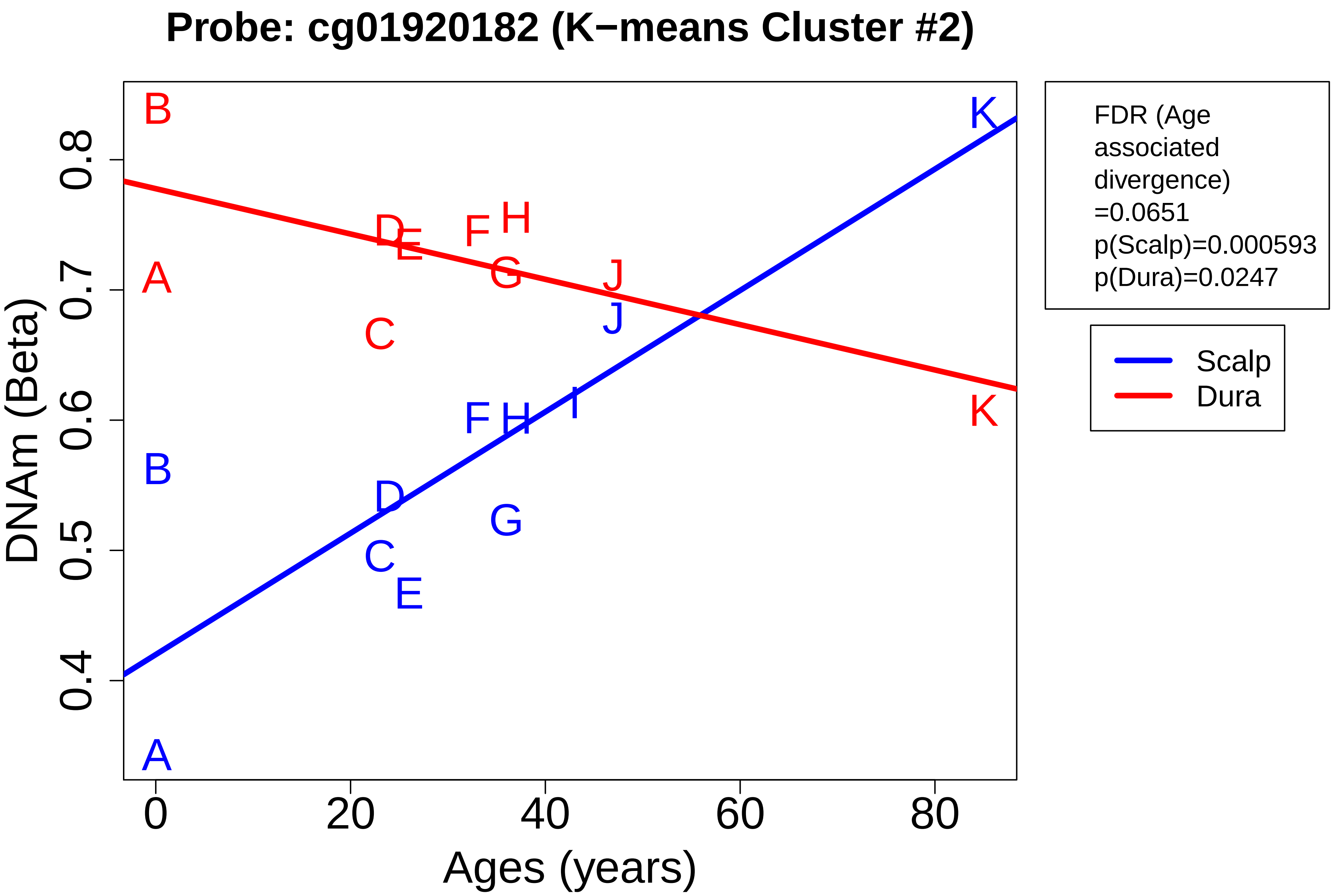

Probe: cg01920182 (K−means Cluster #2)
DNAm (Beta)
0.4
0.5
0.6
0.7
0.8
0
20
40
60
80
Ages (years)
B
A
C
D
E
F
H
G
J
K
J
I
F
H
B
D
G
C
E
A
K
FDR (Age associated divergence) =0.0651
p(Scalp)=0.000593
p(Dura)=0.0247
Scalp
Dura

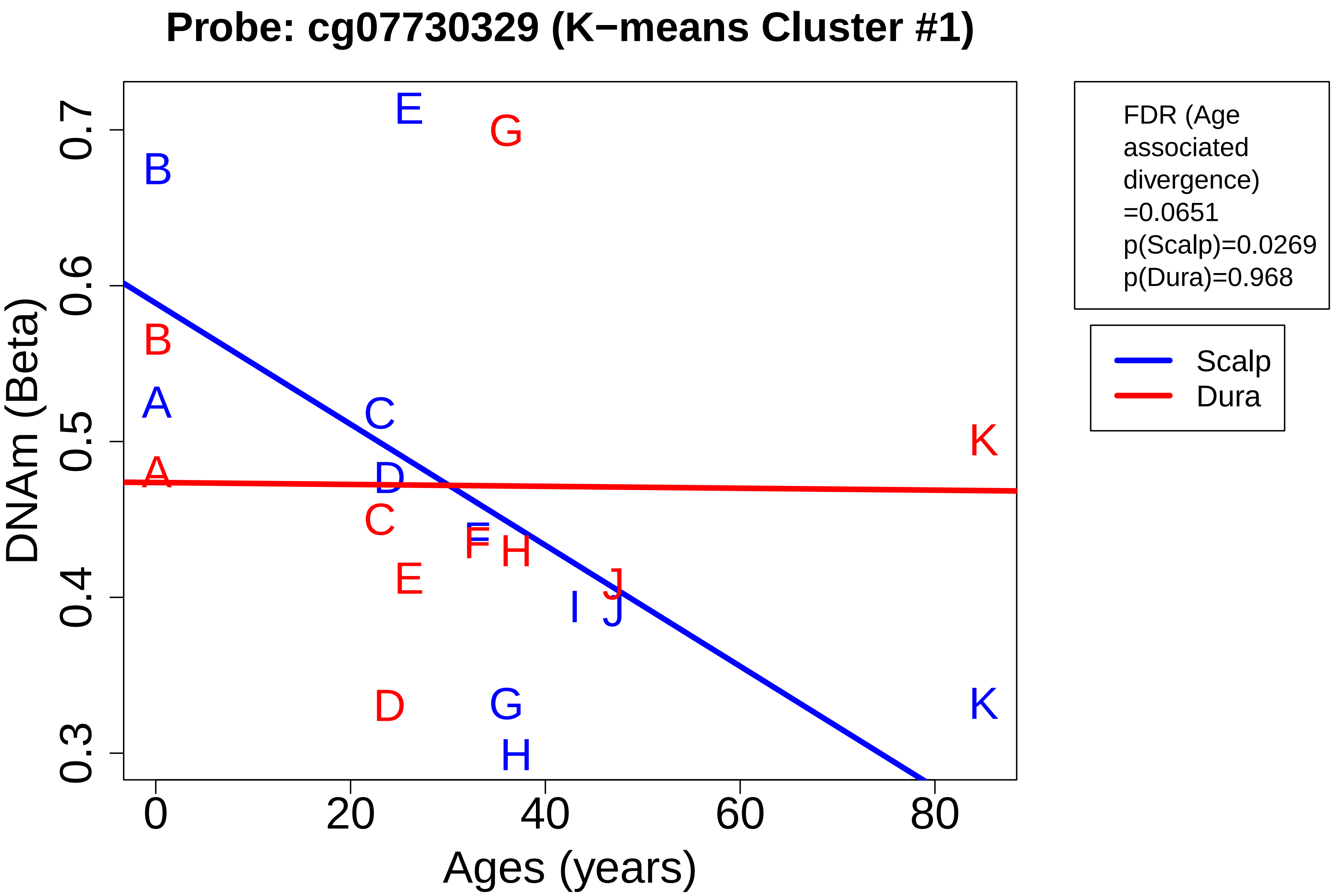
Probe: cg07730329 (K−means Cluster #1)
DNAm (Beta)
0.3
0.4
0.5
0.6
0.7
0
20
40
60
80
Ages (years)
FDR (Age associated divergence) =0.0651
p(Scalp)=0.0269
p(Dura)=0.968
Scalp
Dura
A
B
C
D
E
F
G
H
I
J
K

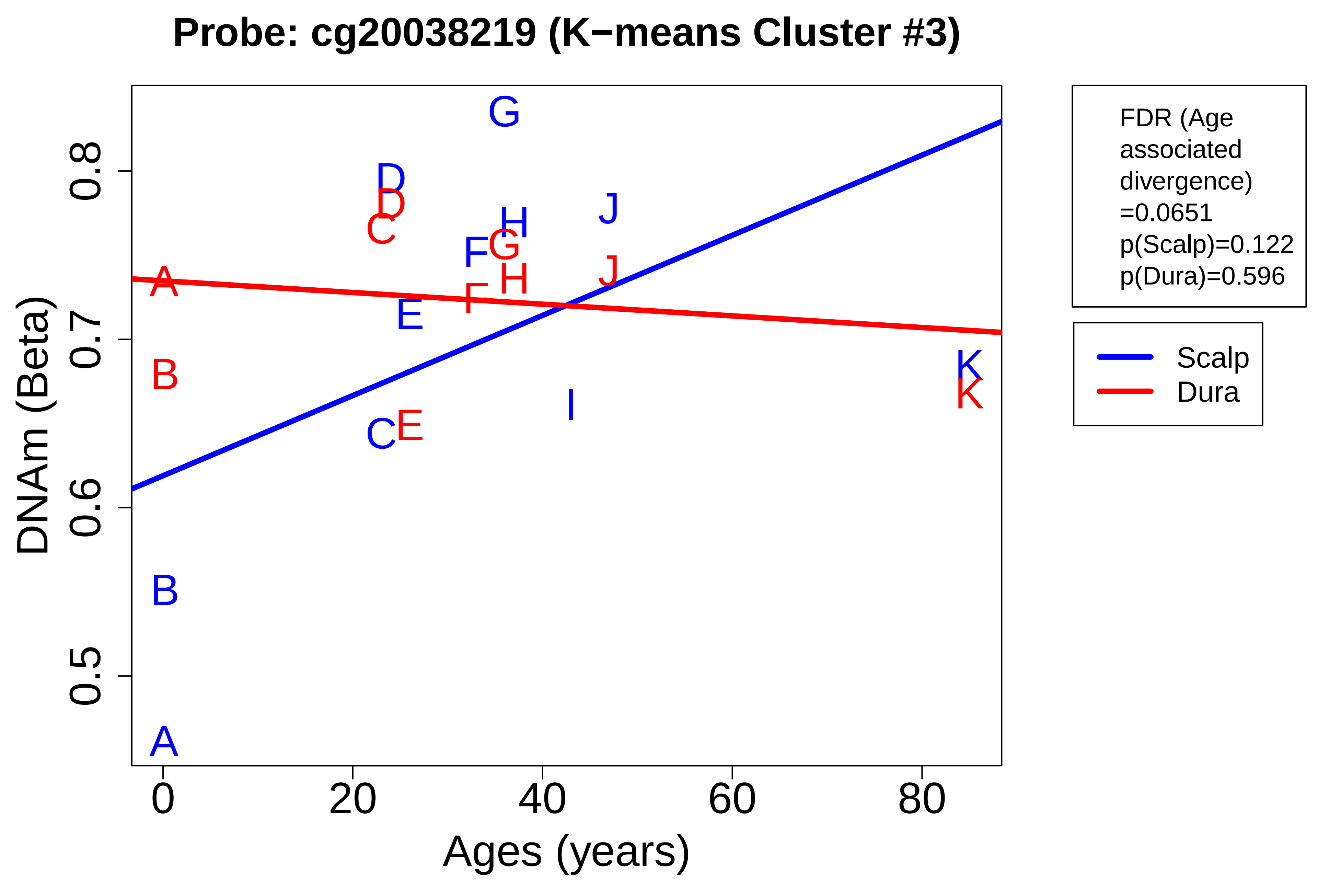
Probe: cg20038219 (K−means Cluster #3)
DNAm (Beta)
Ages (years)
0.5
0.6
0.7
0.8
0
20
40
60
80
FDR (Age associated divergence) =0.0651
p(Scalp)=0.122
p(Dura)=0.596
Scalp
Dura
A
B
C
D
E
F
G
H
I
J
K

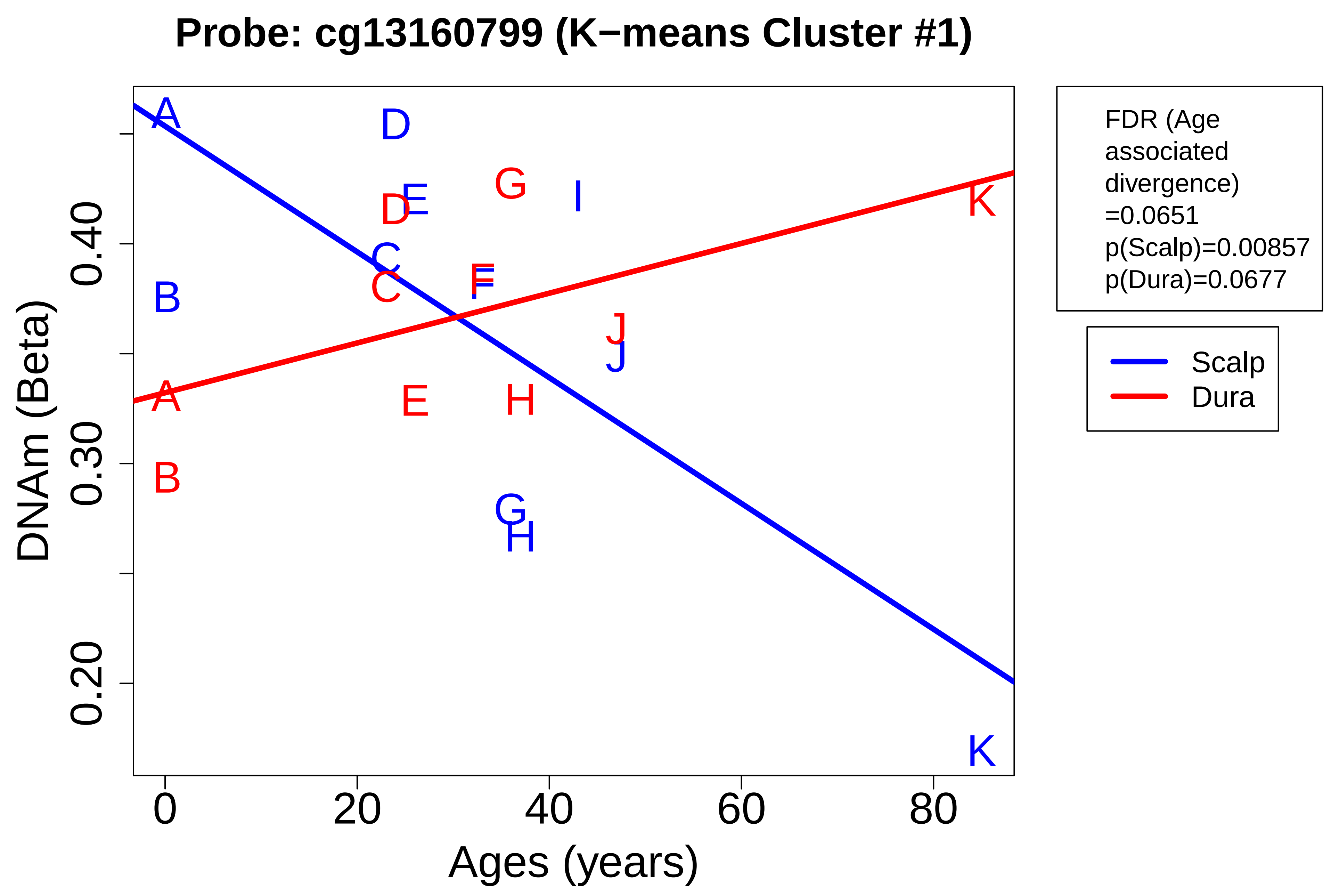
Probe: cg13160799 (K−means Cluster #1)
DNAm (Beta)
0.20
0.30
0.40
0
20
40
60
80
Ages (years)
A
B
C
D
E
F
G
H
I
J
K
FDR (Age associated divergence) =0.0651
p(Scalp)=0.00857
p(Dura)=0.0677
Scalp
Dura

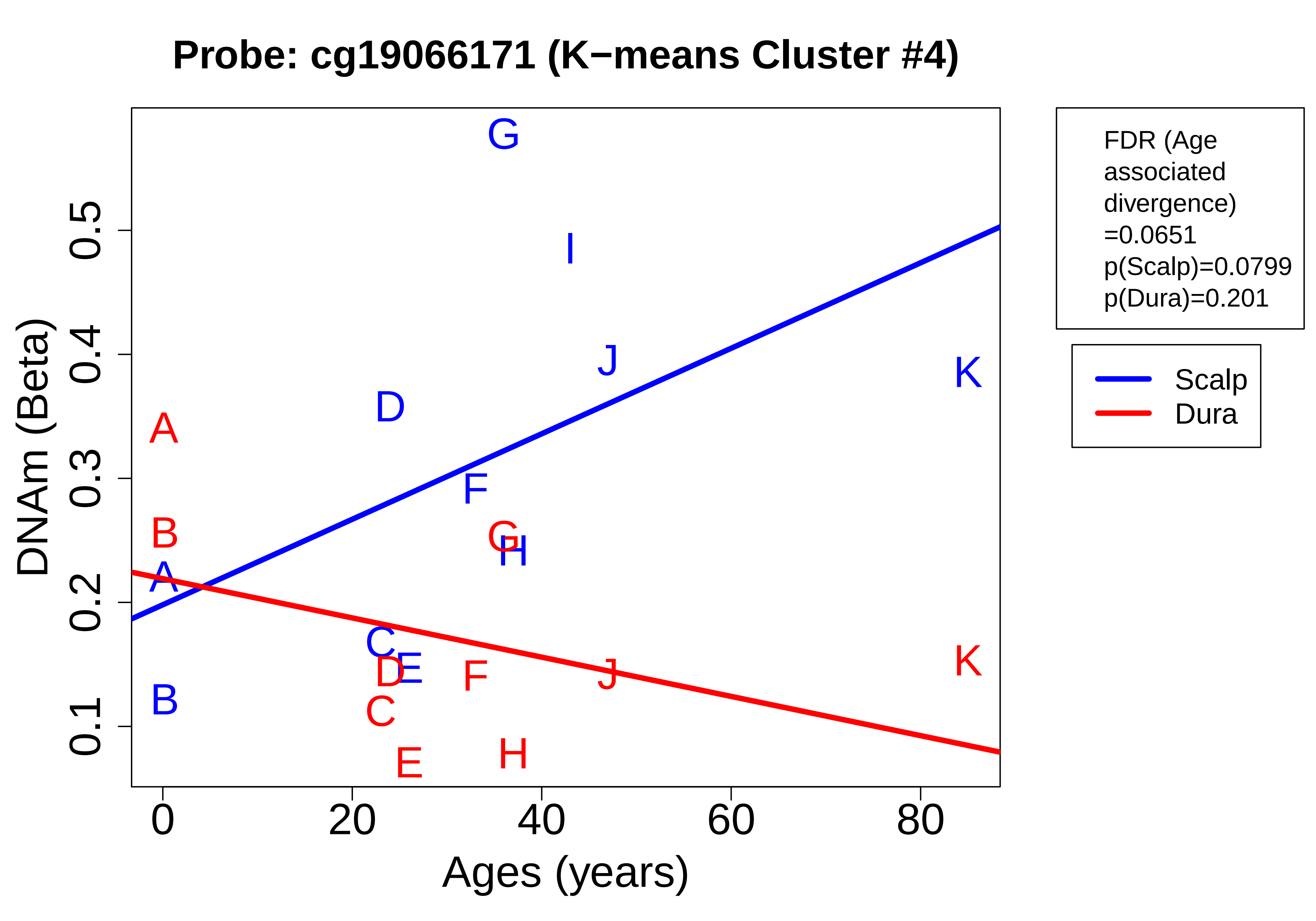
Probe: cg19066171 (K−means Cluster #4)
DNAm (Beta)
0.1
0.2
0.3
0.4
0.5
0
20
40
60
80
Ages (years)
FDR (Age associated divergence) =0.0651
p(Scalp)=0.0799
p(Dura)=0.201
Scalp
Dura

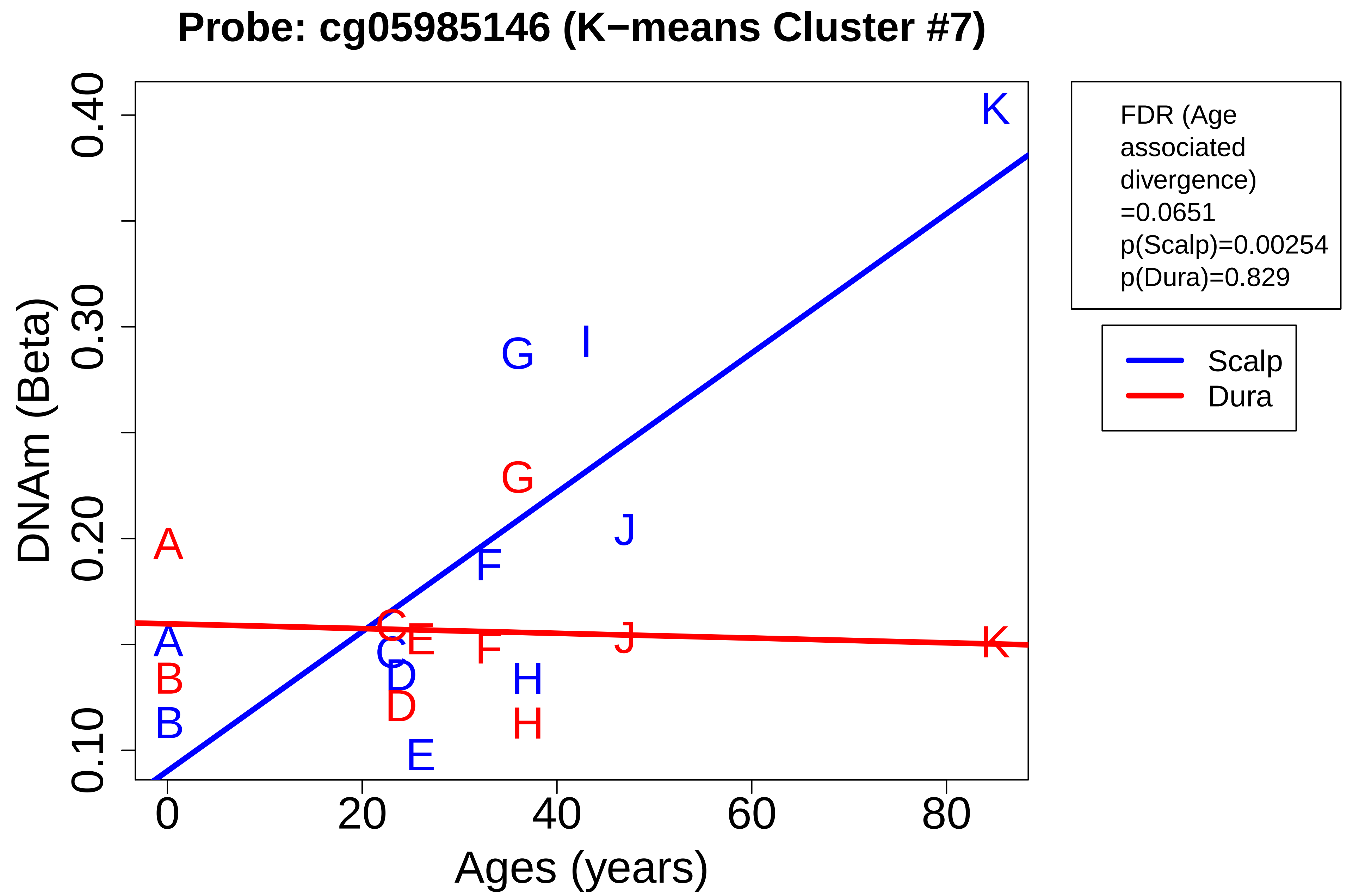

Probe: cg05985146 (K−means Cluster #7)
DNAm (Beta)
0.10
0.20
0.30
0.40
0
20
40
60
80
Ages (years)
FDR (Age associated divergence) =0.0651
p(Scalp)=0.00254
p(Dura)=0.829
Scalp
Dura
A
B
C
D
E
F
G
H
I
J
K

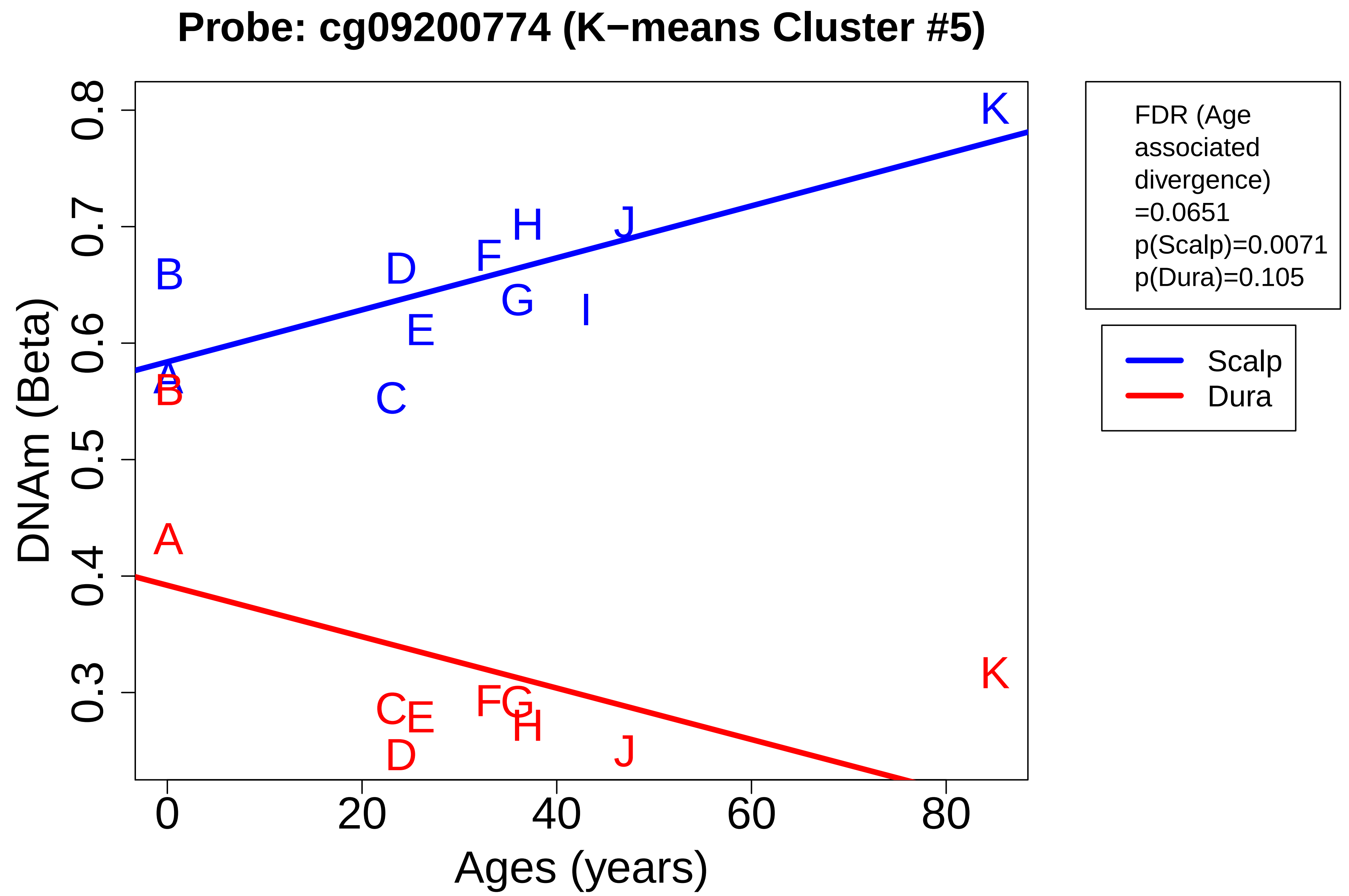
Probe: cg09200774 (K−means Cluster #5)
DNAm (Beta)
0.3
0.4
0.5
0.6
0.7
0.8
Ages (years)
0
20
40
60
80
FDR (Age associated divergence) =0.0651
p(Scalp)=0.0071
p(Dura)=0.105
Scalp
Dura
A
B
C
D
E
F
G
H
I
J
K

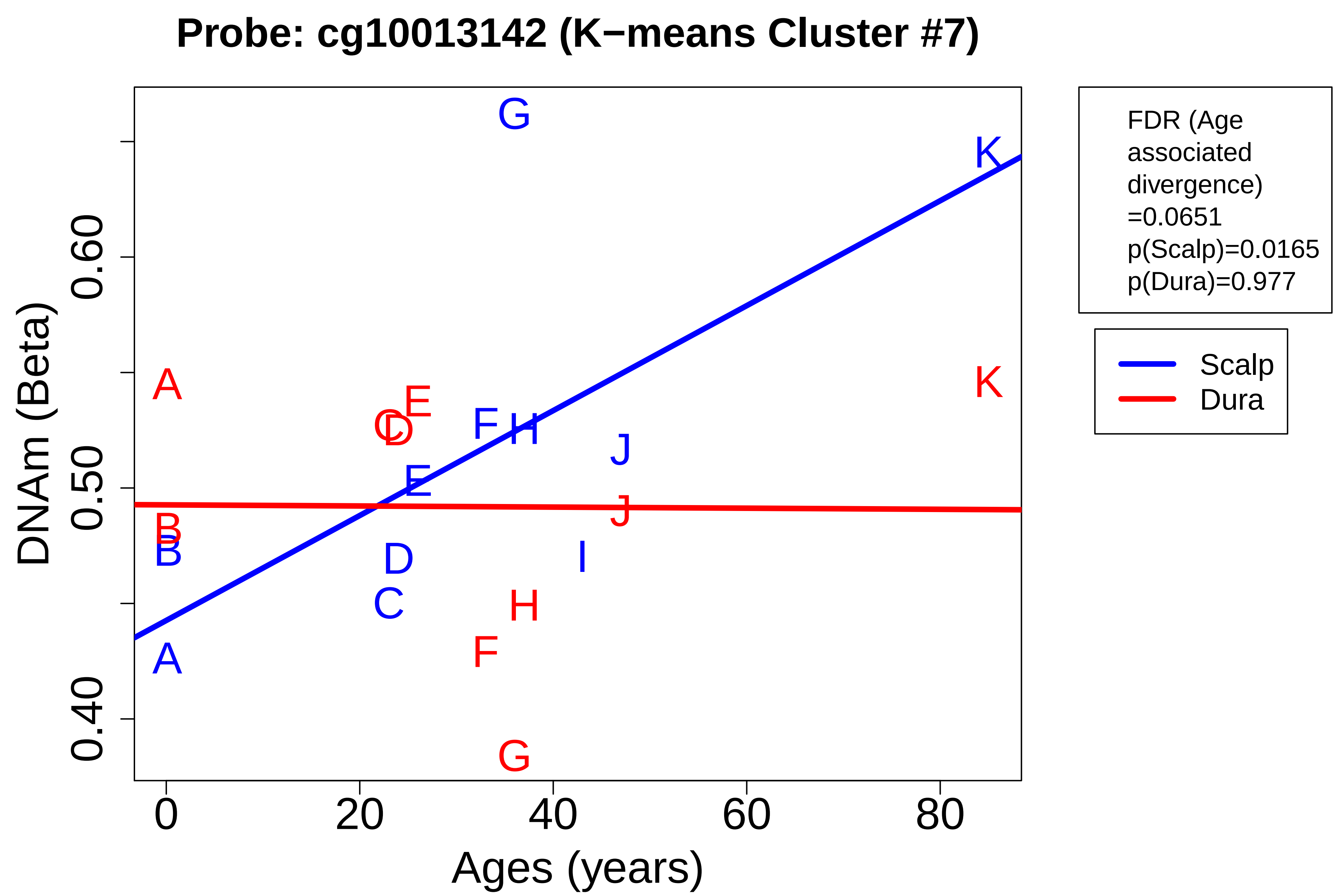
Probe: cg10013142 (K−means Cluster #7)
FDR (Age associated divergence) =0.0651
p(Scalp)=0.0165
p(Dura)=0.977
Scalp
Dura
DNAm (Beta)
0.40
0.50
0.60
0
20
40
60
80
Ages (years)

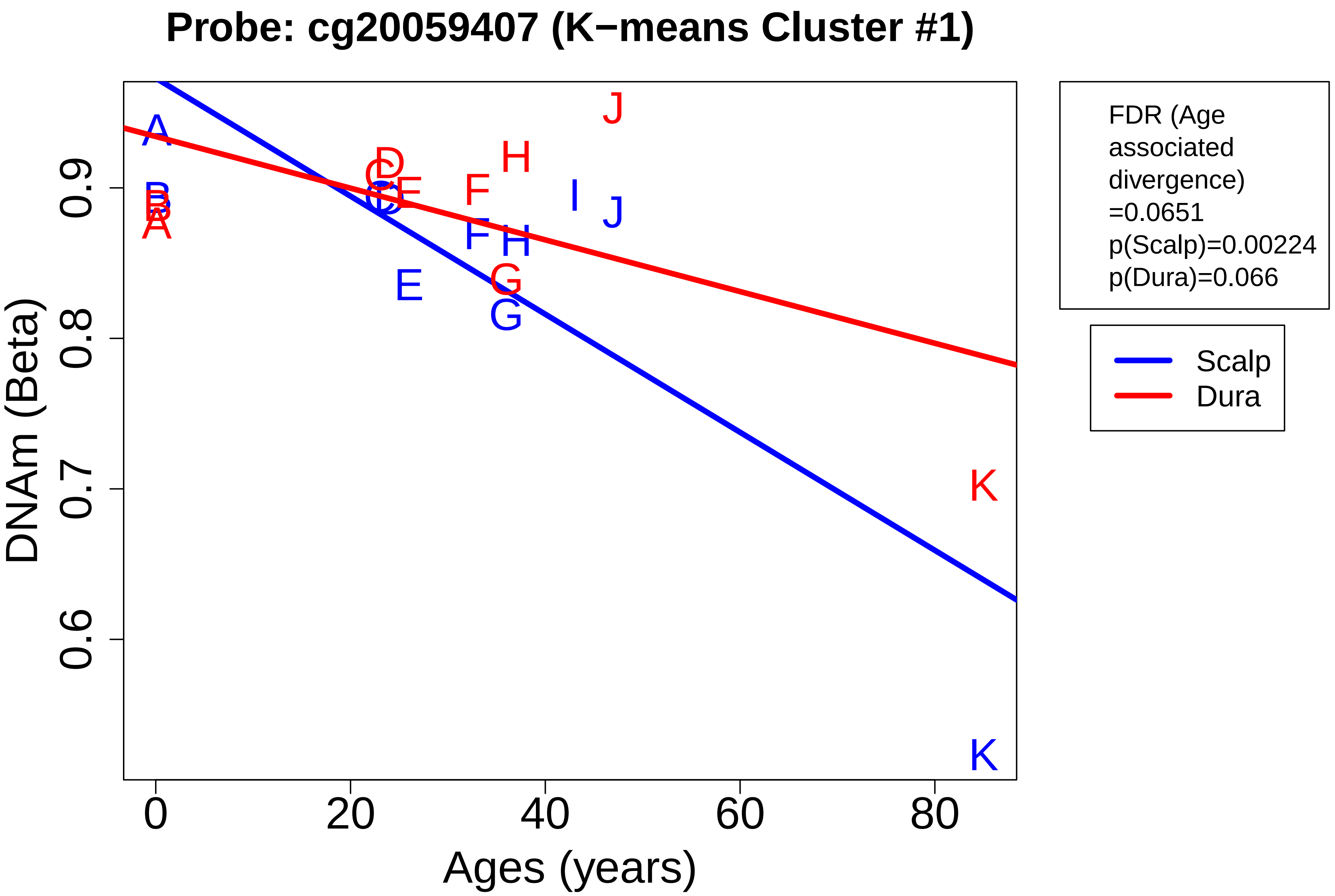

Probe: cg20059407 (K−means Cluster #1)
FDR (Age associated divergence) =0.0651
p(Scalp)=0.00224
p(Dura)=0.066
Scalp
Dura
DNAm (Beta)
0.6
0.7
0.8
0.9
Ages (years)
0
20
40
60
80

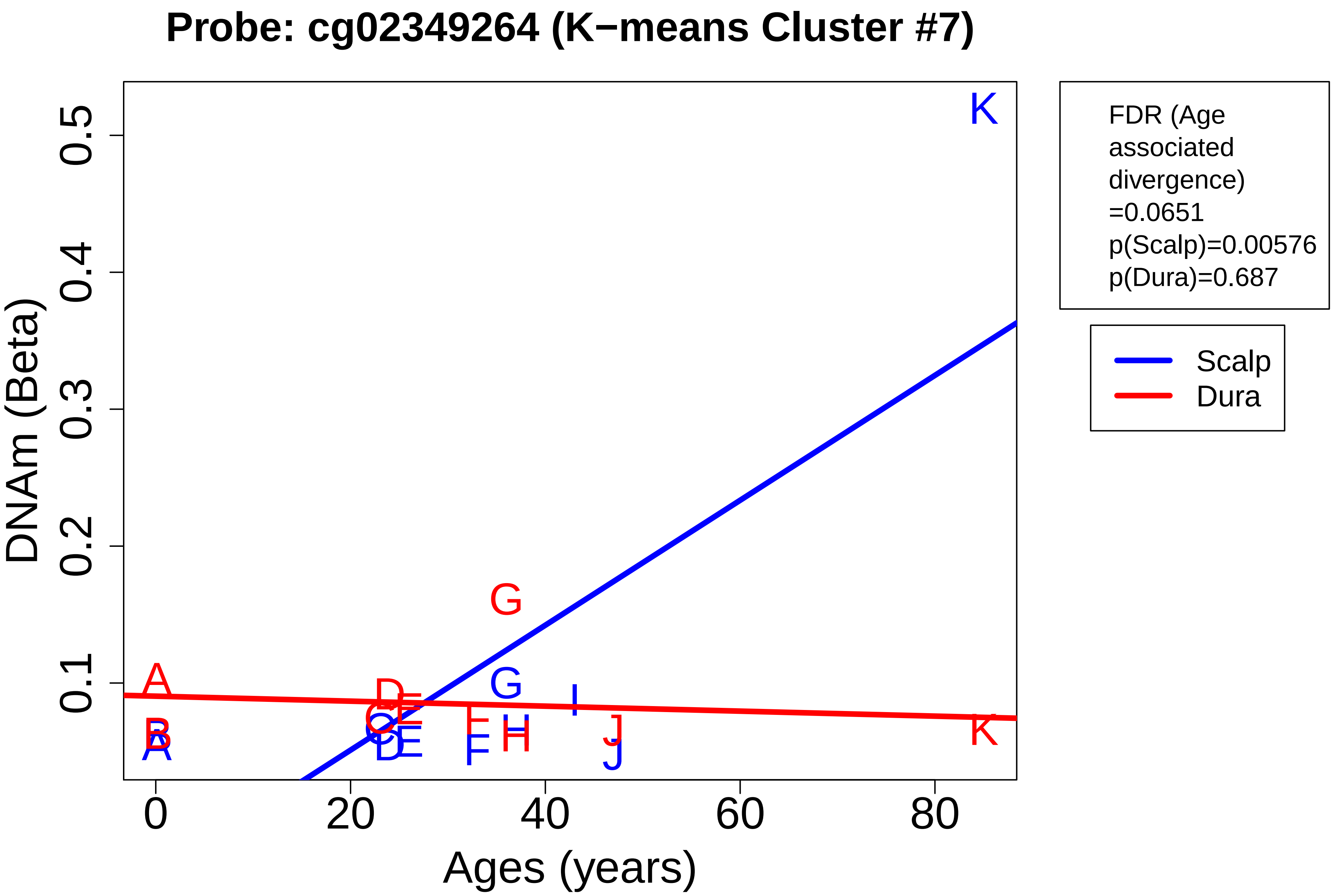

Probe: cg02349264 (K−means Cluster #7)
DNAm (Beta)
0.1
0.2
0.3
0.4
0.5
0
20
40
60
80
Ages (years)
FDR (Age associated divergence) =0.0651
p(Scalp)=0.00576
p(Dura)=0.687
Scalp
Dura

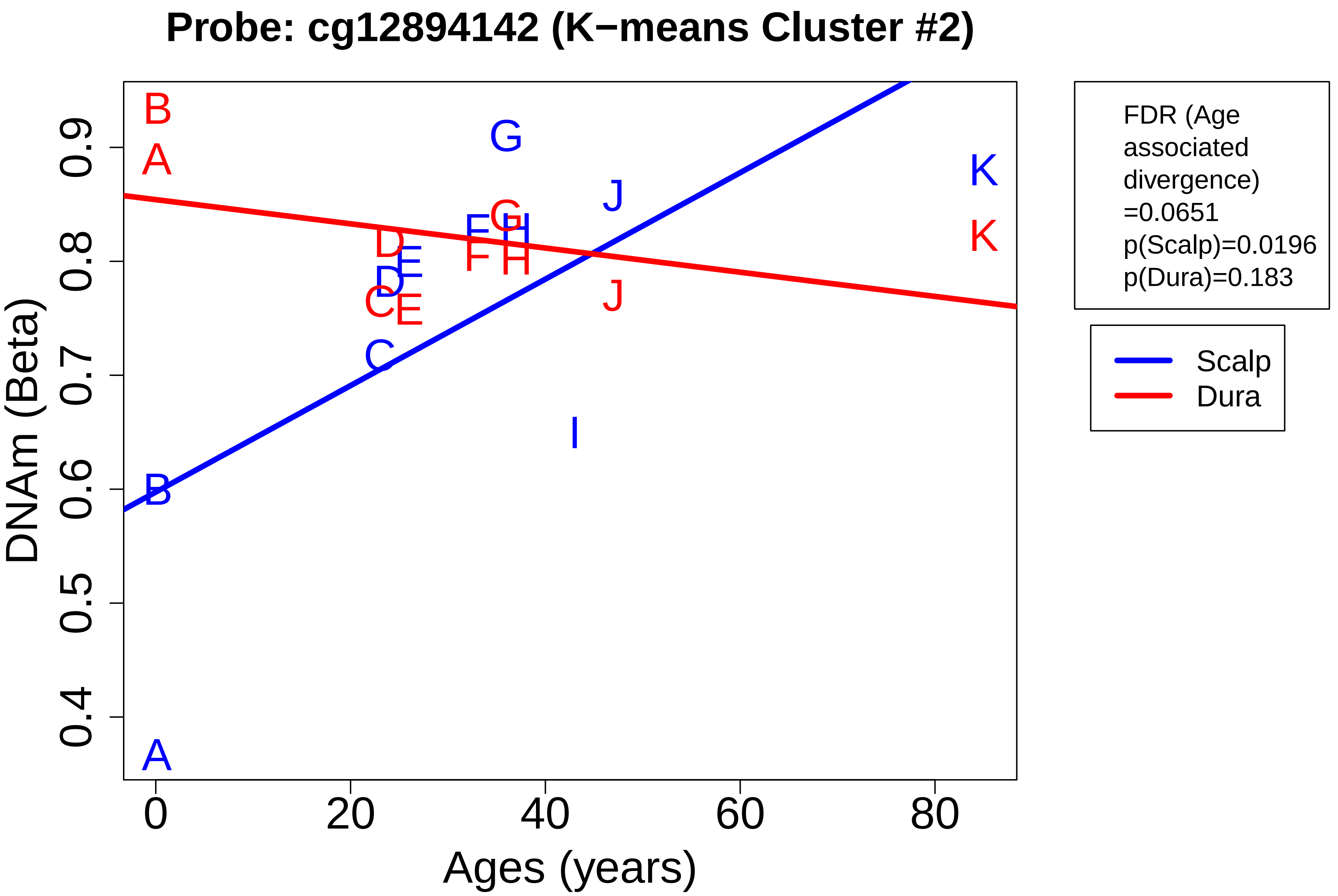
Probe: cg12894142 (K−means Cluster #2)
DNAm (Beta)
0.4
0.5
0.6
0.7
0.8
0.9
0
20
40
60
80
Ages (years)
FDR (Age associated divergence) =0.0651
p(Scalp)=0.0196
p(Dura)=0.183
Scalp
Dura

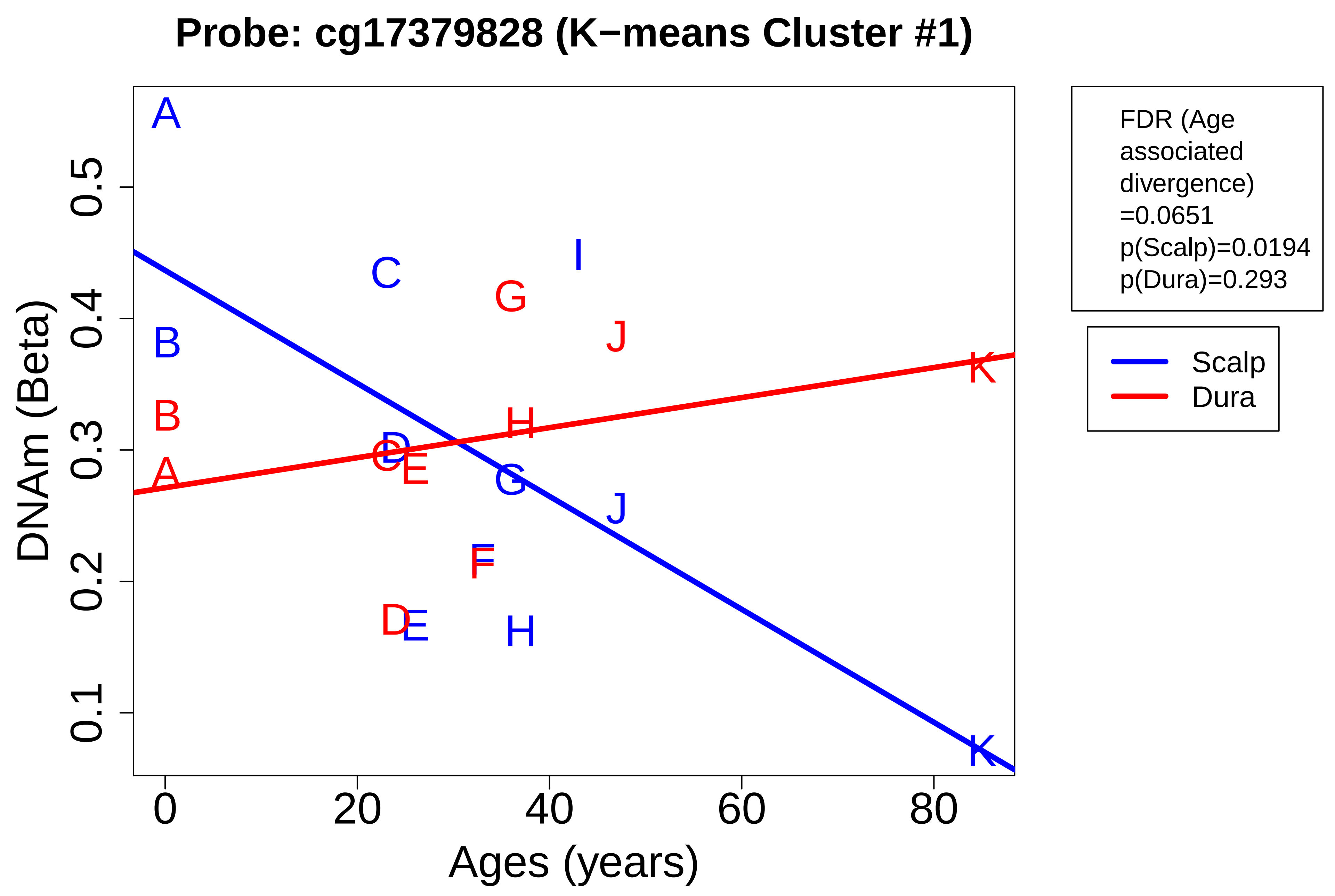

Probe: cg17379828 (K−means Cluster #1)
DNAm (Beta)
Ages (years)
0.1
0.2
0.3
0.4
0.5
0
20
40
60
80
A
B
C
D
E
F
G
H
I
J
K
FDR (Age associated divergence) =0.0651
p(Scalp)=0.0194
p(Dura)=0.293
Scalp
Dura

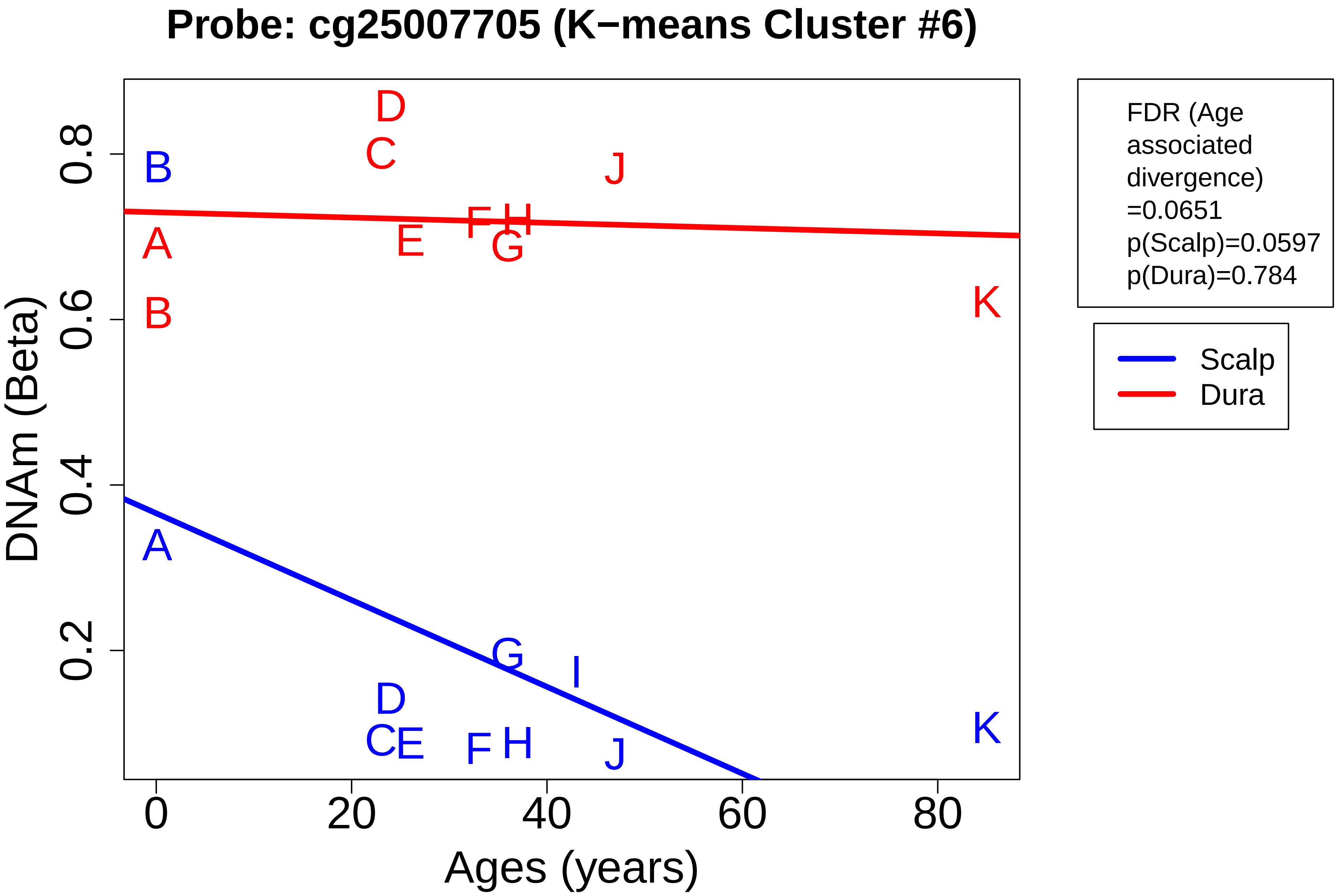

Probe: cg25007705 (K−means Cluster #6)
DNAm (Beta)
0.2
0.4
0.6
0.8
0
20
40
60
80
Ages (years)
FDR (Age associated divergence) =0.0651
p(Scalp)=0.0597
p(Dura)=0.784
Scalp
Dura

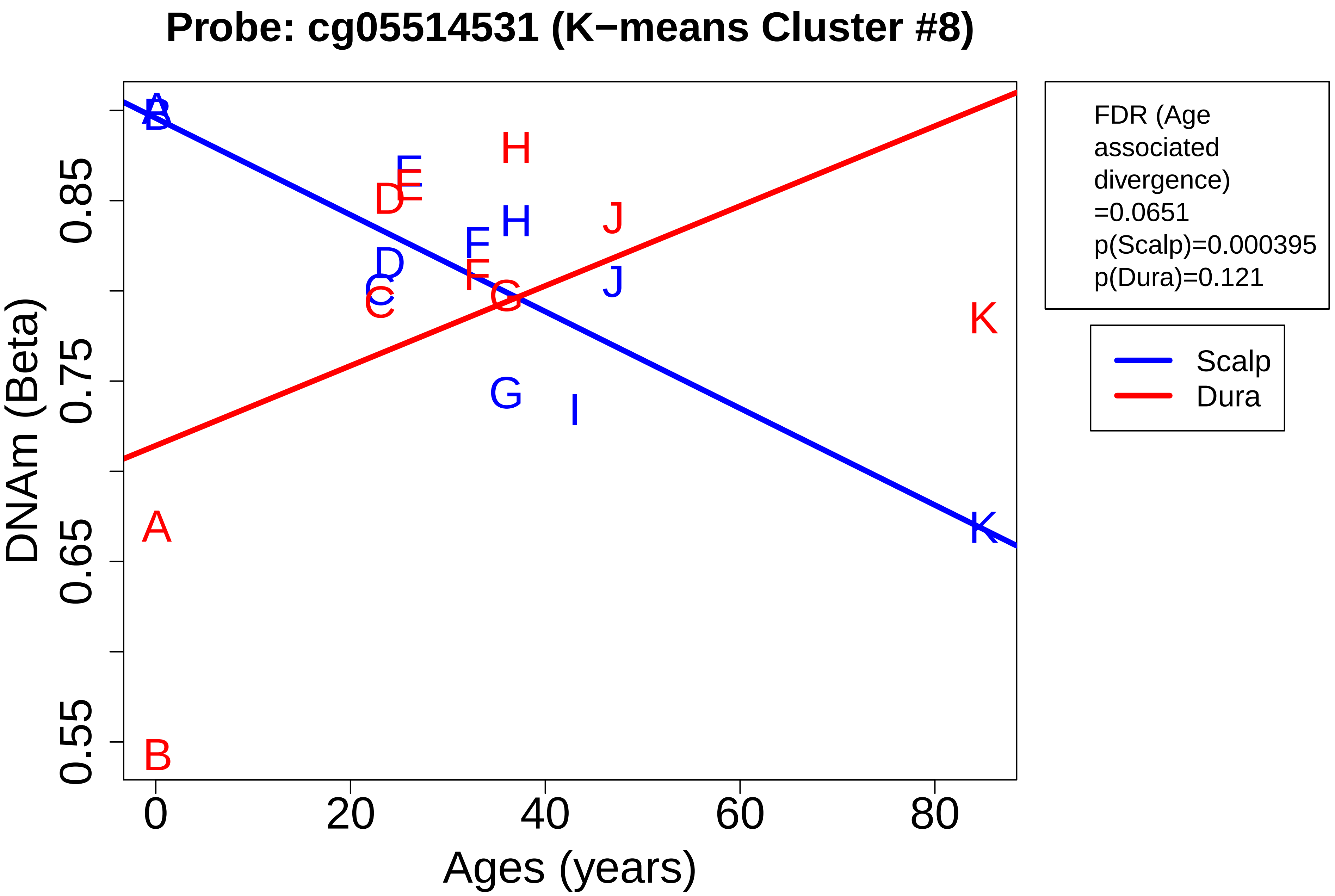

Probe: cg05514531 (K−means Cluster #8)
DNAm (Beta)
0.55
0.65
0.75
0.85
0
20
40
60
80
Ages (years)
FDR (Age associated divergence) =0.0651
p(Scalp)=0.000395
p(Dura)=0.121
Scalp
Dura
A
B
C
D
E
F
G
H
I
J
K

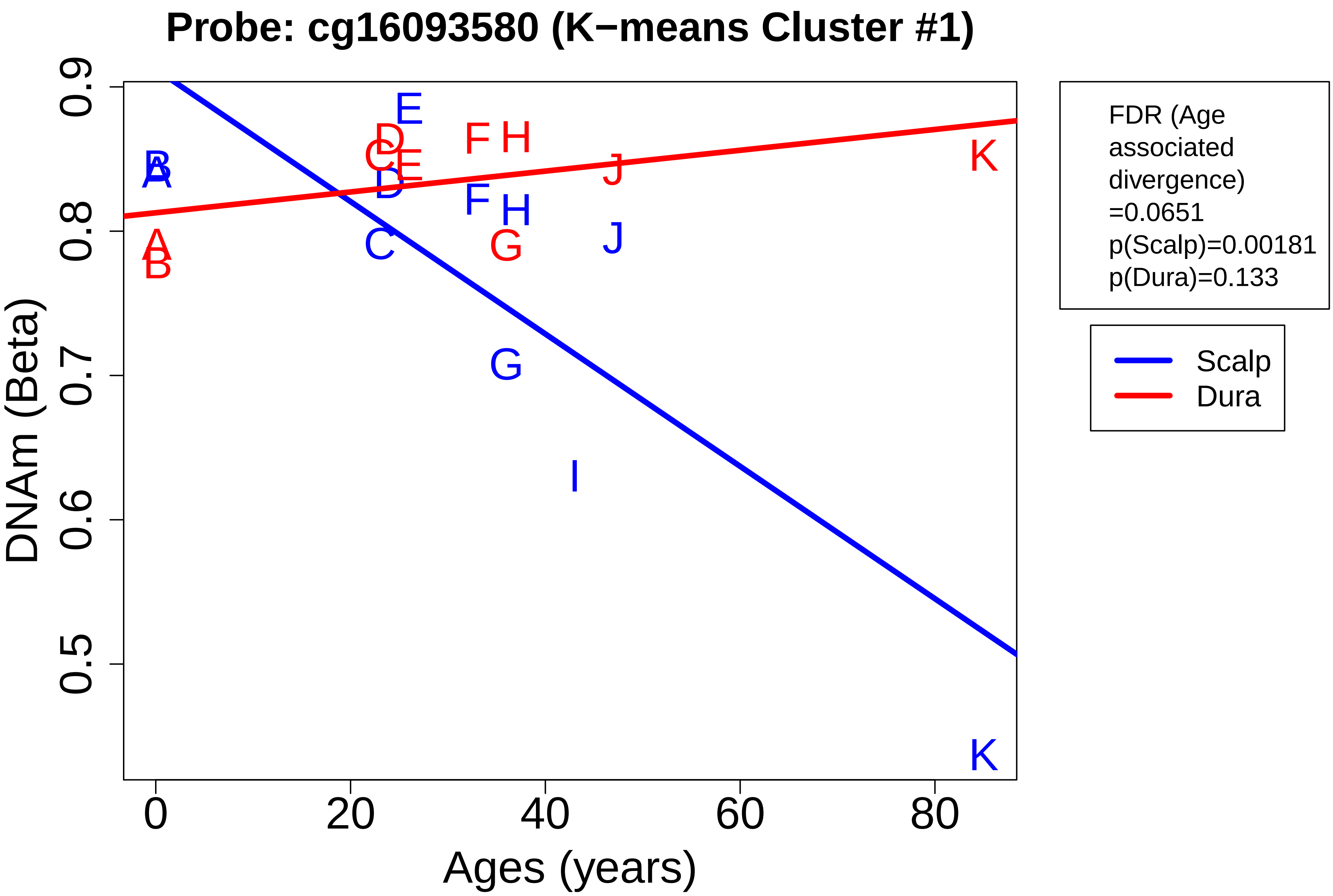
Probe: cg16093580 (K−means Cluster #1)
DNAm (Beta)
0.5
0.6
0.7
0.8
0.9
0
20
40
60
80
Ages (years)
FDR (Age associated divergence) =0.0651
p(Scalp)=0.00181
p(Dura)=0.133
Scalp
Dura

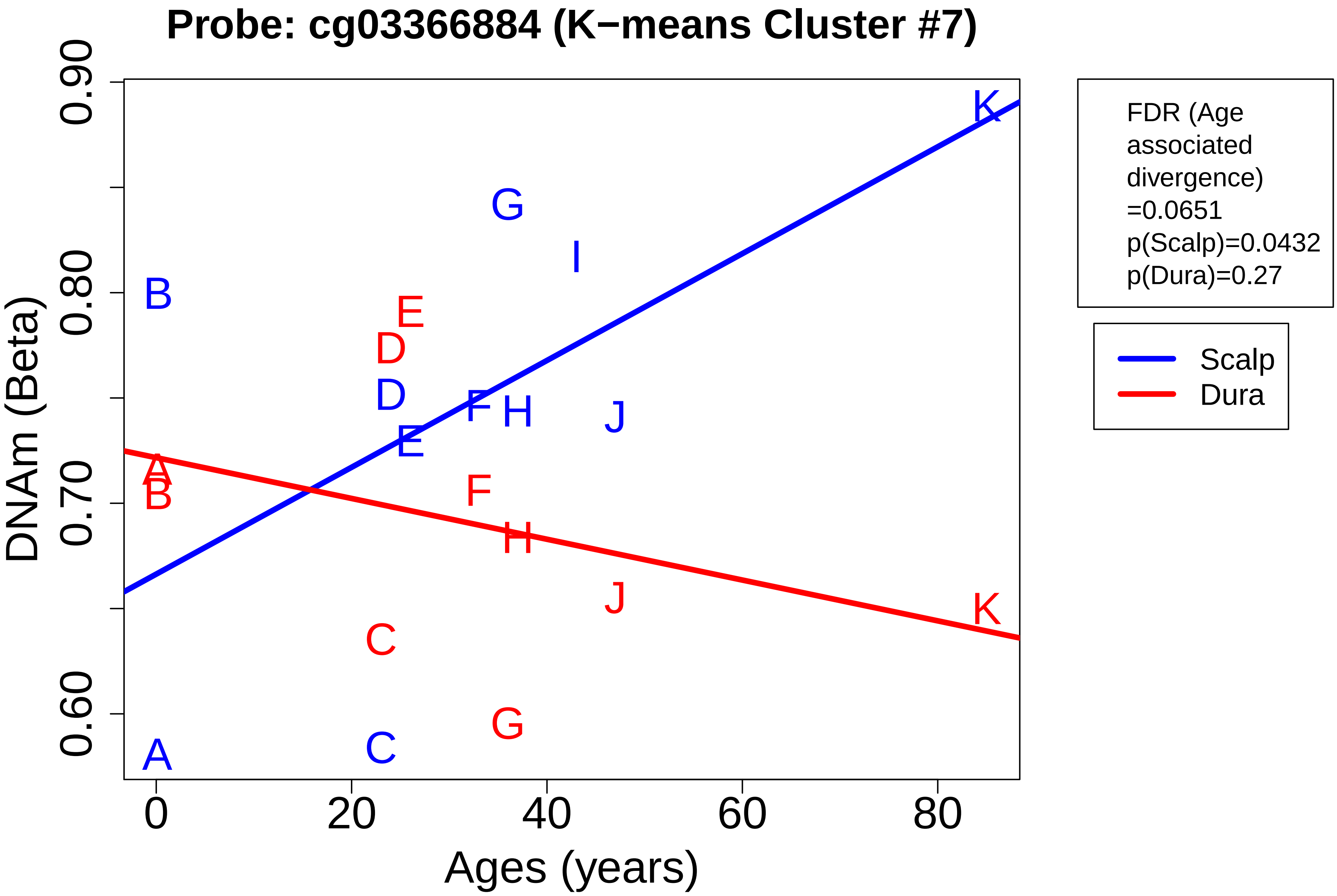
Probe: cg03366884 (K−means Cluster #7)
DNAm (Beta)
0.60
0.70
0.80
0.90
Ages (years)
0
20
40
60
80
FDR (Age associated divergence) =0.0651
p(Scalp)=0.0432
p(Dura)=0.27
Scalp
Dura
A
B
C
D
E
F
G
H
I
J
K

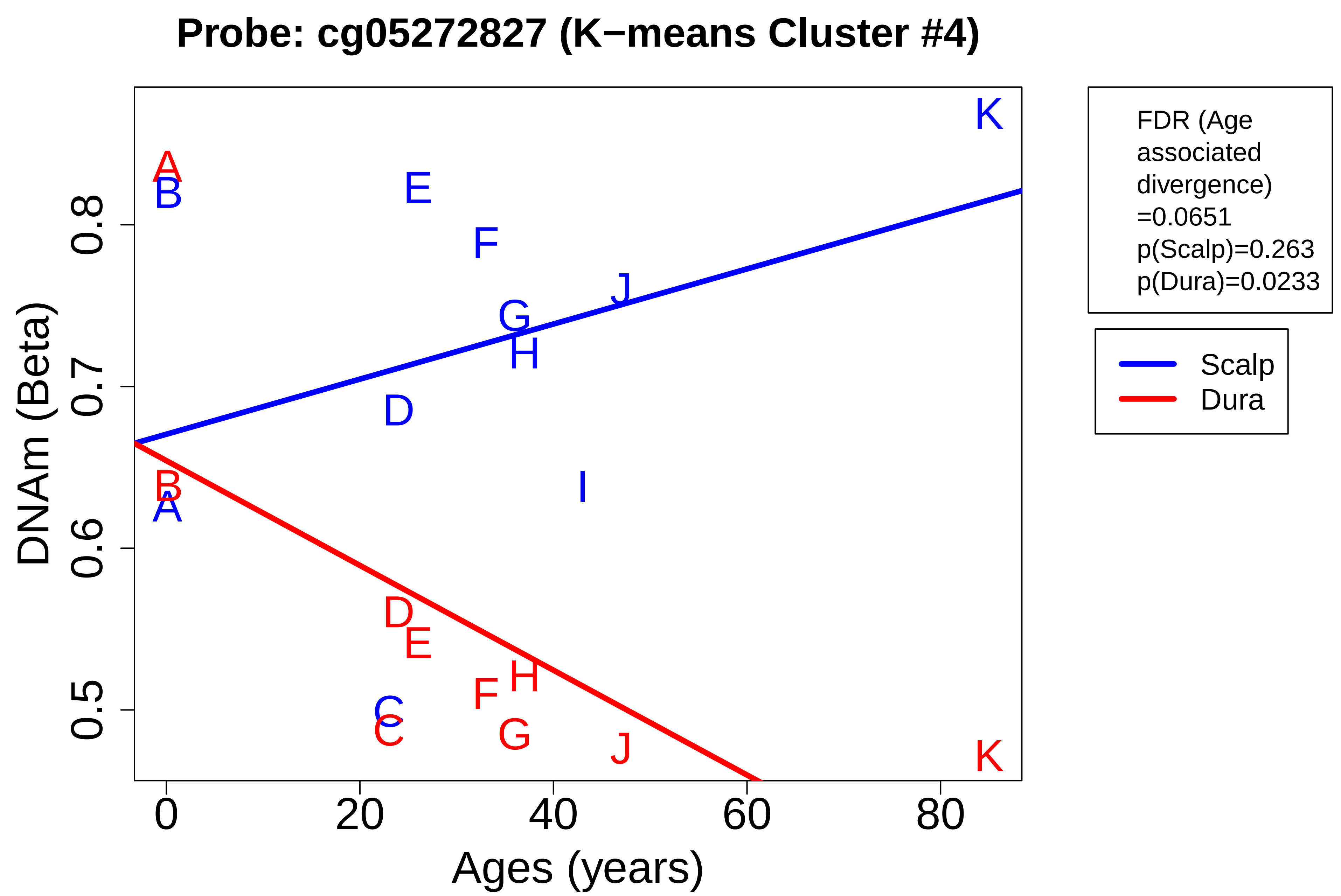

Probe: cg05272827 (K−means Cluster #4)
FDR (Age associated divergence) =0.0651
p(Scalp)=0.263
p(Dura)=0.0233
Scalp
Dura
DNAm (Beta)
0.5
0.6
0.7
0.8
Ages (years)
0
20
40
60
80
A
B
C
D
E
F
G
H
I
J
K

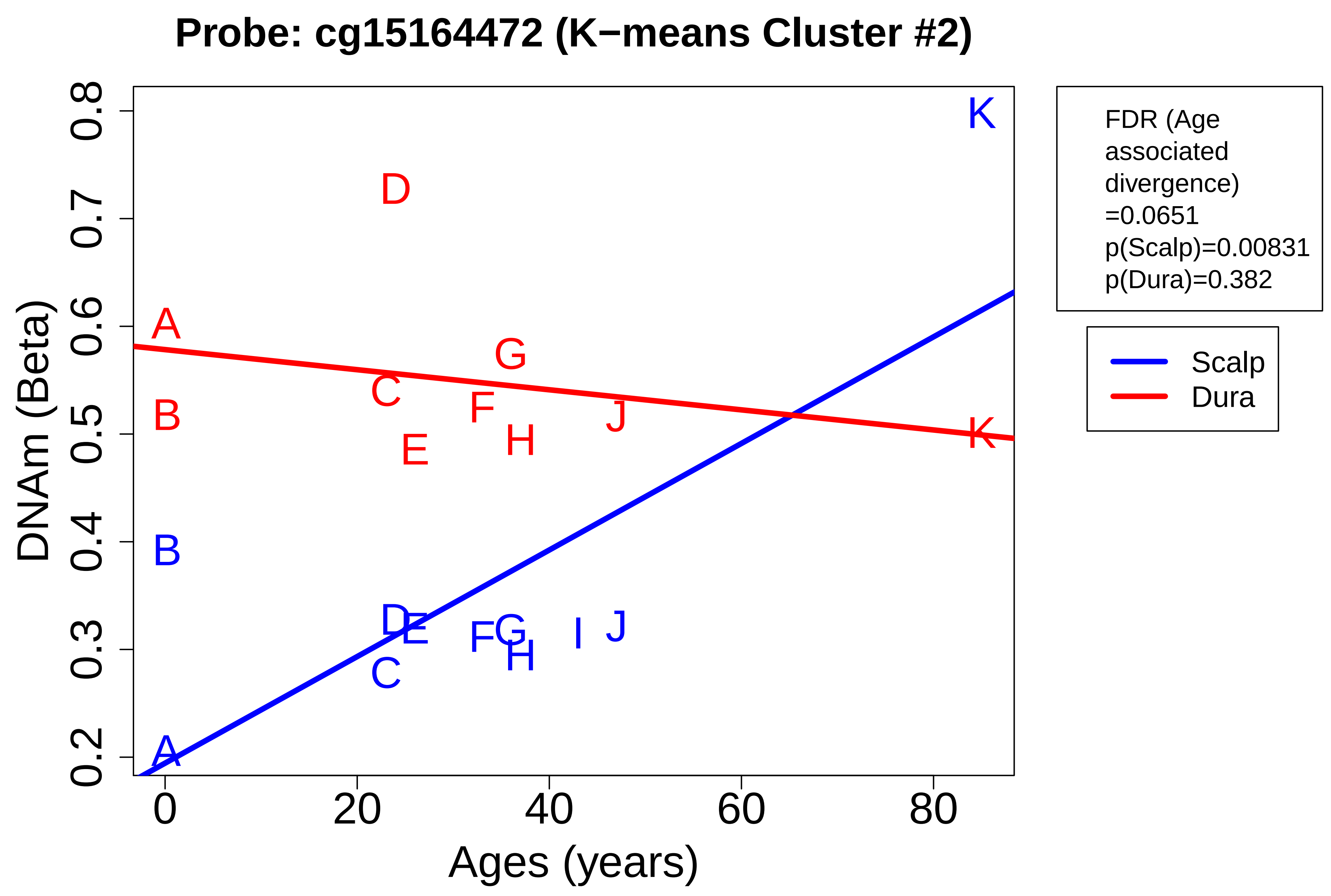
Probe: cg15164472 (K−means Cluster #2)
DNAm (Beta)
Ages (years)
0.2
0.3
0.4
0.5
0.6
0.7
0.8
0
20
40
60
80
FDR (Age associated divergence) =0.0651
p(Scalp)=0.00831
p(Dura)=0.382
Scalp
Dura

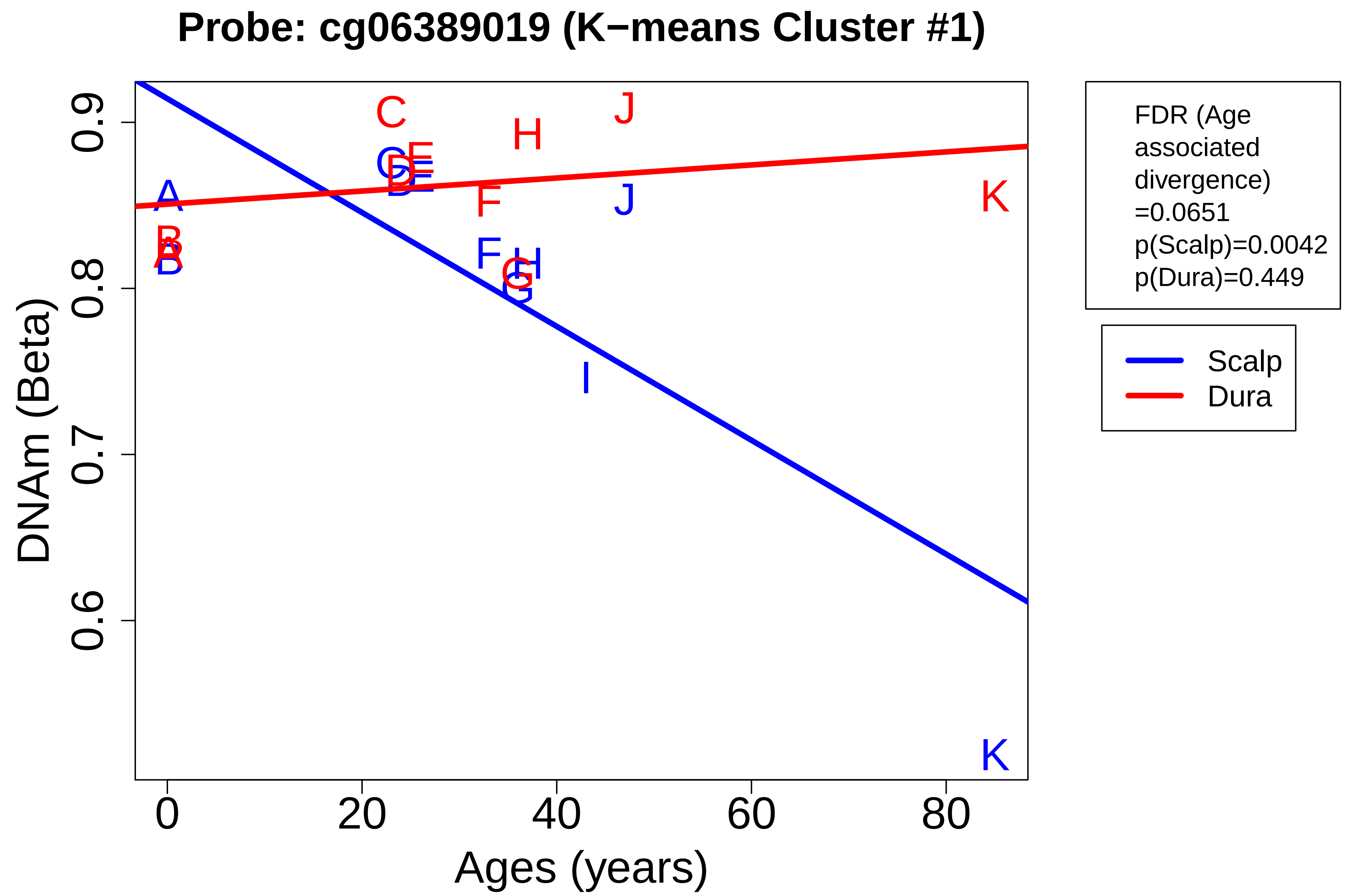

Probe: cg06389019 (K−means Cluster #1)
DNAm (Beta)
0.6
0.7
0.8
0.9
0
20
40
60
80
Ages (years)
FDR (Age associated divergence) =0.0651
p(Scalp)=0.0042
p(Dura)=0.449
Scalp
Dura

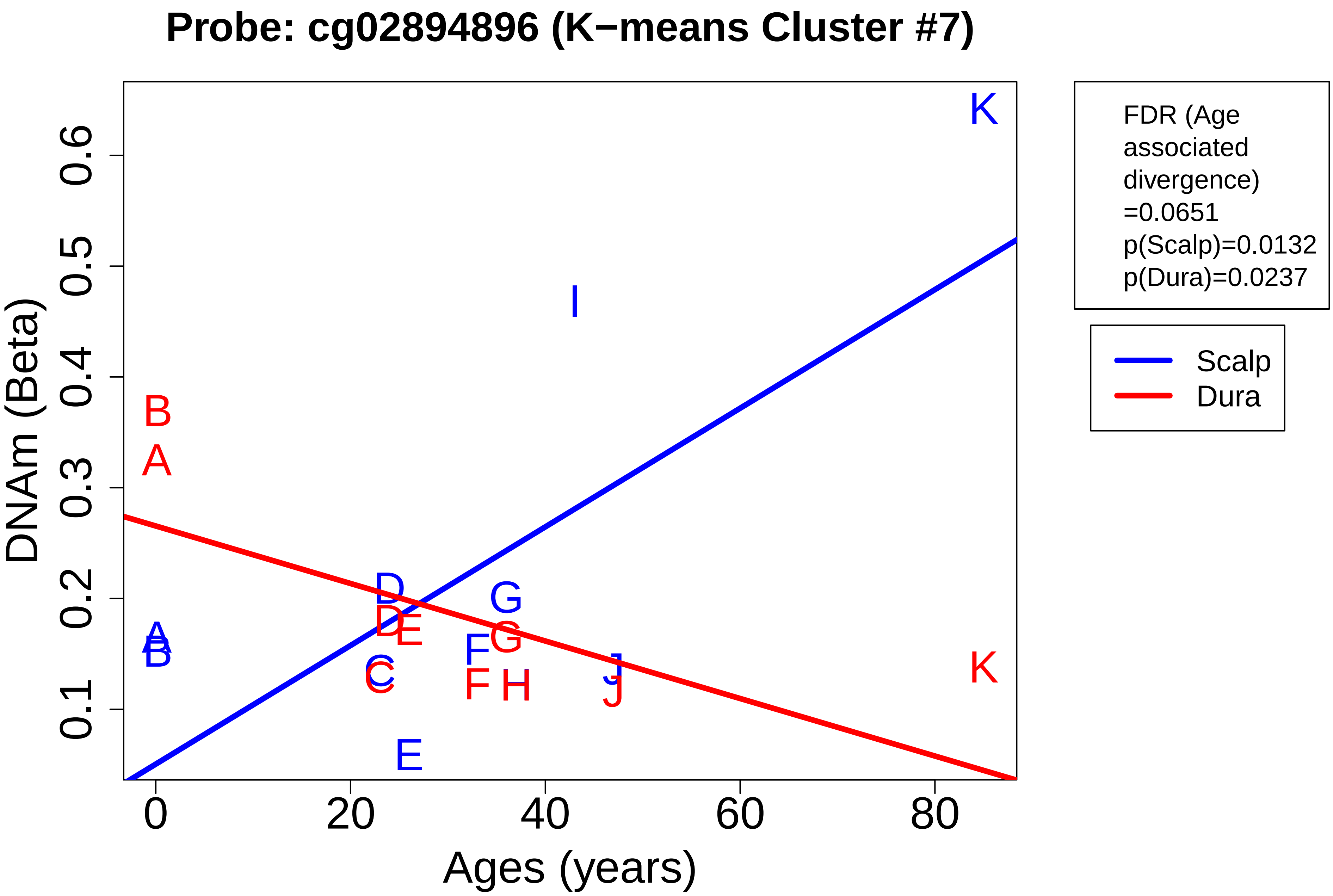
Probe: cg02894896 (K−means Cluster #7)
DNAm (Beta)
Ages (years)
0.1
0.2
0.3
0.4
0.5
0.6
0
20
40
60
80
FDR (Age associated divergence) =0.0651
p(Scalp)=0.0132
p(Dura)=0.0237
Scalp
Dura
A
B
C
D
E
F
G
H
I
J
K

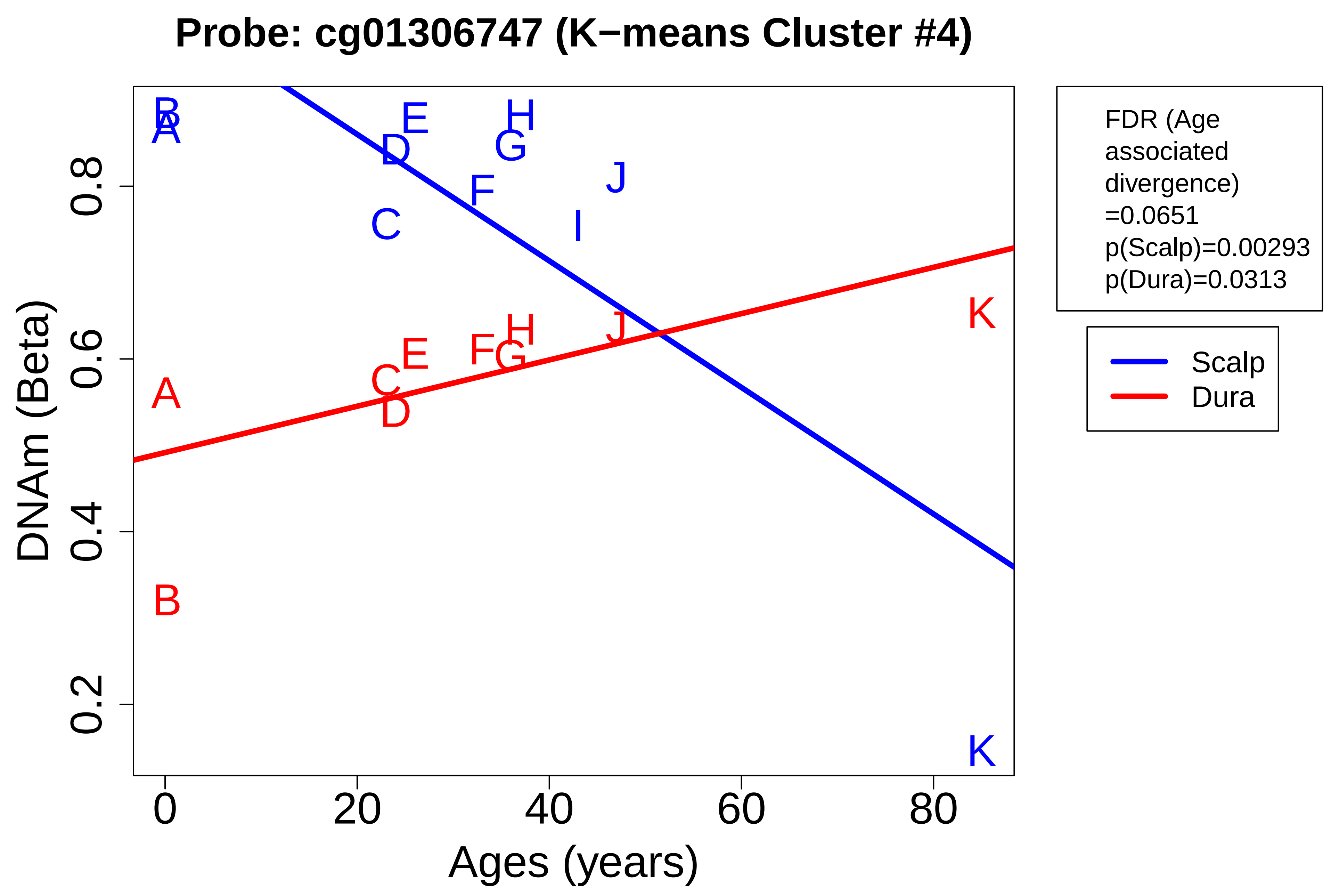

Probe: cg01306747 (K−means Cluster #4)
DNAm (Beta)
0.2
0.4
0.6
0.8
0
20
40
60
80
Ages (years)
FDR (Age associated divergence) =0.0651
p(Scalp)=0.00293
p(Dura)=0.0313
Scalp
Dura
A
B
C
D
E
F
G
H
I
J
K

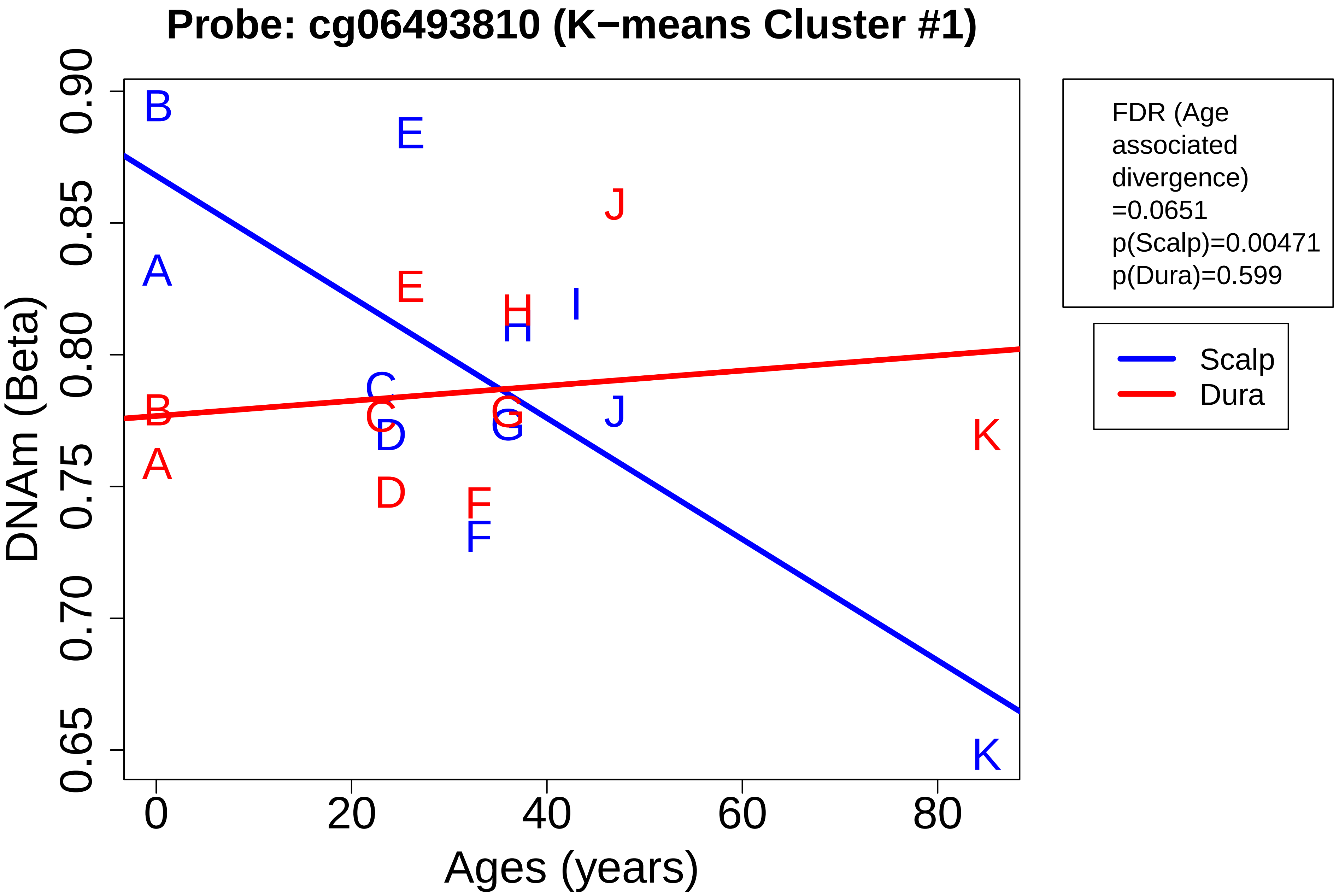

Probe: cg06493810 (K−means Cluster #1)
DNAm (Beta)
0.65
0.70
0.75
0.80
0.85
0.90
Ages (years)
0
20
40
60
80
FDR (Age associated divergence) =0.0651
p(Scalp)=0.00471
p(Dura)=0.599
Scalp
Dura
A
B
C
D
E
F
G
H
I
J
K

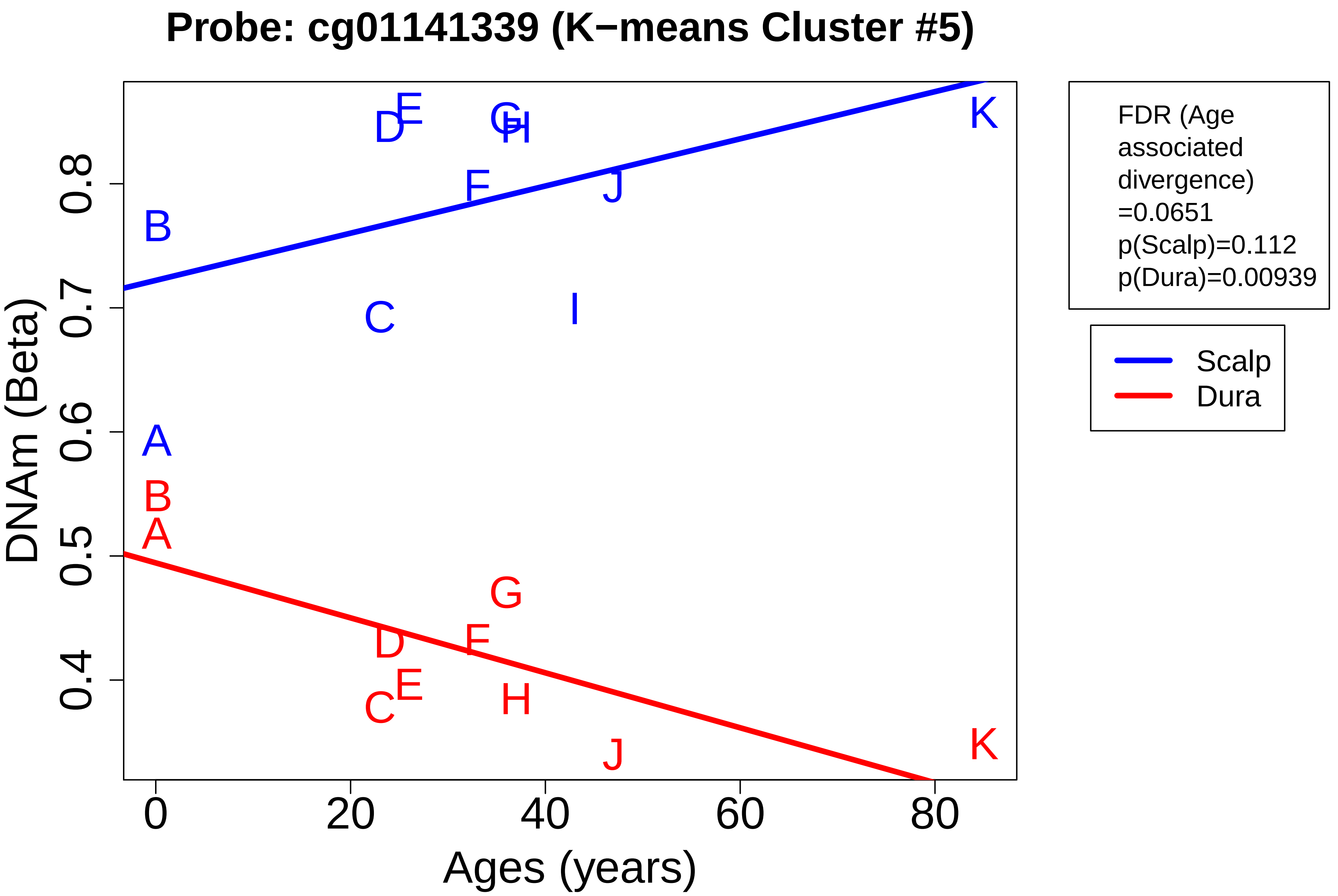
Probe: cg01141339 (K−means Cluster #5)
FDR (Age associated divergence) =0.0651
p(Scalp)=0.112
p(Dura)=0.00939
Scalp
Dura
DNAm (Beta)
0.4
0.5
0.6
0.7
0.8
Ages (years)
0
20
40
60
80
A
B
C
D
E
F
G
H
I
J
K

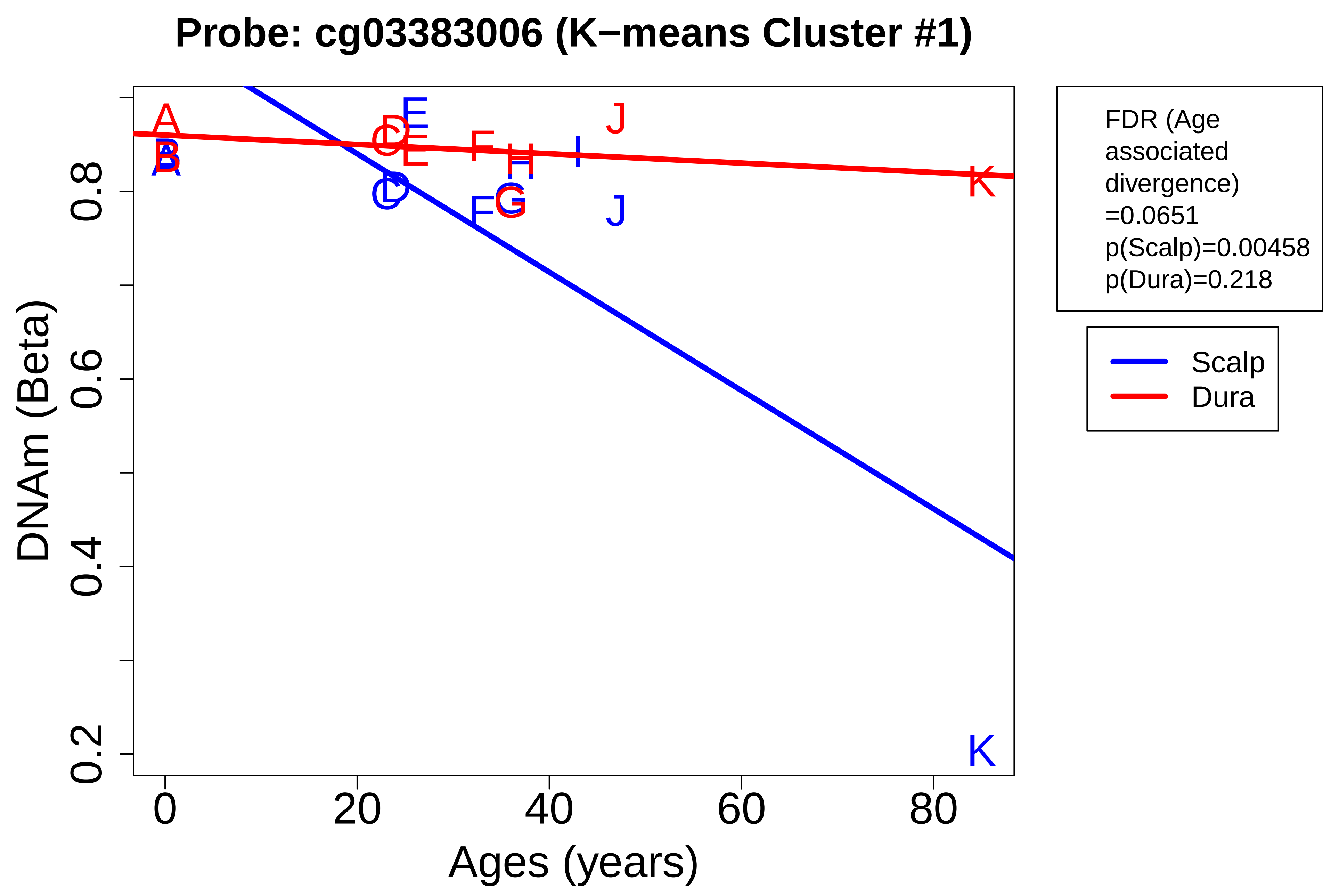

Probe: cg03383006 (K−means Cluster #1)
FDR (Age associated divergence) =0.0651
p(Scalp)=0.00458
p(Dura)=0.218
Scalp
Dura
DNAm (Beta)
0.2
0.4
0.6
0.8
0
20
40
60
80
Ages (years)
A
B
C
D
E
F
G
H
I
J
K

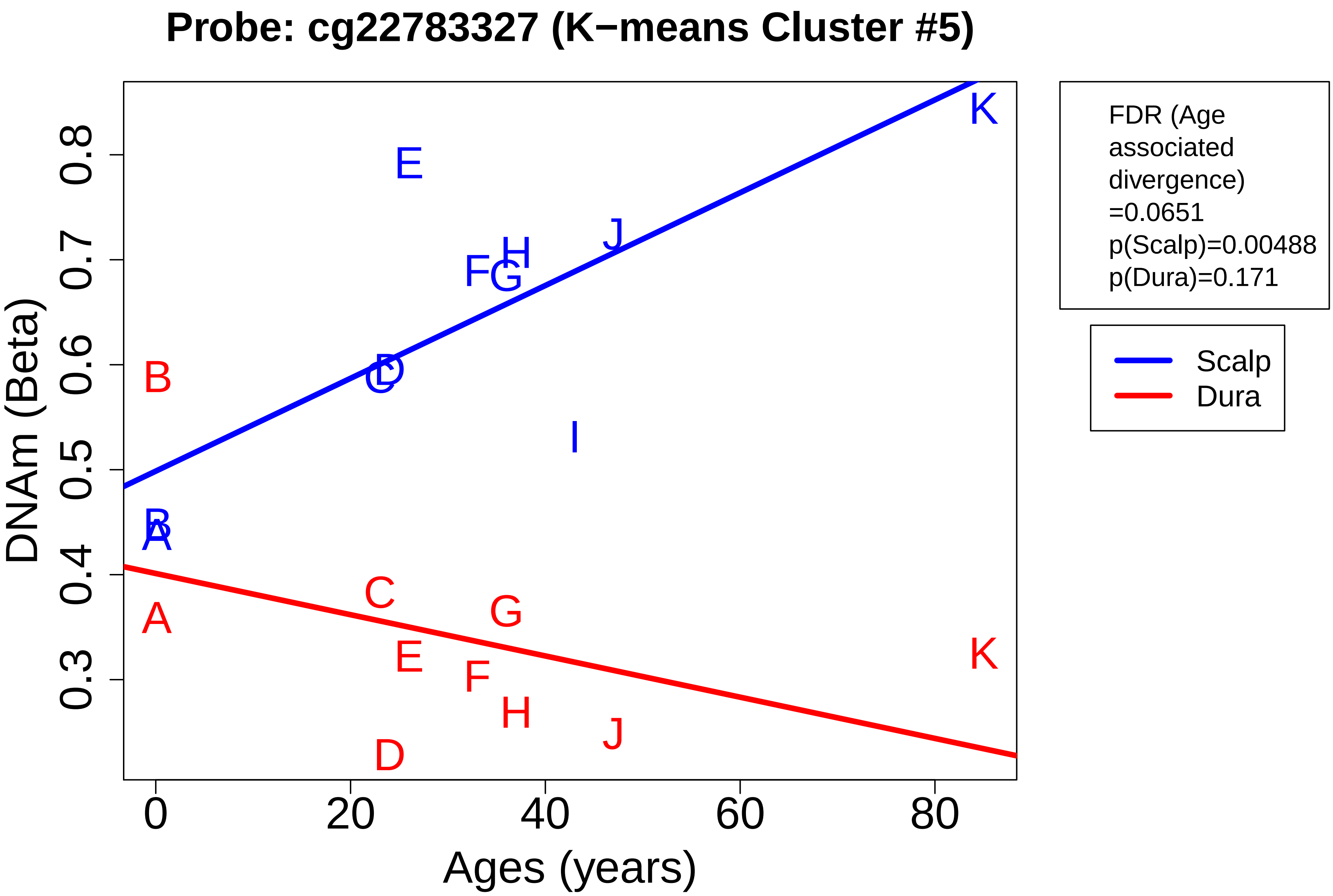
Probe: cg22783327 (K−means Cluster #5)
FDR (Age associated divergence) =0.0651
p(Scalp)=0.00488
p(Dura)=0.171
Scalp
Dura
DNAm (Beta)
Ages (years)
0.3
0.4
0.5
0.6
0.7
0.8
0
20
40
60
80

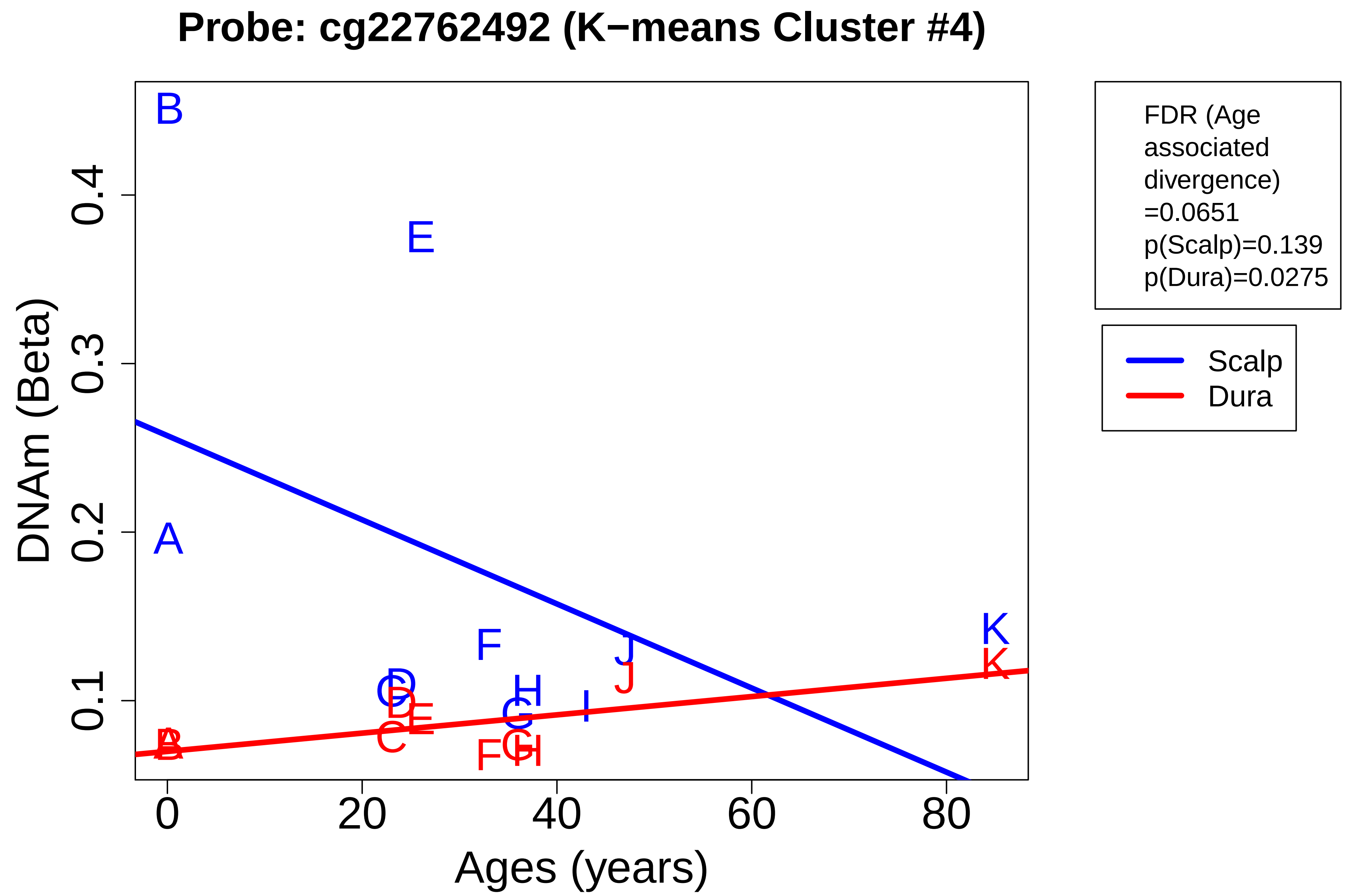

Probe: cg22762492 (K−means Cluster #4)
DNAm (Beta)
Ages (years)
0.1
0.2
0.3
0.4
0
20
40
60
80
FDR (Age associated divergence) =0.0651
p(Scalp)=0.139
p(Dura)=0.0275
Scalp
Dura

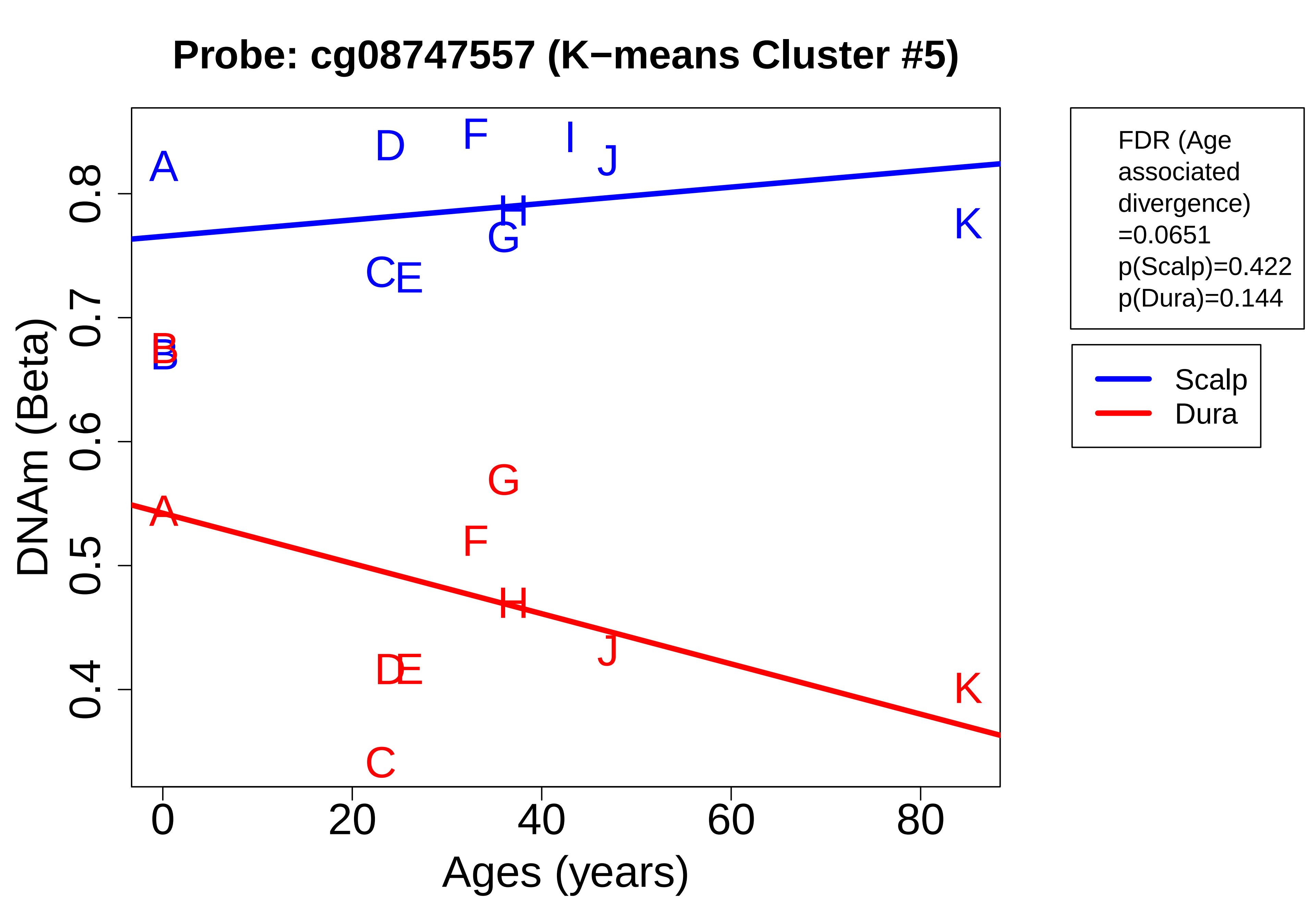

Probe: cg08747557 (K−means Cluster #5)
DNAm (Beta)
0.4
0.5
0.6
0.7
0.8
0
20
40
60
80
Ages (years)
A
B
C
D
E
F
G
H
I
J
K
FDR (Age associated divergence) =0.0651
p(Scalp)=0.422
p(Dura)=0.144
Scalp
Dura

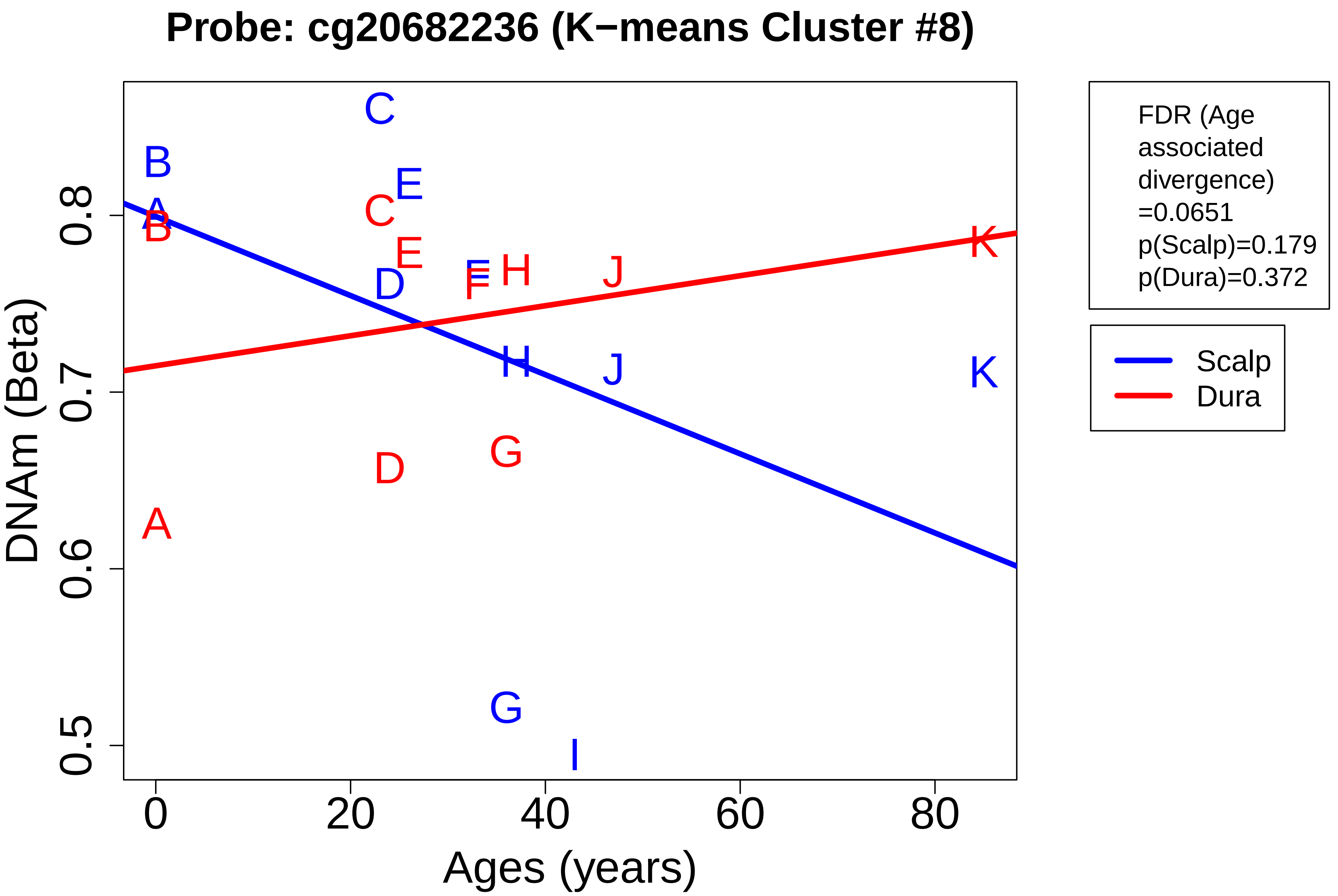

Probe: cg20682236 (K−means Cluster #8)
DNAm (Beta)
Ages (years)
0.5
0.6
0.7
0.8
0
20
40
60
80
FDR (Age associated divergence) =0.0651
p(Scalp)=0.179
p(Dura)=0.372
Scalp
Dura

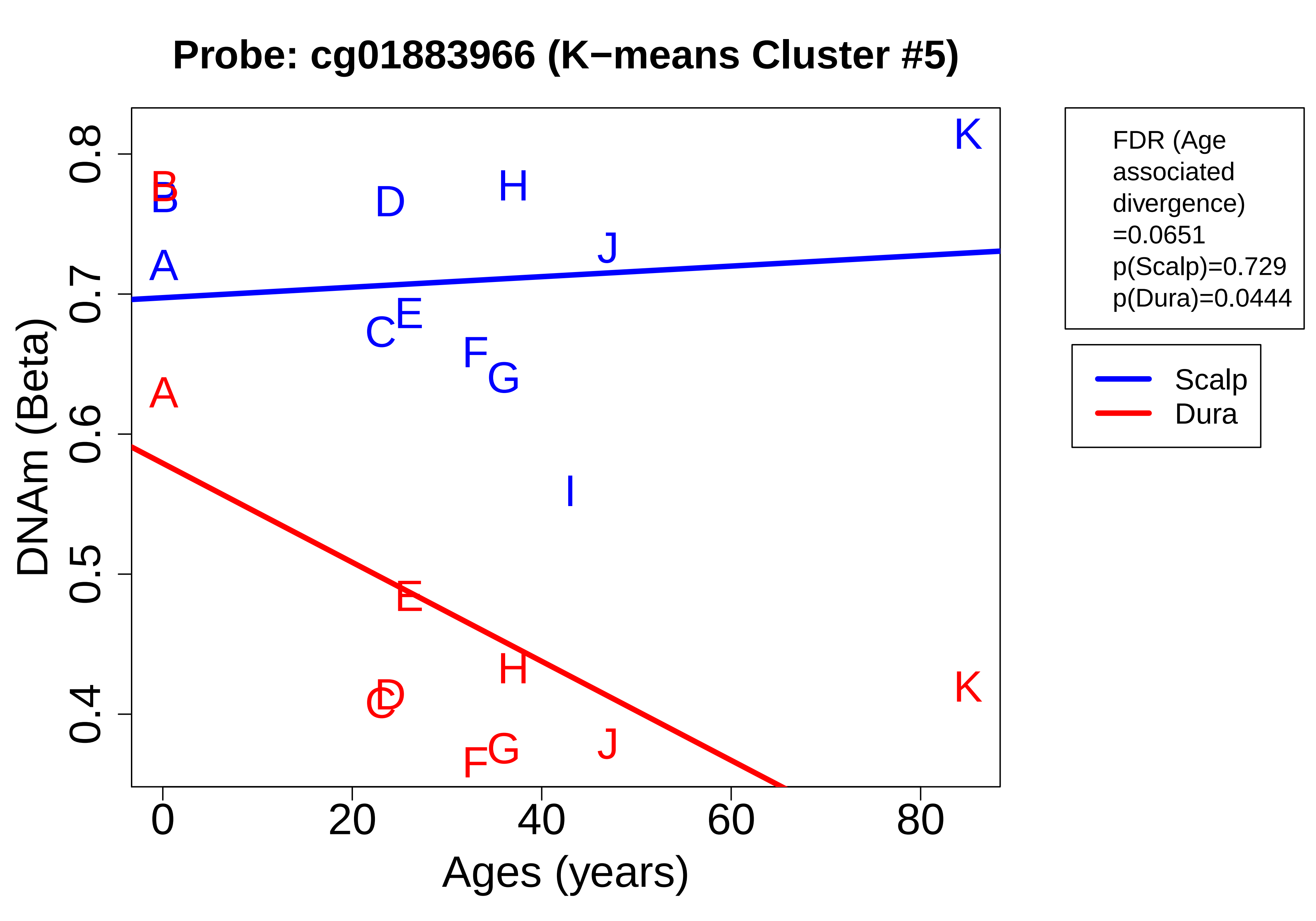

Probe: cg01883966 (K−means Cluster #5)
DNAm (Beta)
0.4
0.5
0.6
0.7
0.8
Ages (years)
0
20
40
60
80
FDR (Age associated divergence) =0.0651
p(Scalp)=0.729
p(Dura)=0.0444
Scalp
Dura

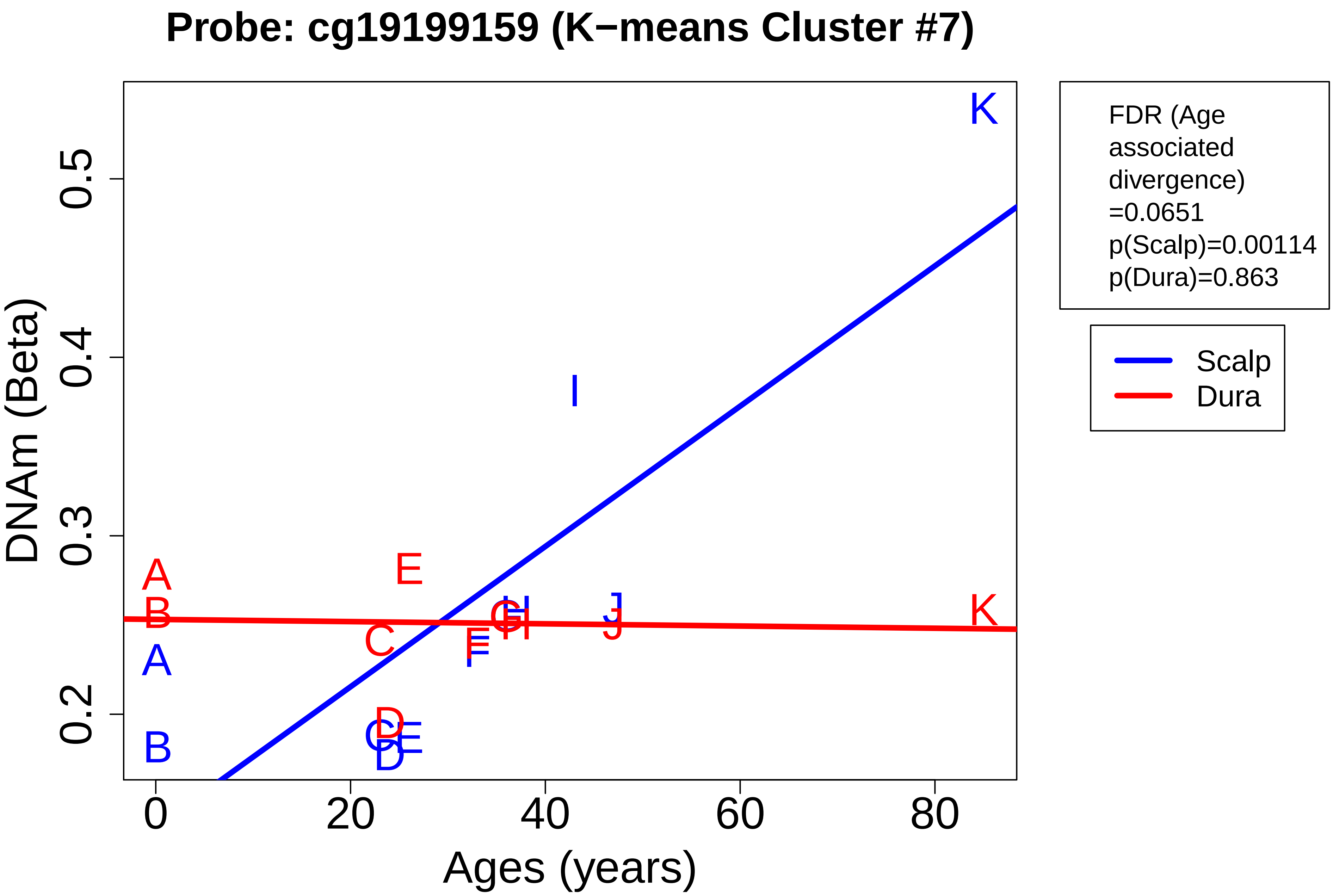

Probe: cg19199159 (K−means Cluster #7)
DNAm (Beta)
0.2
0.3
0.4
0.5
0
20
40
60
80
Ages (years)
FDR (Age associated divergence) =0.0651
p(Scalp)=0.00114
p(Dura)=0.863
Scalp
Dura

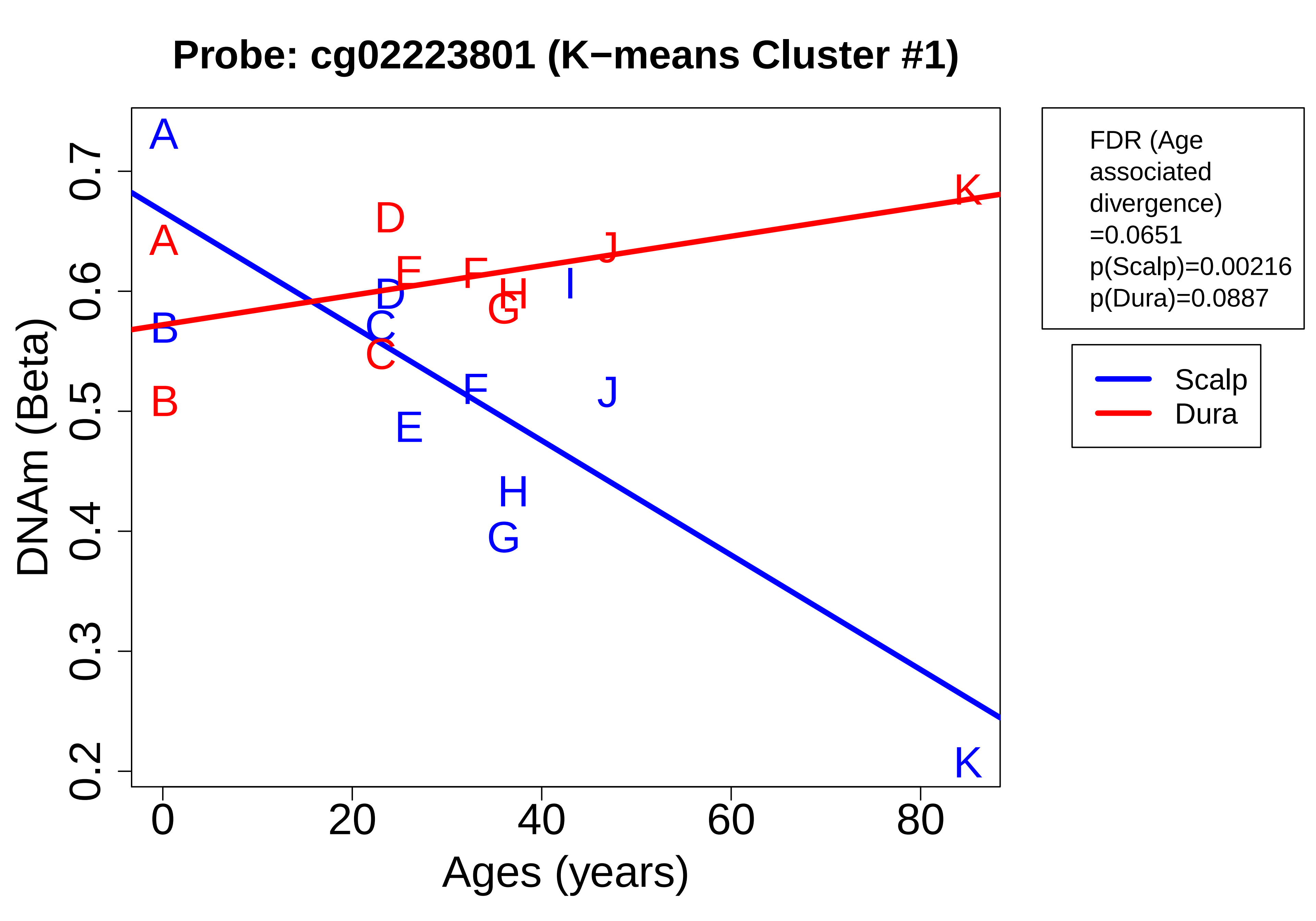

Probe: cg02223801 (K−means Cluster #1)
DNAm (Beta)
Ages (years)
0.2
0.3
0.4
0.5
0.6
0.7
0
20
40
60
80
A
B
C
D
E
F
G
H
I
J
K
FDR (Age associated divergence) =0.0651
p(Scalp)=0.00216
p(Dura)=0.0887
Scalp
Dura

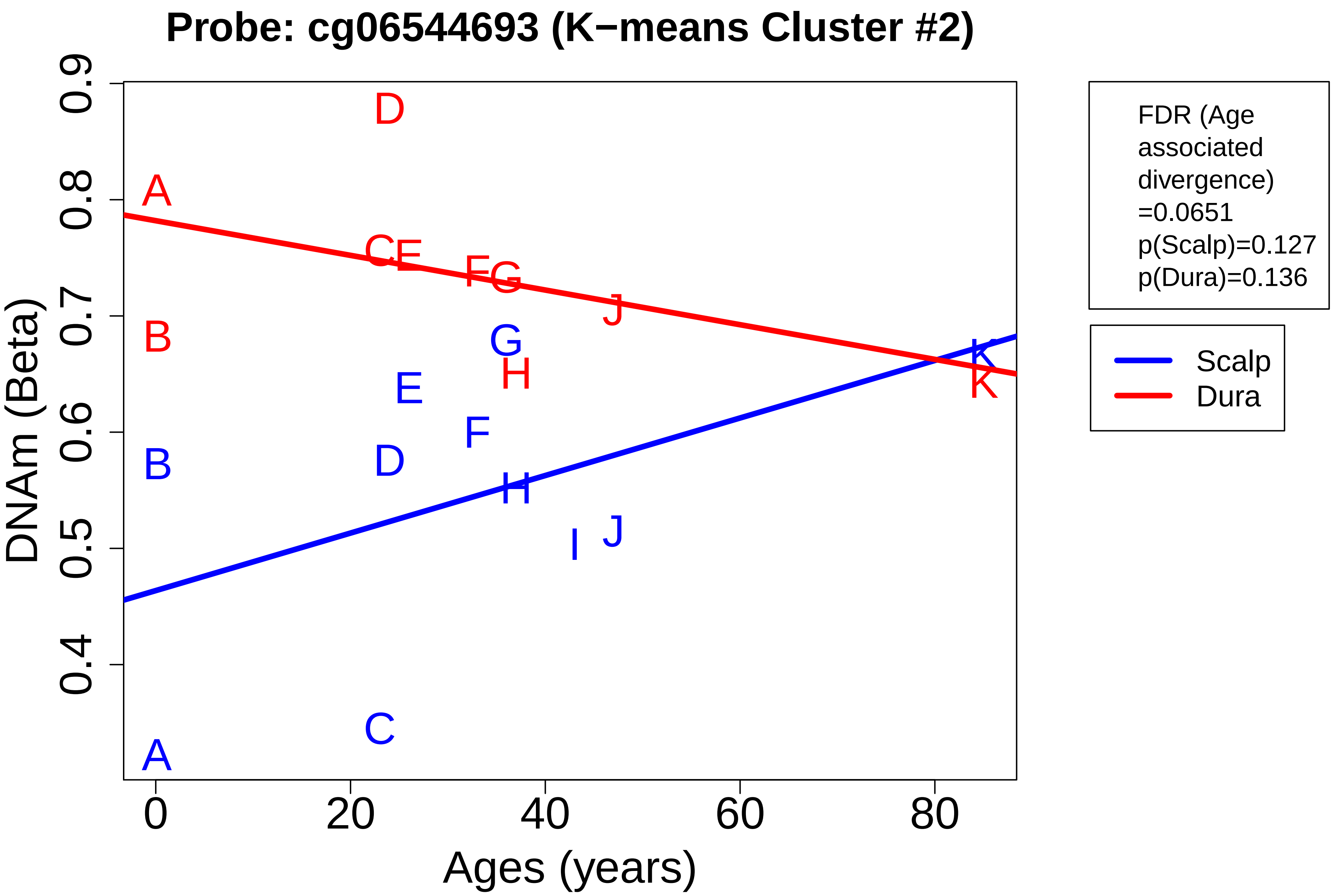
Probe: cg06544693 (K−means Cluster #2)
DNAm (Beta)
0.4
0.5
0.6
0.7
0.8
0.9
Ages (years)
0
20
40
60
80
FDR (Age associated divergence) =0.0651
p(Scalp)=0.127
p(Dura)=0.136
Scalp
Dura

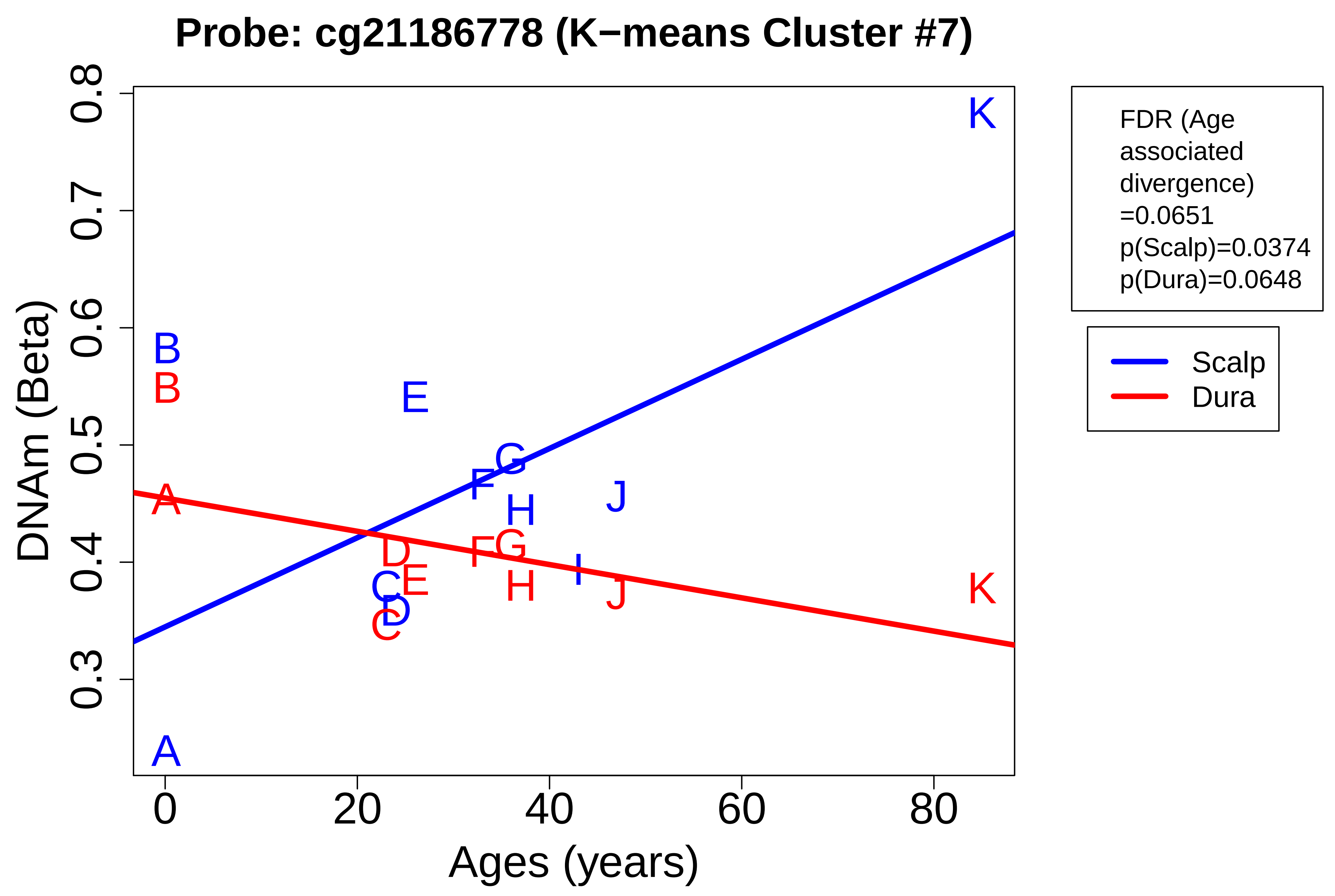

Probe: cg21186778 (K−means Cluster #7)
DNAm (Beta)
0.3
0.4
0.5
0.6
0.7
0.8
0
20
40
60
80
Ages (years)
FDR (Age associated divergence) =0.0651
p(Scalp)=0.0374
p(Dura)=0.0648
Scalp
Dura
A
B
C
D
E
F
G
H
I
J
K

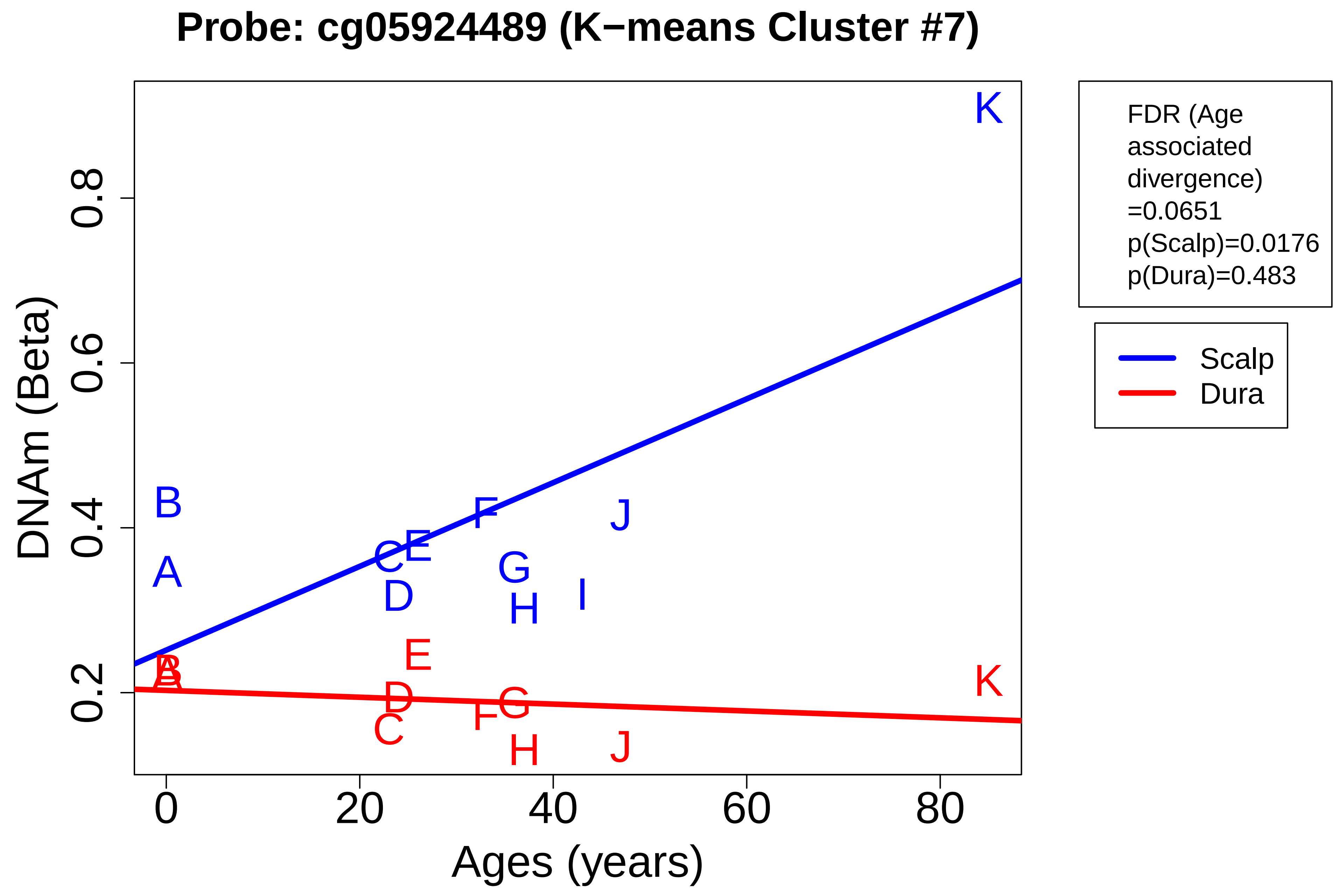

Probe: cg05924489 (K−means Cluster #7)
DNAm (Beta)
0.2
0.4
0.6
0.8
Ages (years)
0
20
40
60
80
FDR (Age associated divergence) =0.0651
p(Scalp)=0.0176
p(Dura)=0.483
Scalp
Dura
A
B
C
D
E
F
G
H
I
J
K

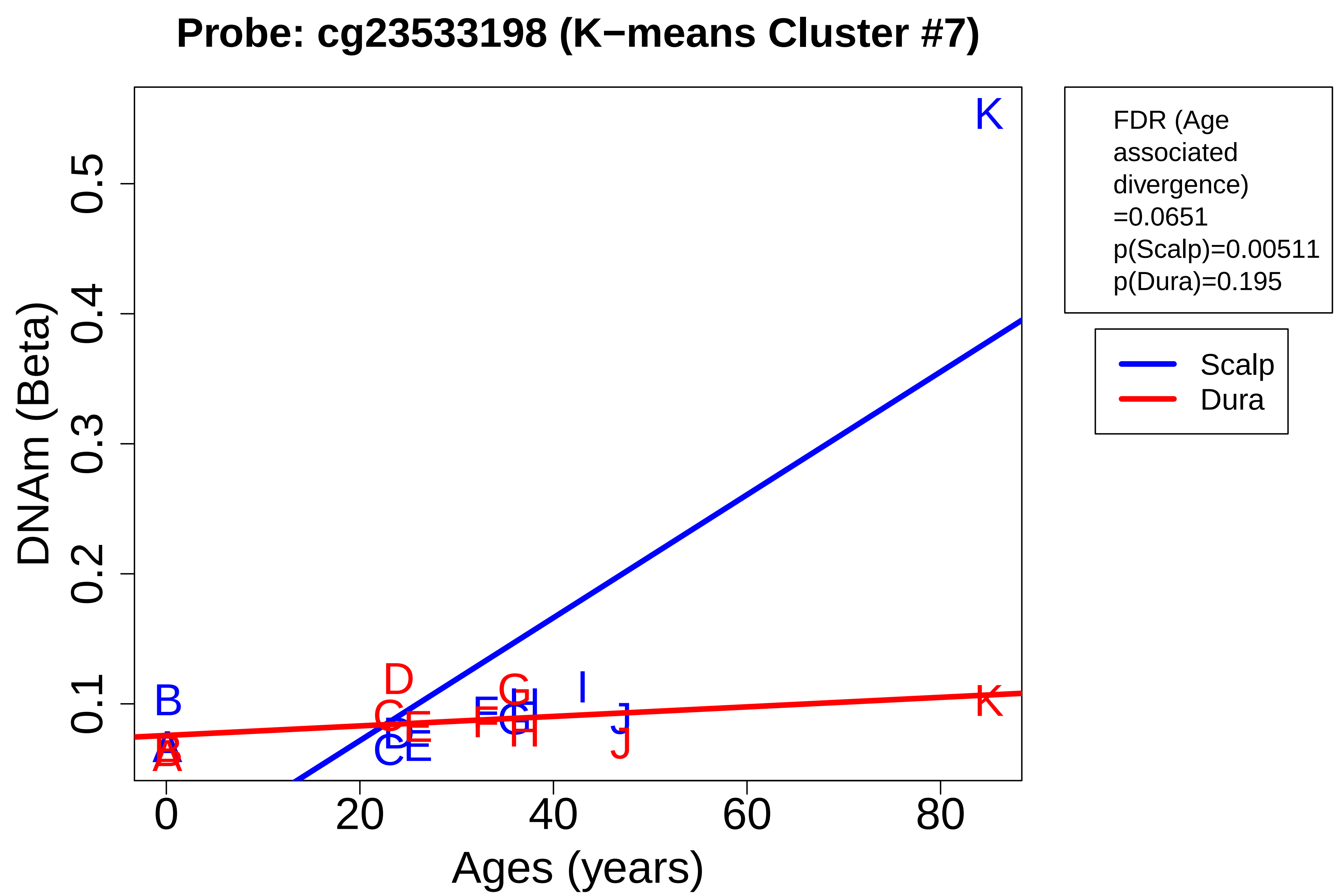
Probe: cg23533198 (K−means Cluster #7)
DNAm (Beta)
0.1
0.2
0.3
0.4
0.5
0
20
40
60
80
Ages (years)
FDR (Age associated divergence) =0.0651
p(Scalp)=0.00511
p(Dura)=0.195
Scalp
Dura

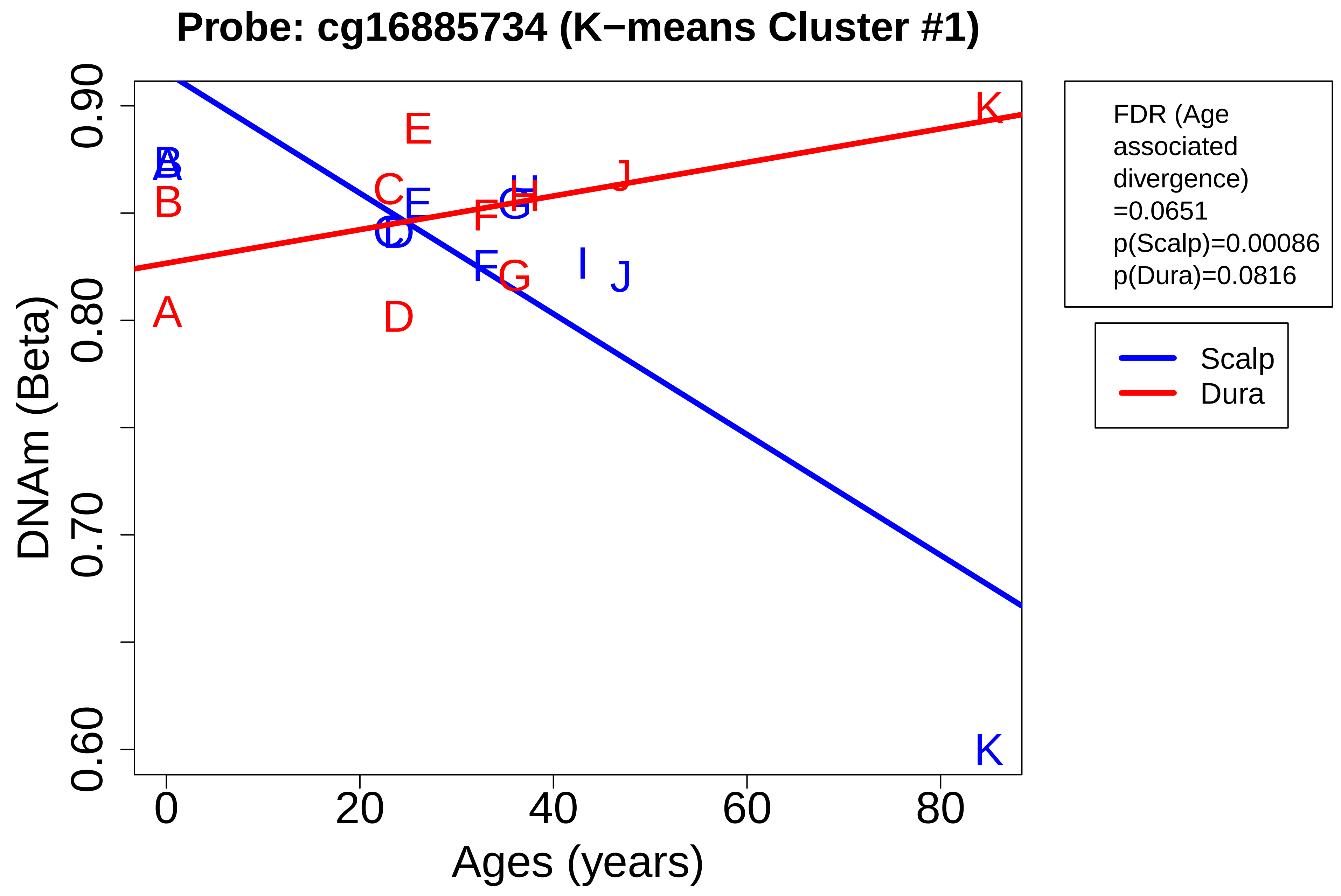

Probe: cg16885734 (K−means Cluster #1)
DNAm (Beta)
0.60
0.70
0.80
0.90
0
20
40
60
80
Ages (years)
FDR (Age associated divergence) =0.0651
p(Scalp)=0.00086
p(Dura)=0.0816
Scalp
Dura

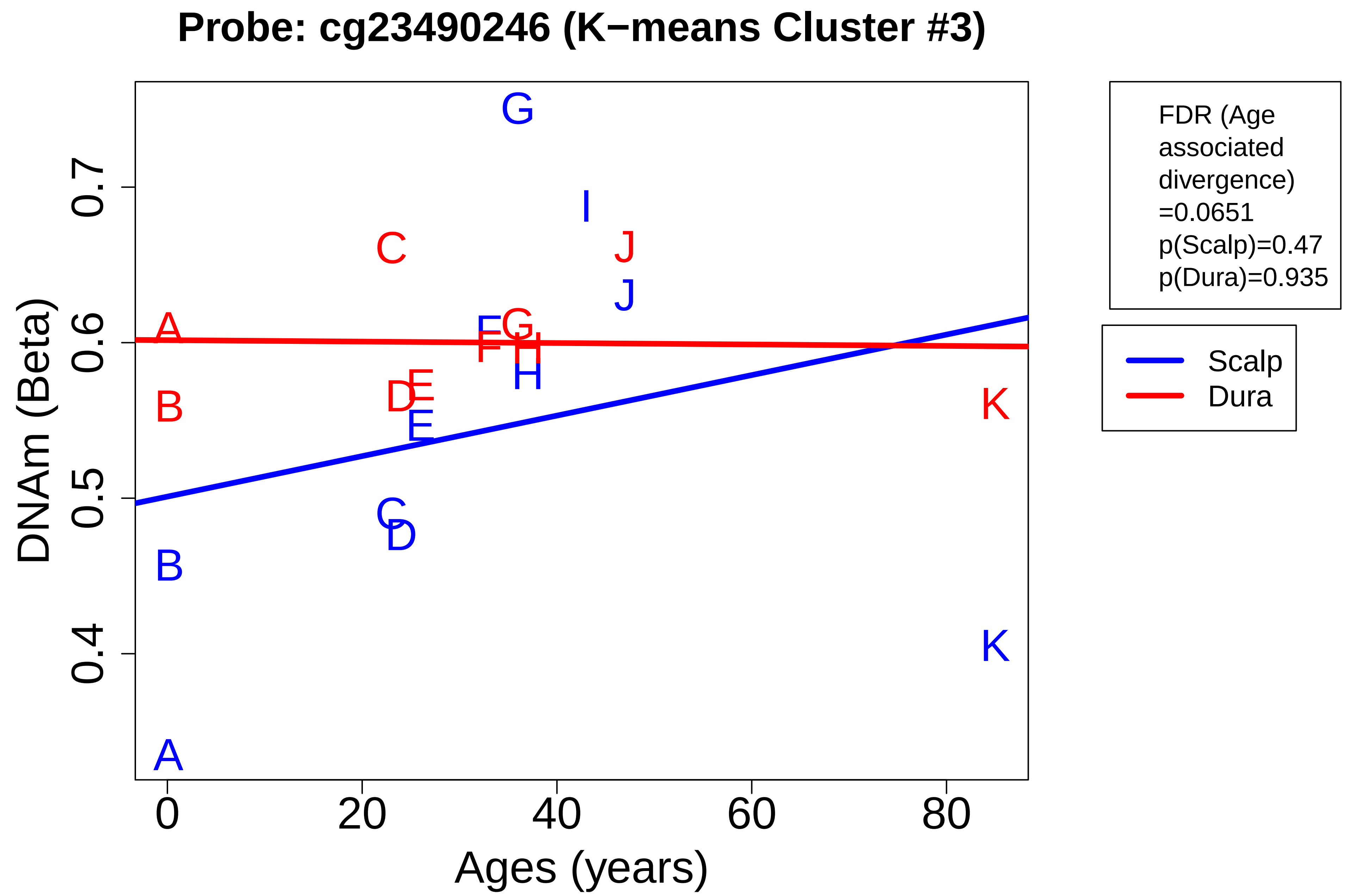

Probe: cg23490246 (K−means Cluster #3)
DNAm (Beta)
0.4
0.5
0.6
0.7
Ages (years)
0
20
40
60
80
A
B
C
D
E
F
G
H
I
J
K
FDR (Age associated divergence) =0.0651
p(Scalp)=0.47
p(Dura)=0.935
Scalp
Dura

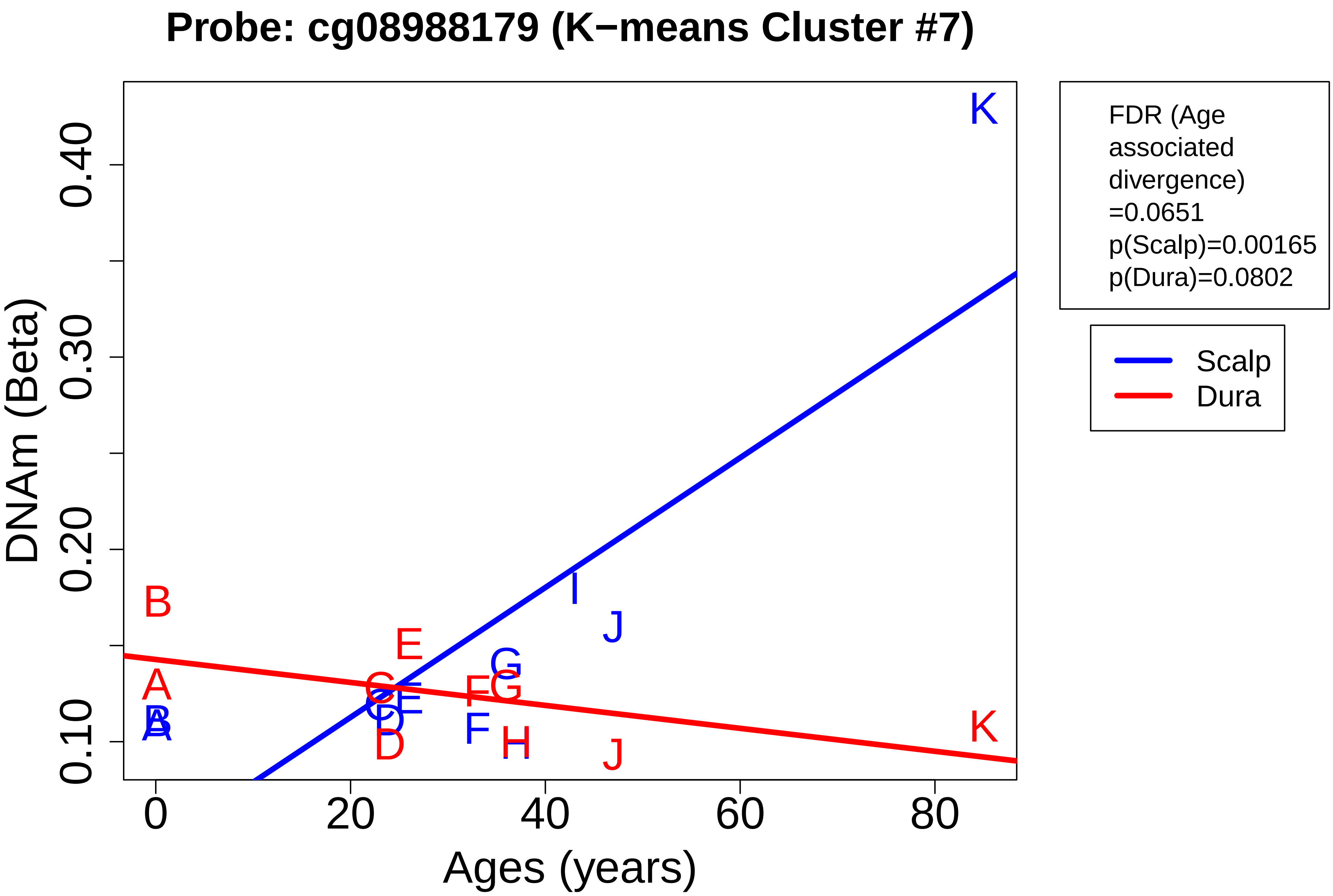

Probe: cg08988179 (K−means Cluster #7)
DNAm (Beta)
0.10
0.20
0.30
0.40
0
20
40
60
80
Ages (years)
FDR (Age associated divergence) =0.0651
p(Scalp)=0.00165
p(Dura)=0.0802
Scalp
Dura

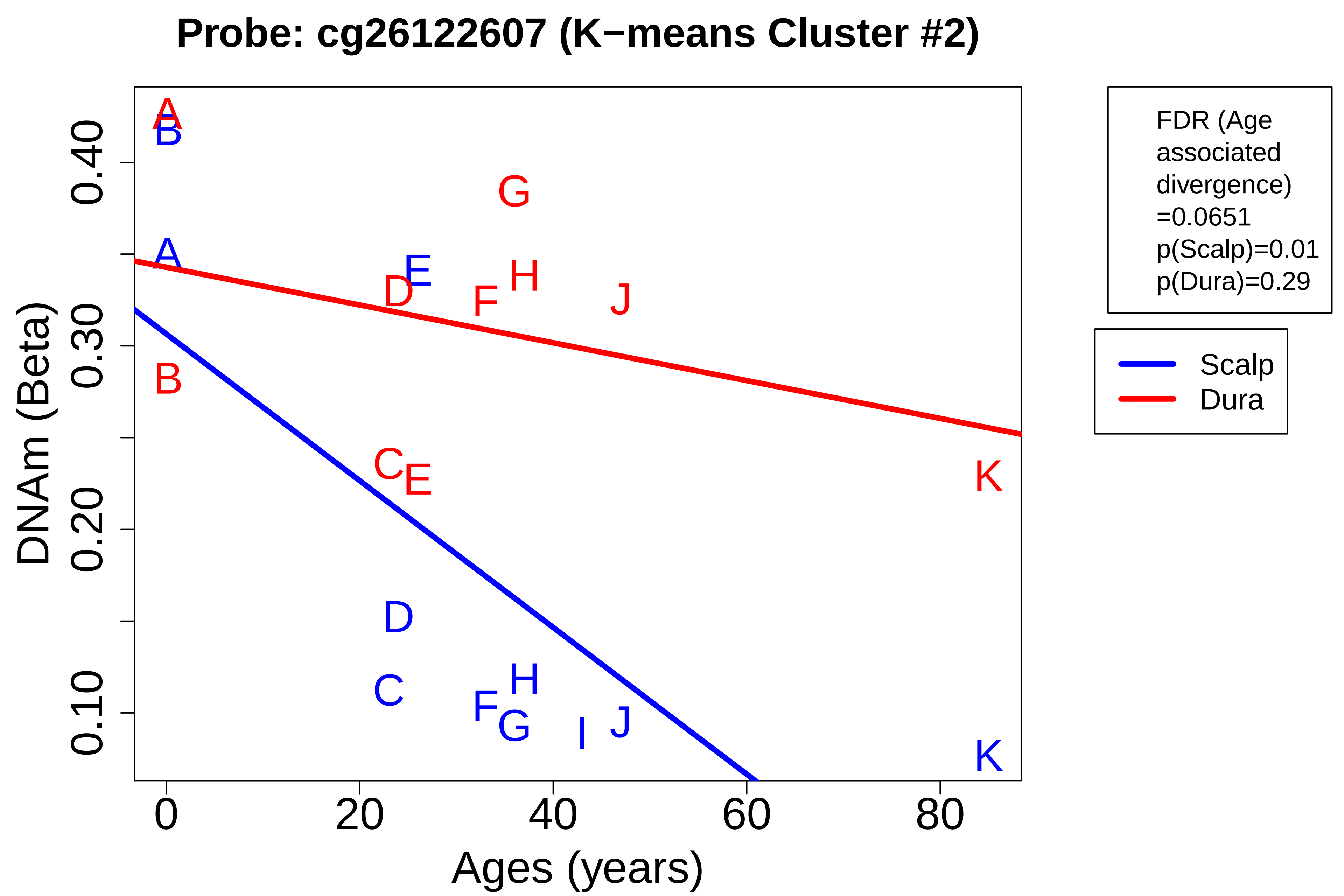

Probe: cg26122607 (K−means Cluster #2)
DNAm (Beta)
0.10
0.20
0.30
0.40
0
20
40
60
80
Ages (years)
A
B
A
B
C
D
E
F
G
H
I
J
K
C
E
D
E
F
G
H
J
K
FDR (Age associated divergence) =0.0651
p(Scalp)=0.01
p(Dura)=0.29
Scalp
Dura

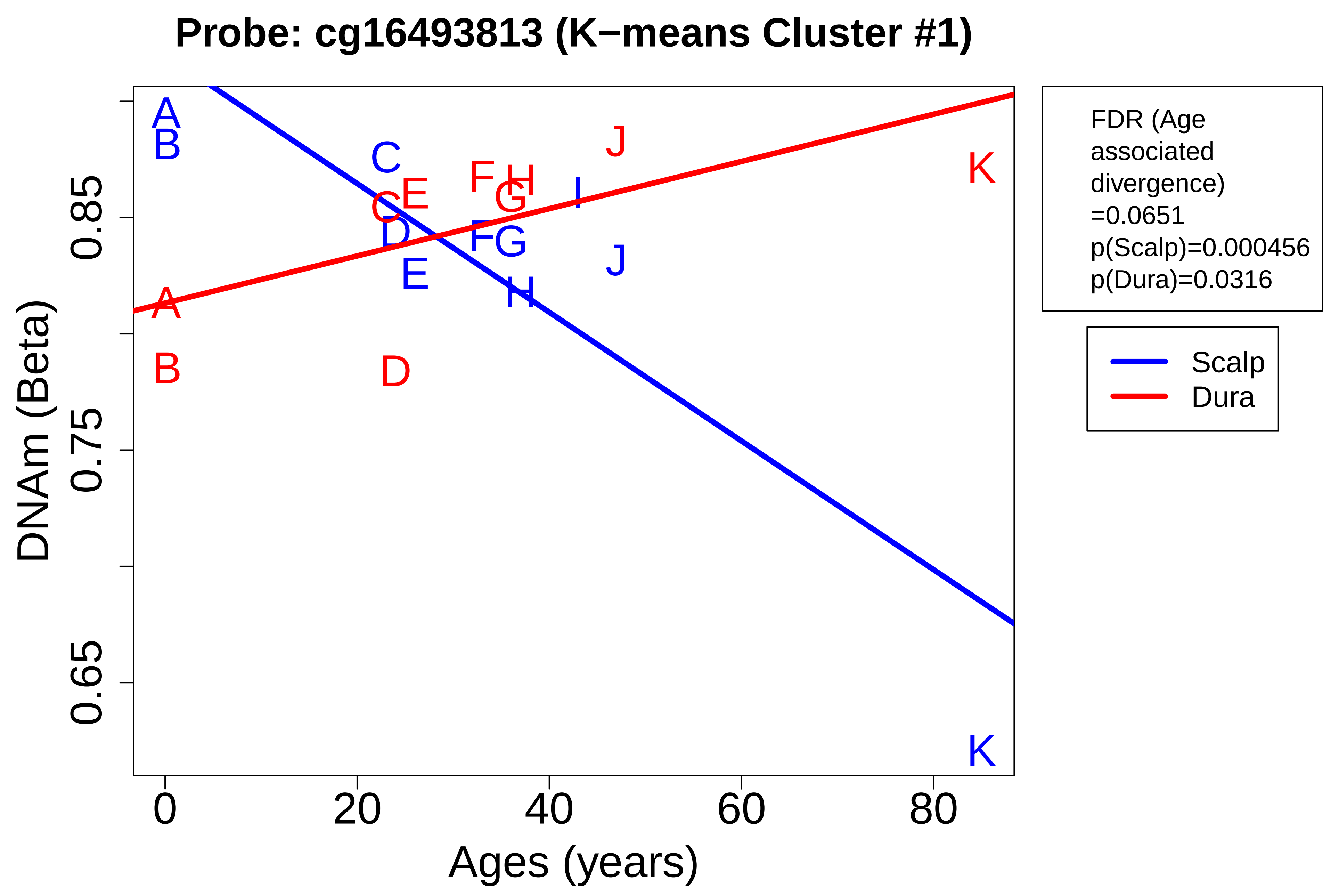

Probe: cg16493813 (K−means Cluster #1)
DNAm (Beta)
0.65
0.75
0.85
0
20
40
60
80
Ages (years)
A
B
C
D
E
F
G
H
I
J
K
FDR (Age associated divergence) =0.0651
p(Scalp)=0.000456
p(Dura)=0.0316
Scalp
Dura

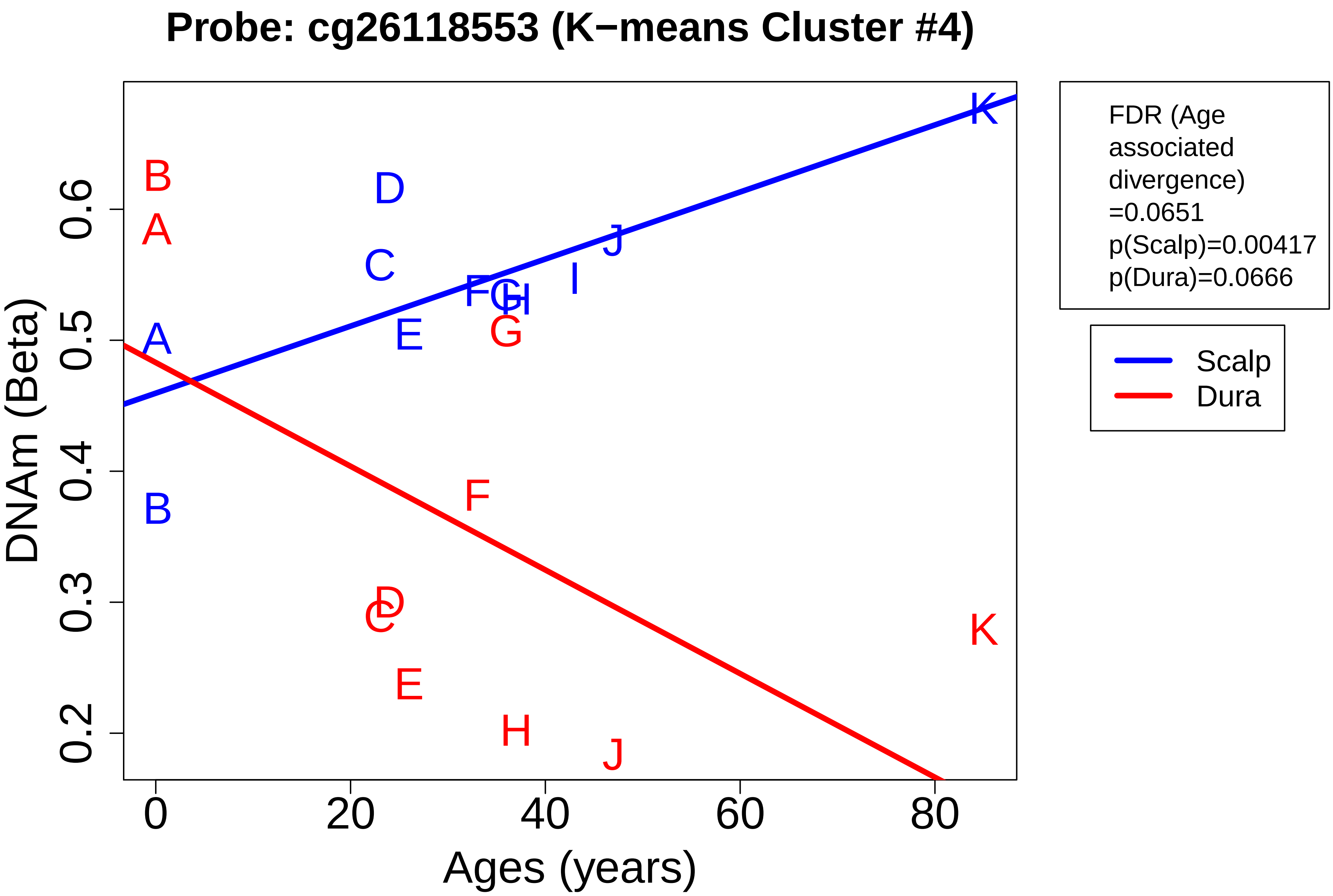

Probe: cg26118553 (K−means Cluster #4)
DNAm (Beta)
0.2
0.3
0.4
0.5
0.6
Ages (years)
0
20
40
60
80
B
A
D
C
F
G
H
I
J
K
E
FDR (Age associated divergence) =0.0651
p(Scalp)=0.00417
p(Dura)=0.0666
Scalp
Dura

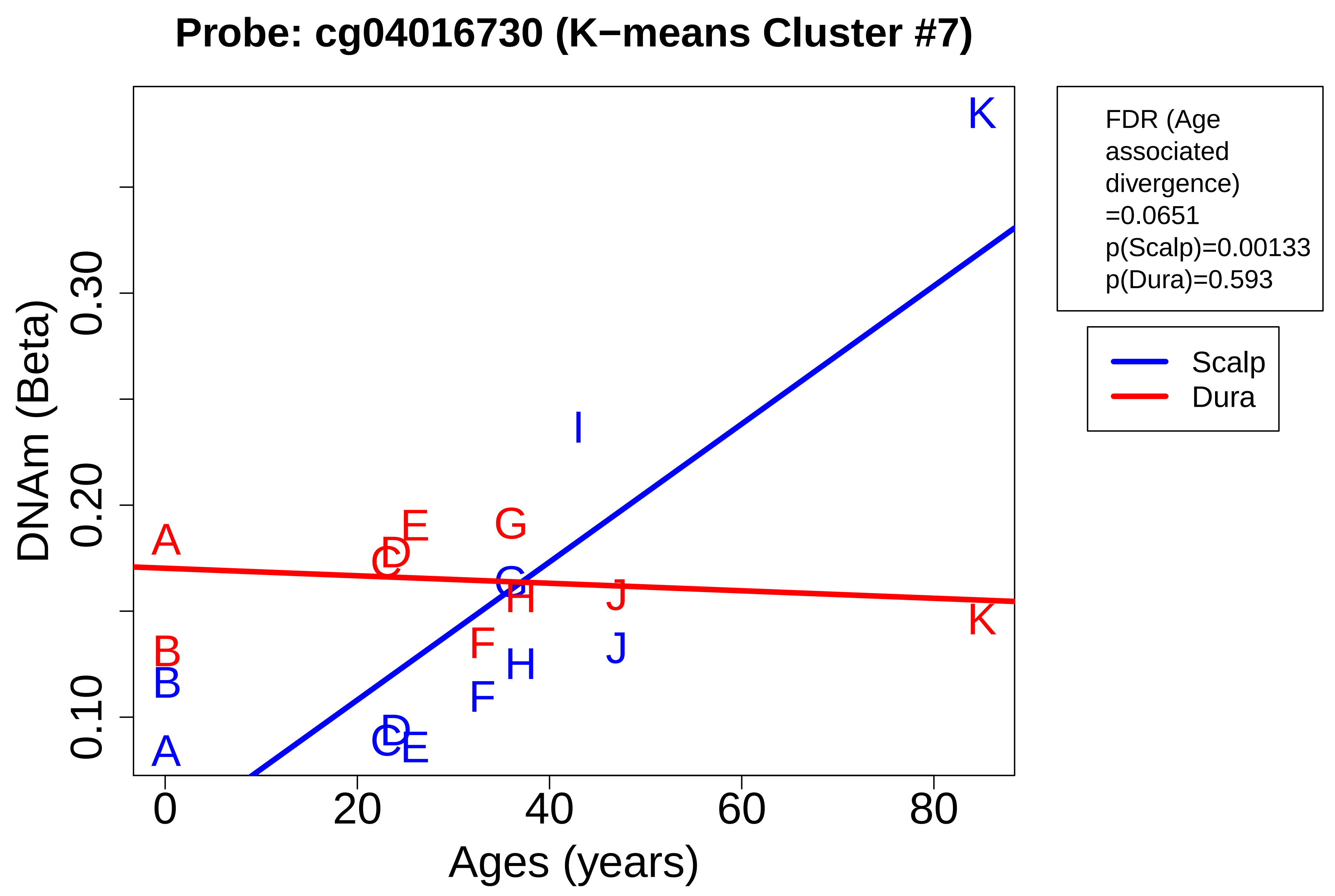
Probe: cg04016730 (K−means Cluster #7)
DNAm (Beta)
0.10
0.20
0.30
Ages (years)
0
20
40
60
80
A
B
C
D
E
F
G
H
I
J
K
FDR (Age associated divergence) =0.0651
p(Scalp)=0.00133
p(Dura)=0.593
Scalp
Dura

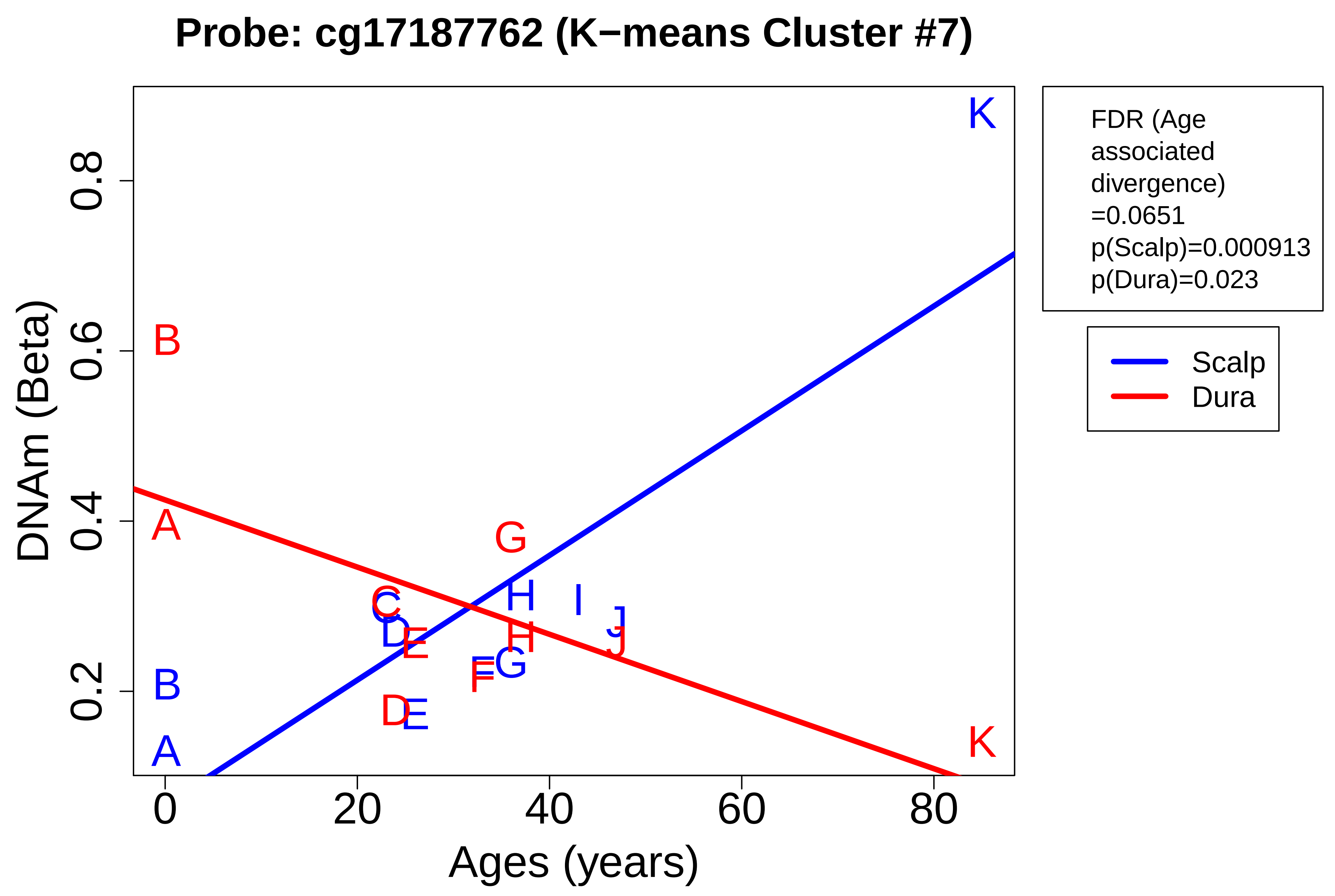

Probe: cg17187762 (K−means Cluster #7)
DNAm (Beta)
0.2
0.4
0.6
0.8
0
20
40
60
80
Ages (years)
A
B
C
D
E
F
G
H
I
J
K
FDR (Age associated divergence) =0.0651
p(Scalp)=0.000913
p(Dura)=0.023
Scalp
Dura

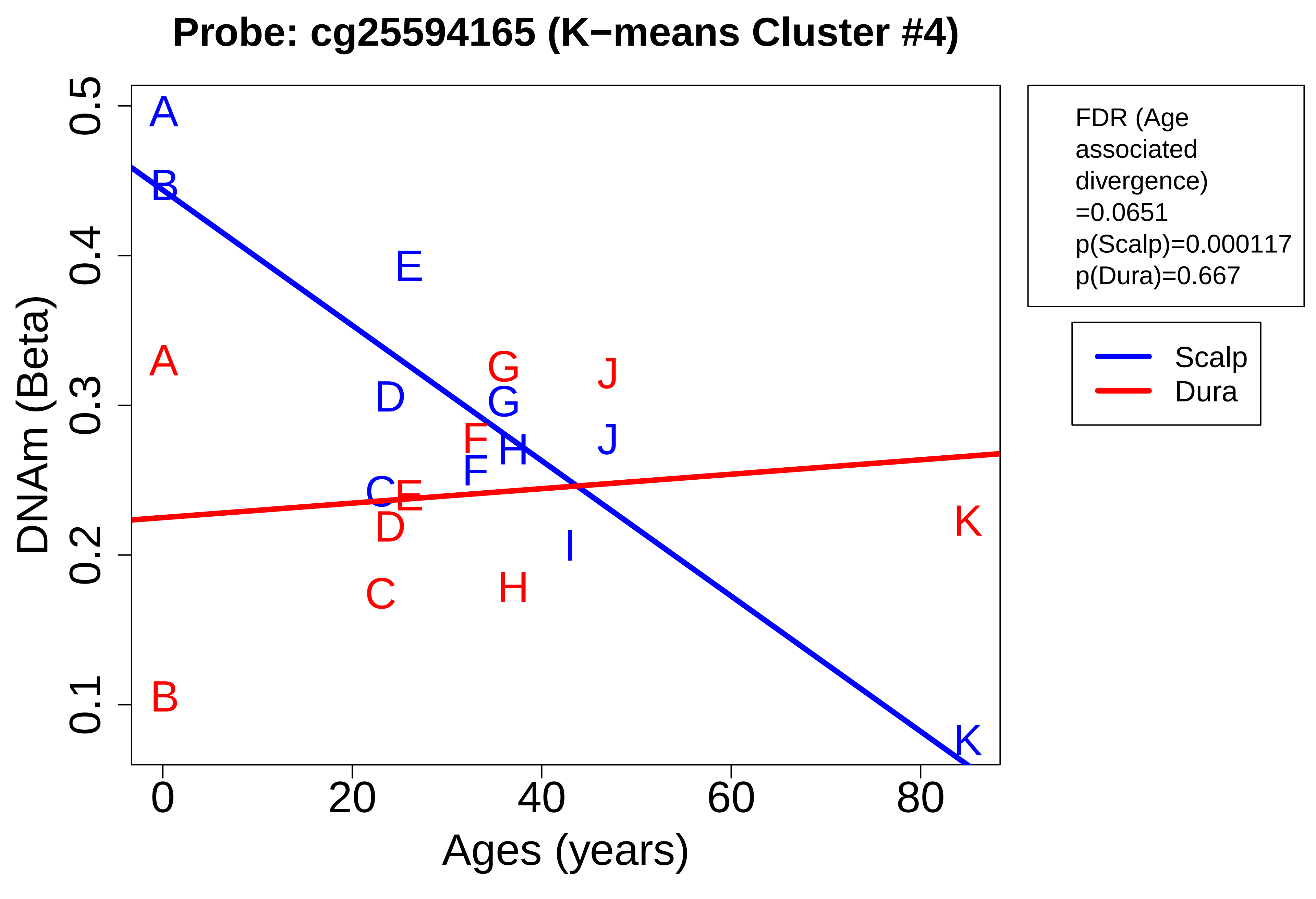
Probe: cg25594165 (K−means Cluster #4)
DNAm (Beta)
Ages (years)
0.1
0.2
0.3
0.4
0.5
0
20
40
60
80
FDR (Age associated divergence) =0.0651
p(Scalp)=0.000117
p(Dura)=0.667
Scalp
Dura

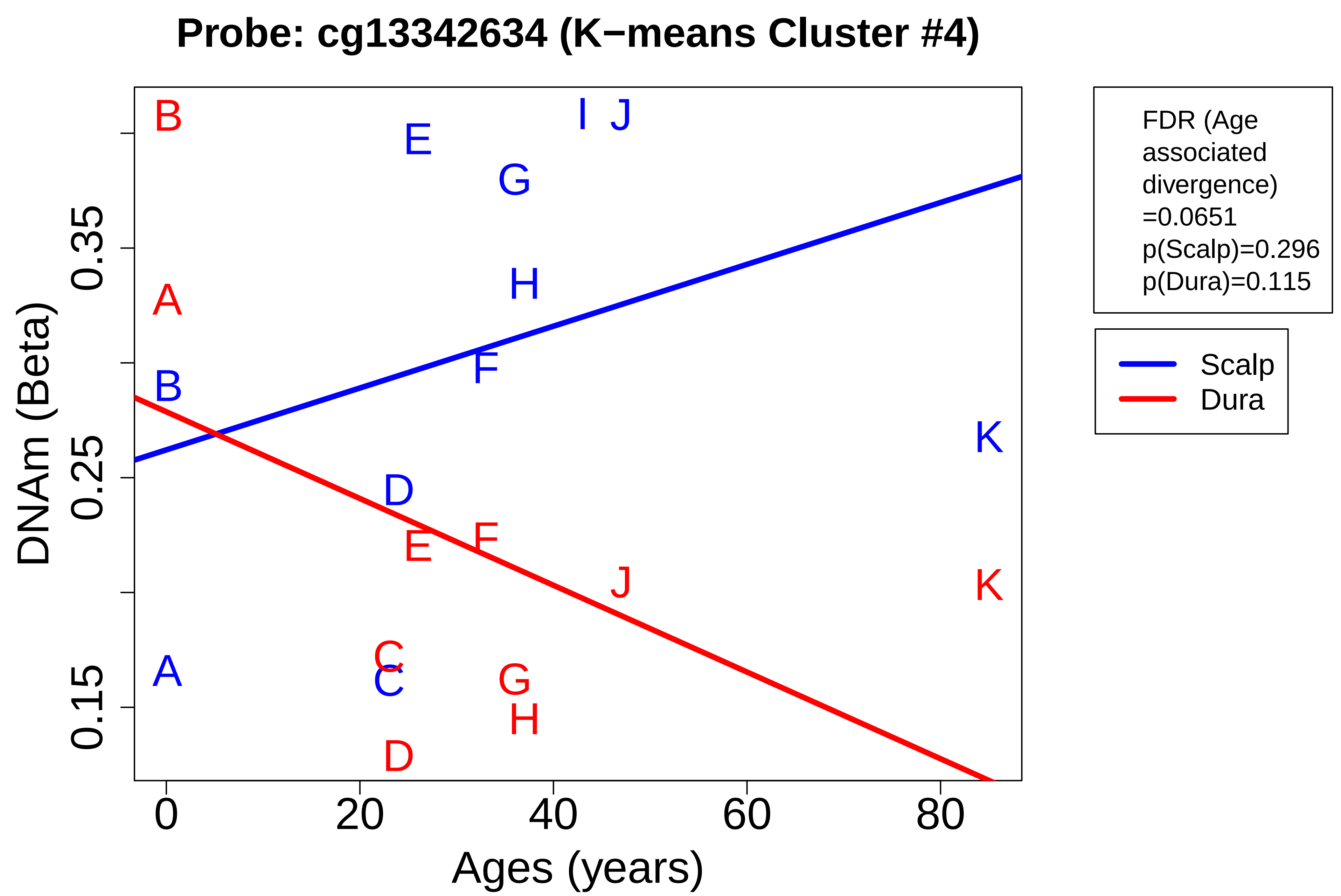

Probe: cg13342634 (K−means Cluster #4)
DNAm (Beta)
0.15
0.25
0.35
Ages (years)
0
20
40
60
80
FDR (Age associated divergence) =0.0651
p(Scalp)=0.296
p(Dura)=0.115
Scalp
Dura

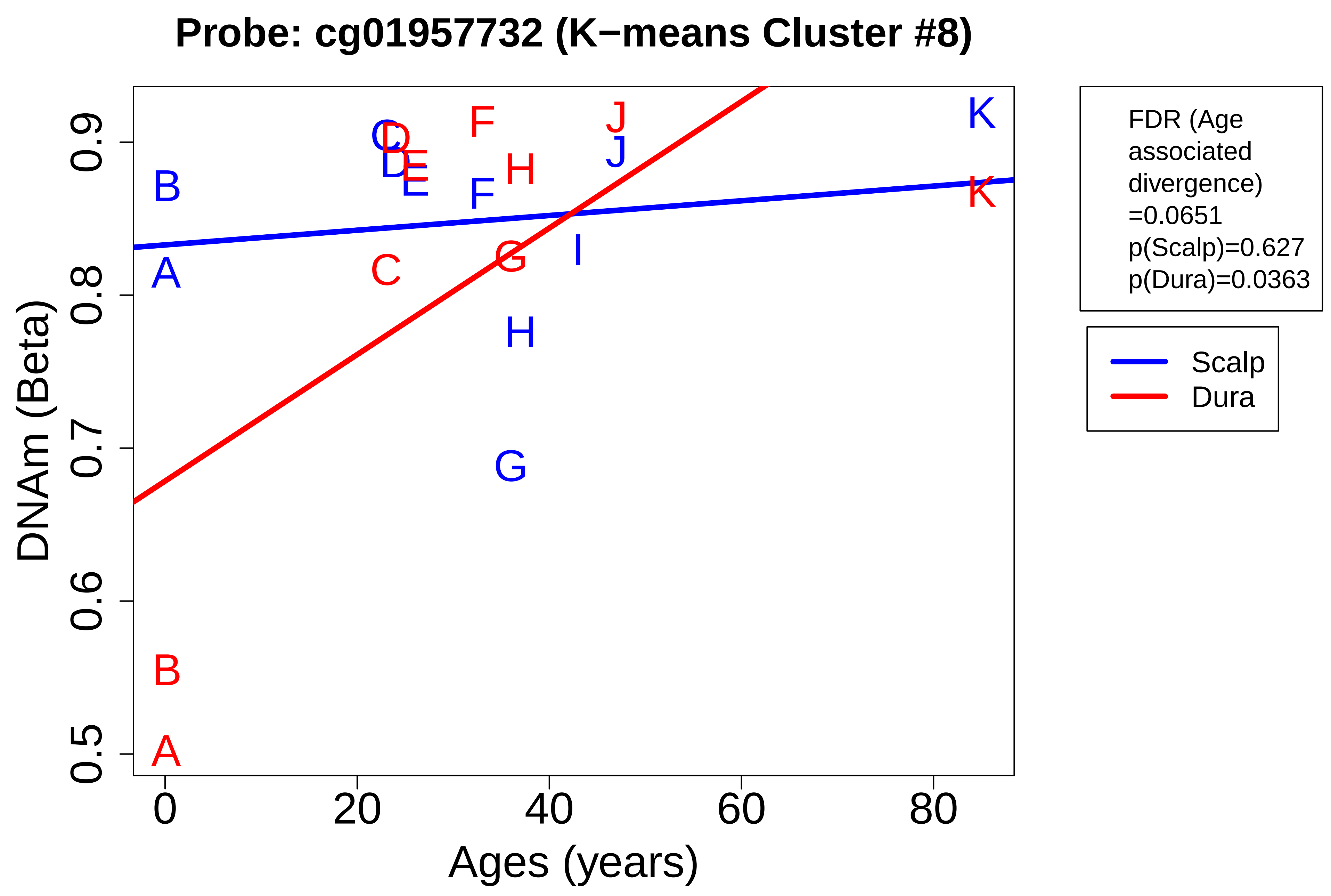

Probe: cg01957732 (K−means Cluster #8)
DNAm (Beta)
0.5
0.6
0.7
0.8
0.9
Ages (years)
0
20
40
60
80
A
B
C
D
E
F
G
H
I
J
K
FDR (Age associated divergence) =0.0651
p(Scalp)=0.627
p(Dura)=0.0363
Scalp
Dura

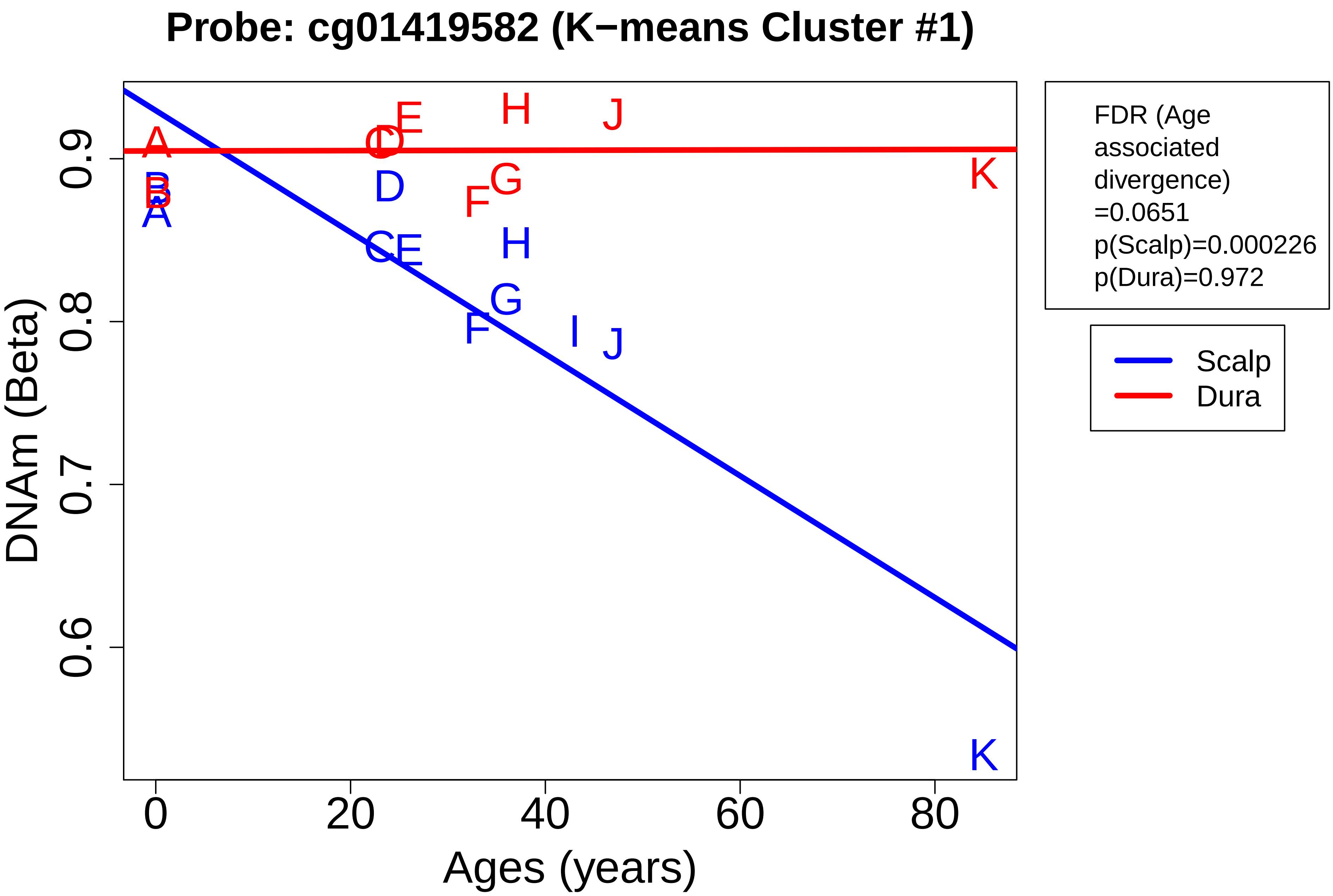
Probe: cg01419582 (K−means Cluster #1)
DNAm (Beta)
0.6
0.7
0.8
0.9
0
20
40
60
80
Ages (years)
A
B
C
D
E
F
G
H
I
J
K
FDR (Age associated divergence) =0.0651
p(Scalp)=0.000226
p(Dura)=0.972
Scalp
Dura

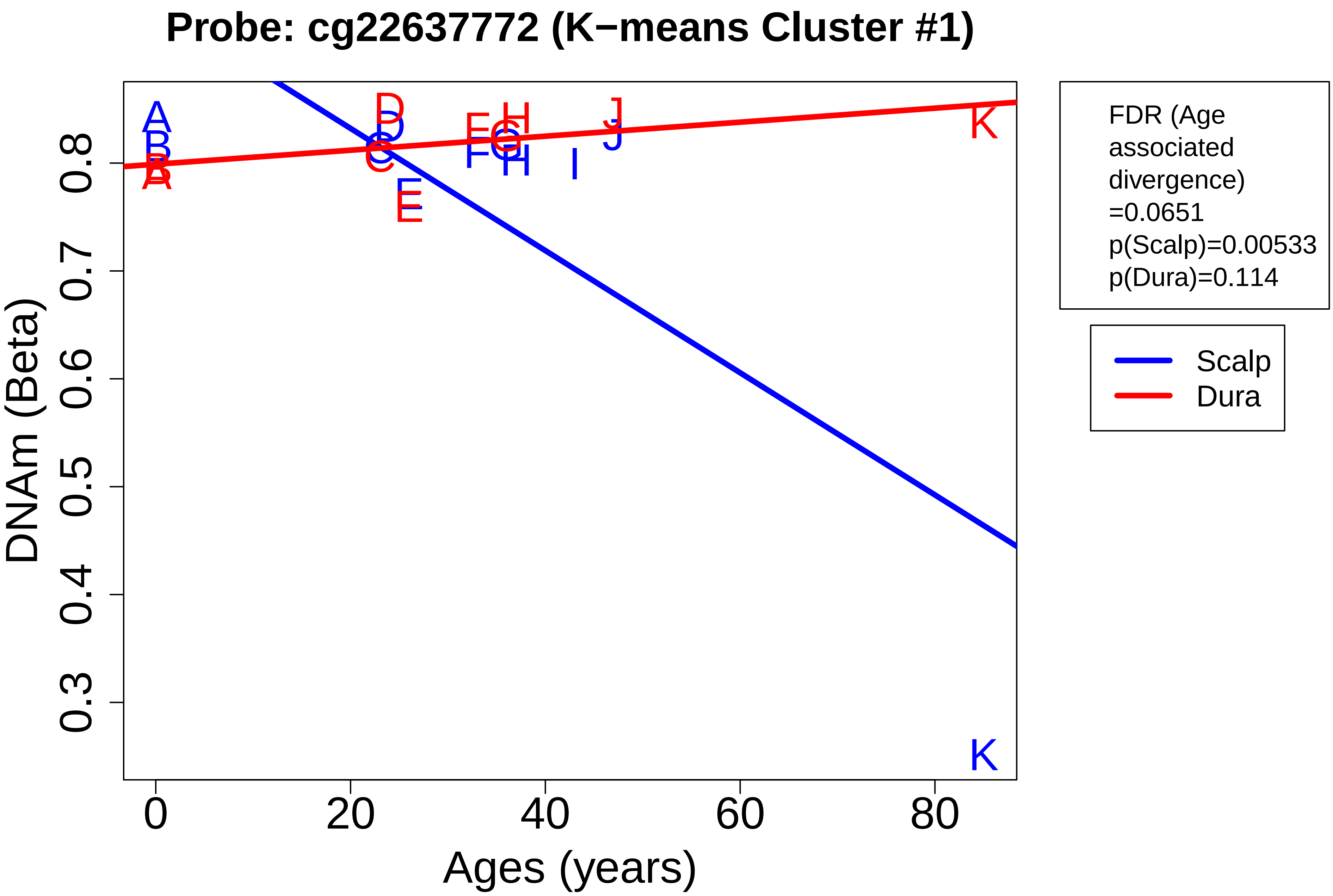

Probe: cg22637772 (K−means Cluster #1)
DNAm (Beta)
0.3
0.4
0.5
0.6
0.7
0.8
0
20
40
60
80
Ages (years)
A
B
C
D
E
F
G
H
I
J
K
FDR (Age associated divergence) =0.0651
p(Scalp)=0.00533
p(Dura)=0.114
Scalp
Dura

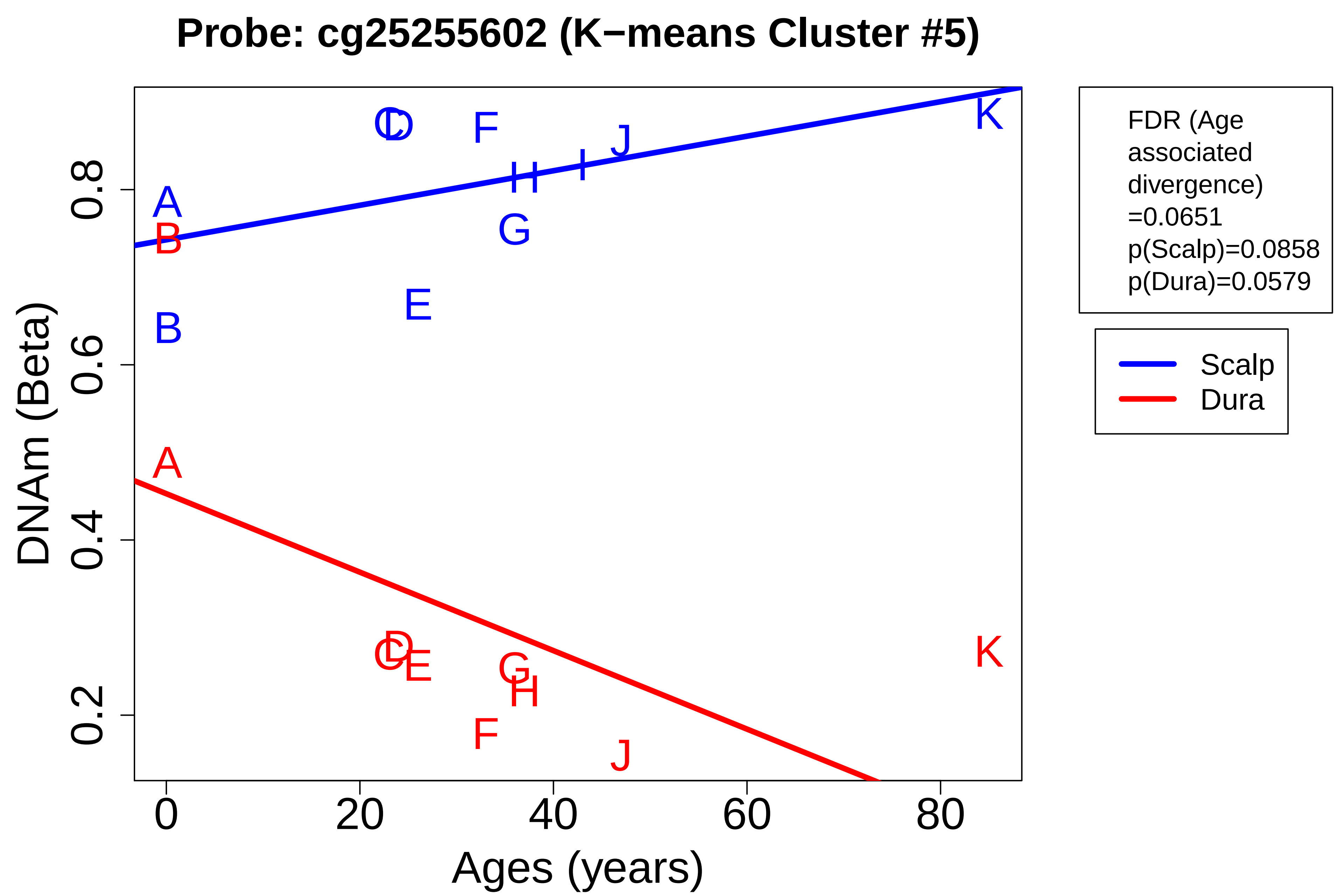

Probe: cg25255602 (K−means Cluster #5)
DNAm (Beta)
Ages (years)
0.2
0.4
0.6
0.8
0
20
40
60
80
FDR (Age associated divergence) =0.0651
p(Scalp)=0.0858
p(Dura)=0.0579
Scalp
Dura
A
B
C
D
E
F
G
H
I
J
K

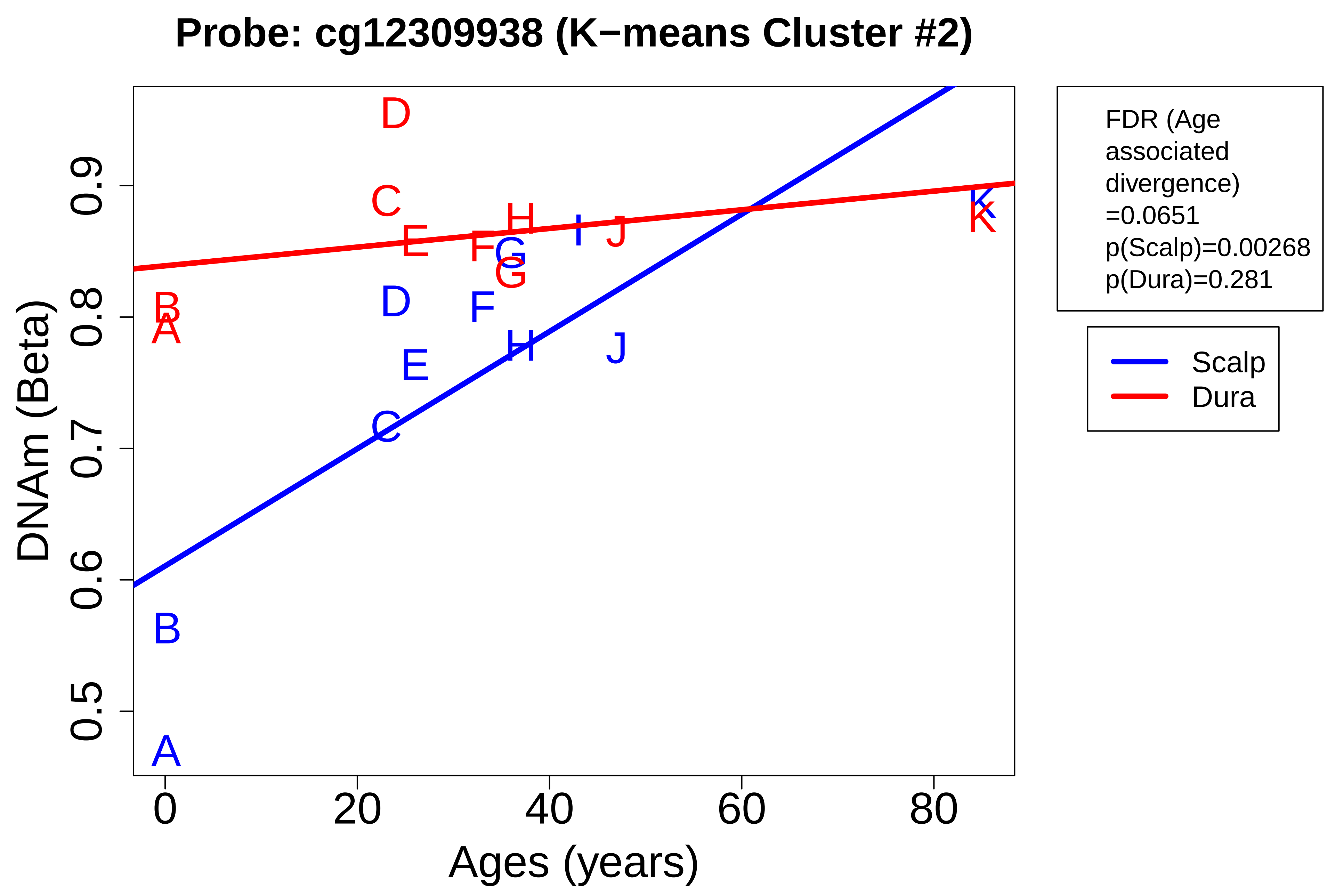

Probe: cg12309938 (K−means Cluster #2)
DNAm (Beta)
Ages (years)
0.5
0.6
0.7
0.8
0.9
0
20
40
60
80
A
B
C
D
E
F
G
H
I
J
K
FDR (Age associated divergence) =0.0651
p(Scalp)=0.00268
p(Dura)=0.281
Scalp
Dura

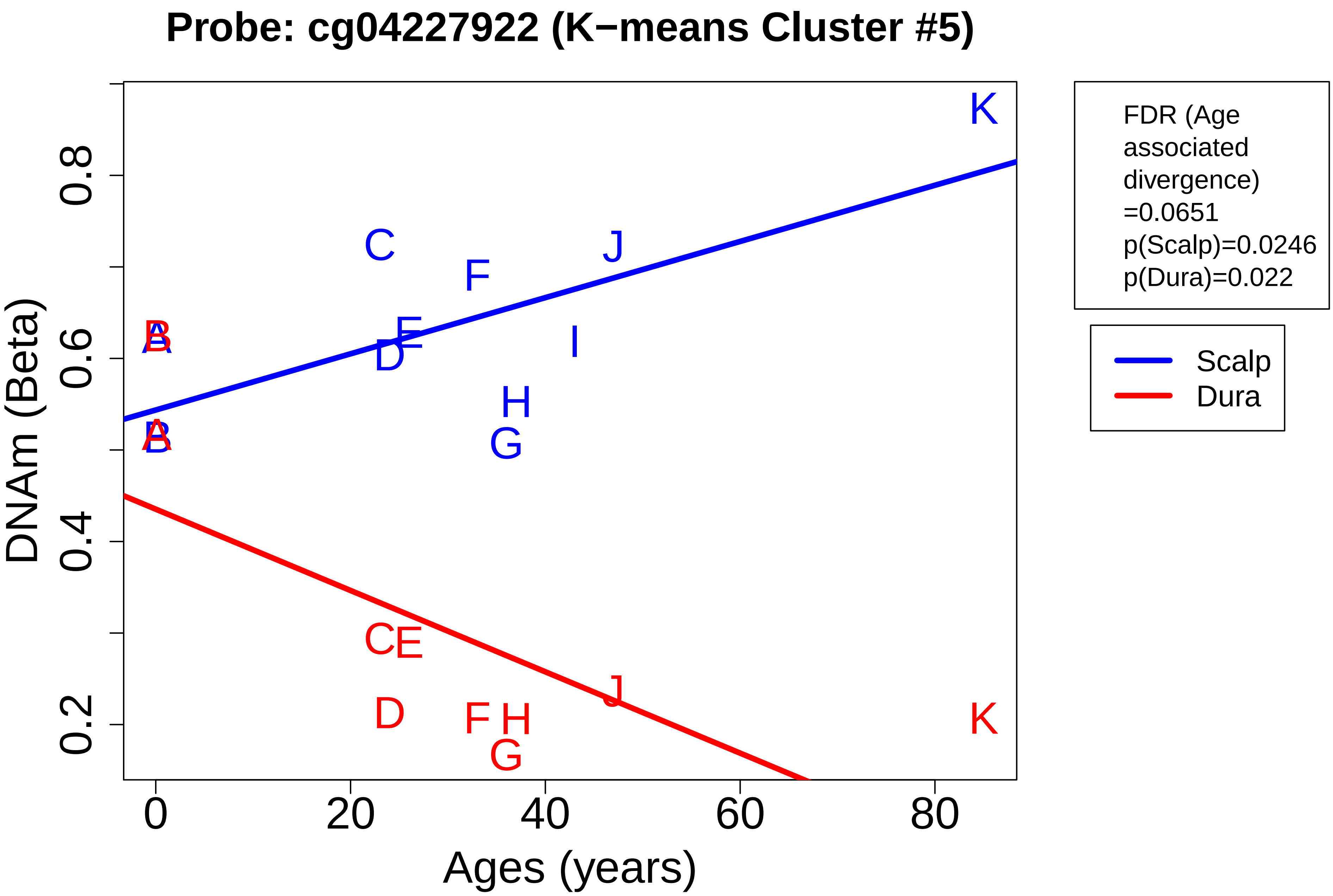

Probe: cg04227922 (K−means Cluster #5)
DNAm (Beta)
0.2
0.4
0.6
0.8
0
20
40
60
80
Ages (years)
FDR (Age associated divergence) =0.0651
p(Scalp)=0.0246
p(Dura)=0.022
Scalp
Dura

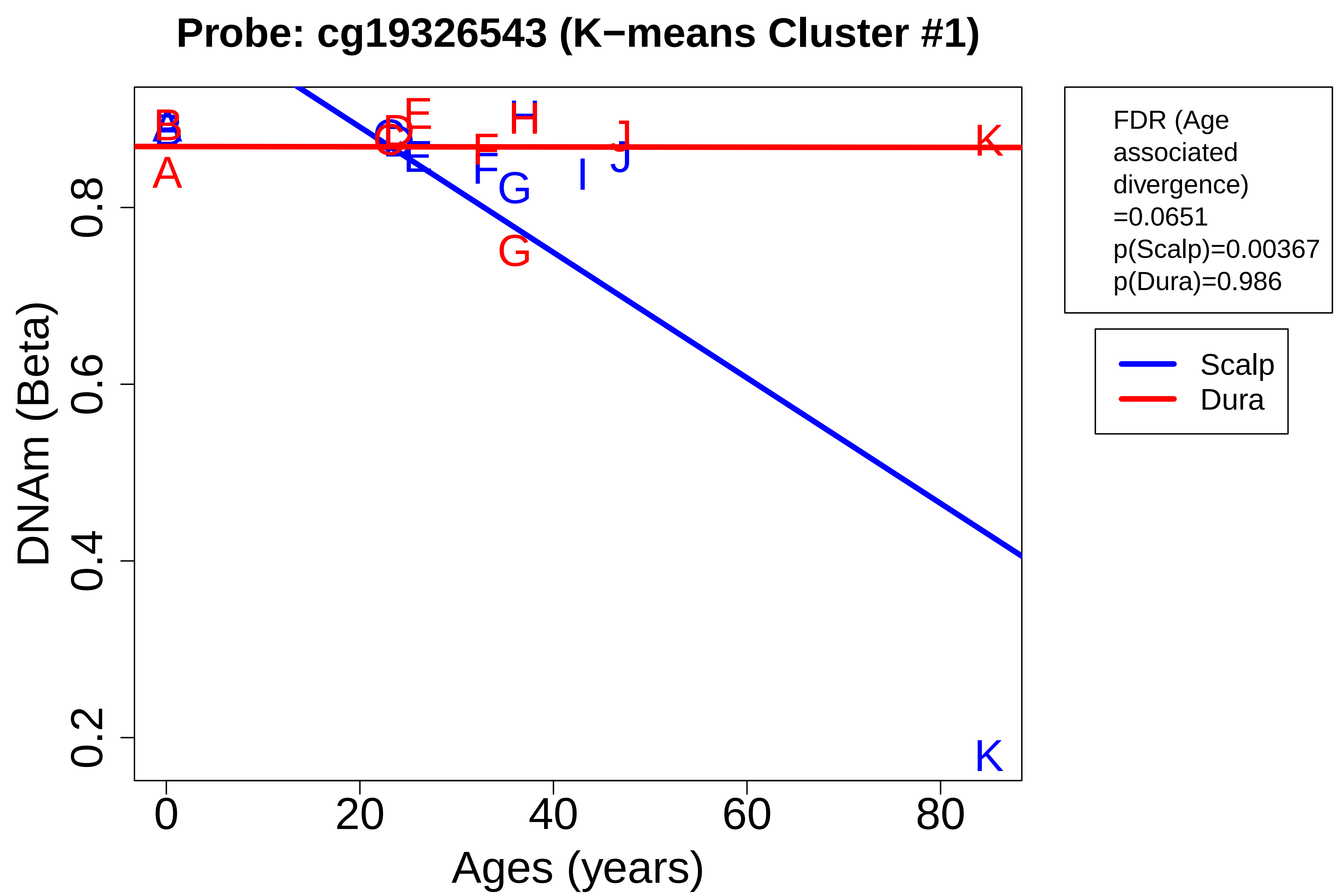

Probe: cg19326543 (K−means Cluster #1)
DNAm (Beta)
Ages (years)
0.2
0.4
0.6
0.8
0
20
40
60
80
A
B
C
D
E
F
G
H
I
J
K
FDR (Age associated divergence) =0.0651
p(Scalp)=0.00367
p(Dura)=0.986
Scalp
Dura

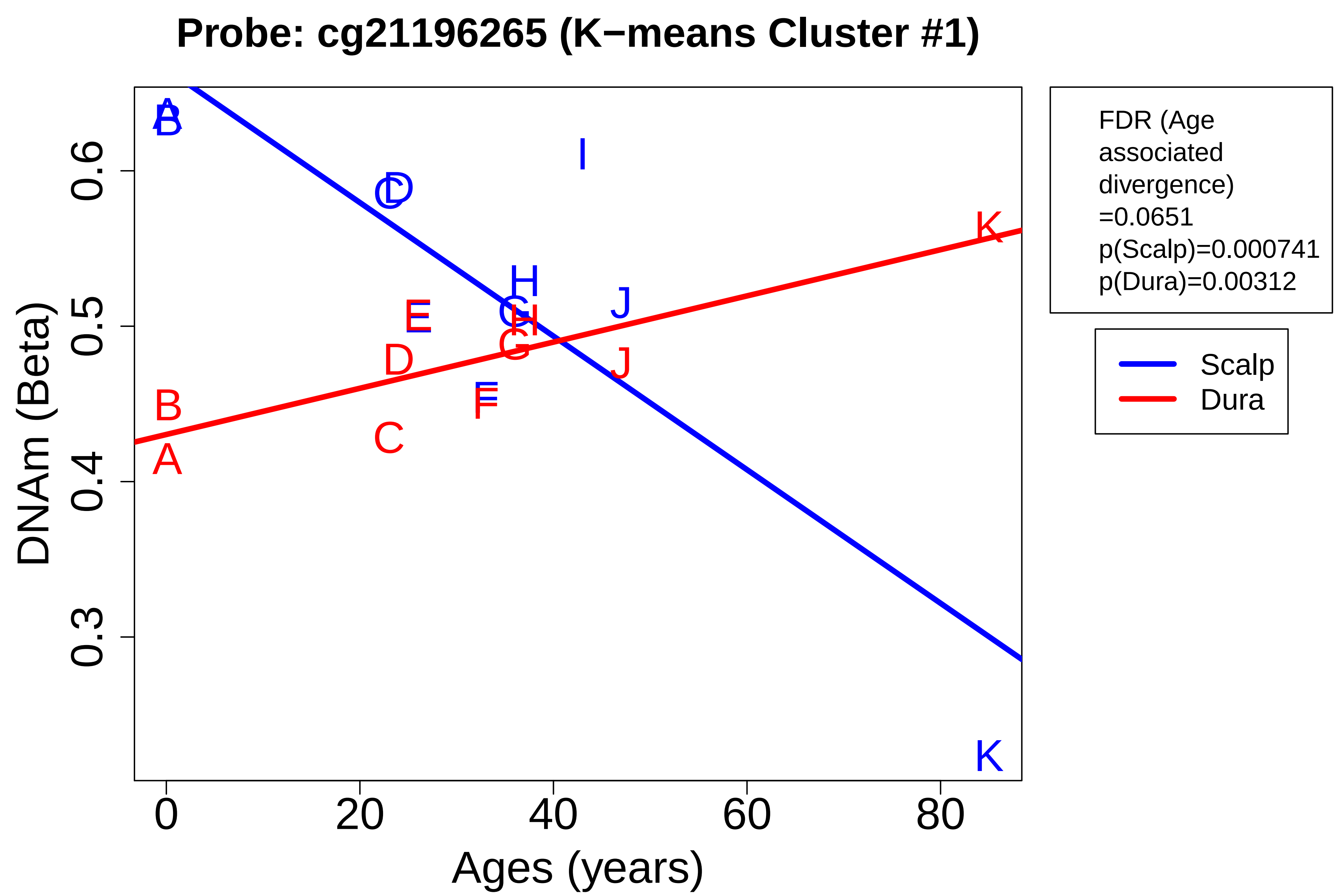
Probe: cg21196265 (K−means Cluster #1)
DNAm (Beta)
Ages (years)
0.3
0.4
0.5
0.6
0
20
40
60
80
FDR (Age associated divergence) =0.0651
p(Scalp)=0.000741
p(Dura)=0.00312
Scalp
Dura

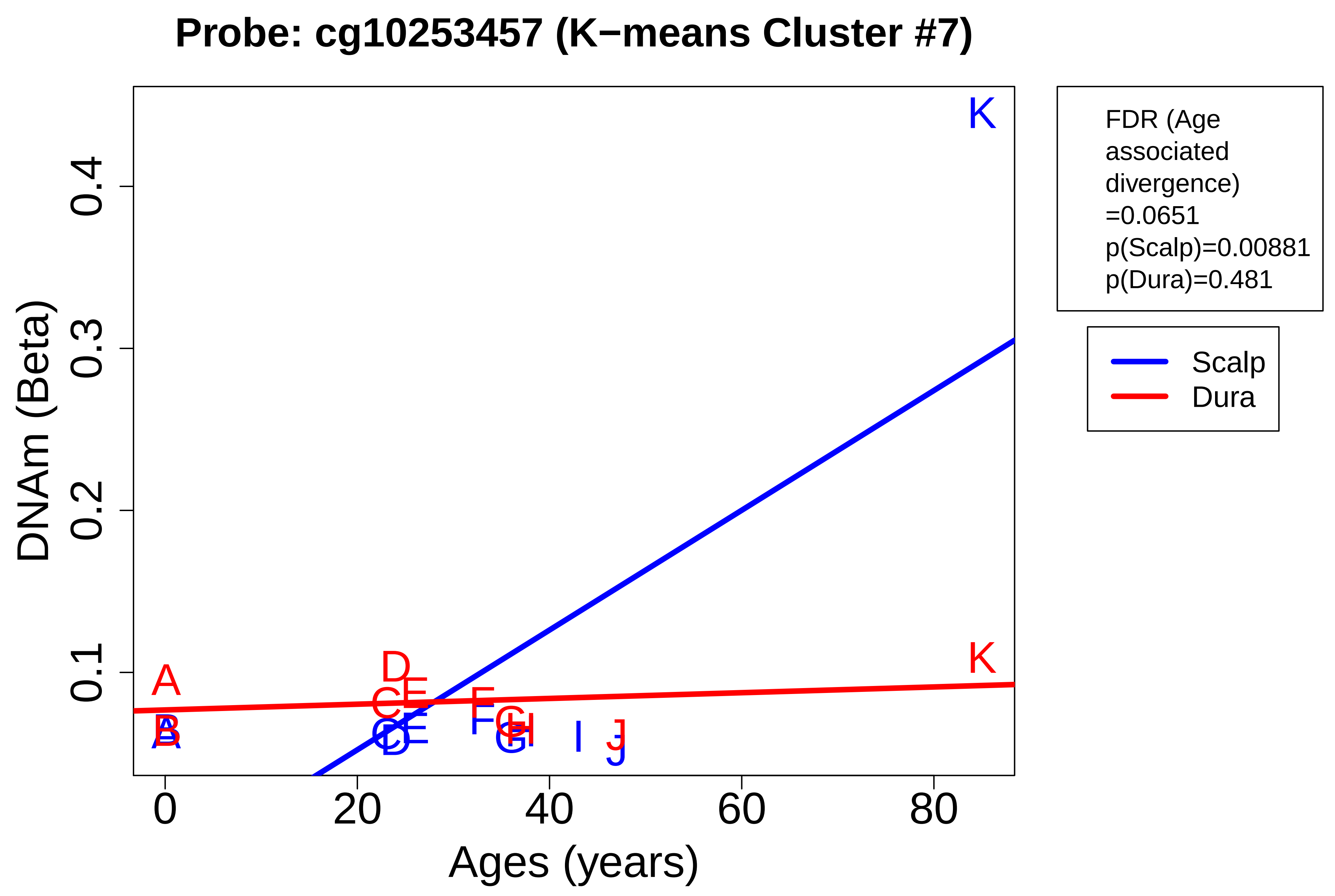
Probe: cg10253457 (K−means Cluster #7)
DNAm (Beta)
0.1
0.2
0.3
0.4
0
20
40
60
80
Ages (years)
A
B
C
D
E
F
G
H
I
J
K
FDR (Age associated divergence) =0.0651
p(Scalp)=0.00881
p(Dura)=0.481
Scalp
Dura

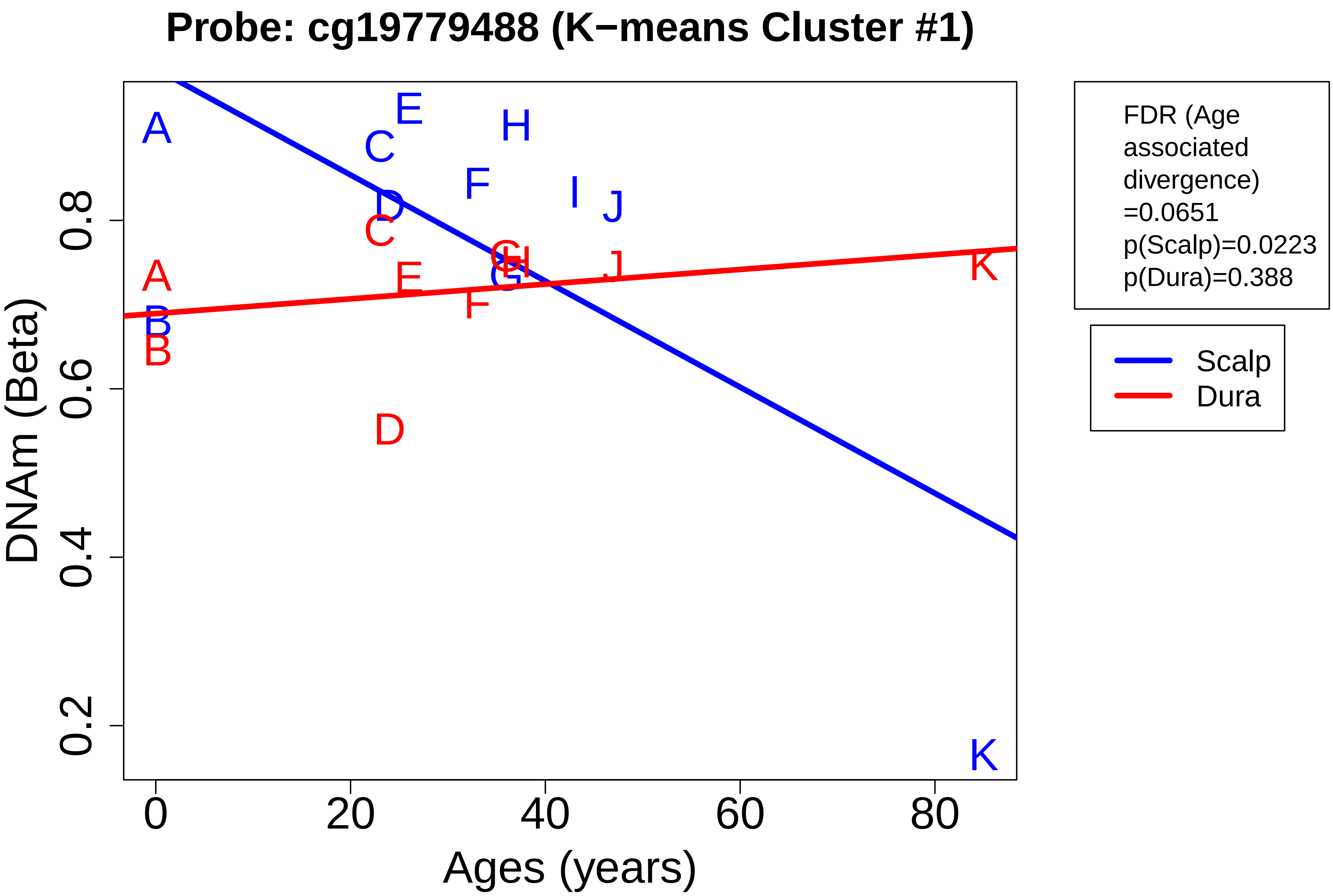

Probe: cg19779488 (K−means Cluster #1)
DNAm (Beta)
Ages (years)
FDR (Age associated divergence) =0.0651
p(Scalp)=0.0223
p(Dura)=0.388
Scalp
Dura

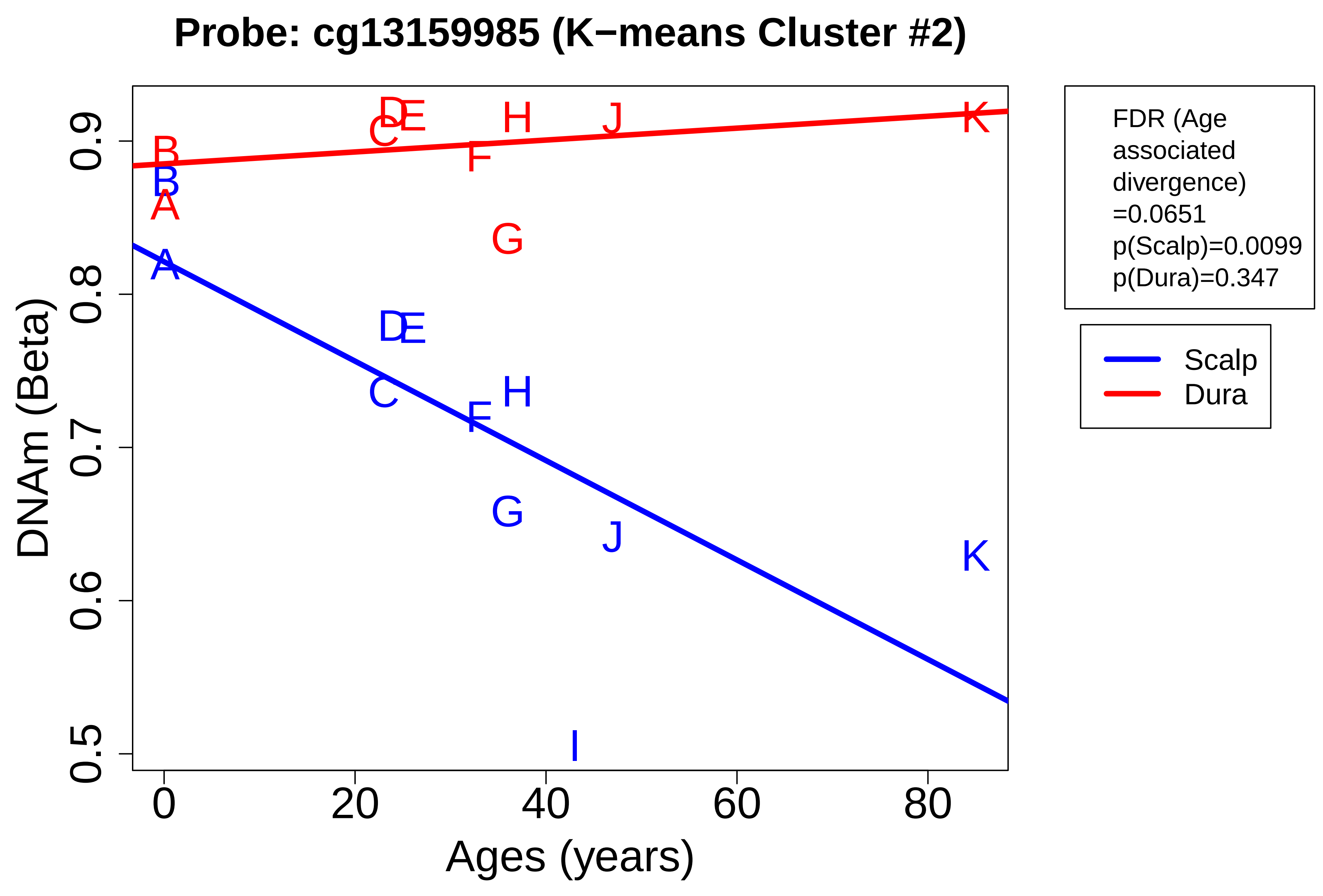
Probe: cg13159985 (K−means Cluster #2)
DNAm (Beta)
Ages (years)
0.5
0.6
0.7
0.8
0.9
0
20
40
60
80
FDR (Age associated divergence) =0.0651
p(Scalp)=0.0099
p(Dura)=0.347
Scalp
Dura

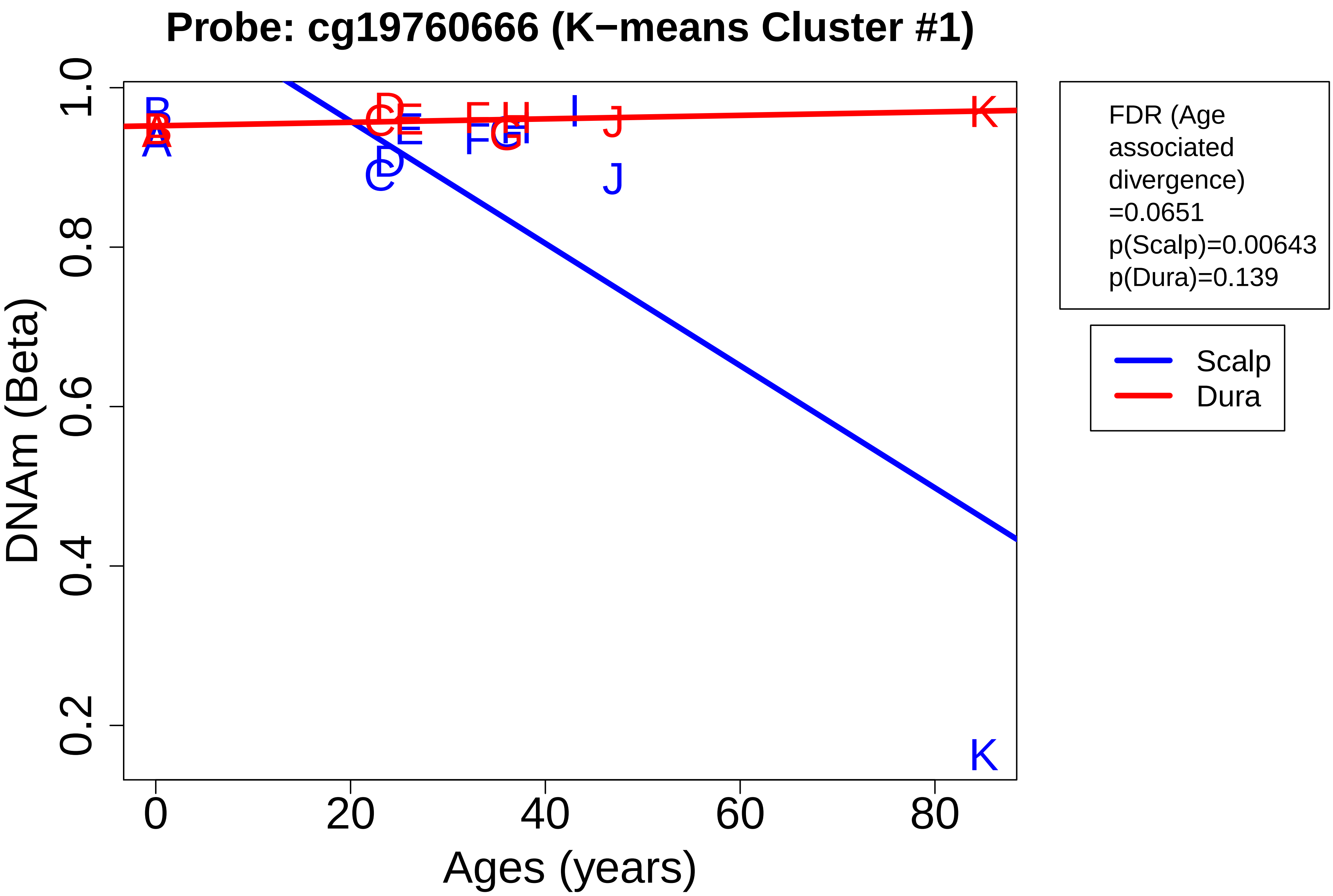

Probe: cg19760666 (K−means Cluster #1)
DNAm (Beta)
1.0
0.8
0.6
0.4
0.2
0
20
40
60
80
Ages (years)
FDR (Age associated divergence) =0.0651
p(Scalp)=0.00643
p(Dura)=0.139
Scalp
Dura

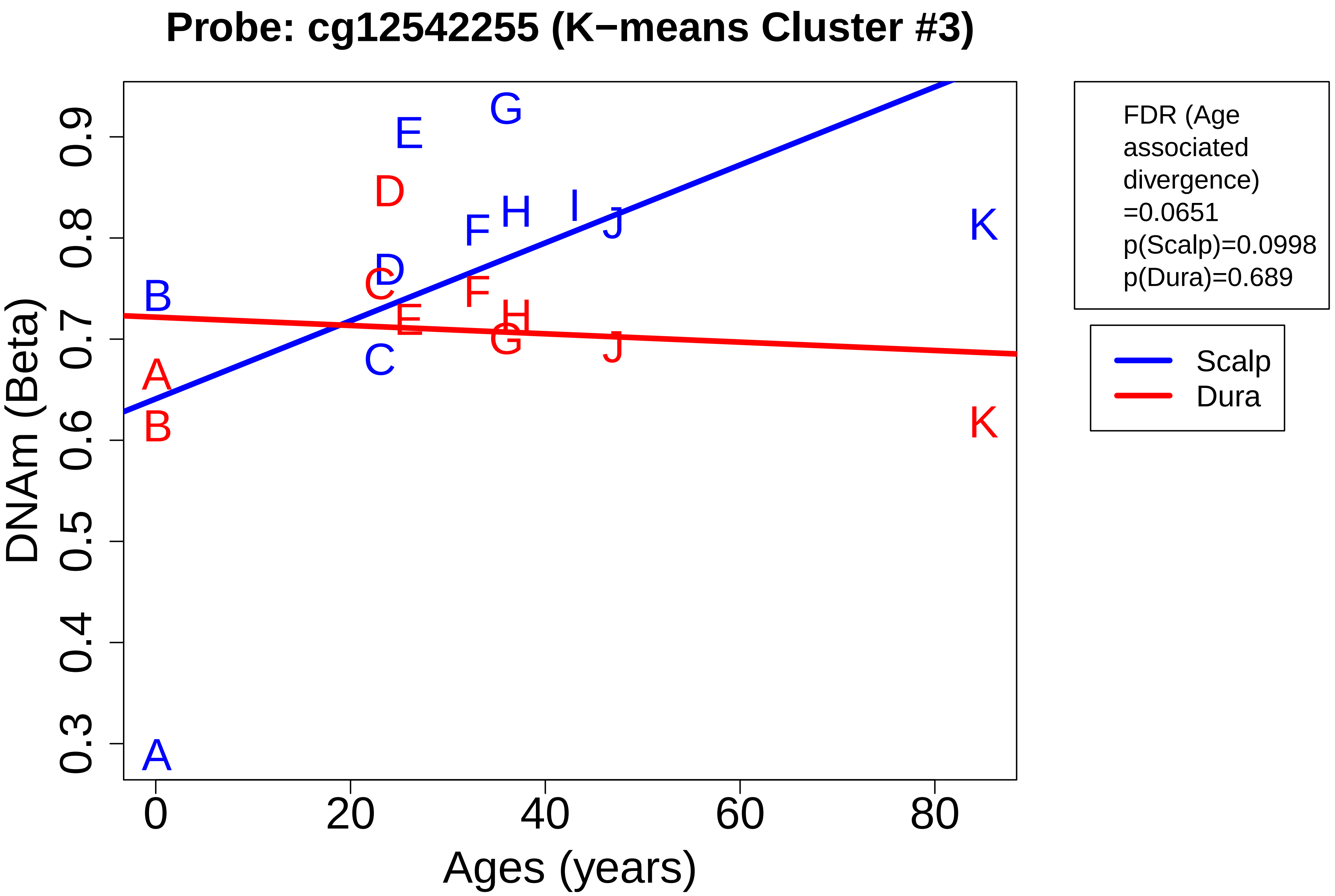
Probe: cg12542255 (K−means Cluster #3)
DNAm (Beta)
0.3
0.4
0.5
0.6
0.7
0.8
0.9
Ages (years)
0
20
40
60
80
FDR (Age associated divergence) =0.0651
p(Scalp)=0.0998
p(Dura)=0.689
Scalp
Dura
A
B
C
D
E
F
G
H
I
J
K

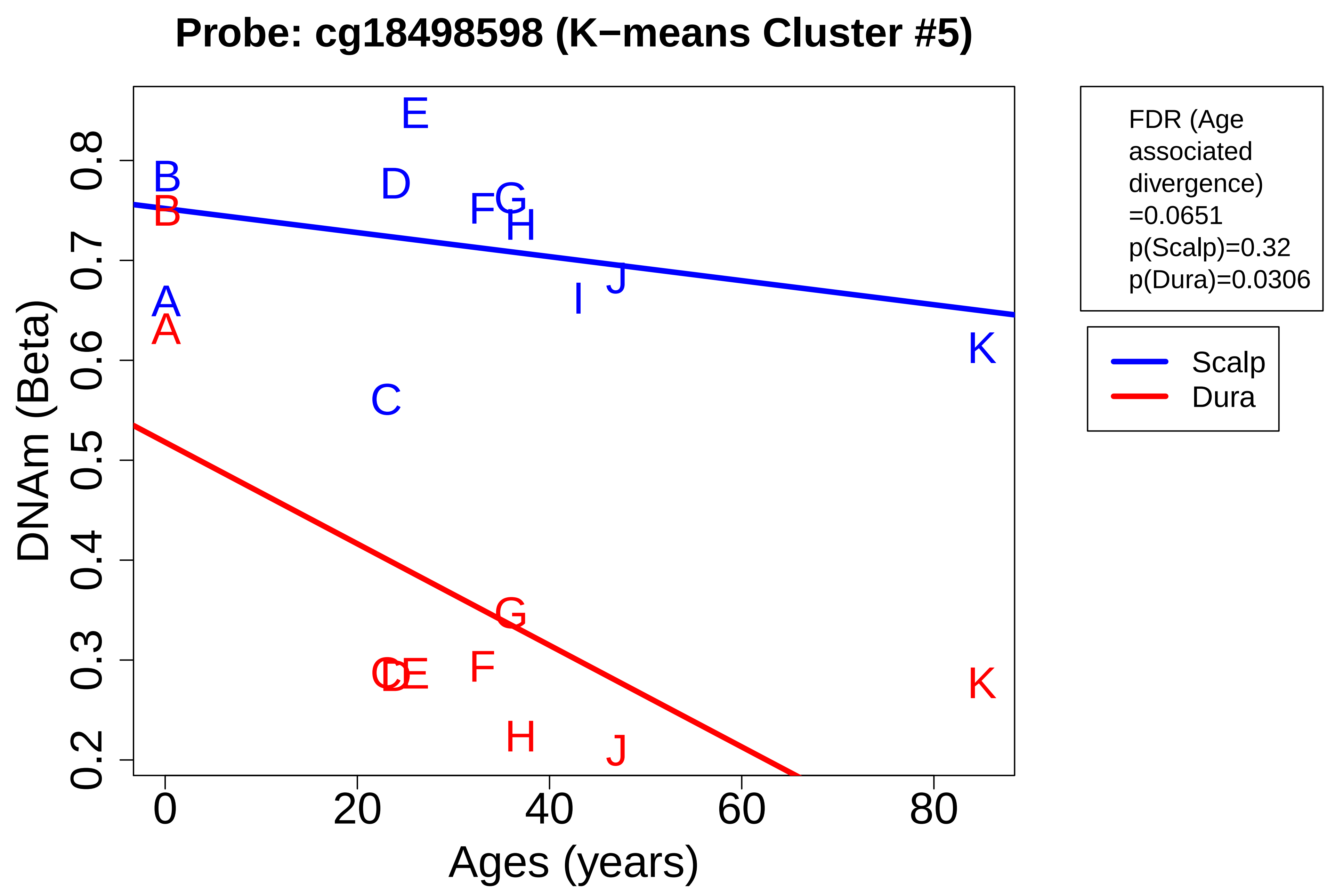

Probe: cg18498598 (K−means Cluster #5)
FDR (Age associated divergence) =0.0651
p(Scalp)=0.32
p(Dura)=0.0306
Scalp
Dura
DNAm (Beta)
Ages (years)
0.2
0.3
0.4
0.5
0.6
0.7
0.8
0
20
40
60
80

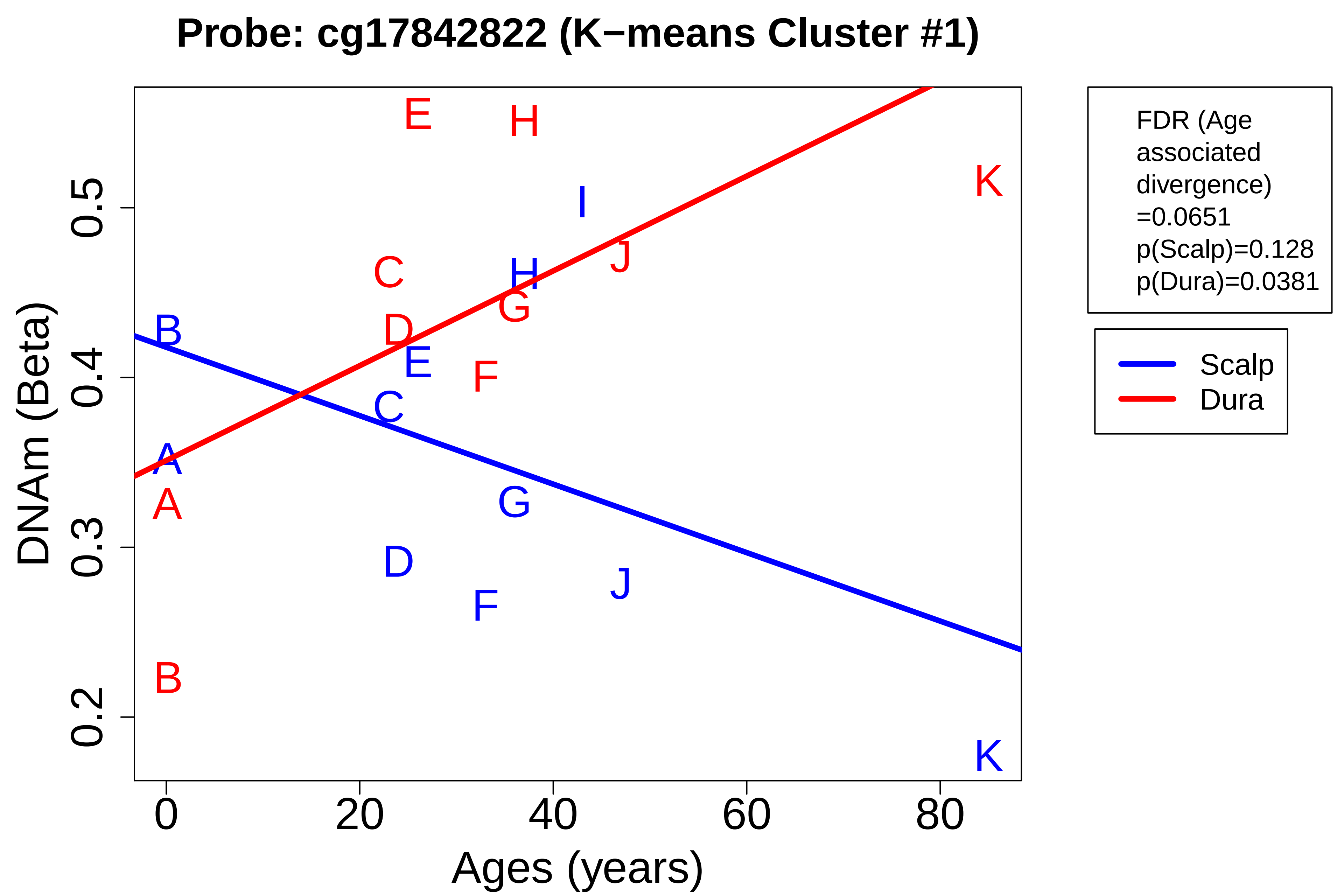
Probe: cg17842822 (K−means Cluster #1)
DNAm (Beta)
0.2
0.3
0.4
0.5
Ages (years)
0
20
40
60
80
FDR (Age associated divergence) =0.0651
p(Scalp)=0.128
p(Dura)=0.0381
Scalp
Dura
A B C D E F G H I J K

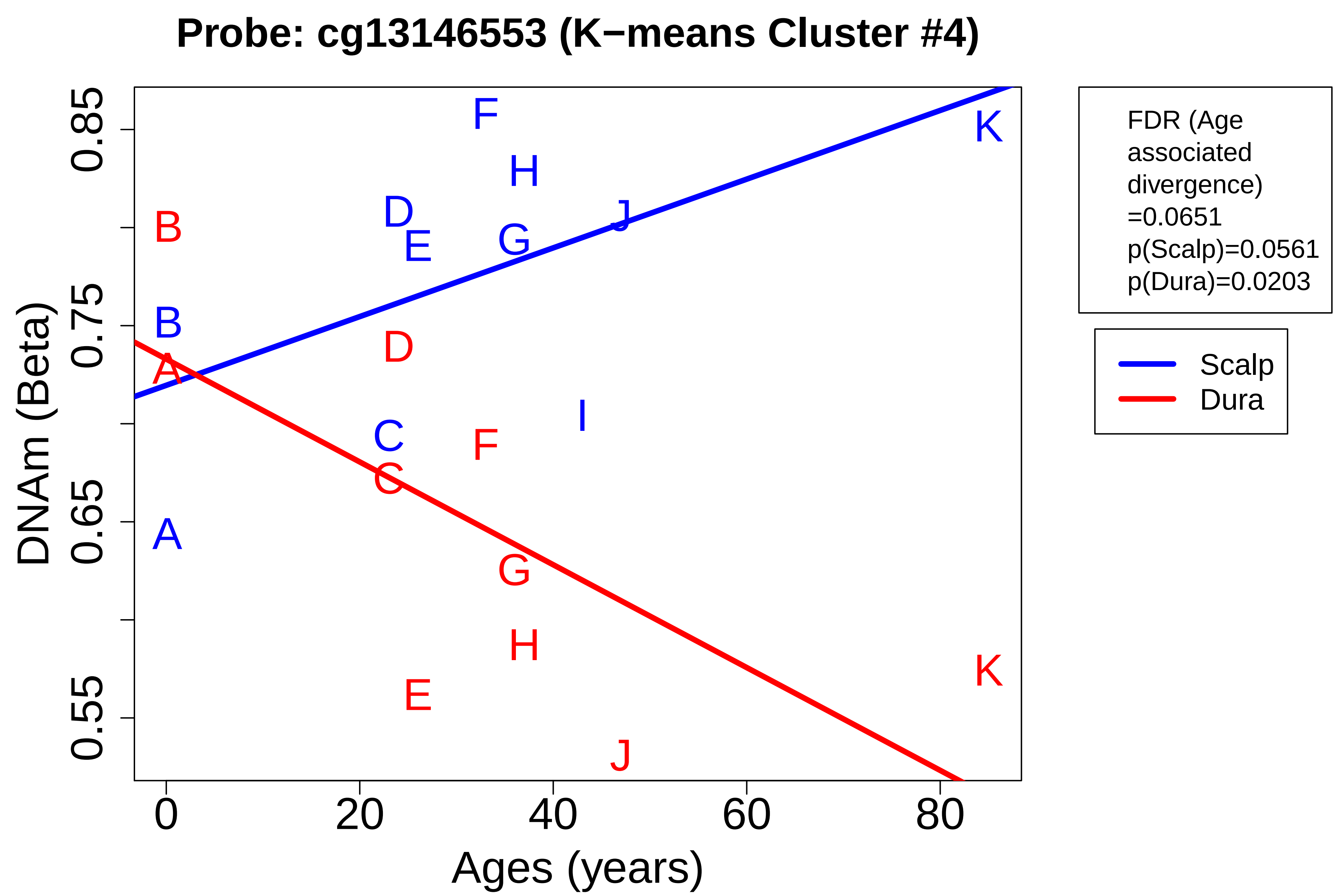

Probe: cg13146553 (K−means Cluster #4)
DNAm (Beta)
0.85
0.75
0.65
0.55
0
20
40
60
80
Ages (years)
FDR (Age associated divergence) =0.0651
p(Scalp)=0.0561
p(Dura)=0.0203
Scalp
Dura

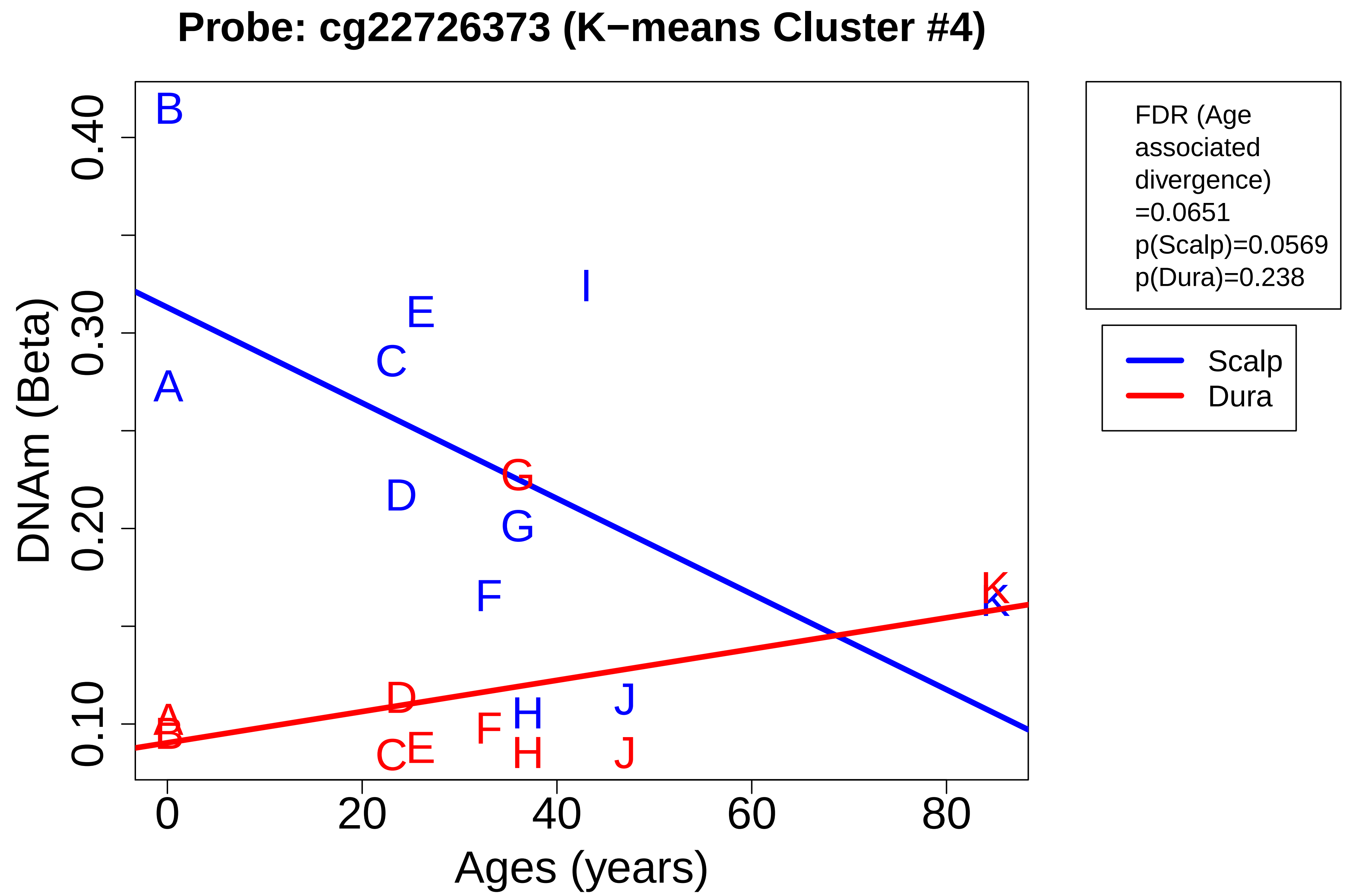

Probe: cg22726373 (K−means Cluster #4)
DNAm (Beta)
0.10
0.20
0.30
0.40
0
20
40
60
80
Ages (years)
A
B
C
D
E
F
G
H
I
J
K
A
B
C
D
E
F
G
H
J
K
FDR (Age associated divergence) =0.0651
p(Scalp)=0.0569
p(Dura)=0.238
Scalp
Dura

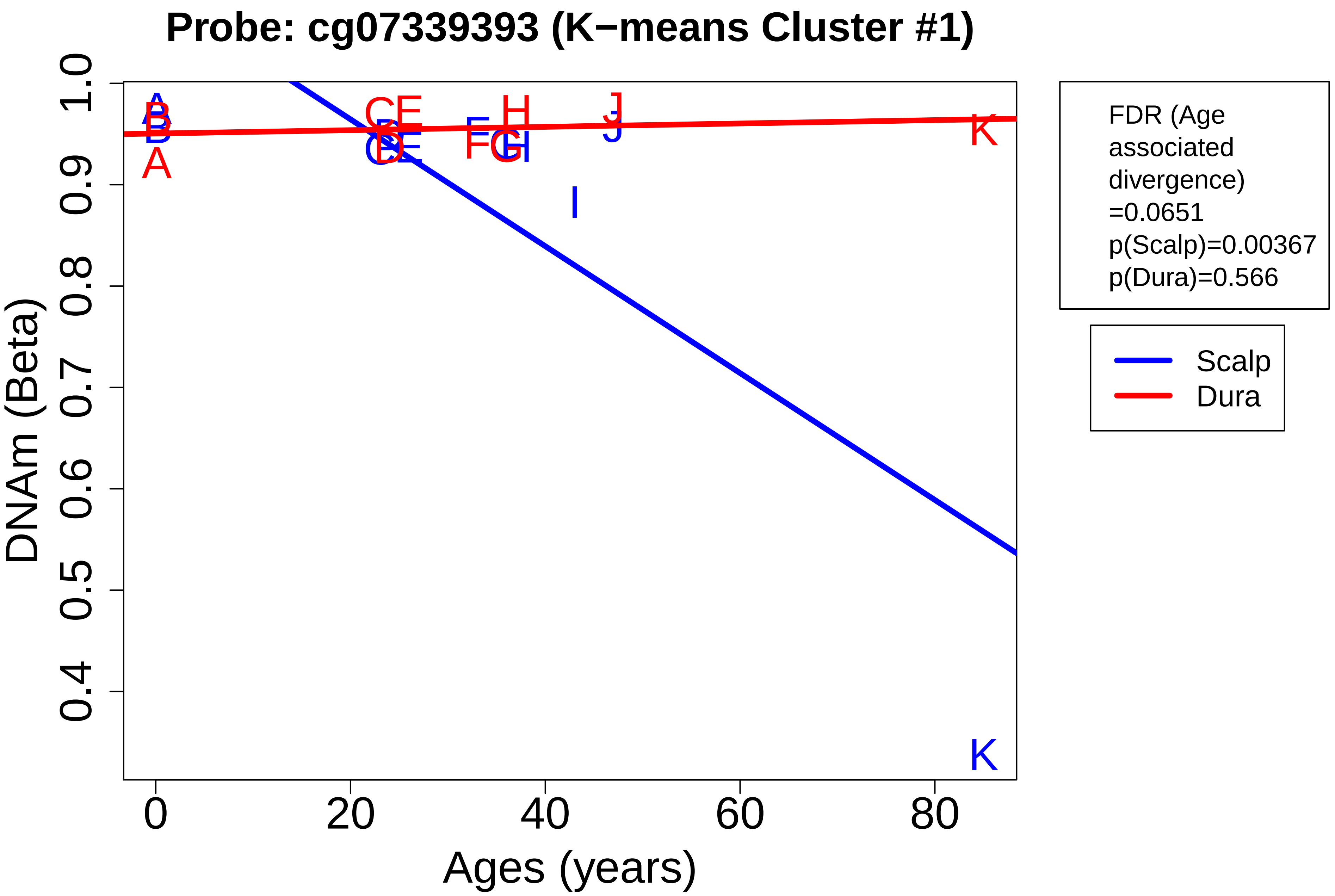

Probe: cg07339393 (K−means Cluster #1)
DNAm (Beta)
Ages (years)
0.4
0.5
0.6
0.7
0.8
0.9
1.0
0
20
40
60
80
FDR (Age associated divergence) =0.0651
p(Scalp)=0.00367
p(Dura)=0.566
Scalp
Dura

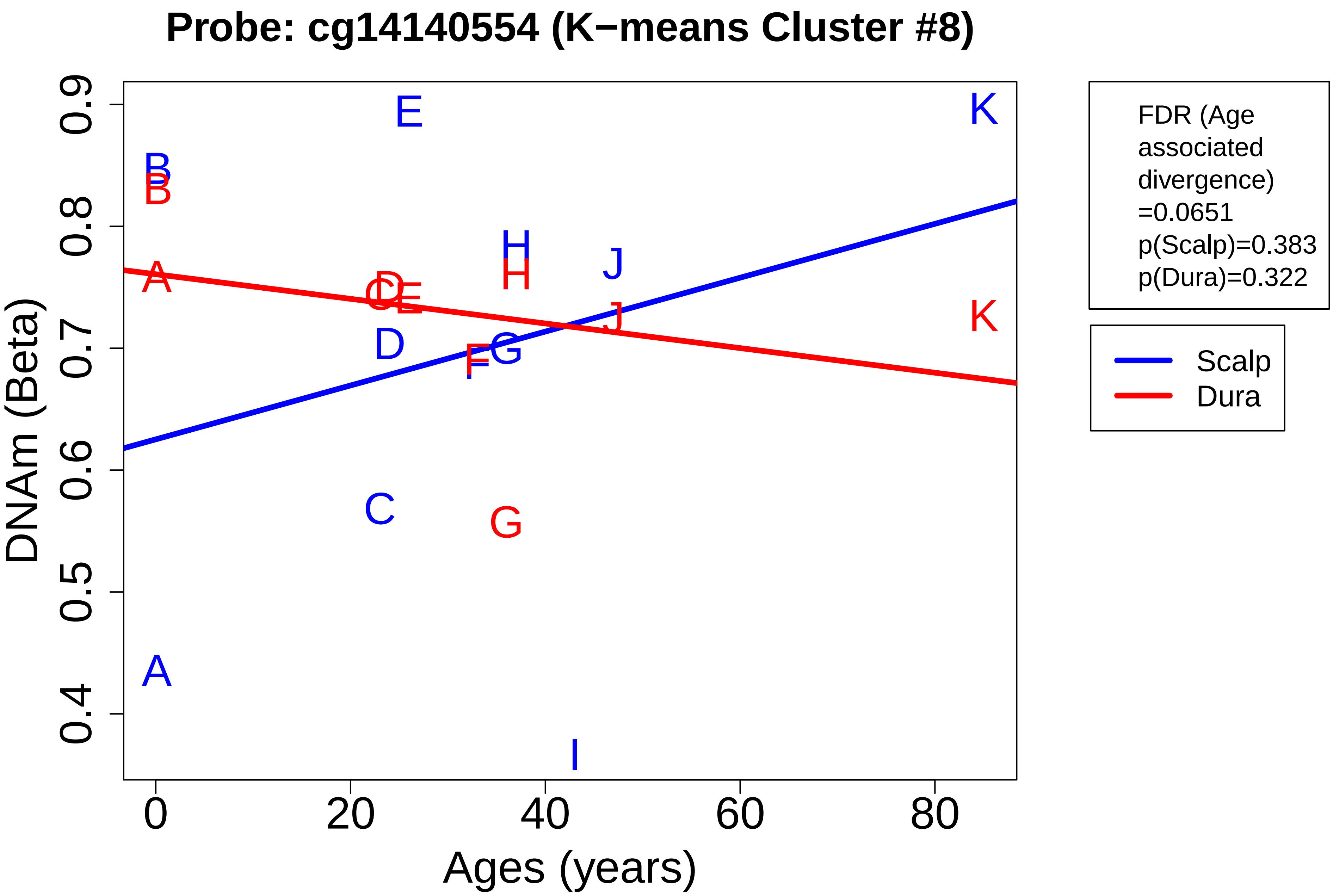
Probe: cg14140554 (K−means Cluster #8)
DNAm (Beta)
Ages (years)
0.4
0.5
0.6
0.7
0.8
0.9
0
20
40
60
80
FDR (Age associated divergence) =0.0651
p(Scalp)=0.383
p(Dura)=0.322
Scalp
Dura
A
B
C
D
E
F
G
H
I
J
K

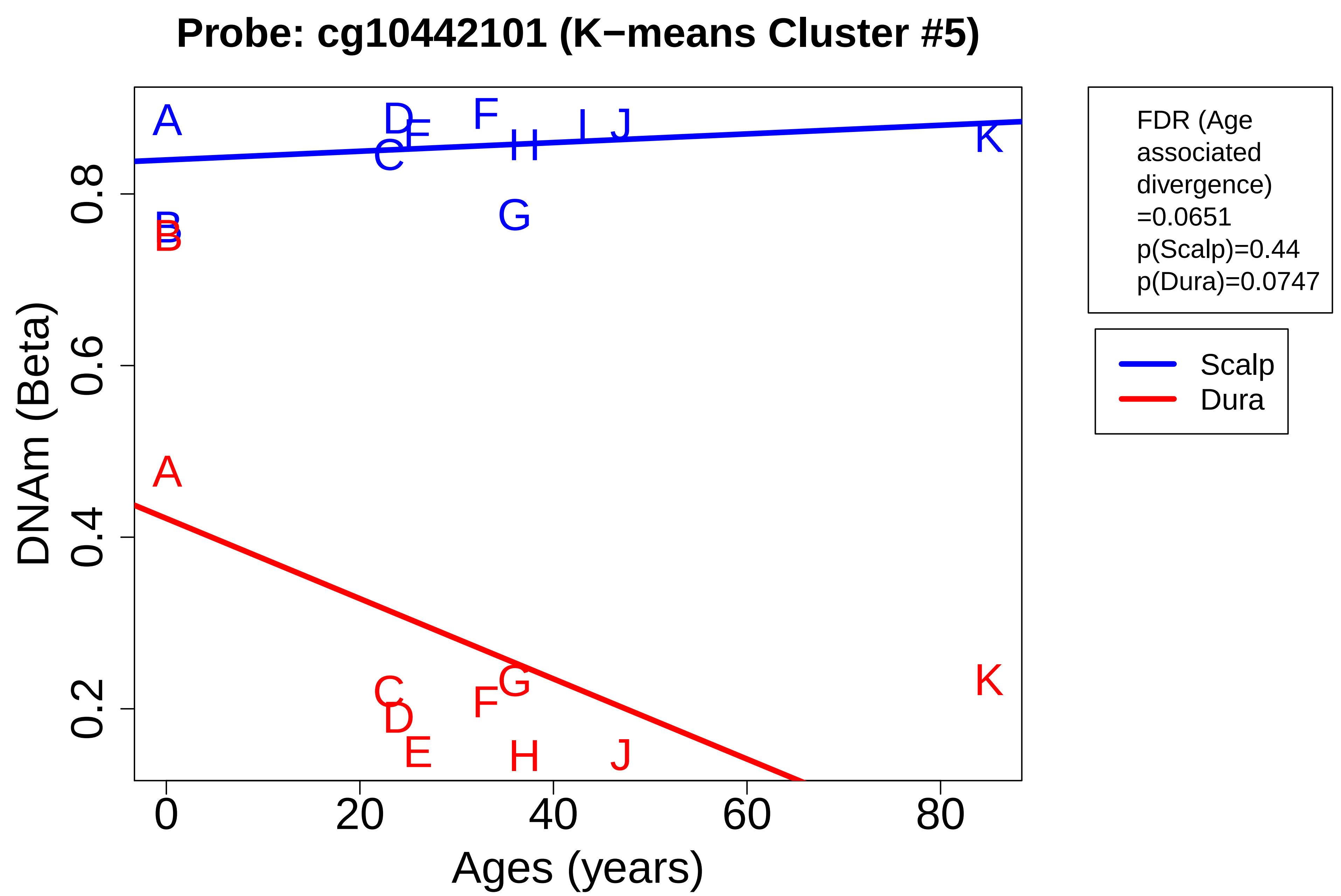
Probe: cg10442101 (K−means Cluster #5)
DNAm (Beta)
0.2
0.4
0.6
0.8
Ages (years)
0
20
40
60
80
A
B
C
D
E
F
G
H
I
J
K
FDR (Age associated divergence) =0.0651
p(Scalp)=0.44
p(Dura)=0.0747
Scalp
Dura

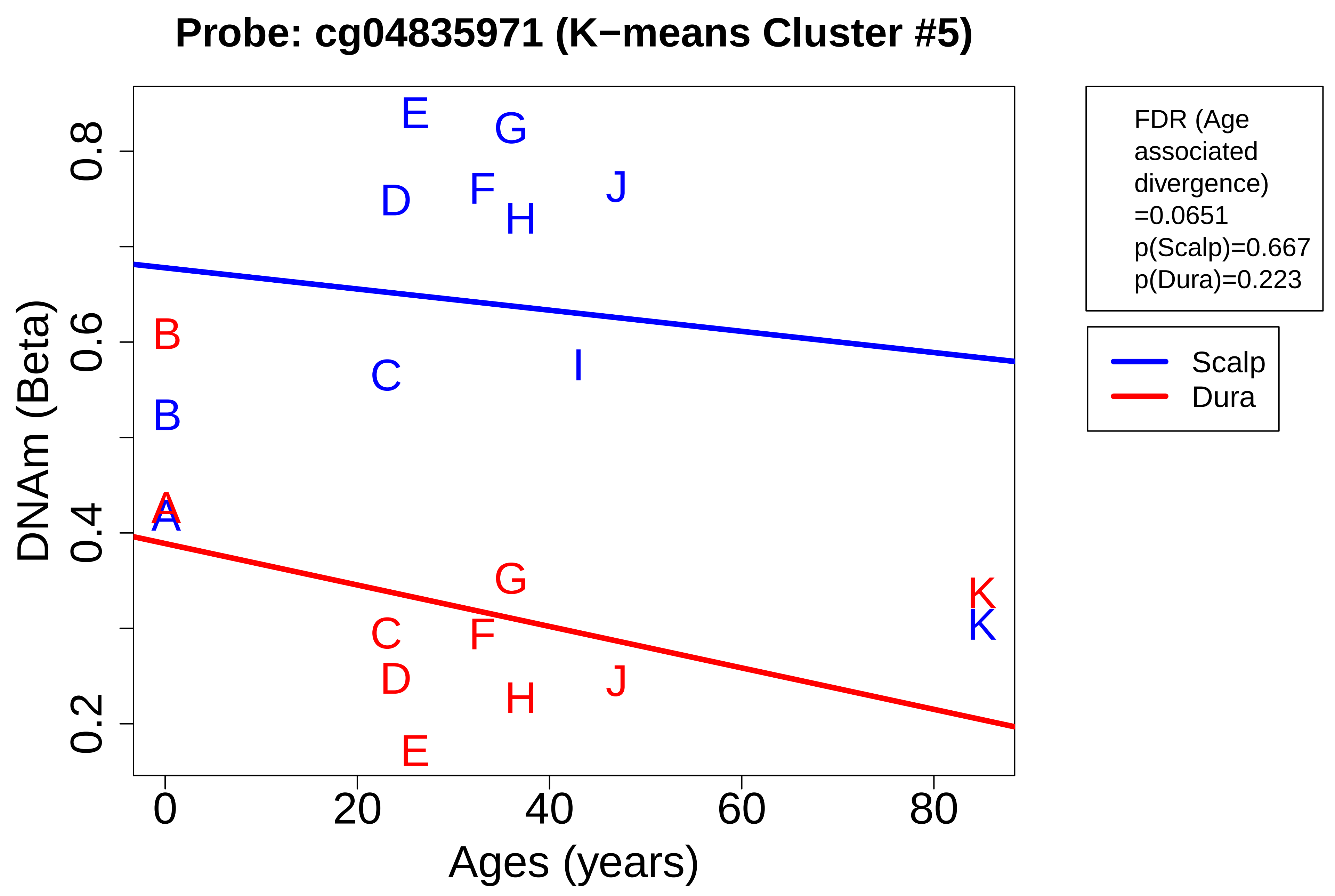

Probe: cg04835971 (K−means Cluster #5)
DNAm (Beta)
0.2
0.4
0.6
0.8
Ages (years)
0
20
40
60
80
FDR (Age associated divergence) =0.0651
p(Scalp)=0.667
p(Dura)=0.223
Scalp
Dura
A
B
C
D
E
F
G
H
I
J
K

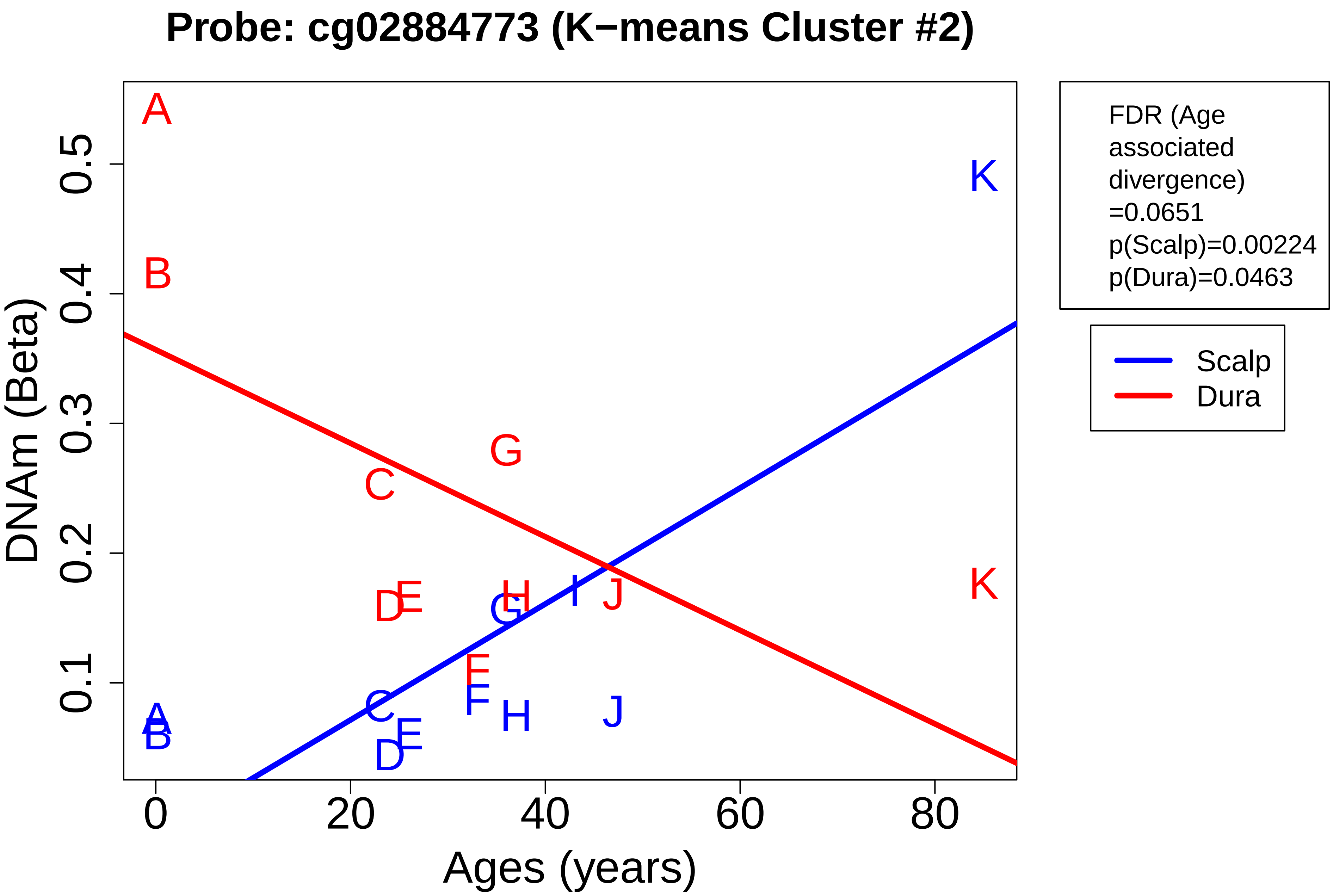

Probe: cg02884773 (K−means Cluster #2)
DNAm (Beta)
0.1
0.2
0.3
0.4
0.5
Ages (years)
0
20
40
60
80
FDR (Age associated divergence) =0.0651
p(Scalp)=0.00224
p(Dura)=0.0463
Scalp
Dura
A
B
C
D
E
F
G
H
I
J
K

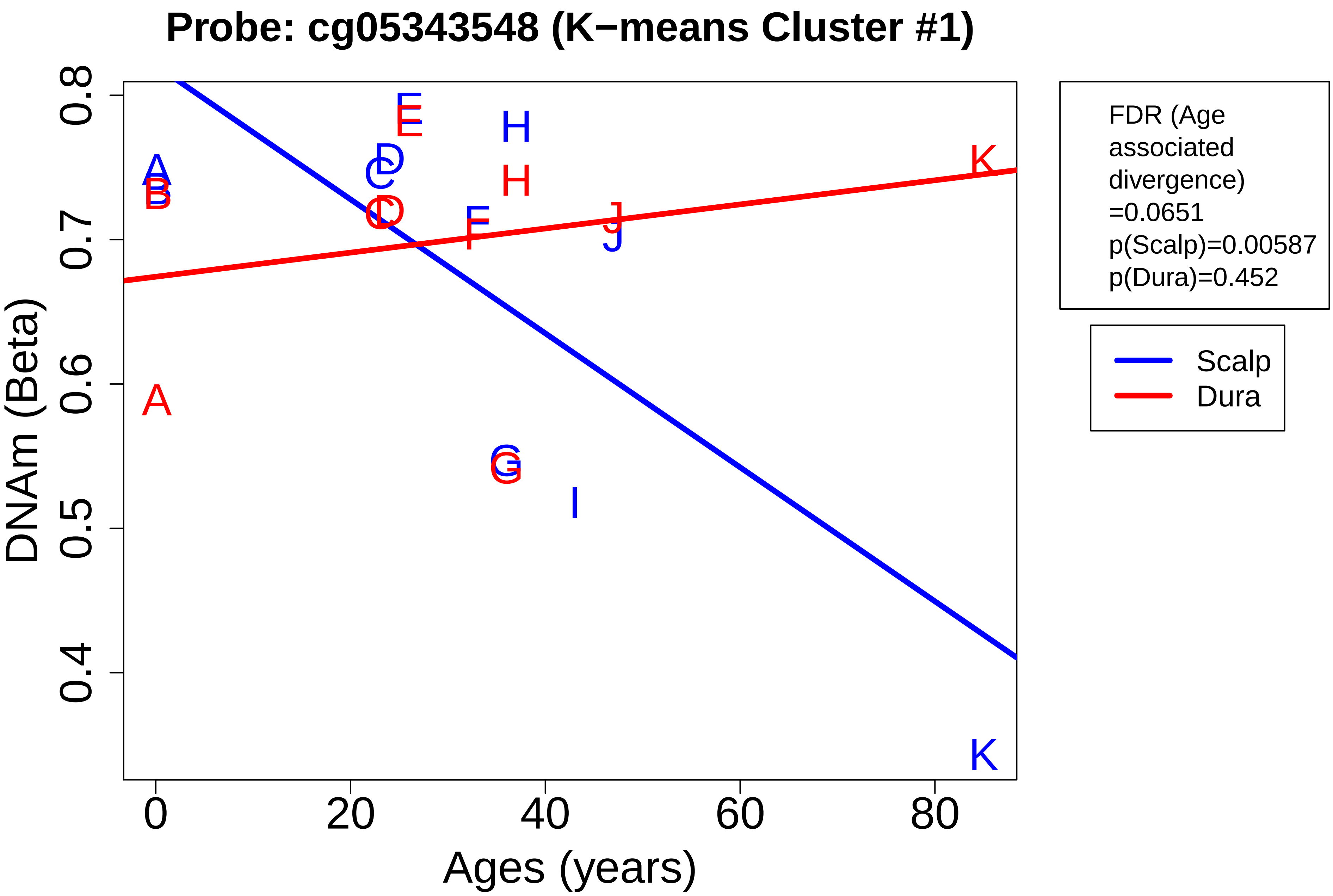

Probe: cg05343548 (K−means Cluster #1)
DNAm (Beta)
0.4
0.5
0.6
0.7
0.8
Ages (years)
0
20
40
60
80
FDR (Age associated divergence) =0.0651
p(Scalp)=0.00587
p(Dura)=0.452
Scalp
Dura

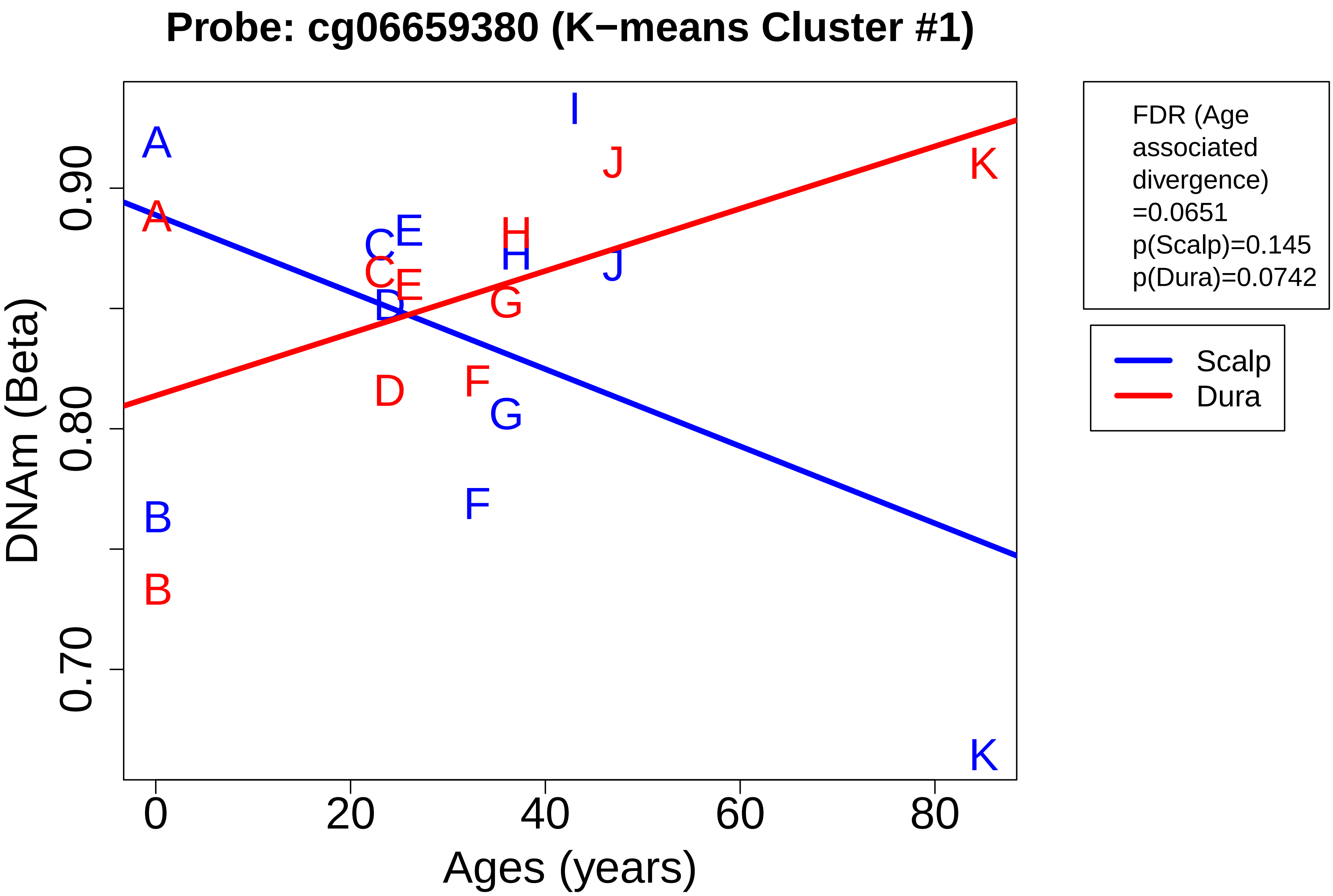
Probe: cg06659380 (K−means Cluster #1)
DNAm (Beta)
0.70
0.80
0.90
Ages (years)
0
20
40
60
80
A
B
C
D
E
F
G
H
I
J
K
FDR (Age associated divergence) =0.0651
p(Scalp)=0.145
p(Dura)=0.0742
Scalp
Dura

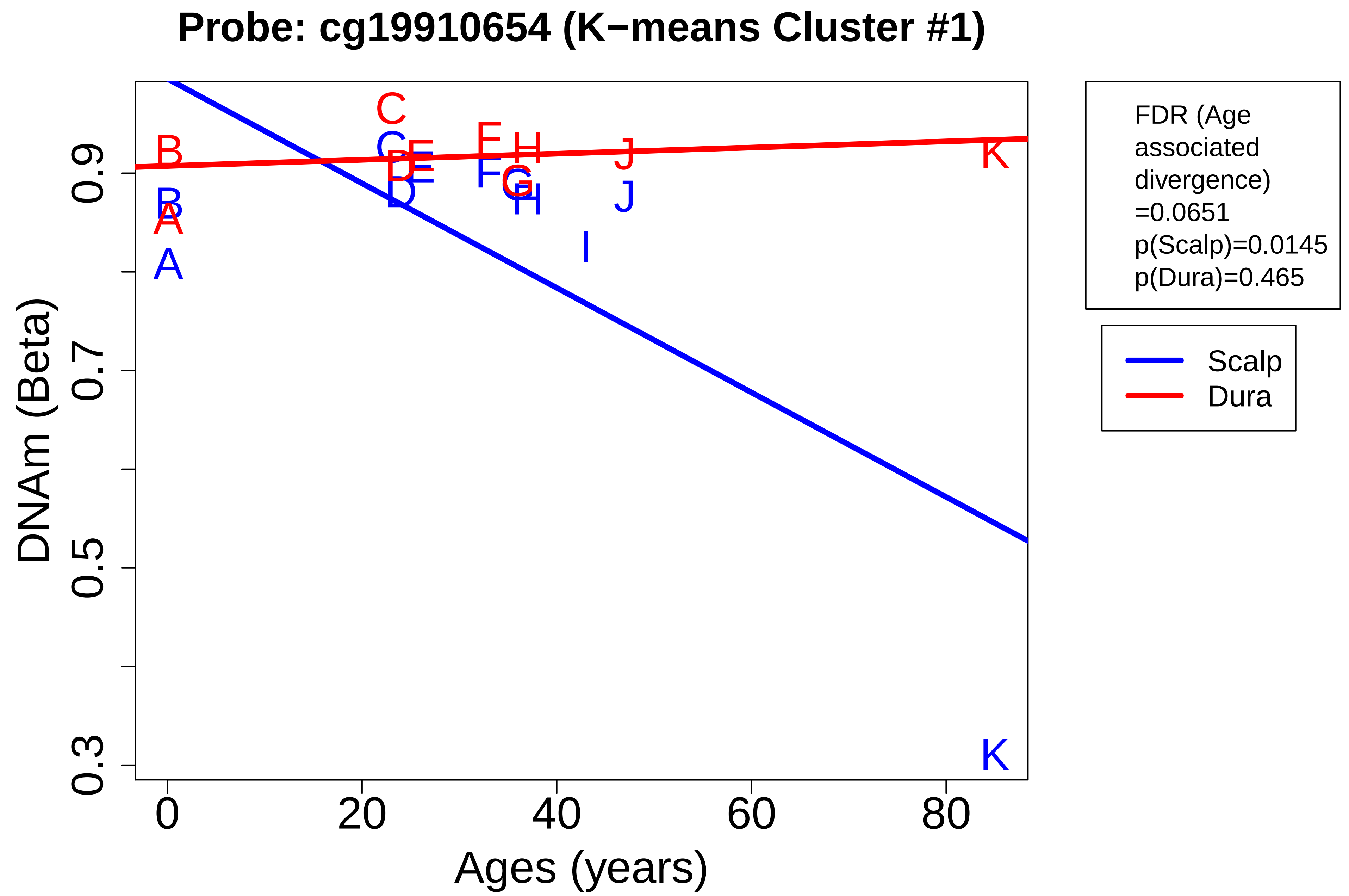

Probe: cg19910654 (K−means Cluster #1)
DNAm (Beta)
0.3
0.5
0.7
0.9
0
20
40
60
80
Ages (years)
FDR (Age associated divergence) =0.0651
p(Scalp)=0.0145
p(Dura)=0.465
Scalp
Dura

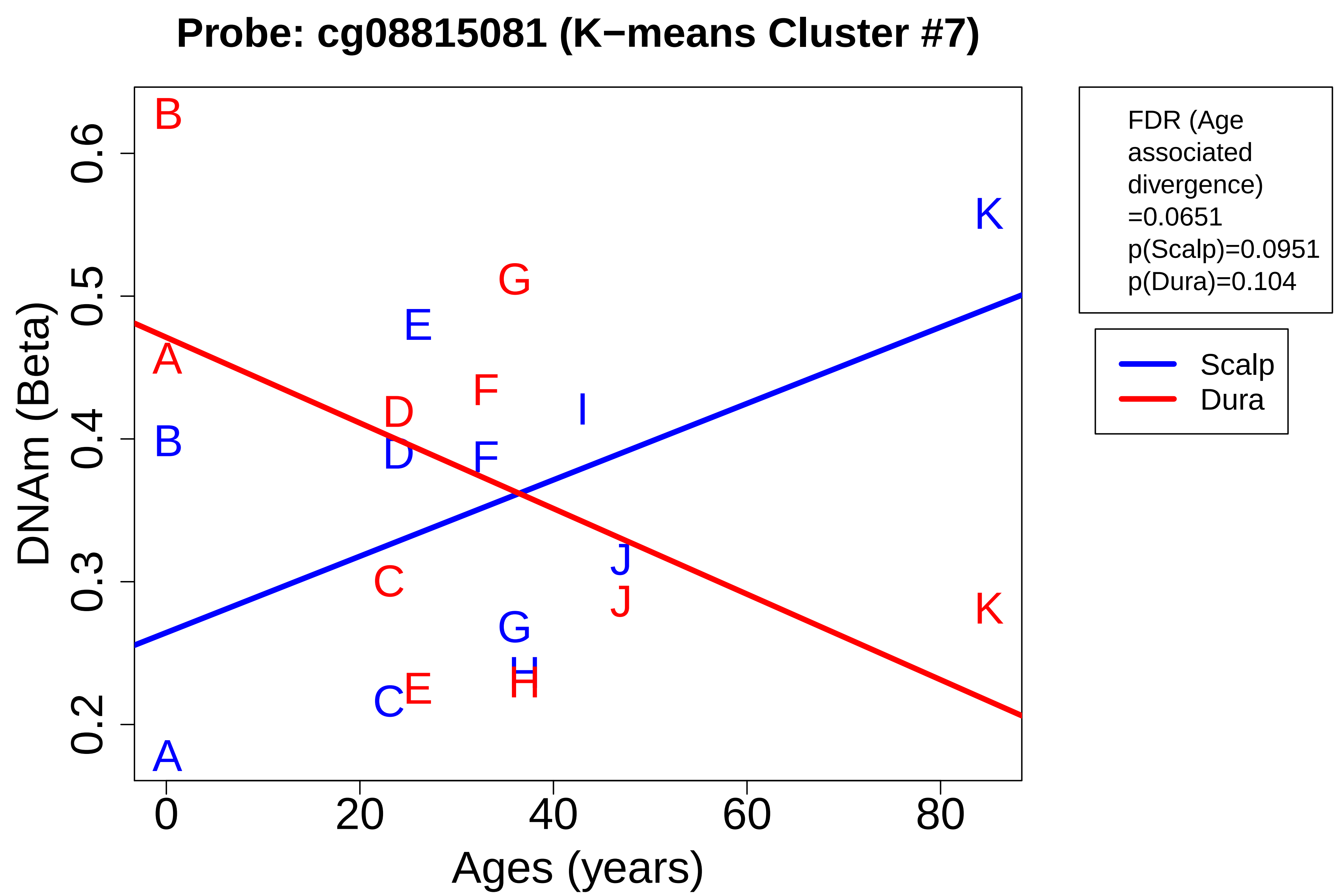
Probe: cg08815081 (K−means Cluster #7)
DNAm (Beta)
0.2
0.3
0.4
0.5
0.6
Ages (years)
0
20
40
60
80
FDR (Age associated divergence) =0.0651
p(Scalp)=0.0951
p(Dura)=0.104
Scalp
Dura
A
B
C
D
E
F
G
H
I
J
K

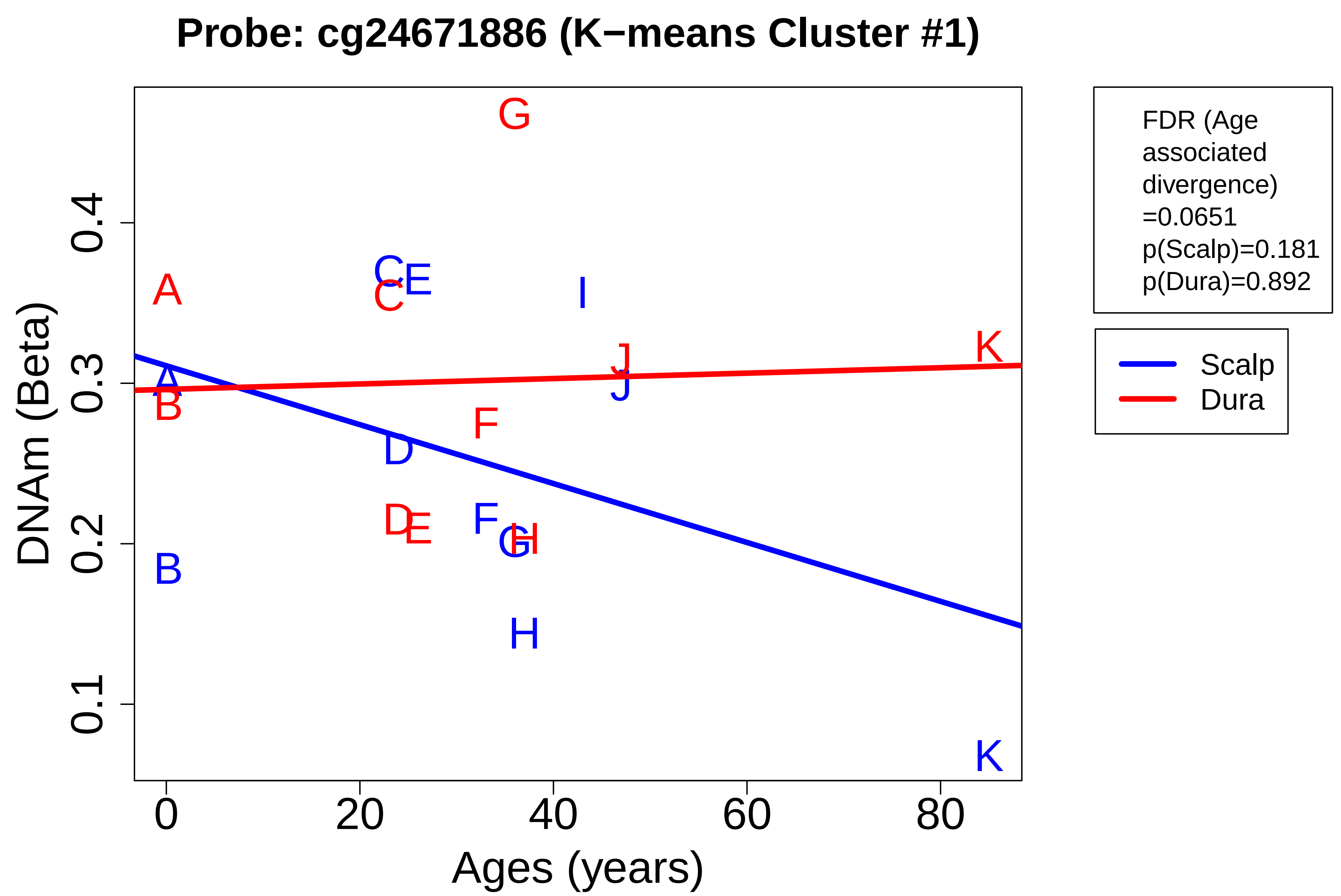

Probe: cg24671886 (K−means Cluster #1)
DNAm (Beta)
0.1
0.2
0.3
0.4
0
20
40
60
80
Ages (years)
A
B
C
D
E
F
G
H
I
J
K
FDR (Age associated divergence) =0.0651
p(Scalp)=0.181
p(Dura)=0.892
Scalp
Dura

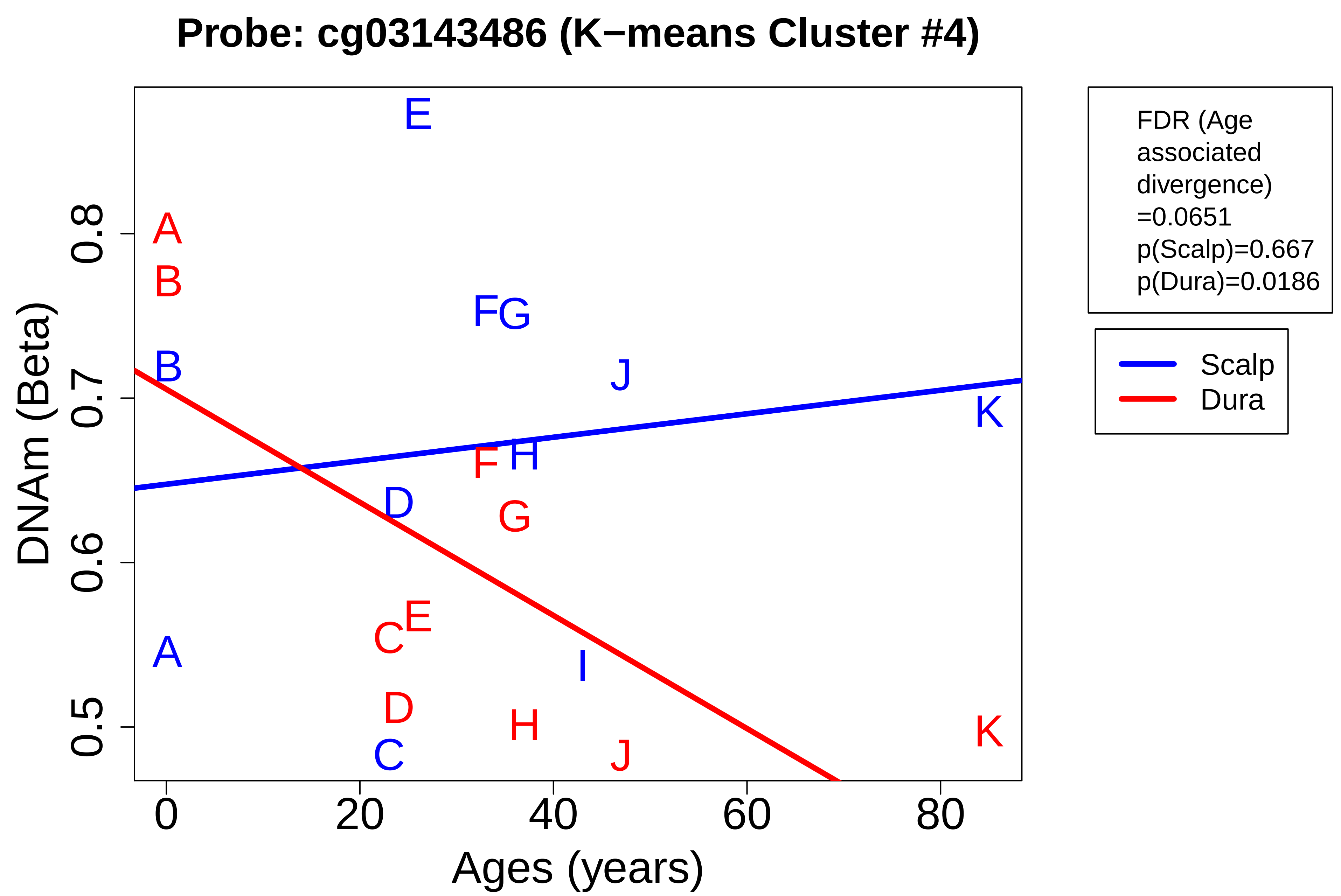
Probe: cg03143486 (K−means Cluster #4)
DNAm (Beta)
0.5
0.6
0.7
0.8
Ages (years)
0
20
40
60
80
A
B
C
D
E
F
G
H
I
J
K
FDR (Age associated divergence) =0.0651
p(Scalp)=0.667
p(Dura)=0.0186
Scalp
Dura

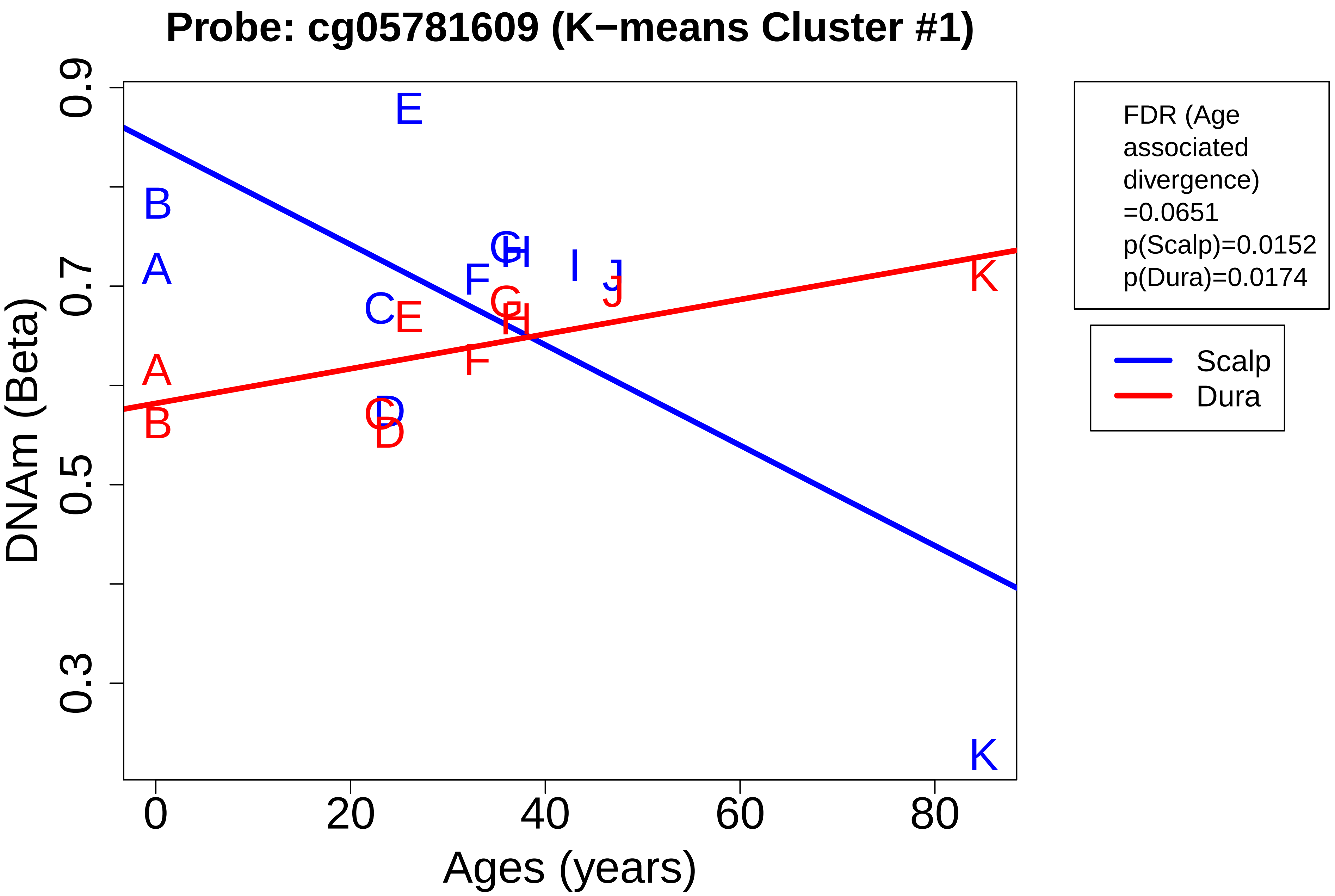

Probe: cg05781609 (K−means Cluster #1)
DNAm (Beta)
0.3
0.5
0.7
0.9
0
20
40
60
80
Ages (years)
FDR (Age associated divergence) =0.0651
p(Scalp)=0.0152
p(Dura)=0.0174
Scalp
Dura
A
B
C
D
E
F
G
H
I
J
K

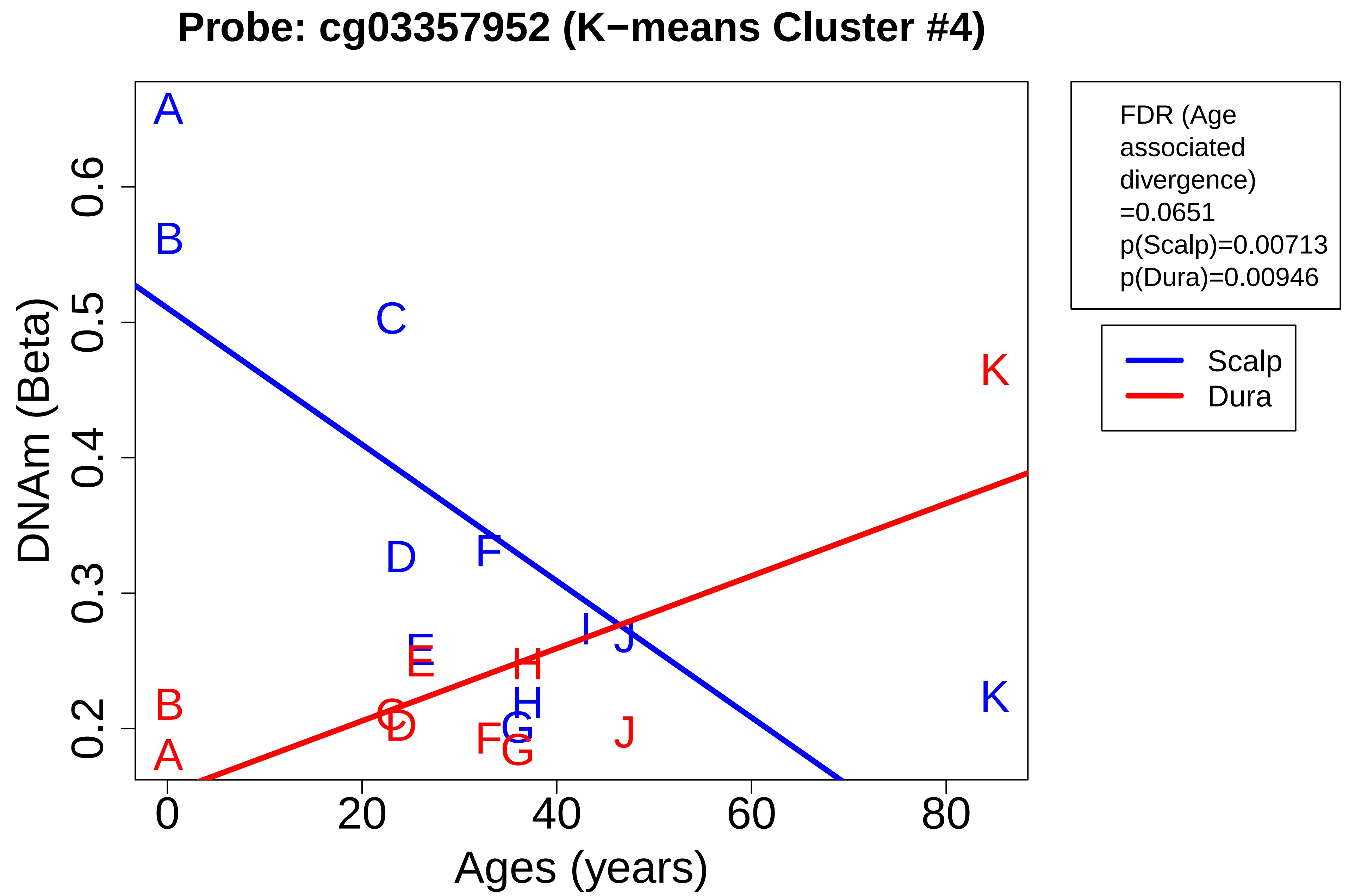

Probe: cg03357952 (K−means Cluster #4)
DNAm (Beta)
0.2
0.3
0.4
0.5
0.6
Ages (years)
0
20
40
60
80
A
B
C
D
E
F
G
H
I
J
K
FDR (Age associated divergence) =0.0651
p(Scalp)=0.00713
p(Dura)=0.00946
Scalp
Dura

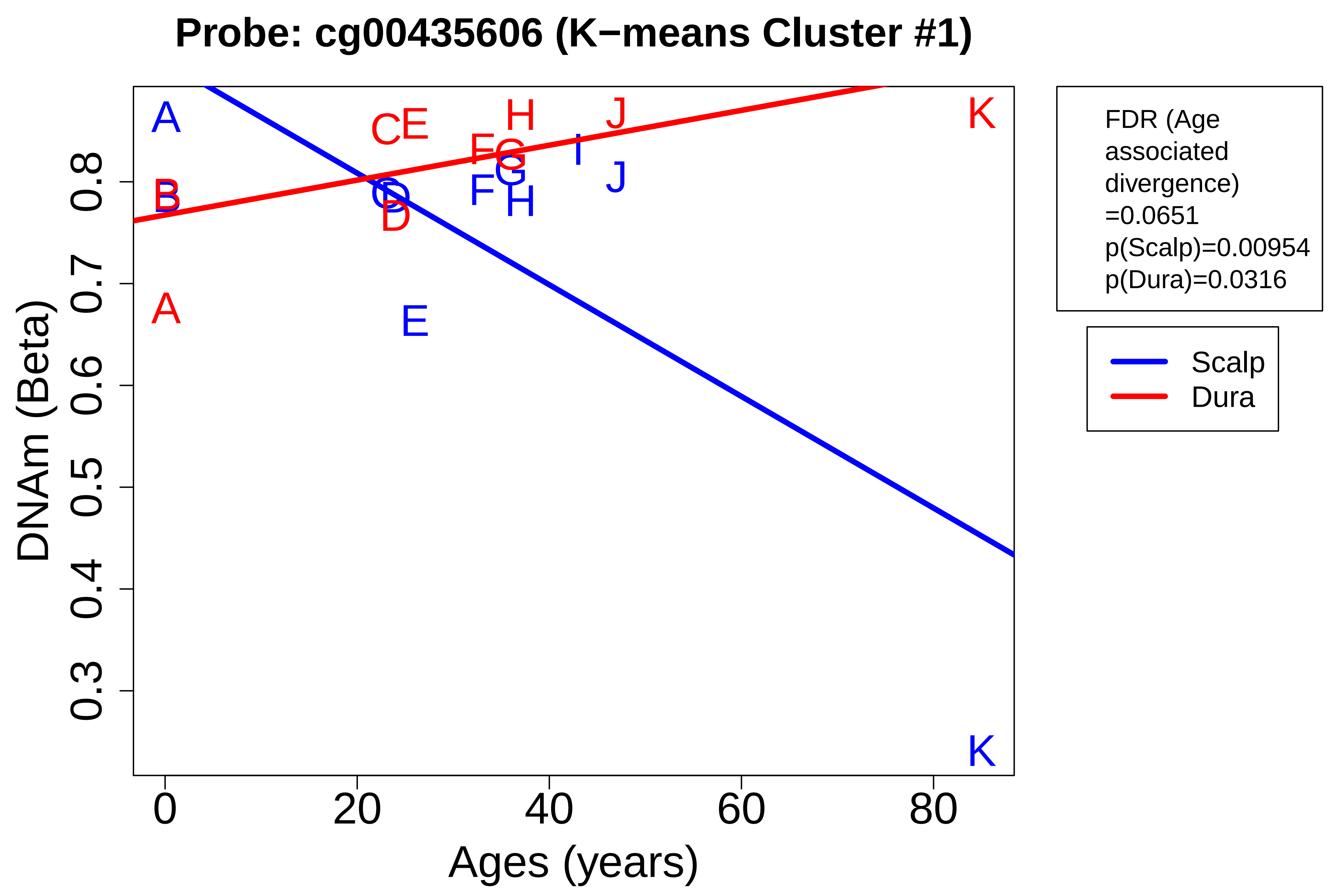

Probe: cg00435606 (K−means Cluster #1)
DNAm (Beta)
0.3
0.4
0.5
0.6
0.7
0.8
0
20
40
60
80
Ages (years)
A
B
C
D
E
F
G
H
I
J
K
FDR (Age associated divergence) =0.0651
p(Scalp)=0.00954
p(Dura)=0.0316
Scalp
Dura

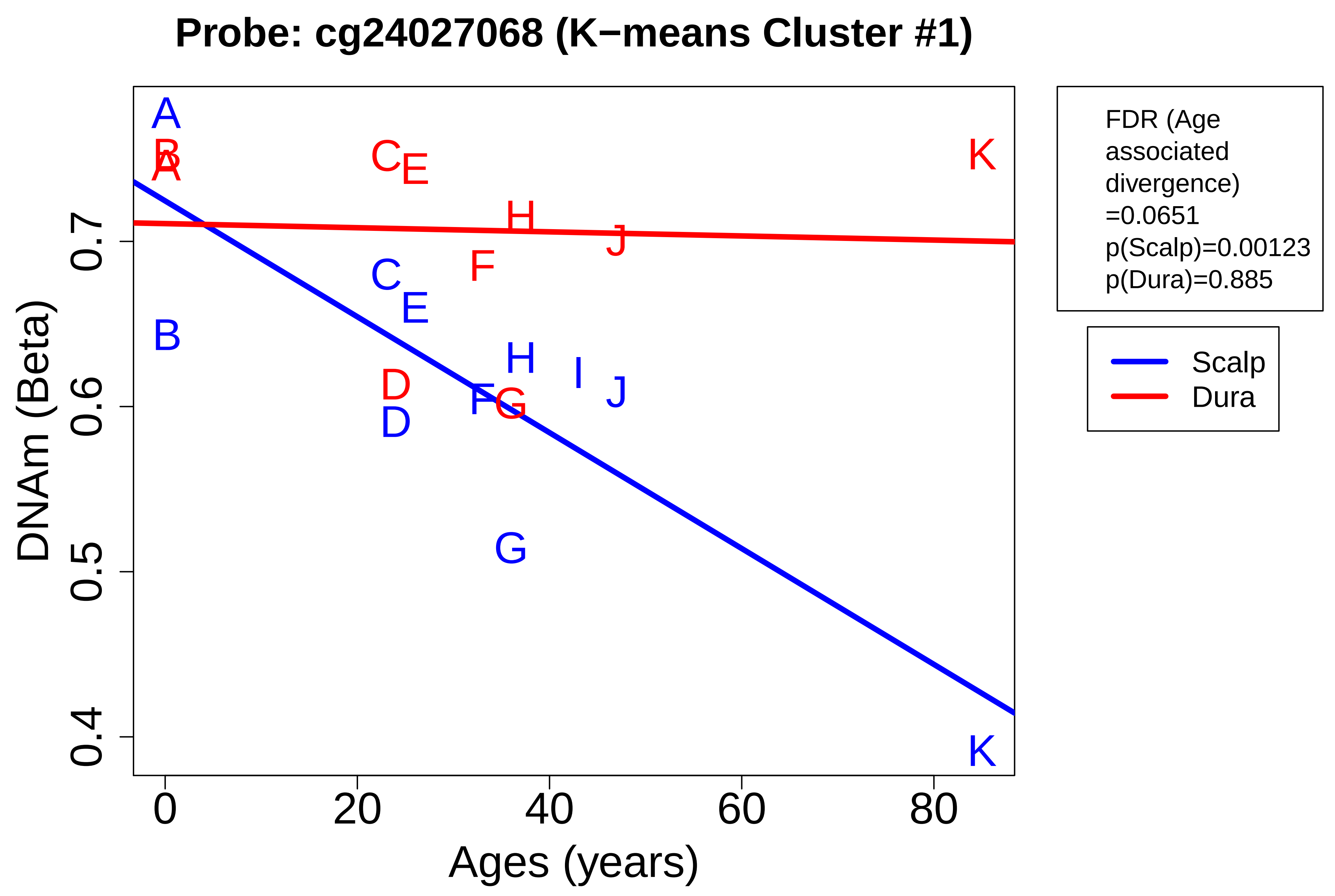

Probe: cg24027068 (K−means Cluster #1)
DNAm (Beta)
Ages (years)
0.4
0.5
0.6
0.7
0
20
40
60
80
FDR (Age associated divergence) =0.0651
p(Scalp)=0.00123
p(Dura)=0.885
Scalp
Dura
A
B
C
D
E
F
G
H
I
J
K

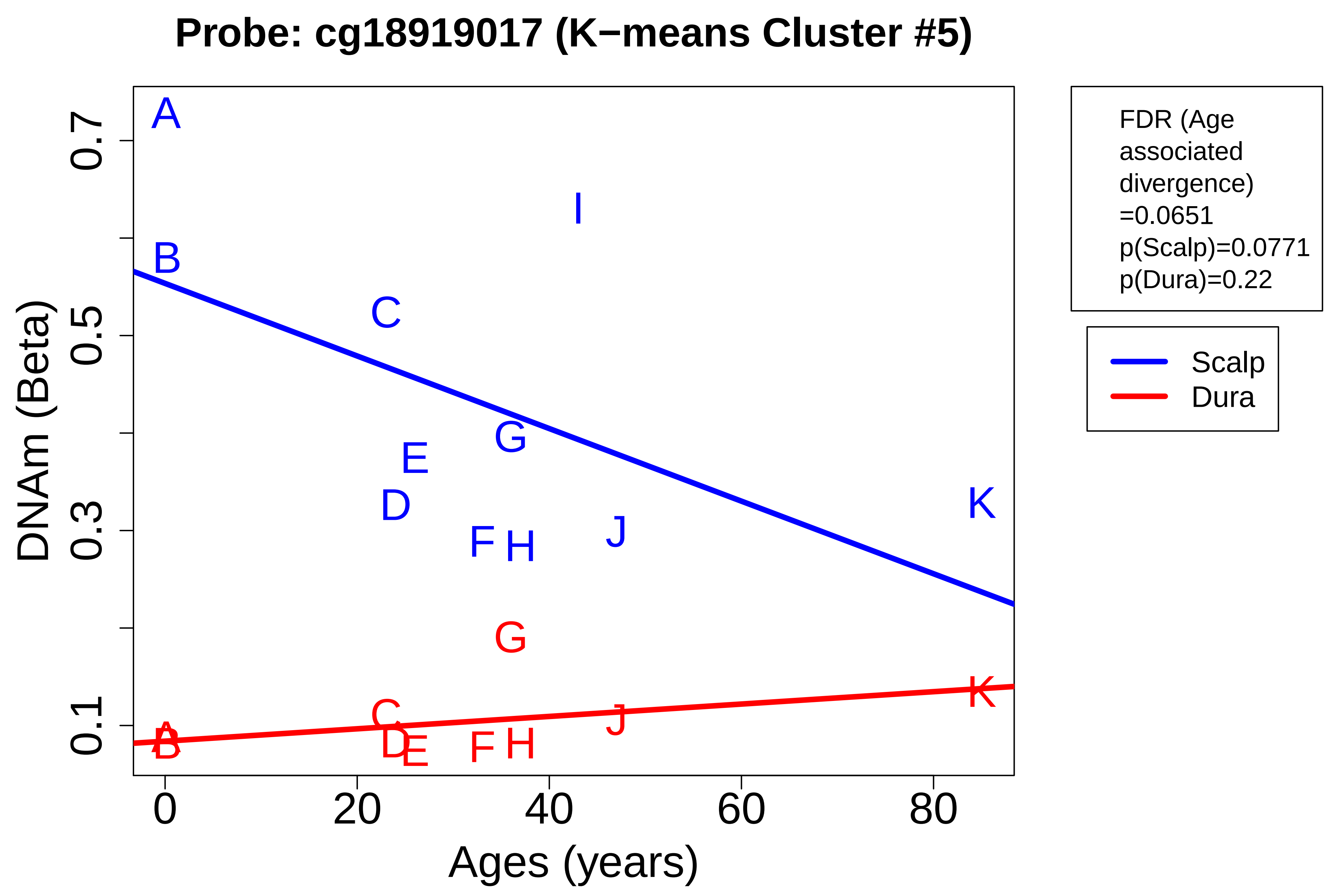
Probe: cg18919017 (K−means Cluster #5)
DNAm (Beta)
0.1
0.3
0.5
0.7
0
20
40
60
80
Ages (years)
A
B
C
D
E
F
G
H
I
J
K
FDR (Age associated divergence) =0.0651
p(Scalp)=0.0771
p(Dura)=0.22
Scalp
Dura

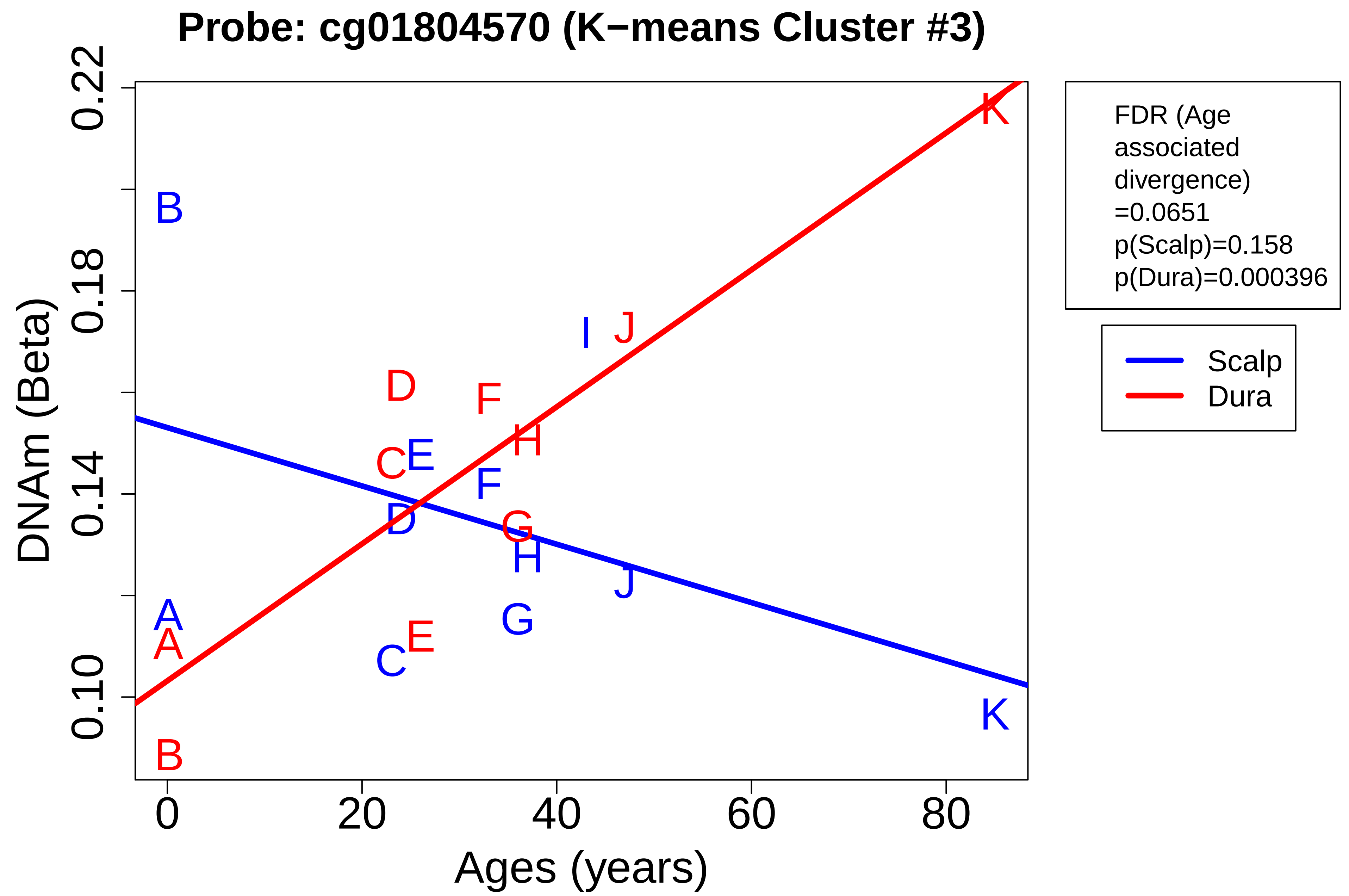
Probe: cg01804570 (K−means Cluster #3)
DNAm (Beta)
0.10
0.14
0.18
0.22
Ages (years)
0
20
40
60
80
FDR (Age associated divergence) =0.0651
p(Scalp)=0.158
p(Dura)=0.000396
Scalp
Dura
A B C D E F G H I J K
A B C D E F G H J K

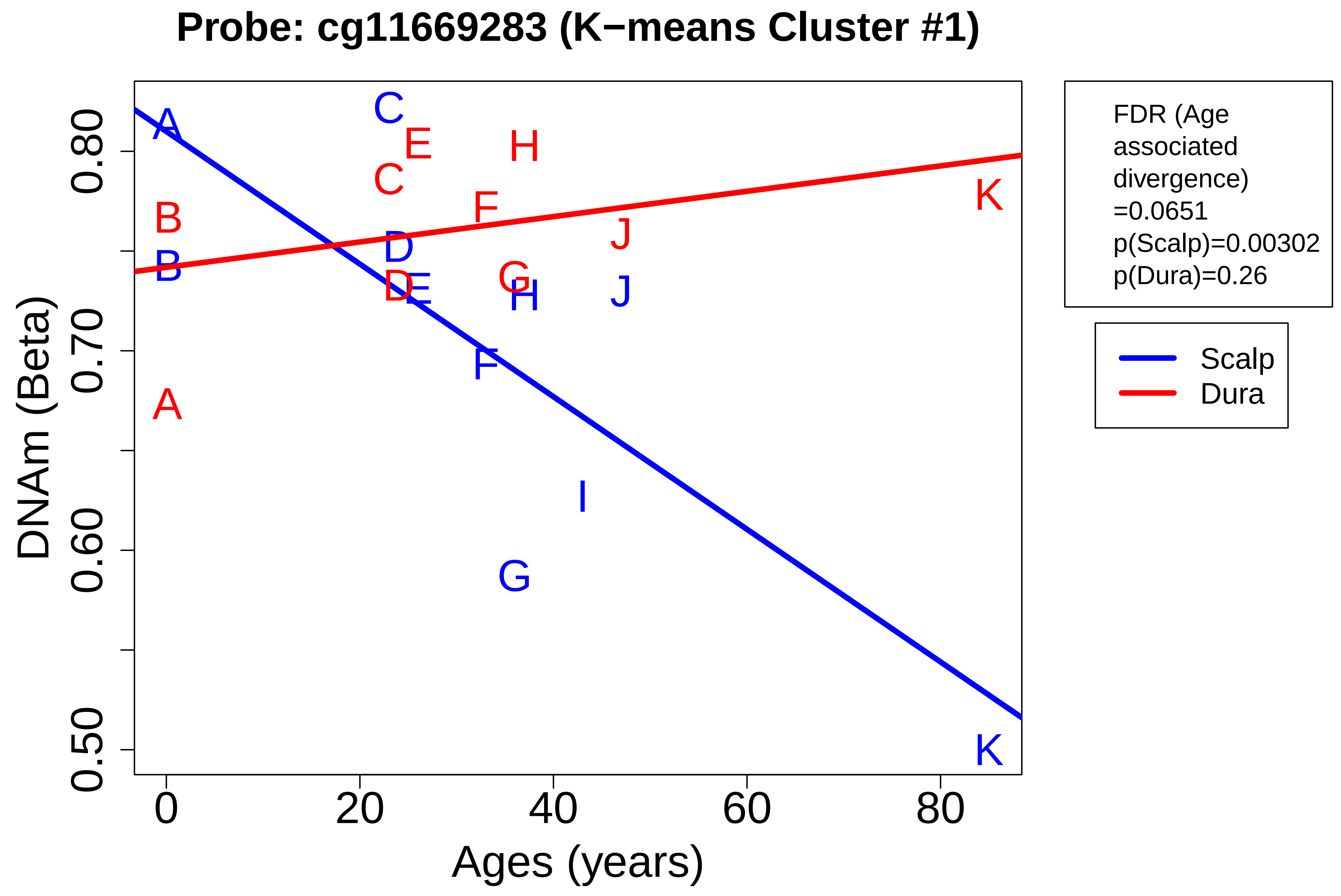

Probe: cg11669283 (K−means Cluster #1)
DNAm (Beta)
0.50
0.60
0.70
0.80
0
20
40
60
80
Ages (years)
FDR (Age associated divergence) =0.0651
p(Scalp)=0.00302
p(Dura)=0.26
Scalp
Dura

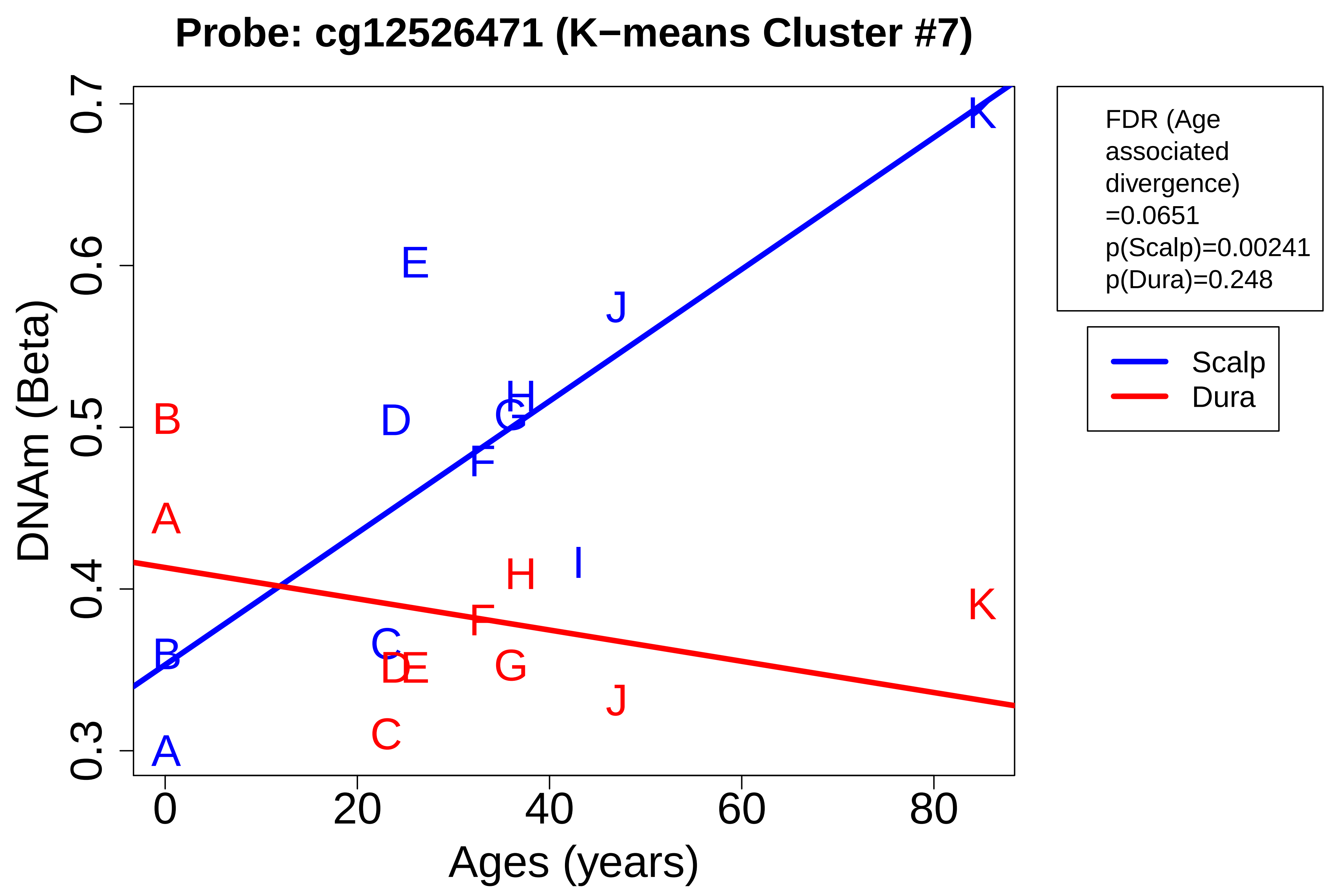

Probe: cg12526471 (K−means Cluster #7)
DNAm (Beta)
0.3
0.4
0.5
0.6
0.7
0
20
40
60
80
Ages (years)
FDR (Age associated divergence) =0.0651
p(Scalp)=0.00241
p(Dura)=0.248
Scalp
Dura
A
B
C
D
E
F
G
H
I
J
K

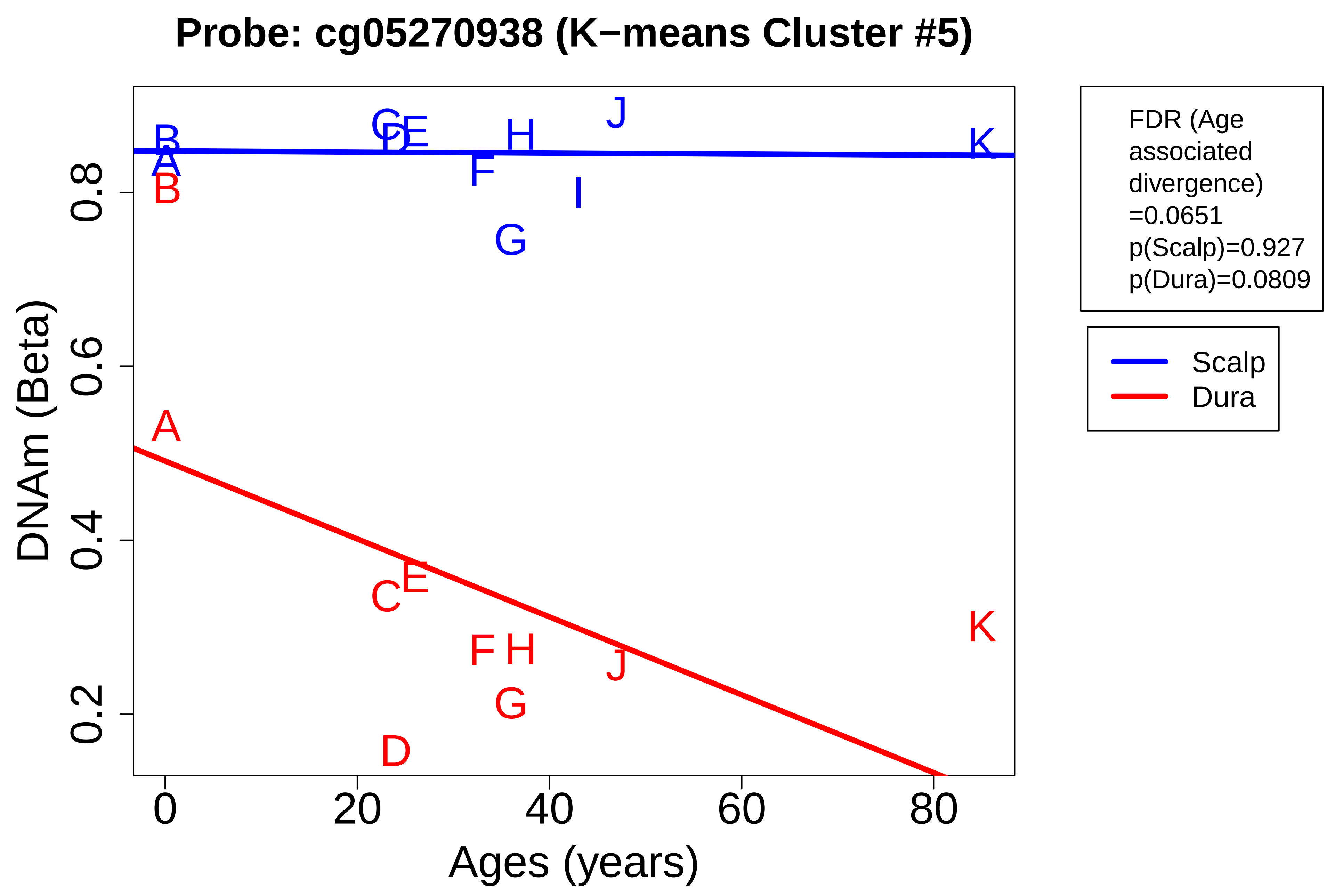

Probe: cg05270938 (K−means Cluster #5)
DNAm (Beta)
0.2
0.4
0.6
0.8
Ages (years)
0
20
40
60
80
FDR (Age associated divergence) =0.0651
p(Scalp)=0.927
p(Dura)=0.0809
Scalp
Dura

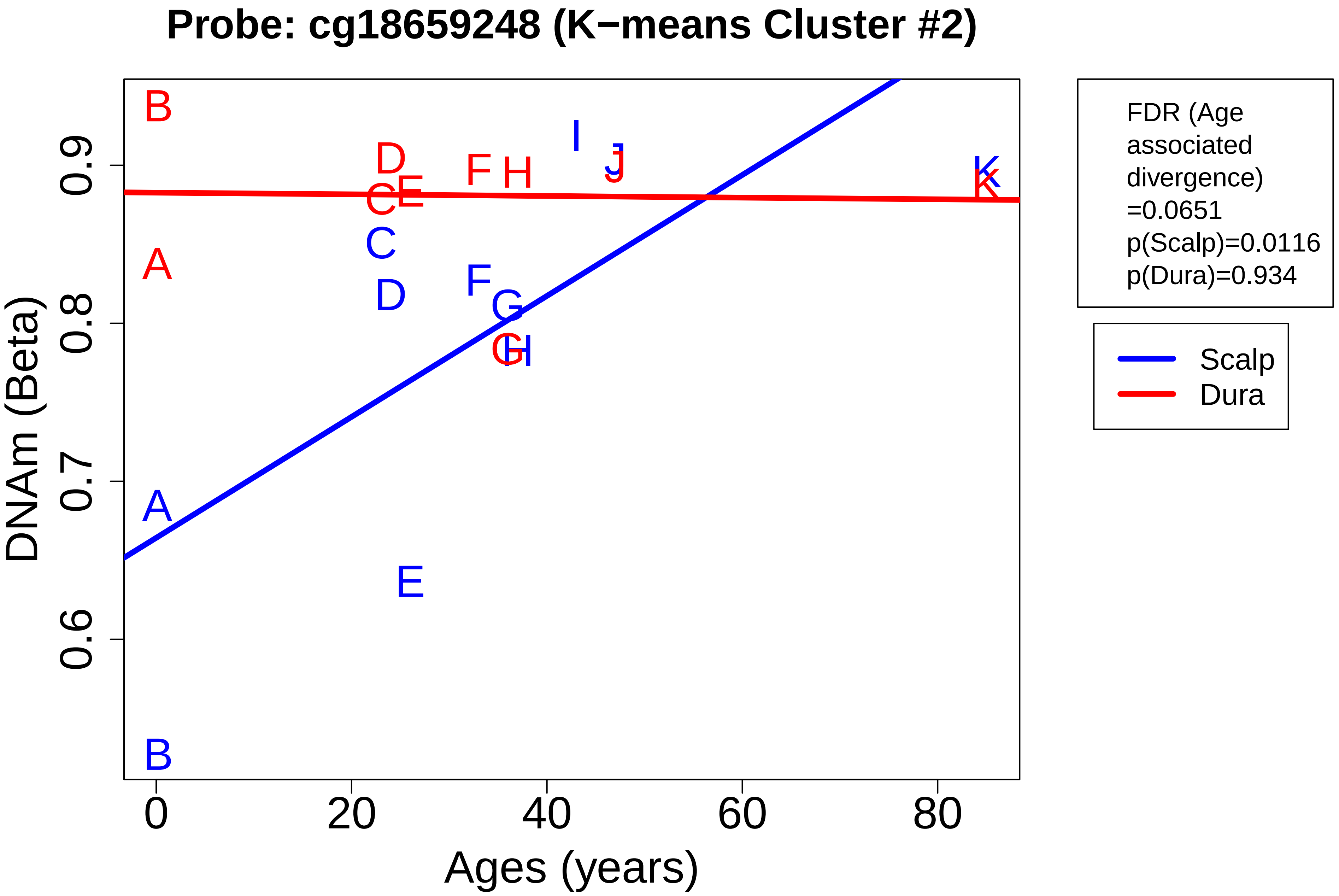

Probe: cg18659248 (K−means Cluster #2)
FDR (Age associated divergence) =0.0651
p(Scalp)=0.0116
p(Dura)=0.934
Scalp
Dura
DNAm (Beta)
Ages (years)
0.6
0.7
0.8
0.9
0
20
40
60
80

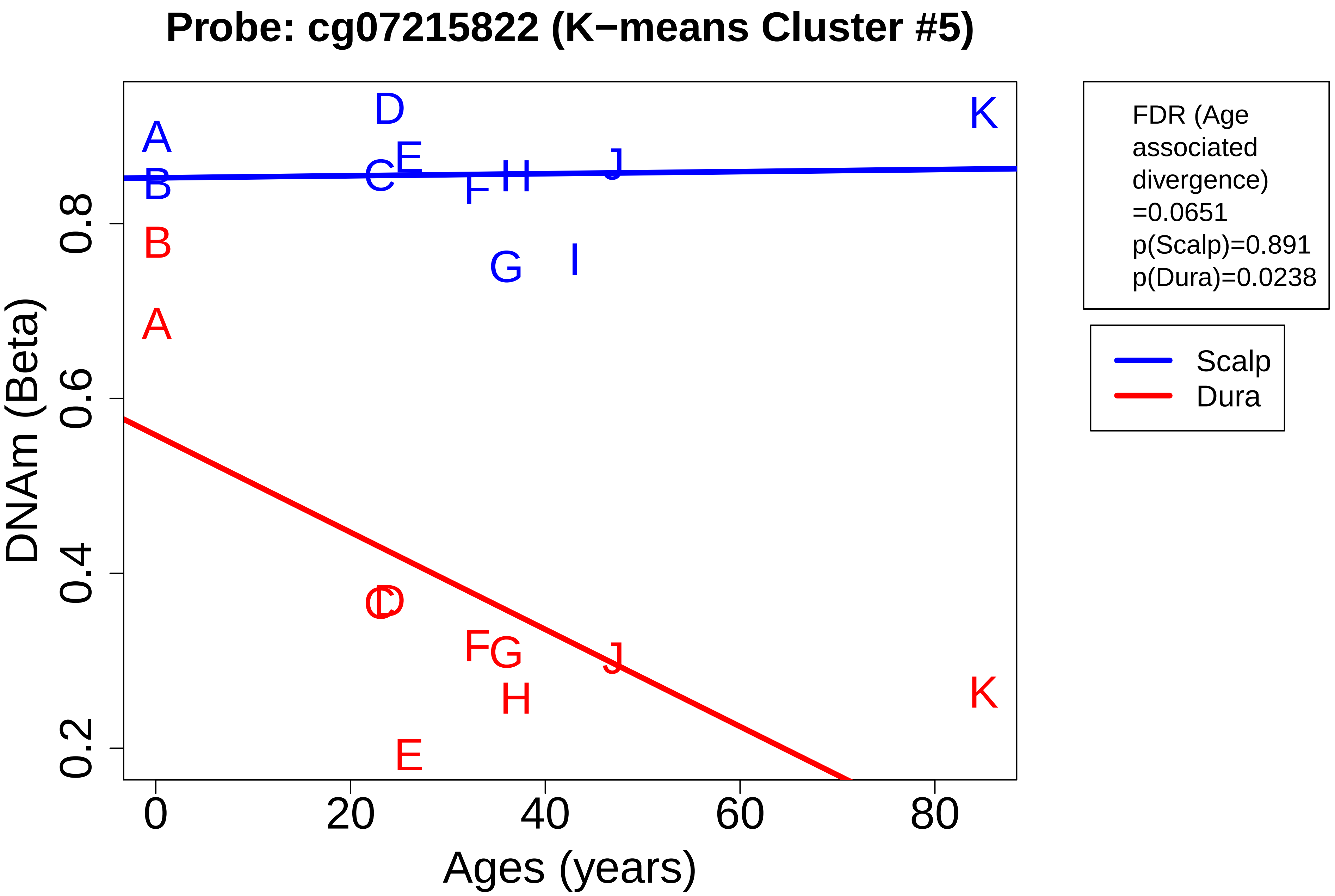
Probe: cg07215822 (K−means Cluster #5)
DNAm (Beta)
Ages (years)
0.2
0.4
0.6
0.8
0
20
40
60
80
FDR (Age associated divergence) =0.0651
p(Scalp)=0.891
p(Dura)=0.0238
Scalp
Dura
A
B
C
D
E
F
G
H
I
J
K

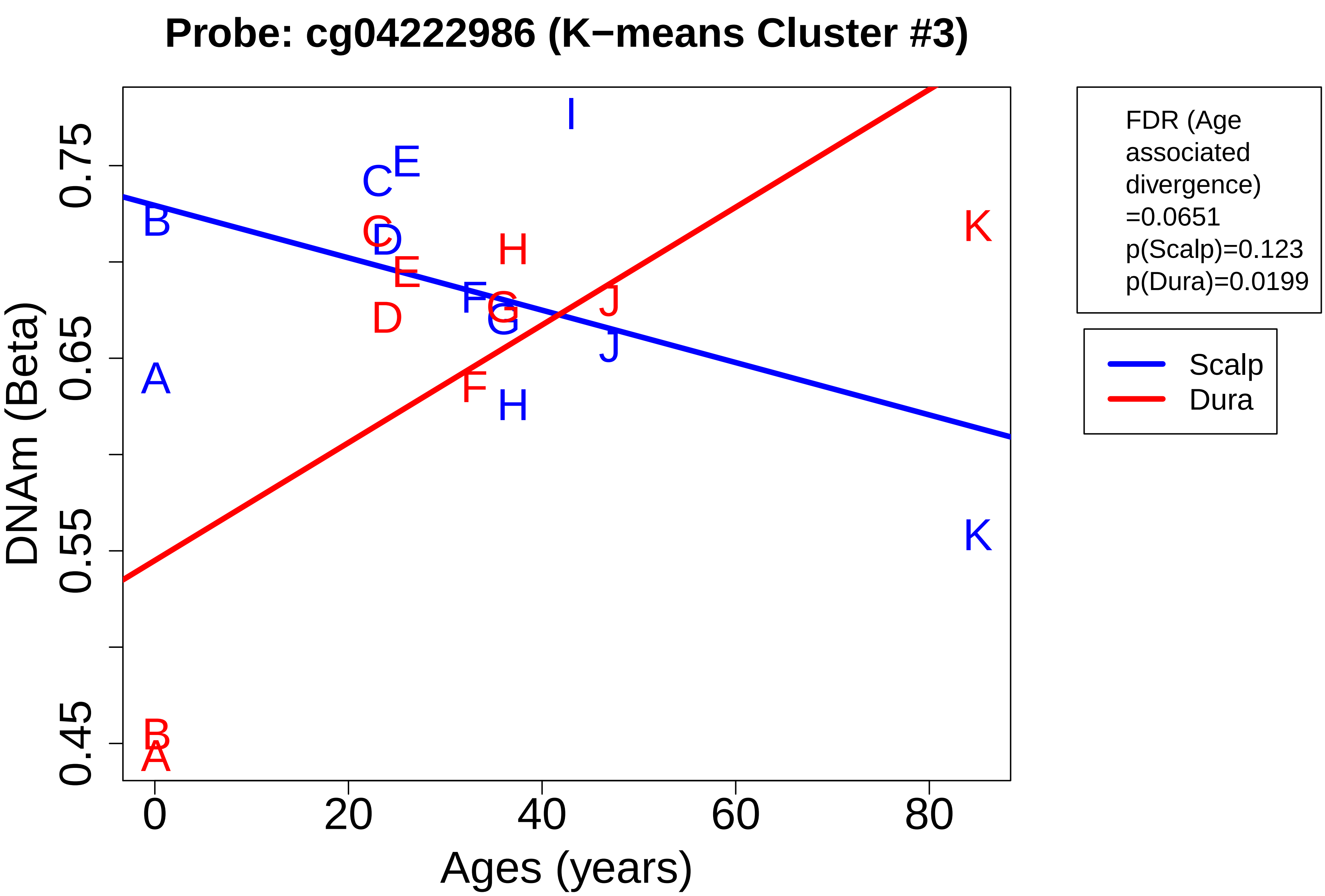

Probe: cg04222986 (K−means Cluster #3)
DNAm (Beta)
0.45
0.55
0.65
0.75
0
20
40
60
80
Ages (years)
FDR (Age associated divergence) =0.0651
p(Scalp)=0.123
p(Dura)=0.0199
Scalp
Dura

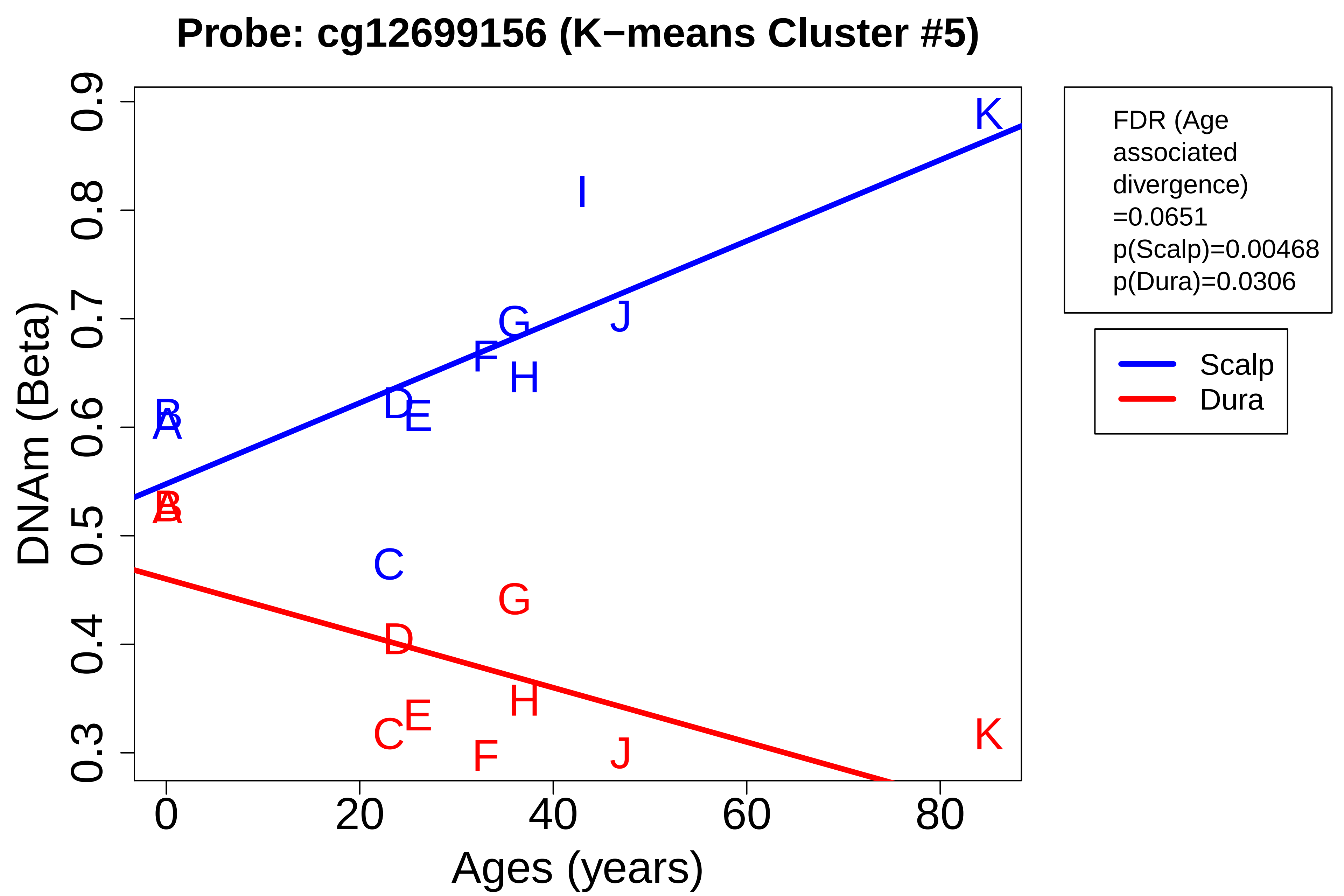
Probe: cg12699156 (K−means Cluster #5)
DNAm (Beta)
0.3
0.4
0.5
0.6
0.7
0.8
0.9
0
20
40
60
80
Ages (years)
FDR (Age associated divergence) =0.0651
p(Scalp)=0.00468
p(Dura)=0.0306
Scalp
Dura
A
B
C
D
E
F
G
H
I
J
K

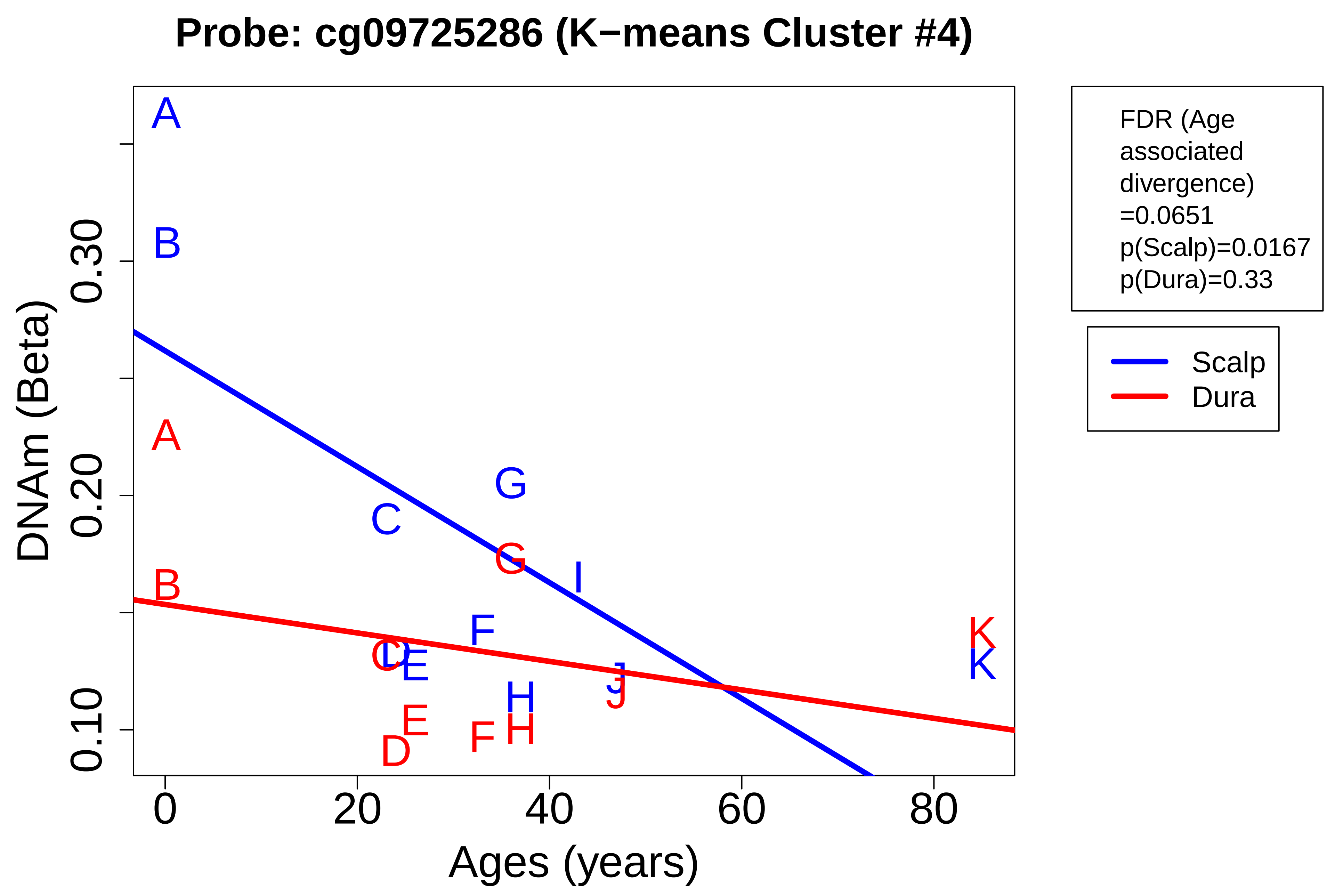
Probe: cg09725286 (K−means Cluster #4)
DNAm (Beta)
0.10
0.20
0.30
Ages (years)
0
20
40
60
80
A
B
A
G
C
G
I
B
F
K
C
D
E
K
J
J
H
E
F
H
D
FDR (Age associated divergence) =0.0651
p(Scalp)=0.0167
p(Dura)=0.33
Scalp
Dura

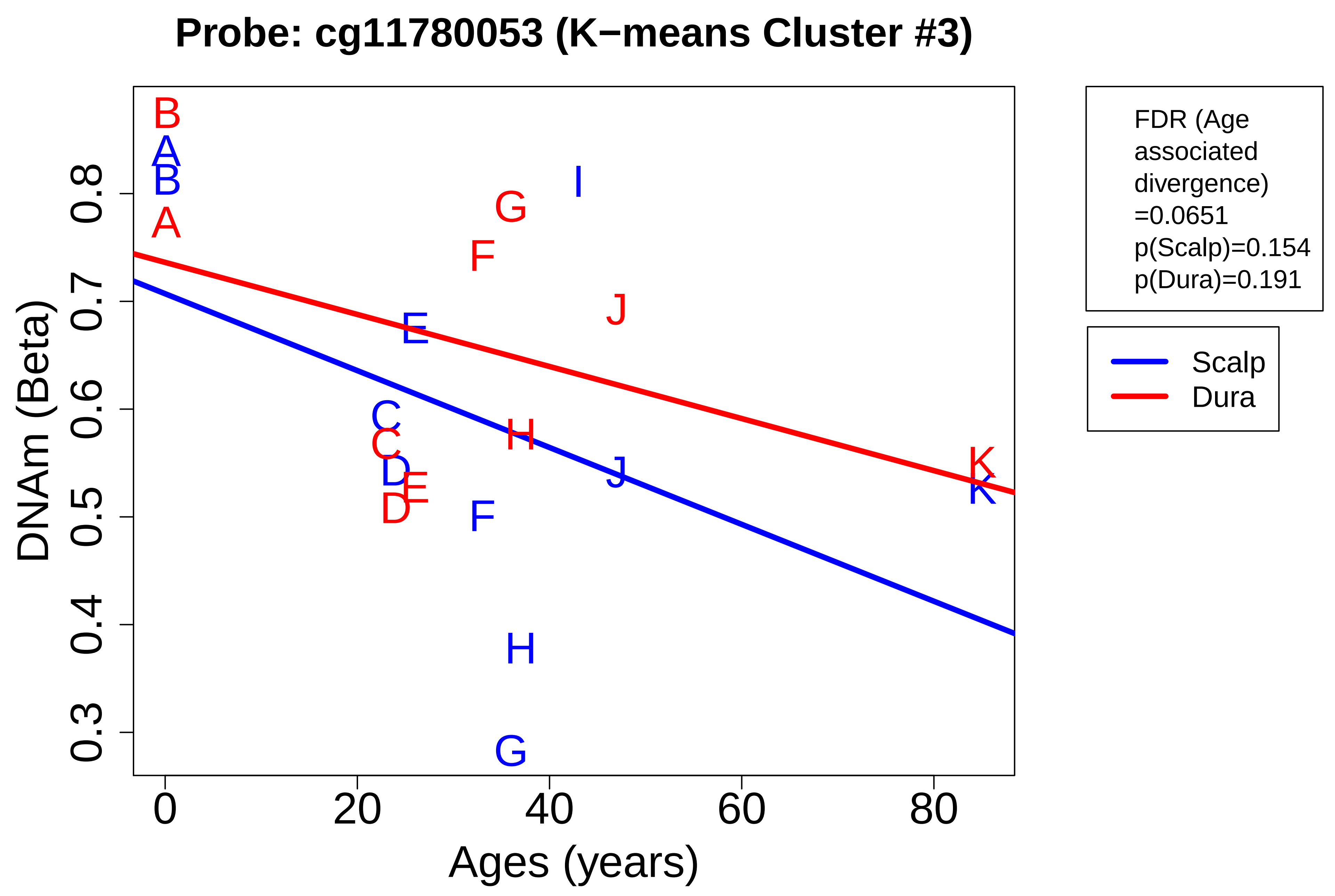

Probe: cg11780053 (K−means Cluster #3)
FDR (Age associated divergence) =0.0651
p(Scalp)=0.154
p(Dura)=0.191
Scalp
Dura
DNAm (Beta)
0.3
0.4
0.5
0.6
0.7
0.8
0
20
40
60
80
Ages (years)
A
B
C
D
E
F
G
H
I
J
K

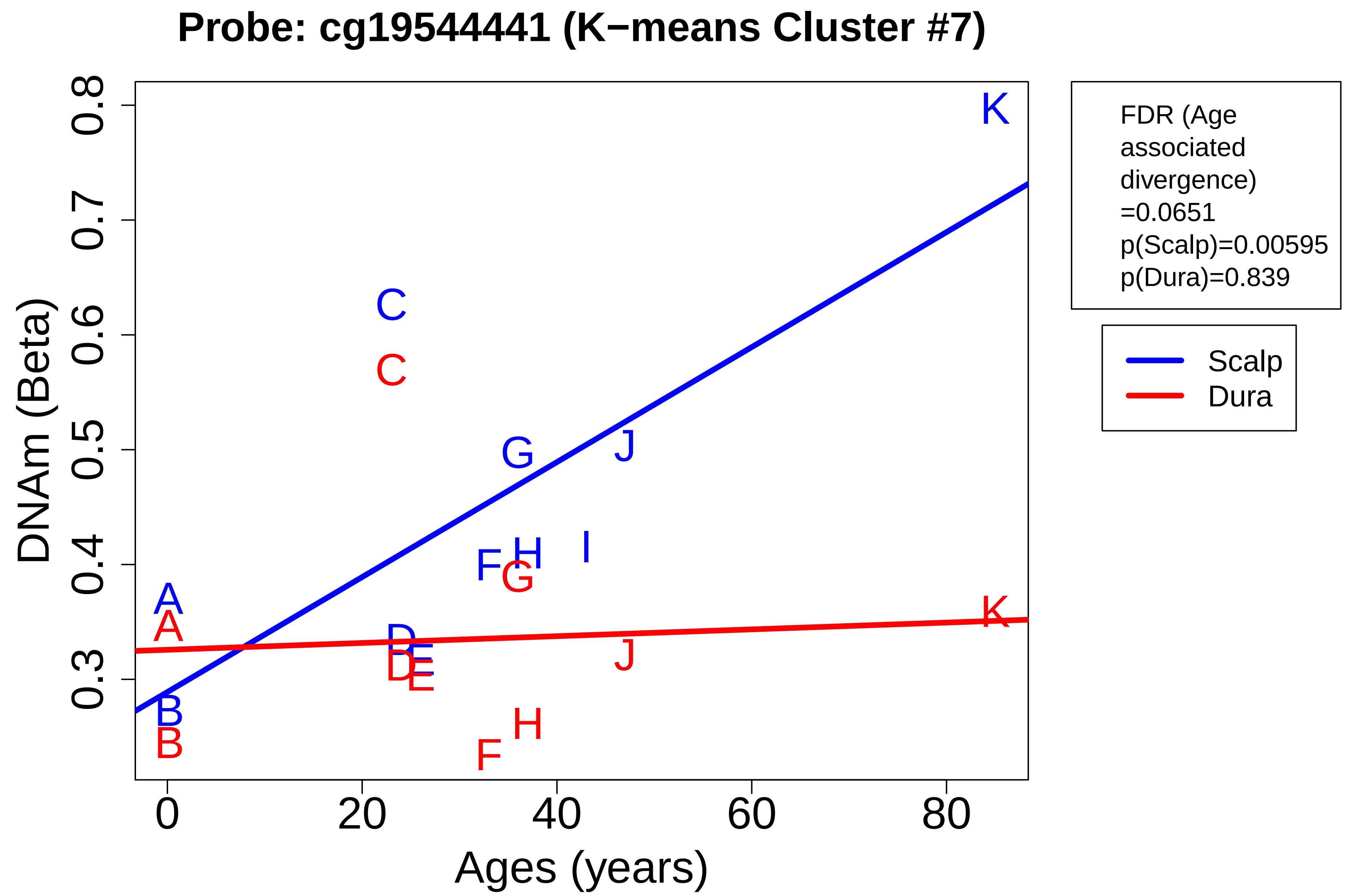

Probe: cg19544441 (K−means Cluster #7)
DNAm (Beta)
Ages (years)
0.3
0.4
0.5
0.6
0.7
0.8
0
20
40
60
80
A
B
C
D
E
F
G
H
I
J
K
FDR (Age associated divergence) =0.0651
p(Scalp)=0.00595
p(Dura)=0.839
Scalp
Dura

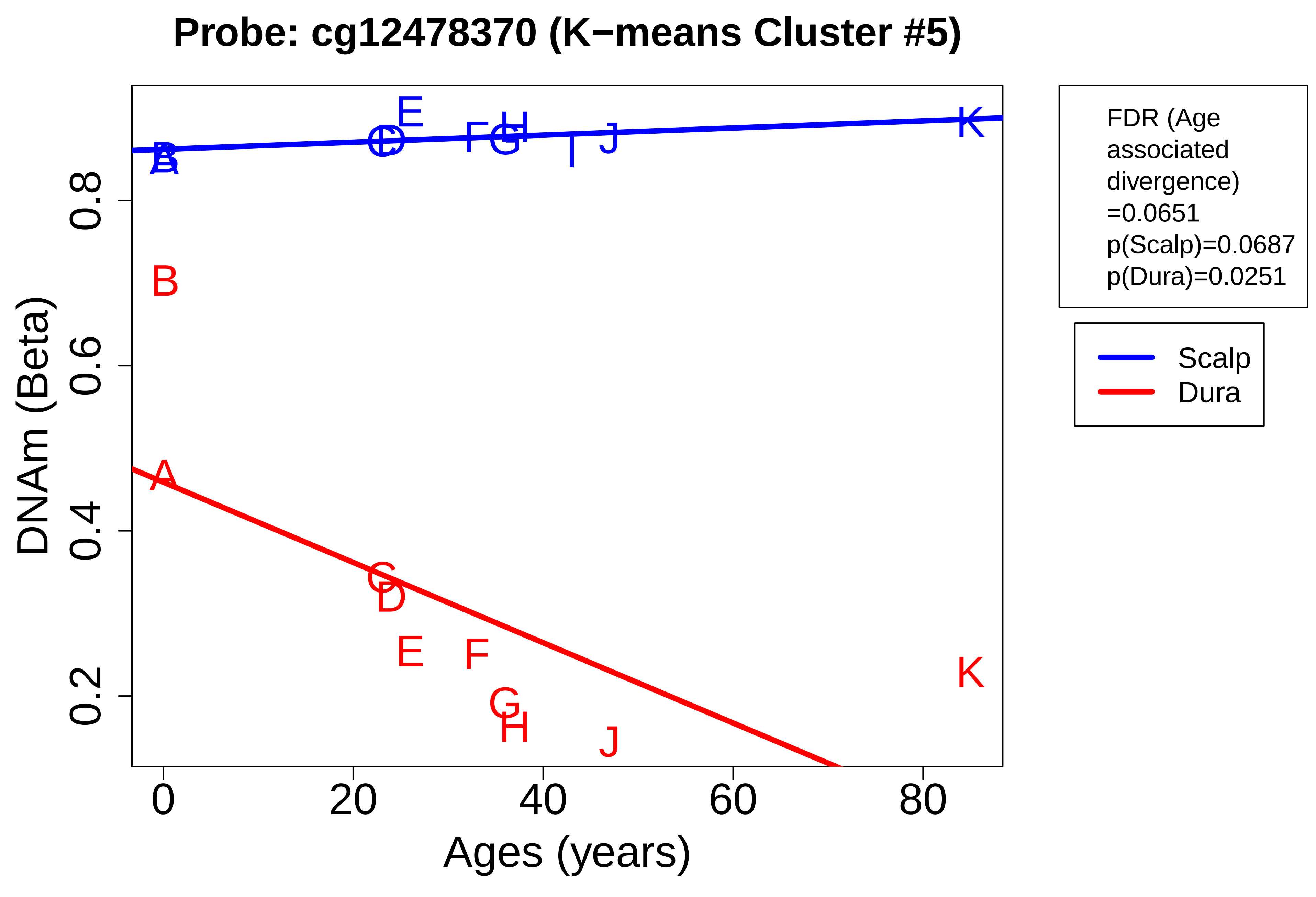

Probe: cg12478370 (K−means Cluster #5)
DNAm (Beta)
0.2
0.4
0.6
0.8
Ages (years)
0
20
40
60
80
FDR (Age associated divergence) =0.0651
p(Scalp)=0.0687
p(Dura)=0.0251
Scalp
Dura

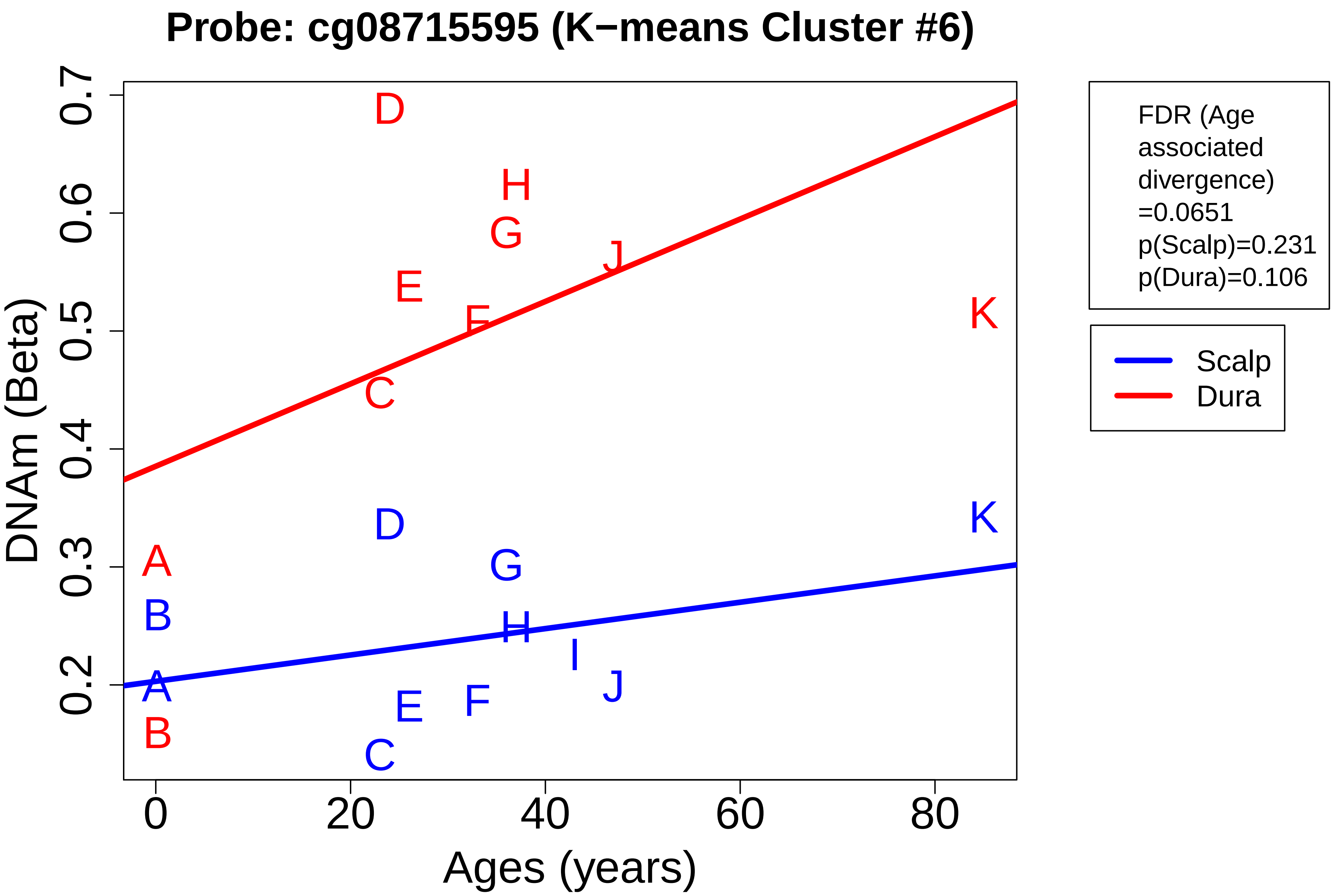

Probe: cg08715595 (K−means Cluster #6)
DNAm (Beta)
0.2
0.3
0.4
0.5
0.6
0.7
0
20
40
60
80
Ages (years)
FDR (Age associated divergence) =0.0651
p(Scalp)=0.231
p(Dura)=0.106
Scalp
Dura

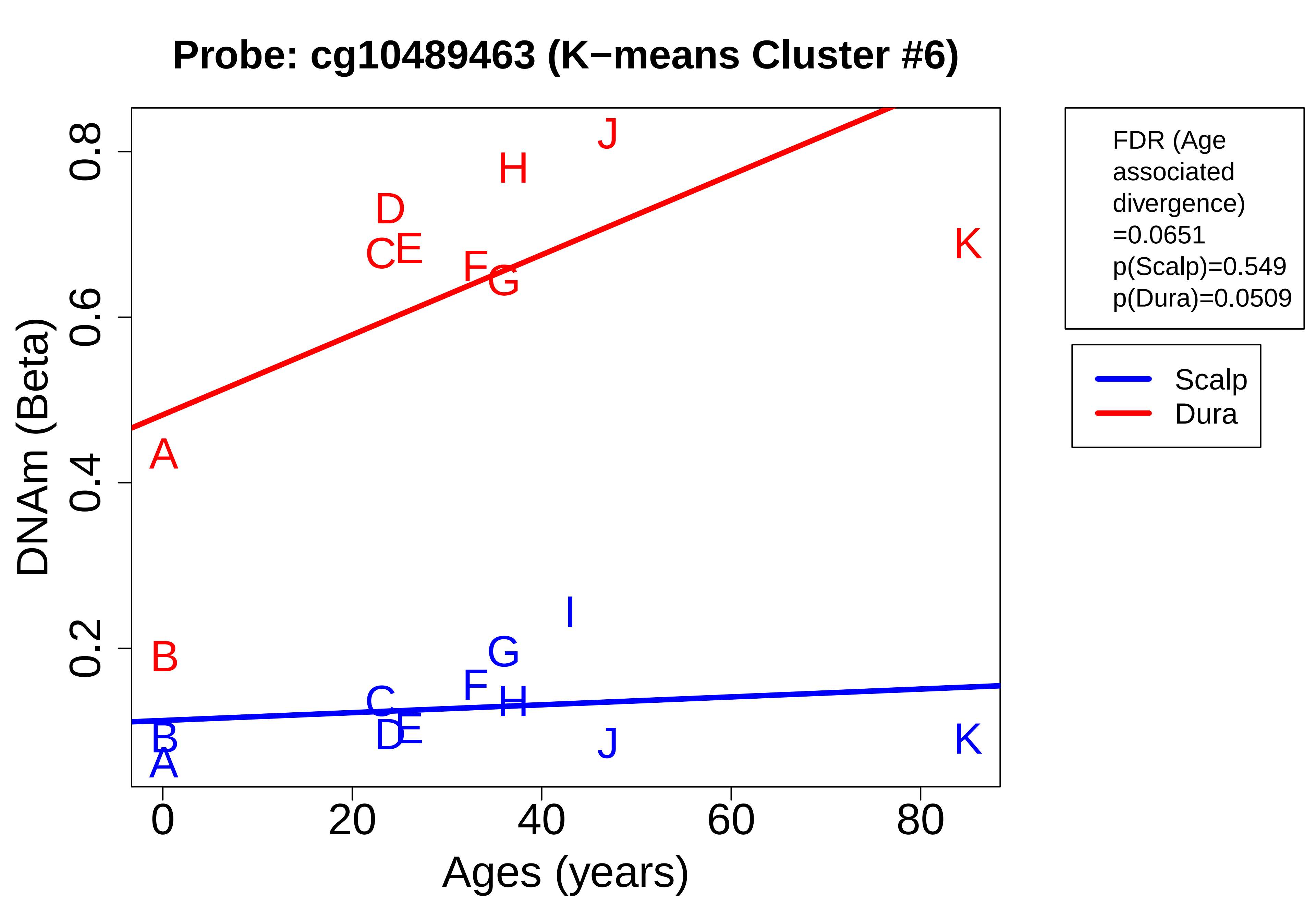

Probe: cg10489463 (K−means Cluster #6)
FDR (Age associated divergence) =0.0651
p(Scalp)=0.549
p(Dura)=0.0509
Scalp
Dura
DNAm (Beta)
0.2
0.4
0.6
0.8
Ages (years)
0
20
40
60
80
A
B
C
D
E
F
G
H
I
J
K

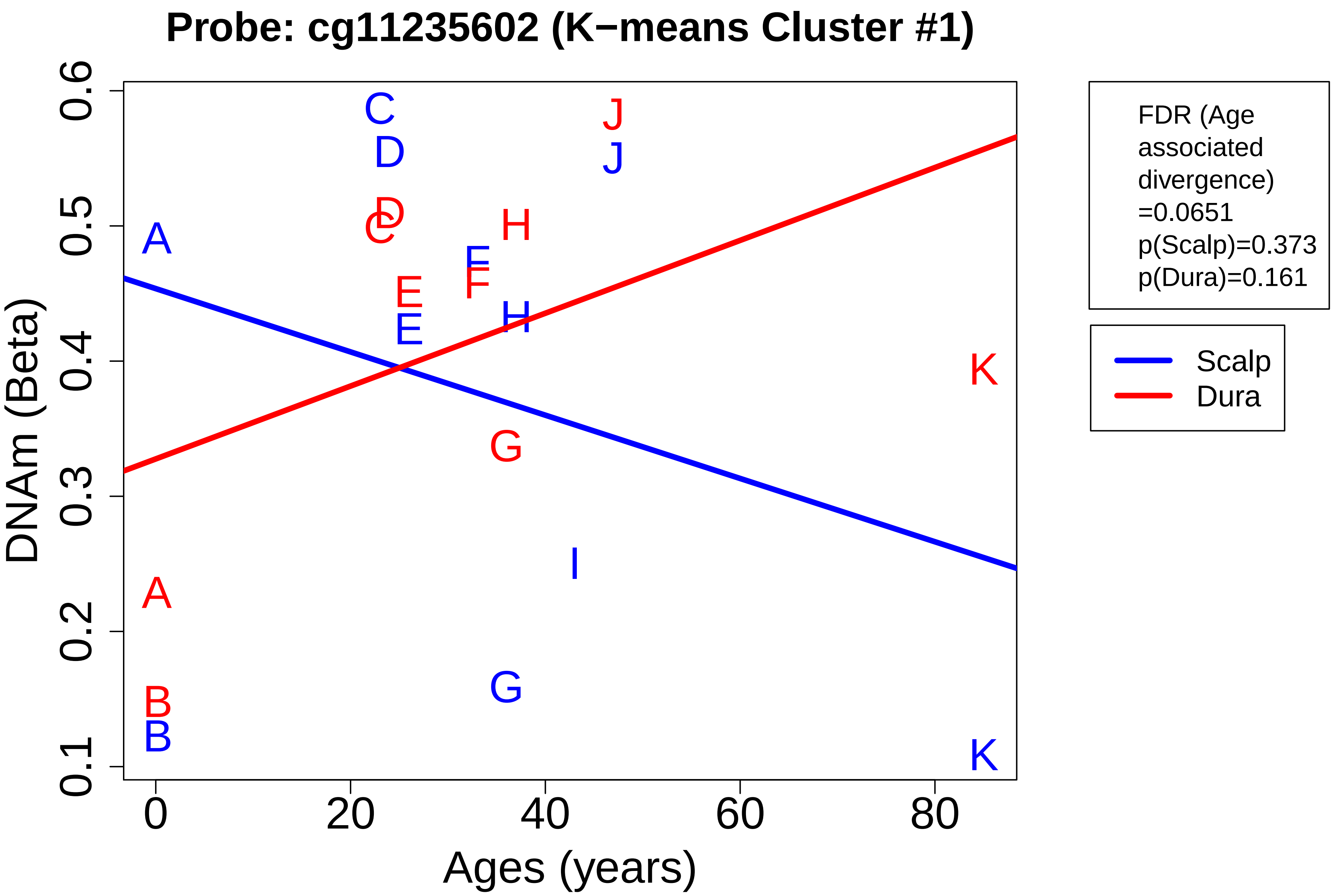

Probe: cg11235602 (K−means Cluster #1)
DNAm (Beta)
0.1
0.2
0.3
0.4
0.5
0.6
Ages (years)
0
20
40
60
80
A
B
C
D
E
F
G
H
I
J
K
FDR (Age associated divergence) =0.0651
p(Scalp)=0.373
p(Dura)=0.161
Scalp
Dura

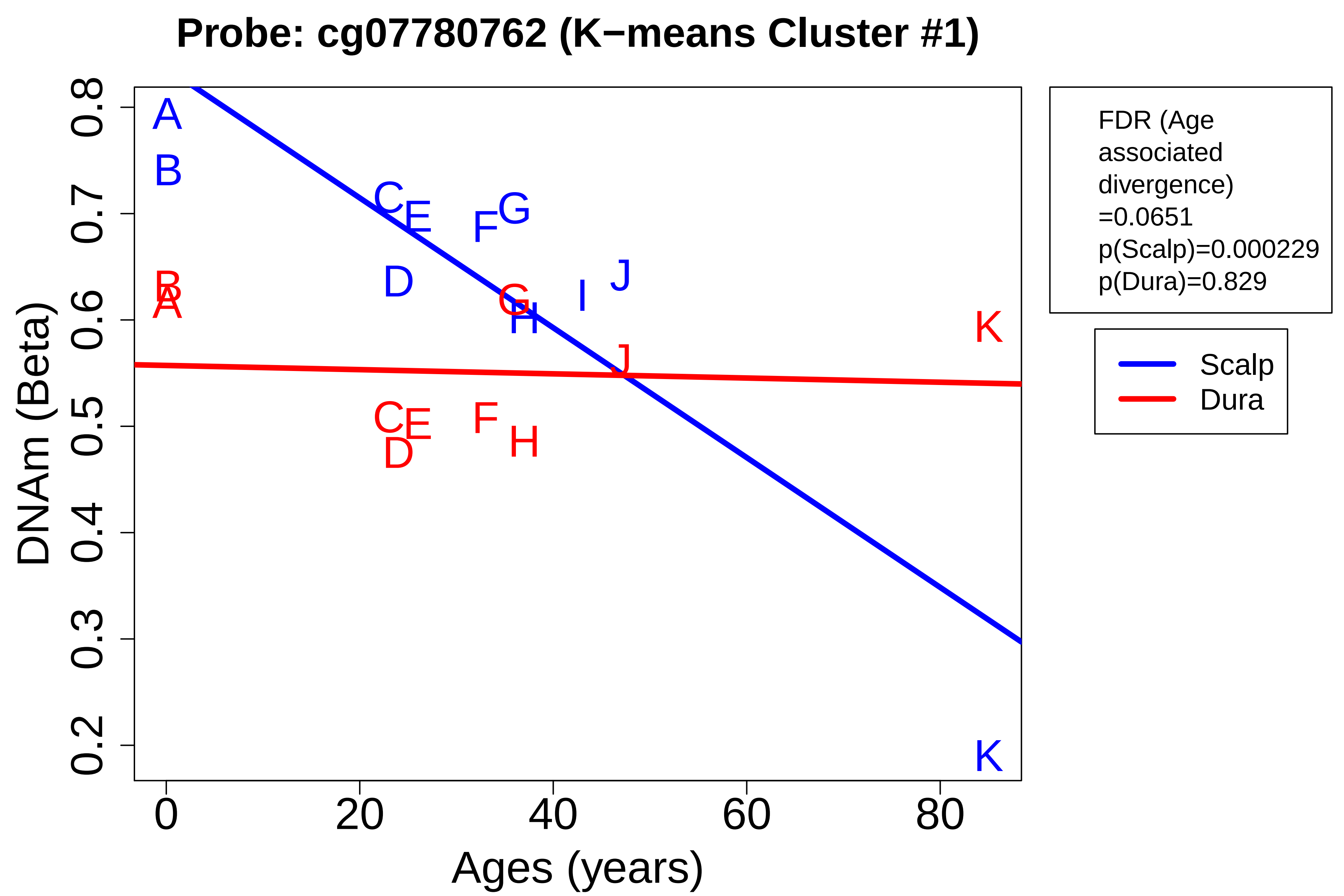
Probe: cg07780762 (K−means Cluster #1)
DNAm (Beta)
0.2
0.3
0.4
0.5
0.6
0.7
0.8
0
20
40
60
80
Ages (years)
A
B
C
D
E
F
G
H
I
J
K
FDR (Age associated divergence) =0.0651
p(Scalp)=0.000229
p(Dura)=0.829
Scalp
Dura

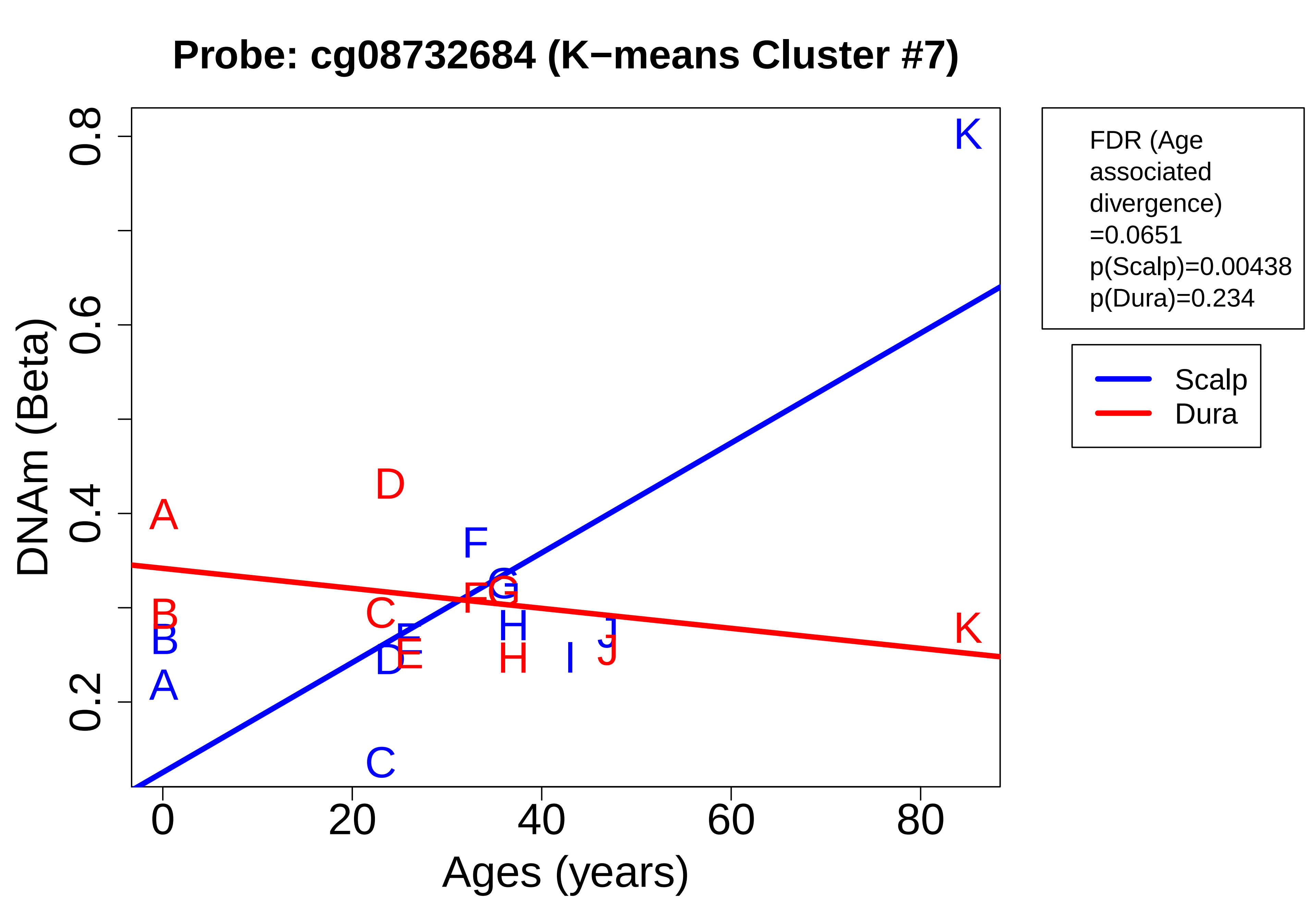

Probe: cg08732684 (K−means Cluster #7)
DNAm (Beta)
0.2
0.4
0.6
0.8
0
20
40
60
80
Ages (years)
A
B
C
D
E
F
G
H
I
J
K
FDR (Age associated divergence) =0.0651
p(Scalp)=0.00438
p(Dura)=0.234
Scalp
Dura

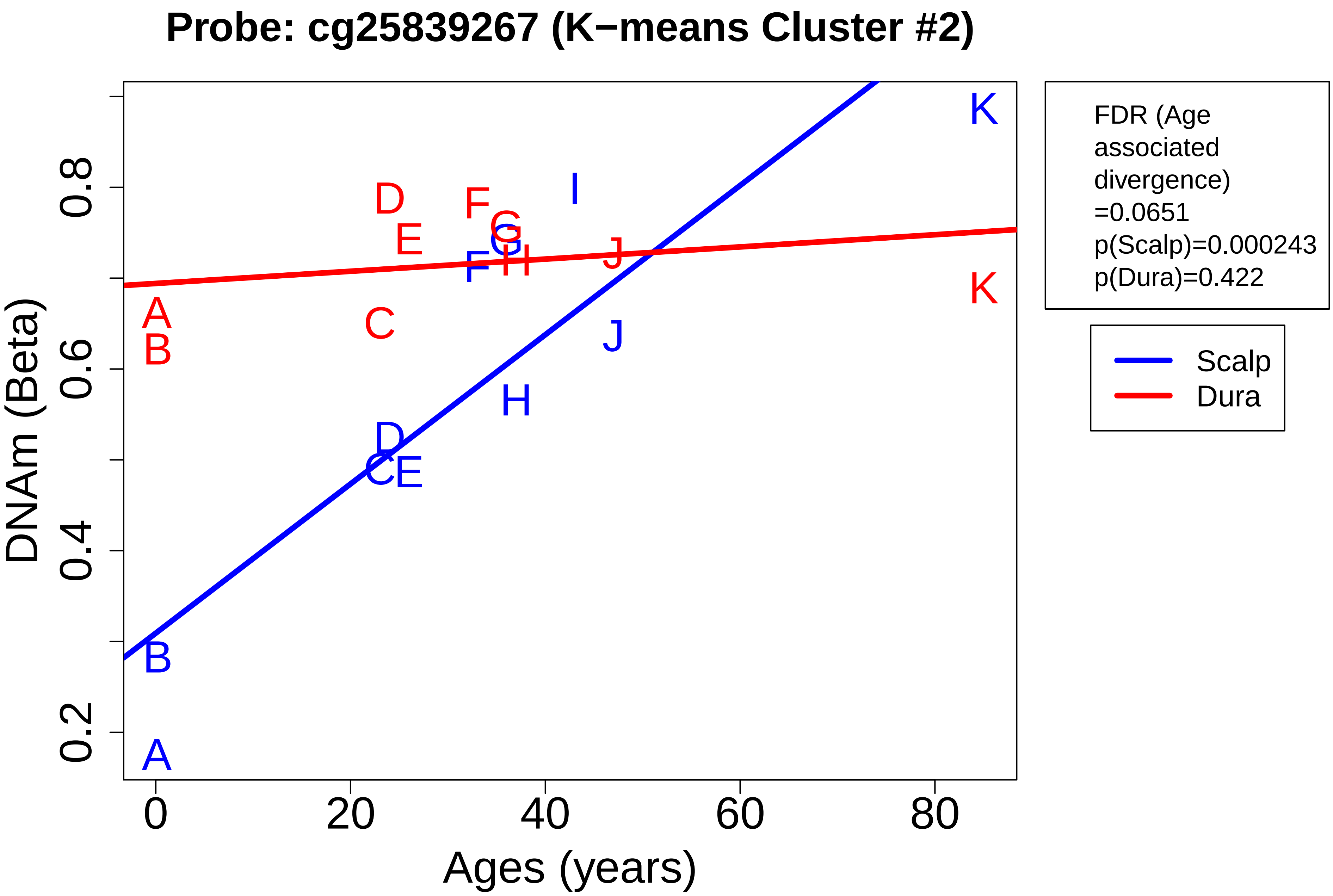

Probe: cg25839267 (K−means Cluster #2)
DNAm (Beta)
0.2
0.4
0.6
0.8
0
20
40
60
80
Ages (years)
FDR (Age associated divergence) =0.0651
p(Scalp)=0.000243
p(Dura)=0.422
Scalp
Dura

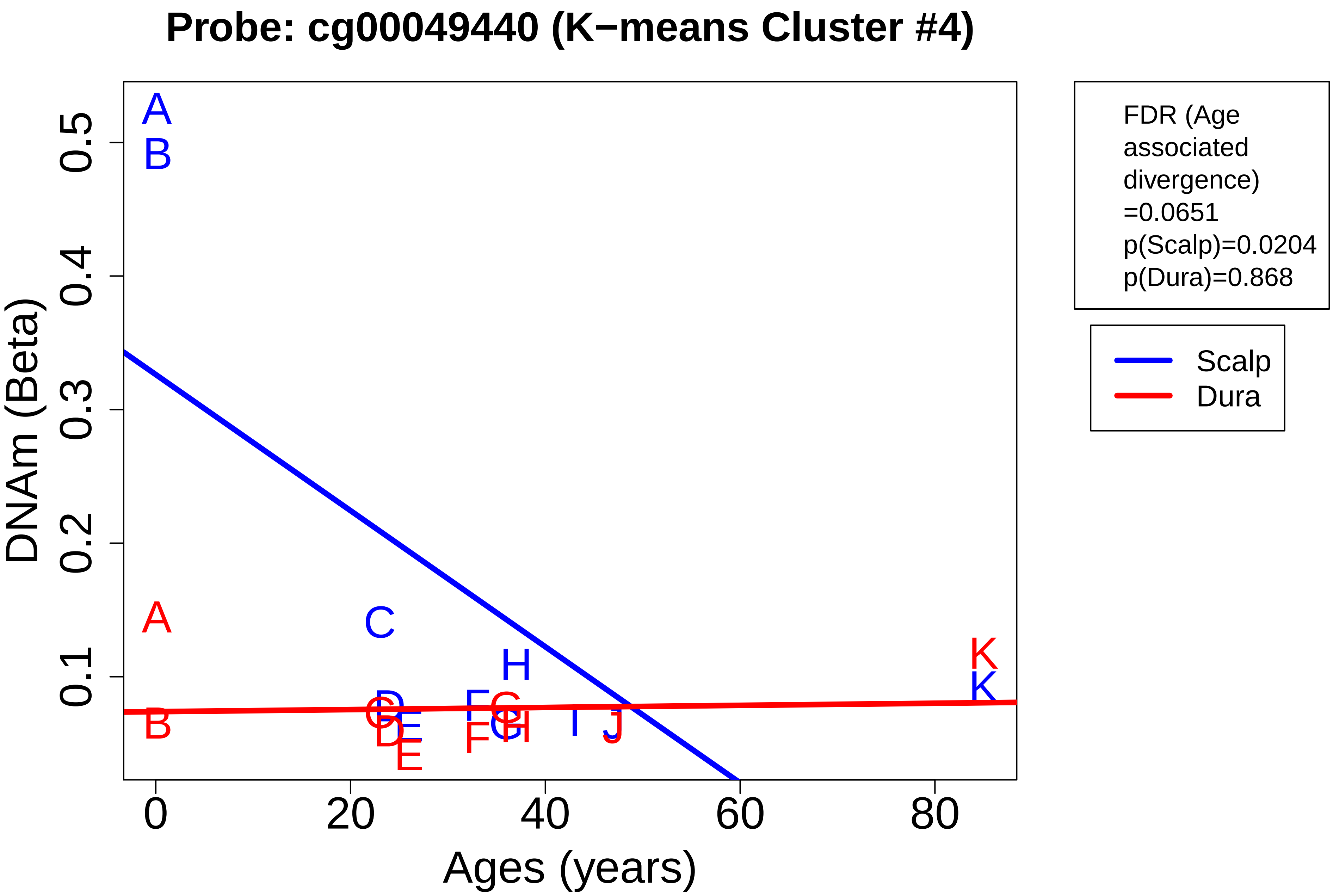

Probe: cg00049440 (K−means Cluster #4)
DNAm (Beta)
0.1
0.2
0.3
0.4
0.5
Ages (years)
0
20
40
60
80
FDR (Age associated divergence) =0.0651
p(Scalp)=0.0204
p(Dura)=0.868
Scalp
Dura

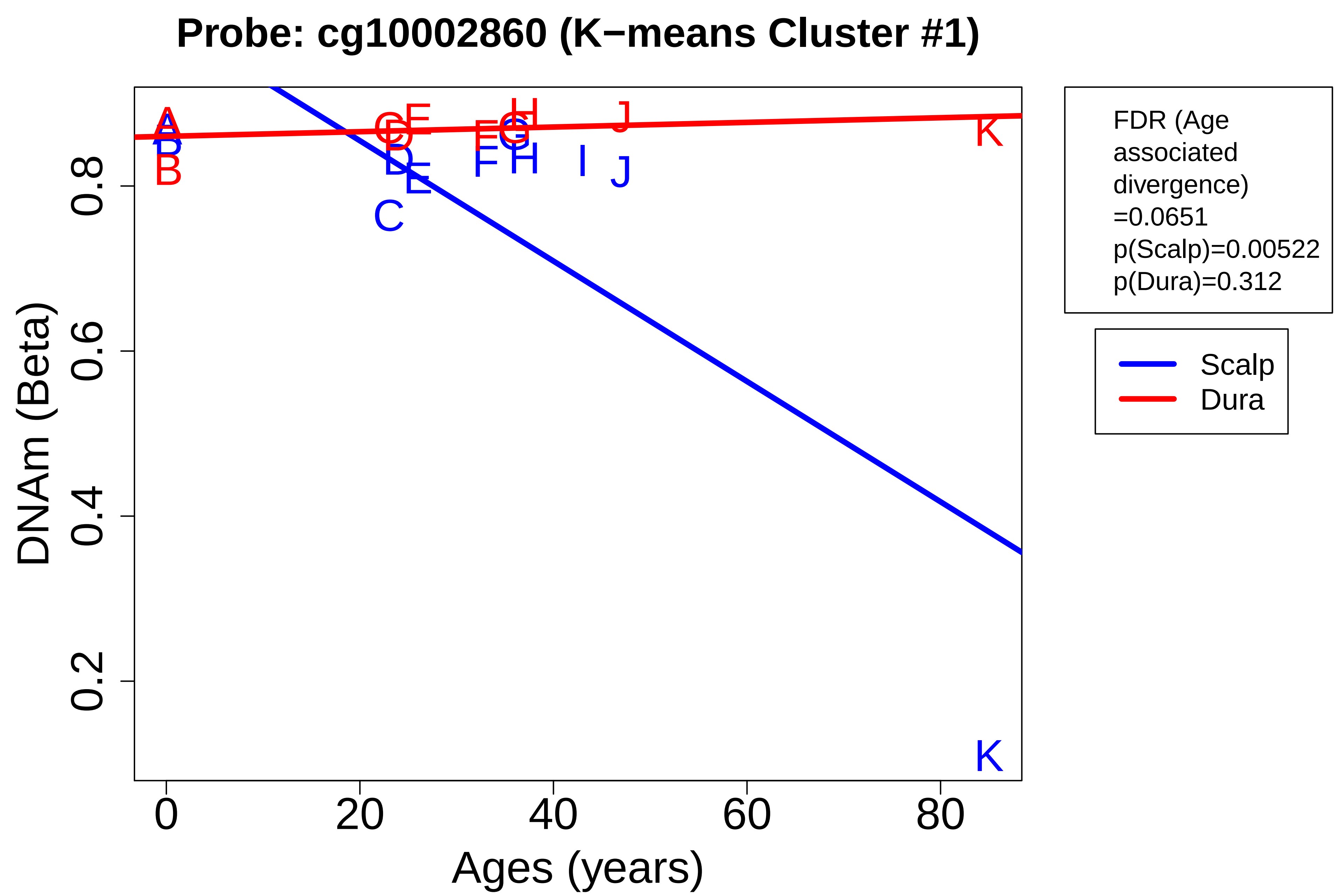

Probe: cg10002860 (K−means Cluster #1)
DNAm (Beta)
0.2
0.4
0.6
0.8
Ages (years)
0
20
40
60
80
A
B
C
D
E
F
G
H
I
J
K
FDR (Age associated divergence) =0.0651
p(Scalp)=0.00522
p(Dura)=0.312
Scalp
Dura

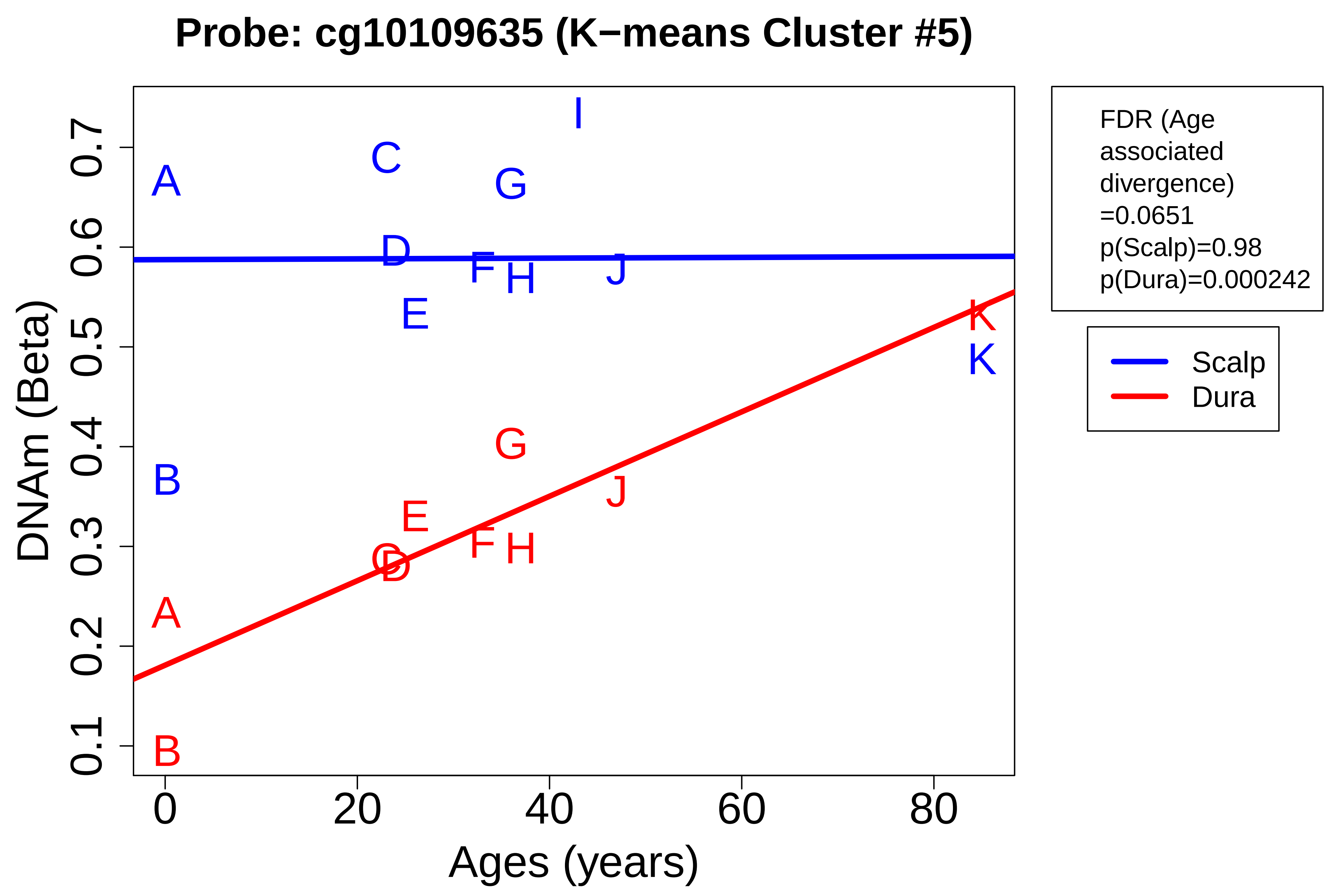

Probe: cg10109635 (K−means Cluster #5)
DNAm (Beta)
0.1
0.2
0.3
0.4
0.5
0.6
0.7
0
20
40
60
80
Ages (years)
A
B
C
D
E
F
G
H
I
J
K
FDR (Age associated divergence) =0.0651
p(Scalp)=0.98
p(Dura)=0.000242
Scalp
Dura

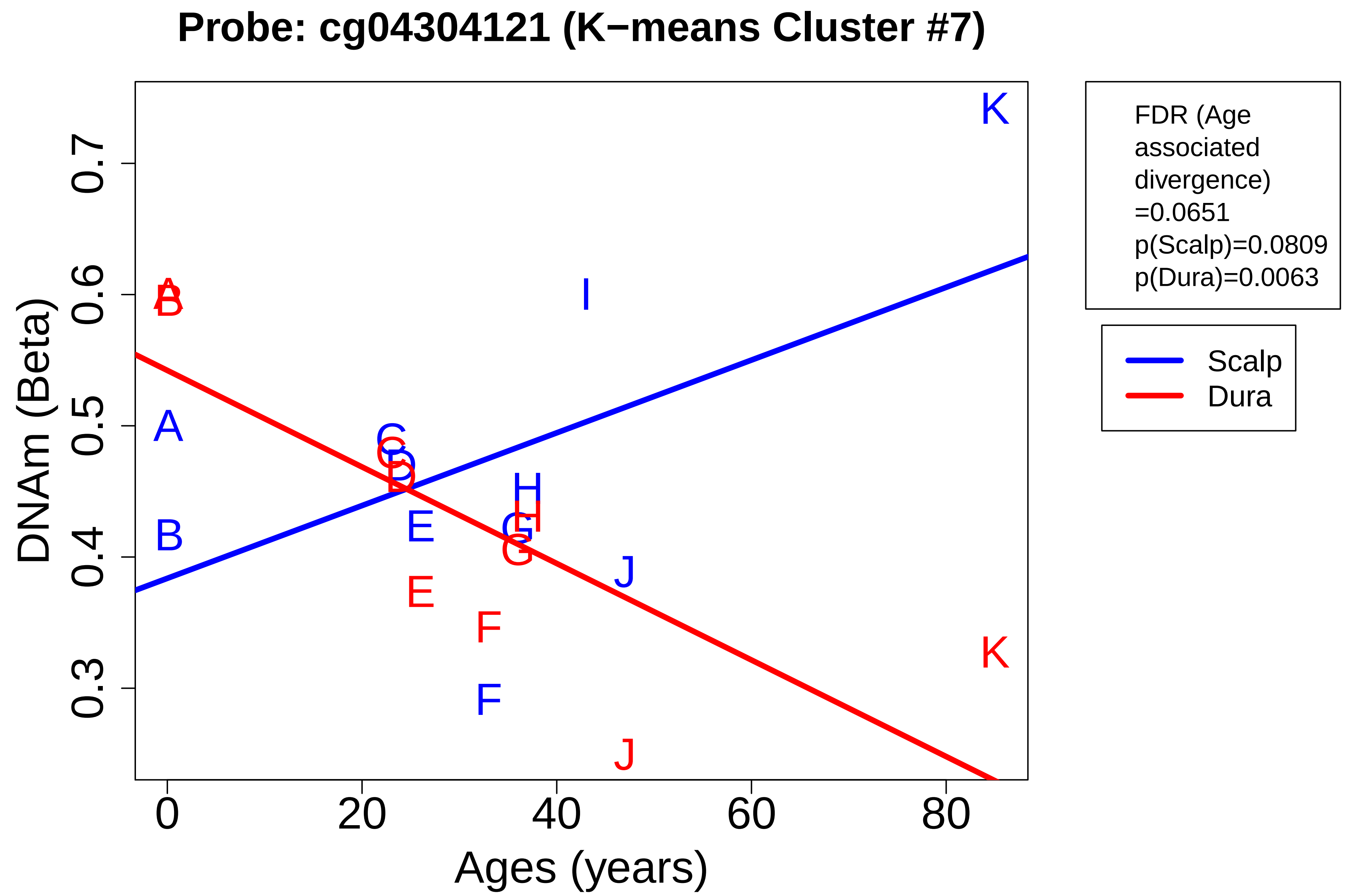

Probe: cg04304121 (K−means Cluster #7)
FDR (Age associated divergence) =0.0651
p(Scalp)=0.0809
p(Dura)=0.0063
Scalp
Dura
DNAm (Beta)
0.3
0.4
0.5
0.6
0.7
0
20
40
60
80
Ages (years)
A
B
C
D
E
F
G
H
I
J
K

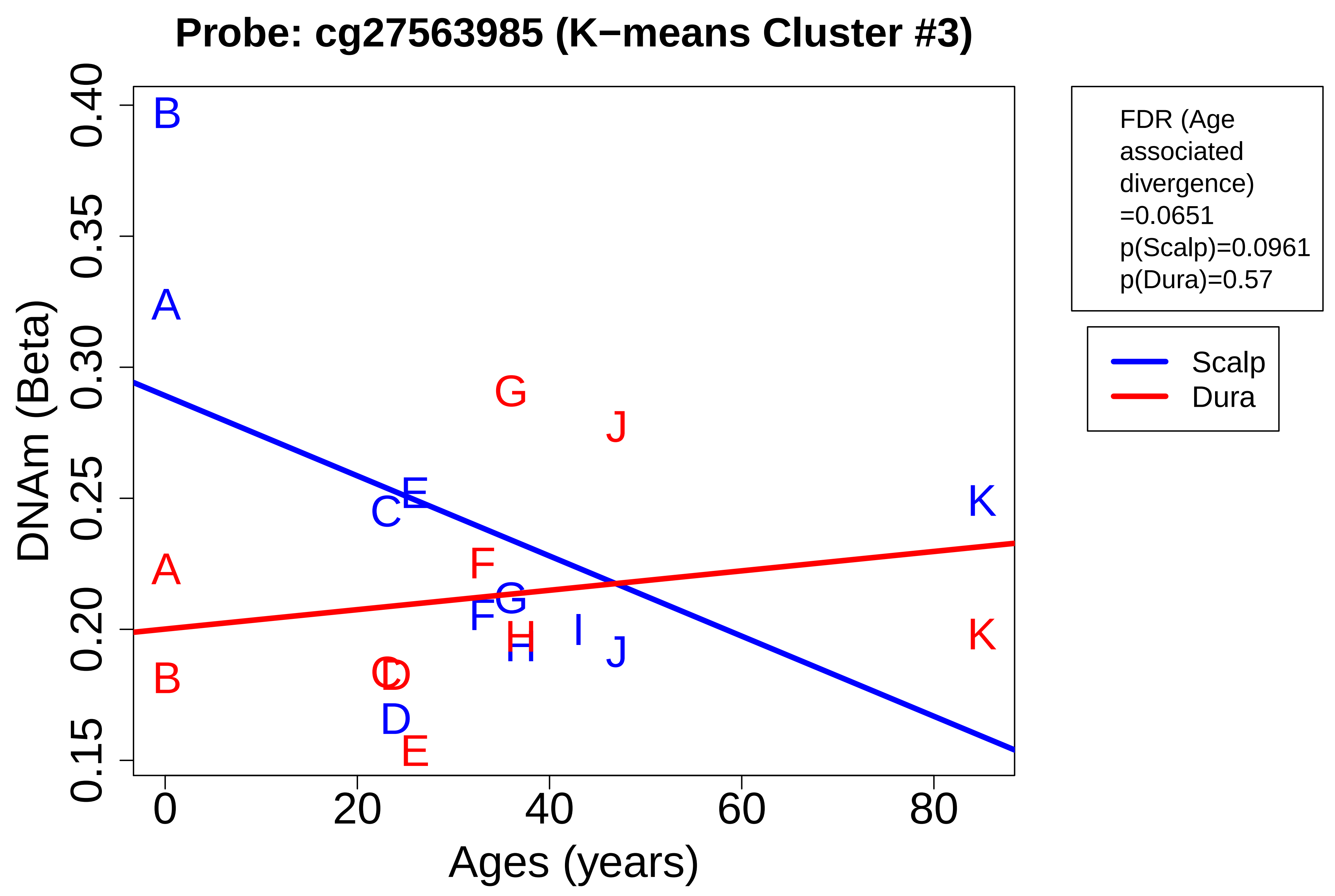
Probe: cg27563985 (K−means Cluster #3)
DNAm (Beta)
0.15
0.20
0.25
0.30
0.35
0.40
0
20
40
60
80
Ages (years)
FDR (Age associated divergence) =0.0651
p(Scalp)=0.0961
p(Dura)=0.57
Scalp
Dura

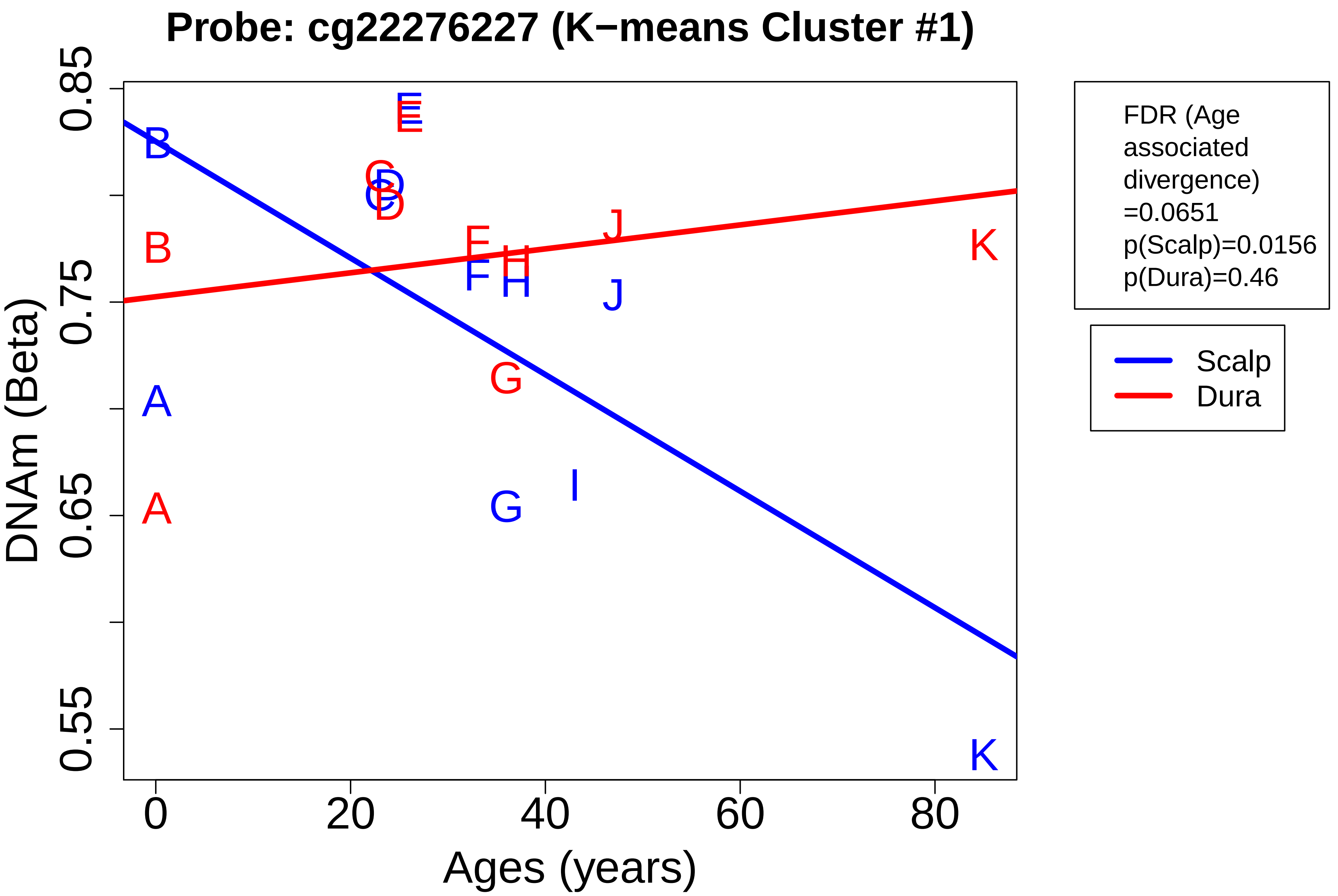
Probe: cg22276227 (K−means Cluster #1)
DNAm (Beta)
0.85
0.75
0.65
0.55
0
20
40
60
80
Ages (years)
FDR (Age associated divergence) =0.0651
p(Scalp)=0.0156
p(Dura)=0.46
Scalp
Dura

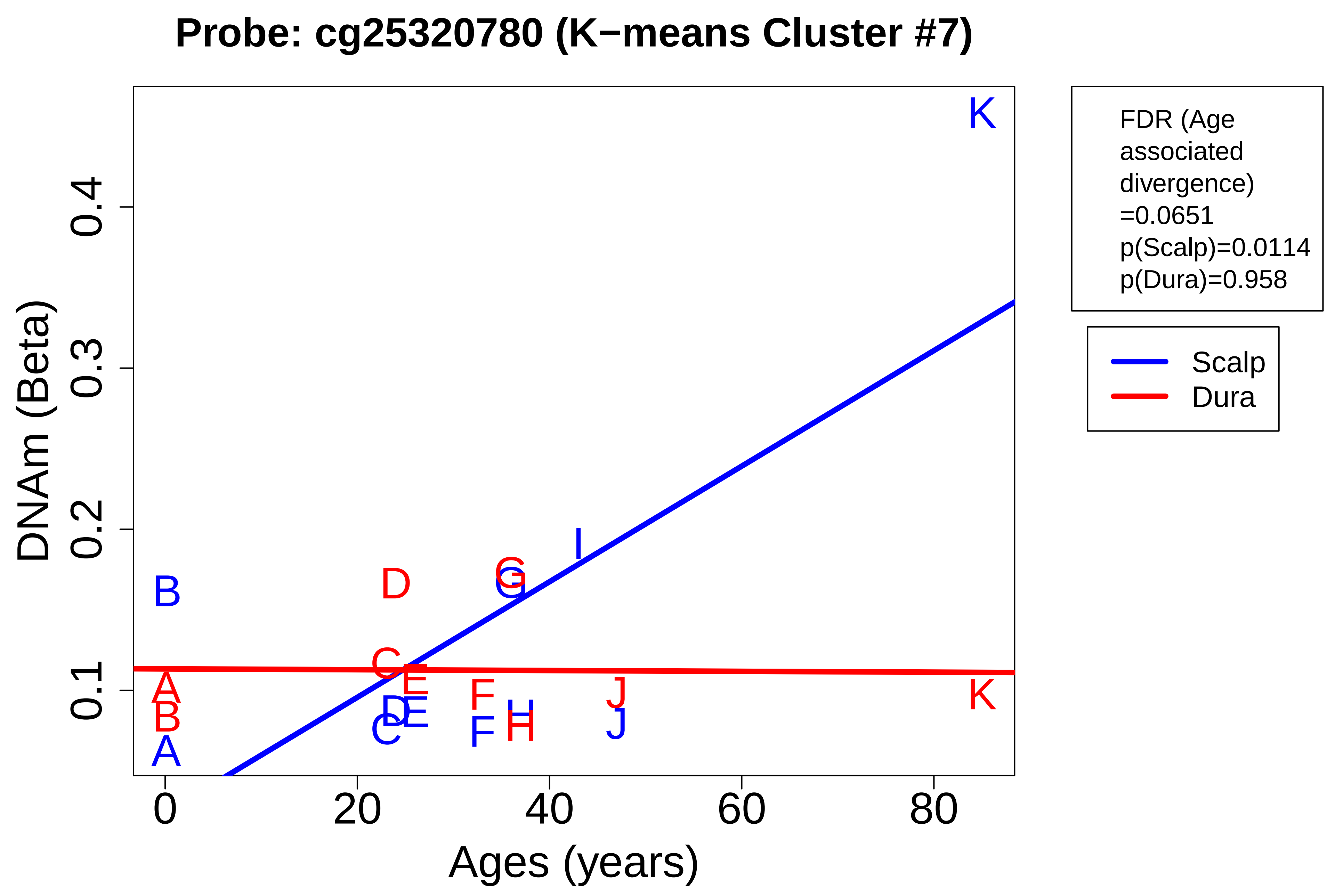
Probe: cg25320780 (K−means Cluster #7)
DNAm (Beta)
0.1
0.2
0.3
0.4
Ages (years)
0
20
40
60
80
FDR (Age associated divergence) =0.0651
p(Scalp)=0.0114
p(Dura)=0.958
Scalp
Dura
A
B
C
D
E
F
G
H
I
J
K

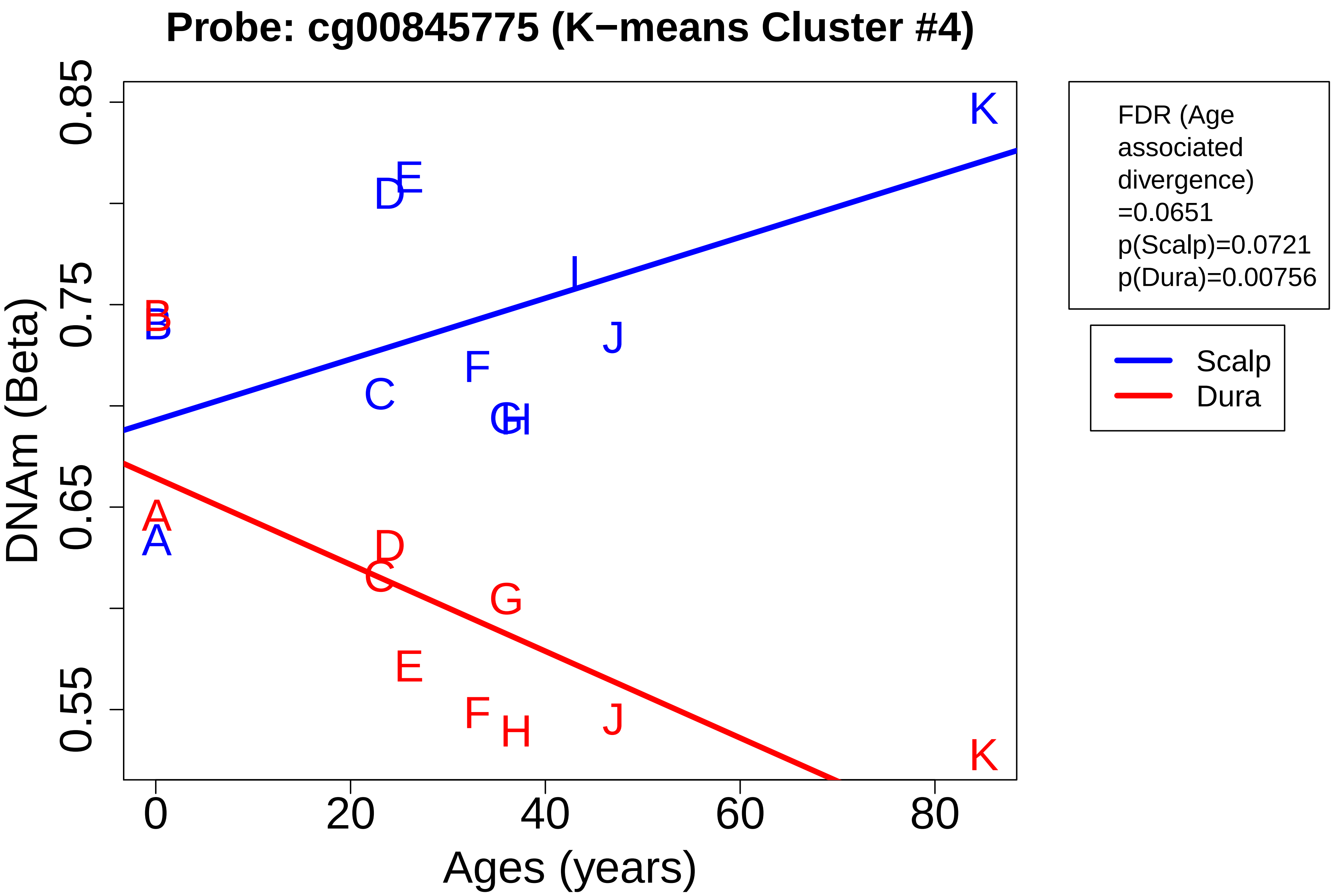

Probe: cg00845775 (K−means Cluster #4)
DNAm (Beta)
0.55
0.65
0.75
0.85
0
20
40
60
80
Ages (years)
FDR (Age associated divergence) =0.0651
p(Scalp)=0.0721
p(Dura)=0.00756
Scalp
Dura

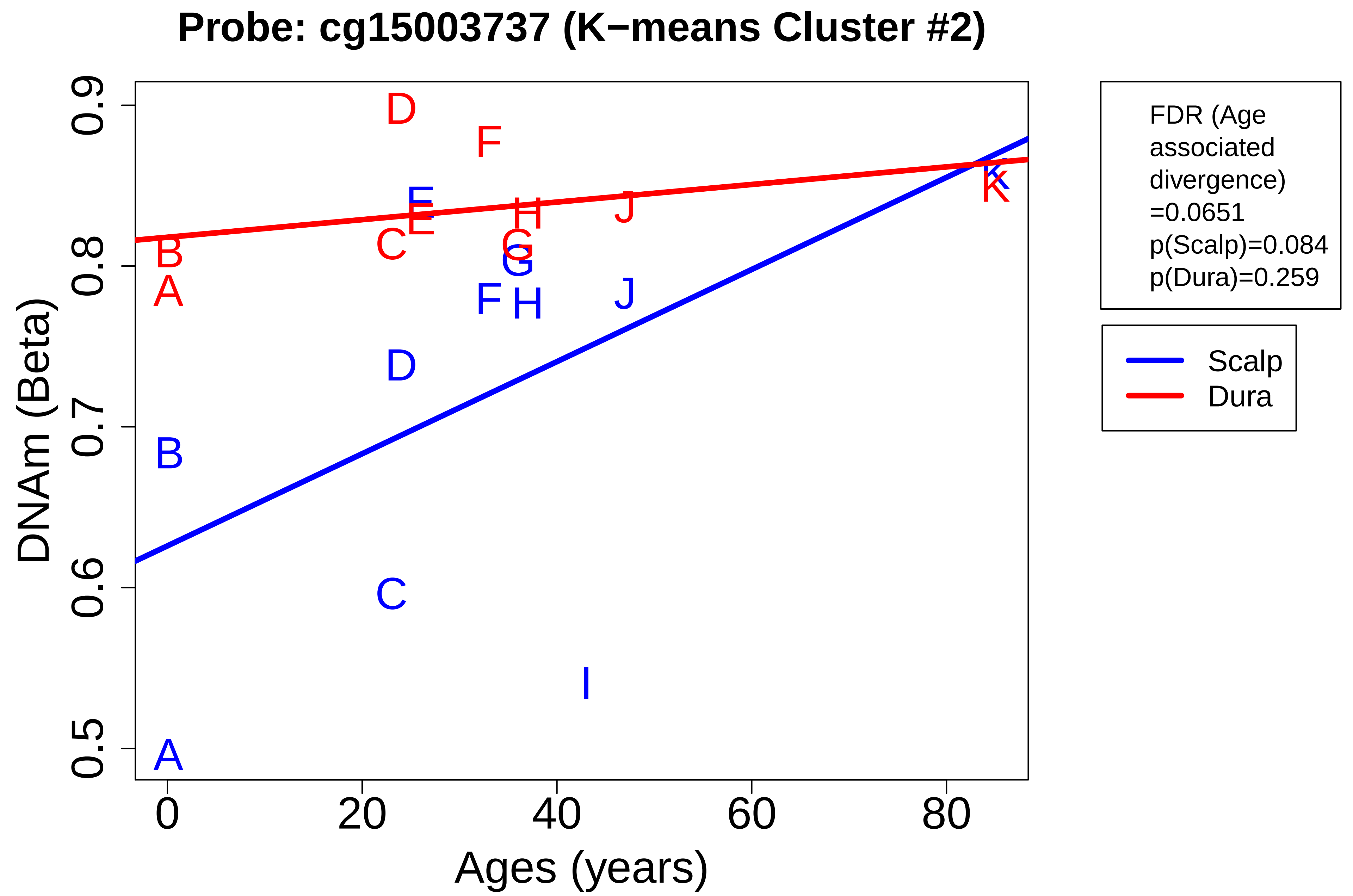

Probe: cg15003737 (K−means Cluster #2)
DNAm (Beta)
0.5
0.6
0.7
0.8
0.9
0
20
40
60
80
Ages (years)
FDR (Age associated divergence) =0.0651
p(Scalp)=0.084
p(Dura)=0.259
Scalp
Dura

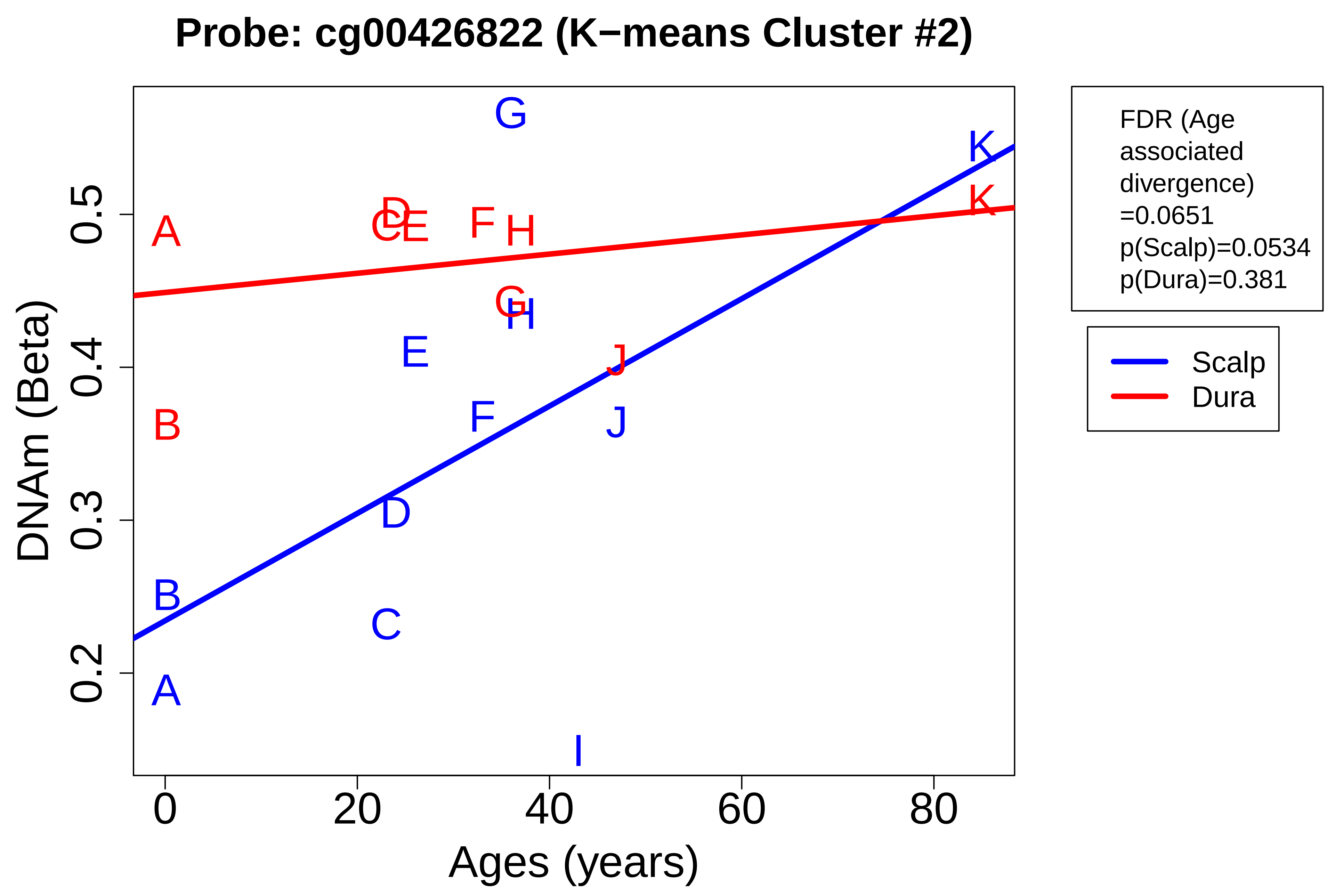
Probe: cg00426822 (K−means Cluster #2)
DNAm (Beta)
0.2
0.3
0.4
0.5
0
20
40
60
80
Ages (years)
FDR (Age associated divergence) =0.0651
p(Scalp)=0.0534
p(Dura)=0.381
Scalp
Dura

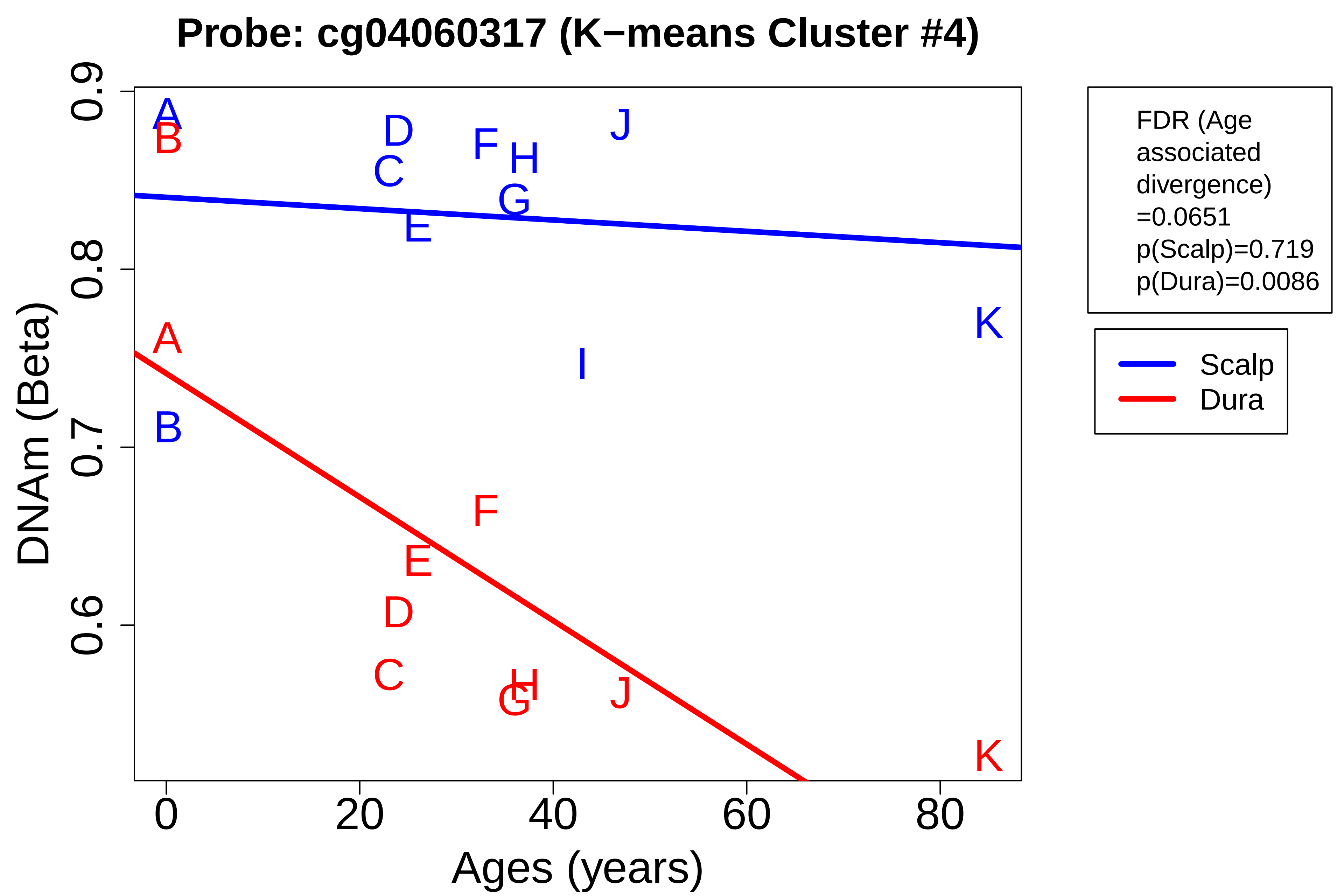
Probe: cg04060317 (K−means Cluster #4)
DNAm (Beta)
0.9
0.8
0.7
0.6
0
20
40
60
80
Ages (years)
A
B
C
D
E
F
G
H
I
J
K
FDR (Age associated divergence) =0.0651
p(Scalp)=0.719
p(Dura)=0.0086
Scalp
Dura

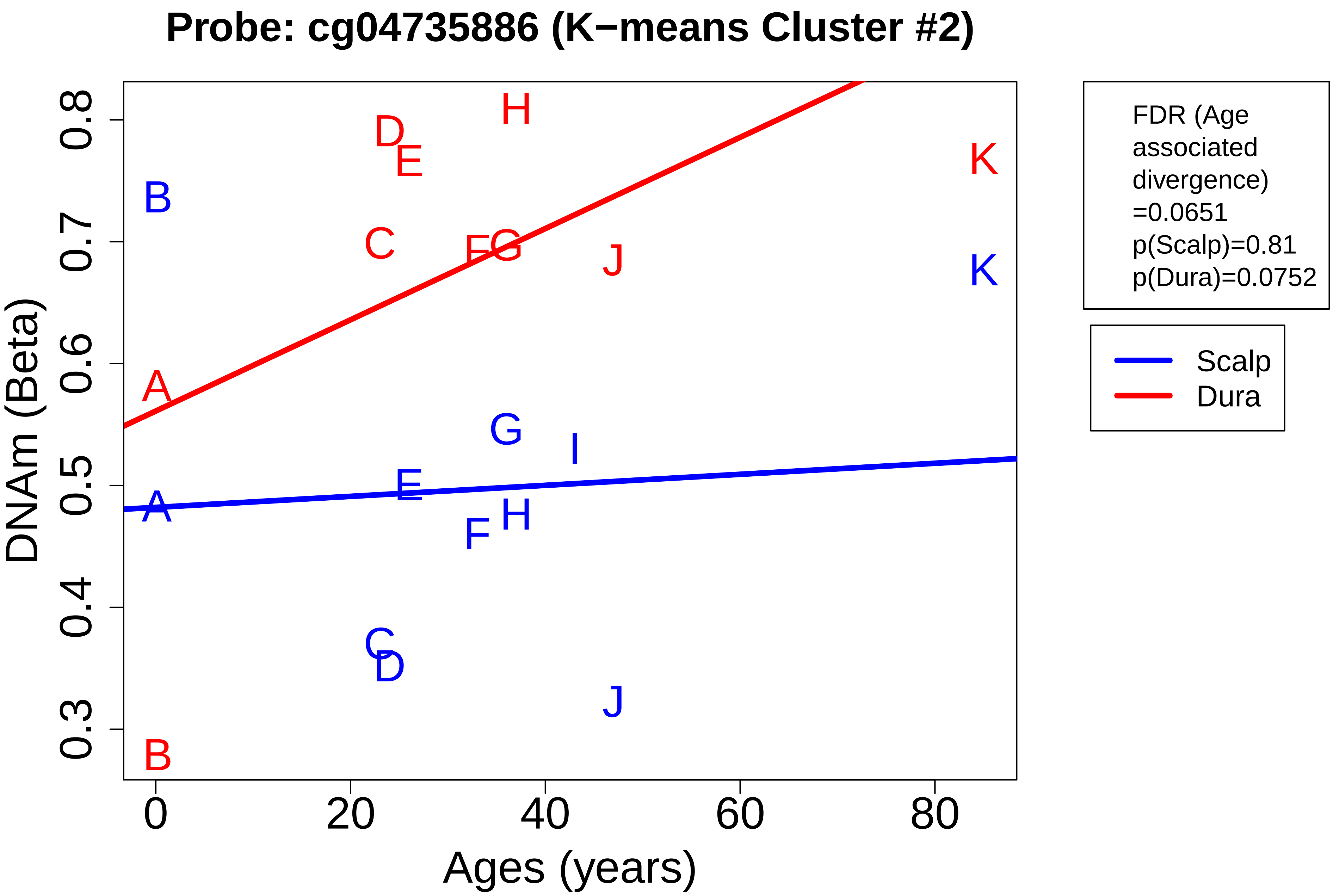

Probe: cg04735886 (K−means Cluster #2)
DNAm (Beta)
Ages (years)
0.3
0.4
0.5
0.6
0.7
0.8
0
20
40
60
80
FDR (Age associated divergence) =0.0651
p(Scalp)=0.81
p(Dura)=0.0752
Scalp
Dura

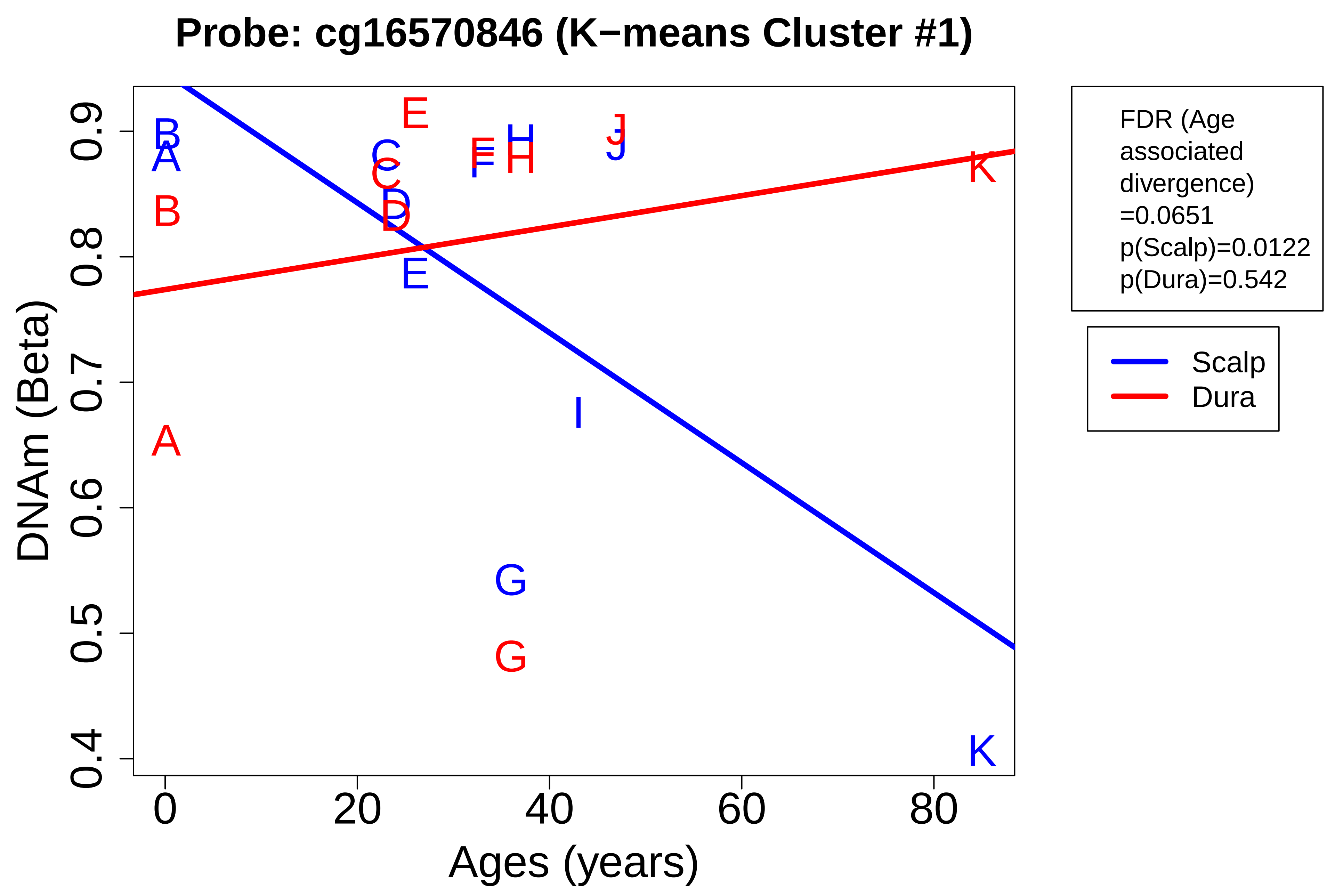

Probe: cg16570846 (K−means Cluster #1)
DNAm (Beta)
0.4
0.5
0.6
0.7
0.8
0.9
0
20
40
60
80
Ages (years)
FDR (Age associated divergence) =0.0651
p(Scalp)=0.0122
p(Dura)=0.542
Scalp
Dura

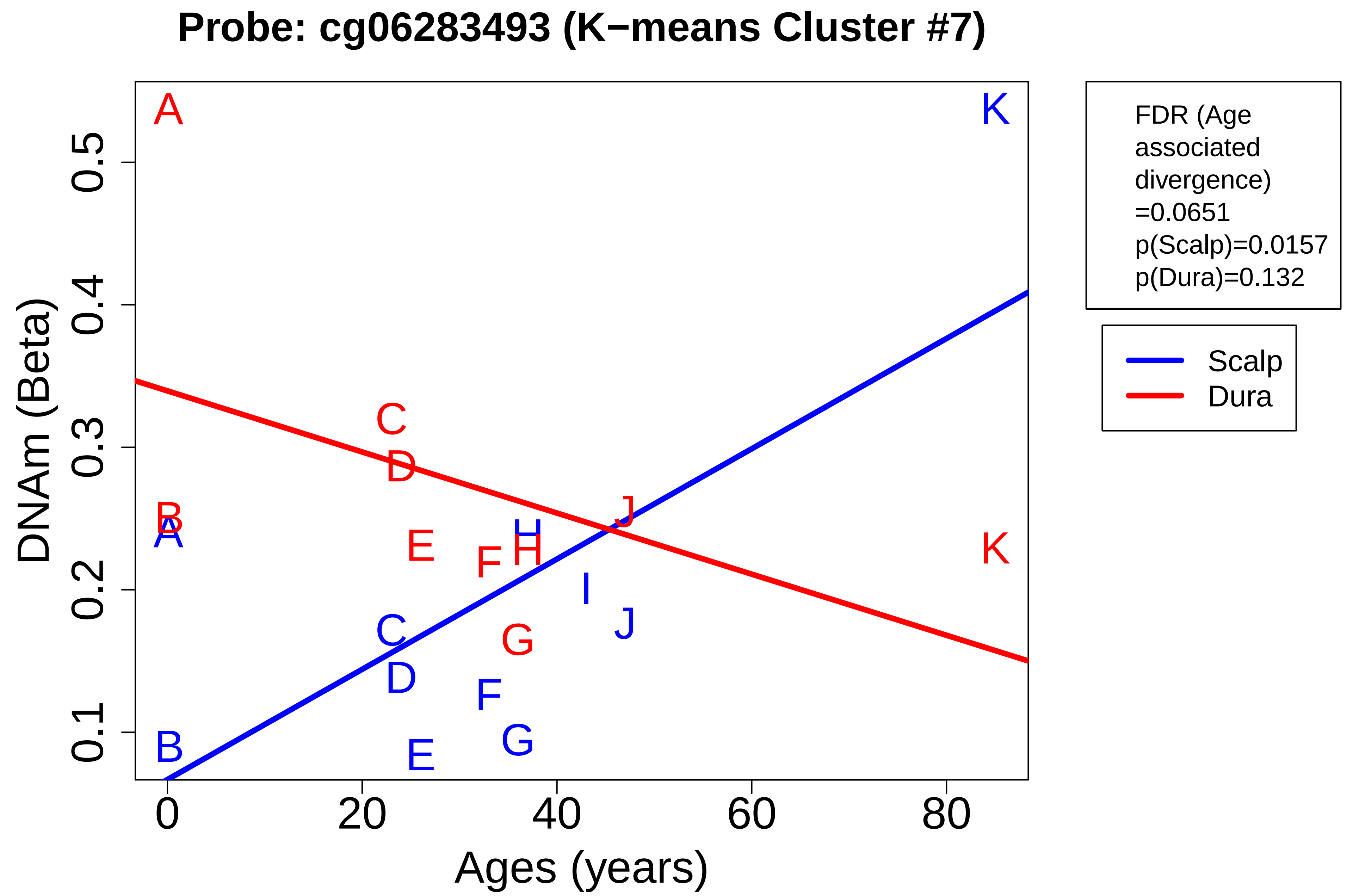
Probe: cg06283493 (K−means Cluster #7)
DNAm (Beta)
Ages (years)
0.1
0.2
0.3
0.4
0.5
0
20
40
60
80
FDR (Age associated divergence) =0.0651
p(Scalp)=0.0157
p(Dura)=0.132
Scalp
Dura

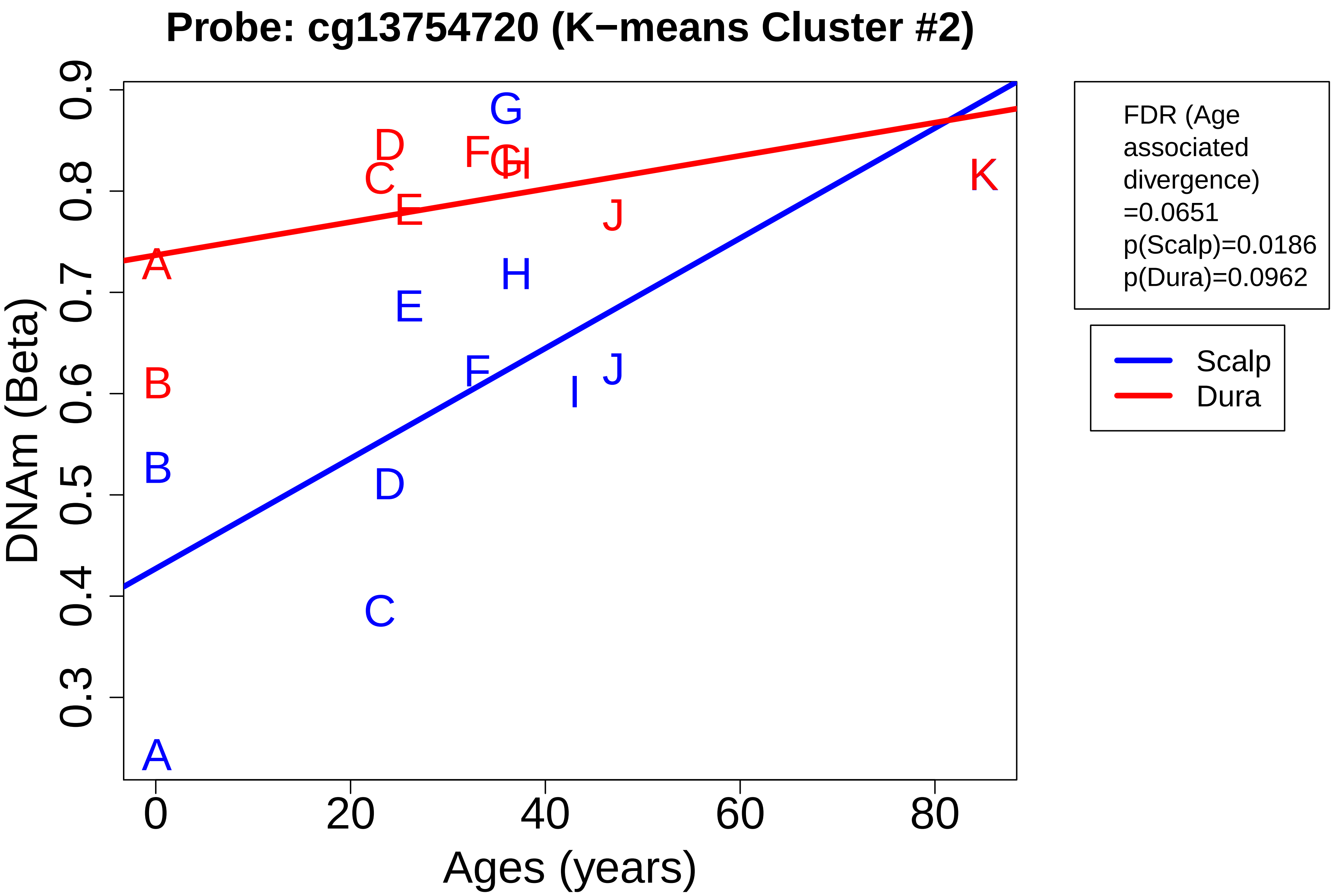

Probe: cg13754720 (K−means Cluster #2)
DNAm (Beta)
0.3
0.4
0.5
0.6
0.7
0.8
0.9
0
20
40
60
80
Ages (years)
FDR (Age associated divergence) =0.0651
p(Scalp)=0.0186
p(Dura)=0.0962
Scalp
Dura

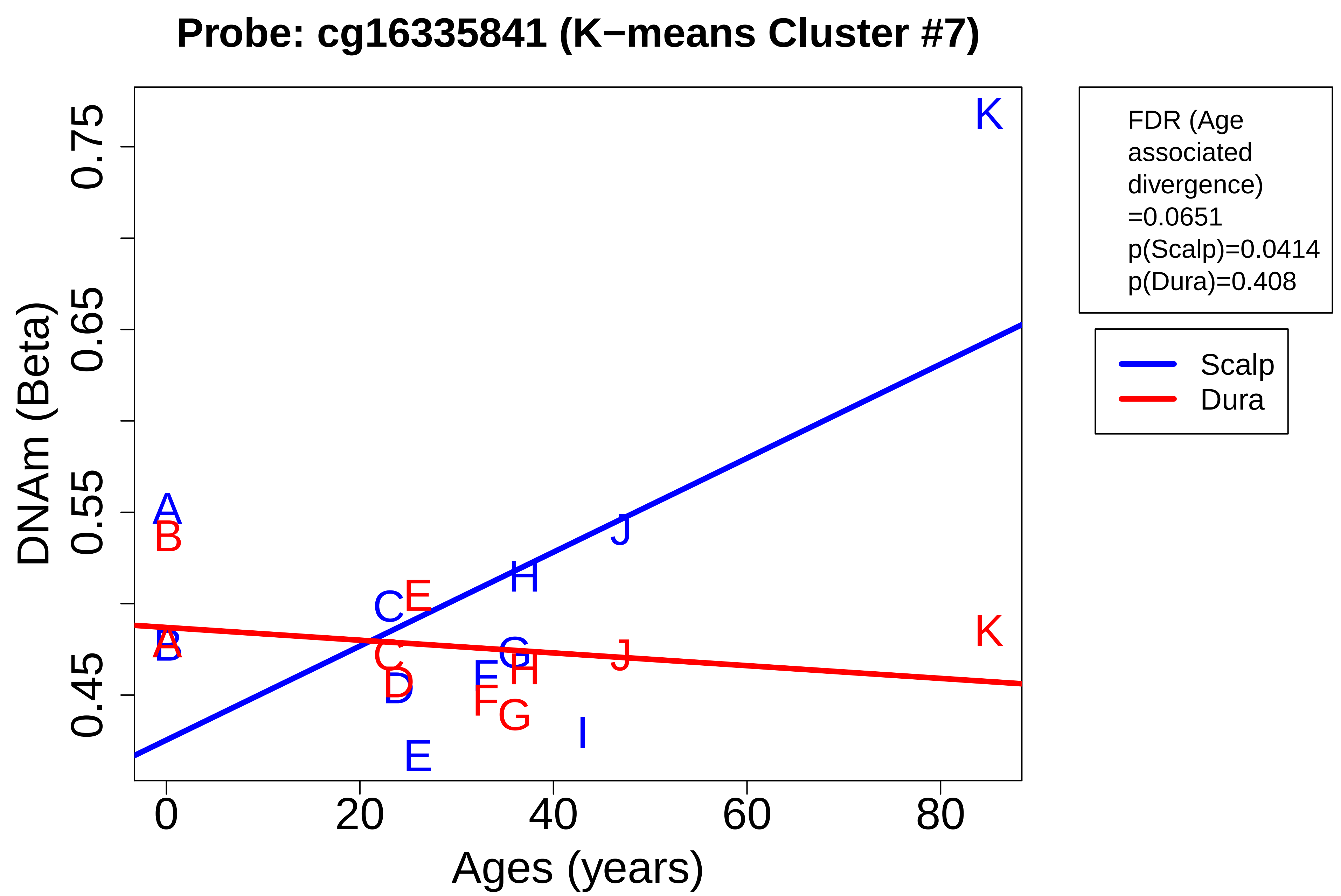
Probe: cg16335841 (K−means Cluster #7)
DNAm (Beta)
Ages (years)
0.45
0.55
0.65
0.75
0
20
40
60
80
FDR (Age associated divergence) =0.0651
p(Scalp)=0.0414
p(Dura)=0.408
Scalp
Dura

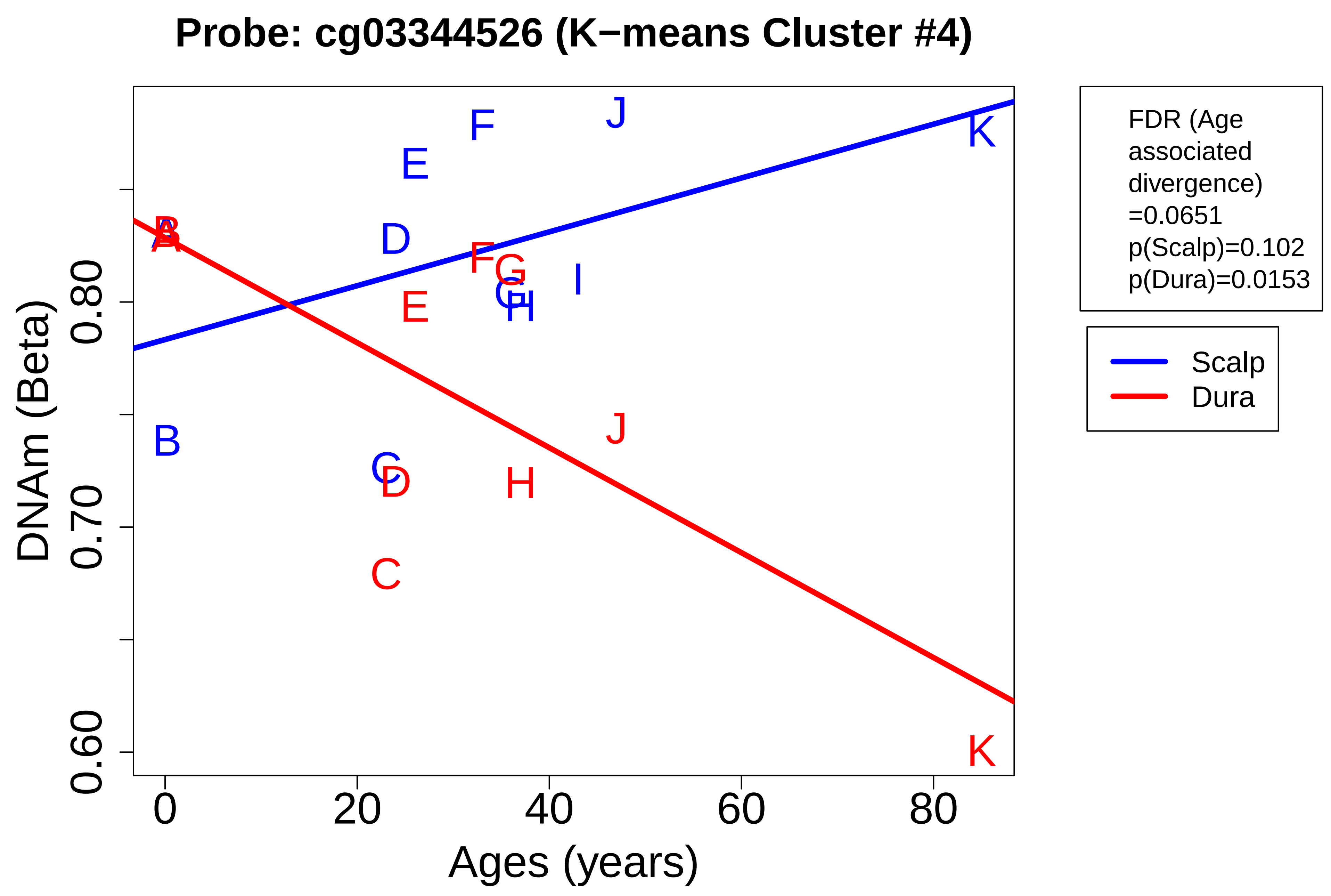
Probe: cg03344526 (K−means Cluster #4)
FDR (Age associated divergence) =0.0651
p(Scalp)=0.102
p(Dura)=0.0153
Scalp
Dura
DNAm (Beta)
0.60
0.70
0.80
0
20
40
60
80
Ages (years)

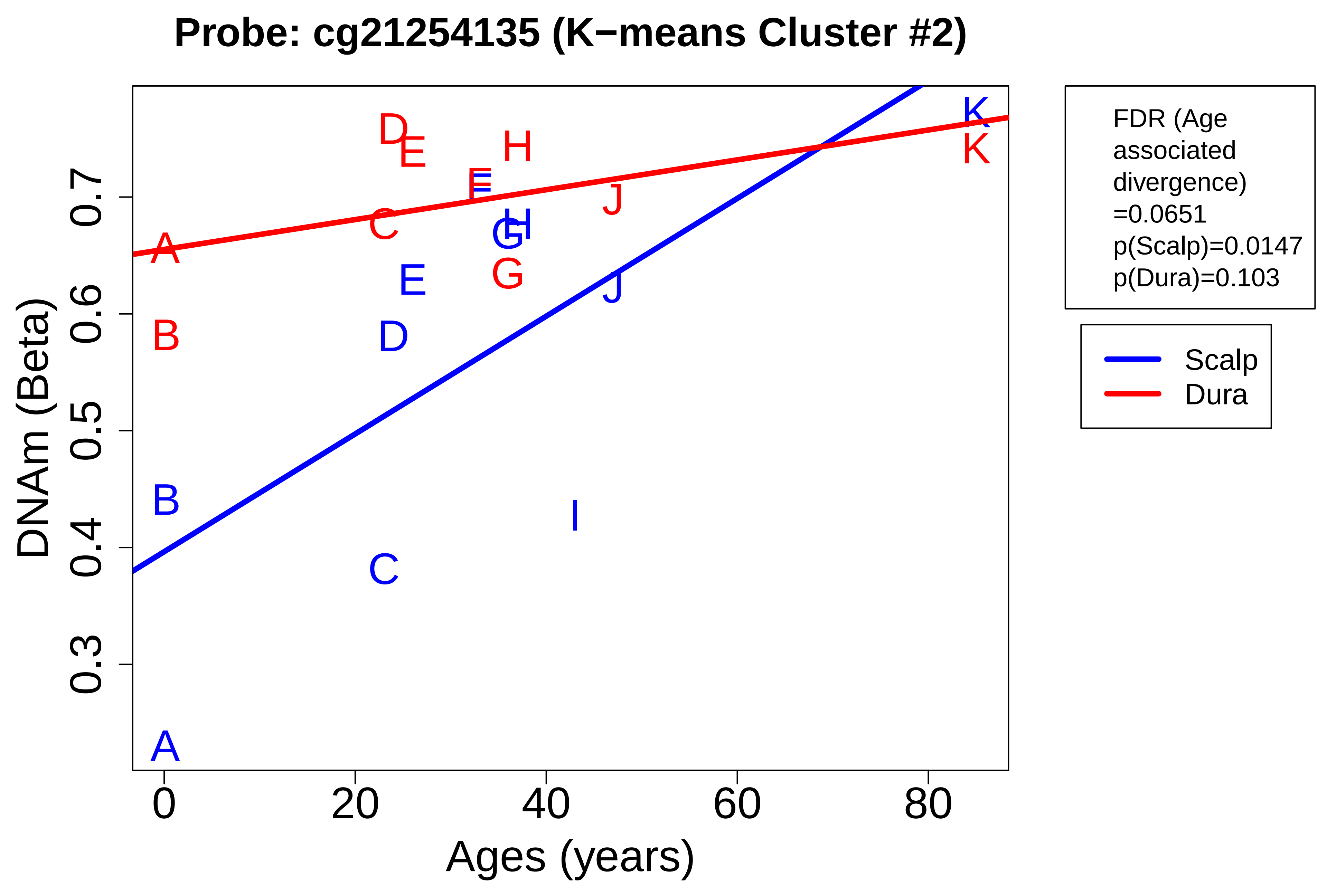

Probe: cg21254135 (K−means Cluster #2)
DNAm (Beta)
0.3
0.4
0.5
0.6
0.7
0
20
40
60
80
Ages (years)
FDR (Age associated divergence) =0.0651
p(Scalp)=0.0147
p(Dura)=0.103
Scalp
Dura
A
B
C
D
E
F
G
H
I
J
K

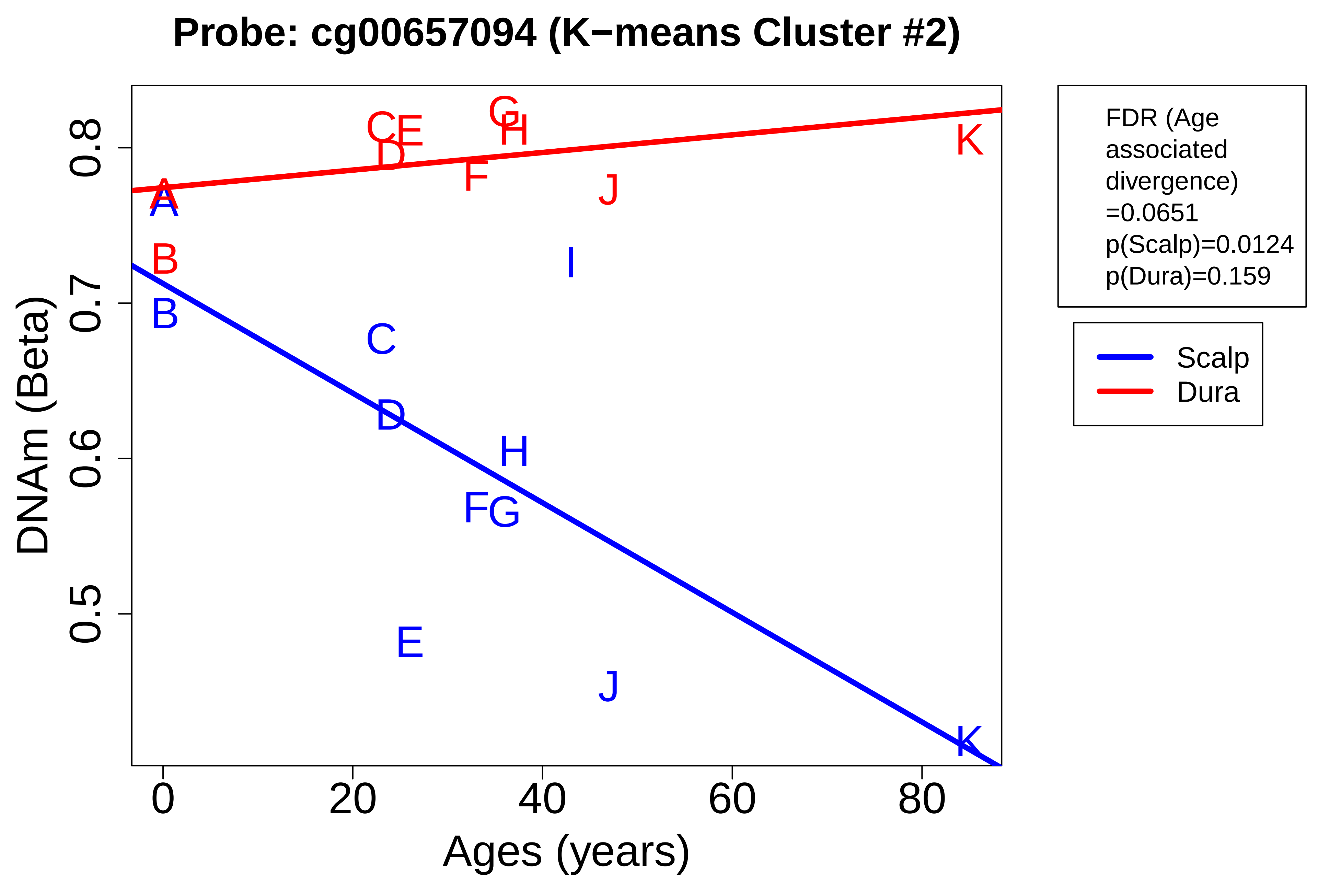

Probe: cg00657094 (K−means Cluster #2)
DNAm (Beta)
0.5
0.6
0.7
0.8
0
20
40
60
80
Ages (years)
A
B
C
D
E
F
G
H
I
J
K
FDR (Age associated divergence) =0.0651
p(Scalp)=0.0124
p(Dura)=0.159
Scalp
Dura

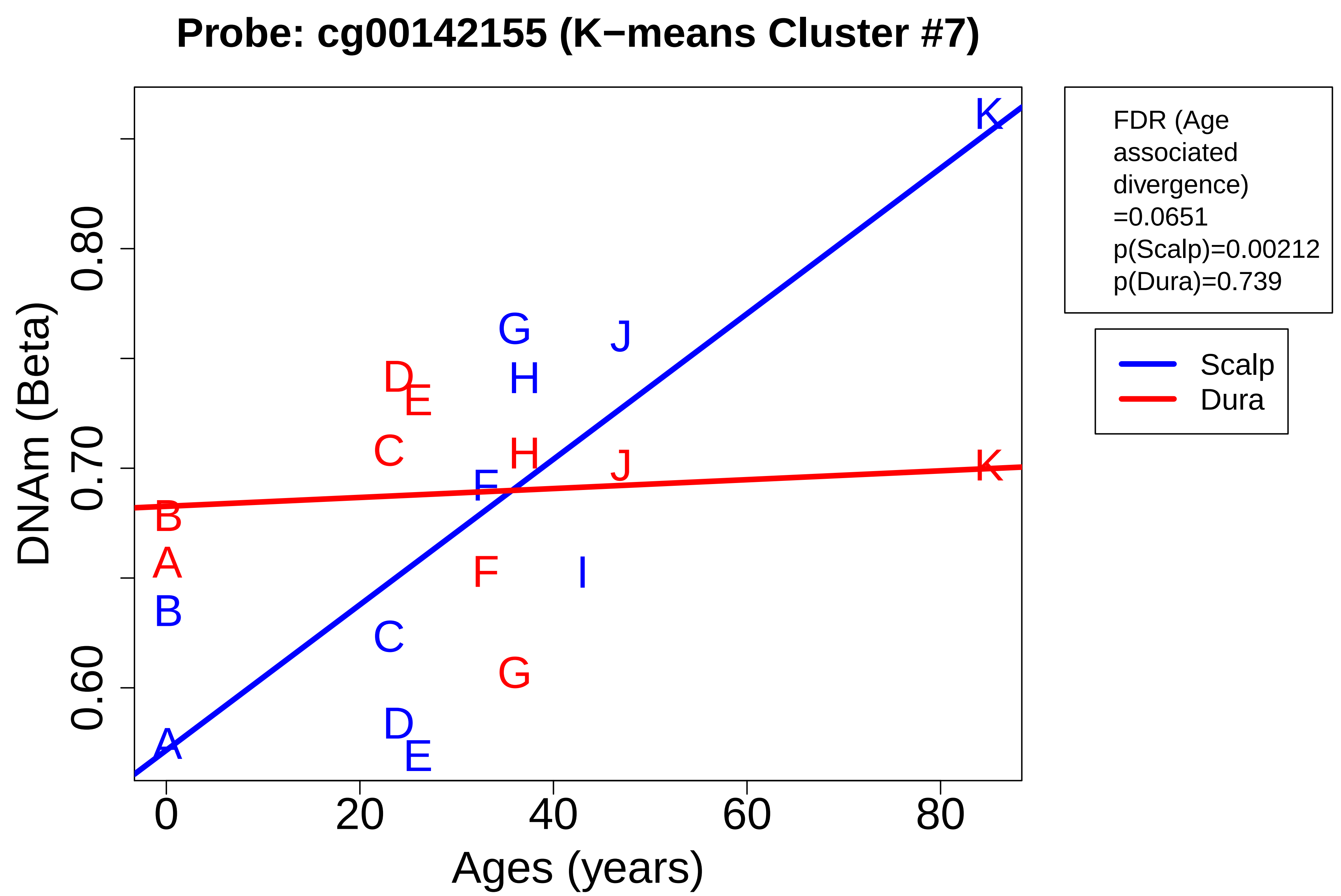

Probe: cg00142155 (K−means Cluster #7)
DNAm (Beta)
0.60
0.70
0.80
Ages (years)
0
20
40
60
80
A
B
C
D
E
F
G
H
I
J
K
FDR (Age associated divergence) =0.0651
p(Scalp)=0.00212
p(Dura)=0.739
Scalp
Dura

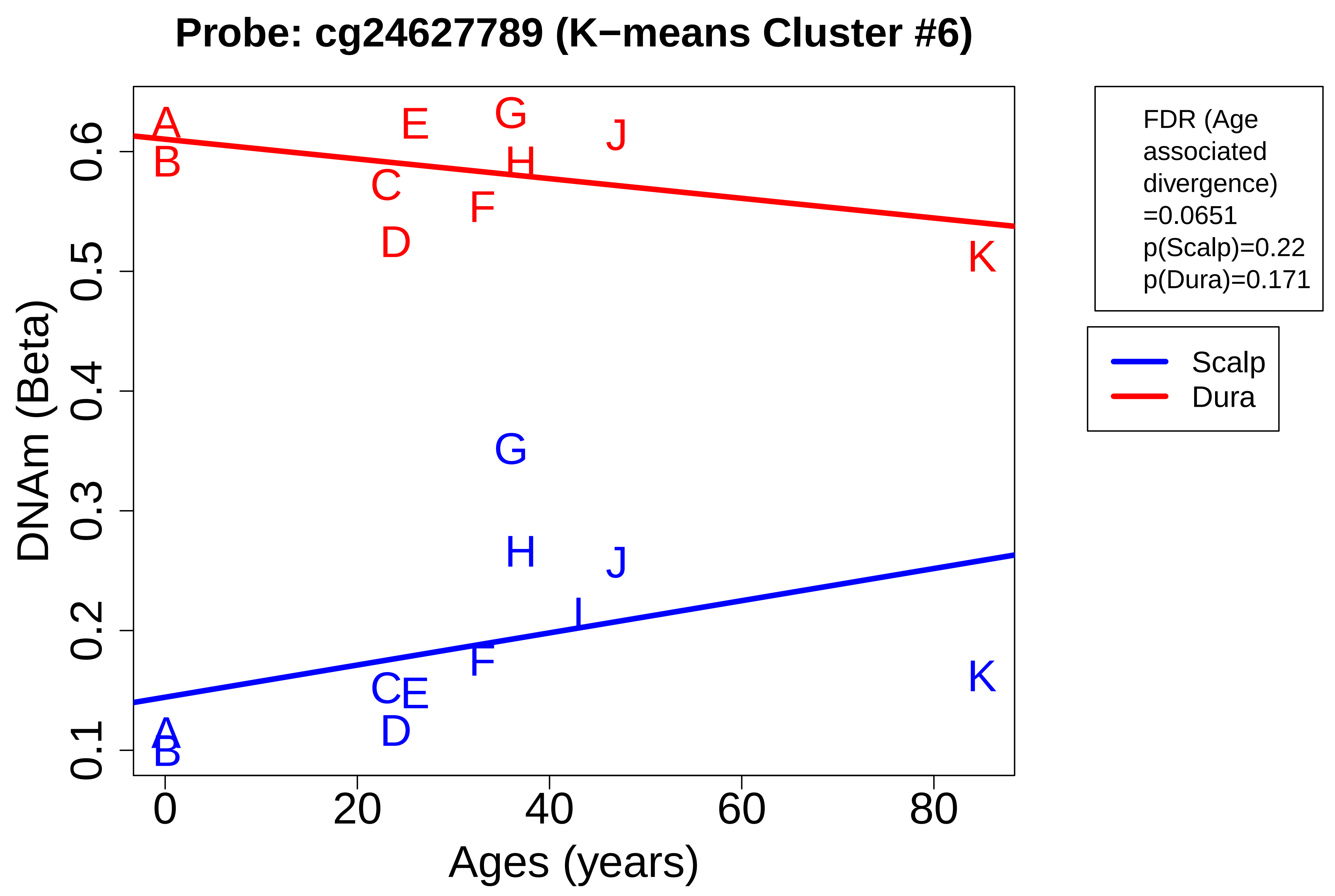

Probe: cg24627789 (K−means Cluster #6)
DNAm (Beta)
0.1
0.2
0.3
0.4
0.5
0.6
Ages (years)
0
20
40
60
80
A
B
C
D
E
F
G
H
I
J
K
FDR (Age associated divergence) =0.0651
p(Scalp)=0.22
p(Dura)=0.171
Scalp
Dura

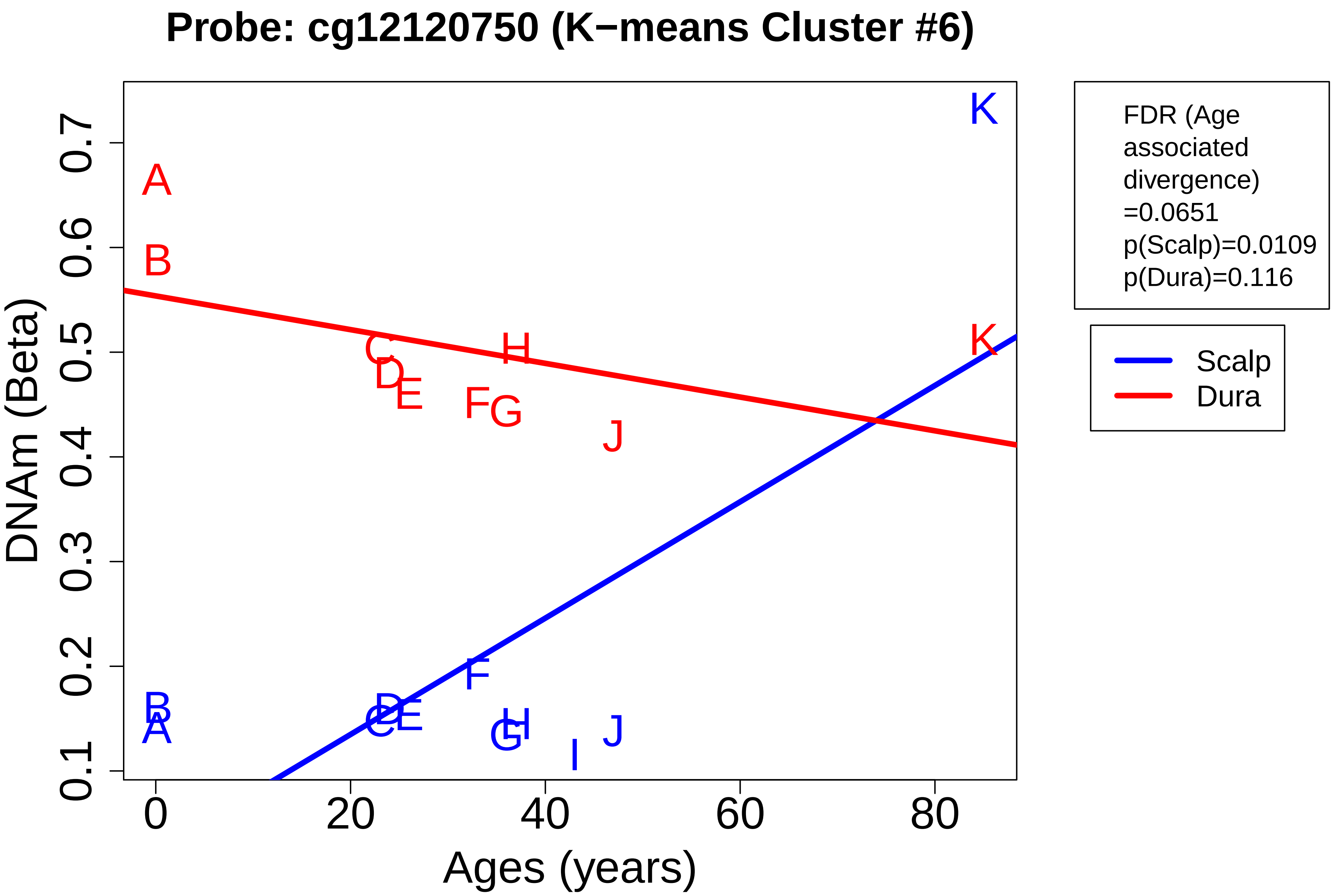

Probe: cg12120750 (K−means Cluster #6)
DNAm (Beta)
0.1
0.2
0.3
0.4
0.5
0.6
0.7
Ages (years)
0
20
40
60
80
A
B
C
D
E
F
G
H
I
J
K
FDR (Age associated divergence) =0.0651
p(Scalp)=0.0109
p(Dura)=0.116
Scalp
Dura

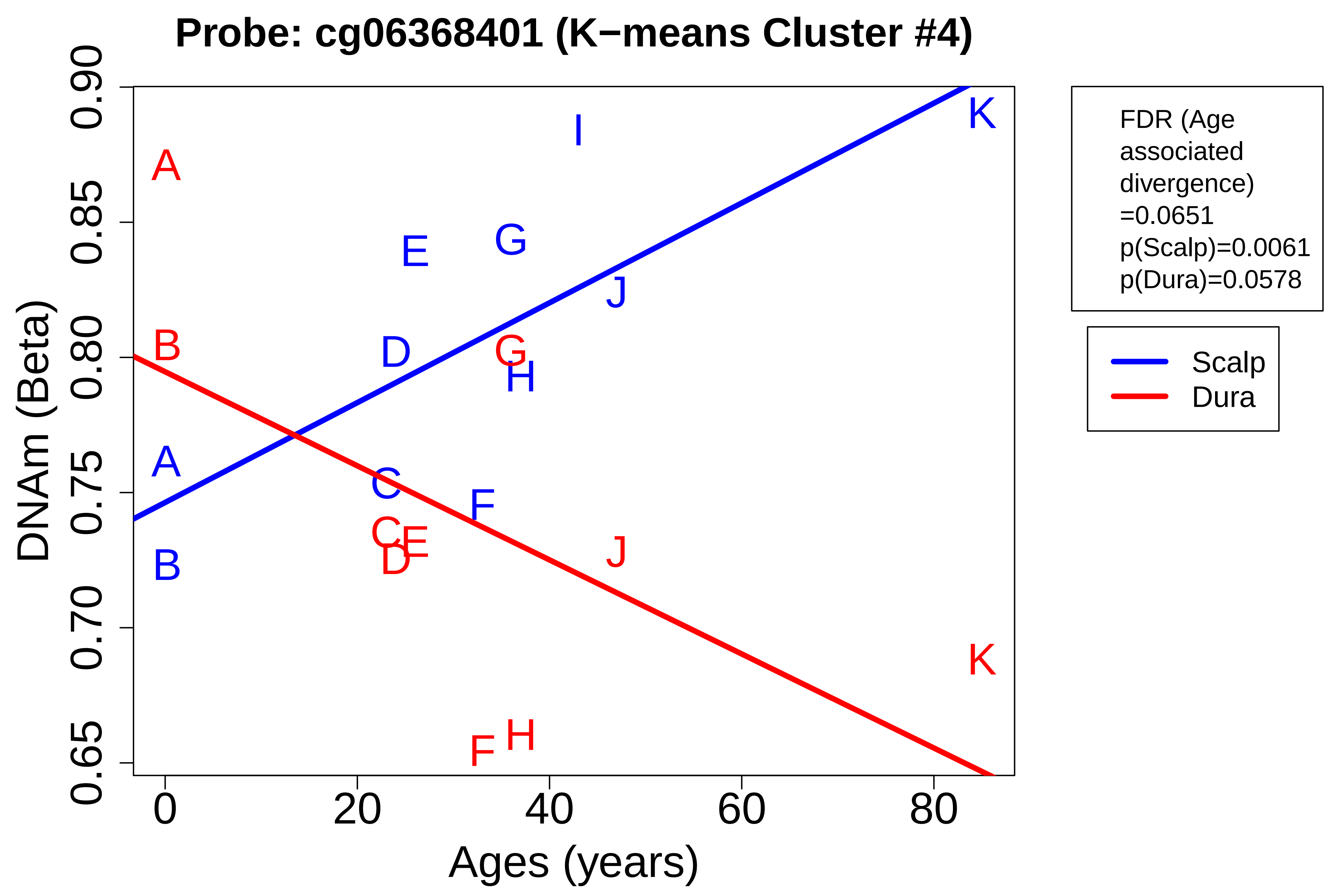
Probe: cg06368401 (K−means Cluster #4)
DNAm (Beta)
0.65
0.70
0.75
0.80
0.85
0.90
0
20
40
60
80
Ages (years)
FDR (Age associated divergence) =0.0651
p(Scalp)=0.0061
p(Dura)=0.0578
Scalp
Dura
A
B
C
D
E
F
G
H
I
J
K

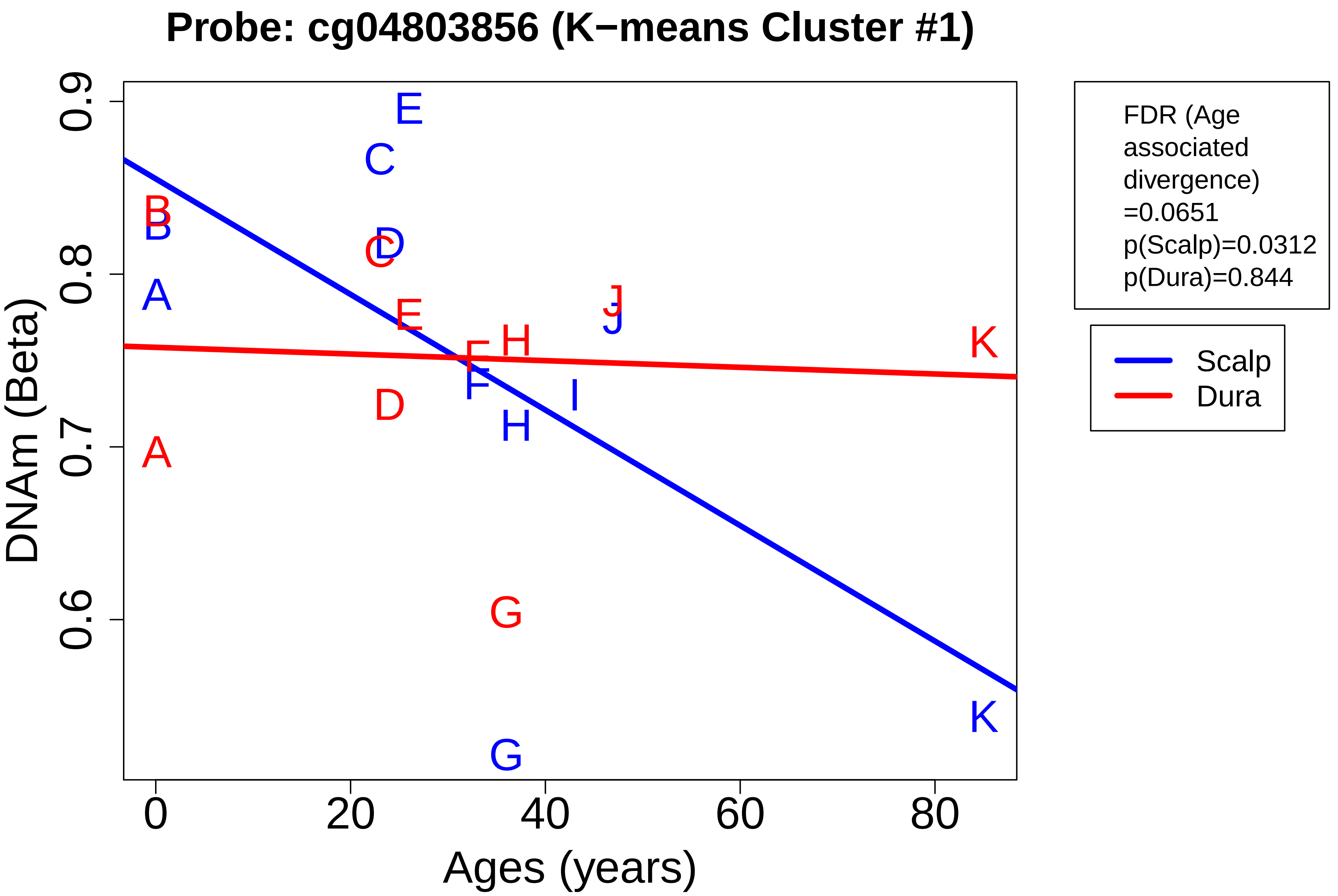
Probe: cg04803856 (K−means Cluster #1)
FDR (Age
associated
divergence)
=0.0651
p(Scalp)=0.0312
p(Dura)=0.844
Scalp
Dura
DNAm (Beta)
0.6
0.7
0.8
0.9
Ages (years)
0
20
40
60
80
A
B
C
D
E
F
G
H
I
J
K

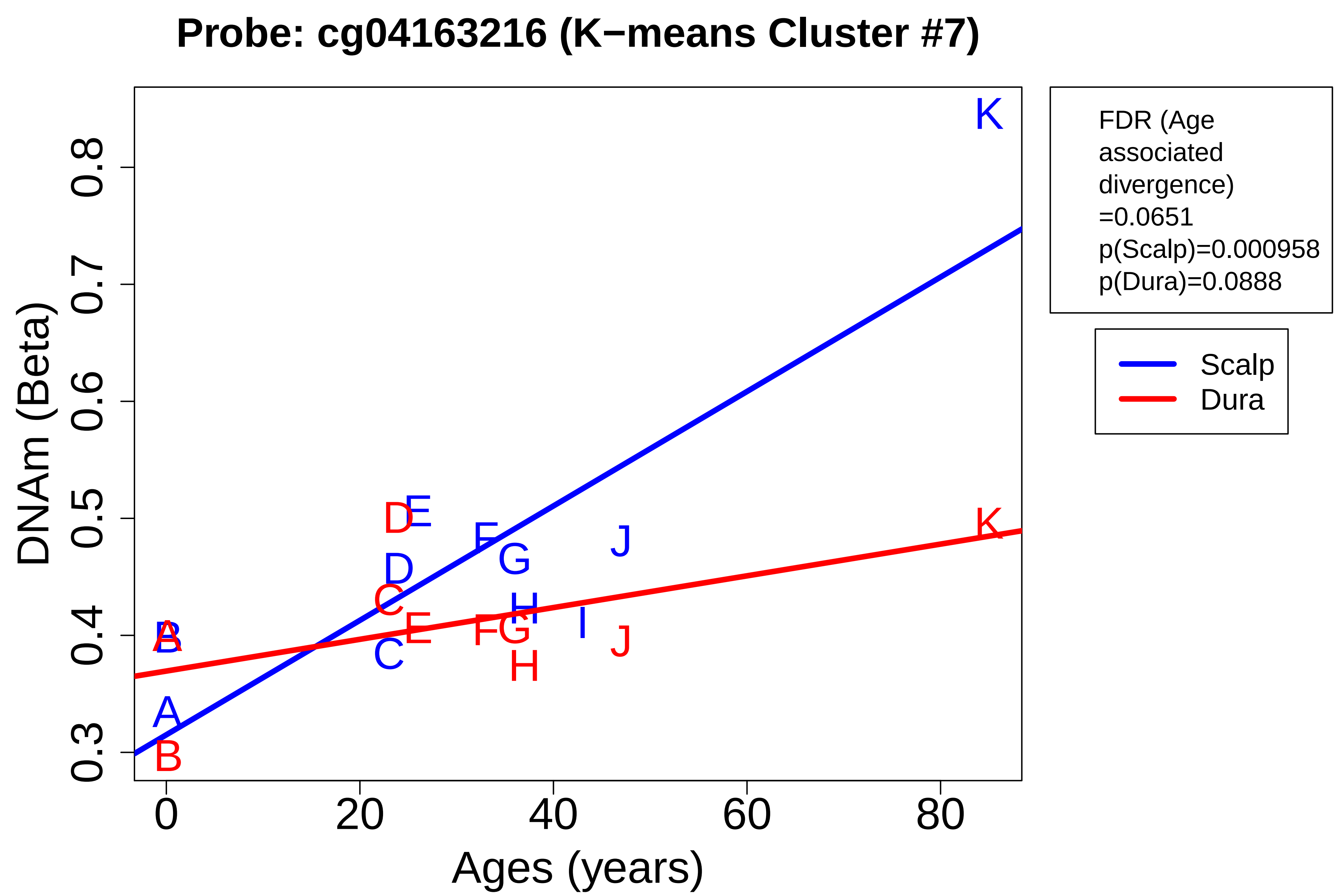
Probe: cg04163216 (K−means Cluster #7)
DNAm (Beta)
0.3
0.4
0.5
0.6
0.7
0.8
0
20
40
60
80
Ages (years)
FDR (Age associated divergence) =0.0651
p(Scalp)=0.000958
p(Dura)=0.0888
Scalp
Dura
A
B
C
D
E
F
G
H
I
J
K

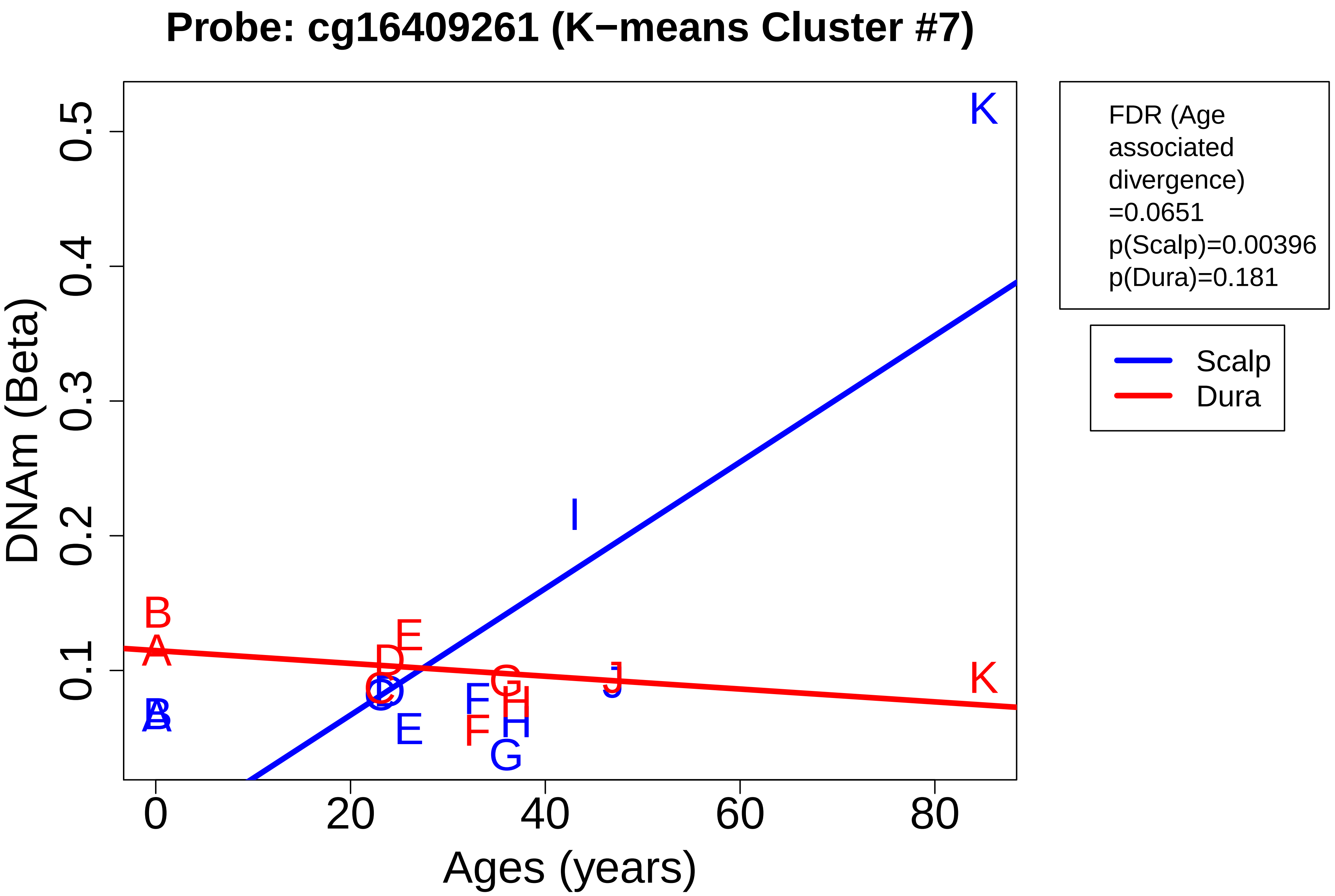

Probe: cg16409261 (K−means Cluster #7)
DNAm (Beta)
0.1
0.2
0.3
0.4
0.5
0
20
40
60
80
Ages (years)
FDR (Age associated divergence) =0.0651
p(Scalp)=0.00396
p(Dura)=0.181
Scalp
Dura
A
B
C
D
E
F
G
H
I
J
K

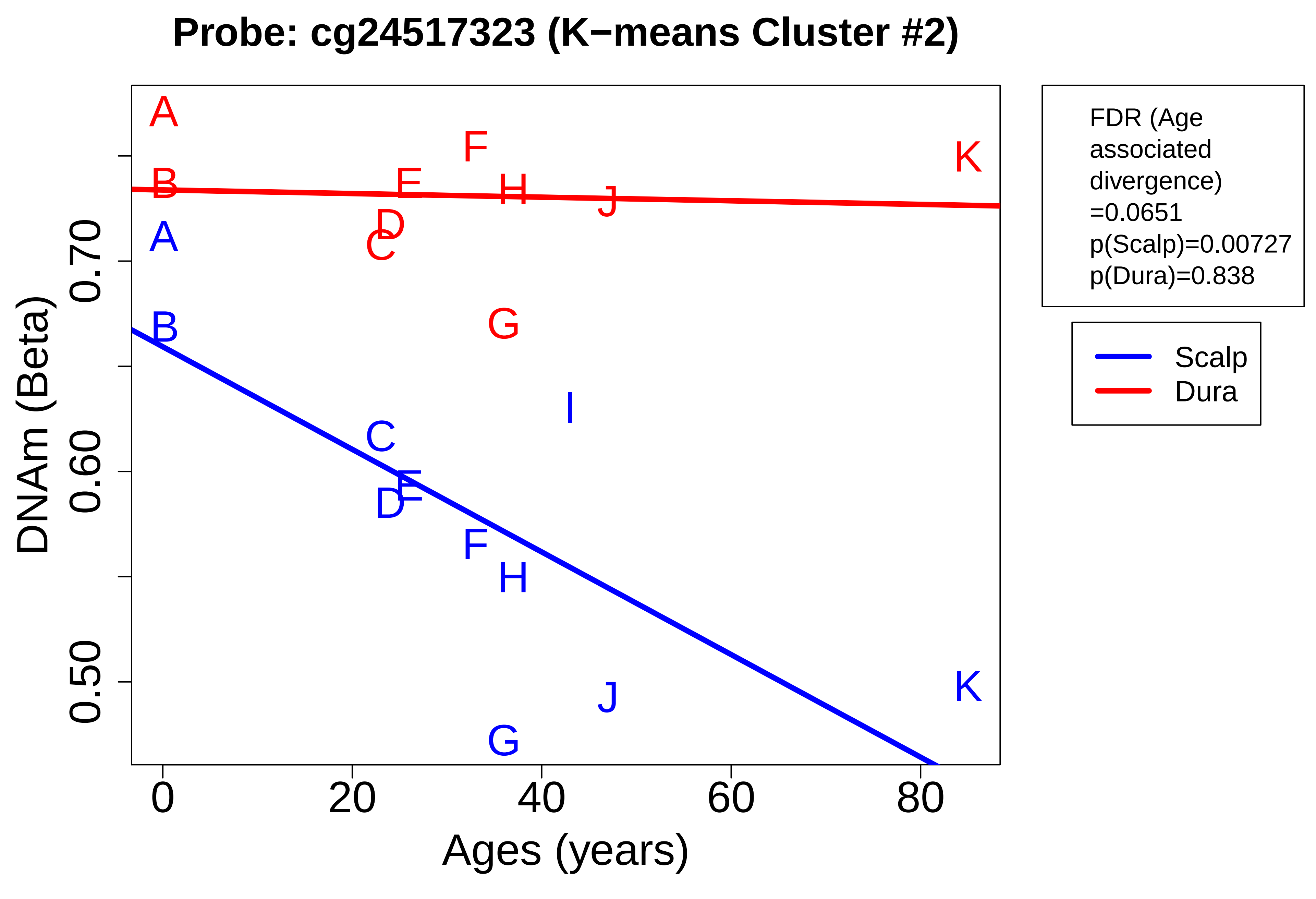

Probe: cg24517323 (K−means Cluster #2)
DNAm (Beta)
0.50
0.60
0.70
Ages (years)
0
20
40
60
80
A
B
C
D
E
F
G
H
I
J
K
FDR (Age associated divergence) =0.0651
p(Scalp)=0.00727
p(Dura)=0.838
Scalp
Dura

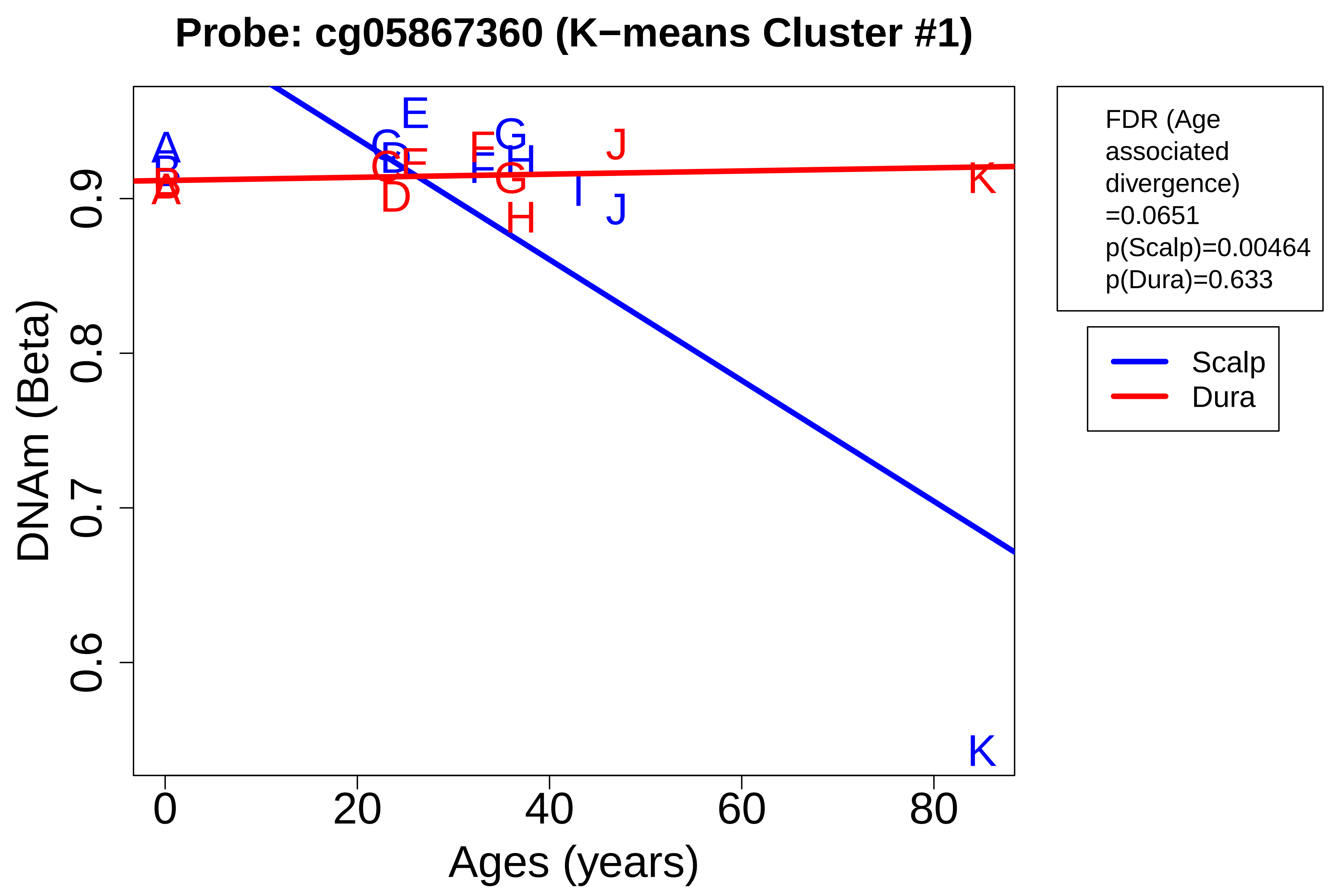

Probe: cg05867360 (K−means Cluster #1)
DNAm (Beta)
0.6
0.7
0.8
0.9
Ages (years)
0
20
40
60
80
FDR (Age associated divergence) =0.0651
p(Scalp)=0.00464
p(Dura)=0.633
Scalp
Dura
A
B
C
D
E
F
G
H
I
J
K

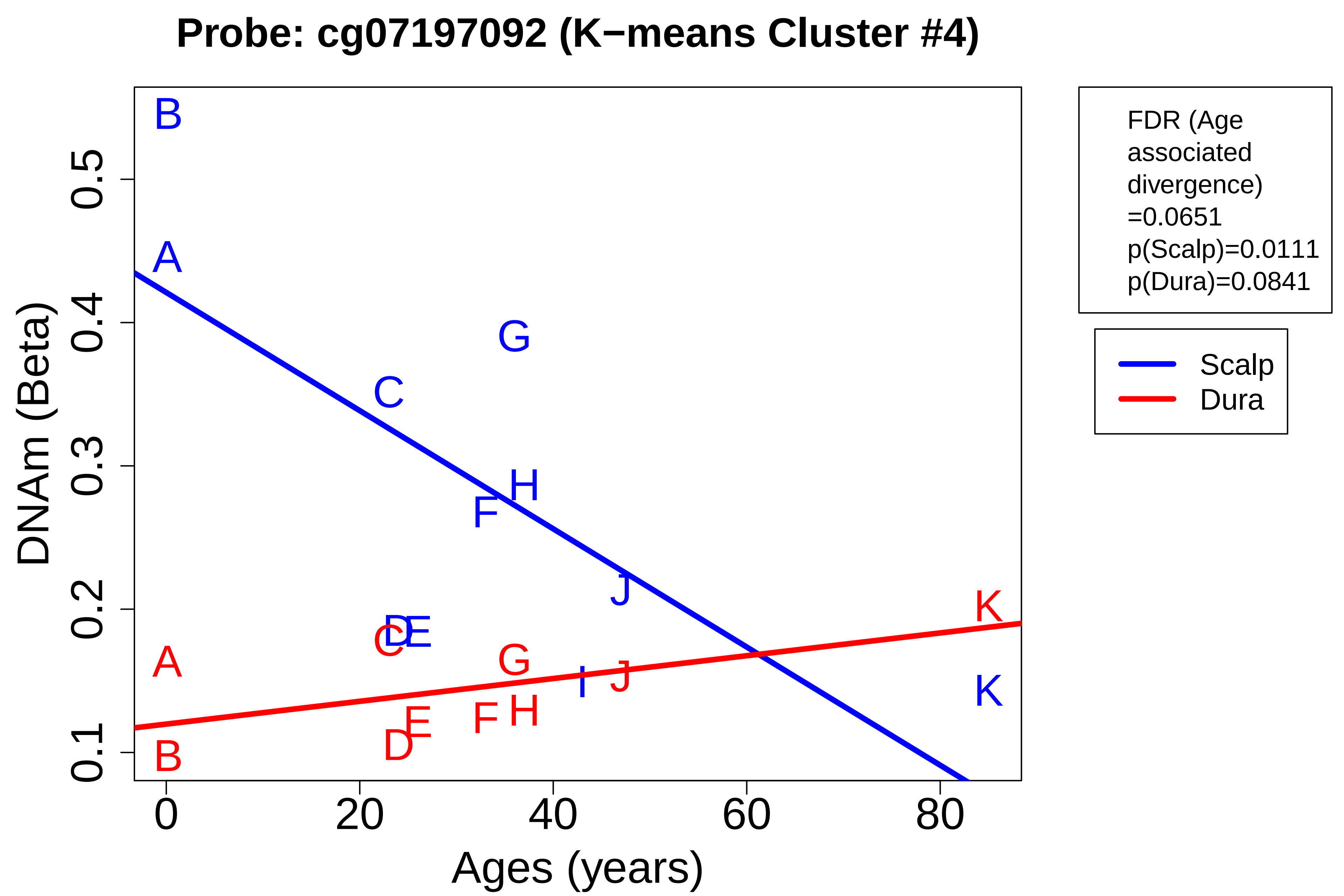

Probe: cg07197092 (K−means Cluster #4)
DNAm (Beta)
0.1
0.2
0.3
0.4
0.5
0
20
40
60
80
Ages (years)
B
A
G
C
H
F
J
DE
C
K
A
G
I
J
K
E
F
H
D
B
FDR (Age associated divergence) =0.0651
p(Scalp)=0.0111
p(Dura)=0.0841
Scalp
Dura

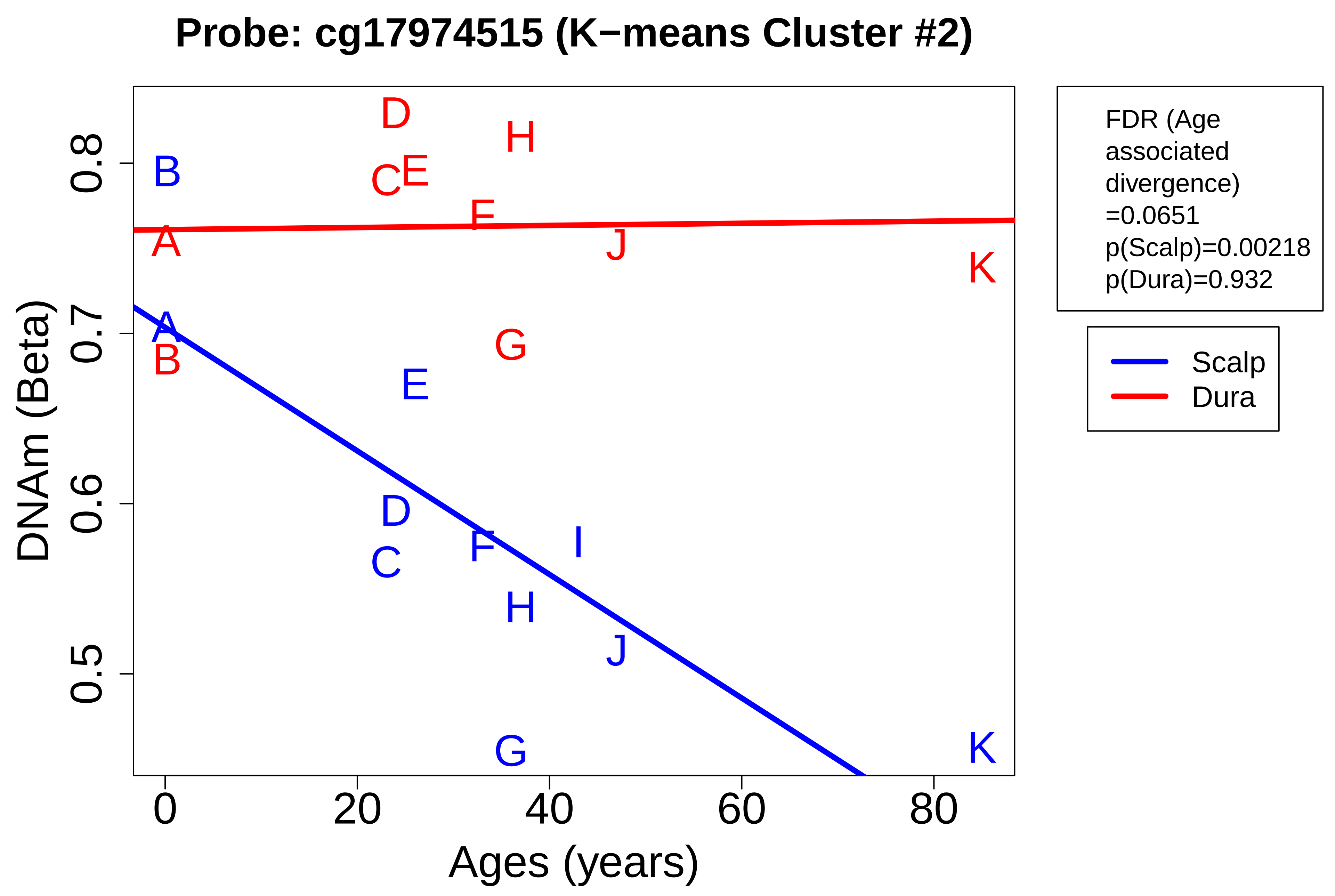

Probe: cg17974515 (K−means Cluster #2)
DNAm (Beta)
0.5
0.6
0.7
0.8
0
20
40
60
80
Ages (years)
FDR (Age associated divergence) =0.0651
p(Scalp)=0.00218
p(Dura)=0.932
Scalp
Dura

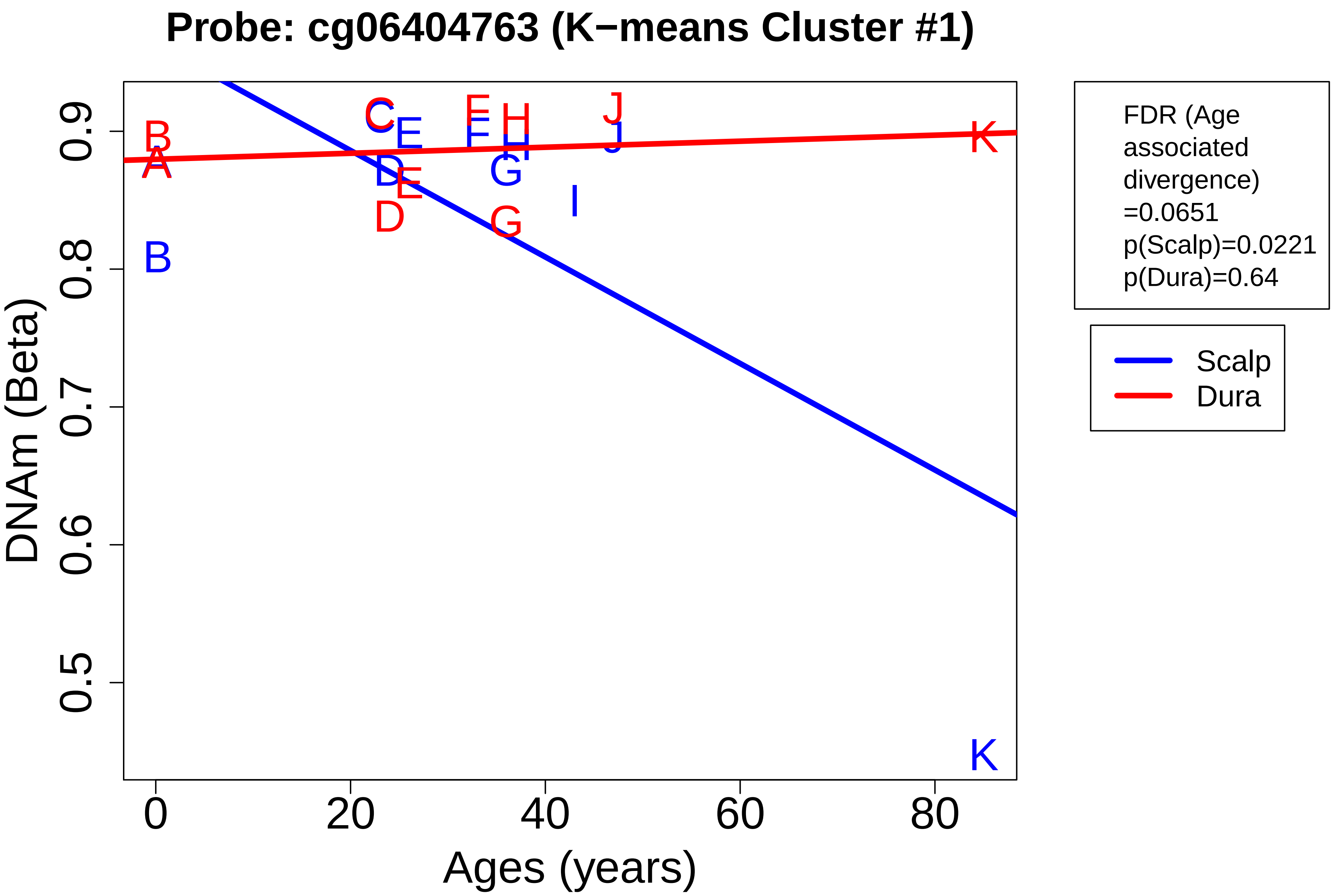

Probe: cg06404763 (K−means Cluster #1)
DNAm (Beta)
Ages (years)
0.5
0.6
0.7
0.8
0.9
0
20
40
60
80
A
B
C
D
E
F
G
H
I
J
K
FDR (Age associated divergence) =0.0651
p(Scalp)=0.0221
p(Dura)=0.64
Scalp
Dura

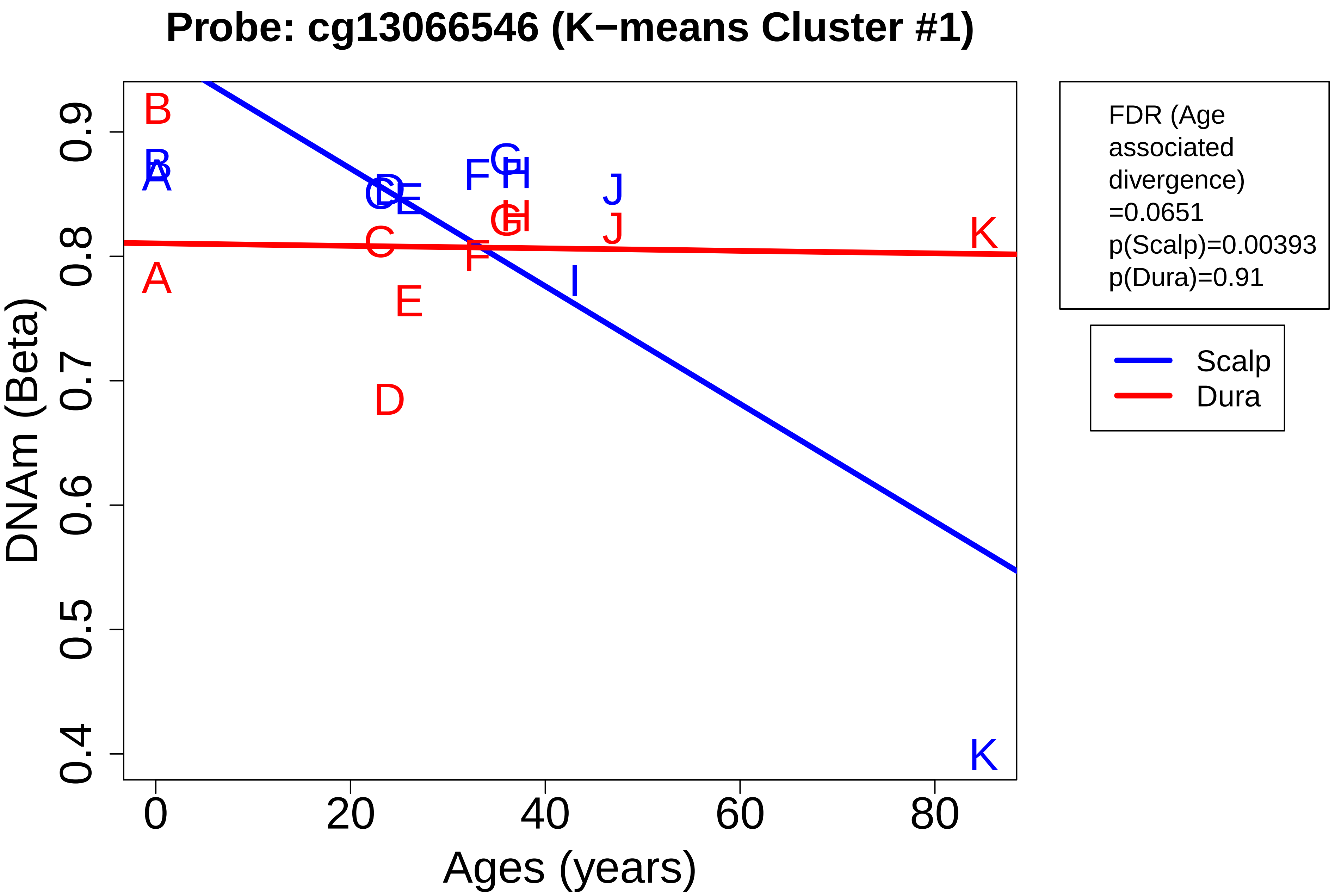
Probe: cg13066546 (K−means Cluster #1)
DNAm (Beta)
0.4
0.5
0.6
0.7
0.8
0.9
0
20
40
60
80
Ages (years)
FDR (Age associated divergence) =0.0651
p(Scalp)=0.00393
p(Dura)=0.91
Scalp
Dura

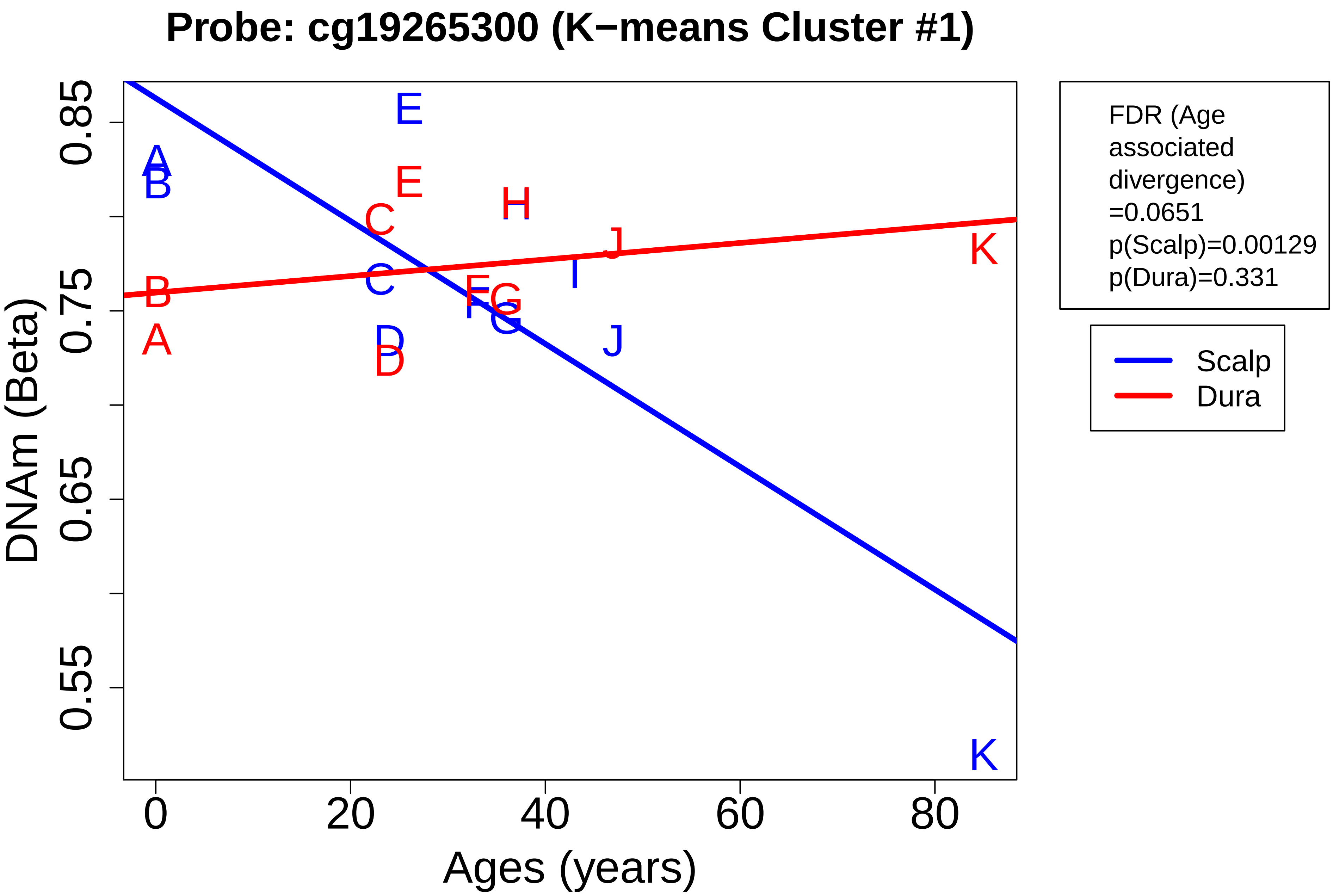
Probe: cg19265300 (K−means Cluster #1)
DNAm (Beta)
0.85
0.75
0.65
0.55
0
20
40
60
80
Ages (years)
A
B
C
D
E
F
G
H
I
J
K
FDR (Age associated divergence) =0.0651
p(Scalp)=0.00129
p(Dura)=0.331
Scalp
Dura

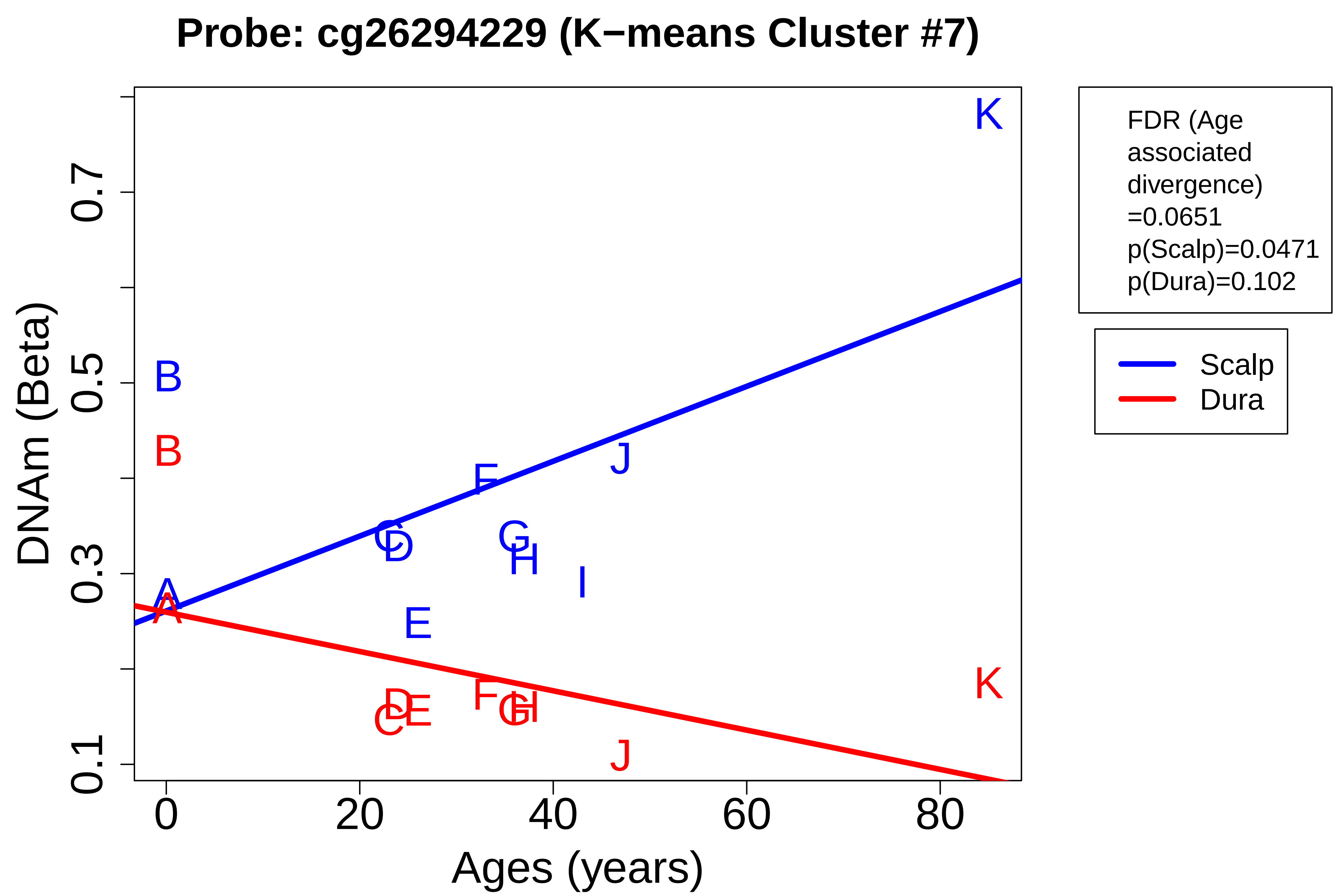

Probe: cg26294229 (K−means Cluster #7)
DNAm (Beta)
0.1
0.3
0.5
0.7
Ages (years)
0
20
40
60
80
FDR (Age associated divergence) =0.0651
p(Scalp)=0.0471
p(Dura)=0.102
Scalp
Dura
A
B
C
D
E
F
G
H
I
J
K

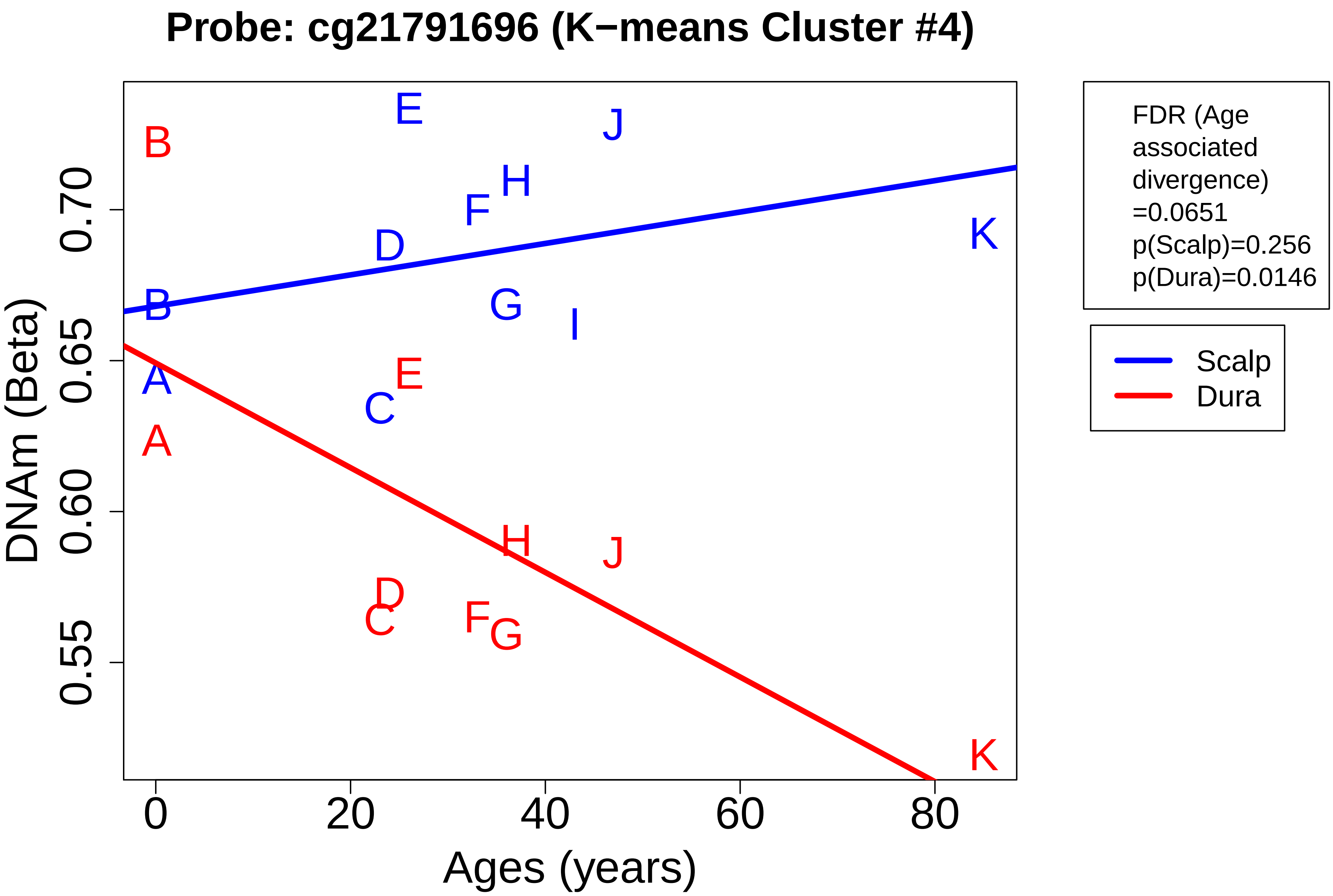

Probe: cg21791696 (K−means Cluster #4)
DNAm (Beta)
0.55
0.60
0.65
0.70
0
20
40
60
80
Ages (years)
FDR (Age associated divergence) =0.0651
p(Scalp)=0.256
p(Dura)=0.0146
Scalp
Dura

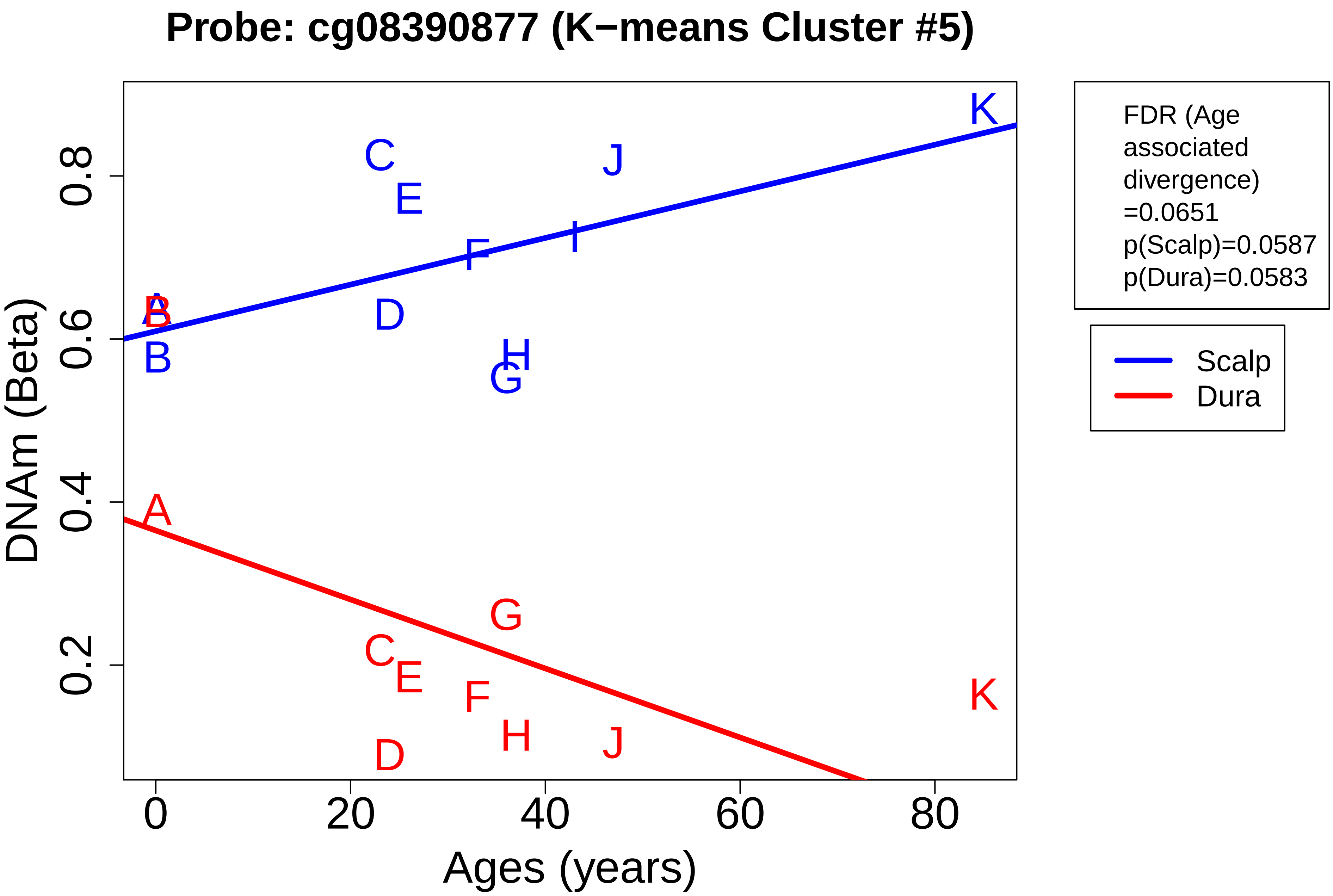

Probe: cg08390877 (K−means Cluster #5)
DNAm (Beta)
0.2
0.4
0.6
0.8
0
20
40
60
80
Ages (years)
FDR (Age associated divergence) =0.0651
p(Scalp)=0.0587
p(Dura)=0.0583
Scalp
Dura

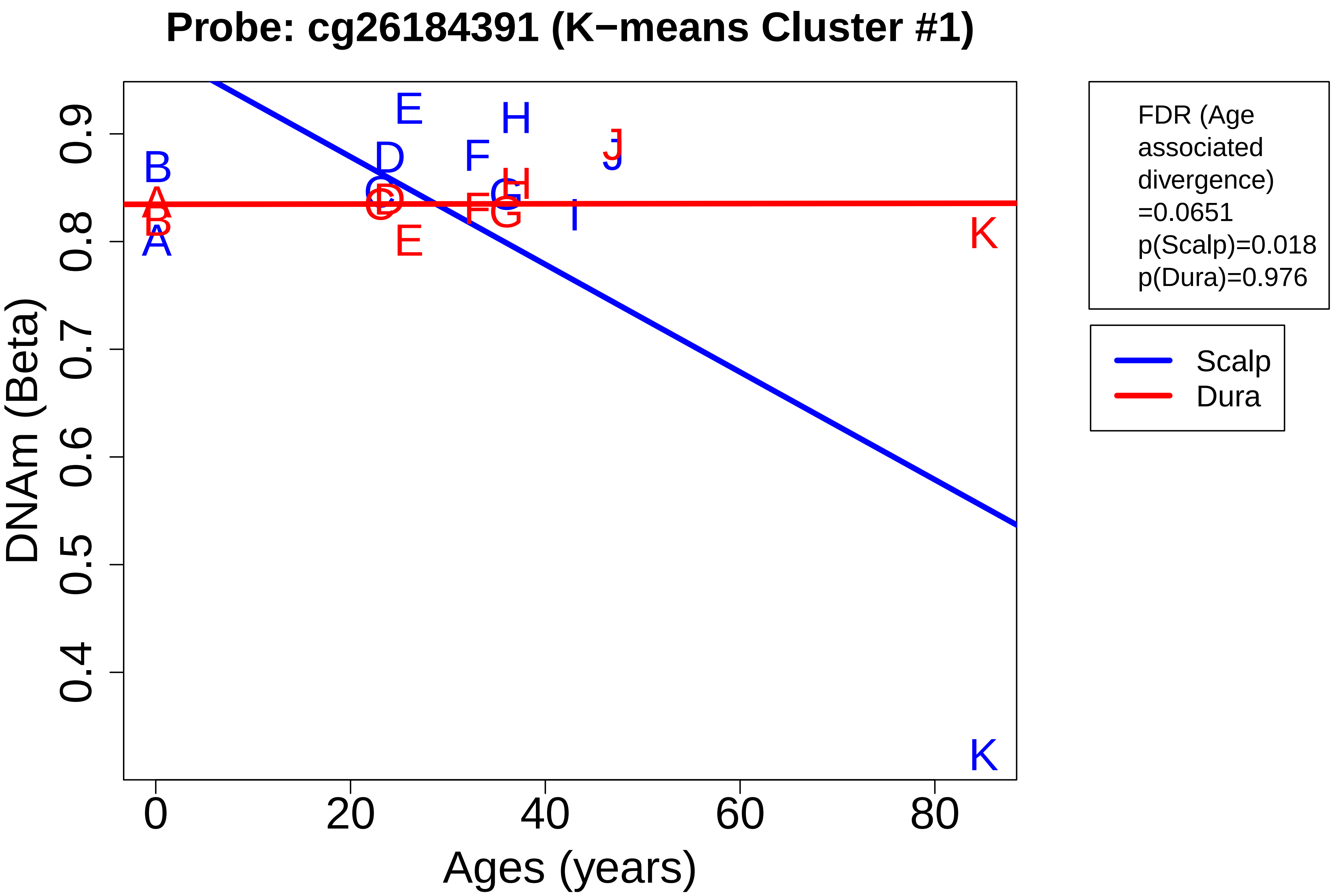

Probe: cg26184391 (K−means Cluster #1)
DNAm (Beta)
Ages (years)
0.4
0.5
0.6
0.7
0.8
0.9
0
20
40
60
80
FDR (Age associated divergence) =0.0651
p(Scalp)=0.018
p(Dura)=0.976
Scalp
Dura

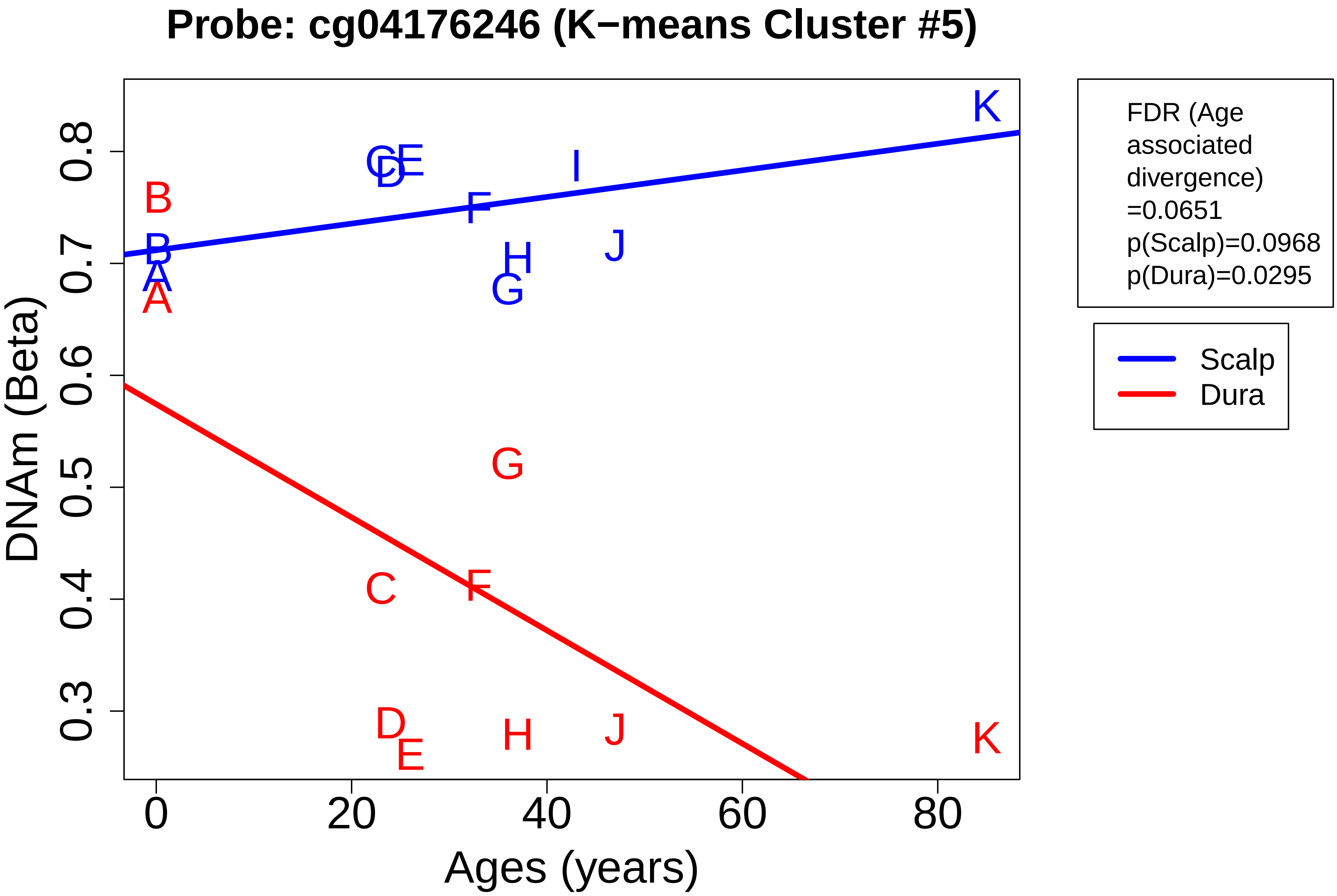

Probe: cg04176246 (K−means Cluster #5)
FDR (Age associated divergence) =0.0651
p(Scalp)=0.0968
p(Dura)=0.0295
Scalp
Dura
DNAm (Beta)
0.3
0.4
0.5
0.6
0.7
0.8
0
20
40
60
80
Ages (years)
A
B
C
D
E
F
G
H
I
J
K

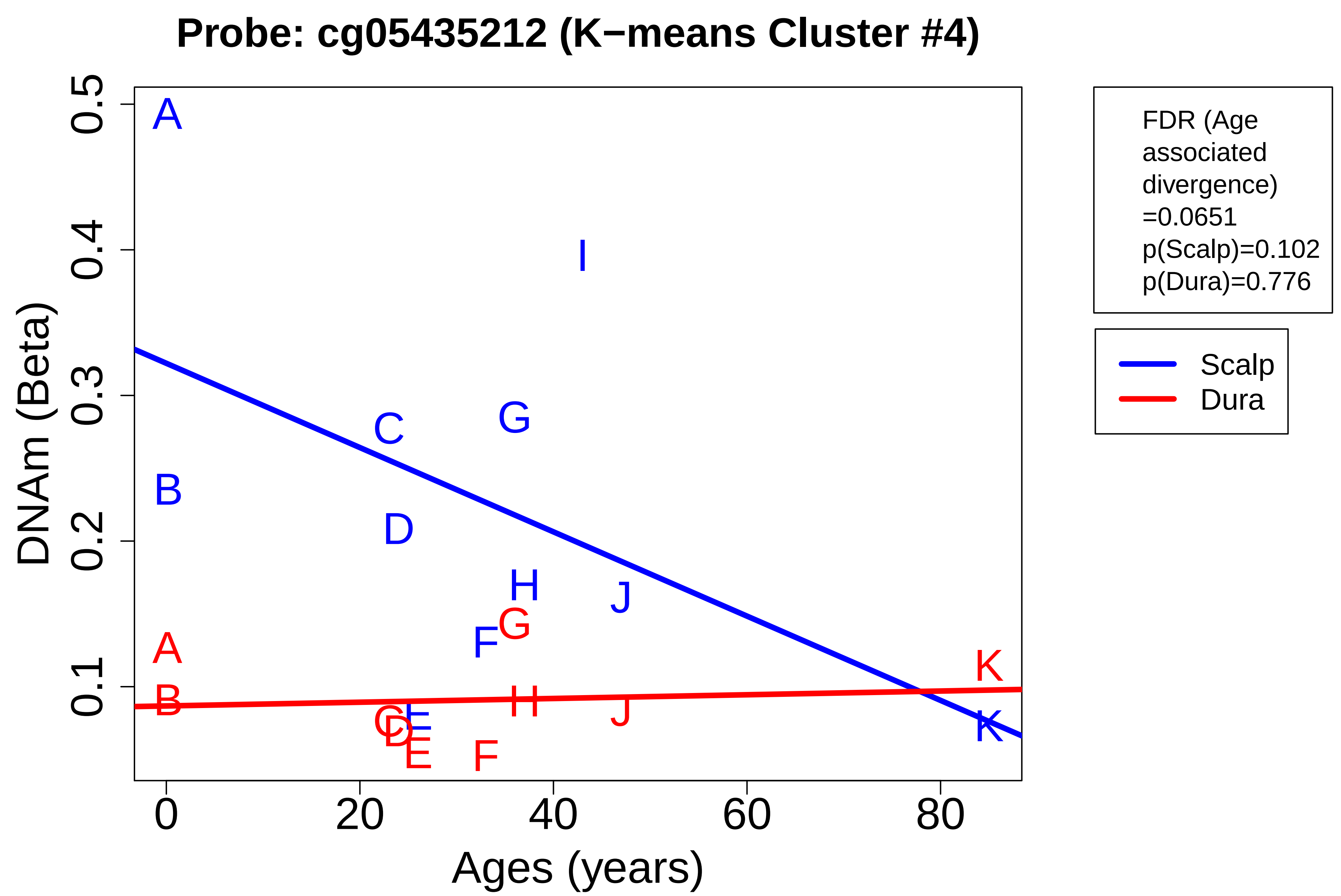

Probe: cg05435212 (K−means Cluster #4)
DNAm (Beta)
0.1
0.2
0.3
0.4
0.5
0
20
40
60
80
Ages (years)
FDR (Age associated divergence) =0.0651
p(Scalp)=0.102
p(Dura)=0.776
Scalp
Dura

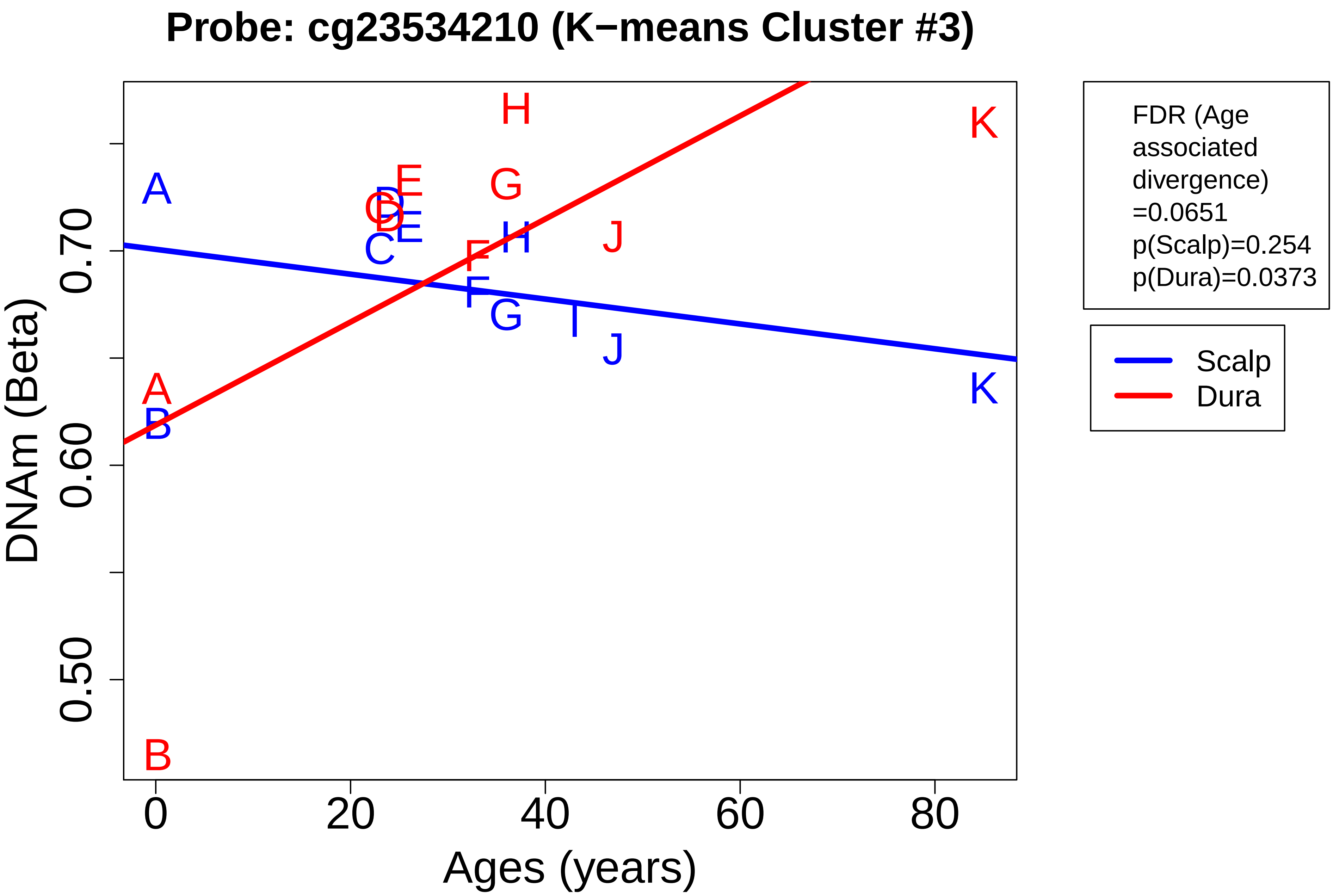

Probe: cg23534210 (K−means Cluster #3)
DNAm (Beta)
0.50
0.60
0.70
0
20
40
60
80
Ages (years)
FDR (Age associated divergence) =0.0651
p(Scalp)=0.254
p(Dura)=0.0373
Scalp
Dura

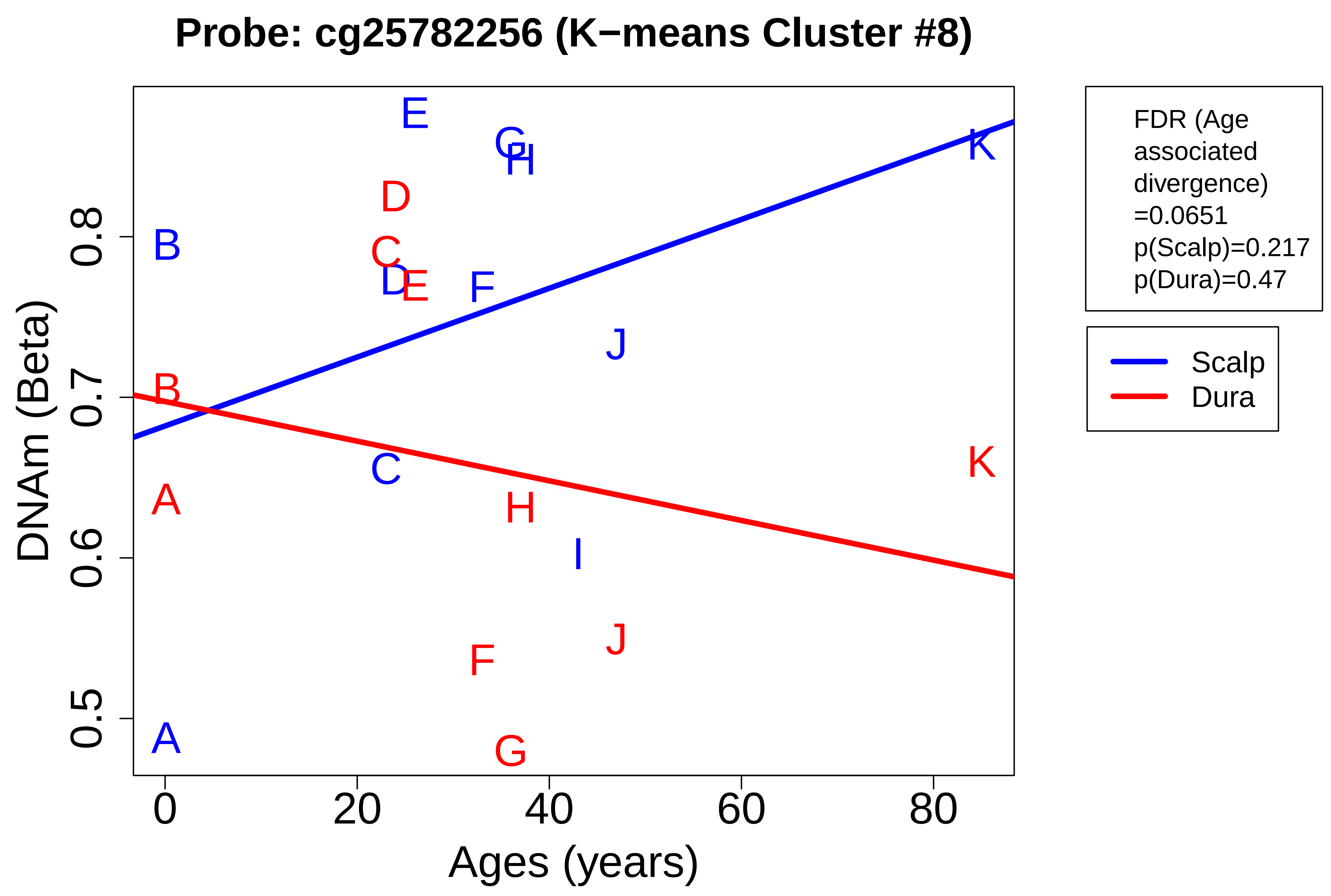

Probe: cg25782256 (K−means Cluster #8)
DNAm (Beta)
0.5
0.6
0.7
0.8
0
20
40
60
80
Ages (years)
FDR (Age associated divergence) =0.0651
p(Scalp)=0.217
p(Dura)=0.47
Scalp
Dura

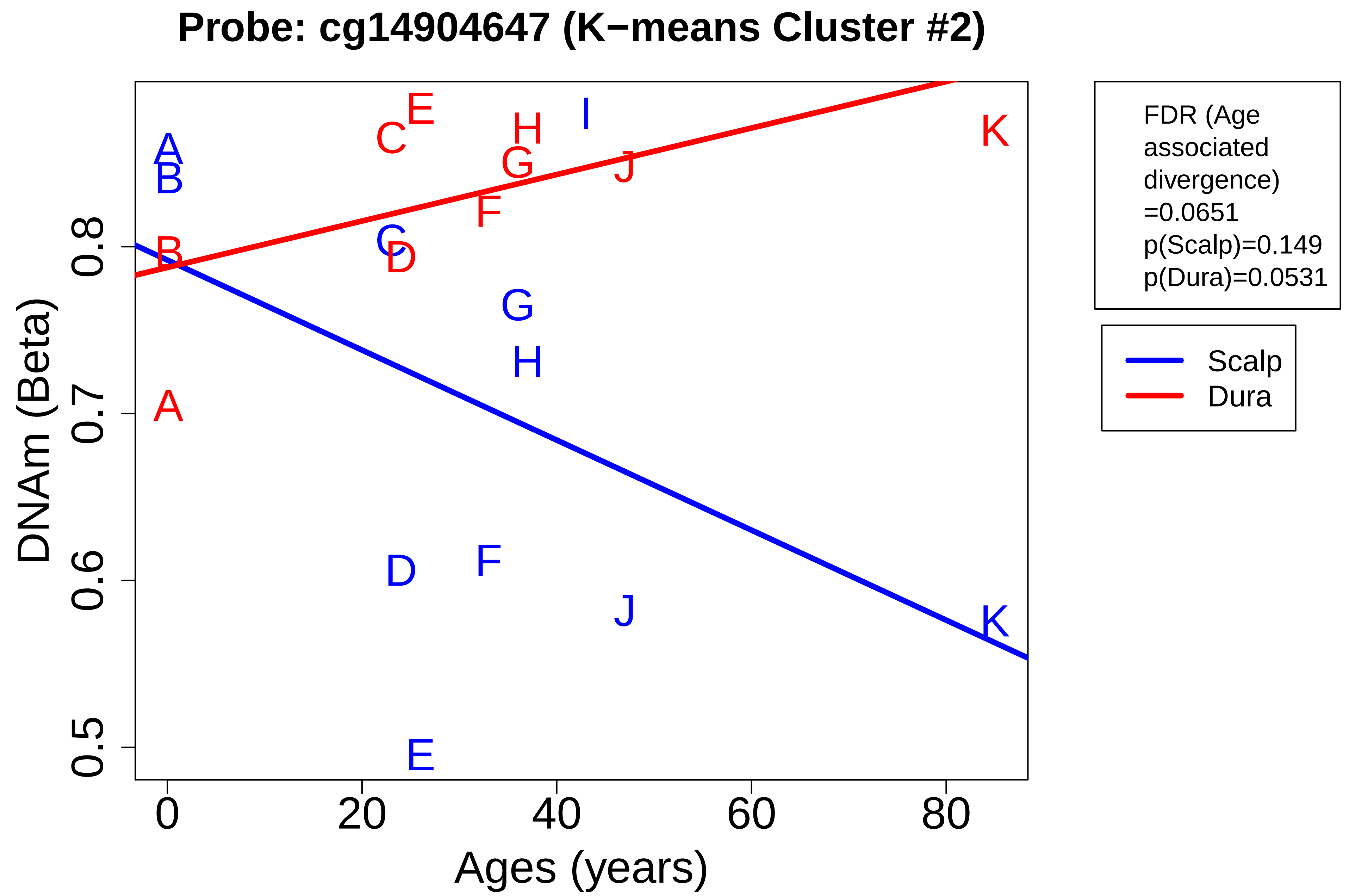

Probe: cg14904647 (K−means Cluster #2)
DNAm (Beta)
0.5
0.6
0.7
0.8
0
20
40
60
80
Ages (years)
A
B
C
D
E
F
G
H
I
J
K
FDR (Age associated divergence) =0.0651
p(Scalp)=0.149
p(Dura)=0.0531
Scalp
Dura

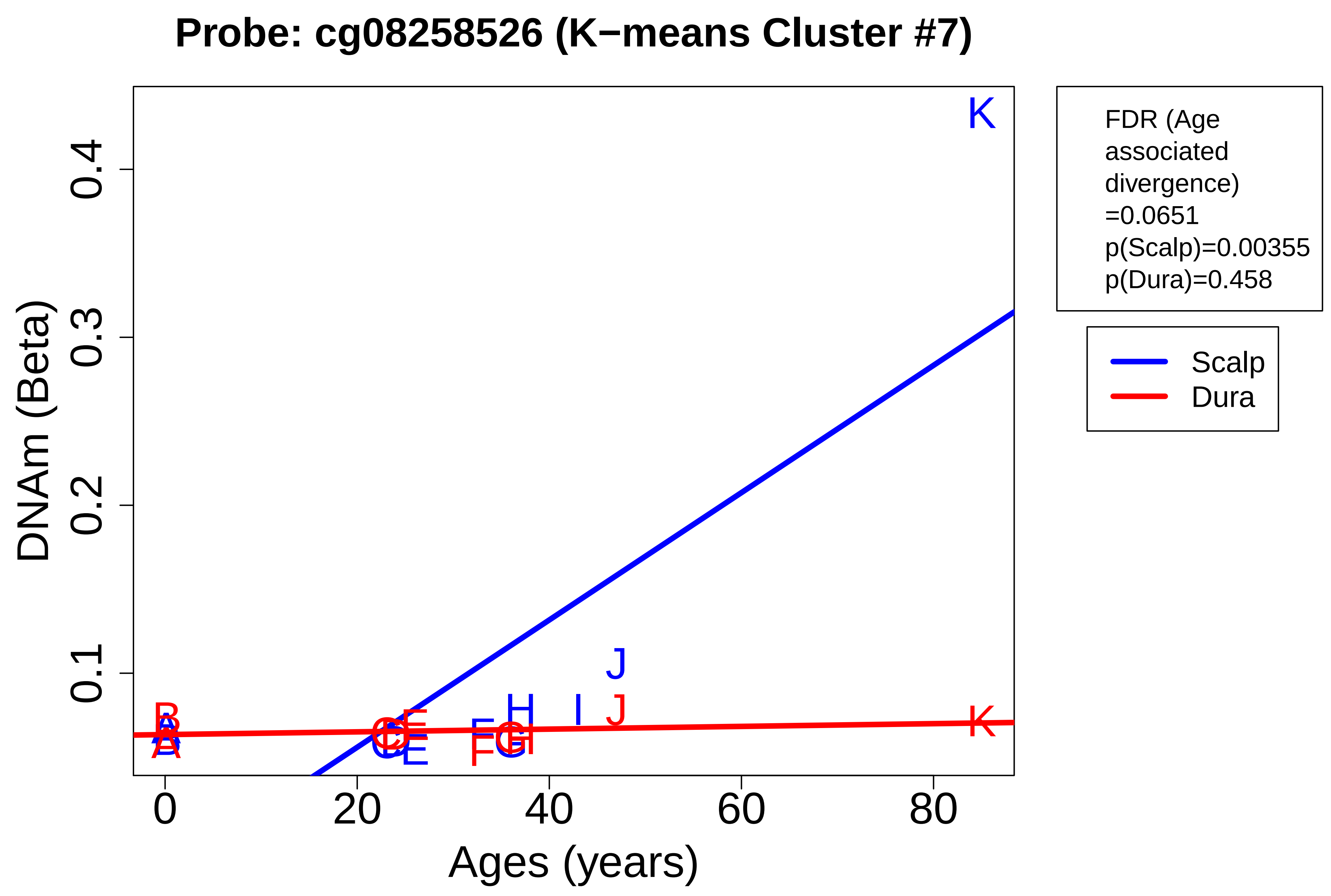

Probe: cg08258526 (K−means Cluster #7)
DNAm (Beta)
0.1
0.2
0.3
0.4
Ages (years)
0
20
40
60
80
FDR (Age associated divergence) =0.0651
p(Scalp)=0.00355
p(Dura)=0.458
Scalp
Dura

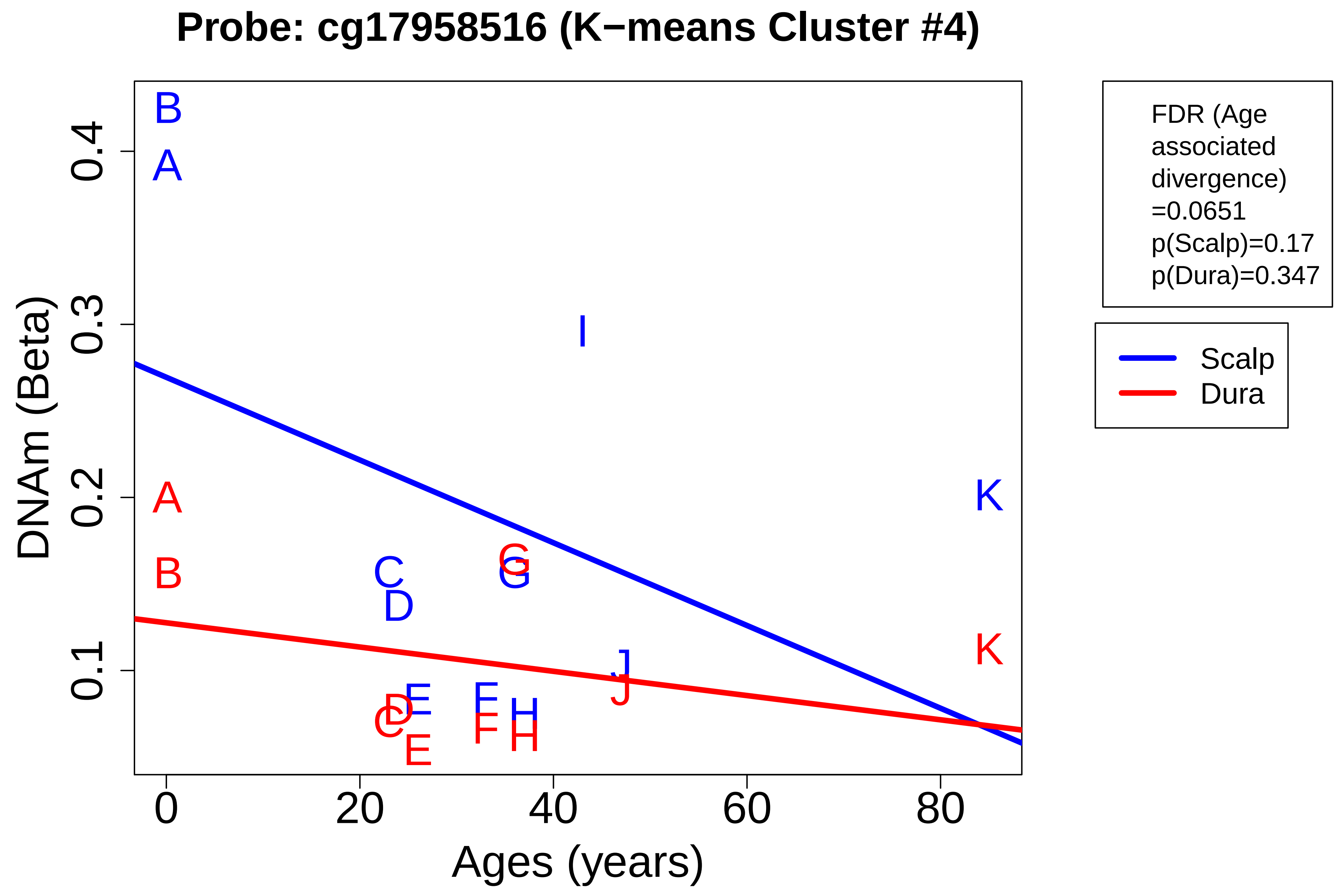

Probe: cg17958516 (K−means Cluster #4)
DNAm (Beta)
0.1
0.2
0.3
0.4
0
20
40
60
80
Ages (years)
FDR (Age associated divergence) =0.0651
p(Scalp)=0.17
p(Dura)=0.347
Scalp
Dura
A
B
C
D
E
F
G
H
I
J
K

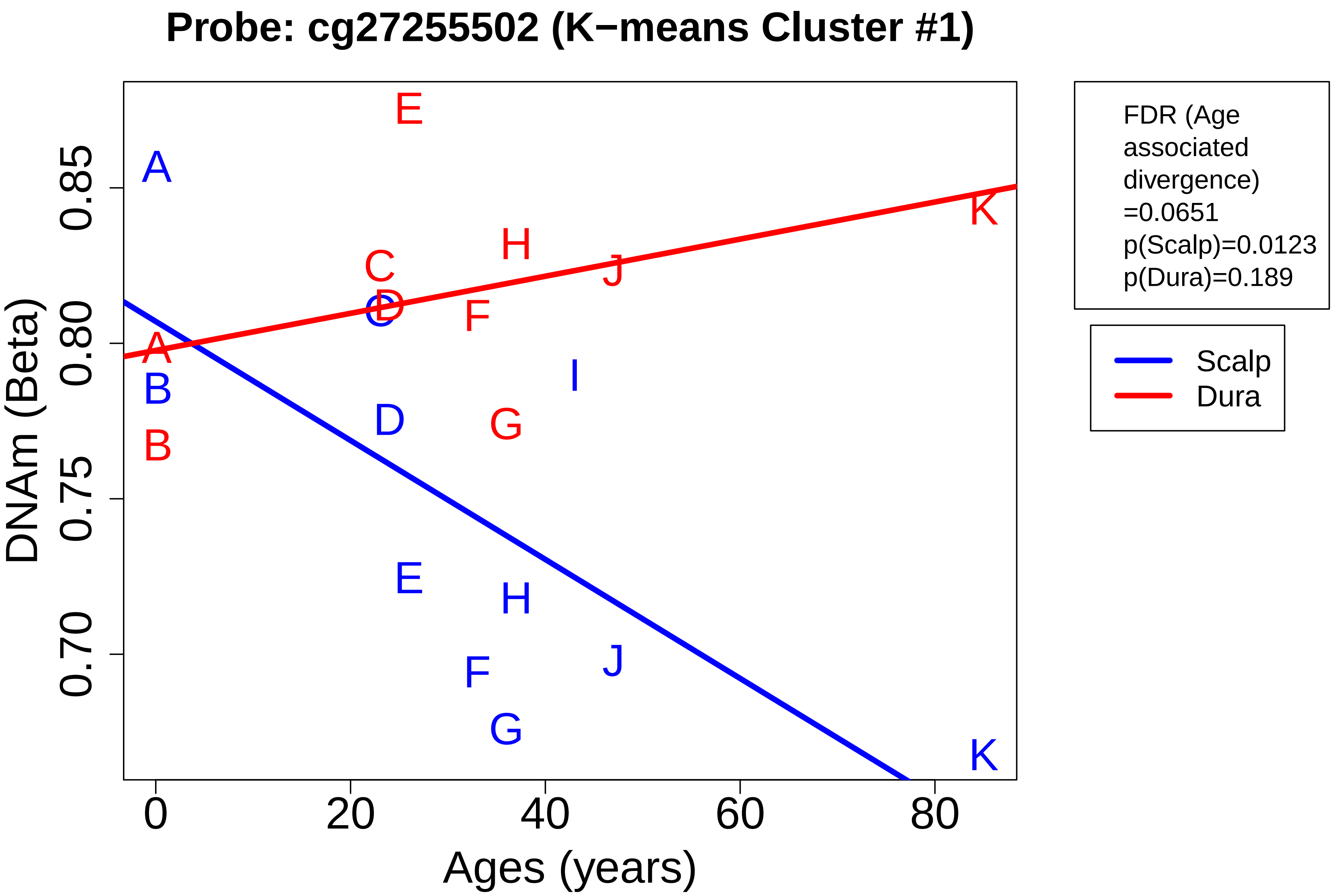

Probe: cg27255502 (K−means Cluster #1)
DNAm (Beta)
0.70
0.75
0.80
0.85
0
20
40
60
80
Ages (years)
A
B
C
D
E
F
G
H
I
J
K
FDR (Age associated divergence) =0.0651
p(Scalp)=0.0123
p(Dura)=0.189
Scalp
Dura

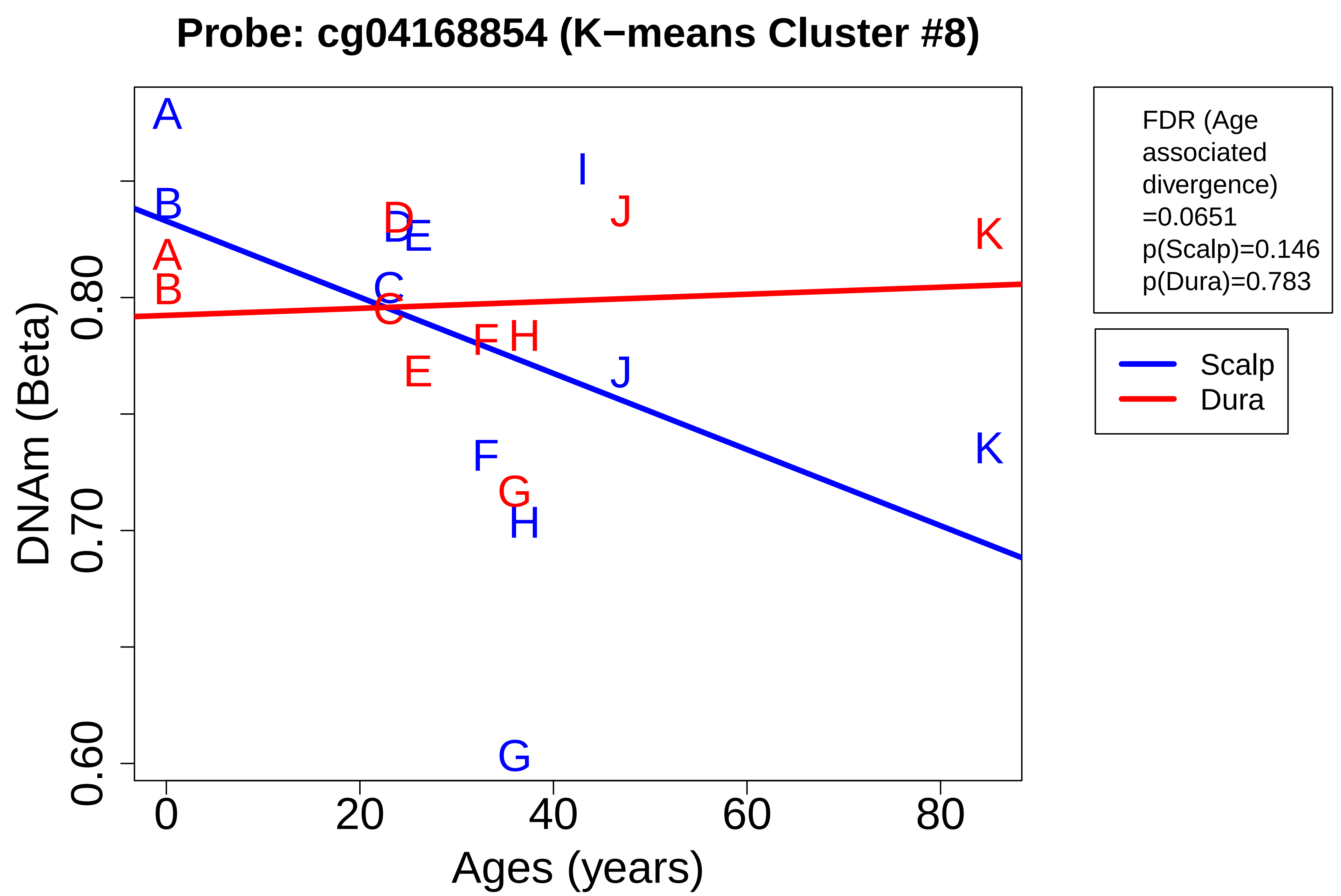

Probe: cg04168854 (K−means Cluster #8)
DNAm (Beta)
0.60
0.70
0.80
Ages (years)
0
20
40
60
80
FDR (Age associated divergence) =0.0651
p(Scalp)=0.146
p(Dura)=0.783
Scalp
Dura

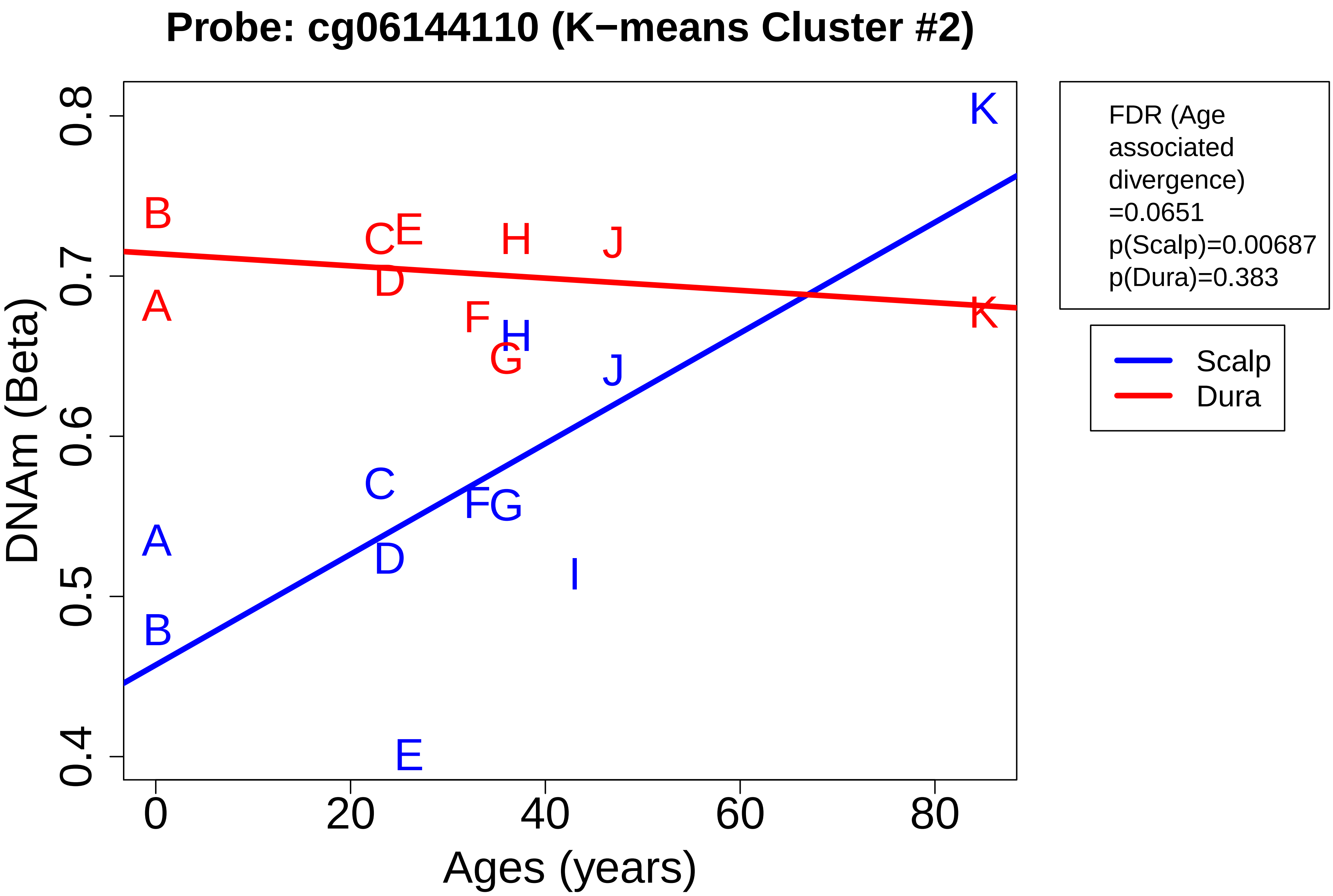

Probe: cg06144110 (K−means Cluster #2)
DNAm (Beta)
0.4
0.5
0.6
0.7
0.8
0
20
40
60
80
Ages (years)
FDR (Age associated divergence) =0.0651
p(Scalp)=0.00687
p(Dura)=0.383
Scalp
Dura

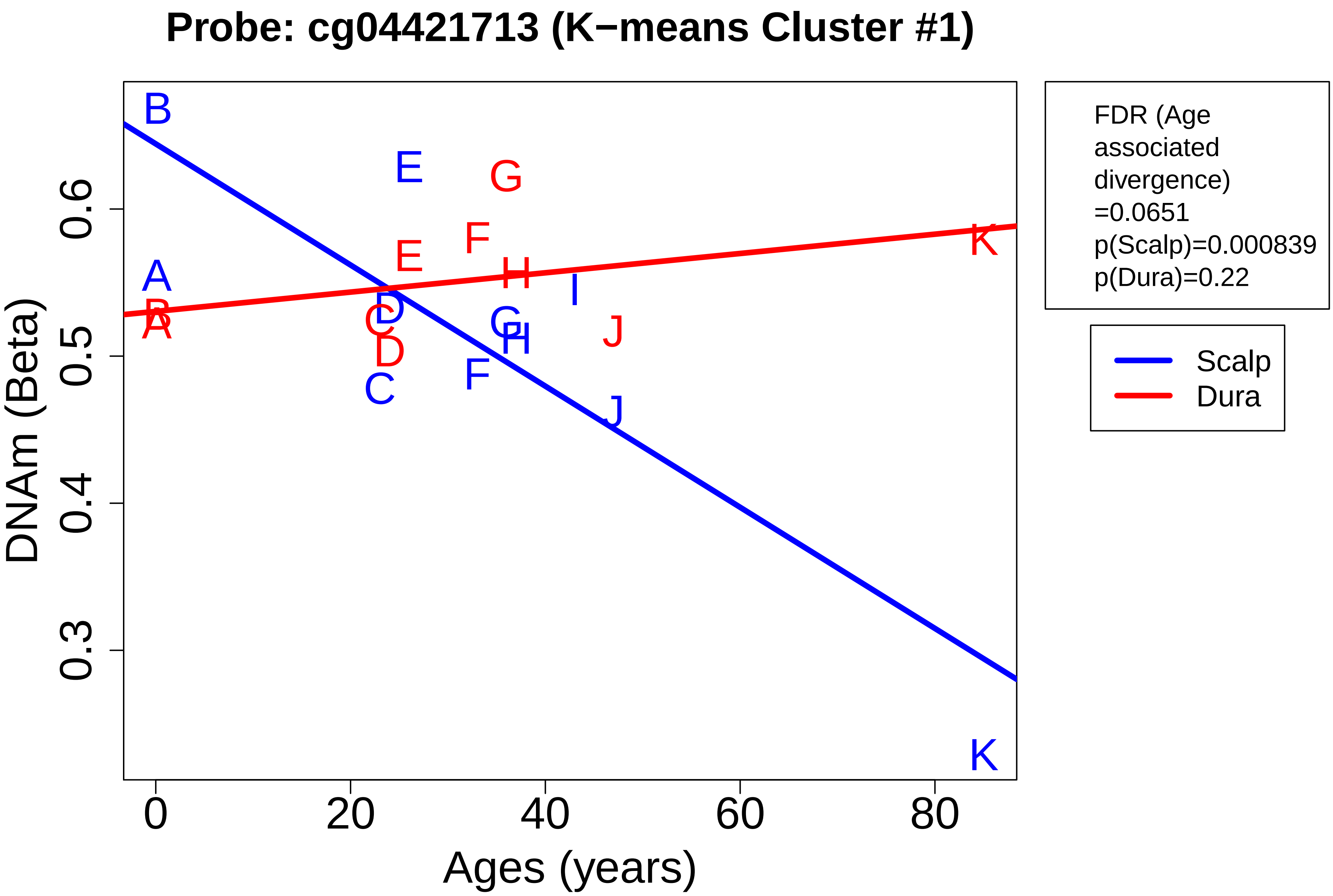

Probe: cg04421713 (K−means Cluster #1)
DNAm (Beta)
0.3
0.4
0.5
0.6
Ages (years)
0
20
40
60
80
FDR (Age associated divergence) =0.0651
p(Scalp)=0.000839
p(Dura)=0.22
Scalp
Dura

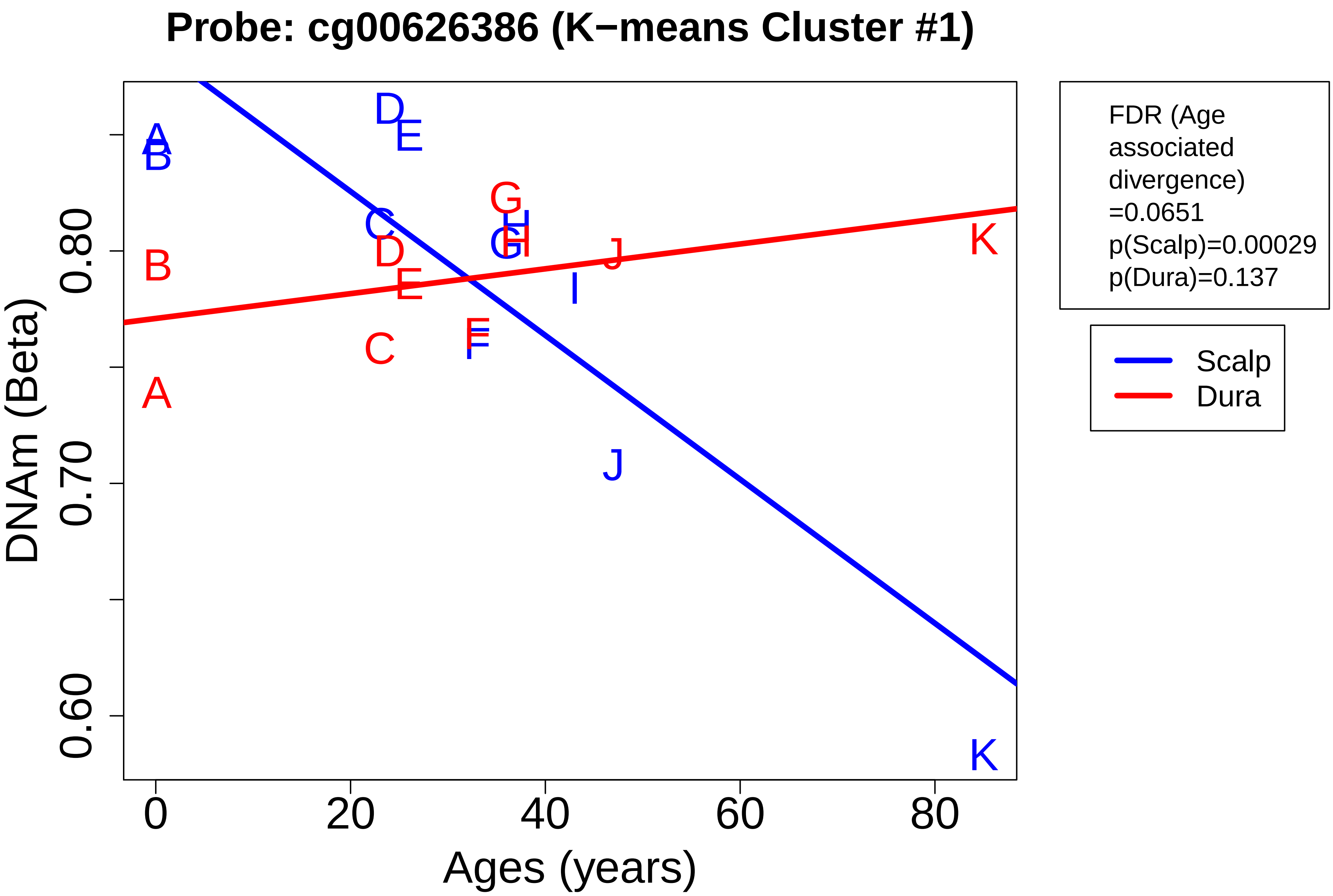
Probe: cg00626386 (K−means Cluster #1)
DNAm (Beta)
0.60
0.70
0.80
0
20
40
60
80
Ages (years)
A
B
C
D
E
F
G
H
I
J
K
FDR (Age associated divergence) =0.0651
p(Scalp)=0.00029
p(Dura)=0.137
Scalp
Dura

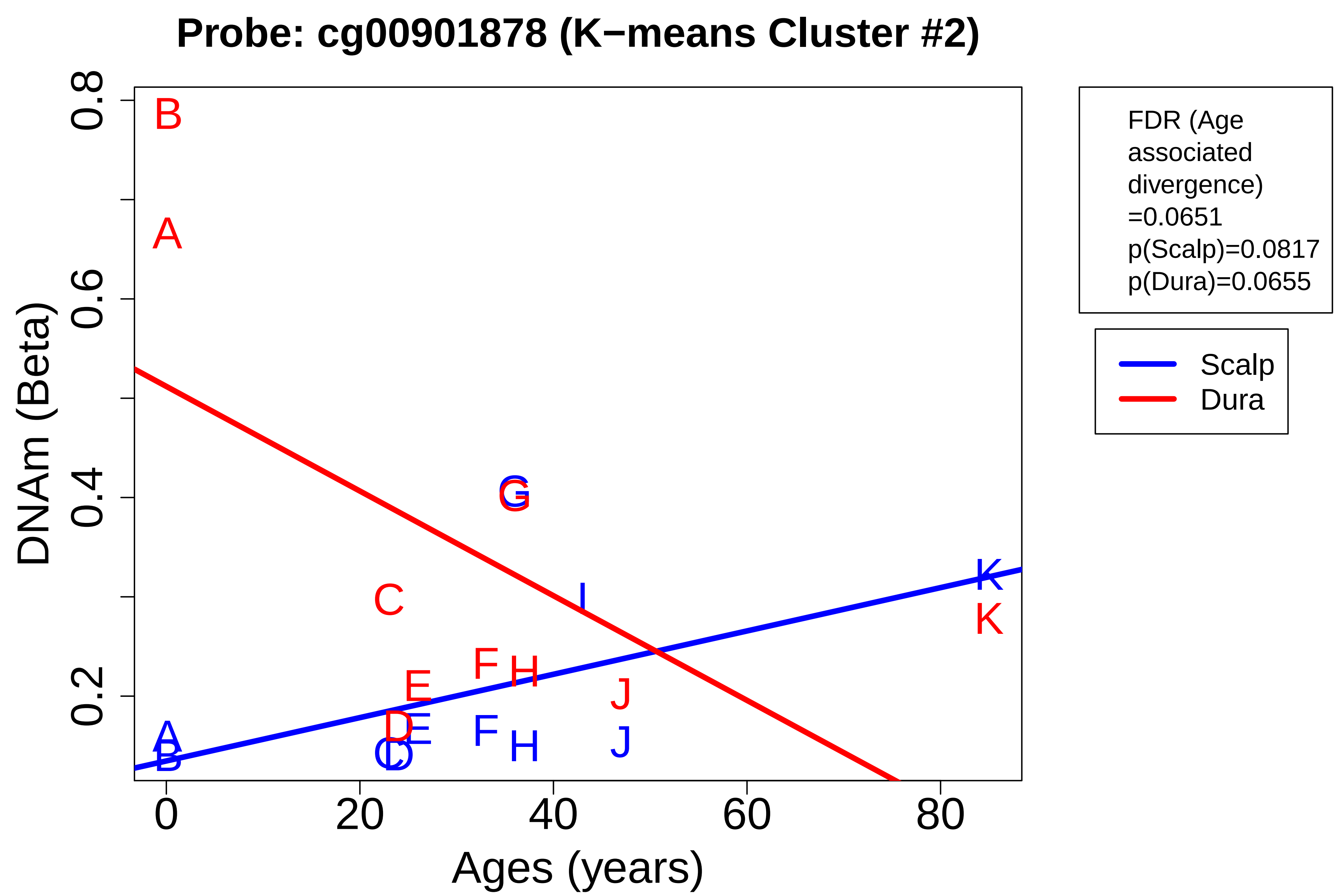
Probe: cg00901878 (K−means Cluster #2)
DNAm (Beta)
0.2
0.4
0.6
0.8
Ages (years)
0
20
40
60
80
FDR (Age associated divergence) =0.0651
p(Scalp)=0.0817
p(Dura)=0.0655
Scalp
Dura
A
B
C
D
E
F
G
H
I
J
K

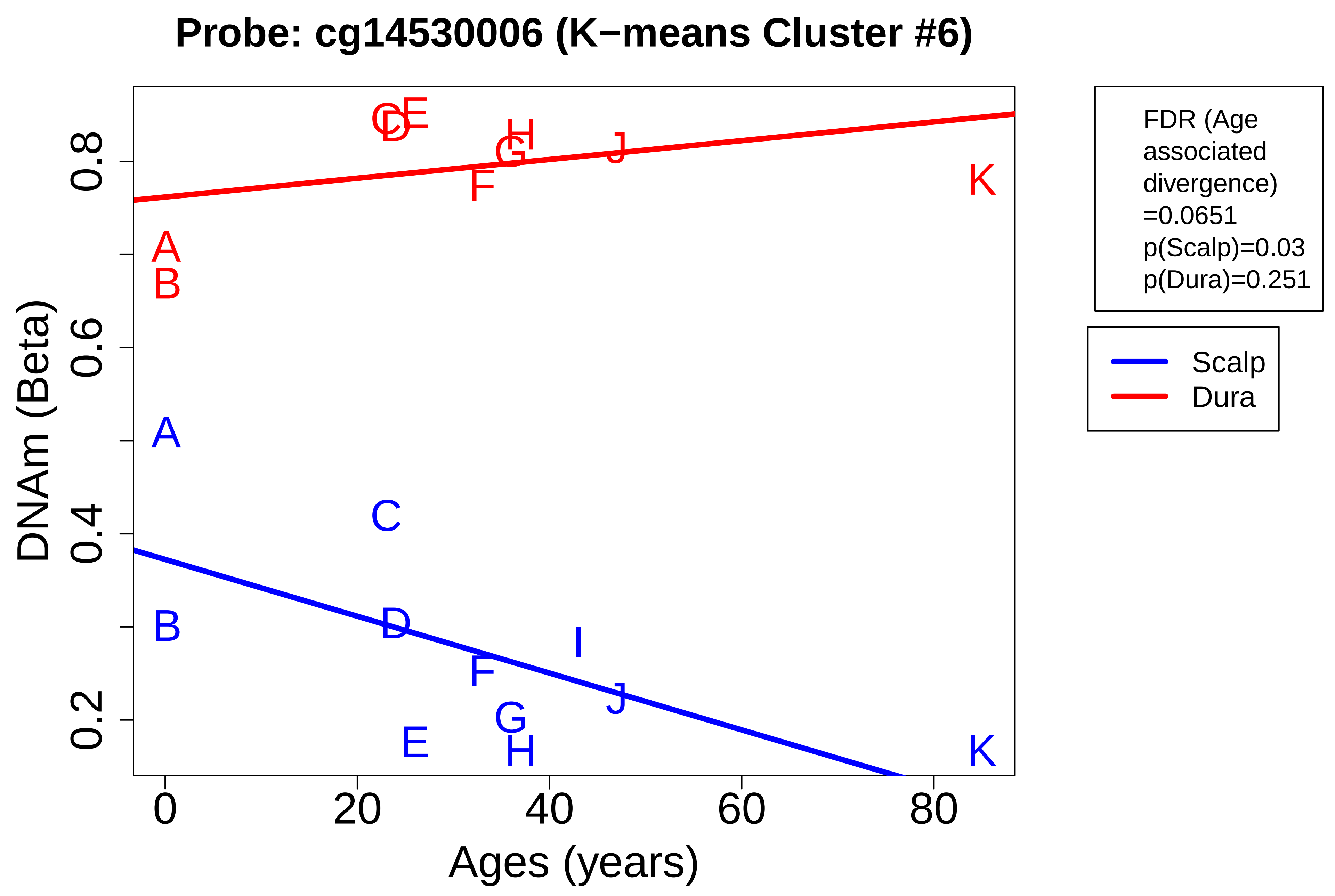

Probe: cg14530006 (K−means Cluster #6)
DNAm (Beta)
0.2
0.4
0.6
0.8
0
20
40
60
80
Ages (years)
A
B
C
D
E
F
G
H
I
J
K
FDR (Age associated divergence) =0.0651
p(Scalp)=0.03
p(Dura)=0.251
Scalp
Dura

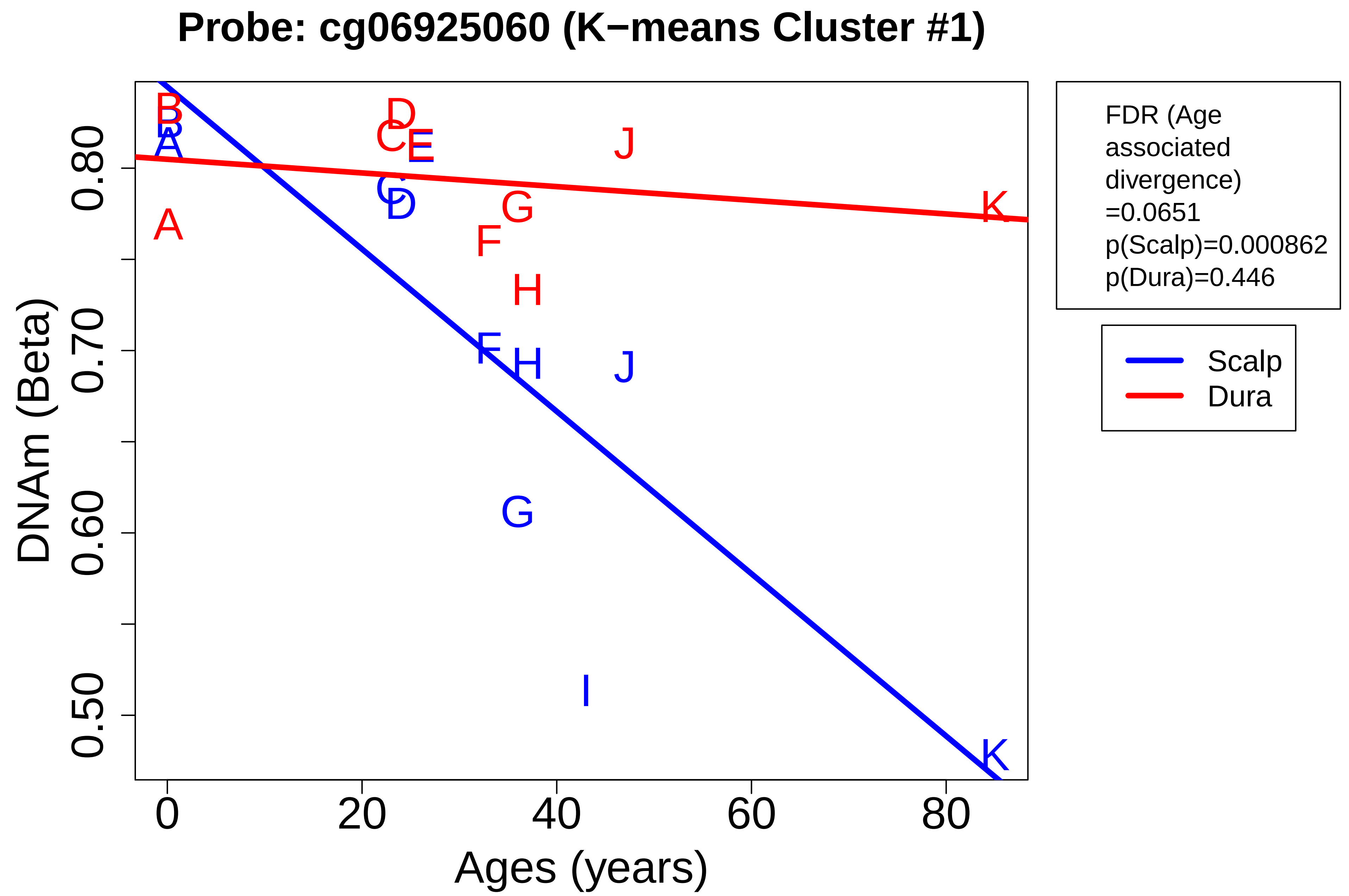

Probe: cg06925060 (K−means Cluster #1)
DNAm (Beta)
0.50
0.60
0.70
0.80
Ages (years)
0
20
40
60
80
FDR (Age associated divergence) =0.0651
p(Scalp)=0.000862
p(Dura)=0.446
Scalp
Dura

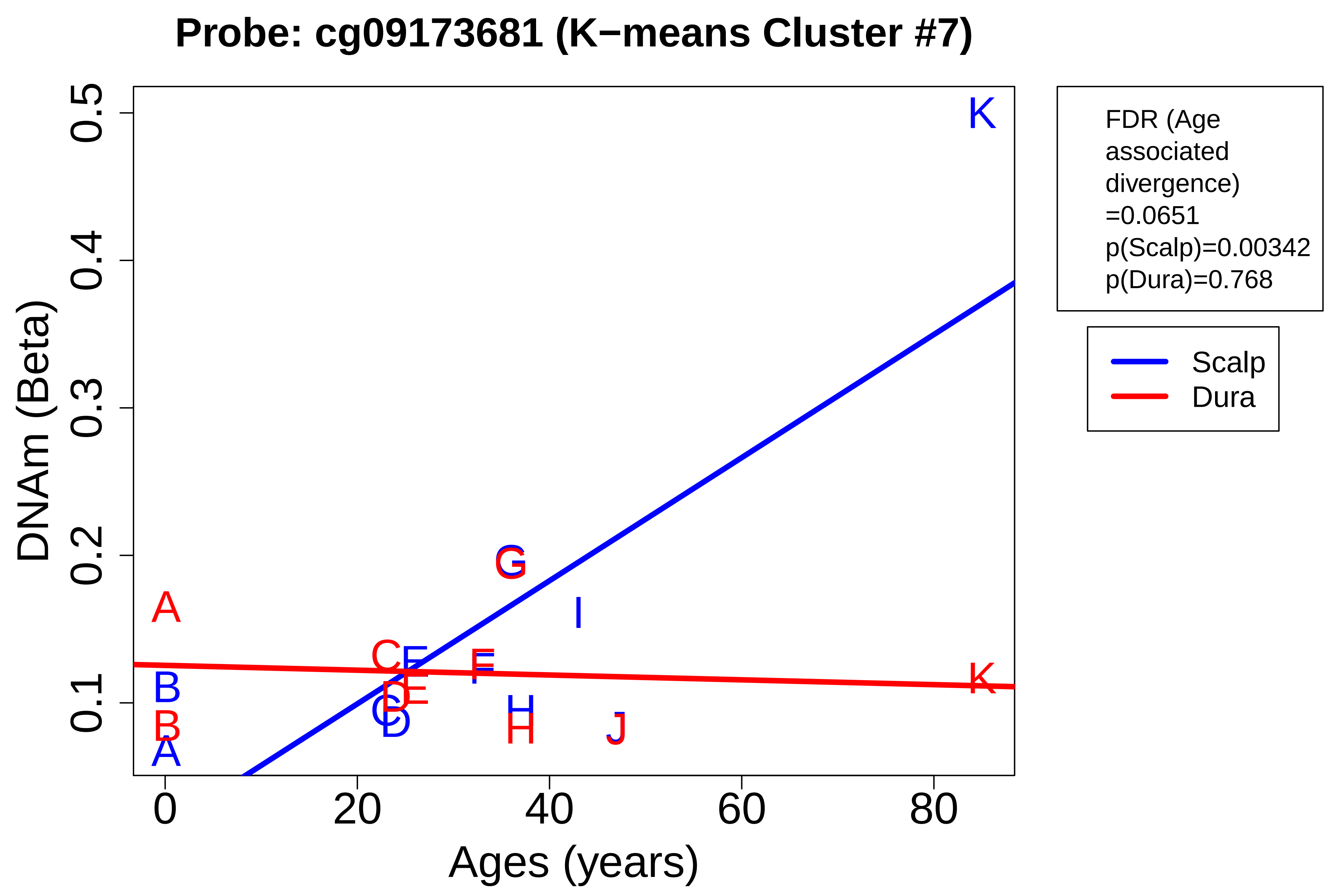

Probe: cg09173681 (K−means Cluster #7)
DNAm (Beta)
Ages (years)
0.1
0.2
0.3
0.4
0.5
0
20
40
60
80
FDR (Age associated divergence) =0.0651
p(Scalp)=0.00342
p(Dura)=0.768
Scalp
Dura

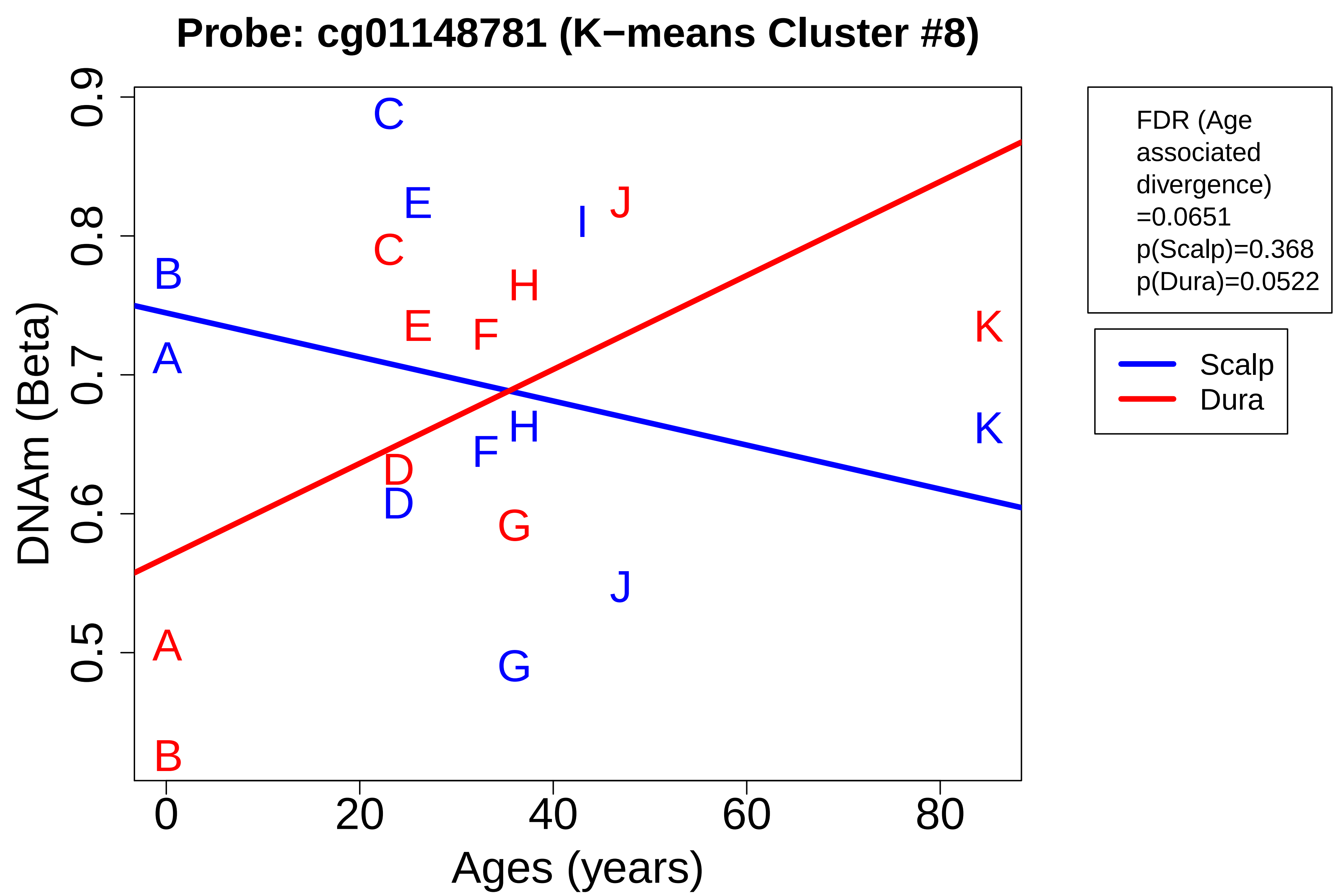

Probe: cg01148781 (K−means Cluster #8)
DNAm (Beta)
0.5
0.6
0.7
0.8
0.9
0
20
40
60
80
Ages (years)
FDR (Age associated divergence) =0.0651
p(Scalp)=0.368
p(Dura)=0.0522
Scalp
Dura

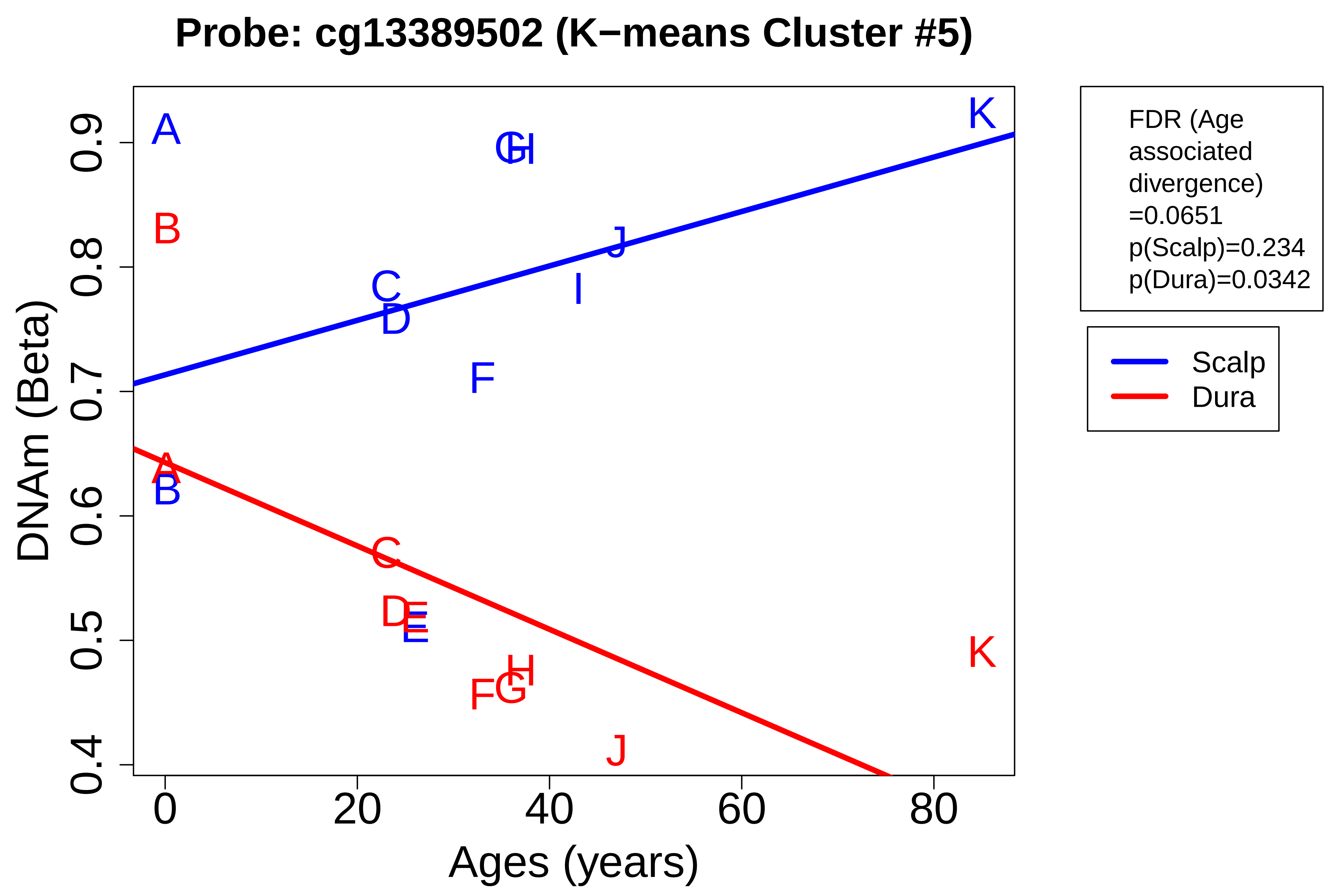

Probe: cg13389502 (K−means Cluster #5)
DNAm (Beta)
0.4
0.5
0.6
0.7
0.8
0.9
0
20
40
60
80
Ages (years)
A
B
C
D
E
F
G
H
I
J
K
FDR (Age associated divergence) =0.0651
p(Scalp)=0.234
p(Dura)=0.0342
Scalp
Dura

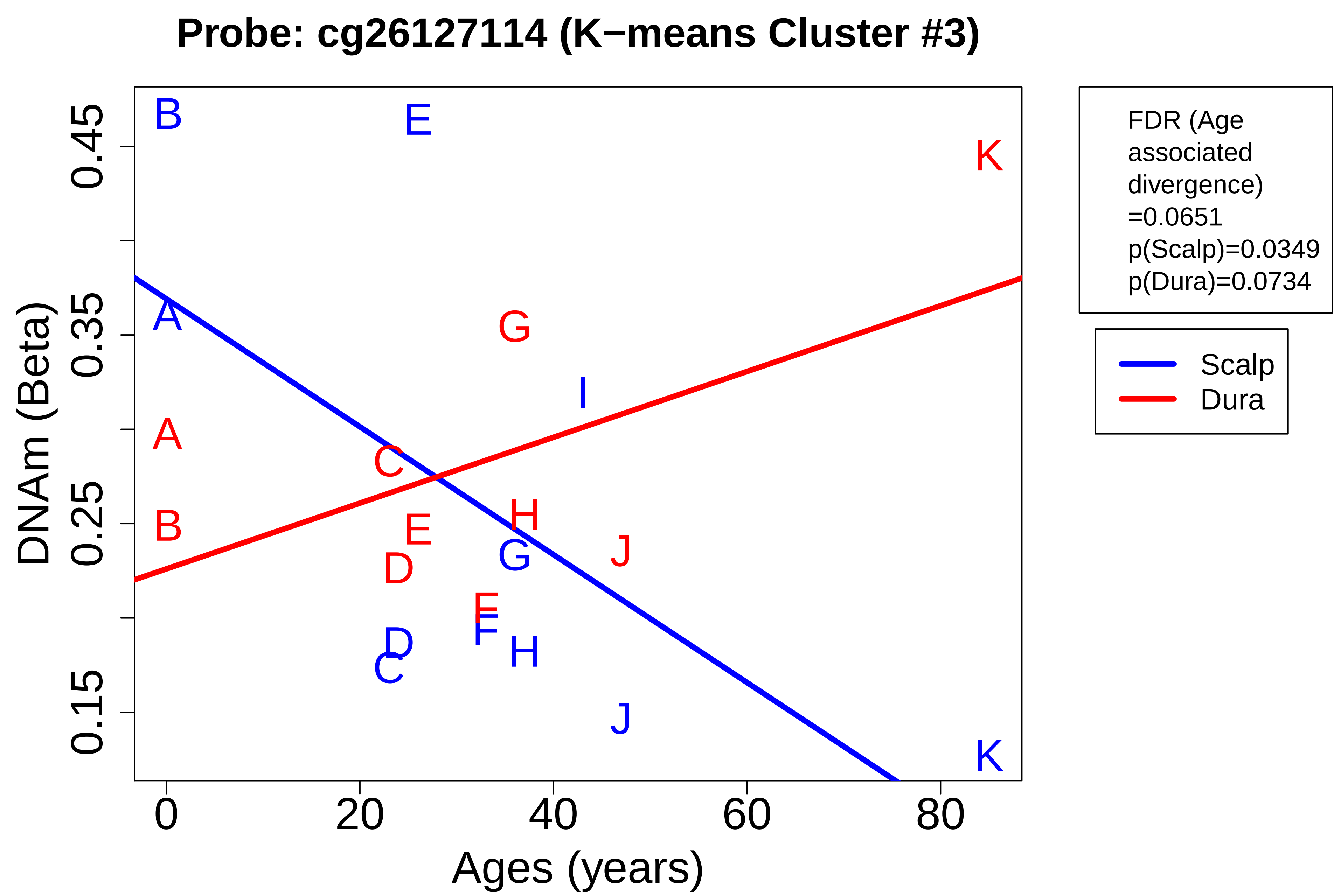
Probe: cg26127114 (K−means Cluster #3)
FDR (Age associated divergence) =0.0651
p(Scalp)=0.0349
p(Dura)=0.0734
Scalp
Dura
DNAm (Beta)
0.15
0.25
0.35
0.45
Ages (years)
0
20
40
60
80
A
B
C
D
E
F
G
H
I
J
K

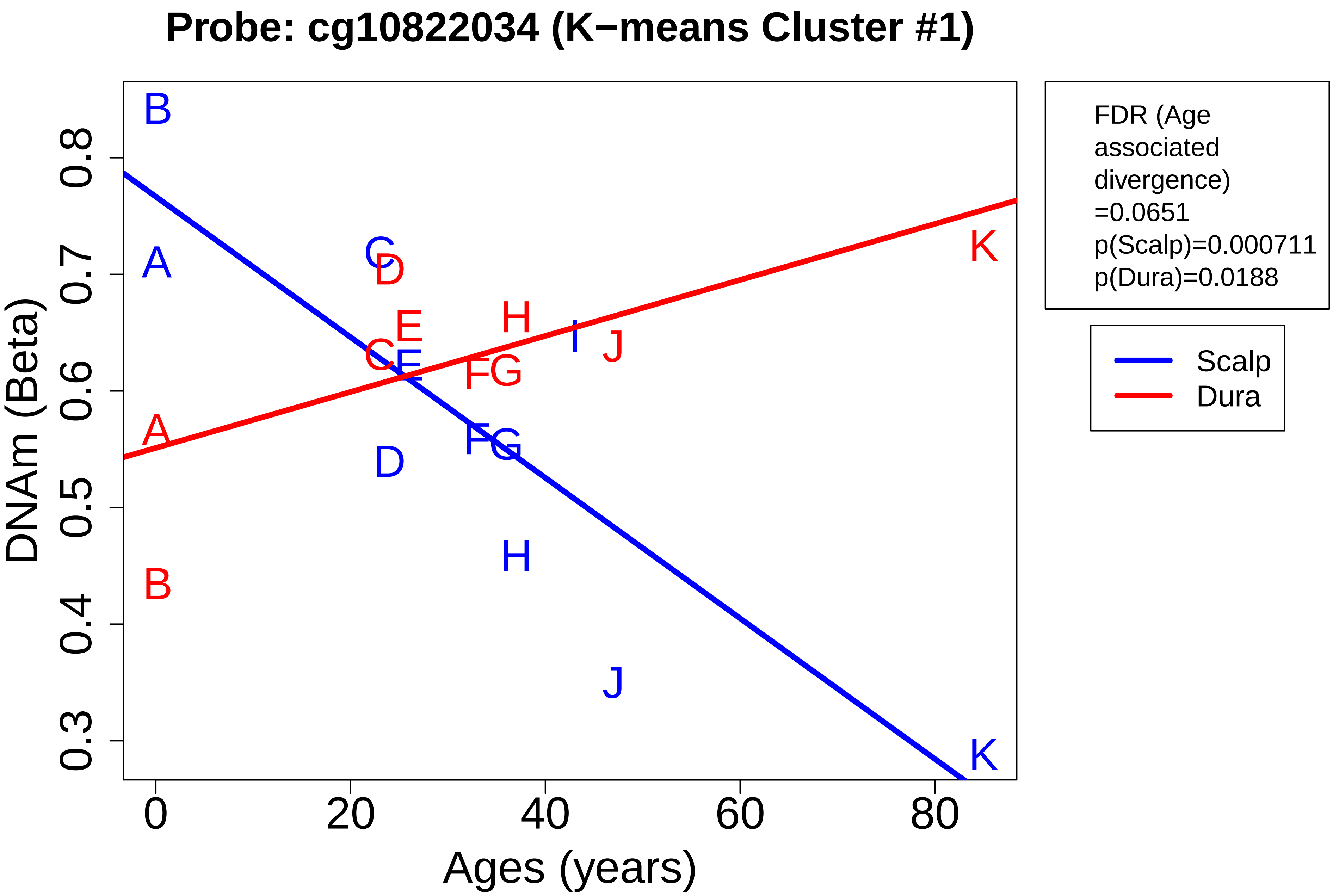

Probe: cg10822034 (K−means Cluster #1)
DNAm (Beta)
Ages (years)
0.3
0.4
0.5
0.6
0.7
0.8
0
20
40
60
80
FDR (Age associated divergence) =0.0651
p(Scalp)=0.000711
p(Dura)=0.0188
Scalp
Dura

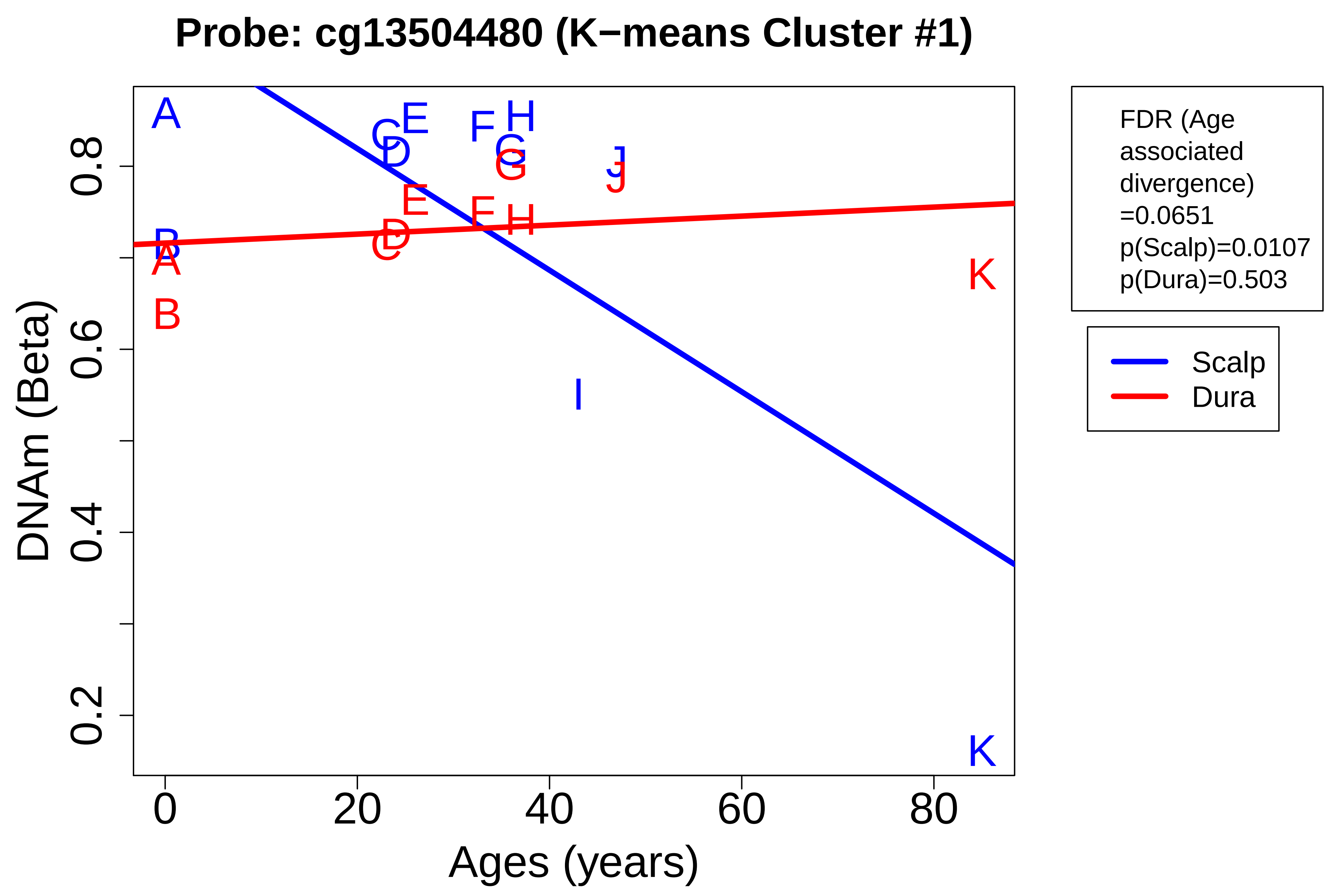

Probe: cg13504480 (K−means Cluster #1)
DNAm (Beta)
Ages (years)
0.2
0.4
0.6
0.8
0
20
40
60
80
A
B
C
D
E
F
G
H
I
J
K
FDR (Age associated divergence) =0.0651
p(Scalp)=0.0107
p(Dura)=0.503
Scalp
Dura

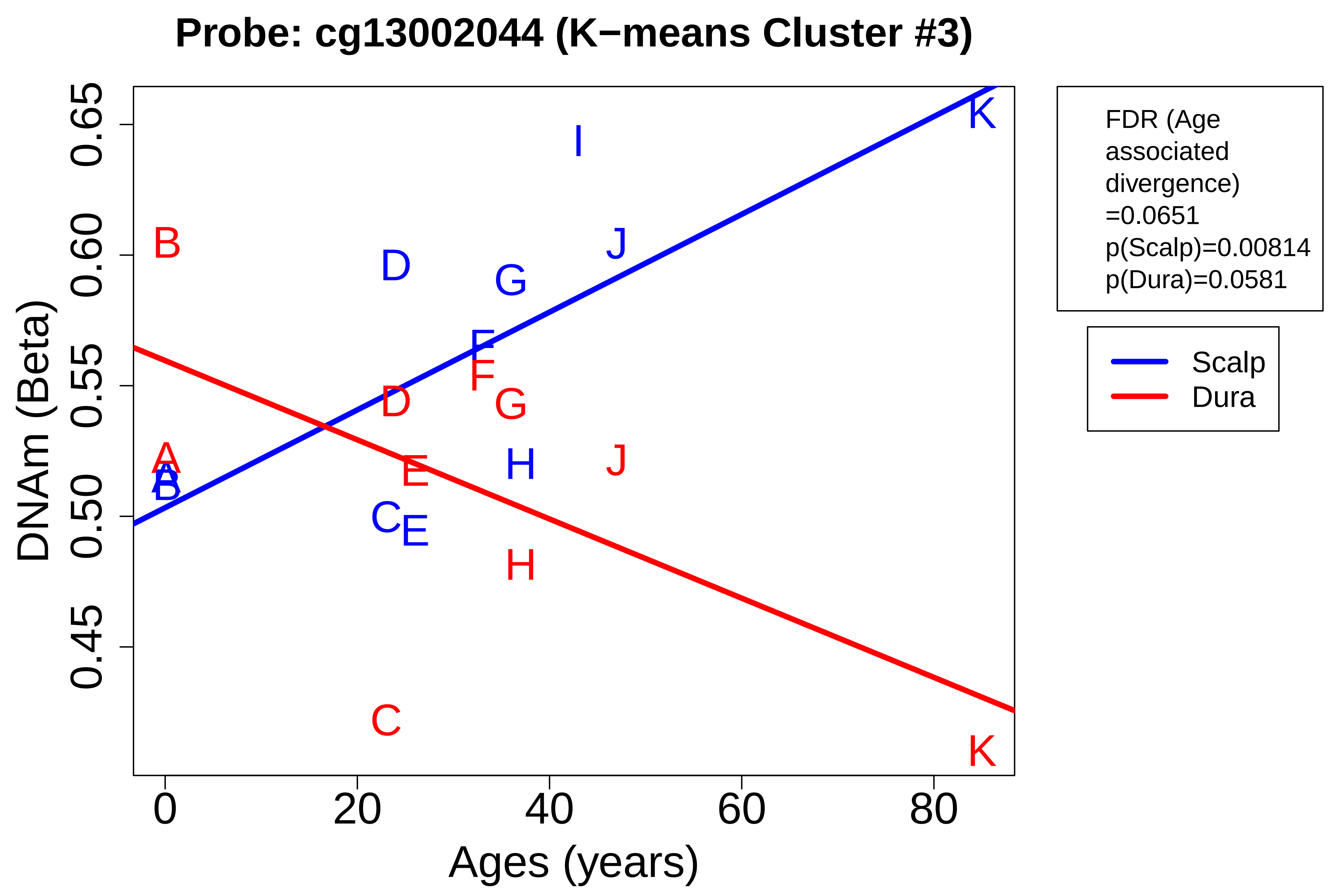

Probe: cg13002044 (K−means Cluster #3)
DNAm (Beta)
0.45
0.50
0.55
0.60
0.65
0
20
40
60
80
Ages (years)
FDR (Age associated divergence) =0.0651
p(Scalp)=0.00814
p(Dura)=0.0581
Scalp
Dura

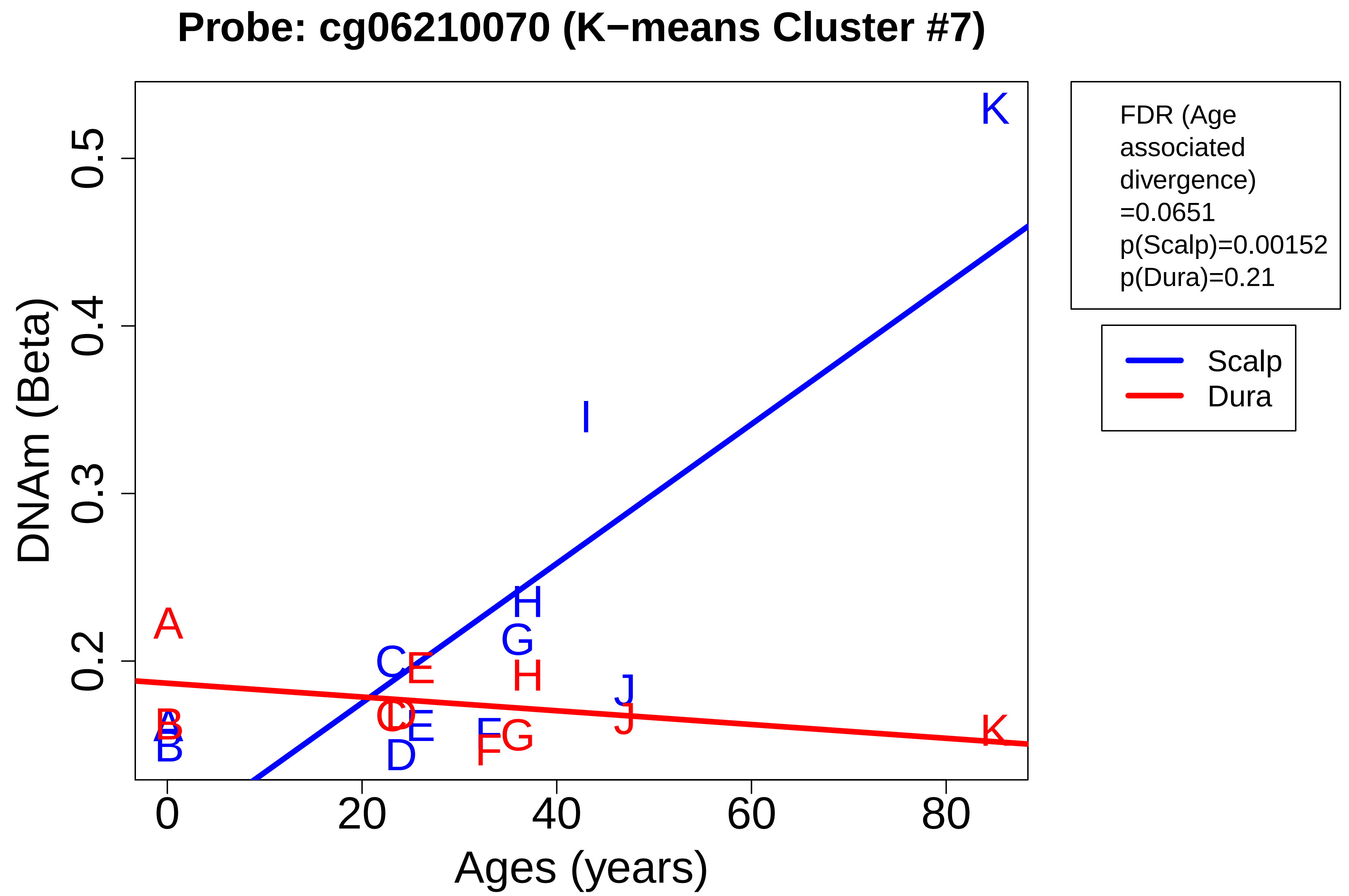

Probe: cg06210070 (K−means Cluster #7)
DNAm (Beta)
0.2
0.3
0.4
0.5
0
20
40
60
80
Ages (years)
FDR (Age associated divergence) =0.0651
p(Scalp)=0.00152
p(Dura)=0.21
Scalp
Dura

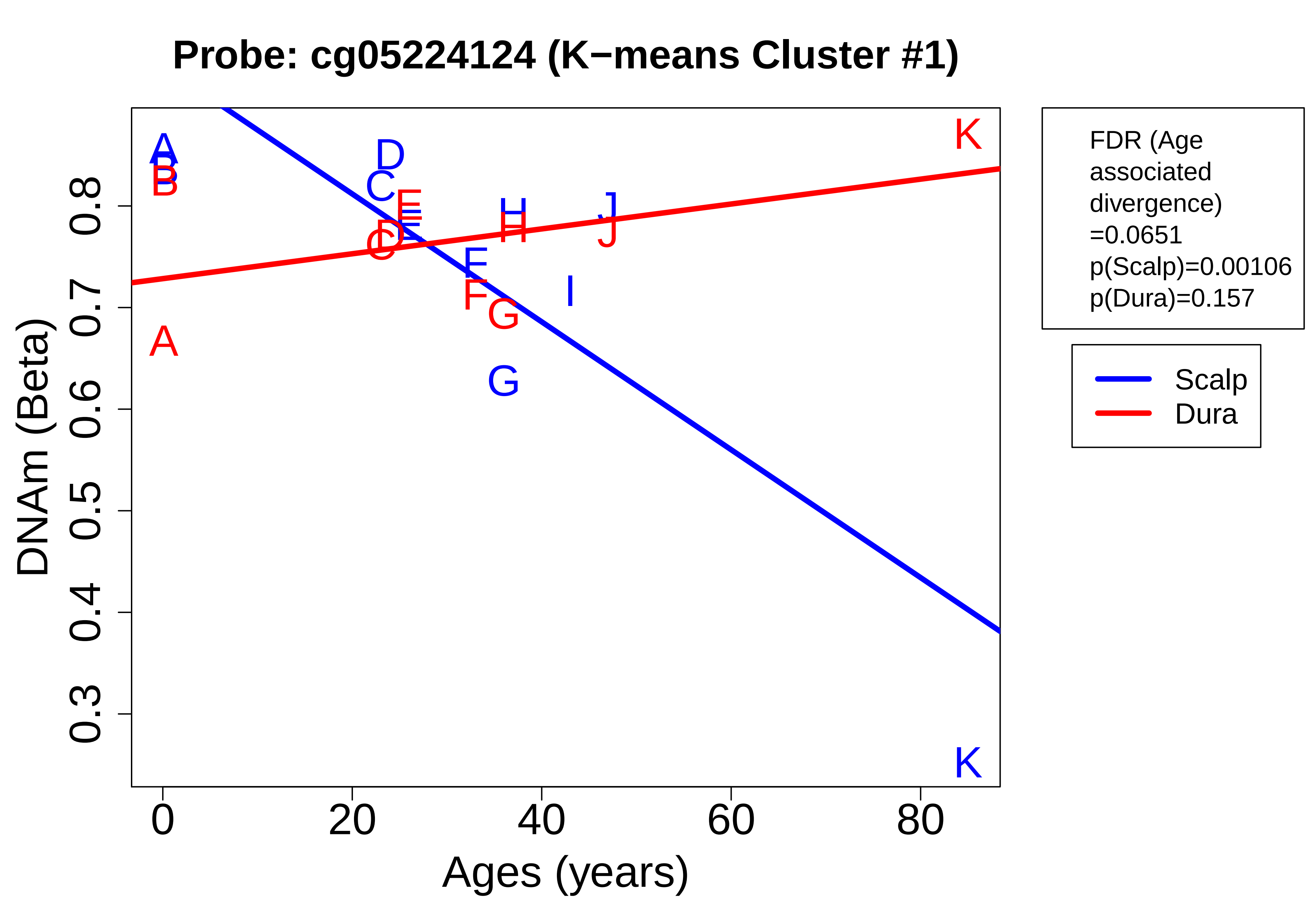

Probe: cg05224124 (K−means Cluster #1)
DNAm (Beta)
0.3
0.4
0.5
0.6
0.7
0.8
0
20
40
60
80
Ages (years)
A
B
C
D
E
F
G
H
I
J
K
FDR (Age associated divergence) =0.0651
p(Scalp)=0.00106
p(Dura)=0.157
Scalp
Dura

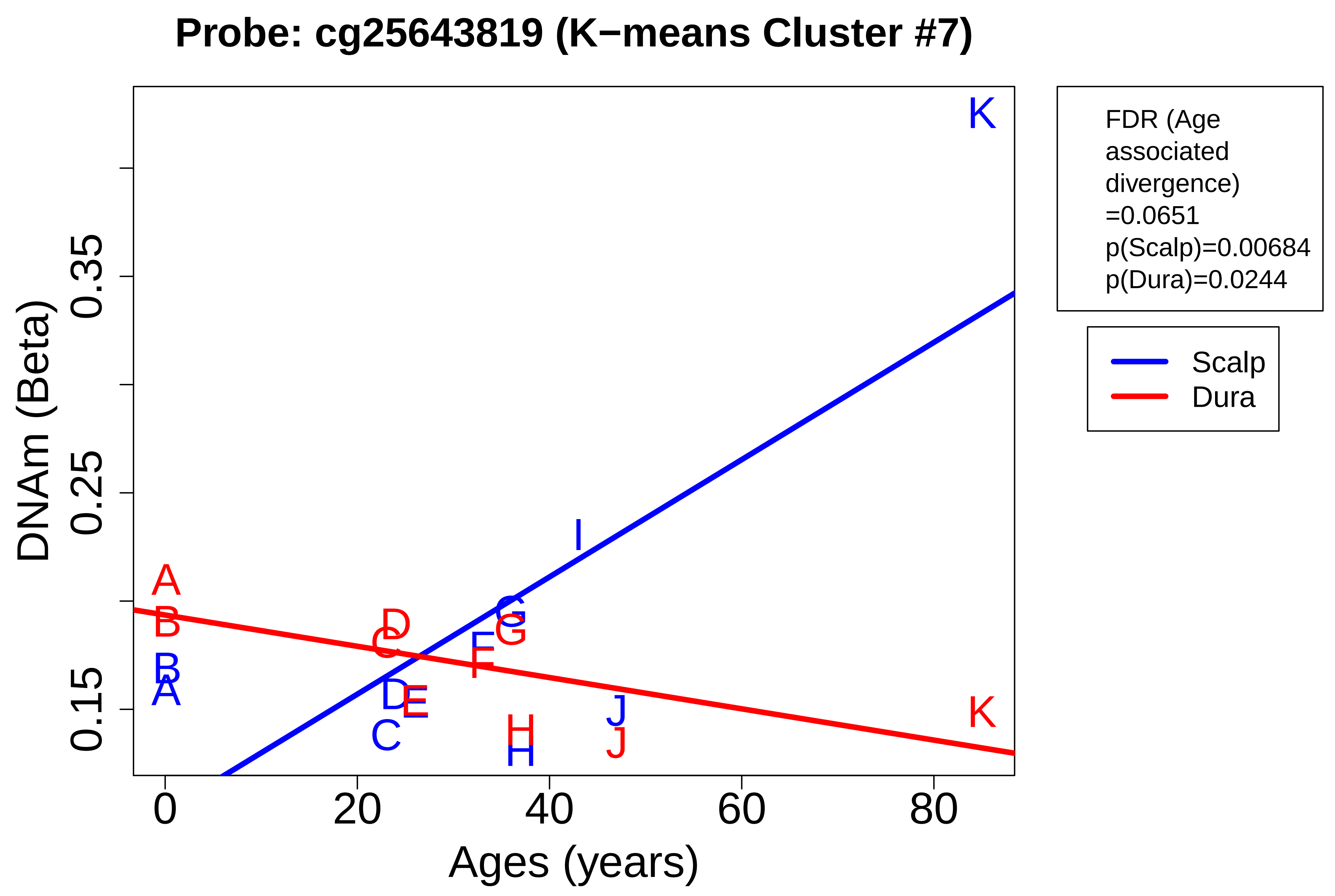

Probe: cg25643819 (K−means Cluster #7)
FDR (Age associated divergence) =0.0651
p(Scalp)=0.00684
p(Dura)=0.0244
Scalp
Dura
DNAm (Beta)
0.15
0.25
0.35
0
20
40
60
80
Ages (years)
A
B
C
D
E
F
G
H
I
J
K

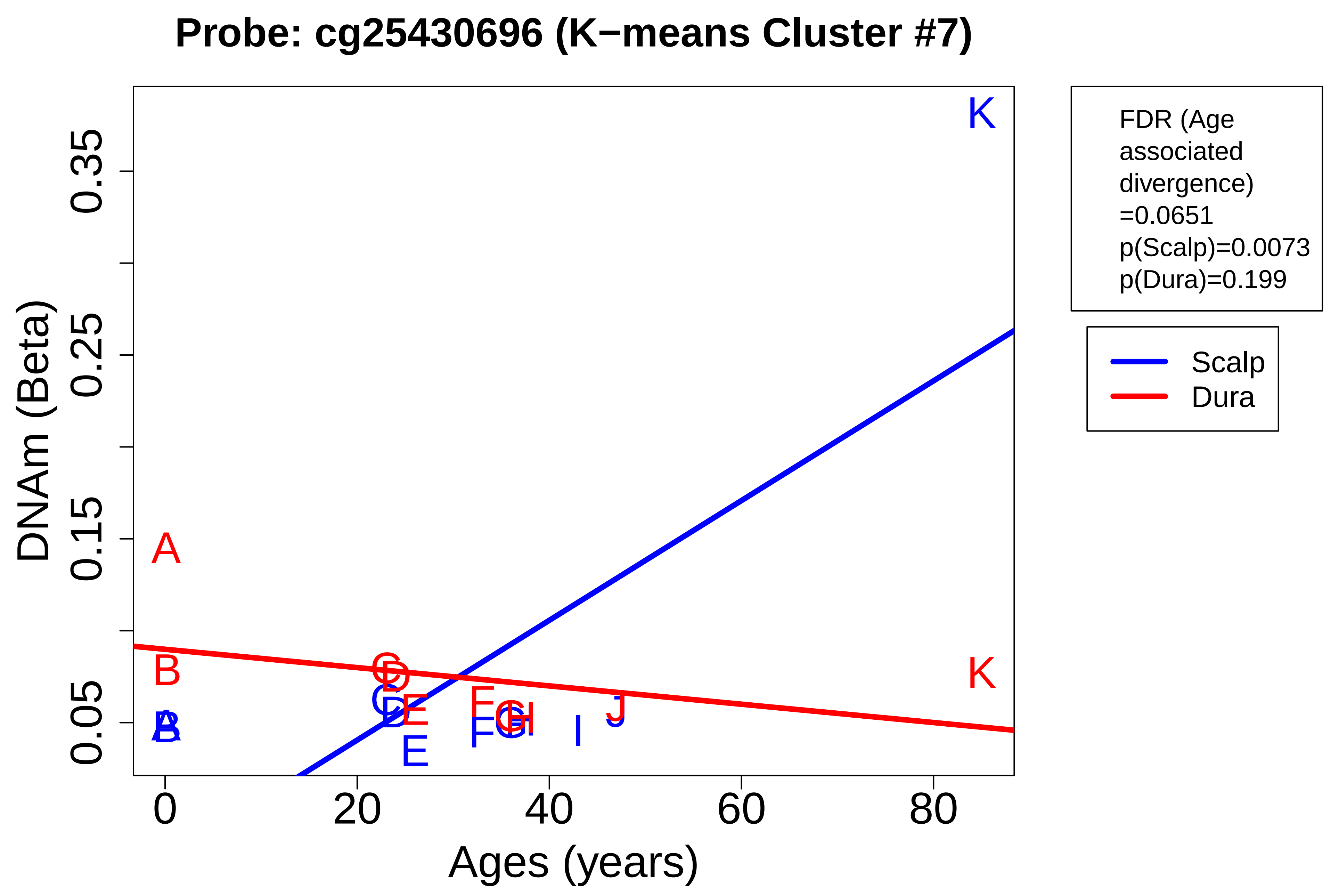
Probe: cg25430696 (K−means Cluster #7)
DNAm (Beta)
0.05
0.15
0.25
0.35
Ages (years)
0
20
40
60
80
FDR (Age associated divergence) =0.0651
p(Scalp)=0.0073
p(Dura)=0.199
Scalp
Dura
A
B
C
D
E
F
G
H
I
J
K

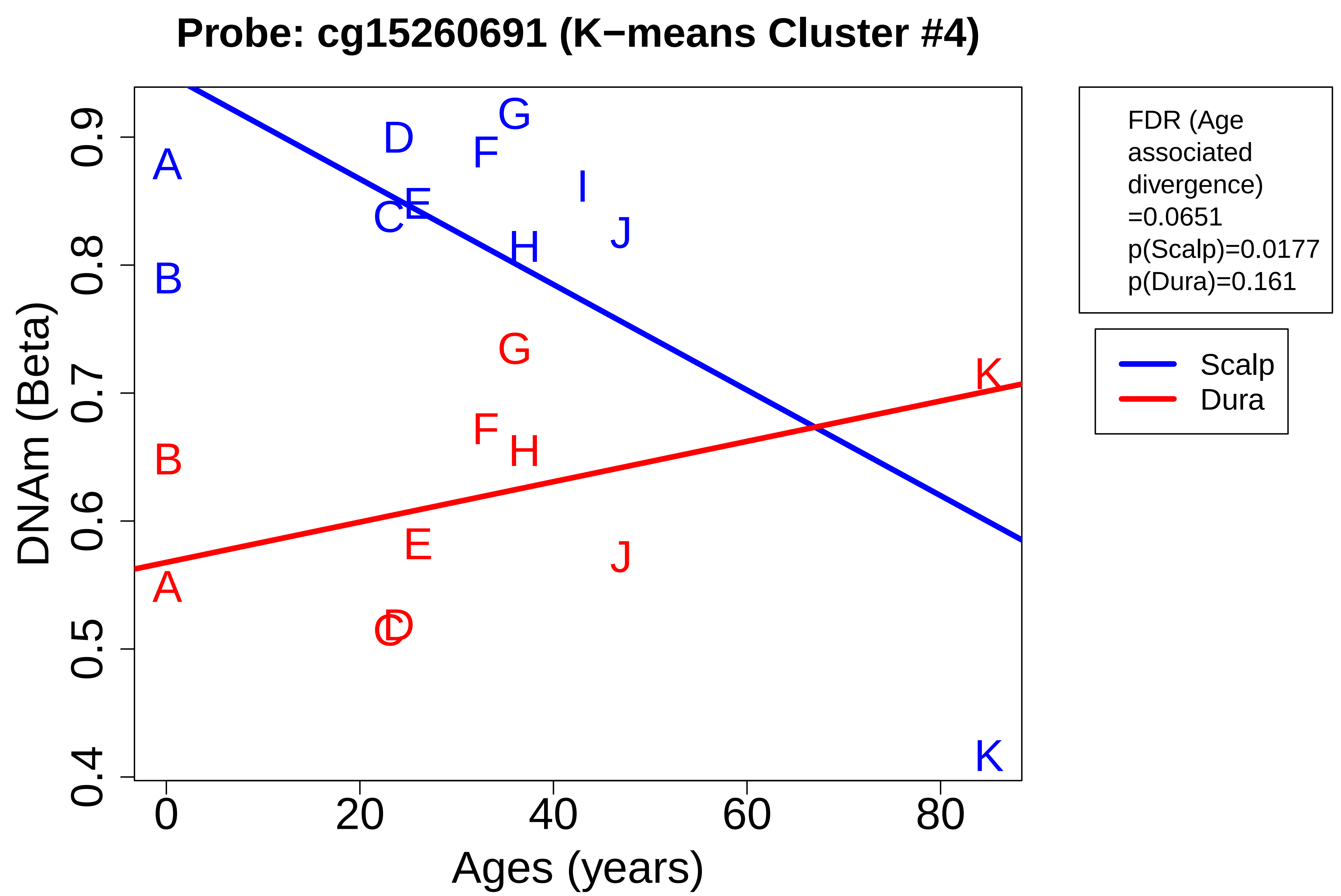

Probe: cg15260691 (K−means Cluster #4)
DNAm (Beta)
0.4
0.5
0.6
0.7
0.8
0.9
0
20
40
60
80
Ages (years)
A
B
C
D
E
F
G
H
I
J
K
A
B
C
D
E
F
G
H
J
K
FDR (Age associated divergence) =0.0651
p(Scalp)=0.0177
p(Dura)=0.161
Scalp
Dura

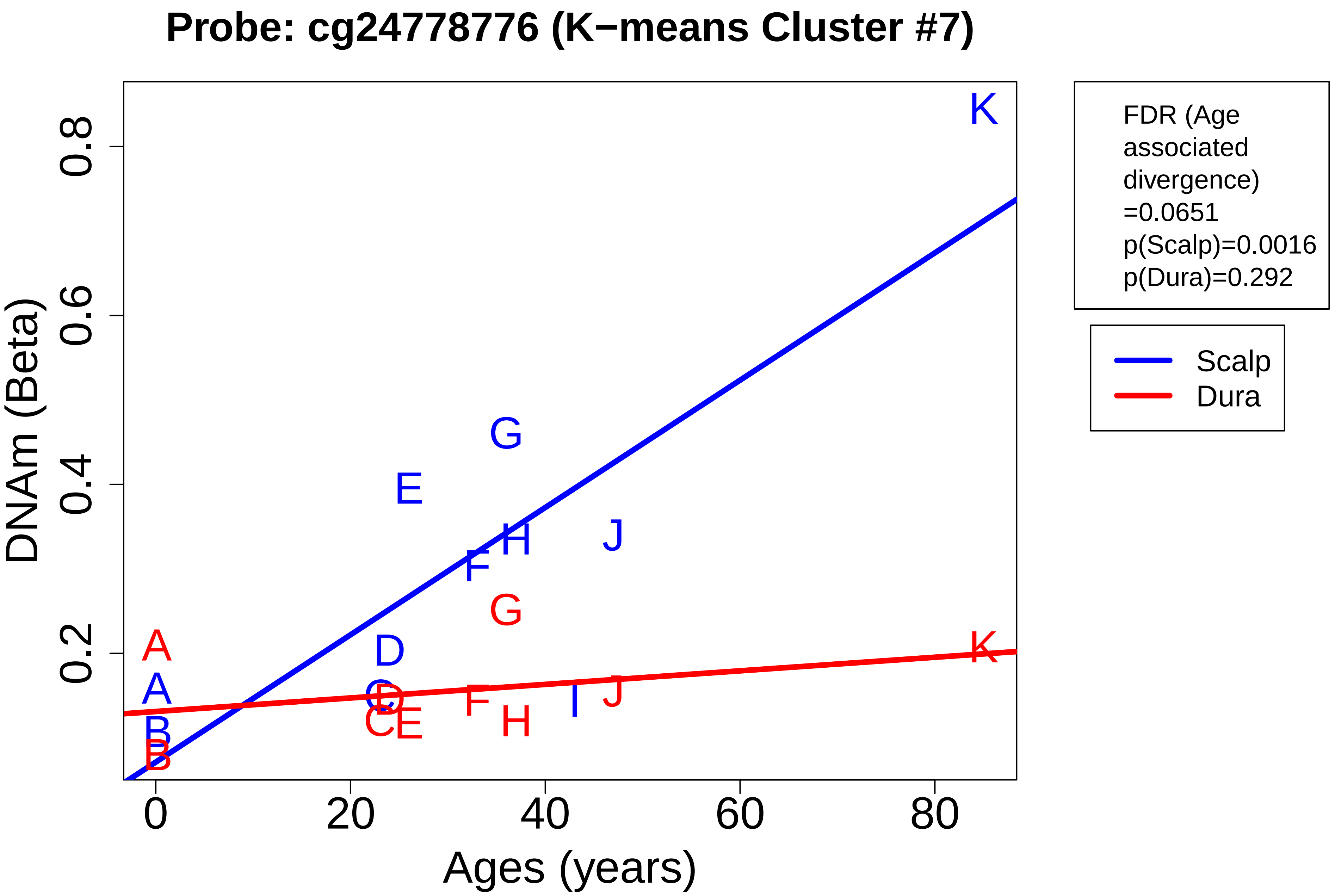
Probe: cg24778776 (K−means Cluster #7)
DNAm (Beta)
0.2
0.4
0.6
0.8
0
20
40
60
80
Ages (years)
FDR (Age associated divergence) =0.0651
p(Scalp)=0.0016
p(Dura)=0.292
Scalp
Dura
A
B
C
D
E
F
G
H
I
J
K

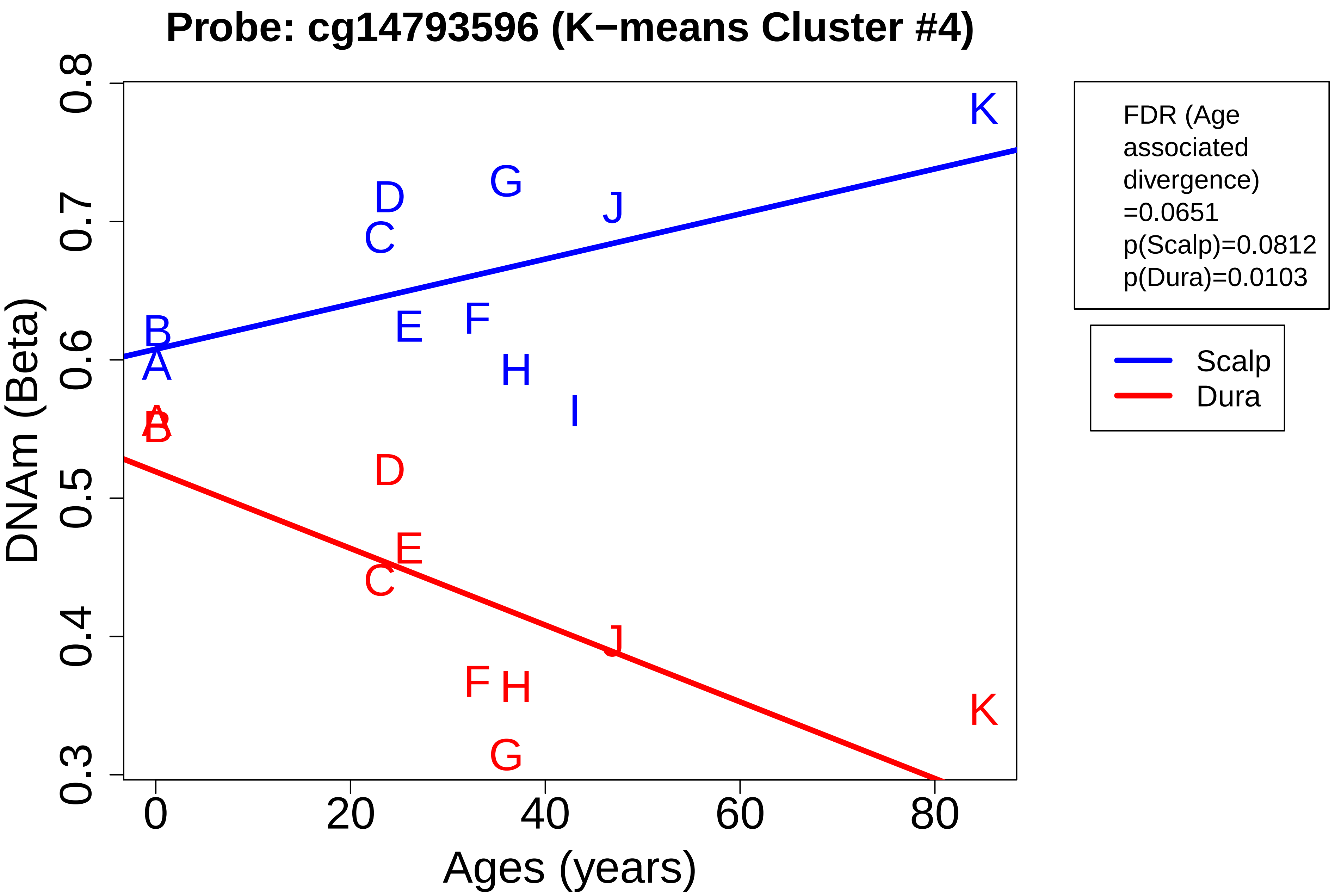

Probe: cg14793596 (K−means Cluster #4)
DNAm (Beta)
0.3
0.4
0.5
0.6
0.7
0.8
0
20
40
60
80
Ages (years)
FDR (Age associated divergence) =0.0651
p(Scalp)=0.0812
p(Dura)=0.0103
Scalp
Dura

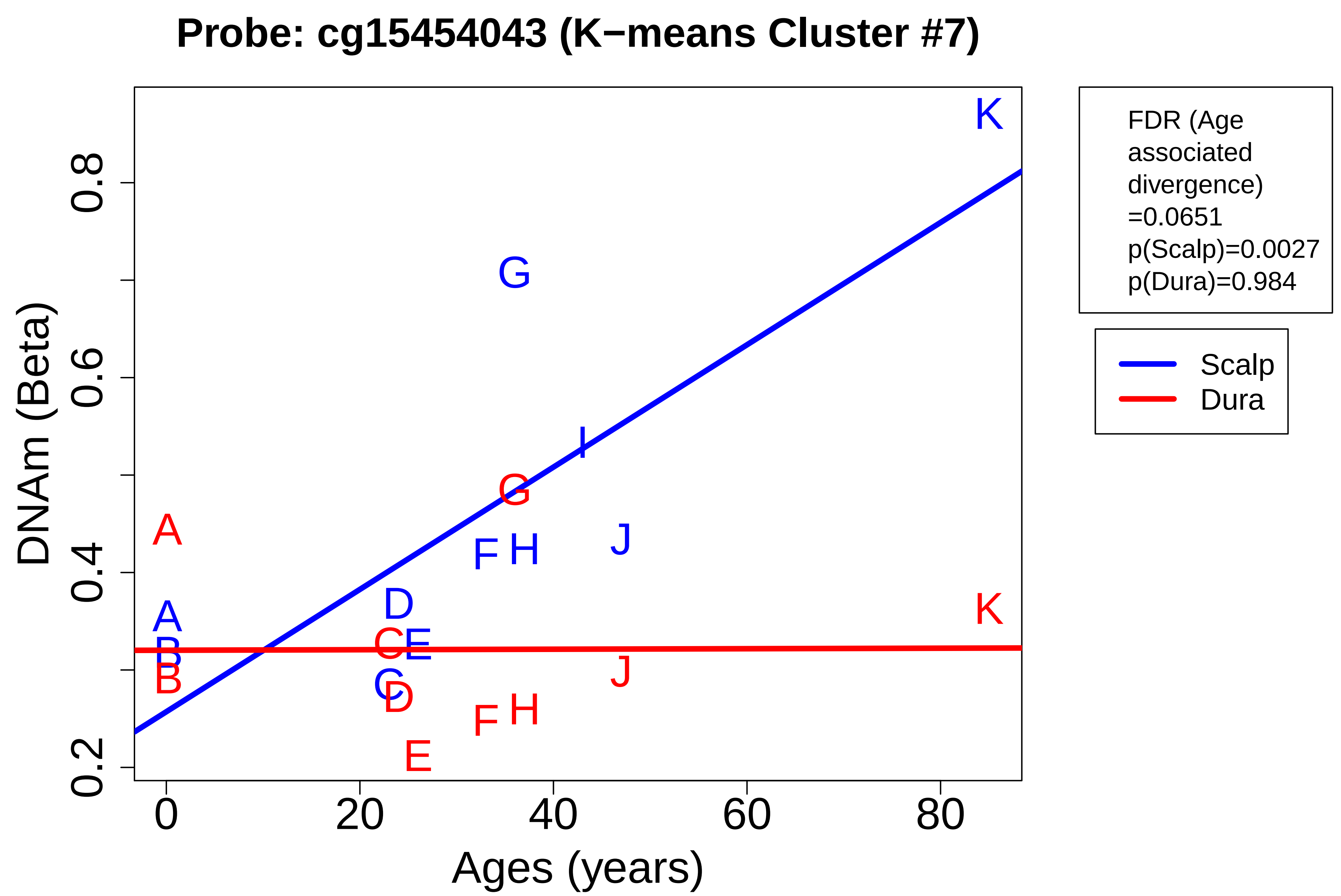

Probe: cg15454043 (K−means Cluster #7)
DNAm (Beta)
0.2
0.4
0.6
0.8
0
20
40
60
80
Ages (years)
A
B
C
D
E
F
G
H
I
J
K
FDR (Age associated divergence) =0.0651
p(Scalp)=0.0027
p(Dura)=0.984
Scalp
Dura

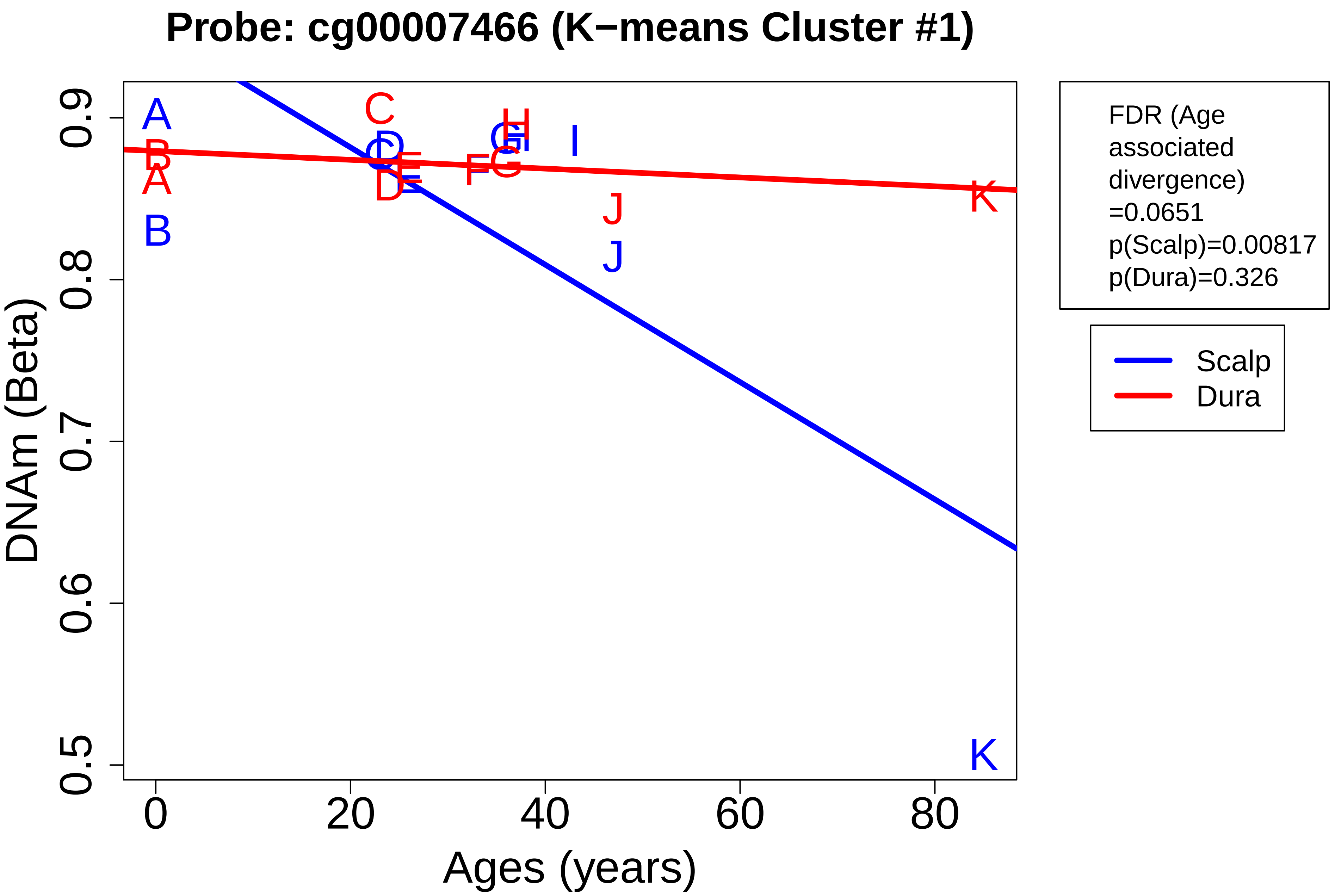

Probe: cg00007466 (K−means Cluster #1)
DNAm (Beta)
0.5
0.6
0.7
0.8
0.9
0
20
40
60
80
Ages (years)
FDR (Age associated divergence) =0.0651
p(Scalp)=0.00817
p(Dura)=0.326
Scalp
Dura
A
B
C
D
E
F
G
H
I
J
K

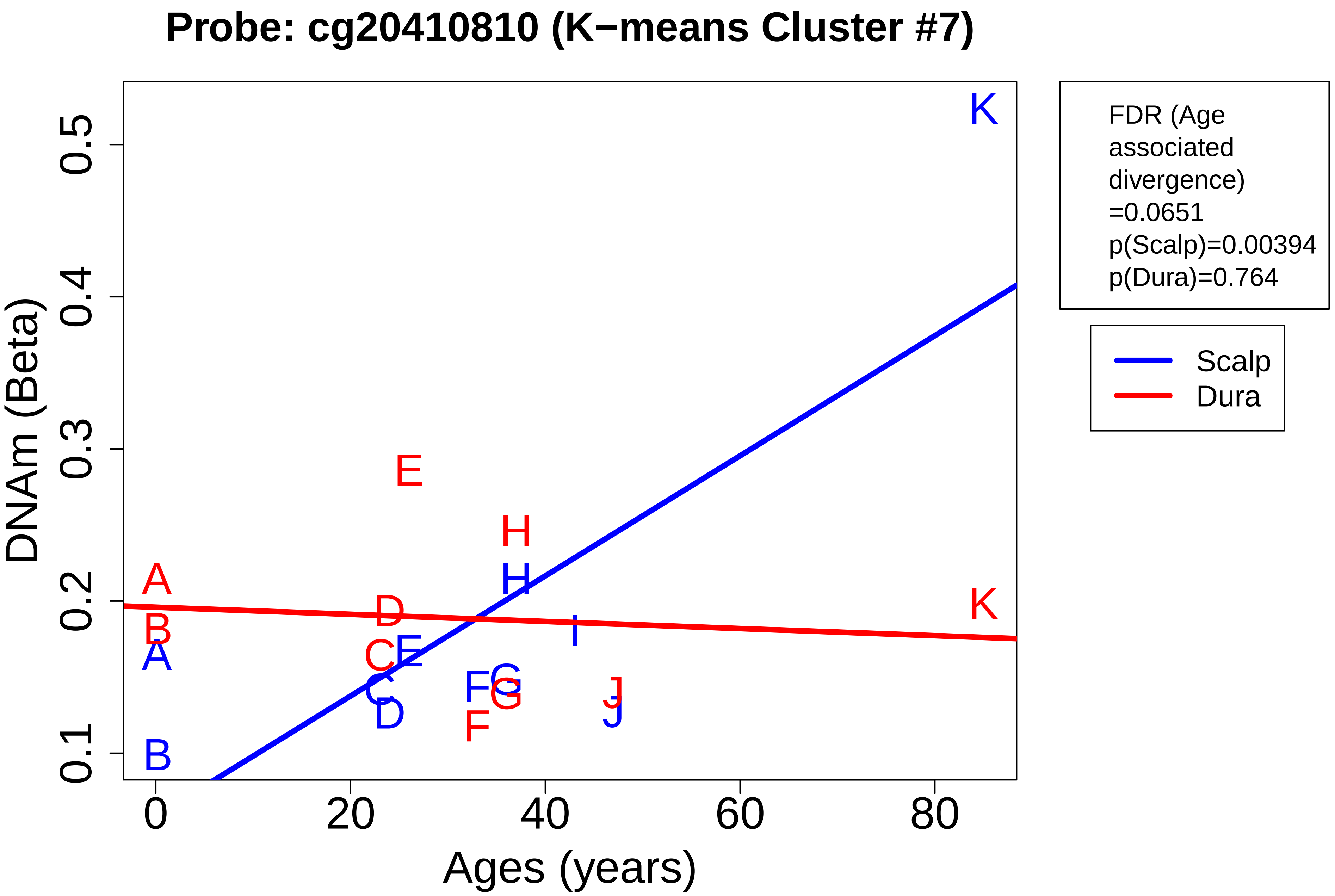

Probe: cg20410810 (K−means Cluster #7)
DNAm (Beta)
0.1
0.2
0.3
0.4
0.5
Ages (years)
0
20
40
60
80
FDR (Age associated divergence) =0.0651
p(Scalp)=0.00394
p(Dura)=0.764
Scalp
Dura
A
B
C
D
E
F
G
H
I
J
K

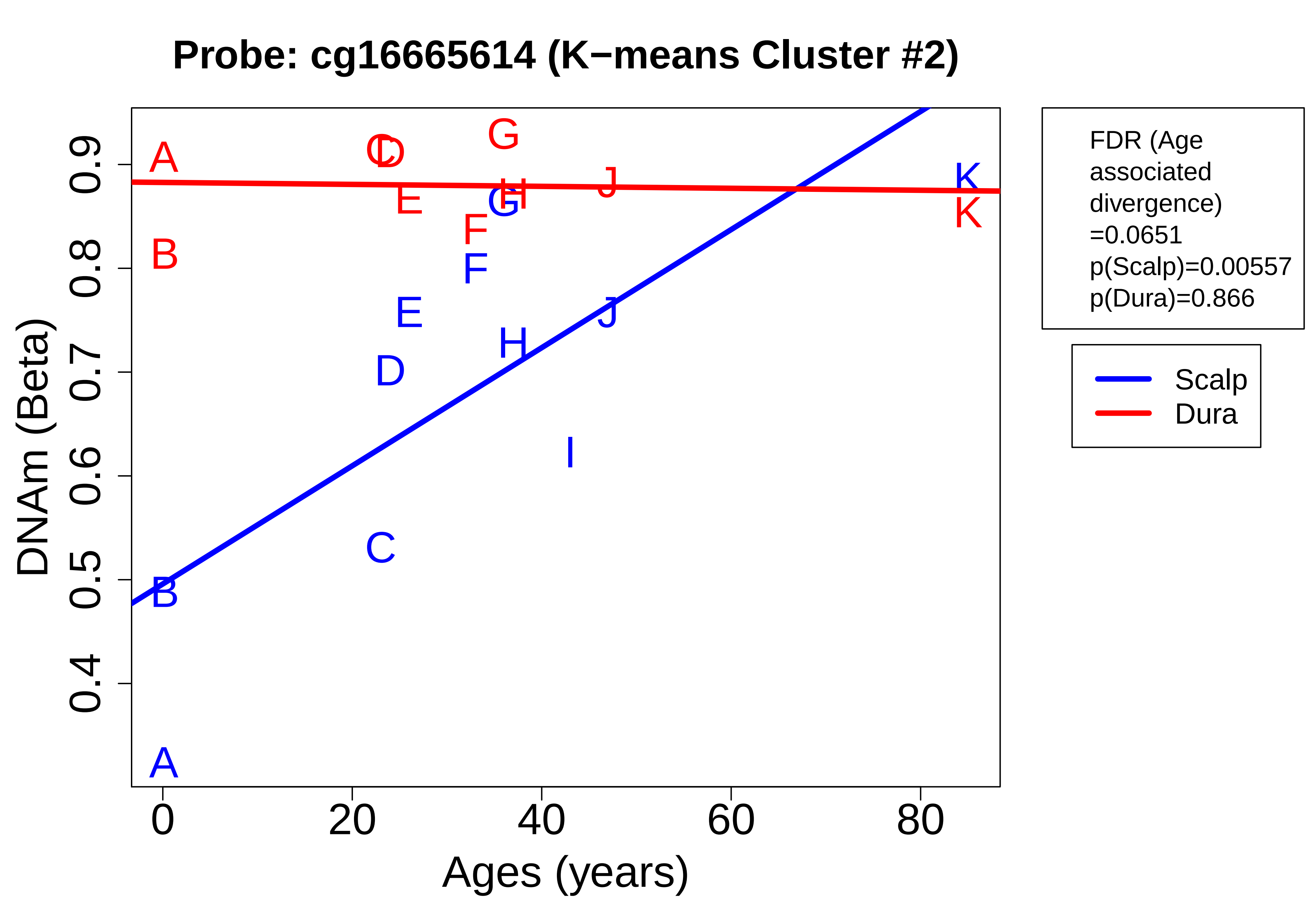

Probe: cg16665614 (K−means Cluster #2)
DNAm (Beta)
0.4
0.5
0.6
0.7
0.8
0.9
0
20
40
60
80
Ages (years)
FDR (Age associated divergence) =0.0651
p(Scalp)=0.00557
p(Dura)=0.866
Scalp
Dura

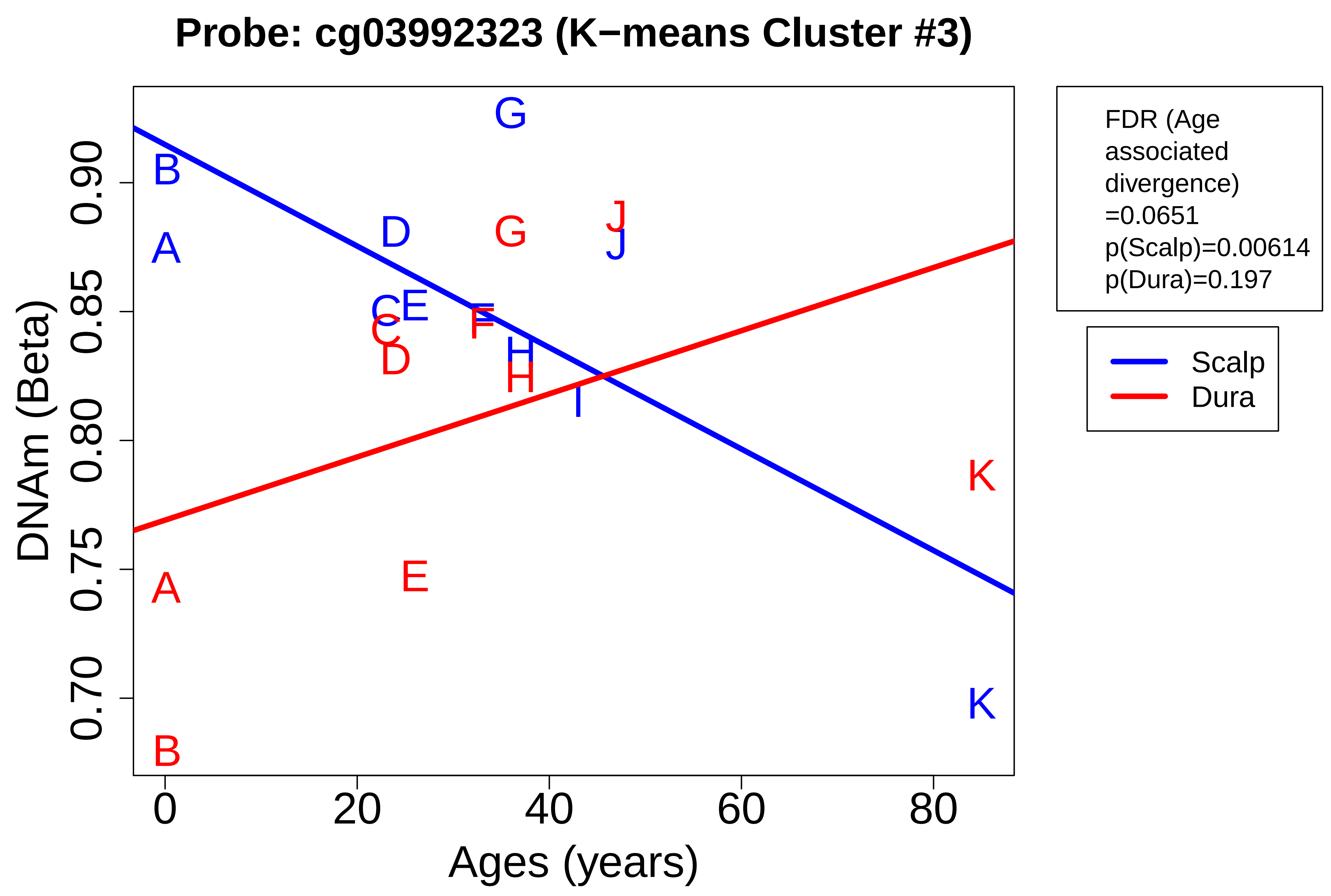

Probe: cg03992323 (K−means Cluster #3)
DNAm (Beta)
0.70
0.75
0.80
0.85
0.90
0
20
40
60
80
Ages (years)
FDR (Age associated divergence) =0.0651
p(Scalp)=0.00614
p(Dura)=0.197
Scalp
Dura

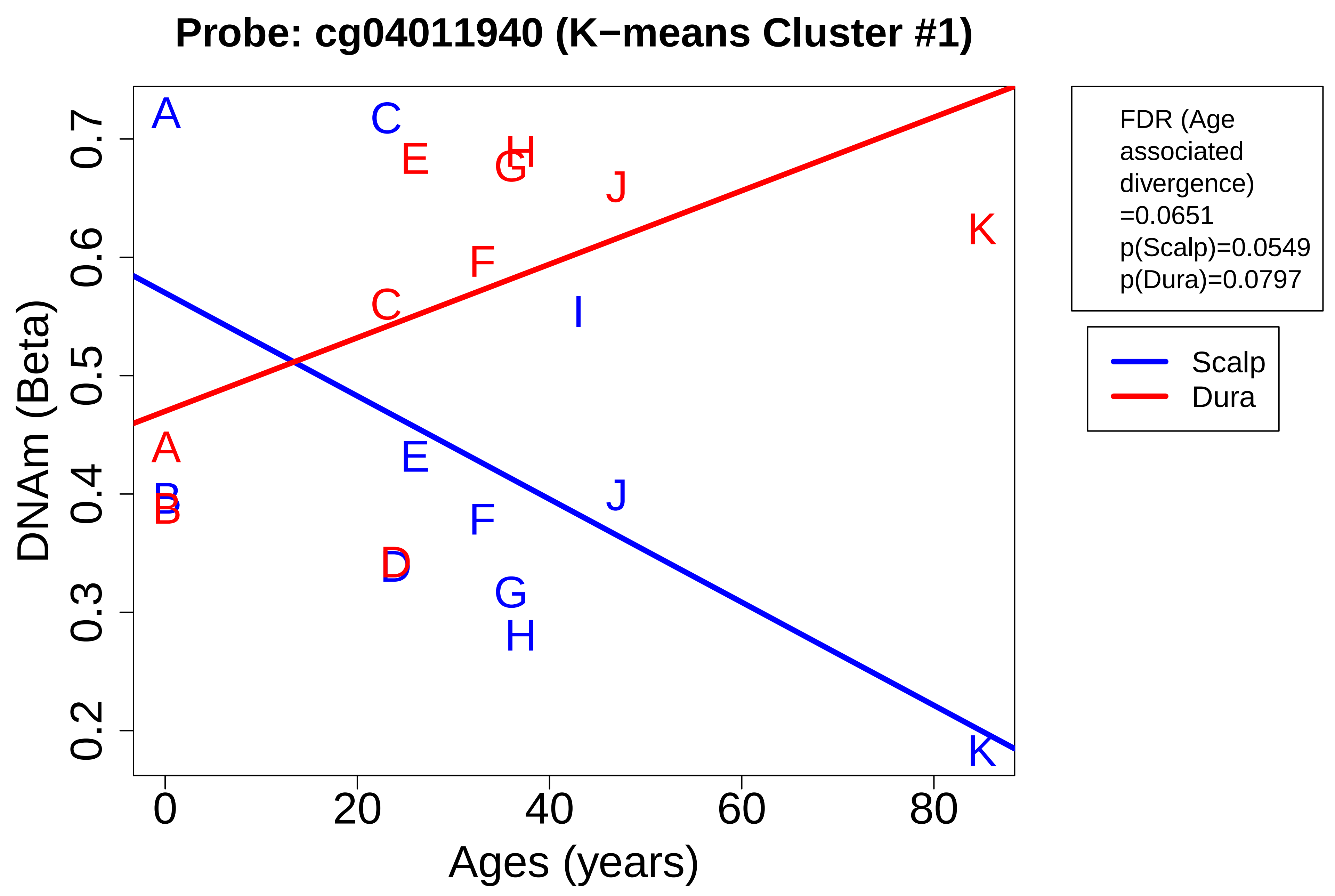
Probe: cg04011940 (K−means Cluster #1)
DNAm (Beta)
Ages (years)
0.2
0.3
0.4
0.5
0.6
0.7
0
20
40
60
80
FDR (Age associated divergence) =0.0651
p(Scalp)=0.0549
p(Dura)=0.0797
Scalp
Dura
A
B
C
D
E
F
G
H
I
J
K

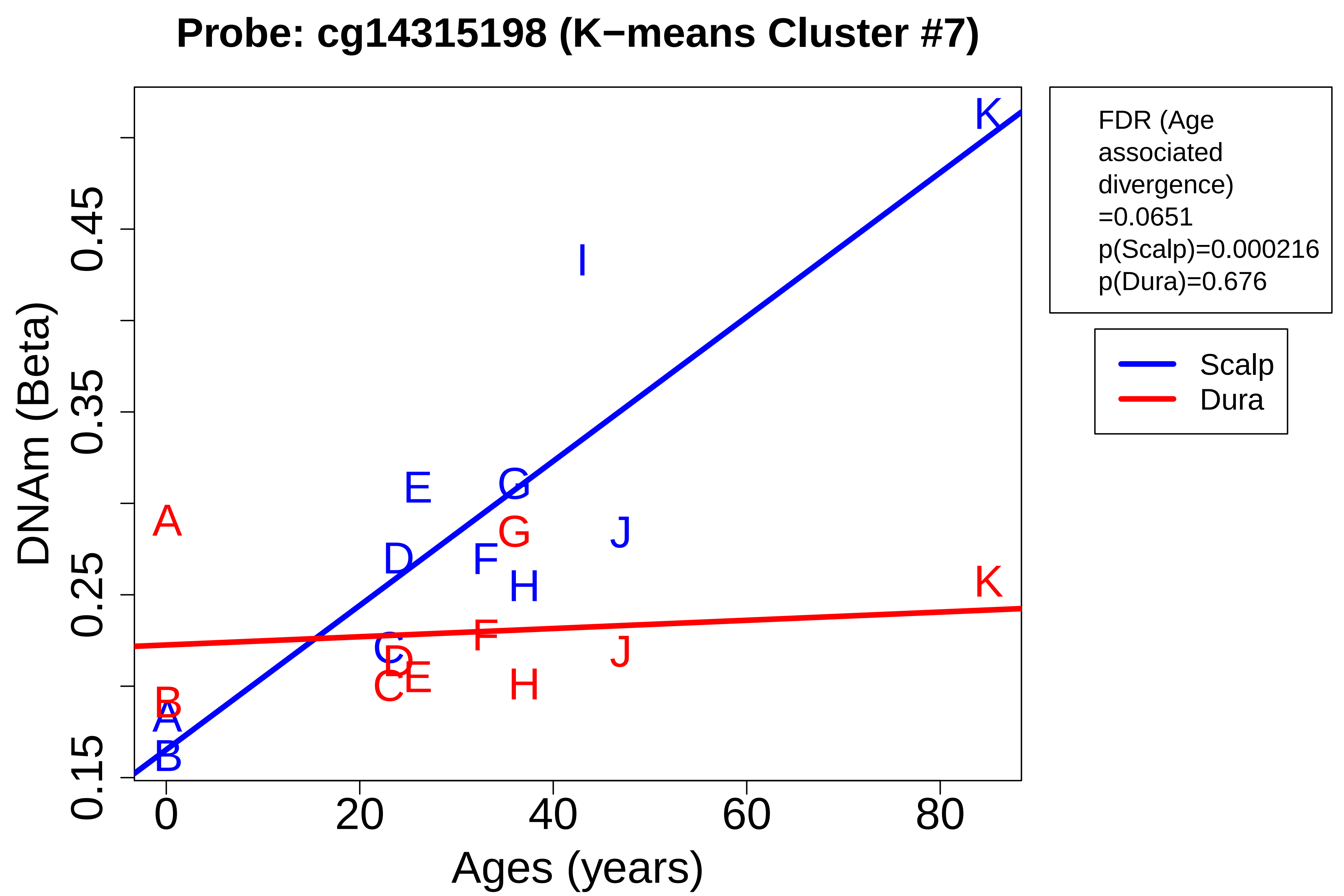
Probe: cg14315198 (K−means Cluster #7)
DNAm (Beta)
0.15
0.25
0.35
0.45
0
20
40
60
80
Ages (years)
FDR (Age associated divergence) =0.0651
p(Scalp)=0.000216
p(Dura)=0.676
Scalp
Dura

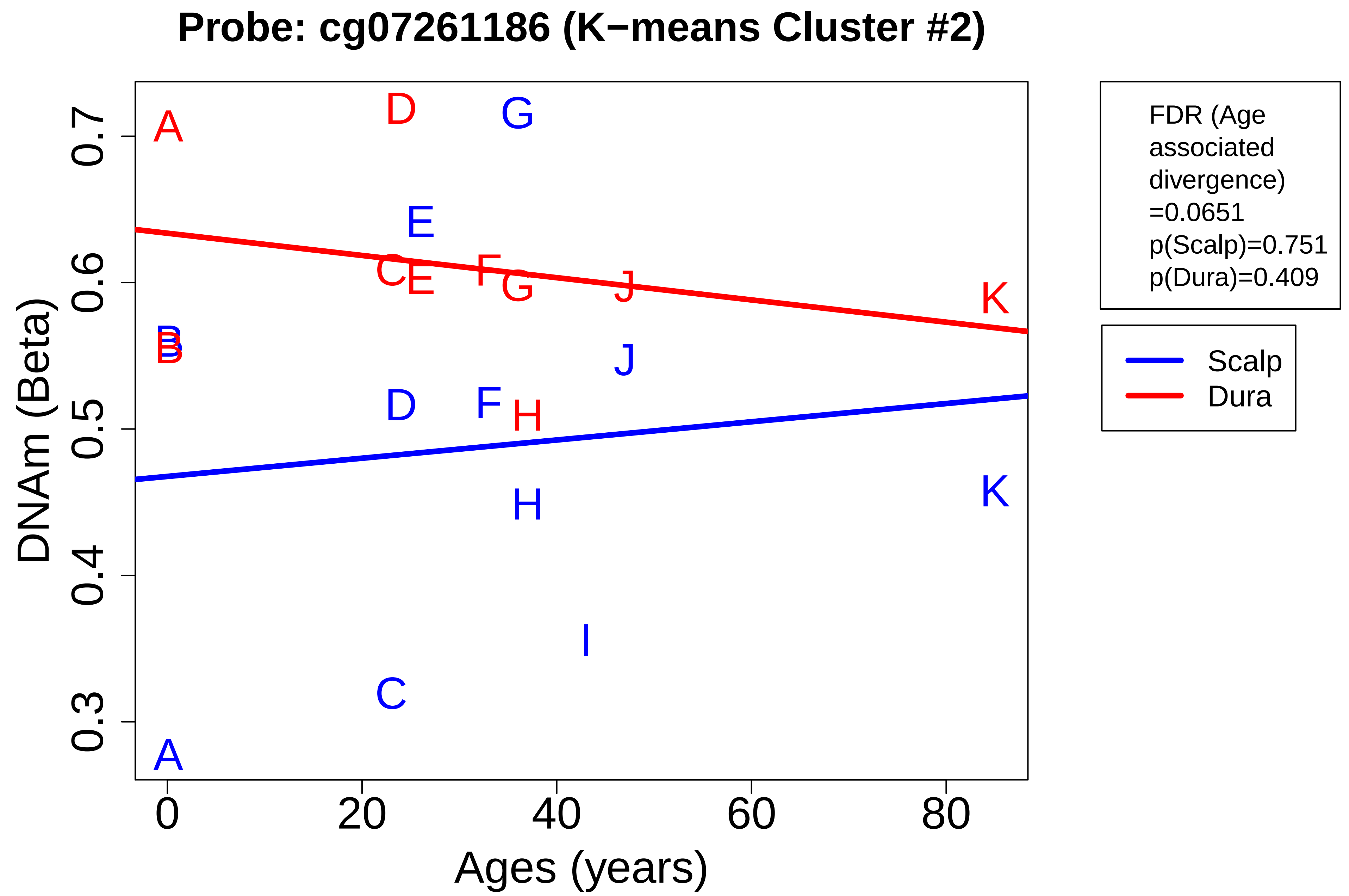

Probe: cg07261186 (K−means Cluster #2)
DNAm (Beta)
0.3
0.4
0.5
0.6
0.7
Ages (years)
0
20
40
60
80
A
B
C
D
E
F
G
H
I
J
K
FDR (Age associated divergence) =0.0651
p(Scalp)=0.751
p(Dura)=0.409
Scalp
Dura

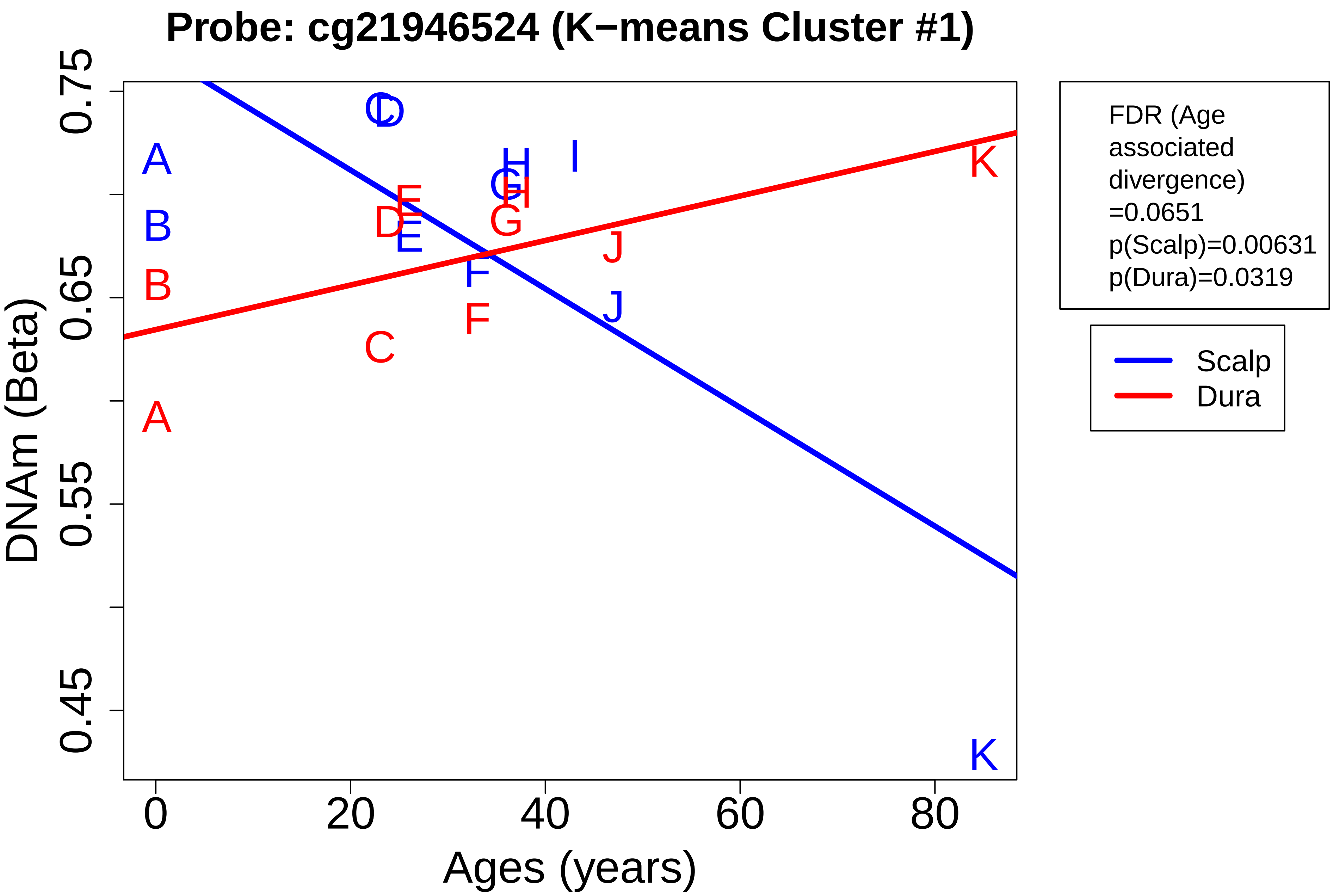
Probe: cg21946524 (K−means Cluster #1)
DNAm (Beta)
0.45
0.55
0.65
0.75
Ages (years)
0
20
40
60
80
FDR (Age associated divergence) =0.0651
p(Scalp)=0.00631
p(Dura)=0.0319
Scalp
Dura
A
B
C
D
E
F
G
H
I
J
K

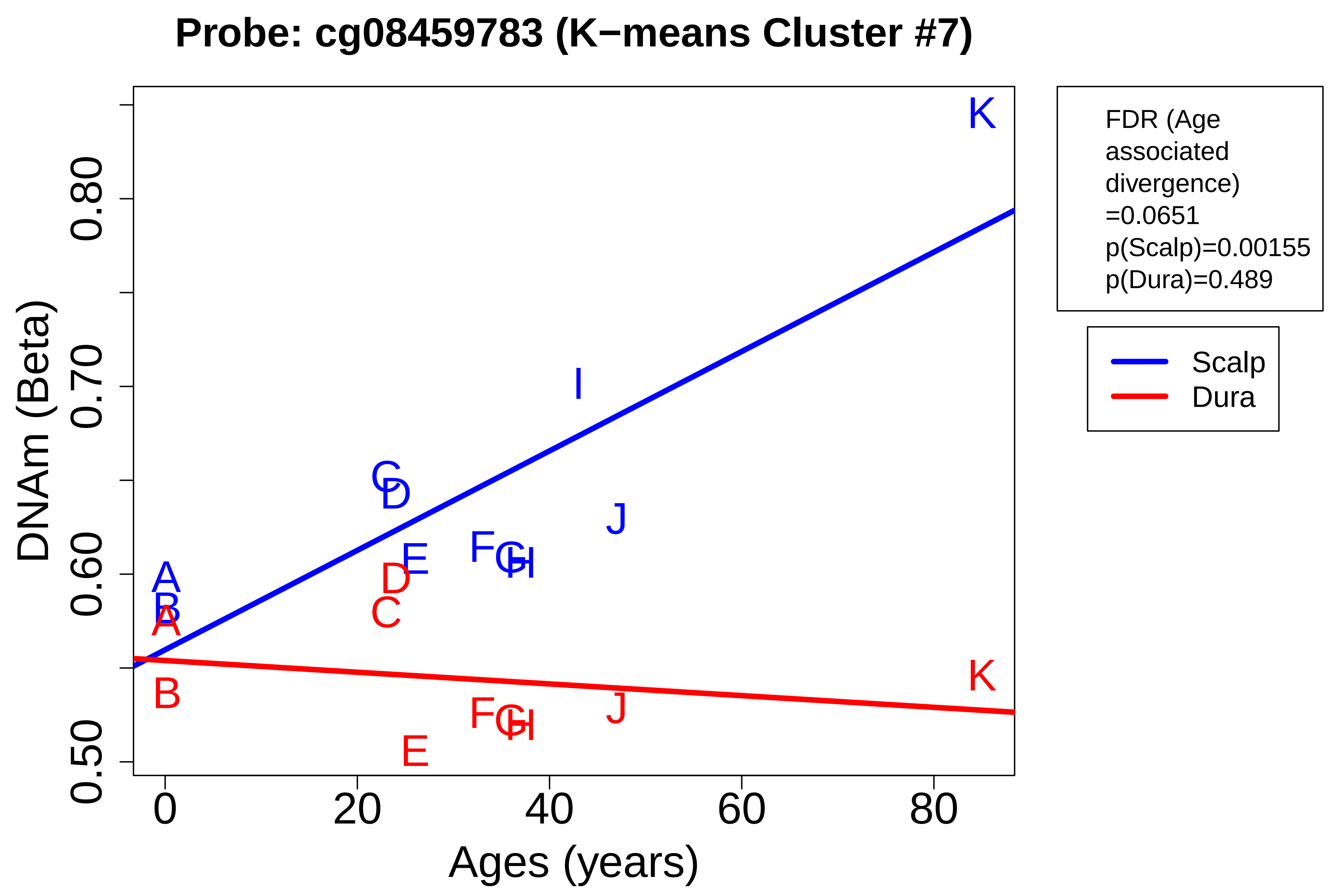
Probe: cg08459783 (K−means Cluster #7)
FDR (Age associated divergence) =0.0651
p(Scalp)=0.00155
p(Dura)=0.489
Scalp
Dura
DNAm (Beta)
0.50
0.60
0.70
0.80
0
20
40
60
80
Ages (years)
A
B
C
D
E
F
G
H
I
J
K

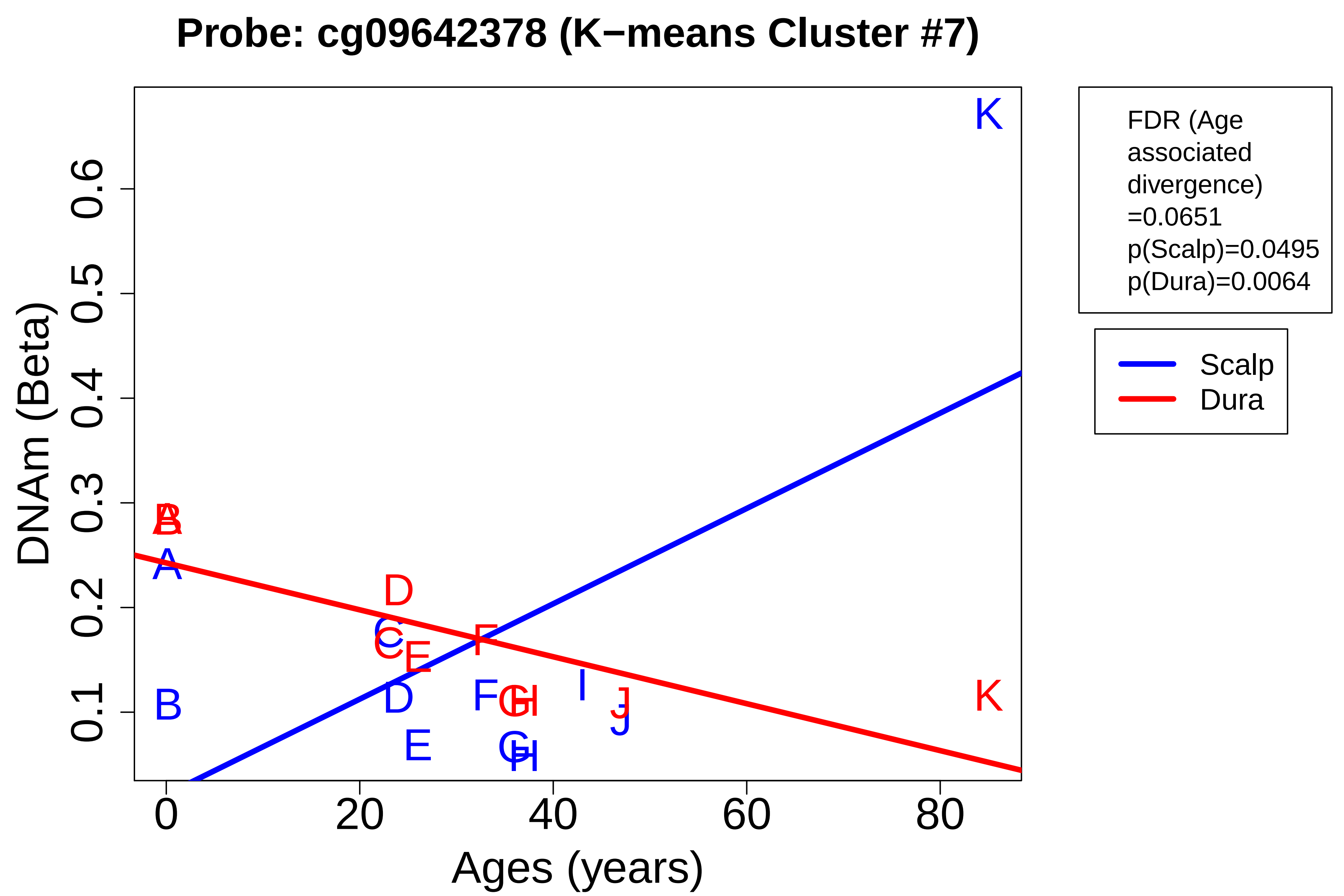

Probe: cg09642378 (K−means Cluster #7)
DNAm (Beta)
0.1
0.2
0.3
0.4
0.5
0.6
Ages (years)
0
20
40
60
80
FDR (Age associated divergence) =0.0651
p(Scalp)=0.0495
p(Dura)=0.0064
Scalp
Dura
A
B
C
D
E
F
G
H
I
J
K

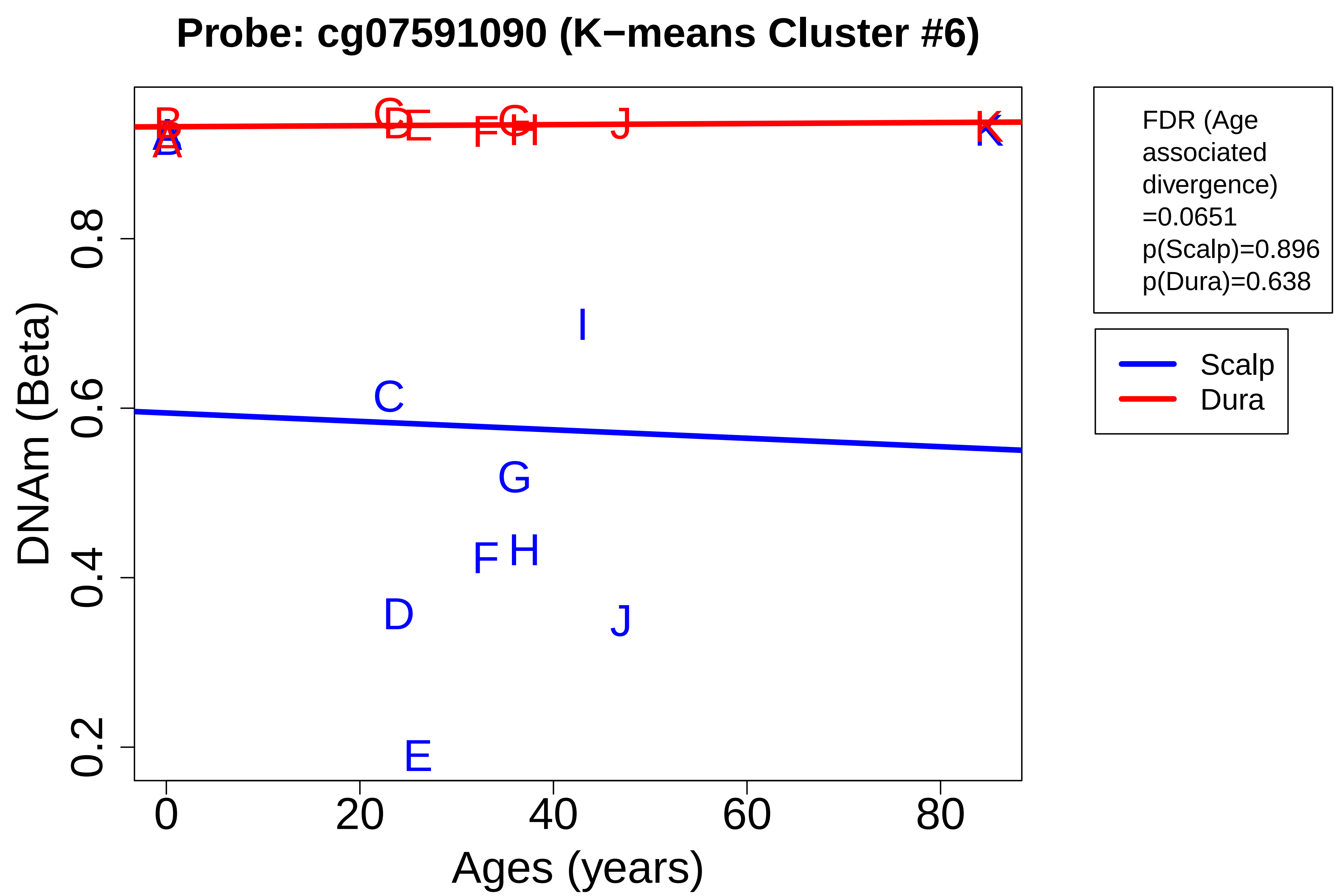
Probe: cg07591090 (K−means Cluster #6)
DNAm (Beta)
0.2
0.4
0.6
0.8
0
20
40
60
80
Ages (years)
FDR (Age associated divergence) =0.0651
p(Scalp)=0.896
p(Dura)=0.638
Scalp
Dura
A
B
C
D
E
F
G
H
I
J
K

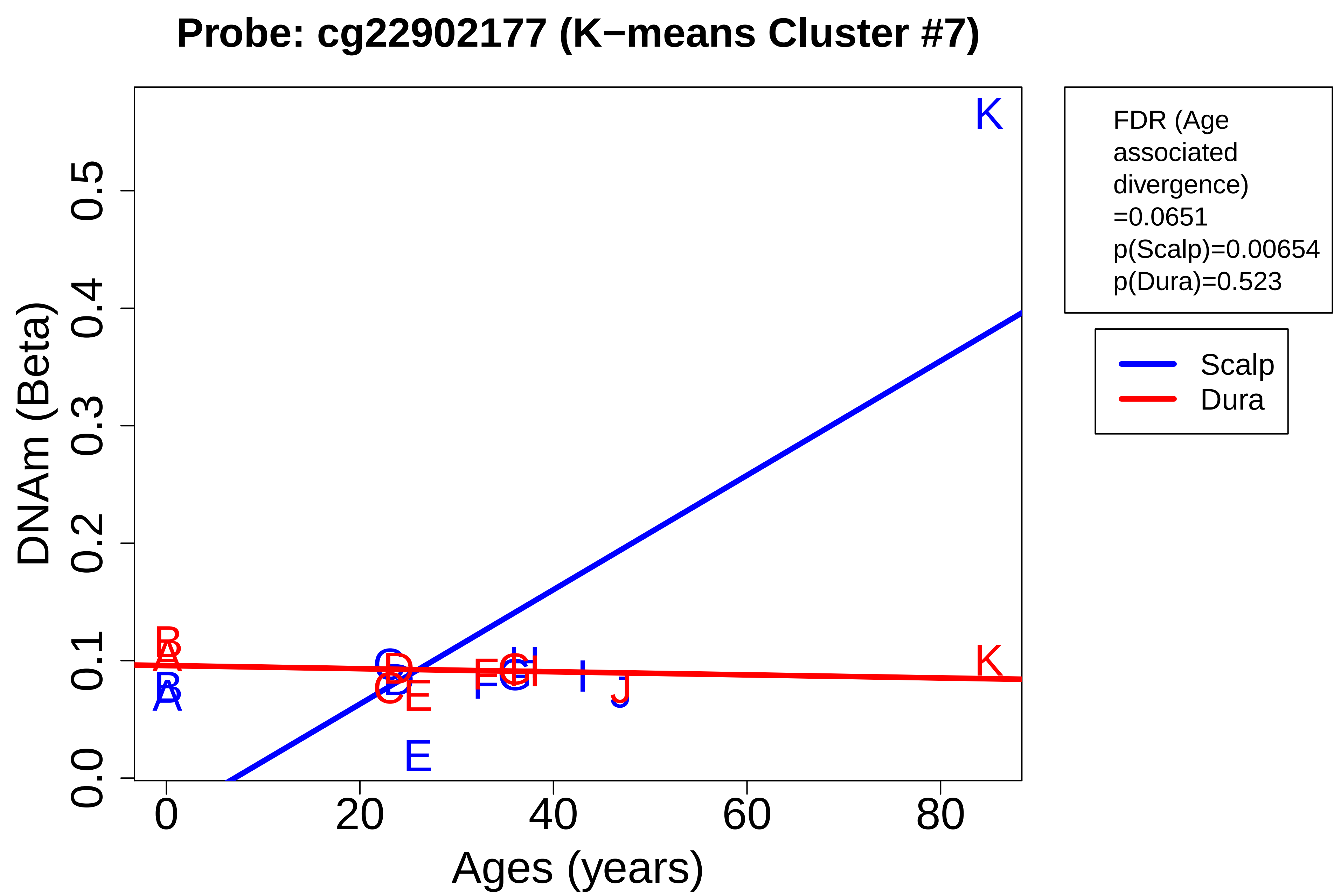

Probe: cg22902177 (K−means Cluster #7)
DNAm (Beta)
0.0
0.1
0.2
0.3
0.4
0.5
0
20
40
60
80
Ages (years)
FDR (Age associated divergence) =0.0651
p(Scalp)=0.00654
p(Dura)=0.523
Scalp
Dura

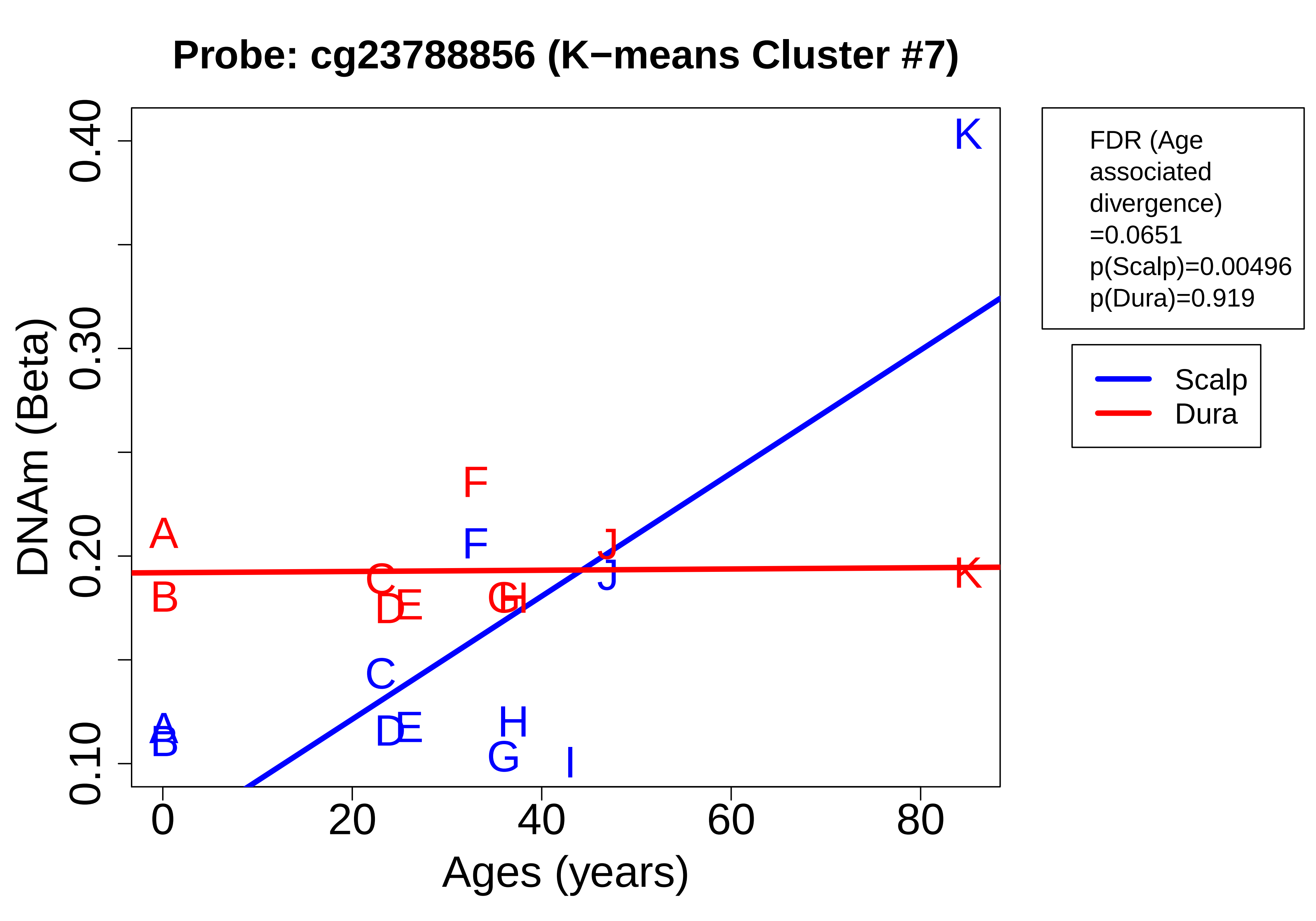
Probe: cg23788856 (K−means Cluster #7)
DNAm (Beta)
0.10
0.20
0.30
0.40
0
20
40
60
80
Ages (years)
A
B
C
D
E
F
G
H
I
J
K
FDR (Age associated divergence) =0.0651
p(Scalp)=0.00496
p(Dura)=0.919
Scalp
Dura

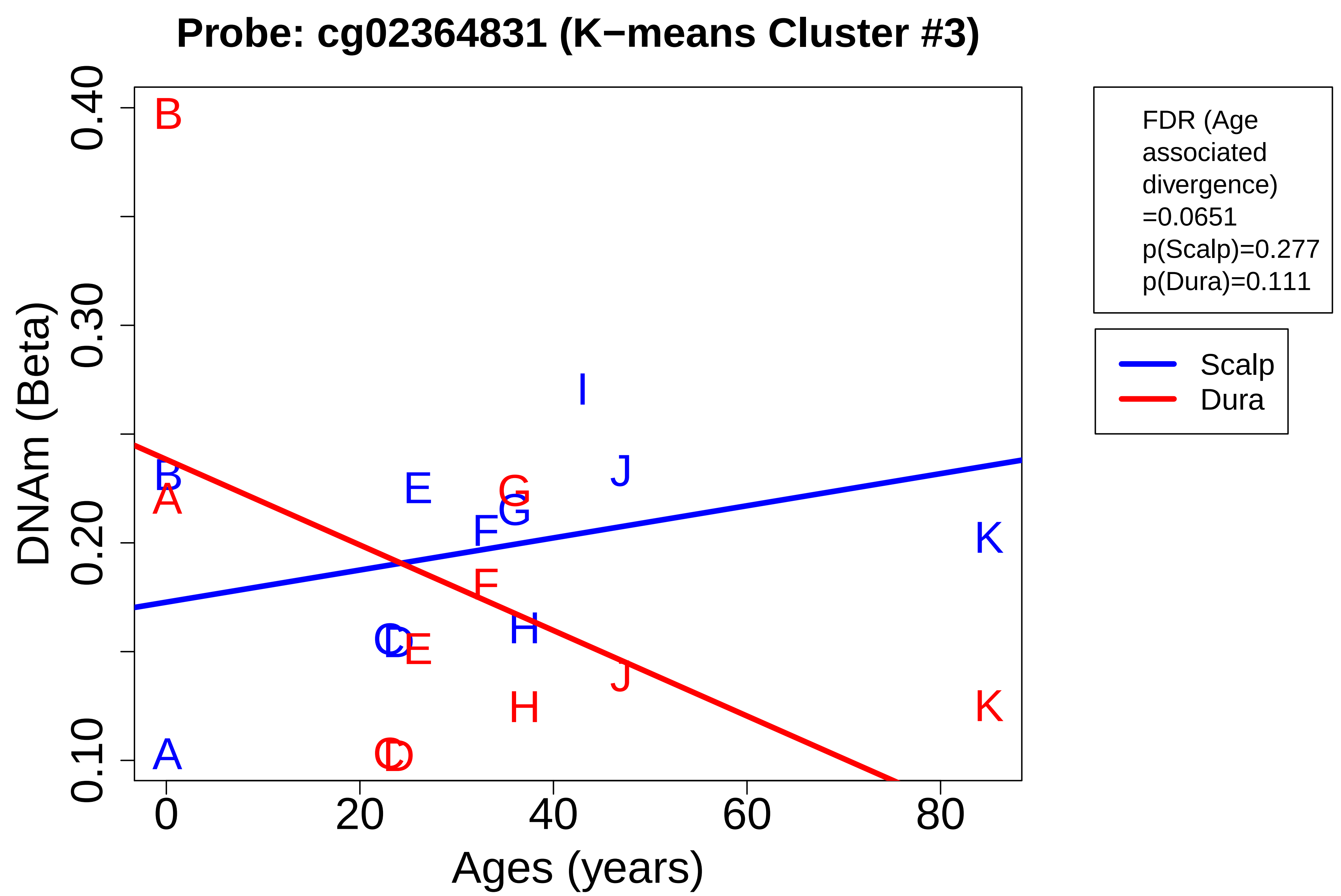

Probe: cg02364831 (K−means Cluster #3)
DNAm (Beta)
0.10
0.20
0.30
0.40
0
20
40
60
80
Ages (years)
FDR (Age associated divergence) =0.0651
p(Scalp)=0.277
p(Dura)=0.111
Scalp
Dura
A
B
C
D
E
F
G
H
I
J
K

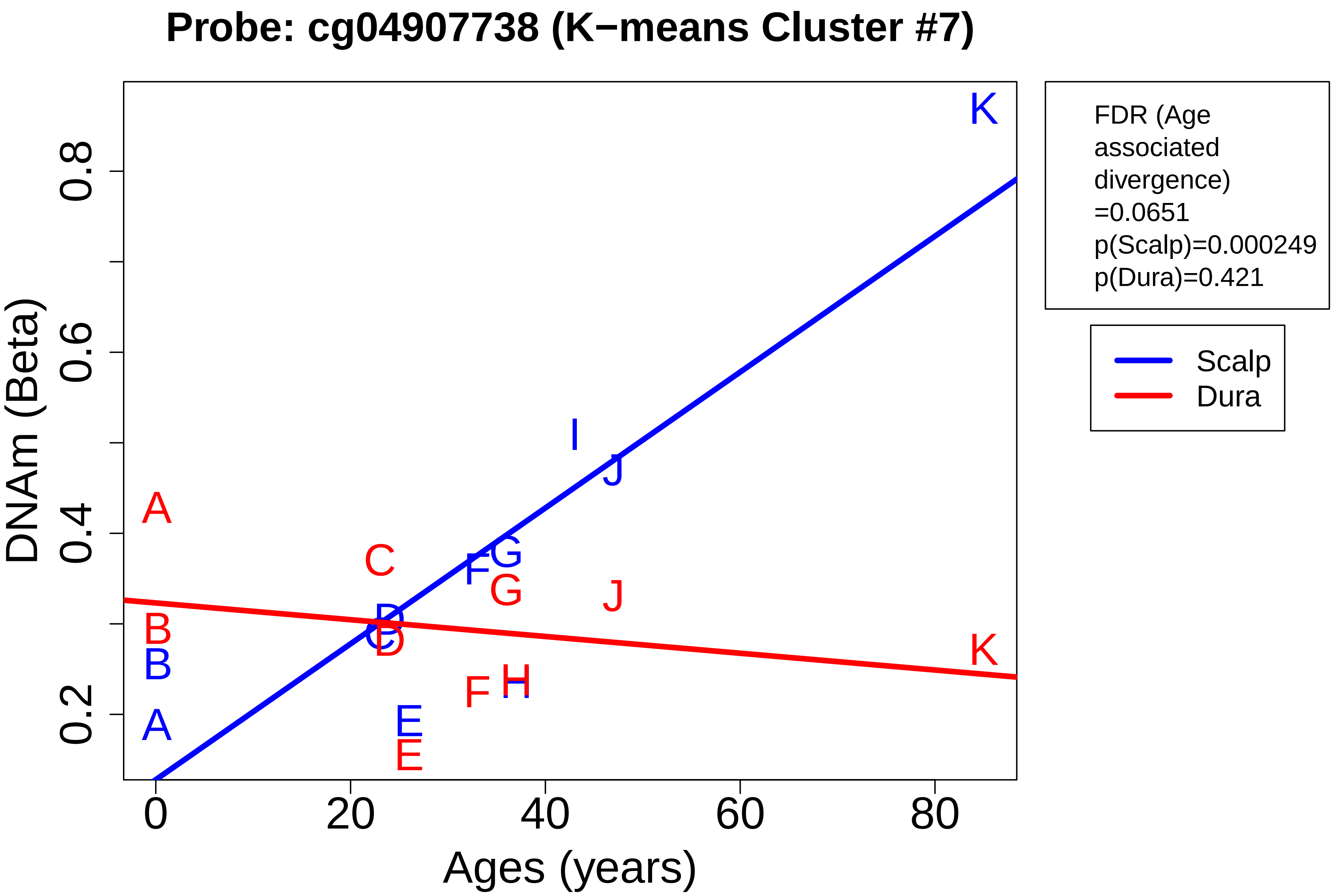

Probe: cg04907738 (K−means Cluster #7)
DNAm (Beta)
0.2
0.4
0.6
0.8
Ages (years)
0
20
40
60
80
FDR (Age associated divergence) =0.0651
p(Scalp)=0.000249
p(Dura)=0.421
Scalp
Dura
A
B
C
D
E
F
G
H
I
J
K

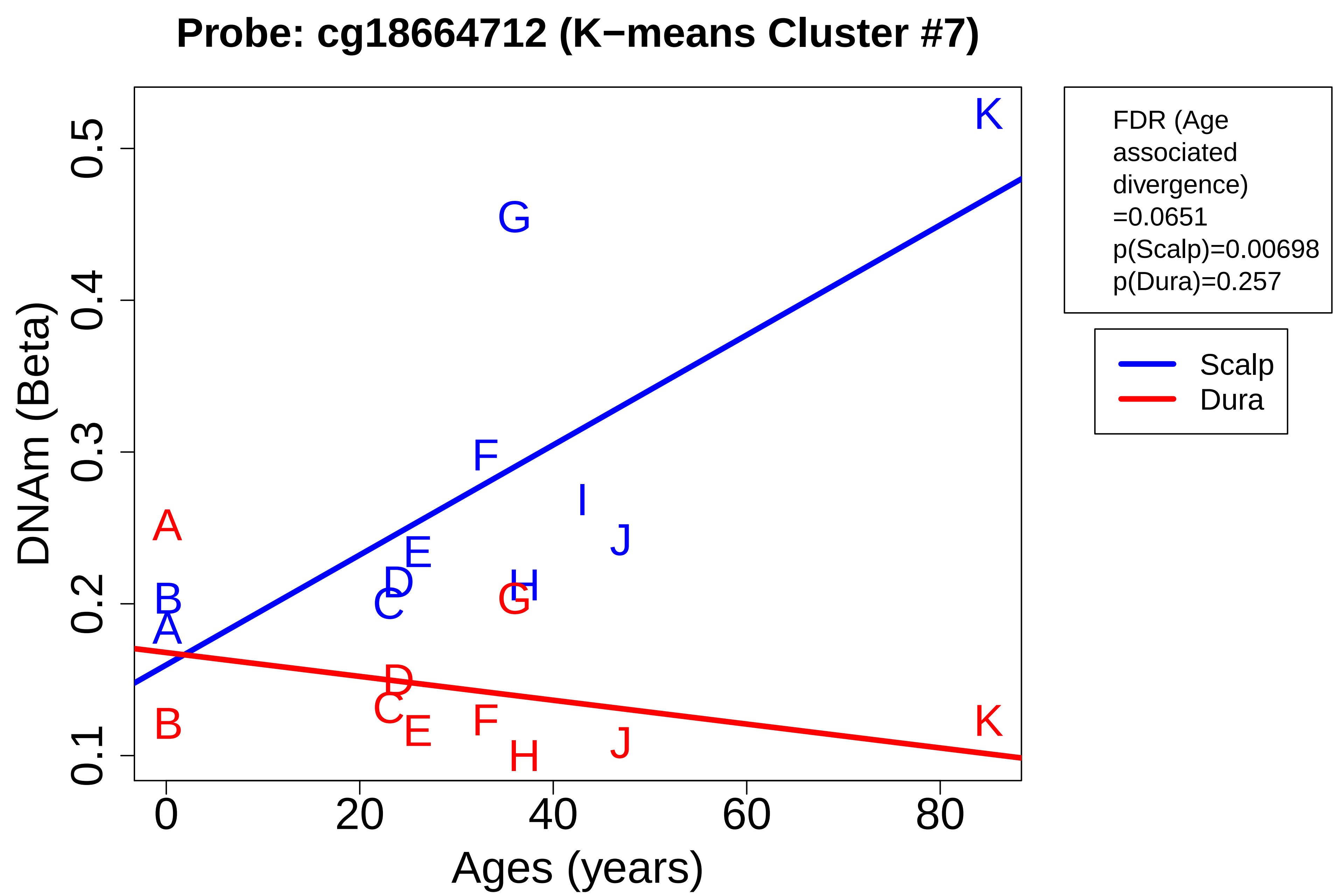
Probe: cg18664712 (K−means Cluster #7)
FDR (Age associated divergence) =0.0651
p(Scalp)=0.00698
p(Dura)=0.257
Scalp
Dura
DNAm (Beta)
0.1
0.2
0.3
0.4
0.5
0
20
40
60
80
Ages (years)
A
B
C
D
E
F
G
H
I
J
K

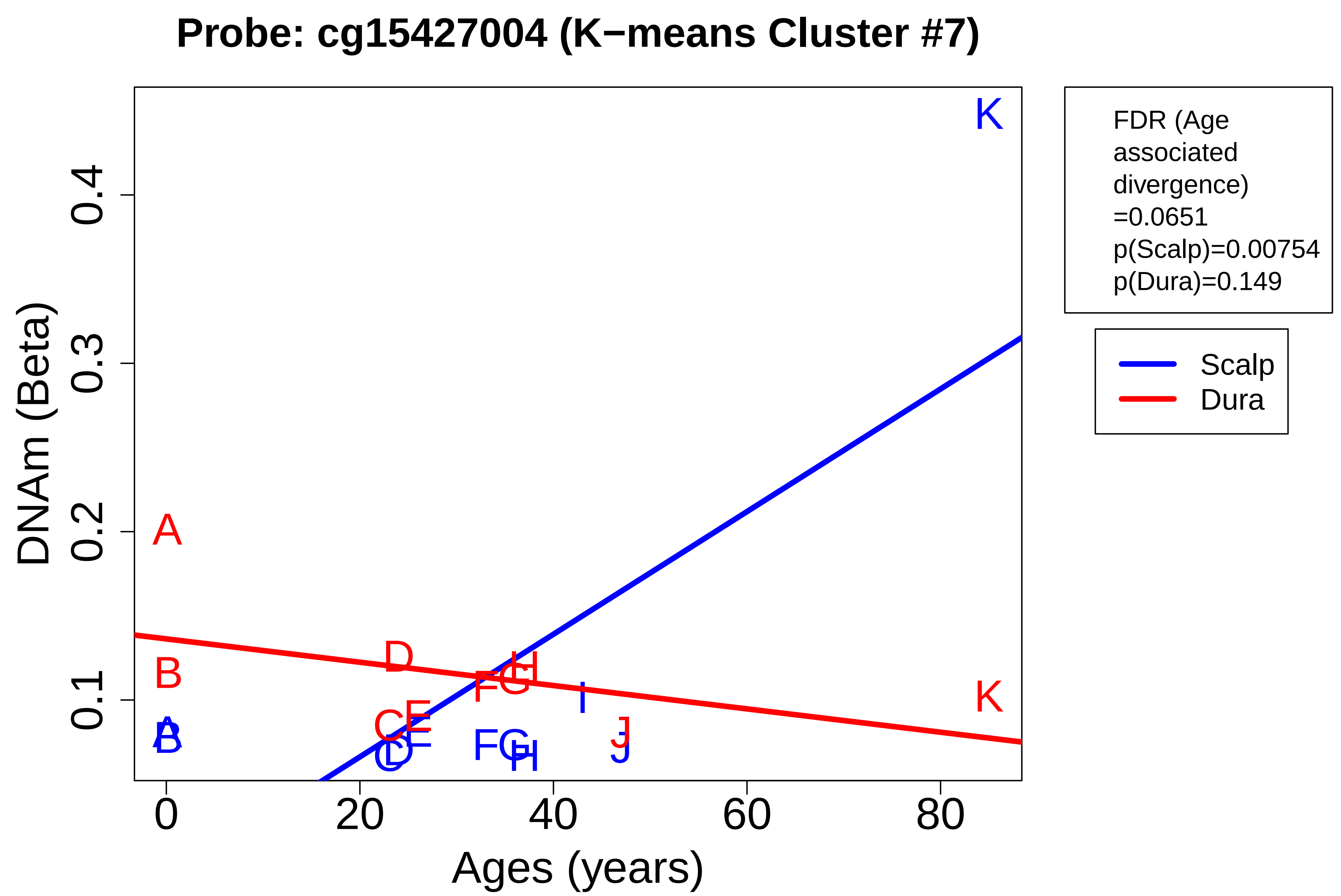

Probe: cg15427004 (K−means Cluster #7)
DNAm (Beta)
0.1
0.2
0.3
0.4
0
20
40
60
80
Ages (years)
FDR (Age associated divergence) =0.0651
p(Scalp)=0.00754
p(Dura)=0.149
Scalp
Dura

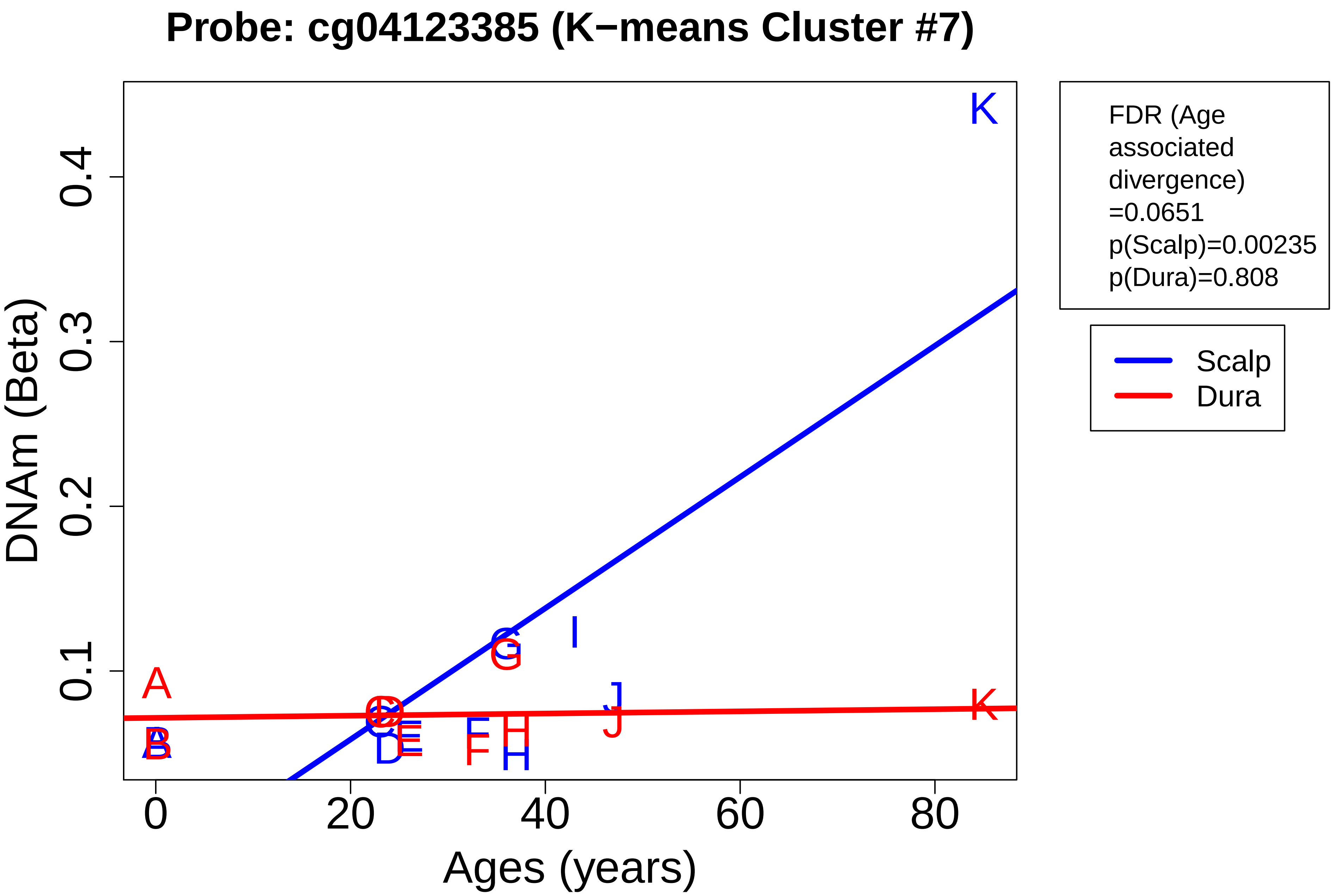

Probe: cg04123385 (K−means Cluster #7)
DNAm (Beta)
0.1
0.2
0.3
0.4
0
20
40
60
80
Ages (years)
A
B
C
D
E
F
G
H
I
J
K
FDR (Age associated divergence) =0.0651
p(Scalp)=0.00235
p(Dura)=0.808
Scalp
Dura

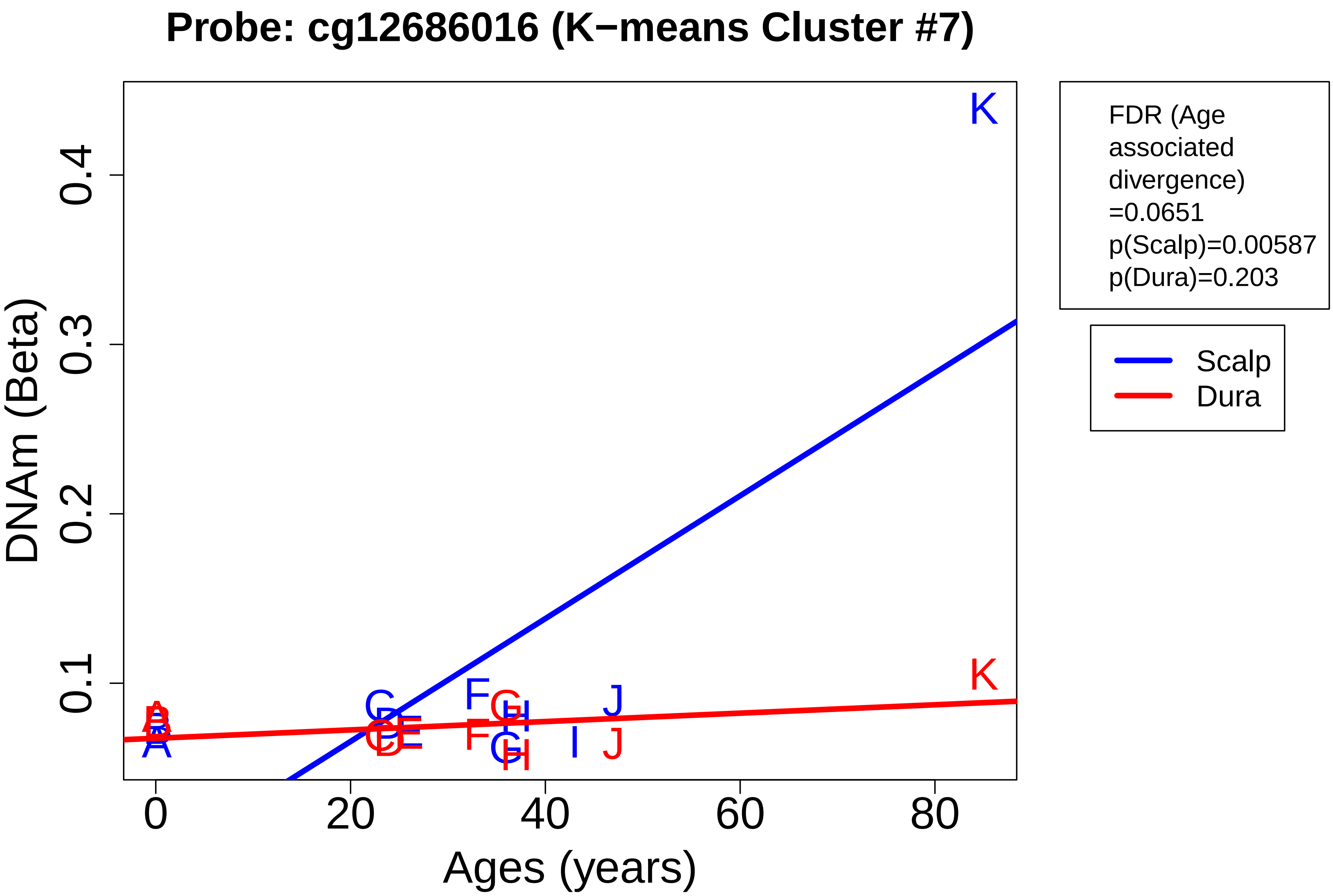

Probe: cg12686016 (K−means Cluster #7)
DNAm (Beta)
Ages (years)
0.1
0.2
0.3
0.4
0
20
40
60
80
FDR (Age associated divergence) =0.0651
p(Scalp)=0.00587
p(Dura)=0.203
Scalp
Dura
A B C D E F G H I J K
A B C D E F G H I J K

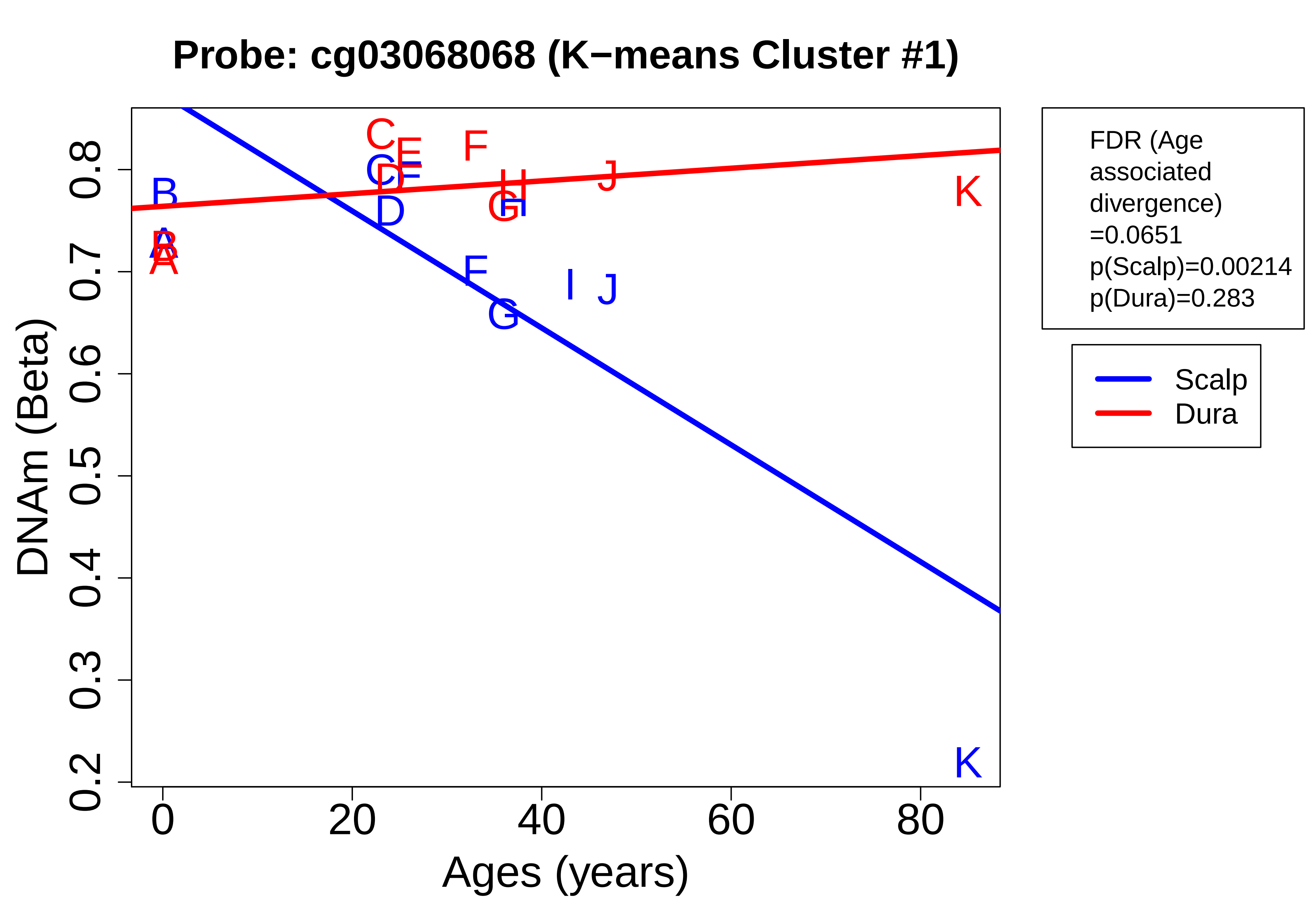
Probe: cg03068068 (K−means Cluster #1)
FDR (Age associated divergence) =0.0651
p(Scalp)=0.00214
p(Dura)=0.283
Scalp
Dura
DNAm (Beta)
Ages (years)
0.2
0.3
0.4
0.5
0.6
0.7
0.8
0
20
40
60
80

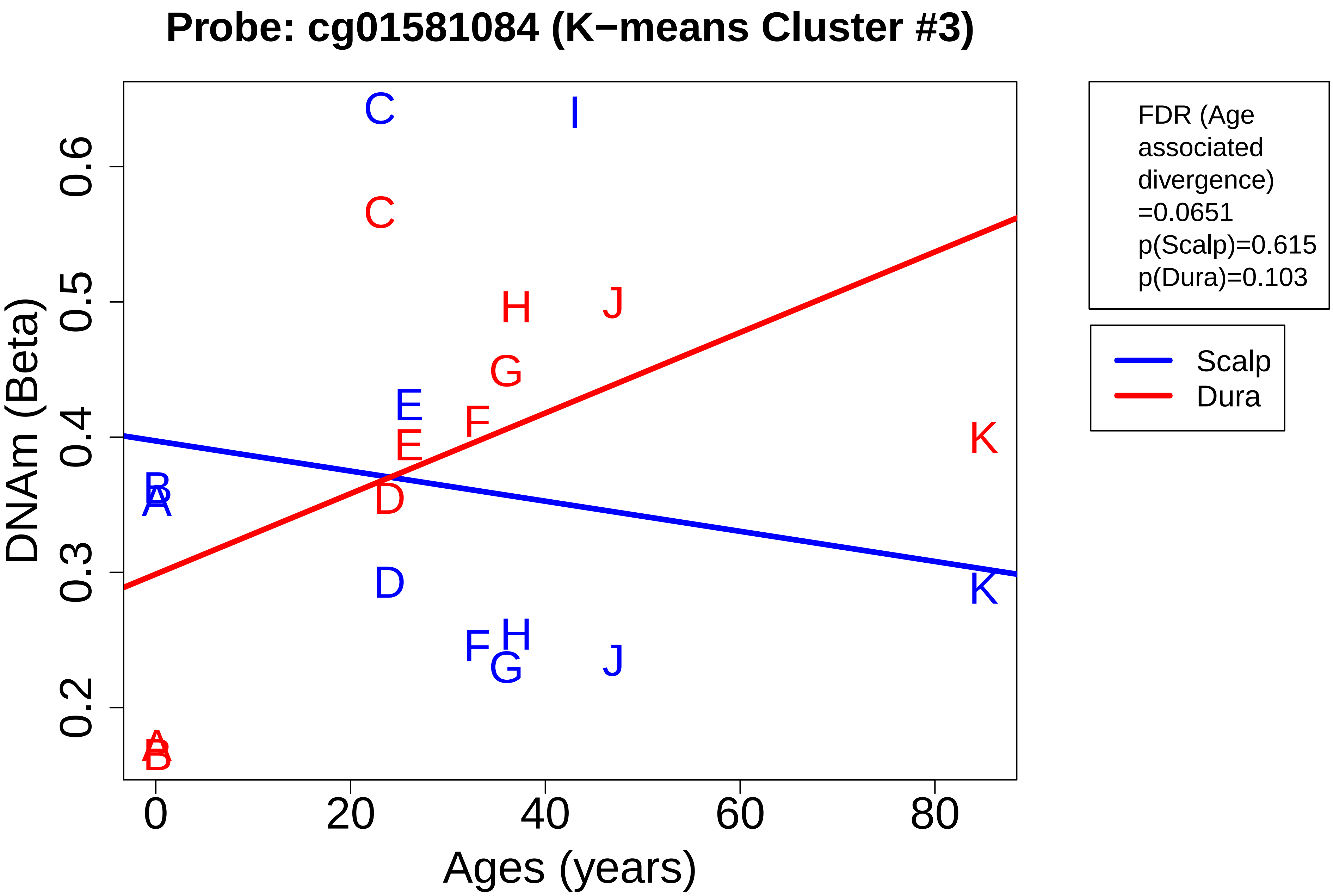

Probe: cg01581084 (K−means Cluster #3)
DNAm (Beta)
0.2
0.3
0.4
0.5
0.6
Ages (years)
0
20
40
60
80
FDR (Age associated divergence) =0.0651
p(Scalp)=0.615
p(Dura)=0.103
Scalp
Dura

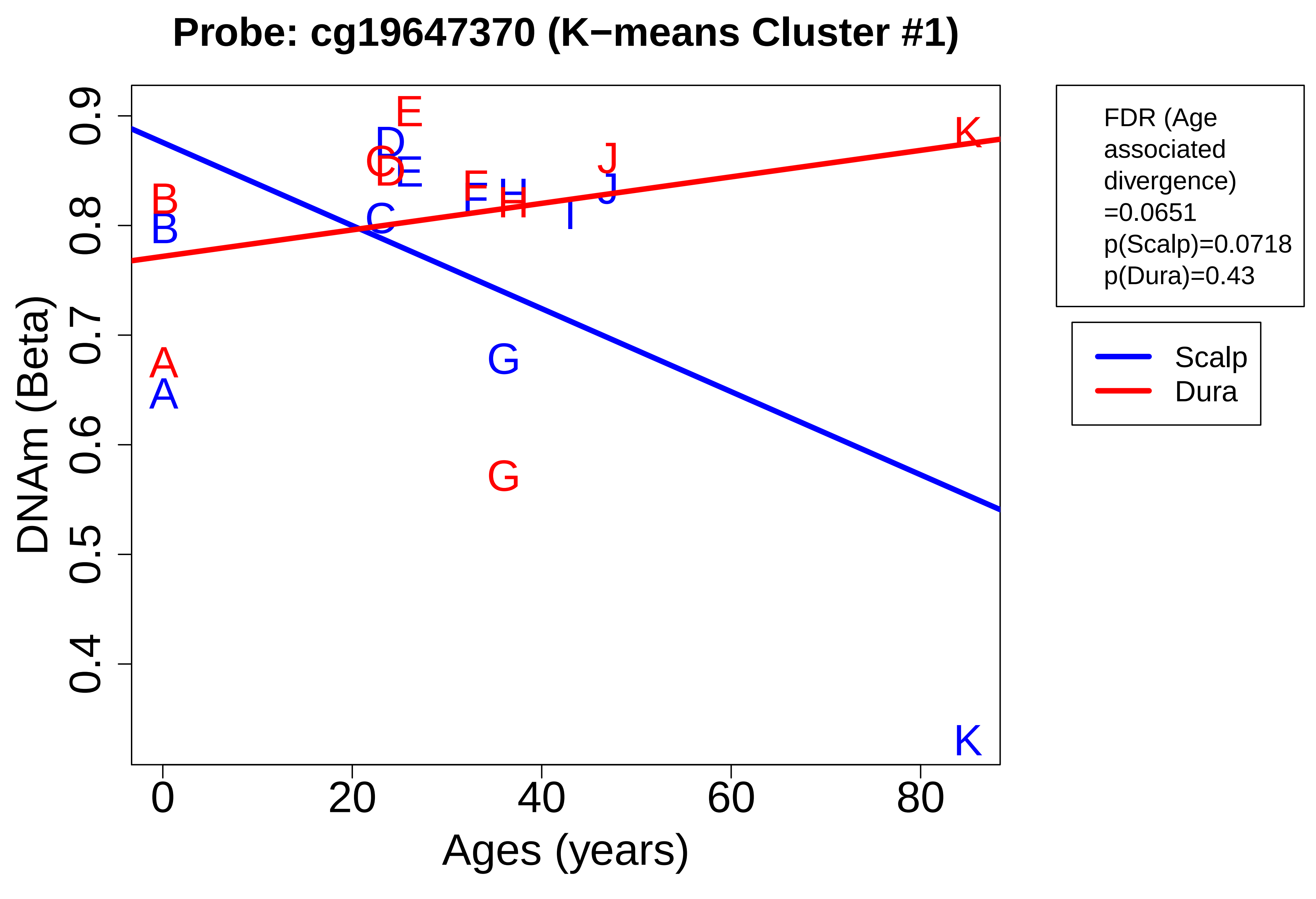

Probe: cg19647370 (K−means Cluster #1)
DNAm (Beta)
0.4
0.5
0.6
0.7
0.8
0.9
0
20
40
60
80
Ages (years)
FDR (Age associated divergence) =0.0651
p(Scalp)=0.0718
p(Dura)=0.43
Scalp
Dura

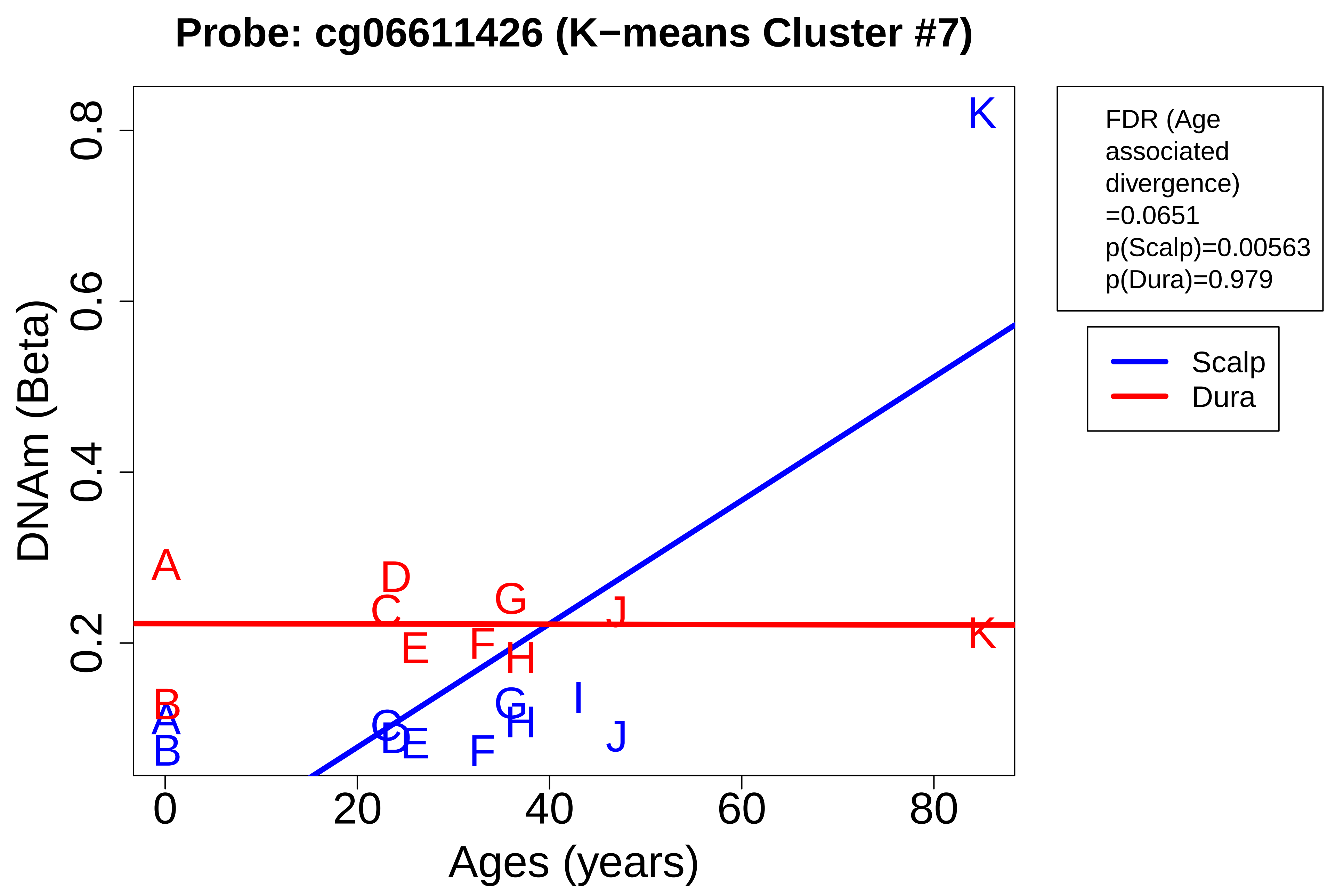

Probe: cg06611426 (K−means Cluster #7)
DNAm (Beta)
0.2
0.4
0.6
0.8
Ages (years)
0
20
40
60
80
FDR (Age associated divergence) =0.0651
p(Scalp)=0.00563
p(Dura)=0.979
Scalp
Dura

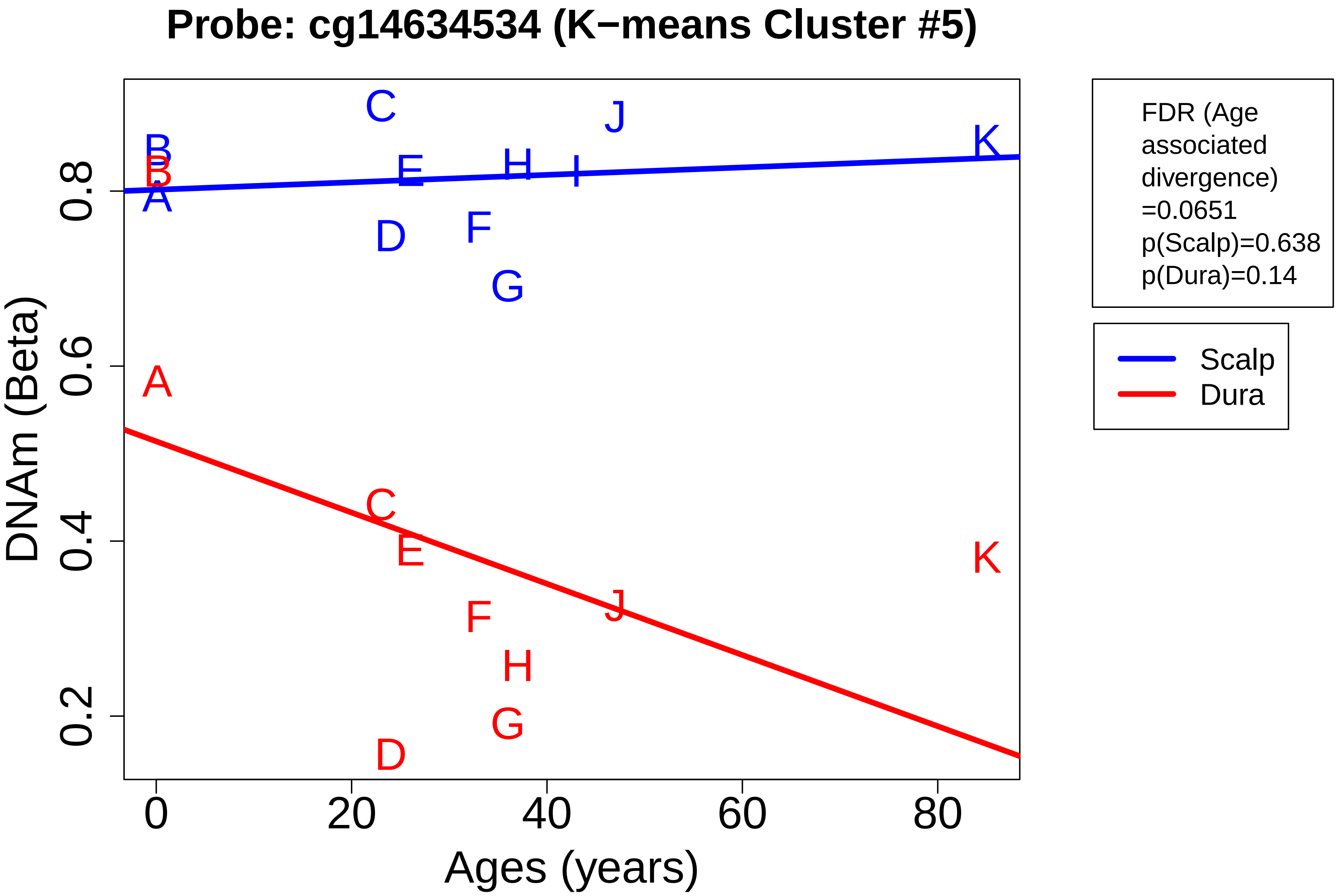
Probe: cg14634534 (K−means Cluster #5)
DNAm (Beta)
0.2
0.4
0.6
0.8
0
20
40
60
80
Ages (years)
A
B
C
D
E
F
G
H
I
J
K
FDR (Age associated divergence) =0.0651
p(Scalp)=0.638
p(Dura)=0.14
Scalp
Dura

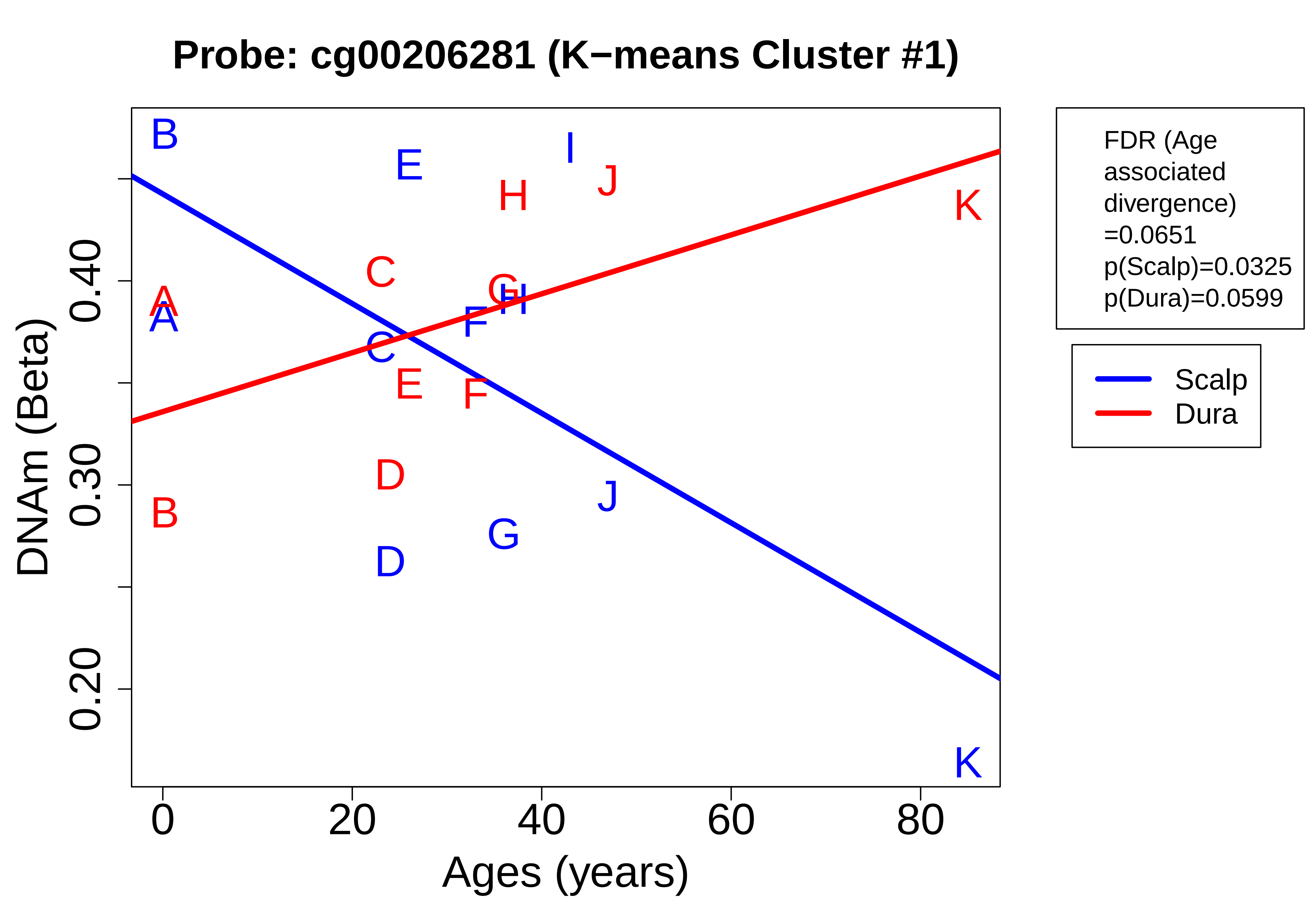

Probe: cg00206281 (K−means Cluster #1)
DNAm (Beta)
0.20
0.30
0.40
0
20
40
60
80
Ages (years)
B
E
I
J
H
K
C
G
H
A
A
F
C
E
F
D
J
B
G
D
K
FDR (Age associated divergence) =0.0651
p(Scalp)=0.0325
p(Dura)=0.0599
Scalp
Dura

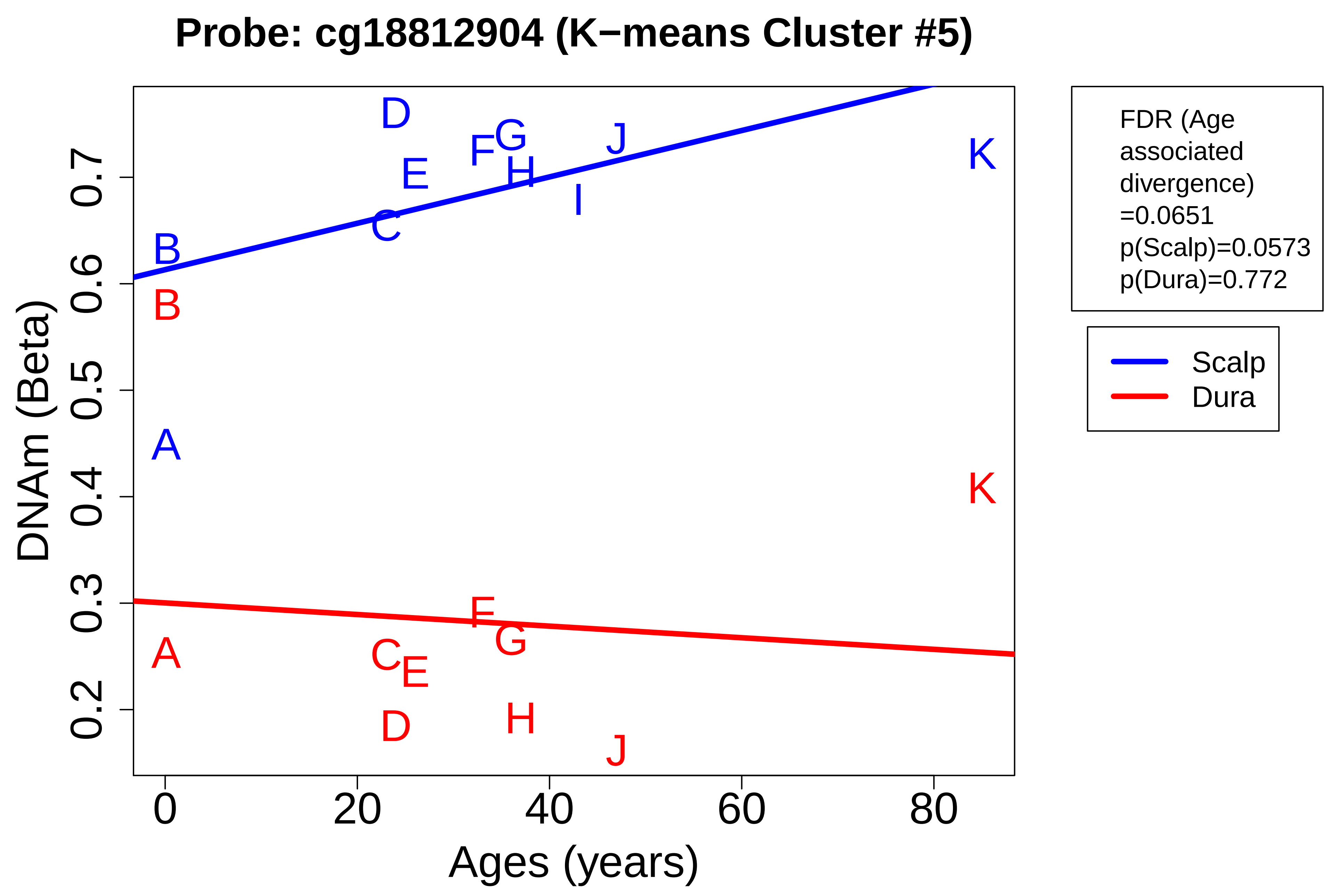

Probe: cg18812904 (K−means Cluster #5)
DNAm (Beta)
0.2
0.3
0.4
0.5
0.6
0.7
0
20
40
60
80
Ages (years)
A
B
C
D
E
F
G
H
I
J
K
FDR (Age associated divergence) =0.0651
p(Scalp)=0.0573
p(Dura)=0.772
Scalp
Dura

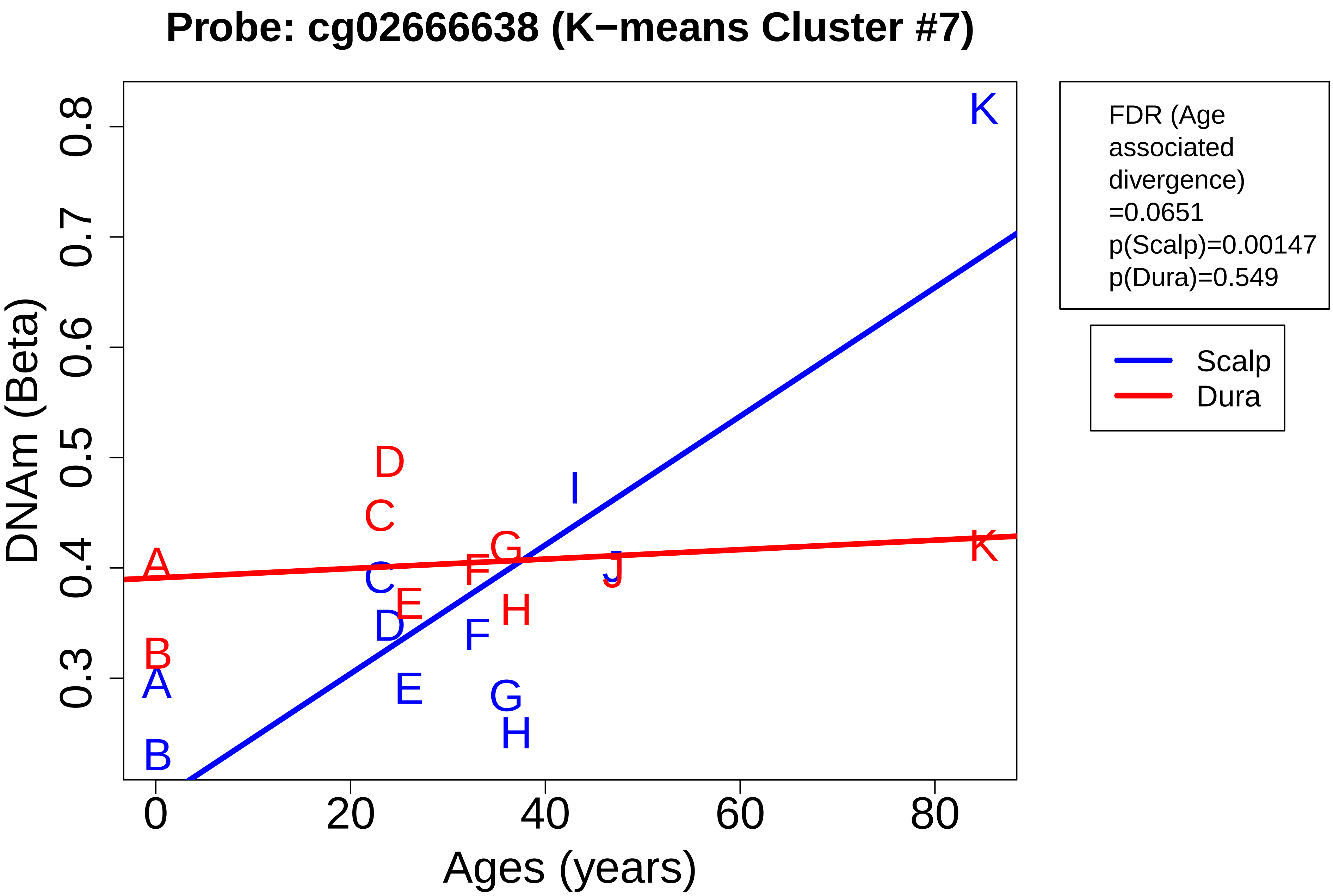

Probe: cg02666638 (K−means Cluster #7)
DNAm (Beta)
0.3
0.4
0.5
0.6
0.7
0.8
Ages (years)
0
20
40
60
80
FDR (Age associated divergence) =0.0651
p(Scalp)=0.00147
p(Dura)=0.549
Scalp
Dura
A
B
C
D
E
F
G
H
I
J
K

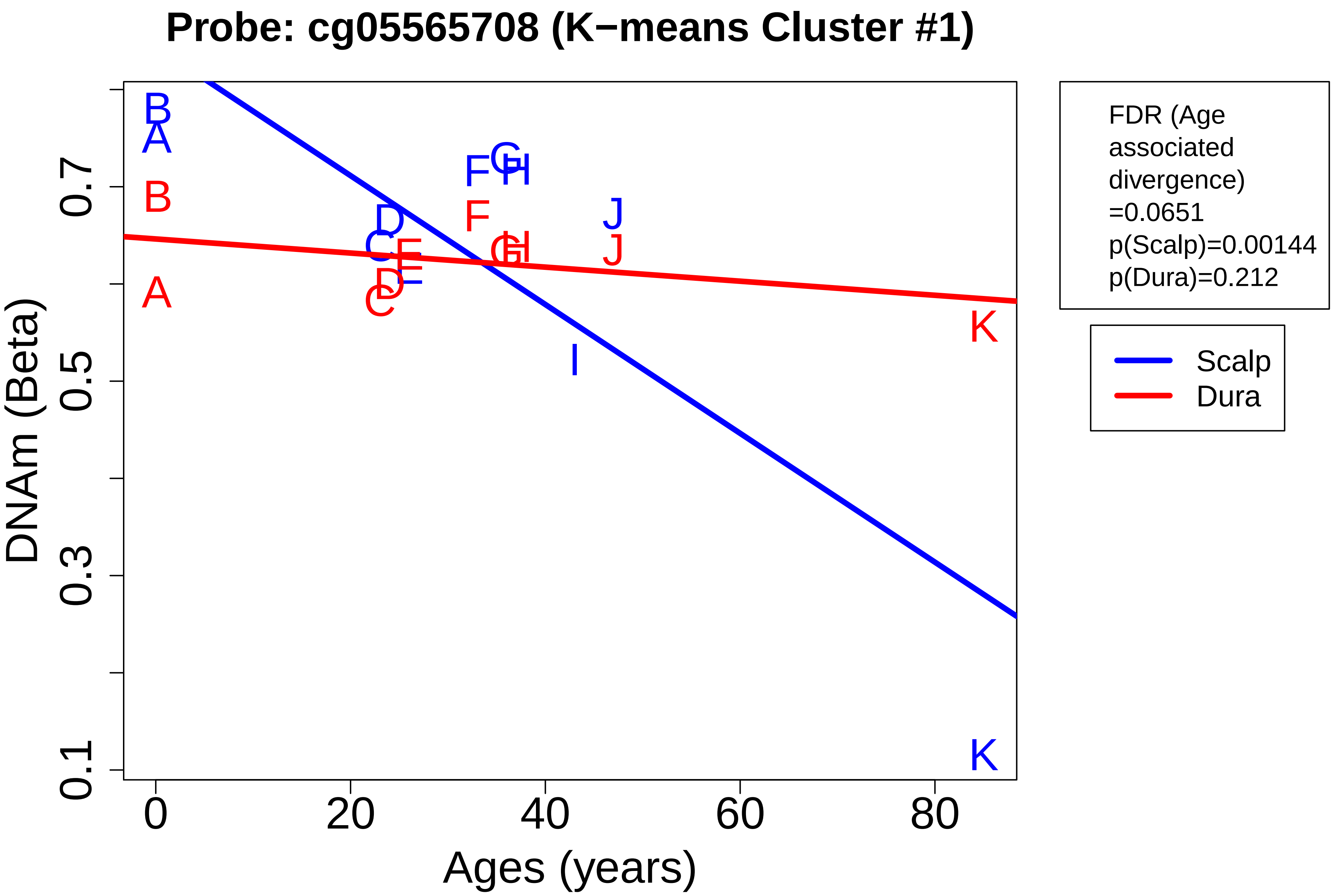

Probe: cg05565708 (K−means Cluster #1)
DNAm (Beta)
0.1
0.3
0.5
0.7
0
20
40
60
80
Ages (years)
FDR (Age associated divergence) =0.0651
p(Scalp)=0.00144
p(Dura)=0.212
Scalp
Dura

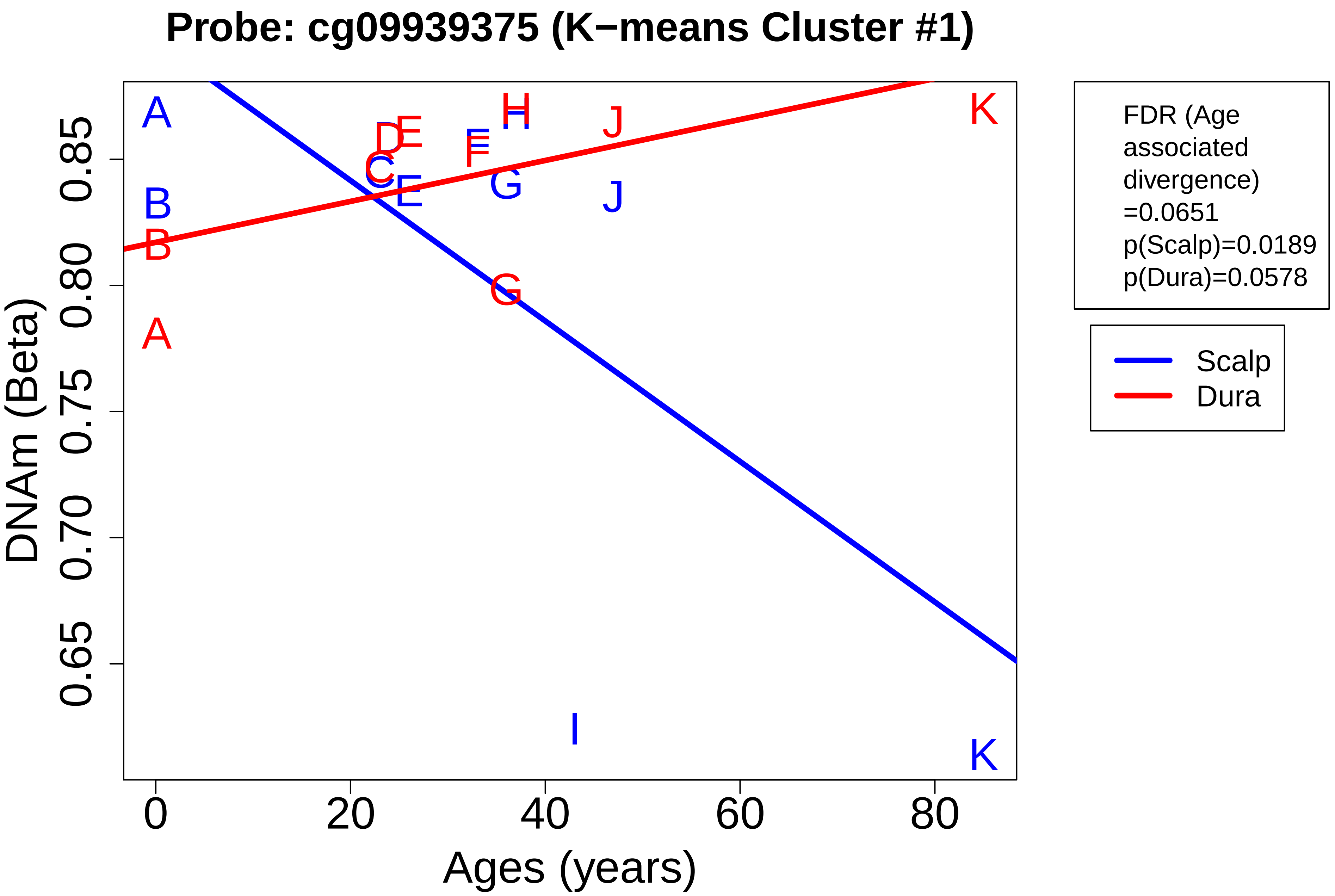

Probe: cg09939375 (K−means Cluster #1)
DNAm (Beta)
0.65
0.70
0.75
0.80
0.85
Ages (years)
0
20
40
60
80
A
B
C
D
E
F
G
H
I
J
K
FDR (Age associated divergence) =0.0651
p(Scalp)=0.0189
p(Dura)=0.0578
Scalp
Dura

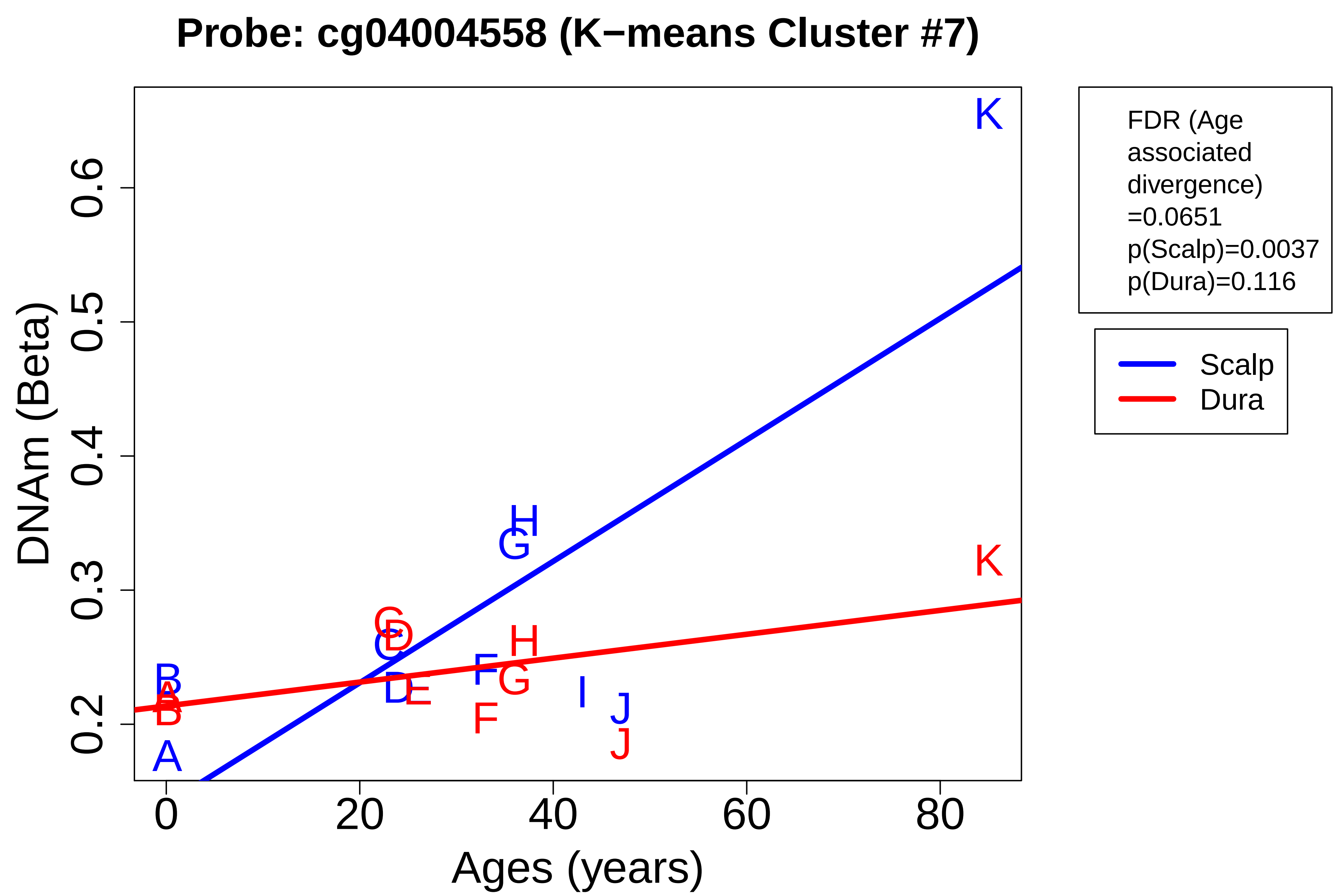
Probe: cg04004558 (K−means Cluster #7)
DNAm (Beta)
0.2
0.3
0.4
0.5
0.6
Ages (years)
0
20
40
60
80
FDR (Age associated divergence) =0.0651
p(Scalp)=0.0037
p(Dura)=0.116
Scalp
Dura

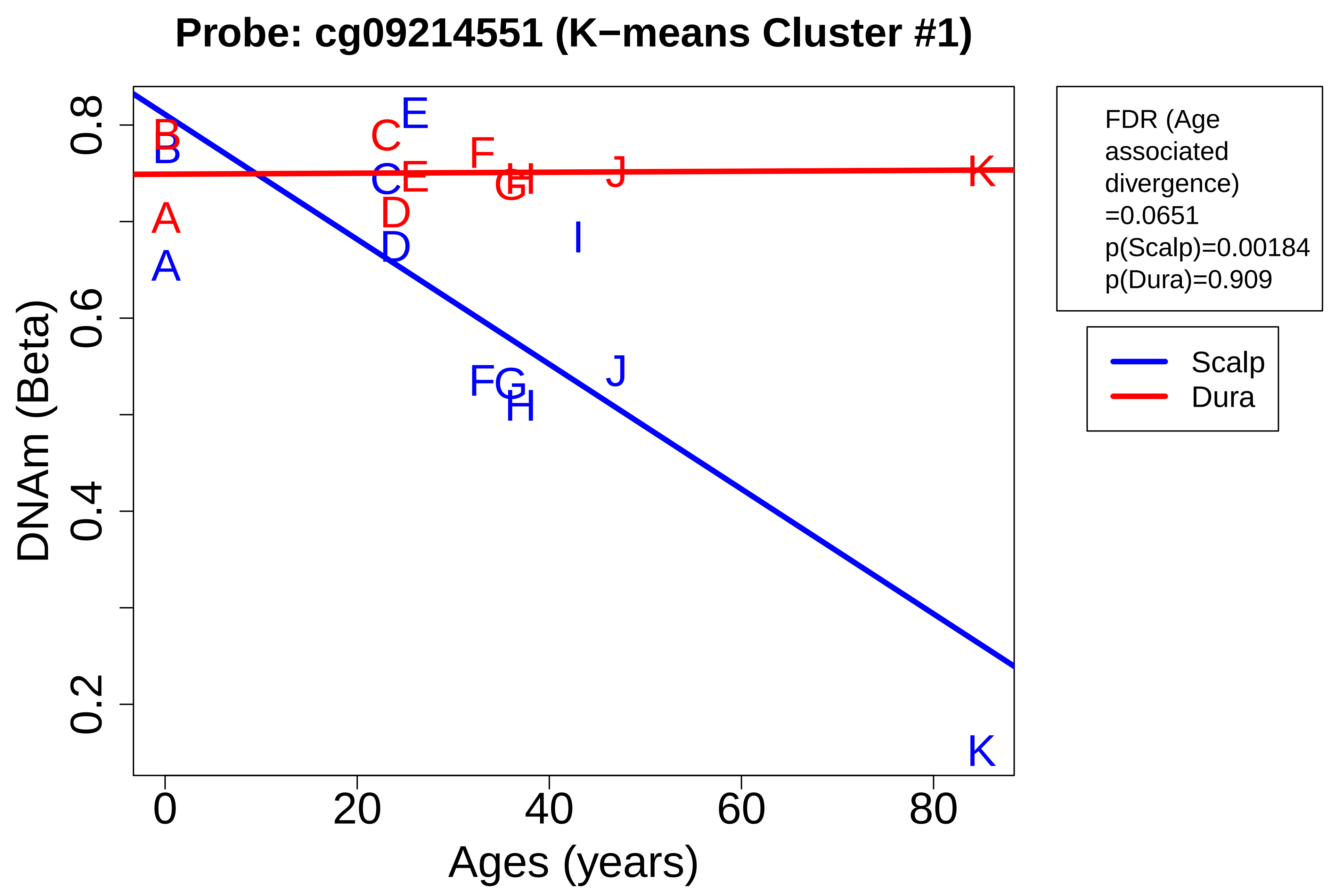
Probe: cg09214551 (K−means Cluster #1)
DNAm (Beta)
0.2
0.4
0.6
0.8
Ages (years)
0
20
40
60
80
FDR (Age associated divergence) =0.0651
p(Scalp)=0.00184
p(Dura)=0.909
Scalp
Dura
A
B
C
D
E
F
G
H
I
J
K

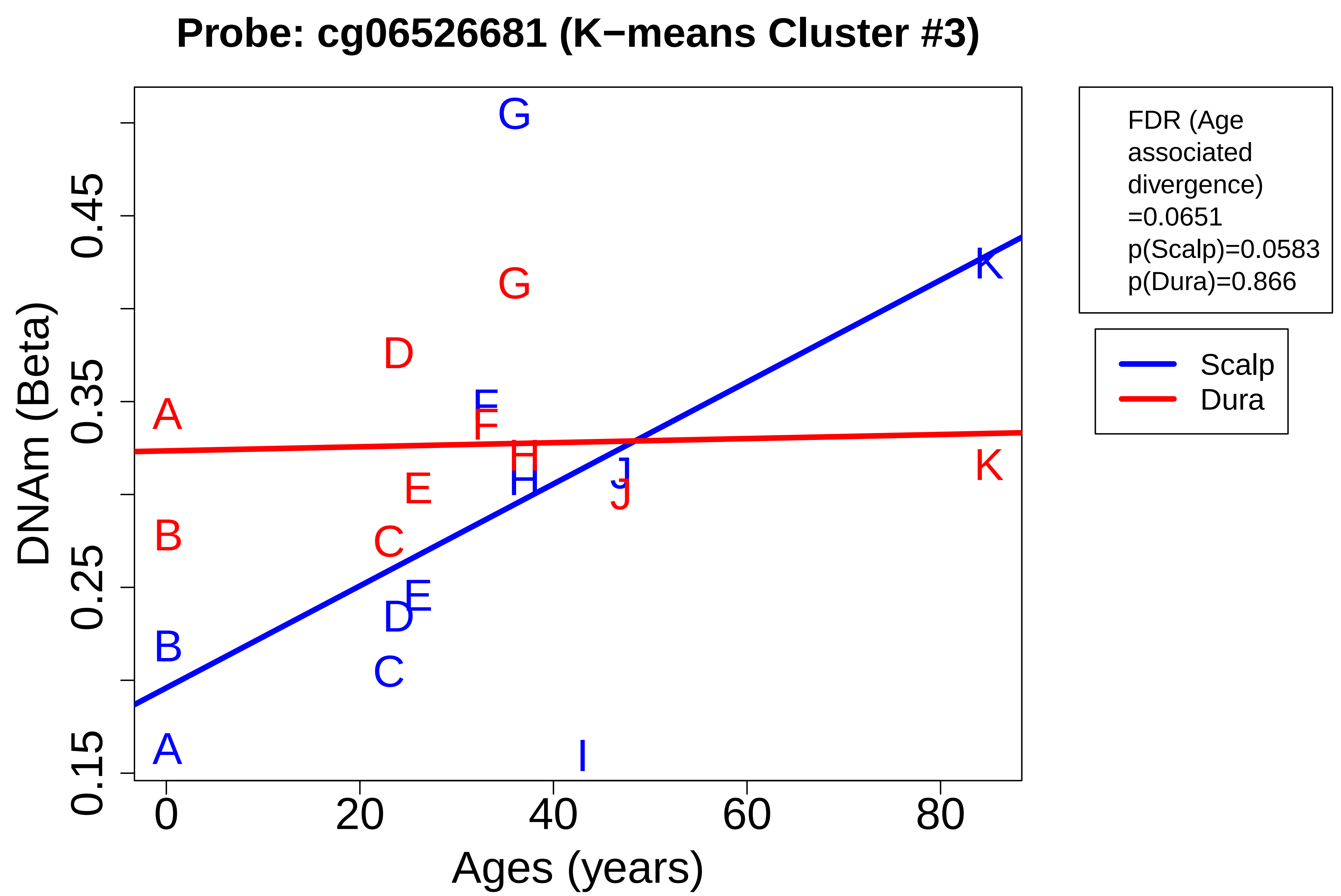

Probe: cg06526681 (K−means Cluster #3)
DNAm (Beta)
0.15
0.25
0.35
0.45
0
20
40
60
80
Ages (years)
A
B
C
D
E
F
G
H
I
J
K
FDR (Age associated divergence) =0.0651
p(Scalp)=0.0583
p(Dura)=0.866
Scalp
Dura

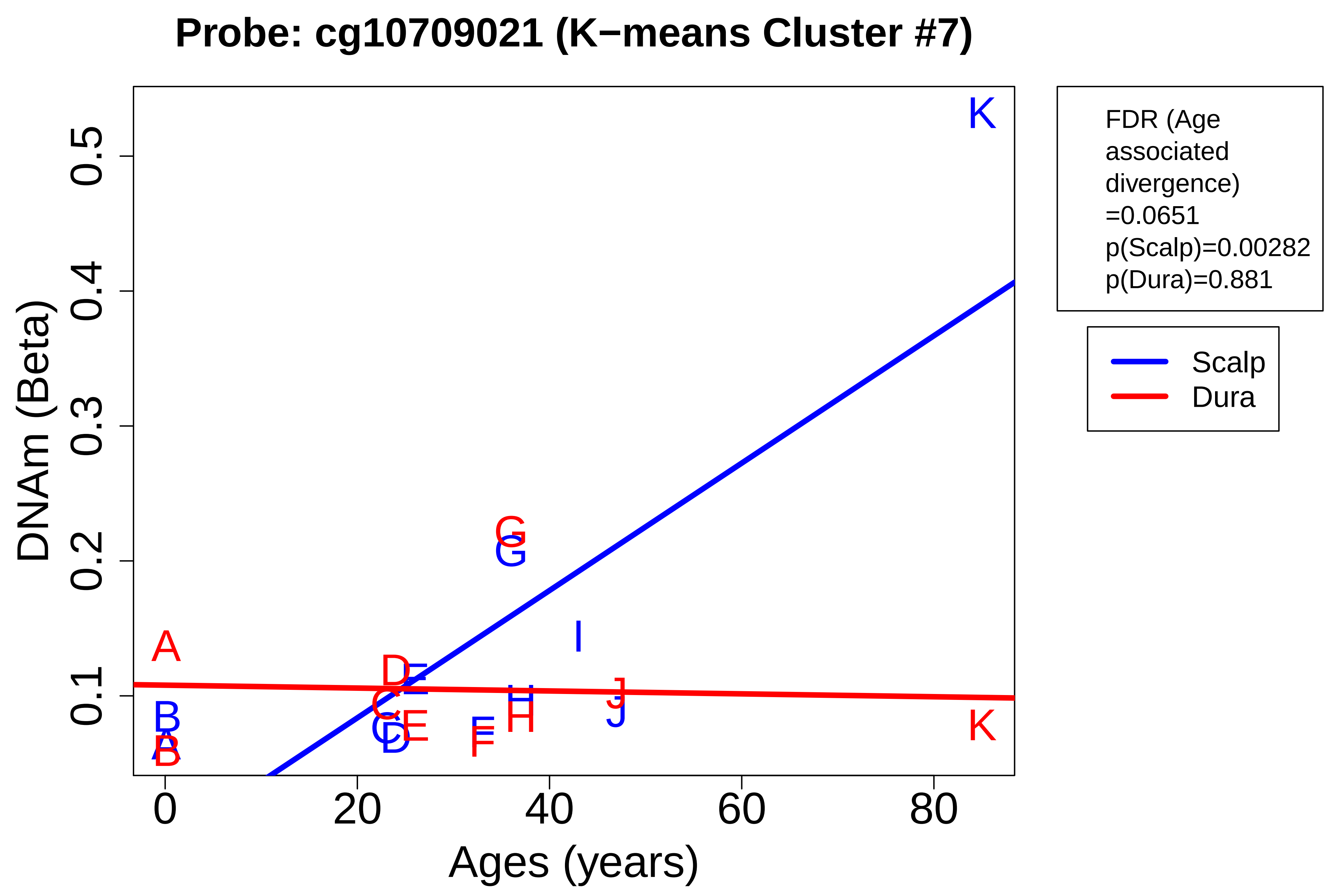
Probe: cg10709021 (K−means Cluster #7)
DNAm (Beta)
0.1
0.2
0.3
0.4
0.5
0
20
40
60
80
Ages (years)
A
B
C
D
E
F
G
H
I
J
K
FDR (Age associated divergence) =0.0651
p(Scalp)=0.00282
p(Dura)=0.881
Scalp
Dura

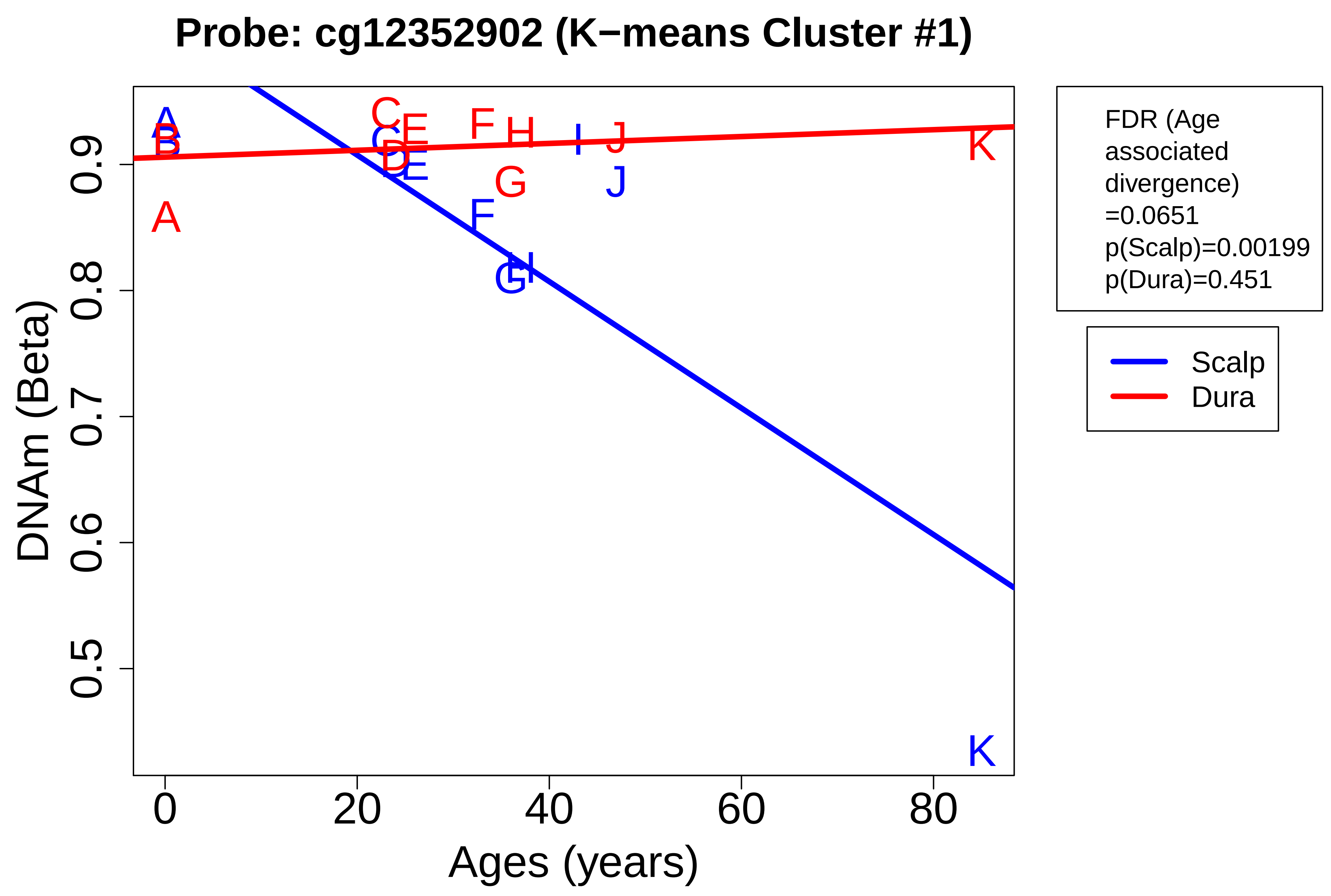

Probe: cg12352902 (K−means Cluster #1)
DNAm (Beta)
0.5
0.6
0.7
0.8
0.9
0
20
40
60
80
Ages (years)
FDR (Age associated divergence) =0.0651
p(Scalp)=0.00199
p(Dura)=0.451
Scalp
Dura

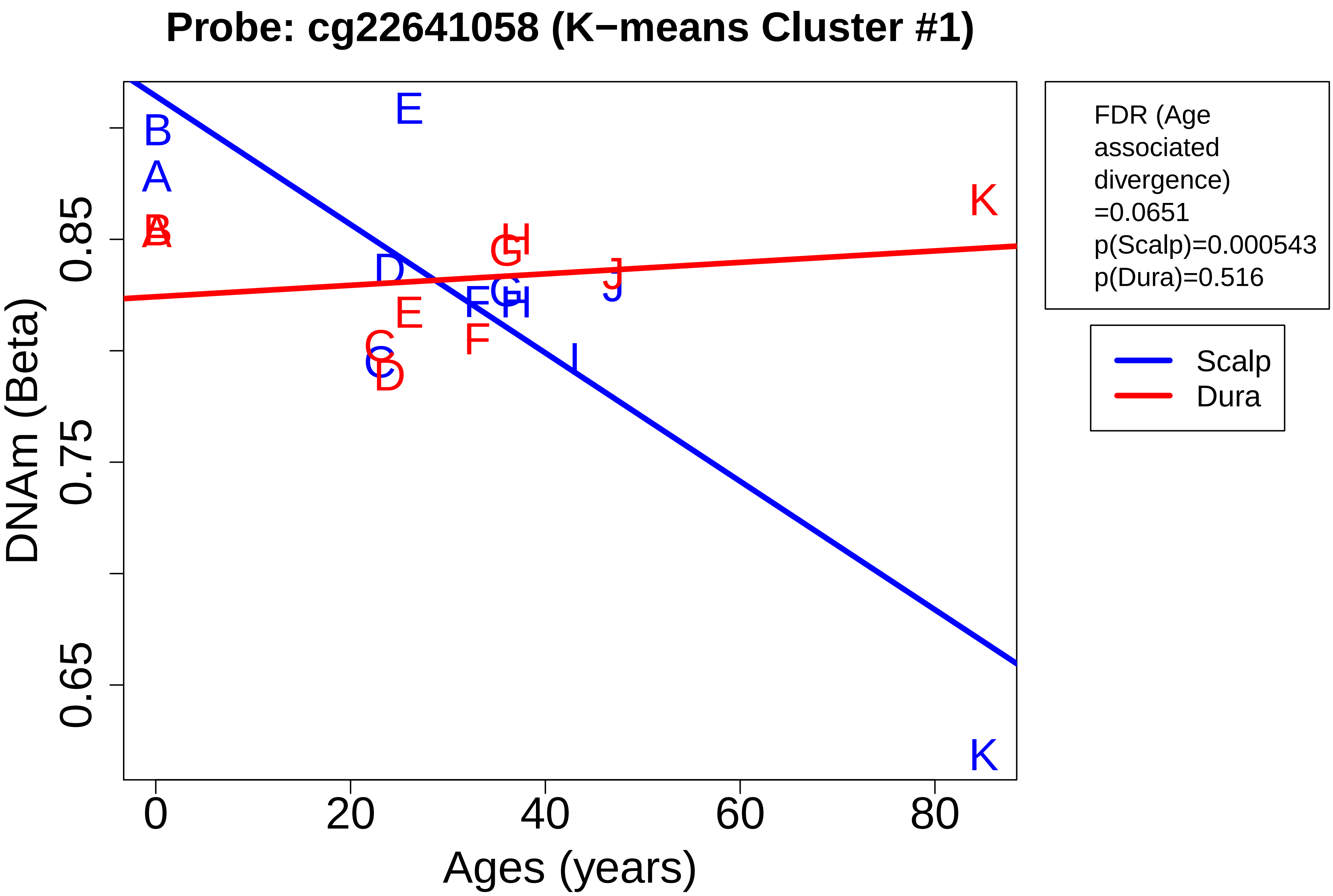

Probe: cg22641058 (K−means Cluster #1)
FDR (Age associated divergence) =0.0651
p(Scalp)=0.000543
p(Dura)=0.516
Scalp
Dura
DNAm (Beta)
0.65
0.75
0.85
0
20
40
60
80
Ages (years)

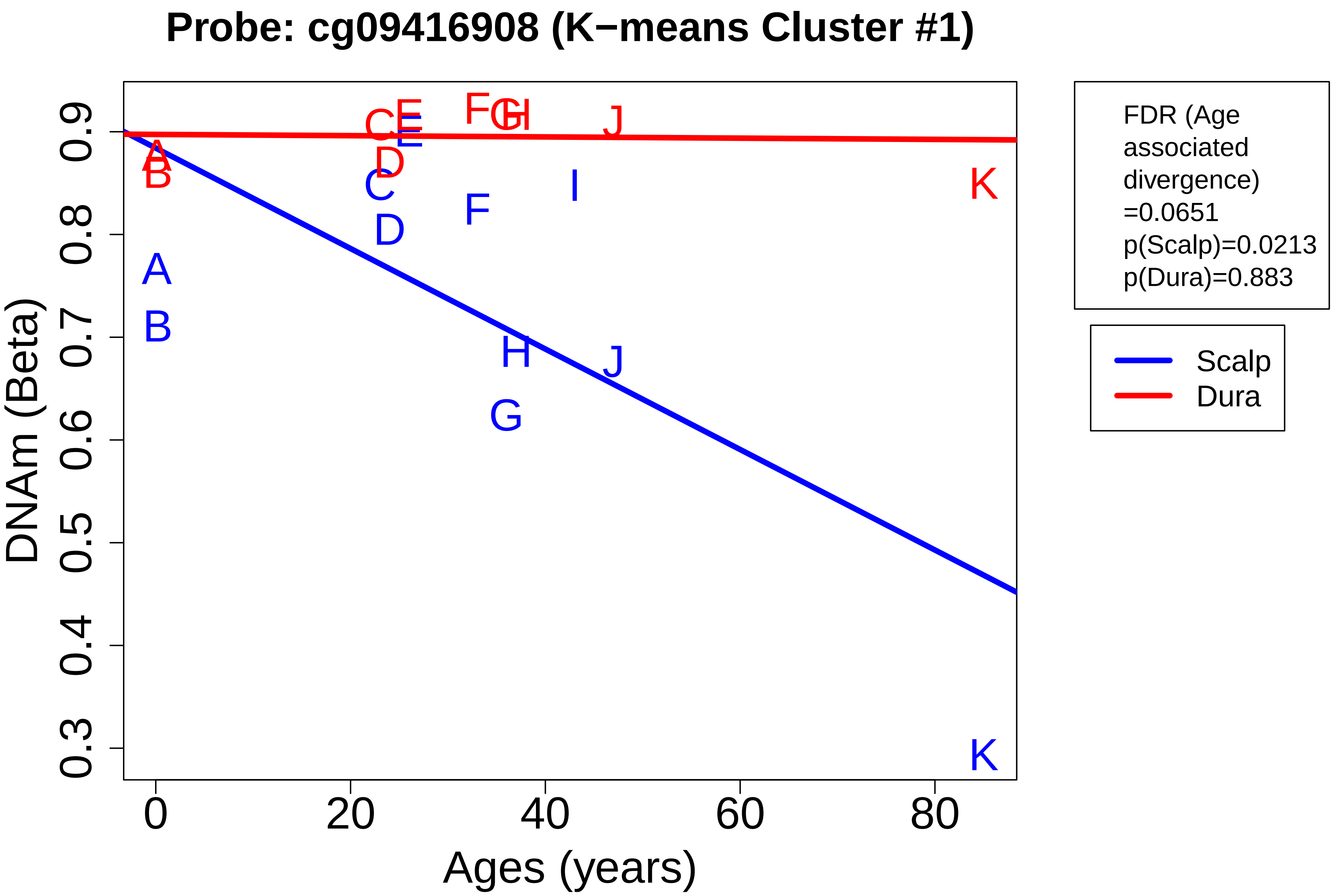

Probe: cg09416908 (K−means Cluster #1)
DNAm (Beta)
0.3
0.4
0.5
0.6
0.7
0.8
0.9
0
20
40
60
80
Ages (years)
A
B
C
D
E
F
G
H
I
J
K
FDR (Age associated divergence) =0.0651
p(Scalp)=0.0213
p(Dura)=0.883
Scalp
Dura

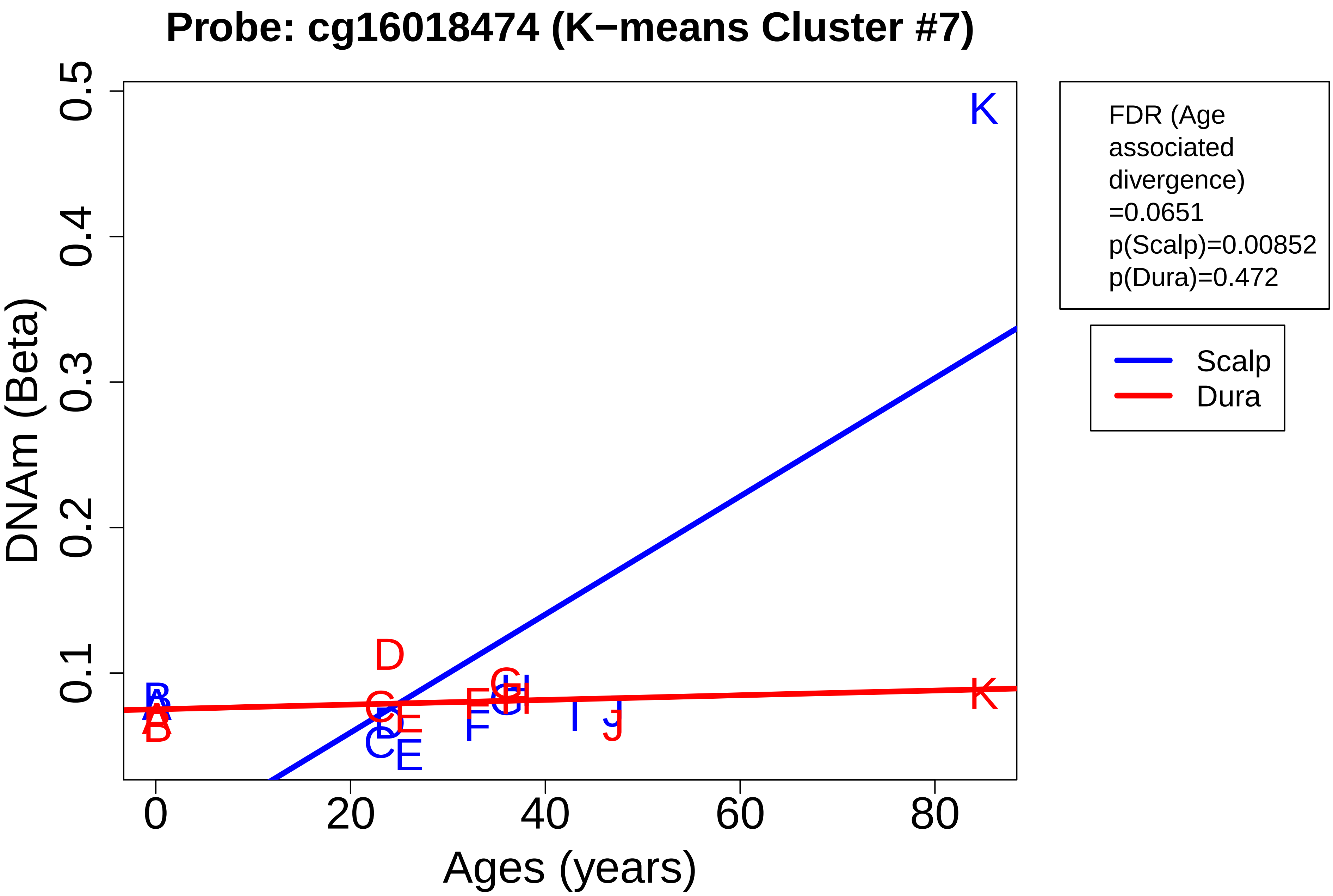

Probe: cg16018474 (K−means Cluster #7)
DNAm (Beta)
0.1
0.2
0.3
0.4
0.5
0
20
40
60
80
Ages (years)
FDR (Age associated divergence) =0.0651
p(Scalp)=0.00852
p(Dura)=0.472
Scalp
Dura

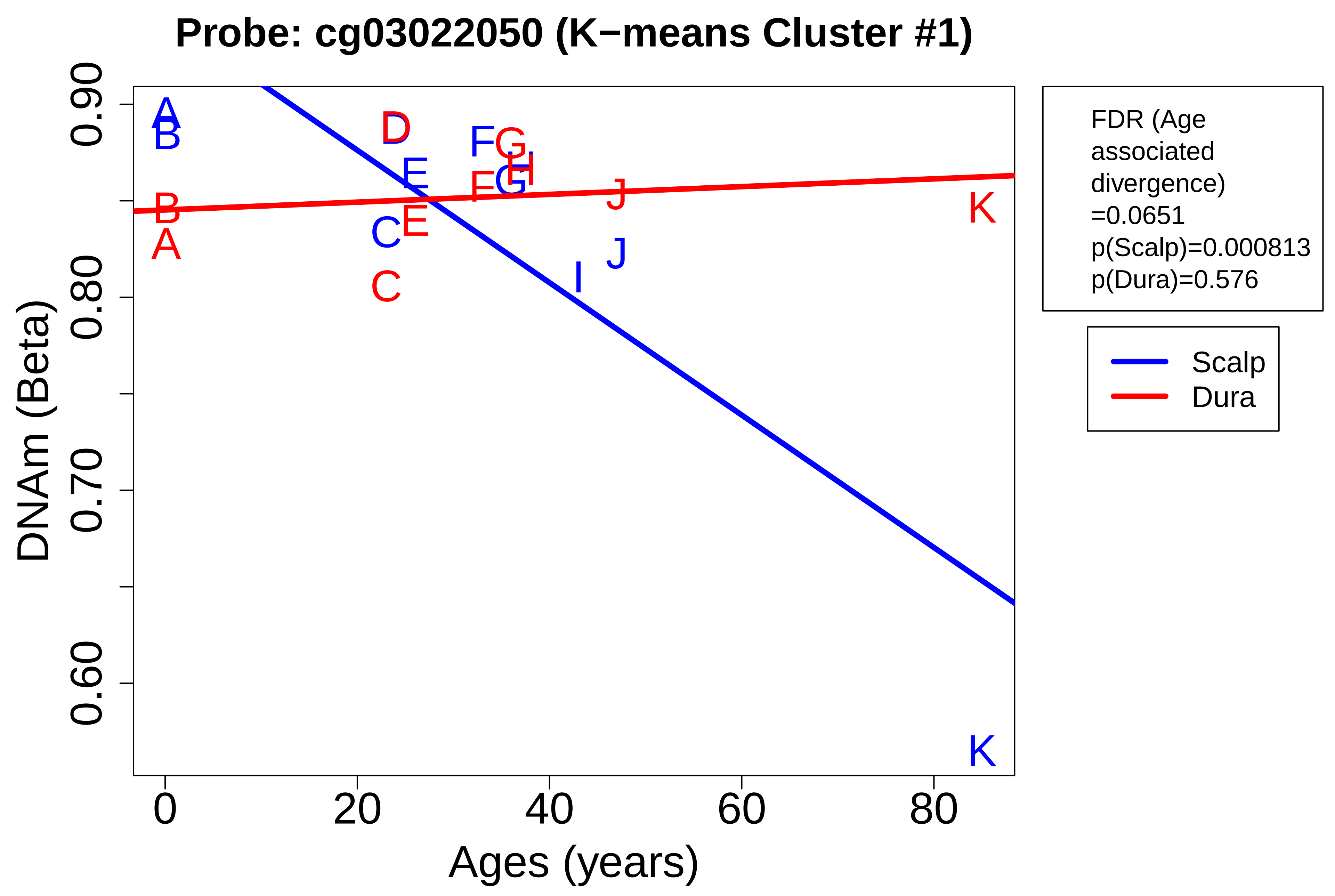
Probe: cg03022050 (K−means Cluster #1)
DNAm (Beta)
0.60
0.70
0.80
0.90
Ages (years)
0
20
40
60
80
FDR (Age associated divergence) =0.0651
p(Scalp)=0.000813
p(Dura)=0.576
Scalp
Dura
A
B
C
D
E
F
G
H
I
J
K

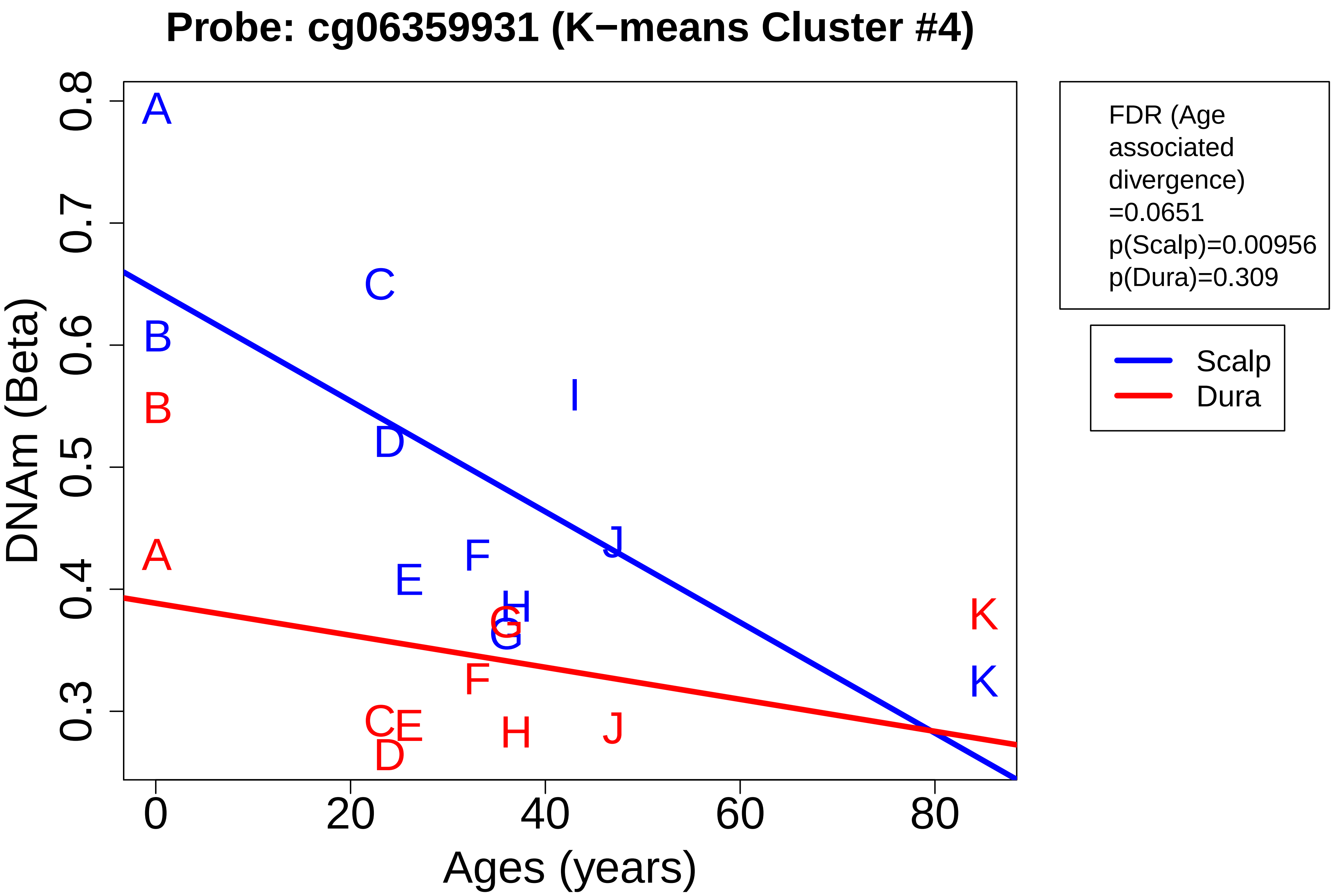
Probe: cg06359931 (K−means Cluster #4)
DNAm (Beta)
0.3
0.4
0.5
0.6
0.7
0.8
0
20
40
60
80
Ages (years)
A
B
C
D
E
F
G
H
I
J
K
A
B
CE
D
F
G
H
J
K
FDR (Age associated divergence) =0.0651
p(Scalp)=0.00956
p(Dura)=0.309
Scalp
Dura

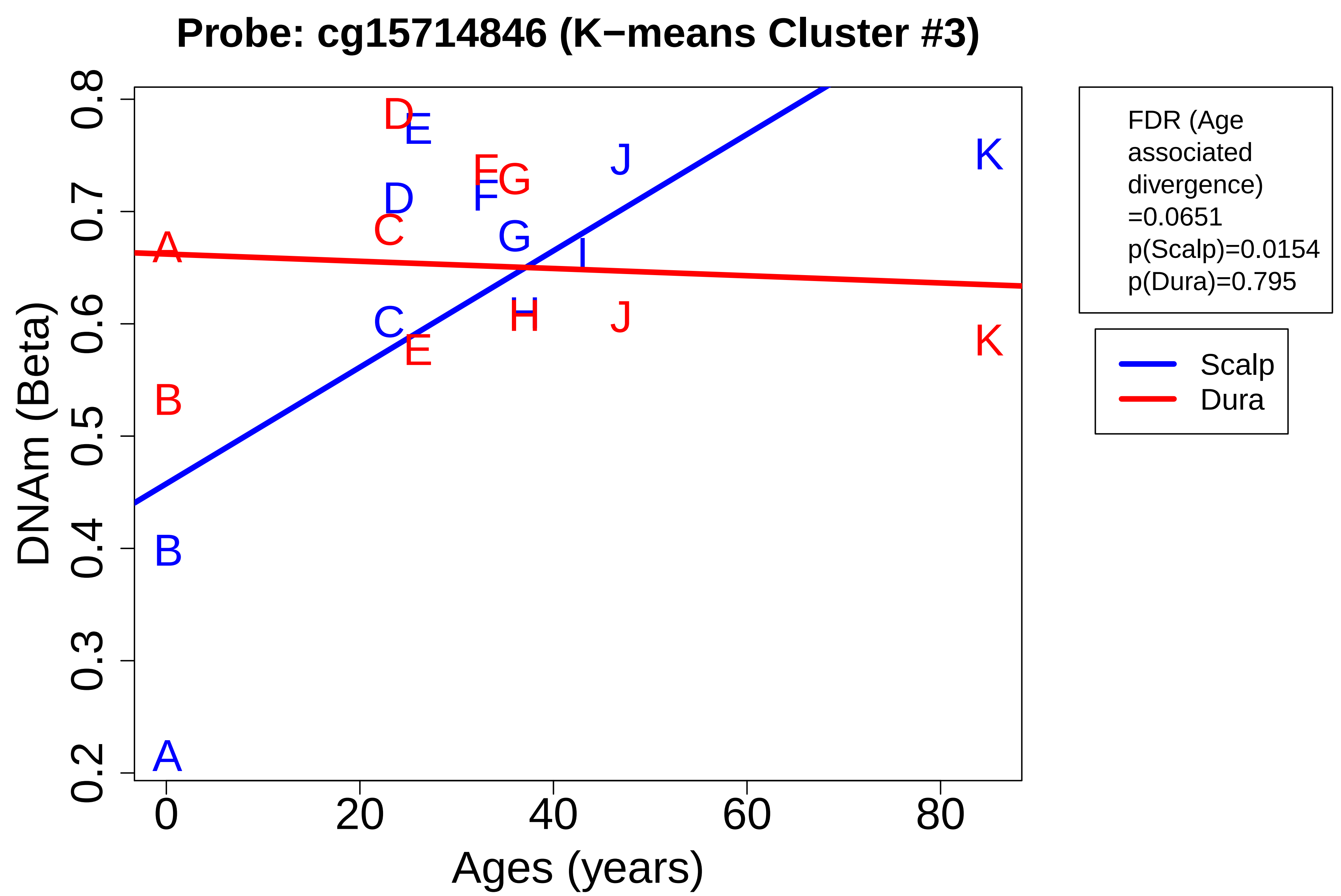

Probe: cg15714846 (K−means Cluster #3)
DNAm (Beta)
0.2
0.3
0.4
0.5
0.6
0.7
0.8
Ages (years)
0
20
40
60
80
A
B
C
D
E
F
G
H
I
J
K
FDR (Age associated divergence) =0.0651
p(Scalp)=0.0154
p(Dura)=0.795
Scalp
Dura

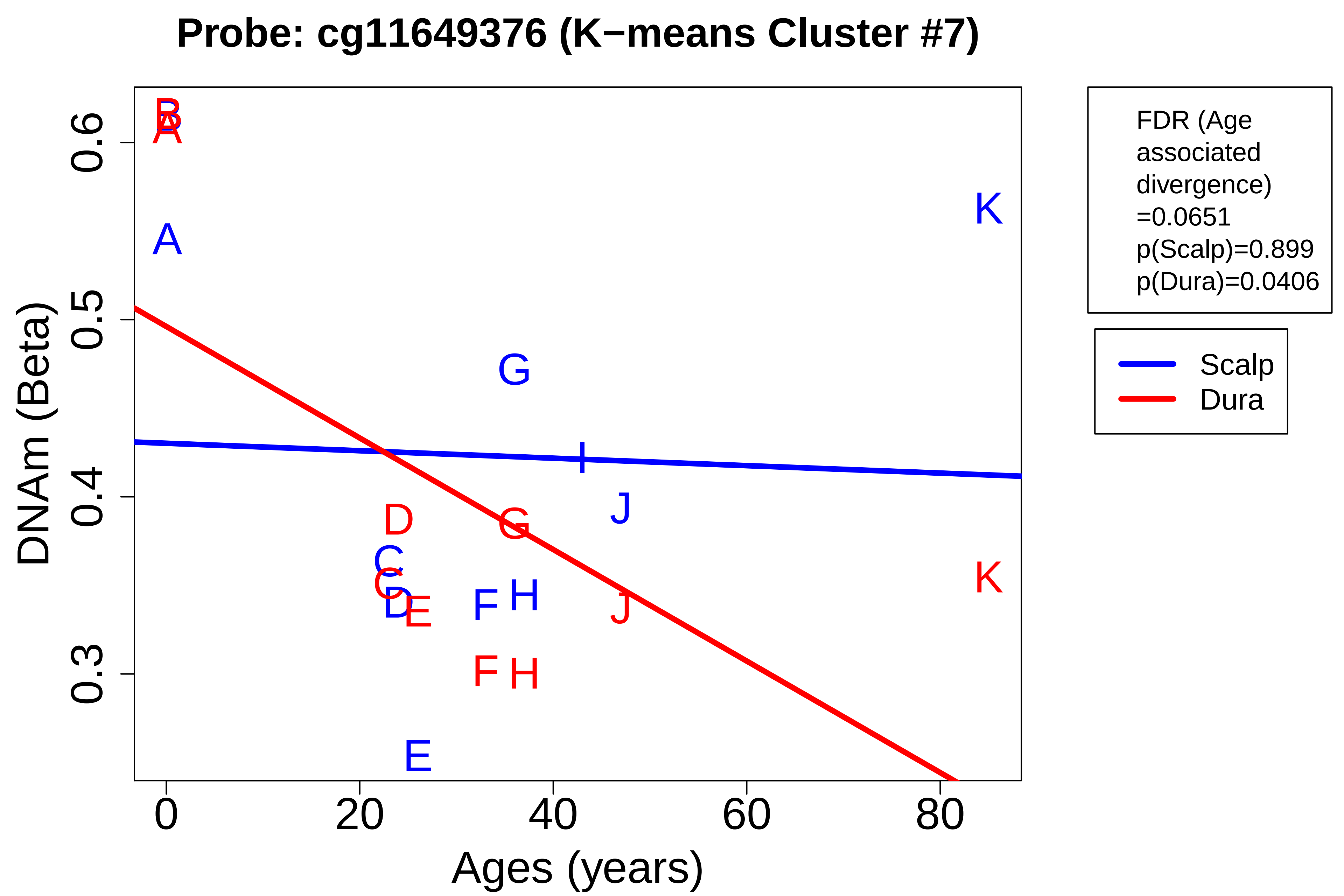

Probe: cg11649376 (K−means Cluster #7)
DNAm (Beta)
0.3
0.4
0.5
0.6
0
20
40
60
80
Ages (years)
A
B
C
D
E
F
G
H
J
K
FDR (Age associated divergence) =0.0651
p(Scalp)=0.899
p(Dura)=0.0406
Scalp
Dura

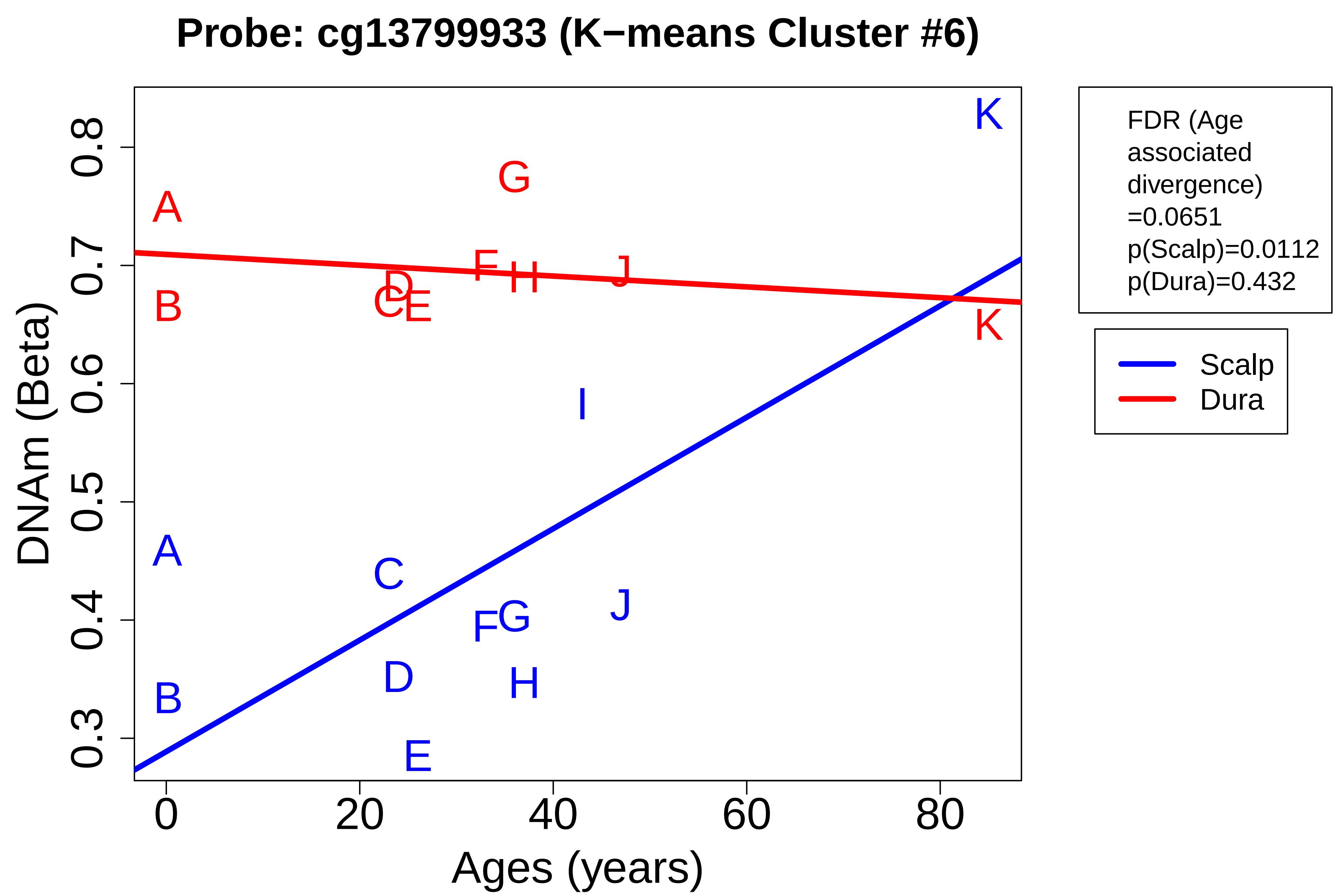
Probe: cg13799933 (K−means Cluster #6)
DNAm (Beta)
Ages (years)
0.3
0.4
0.5
0.6
0.7
0.8
0
20
40
60
80
FDR (Age associated divergence) =0.0651
p(Scalp)=0.0112
p(Dura)=0.432
Scalp
Dura

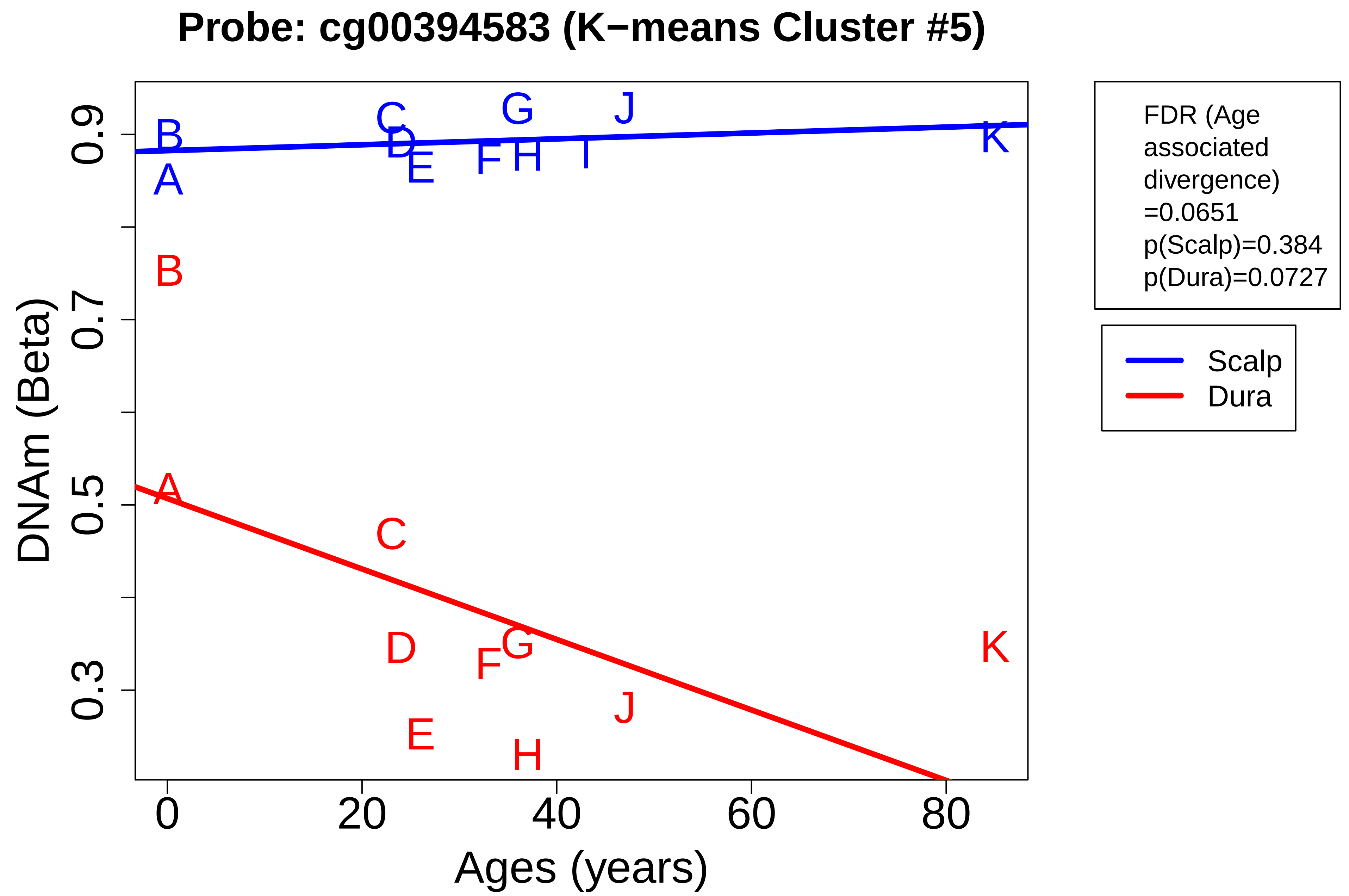

Probe: cg00394583 (K−means Cluster #5)
DNAm (Beta)
0.9
0.7
0.5
0.3
0
20
40
60
80
Ages (years)
A
B
C
D
E
F
G
H
I
J
K
FDR (Age associated divergence) =0.0651
p(Scalp)=0.384
p(Dura)=0.0727
Scalp
Dura

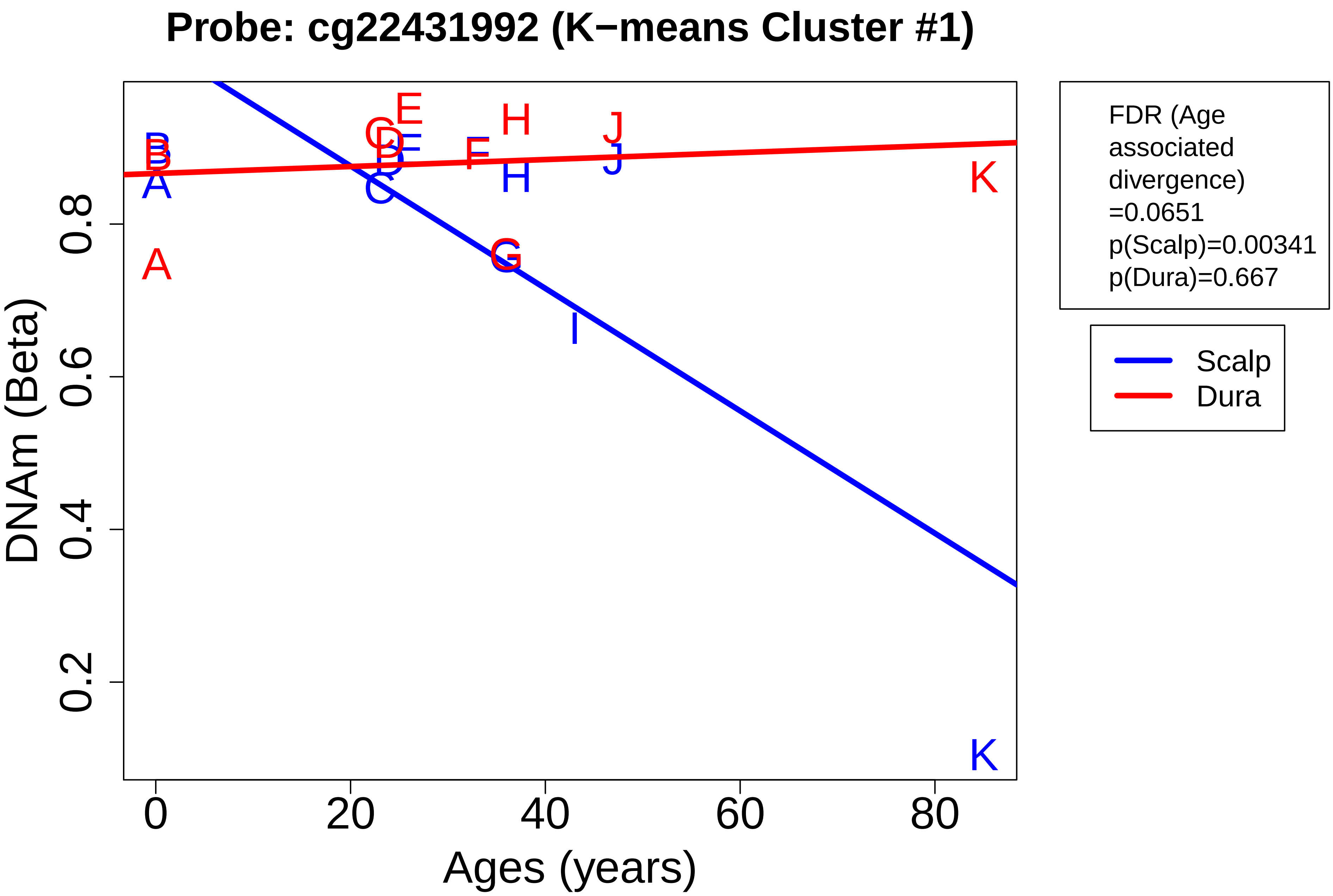
Probe: cg22431992 (K−means Cluster #1)
DNAm (Beta)
Ages (years)
0.2
0.4
0.6
0.8
0
20
40
60
80
A
B
C
D
E
F
G
H
I
J
K
FDR (Age associated divergence) =0.0651
p(Scalp)=0.00341
p(Dura)=0.667
Scalp
Dura

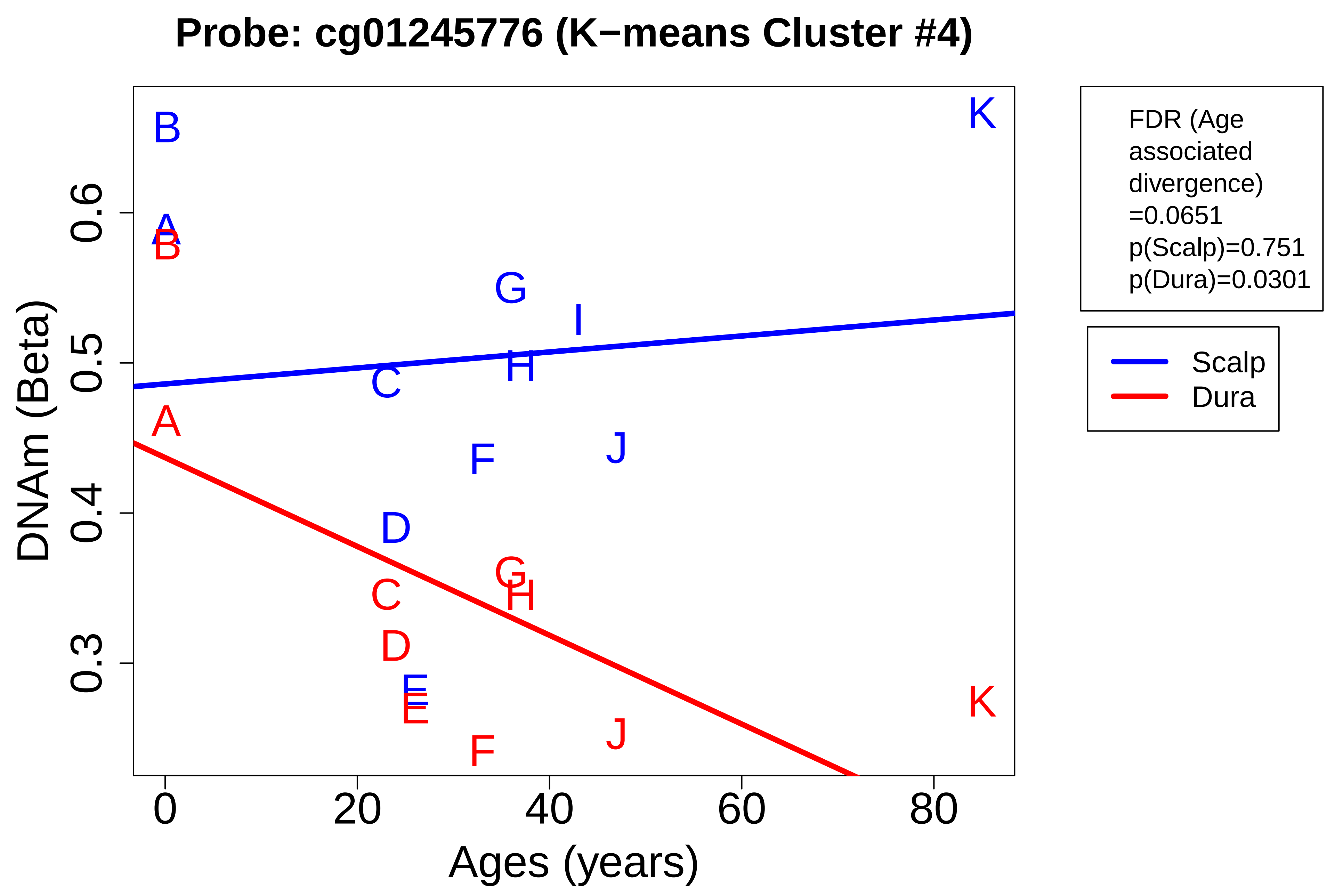

Probe: cg01245776 (K−means Cluster #4)
DNAm (Beta)
0.3
0.4
0.5
0.6
0
20
40
60
80
Ages (years)
A
B
C
D
E
F
G
H
I
J
K
FDR (Age associated divergence) =0.0651
p(Scalp)=0.751
p(Dura)=0.0301
Scalp
Dura

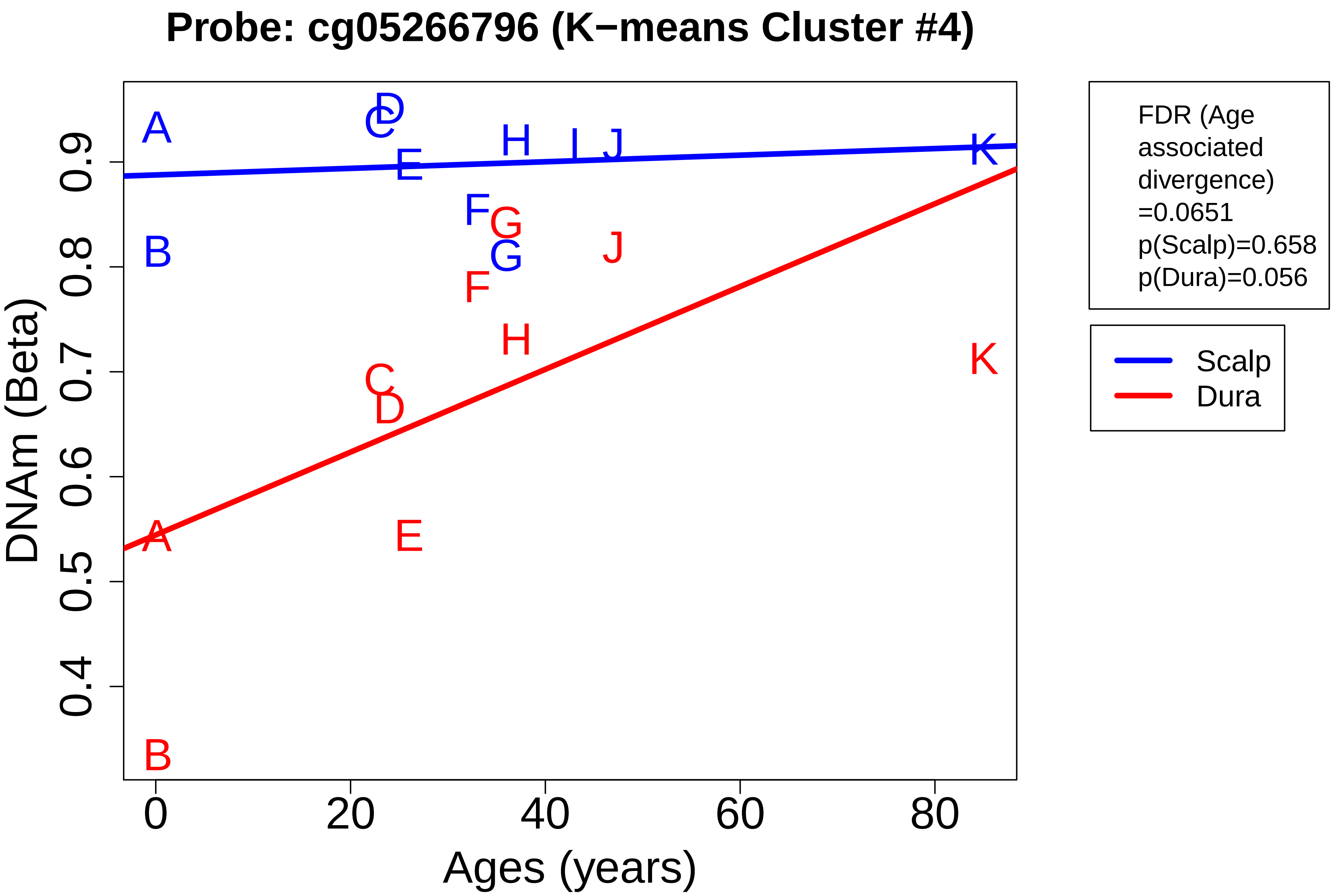
Probe: cg05266796 (K−means Cluster #4)
DNAm (Beta)
Ages (years)
0.4
0.5
0.6
0.7
0.8
0.9
0
20
40
60
80
A
B
C
D
E
F
G
H
I
J
K
FDR (Age associated divergence) =0.0651
p(Scalp)=0.658
p(Dura)=0.056
Scalp
Dura

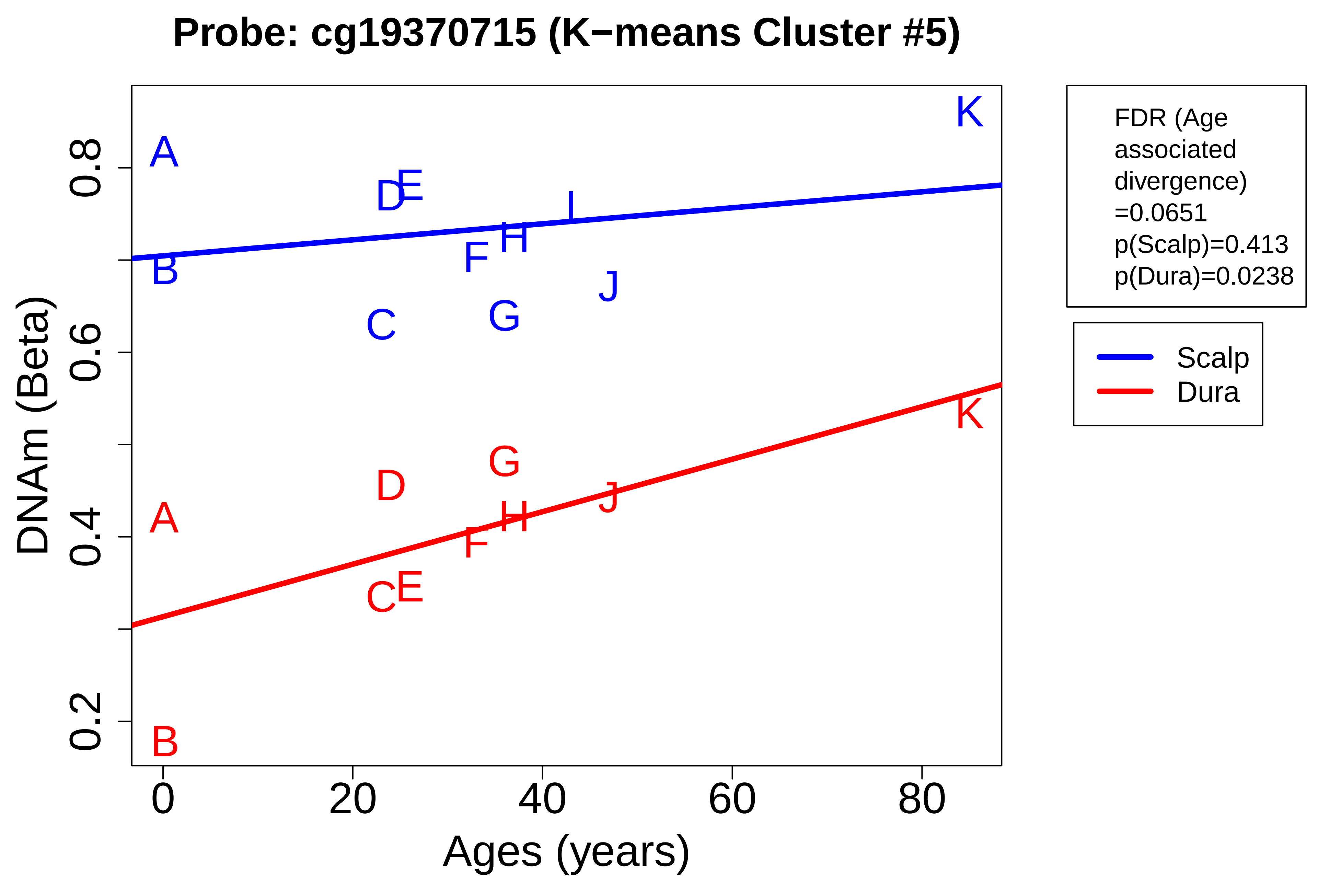

Probe: cg19370715 (K−means Cluster #5)
DNAm (Beta)
0.2
0.4
0.6
0.8
0
20
40
60
80
Ages (years)
FDR (Age associated divergence) =0.0651
p(Scalp)=0.413
p(Dura)=0.0238
Scalp
Dura

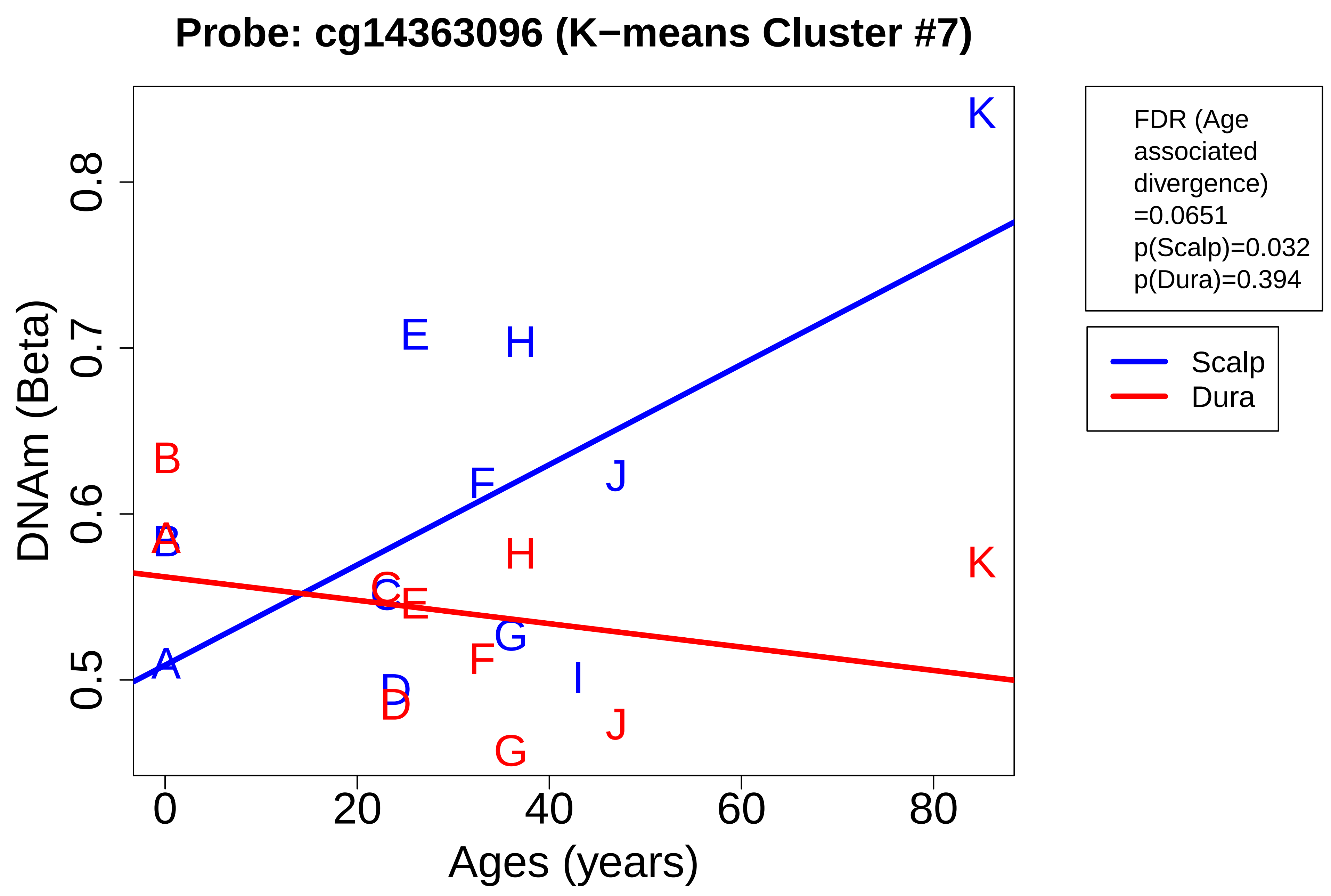

Probe: cg14363096 (K−means Cluster #7)
DNAm (Beta)
0.5
0.6
0.7
0.8
Ages (years)
0
20
40
60
80
FDR (Age associated divergence) =0.0651
p(Scalp)=0.032
p(Dura)=0.394
Scalp
Dura
A
B
C
D
E
F
G
H
I
J
K

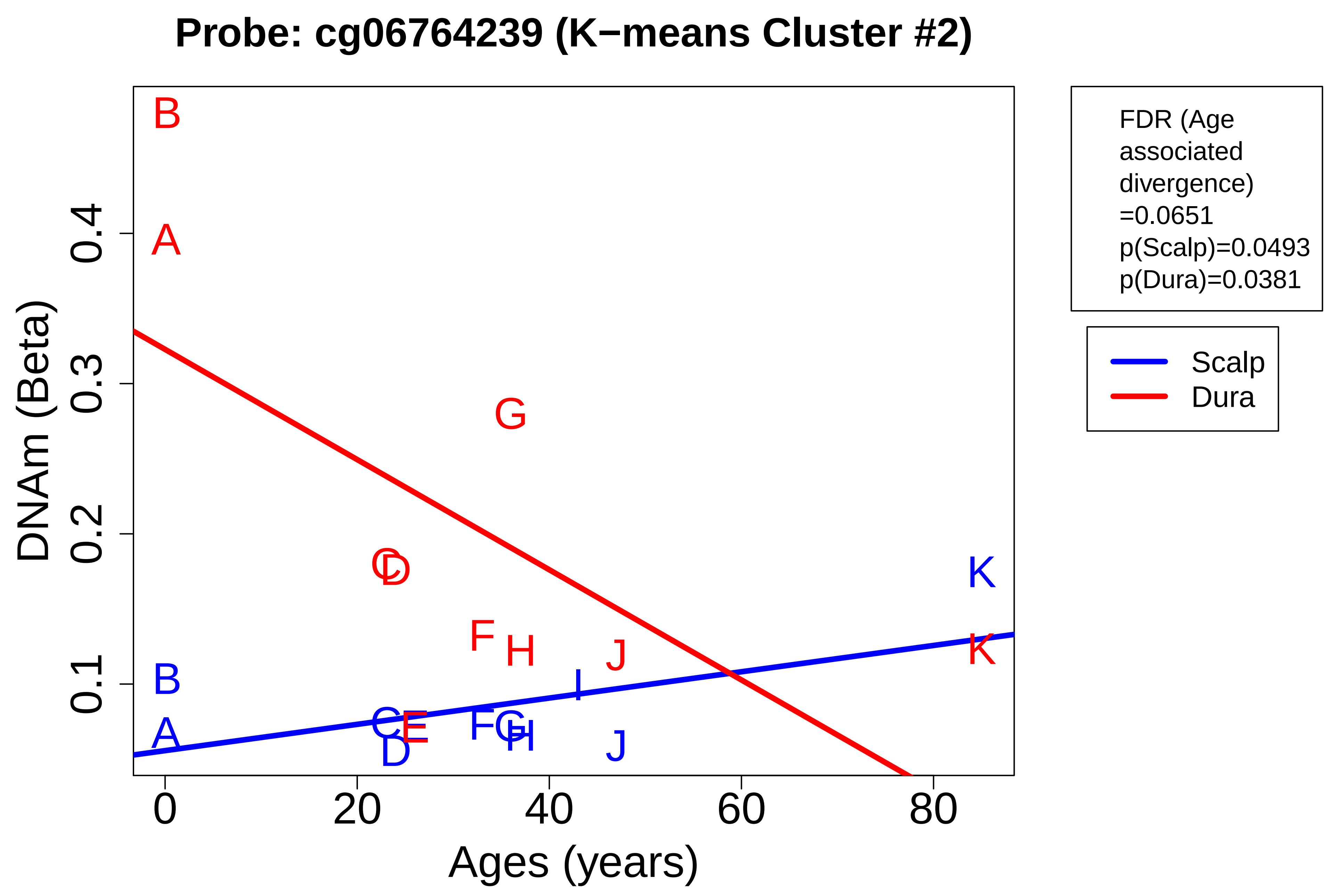

Probe: cg06764239 (K−means Cluster #2)
DNAm (Beta)
0.1
0.2
0.3
0.4
0
20
40
60
80
Ages (years)
FDR (Age associated divergence) =0.0651
p(Scalp)=0.0493
p(Dura)=0.0381
Scalp
Dura
A
B
C
D
E
F
G
H
I
J
K

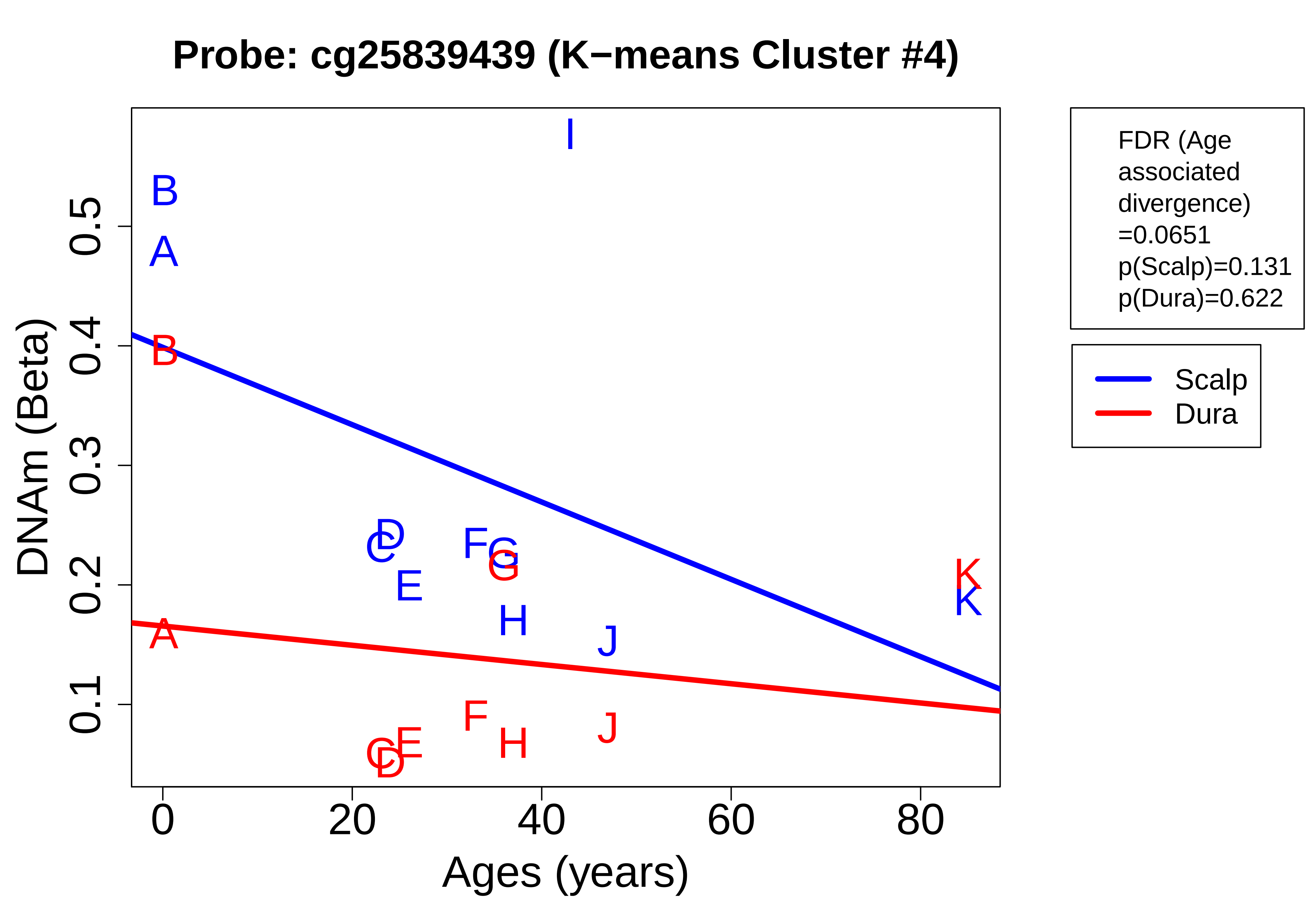

Probe: cg25839439 (K−means Cluster #4)
DNAm (Beta)
0.1
0.2
0.3
0.4
0.5
0
20
40
60
80
Ages (years)
FDR (Age associated divergence) =0.0651
p(Scalp)=0.131
p(Dura)=0.622
Scalp
Dura

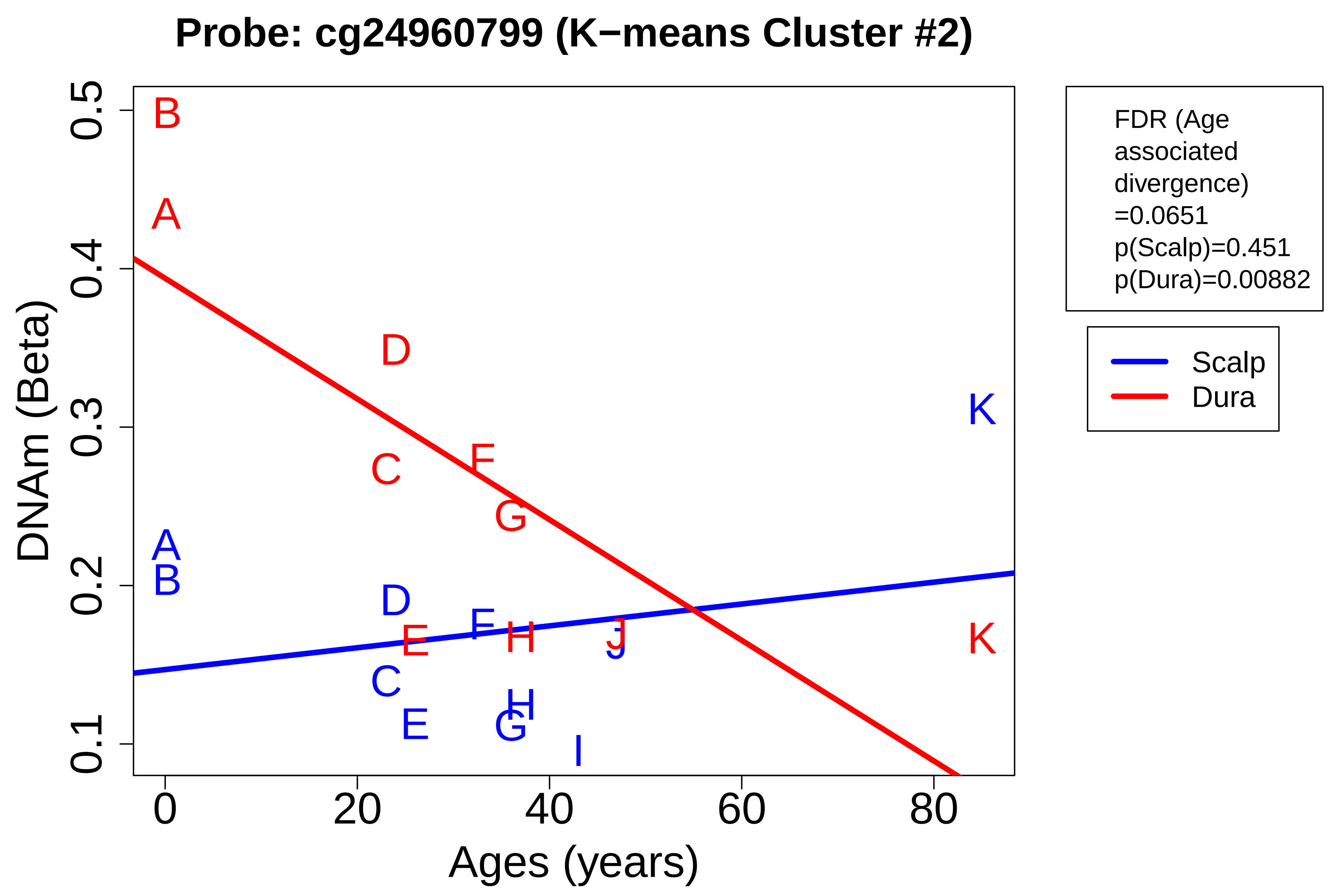

Probe: cg24960799 (K−means Cluster #2)
DNAm (Beta)
Ages (years)
0.1
0.2
0.3
0.4
0.5
0
20
40
60
80
FDR (Age associated divergence) =0.0651
p(Scalp)=0.451
p(Dura)=0.00882
Scalp
Dura

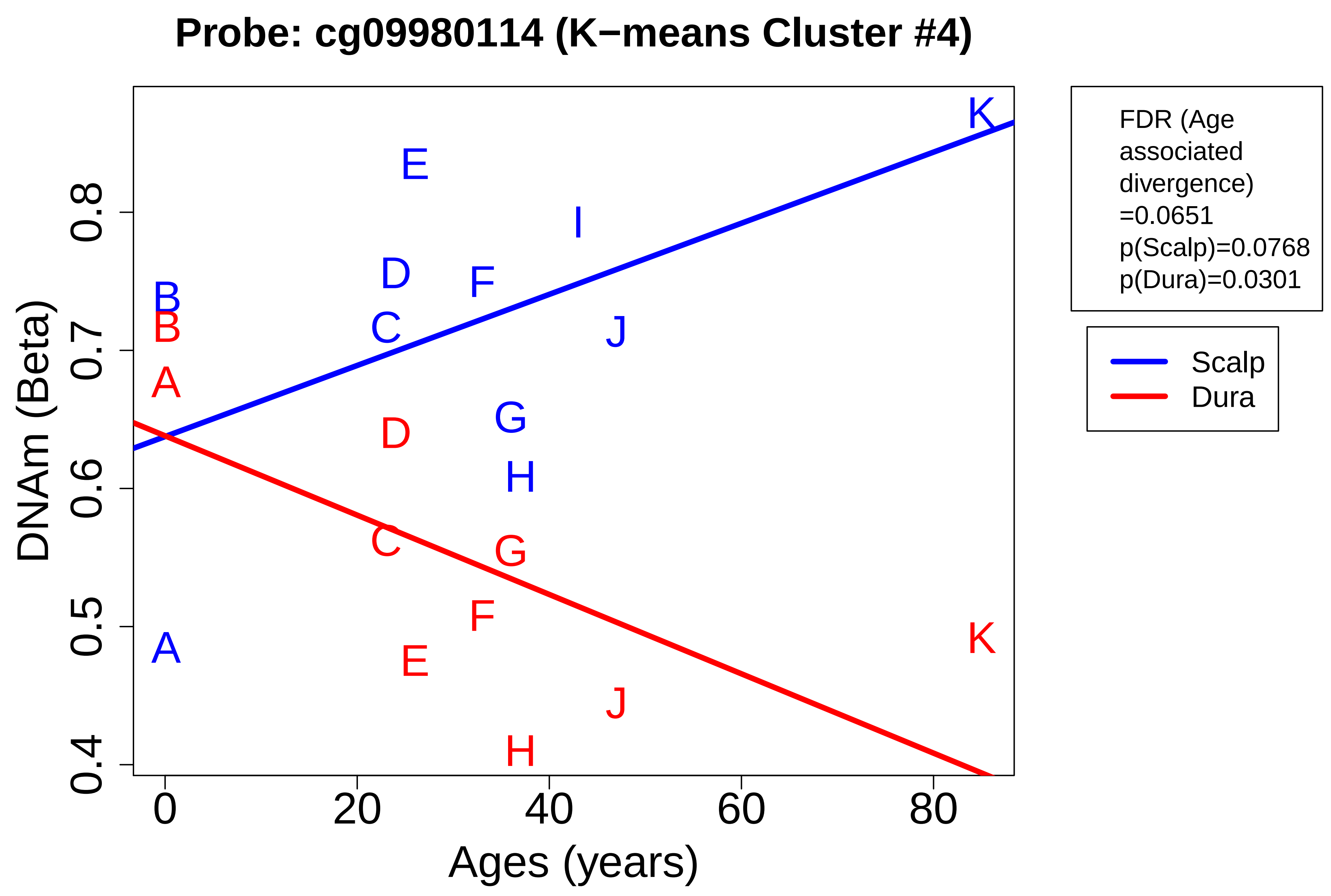

Probe: cg09980114 (K−means Cluster #4)
FDR (Age associated divergence) =0.0651
p(Scalp)=0.0768
p(Dura)=0.0301
Scalp
Dura
DNAm (Beta)
0.4
0.5
0.6
0.7
0.8
0
20
40
60
80
Ages (years)
A
B
C
D
E
F
G
H
I
J
K

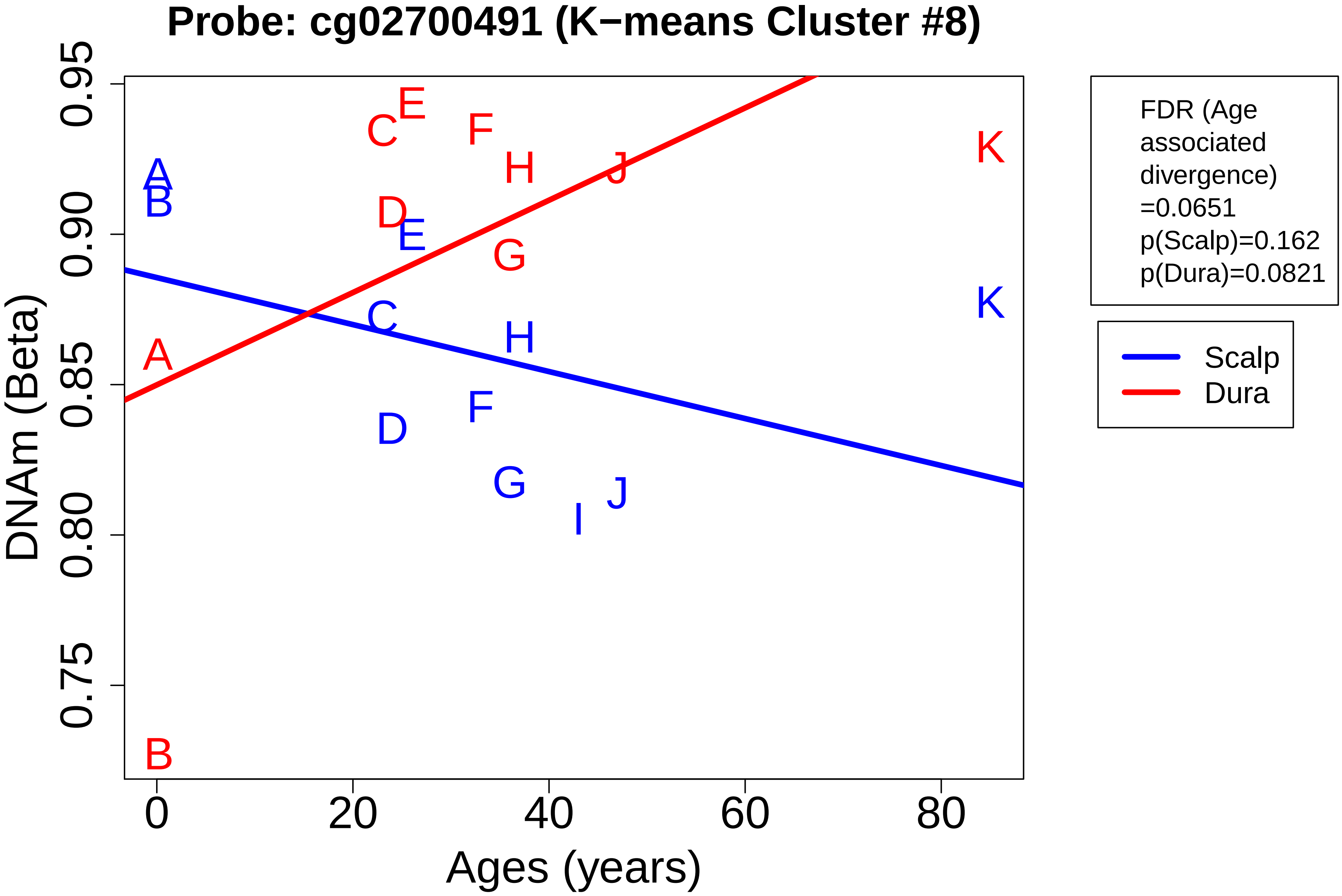

Probe: cg02700491 (K−means Cluster #8)
DNAm (Beta)
0.75
0.80
0.85
0.90
0.95
0
20
40
60
80
Ages (years)
A
B
C
D
E
F
G
H
I
J
K
A
B
C
D
E
F
G
H
J
K
FDR (Age associated divergence) =0.0651
p(Scalp)=0.162
p(Dura)=0.0821
Scalp
Dura

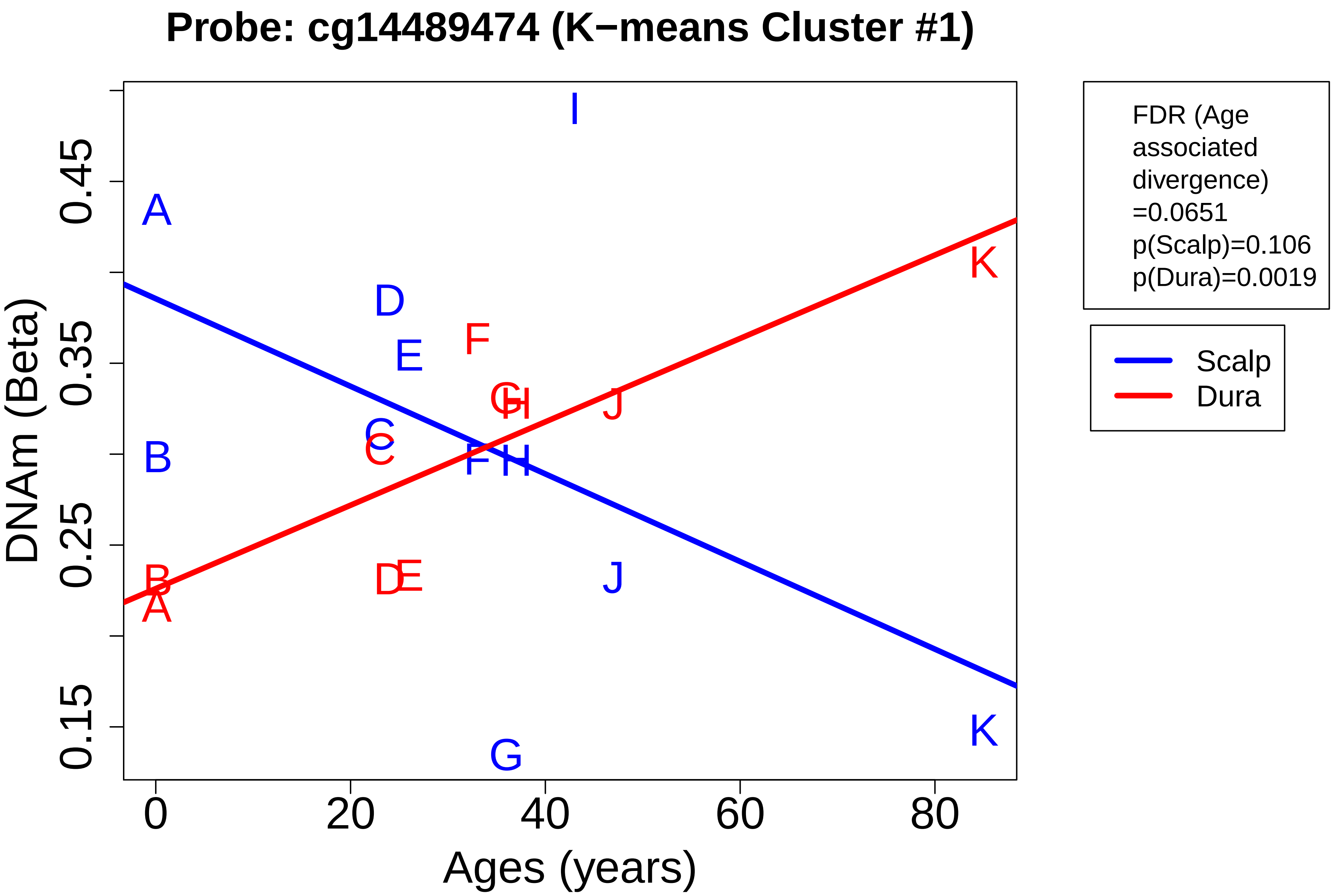

Probe: cg14489474 (K−means Cluster #1)
DNAm (Beta)
0.15
0.25
0.35
0.45
0
20
40
60
80
Ages (years)
FDR (Age associated divergence) =0.0651
p(Scalp)=0.106
p(Dura)=0.0019
Scalp
Dura
A
B
C
D
E
F
G
H
I
J
K

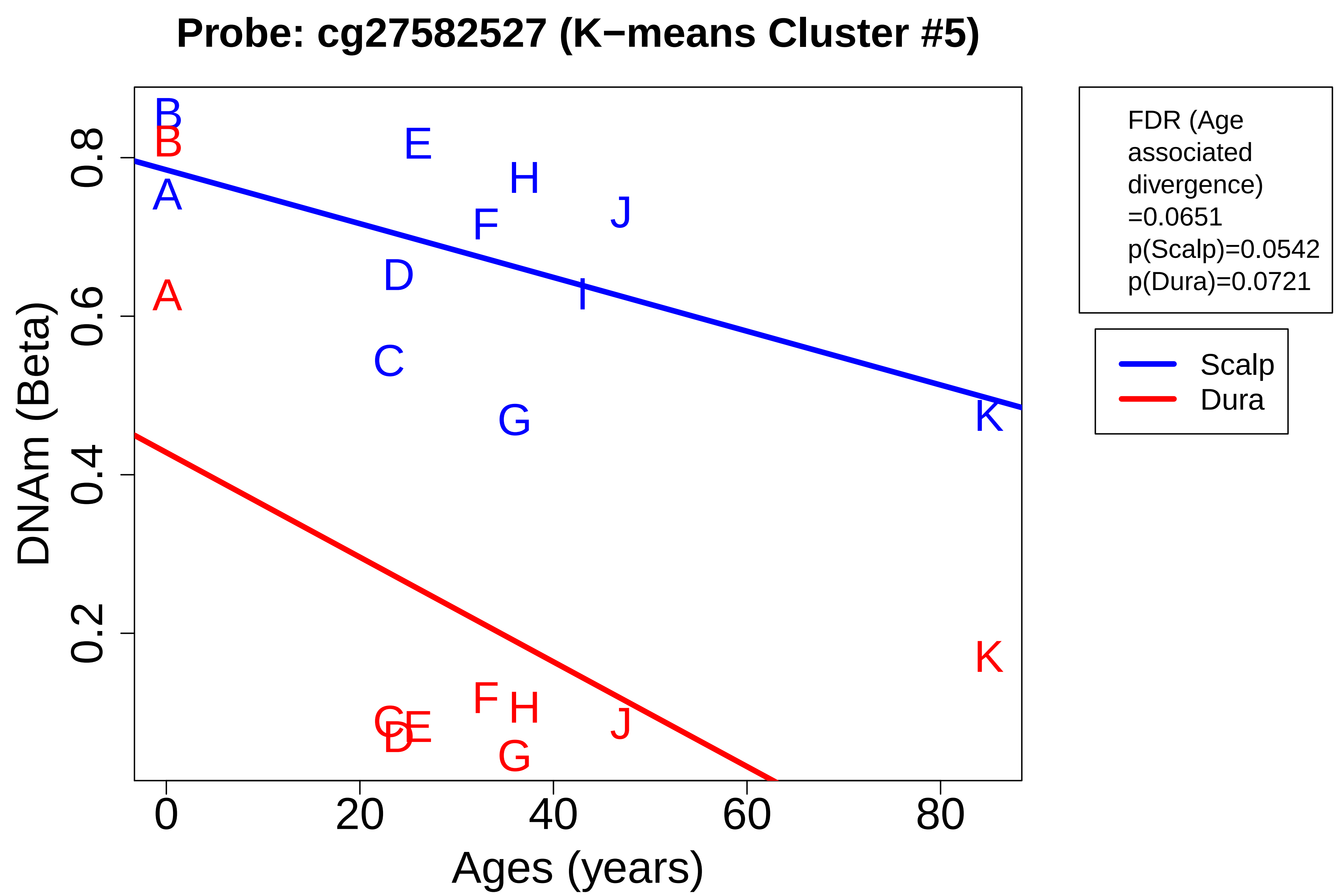

Probe: cg27582527 (K−means Cluster #5)
DNAm (Beta)
0.2
0.4
0.6
0.8
Ages (years)
0
20
40
60
80
FDR (Age associated divergence) =0.0651
p(Scalp)=0.0542
p(Dura)=0.0721
Scalp
Dura

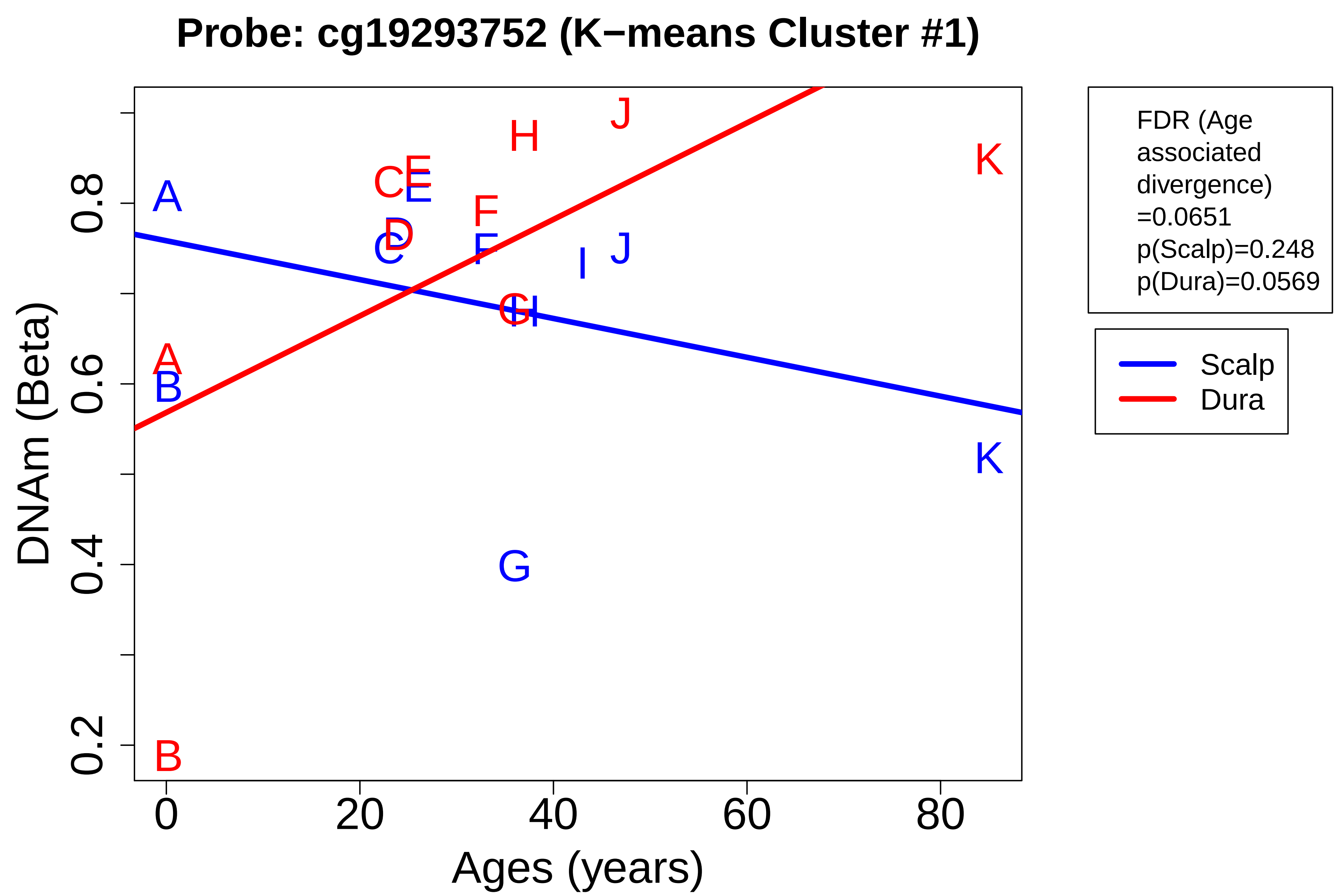
Probe: cg19293752 (K−means Cluster #1)
DNAm (Beta)
Ages (years)
0.2
0.4
0.6
0.8
0
20
40
60
80
FDR (Age associated divergence) =0.0651
p(Scalp)=0.248
p(Dura)=0.0569
Scalp
Dura

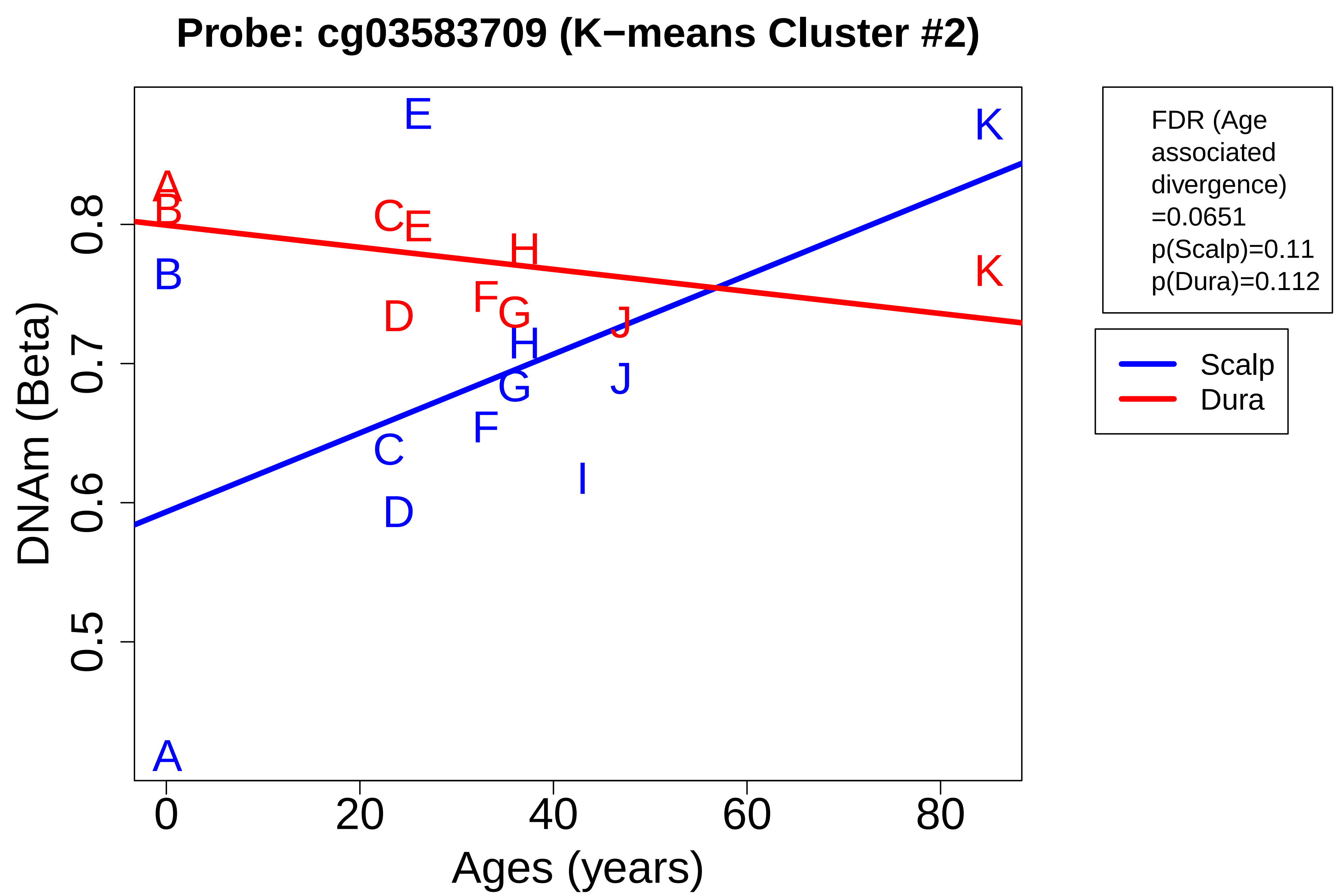
Probe: cg03583709 (K−means Cluster #2)
DNAm (Beta)
0.5
0.6
0.7
0.8
Ages (years)
0
20
40
60
80
FDR (Age associated divergence) =0.0651
p(Scalp)=0.11
p(Dura)=0.112
Scalp
Dura

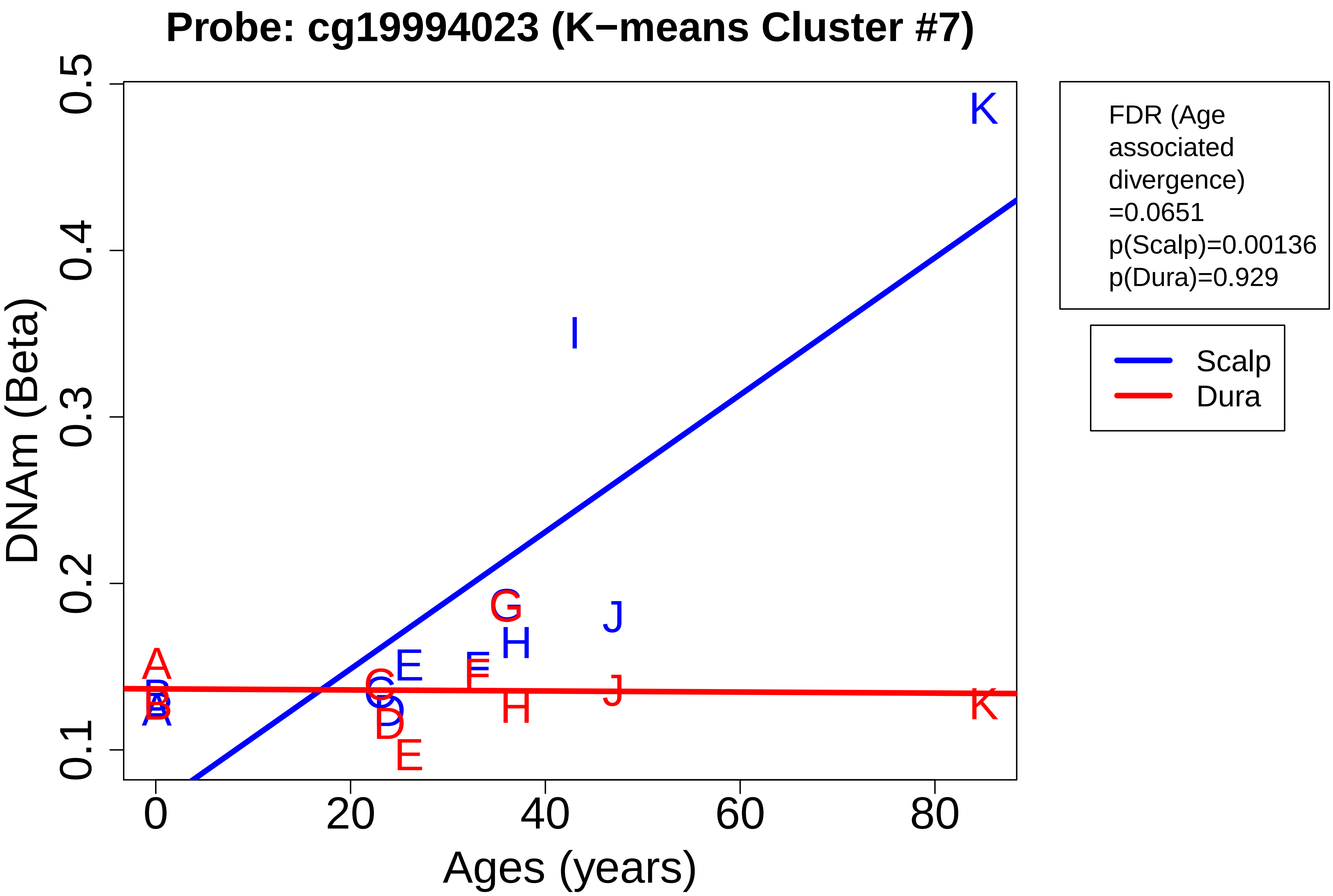

Probe: cg19994023 (K−means Cluster #7)
DNAm (Beta)
0.1
0.2
0.3
0.4
0.5
0
20
40
60
80
Ages (years)
FDR (Age associated divergence) =0.0651
p(Scalp)=0.00136
p(Dura)=0.929
Scalp
Dura

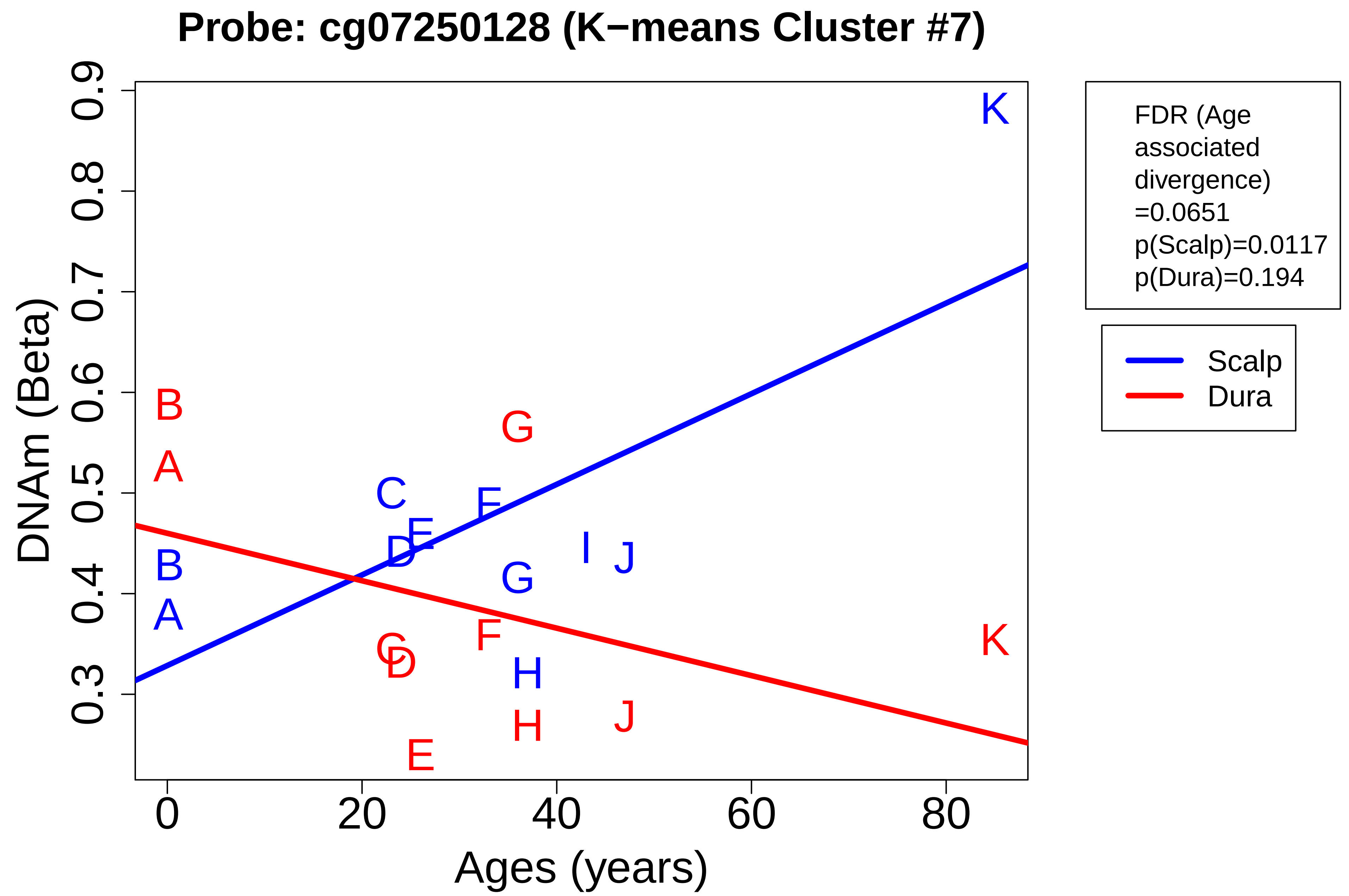

Probe: cg07250128 (K−means Cluster #7)
DNAm (Beta)
0.3
0.4
0.5
0.6
0.7
0.8
0.9
Ages (years)
0
20
40
60
80
FDR (Age associated divergence) =0.0651
p(Scalp)=0.0117
p(Dura)=0.194
Scalp
Dura

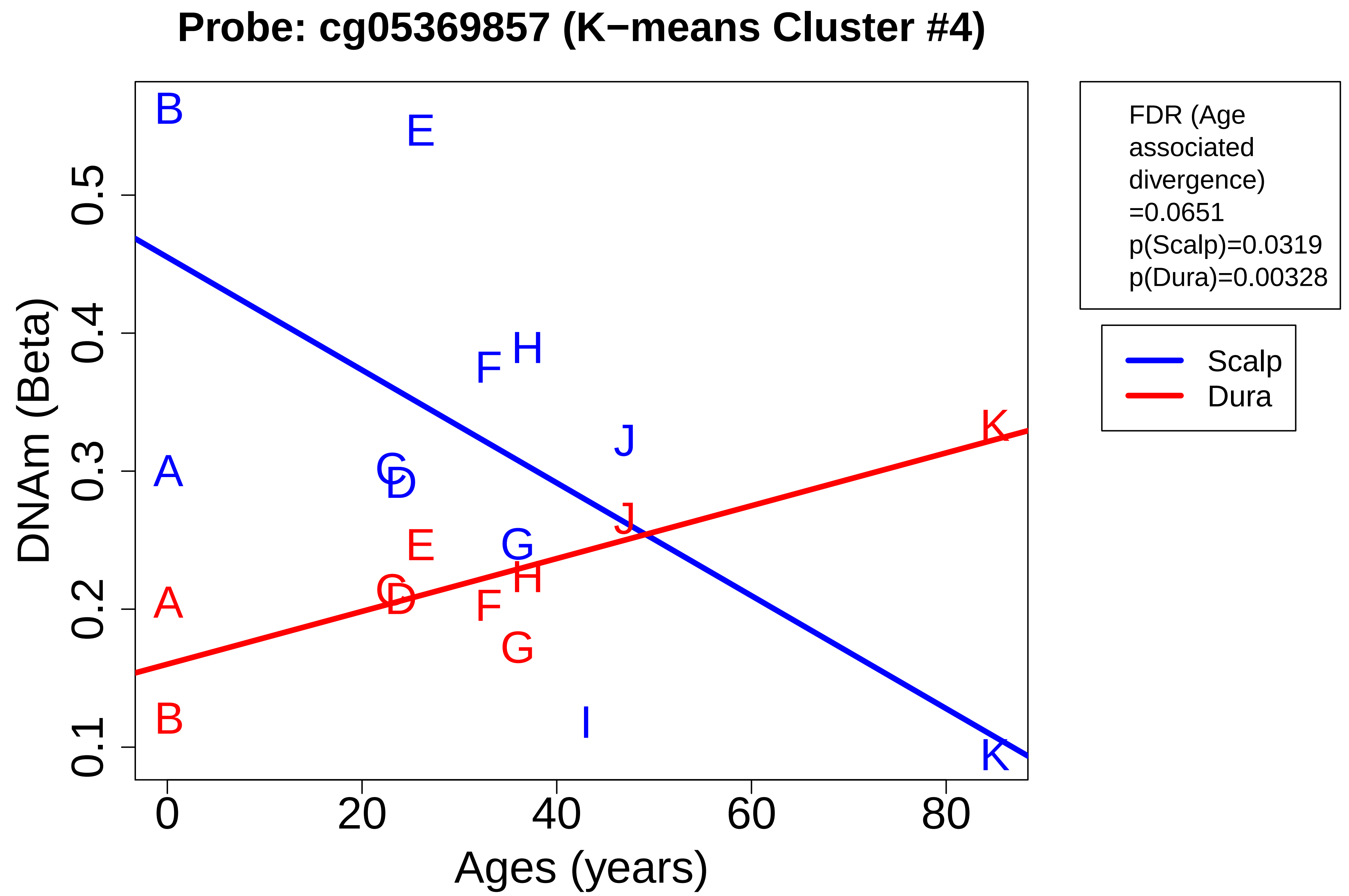

Probe: cg05369857 (K−means Cluster #4)
FDR (Age associated divergence) =0.0651
p(Scalp)=0.0319
p(Dura)=0.00328
Scalp
Dura
DNAm (Beta)
0.1
0.2
0.3
0.4
0.5
0
20
40
60
80
Ages (years)
A
B
C
D
E
F
G
H
I
J
K

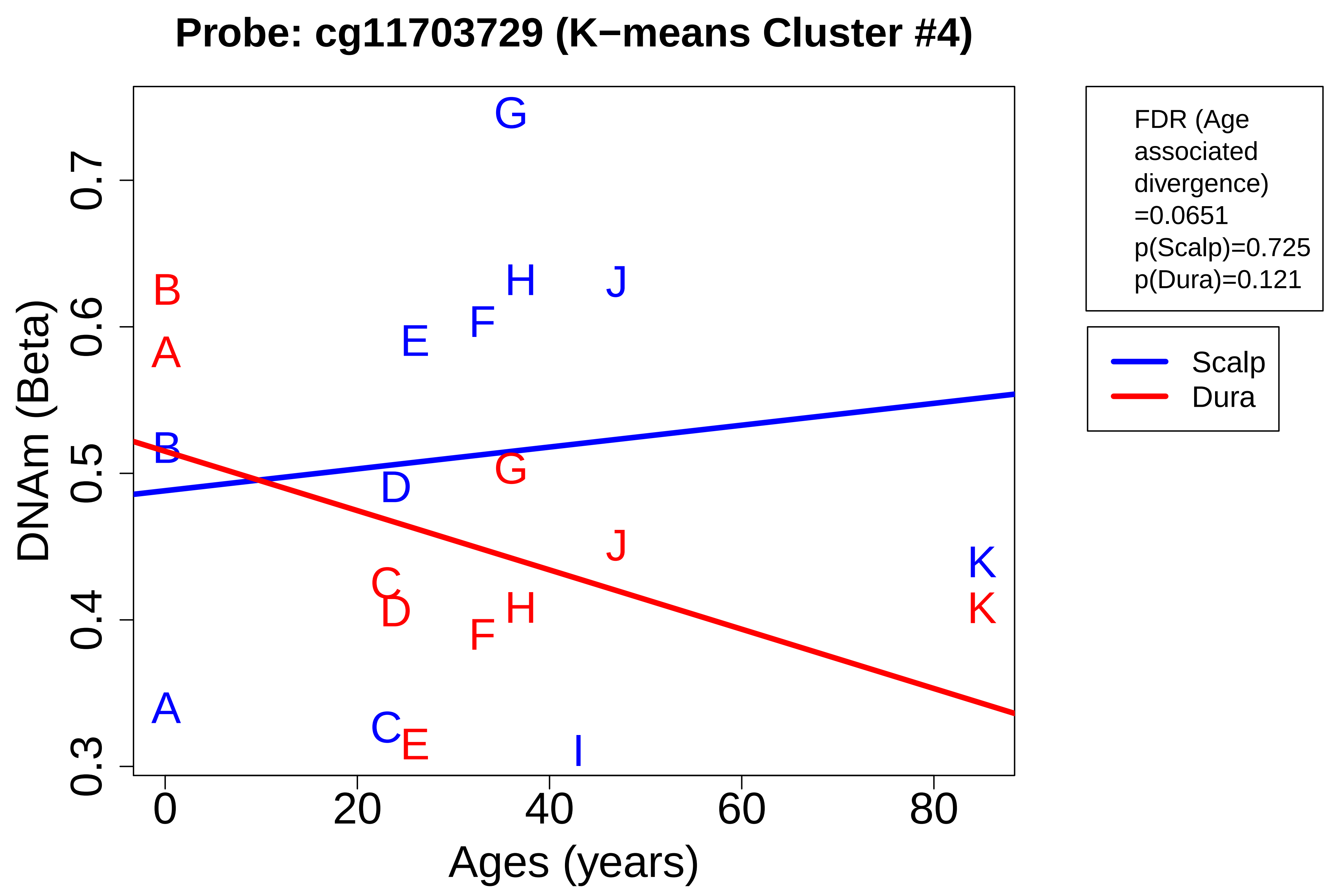

Probe: cg11703729 (K−means Cluster #4)
DNAm (Beta)
Ages (years)
0.3
0.4
0.5
0.6
0.7
0
20
40
60
80
FDR (Age associated divergence) =0.0651
p(Scalp)=0.725
p(Dura)=0.121
Scalp
Dura

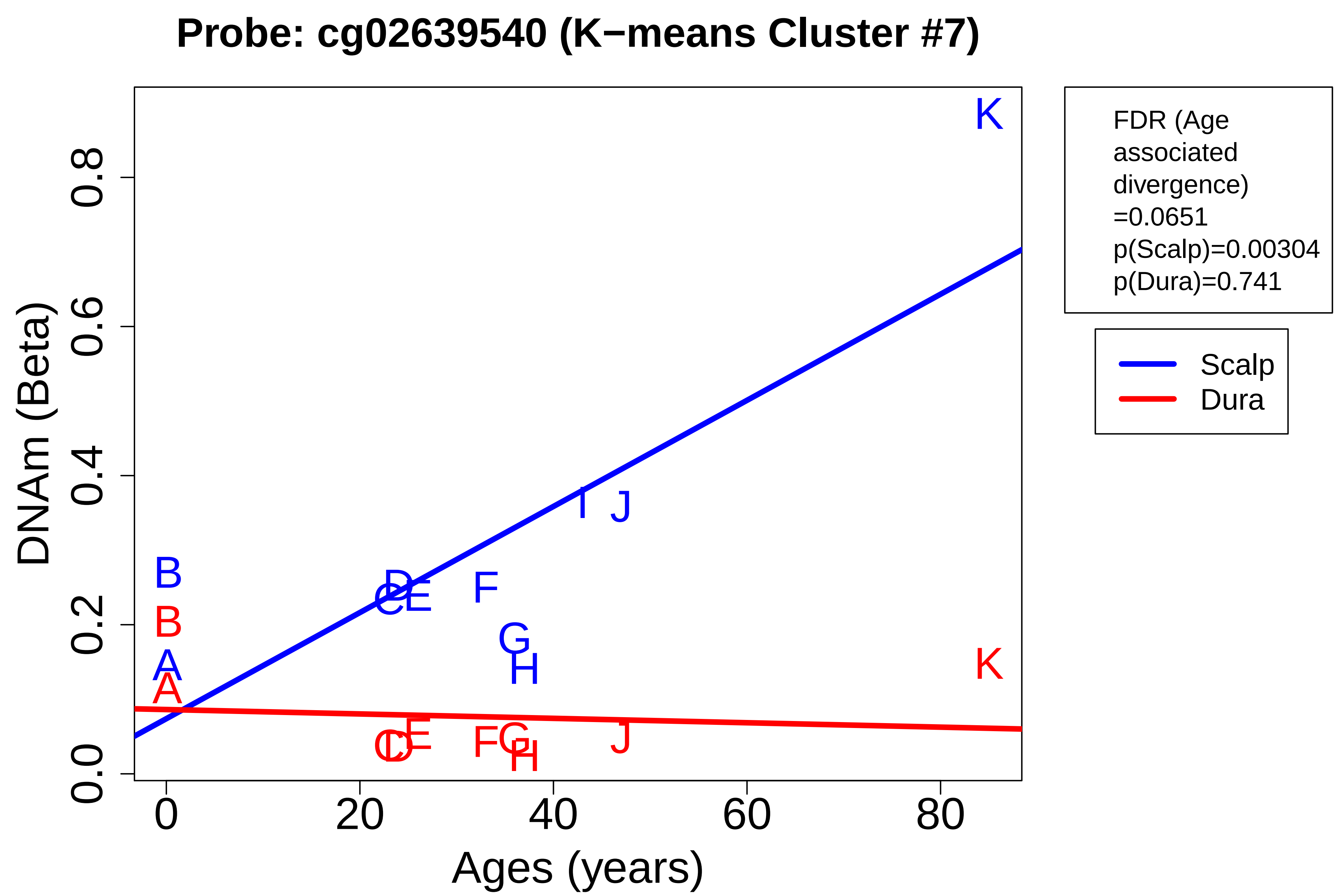
Probe: cg02639540 (K−means Cluster #7)
FDR (Age associated divergence) =0.0651
p(Scalp)=0.00304
p(Dura)=0.741
Scalp
Dura
DNAm (Beta)
0.0
0.2
0.4
0.6
0.8
0
20
40
60
80
Ages (years)
A
B
CDE
F
G
H
I
J
K
A
B
CD
E
F
G
H
J
K

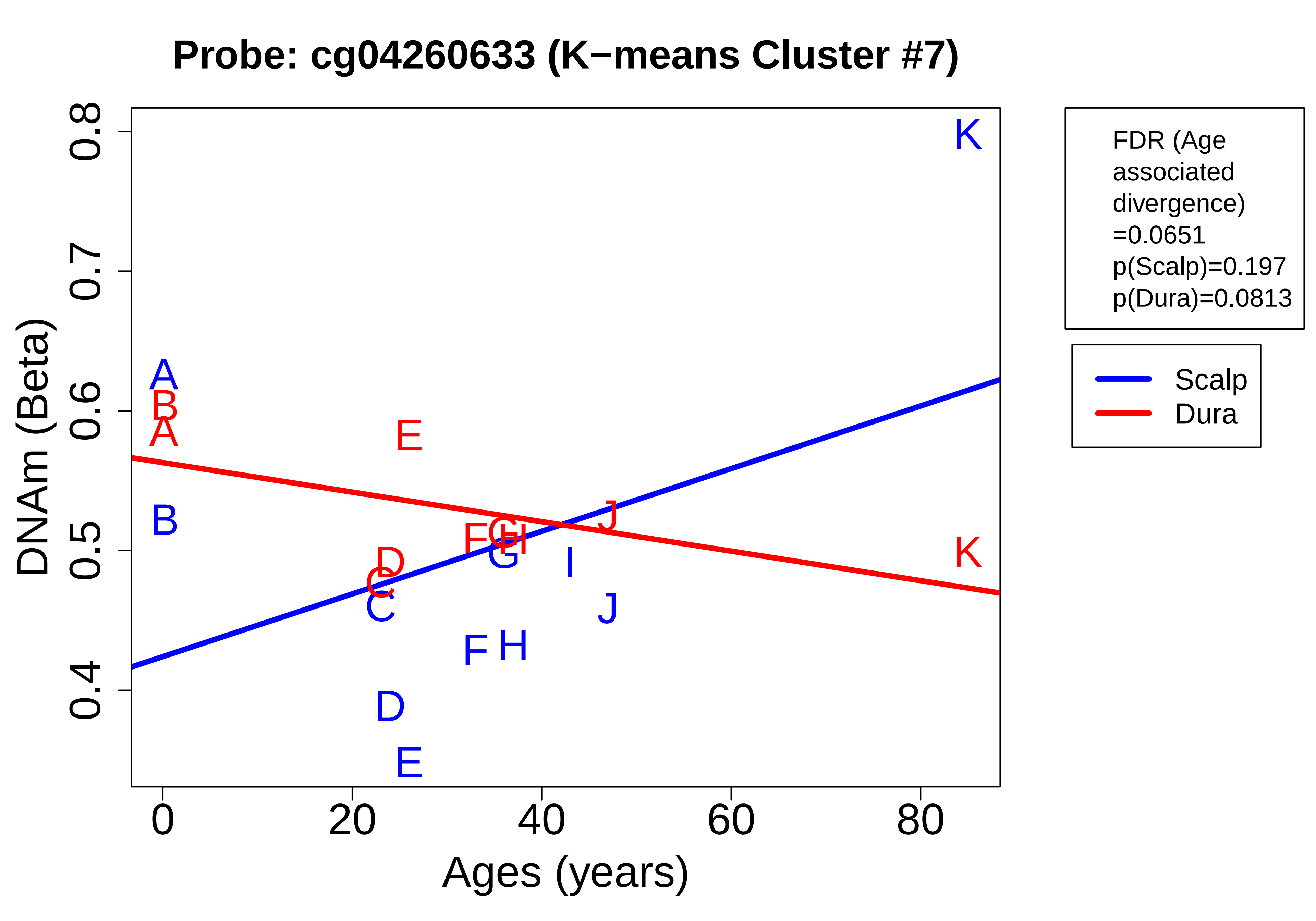
Probe: cg04260633 (K−means Cluster #7)
DNAm (Beta)
Ages (years)
0.4
0.5
0.6
0.7
0.8
0
20
40
60
80
FDR (Age associated divergence) =0.0651
p(Scalp)=0.197
p(Dura)=0.0813
Scalp
Dura
A
B
C
D
E
F
G
H
I
J
K

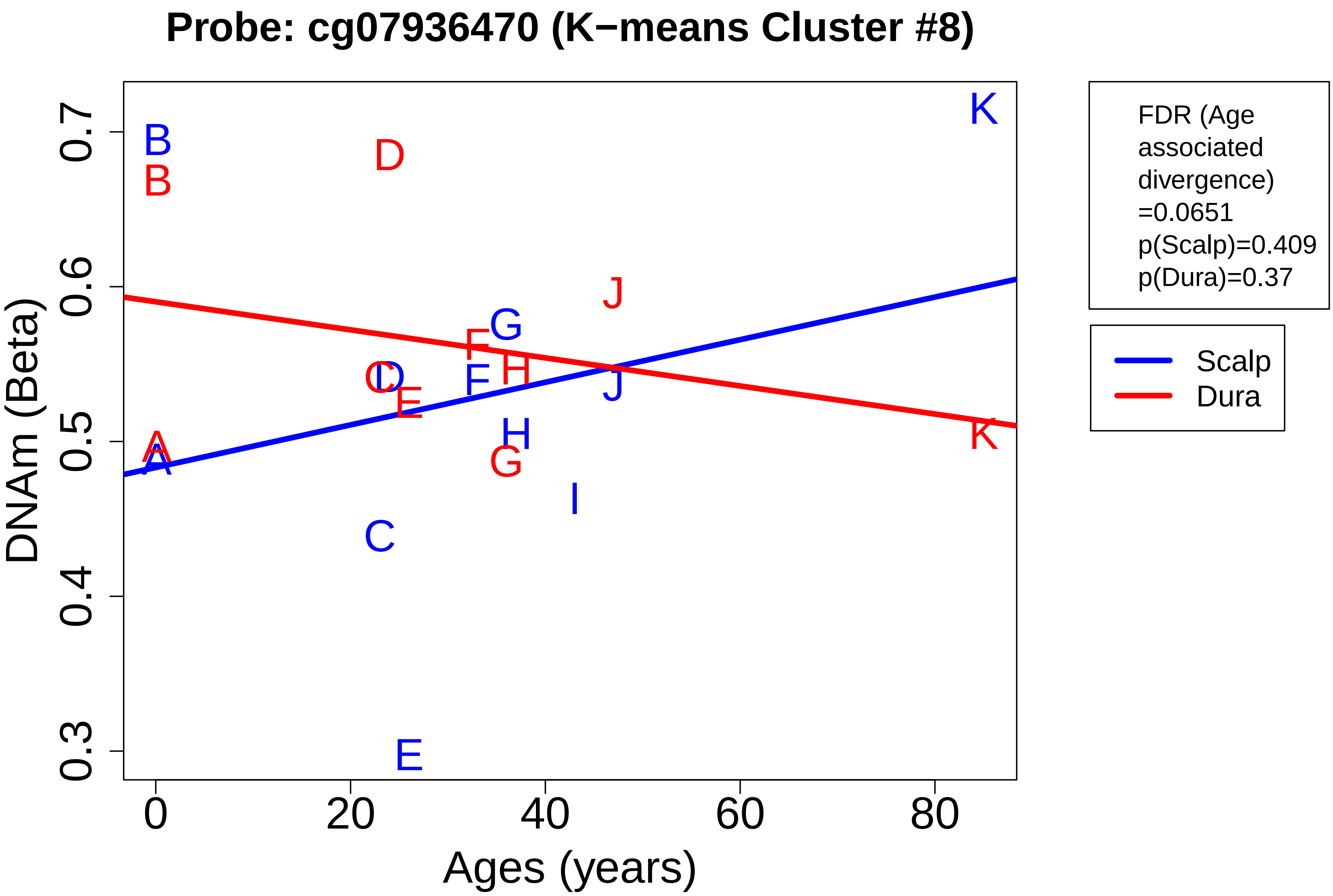

Probe: cg07936470 (K−means Cluster #8)
DNAm (Beta)
0.3
0.4
0.5
0.6
0.7
0
20
40
60
80
Ages (years)
FDR (Age associated divergence) =0.0651
p(Scalp)=0.409
p(Dura)=0.37
Scalp
Dura

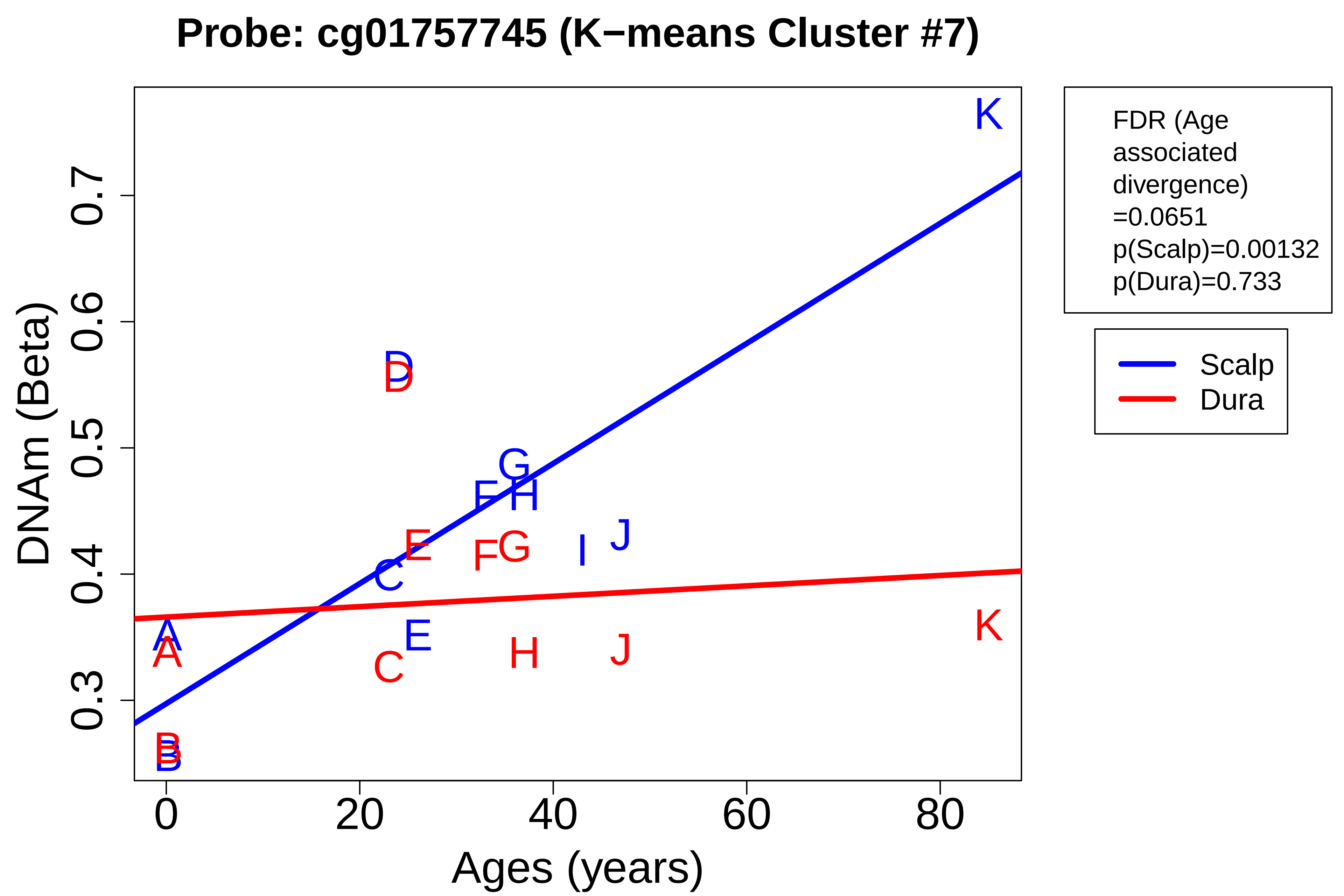

Probe: cg01757745 (K−means Cluster #7)
DNAm (Beta)
0.3
0.4
0.5
0.6
0.7
0
20
40
60
80
Ages (years)
FDR (Age associated divergence) =0.0651
p(Scalp)=0.00132
p(Dura)=0.733
Scalp
Dura
A
B
C
D
E
F
G
H
I
J
K

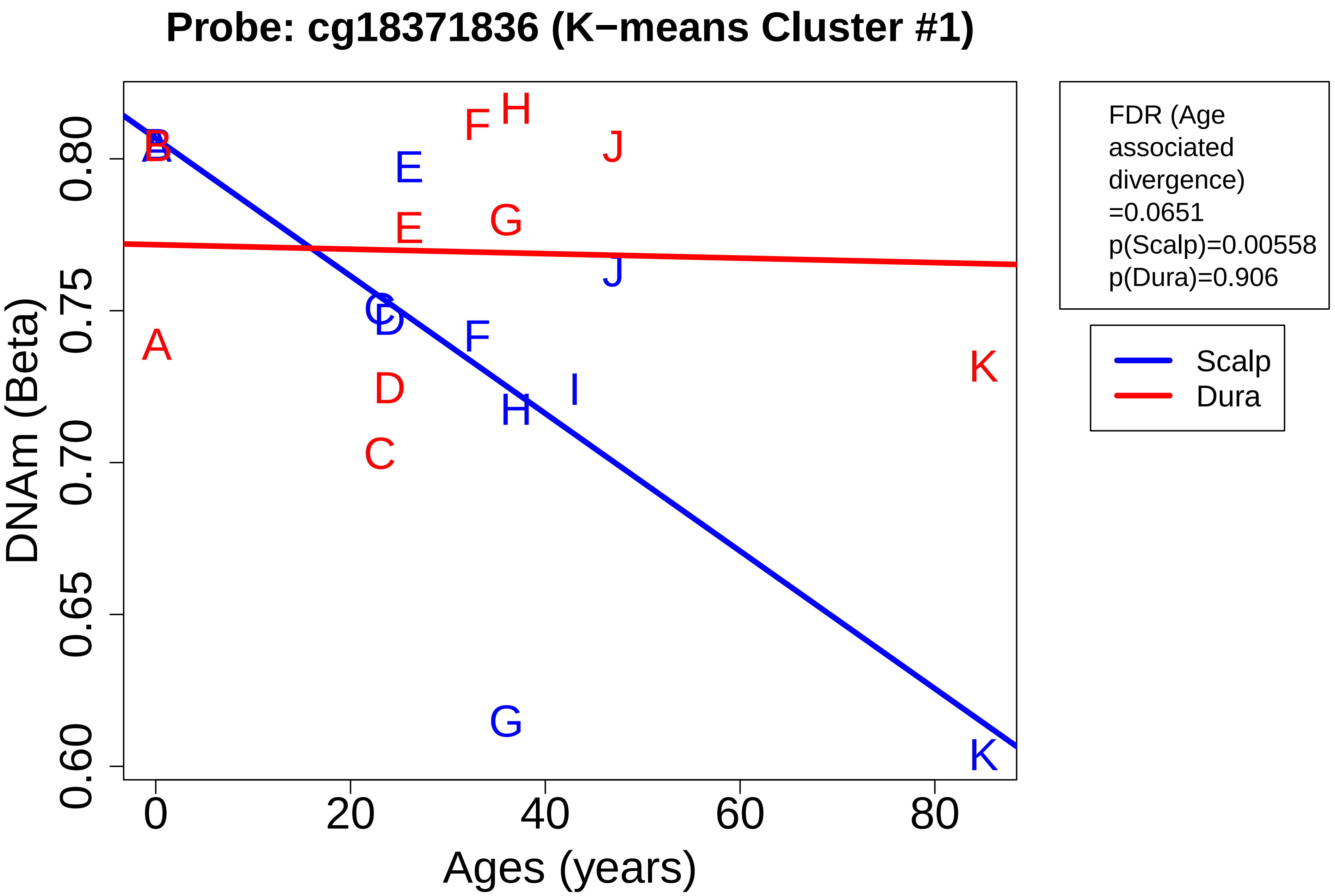

Probe: cg18371836 (K−means Cluster #1)
DNAm (Beta)
0.60
0.65
0.70
0.75
0.80
0
20
40
60
80
Ages (years)
A
B
C
D
E
F
G
H
I
J
K
FDR (Age associated divergence) =0.0651
p(Scalp)=0.00558
p(Dura)=0.906
Scalp
Dura

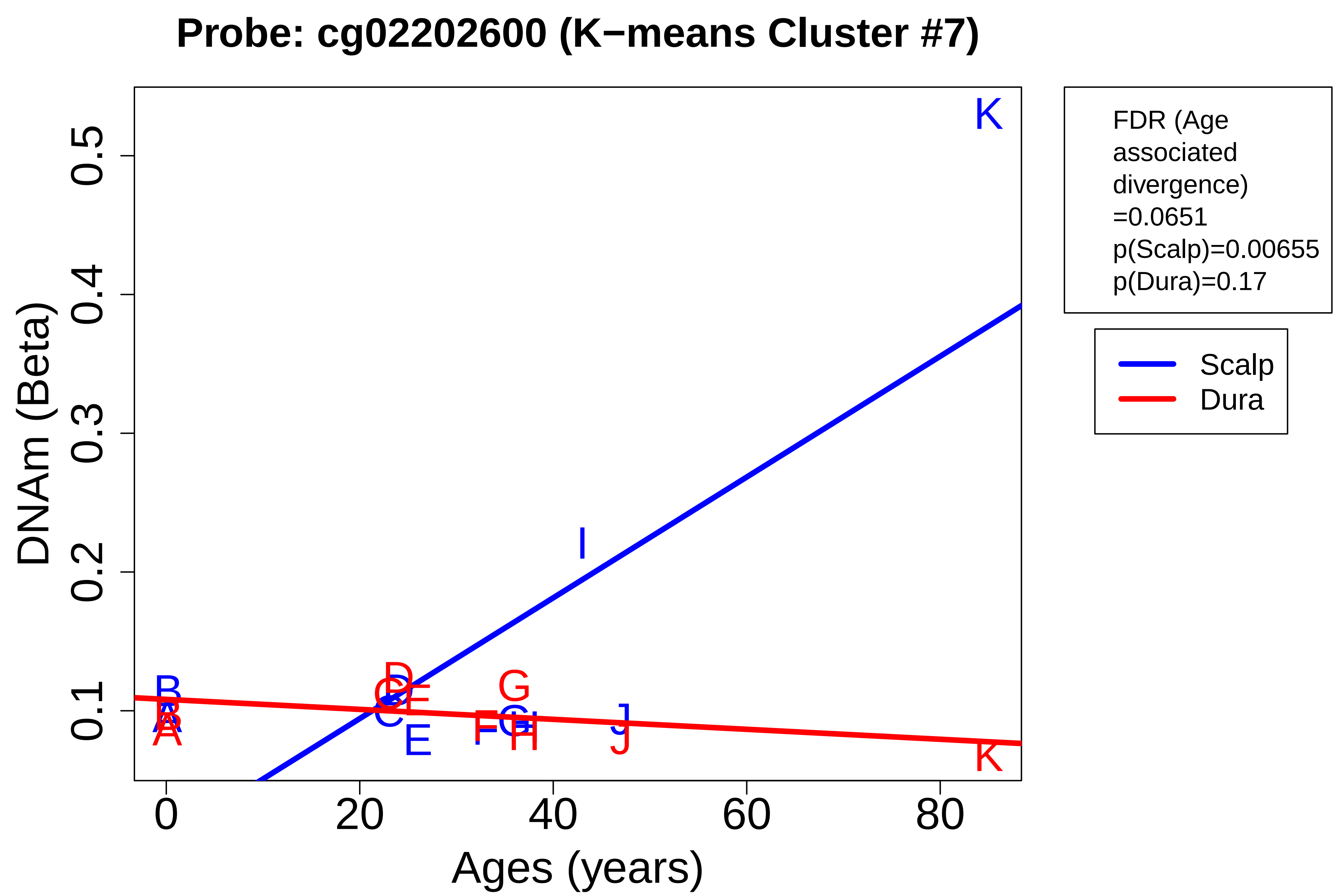
Probe: cg02202600 (K−means Cluster #7)
DNAm (Beta)
0.1
0.2
0.3
0.4
0.5
Ages (years)
0
20
40
60
80
FDR (Age associated divergence) =0.0651
p(Scalp)=0.00655
p(Dura)=0.17
Scalp
Dura

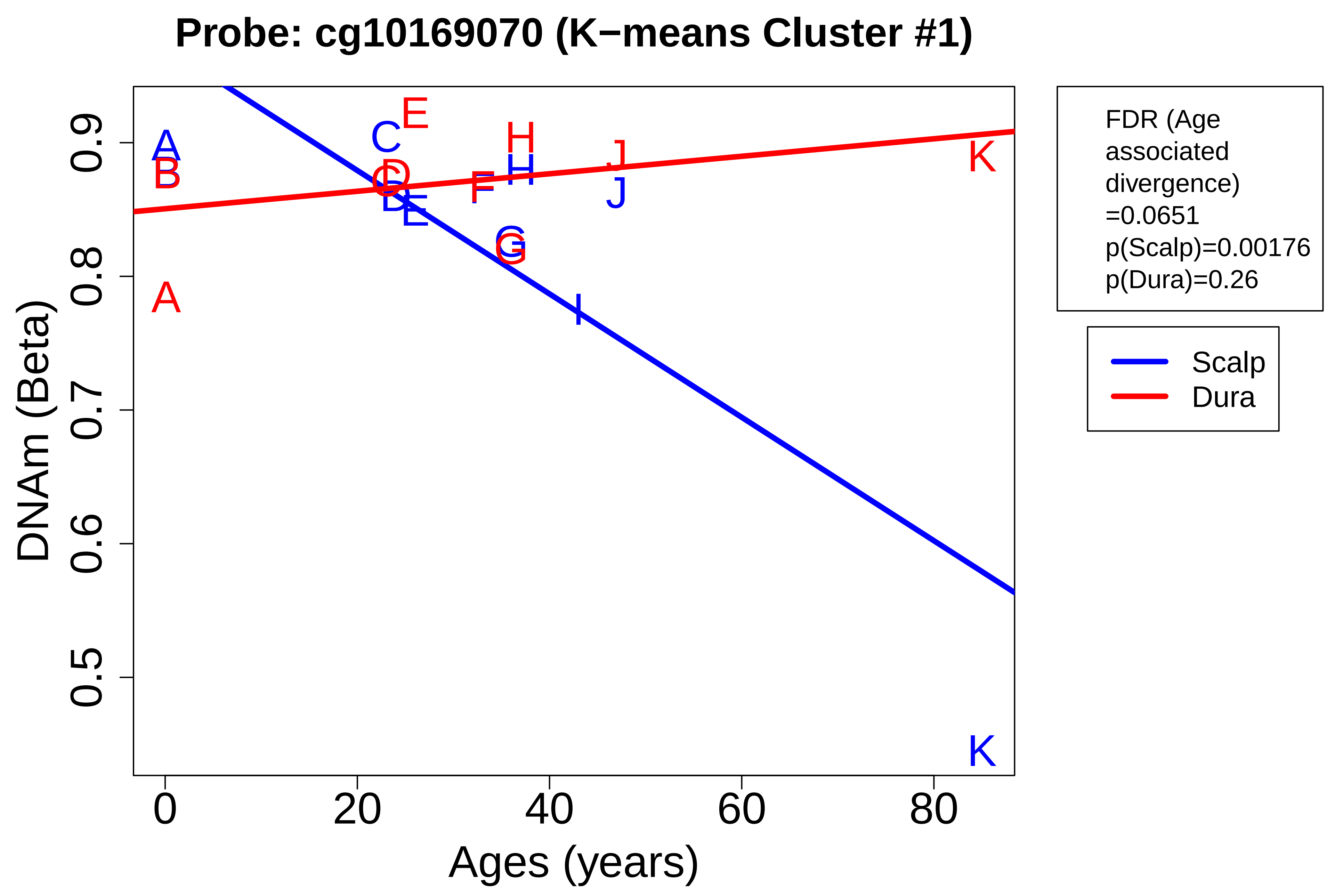

Probe: cg10169070 (K−means Cluster #1)
DNAm (Beta)
Ages (years)
0.5
0.6
0.7
0.8
0.9
0
20
40
60
80
FDR (Age associated divergence) =0.0651
p(Scalp)=0.00176
p(Dura)=0.26
Scalp
Dura

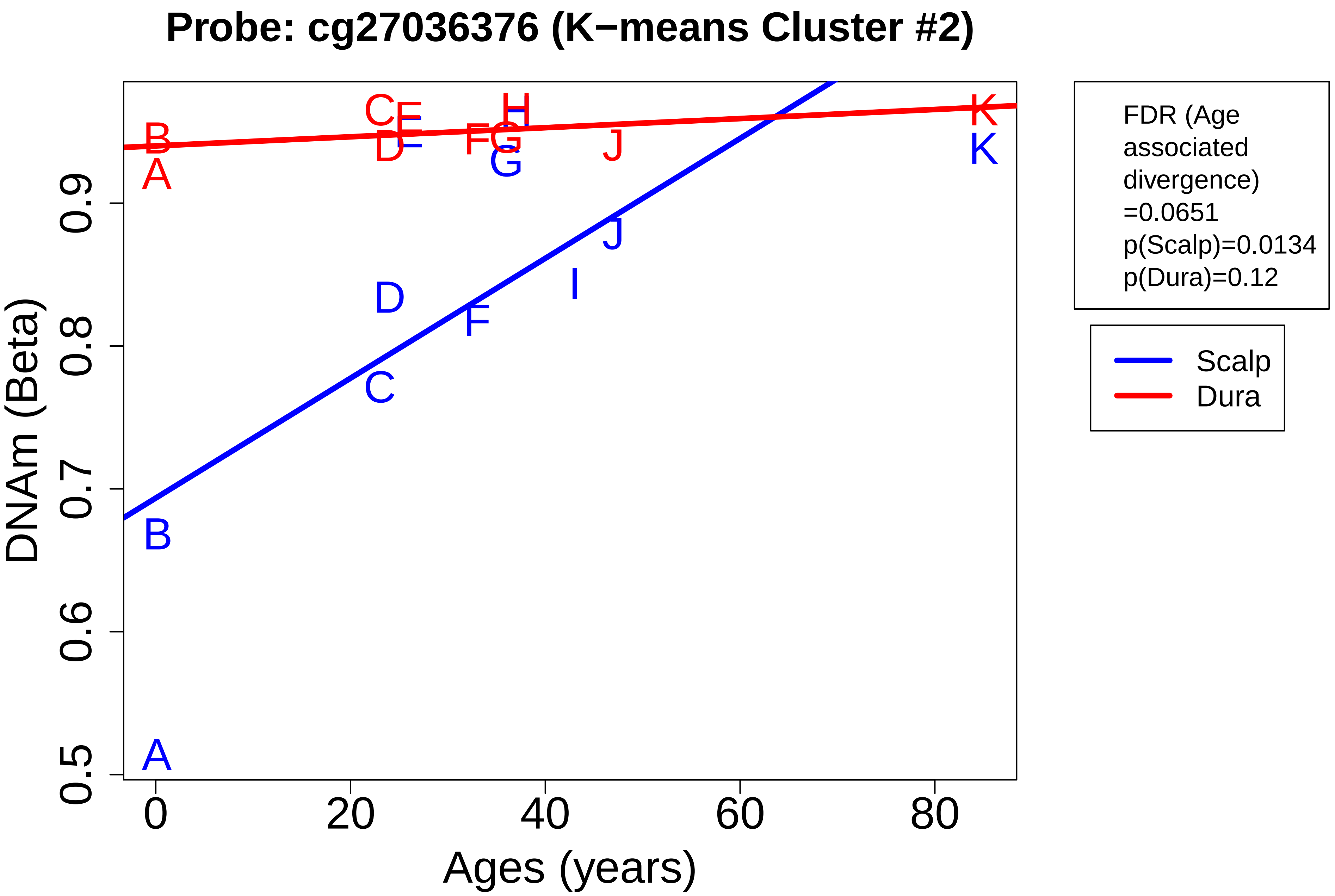
Probe: cg27036376 (K−means Cluster #2)
DNAm (Beta)
0.5
0.6
0.7
0.8
0.9
Ages (years)
0
20
40
60
80
A
B
C
D
E
F
G
H
I
J
K
FDR (Age associated divergence) =0.0651
p(Scalp)=0.0134
p(Dura)=0.12
Scalp
Dura

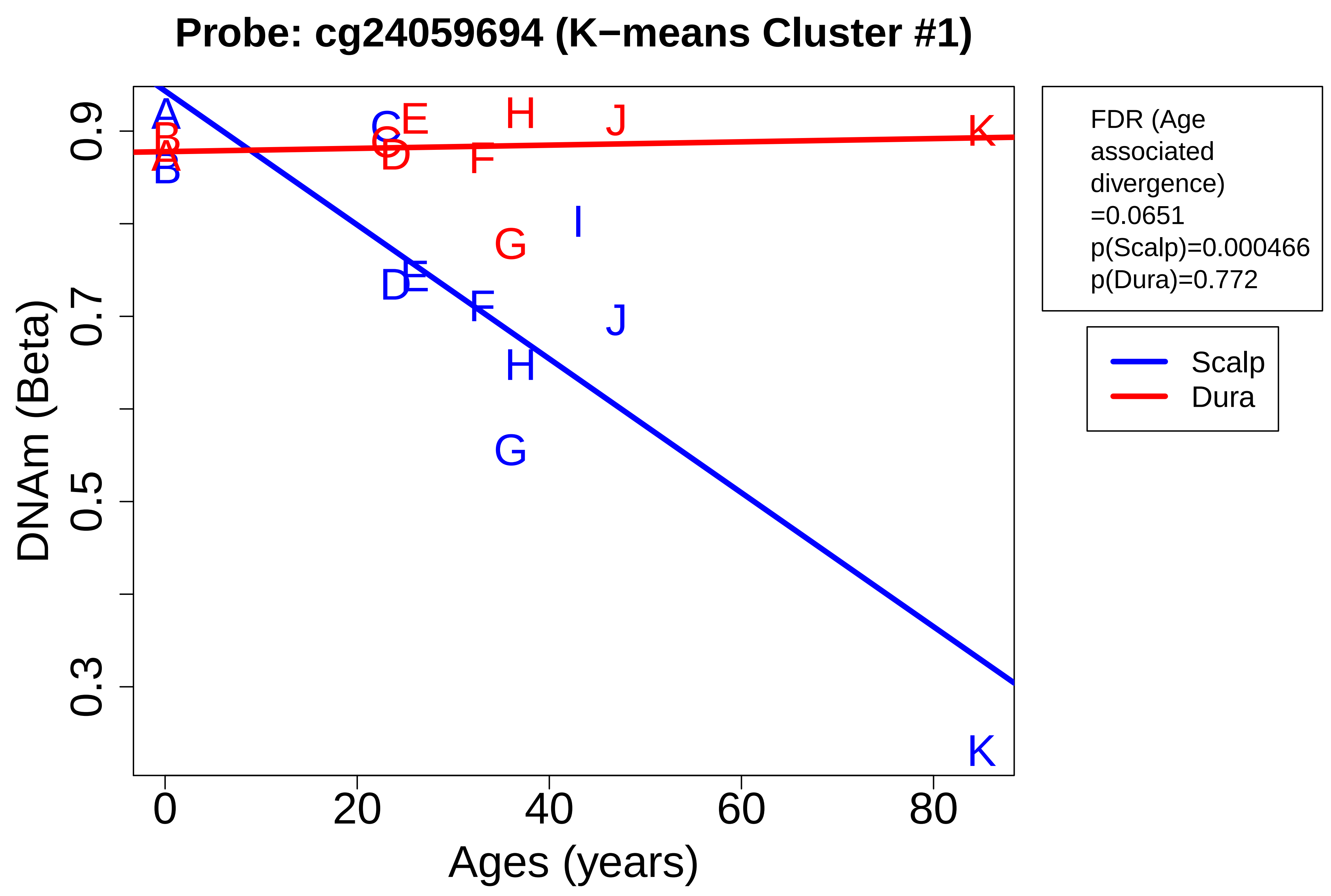

Probe: cg24059694 (K−means Cluster #1)
DNAm (Beta)
0.3
0.5
0.7
0.9
Ages (years)
0
20
40
60
80
A
B
C
D
E
F
G
H
I
J
K
FDR (Age associated divergence) =0.0651
p(Scalp)=0.000466
p(Dura)=0.772
Scalp
Dura

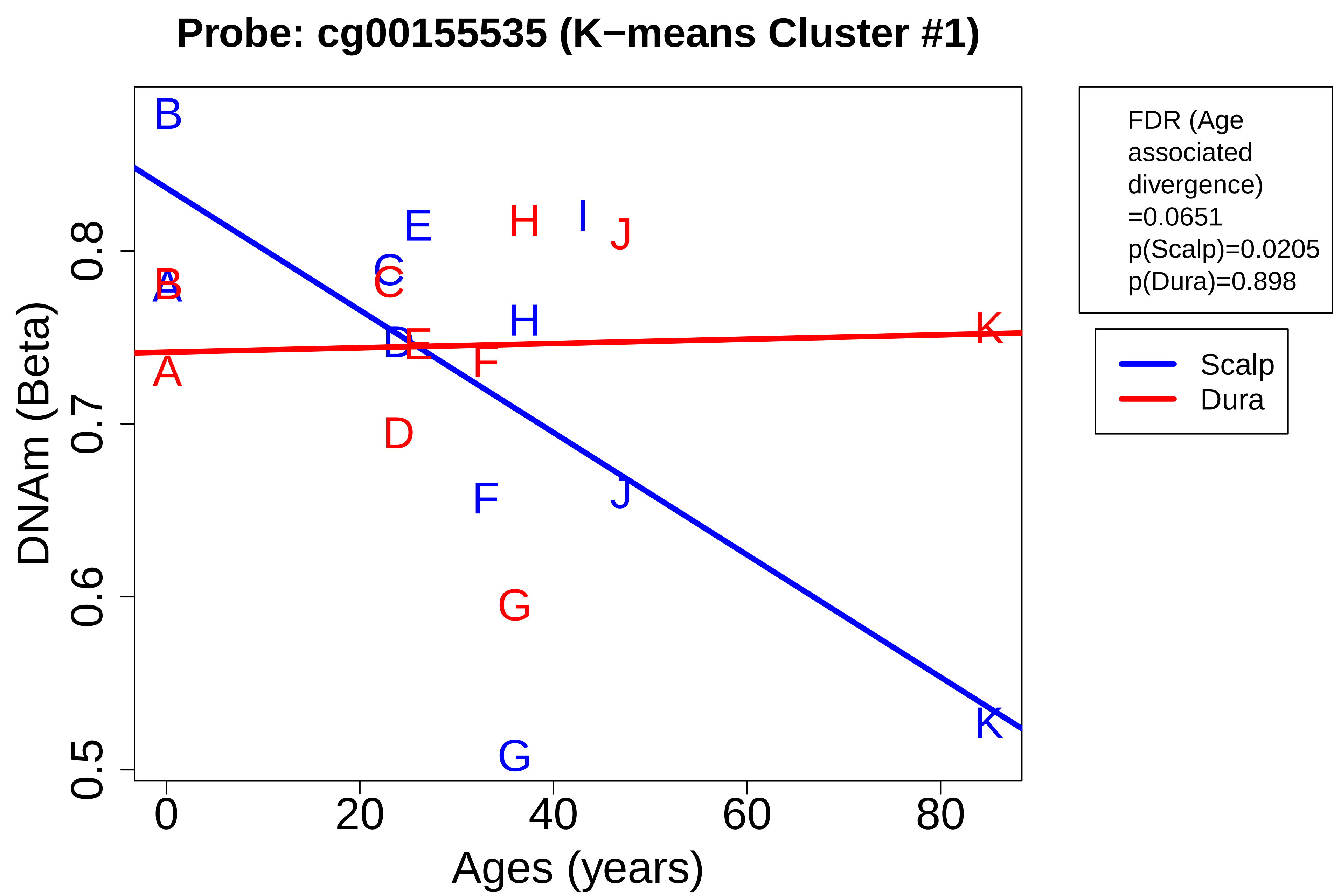
Probe: cg00155535 (K−means Cluster #1)
DNAm (Beta)
0.5
0.6
0.7
0.8
0
20
40
60
80
Ages (years)
A
B
C
D
E
F
G
H
I
J
K
FDR (Age associated divergence) =0.0651
p(Scalp)=0.0205
p(Dura)=0.898
Scalp
Dura

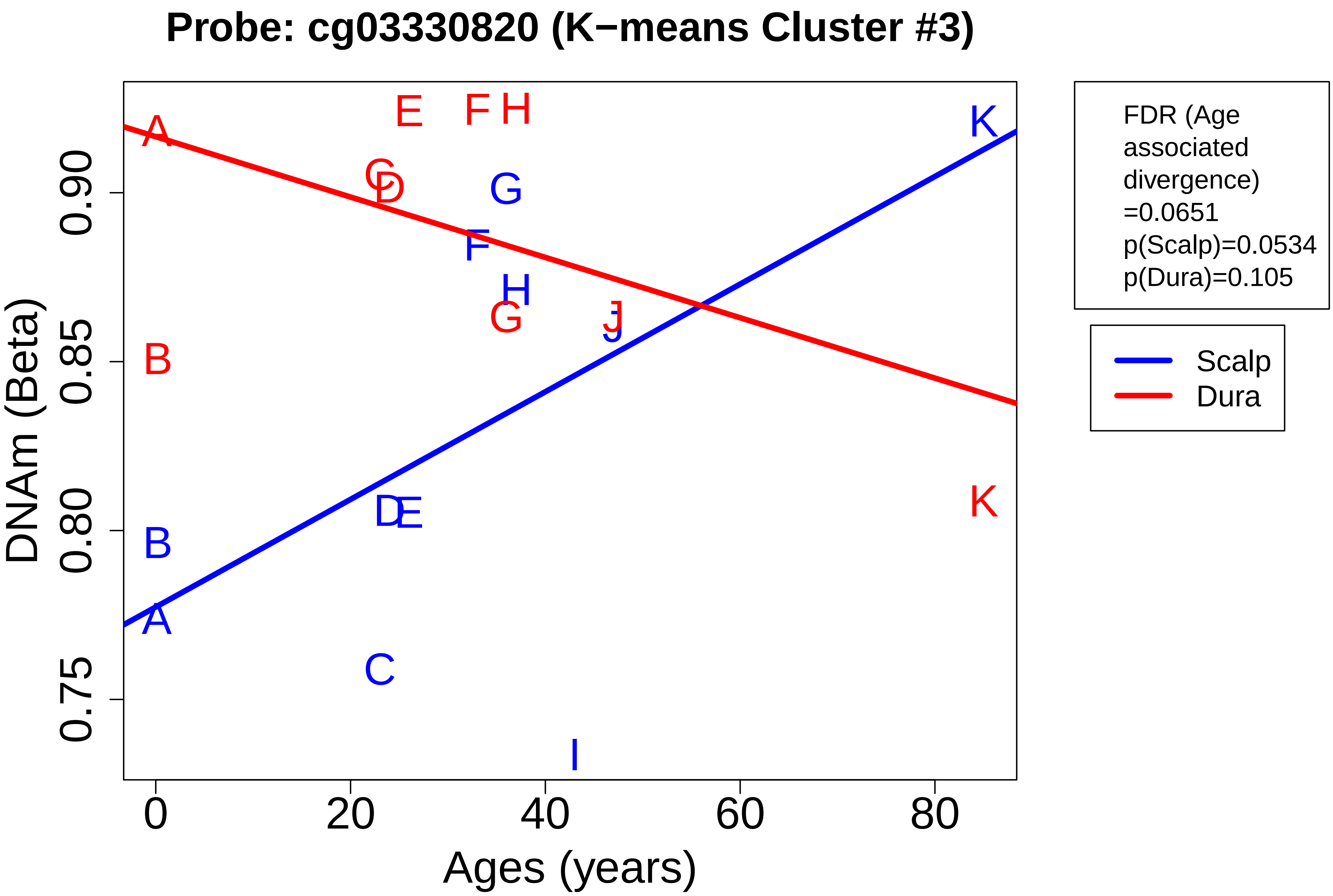

Probe: cg03330820 (K−means Cluster #3)
DNAm (Beta)
0.75
0.80
0.85
0.90
Ages (years)
0
20
40
60
80
FDR (Age associated divergence) =0.0651
p(Scalp)=0.0534
p(Dura)=0.105
Scalp
Dura

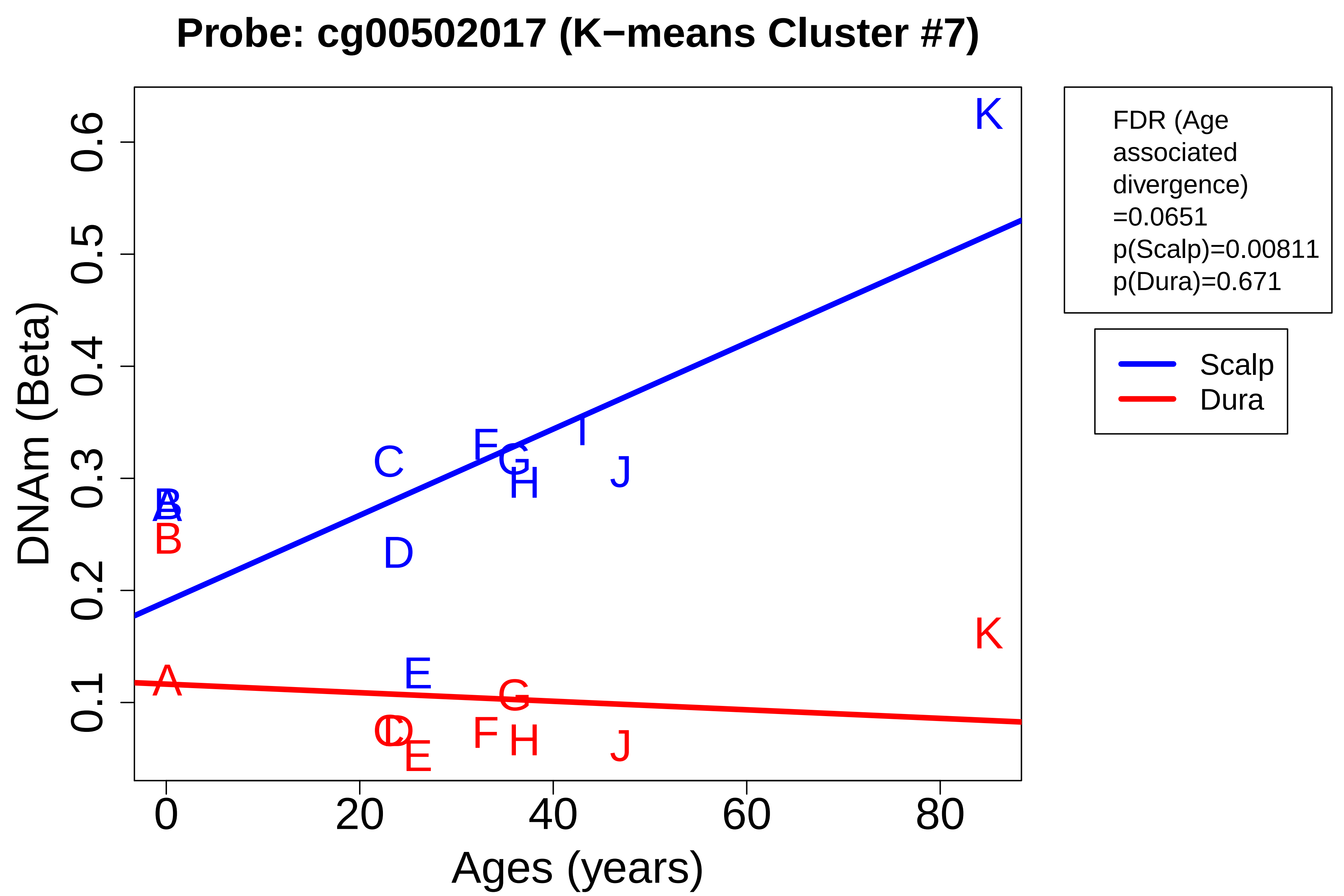

Probe: cg00502017 (K−means Cluster #7)
DNAm (Beta)
0.1
0.2
0.3
0.4
0.5
0.6
Ages (years)
0
20
40
60
80
FDR (Age associated divergence) =0.0651
p(Scalp)=0.00811
p(Dura)=0.671
Scalp
Dura

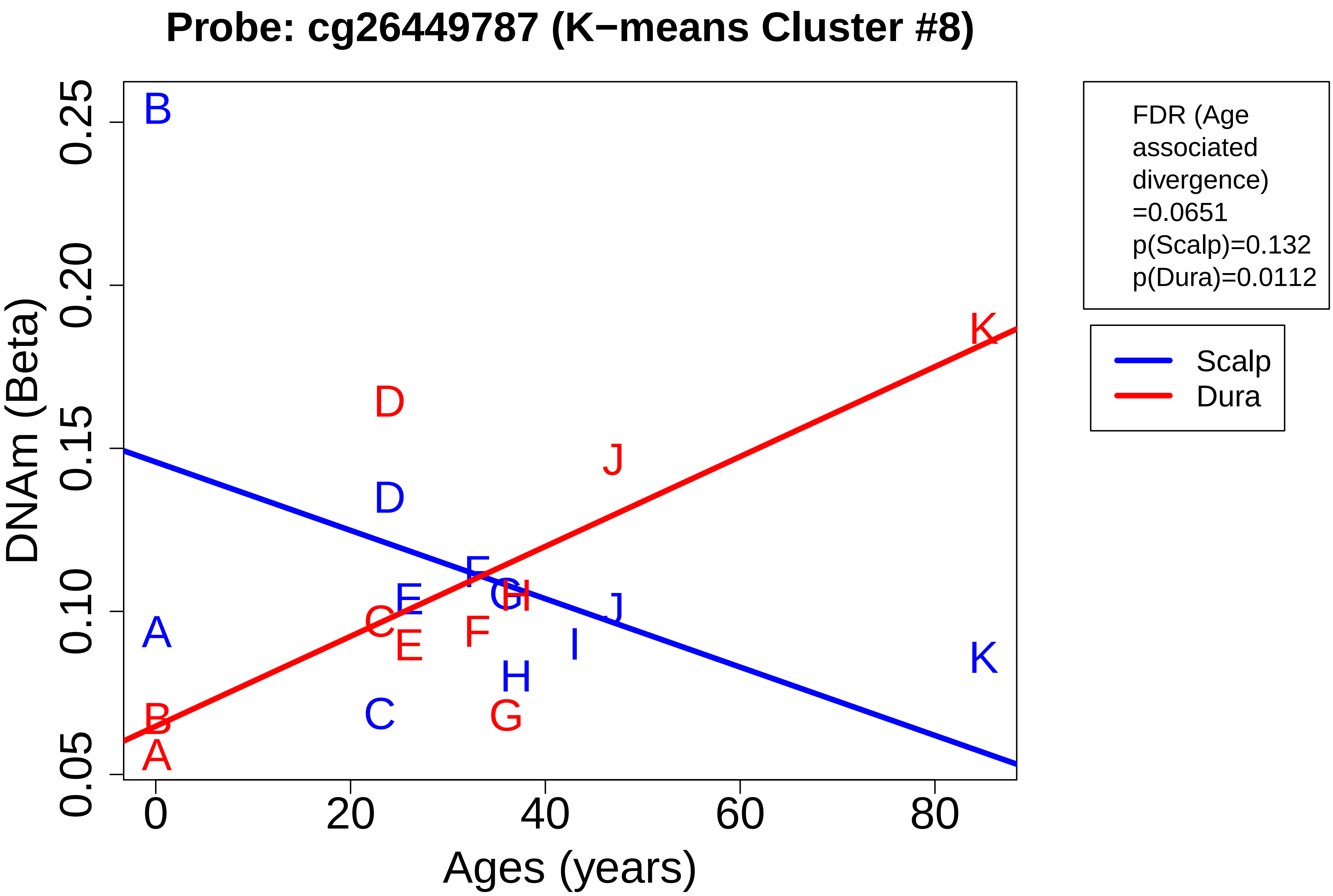
Probe: cg26449787 (K−means Cluster #8)
DNAm (Beta)
0.05
0.10
0.15
0.20
0.25
0
20
40
60
80
Ages (years)
FDR (Age associated divergence) =0.0651
p(Scalp)=0.132
p(Dura)=0.0112
Scalp
Dura
A
B
C
D
E
F
G
H
I
J
K

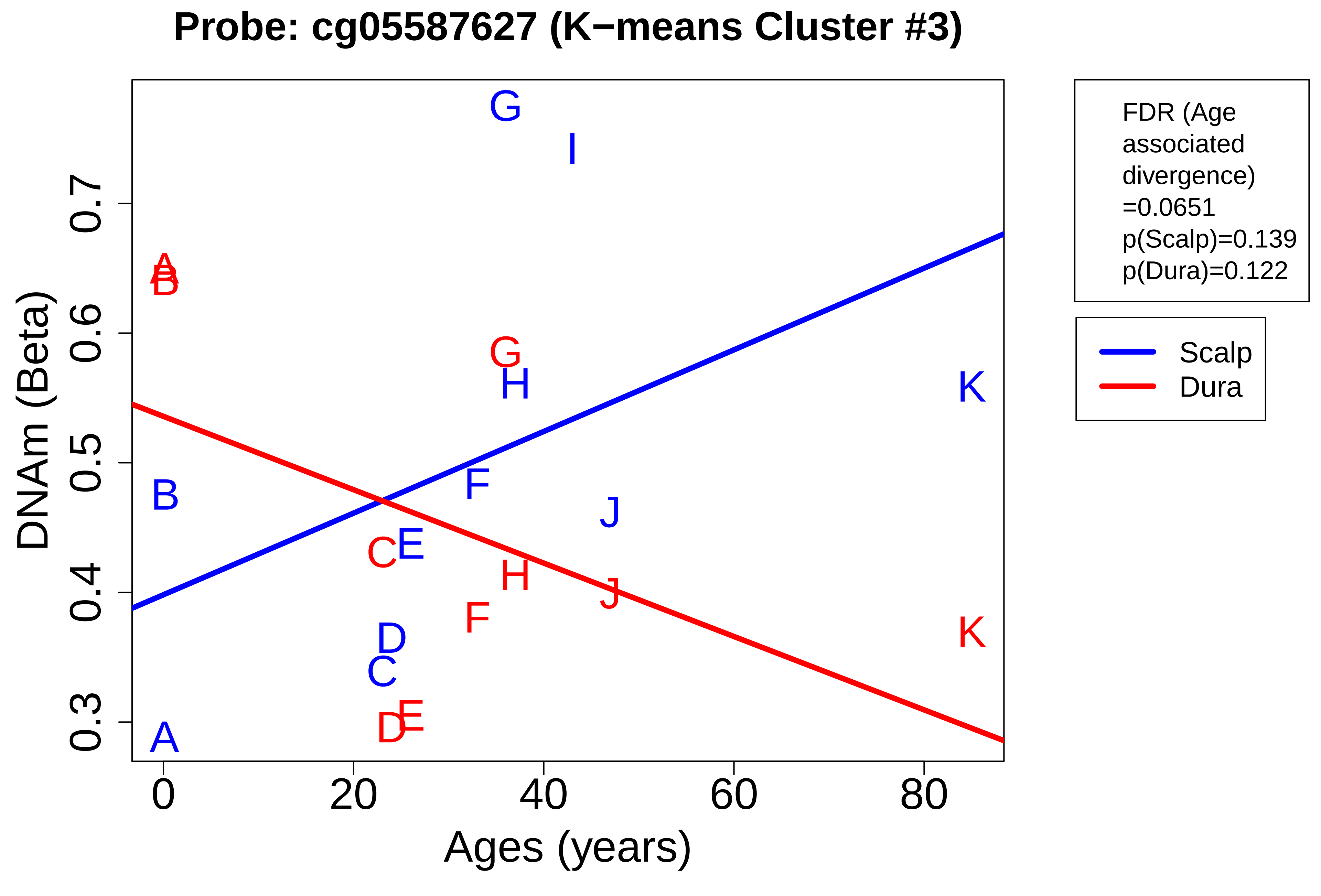

Probe: cg05587627 (K−means Cluster #3)
FDR (Age associated divergence) =0.0651
p(Scalp)=0.139
p(Dura)=0.122
Scalp
Dura
DNAm (Beta)
0.3
0.4
0.5
0.6
0.7
0
20
40
60
80
Ages (years)

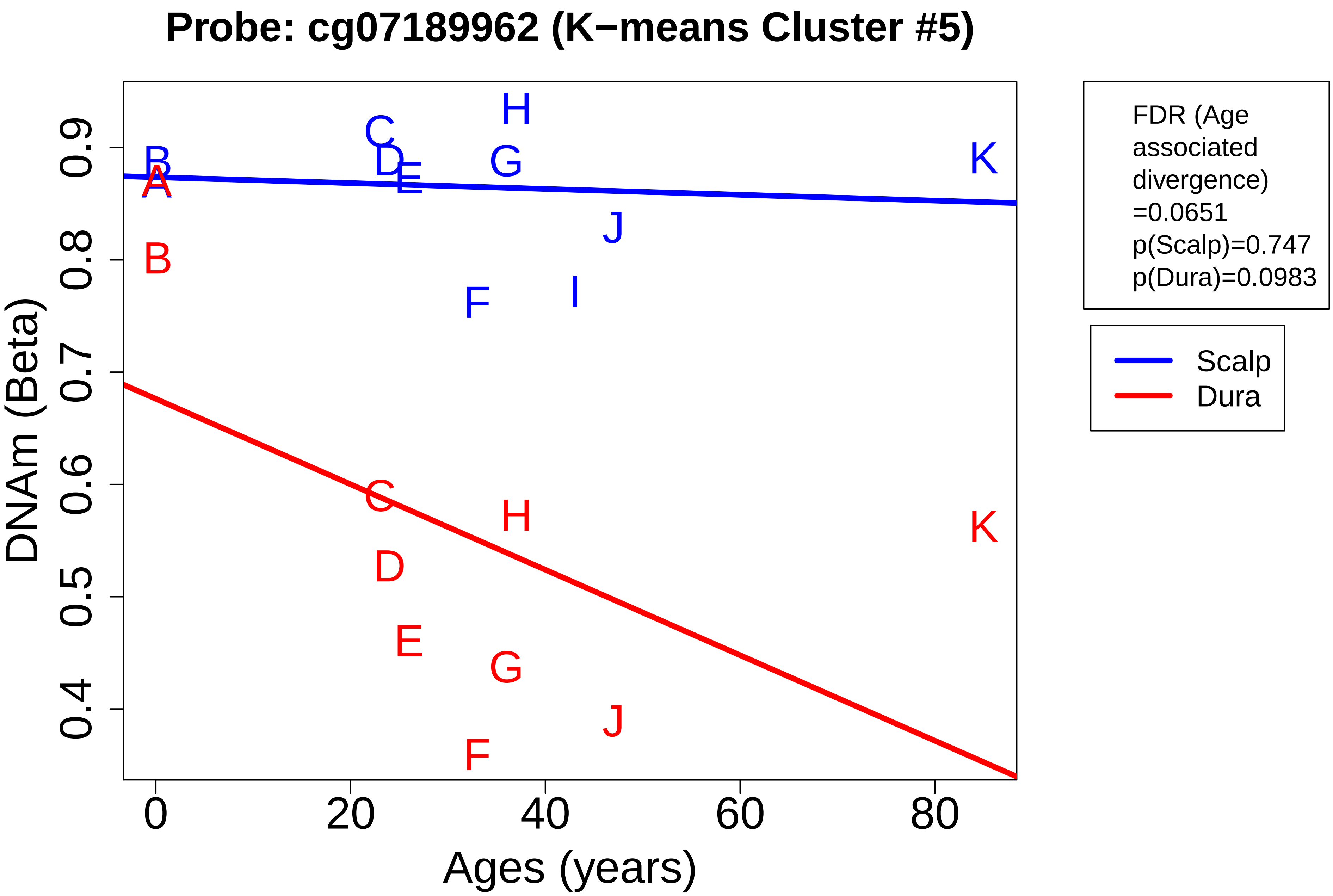

Probe: cg07189962 (K−means Cluster #5)
DNAm (Beta)
0.4
0.5
0.6
0.7
0.8
0.9
0
20
40
60
80
Ages (years)
FDR (Age associated divergence) =0.0651
p(Scalp)=0.747
p(Dura)=0.0983
Scalp
Dura

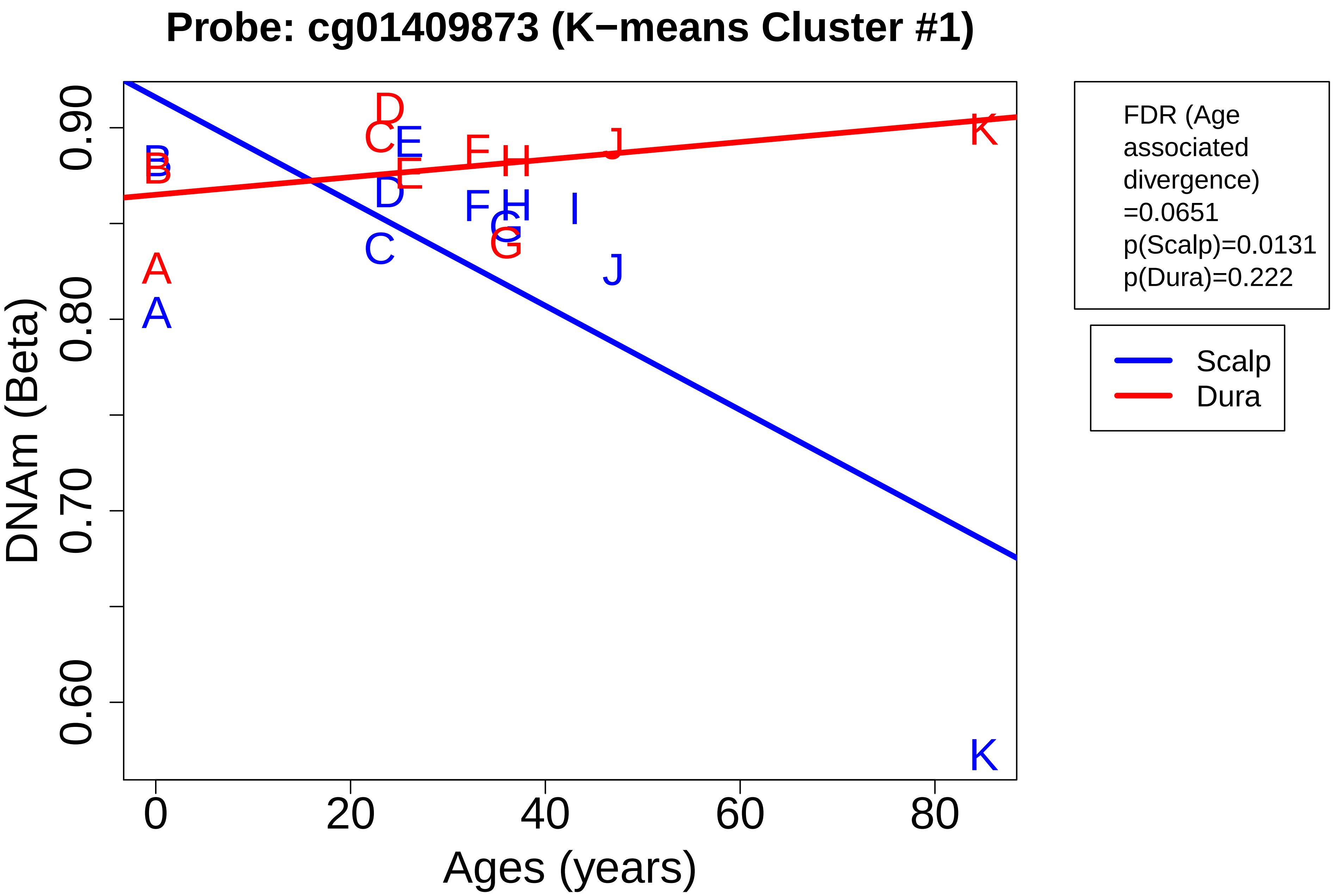

Probe: cg01409873 (K−means Cluster #1)
DNAm (Beta)
0.60
0.70
0.80
0.90
Ages (years)
0
20
40
60
80
FDR (Age associated divergence) =0.0651
p(Scalp)=0.0131
p(Dura)=0.222
Scalp
Dura
A
B
C
D
E
F
G
H
I
J
K

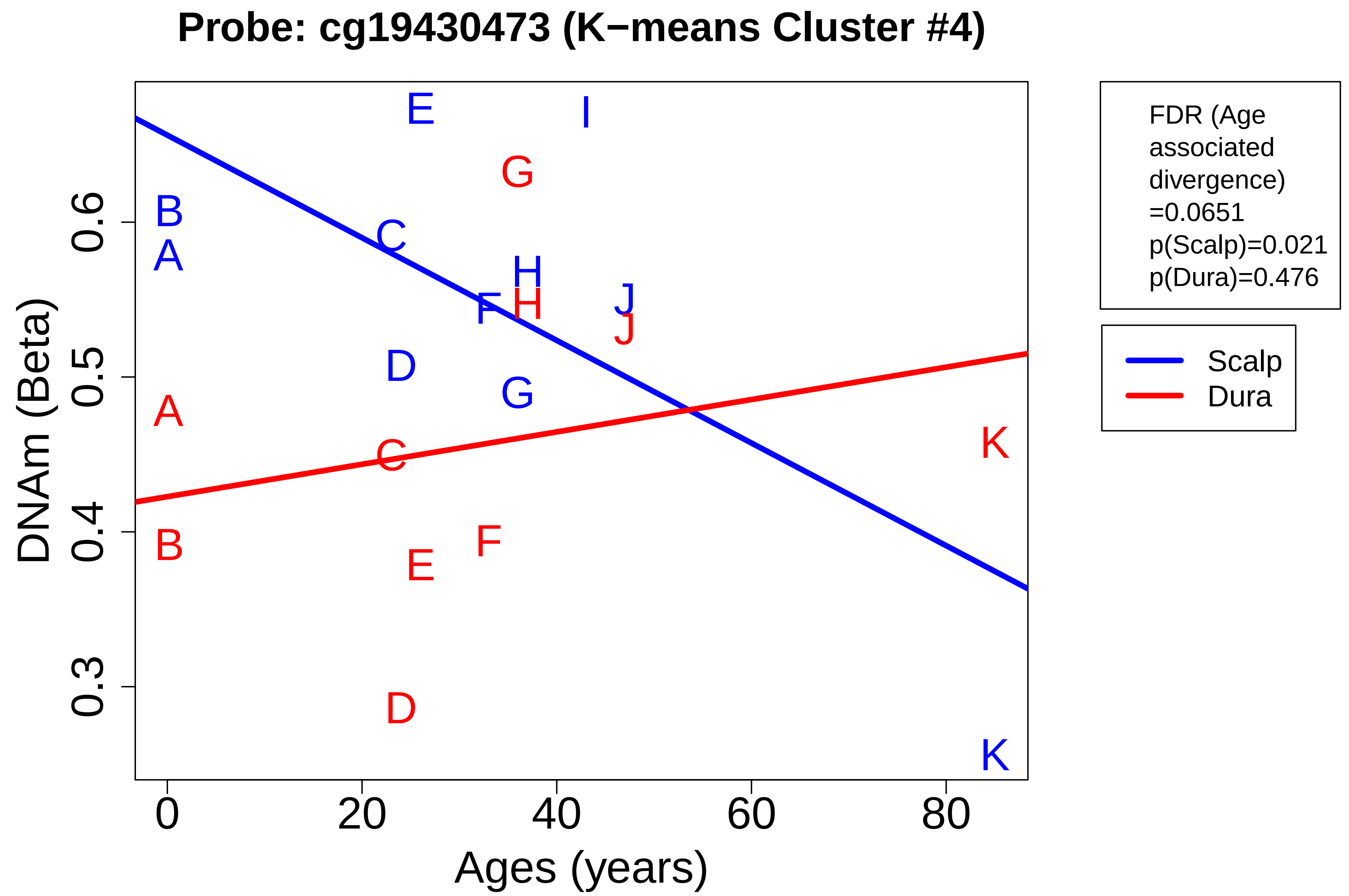
Probe: cg19430473 (K−means Cluster #4)
DNAm (Beta)
0.3
0.4
0.5
0.6
Ages (years)
0
20
40
60
80
A
B
C
D
E
F
G
H
I
J
K
FDR (Age associated divergence) =0.0651
p(Scalp)=0.021
p(Dura)=0.476
Scalp
Dura

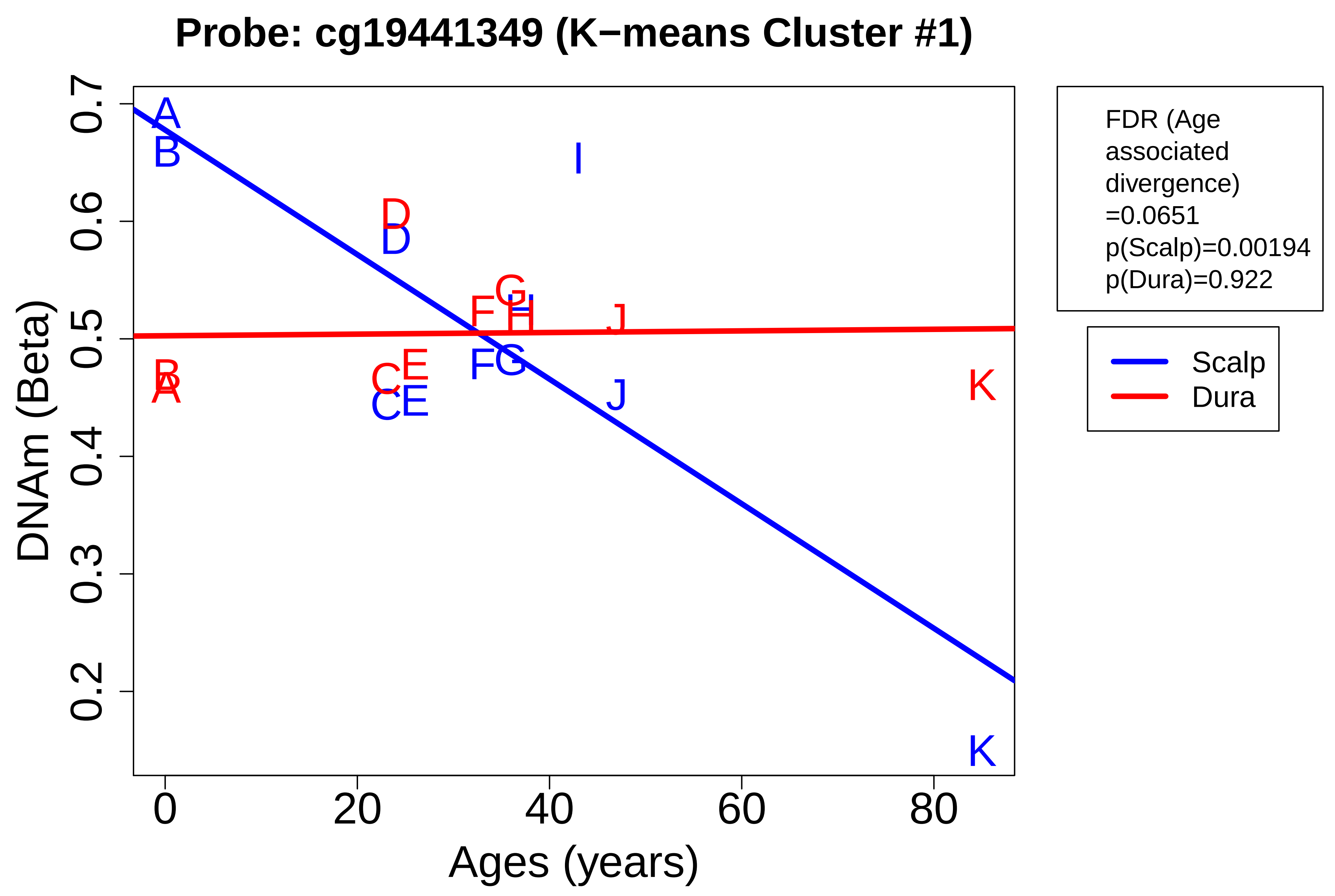

Probe: cg19441349 (K−means Cluster #1)
DNAm (Beta)
0.2
0.3
0.4
0.5
0.6
0.7
Ages (years)
0
20
40
60
80
A
B
C
D
E
F
G
H
I
J
K
FDR (Age associated divergence) =0.0651
p(Scalp)=0.00194
p(Dura)=0.922
Scalp
Dura

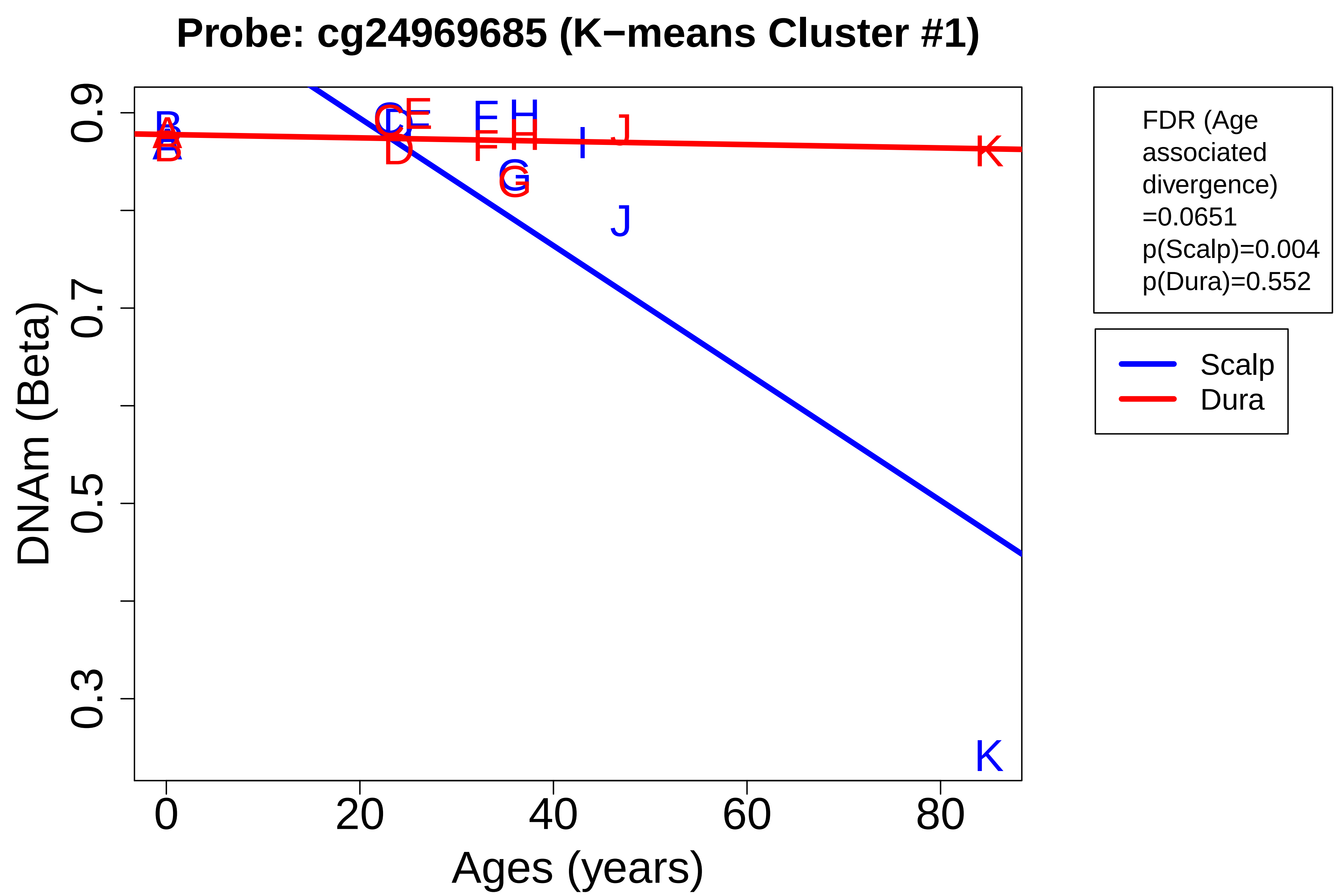

Probe: cg24969685 (K−means Cluster #1)
DNAm (Beta)
0.3
0.5
0.7
0.9
Ages (years)
0
20
40
60
80
FDR (Age associated divergence) =0.0651
p(Scalp)=0.004
p(Dura)=0.552
Scalp
Dura

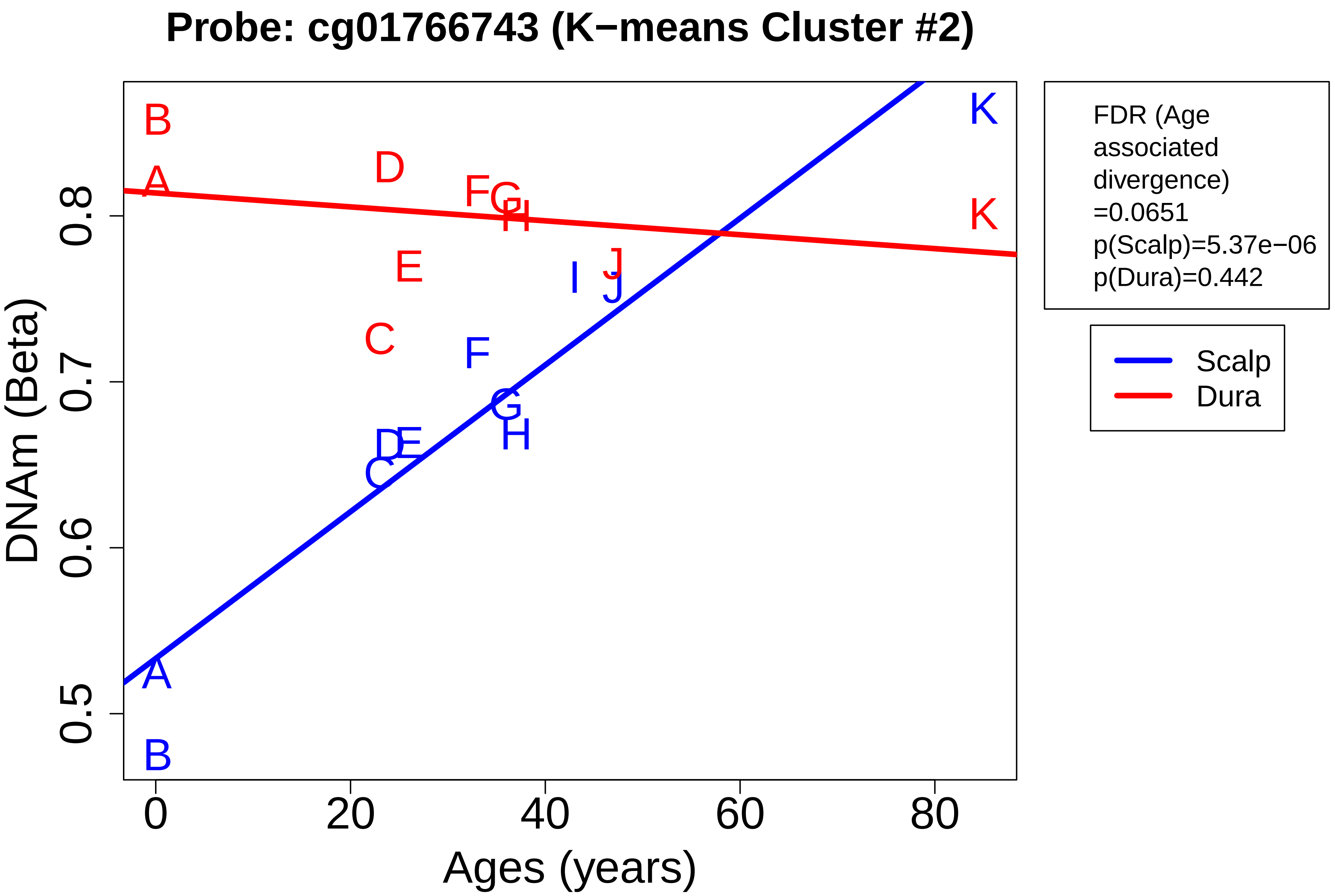
Probe: cg01766743 (K−means Cluster #2)
DNAm (Beta)
0.5
0.6
0.7
0.8
Ages (years)
0
20
40
60
80
FDR (Age associated divergence) =0.0651
p(Scalp)=5.37e−06
p(Dura)=0.442
Scalp
Dura

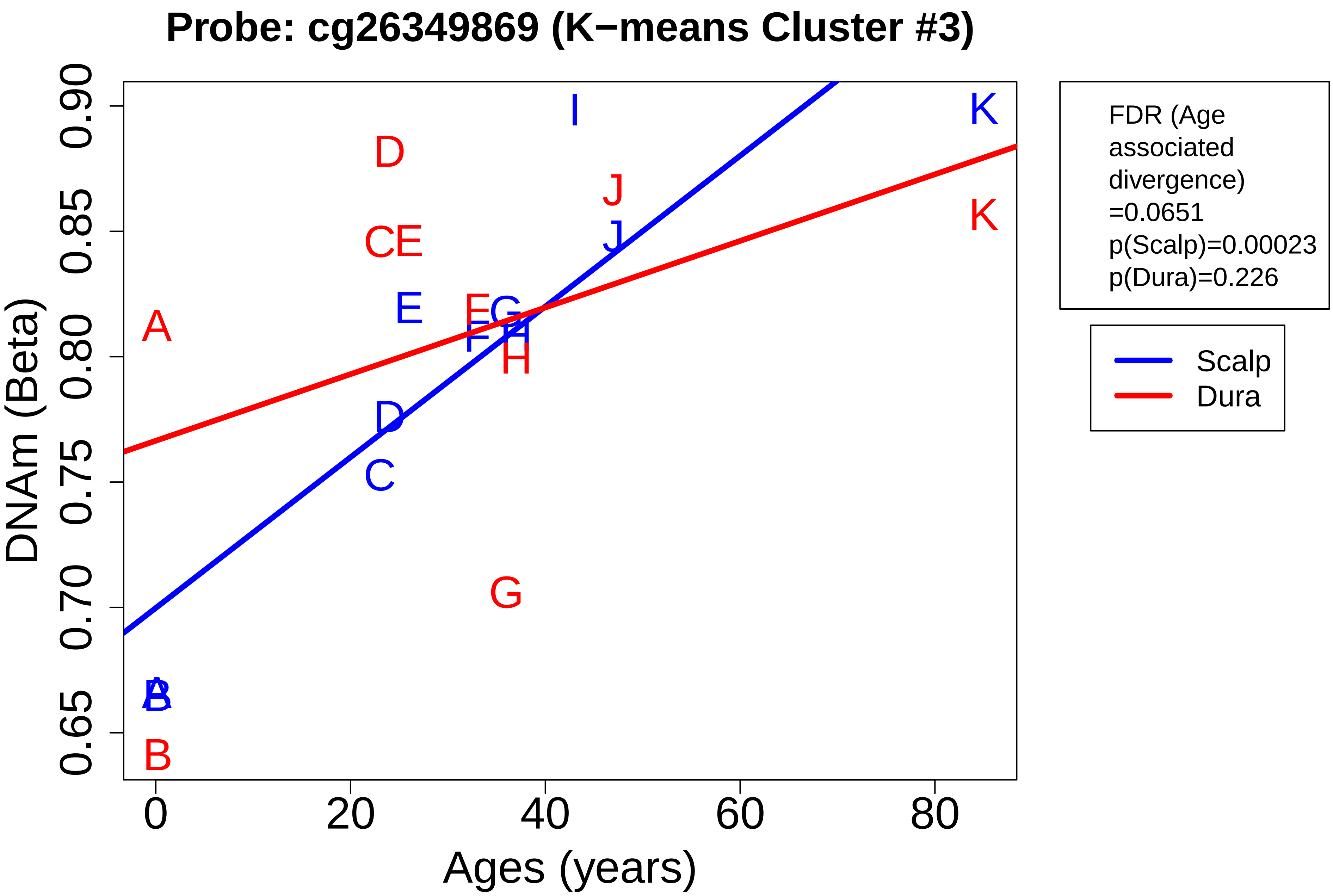

Probe: cg26349869 (K−means Cluster #3)
DNAm (Beta)
0.65
0.70
0.75
0.80
0.85
0.90
0
20
40
60
80
Ages (years)
FDR (Age associated divergence) =0.0651
p(Scalp)=0.00023
p(Dura)=0.226
Scalp
Dura

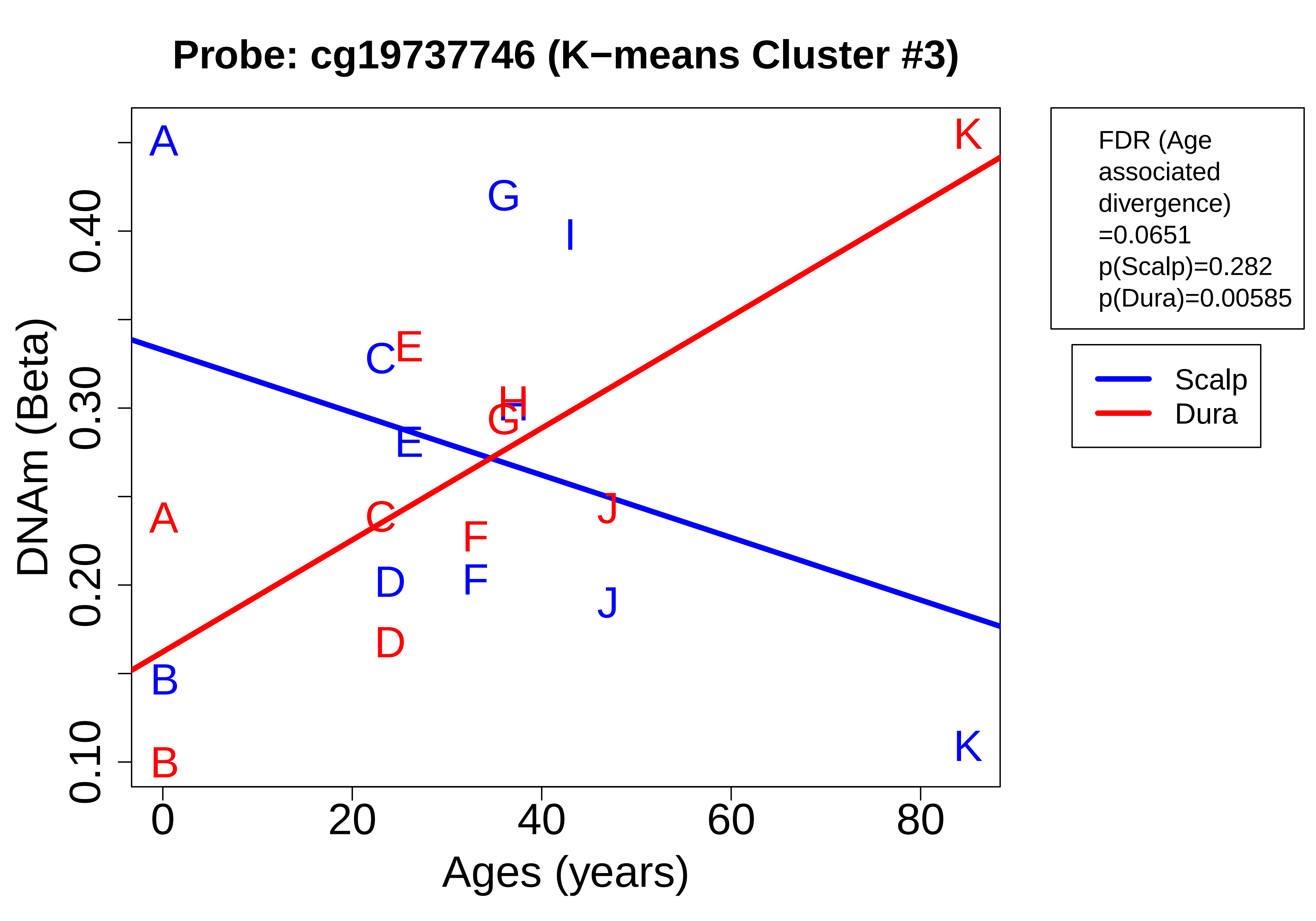

Probe: cg19737746 (K−means Cluster #3)
DNAm (Beta)
0.10
0.20
0.30
0.40
0
20
40
60
80
Ages (years)
A
B
C
D
E
F
G
H
I
J
K
FDR (Age associated divergence) =0.0651
p(Scalp)=0.282
p(Dura)=0.00585
Scalp
Dura

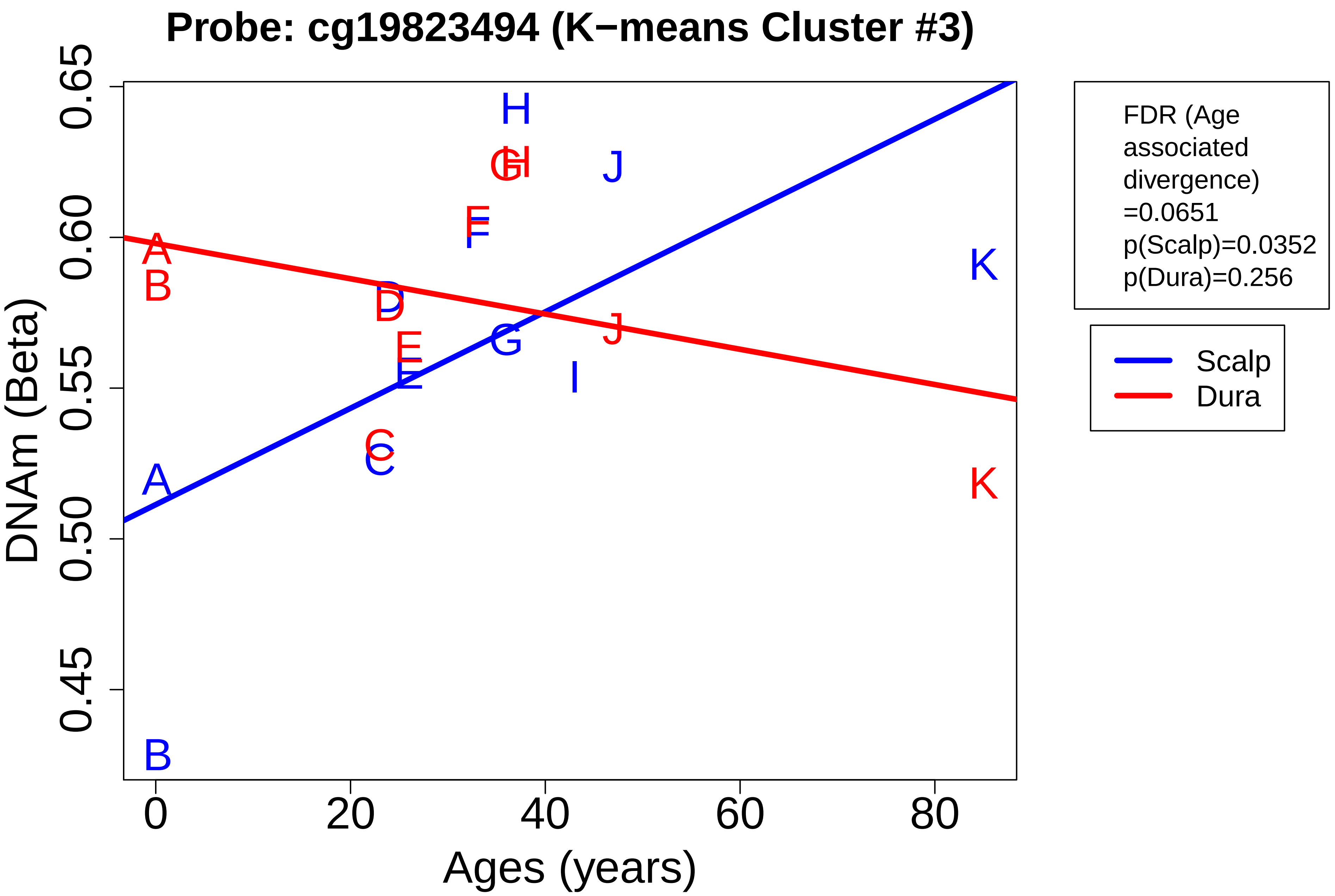

Probe: cg19823494 (K−means Cluster #3)
FDR (Age associated divergence) =0.0651
p(Scalp)=0.0352
p(Dura)=0.256
Scalp
Dura
DNAm (Beta)
0.45
0.50
0.55
0.60
0.65
Ages (years)
0
20
40
60
80
A
B
C
D
E
F
G
H
I
J
K

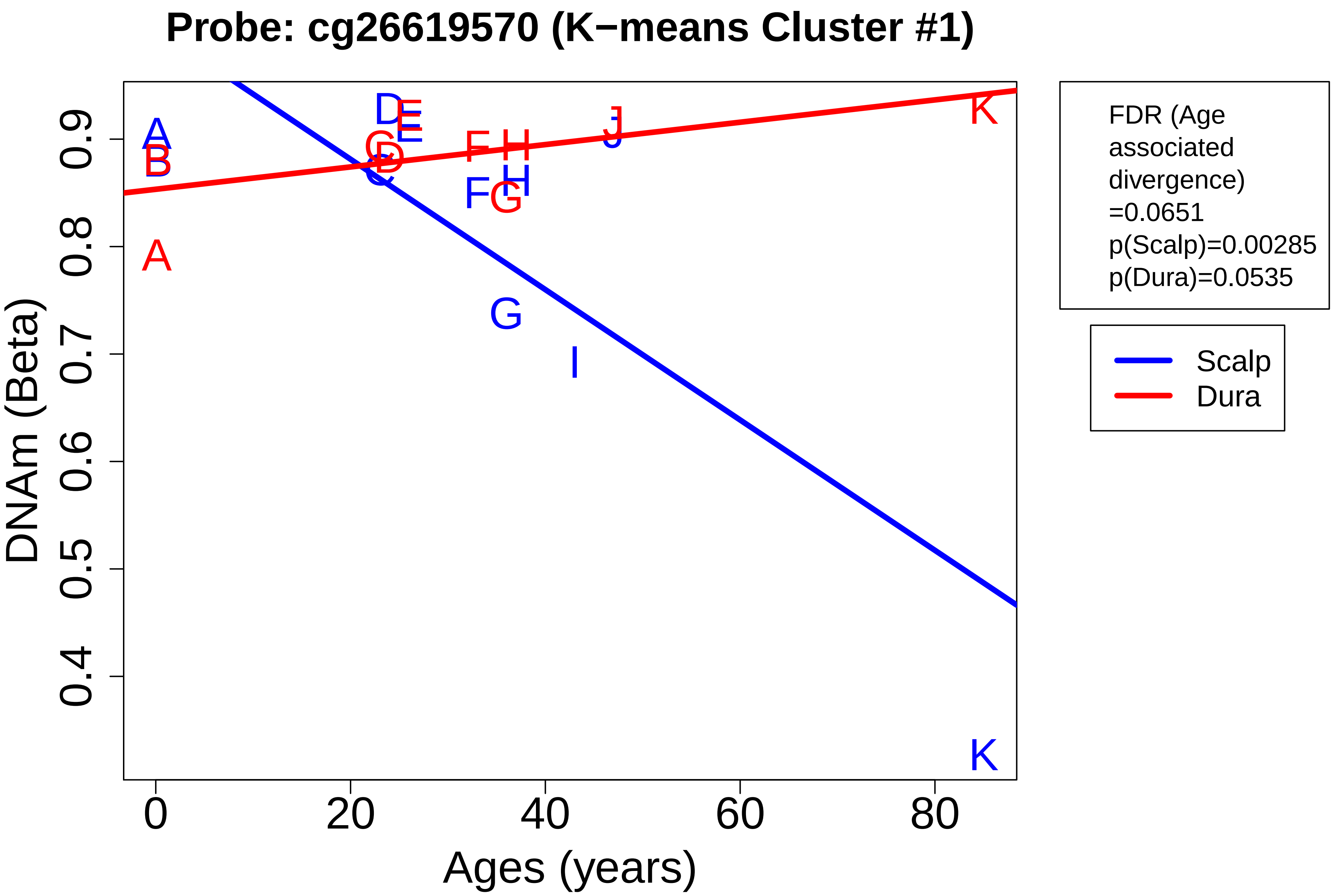

Probe: cg26619570 (K−means Cluster #1)
DNAm (Beta)
0.4
0.5
0.6
0.7
0.8
0.9
0
20
40
60
80
Ages (years)
FDR (Age associated divergence) =0.0651
p(Scalp)=0.00285
p(Dura)=0.0535
Scalp
Dura
A
B
C
D
E
F
G
H
I
J
K

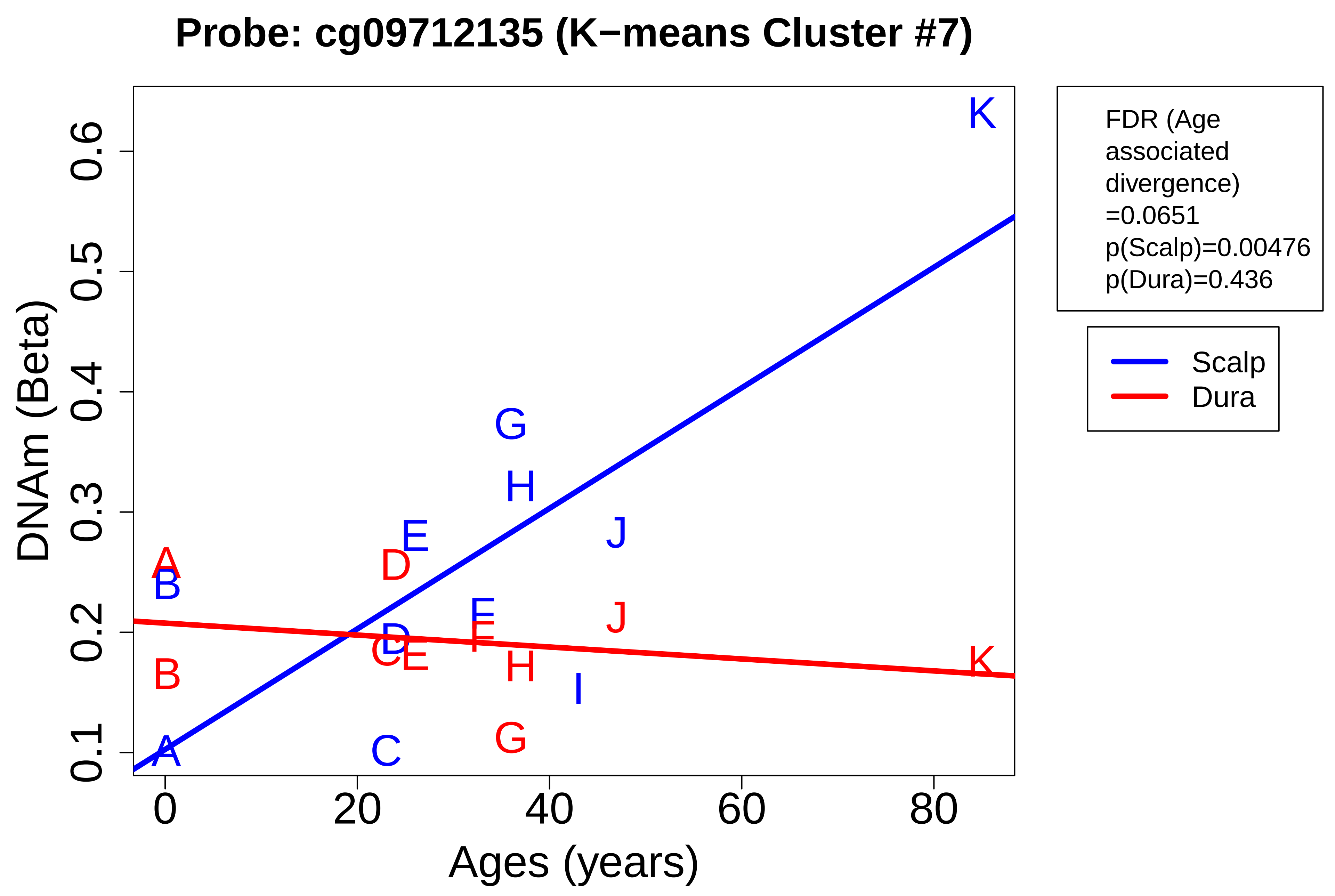

Probe: cg09712135 (K−means Cluster #7)
DNAm (Beta)
0.1
0.2
0.3
0.4
0.5
0.6
0
20
40
60
80
Ages (years)
A
B
C
D
E
F
G
H
I
J
K
FDR (Age associated divergence) =0.0651
p(Scalp)=0.00476
p(Dura)=0.436
Scalp
Dura

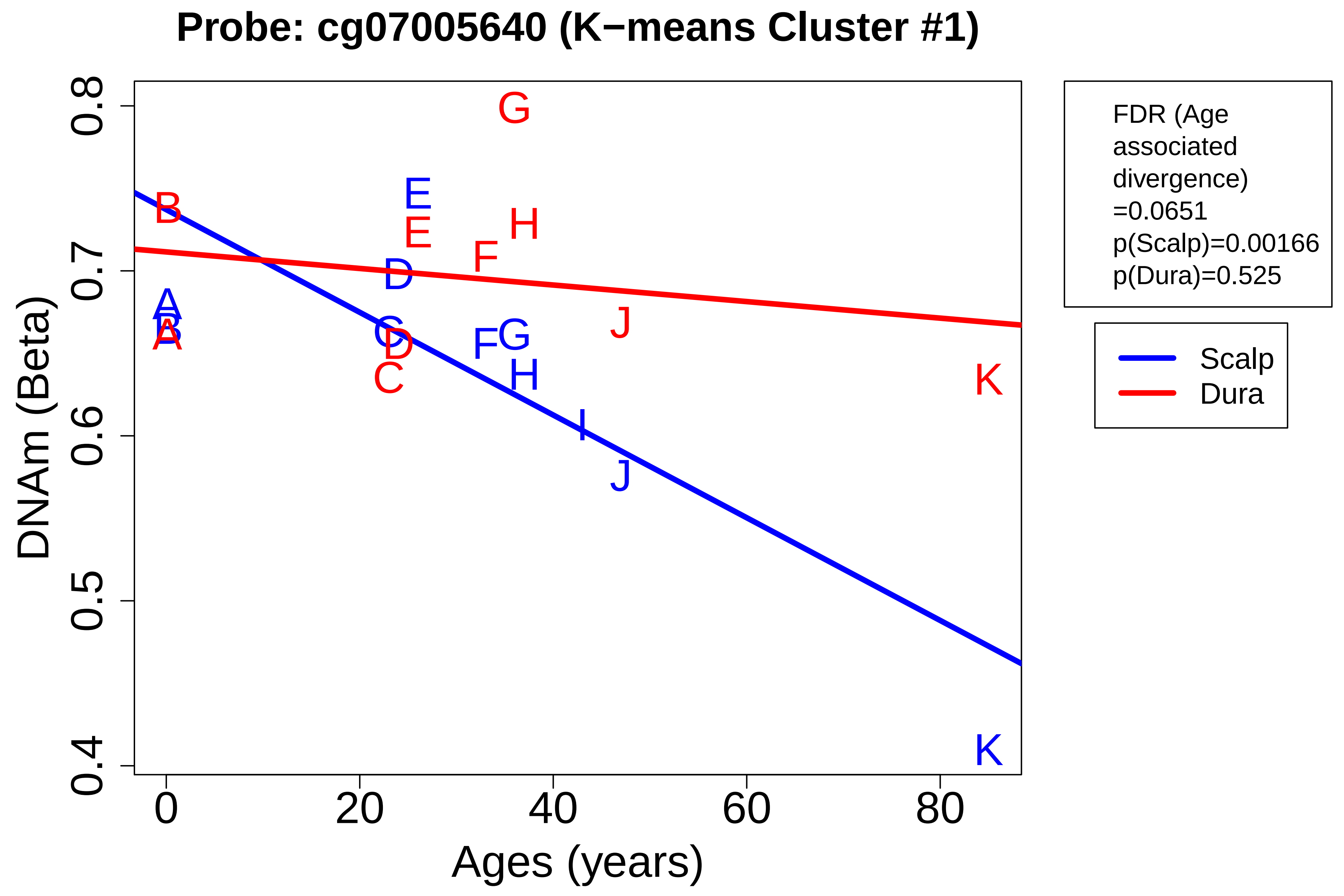

Probe: cg07005640 (K−means Cluster #1)
DNAm (Beta)
0.4
0.5
0.6
0.7
0.8
0
20
40
60
80
Ages (years)
FDR (Age associated divergence) =0.0651
p(Scalp)=0.00166
p(Dura)=0.525
Scalp
Dura

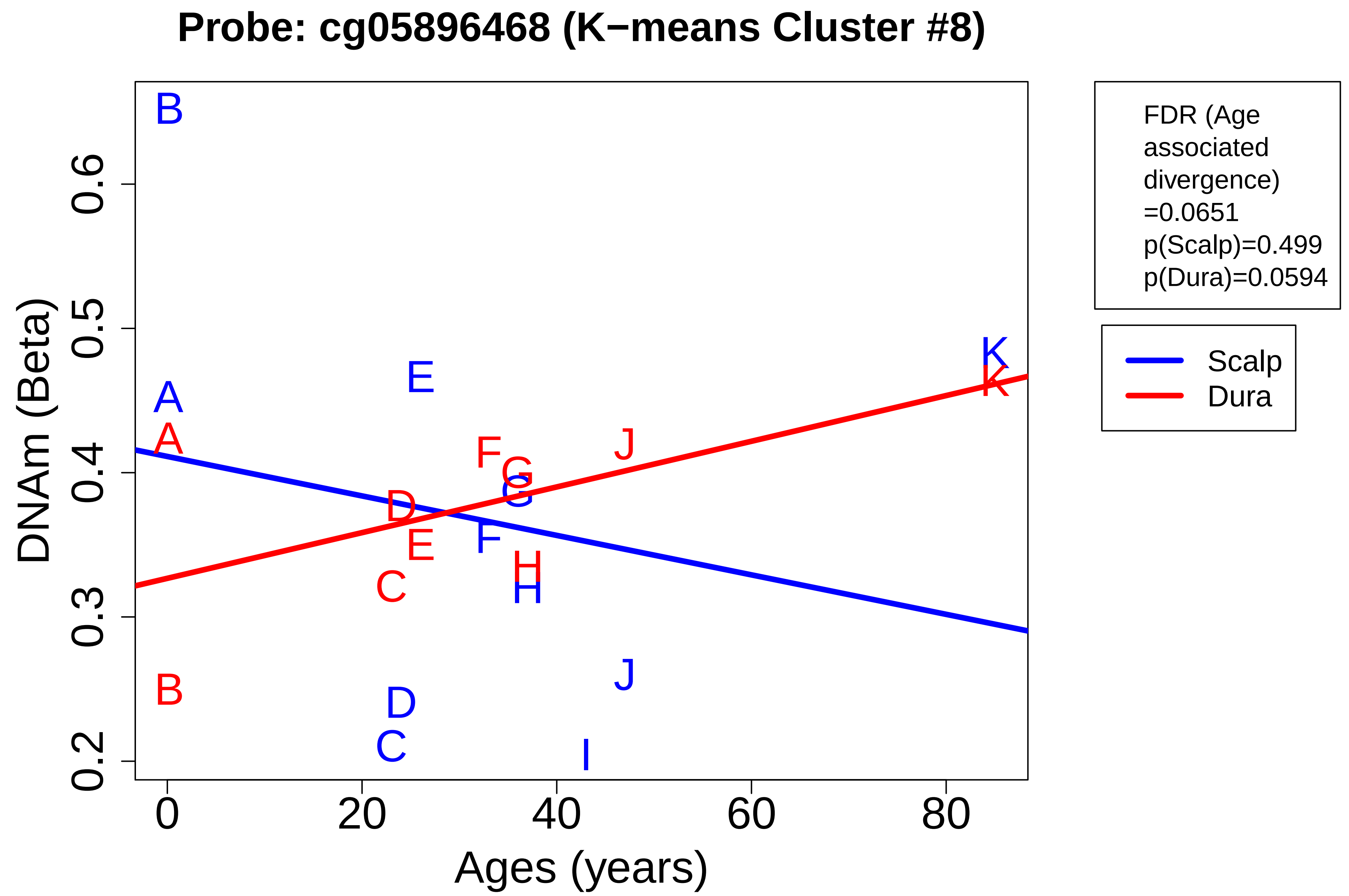
Probe: cg05896468 (K−means Cluster #8)
FDR (Age associated divergence) =0.0651
p(Scalp)=0.499
p(Dura)=0.0594
Scalp
Dura
DNAm (Beta)
Ages (years)
0.2
0.3
0.4
0.5
0.6
0
20
40
60
80
A
B
C
D
E
F
G
H
I
J
K

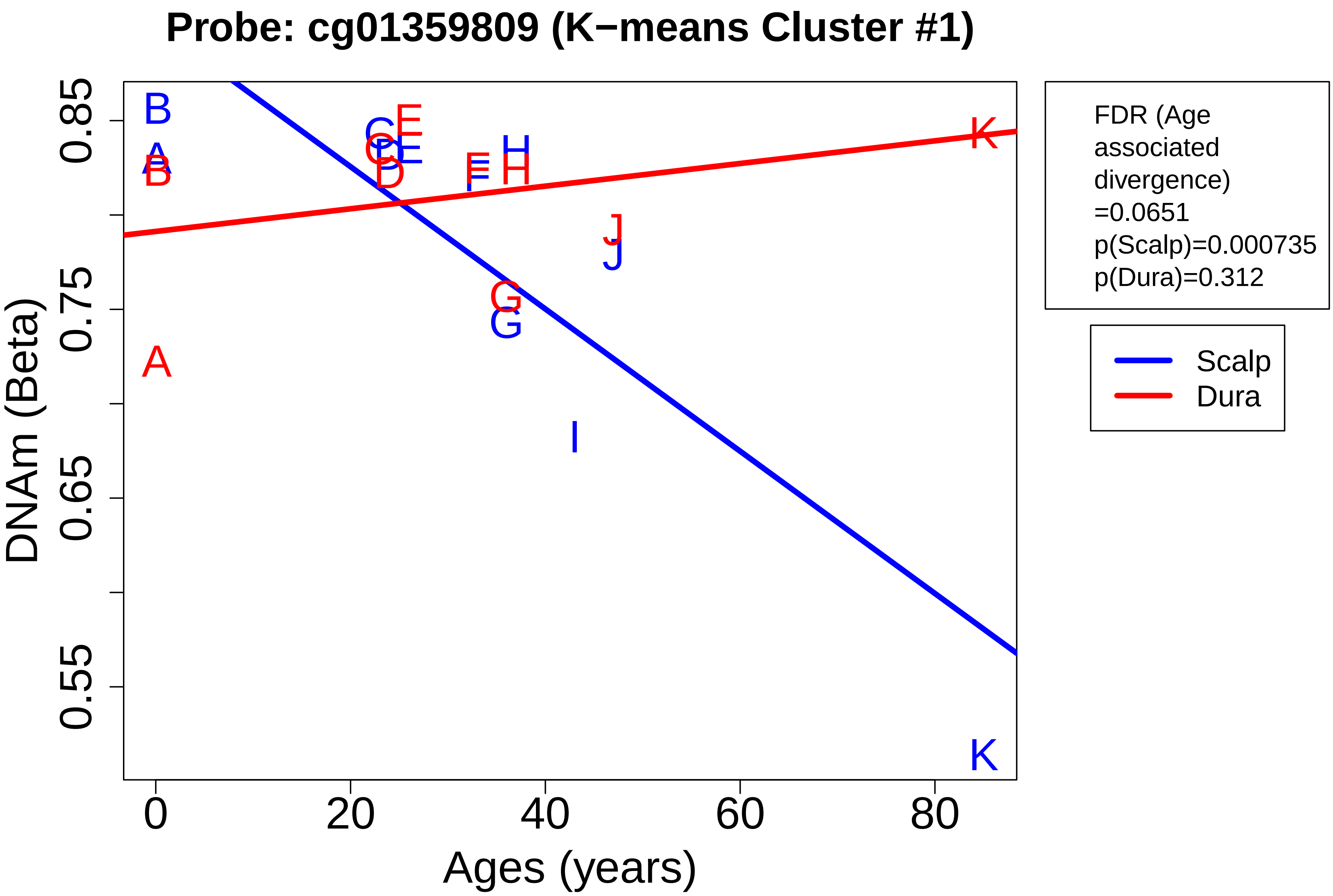

Probe: cg01359809 (K−means Cluster #1)
DNAm (Beta)
0.55
0.65
0.75
0.85
Ages (years)
0
20
40
60
80
FDR (Age associated divergence) =0.0651
p(Scalp)=0.000735
p(Dura)=0.312
Scalp
Dura

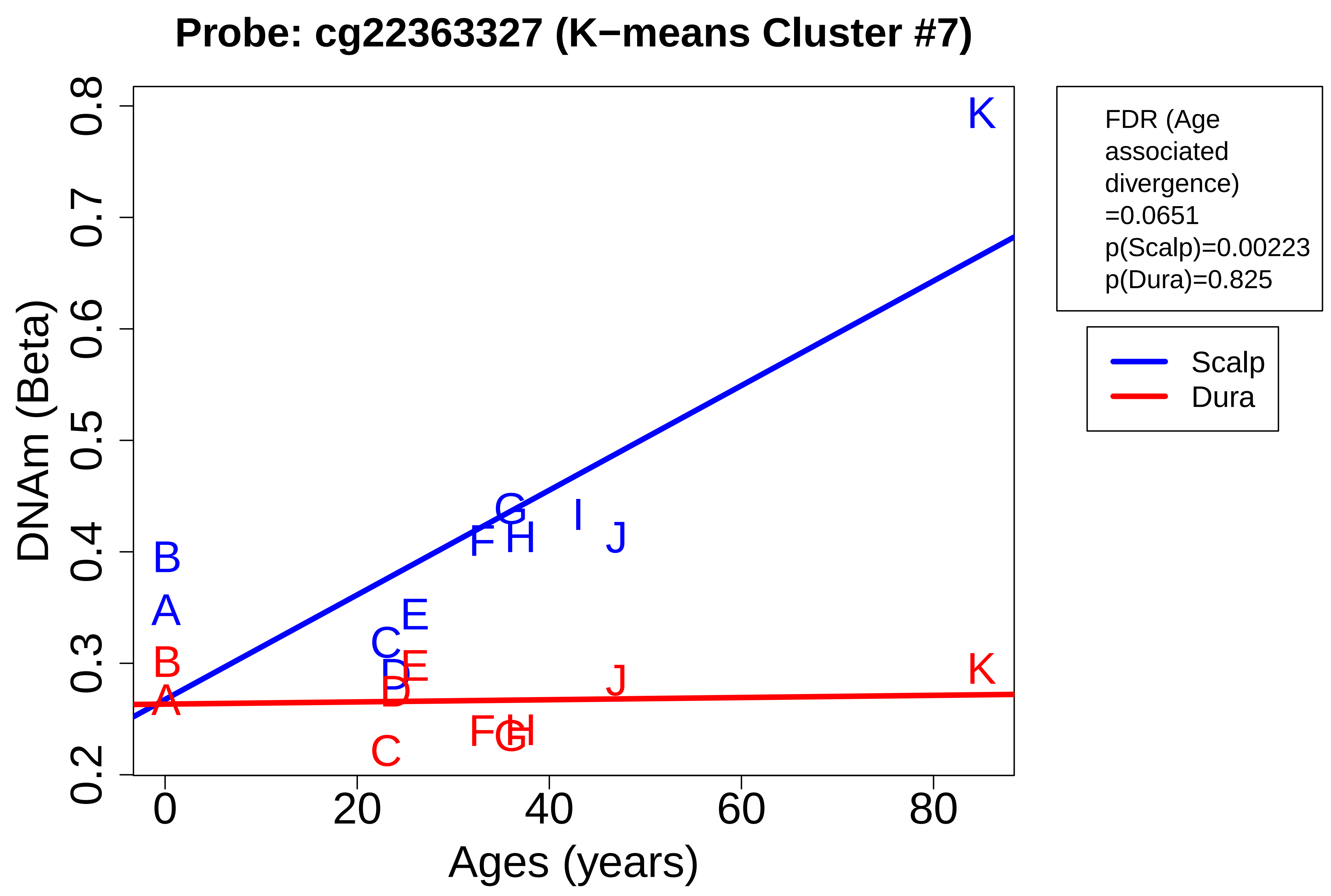

Probe: cg22363327 (K−means Cluster #7)
DNAm (Beta)
0.2
0.3
0.4
0.5
0.6
0.7
0.8
Ages (years)
0
20
40
60
80
FDR (Age associated divergence) =0.0651
p(Scalp)=0.00223
p(Dura)=0.825
Scalp
Dura

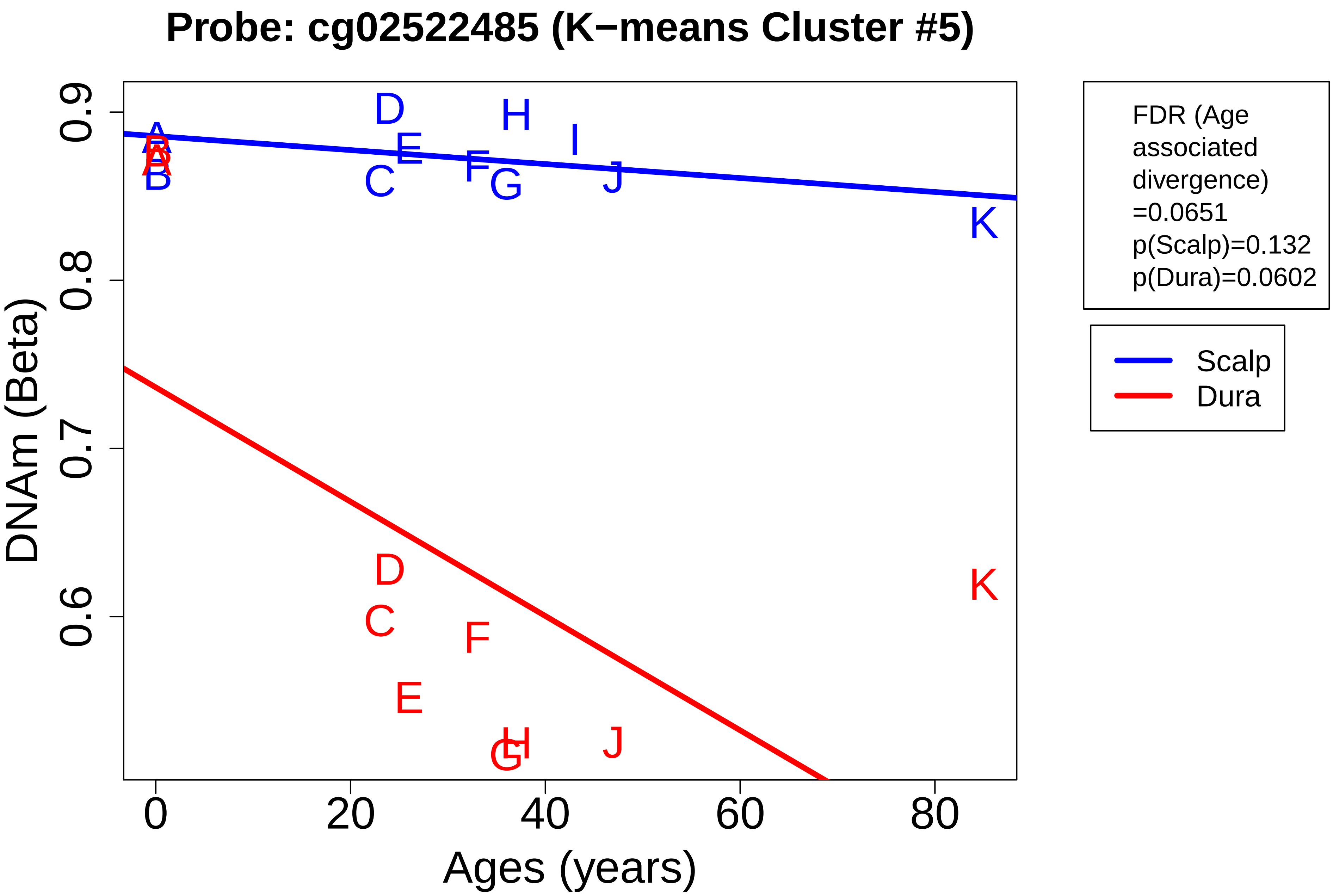

Probe: cg02522485 (K−means Cluster #5)
DNAm (Beta)
0.6
0.7
0.8
0.9
Ages (years)
0
20
40
60
80
FDR (Age associated divergence) =0.0651
p(Scalp)=0.132
p(Dura)=0.0602
Scalp
Dura

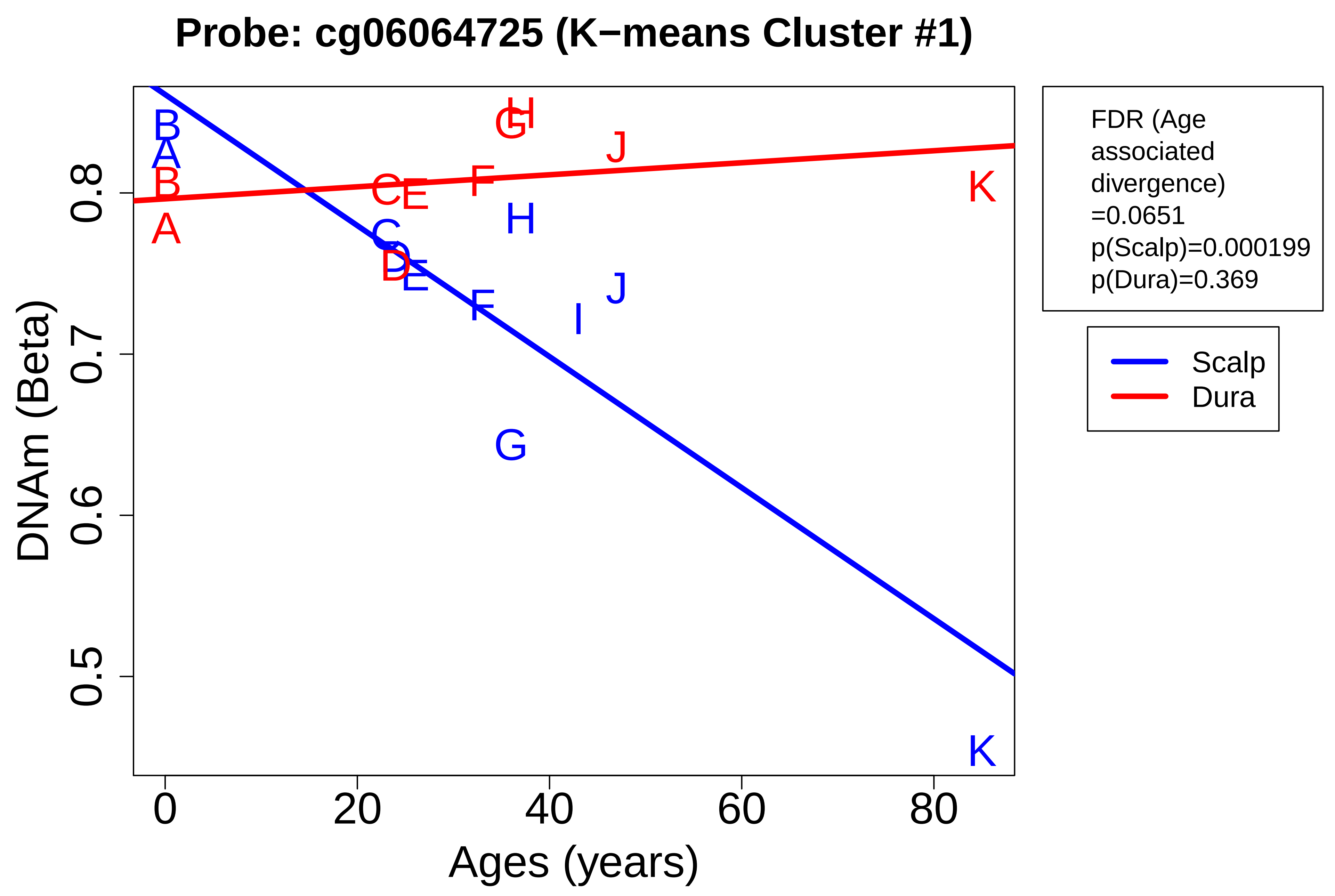

Probe: cg06064725 (K−means Cluster #1)
DNAm (Beta)
0.5
0.6
0.7
0.8
0
20
40
60
80
Ages (years)
A
B
C
D
E
F
G
H
I
J
K
A
B
C
D
E
F
G
H
J
K
FDR (Age associated divergence) =0.0651
p(Scalp)=0.000199
p(Dura)=0.369
Scalp
Dura

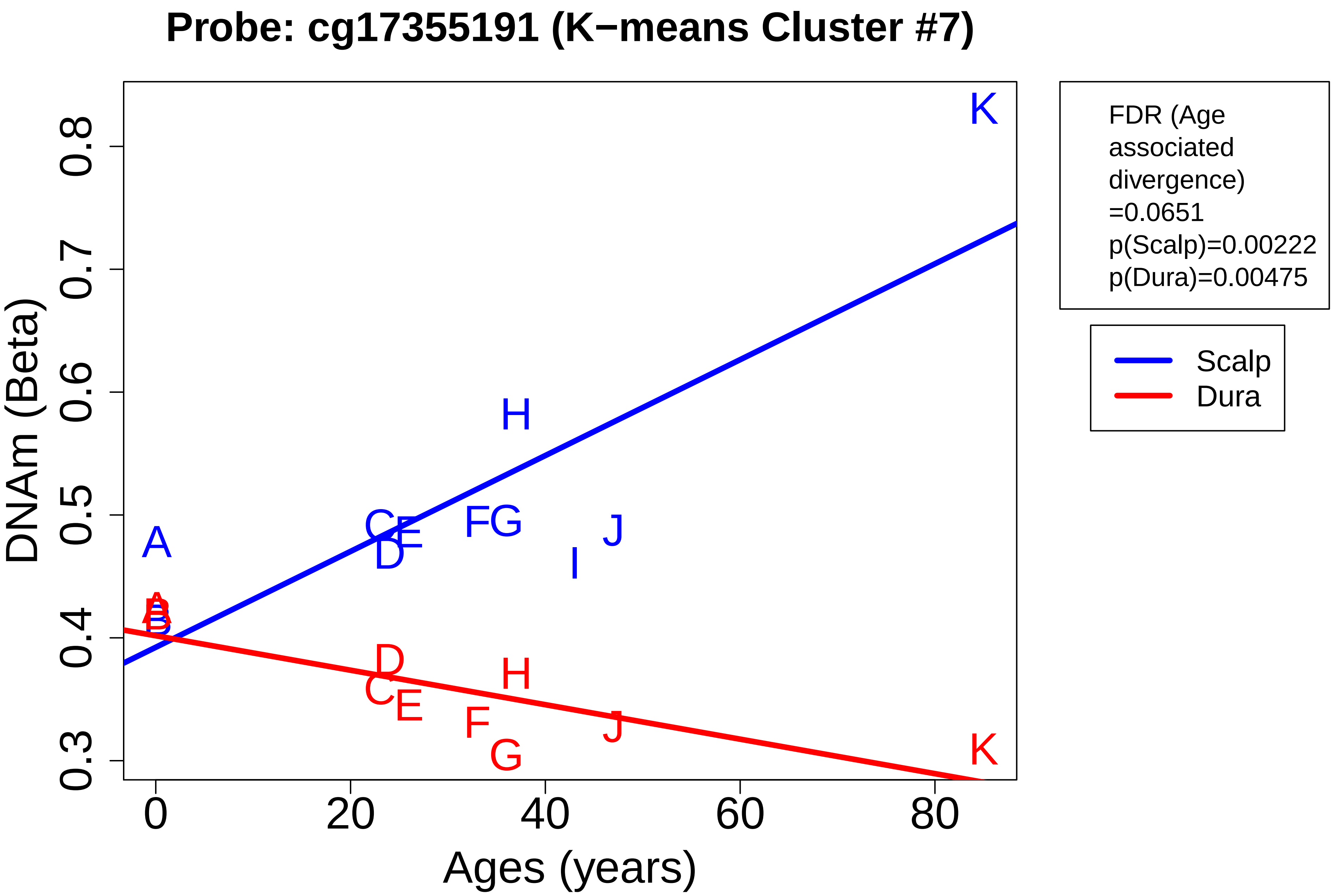

Probe: cg17355191 (K−means Cluster #7)
FDR (Age associated divergence) =0.0651
p(Scalp)=0.00222
p(Dura)=0.00475
Scalp
Dura
DNAm (Beta)
0.3
0.4
0.5
0.6
0.7
0.8
0
20
40
60
80
Ages (years)
A
B
C
D
E
F
G
H
I
J
K

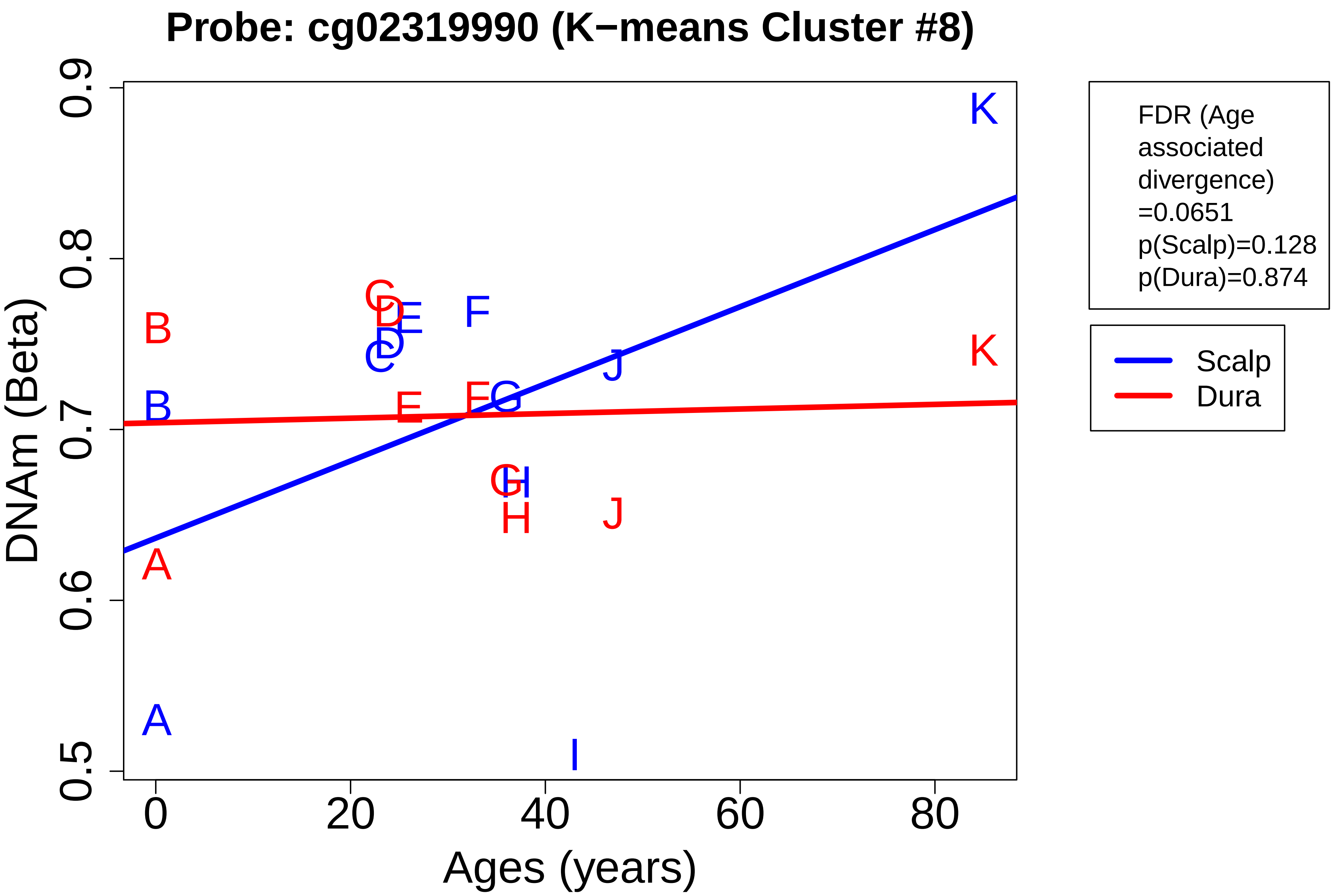
Probe: cg02319990 (K−means Cluster #8)
DNAm (Beta)
0.5
0.6
0.7
0.8
0.9
0
20
40
60
80
Ages (years)
A
B
C
D
E
F
G
H
I
J
K
FDR (Age associated divergence) =0.0651
p(Scalp)=0.128
p(Dura)=0.874
Scalp
Dura

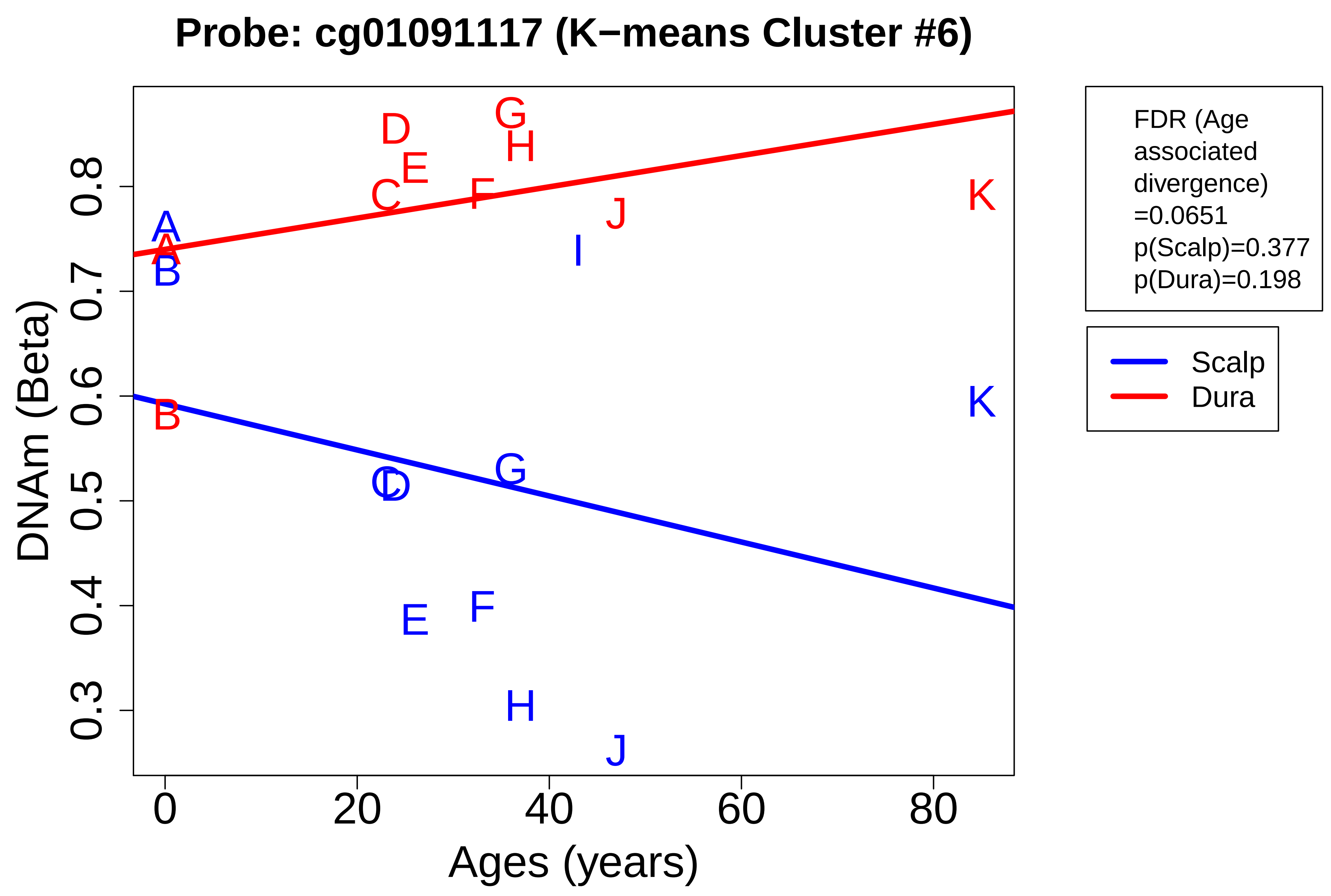

Probe: cg01091117 (K−means Cluster #6)
DNAm (Beta)
0.3
0.4
0.5
0.6
0.7
0.8
0
20
40
60
80
Ages (years)
FDR (Age associated divergence) =0.0651
p(Scalp)=0.377
p(Dura)=0.198
Scalp
Dura
A
B
C
D
E
F
G
H
I
J
K

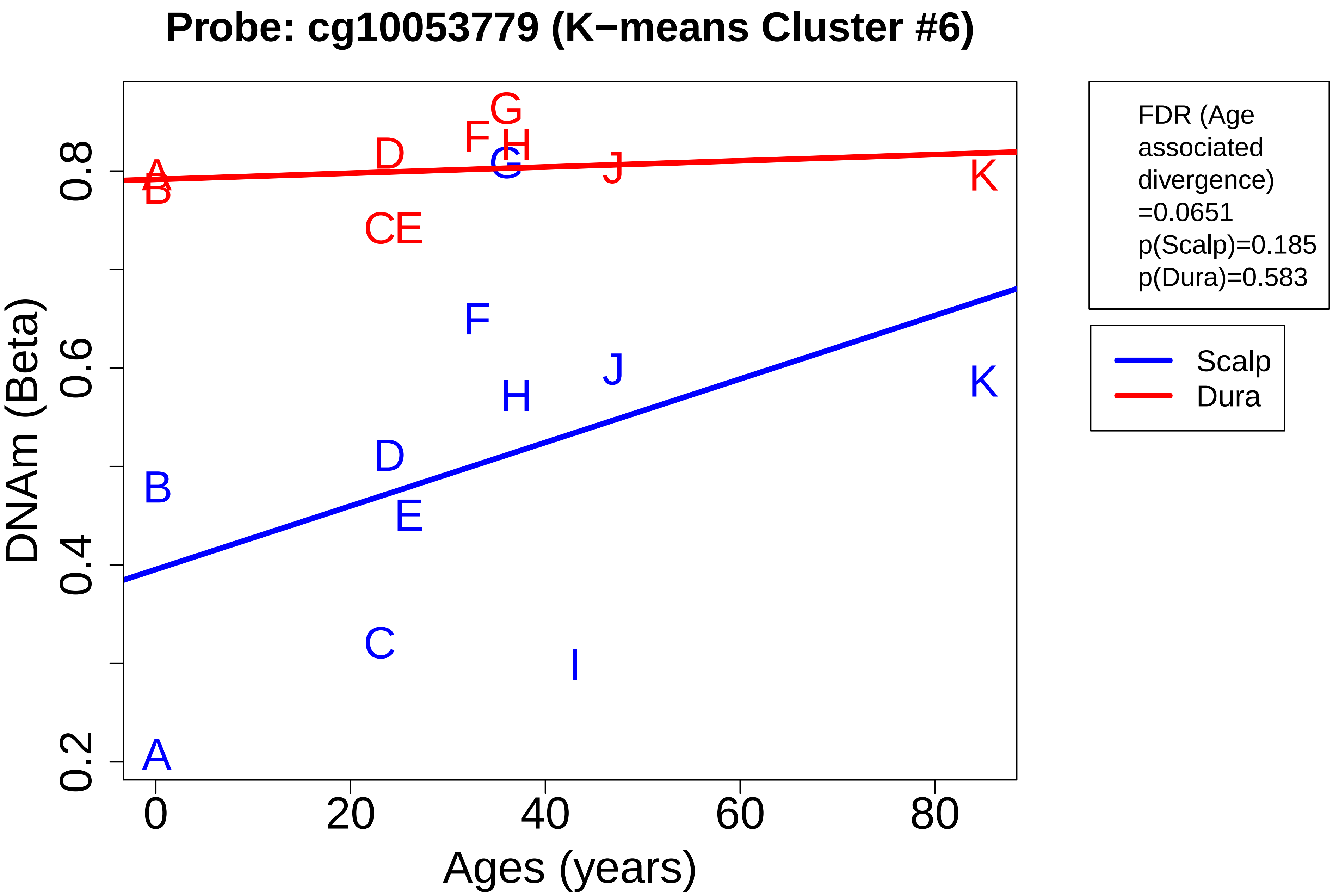

Probe: cg10053779 (K−means Cluster #6)
DNAm (Beta)
0.2
0.4
0.6
0.8
0
20
40
60
80
Ages (years)
FDR (Age associated divergence) =0.0651
p(Scalp)=0.185
p(Dura)=0.583
Scalp
Dura

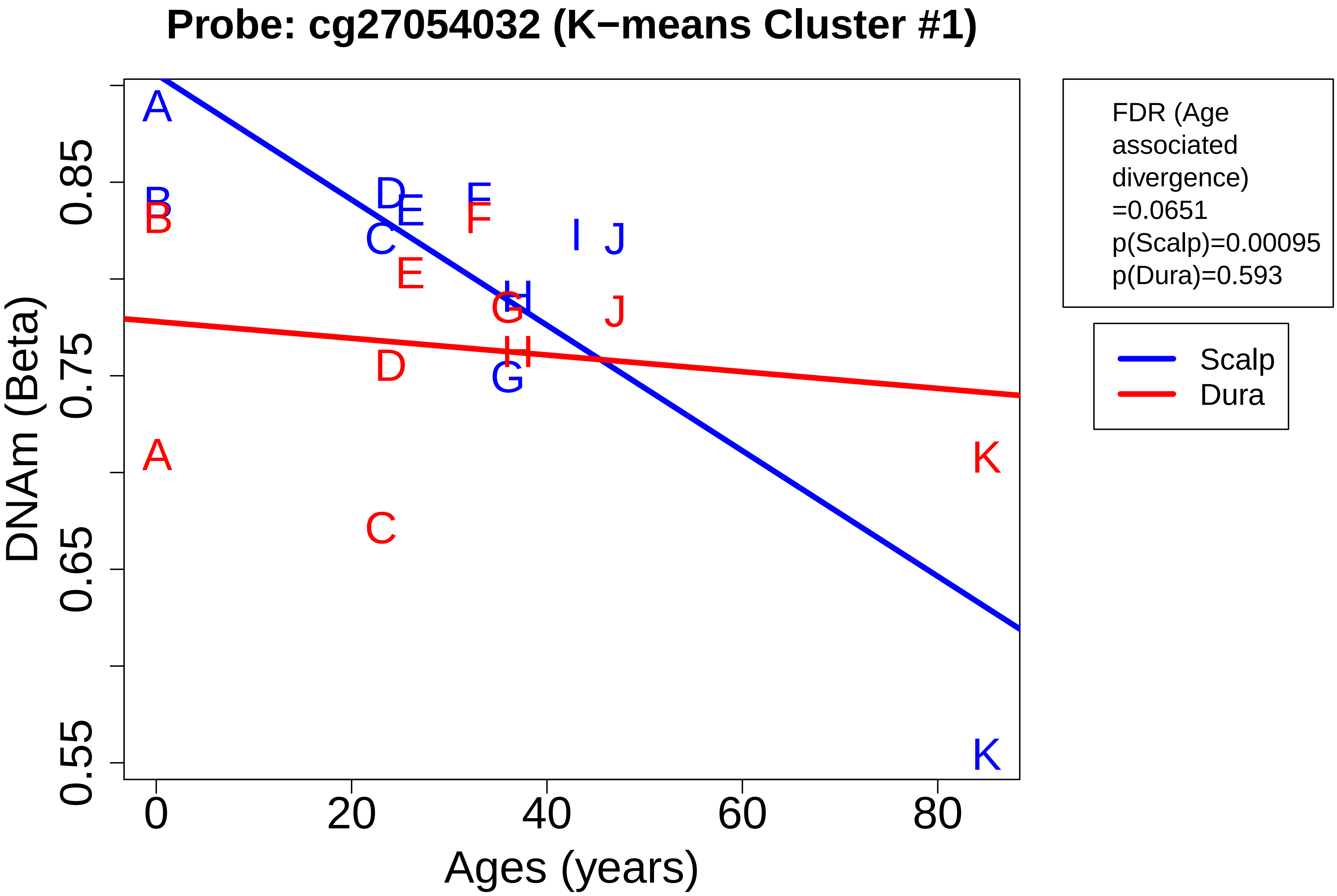

Probe: cg27054032 (K−means Cluster #1)
FDR (Age associated divergence) =0.0651
p(Scalp)=0.00095
p(Dura)=0.593
Scalp
Dura
DNAm (Beta)
0.55
0.65
0.75
0.85
0
20
40
60
80
Ages (years)

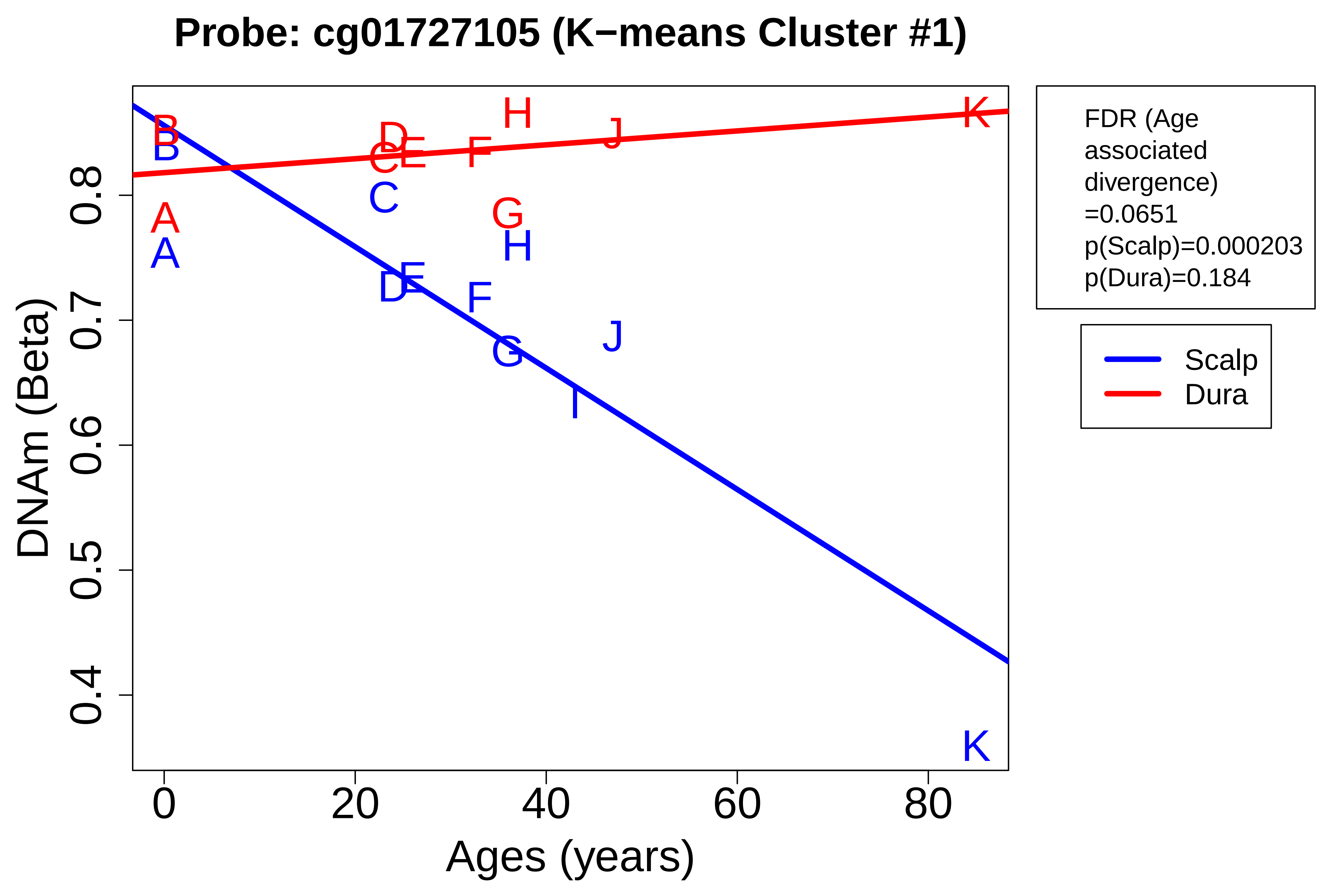

Probe: cg01727105 (K−means Cluster #1)
FDR (Age associated divergence) =0.0651
p(Scalp)=0.000203
p(Dura)=0.184
Scalp
Dura
DNAm (Beta)
0.4
0.5
0.6
0.7
0.8
0
20
40
60
80
Ages (years)
A
B
C
D
E
F
G
H
I
J
K

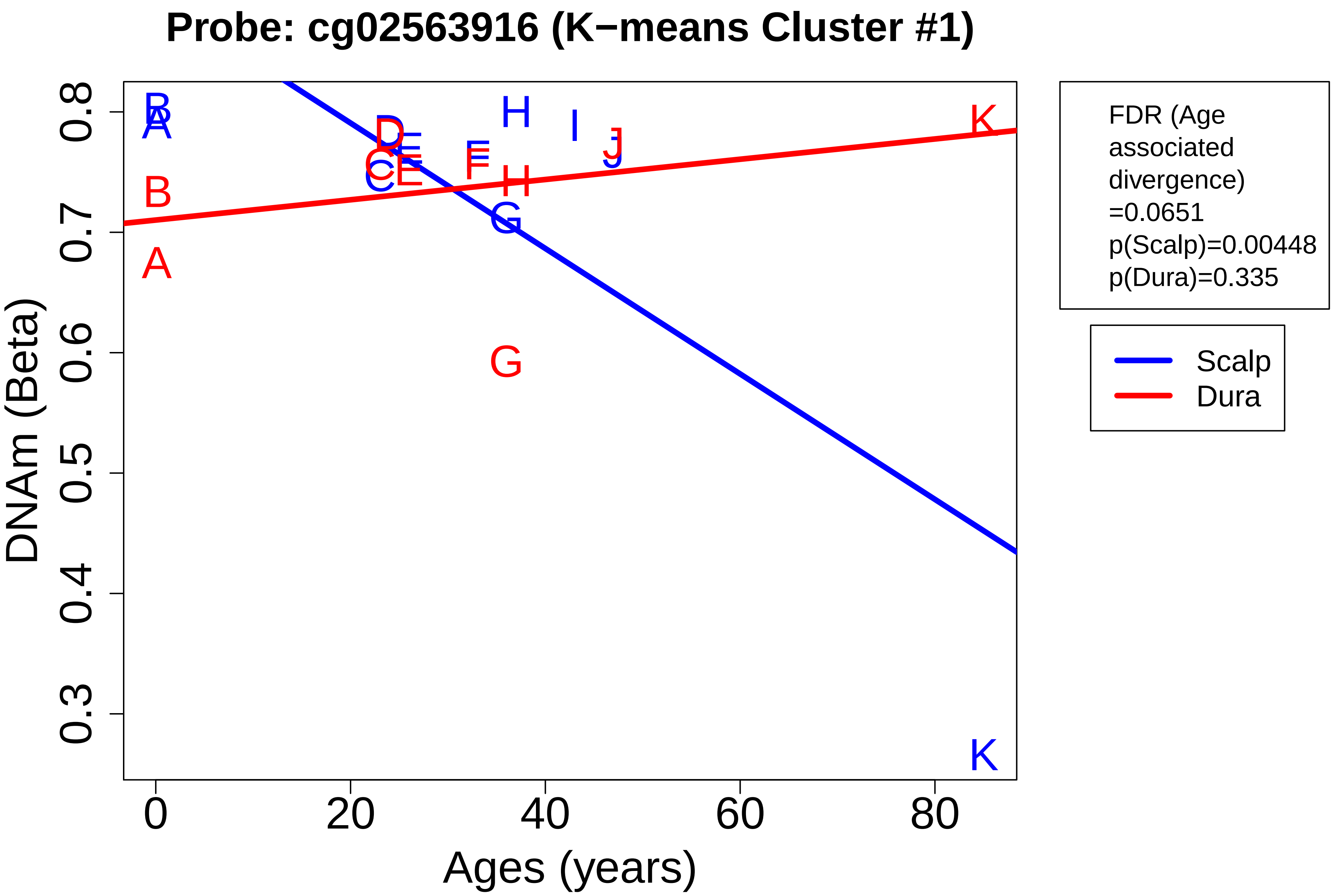
Probe: cg02563916 (K−means Cluster #1)
DNAm (Beta)
0.3
0.4
0.5
0.6
0.7
0.8
0
20
40
60
80
Ages (years)
B
A
D
E
C
F
H
I
J
G
K
FDR (Age associated divergence) =0.0651
p(Scalp)=0.00448
p(Dura)=0.335
Scalp
Dura

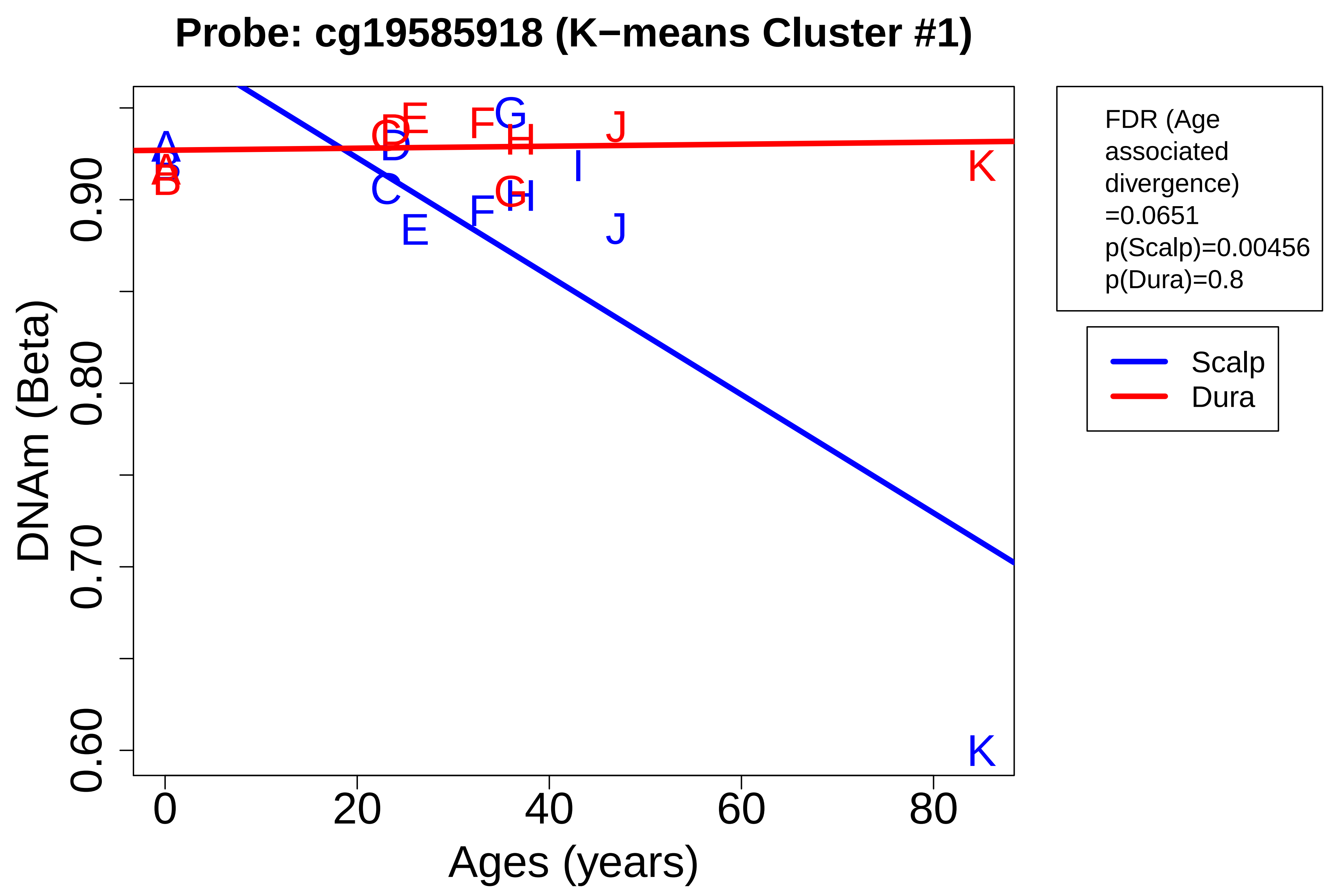

Probe: cg19585918 (K−means Cluster #1)
DNAm (Beta)
0.60
0.70
0.80
0.90
0
20
40
60
80
Ages (years)
A
B
C
D
E
F
G
H
I
J
K
FDR (Age associated divergence) =0.0651
p(Scalp)=0.00456
p(Dura)=0.8
Scalp
Dura

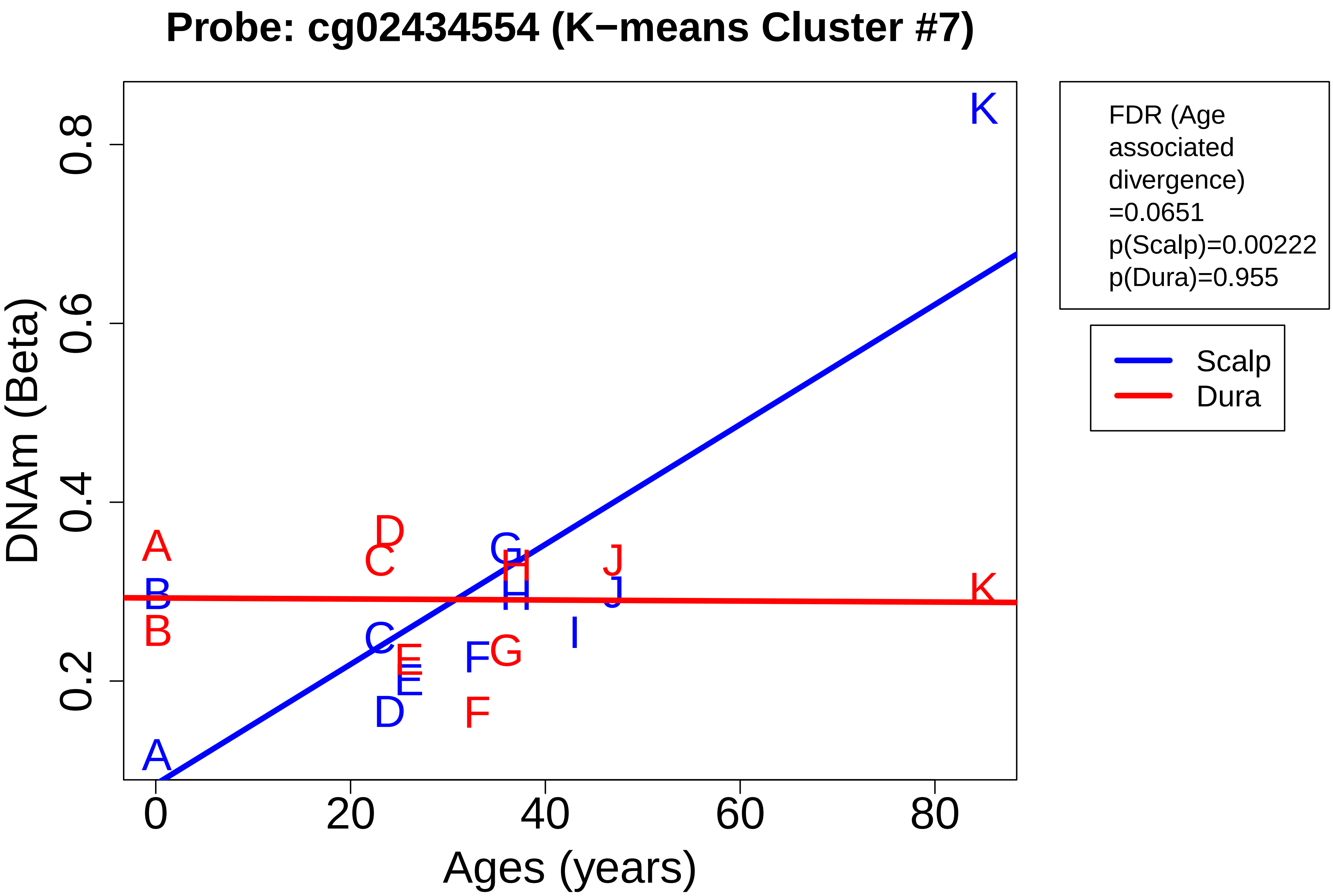

Probe: cg02434554 (K−means Cluster #7)
DNAm (Beta)
0.2
0.4
0.6
0.8
Ages (years)
0
20
40
60
80
FDR (Age associated divergence) =0.0651
p(Scalp)=0.00222
p(Dura)=0.955
Scalp
Dura

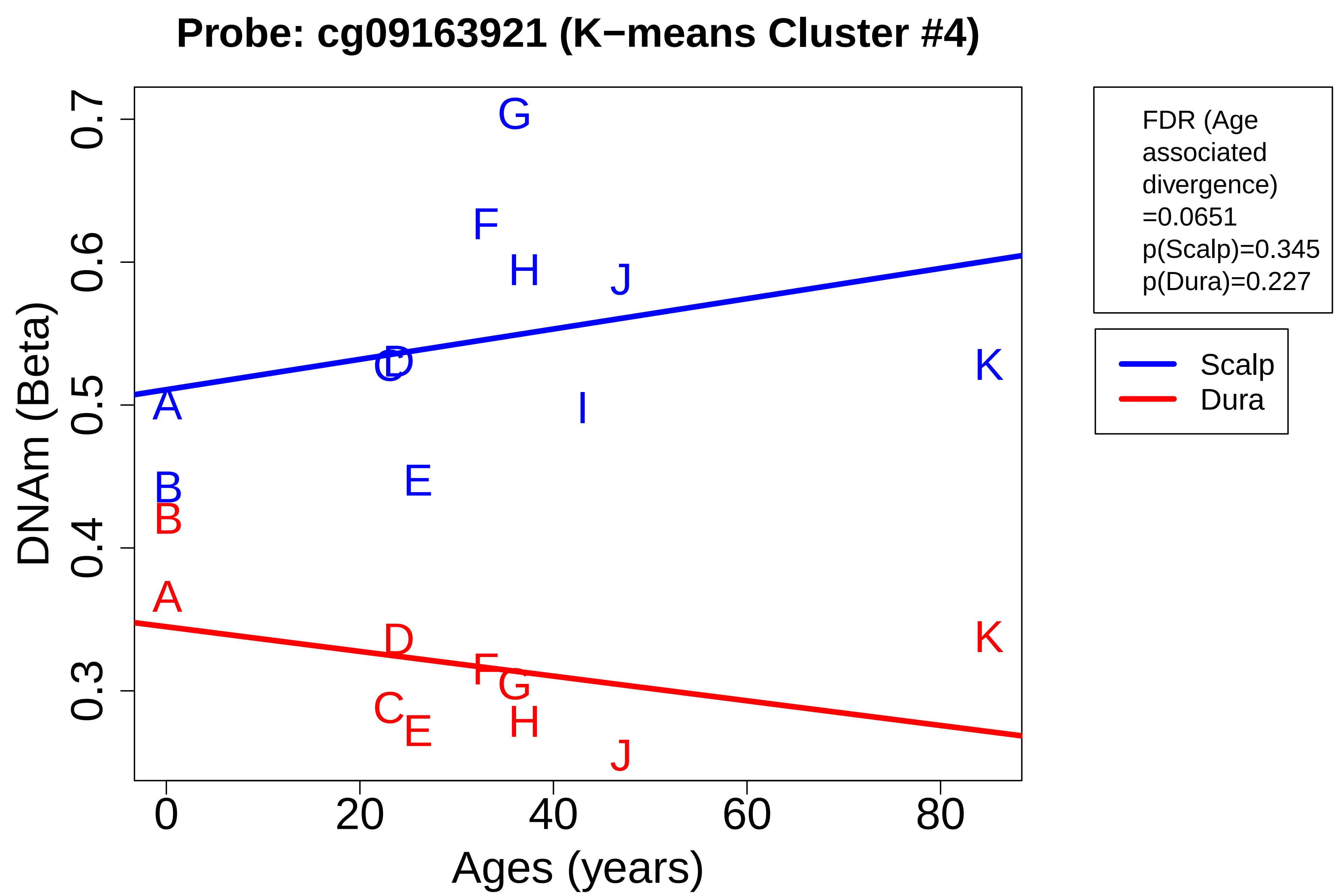

Probe: cg09163921 (K−means Cluster #4)
DNAm (Beta)
0.3
0.4
0.5
0.6
0.7
Ages (years)
0
20
40
60
80
A
B
C
D
E
F
G
H
I
J
K
FDR (Age associated divergence) =0.0651
p(Scalp)=0.345
p(Dura)=0.227
Scalp
Dura

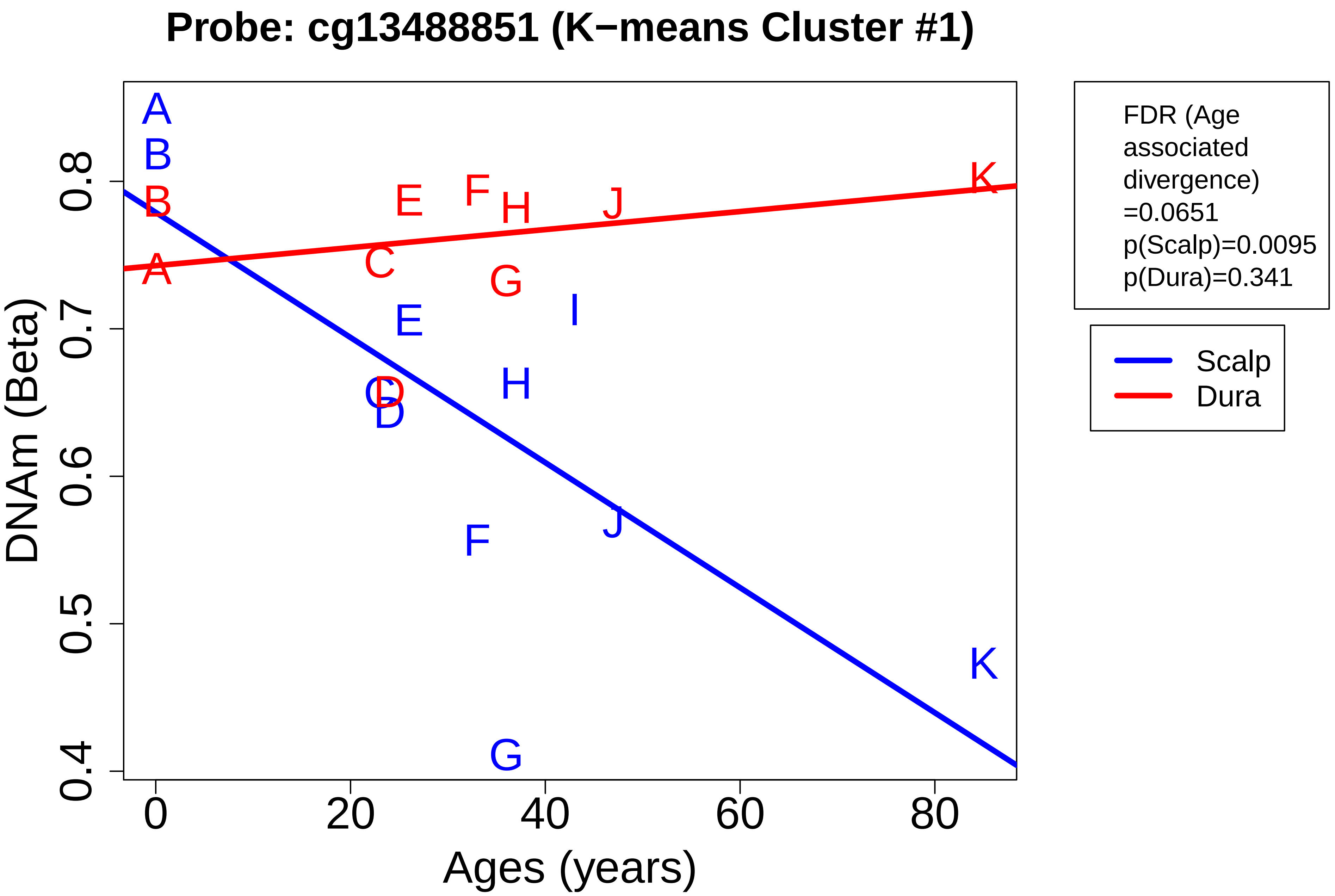

Probe: cg13488851 (K−means Cluster #1)
DNAm (Beta)
0.4
0.5
0.6
0.7
0.8
0
20
40
60
80
Ages (years)
A
B
B
A
E
F
H
J
K
C
G
E
I
C
D
D
H
F
J
K
G
FDR (Age associated divergence) =0.0651
p(Scalp)=0.0095
p(Dura)=0.341
Scalp
Dura

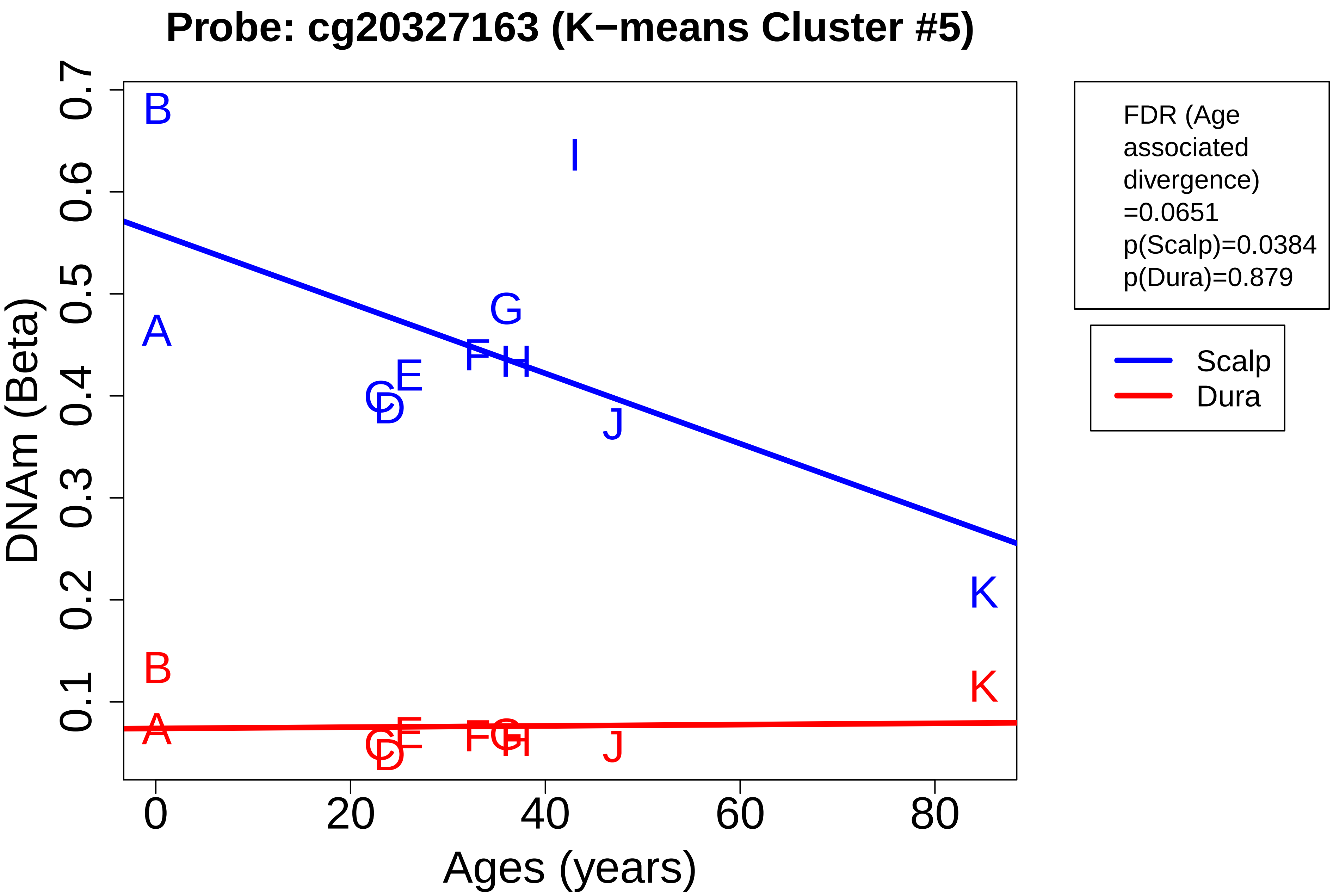

Probe: cg20327163 (K−means Cluster #5)
DNAm (Beta)
0.1
0.2
0.3
0.4
0.5
0.6
0.7
Ages (years)
0
20
40
60
80
FDR (Age associated divergence) =0.0651
p(Scalp)=0.0384
p(Dura)=0.879
Scalp
Dura
A
B
C
D
E
F
G
H
I
J
K

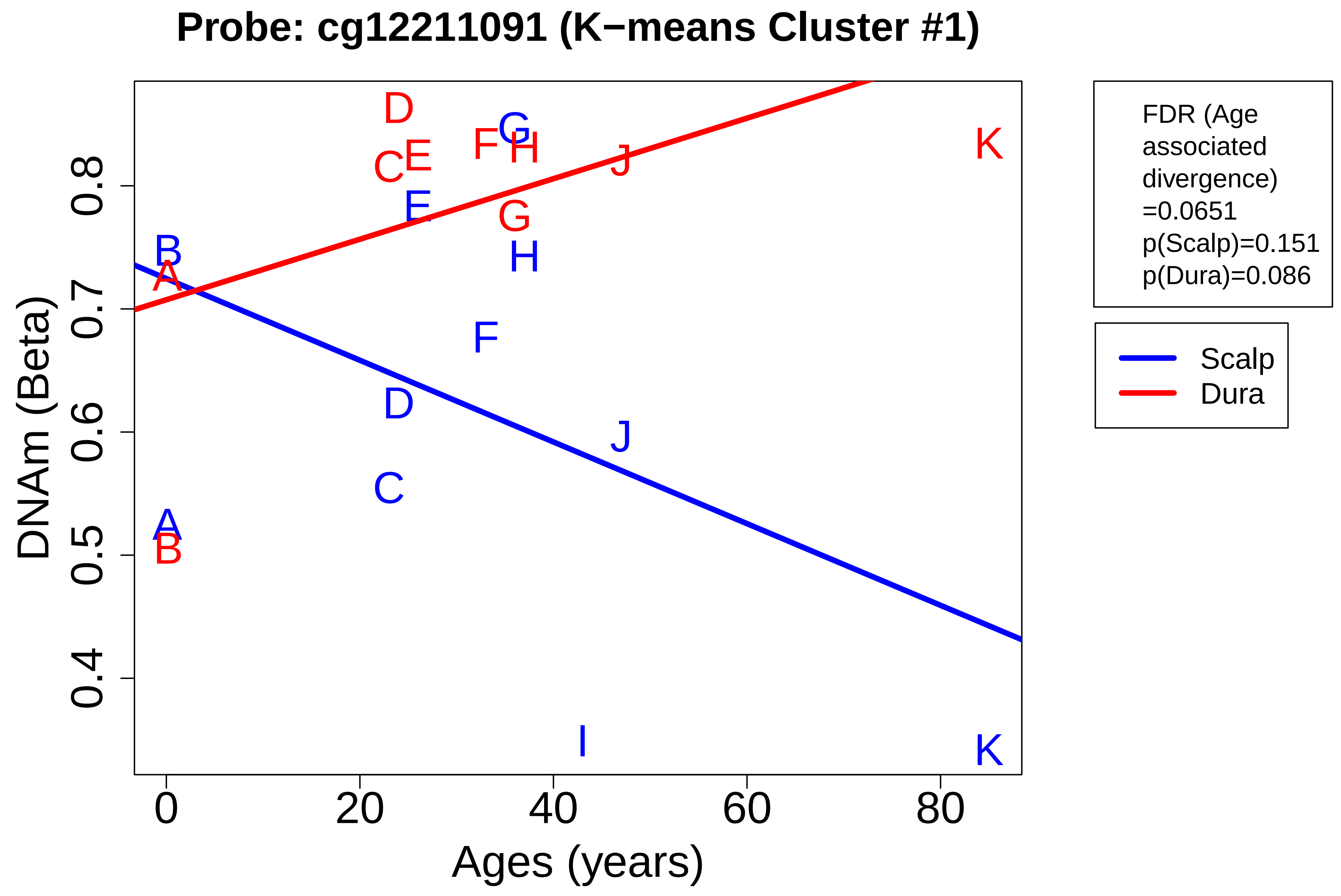
Probe: cg12211091 (K−means Cluster #1)
DNAm (Beta)
0.4
0.5
0.6
0.7
0.8
0
20
40
60
80
Ages (years)
A
B
C
D
E
F
G
H
I
J
K
FDR (Age associated divergence) =0.0651
p(Scalp)=0.151
p(Dura)=0.086
Scalp
Dura

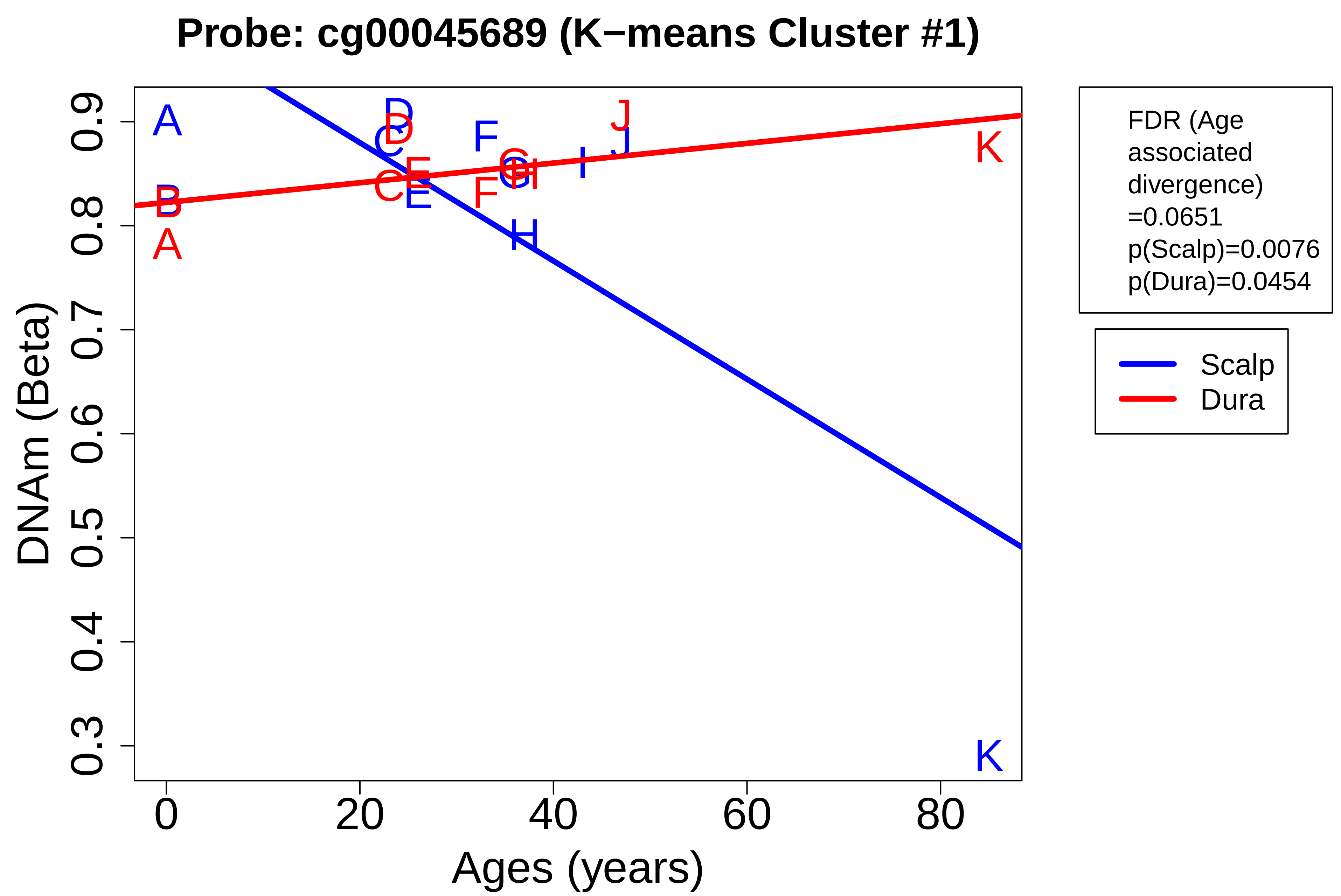
Probe: cg00045689 (K−means Cluster #1)
DNAm (Beta)
0.3
0.4
0.5
0.6
0.7
0.8
0.9
0
20
40
60
80
Ages (years)
A
B
C
D
E
F
G
H
I
J
K
FDR (Age associated divergence) =0.0651
p(Scalp)=0.0076
p(Dura)=0.0454
Scalp
Dura

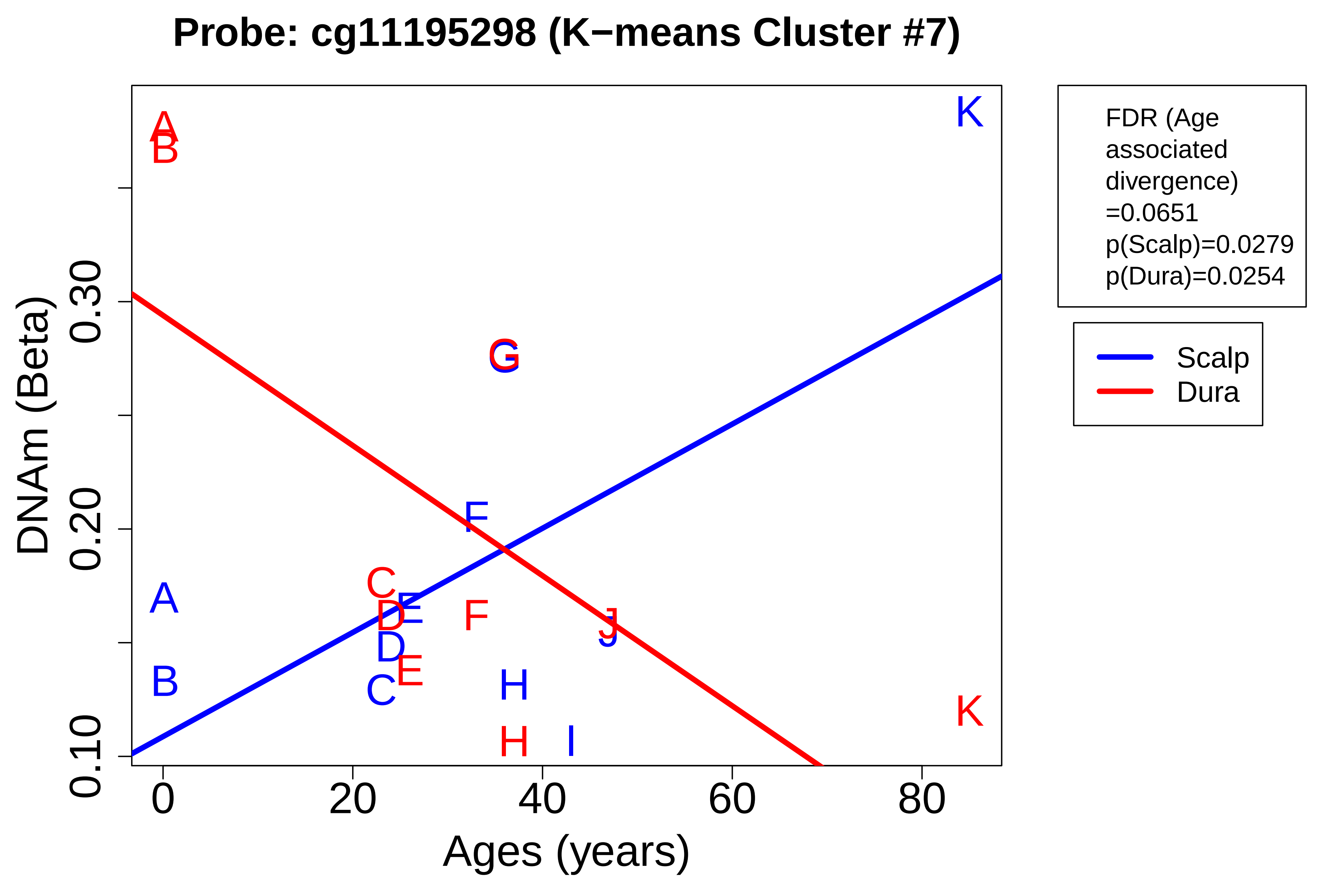

Probe: cg11195298 (K−means Cluster #7)
DNAm (Beta)
0.10
0.20
0.30
0
20
40
60
80
Ages (years)
FDR (Age associated divergence) =0.0651
p(Scalp)=0.0279
p(Dura)=0.0254
Scalp
Dura
A
B
C
D
E
F
G
H
I
J
K

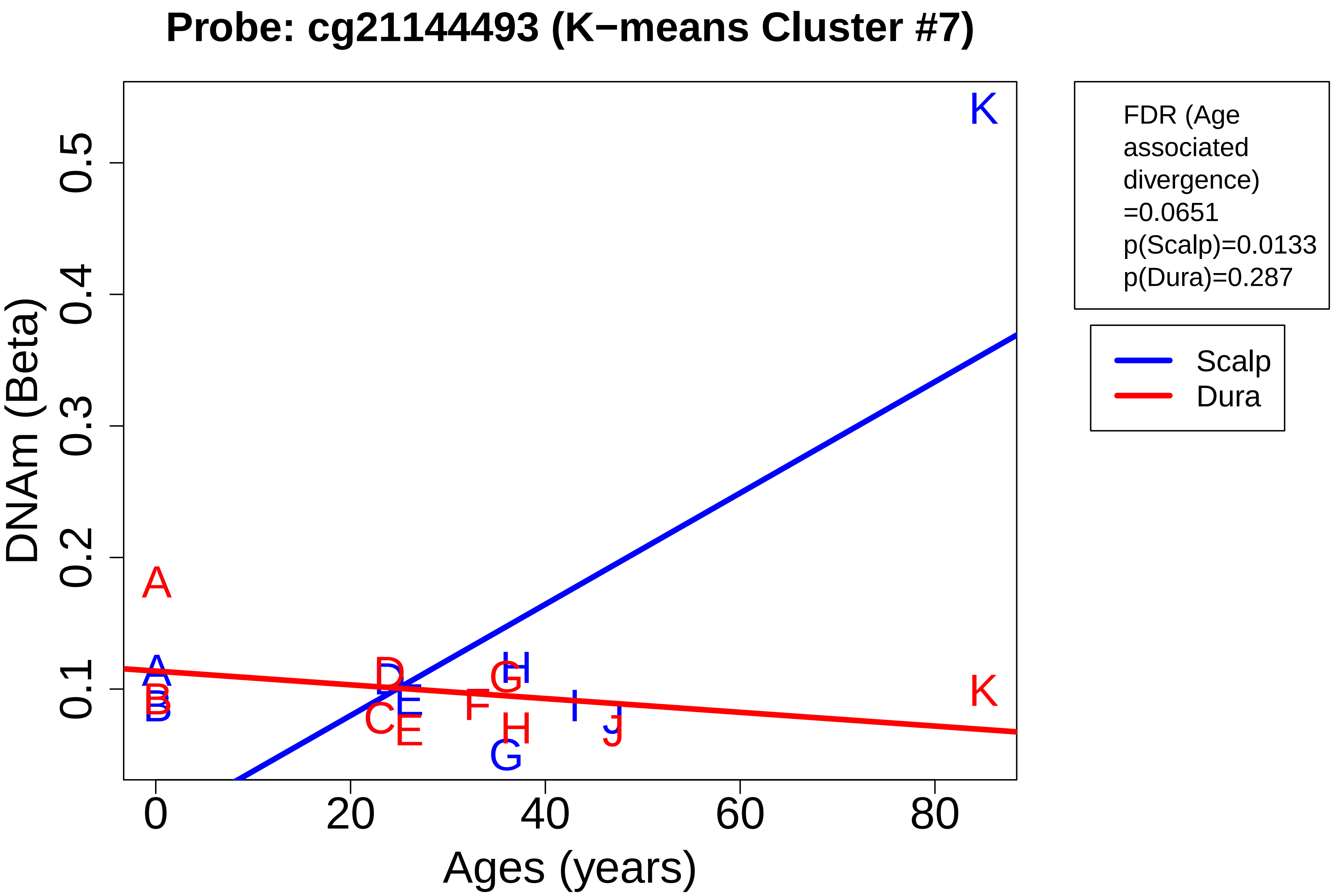

Probe: cg21144493 (K−means Cluster #7)
DNAm (Beta)
0.1
0.2
0.3
0.4
0.5
0
20
40
60
80
Ages (years)
FDR (Age associated divergence) =0.0651
p(Scalp)=0.0133
p(Dura)=0.287
Scalp
Dura

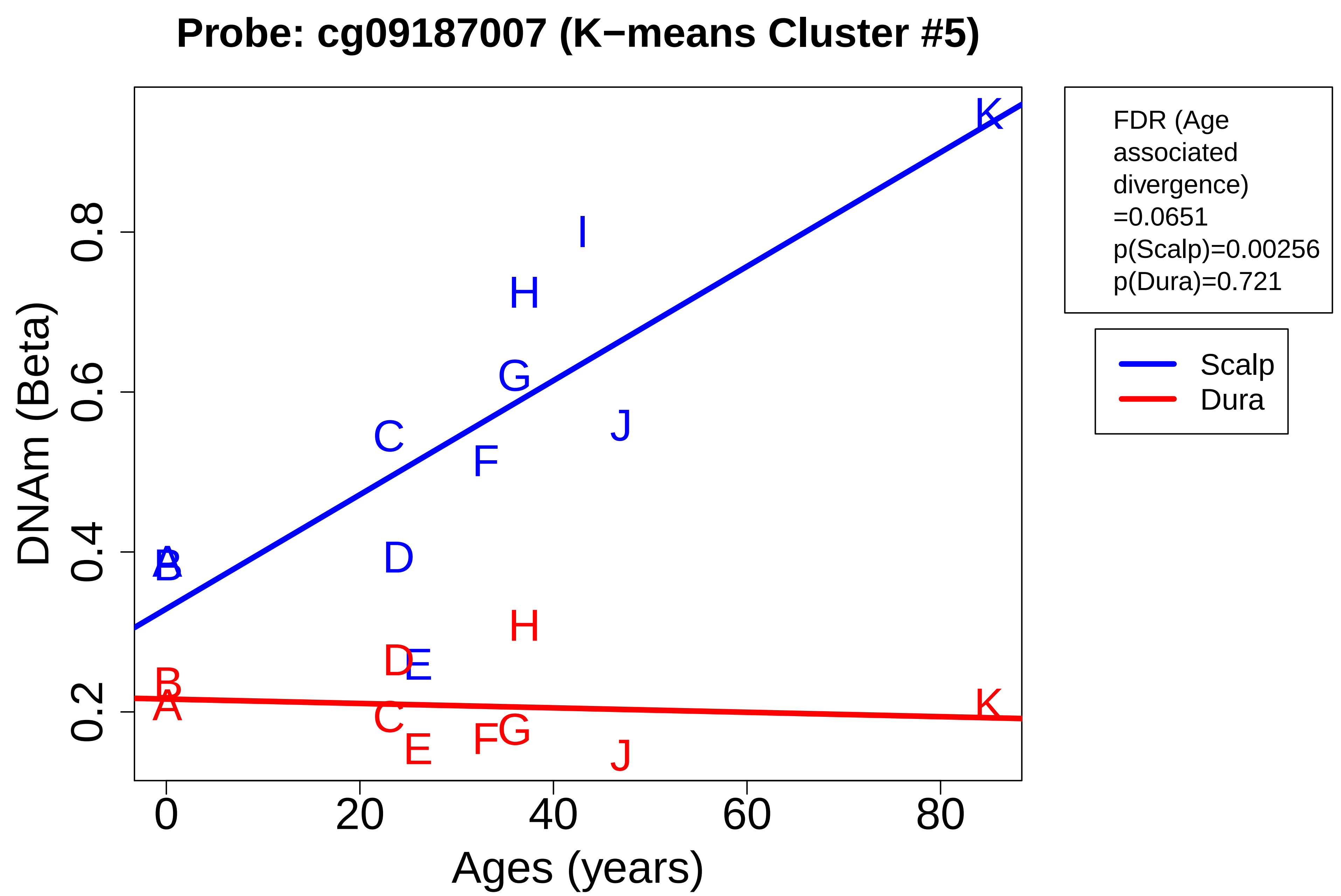

Probe: cg09187007 (K−means Cluster #5)
DNAm (Beta)
0.2
0.4
0.6
0.8
0
20
40
60
80
Ages (years)
FDR (Age associated divergence) =0.0651
p(Scalp)=0.00256
p(Dura)=0.721
Scalp
Dura

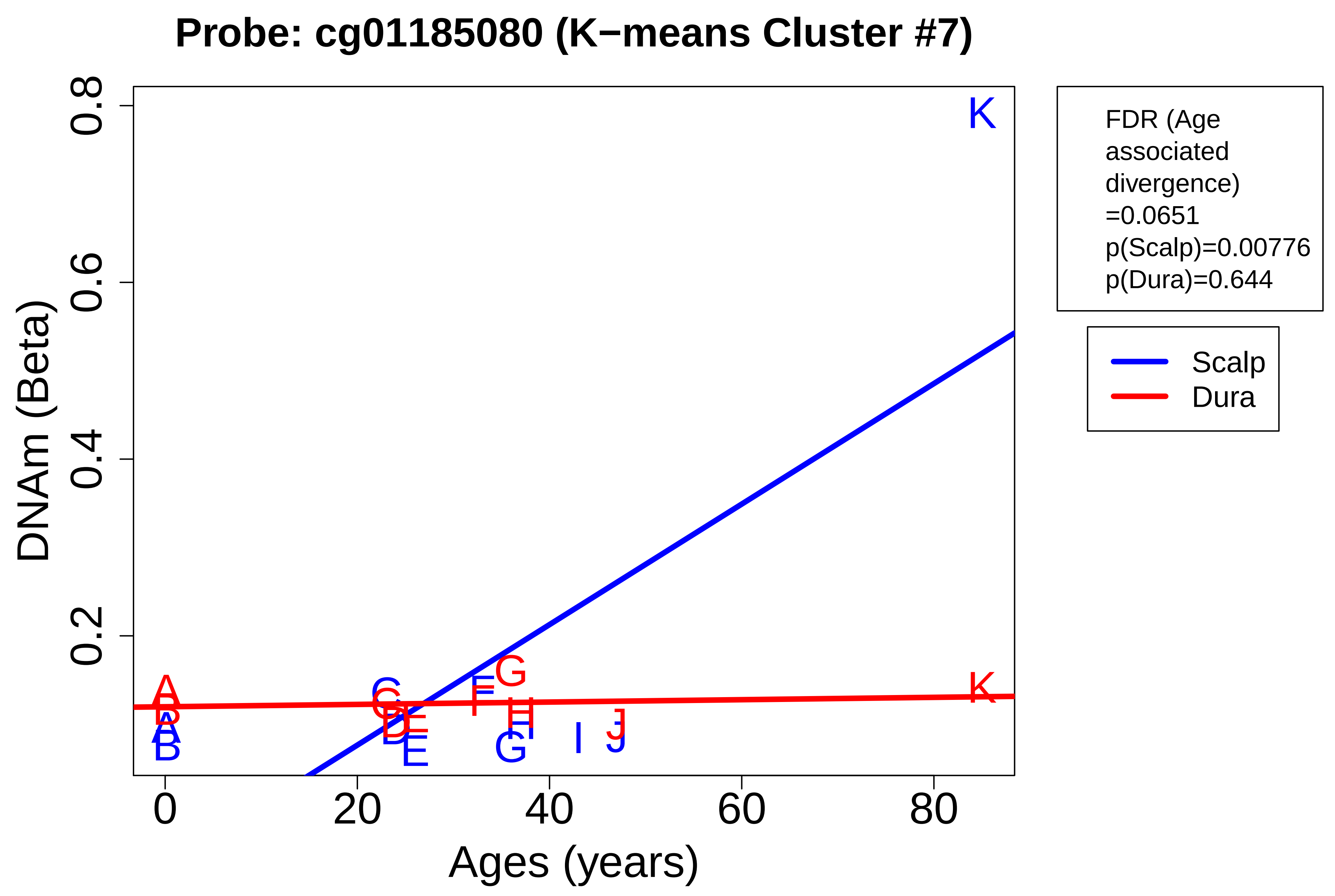

Probe: cg01185080 (K−means Cluster #7)
DNAm (Beta)
0.2
0.4
0.6
0.8
Ages (years)
0
20
40
60
80
FDR (Age associated divergence) =0.0651
p(Scalp)=0.00776
p(Dura)=0.644
Scalp
Dura
A
B
C
D
E
F
G
H
I
J
K

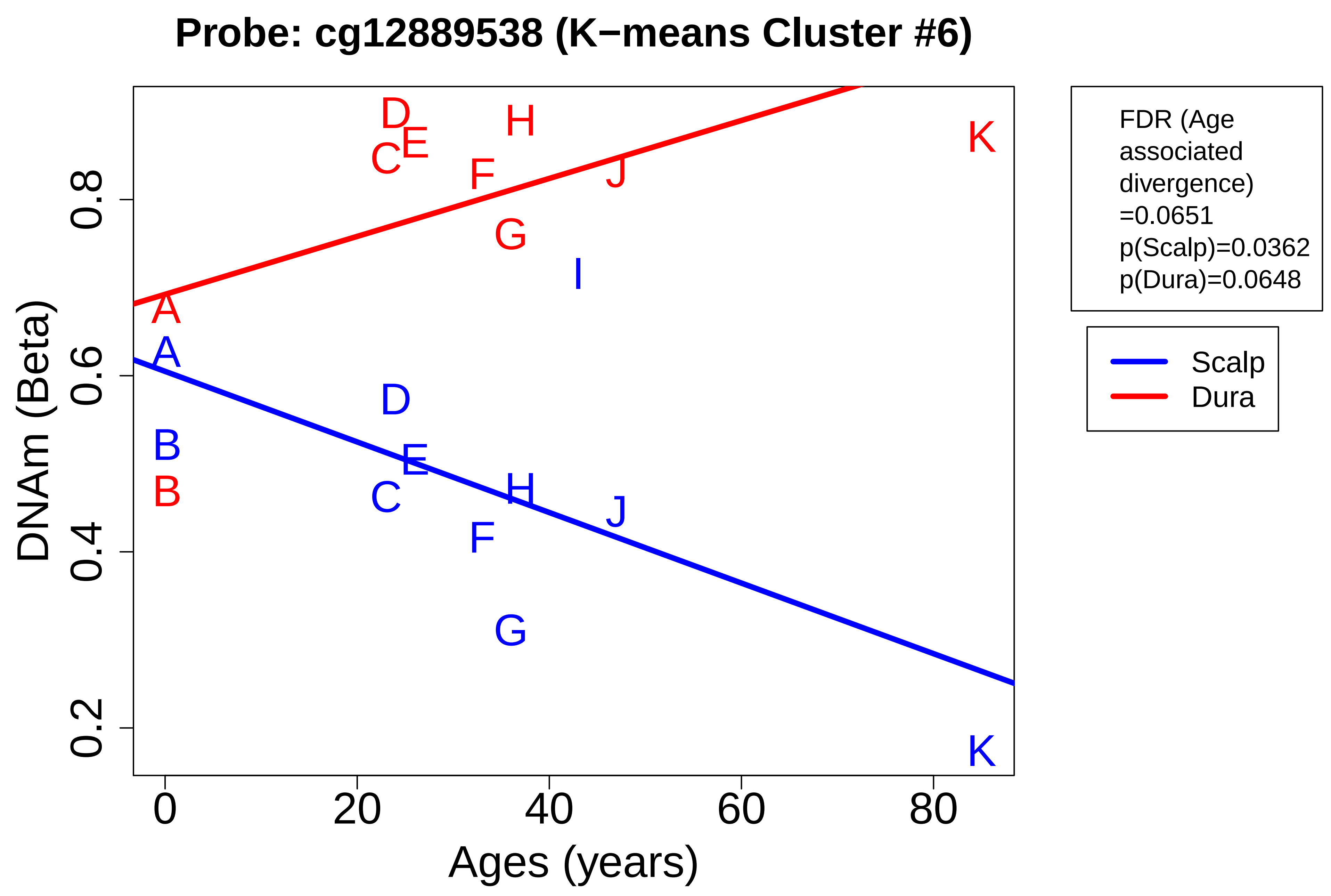

Probe: cg12889538 (K−means Cluster #6)
FDR (Age associated divergence) =0.0651
p(Scalp)=0.0362
p(Dura)=0.0648
Scalp
Dura
DNAm (Beta)
0.2
0.4
0.6
0.8
0
20
40
60
80
Ages (years)
A
B
C
D
E
F
G
H
I
J
K

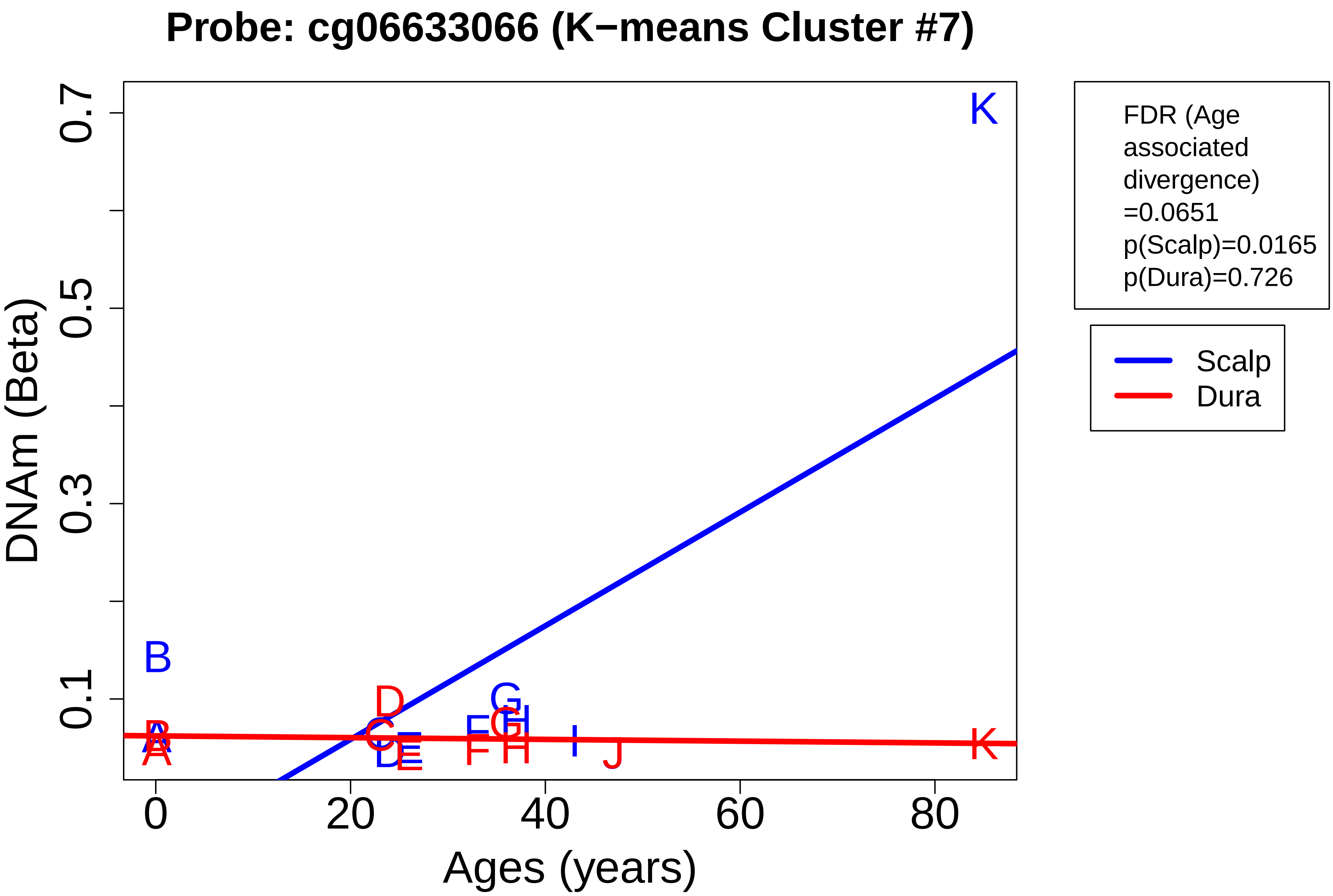
Probe: cg06633066 (K−means Cluster #7)
DNAm (Beta)
0.1
0.3
0.5
0.7
Ages (years)
0
20
40
60
80
FDR (Age associated divergence) =0.0651
p(Scalp)=0.0165
p(Dura)=0.726
Scalp
Dura
A
B
C
D
E
F
G
H
I
J
K

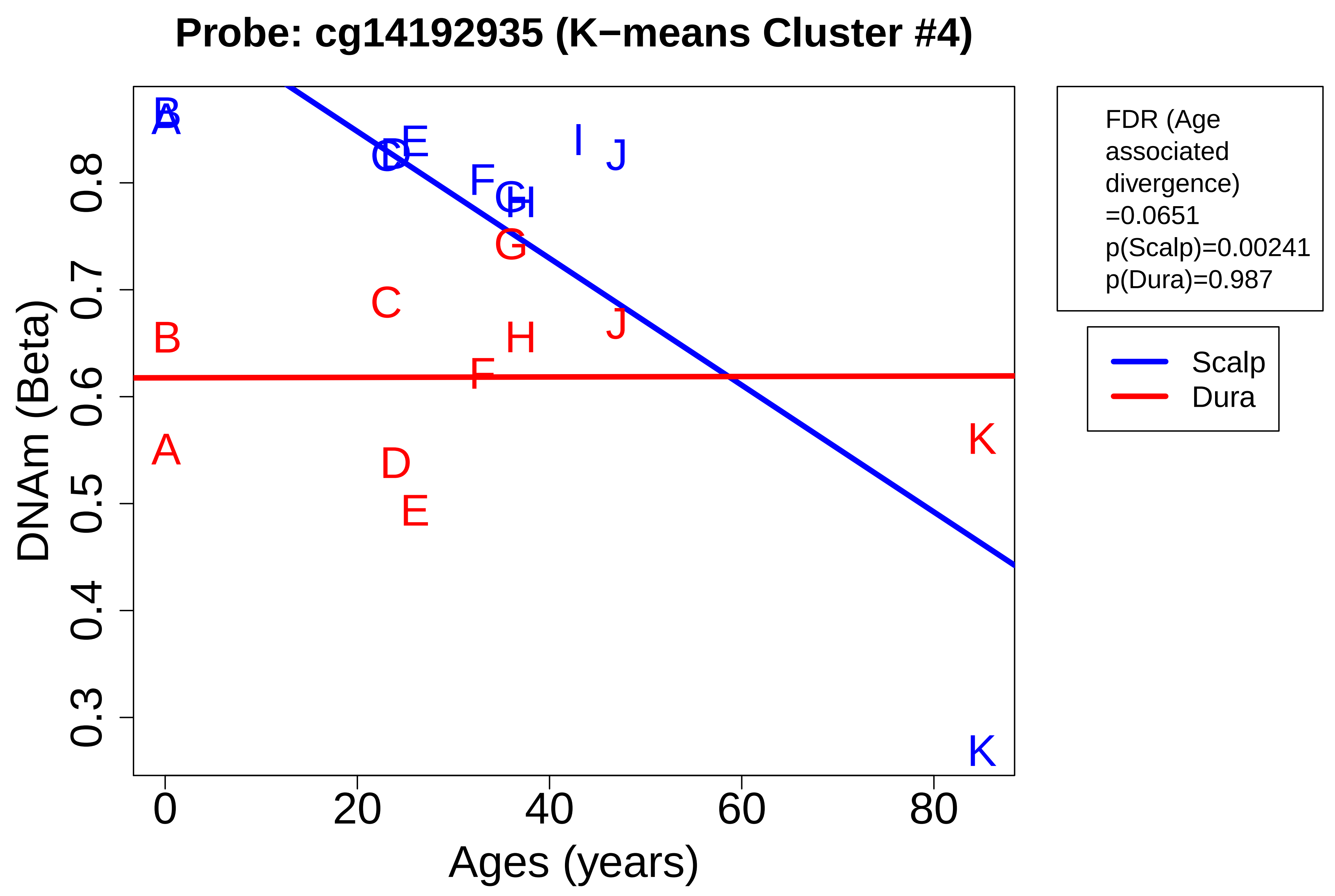

Probe: cg14192935 (K−means Cluster #4)
DNAm (Beta)
0.3
0.4
0.5
0.6
0.7
0.8
0
20
40
60
80
Ages (years)
FDR (Age associated divergence) =0.0651
p(Scalp)=0.00241
p(Dura)=0.987
Scalp
Dura

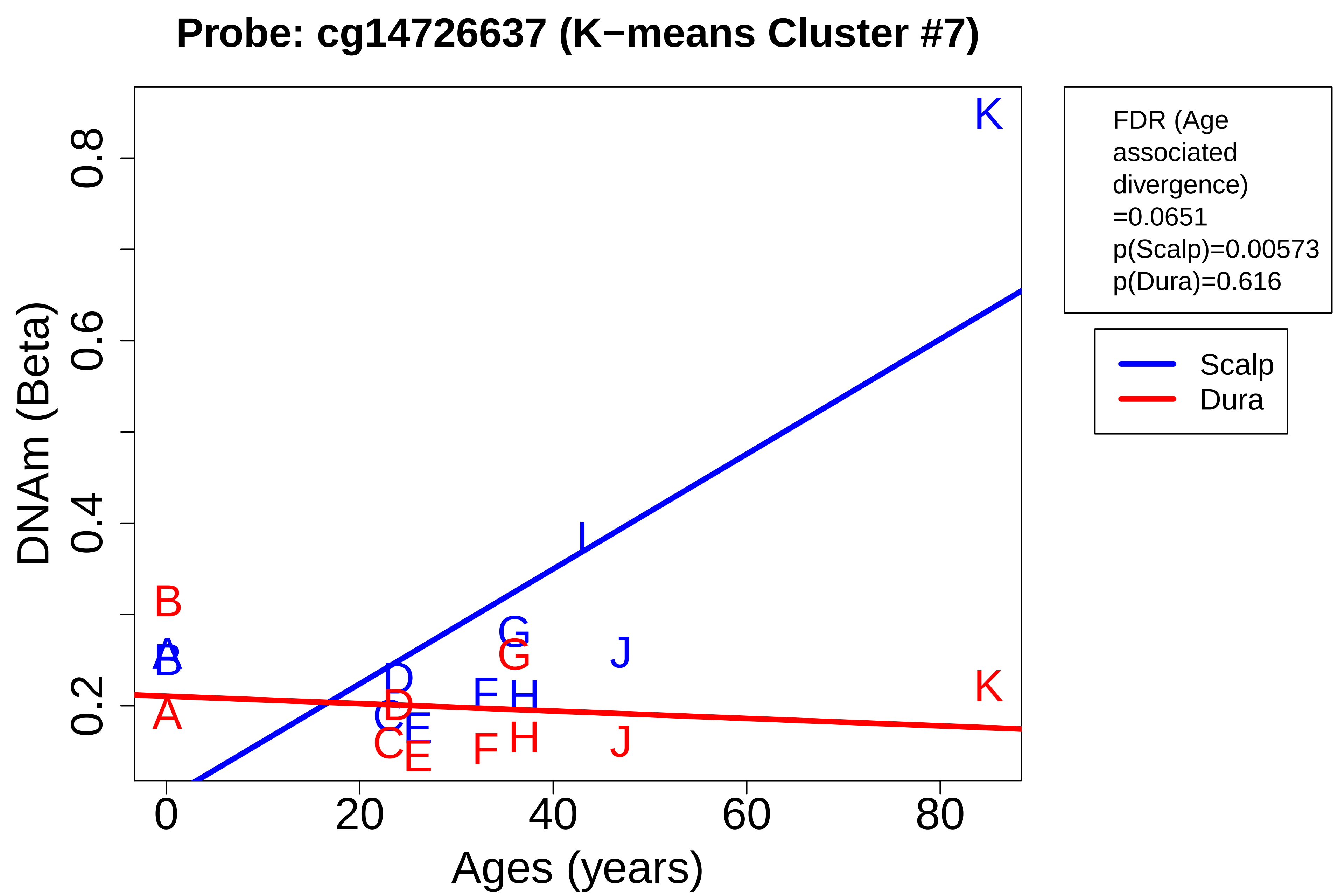

Probe: cg14726637 (K−means Cluster #7)
DNAm (Beta)
0.2
0.4
0.6
0.8
0
20
40
60
80
Ages (years)
A
B
C
D
E
F
G
H
I
J
K
FDR (Age associated divergence) =0.0651
p(Scalp)=0.00573
p(Dura)=0.616
Scalp
Dura

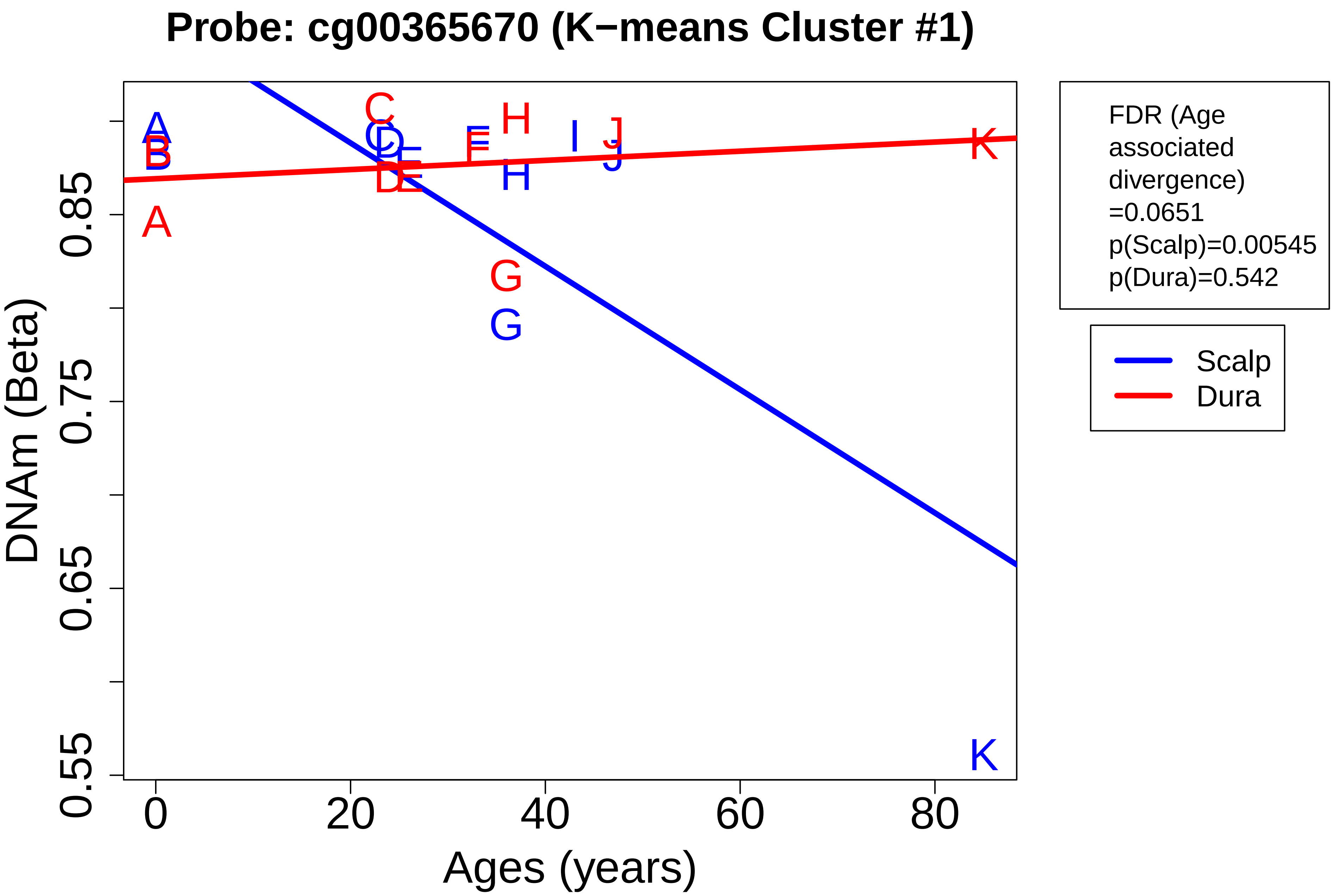

Probe: cg00365670 (K−means Cluster #1)
DNAm (Beta)
0.55
0.65
0.75
0.85
0
20
40
60
80
Ages (years)
FDR (Age associated divergence) =0.0651
p(Scalp)=0.00545
p(Dura)=0.542
Scalp
Dura

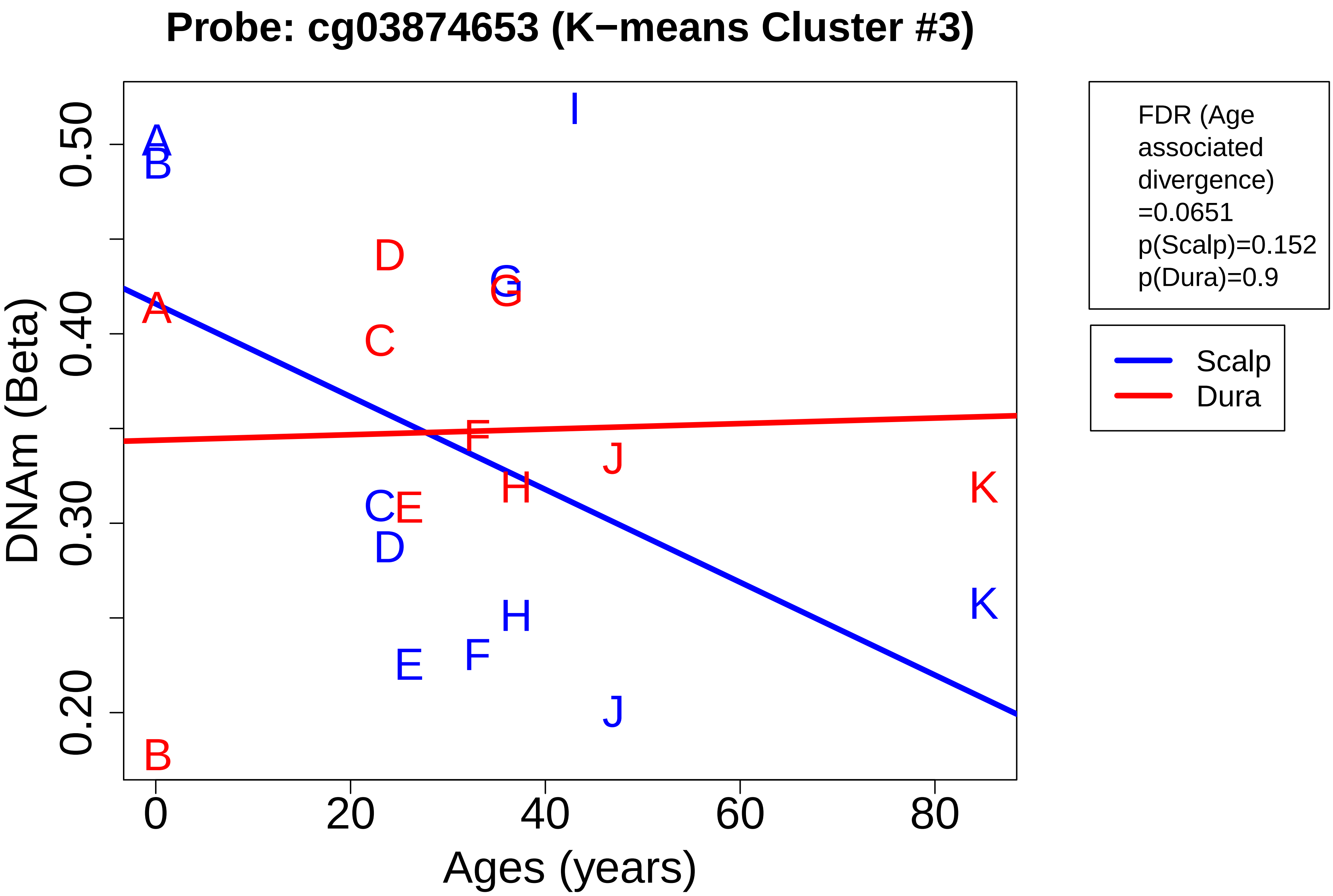

Probe: cg03874653 (K−means Cluster #3)
FDR (Age associated divergence) =0.0651
p(Scalp)=0.152
p(Dura)=0.9
Scalp
Dura
DNAm (Beta)
0.20
0.30
0.40
0.50
0
20
40
60
80
Ages (years)
A
B
C
D
E
F
G
H
I
J
K

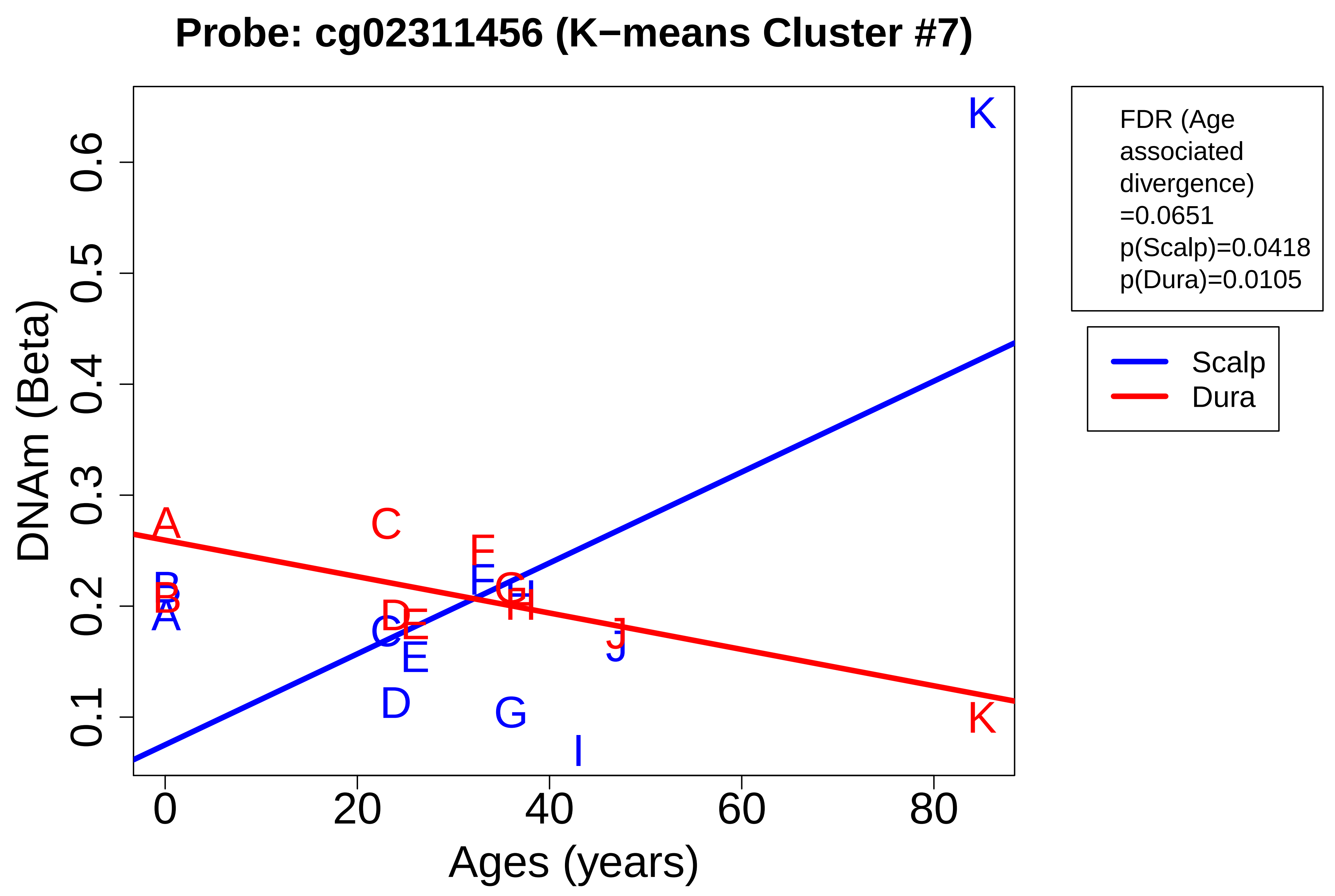

Probe: cg02311456 (K−means Cluster #7)
DNAm (Beta)
0.1
0.2
0.3
0.4
0.5
0.6
Ages (years)
0
20
40
60
80
FDR (Age associated divergence) =0.0651
p(Scalp)=0.0418
p(Dura)=0.0105
Scalp
Dura
A
B
C
D
E
F
G
H
I
J
K

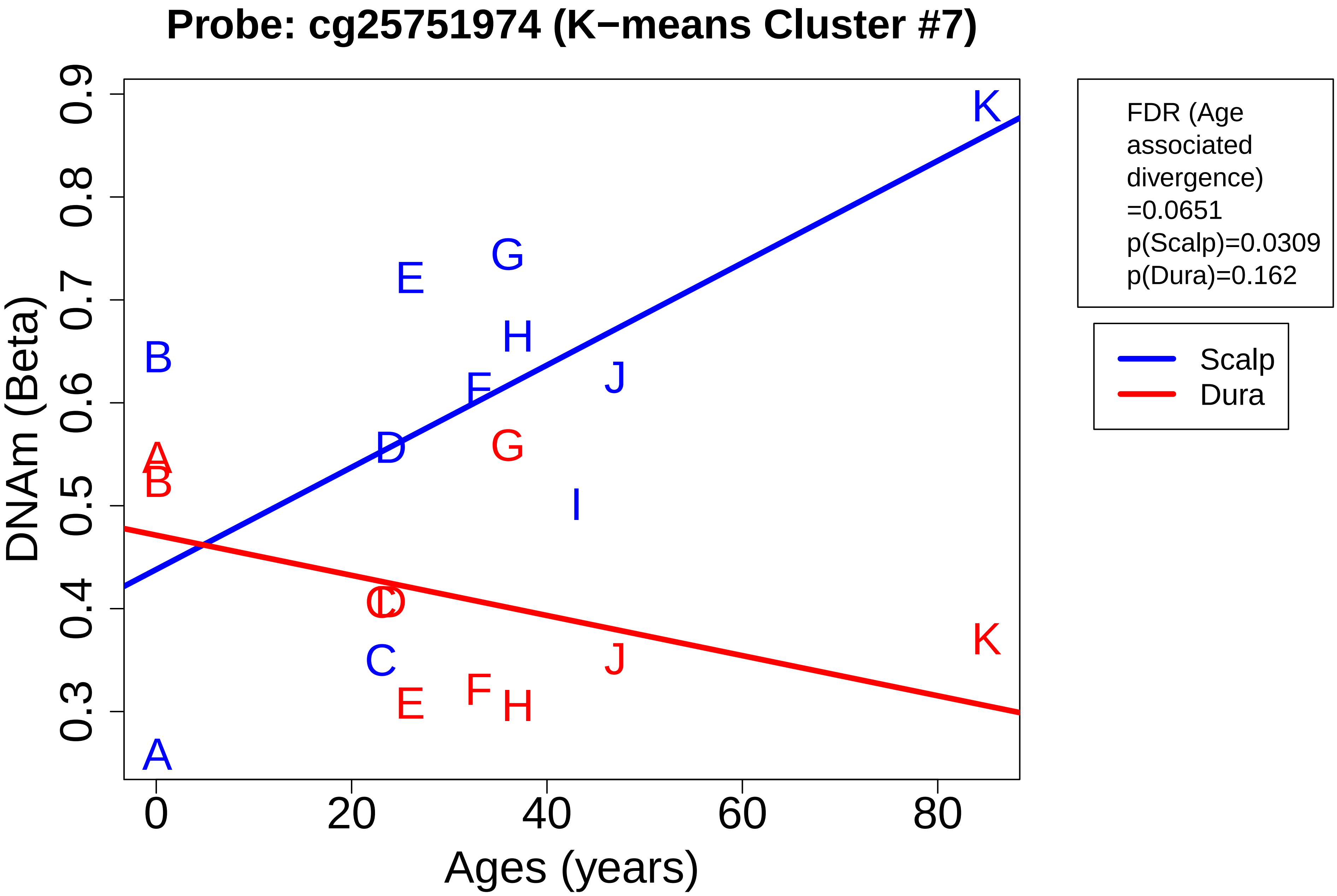

Probe: cg25751974 (K−means Cluster #7)
DNAm (Beta)
0.3
0.4
0.5
0.6
0.7
0.8
0.9
0
20
40
60
80
Ages (years)
A
B
C
D
E
F
G
H
I
J
K
A
B
C
D
E
F
G
H
J
K
FDR (Age associated divergence) =0.0651
p(Scalp)=0.0309
p(Dura)=0.162
Scalp
Dura

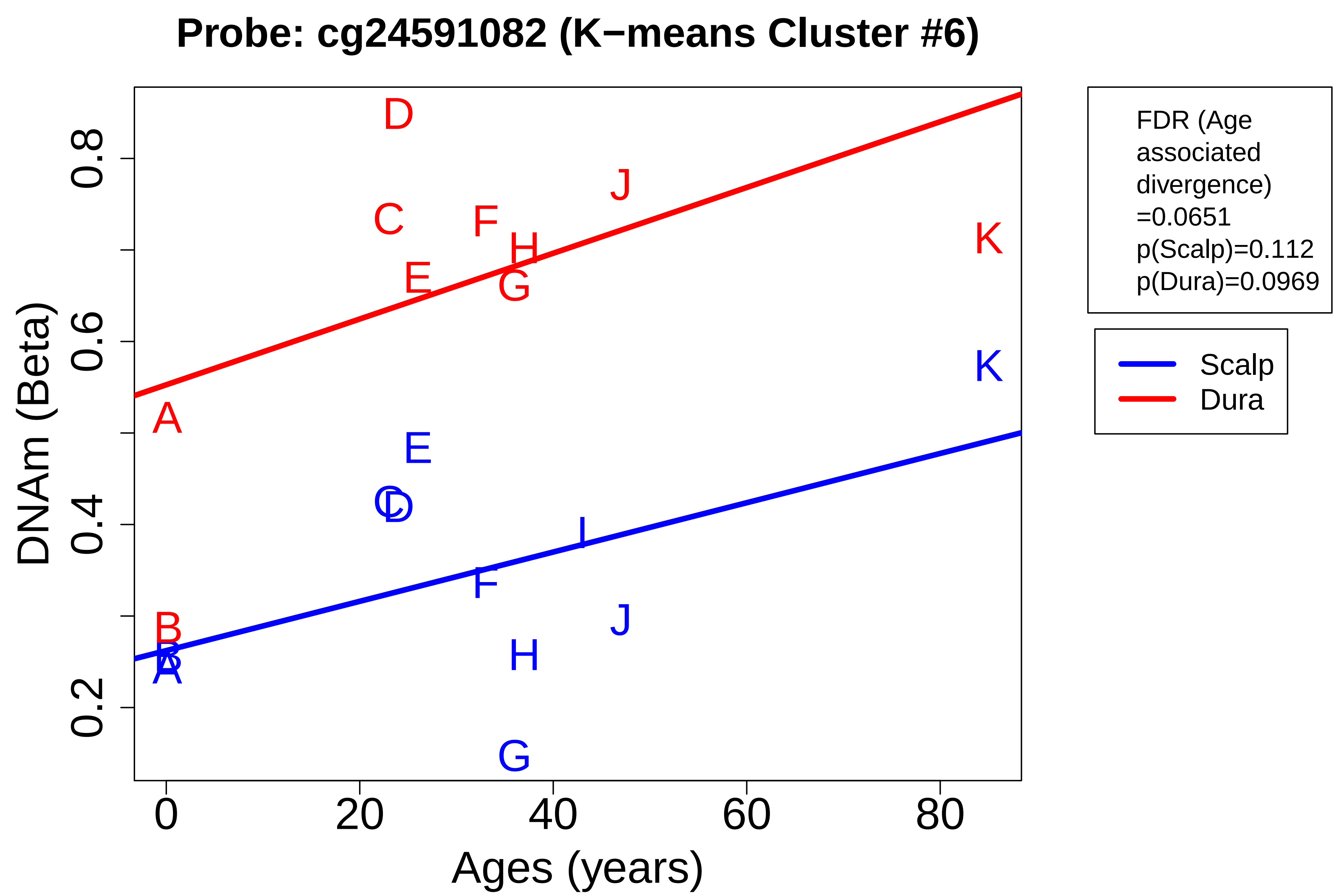

Probe: cg24591082 (K−means Cluster #6)
DNAm (Beta)
0.2
0.4
0.6
0.8
0
20
40
60
80
Ages (years)
A
B
C
D
E
F
G
H
I
J
K
FDR (Age associated divergence) =0.0651
p(Scalp)=0.112
p(Dura)=0.0969
Scalp
Dura

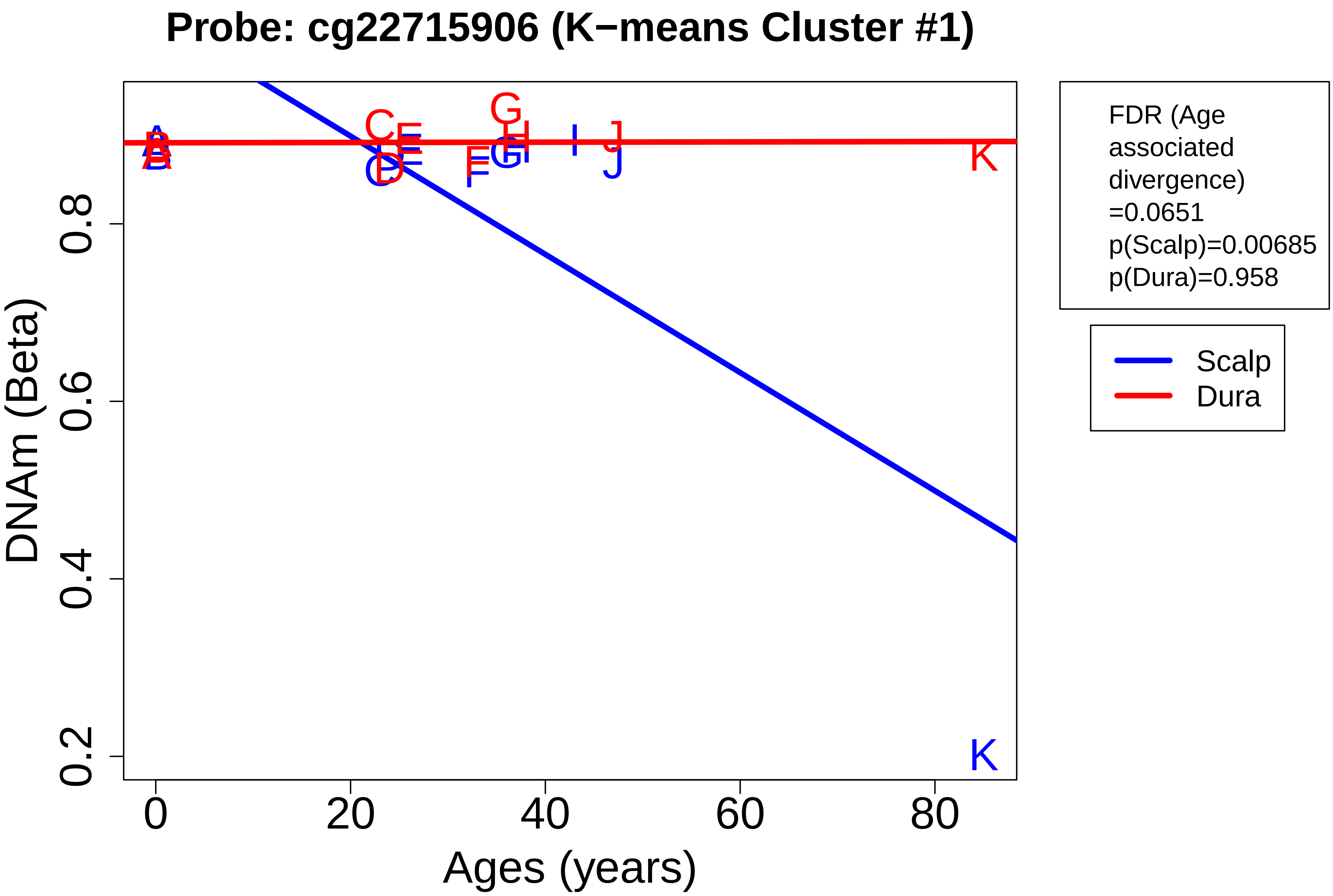

Probe: cg22715906 (K−means Cluster #1)
DNAm (Beta)
Ages (years)
0.2
0.4
0.6
0.8
0
20
40
60
80
FDR (Age associated divergence) =0.0651
p(Scalp)=0.00685
p(Dura)=0.958
Scalp
Dura

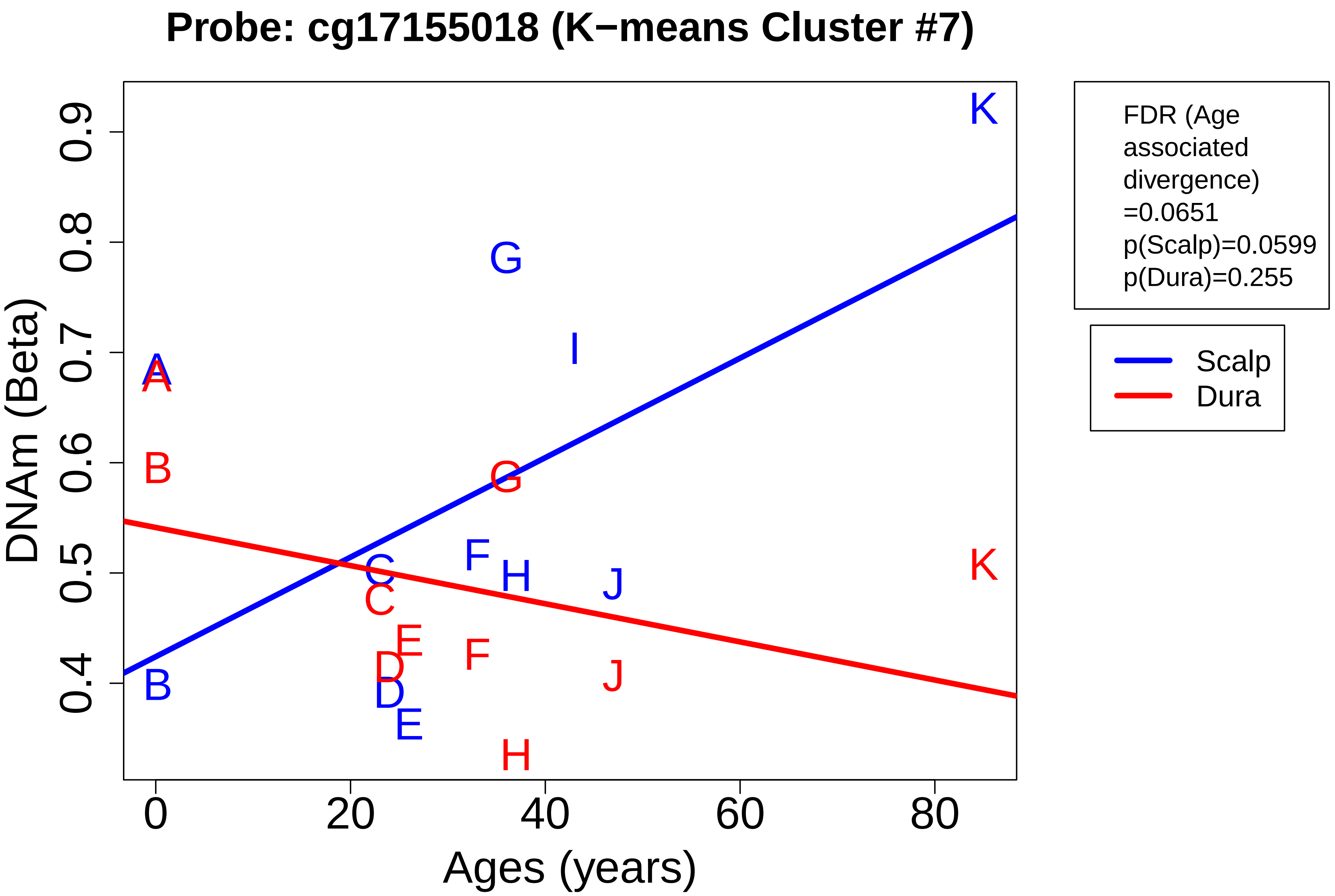

Probe: cg17155018 (K−means Cluster #7)
DNAm (Beta)
0.4
0.5
0.6
0.7
0.8
0.9
Ages (years)
0
20
40
60
80
A
B
C
D
E
F
G
H
I
J
K
FDR (Age associated divergence) =0.0651
p(Scalp)=0.0599
p(Dura)=0.255
Scalp
Dura

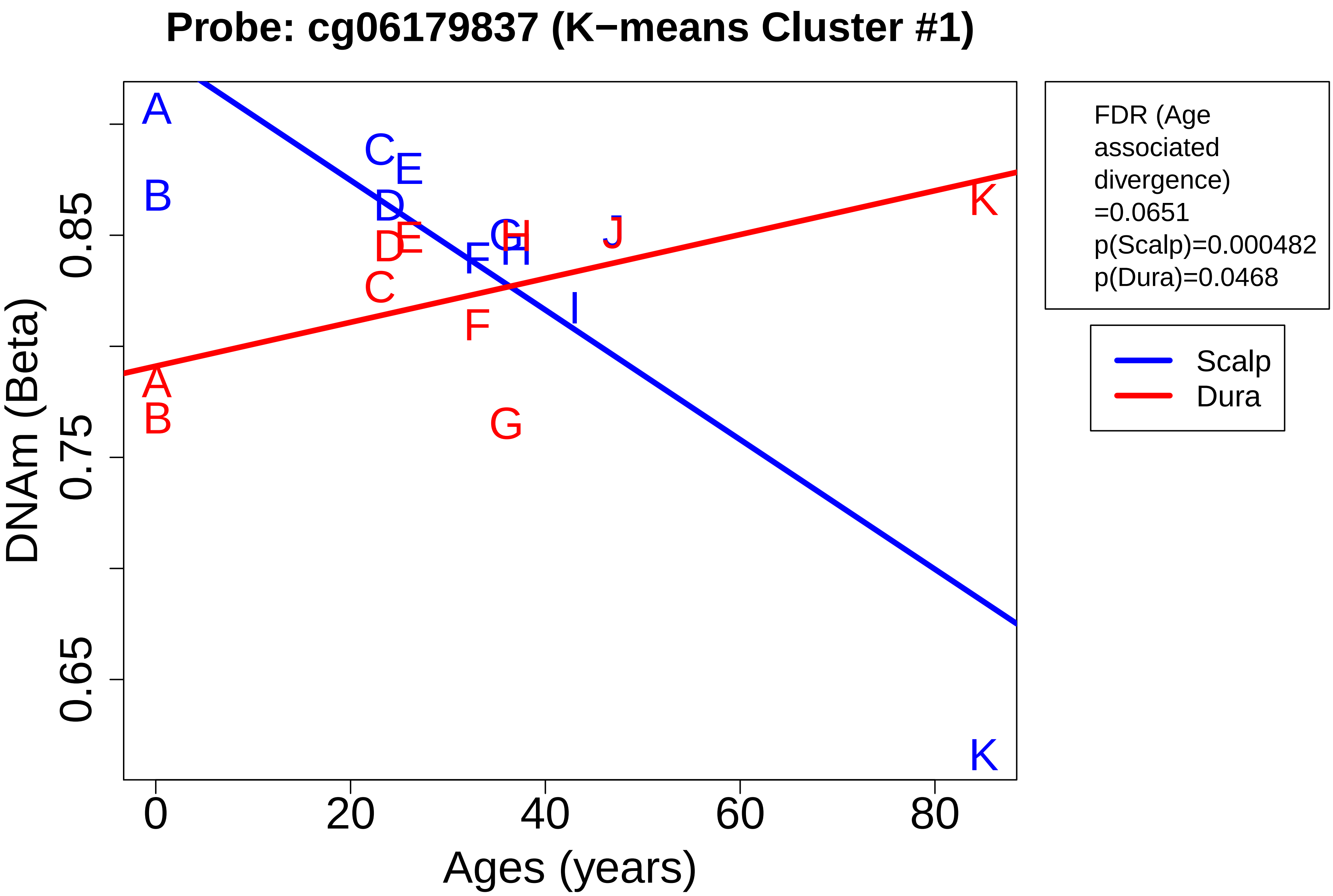

Probe: cg06179837 (K−means Cluster #1)
DNAm (Beta)
0.65
0.75
0.85
Ages (years)
0
20
40
60
80
FDR (Age
associated
divergence)
=0.0651
p(Scalp)=0.000482
p(Dura)=0.0468
Scalp
Dura
A
B
C
D
E
F
G
H
I
J
K

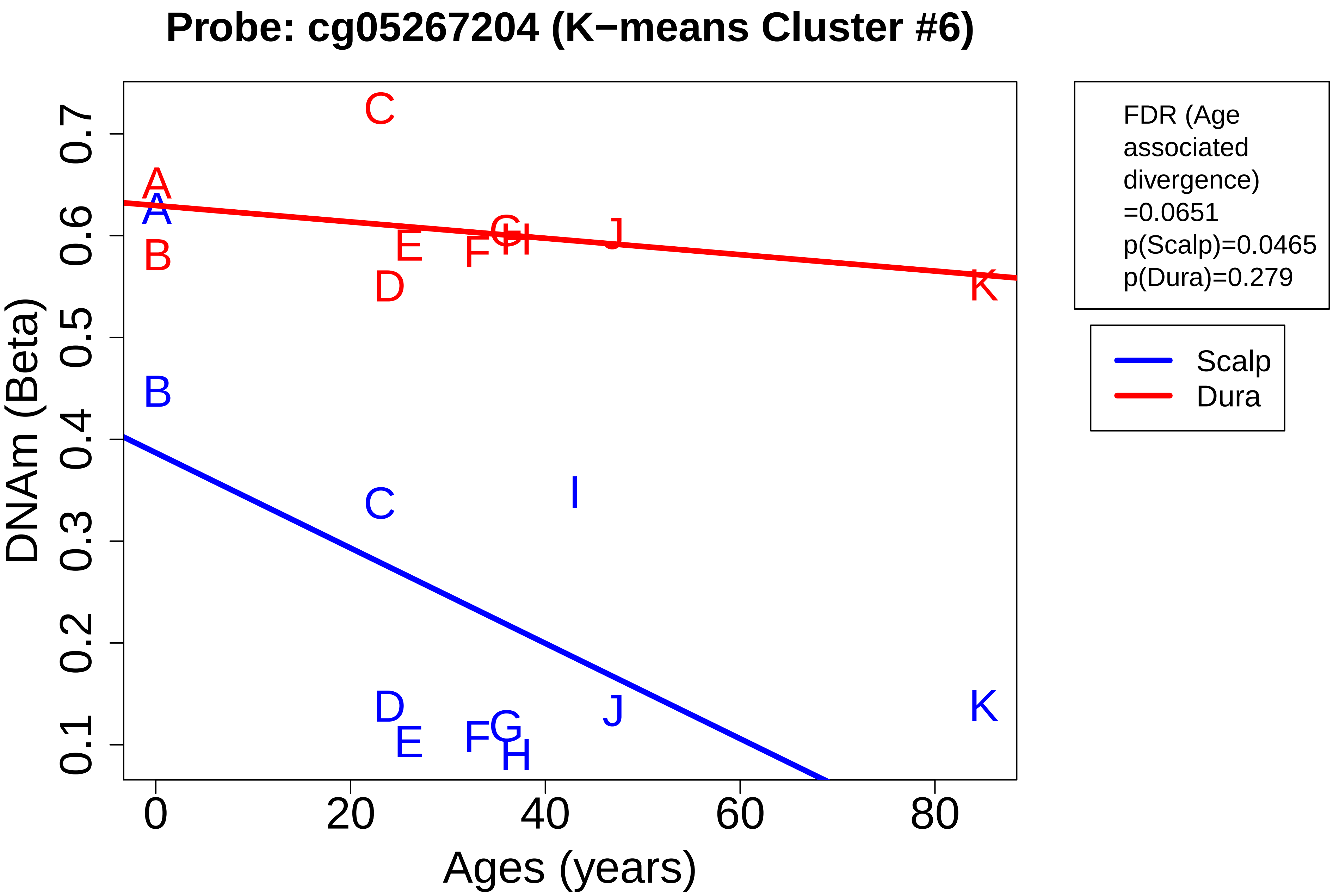
Probe: cg05267204 (K−means Cluster #6)
DNAm (Beta)
0.1
0.2
0.3
0.4
0.5
0.6
0.7
Ages (years)
0
20
40
60
80
A
B
C
D
E
F
G
H
I
J
K
FDR (Age associated divergence) =0.0651
p(Scalp)=0.0465
p(Dura)=0.279
Scalp
Dura

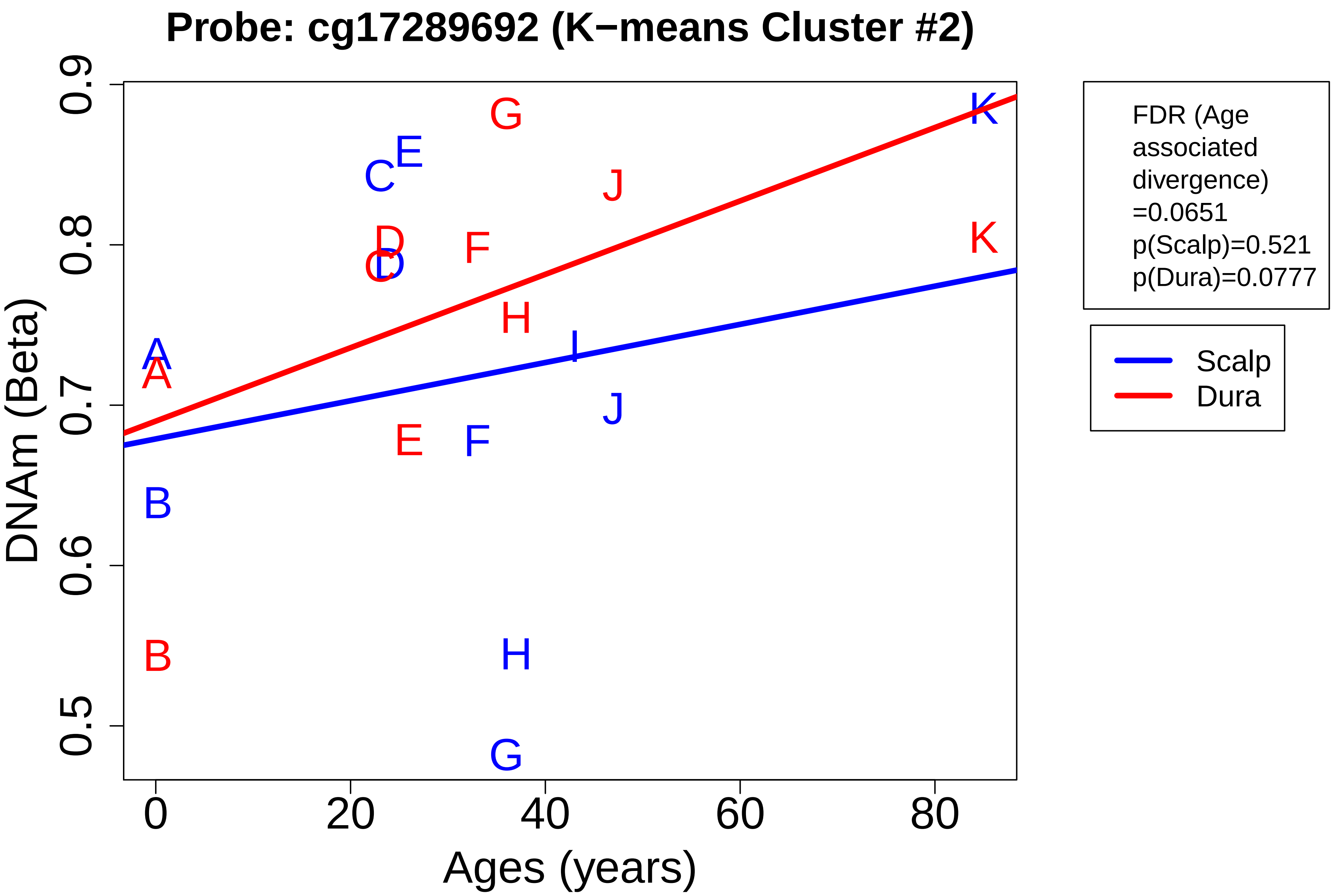

Probe: cg17289692 (K−means Cluster #2)
DNAm (Beta)
0.5
0.6
0.7
0.8
0.9
0
20
40
60
80
Ages (years)
FDR (Age associated divergence) =0.0651
p(Scalp)=0.521
p(Dura)=0.0777
Scalp
Dura

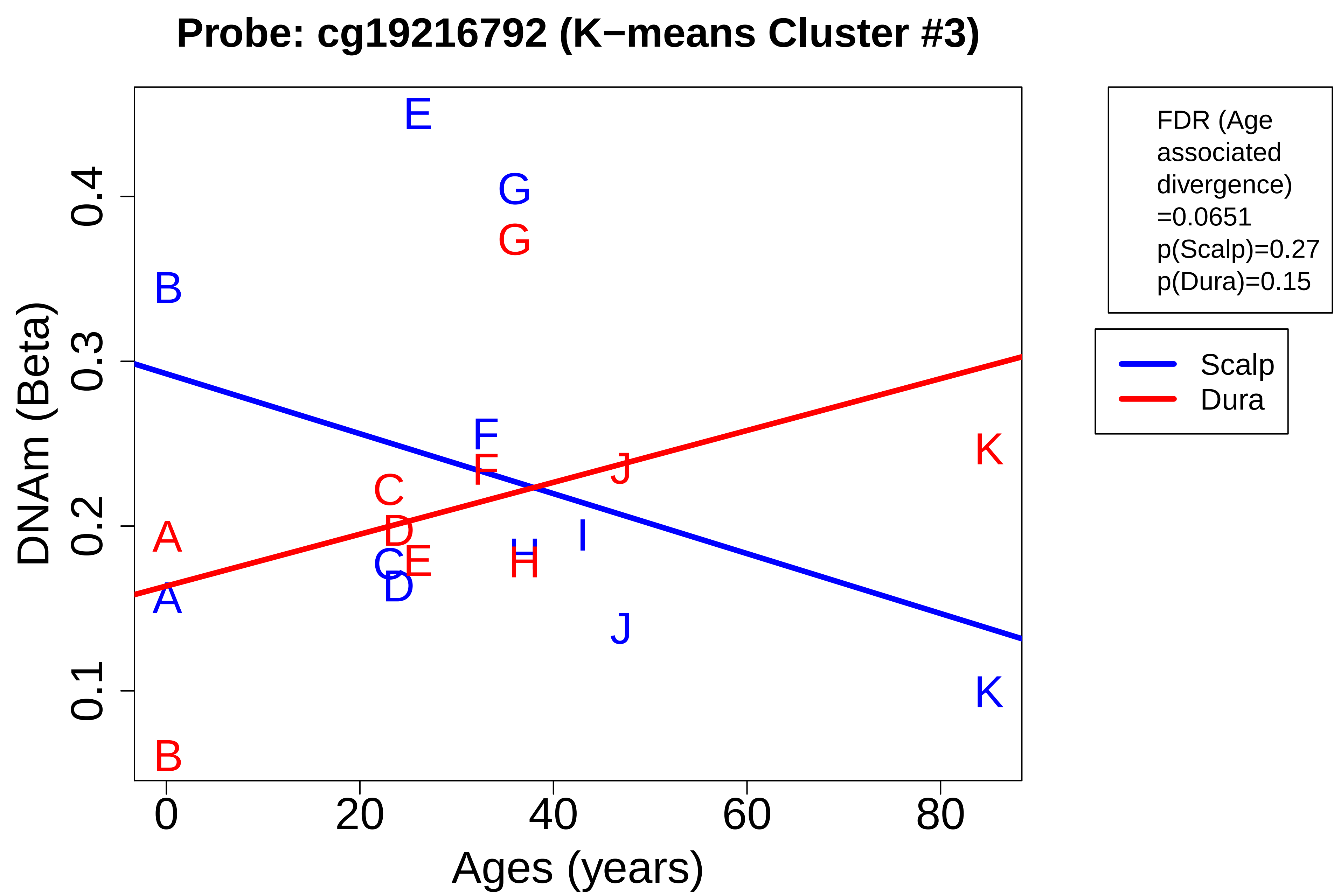
Probe: cg19216792 (K−means Cluster #3)
DNAm (Beta)
0.1
0.2
0.3
0.4
0
20
40
60
80
Ages (years)
A
B
C
D
E
F
G
H
I
J
K
A
B
C
D
E
F
G
H
J
K
FDR (Age associated divergence) =0.0651
p(Scalp)=0.27
p(Dura)=0.15
Scalp
Dura

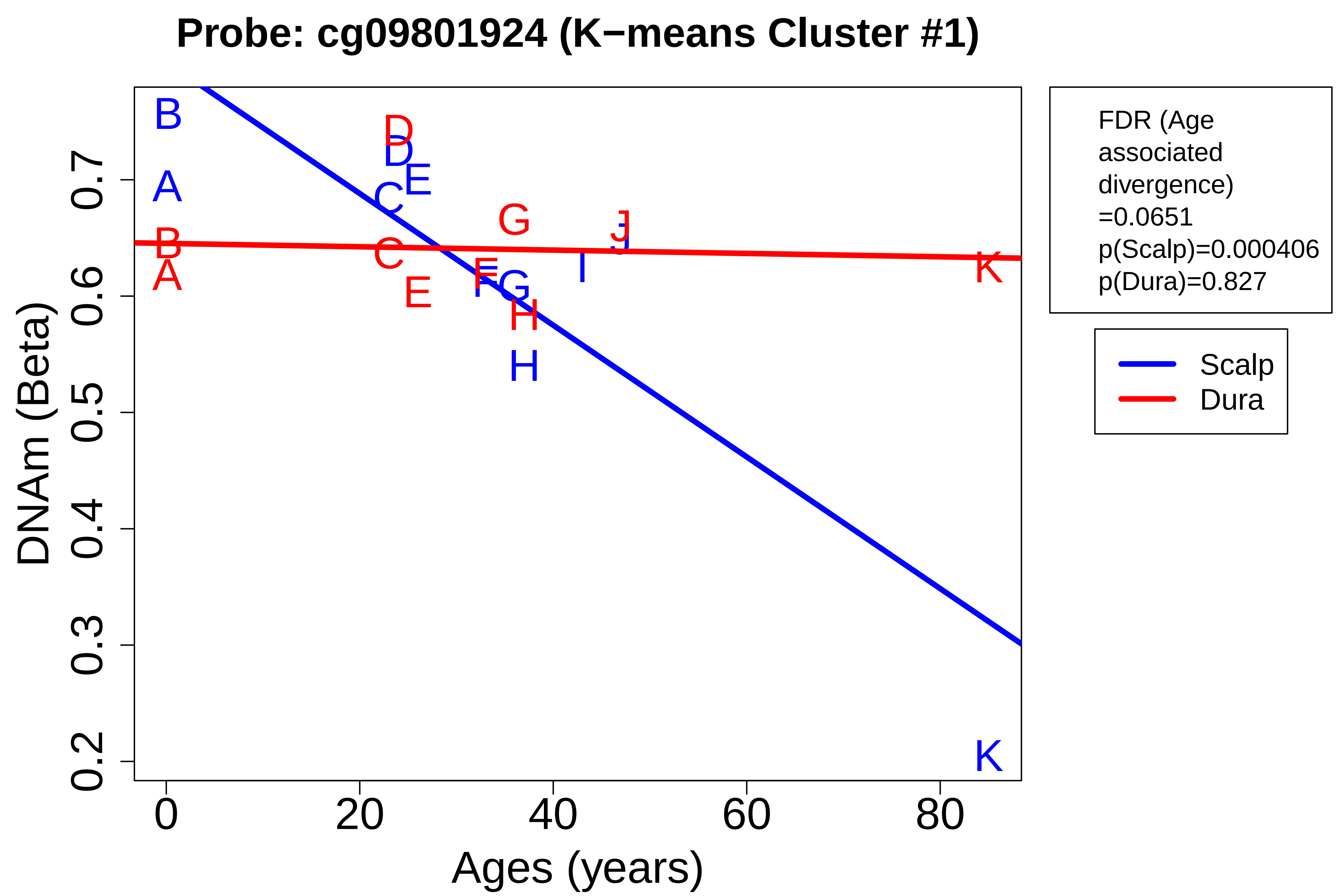

Probe: cg09801924 (K−means Cluster #1)
DNAm (Beta)
0.2
0.3
0.4
0.5
0.6
0.7
0
20
40
60
80
Ages (years)
FDR (Age associated divergence) =0.0651
p(Scalp)=0.000406
p(Dura)=0.827
Scalp
Dura

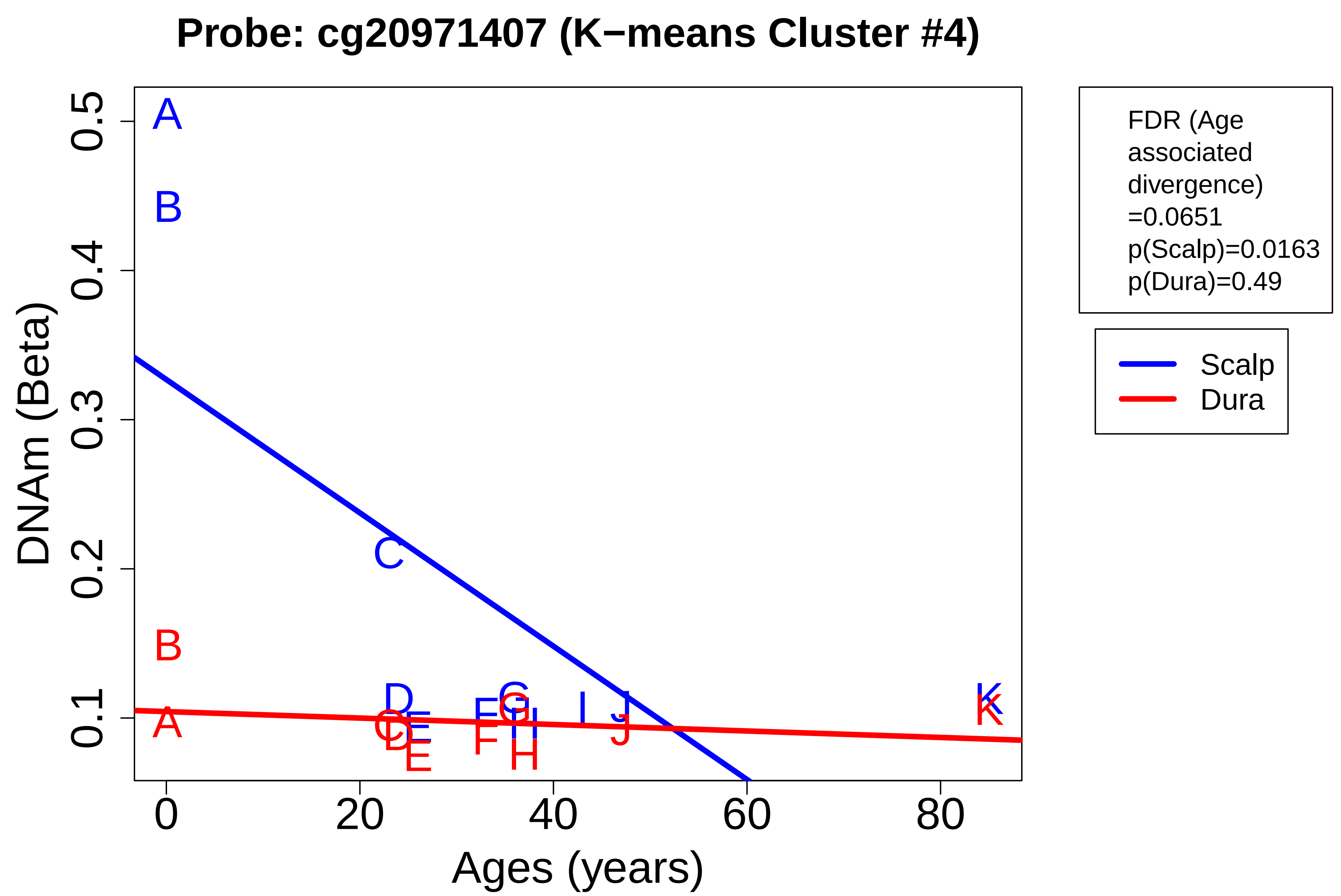

Probe: cg20971407 (K−means Cluster #4)
DNAm (Beta)
0.1
0.2
0.3
0.4
0.5
0
20
40
60
80
Ages (years)
A
B
C
D
E
F
G
H
I
J
K
FDR (Age associated divergence) =0.0651
p(Scalp)=0.0163
p(Dura)=0.49
Scalp
Dura

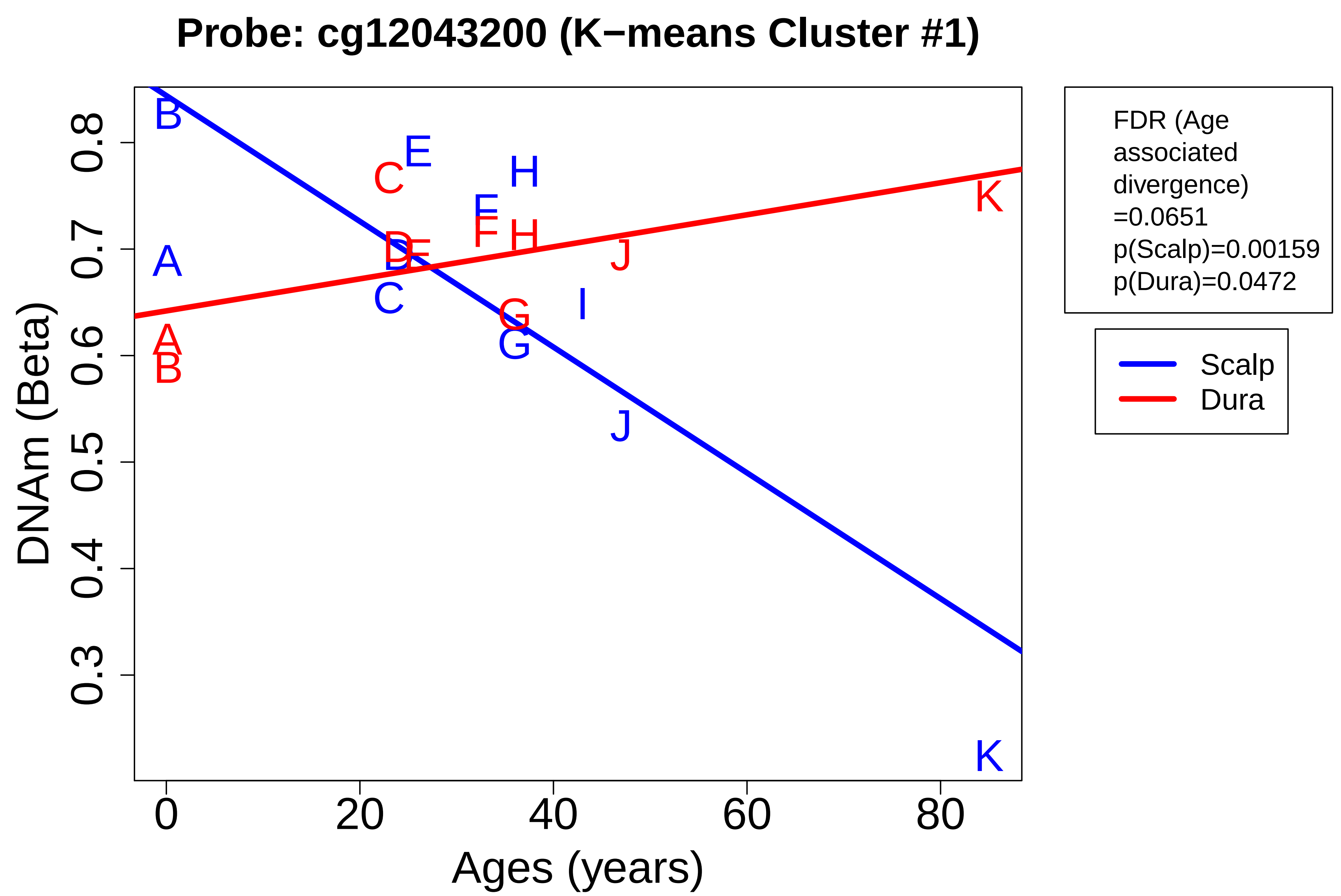

Probe: cg12043200 (K−means Cluster #1)
DNAm (Beta)
Ages (years)
0.3
0.4
0.5
0.6
0.7
0.8
0
20
40
60
80
FDR (Age associated divergence) =0.0651
p(Scalp)=0.00159
p(Dura)=0.0472
Scalp
Dura

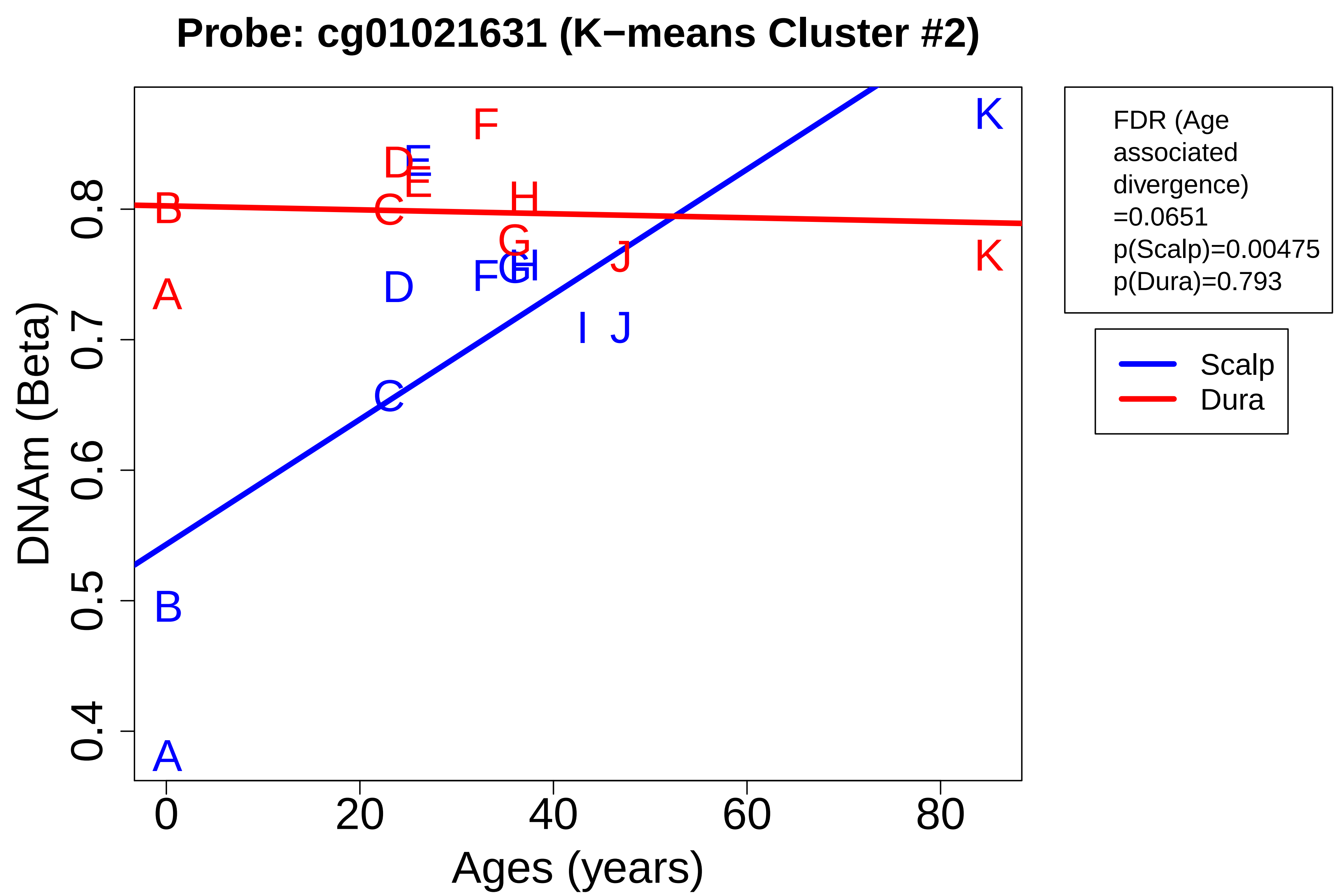

Probe: cg01021631 (K−means Cluster #2)
FDR (Age associated divergence) =0.0651
p(Scalp)=0.00475
p(Dura)=0.793
Scalp
Dura
DNAm (Beta)
0.4
0.5
0.6
0.7
0.8
0
20
40
60
80
Ages (years)
A
B
C
D
E
F
G
H
I
J
K

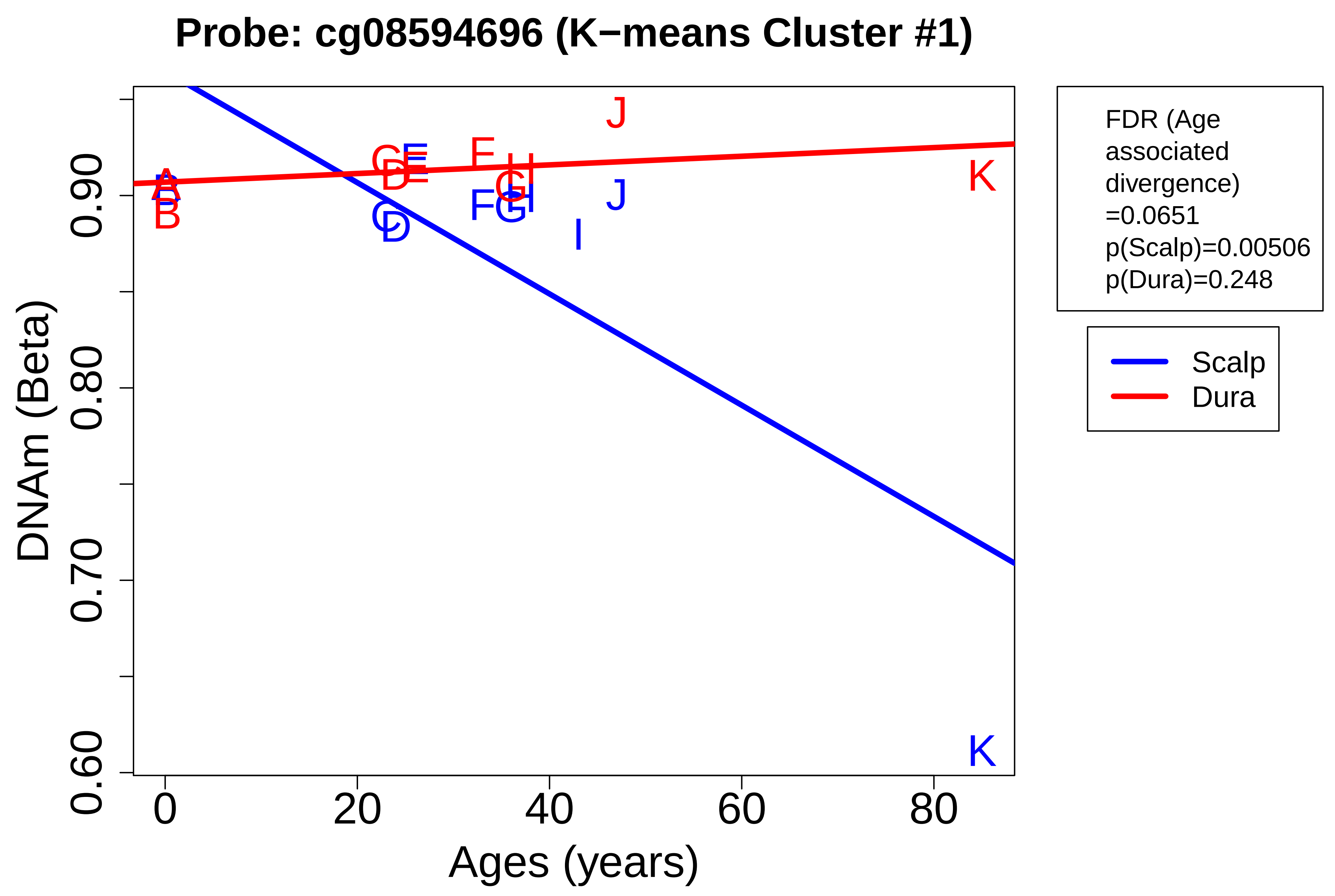
Probe: cg08594696 (K−means Cluster #1)
DNAm (Beta)
0.60
0.70
0.80
0.90
0
20
40
60
80
Ages (years)
FDR (Age associated divergence) =0.0651
p(Scalp)=0.00506
p(Dura)=0.248
Scalp
Dura

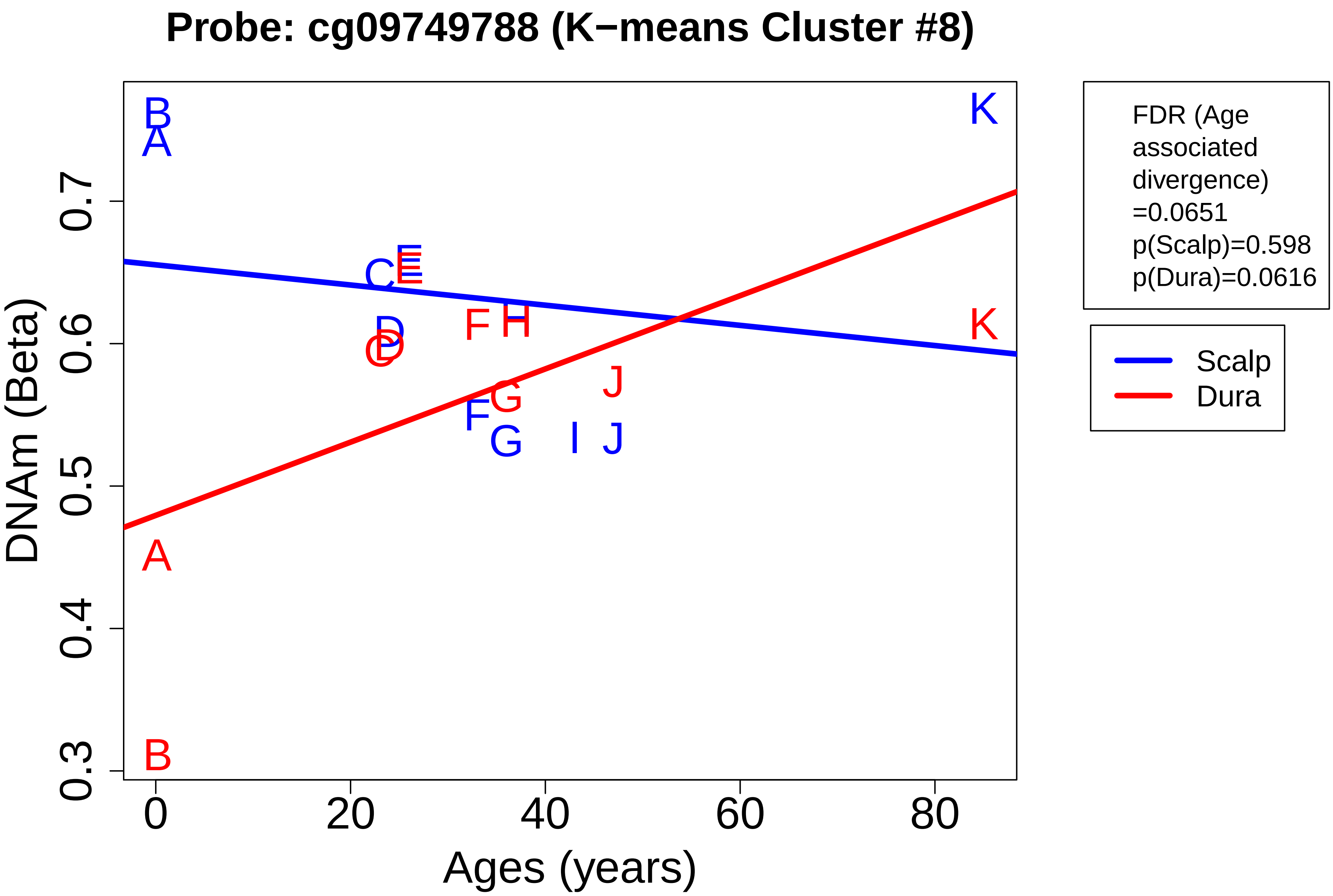
Probe: cg09749788 (K−means Cluster #8)
DNAm (Beta)
0.3
0.4
0.5
0.6
0.7
Ages (years)
0
20
40
60
80
FDR (Age associated divergence) =0.0651
p(Scalp)=0.598
p(Dura)=0.0616
Scalp
Dura
A
B
C
D
E
F
G
H
I
J
K

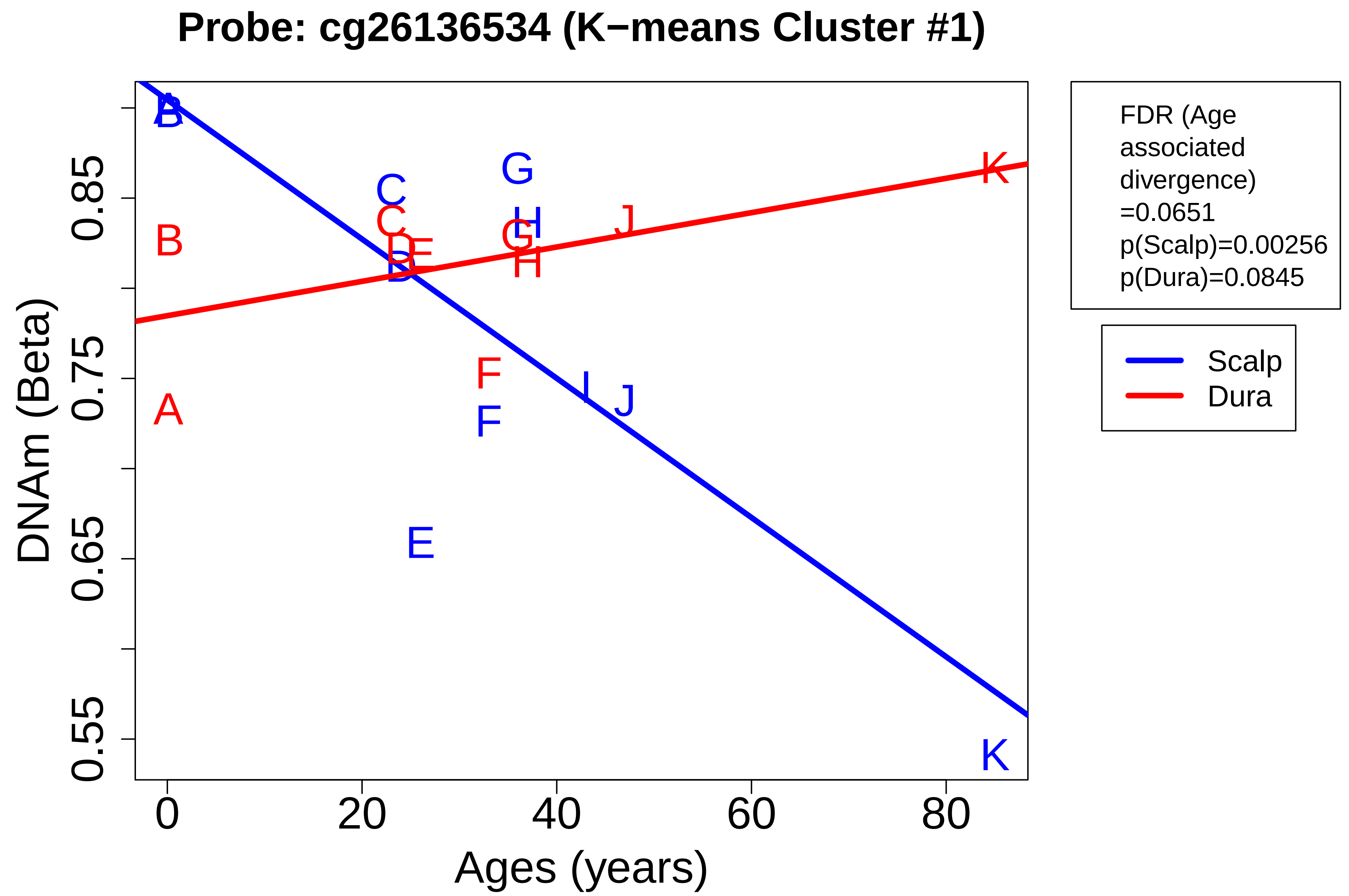
Probe: cg26136534 (K−means Cluster #1)
DNAm (Beta)
0.55
0.65
0.75
0.85
0
20
40
60
80
Ages (years)
FDR (Age associated divergence) =0.0651
p(Scalp)=0.00256
p(Dura)=0.0845
Scalp
Dura

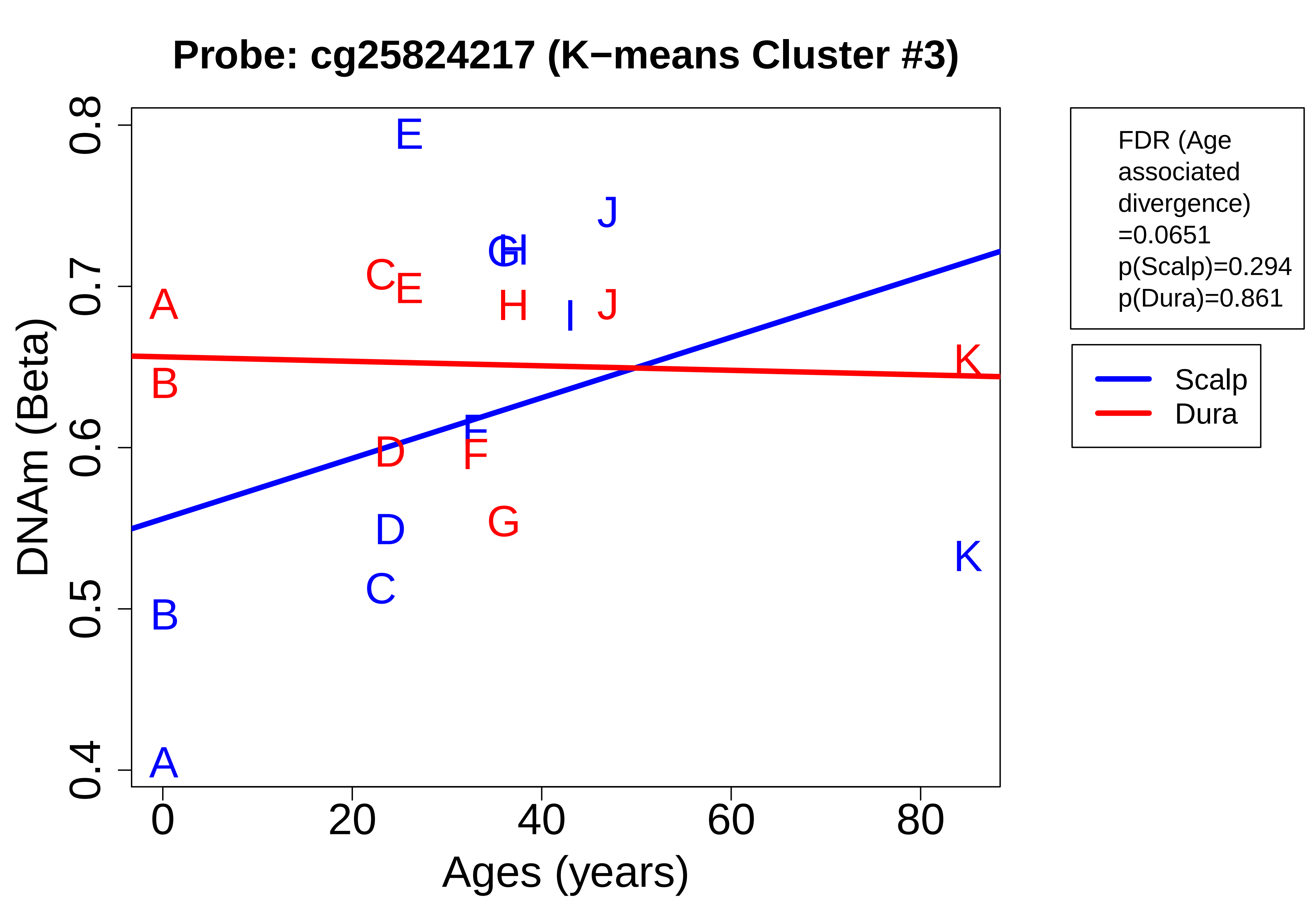

Probe: cg25824217 (K−means Cluster #3)
DNAm (Beta)
0.4
0.5
0.6
0.7
0.8
Ages (years)
0
20
40
60
80
FDR (Age associated divergence) =0.0651
p(Scalp)=0.294
p(Dura)=0.861
Scalp
Dura

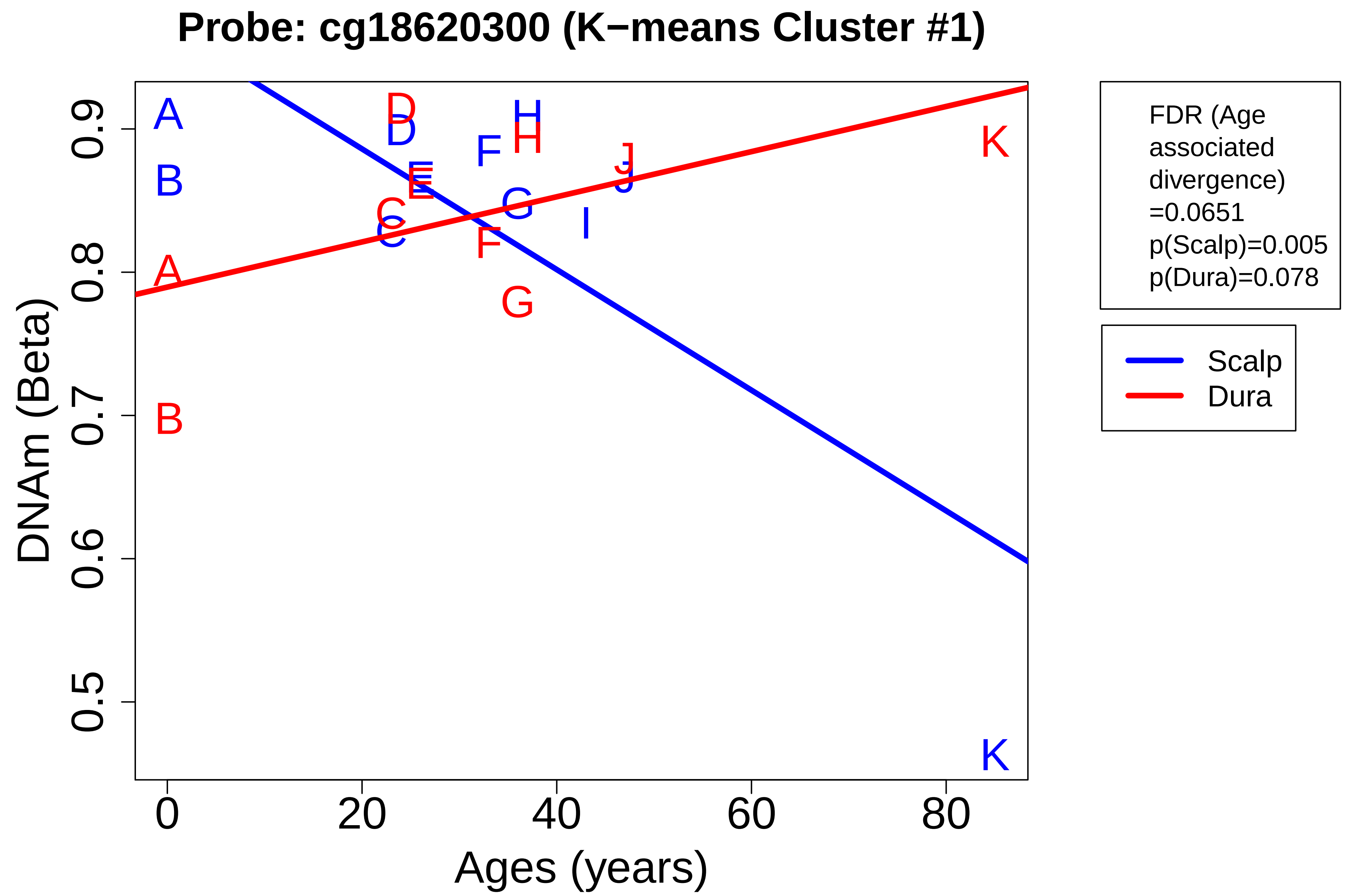

Probe: cg18620300 (K−means Cluster #1)
DNAm (Beta)
0.5
0.6
0.7
0.8
0.9
0
20
40
60
80
Ages (years)
A
B
C
D
E
F
G
H
I
J
K
FDR (Age associated divergence) =0.0651
p(Scalp)=0.005
p(Dura)=0.078
Scalp
Dura

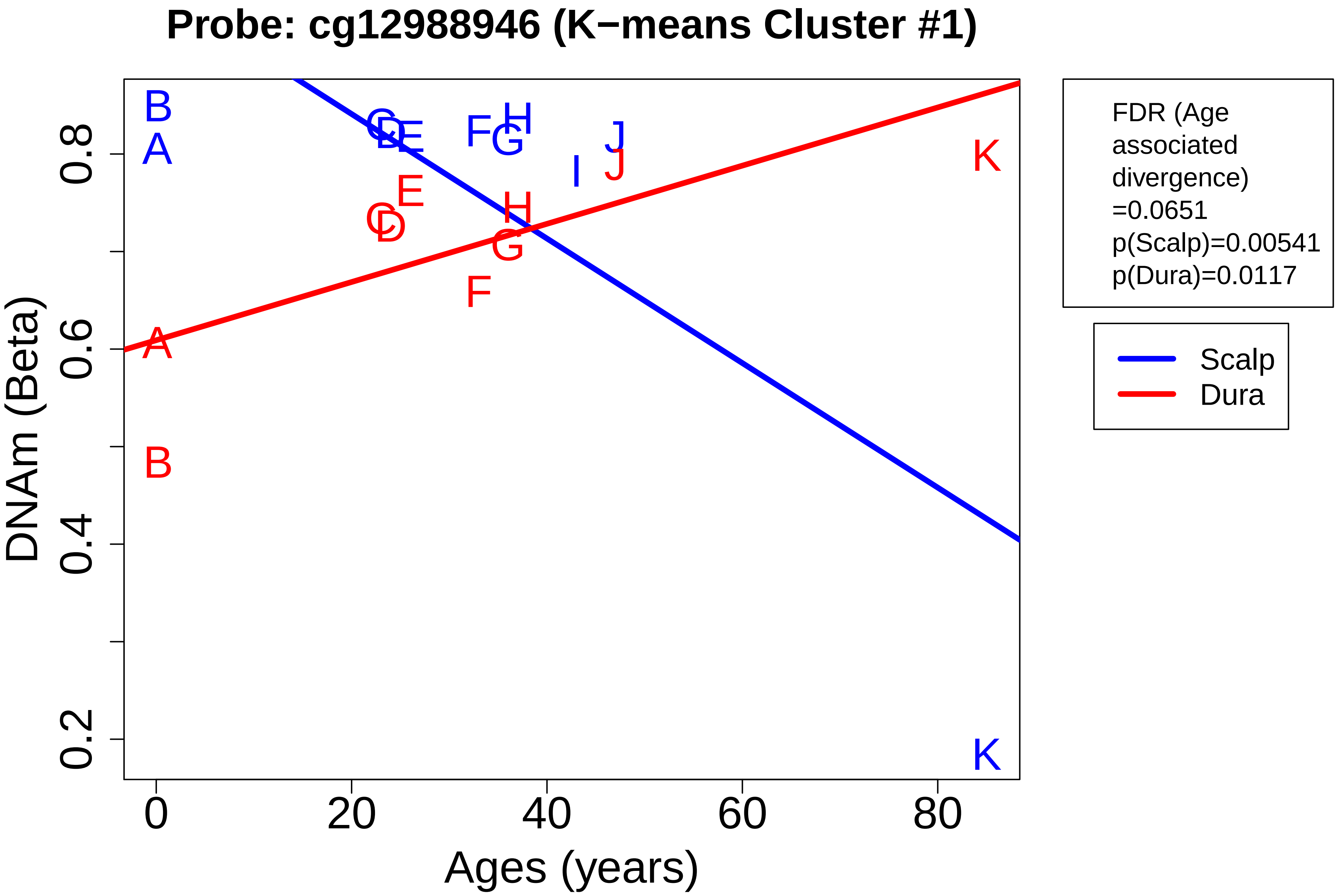
Probe: cg12988946 (K−means Cluster #1)
DNAm (Beta)
0.2
0.4
0.6
0.8
0
20
40
60
80
Ages (years)
A
B
C
D
E
F
G
H
I
J
K
FDR (Age associated divergence) =0.0651
p(Scalp)=0.00541
p(Dura)=0.0117
Scalp
Dura

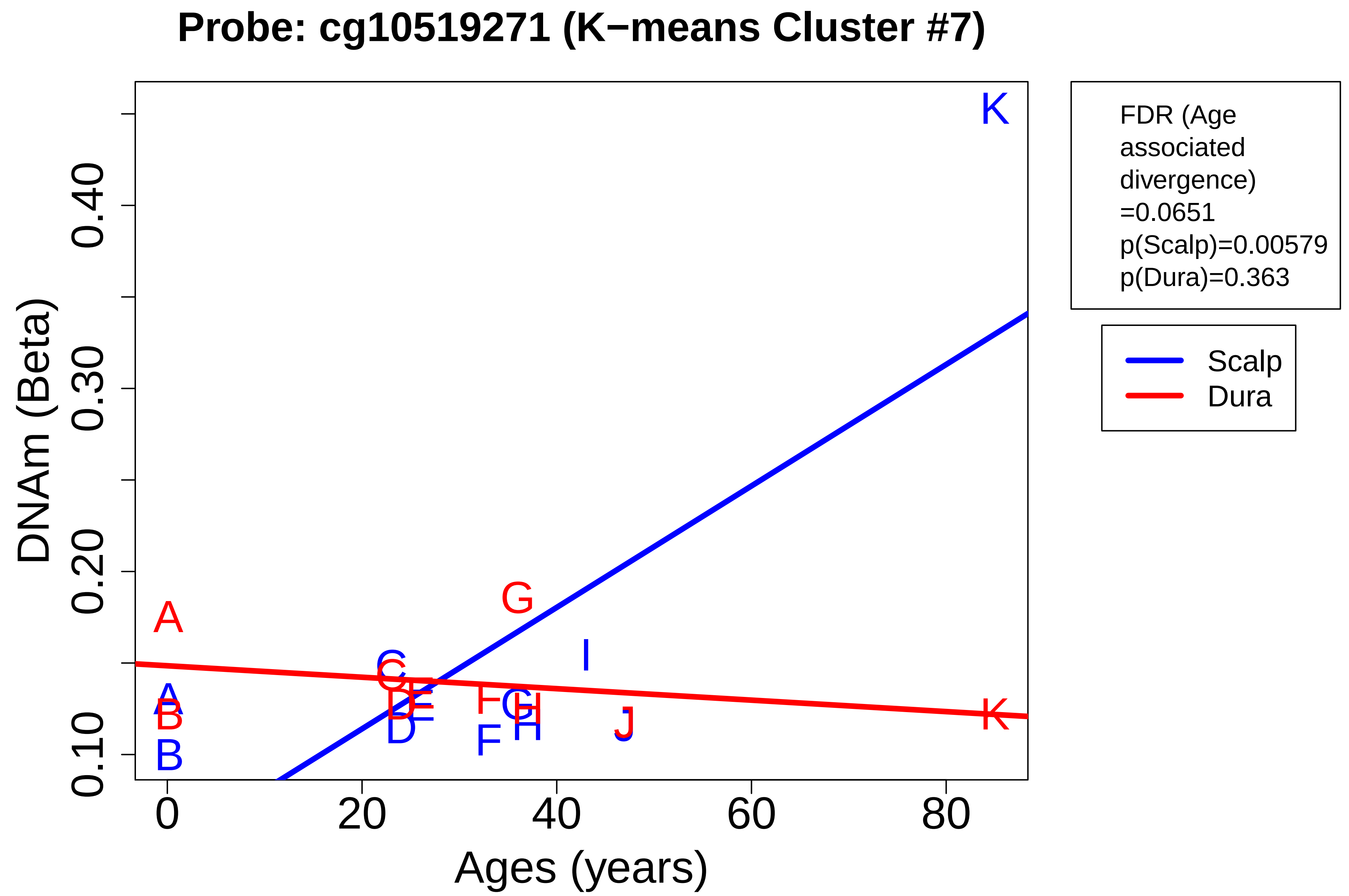

Probe: cg10519271 (K−means Cluster #7)
DNAm (Beta)
Ages (years)
0.10
0.20
0.30
0.40
0
20
40
60
80
FDR (Age associated divergence) =0.0651
p(Scalp)=0.00579
p(Dura)=0.363
Scalp
Dura

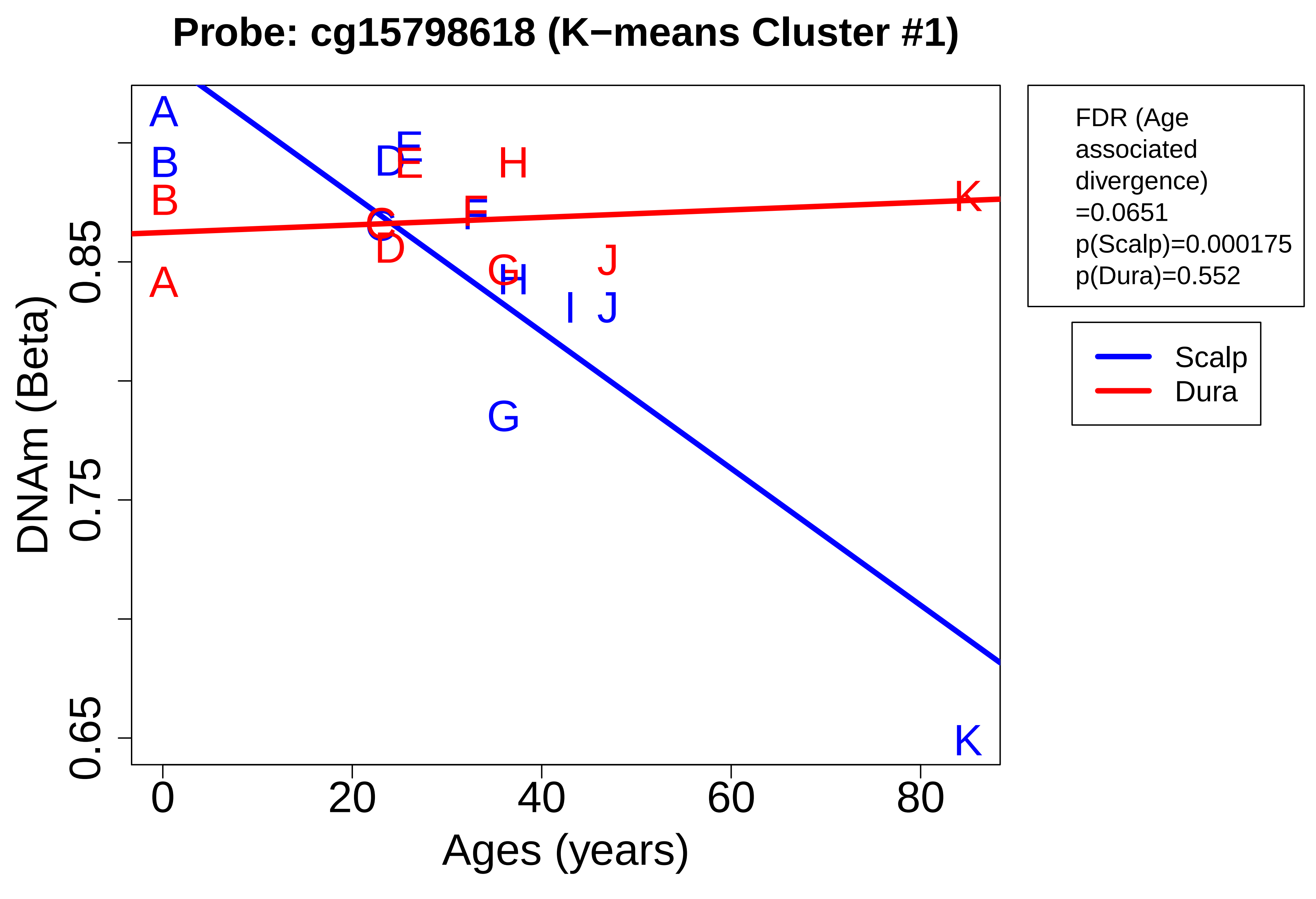
Probe: cg15798618 (K−means Cluster #1)
DNAm (Beta)
0.65
0.75
0.85
0
20
40
60
80
Ages (years)
A
B
C
D
E
F
G
H
I
J
K
FDR (Age associated divergence) =0.0651
p(Scalp)=0.000175
p(Dura)=0.552
Scalp
Dura

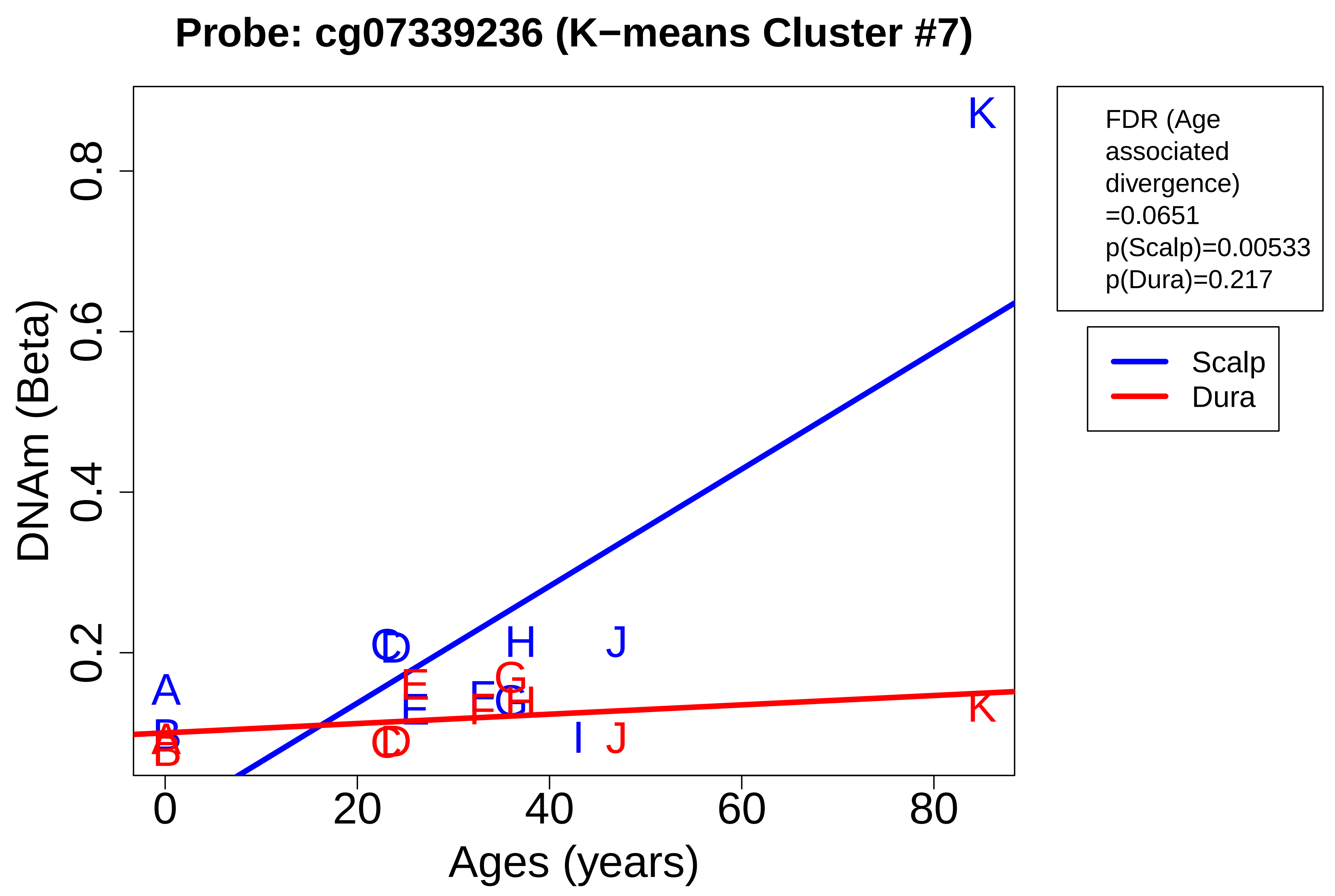
Probe: cg07339236 (K−means Cluster #7)
DNAm (Beta)
0.2
0.4
0.6
0.8
0
20
40
60
80
Ages (years)
FDR (Age associated divergence) =0.0651
p(Scalp)=0.00533
p(Dura)=0.217
Scalp
Dura
A
B
C
D
E
F
G
H
I
J
K

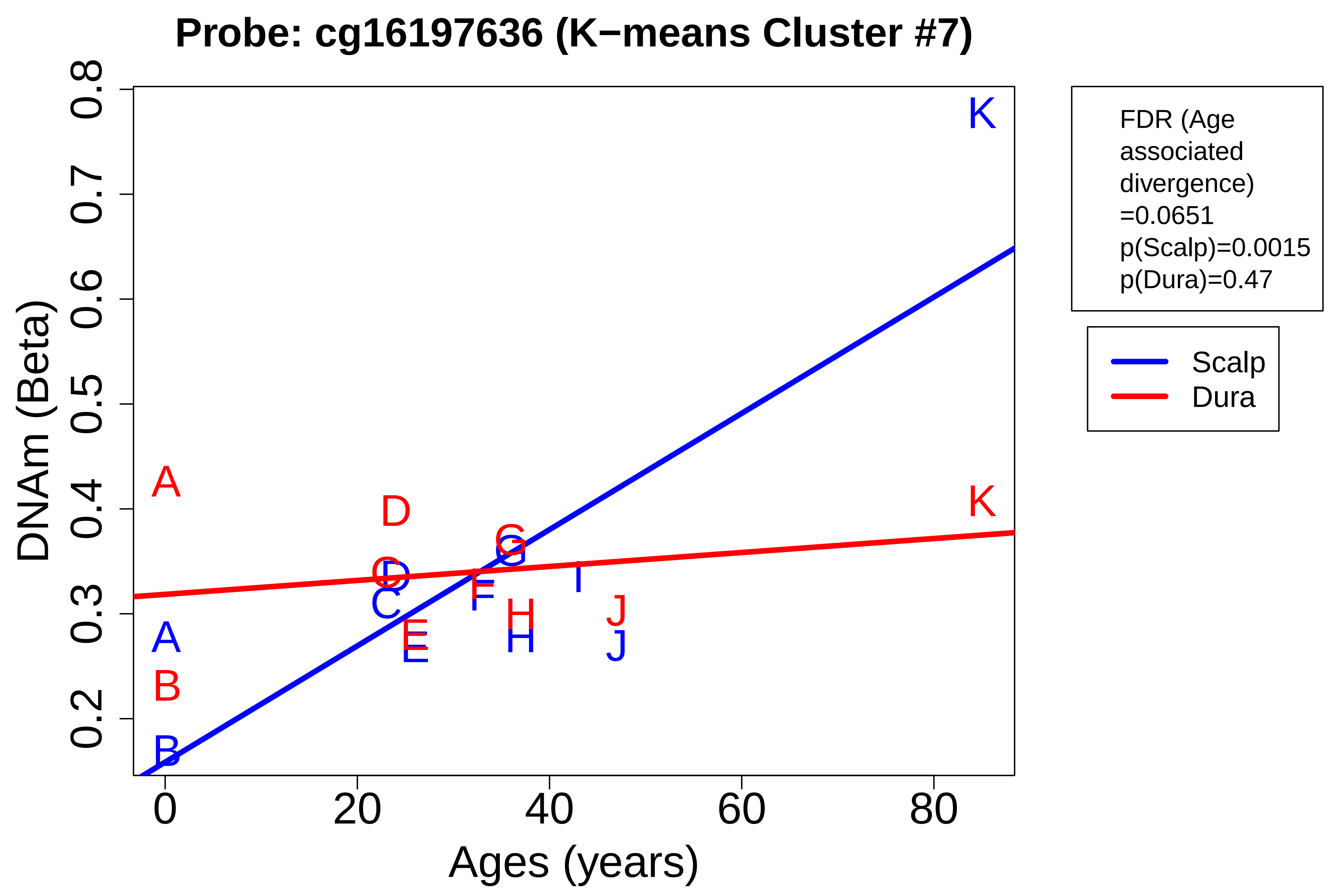

Probe: cg16197636 (K−means Cluster #7)
DNAm (Beta)
0.2
0.3
0.4
0.5
0.6
0.7
0.8
0
20
40
60
80
Ages (years)
FDR (Age associated divergence) =0.0651
p(Scalp)=0.0015
p(Dura)=0.47
Scalp
Dura
A
B
C
D
E
F
G
H
I
J
K

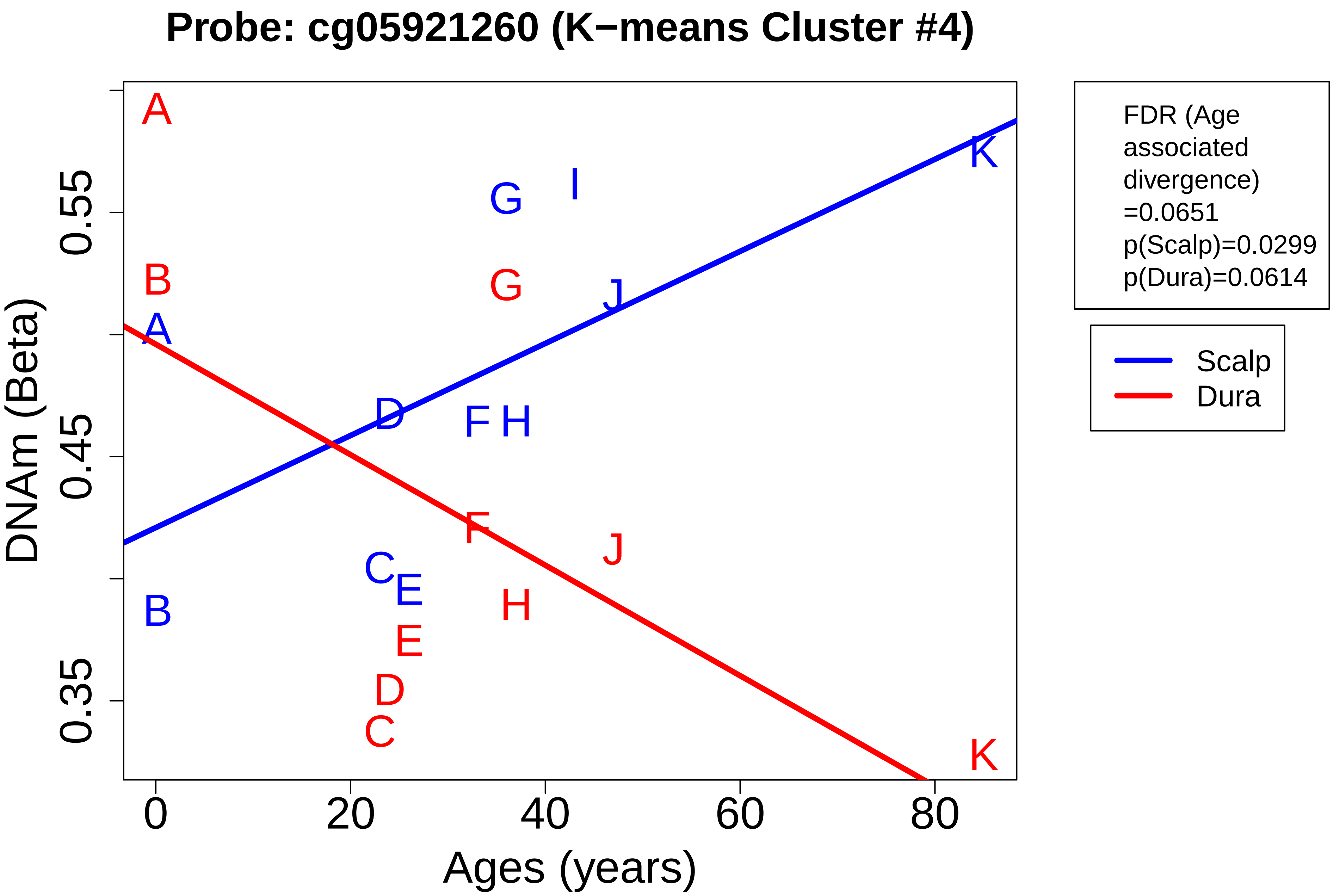

Probe: cg05921260 (K−means Cluster #4)
FDR (Age associated divergence) =0.0651
p(Scalp)=0.0299
p(Dura)=0.0614
Scalp
Dura
DNAm (Beta)
0.35
0.45
0.55
0
20
40
60
80
Ages (years)
A
B
C
D
E
F
G
H
I
J
K

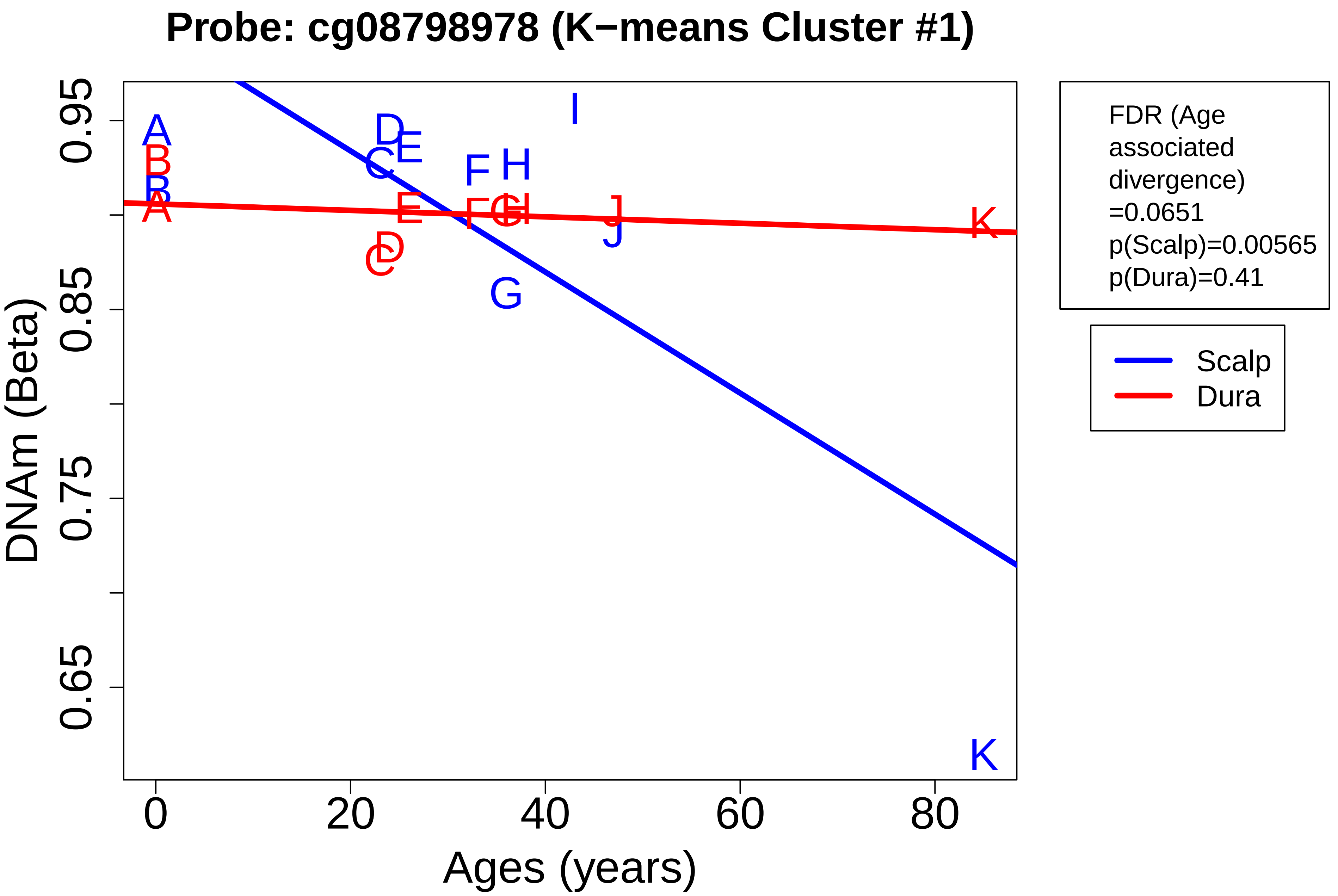

Probe: cg08798978 (K−means Cluster #1)
DNAm (Beta)
0.65
0.75
0.85
0.95
0
20
40
60
80
Ages (years)
A
B
C
D
E
F
G
H
I
J
K
FDR (Age associated divergence) =0.0651
p(Scalp)=0.00565
p(Dura)=0.41
Scalp
Dura

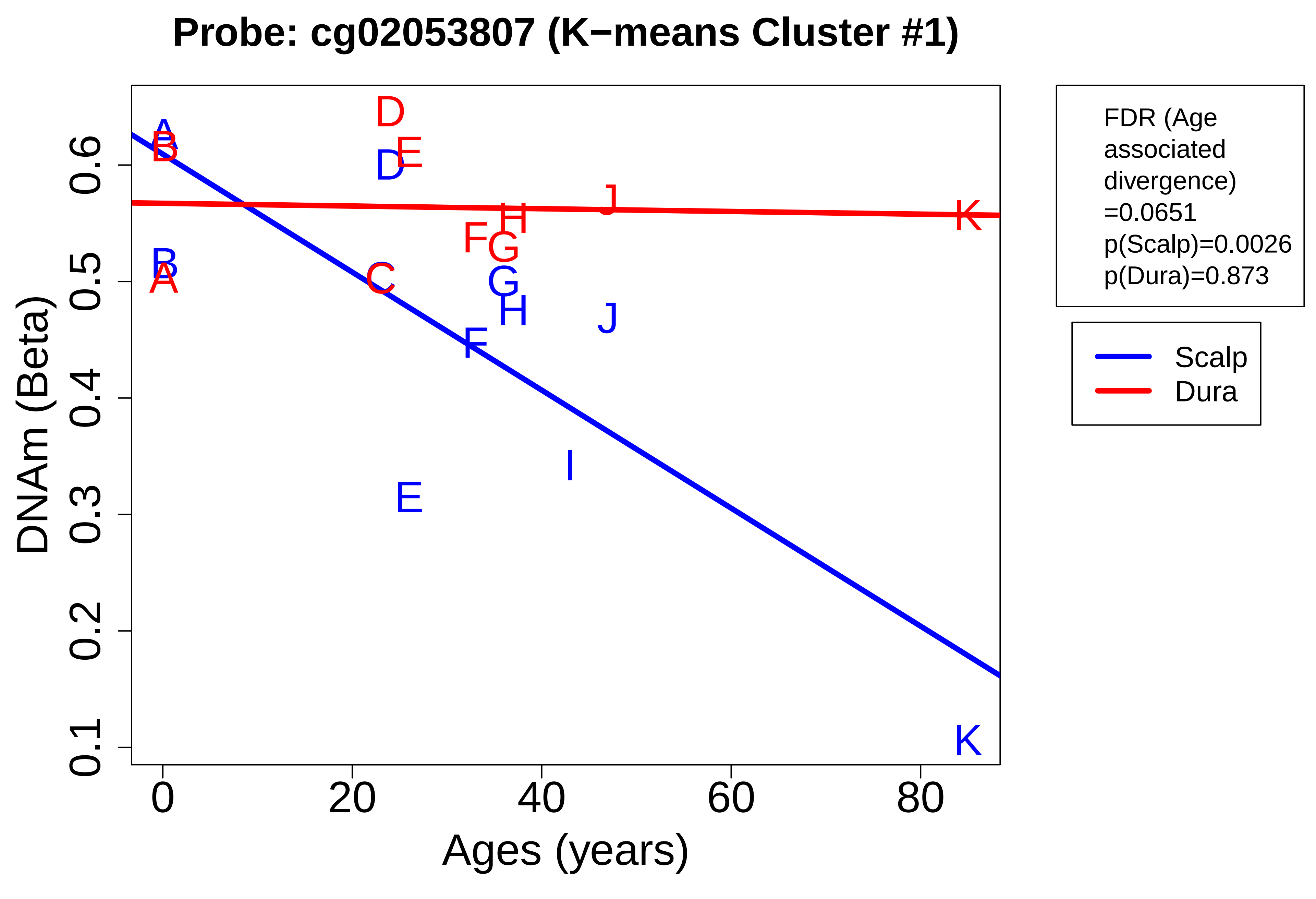

Probe: cg02053807 (K−means Cluster #1)
DNAm (Beta)
Ages (years)
0.1
0.2
0.3
0.4
0.5
0.6
0
20
40
60
80
A
B
C
D
E
F
G
H
I
J
K
FDR (Age associated divergence) =0.0651
p(Scalp)=0.0026
p(Dura)=0.873
Scalp
Dura

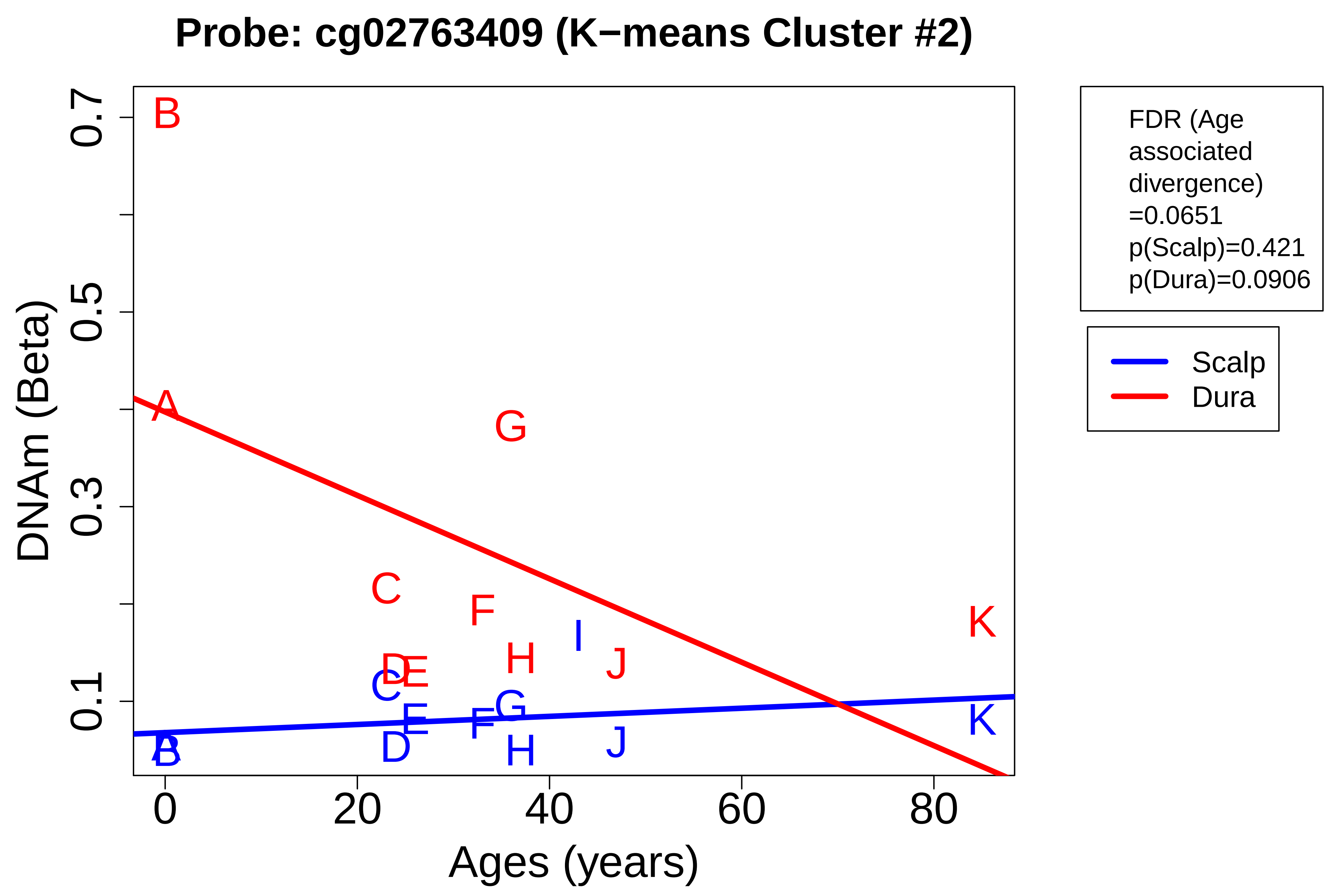

Probe: cg02763409 (K−means Cluster #2)
DNAm (Beta)
0.1
0.3
0.5
0.7
Ages (years)
0
20
40
60
80
FDR (Age associated divergence) =0.0651
p(Scalp)=0.421
p(Dura)=0.0906
Scalp
Dura

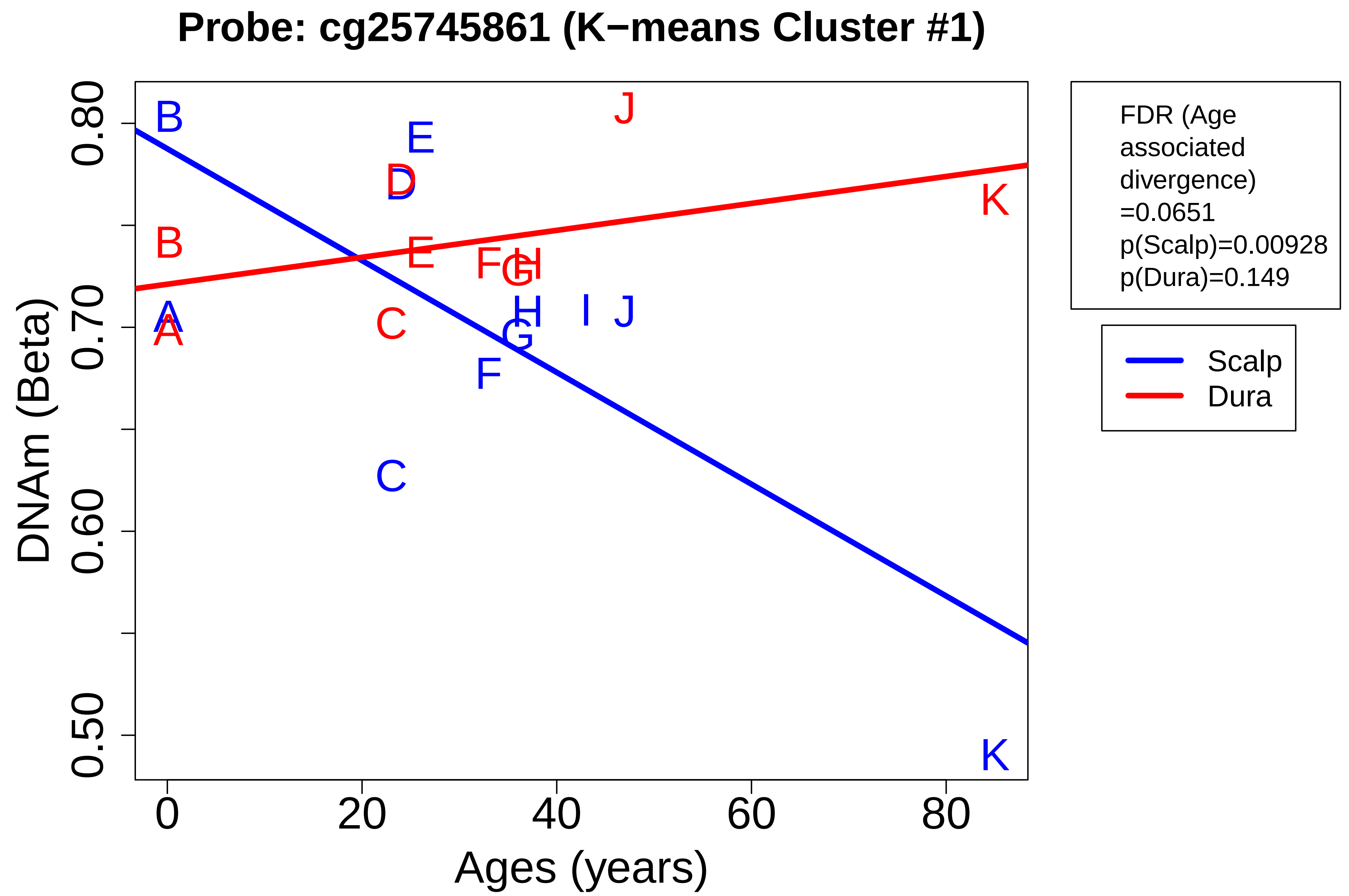

Probe: cg25745861 (K−means Cluster #1)
DNAm (Beta)
0.50
0.60
0.70
0.80
0
20
40
60
80
Ages (years)
A B C D E F G H I J K
A B C D E F G H J K
FDR (Age associated divergence) =0.0651
p(Scalp)=0.00928
p(Dura)=0.149
Scalp
Dura

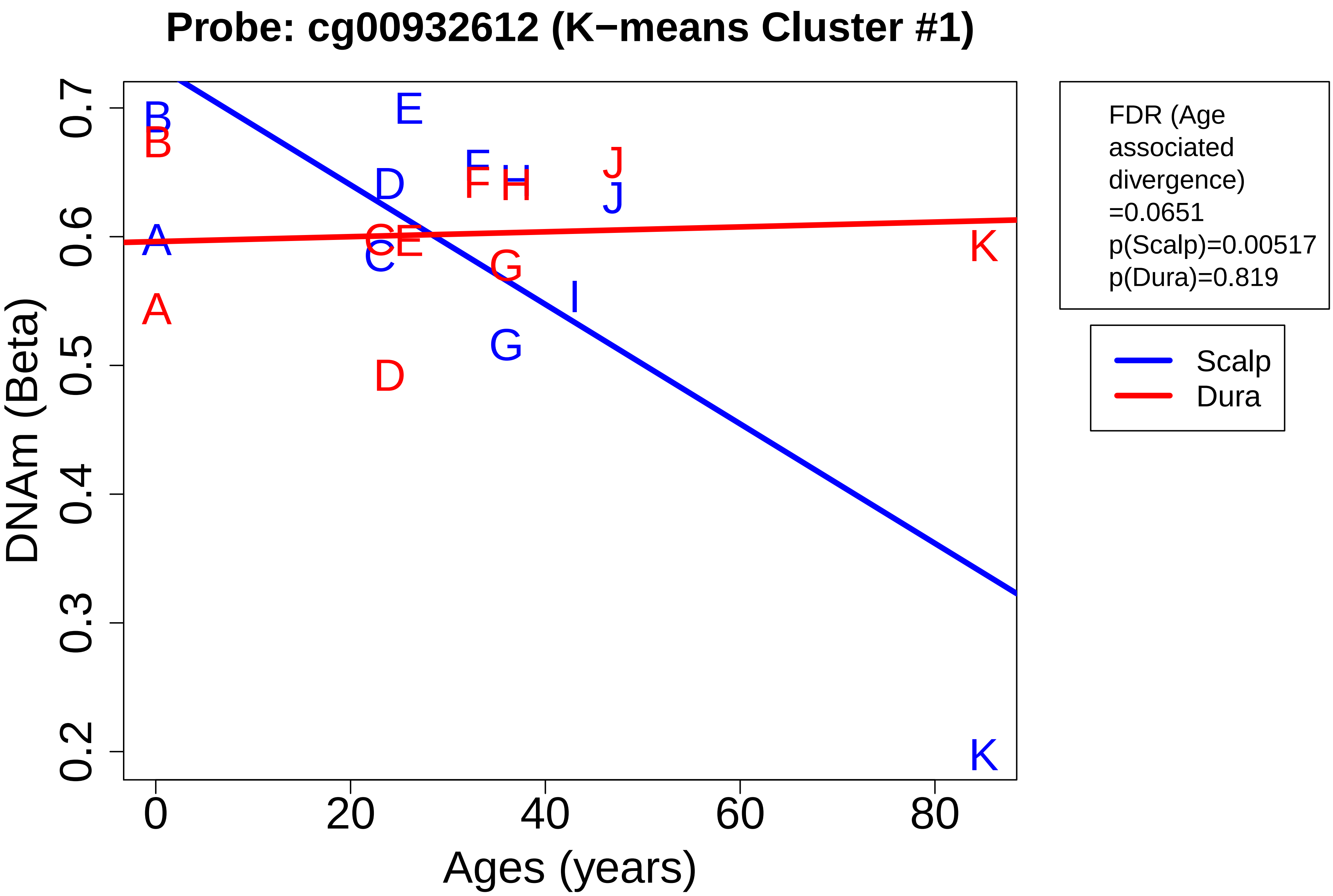
Probe: cg00932612 (K−means Cluster #1)
DNAm (Beta)
Ages (years)
FDR (Age associated divergence) =0.0651
p(Scalp)=0.00517
p(Dura)=0.819
Scalp
Dura

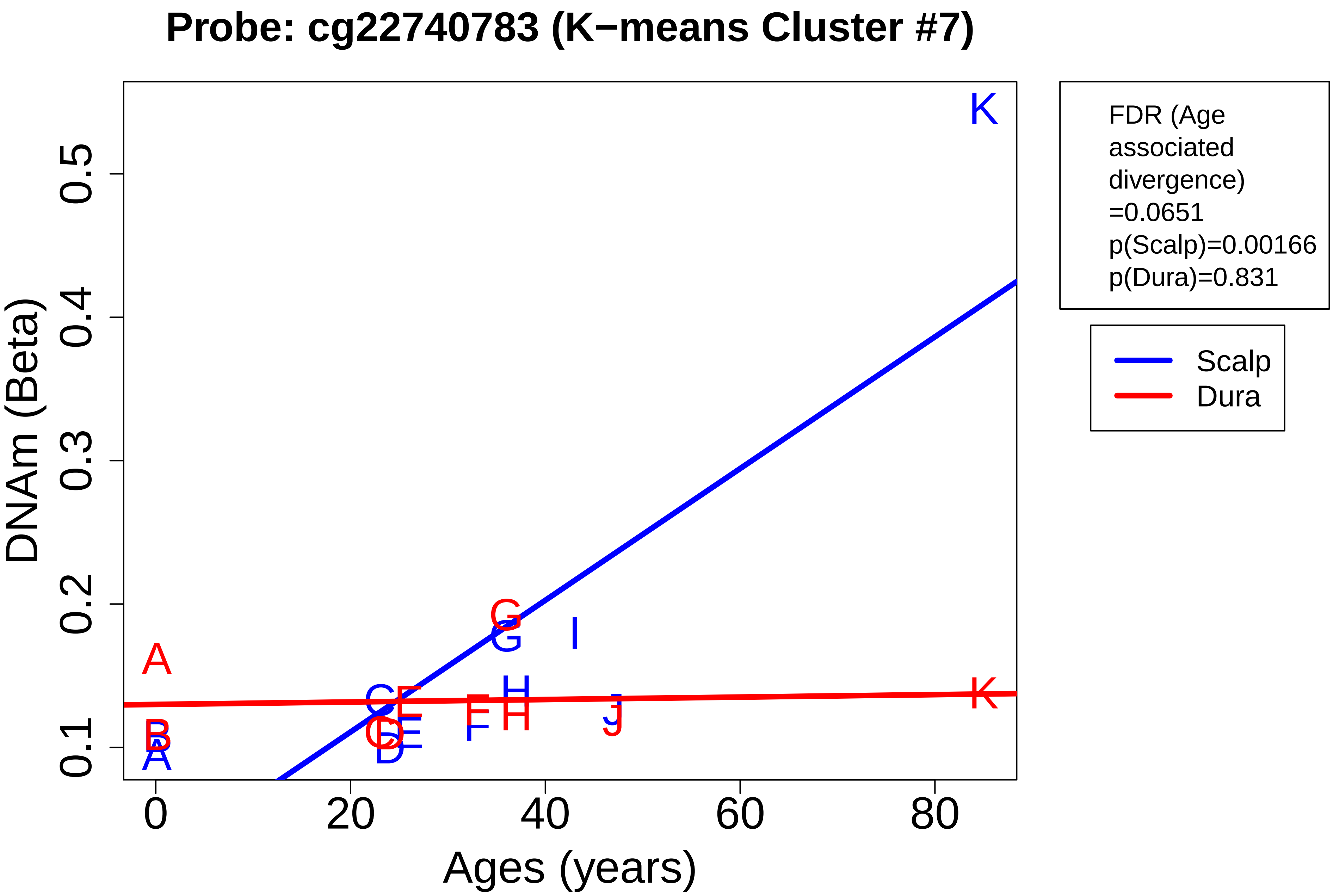
Probe: cg22740783 (K−means Cluster #7)
DNAm (Beta)
0.1
0.2
0.3
0.4
0.5
Ages (years)
0
20
40
60
80
FDR (Age associated divergence) =0.0651
p(Scalp)=0.00166
p(Dura)=0.831
Scalp
Dura
A
B
C
D
E
F
G
H
I
J
K

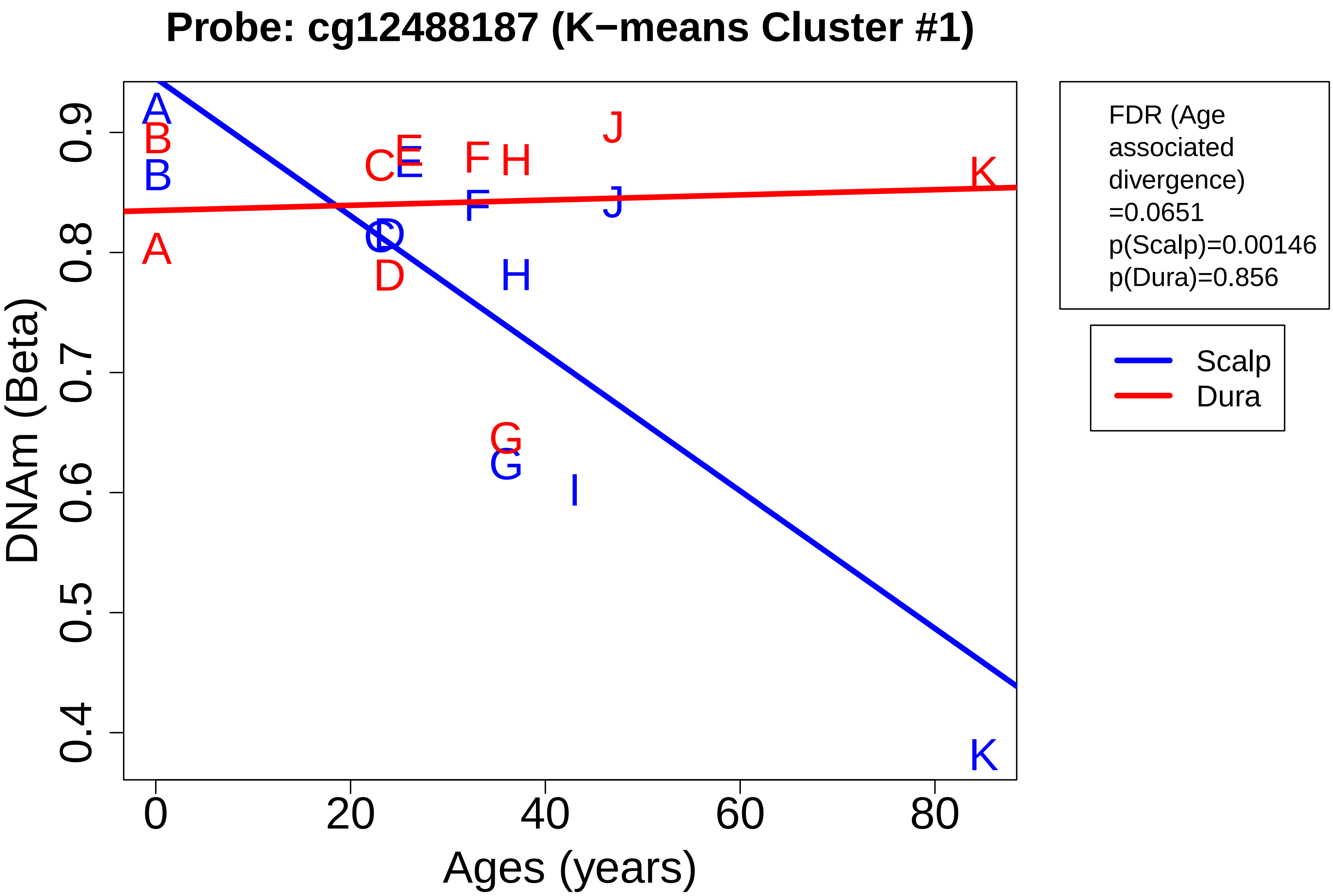

Probe: cg12488187 (K−means Cluster #1)
DNAm (Beta)
0.4
0.5
0.6
0.7
0.8
0.9
0
20
40
60
80
Ages (years)
FDR (Age associated divergence) =0.0651
p(Scalp)=0.00146
p(Dura)=0.856
Scalp
Dura

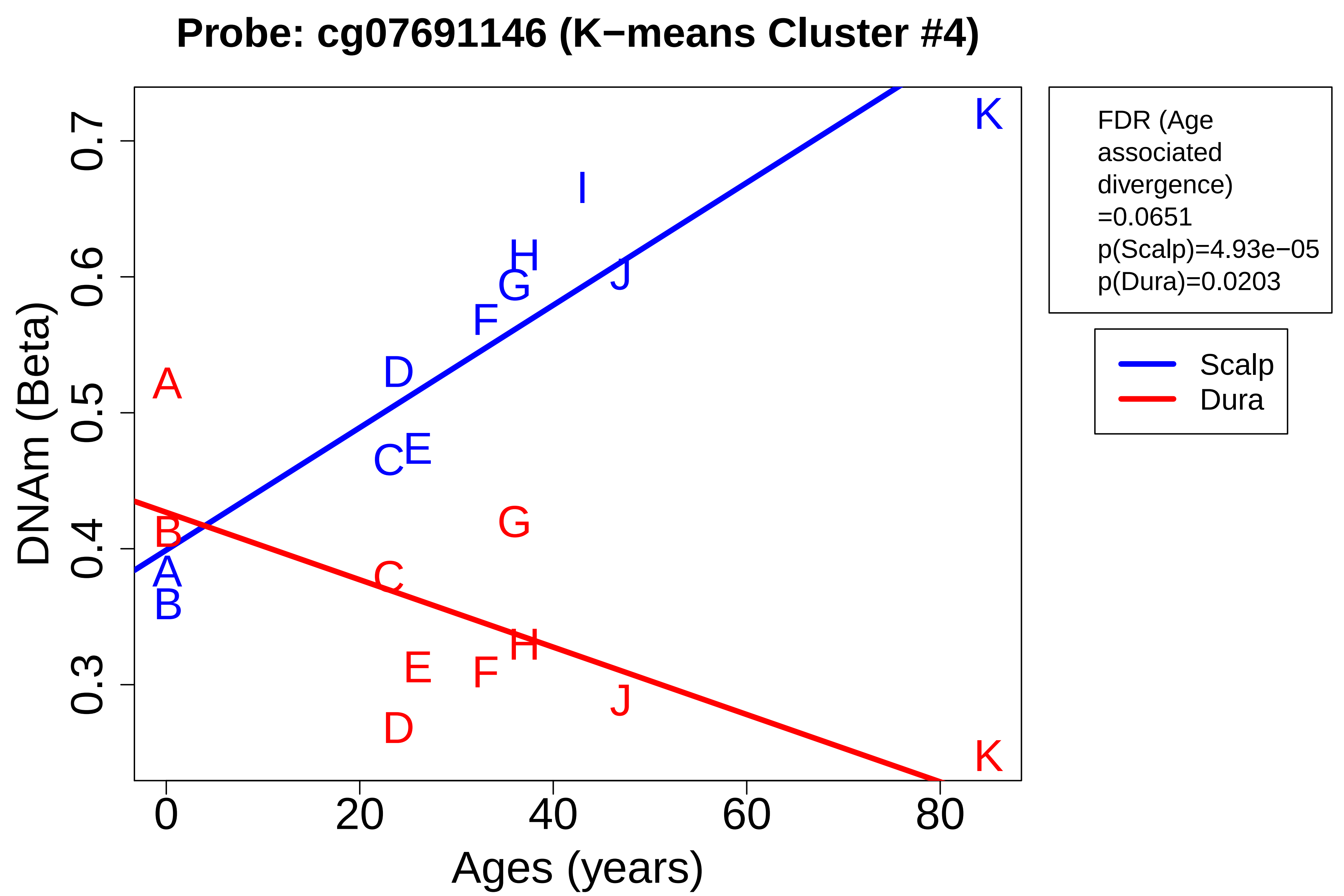
Probe: cg07691146 (K−means Cluster #4)
DNAm (Beta)
0.3
0.4
0.5
0.6
0.7
0
20
40
60
80
Ages (years)
A
B
C
D
E
F
G
H
I
J
K
FDR (Age associated divergence) =0.0651
p(Scalp)=4.93e−05
p(Dura)=0.0203
Scalp
Dura

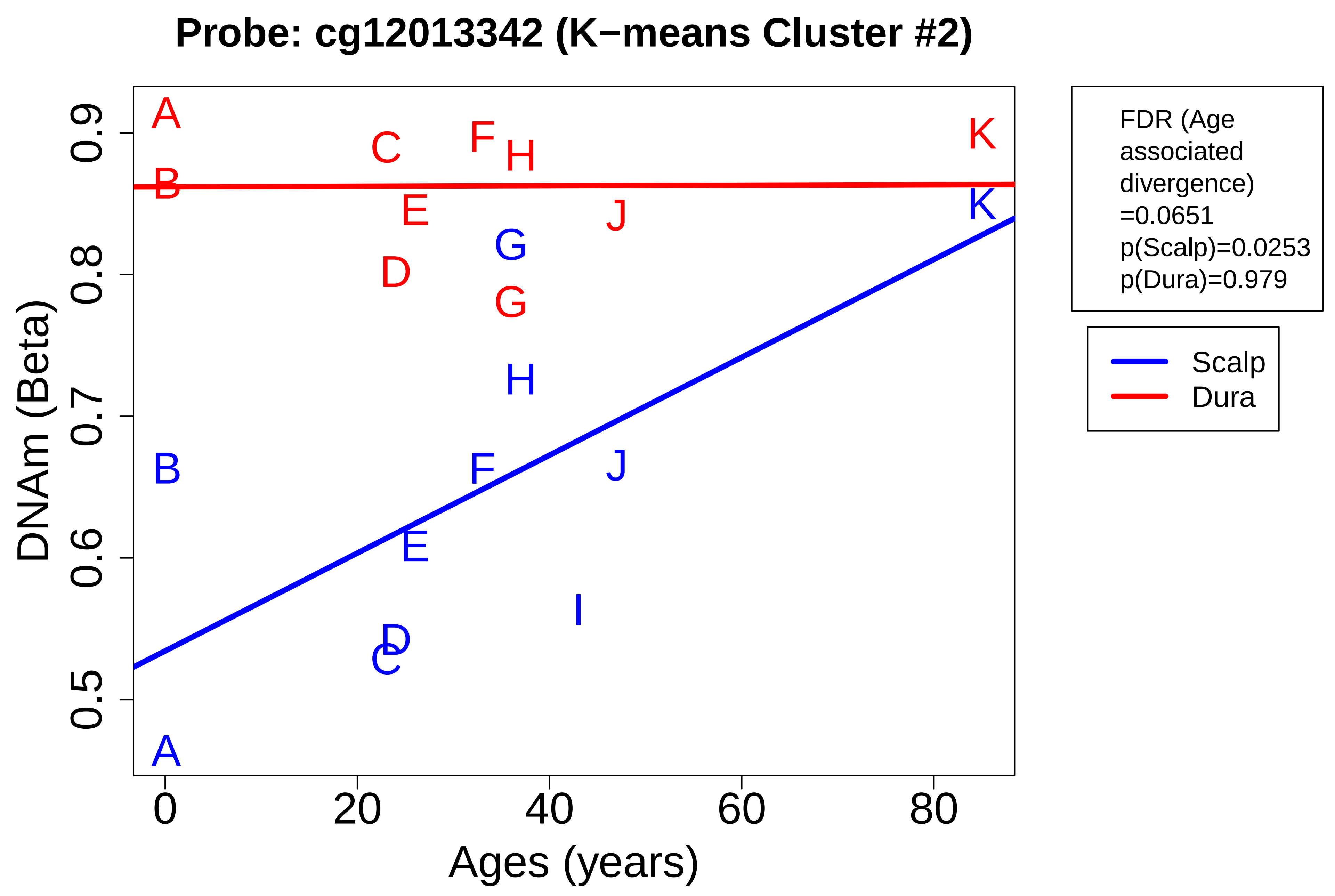

Probe: cg12013342 (K−means Cluster #2)
DNAm (Beta)
0.5
0.6
0.7
0.8
0.9
0
20
40
60
80
Ages (years)
A
B
C
D
E
F
G
H
I
J
K
FDR (Age associated divergence) =0.0651
p(Scalp)=0.0253
p(Dura)=0.979
Scalp
Dura

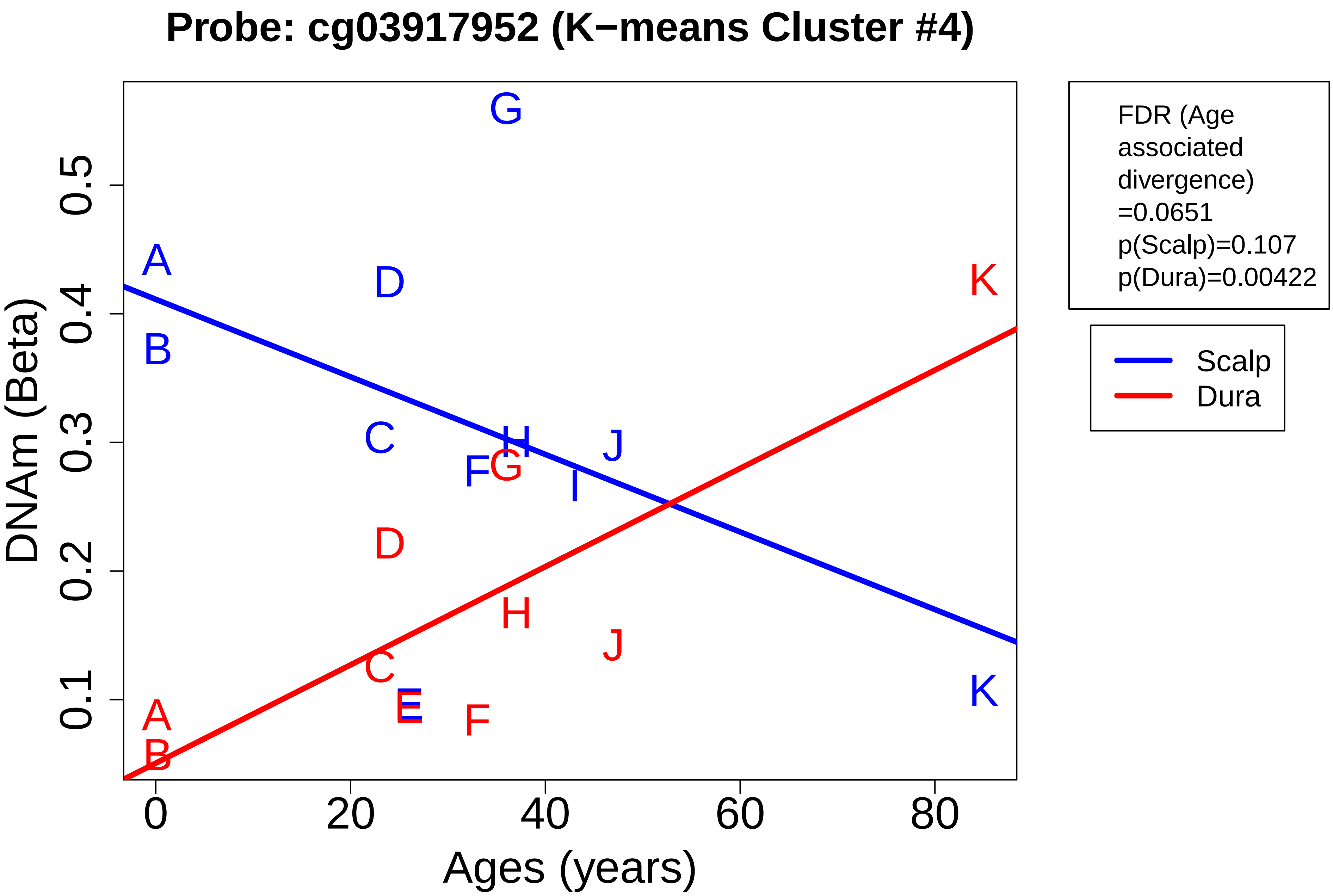

Probe: cg03917952 (K−means Cluster #4)
DNAm (Beta)
Ages (years)
0.1
0.2
0.3
0.4
0.5
0
20
40
60
80
FDR (Age associated divergence) =0.0651
p(Scalp)=0.107
p(Dura)=0.00422
Scalp
Dura

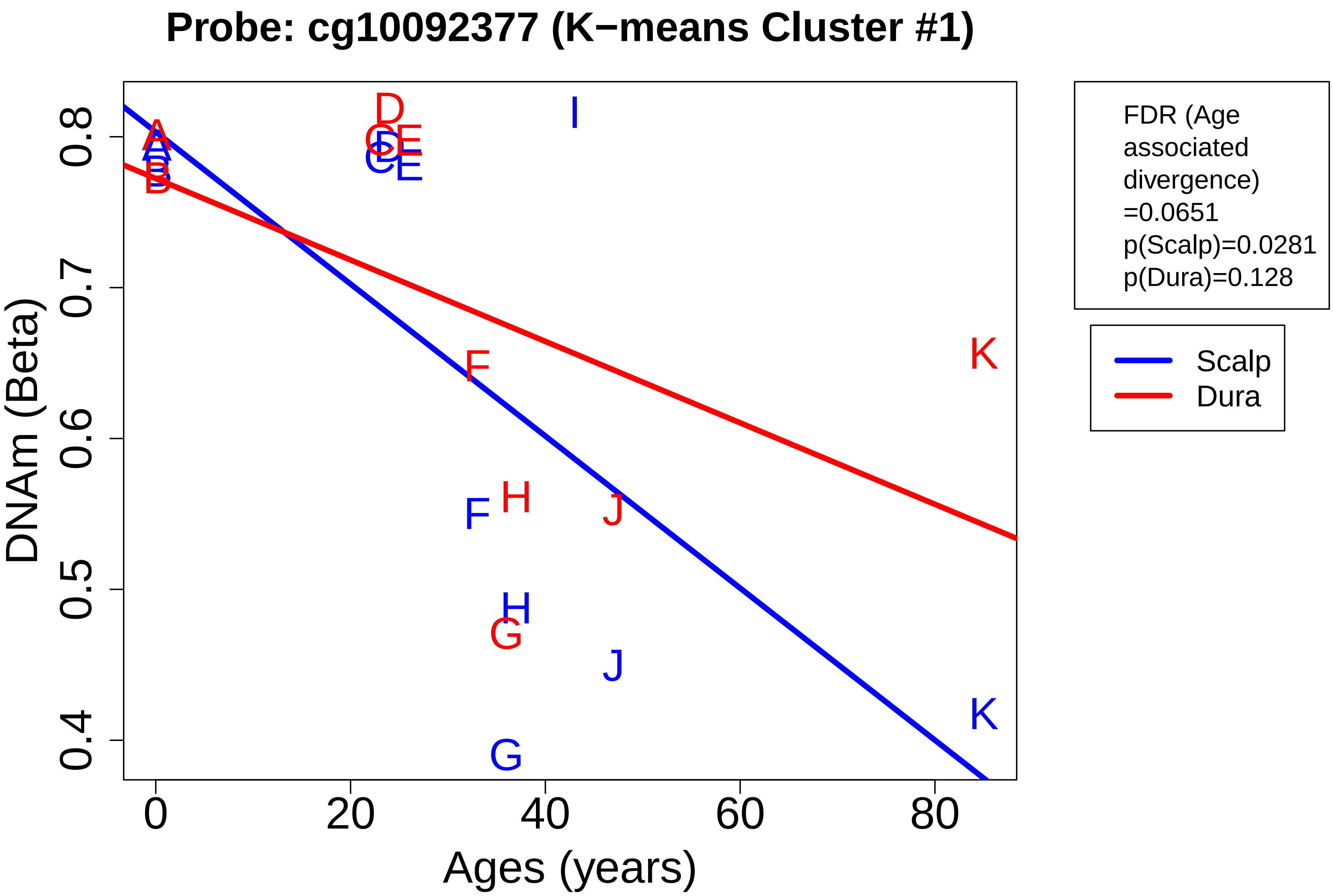
Probe: cg10092377 (K−means Cluster #1)
FDR (Age associated divergence) =0.0651
p(Scalp)=0.0281
p(Dura)=0.128
Scalp
Dura
DNAm (Beta)
0.4
0.5
0.6
0.7
0.8
0
20
40
60
80
Ages (years)
A
B
C
D
E
F
G
H
I
J
K

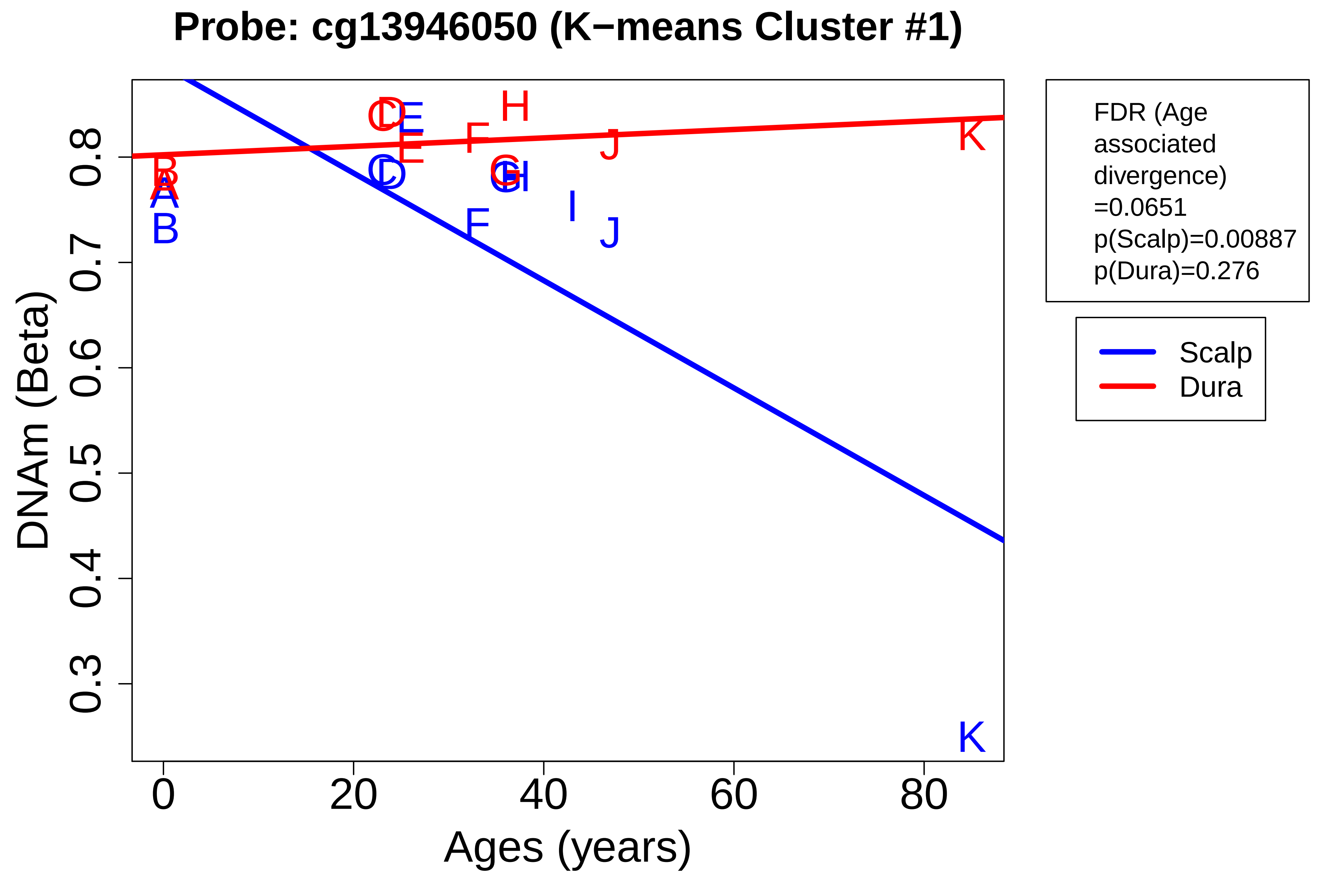
Probe: cg13946050 (K−means Cluster #1)
DNAm (Beta)
0.3
0.4
0.5
0.6
0.7
0.8
Ages (years)
0
20
40
60
80
FDR (Age associated divergence) =0.0651
p(Scalp)=0.00887
p(Dura)=0.276
Scalp
Dura

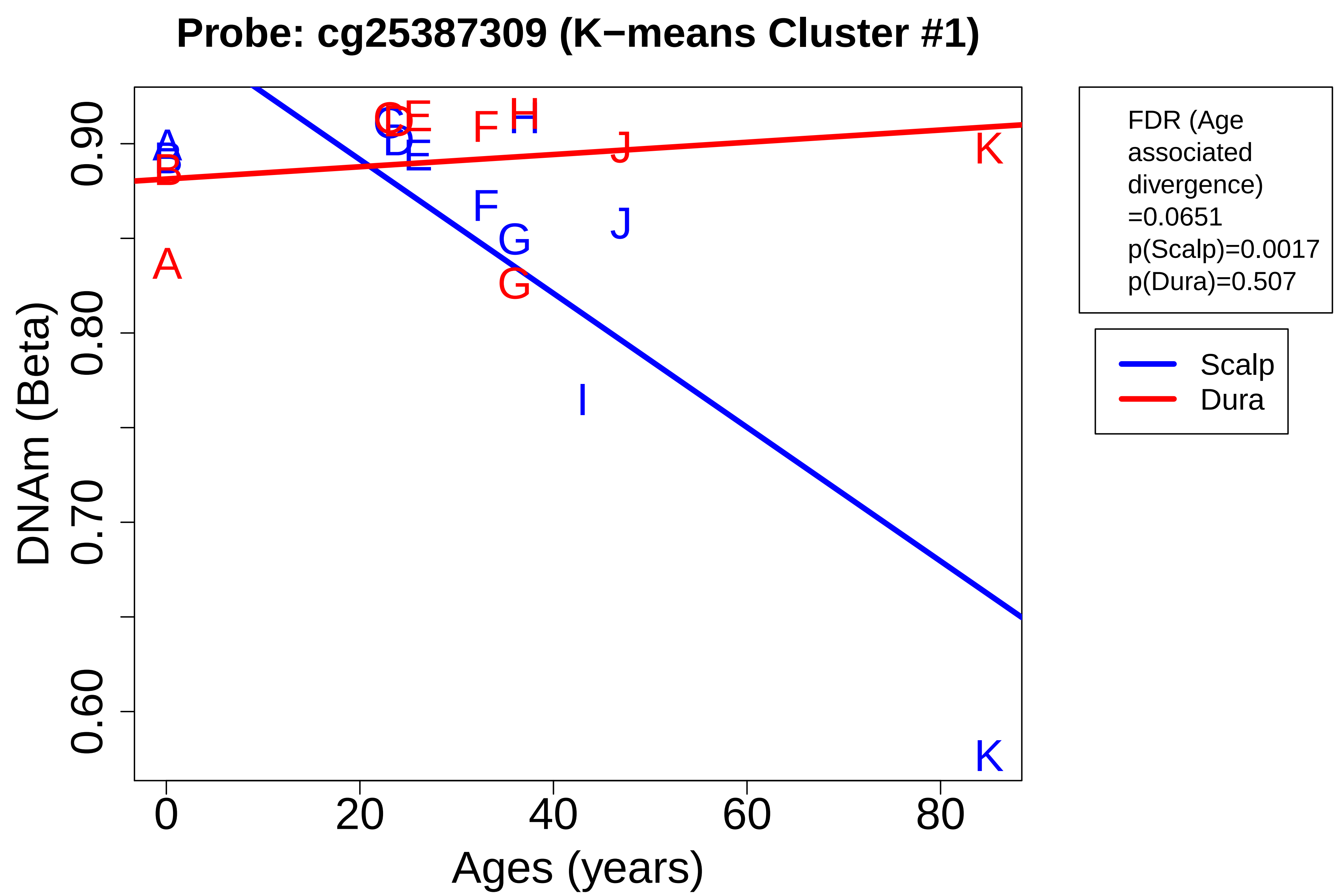

Probe: cg25387309 (K−means Cluster #1)
DNAm (Beta)
0.60
0.70
0.80
0.90
Ages (years)
0
20
40
60
80
FDR (Age associated divergence) =0.0651
p(Scalp)=0.0017
p(Dura)=0.507
Scalp
Dura

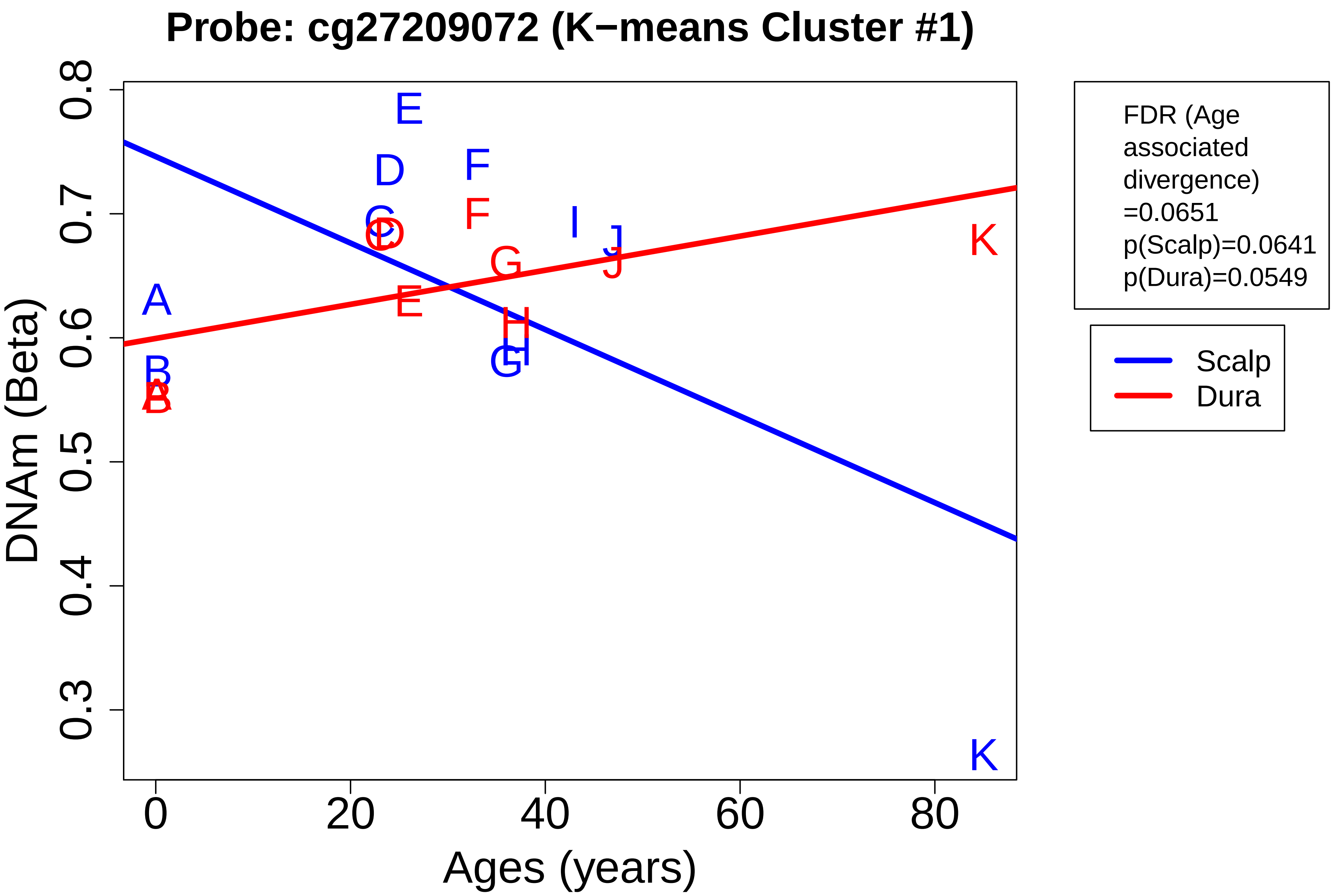
Probe: cg27209072 (K−means Cluster #1)
DNAm (Beta)
0.3
0.4
0.5
0.6
0.7
0.8
Ages (years)
0
20
40
60
80
A
B
C
D
E
F
G
H
I
J
K
FDR (Age associated divergence) =0.0651
p(Scalp)=0.0641
p(Dura)=0.0549
Scalp
Dura

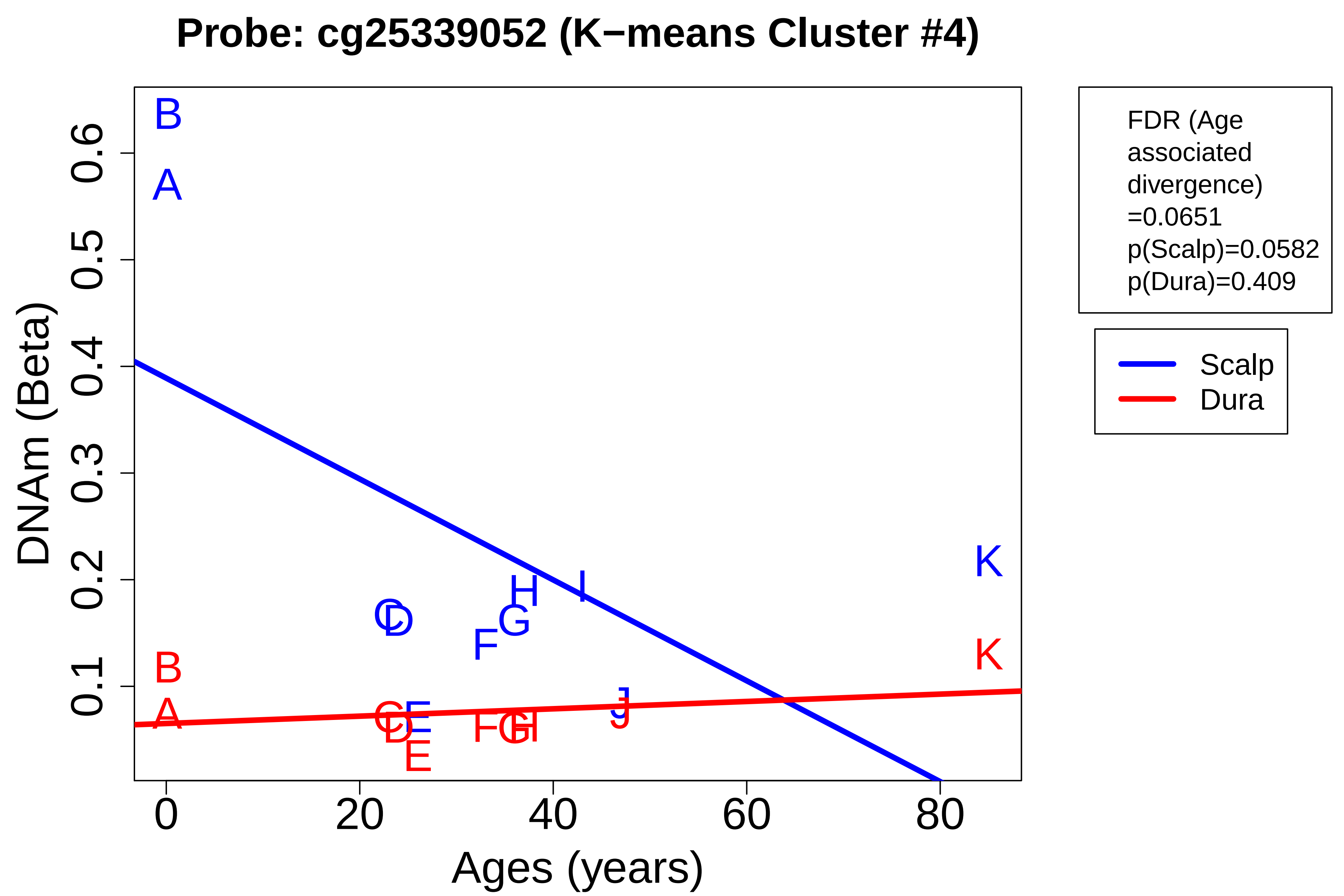

Probe: cg25339052 (K−means Cluster #4)
DNAm (Beta)
Ages (years)
0.1
0.2
0.3
0.4
0.5
0.6
0
20
40
60
80
FDR (Age associated divergence) =0.0651
p(Scalp)=0.0582
p(Dura)=0.409
Scalp
Dura

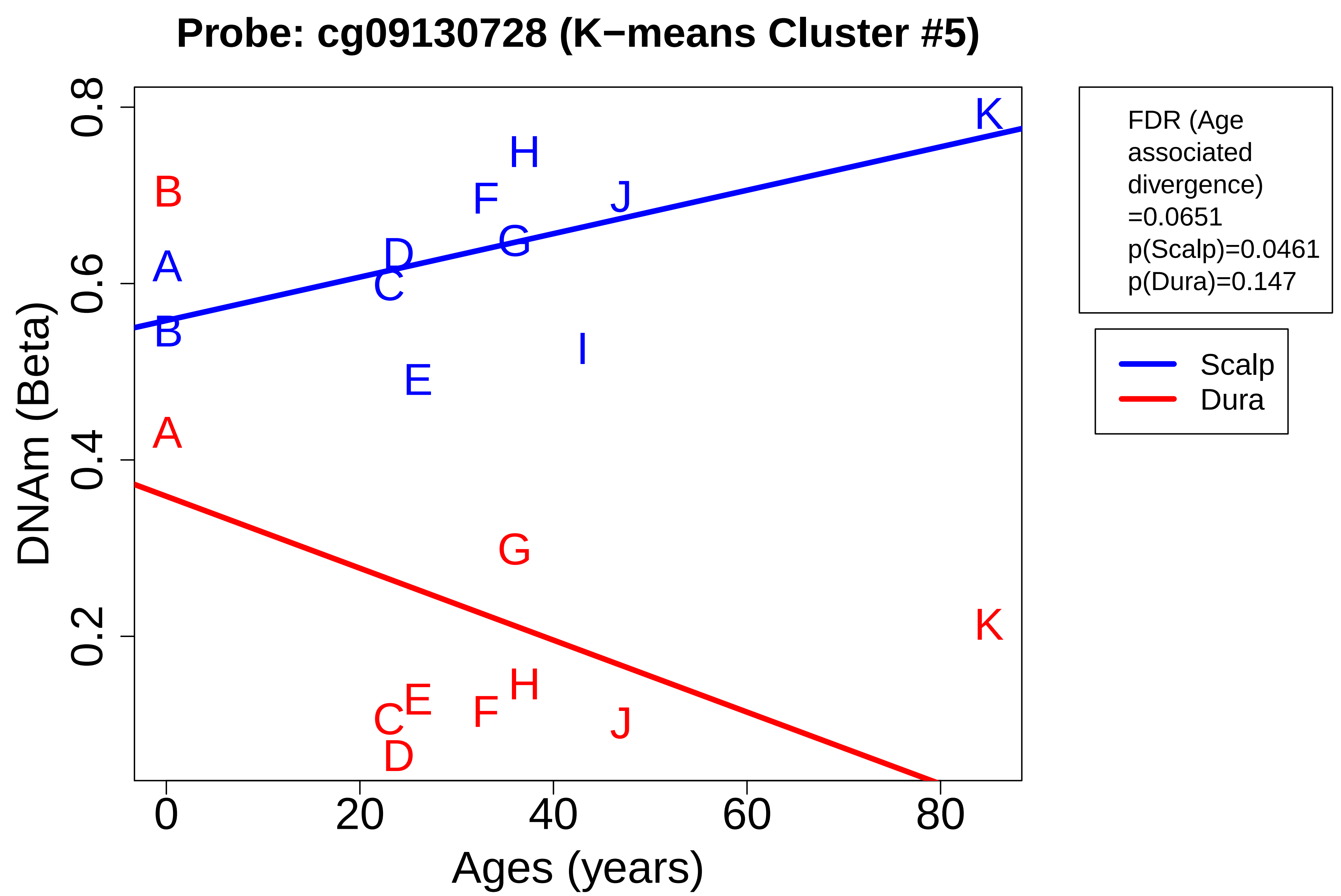

Probe: cg09130728 (K−means Cluster #5)
DNAm (Beta)
0.2
0.4
0.6
0.8
Ages (years)
0
20
40
60
80
A
B
C
D
E
F
G
H
I
J
K
FDR (Age associated divergence) =0.0651
p(Scalp)=0.0461
p(Dura)=0.147
Scalp
Dura

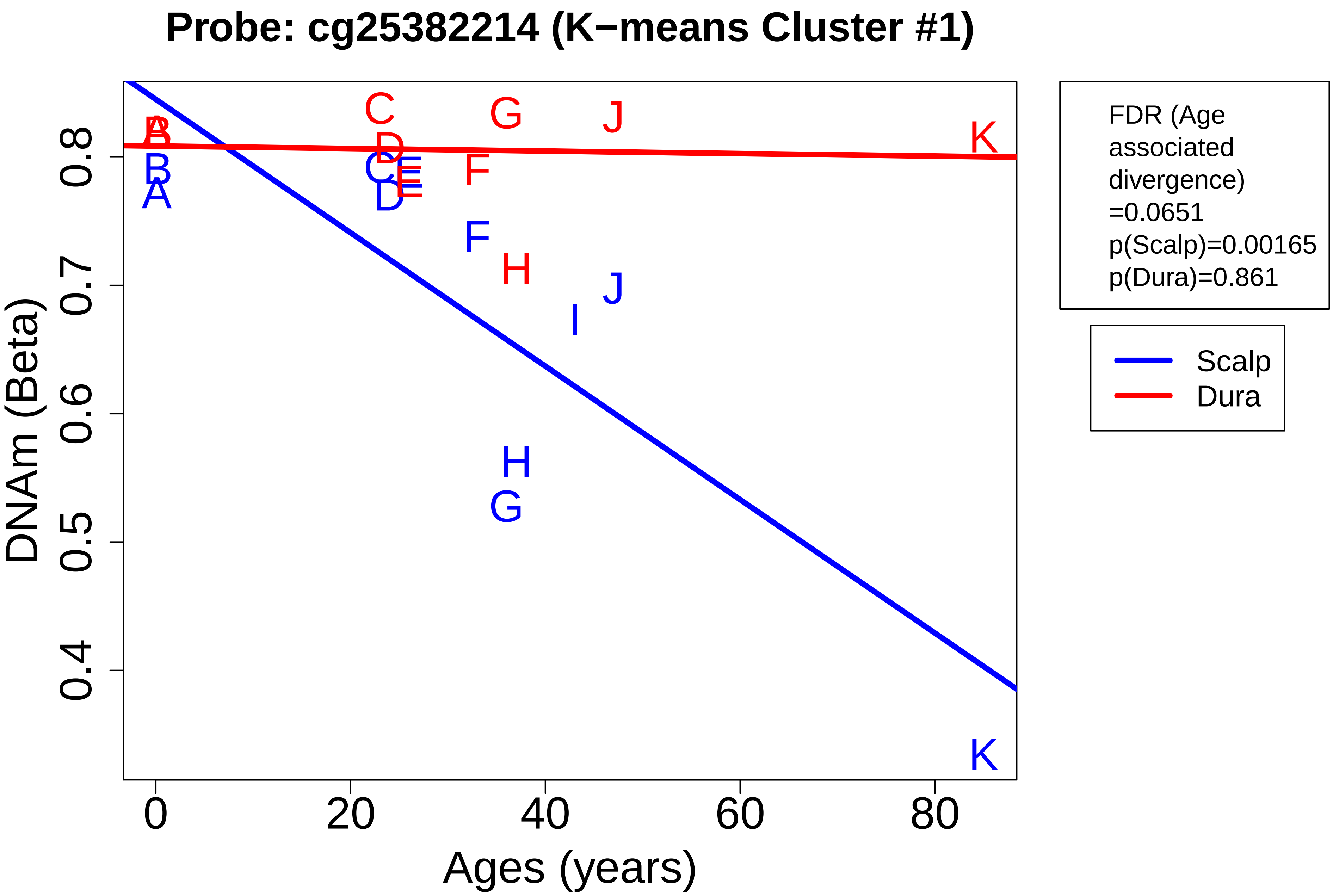
Probe: cg25382214 (K−means Cluster #1)
DNAm (Beta)
0.4
0.5
0.6
0.7
0.8
0
20
40
60
80
Ages (years)
FDR (Age associated divergence) =0.0651
p(Scalp)=0.00165
p(Dura)=0.861
Scalp
Dura

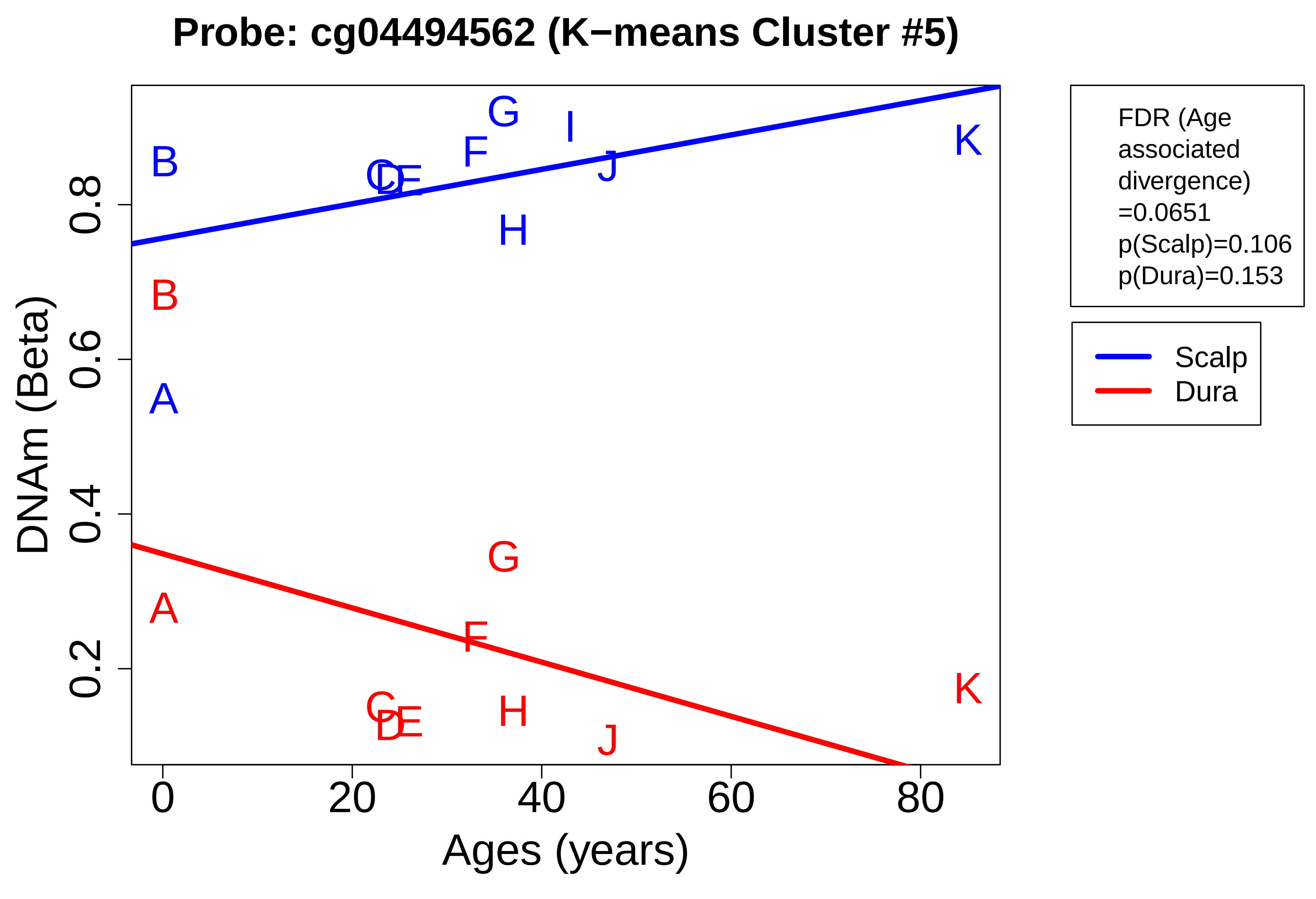
Probe: cg04494562 (K−means Cluster #5)
DNAm (Beta)
0.2
0.4
0.6
0.8
Ages (years)
0
20
40
60
80
A
B
C
D
E
F
G
H
I
J
K
FDR (Age associated divergence) =0.0651
p(Scalp)=0.106
p(Dura)=0.153
Scalp
Dura

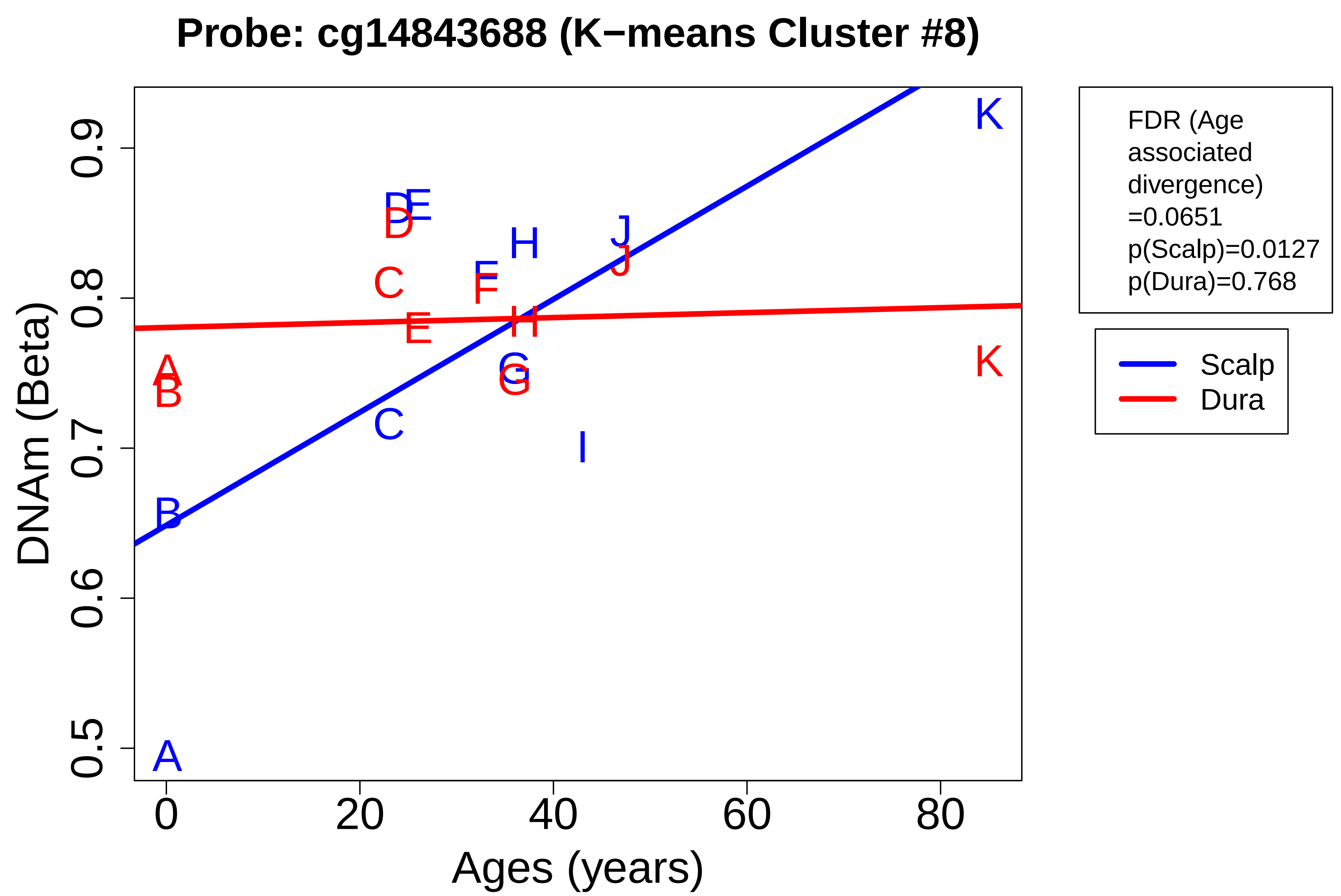
Probe: cg14843688 (K−means Cluster #8)
DNAm (Beta)
Ages (years)
0.5
0.6
0.7
0.8
0.9
0
20
40
60
80
A
B
C
D
E
F
G
H
I
J
K
FDR (Age associated divergence) =0.0651
p(Scalp)=0.0127
p(Dura)=0.768
Scalp
Dura

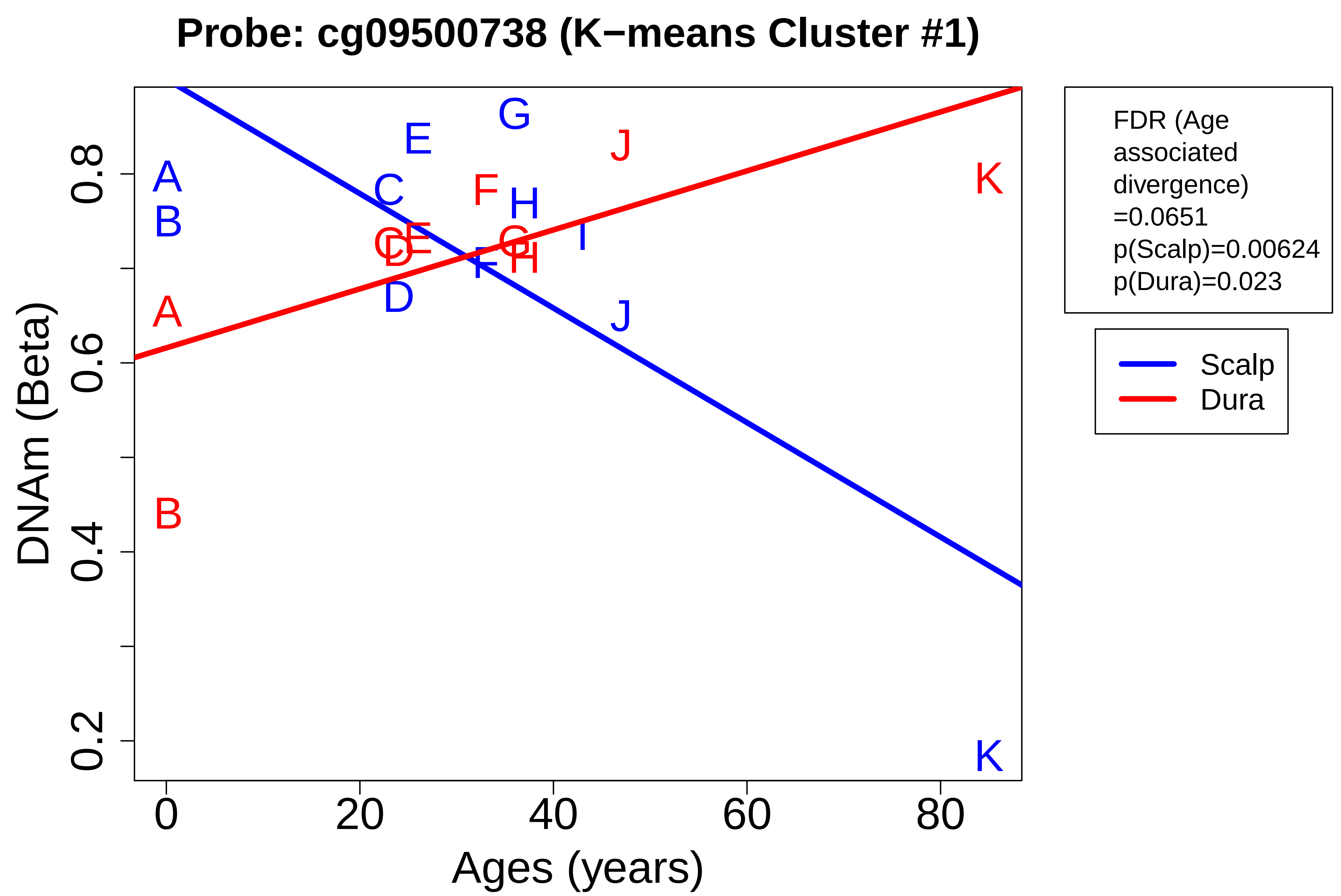

Probe: cg09500738 (K−means Cluster #1)
DNAm (Beta)
0.2
0.4
0.6
0.8
Ages (years)
0
20
40
60
80
A
B
C
D
E
F
G
H
I
J
K
FDR (Age associated divergence) =0.0651
p(Scalp)=0.00624
p(Dura)=0.023
Scalp
Dura

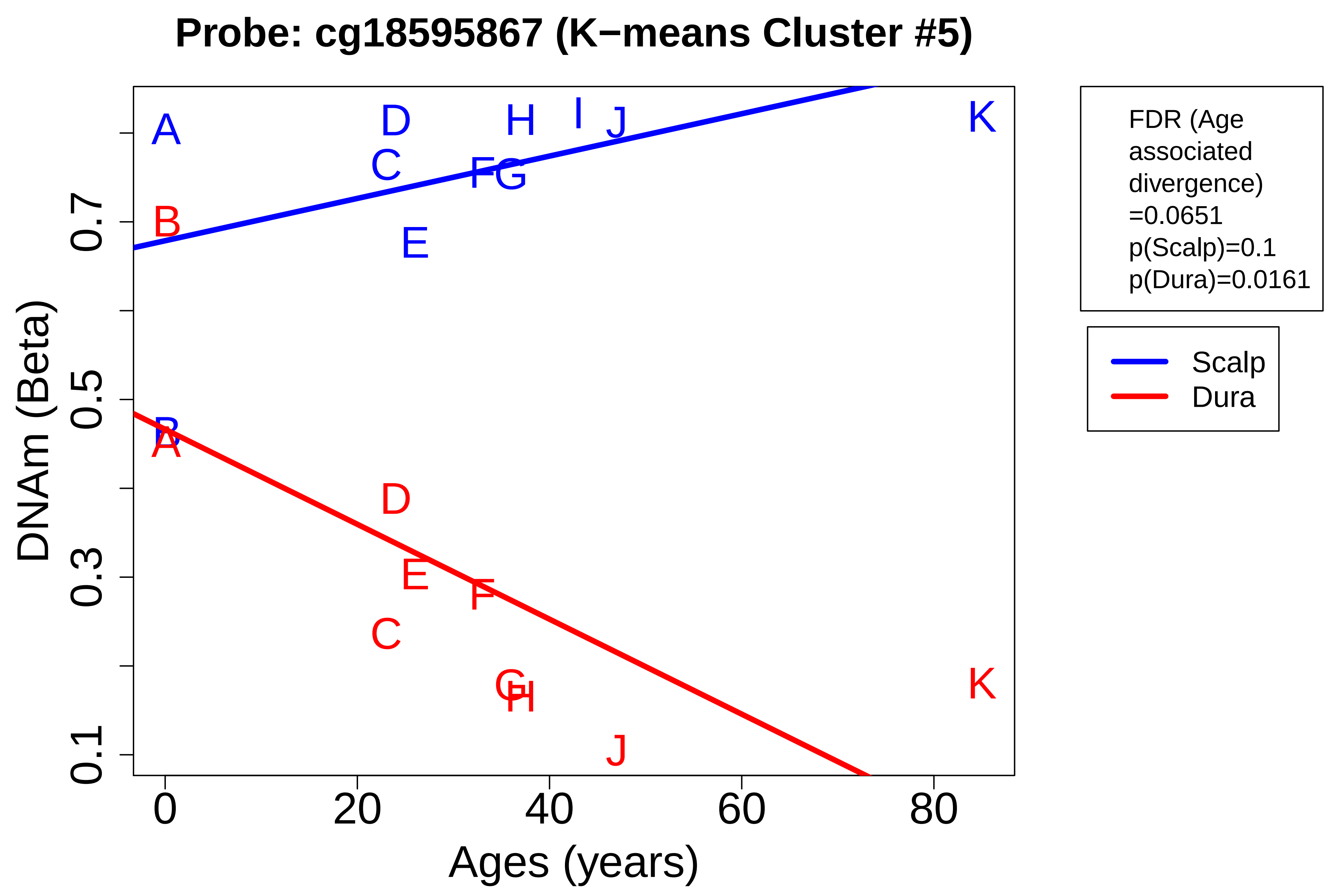

Probe: cg18595867 (K−means Cluster #5)
FDR (Age associated divergence) =0.0651
p(Scalp)=0.1
p(Dura)=0.0161
Scalp
Dura
DNAm (Beta)
Ages (years)
0.1
0.3
0.5
0.7
0
20
40
60
80

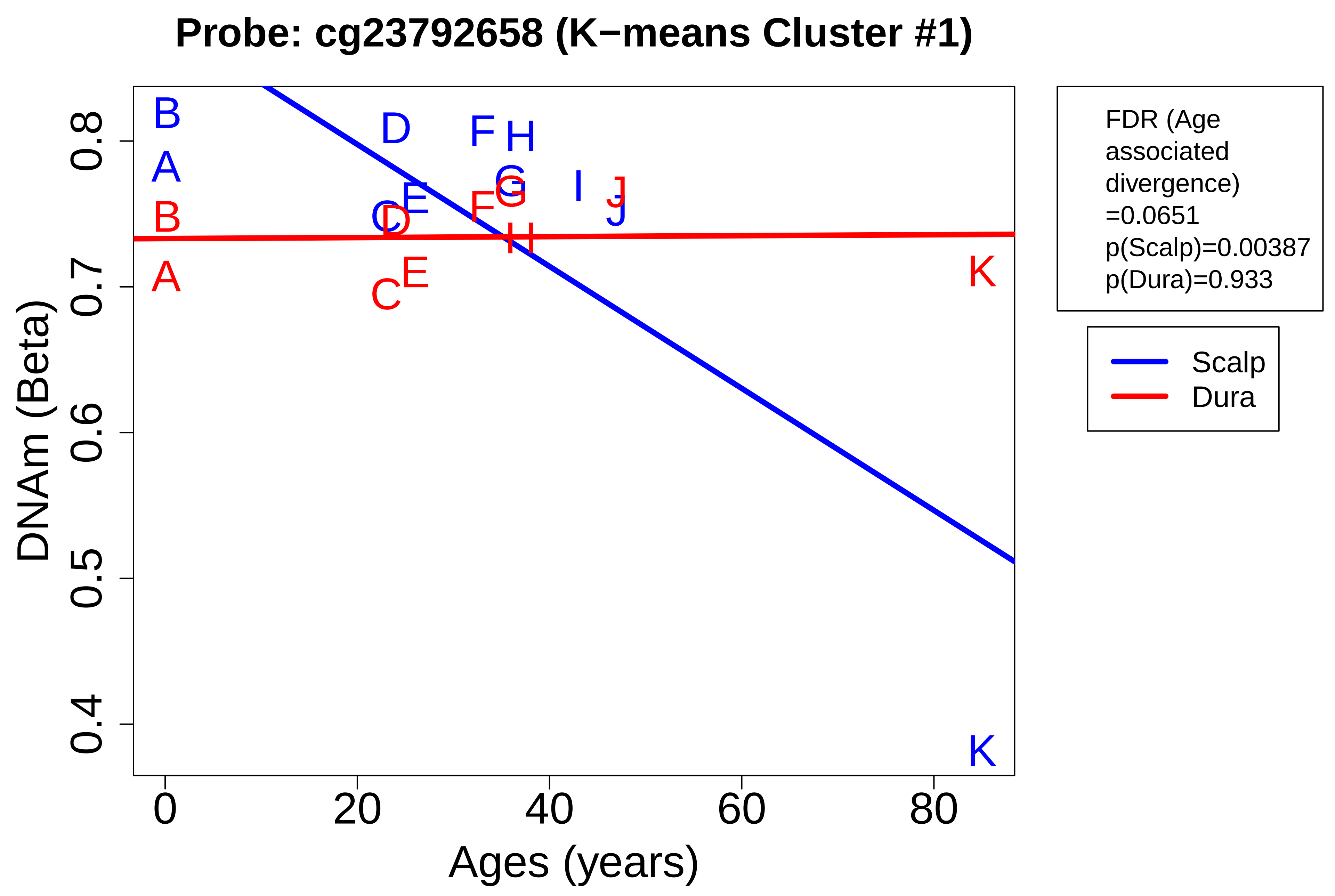

Probe: cg23792658 (K−means Cluster #1)
DNAm (Beta)
0.4
0.5
0.6
0.7
0.8
0
20
40
60
80
Ages (years)
FDR (Age associated divergence) =0.0651
p(Scalp)=0.00387
p(Dura)=0.933
Scalp
Dura

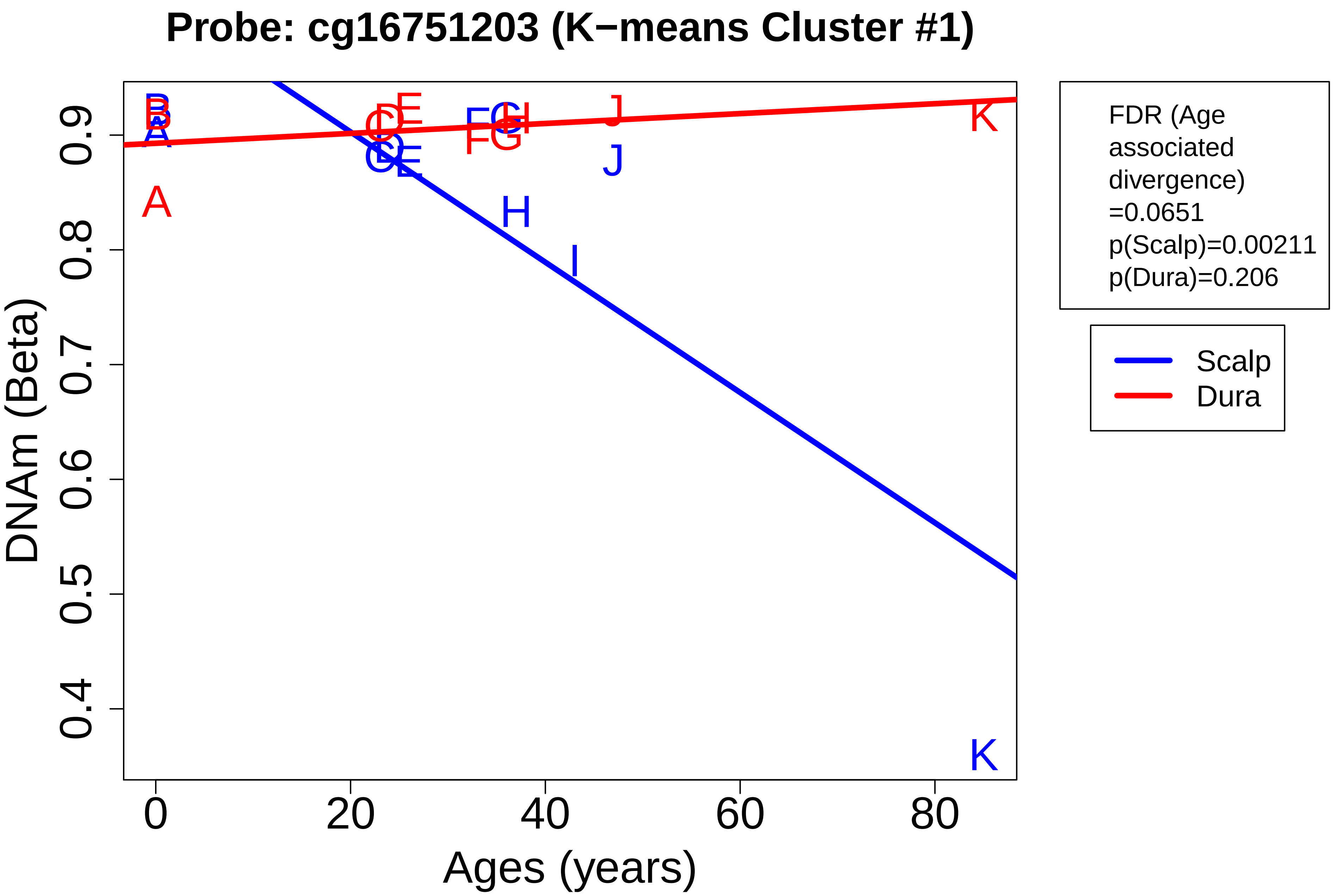
Probe: cg16751203 (K−means Cluster #1)
DNAm (Beta)
0.4
0.5
0.6
0.7
0.8
0.9
0
20
40
60
80
Ages (years)
FDR (Age associated divergence) =0.0651
p(Scalp)=0.00211
p(Dura)=0.206
Scalp
Dura

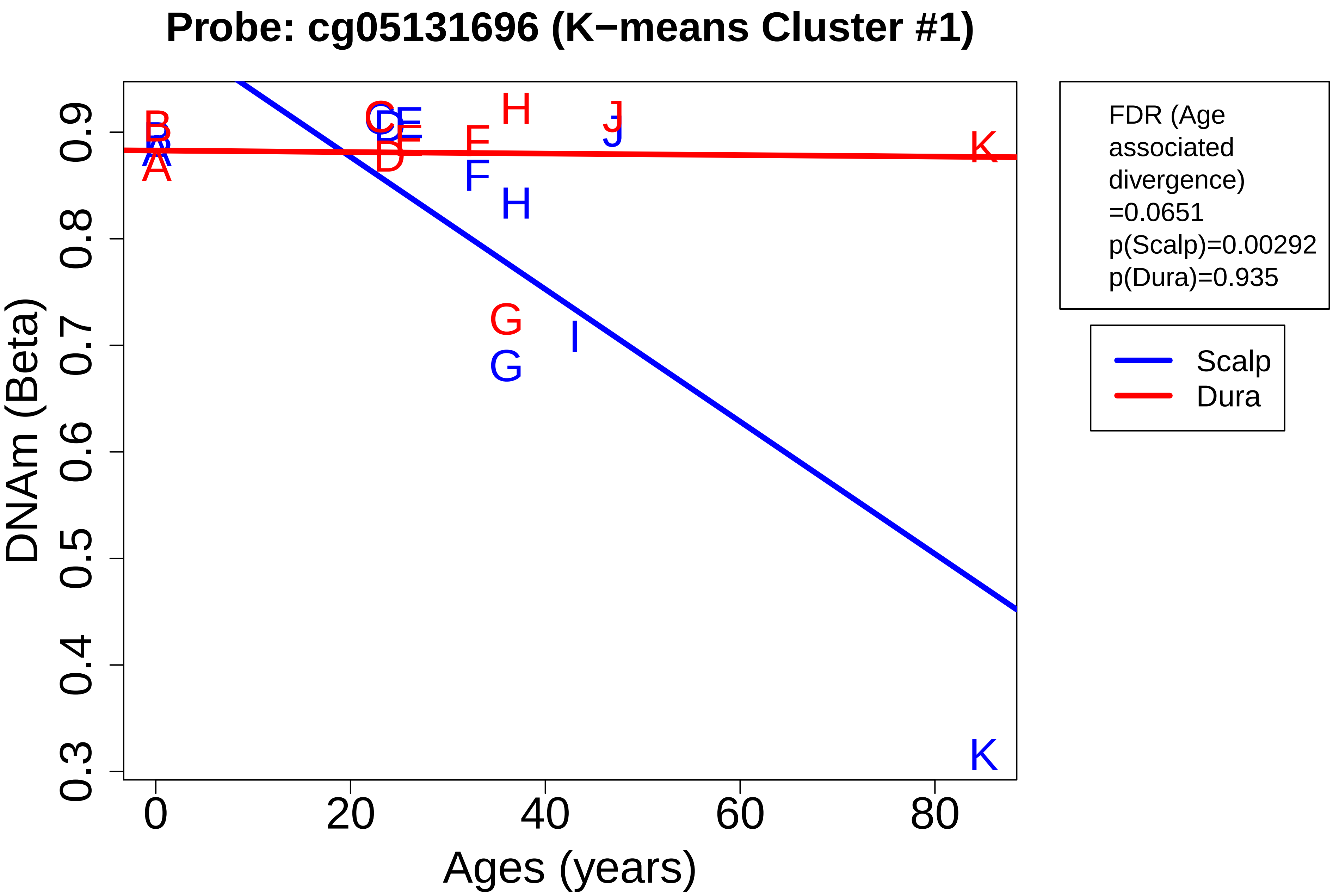

Probe: cg05131696 (K−means Cluster #1)
FDR (Age associated divergence) =0.0651
p(Scalp)=0.00292
p(Dura)=0.935
Scalp
Dura
DNAm (Beta)
Ages (years)
0.3
0.4
0.5
0.6
0.7
0.8
0.9
0
20
40
60
80

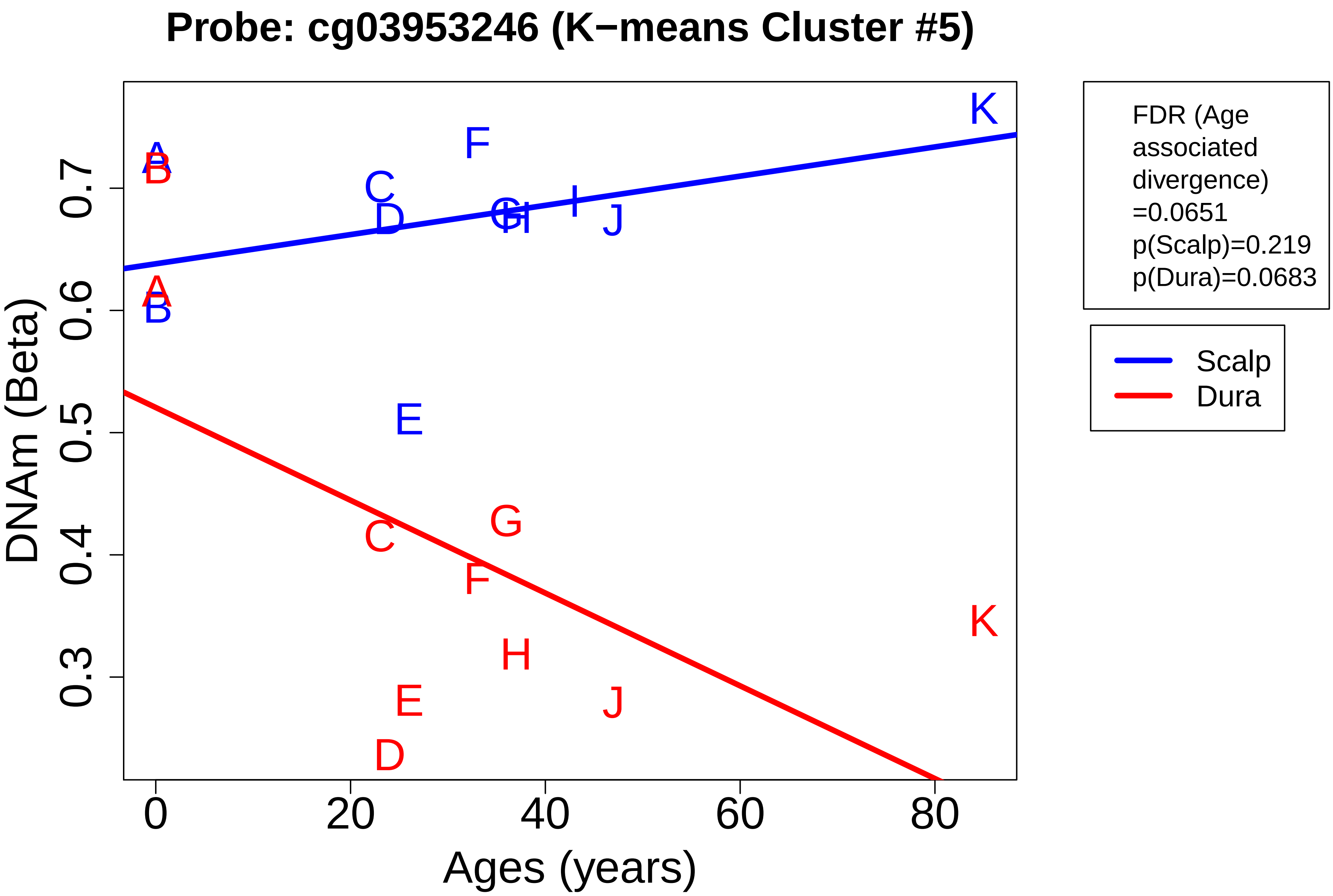

Probe: cg03953246 (K−means Cluster #5)
DNAm (Beta)
0.3
0.4
0.5
0.6
0.7
Ages (years)
0
20
40
60
80
A
B
C
D
E
F
G
H
I
J
K
FDR (Age associated divergence) =0.0651
p(Scalp)=0.219
p(Dura)=0.0683
Scalp
Dura

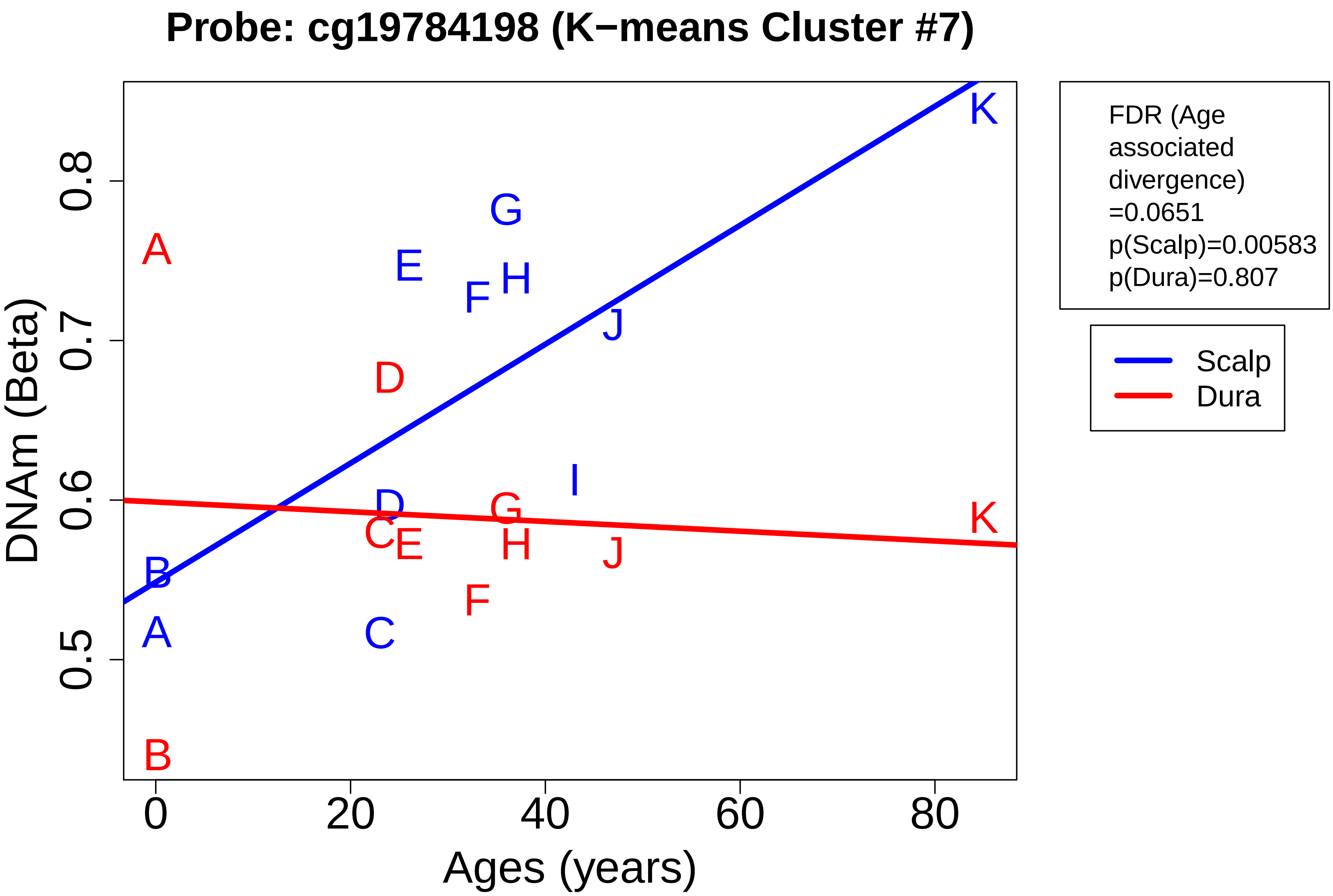
Probe: cg19784198 (K−means Cluster #7)
DNAm (Beta)
0.5
0.6
0.7
0.8
Ages (years)
0
20
40
60
80
A
B
C
D
E
F
G
H
I
J
K
FDR (Age associated divergence) =0.0651
p(Scalp)=0.00583
p(Dura)=0.807
Scalp
Dura

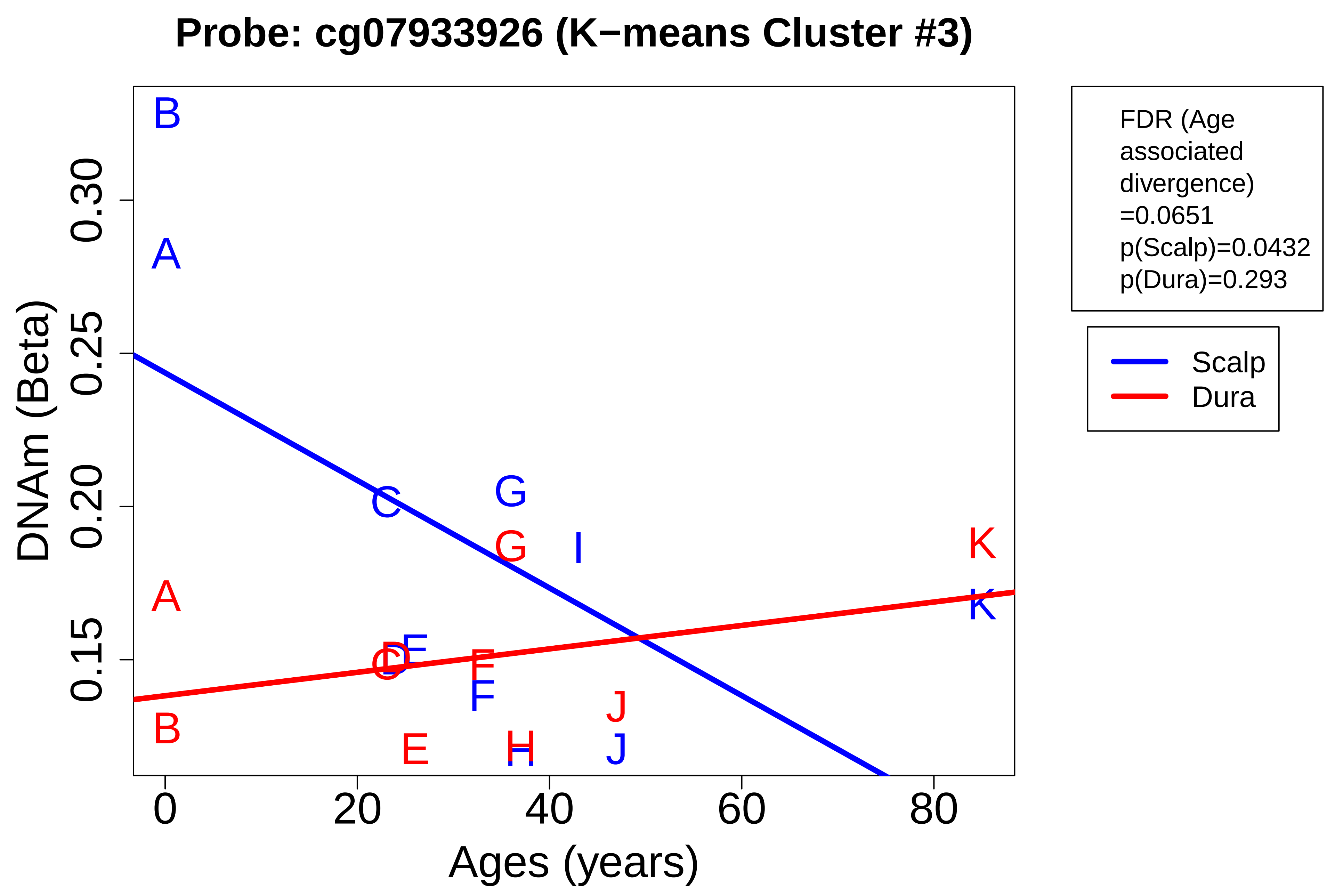

Probe: cg07933926 (K−means Cluster #3)
DNAm (Beta)
0.15
0.20
0.25
0.30
Ages (years)
0
20
40
60
80
FDR (Age associated divergence) =0.0651
p(Scalp)=0.0432
p(Dura)=0.293
Scalp
Dura

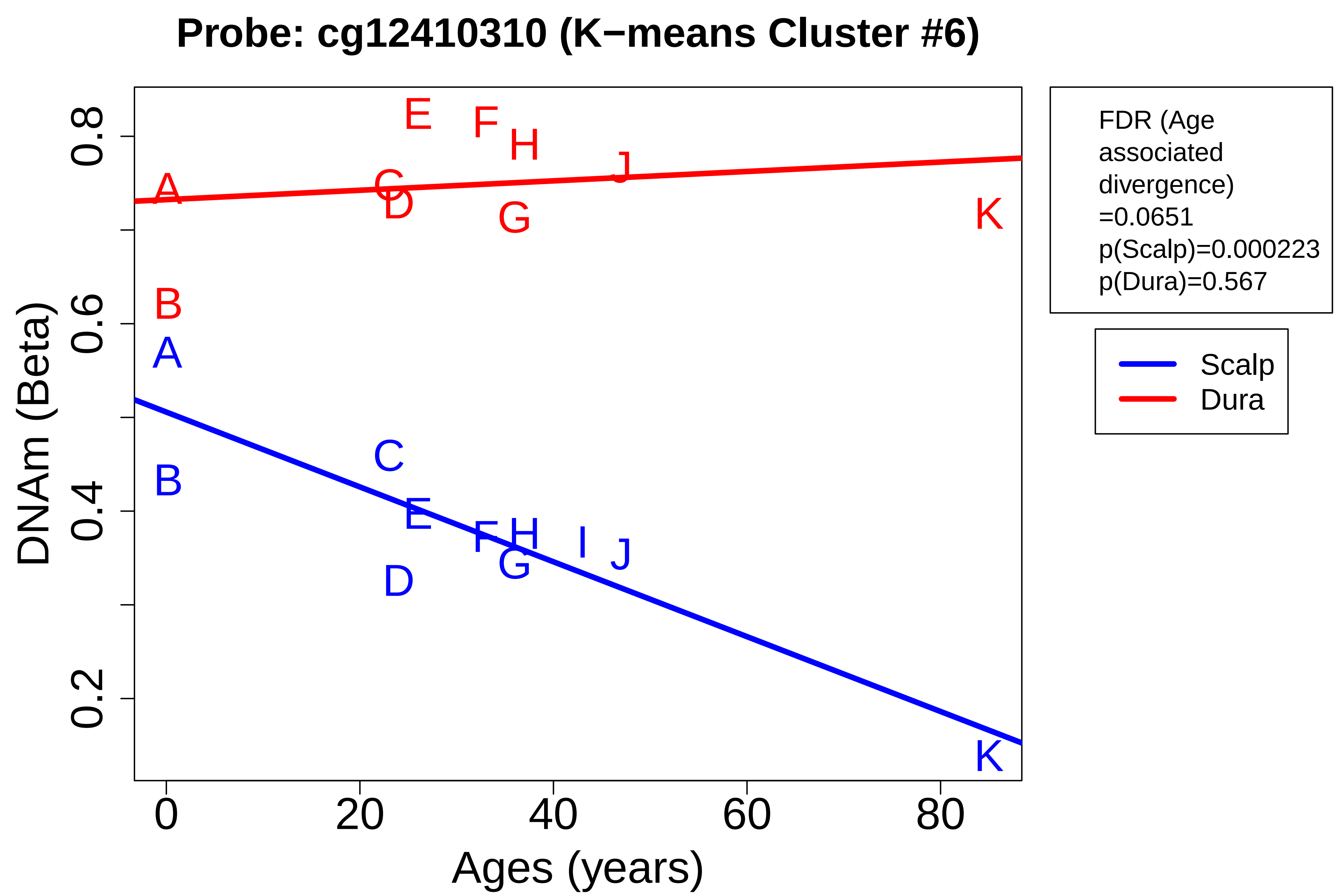

Probe: cg12410310 (K−means Cluster #6)
DNAm (Beta)
0.2
0.4
0.6
0.8
0
20
40
60
80
Ages (years)
FDR (Age associated divergence) =0.0651
p(Scalp)=0.000223
p(Dura)=0.567
Scalp
Dura

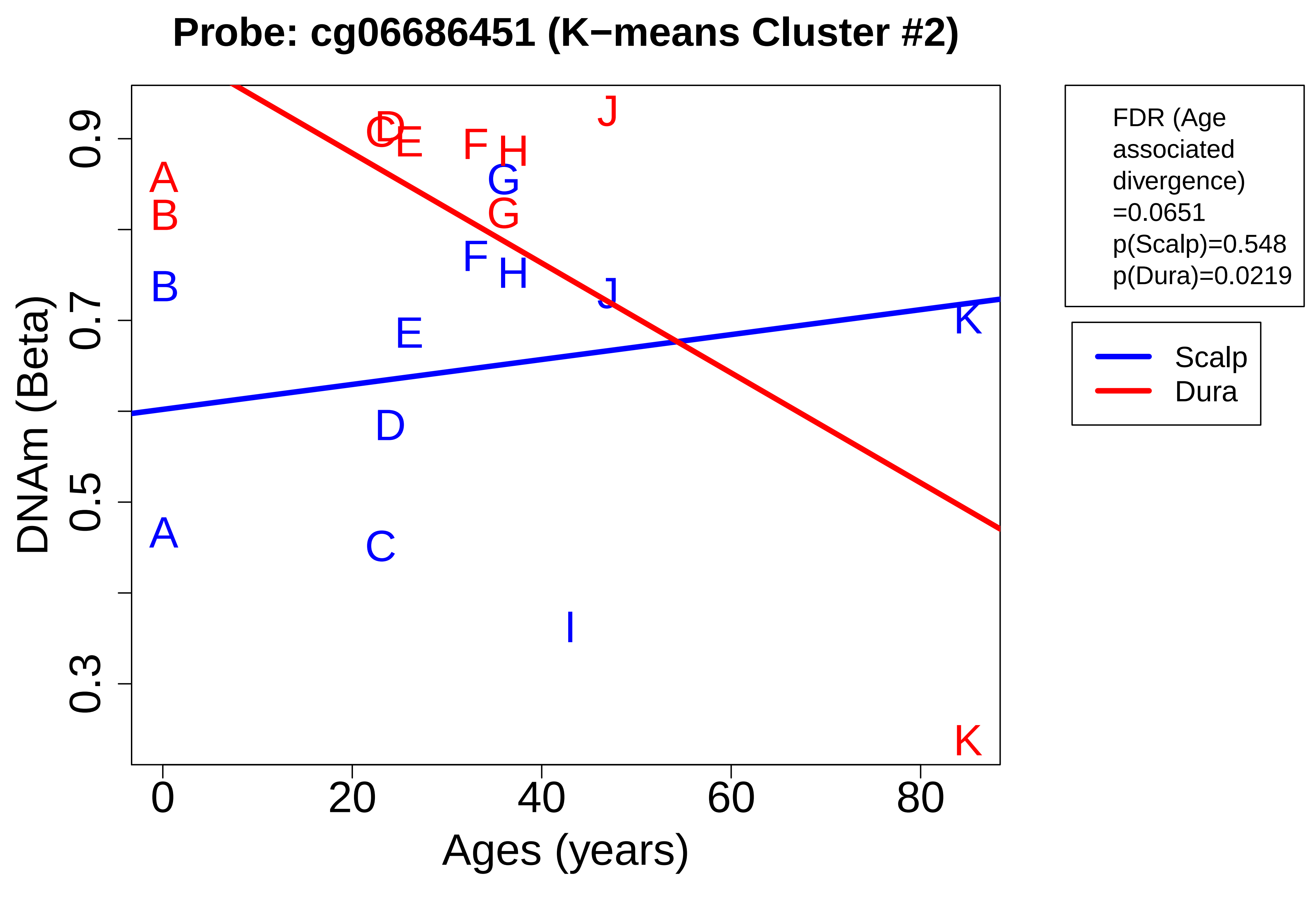

Probe: cg06686451 (K−means Cluster #2)
DNAm (Beta)
0.3
0.5
0.7
0.9
0
20
40
60
80
Ages (years)
A
B
C
D
E
F
G
H
I
J
K
FDR (Age associated divergence) =0.0651
p(Scalp)=0.548
p(Dura)=0.0219
Scalp
Dura

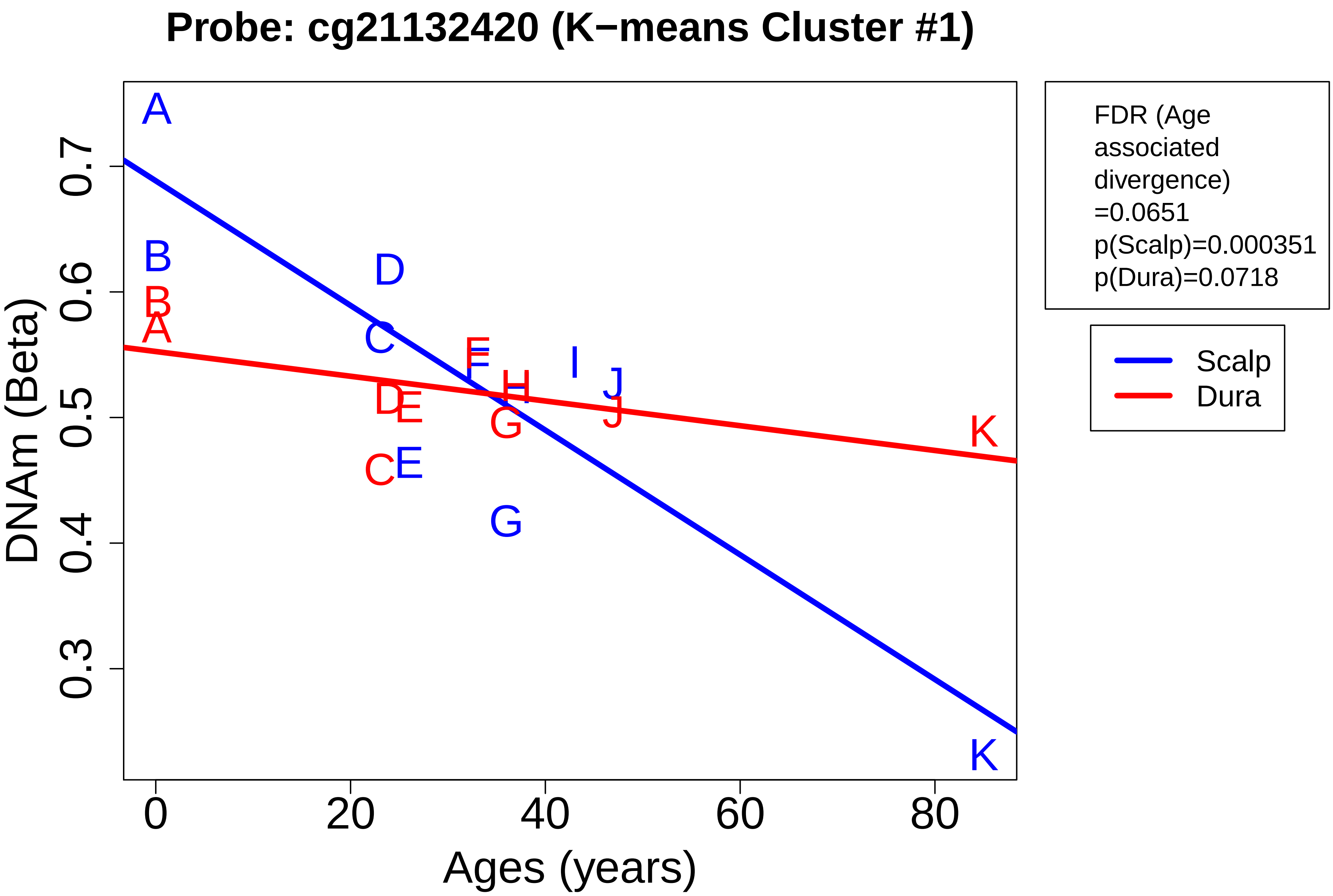

Probe: cg21132420 (K−means Cluster #1)
DNAm (Beta)
0.3
0.4
0.5
0.6
0.7
0
20
40
60
80
Ages (years)
FDR (Age associated divergence) =0.0651
p(Scalp)=0.000351
p(Dura)=0.0718
Scalp
Dura

Probe: cg25852019 (K−means Cluster #1)

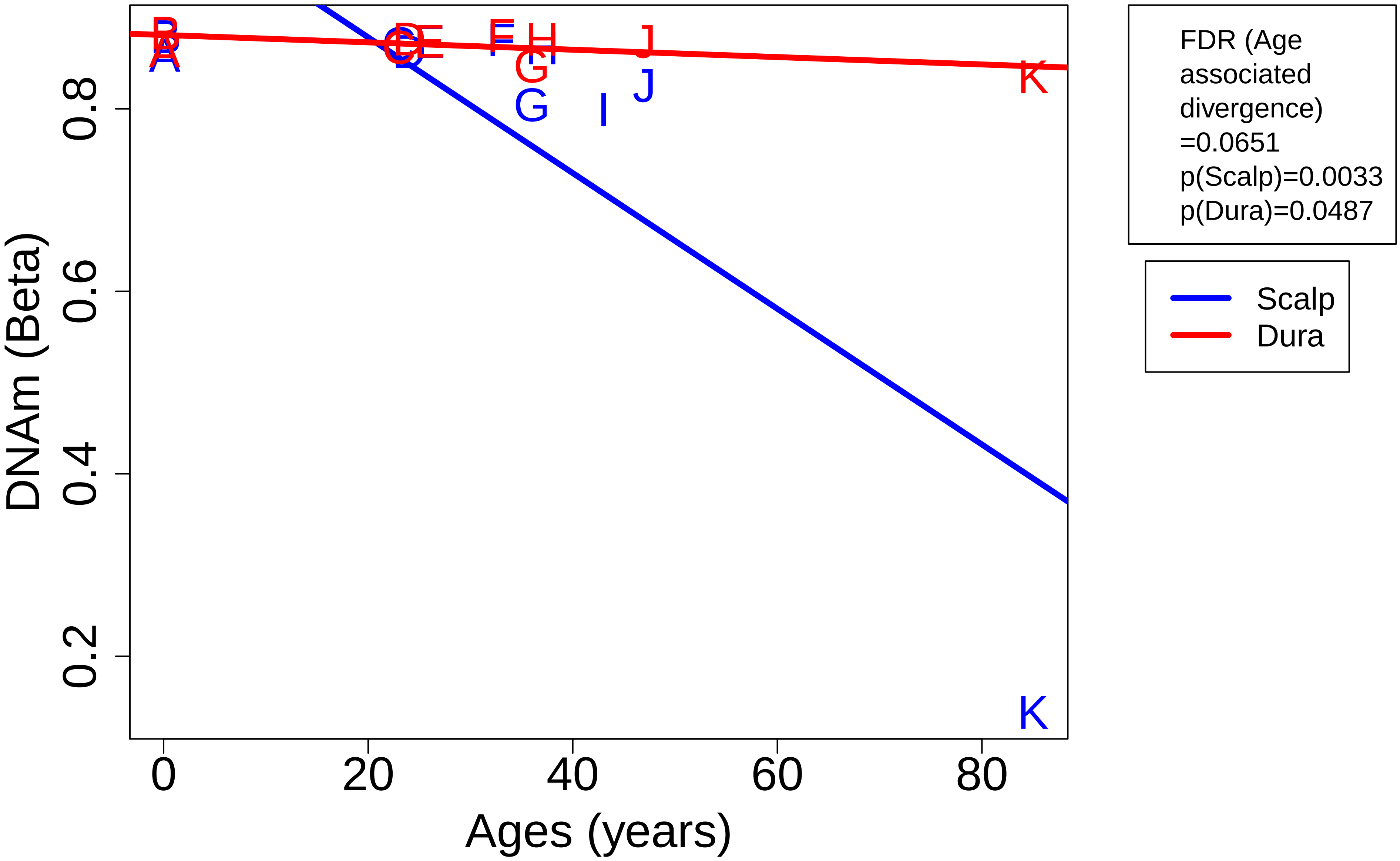

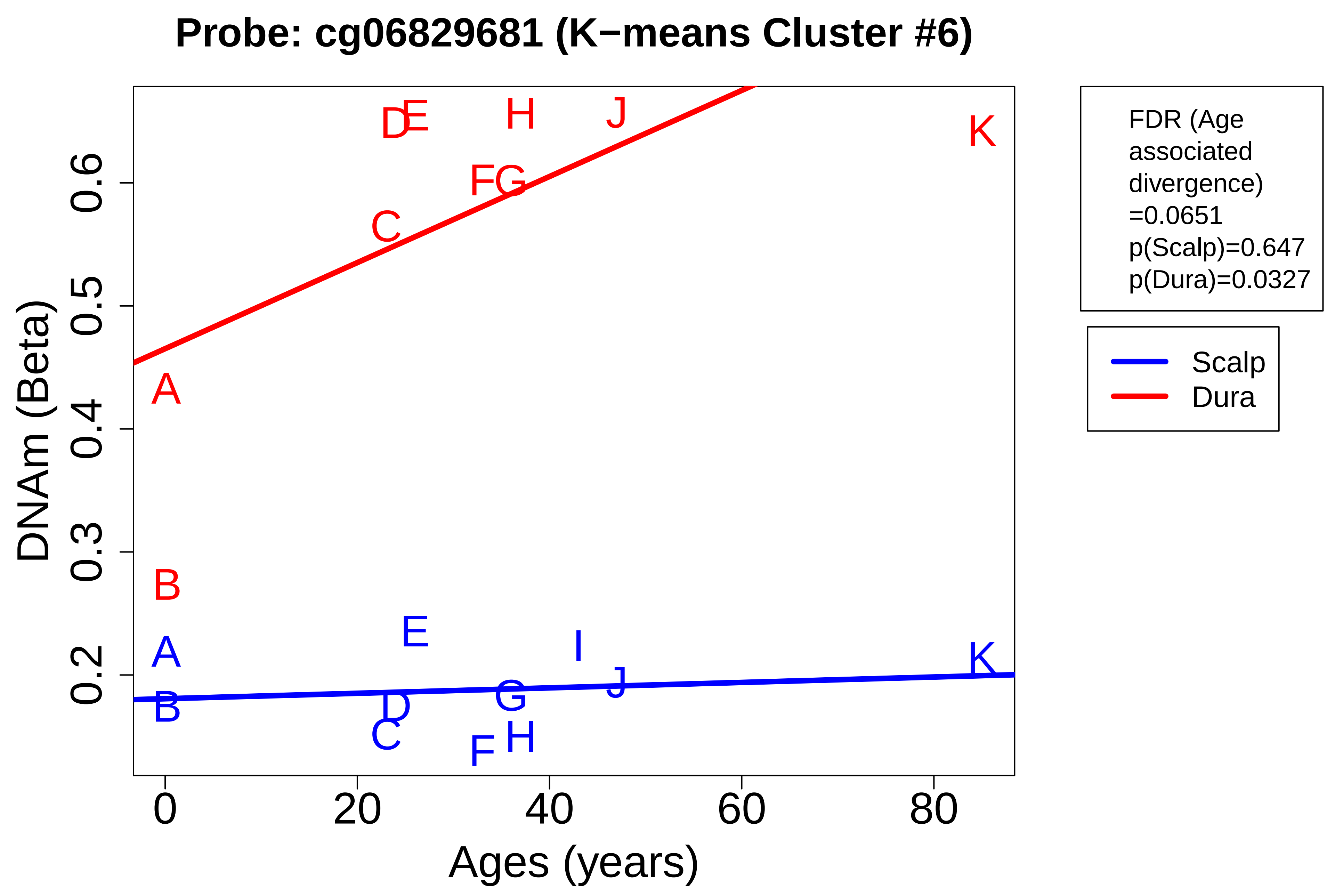
Probe: cg06829681 (K−means Cluster #6)
DNAm (Beta)
0.2
0.3
0.4
0.5
0.6
Ages (years)
0
20
40
60
80
FDR (Age associated divergence) =0.0651
p(Scalp)=0.647
p(Dura)=0.0327
Scalp
Dura
A
B
C
D
E
F
G
H
I
J
K

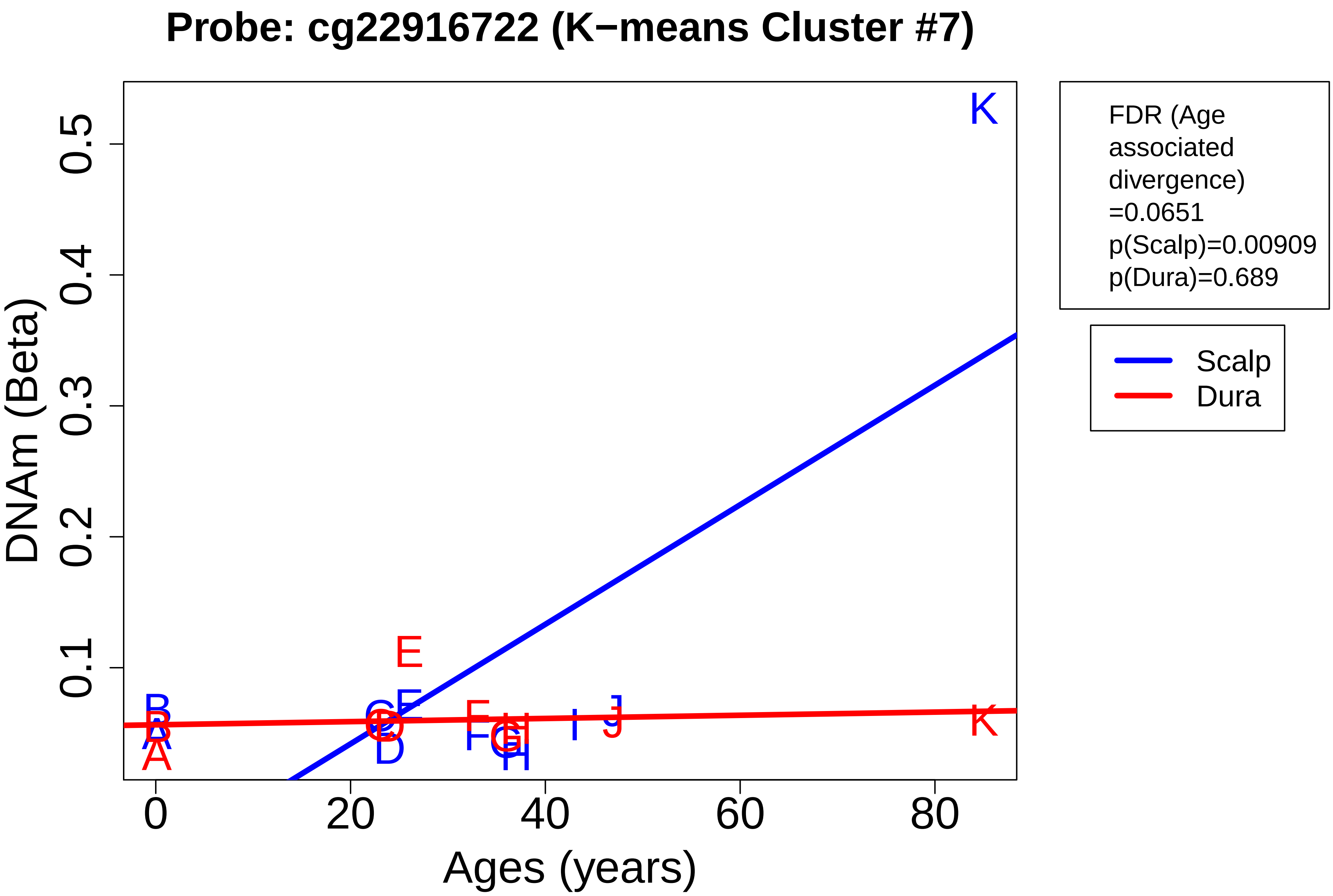

Probe: cg22916722 (K−means Cluster #7)
DNAm (Beta)
0.1
0.2
0.3
0.4
0.5
0
20
40
60
80
Ages (years)
FDR (Age associated divergence) =0.0651
p(Scalp)=0.00909
p(Dura)=0.689
Scalp
Dura

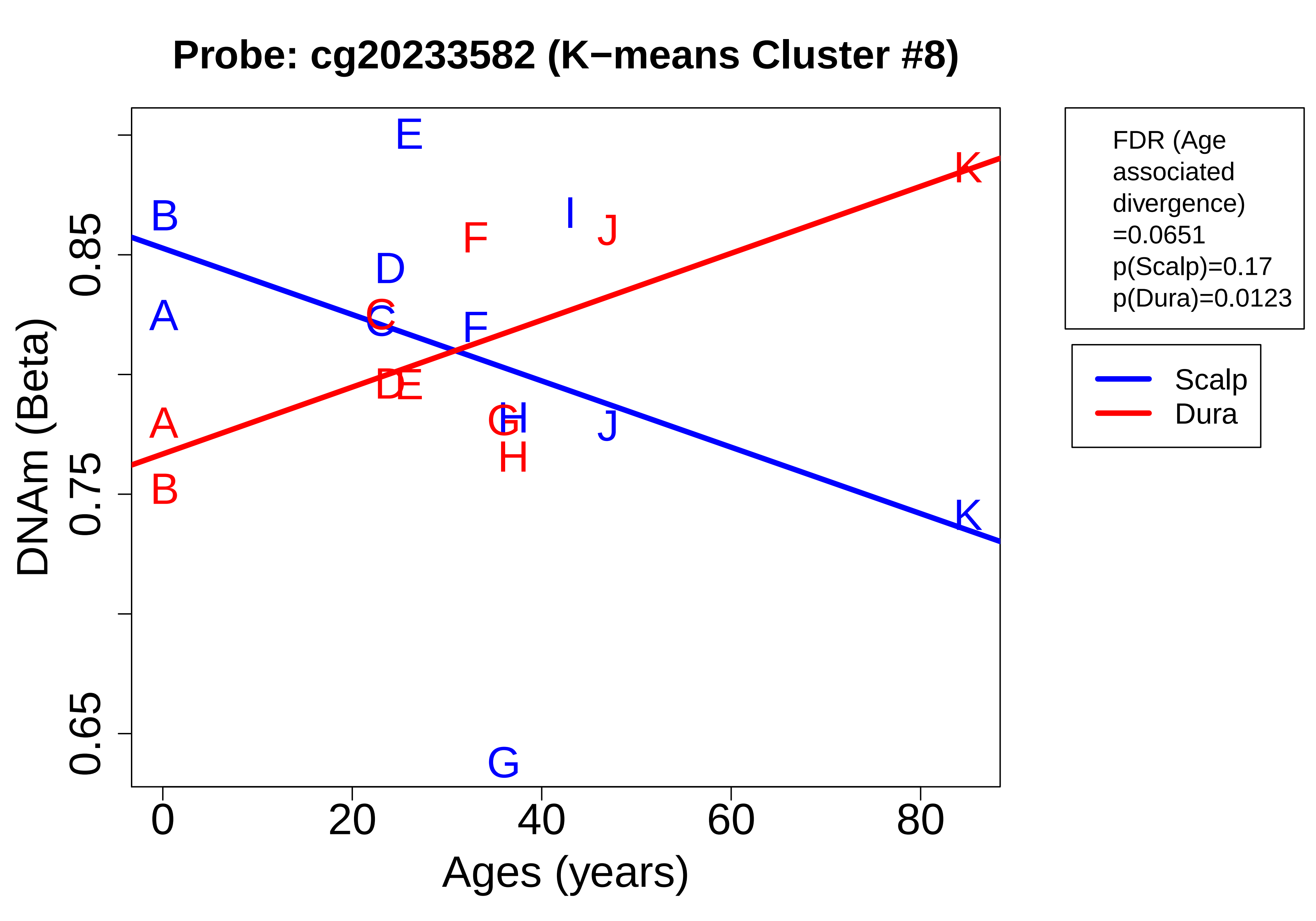

Probe: cg20233582 (K−means Cluster #8)
DNAm (Beta)
0.65
0.75
0.85
Ages (years)
0
20
40
60
80
FDR (Age associated divergence) =0.0651
p(Scalp)=0.17
p(Dura)=0.0123
Scalp
Dura

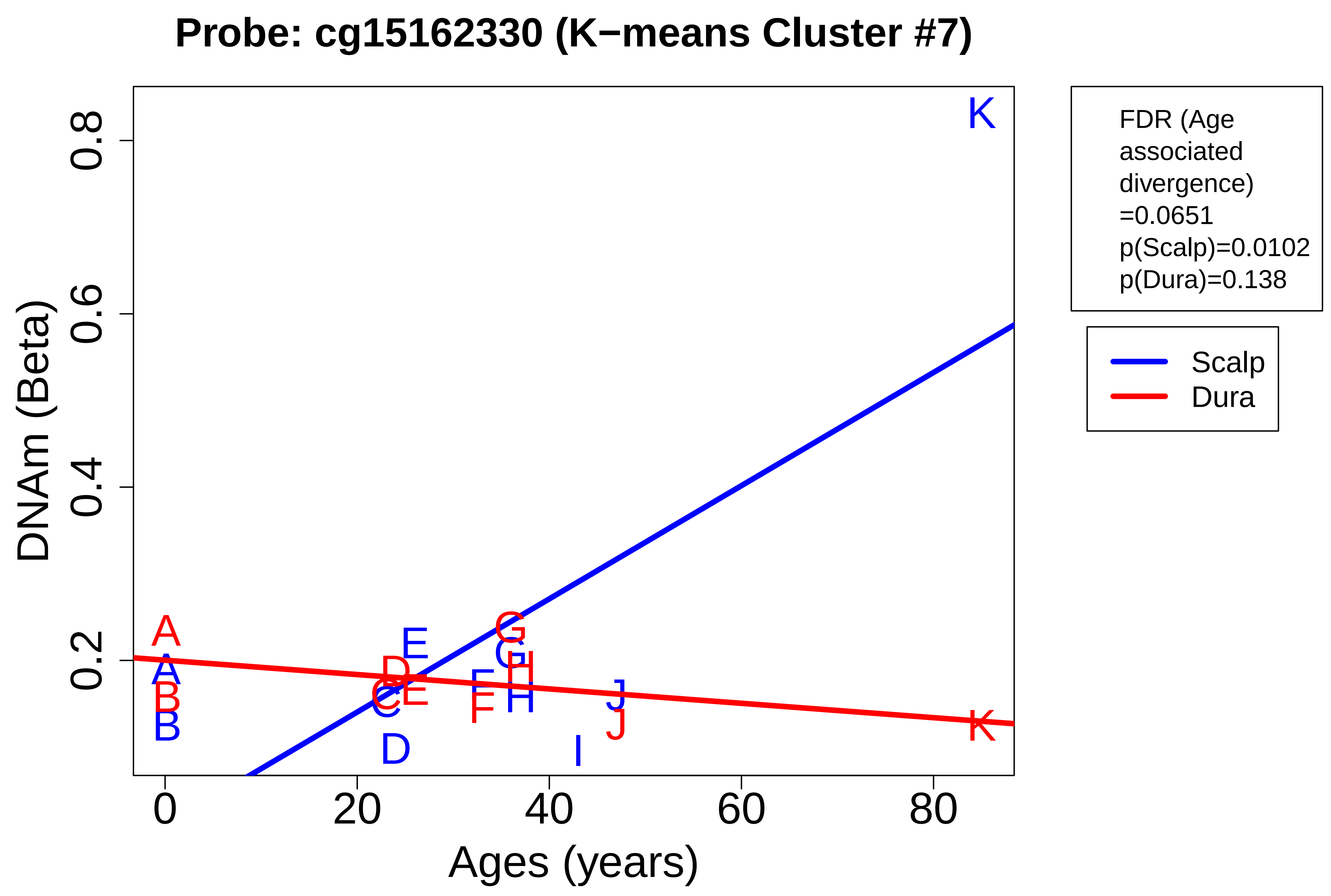

Probe: cg15162330 (K−means Cluster #7)
DNAm (Beta)
0.2
0.4
0.6
0.8
Ages (years)
0
20
40
60
80
FDR (Age associated divergence) =0.0651
p(Scalp)=0.0102
p(Dura)=0.138
Scalp
Dura

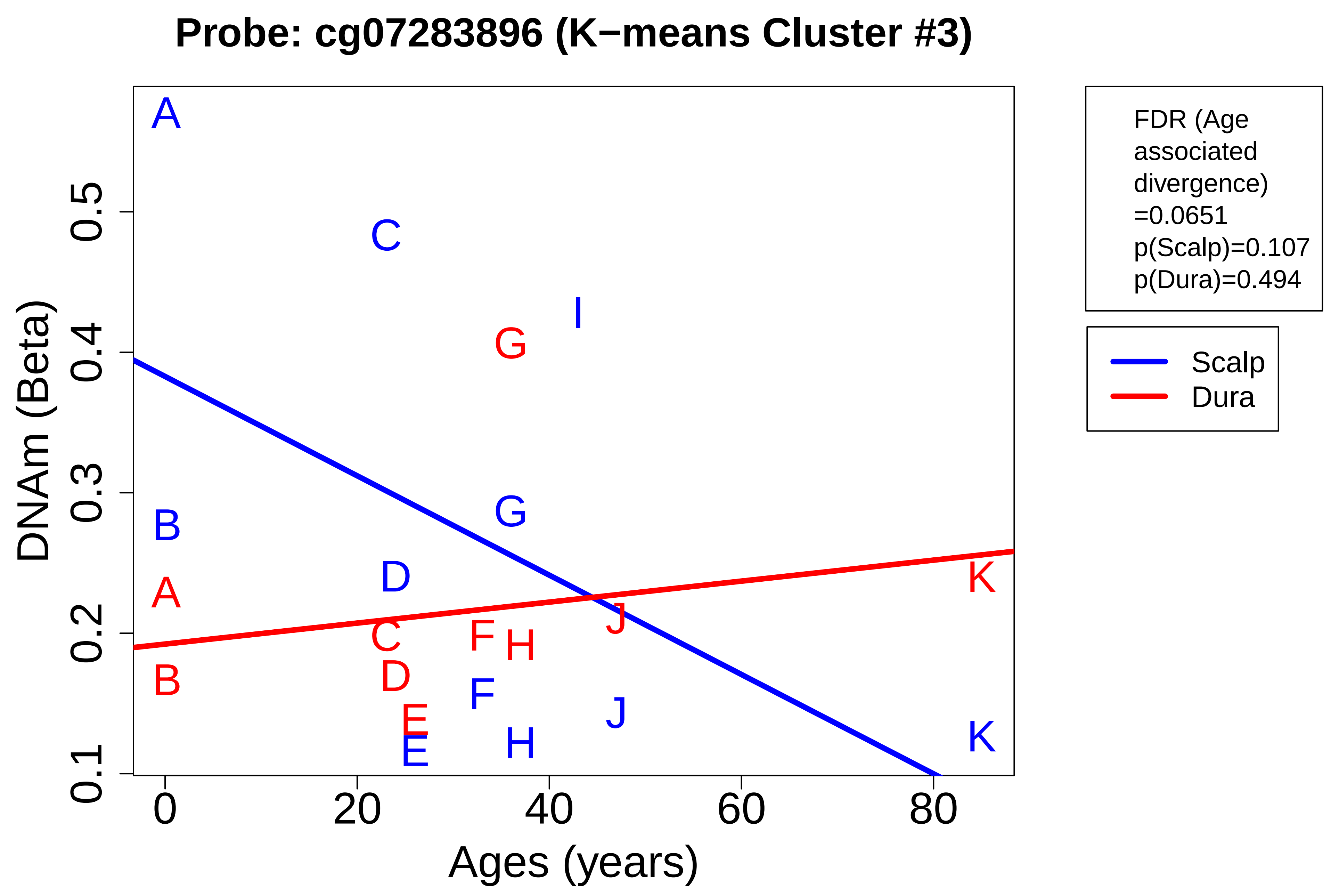
Probe: cg07283896 (K−means Cluster #3)
DNAm (Beta)
0.1
0.2
0.3
0.4
0.5
0
20
40
60
80
Ages (years)
A
B
C
D
E
F
G
H
I
J
K
FDR (Age associated divergence) =0.0651
p(Scalp)=0.107
p(Dura)=0.494
Scalp
Dura

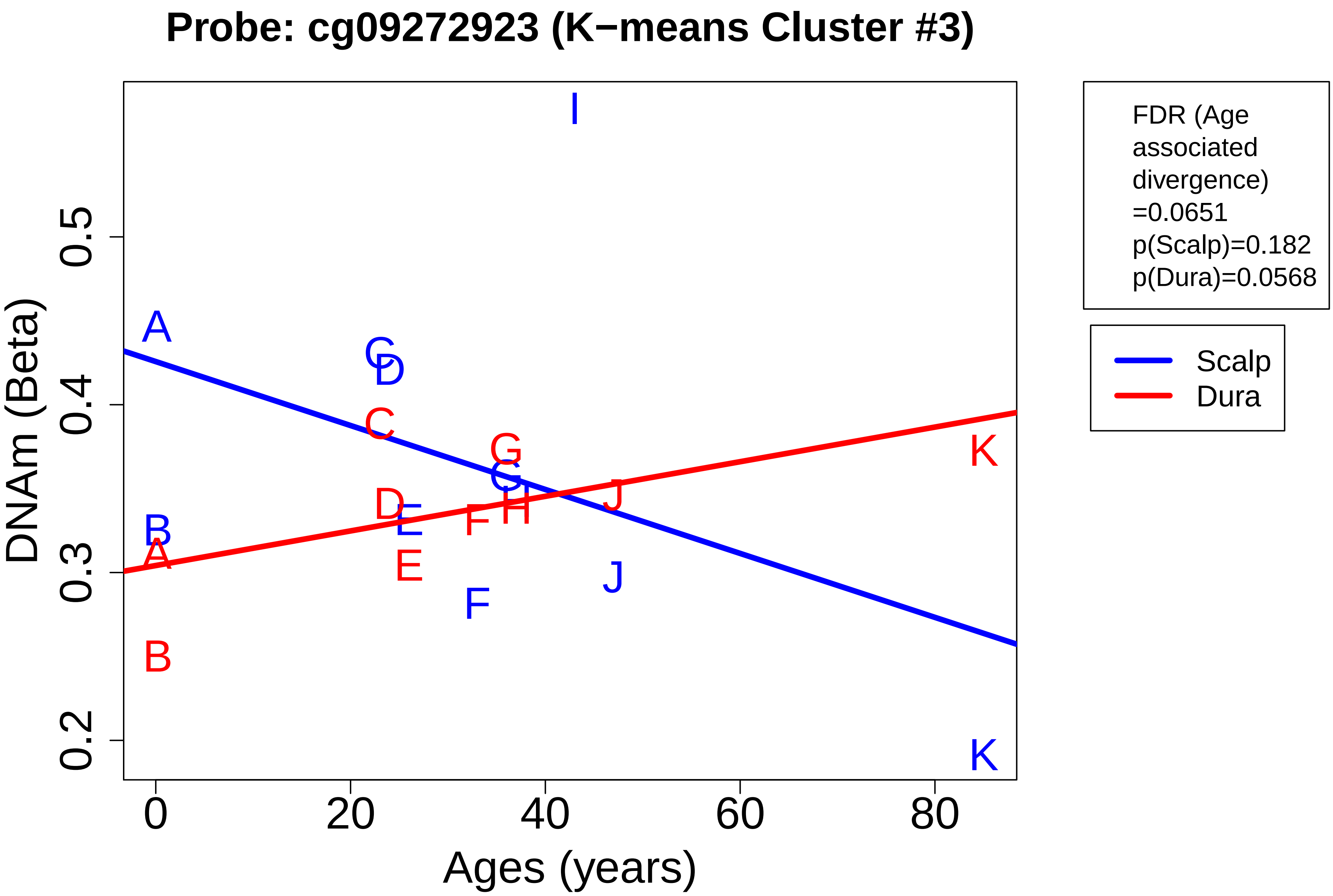

Probe: cg09272923 (K−means Cluster #3)
DNAm (Beta)
0.2
0.3
0.4
0.5
0
20
40
60
80
Ages (years)
A
B
C
D
E
F
G
H
I
J
K
FDR (Age associated divergence) =0.0651
p(Scalp)=0.182
p(Dura)=0.0568
Scalp
Dura

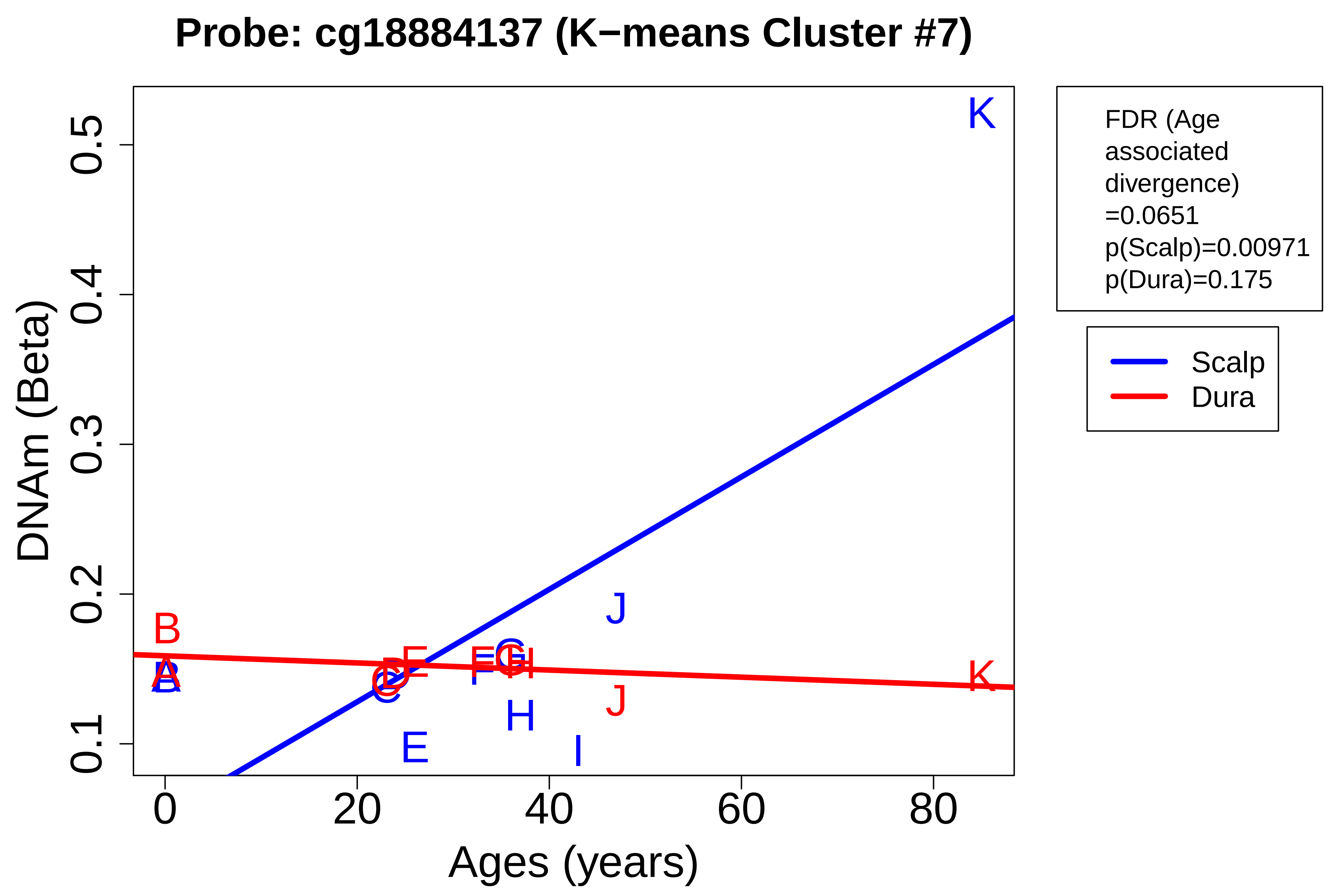

Probe: cg18884137 (K−means Cluster #7)
DNAm (Beta)
0.1
0.2
0.3
0.4
0.5
0
20
40
60
80
Ages (years)
A
B
C
D
E
F
G
H
I
J
K
FDR (Age associated divergence) =0.0651
p(Scalp)=0.00971
p(Dura)=0.175
Scalp
Dura

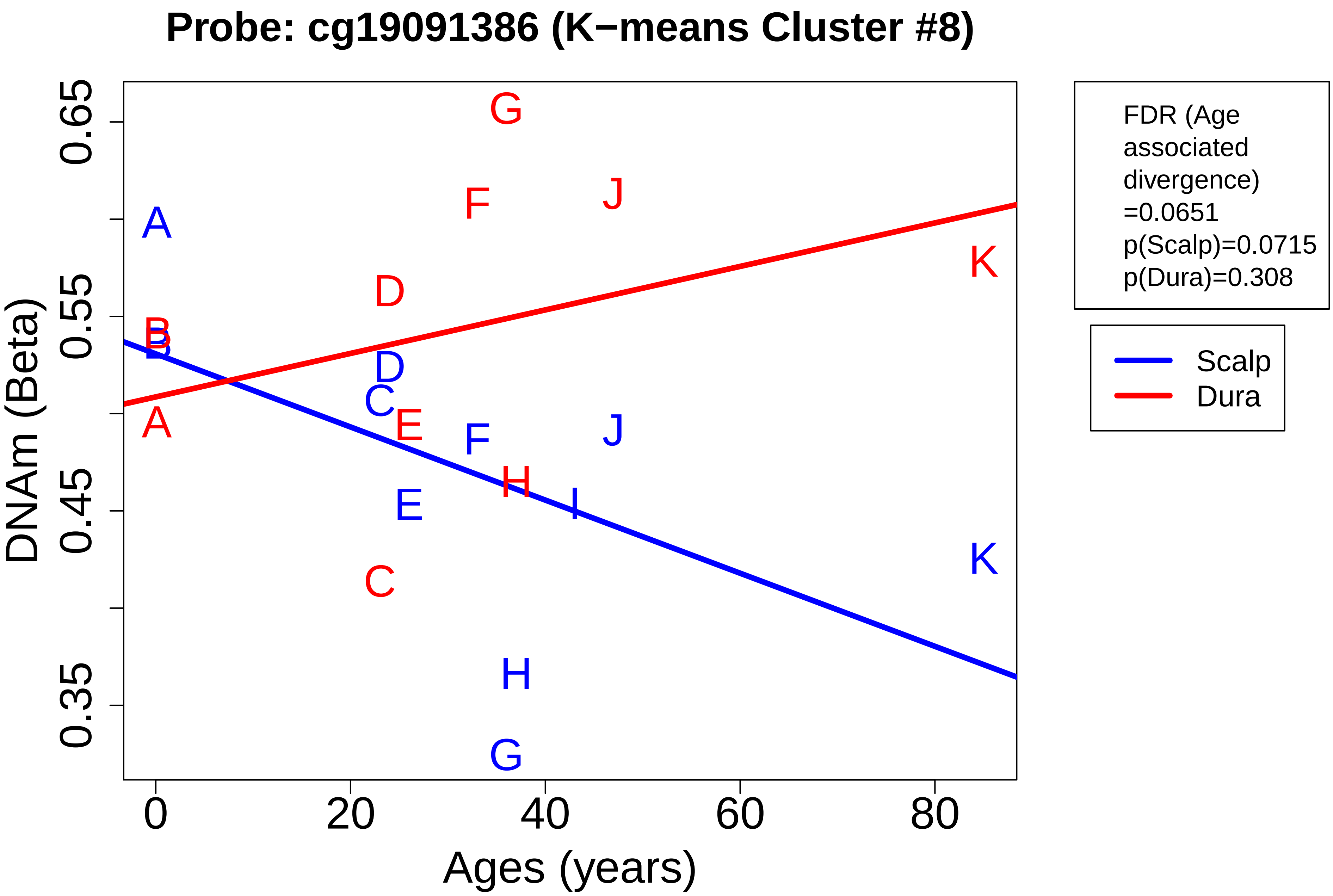
Probe: cg19091386 (K−means Cluster #8)
DNAm (Beta)
0.35
0.45
0.55
0.65
Ages (years)
0
20
40
60
80
A
B
C
D
E
F
G
H
I
J
K
FDR (Age associated divergence) =0.0651
p(Scalp)=0.0715
p(Dura)=0.308
Scalp
Dura

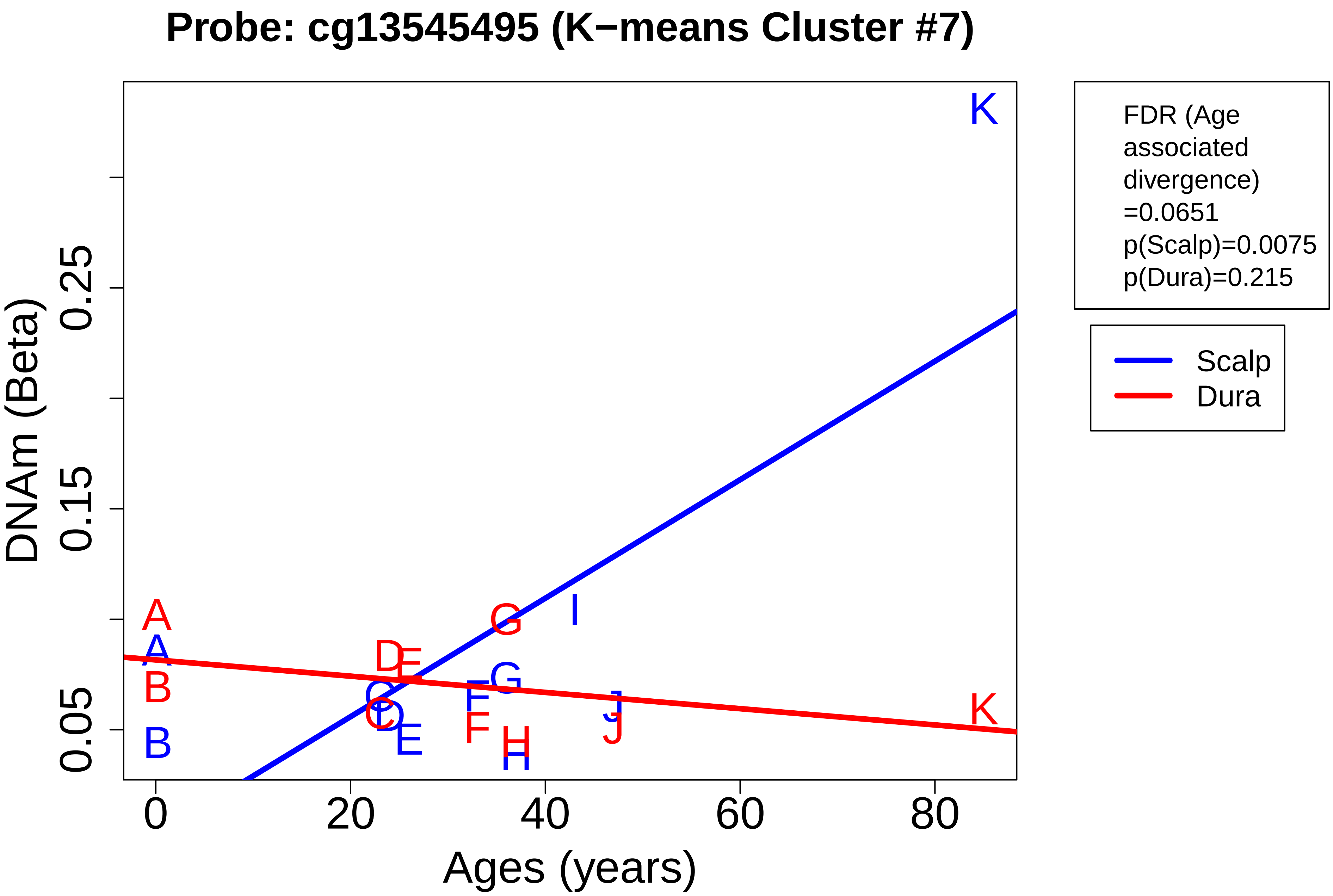

Probe: cg13545495 (K−means Cluster #7)
DNAm (Beta)
0.05
0.15
0.25
0
20
40
60
80
Ages (years)
FDR (Age associated divergence) =0.0651
p(Scalp)=0.0075
p(Dura)=0.215
Scalp
Dura

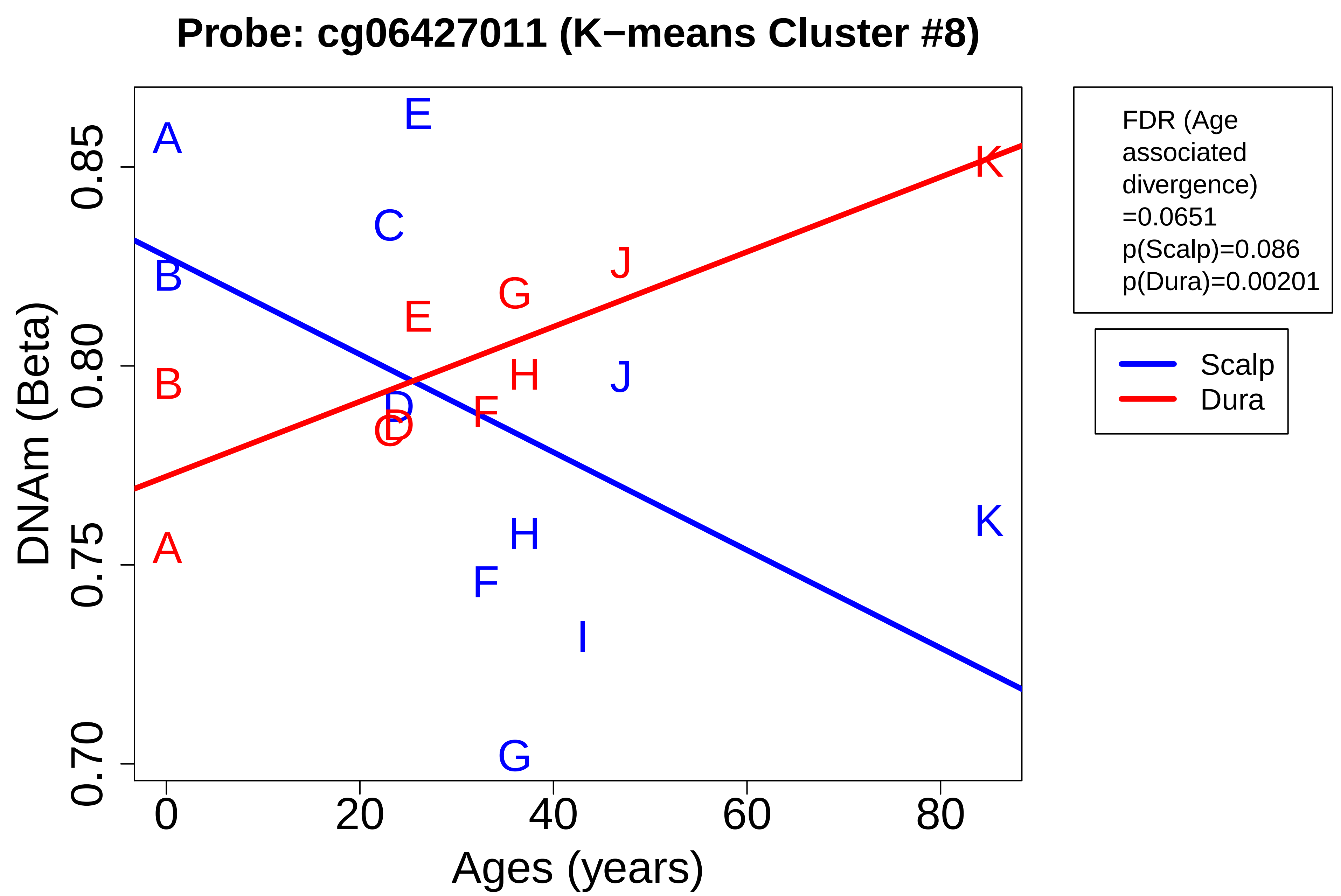

Probe: cg06427011 (K−means Cluster #8)
DNAm (Beta)
0.70
0.75
0.80
0.85
0
20
40
60
80
Ages (years)
A
B
C
D
E
F
G
H
I
J
K
FDR (Age associated divergence) =0.0651
p(Scalp)=0.086
p(Dura)=0.00201
Scalp
Dura

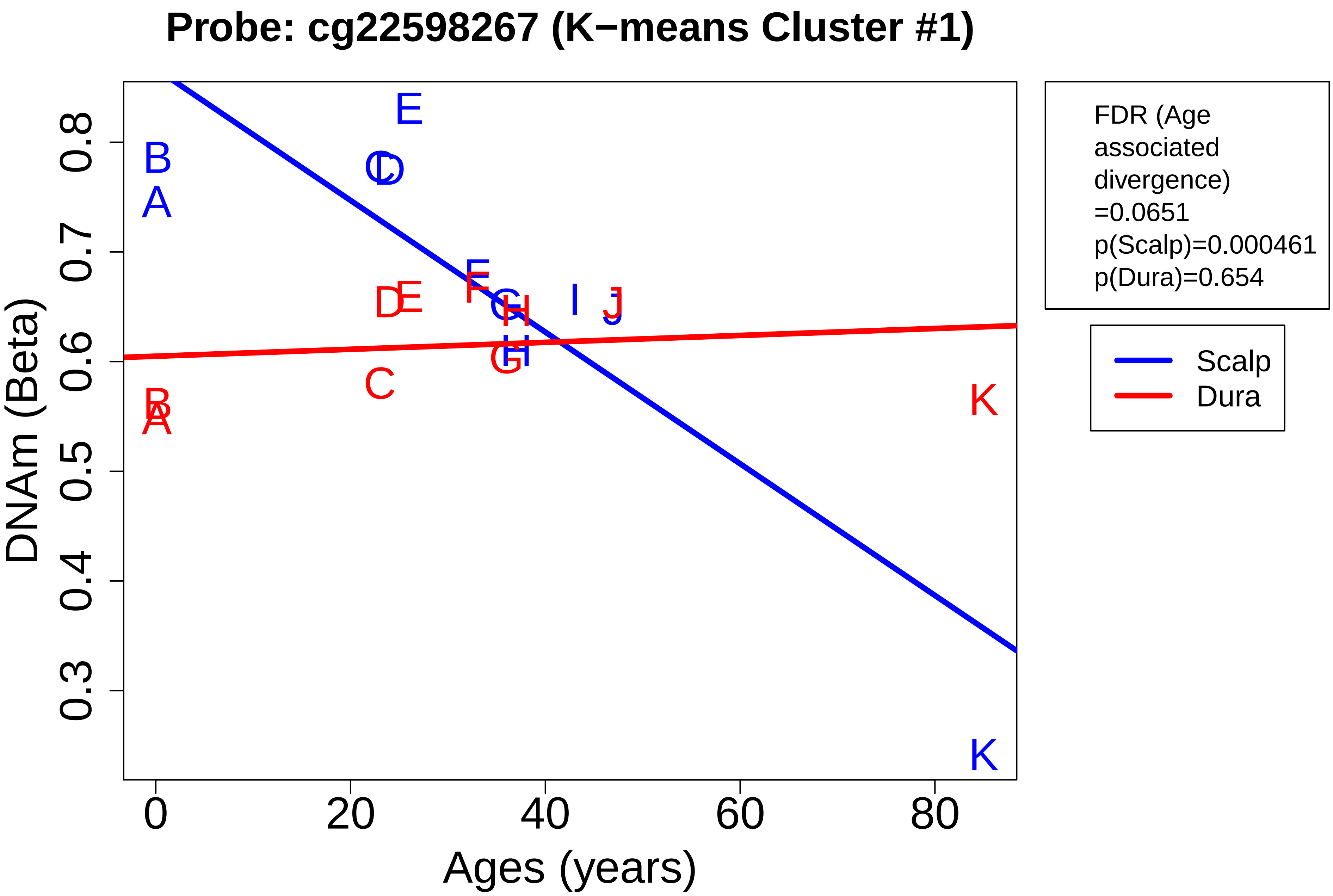

Probe: cg22598267 (K−means Cluster #1)
DNAm (Beta)
0.3
0.4
0.5
0.6
0.7
0.8
0
20
40
60
80
Ages (years)
FDR (Age associated divergence) =0.0651
p(Scalp)=0.000461
p(Dura)=0.654
Scalp
Dura

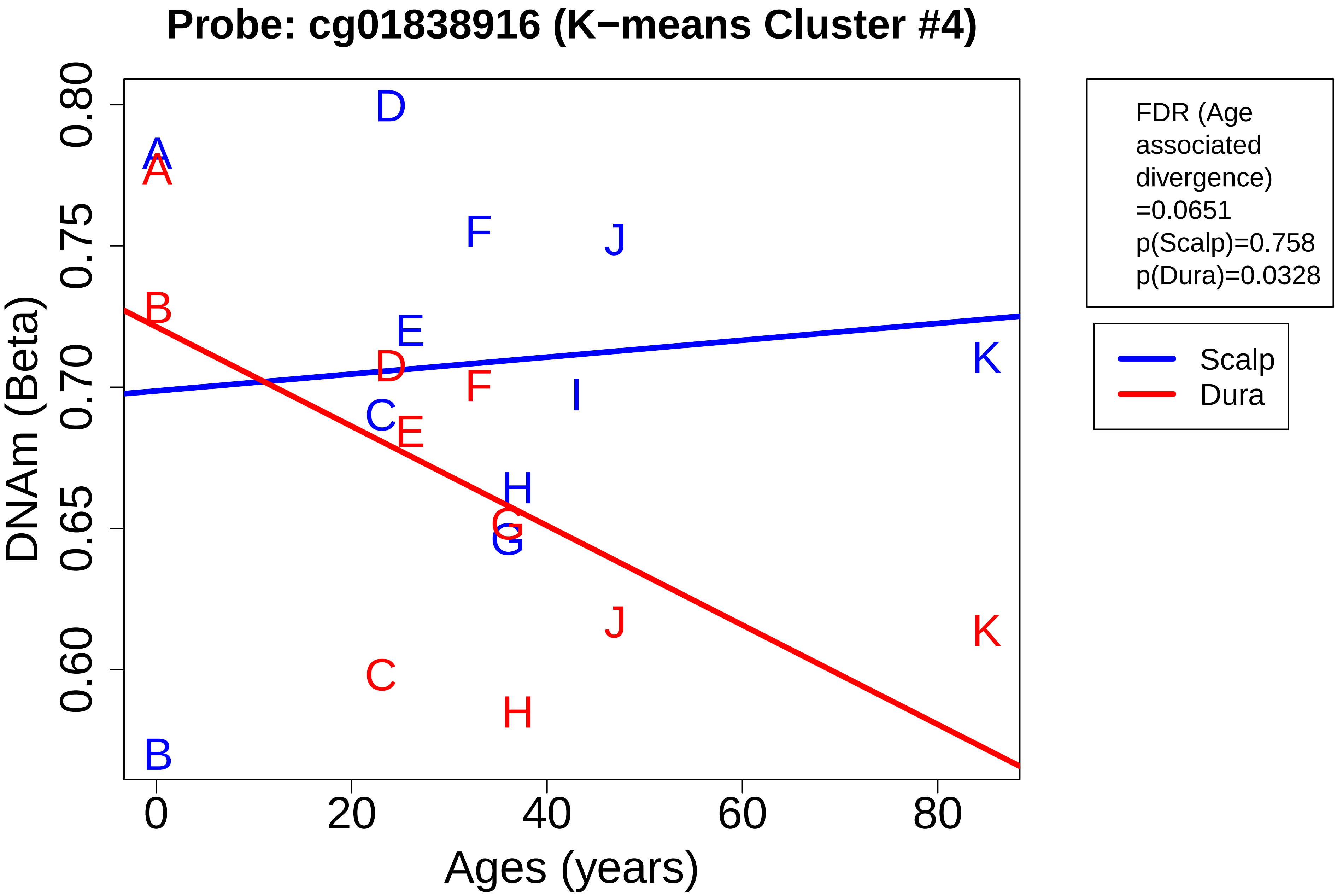

Probe: cg01838916 (K−means Cluster #4)
DNAm (Beta)
0.60
0.65
0.70
0.75
0.80
Ages (years)
0
20
40
60
80
A
B
C
D
E
F
G
H
I
J
K
A
B
C
D
E
F
G
H
J
K
FDR (Age associated divergence) =0.0651
p(Scalp)=0.758
p(Dura)=0.0328
Scalp
Dura

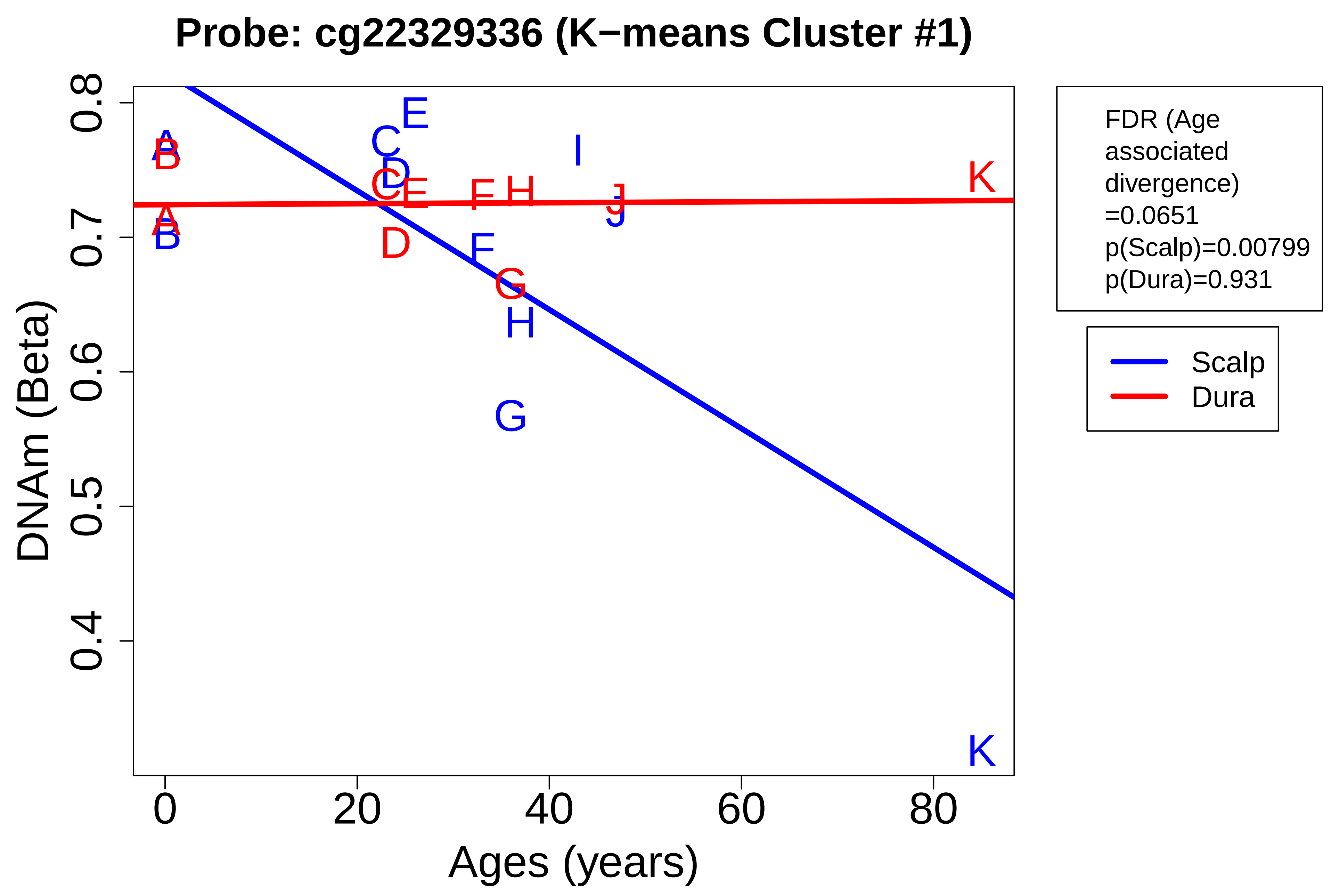

Probe: cg22329336 (K−means Cluster #1)
DNAm (Beta)
0.8
0.7
0.6
0.5
0.4
0
20
40
60
80
Ages (years)
A
B
C
D
E
F
G
H
I
J
K
FDR (Age associated divergence) =0.0651
p(Scalp)=0.00799
p(Dura)=0.931
Scalp
Dura

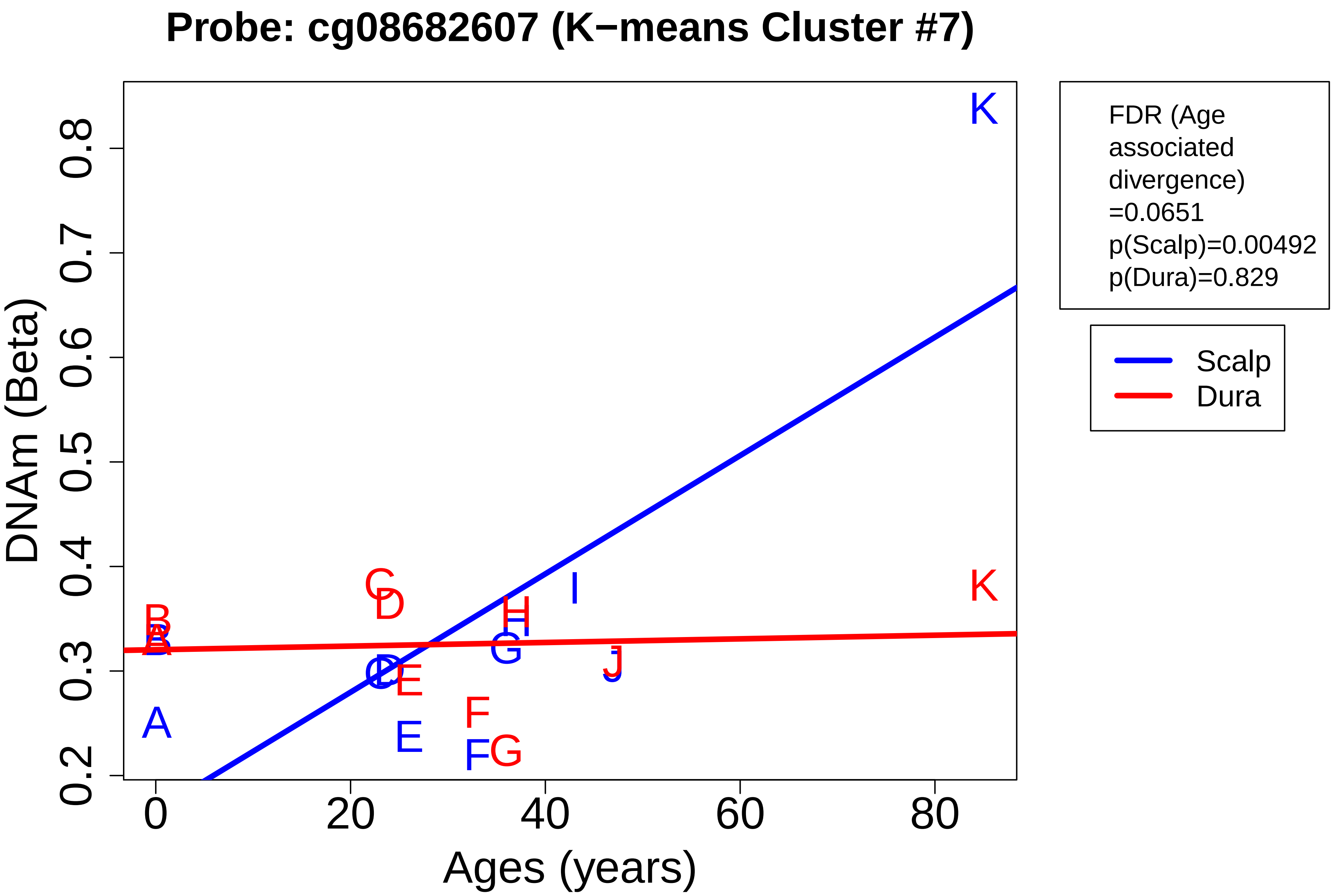
Probe: cg08682607 (K−means Cluster #7)
DNAm (Beta)
Ages (years)
0.2
0.3
0.4
0.5
0.6
0.7
0.8
0
20
40
60
80
FDR (Age associated divergence) =0.0651
p(Scalp)=0.00492
p(Dura)=0.829
Scalp
Dura

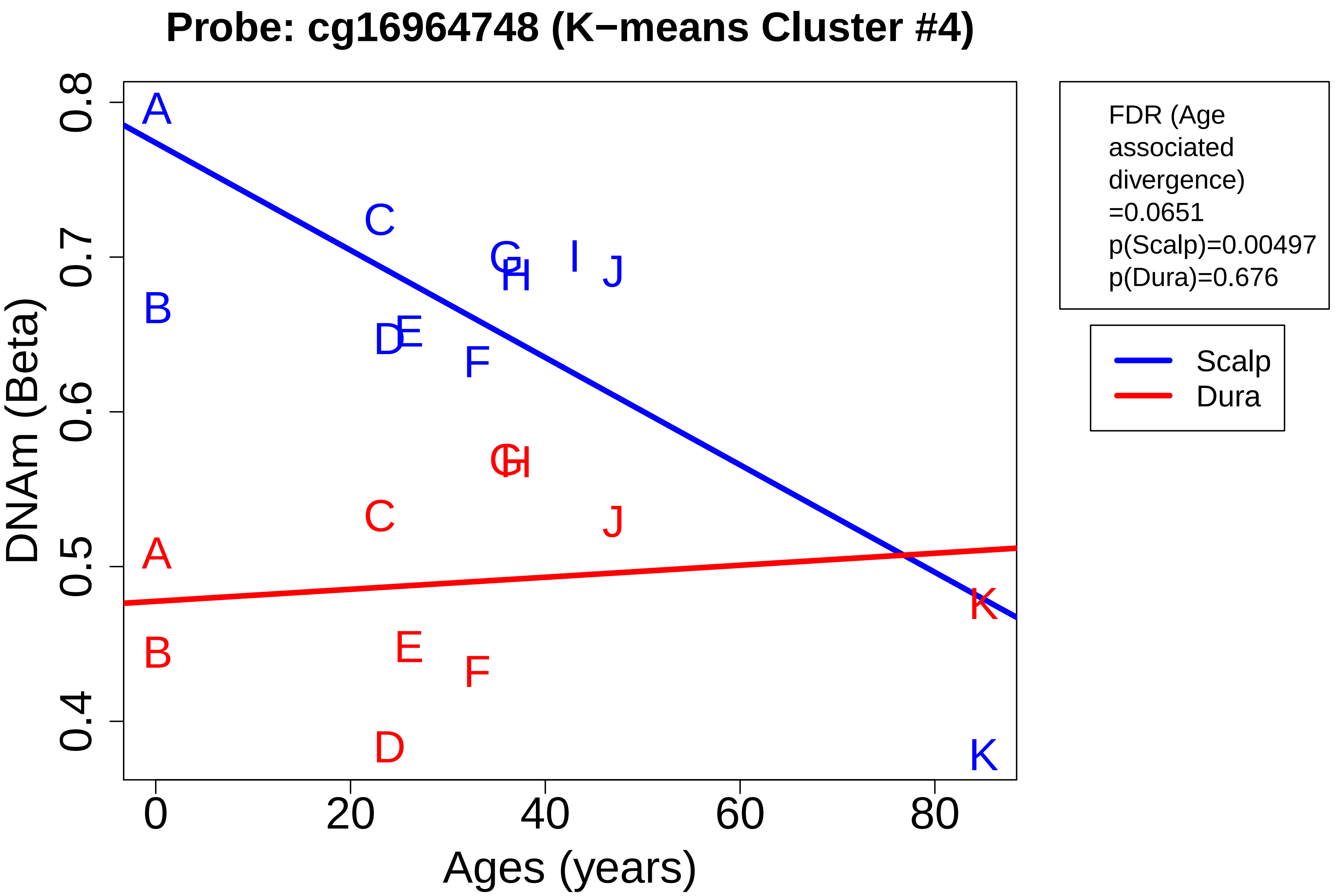

Probe: cg16964748 (K−means Cluster #4)
DNAm (Beta)
0.4
0.5
0.6
0.7
0.8
0
20
40
60
80
Ages (years)
A
B
C
D
E
F
G
H
I
J
K
FDR (Age associated divergence) =0.0651
p(Scalp)=0.00497
p(Dura)=0.676
Scalp
Dura

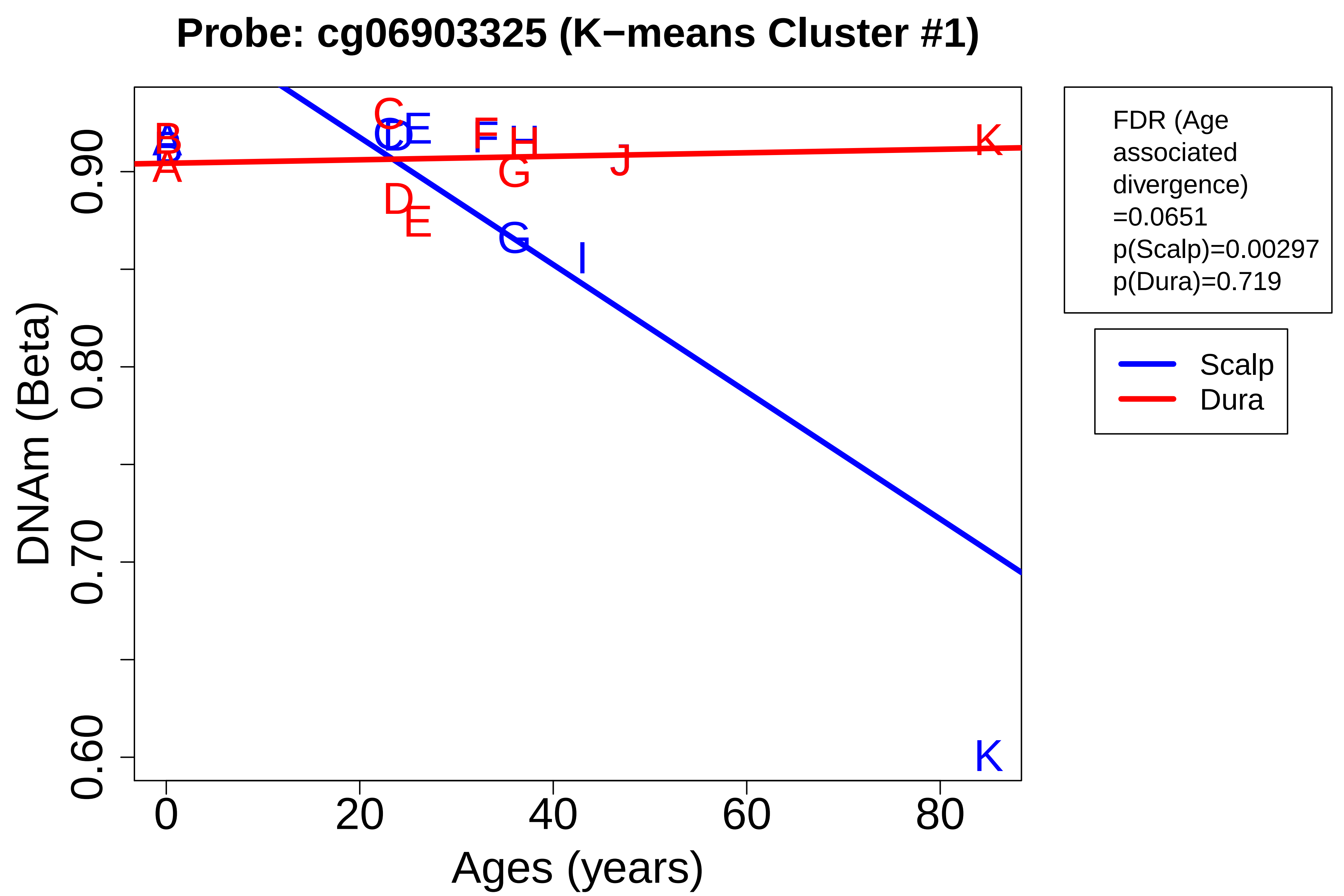

Probe: cg06903325 (K−means Cluster #1)
DNAm (Beta)
0.60
0.70
0.80
0.90
Ages (years)
0
20
40
60
80
FDR (Age associated divergence) =0.0651
p(Scalp)=0.00297
p(Dura)=0.719
Scalp
Dura
A
B
C
D
E
F
G
H
I
J
K

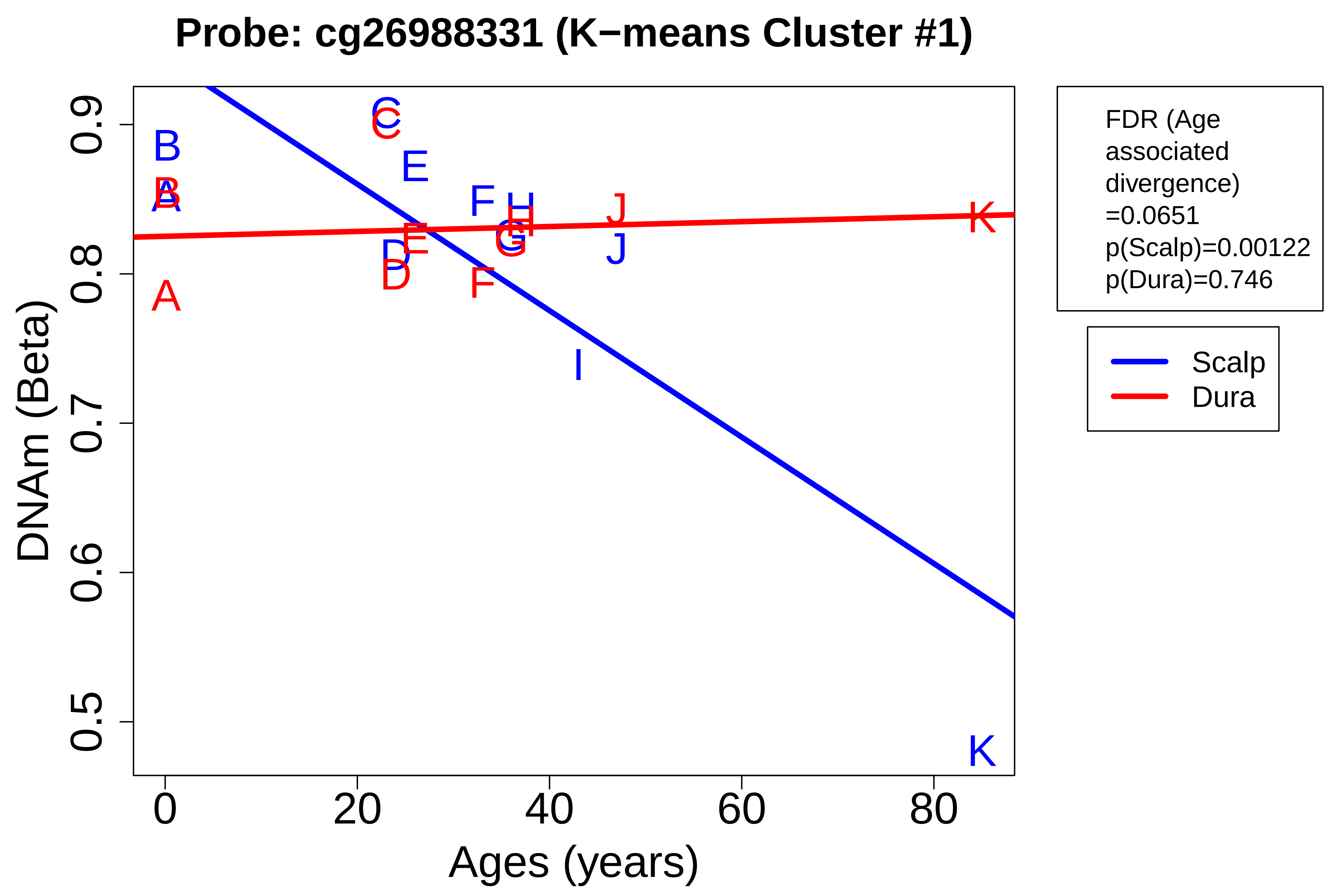
Probe: cg26988331 (K−means Cluster #1)
DNAm (Beta)
Ages (years)
0.5
0.6
0.7
0.8
0.9
0
20
40
60
80
A
B
C
D
E
F
G
H
I
J
K
FDR (Age associated divergence) =0.0651
p(Scalp)=0.00122
p(Dura)=0.746
Scalp
Dura

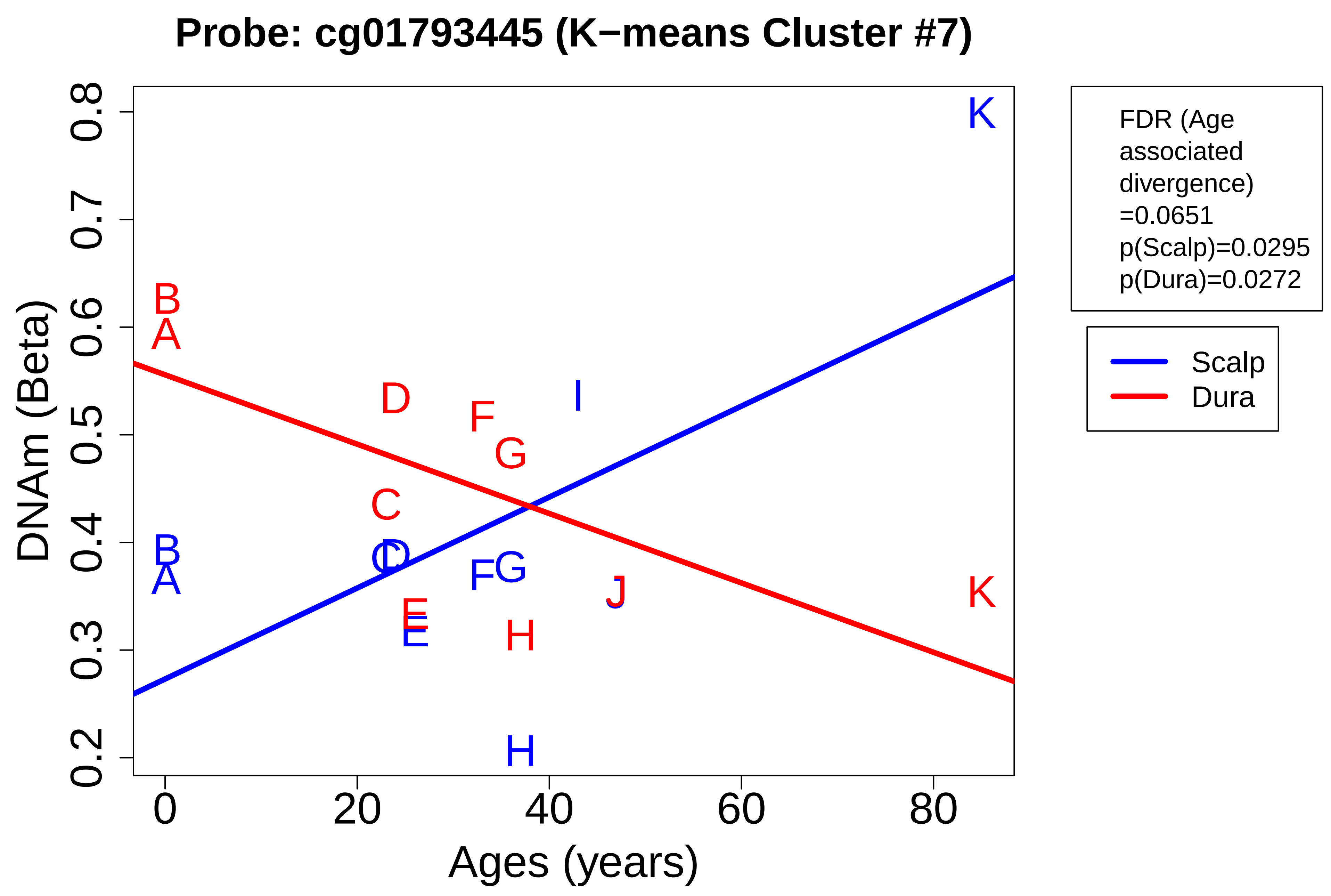

Probe: cg01793445 (K−means Cluster #7)
DNAm (Beta)
0.2
0.3
0.4
0.5
0.6
0.7
0.8
Ages (years)
0
20
40
60
80
FDR (Age associated divergence) =0.0651
p(Scalp)=0.0295
p(Dura)=0.0272
Scalp
Dura
A
B
C
D
E
F
G
H
I
J
K

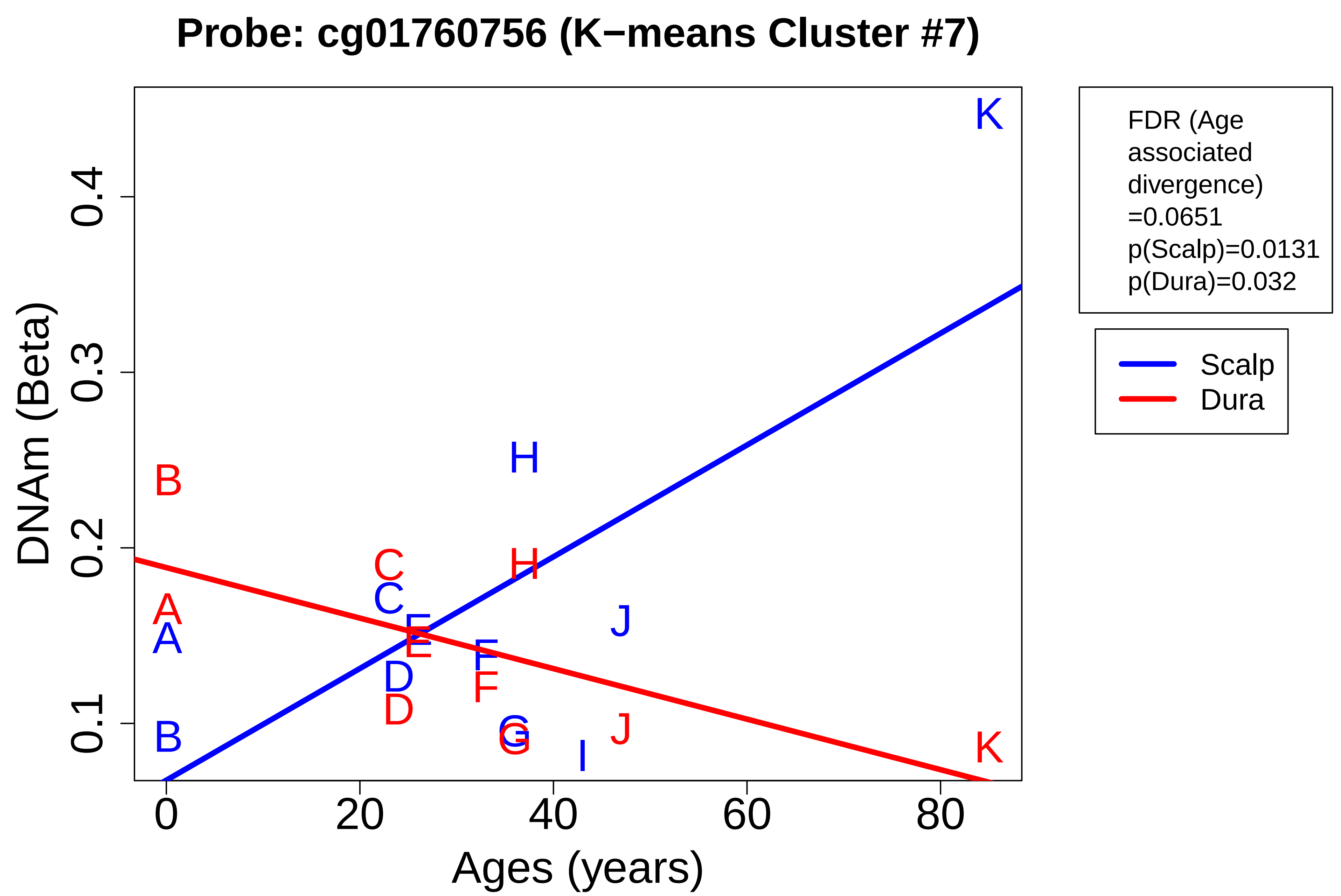

Probe: cg01760756 (K−means Cluster #7)
DNAm (Beta)
0.1
0.2
0.3
0.4
0
20
40
60
80
Ages (years)
FDR (Age associated divergence) =0.0651
p(Scalp)=0.0131
p(Dura)=0.032
Scalp
Dura

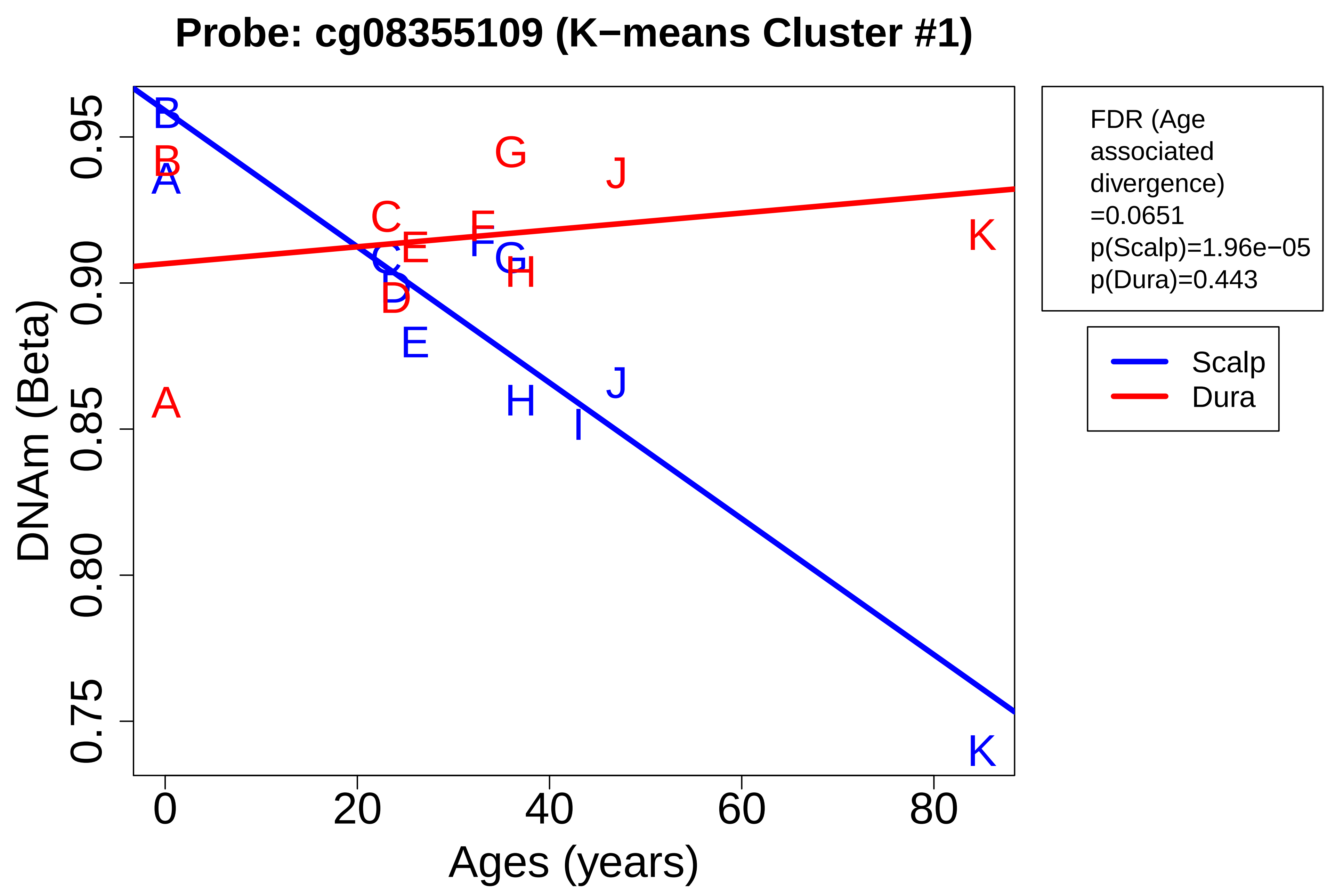
Probe: cg08355109 (K−means Cluster #1)
DNAm (Beta)
0.75
0.80
0.85
0.90
0.95
0
20
40
60
80
Ages (years)
FDR (Age associated divergence) =0.0651
p(Scalp)=1.96e−05
p(Dura)=0.443
Scalp
Dura
A
B
C
D
E
F
G
H
I
J
K

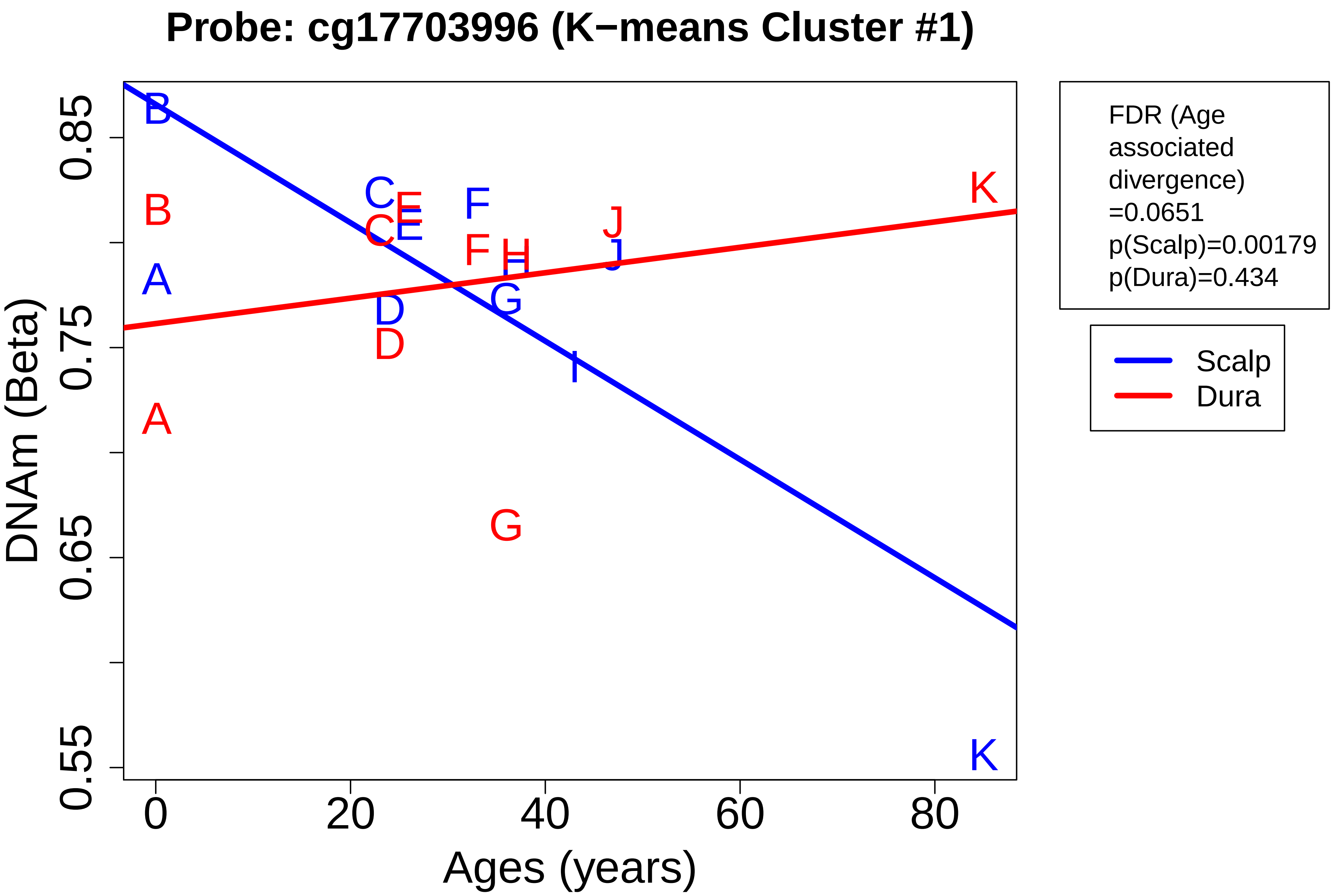

Probe: cg17703996 (K−means Cluster #1)
DNAm (Beta)
0.55
0.65
0.75
0.85
Ages (years)
0
20
40
60
80
FDR (Age associated divergence) =0.0651
p(Scalp)=0.00179
p(Dura)=0.434
Scalp
Dura
A
B
C
D
E
F
G
H
I
J
K

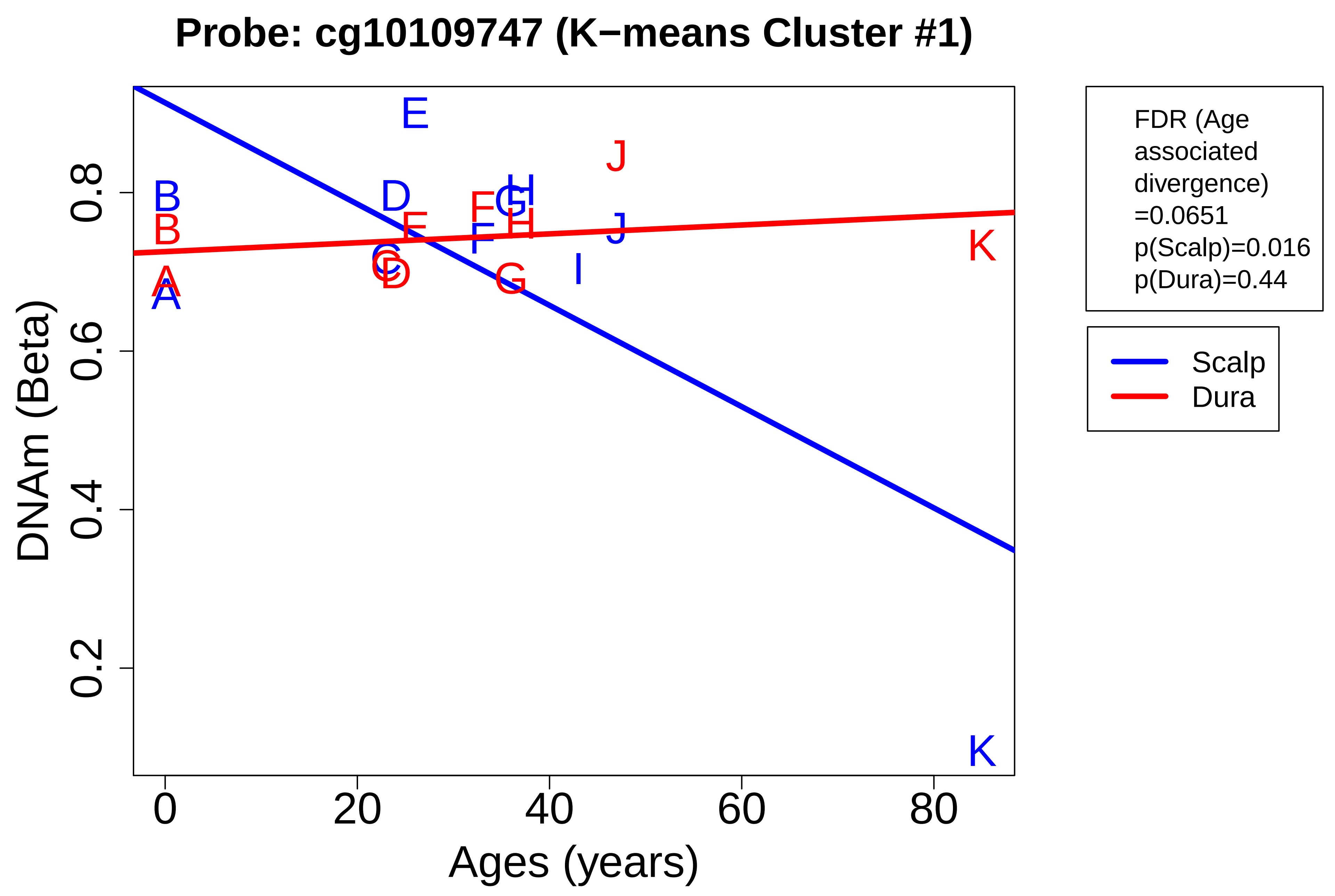
Probe: cg10109747 (K−means Cluster #1)
DNAm (Beta)
0.2
0.4
0.6
0.8
0
20
40
60
80
Ages (years)
A
B
C
D
E
F
G
H
I
J
K
FDR (Age associated divergence) =0.0651
p(Scalp)=0.016
p(Dura)=0.44
Scalp
Dura

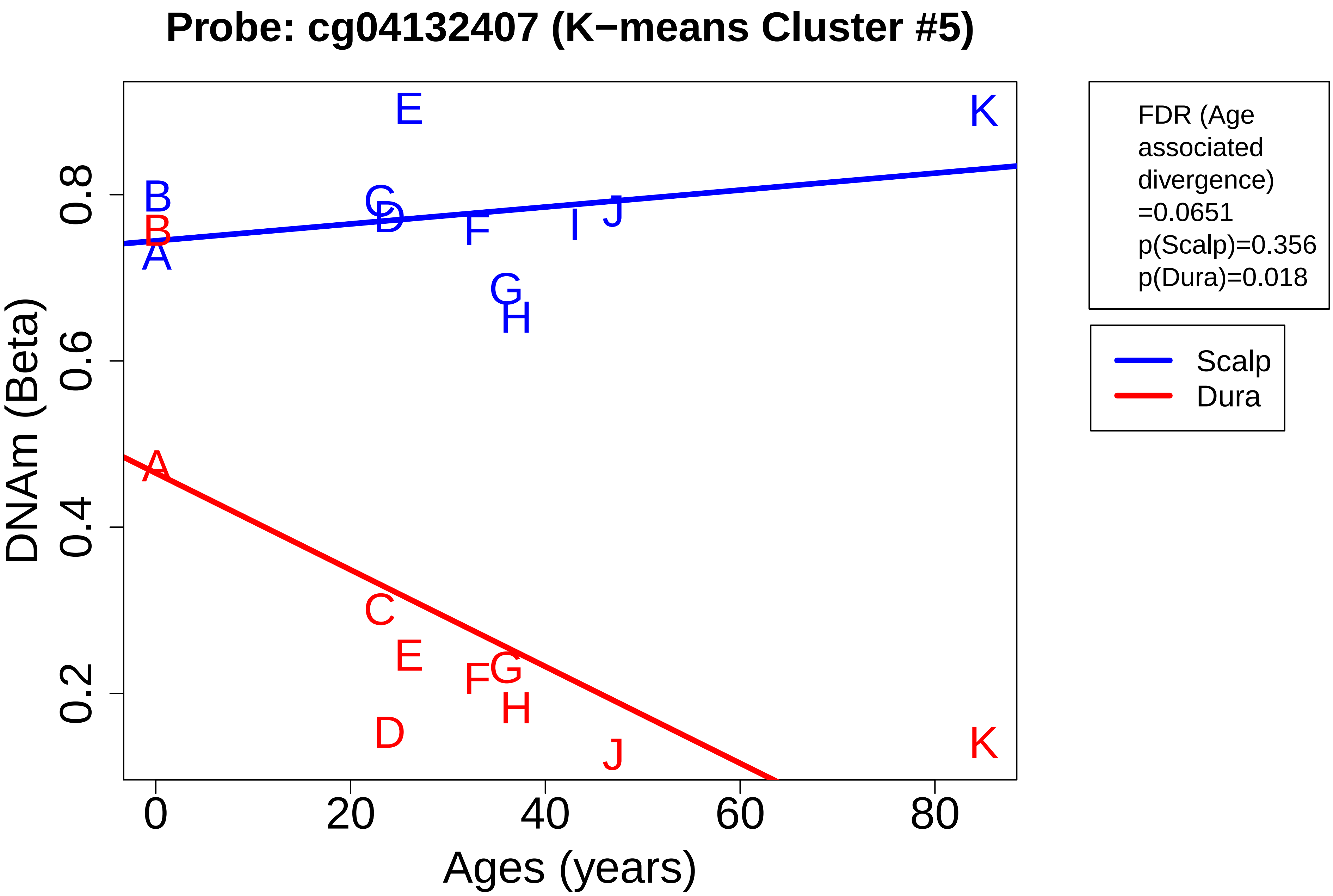
Probe: cg04132407 (K−means Cluster #5)
DNAm (Beta)
0.2
0.4
0.6
0.8
Ages (years)
0
20
40
60
80
FDR (Age associated divergence) =0.0651
p(Scalp)=0.356
p(Dura)=0.018
Scalp
Dura
A
B
C
D
E
F
G
H
I
J
K

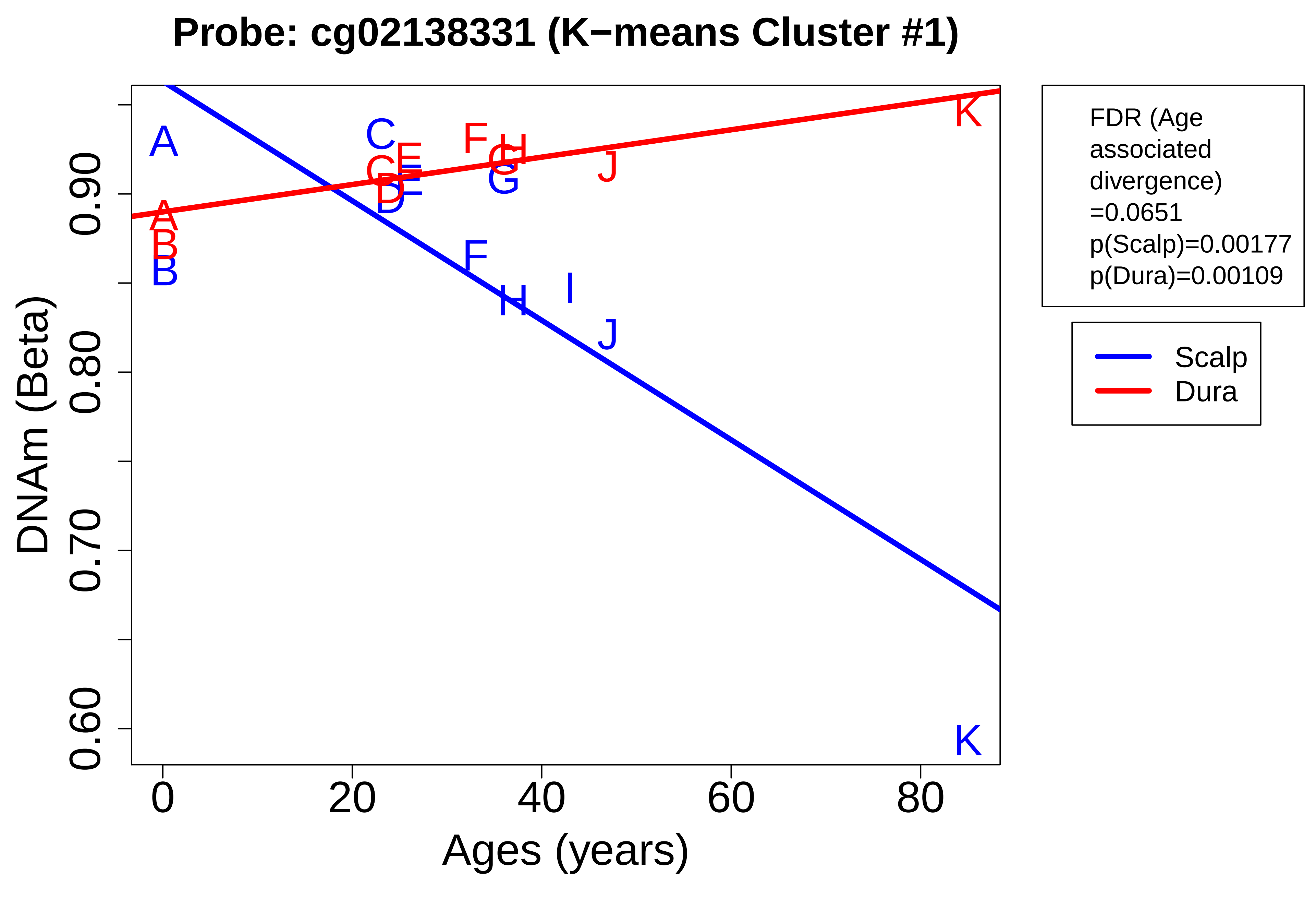

Probe: cg02138331 (K−means Cluster #1)
DNAm (Beta)
0.60
0.70
0.80
0.90
0
20
40
60
80
Ages (years)
FDR (Age associated divergence) =0.0651
p(Scalp)=0.00177
p(Dura)=0.00109
Scalp
Dura

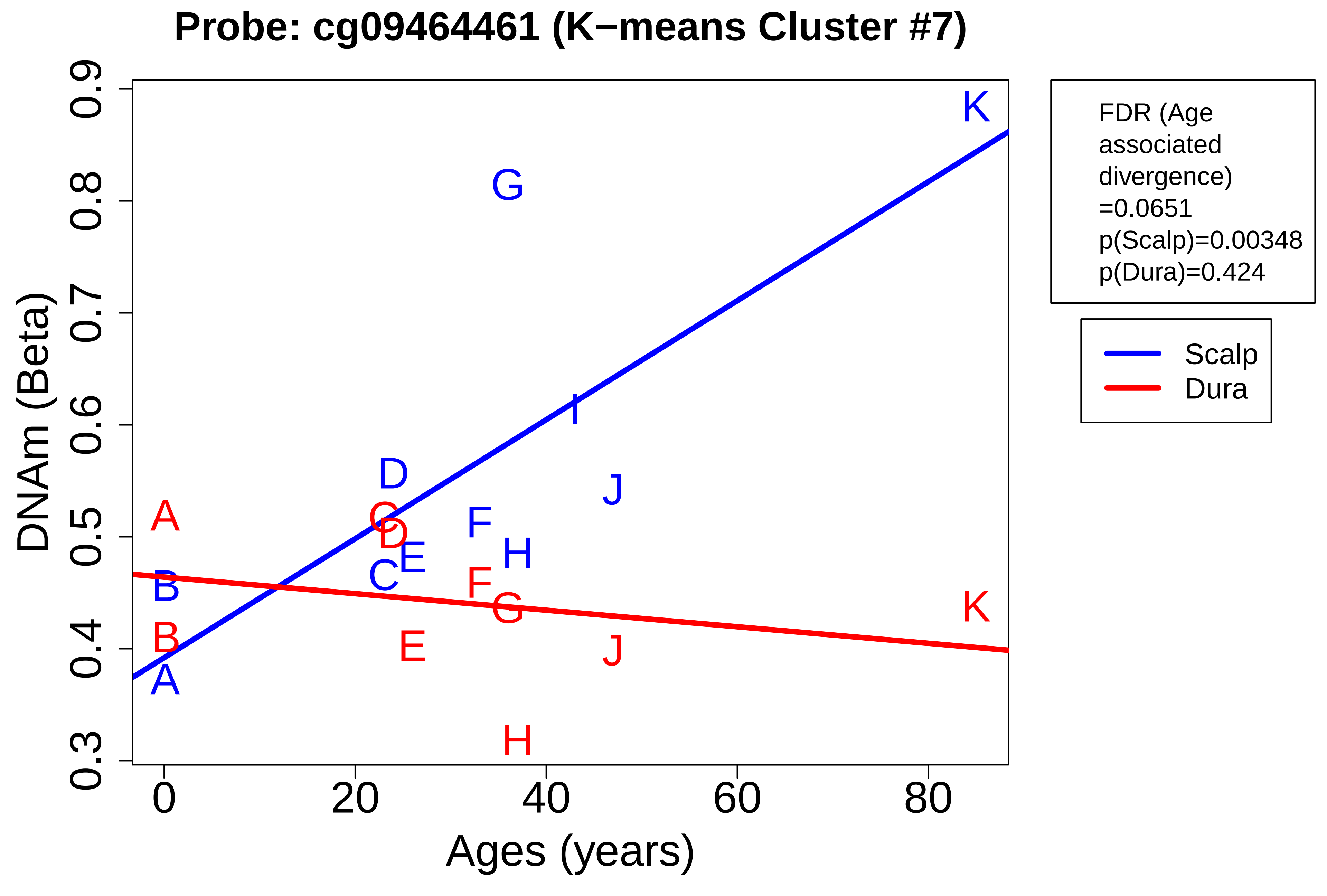

Probe: cg09464461 (K−means Cluster #7)
DNAm (Beta)
0.3
0.4
0.5
0.6
0.7
0.8
0.9
0
20
40
60
80
Ages (years)
FDR (Age associated divergence) =0.0651
p(Scalp)=0.00348
p(Dura)=0.424
Scalp
Dura
A
B
C
D
E
F
G
H
I
J
K

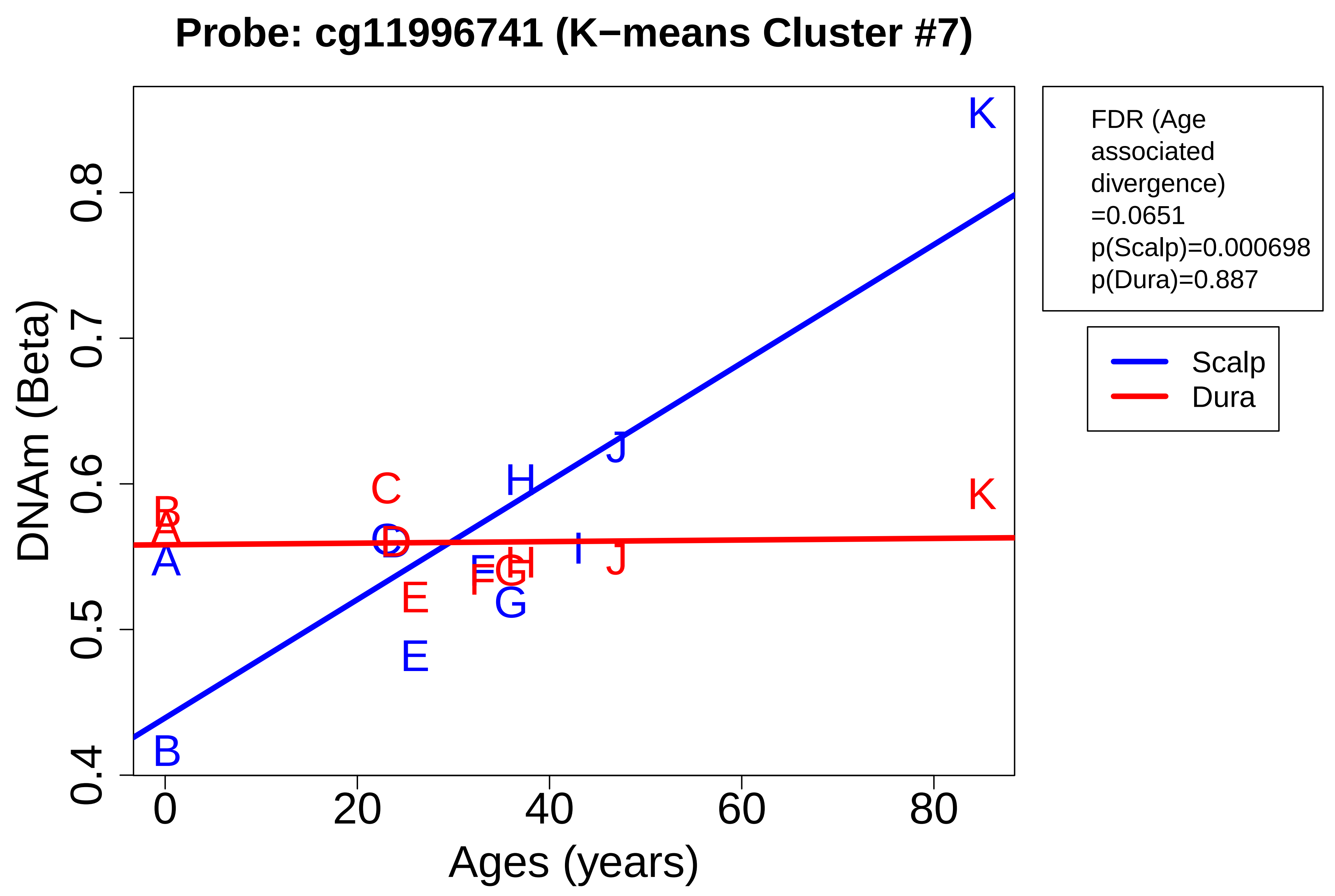

Probe: cg11996741 (K−means Cluster #7)
FDR (Age associated divergence) =0.0651
p(Scalp)=0.000698
p(Dura)=0.887
Scalp
Dura
DNAm (Beta)
0.4
0.5
0.6
0.7
0.8
0
20
40
60
80
Ages (years)

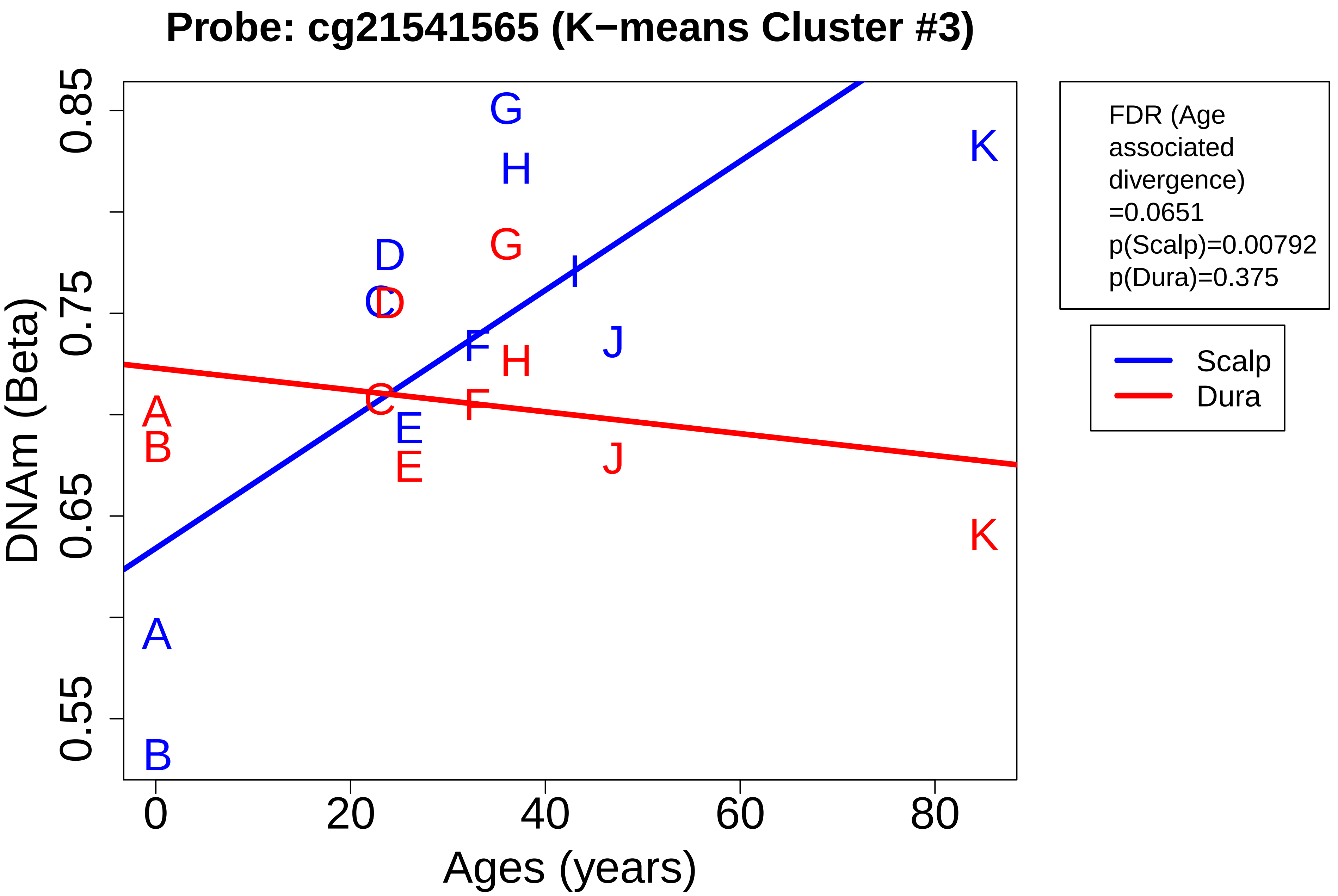

Probe: cg21541565 (K−means Cluster #3)
DNAm (Beta)
0.55
0.65
0.75
0.85
Ages (years)
0
20
40
60
80
A
B
C
D
E
F
G
H
I
J
K
FDR (Age associated divergence) =0.0651
p(Scalp)=0.00792
p(Dura)=0.375
Scalp
Dura

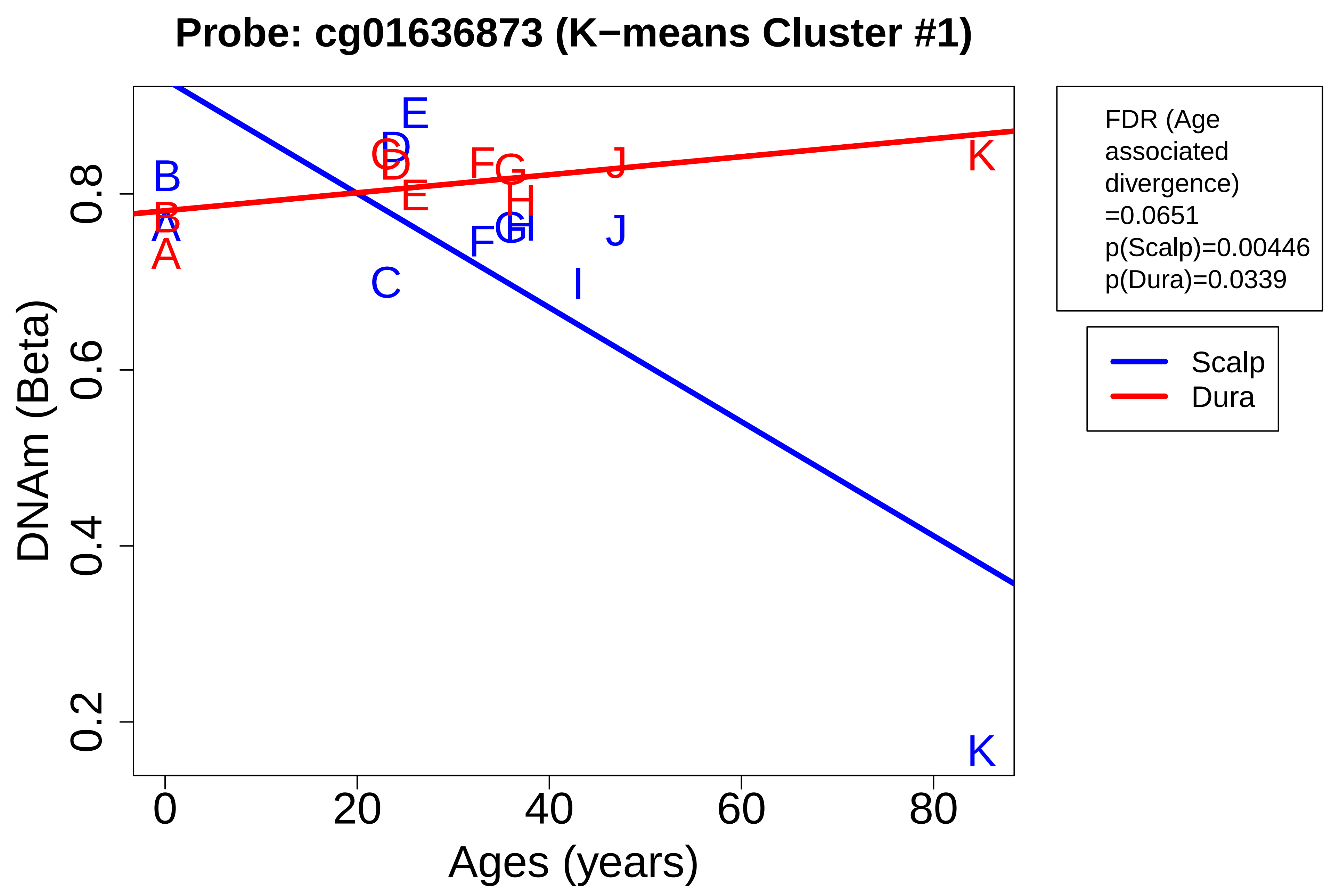

Probe: cg01636873 (K−means Cluster #1)
DNAm (Beta)
0.2
0.4
0.6
0.8
0
20
40
60
80
Ages (years)
FDR (Age associated divergence) =0.0651
p(Scalp)=0.00446
p(Dura)=0.0339
Scalp
Dura

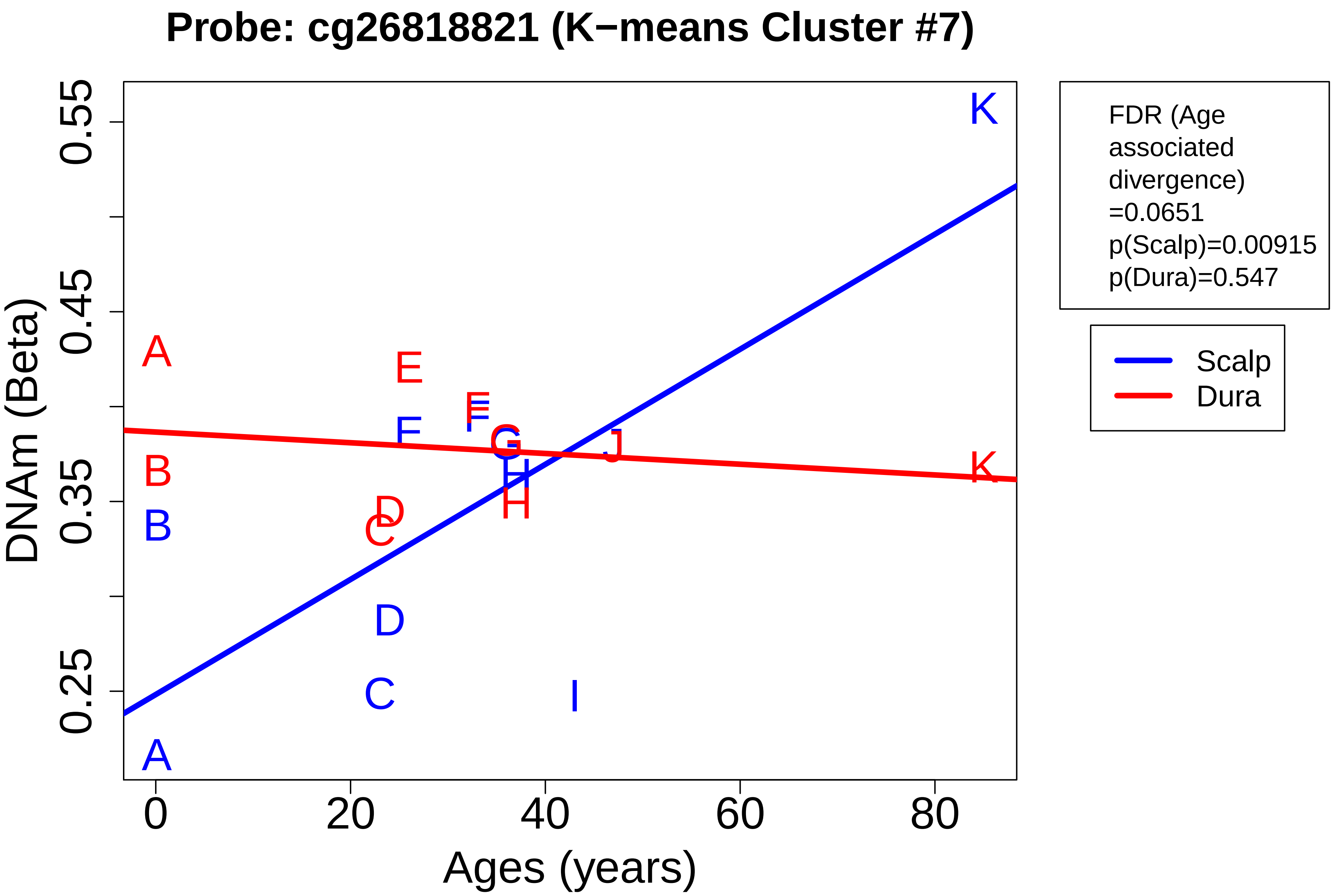
Probe: cg26818821 (K−means Cluster #7)
DNAm (Beta)
0.25
0.35
0.45
0.55
Ages (years)
0
20
40
60
80
A
B
C
D
E
F
G
H
I
J
K
FDR (Age associated divergence) =0.0651
p(Scalp)=0.00915
p(Dura)=0.547
Scalp
Dura

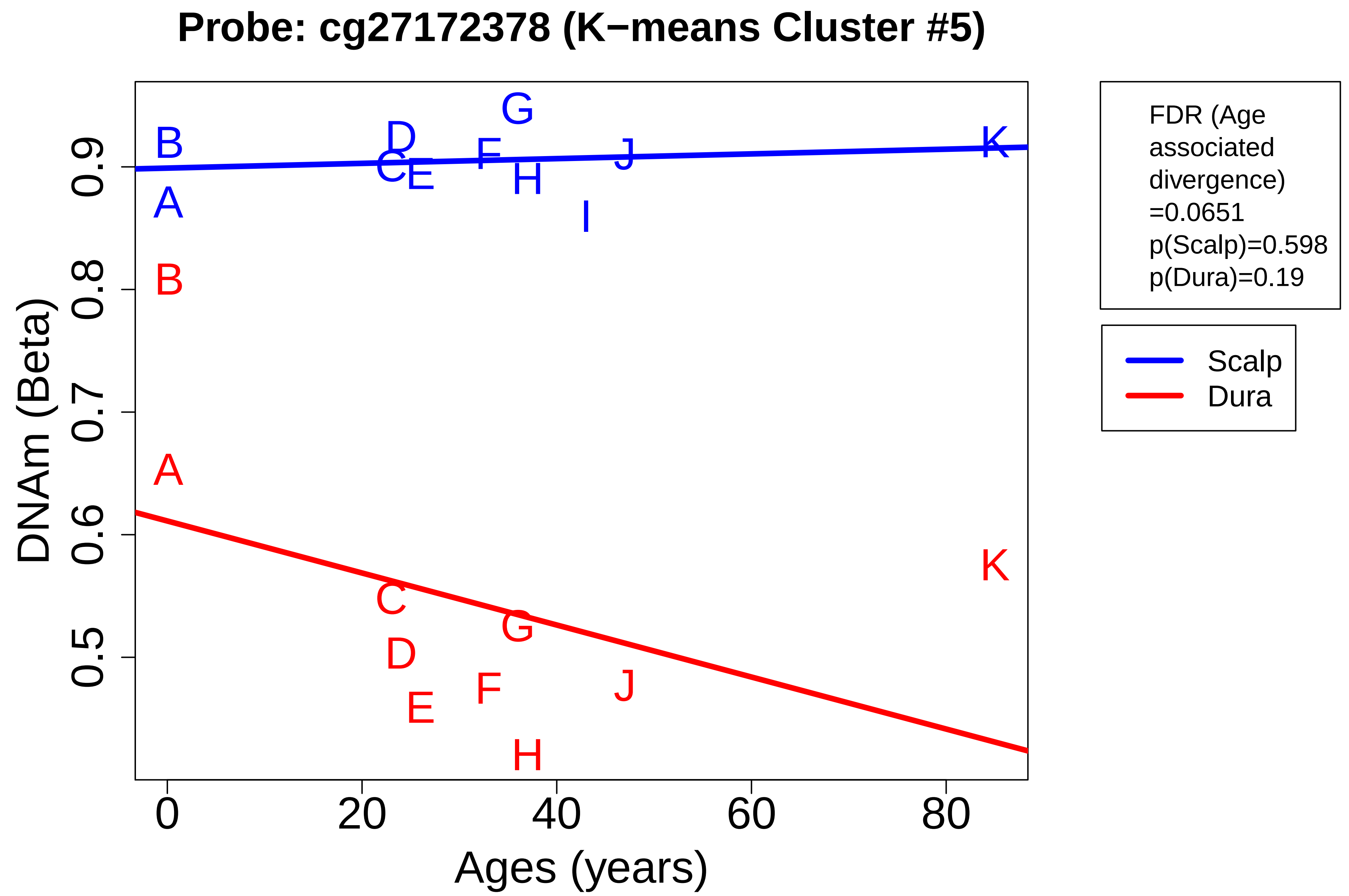

Probe: cg27172378 (K−means Cluster #5)
DNAm (Beta)
0.5
0.6
0.7
0.8
0.9
0
20
40
60
80
Ages (years)
B
A
C
D
E
F
G
H
I
J
K
FDR (Age associated divergence) =0.0651
p(Scalp)=0.598
p(Dura)=0.19
Scalp
Dura

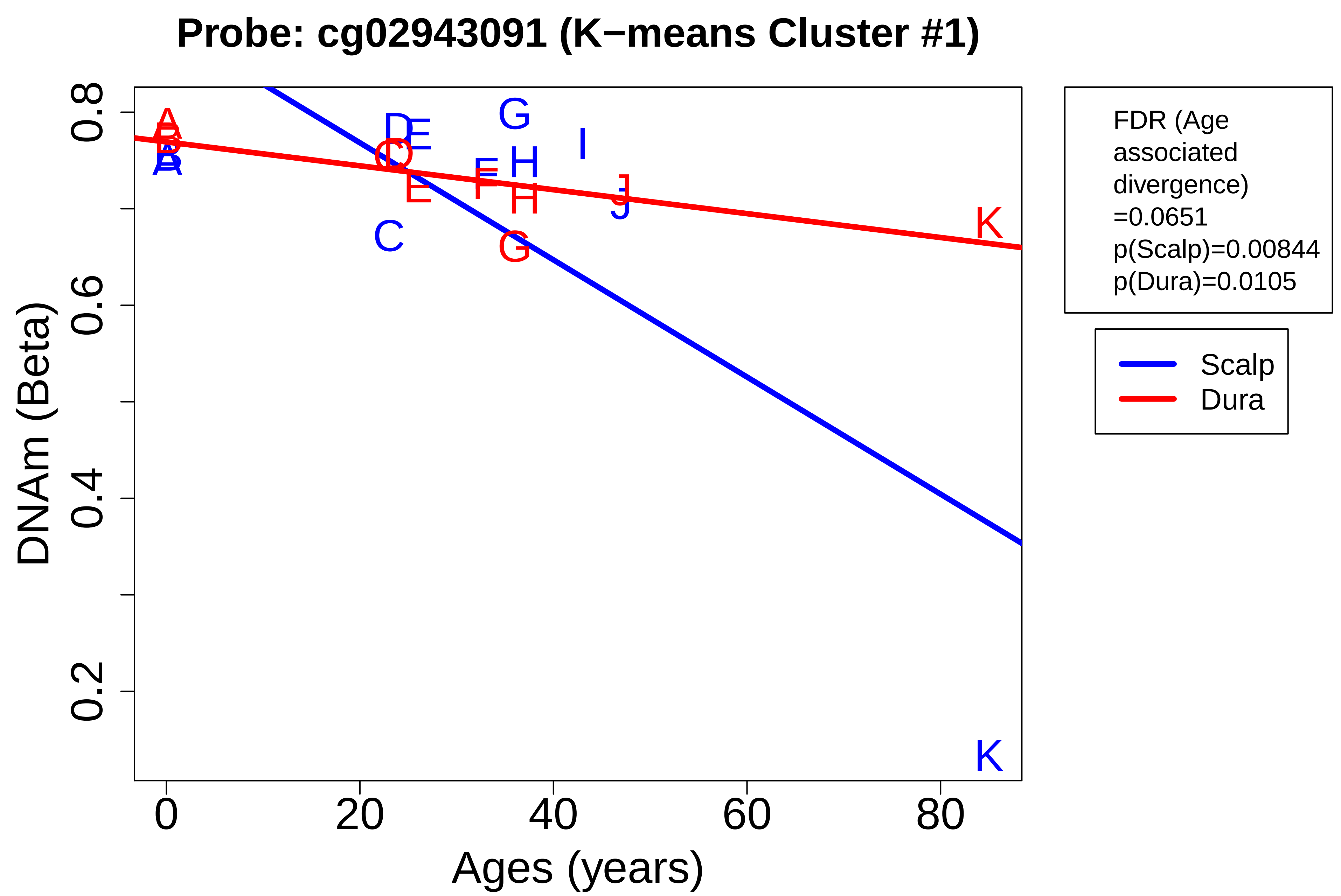

Probe: cg02943091 (K−means Cluster #1)
DNAm (Beta)
Ages (years)
0.2
0.4
0.6
0.8
0
20
40
60
80
FDR (Age associated divergence) =0.0651
p(Scalp)=0.00844
p(Dura)=0.0105
Scalp
Dura

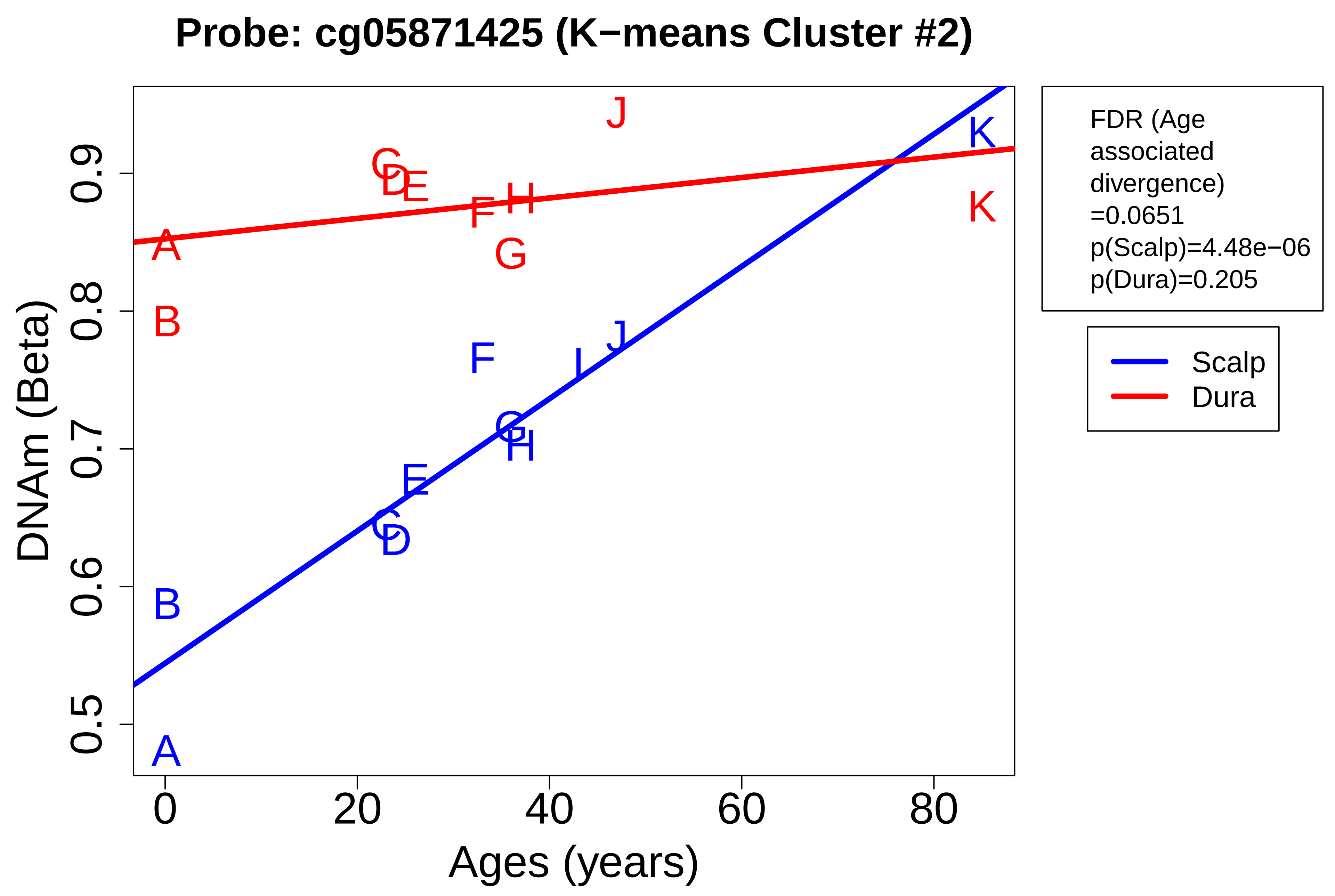

Probe: cg05871425 (K−means Cluster #2)
DNAm (Beta)
0.5
0.6
0.7
0.8
0.9
0
20
40
60
80
Ages (years)
A
B
C
D
E
F
G
H
I
J
K
FDR (Age associated divergence) =0.0651
p(Scalp)=4.48e−06
p(Dura)=0.205
Scalp
Dura

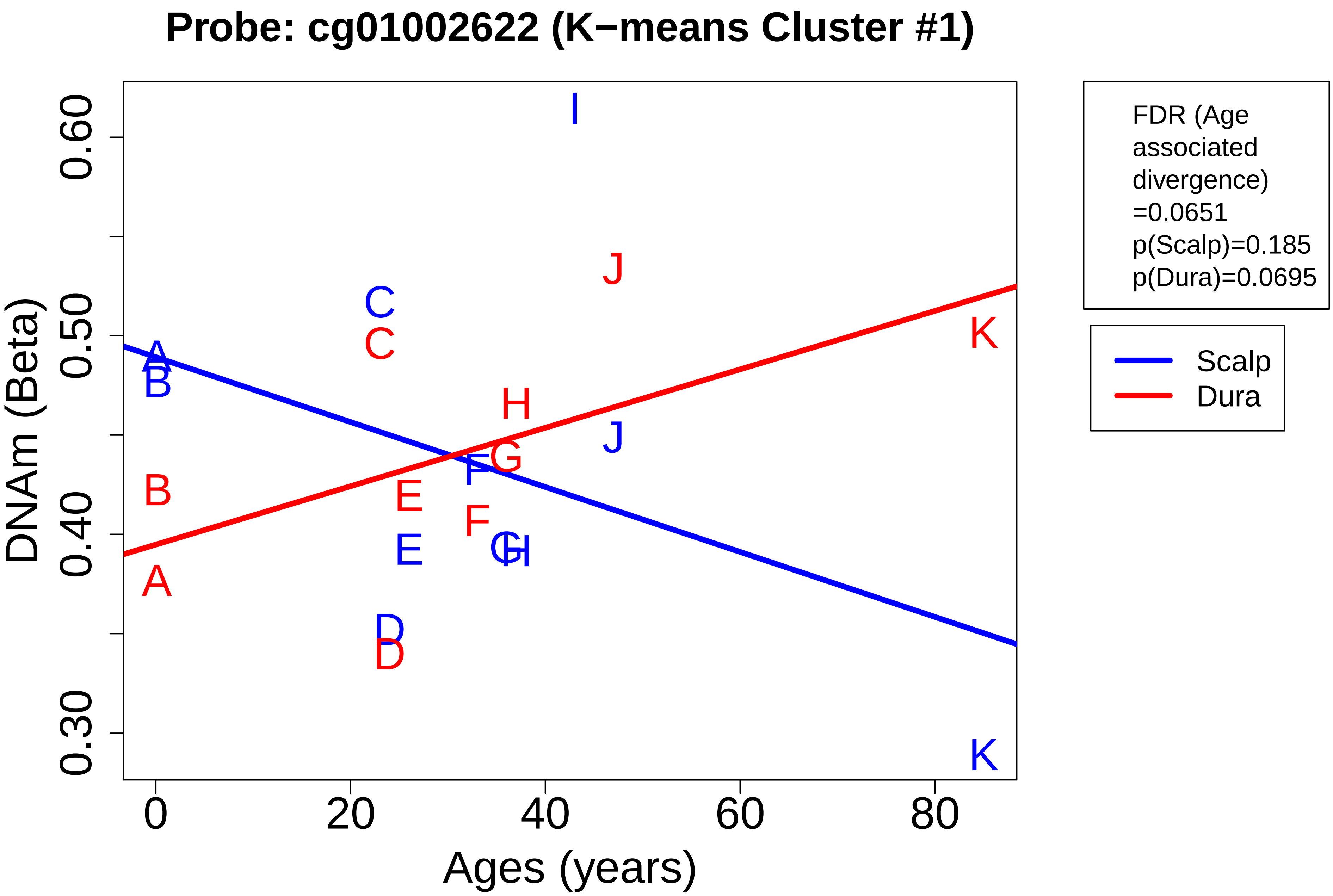

Probe: cg01002622 (K−means Cluster #1)
FDR (Age associated divergence) =0.0651
p(Scalp)=0.185
p(Dura)=0.0695
Scalp
Dura
DNAm (Beta)
0.30
0.40
0.50
0.60
0
20
40
60
80
Ages (years)
A
B
C
D
E
F
G
H
I
J
K

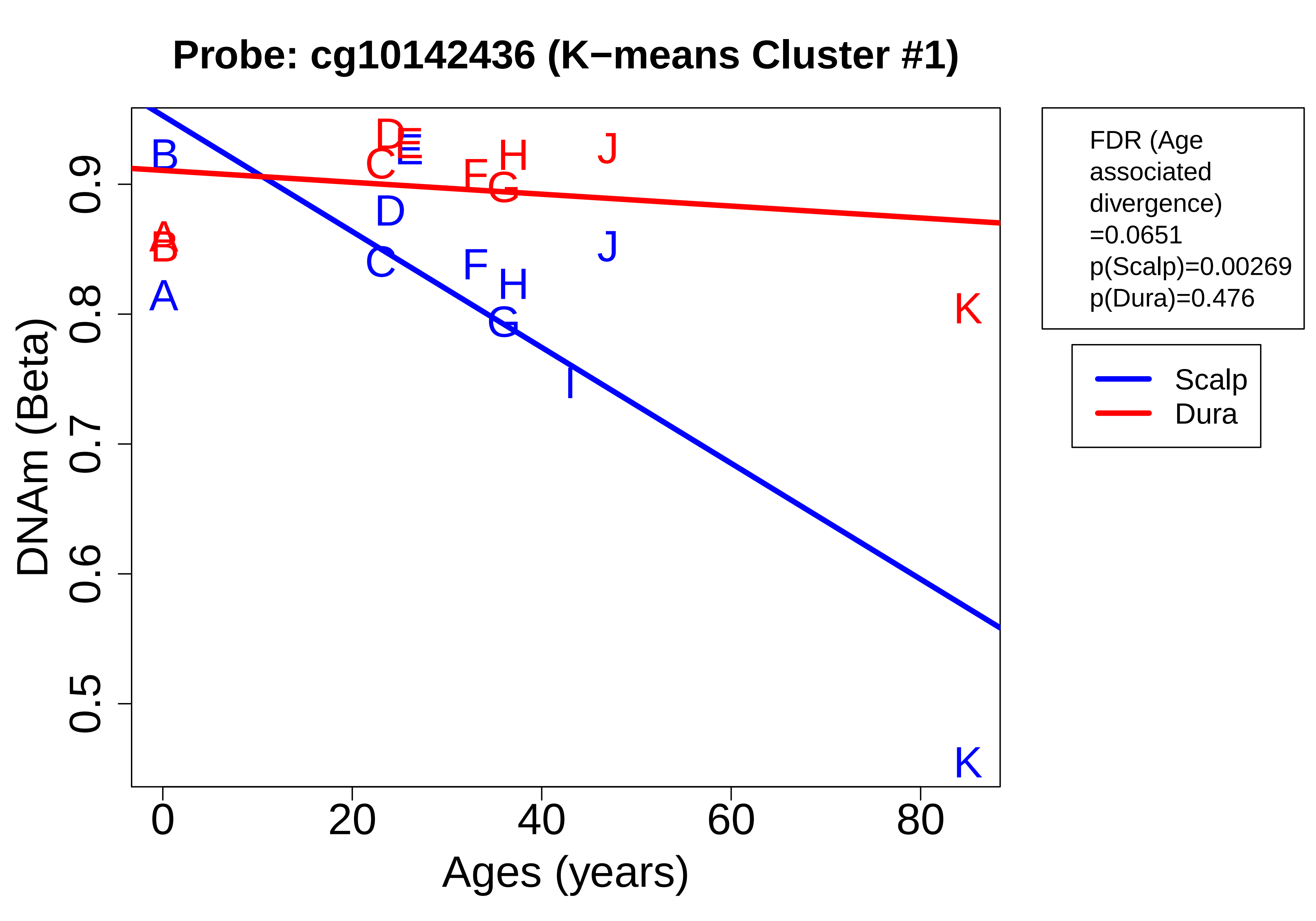
Probe: cg10142436 (K−means Cluster #1)
DNAm (Beta)
0.5
0.6
0.7
0.8
0.9
0
20
40
60
80
Ages (years)
A
B
C
D
E
F
G
H
I
J
K
FDR (Age associated divergence) =0.0651
p(Scalp)=0.00269
p(Dura)=0.476
Scalp
Dura

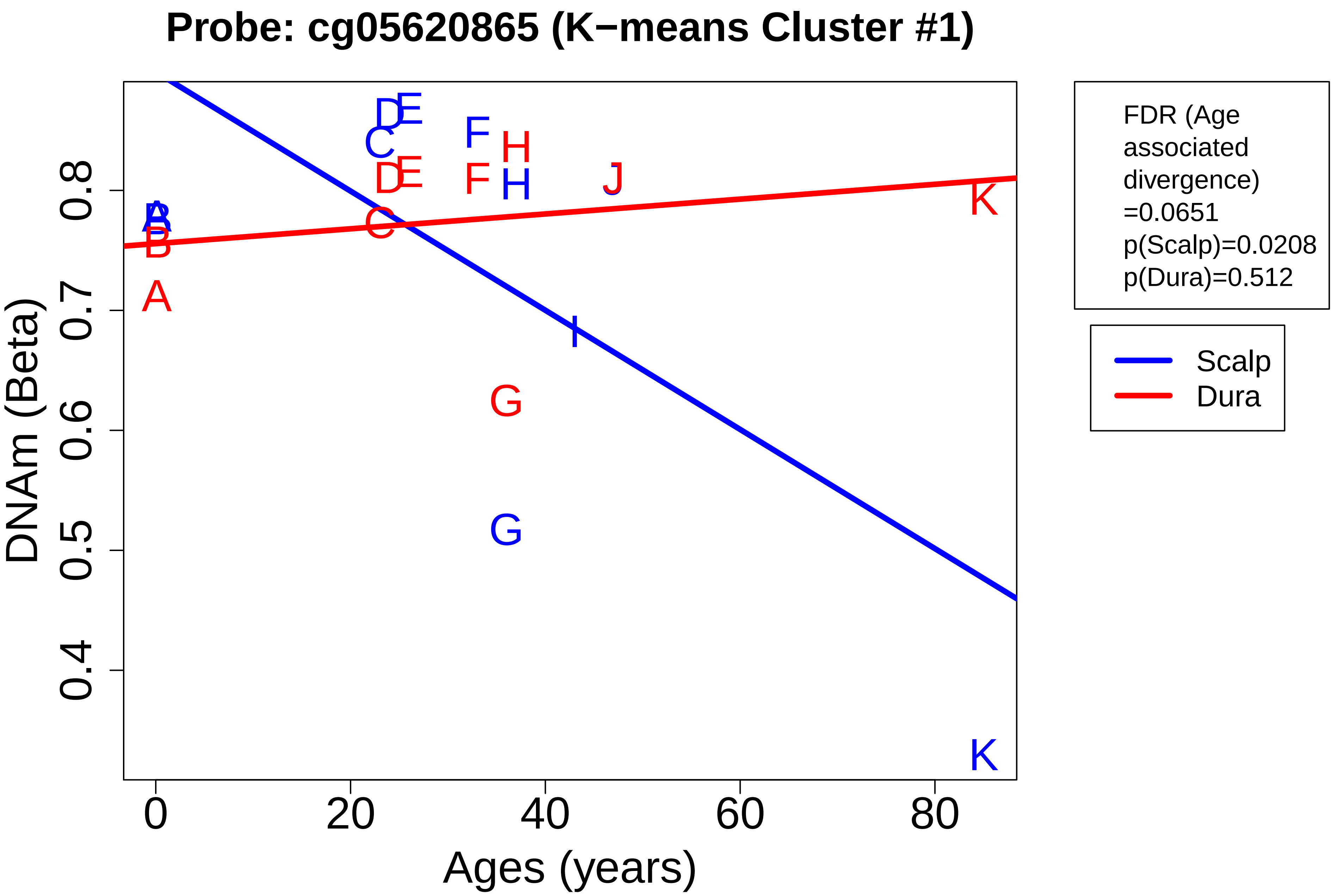

Probe: cg05620865 (K−means Cluster #1)
DNAm (Beta)
0.4
0.5
0.6
0.7
0.8
Ages (years)
0
20
40
60
80
FDR (Age associated divergence) =0.0651
p(Scalp)=0.0208
p(Dura)=0.512
Scalp
Dura
A
B
C
D
E
F
G
H
I
J
K

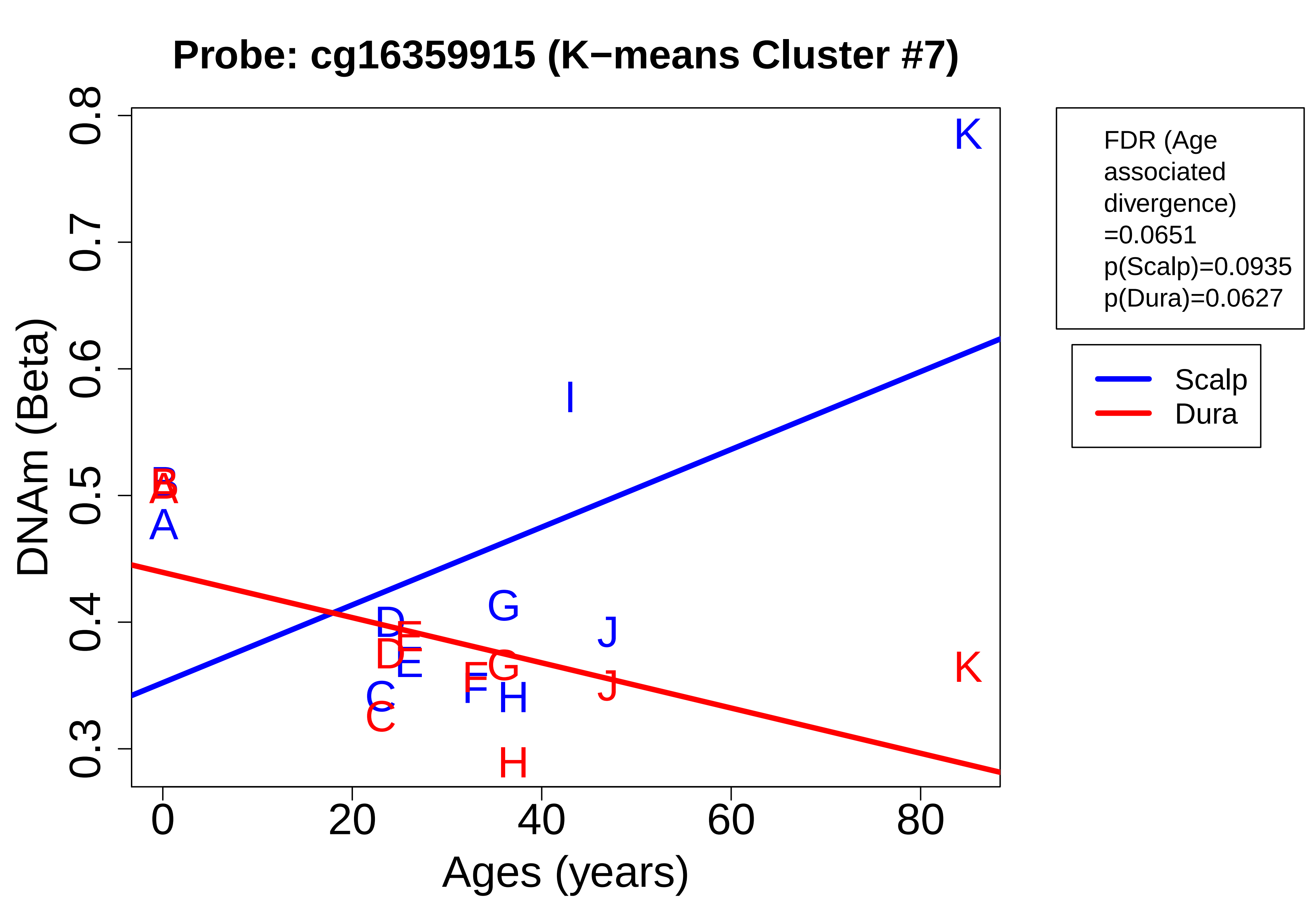

Probe: cg16359915 (K−means Cluster #7)
DNAm (Beta)
Ages (years)
0.3
0.4
0.5
0.6
0.7
0.8
0
20
40
60
80
FDR (Age associated divergence) =0.0651
p(Scalp)=0.0935
p(Dura)=0.0627
Scalp
Dura
A
B
C
D
E
F
G
H
I
J
K

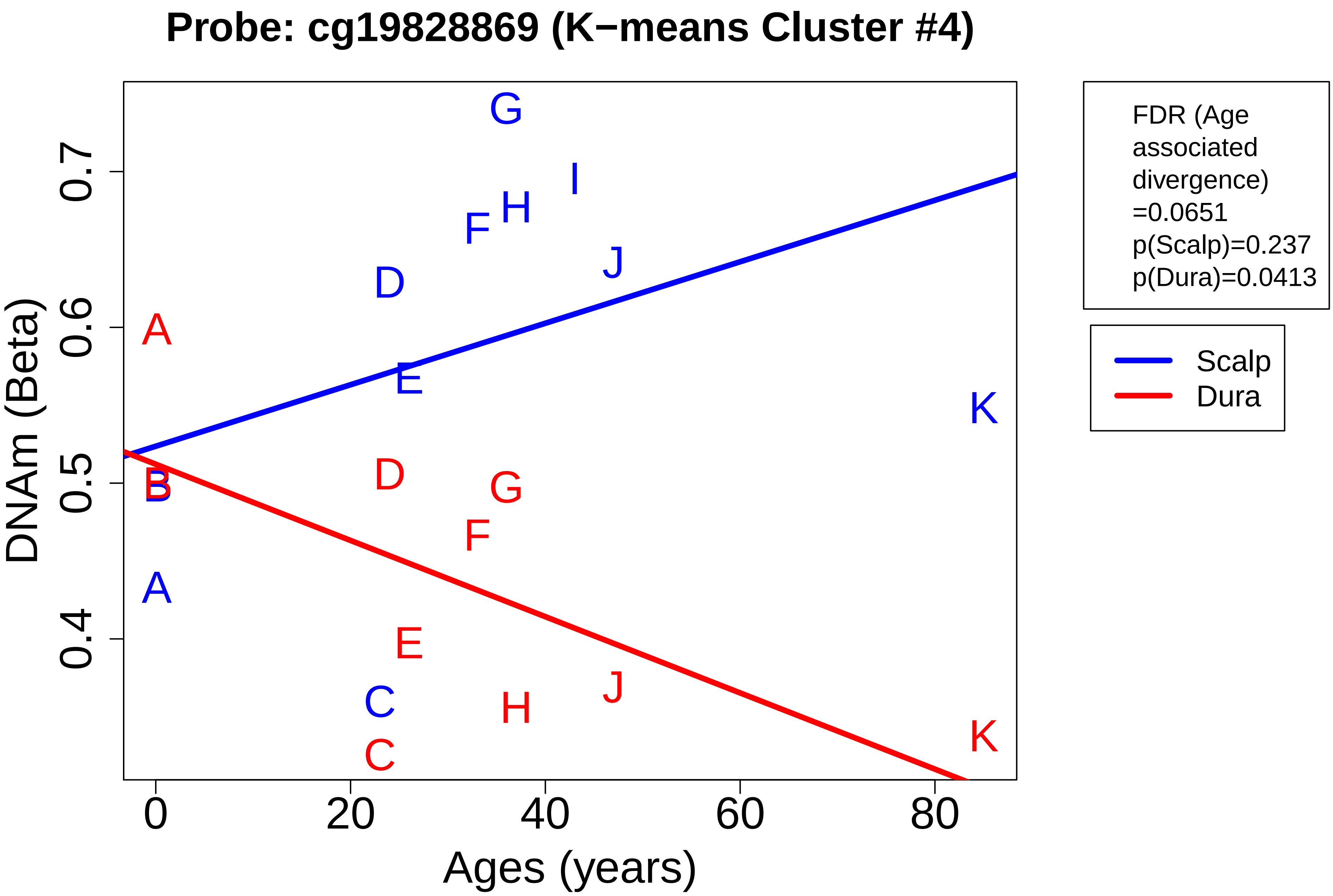
Probe: cg19828869 (K−means Cluster #4)
DNAm (Beta)
0.4
0.5
0.6
0.7
Ages (years)
0
20
40
60
80
FDR (Age associated divergence) =0.0651
p(Scalp)=0.237
p(Dura)=0.0413
Scalp
Dura
A
B
C
D
E
F
G
H
I
J
K

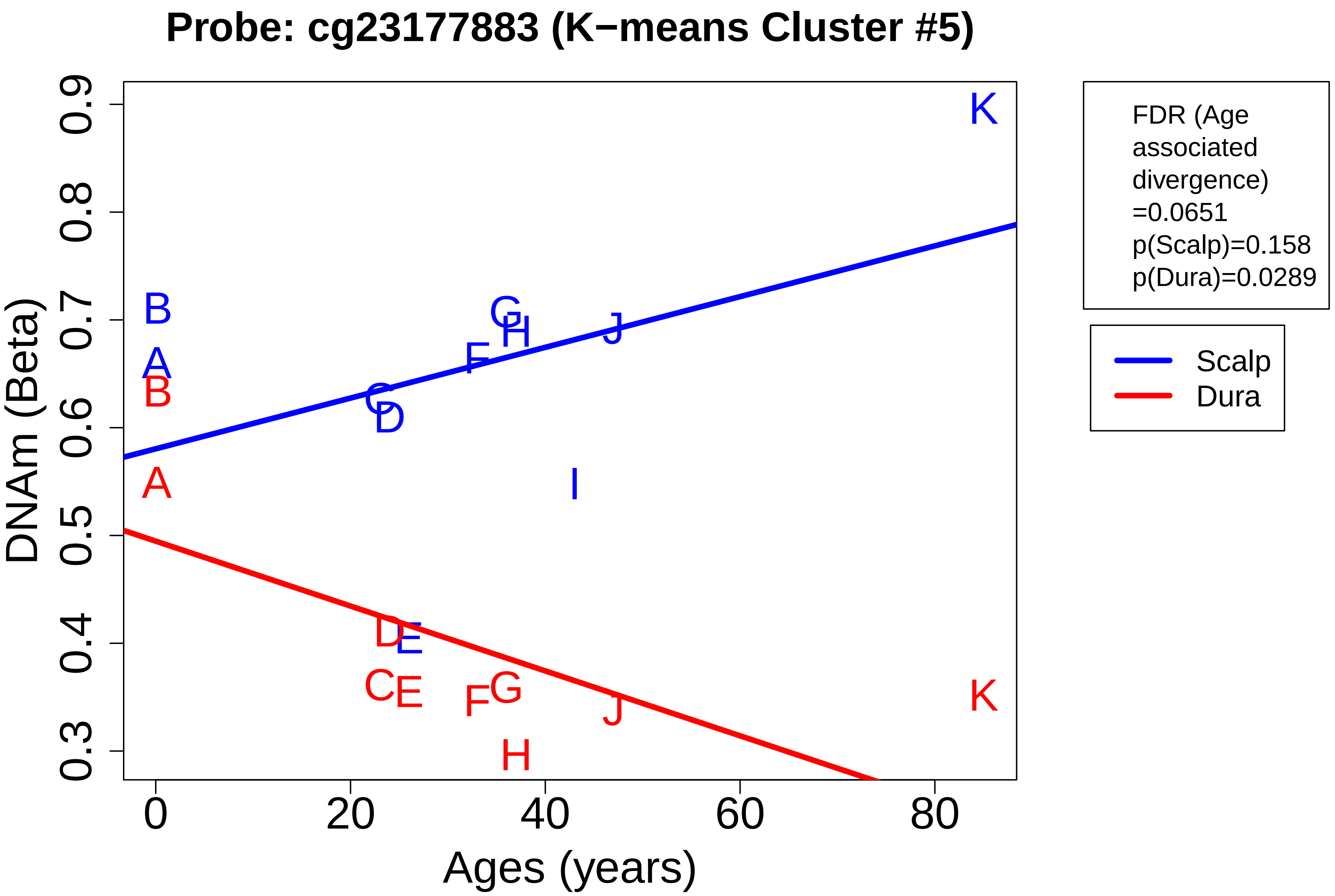

Probe: cg23177883 (K−means Cluster #5)
DNAm (Beta)
0.3
0.4
0.5
0.6
0.7
0.8
0.9
0
20
40
60
80
Ages (years)
FDR (Age associated divergence) =0.0651
p(Scalp)=0.158
p(Dura)=0.0289
Scalp
Dura
A
B
C
D
E
F
G
H
I
J
K

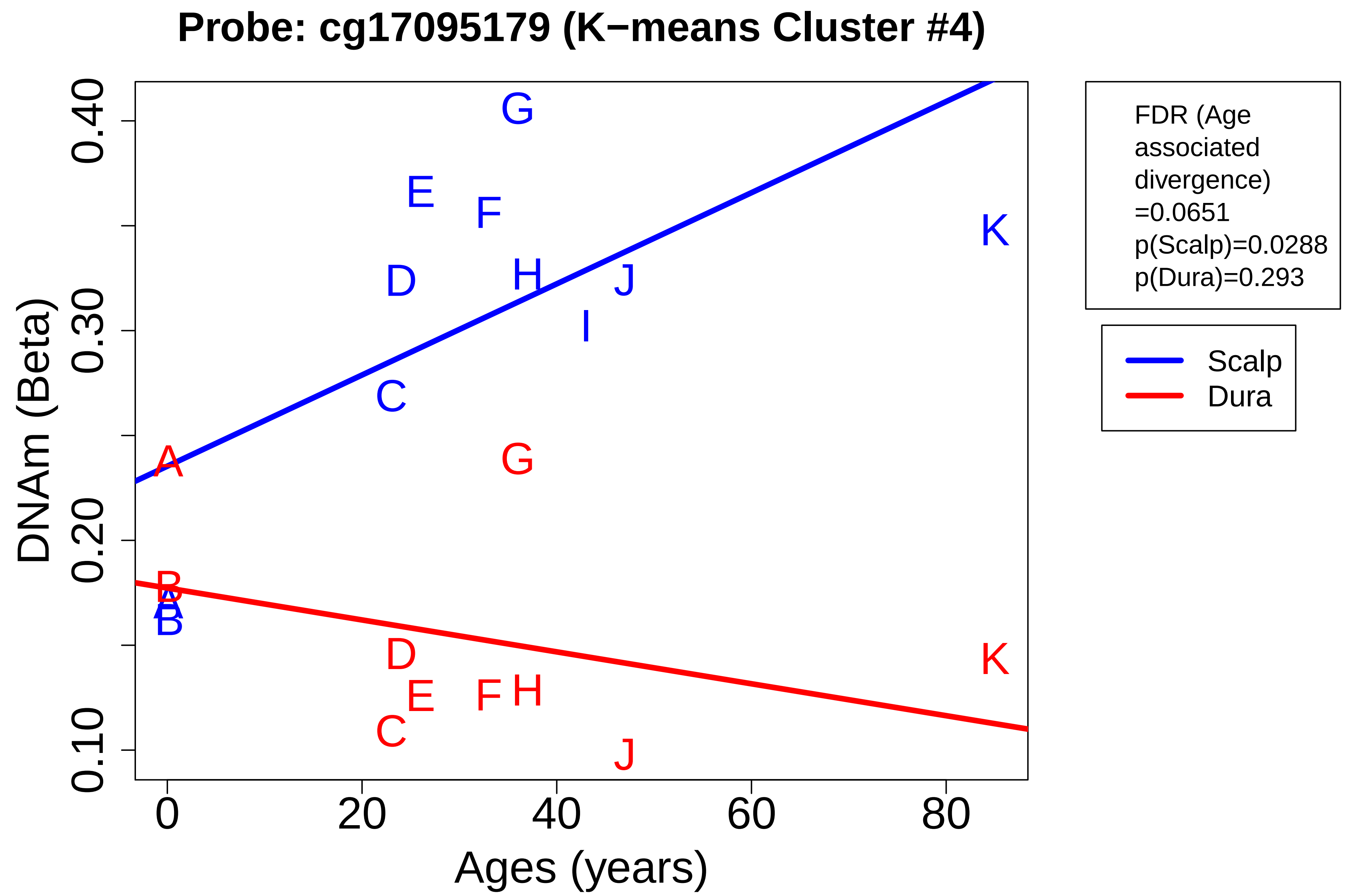

Probe: cg17095179 (K−means Cluster #4)
DNAm (Beta)
0.10
0.20
0.30
0.40
Ages (years)
0
20
40
60
80
FDR (Age associated divergence) =0.0651
p(Scalp)=0.0288
p(Dura)=0.293
Scalp
Dura

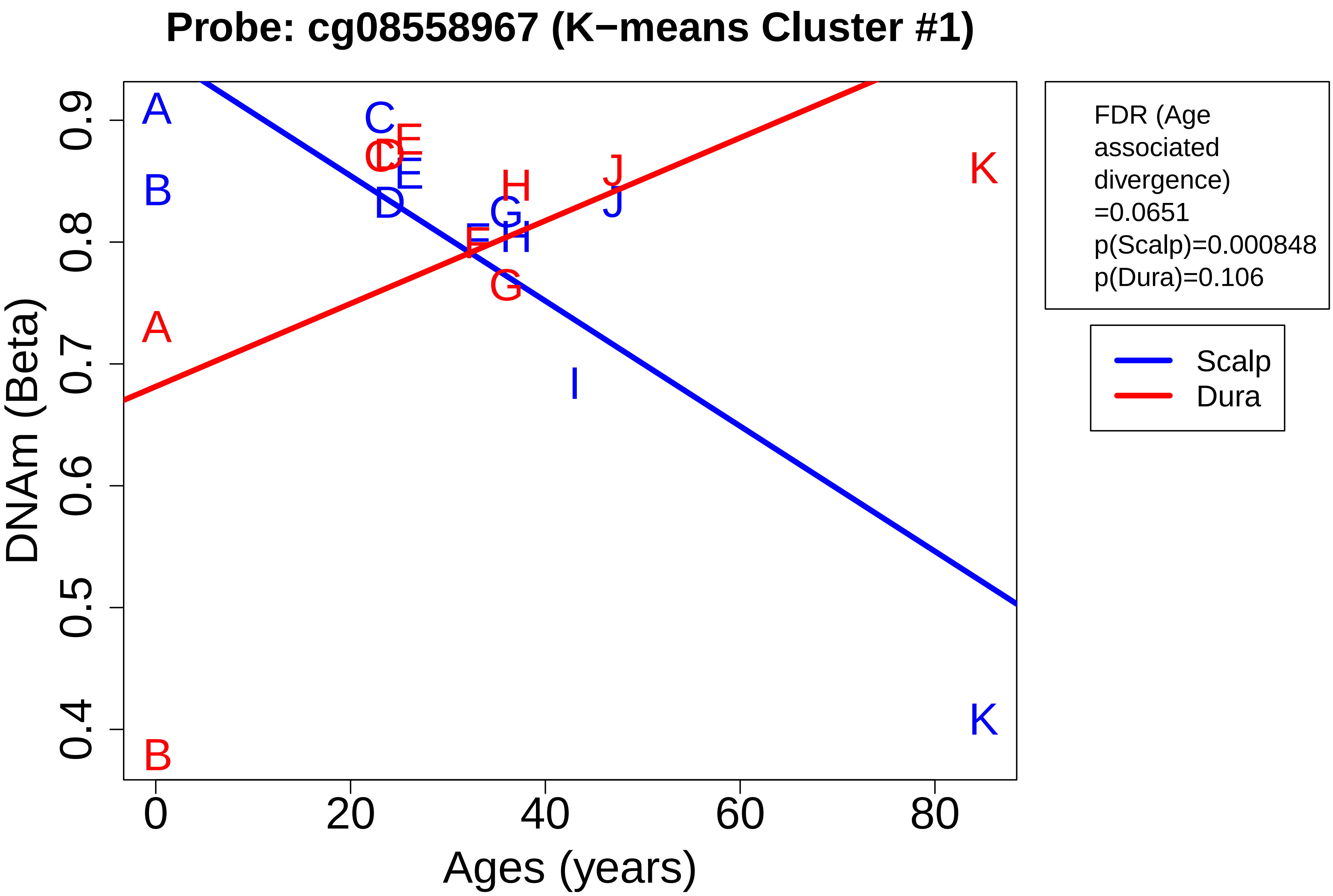

Probe: cg08558967 (K−means Cluster #1)
DNAm (Beta)
0.4
0.5
0.6
0.7
0.8
0.9
0
20
40
60
80
Ages (years)
A
B
C
D
E
F
G
H
I
J
K
FDR (Age associated divergence) =0.0651
p(Scalp)=0.000848
p(Dura)=0.106
Scalp
Dura

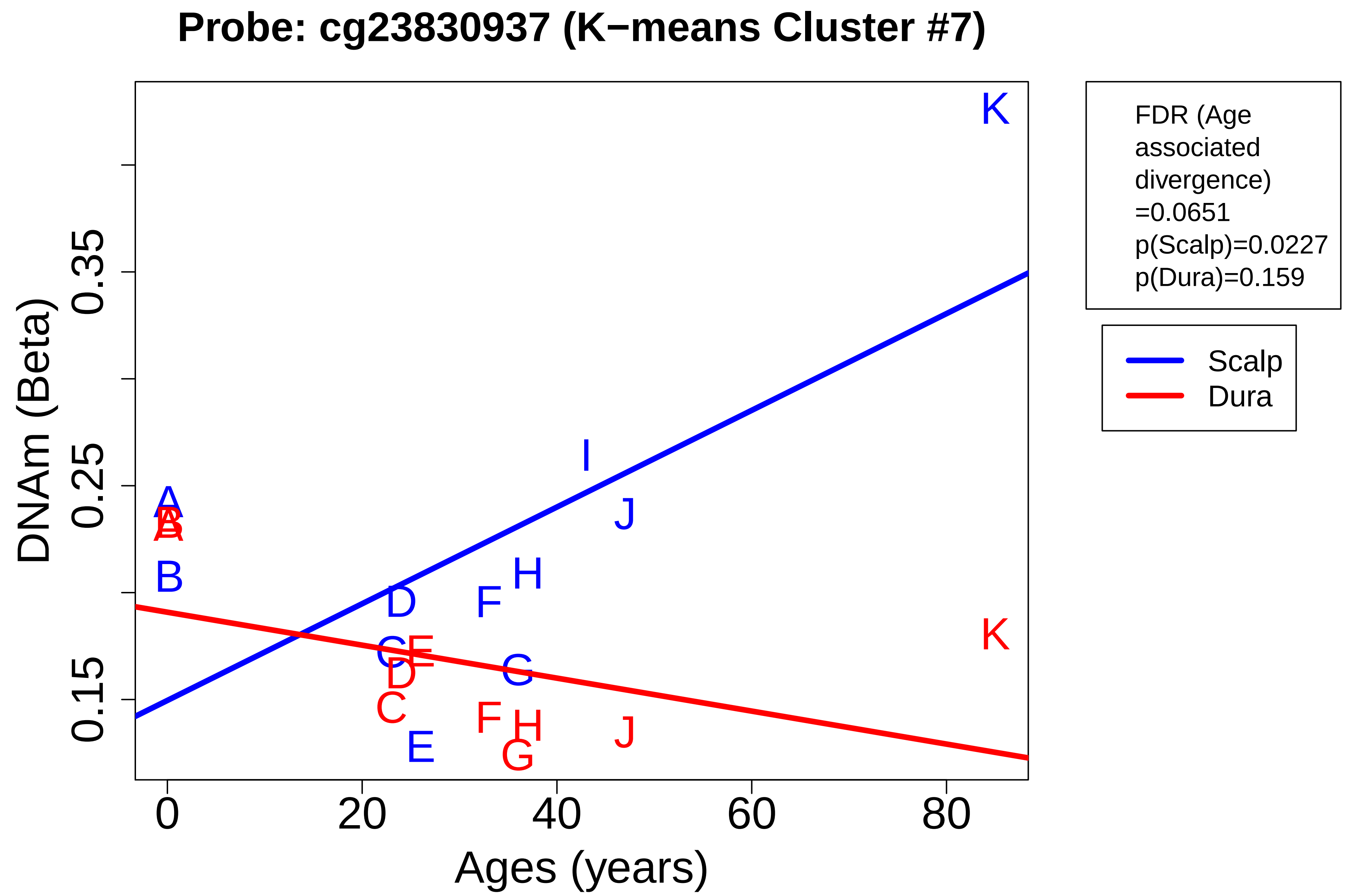
Probe: cg23830937 (K−means Cluster #7)
FDR (Age associated divergence) =0.0651
p(Scalp)=0.0227
p(Dura)=0.159
Scalp
Dura
DNAm (Beta)
0.15
0.25
0.35
0
20
40
60
80
Ages (years)

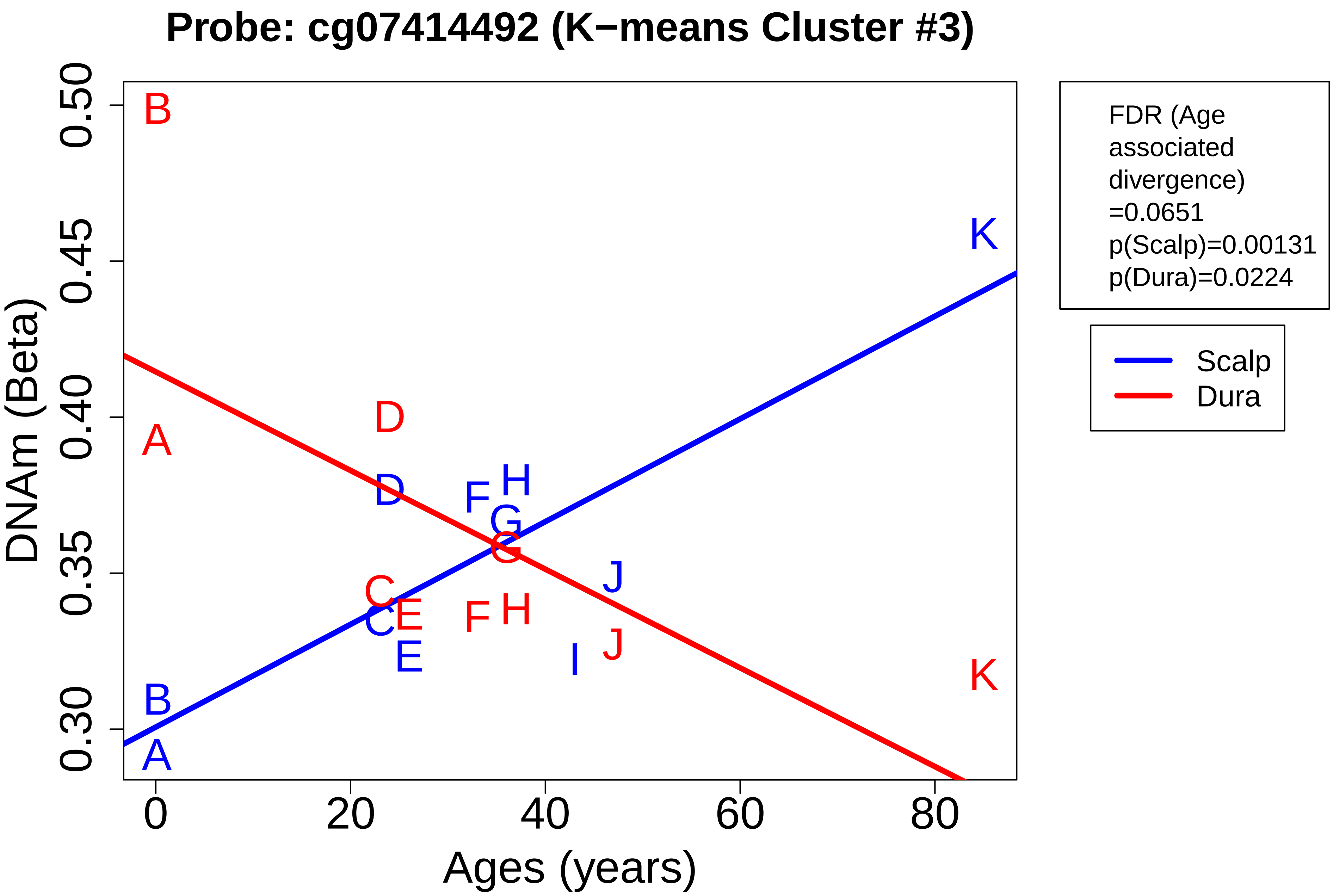

Probe: cg07414492 (K−means Cluster #3)
DNAm (Beta)
0.30
0.35
0.40
0.45
0.50
Ages (years)
0
20
40
60
80
A
B
C
D
E
F
G
H
I
J
K
FDR (Age associated divergence) =0.0651
p(Scalp)=0.00131
p(Dura)=0.0224
Scalp
Dura

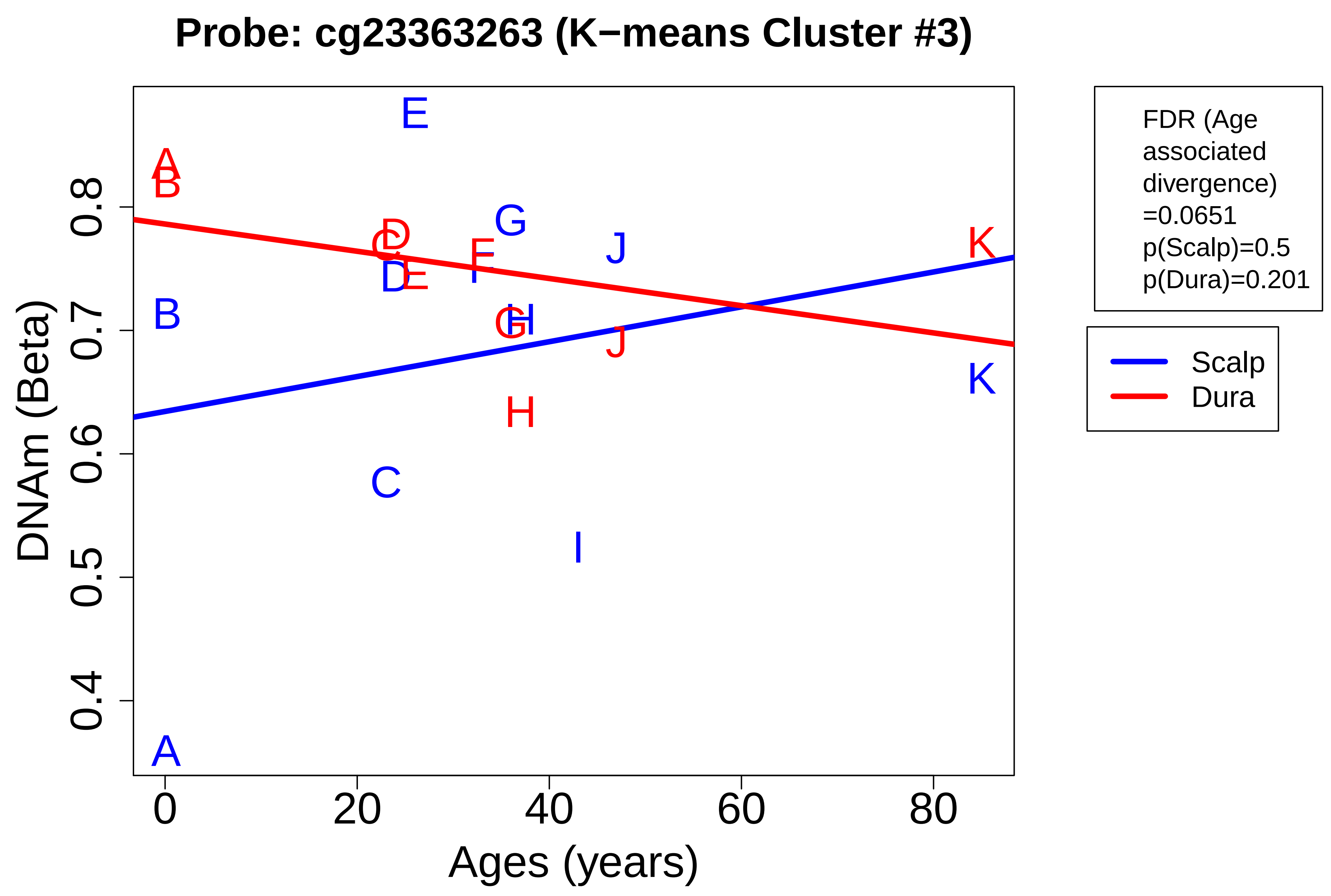
Probe: cg23363263 (K−means Cluster #3)
DNAm (Beta)
0.4
0.5
0.6
0.7
0.8
0
20
40
60
80
Ages (years)
FDR (Age associated divergence) =0.0651
p(Scalp)=0.5
p(Dura)=0.201
Scalp
Dura

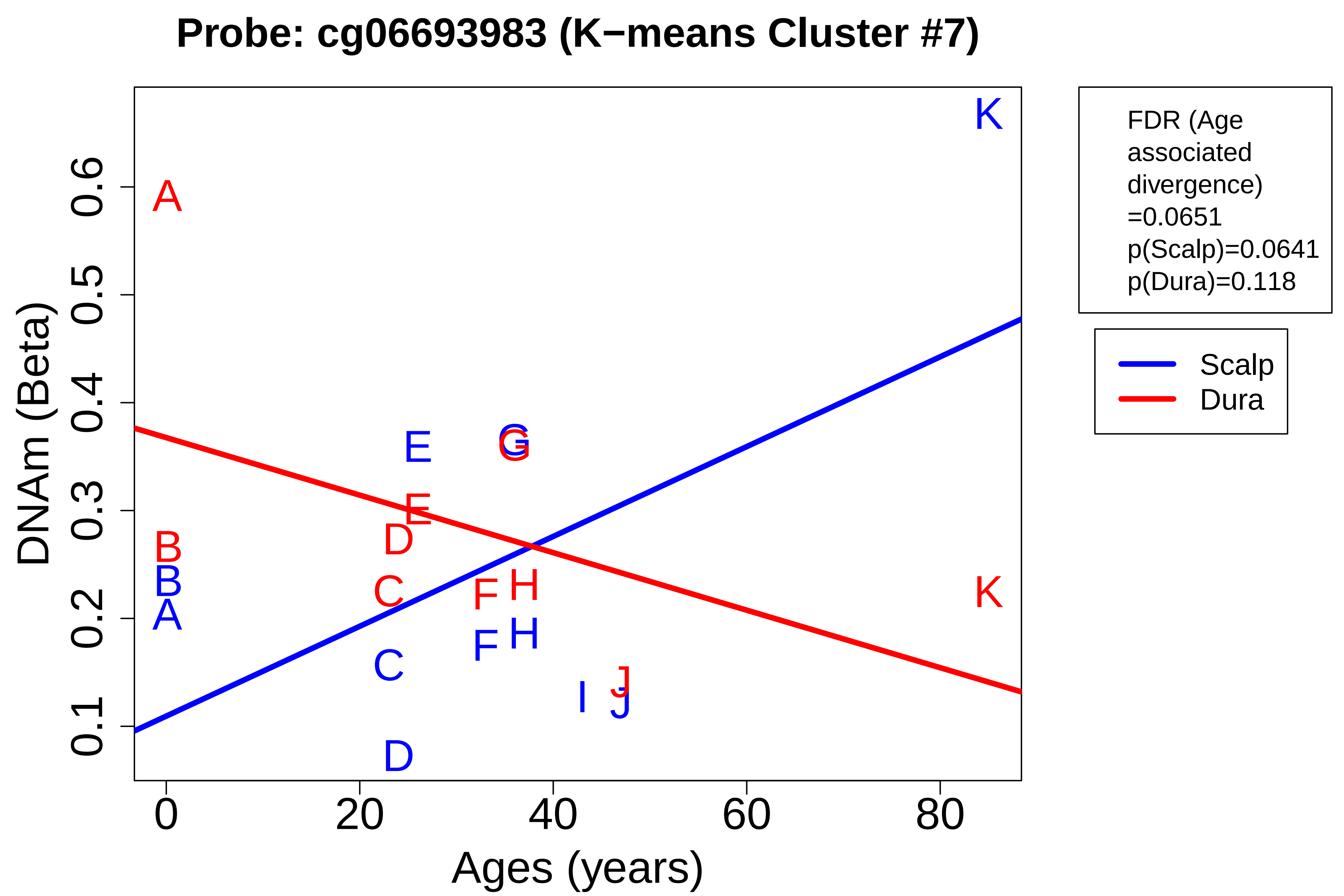

Probe: cg06693983 (K−means Cluster #7)
DNAm (Beta)
0.1
0.2
0.3
0.4
0.5
0.6
Ages (years)
0
20
40
60
80
FDR (Age associated divergence) =0.0651
p(Scalp)=0.0641
p(Dura)=0.118
Scalp
Dura
A
B
C
D
E
F
G
H
I
J
K

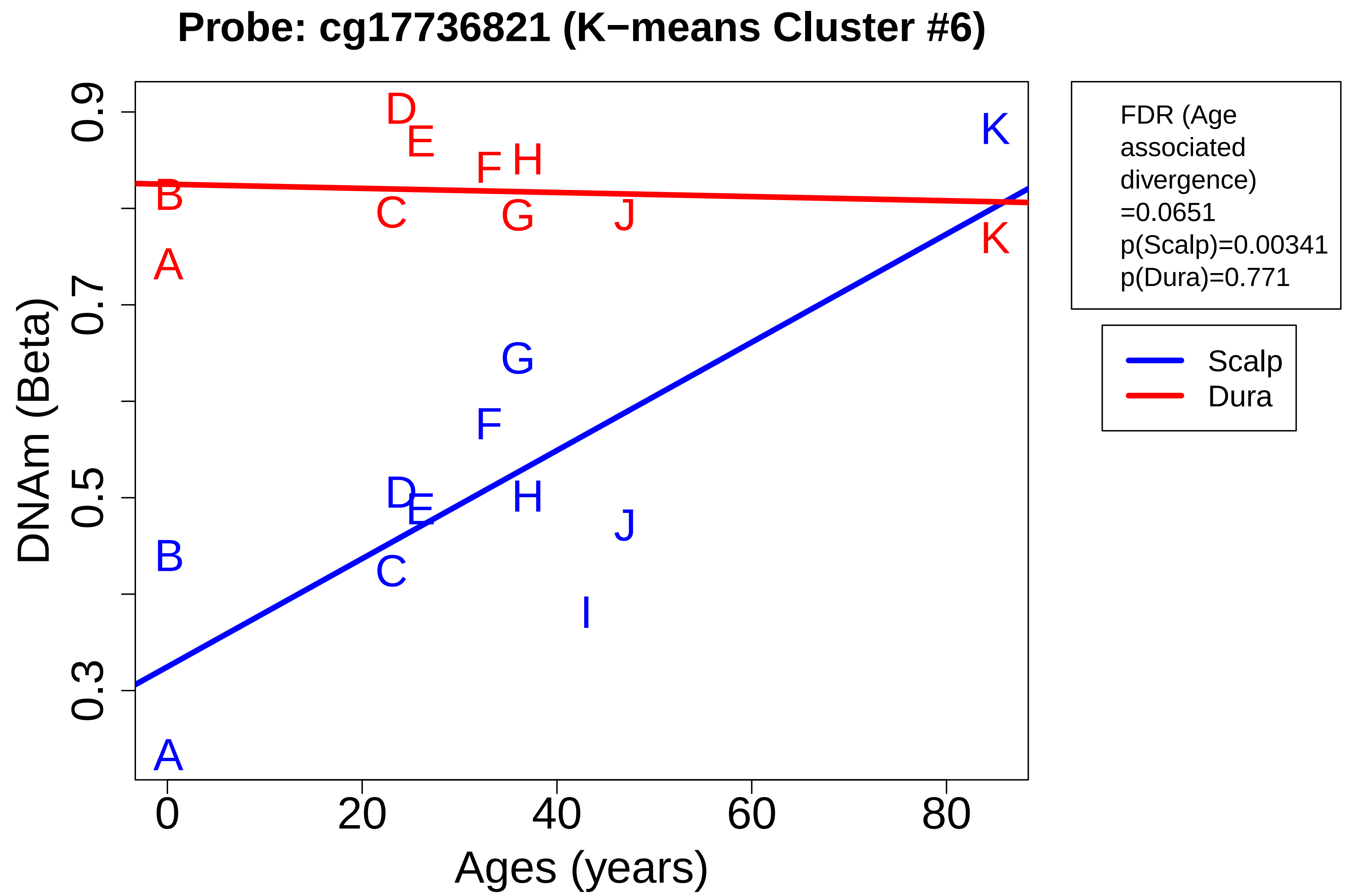

Probe: cg17736821 (K−means Cluster #6)
DNAm (Beta)
0.3
0.5
0.7
0.9
0
20
40
60
80
Ages (years)
A
B
C
D
E
F
G
H
I
J
K
FDR (Age associated divergence) =0.0651
p(Scalp)=0.00341
p(Dura)=0.771
Scalp
Dura

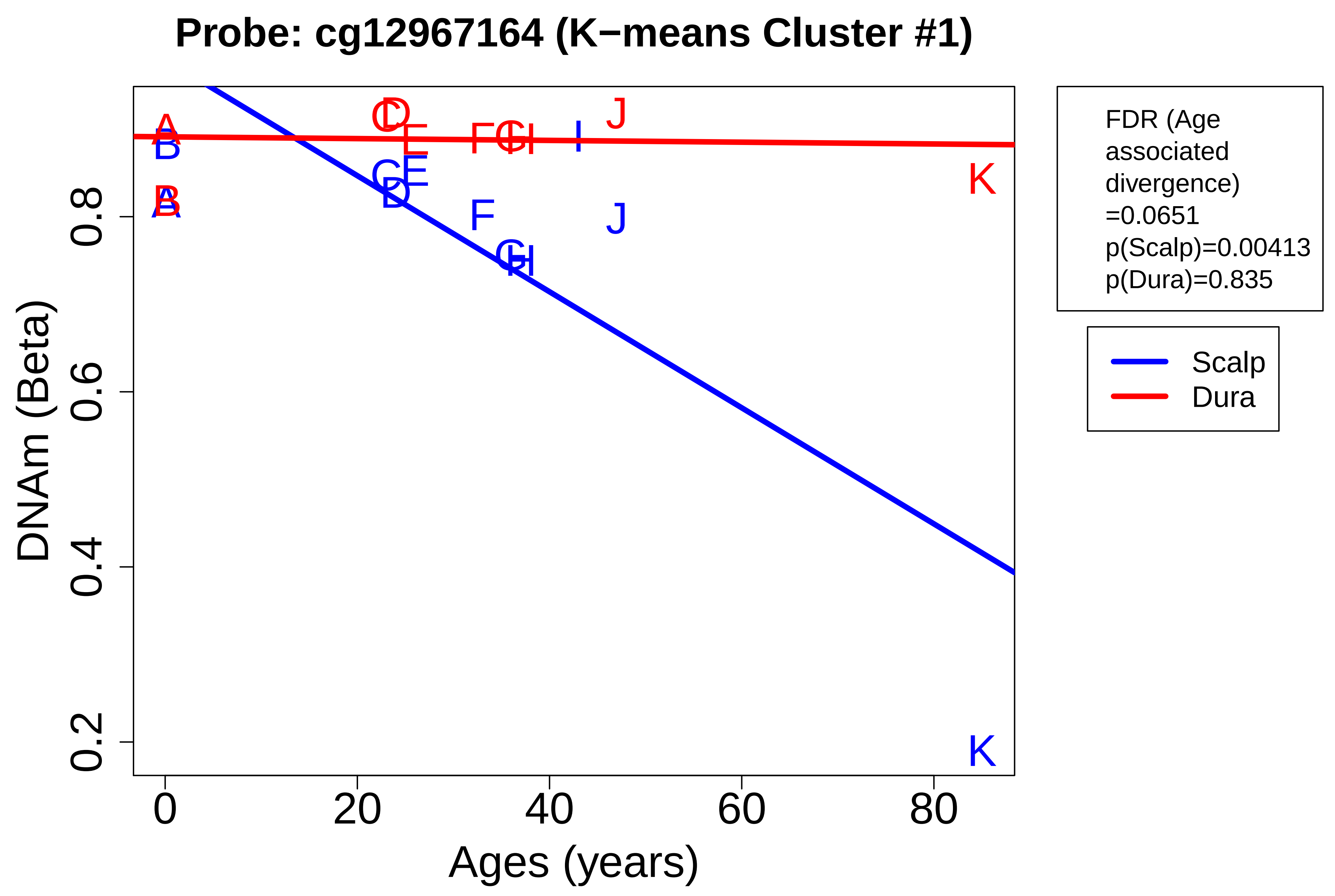

Probe: cg12967164 (K−means Cluster #1)
DNAm (Beta)
0.2
0.4
0.6
0.8
0
20
40
60
80
Ages (years)
A
B
C
D
E
F
G
H
I
J
K
FDR (Age associated divergence) =0.0651
p(Scalp)=0.00413
p(Dura)=0.835
Scalp
Dura

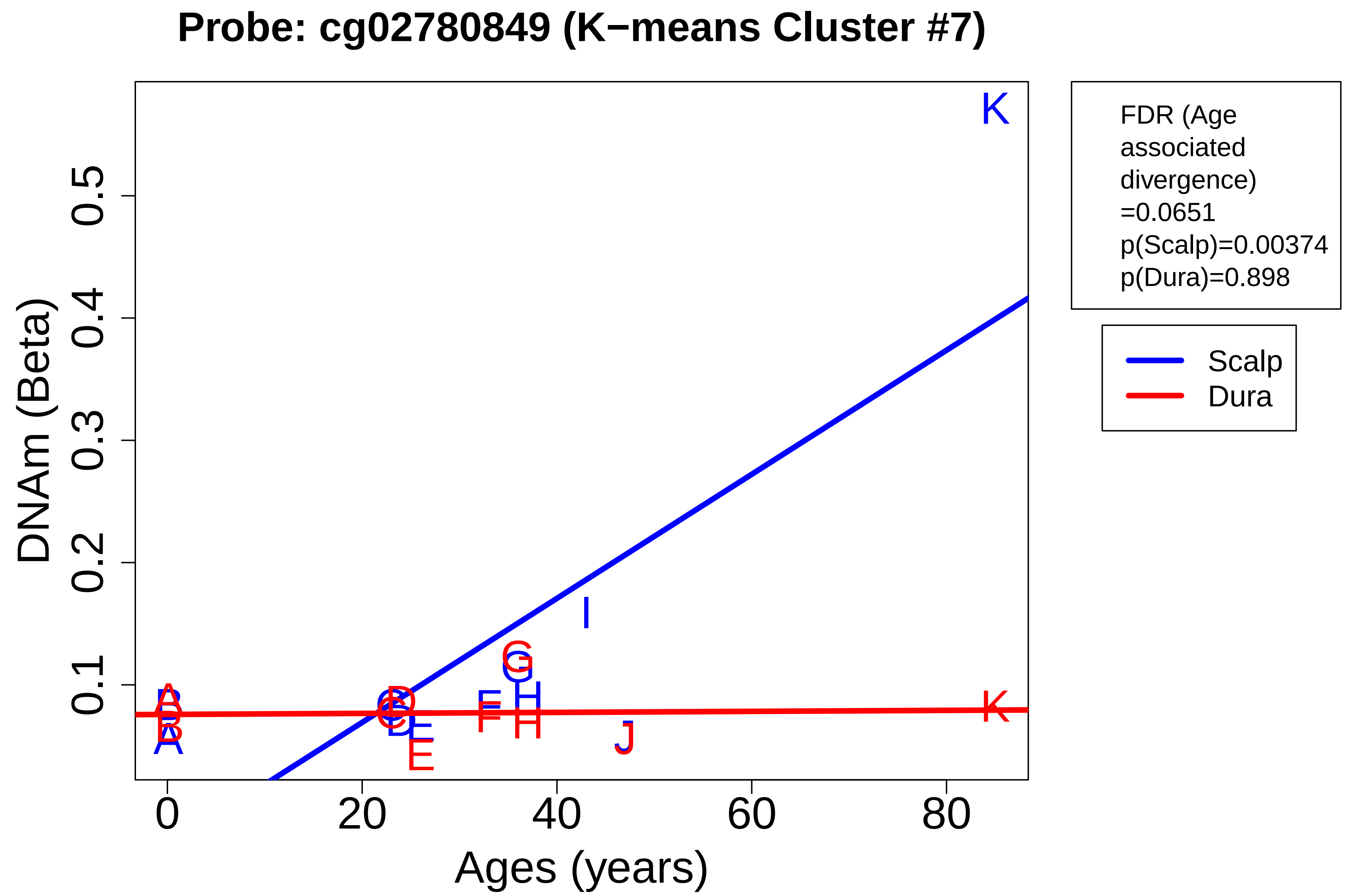

Probe: cg02780849 (K−means Cluster #7)
DNAm (Beta)
0.1
0.2
0.3
0.4
0.5
Ages (years)
0
20
40
60
80
FDR (Age associated divergence) =0.0651
p(Scalp)=0.00374
p(Dura)=0.898
Scalp
Dura

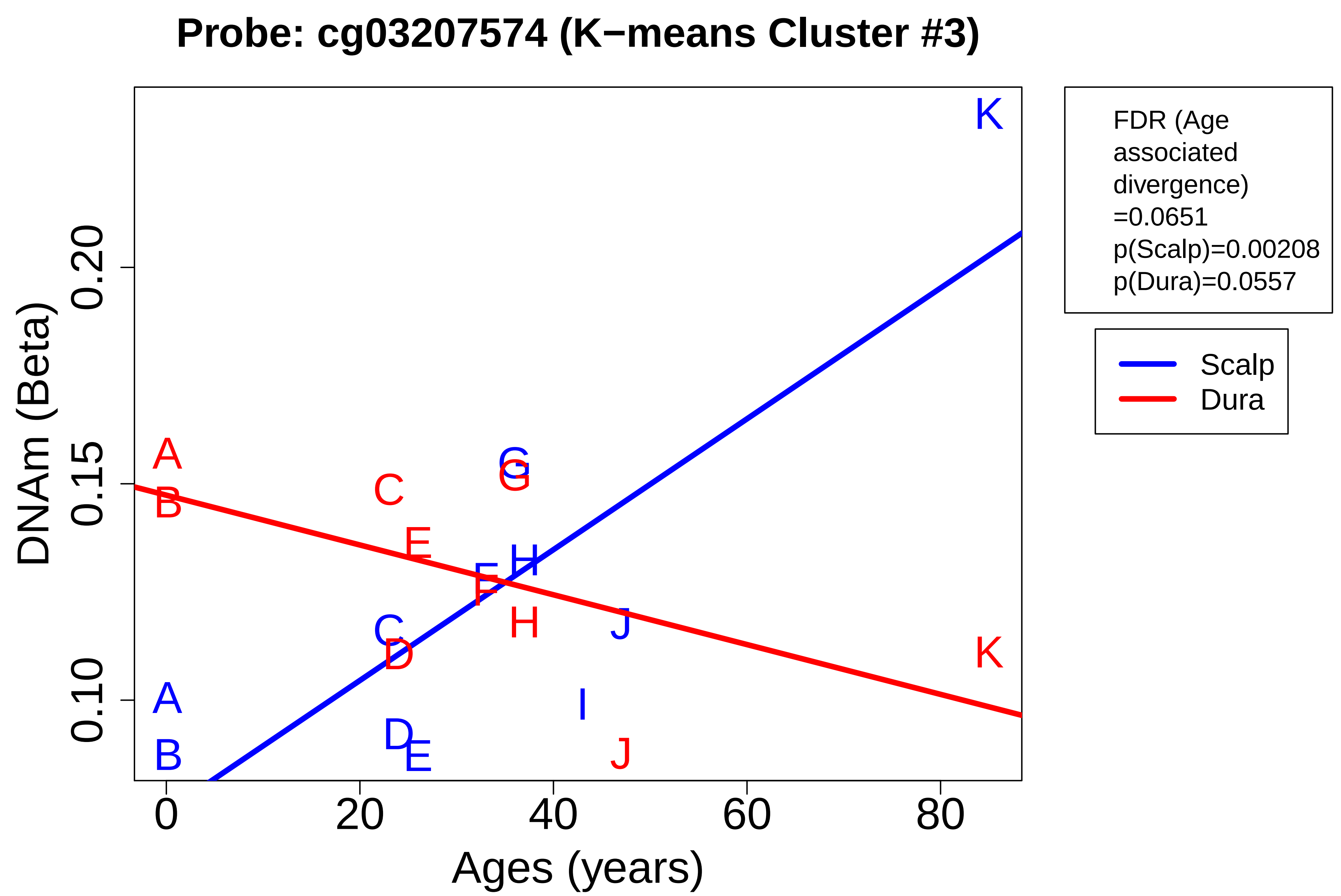

Probe: cg03207574 (K−means Cluster #3)
DNAm (Beta)
0.10
0.15
0.20
Ages (years)
0
20
40
60
80
FDR (Age associated divergence) =0.0651
p(Scalp)=0.00208
p(Dura)=0.0557
Scalp
Dura
A
B
C
D
E
F
G
H
I
J
K

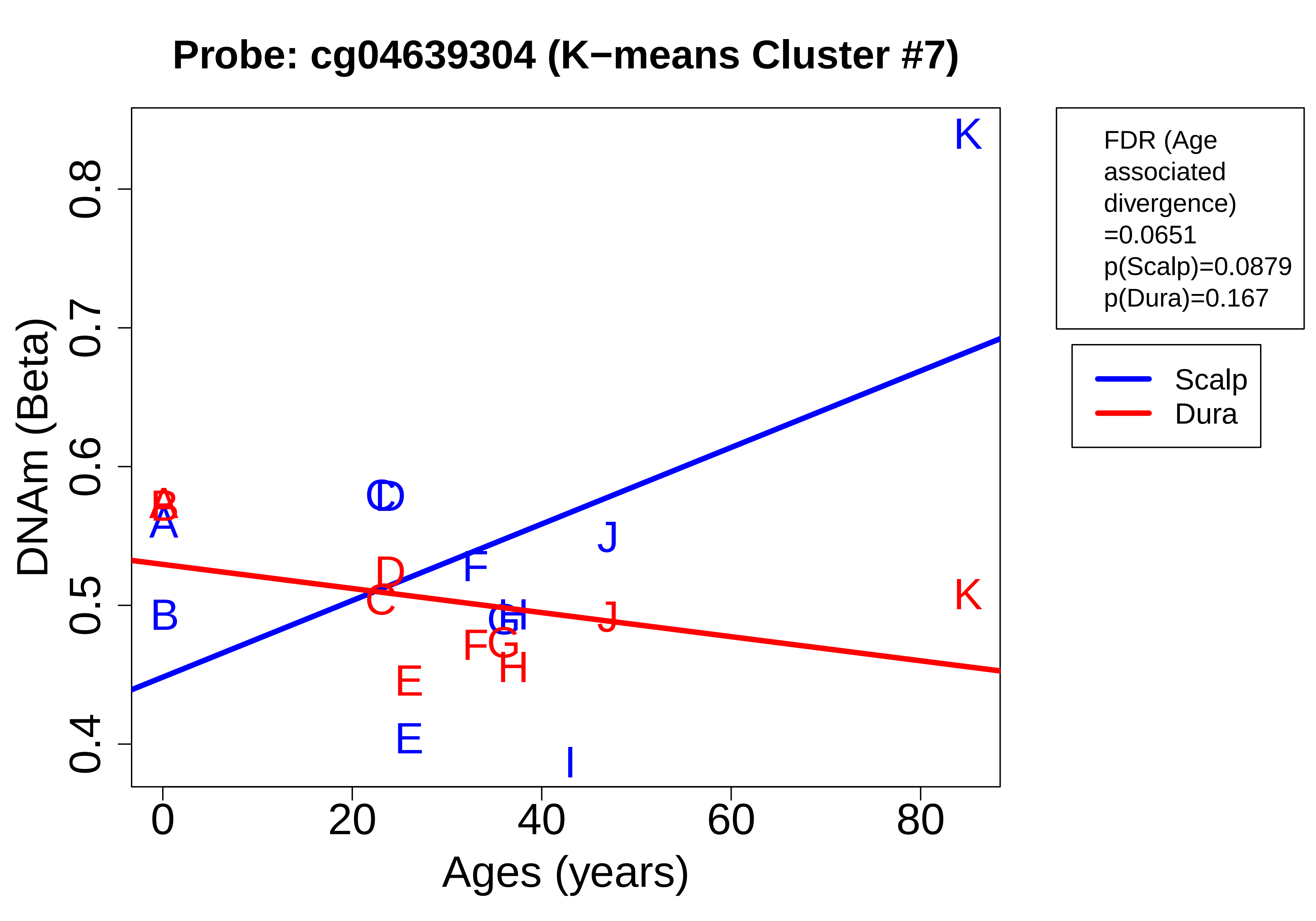
Probe: cg04639304 (K−means Cluster #7)
DNAm (Beta)
0.4
0.5
0.6
0.7
0.8
Ages (years)
0
20
40
60
80
FDR (Age associated divergence) =0.0651
p(Scalp)=0.0879
p(Dura)=0.167
Scalp
Dura
A
B
C
D
E
F
G
H
I
J
K

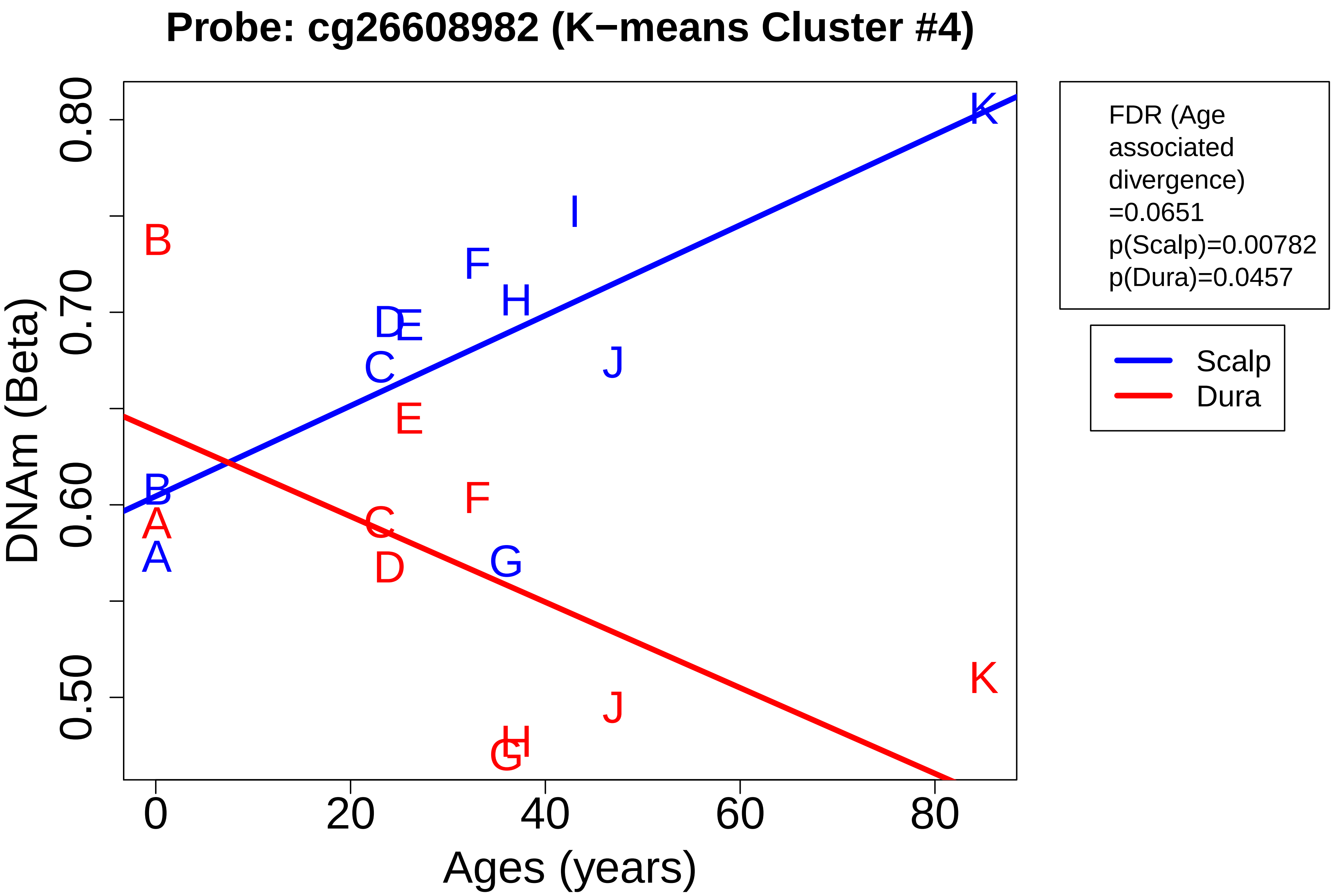

Probe: cg26608982 (K−means Cluster #4)
DNAm (Beta)
0.50
0.60
0.70
0.80
Ages (years)
0
20
40
60
80
A
B
C
D
E
F
G
H
I
J
K
FDR (Age associated divergence) =0.0651
p(Scalp)=0.00782
p(Dura)=0.0457
Scalp
Dura

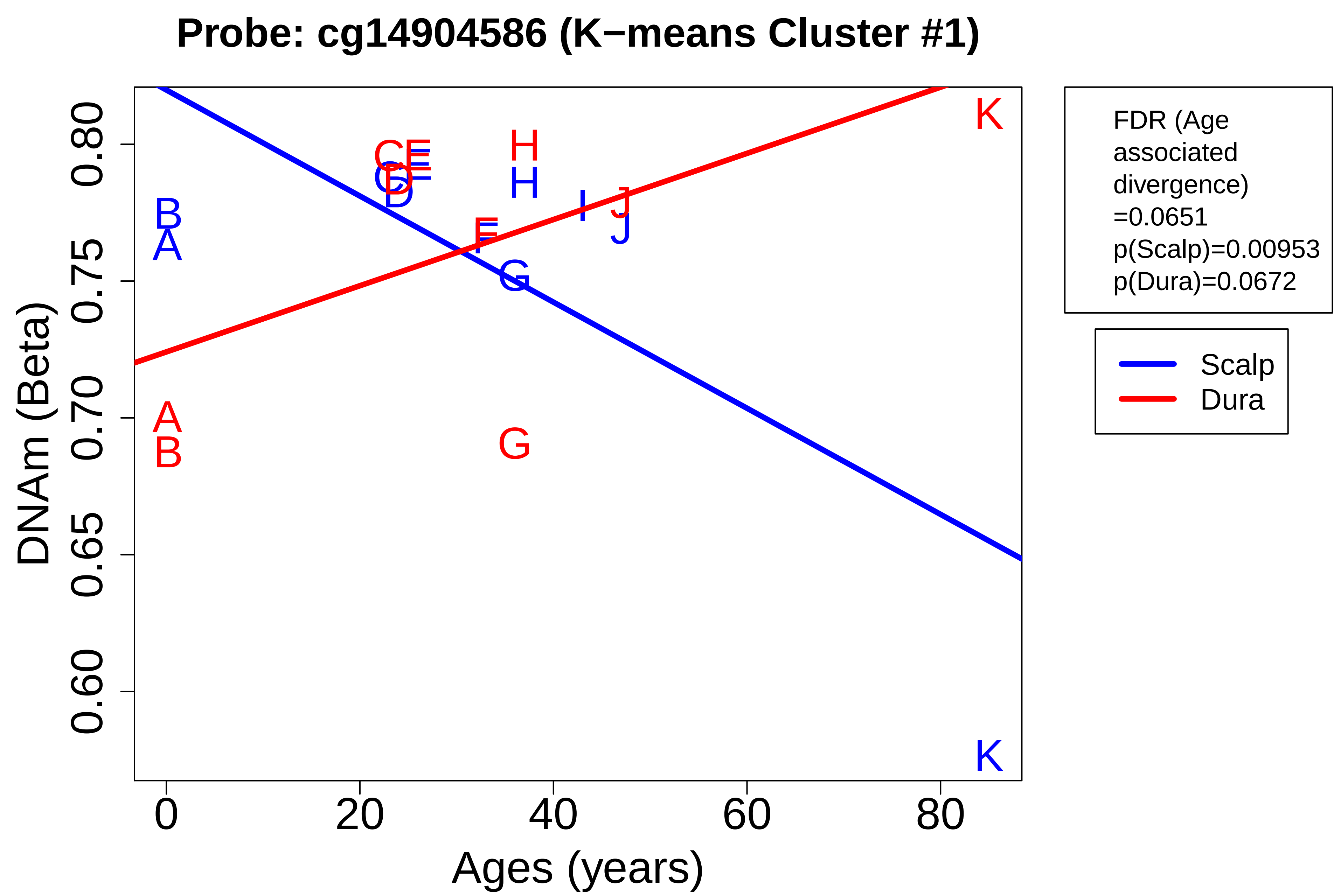

Probe: cg14904586 (K−means Cluster #1)
DNAm (Beta)
0.60
0.65
0.70
0.75
0.80
0
20
40
60
80
Ages (years)
FDR (Age associated divergence) =0.0651
p(Scalp)=0.00953
p(Dura)=0.0672
Scalp
Dura

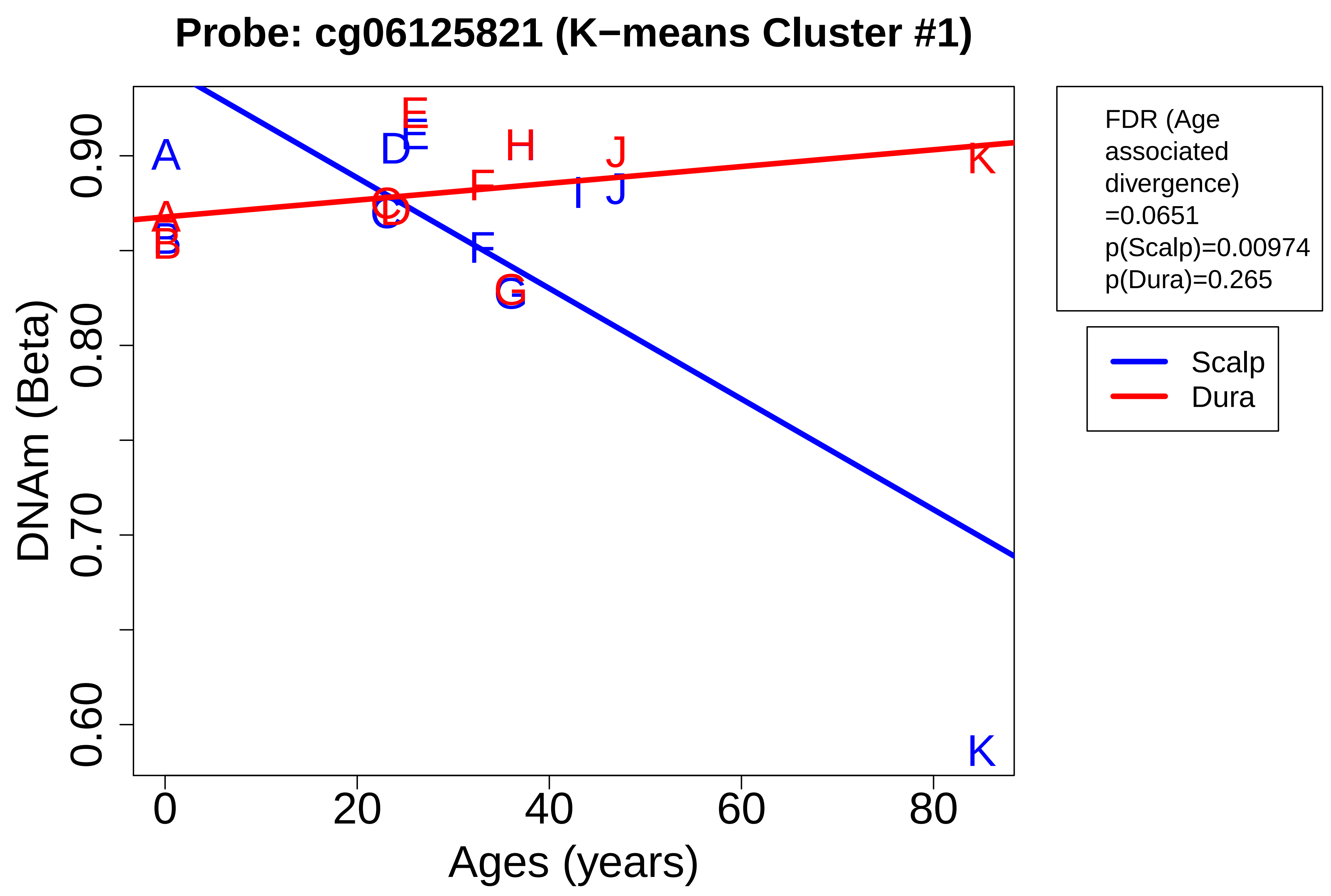

Probe: cg06125821 (K−means Cluster #1)
DNAm (Beta)
0.60
0.70
0.80
0.90
Ages (years)
0
20
40
60
80
FDR (Age associated divergence) =0.0651
p(Scalp)=0.00974
p(Dura)=0.265
Scalp
Dura
A
B
C
D
E
F
G
H
I
J
K

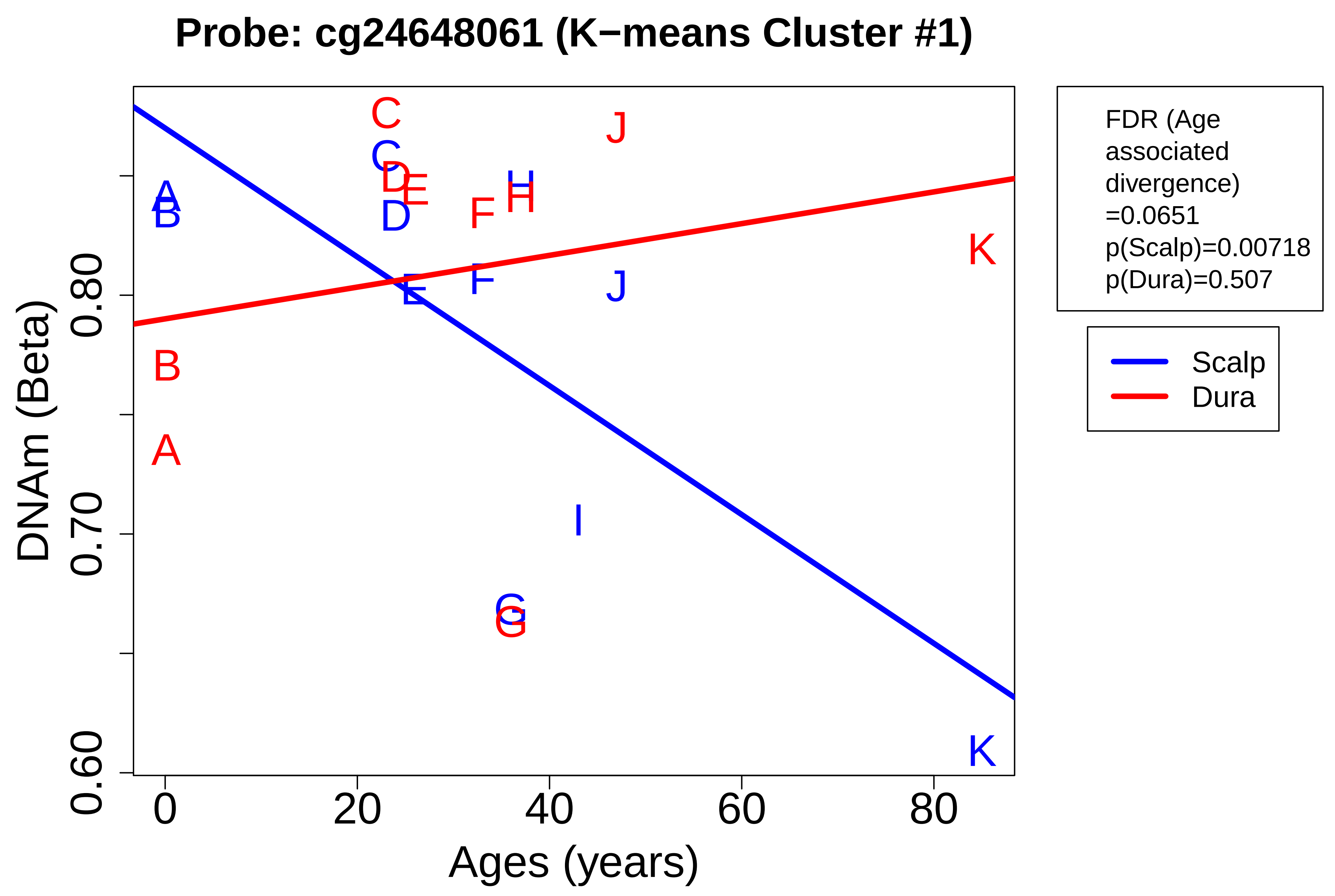

Probe: cg24648061 (K−means Cluster #1)
DNAm (Beta)
0.60
0.70
0.80
0
20
40
60
80
Ages (years)
FDR (Age associated divergence) =0.0651
p(Scalp)=0.00718
p(Dura)=0.507
Scalp
Dura

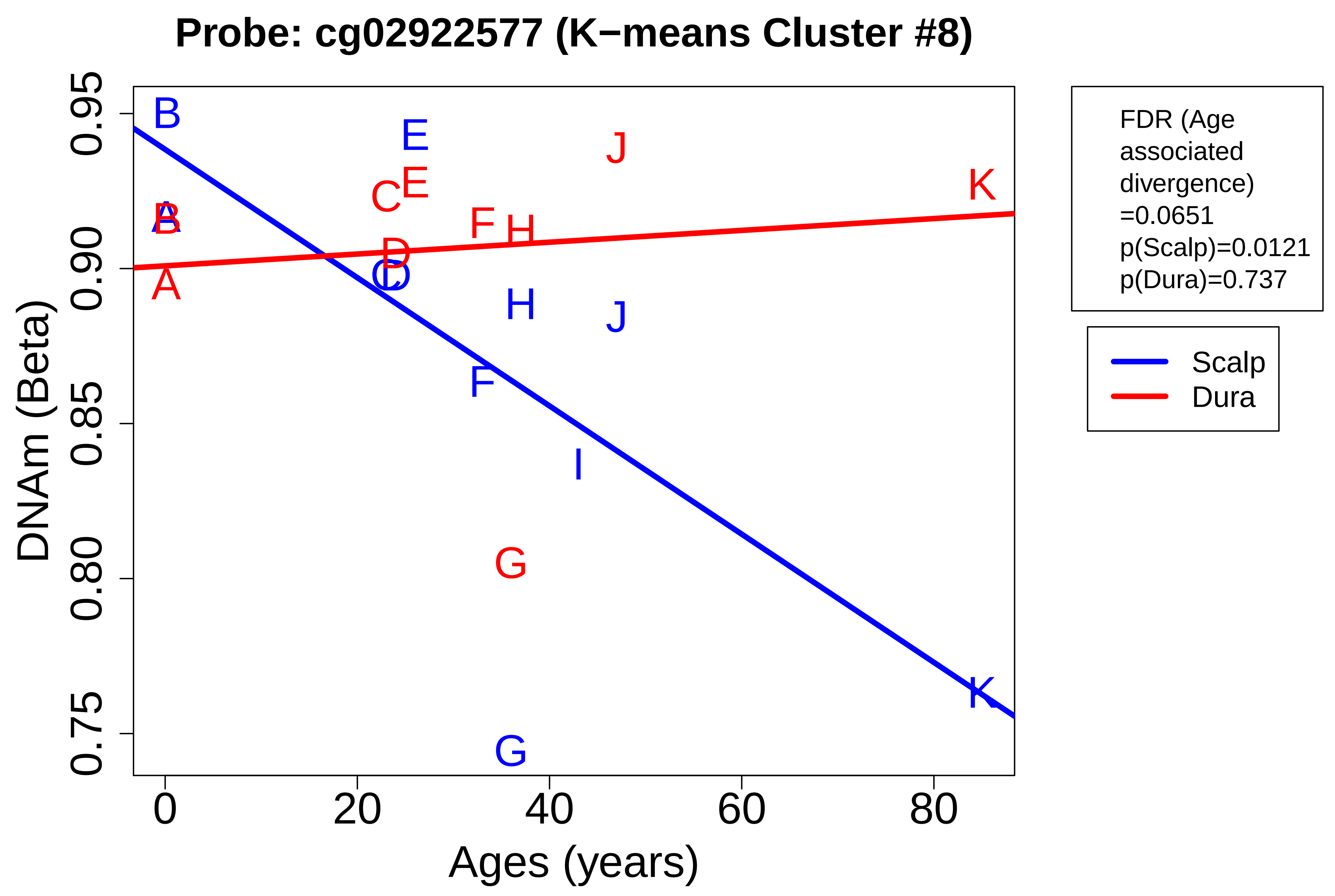

Probe: cg02922577 (K−means Cluster #8)
DNAm (Beta)
Ages (years)
0.75
0.80
0.85
0.90
0.95
0
20
40
60
80
FDR (Age associated divergence) =0.0651
p(Scalp)=0.0121
p(Dura)=0.737
Scalp
Dura

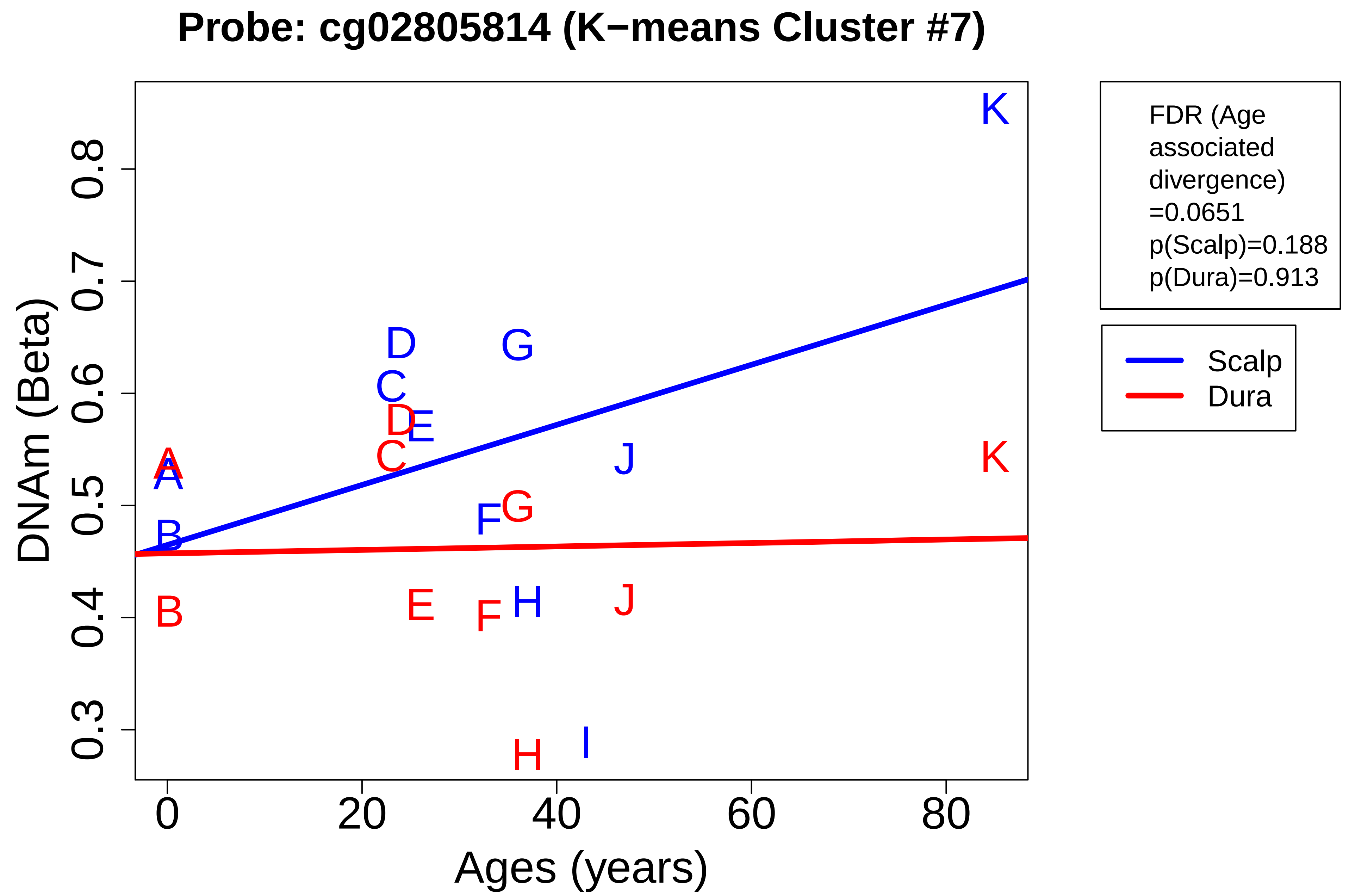

Probe: cg02805814 (K−means Cluster #7)
DNAm (Beta)
0.3
0.4
0.5
0.6
0.7
0.8
0
20
40
60
80
Ages (years)
FDR (Age associated divergence) =0.0651
p(Scalp)=0.188
p(Dura)=0.913
Scalp
Dura
A
B
C
D
E
F
G
H
I
J
K

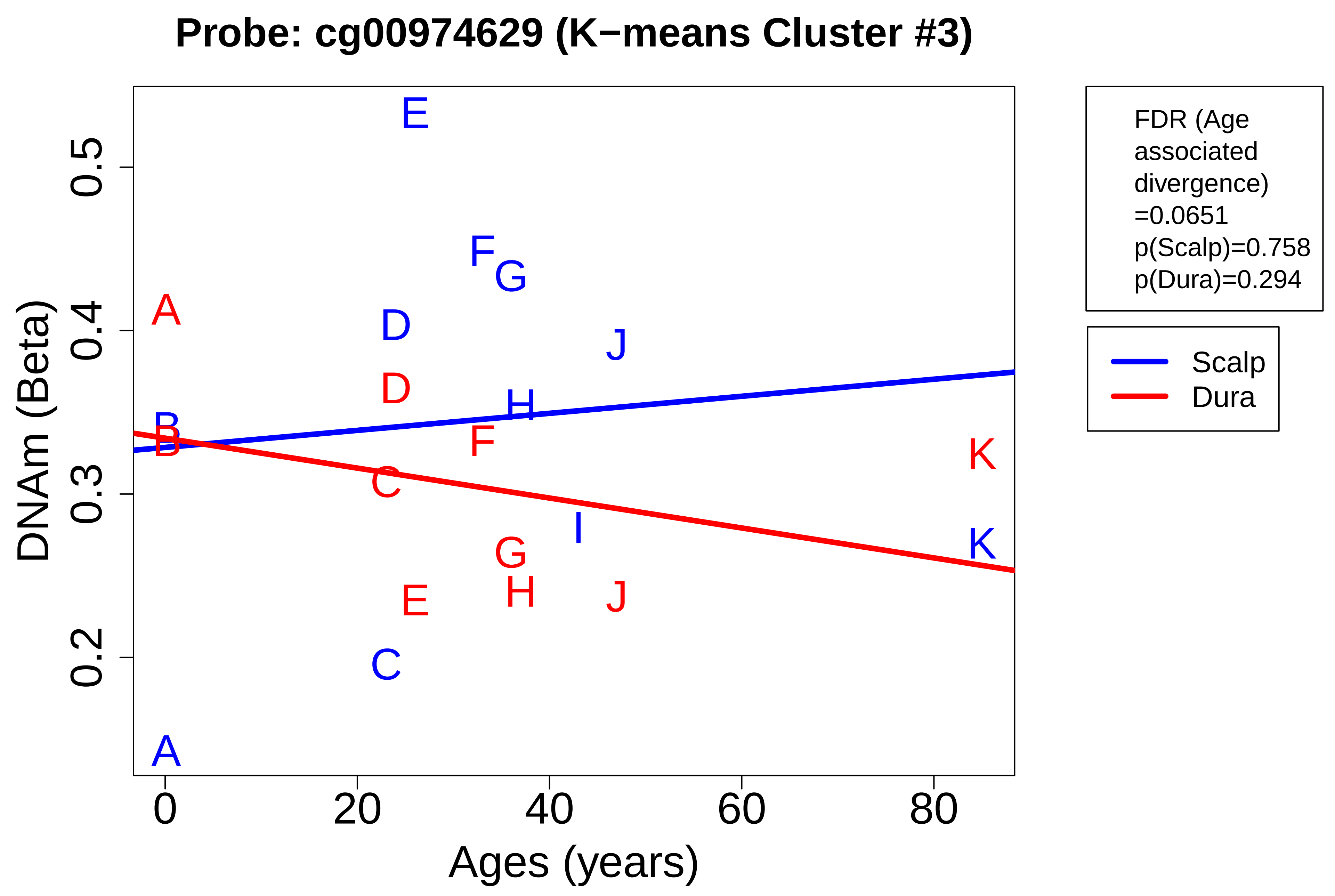

Probe: cg00974629 (K−means Cluster #3)
FDR (Age associated divergence) =0.0651
p(Scalp)=0.758
p(Dura)=0.294
Scalp
Dura
DNAm (Beta)
0.2
0.3
0.4
0.5
0
20
40
60
80
Ages (years)
A
B
C
D
E
F
G
H
I
J
K

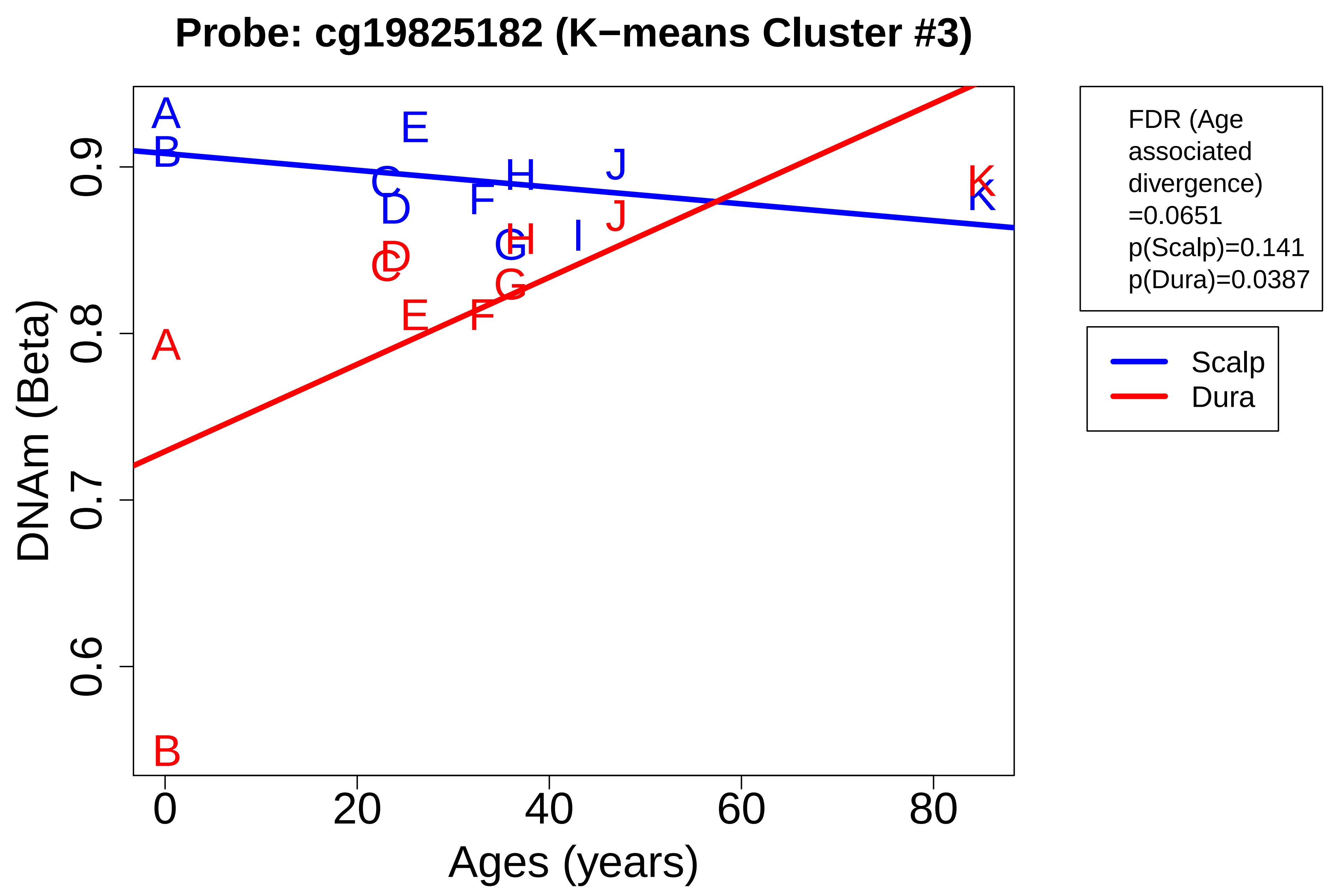
Probe: cg19825182 (K−means Cluster #3)
DNAm (Beta)
0.6
0.7
0.8
0.9
Ages (years)
0
20
40
60
80
A
B
C
D
E
F
G
H
I
J
K
FDR (Age associated divergence) =0.0651
p(Scalp)=0.141
p(Dura)=0.0387
Scalp
Dura

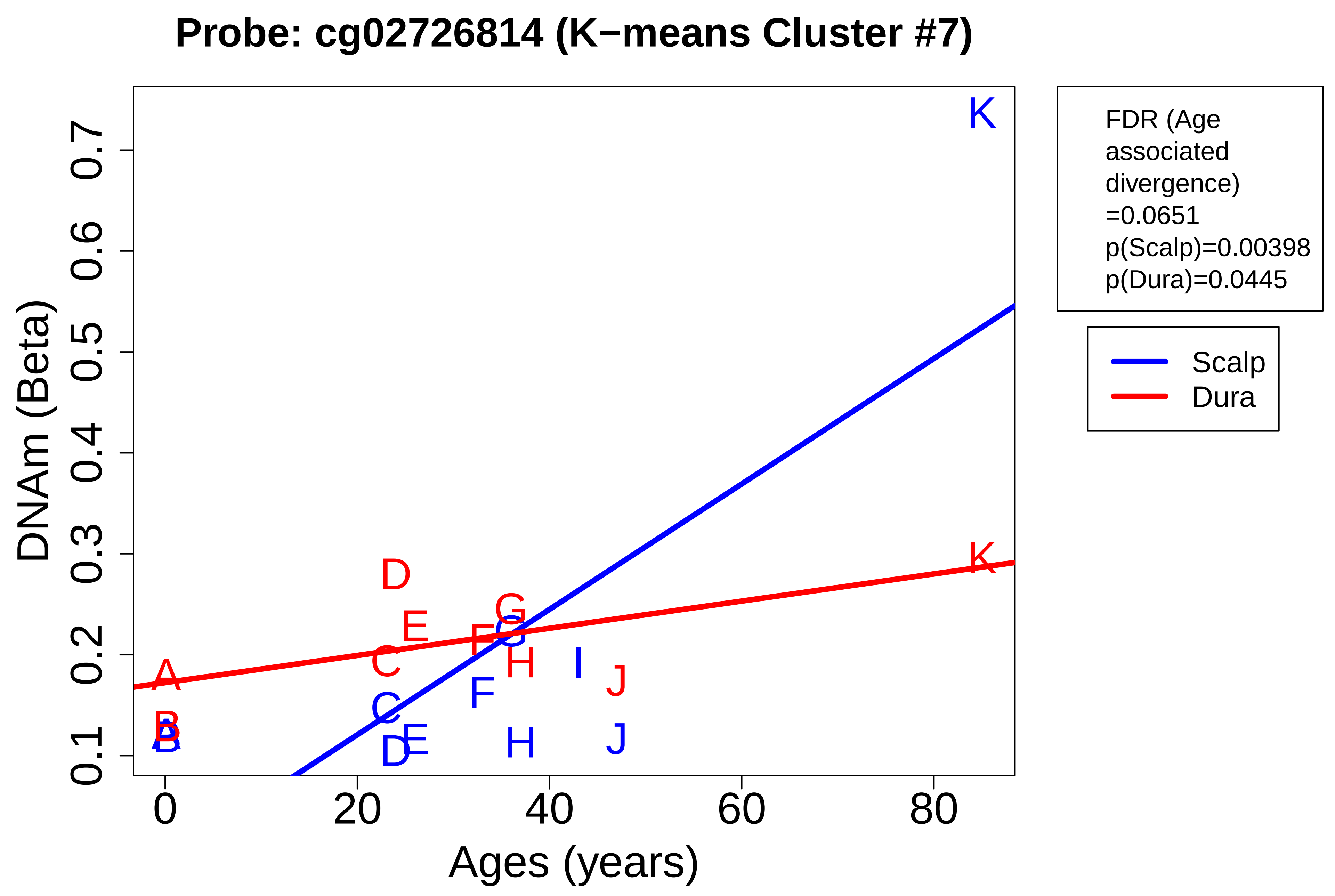
Probe: cg02726814 (K−means Cluster #7)
DNAm (Beta)
0.1
0.2
0.3
0.4
0.5
0.6
0.7
Ages (years)
0
20
40
60
80
FDR (Age associated divergence) =0.0651
p(Scalp)=0.00398
p(Dura)=0.0445
Scalp
Dura

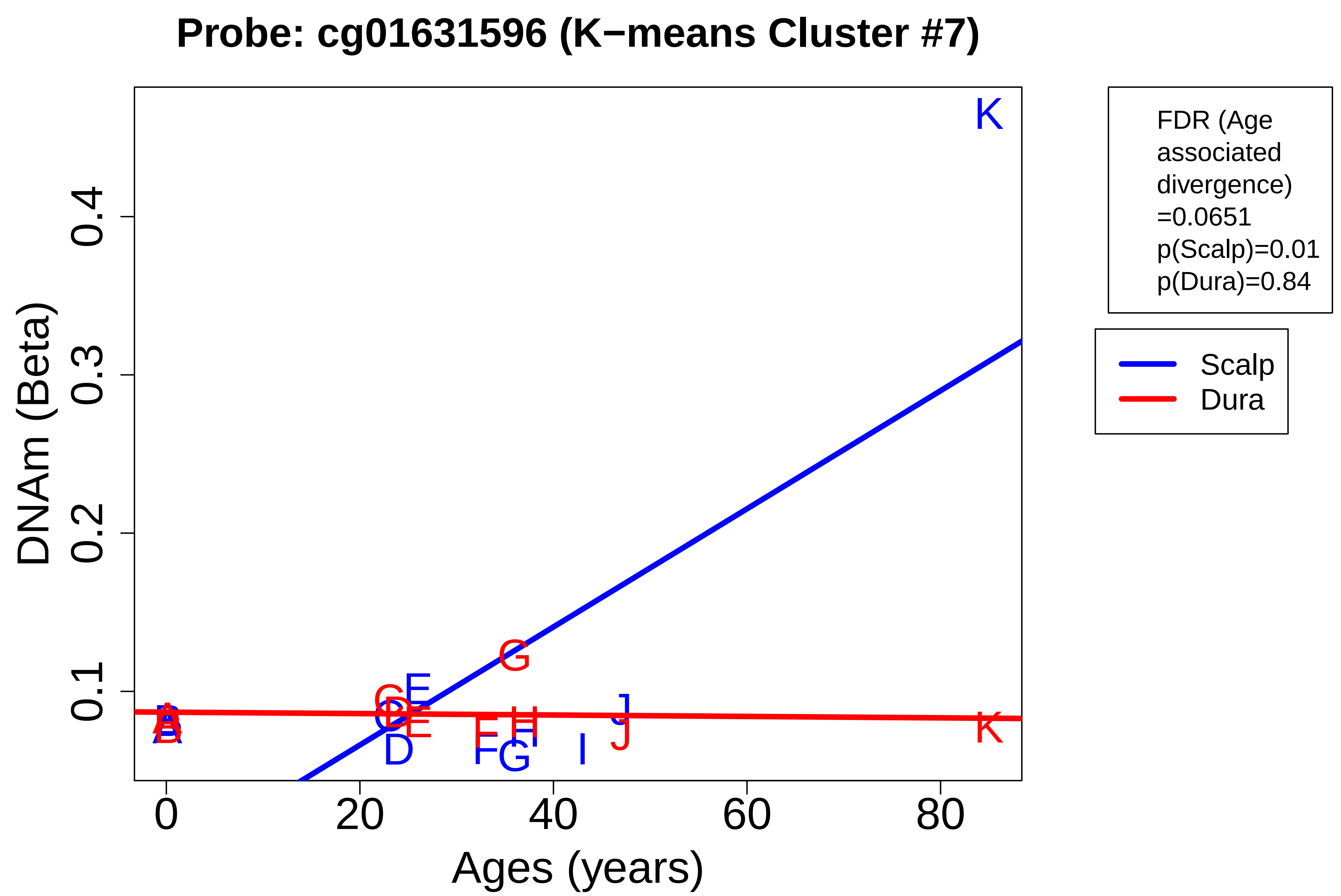
Probe: cg01631596 (K−means Cluster #7)
DNAm (Beta)
0.1
0.2
0.3
0.4
Ages (years)
0
20
40
60
80
FDR (Age associated divergence) =0.0651
p(Scalp)=0.01
p(Dura)=0.84
Scalp
Dura
A
B
C
D
E
F
G
H
I
J
K

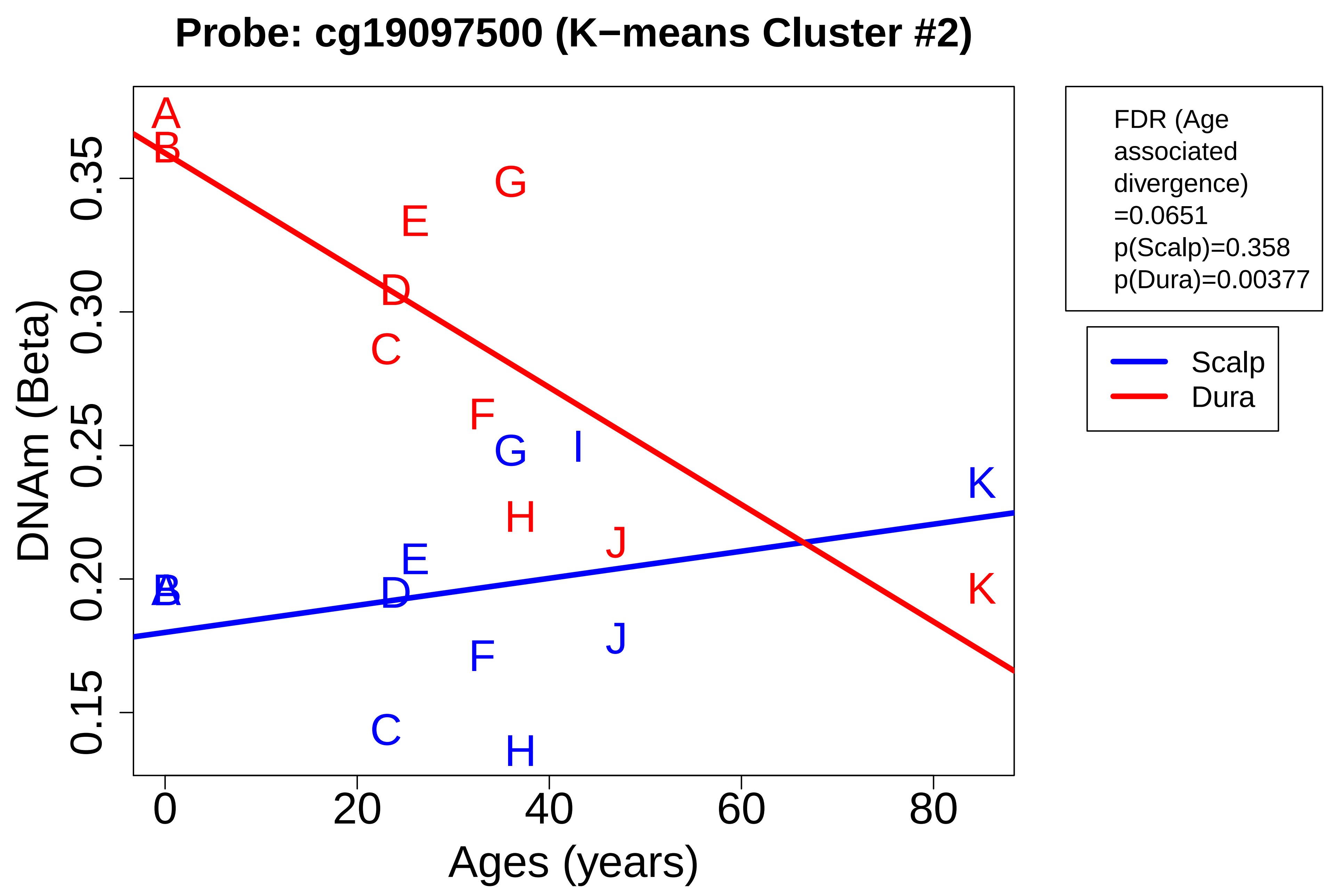

Probe: cg19097500 (K−means Cluster #2)
DNAm (Beta)
0.15
0.20
0.25
0.30
0.35
Ages (years)
0
20
40
60
80
FDR (Age associated divergence) =0.0651
p(Scalp)=0.358
p(Dura)=0.00377
Scalp
Dura
A
B
C
D
E
F
G
H
I
J
K

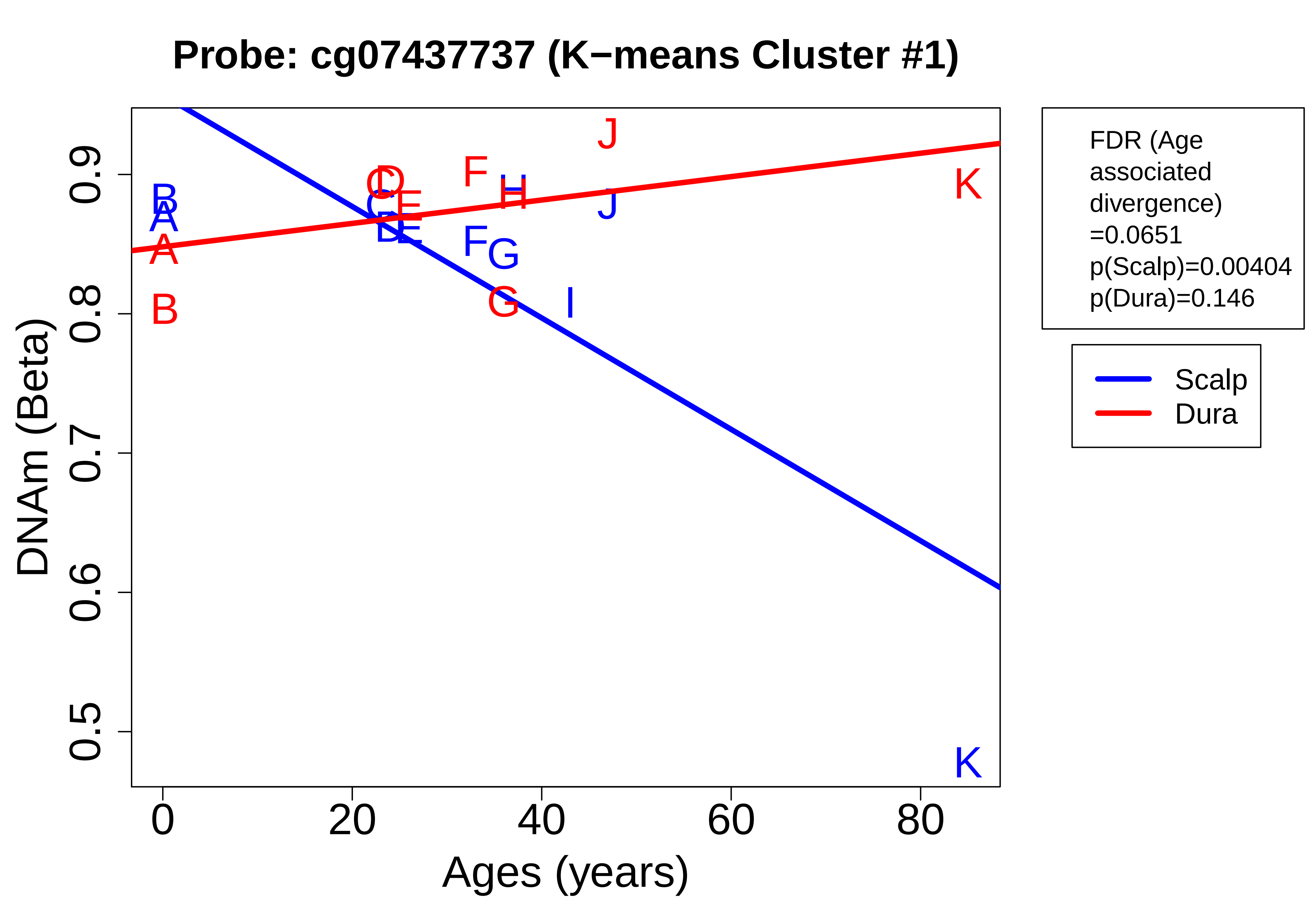

Probe: cg07437737 (K−means Cluster #1)
DNAm (Beta)
0.5
0.6
0.7
0.8
0.9
0
20
40
60
80
Ages (years)
A
B
C
D
E
F
G
H
I
J
K
FDR (Age associated divergence) =0.0651
p(Scalp)=0.00404
p(Dura)=0.146
Scalp
Dura

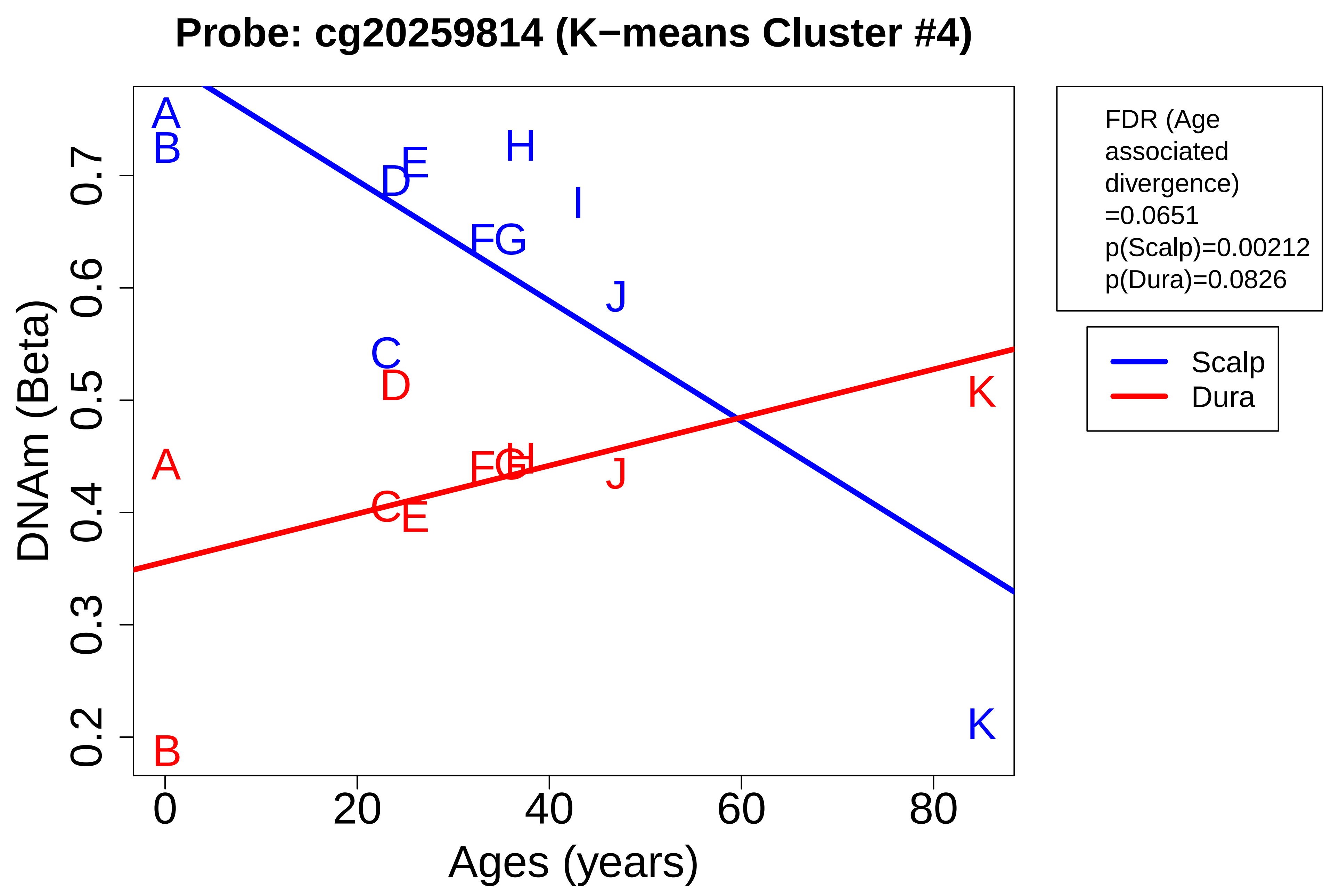

Probe: cg20259814 (K−means Cluster #4)
FDR (Age associated divergence) =0.0651
p(Scalp)=0.00212
p(Dura)=0.0826
Scalp
Dura
DNAm (Beta)
0.2
0.3
0.4
0.5
0.6
0.7
Ages (years)
0
20
40
60
80
A
B
C
D
E
F
G
H
I
J
K

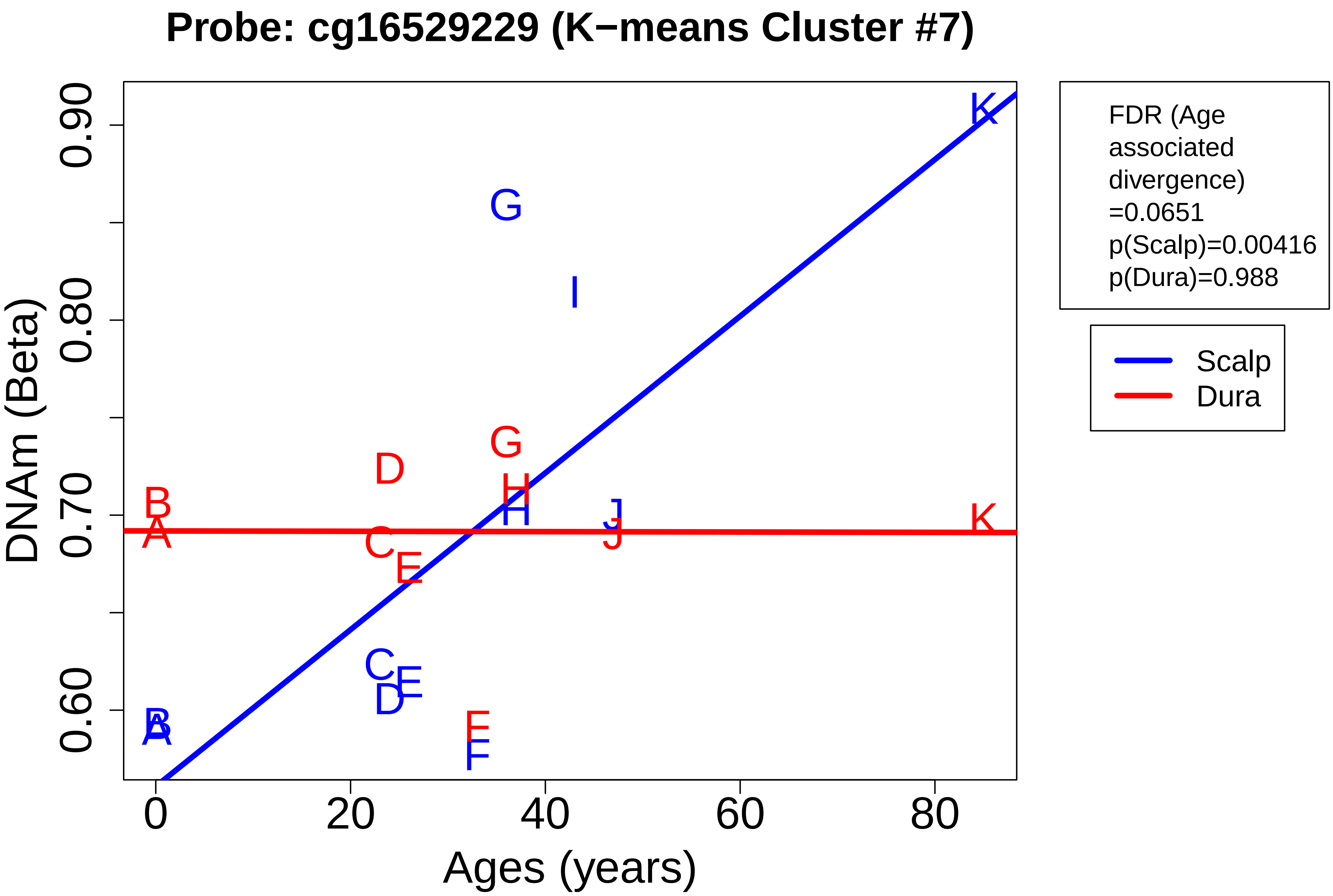

Probe: cg16529229 (K−means Cluster #7)
DNAm (Beta)
0.60
0.70
0.80
0.90
0
20
40
60
80
Ages (years)
FDR (Age associated divergence) =0.0651
p(Scalp)=0.00416
p(Dura)=0.988
Scalp
Dura

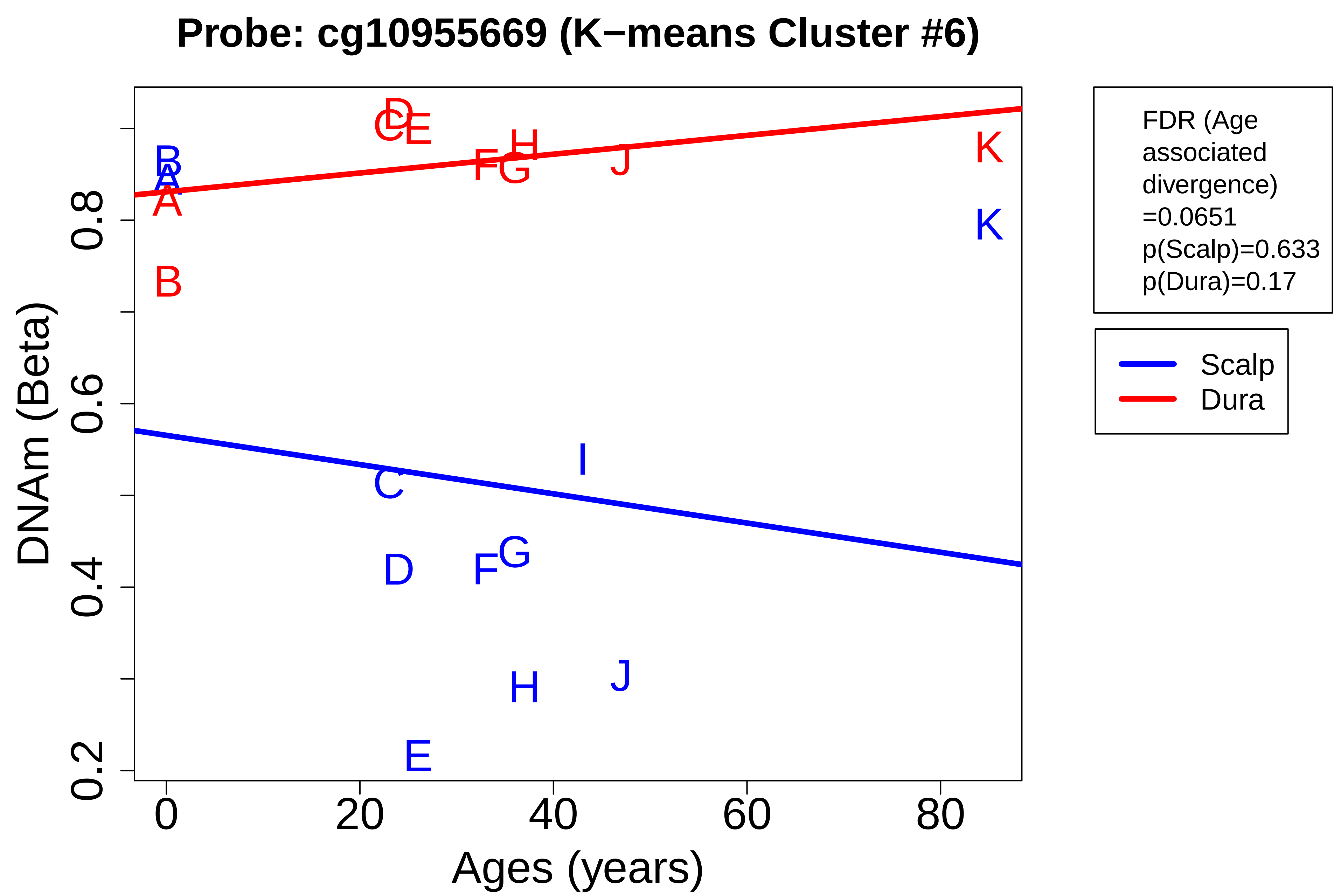

Probe: cg10955669 (K−means Cluster #6)
DNAm (Beta)
Ages (years)
0.2
0.4
0.6
0.8
0
20
40
60
80
FDR (Age associated divergence) =0.0651
p(Scalp)=0.633
p(Dura)=0.17
Scalp
Dura

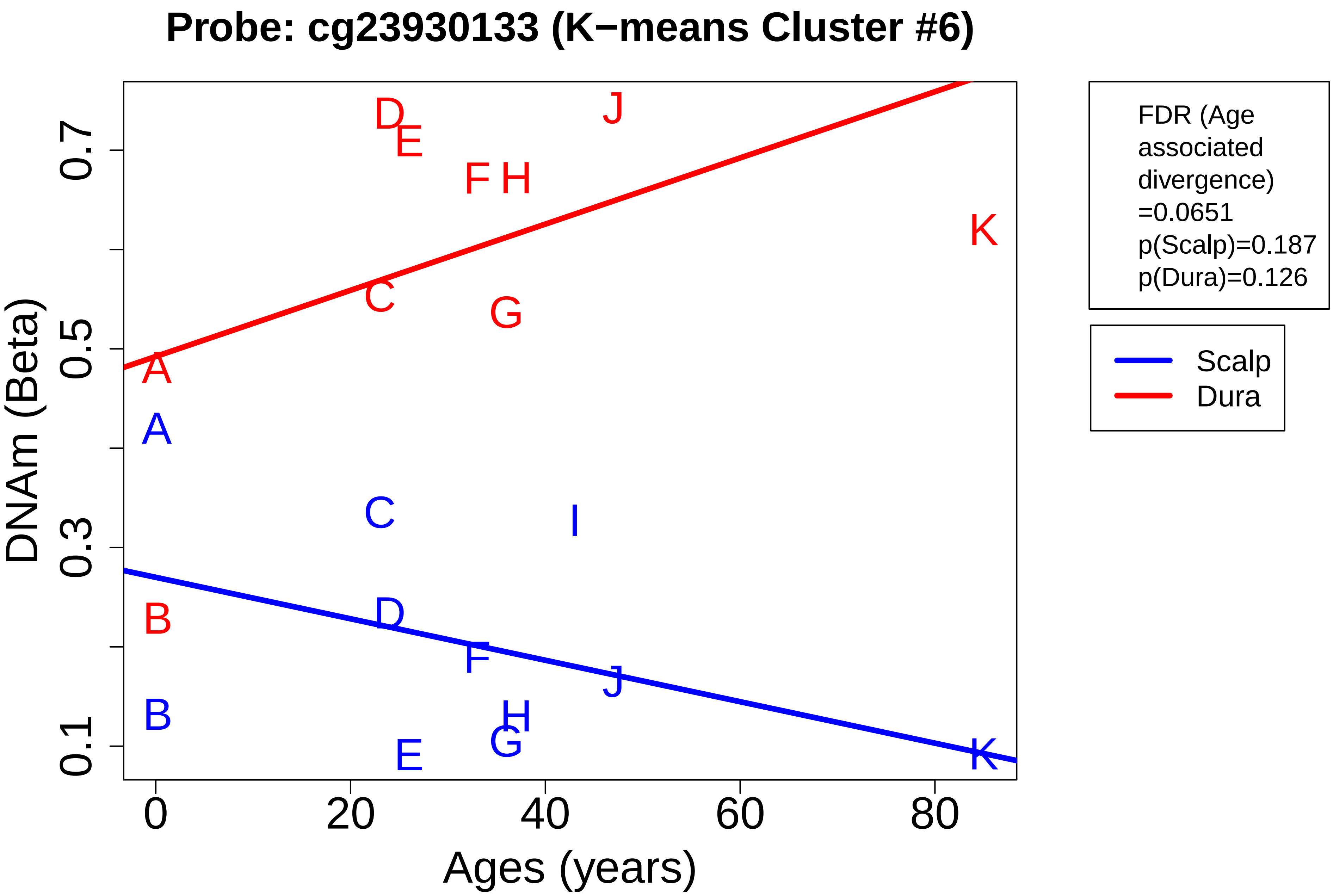
Probe: cg23930133 (K−means Cluster #6)
DNAm (Beta)
0.1
0.3
0.5
0.7
Ages (years)
0
20
40
60
80
A
B
C
D
E
F
G
H
I
J
K
FDR (Age associated divergence) =0.0651
p(Scalp)=0.187
p(Dura)=0.126
Scalp
Dura

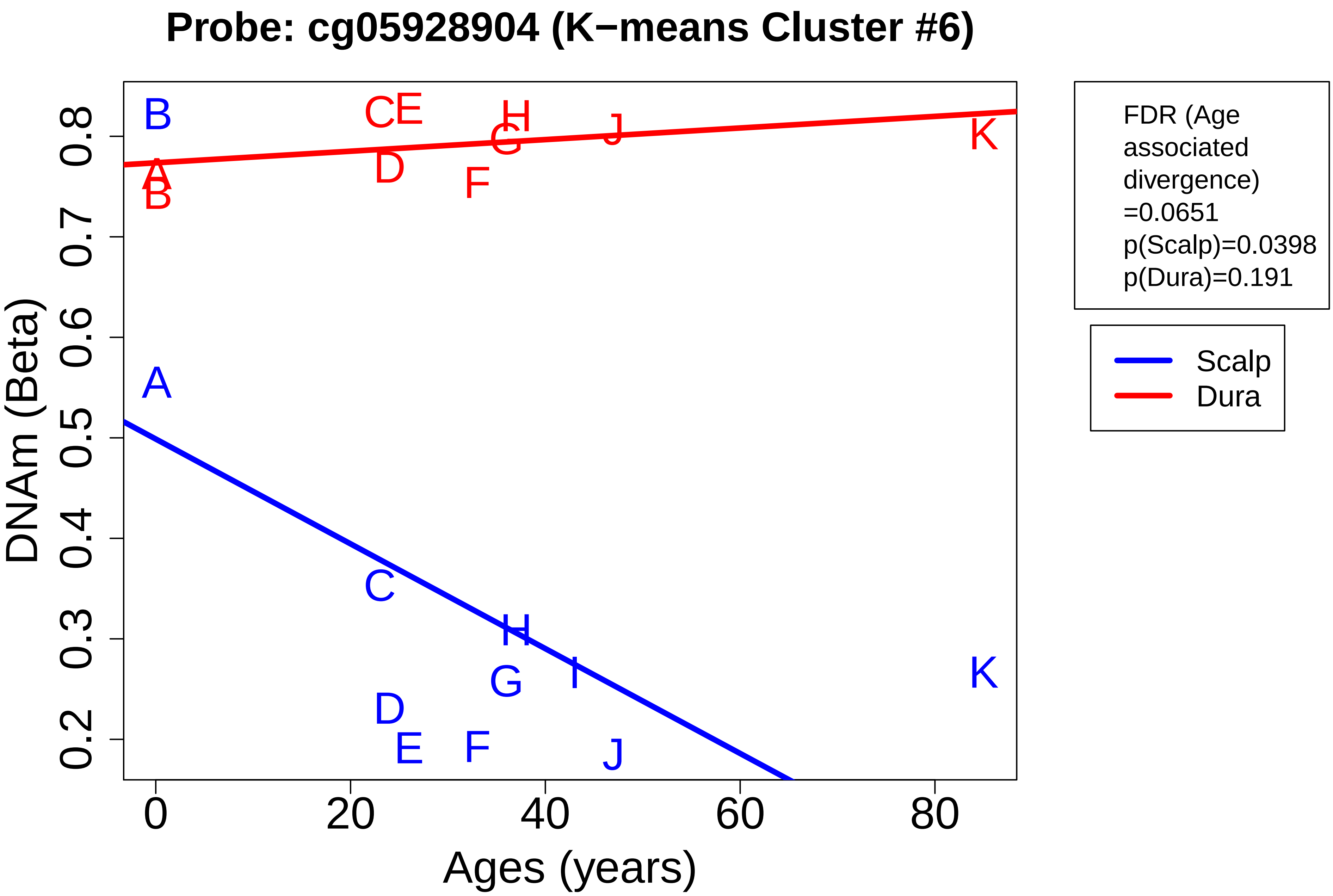

Probe: cg05928904 (K−means Cluster #6)
DNAm (Beta)
0.2
0.3
0.4
0.5
0.6
0.7
0.8
Ages (years)
0
20
40
60
80
FDR (Age associated divergence) =0.0651
p(Scalp)=0.0398
p(Dura)=0.191
Scalp
Dura

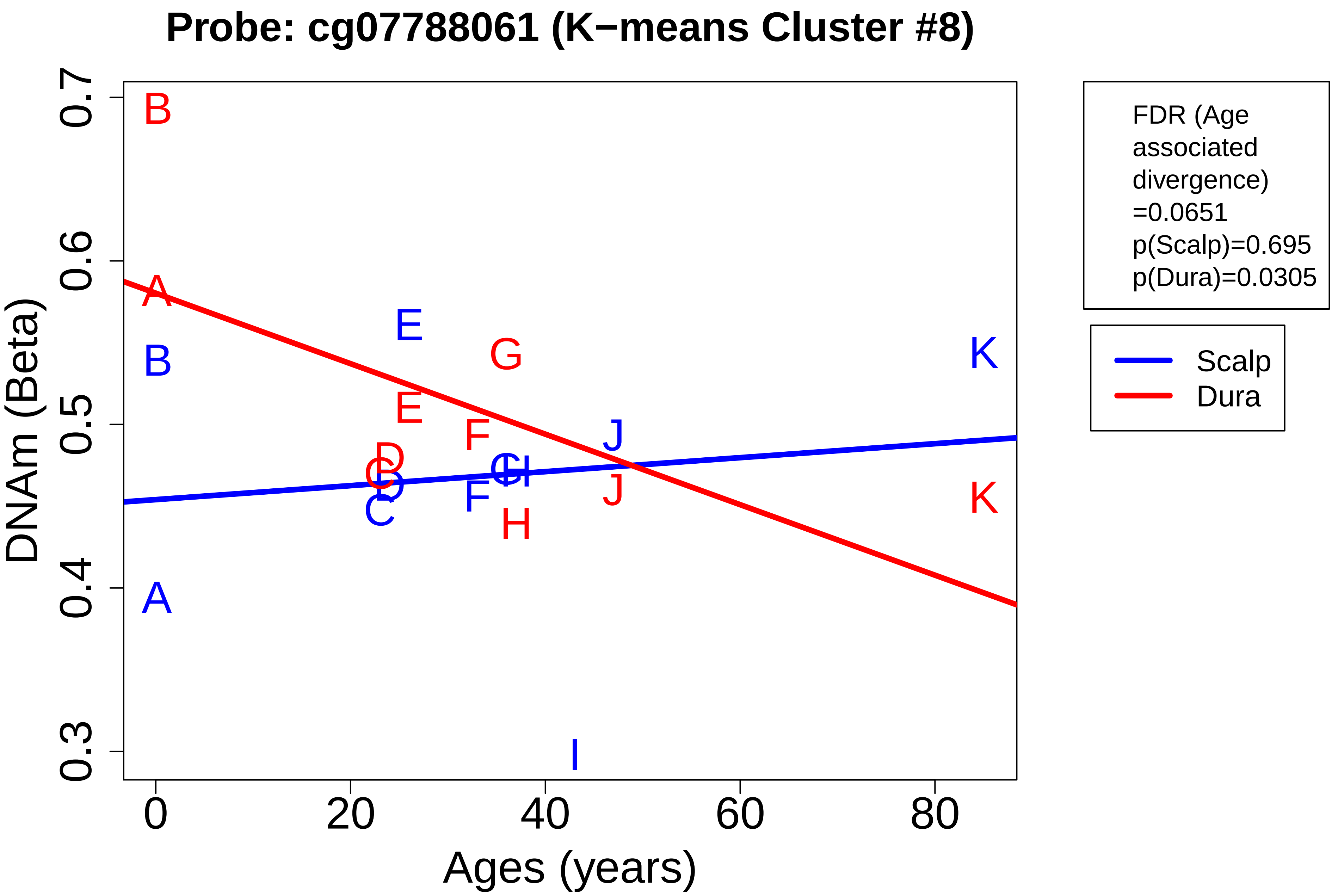
Probe: cg07788061 (K−means Cluster #8)
DNAm (Beta)
0.3
0.4
0.5
0.6
0.7
0
20
40
60
80
Ages (years)
FDR (Age associated divergence) =0.0651
p(Scalp)=0.695
p(Dura)=0.0305
Scalp
Dura

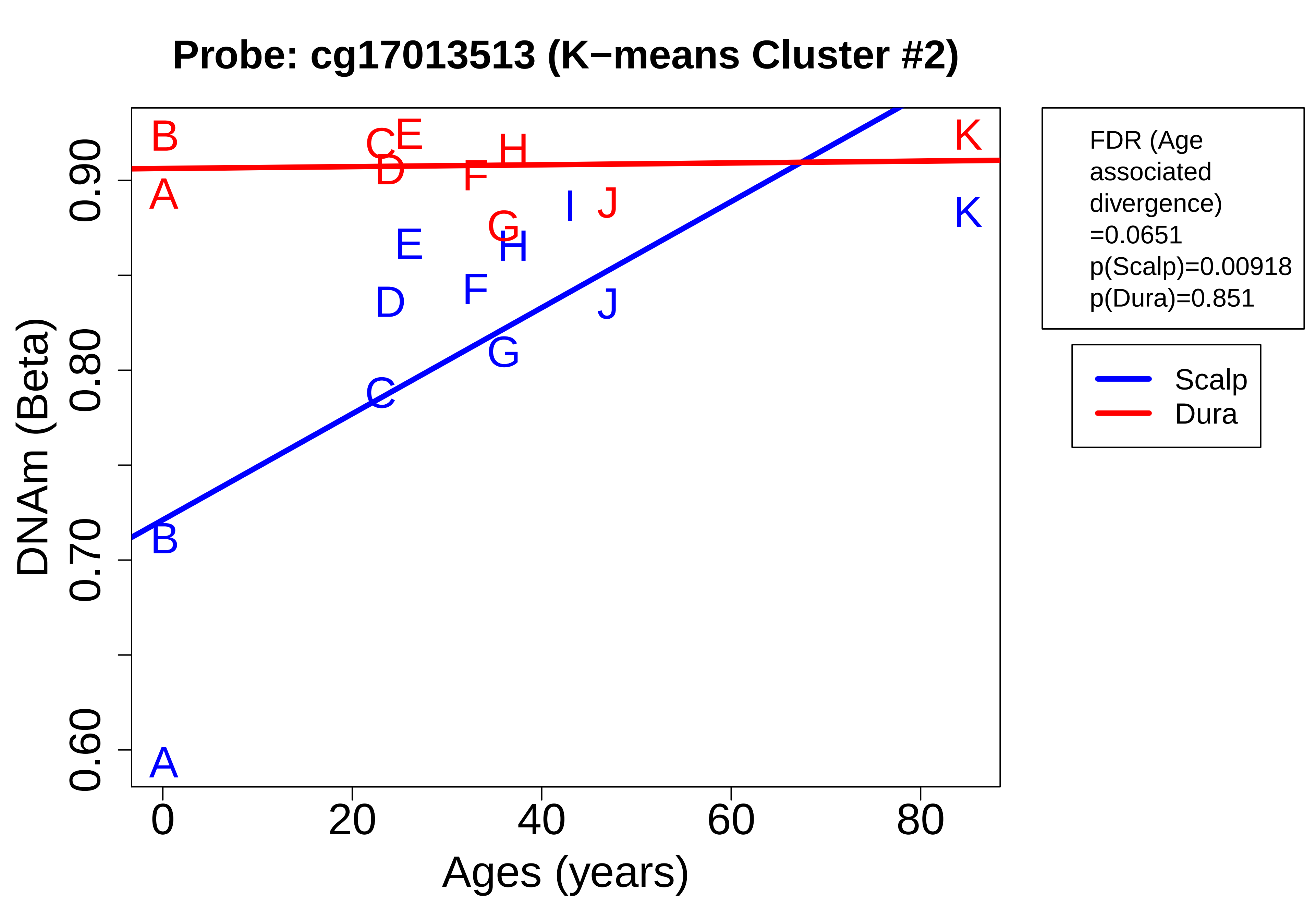
Probe: cg17013513 (K−means Cluster #2)
FDR (Age associated divergence) =0.0651
p(Scalp)=0.00918
p(Dura)=0.851
Scalp
Dura
DNAm (Beta)
0.60
0.70
0.80
0.90
0
20
40
60
80
Ages (years)
A
B
C
D
E
F
G
H
I
J
K

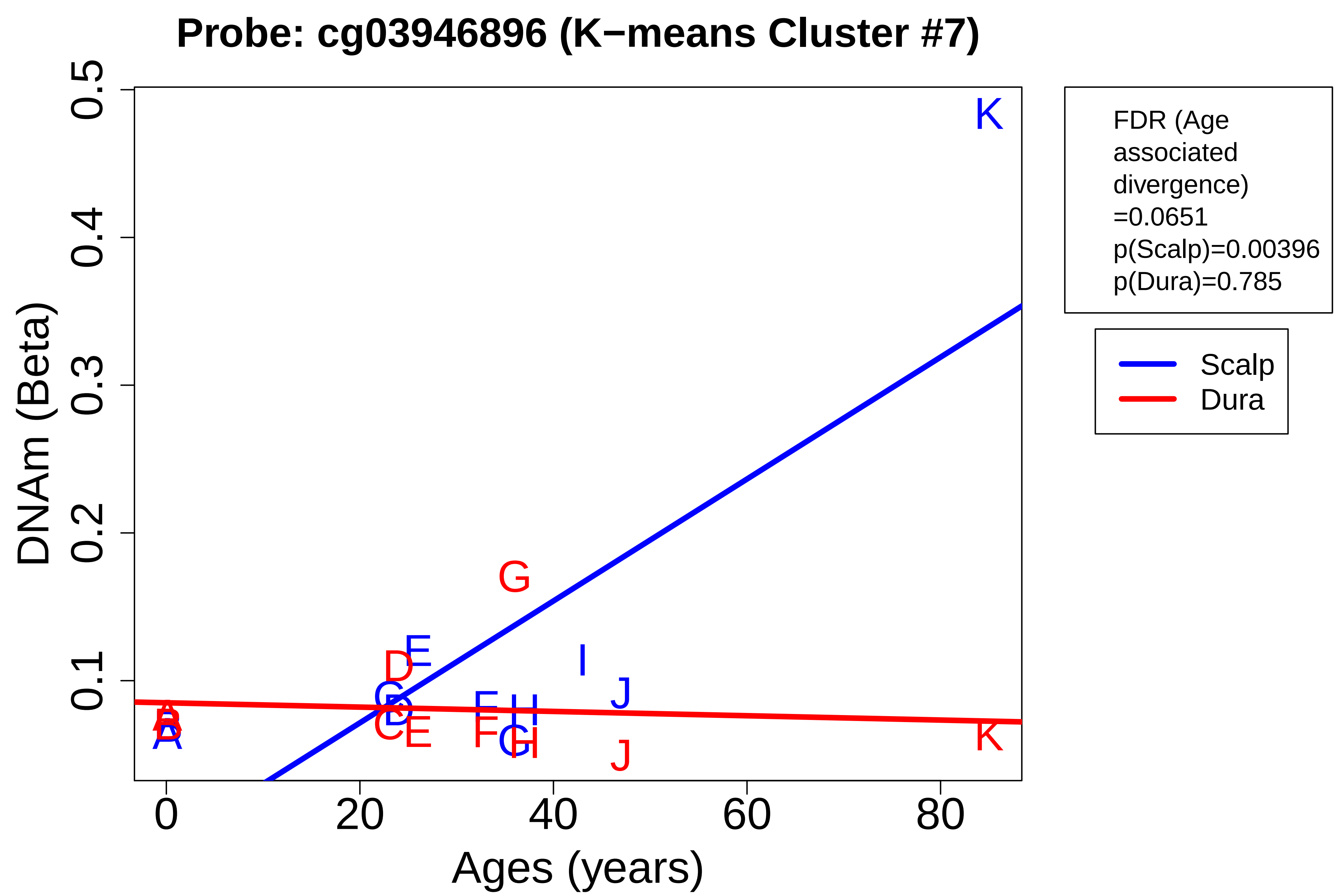
Probe: cg03946896 (K−means Cluster #7)
DNAm (Beta)
0.1
0.2
0.3
0.4
0.5
0
20
40
60
80
Ages (years)
FDR (Age associated divergence) =0.0651
p(Scalp)=0.00396
p(Dura)=0.785
Scalp
Dura

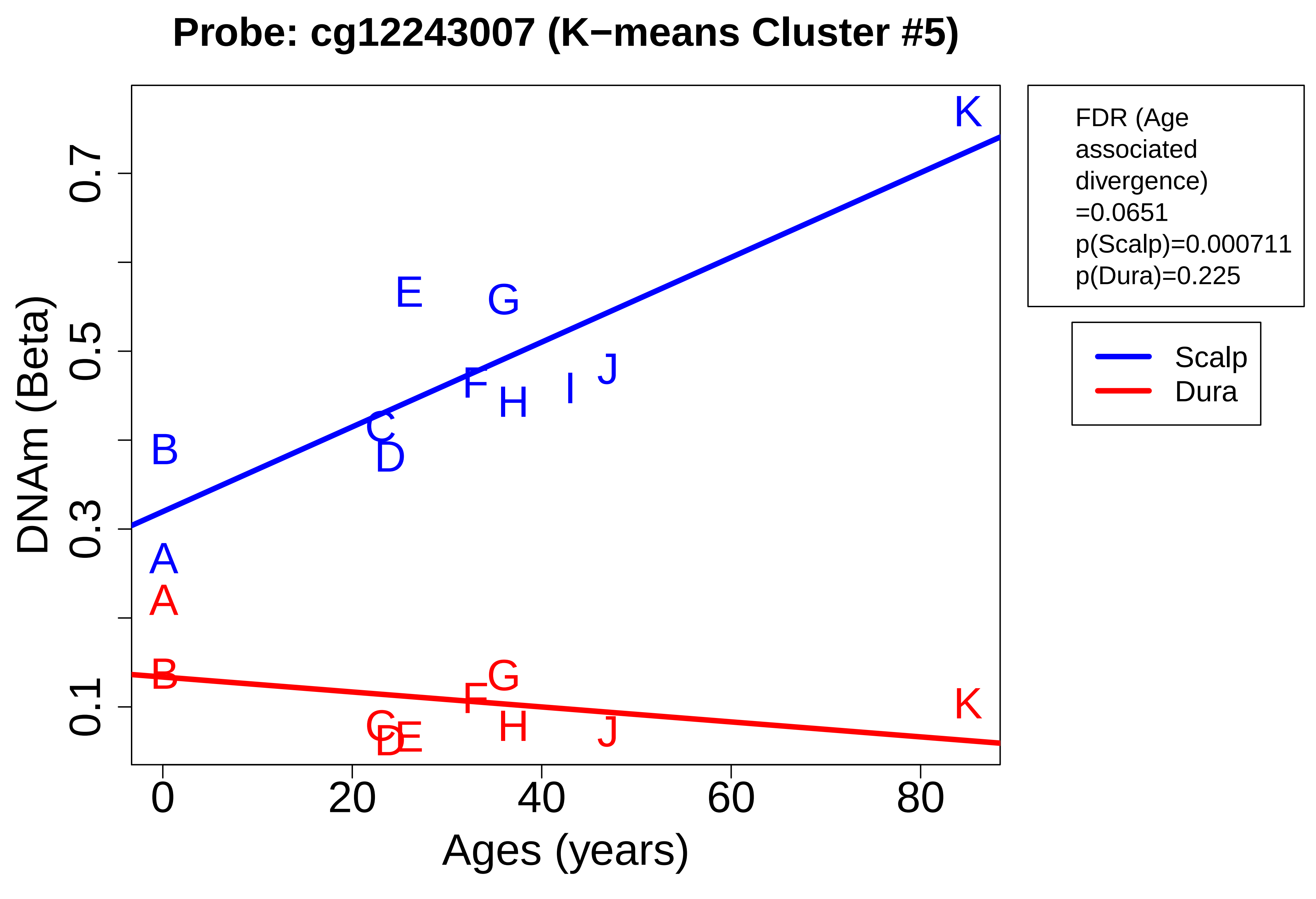

Probe: cg12243007 (K−means Cluster #5)
DNAm (Beta)
0.1
0.3
0.5
0.7
Ages (years)
0
20
40
60
80
A
B
C
D
E
F
G
H
I
J
K
FDR (Age associated divergence) =0.0651
p(Scalp)=0.000711
p(Dura)=0.225
Scalp
Dura

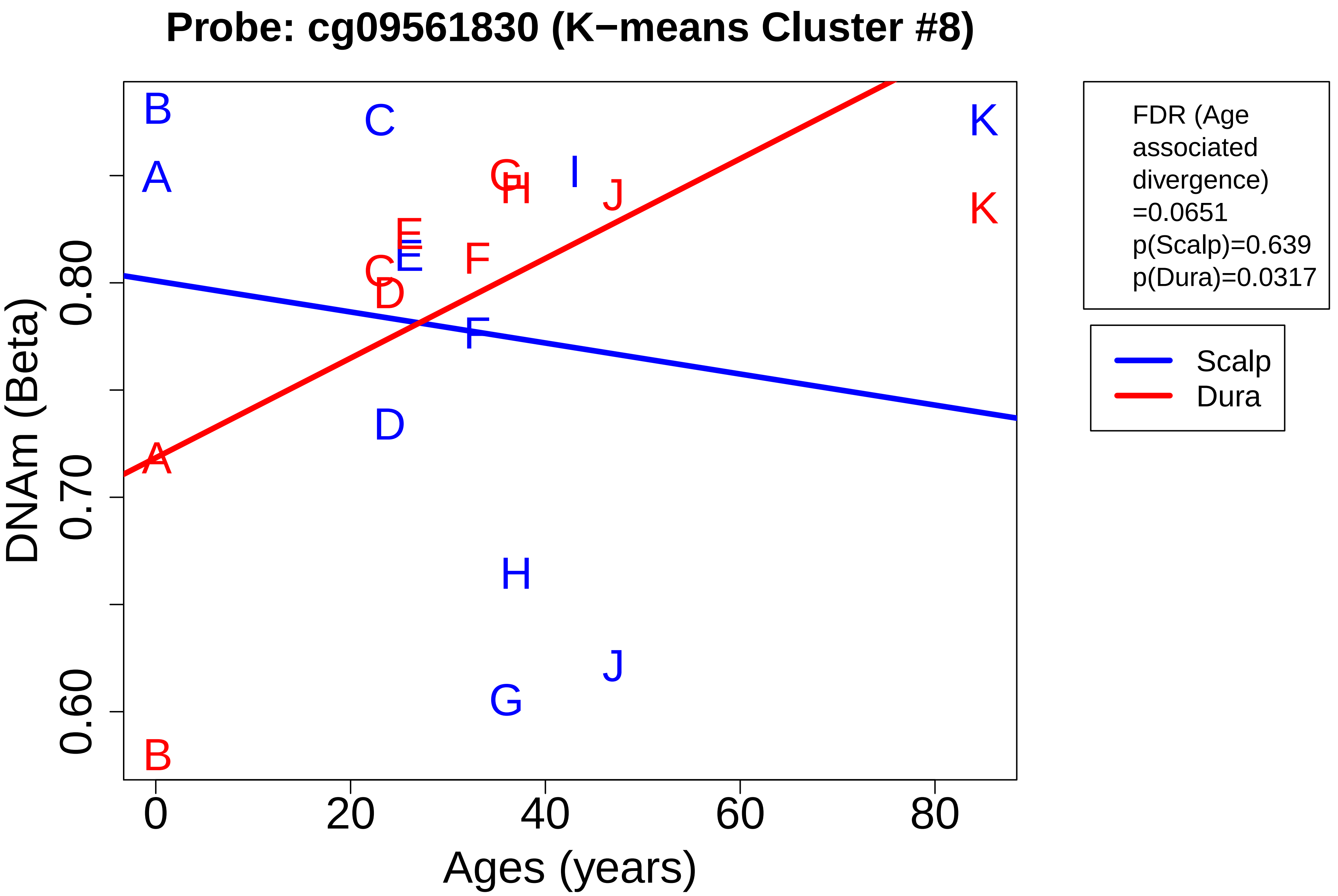

Probe: cg09561830 (K−means Cluster #8)
FDR (Age associated divergence) =0.0651
p(Scalp)=0.639
p(Dura)=0.0317
Scalp
Dura
DNAm (Beta)
0.60
0.70
0.80
0
20
40
60
80
Ages (years)

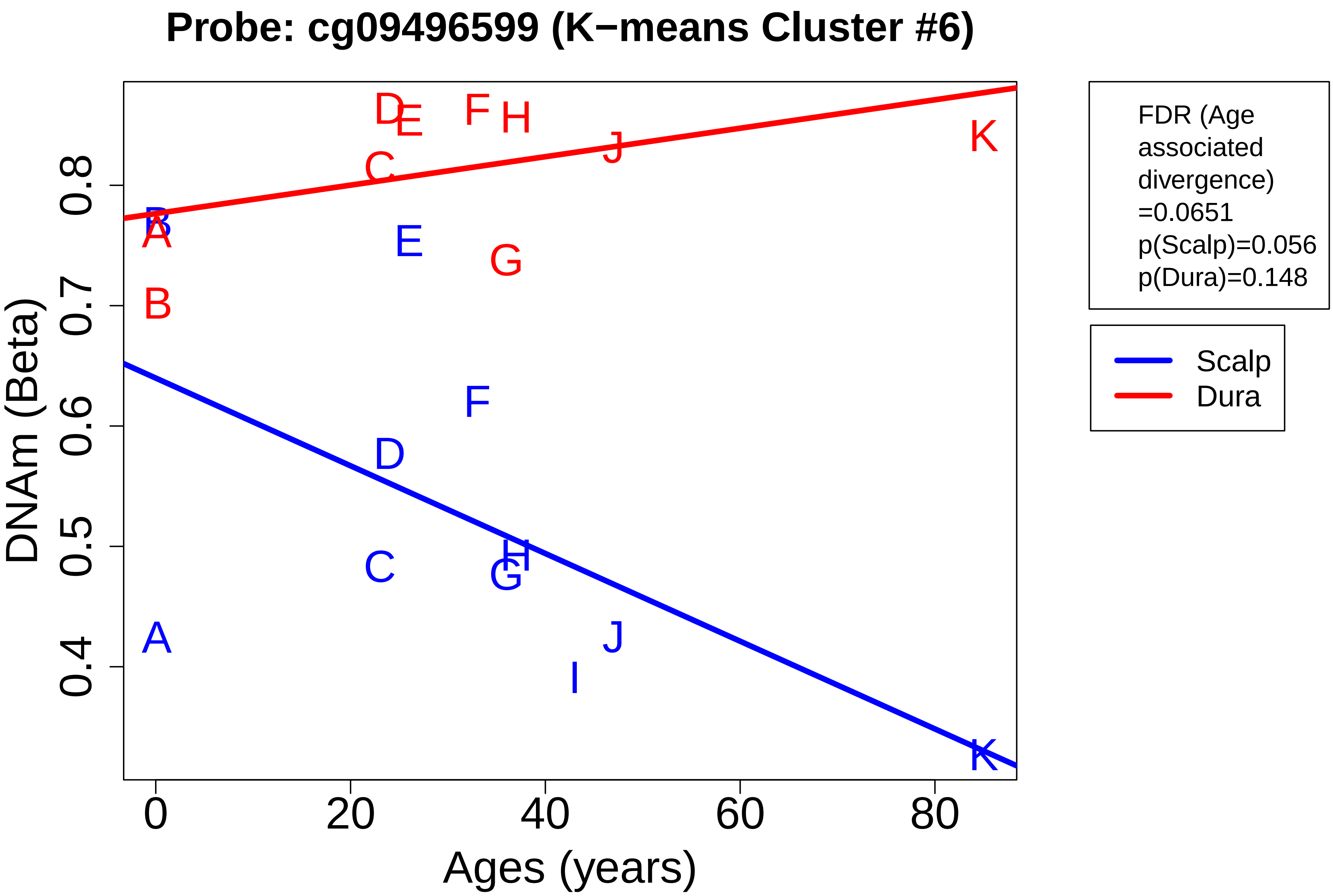
Probe: cg09496599 (K−means Cluster #6)
DNAm (Beta)
0.4
0.5
0.6
0.7
0.8
0
20
40
60
80
Ages (years)
FDR (Age associated divergence) =0.0651
p(Scalp)=0.056
p(Dura)=0.148
Scalp
Dura

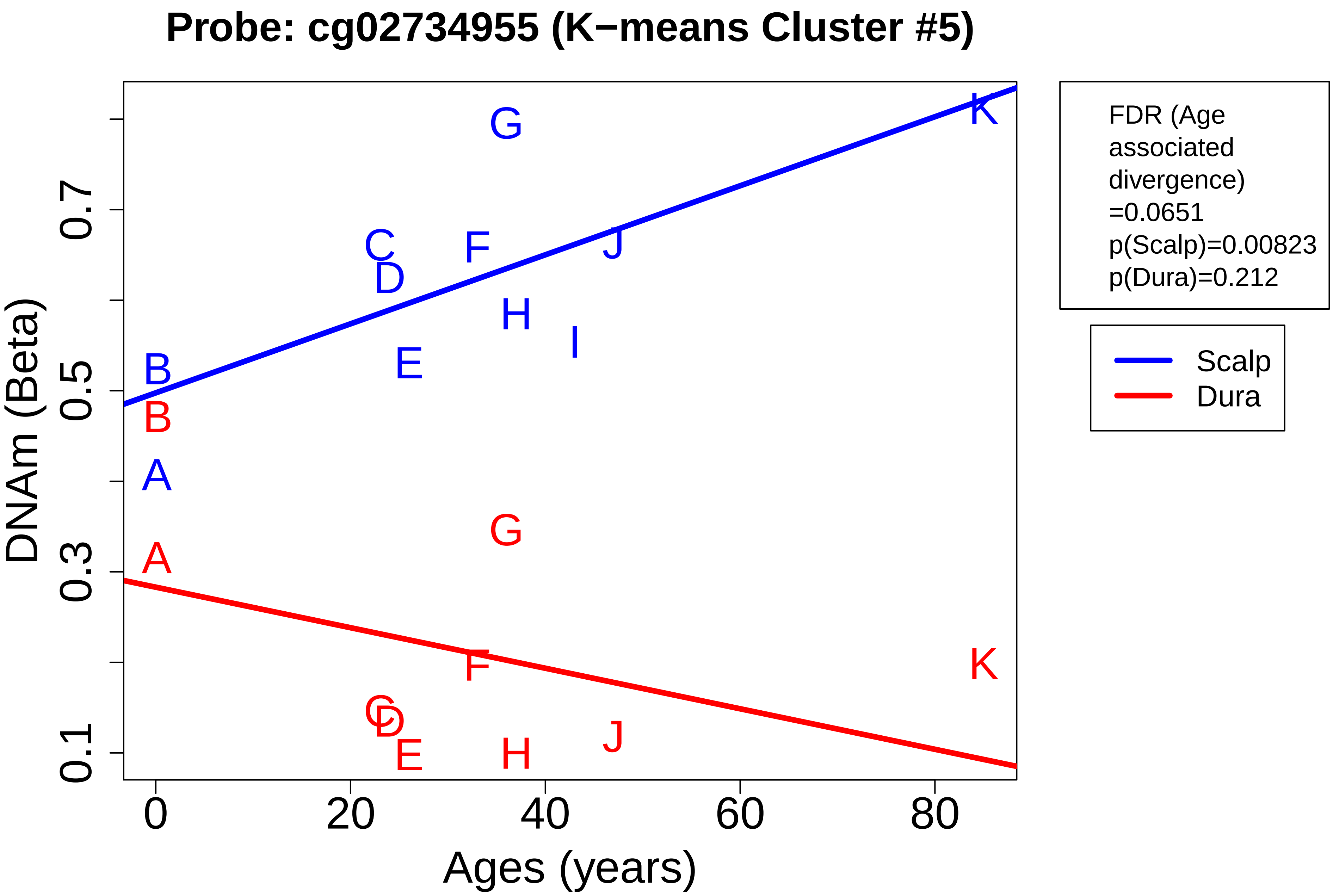
Probe: cg02734955 (K−means Cluster #5)
DNAm (Beta)
Ages (years)
0.1
0.3
0.5
0.7
0
20
40
60
80
FDR (Age associated divergence) =0.0651
p(Scalp)=0.00823
p(Dura)=0.212
Scalp
Dura
A
B
C
D
E
F
G
H
I
J
K

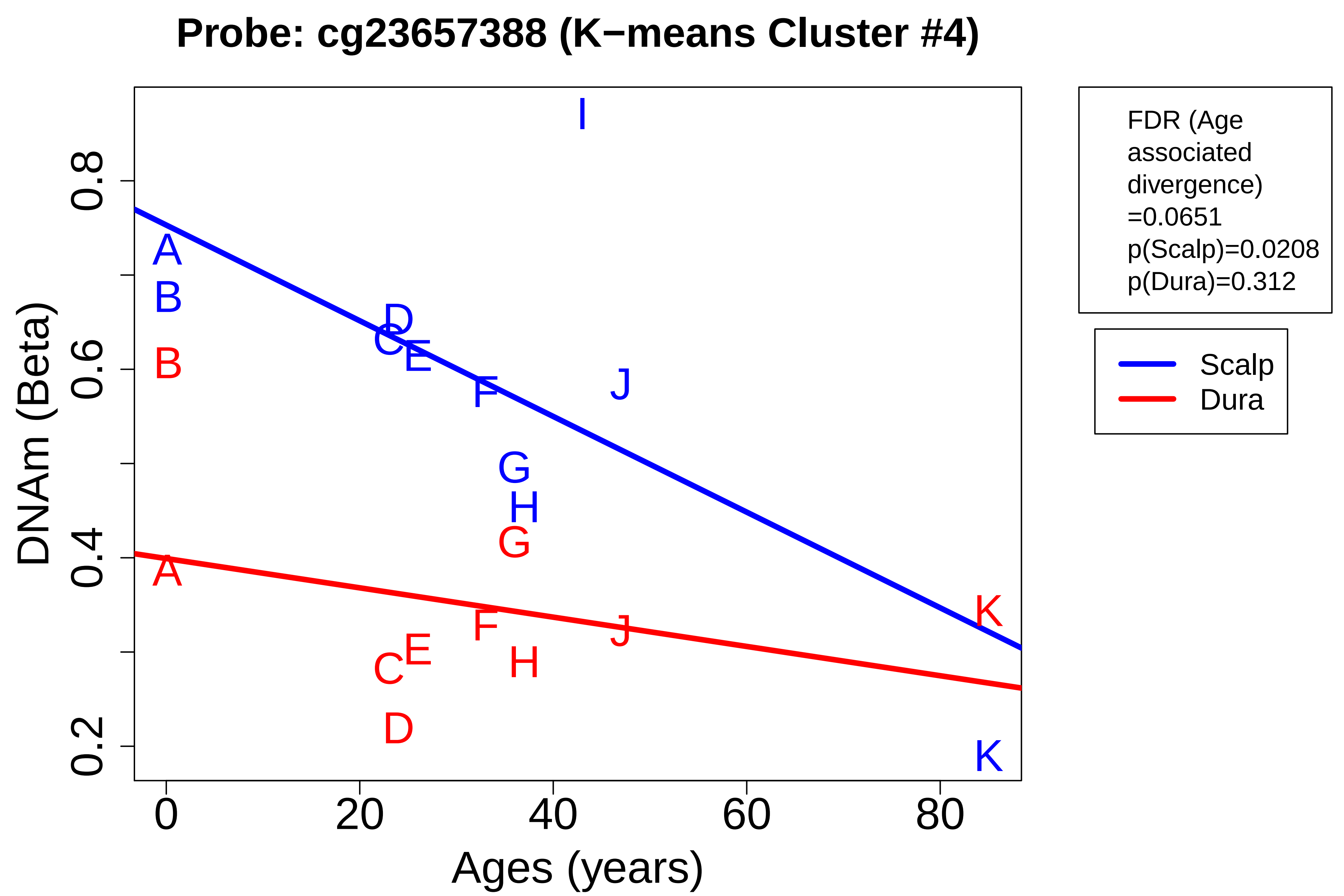
Probe: cg23657388 (K−means Cluster #4)
FDR (Age associated divergence) =0.0651
p(Scalp)=0.0208
p(Dura)=0.312
Scalp
Dura
DNAm (Beta)
0.2
0.4
0.6
0.8
Ages (years)
0
20
40
60
80
A
B
C
D
E
F
G
H
I
J
K

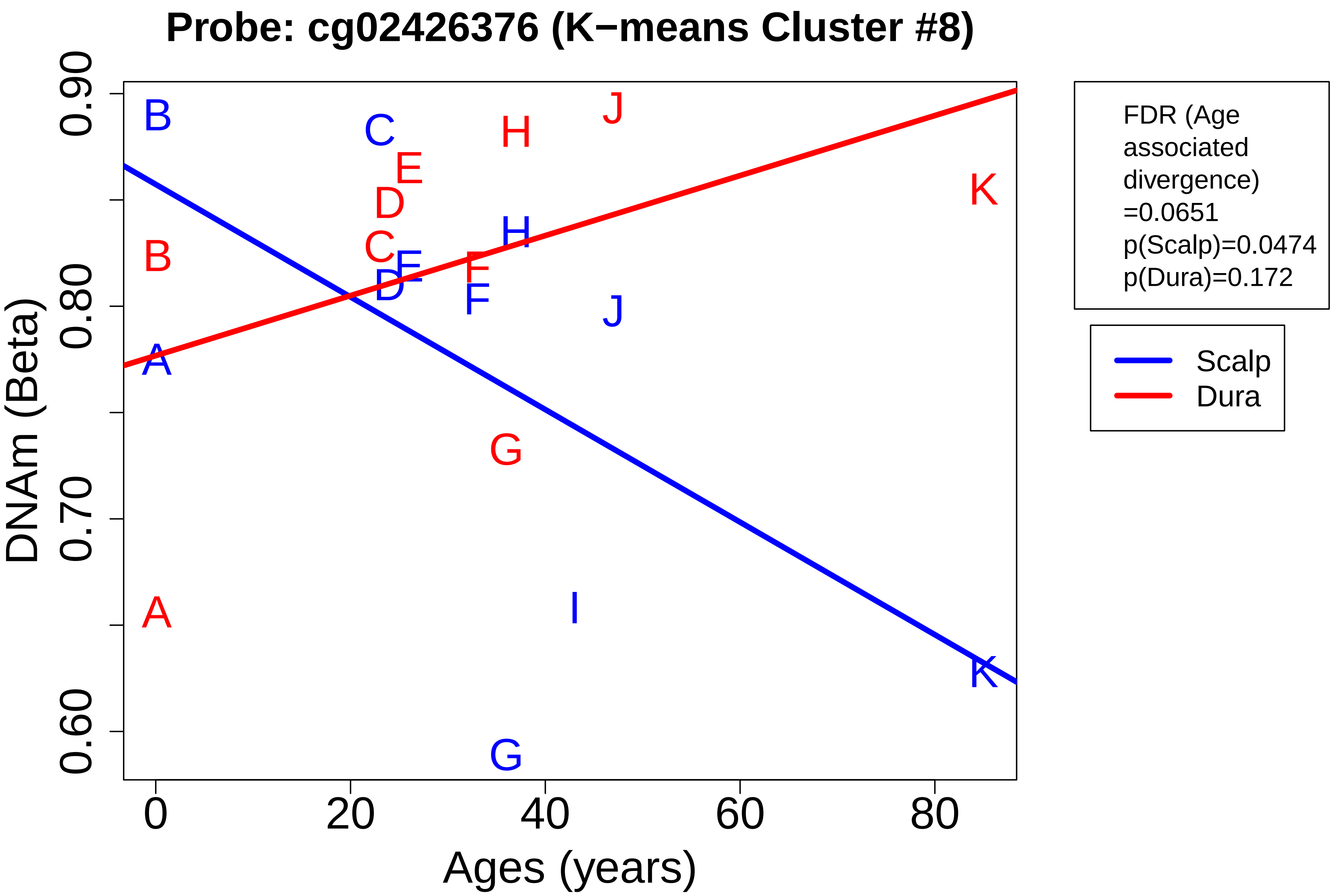
Probe: cg02426376 (K−means Cluster #8)
DNAm (Beta)
0.60
0.70
0.80
0.90
0
20
40
60
80
Ages (years)
FDR (Age associated divergence) =0.0651
p(Scalp)=0.0474
p(Dura)=0.172
Scalp
Dura
A
B
C
D
E
F
G
H
I
J
K

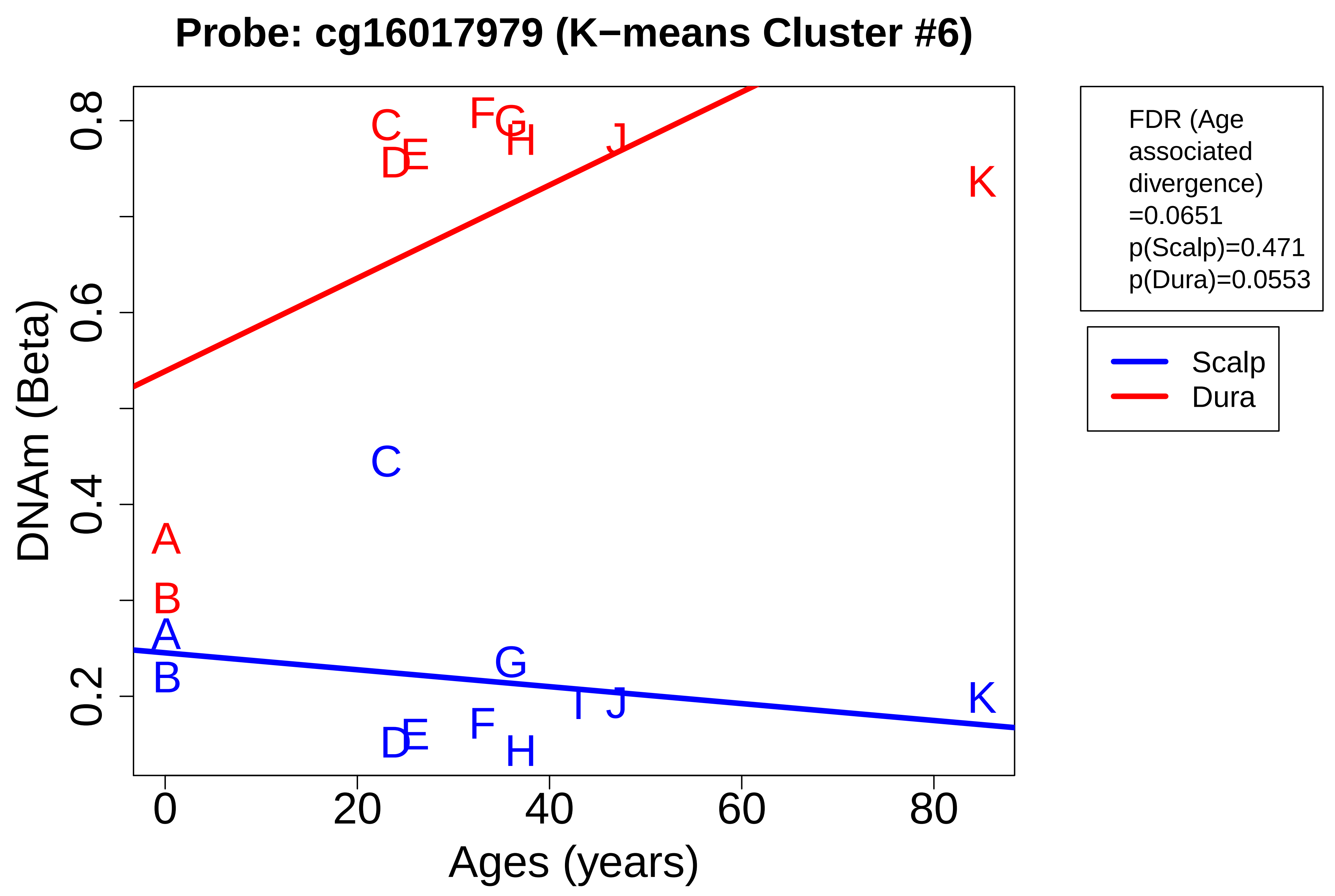
Probe: cg16017979 (K−means Cluster #6)
DNAm (Beta)
0.2
0.4
0.6
0.8
Ages (years)
0
20
40
60
80
A
B
C
D
E
F
G
H
I
J
K
FDR (Age associated divergence) =0.0651
p(Scalp)=0.471
p(Dura)=0.0553
Scalp
Dura

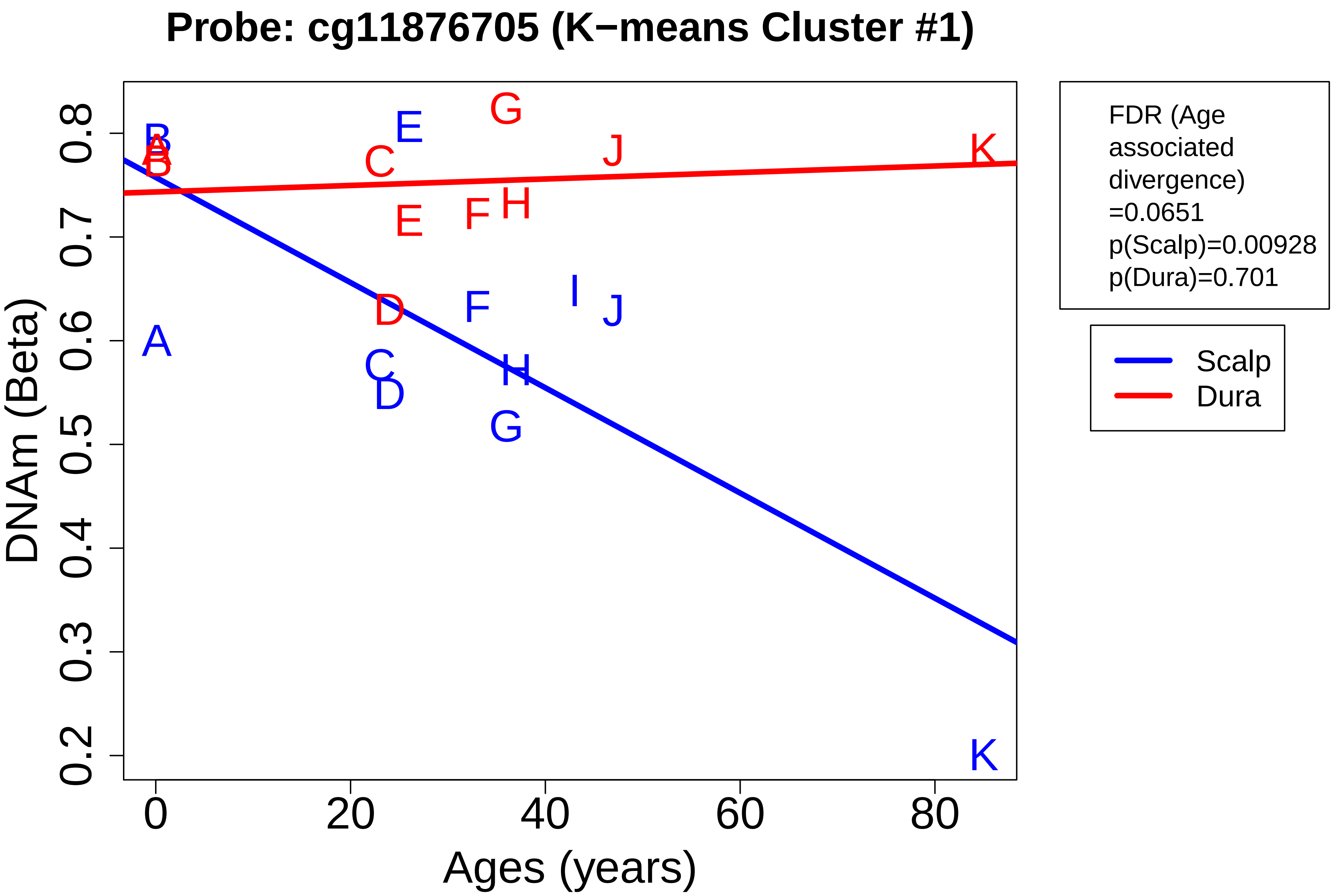

Probe: cg11876705 (K−means Cluster #1)
DNAm (Beta)
0.2
0.3
0.4
0.5
0.6
0.7
0.8
Ages (years)
0
20
40
60
80
FDR (Age associated divergence) =0.0651
p(Scalp)=0.00928
p(Dura)=0.701
Scalp
Dura
A
B
C
D
E
F
G
H
I
J
K

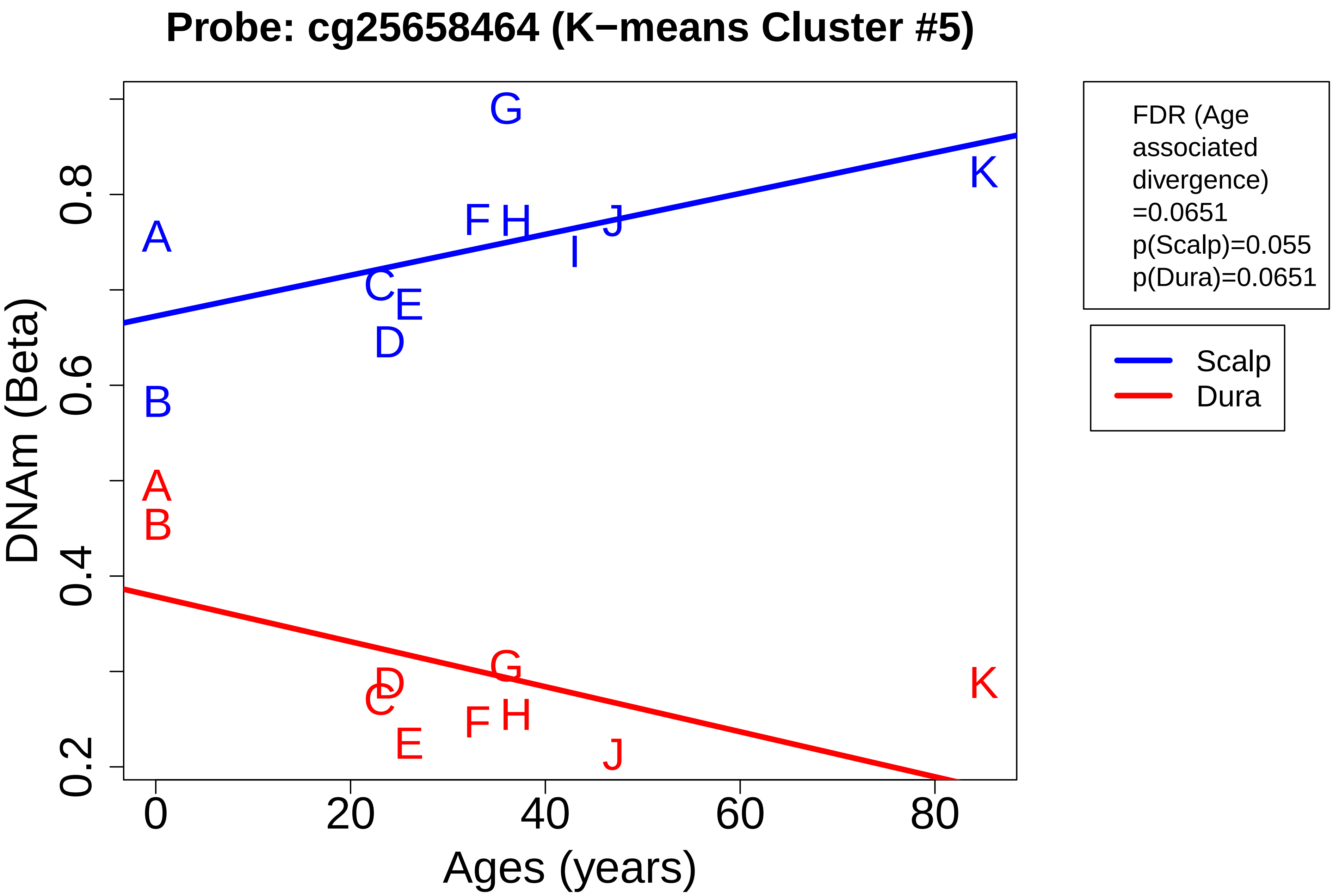
Probe: cg25658464 (K−means Cluster #5)
DNAm (Beta)
0.2
0.4
0.6
0.8
Ages (years)
0
20
40
60
80
A
B
C
D
E
F
G
H
I
J
K
A
B
C
D
E
F
G
H
J
K
FDR (Age associated divergence) =0.0651
p(Scalp)=0.055
p(Dura)=0.0651
Scalp
Dura

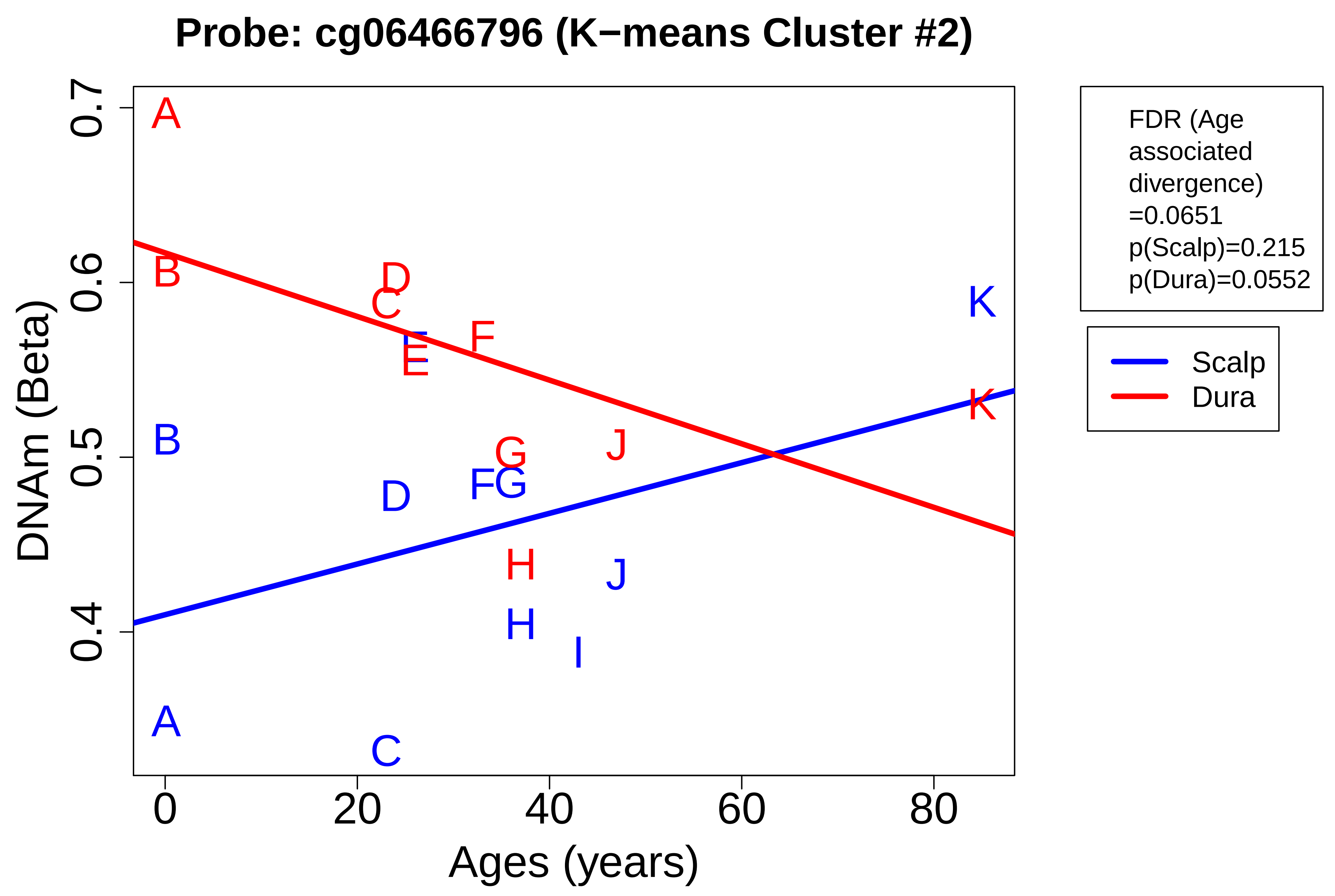

Probe: cg06466796 (K−means Cluster #2)
DNAm (Beta)
0.4
0.5
0.6
0.7
Ages (years)
0
20
40
60
80
FDR (Age associated divergence) =0.0651
p(Scalp)=0.215
p(Dura)=0.0552
Scalp
Dura
A
B
C
D
E
F
G
H
I
J
K

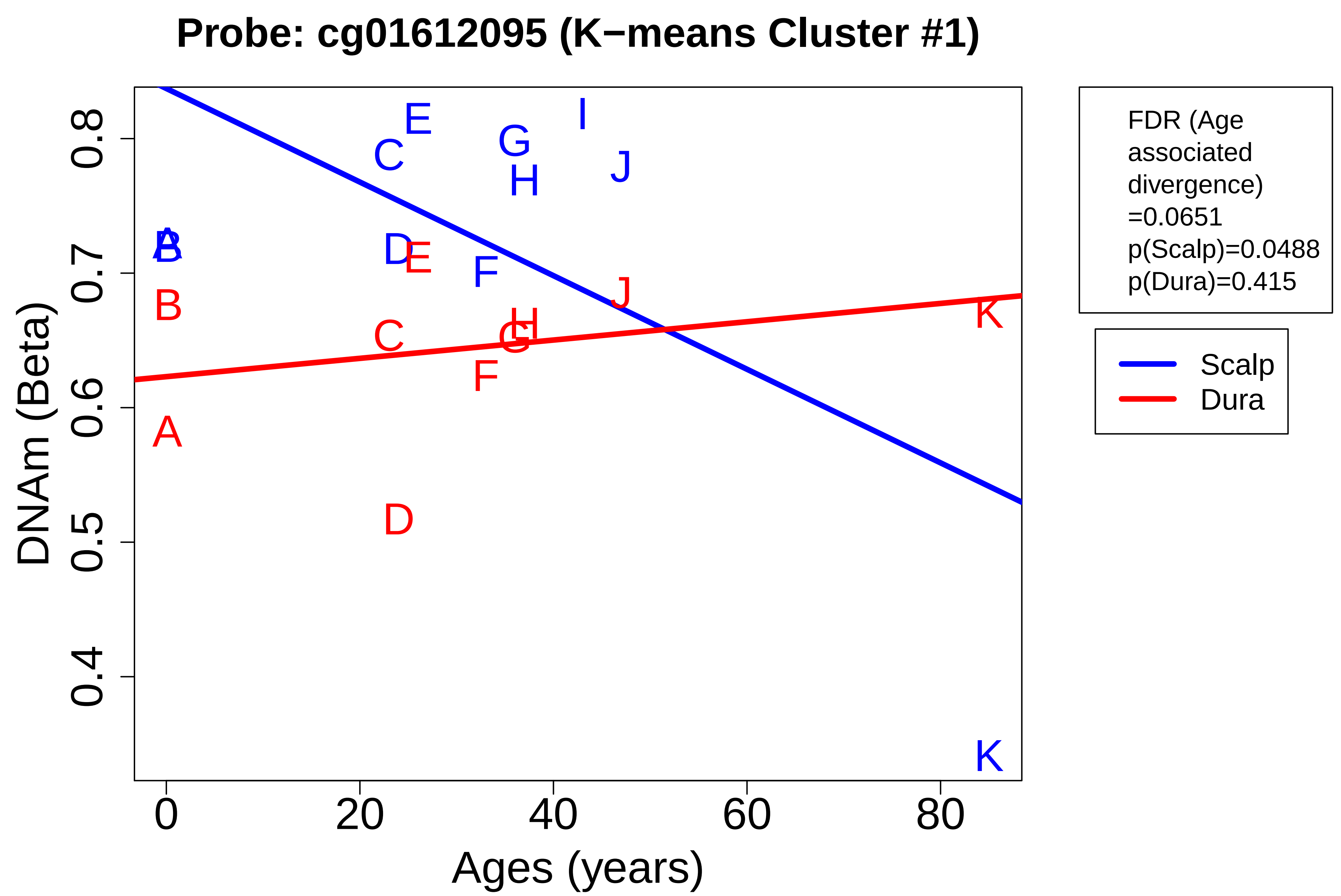
Probe: cg01612095 (K−means Cluster #1)
DNAm (Beta)
Ages (years)
0.4
0.5
0.6
0.7
0.8
0
20
40
60
80
FDR (Age associated divergence) =0.0651
p(Scalp)=0.0488
p(Dura)=0.415
Scalp
Dura

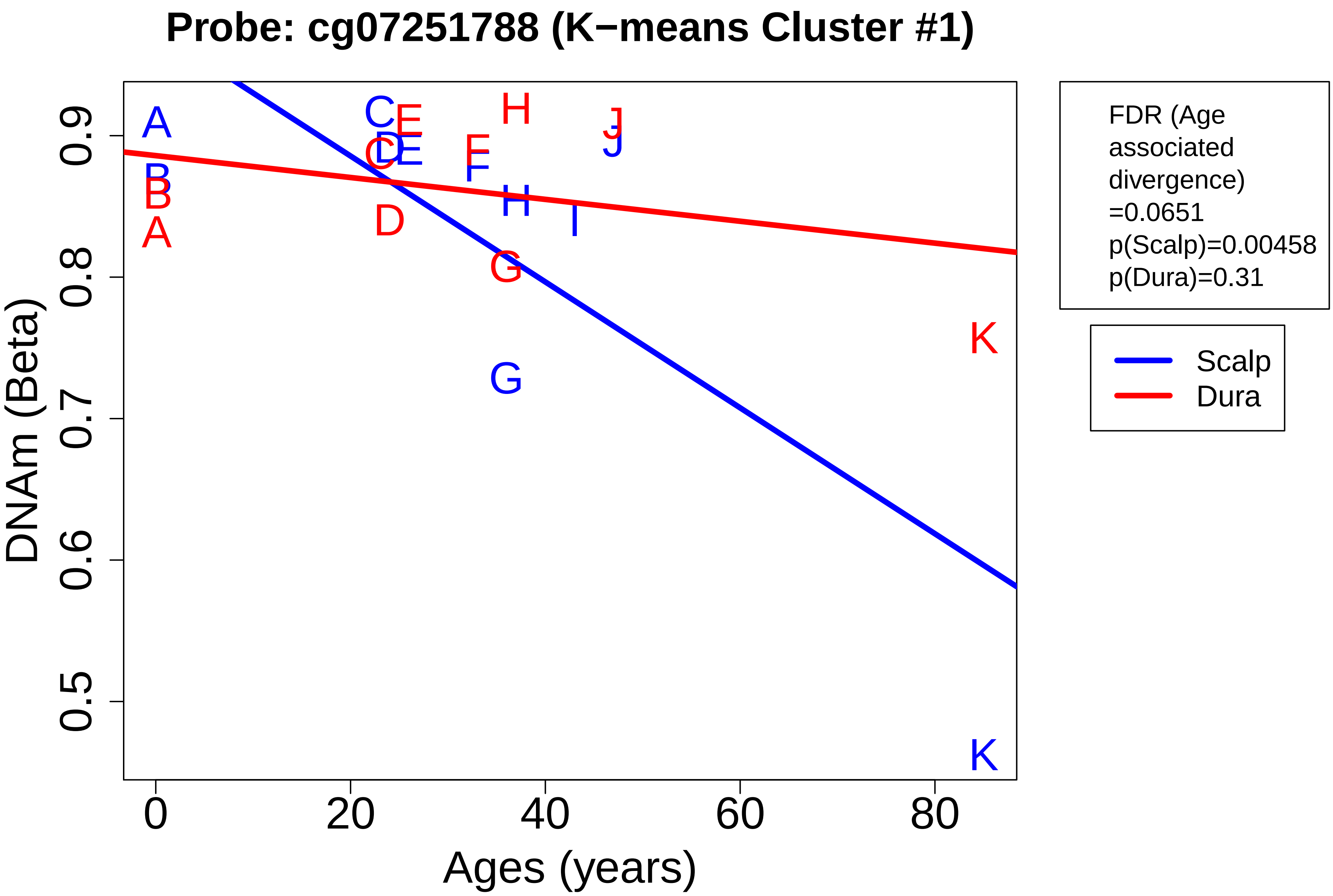

Probe: cg07251788 (K−means Cluster #1)
DNAm (Beta)
0.5
0.6
0.7
0.8
0.9
0
20
40
60
80
Ages (years)
FDR (Age associated divergence) =0.0651
p(Scalp)=0.00458
p(Dura)=0.31
Scalp
Dura

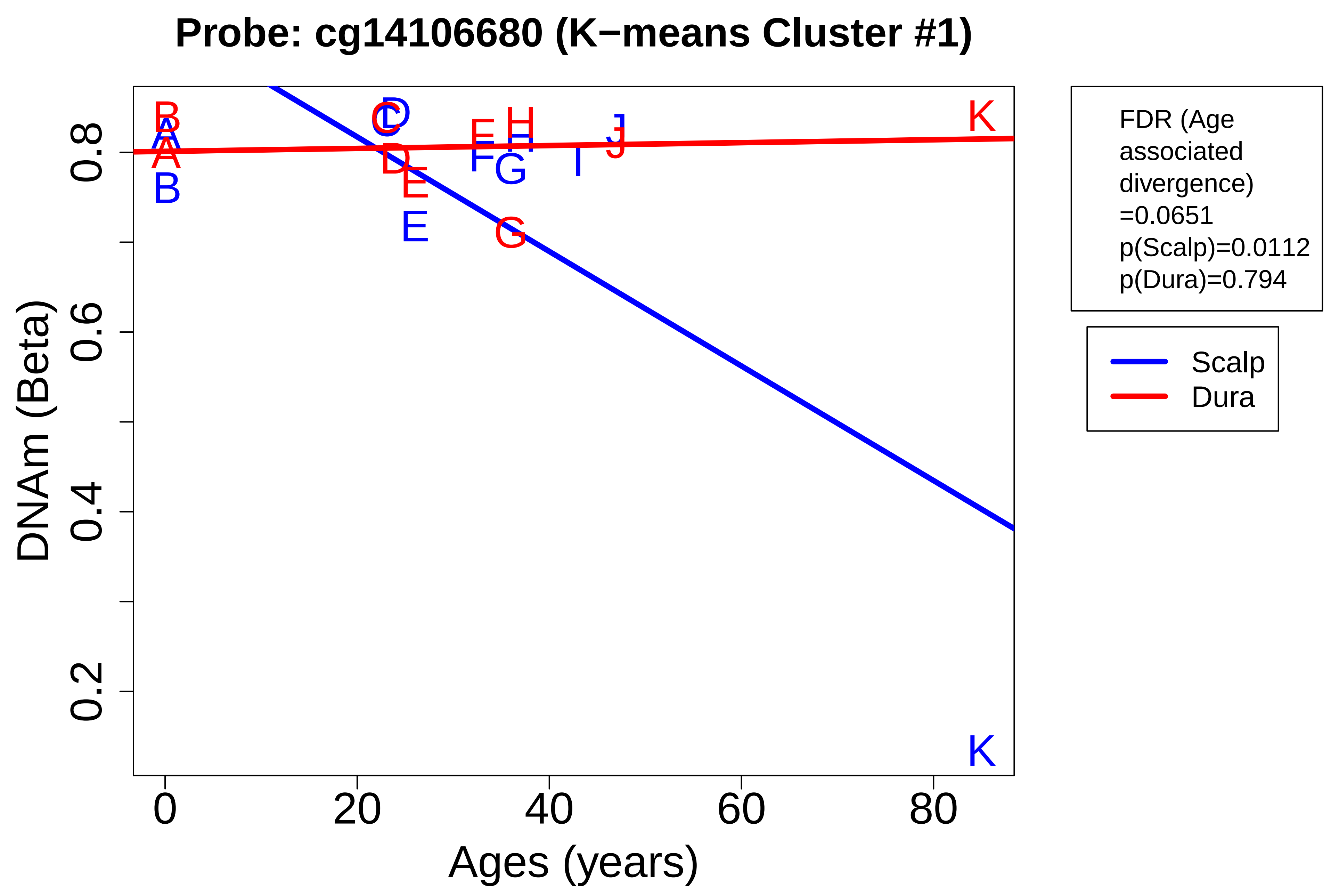

Probe: cg14106680 (K−means Cluster #1)
DNAm (Beta)
0.2
0.4
0.6
0.8
0
20
40
60
80
Ages (years)
FDR (Age associated divergence) =0.0651
p(Scalp)=0.0112
p(Dura)=0.794
Scalp
Dura

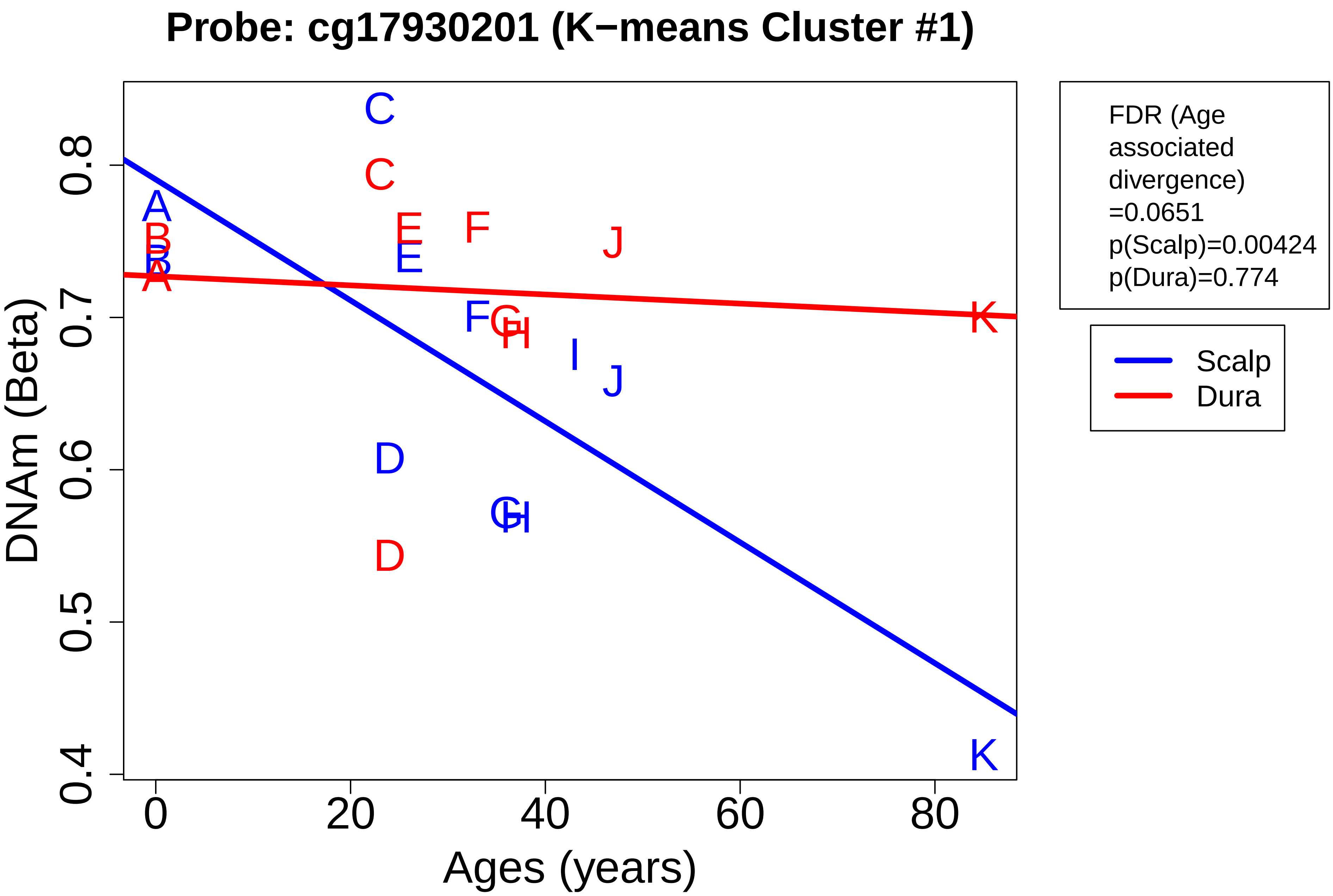

Probe: cg17930201 (K−means Cluster #1)
DNAm (Beta)
Ages (years)
0.4
0.5
0.6
0.7
0.8
0
20
40
60
80
FDR (Age associated divergence) =0.0651
p(Scalp)=0.00424
p(Dura)=0.774
Scalp
Dura

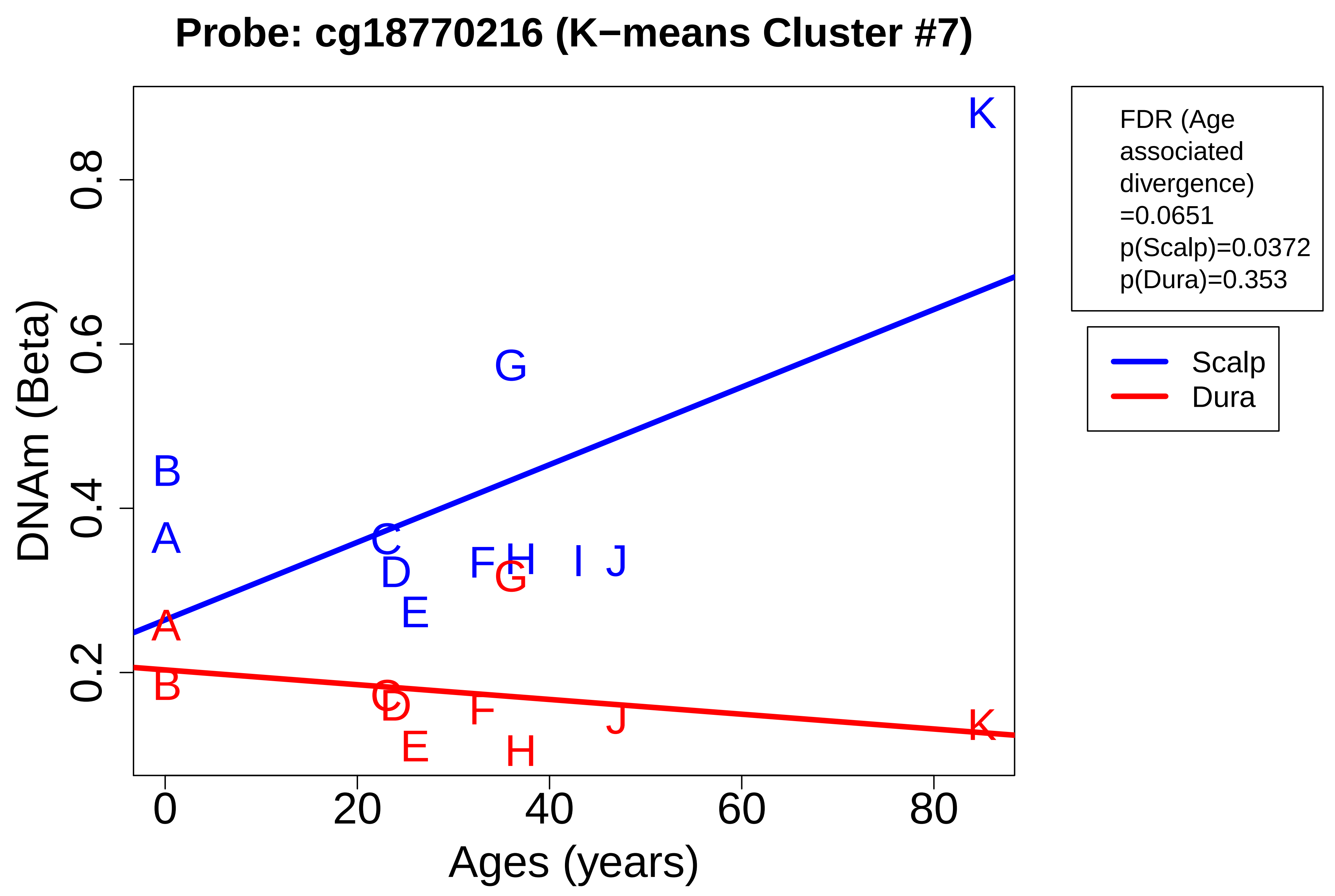

Probe: cg18770216 (K−means Cluster #7)
DNAm (Beta)
0.2
0.4
0.6
0.8
0
20
40
60
80
Ages (years)
FDR (Age associated divergence) =0.0651
p(Scalp)=0.0372
p(Dura)=0.353
Scalp
Dura

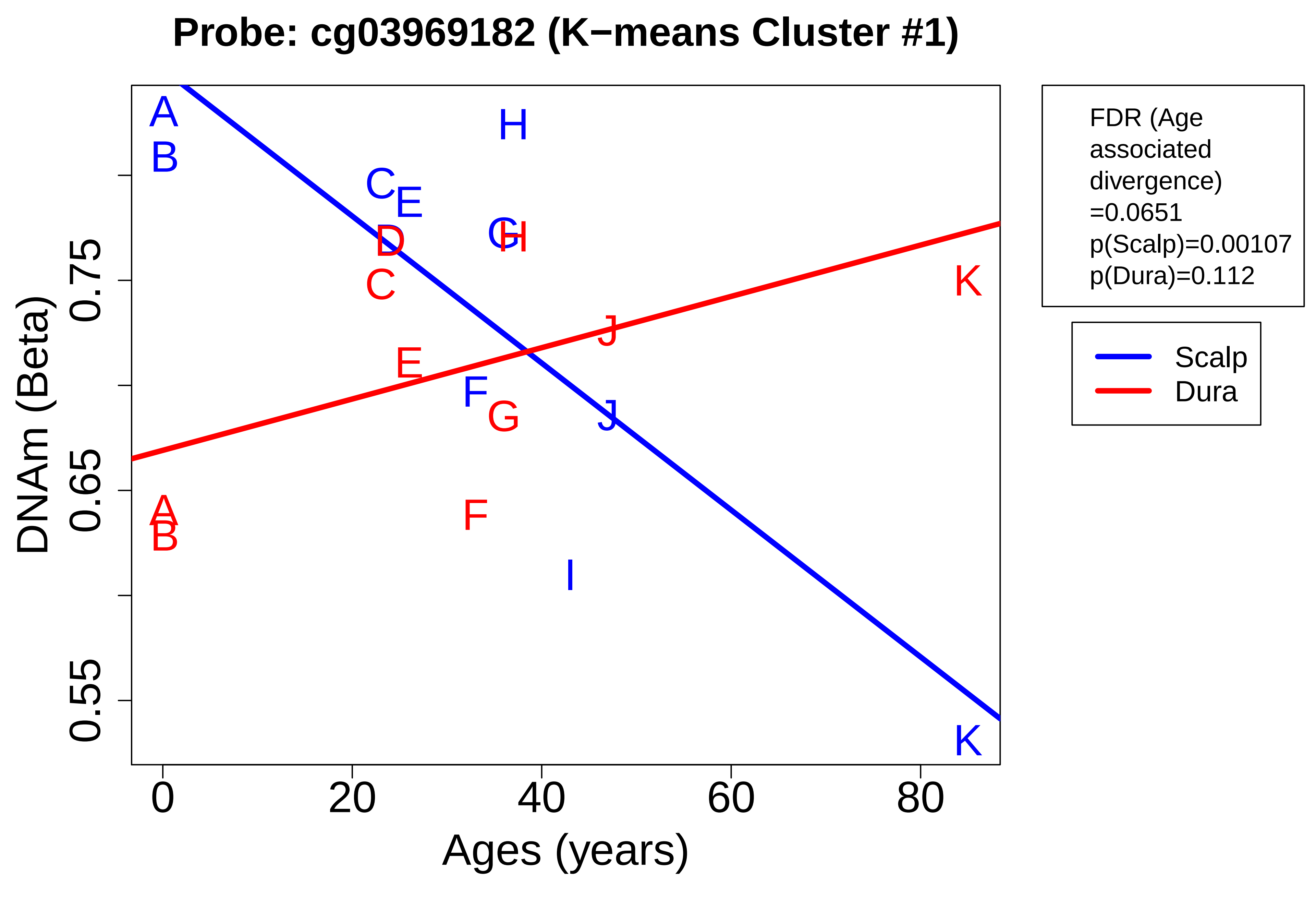

Probe: cg03969182 (K−means Cluster #1)
DNAm (Beta)
0.55
0.65
0.75
0
20
40
60
80
Ages (years)
A
B
C
D
E
F
G
H
I
J
K
FDR (Age associated divergence) =0.0651
p(Scalp)=0.00107
p(Dura)=0.112
Scalp
Dura

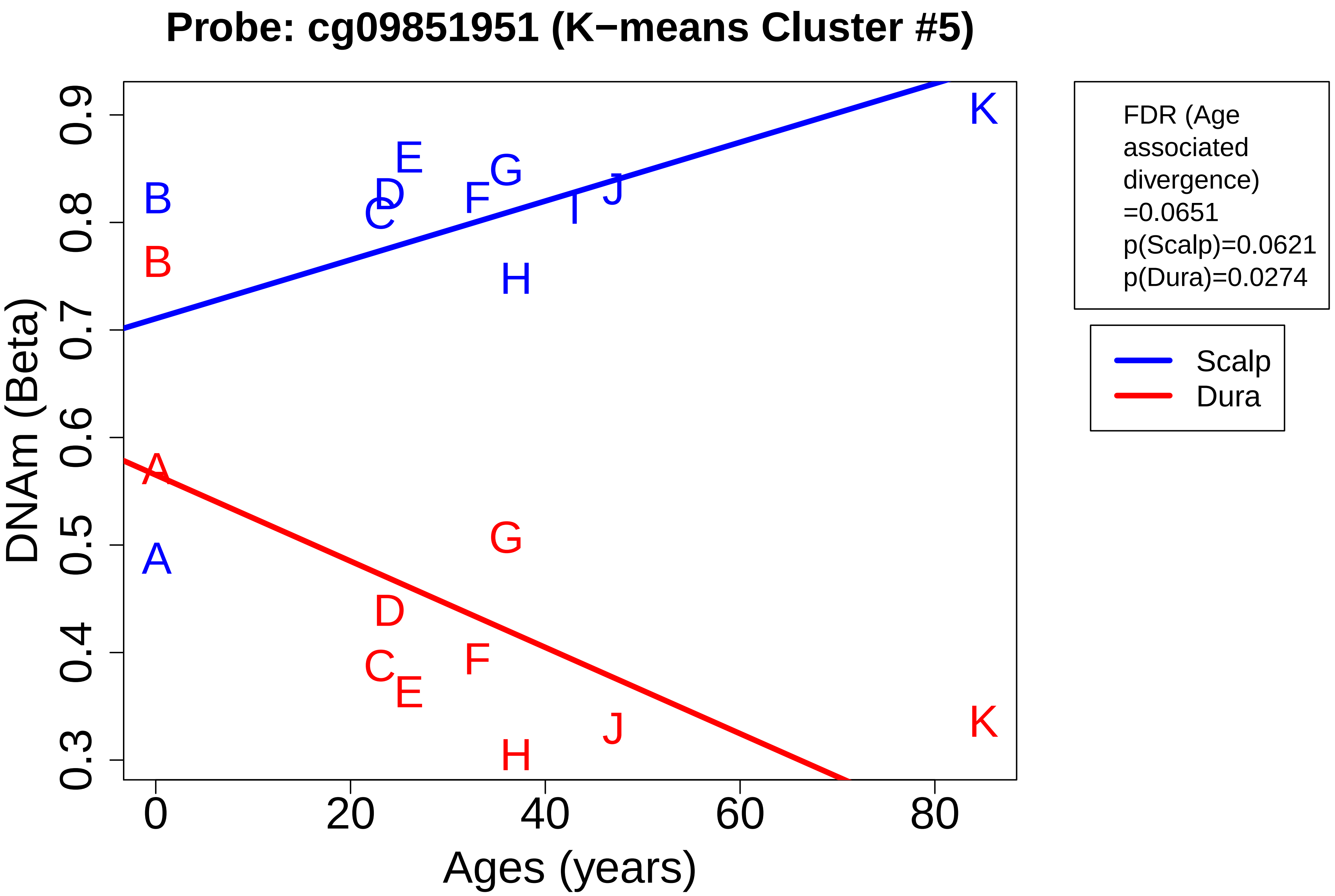

Probe: cg09851951 (K−means Cluster #5)
DNAm (Beta)
Ages (years)
0.3
0.4
0.5
0.6
0.7
0.8
0.9
0
20
40
60
80
FDR (Age associated divergence) =0.0651
p(Scalp)=0.0621
p(Dura)=0.0274
Scalp
Dura
A
B
C
D
E
F
G
H
I
J
K

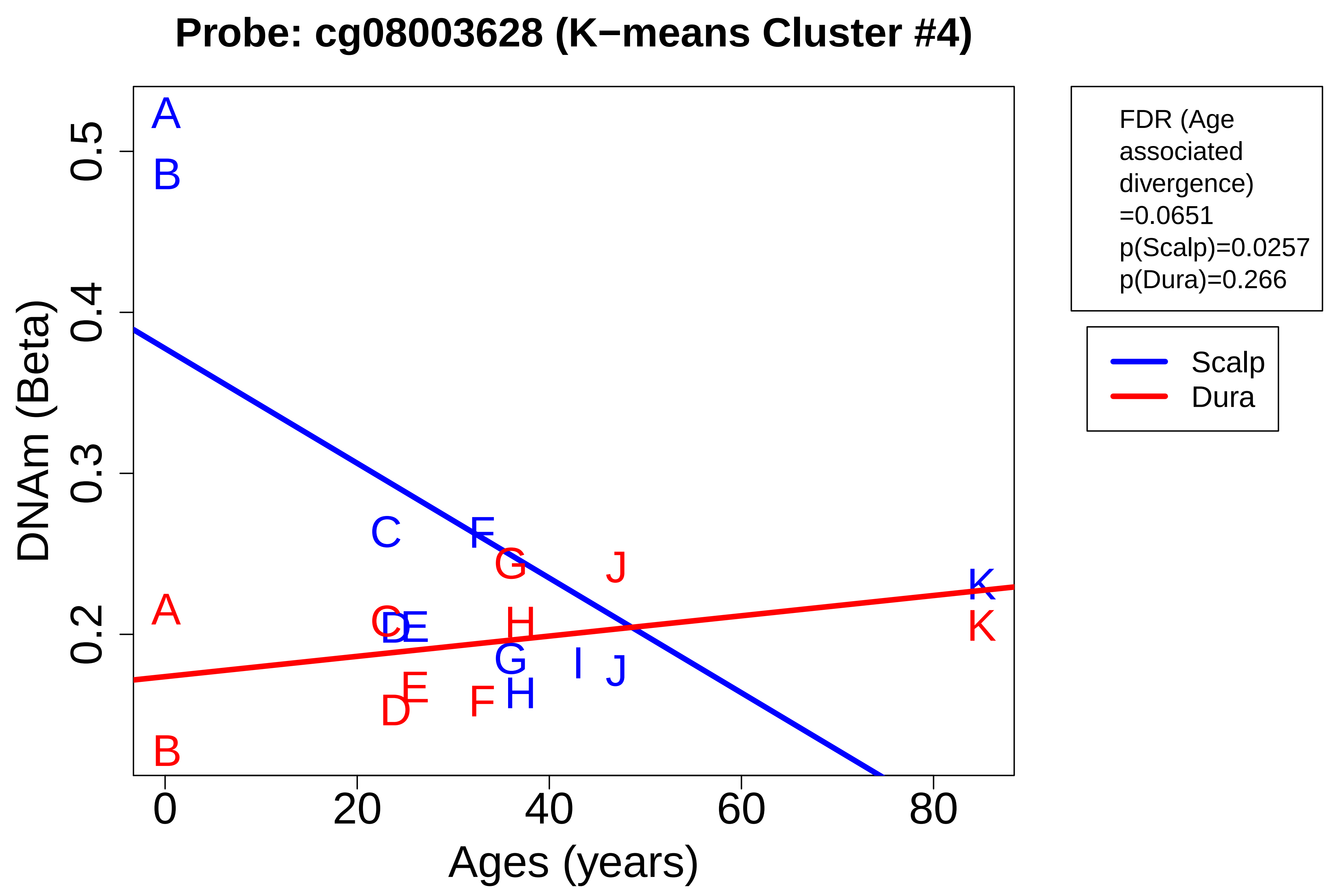
Probe: cg08003628 (K−means Cluster #4)
DNAm (Beta)
0.2
0.3
0.4
0.5
Ages (years)
0
20
40
60
80
FDR (Age associated divergence) =0.0651
p(Scalp)=0.0257
p(Dura)=0.266
Scalp
Dura

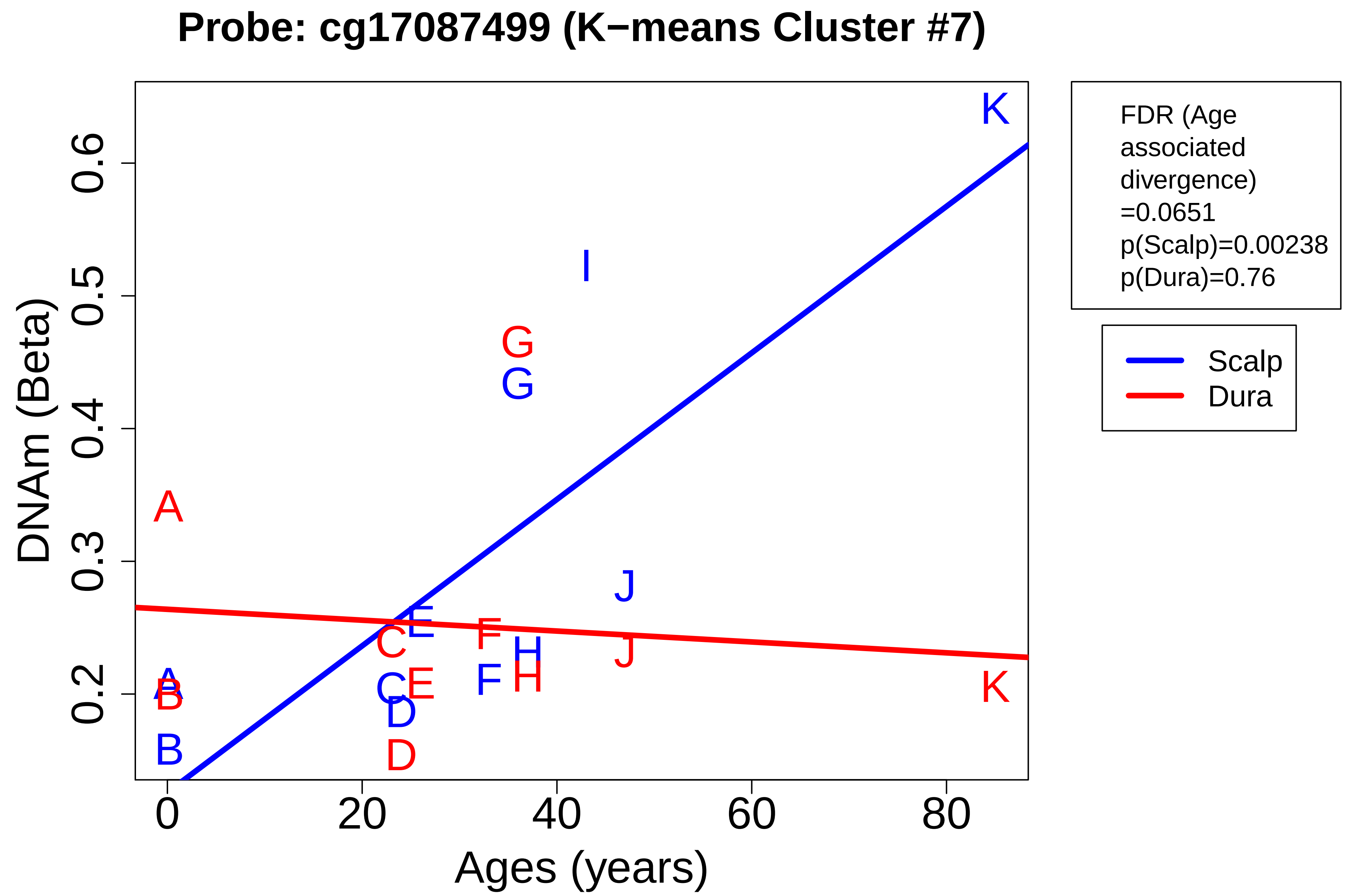

Probe: cg17087499 (K−means Cluster #7)
DNAm (Beta)
0.2
0.3
0.4
0.5
0.6
0
20
40
60
80
Ages (years)
FDR (Age associated divergence) =0.0651
p(Scalp)=0.00238
p(Dura)=0.76
Scalp
Dura
A
B
C
D
E
F
G
H
I
J
K

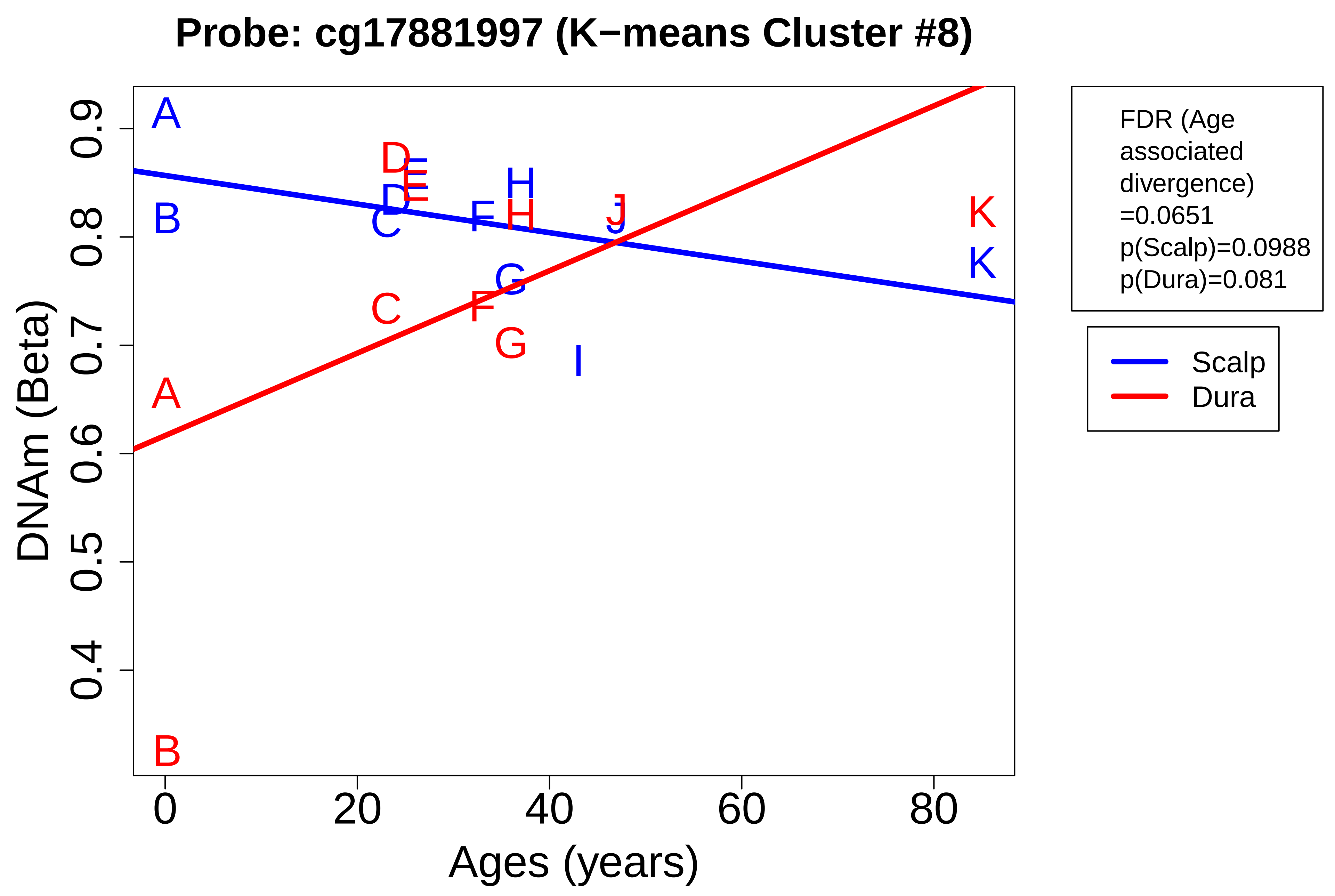

Probe: cg17881997 (K−means Cluster #8)
DNAm (Beta)
0.4
0.5
0.6
0.7
0.8
0.9
0
20
40
60
80
Ages (years)
A
B
C
D
E
F
G
H
I
J
K
FDR (Age associated divergence) =0.0651
p(Scalp)=0.0988
p(Dura)=0.081
Scalp
Dura

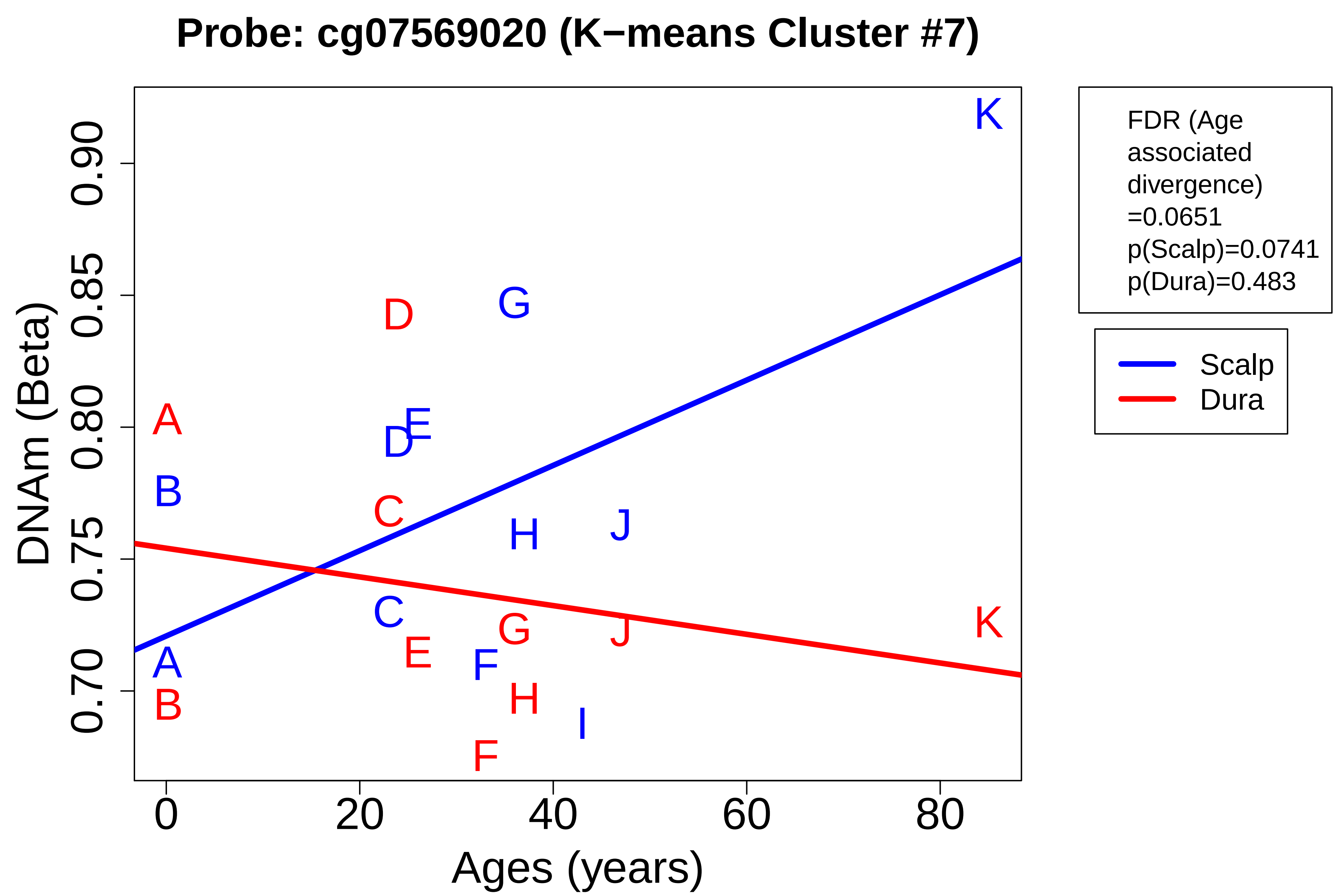

Probe: cg07569020 (K−means Cluster #7)
DNAm (Beta)
0.70
0.75
0.80
0.85
0.90
Ages (years)
0
20
40
60
80
FDR (Age associated divergence) =0.0651
p(Scalp)=0.0741
p(Dura)=0.483
Scalp
Dura
A
B
C
D
E
F
G
H
I
J
K

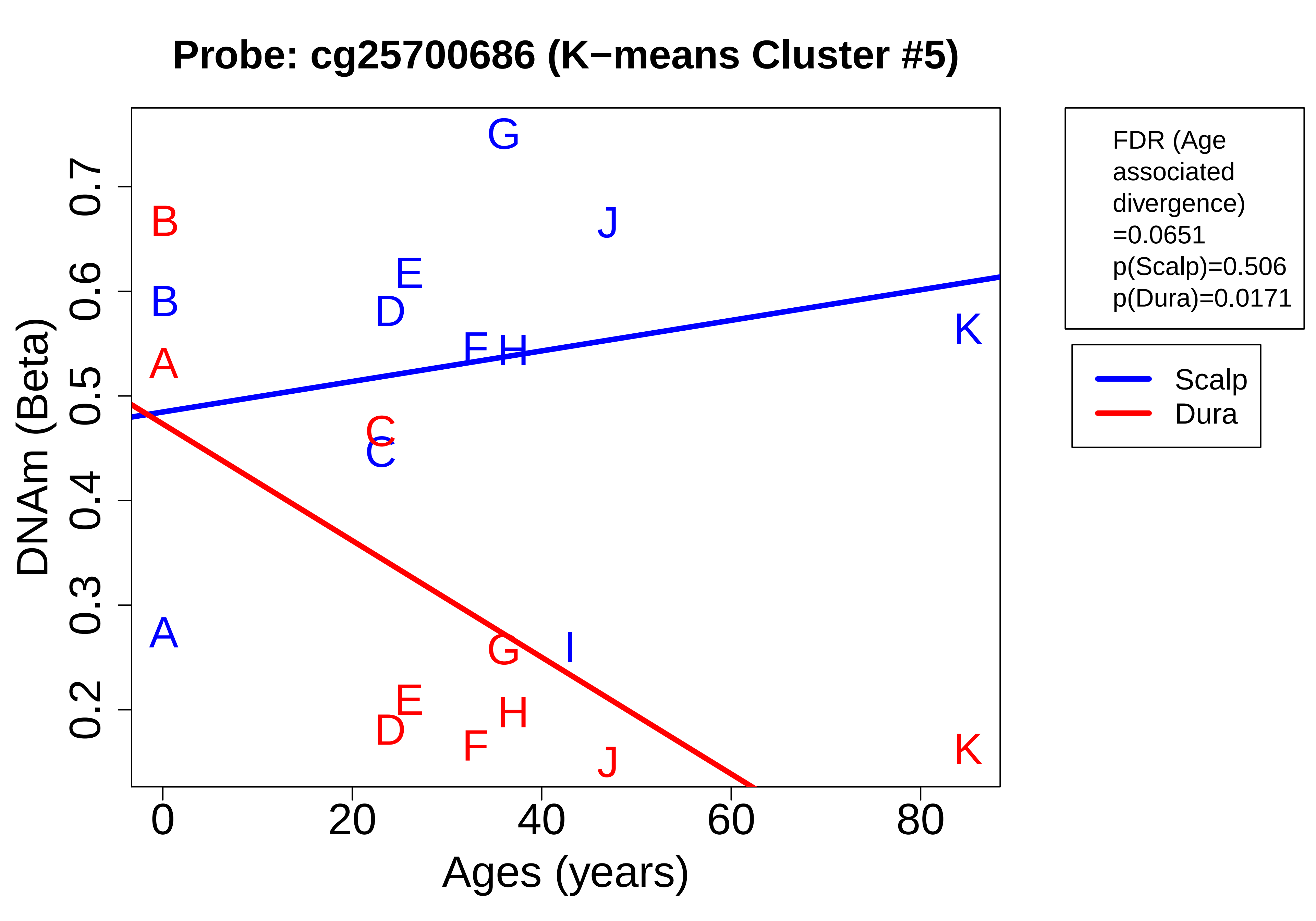
Probe: cg25700686 (K−means Cluster #5)
DNAm (Beta)
Ages (years)
0.2
0.3
0.4
0.5
0.6
0.7
0
20
40
60
80
FDR (Age associated divergence) =0.0651
p(Scalp)=0.506
p(Dura)=0.0171
Scalp
Dura
A
B
C
D
E
F
G
H
I
J
K

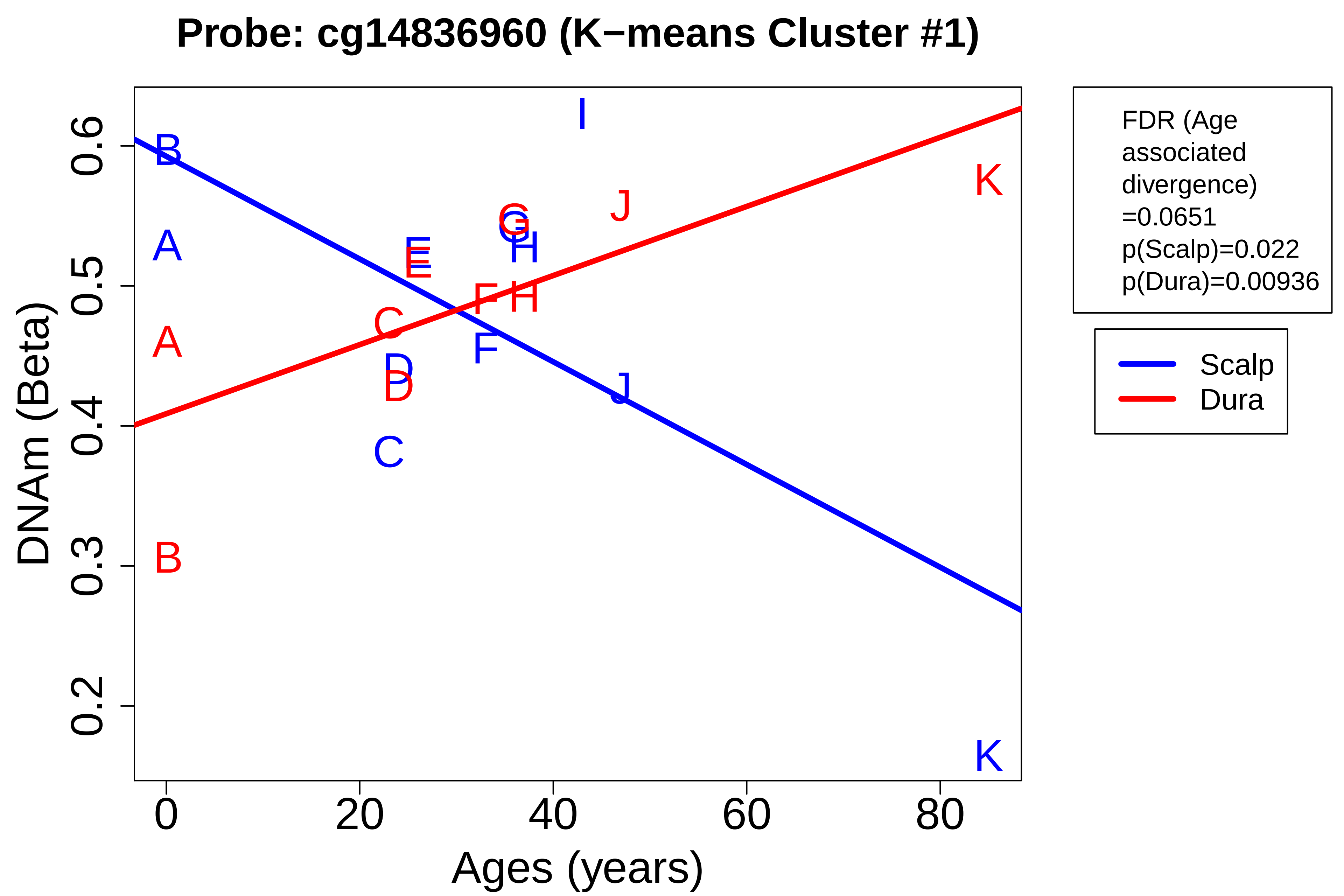
Probe: cg14836960 (K−means Cluster #1)
DNAm (Beta)
0.2
0.3
0.4
0.5
0.6
Ages (years)
0
20
40
60
80
FDR (Age associated divergence) =0.0651
p(Scalp)=0.022
p(Dura)=0.00936
Scalp
Dura
A
B
C
D
E
F
G
H
I
J
K

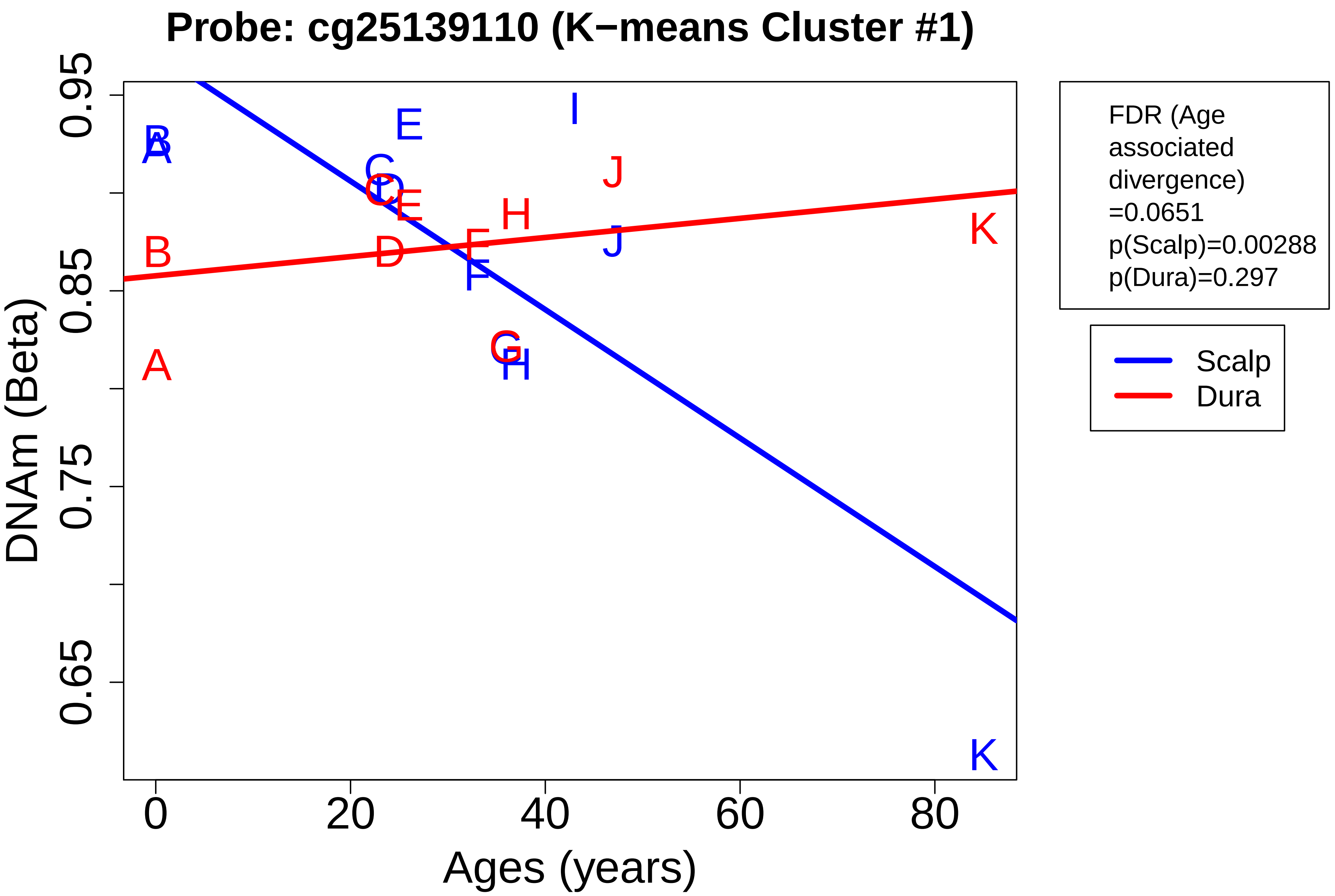

Probe: cg25139110 (K−means Cluster #1)
DNAm (Beta)
0.95
0.85
0.75
0.65
0
20
40
60
80
Ages (years)
FDR (Age associated divergence) =0.0651
p(Scalp)=0.00288
p(Dura)=0.297
Scalp
Dura

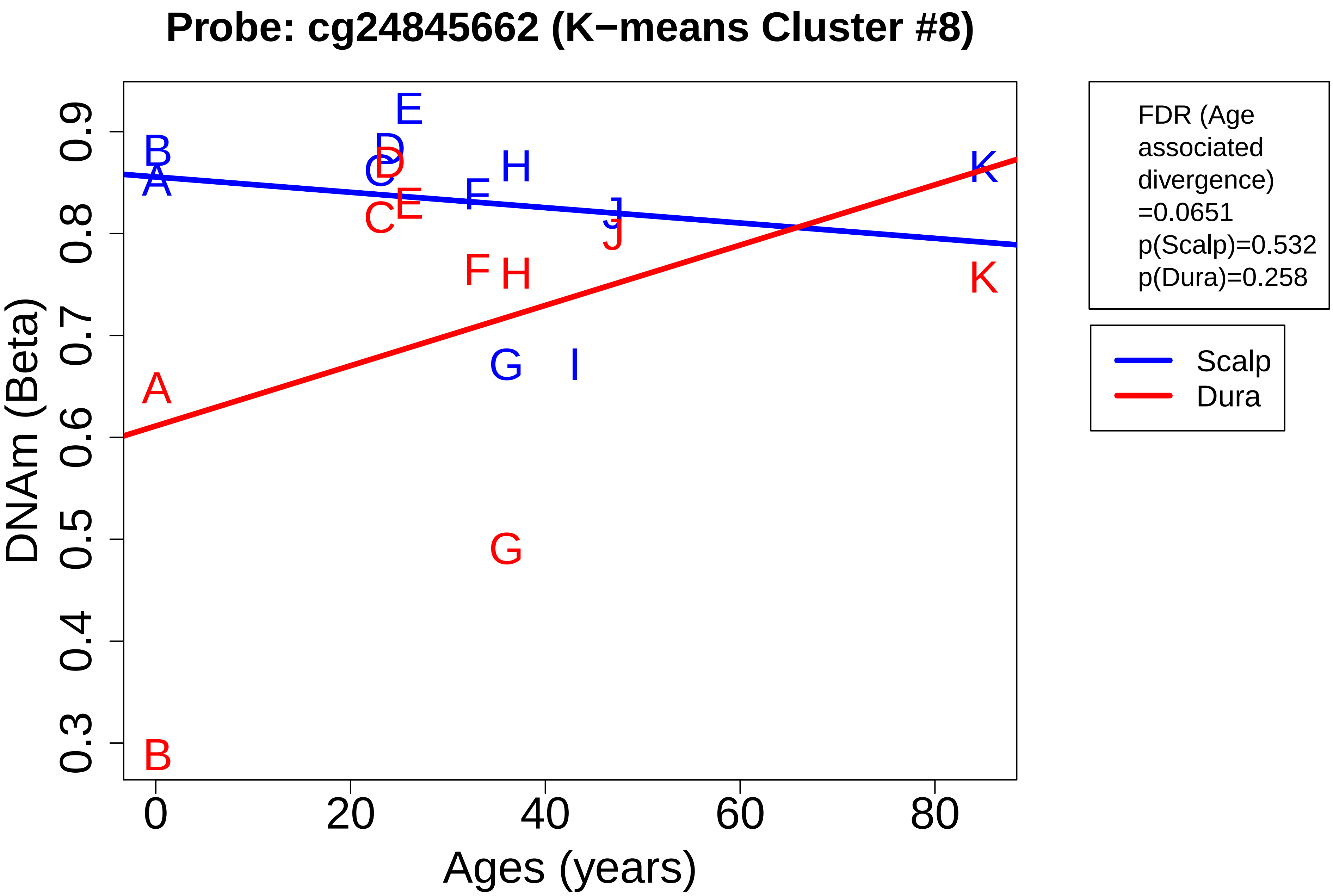

Probe: cg24845662 (K−means Cluster #8)
DNAm (Beta)
0.3
0.4
0.5
0.6
0.7
0.8
0.9
0
20
40
60
80
Ages (years)
A
B
C
D
E
F
G
H
I
J
K
FDR (Age associated divergence) =0.0651
p(Scalp)=0.532
p(Dura)=0.258
Scalp
Dura

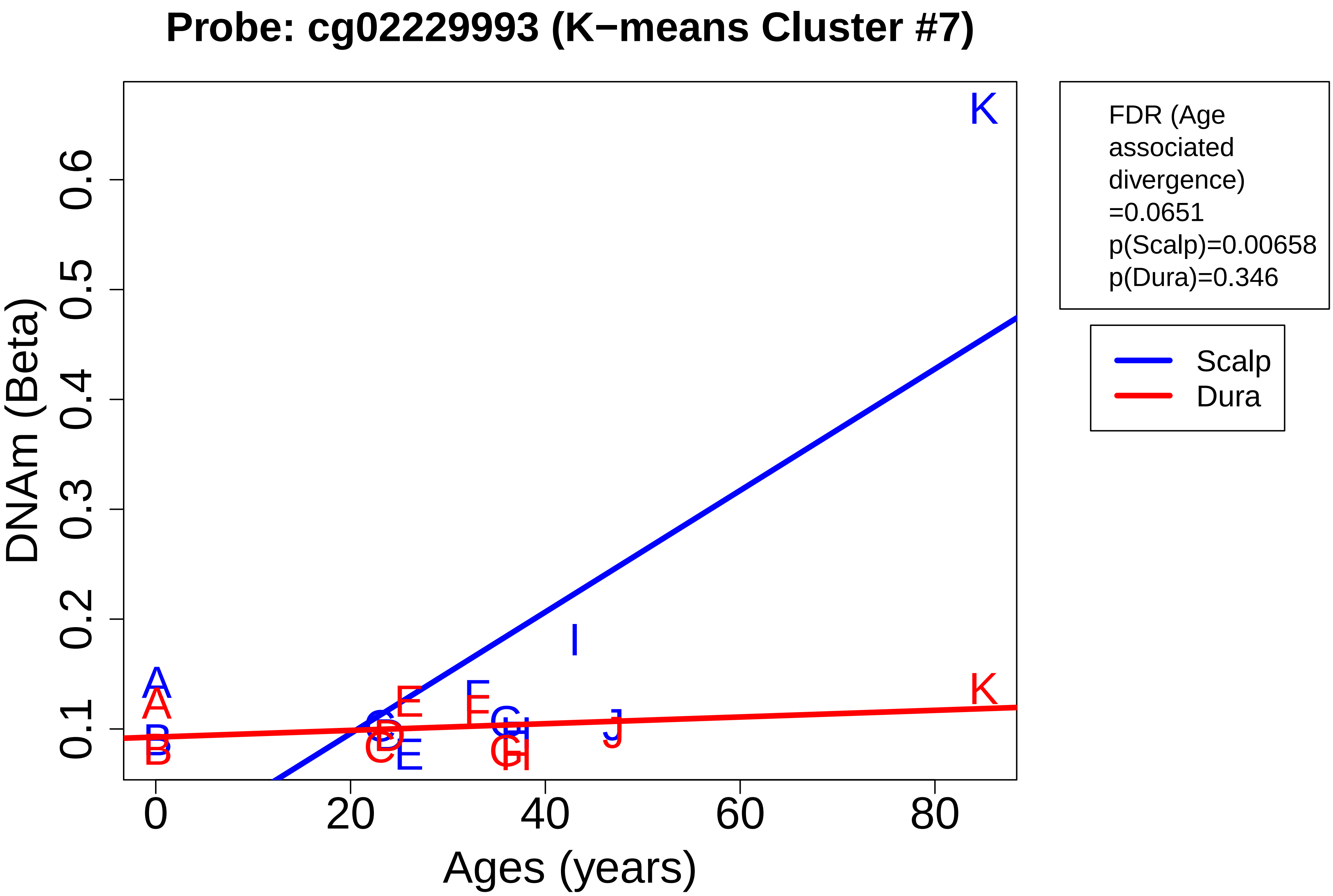

Probe: cg02229993 (K−means Cluster #7)
FDR (Age associated divergence) =0.0651
p(Scalp)=0.00658
p(Dura)=0.346
Scalp
Dura
DNAm (Beta)
0.1
0.2
0.3
0.4
0.5
0.6
Ages (years)
0
20
40
60
80
A
B
C
D
E
F
G
H
I
J
K

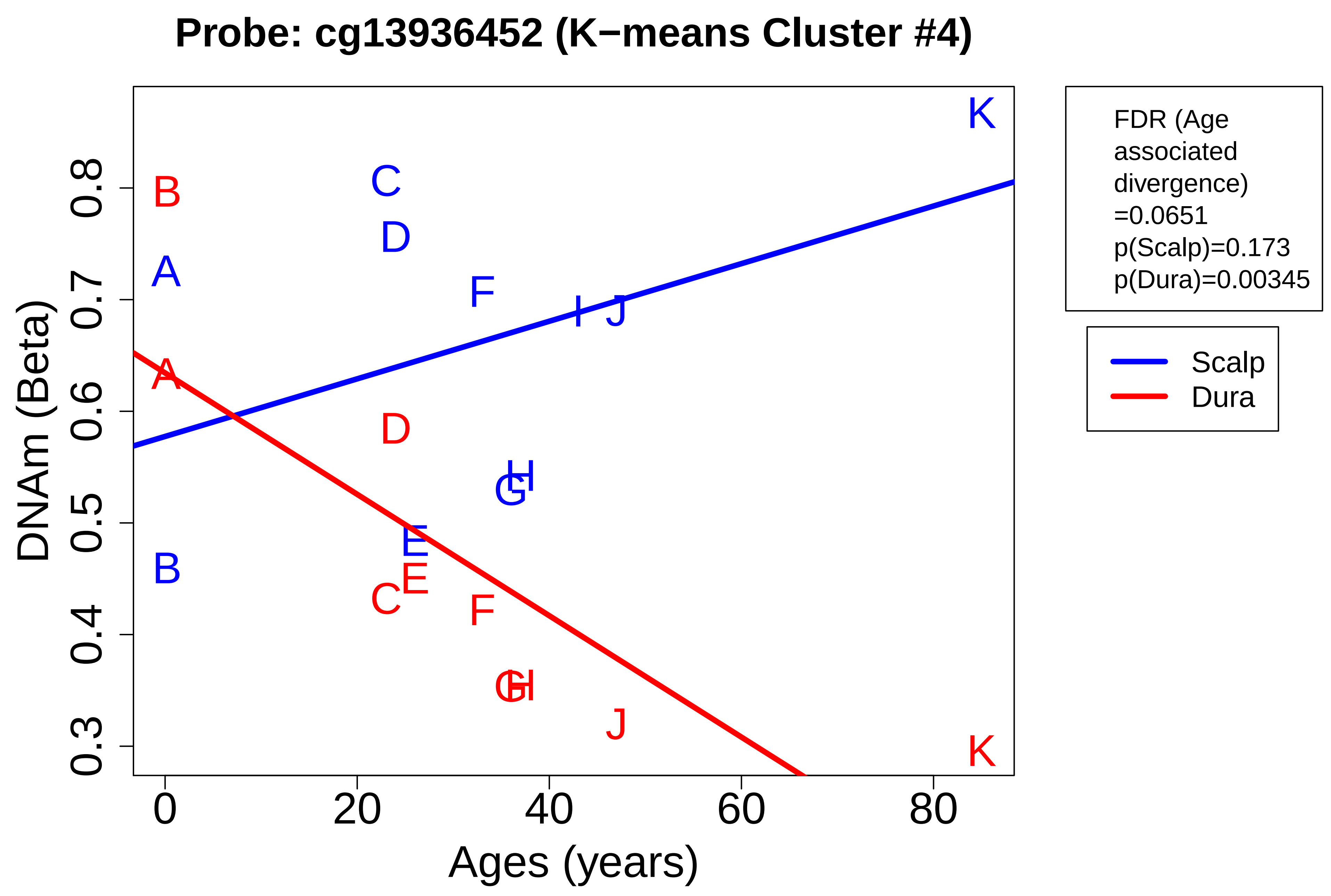
Probe: cg13936452 (K−means Cluster #4)
DNAm (Beta)
0.3
0.4
0.5
0.6
0.7
0.8
0
20
40
60
80
Ages (years)
B
A
C
D
F
I
J
K
A
D
H
G
E
B
E
C
F
G
H
J
K
FDR (Age associated divergence) =0.0651
p(Scalp)=0.173
p(Dura)=0.00345
Scalp
Dura

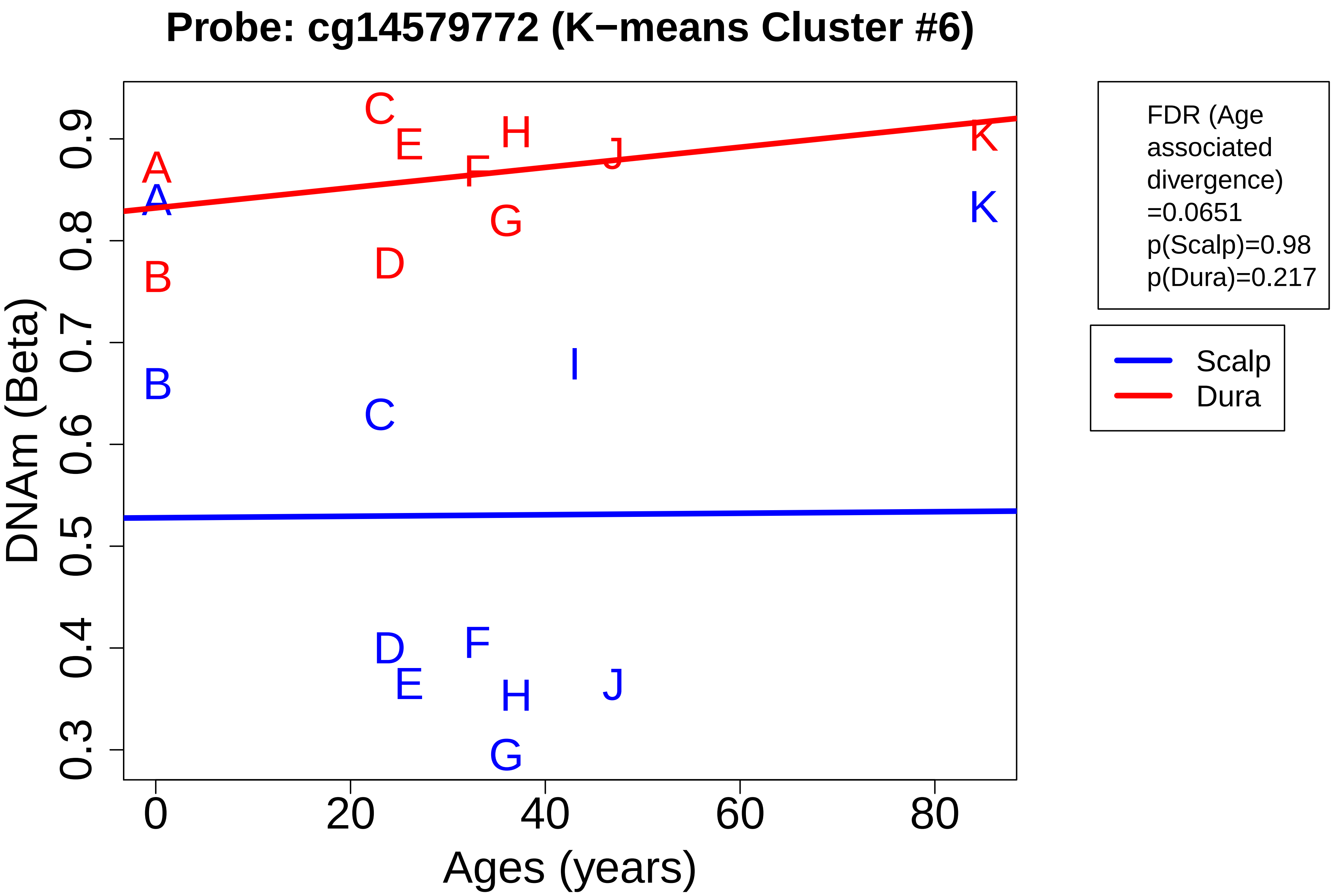

Probe: cg14579772 (K−means Cluster #6)
DNAm (Beta)
0.3
0.4
0.5
0.6
0.7
0.8
0.9
0
20
40
60
80
Ages (years)
A
B
C
D
E
F
G
H
I
J
K
A
B
C
D
E
F
G
H
J
K
FDR (Age associated divergence) =0.0651
p(Scalp)=0.98
p(Dura)=0.217
Scalp
Dura

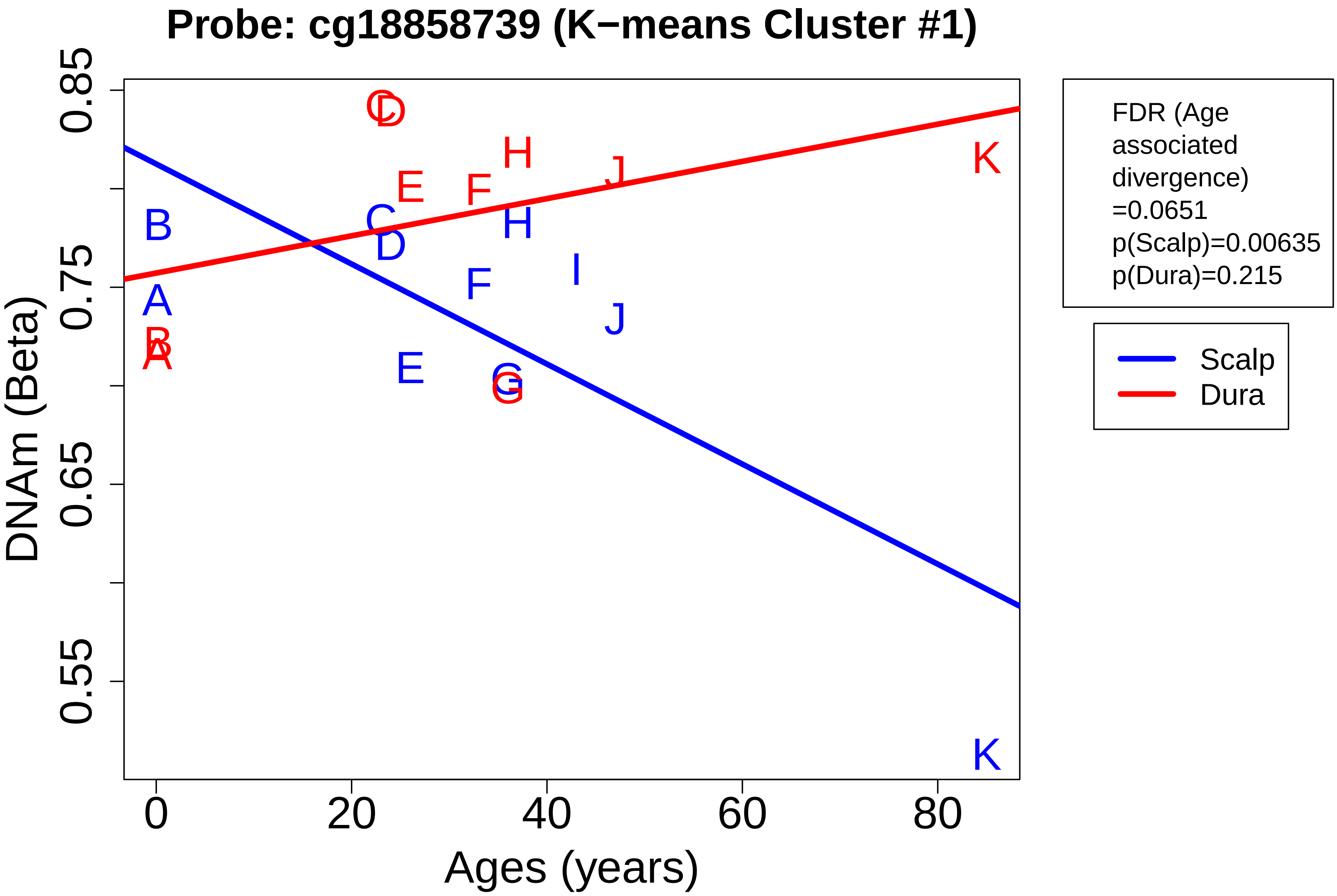
Probe: cg18858739 (K−means Cluster #1)
DNAm (Beta)
0.55
0.65
0.75
0.85
0
20
40
60
80
Ages (years)
FDR (Age associated divergence) =0.0651
p(Scalp)=0.00635
p(Dura)=0.215
Scalp
Dura
A
B
C
D
E
F
G
H
I
J
K

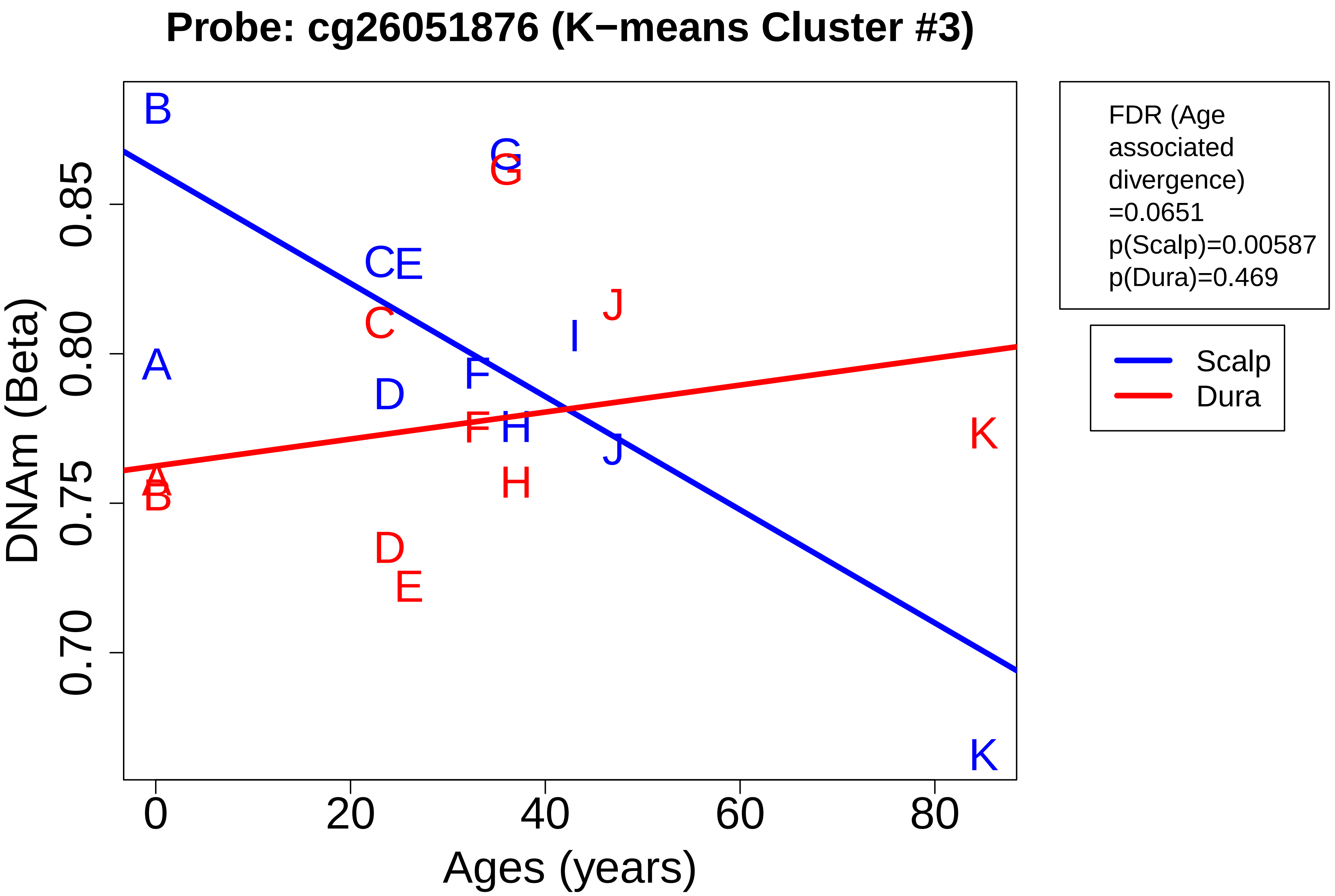

Probe: cg26051876 (K−means Cluster #3)
FDR (Age associated divergence) =0.0651
p(Scalp)=0.00587
p(Dura)=0.469
Scalp
Dura
DNAm (Beta)
0.70
0.75
0.80
0.85
0
20
40
60
80
Ages (years)

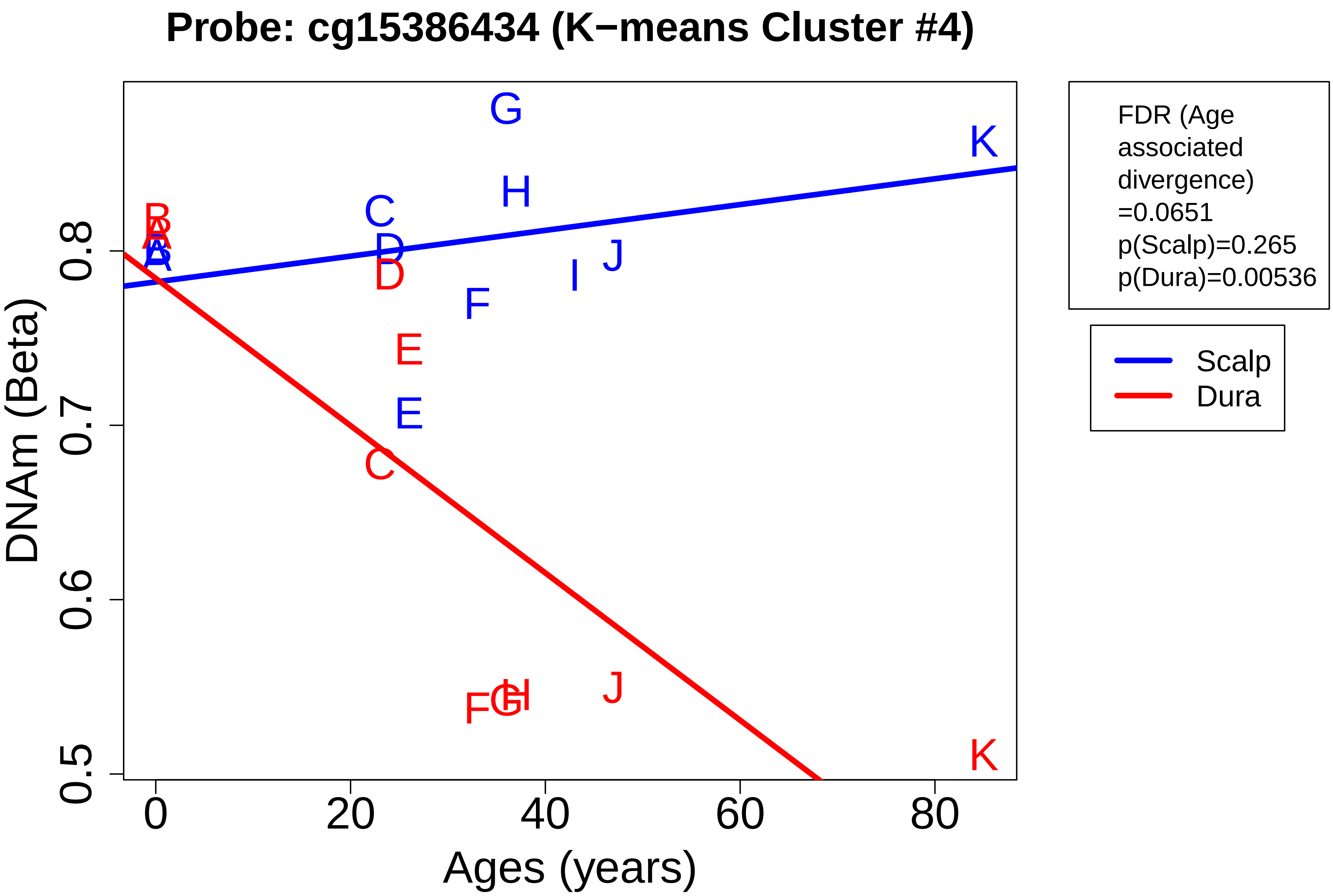

Probe: cg15386434 (K−means Cluster #4)
DNAm (Beta)
Ages (years)
0.5
0.6
0.7
0.8
0
20
40
60
80
FDR (Age associated divergence) =0.0651
p(Scalp)=0.265
p(Dura)=0.00536
Scalp
Dura

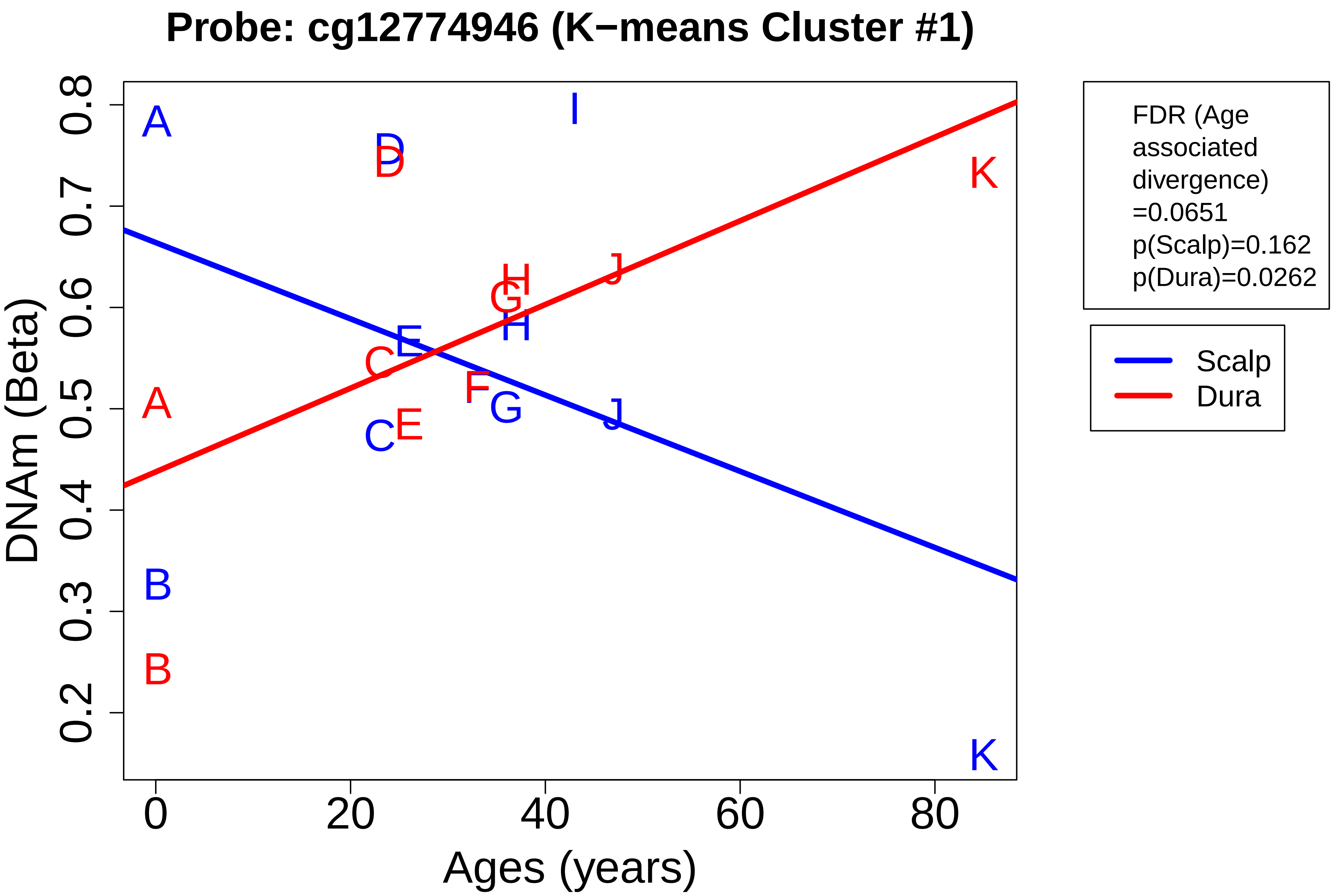

Probe: cg12774946 (K−means Cluster #1)
DNAm (Beta)
0.2
0.3
0.4
0.5
0.6
0.7
0.8
0
20
40
60
80
Ages (years)
A
B
C
D
E
F
G
H
I
J
K
FDR (Age associated divergence) =0.0651
p(Scalp)=0.162
p(Dura)=0.0262
Scalp
Dura

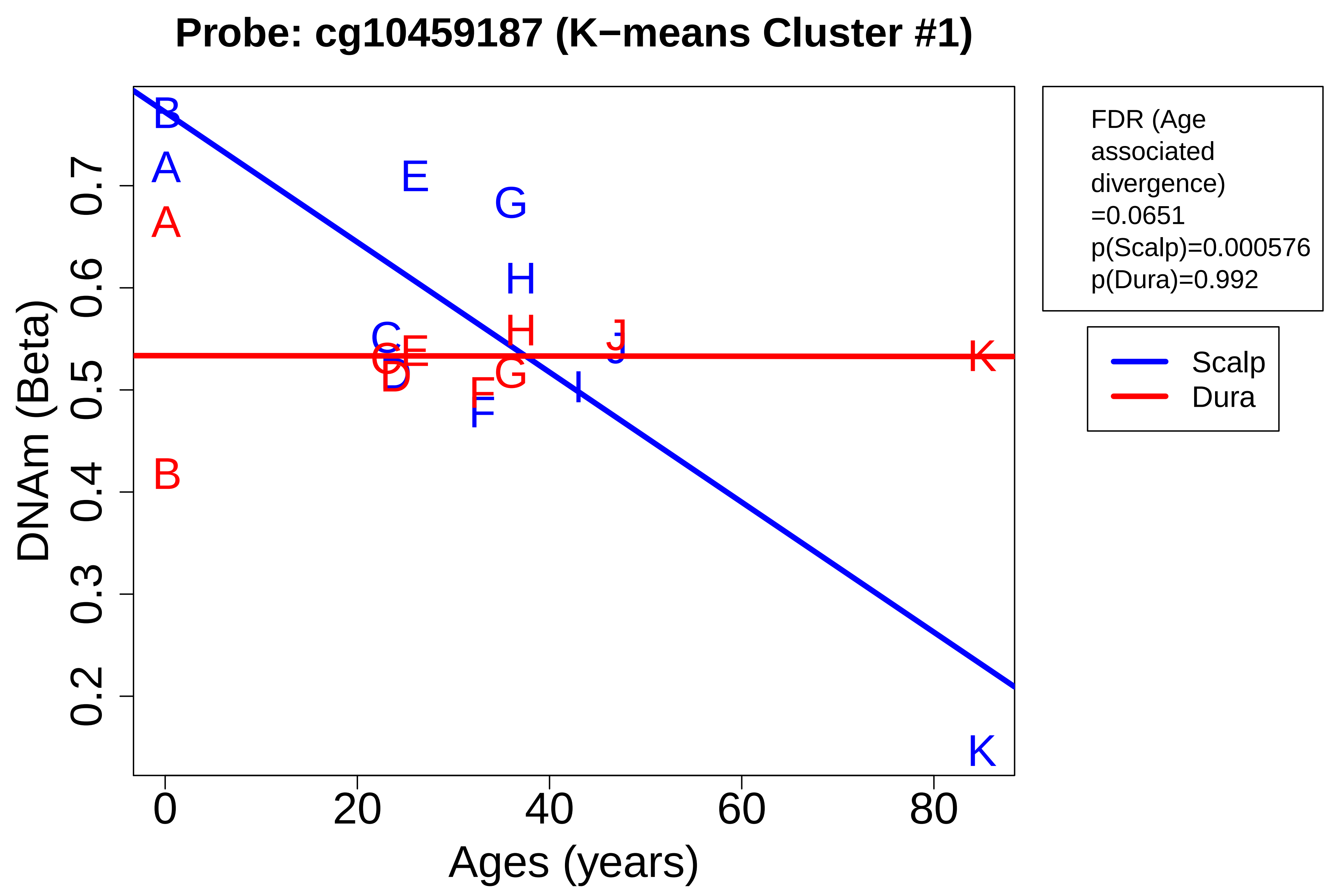

Probe: cg10459187 (K−means Cluster #1)
DNAm (Beta)
0.2
0.3
0.4
0.5
0.6
0.7
Ages (years)
0
20
40
60
80
FDR (Age associated divergence) =0.0651
p(Scalp)=0.000576
p(Dura)=0.992
Scalp
Dura

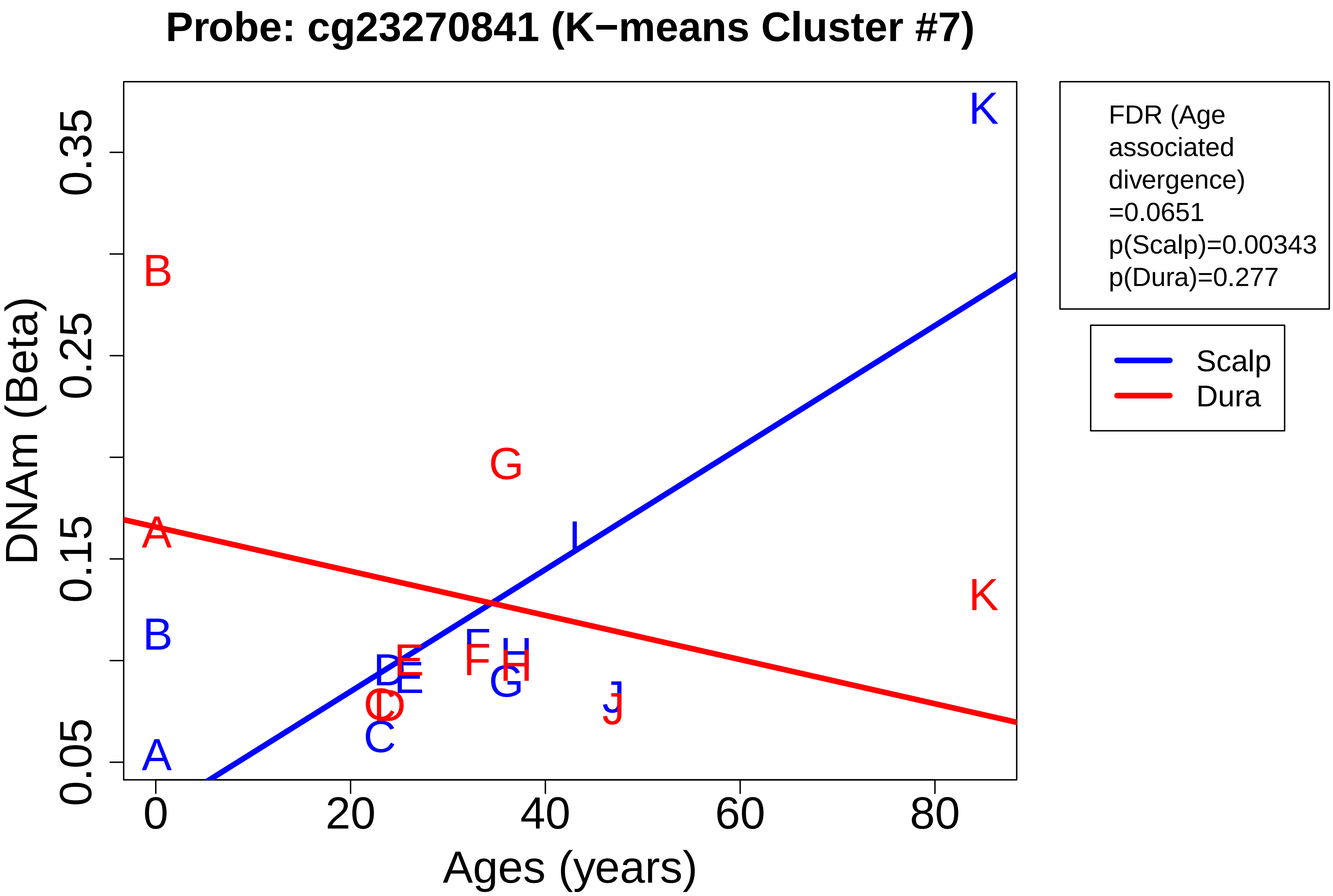

Probe: cg23270841 (K−means Cluster #7)
FDR (Age associated divergence) =0.0651
p(Scalp)=0.00343
p(Dura)=0.277
Scalp
Dura
DNAm (Beta)
0.05
0.15
0.25
0.35
0
20
40
60
80
Ages (years)

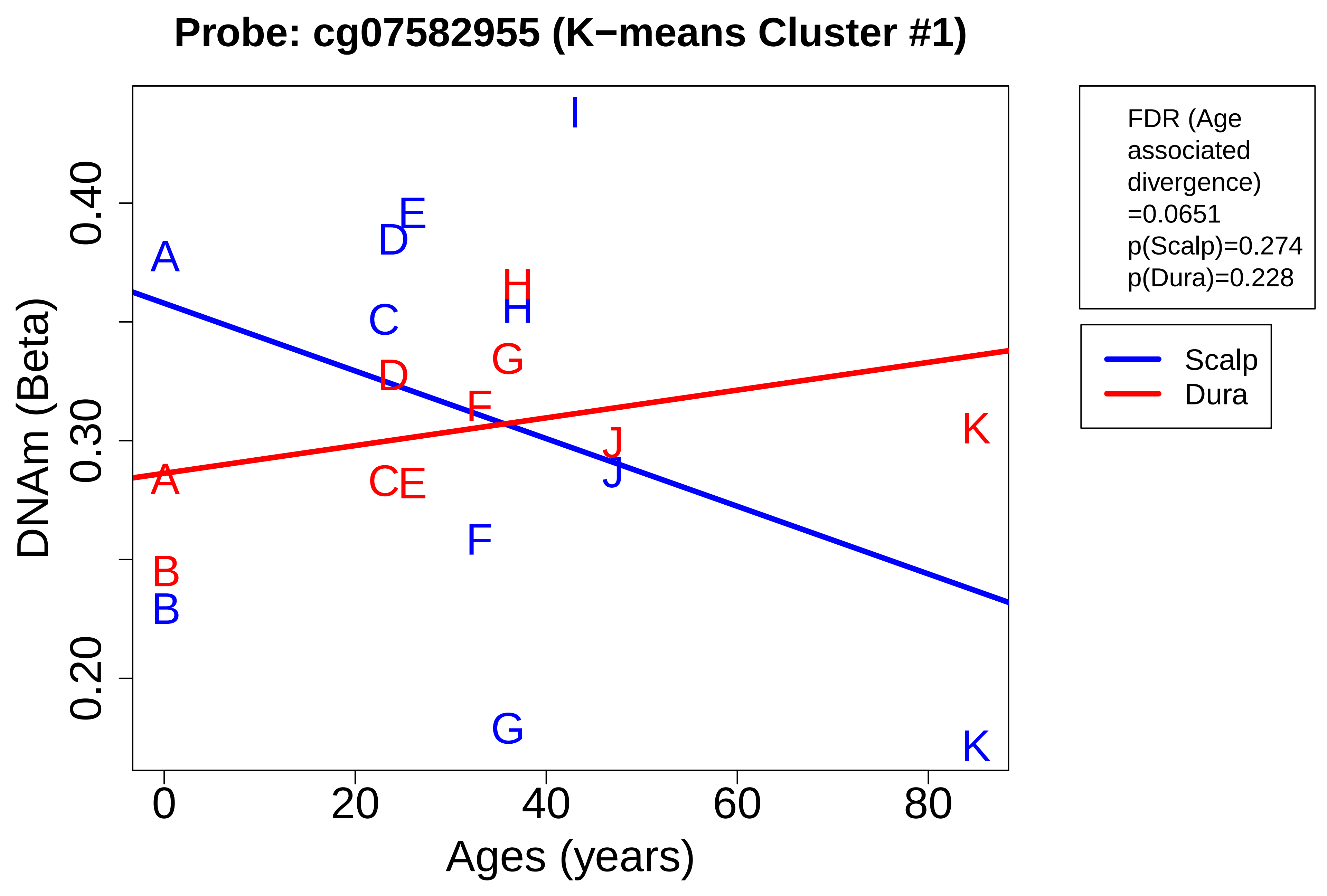

Probe: cg07582955 (K−means Cluster #1)
DNAm (Beta)
0.20
0.30
0.40
0
20
40
60
80
Ages (years)
FDR (Age associated divergence) =0.0651
p(Scalp)=0.274
p(Dura)=0.228
Scalp
Dura
A
B
C
D
E
F
G
H
I
J
K

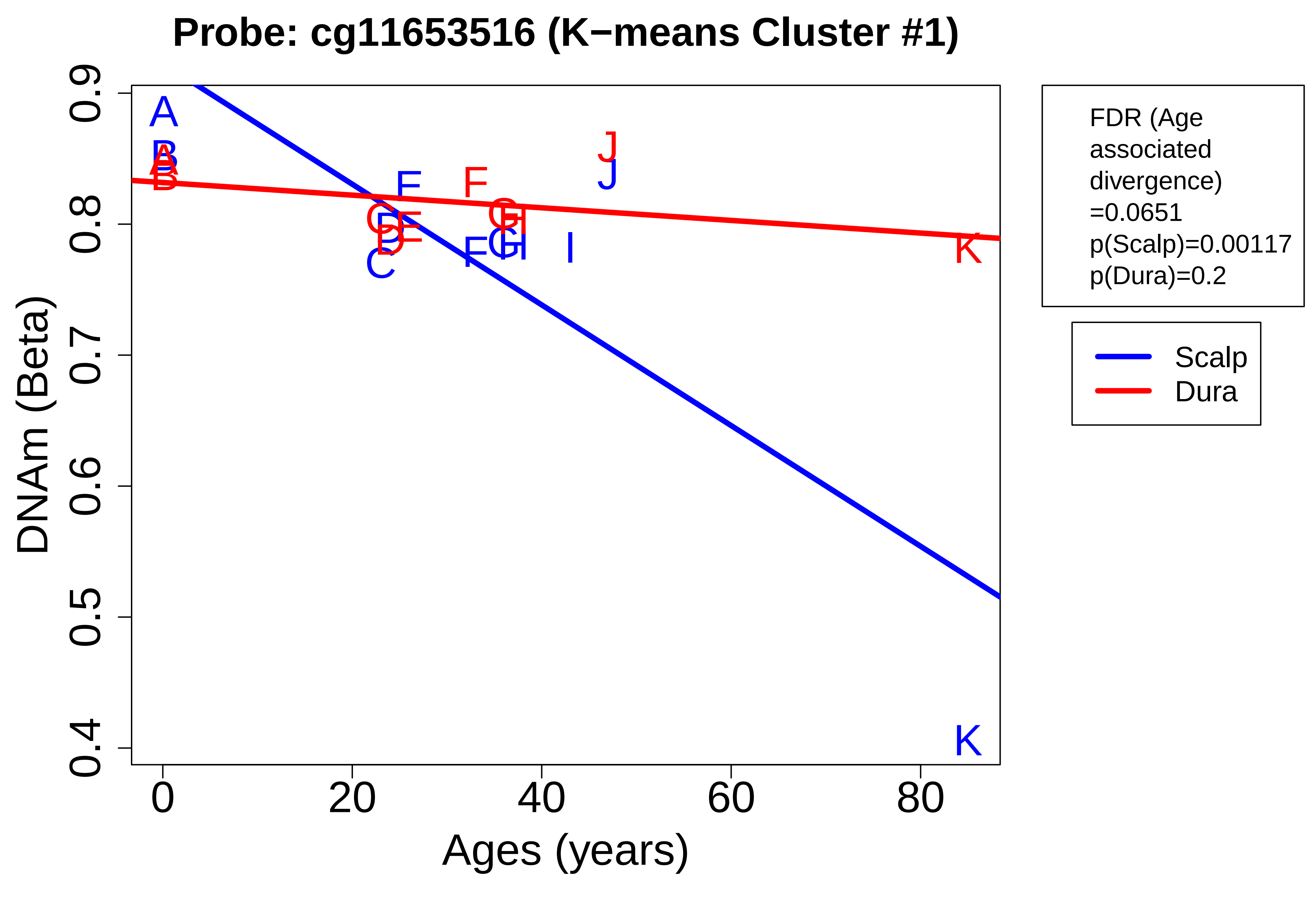

Probe: cg11653516 (K−means Cluster #1)
DNAm (Beta)
0.4
0.5
0.6
0.7
0.8
0.9
0
20
40
60
80
Ages (years)
A
B
C
D
E
F
G
H
I
J
K
FDR (Age associated divergence) =0.0651
p(Scalp)=0.00117
p(Dura)=0.2
Scalp
Dura

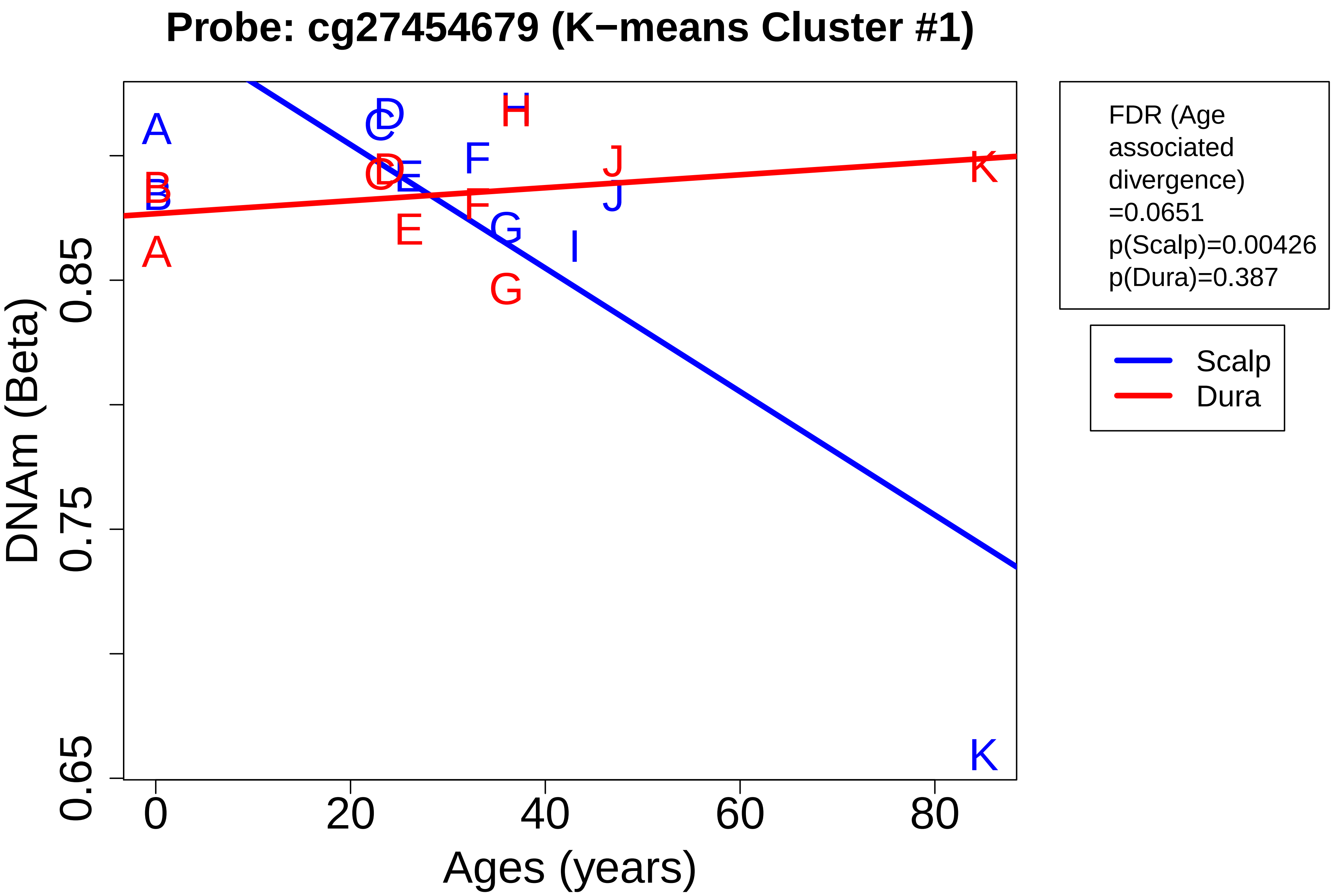
Probe: cg27454679 (K−means Cluster #1)
DNAm (Beta)
0.65
0.75
0.85
0
20
40
60
80
Ages (years)
A
B
C
D
E
F
G
H
I
J
K
FDR (Age associated divergence) =0.0651
p(Scalp)=0.00426
p(Dura)=0.387
Scalp
Dura

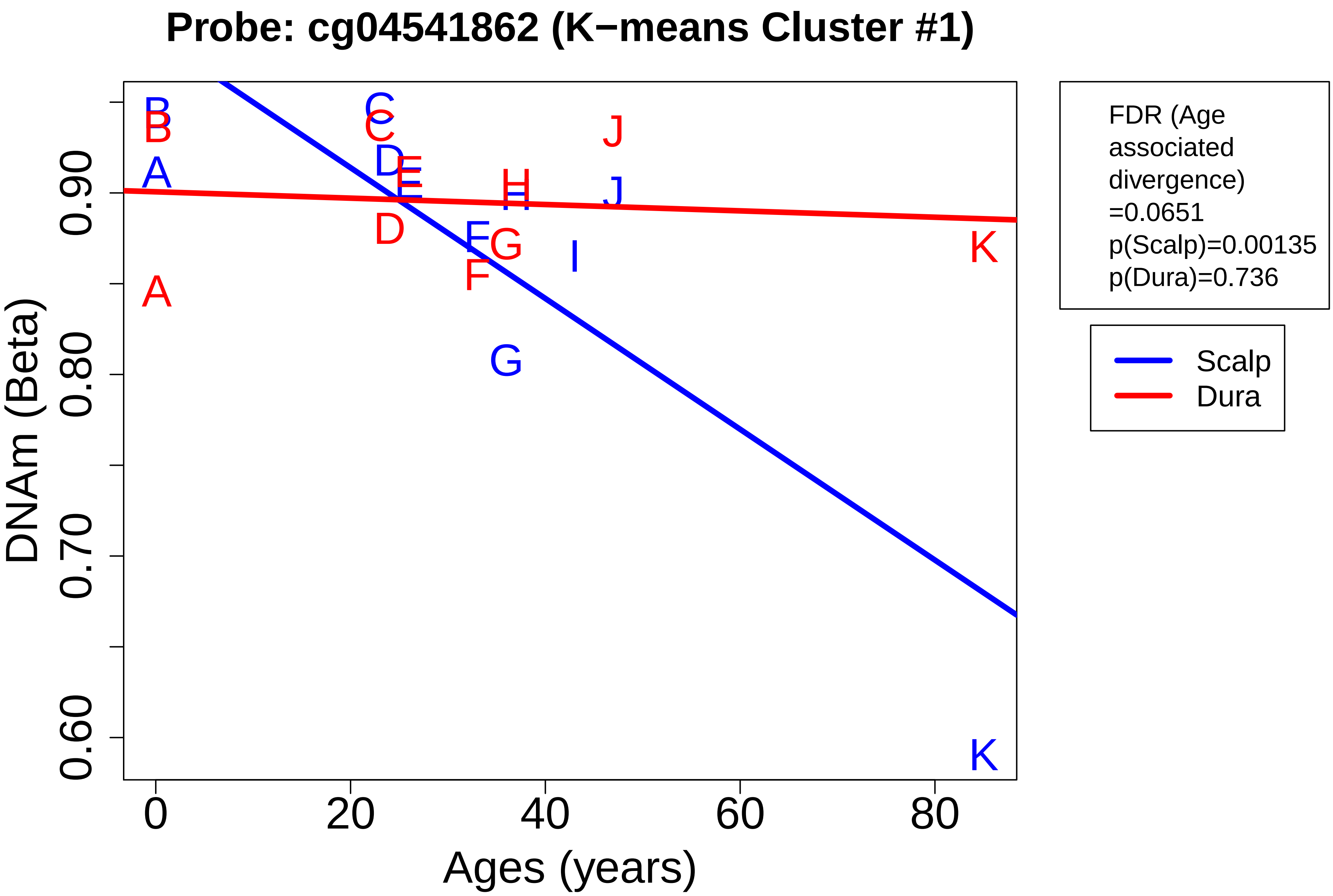
Probe: cg04541862 (K−means Cluster #1)
DNAm (Beta)
0.60
0.70
0.80
0.90
Ages (years)
0
20
40
60
80
A
B
C
D
E
F
G
H
I
J
K
FDR (Age associated divergence) =0.0651
p(Scalp)=0.00135
p(Dura)=0.736
Scalp
Dura

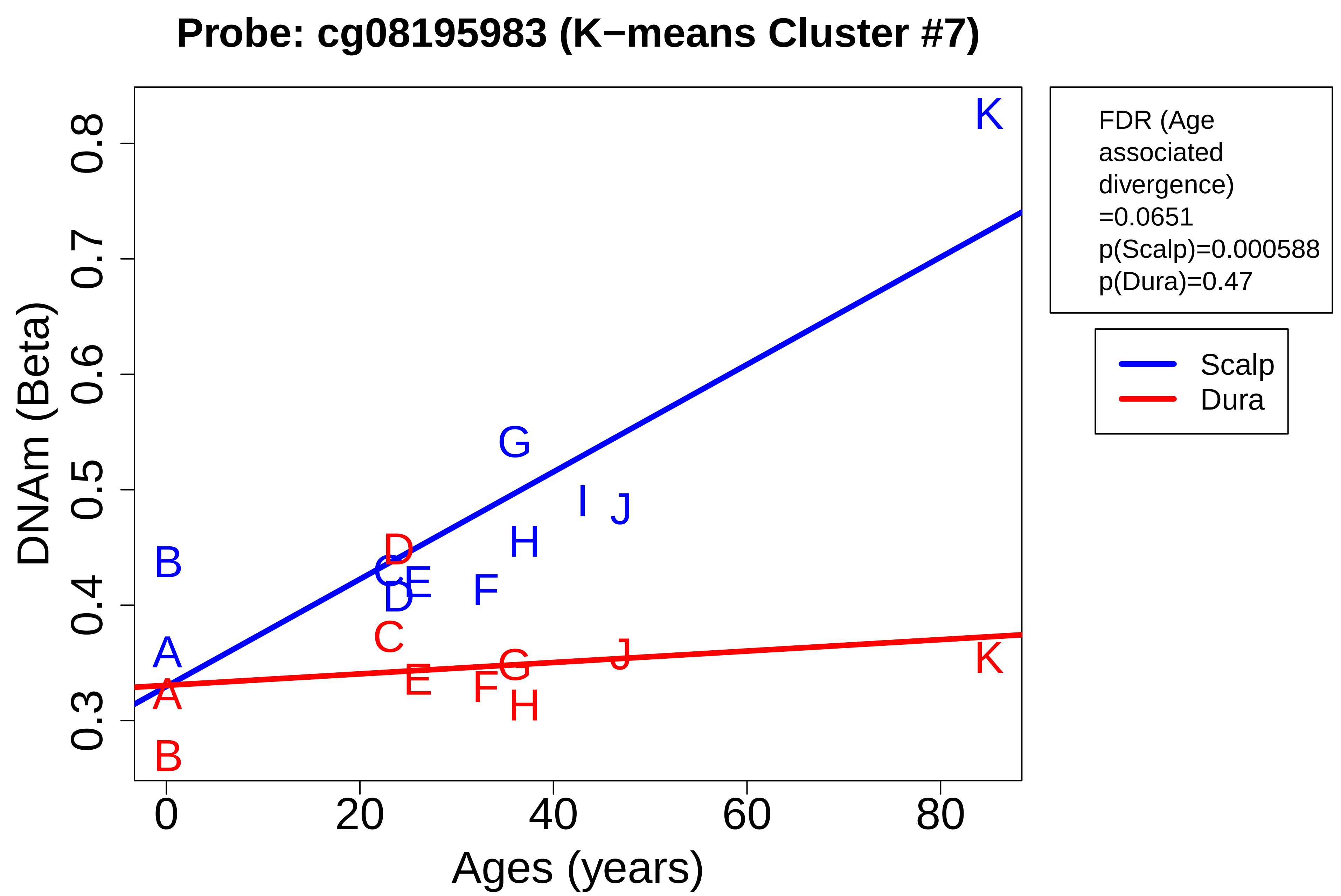

Probe: cg08195983 (K−means Cluster #7)
DNAm (Beta)
0.3
0.4
0.5
0.6
0.7
0.8
Ages (years)
0
20
40
60
80
FDR (Age associated divergence) =0.0651
p(Scalp)=0.000588
p(Dura)=0.47
Scalp
Dura
A
B
C
D
E
F
G
H
I
J
K

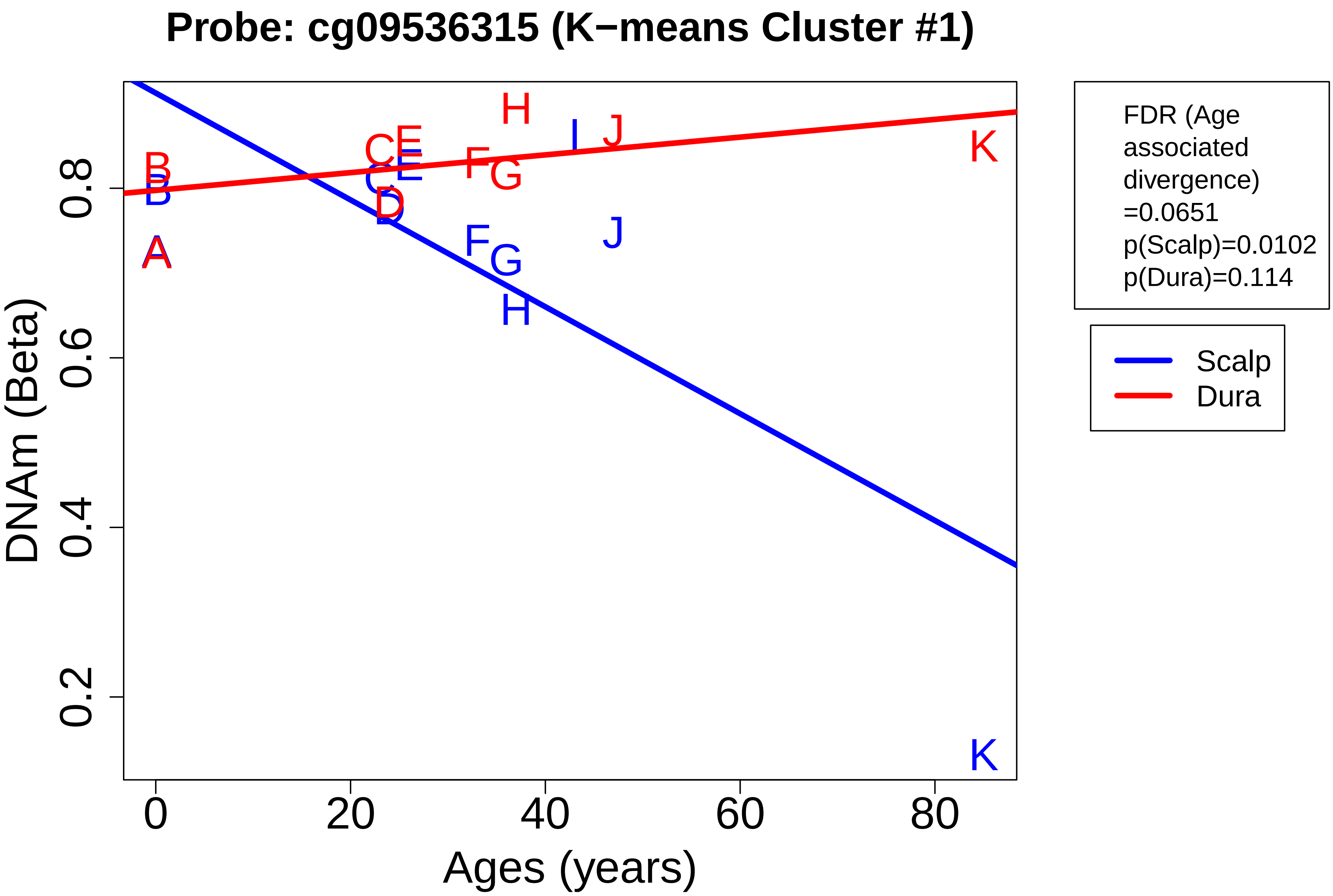

Probe: cg09536315 (K−means Cluster #1)
FDR (Age associated divergence) =0.0651
p(Scalp)=0.0102
p(Dura)=0.114
Scalp
Dura
DNAm (Beta)
0.2
0.4
0.6
0.8
Ages (years)
0
20
40
60
80

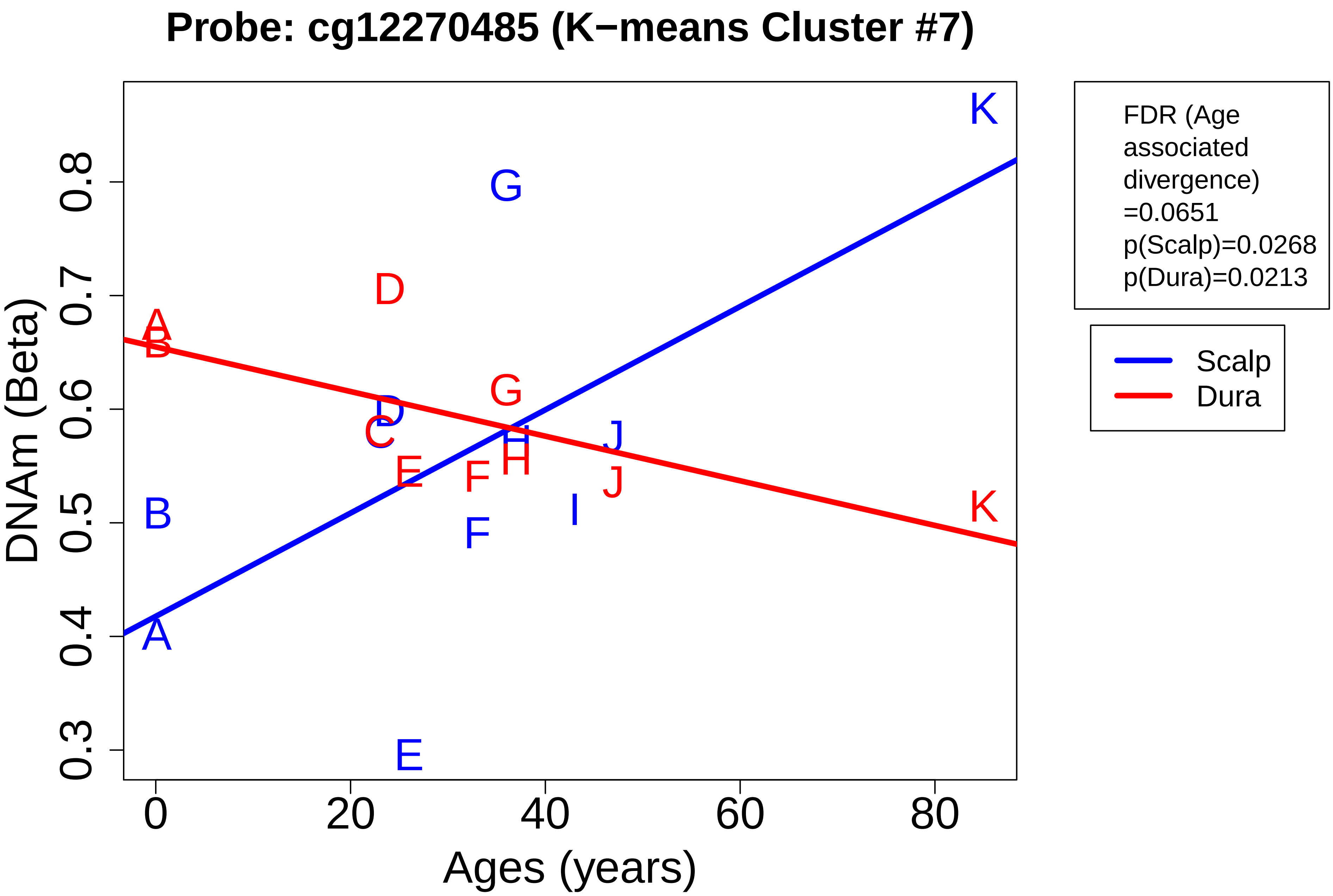

Probe: cg12270485 (K−means Cluster #7)
DNAm (Beta)
0.3
0.4
0.5
0.6
0.7
0.8
Ages (years)
0
20
40
60
80
FDR (Age associated divergence) =0.0651
p(Scalp)=0.0268
p(Dura)=0.0213
Scalp
Dura

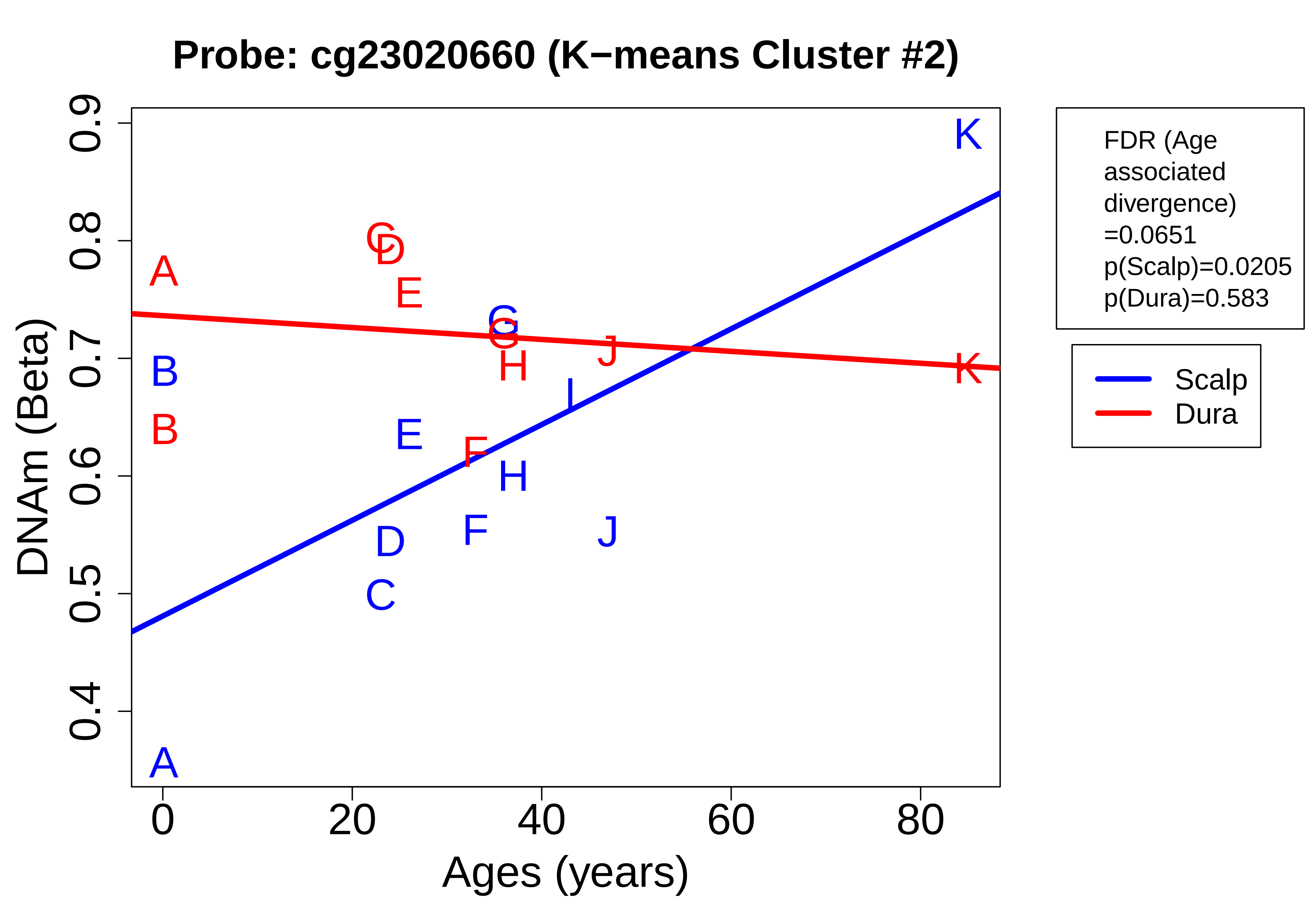
Probe: cg23020660 (K−means Cluster #2)
DNAm (Beta)
0.4
0.5
0.6
0.7
0.8
0.9
Ages (years)
0
20
40
60
80
FDR (Age associated divergence) =0.0651
p(Scalp)=0.0205
p(Dura)=0.583
Scalp
Dura
A
B
C
D
E
F
G
H
I
J
K

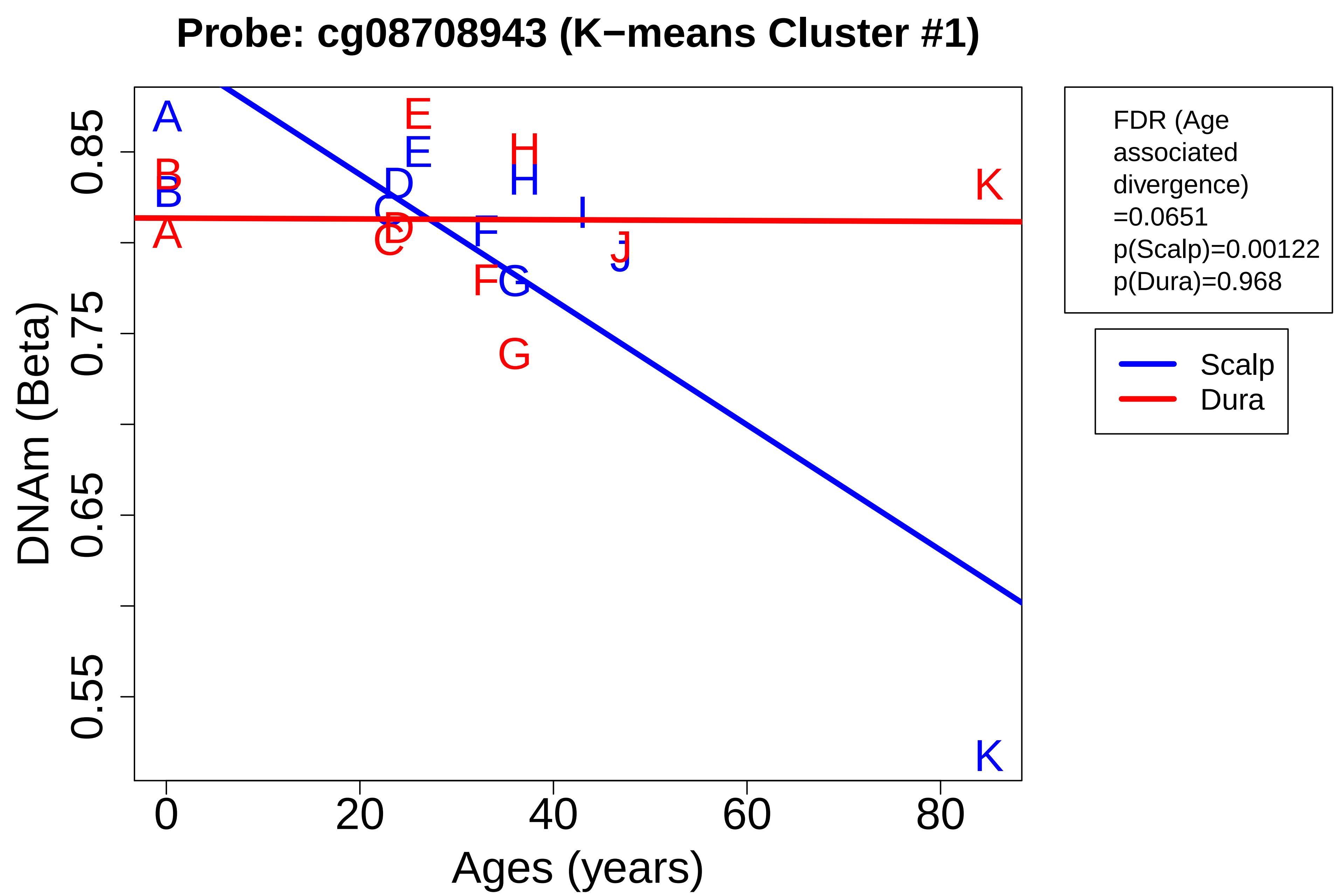
Probe: cg08708943 (K−means Cluster #1)
DNAm (Beta)
0.55
0.65
0.75
0.85
Ages (years)
0
20
40
60
80
A
B
C
D
E
F
G
H
I
J
K
FDR (Age associated divergence) =0.0651
p(Scalp)=0.00122
p(Dura)=0.968
Scalp
Dura

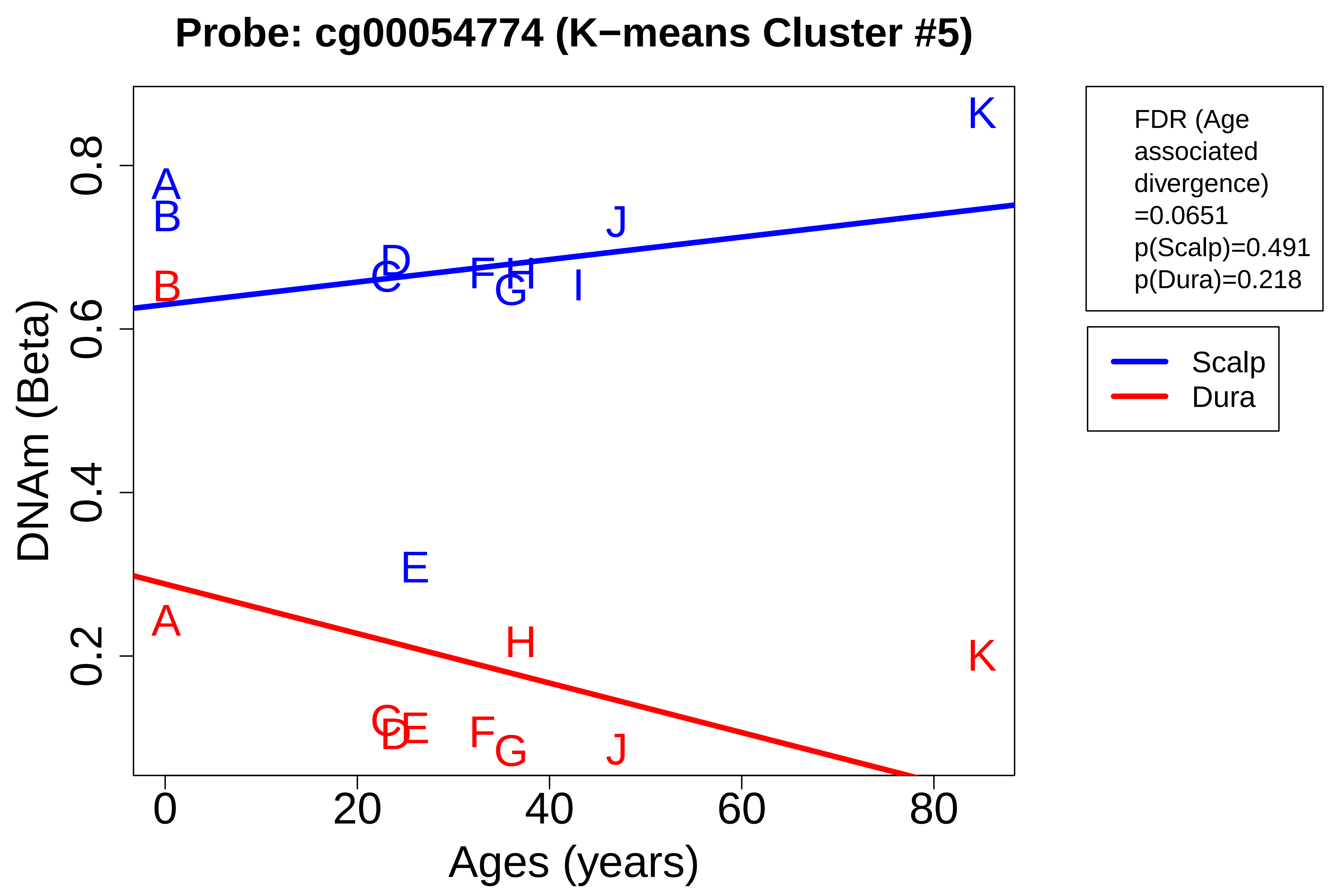

Probe: cg00054774 (K−means Cluster #5)
DNAm (Beta)
Ages (years)
0.2
0.4
0.6
0.8
0
20
40
60
80
A
B
C
D
E
F
G
H
I
J
K
FDR (Age associated divergence) =0.0651
p(Scalp)=0.491
p(Dura)=0.218
Scalp
Dura

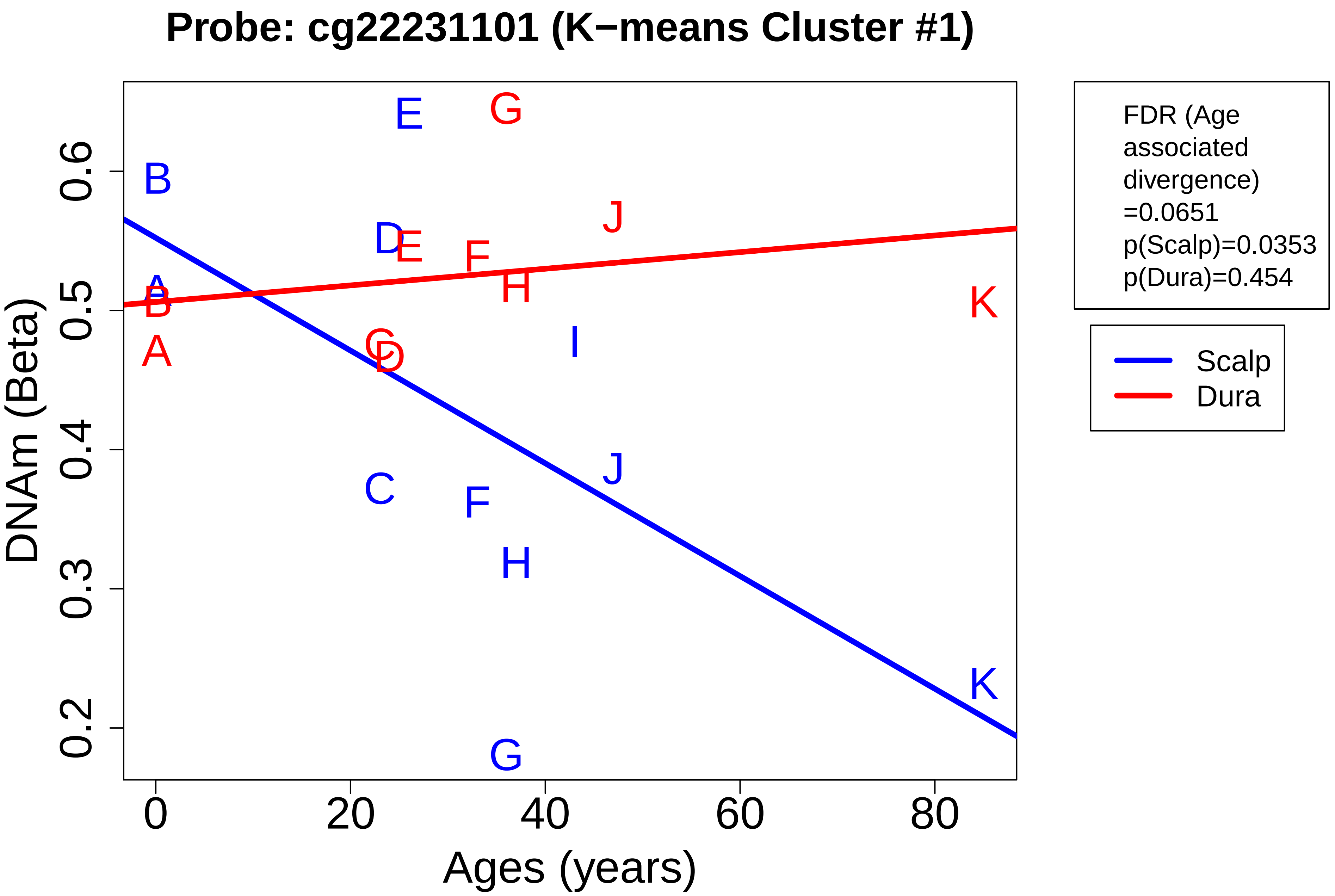

Probe: cg22231101 (K−means Cluster #1)
FDR (Age associated divergence) =0.0651
p(Scalp)=0.0353
p(Dura)=0.454
Scalp
Dura
DNAm (Beta)
0.2
0.3
0.4
0.5
0.6
Ages (years)
0
20
40
60
80

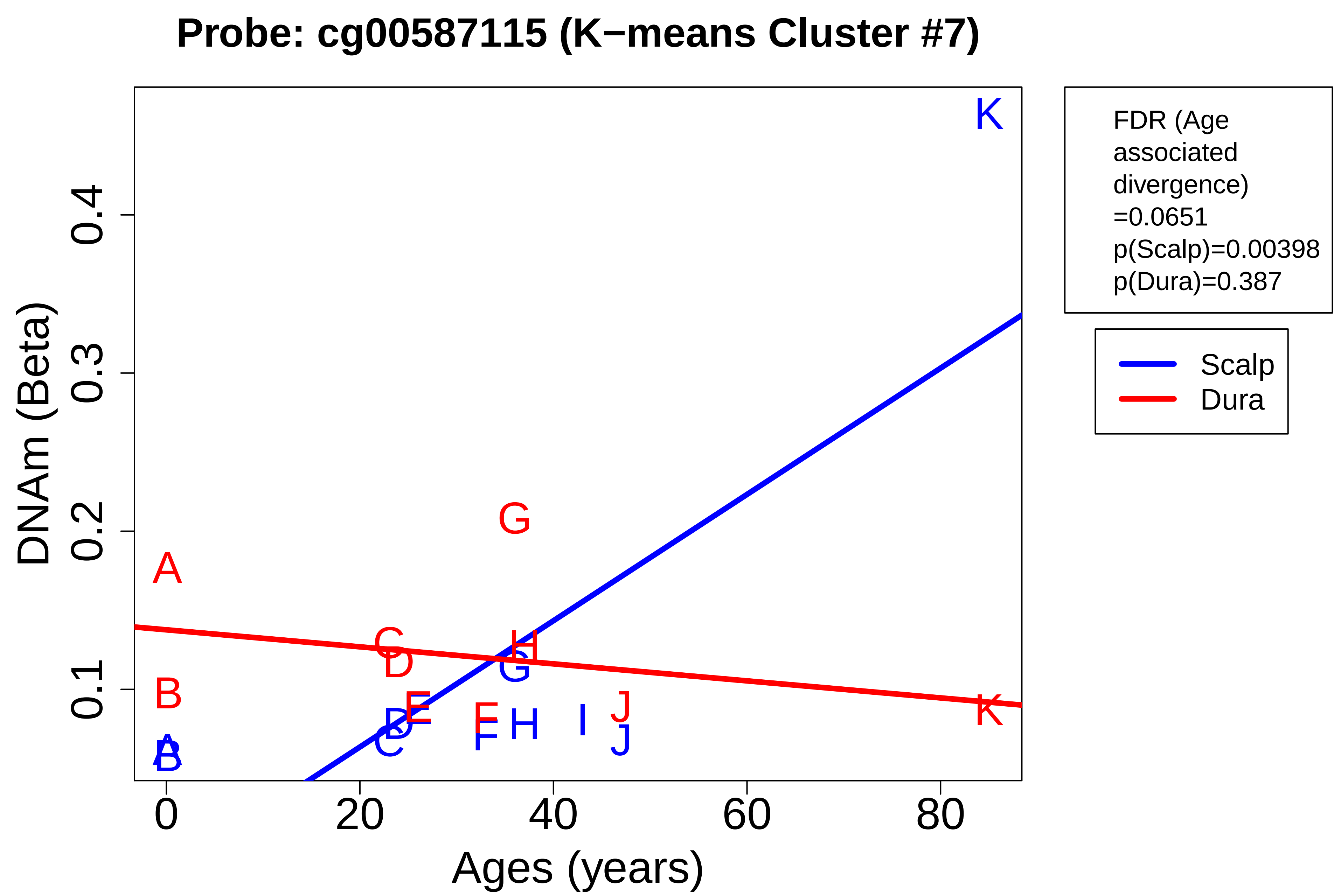
Probe: cg00587115 (K−means Cluster #7)
DNAm (Beta)
0.1
0.2
0.3
0.4
0
20
40
60
80
Ages (years)
FDR (Age associated divergence) =0.0651
p(Scalp)=0.00398
p(Dura)=0.387
Scalp
Dura

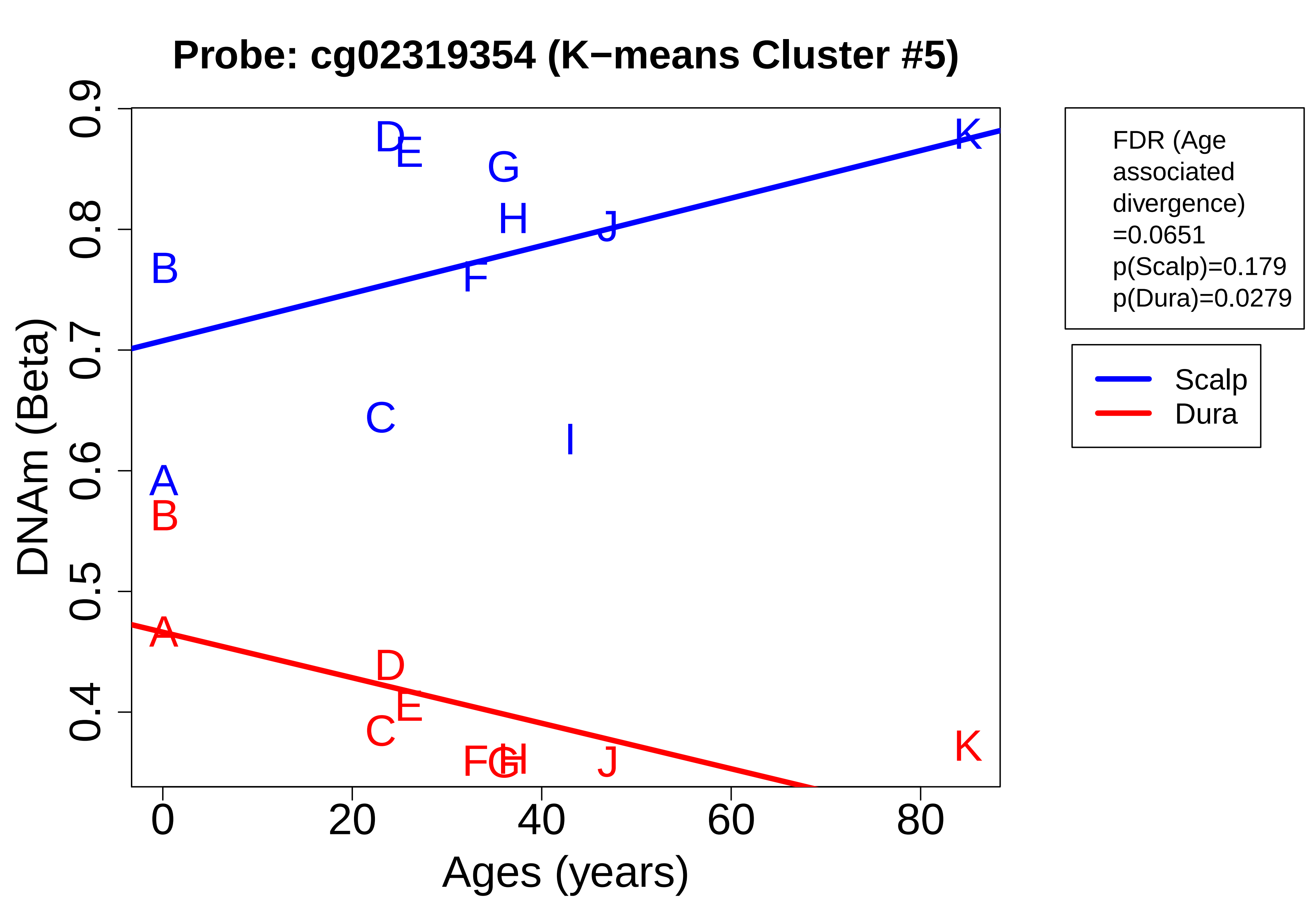

Probe: cg02319354 (K−means Cluster #5)
DNAm (Beta)
Ages (years)
0.4
0.5
0.6
0.7
0.8
0.9
0
20
40
60
80
FDR (Age associated divergence) =0.0651
p(Scalp)=0.179
p(Dura)=0.0279
Scalp
Dura
A
B
C
D
E
F
G
H
I
J
K

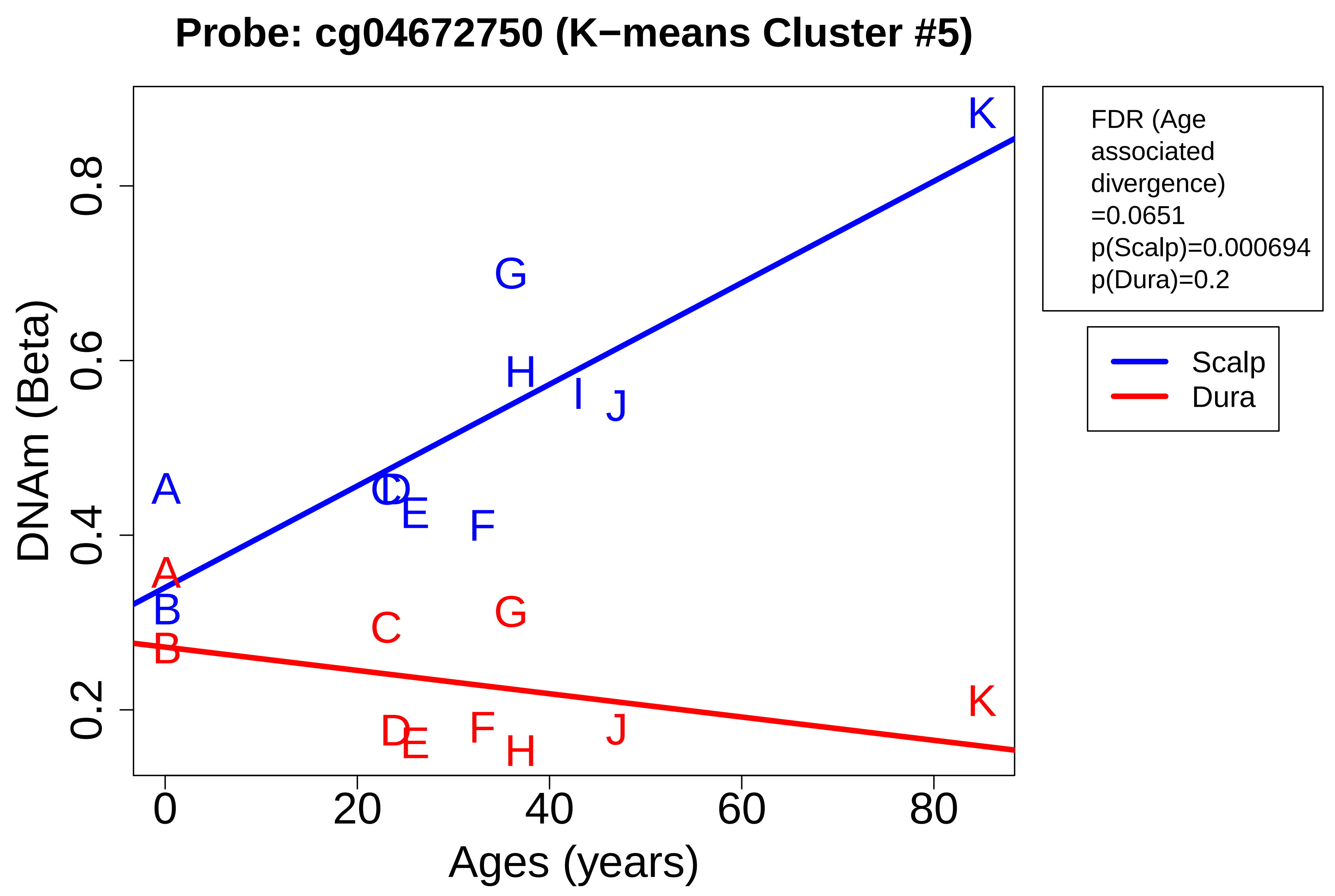

Probe: cg04672750 (K−means Cluster #5)
FDR (Age associated divergence) =0.0651
p(Scalp)=0.000694
p(Dura)=0.2
Scalp
Dura
DNAm (Beta)
0.2
0.4
0.6
0.8
0
20
40
60
80
Ages (years)
A
B
C
D
E
F
G
H
I
J
K

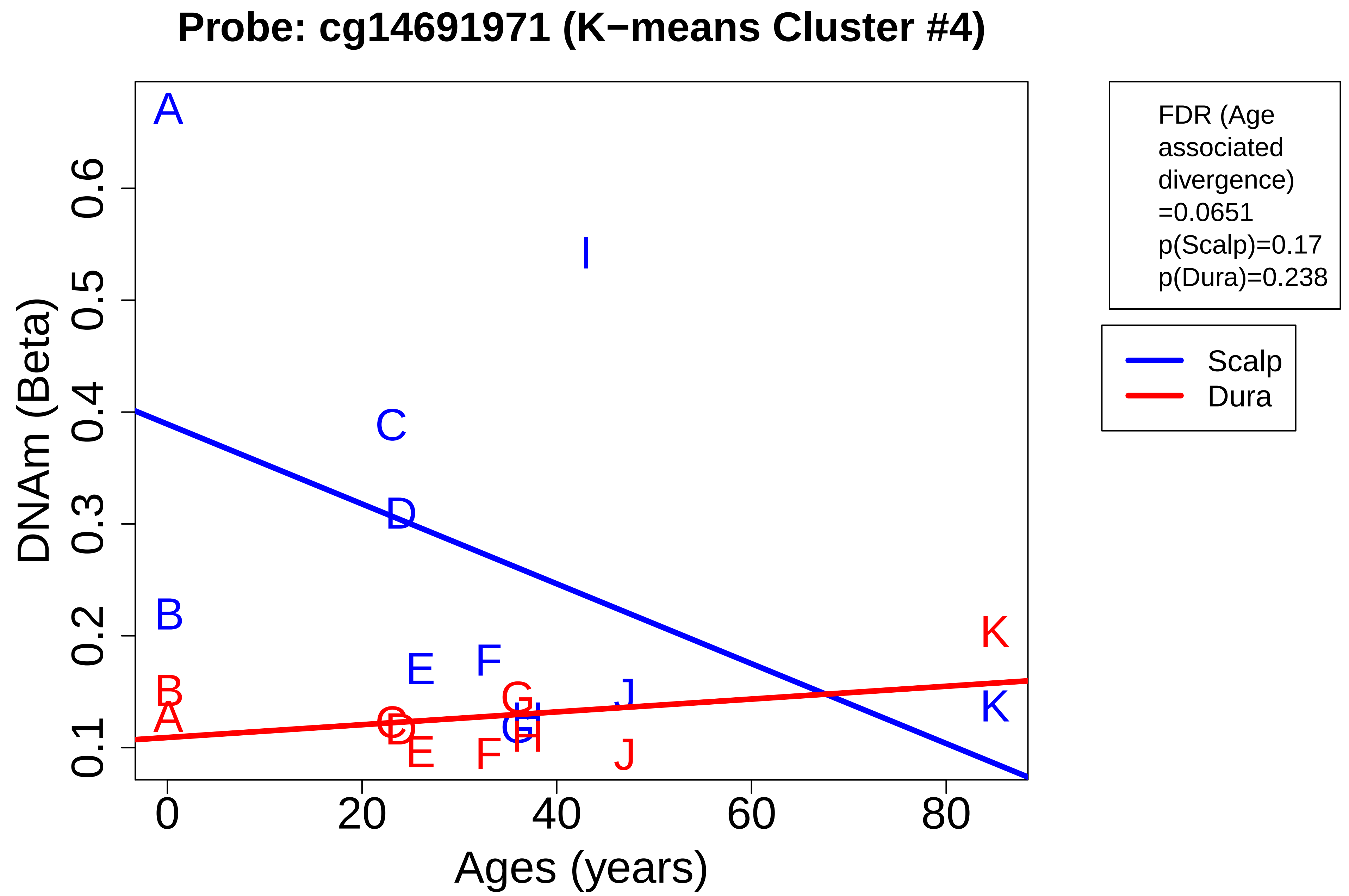

Probe: cg14691971 (K−means Cluster #4)
DNAm (Beta)
Ages (years)
0.1
0.2
0.3
0.4
0.5
0.6
0
20
40
60
80
A
B
C
D
E
F
G
H
I
J
K
FDR (Age associated divergence) =0.0651
p(Scalp)=0.17
p(Dura)=0.238
Scalp
Dura

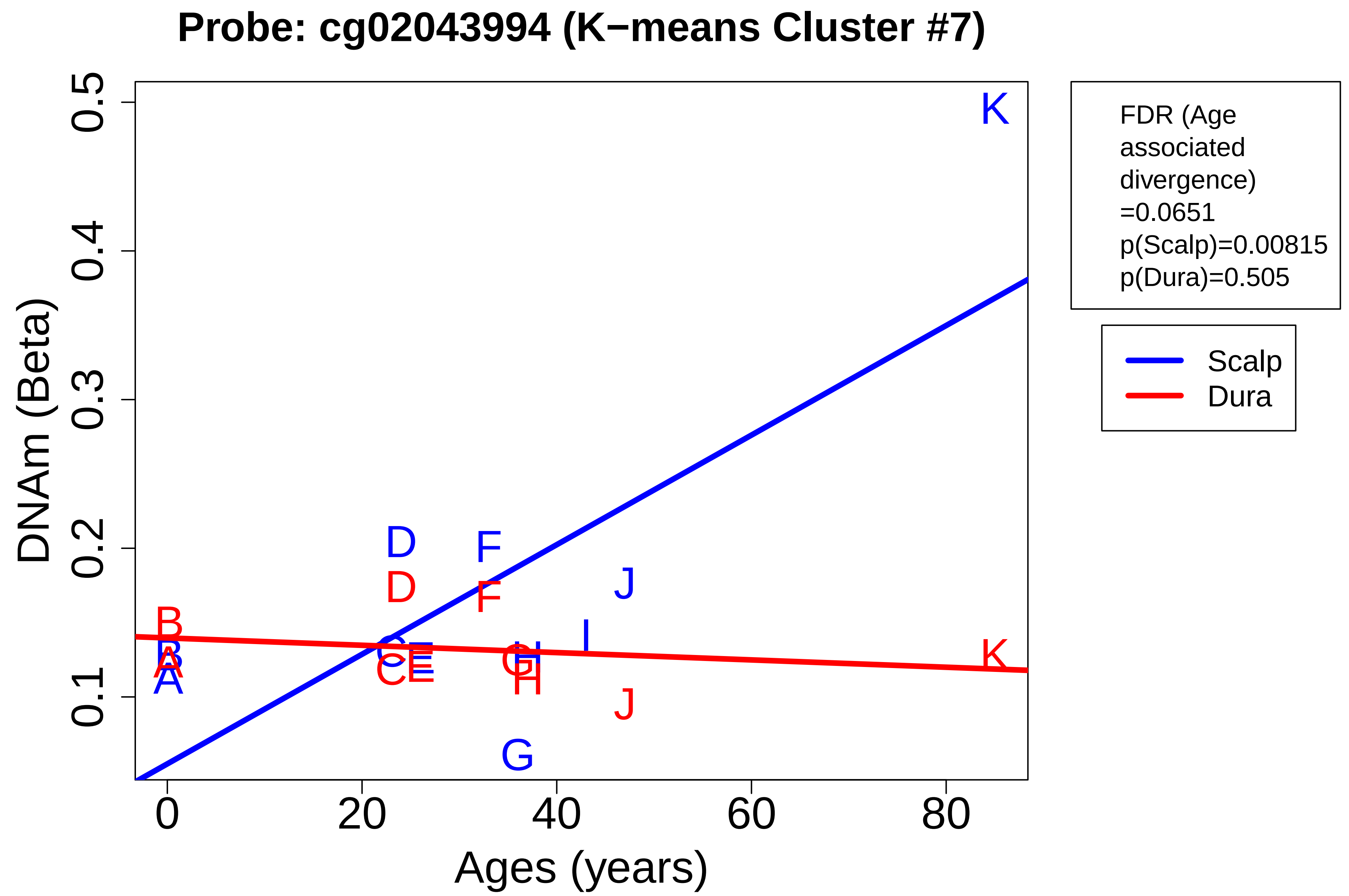

Probe: cg02043994 (K−means Cluster #7)
DNAm (Beta)
0.1
0.2
0.3
0.4
0.5
Ages (years)
0
20
40
60
80
FDR (Age associated divergence) =0.0651
p(Scalp)=0.00815
p(Dura)=0.505
Scalp
Dura

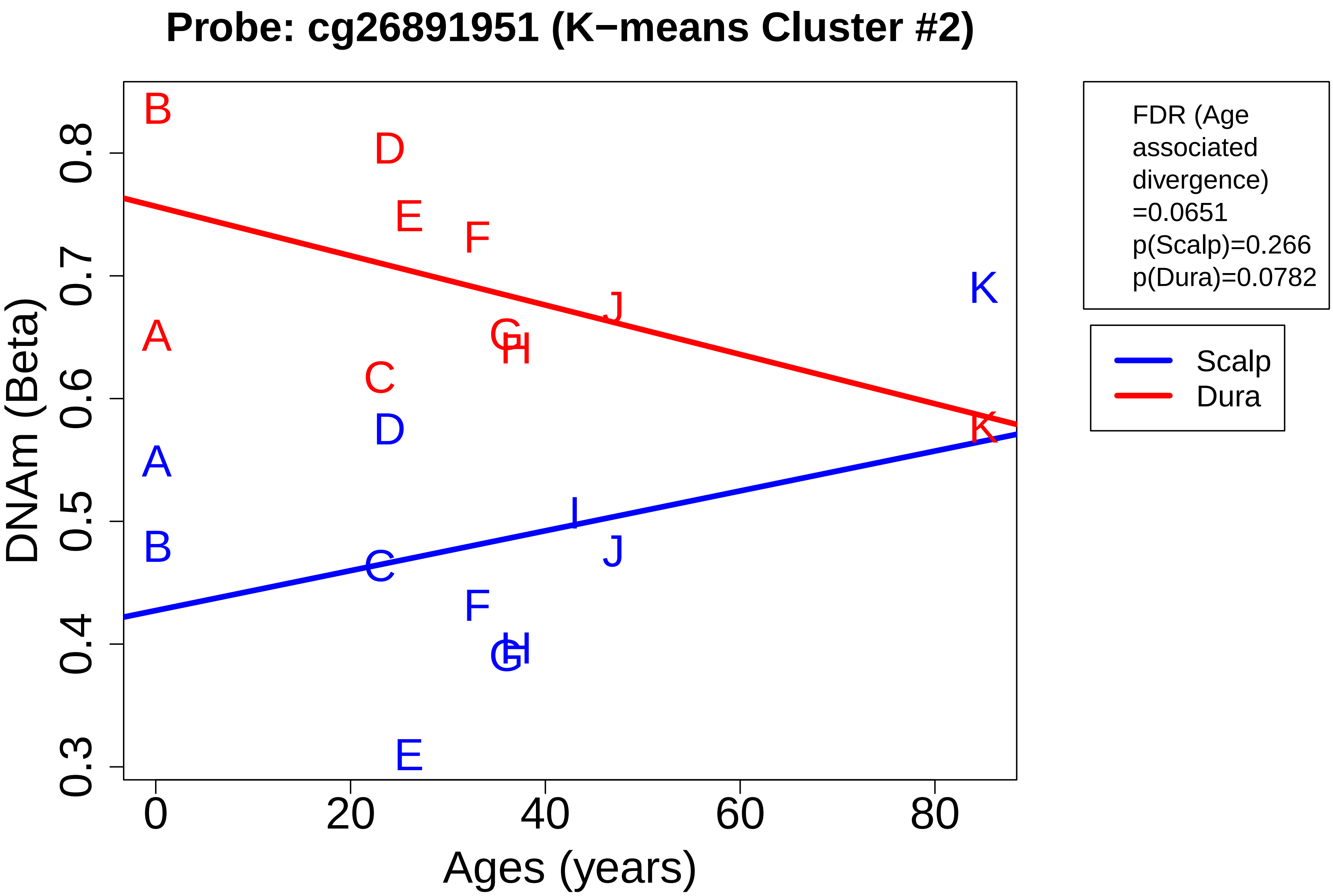
Probe: cg26891951 (K−means Cluster #2)
DNAm (Beta)
0.3
0.4
0.5
0.6
0.7
0.8
0
20
40
60
80
Ages (years)
FDR (Age associated divergence) =0.0651
p(Scalp)=0.266
p(Dura)=0.0782
Scalp
Dura

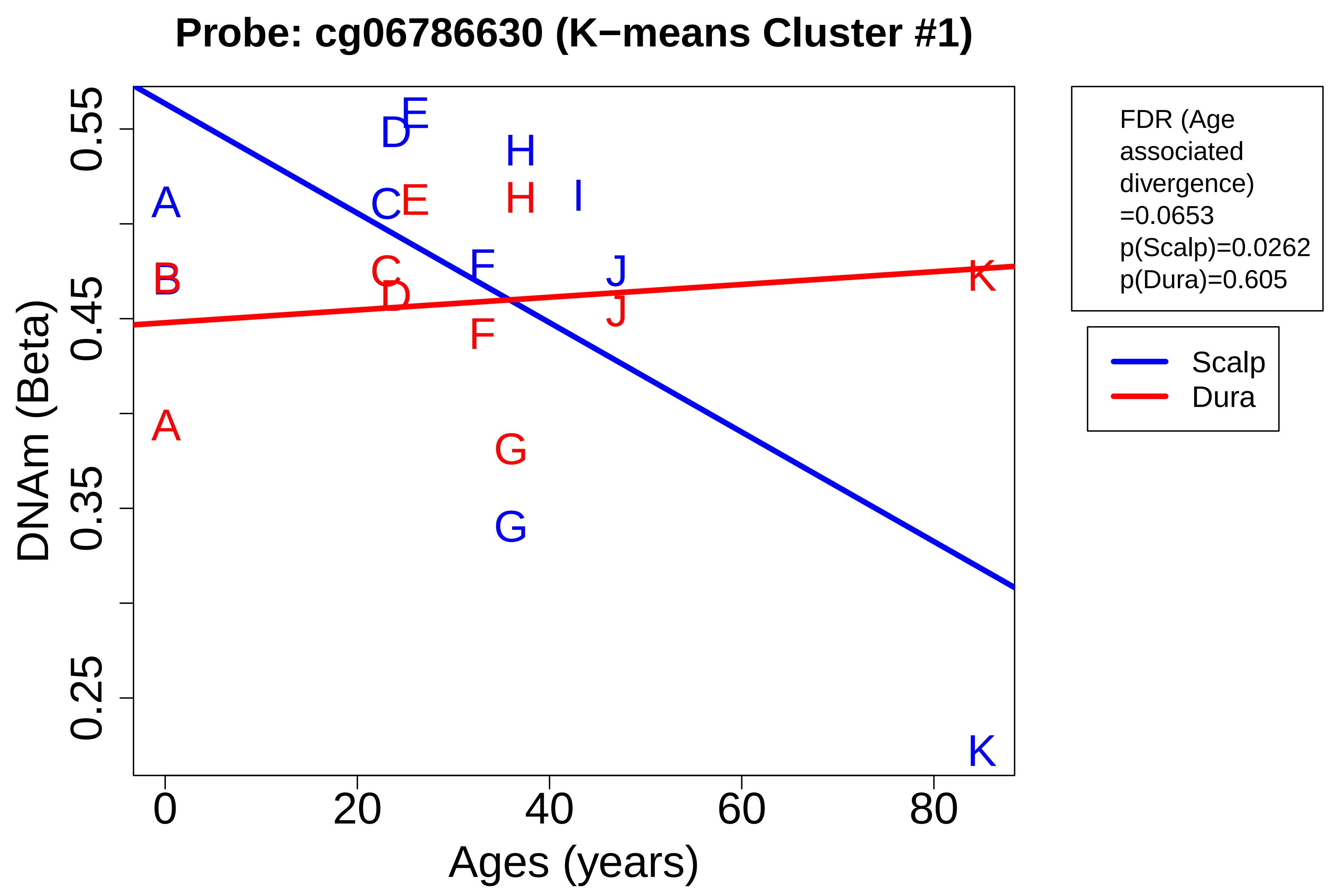

Probe: cg06786630 (K−means Cluster #1)
DNAm (Beta)
0.25
0.35
0.45
0.55
Ages (years)
0
20
40
60
80
A
B
C
D
E
F
G
H
I
J
K
FDR (Age associated divergence) =0.0653
p(Scalp)=0.0262
p(Dura)=0.605
Scalp
Dura

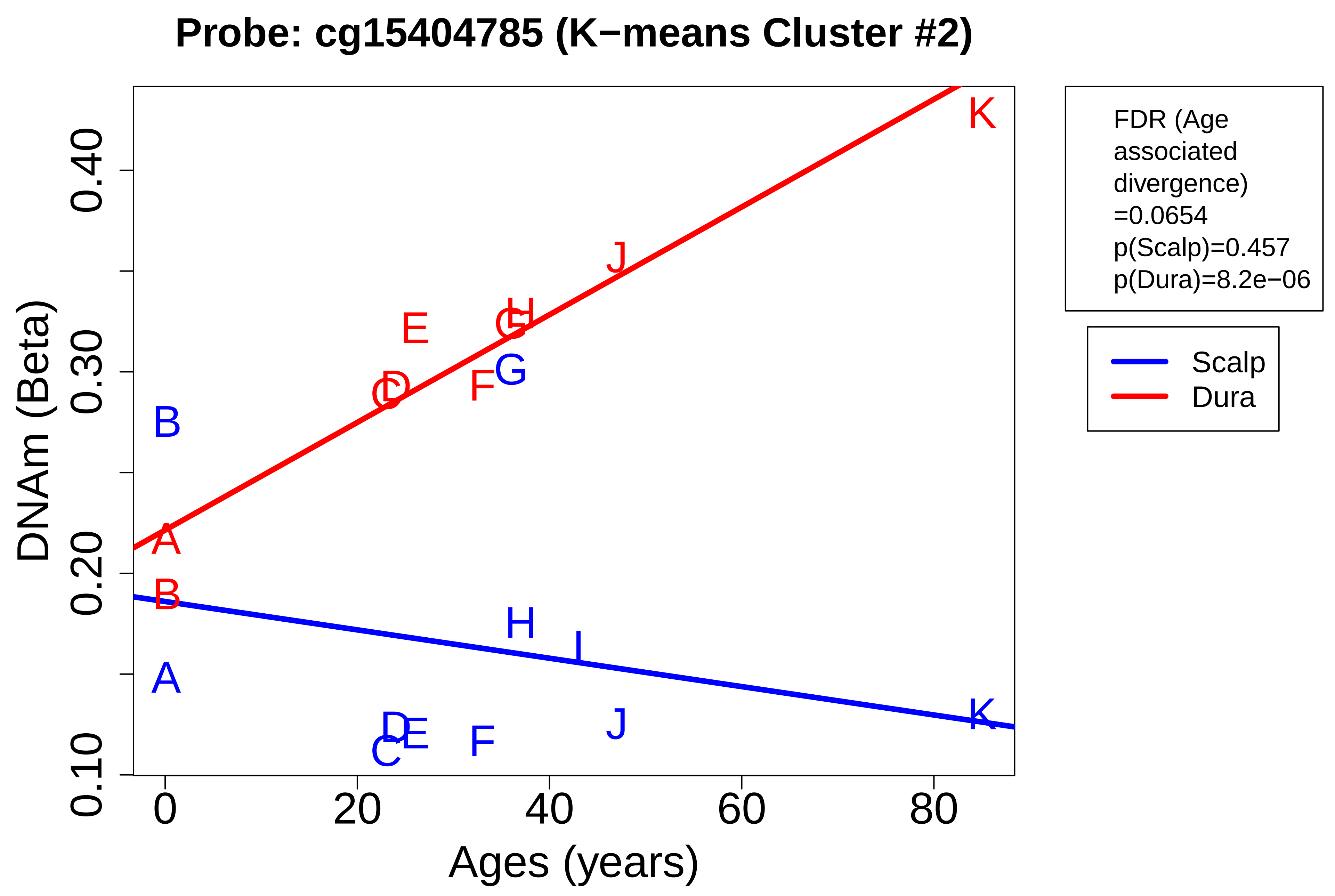

Probe: cg15404785 (K−means Cluster #2)
DNAm (Beta)
0.10
0.20
0.30
0.40
Ages (years)
0
20
40
60
80
FDR (Age associated divergence) =0.0654
p(Scalp)=0.457
p(Dura)=8.2e−06
Scalp
Dura

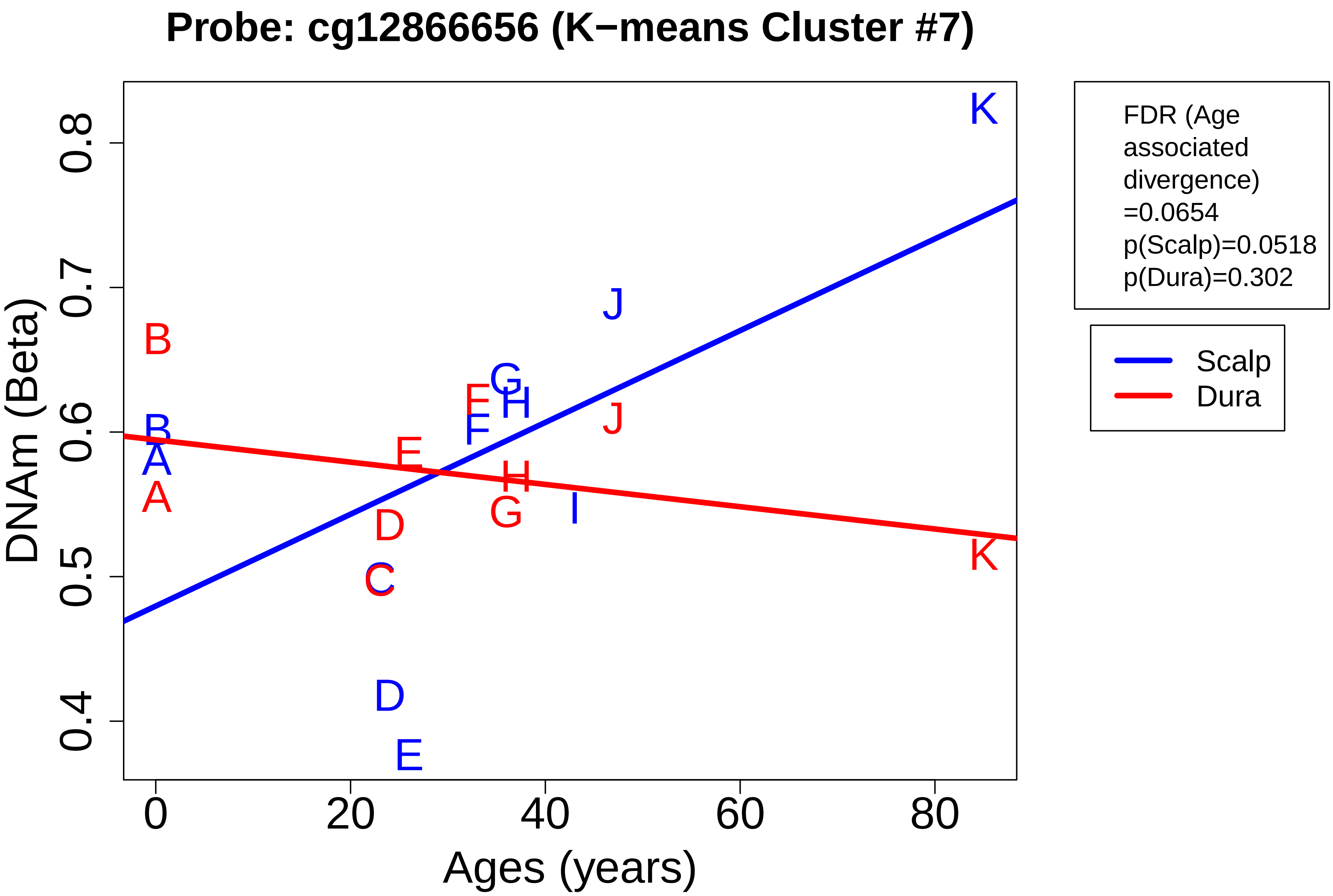
Probe: cg12866656 (K−means Cluster #7)
DNAm (Beta)
0.4
0.5
0.6
0.7
0.8
0
20
40
60
80
Ages (years)
FDR (Age associated divergence) =0.0654
p(Scalp)=0.0518
p(Dura)=0.302
Scalp
Dura
A
B
C
D
E
F
G
H
I
J
K

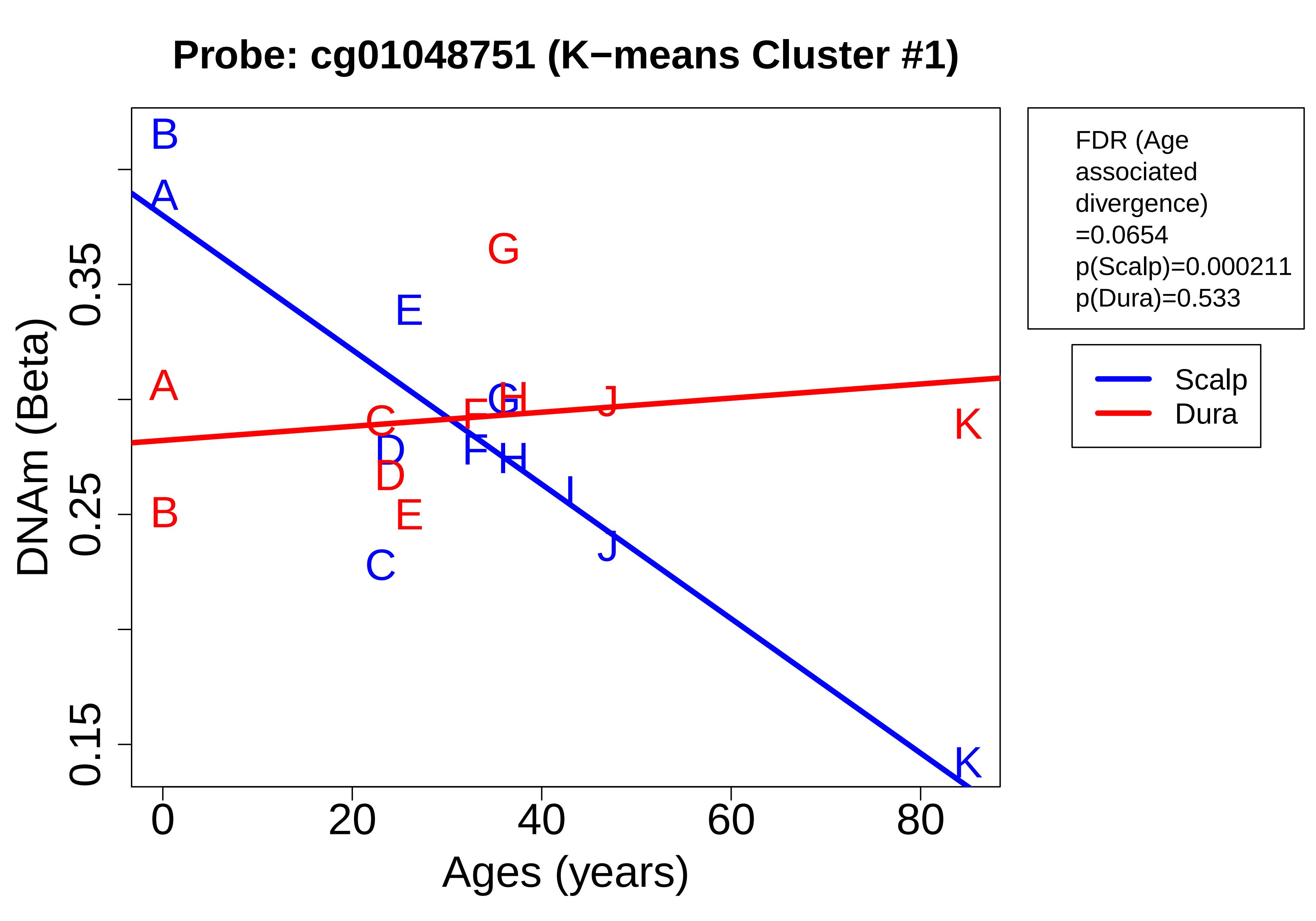
Probe: cg01048751 (K−means Cluster #1)
DNAm (Beta)
0.15
0.25
0.35
Ages (years)
0
20
40
60
80
FDR (Age associated divergence) =0.0654
p(Scalp)=0.000211
p(Dura)=0.533
Scalp
Dura

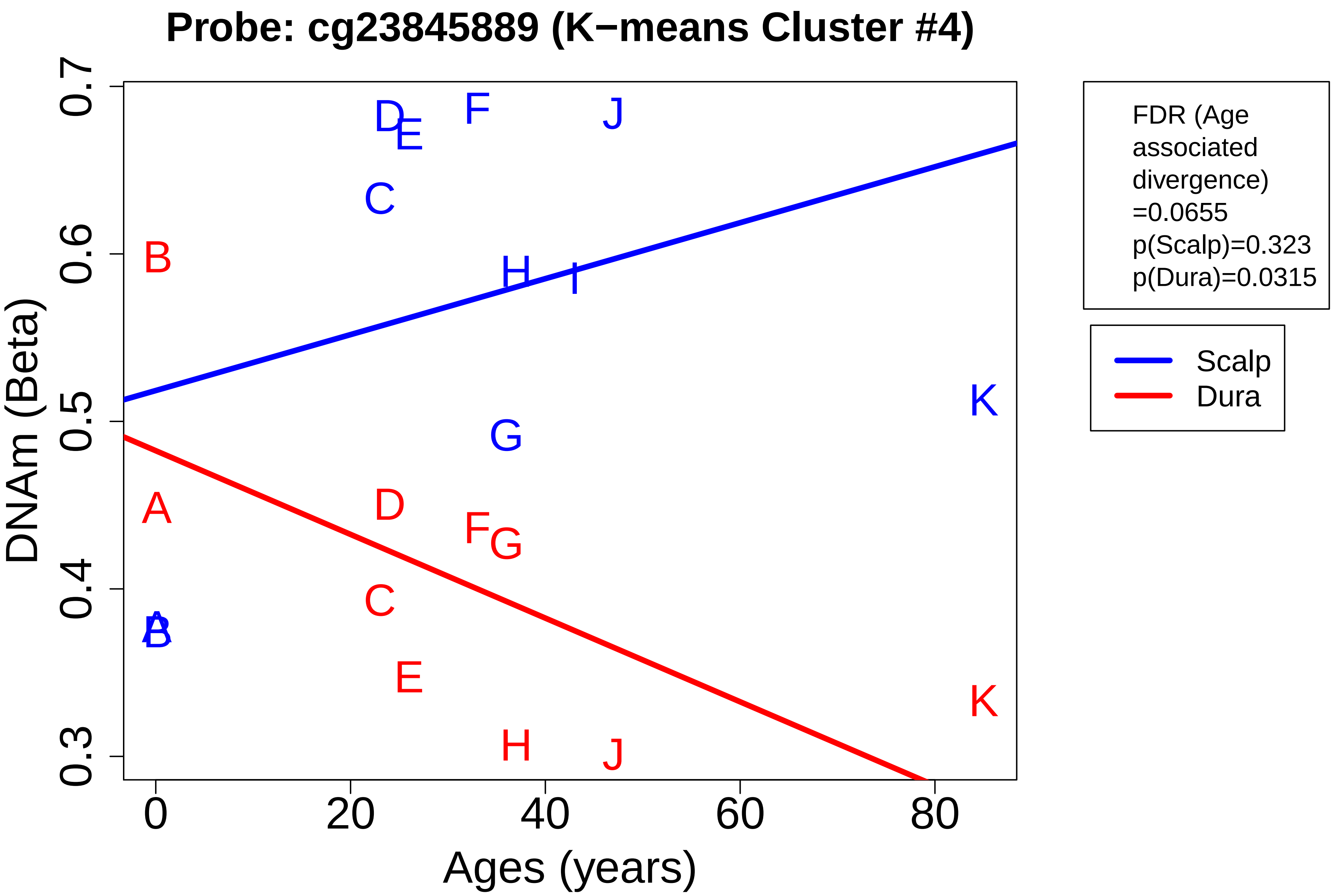

Probe: cg23845889 (K−means Cluster #4)
DNAm (Beta)
0.3
0.4
0.5
0.6
0.7
Ages (years)
0
20
40
60
80
FDR (Age associated divergence) =0.0655
p(Scalp)=0.323
p(Dura)=0.0315
Scalp
Dura
A
B
C
D
E
F
G
H
I
J
K

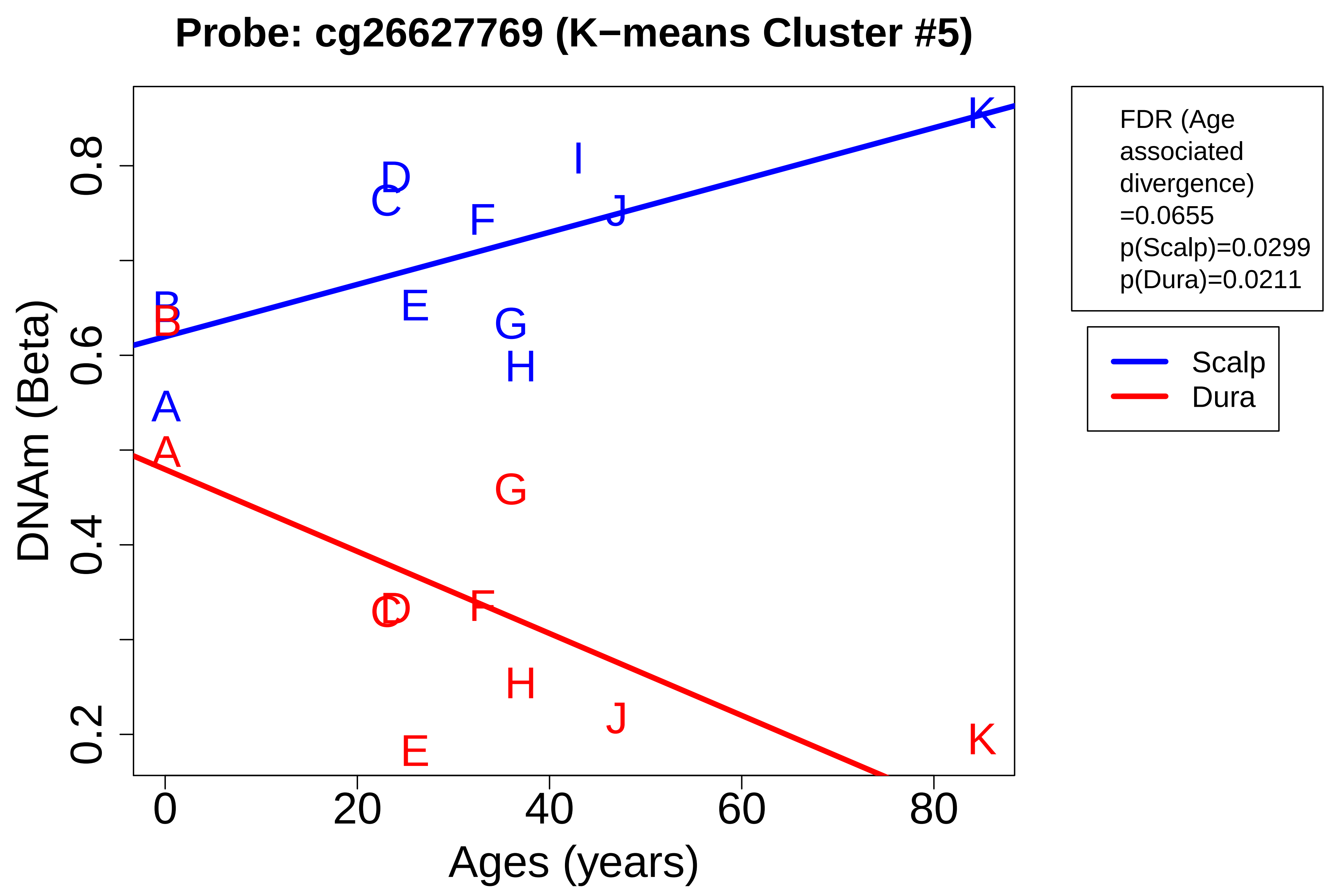

Probe: cg26627769 (K−means Cluster #5)
DNAm (Beta)
0.2
0.4
0.6
0.8
Ages (years)
0
20
40
60
80
FDR (Age associated divergence) =0.0655
p(Scalp)=0.0299
p(Dura)=0.0211
Scalp
Dura

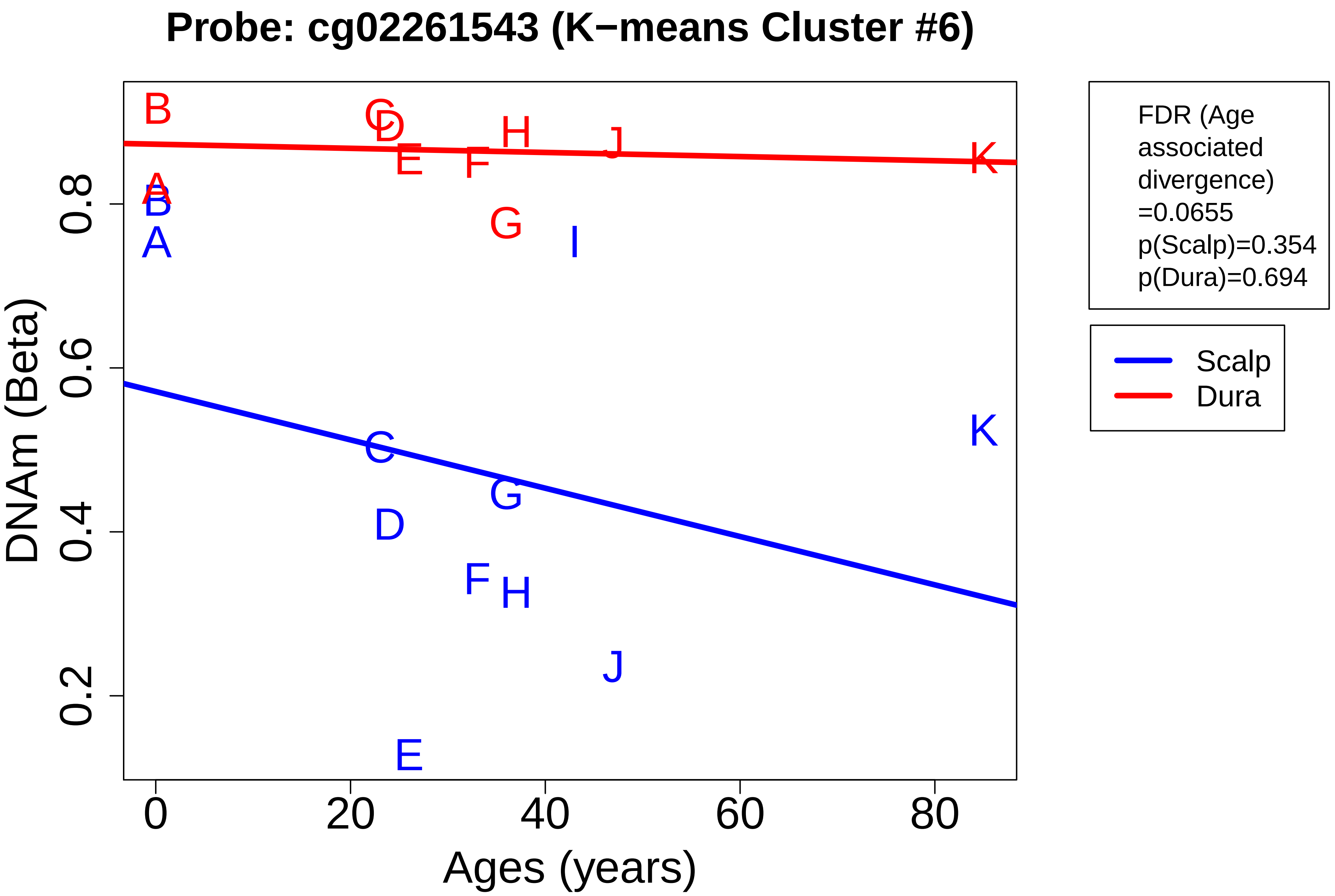

Probe: cg02261543 (K−means Cluster #6)
FDR (Age associated divergence) =0.0655
p(Scalp)=0.354
p(Dura)=0.694
Scalp
Dura
DNAm (Beta)
0.2
0.4
0.6
0.8
Ages (years)
0
20
40
60
80

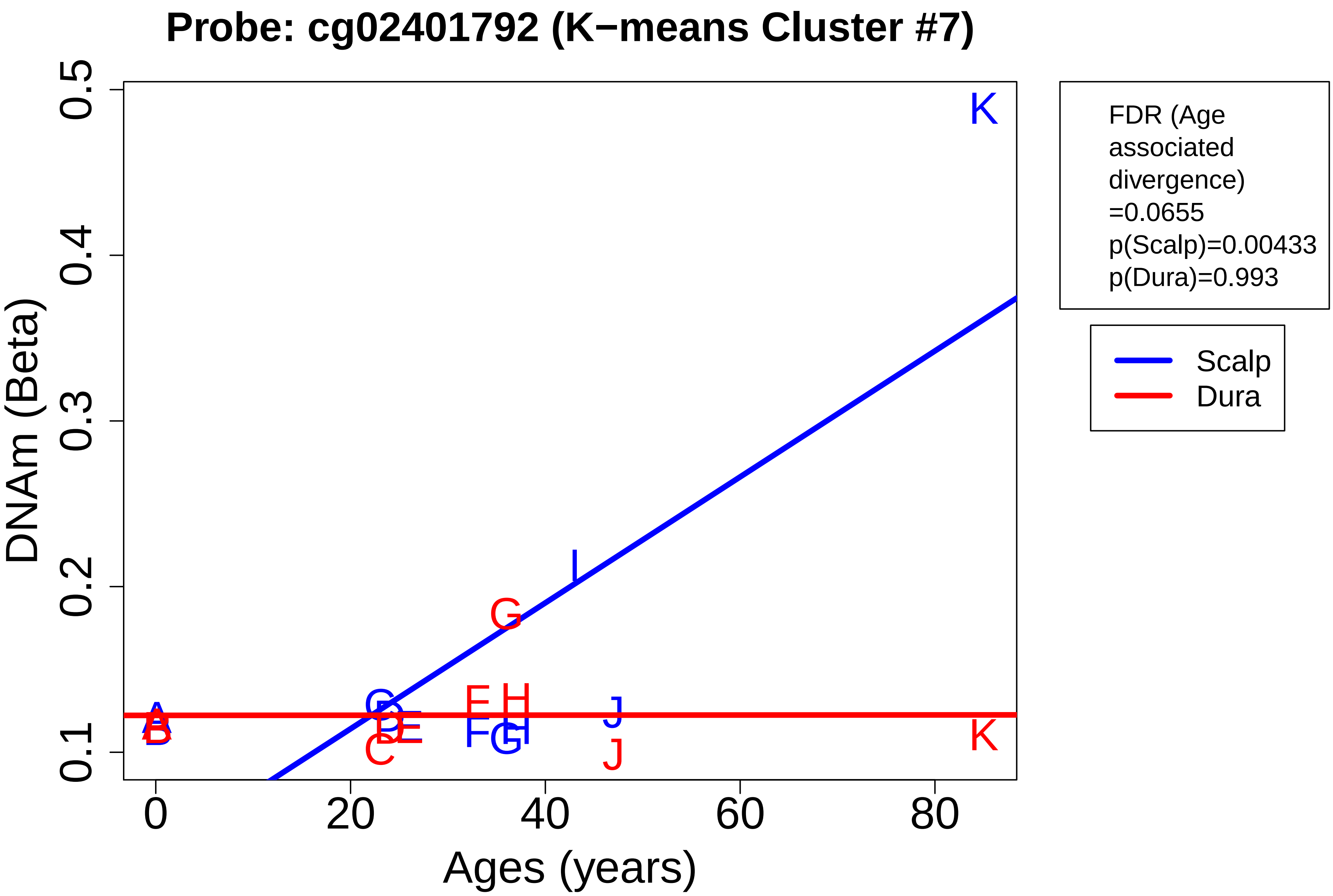
Probe: cg02401792 (K−means Cluster #7)
DNAm (Beta)
Ages (years)
0.1
0.2
0.3
0.4
0.5
0
20
40
60
80
FDR (Age associated divergence) =0.0655
p(Scalp)=0.00433
p(Dura)=0.993
Scalp
Dura
A
B
C
D
E
F
G
H
I
J
K

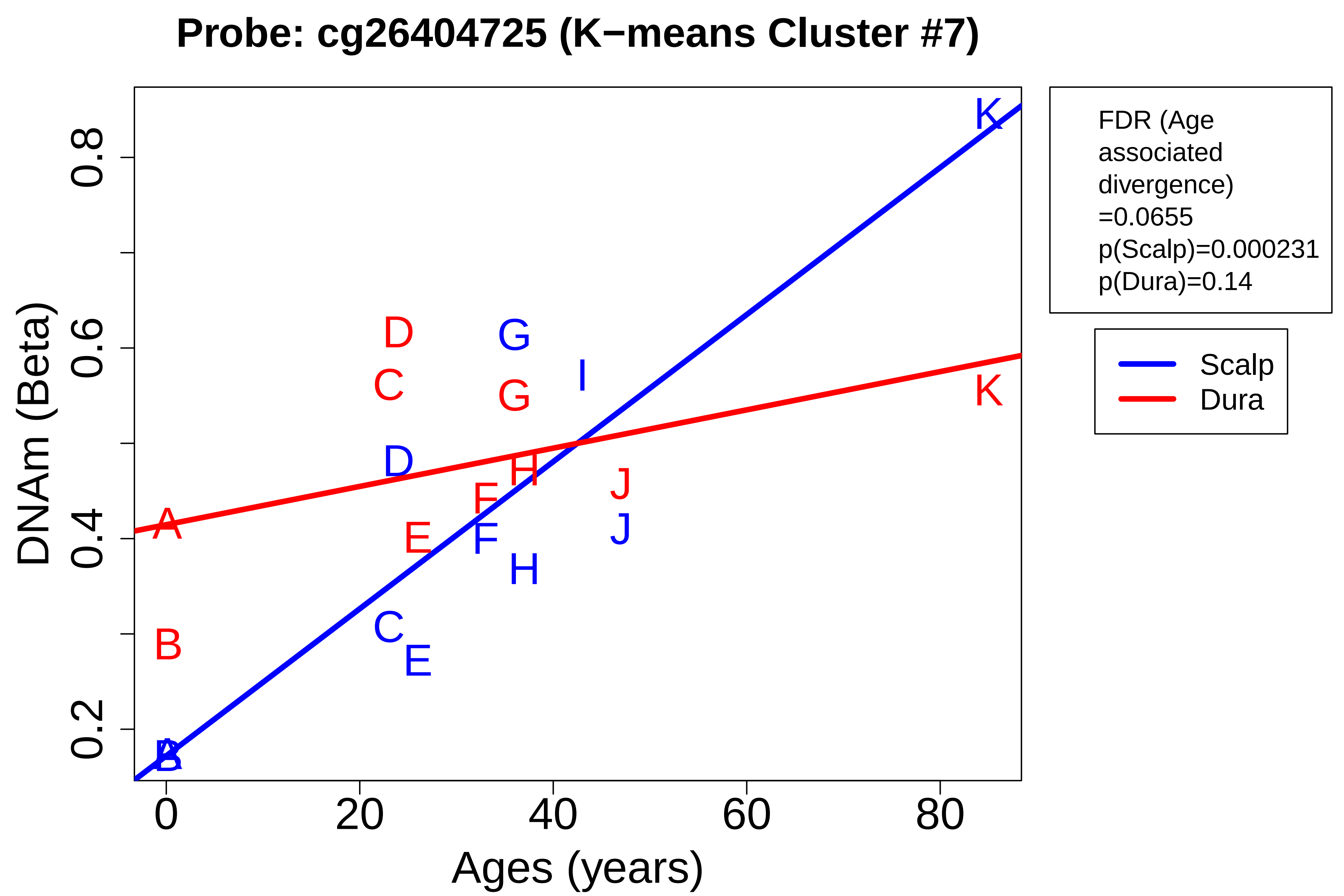
Probe: cg26404725 (K−means Cluster #7)
DNAm (Beta)
0.2
0.4
0.6
0.8
Ages (years)
0
20
40
60
80
FDR (Age associated divergence) =0.0655
p(Scalp)=0.000231
p(Dura)=0.14
Scalp
Dura

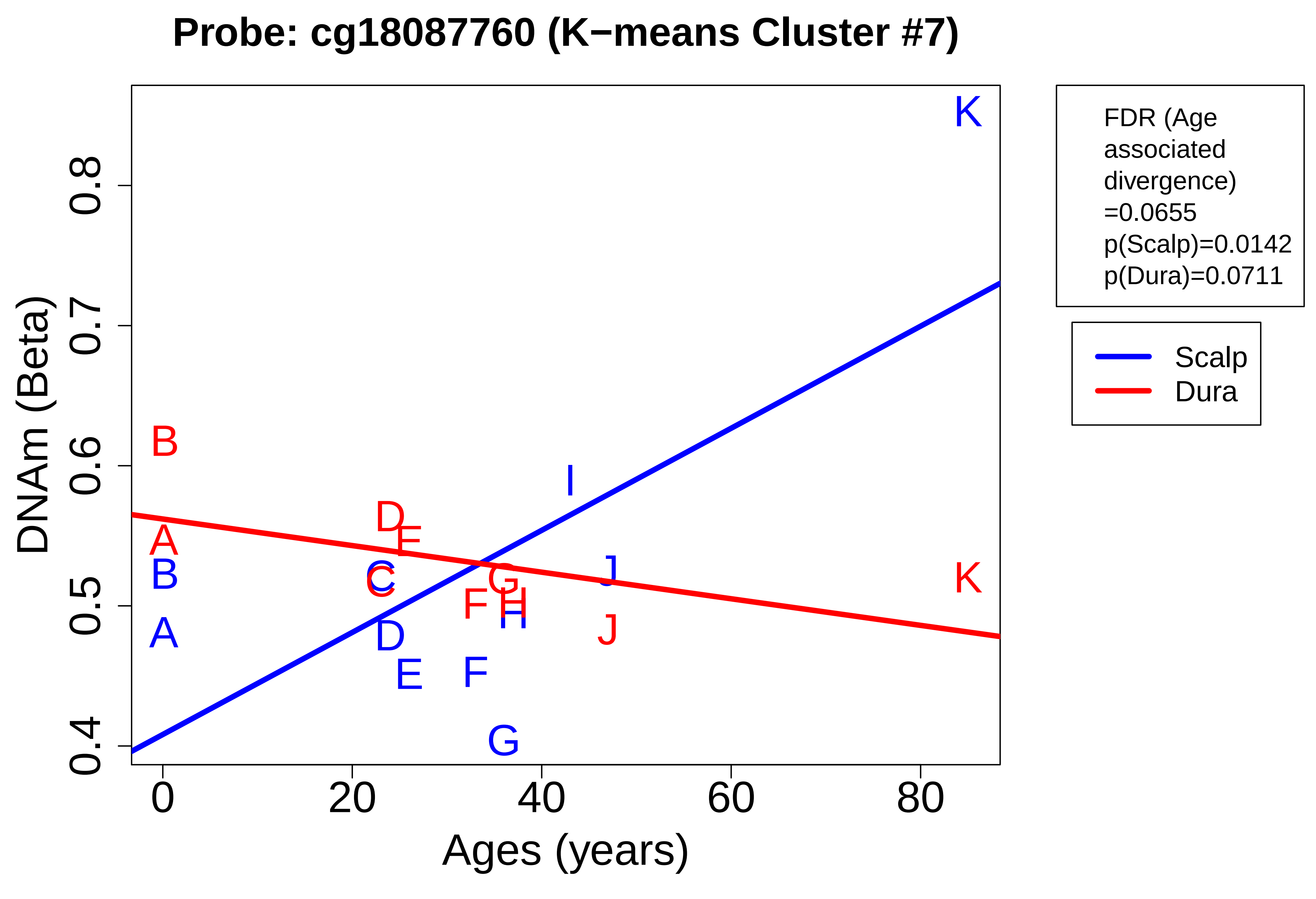

Probe: cg18087760 (K−means Cluster #7)
DNAm (Beta)
Ages (years)
0.4
0.5
0.6
0.7
0.8
0
20
40
60
80
A
B
C
D
E
F
G
H
I
J
K
FDR (Age associated divergence) =0.0655
p(Scalp)=0.0142
p(Dura)=0.0711
Scalp
Dura

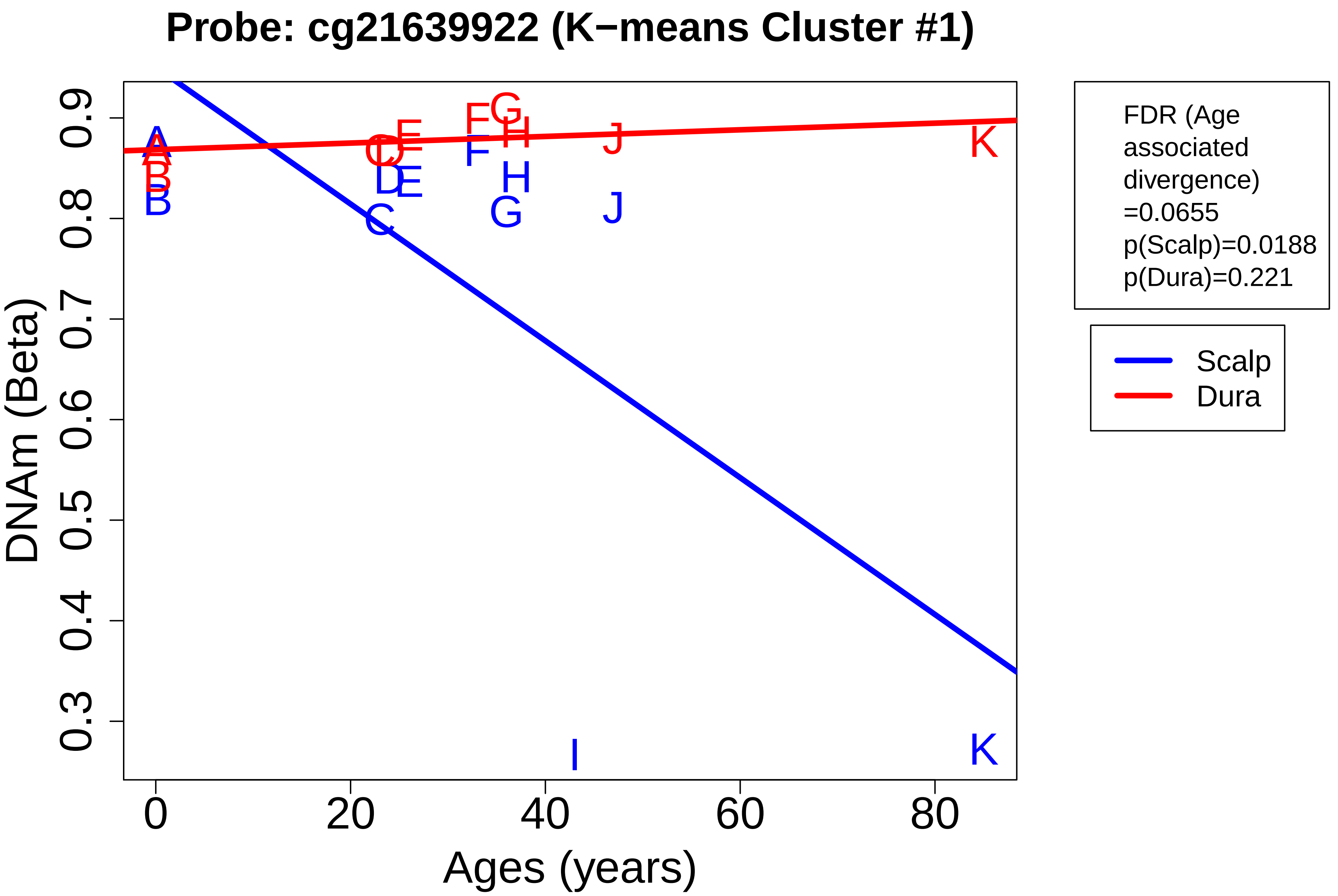

Probe: cg21639922 (K−means Cluster #1)
FDR (Age associated divergence) =0.0655
p(Scalp)=0.0188
p(Dura)=0.221
Scalp
Dura
DNAm (Beta)
Ages (years)
0.3
0.4
0.5
0.6
0.7
0.8
0.9
0
20
40
60
80

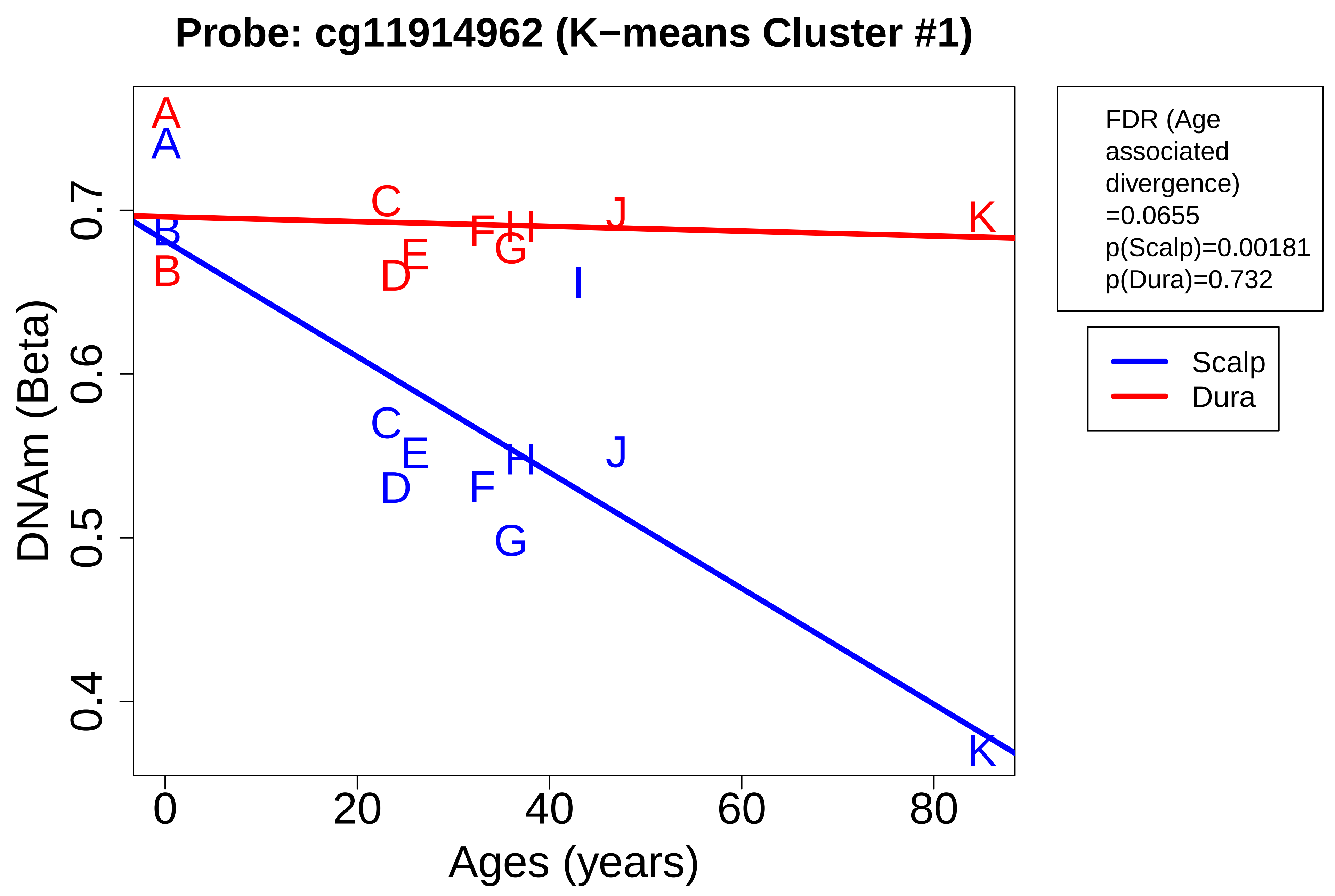

Probe: cg11914962 (K−means Cluster #1)
DNAm (Beta)
0.4
0.5
0.6
0.7
0
20
40
60
80
Ages (years)
FDR (Age associated divergence) =0.0655
p(Scalp)=0.00181
p(Dura)=0.732
Scalp
Dura

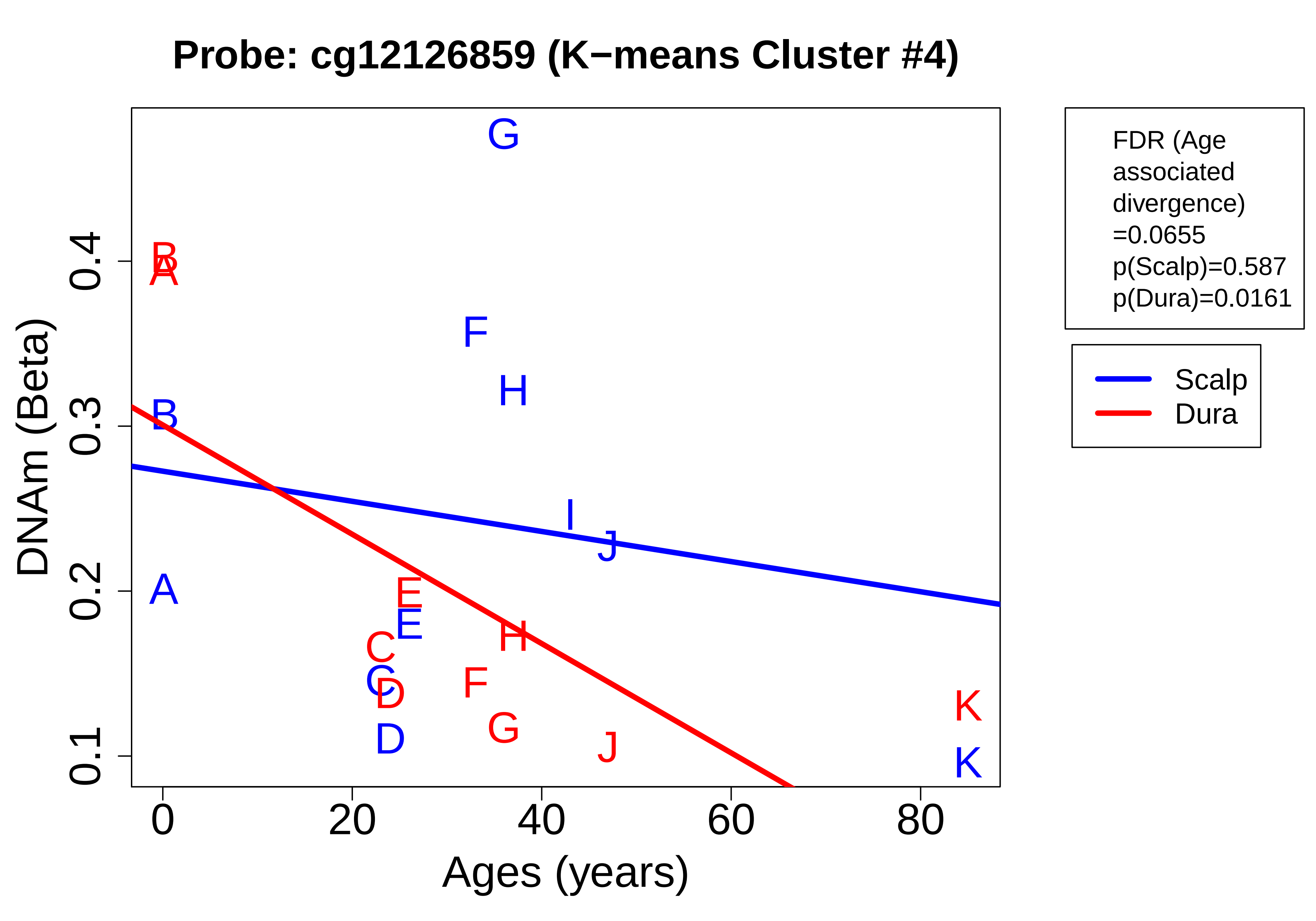

Probe: cg12126859 (K−means Cluster #4)
DNAm (Beta)
0.1
0.2
0.3
0.4
0
20
40
60
80
Ages (years)
FDR (Age associated divergence) =0.0655
p(Scalp)=0.587
p(Dura)=0.0161
Scalp
Dura
A
B
C
D
E
F
G
H
I
J
K

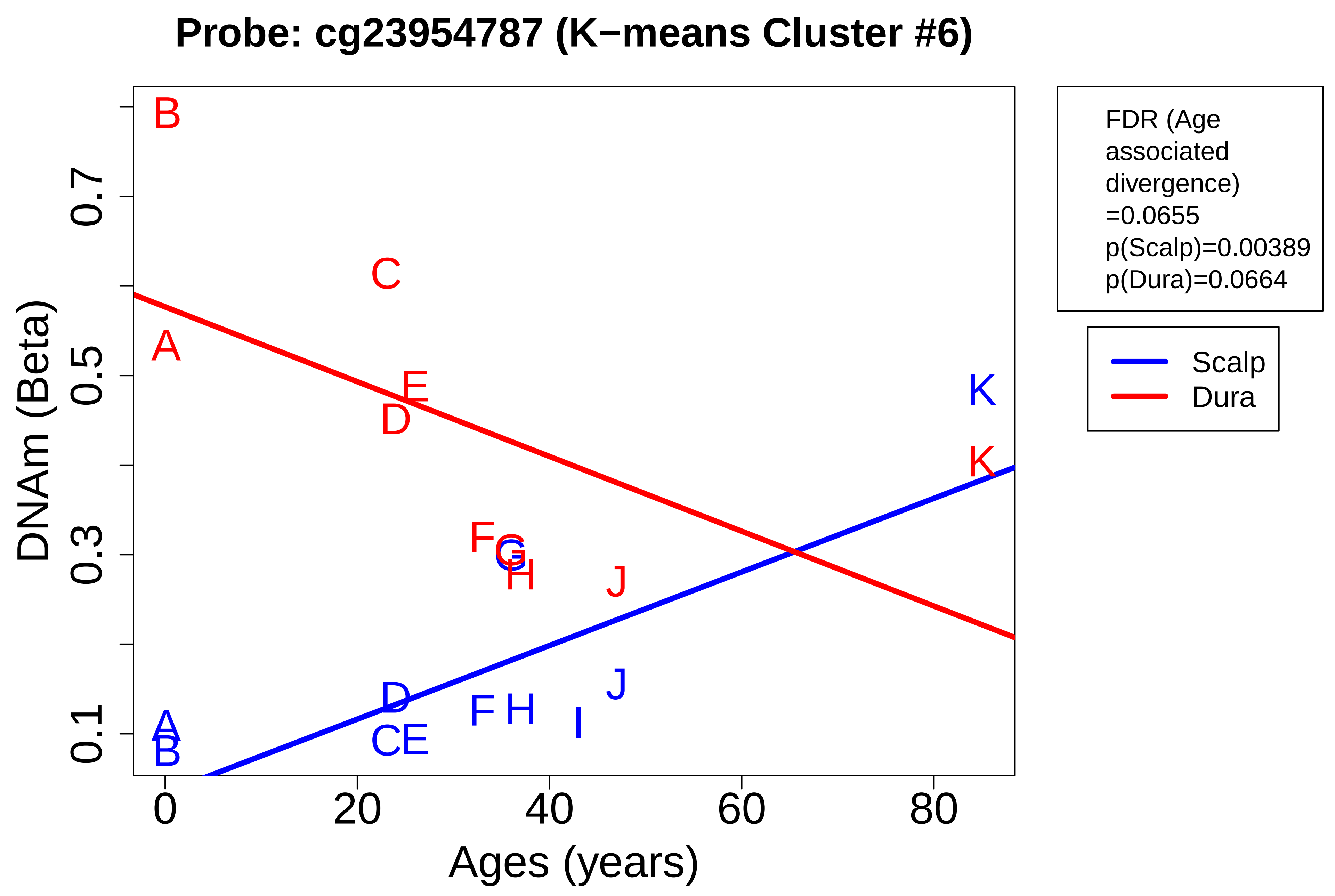
Probe: cg23954787 (K−means Cluster #6)
DNAm (Beta)
0.1
0.3
0.5
0.7
Ages (years)
0
20
40
60
80
FDR (Age associated divergence) =0.0655
p(Scalp)=0.00389
p(Dura)=0.0664
Scalp
Dura
A
B
C
D
E
F
G
H
I
J
K

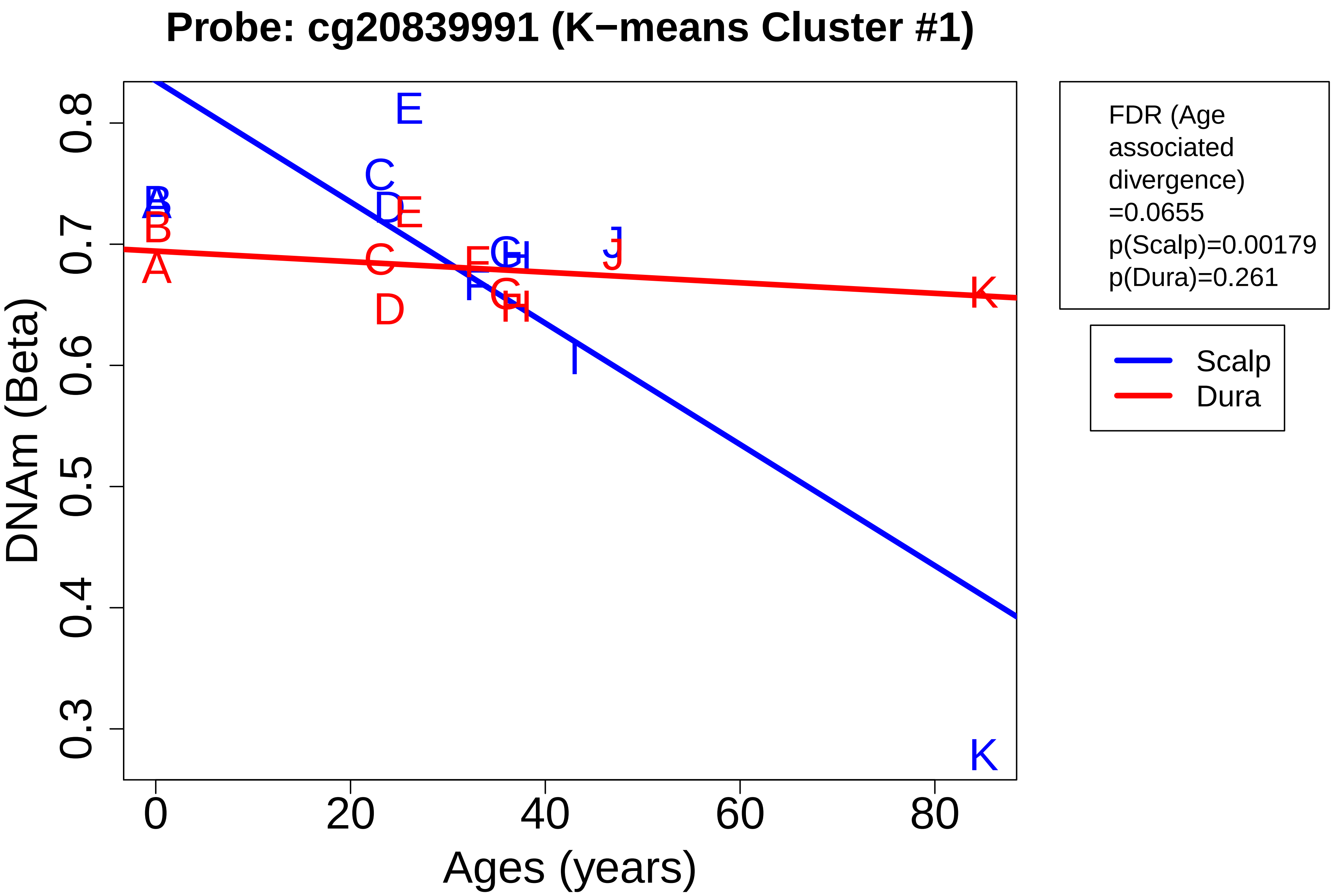
Probe: cg20839991 (K−means Cluster #1)
DNAm (Beta)
0.3
0.4
0.5
0.6
0.7
0.8
0
20
40
60
80
Ages (years)
FDR (Age associated divergence) =0.0655
p(Scalp)=0.00179
p(Dura)=0.261
Scalp
Dura

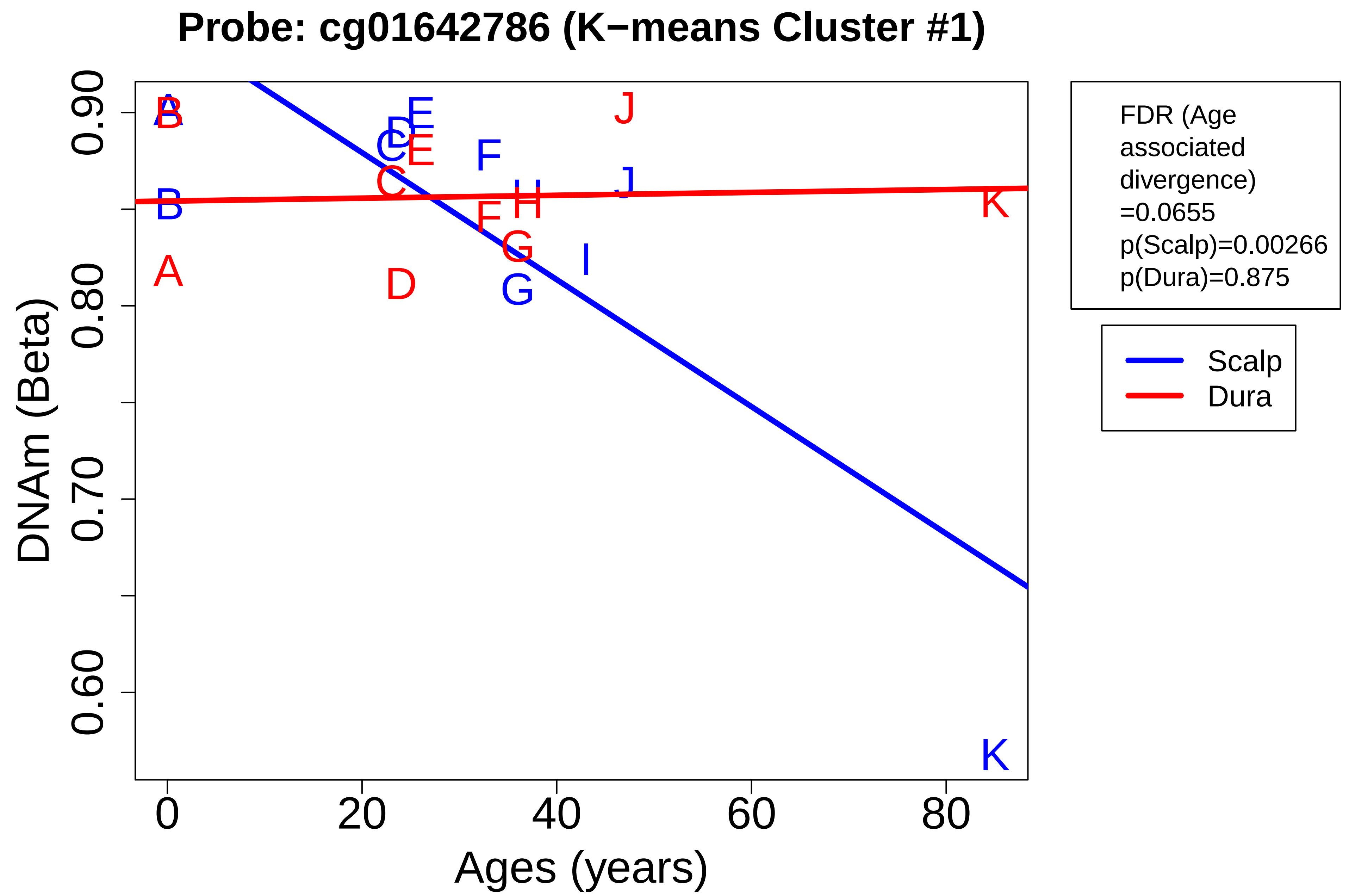
Probe: cg01642786 (K−means Cluster #1)
DNAm (Beta)
0.60
0.70
0.80
0.90
Ages (years)
0
20
40
60
80
FDR (Age associated divergence) =0.0655
p(Scalp)=0.00266
p(Dura)=0.875
Scalp
Dura

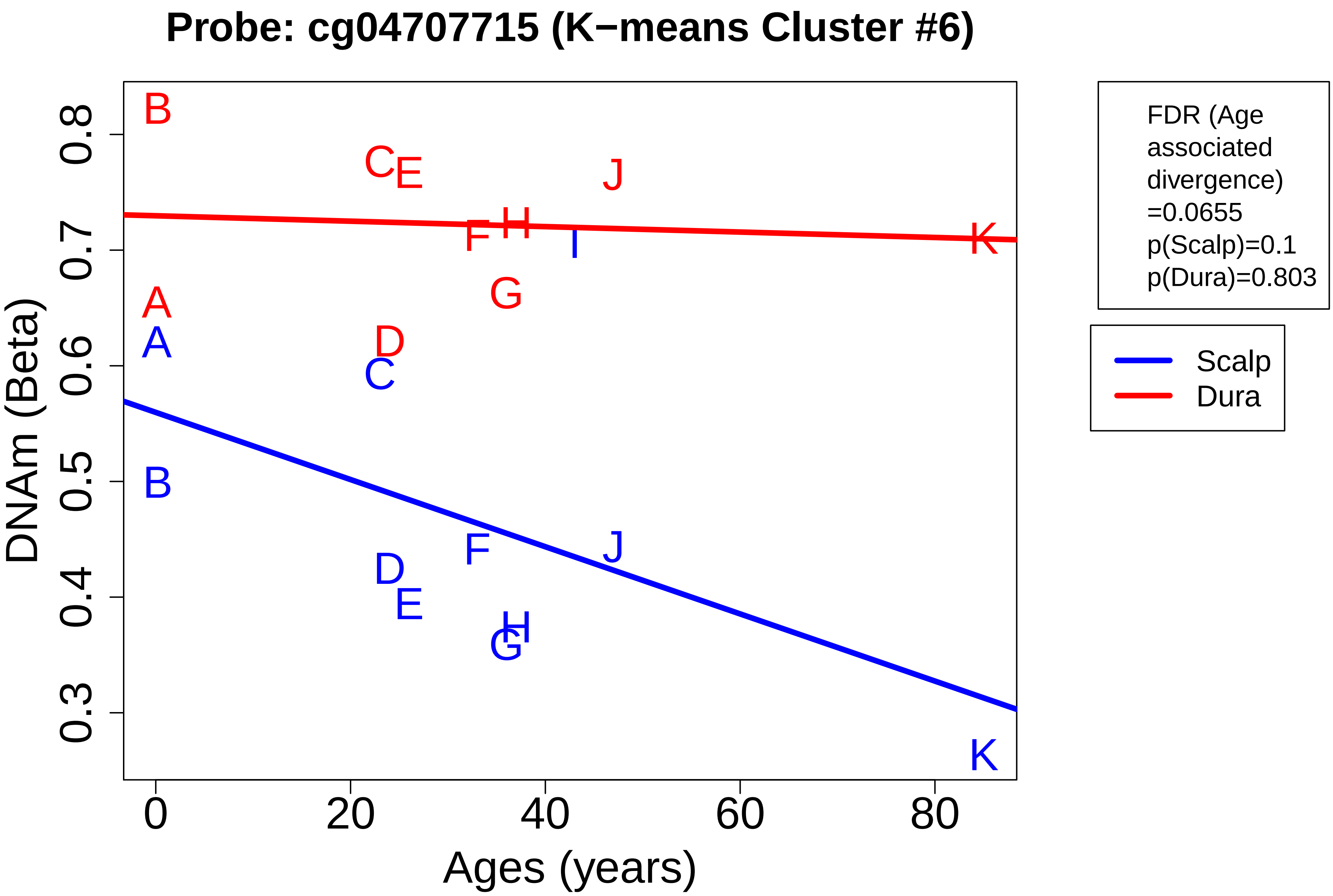

Probe: cg04707715 (K−means Cluster #6)
FDR (Age associated divergence) =0.0655
p(Scalp)=0.1
p(Dura)=0.803
Scalp
Dura
DNAm (Beta)
0.3
0.4
0.5
0.6
0.7
0.8
0
20
40
60
80
Ages (years)
A
B
C
D
E
F
G
H
I
J
K

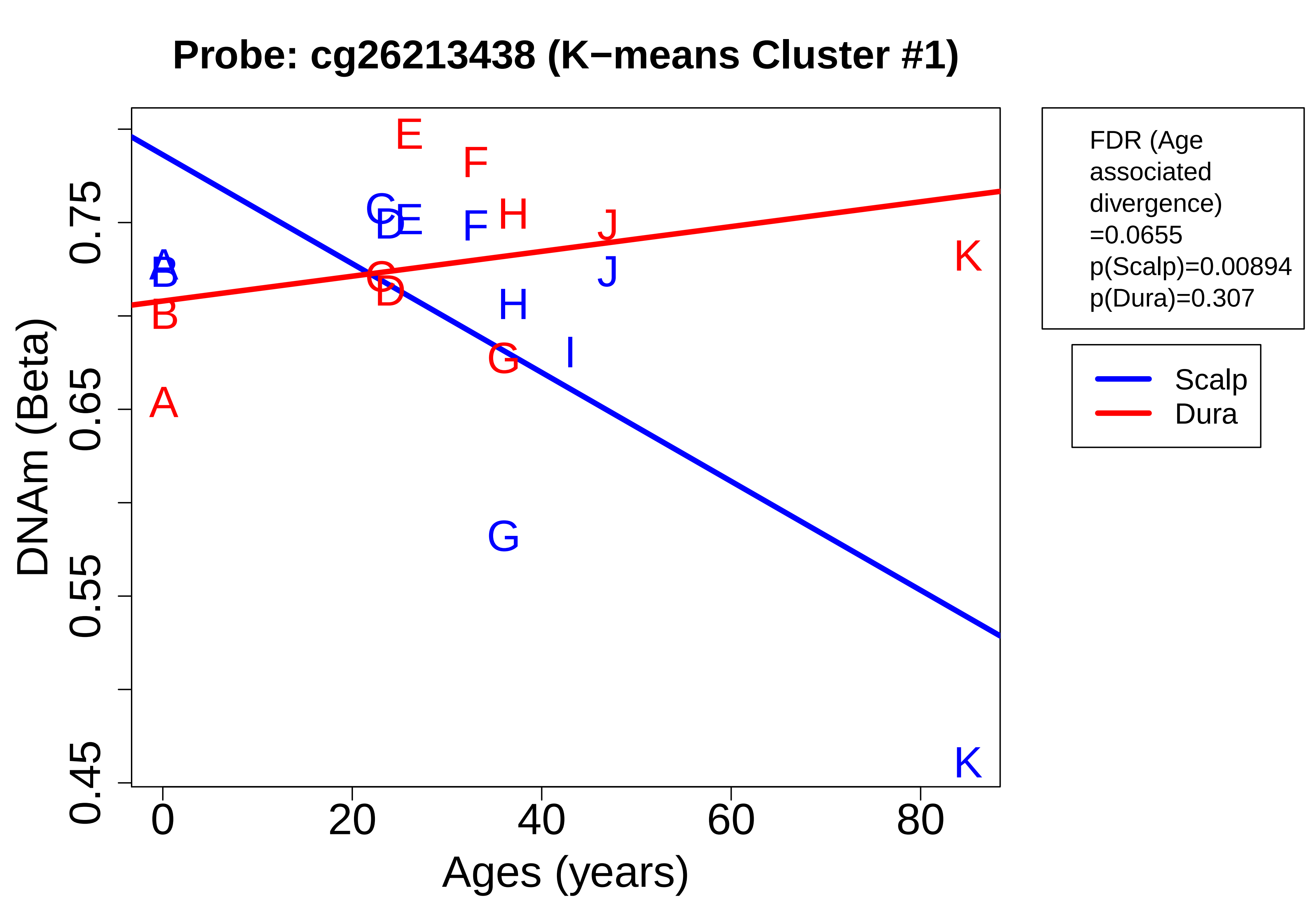

Probe: cg26213438 (K−means Cluster #1)
DNAm (Beta)
0.45
0.55
0.65
0.75
0
20
40
60
80
Ages (years)
FDR (Age associated divergence) =0.0655
p(Scalp)=0.00894
p(Dura)=0.307
Scalp
Dura

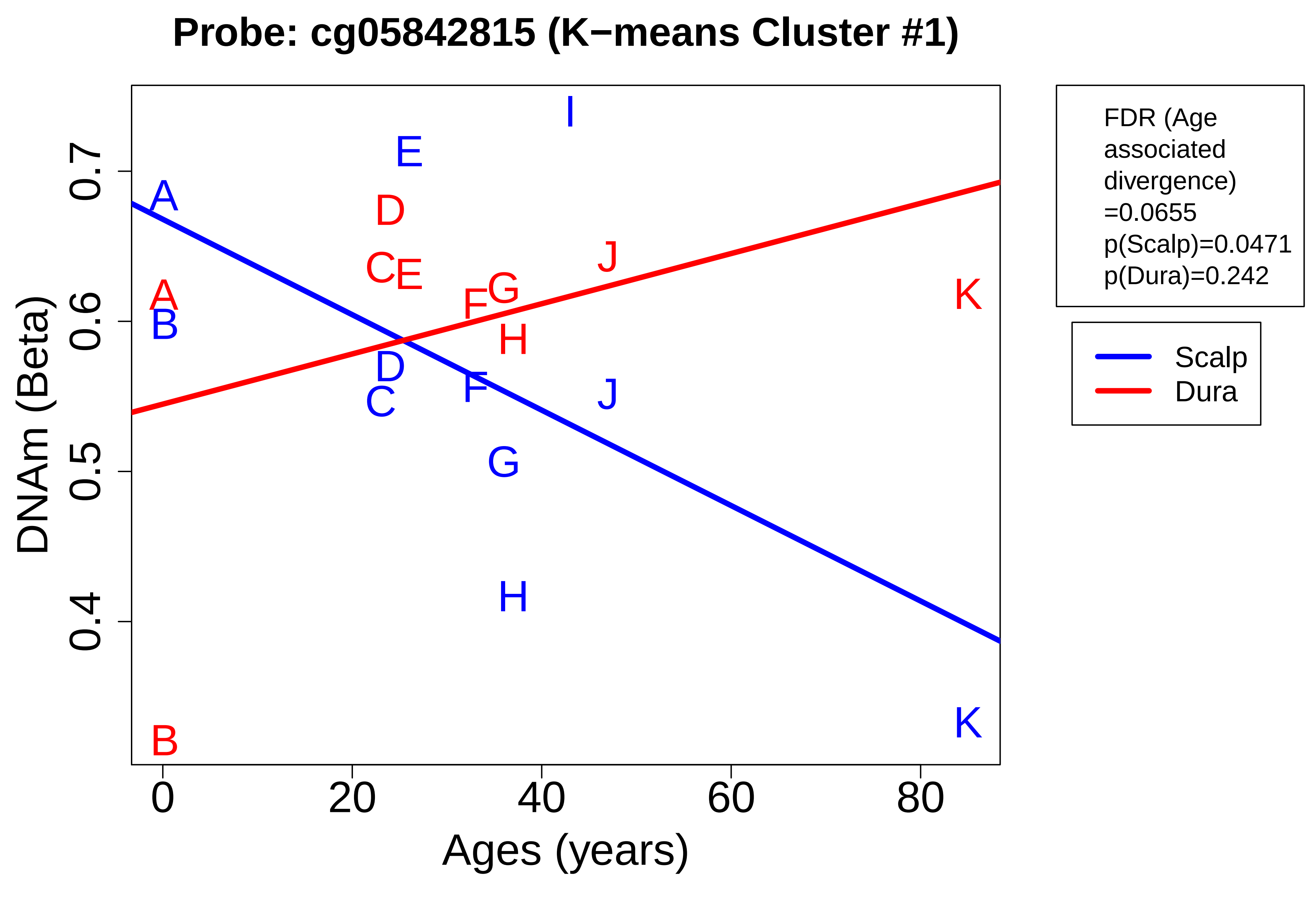

Probe: cg05842815 (K−means Cluster #1)
DNAm (Beta)
0.4
0.5
0.6
0.7
Ages (years)
0
20
40
60
80
FDR (Age associated divergence) =0.0655
p(Scalp)=0.0471
p(Dura)=0.242
Scalp
Dura

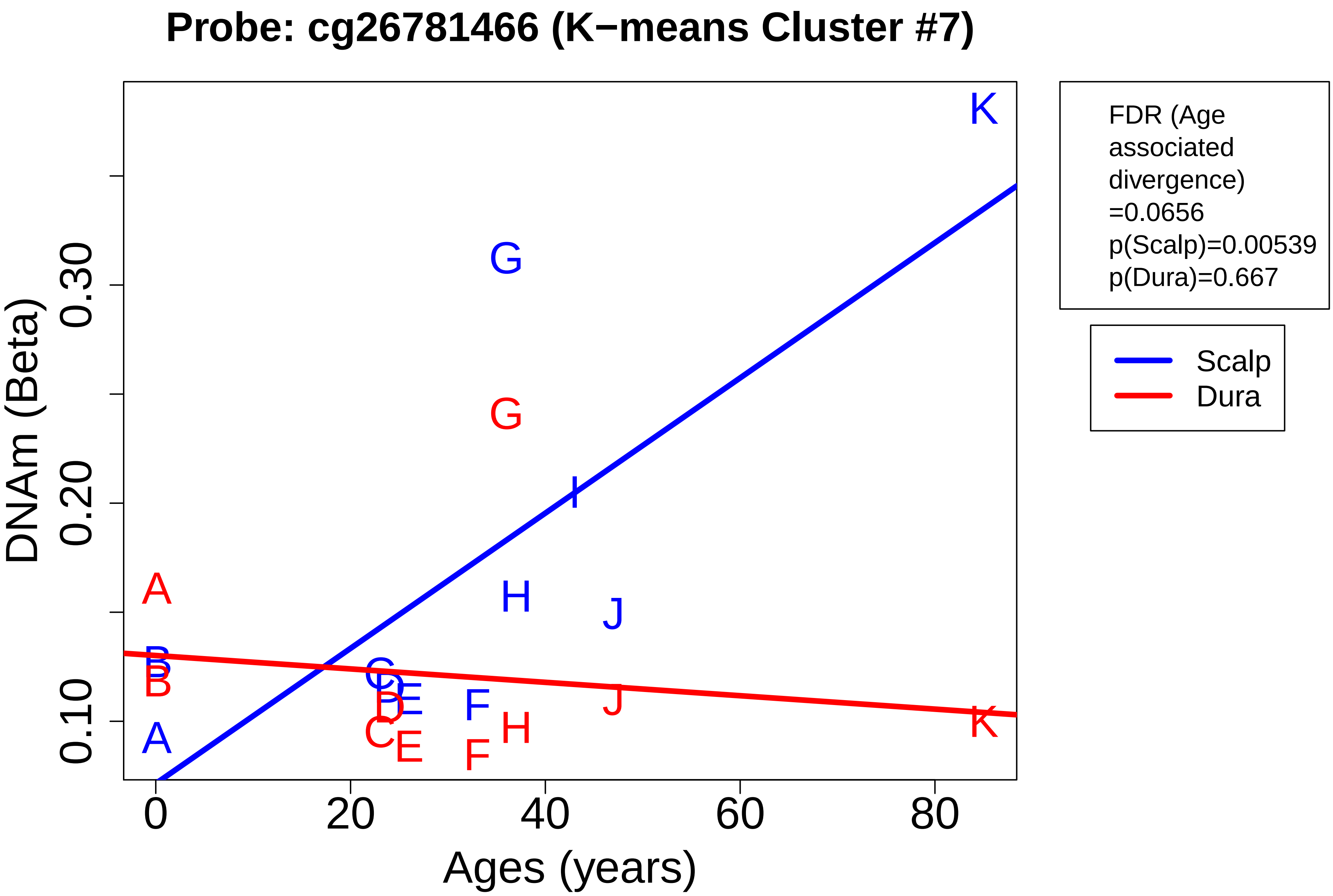

Probe: cg26781466 (K−means Cluster #7)
DNAm (Beta)
0.10
0.20
0.30
0
20
40
60
80
Ages (years)
FDR (Age associated divergence) =0.0656
p(Scalp)=0.00539
p(Dura)=0.667
Scalp
Dura

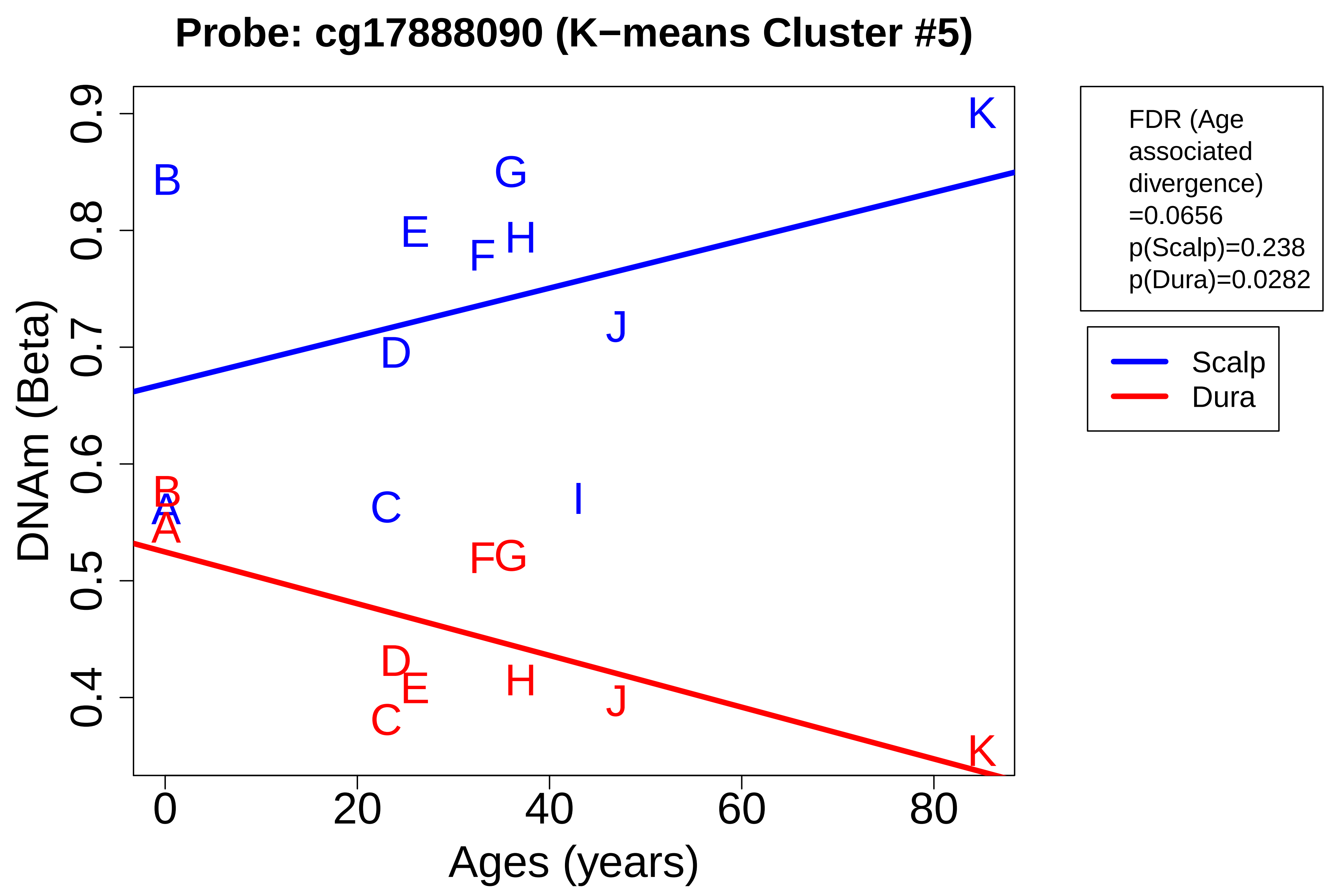
Probe: cg17888090 (K−means Cluster #5)
DNAm (Beta)
Ages (years)
0.4
0.5
0.6
0.7
0.8
0.9
0
20
40
60
80
FDR (Age associated divergence) =0.0656
p(Scalp)=0.238
p(Dura)=0.0282
Scalp
Dura
A
B
C
D
E
F
G
H
I
J
K

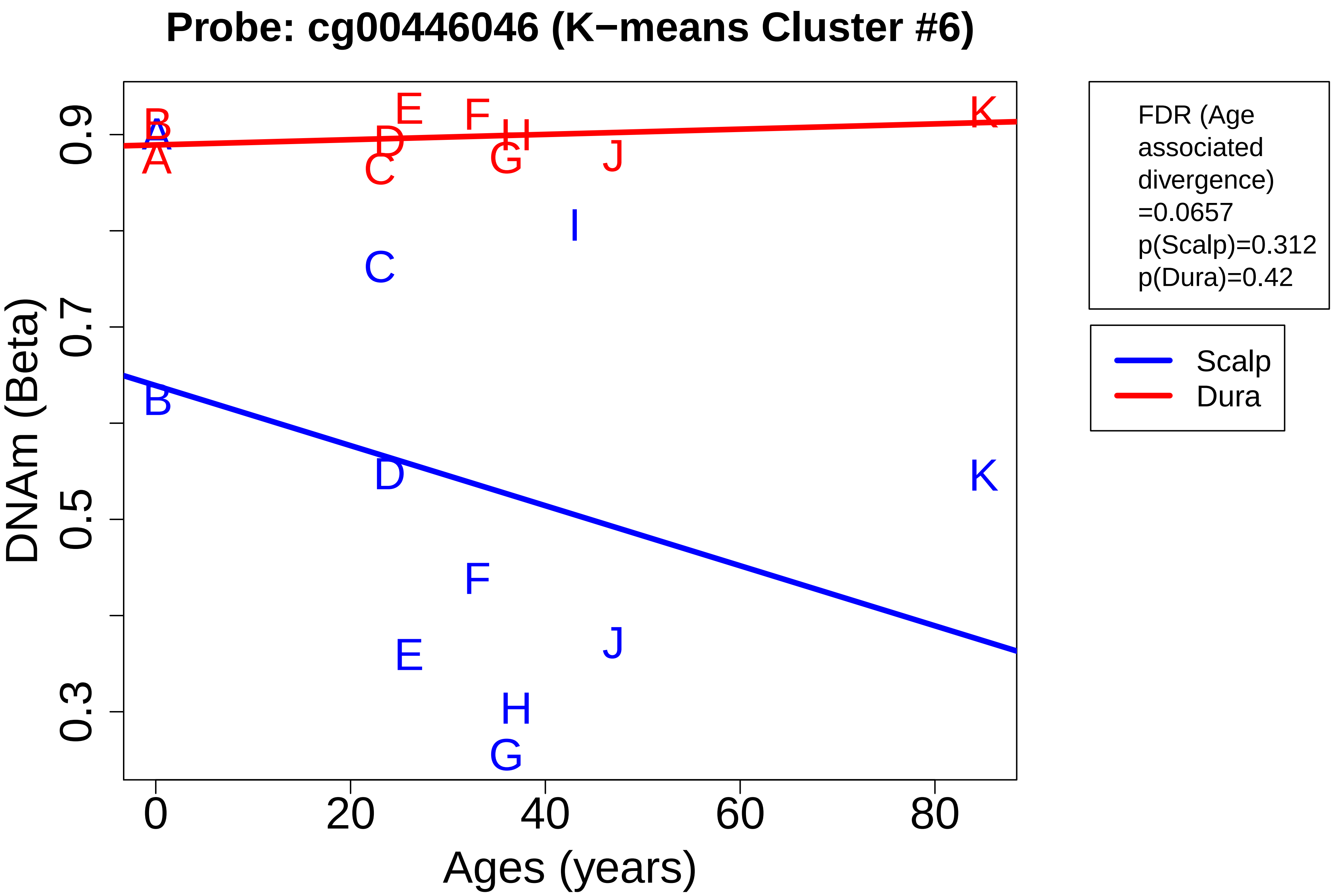

Probe: cg00446046 (K−means Cluster #6)
DNAm (Beta)
0.3
0.5
0.7
0.9
Ages (years)
0
20
40
60
80
FDR (Age associated divergence) =0.0657
p(Scalp)=0.312
p(Dura)=0.42
Scalp
Dura
A
B
C
D
E
F
G
H
I
J
K

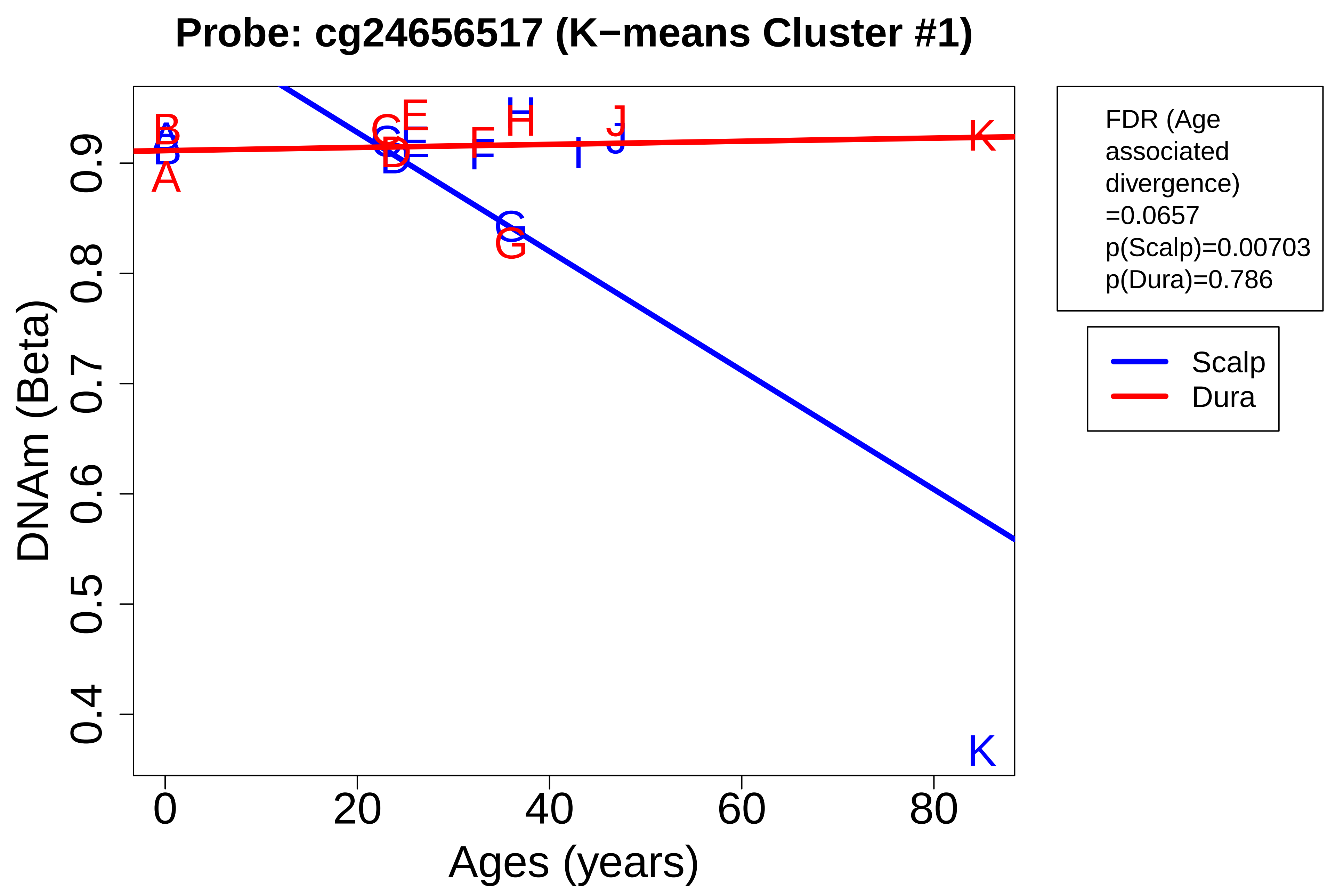

Probe: cg24656517 (K−means Cluster #1)
DNAm (Beta)
Ages (years)
0.4
0.5
0.6
0.7
0.8
0.9
0
20
40
60
80
A
B
C
D
E
F
G
H
I
J
K
FDR (Age associated divergence) =0.0657
p(Scalp)=0.00703
p(Dura)=0.786
Scalp
Dura

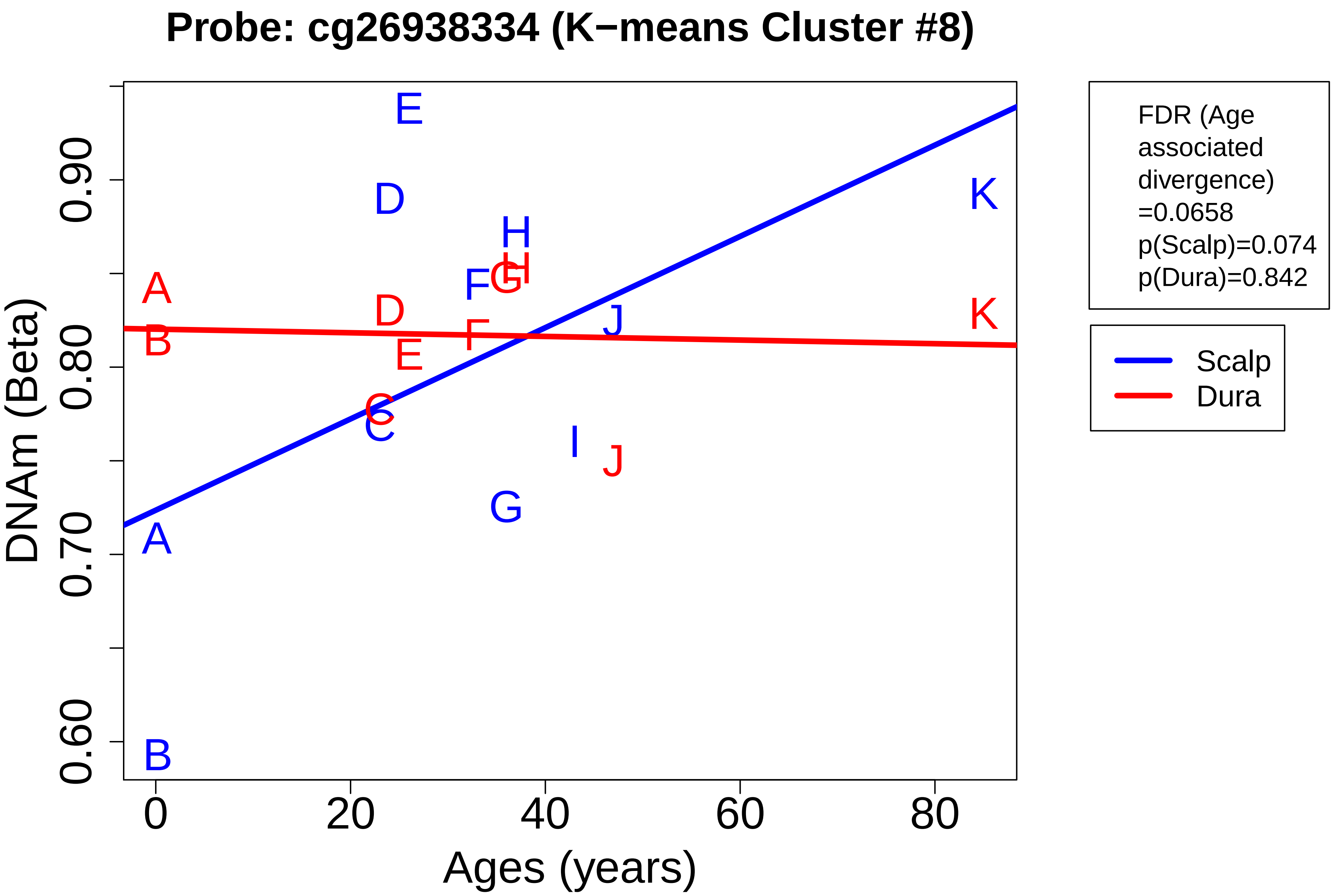

Probe: cg26938334 (K−means Cluster #8)
DNAm (Beta)
0.60
0.70
0.80
0.90
0
20
40
60
80
Ages (years)
FDR (Age associated divergence) =0.0658
p(Scalp)=0.074
p(Dura)=0.842
Scalp
Dura
A
B
C
D
E
F
G
H
I
J
K

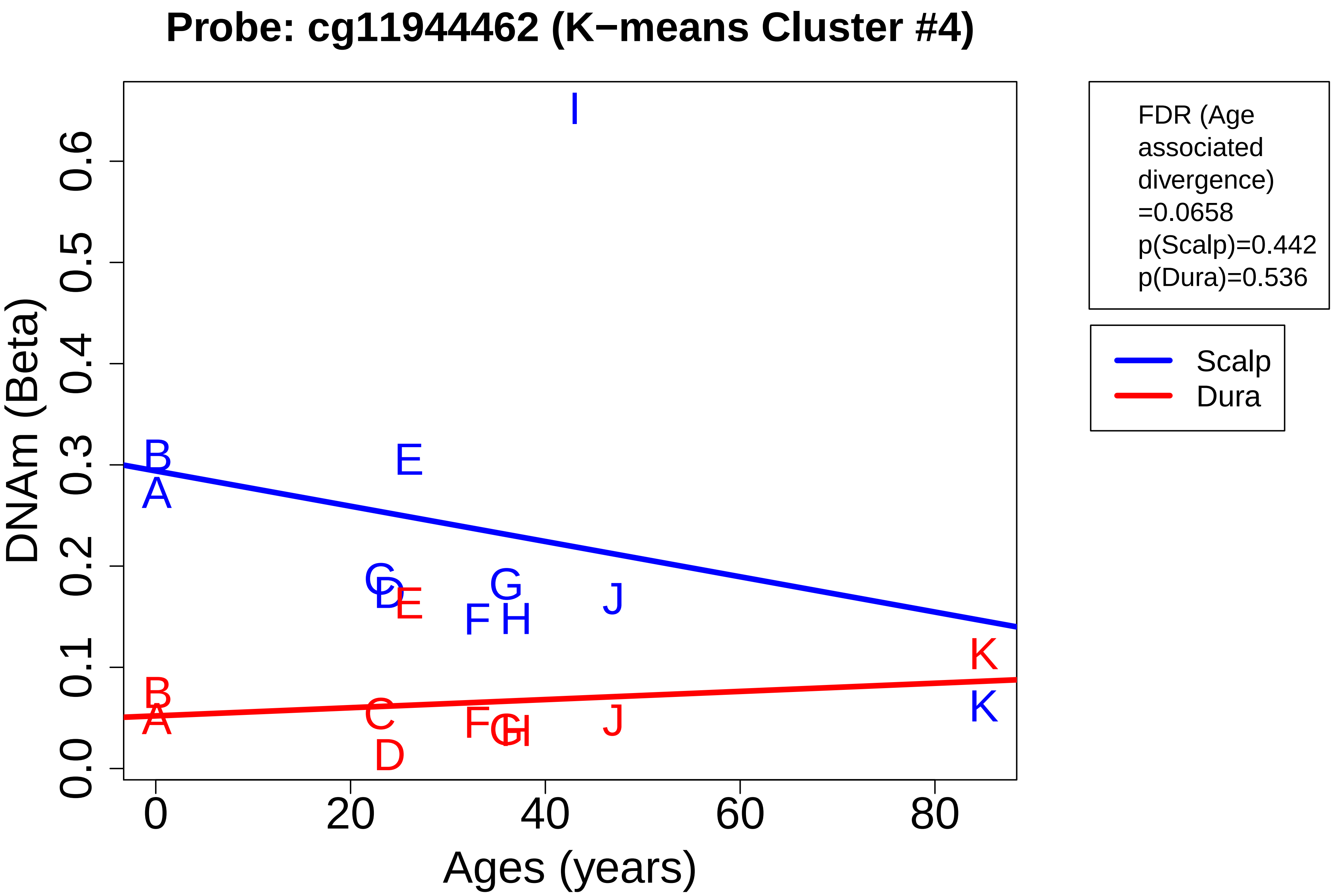
Probe: cg11944462 (K−means Cluster #4)
FDR (Age associated divergence) =0.0658
p(Scalp)=0.442
p(Dura)=0.536
Scalp
Dura
DNAm (Beta)
0.0
0.1
0.2
0.3
0.4
0.5
0.6
Ages (years)
0
20
40
60
80
A
B
C
D
E
F
G
H
I
J
K

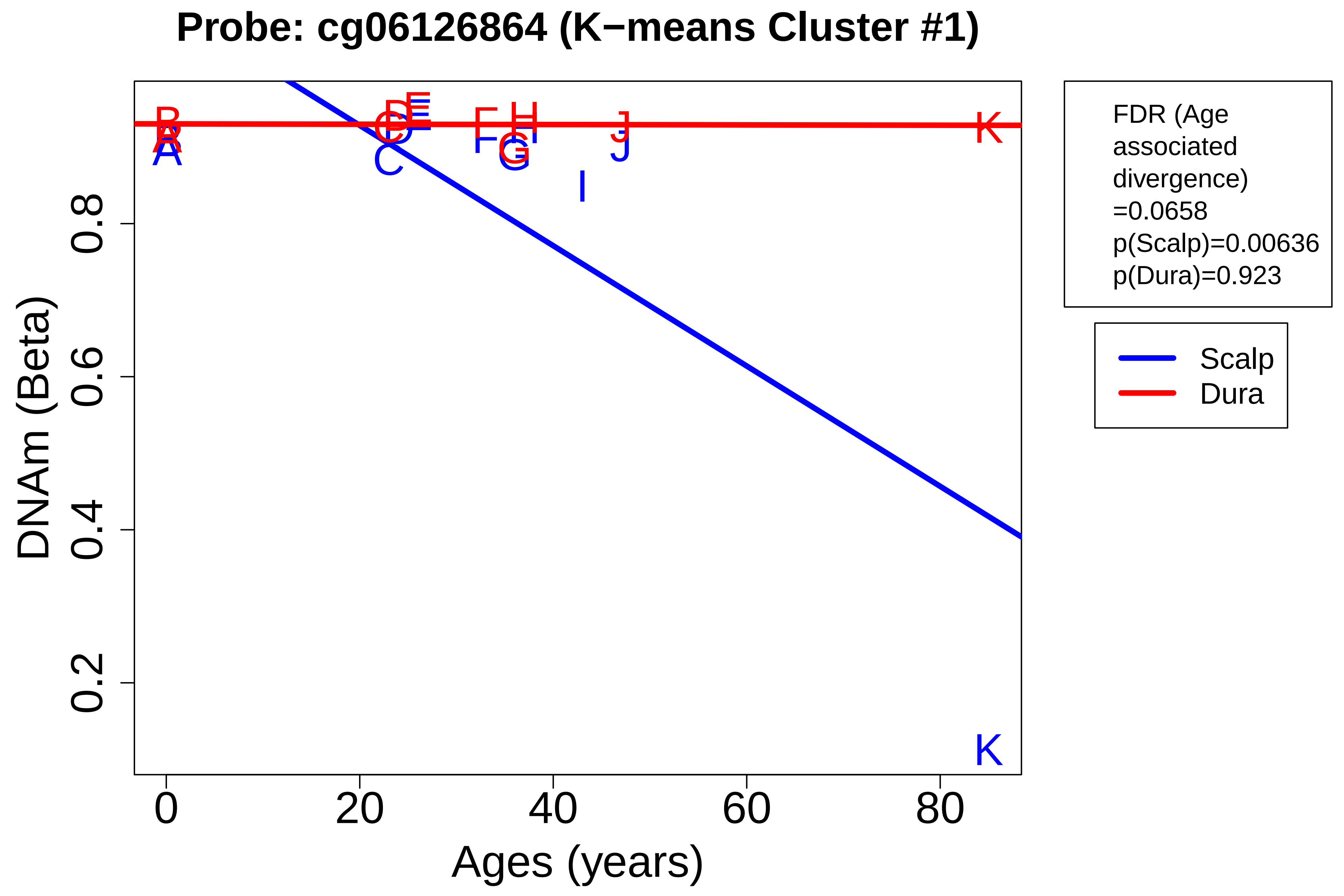

Probe: cg06126864 (K−means Cluster #1)
FDR (Age associated divergence) =0.0658
p(Scalp)=0.00636
p(Dura)=0.923
Scalp
Dura
DNAm (Beta)
0.2
0.4
0.6
0.8
Ages (years)
0
20
40
60
80
A
B
C
D
E
F
G
H
I
J
K

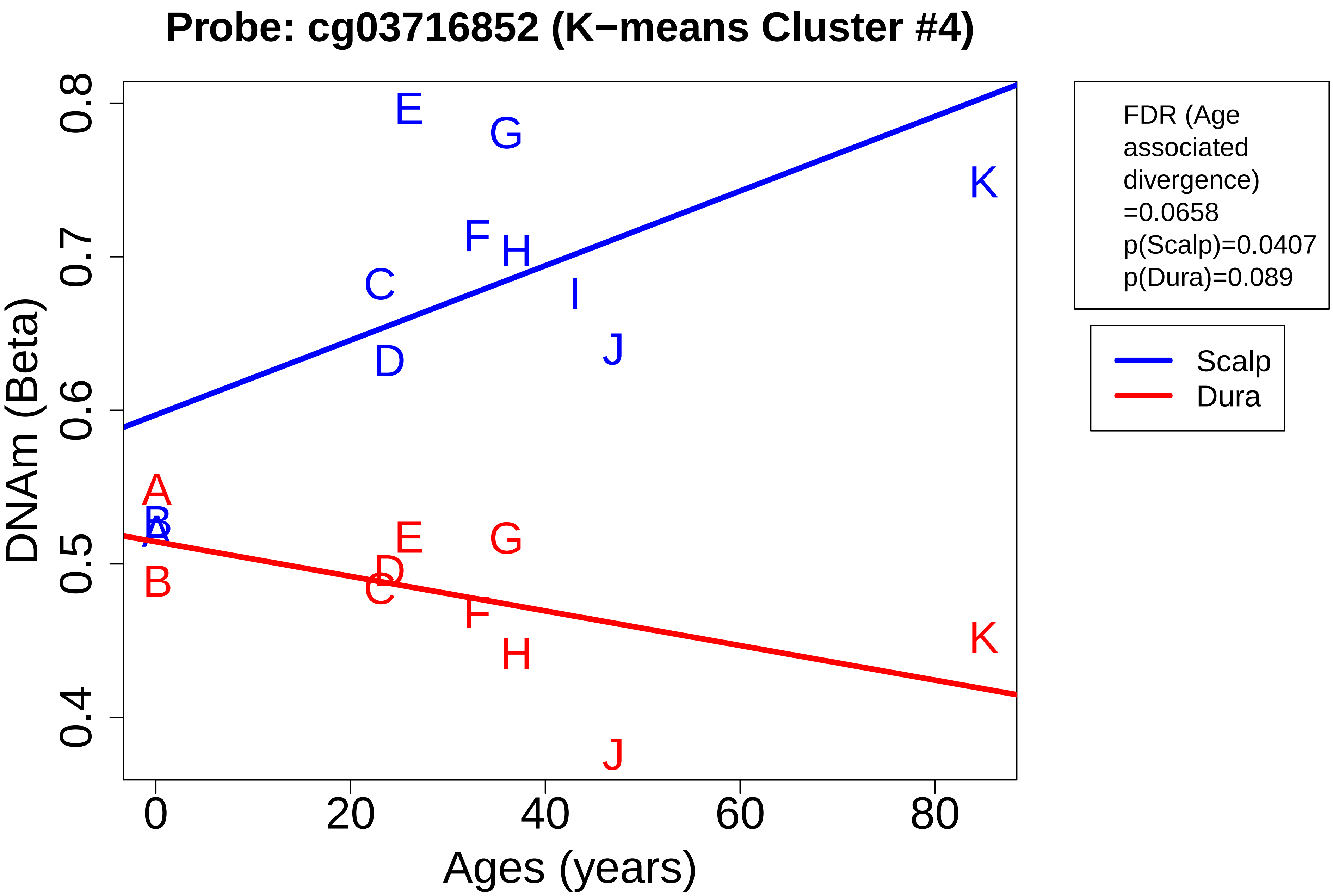
Probe: cg03716852 (K−means Cluster #4)
DNAm (Beta)
Ages (years)
0.4
0.5
0.6
0.7
0.8
0
20
40
60
80
FDR (Age associated divergence) =0.0658
p(Scalp)=0.0407
p(Dura)=0.089
Scalp
Dura
A
B
C
D
E
F
G
H
I
J
K

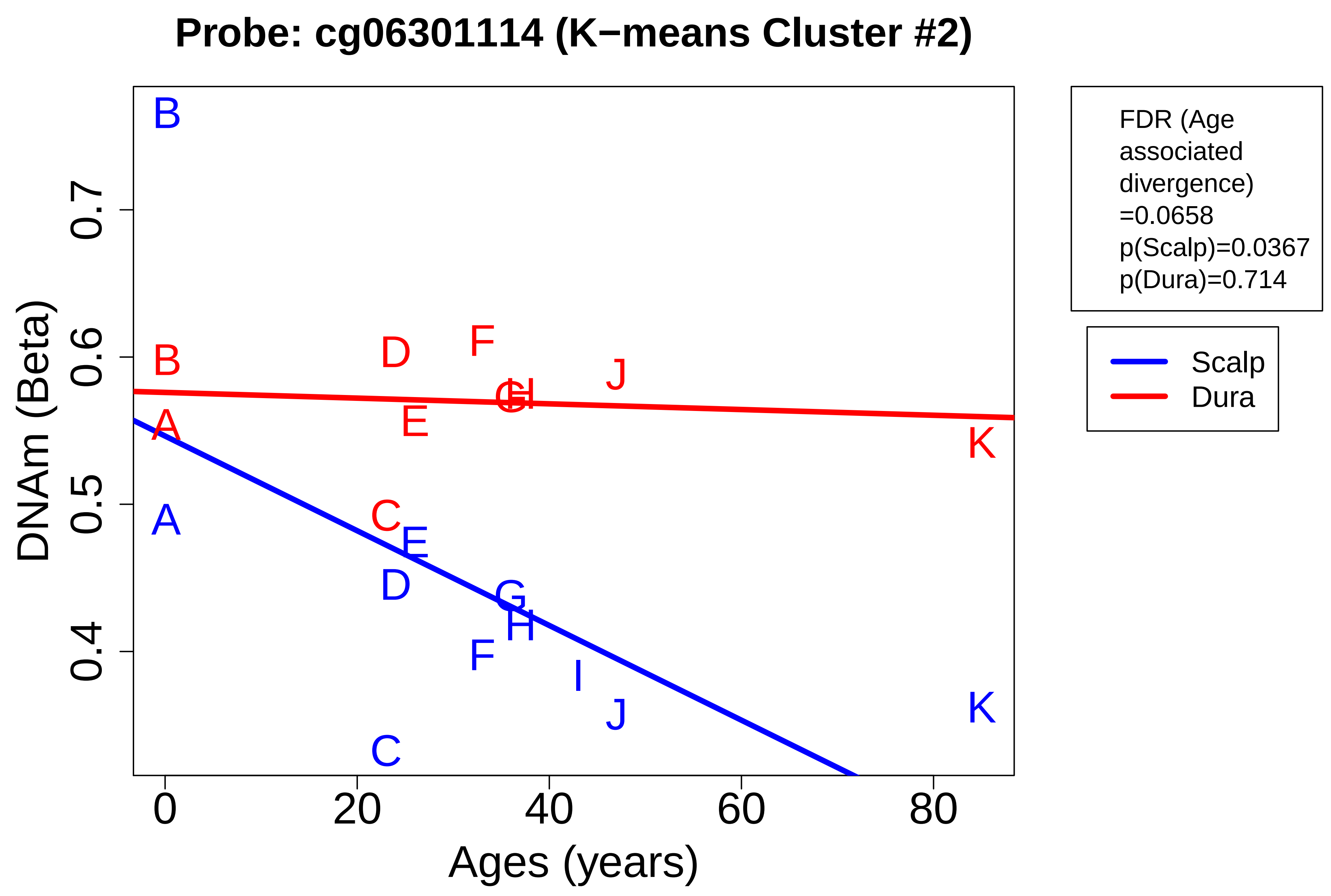
Probe: cg06301114 (K−means Cluster #2)
DNAm (Beta)
0.4
0.5
0.6
0.7
Ages (years)
0
20
40
60
80
FDR (Age associated divergence) =0.0658
p(Scalp)=0.0367
p(Dura)=0.714
Scalp
Dura

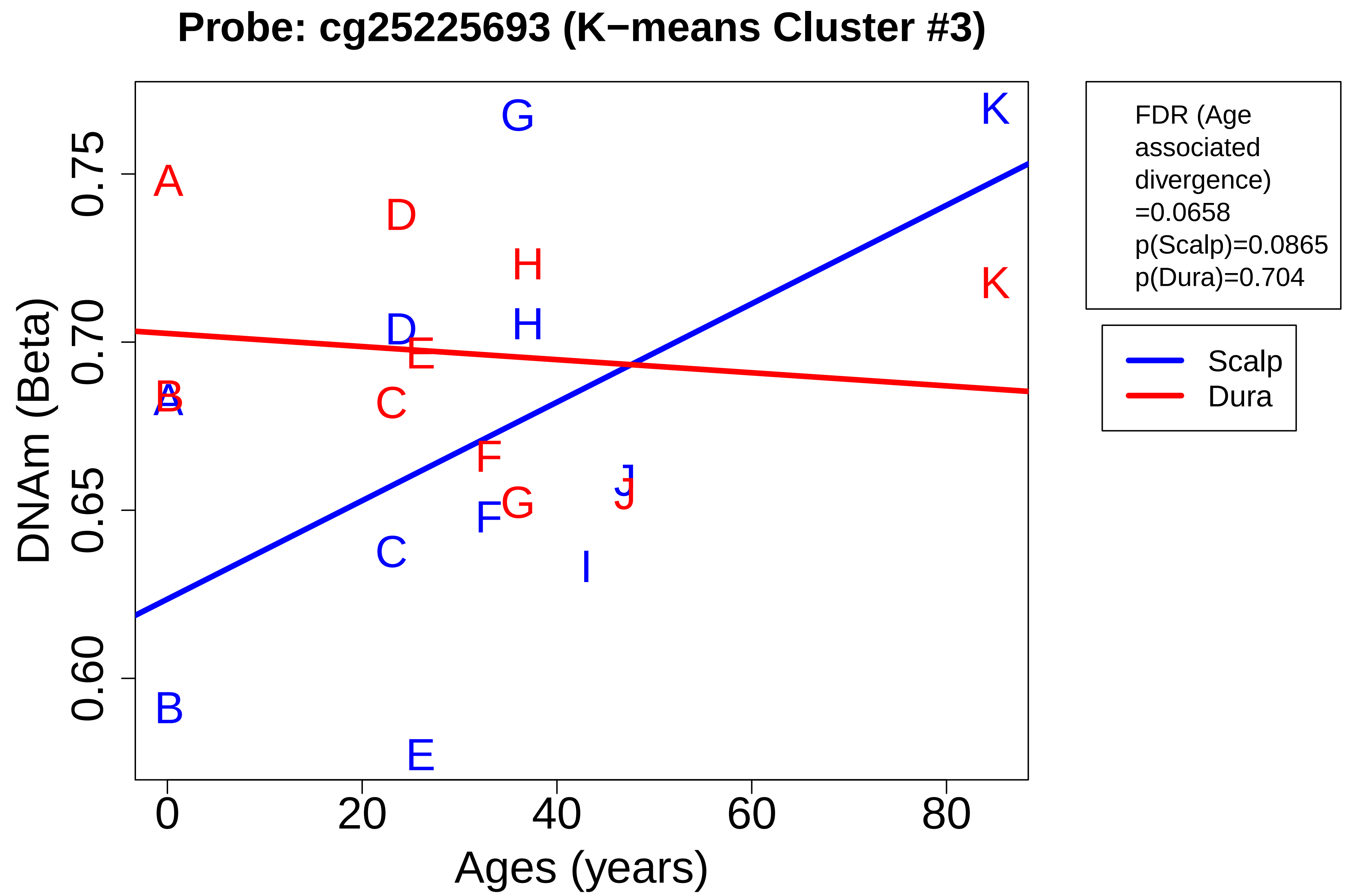
Probe: cg25225693 (K−means Cluster #3)
DNAm (Beta)
0.60
0.65
0.70
0.75
Ages (years)
0
20
40
60
80
FDR (Age associated divergence) =0.0658
p(Scalp)=0.0865
p(Dura)=0.704
Scalp
Dura

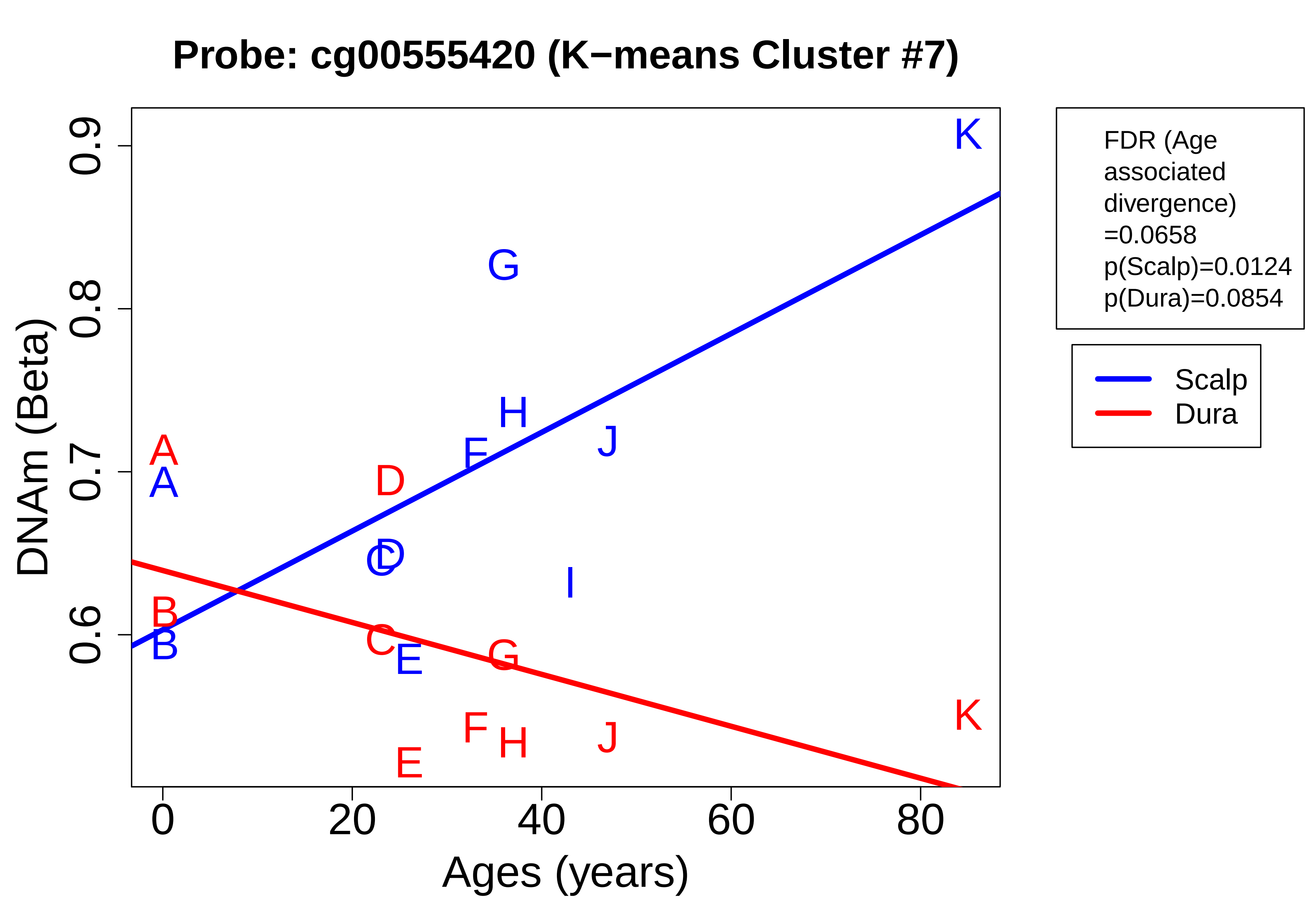

Probe: cg00555420 (K−means Cluster #7)
DNAm (Beta)
0.6
0.7
0.8
0.9
Ages (years)
0
20
40
60
80
A
B
C
D
E
F
G
H
I
J
K
FDR (Age associated divergence) =0.0658
p(Scalp)=0.0124
p(Dura)=0.0854
Scalp
Dura

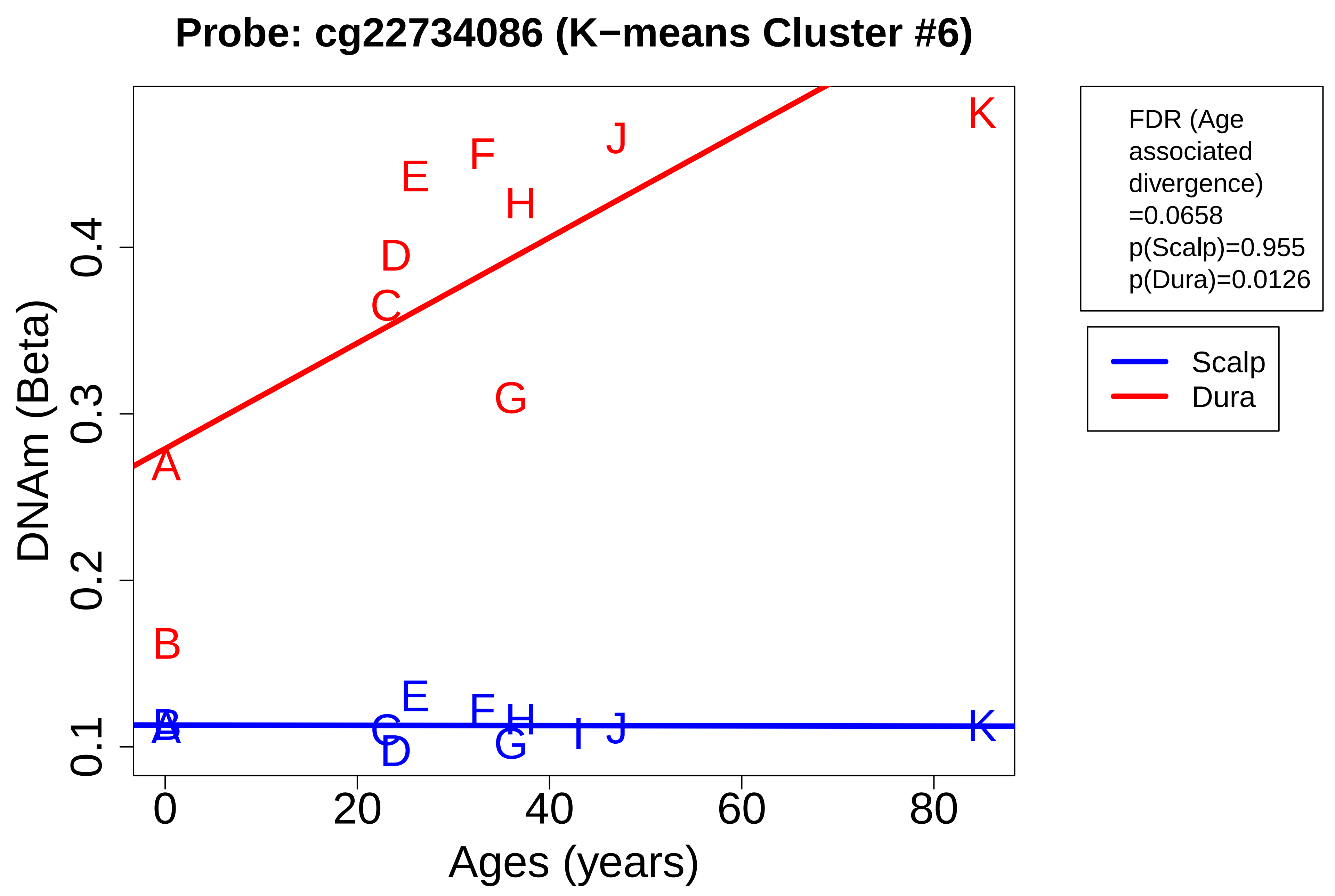

Probe: cg22734086 (K−means Cluster #6)
FDR (Age associated divergence) =0.0658
p(Scalp)=0.955
p(Dura)=0.0126
Scalp
Dura
DNAm (Beta)
0.1
0.2
0.3
0.4
0
20
40
60
80
Ages (years)

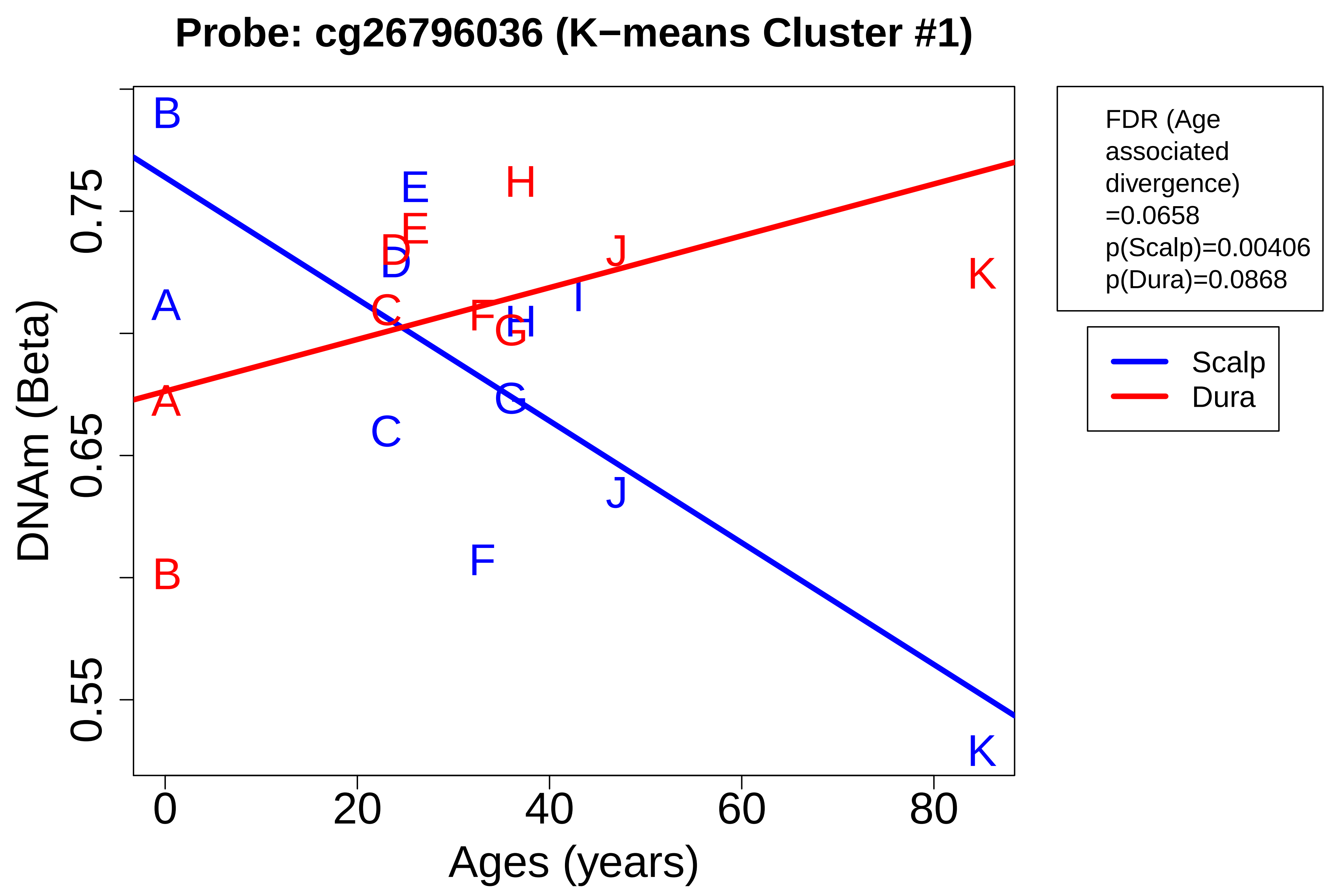
Probe: cg26796036 (K−means Cluster #1)
DNAm (Beta)
0.55
0.65
0.75
0
20
40
60
80
Ages (years)
FDR (Age associated divergence) =0.0658
p(Scalp)=0.00406
p(Dura)=0.0868
Scalp
Dura

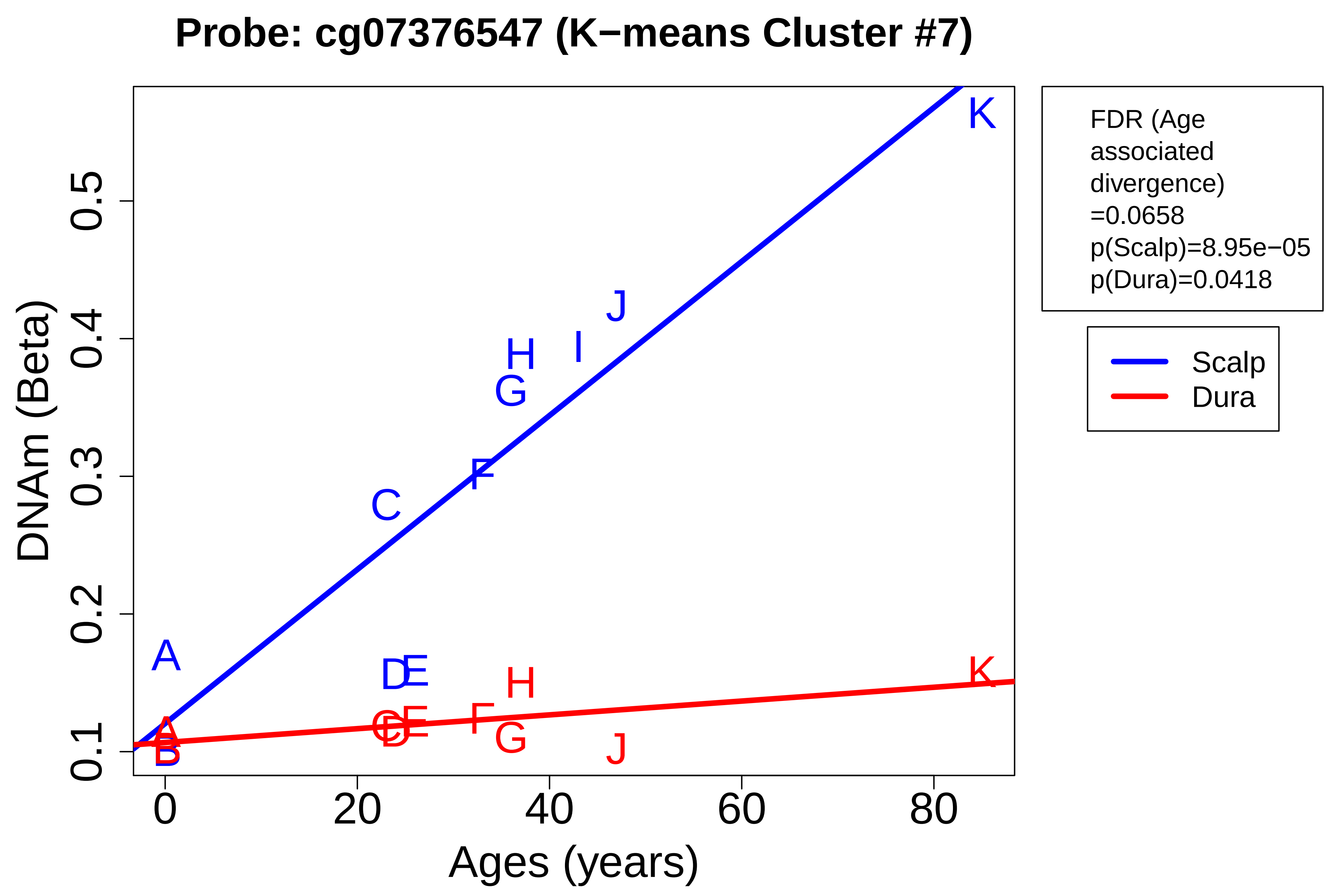
Probe: cg07376547 (K−means Cluster #7)
DNAm (Beta)
Ages (years)
0.1
0.2
0.3
0.4
0.5
0
20
40
60
80
FDR (Age associated divergence) =0.0658
p(Scalp)=8.95e−05
p(Dura)=0.0418
Scalp
Dura

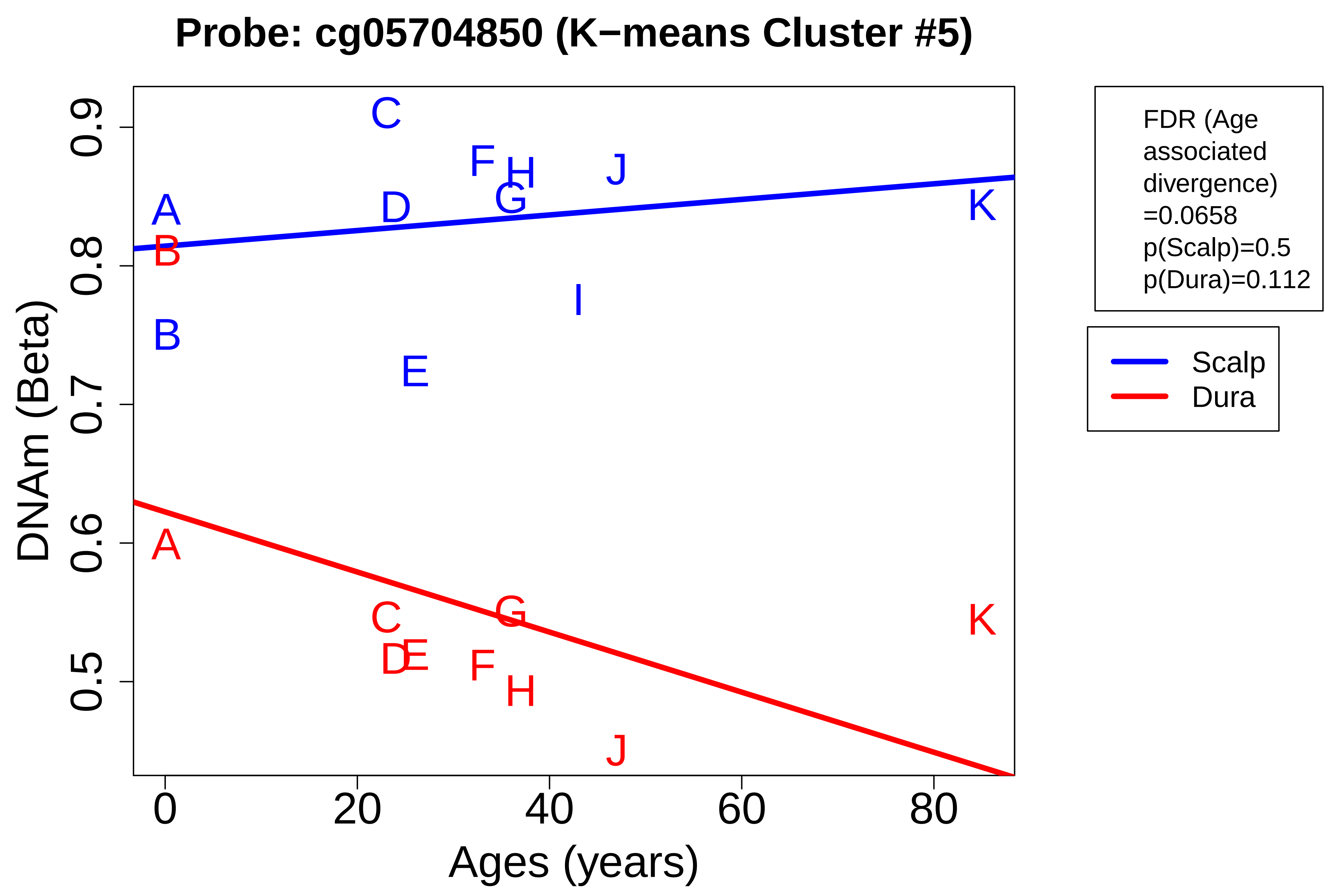

Probe: cg05704850 (K−means Cluster #5)
DNAm (Beta)
0.5
0.6
0.7
0.8
0.9
0
20
40
60
80
Ages (years)
A
B
C
D
E
F
G
H
I
J
K
FDR (Age associated divergence) =0.0658
p(Scalp)=0.5
p(Dura)=0.112
Scalp
Dura

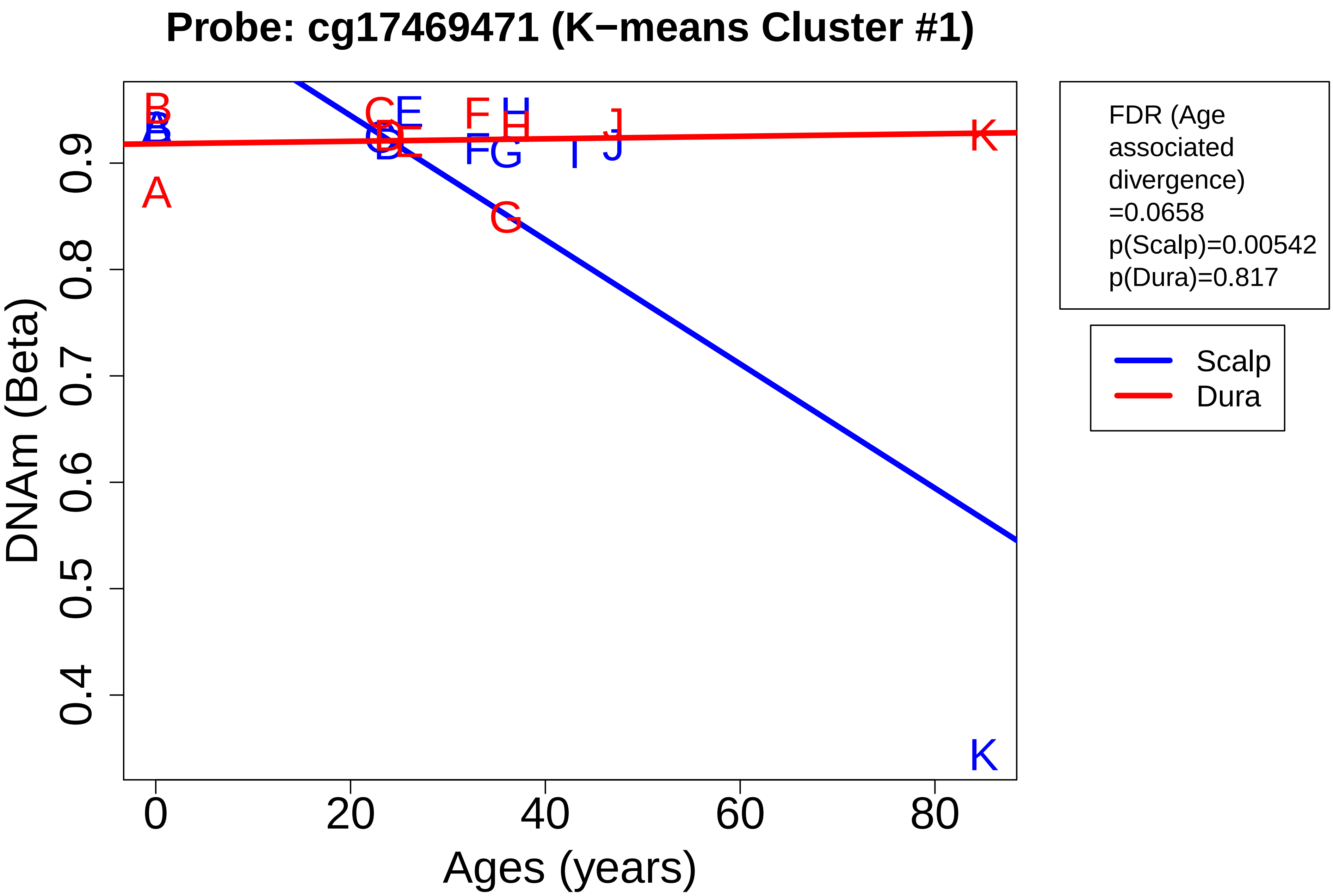

Probe: cg17469471 (K−means Cluster #1)
DNAm (Beta)
0.4
0.5
0.6
0.7
0.8
0.9
Ages (years)
0
20
40
60
80
FDR (Age associated divergence) =0.0658
p(Scalp)=0.00542
p(Dura)=0.817
Scalp
Dura

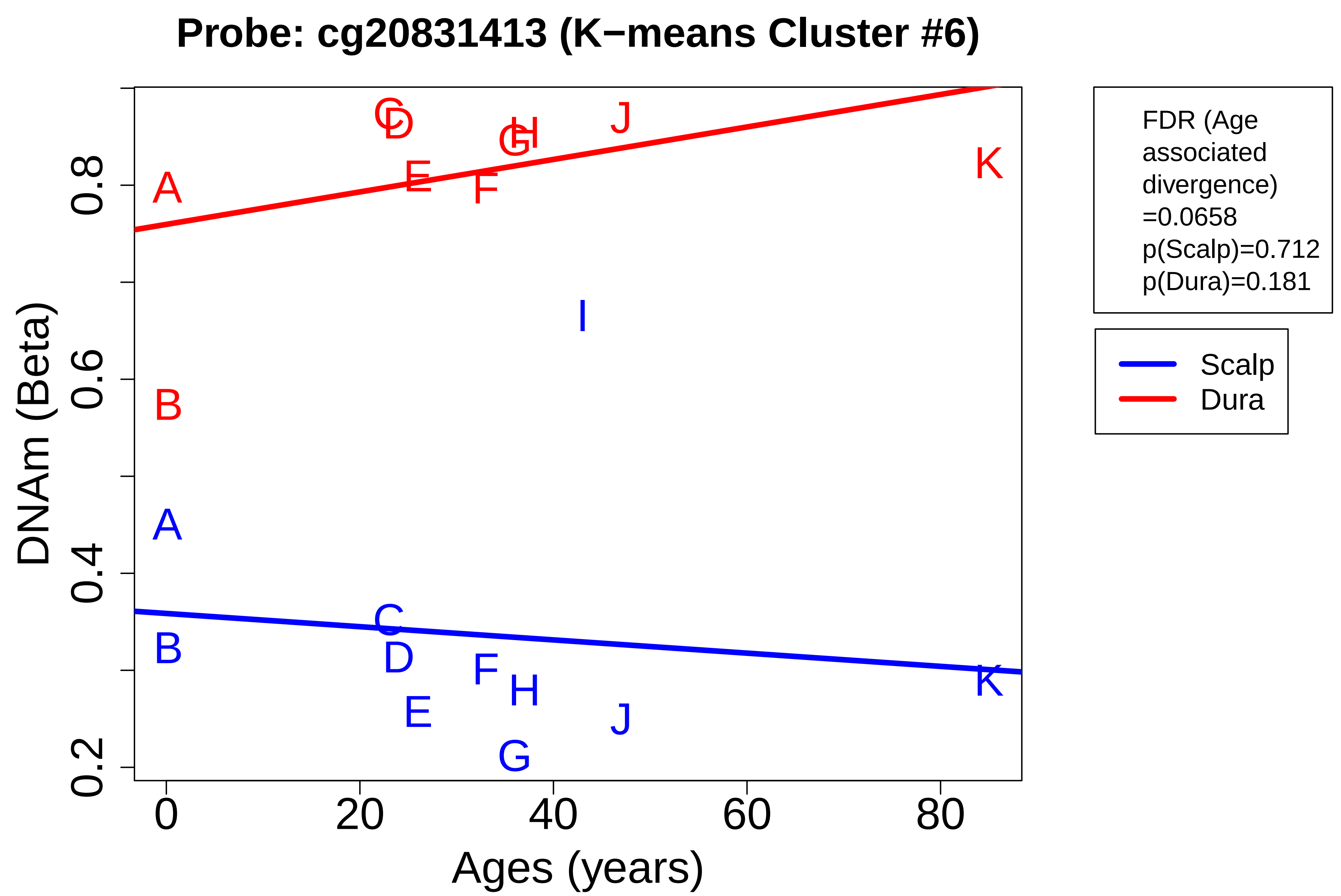

Probe: cg20831413 (K−means Cluster #6)
DNAm (Beta)
0.2
0.4
0.6
0.8
0
20
40
60
80
Ages (years)
A
B
C
D
E
F
G
H
I
J
K
FDR (Age associated divergence) =0.0658
p(Scalp)=0.712
p(Dura)=0.181
Scalp
Dura

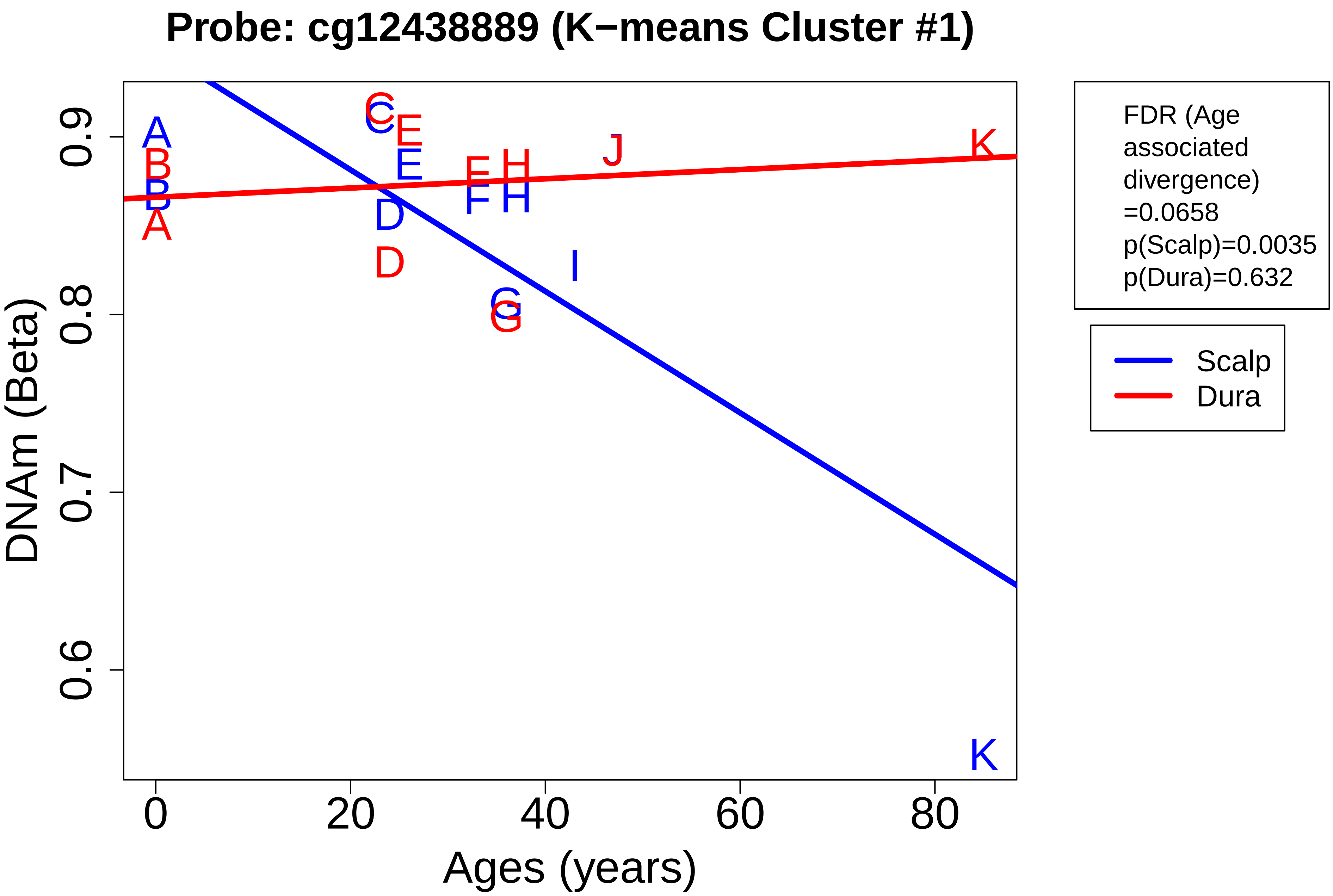
Probe: cg12438889 (K−means Cluster #1)
DNAm (Beta)
0.6
0.7
0.8
0.9
0
20
40
60
80
Ages (years)
A
B
C
D
E
F
G
H
I
J
K
FDR (Age associated divergence) =0.0658
p(Scalp)=0.0035
p(Dura)=0.632
Scalp
Dura

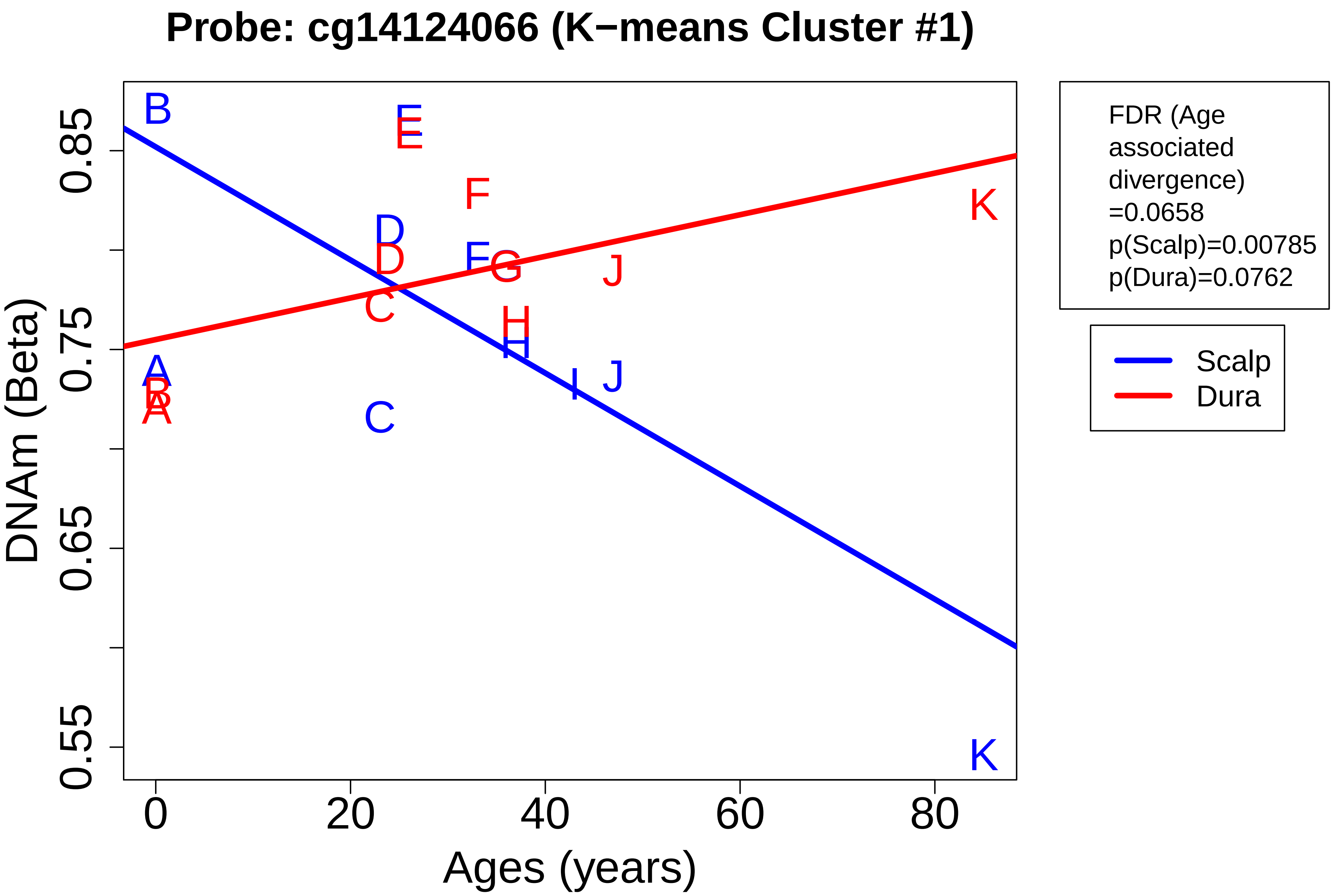
Probe: cg14124066 (K−means Cluster #1)
DNAm (Beta)
0.55
0.65
0.75
0.85
0
20
40
60
80
Ages (years)
A
B
C
D
E
F
G
H
I
J
K
FDR (Age associated divergence) =0.0658
p(Scalp)=0.00785
p(Dura)=0.0762
Scalp
Dura

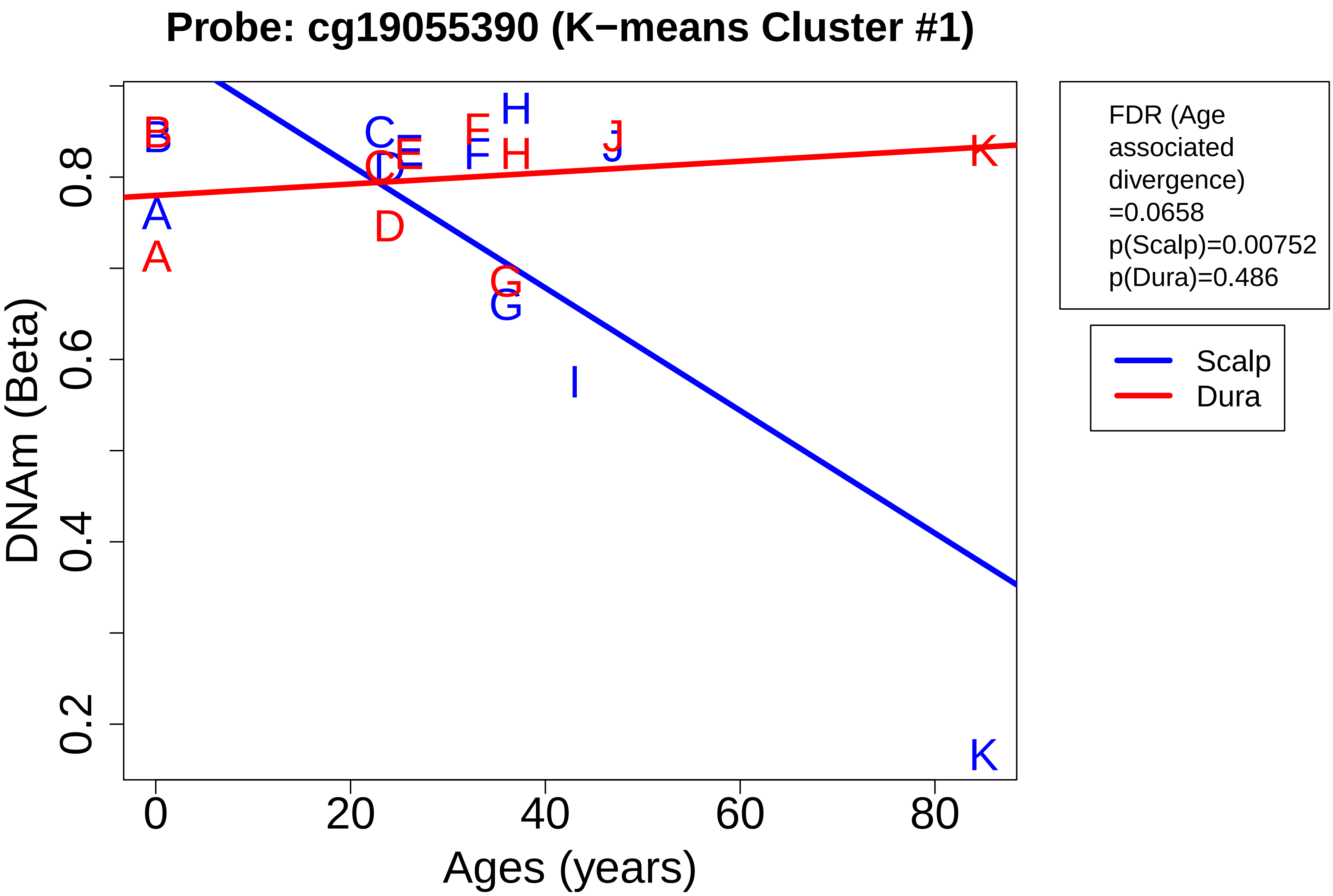
Probe: cg19055390 (K−means Cluster #1)
DNAm (Beta)
0.2
0.4
0.6
0.8
0
20
40
60
80
Ages (years)
FDR (Age associated divergence) =0.0658
p(Scalp)=0.00752
p(Dura)=0.486
Scalp
Dura

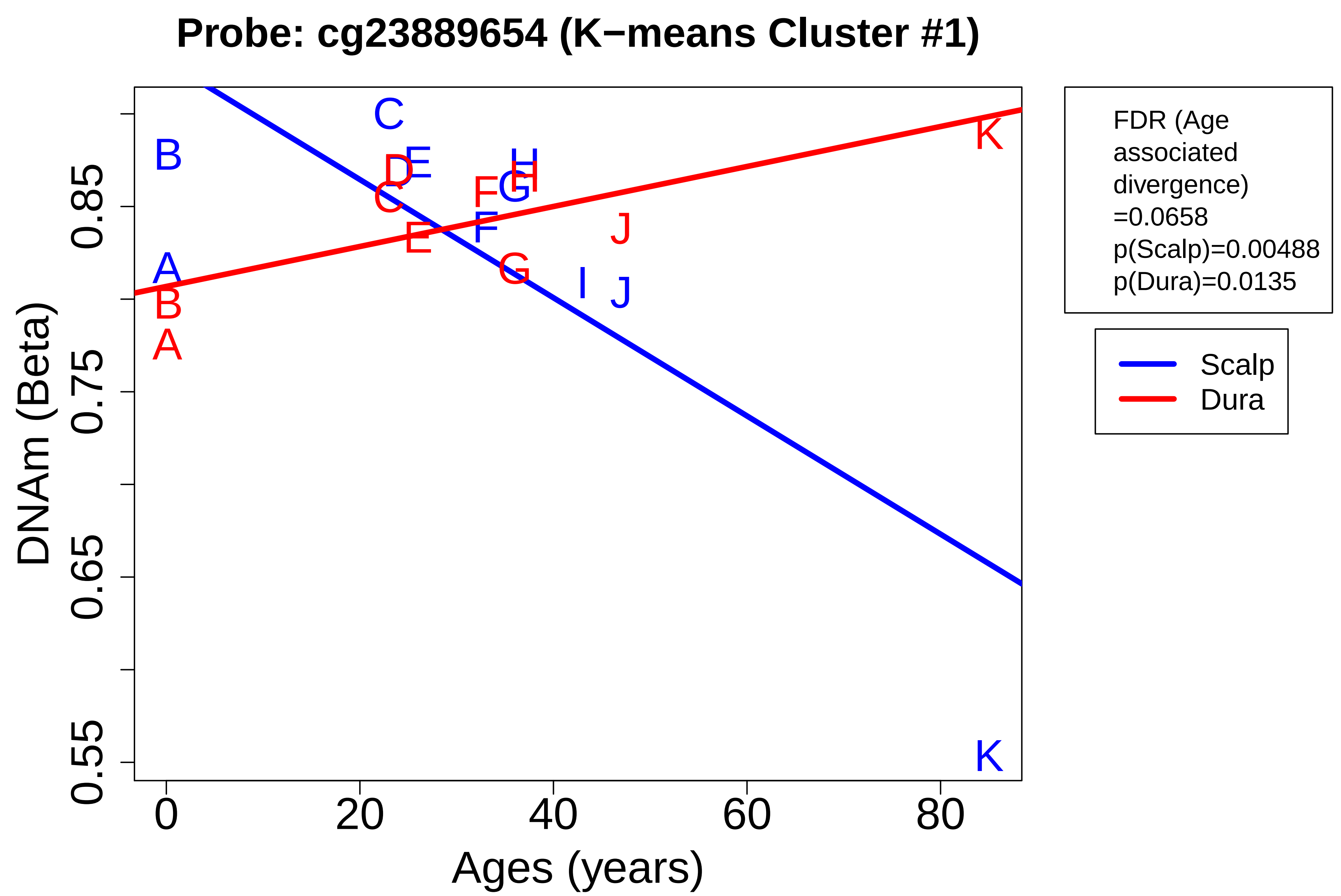

Probe: cg23889654 (K−means Cluster #1)
DNAm (Beta)
0.55
0.65
0.75
0.85
0
20
40
60
80
Ages (years)
A
B
C
D
E
F
G
H
I
J
K
FDR (Age associated divergence) =0.0658
p(Scalp)=0.00488
p(Dura)=0.0135
Scalp
Dura

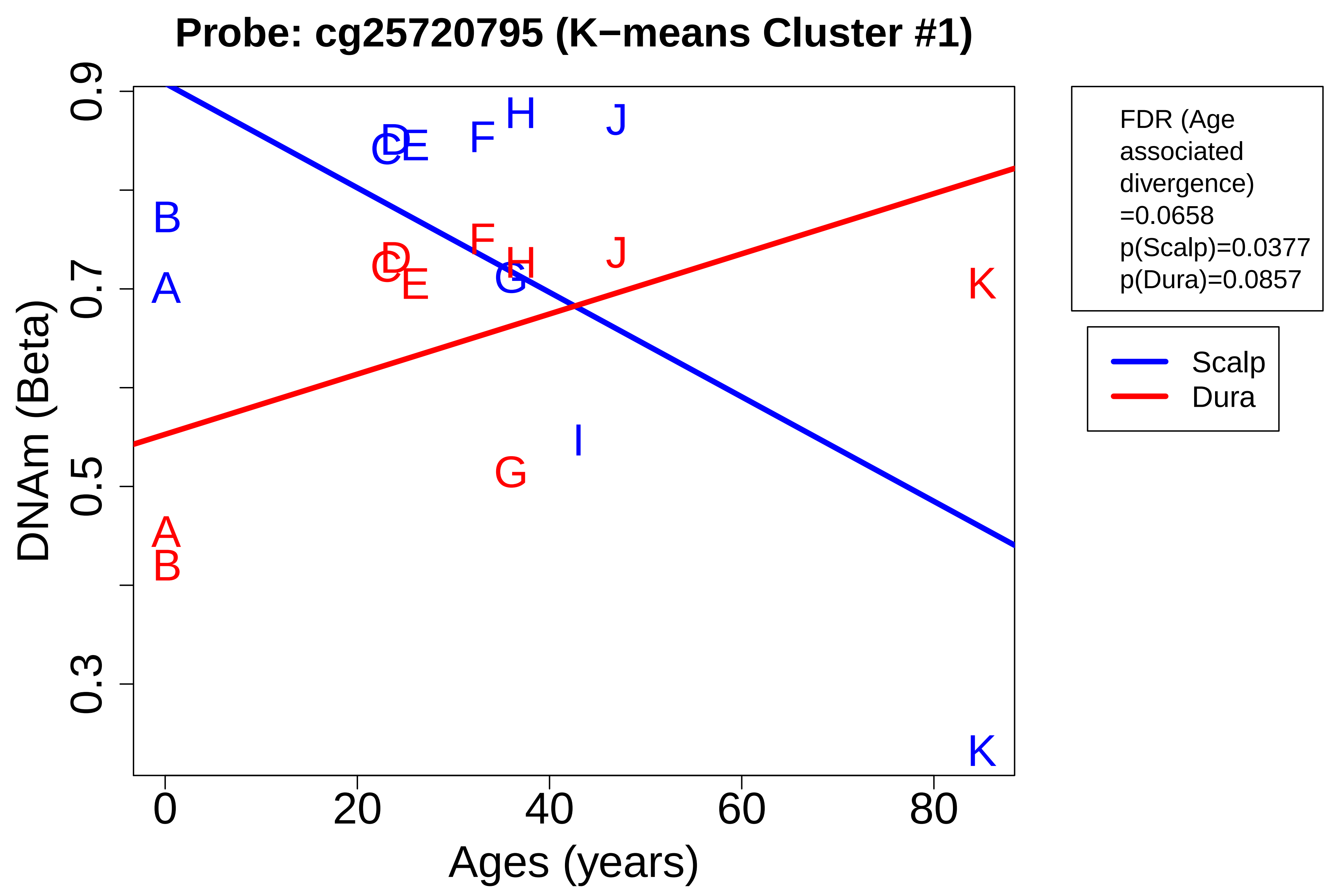

Probe: cg25720795 (K−means Cluster #1)
DNAm (Beta)
0.9
0.7
0.5
0.3
0
20
40
60
80
Ages (years)
A
B
C
D
E
F
G
H
I
J
K
FDR (Age associated divergence) =0.0658
p(Scalp)=0.0377
p(Dura)=0.0857
Scalp
Dura

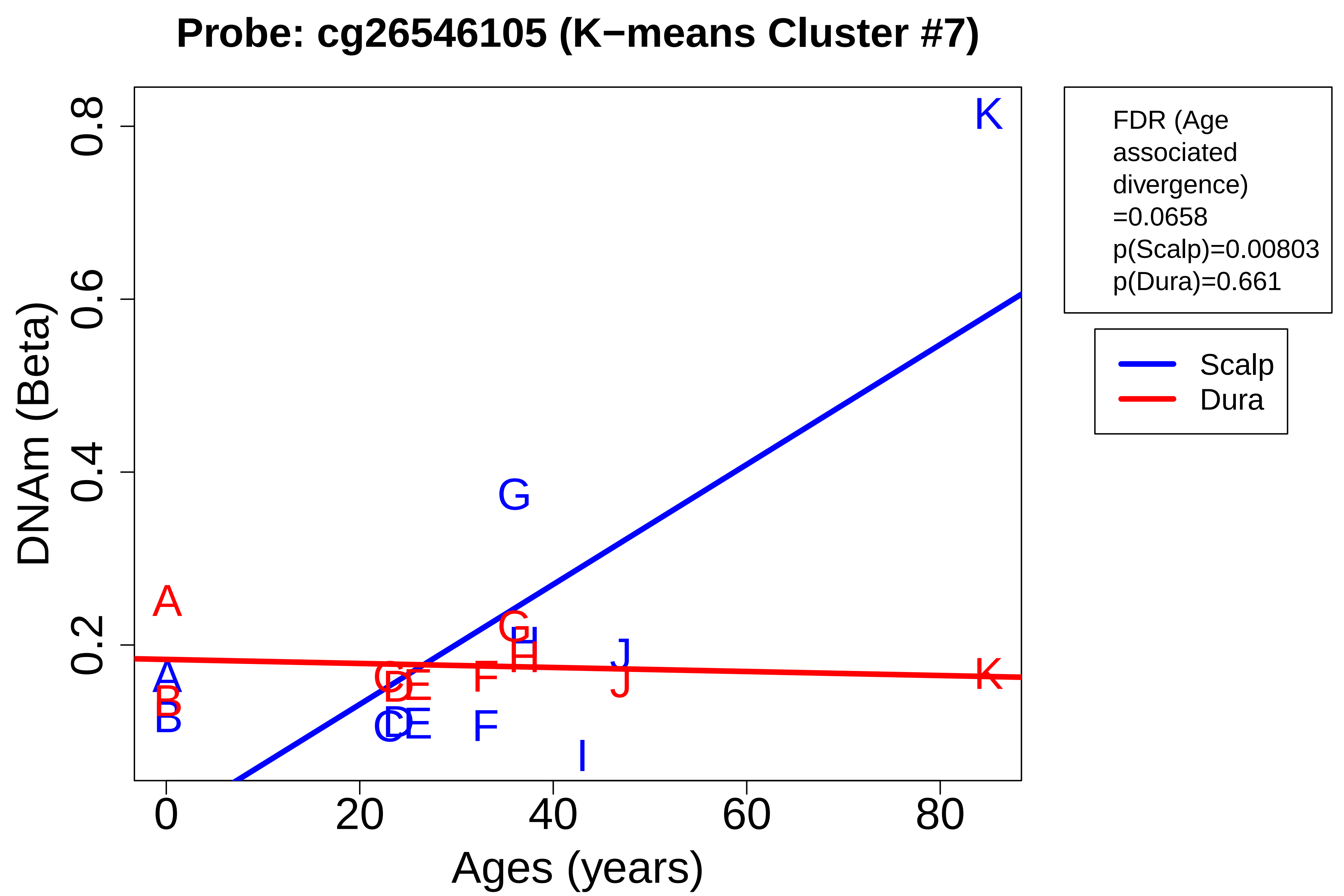

Probe: cg26546105 (K−means Cluster #7)
DNAm (Beta)
0.2
0.4
0.6
0.8
0
20
40
60
80
Ages (years)
FDR (Age associated divergence) =0.0658
p(Scalp)=0.00803
p(Dura)=0.661
Scalp
Dura

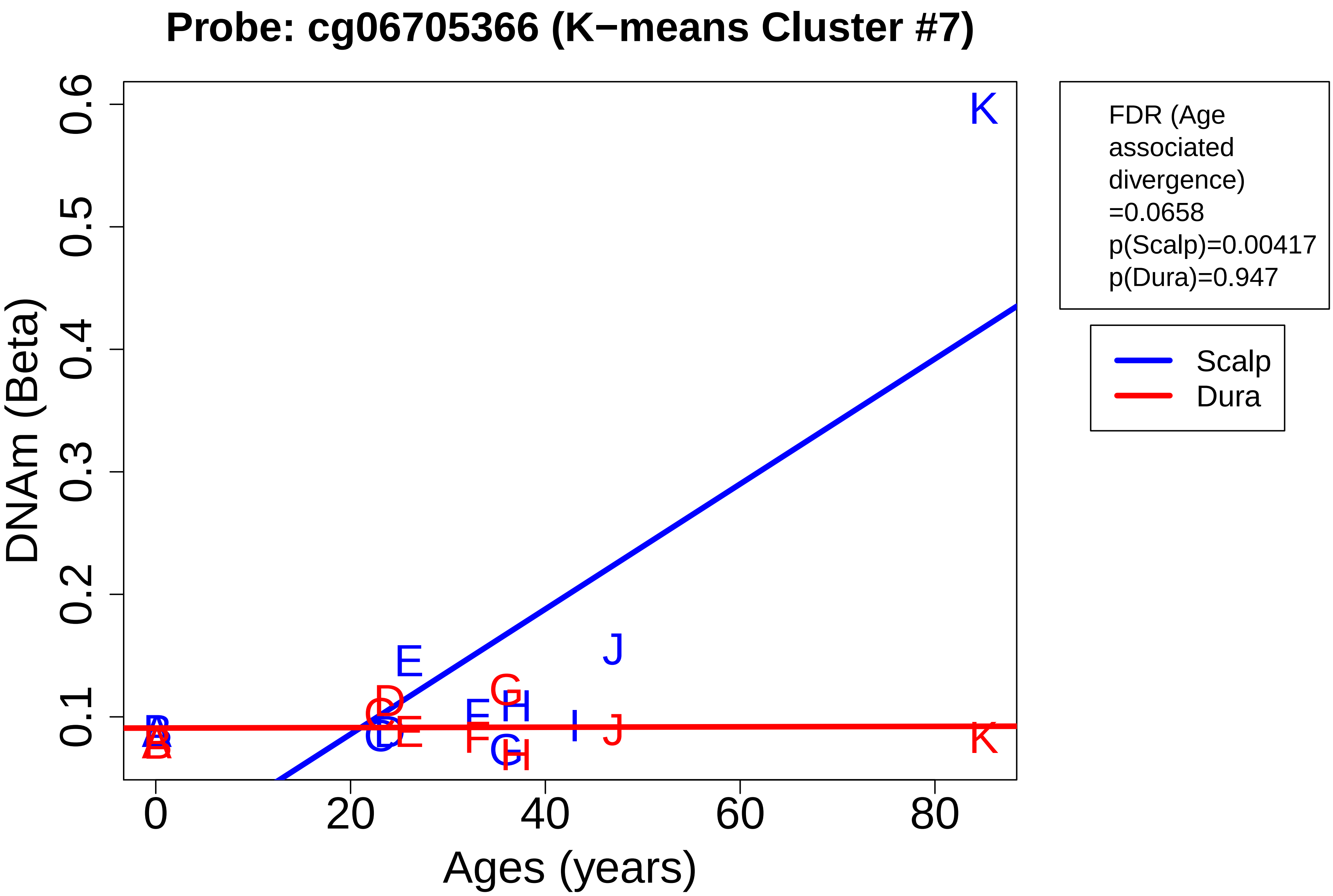

Probe: cg06705366 (K−means Cluster #7)
DNAm (Beta)
0.1
0.2
0.3
0.4
0.5
0.6
Ages (years)
0
20
40
60
80
FDR (Age associated divergence) =0.0658
p(Scalp)=0.00417
p(Dura)=0.947
Scalp
Dura

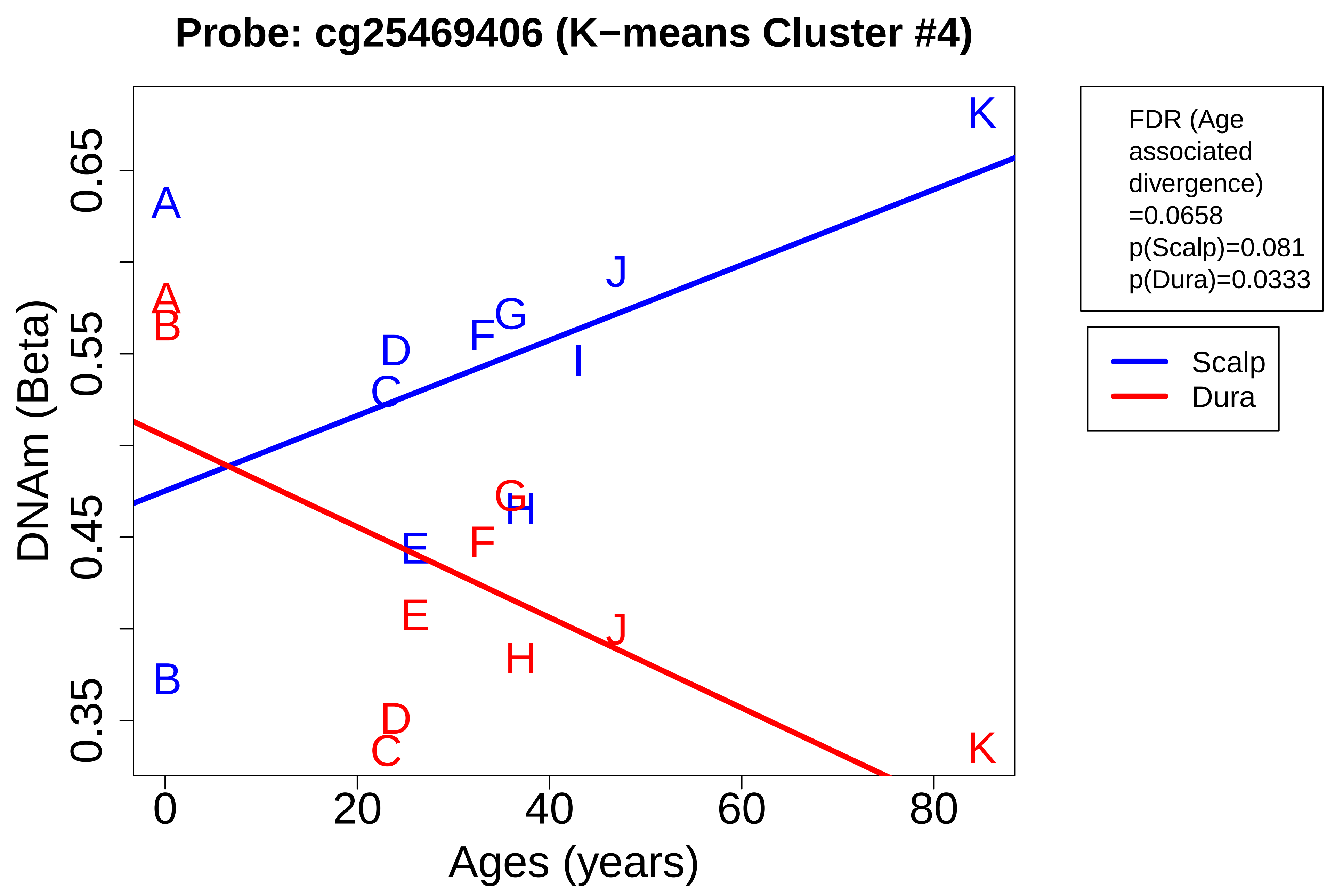

Probe: cg25469406 (K−means Cluster #4)
FDR (Age associated divergence) =0.0658
p(Scalp)=0.081
p(Dura)=0.0333
Scalp
Dura
DNAm (Beta)
0.35
0.45
0.55
0.65
Ages (years)
0
20
40
60
80
A B C D E F G H I J K

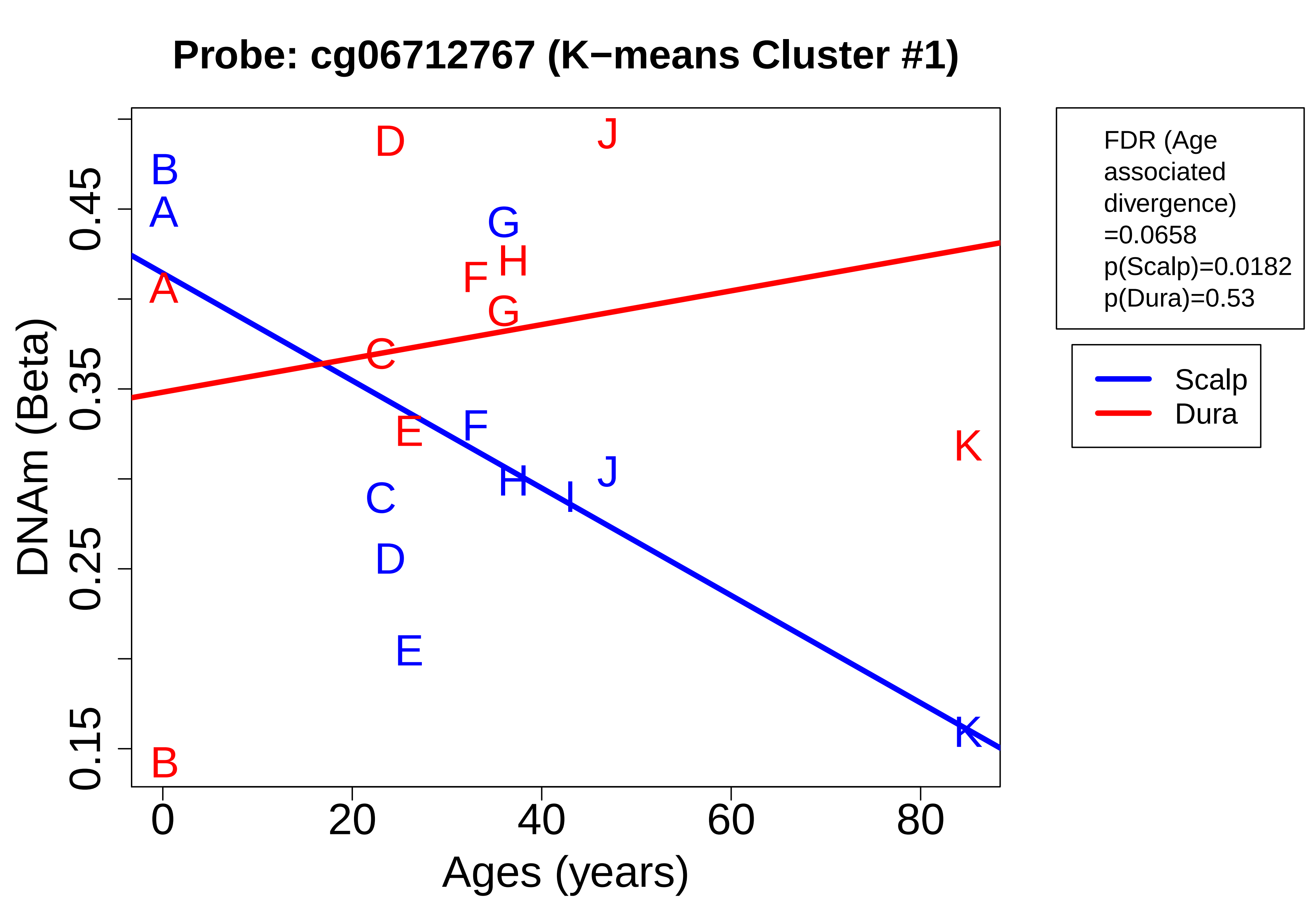

Probe: cg06712767 (K−means Cluster #1)
DNAm (Beta)
0.15
0.25
0.35
0.45
0
20
40
60
80
Ages (years)
A
B
C
D
E
F
G
H
I
J
K
FDR (Age associated divergence) =0.0658
p(Scalp)=0.0182
p(Dura)=0.53
Scalp
Dura

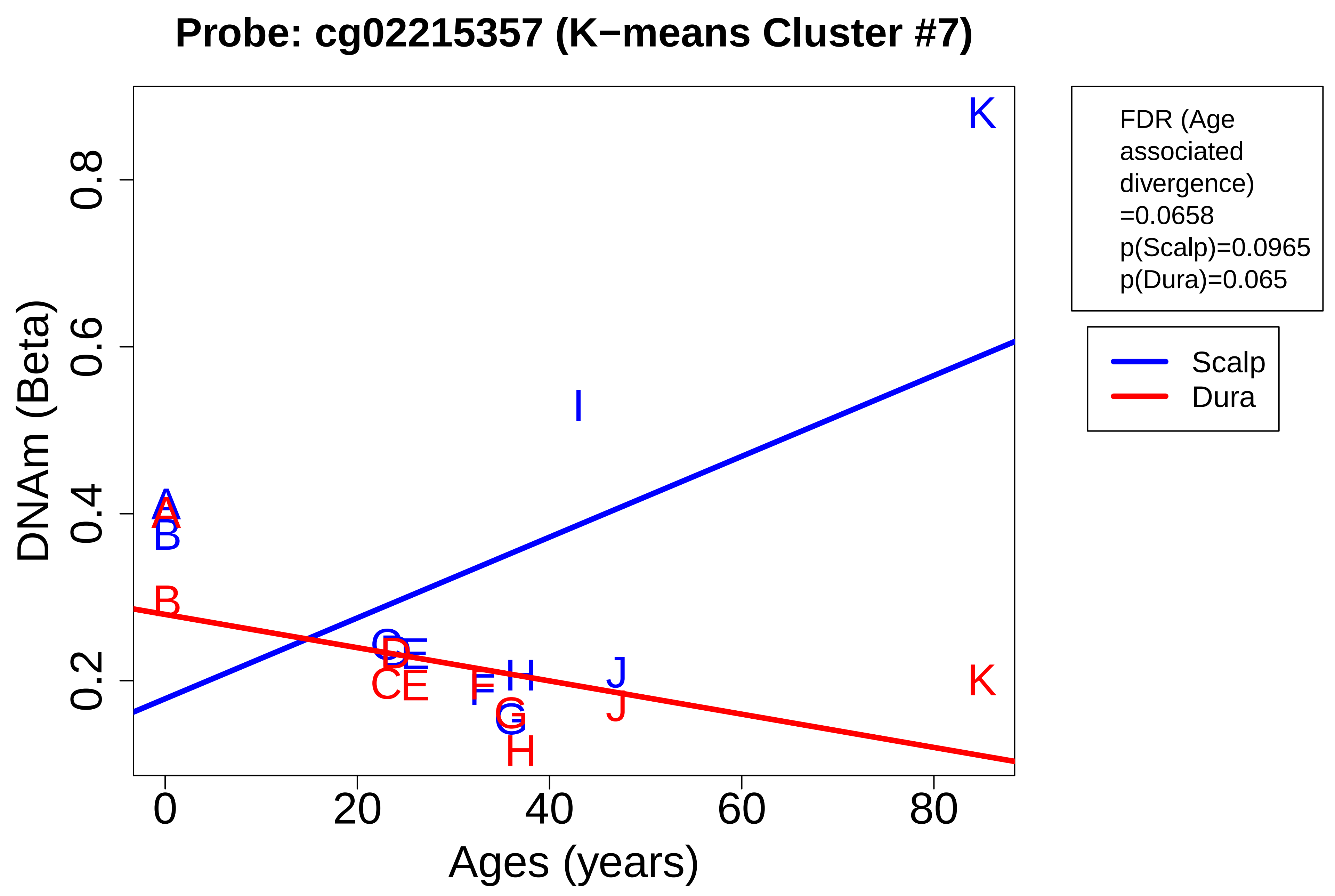

Probe: cg02215357 (K−means Cluster #7)
DNAm (Beta)
0.2
0.4
0.6
0.8
0
20
40
60
80
Ages (years)
FDR (Age associated divergence) =0.0658
p(Scalp)=0.0965
p(Dura)=0.065
Scalp
Dura
A
B
C
D
E
F
G
H
I
J
K

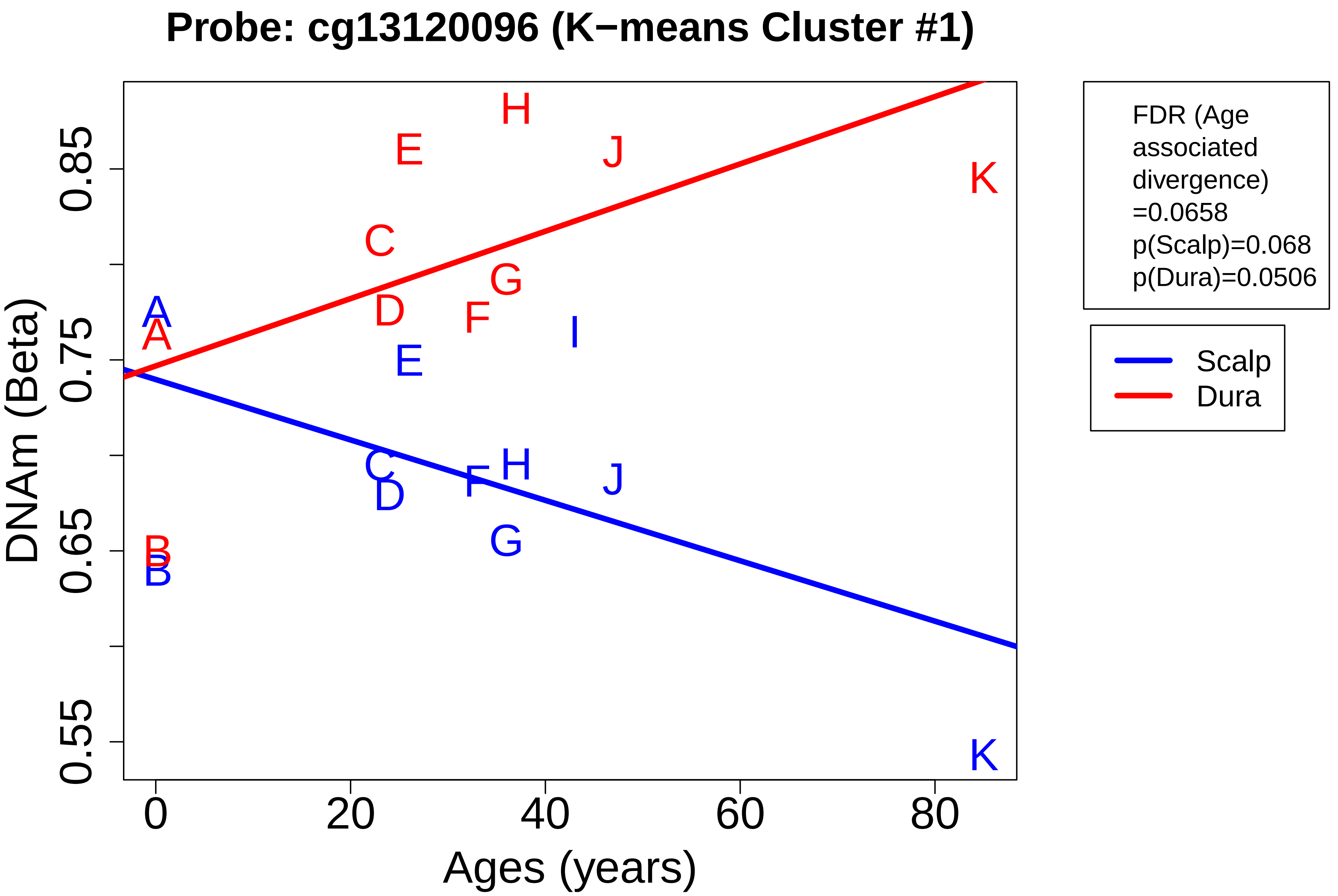
Probe: cg13120096 (K−means Cluster #1)
FDR (Age associated divergence) =0.0658
p(Scalp)=0.068
p(Dura)=0.0506
Scalp
Dura
DNAm (Beta)
0.55
0.65
0.75
0.85
0
20
40
60
80
Ages (years)
A
B
C
D
E
F
G
H
I
J
K

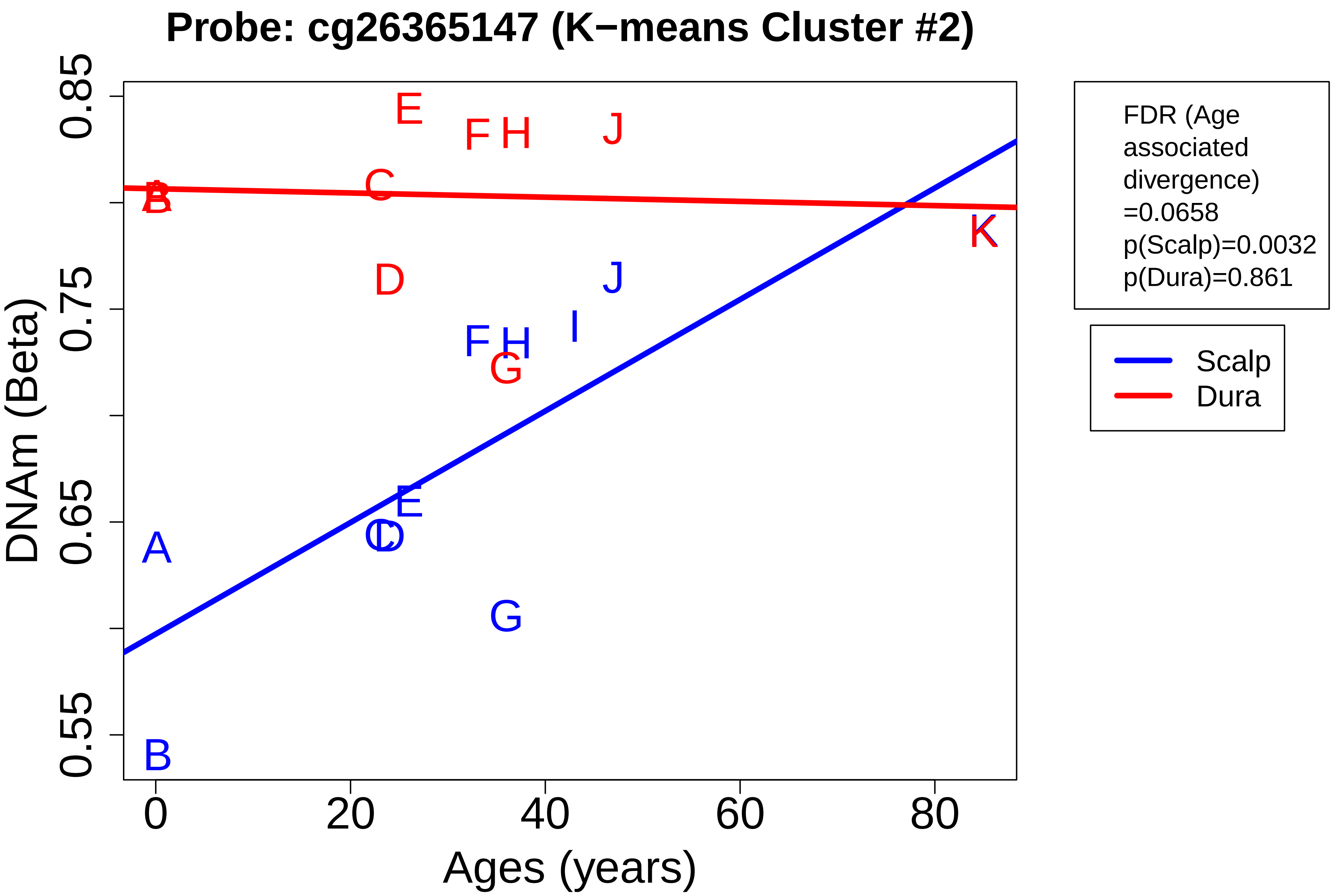

Probe: cg26365147 (K−means Cluster #2)
DNAm (Beta)
0.55
0.65
0.75
0.85
0
20
40
60
80
Ages (years)
FDR (Age associated divergence) =0.0658
p(Scalp)=0.0032
p(Dura)=0.861
Scalp
Dura

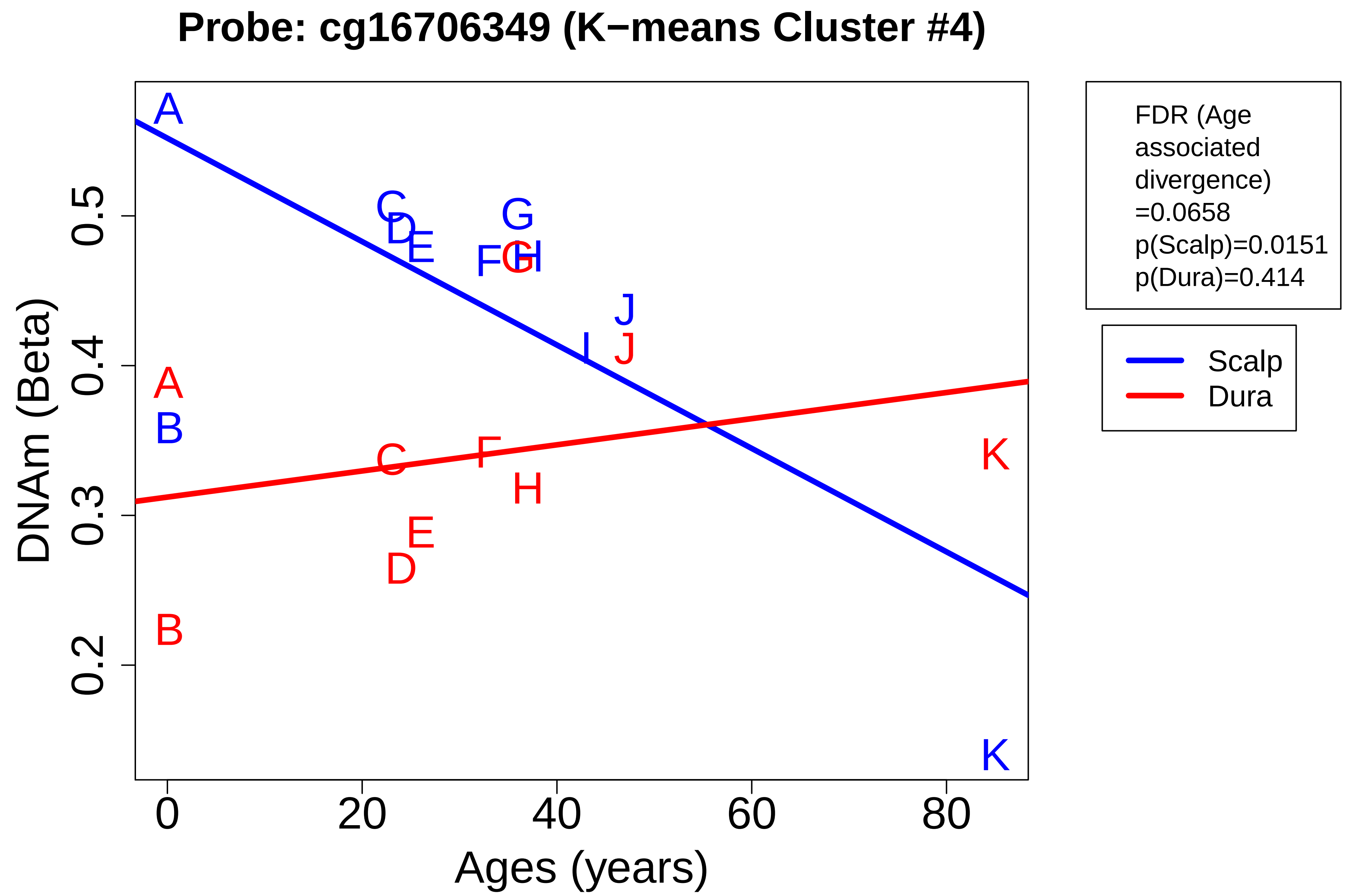
Probe: cg16706349 (K−means Cluster #4)
FDR (Age associated divergence) =0.0658
p(Scalp)=0.0151
p(Dura)=0.414
Scalp
Dura
DNAm (Beta)
0.2
0.3
0.4
0.5
0
20
40
60
80
Ages (years)
A
B
C
D
E
F
G
H
I
J
K

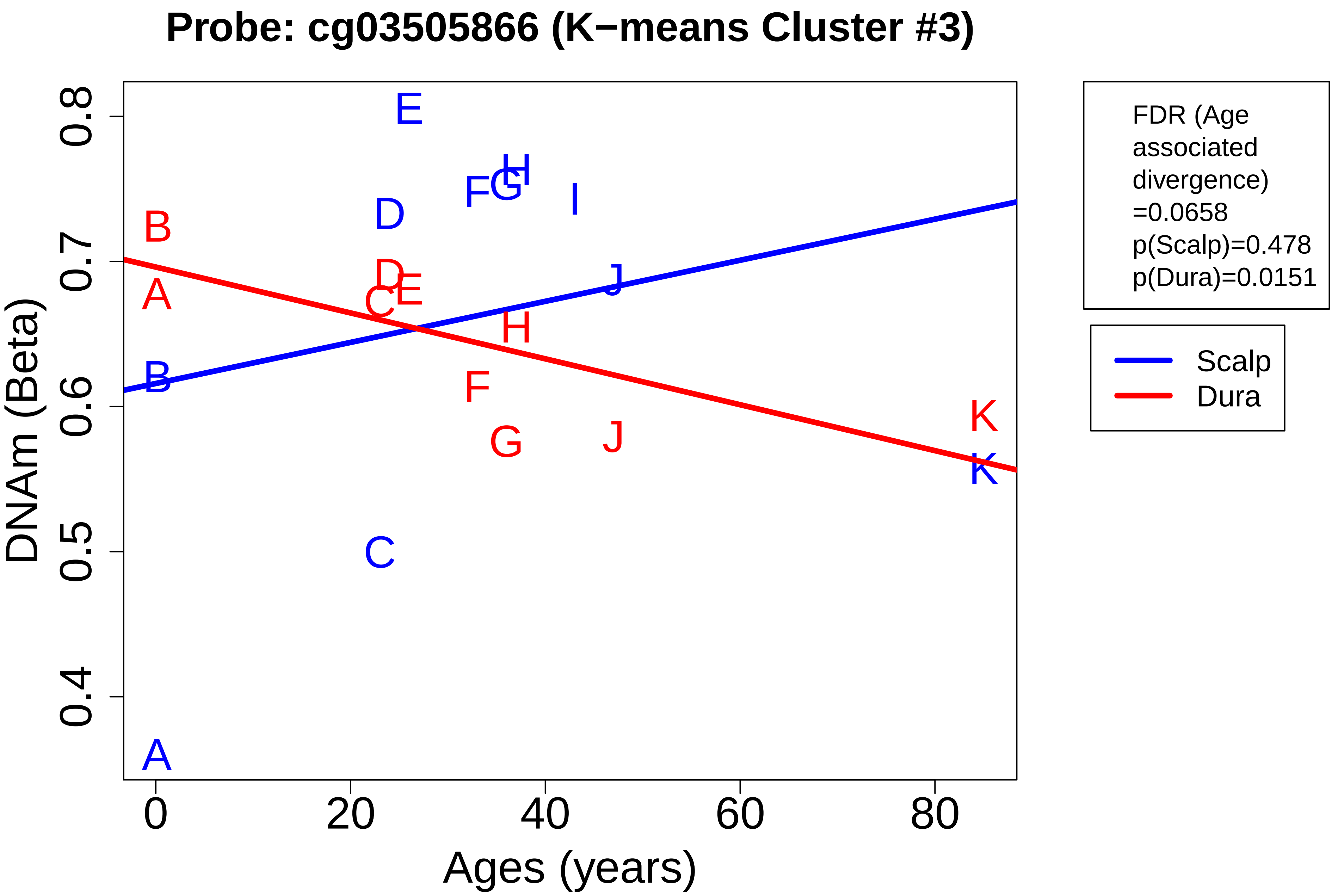

Probe: cg03505866 (K−means Cluster #3)
FDR (Age associated divergence) =0.0658
p(Scalp)=0.478
p(Dura)=0.0151
Scalp
Dura
DNAm (Beta)
0.4
0.5
0.6
0.7
0.8
Ages (years)
0
20
40
60
80
A
B
C
D
E
F
G
H
I
J
K

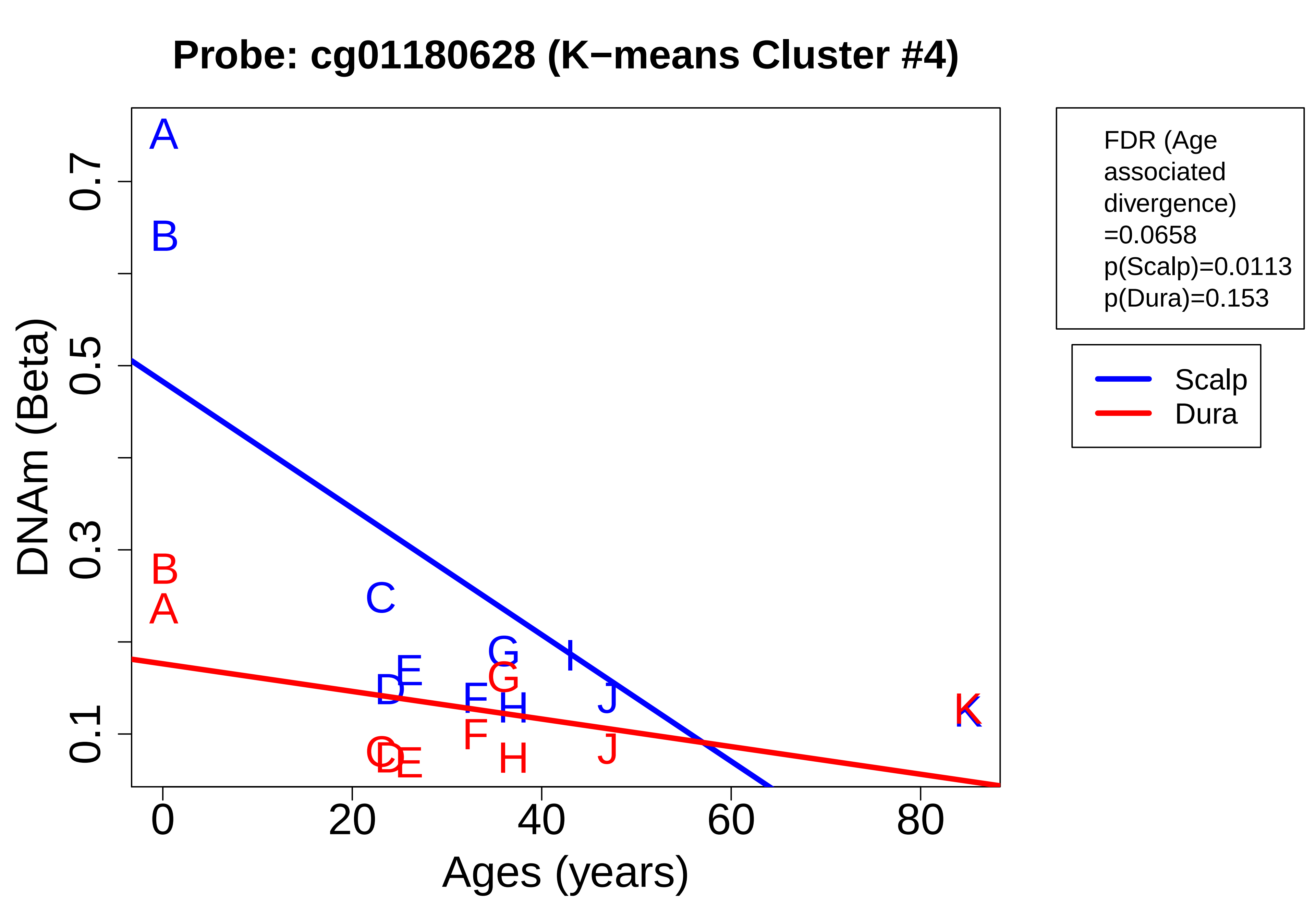
Probe: cg01180628 (K−means Cluster #4)
DNAm (Beta)
0.1
0.3
0.5
0.7
Ages (years)
0
20
40
60
80
A
B
C
D
E
F
G
H
I
J
K
FDR (Age associated divergence) =0.0658
p(Scalp)=0.0113
p(Dura)=0.153
Scalp
Dura

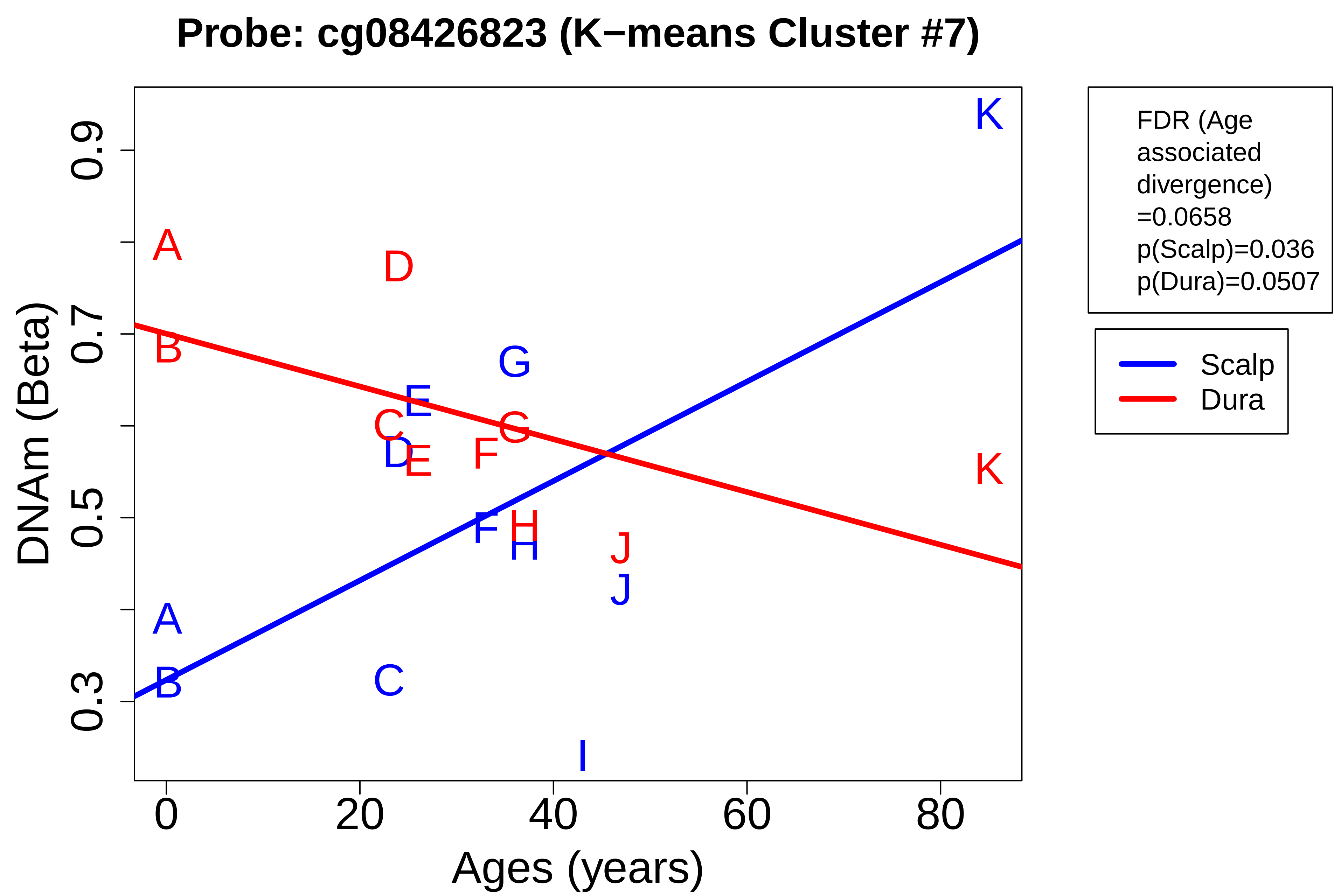

Probe: cg08426823 (K−means Cluster #7)
DNAm (Beta)
0.3
0.5
0.7
0.9
Ages (years)
0
20
40
60
80
A
B
C
D
E
F
G
H
I
J
K
A
B
C
D
E
F
G
H
J
K
FDR (Age associated divergence) =0.0658
p(Scalp)=0.036
p(Dura)=0.0507
Scalp
Dura

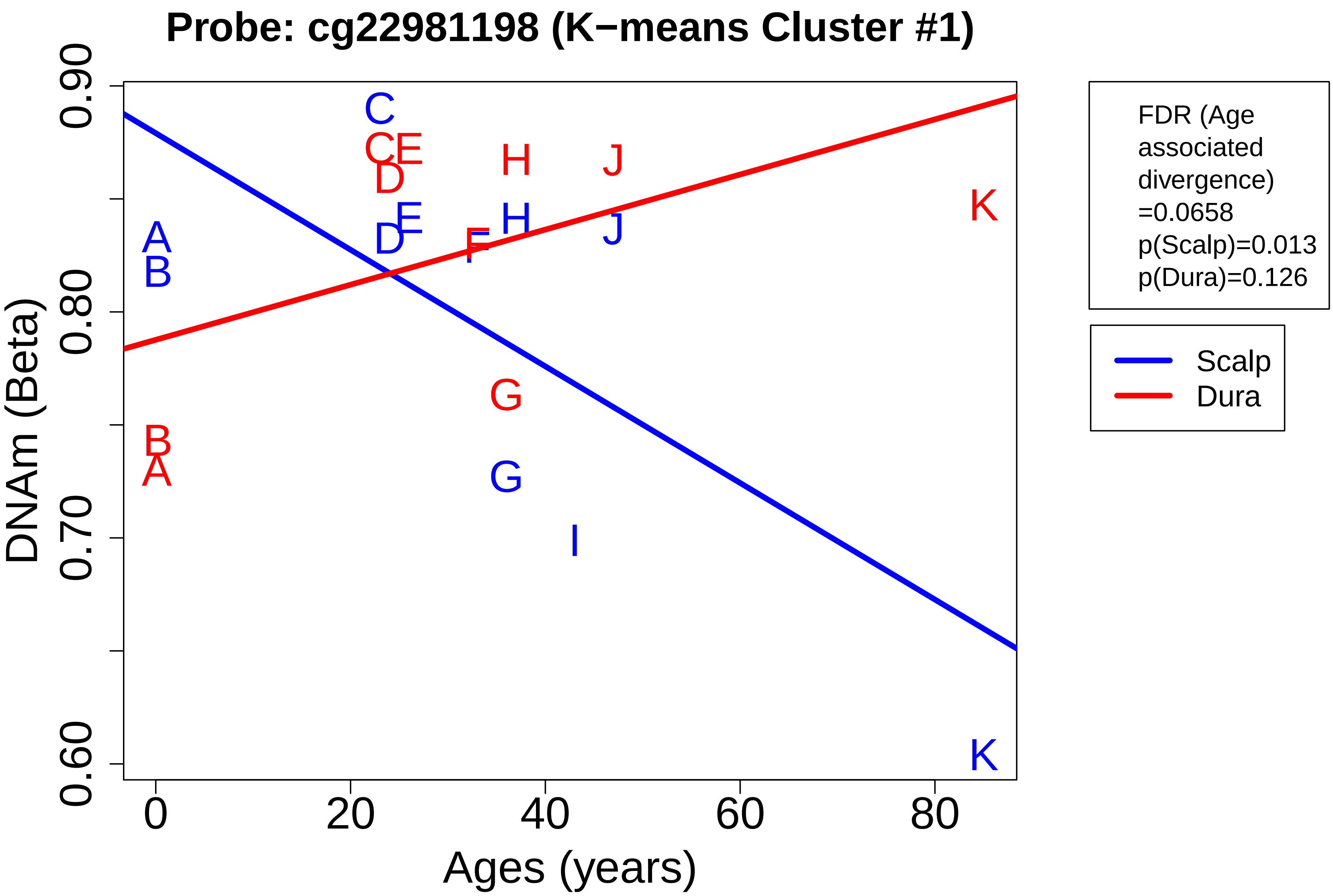
Probe: cg22981198 (K−means Cluster #1)
DNAm (Beta)
0.60
0.70
0.80
0.90
Ages (years)
0
20
40
60
80
FDR (Age associated divergence) =0.0658
p(Scalp)=0.013
p(Dura)=0.126
Scalp
Dura
A
B
C
D
E
F
G
H
I
J
K

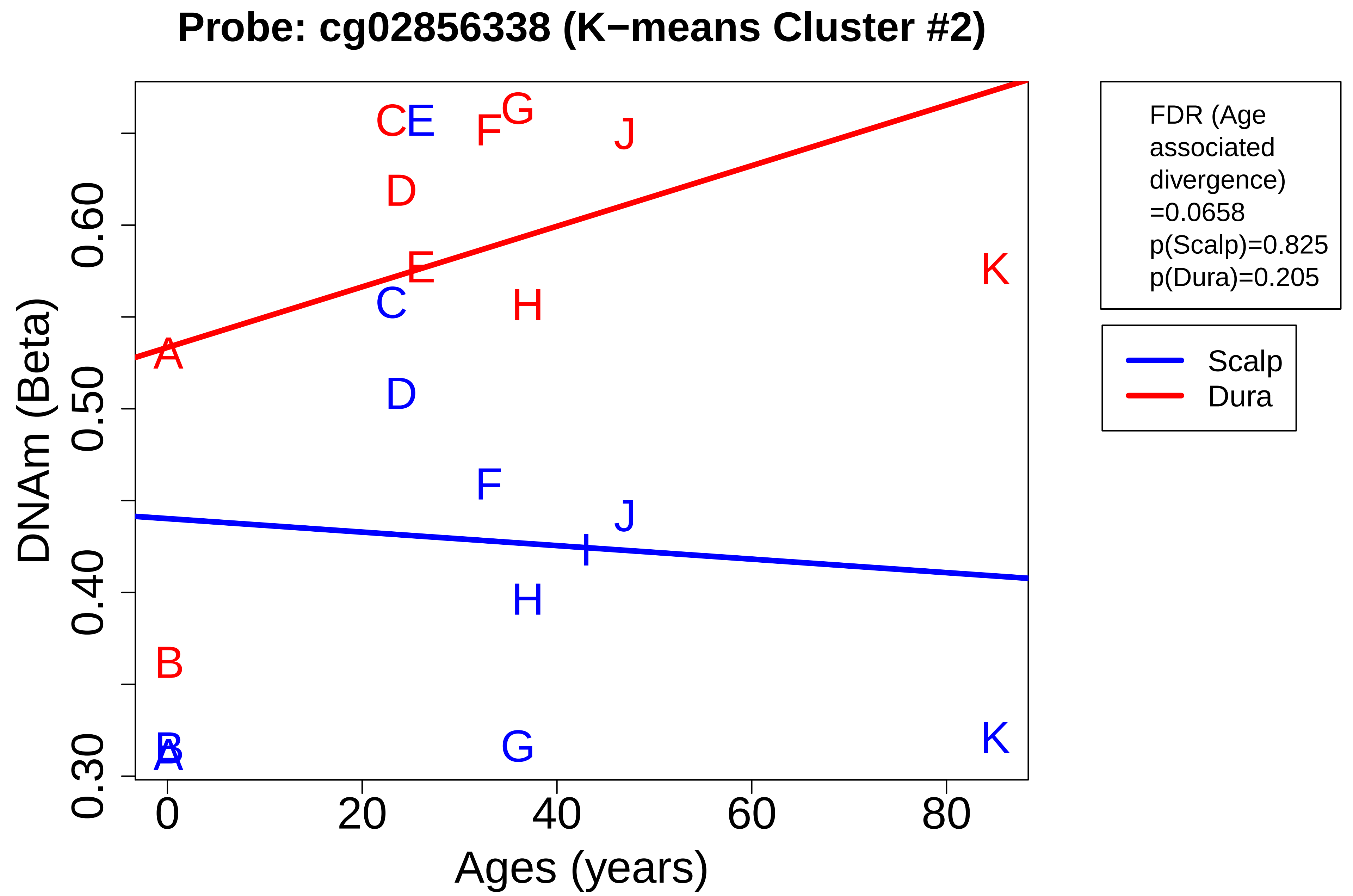
Probe: cg02856338 (K−means Cluster #2)
DNAm (Beta)
0.30
0.40
0.50
0.60
0
20
40
60
80
Ages (years)
FDR (Age associated divergence) =0.0658
p(Scalp)=0.825
p(Dura)=0.205
Scalp
Dura
A
B
C
D
E
F
G
H
J
K

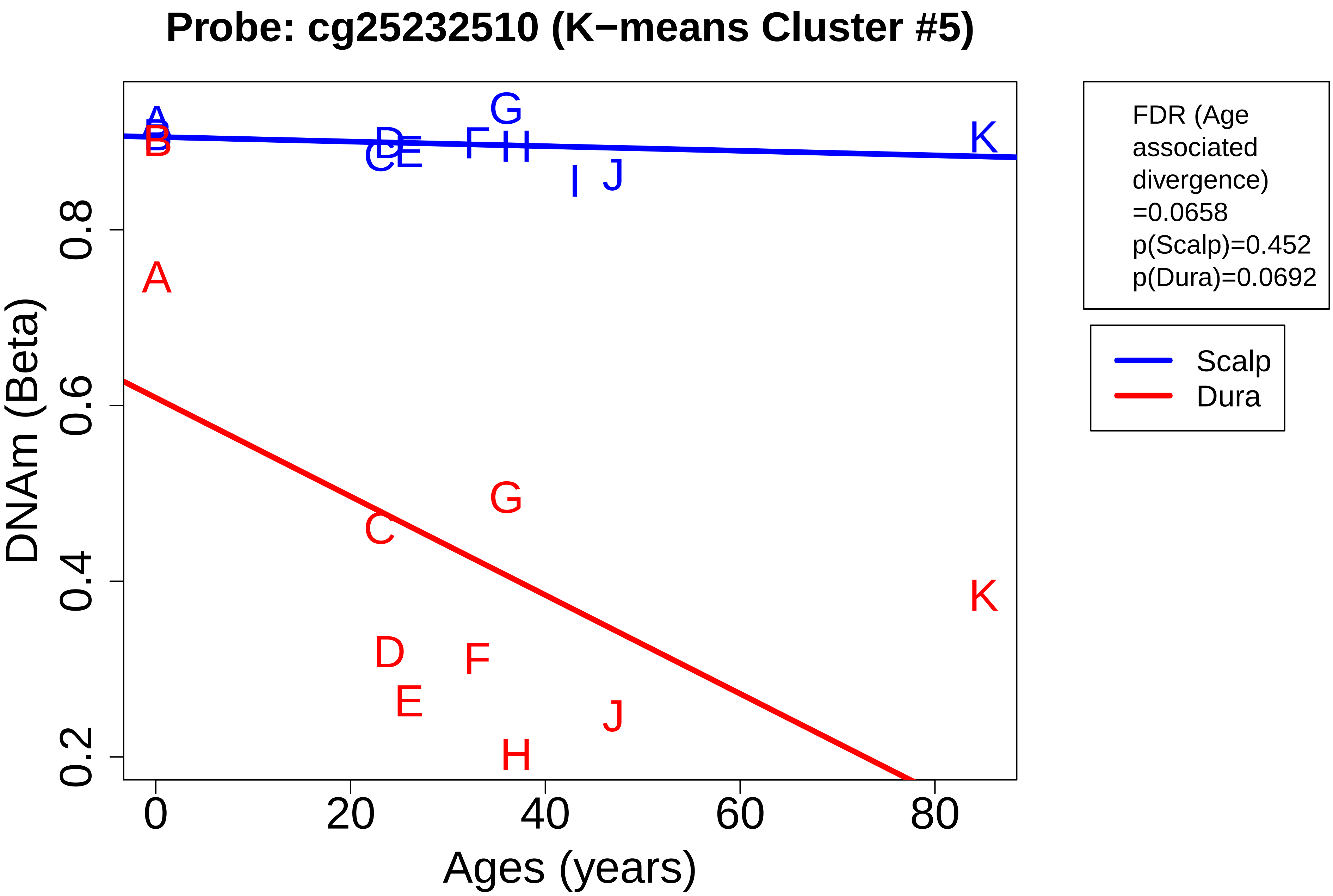
Probe: cg25232510 (K−means Cluster #5)
FDR (Age associated divergence) =0.0658
p(Scalp)=0.452
p(Dura)=0.0692
Scalp
Dura
DNAm (Beta)
0.2
0.4
0.6
0.8
Ages (years)
0
20
40
60
80
A
B
C
D
E
F
G
H
I
J
K

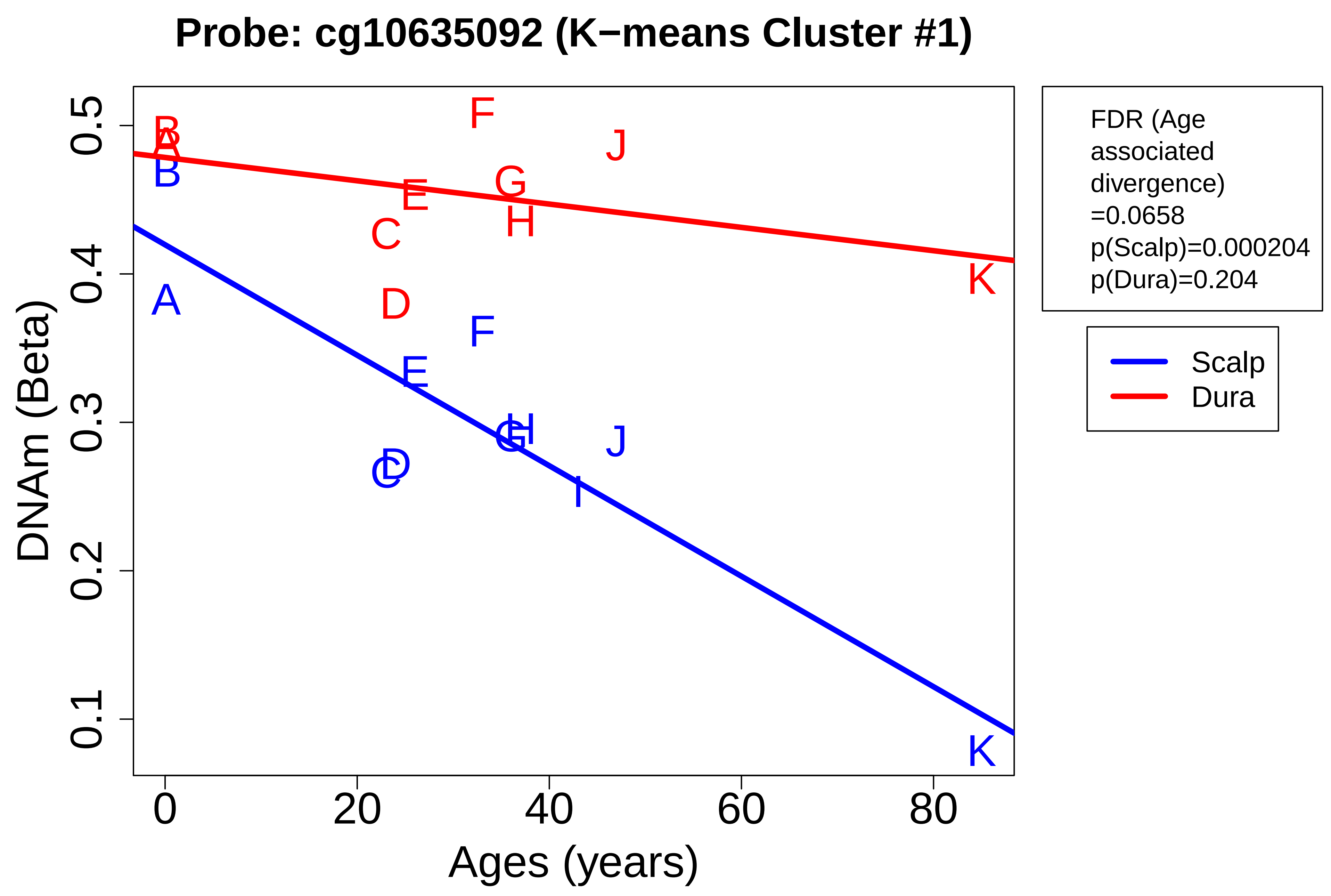

Probe: cg10635092 (K−means Cluster #1)
DNAm (Beta)
0.1
0.2
0.3
0.4
0.5
Ages (years)
0
20
40
60
80
FDR (Age associated divergence) =0.0658
p(Scalp)=0.000204
p(Dura)=0.204
Scalp
Dura
A
B
C
D
E
F
G
H
I
J
K

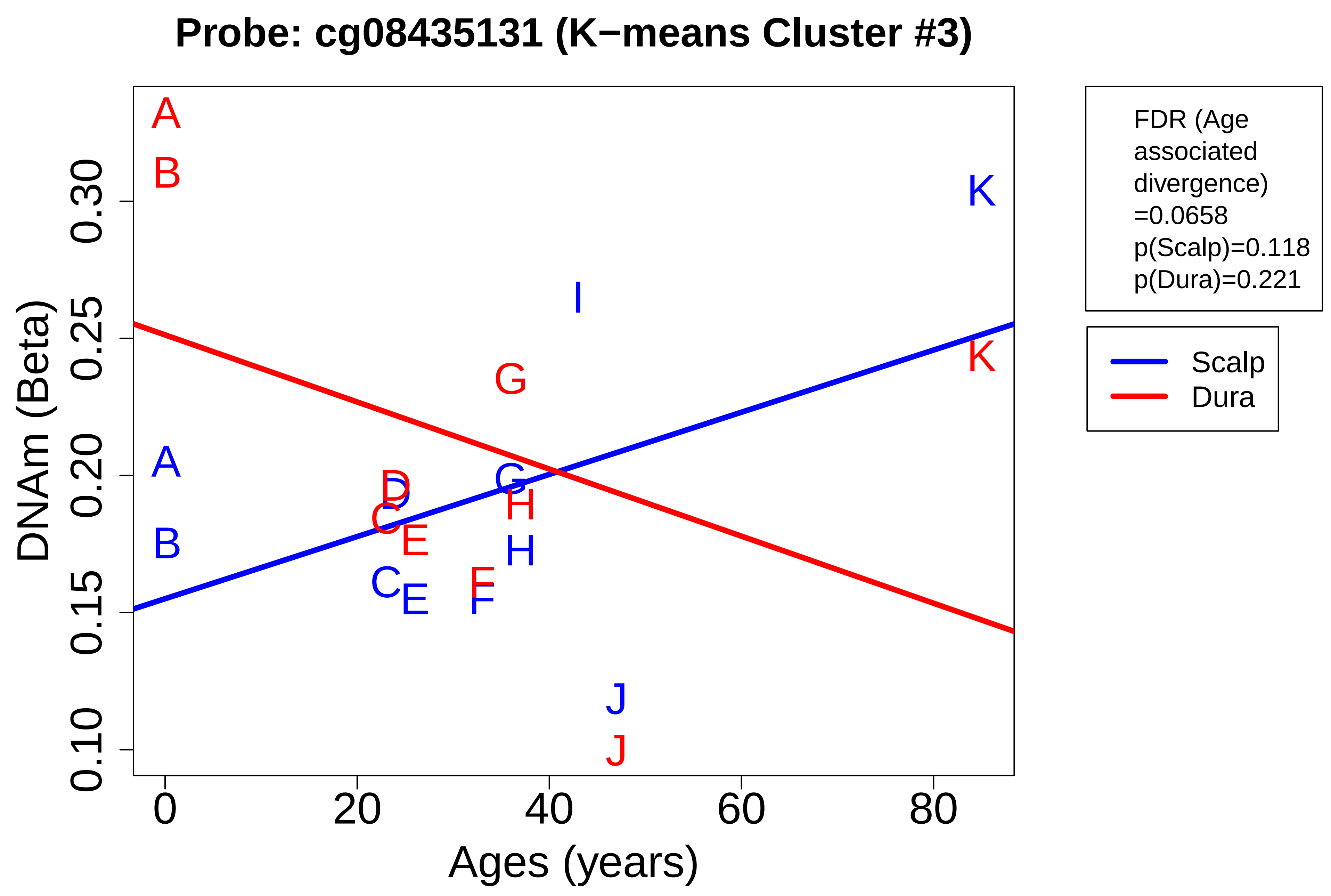

Probe: cg08435131 (K−means Cluster #3)
DNAm (Beta)
0.10
0.15
0.20
0.25
0.30
0
20
40
60
80
Ages (years)
FDR (Age associated divergence) =0.0658
p(Scalp)=0.118
p(Dura)=0.221
Scalp
Dura
A
B
C
D
E
F
G
H
I
J
K

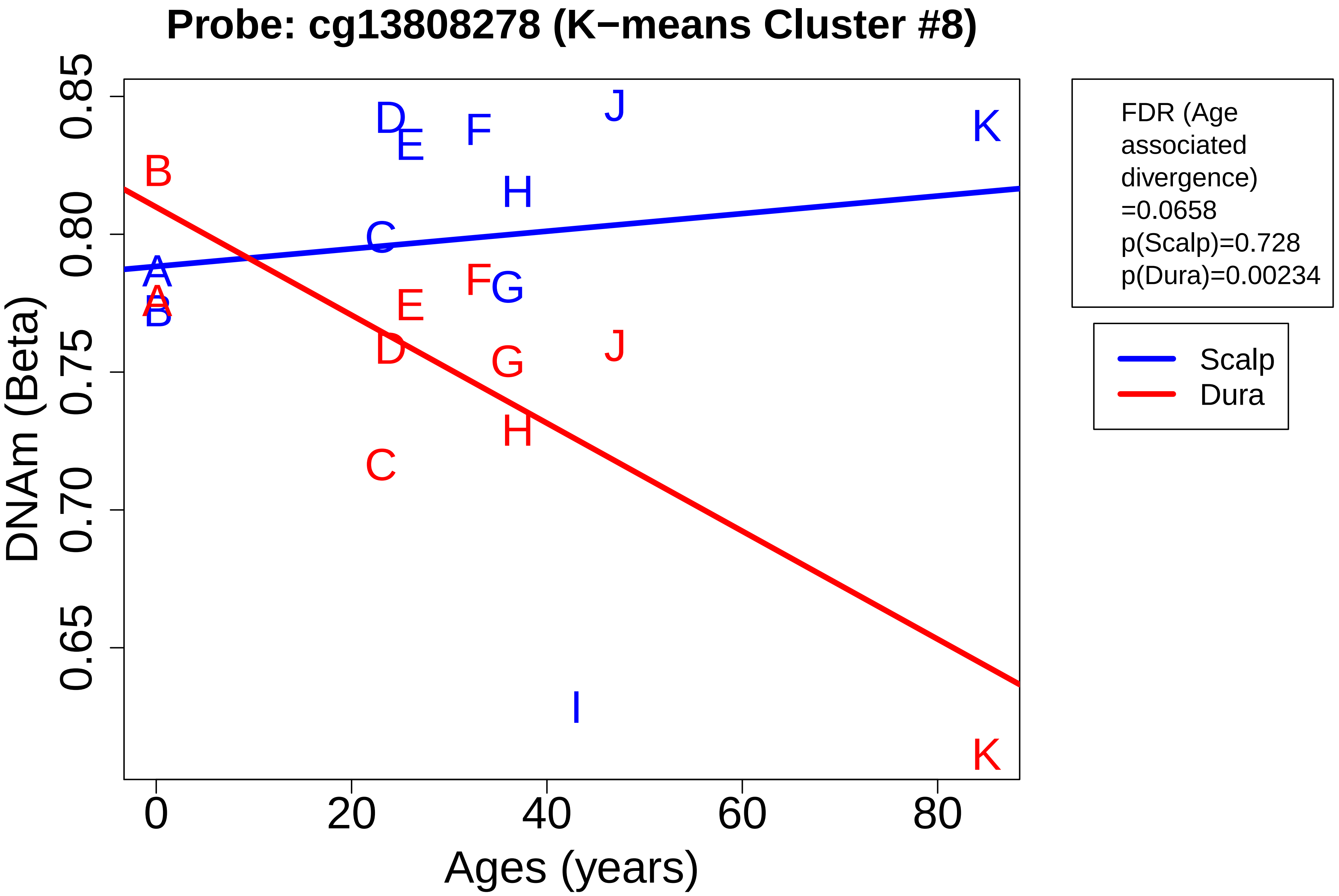

Probe: cg13808278 (K−means Cluster #8)
DNAm (Beta)
0.65
0.70
0.75
0.80
0.85
Ages (years)
0
20
40
60
80
FDR (Age associated divergence) =0.0658
p(Scalp)=0.728
p(Dura)=0.00234
Scalp
Dura
A
B
C
D
E
F
G
H
I
J
K

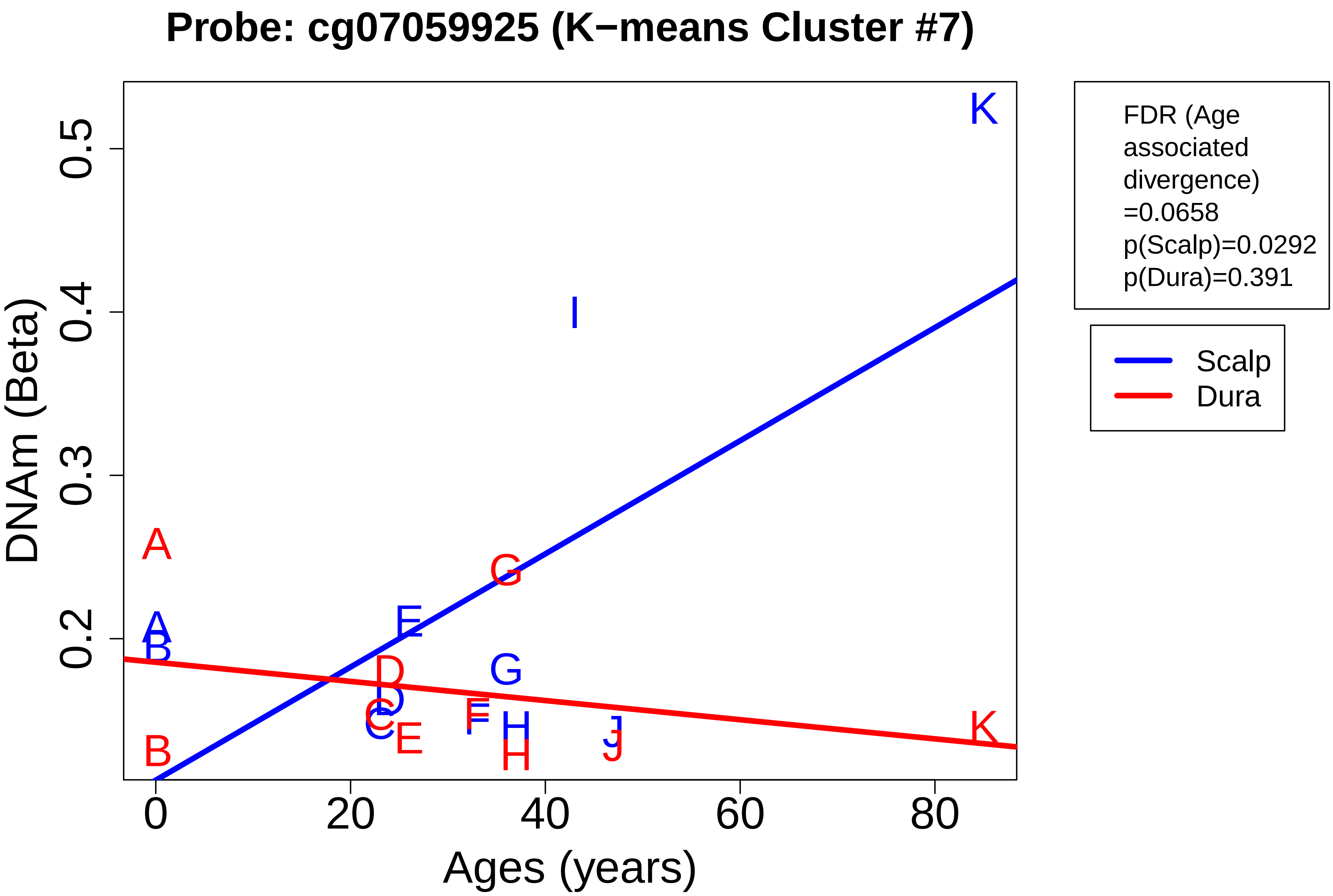
Probe: cg07059925 (K−means Cluster #7)
DNAm (Beta)
Ages (years)
0.2
0.3
0.4
0.5
0
20
40
60
80
FDR (Age associated divergence) =0.0658
p(Scalp)=0.0292
p(Dura)=0.391
Scalp
Dura
A
B
C
D
E
F
G
H
I
J
K

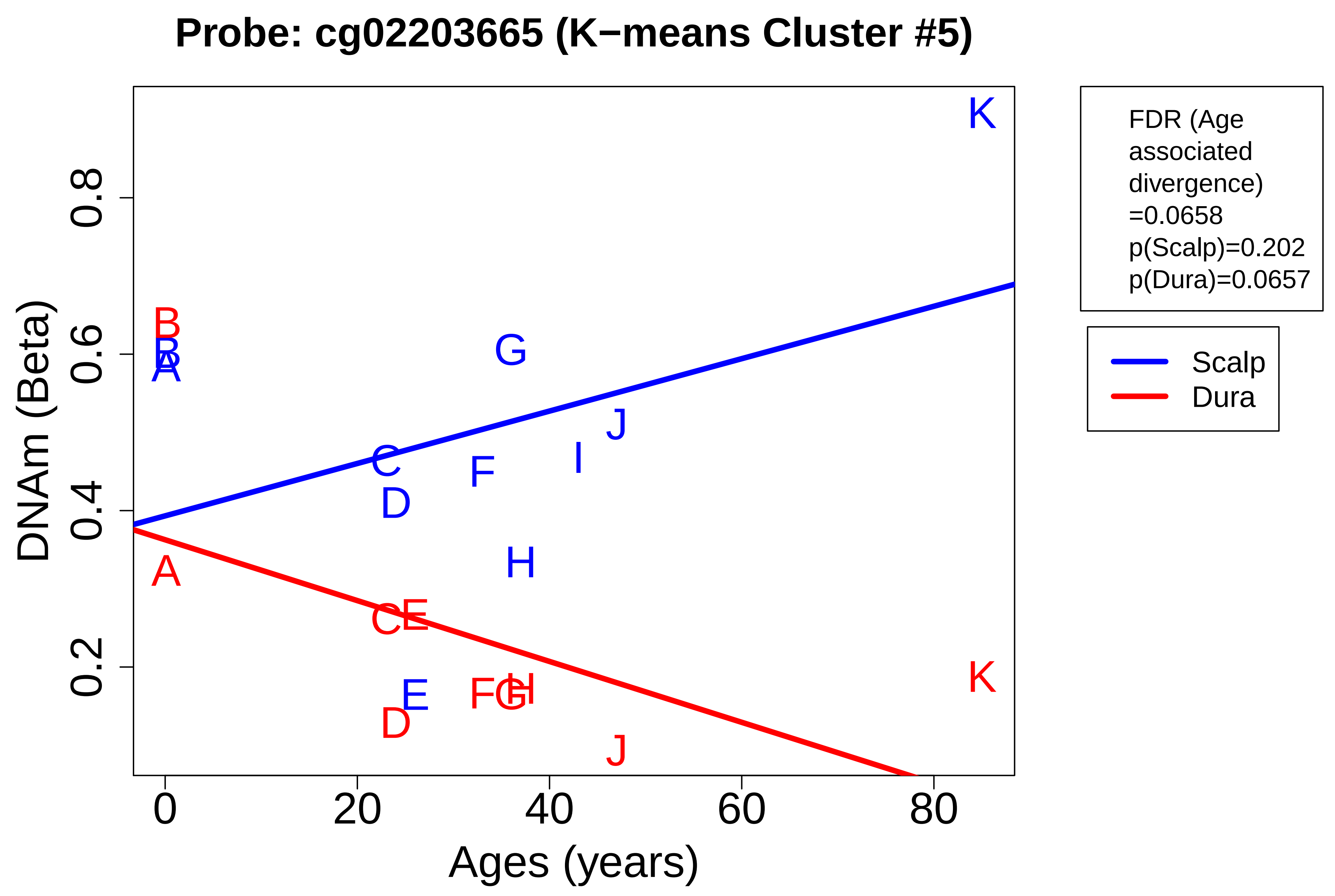

Probe: cg02203665 (K−means Cluster #5)
DNAm (Beta)
0.2
0.4
0.6
0.8
0
20
40
60
80
Ages (years)
A
B
C
D
E
F
G
H
I
J
K
FDR (Age associated divergence) =0.0658
p(Scalp)=0.202
p(Dura)=0.0657
Scalp
Dura

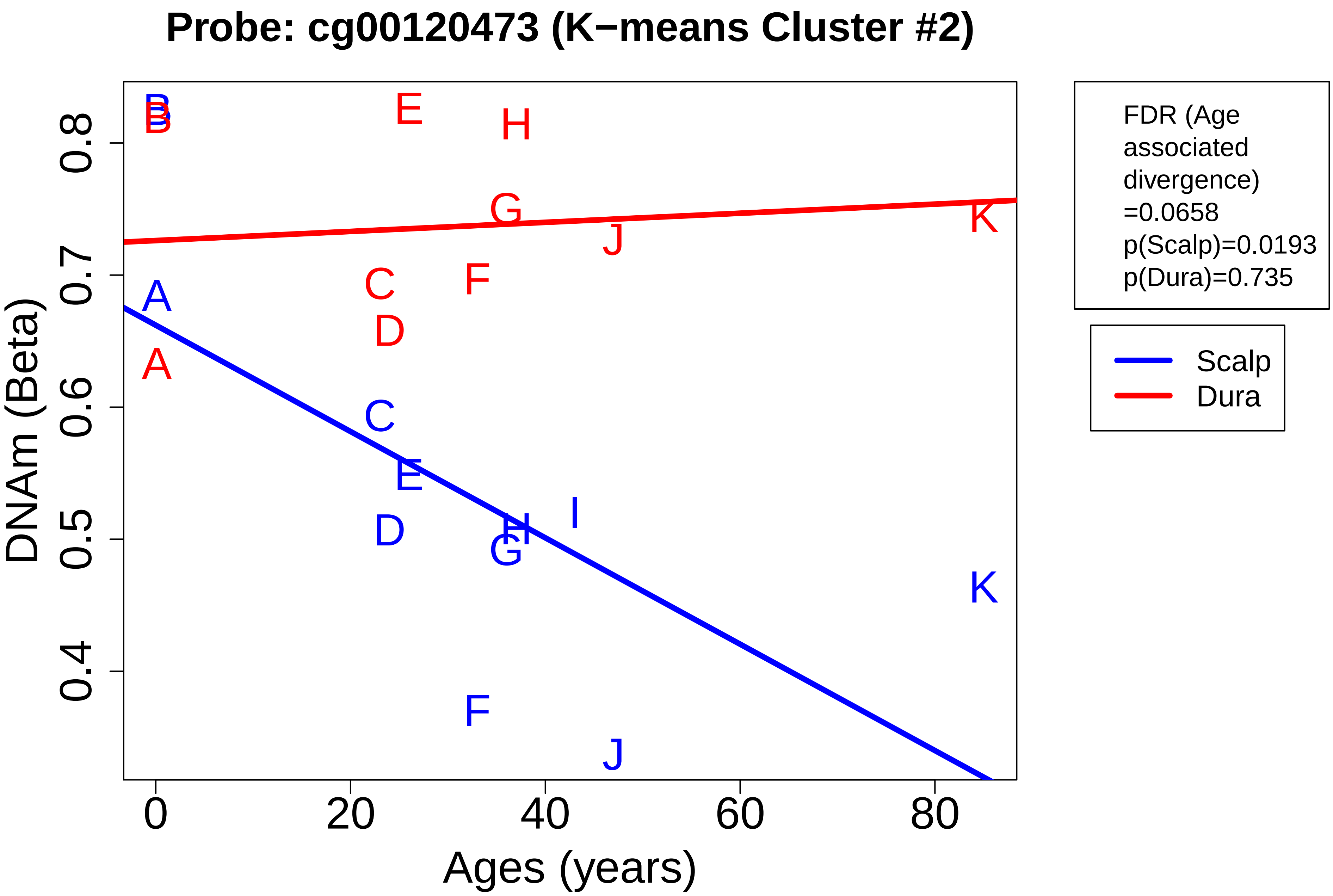

Probe: cg00120473 (K−means Cluster #2)
DNAm (Beta)
0.4
0.5
0.6
0.7
0.8
0
20
40
60
80
Ages (years)
FDR (Age associated divergence) =0.0658
p(Scalp)=0.0193
p(Dura)=0.735
Scalp
Dura

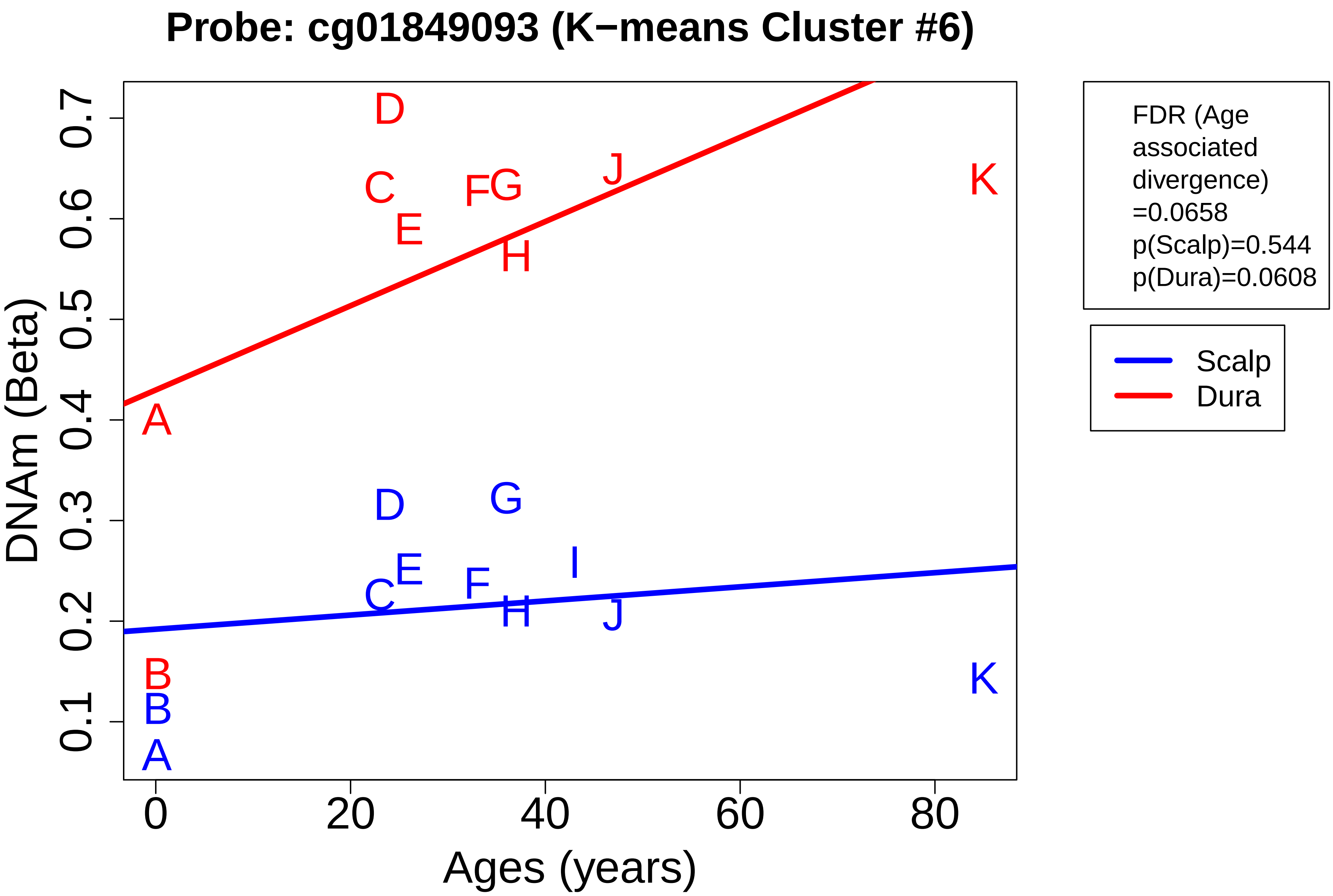
Probe: cg01849093 (K−means Cluster #6)
DNAm (Beta)
0.1
0.2
0.3
0.4
0.5
0.6
0.7
Ages (years)
0
20
40
60
80
FDR (Age associated divergence) =0.0658
p(Scalp)=0.544
p(Dura)=0.0608
Scalp
Dura
A
B
C
D
E
F
G
H
I
J
K

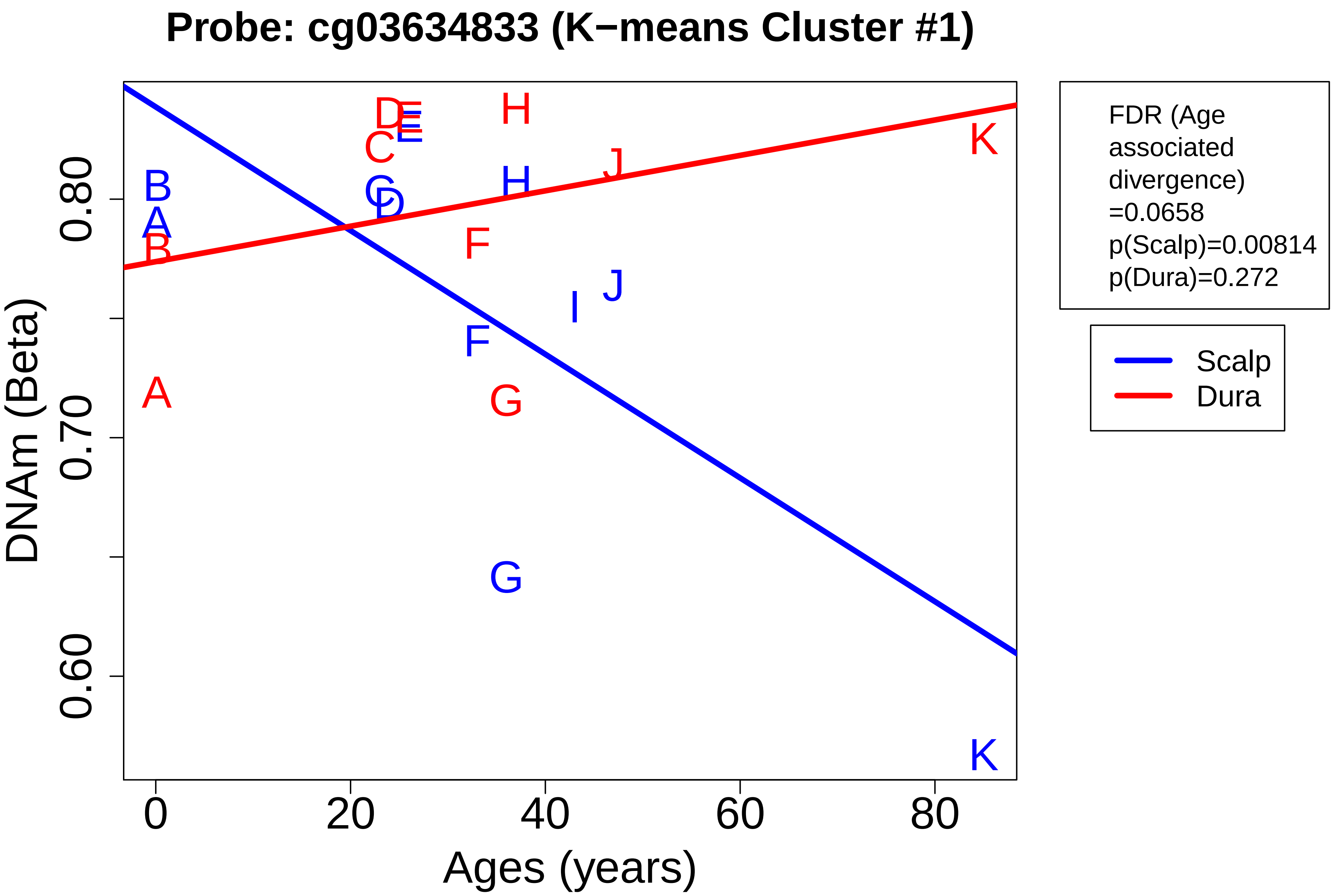
Probe: cg03634833 (K−means Cluster #1)
DNAm (Beta)
0.60
0.70
0.80
Ages (years)
0
20
40
60
80
FDR (Age associated divergence) =0.0658
p(Scalp)=0.00814
p(Dura)=0.272
Scalp
Dura
A
B
C
D
E
F
G
H
I
J
K

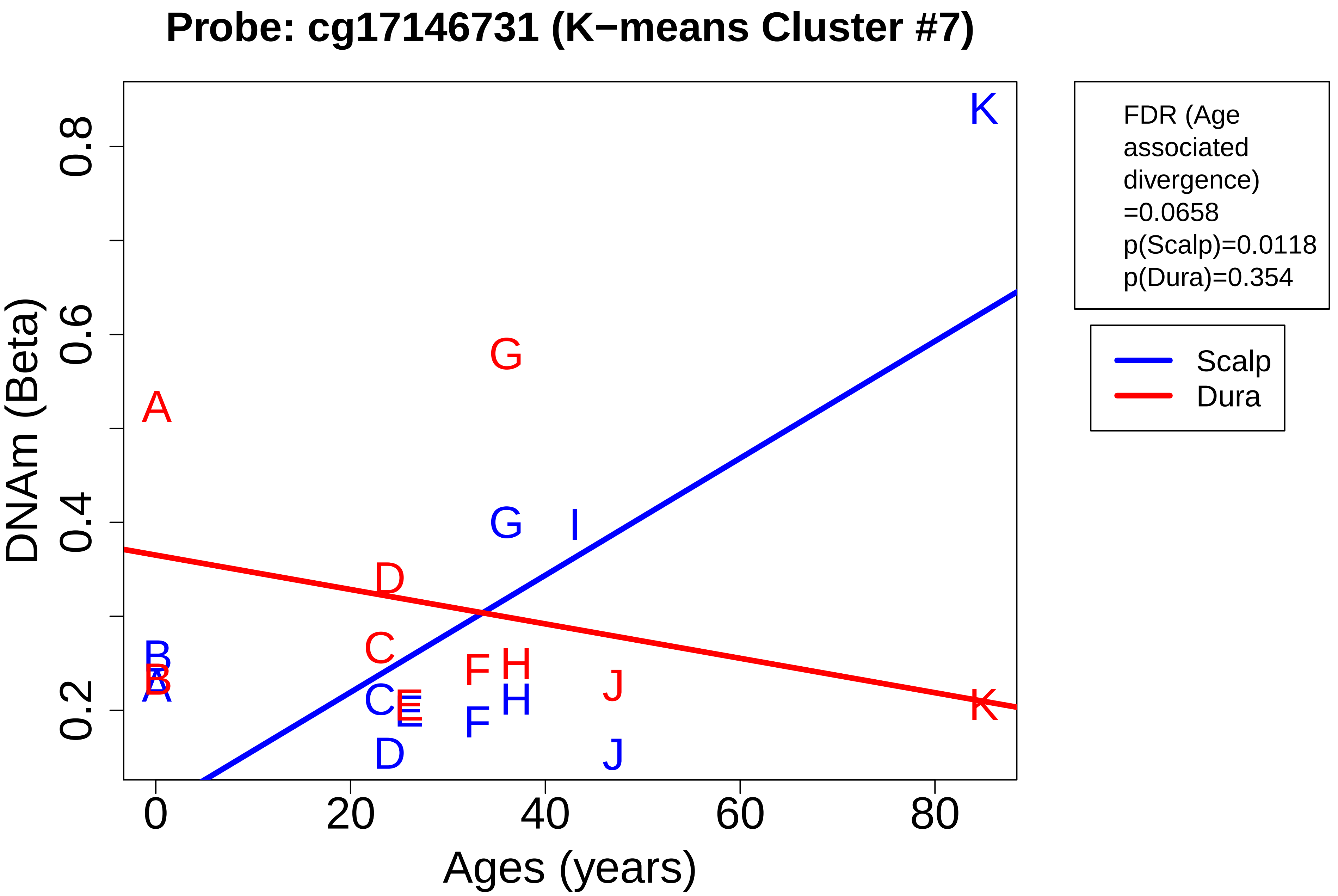
Probe: cg17146731 (K−means Cluster #7)
DNAm (Beta)
0.2
0.4
0.6
0.8
0
20
40
60
80
Ages (years)
A
B
C
D
E
F
G
H
I
J
K
FDR (Age associated divergence) =0.0658
p(Scalp)=0.0118
p(Dura)=0.354
Scalp
Dura

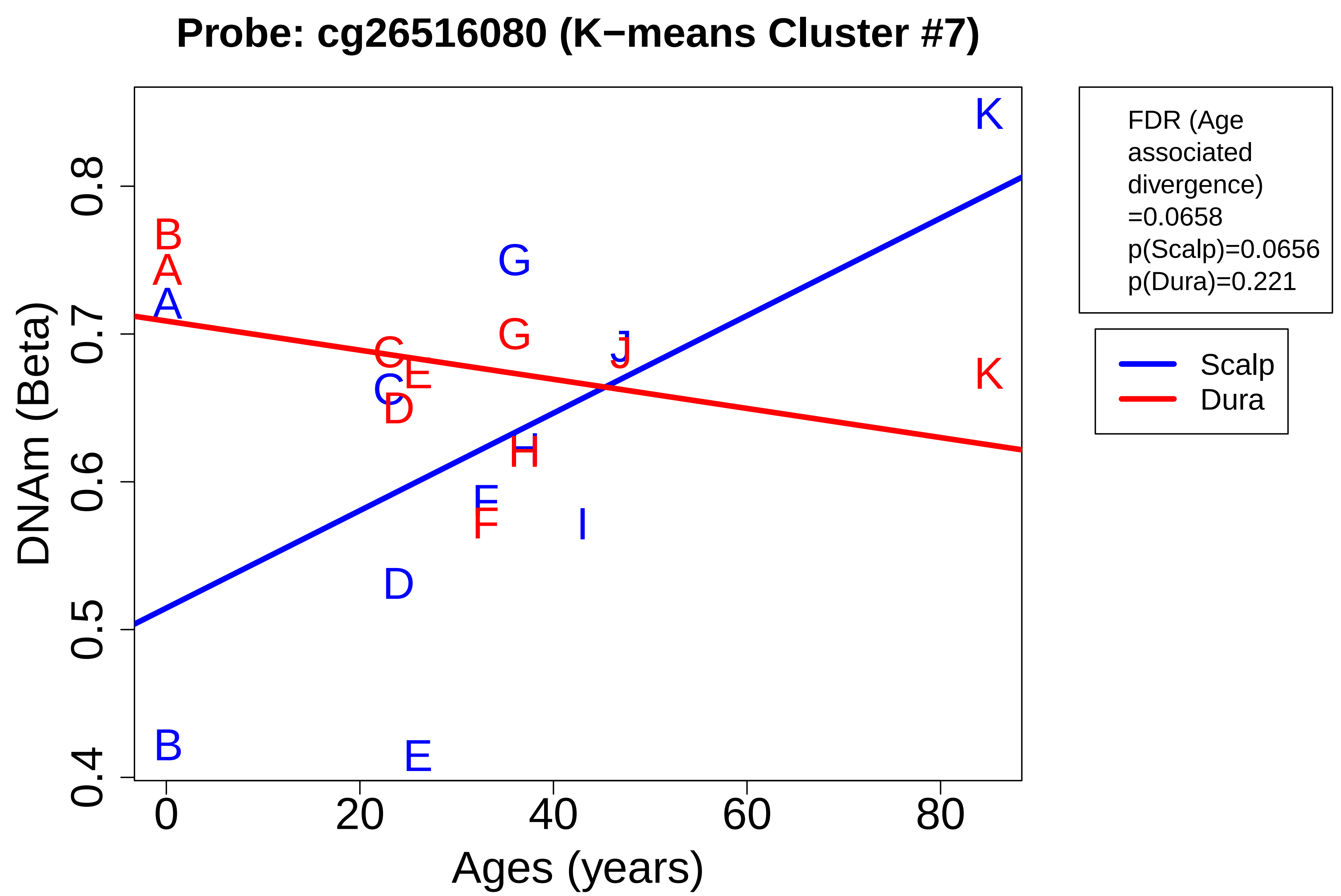

Probe: cg26516080 (K−means Cluster #7)
DNAm (Beta)
0.4
0.5
0.6
0.7
0.8
0
20
40
60
80
Ages (years)
FDR (Age associated divergence) =0.0658
p(Scalp)=0.0656
p(Dura)=0.221
Scalp
Dura
A
B
C
D
E
F
G
H
I
J
K

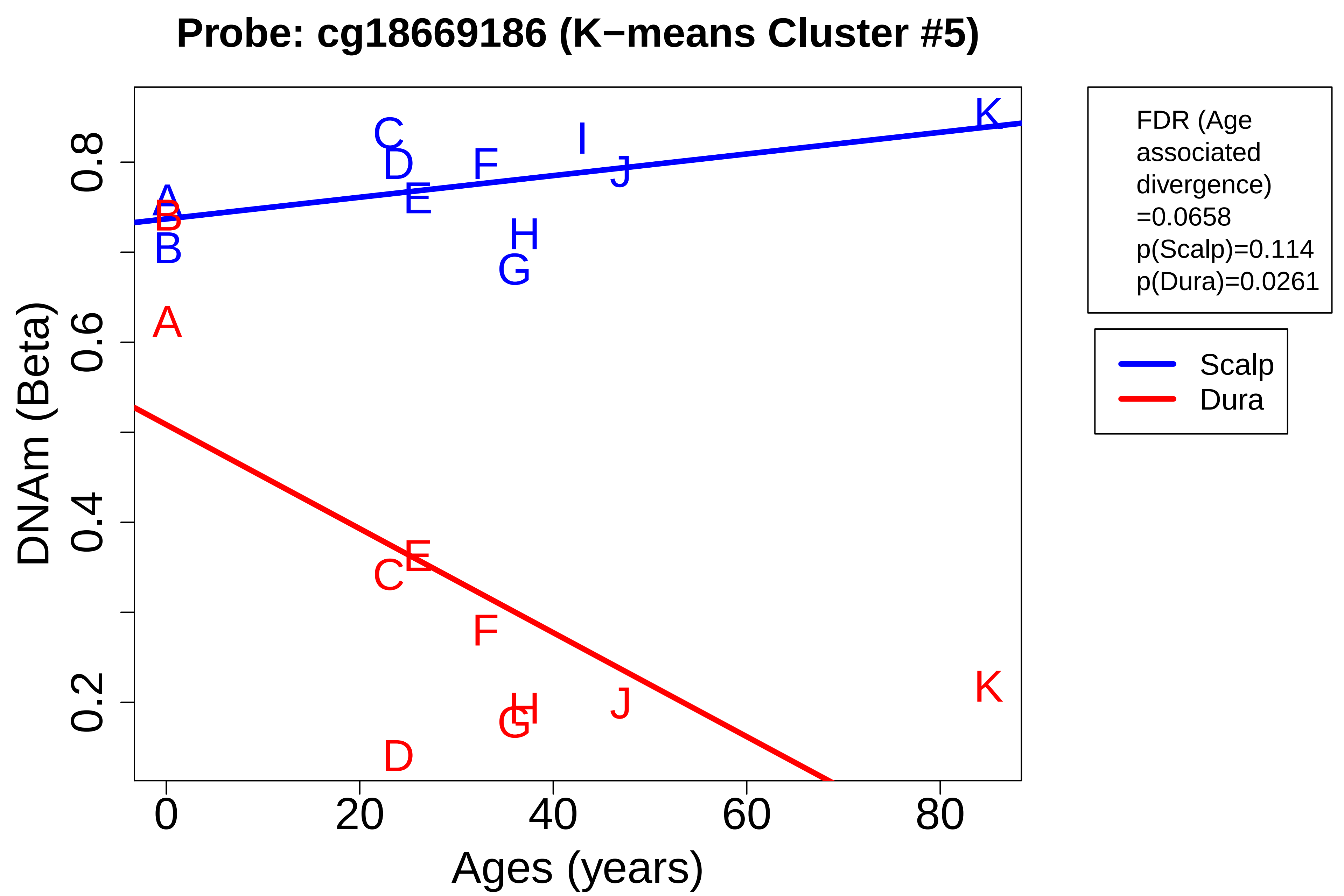
Probe: cg18669186 (K−means Cluster #5)
DNAm (Beta)
Ages (years)
0.2
0.4
0.6
0.8
0
20
40
60
80
FDR (Age associated divergence) =0.0658
p(Scalp)=0.114
p(Dura)=0.0261
Scalp
Dura
A B C D E F G H I J K

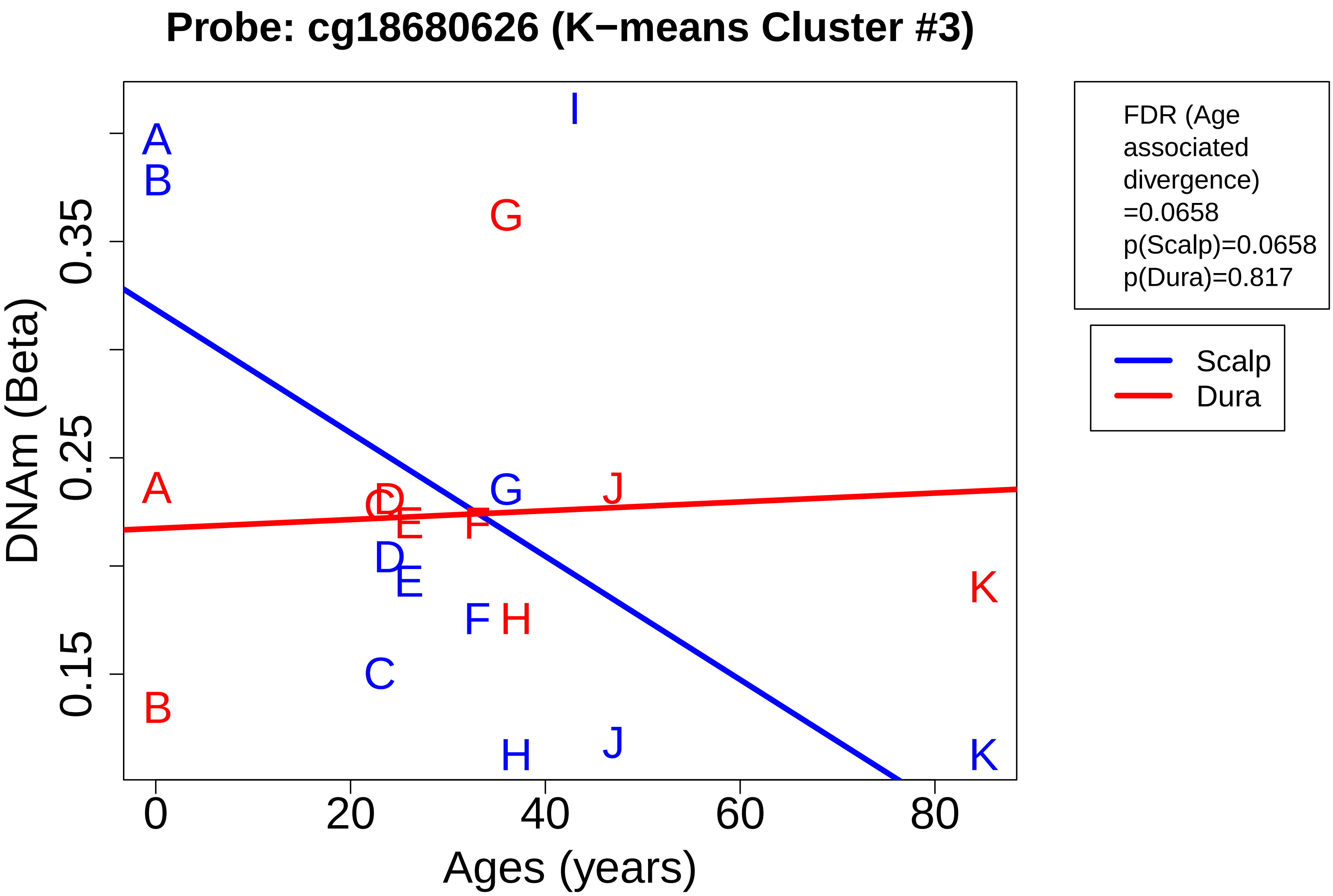
Probe: cg18680626 (K−means Cluster #3)
FDR (Age associated divergence) =0.0658
p(Scalp)=0.0658
p(Dura)=0.817
Scalp
Dura
DNAm (Beta)
0.15
0.25
0.35
Ages (years)
0
20
40
60
80
A
B
C
D
E
F
G
H
I
J
K

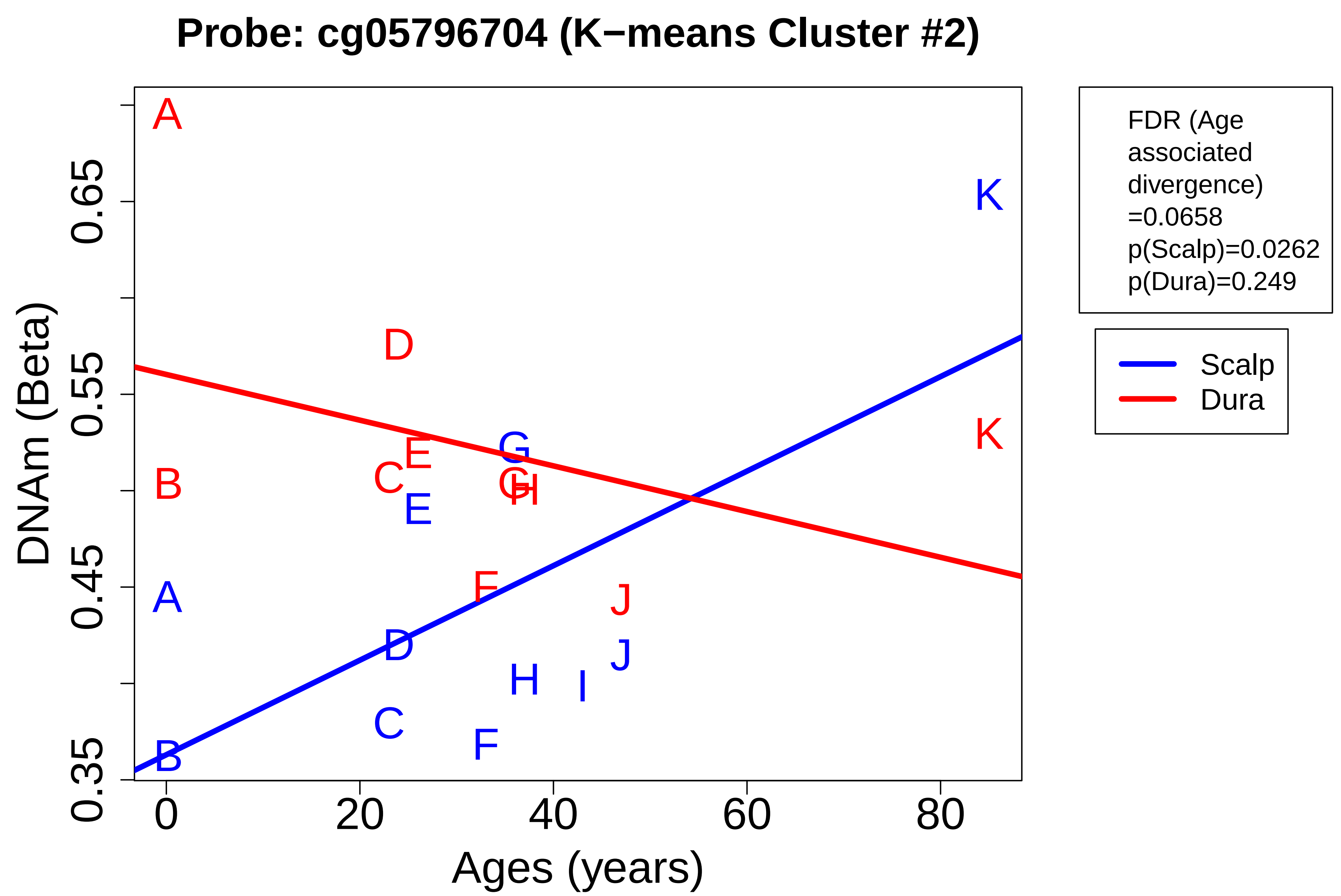

Probe: cg05796704 (K−means Cluster #2)
DNAm (Beta)
0.35
0.45
0.55
0.65
0
20
40
60
80
Ages (years)
FDR (Age associated divergence) =0.0658
p(Scalp)=0.0262
p(Dura)=0.249
Scalp
Dura
A
B
C
D
E
F
G
H
I
J
K

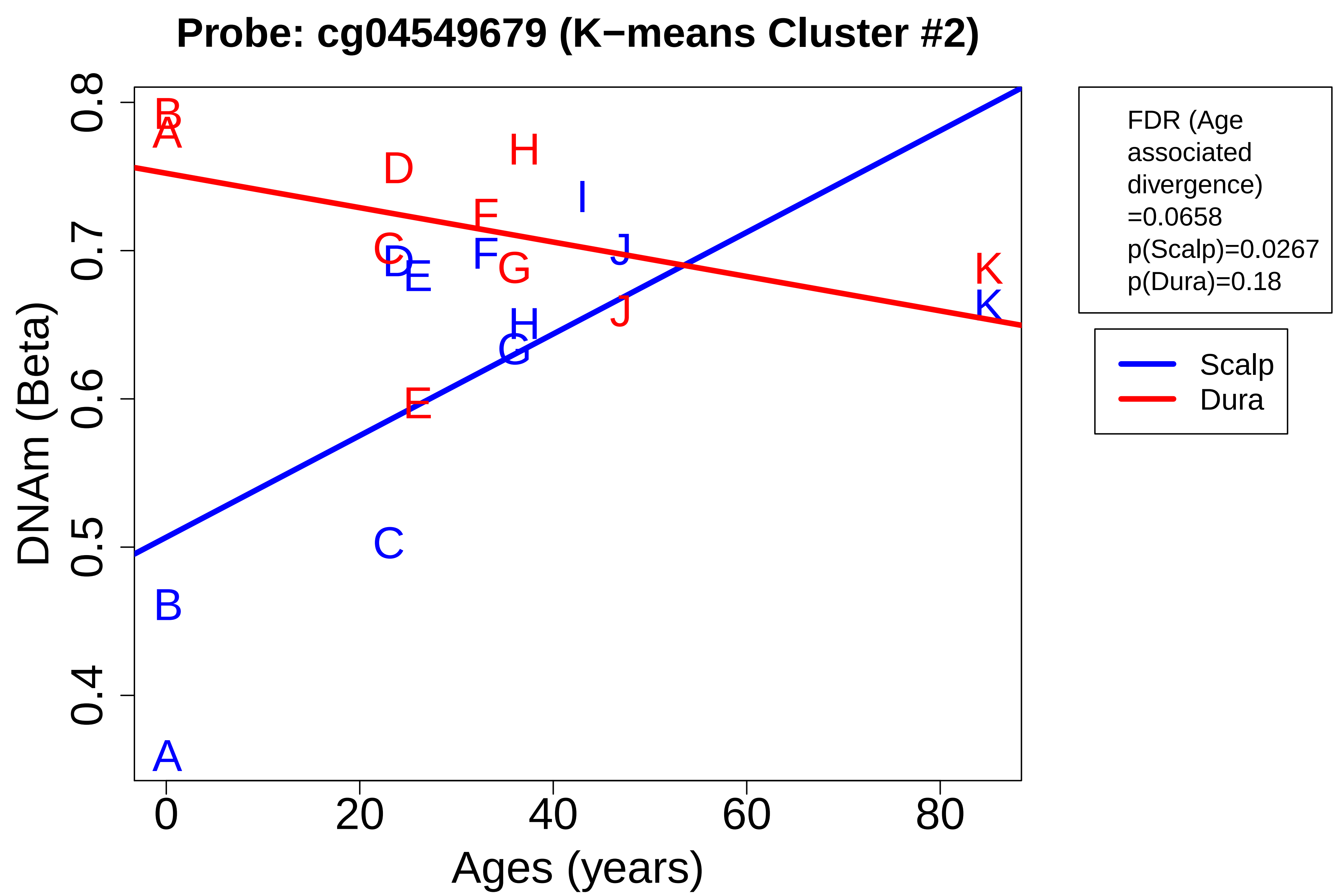

Probe: cg04549679 (K−means Cluster #2)
DNAm (Beta)
0.4
0.5
0.6
0.7
0.8
0
20
40
60
80
Ages (years)
FDR (Age associated divergence) =0.0658
p(Scalp)=0.0267
p(Dura)=0.18
Scalp
Dura
A
B
C
D
E
F
G
H
I
J
K

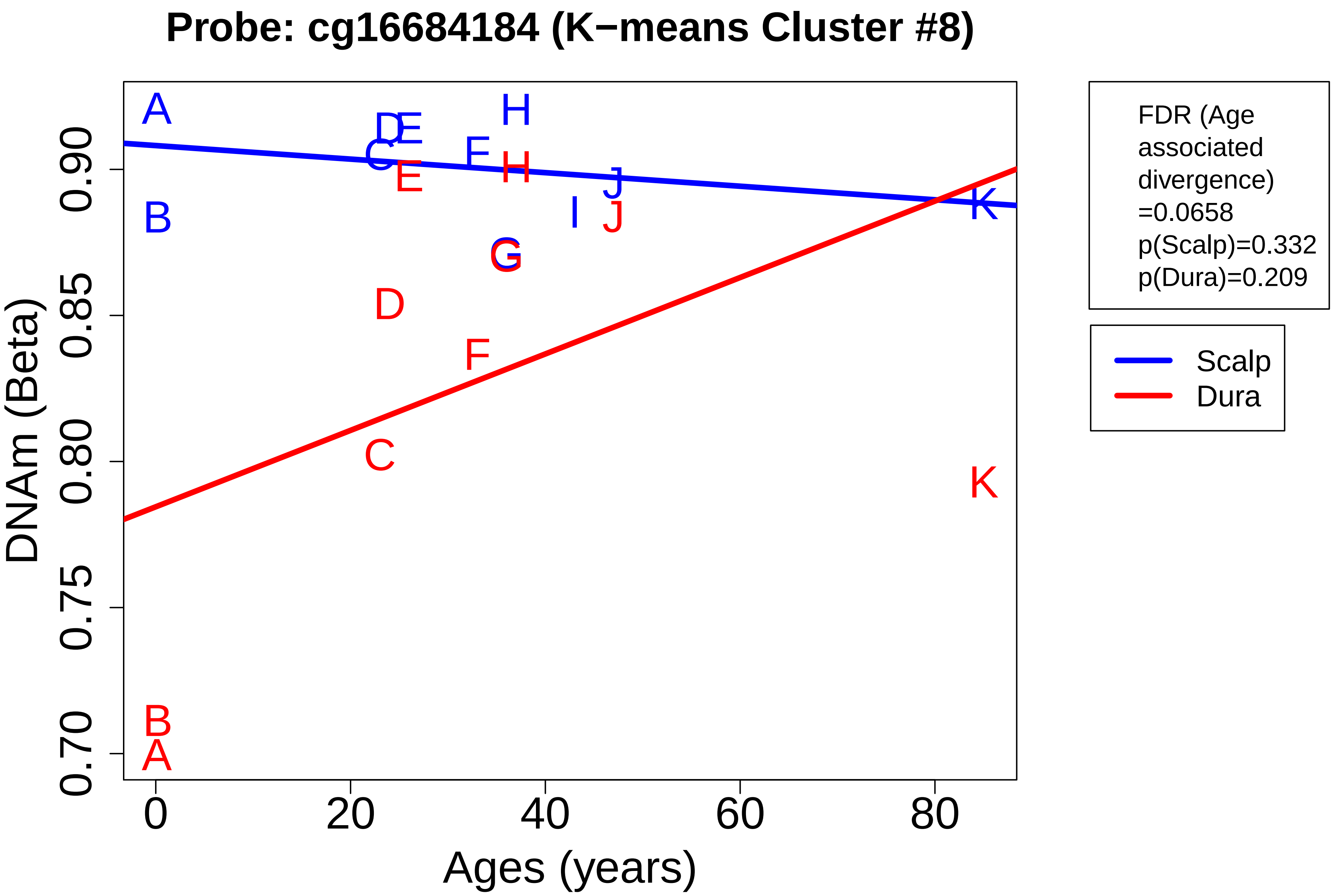

Probe: cg16684184 (K−means Cluster #8)
DNAm (Beta)
0.70
0.75
0.80
0.85
0.90
0
20
40
60
80
Ages (years)
A
B
C
D
E
F
G
H
I
J
K
FDR (Age associated divergence) =0.0658
p(Scalp)=0.332
p(Dura)=0.209
Scalp
Dura

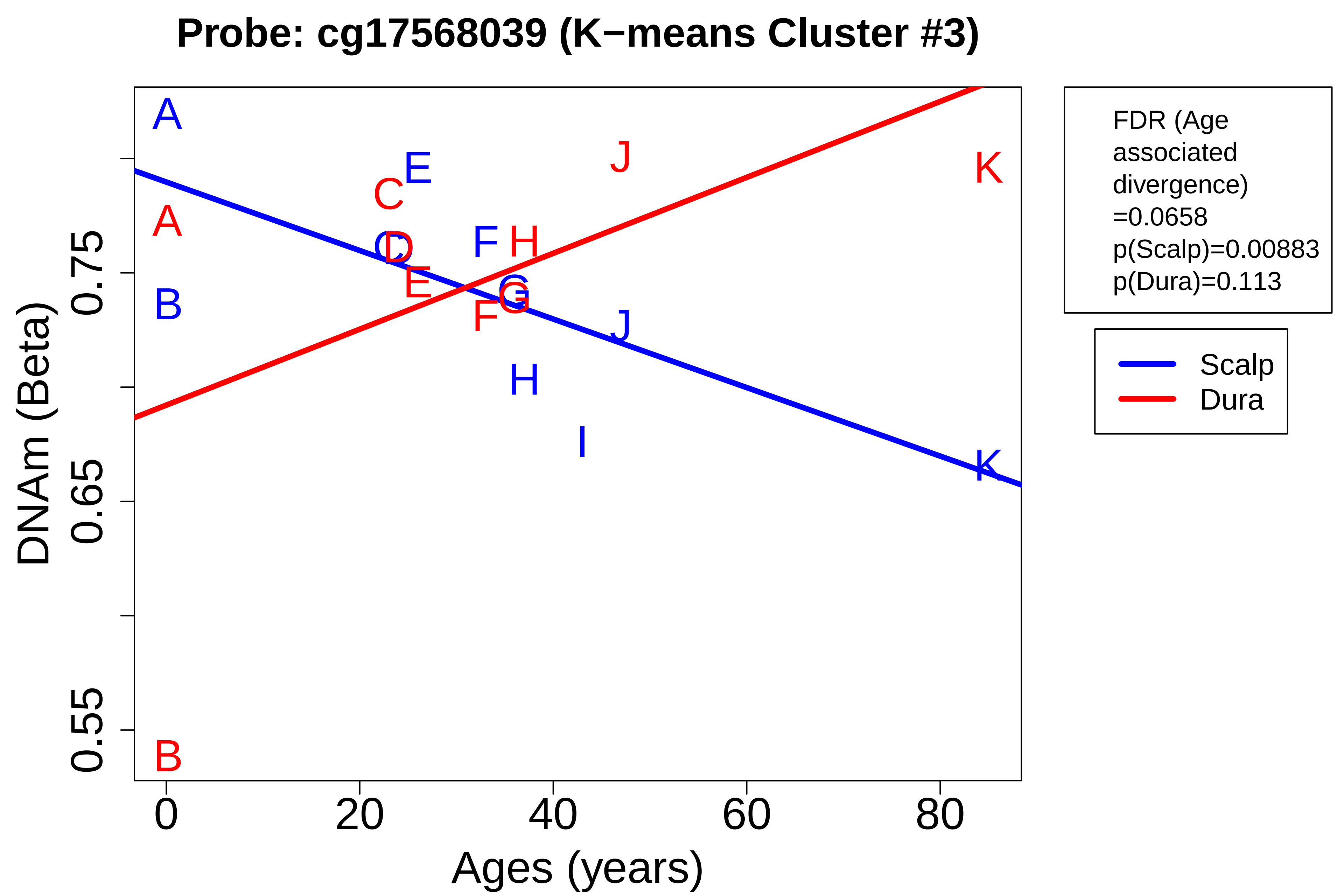

Probe: cg17568039 (K−means Cluster #3)
FDR (Age associated divergence) =0.0658
p(Scalp)=0.00883
p(Dura)=0.113
Scalp
Dura
DNAm (Beta)
0.55
0.65
0.75
0
20
40
60
80
Ages (years)
A
B
C
D
E
F
G
H
I
J
K

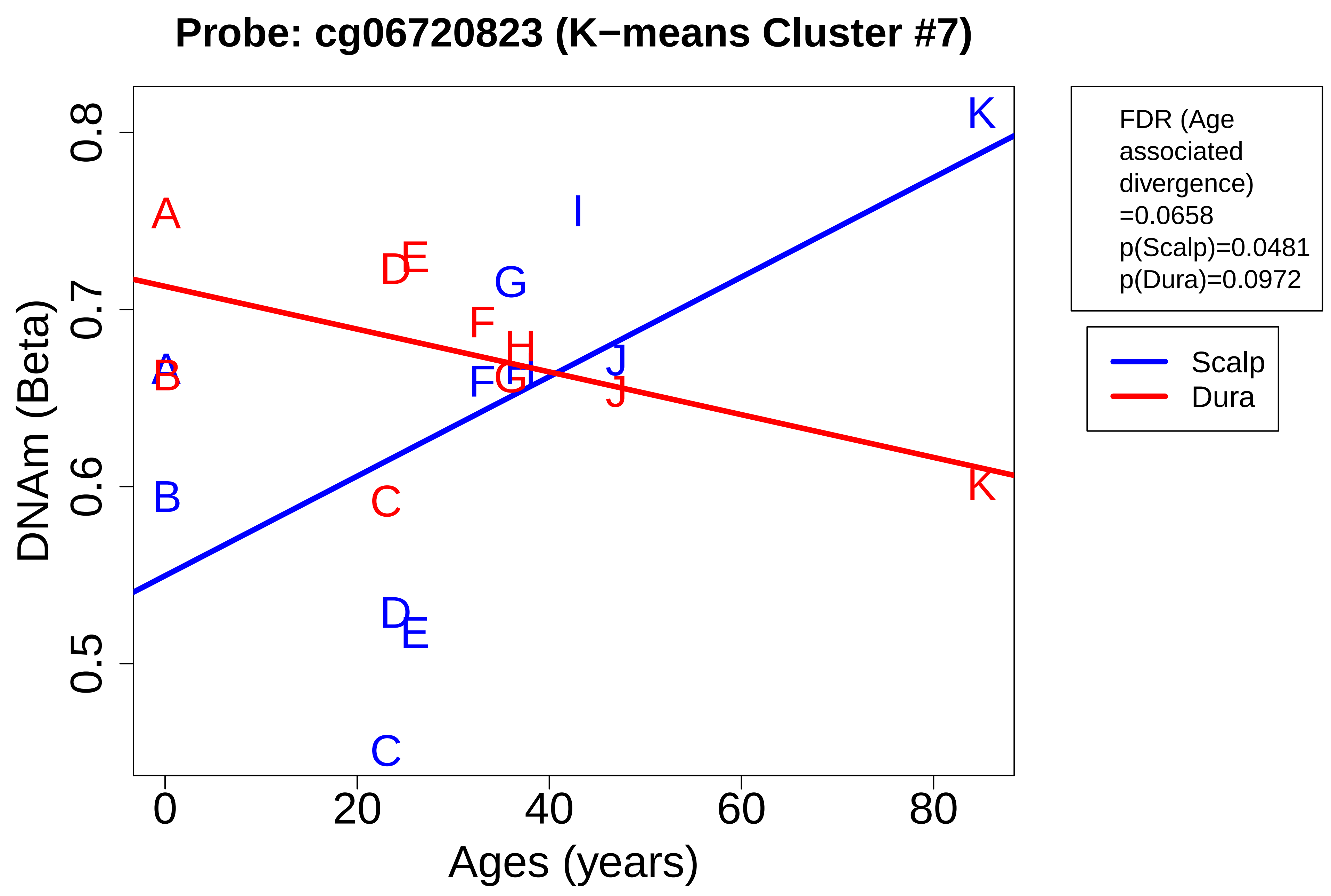

Probe: cg06720823 (K−means Cluster #7)
DNAm (Beta)
0.5
0.6
0.7
0.8
0
20
40
60
80
Ages (years)
A
B
C
D
E
F
G
H
I
J
K
FDR (Age associated divergence) =0.0658
p(Scalp)=0.0481
p(Dura)=0.0972
Scalp
Dura

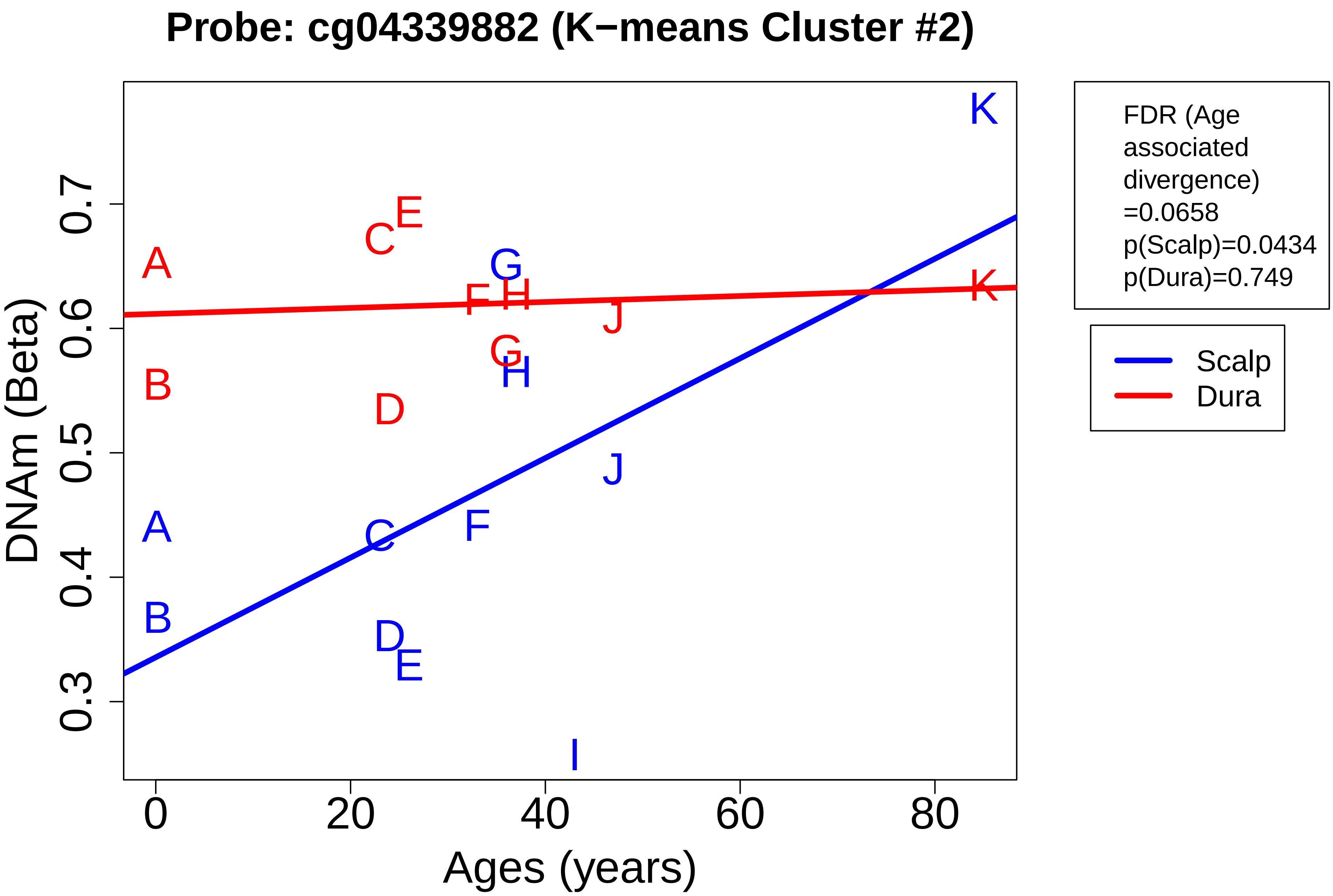
Probe: cg04339882 (K−means Cluster #2)
DNAm (Beta)
0.3
0.4
0.5
0.6
0.7
Ages (years)
0
20
40
60
80
A
B
C
D
E
F
G
H
I
J
K
FDR (Age associated divergence) =0.0658
p(Scalp)=0.0434
p(Dura)=0.749
Scalp
Dura

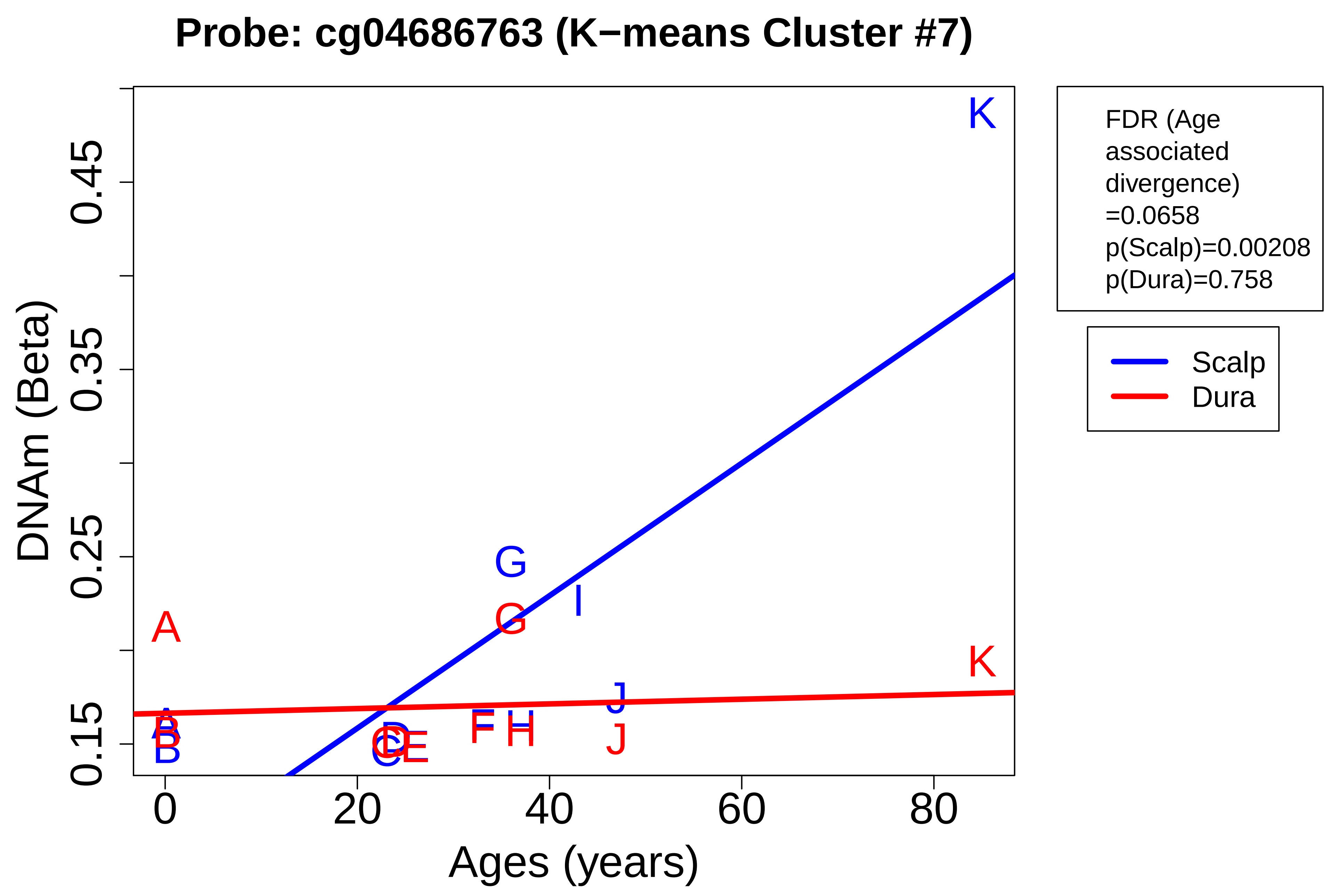

Probe: cg04686763 (K−means Cluster #7)
DNAm (Beta)
0.15
0.25
0.35
0.45
Ages (years)
0
20
40
60
80
FDR (Age associated divergence) =0.0658
p(Scalp)=0.00208
p(Dura)=0.758
Scalp
Dura

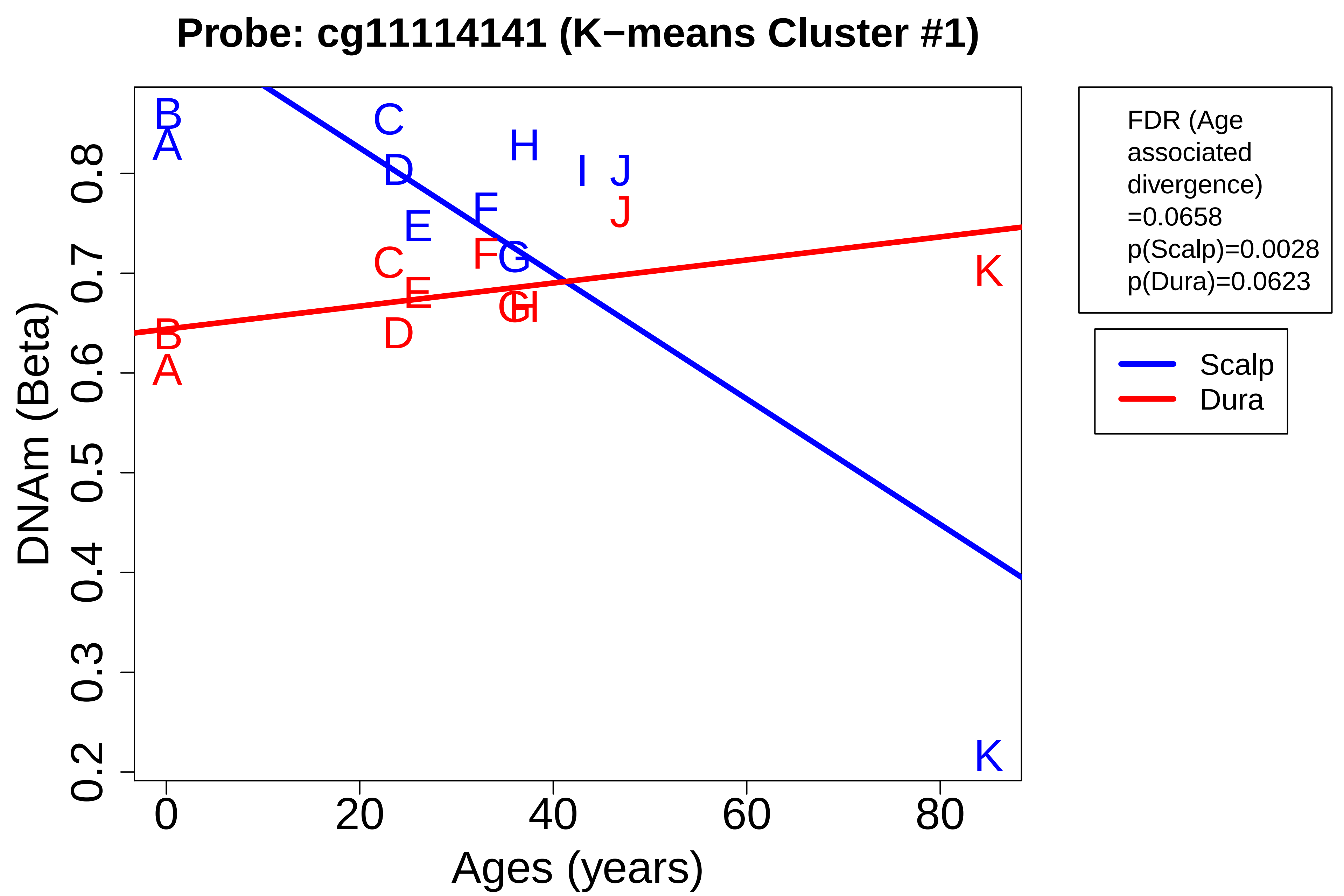

Probe: cg11114141 (K−means Cluster #1)
DNAm (Beta)
Ages (years)
0.2
0.3
0.4
0.5
0.6
0.7
0.8
0
20
40
60
80
FDR (Age associated divergence) =0.0658
p(Scalp)=0.0028
p(Dura)=0.0623
Scalp
Dura

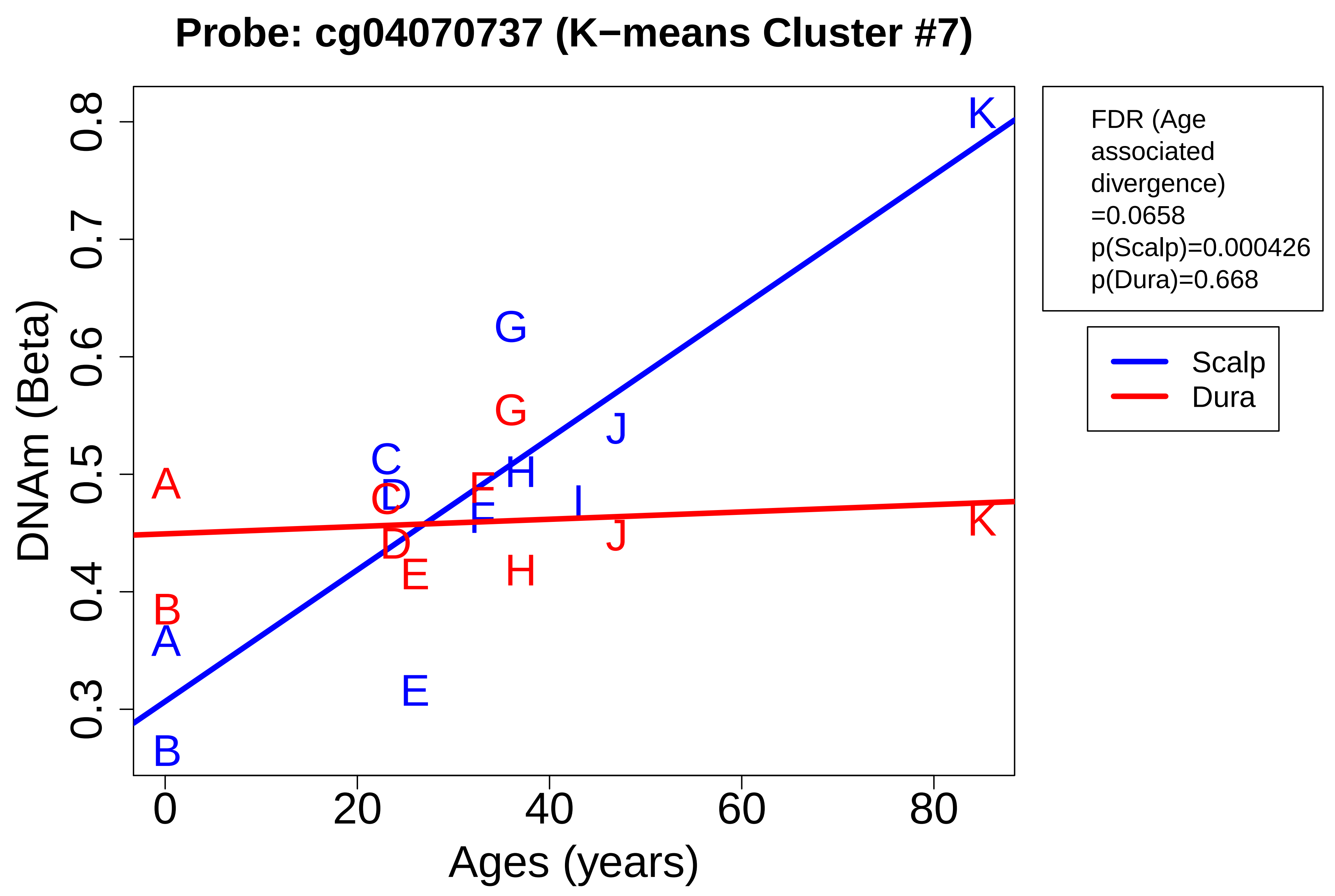

Probe: cg04070737 (K−means Cluster #7)
DNAm (Beta)
0.3
0.4
0.5
0.6
0.7
0.8
0
20
40
60
80
Ages (years)
FDR (Age associated divergence) =0.0658
p(Scalp)=0.000426
p(Dura)=0.668
Scalp
Dura

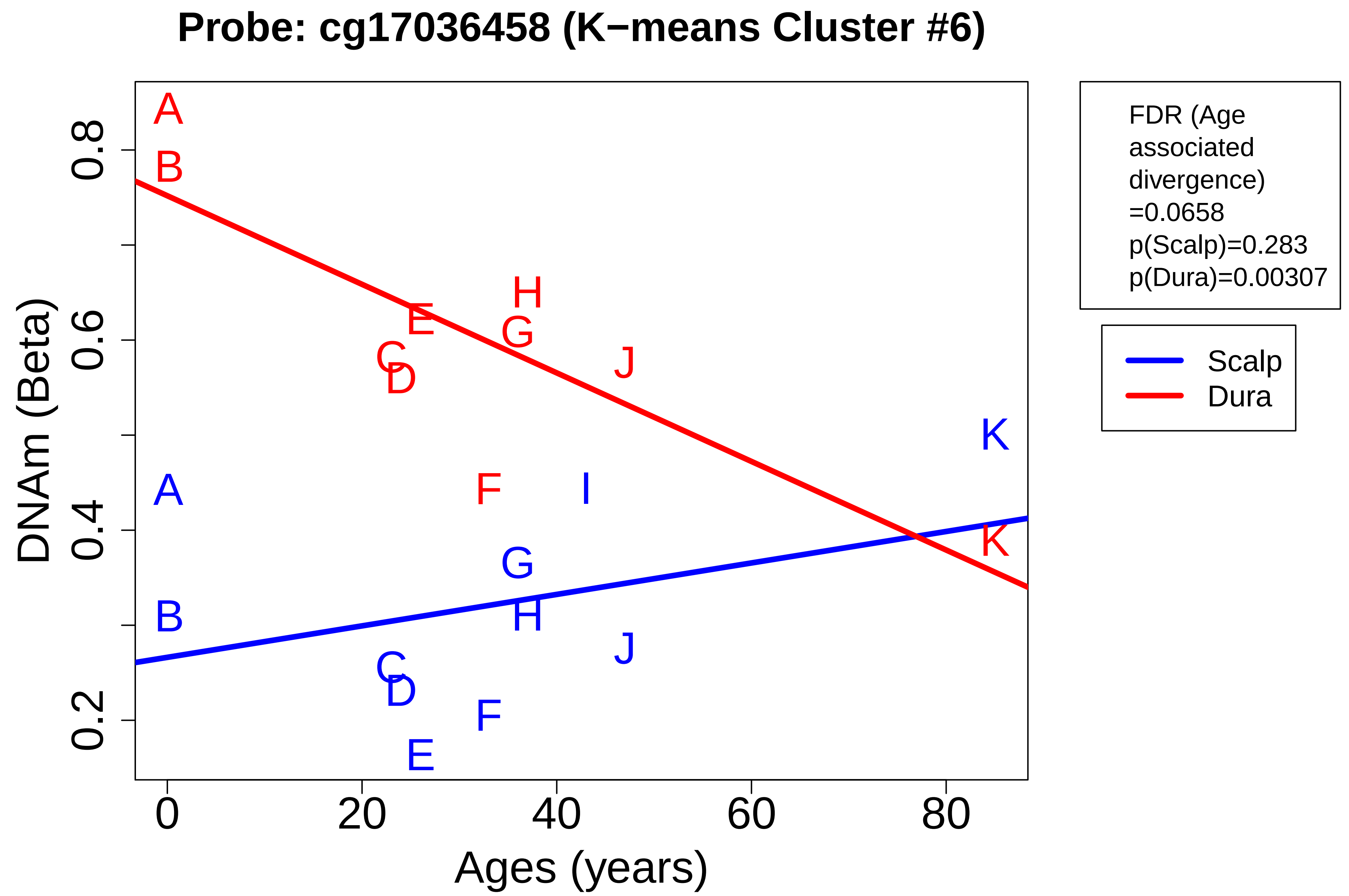

Probe: cg17036458 (K−means Cluster #6)
FDR (Age associated divergence) =0.0658
p(Scalp)=0.283
p(Dura)=0.00307
Scalp
Dura
DNAm (Beta)
0.2
0.4
0.6
0.8
0
20
40
60
80
Ages (years)
A
B
C
D
E
F
G
H
I
J
K

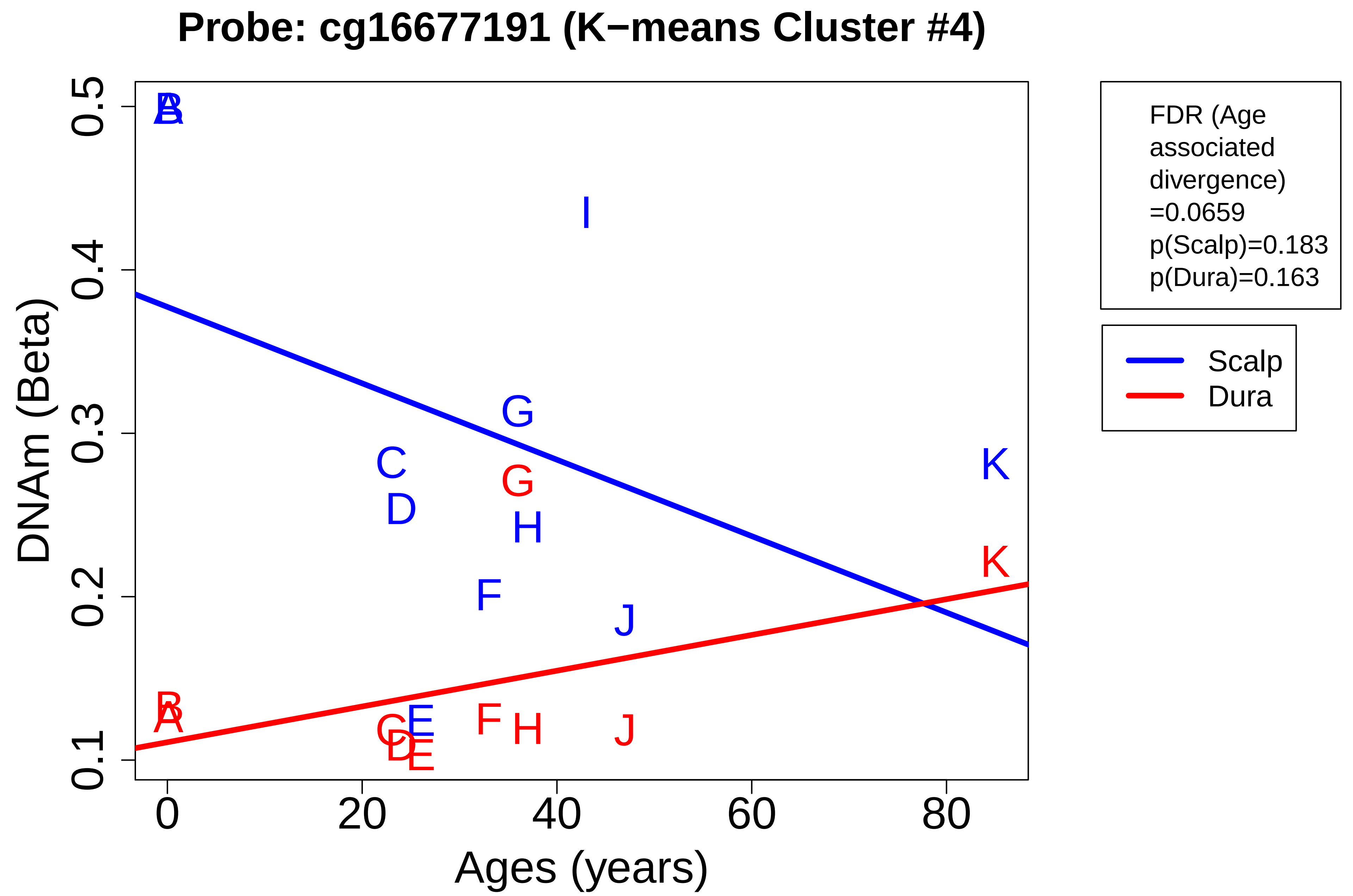

Probe: cg16677191 (K−means Cluster #4)
DNAm (Beta)
0.1
0.2
0.3
0.4
0.5
0
20
40
60
80
Ages (years)
A
B
C
D
E
F
G
H
I
J
K
FDR (Age associated divergence) =0.0659
p(Scalp)=0.183
p(Dura)=0.163
Scalp
Dura

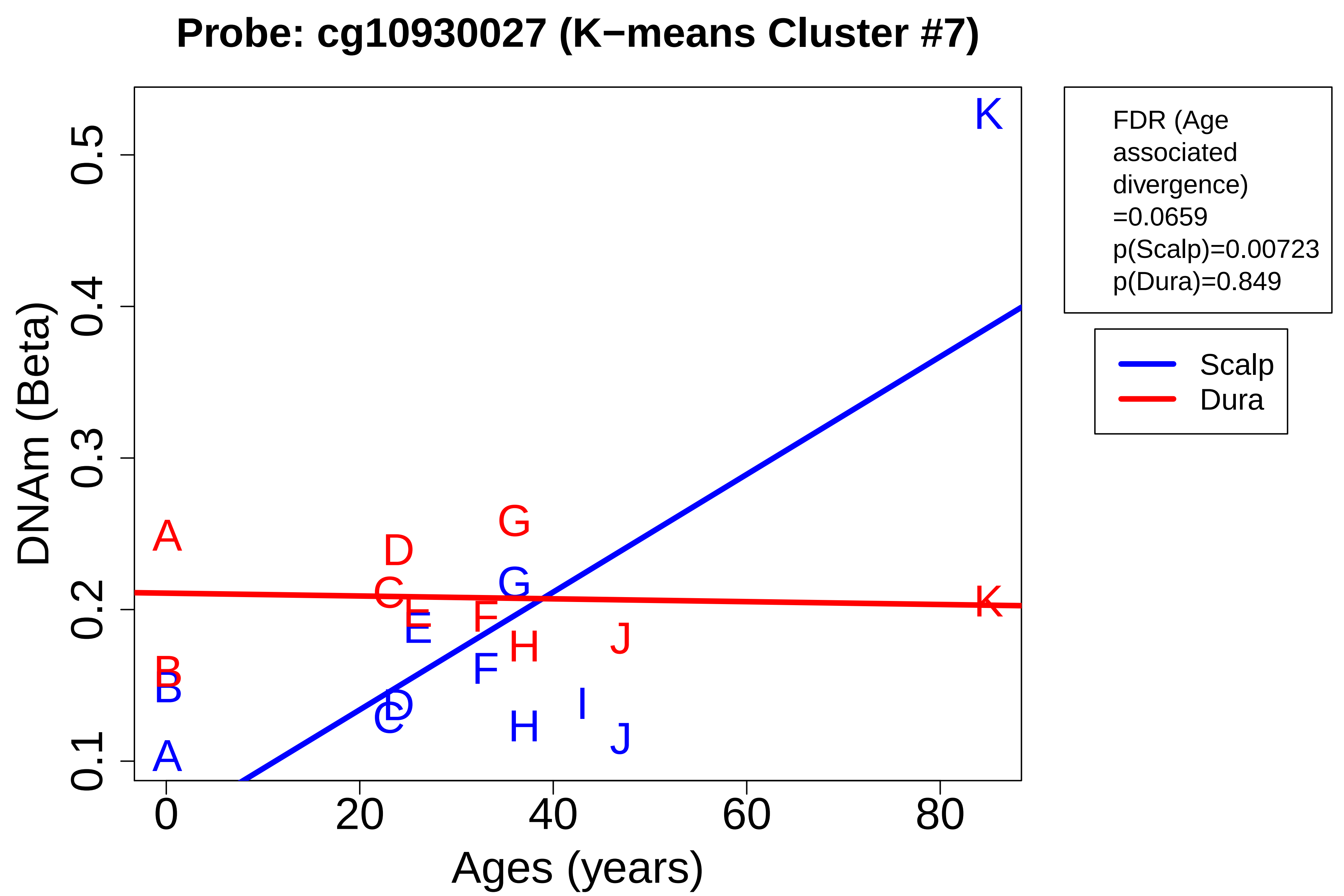

Probe: cg10930027 (K−means Cluster #7)
DNAm (Beta)
0.1
0.2
0.3
0.4
0.5
0
20
40
60
80
Ages (years)
FDR (Age associated divergence) =0.0659
p(Scalp)=0.00723
p(Dura)=0.849
Scalp
Dura
A
B
C
D
E
F
G
H
I
J
K

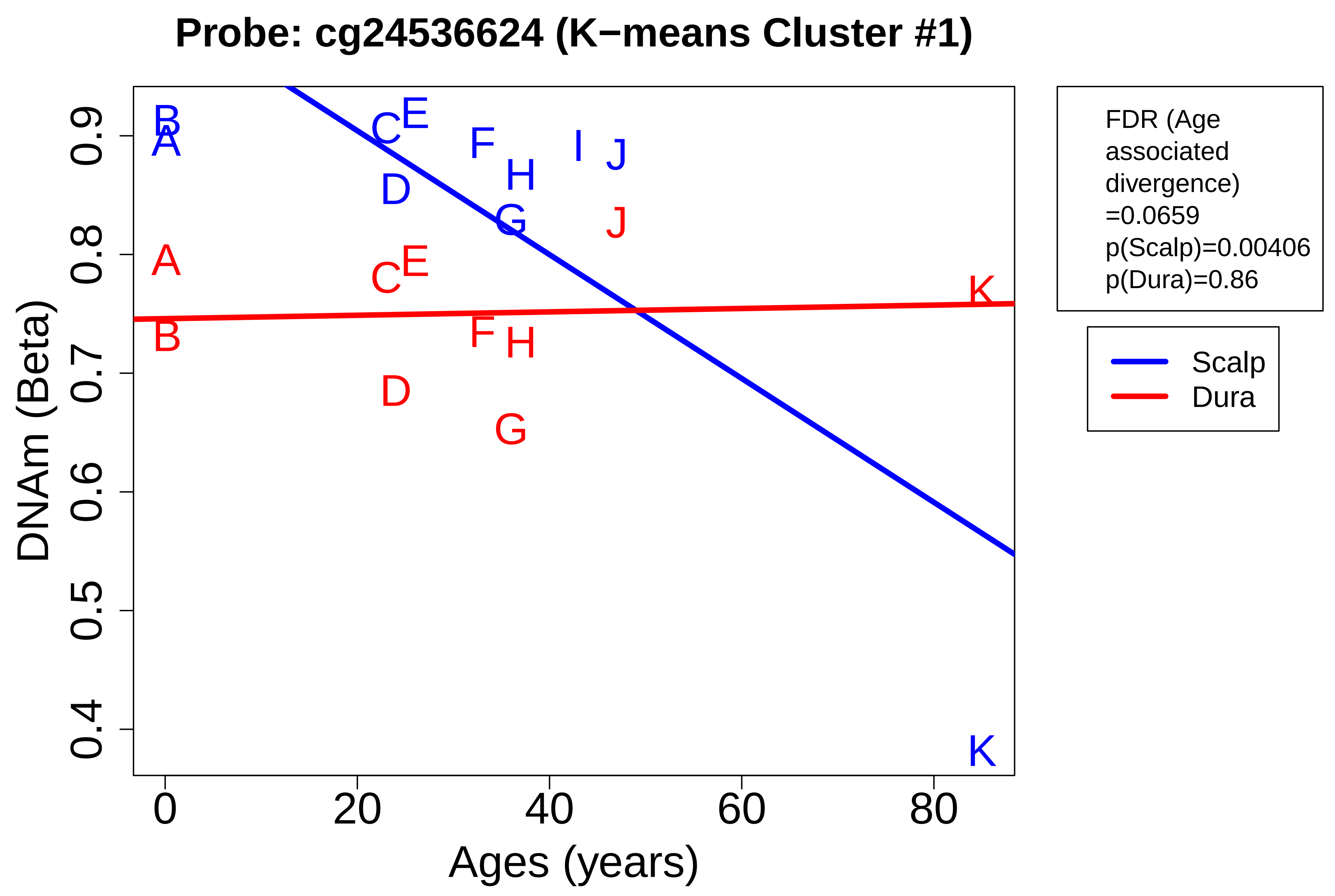
Probe: cg24536624 (K−means Cluster #1)
DNAm (Beta)
Ages (years)
0.4
0.5
0.6
0.7
0.8
0.9
0
20
40
60
80
A
B
C
D
E
F
G
H
I
J
K
FDR (Age associated divergence) =0.0659
p(Scalp)=0.00406
p(Dura)=0.86
Scalp
Dura

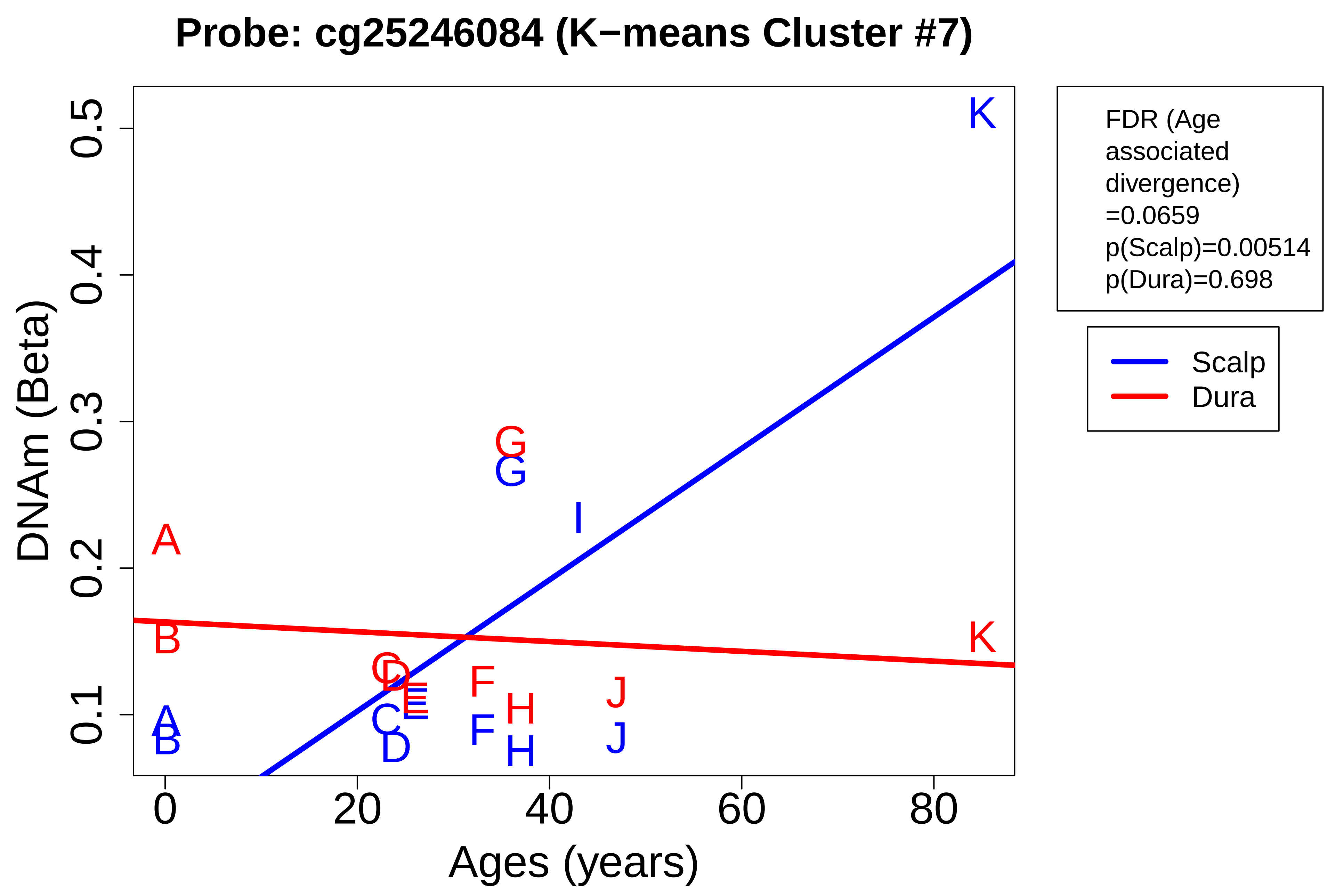

Probe: cg25246084 (K−means Cluster #7)
DNAm (Beta)
0.1
0.2
0.3
0.4
0.5
0
20
40
60
80
Ages (years)
A
B
C
D
E
F
G
H
I
J
K
FDR (Age associated divergence) =0.0659
p(Scalp)=0.00514
p(Dura)=0.698
Scalp
Dura

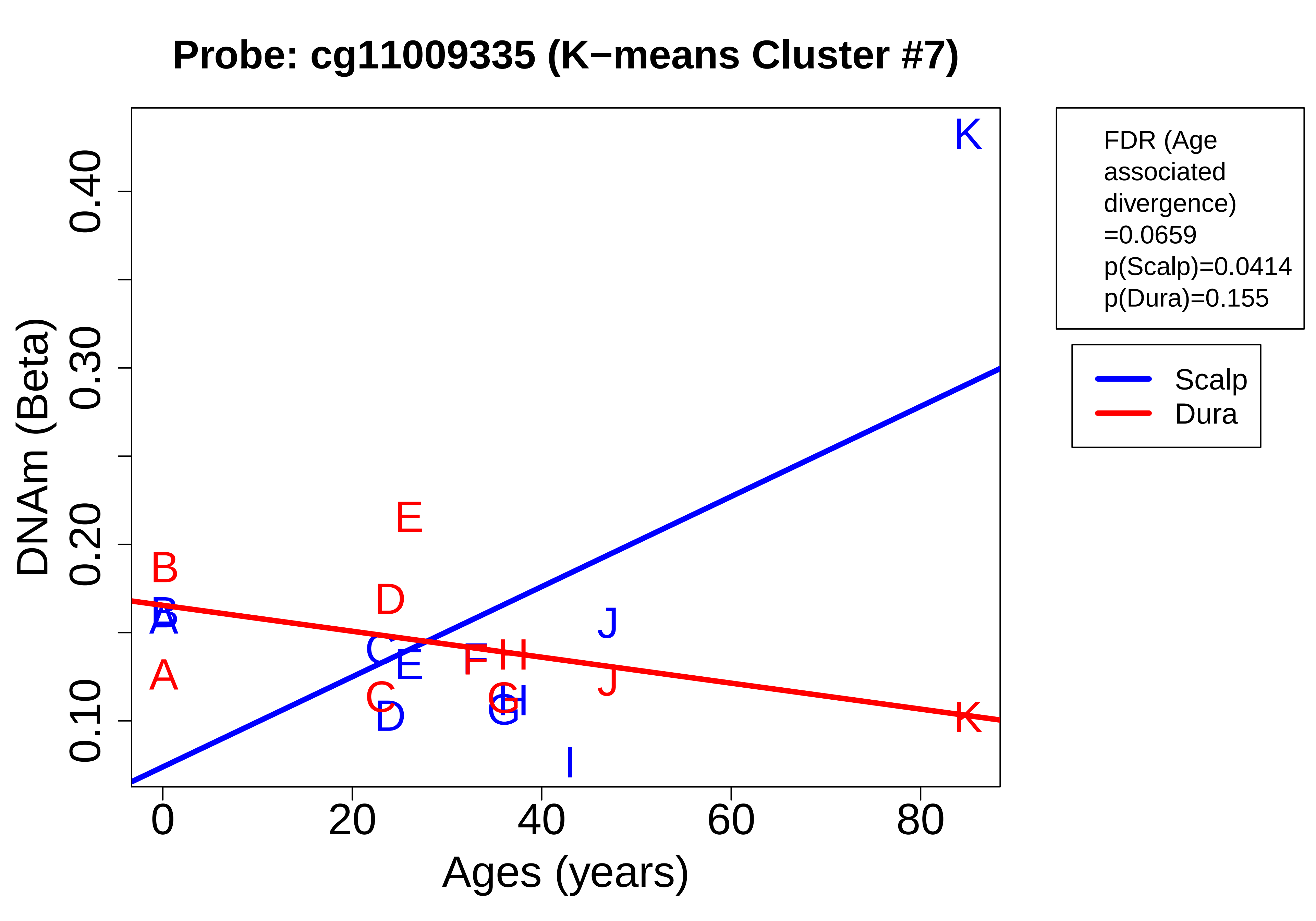

Probe: cg11009335 (K−means Cluster #7)
FDR (Age associated divergence) =0.0659
p(Scalp)=0.0414
p(Dura)=0.155
Scalp
Dura
DNAm (Beta)
0.10
0.20
0.30
0.40
0
20
40
60
80
Ages (years)
A
B
C
D
E
F
G
H
I
J
K

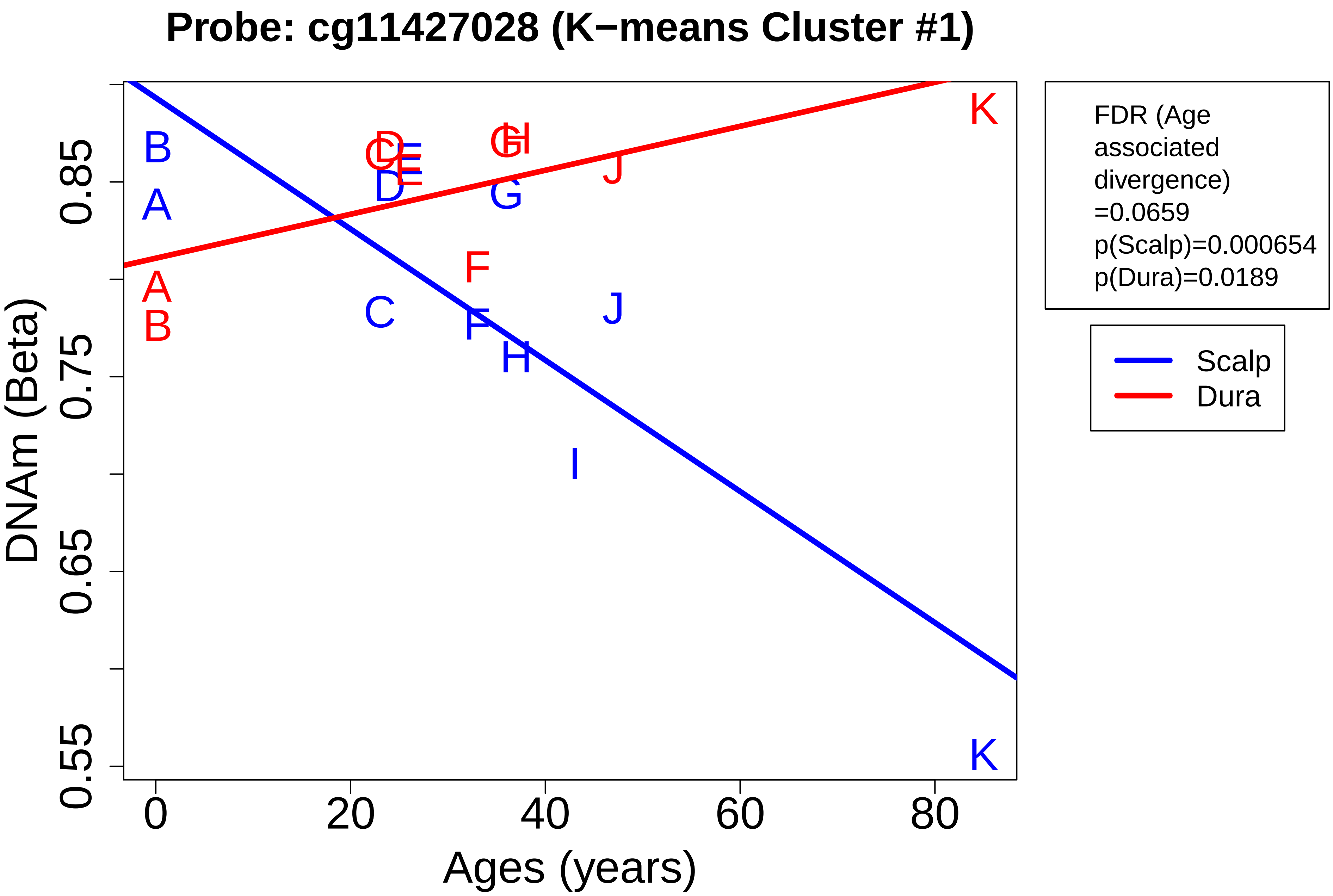

Probe: cg11427028 (K−means Cluster #1)
DNAm (Beta)
0.55
0.65
0.75
0.85
Ages (years)
0
20
40
60
80
FDR (Age associated divergence) =0.0659
p(Scalp)=0.000654
p(Dura)=0.0189
Scalp
Dura
A
B
C
D
E
F
G
H
I
J
K

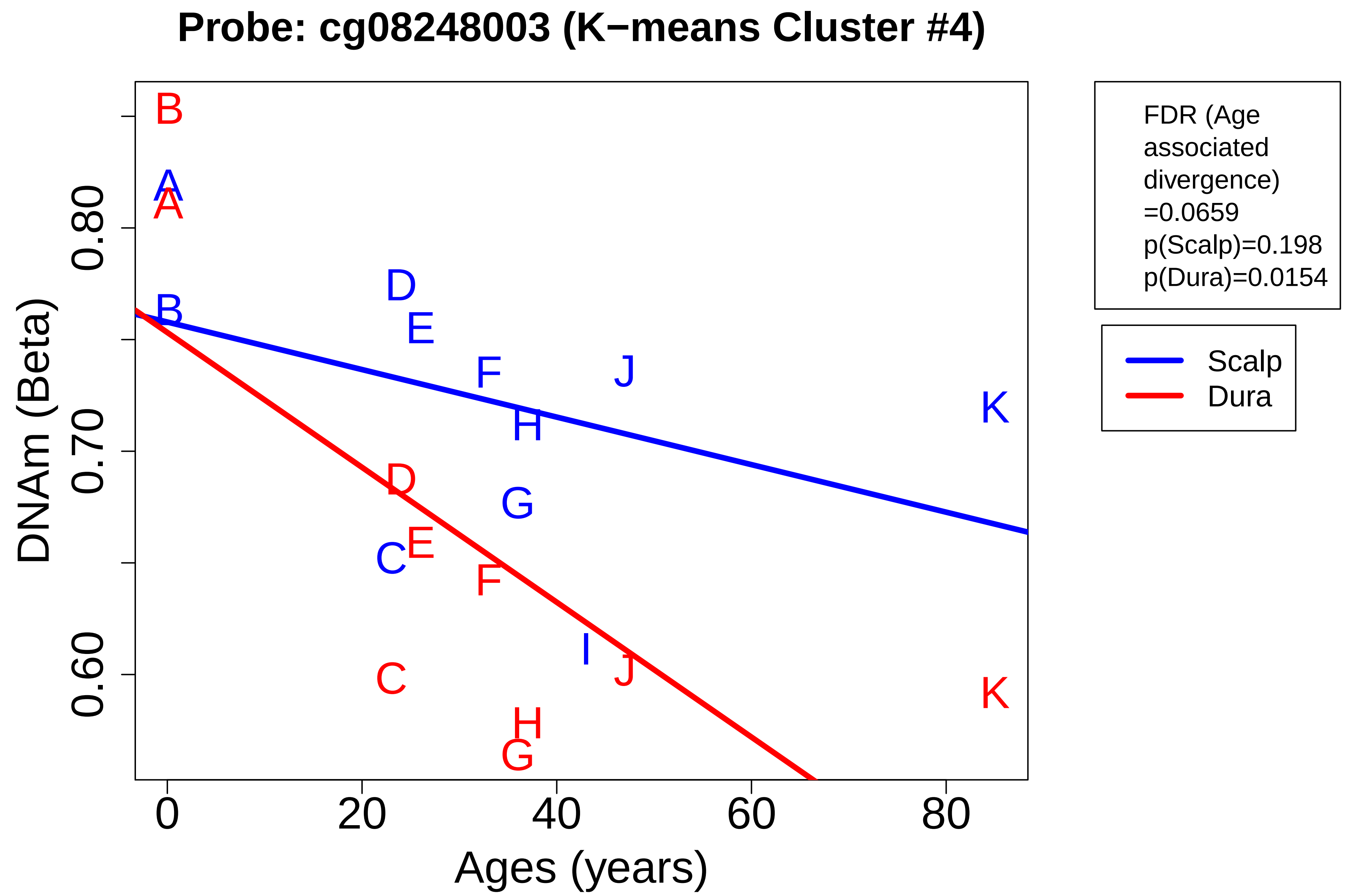
Probe: cg08248003 (K−means Cluster #4)
DNAm (Beta)
0.60
0.70
0.80
Ages (years)
0
20
40
60
80
A
B
C
D
E
F
G
H
I
J
K
FDR (Age associated divergence) =0.0659
p(Scalp)=0.198
p(Dura)=0.0154
Scalp
Dura

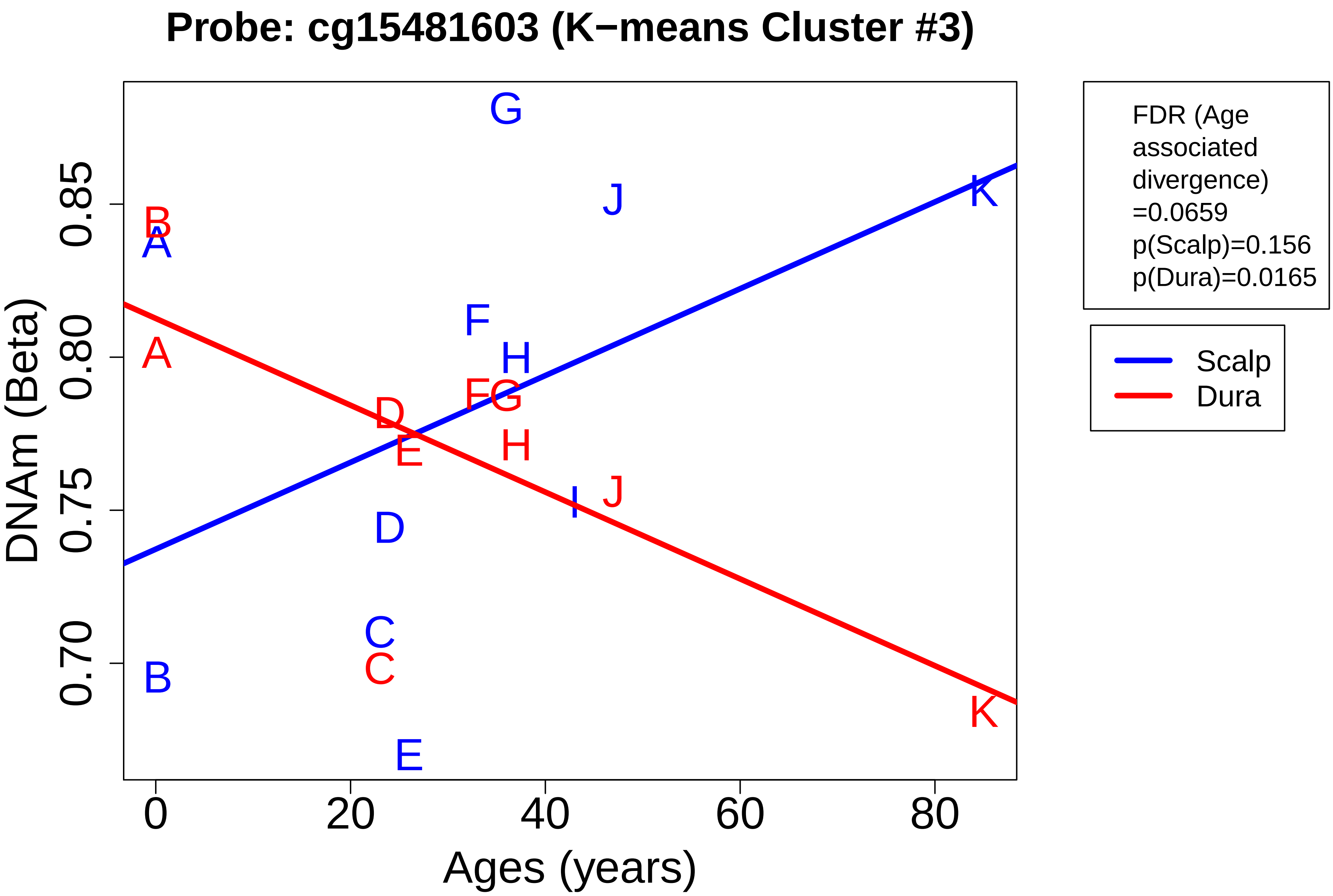

Probe: cg15481603 (K−means Cluster #3)
FDR (Age associated divergence) =0.0659
p(Scalp)=0.156
p(Dura)=0.0165
Scalp
Dura
DNAm (Beta)
0.70
0.75
0.80
0.85
0
20
40
60
80
Ages (years)

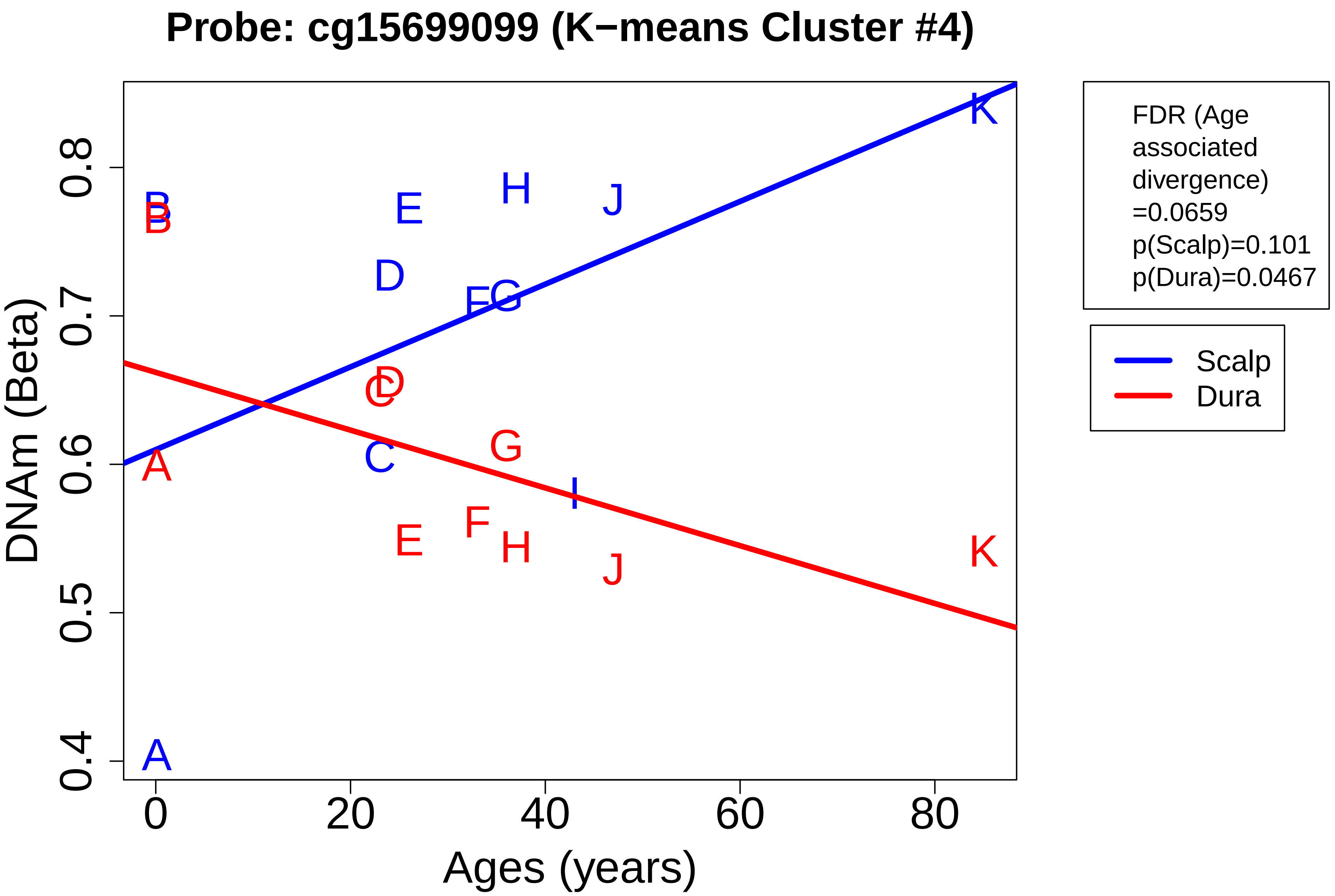

Probe: cg15699099 (K−means Cluster #4)
DNAm (Beta)
0.4
0.5
0.6
0.7
0.8
0
20
40
60
80
Ages (years)
FDR (Age associated divergence) =0.0659
p(Scalp)=0.101
p(Dura)=0.0467
Scalp
Dura
A
B
C
D
E
F
G
H
I
J
K

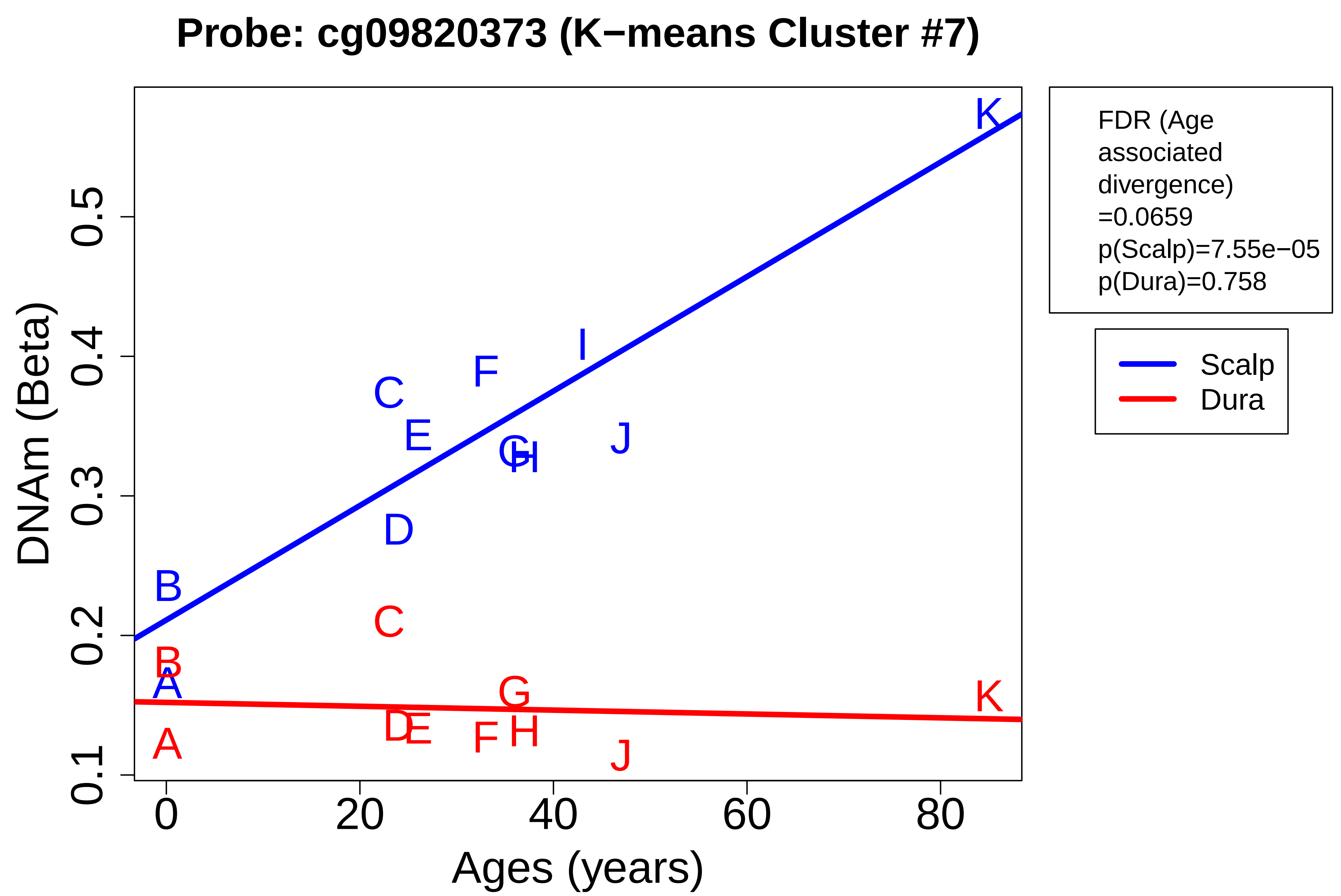

Probe: cg09820373 (K−means Cluster #7)
DNAm (Beta)
0.1
0.2
0.3
0.4
0.5
0
20
40
60
80
Ages (years)
FDR (Age associated divergence) =0.0659
p(Scalp)=7.55e−05
p(Dura)=0.758
Scalp
Dura
A
B
C
D
E
F
G
H
I
J
K

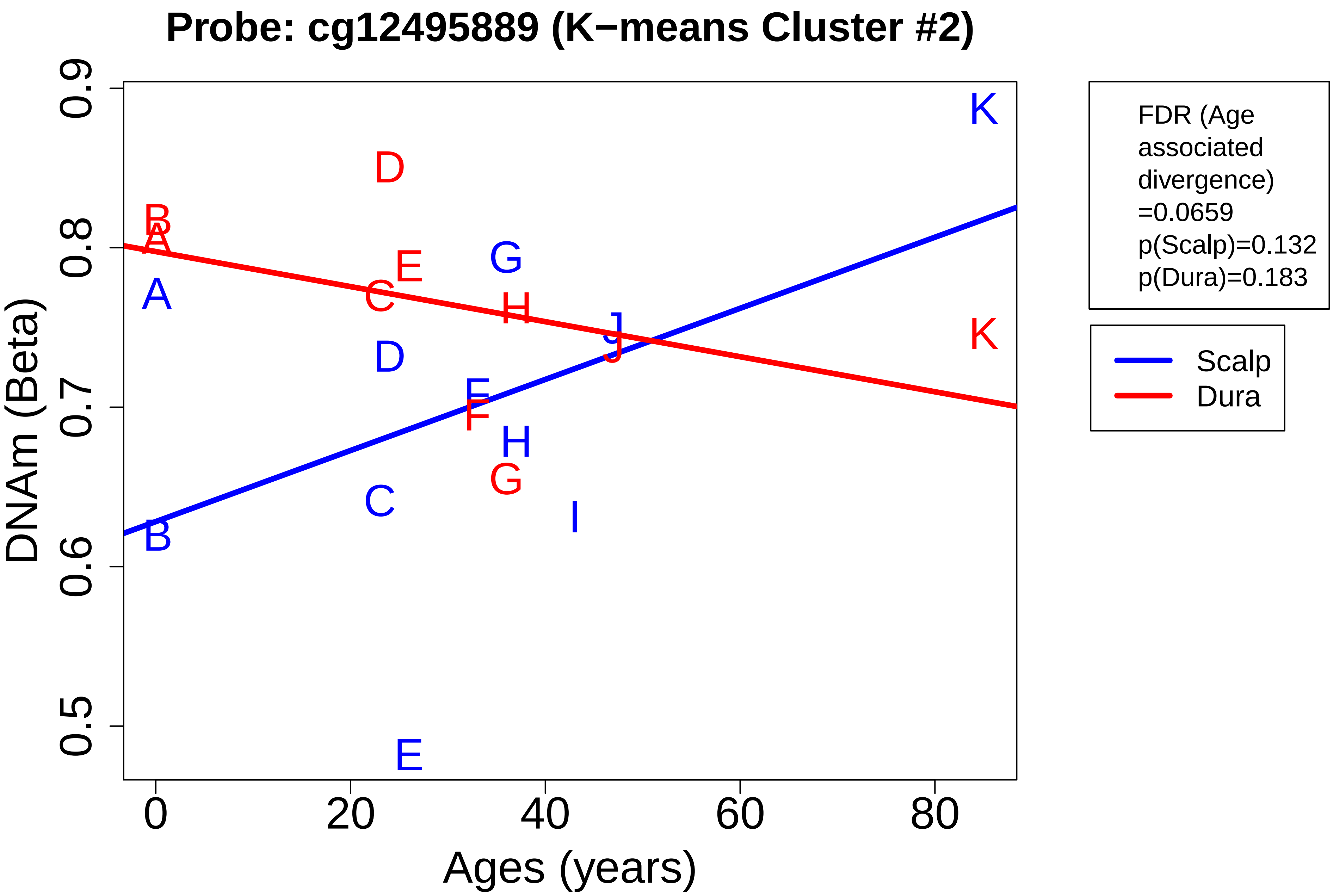

Probe: cg12495889 (K−means Cluster #2)
DNAm (Beta)
0.5
0.6
0.7
0.8
0.9
Ages (years)
0
20
40
60
80
FDR (Age associated divergence) =0.0659
p(Scalp)=0.132
p(Dura)=0.183
Scalp
Dura

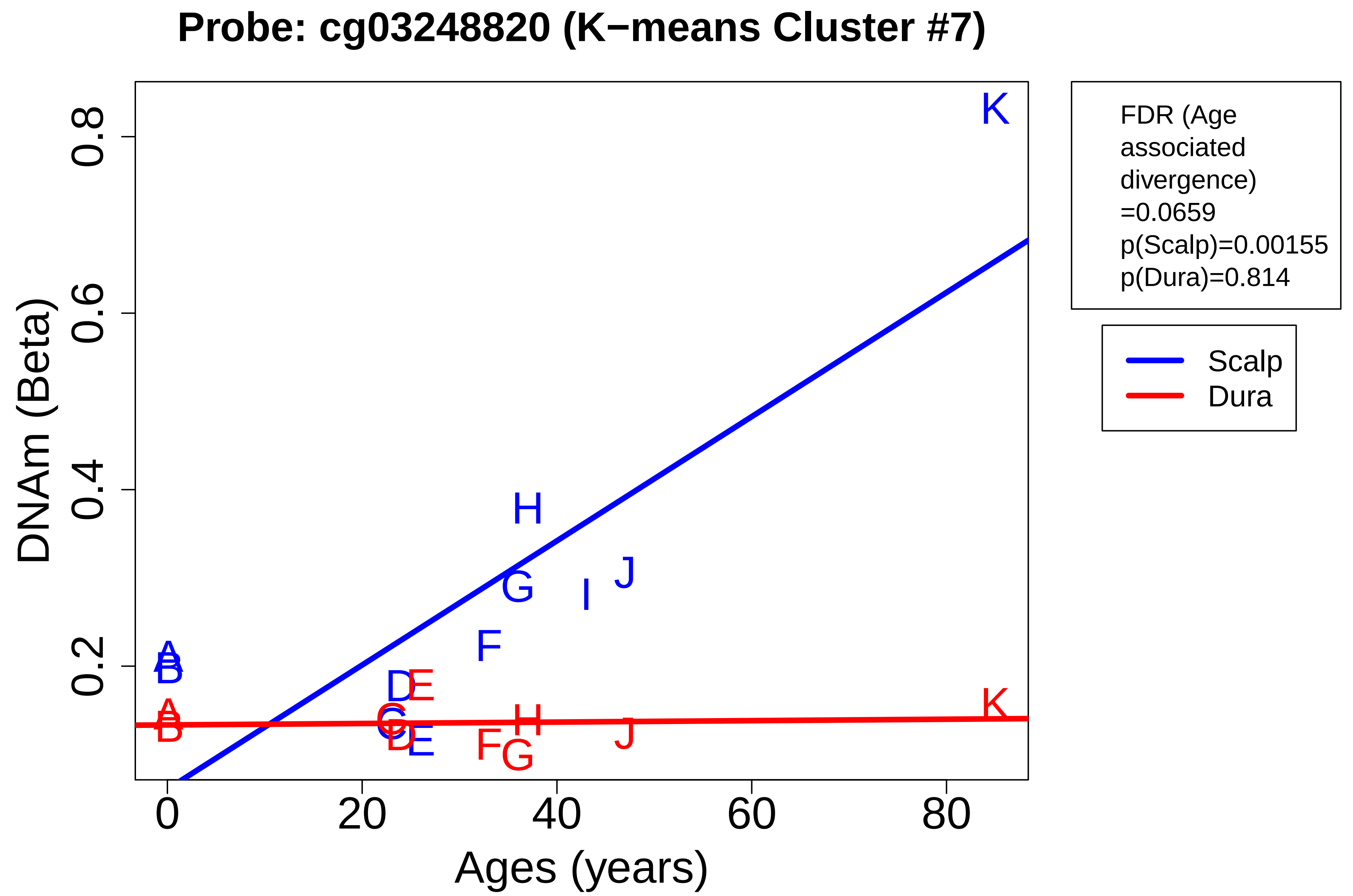
Probe: cg03248820 (K−means Cluster #7)
DNAm (Beta)
0.2
0.4
0.6
0.8
Ages (years)
0
20
40
60
80
FDR (Age associated divergence) =0.0659
p(Scalp)=0.00155
p(Dura)=0.814
Scalp
Dura
A
B
C
D
E
F
G
H
I
J
K

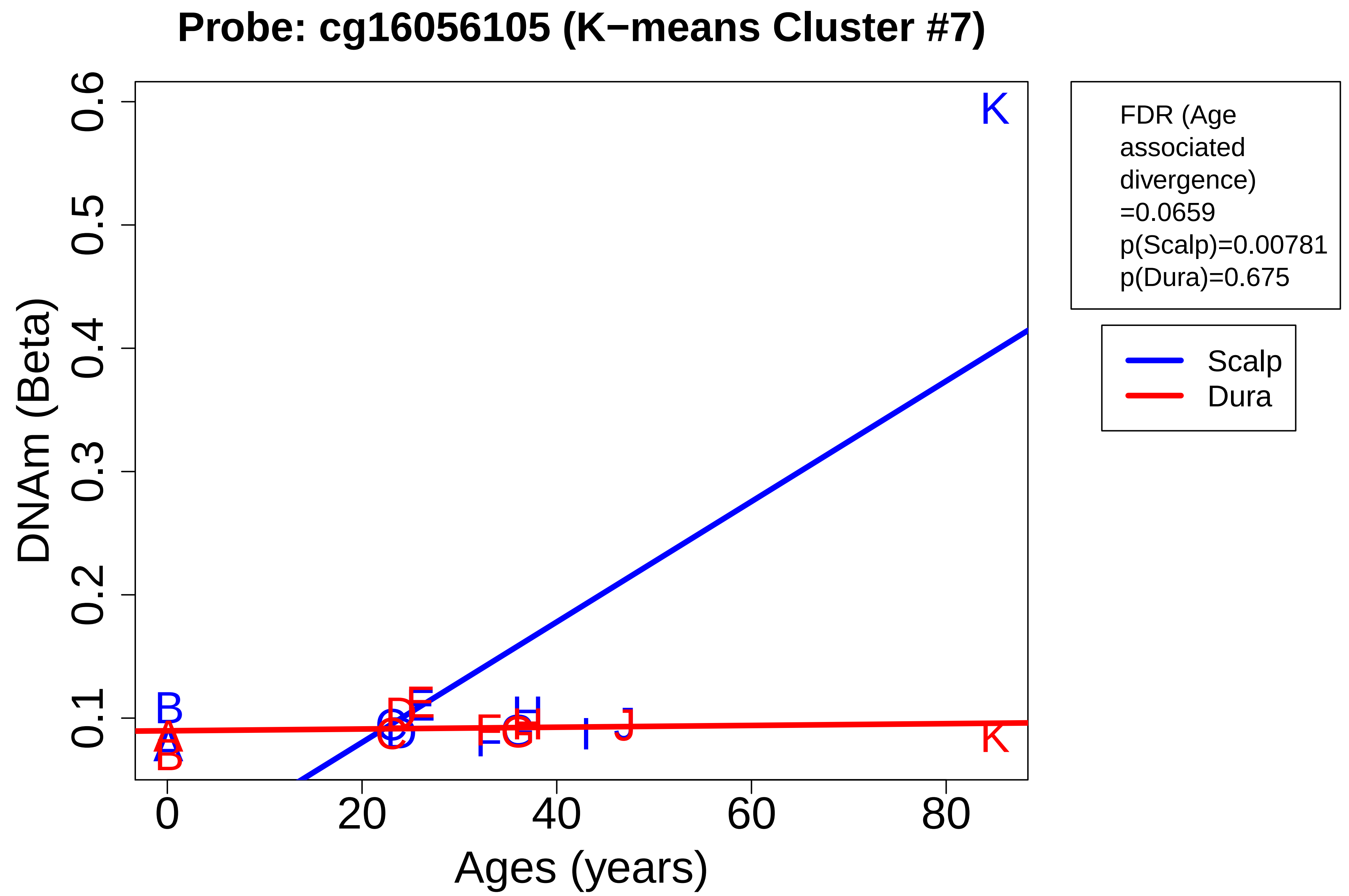

Probe: cg16056105 (K−means Cluster #7)
FDR (Age associated divergence) =0.0659
p(Scalp)=0.00781
p(Dura)=0.675
Scalp
Dura
DNAm (Beta)
Ages (years)
0.1
0.2
0.3
0.4
0.5
0.6
0
20
40
60
80

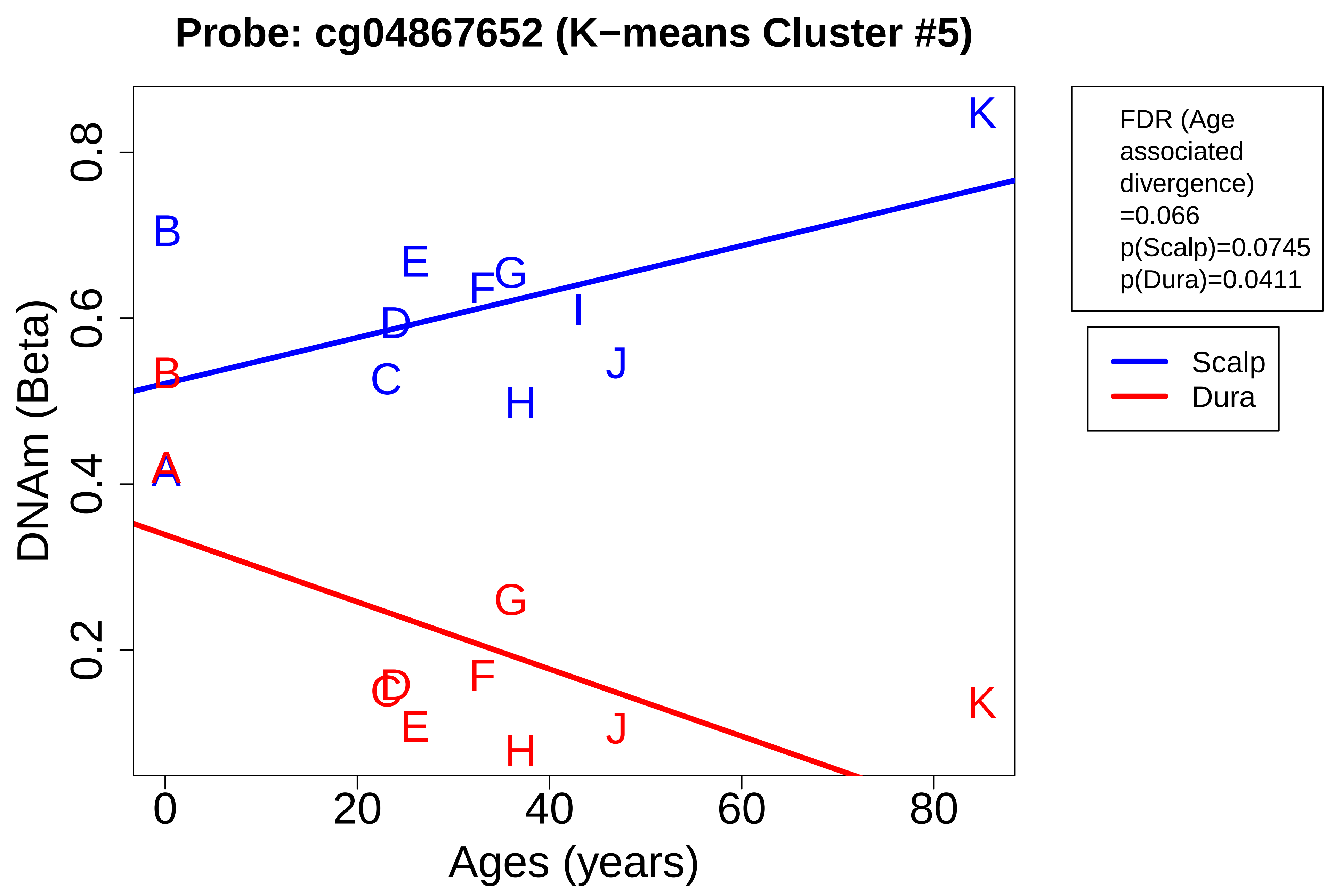
Probe: cg04867652 (K−means Cluster #5)
DNAm (Beta)
0.2
0.4
0.6
0.8
0
20
40
60
80
Ages (years)
A
B
C
D
E
F
G
H
I
J
K
FDR (Age associated divergence) =0.066
p(Scalp)=0.0745
p(Dura)=0.0411
Scalp
Dura

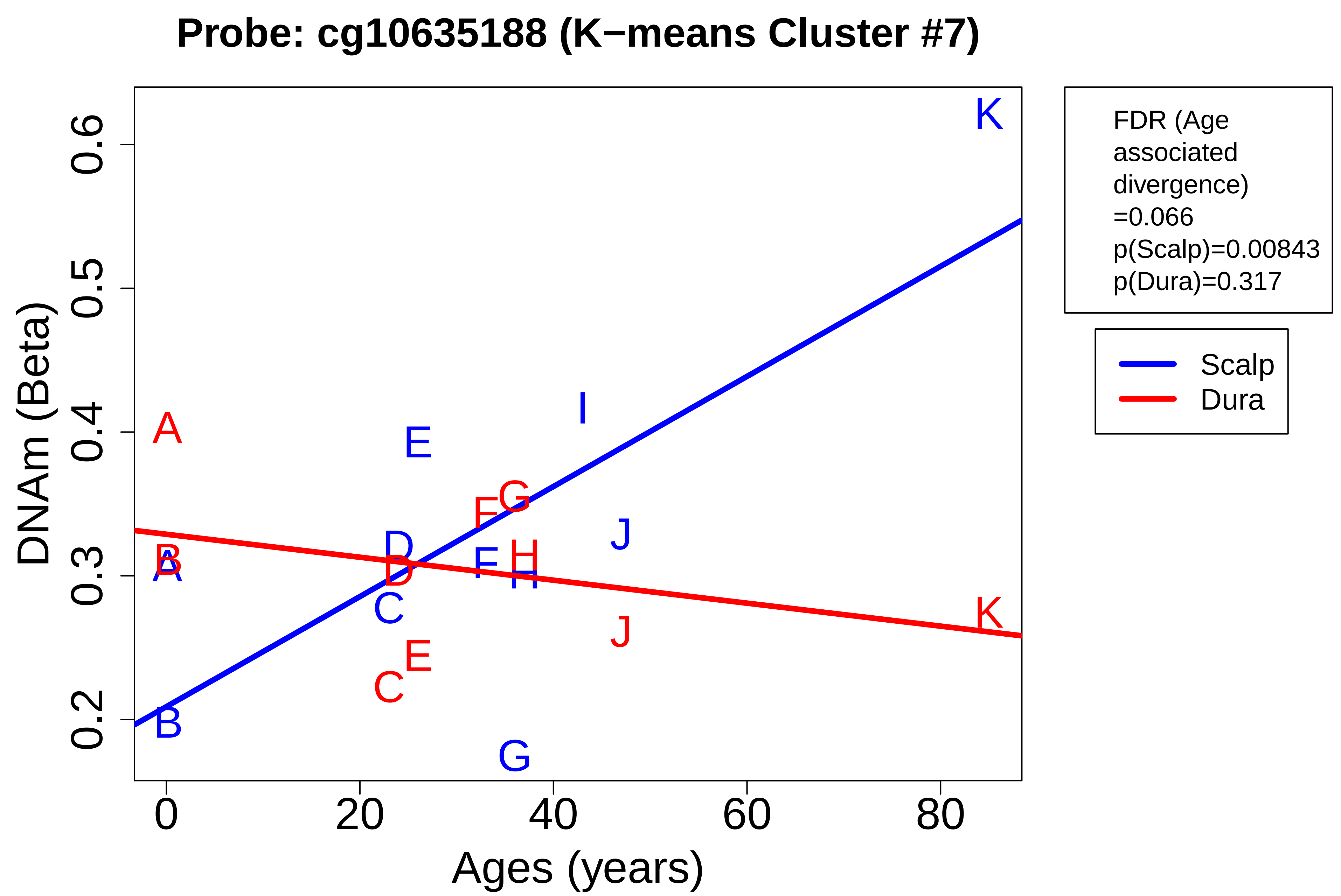

Probe: cg10635188 (K−means Cluster #7)
DNAm (Beta)
0.2
0.3
0.4
0.5
0.6
Ages (years)
0
20
40
60
80
A
B
C
D
E
F
G
H
I
J
K
FDR (Age associated divergence) =0.066
p(Scalp)=0.00843
p(Dura)=0.317
Scalp
Dura

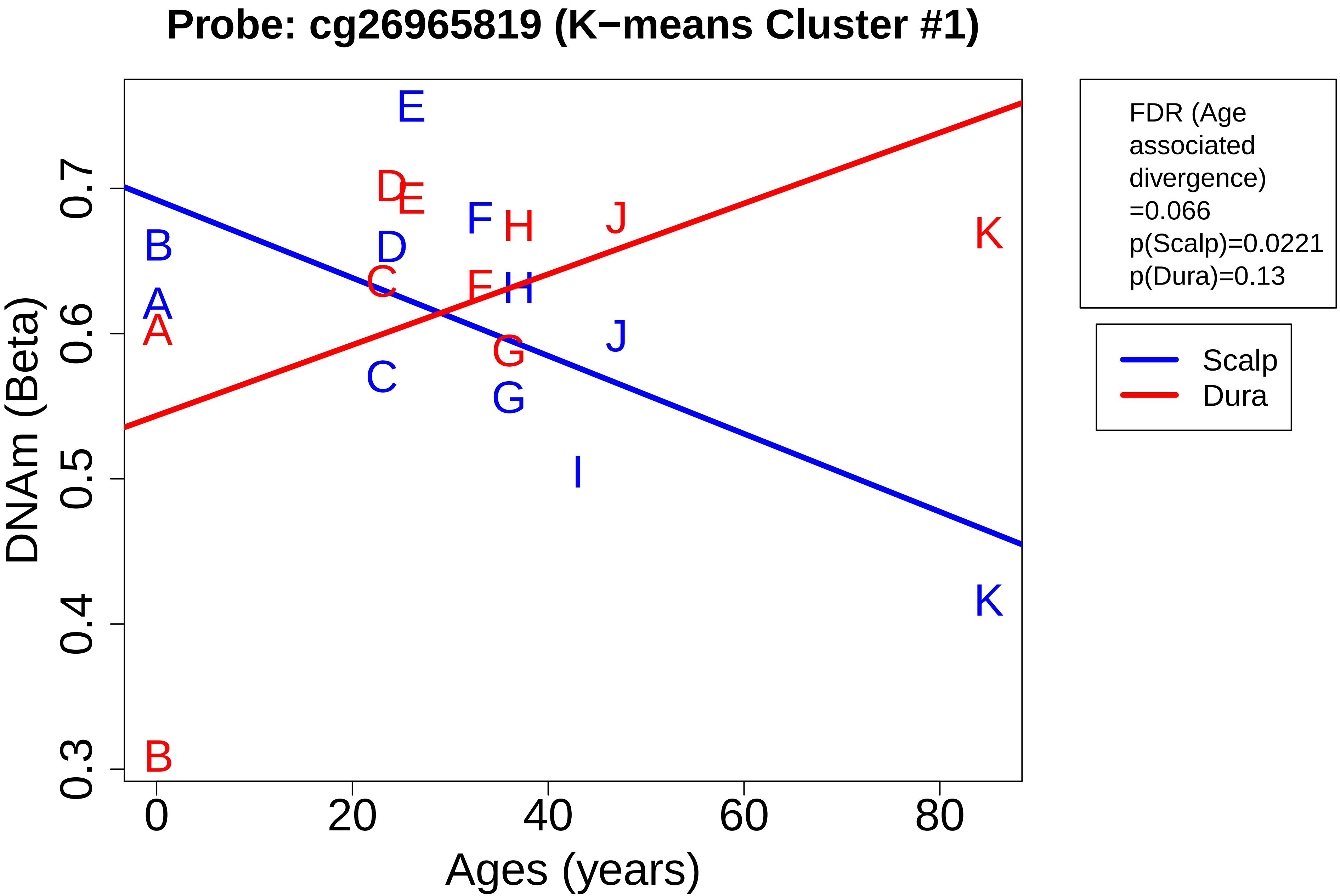

Probe: cg26965819 (K−means Cluster #1)
DNAm (Beta)
0.3
0.4
0.5
0.6
0.7
0
20
40
60
80
Ages (years)
A
B
C
D
E
F
G
H
I
J
K
FDR (Age associated divergence) =0.066
p(Scalp)=0.0221
p(Dura)=0.13
Scalp
Dura

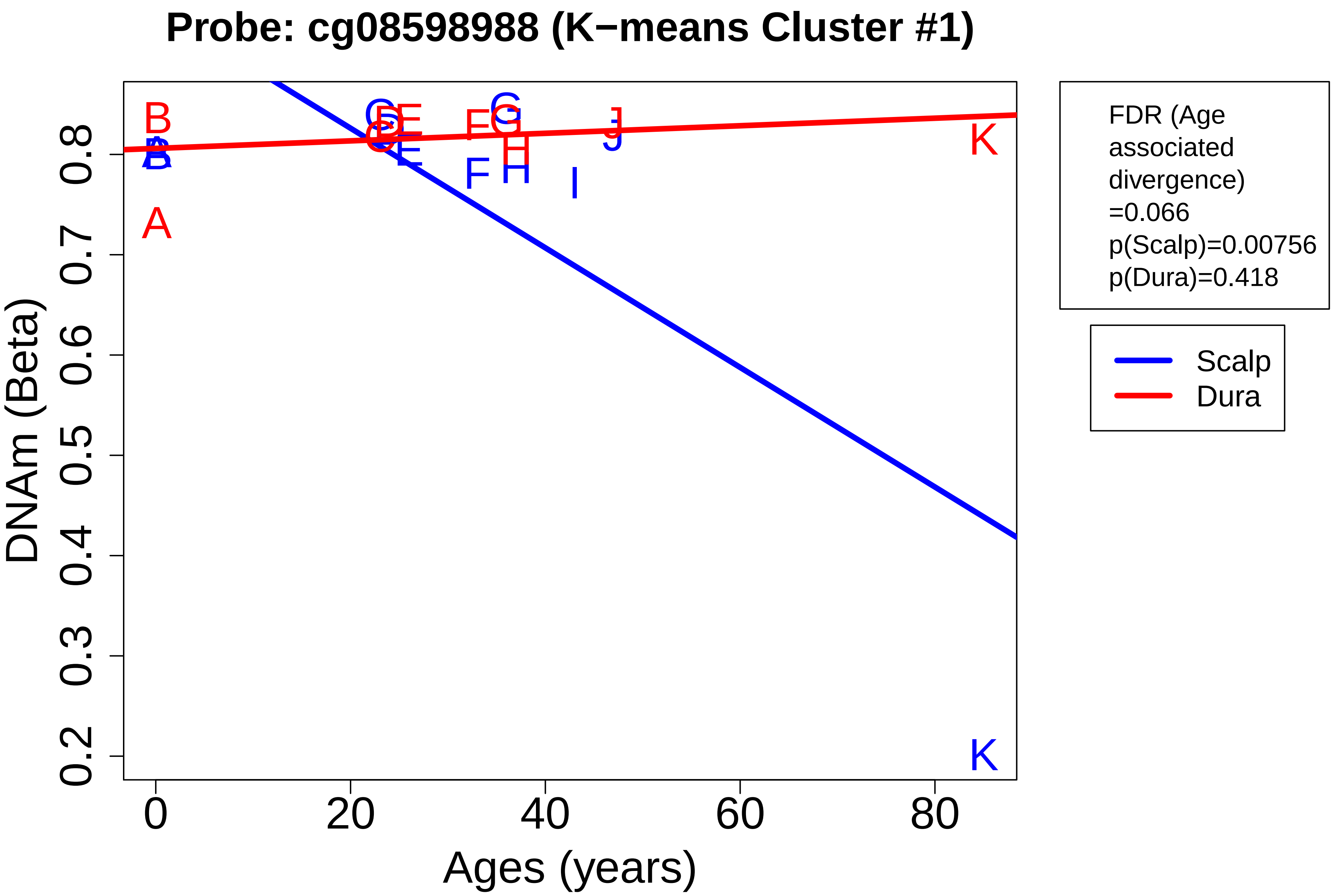
Probe: cg08598988 (K−means Cluster #1)
DNAm (Beta)
Ages (years)
0.2
0.3
0.4
0.5
0.6
0.7
0.8
0
20
40
60
80
FDR (Age associated divergence) =0.066
p(Scalp)=0.00756
p(Dura)=0.418
Scalp
Dura

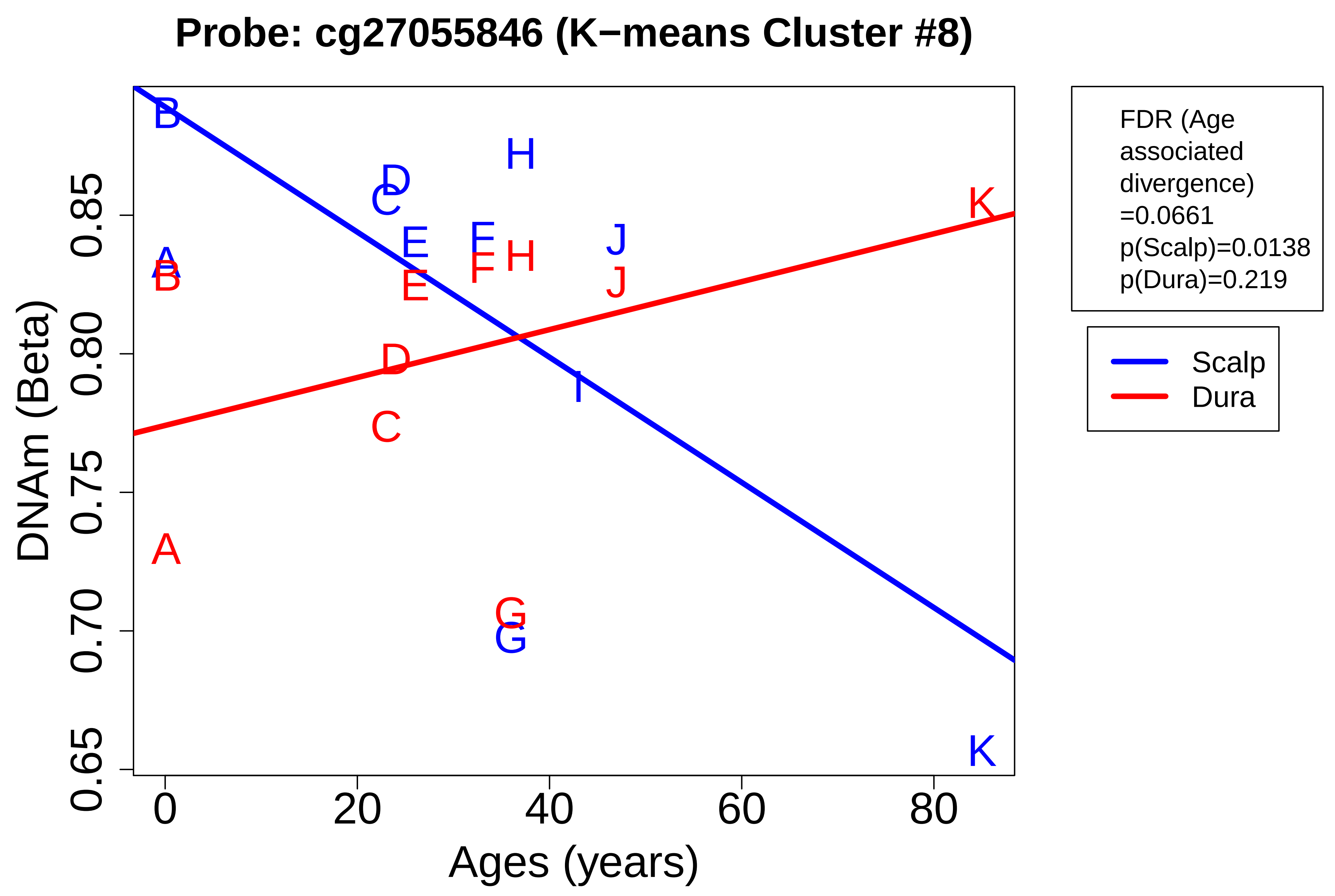

Probe: cg27055846 (K−means Cluster #8)
FDR (Age associated divergence) =0.0661
p(Scalp)=0.0138
p(Dura)=0.219
Scalp
Dura
DNAm (Beta)
0.65
0.70
0.75
0.80
0.85
0
20
40
60
80
Ages (years)
A
B
C
D
E
F
G
H
I
J
K

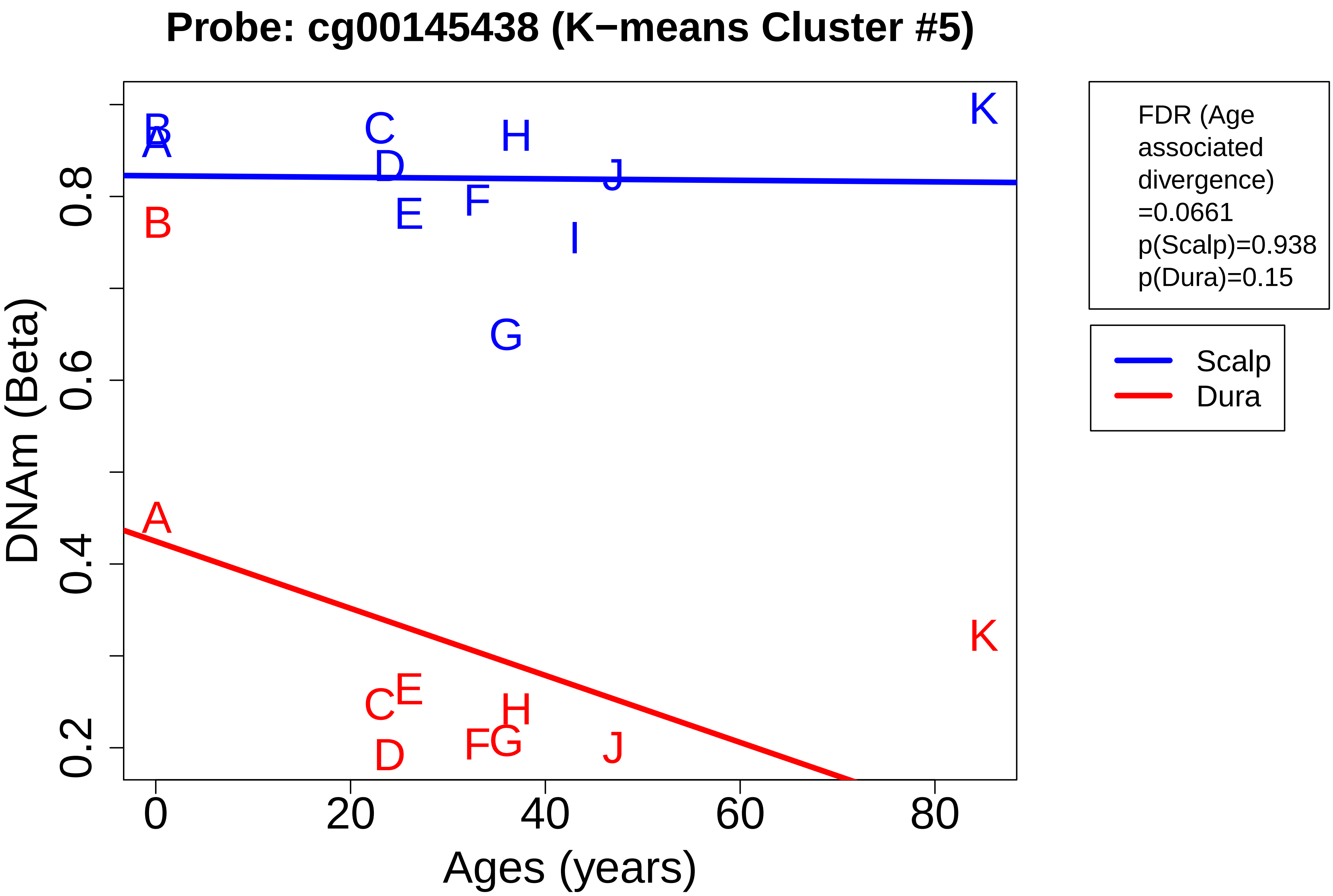

Probe: cg00145438 (K−means Cluster #5)
DNAm (Beta)
0.2
0.4
0.6
0.8
0
20
40
60
80
Ages (years)
A
B
C
D
E
F
G
H
I
J
K
FDR (Age associated divergence) =0.0661
p(Scalp)=0.938
p(Dura)=0.15
Scalp
Dura

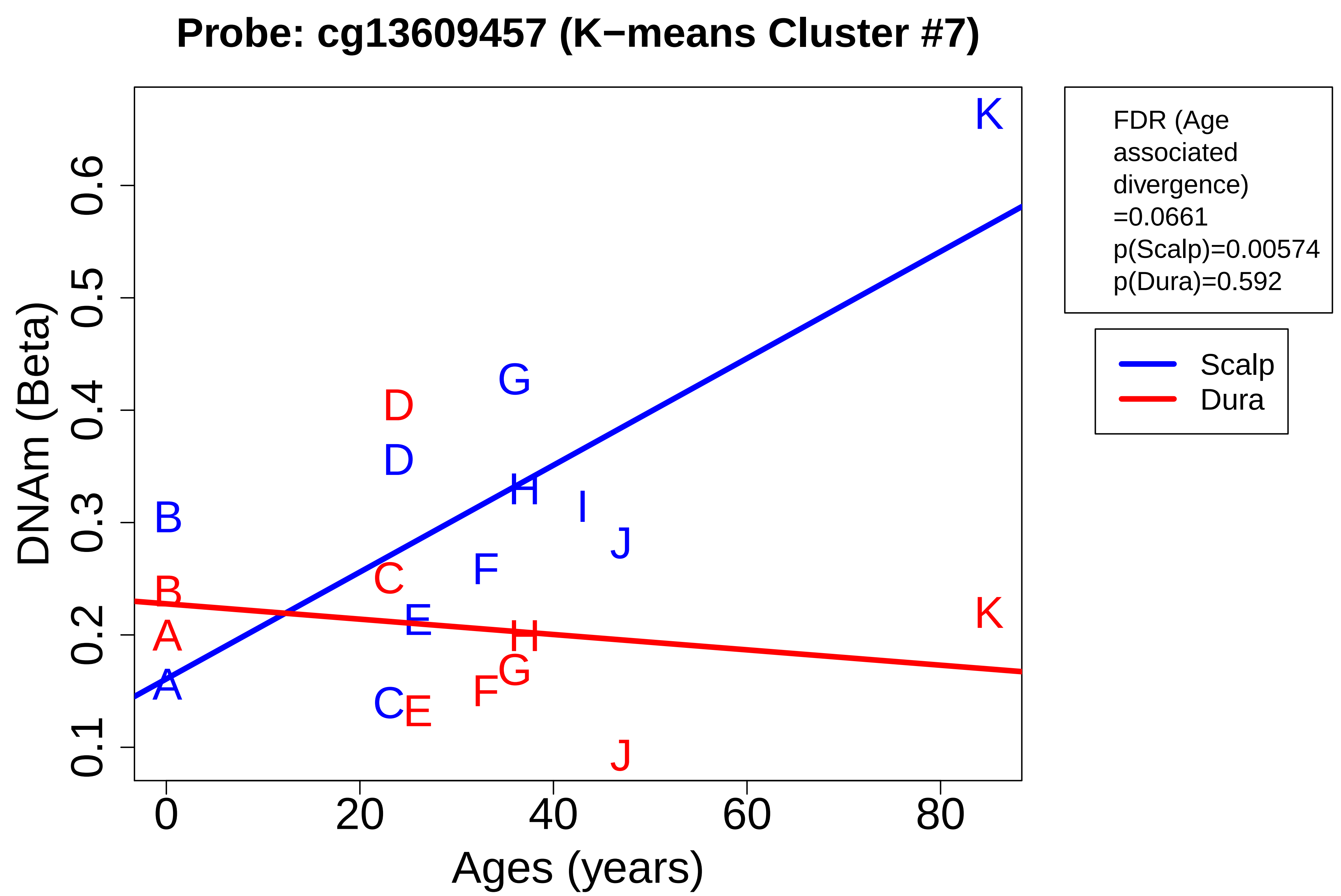

Probe: cg13609457 (K−means Cluster #7)
DNAm (Beta)
0.1
0.2
0.3
0.4
0.5
0.6
0
20
40
60
80
Ages (years)
FDR (Age associated divergence) =0.0661
p(Scalp)=0.00574
p(Dura)=0.592
Scalp
Dura

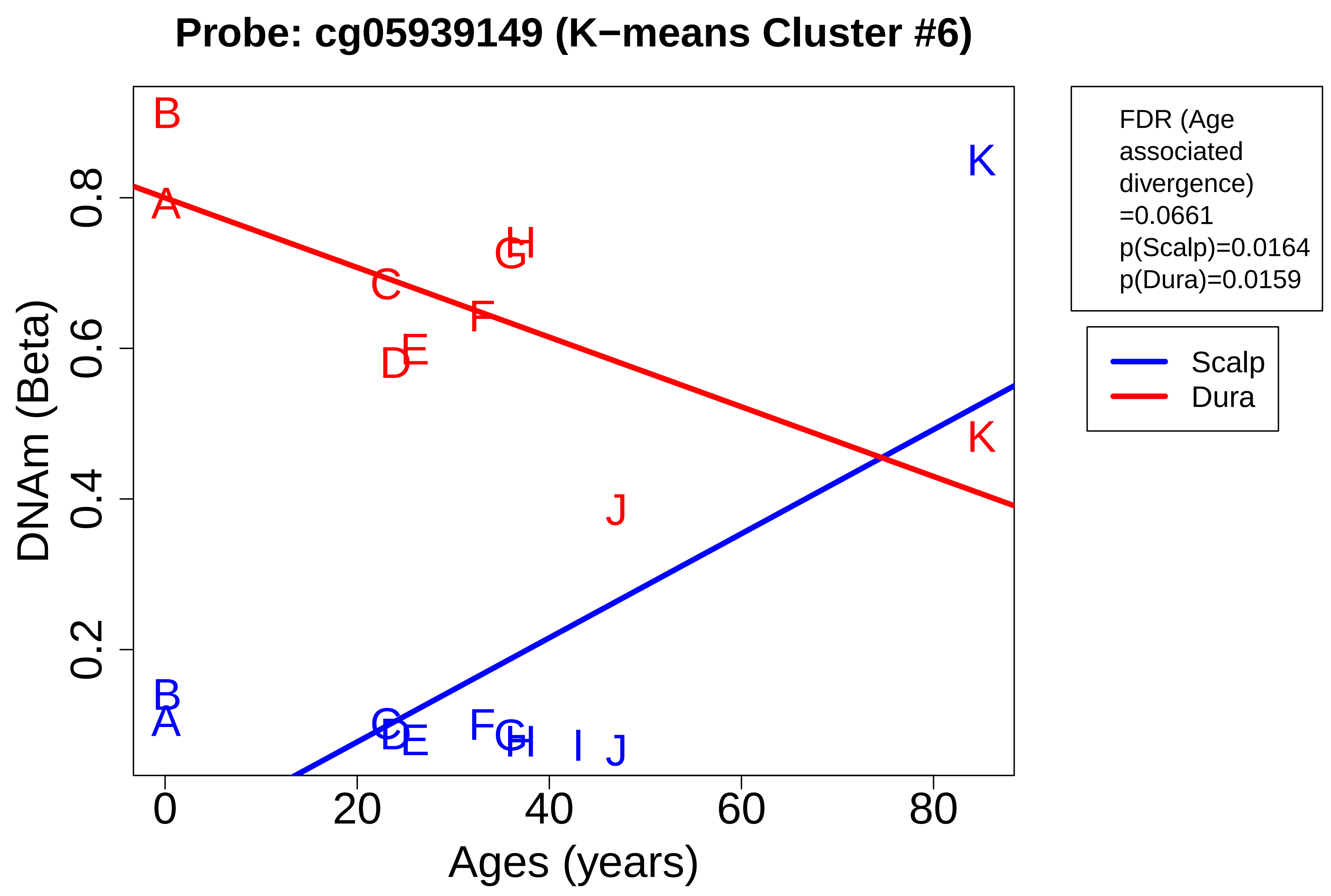

Probe: cg05939149 (K−means Cluster #6)
DNAm (Beta)
0.2
0.4
0.6
0.8
0
20
40
60
80
Ages (years)
FDR (Age associated divergence) =0.0661
p(Scalp)=0.0164
p(Dura)=0.0159
Scalp
Dura
A
B
C
D
E
F
G
H
I
J
K

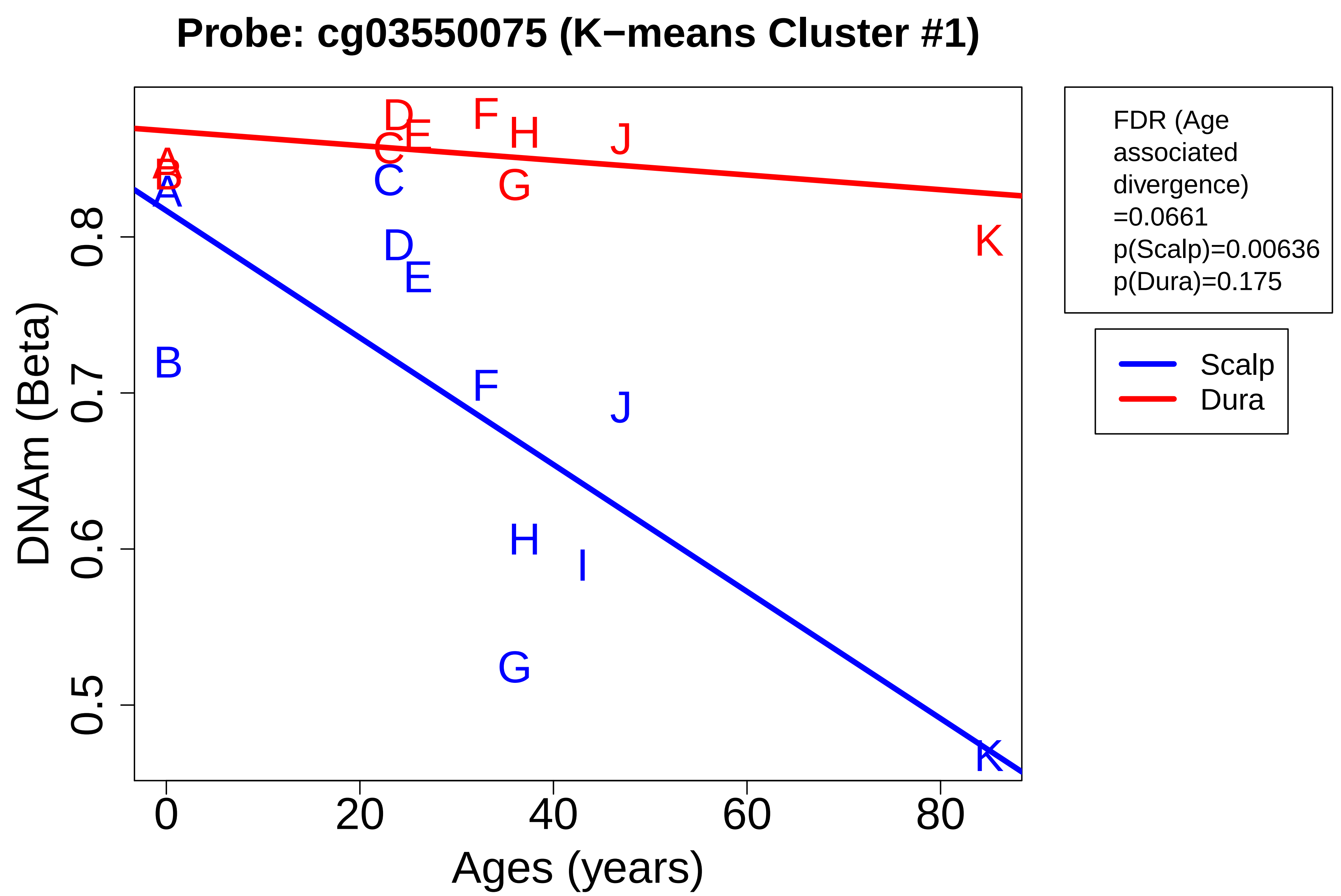

Probe: cg03550075 (K−means Cluster #1)
DNAm (Beta)
0.5
0.6
0.7
0.8
0
20
40
60
80
Ages (years)
A
B
C
D
E
F
G
H
I
J
K
FDR (Age associated divergence) =0.0661
p(Scalp)=0.00636
p(Dura)=0.175
Scalp
Dura

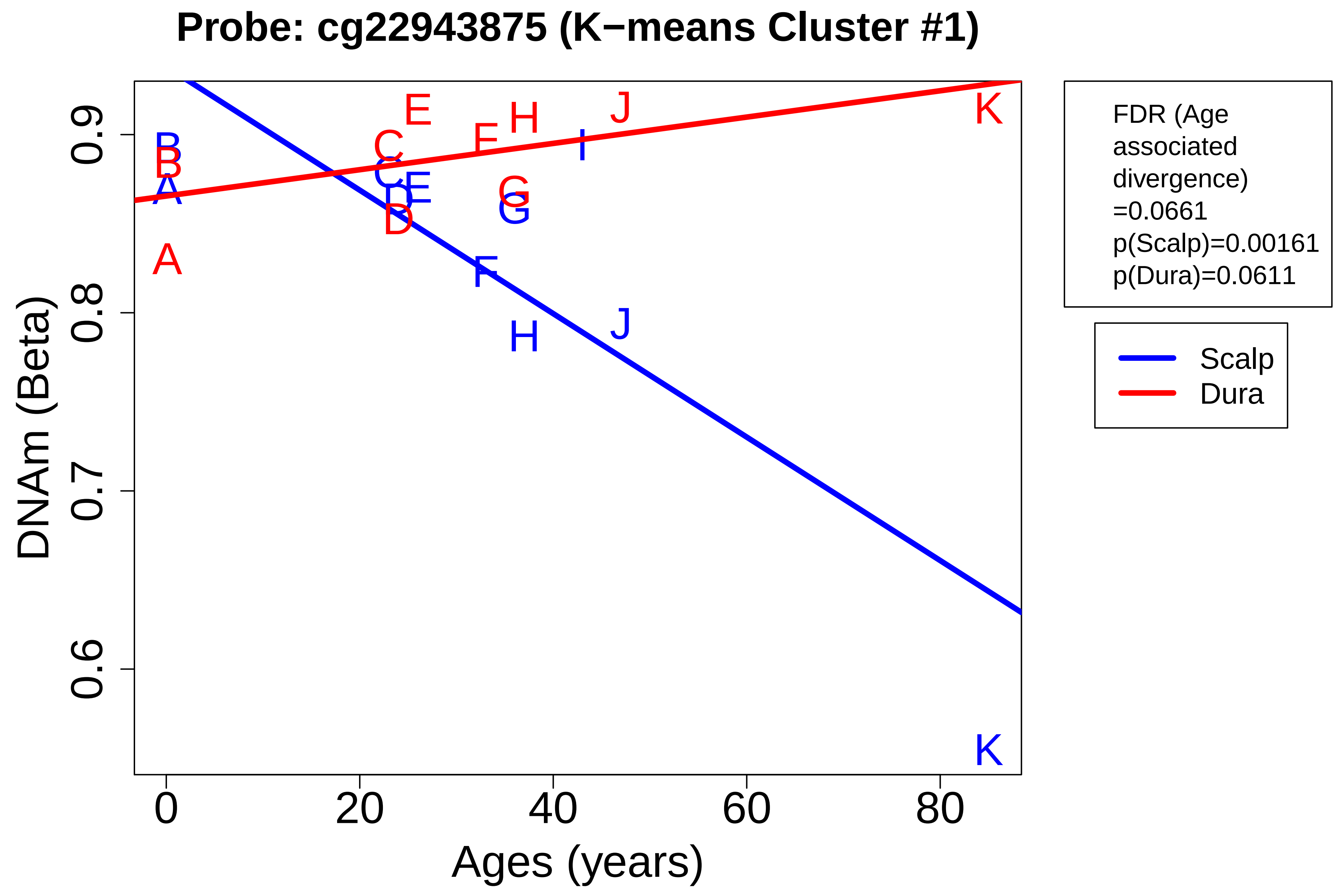

Probe: cg22943875 (K−means Cluster #1)
DNAm (Beta)
0.6
0.7
0.8
0.9
Ages (years)
0
20
40
60
80
A
B
C
D
E
F
G
H
I
J
K
A
B
C
D
E
F
G
H
J
K
FDR (Age associated divergence) =0.0661
p(Scalp)=0.00161
p(Dura)=0.0611
Scalp
Dura

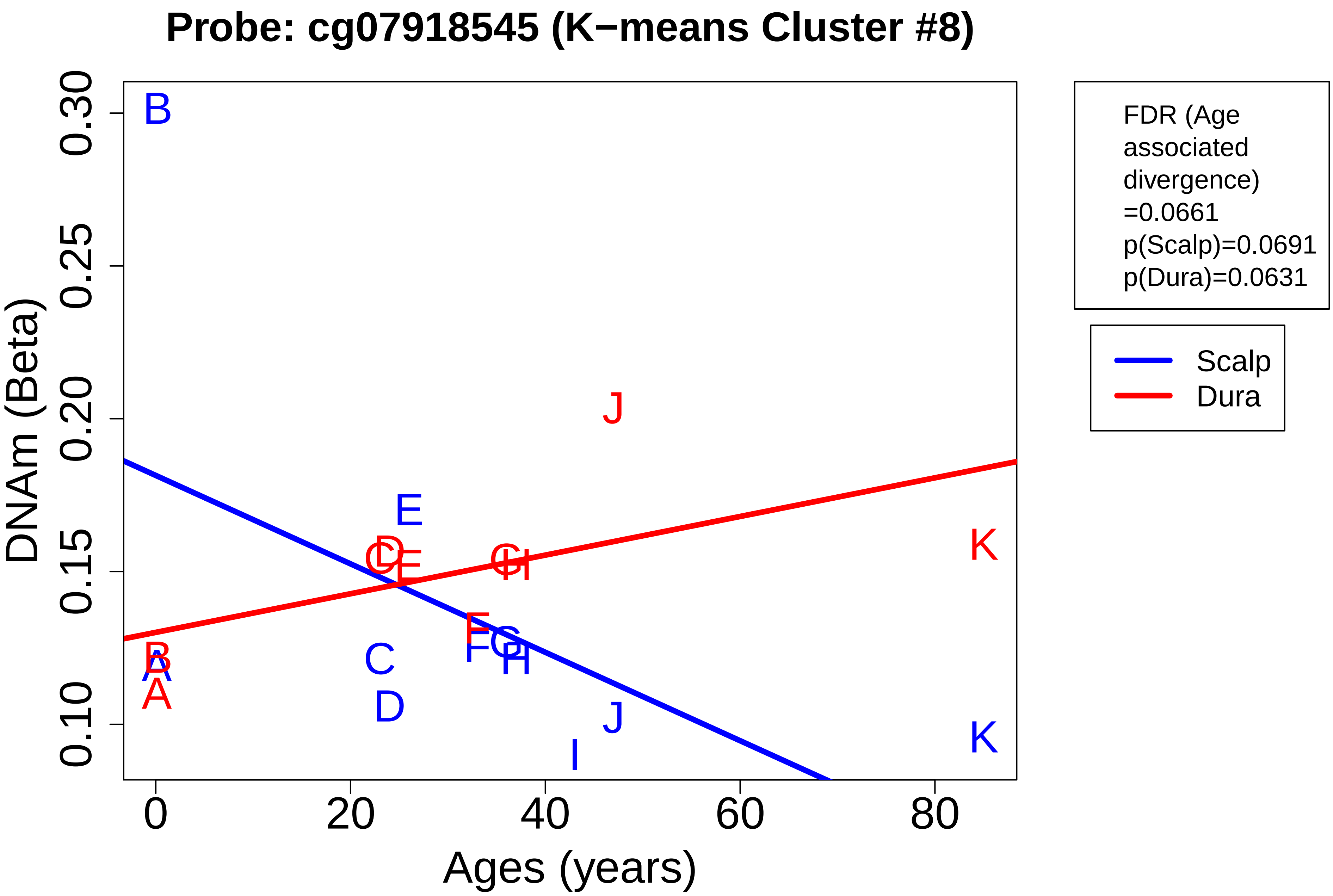
Probe: cg07918545 (K−means Cluster #8)
DNAm (Beta)
0.10
0.15
0.20
0.25
0.30
0
20
40
60
80
Ages (years)
FDR (Age associated divergence) =0.0661
p(Scalp)=0.0691
p(Dura)=0.0631
Scalp
Dura
A
B
C
D
E
F
G
H
I
J
K

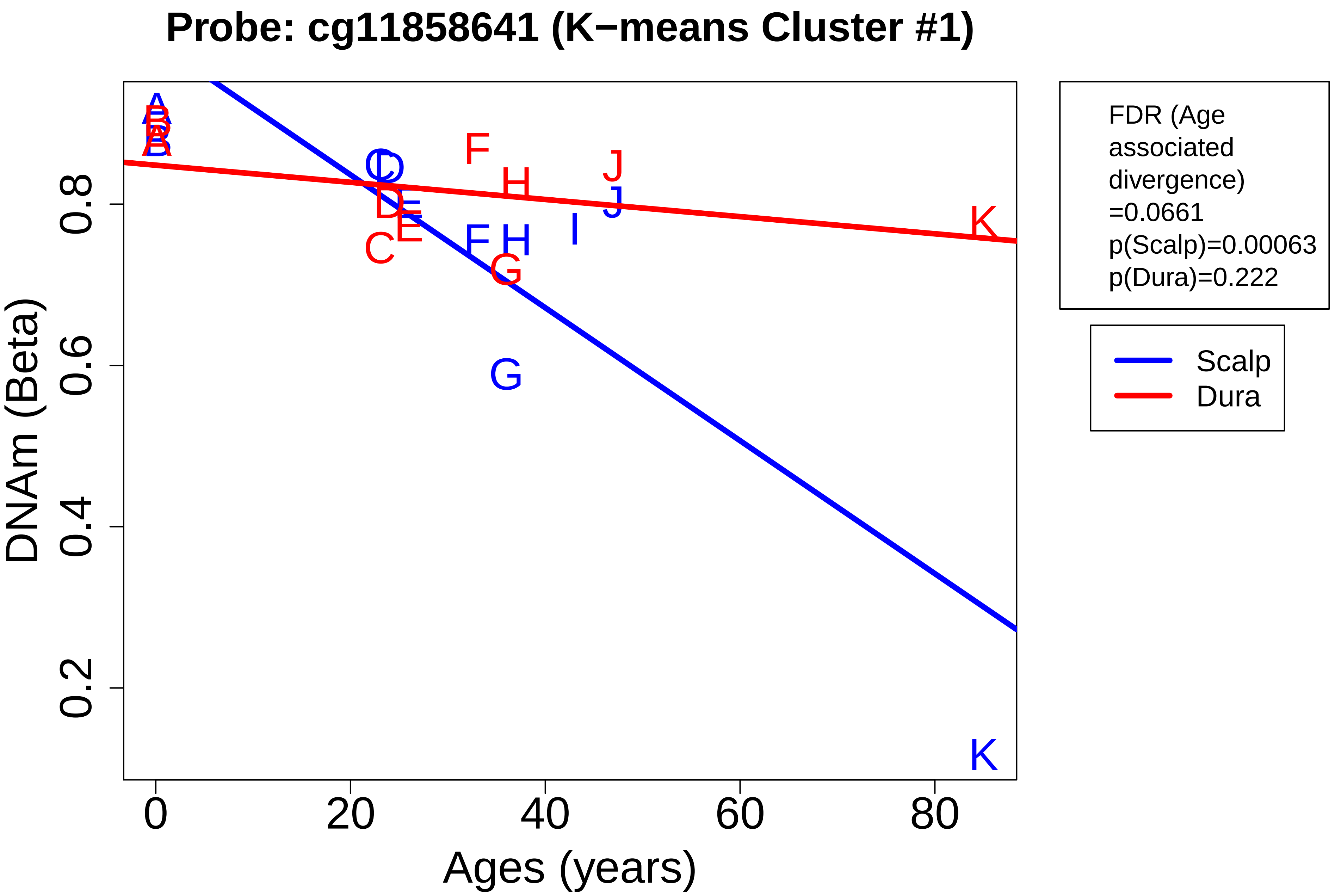

Probe: cg11858641 (K−means Cluster #1)
FDR (Age associated divergence) =0.0661
p(Scalp)=0.00063
p(Dura)=0.222
Scalp
Dura
DNAm (Beta)
0.2
0.4
0.6
0.8
0
20
40
60
80
Ages (years)

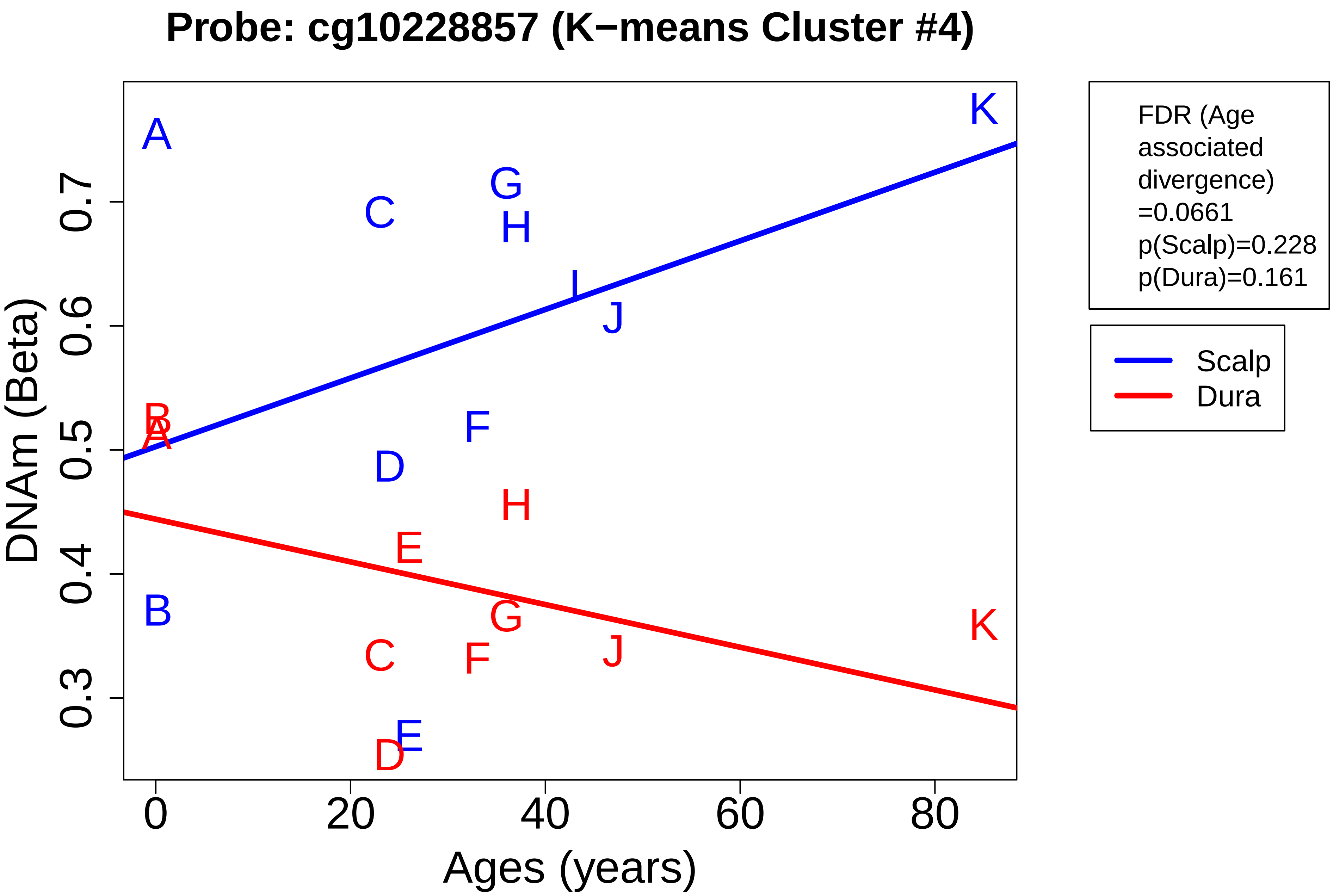
Probe: cg10228857 (K−means Cluster #4)
DNAm (Beta)
0.3
0.4
0.5
0.6
0.7
0
20
40
60
80
Ages (years)
FDR (Age associated divergence) =0.0661
p(Scalp)=0.228
p(Dura)=0.161
Scalp
Dura

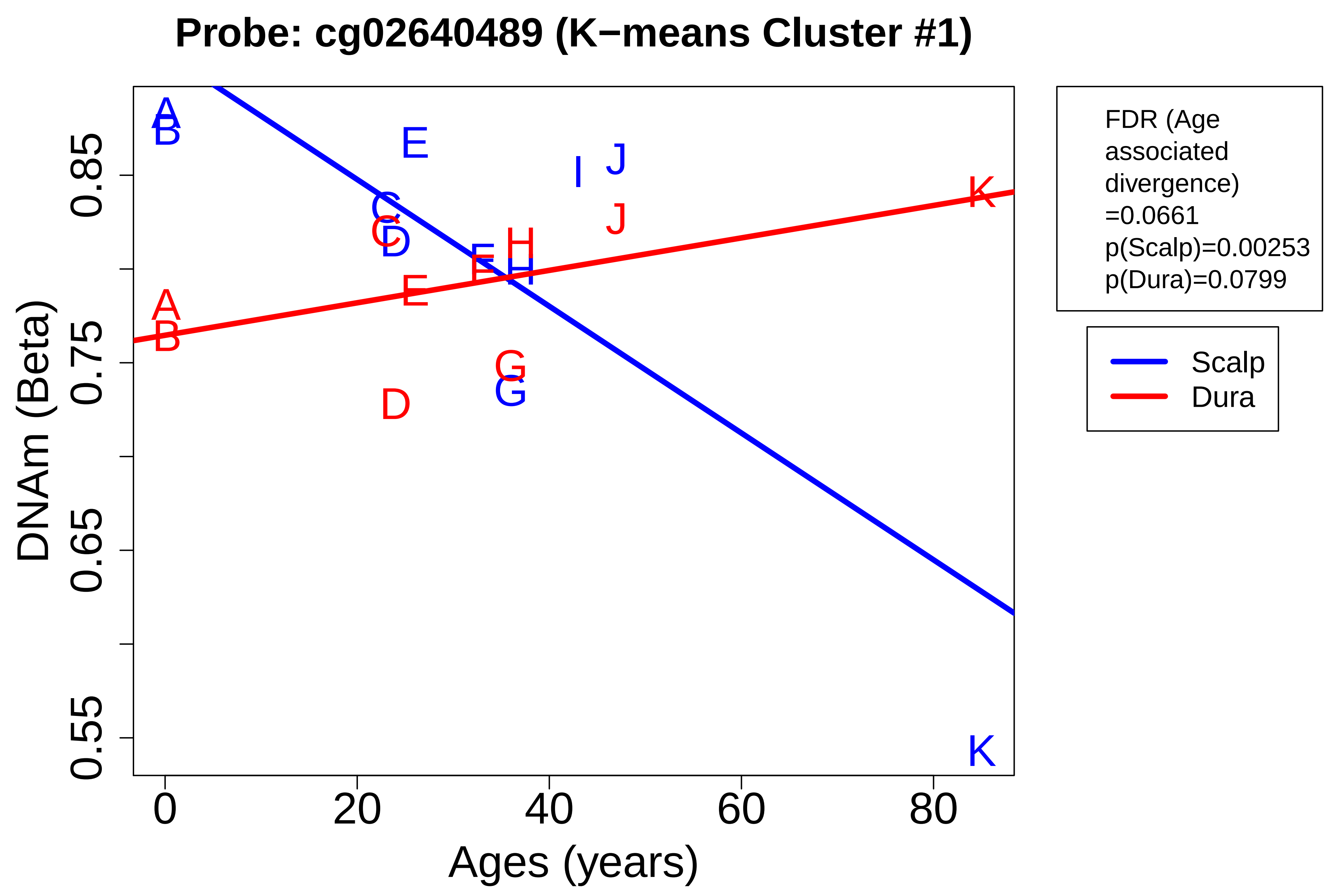
Probe: cg02640489 (K−means Cluster #1)
DNAm (Beta)
0.55
0.65
0.75
0.85
0
20
40
60
80
Ages (years)
A
B
C
D
E
F
G
H
I
J
K
FDR (Age associated divergence) =0.0661
p(Scalp)=0.00253
p(Dura)=0.0799
Scalp
Dura

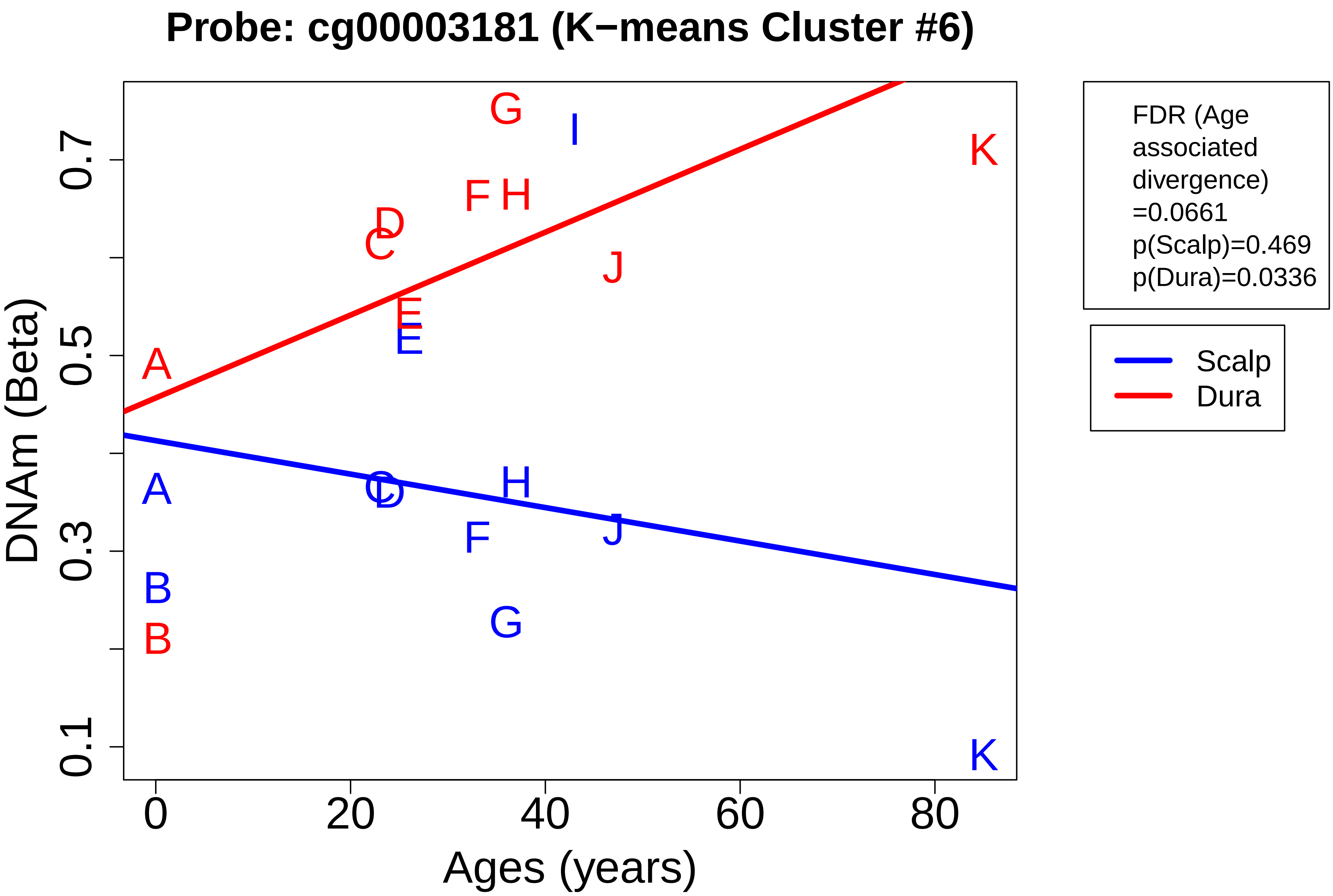
Probe: cg00003181 (K−means Cluster #6)
DNAm (Beta)
Ages (years)
0.1
0.3
0.5
0.7
0
20
40
60
80
A
B
C
D
E
F
G
H
I
J
K
FDR (Age associated divergence) =0.0661
p(Scalp)=0.469
p(Dura)=0.0336
Scalp
Dura

Probe: cg00399951 (K−means Cluster #4)

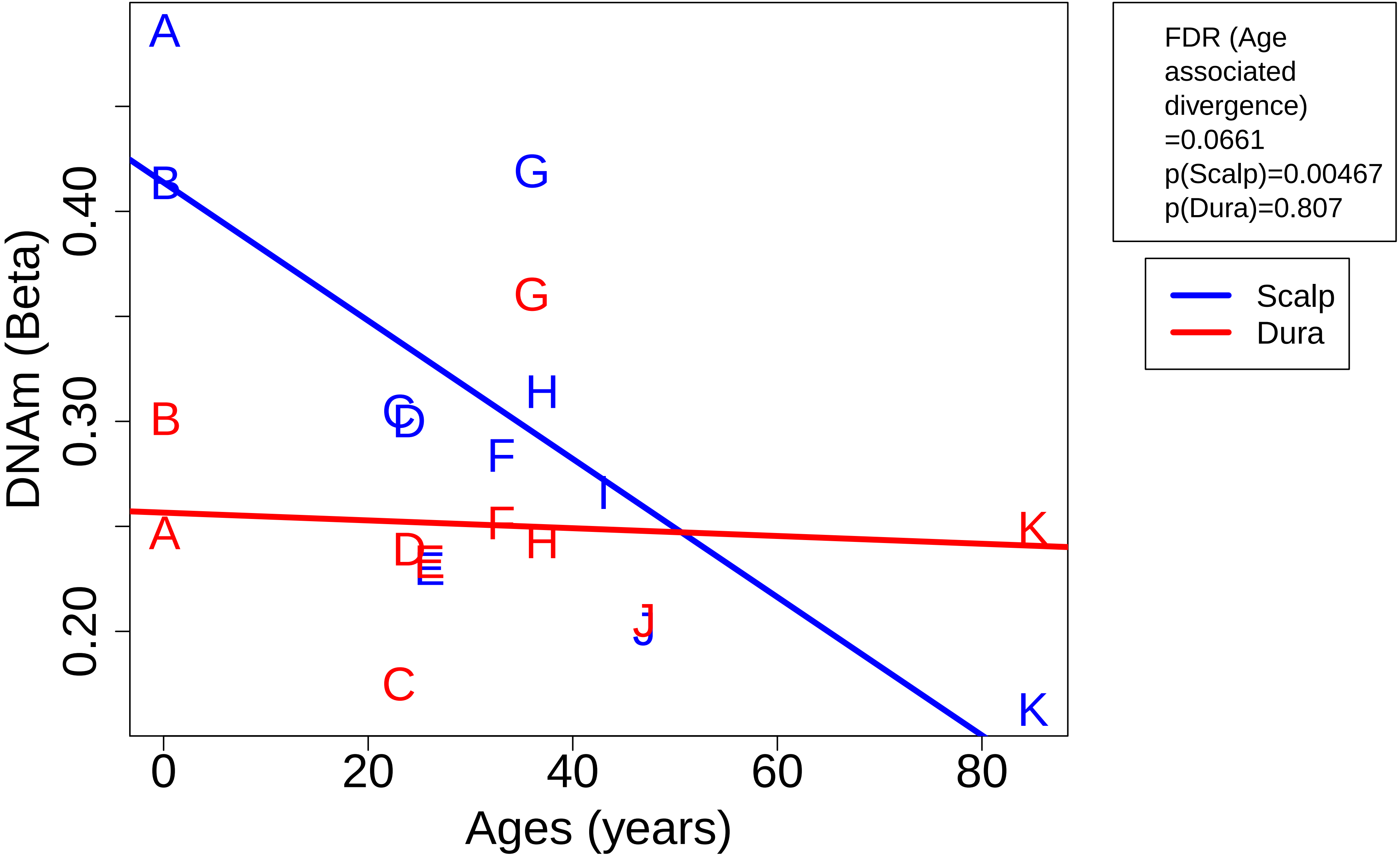

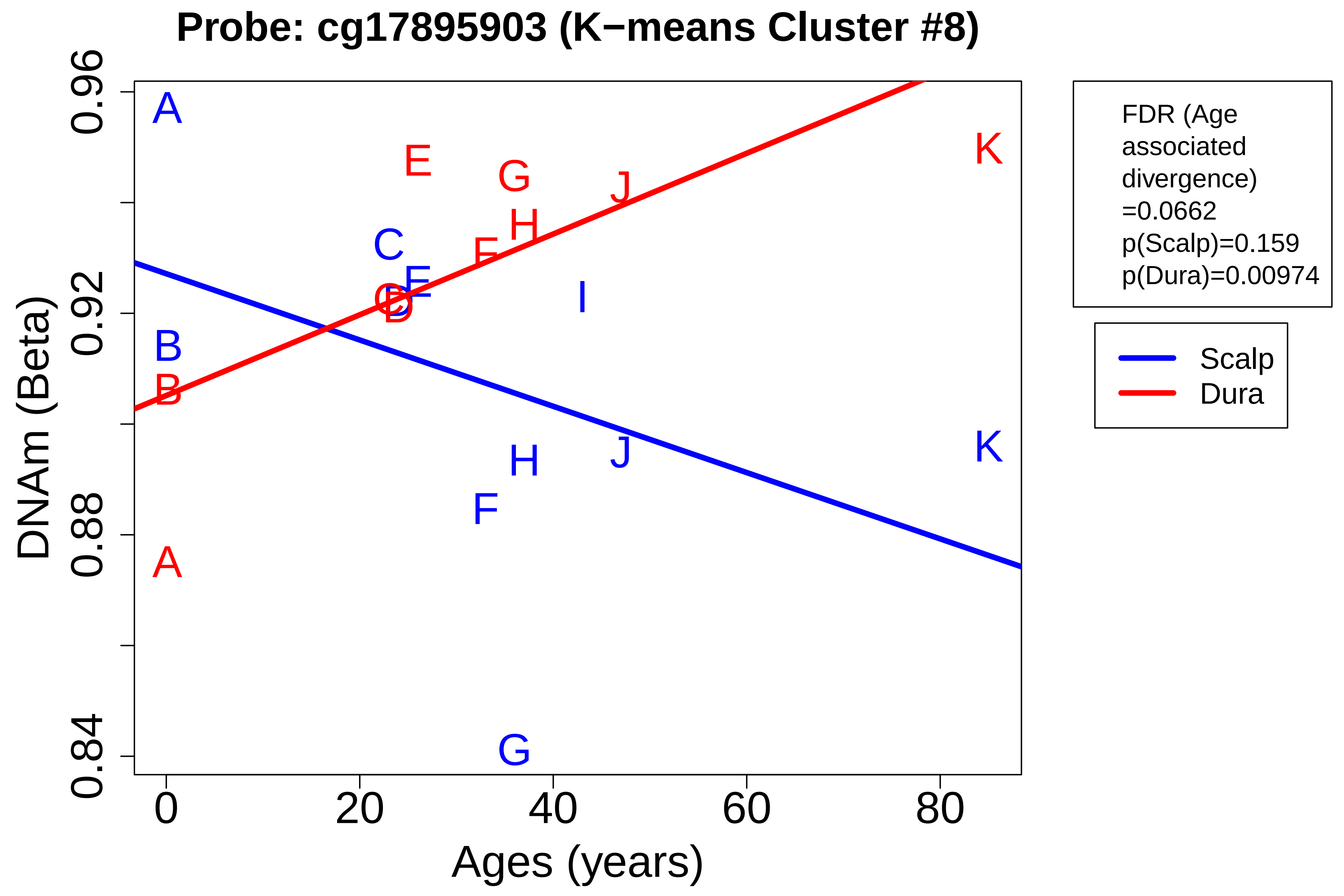
Probe: cg17895903 (K−means Cluster #8)
DNAm (Beta)
0.96
0.92
0.88
0.84
0
20
40
60
80
Ages (years)
A
B
C
D
E
F
G
H
I
J
K
FDR (Age associated divergence) =0.0662
p(Scalp)=0.159
p(Dura)=0.00974
Scalp
Dura

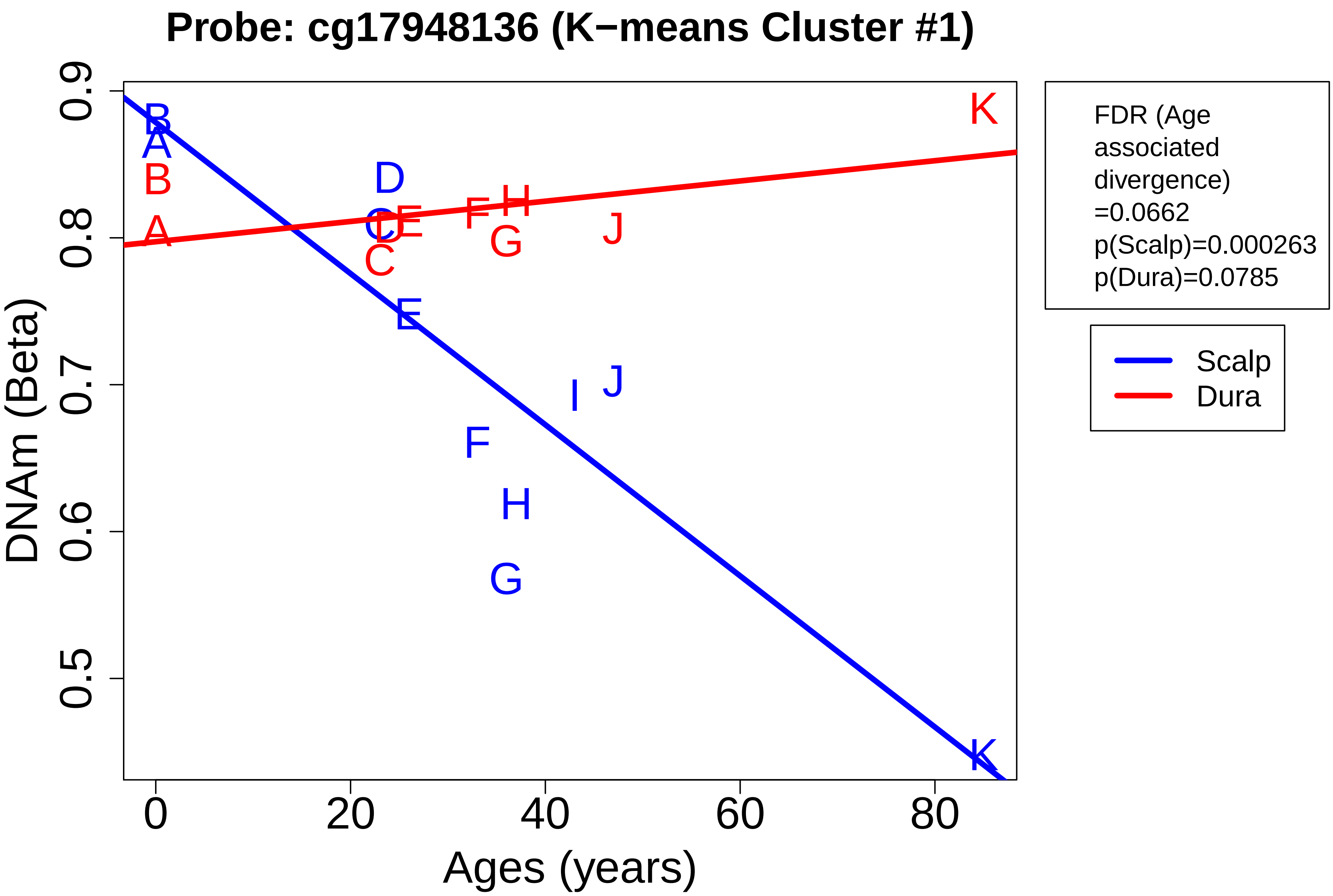
Probe: cg17948136 (K−means Cluster #1)
DNAm (Beta)
0.5
0.6
0.7
0.8
0.9
0
20
40
60
80
Ages (years)
FDR (Age associated divergence) =0.0662
p(Scalp)=0.000263
p(Dura)=0.0785
Scalp
Dura

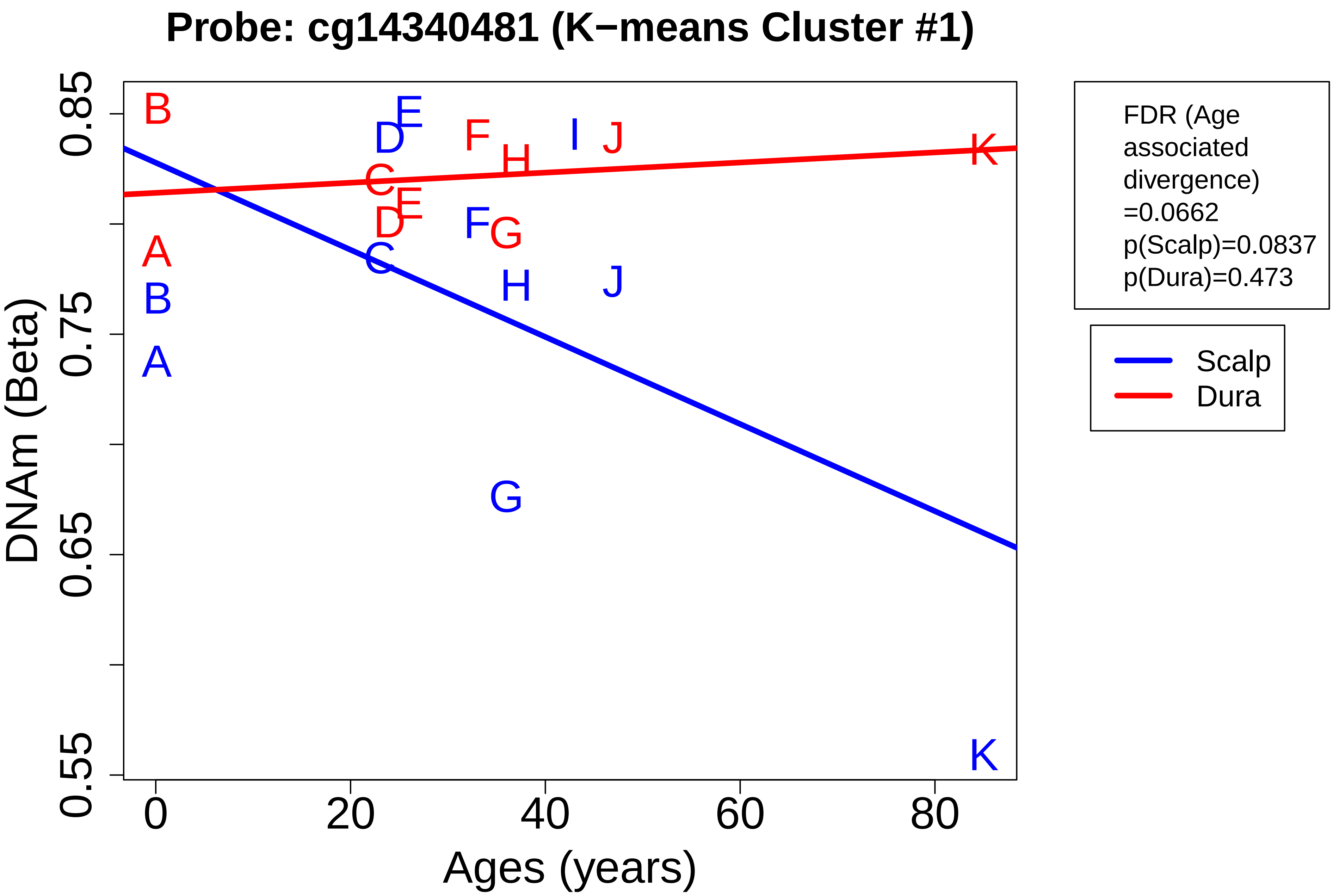

Probe: cg14340481 (K−means Cluster #1)
DNAm (Beta)
0.55
0.65
0.75
0.85
0
20
40
60
80
Ages (years)
A
B
C
D
E
F
G
H
I
J
K
FDR (Age associated divergence) =0.0662
p(Scalp)=0.0837
p(Dura)=0.473
Scalp
Dura

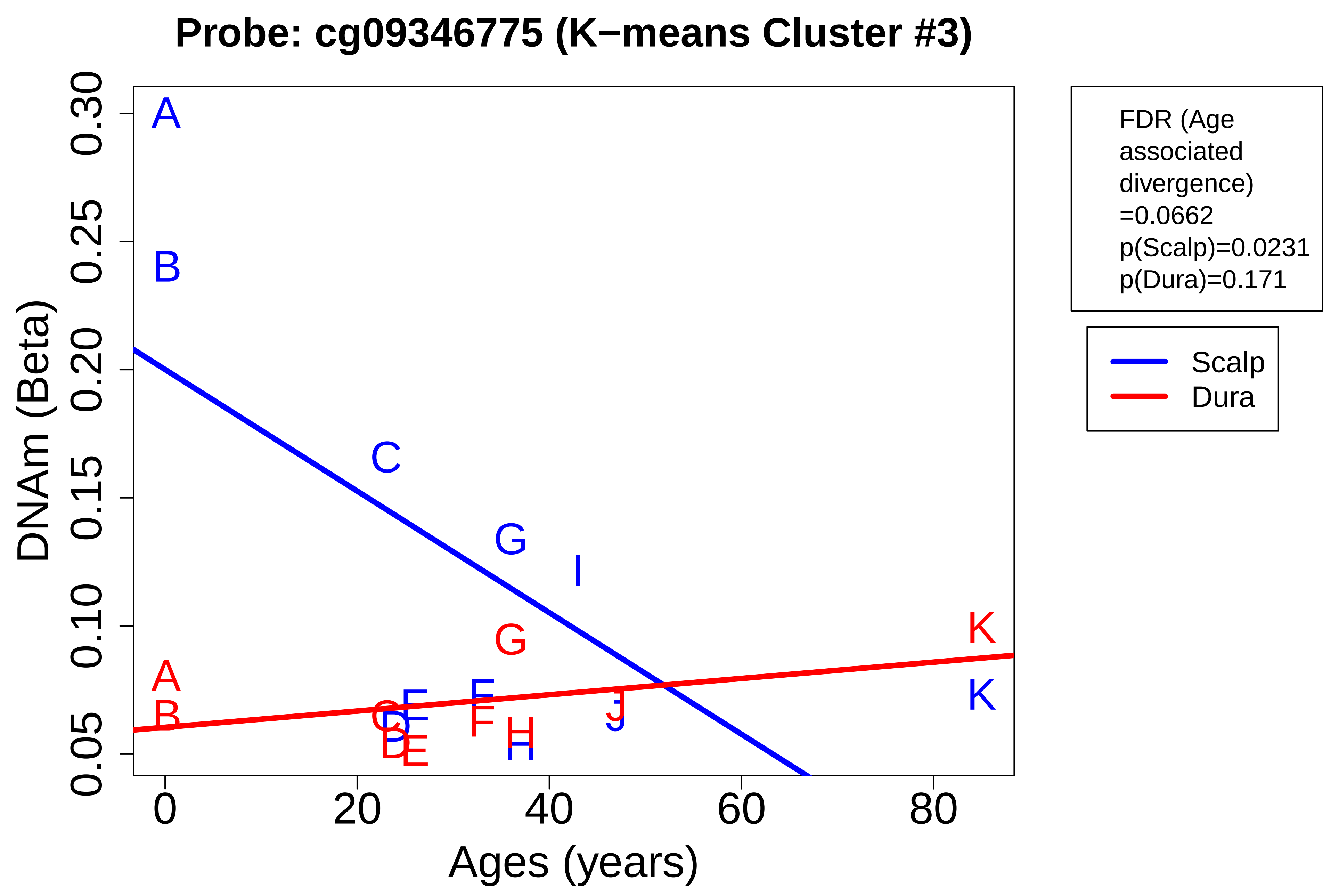

Probe: cg09346775 (K−means Cluster #3)
DNAm (Beta)
0.05
0.10
0.15
0.20
0.25
0.30
Ages (years)
0
20
40
60
80
FDR (Age associated divergence) =0.0662
p(Scalp)=0.0231
p(Dura)=0.171
Scalp
Dura

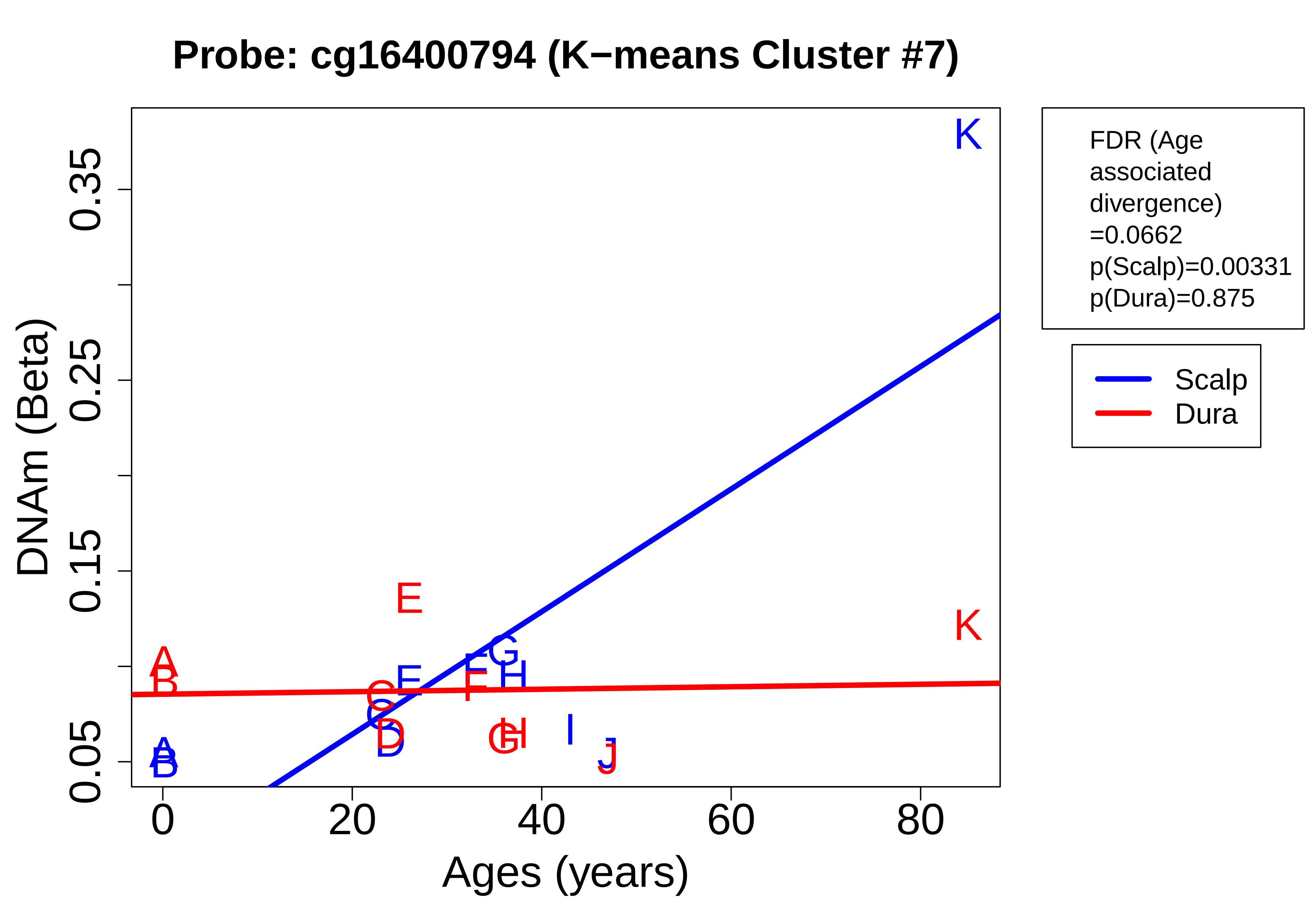

Probe: cg16400794 (K−means Cluster #7)
DNAm (Beta)
0.05
0.15
0.25
0.35
0
20
40
60
80
Ages (years)
FDR (Age associated divergence) =0.0662
p(Scalp)=0.00331
p(Dura)=0.875
Scalp
Dura

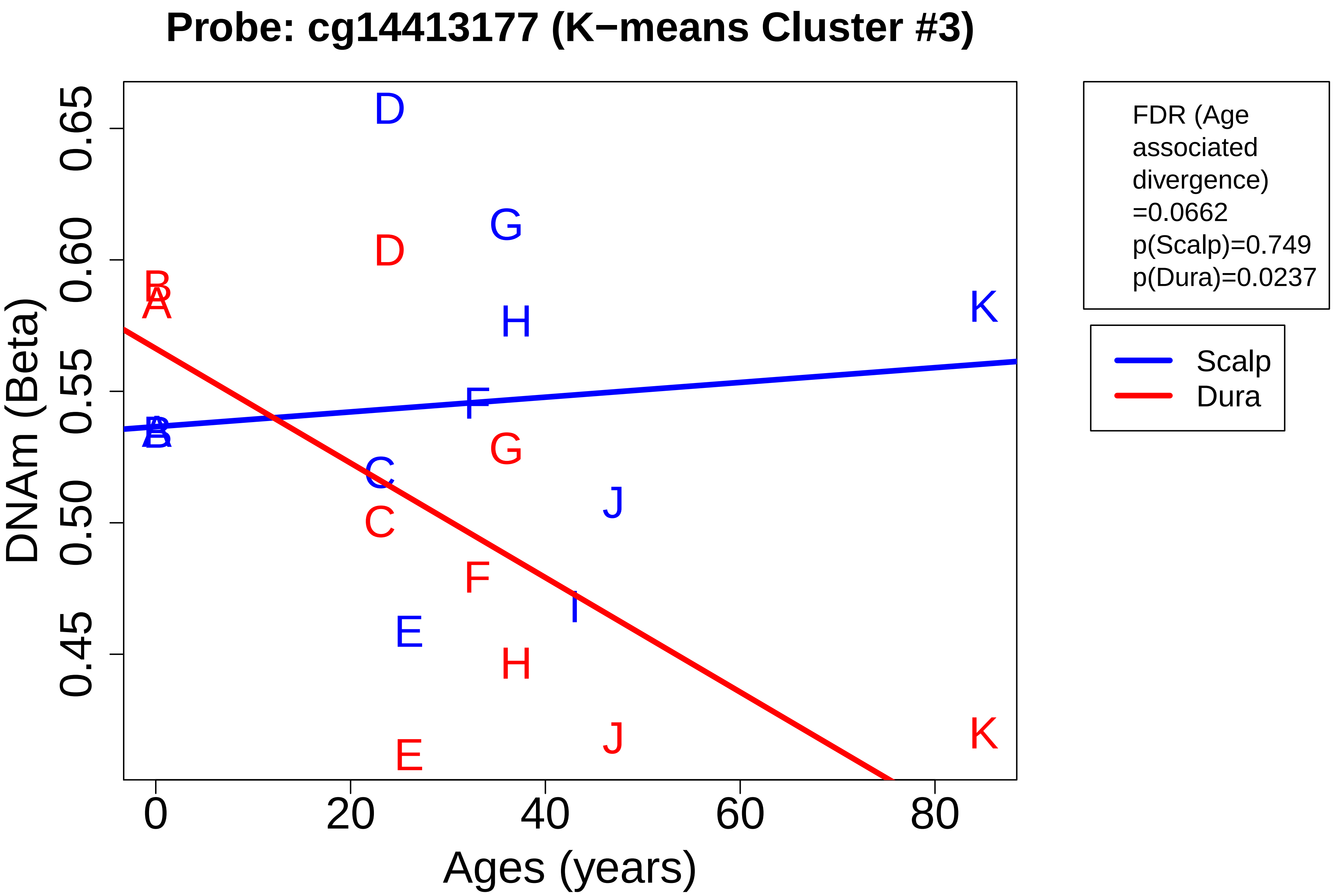

Probe: cg14413177 (K−means Cluster #3)
DNAm (Beta)
0.45
0.50
0.55
0.60
0.65
Ages (years)
0
20
40
60
80
FDR (Age associated divergence) =0.0662
p(Scalp)=0.749
p(Dura)=0.0237
Scalp
Dura

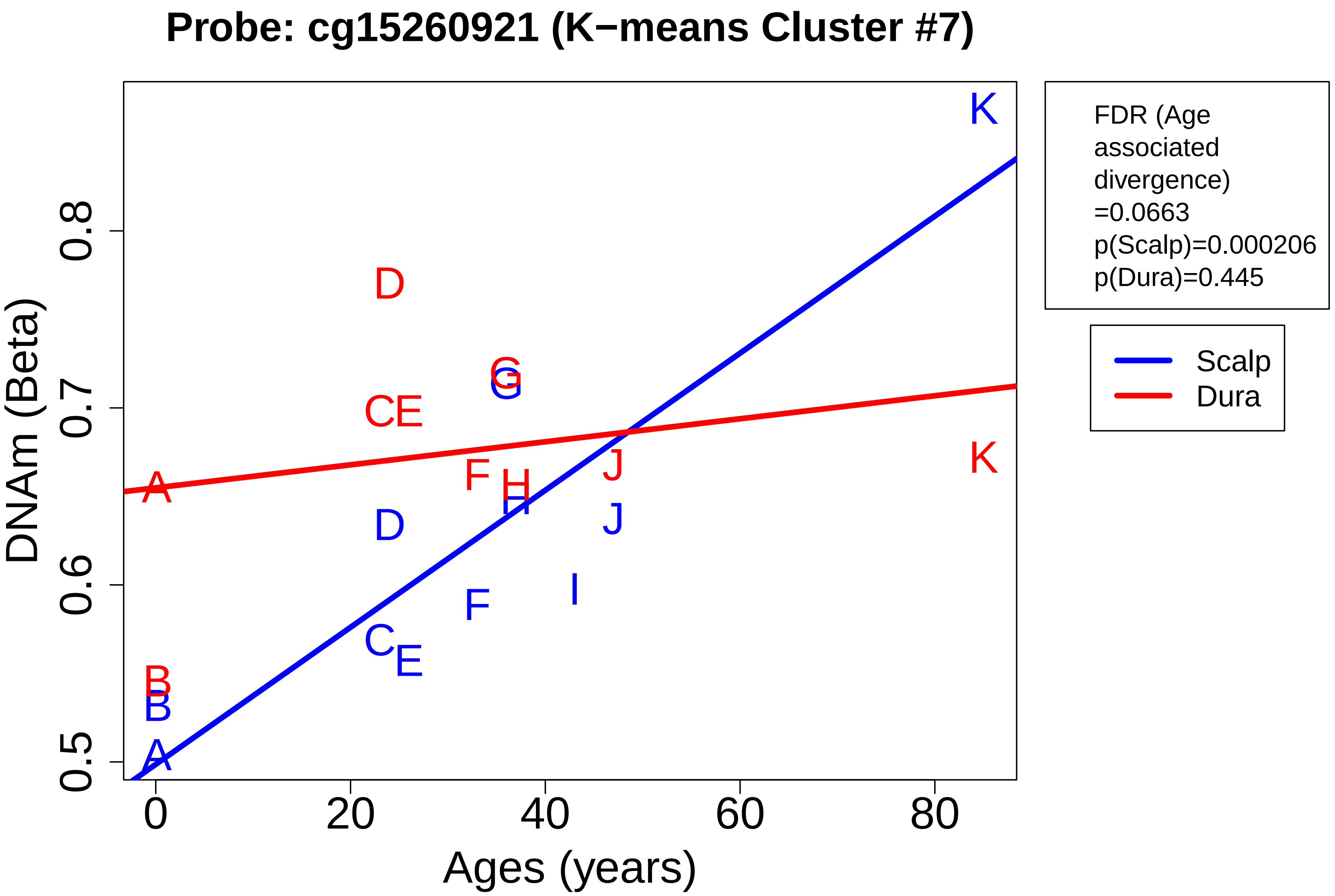

Probe: cg15260921 (K−means Cluster #7)
FDR (Age associated divergence) =0.0663
p(Scalp)=0.000206
p(Dura)=0.445
Scalp
Dura
DNAm (Beta)
0.5
0.6
0.7
0.8
0
20
40
60
80
Ages (years)

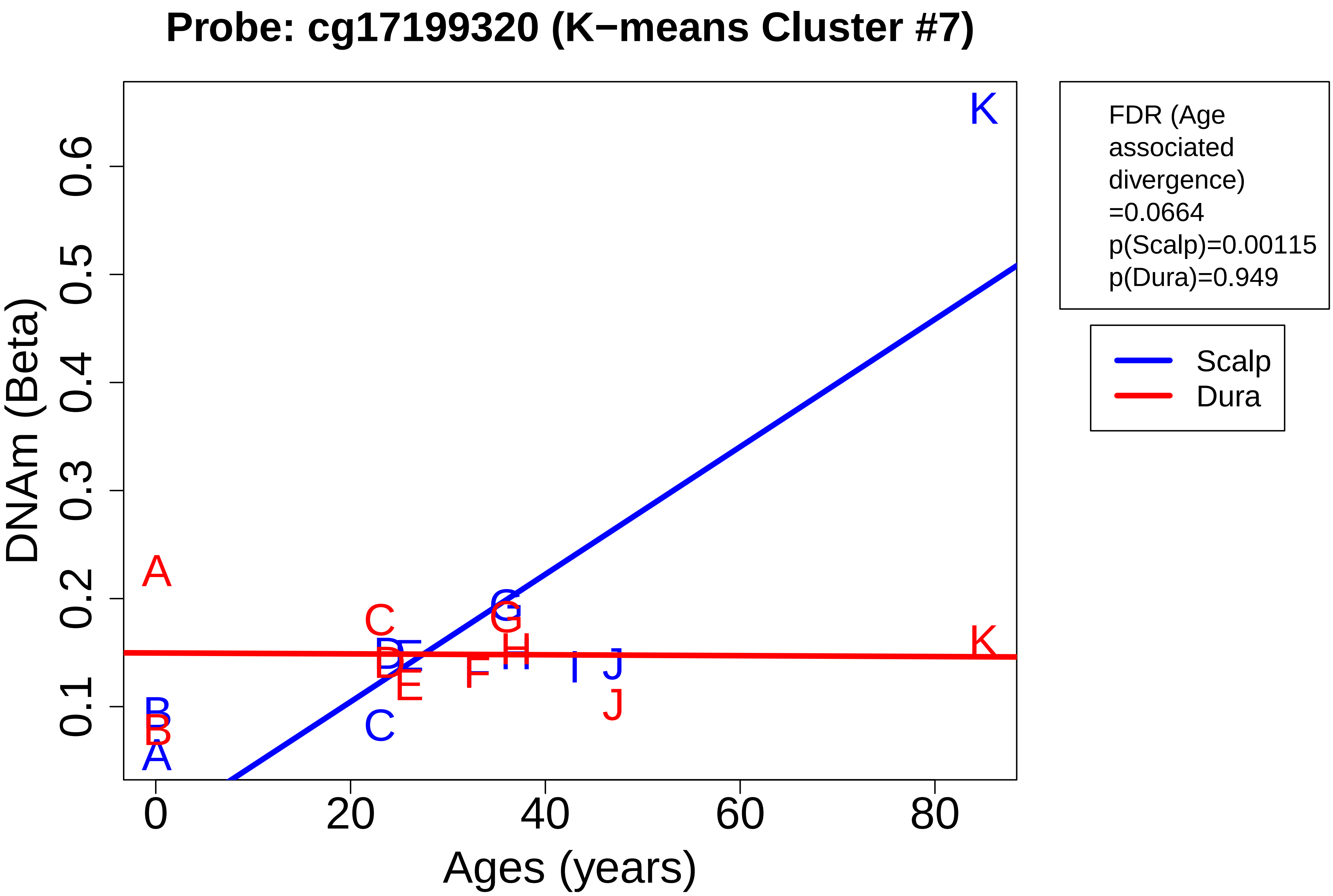

Probe: cg17199320 (K−means Cluster #7)
FDR (Age associated divergence) =0.0664
p(Scalp)=0.00115
p(Dura)=0.949
Scalp
Dura
DNAm (Beta)
0.1
0.2
0.3
0.4
0.5
0.6
Ages (years)
0
20
40
60
80
A
B
C
D
E
F
G
H
I
J
K

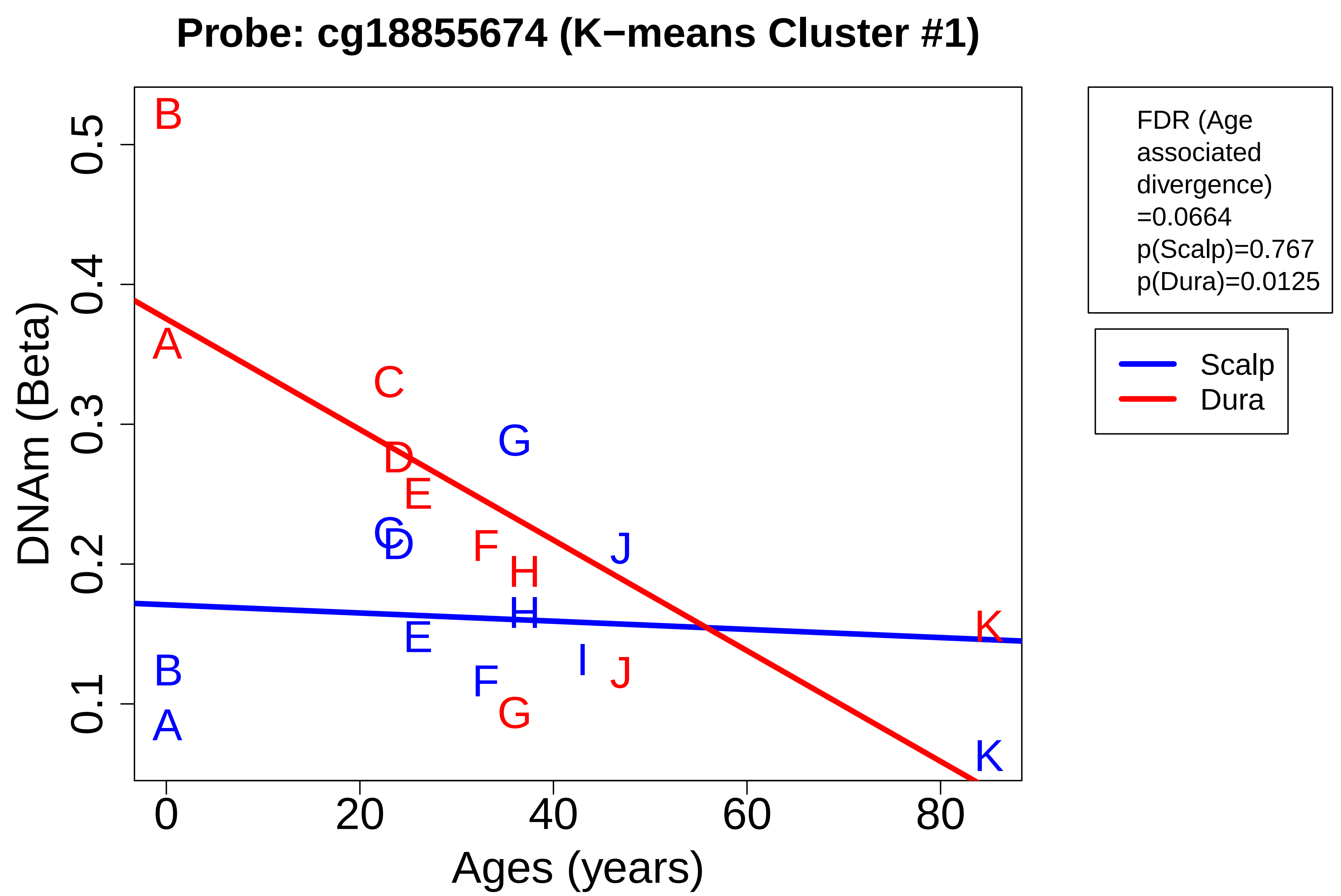
Probe: cg18855674 (K−means Cluster #1)
DNAm (Beta)
Ages (years)
0.1
0.2
0.3
0.4
0.5
0
20
40
60
80
FDR (Age associated divergence) =0.0664
p(Scalp)=0.767
p(Dura)=0.0125
Scalp
Dura
A
B
C
D
E
F
G
H
I
J
K

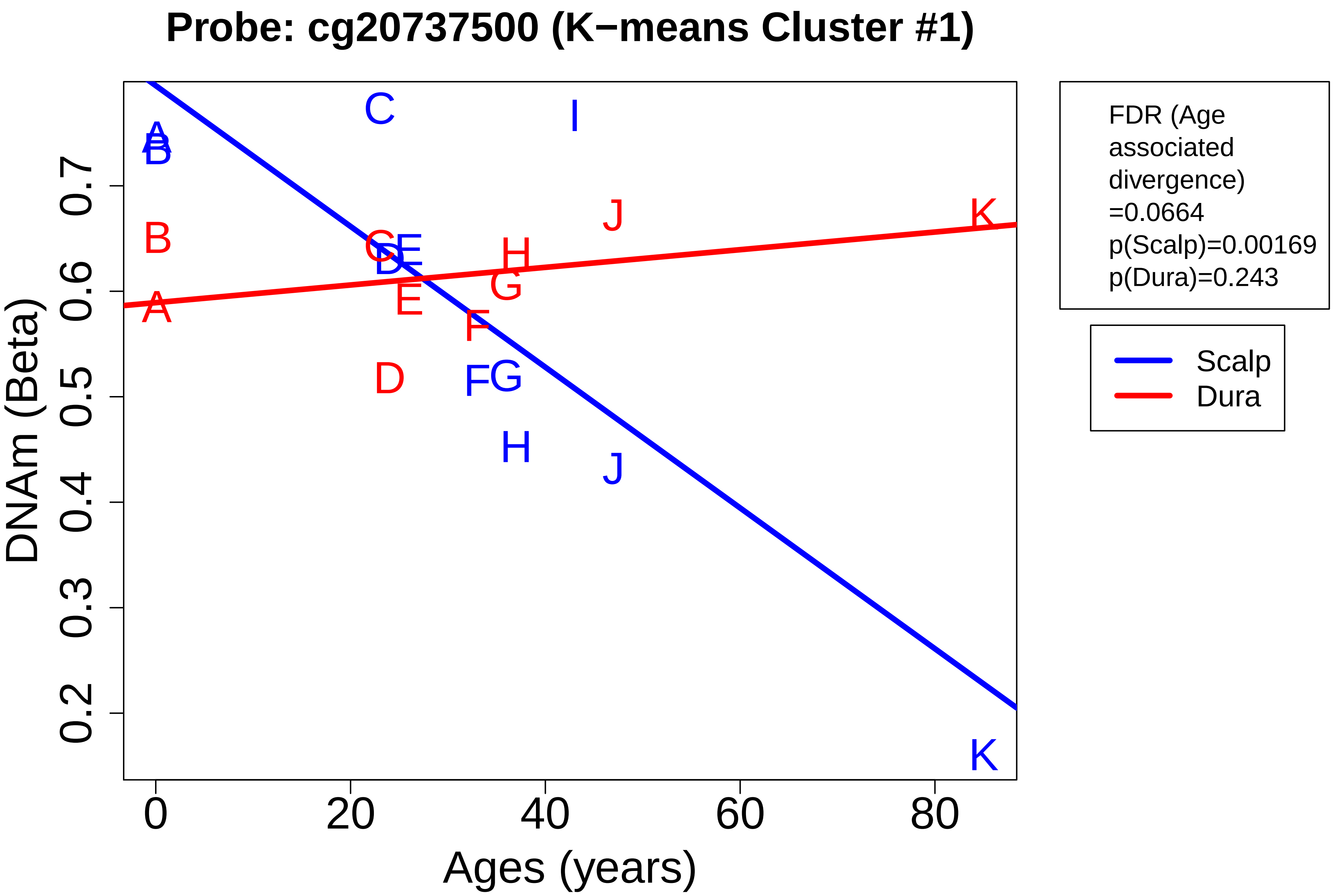

Probe: cg20737500 (K−means Cluster #1)
DNAm (Beta)
0.2
0.3
0.4
0.5
0.6
0.7
0
20
40
60
80
Ages (years)
FDR (Age associated divergence) =0.0664
p(Scalp)=0.00169
p(Dura)=0.243
Scalp
Dura

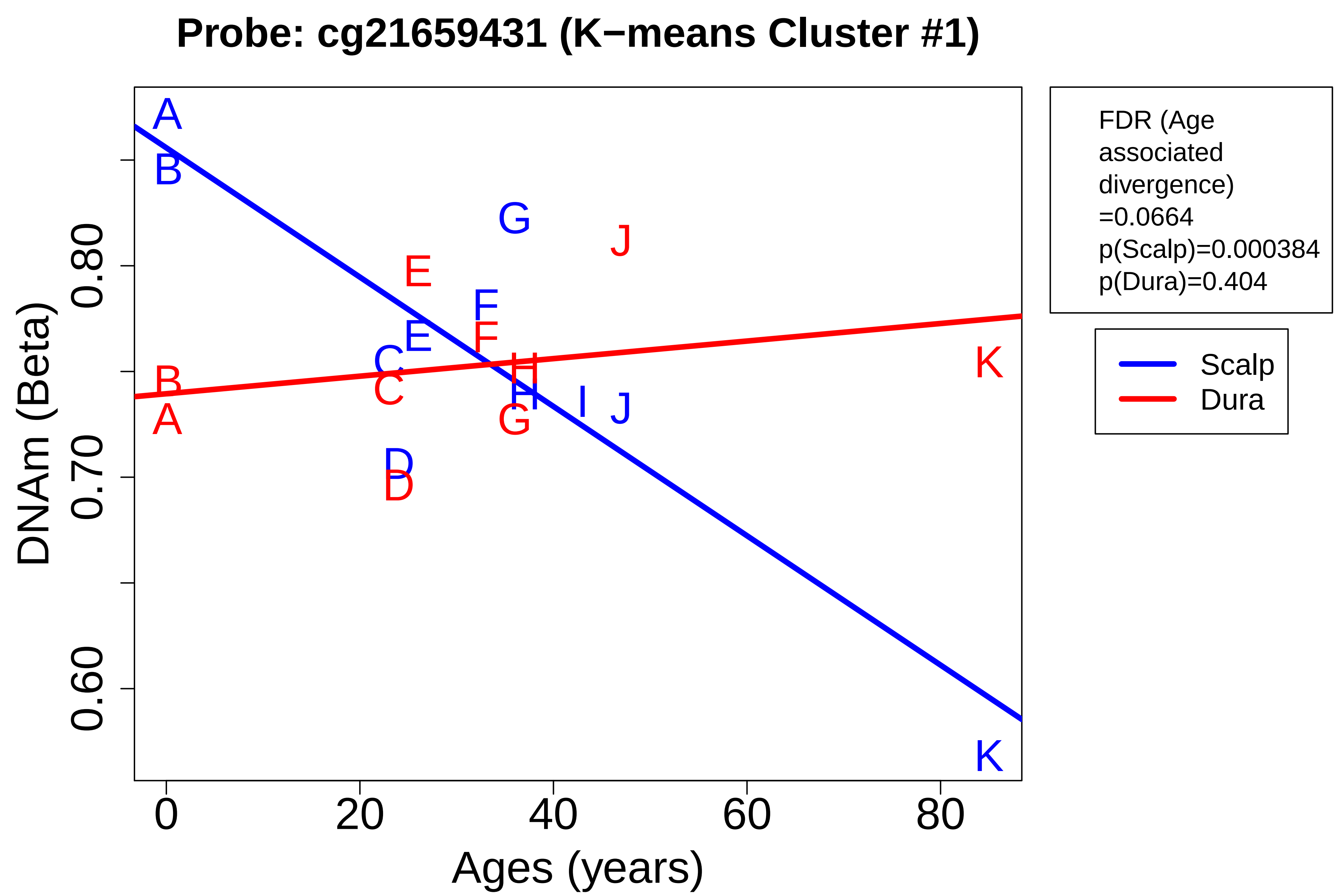

Probe: cg21659431 (K−means Cluster #1)
DNAm (Beta)
0.60
0.70
0.80
Ages (years)
0
20
40
60
80
A
B
C
D
E
F
G
H
I
J
K
FDR (Age associated divergence) =0.0664
p(Scalp)=0.000384
p(Dura)=0.404
Scalp
Dura

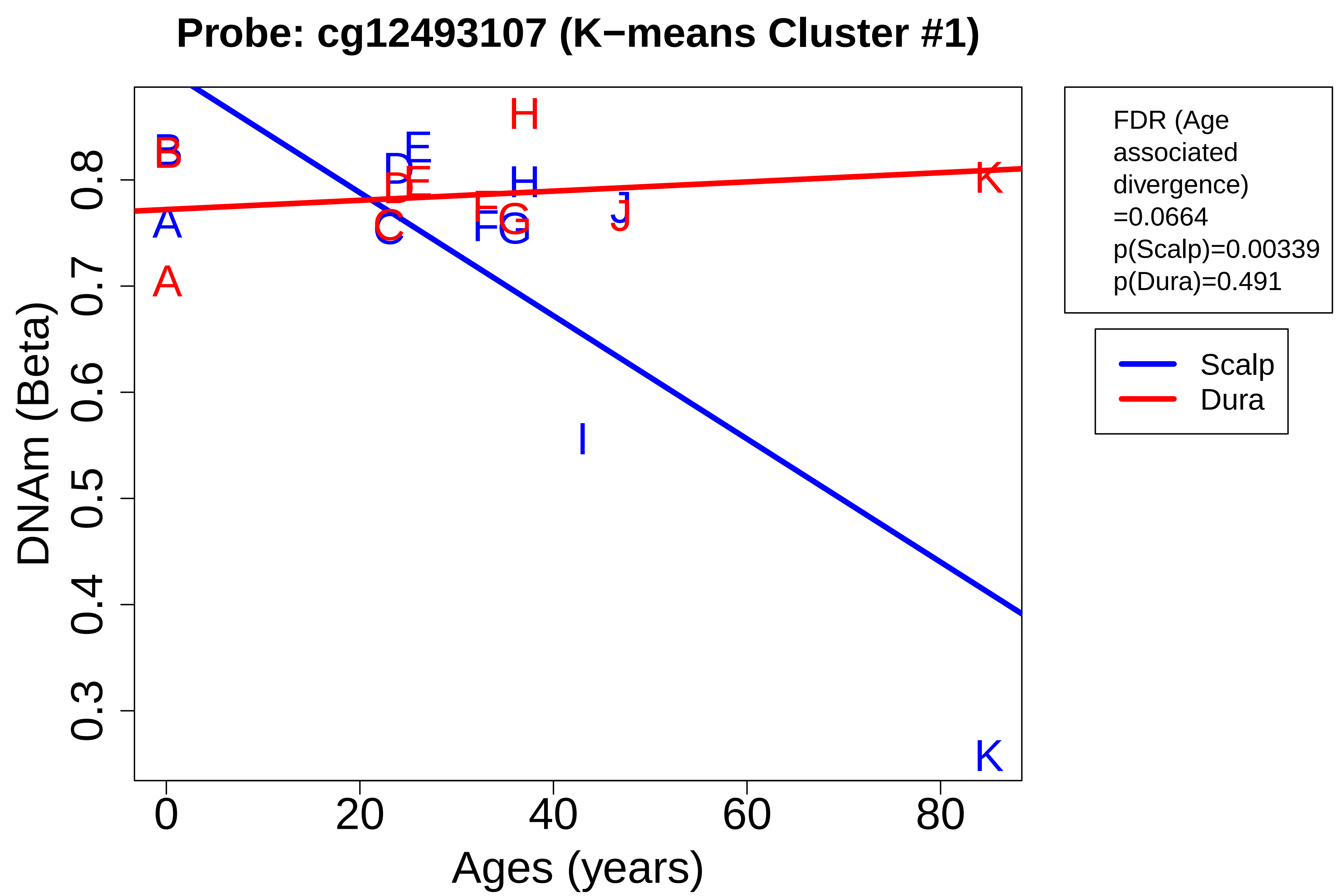

Probe: cg12493107 (K−means Cluster #1)
DNAm (Beta)
0.3
0.4
0.5
0.6
0.7
0.8
0
20
40
60
80
Ages (years)
A
B
C
D
E
F
G
H
I
J
K
FDR (Age associated divergence) =0.0664
p(Scalp)=0.00339
p(Dura)=0.491
Scalp
Dura

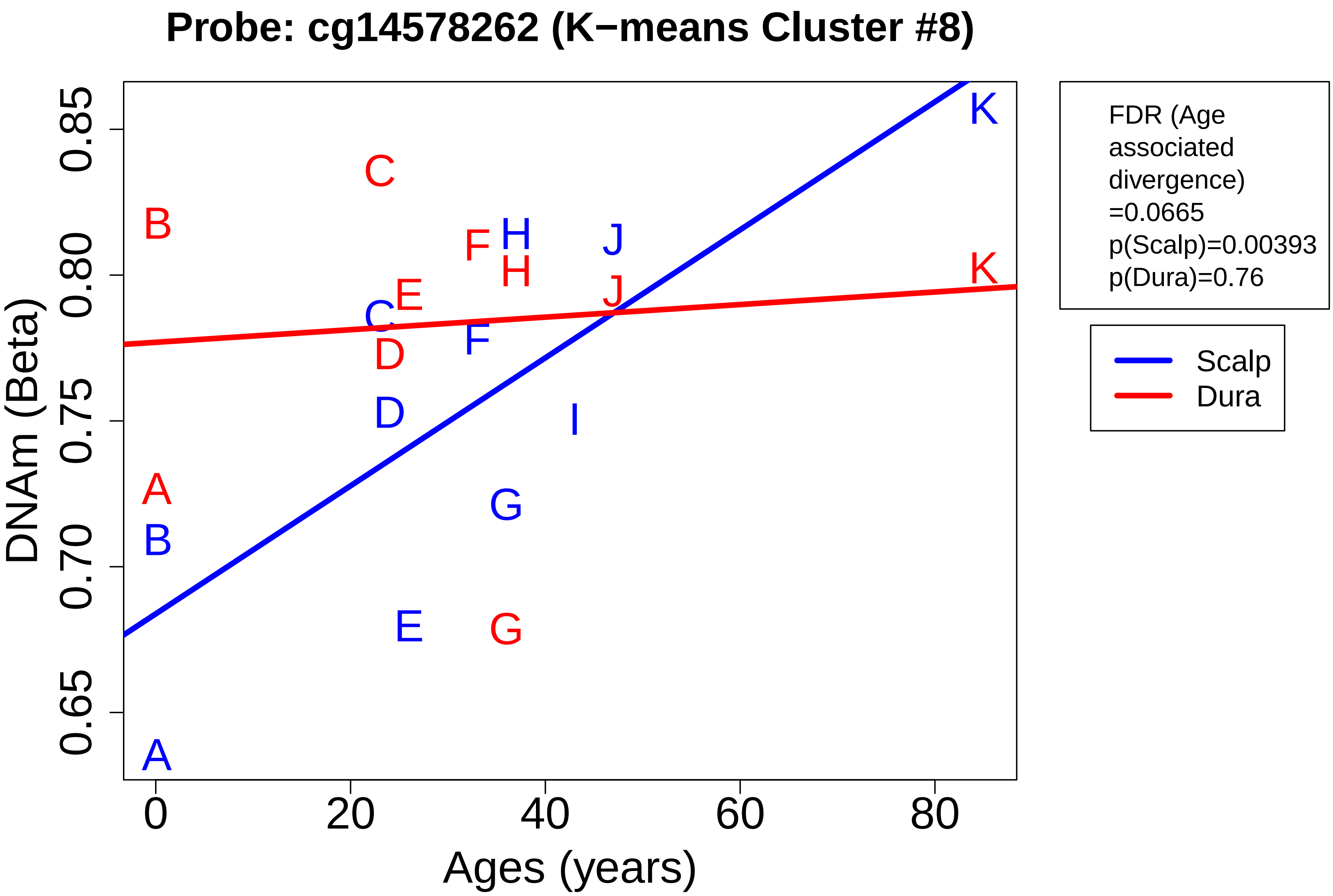

Probe: cg14578262 (K−means Cluster #8)
DNAm (Beta)
0.65
0.70
0.75
0.80
0.85
Ages (years)
0
20
40
60
80
A
B
C
D
E
F
G
H
I
J
K
FDR (Age associated divergence) =0.0665
p(Scalp)=0.00393
p(Dura)=0.76
Scalp
Dura

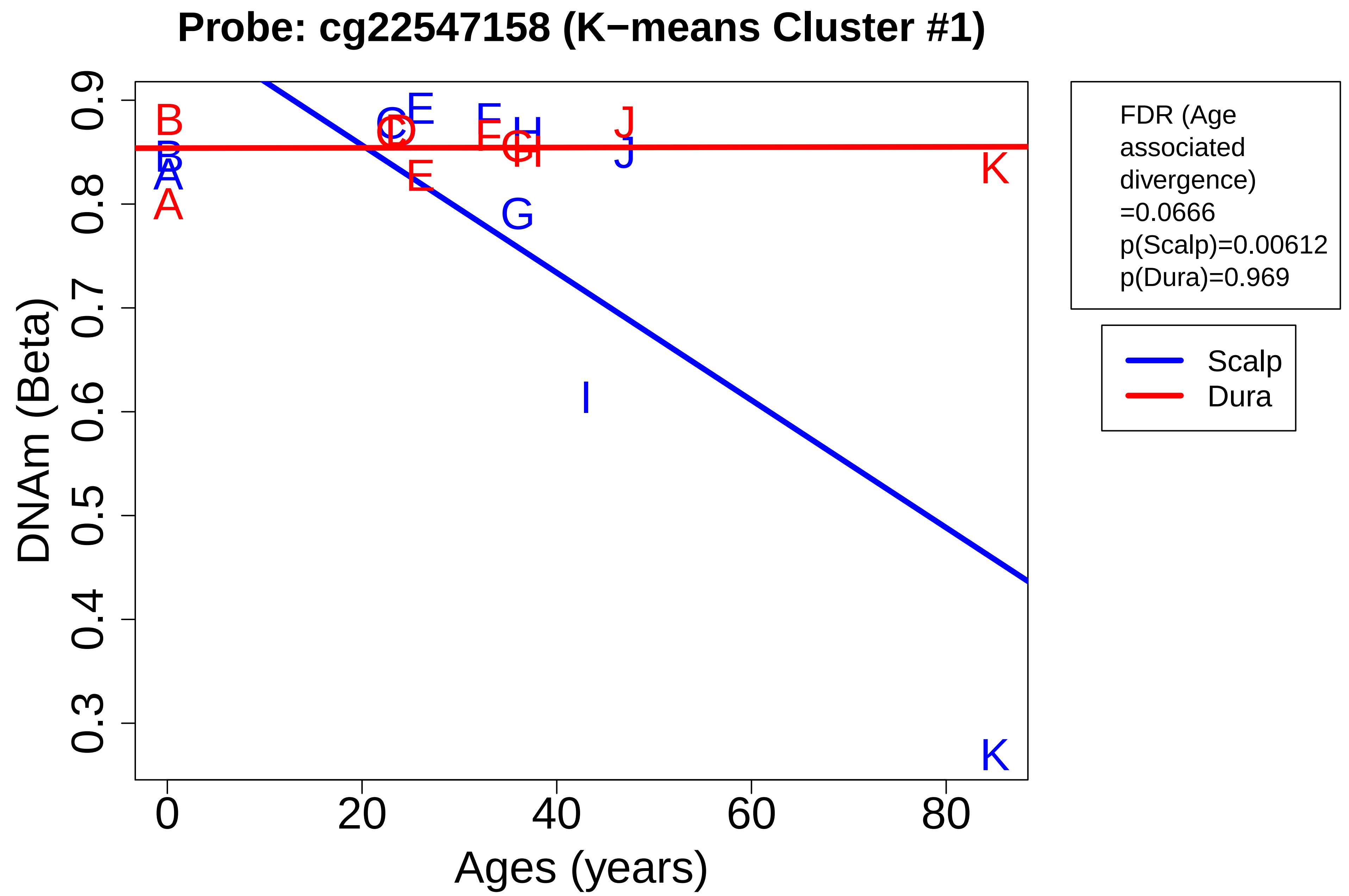

Probe: cg22547158 (K−means Cluster #1)
DNAm (Beta)
Ages (years)
FDR (Age associated divergence) =0.0666
p(Scalp)=0.00612
p(Dura)=0.969
Scalp
Dura

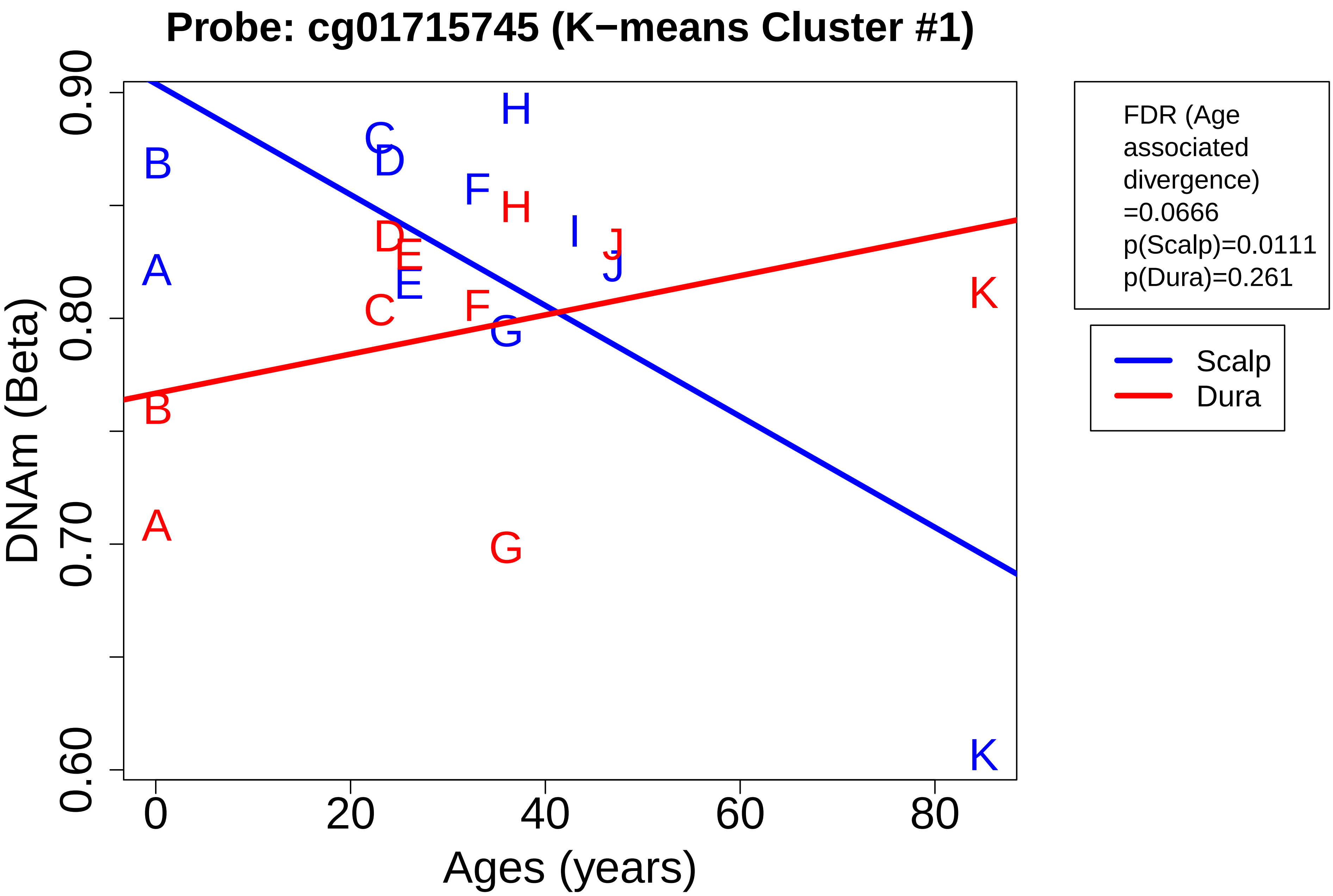

Probe: cg01715745 (K−means Cluster #1)
DNAm (Beta)
0.60
0.70
0.80
0.90
Ages (years)
0
20
40
60
80
A
B
C
D
E
F
G
H
I
J
K
FDR (Age associated divergence) =0.0666
p(Scalp)=0.0111
p(Dura)=0.261
Scalp
Dura

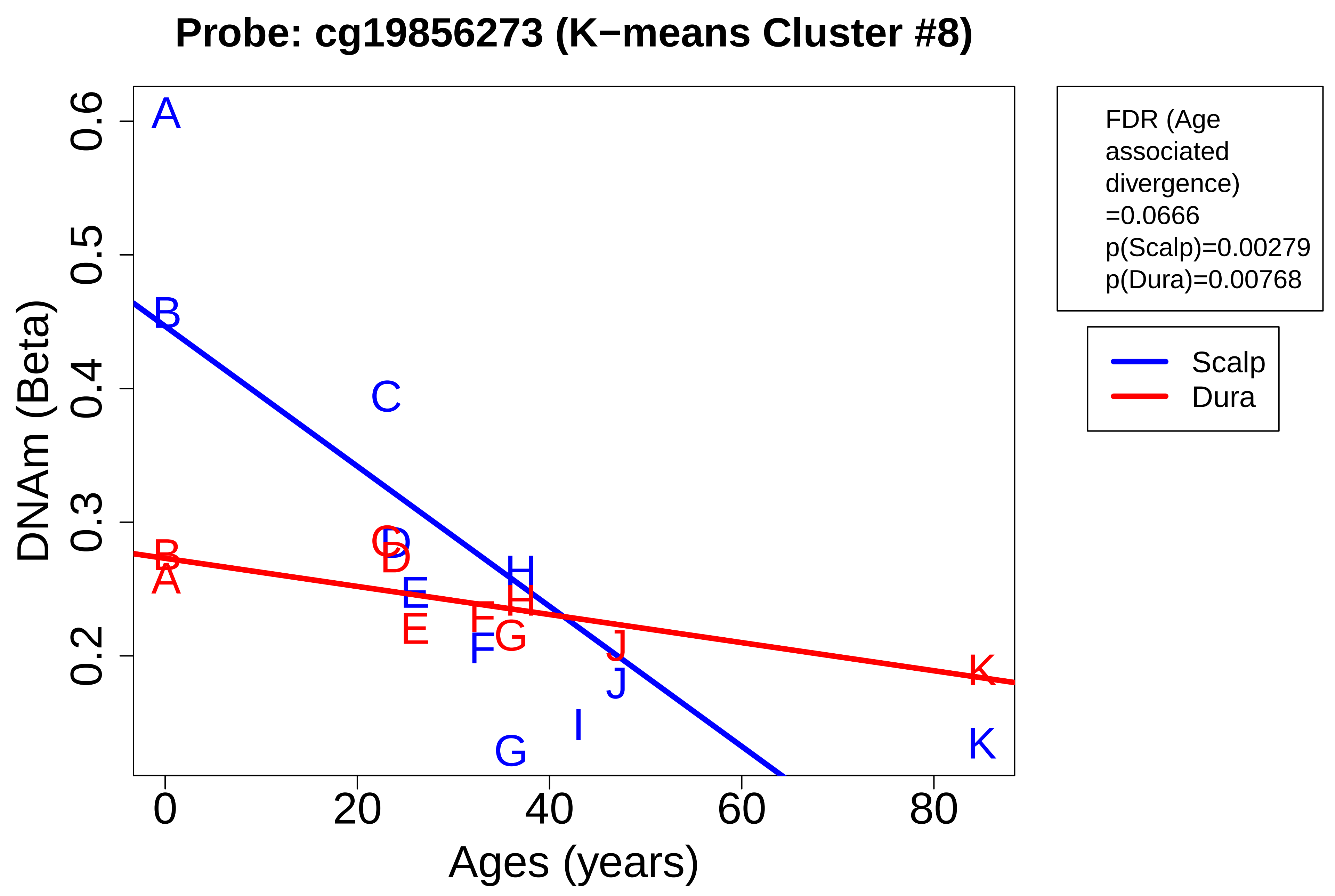

Probe: cg19856273 (K−means Cluster #8)
DNAm (Beta)
0.2
0.3
0.4
0.5
0.6
Ages (years)
0
20
40
60
80
FDR (Age associated divergence) =0.0666
p(Scalp)=0.00279
p(Dura)=0.00768
Scalp
Dura

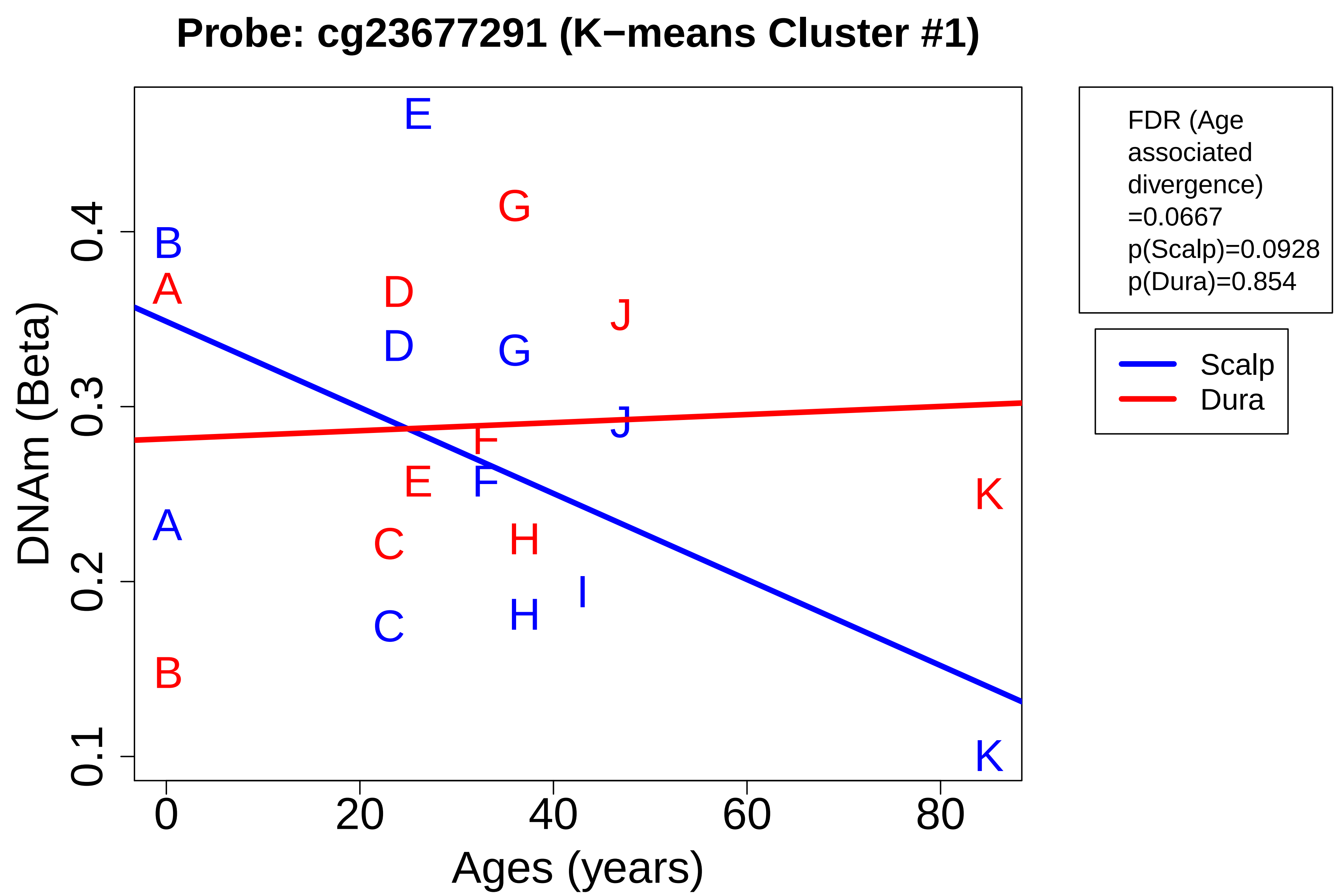

Probe: cg23677291 (K−means Cluster #1)
DNAm (Beta)
Ages (years)
0.1
0.2
0.3
0.4
0
20
40
60
80
FDR (Age associated divergence) =0.0667
p(Scalp)=0.0928
p(Dura)=0.854
Scalp
Dura

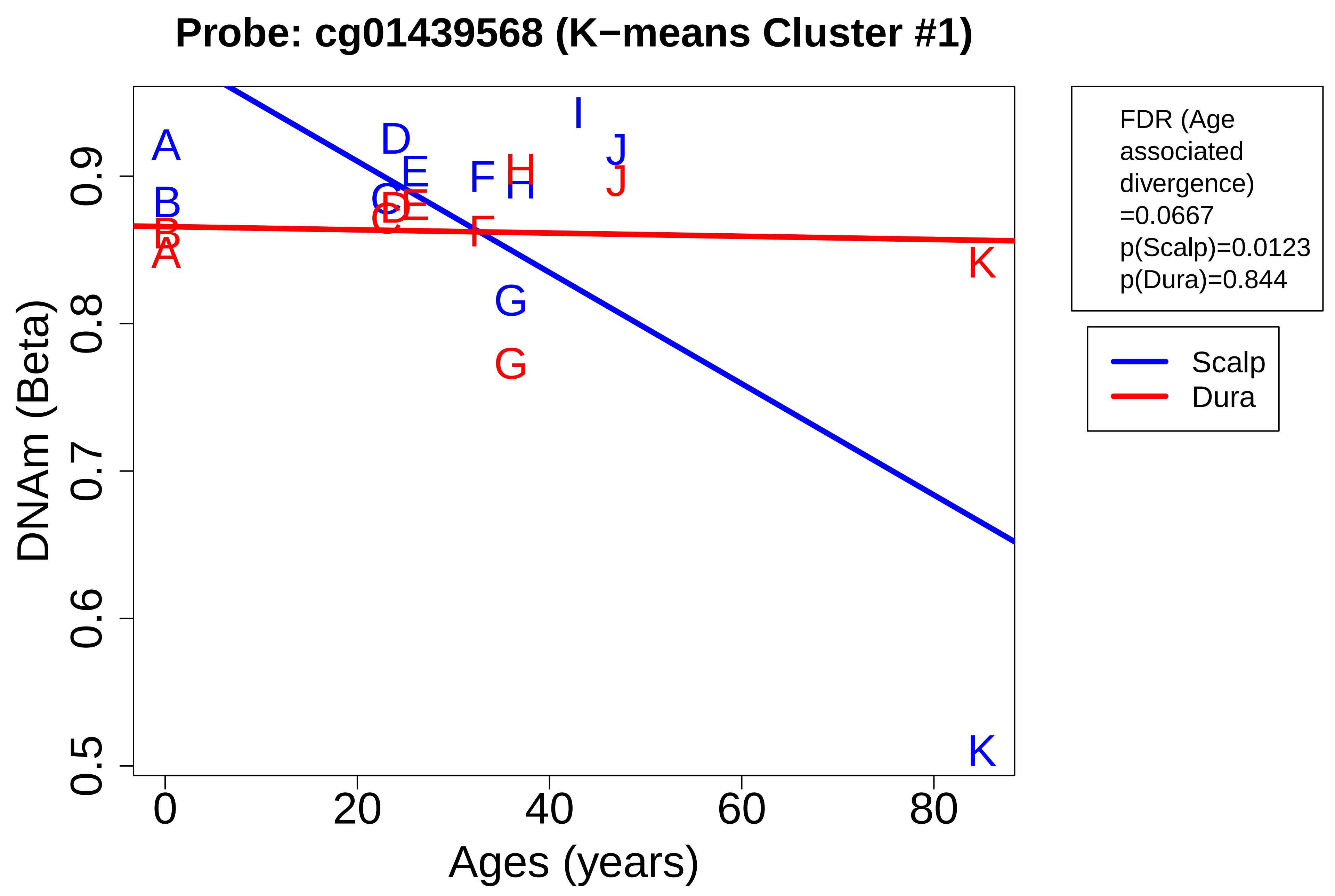

Probe: cg01439568 (K−means Cluster #1)
DNAm (Beta)
Ages (years)
0.5
0.6
0.7
0.8
0.9
0
20
40
60
80
FDR (Age associated divergence) =0.0667
p(Scalp)=0.0123
p(Dura)=0.844
Scalp
Dura

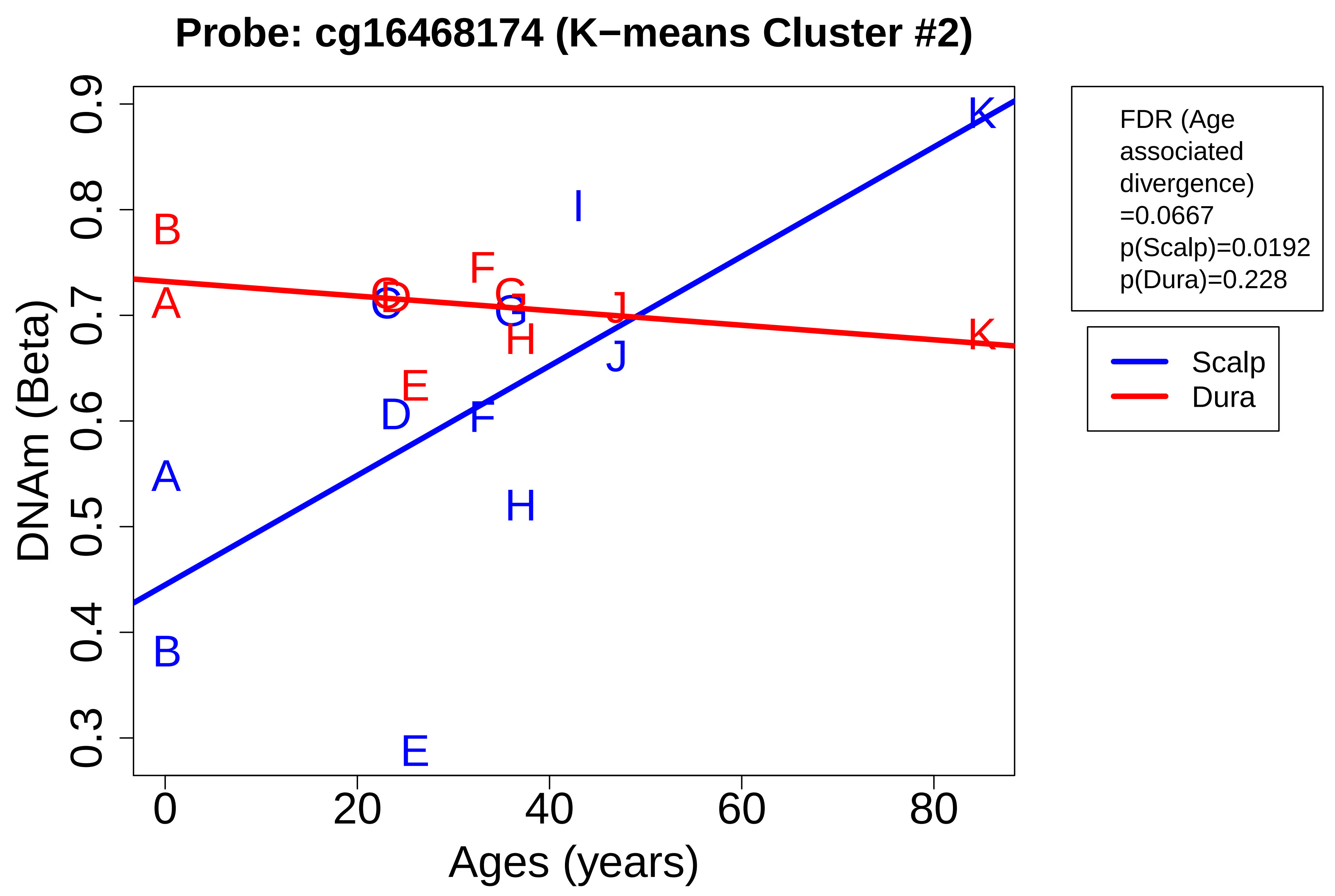

Probe: cg16468174 (K−means Cluster #2)
DNAm (Beta)
0.3
0.4
0.5
0.6
0.7
0.8
0.9
0
20
40
60
80
Ages (years)
FDR (Age associated divergence) =0.0667
p(Scalp)=0.0192
p(Dura)=0.228
Scalp
Dura

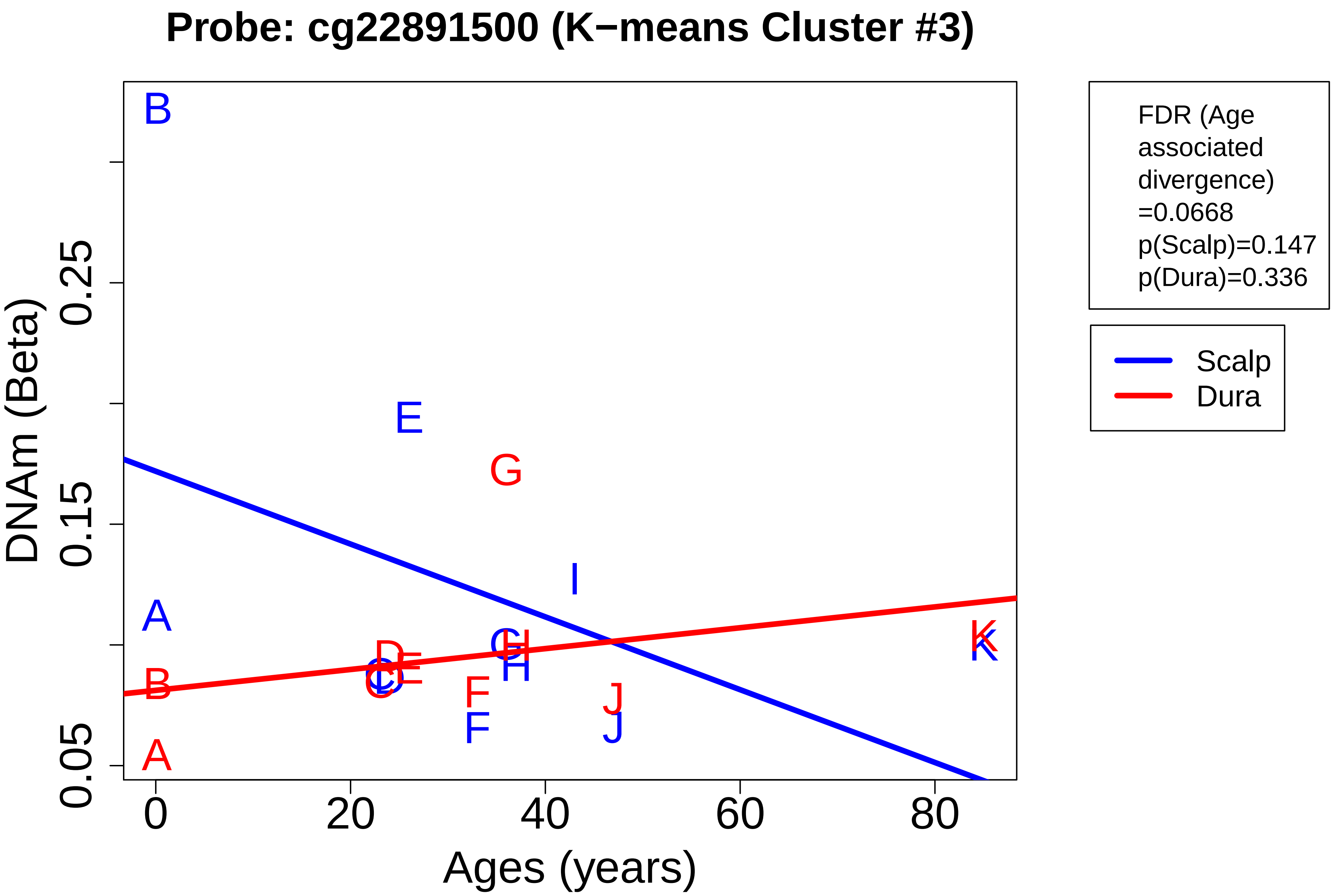
Probe: cg22891500 (K−means Cluster #3)
DNAm (Beta)
0.05
0.15
0.25
0
20
40
60
80
Ages (years)
FDR (Age associated divergence) =0.0668
p(Scalp)=0.147
p(Dura)=0.336
Scalp
Dura

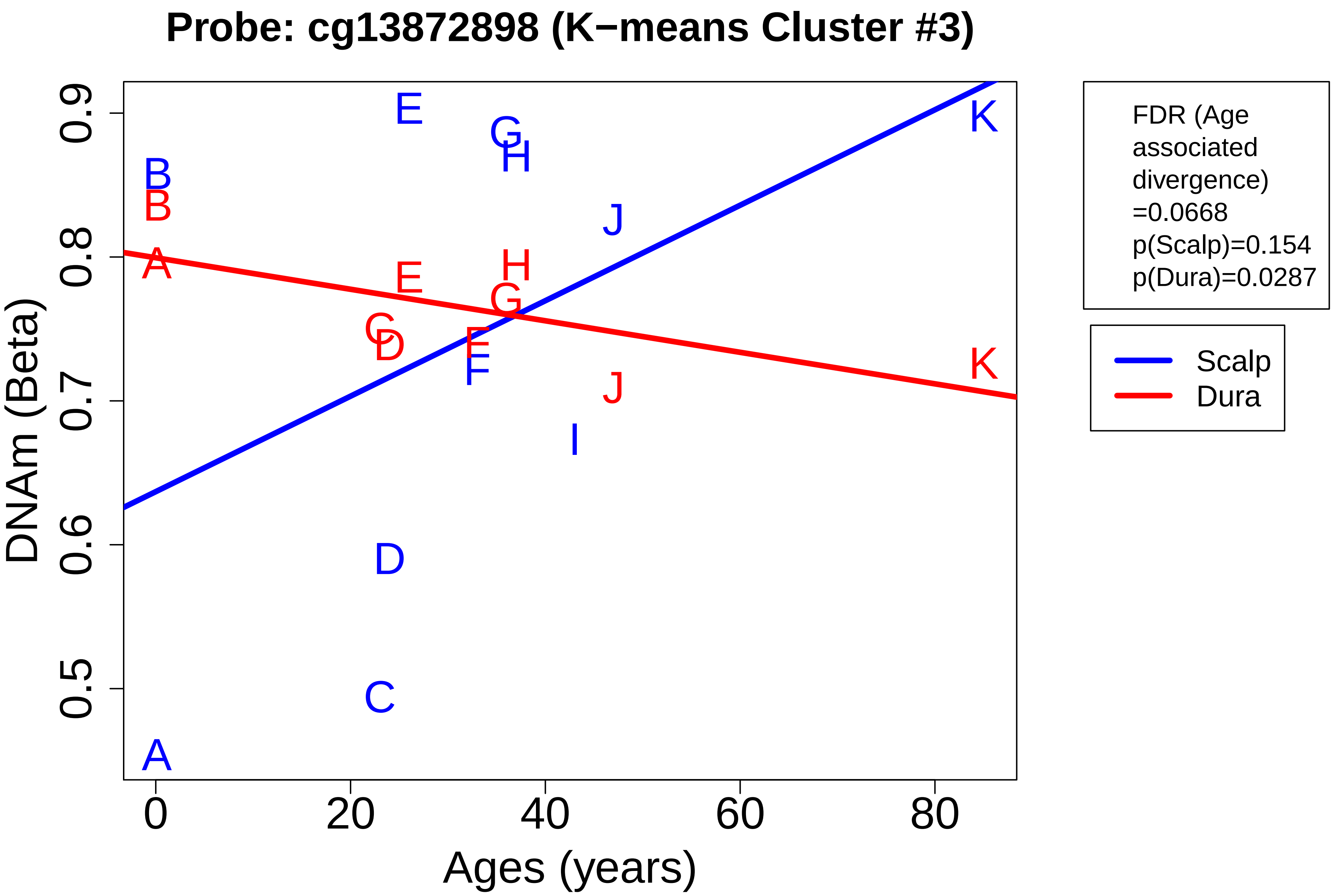

Probe: cg13872898 (K−means Cluster #3)
DNAm (Beta)
0.5
0.6
0.7
0.8
0.9
0
20
40
60
80
Ages (years)
A
B
C
D
E
F
G
H
I
J
K
FDR (Age associated divergence) =0.0668
p(Scalp)=0.154
p(Dura)=0.0287
Scalp
Dura

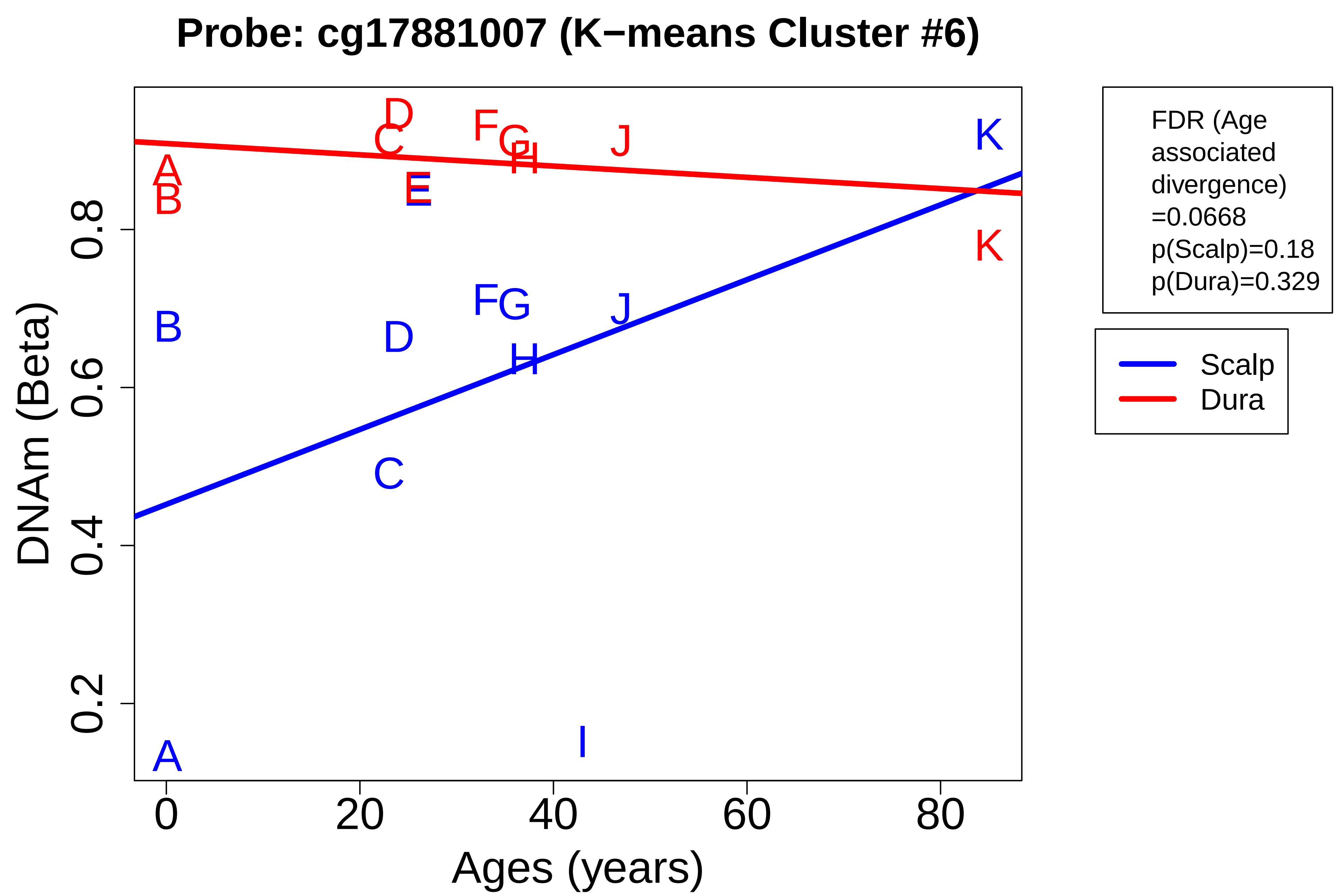
Probe: cg17881007 (K−means Cluster #6)
DNAm (Beta)
0.2
0.4
0.6
0.8
0
20
40
60
80
Ages (years)
A
B
C
D
E
F
G
H
I
J
K
FDR (Age associated divergence) =0.0668
p(Scalp)=0.18
p(Dura)=0.329
Scalp
Dura

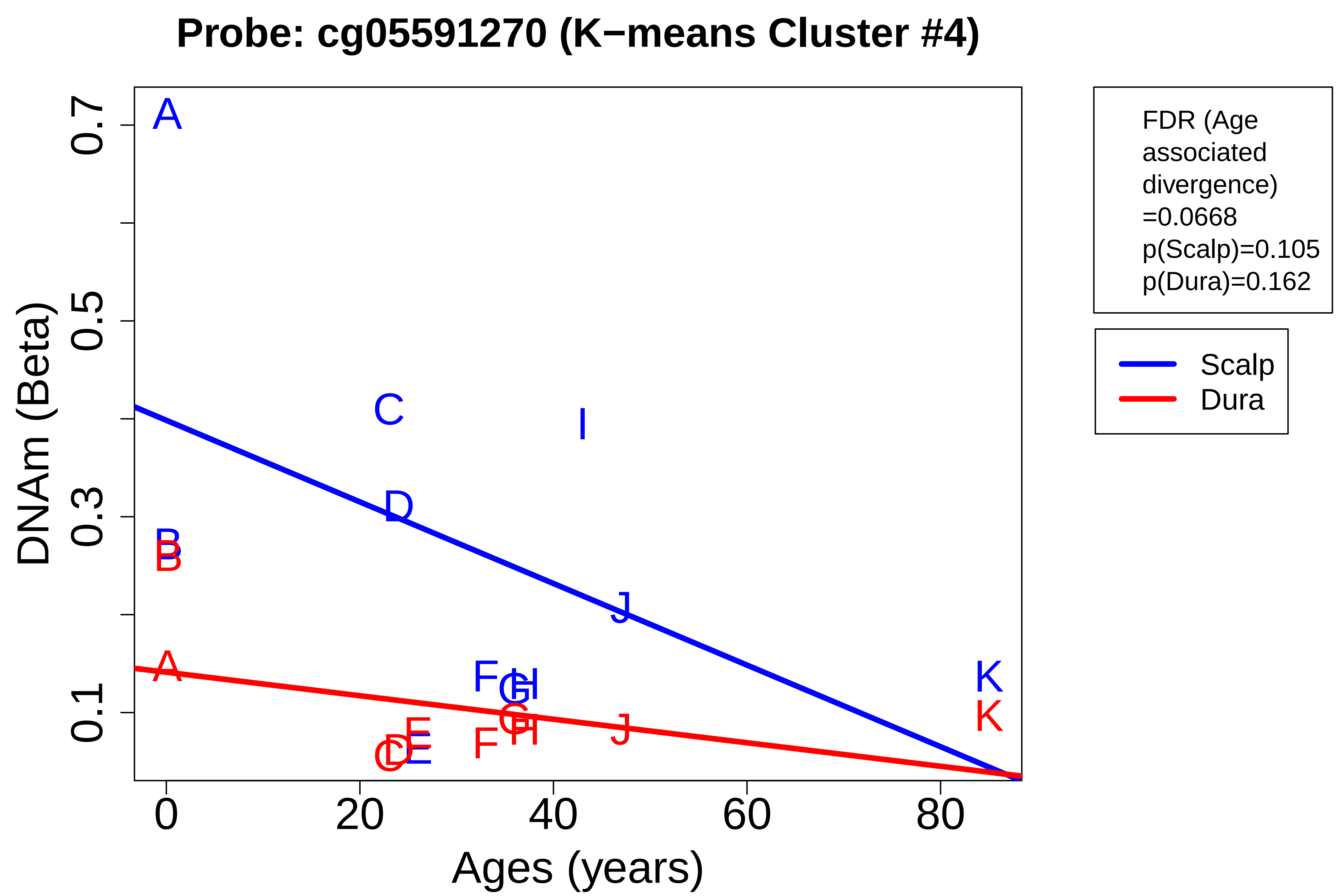

Probe: cg05591270 (K−means Cluster #4)
DNAm (Beta)
0.1
0.3
0.5
0.7
Ages (years)
0
20
40
60
80
FDR (Age associated divergence) =0.0668
p(Scalp)=0.105
p(Dura)=0.162
Scalp
Dura

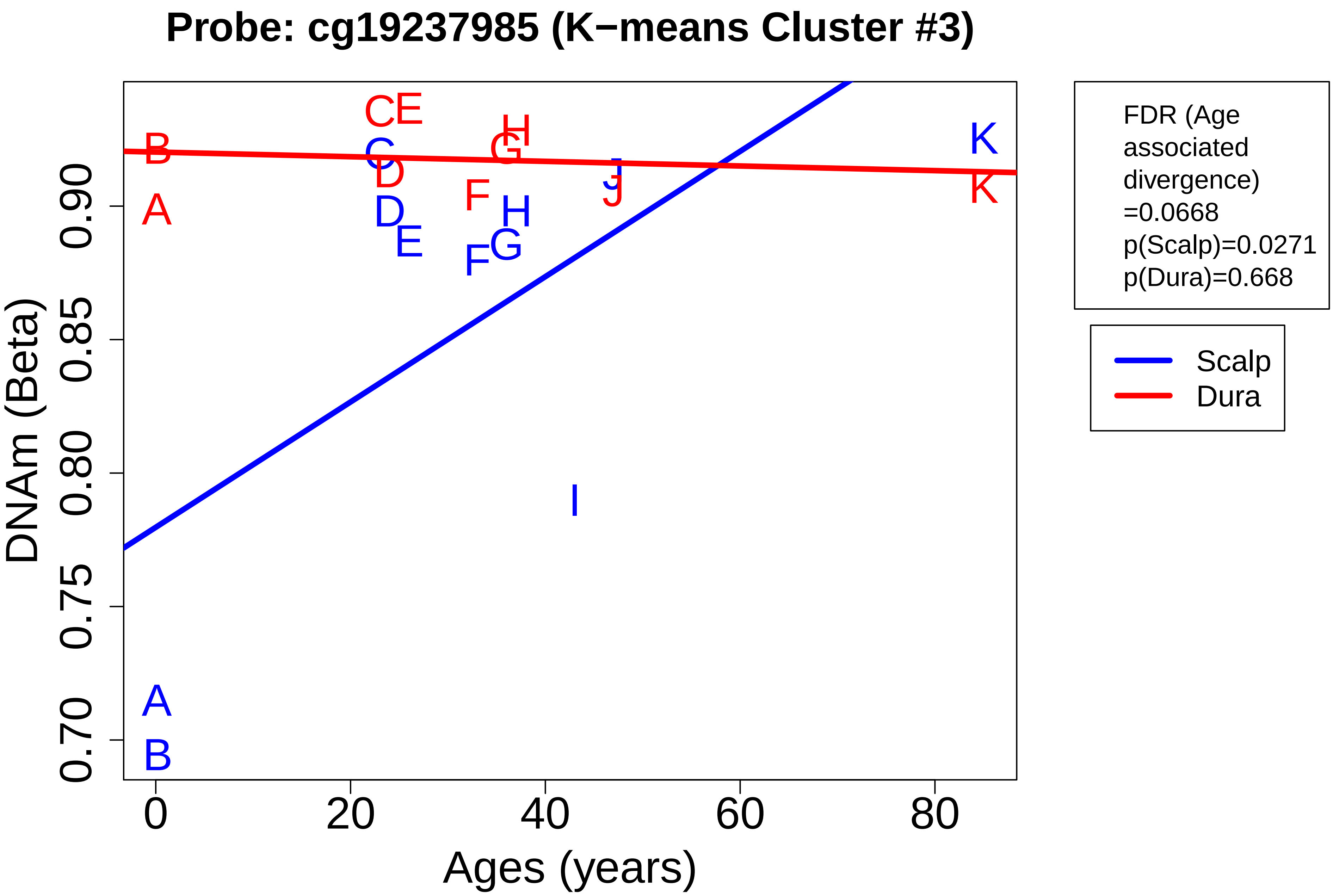

Probe: cg19237985 (K−means Cluster #3)
DNAm (Beta)
0.70
0.75
0.80
0.85
0.90
0
20
40
60
80
Ages (years)
FDR (Age associated divergence) =0.0668
p(Scalp)=0.0271
p(Dura)=0.668
Scalp
Dura
A
B
C
D
E
F
G
H
I
J
K

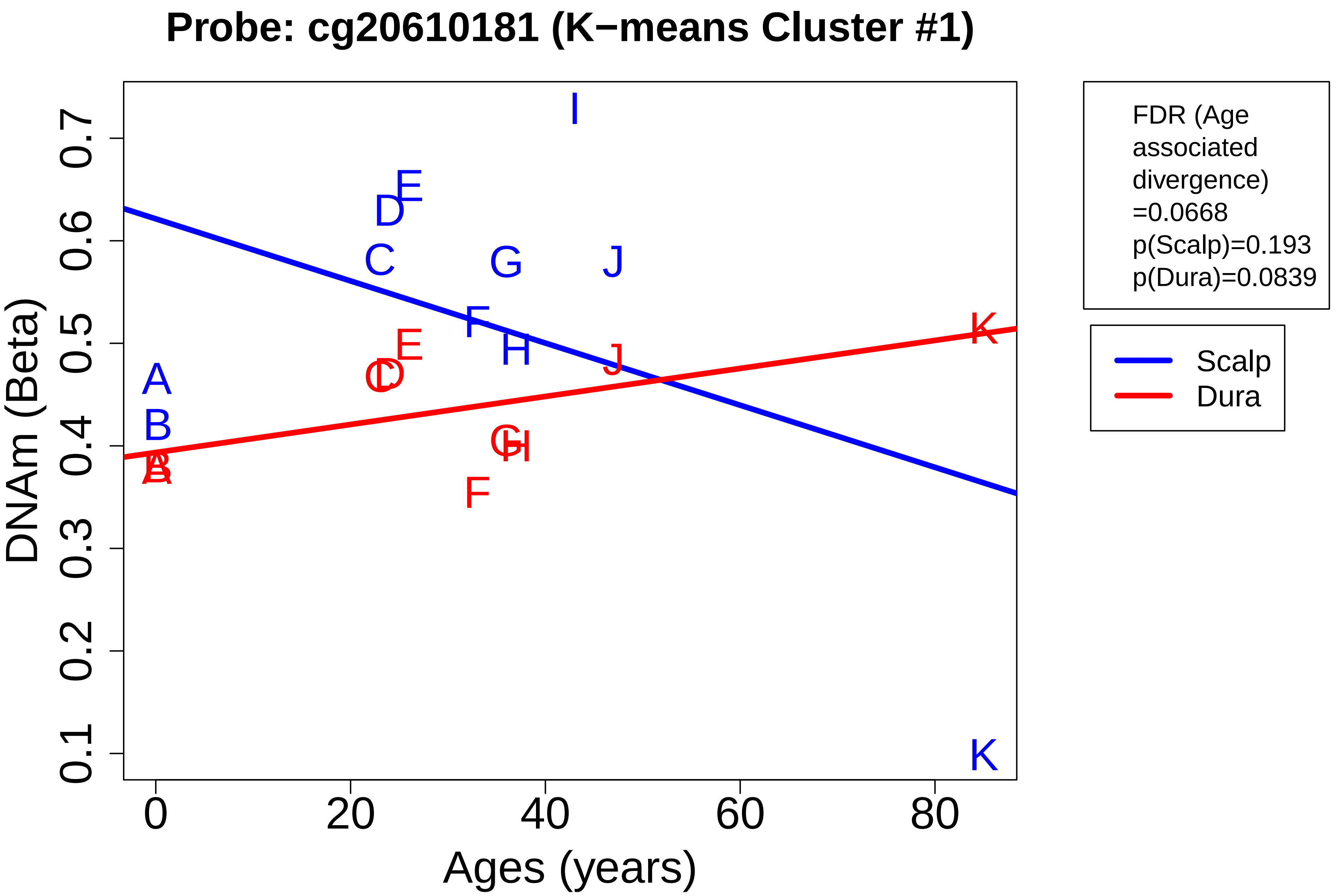
Probe: cg20610181 (K−means Cluster #1)
DNAm (Beta)
0.1
0.2
0.3
0.4
0.5
0.6
0.7
0
20
40
60
80
Ages (years)
FDR (Age associated divergence) =0.0668
p(Scalp)=0.193
p(Dura)=0.0839
Scalp
Dura

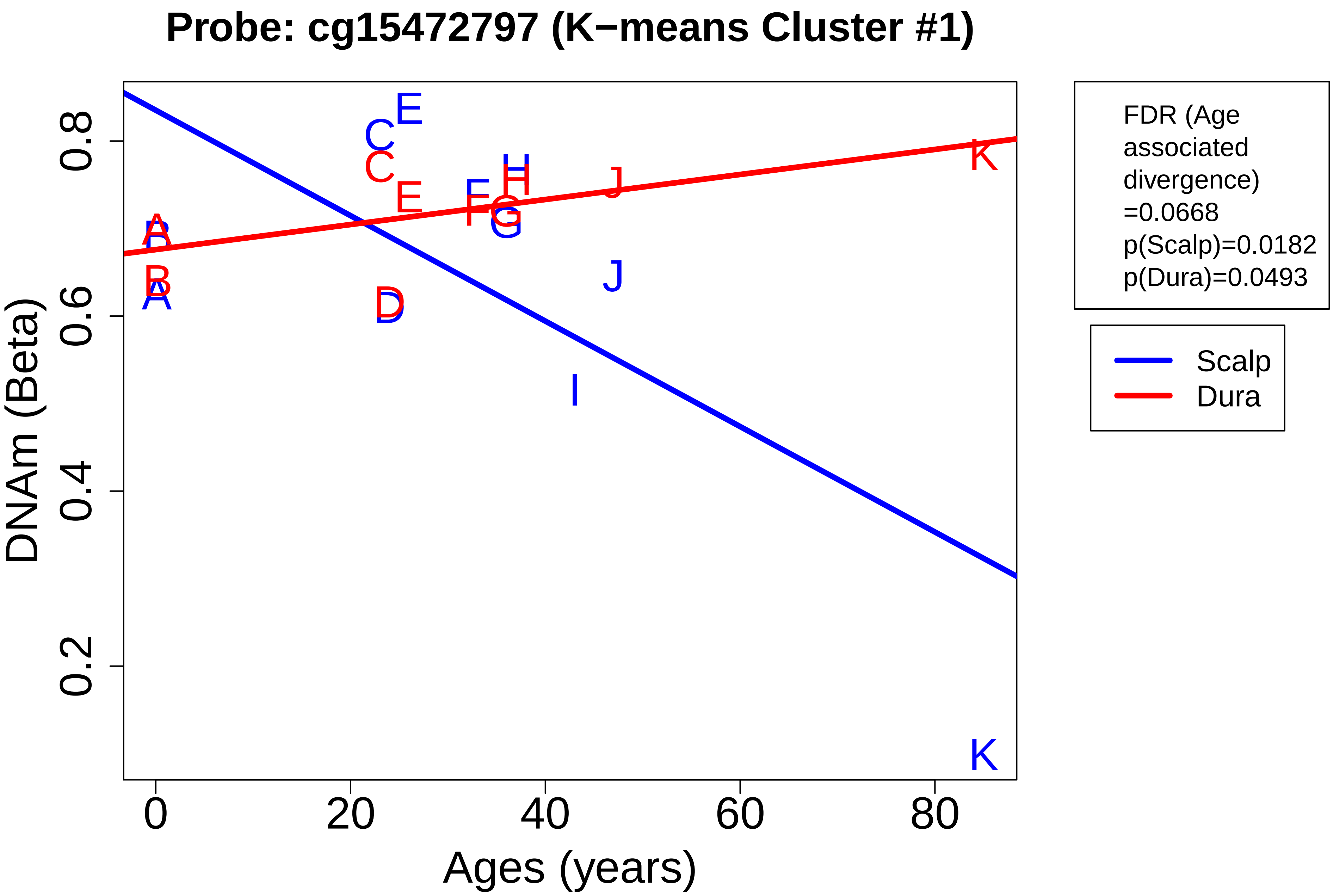

Probe: cg15472797 (K−means Cluster #1)
DNAm (Beta)
0.2
0.4
0.6
0.8
0
20
40
60
80
Ages (years)
FDR (Age associated divergence) =0.0668
p(Scalp)=0.0182
p(Dura)=0.0493
Scalp
Dura

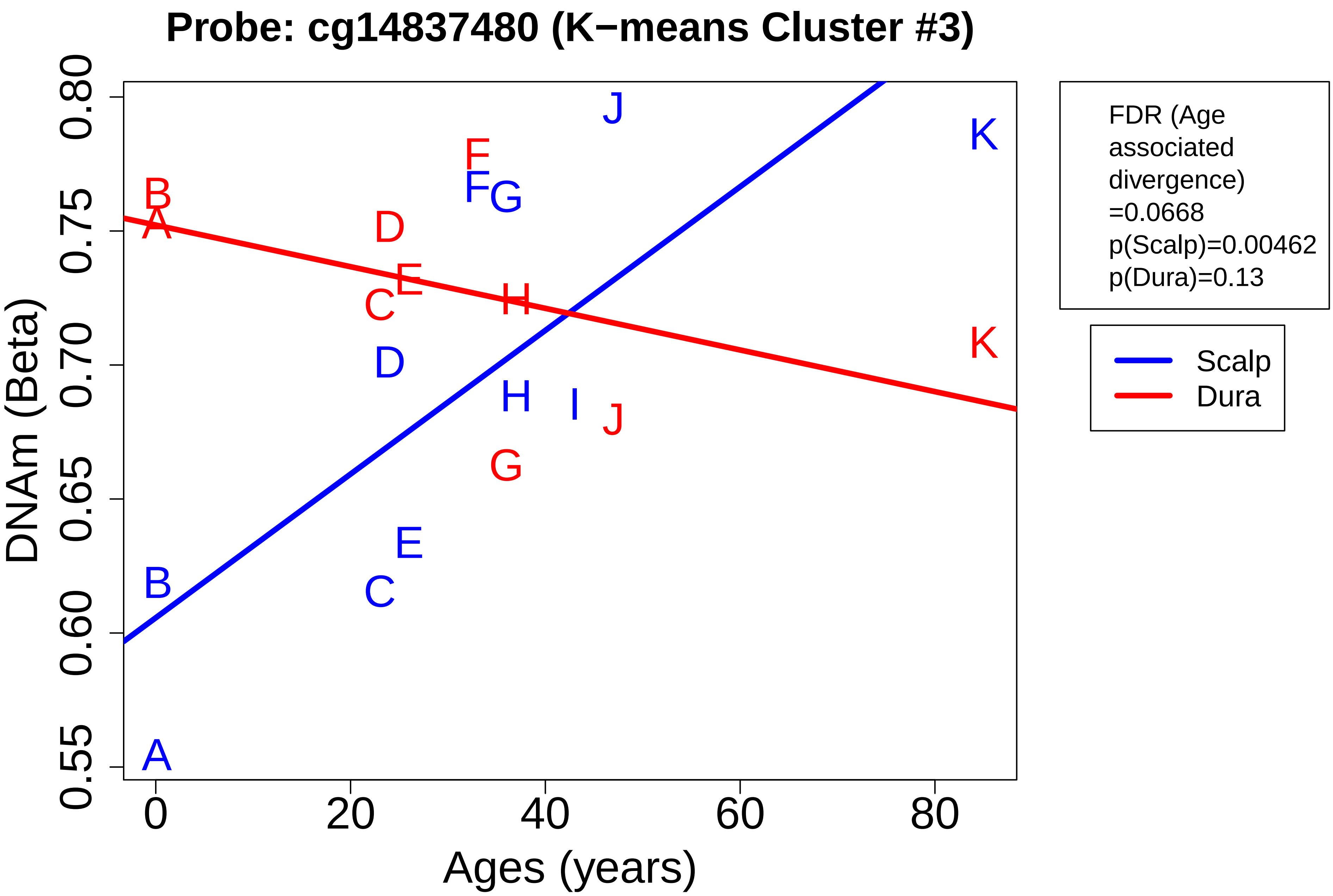

Probe: cg14837480 (K−means Cluster #3)
DNAm (Beta)
0.55
0.60
0.65
0.70
0.75
0.80
Ages (years)
0
20
40
60
80
A
B
C
D
E
F
G
H
I
J
K
FDR (Age associated divergence) =0.0668
p(Scalp)=0.00462
p(Dura)=0.13
Scalp
Dura

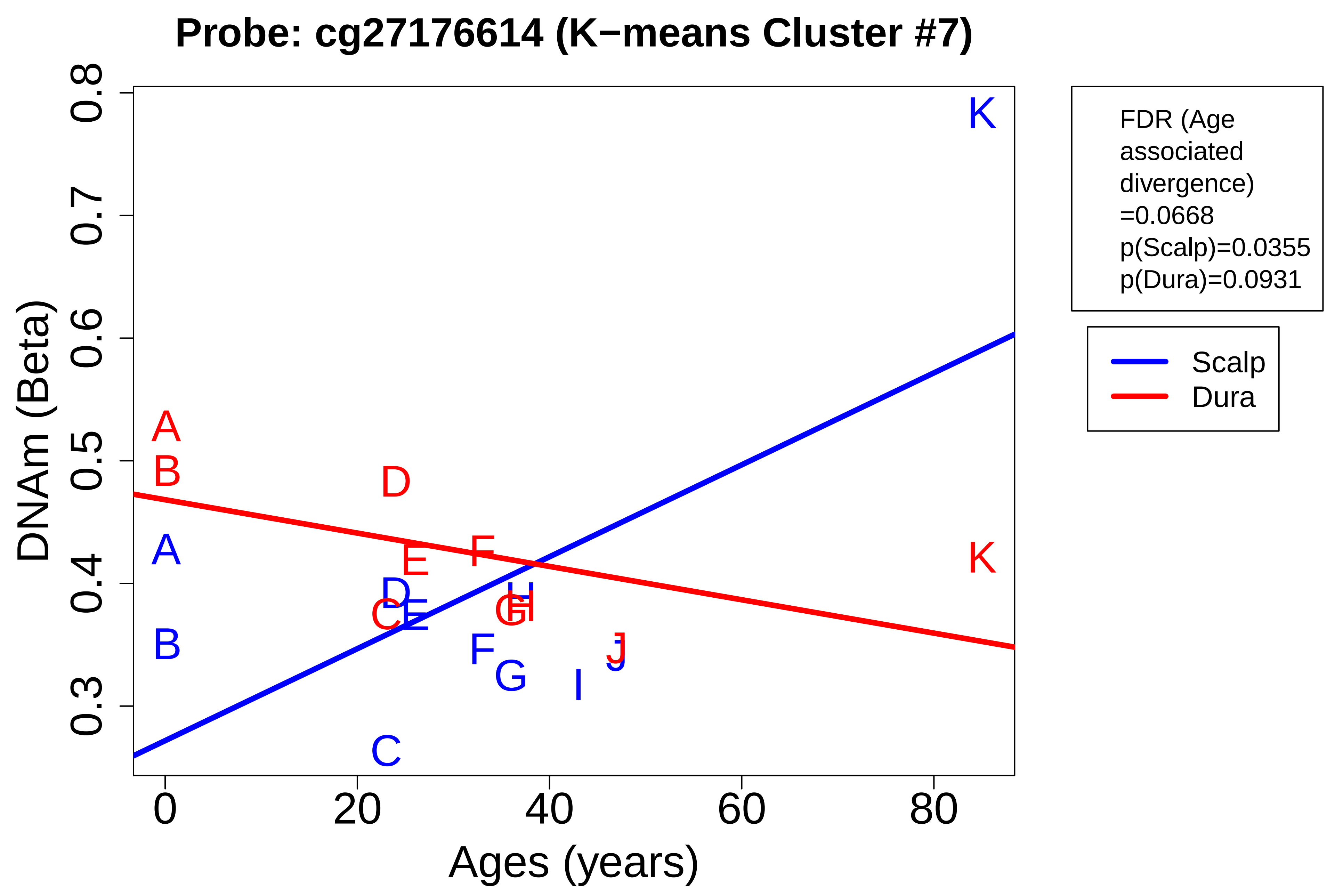

Probe: cg27176614 (K−means Cluster #7)
DNAm (Beta)
0.3
0.4
0.5
0.6
0.7
0.8
0
20
40
60
80
Ages (years)
A
B
C
D
E
F
G
H
I
J
K
FDR (Age associated divergence) =0.0668
p(Scalp)=0.0355
p(Dura)=0.0931
Scalp
Dura

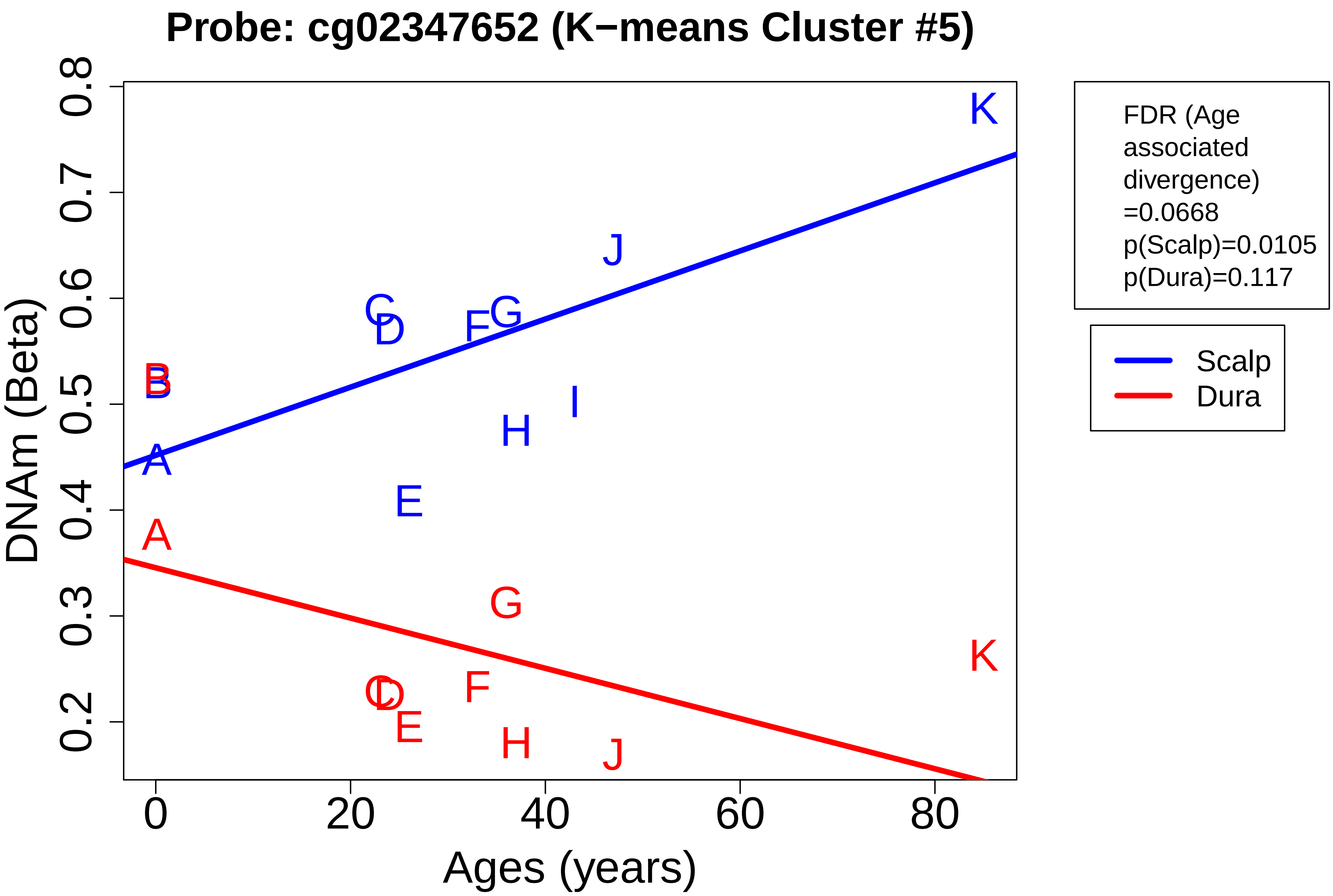
Probe: cg02347652 (K−means Cluster #5)
DNAm (Beta)
0.2
0.3
0.4
0.5
0.6
0.7
0.8
Ages (years)
0
20
40
60
80
A
B
C
D
E
F
G
H
I
J
K
FDR (Age associated divergence) =0.0668
p(Scalp)=0.0105
p(Dura)=0.117
Scalp
Dura

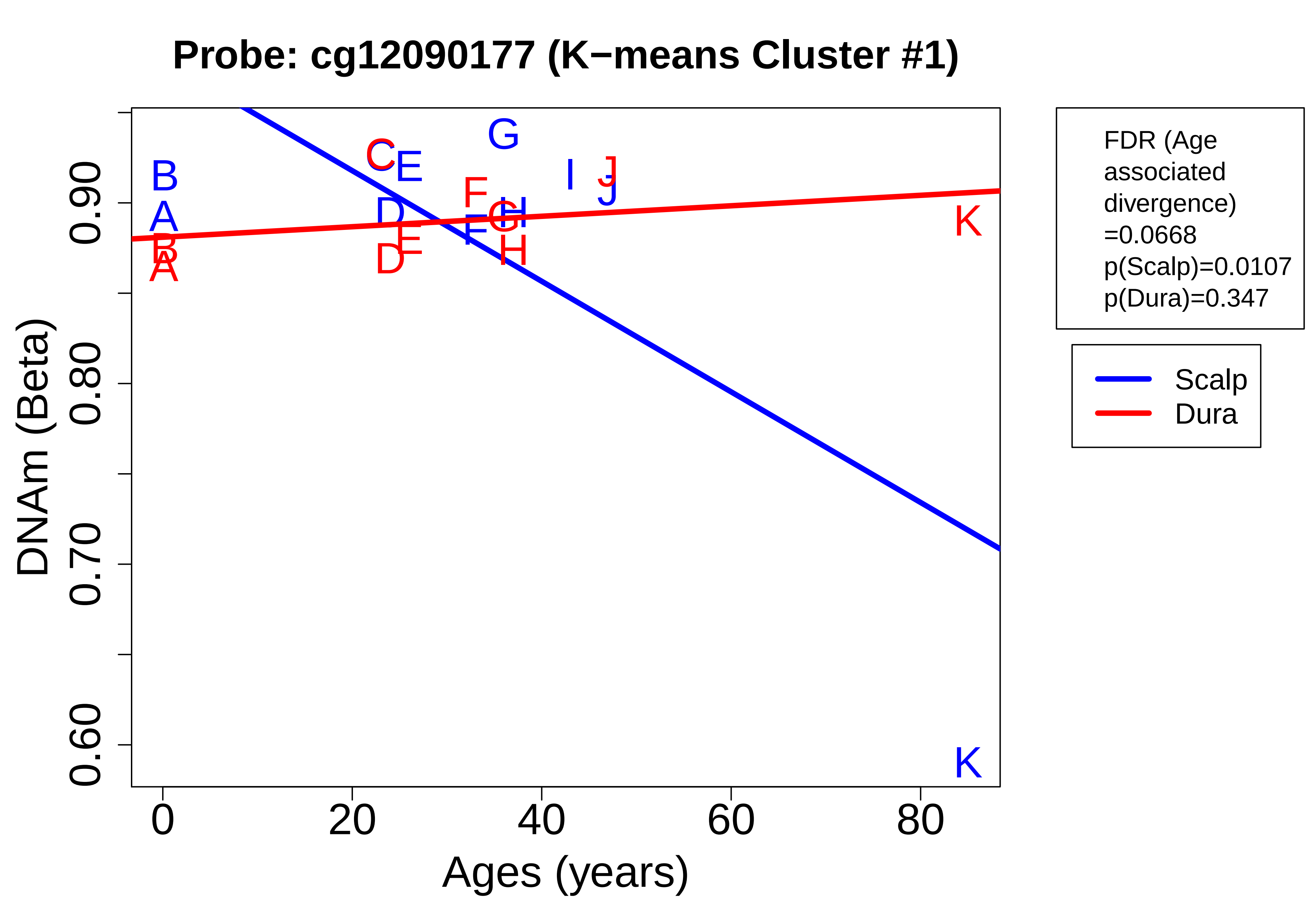
Probe: cg12090177 (K−means Cluster #1)
FDR (Age associated divergence) =0.0668
p(Scalp)=0.0107
p(Dura)=0.347
Scalp
Dura
DNAm (Beta)
0.60
0.70
0.80
0.90
0
20
40
60
80
Ages (years)
A
B
C
D
E
F
G
H
I
J
K

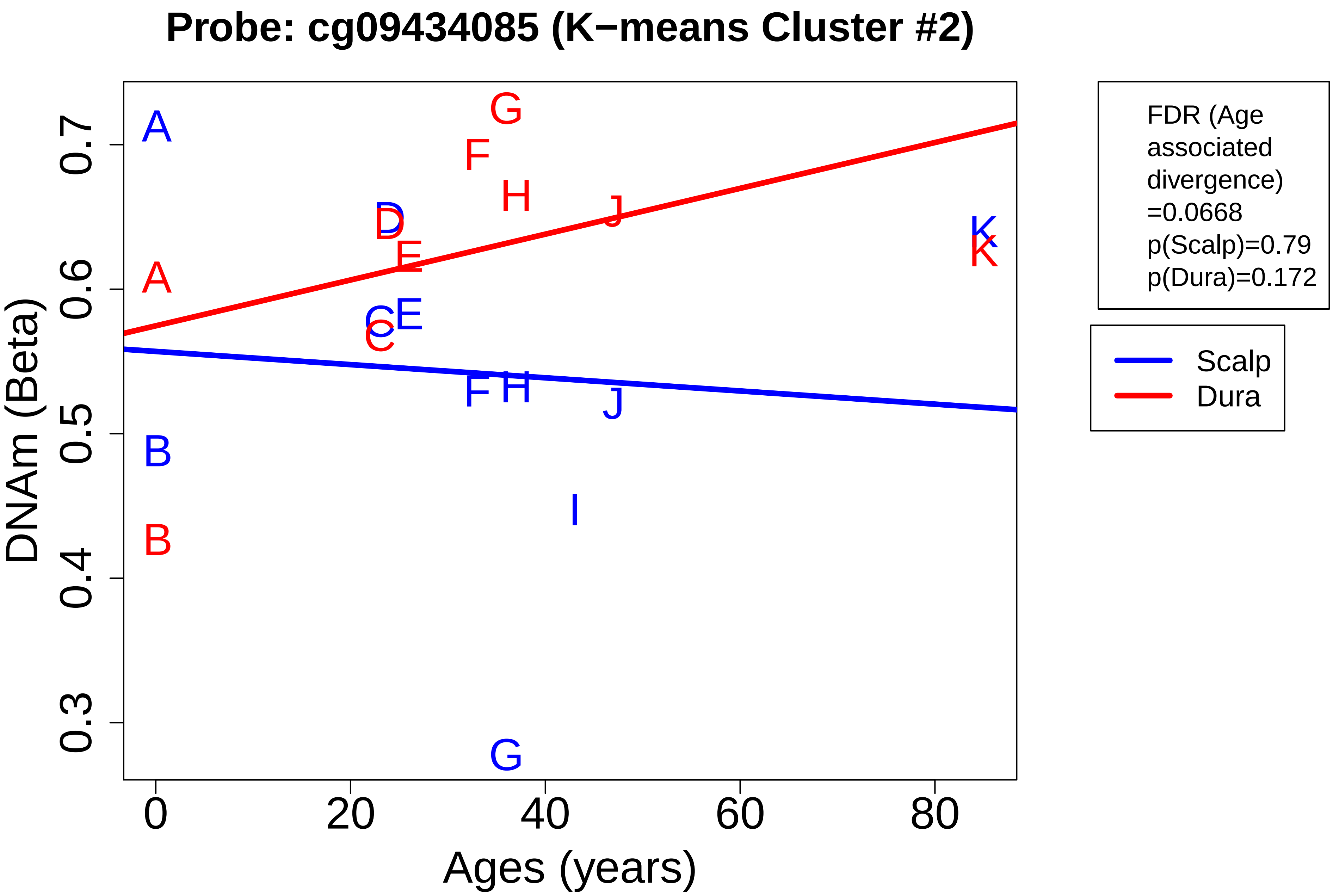

Probe: cg09434085 (K−means Cluster #2)
DNAm (Beta)
0.3
0.4
0.5
0.6
0.7
Ages (years)
0
20
40
60
80
FDR (Age associated divergence) =0.0668
p(Scalp)=0.79
p(Dura)=0.172
Scalp
Dura
A
B
C
D
E
F
G
H
I
J
K

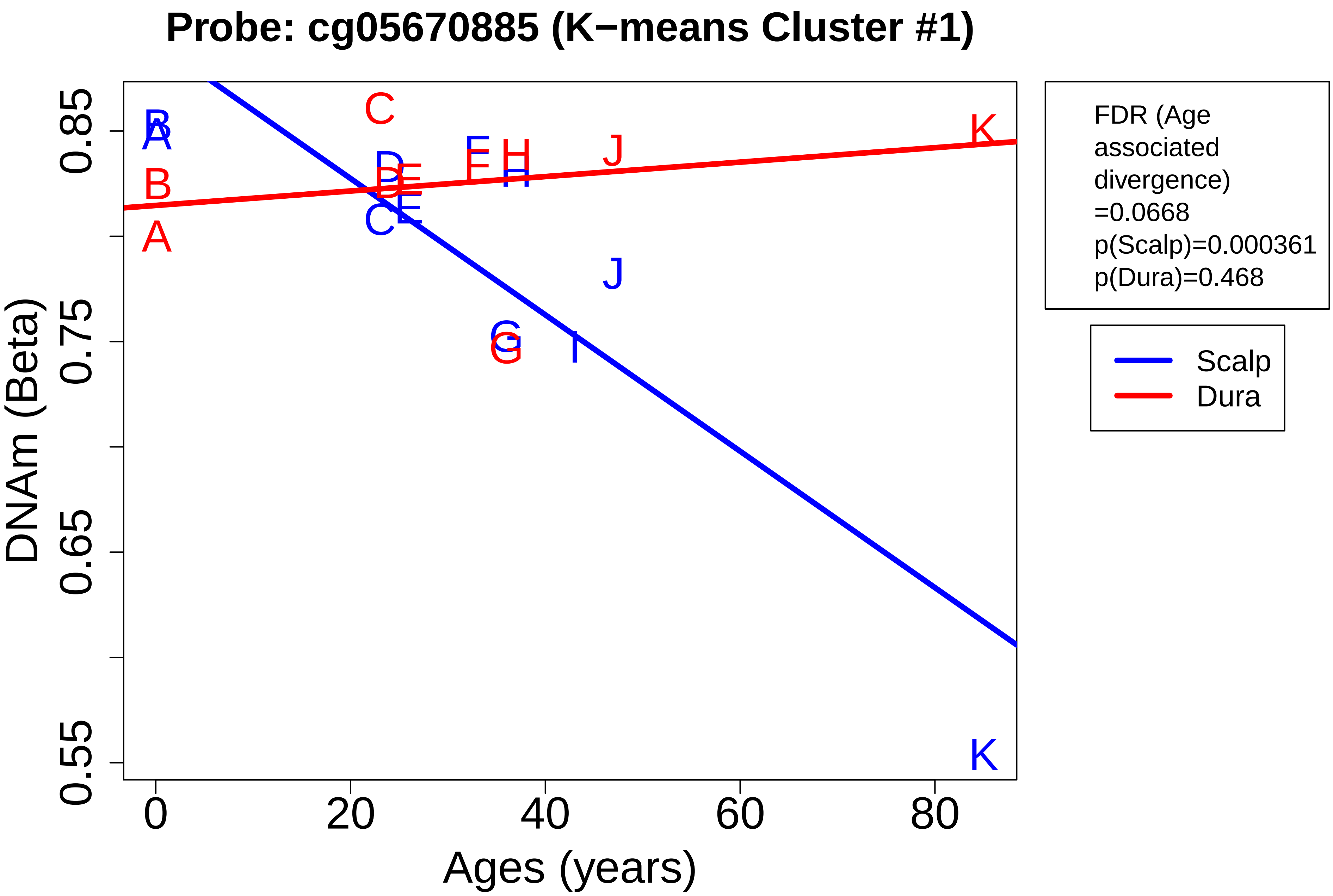
Probe: cg05670885 (K−means Cluster #1)
DNAm (Beta)
0.55
0.65
0.75
0.85
0
20
40
60
80
Ages (years)
FDR (Age associated divergence) =0.0668
p(Scalp)=0.000361
p(Dura)=0.468
Scalp
Dura
A
B
C
D
E
F
G
H
I
J
K

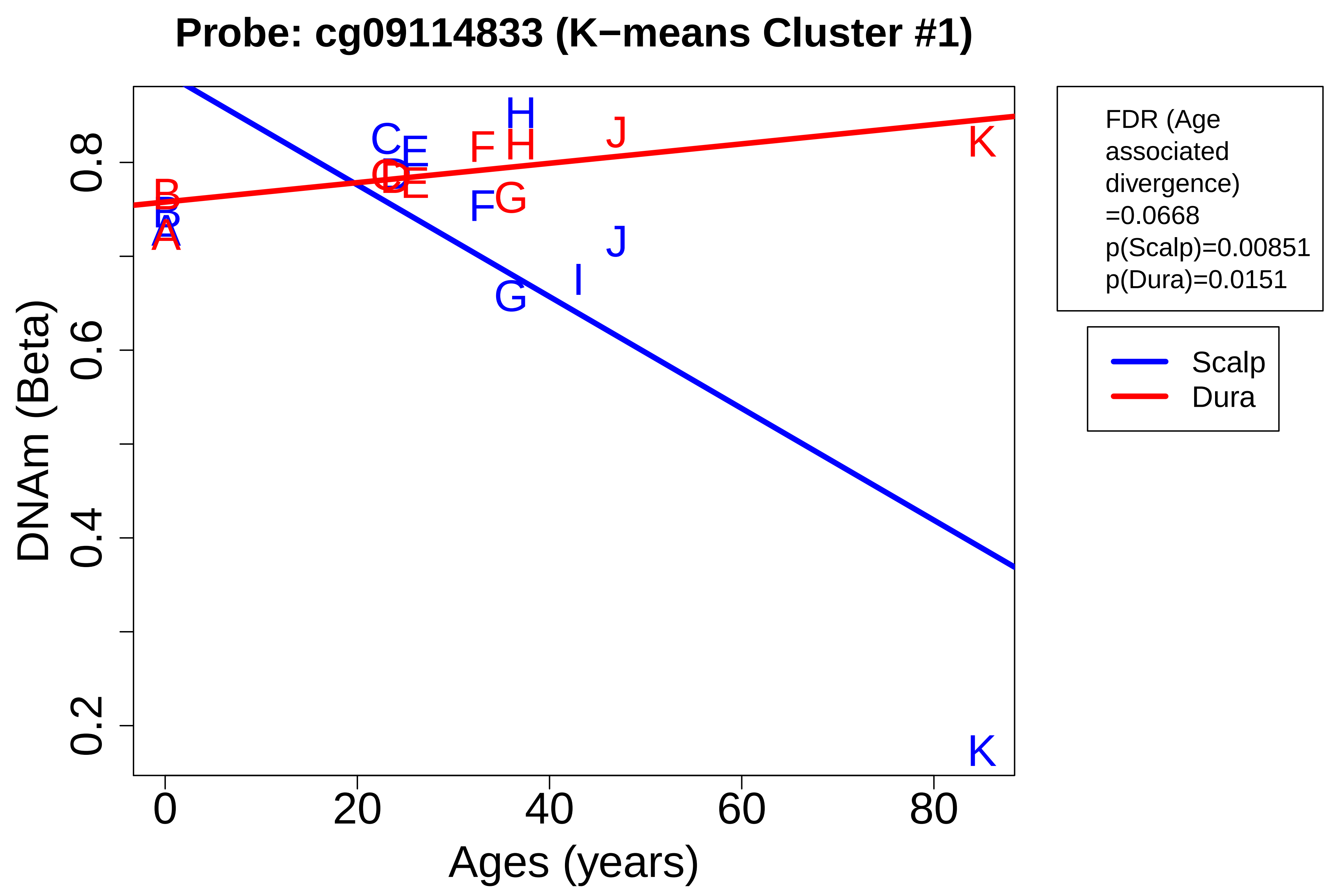

Probe: cg09114833 (K−means Cluster #1)
DNAm (Beta)
0.2
0.4
0.6
0.8
0
20
40
60
80
Ages (years)
A
B
C
D
E
F
G
H
I
J
K
FDR (Age associated divergence) =0.0668
p(Scalp)=0.00851
p(Dura)=0.0151
Scalp
Dura

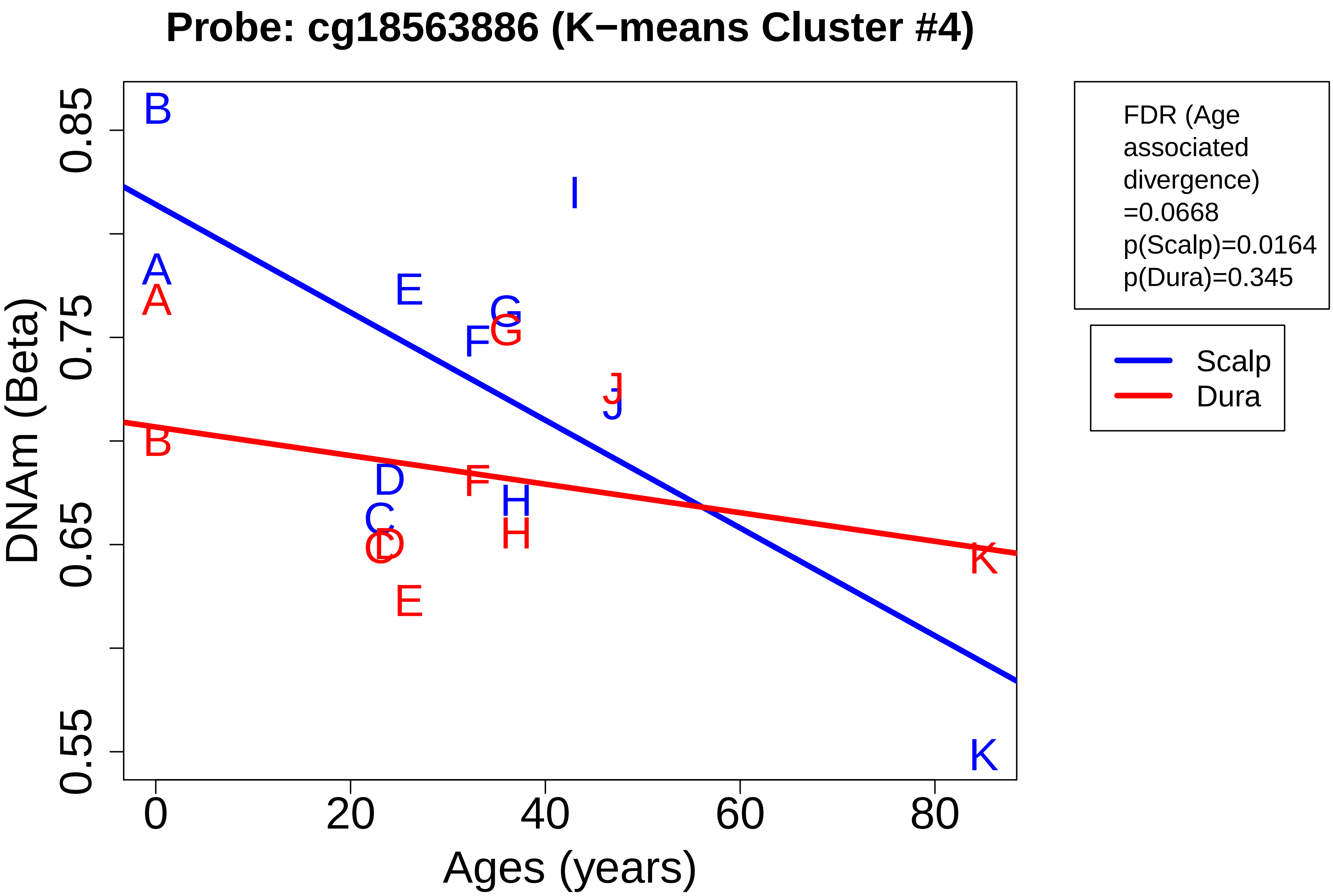

Probe: cg18563886 (K−means Cluster #4)
DNAm (Beta)
0.55
0.65
0.75
0.85
Ages (years)
0
20
40
60
80
FDR (Age associated divergence) =0.0668
p(Scalp)=0.0164
p(Dura)=0.345
Scalp
Dura
A
B
C
D
E
F
G
H
I
J
K

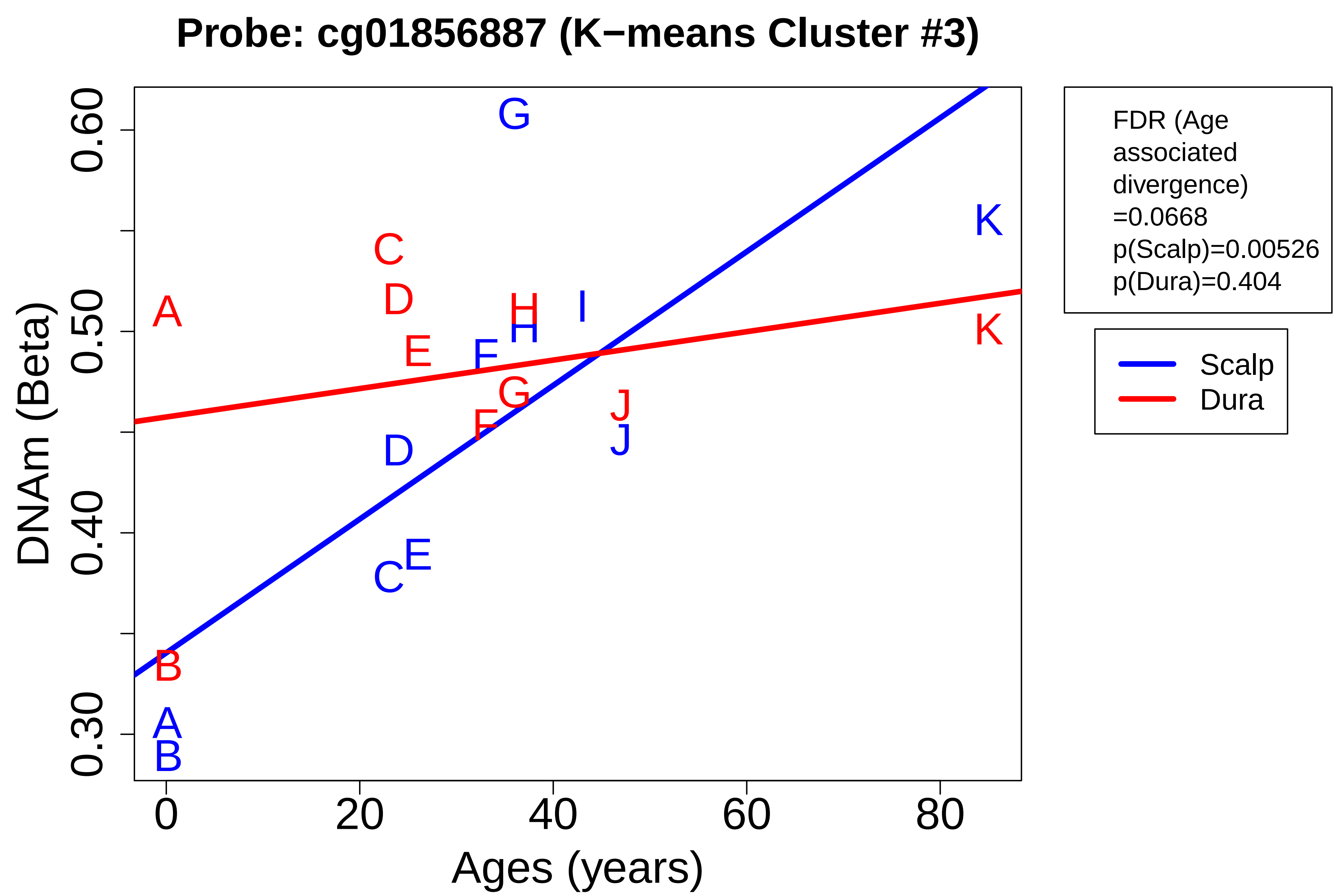

Probe: cg01856887 (K−means Cluster #3)
DNAm (Beta)
0.30
0.40
0.50
0.60
Ages (years)
0
20
40
60
80
A
B
C
D
E
F
G
H
I
J
K
FDR (Age associated divergence) =0.0668
p(Scalp)=0.00526
p(Dura)=0.404
Scalp
Dura

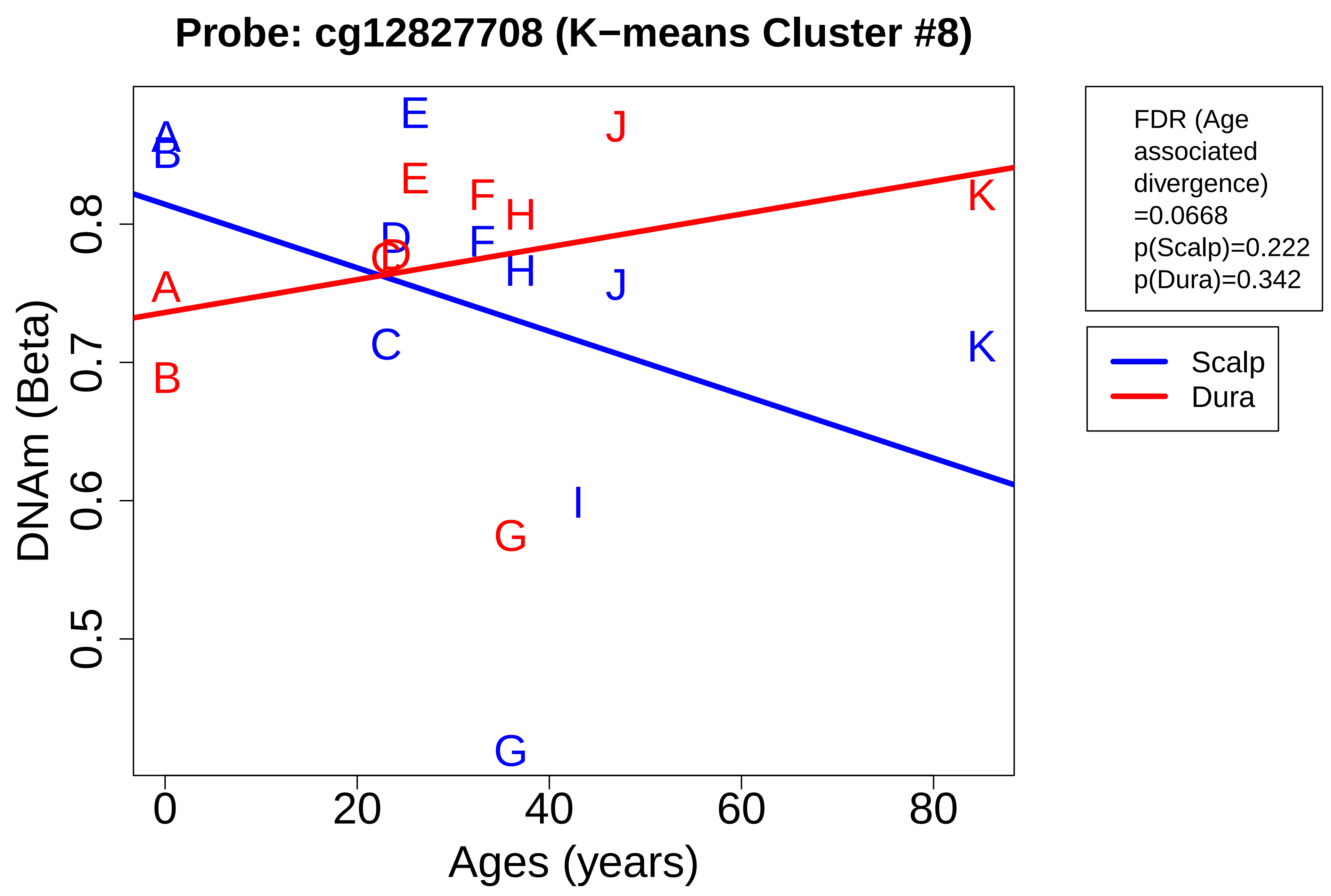

Probe: cg12827708 (K−means Cluster #8)
FDR (Age associated divergence) =0.0668
p(Scalp)=0.222
p(Dura)=0.342
Scalp
Dura
DNAm (Beta)
0.5
0.6
0.7
0.8
0
20
40
60
80
Ages (years)
A
B
C
D
E
F
G
H
I
J
K

Probe: cg18221862 (K−means Cluster #7)

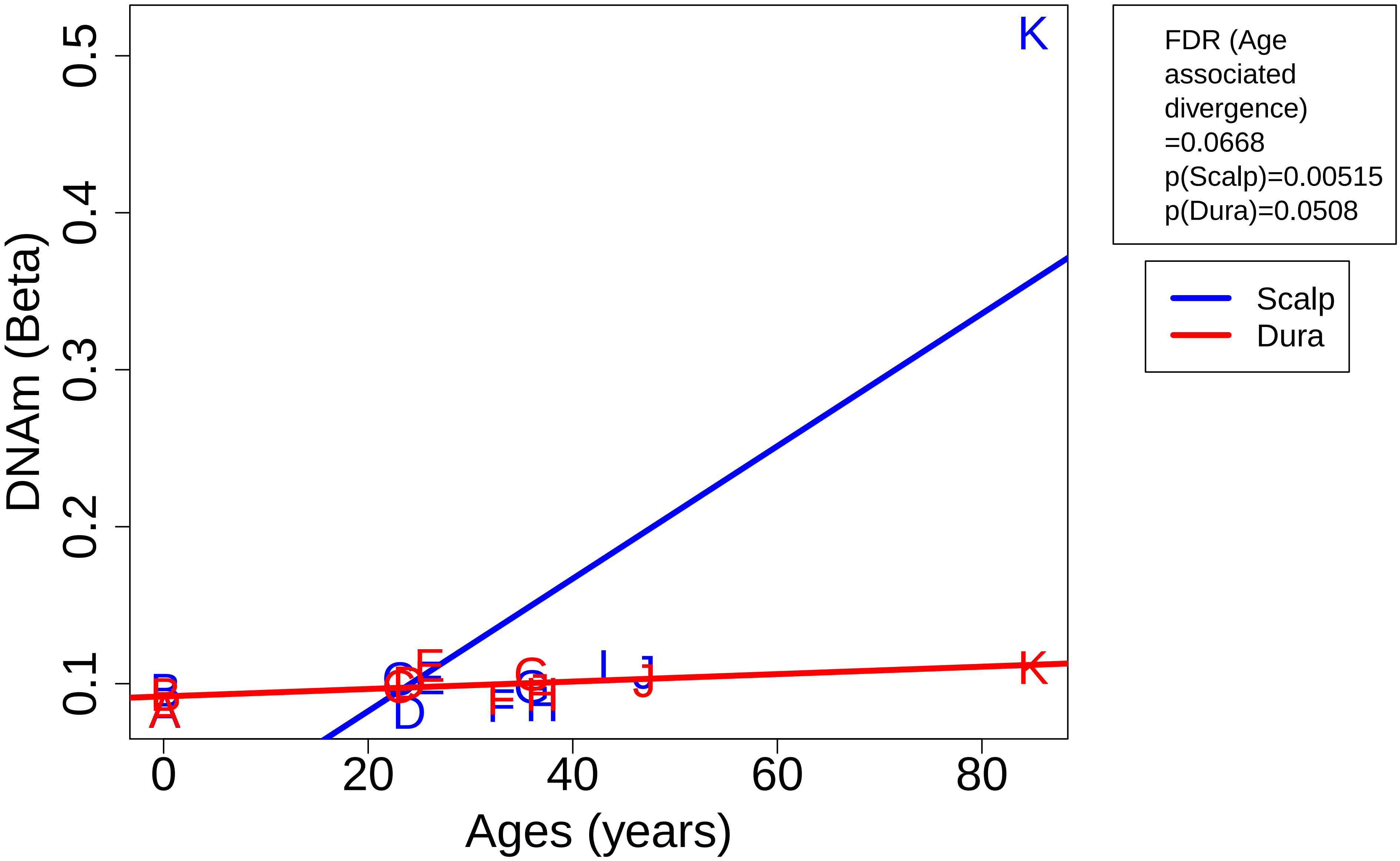

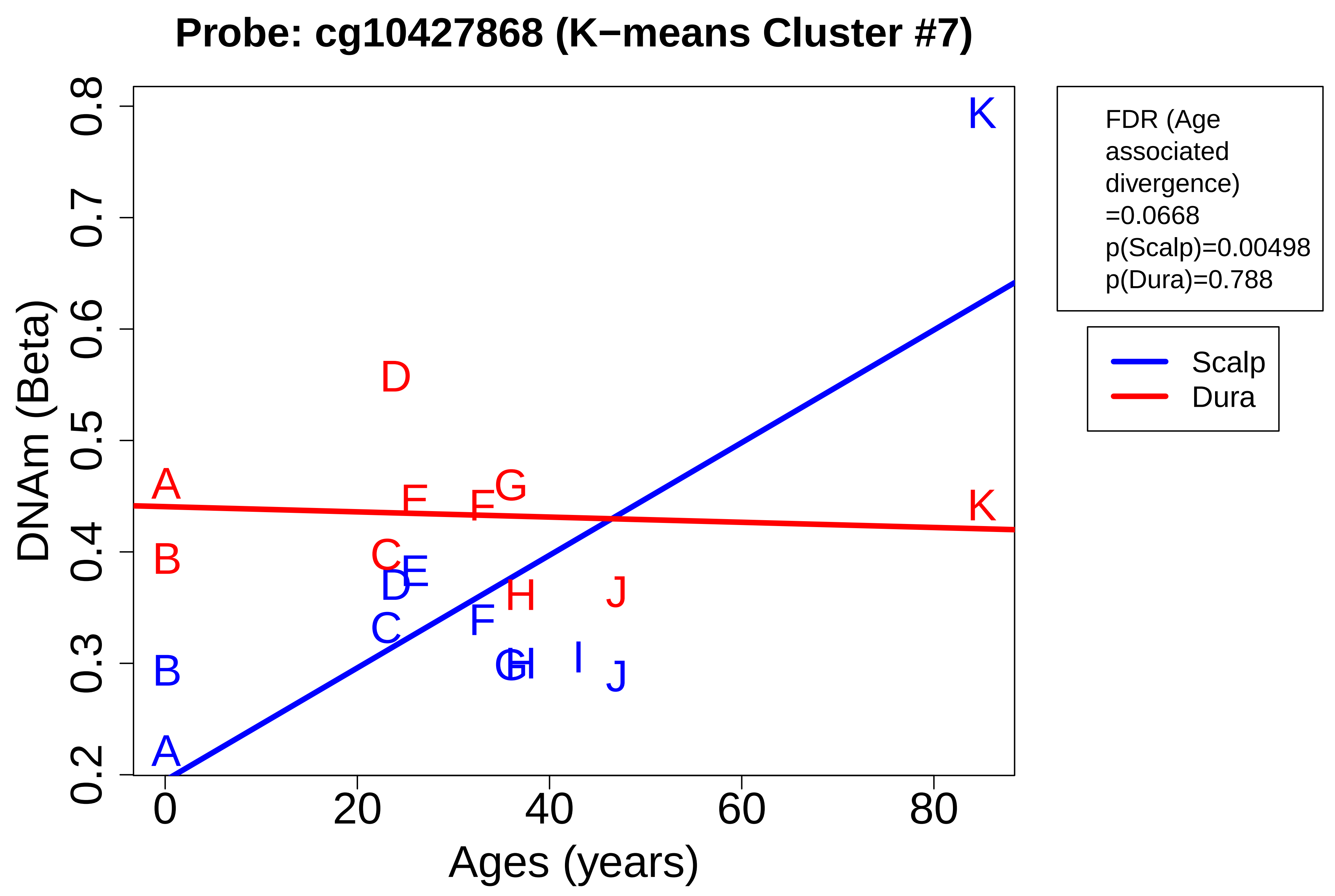

Probe: cg10427868 (K−means Cluster #7)
DNAm (Beta)
0.2
0.3
0.4
0.5
0.6
0.7
0.8
Ages (years)
0
20
40
60
80
FDR (Age associated divergence) =0.0668
p(Scalp)=0.00498
p(Dura)=0.788
Scalp
Dura

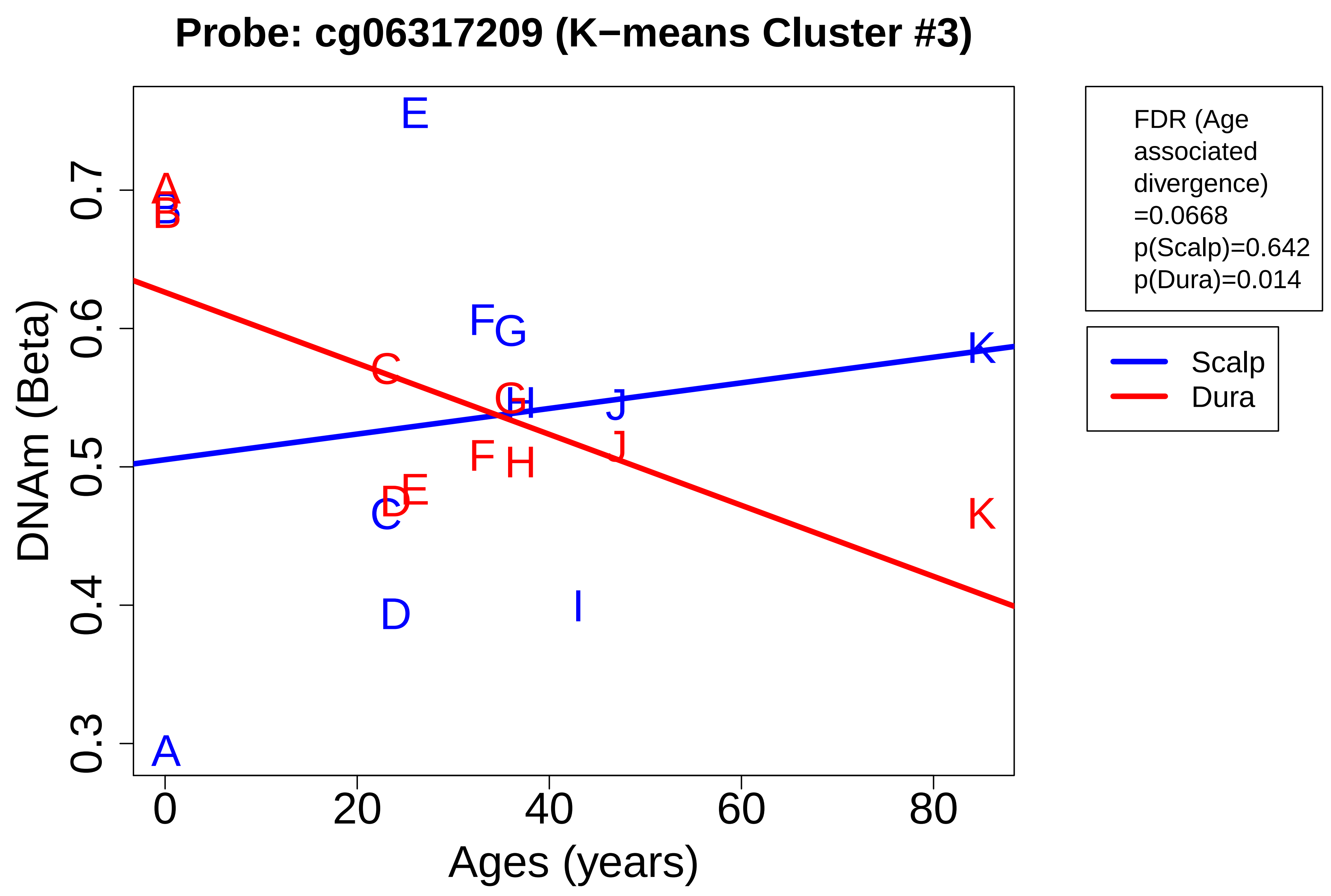

Probe: cg06317209 (K−means Cluster #3)
FDR (Age associated divergence) =0.0668
p(Scalp)=0.642
p(Dura)=0.014
Scalp
Dura
DNAm (Beta)
Ages (years)
0.3
0.4
0.5
0.6
0.7
0
20
40
60
80

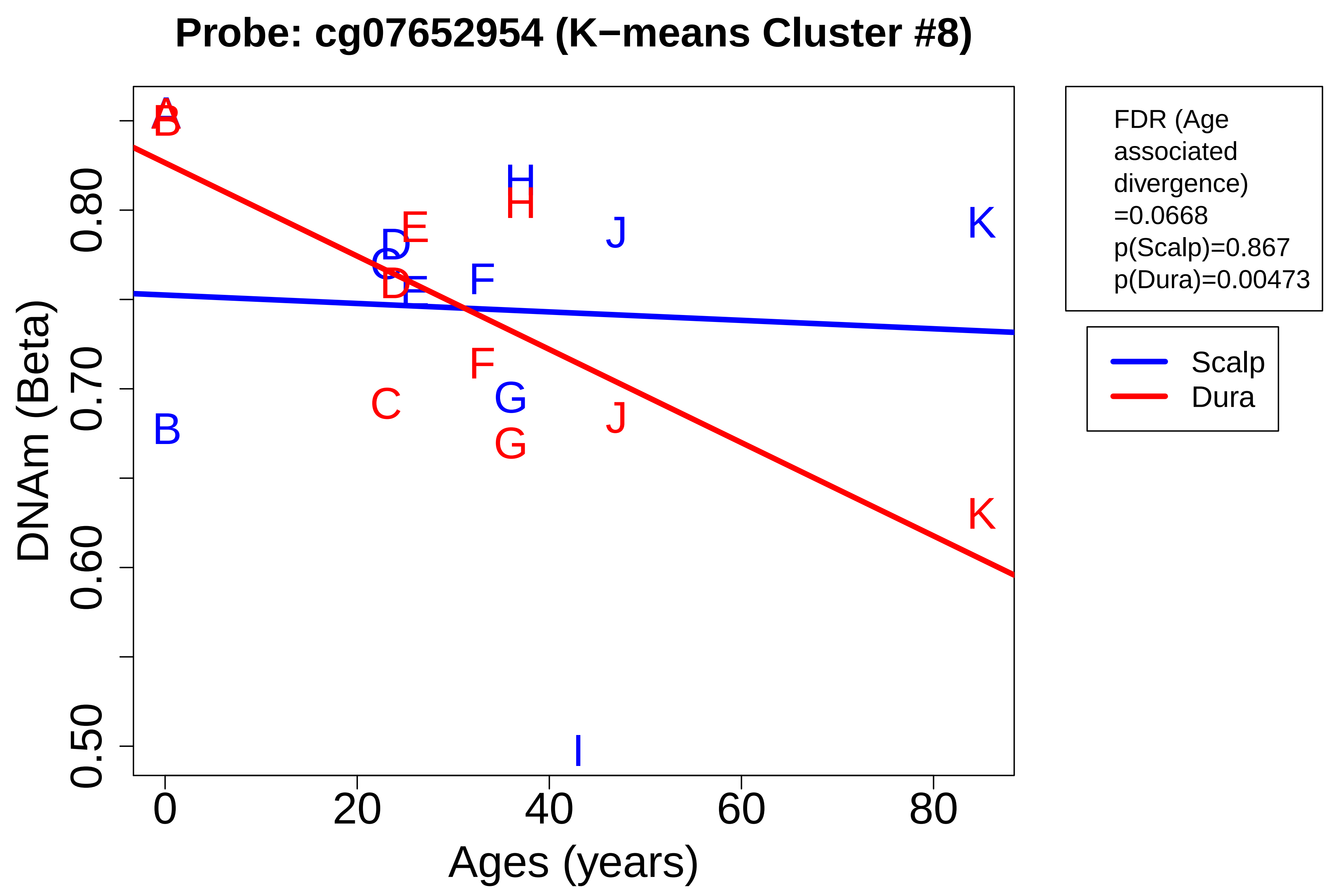

Probe: cg07652954 (K−means Cluster #8)
DNAm (Beta)
0.50
0.60
0.70
0.80
0
20
40
60
80
Ages (years)
FDR (Age associated divergence) =0.0668
p(Scalp)=0.867
p(Dura)=0.00473
Scalp
Dura

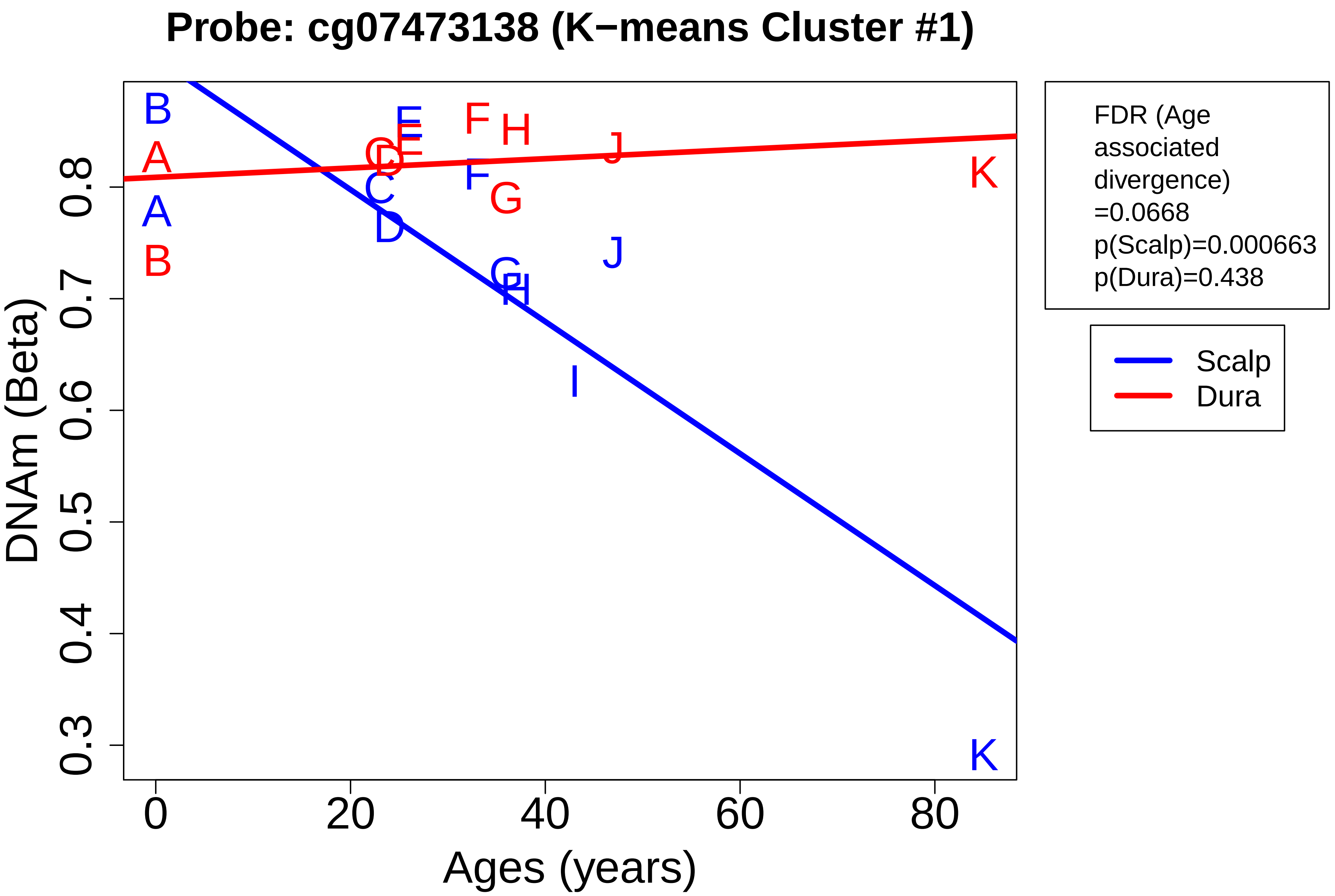

Probe: cg07473138 (K−means Cluster #1)
DNAm (Beta)
Ages (years)
0.3
0.4
0.5
0.6
0.7
0.8
0
20
40
60
80
FDR (Age associated divergence) =0.0668
p(Scalp)=0.000663
p(Dura)=0.438
Scalp
Dura

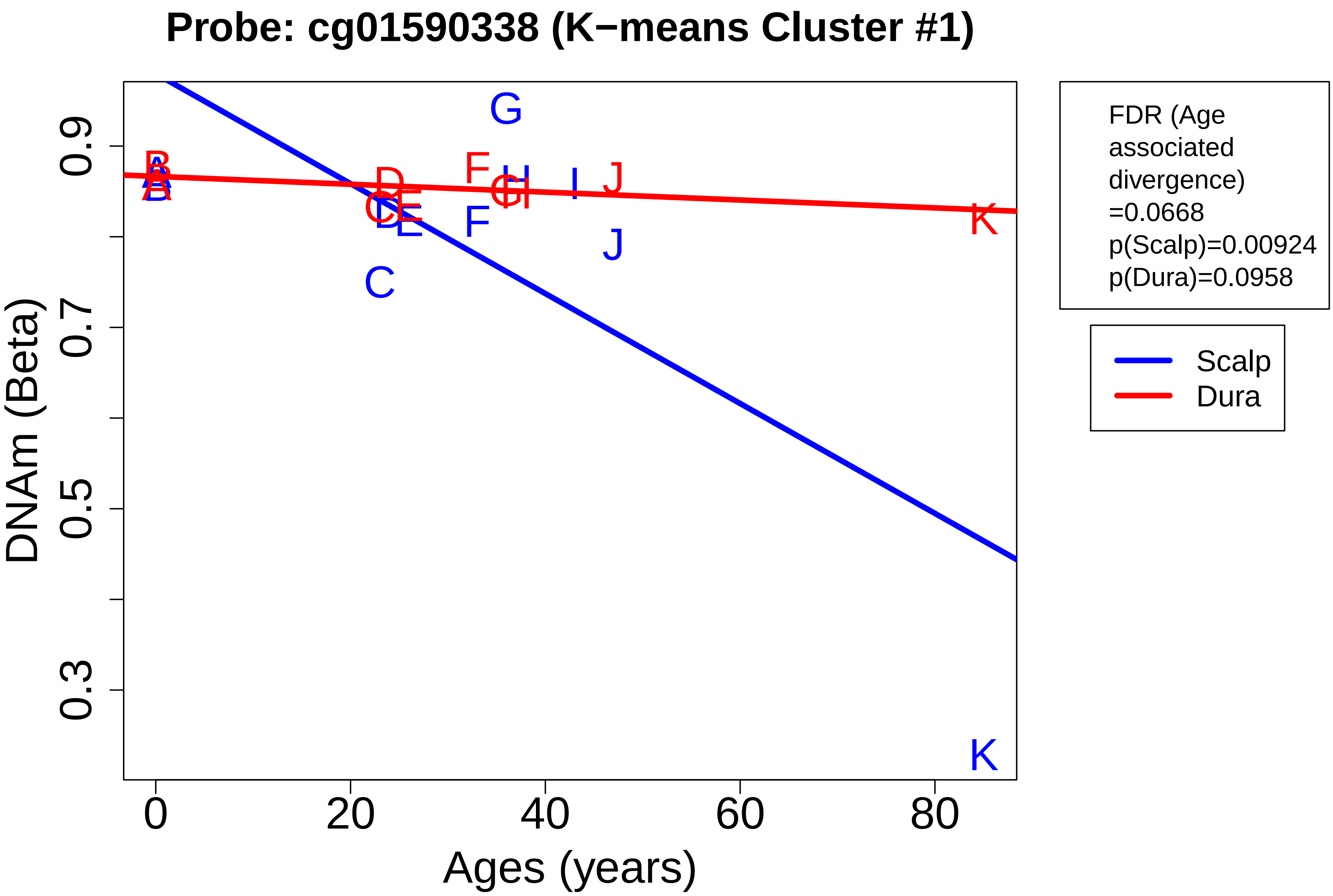

Probe: cg01590338 (K−means Cluster #1)
DNAm (Beta)
0.3
0.5
0.7
0.9
0
20
40
60
80
Ages (years)
FDR (Age associated divergence) =0.0668
p(Scalp)=0.00924
p(Dura)=0.0958
Scalp
Dura

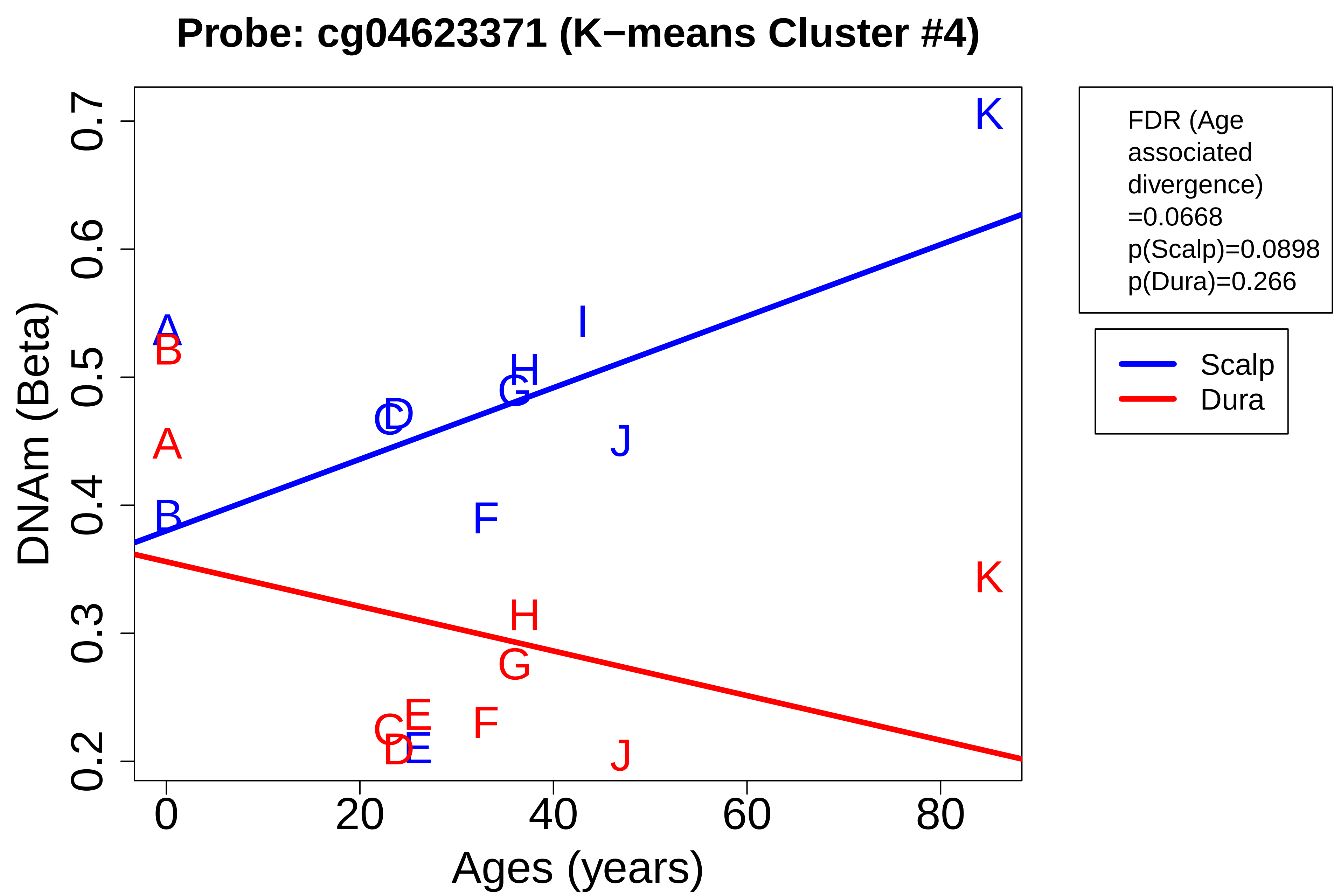

Probe: cg04623371 (K−means Cluster #4)
DNAm (Beta)
0.2
0.3
0.4
0.5
0.6
0.7
0
20
40
60
80
Ages (years)
FDR (Age associated divergence) =0.0668
p(Scalp)=0.0898
p(Dura)=0.266
Scalp
Dura

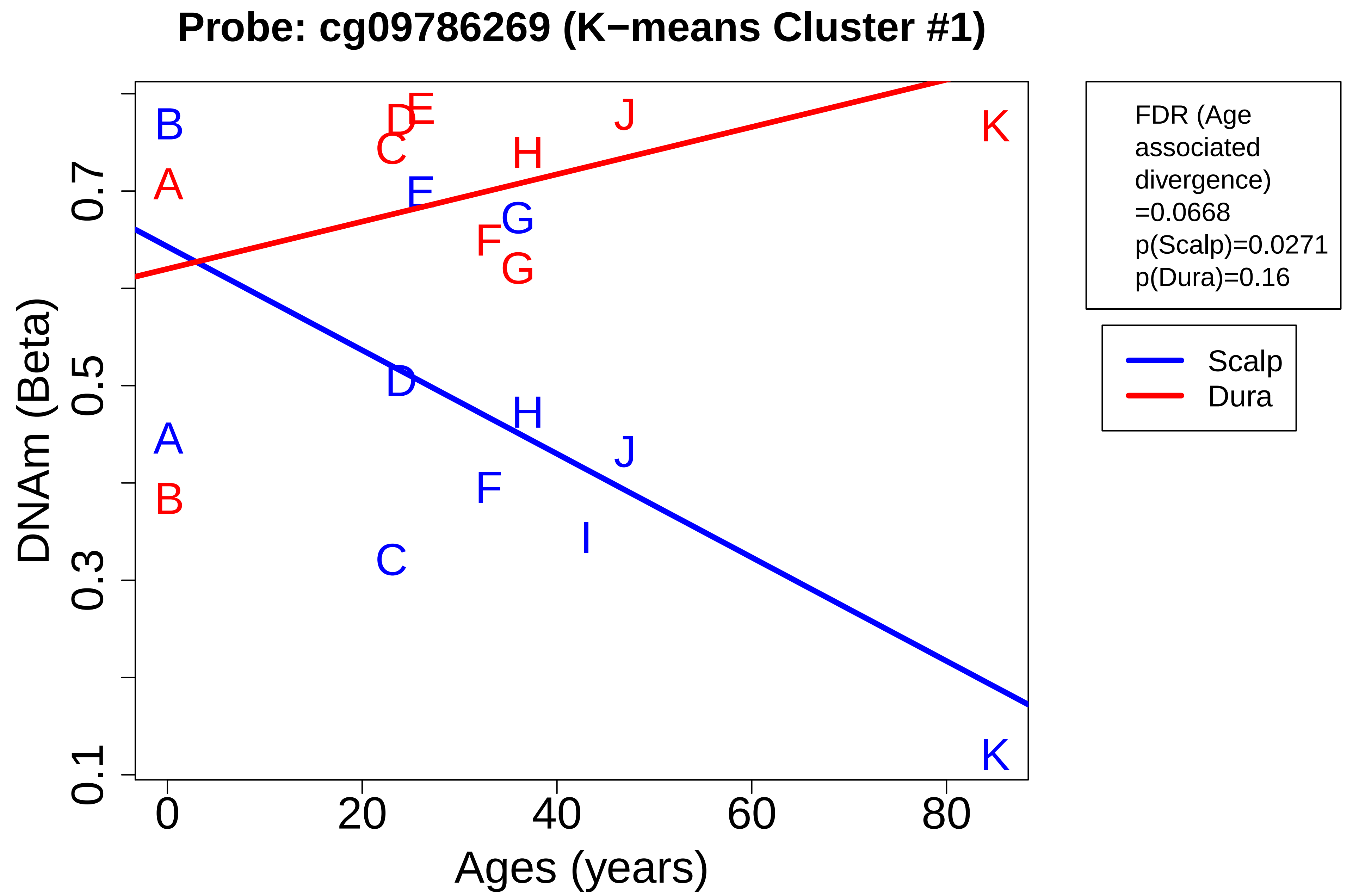

Probe: cg09786269 (K−means Cluster #1)
DNAm (Beta)
0.1
0.3
0.5
0.7
0
20
40
60
80
Ages (years)
A
B
C
D
E
F
G
H
I
J
K
FDR (Age associated divergence) =0.0668
p(Scalp)=0.0271
p(Dura)=0.16
Scalp
Dura

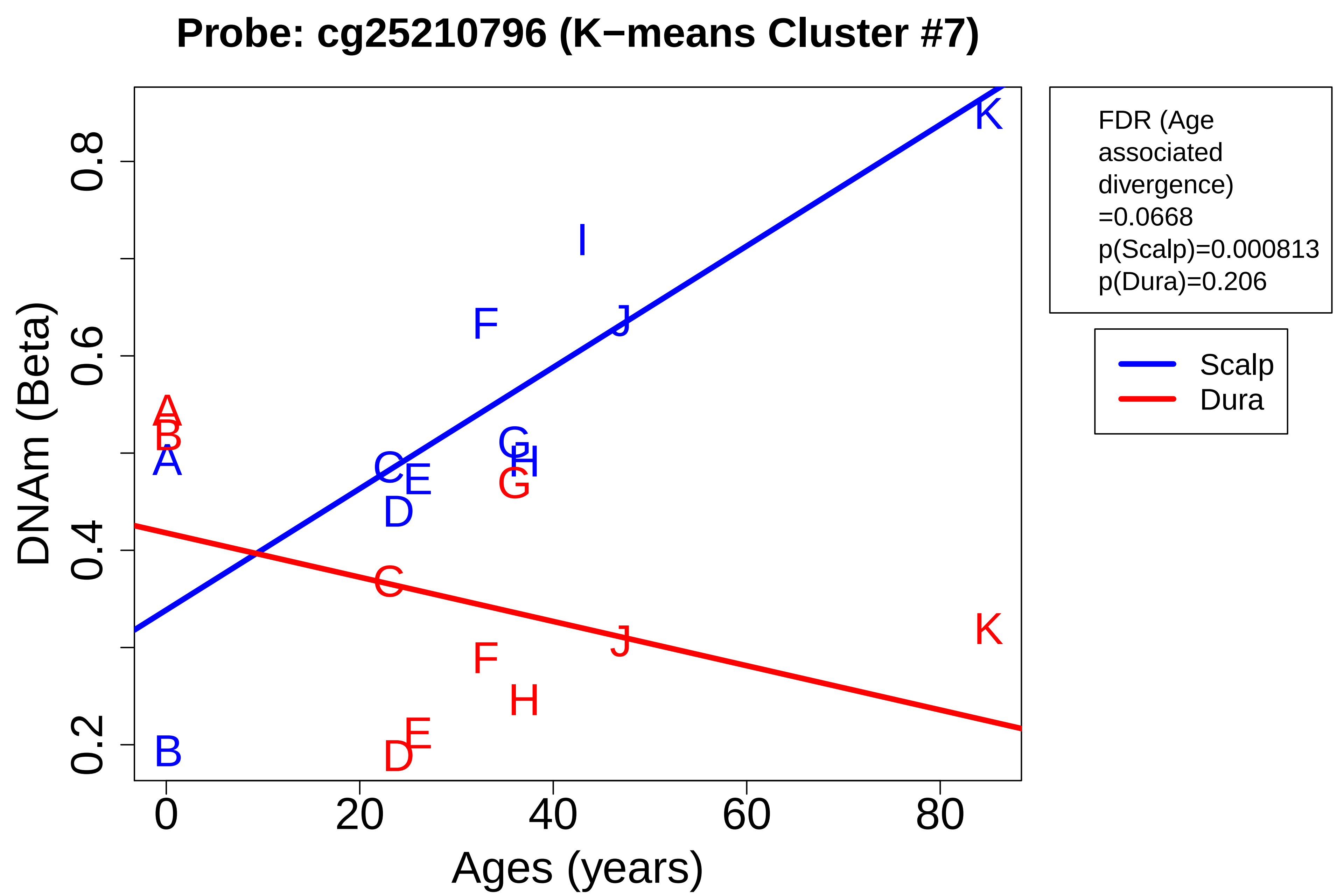

Probe: cg25210796 (K−means Cluster #7)
DNAm (Beta)
0.2
0.4
0.6
0.8
Ages (years)
0
20
40
60
80
FDR (Age associated divergence) =0.0668
p(Scalp)=0.000813
p(Dura)=0.206
Scalp
Dura
A
B
C
D
E
F
G
H
I
J
K

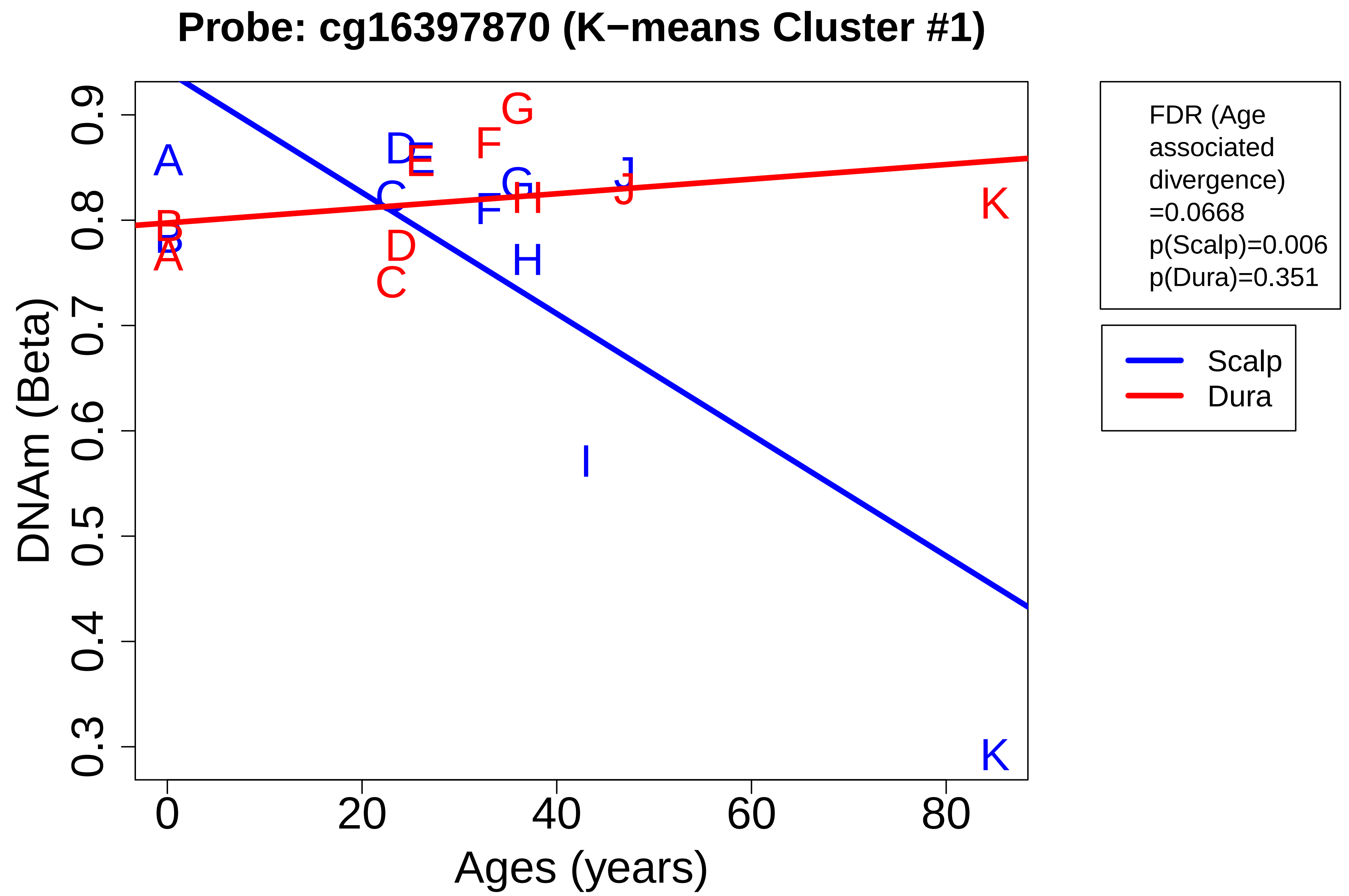

Probe: cg16397870 (K−means Cluster #1)
DNAm (Beta)
0.3
0.4
0.5
0.6
0.7
0.8
0.9
0
20
40
60
80
Ages (years)
FDR (Age associated divergence) =0.0668
p(Scalp)=0.006
p(Dura)=0.351
Scalp
Dura

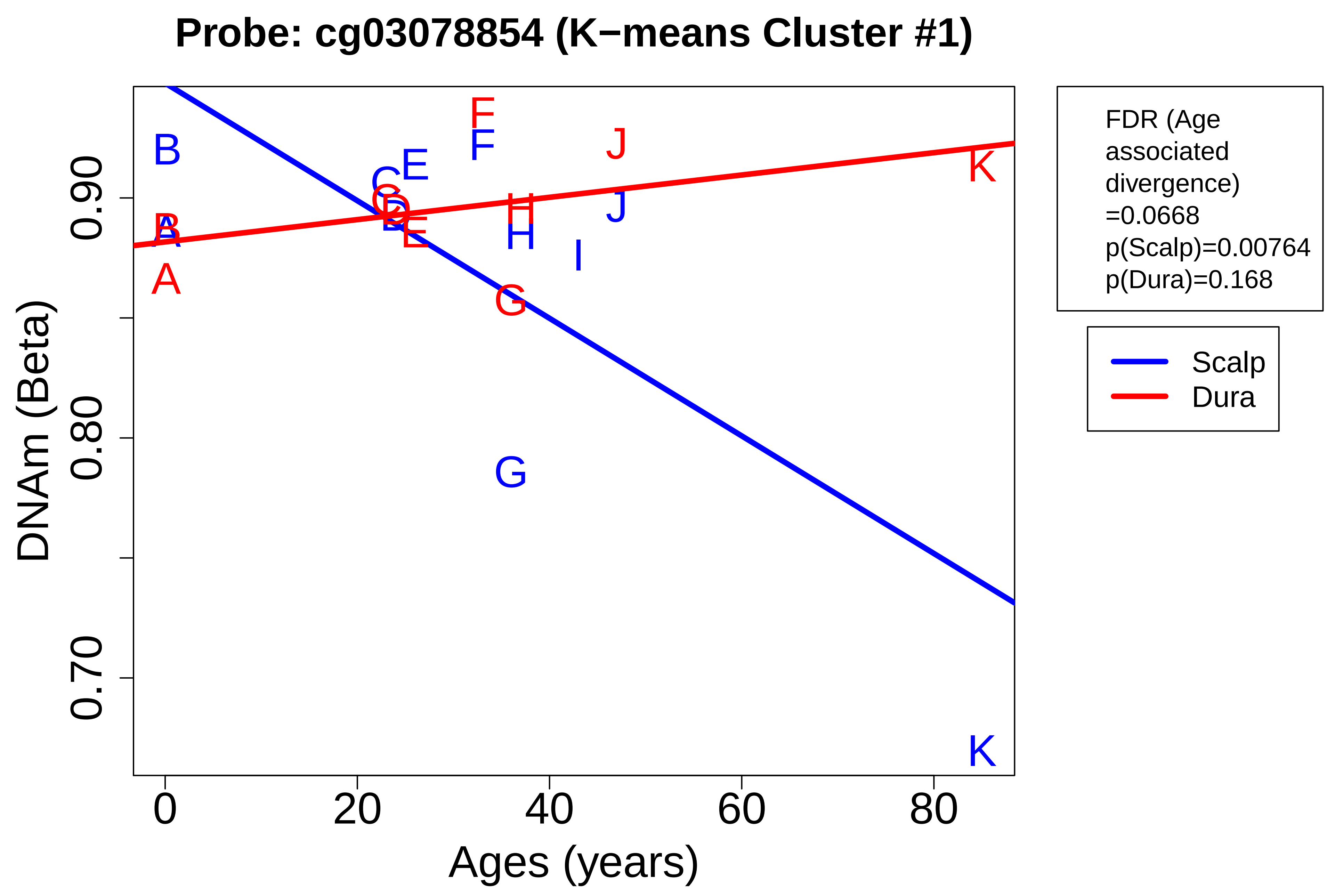

Probe: cg03078854 (K−means Cluster #1)
FDR (Age associated divergence) =0.0668
p(Scalp)=0.00764
p(Dura)=0.168
Scalp
Dura
DNAm (Beta)
0.70
0.80
0.90
Ages (years)
0
20
40
60
80
A
B
C
D
E
F
G
H
I
J
K

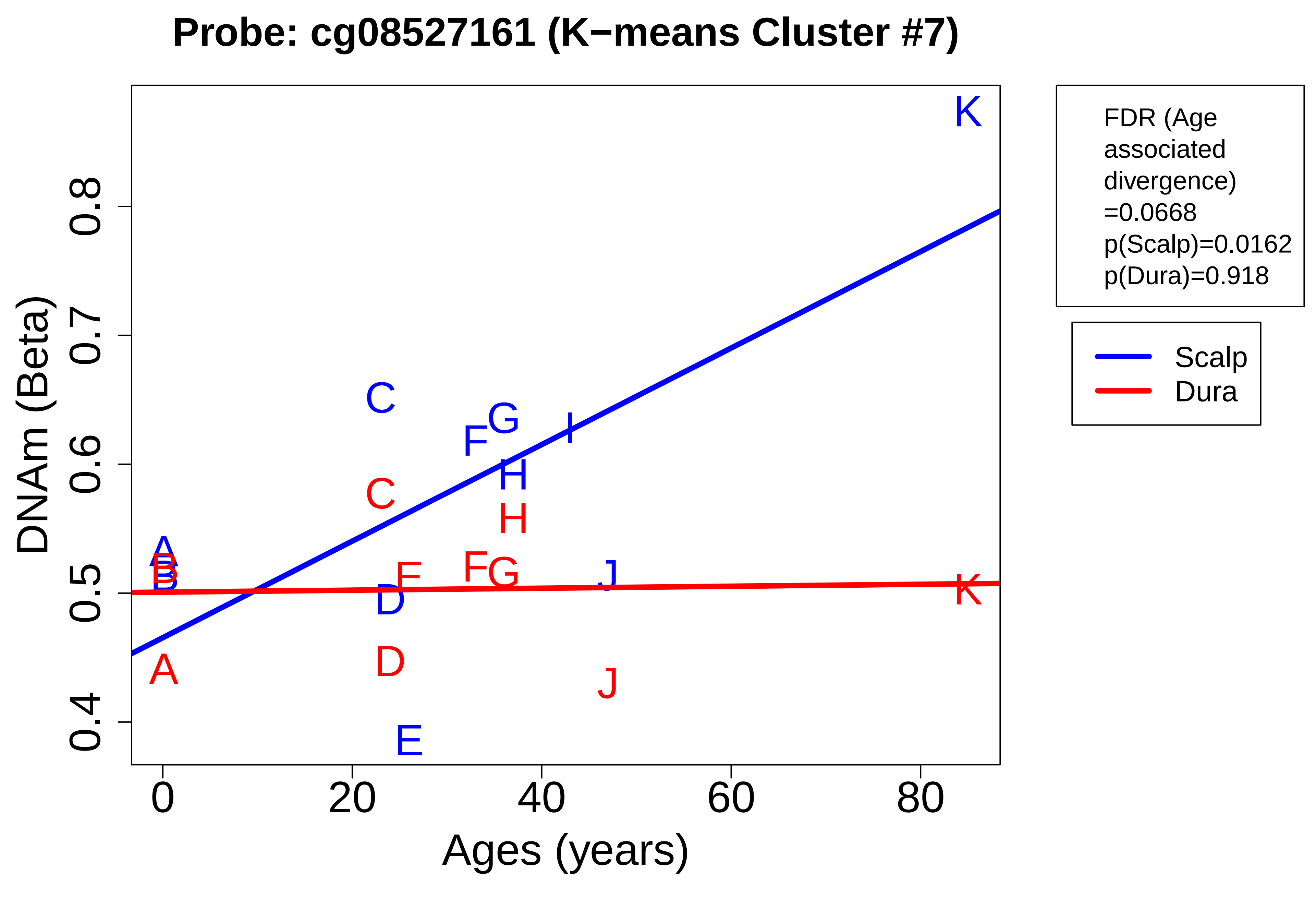
Probe: cg08527161 (K−means Cluster #7)
DNAm (Beta)
0.4
0.5
0.6
0.7
0.8
Ages (years)
0
20
40
60
80
FDR (Age associated divergence) =0.0668
p(Scalp)=0.0162
p(Dura)=0.918
Scalp
Dura
A
B
C
D
E
F
G
H
I
J
K

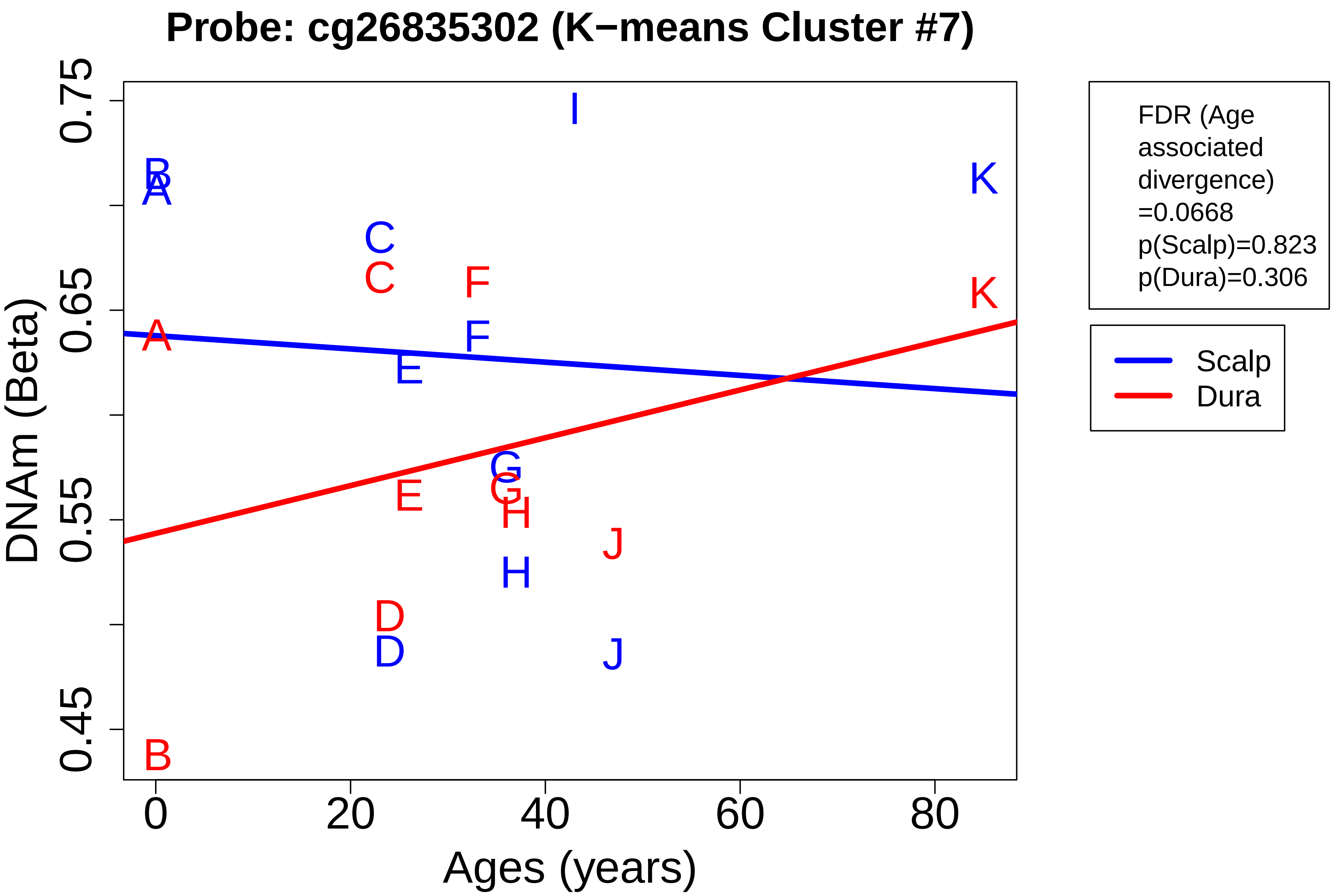
Probe: cg26835302 (K−means Cluster #7)
DNAm (Beta)
0.45
0.55
0.65
0.75
Ages (years)
0
20
40
60
80
FDR (Age associated divergence) =0.0668
p(Scalp)=0.823
p(Dura)=0.306
Scalp
Dura

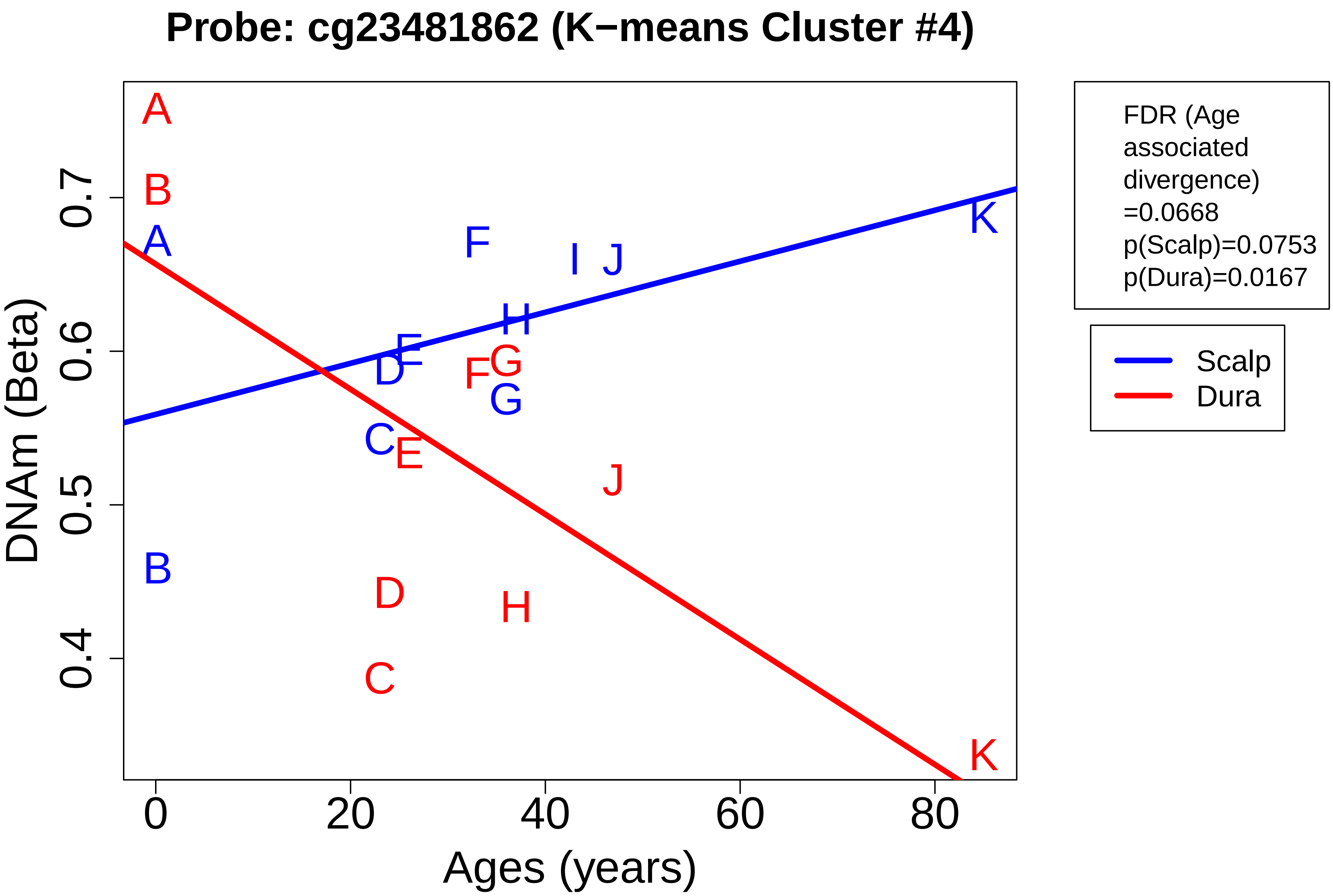
Probe: cg23481862 (K−means Cluster #4)
DNAm (Beta)
0.4
0.5
0.6
0.7
0
20
40
60
80
Ages (years)
FDR (Age associated divergence) =0.0668
p(Scalp)=0.0753
p(Dura)=0.0167
Scalp
Dura
A
B
C
D
E
F
G
H
I
J
K

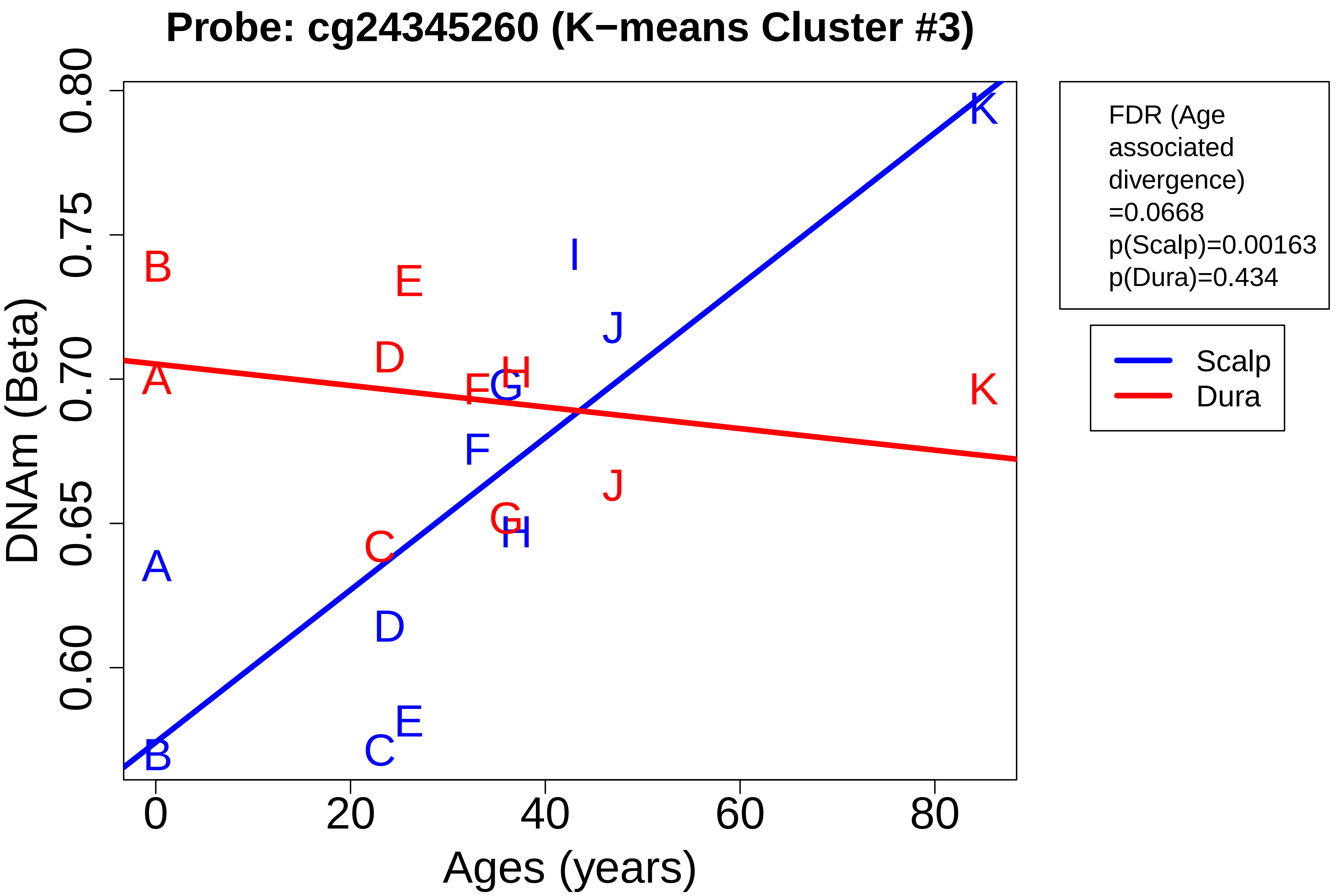
Probe: cg24345260 (K−means Cluster #3)
DNAm (Beta)
0.60
0.65
0.70
0.75
0.80
0
20
40
60
80
Ages (years)
A B C D E F G H I J K
FDR (Age associated divergence) =0.0668
p(Scalp)=0.00163
p(Dura)=0.434
Scalp
Dura

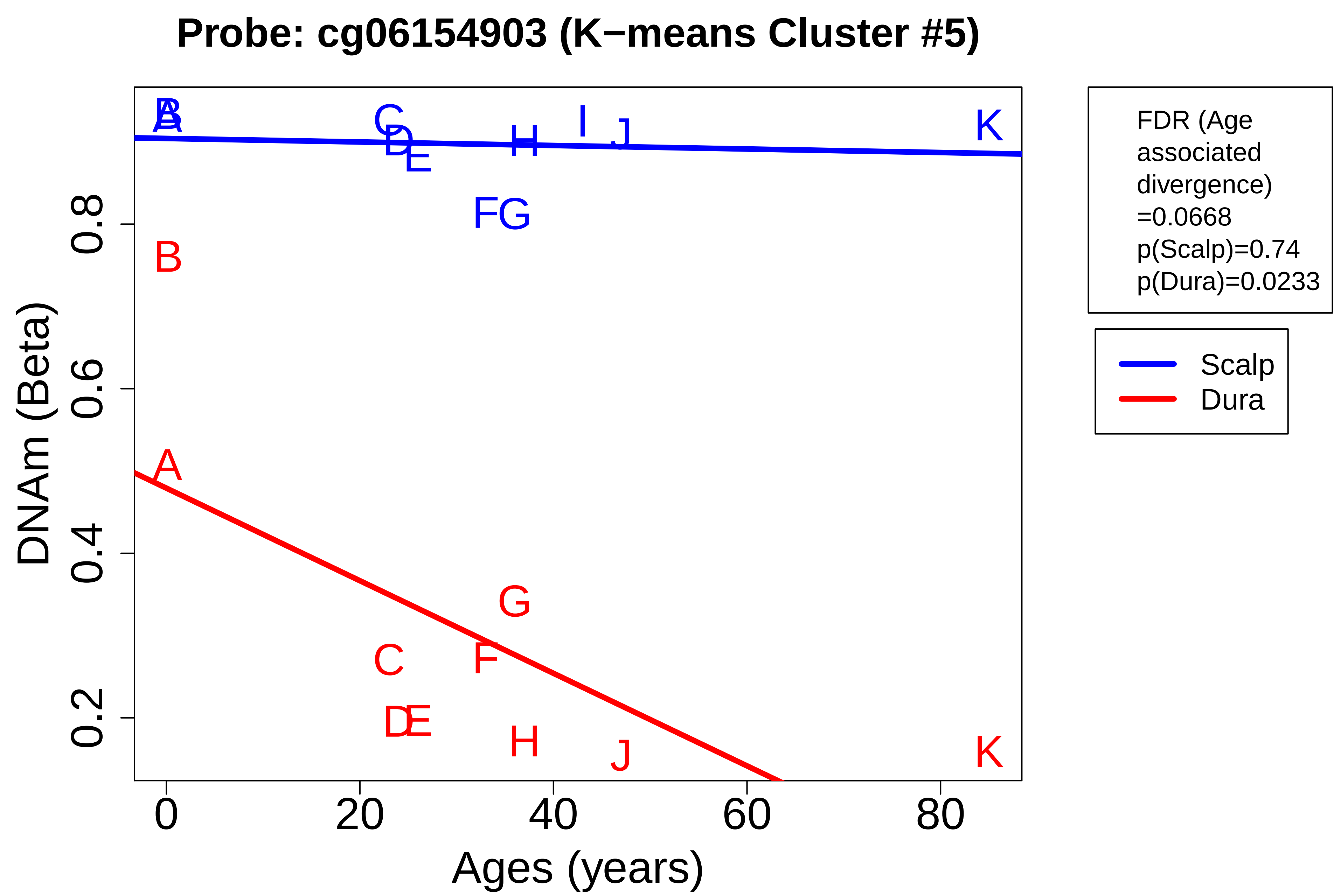

Probe: cg06154903 (K−means Cluster #5)
DNAm (Beta)
0.2
0.4
0.6
0.8
0
20
40
60
80
Ages (years)
FDR (Age associated divergence) =0.0668
p(Scalp)=0.74
p(Dura)=0.0233
Scalp
Dura

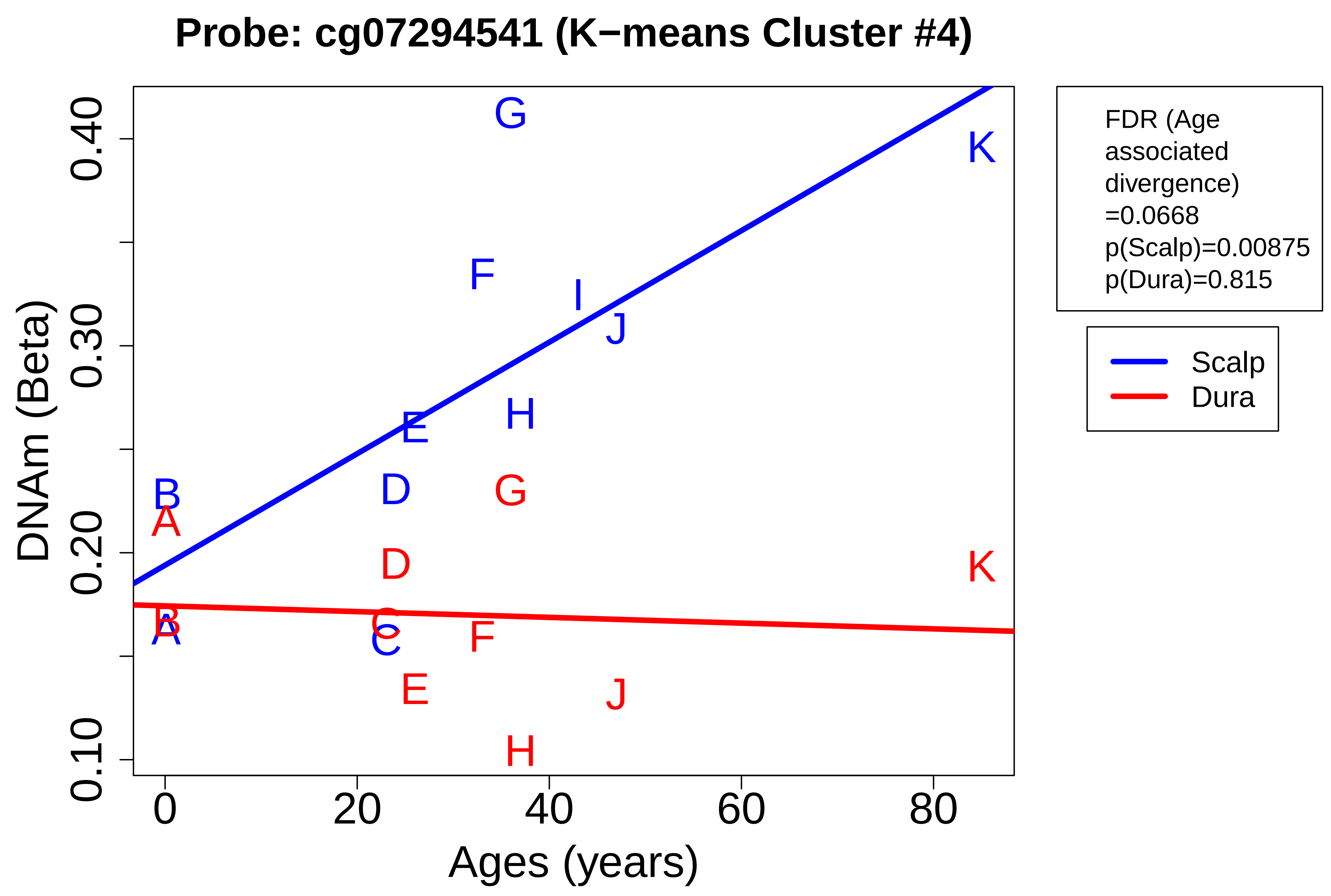

Probe: cg07294541 (K−means Cluster #4)
DNAm (Beta)
0.10
0.20
0.30
0.40
Ages (years)
0
20
40
60
80
A
B
C
D
E
F
G
H
I
J
K
FDR (Age associated divergence) =0.0668
p(Scalp)=0.00875
p(Dura)=0.815
Scalp
Dura

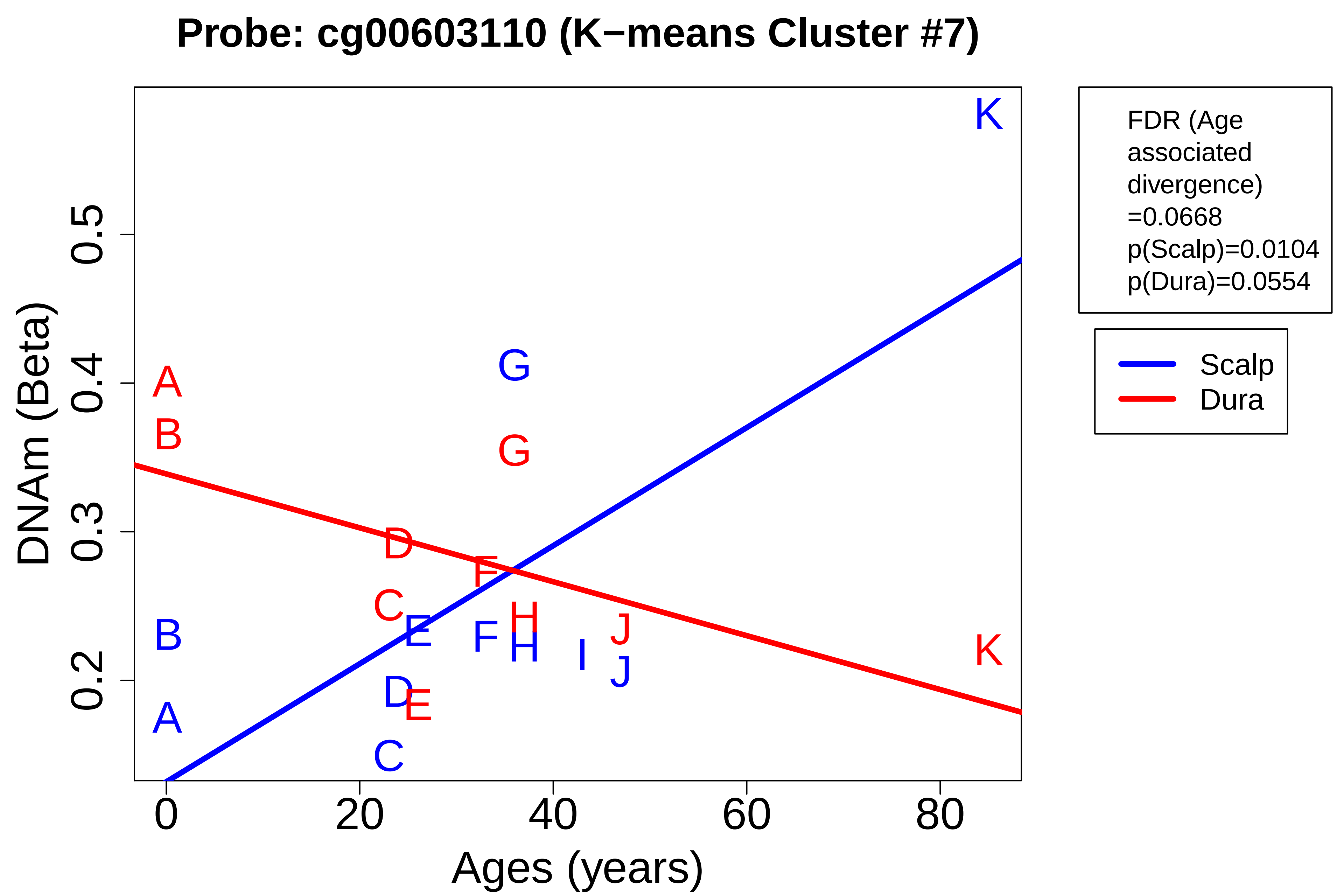

Probe: cg00603110 (K−means Cluster #7)
DNAm (Beta)
0.2
0.3
0.4
0.5
Ages (years)
0
20
40
60
80
FDR (Age associated divergence) =0.0668
p(Scalp)=0.0104
p(Dura)=0.0554
Scalp
Dura
A
B
C
D
E
F
G
H
I
J
K

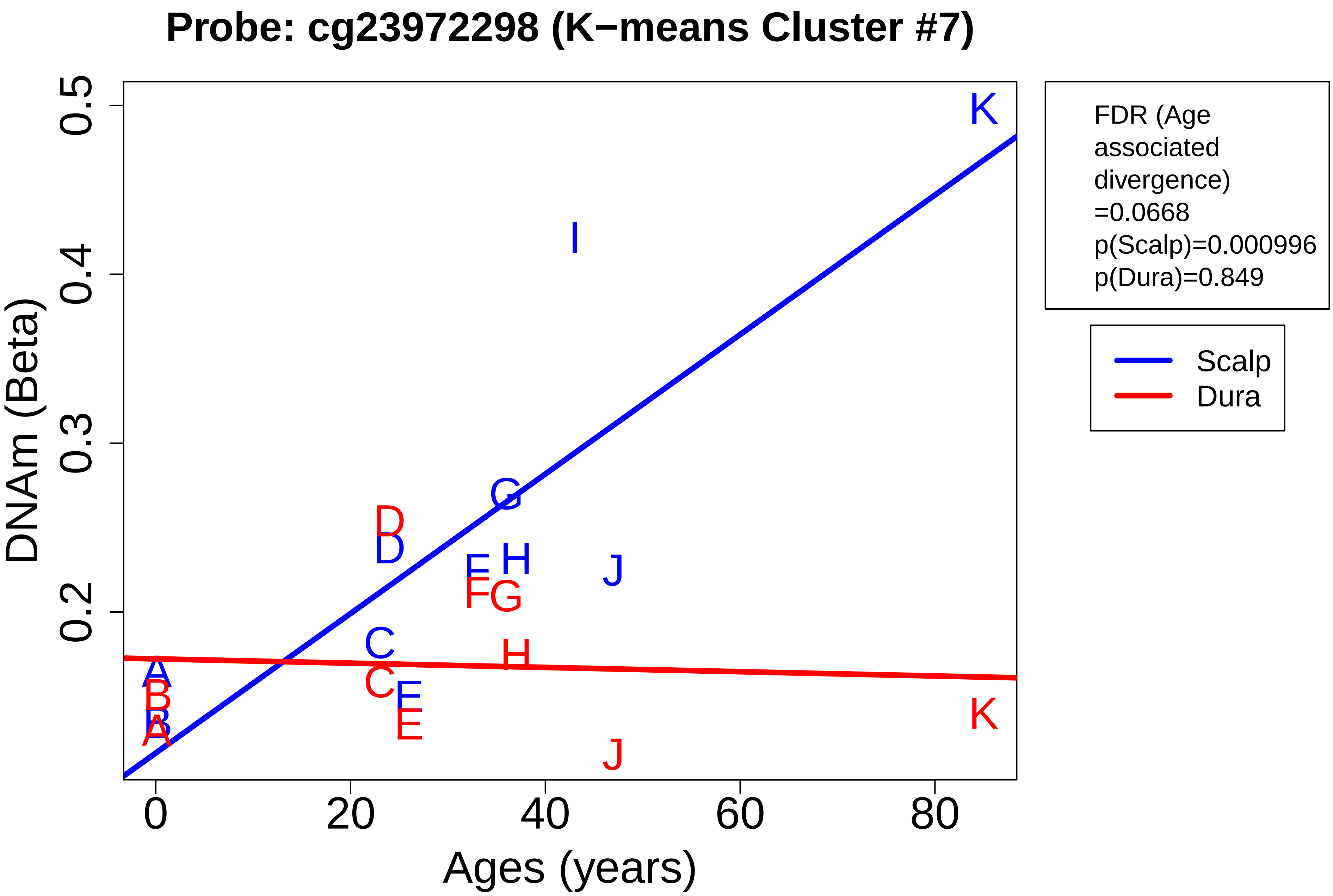
Probe: cg23972298 (K−means Cluster #7)
DNAm (Beta)
0.2
0.3
0.4
0.5
0
20
40
60
80
Ages (years)
A
B
C
D
E
F
G
H
I
J
K
FDR (Age associated divergence) =0.0668
p(Scalp)=0.000996
p(Dura)=0.849
Scalp
Dura

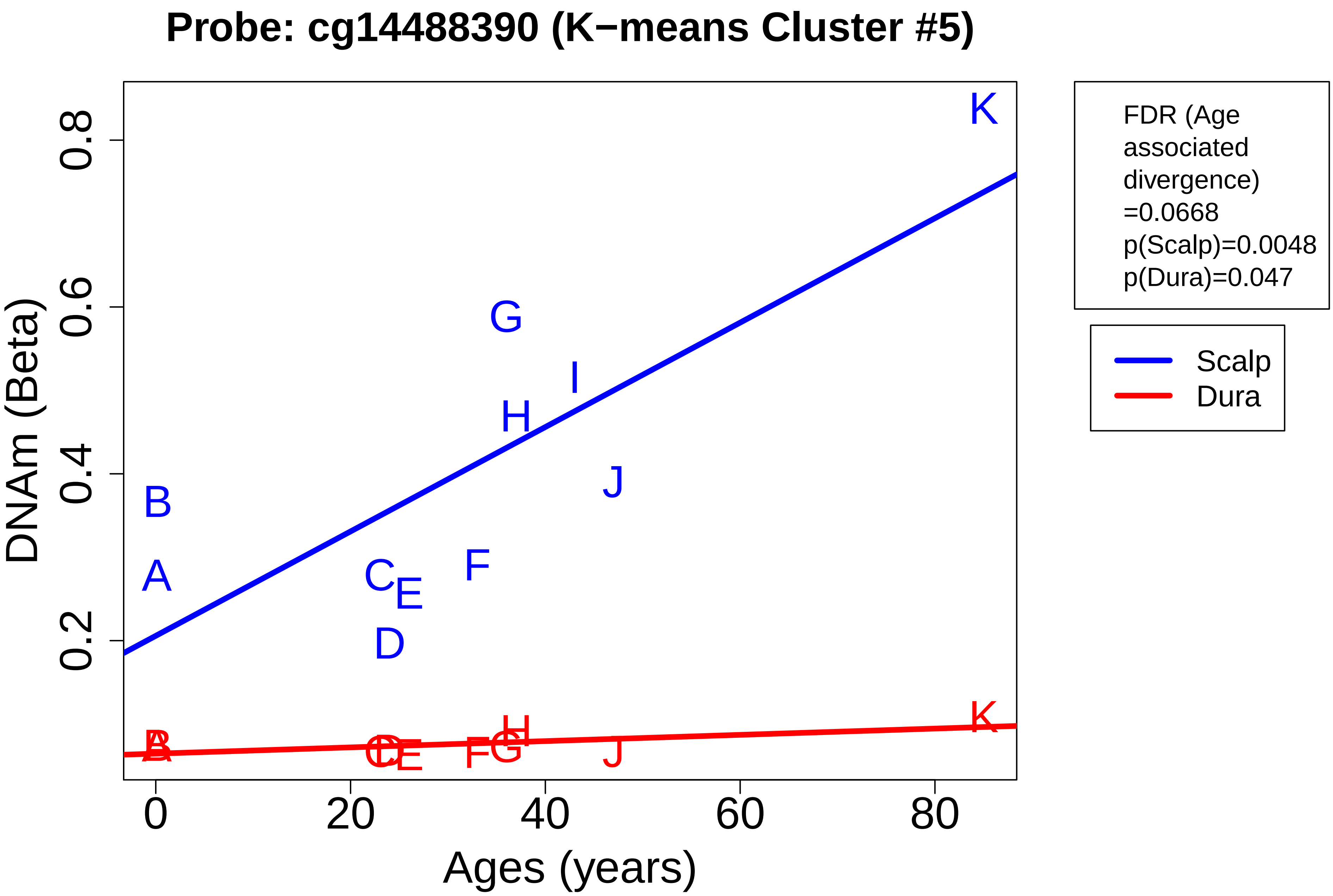

Probe: cg14488390 (K−means Cluster #5)
DNAm (Beta)
0.2
0.4
0.6
0.8
Ages (years)
0
20
40
60
80
FDR (Age associated divergence) =0.0668
p(Scalp)=0.0048
p(Dura)=0.047
Scalp
Dura

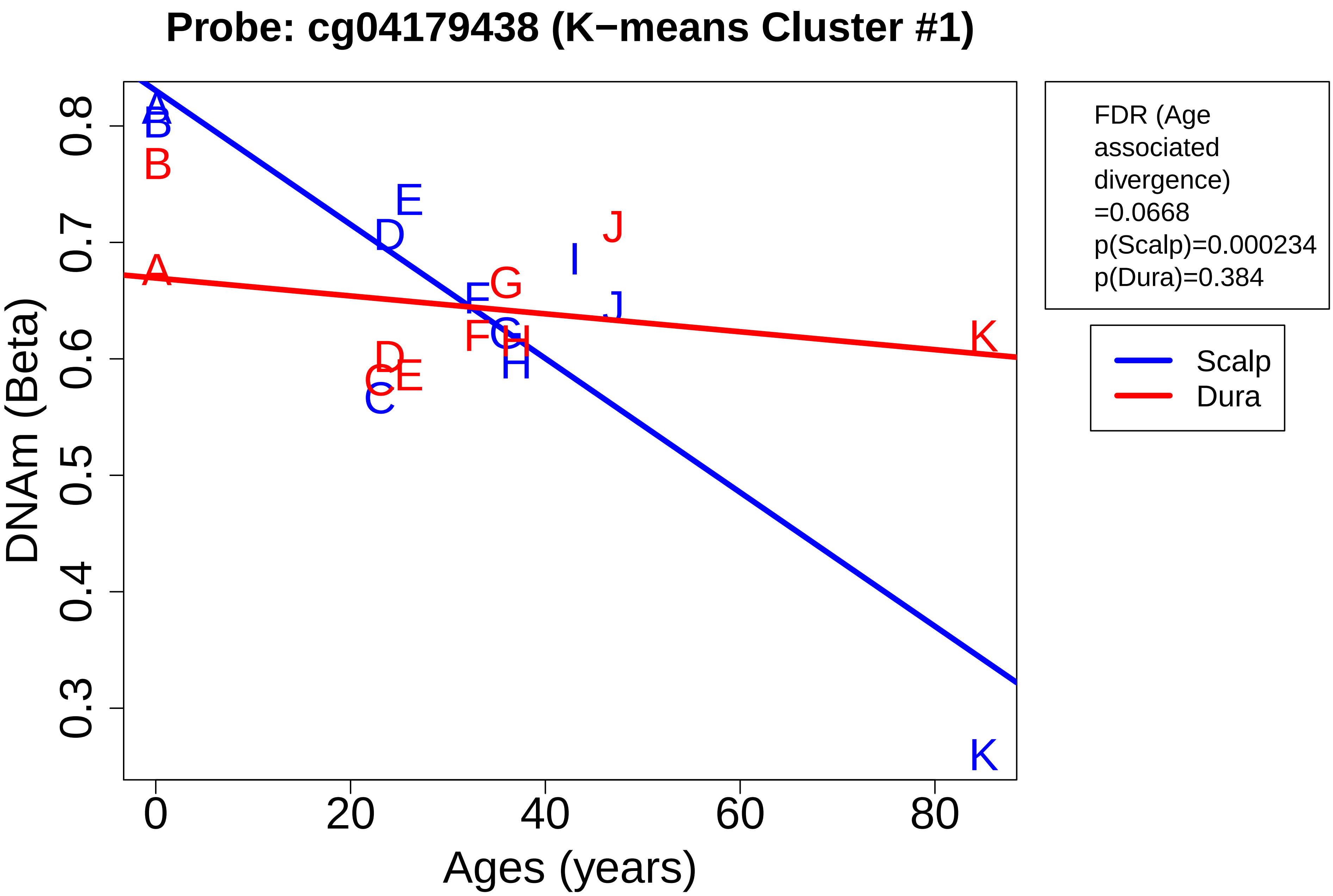

Probe: cg04179438 (K−means Cluster #1)
DNAm (Beta)
0.3
0.4
0.5
0.6
0.7
0.8
0
20
40
60
80
Ages (years)
A
B
C
D
E
F
G
H
I
J
K
FDR (Age associated divergence) =0.0668
p(Scalp)=0.000234
p(Dura)=0.384
Scalp
Dura

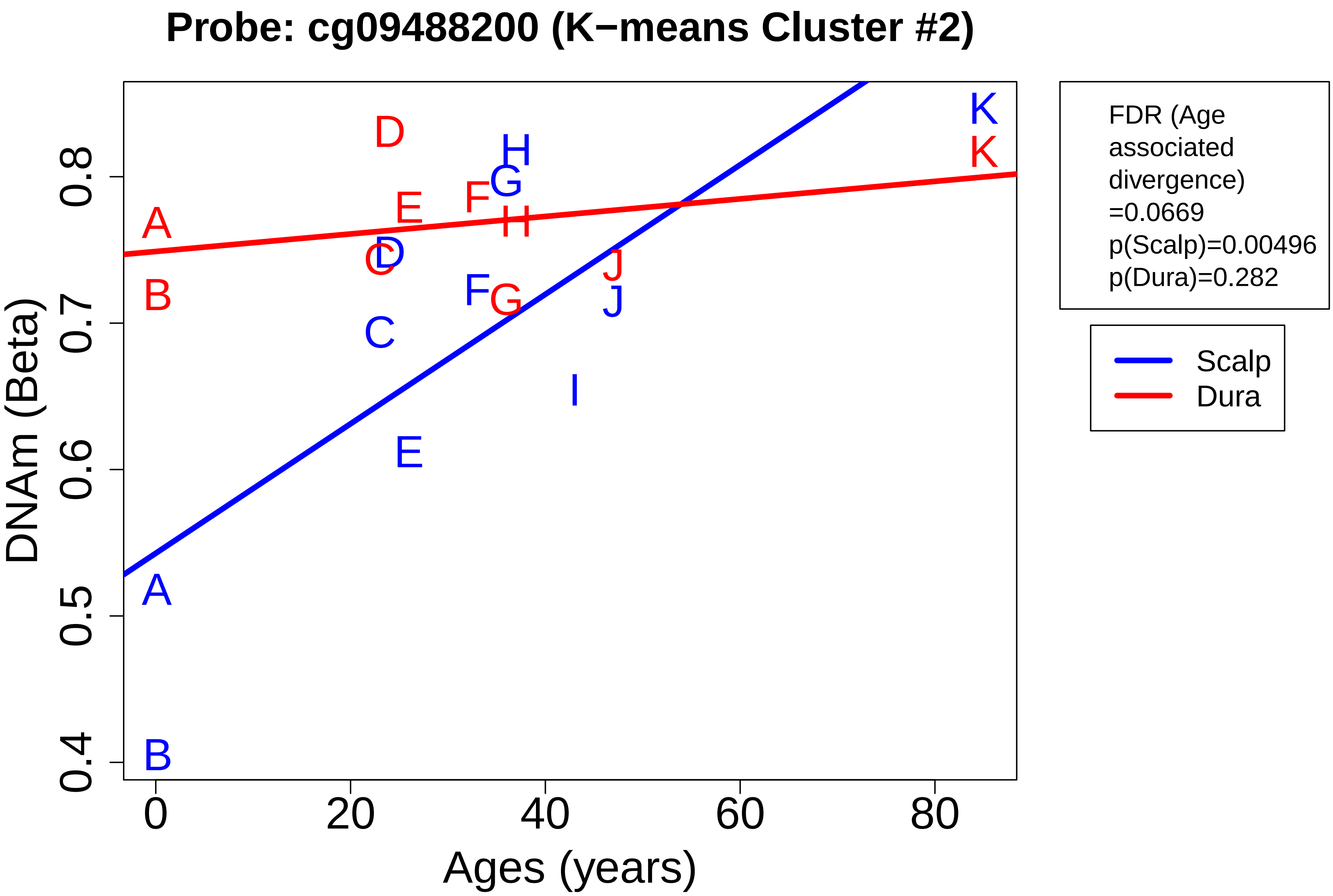

Probe: cg09488200 (K−means Cluster #2)
DNAm (Beta)
0.4
0.5
0.6
0.7
0.8
0
20
40
60
80
Ages (years)
A
B
C
D
E
F
G
H
I
J
K
FDR (Age associated divergence) =0.0669
p(Scalp)=0.00496
p(Dura)=0.282
Scalp
Dura

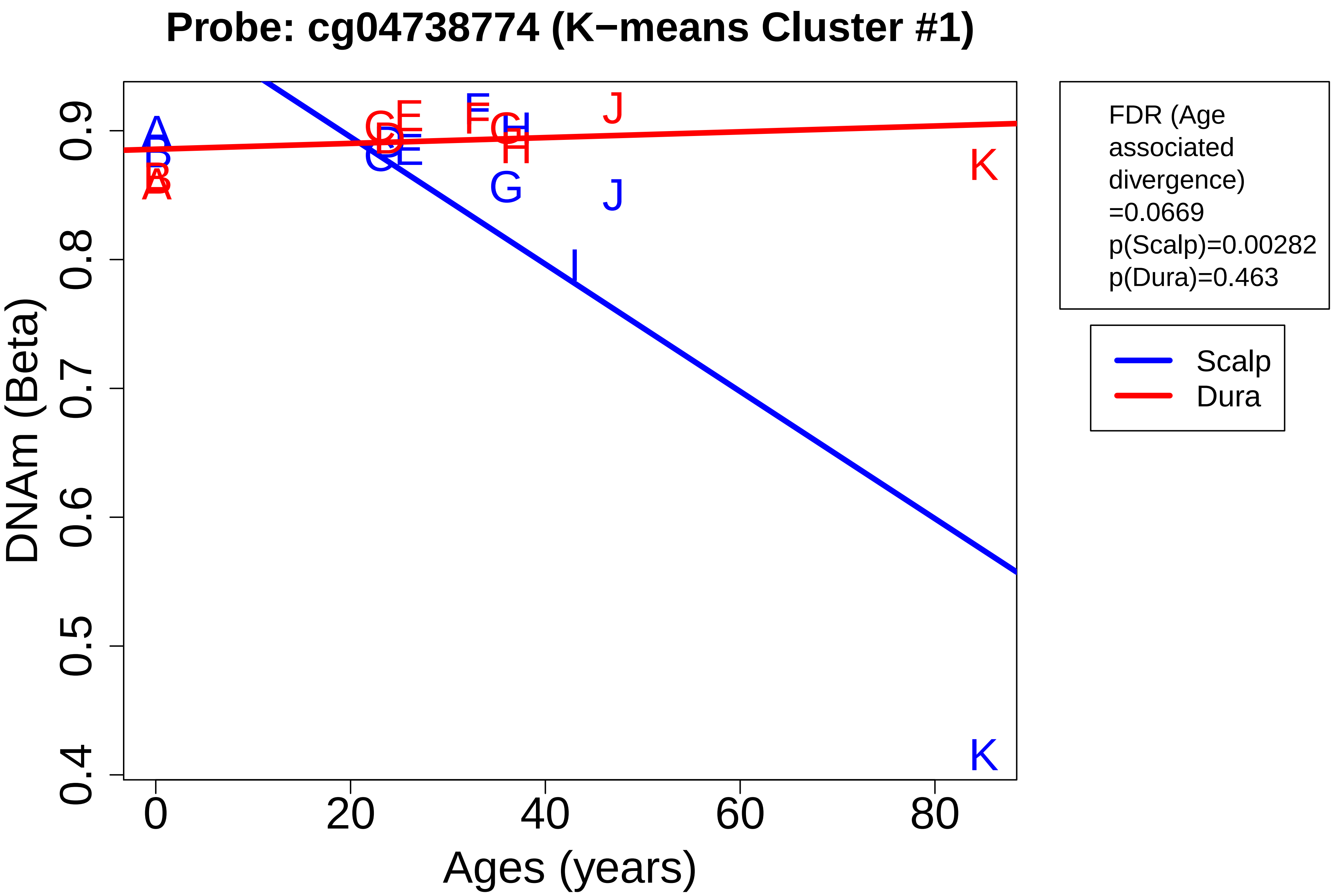

Probe: cg04738774 (K−means Cluster #1)
DNAm (Beta)
0.4
0.5
0.6
0.7
0.8
0.9
0
20
40
60
80
Ages (years)
A
B
C
D
E
F
G
H
I
J
K
FDR (Age associated divergence) =0.0669
p(Scalp)=0.00282
p(Dura)=0.463
Scalp
Dura

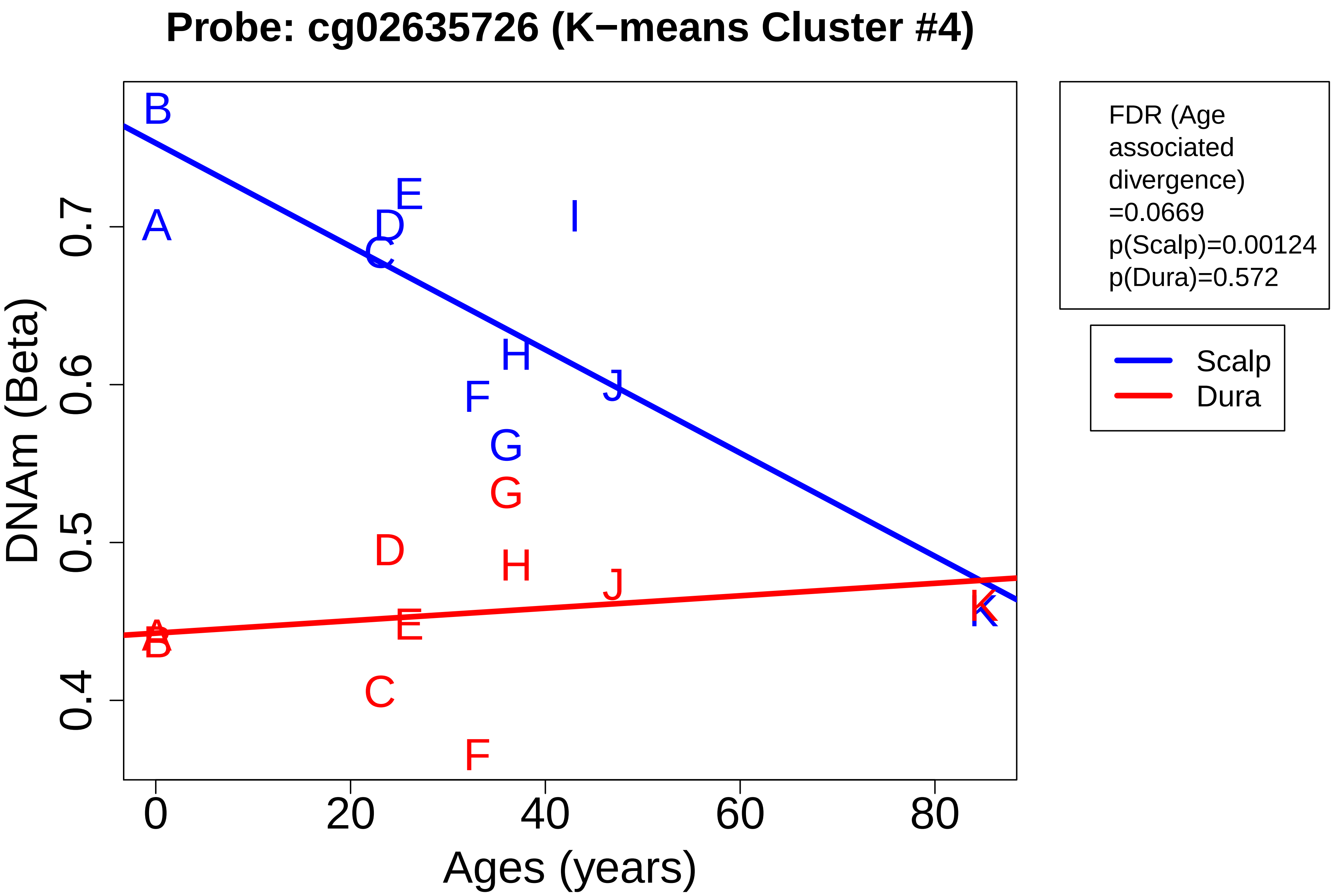

Probe: cg02635726 (K−means Cluster #4)
DNAm (Beta)
0.4
0.5
0.6
0.7
Ages (years)
0
20
40
60
80
FDR (Age associated divergence) =0.0669
p(Scalp)=0.00124
p(Dura)=0.572
Scalp
Dura

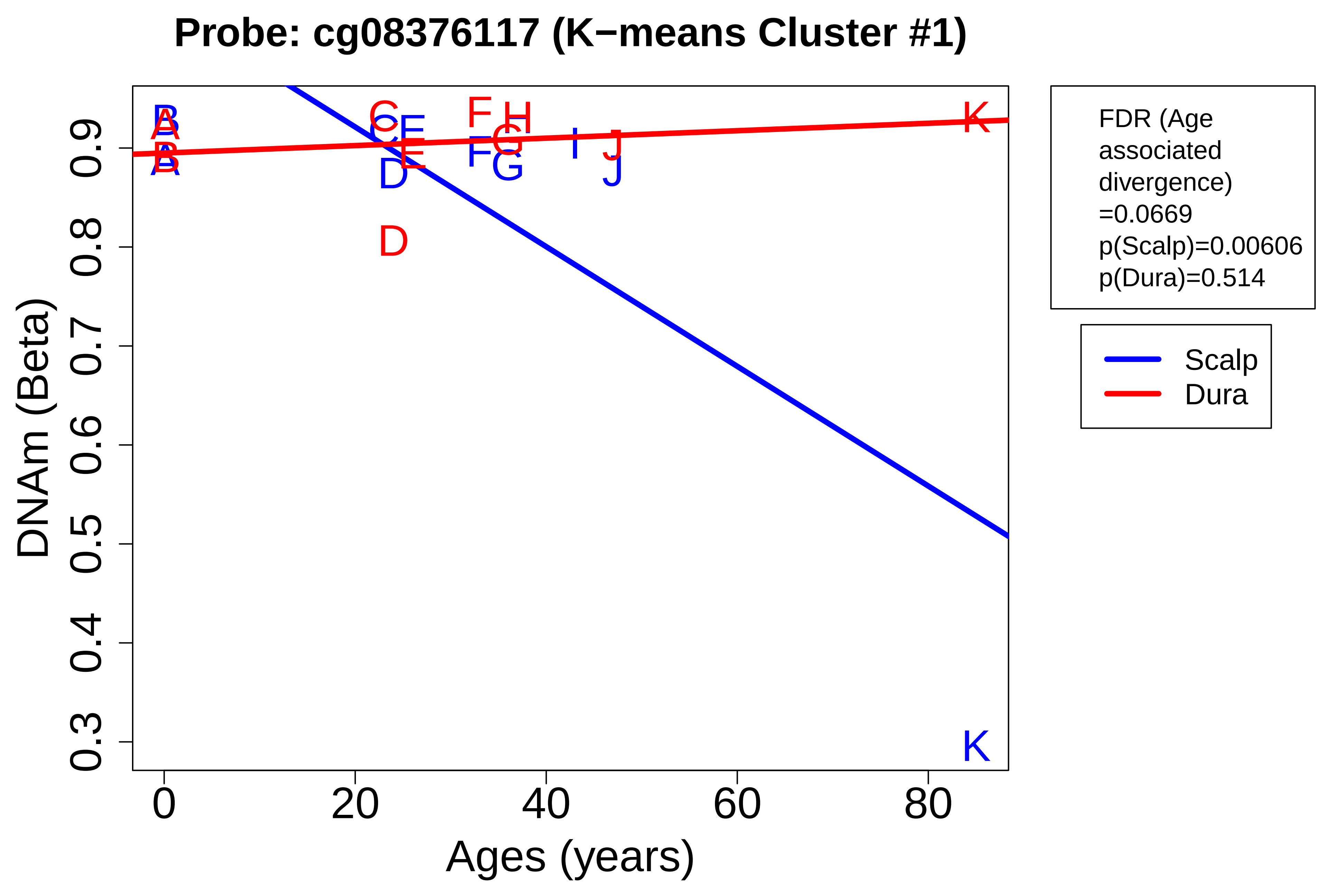

Probe: cg08376117 (K−means Cluster #1)
DNAm (Beta)
Ages (years)
0.3
0.4
0.5
0.6
0.7
0.8
0.9
0
20
40
60
80
FDR (Age associated divergence) =0.0669
p(Scalp)=0.00606
p(Dura)=0.514
Scalp
Dura

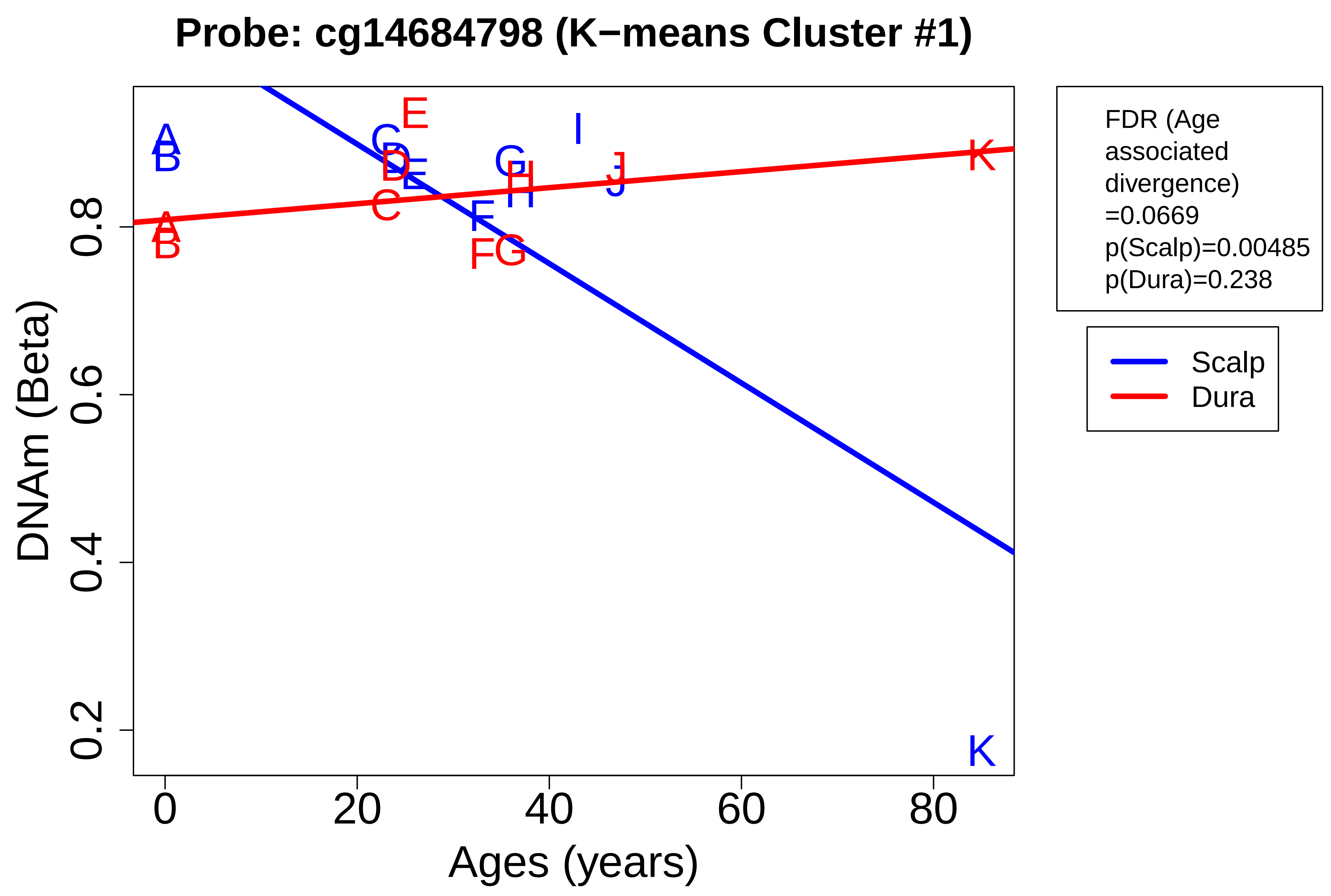

Probe: cg14684798 (K−means Cluster #1)
DNAm (Beta)
0.2
0.4
0.6
0.8
0
20
40
60
80
Ages (years)
A
B
C
D
E
F
G
H
I
J
K
FDR (Age associated divergence) =0.0669
p(Scalp)=0.00485
p(Dura)=0.238
Scalp
Dura

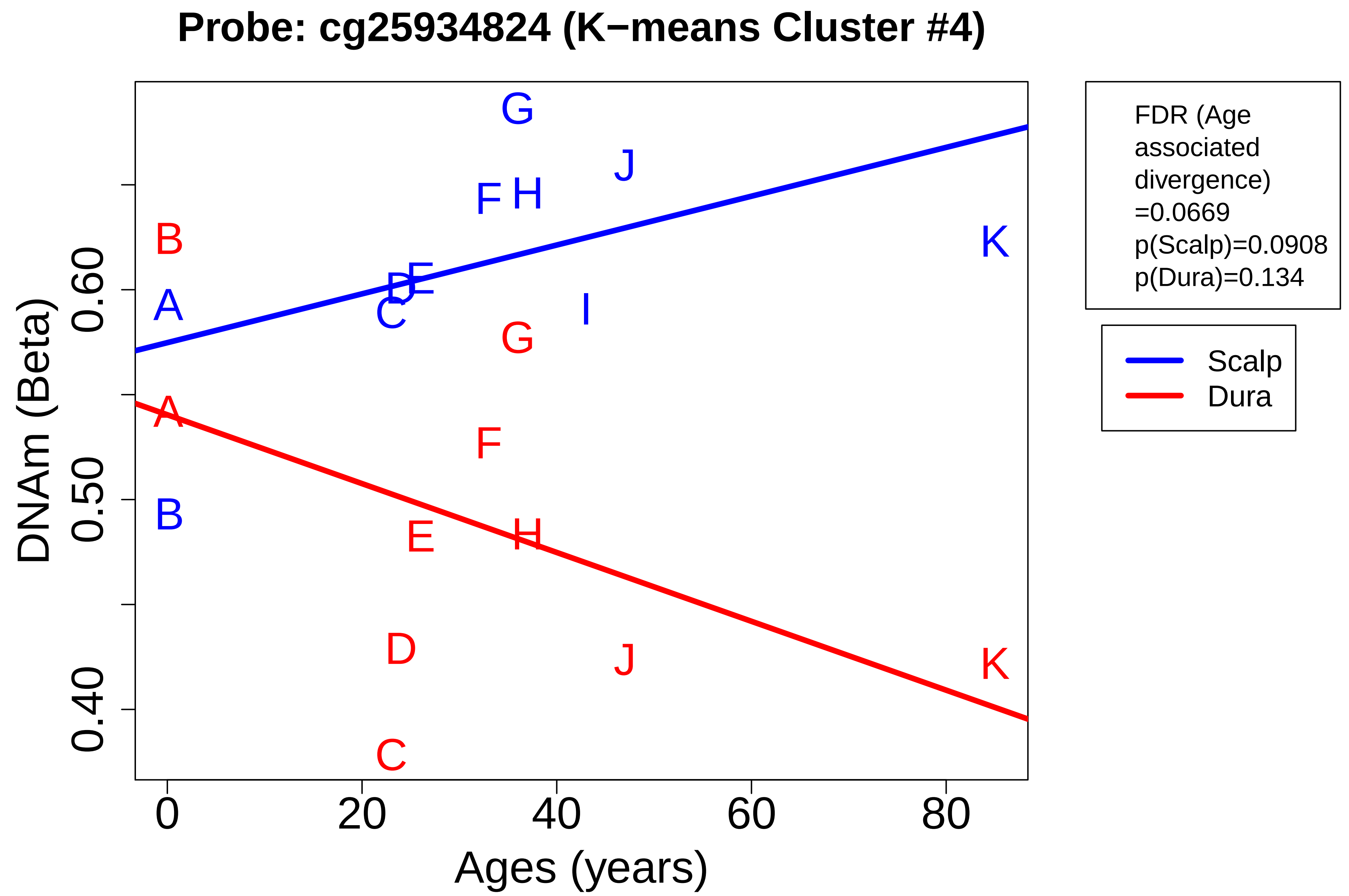
Probe: cg25934824 (K−means Cluster #4)
DNAm (Beta)
0.40
0.50
0.60
Ages (years)
0
20
40
60
80
FDR (Age associated divergence) =0.0669
p(Scalp)=0.0908
p(Dura)=0.134
Scalp
Dura

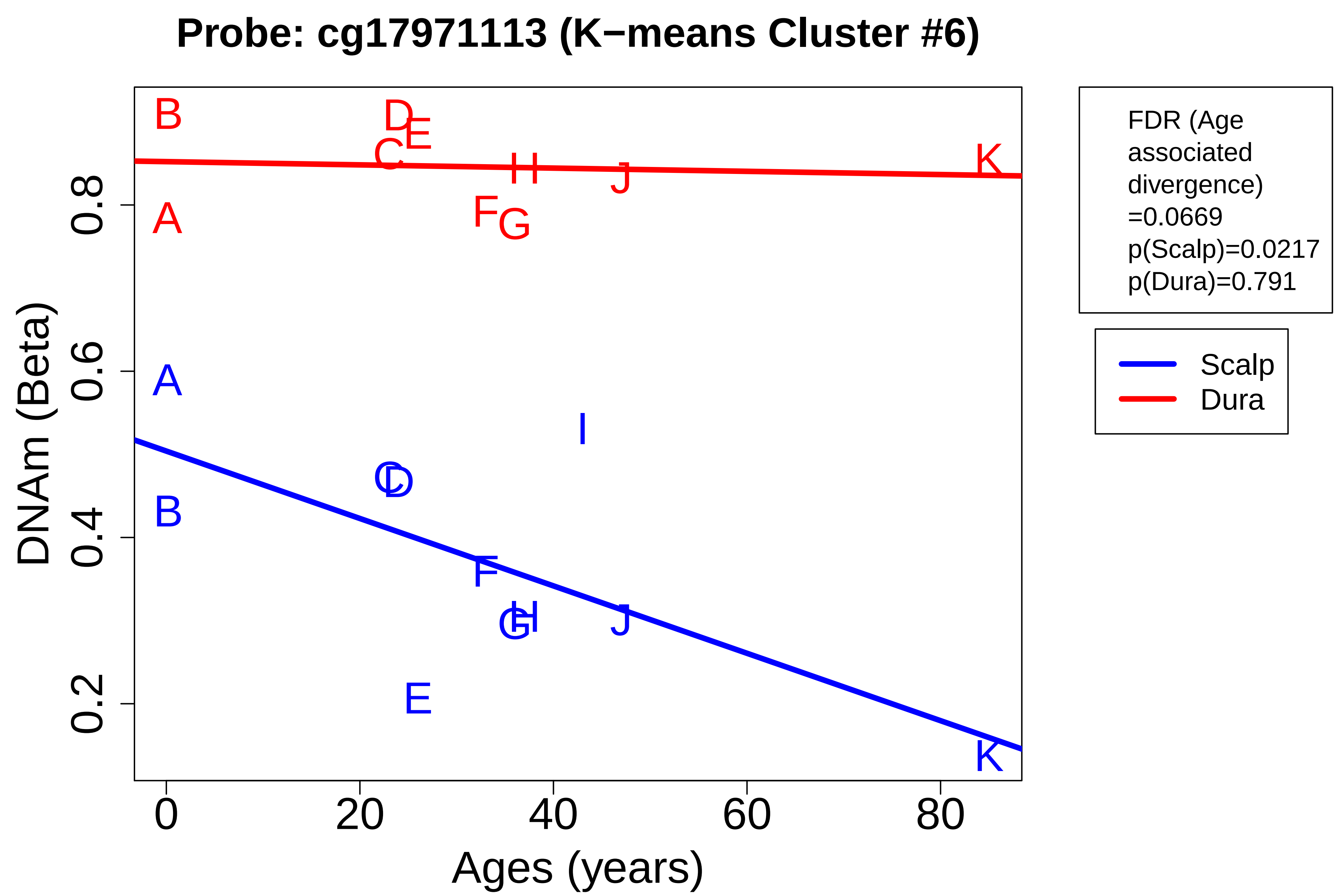

Probe: cg17971113 (K−means Cluster #6)
DNAm (Beta)
0.2
0.4
0.6
0.8
0
20
40
60
80
Ages (years)
FDR (Age associated divergence) =0.0669
p(Scalp)=0.0217
p(Dura)=0.791
Scalp
Dura

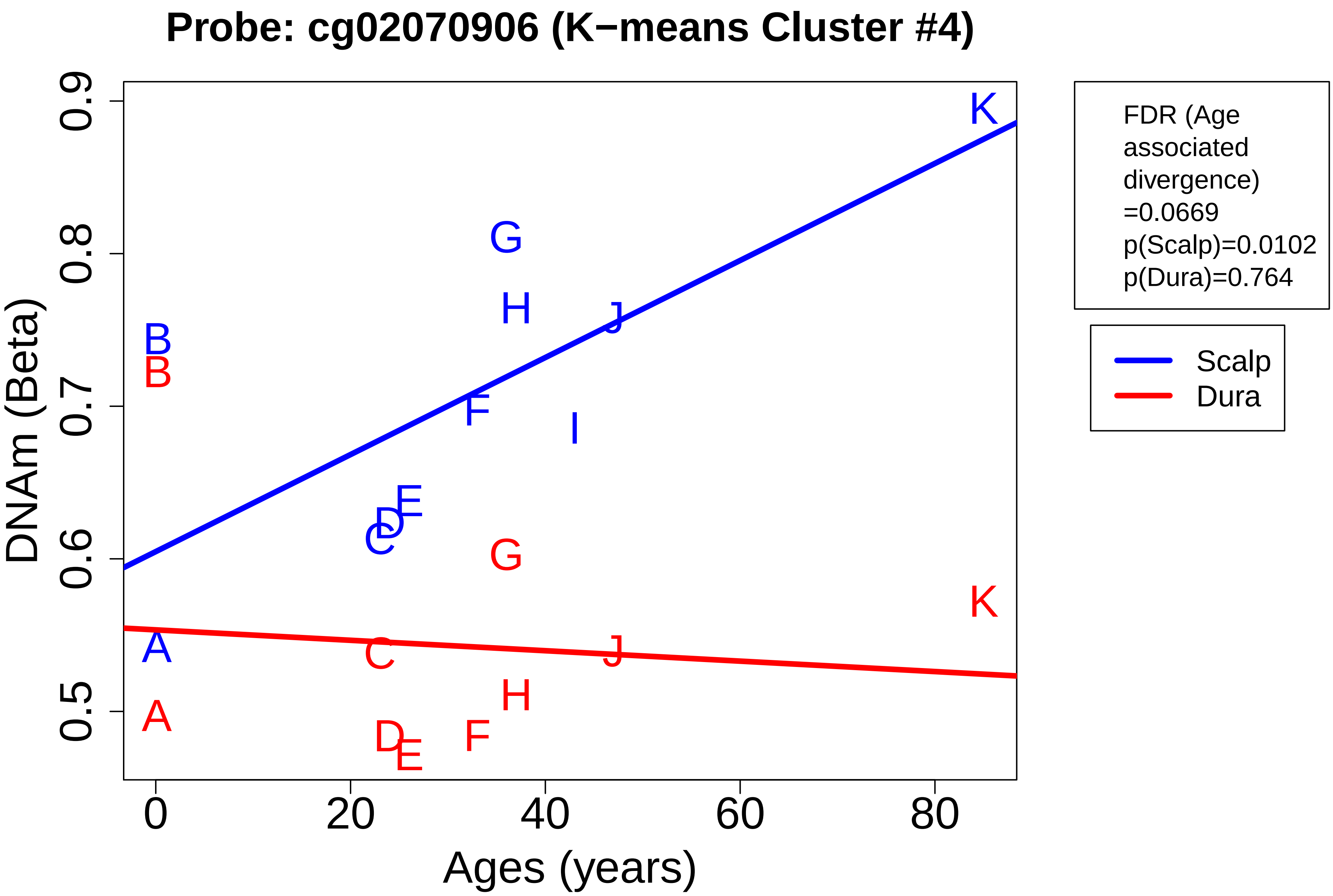

Probe: cg02070906 (K−means Cluster #4)
DNAm (Beta)
0.5
0.6
0.7
0.8
0.9
0
20
40
60
80
Ages (years)
FDR (Age associated divergence) =0.0669
p(Scalp)=0.0102
p(Dura)=0.764
Scalp
Dura
A
B
C
D
E
F
G
H
I
J
K

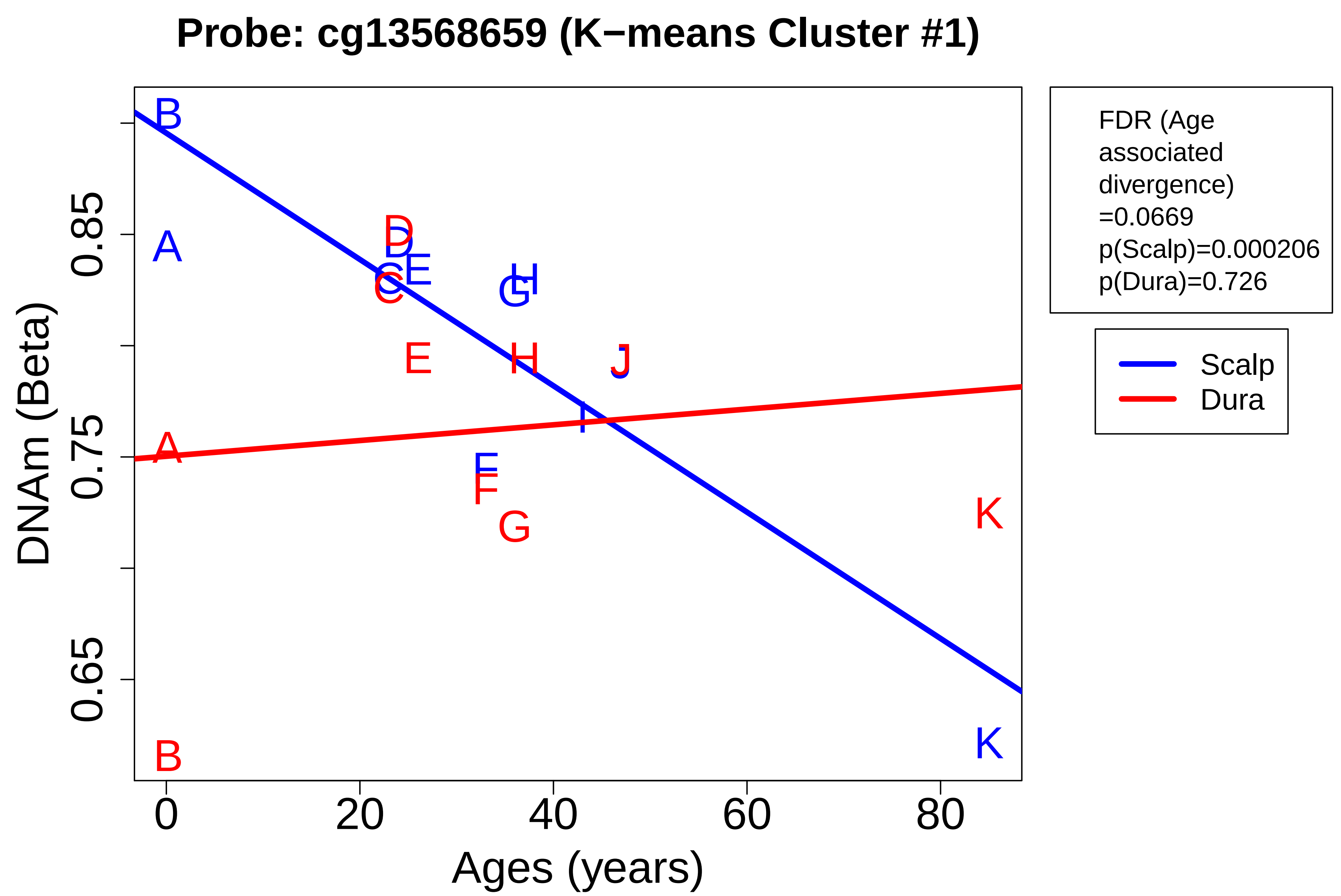
Probe: cg13568659 (K−means Cluster #1)
DNAm (Beta)
0.85
0.75
0.65
0
20
40
60
80
Ages (years)
A
B
C
D
E
F
G
H
I
J
K
FDR (Age associated divergence) =0.0669
p(Scalp)=0.000206
p(Dura)=0.726
Scalp
Dura

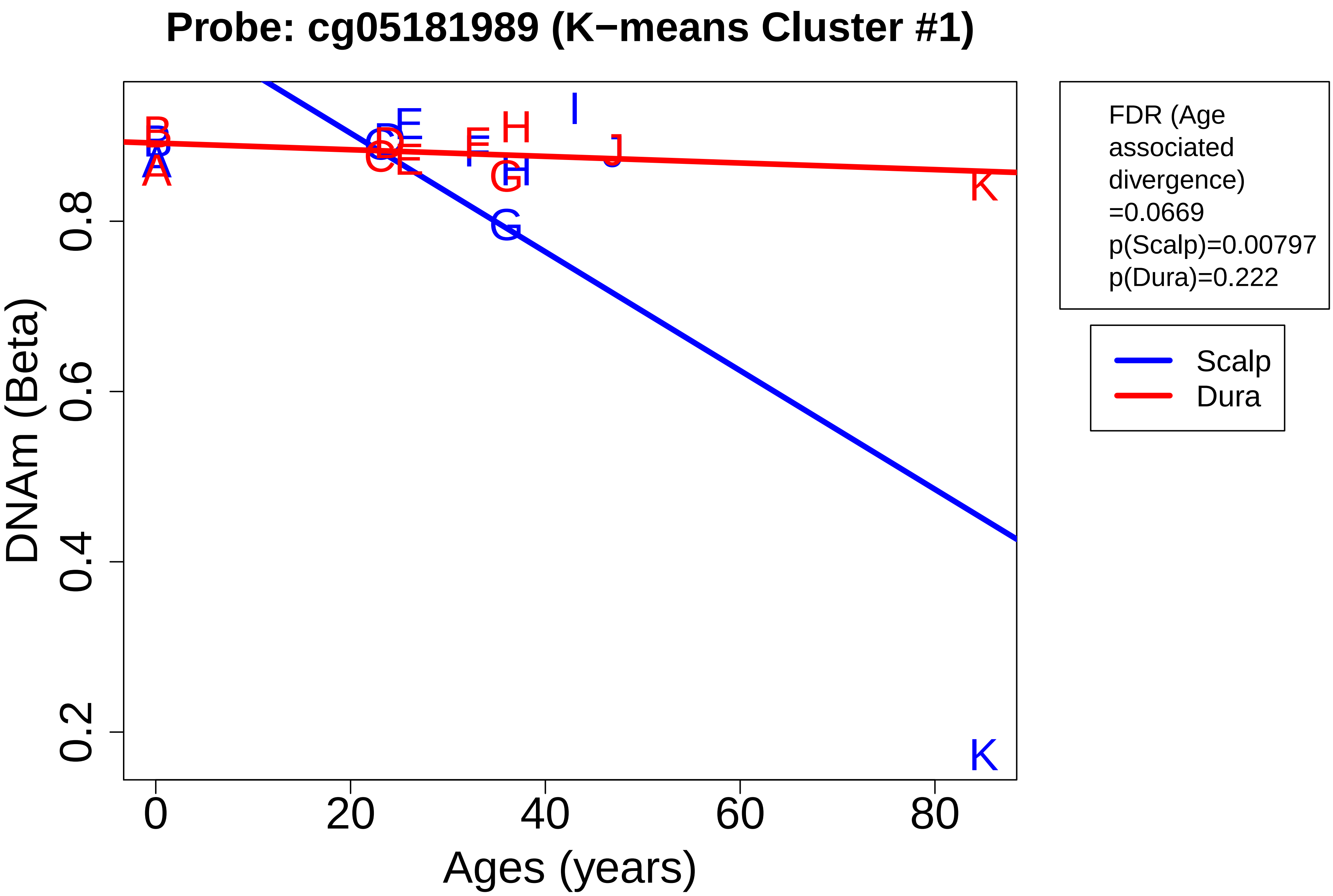

Probe: cg05181989 (K−means Cluster #1)
FDR (Age associated divergence) =0.0669
p(Scalp)=0.00797
p(Dura)=0.222
Scalp
Dura
DNAm (Beta)
Ages (years)
0.2
0.4
0.6
0.8
0
20
40
60
80
A
B
C
D
E
F
G
H
I
J
K

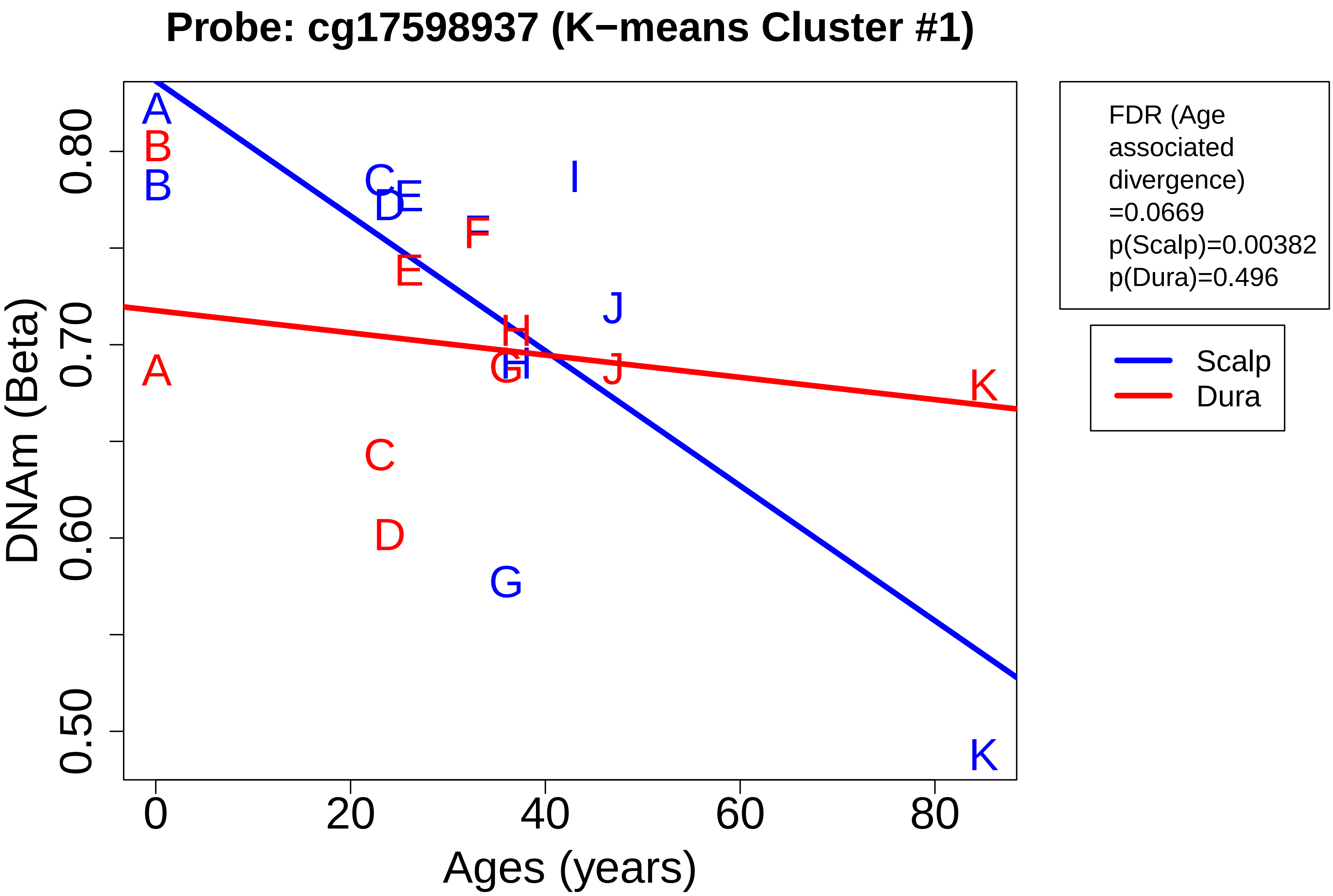

Probe: cg17598937 (K−means Cluster #1)
DNAm (Beta)
0.50
0.60
0.70
0.80
Ages (years)
0
20
40
60
80
FDR (Age associated divergence) =0.0669
p(Scalp)=0.00382
p(Dura)=0.496
Scalp
Dura

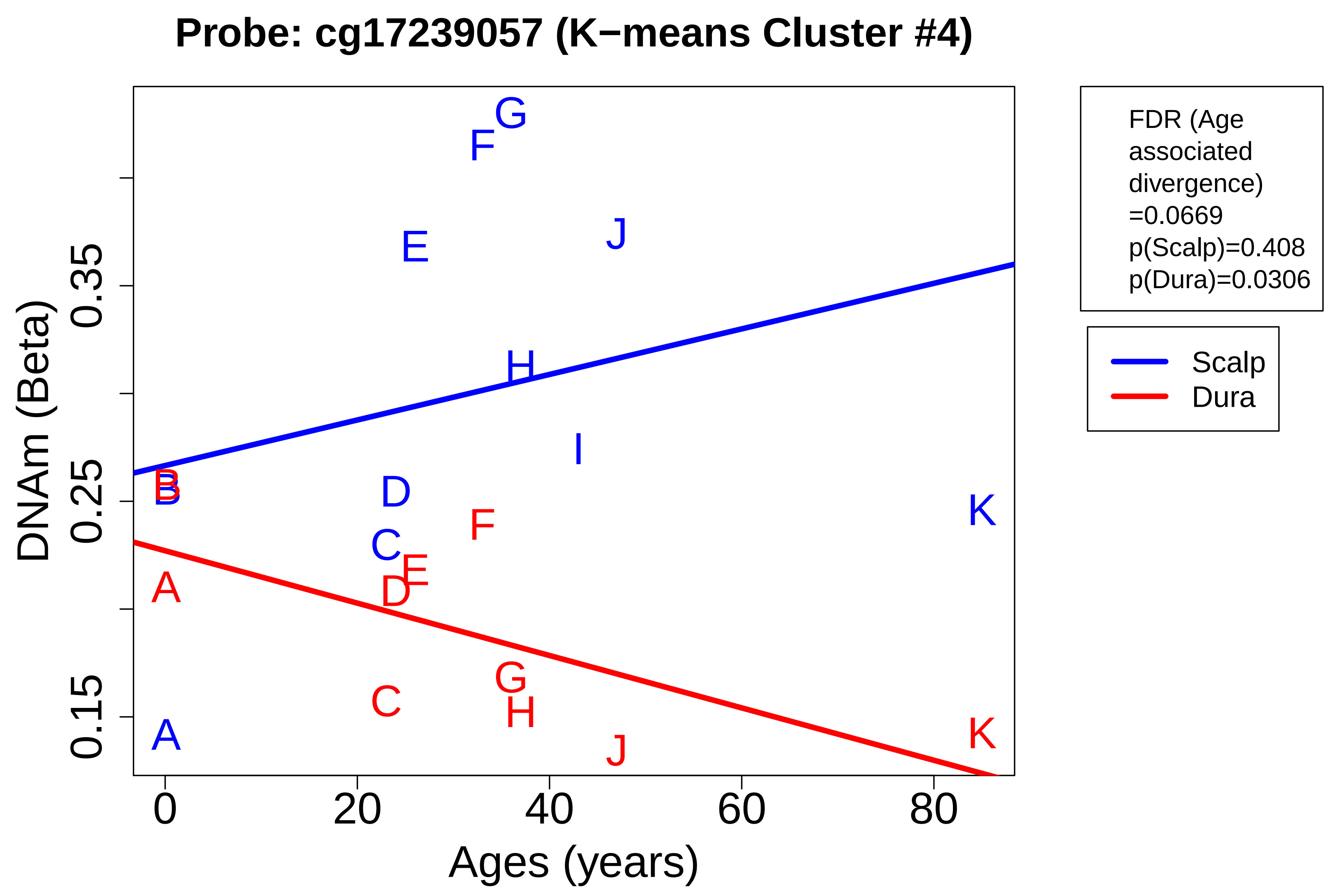
Probe: cg17239057 (K−means Cluster #4)
FDR (Age associated divergence) =0.0669
p(Scalp)=0.408
p(Dura)=0.0306
Scalp
Dura
DNAm (Beta)
0.15
0.25
0.35
Ages (years)
0
20
40
60
80
A
B
C
D
E
F
G
H
I
J
K

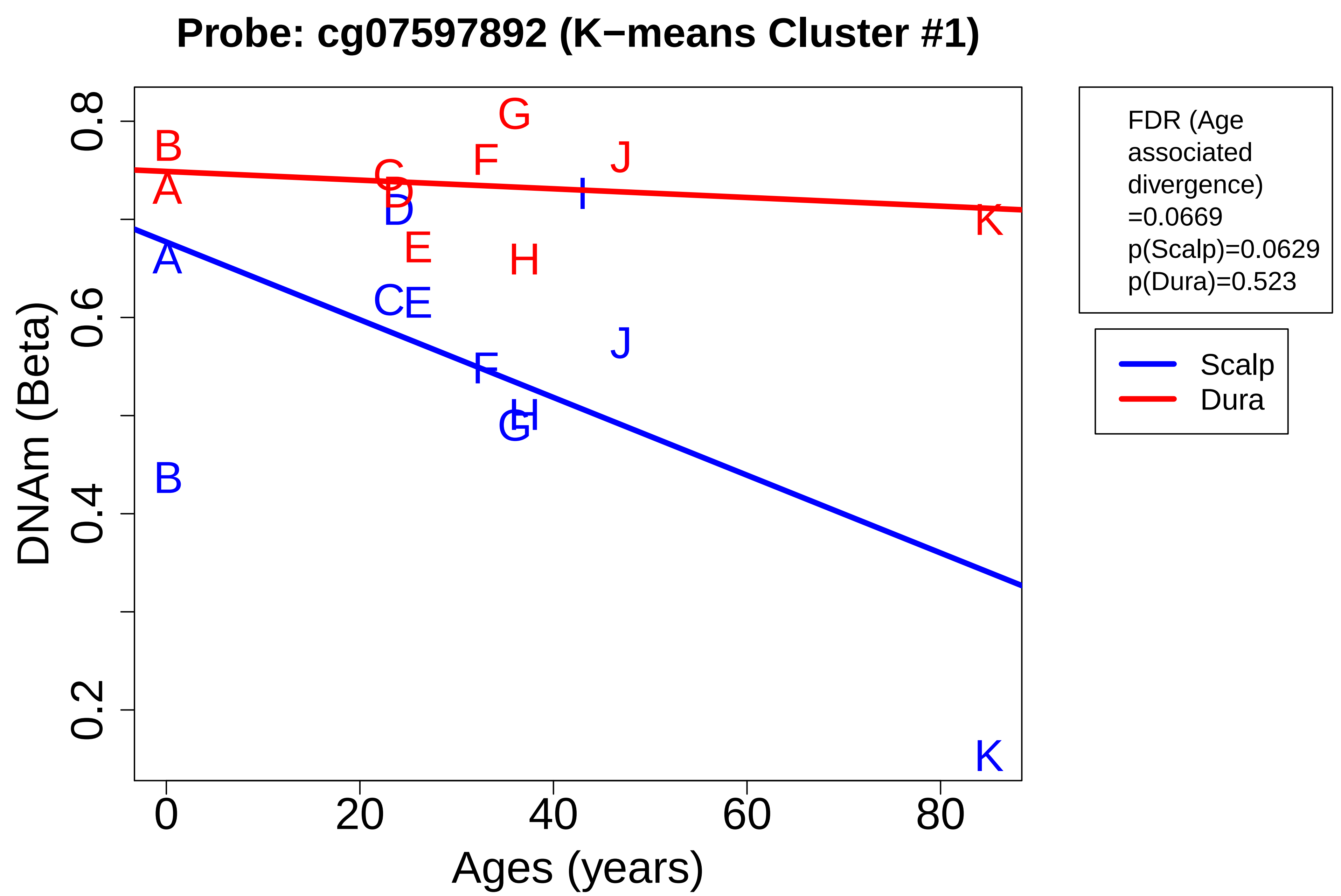

Probe: cg07597892 (K−means Cluster #1)
DNAm (Beta)
0.2
0.4
0.6
0.8
0
20
40
60
80
Ages (years)
FDR (Age associated divergence) =0.0669
p(Scalp)=0.0629
p(Dura)=0.523
Scalp
Dura

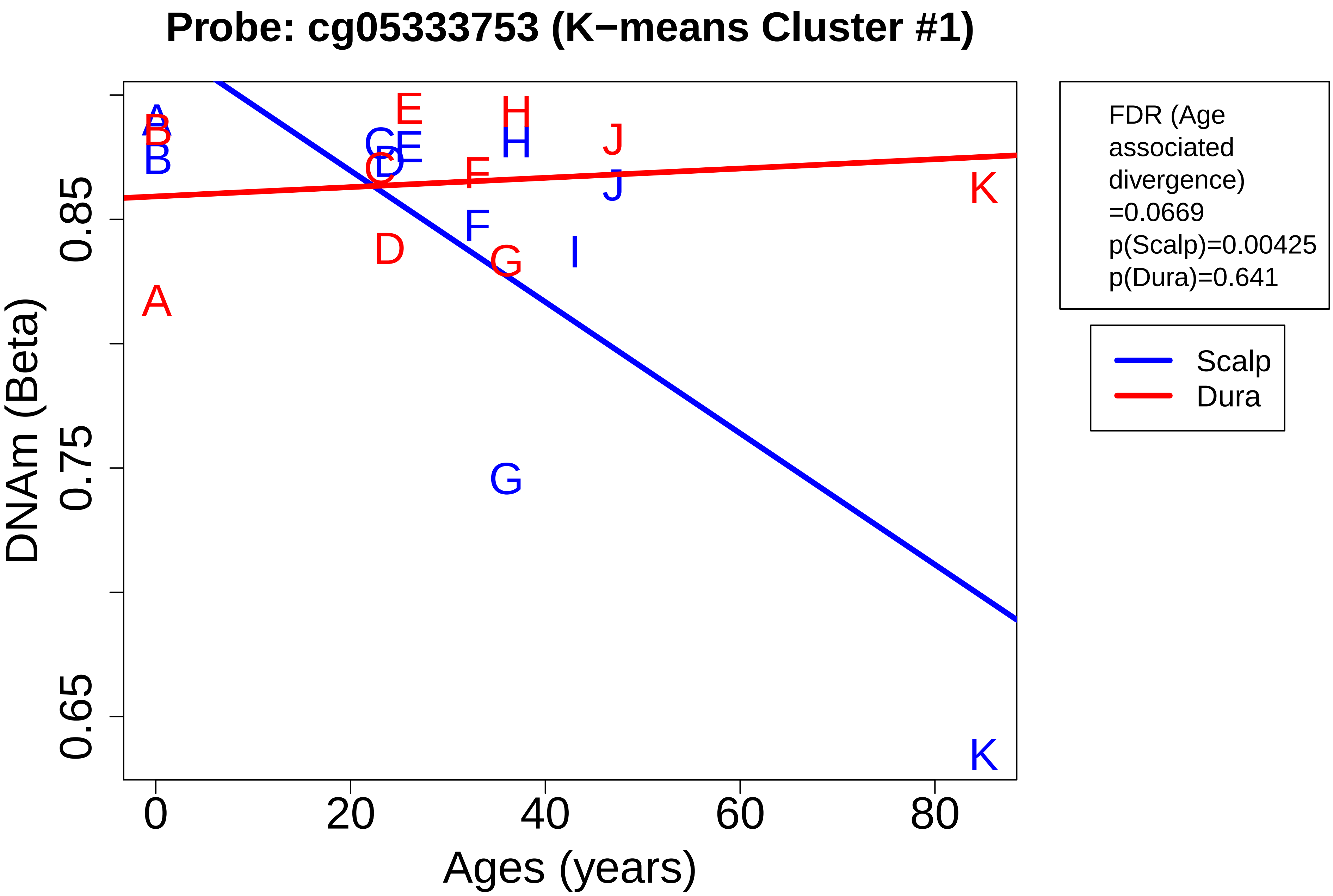

Probe: cg05333753 (K−means Cluster #1)
DNAm (Beta)
0.65
0.75
0.85
Ages (years)
0
20
40
60
80
FDR (Age associated divergence) =0.0669
p(Scalp)=0.00425
p(Dura)=0.641
Scalp
Dura

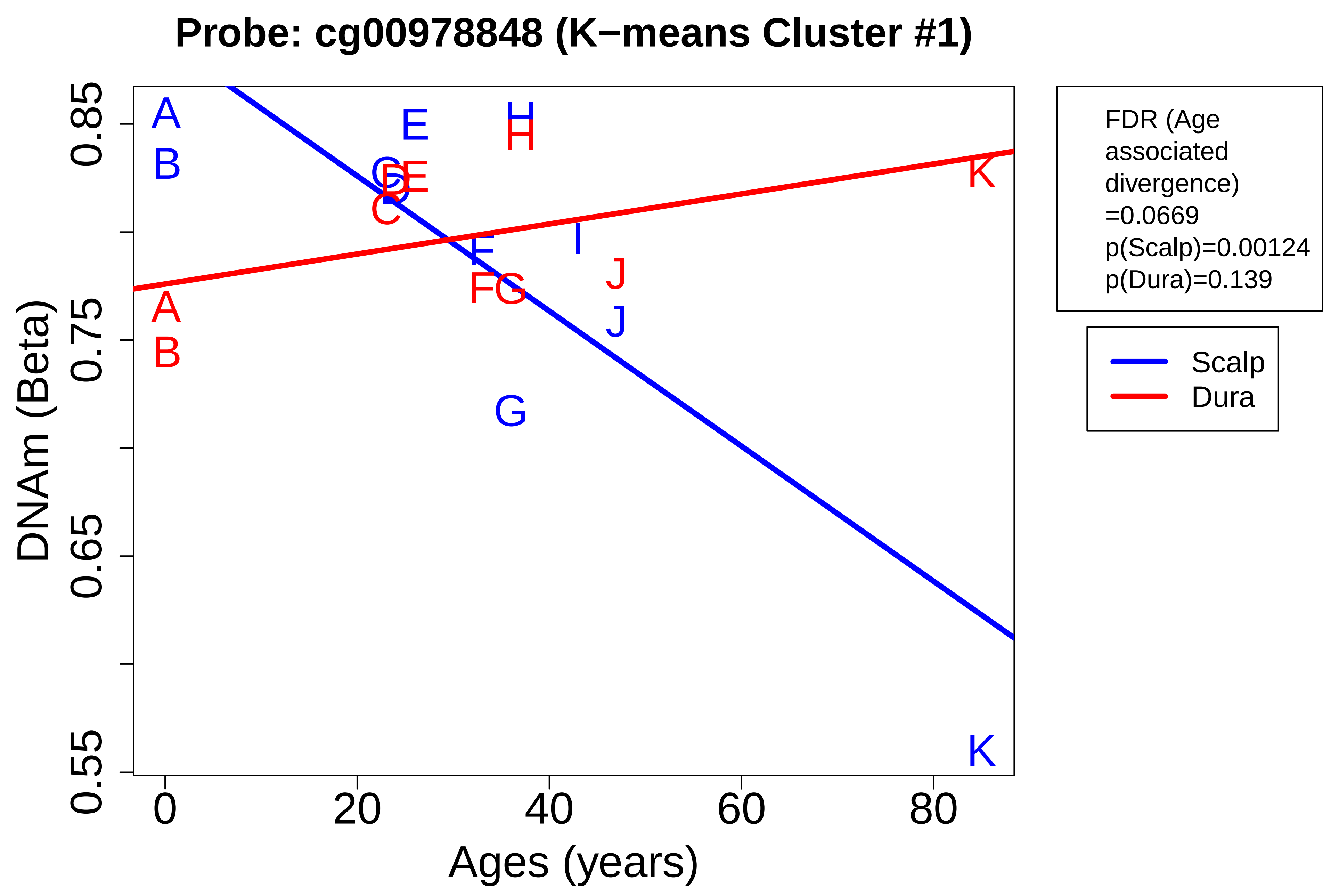
Probe: cg00978848 (K−means Cluster #1)
DNAm (Beta)
0.55
0.65
0.75
0.85
0
20
40
60
80
Ages (years)
A
B
C
D
E
F
G
H
I
J
K
FDR (Age associated divergence) =0.0669
p(Scalp)=0.00124
p(Dura)=0.139
Scalp
Dura

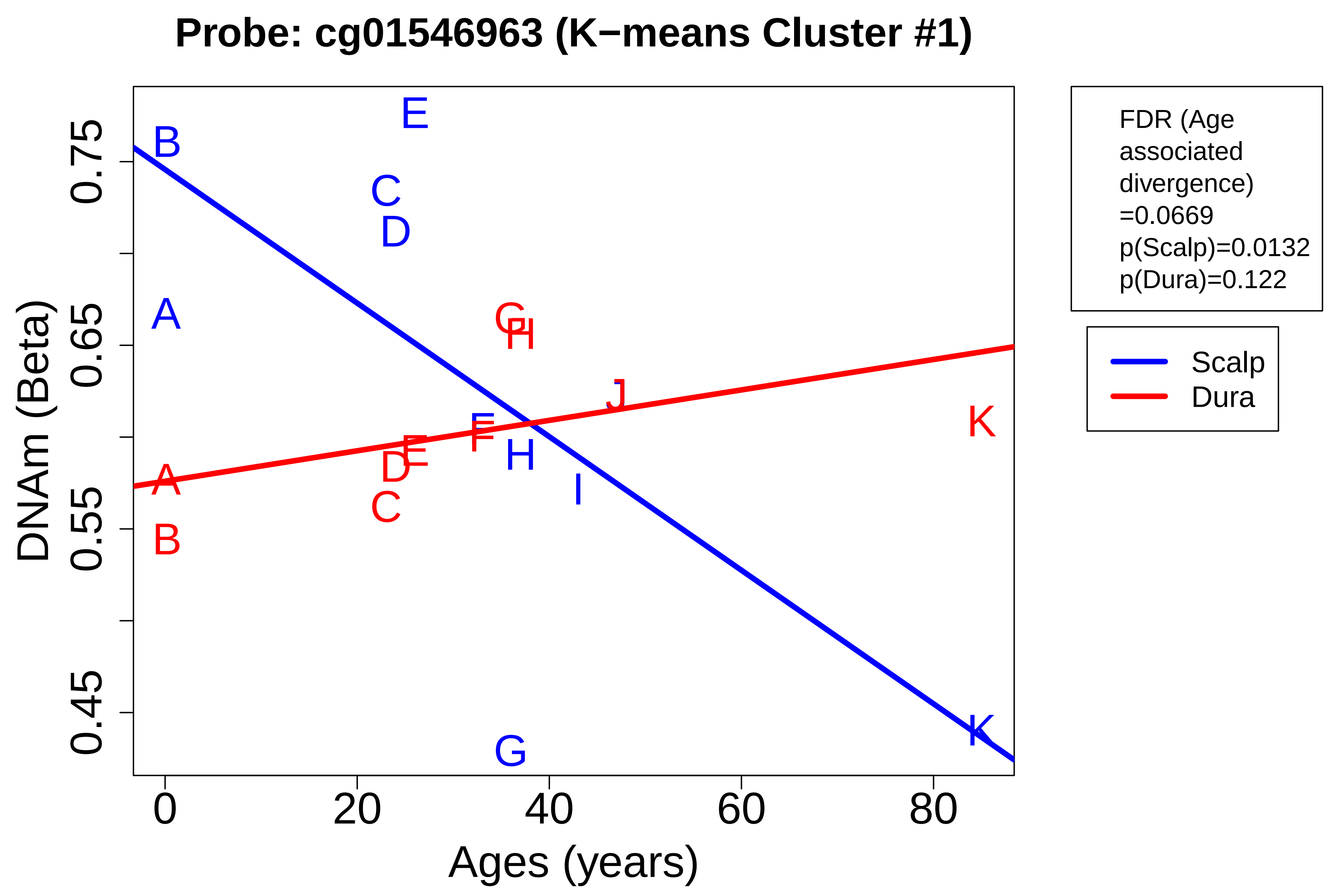

Probe: cg01546963 (K−means Cluster #1)
FDR (Age associated divergence) =0.0669
p(Scalp)=0.0132
p(Dura)=0.122
Scalp
Dura
DNAm (Beta)
0.45
0.55
0.65
0.75
0
20
40
60
80
Ages (years)
A
B
C
D
E
F
G
H
I
J
K

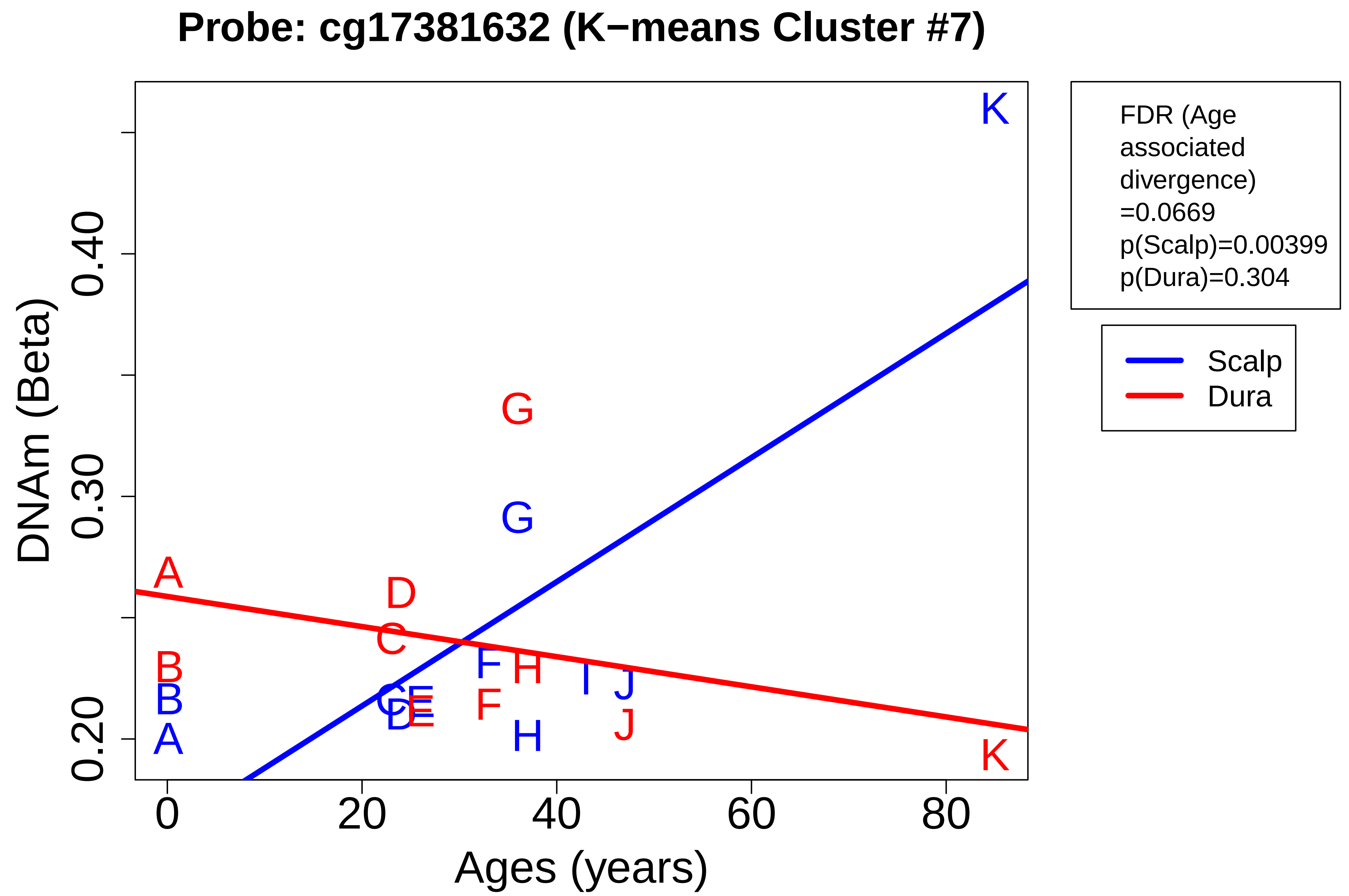
Probe: cg17381632 (K−means Cluster #7)
DNAm (Beta)
0.20
0.30
0.40
0
20
40
60
80
Ages (years)
FDR (Age associated divergence) =0.0669
p(Scalp)=0.00399
p(Dura)=0.304
Scalp
Dura
A
B
C
D
E
F
G
H
I
J
K

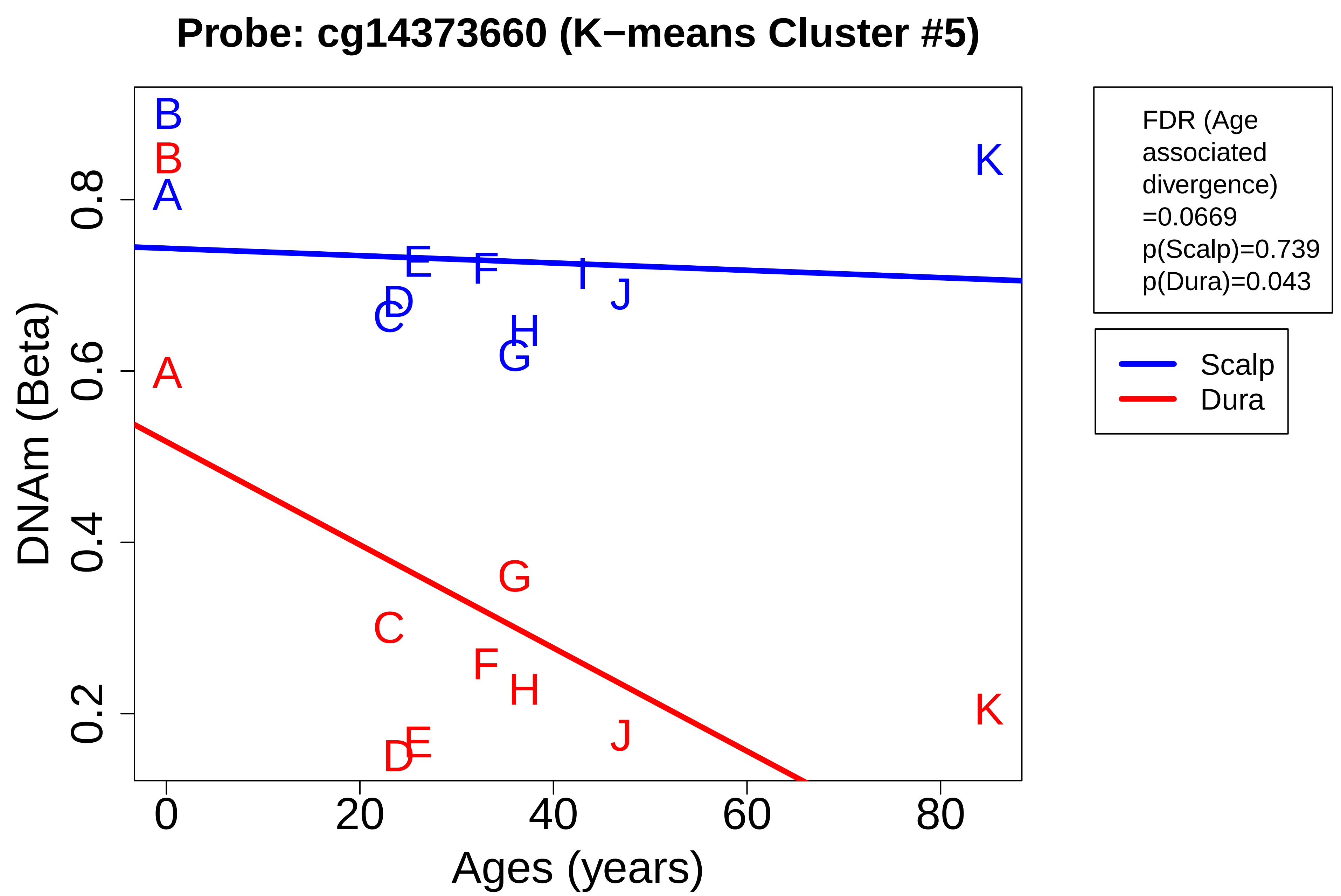

Probe: cg14373660 (K−means Cluster #5)
FDR (Age associated divergence) =0.0669
p(Scalp)=0.739
p(Dura)=0.043
Scalp
Dura
DNAm (Beta)
0.2
0.4
0.6
0.8
0
20
40
60
80
Ages (years)
A
B
C
D
E
F
G
H
I
J
K

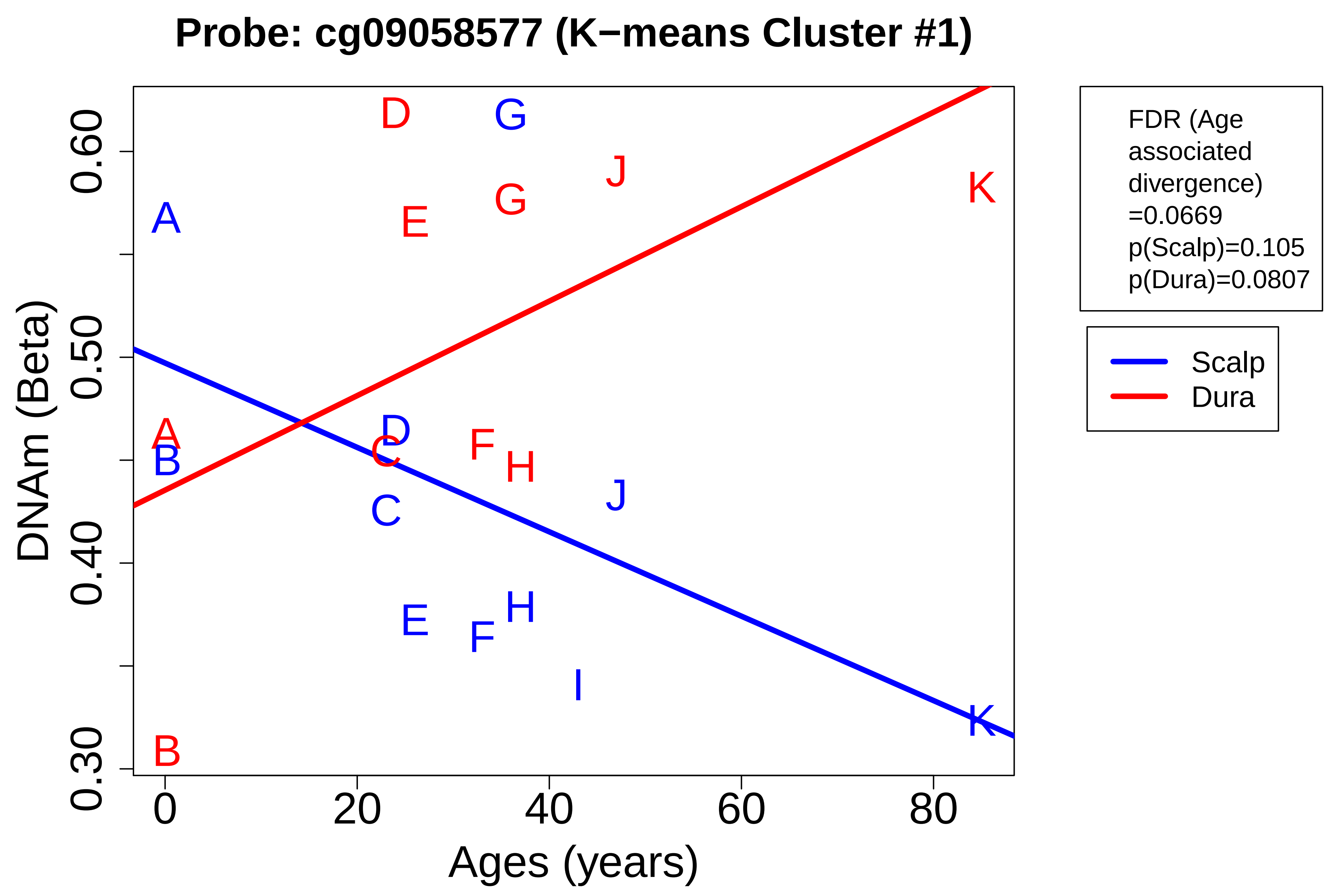

Probe: cg09058577 (K−means Cluster #1)
DNAm (Beta)
0.30
0.40
0.50
0.60
0
20
40
60
80
Ages (years)
A
B
C
D
E
F
G
H
I
J
K
FDR (Age associated divergence) =0.0669
p(Scalp)=0.105
p(Dura)=0.0807
Scalp
Dura

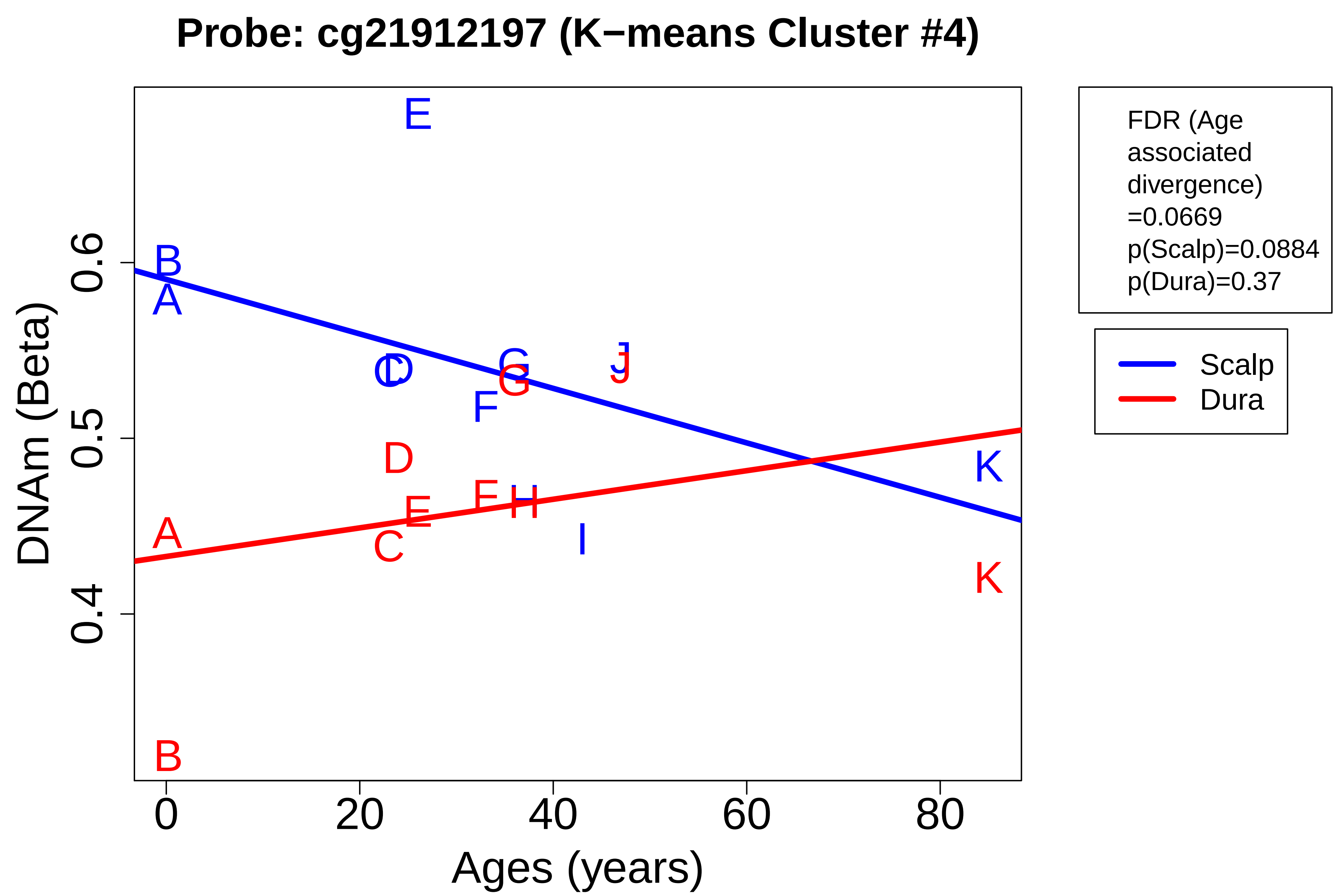

Probe: cg21912197 (K−means Cluster #4)
DNAm (Beta)
0.4
0.5
0.6
0
20
40
60
80
Ages (years)
A
B
C
D
E
F
G
H
I
J
K
FDR (Age associated divergence) =0.0669
p(Scalp)=0.0884
p(Dura)=0.37
Scalp
Dura

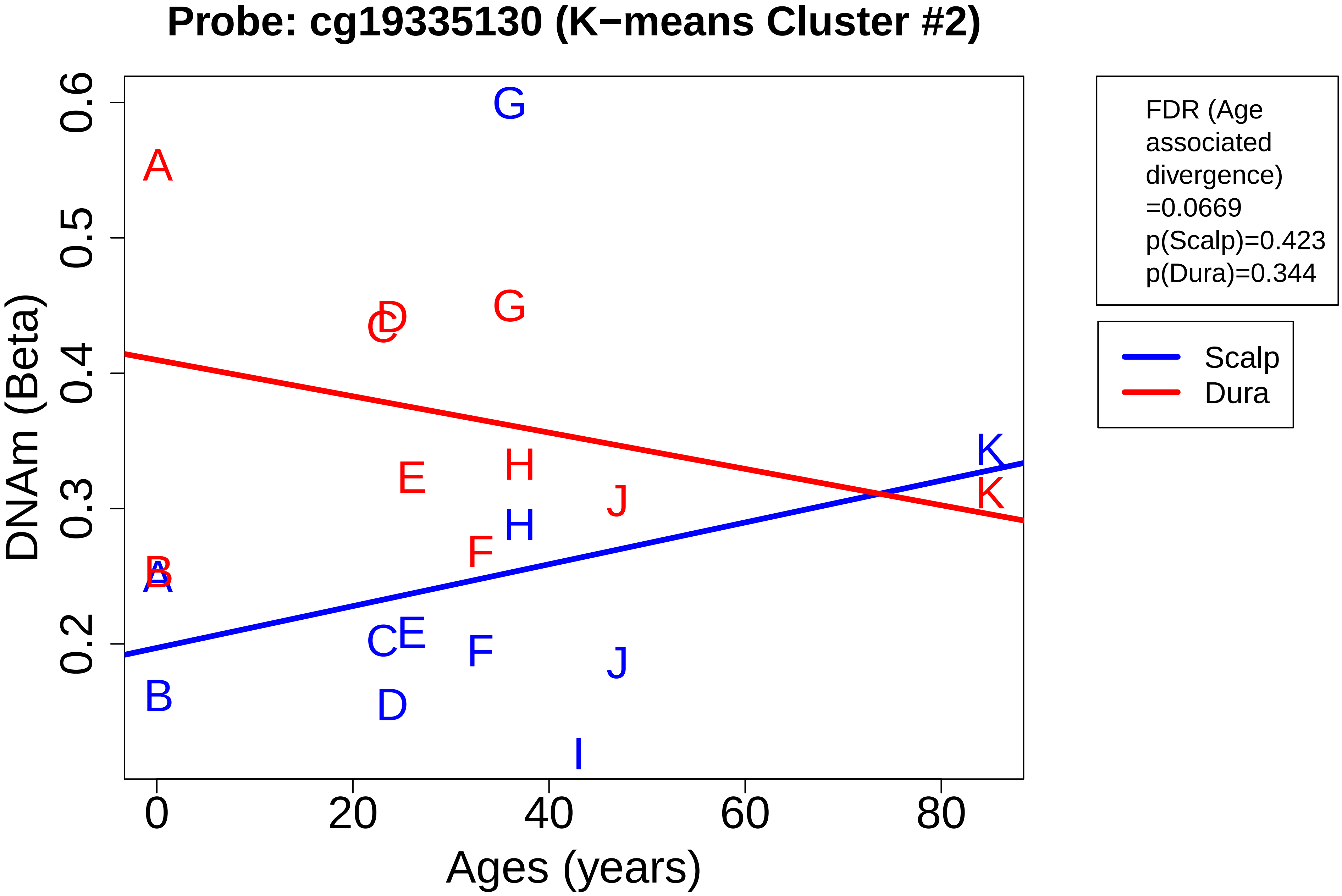

Probe: cg19335130 (K−means Cluster #2)
DNAm (Beta)
0.2
0.3
0.4
0.5
0.6
Ages (years)
0
20
40
60
80
A
B
C
D
E
F
G
H
I
J
K
FDR (Age associated divergence) =0.0669
p(Scalp)=0.423
p(Dura)=0.344
Scalp
Dura

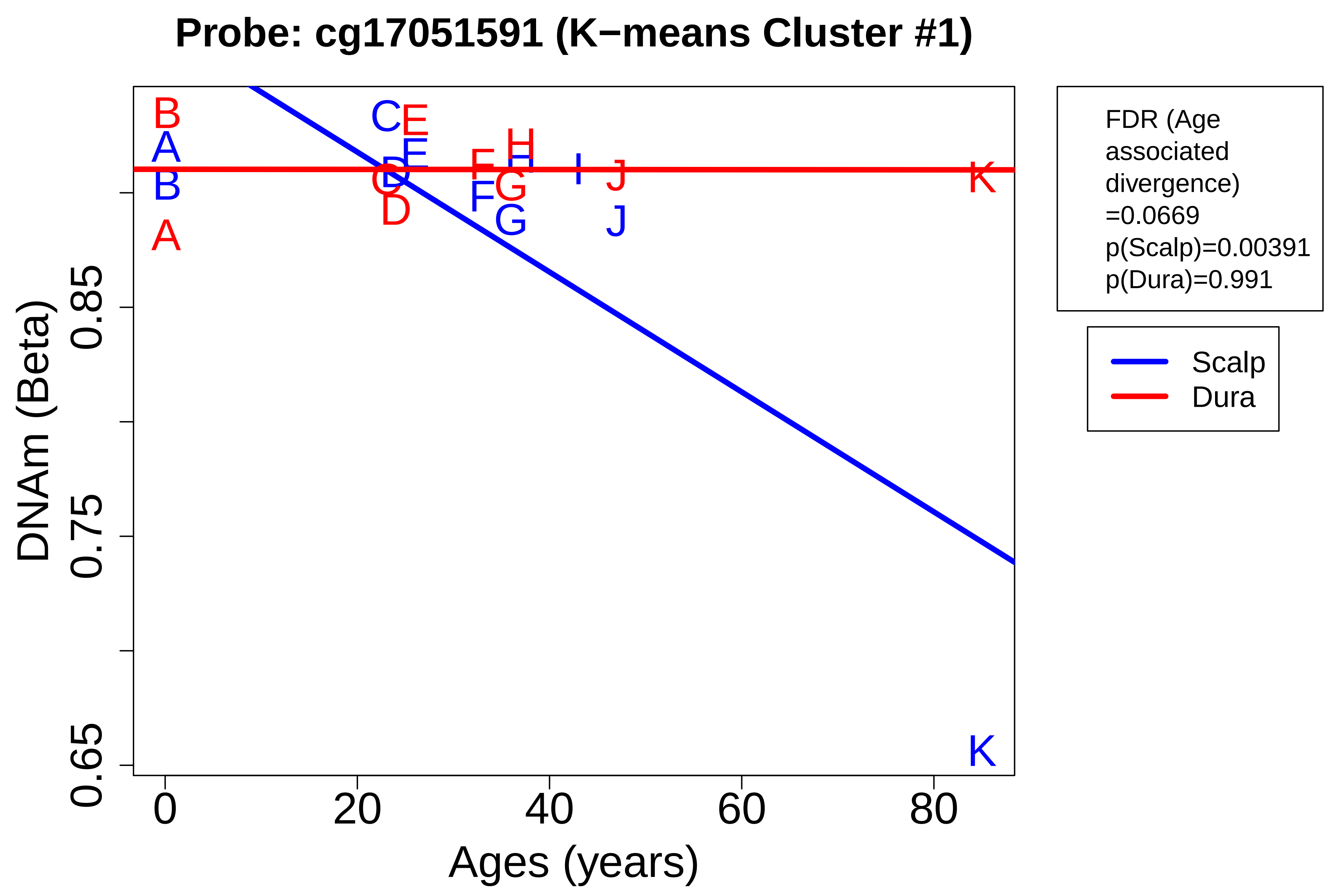

Probe: cg17051591 (K−means Cluster #1)
DNAm (Beta)
0.65
0.75
0.85
Ages (years)
0
20
40
60
80
FDR (Age associated divergence) =0.0669
p(Scalp)=0.00391
p(Dura)=0.991
Scalp
Dura

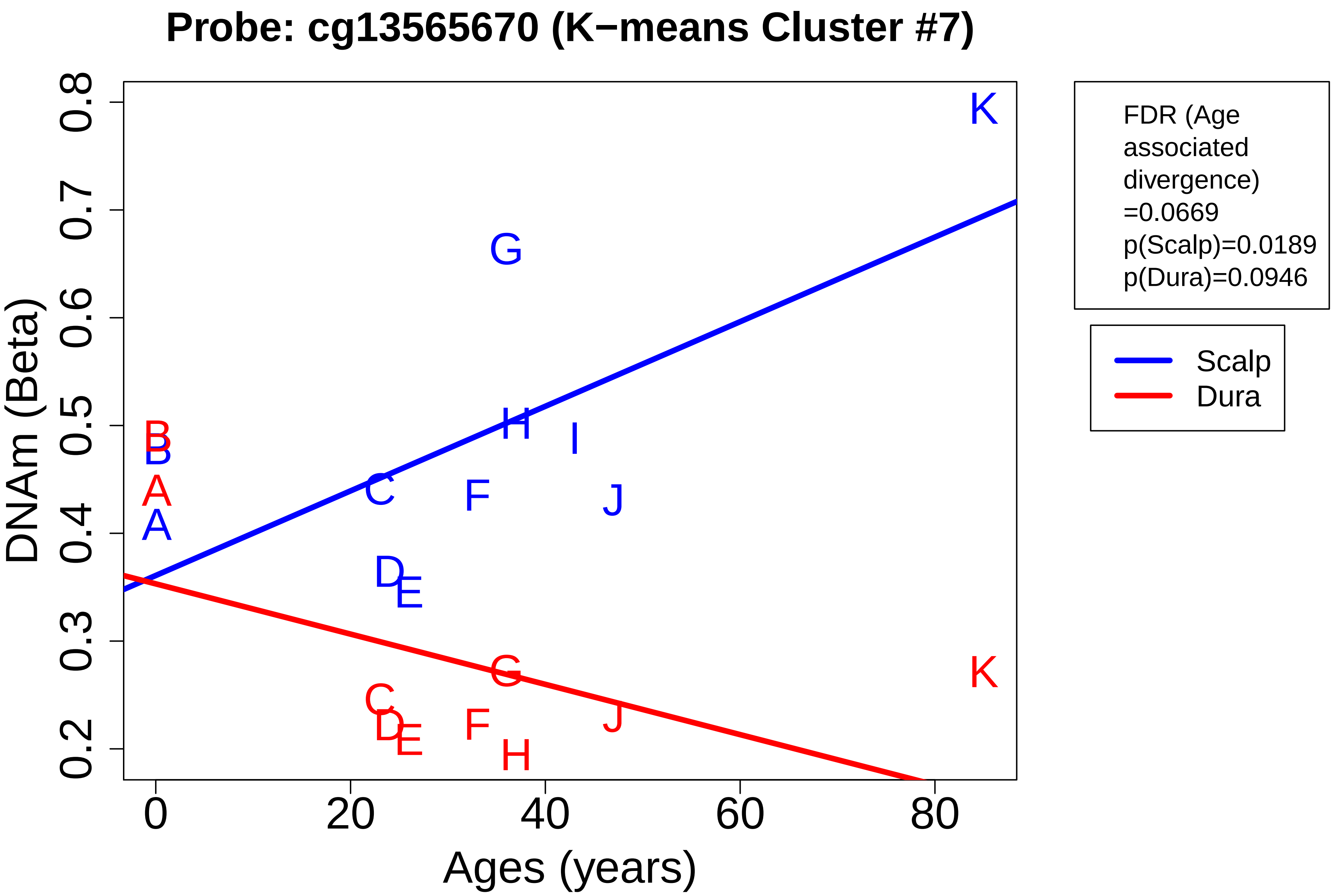

Probe: cg13565670 (K−means Cluster #7)
DNAm (Beta)
Ages (years)
0.2
0.3
0.4
0.5
0.6
0.7
0.8
0
20
40
60
80
FDR (Age associated divergence) =0.0669
p(Scalp)=0.0189
p(Dura)=0.0946
Scalp
Dura
A
B
C
D
E
F
G
H
I
J
K

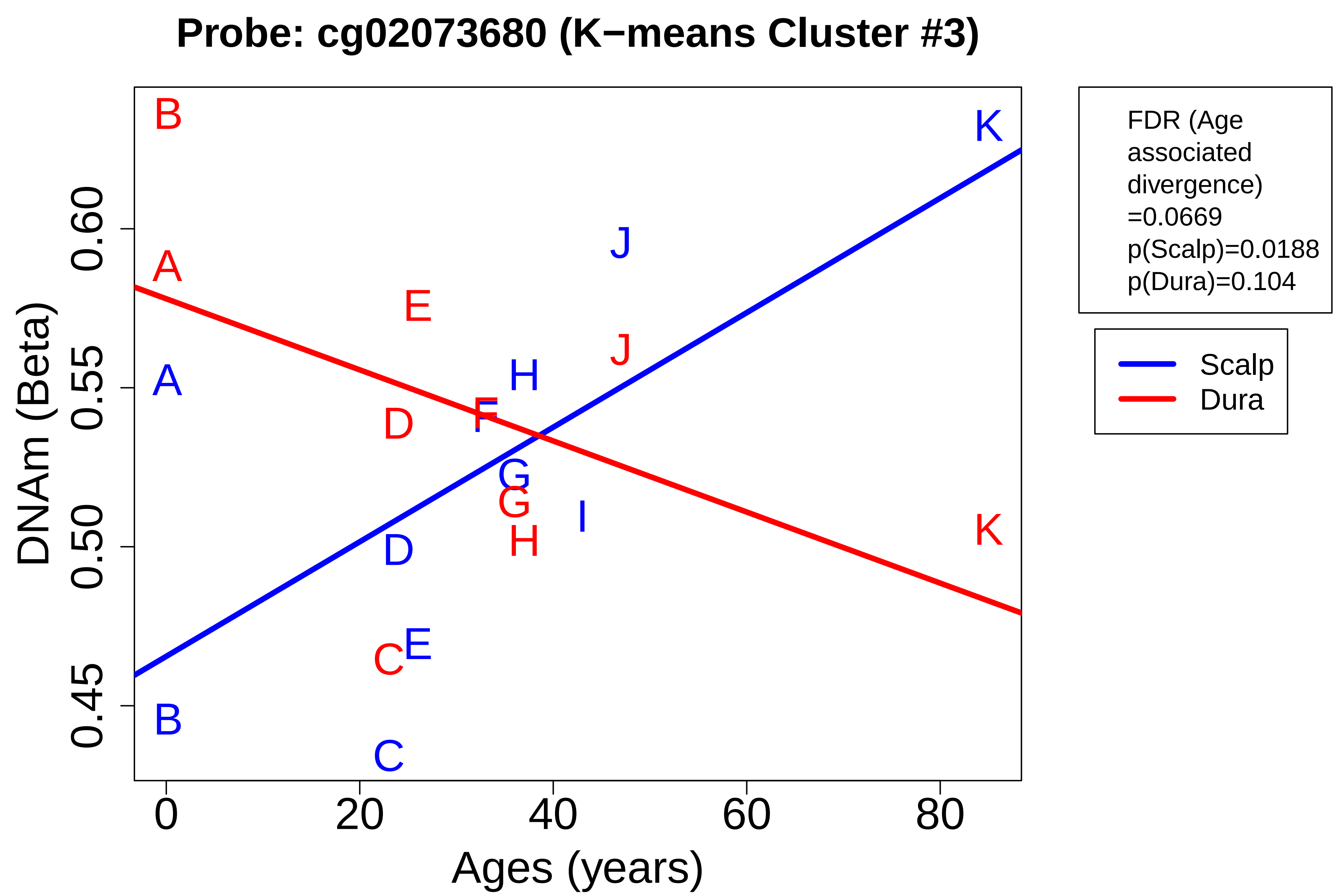
Probe: cg02073680 (K−means Cluster #3)
DNAm (Beta)
0.45
0.50
0.55
0.60
Ages (years)
0
20
40
60
80
B
A
E
D
F
H
G
J
I
K
C
FDR (Age associated divergence) =0.0669
p(Scalp)=0.0188
p(Dura)=0.104
Scalp
Dura

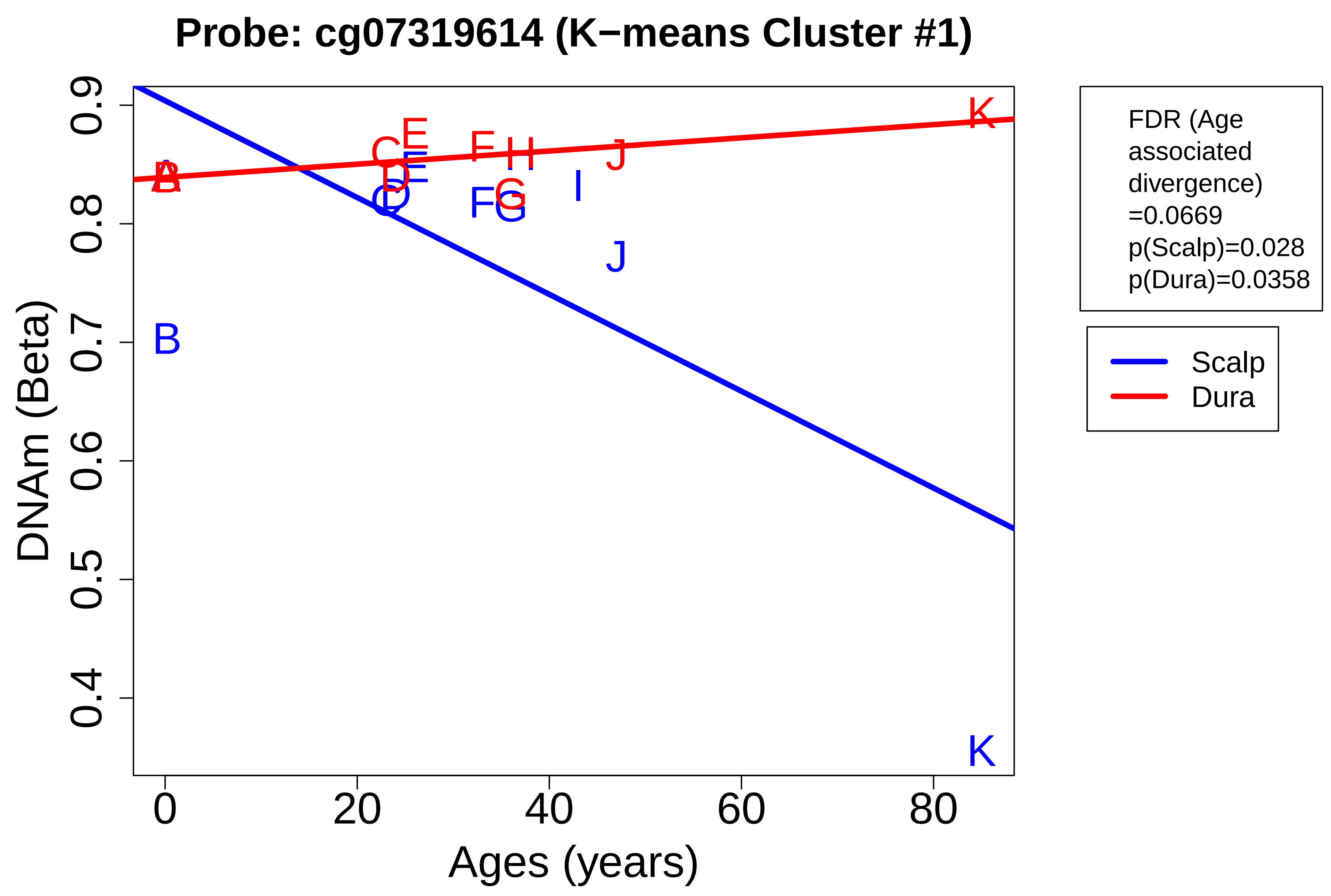

Probe: cg07319614 (K−means Cluster #1)
DNAm (Beta)
0.4
0.5
0.6
0.7
0.8
0.9
0
20
40
60
80
Ages (years)
A
B
C
D
E
F
G
H
I
J
K
FDR (Age associated divergence) =0.0669
p(Scalp)=0.028
p(Dura)=0.0358
Scalp
Dura

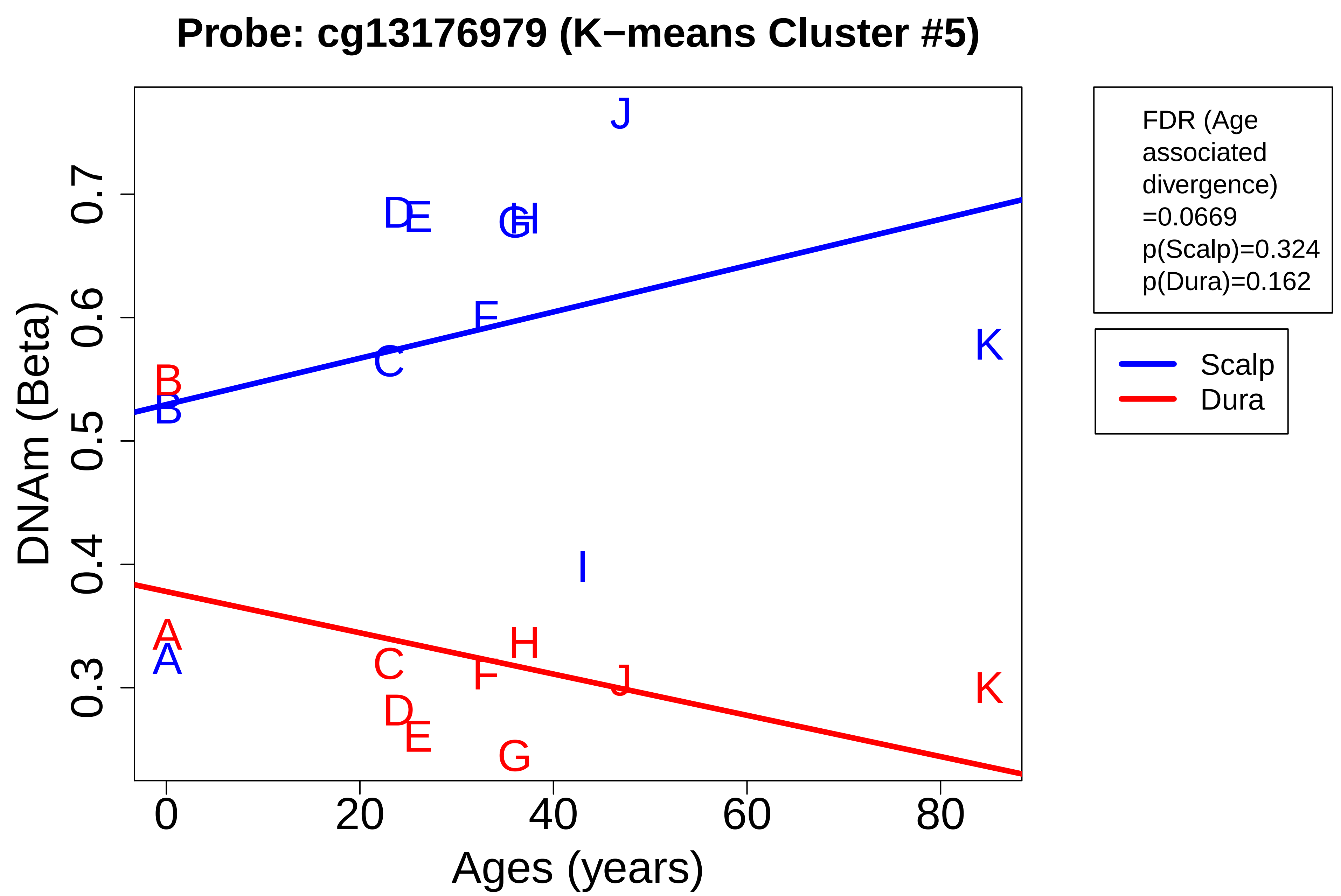
Probe: cg13176979 (K−means Cluster #5)
DNAm (Beta)
0.3
0.4
0.5
0.6
0.7
0
20
40
60
80
Ages (years)
FDR (Age associated divergence) =0.0669
p(Scalp)=0.324
p(Dura)=0.162
Scalp
Dura

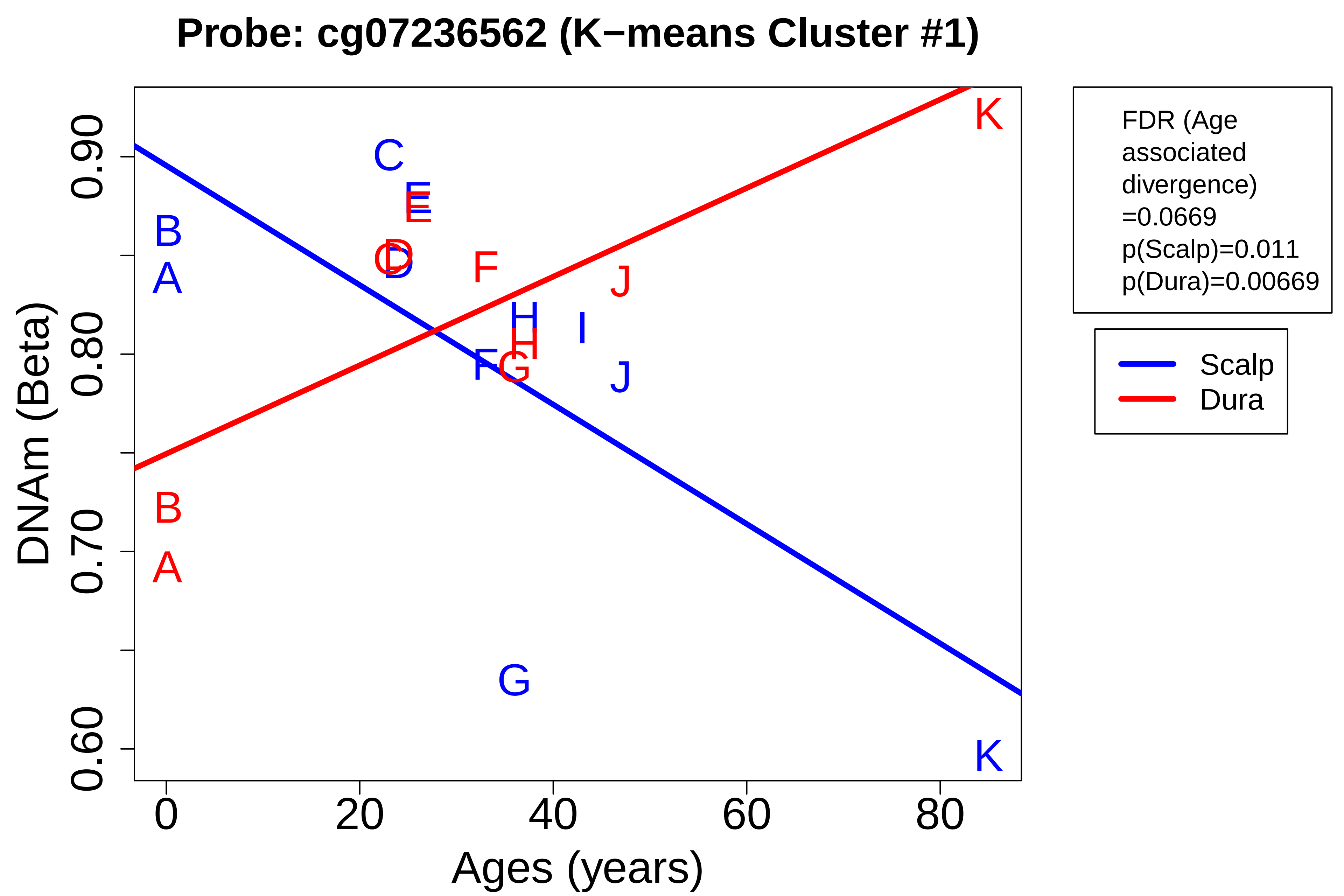

Probe: cg07236562 (K−means Cluster #1)
DNAm (Beta)
0.60
0.70
0.80
0.90
Ages (years)
0
20
40
60
80
FDR (Age associated divergence) =0.0669
p(Scalp)=0.011
p(Dura)=0.00669
Scalp
Dura
A
B
C
D
E
F
G
H
I
J
K

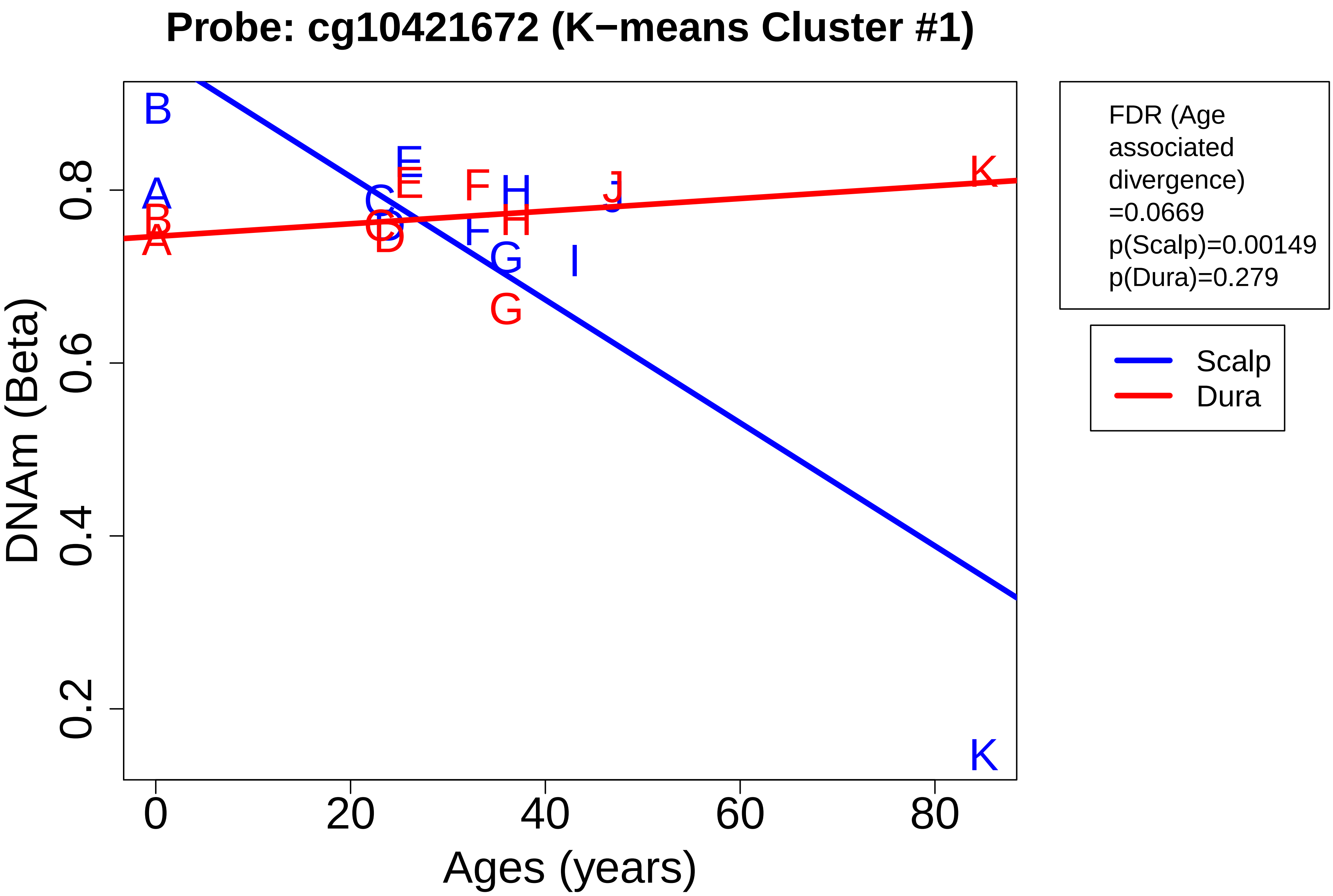
Probe: cg10421672 (K−means Cluster #1)
DNAm (Beta)
0.2
0.4
0.6
0.8
0
20
40
60
80
Ages (years)
FDR (Age associated divergence) =0.0669
p(Scalp)=0.00149
p(Dura)=0.279
Scalp
Dura

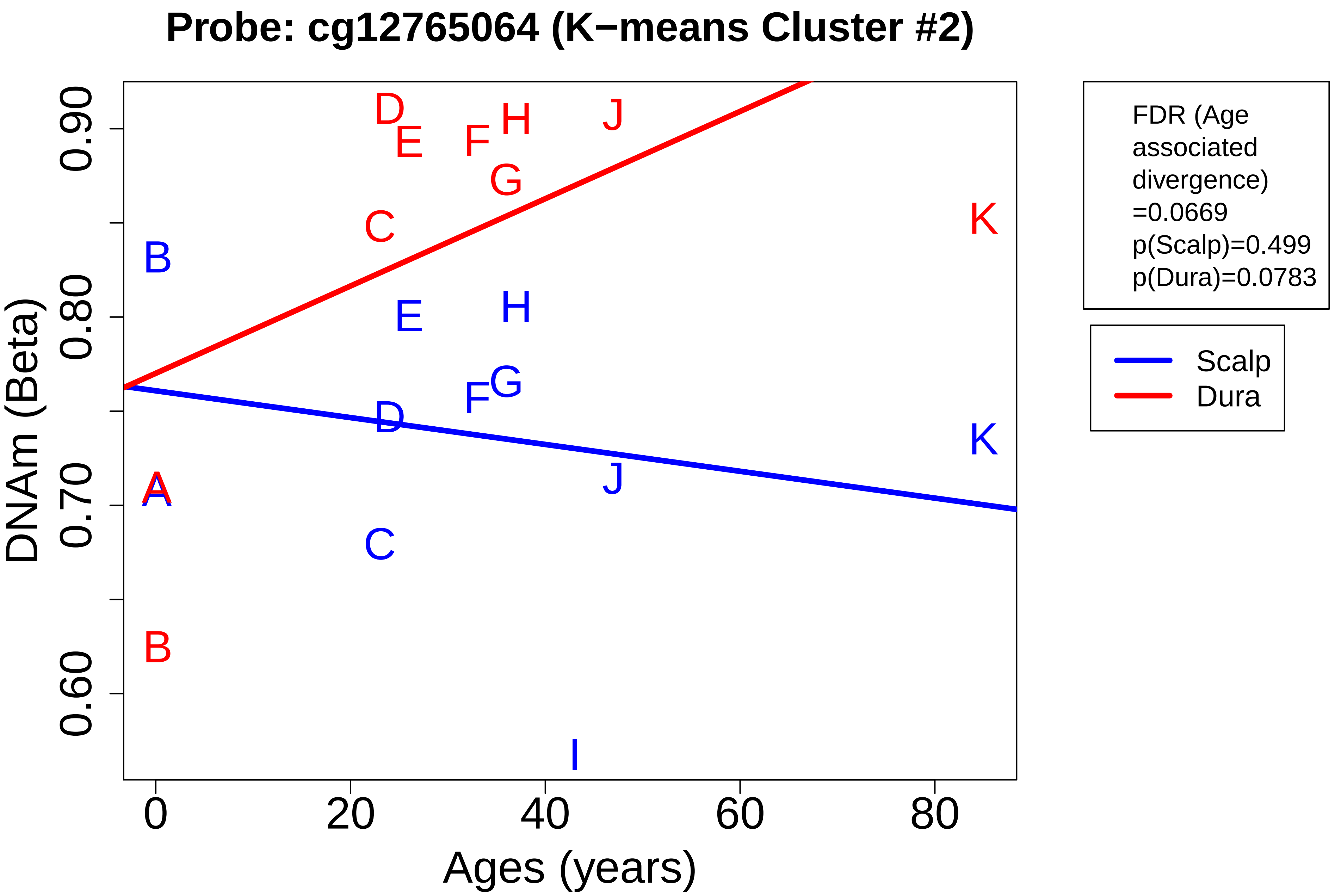
Probe: cg12765064 (K−means Cluster #2)
DNAm (Beta)
0.60
0.70
0.80
0.90
0
20
40
60
80
Ages (years)
A
B
C
D
E
F
G
H
I
J
K
FDR (Age associated divergence) =0.0669
p(Scalp)=0.499
p(Dura)=0.0783
Scalp
Dura

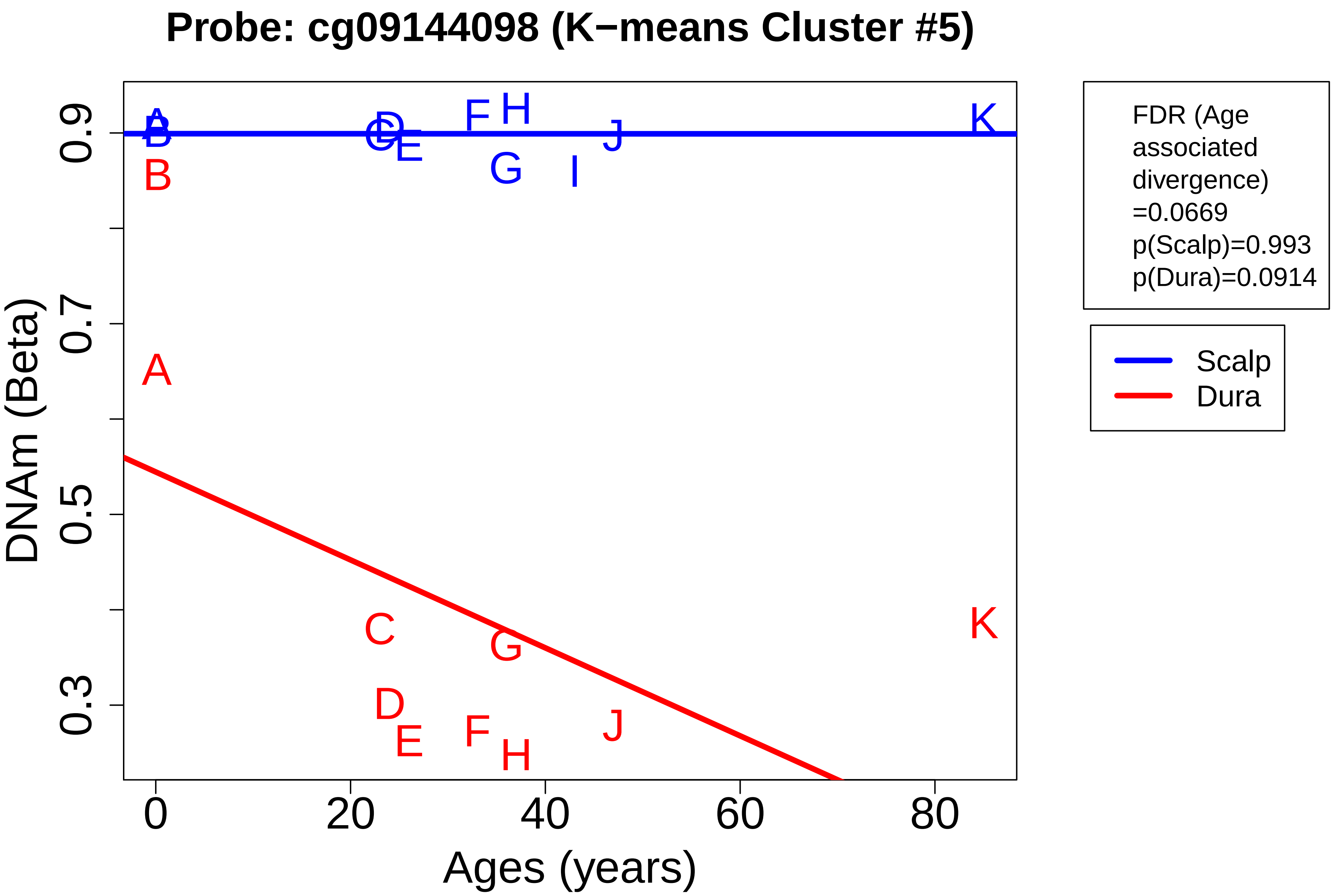

Probe: cg09144098 (K−means Cluster #5)
FDR (Age associated divergence) =0.0669
p(Scalp)=0.993
p(Dura)=0.0914
Scalp
Dura
DNAm (Beta)
0.3
0.5
0.7
0.9
0
20
40
60
80
Ages (years)
A
B
C
D
E
F
G
H
I
J
K

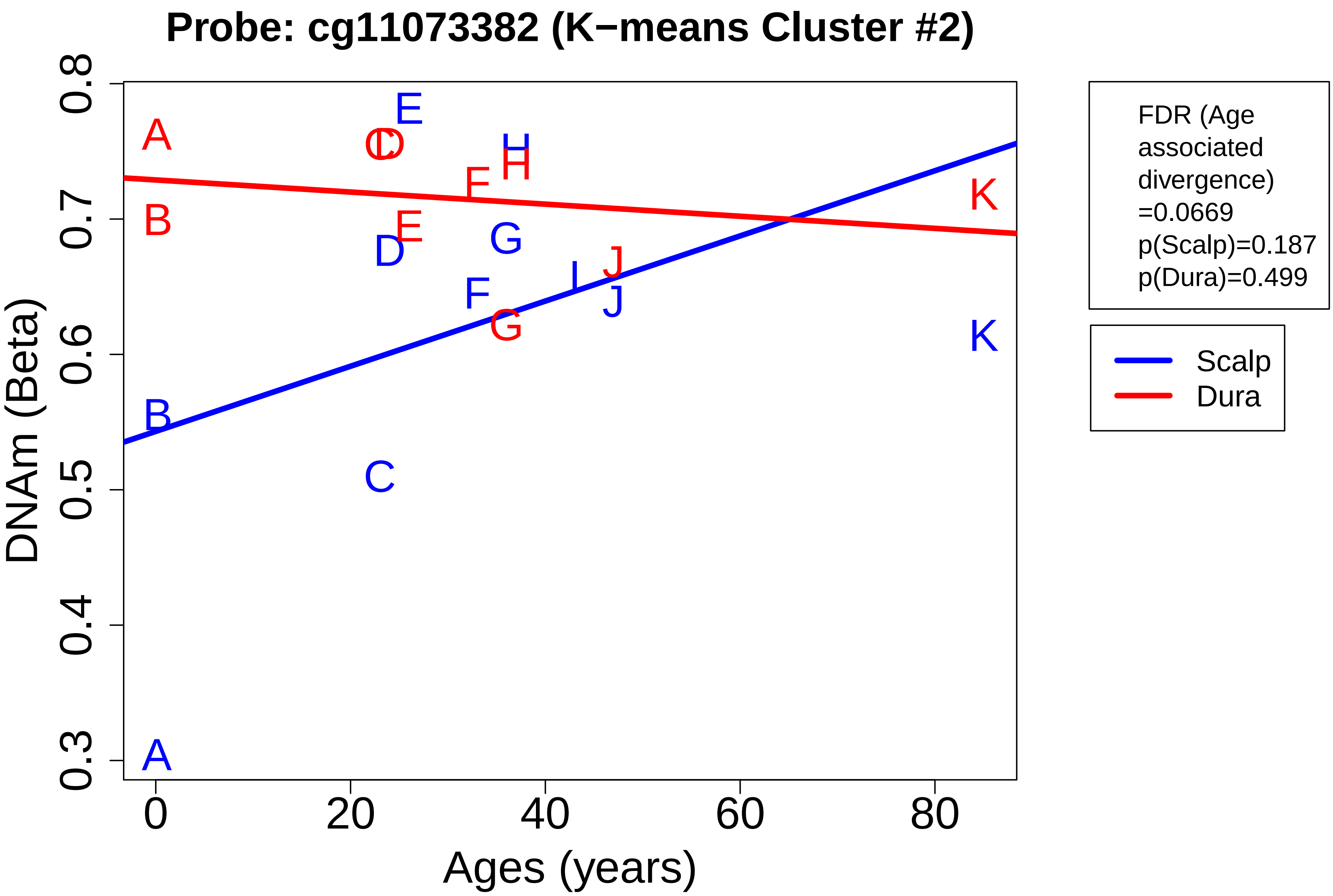

Probe: cg11073382 (K−means Cluster #2)
DNAm (Beta)
0.3
0.4
0.5
0.6
0.7
0.8
Ages (years)
0
20
40
60
80
FDR (Age associated divergence) =0.0669
p(Scalp)=0.187
p(Dura)=0.499
Scalp
Dura

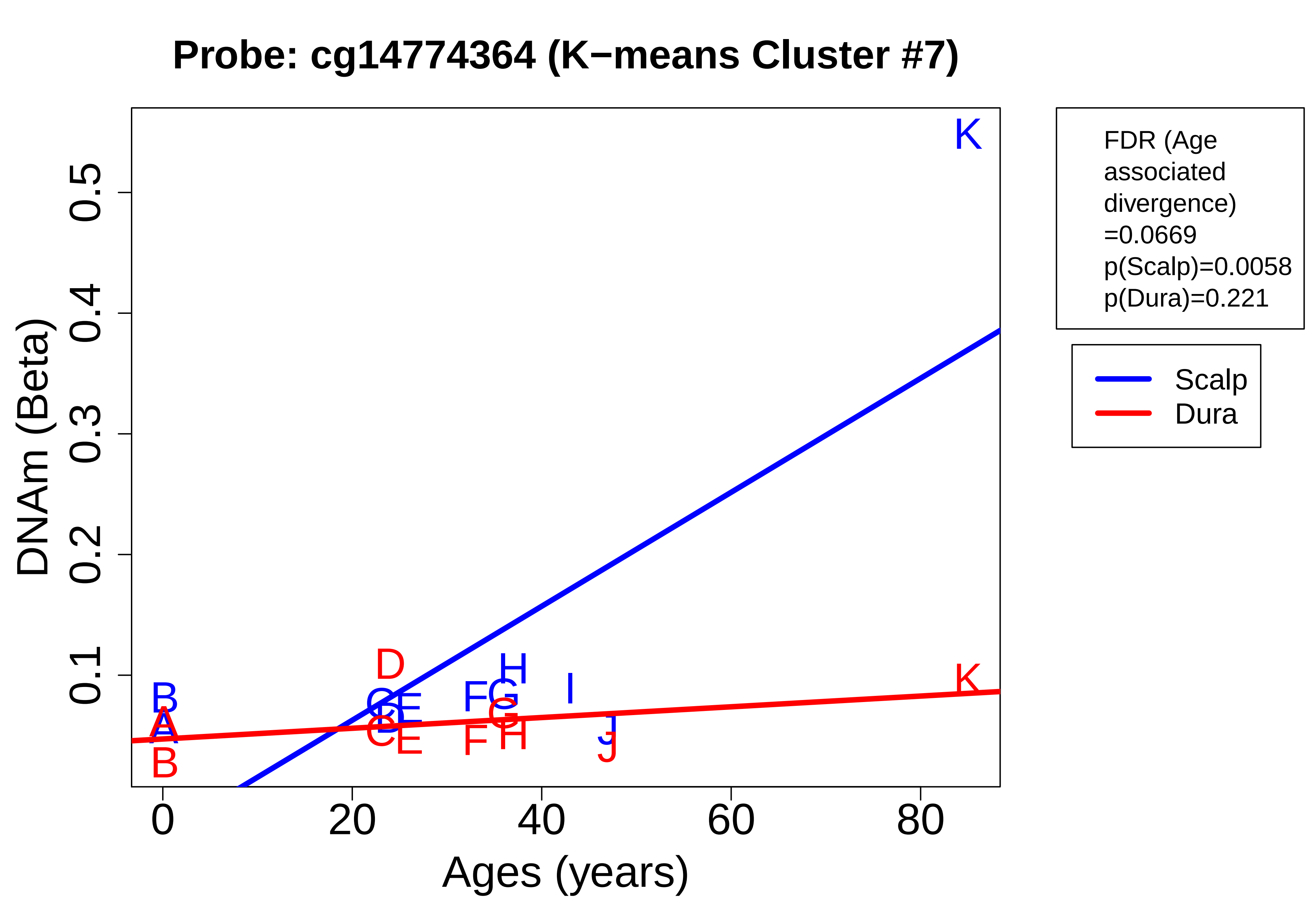
Probe: cg14774364 (K−means Cluster #7)
DNAm (Beta)
0.1
0.2
0.3
0.4
0.5
0
20
40
60
80
Ages (years)
FDR (Age associated divergence) =0.0669
p(Scalp)=0.0058
p(Dura)=0.221
Scalp
Dura

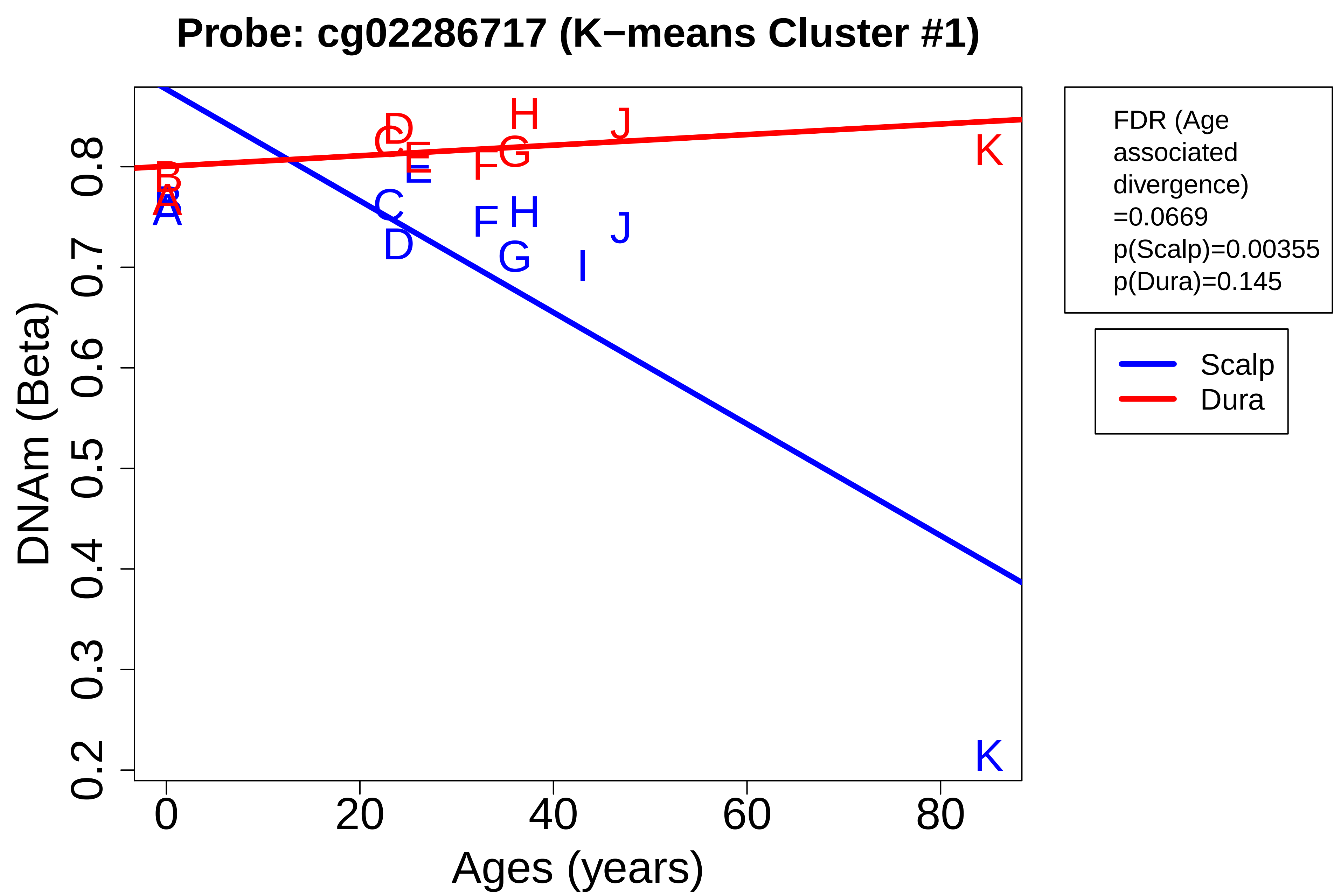

Probe: cg02286717 (K−means Cluster #1)
DNAm (Beta)
Ages (years)
0.2
0.3
0.4
0.5
0.6
0.7
0.8
0
20
40
60
80
FDR (Age associated divergence) =0.0669
p(Scalp)=0.00355
p(Dura)=0.145
Scalp
Dura
A
B
C
D
E
F
G
H
I
J
K

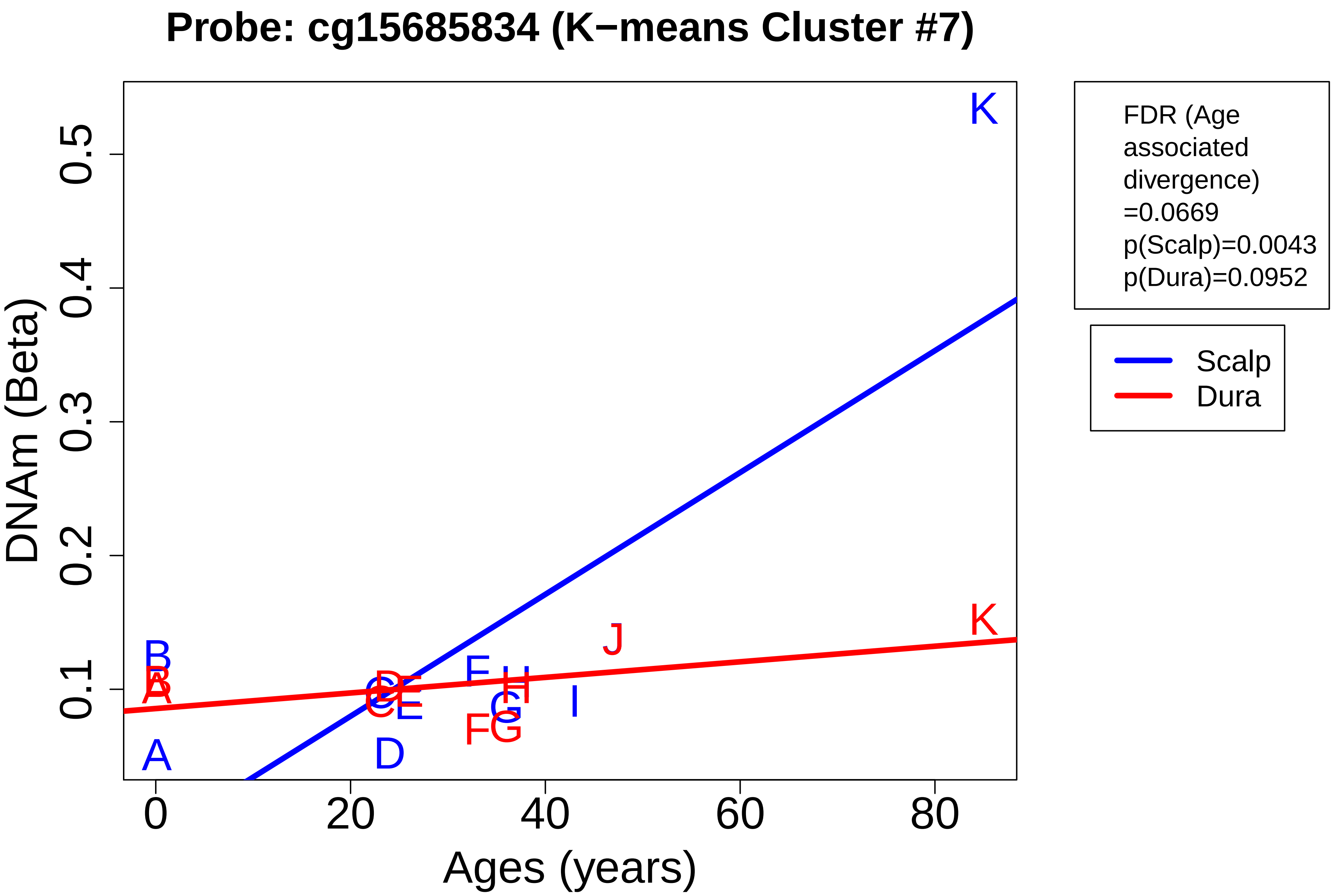

Probe: cg15685834 (K−means Cluster #7)
DNAm (Beta)
0.1
0.2
0.3
0.4
0.5
Ages (years)
0
20
40
60
80
FDR (Age associated divergence) =0.0669
p(Scalp)=0.0043
p(Dura)=0.0952
Scalp
Dura

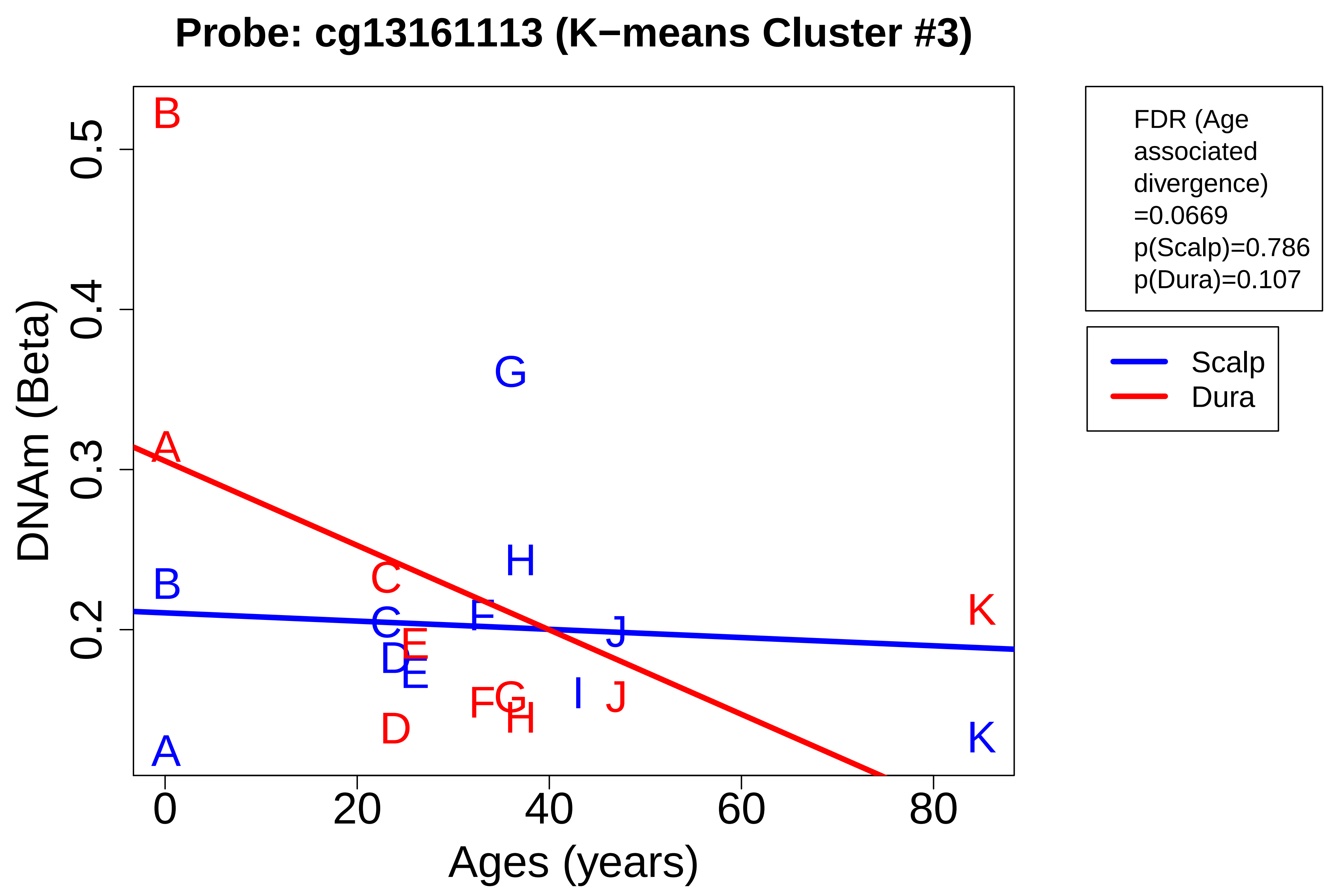

Probe: cg13161113 (K−means Cluster #3)
DNAm (Beta)
0.2
0.3
0.4
0.5
0
20
40
60
80
Ages (years)
A
B
C
D
E
F
G
H
I
J
K
FDR (Age associated divergence) =0.0669
p(Scalp)=0.786
p(Dura)=0.107
Scalp
Dura

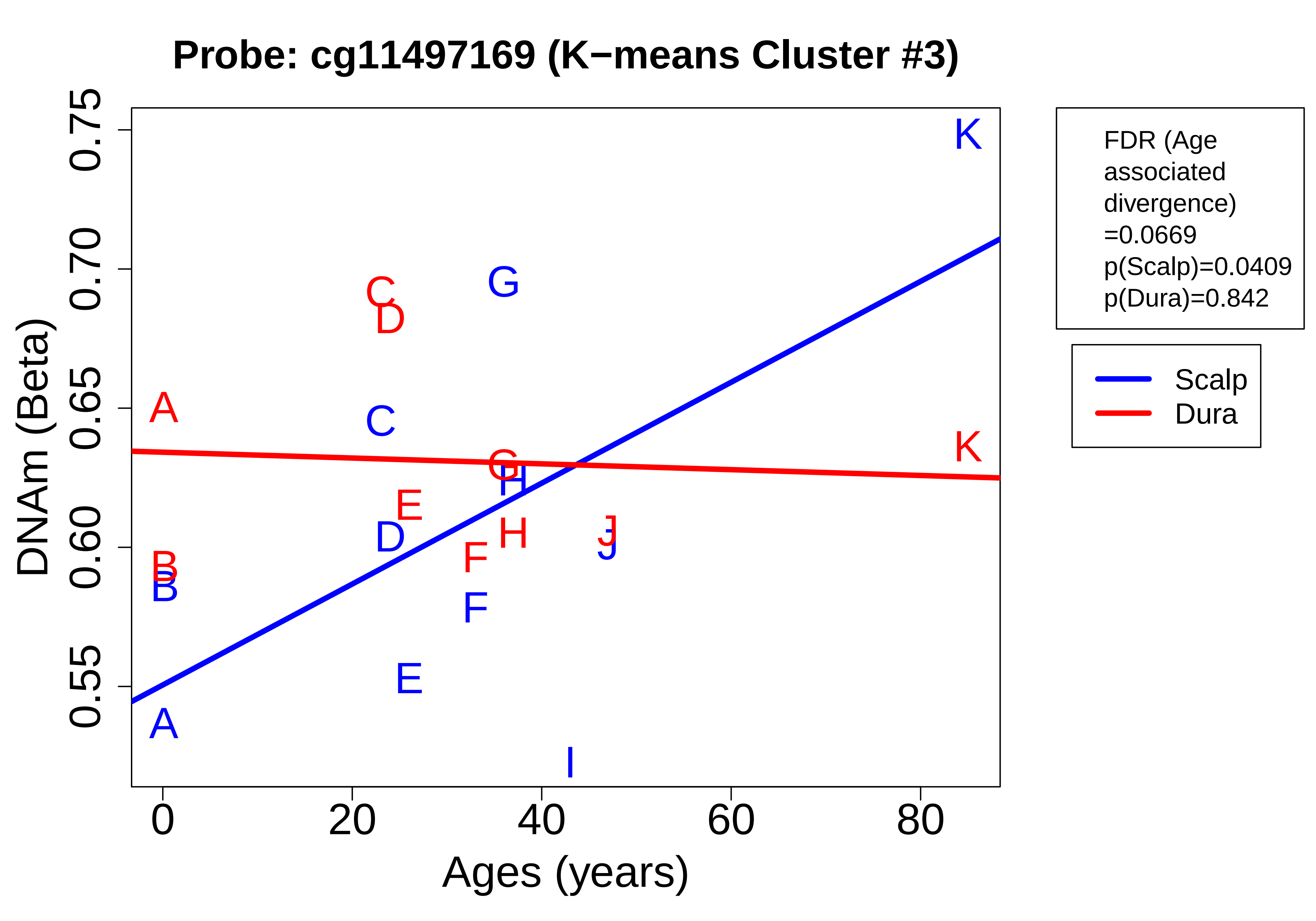

Probe: cg11497169 (K−means Cluster #3)
DNAm (Beta)
0.55
0.60
0.65
0.70
0.75
Ages (years)
0
20
40
60
80
A
B
C
D
E
F
G
H
I
J
K
FDR (Age associated divergence) =0.0669
p(Scalp)=0.0409
p(Dura)=0.842
Scalp
Dura

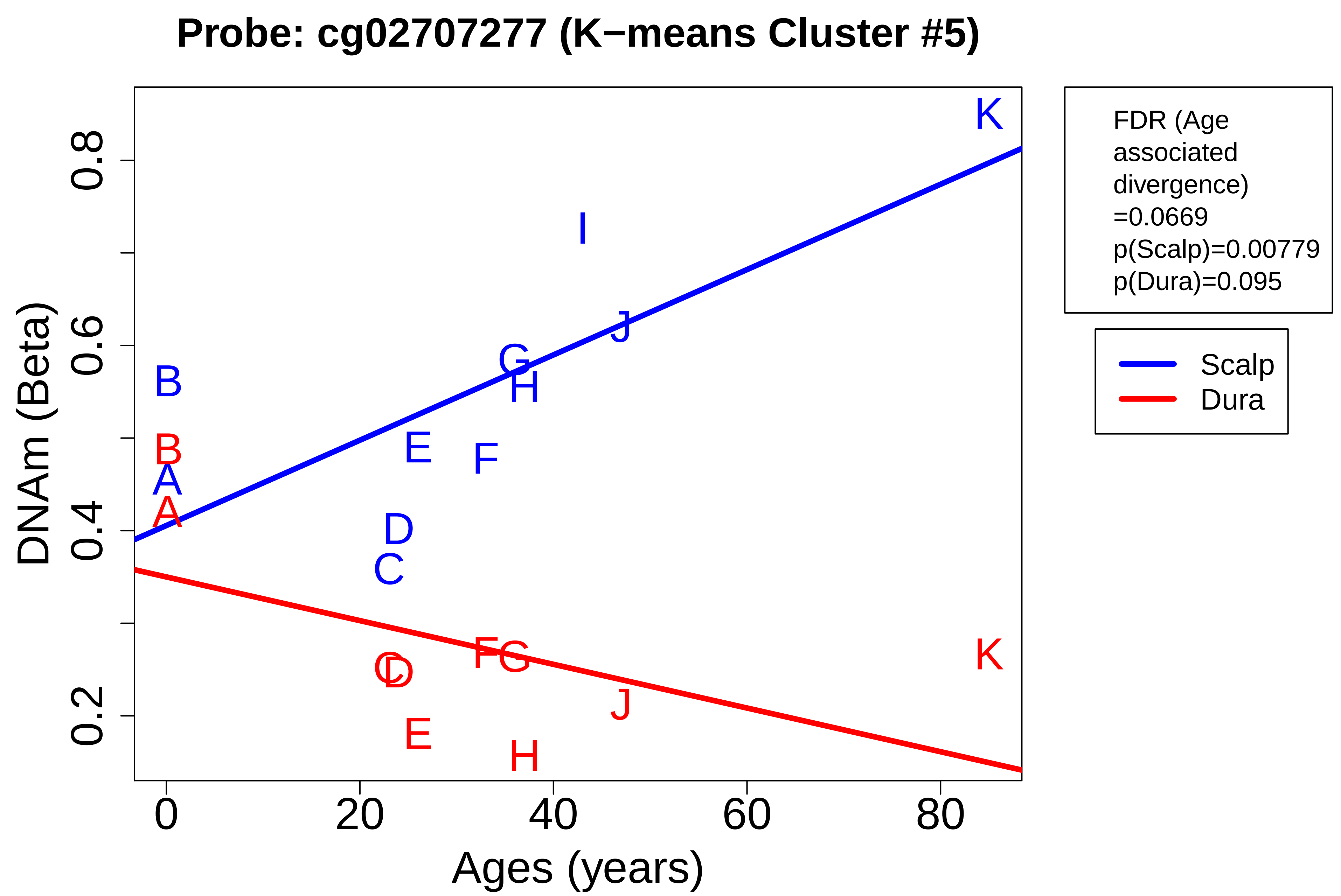

Probe: cg02707277 (K−means Cluster #5)
DNAm (Beta)
0.2
0.4
0.6
0.8
Ages (years)
0
20
40
60
80
FDR (Age associated divergence) =0.0669
p(Scalp)=0.00779
p(Dura)=0.095
Scalp
Dura
A
B
C
D
E
F
G
H
I
J
K

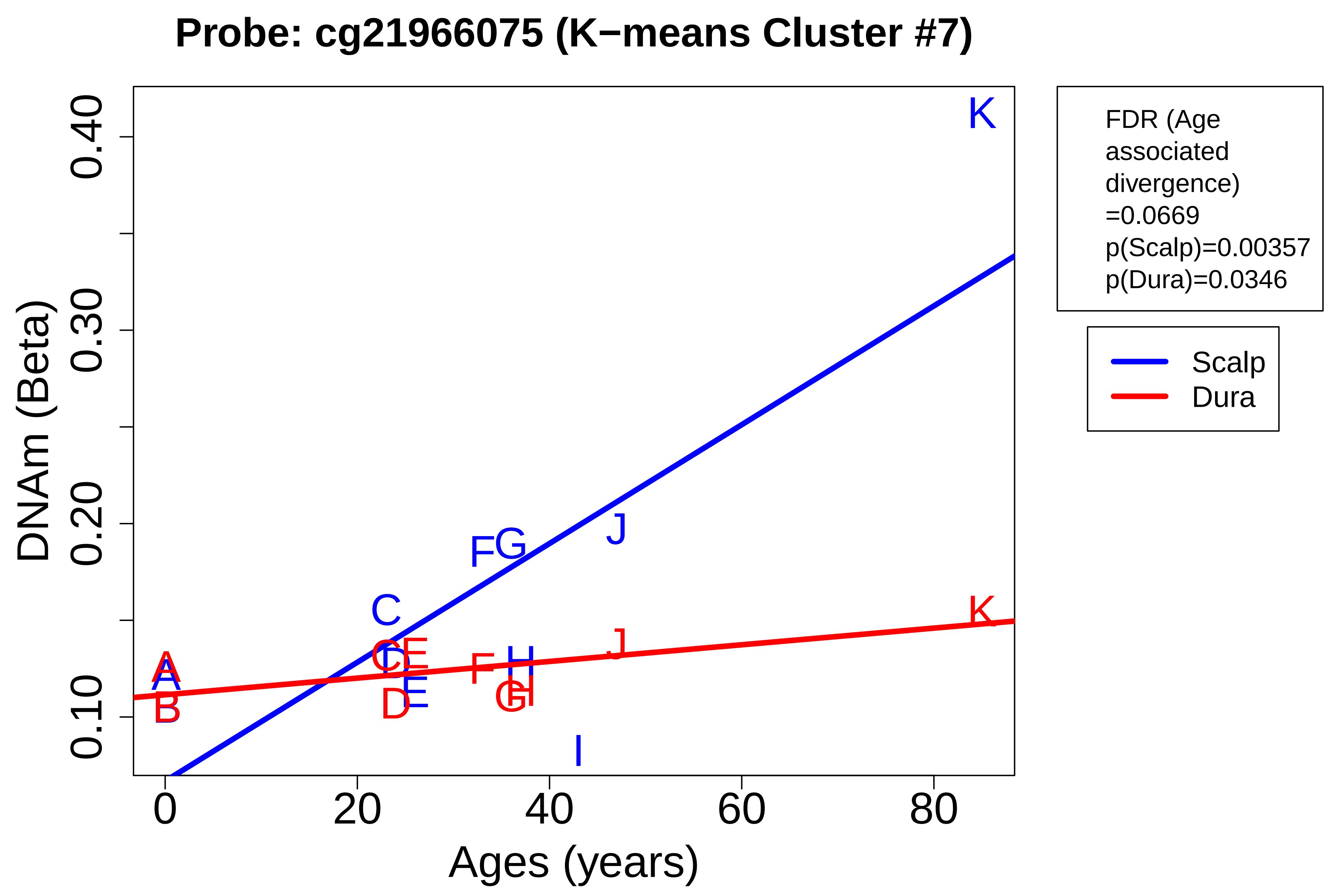

Probe: cg21966075 (K−means Cluster #7)
DNAm (Beta)
0.10
0.20
0.30
0.40
0
20
40
60
80
Ages (years)
FDR (Age associated divergence) =0.0669
p(Scalp)=0.00357
p(Dura)=0.0346
Scalp
Dura

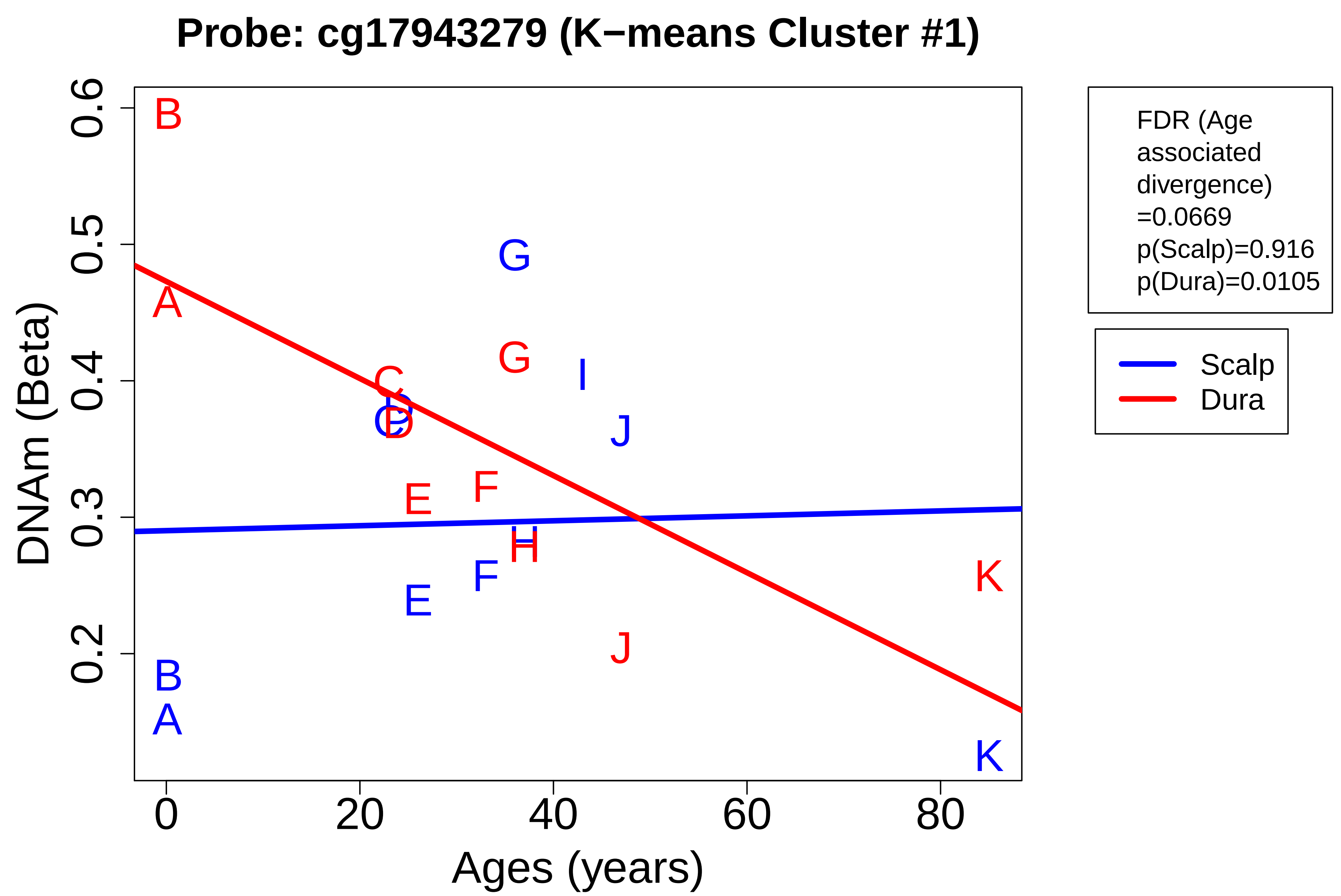
Probe: cg17943279 (K−means Cluster #1)
DNAm (Beta)
0.2
0.3
0.4
0.5
0.6
Ages (years)
0
20
40
60
80
FDR (Age associated divergence) =0.0669
p(Scalp)=0.916
p(Dura)=0.0105
Scalp
Dura

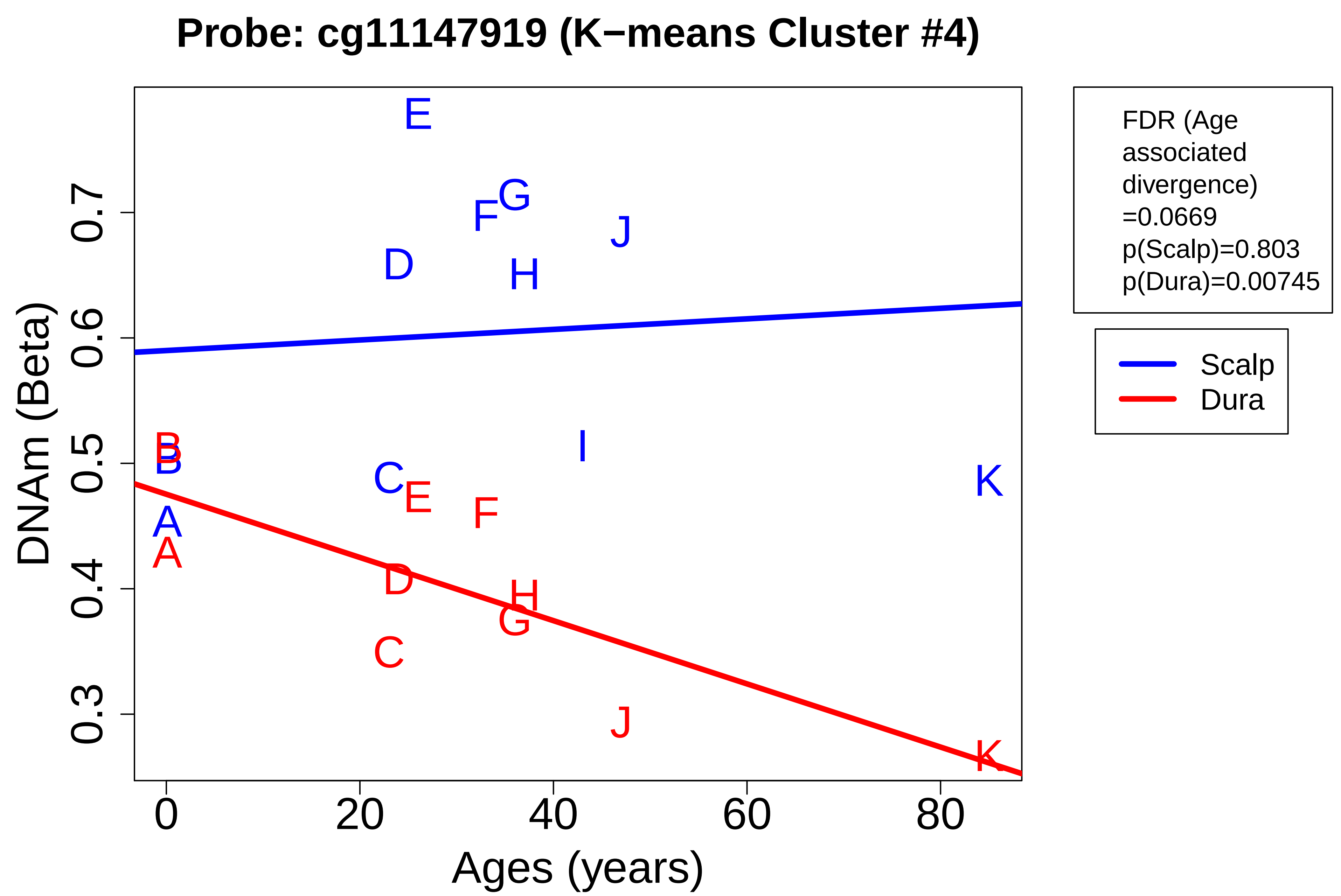

Probe: cg11147919 (K−means Cluster #4)
DNAm (Beta)
0.3
0.4
0.5
0.6
0.7
0
20
40
60
80
Ages (years)
FDR (Age associated divergence) =0.0669
p(Scalp)=0.803
p(Dura)=0.00745
Scalp
Dura

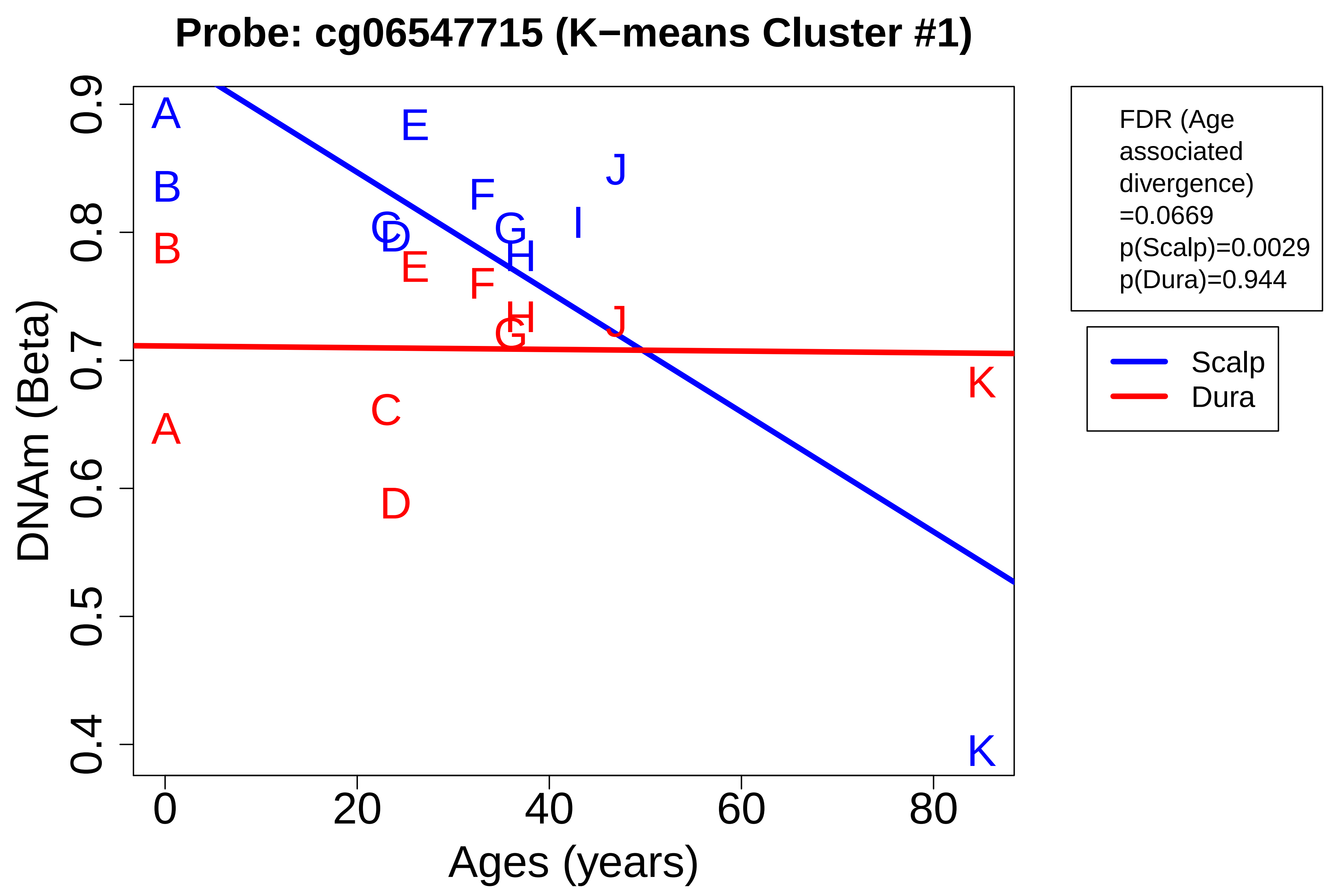

Probe: cg06547715 (K−means Cluster #1)
DNAm (Beta)
0.4
0.5
0.6
0.7
0.8
0.9
0
20
40
60
80
Ages (years)
A
B
C
D
E
F
G
H
I
J
K
FDR (Age associated divergence) =0.0669
p(Scalp)=0.0029
p(Dura)=0.944
Scalp
Dura

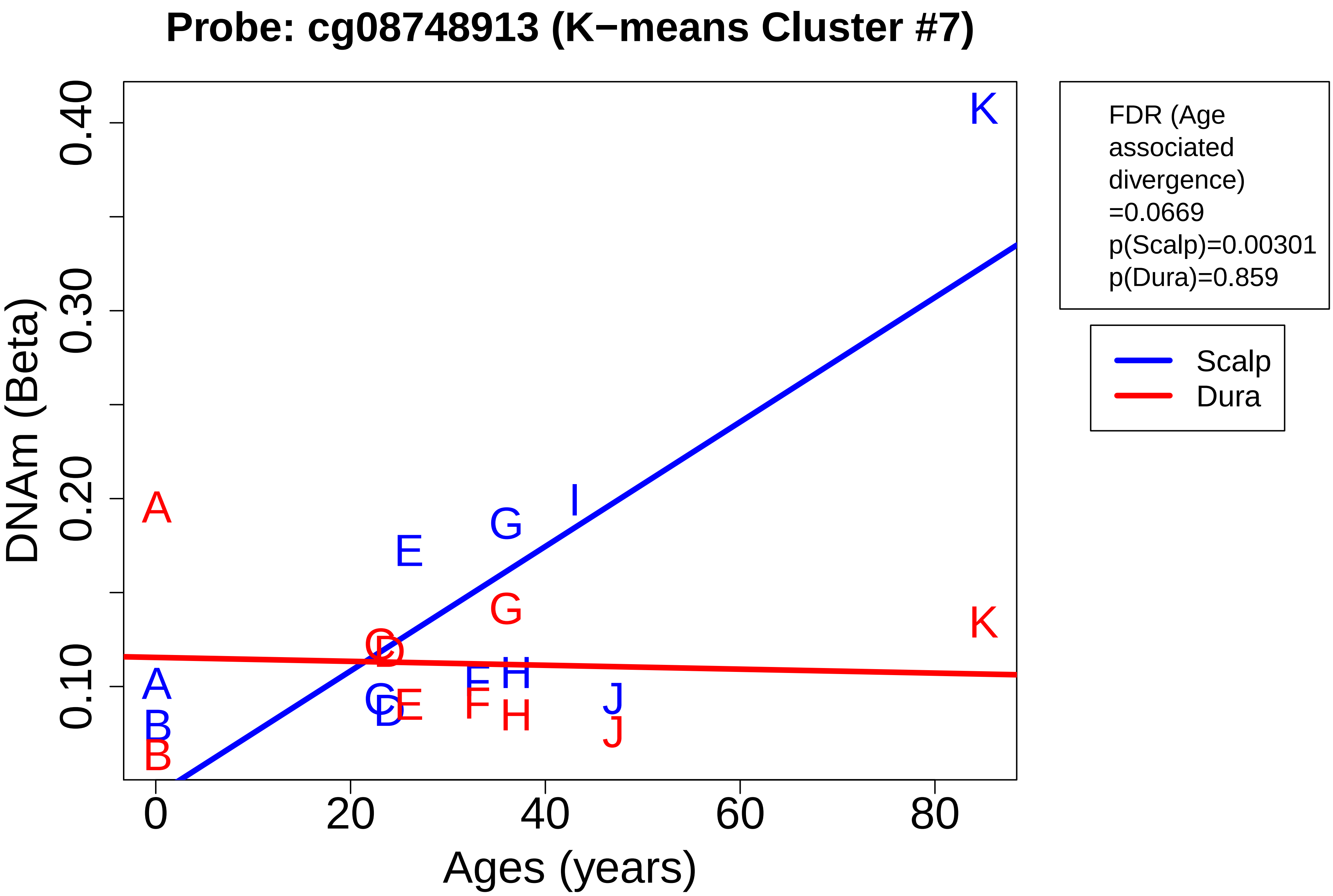

Probe: cg08748913 (K−means Cluster #7)
FDR (Age associated divergence) =0.0669
p(Scalp)=0.00301
p(Dura)=0.859
Scalp
Dura
DNAm (Beta)
0.10
0.20
0.30
0.40
0
20
40
60
80
Ages (years)
A
B
C
D
E
F
G
H
I
J
K

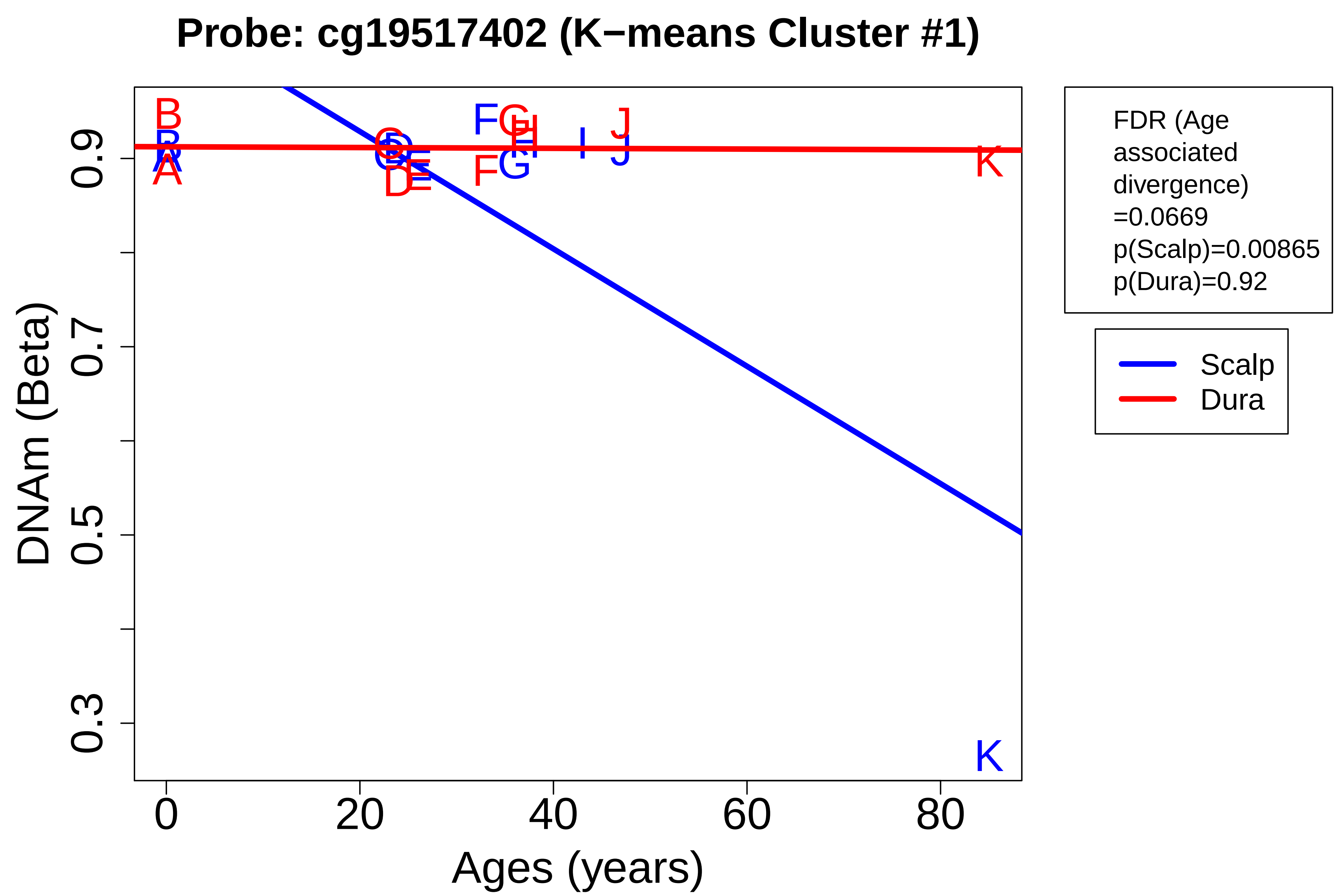

Probe: cg19517402 (K−means Cluster #1)
DNAm (Beta)
0.3
0.5
0.7
0.9
0
20
40
60
80
Ages (years)
FDR (Age associated divergence) =0.0669
p(Scalp)=0.00865
p(Dura)=0.92
Scalp
Dura

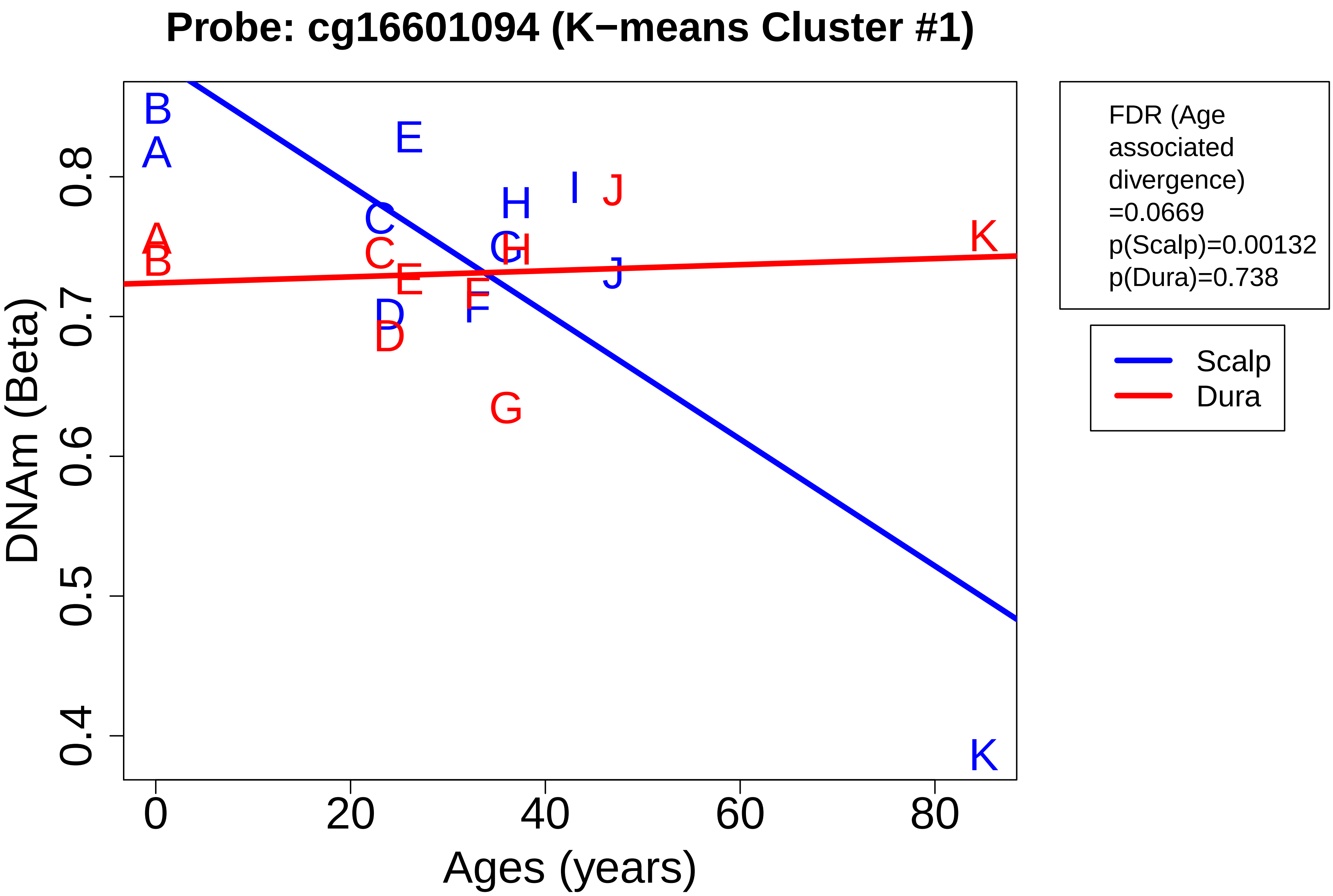
Probe: cg16601094 (K−means Cluster #1)
DNAm (Beta)
0.4
0.5
0.6
0.7
0.8
0
20
40
60
80
Ages (years)
FDR (Age associated divergence) =0.0669
p(Scalp)=0.00132
p(Dura)=0.738
Scalp
Dura
A
B
C
D
E
F
G
H
I
J
K

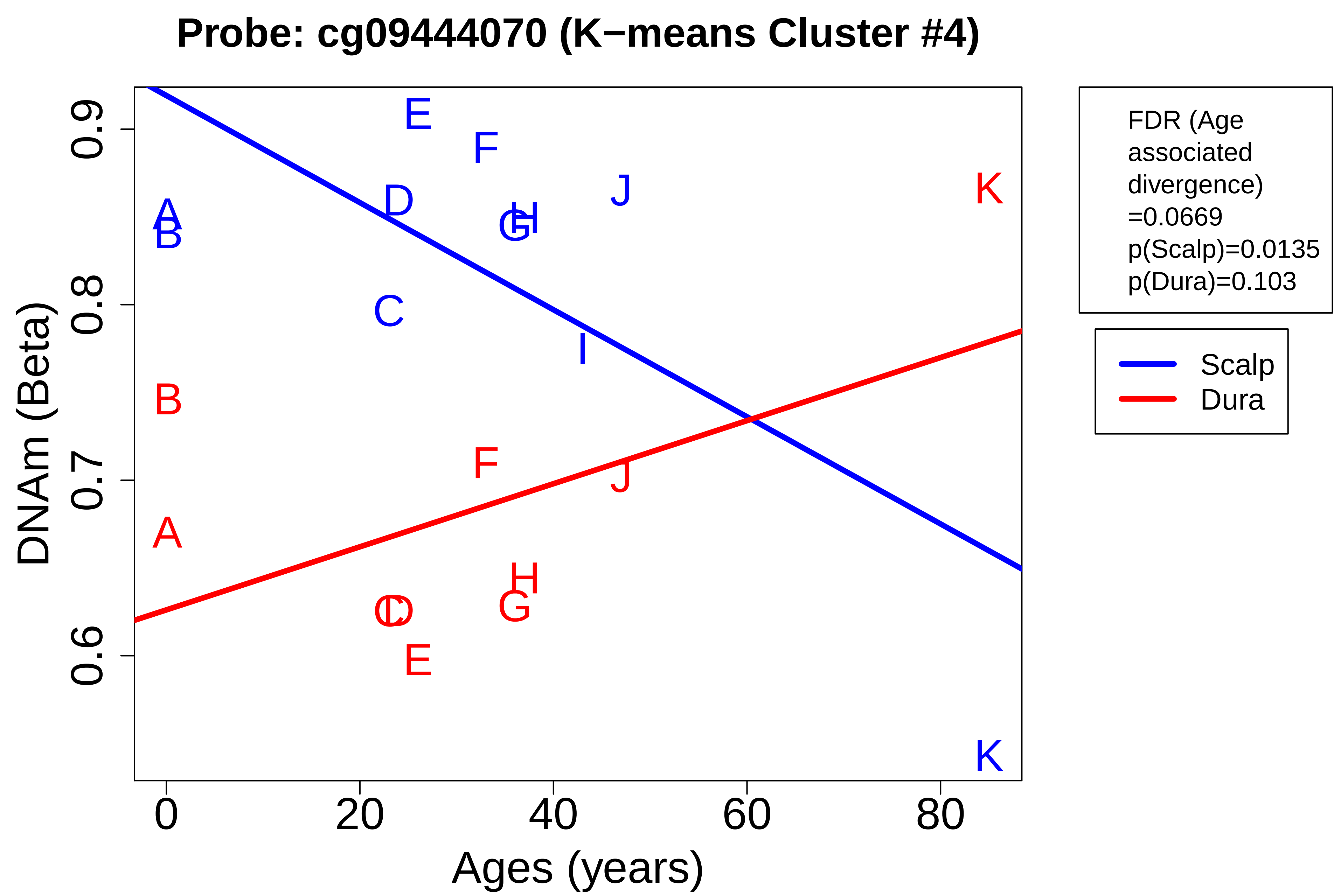

Probe: cg09444070 (K−means Cluster #4)
DNAm (Beta)
0.6
0.7
0.8
0.9
0
20
40
60
80
Ages (years)
E
F
J
K
A
B
D
G
H
C
I
B
F
J
A
H
C
D
G
E
K
FDR (Age associated divergence) =0.0669
p(Scalp)=0.0135
p(Dura)=0.103
Scalp
Dura

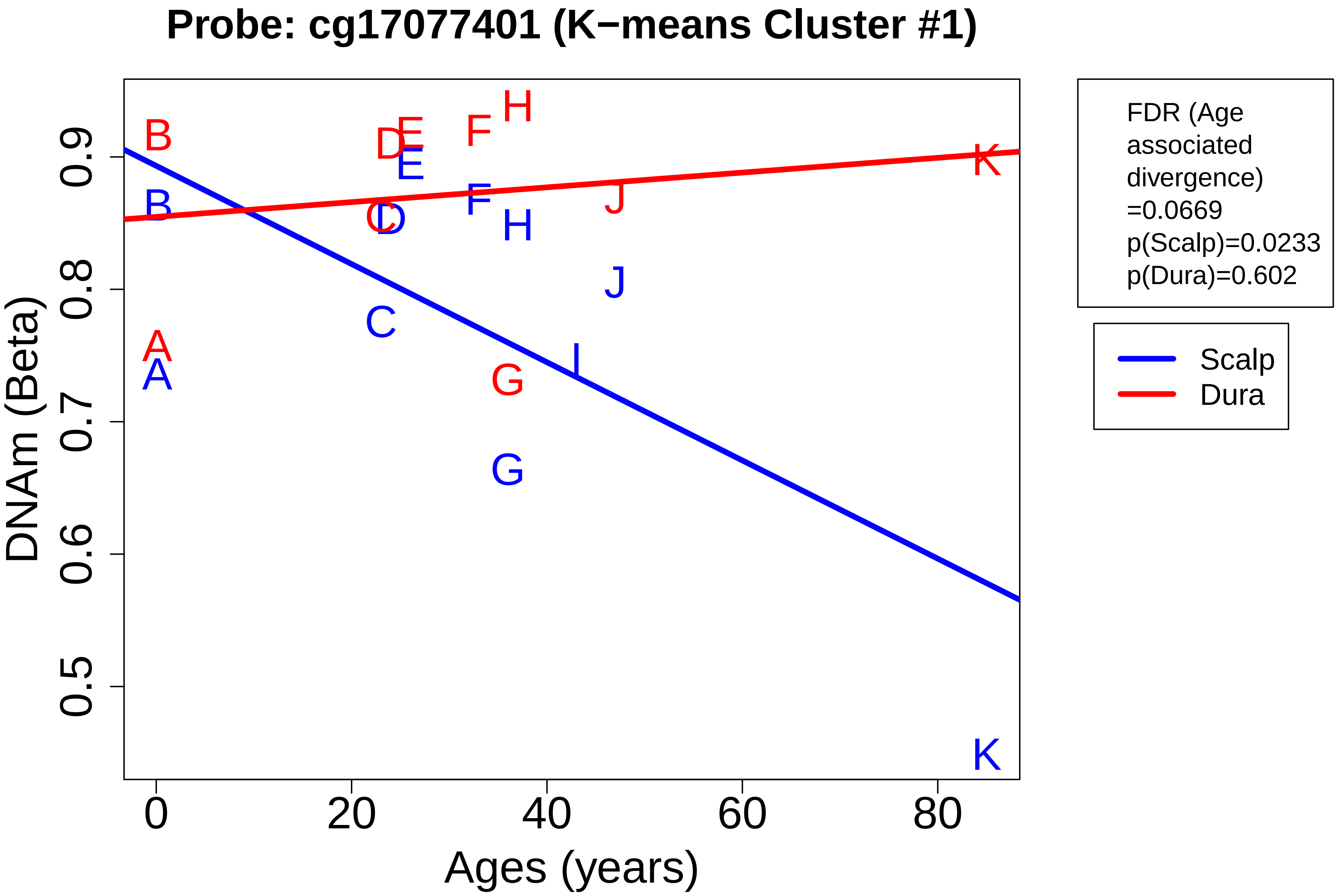

Probe: cg17077401 (K−means Cluster #1)
DNAm (Beta)
0.5
0.6
0.7
0.8
0.9
0
20
40
60
80
Ages (years)
A
B
C
D
E
F
G
H
I
J
K
FDR (Age associated divergence) =0.0669
p(Scalp)=0.0233
p(Dura)=0.602
Scalp
Dura

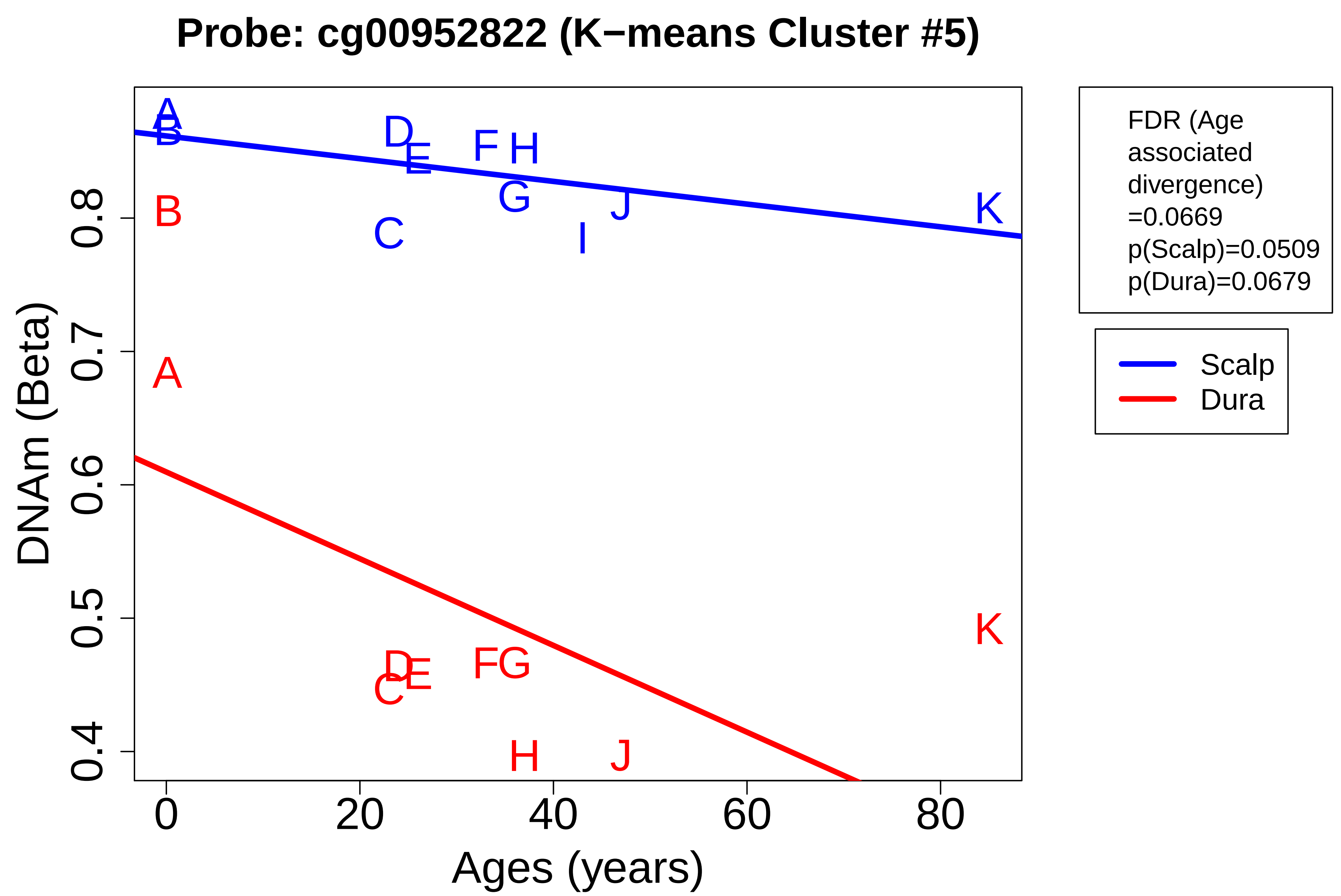
Probe: cg00952822 (K−means Cluster #5)
FDR (Age associated divergence) =0.0669
p(Scalp)=0.0509
p(Dura)=0.0679
Scalp
Dura
DNAm (Beta)
0.4
0.5
0.6
0.7
0.8
Ages (years)
0
20
40
60
80

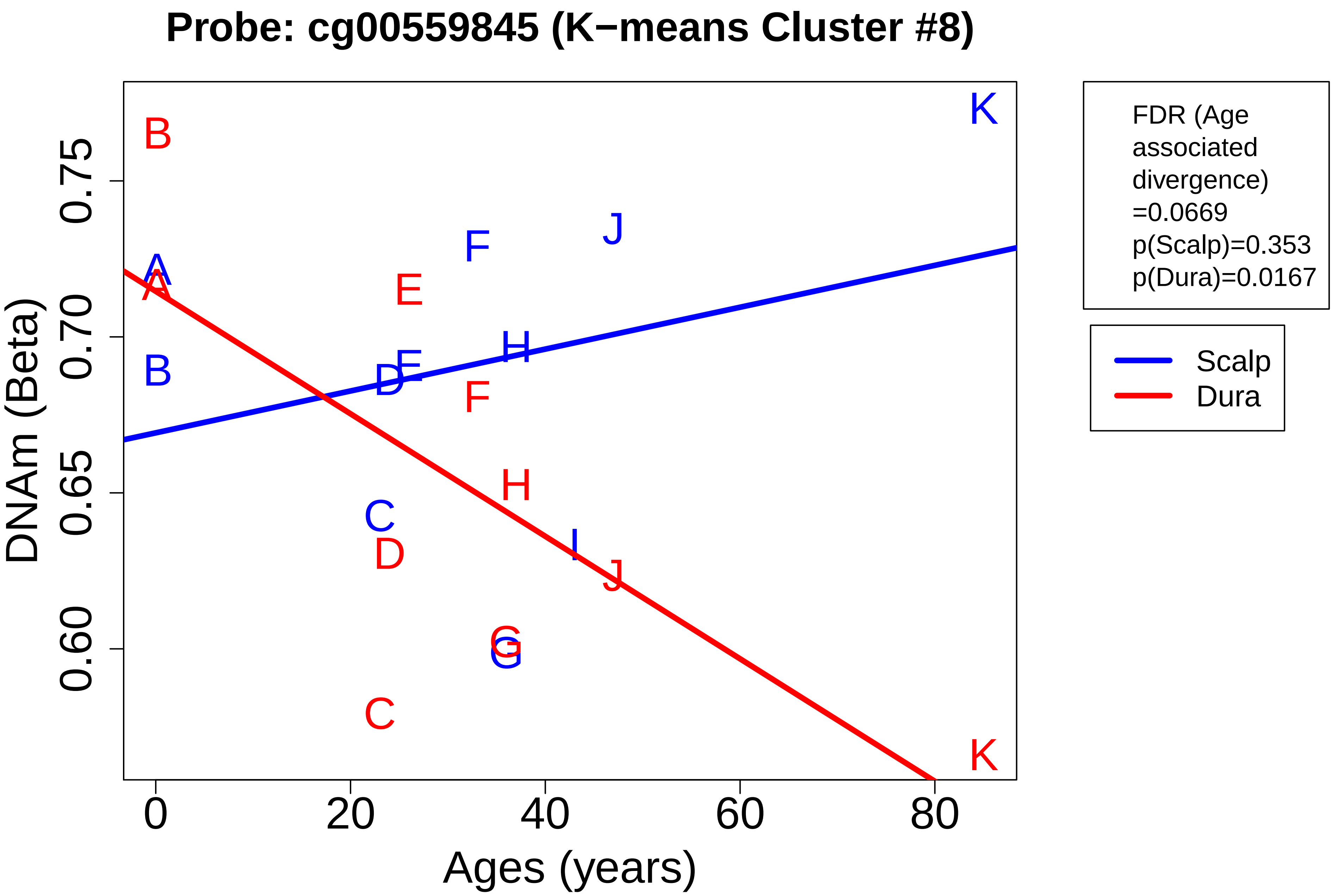

Probe: cg00559845 (K−means Cluster #8)
DNAm (Beta)
0.60
0.65
0.70
0.75
Ages (years)
0
20
40
60
80
FDR (Age associated divergence) =0.0669
p(Scalp)=0.353
p(Dura)=0.0167
Scalp
Dura

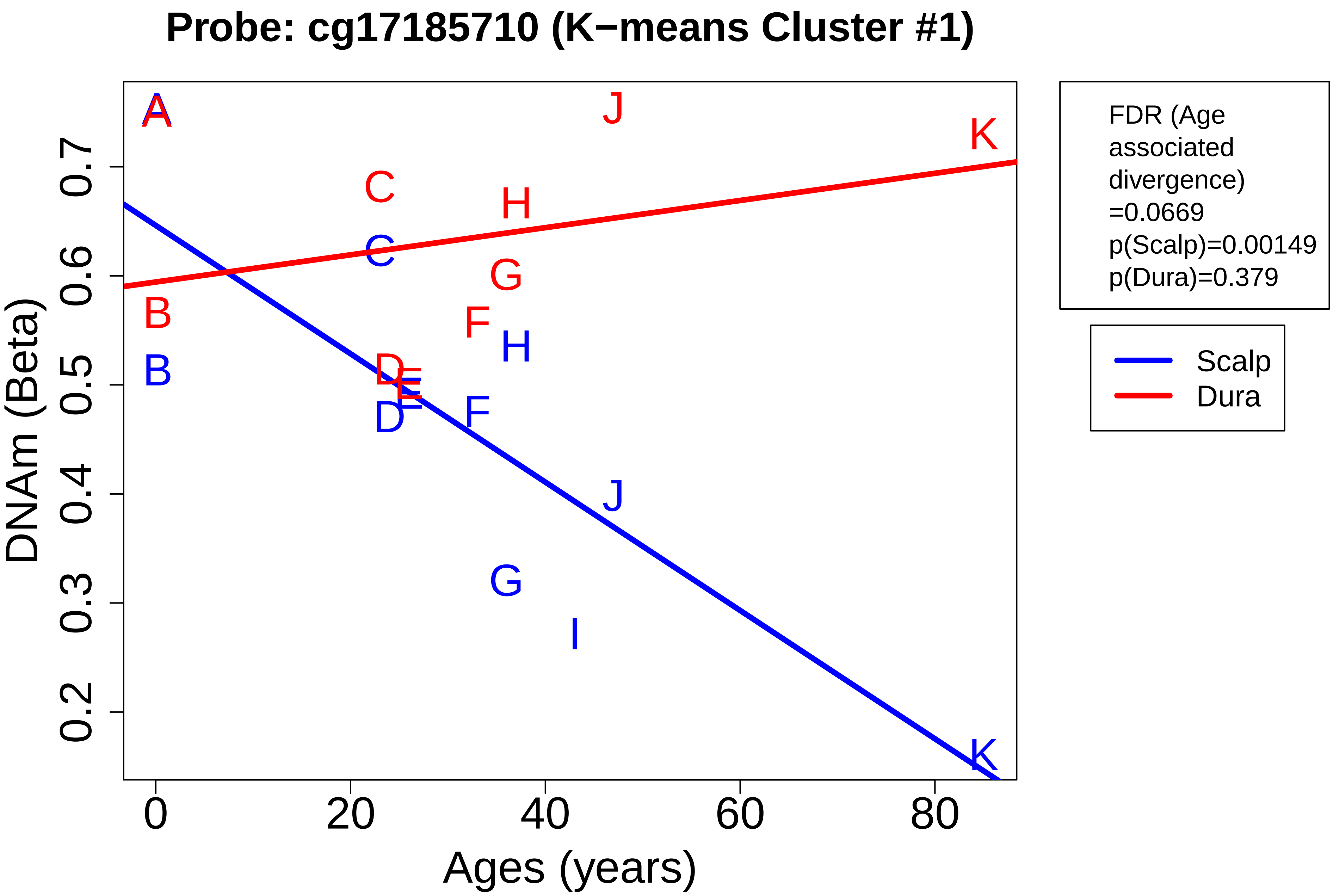

Probe: cg17185710 (K−means Cluster #1)
DNAm (Beta)
0.2
0.3
0.4
0.5
0.6
0.7
Ages (years)
0
20
40
60
80
FDR (Age associated divergence) =0.0669
p(Scalp)=0.00149
p(Dura)=0.379
Scalp
Dura

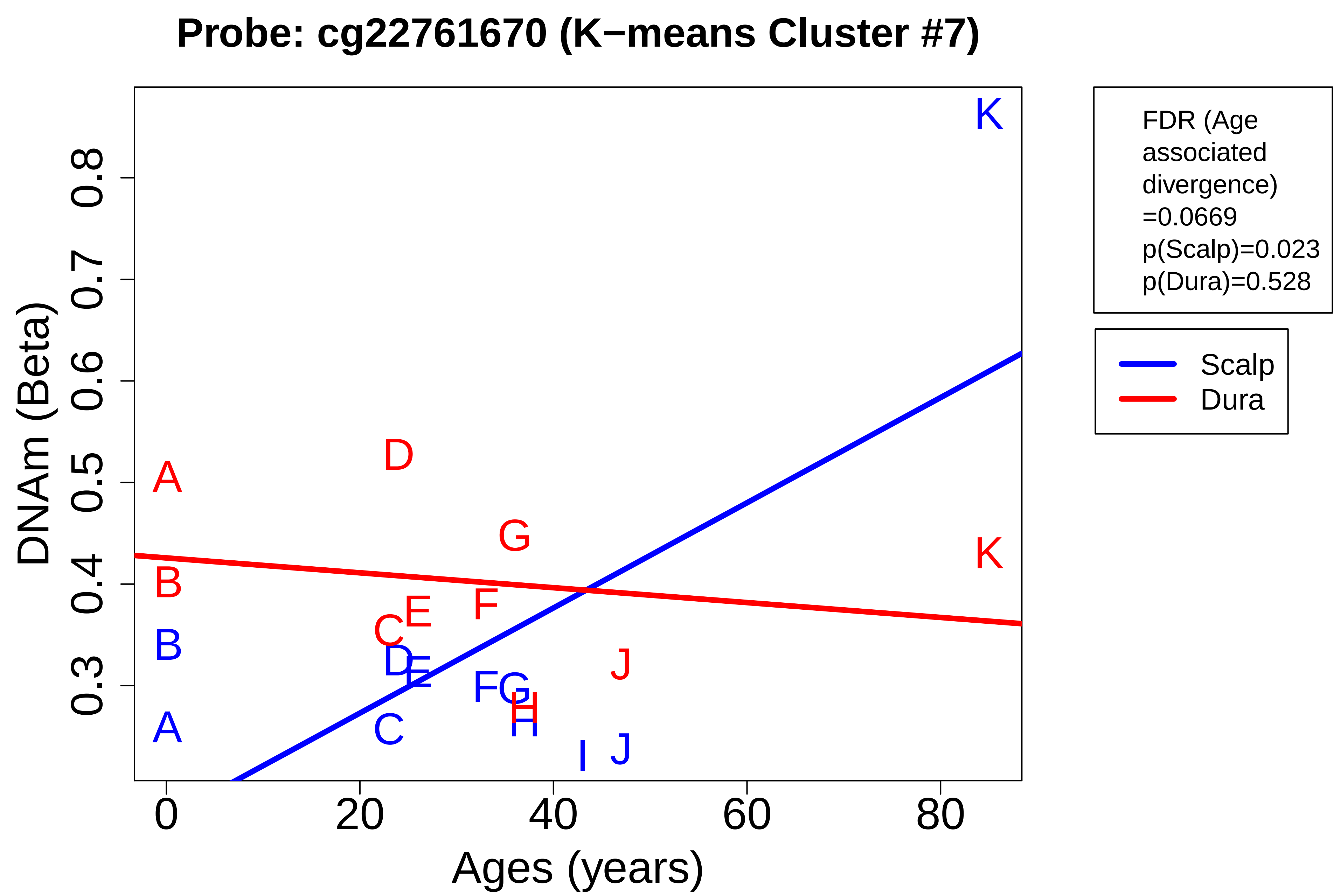

Probe: cg22761670 (K−means Cluster #7)
FDR (Age associated divergence) =0.0669
p(Scalp)=0.023
p(Dura)=0.528
Scalp
Dura
DNAm (Beta)
0.3
0.4
0.5
0.6
0.7
0.8
0
20
40
60
80
Ages (years)
A
B
C
D
E
F
G
H
I
J
K

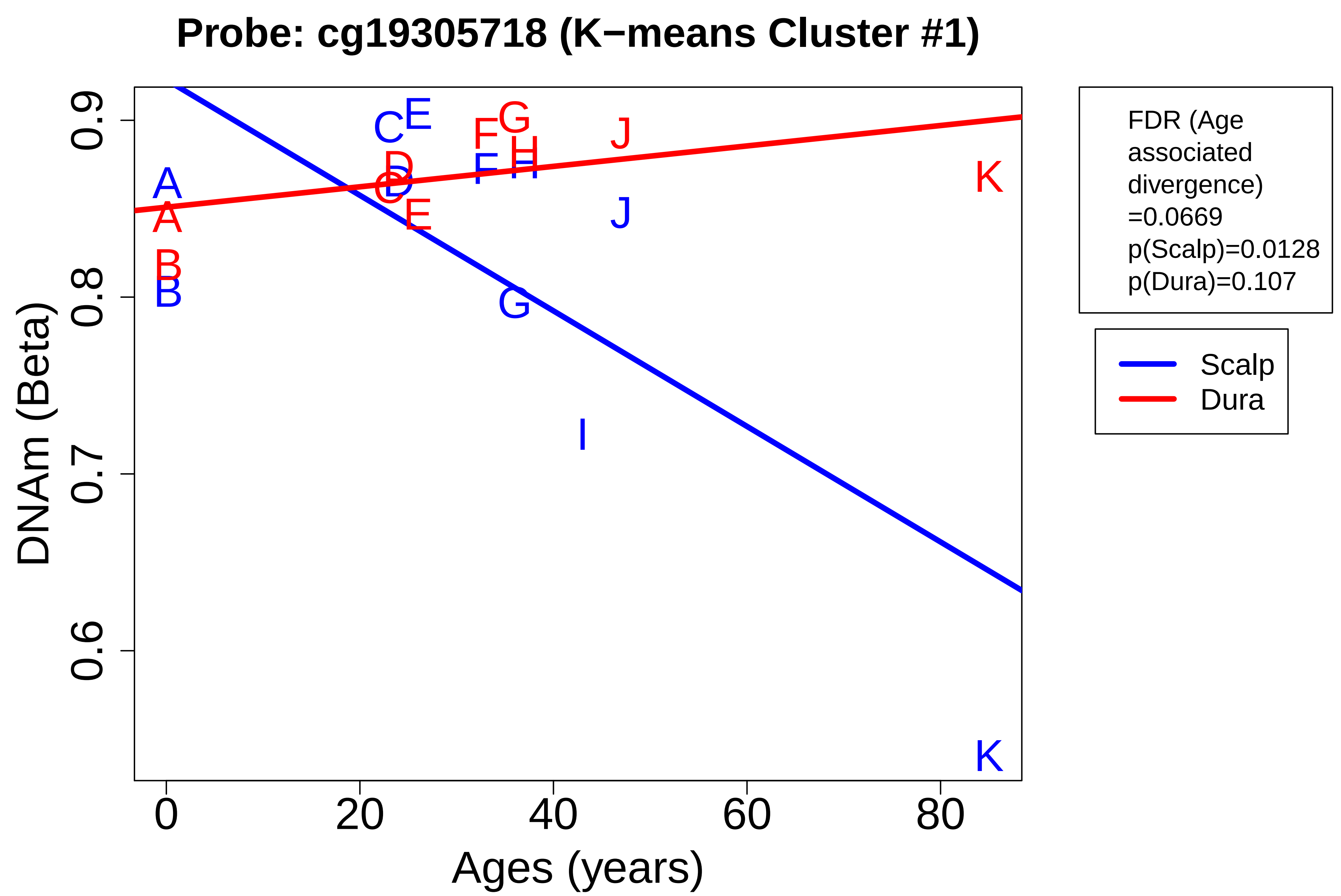
Probe: cg19305718 (K−means Cluster #1)
DNAm (Beta)
0.6
0.7
0.8
0.9
Ages (years)
0
20
40
60
80
A
B
C
D
E
F
G
H
I
J
K
FDR (Age associated divergence) =0.0669
p(Scalp)=0.0128
p(Dura)=0.107
Scalp
Dura

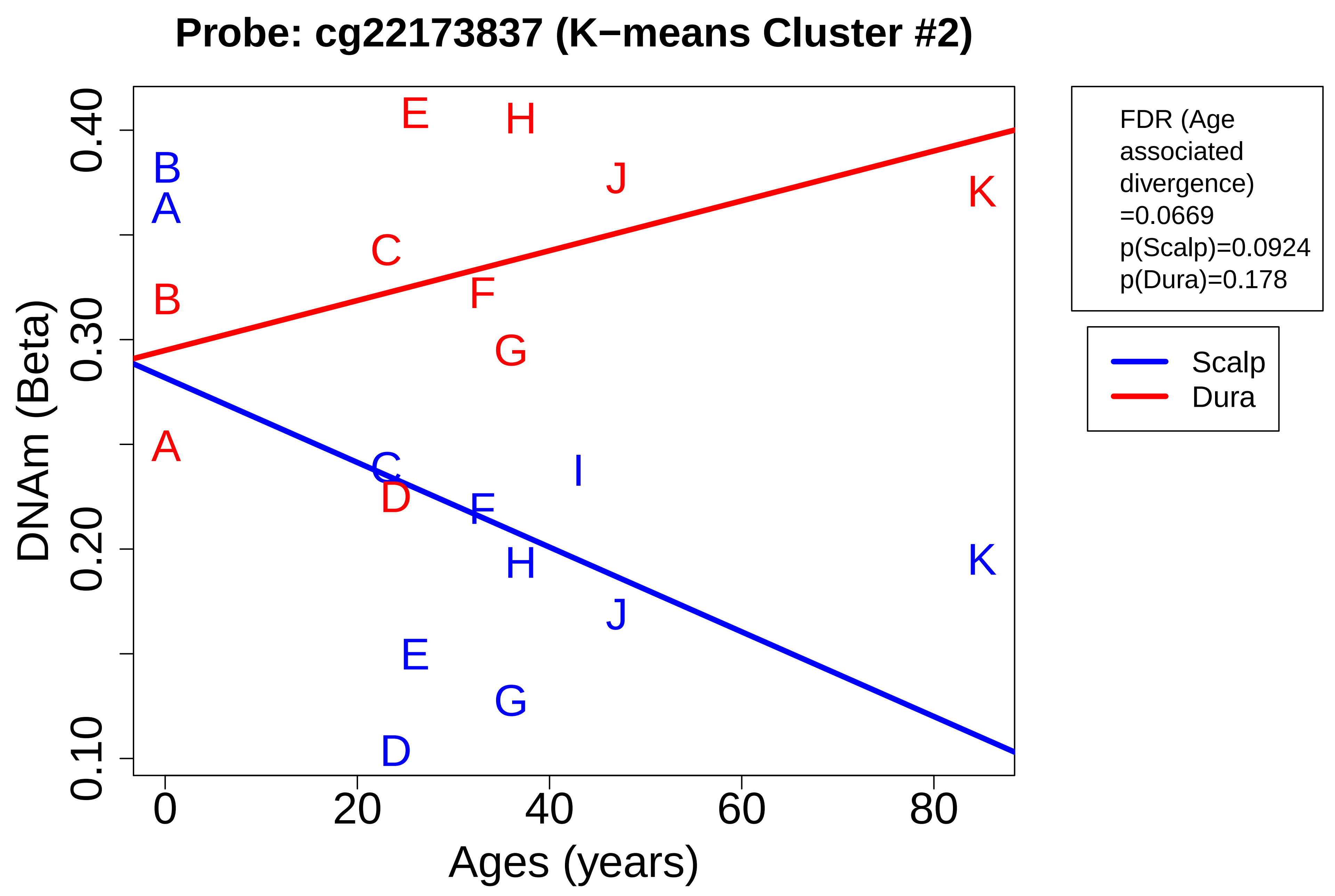

Probe: cg22173837 (K−means Cluster #2)
DNAm (Beta)
0.10
0.20
0.30
0.40
0
20
40
60
80
Ages (years)
FDR (Age associated divergence) =0.0669
p(Scalp)=0.0924
p(Dura)=0.178
Scalp
Dura

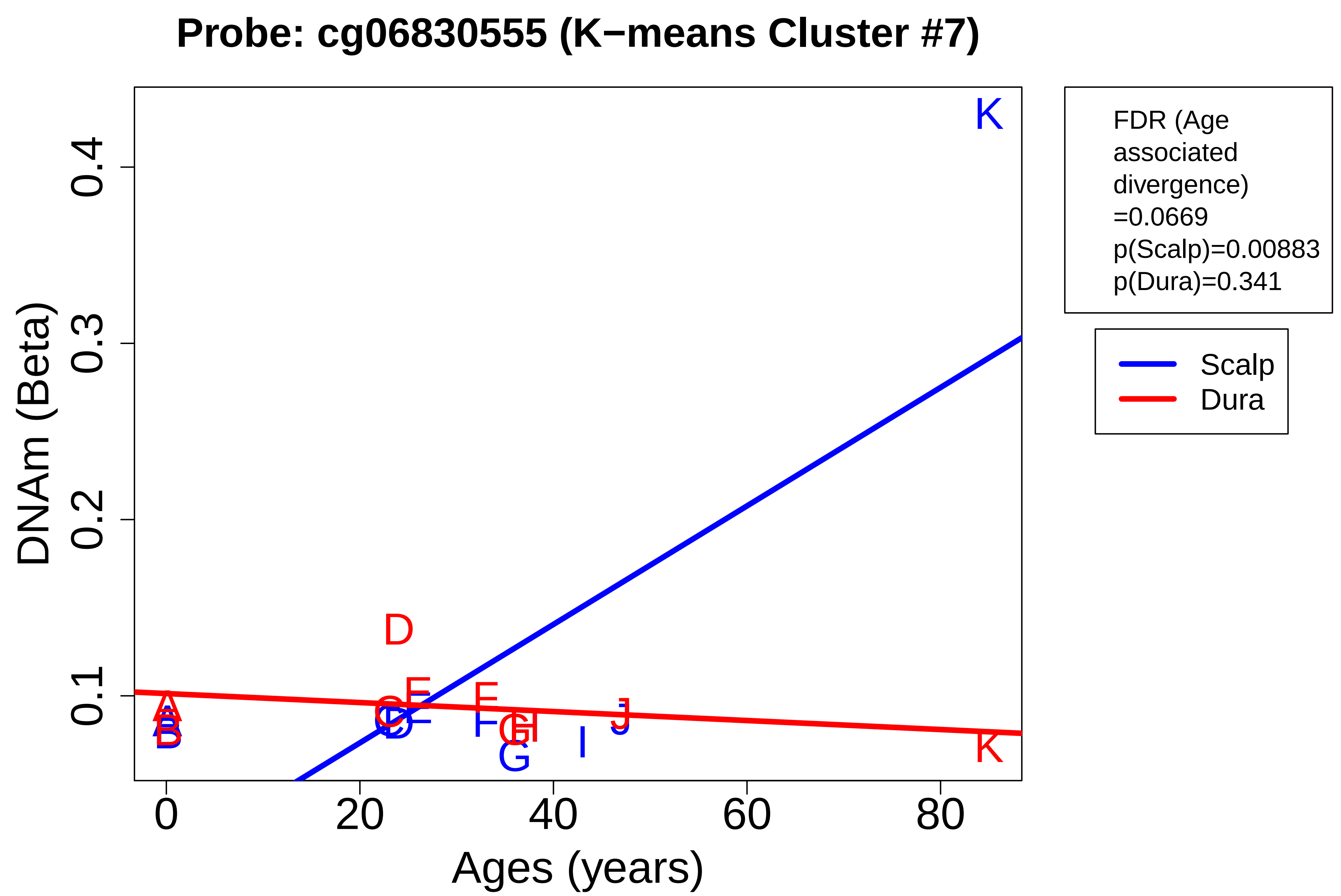

Probe: cg06830555 (K−means Cluster #7)
DNAm (Beta)
0.1
0.2
0.3
0.4
Ages (years)
0
20
40
60
80
FDR (Age associated divergence) =0.0669
p(Scalp)=0.00883
p(Dura)=0.341
Scalp
Dura

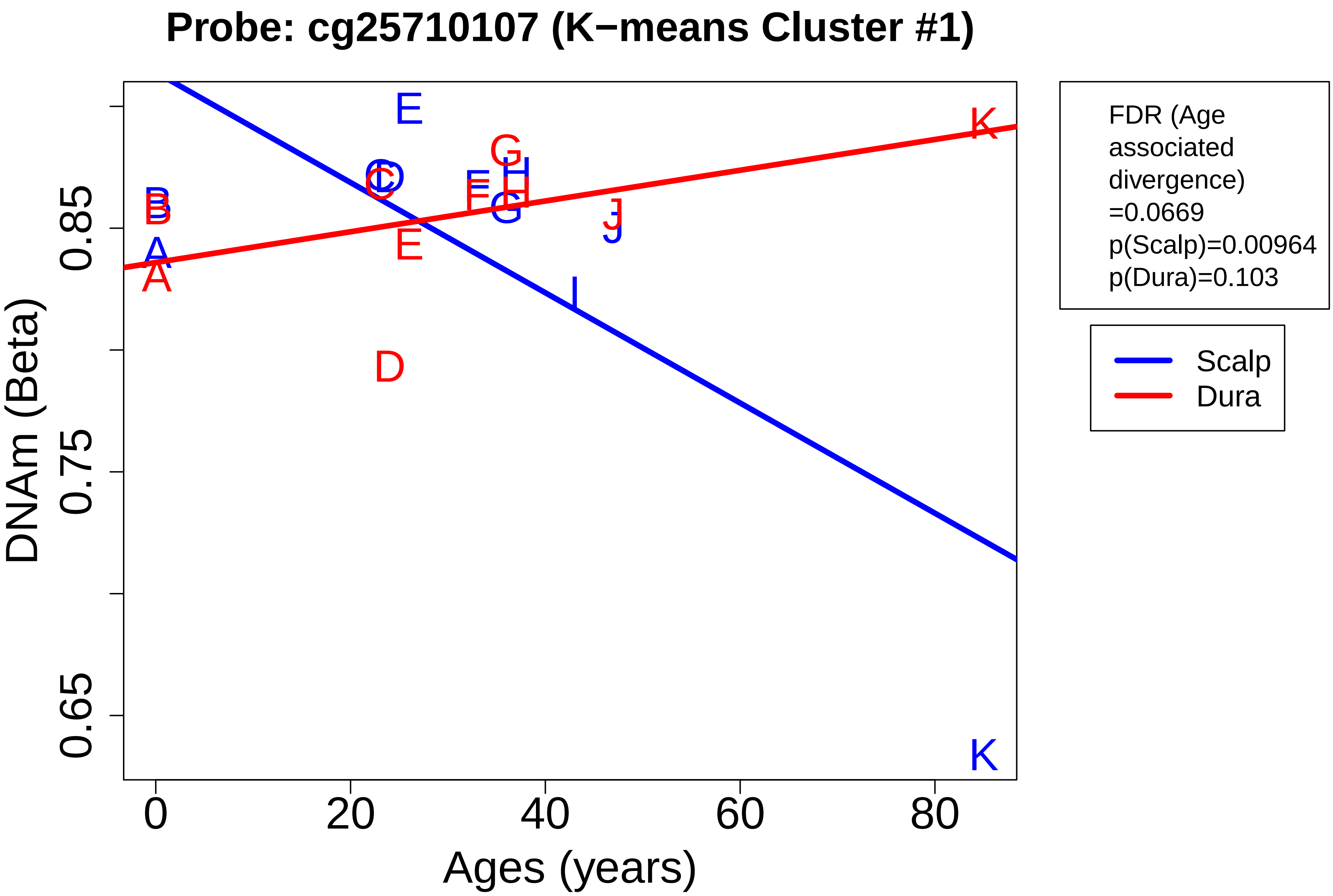

Probe: cg25710107 (K−means Cluster #1)
FDR (Age associated divergence) =0.0669
p(Scalp)=0.00964
p(Dura)=0.103
Scalp
Dura
DNAm (Beta)
0.65
0.75
0.85
Ages (years)
0
20
40
60
80
A
B
C
D
E
F
G
H
I
J
K

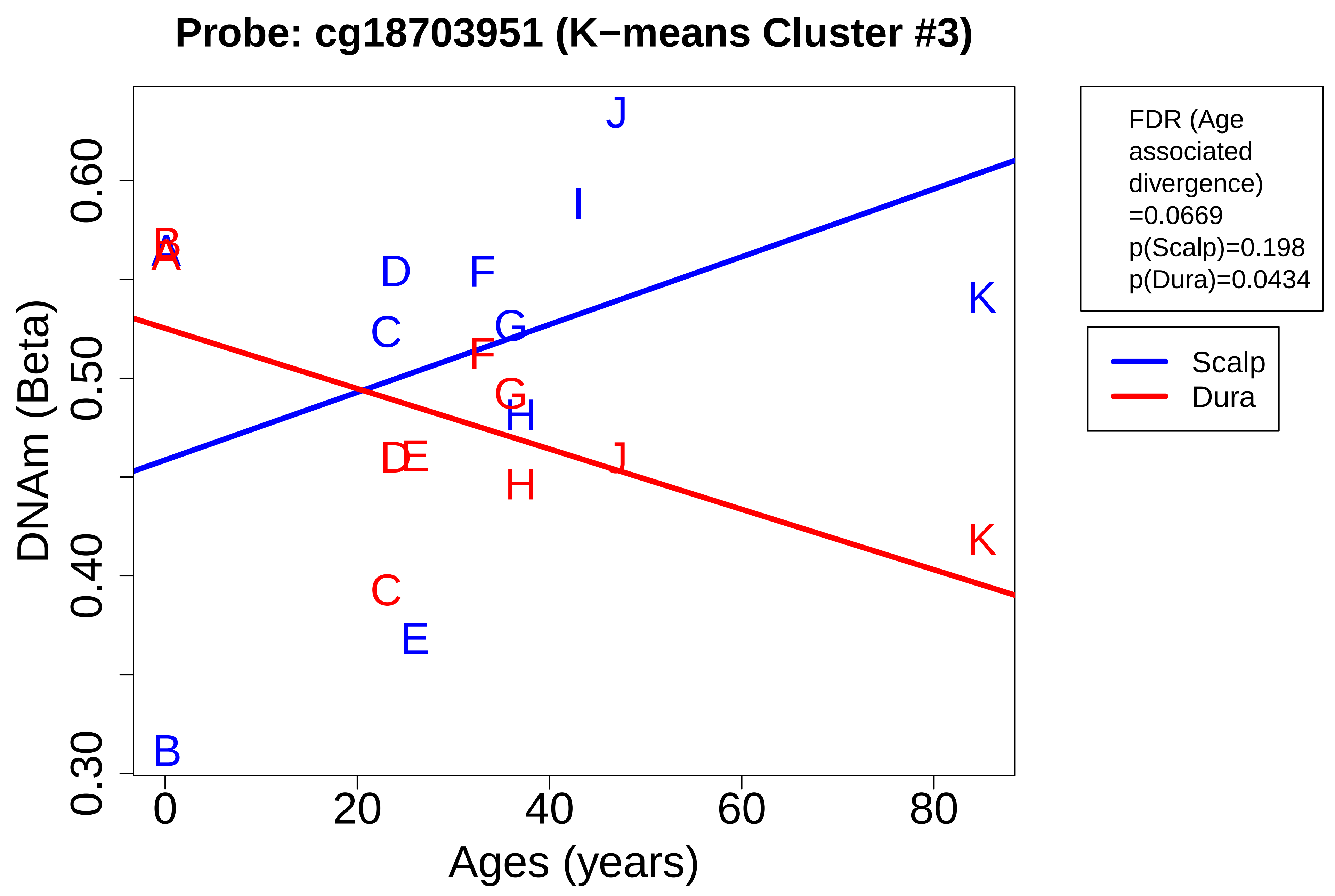

Probe: cg18703951 (K−means Cluster #3)
DNAm (Beta)
0.30
0.40
0.50
0.60
0
20
40
60
80
Ages (years)
FDR (Age associated divergence) =0.0669
p(Scalp)=0.198
p(Dura)=0.0434
Scalp
Dura

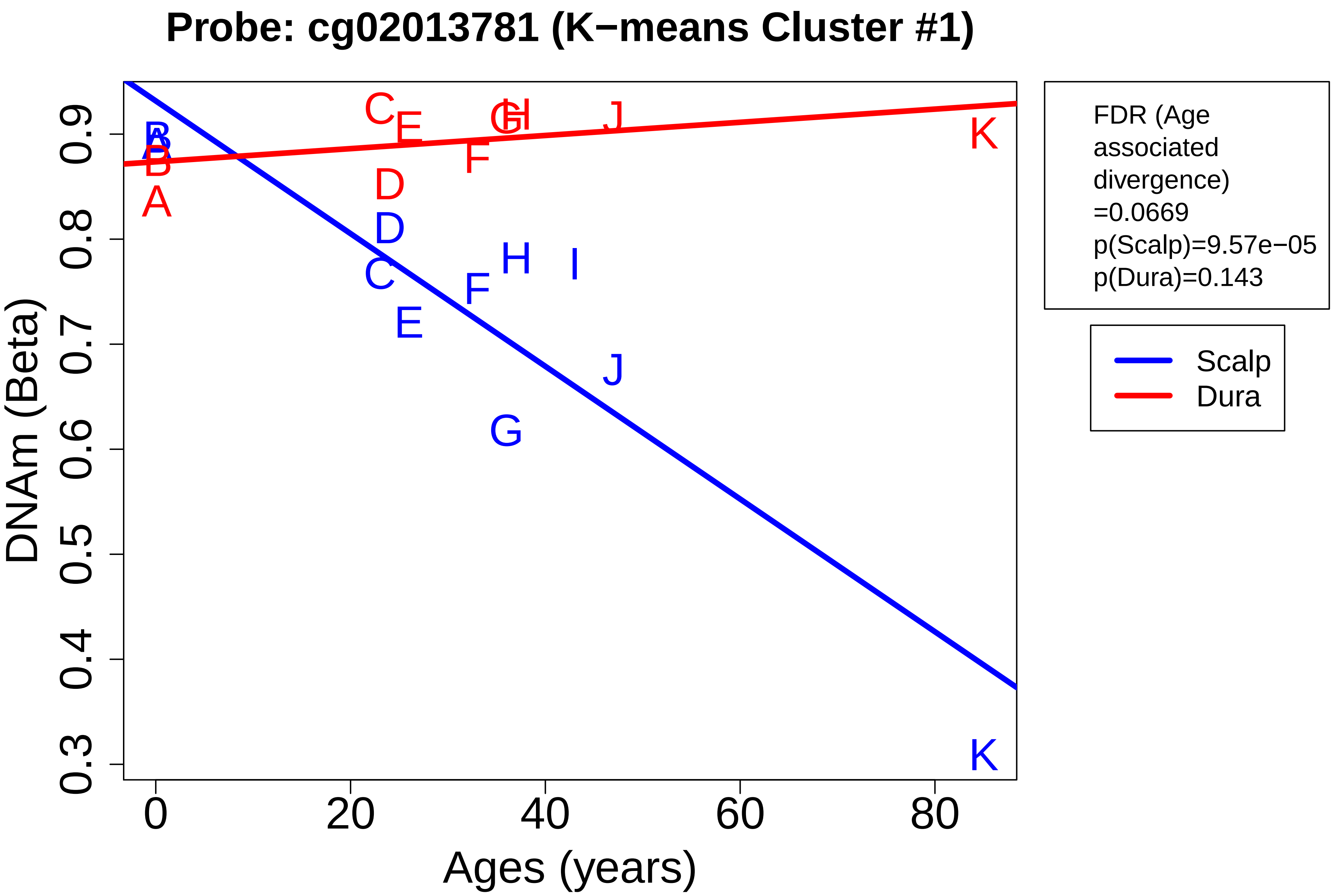

Probe: cg02013781 (K−means Cluster #1)
DNAm (Beta)
0.3
0.4
0.5
0.6
0.7
0.8
0.9
0
20
40
60
80
Ages (years)
FDR (Age associated divergence) =0.0669
p(Scalp)=9.57e−05
p(Dura)=0.143
Scalp
Dura

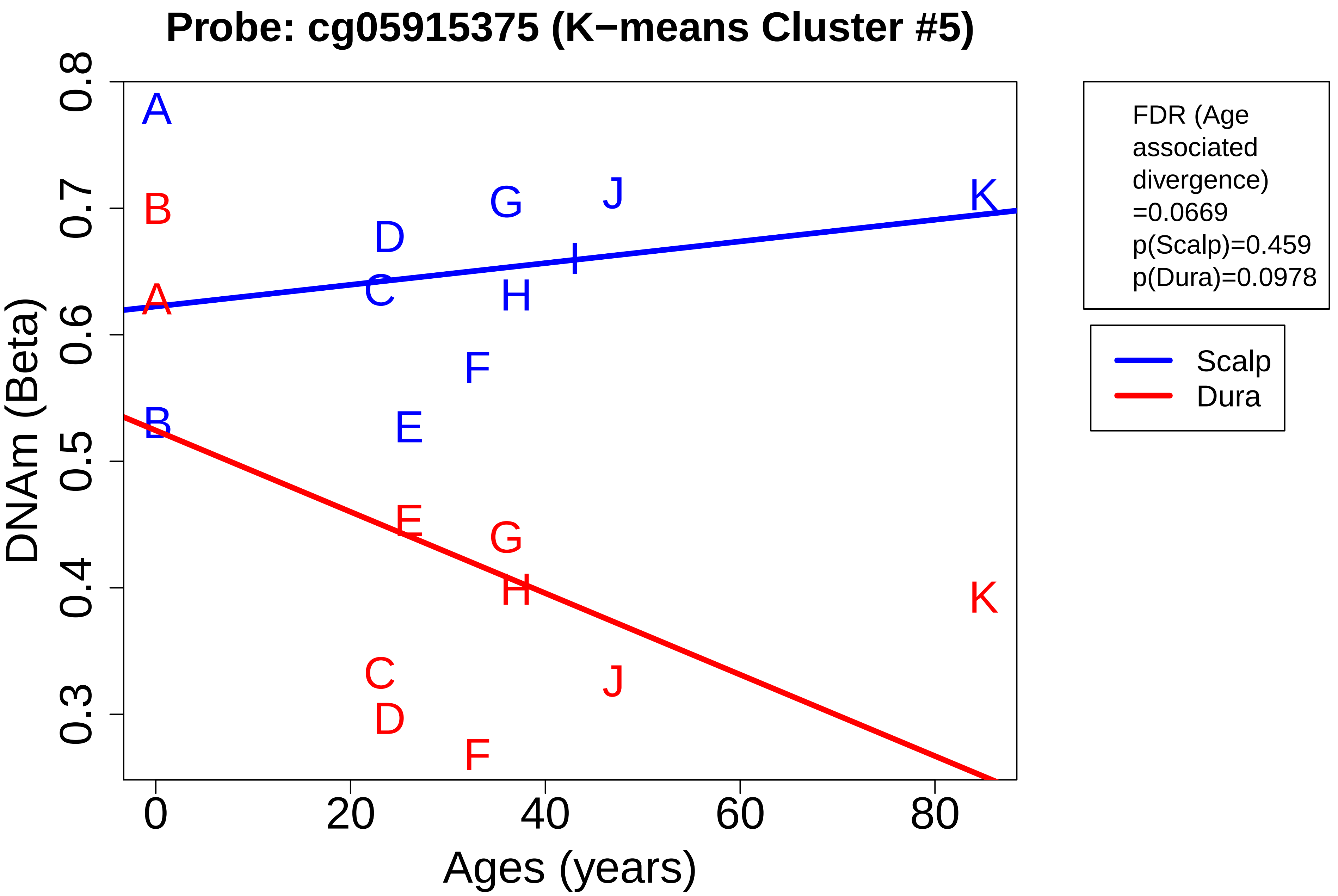

Probe: cg05915375 (K−means Cluster #5)
DNAm (Beta)
0.3
0.4
0.5
0.6
0.7
0.8
0
20
40
60
80
Ages (years)
A
B
C
D
E
F
G
H
I
J
K
FDR (Age associated divergence) =0.0669
p(Scalp)=0.459
p(Dura)=0.0978
Scalp
Dura

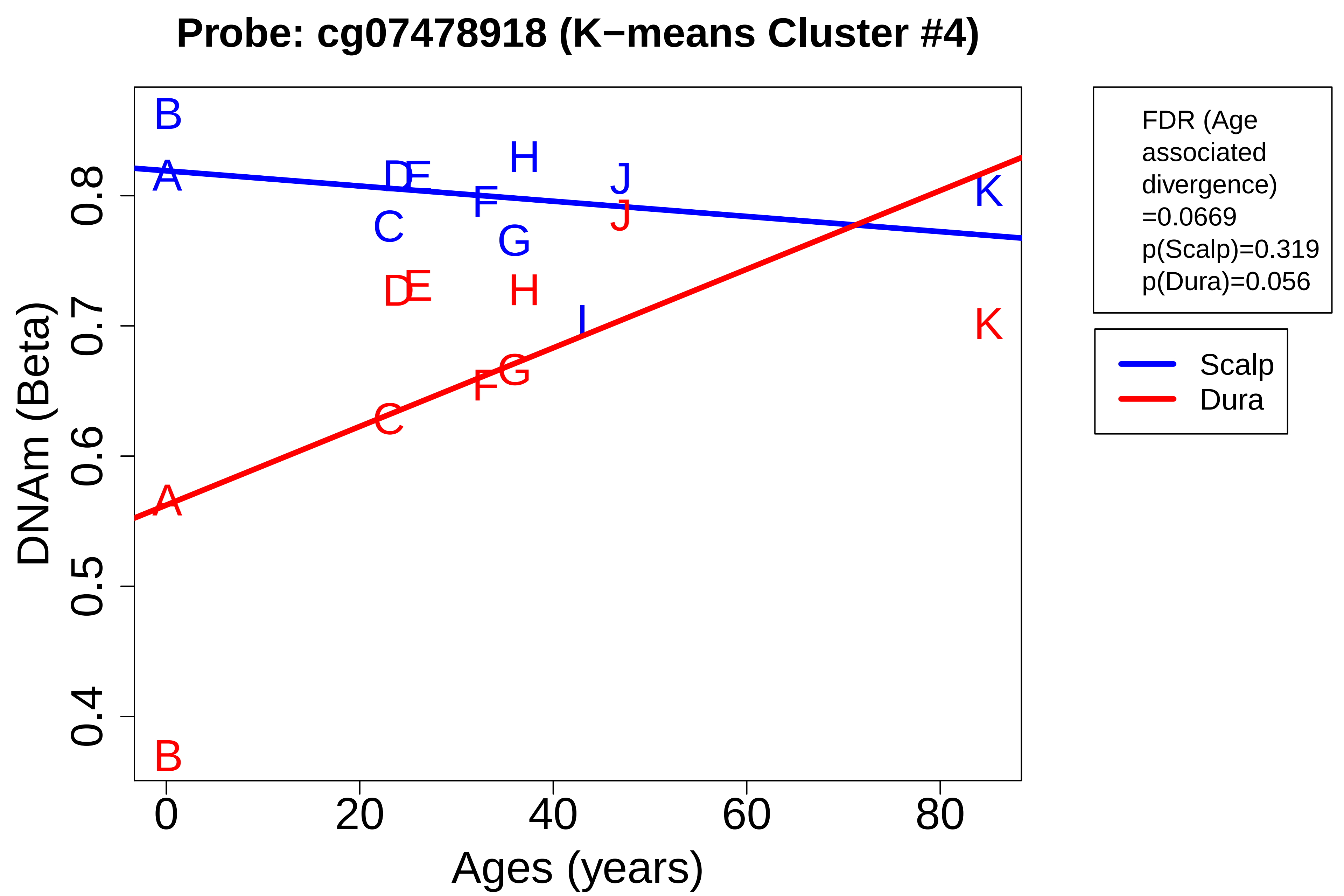

Probe: cg07478918 (K−means Cluster #4)
DNAm (Beta)
0.4
0.5
0.6
0.7
0.8
Ages (years)
0
20
40
60
80
A
B
C
D
E
F
G
H
I
J
K
FDR (Age associated divergence) =0.0669
p(Scalp)=0.319
p(Dura)=0.056
Scalp
Dura

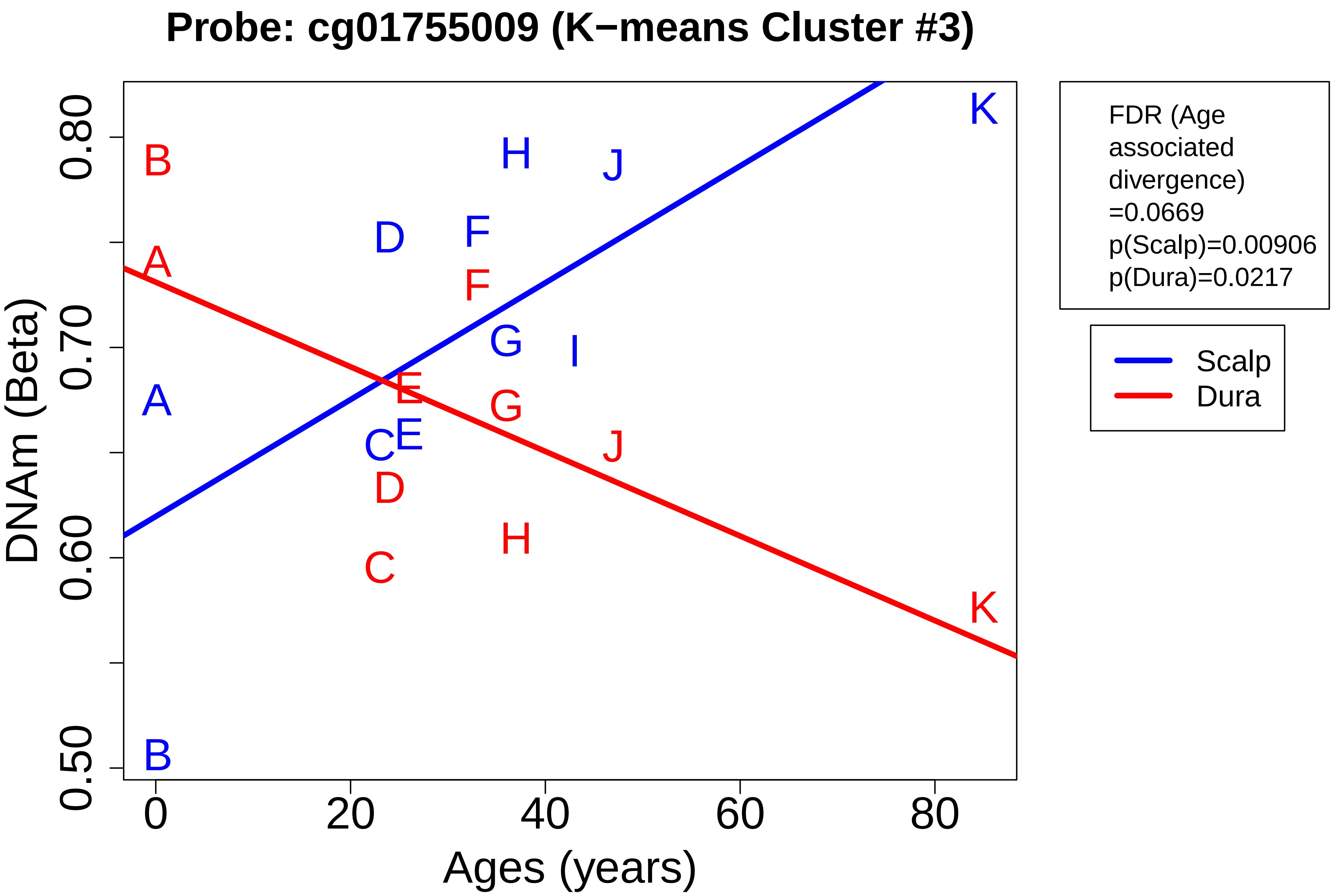

Probe: cg01755009 (K−means Cluster #3)
DNAm (Beta)
0.50
0.60
0.70
0.80
0
20
40
60
80
Ages (years)
A
B
C
D
E
F
G
H
I
J
K
FDR (Age associated divergence) =0.0669
p(Scalp)=0.00906
p(Dura)=0.0217
Scalp
Dura

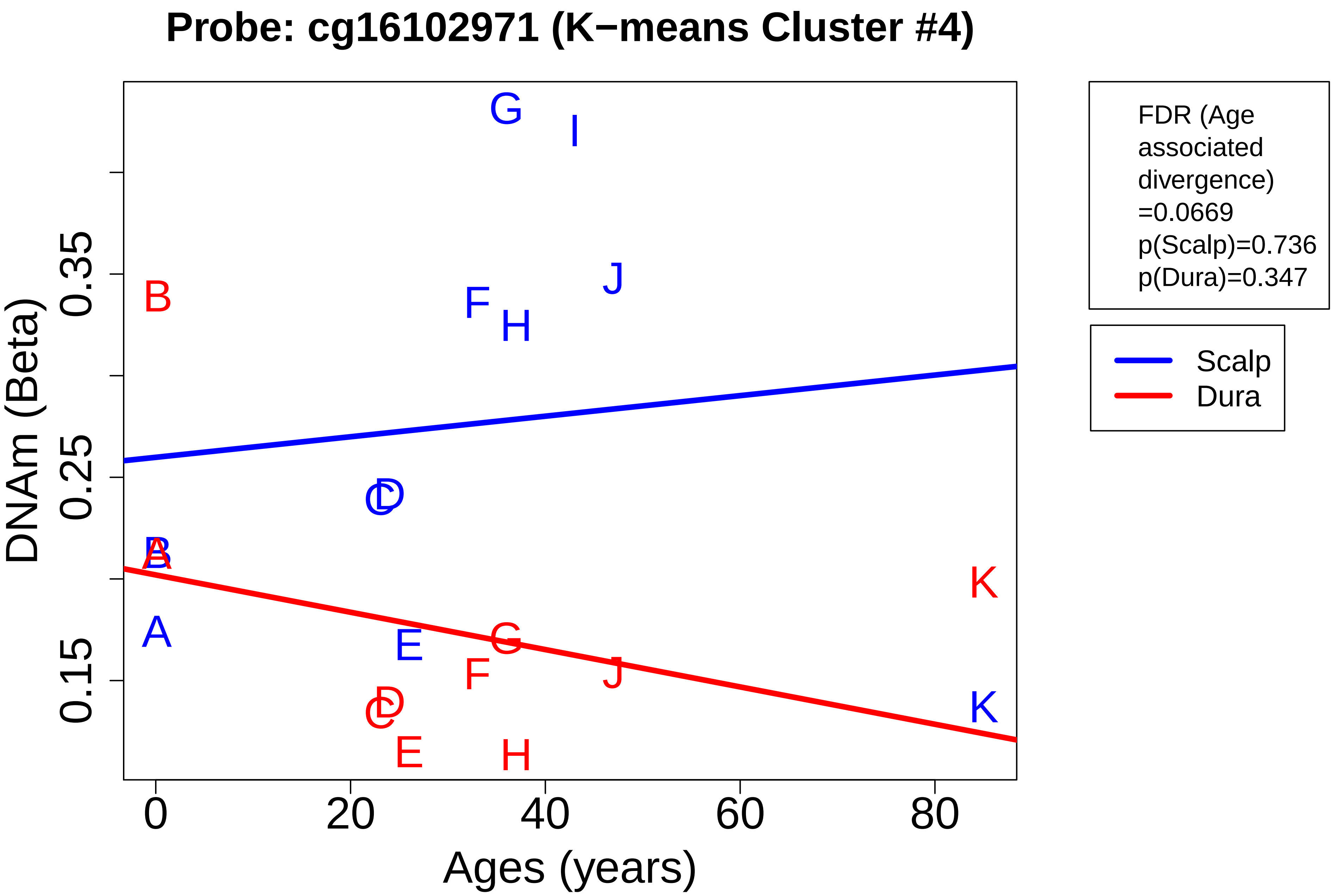

Probe: cg16102971 (K−means Cluster #4)
DNAm (Beta)
0.15
0.25
0.35
Ages (years)
0
20
40
60
80
FDR (Age associated divergence) =0.0669
p(Scalp)=0.736
p(Dura)=0.347
Scalp
Dura

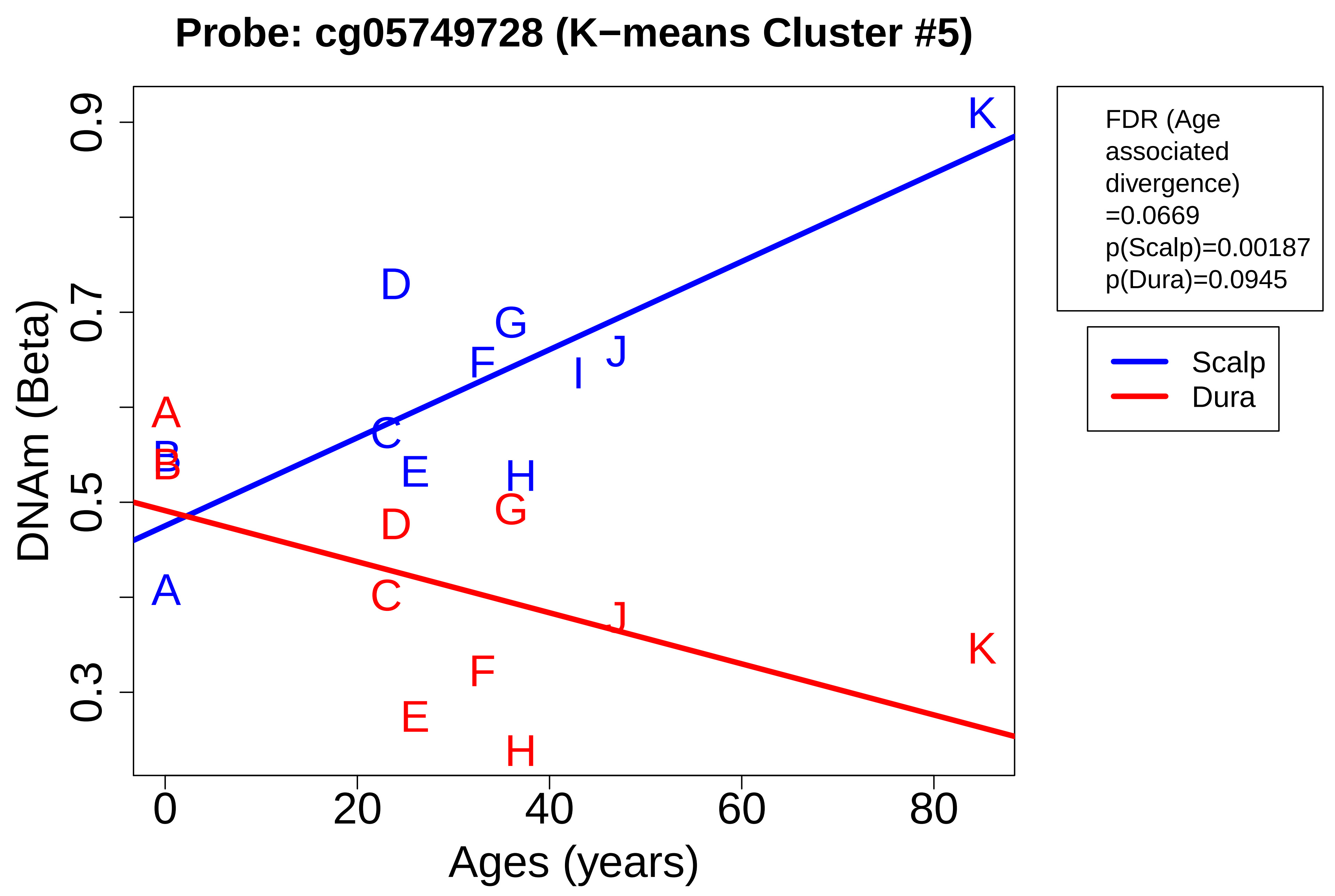

Probe: cg05749728 (K−means Cluster #5)
DNAm (Beta)
0.3
0.5
0.7
0.9
0
20
40
60
80
Ages (years)
A
B
C
D
E
F
G
H
I
J
K
FDR (Age associated divergence) =0.0669
p(Scalp)=0.00187
p(Dura)=0.0945
Scalp
Dura

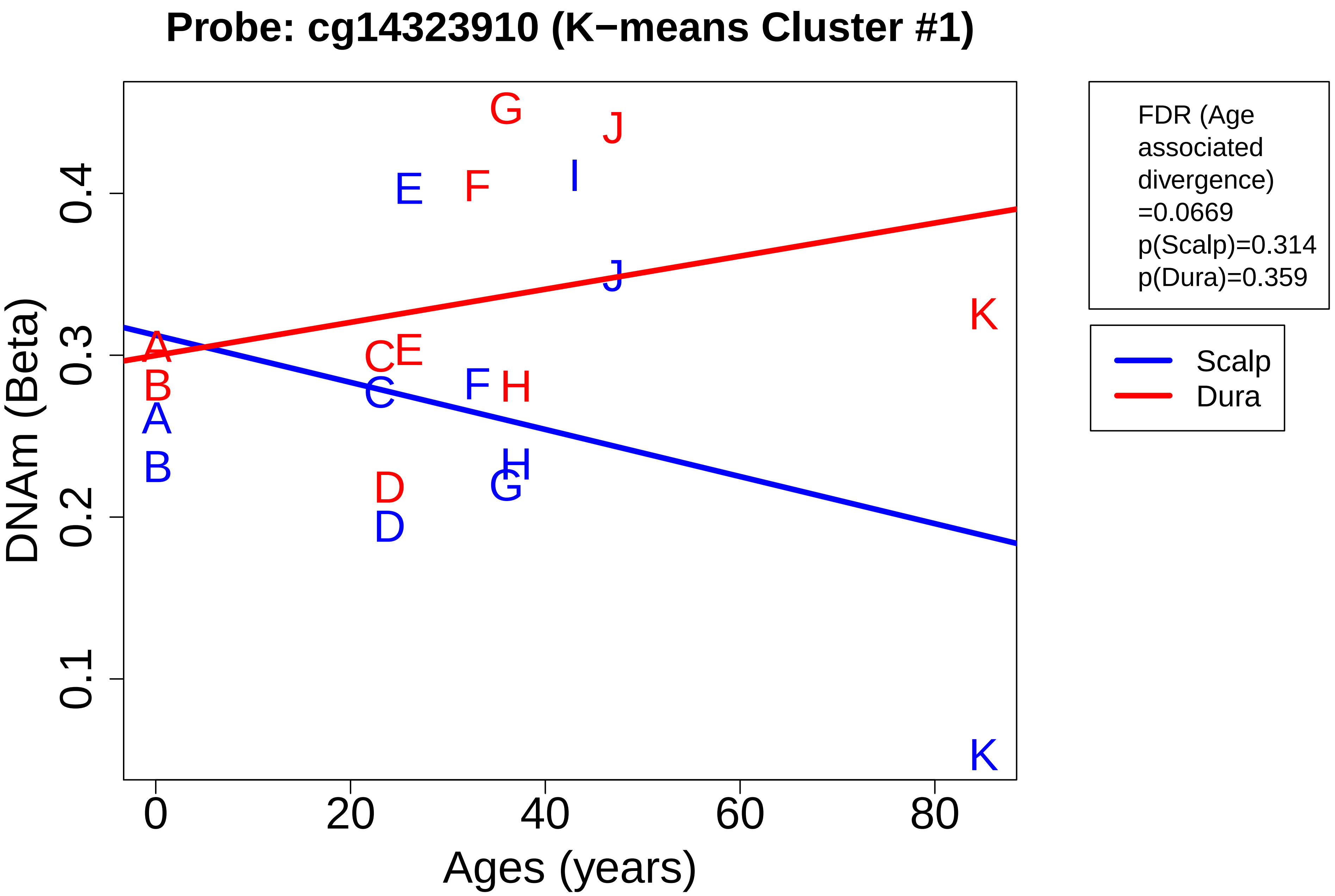

Probe: cg14323910 (K−means Cluster #1)
DNAm (Beta)
0.1
0.2
0.3
0.4
Ages (years)
0
20
40
60
80
FDR (Age associated divergence) =0.0669
p(Scalp)=0.314
p(Dura)=0.359
Scalp
Dura
A
B
C
D
E
F
G
H
I
J
K

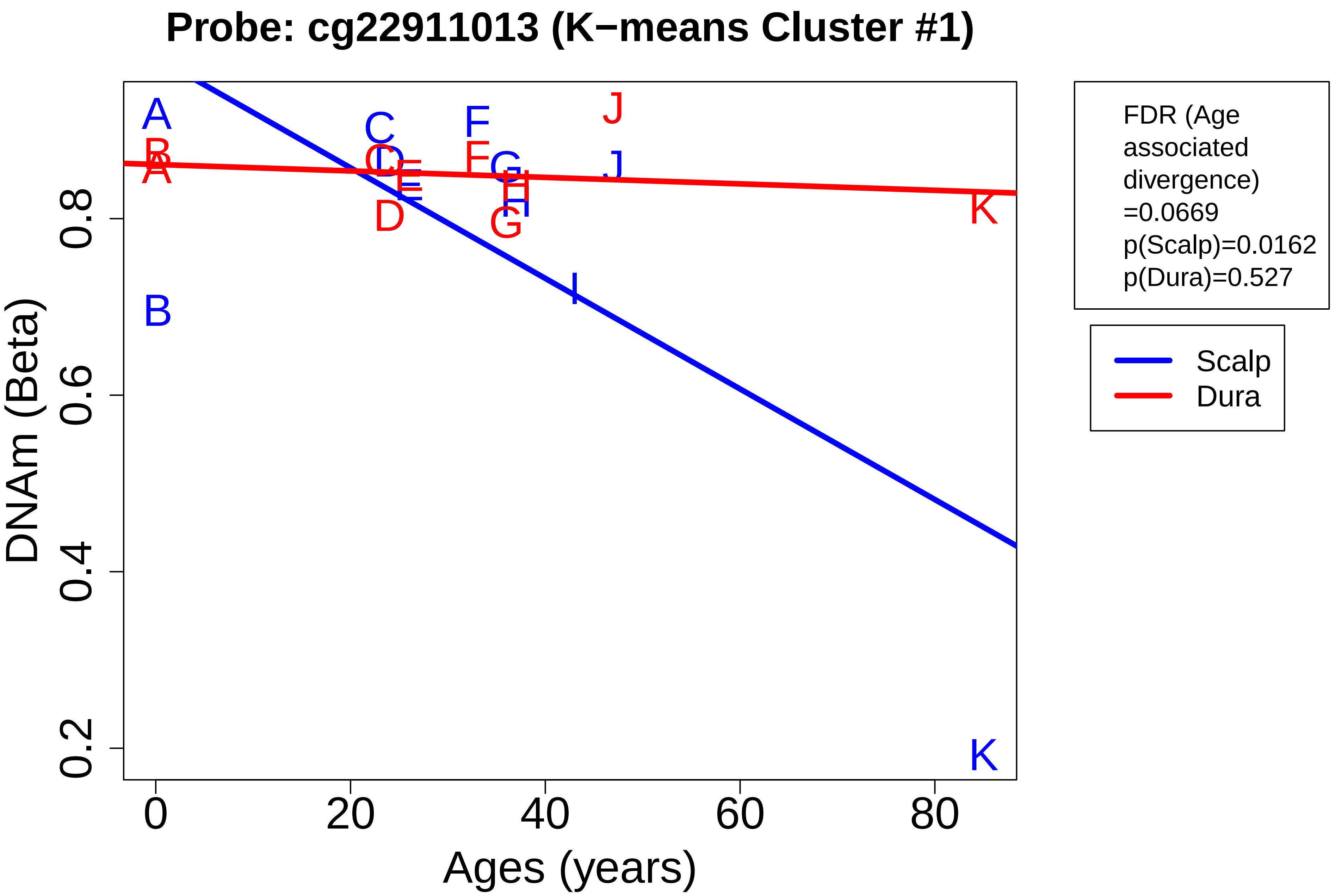
Probe: cg22911013 (K−means Cluster #1)
DNAm (Beta)
0.2
0.4
0.6
0.8
0
20
40
60
80
Ages (years)
A
B
C
D
E
F
G
H
I
J
K
FDR (Age associated divergence) =0.0669
p(Scalp)=0.0162
p(Dura)=0.527
Scalp
Dura

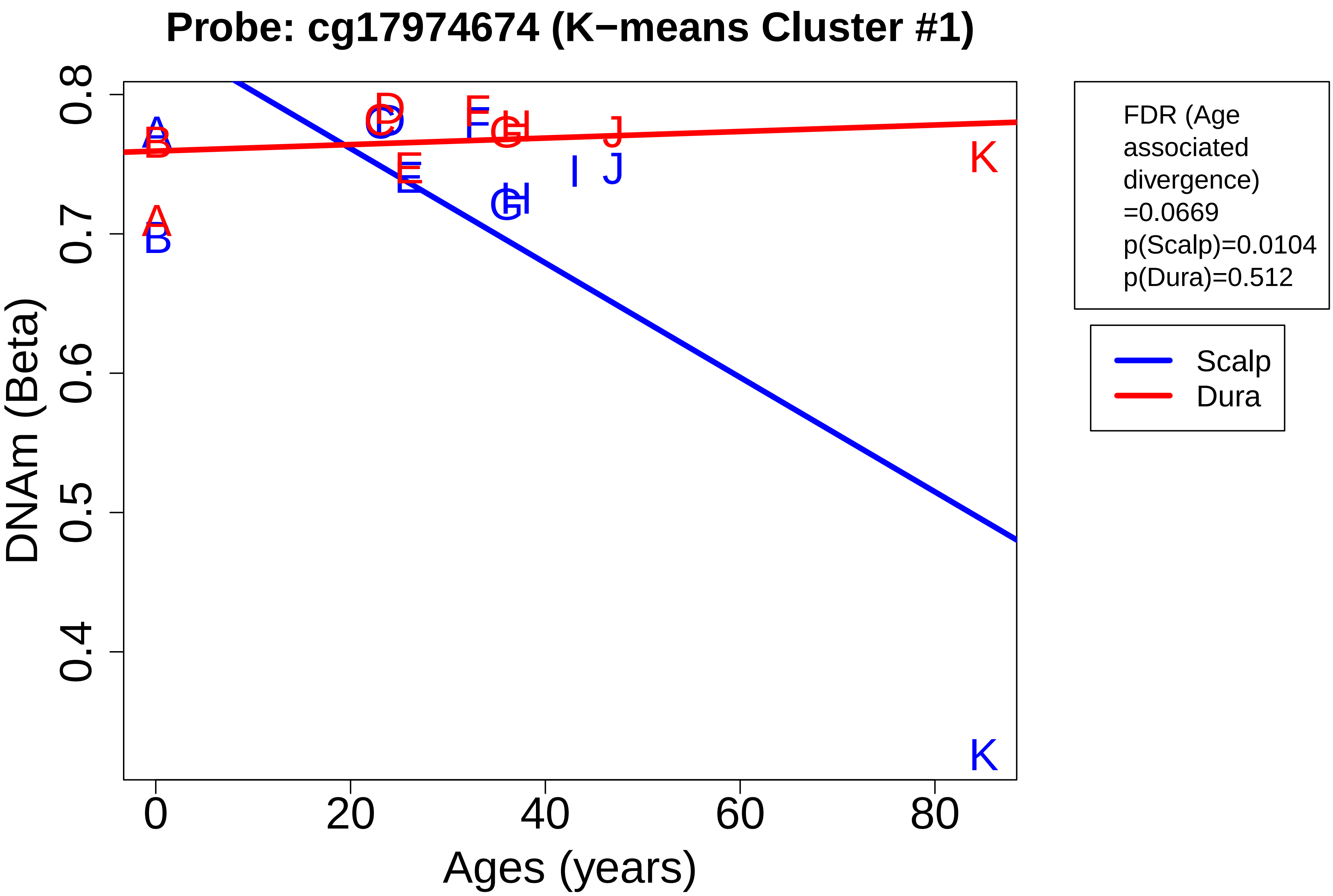

Probe: cg17974674 (K−means Cluster #1)
DNAm (Beta)
0.4
0.5
0.6
0.7
0.8
0
20
40
60
80
Ages (years)
FDR (Age associated divergence) =0.0669
p(Scalp)=0.0104
p(Dura)=0.512
Scalp
Dura

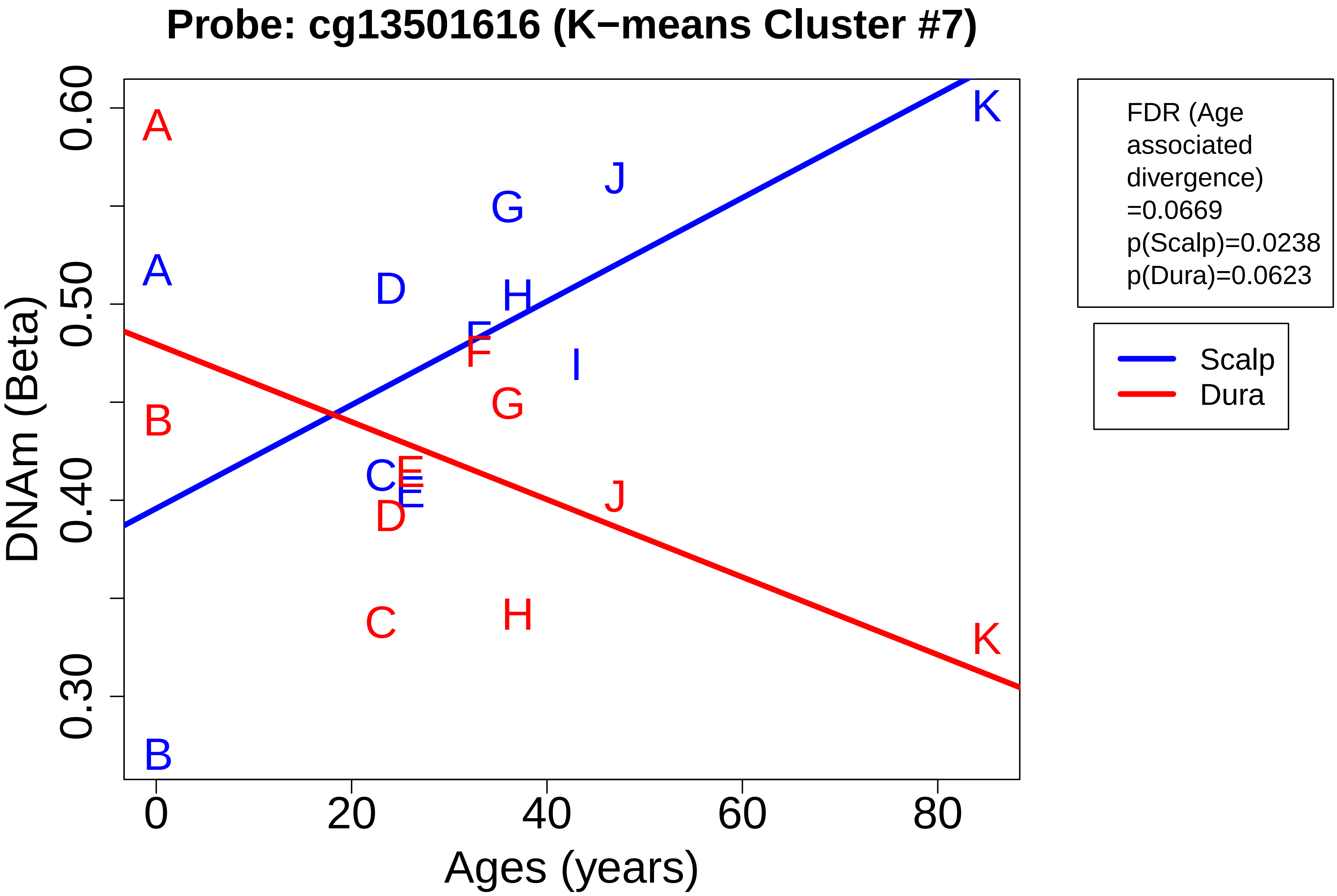

Probe: cg13501616 (K−means Cluster #7)
DNAm (Beta)
0.30
0.40
0.50
0.60
0
20
40
60
80
Ages (years)
A
B
C
D
E
F
G
H
I
J
K
FDR (Age associated divergence) =0.0669
p(Scalp)=0.0238
p(Dura)=0.0623
Scalp
Dura

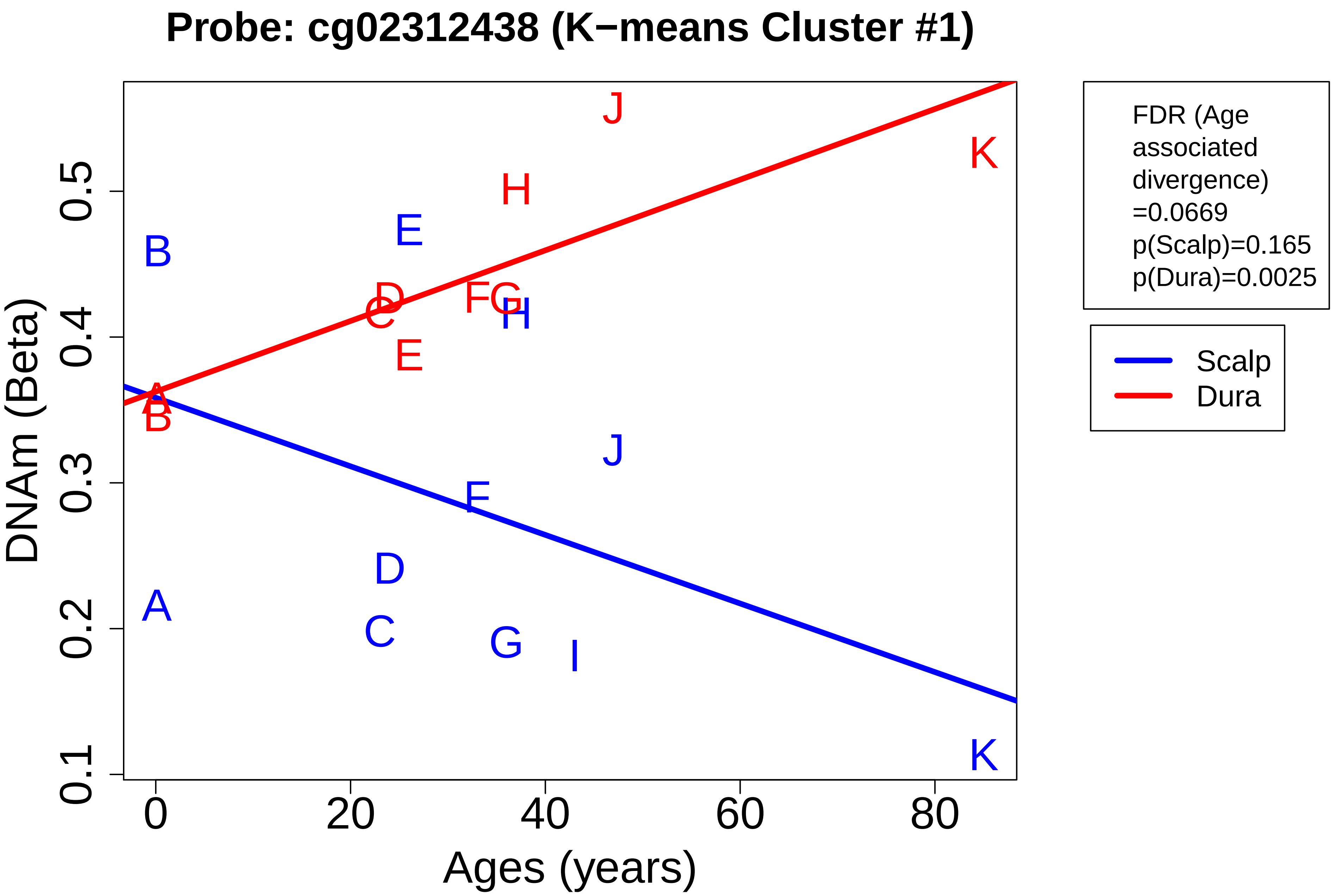
Probe: cg02312438 (K−means Cluster #1)
DNAm (Beta)
0.1
0.2
0.3
0.4
0.5
Ages (years)
0
20
40
60
80
FDR (Age associated divergence) =0.0669
p(Scalp)=0.165
p(Dura)=0.0025
Scalp
Dura
A
B
C
D
E
F
G
H
I
J
K

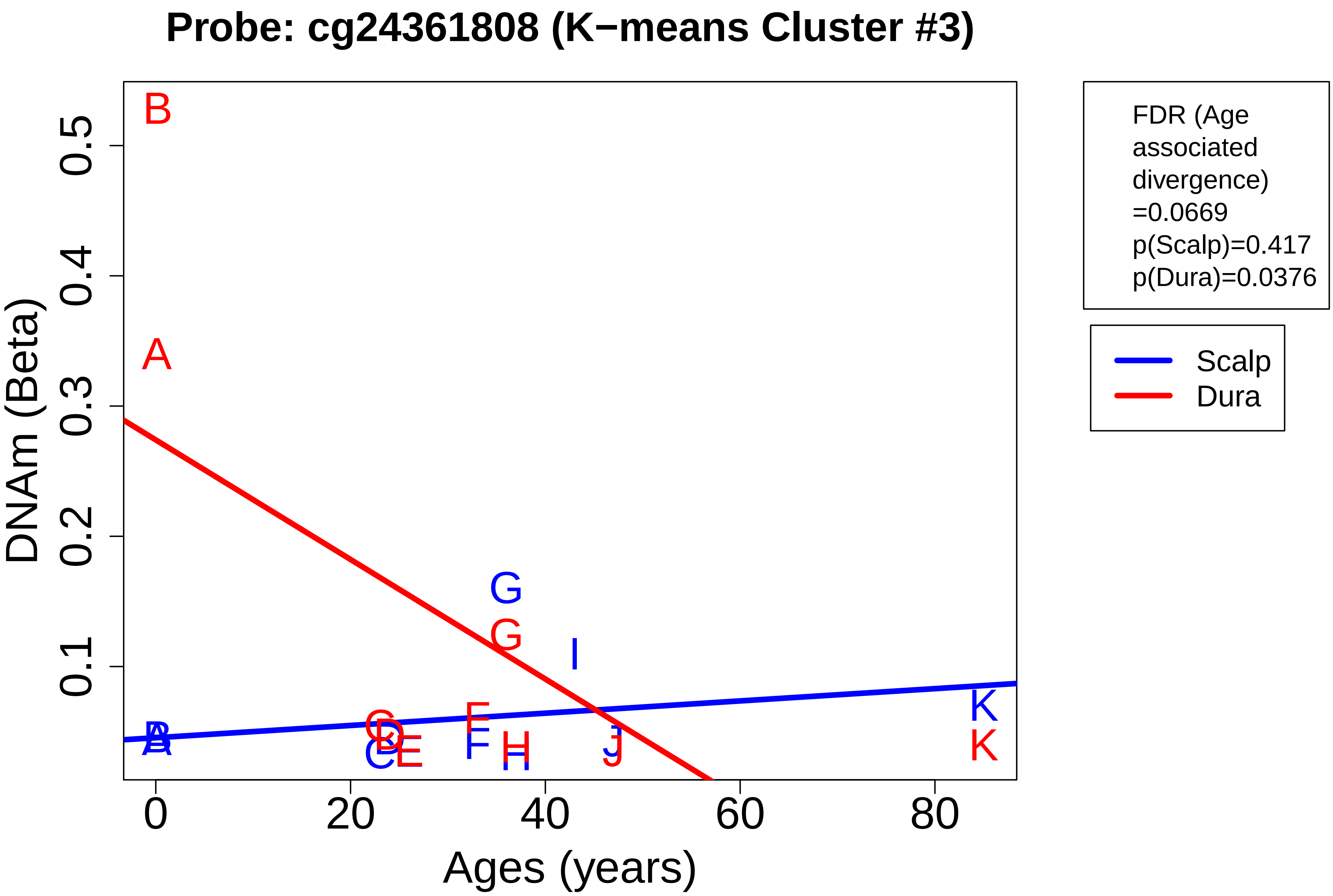

Probe: cg24361808 (K−means Cluster #3)
DNAm (Beta)
0.1
0.2
0.3
0.4
0.5
0
20
40
60
80
Ages (years)
FDR (Age associated divergence) =0.0669
p(Scalp)=0.417
p(Dura)=0.0376
Scalp
Dura

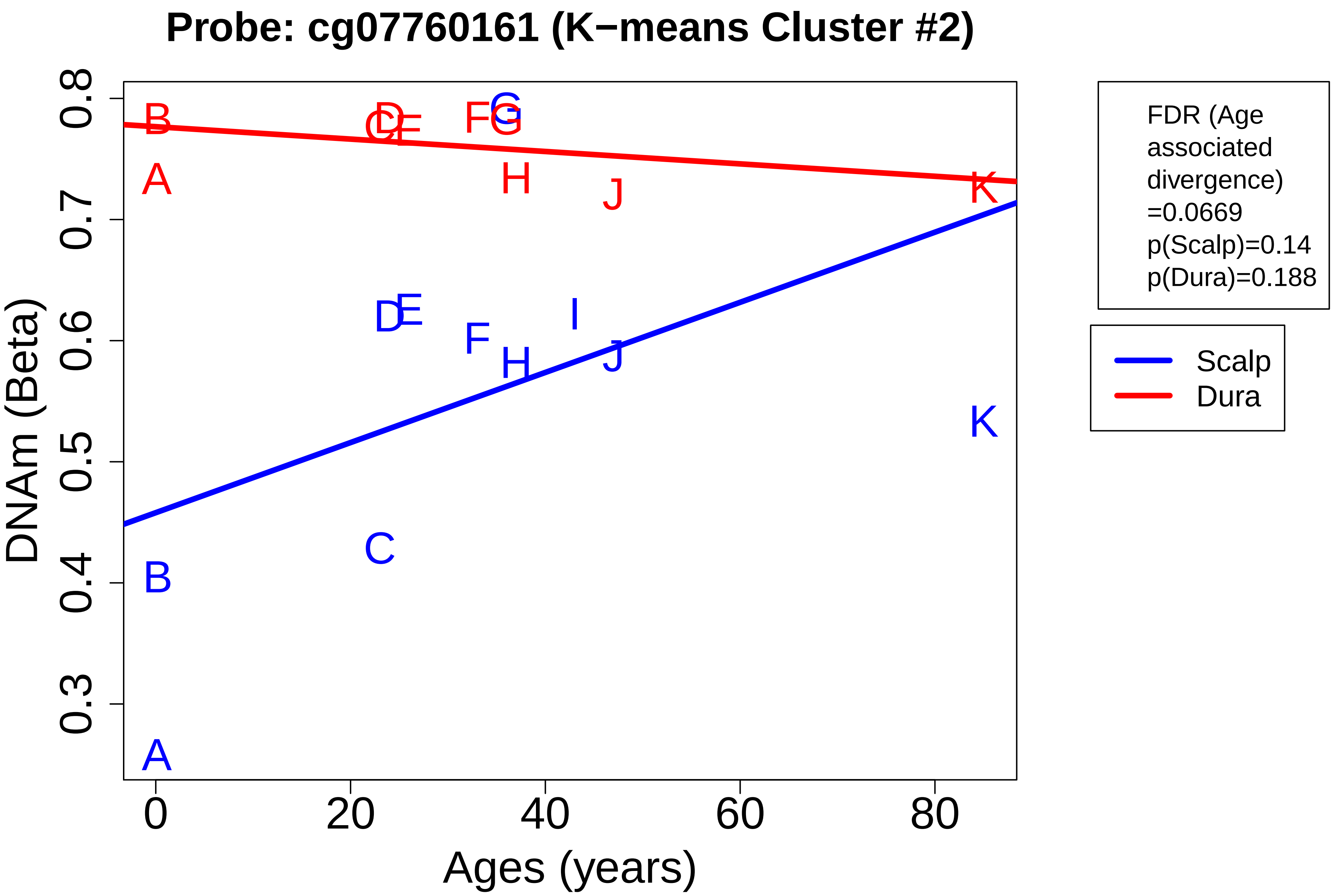

Probe: cg07760161 (K−means Cluster #2)
DNAm (Beta)
0.3
0.4
0.5
0.6
0.7
0.8
Ages (years)
0
20
40
60
80
A
B
C
D
E
F
G
H
I
J
K
FDR (Age associated divergence) =0.0669
p(Scalp)=0.14
p(Dura)=0.188
Scalp
Dura

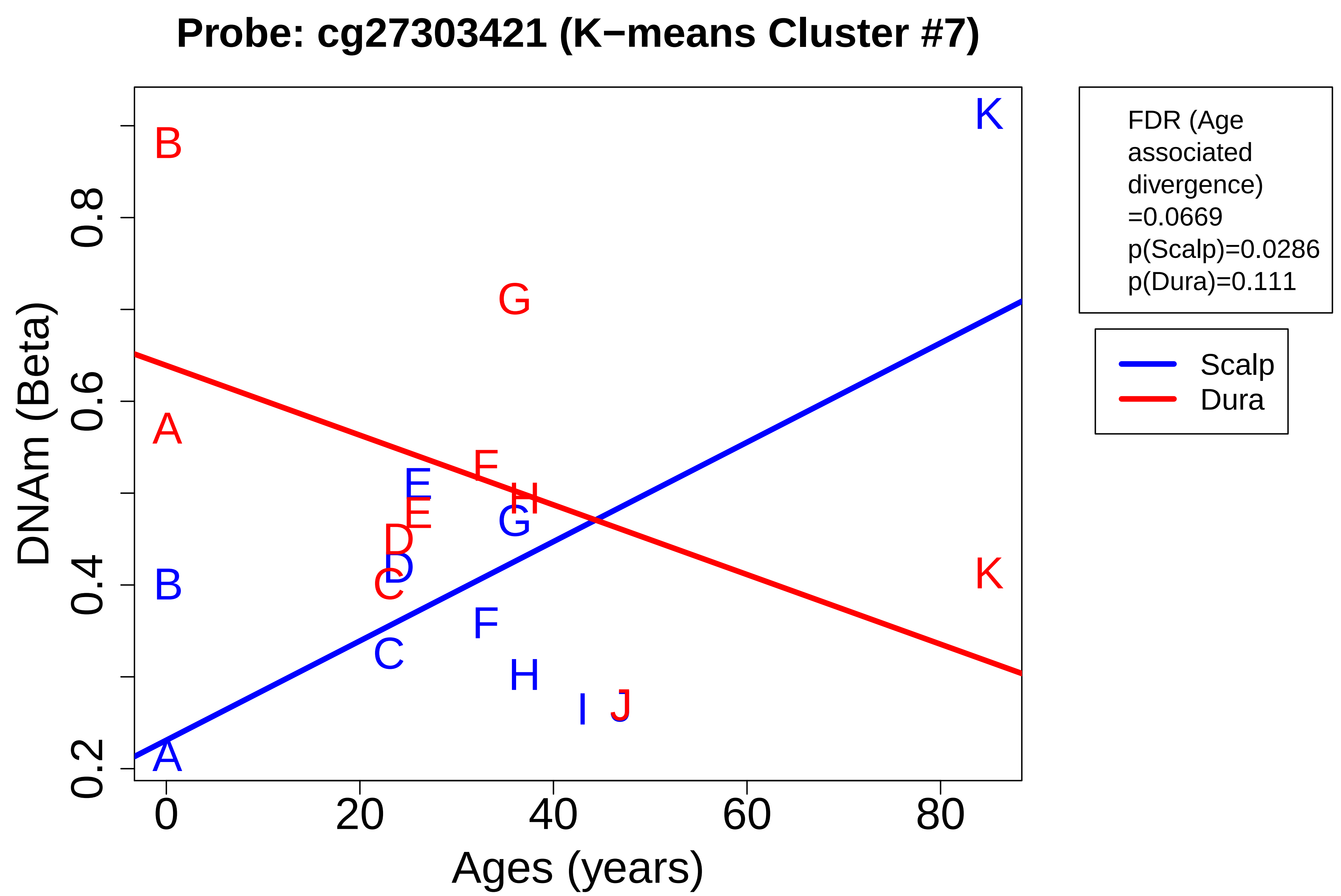

Probe: cg27303421 (K−means Cluster #7)
DNAm (Beta)
0.2
0.4
0.6
0.8
0
20
40
60
80
Ages (years)
FDR (Age associated divergence) =0.0669
p(Scalp)=0.0286
p(Dura)=0.111
Scalp
Dura

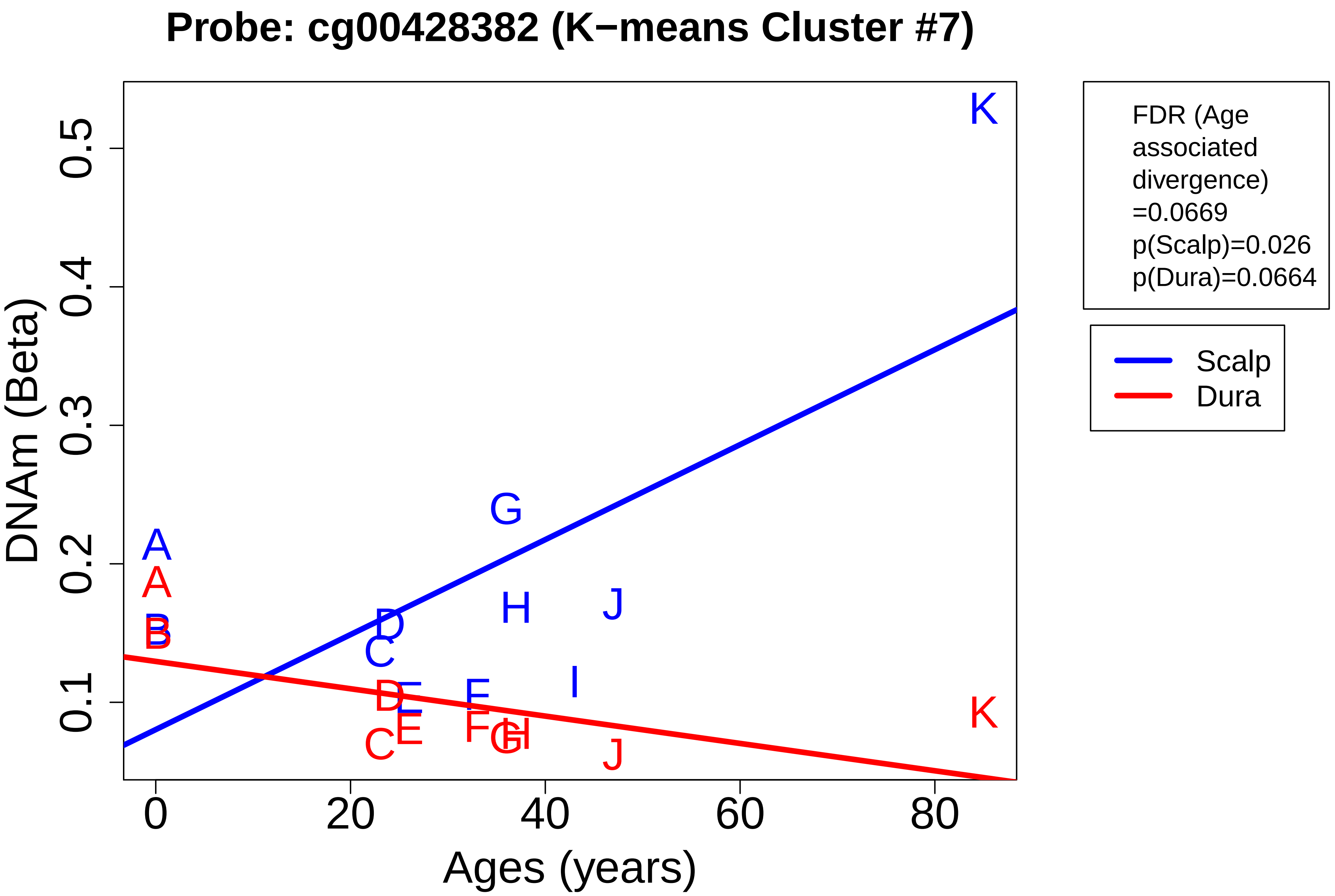
Probe: cg00428382 (K−means Cluster #7)
DNAm (Beta)
0.1
0.2
0.3
0.4
0.5
0
20
40
60
80
Ages (years)
A
B
C
D
E
F
G
H
I
J
K
FDR (Age associated divergence) =0.0669
p(Scalp)=0.026
p(Dura)=0.0664
Scalp
Dura

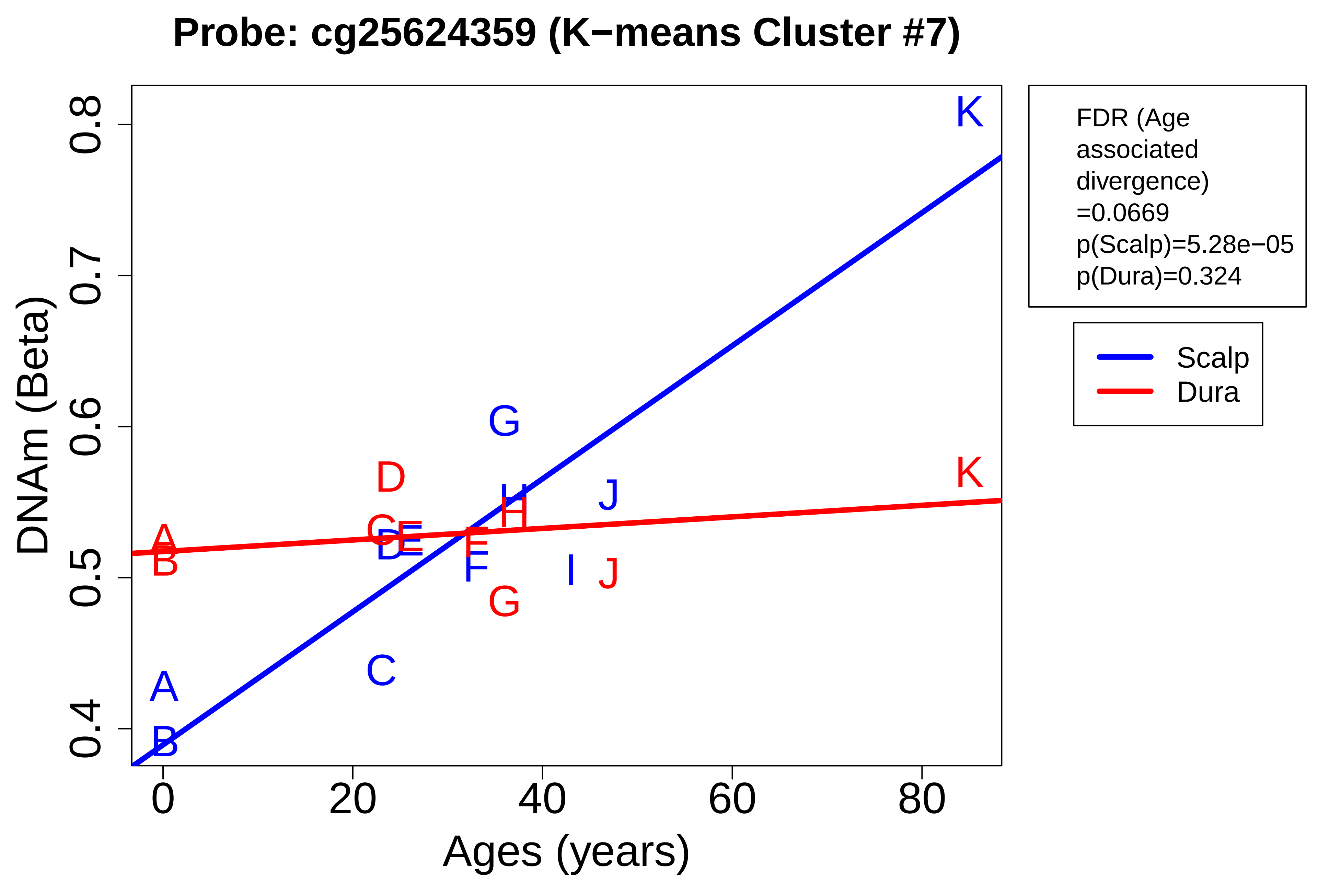

Probe: cg25624359 (K−means Cluster #7)
DNAm (Beta)
Ages (years)
0.4
0.5
0.6
0.7
0.8
0
20
40
60
80
FDR (Age associated divergence) =0.0669
p(Scalp)=5.28e−05
p(Dura)=0.324
Scalp
Dura

Probe: cg03583311 (K−means Cluster #4)

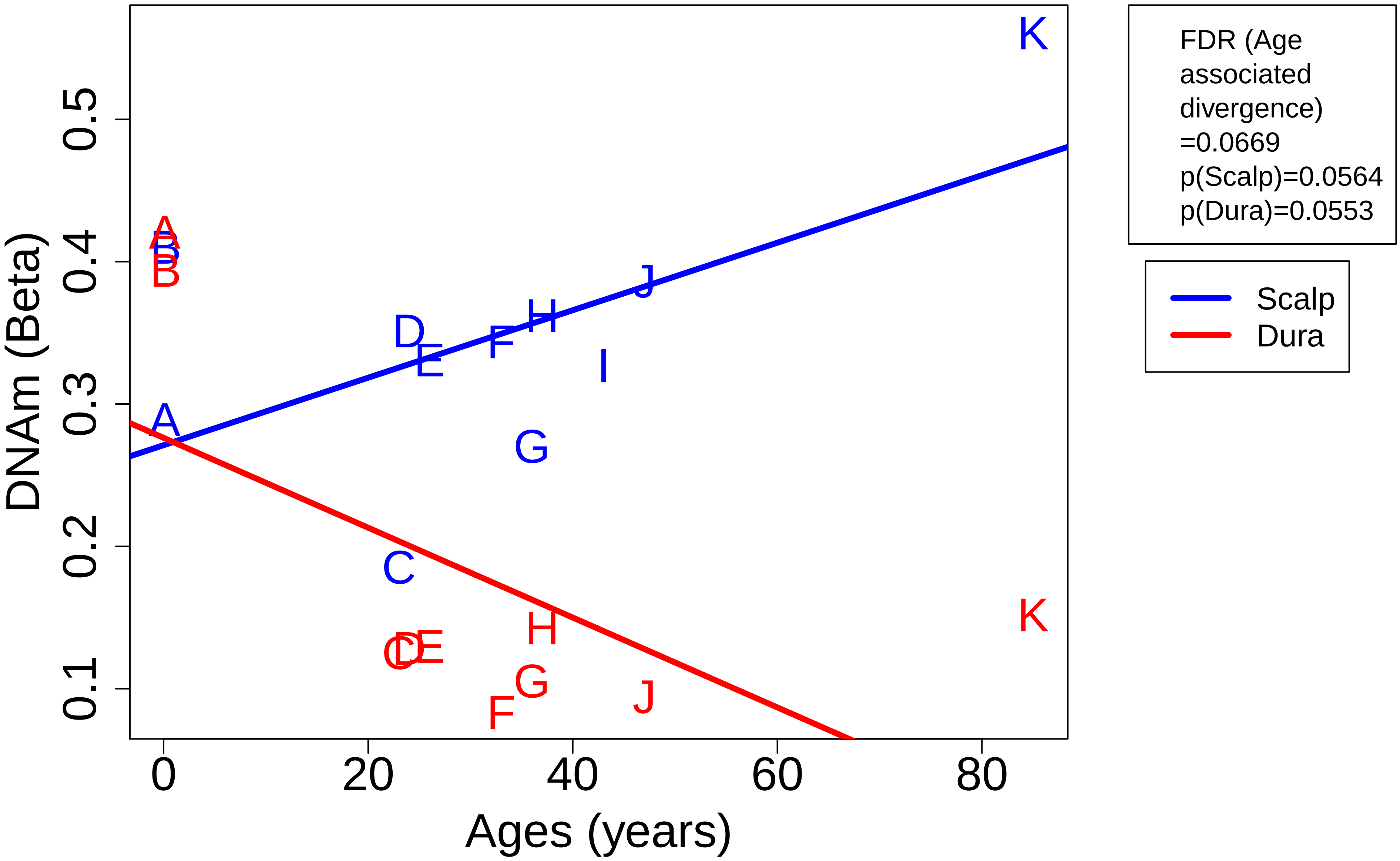

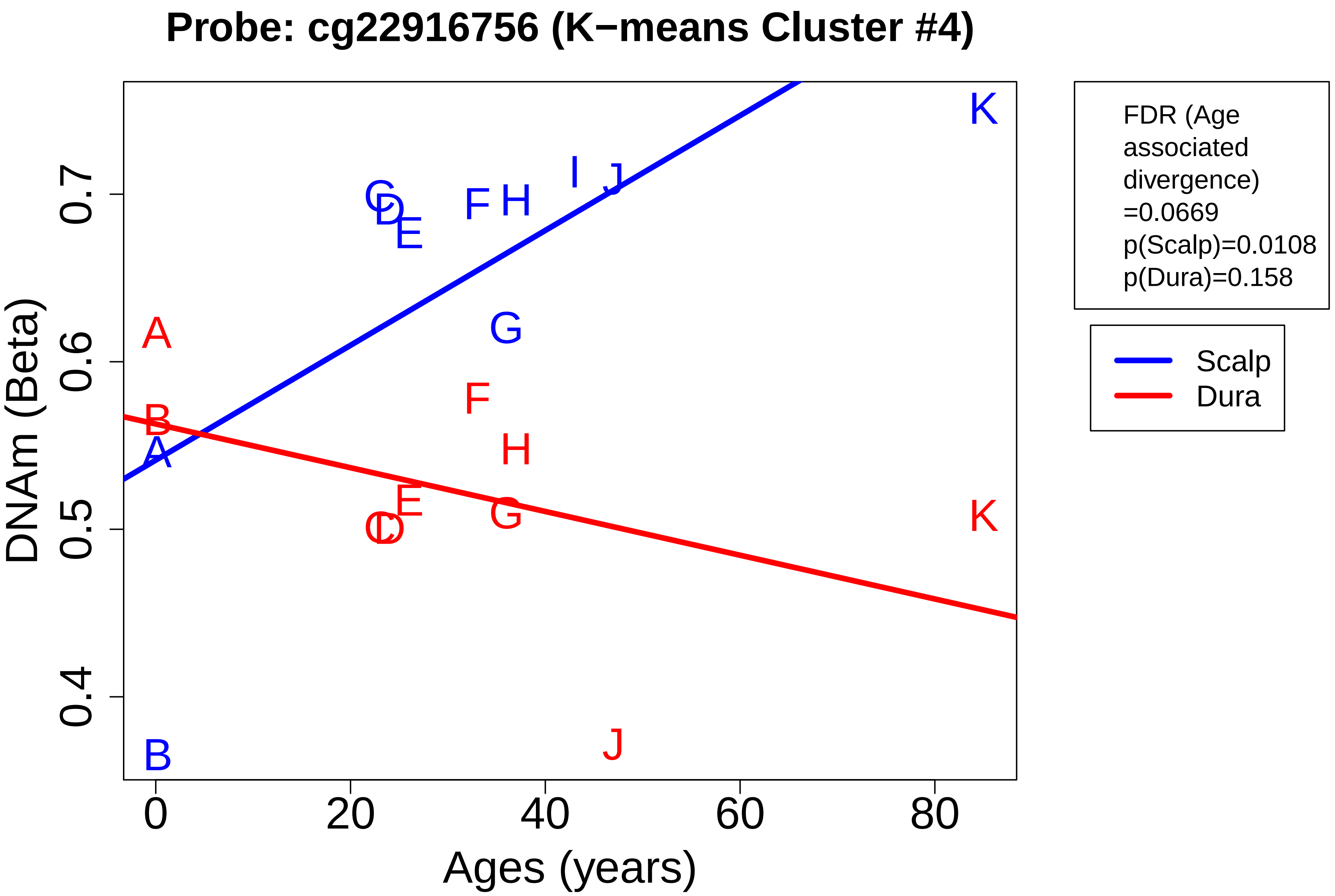

Probe: cg22916756 (K−means Cluster #4)
DNAm (Beta)
0.4
0.5
0.6
0.7
0
20
40
60
80
Ages (years)
FDR (Age associated divergence) =0.0669
p(Scalp)=0.0108
p(Dura)=0.158
Scalp
Dura

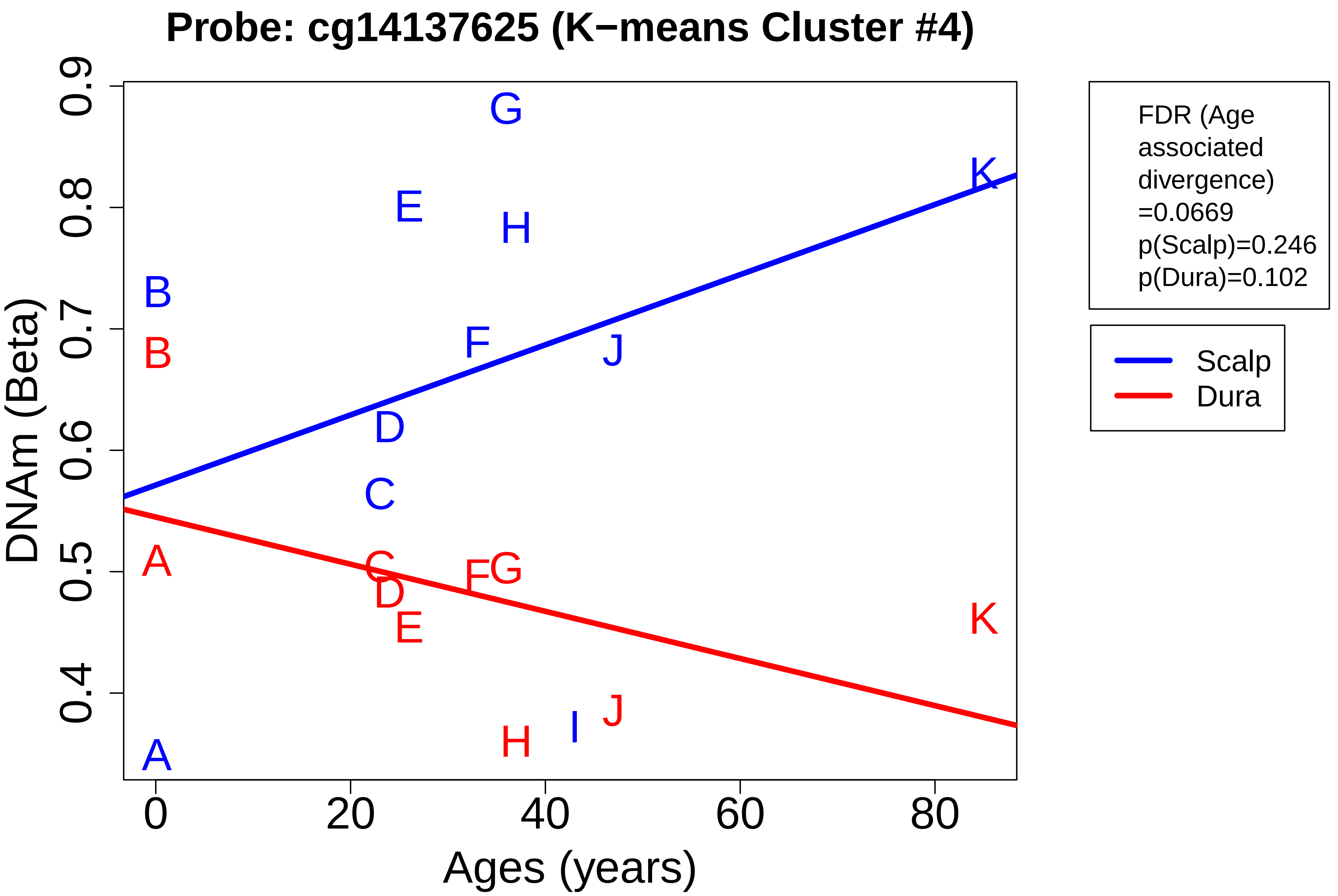

Probe: cg14137625 (K−means Cluster #4)
DNAm (Beta)
0.4
0.5
0.6
0.7
0.8
0.9
0
20
40
60
80
Ages (years)
FDR (Age associated divergence) =0.0669
p(Scalp)=0.246
p(Dura)=0.102
Scalp
Dura

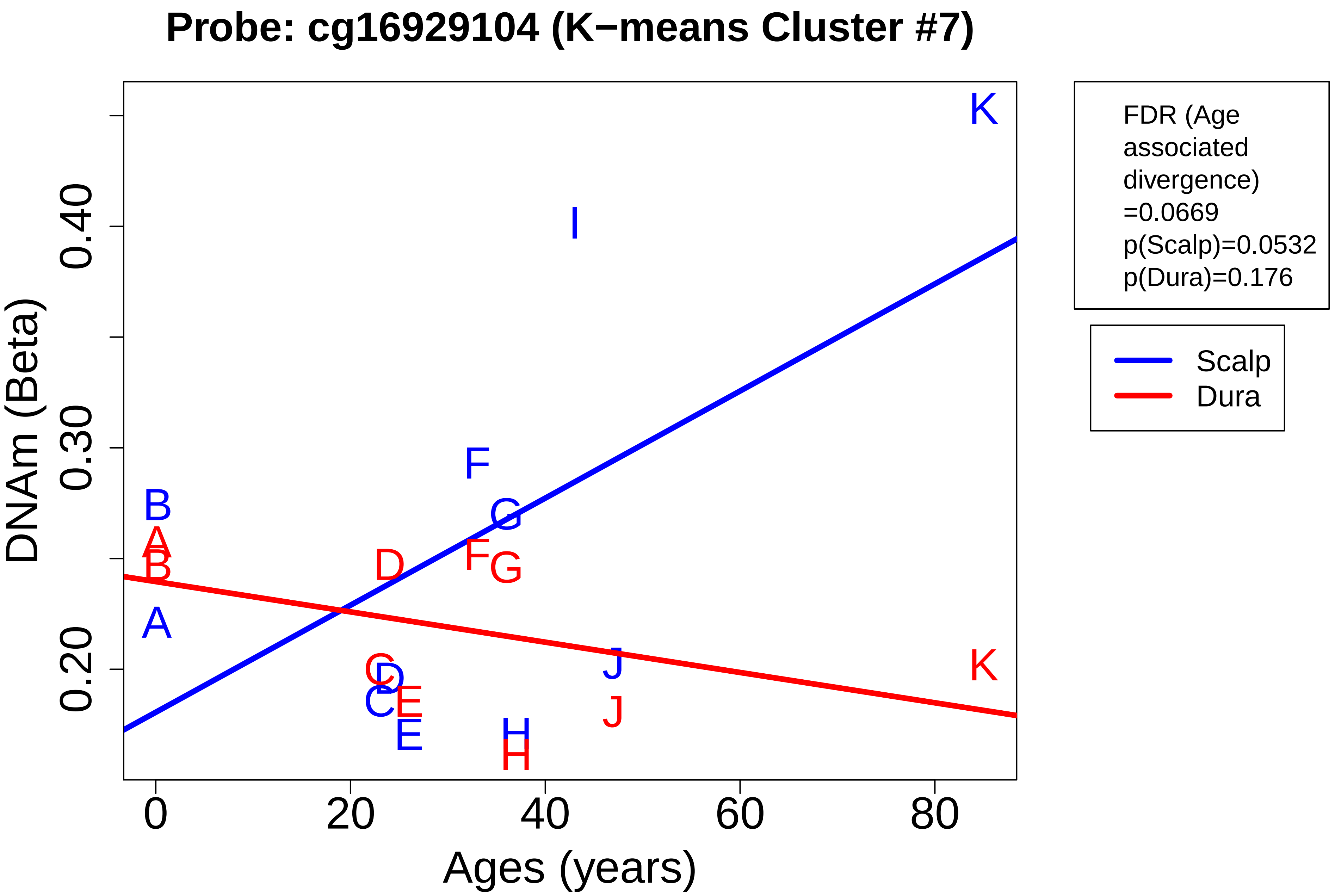

Probe: cg16929104 (K−means Cluster #7)
DNAm (Beta)
0.20
0.30
0.40
0
20
40
60
80
Ages (years)
FDR (Age associated divergence) =0.0669
p(Scalp)=0.0532
p(Dura)=0.176
Scalp
Dura

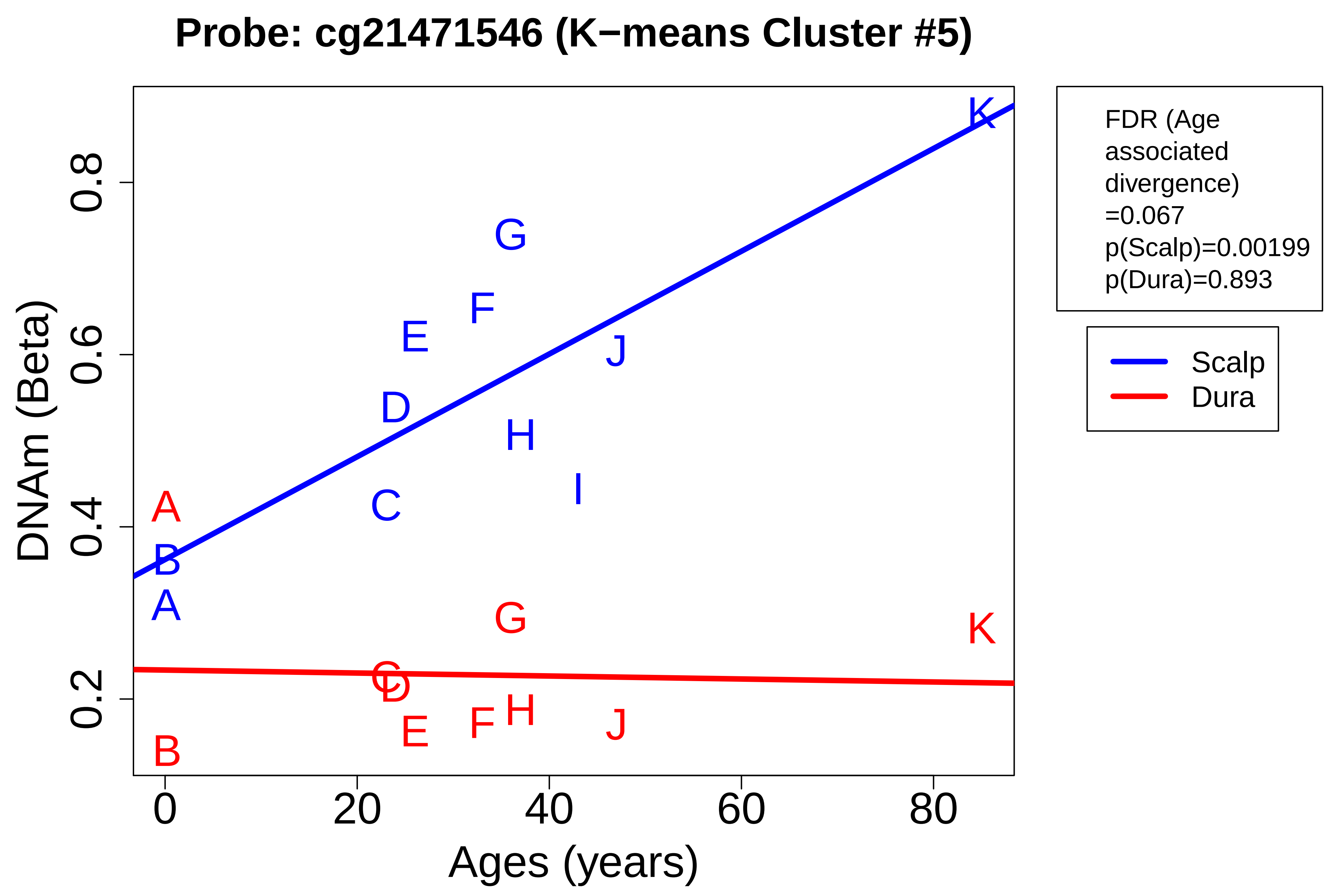

Probe: cg21471546 (K−means Cluster #5)
DNAm (Beta)
0.2
0.4
0.6
0.8
0
20
40
60
80
Ages (years)
A
B
C
D
E
F
G
H
I
J
K
FDR (Age associated divergence) =0.067
p(Scalp)=0.00199
p(Dura)=0.893
Scalp
Dura

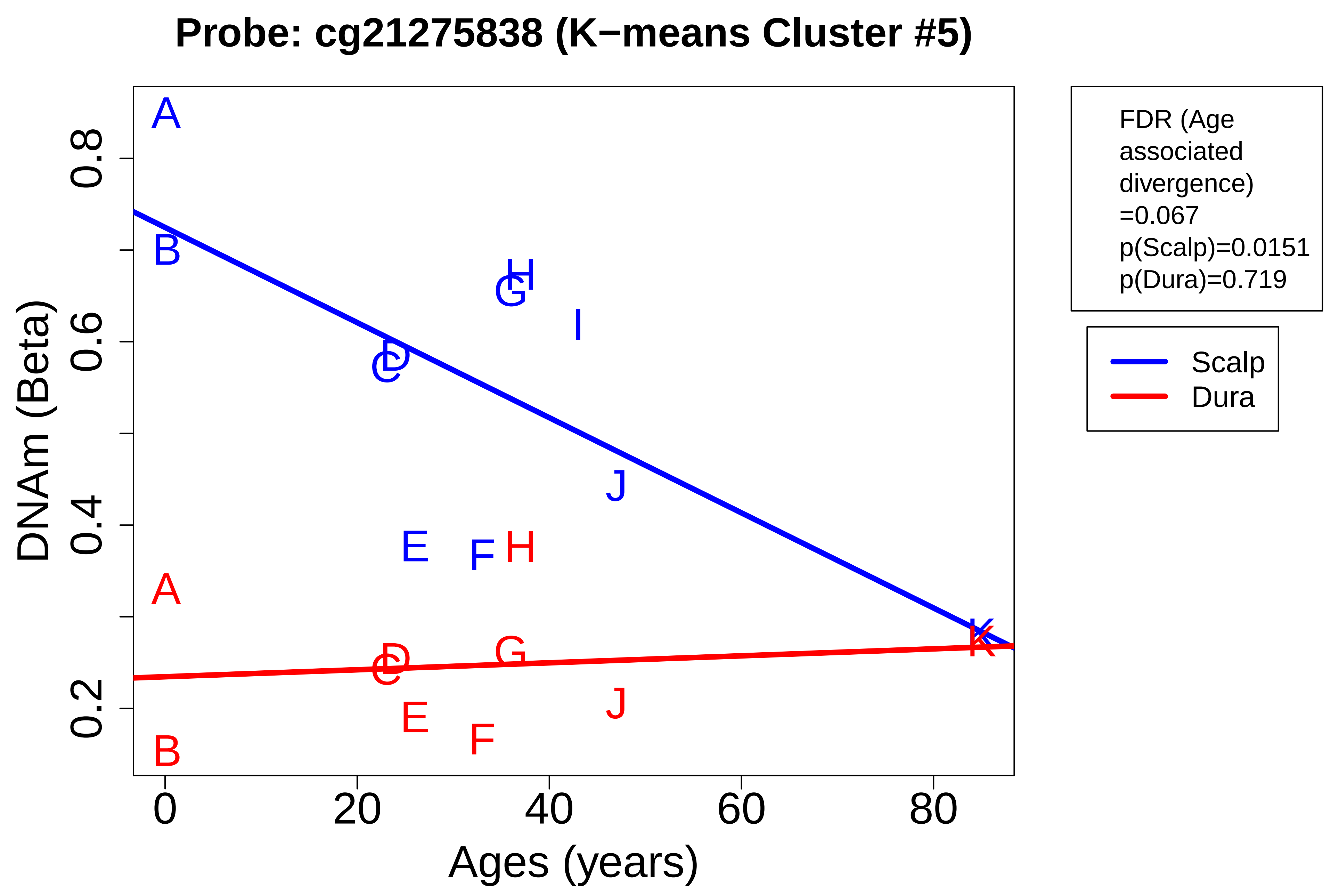
Probe: cg21275838 (K−means Cluster #5)
DNAm (Beta)
Ages (years)
0.2
0.4
0.6
0.8
0
20
40
60
80
A
B
C
D
E
F
G
H
I
J
K
FDR (Age associated divergence) =0.067
p(Scalp)=0.0151
p(Dura)=0.719
Scalp
Dura

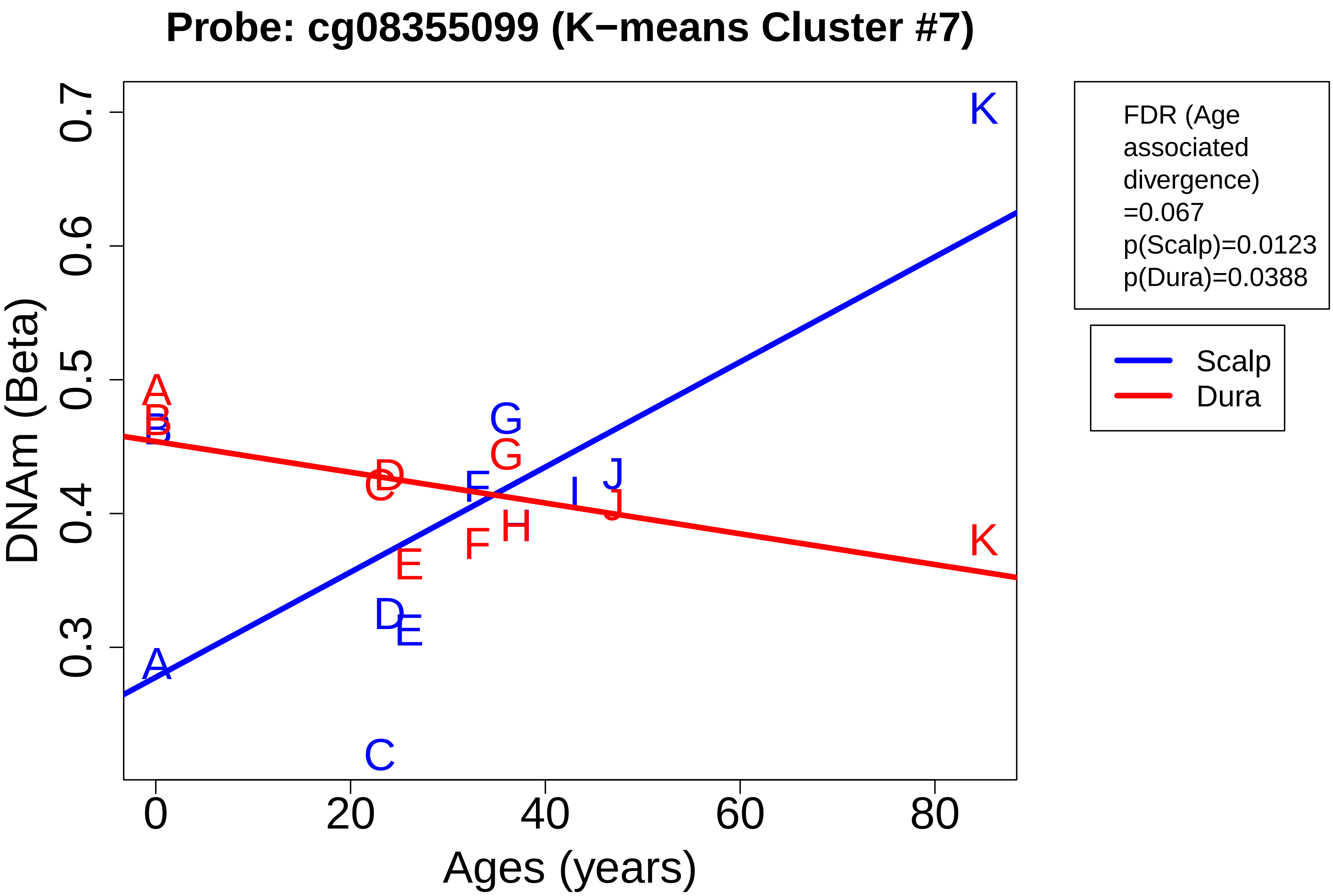

Probe: cg08355099 (K−means Cluster #7)
DNAm (Beta)
0.3
0.4
0.5
0.6
0.7
Ages (years)
0
20
40
60
80
FDR (Age associated divergence) =0.067
p(Scalp)=0.0123
p(Dura)=0.0388
Scalp
Dura

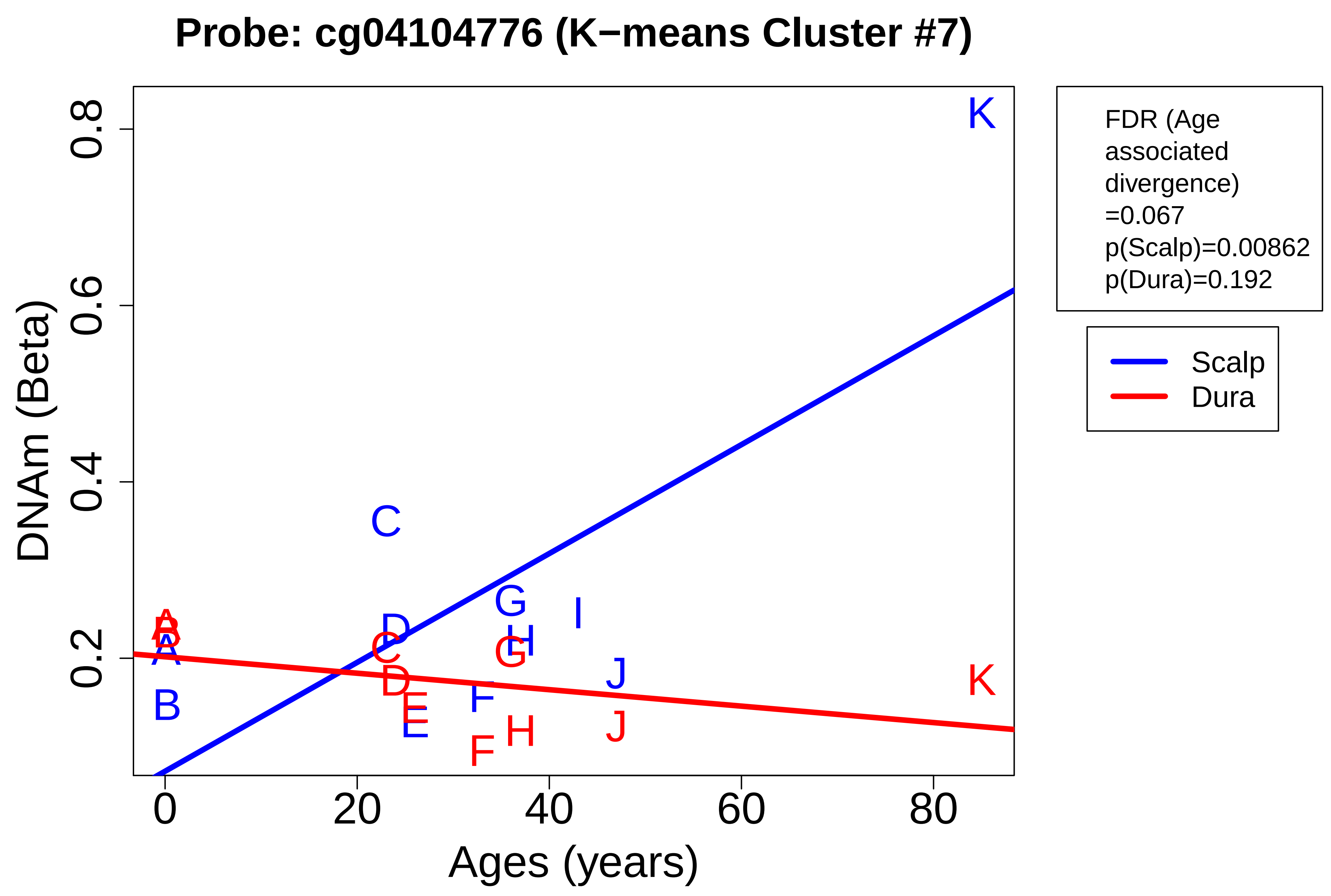

Probe: cg04104776 (K−means Cluster #7)
DNAm (Beta)
0.2
0.4
0.6
0.8
0
20
40
60
80
Ages (years)
A
B
C
D
E
F
G
H
I
J
K
FDR (Age associated divergence) =0.067
p(Scalp)=0.00862
p(Dura)=0.192
Scalp
Dura

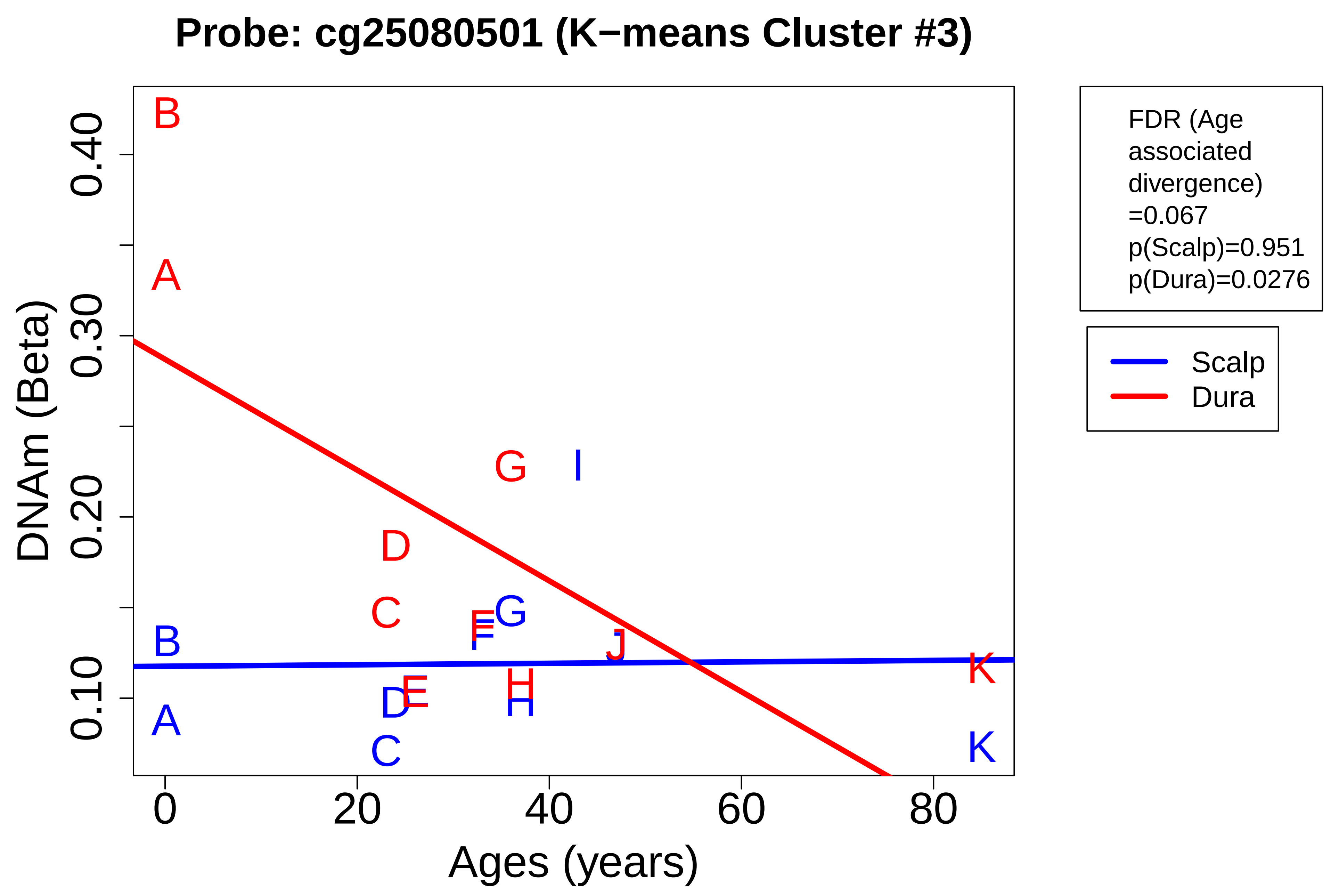
Probe: cg25080501 (K−means Cluster #3)
DNAm (Beta)
0.10
0.20
0.30
0.40
Ages (years)
0
20
40
60
80
FDR (Age associated divergence) =0.067
p(Scalp)=0.951
p(Dura)=0.0276
Scalp
Dura

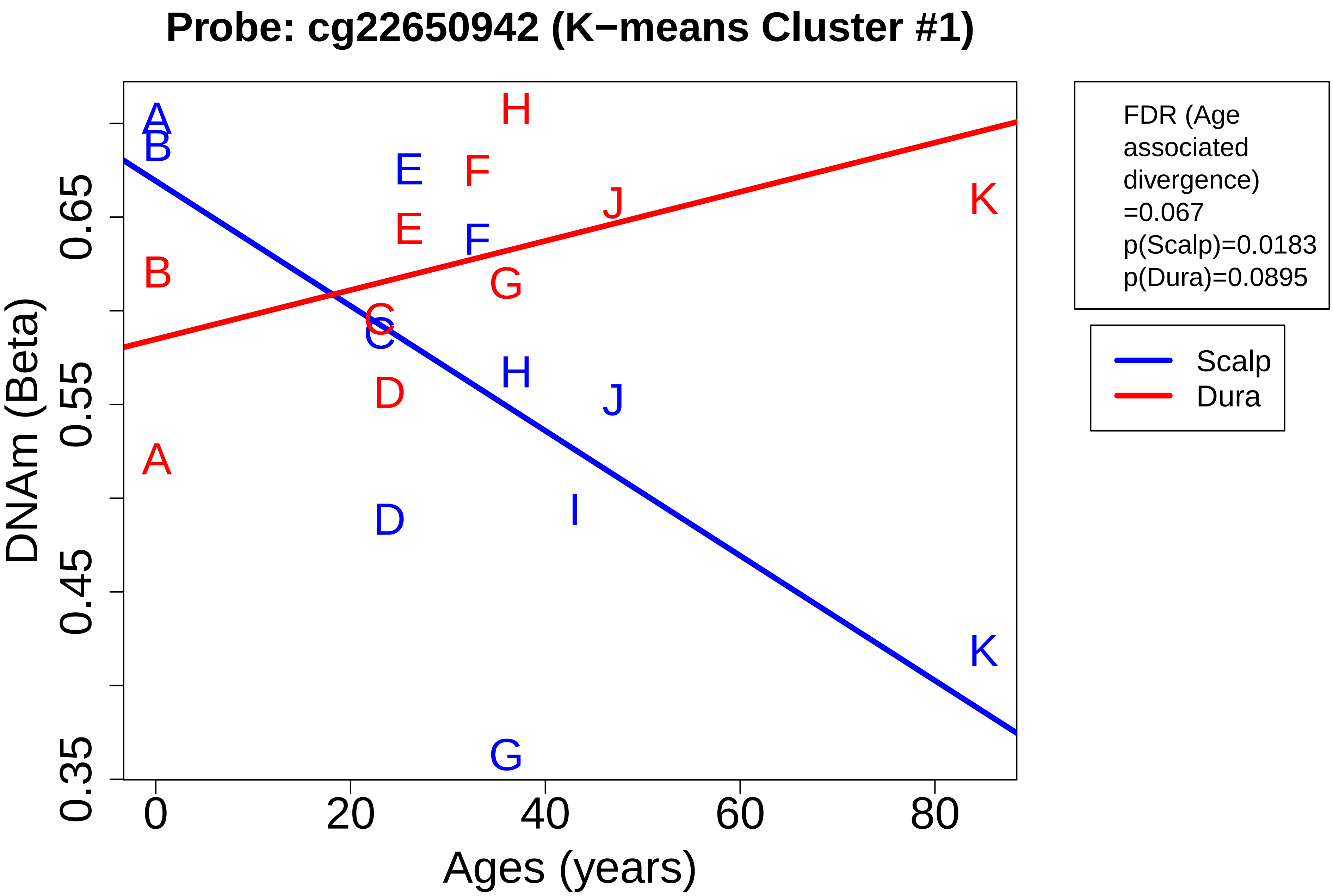
Probe: cg22650942 (K−means Cluster #1)
DNAm (Beta)
0.35
0.45
0.55
0.65
0
20
40
60
80
Ages (years)
A
B
C
D
E
F
G
H
I
J
K
FDR (Age associated divergence) =0.067
p(Scalp)=0.0183
p(Dura)=0.0895
Scalp
Dura

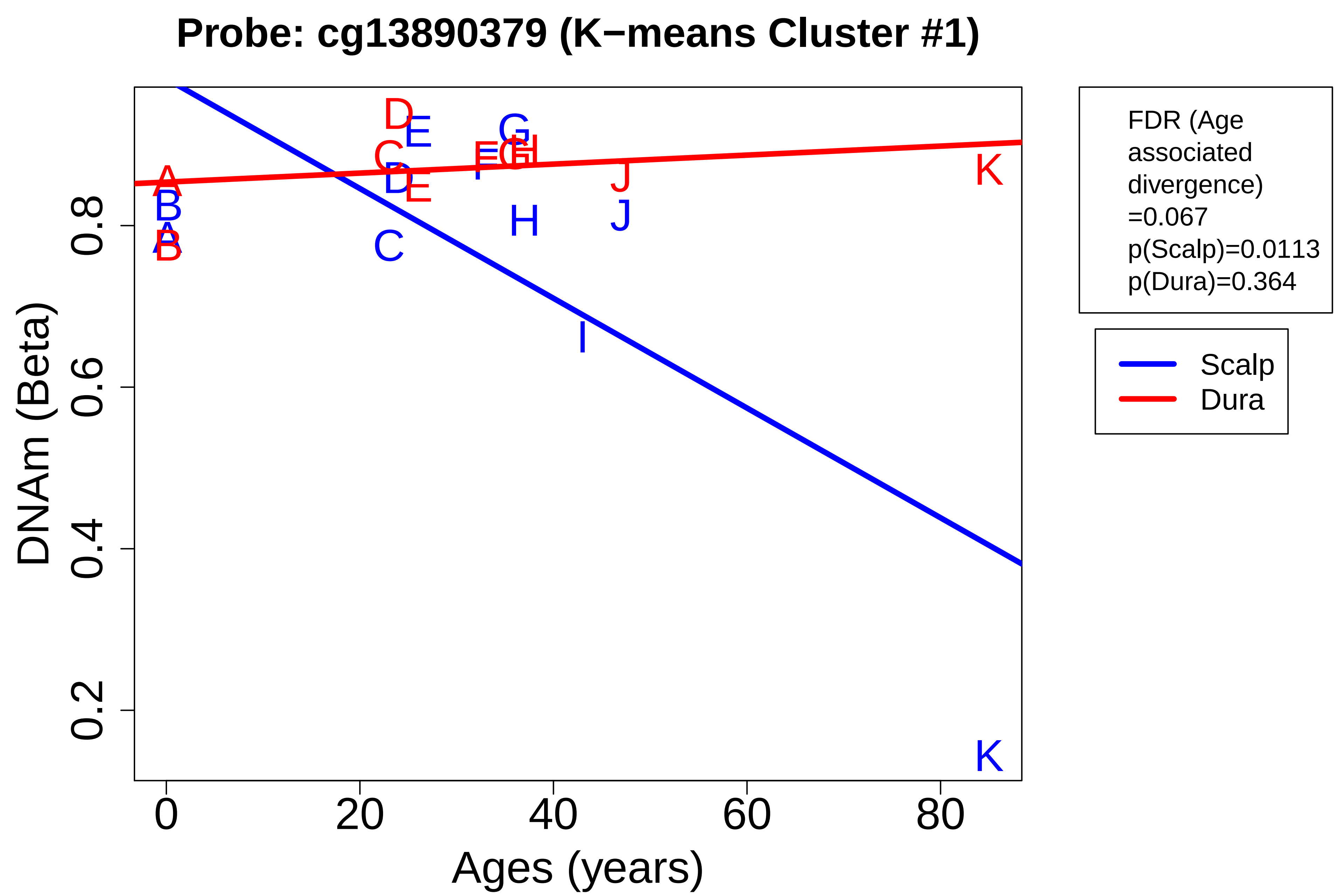

Probe: cg13890379 (K−means Cluster #1)
DNAm (Beta)
0.2
0.4
0.6
0.8
0
20
40
60
80
Ages (years)
FDR (Age associated divergence) =0.067
p(Scalp)=0.0113
p(Dura)=0.364
Scalp
Dura

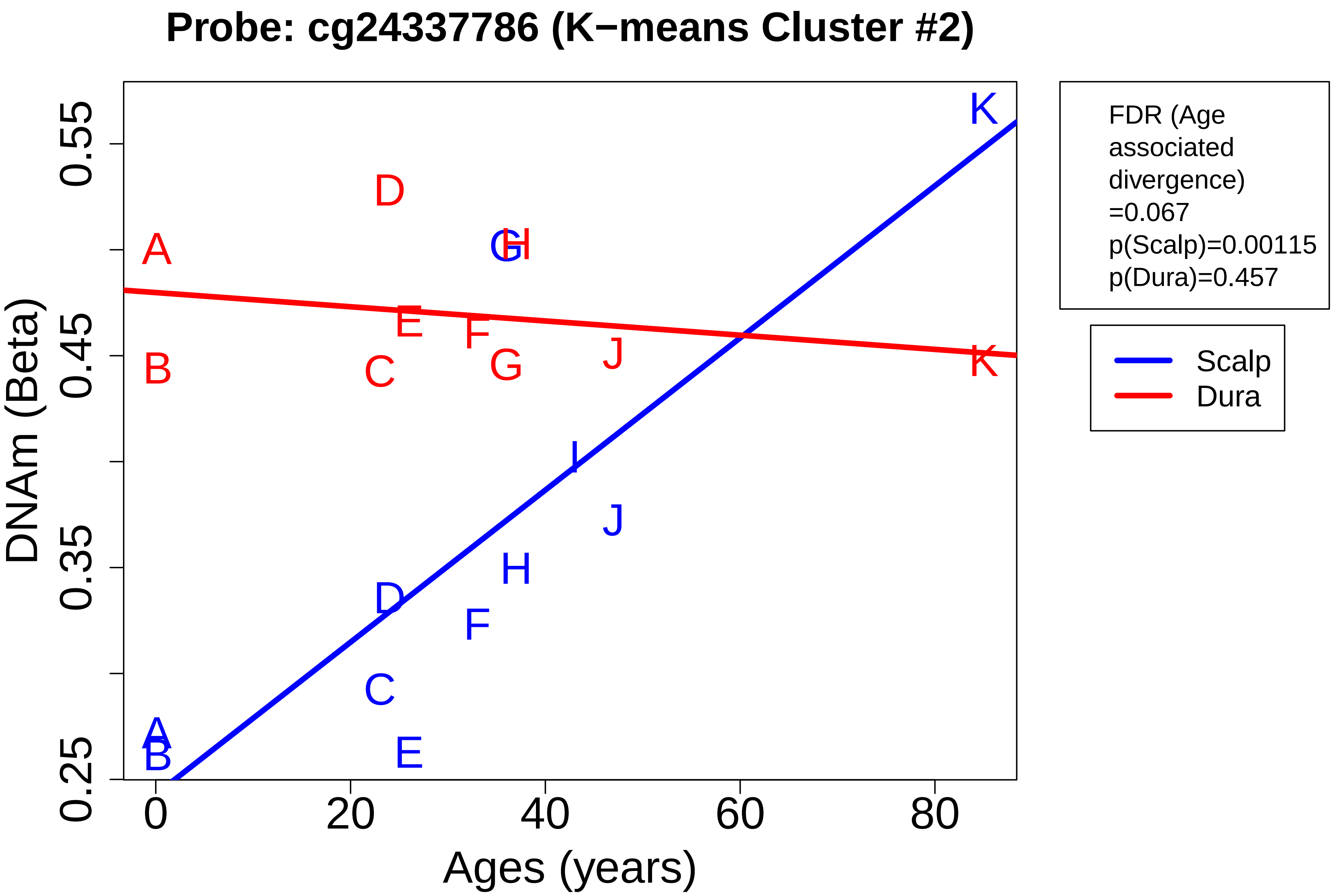
Probe: cg24337786 (K−means Cluster #2)
DNAm (Beta)
0.25
0.35
0.45
0.55
Ages (years)
0
20
40
60
80
A
B
C
D
E
F
G
H
I
J
K
FDR (Age associated divergence) =0.067
p(Scalp)=0.00115
p(Dura)=0.457
Scalp
Dura

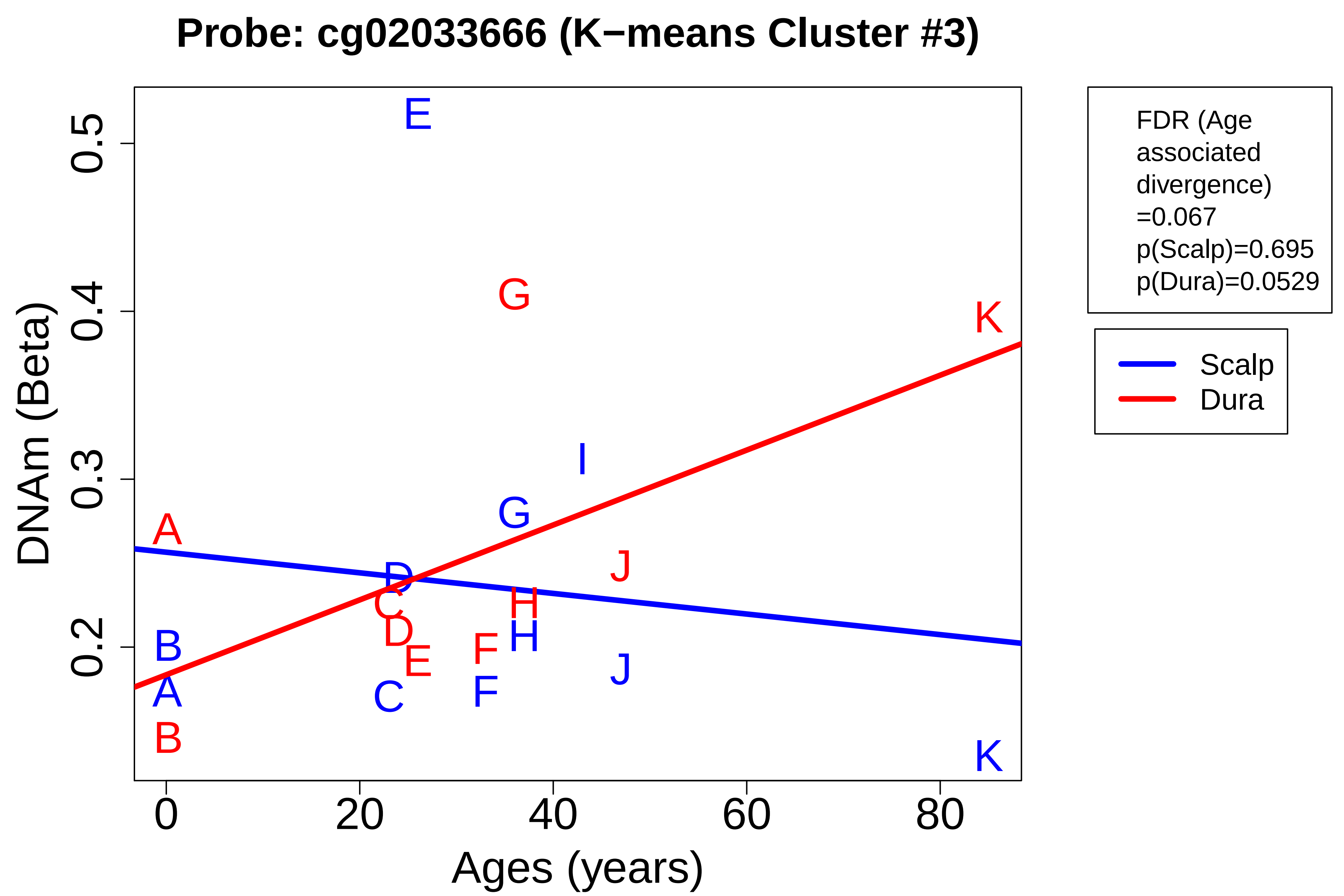

Probe: cg02033666 (K−means Cluster #3)
DNAm (Beta)
Ages (years)
0.2
0.3
0.4
0.5
0
20
40
60
80
FDR (Age associated divergence) =0.067
p(Scalp)=0.695
p(Dura)=0.0529
Scalp
Dura
A
B
C
D
E
F
G
H
I
J
K

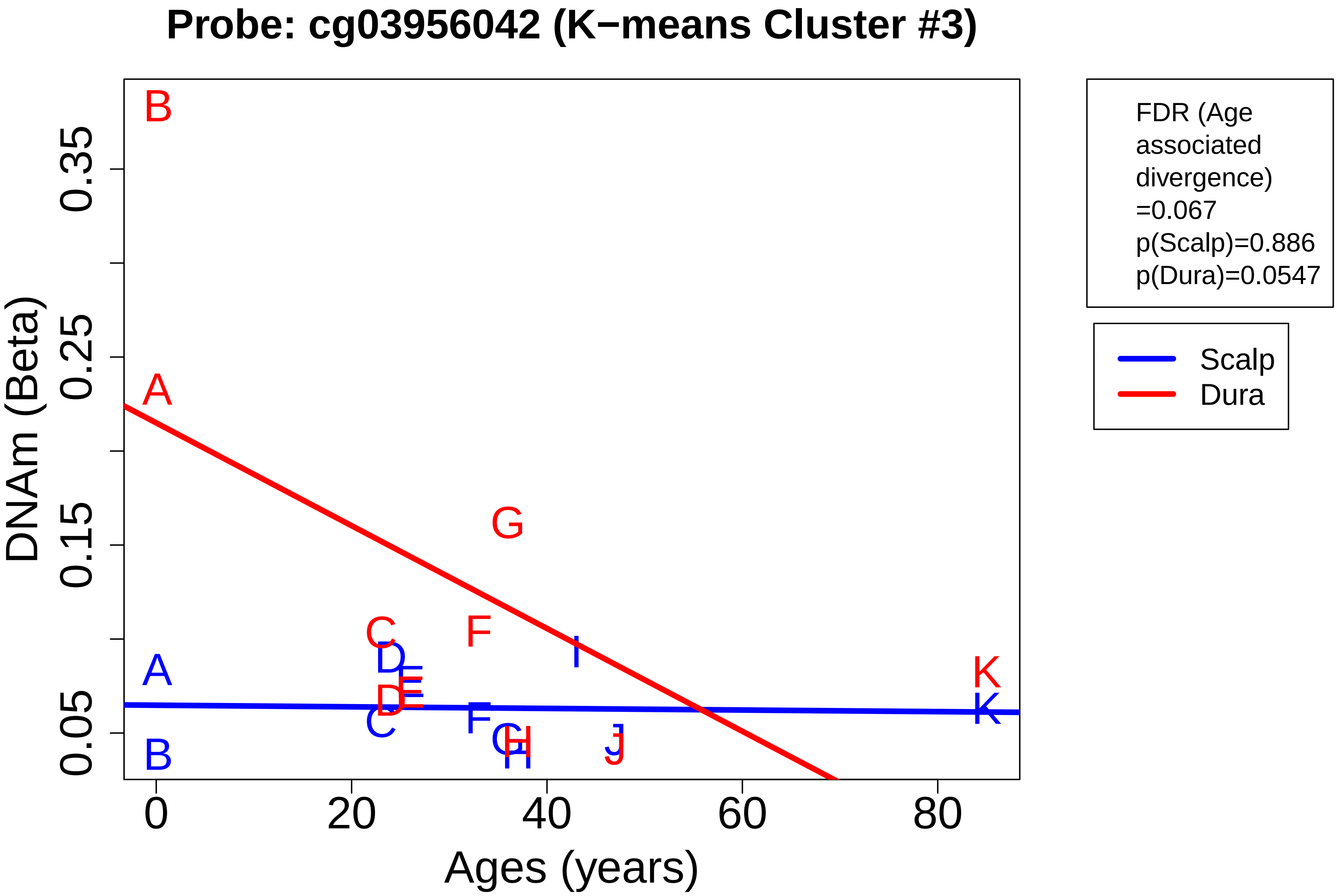

Probe: cg03956042 (K−means Cluster #3)
DNAm (Beta)
0.05
0.15
0.25
0.35
Ages (years)
0
20
40
60
80
A
B
C
D
E
F
G
H
I
J
K
FDR (Age associated divergence) =0.067
p(Scalp)=0.886
p(Dura)=0.0547
Scalp
Dura

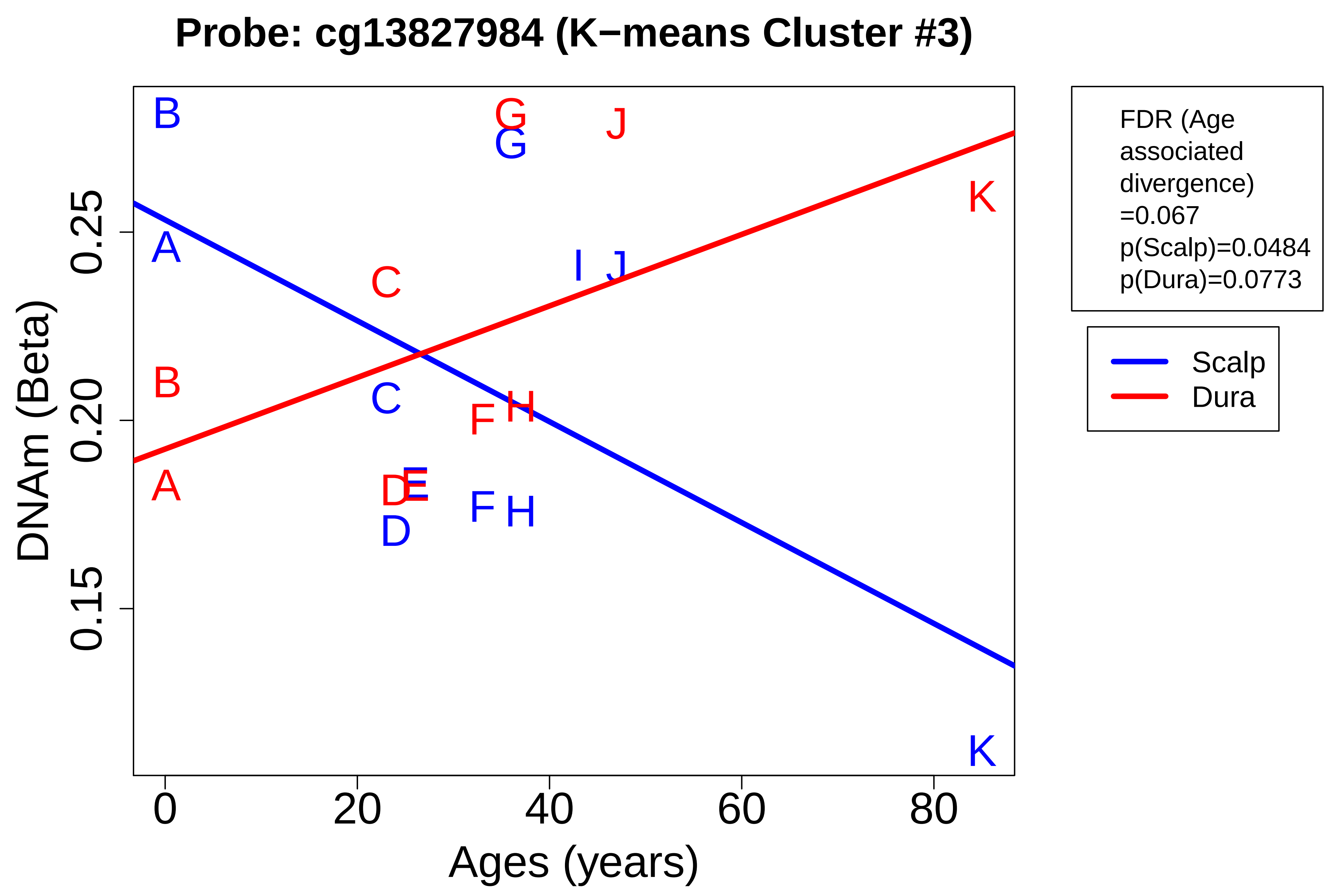

Probe: cg13827984 (K−means Cluster #3)
DNAm (Beta)
0.15
0.20
0.25
0
20
40
60
80
Ages (years)
A
B
C
D
E
F
G
H
I
J
K
FDR (Age associated divergence) =0.067
p(Scalp)=0.0484
p(Dura)=0.0773
Scalp
Dura

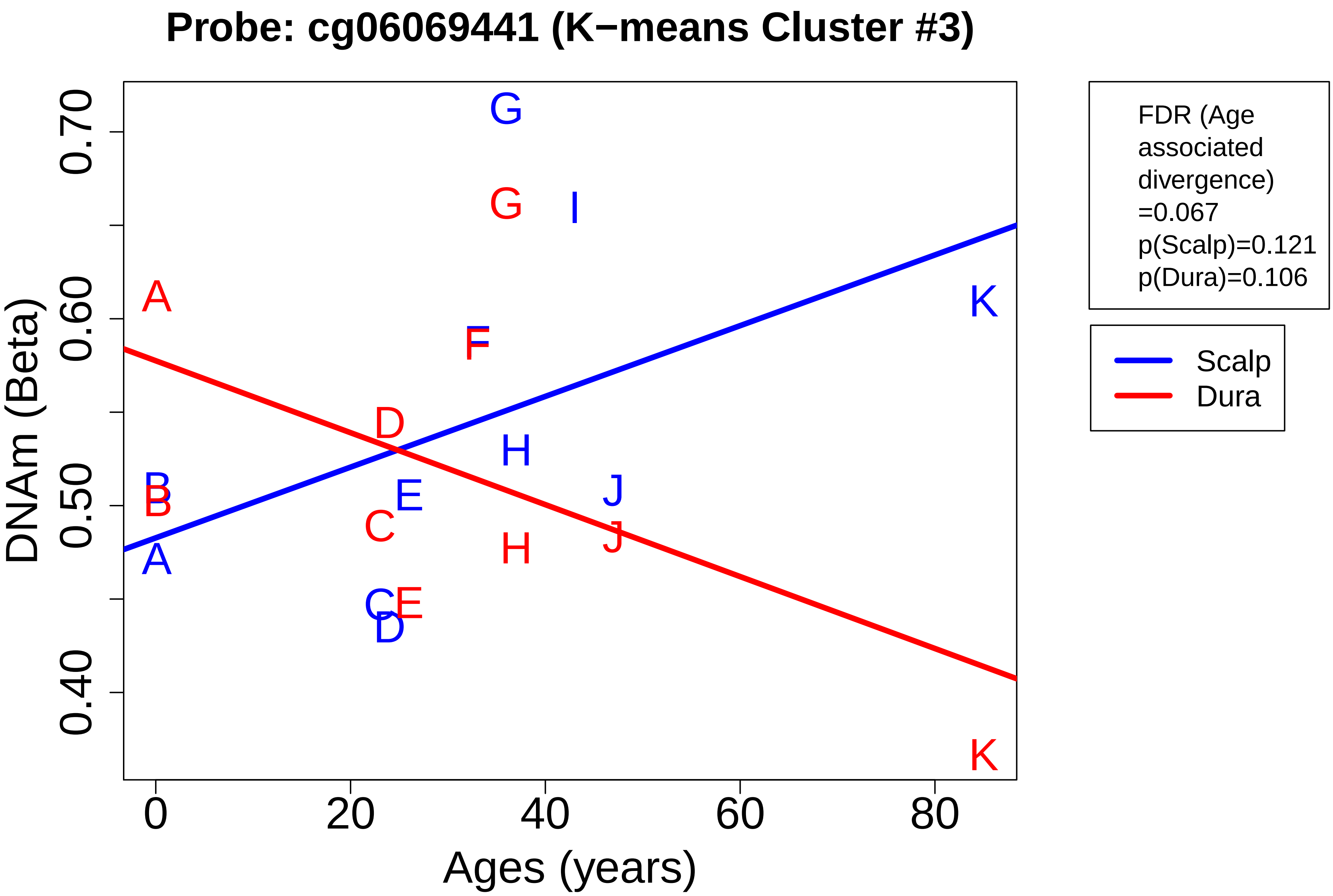
Probe: cg06069441 (K−means Cluster #3)
DNAm (Beta)
0.40
0.50
0.60
0.70
0
20
40
60
80
Ages (years)
A
B
C
D
E
F
G
H
I
J
K
FDR (Age associated divergence) =0.067
p(Scalp)=0.121
p(Dura)=0.106
Scalp
Dura

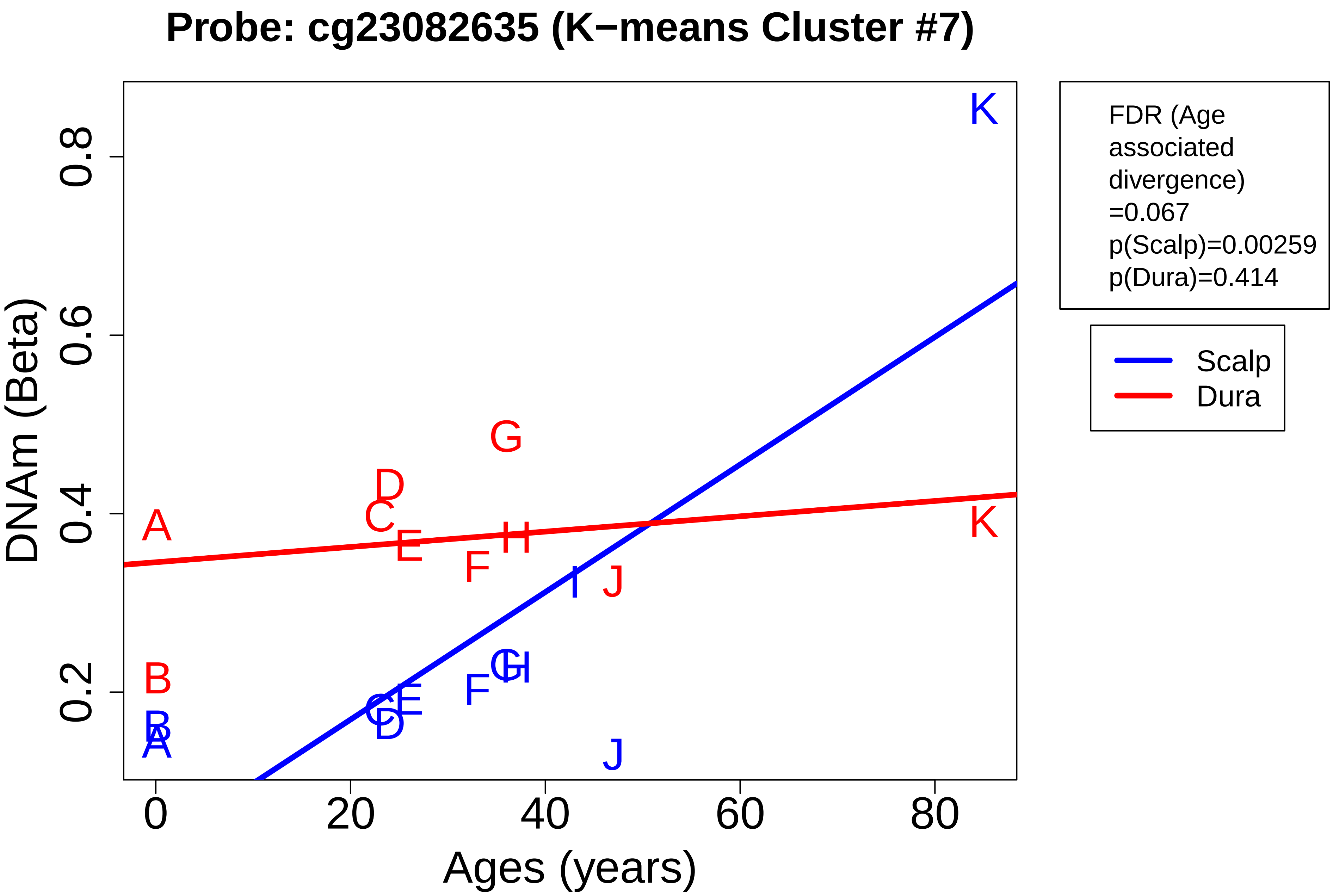
Probe: cg23082635 (K−means Cluster #7)
DNAm (Beta)
0.2
0.4
0.6
0.8
Ages (years)
0
20
40
60
80
FDR (Age associated divergence) =0.067
p(Scalp)=0.00259
p(Dura)=0.414
Scalp
Dura
A
B
C
D
E
F
G
H
I
J
K

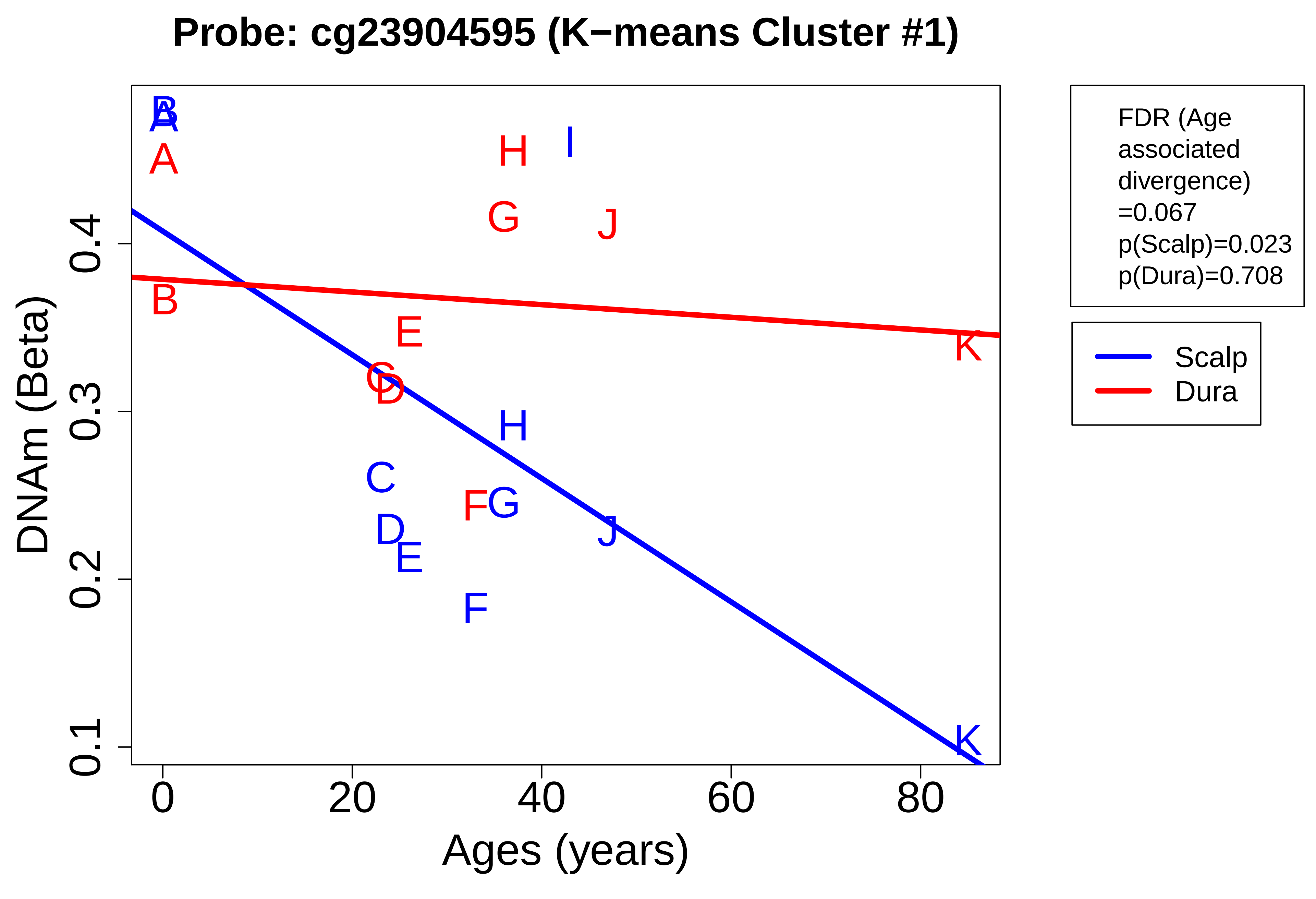

Probe: cg23904595 (K−means Cluster #1)
DNAm (Beta)
0.1
0.2
0.3
0.4
0
20
40
60
80
Ages (years)
A
B
C
D
E
F
G
H
I
J
K
FDR (Age associated divergence) =0.067
p(Scalp)=0.023
p(Dura)=0.708
Scalp
Dura

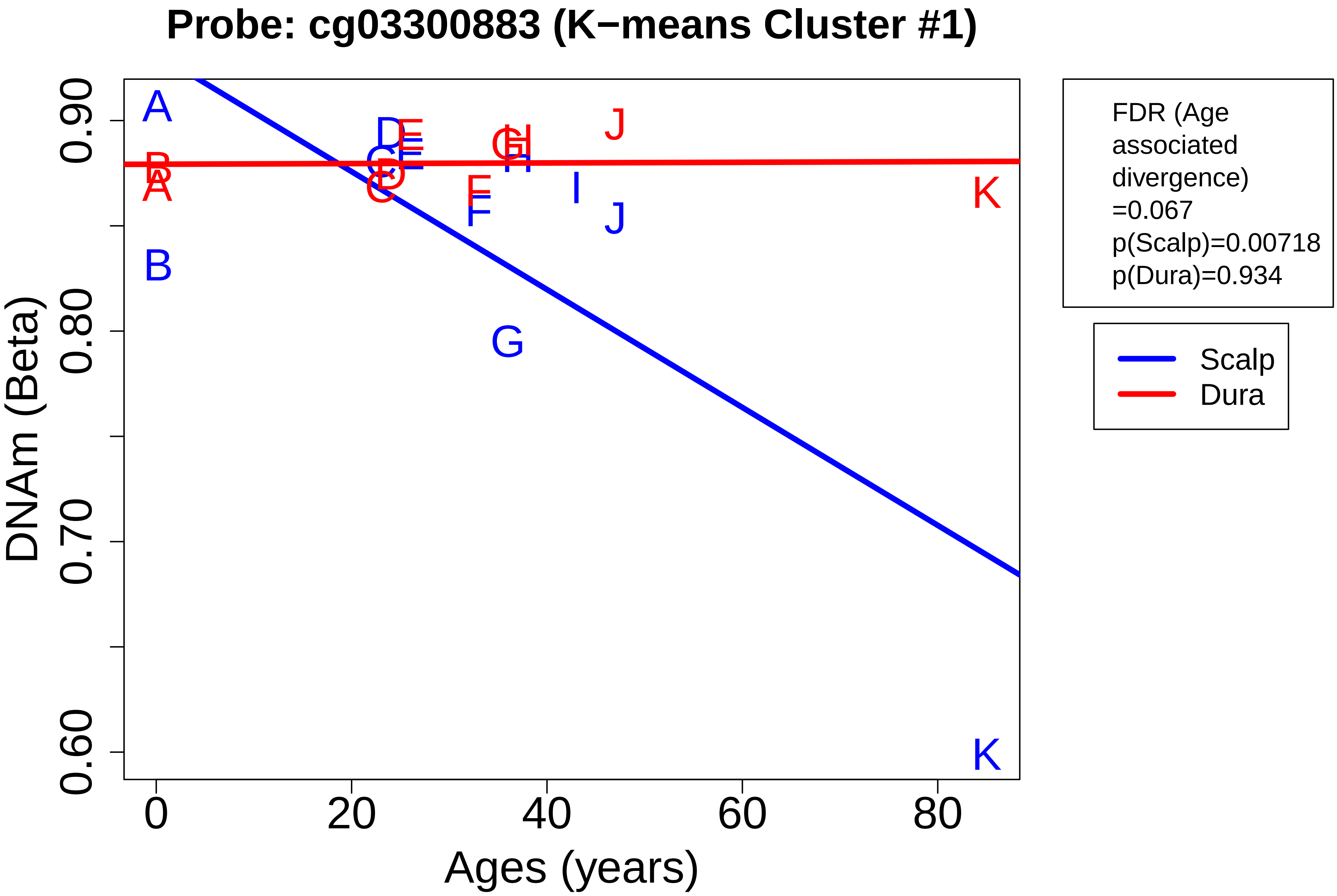

Probe: cg03300883 (K−means Cluster #1)
DNAm (Beta)
0.60
0.70
0.80
0.90
Ages (years)
0
20
40
60
80
FDR (Age associated divergence) =0.067
p(Scalp)=0.00718
p(Dura)=0.934
Scalp
Dura
A
B
C
D
E
F
G
H
I
J
K

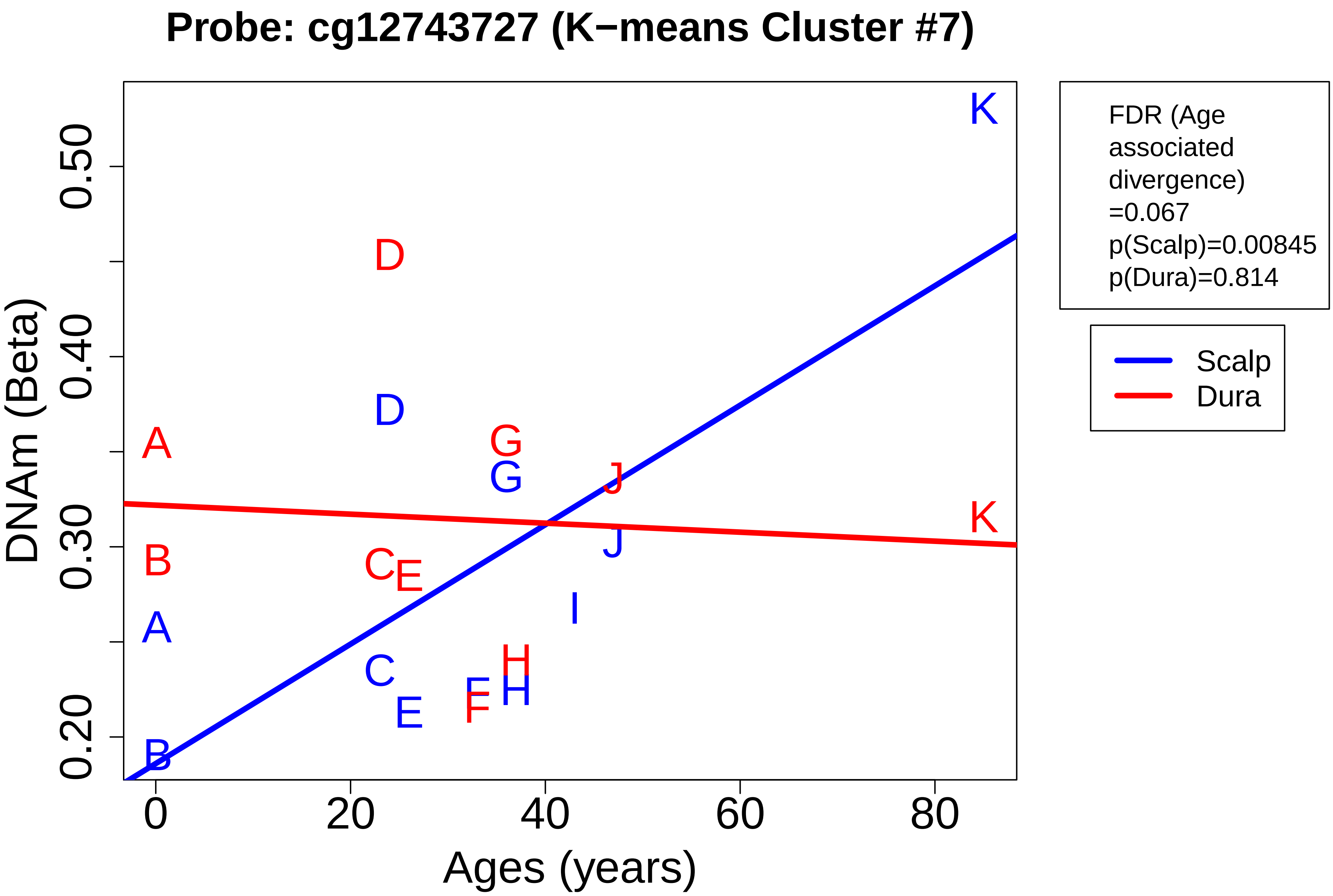

Probe: cg12743727 (K−means Cluster #7)
DNAm (Beta)
0.20
0.30
0.40
0.50
0
20
40
60
80
Ages (years)
FDR (Age associated divergence) =0.067
p(Scalp)=0.00845
p(Dura)=0.814
Scalp
Dura
A
B
C
D
E
F
G
H
I
J
K

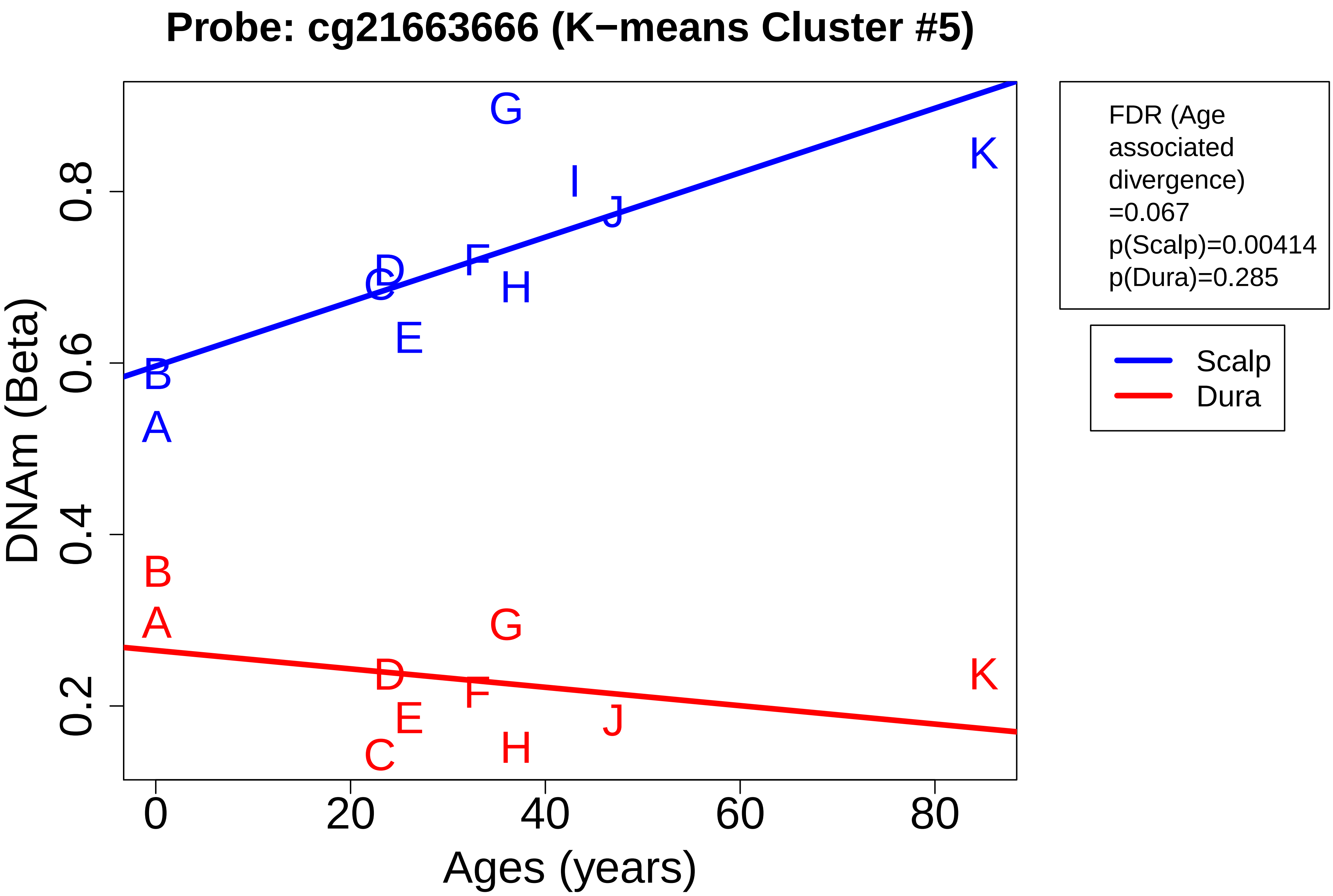

Probe: cg21663666 (K−means Cluster #5)
FDR (Age associated divergence) =0.067
p(Scalp)=0.00414
p(Dura)=0.285
Scalp
Dura
DNAm (Beta)
0.2
0.4
0.6
0.8
Ages (years)
0
20
40
60
80
A B C D E F G H I J K

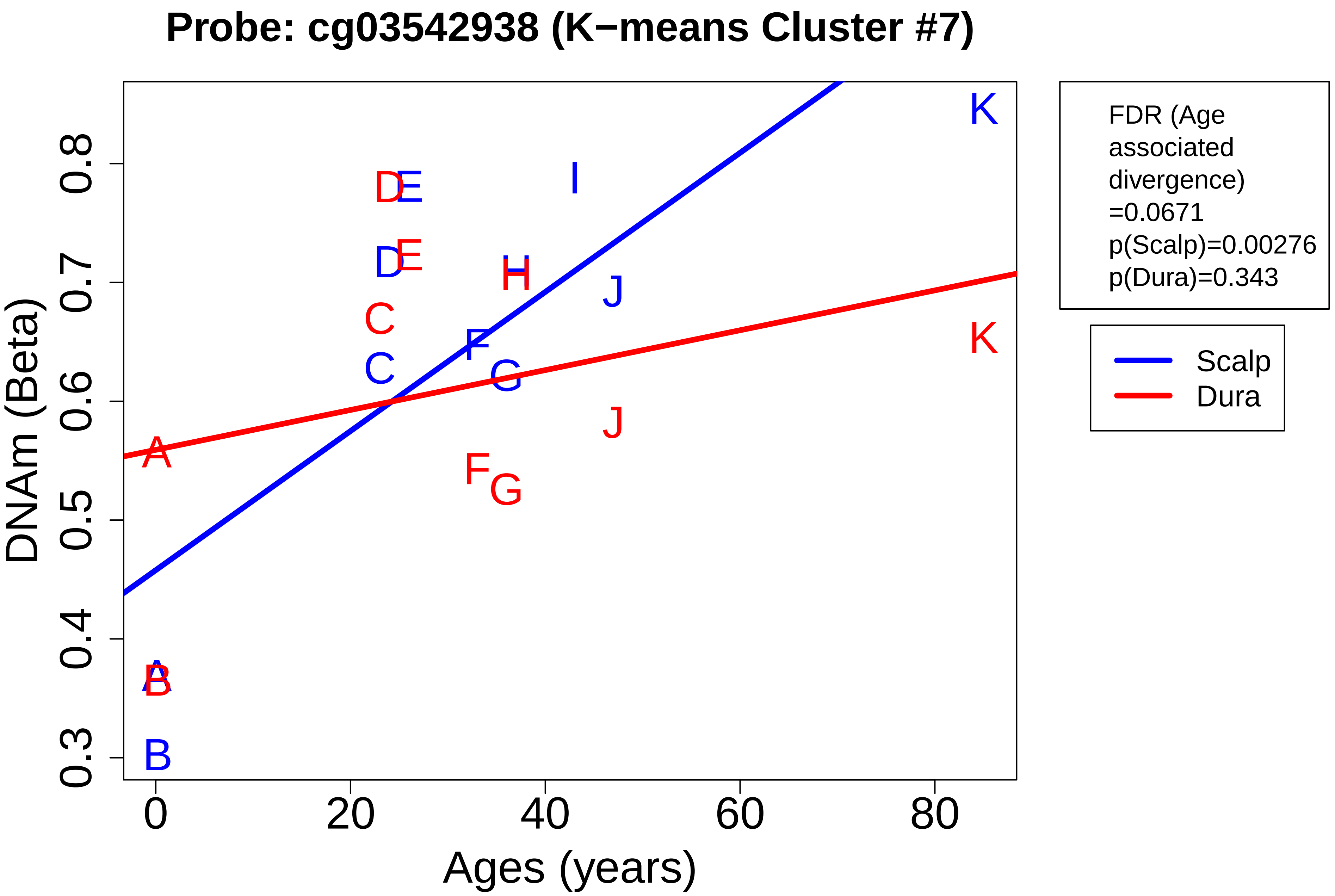
Probe: cg03542938 (K−means Cluster #7)
DNAm (Beta)
0.3
0.4
0.5
0.6
0.7
0.8
0
20
40
60
80
Ages (years)
FDR (Age associated divergence) =0.0671
p(Scalp)=0.00276
p(Dura)=0.343
Scalp
Dura

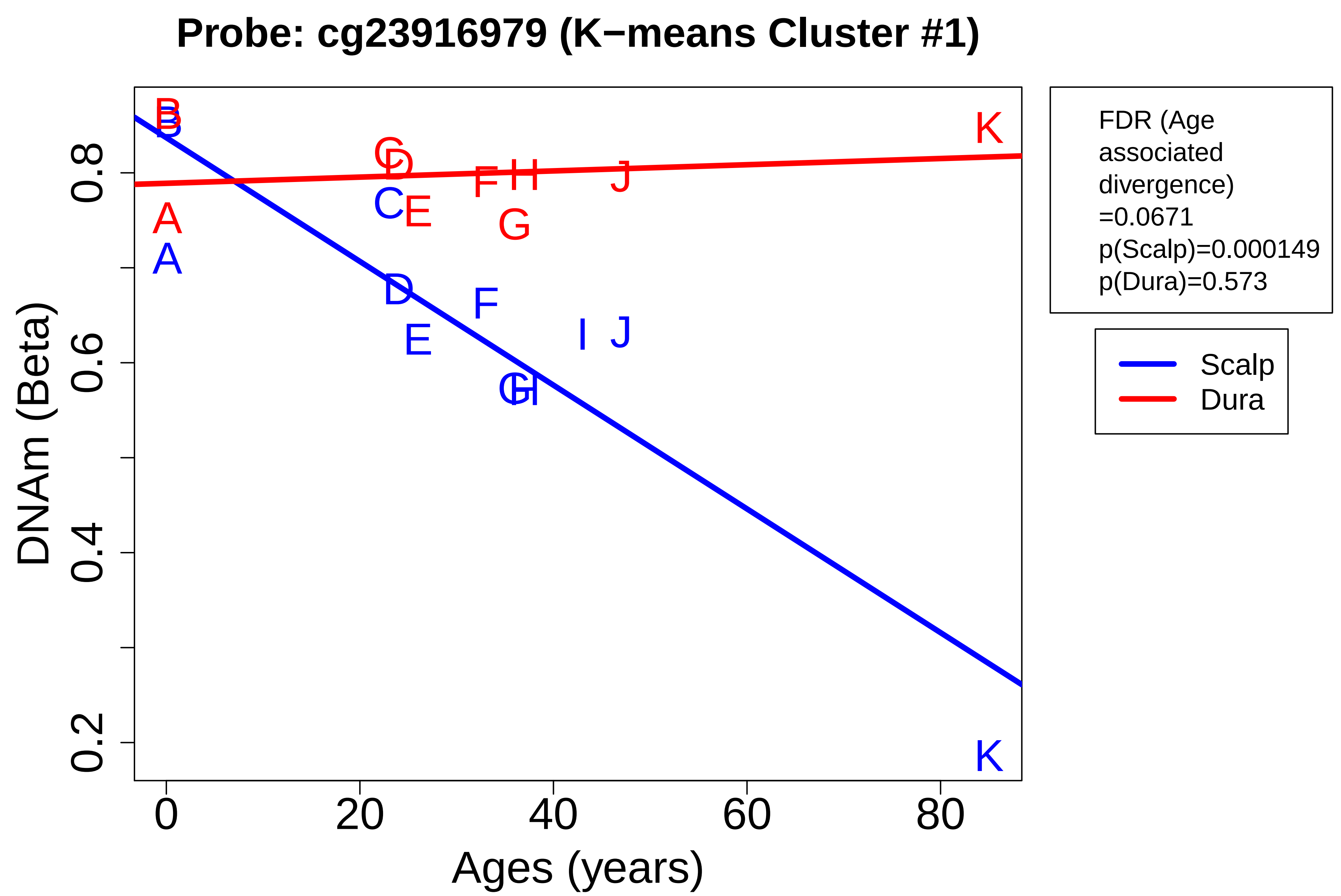
Probe: cg23916979 (K−means Cluster #1)
DNAm (Beta)
0.2
0.4
0.6
0.8
Ages (years)
0
20
40
60
80
FDR (Age associated divergence) =0.0671
p(Scalp)=0.000149
p(Dura)=0.573
Scalp
Dura
A
B
C
D
E
F
G
H
I
J
K

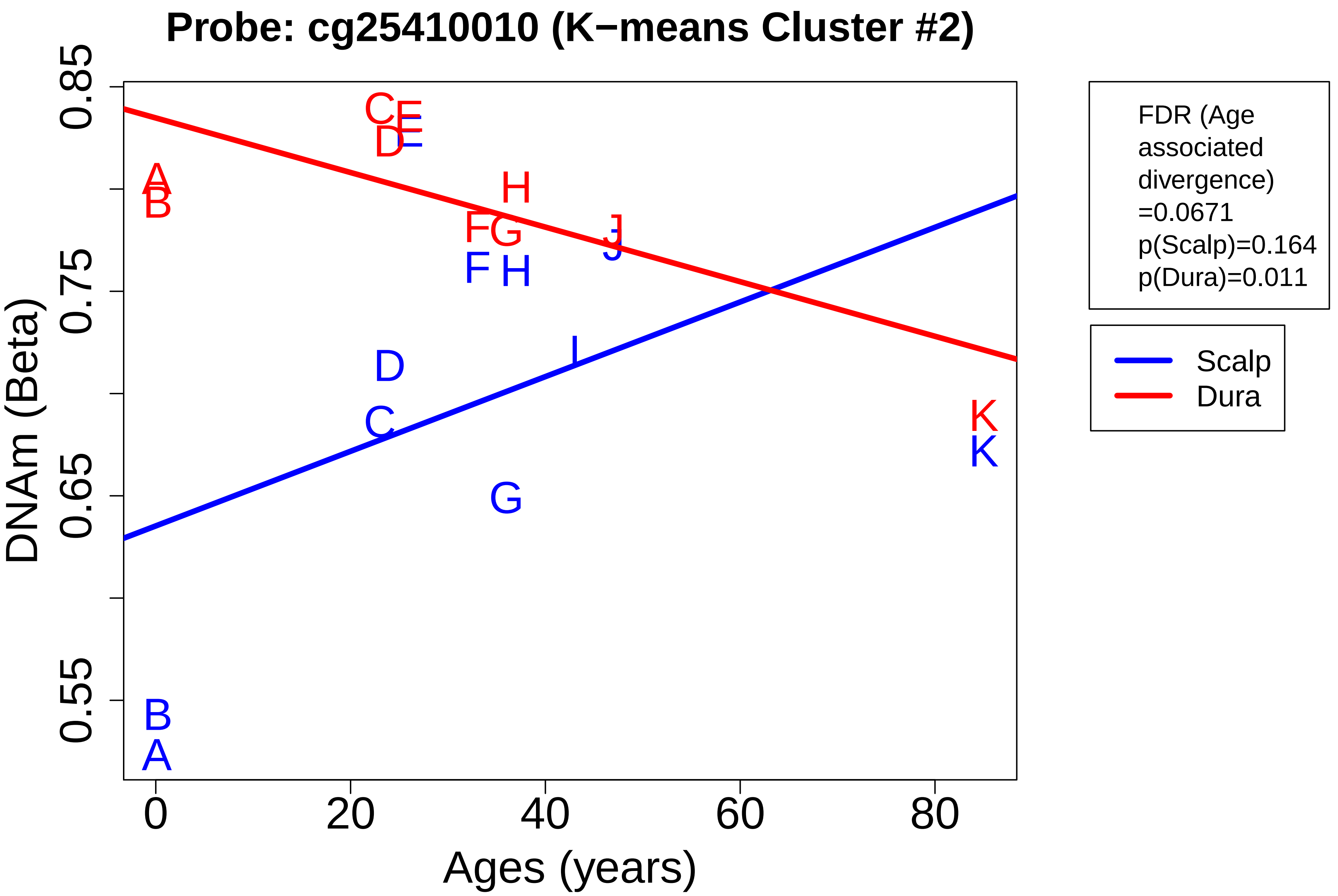

Probe: cg25410010 (K−means Cluster #2)
DNAm (Beta)
0.55
0.65
0.75
0.85
Ages (years)
0
20
40
60
80
FDR (Age associated divergence) =0.0671
p(Scalp)=0.164
p(Dura)=0.011
Scalp
Dura

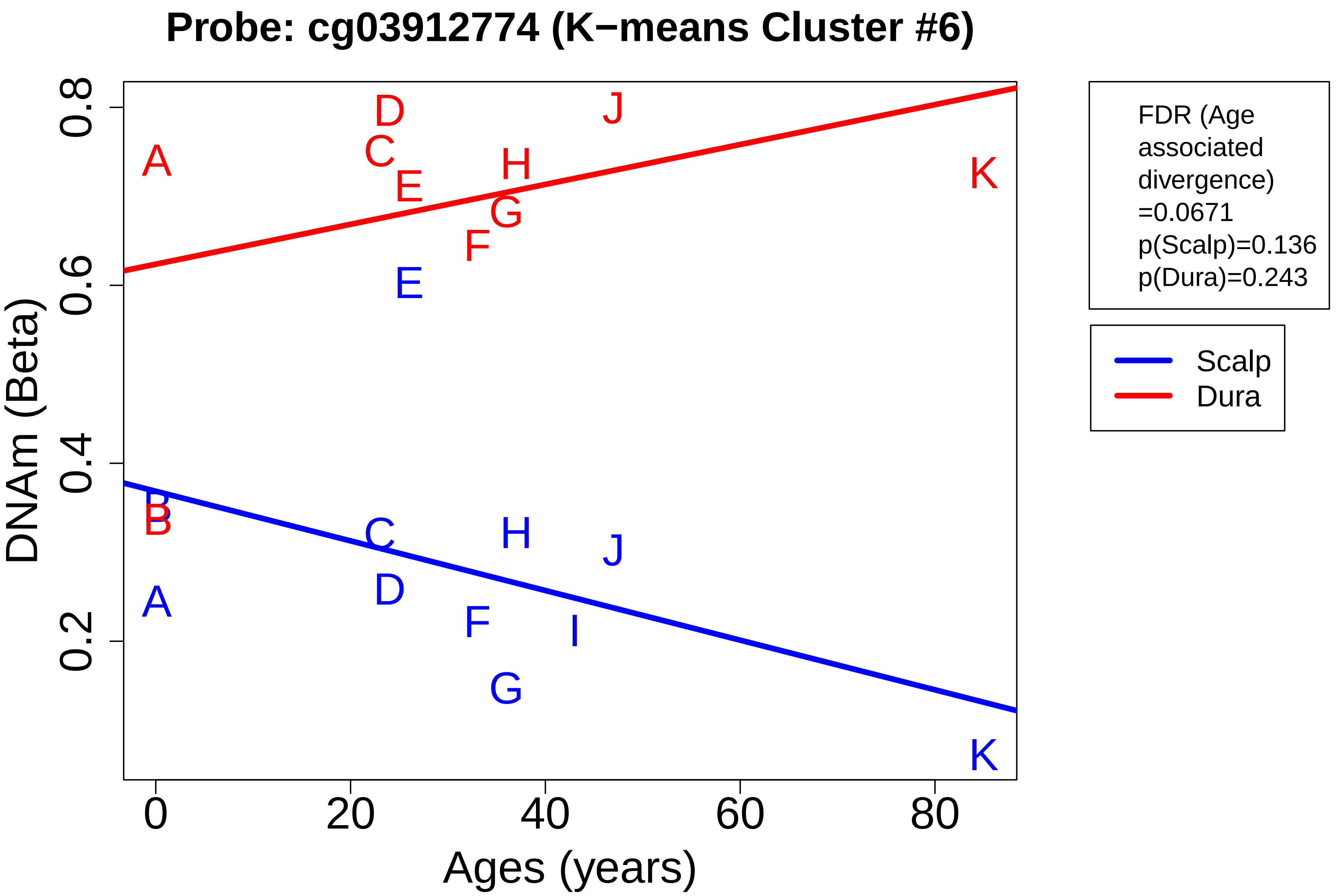

Probe: cg03912774 (K−means Cluster #6)
DNAm (Beta)
0.2
0.4
0.6
0.8
Ages (years)
0
20
40
60
80
FDR (Age associated divergence) =0.0671
p(Scalp)=0.136
p(Dura)=0.243
Scalp
Dura
A
B
C
D
E
F
G
H
I
J
K

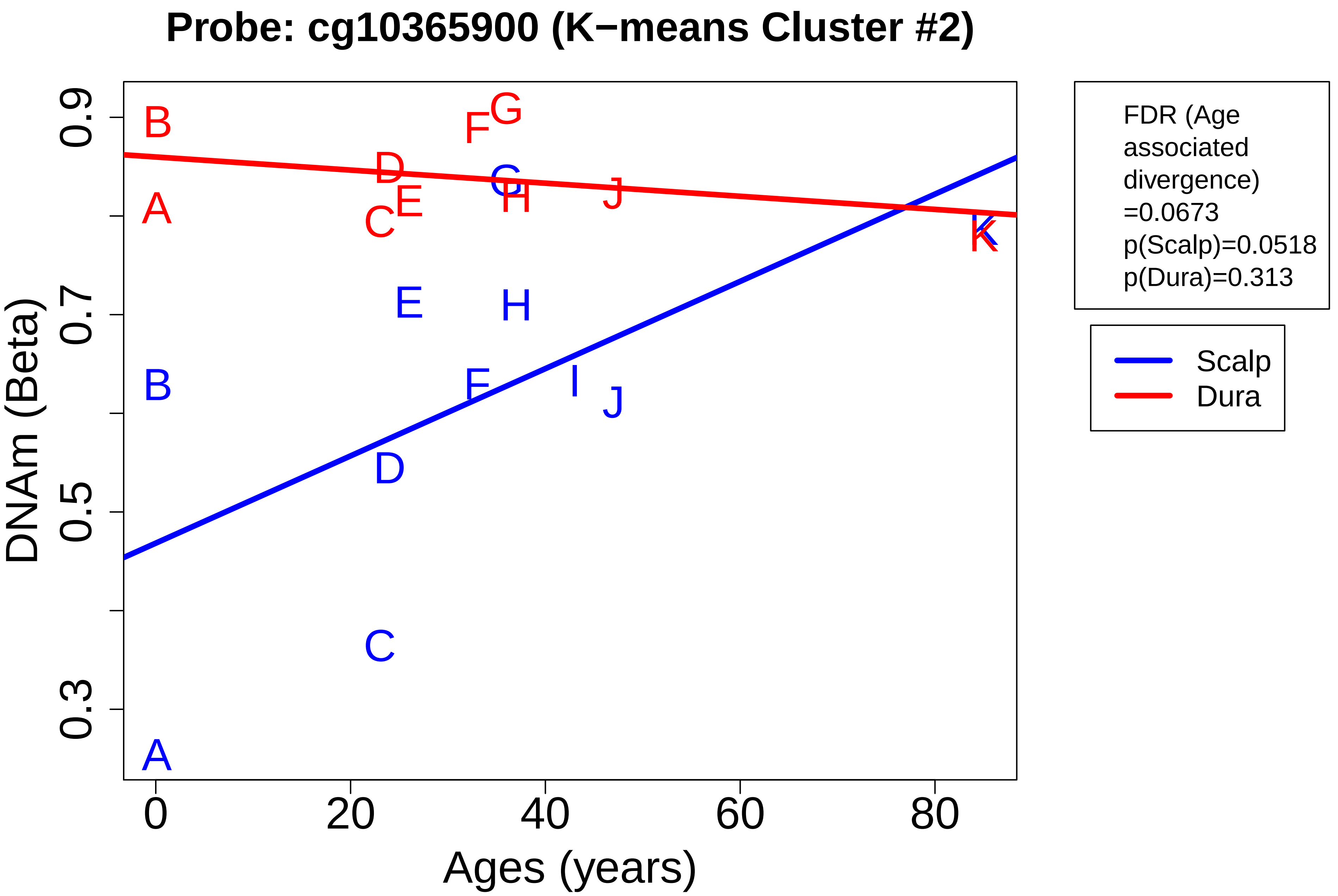

Probe: cg10365900 (K−means Cluster #2)
DNAm (Beta)
0.3
0.5
0.7
0.9
Ages (years)
0
20
40
60
80
FDR (Age associated divergence) =0.0673
p(Scalp)=0.0518
p(Dura)=0.313
Scalp
Dura

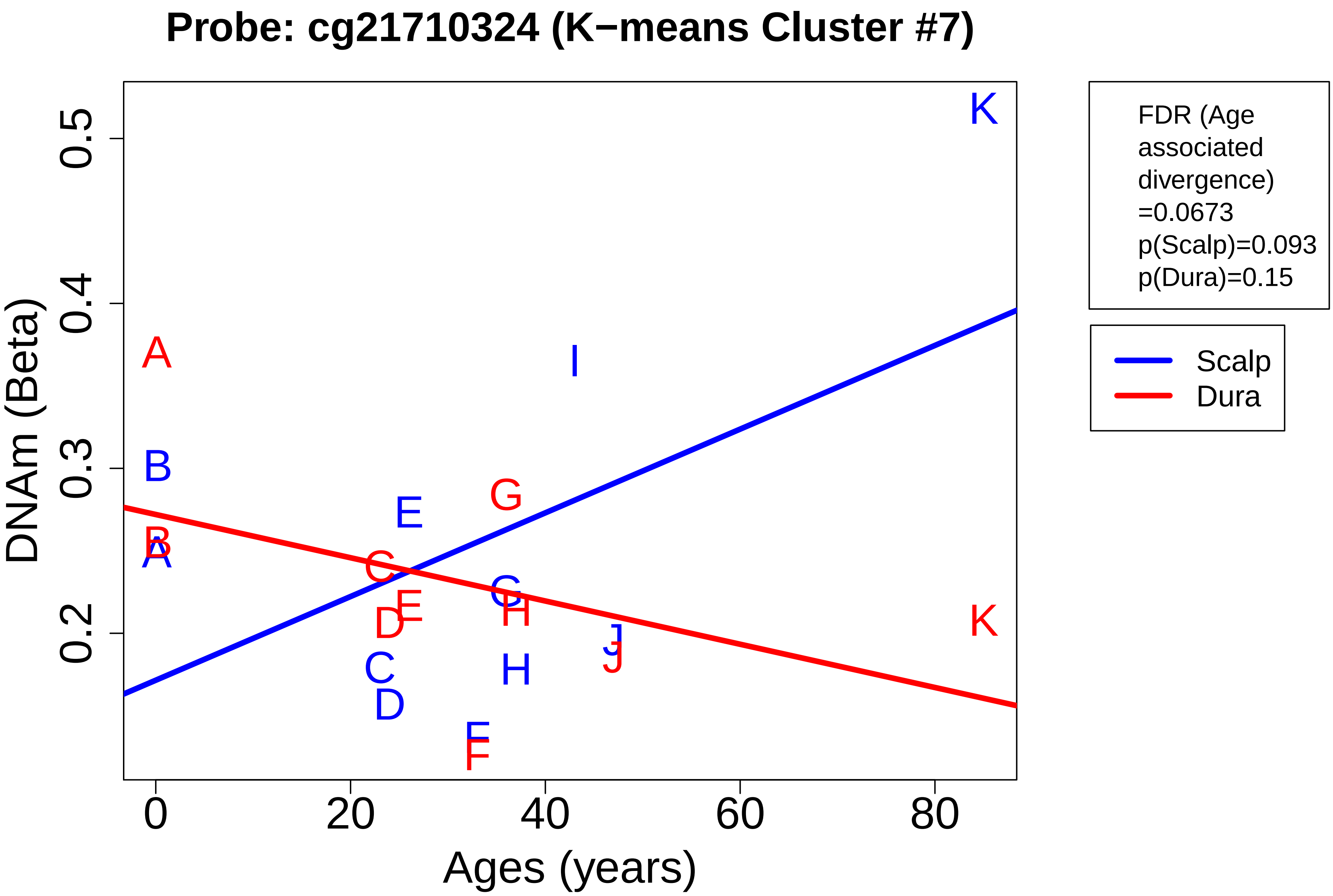

Probe: cg21710324 (K−means Cluster #7)
DNAm (Beta)
0.2
0.3
0.4
0.5
0
20
40
60
80
Ages (years)
FDR (Age associated divergence) =0.0673
p(Scalp)=0.093
p(Dura)=0.15
Scalp
Dura

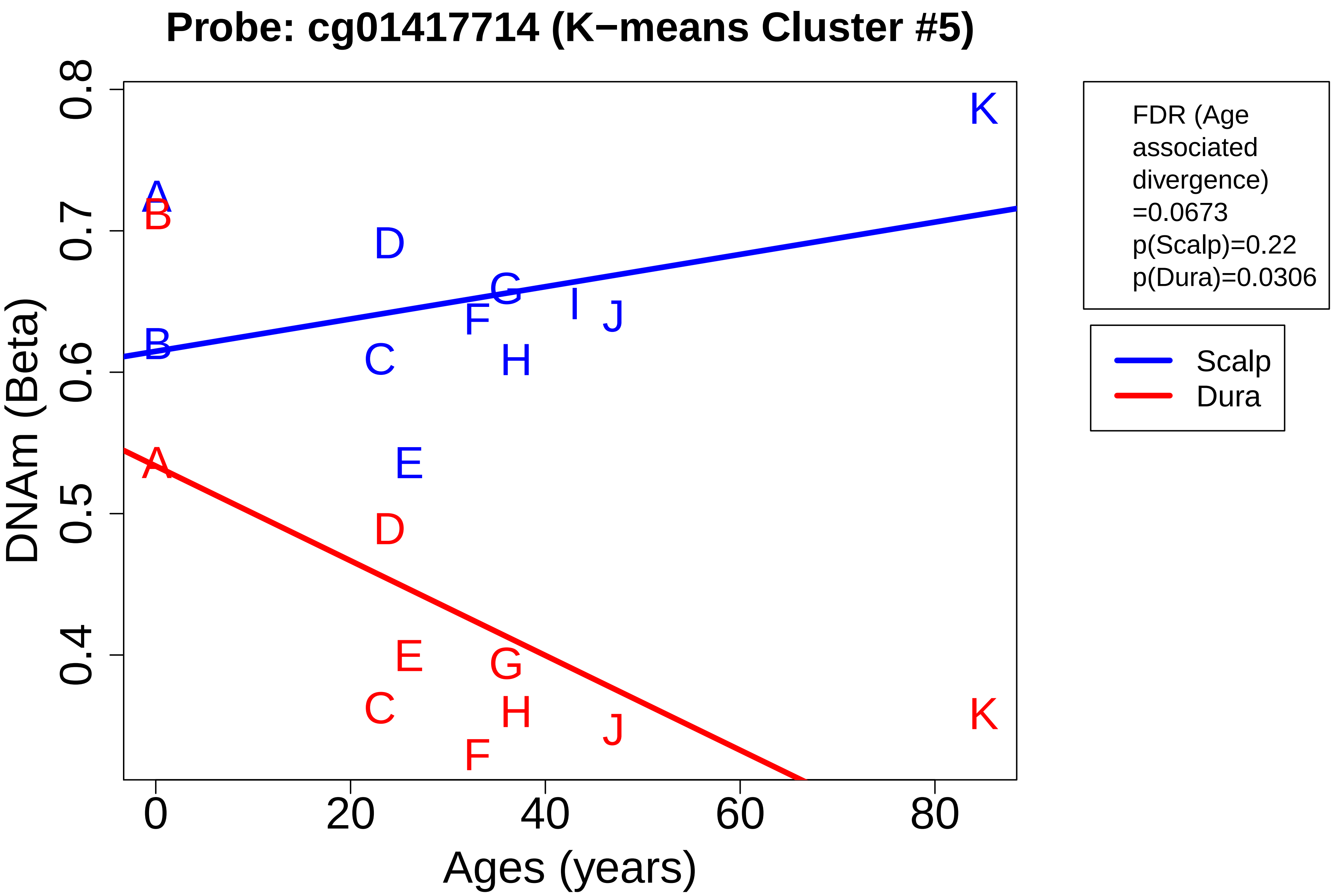

Probe: cg01417714 (K−means Cluster #5)
DNAm (Beta)
0.4
0.5
0.6
0.7
0.8
0
20
40
60
80
Ages (years)
A
B
C
D
E
F
G
H
I
J
K
FDR (Age associated divergence) =0.0673
p(Scalp)=0.22
p(Dura)=0.0306
Scalp
Dura

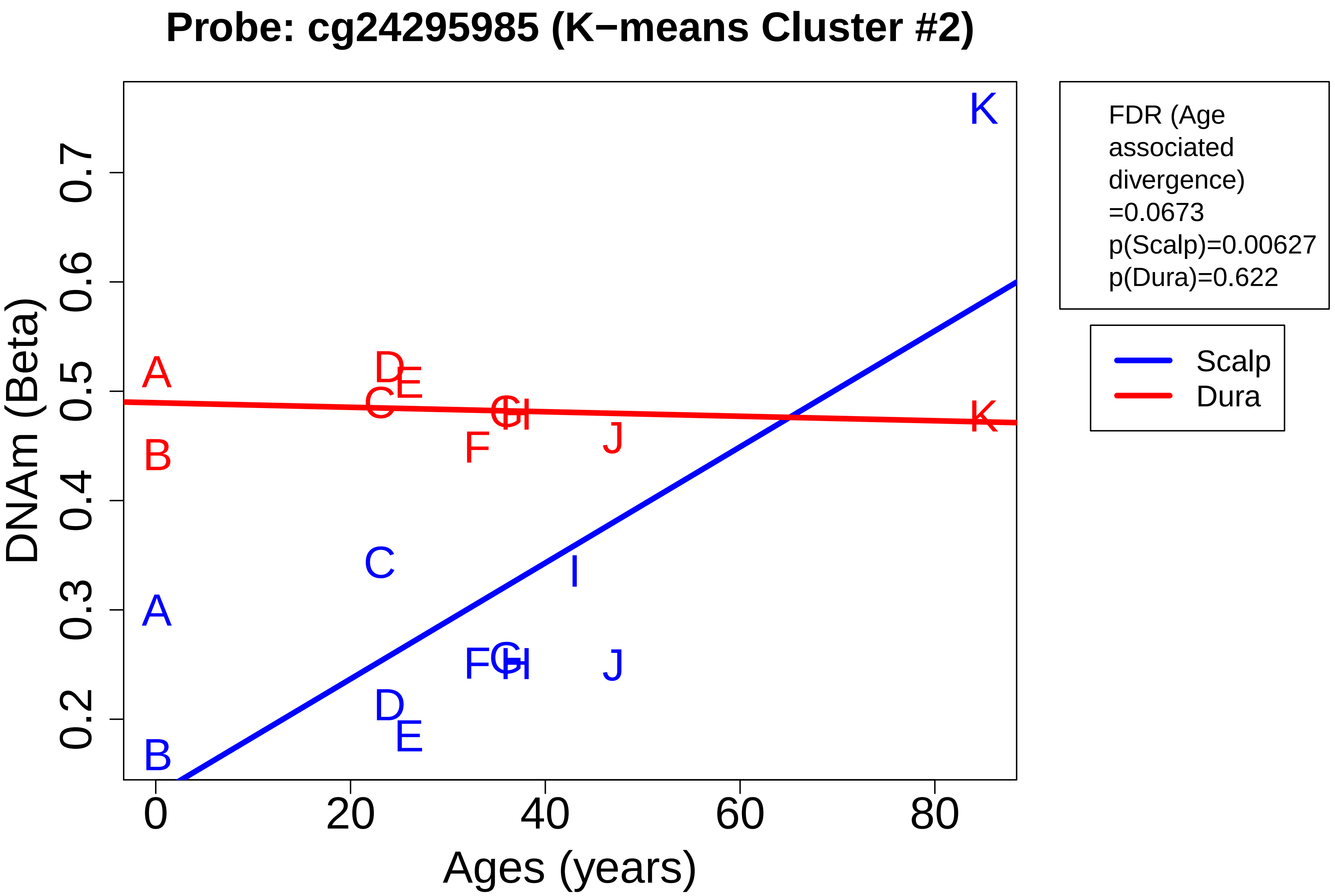

Probe: cg24295985 (K−means Cluster #2)
DNAm (Beta)
0.2
0.3
0.4
0.5
0.6
0.7
Ages (years)
0
20
40
60
80
FDR (Age associated divergence) =0.0673
p(Scalp)=0.00627
p(Dura)=0.622
Scalp
Dura

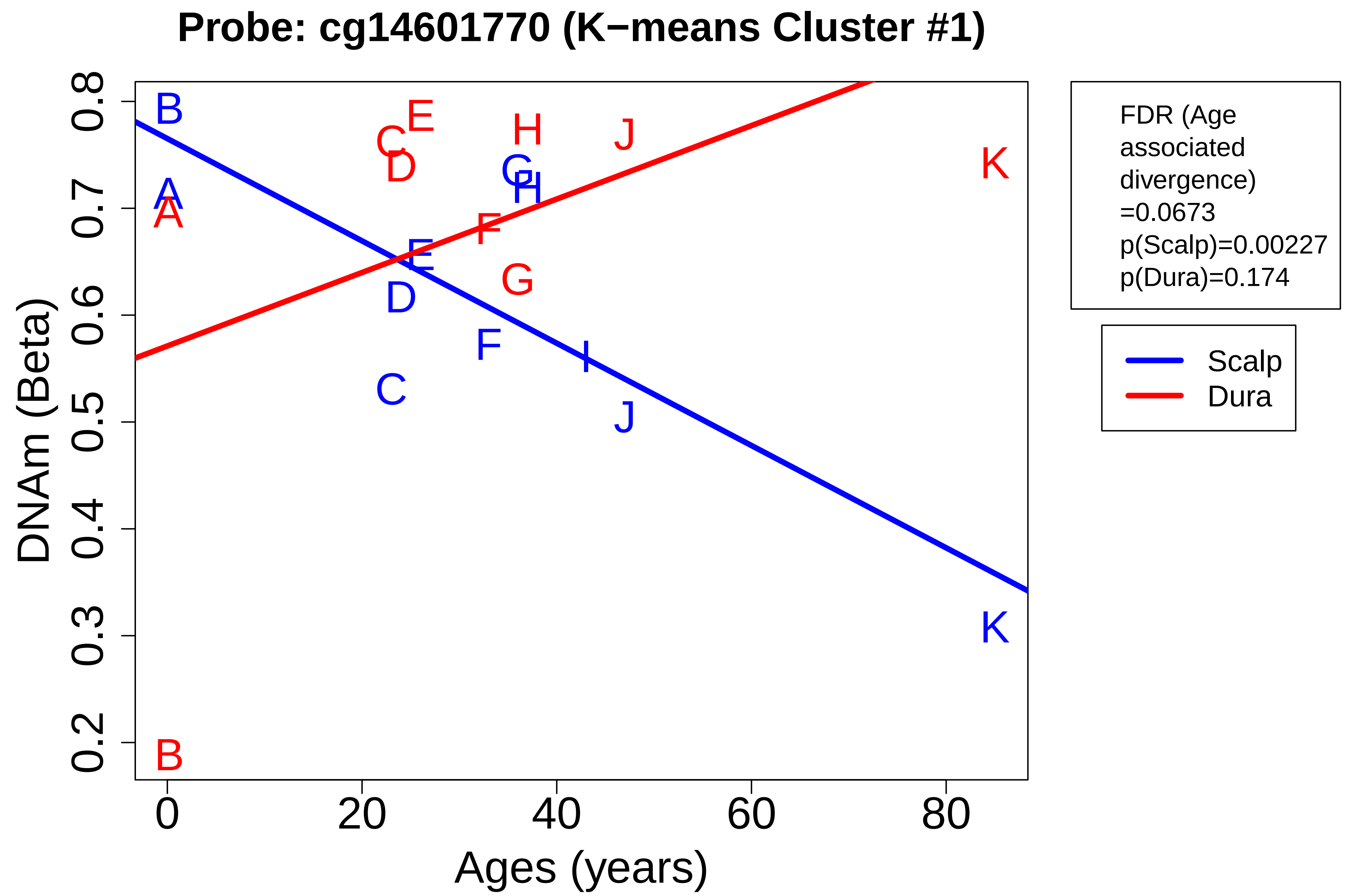

Probe: cg14601770 (K−means Cluster #1)
DNAm (Beta)
Ages (years)
0.2
0.3
0.4
0.5
0.6
0.7
0.8
0
20
40
60
80
FDR (Age associated divergence) =0.0673
p(Scalp)=0.00227
p(Dura)=0.174
Scalp
Dura

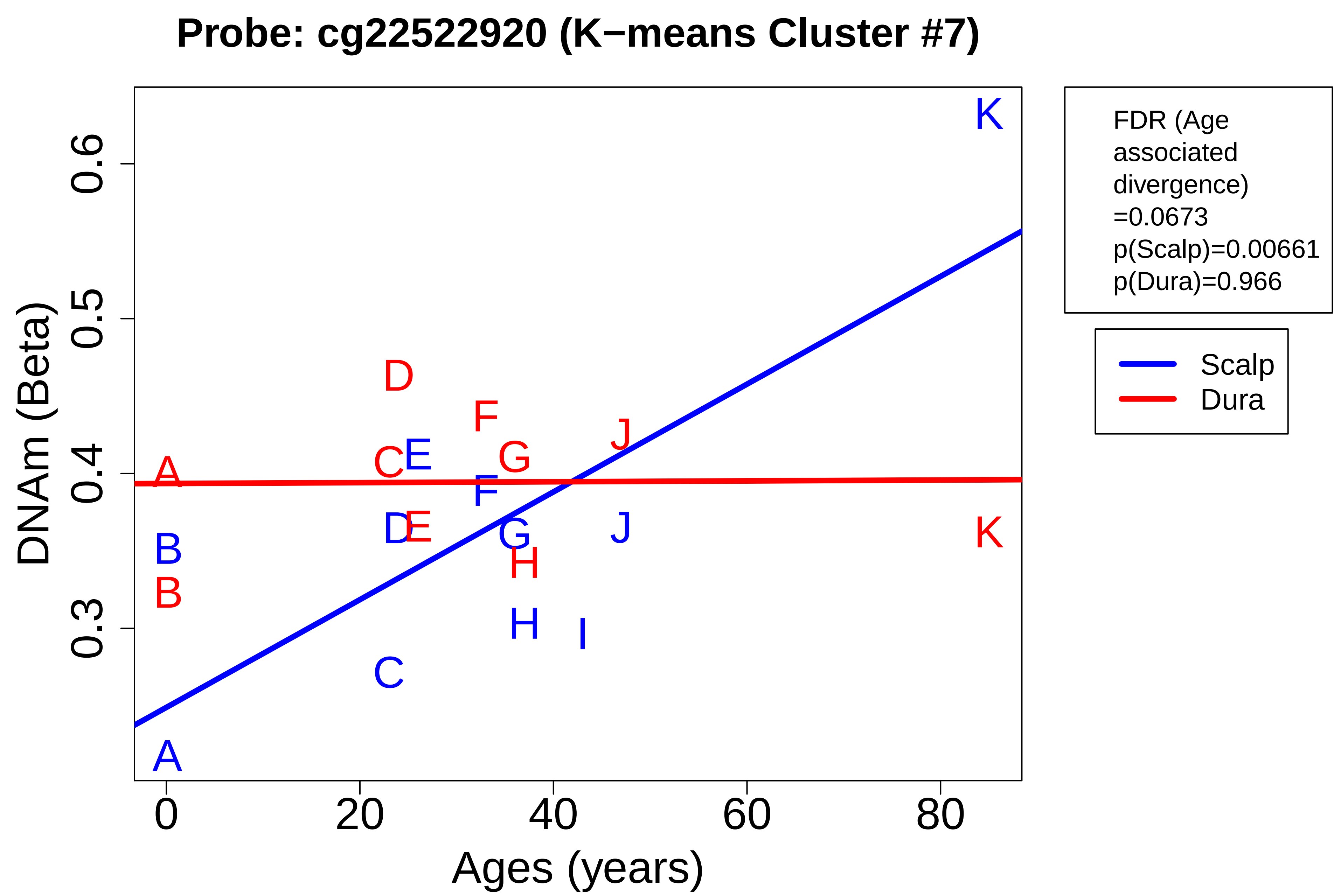

Probe: cg22522920 (K−means Cluster #7)
DNAm (Beta)
0.3
0.4
0.5
0.6
Ages (years)
0
20
40
60
80
A
B
C
D
E
F
G
H
I
J
K
FDR (Age associated divergence) =0.0673
p(Scalp)=0.00661
p(Dura)=0.966
Scalp
Dura

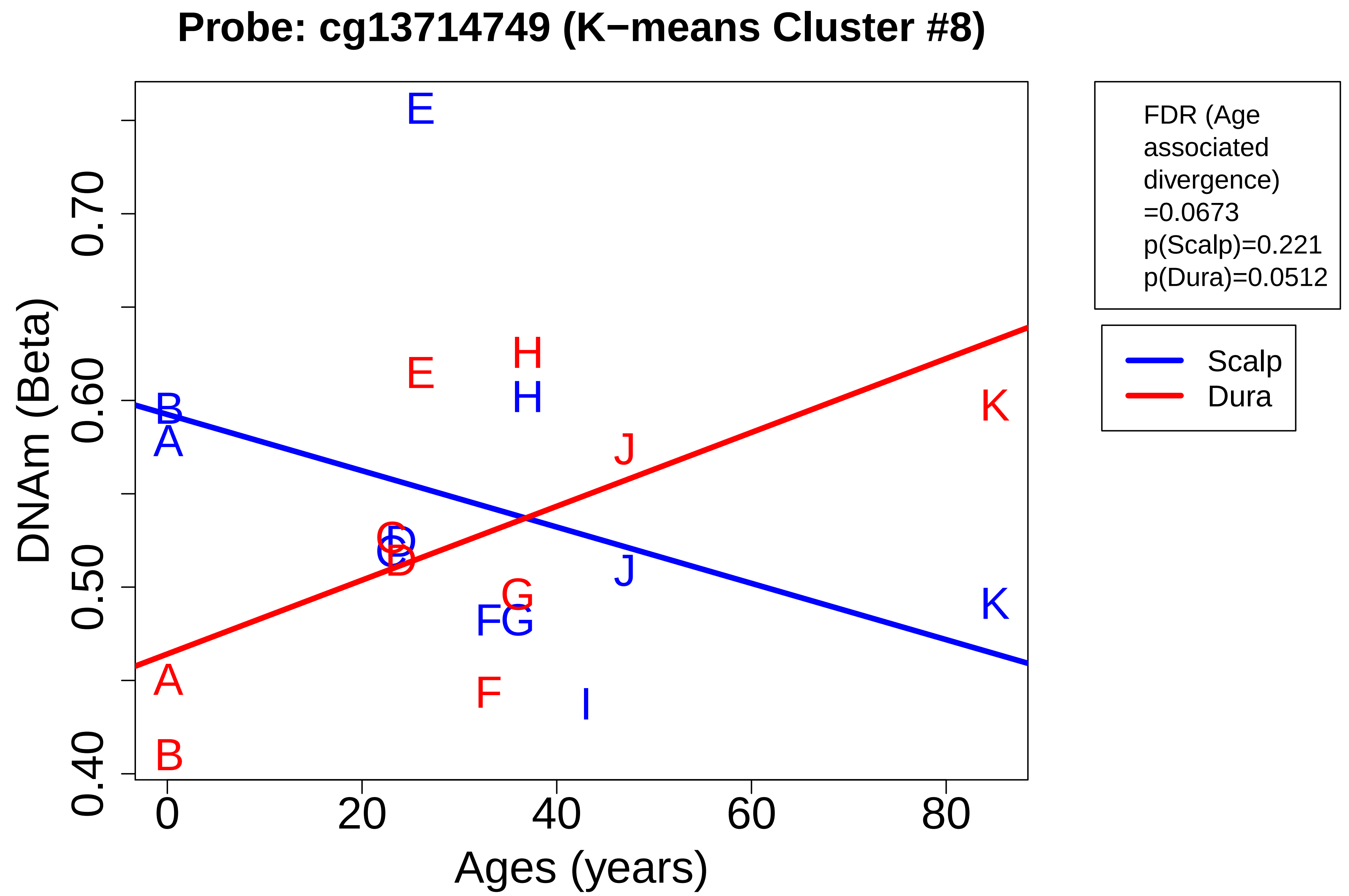

Probe: cg13714749 (K−means Cluster #8)
DNAm (Beta)
0.40
0.50
0.60
0.70
0
20
40
60
80
Ages (years)
A
B
C
D
E
F
G
H
I
J
K
FDR (Age associated divergence) =0.0673
p(Scalp)=0.221
p(Dura)=0.0512
Scalp
Dura

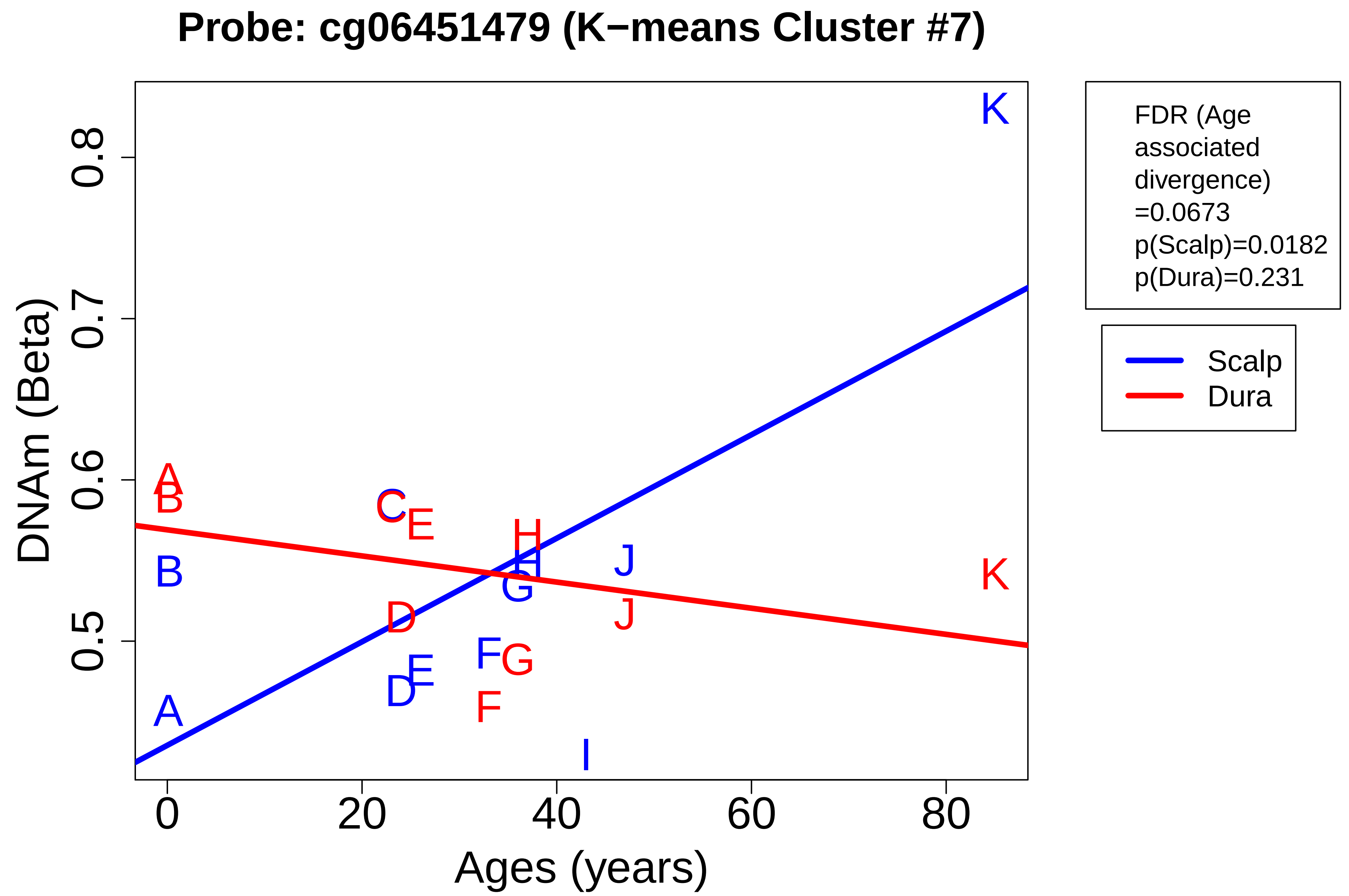

Probe: cg06451479 (K−means Cluster #7)
DNAm (Beta)
0.5
0.6
0.7
0.8
0
20
40
60
80
Ages (years)
FDR (Age associated divergence) =0.0673
p(Scalp)=0.0182
p(Dura)=0.231
Scalp
Dura

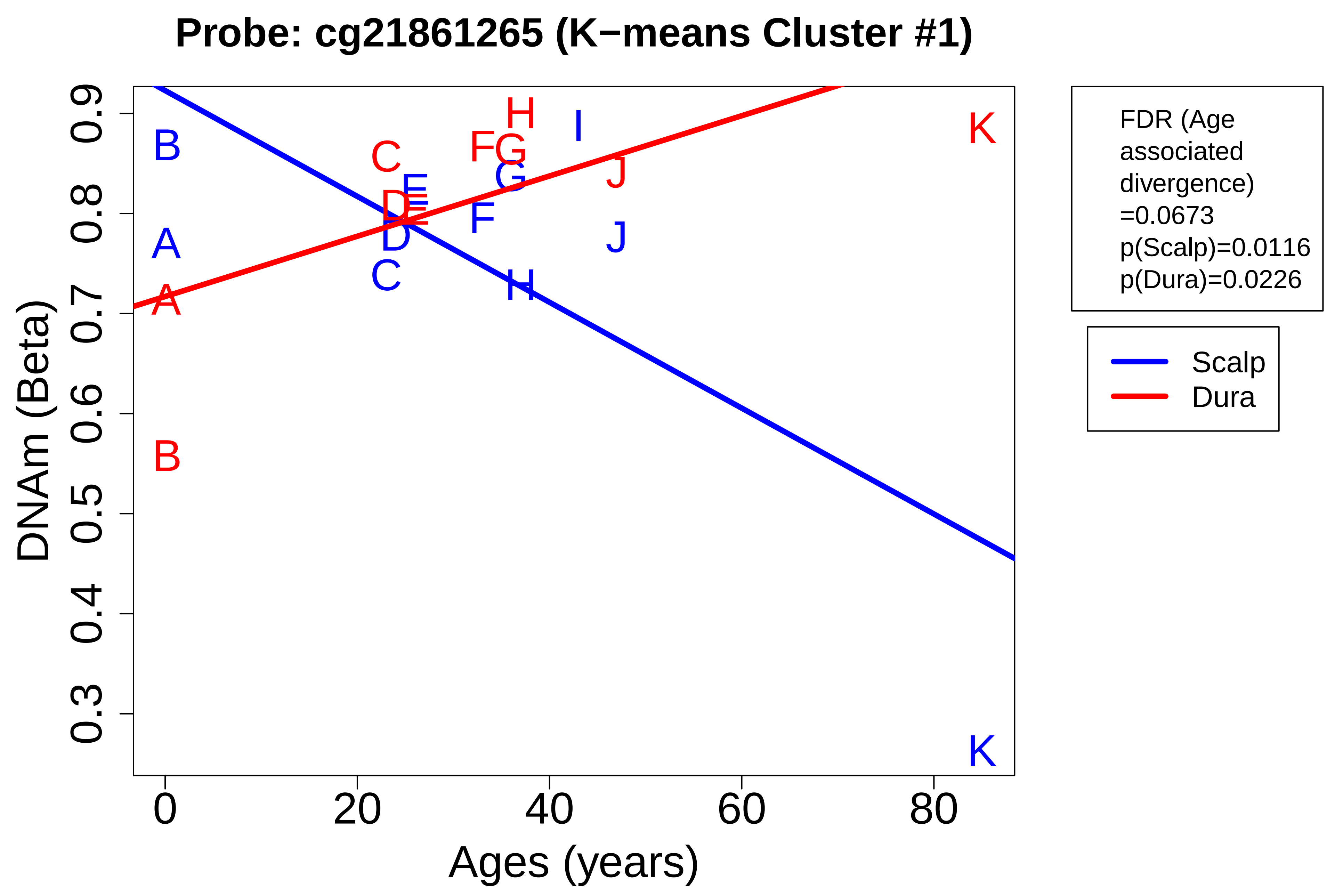

Probe: cg21861265 (K−means Cluster #1)
DNAm (Beta)
Ages (years)
0.3
0.4
0.5
0.6
0.7
0.8
0.9
0
20
40
60
80
FDR (Age associated divergence) =0.0673
p(Scalp)=0.0116
p(Dura)=0.0226
Scalp
Dura
A
B
C
D
E
F
G
H
I
J
K

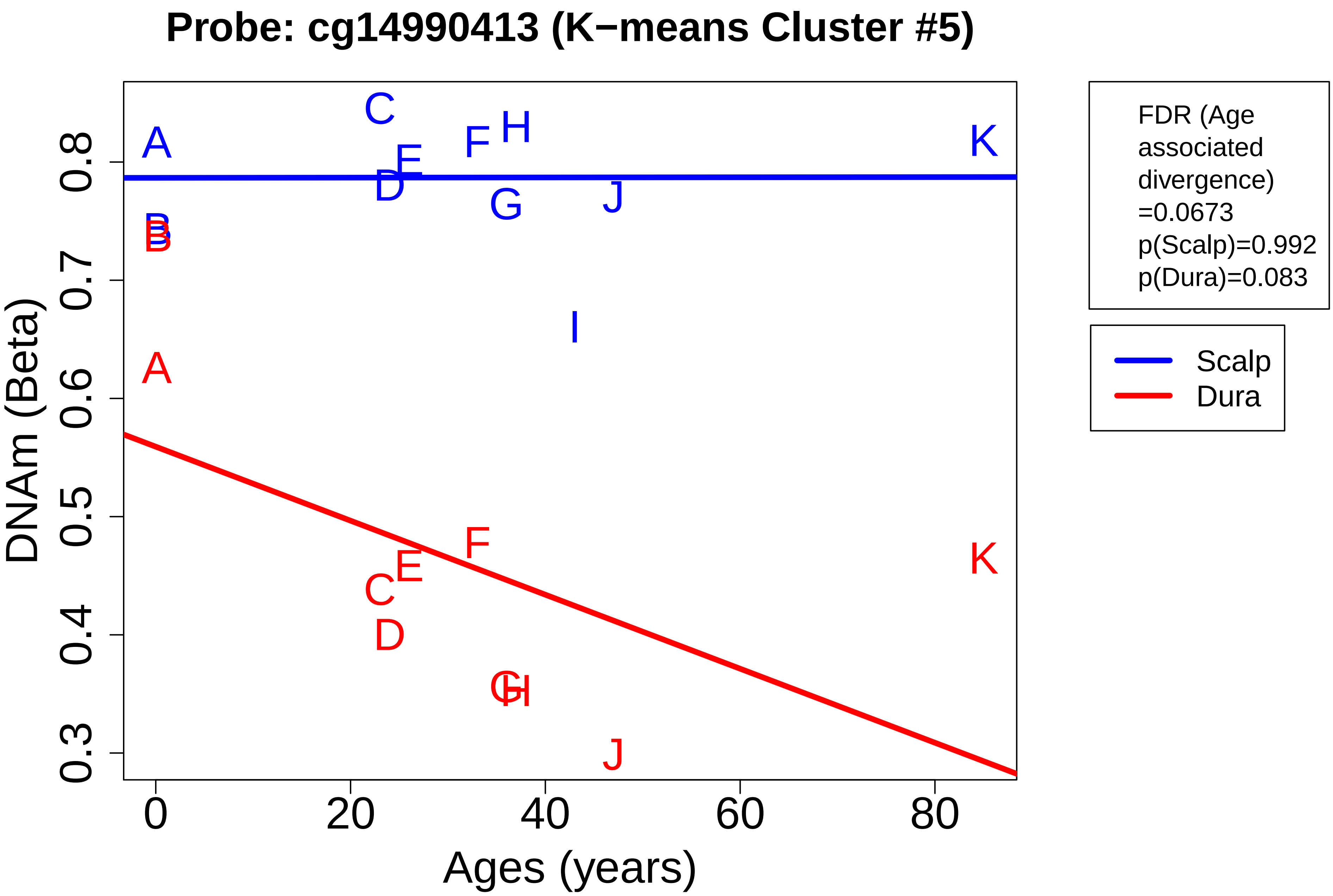

Probe: cg14990413 (K−means Cluster #5)
DNAm (Beta)
0.3
0.4
0.5
0.6
0.7
0.8
0
20
40
60
80
Ages (years)
A
B
C
D
E
F
G
H
I
J
K
FDR (Age associated divergence) =0.0673
p(Scalp)=0.992
p(Dura)=0.083
Scalp
Dura

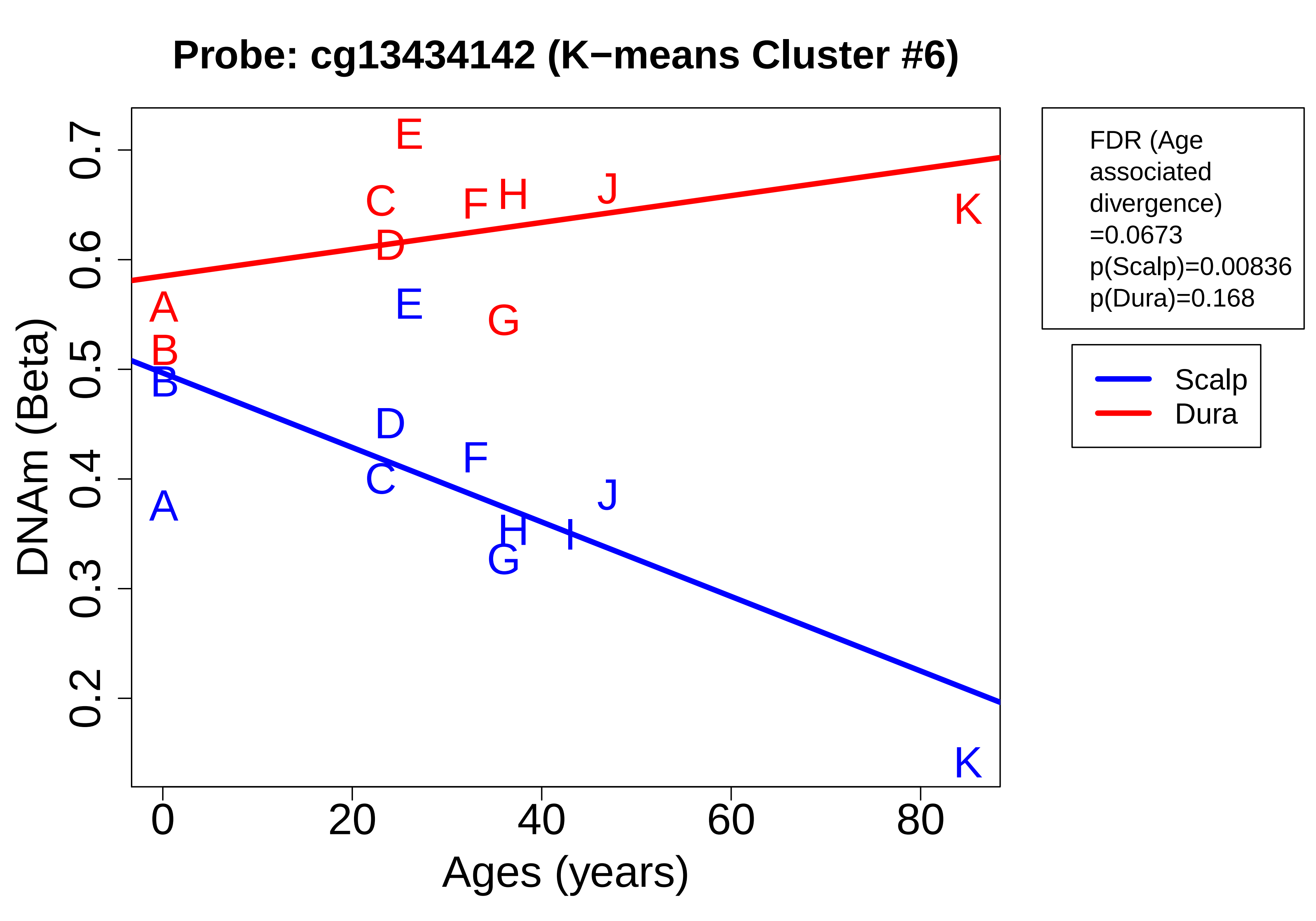
Probe: cg13434142 (K−means Cluster #6)
DNAm (Beta)
Ages (years)
0.2
0.3
0.4
0.5
0.6
0.7
0
20
40
60
80
FDR (Age associated divergence) =0.0673
p(Scalp)=0.00836
p(Dura)=0.168
Scalp
Dura
A
B
C
D
E
F
G
H
I
J
K

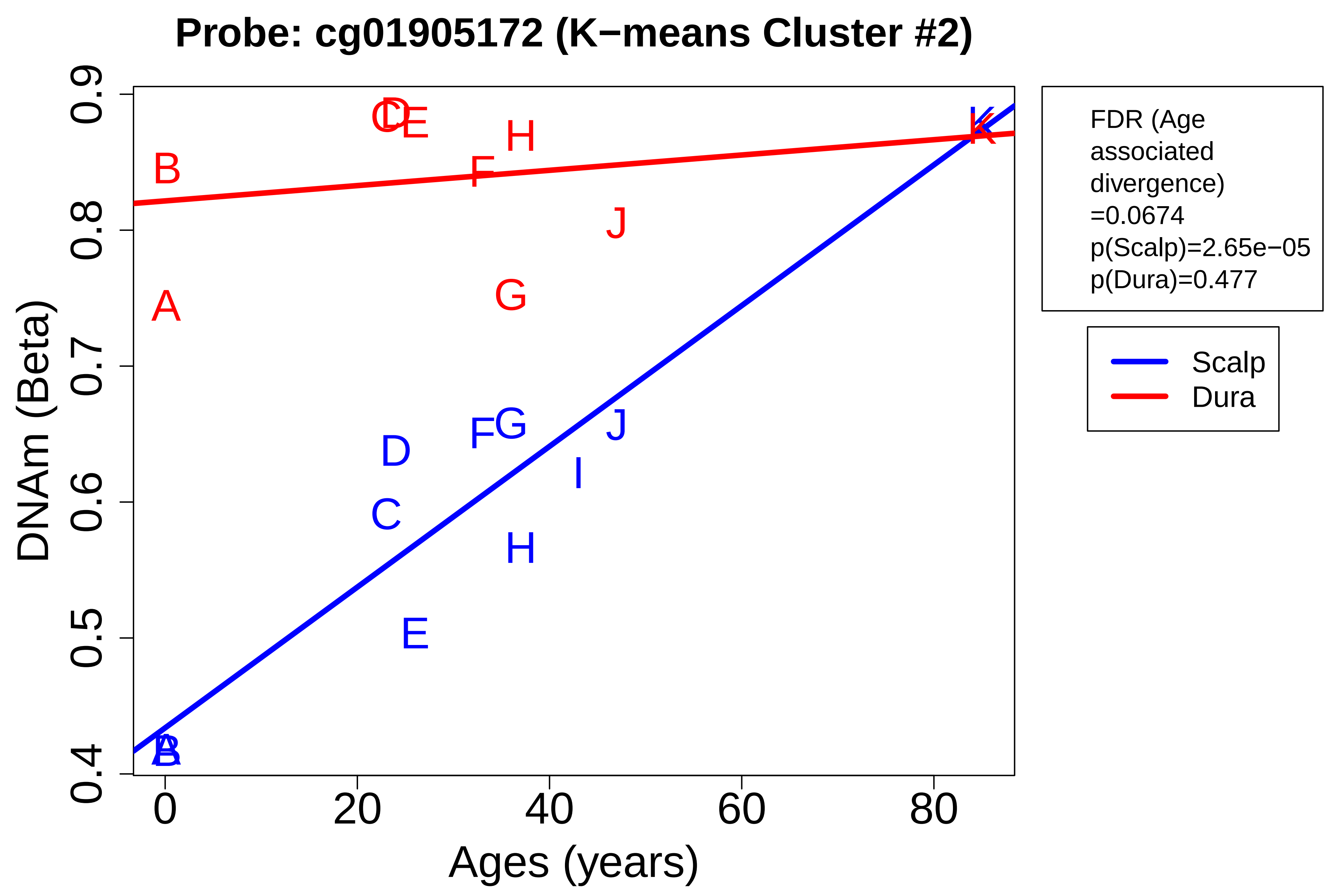
Probe: cg01905172 (K−means Cluster #2)
DNAm (Beta)
0.4
0.5
0.6
0.7
0.8
0.9
Ages (years)
0
20
40
60
80
FDR (Age associated divergence) =0.0674
p(Scalp)=2.65e−05
p(Dura)=0.477
Scalp
Dura

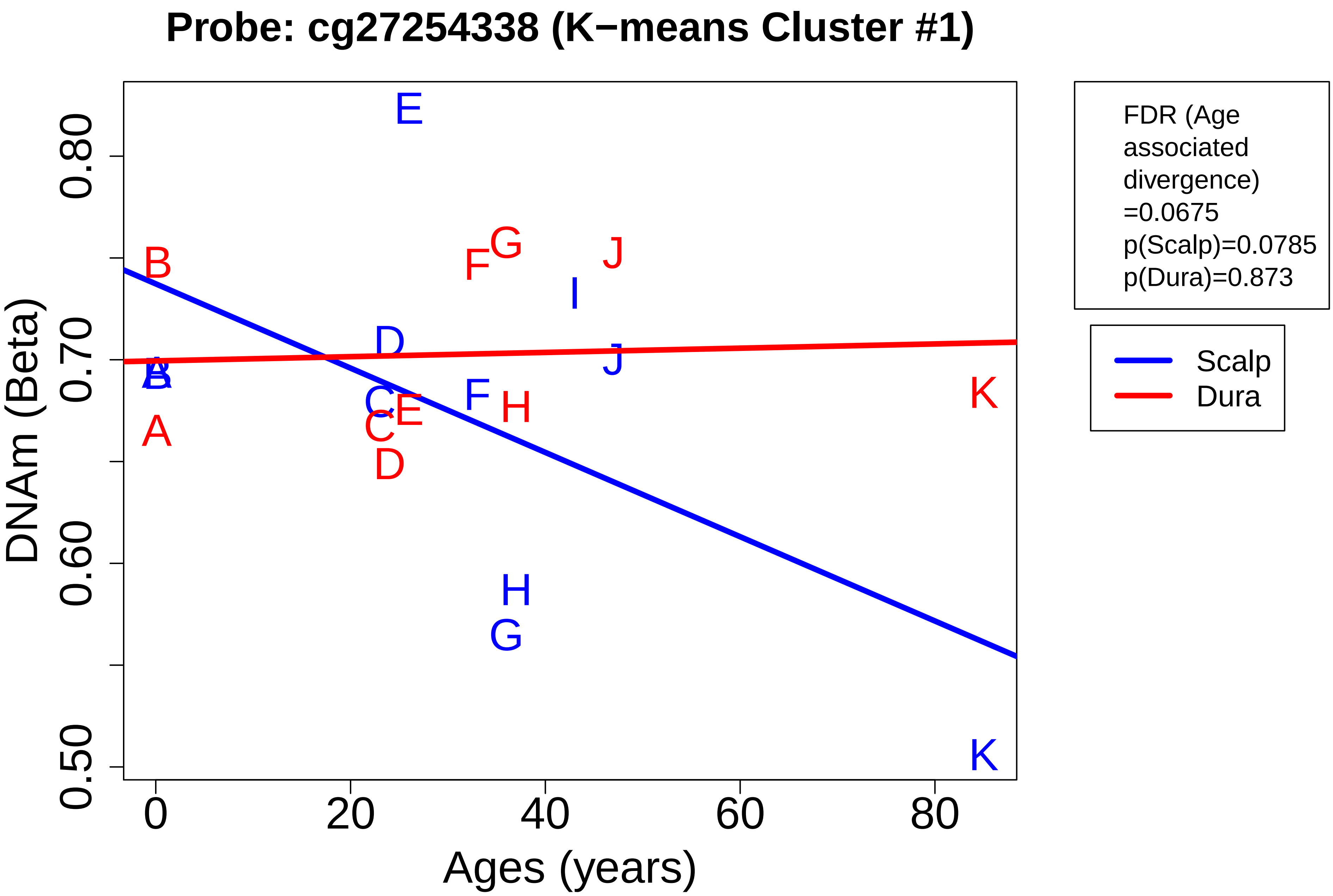

Probe: cg27254338 (K−means Cluster #1)
DNAm (Beta)
0.50
0.60
0.70
0.80
0
20
40
60
80
Ages (years)
FDR (Age associated divergence) =0.0675
p(Scalp)=0.0785
p(Dura)=0.873
Scalp
Dura

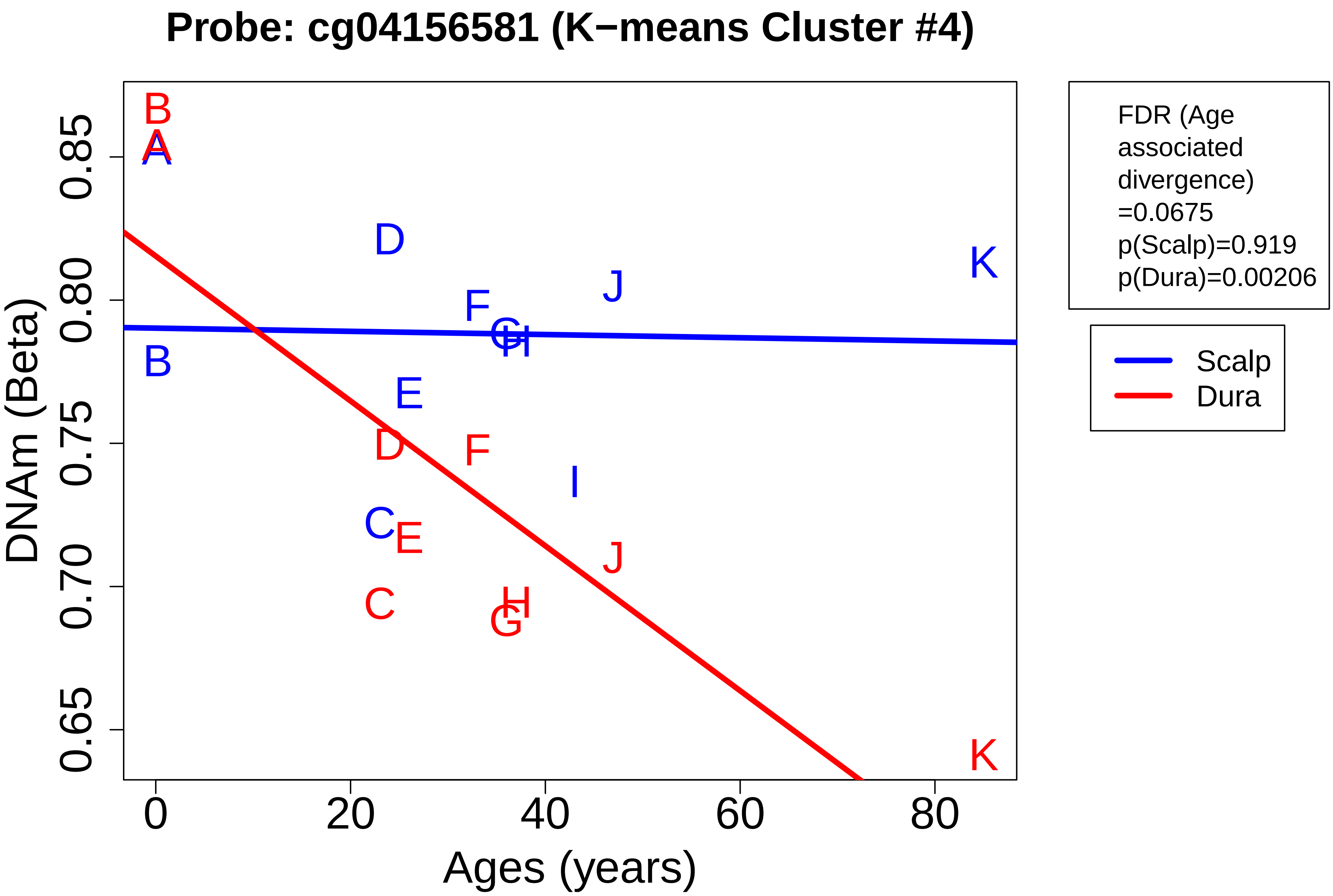

Probe: cg04156581 (K−means Cluster #4)
DNAm (Beta)
0.65
0.70
0.75
0.80
0.85
Ages (years)
0
20
40
60
80
A
B
C
D
E
F
G
H
I
J
K
FDR (Age associated divergence) =0.0675
p(Scalp)=0.919
p(Dura)=0.00206
Scalp
Dura

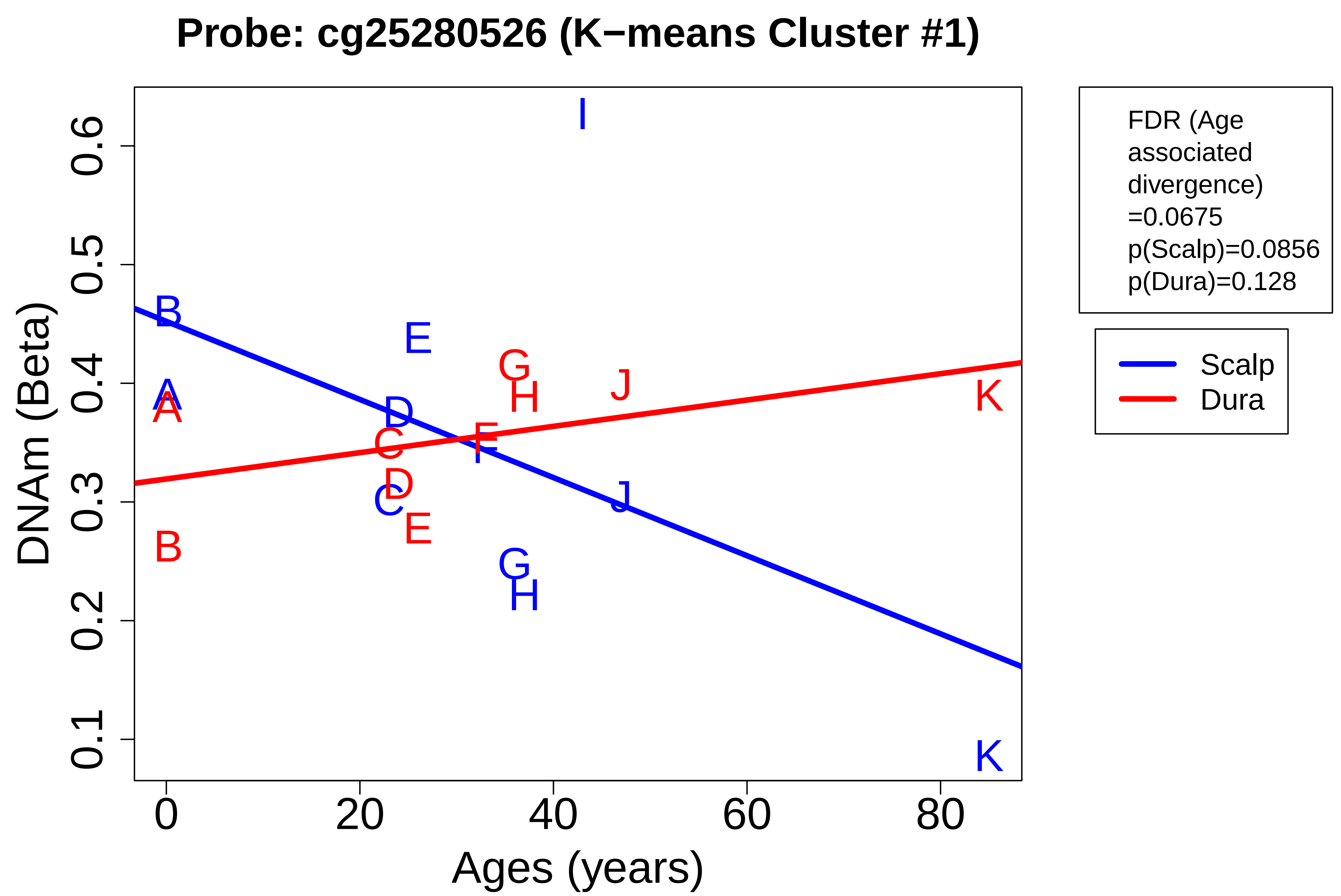

Probe: cg25280526 (K−means Cluster #1)
DNAm (Beta)
0.1
0.2
0.3
0.4
0.5
0.6
0
20
40
60
80
Ages (years)
A
B
C
D
E
F
G
H
I
J
K
FDR (Age associated divergence) =0.0675
p(Scalp)=0.0856
p(Dura)=0.128
Scalp
Dura

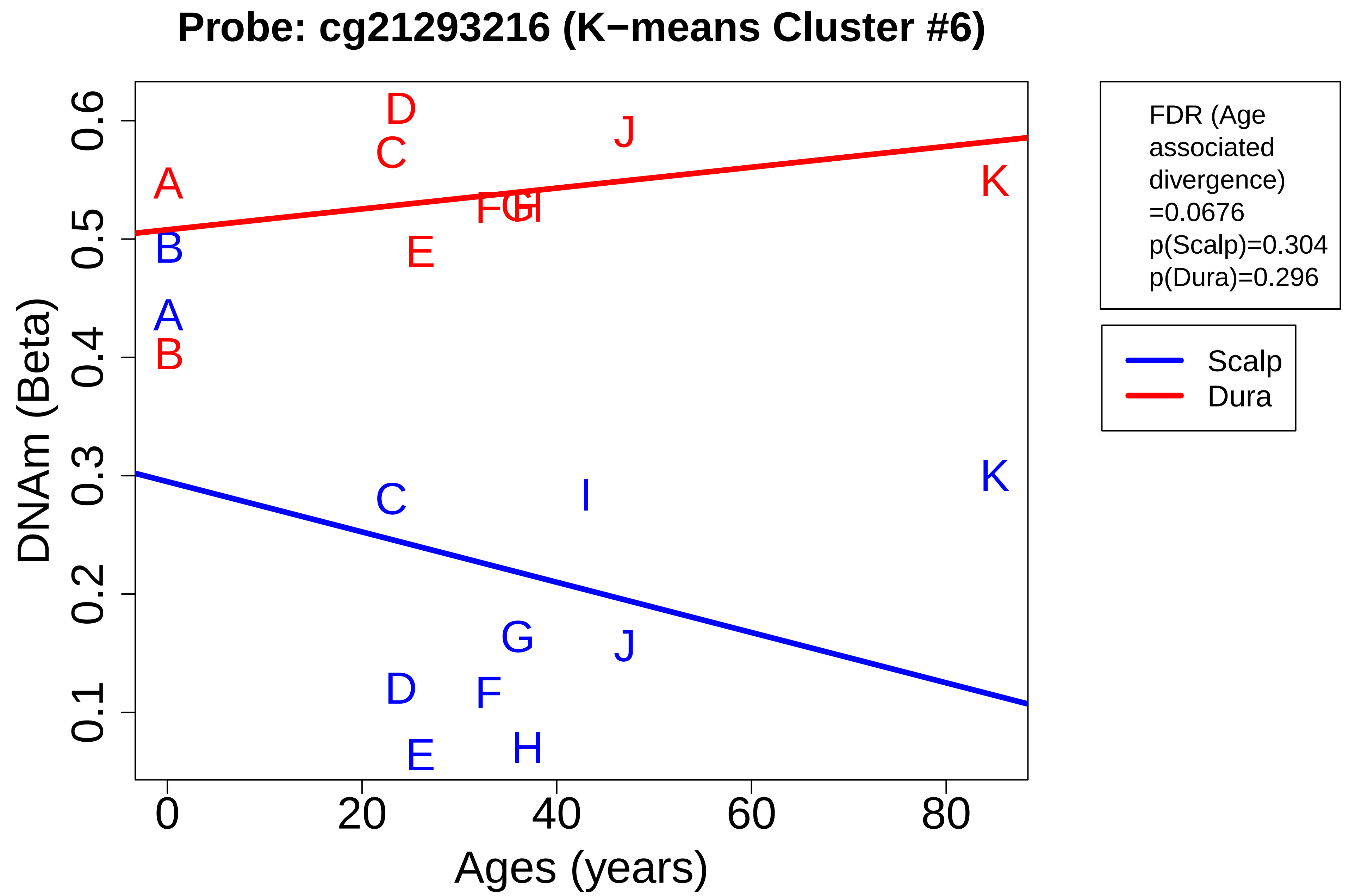
Probe: cg21293216 (K−means Cluster #6)
DNAm (Beta)
0.1
0.2
0.3
0.4
0.5
0.6
0
20
40
60
80
Ages (years)
FDR (Age associated divergence) =0.0676
p(Scalp)=0.304
p(Dura)=0.296
Scalp
Dura
A
B
C
D
E
F
G
H
I
J
K

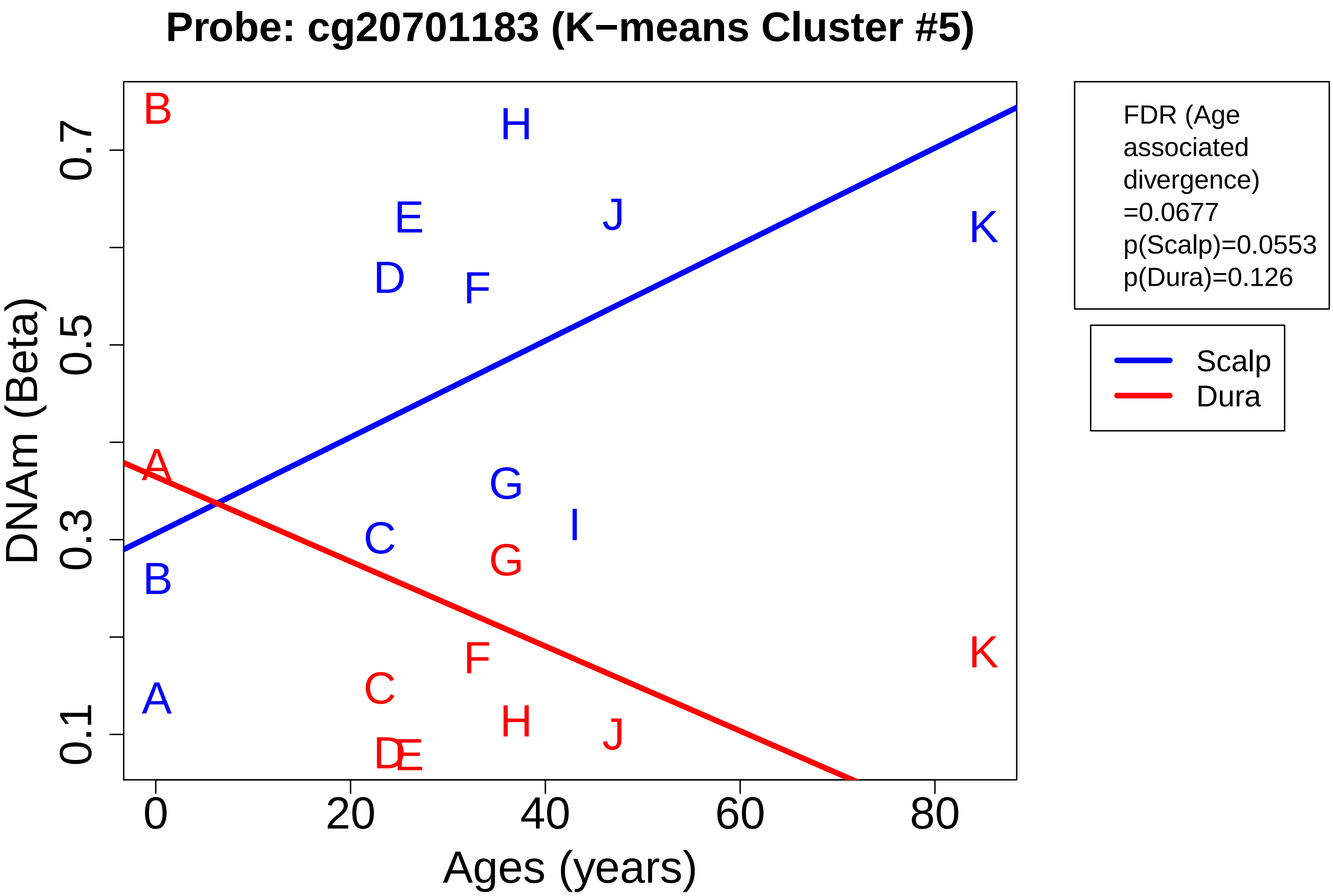

Probe: cg20701183 (K−means Cluster #5)
DNAm (Beta)
Ages (years)
0.1
0.3
0.5
0.7
0
20
40
60
80
FDR (Age associated divergence) =0.0677
p(Scalp)=0.0553
p(Dura)=0.126
Scalp
Dura
A
B
C
D
E
F
G
H
I
J
K

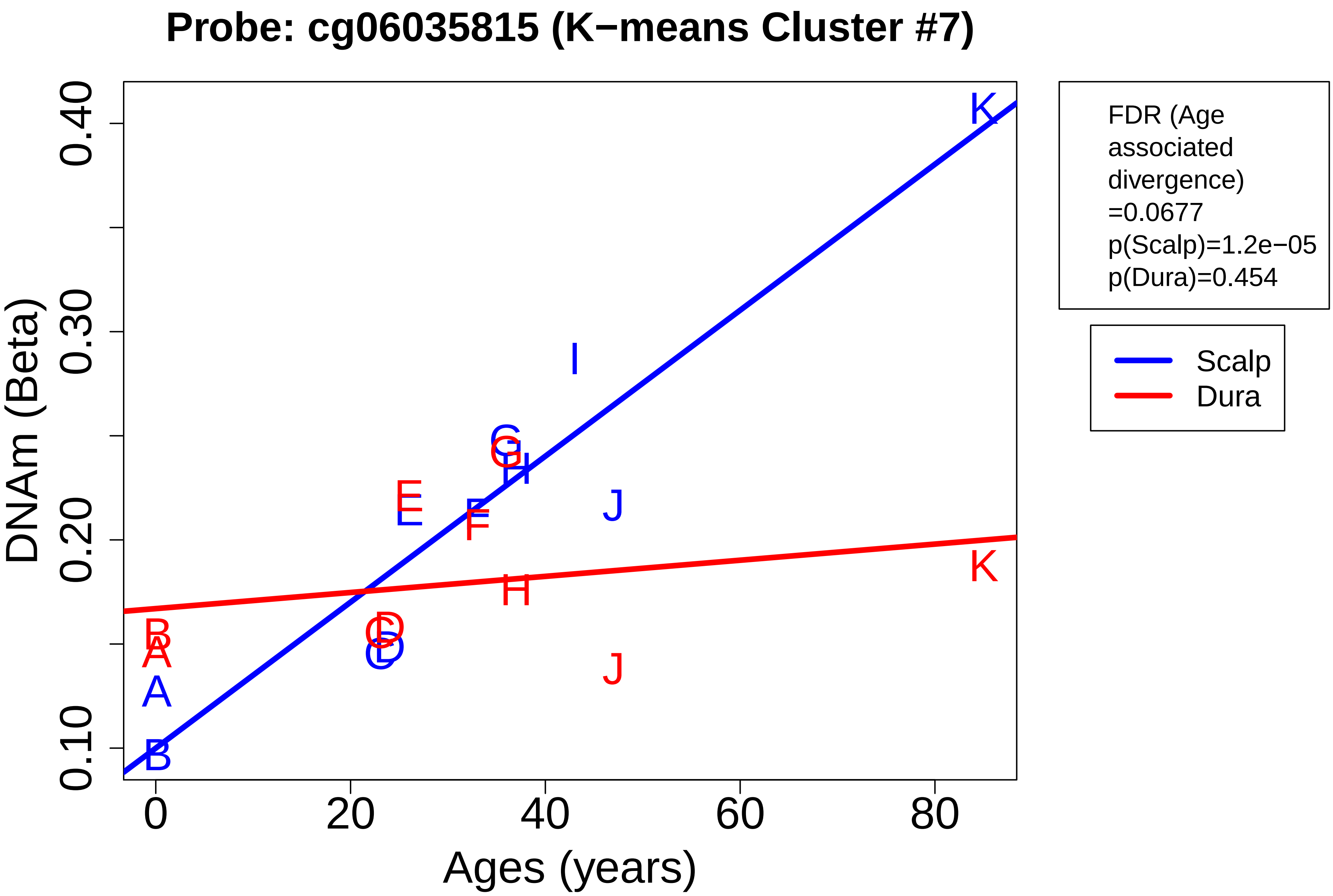

Probe: cg06035815 (K−means Cluster #7)
DNAm (Beta)
0.10
0.20
0.30
0.40
0
20
40
60
80
Ages (years)
FDR (Age associated divergence) =0.0677
p(Scalp)=1.2e−05
p(Dura)=0.454
Scalp
Dura
A
B
C
D
E
F
G
H
I
J
K

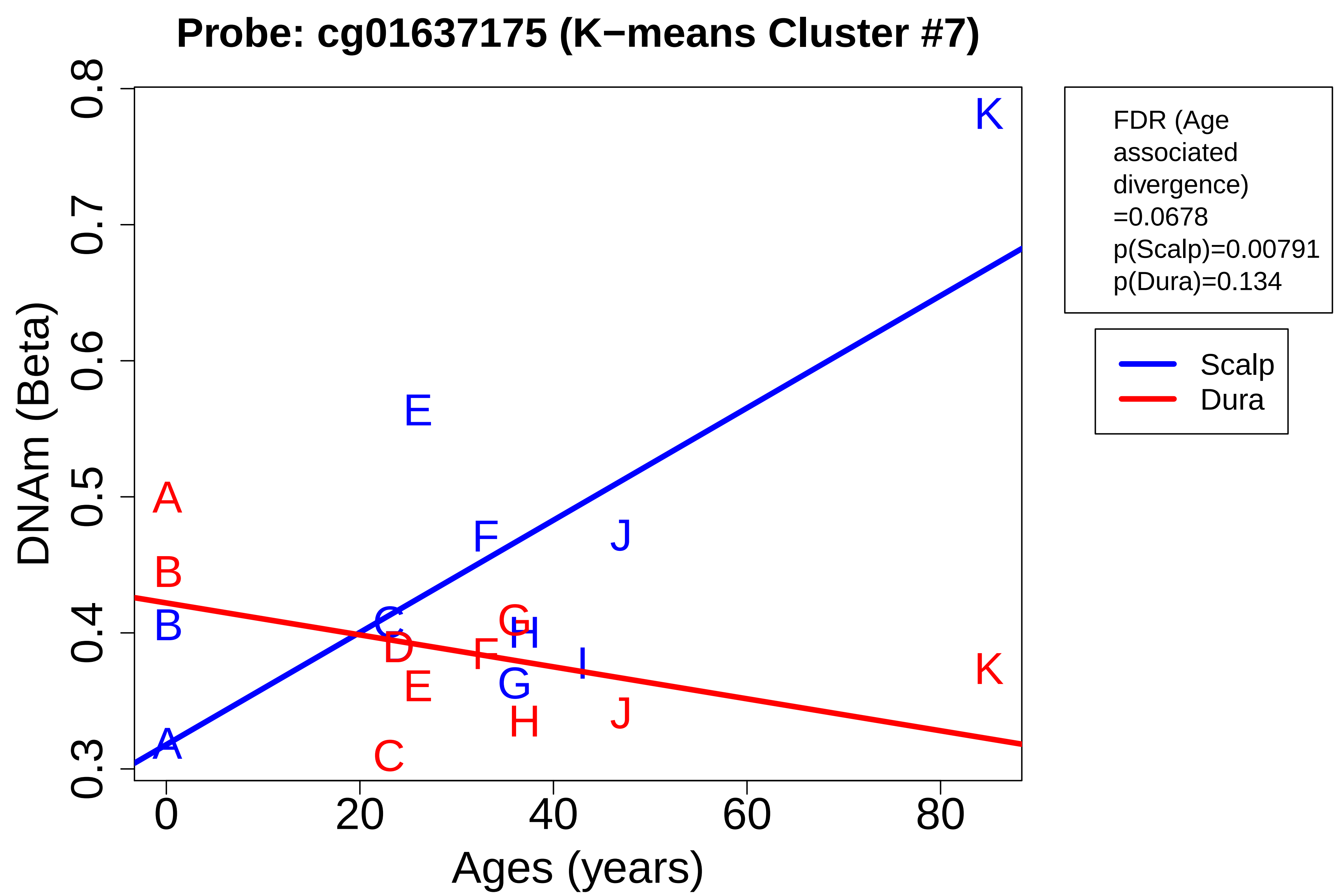
Probe: cg01637175 (K−means Cluster #7)
DNAm (Beta)
0.3
0.4
0.5
0.6
0.7
0.8
Ages (years)
0
20
40
60
80
A
B
C
D
E
F
G
H
I
J
K
FDR (Age associated divergence) =0.0678
p(Scalp)=0.00791
p(Dura)=0.134
Scalp
Dura

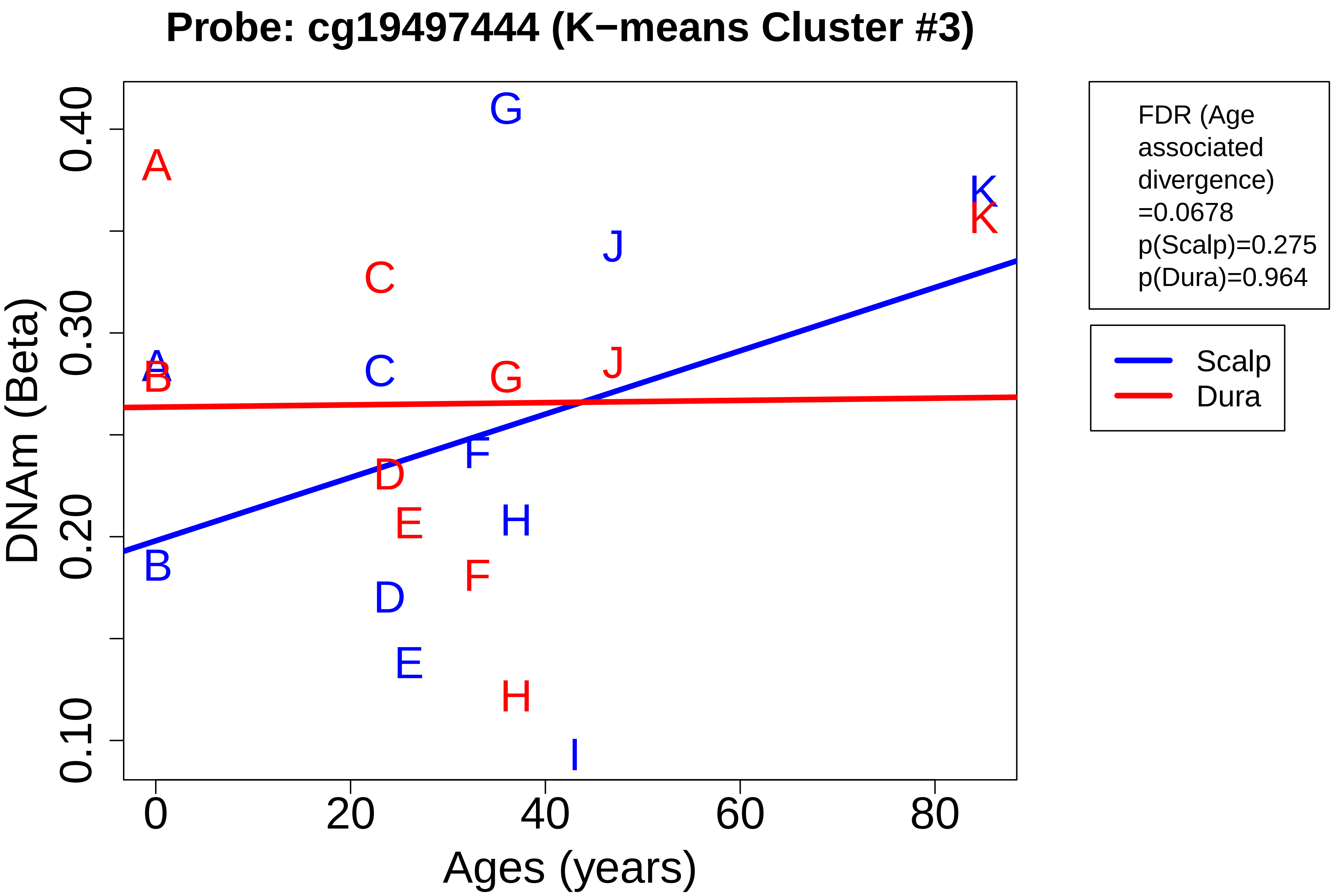

Probe: cg19497444 (K−means Cluster #3)
DNAm (Beta)
0.10
0.20
0.30
0.40
0
20
40
60
80
Ages (years)
FDR (Age associated divergence) =0.0678
p(Scalp)=0.275
p(Dura)=0.964
Scalp
Dura

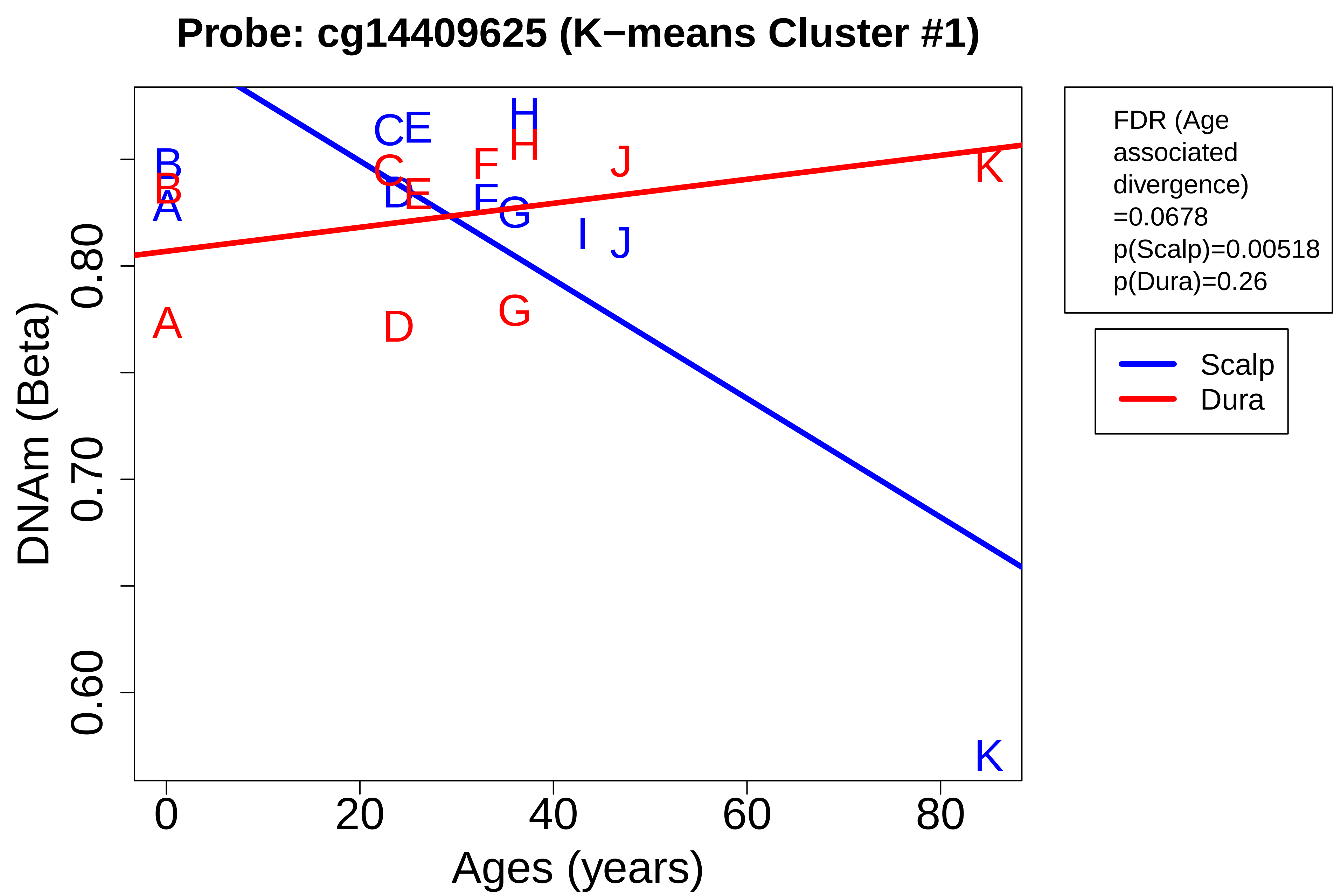

Probe: cg14409625 (K−means Cluster #1)
DNAm (Beta)
0.60
0.70
0.80
0
20
40
60
80
Ages (years)
A
B
C
D
E
F
G
H
I
J
K
FDR (Age associated divergence) =0.0678
p(Scalp)=0.00518
p(Dura)=0.26
Scalp
Dura

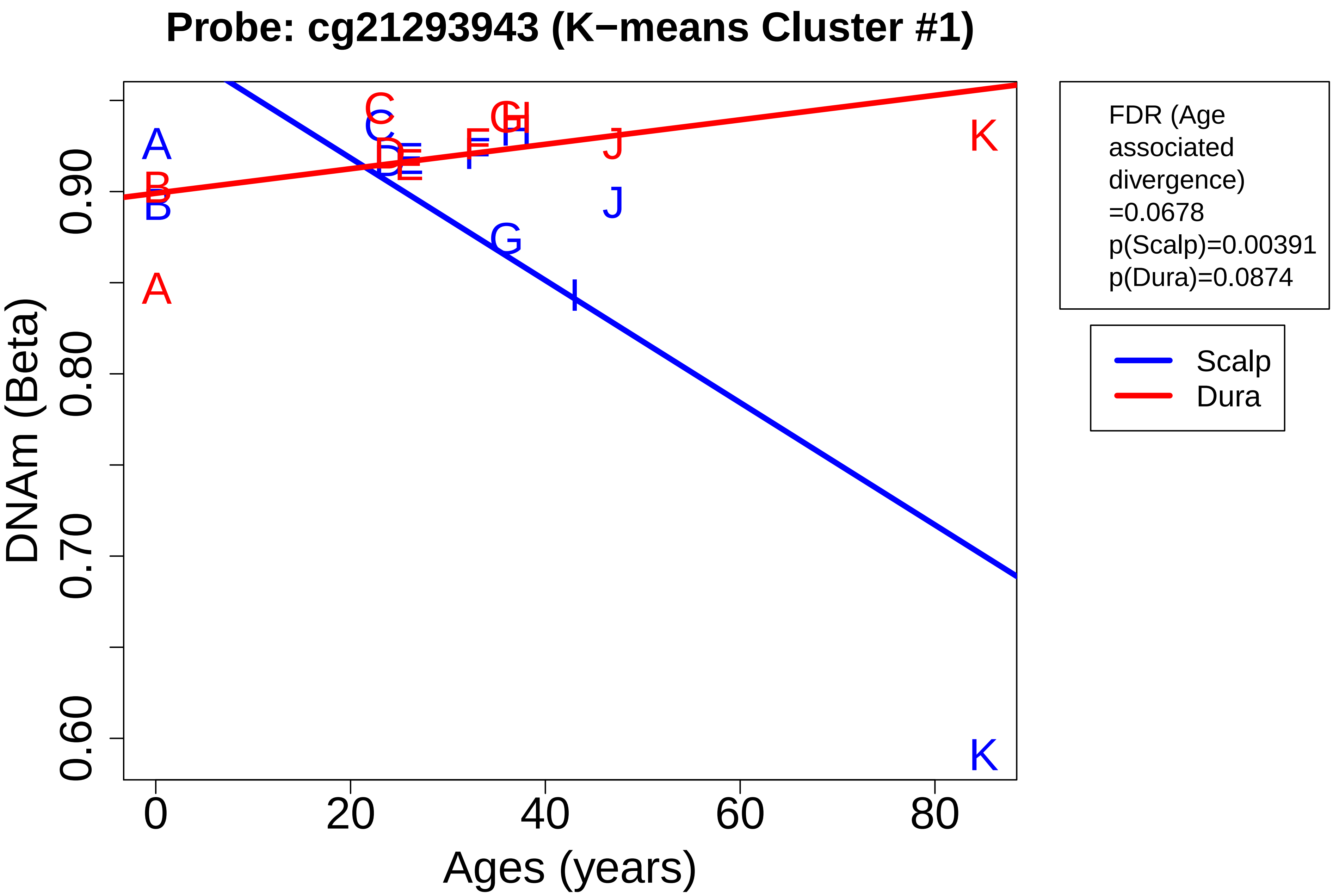

Probe: cg21293943 (K−means Cluster #1)
DNAm (Beta)
0.60
0.70
0.80
0.90
Ages (years)
0
20
40
60
80
A
B
C
D
E
F
G
H
I
J
K
FDR (Age associated divergence) =0.0678
p(Scalp)=0.00391
p(Dura)=0.0874
Scalp
Dura

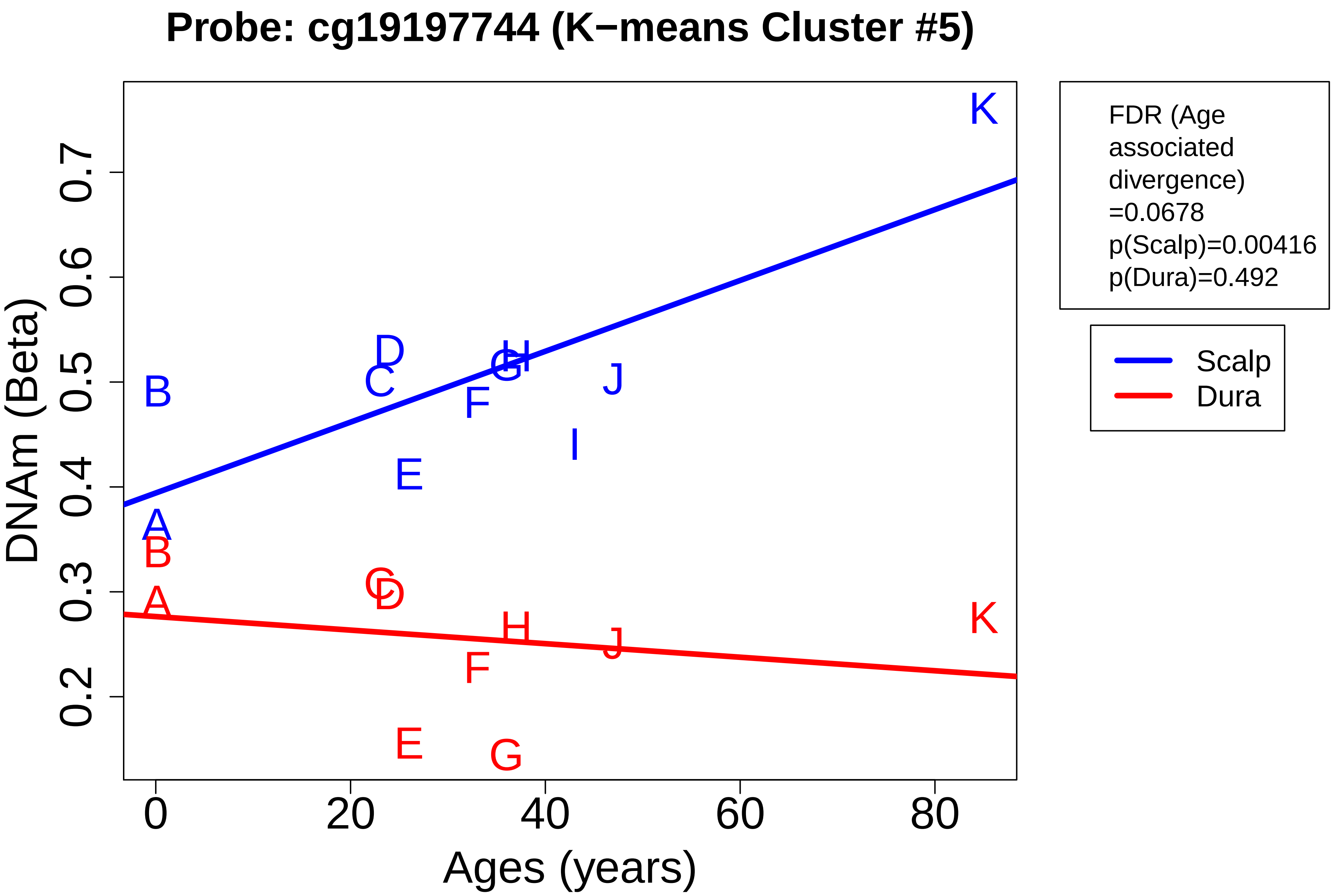

Probe: cg19197744 (K−means Cluster #5)
FDR (Age associated divergence) =0.0678
p(Scalp)=0.00416
p(Dura)=0.492
Scalp
Dura
DNAm (Beta)
0.2
0.3
0.4
0.5
0.6
0.7
Ages (years)
0
20
40
60
80
A
B
C
D
E
F
G
H
I
J
K

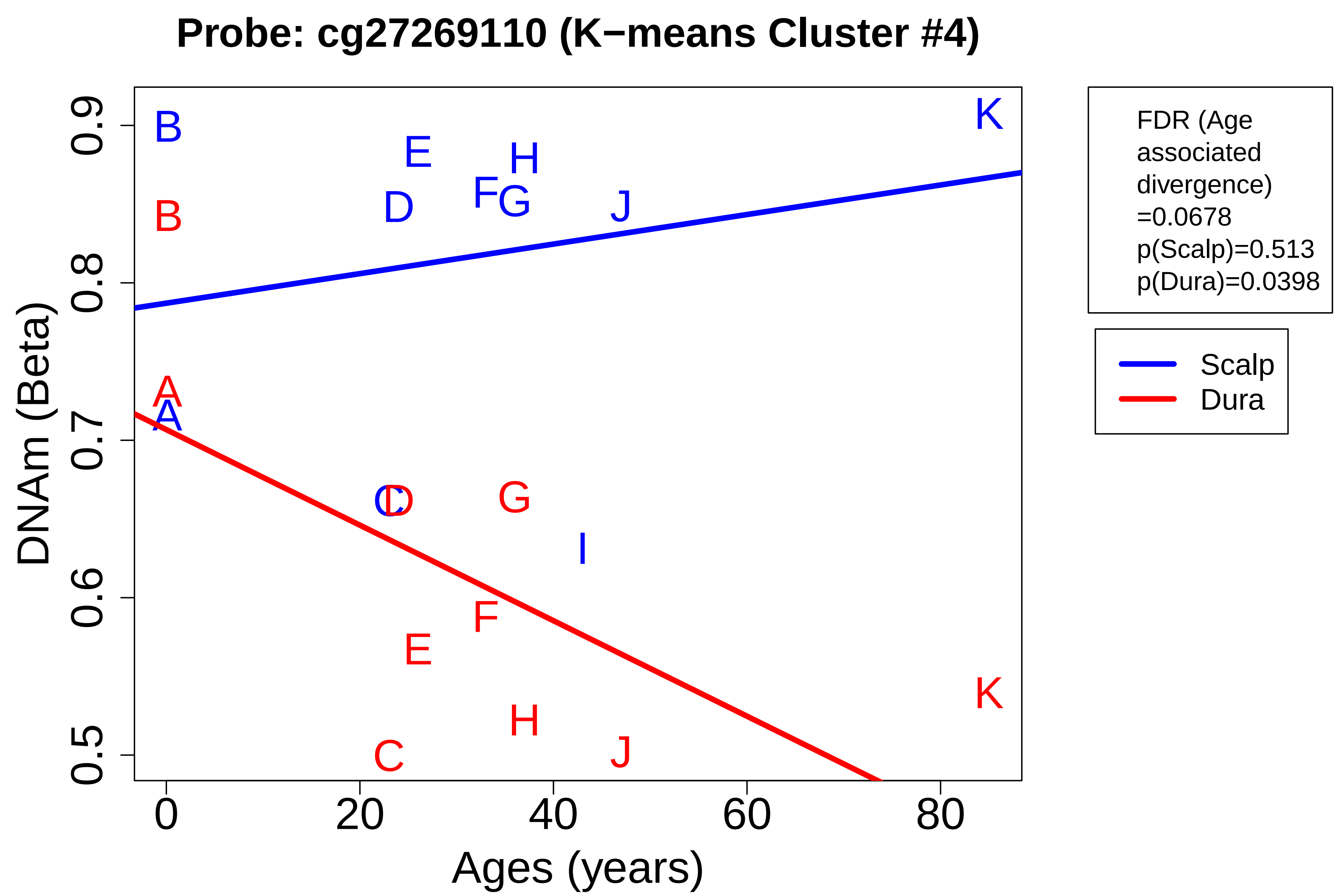

Probe: cg27269110 (K−means Cluster #4)
DNAm (Beta)
0.5
0.6
0.7
0.8
0.9
0
20
40
60
80
Ages (years)
B
E
H
F
G
D
J
K
B
A
A
C
D
G
I
F
E
H
K
C
J
FDR (Age associated divergence) =0.0678
p(Scalp)=0.513
p(Dura)=0.0398
Scalp
Dura

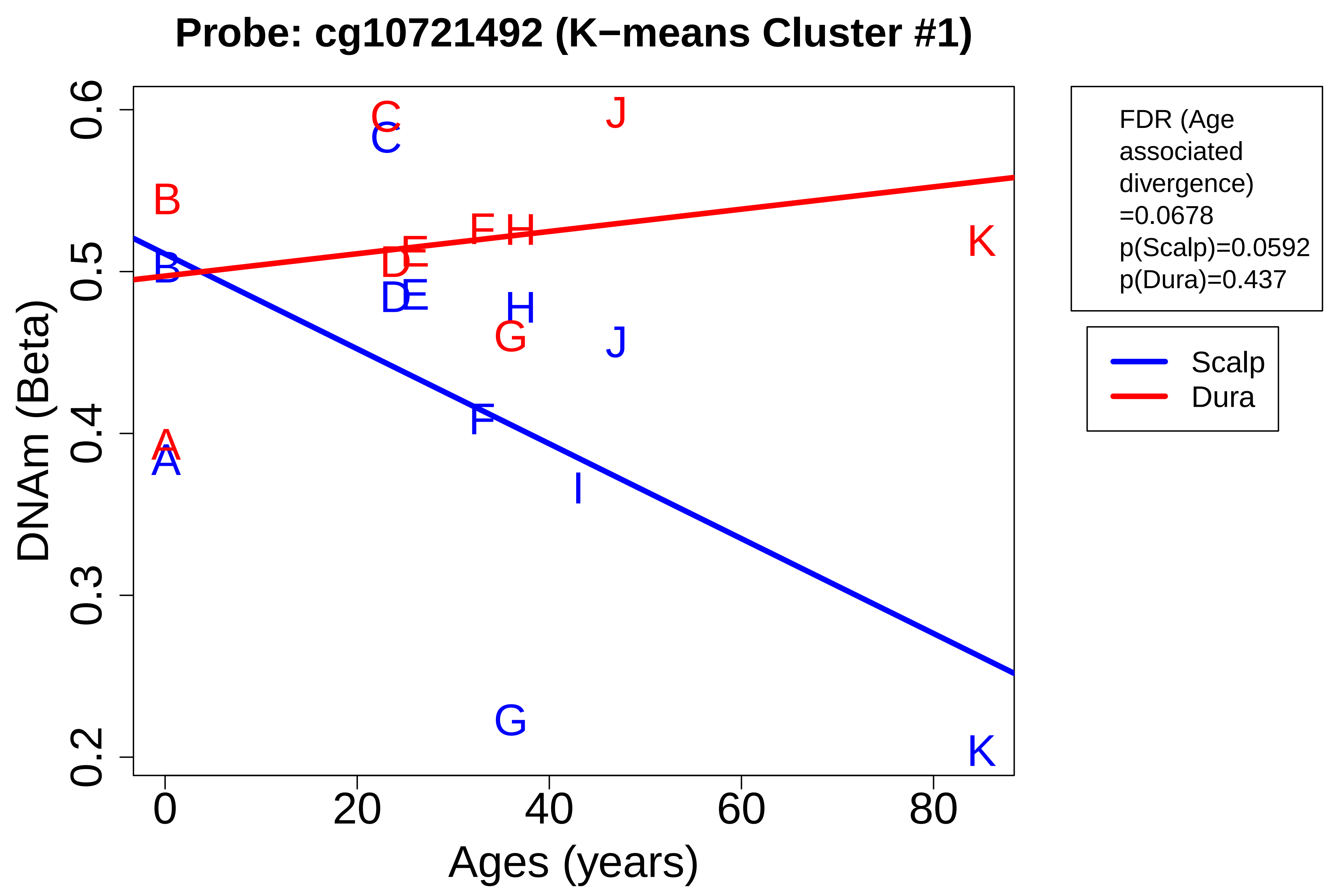

Probe: cg10721492 (K−means Cluster #1)
DNAm (Beta)
0.2
0.3
0.4
0.5
0.6
Ages (years)
0
20
40
60
80
FDR (Age associated divergence) =0.0678
p(Scalp)=0.0592
p(Dura)=0.437
Scalp
Dura

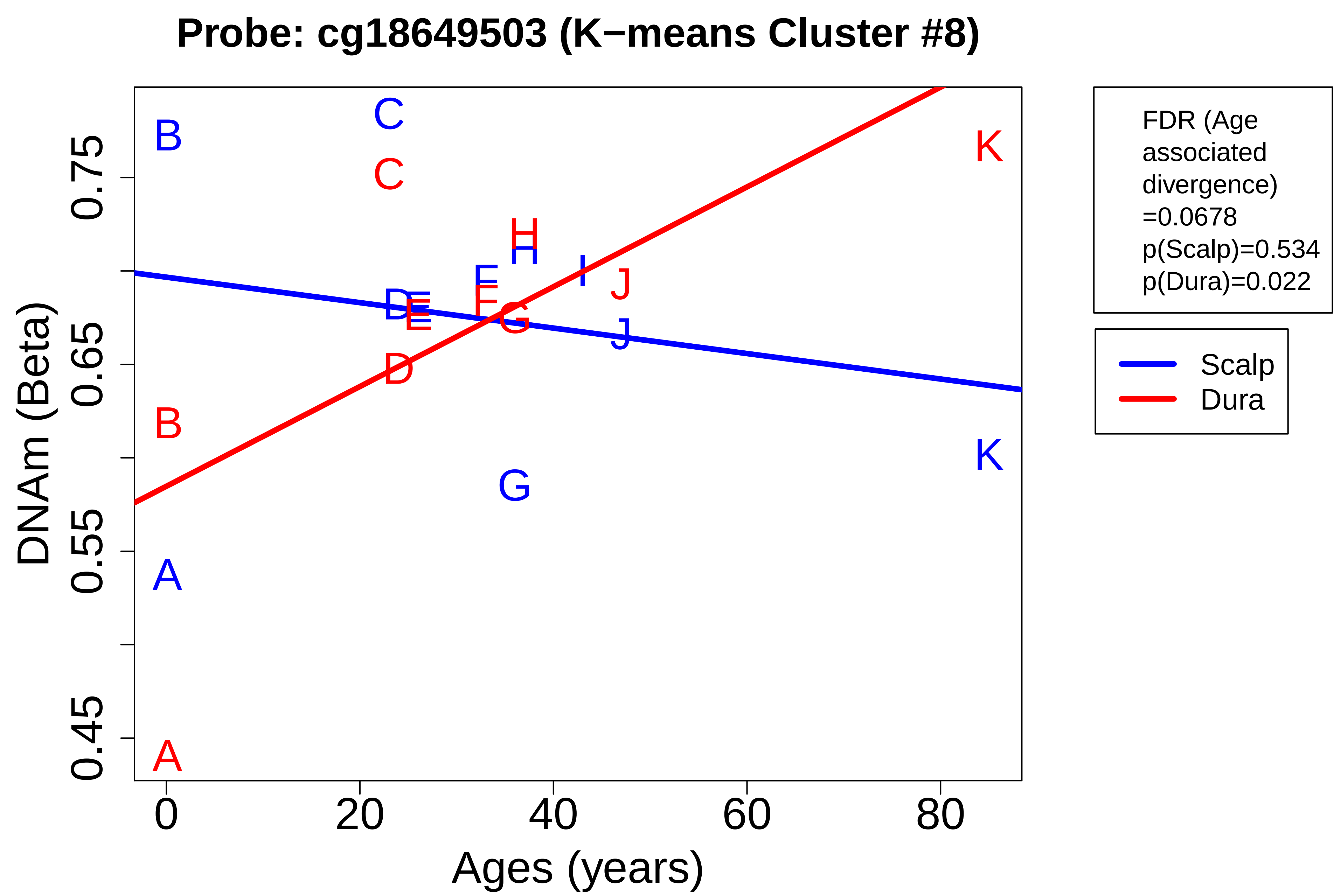
Probe: cg18649503 (K−means Cluster #8)
DNAm (Beta)
0.45
0.55
0.65
0.75
Ages (years)
0
20
40
60
80
FDR (Age associated divergence) =0.0678
p(Scalp)=0.534
p(Dura)=0.022
Scalp
Dura
A
B
C
D
E
F
G
H
I
J
K

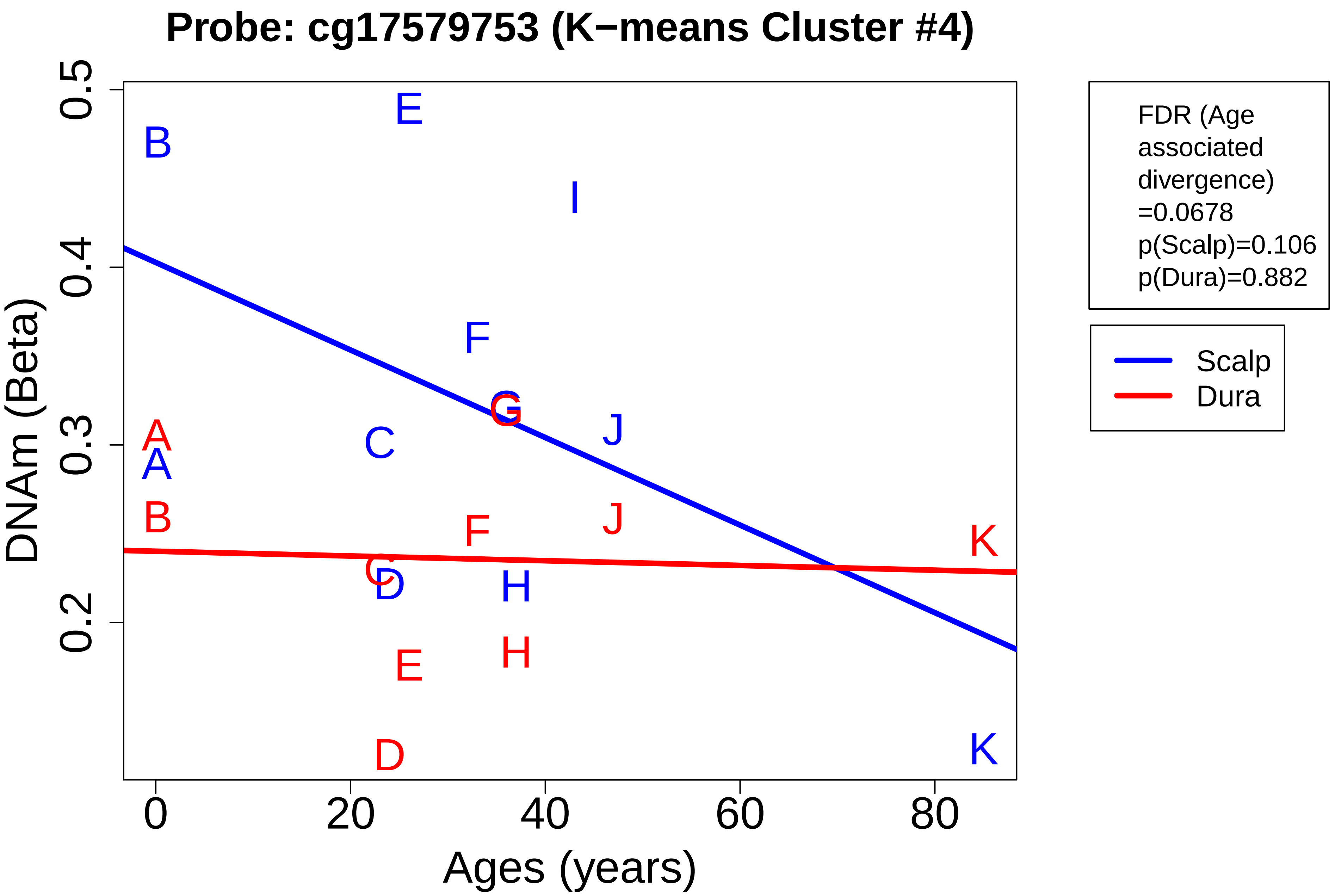

Probe: cg17579753 (K−means Cluster #4)
FDR (Age associated divergence) =0.0678
p(Scalp)=0.106
p(Dura)=0.882
Scalp
Dura
DNAm (Beta)
0.2
0.3
0.4
0.5
Ages (years)
0
20
40
60
80
A
B
C
D
E
F
G
H
I
J
K

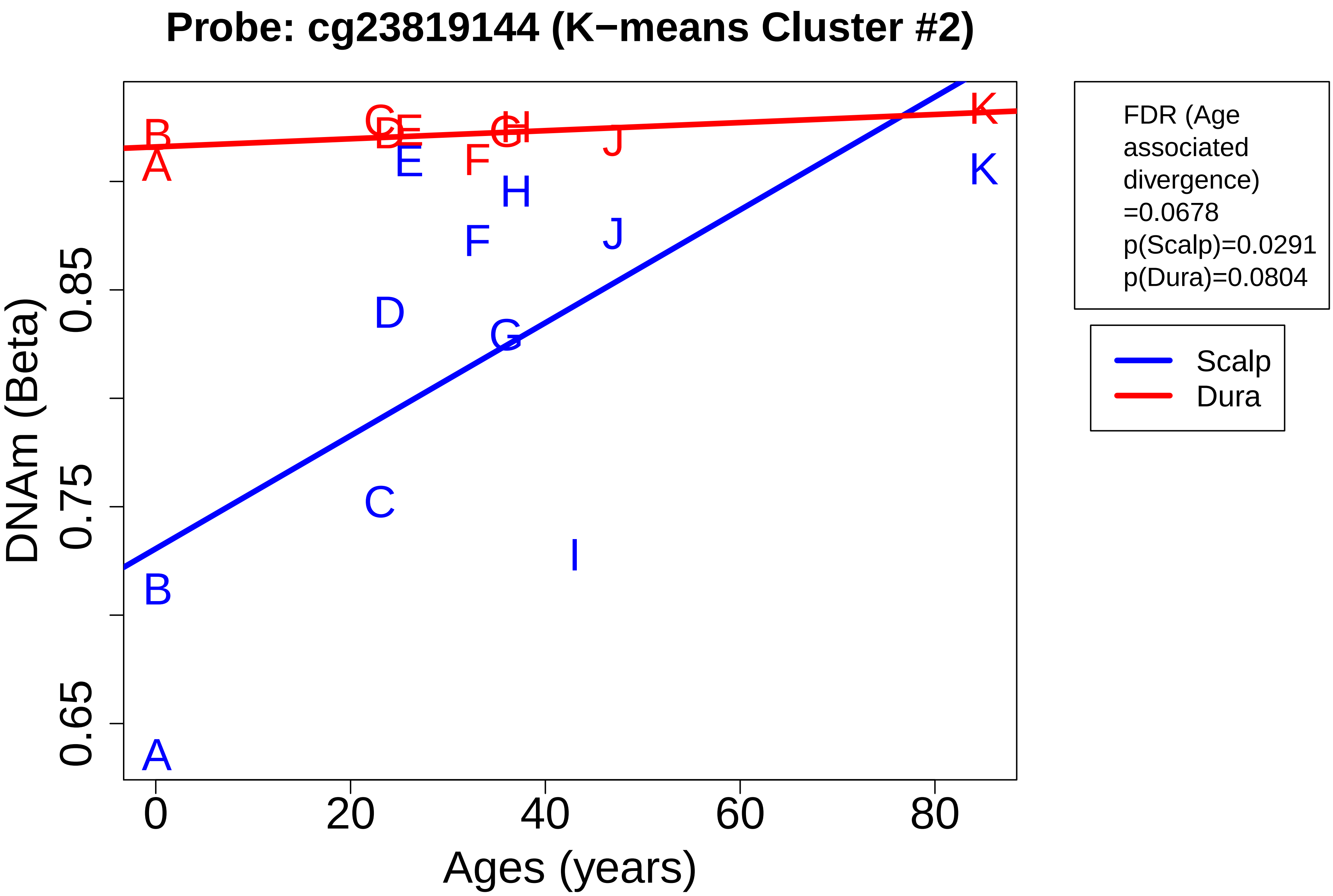

Probe: cg23819144 (K−means Cluster #2)
DNAm (Beta)
0.65
0.75
0.85
Ages (years)
0
20
40
60
80
FDR (Age associated divergence) =0.0678
p(Scalp)=0.0291
p(Dura)=0.0804
Scalp
Dura

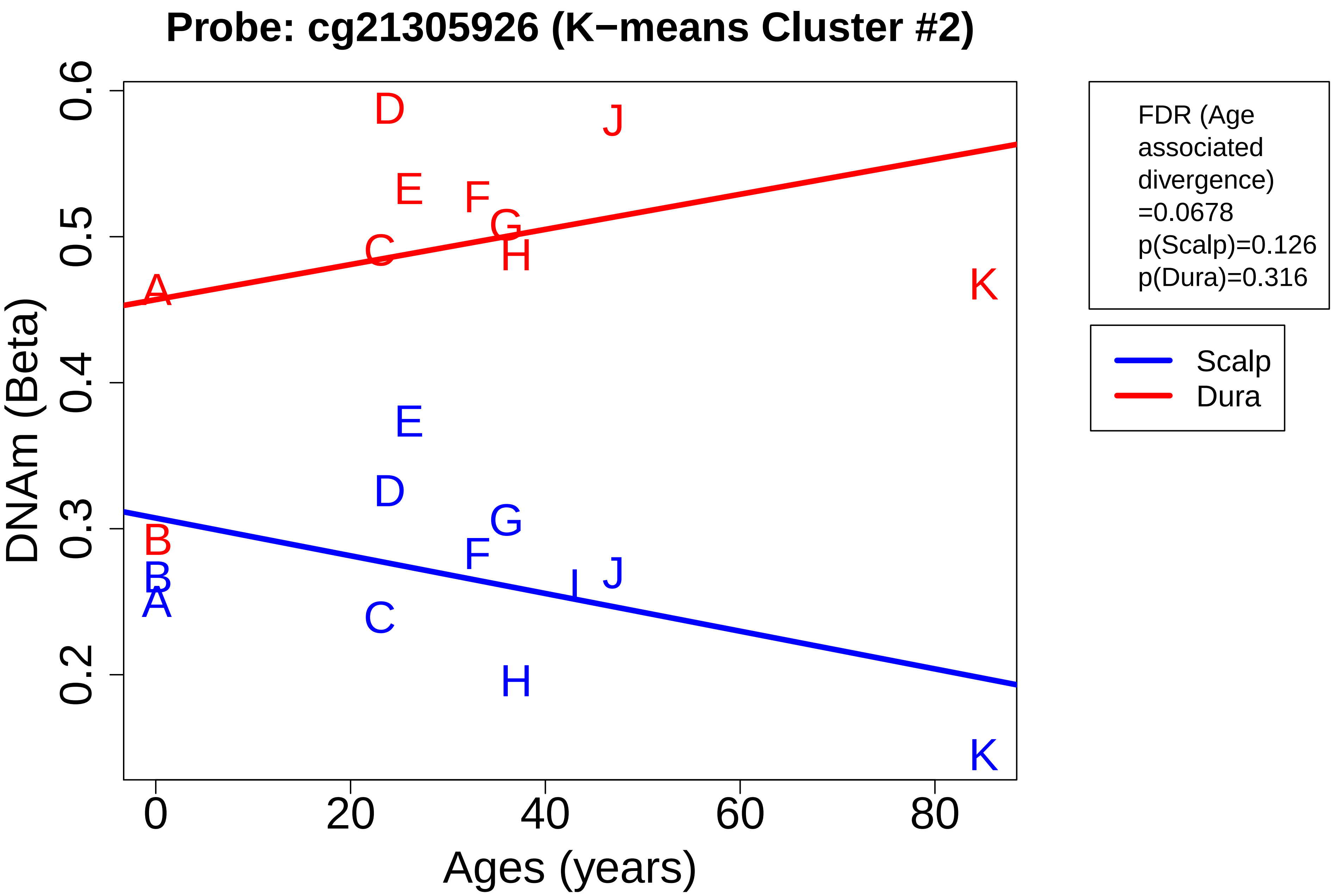

Probe: cg21305926 (K−means Cluster #2)
DNAm (Beta)
0.2
0.3
0.4
0.5
0.6
0
20
40
60
80
Ages (years)
A
B
C
D
E
F
G
H
I
J
K
FDR (Age associated divergence) =0.0678
p(Scalp)=0.126
p(Dura)=0.316
Scalp
Dura

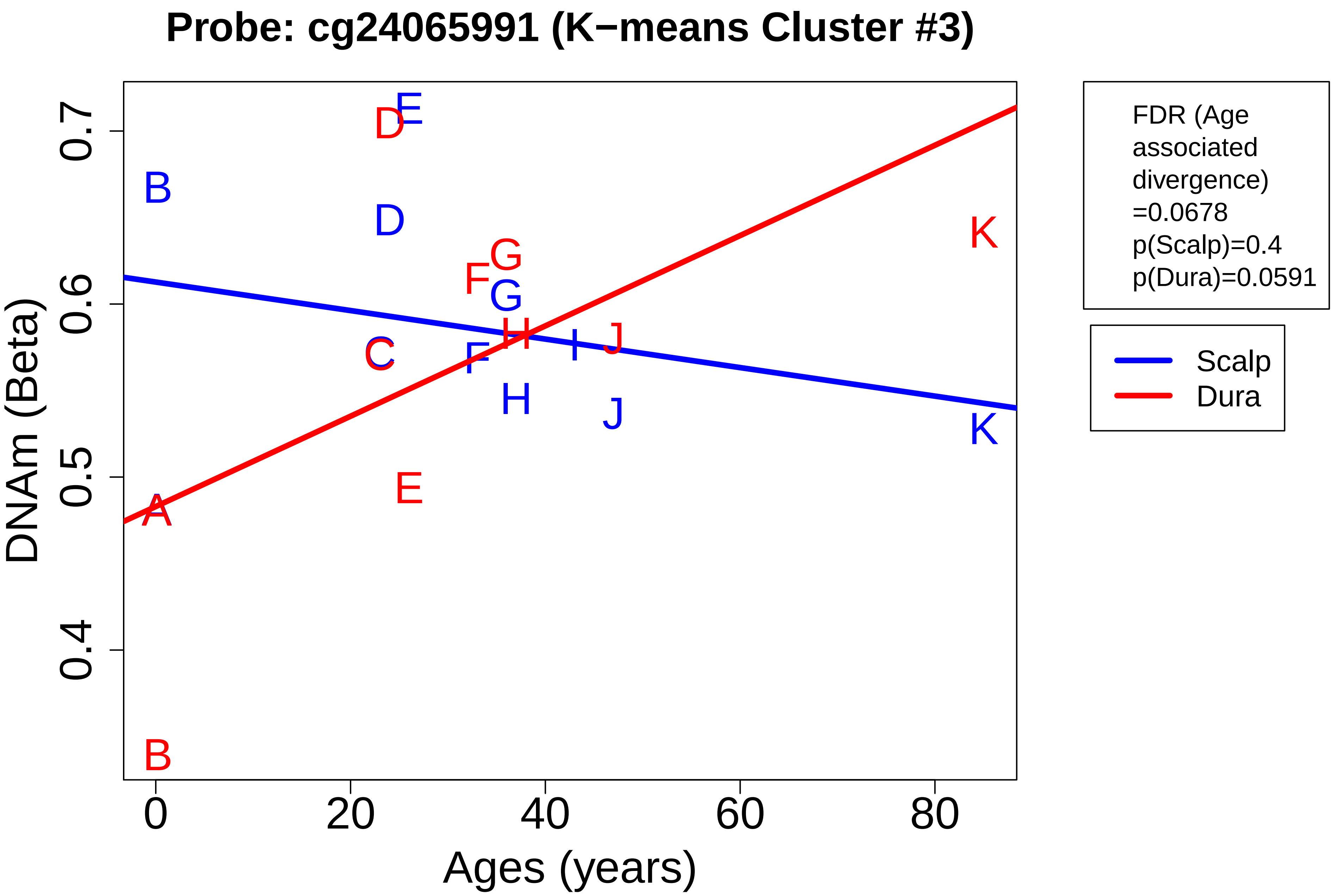
Probe: cg24065991 (K−means Cluster #3)
DNAm (Beta)
Ages (years)
0.4
0.5
0.6
0.7
0
20
40
60
80
FDR (Age associated divergence) =0.0678
p(Scalp)=0.4
p(Dura)=0.0591
Scalp
Dura
A
B
C
D
E
F
G
H
I
J
K

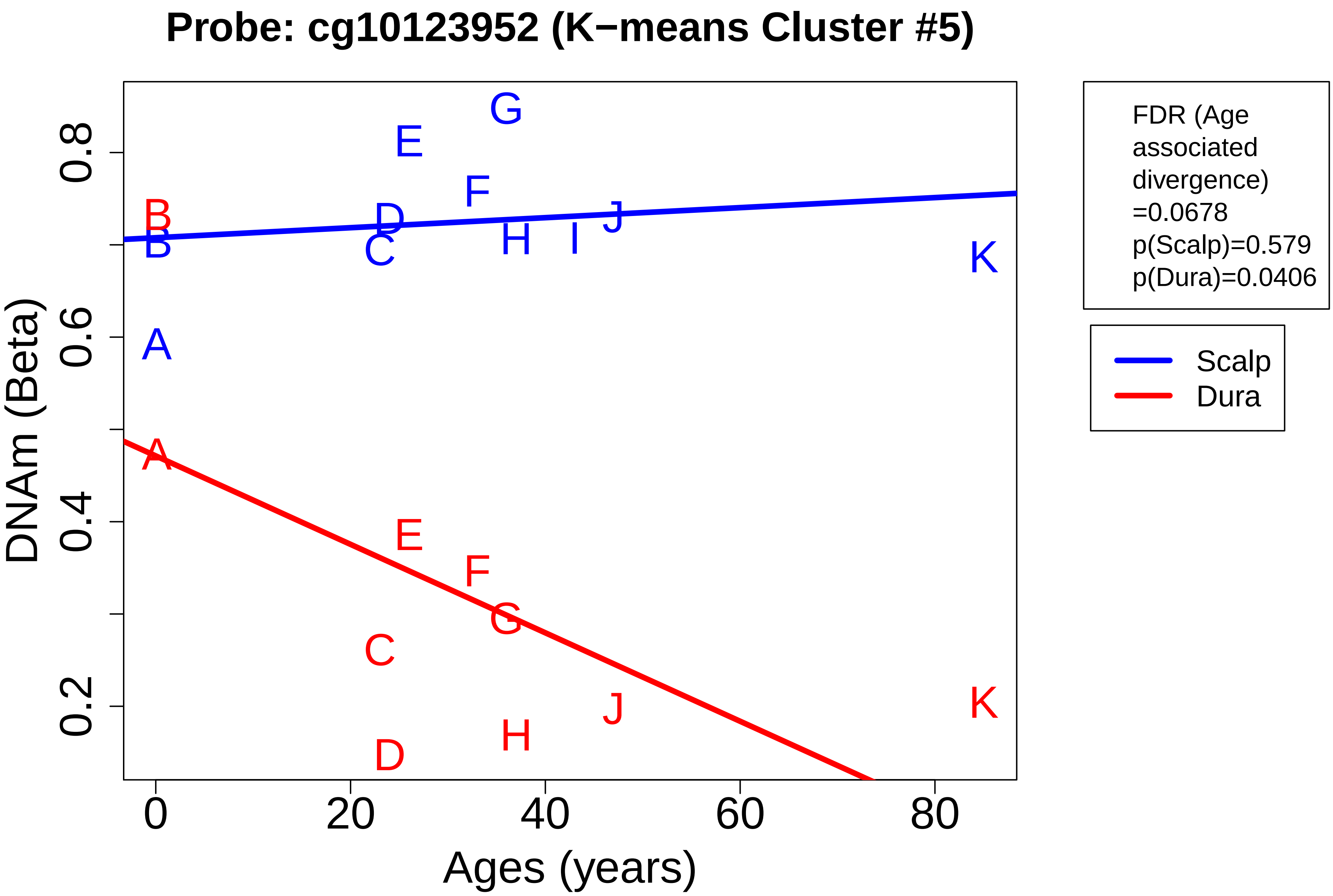
Probe: cg10123952 (K−means Cluster #5)
DNAm (Beta)
0.2
0.4
0.6
0.8
0
20
40
60
80
Ages (years)
FDR (Age associated divergence) =0.0678
p(Scalp)=0.579
p(Dura)=0.0406
Scalp
Dura

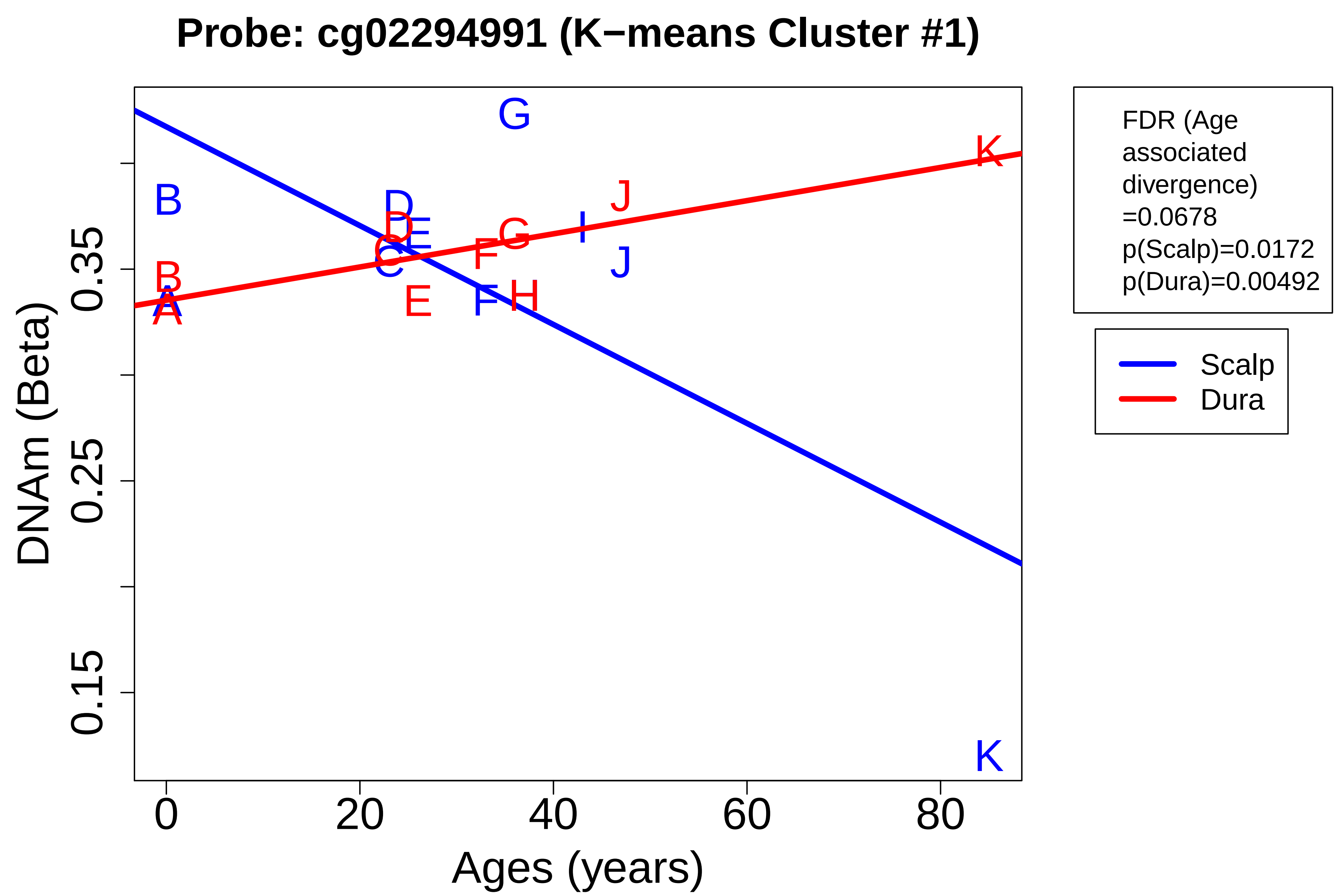

Probe: cg02294991 (K−means Cluster #1)
DNAm (Beta)
0.15
0.25
0.35
Ages (years)
0
20
40
60
80
FDR (Age associated divergence) =0.0678
p(Scalp)=0.0172
p(Dura)=0.00492
Scalp
Dura
A
B
C
D
E
F
G
H
I
J
K

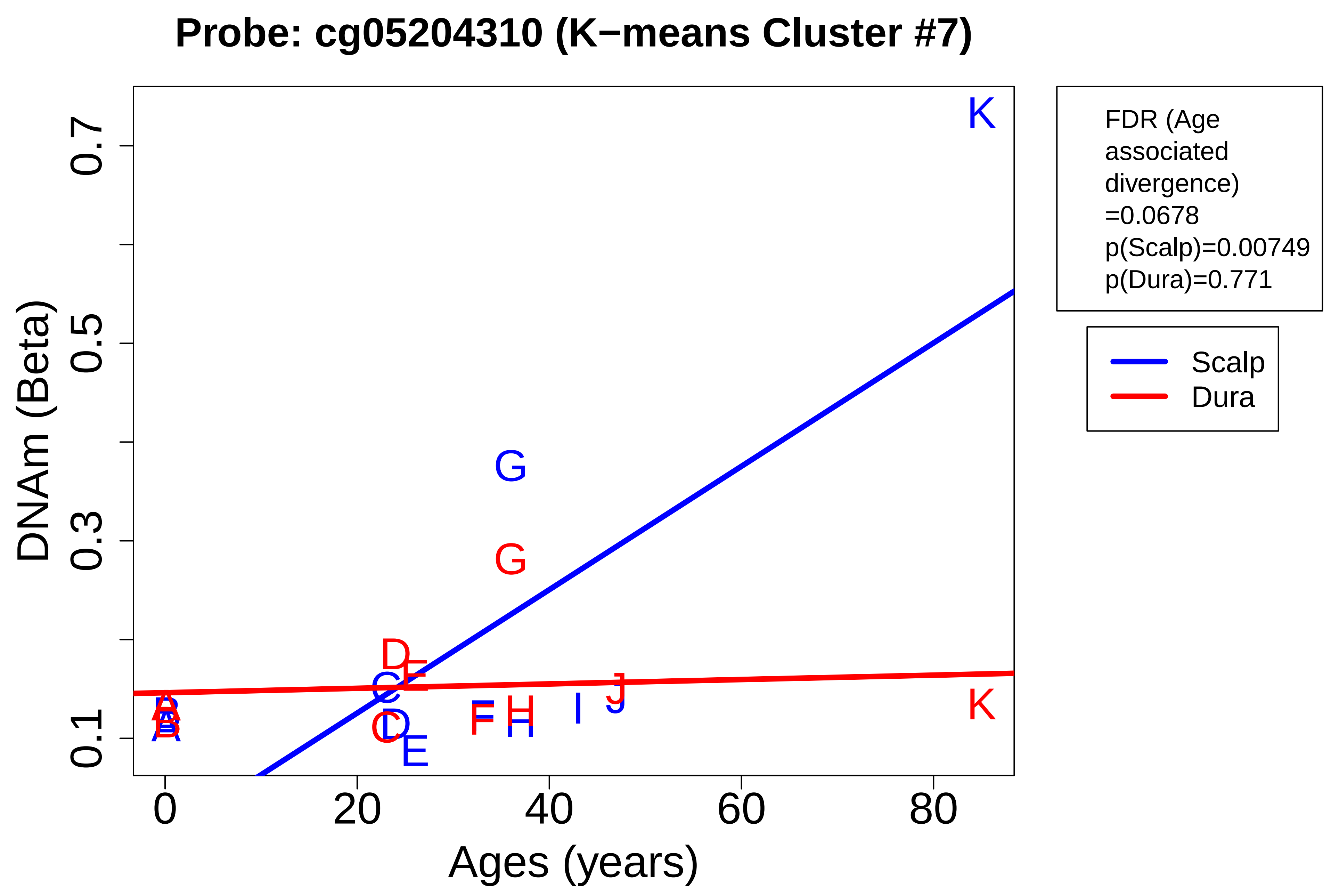
Probe: cg05204310 (K−means Cluster #7)
DNAm (Beta)
0.1
0.3
0.5
0.7
Ages (years)
0
20
40
60
80
FDR (Age associated divergence) =0.0678
p(Scalp)=0.00749
p(Dura)=0.771
Scalp
Dura
A B C D E F G H I J K

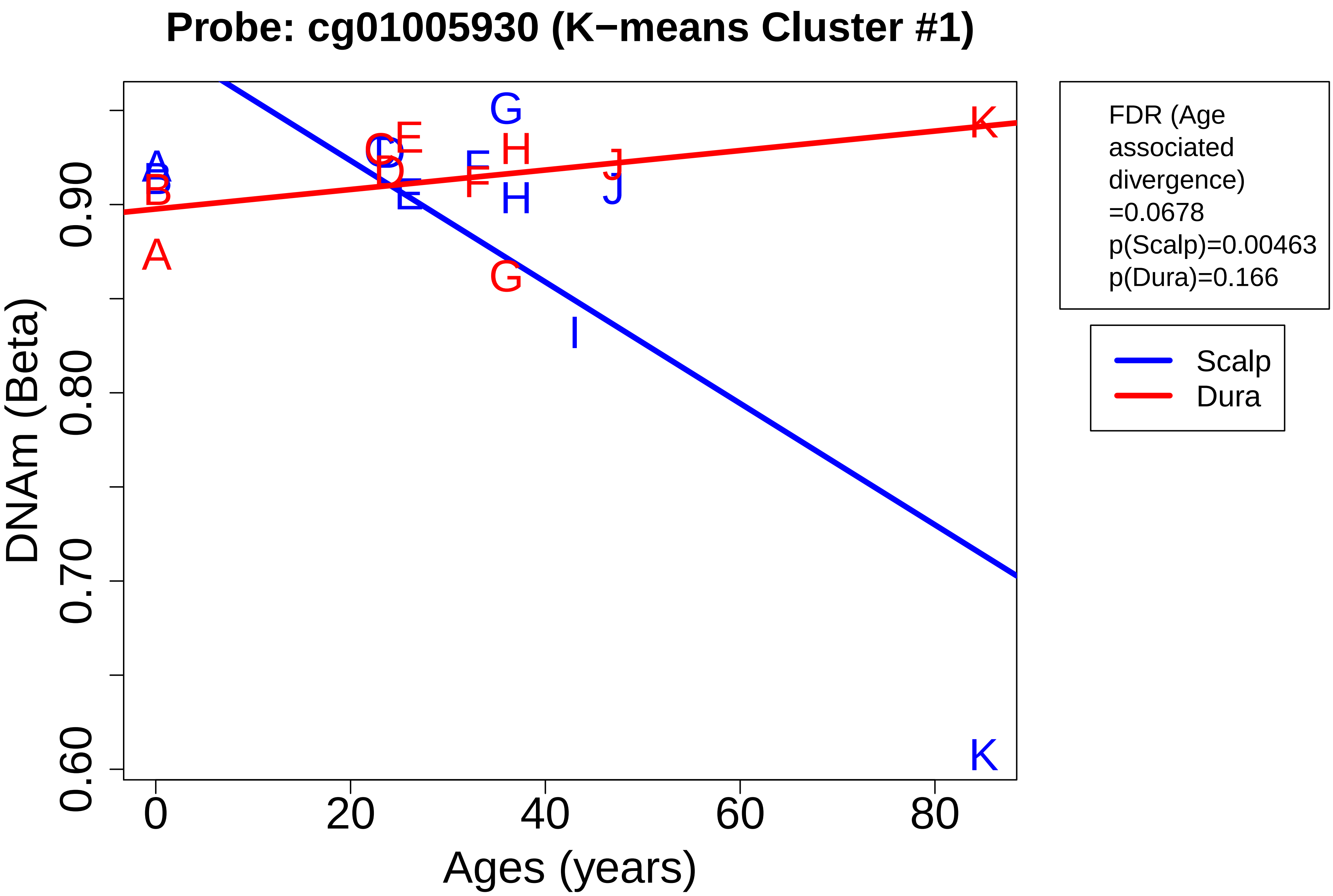

Probe: cg01005930 (K−means Cluster #1)
DNAm (Beta)
0.60
0.70
0.80
0.90
0
20
40
60
80
Ages (years)
FDR (Age associated divergence) =0.0678
p(Scalp)=0.00463
p(Dura)=0.166
Scalp
Dura

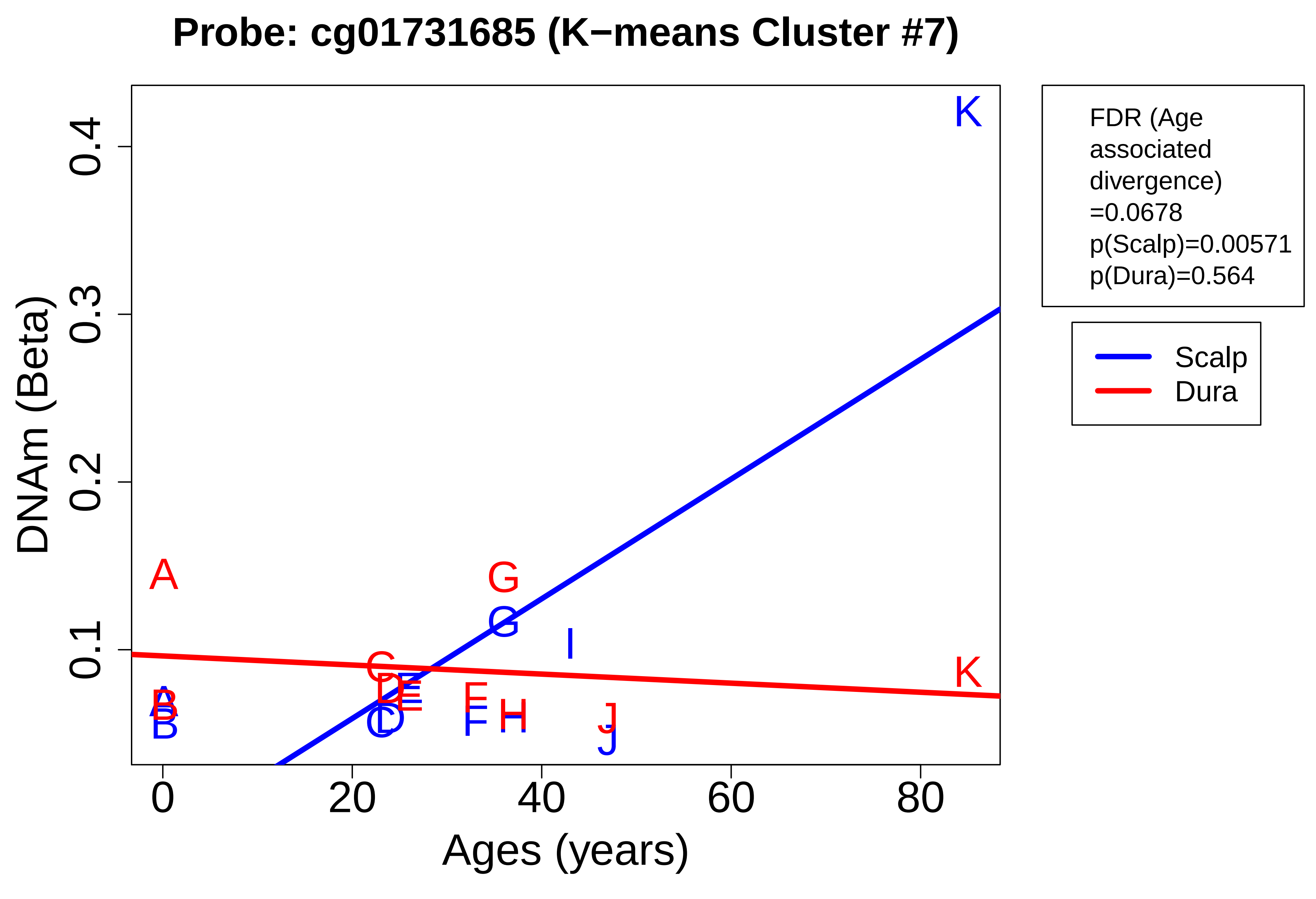

Probe: cg01731685 (K−means Cluster #7)
DNAm (Beta)
0.1
0.2
0.3
0.4
0
20
40
60
80
Ages (years)
FDR (Age associated divergence) =0.0678
p(Scalp)=0.00571
p(Dura)=0.564
Scalp
Dura

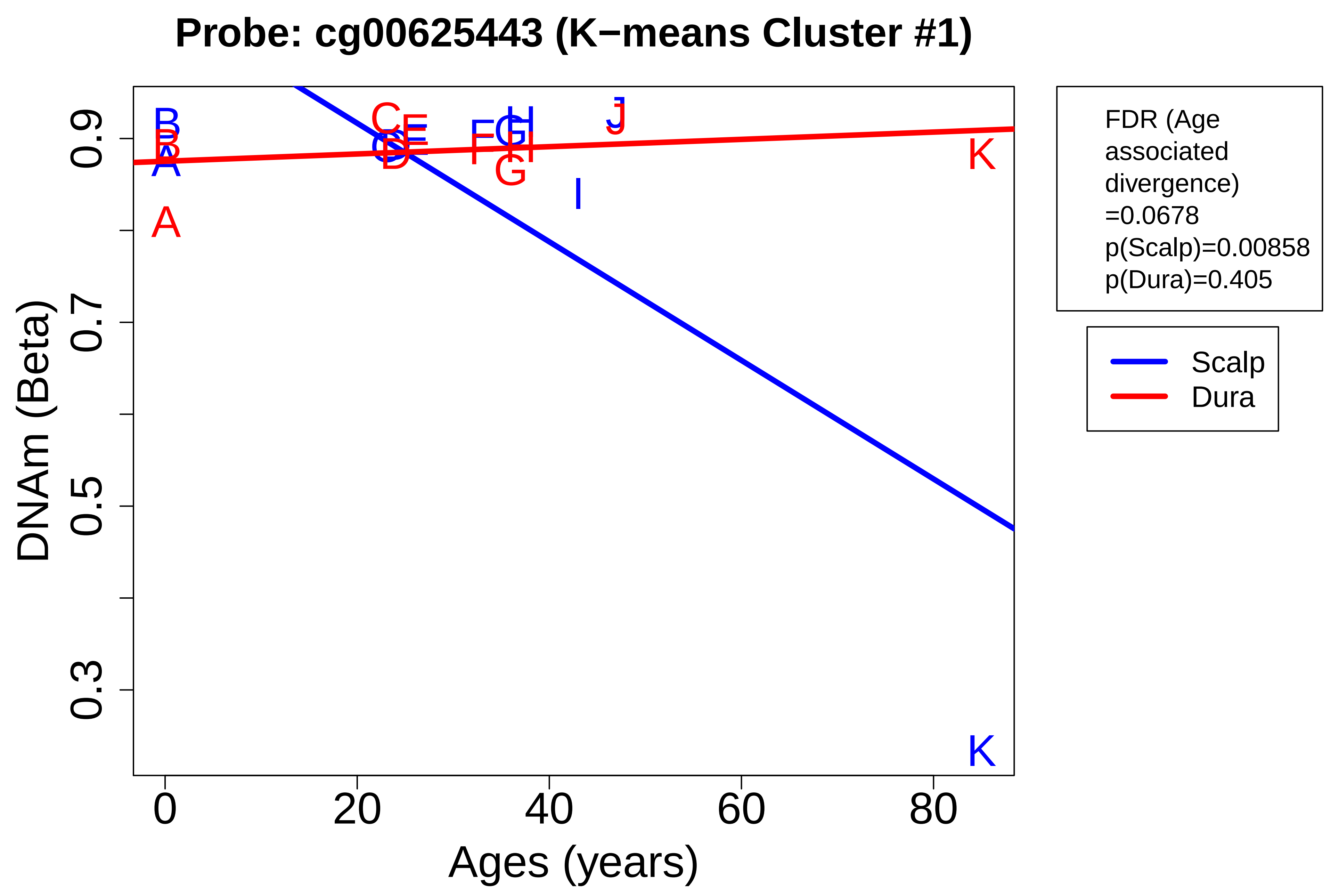

Probe: cg00625443 (K−means Cluster #1)
DNAm (Beta)
0.3
0.5
0.7
0.9
0
20
40
60
80
Ages (years)
FDR (Age associated divergence) =0.0678
p(Scalp)=0.00858
p(Dura)=0.405
Scalp
Dura
A
B
C
D
E
F
G
H
I
J
K

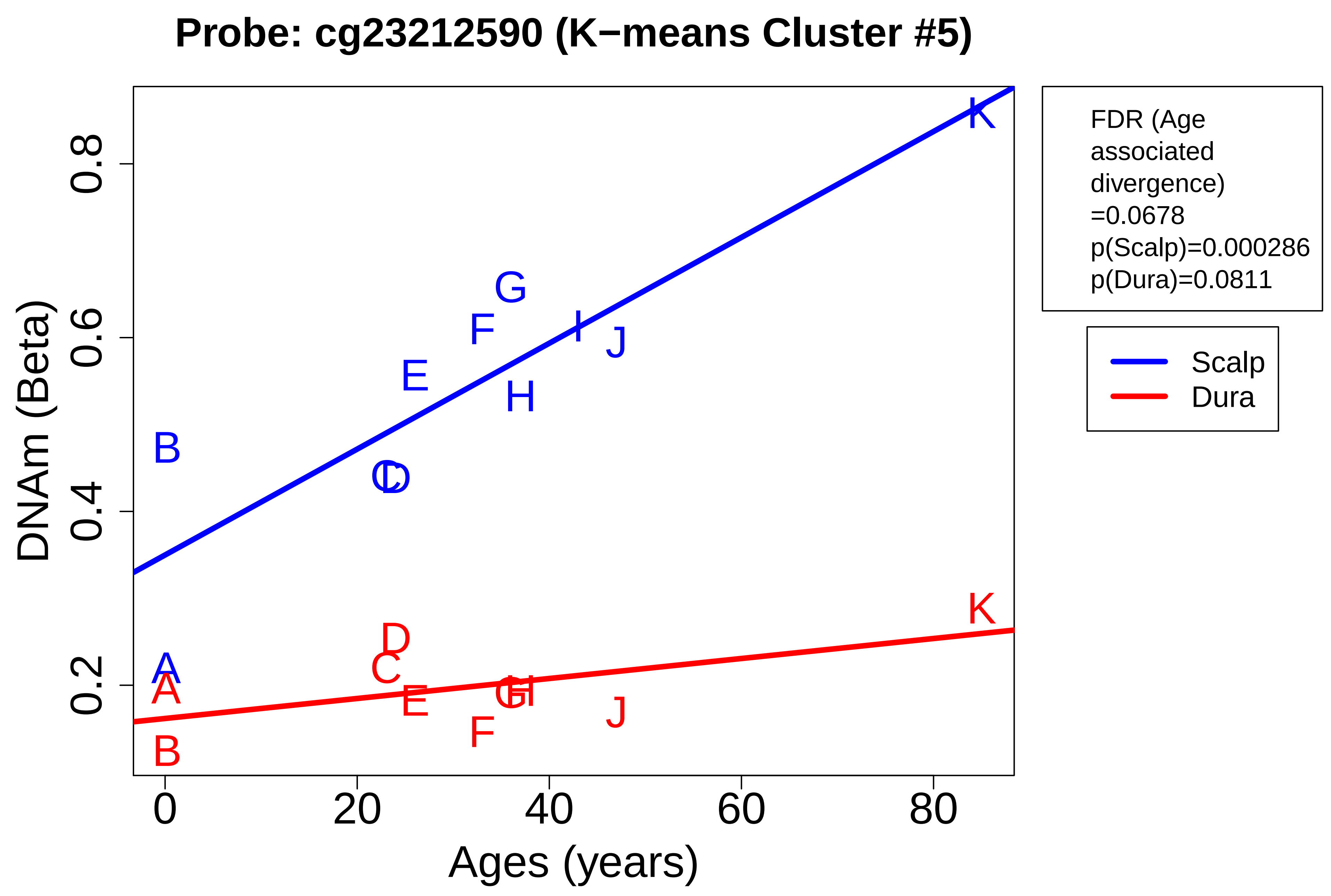

Probe: cg23212590 (K−means Cluster #5)
FDR (Age associated divergence) =0.0678
p(Scalp)=0.000286
p(Dura)=0.0811
Scalp
Dura
DNAm (Beta)
0.2
0.4
0.6
0.8
0
20
40
60
80
Ages (years)
A
B
C
D
E
F
G
H
I
J
K

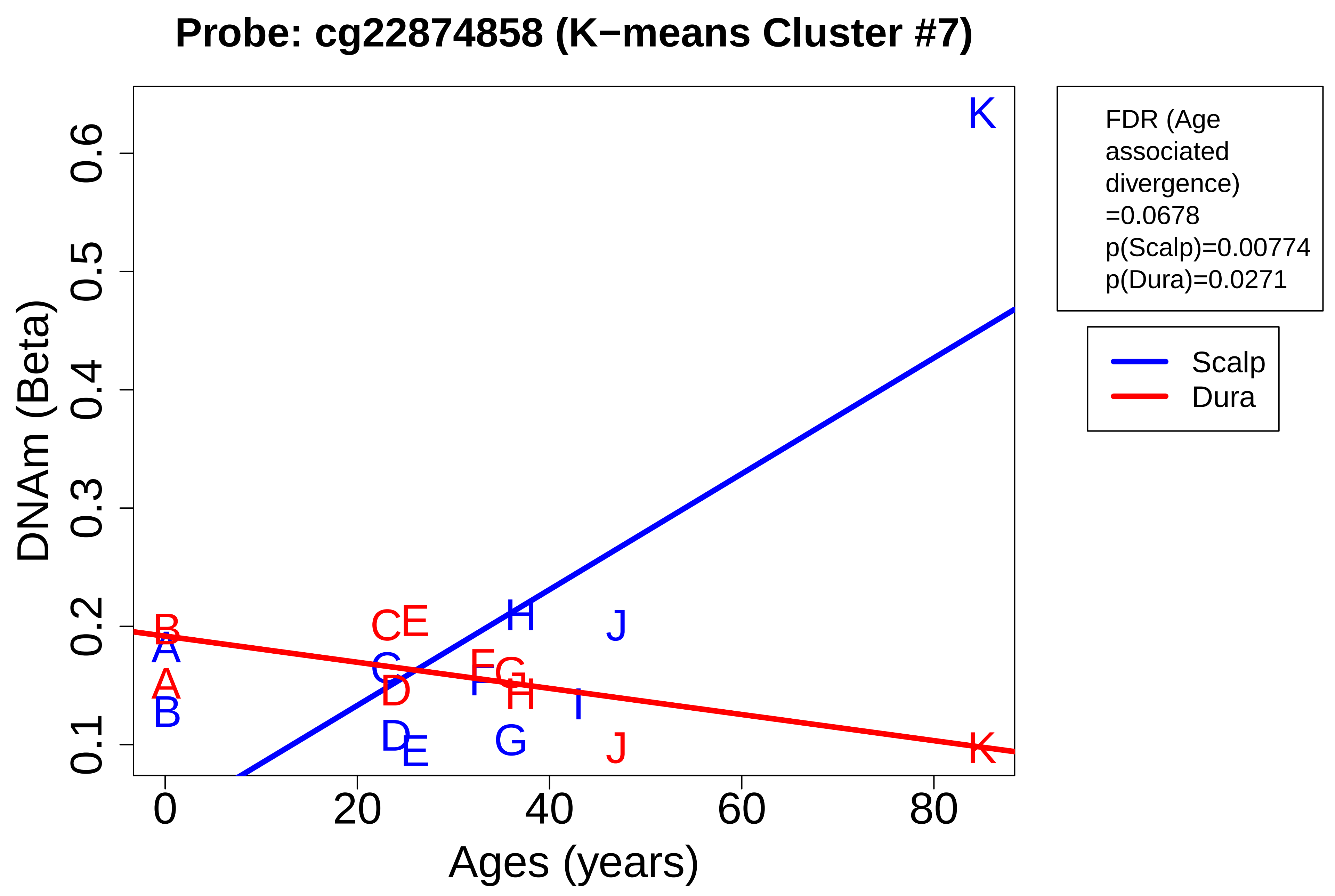

Probe: cg22874858 (K−means Cluster #7)
DNAm (Beta)
0.1
0.2
0.3
0.4
0.5
0.6
Ages (years)
0
20
40
60
80
FDR (Age associated divergence) =0.0678
p(Scalp)=0.00774
p(Dura)=0.0271
Scalp
Dura

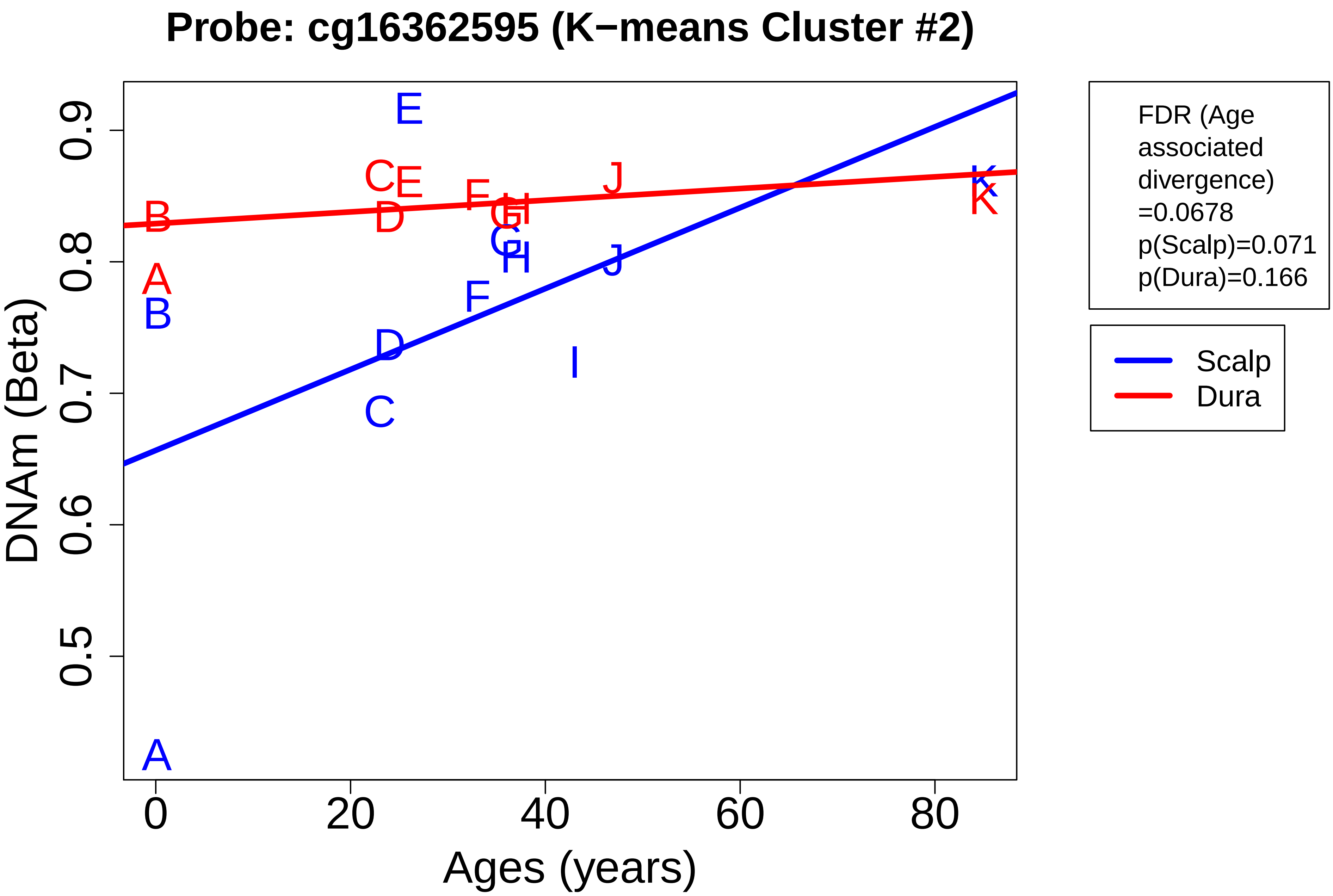

Probe: cg16362595 (K−means Cluster #2)
DNAm (Beta)
0.5
0.6
0.7
0.8
0.9
0
20
40
60
80
Ages (years)
FDR (Age associated divergence) =0.0678
p(Scalp)=0.071
p(Dura)=0.166
Scalp
Dura

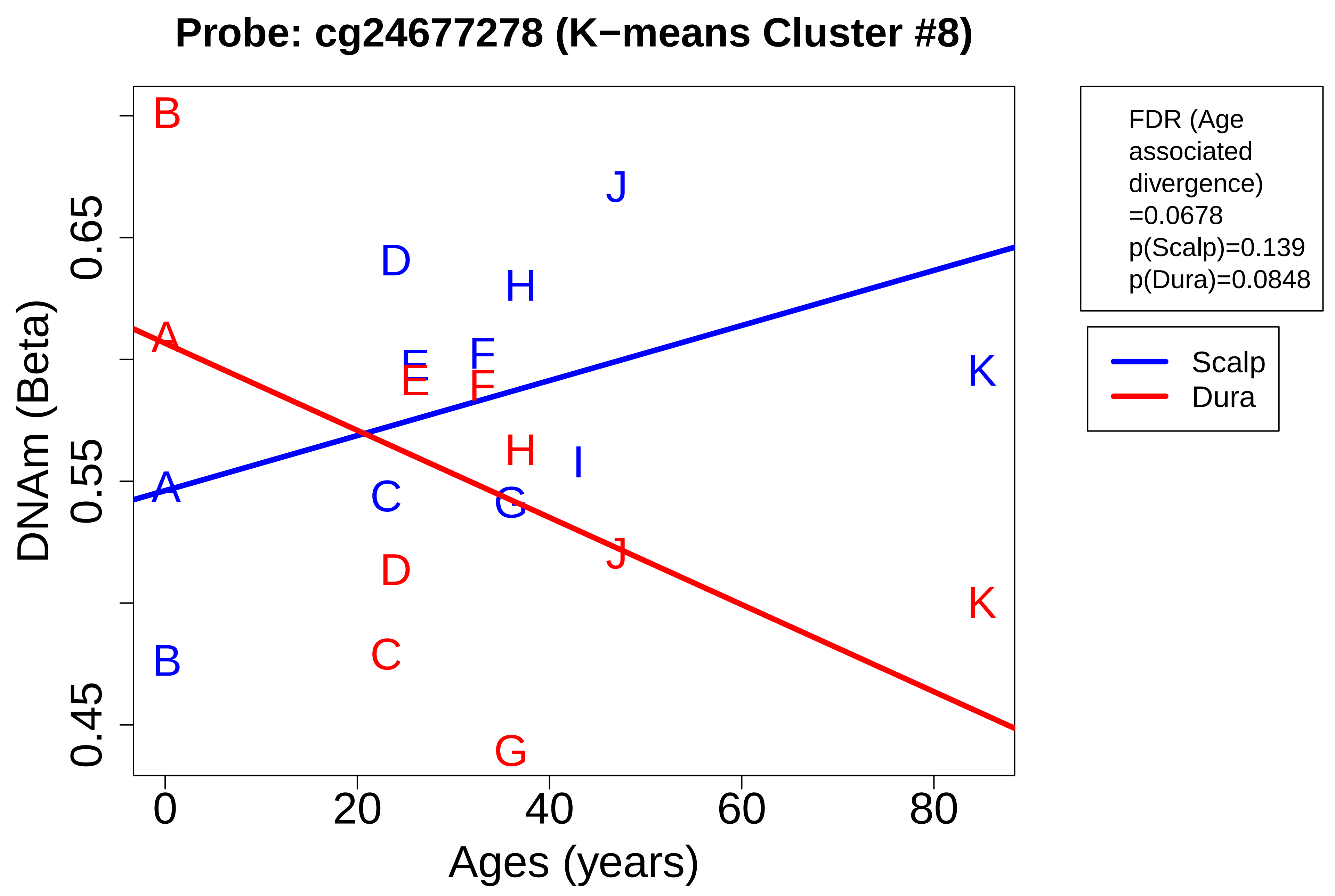

Probe: cg24677278 (K−means Cluster #8)
DNAm (Beta)
0.45
0.55
0.65
Ages (years)
0
20
40
60
80
FDR (Age associated divergence) =0.0678
p(Scalp)=0.139
p(Dura)=0.0848
Scalp
Dura
A
B
C
D
E
F
G
H
I
J
K

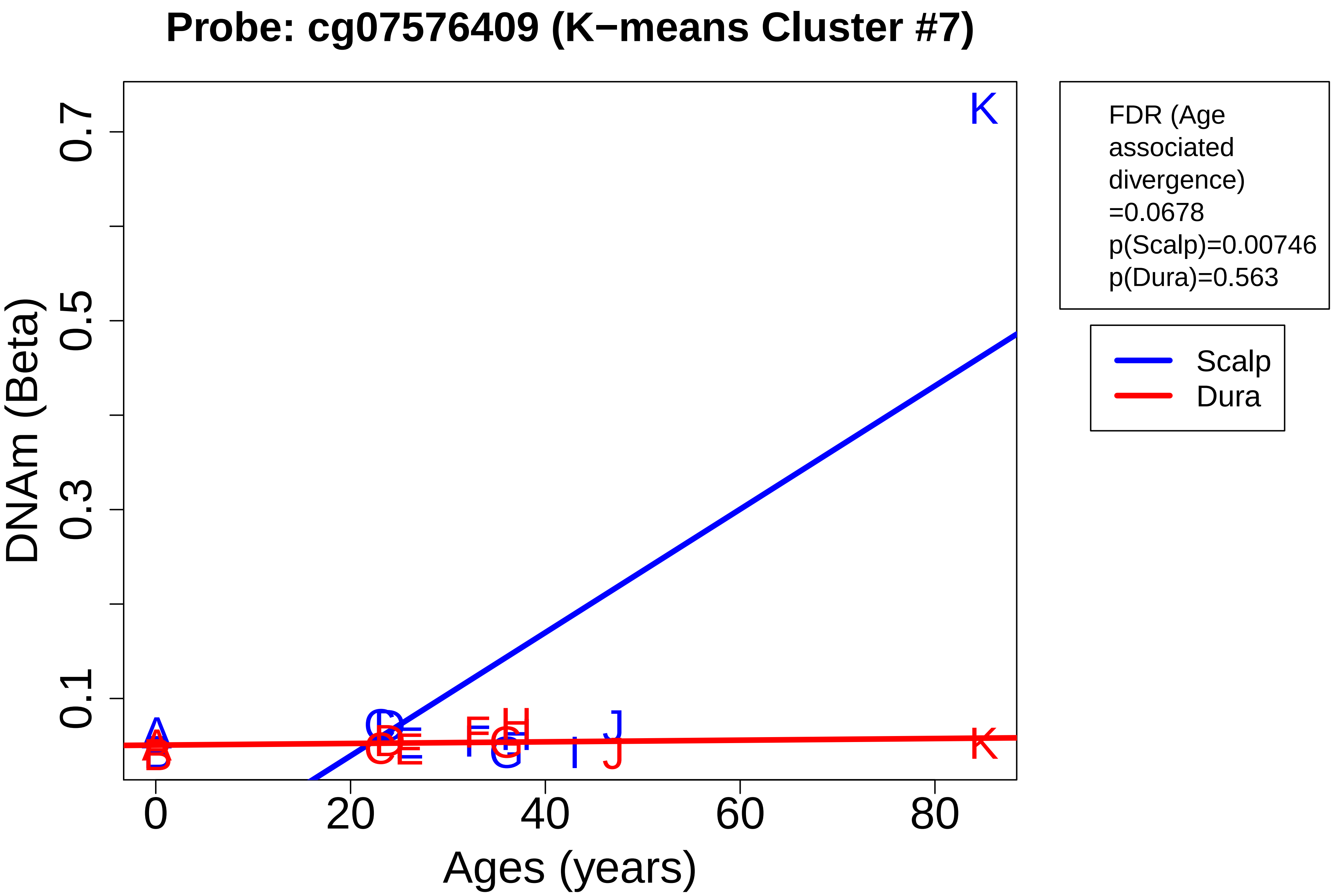

Probe: cg07576409 (K−means Cluster #7)
DNAm (Beta)
0.1
0.3
0.5
0.7
Ages (years)
0
20
40
60
80
FDR (Age associated divergence) =0.0678
p(Scalp)=0.00746
p(Dura)=0.563
Scalp
Dura

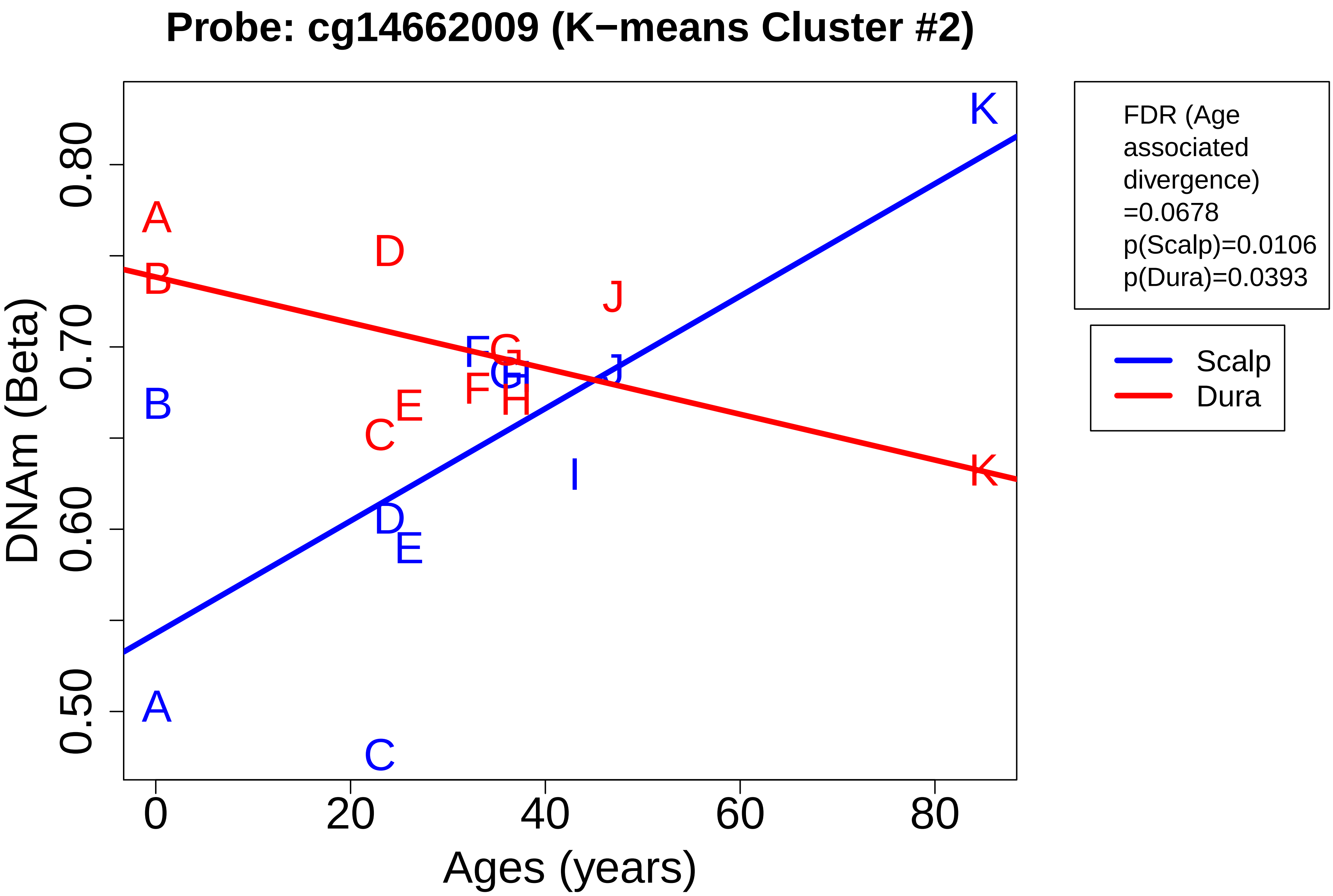
Probe: cg14662009 (K−means Cluster #2)
DNAm (Beta)
0.50
0.60
0.70
0.80
0
20
40
60
80
Ages (years)
A
B
C
D
E
F
G
H
I
J
K
FDR (Age associated divergence) =0.0678
p(Scalp)=0.0106
p(Dura)=0.0393
Scalp
Dura

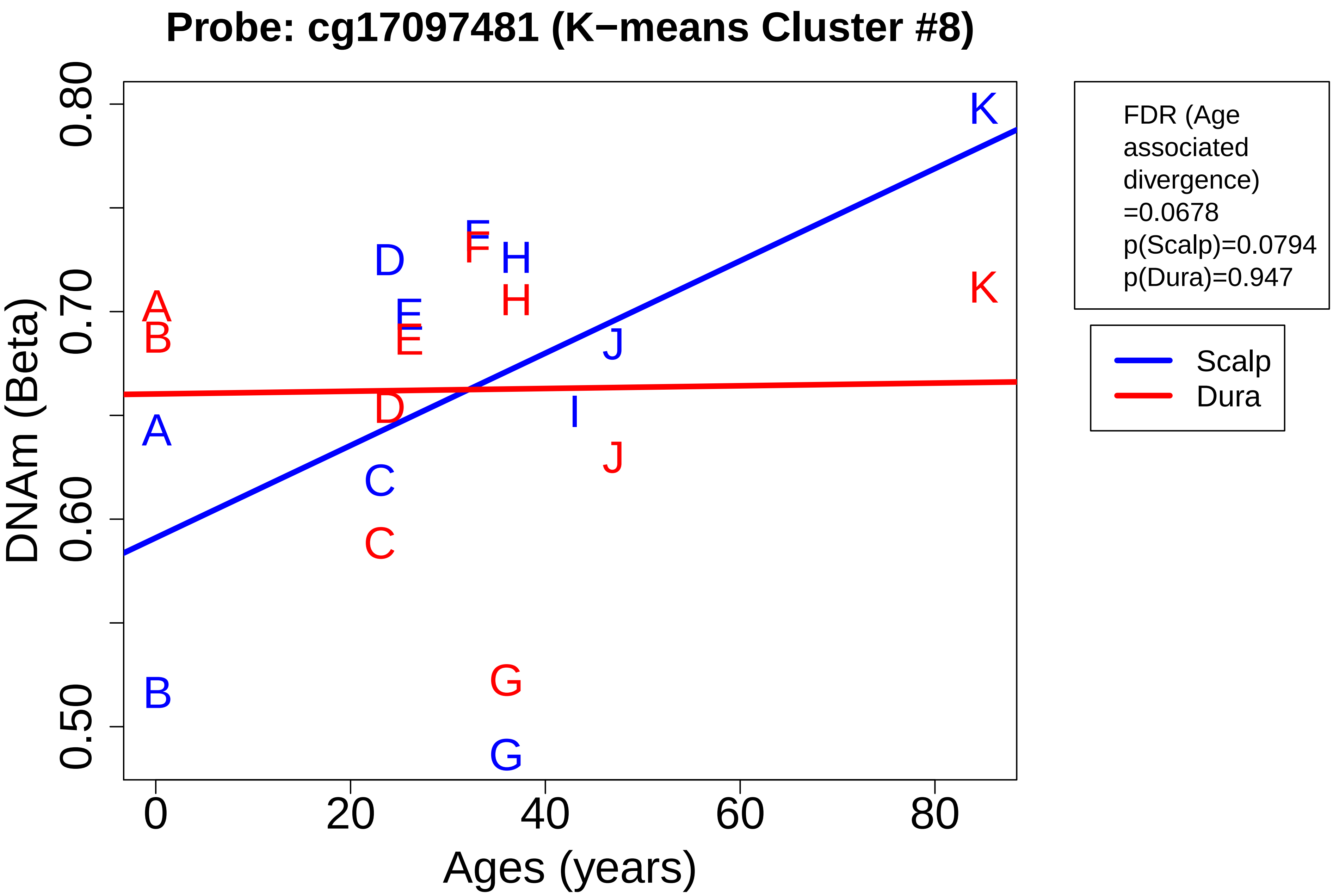

Probe: cg17097481 (K−means Cluster #8)
DNAm (Beta)
0.50
0.60
0.70
0.80
Ages (years)
0
20
40
60
80
A
B
C
D
E
F
G
H
I
J
K
FDR (Age associated divergence) =0.0678
p(Scalp)=0.0794
p(Dura)=0.947
Scalp
Dura

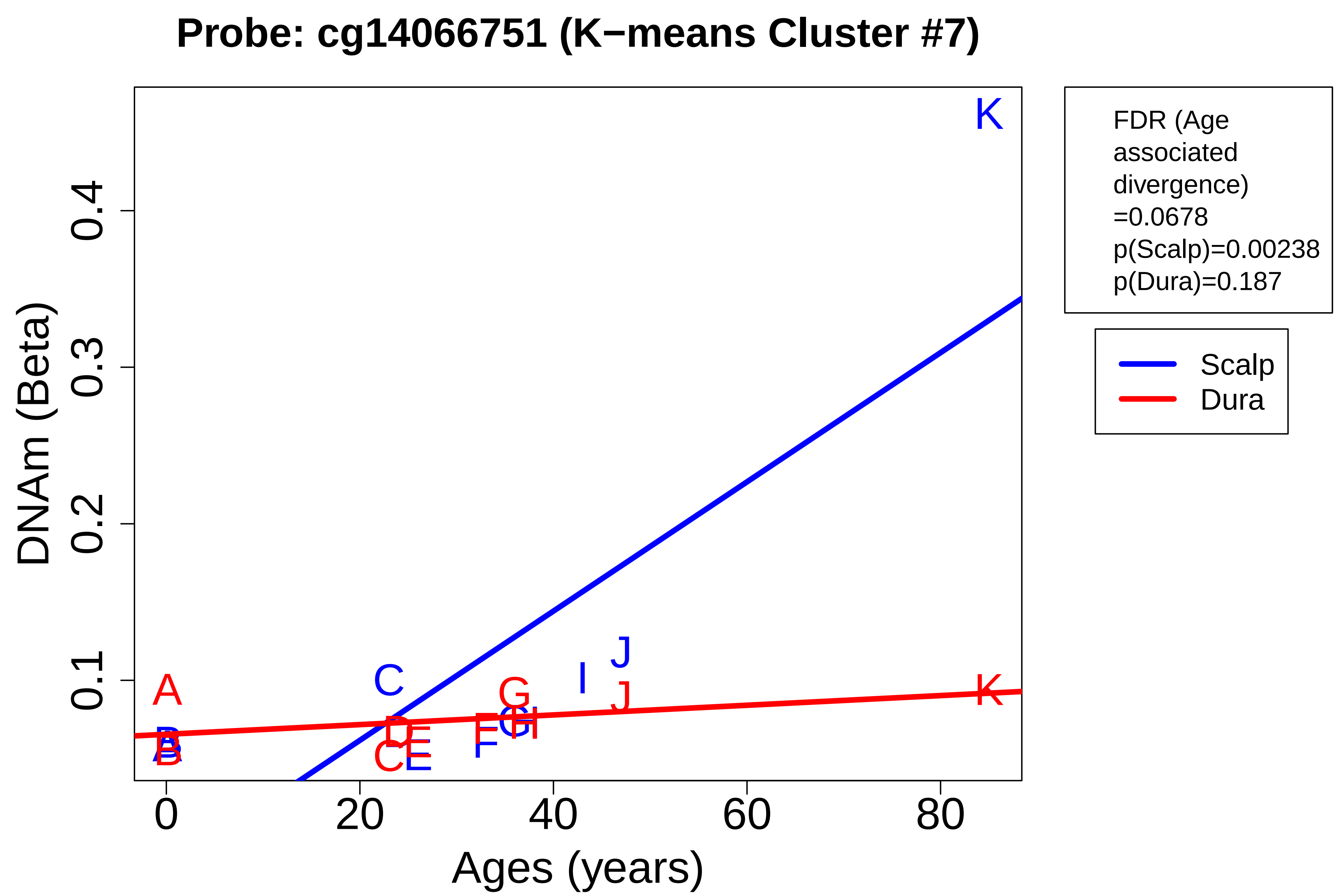

Probe: cg14066751 (K−means Cluster #7)
FDR (Age associated divergence) =0.0678
p(Scalp)=0.00238
p(Dura)=0.187
Scalp
Dura
DNAm (Beta)
0.1
0.2
0.3
0.4
0
20
40
60
80
Ages (years)

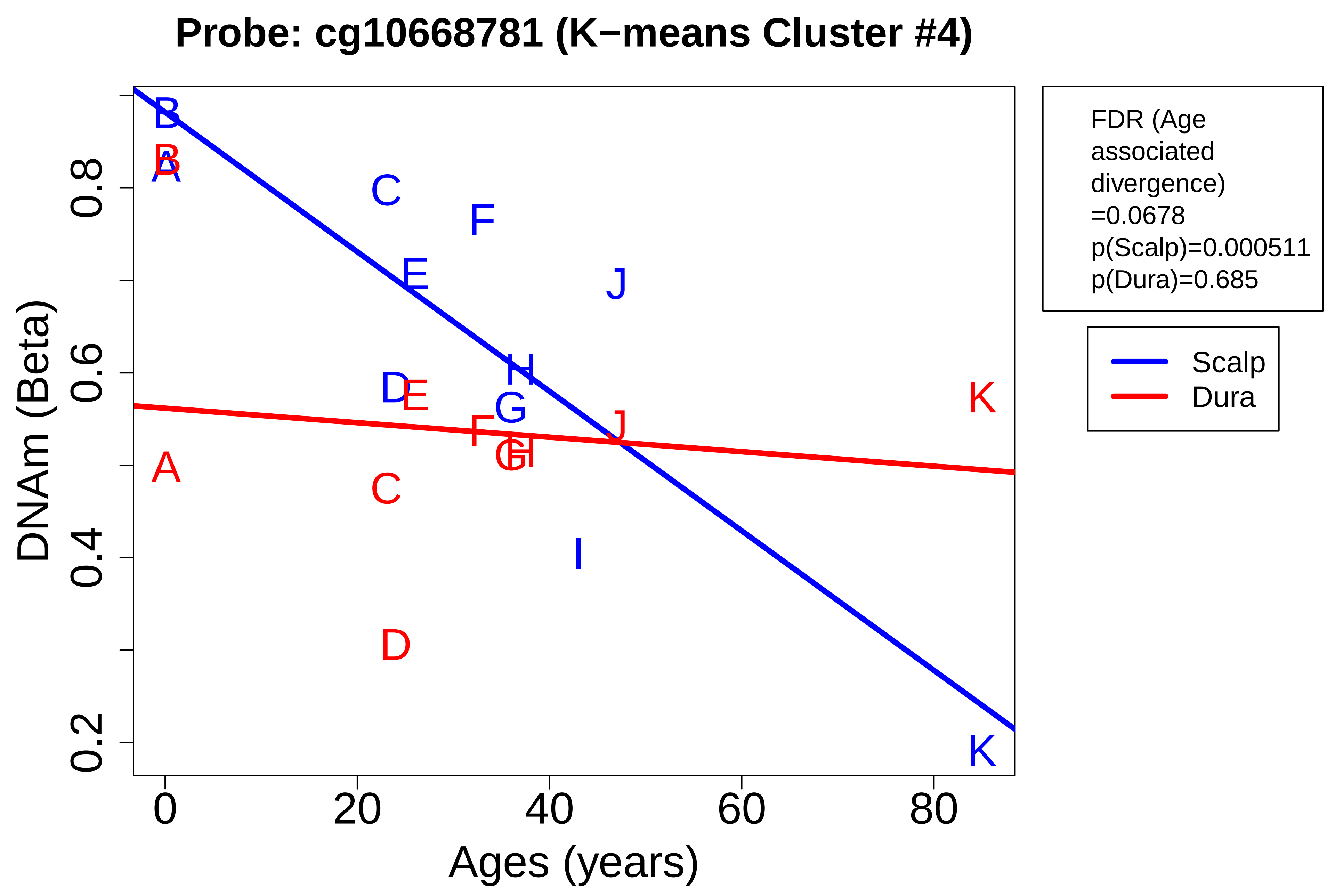

Probe: cg10668781 (K−means Cluster #4)
DNAm (Beta)
0.2
0.4
0.6
0.8
Ages (years)
0
20
40
60
80
FDR (Age associated divergence) =0.0678
p(Scalp)=0.000511
p(Dura)=0.685
Scalp
Dura
A
B
C
D
E
F
G
H
I
J
K

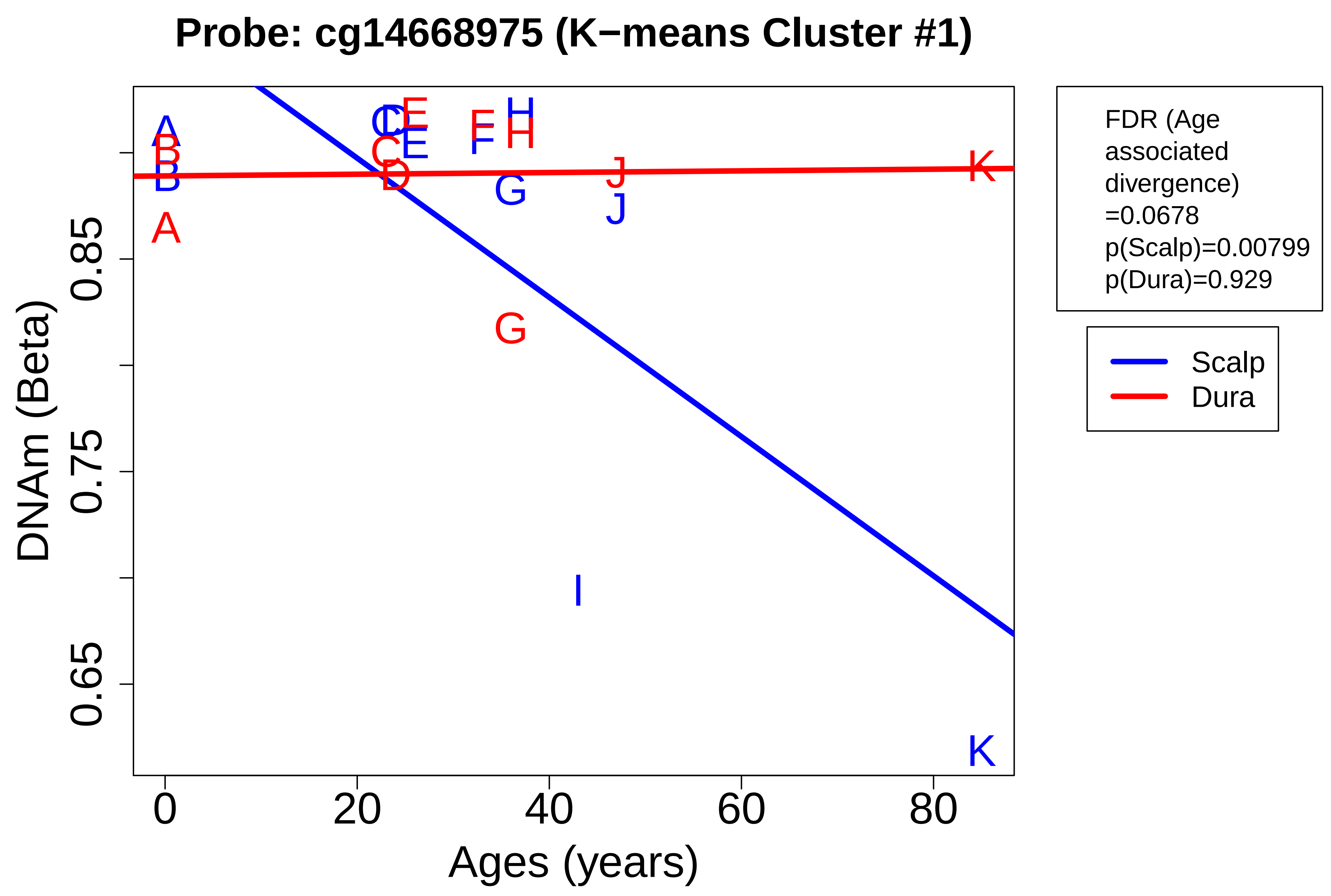

Probe: cg14668975 (K−means Cluster #1)
DNAm (Beta)
0.65
0.75
0.85
Ages (years)
0
20
40
60
80
FDR (Age associated divergence) =0.0678
p(Scalp)=0.00799
p(Dura)=0.929
Scalp
Dura
A
B
C
D
E
F
G
H
I
J
K

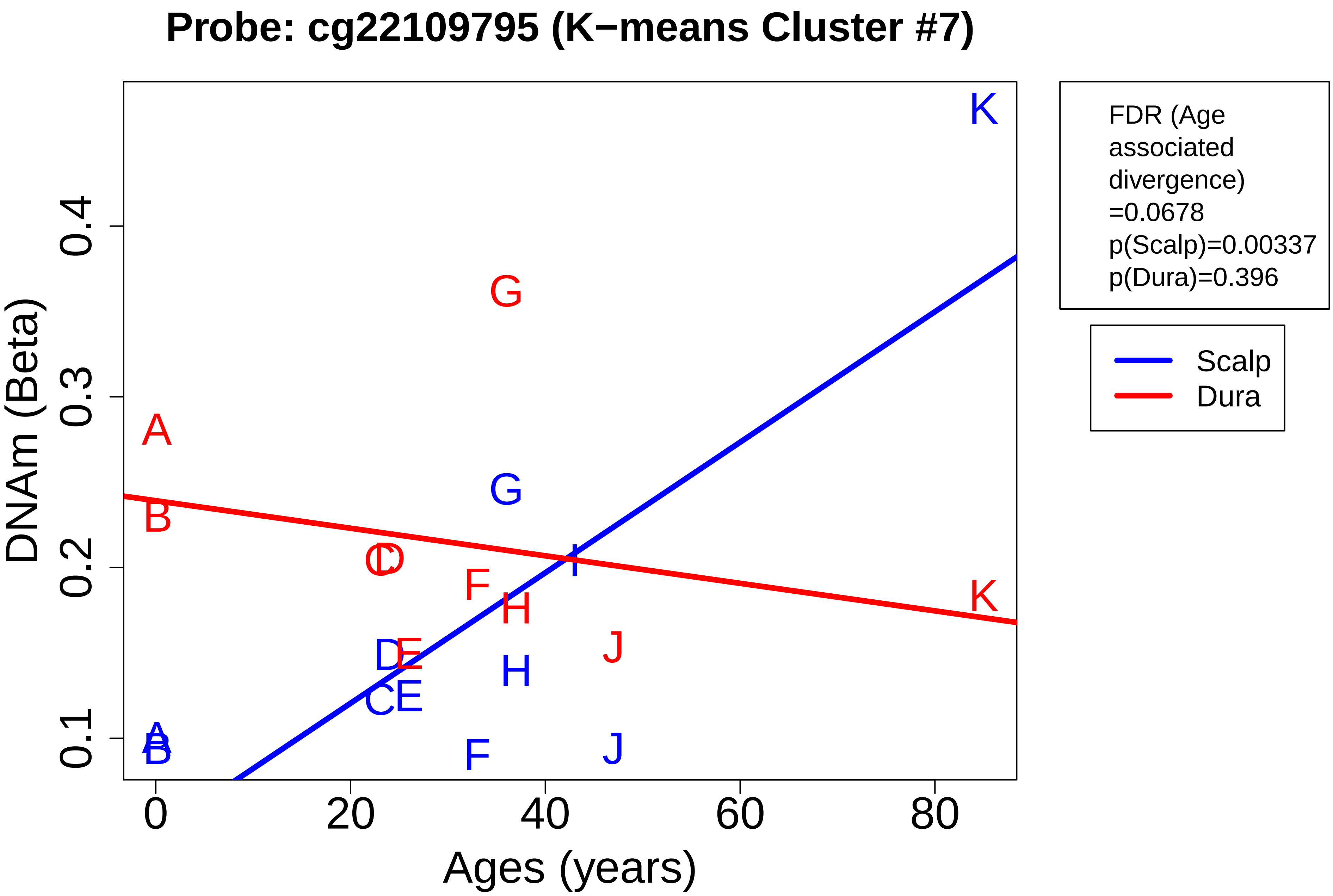

Probe: cg22109795 (K−means Cluster #7)
DNAm (Beta)
0.1
0.2
0.3
0.4
Ages (years)
0
20
40
60
80
FDR (Age associated divergence) =0.0678
p(Scalp)=0.00337
p(Dura)=0.396
Scalp
Dura

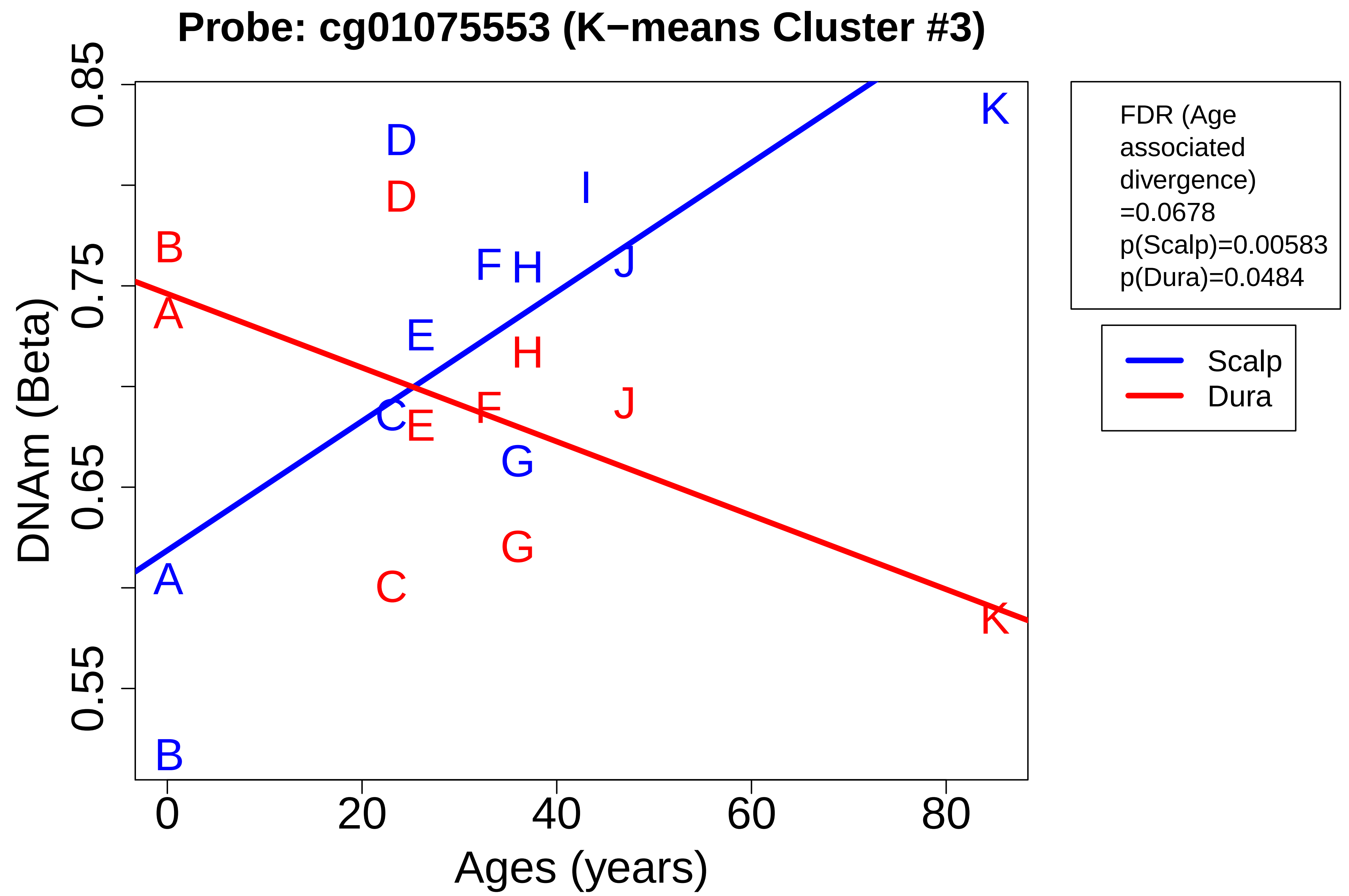

Probe: cg01075553 (K−means Cluster #3)
DNAm (Beta)
0.55
0.65
0.75
0.85
0
20
40
60
80
Ages (years)
FDR (Age associated divergence) =0.0678
p(Scalp)=0.00583
p(Dura)=0.0484
Scalp
Dura

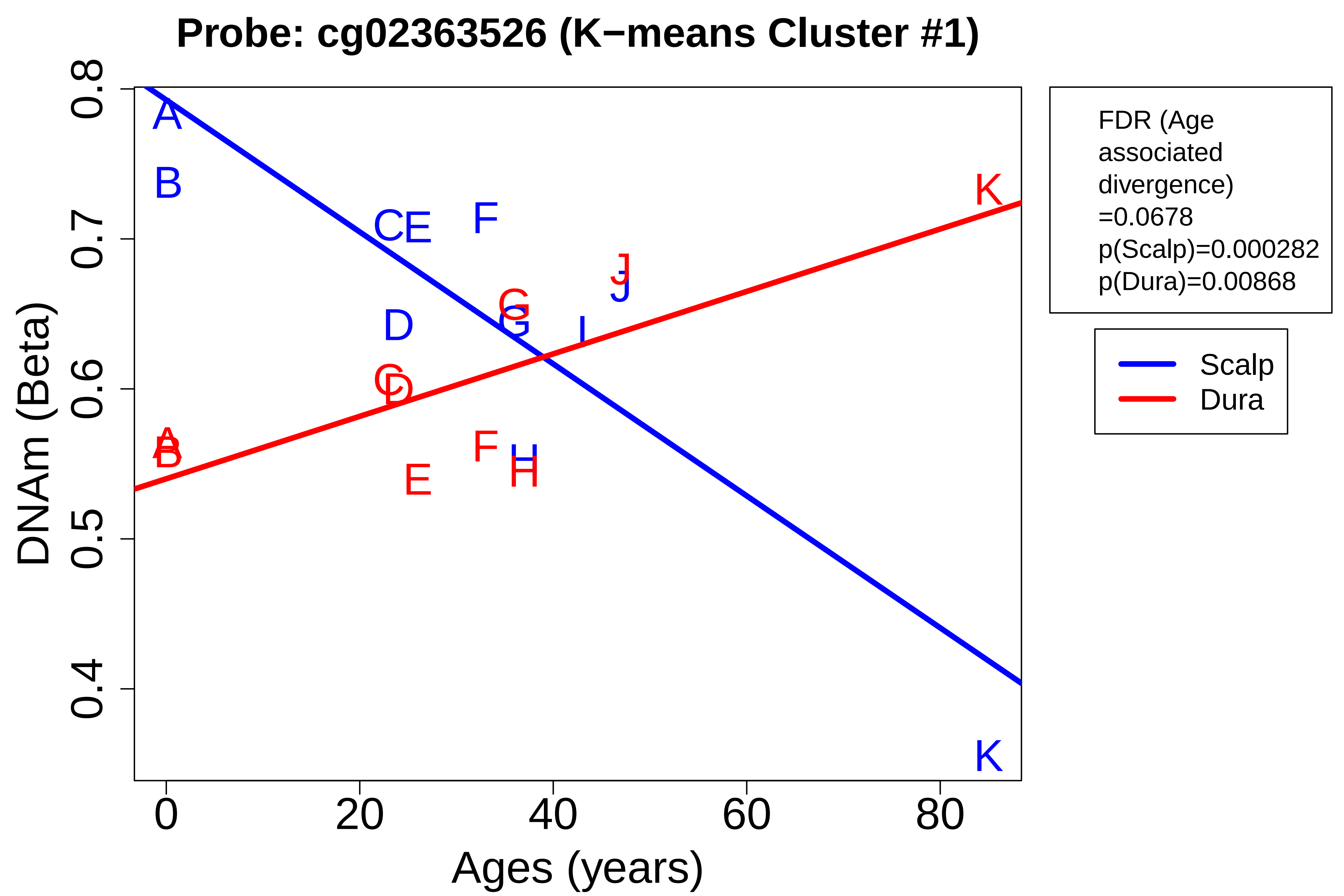

Probe: cg02363526 (K−means Cluster #1)
DNAm (Beta)
0.4
0.5
0.6
0.7
0.8
0
20
40
60
80
Ages (years)
A
B
CE
F
D
G
H
I
J
K
FDR (Age associated divergence) =0.0678
p(Scalp)=0.000282
p(Dura)=0.00868
Scalp
Dura

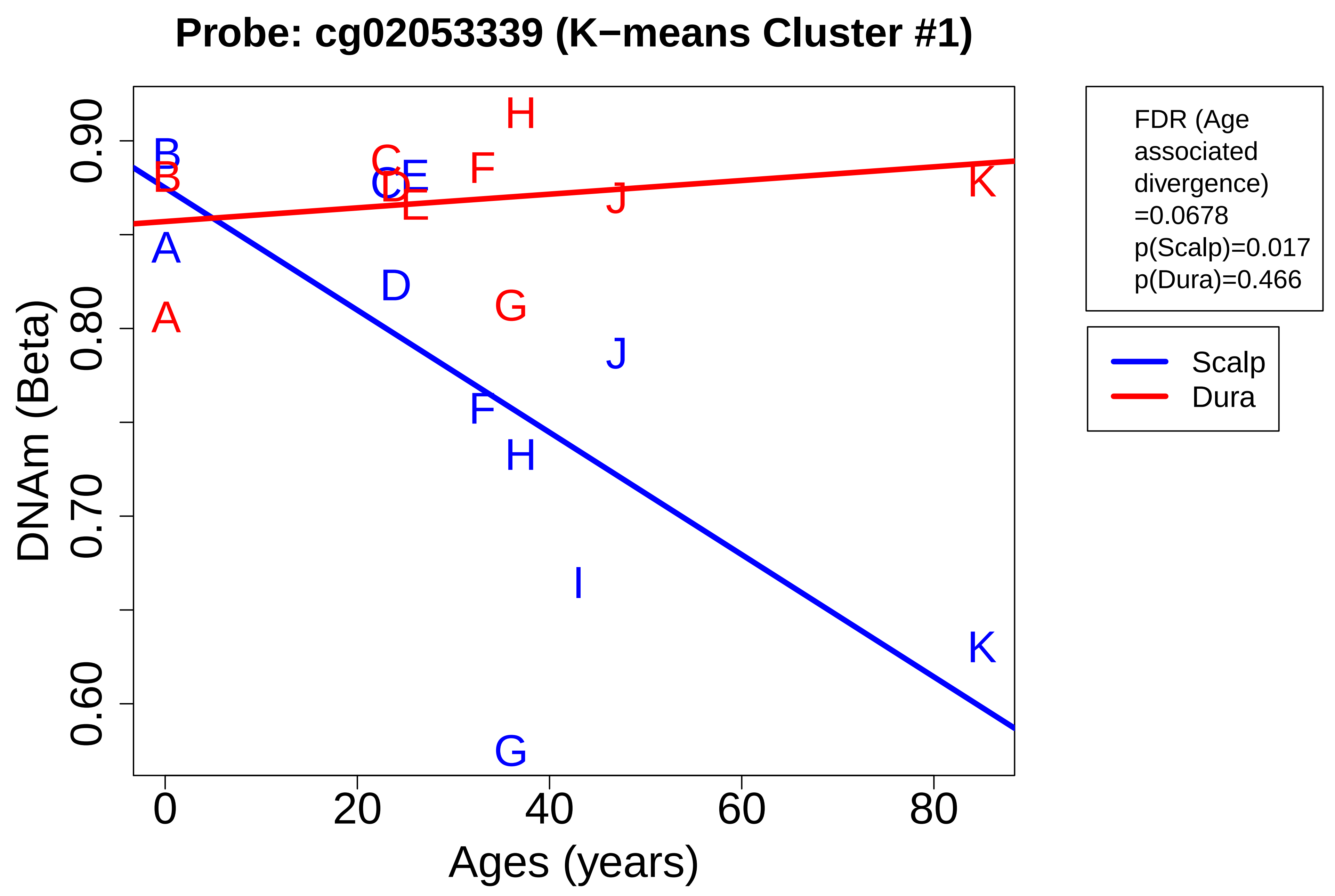

Probe: cg02053339 (K−means Cluster #1)
DNAm (Beta)
0.60
0.70
0.80
0.90
0
20
40
60
80
Ages (years)
FDR (Age associated divergence) =0.0678
p(Scalp)=0.017
p(Dura)=0.466
Scalp
Dura

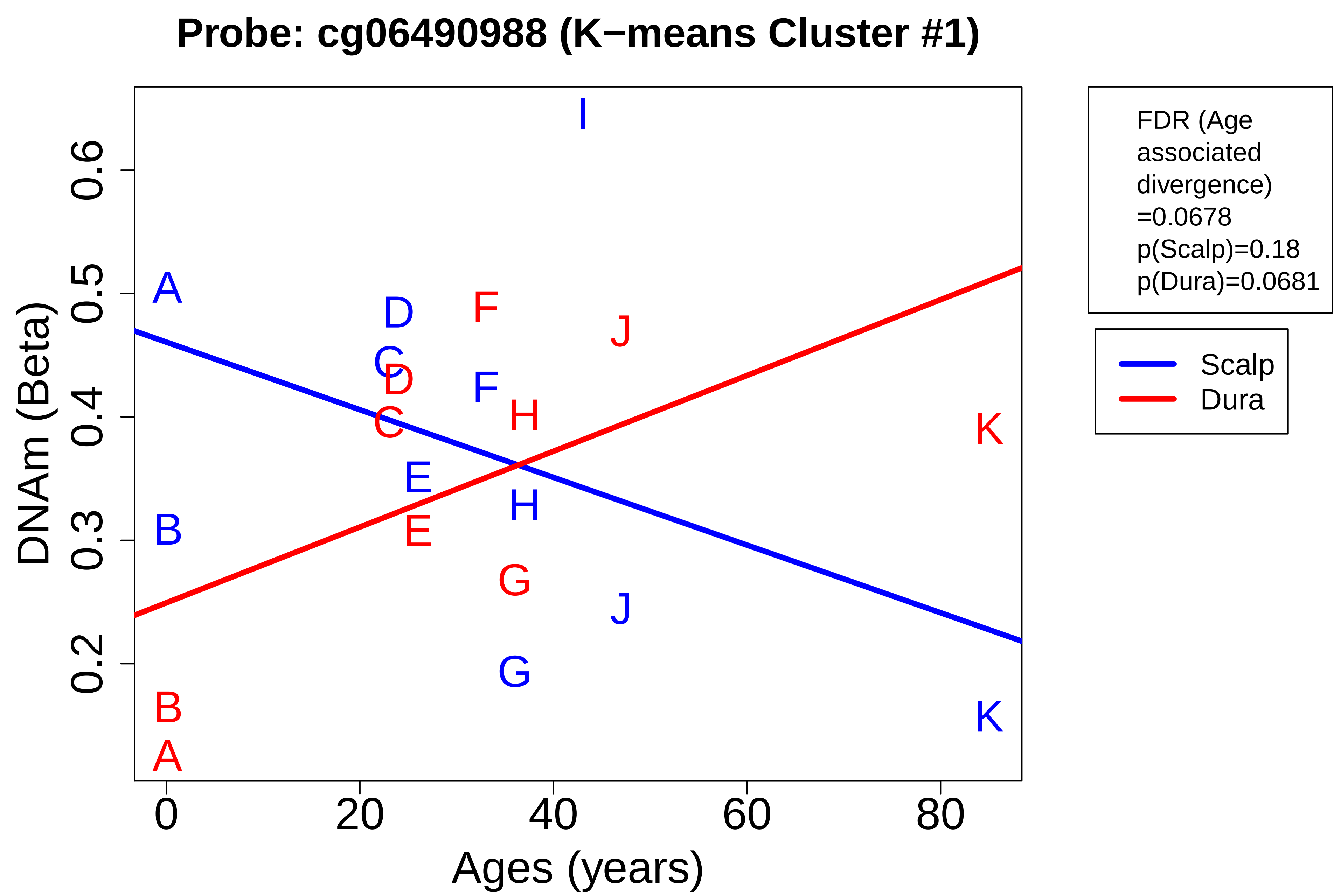

Probe: cg06490988 (K−means Cluster #1)
DNAm (Beta)
Ages (years)
0.2
0.3
0.4
0.5
0.6
0
20
40
60
80
FDR (Age associated divergence) =0.0678
p(Scalp)=0.18
p(Dura)=0.0681
Scalp
Dura

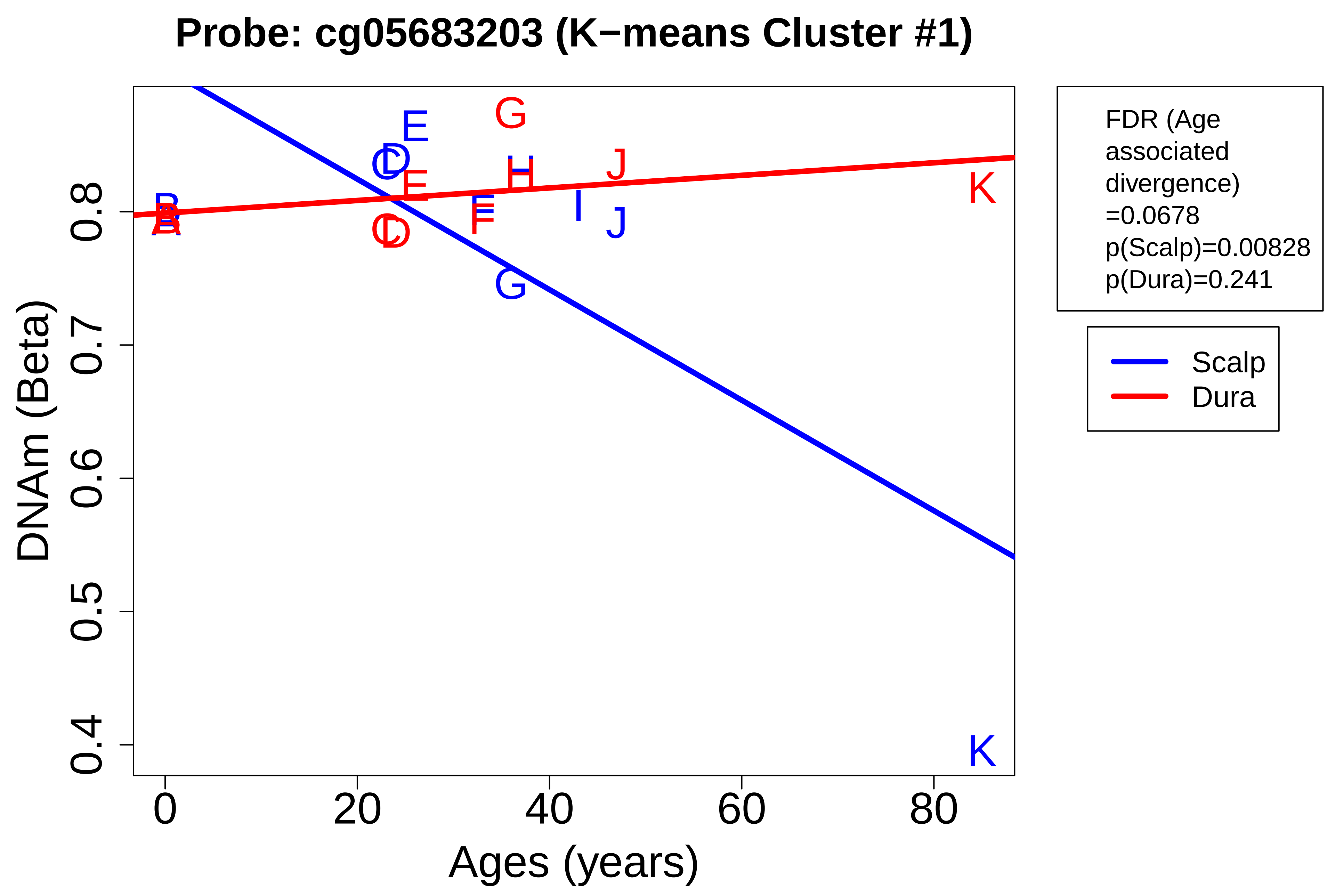

Probe: cg05683203 (K−means Cluster #1)
FDR (Age associated divergence) =0.0678
p(Scalp)=0.00828
p(Dura)=0.241
Scalp
Dura
DNAm (Beta)
Ages (years)
0.4
0.5
0.6
0.7
0.8
0
20
40
60
80

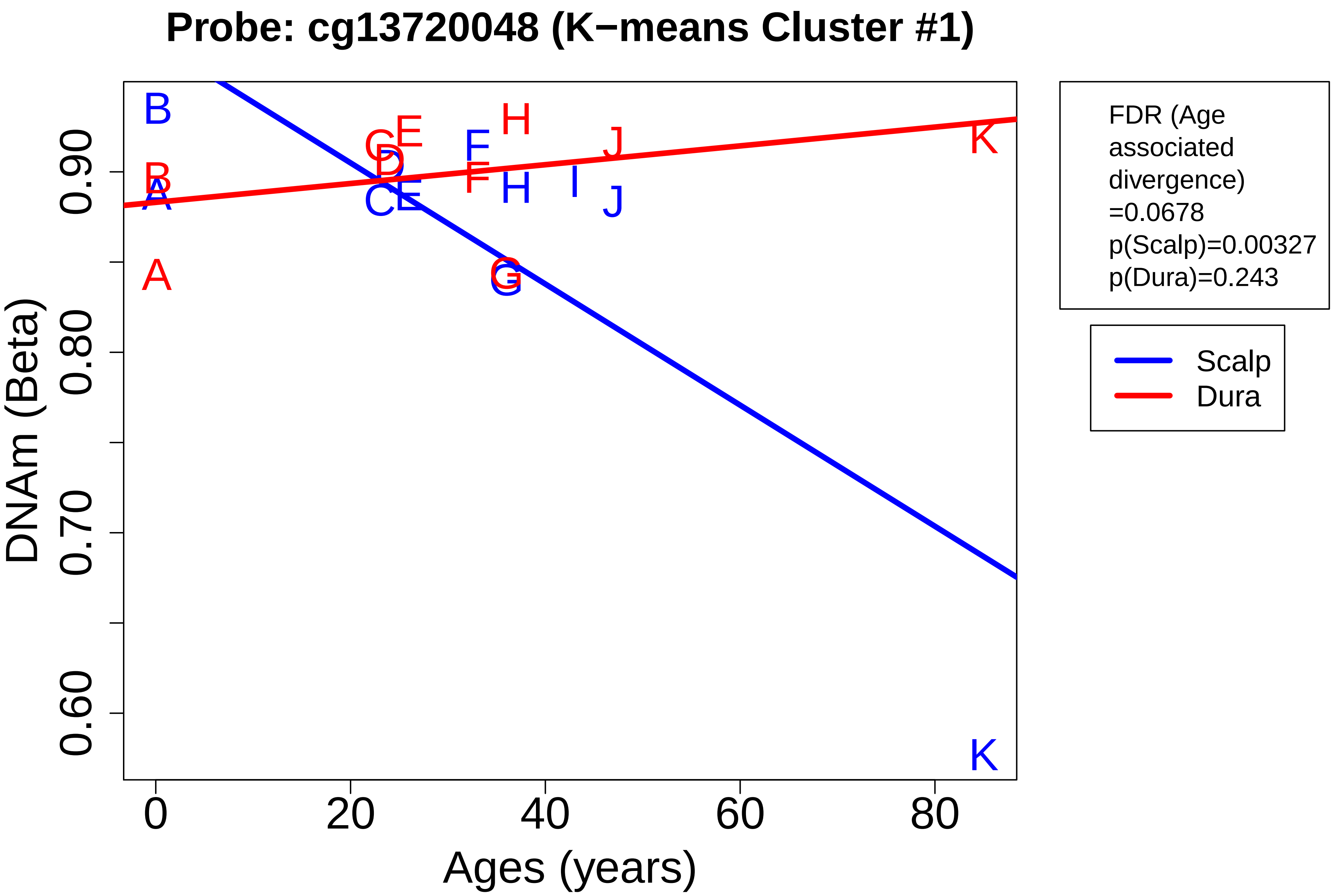

Probe: cg13720048 (K−means Cluster #1)
DNAm (Beta)
0.60
0.70
0.80
0.90
Ages (years)
0
20
40
60
80
FDR (Age associated divergence) =0.0678
p(Scalp)=0.00327
p(Dura)=0.243
Scalp
Dura

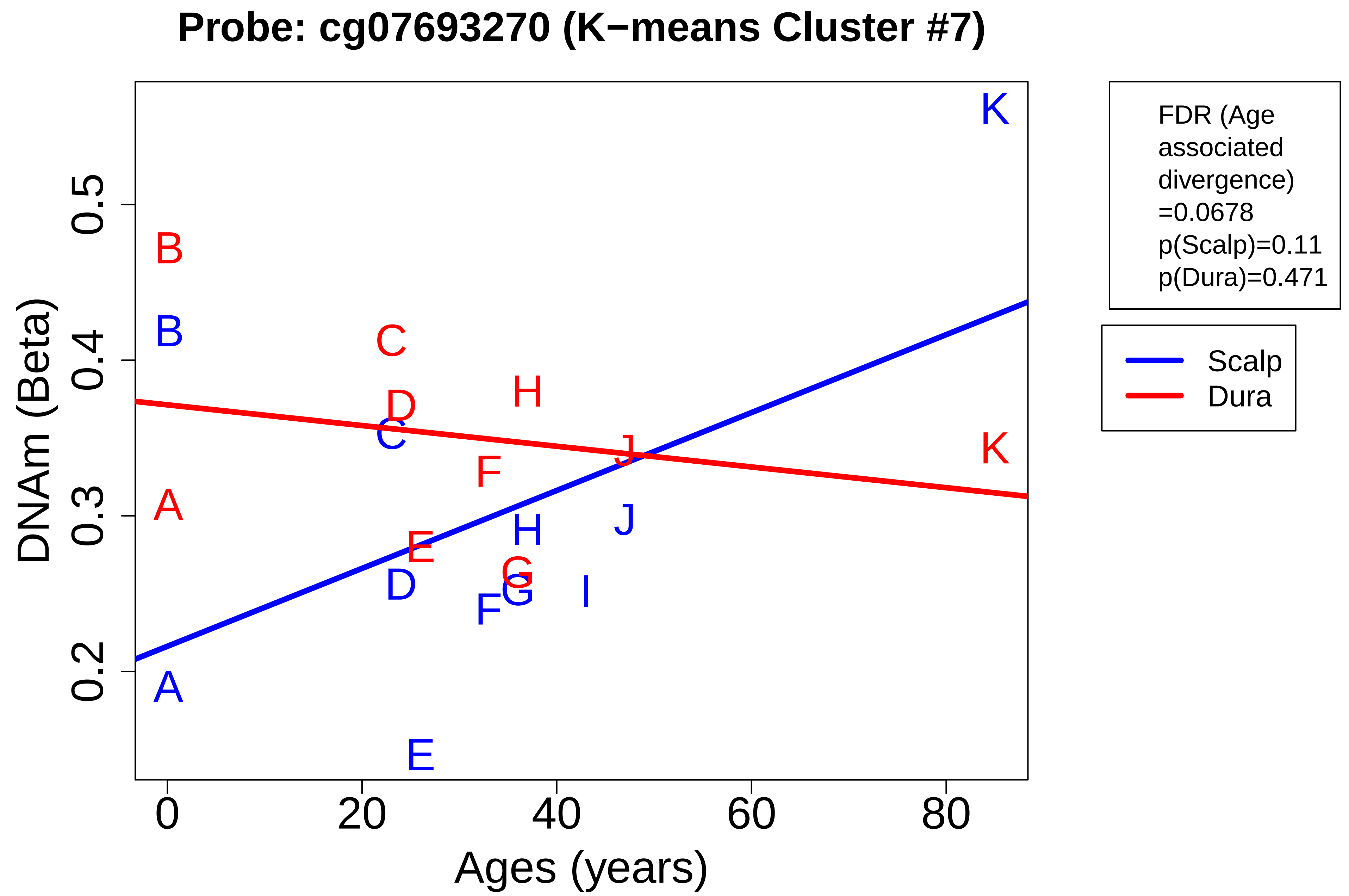

Probe: cg07693270 (K−means Cluster #7)
DNAm (Beta)
Ages (years)
0.2
0.3
0.4
0.5
0
20
40
60
80
FDR (Age associated divergence) =0.0678
p(Scalp)=0.11
p(Dura)=0.471
Scalp
Dura

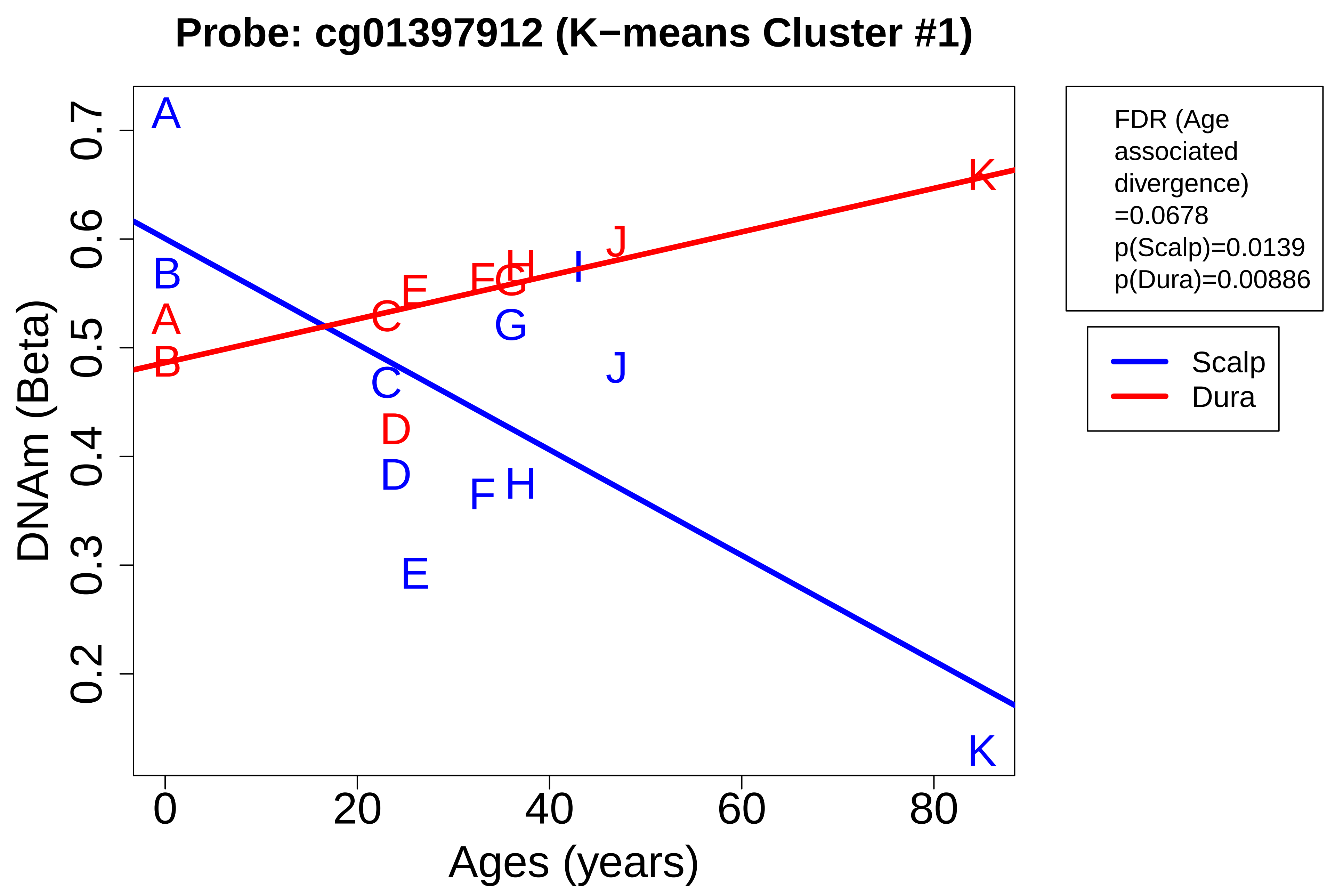

Probe: cg01397912 (K−means Cluster #1)
FDR (Age associated divergence) =0.0678
p(Scalp)=0.0139
p(Dura)=0.00886
Scalp
Dura
DNAm (Beta)
0.2
0.3
0.4
0.5
0.6
0.7
0
20
40
60
80
Ages (years)
A
B
C
D
E
F
G
H
I
J
K

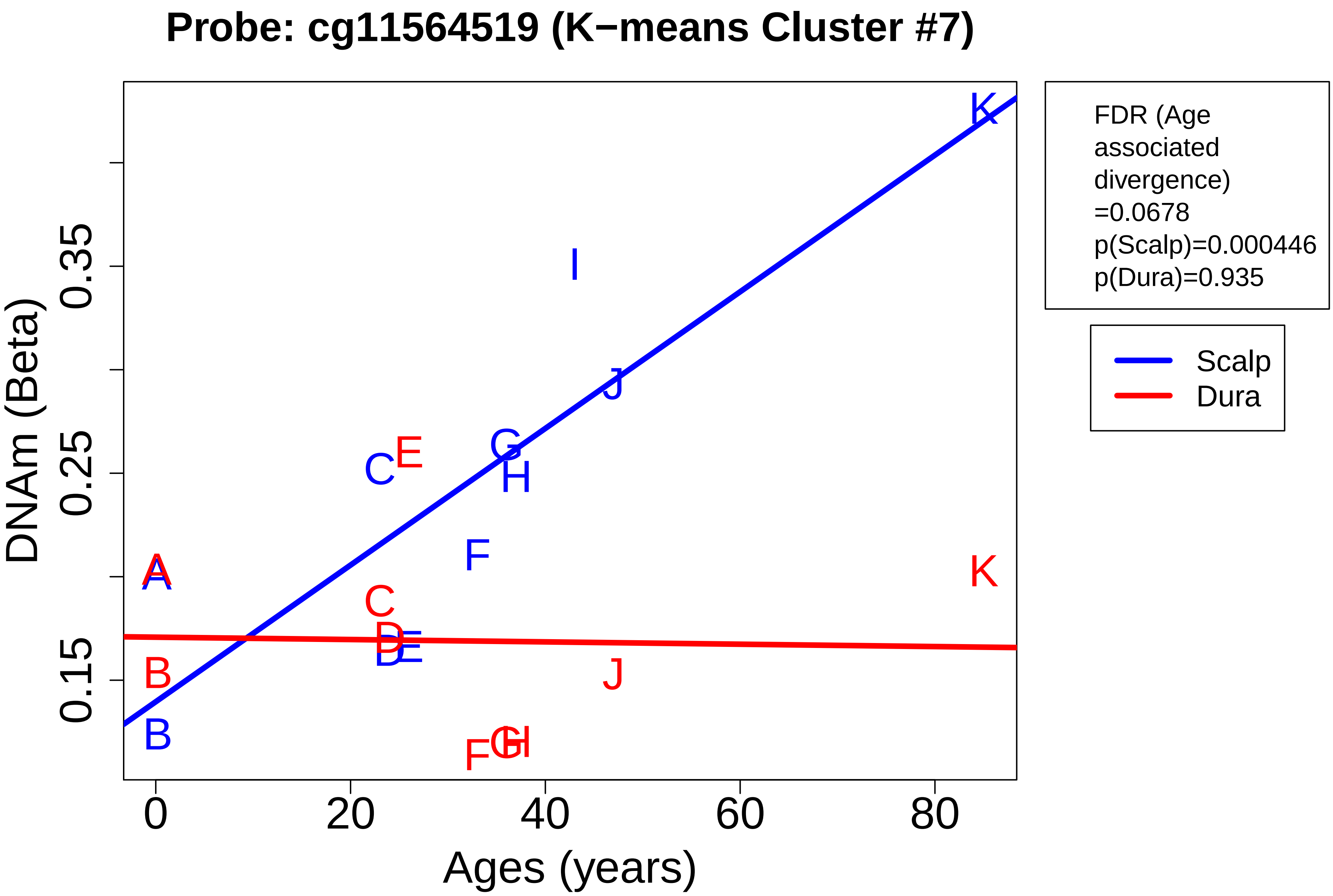

Probe: cg11564519 (K−means Cluster #7)
DNAm (Beta)
0.15
0.25
0.35
Ages (years)
0
20
40
60
80
FDR (Age associated divergence) =0.0678
p(Scalp)=0.000446
p(Dura)=0.935
Scalp
Dura

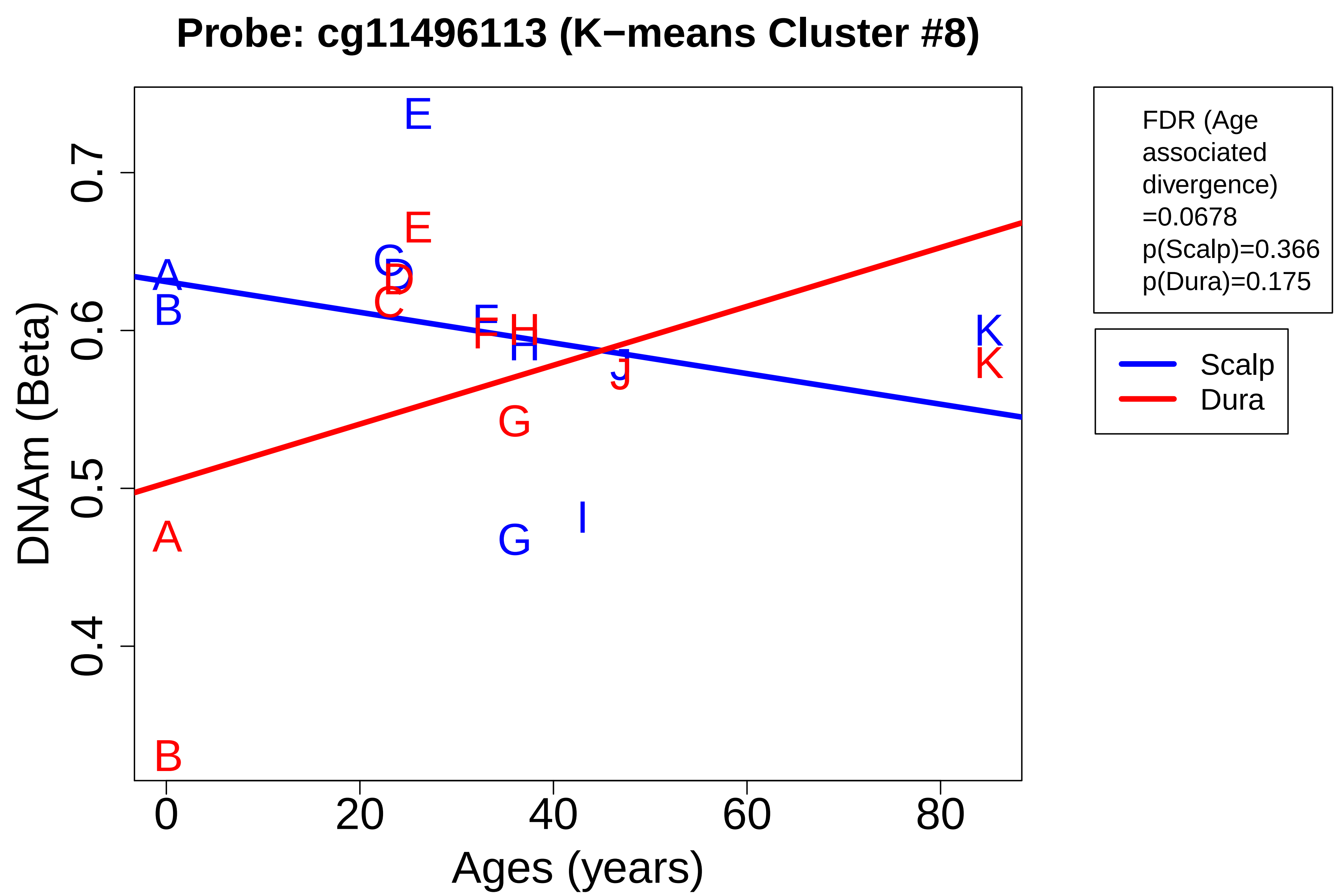

Probe: cg11496113 (K−means Cluster #8)
DNAm (Beta)
Ages (years)
0.4
0.5
0.6
0.7
0
20
40
60
80
FDR (Age associated divergence) =0.0678
p(Scalp)=0.366
p(Dura)=0.175
Scalp
Dura

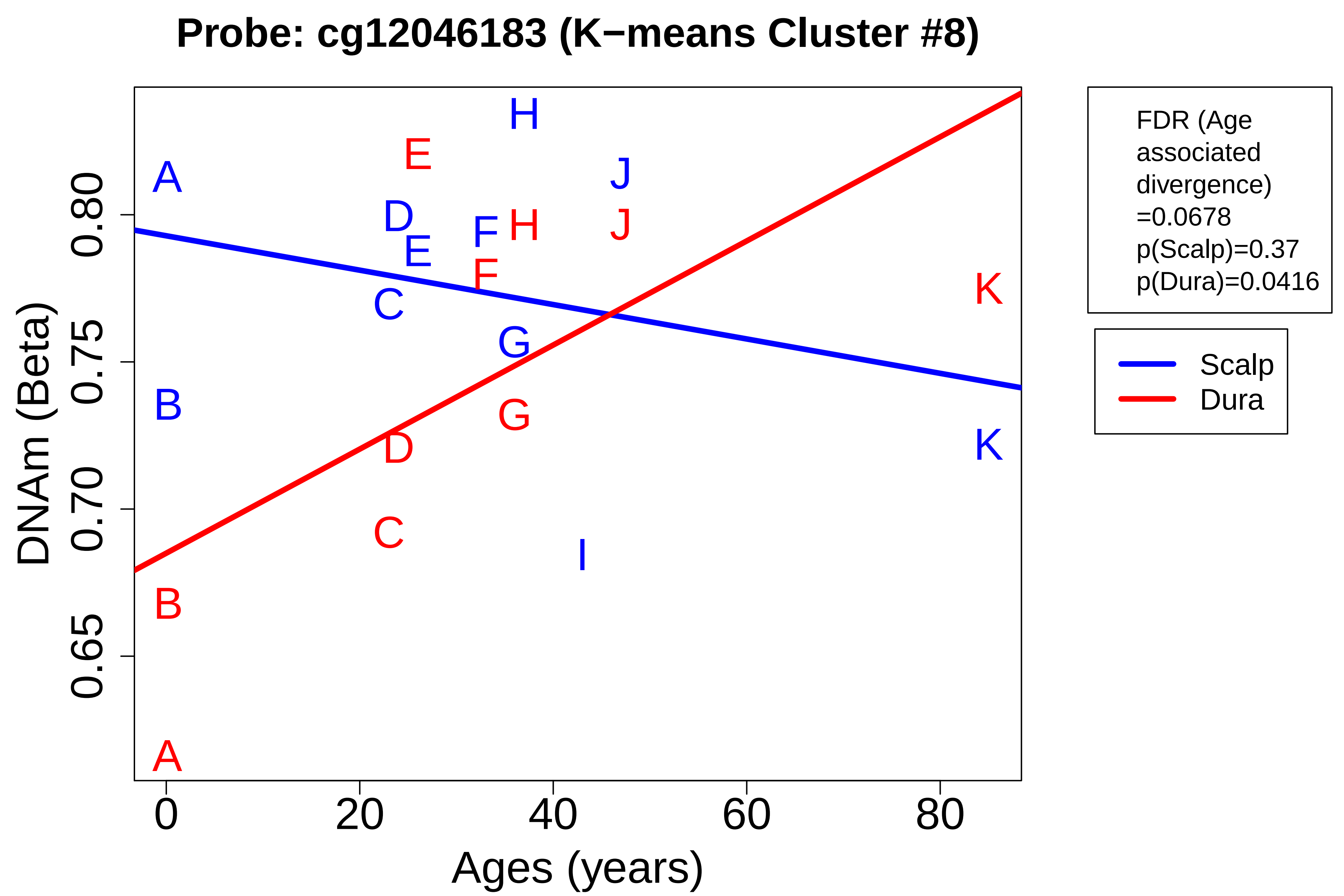

Probe: cg12046183 (K−means Cluster #8)
DNAm (Beta)
0.65
0.70
0.75
0.80
Ages (years)
0
20
40
60
80
A
B
C
D
E
F
G
H
I
J
K
FDR (Age associated divergence) =0.0678
p(Scalp)=0.37
p(Dura)=0.0416
Scalp
Dura

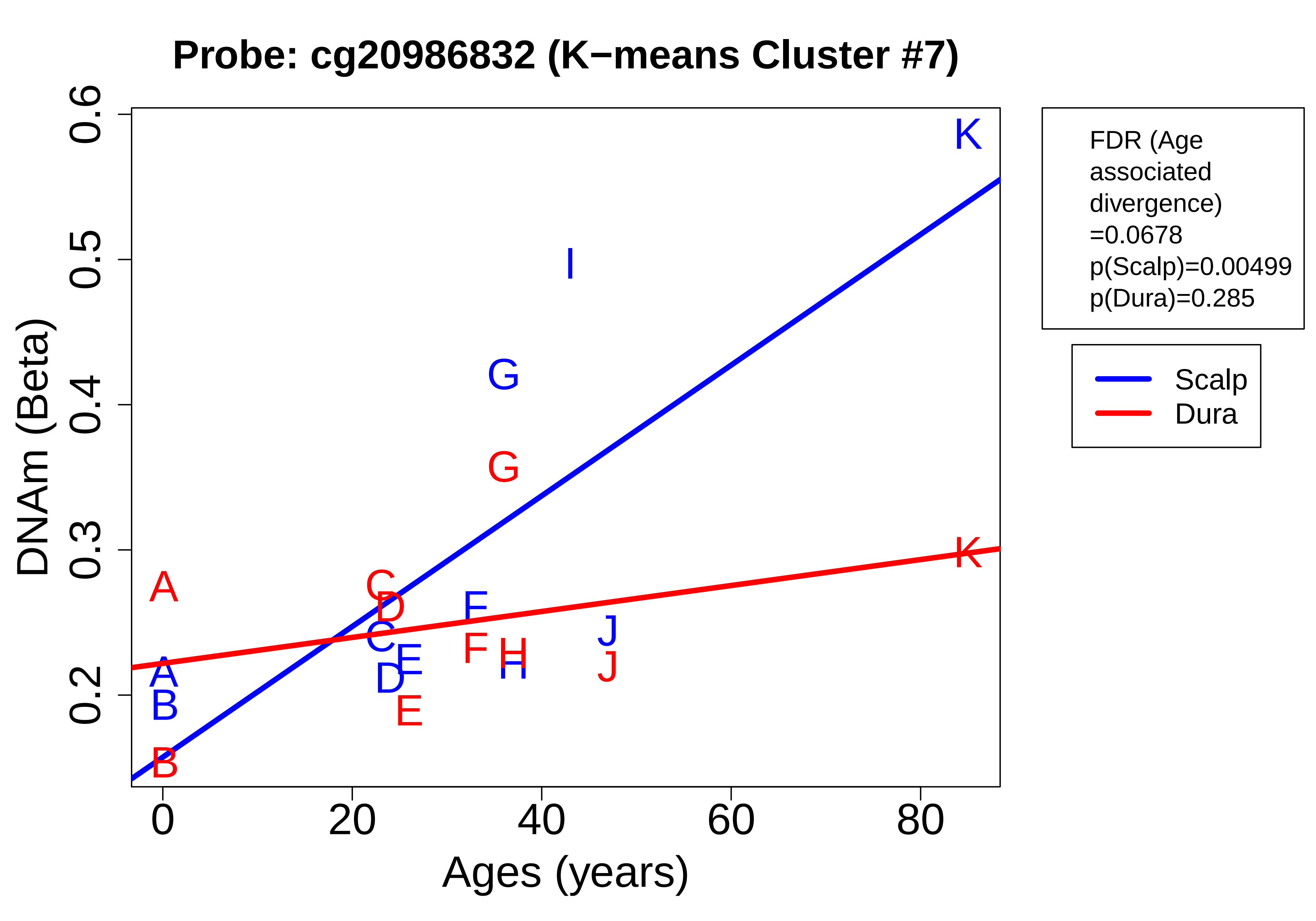

Probe: cg20986832 (K−means Cluster #7)
DNAm (Beta)
0.2
0.3
0.4
0.5
0.6
0
20
40
60
80
Ages (years)
FDR (Age associated divergence) =0.0678
p(Scalp)=0.00499
p(Dura)=0.285
Scalp
Dura

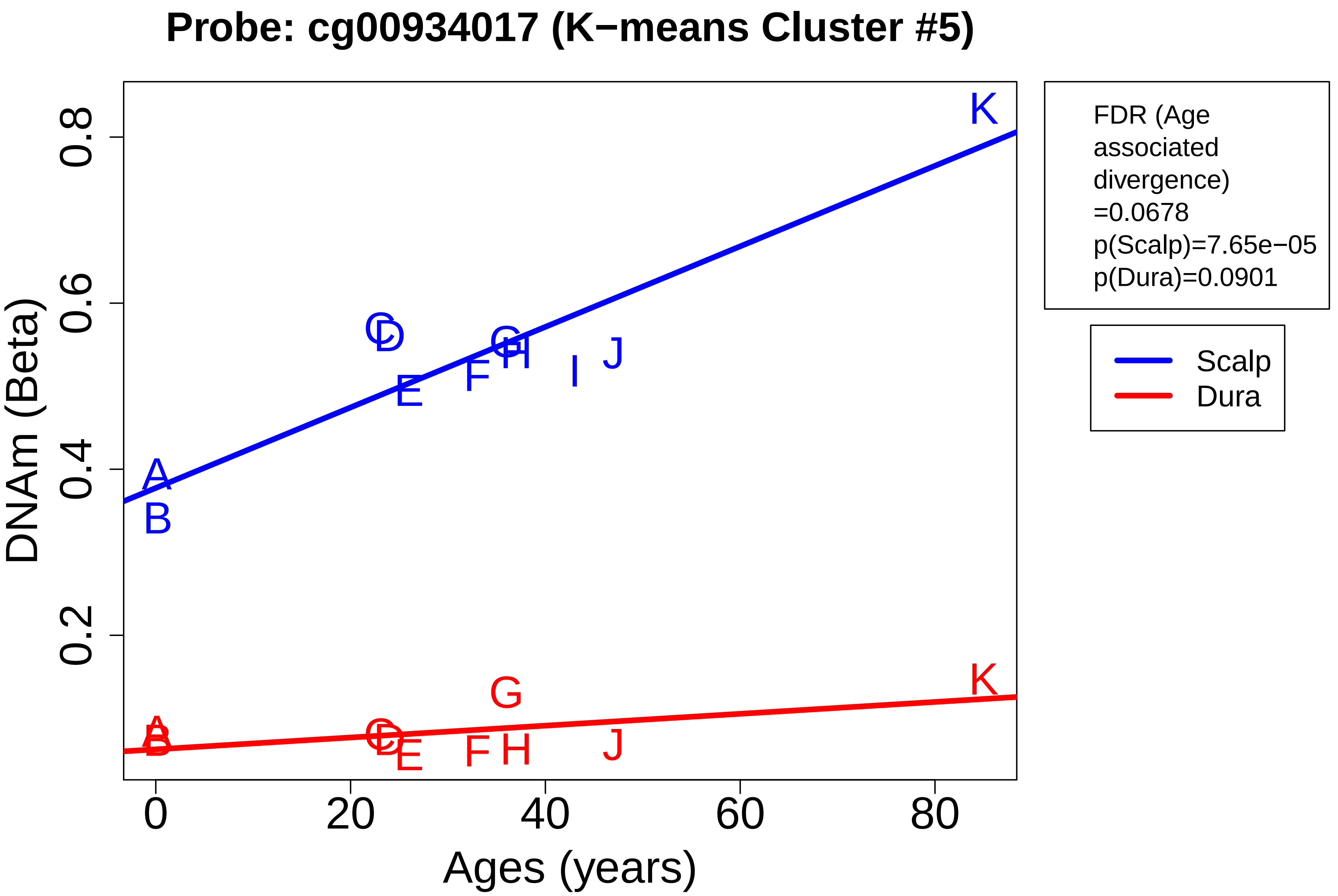

Probe: cg00934017 (K−means Cluster #5)
DNAm (Beta)
0.2
0.4
0.6
0.8
0
20
40
60
80
Ages (years)
FDR (Age associated divergence) =0.0678
p(Scalp)=7.65e−05
p(Dura)=0.0901
Scalp
Dura

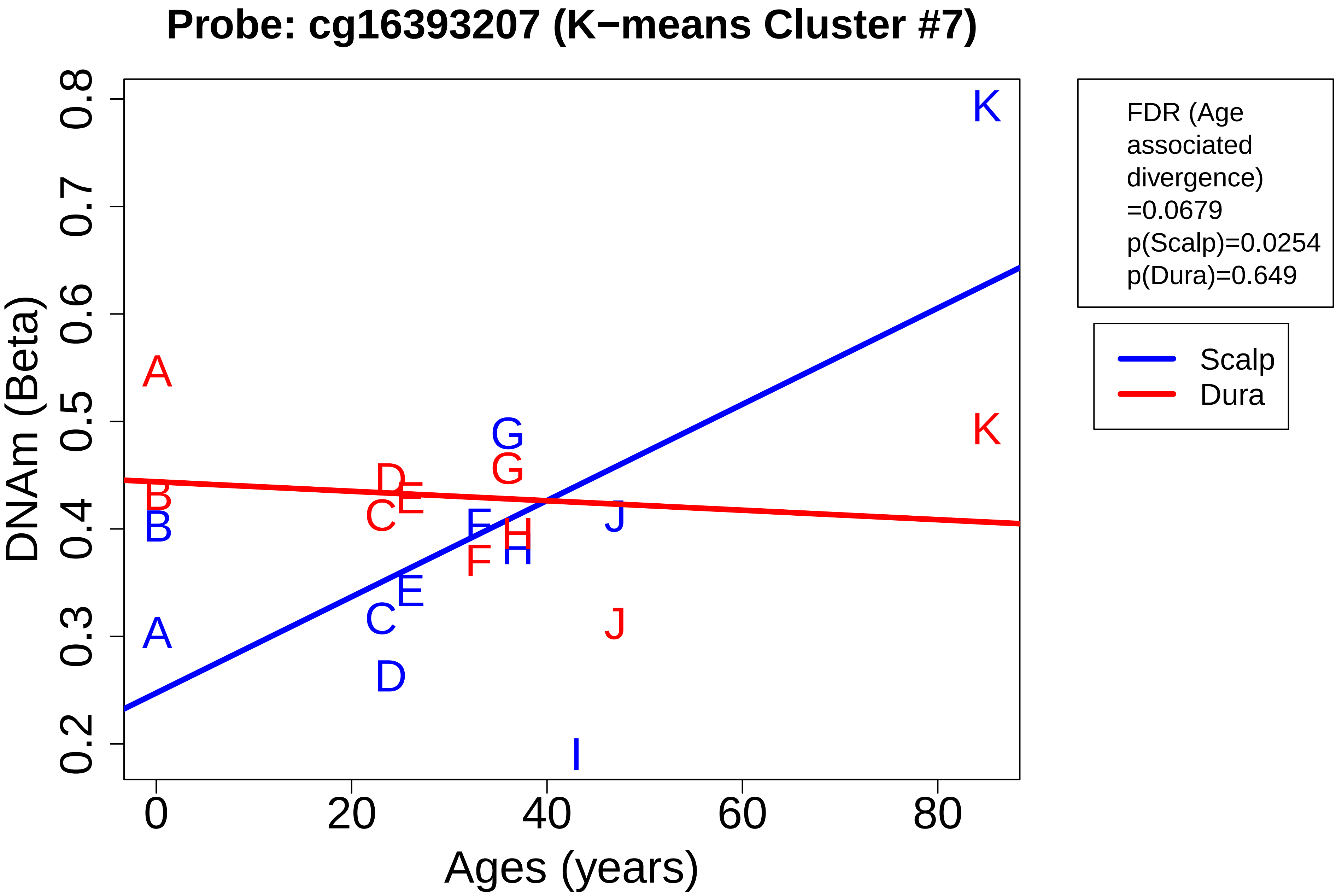

Probe: cg16393207 (K−means Cluster #7)
DNAm (Beta)
0.2
0.3
0.4
0.5
0.6
0.7
0.8
Ages (years)
0
20
40
60
80
FDR (Age associated divergence) =0.0679
p(Scalp)=0.0254
p(Dura)=0.649
Scalp
Dura
A
B
C
D
E
F
G
H
I
J
K

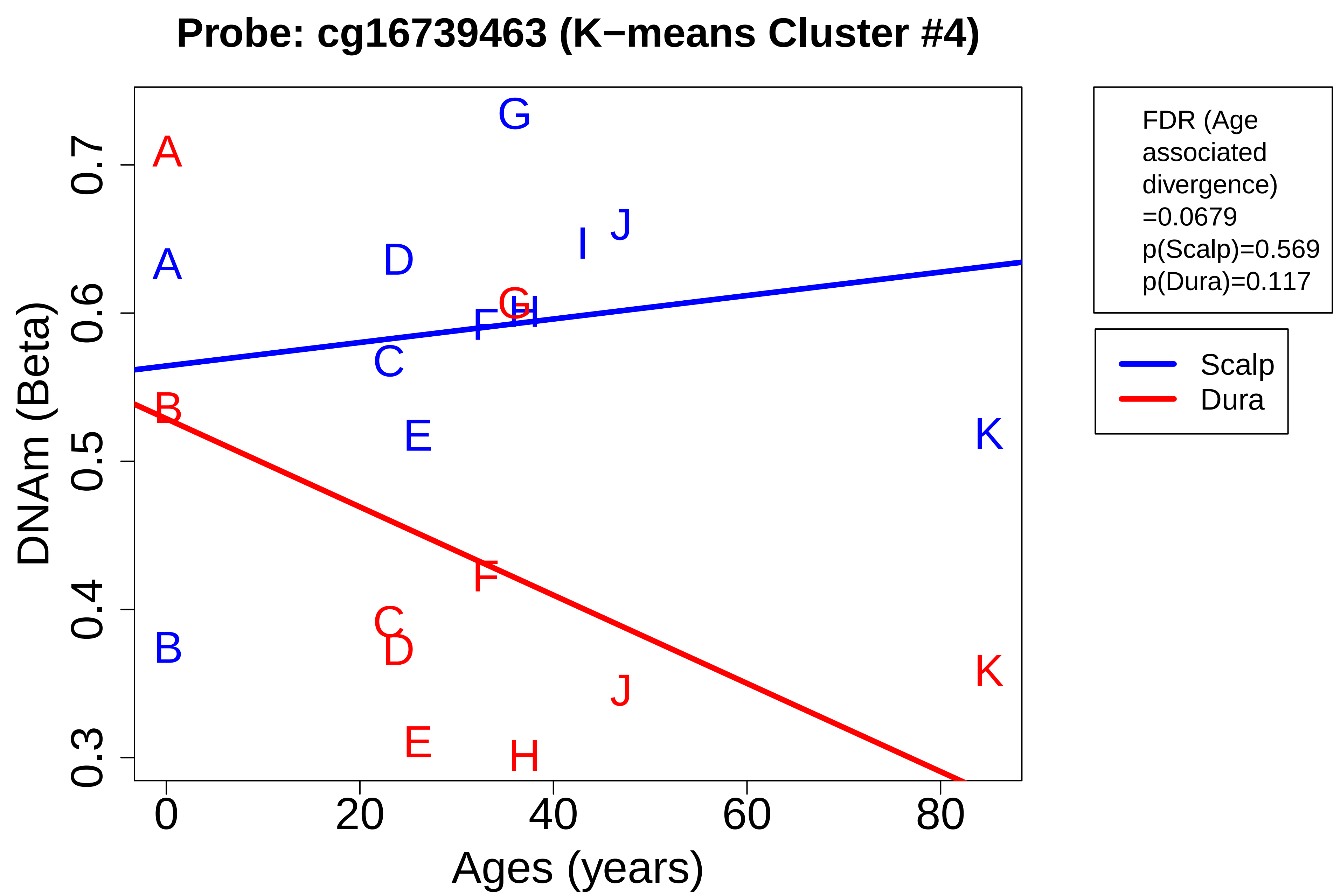

Probe: cg16739463 (K−means Cluster #4)
DNAm (Beta)
0.3
0.4
0.5
0.6
0.7
0
20
40
60
80
Ages (years)
A
B
C
D
E
F
G
H
I
J
K
FDR (Age associated divergence) =0.0679
p(Scalp)=0.569
p(Dura)=0.117
Scalp
Dura

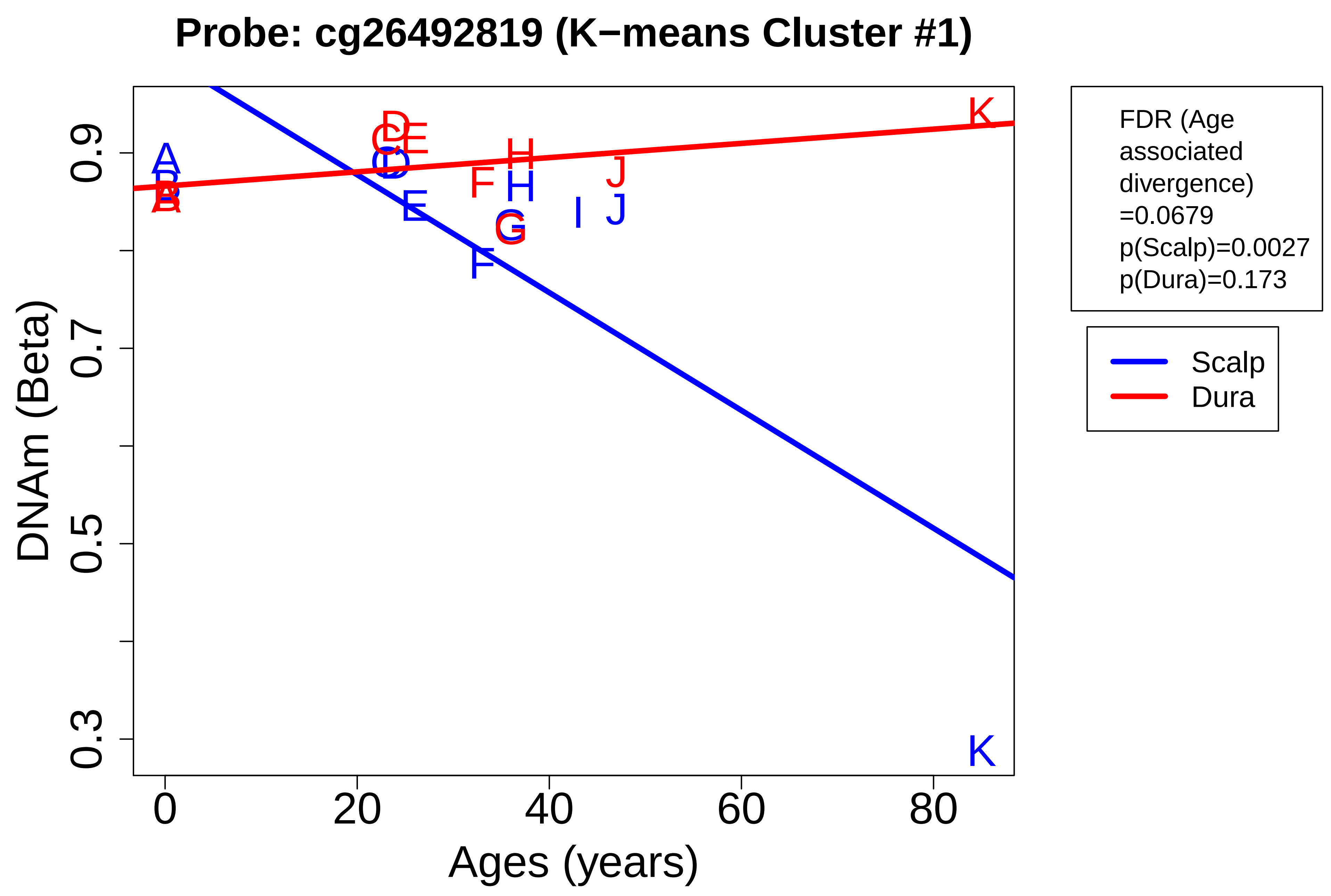
Probe: cg26492819 (K−means Cluster #1)
FDR (Age associated divergence) =0.0679
p(Scalp)=0.0027
p(Dura)=0.173
Scalp
Dura
DNAm (Beta)
0.3
0.5
0.7
0.9
0
20
40
60
80
Ages (years)
A
B
C
D
E
F
G
H
I
J
K

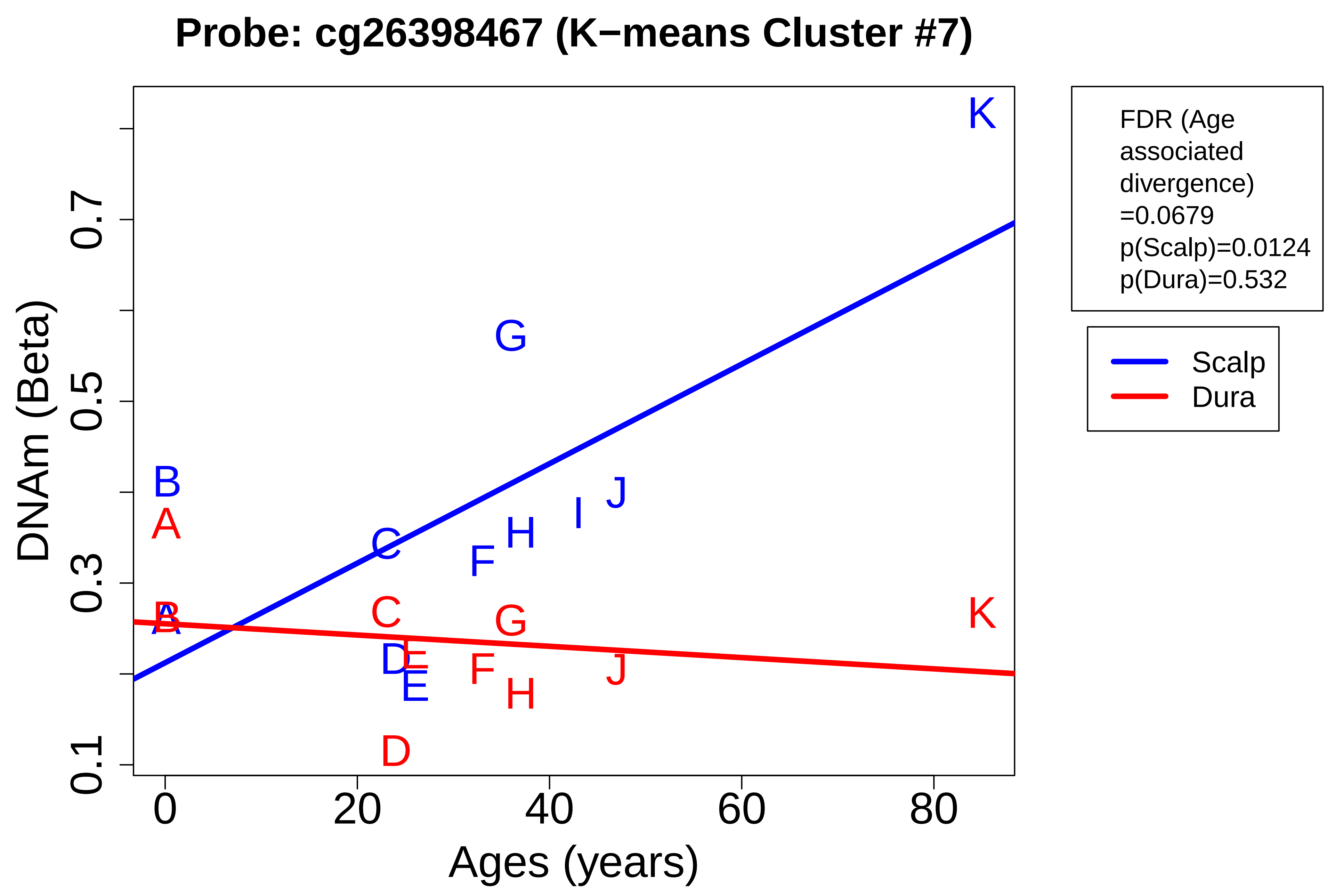

Probe: cg26398467 (K−means Cluster #7)
DNAm (Beta)
0.1
0.3
0.5
0.7
Ages (years)
0
20
40
60
80
FDR (Age associated divergence) =0.0679
p(Scalp)=0.0124
p(Dura)=0.532
Scalp
Dura

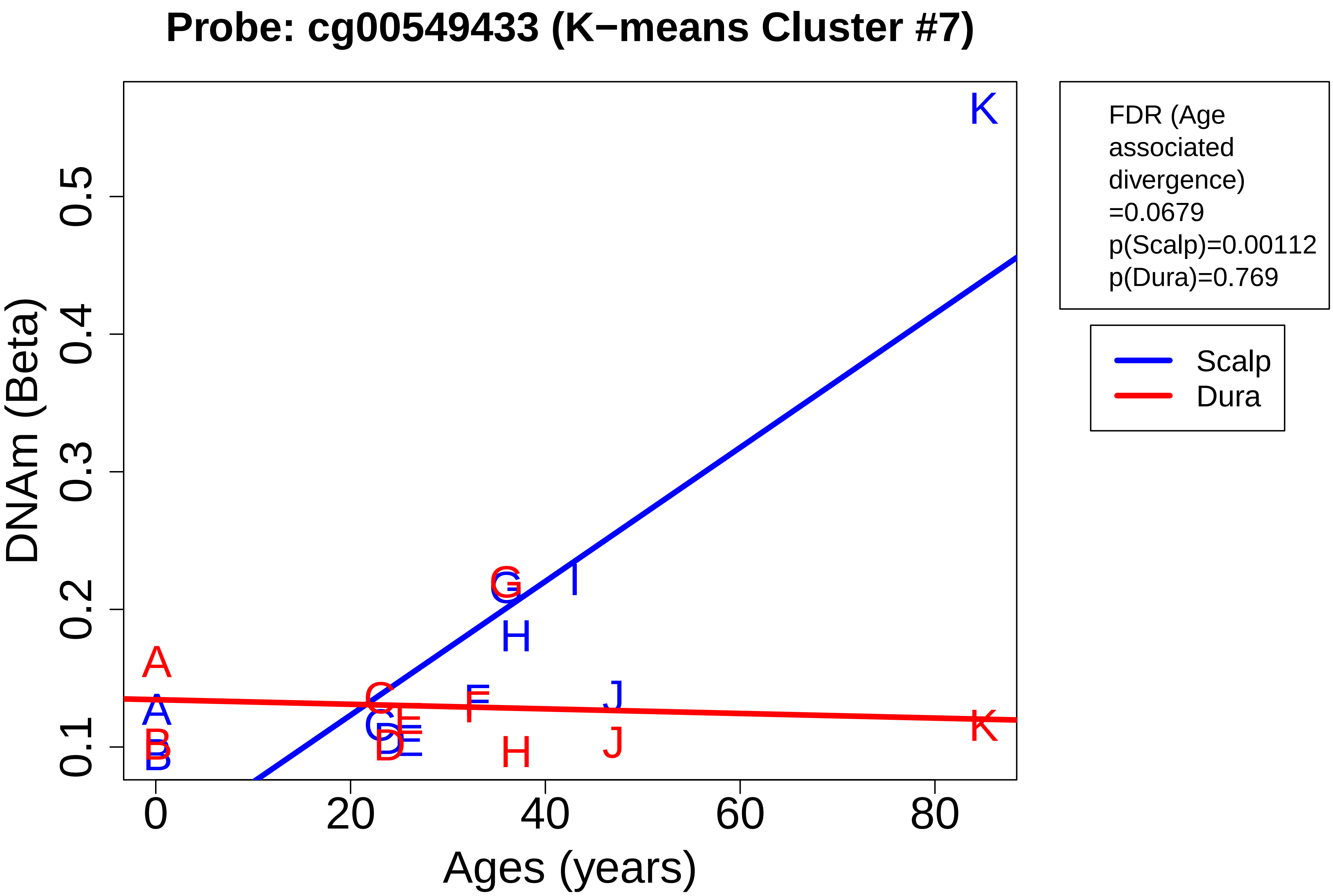

Probe: cg00549433 (K−means Cluster #7)
DNAm (Beta)
0.1
0.2
0.3
0.4
0.5
0
20
40
60
80
Ages (years)
FDR (Age associated divergence) =0.0679
p(Scalp)=0.00112
p(Dura)=0.769
Scalp
Dura

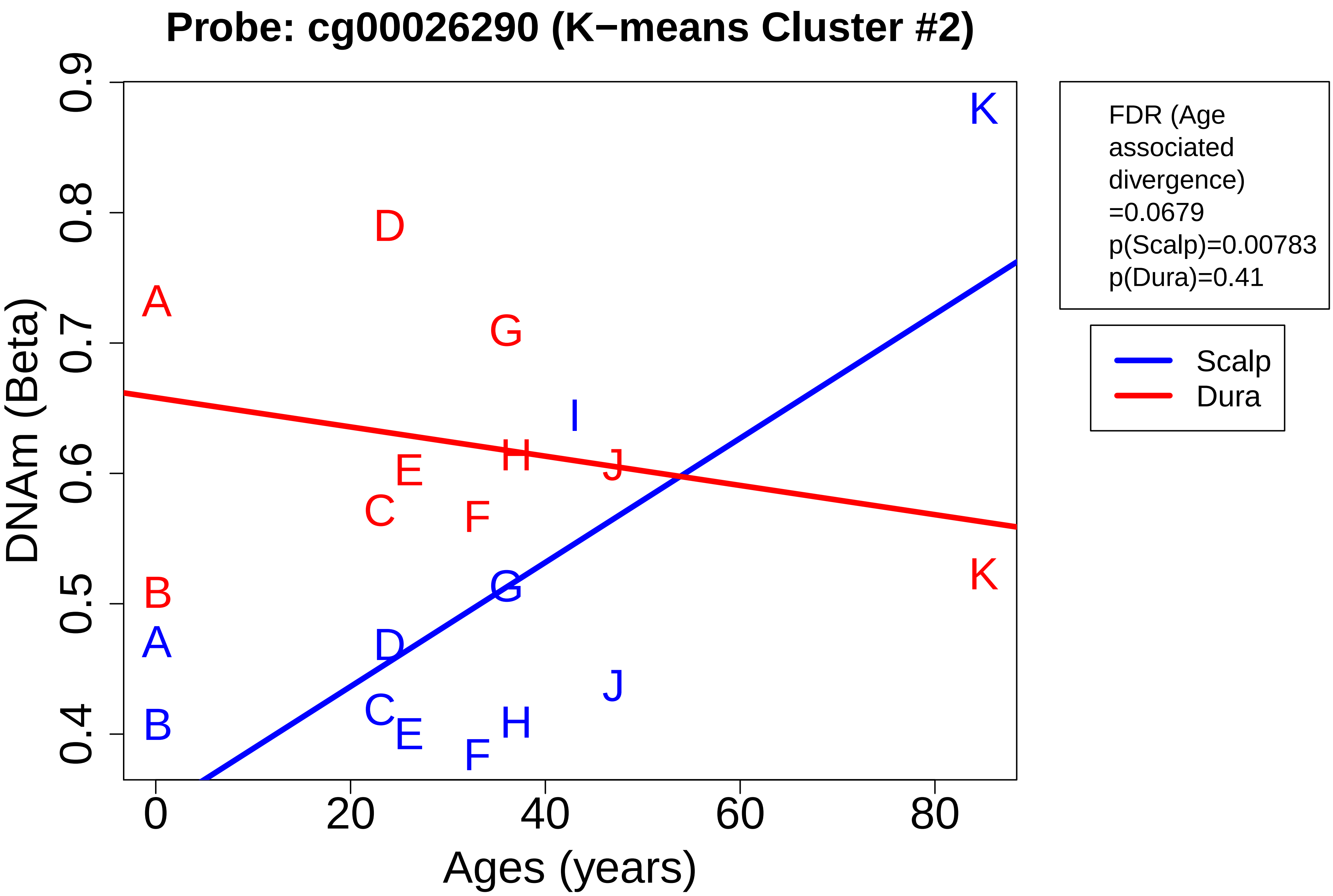

Probe: cg00026290 (K−means Cluster #2)
DNAm (Beta)
0.4
0.5
0.6
0.7
0.8
0.9
Ages (years)
0
20
40
60
80
FDR (Age associated divergence) =0.0679
p(Scalp)=0.00783
p(Dura)=0.41
Scalp
Dura
A
B
C
D
E
F
G
H
I
J
K

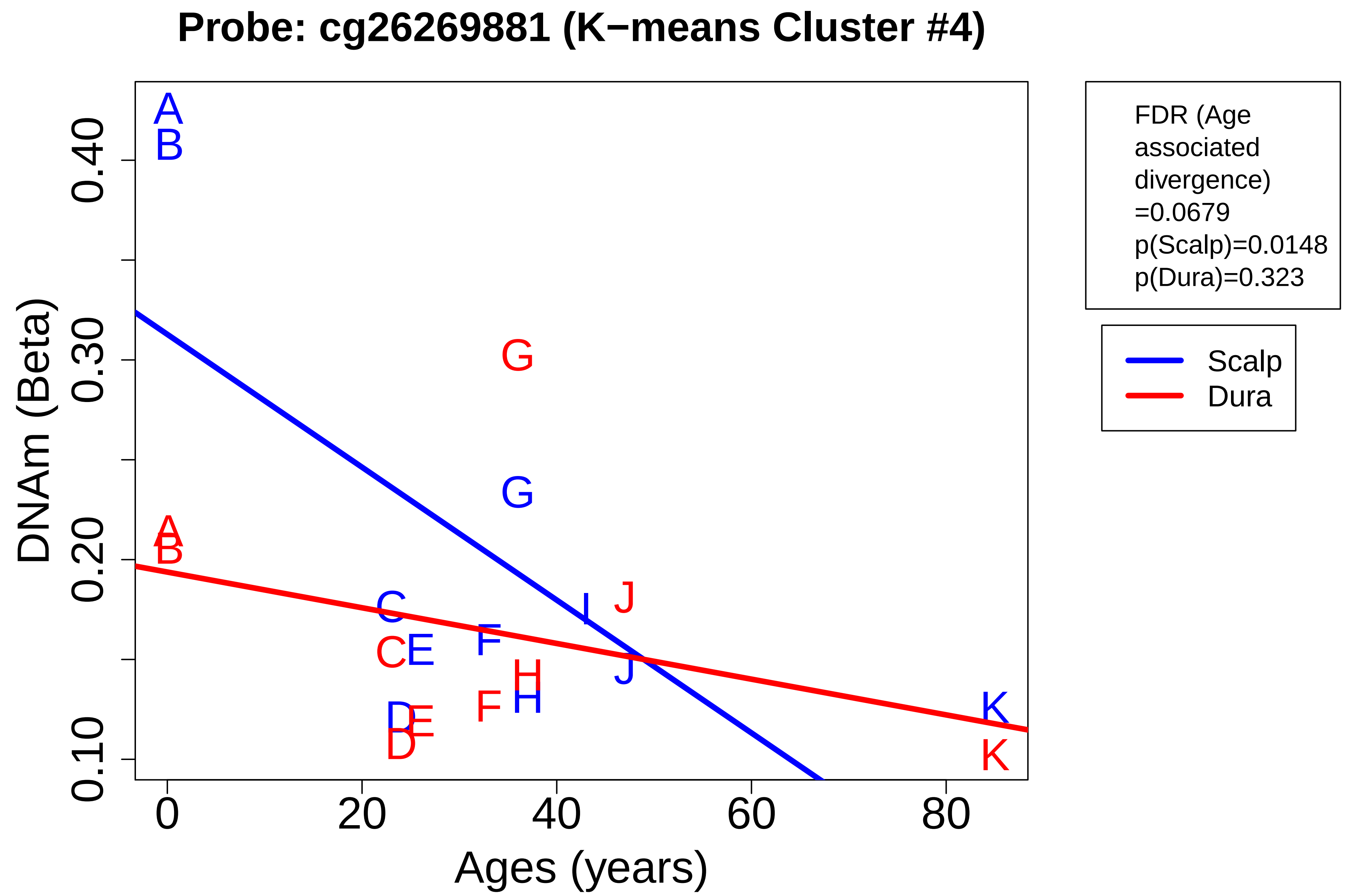

Probe: cg26269881 (K−means Cluster #4)
DNAm (Beta)
0.10
0.20
0.30
0.40
0
20
40
60
80
Ages (years)
A
B
C
D
E
F
G
H
I
J
K
FDR (Age associated divergence) =0.0679
p(Scalp)=0.0148
p(Dura)=0.323
Scalp
Dura

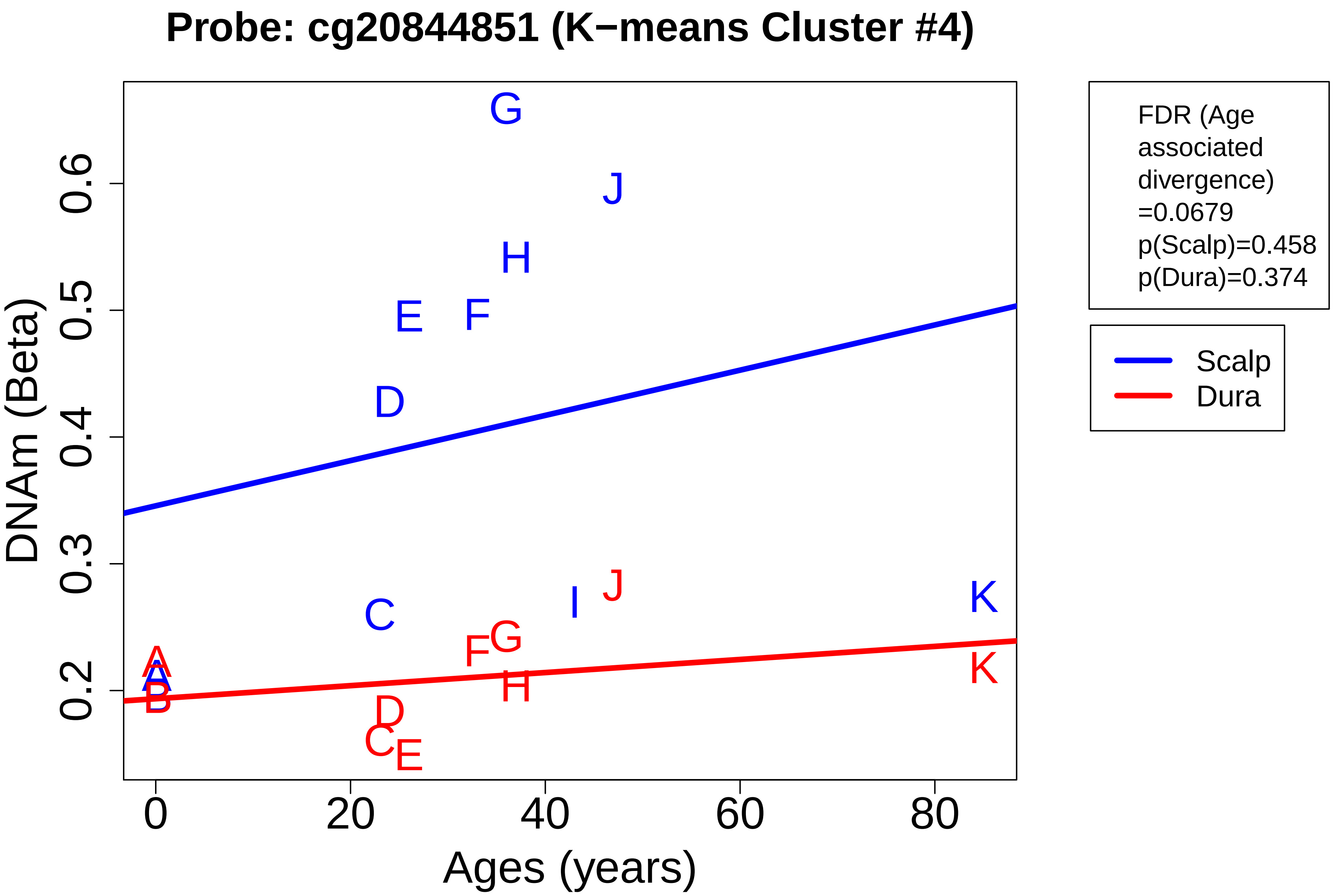

Probe: cg20844851 (K−means Cluster #4)
DNAm (Beta)
0.2
0.3
0.4
0.5
0.6
0
20
40
60
80
Ages (years)
FDR (Age associated divergence) =0.0679
p(Scalp)=0.458
p(Dura)=0.374
Scalp
Dura
A B C D E F G H I J K
A B C D E F G H J K

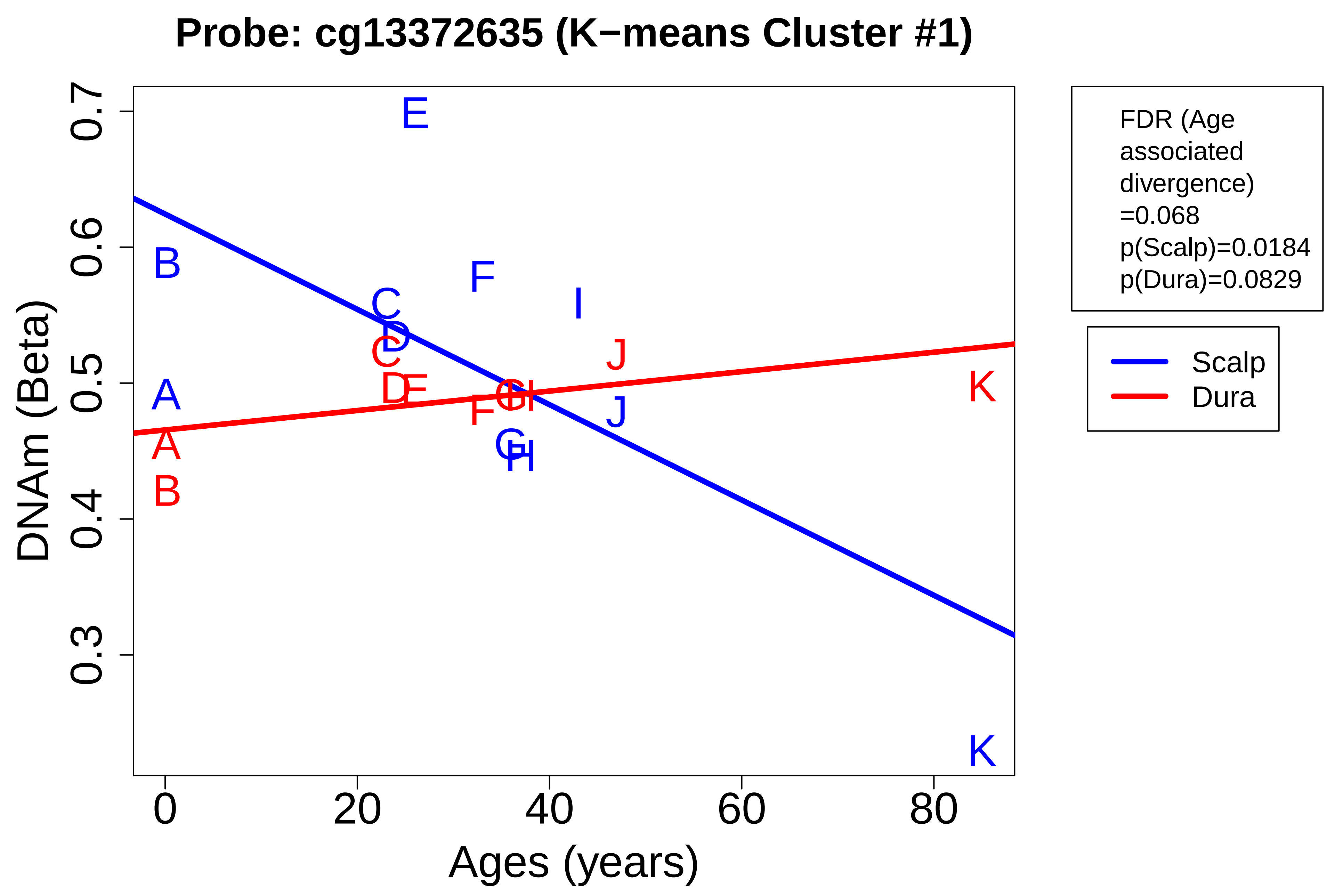

Probe: cg13372635 (K−means Cluster #1)
DNAm (Beta)
0.3
0.4
0.5
0.6
0.7
0
20
40
60
80
Ages (years)
FDR (Age associated divergence) =0.068
p(Scalp)=0.0184
p(Dura)=0.0829
Scalp
Dura

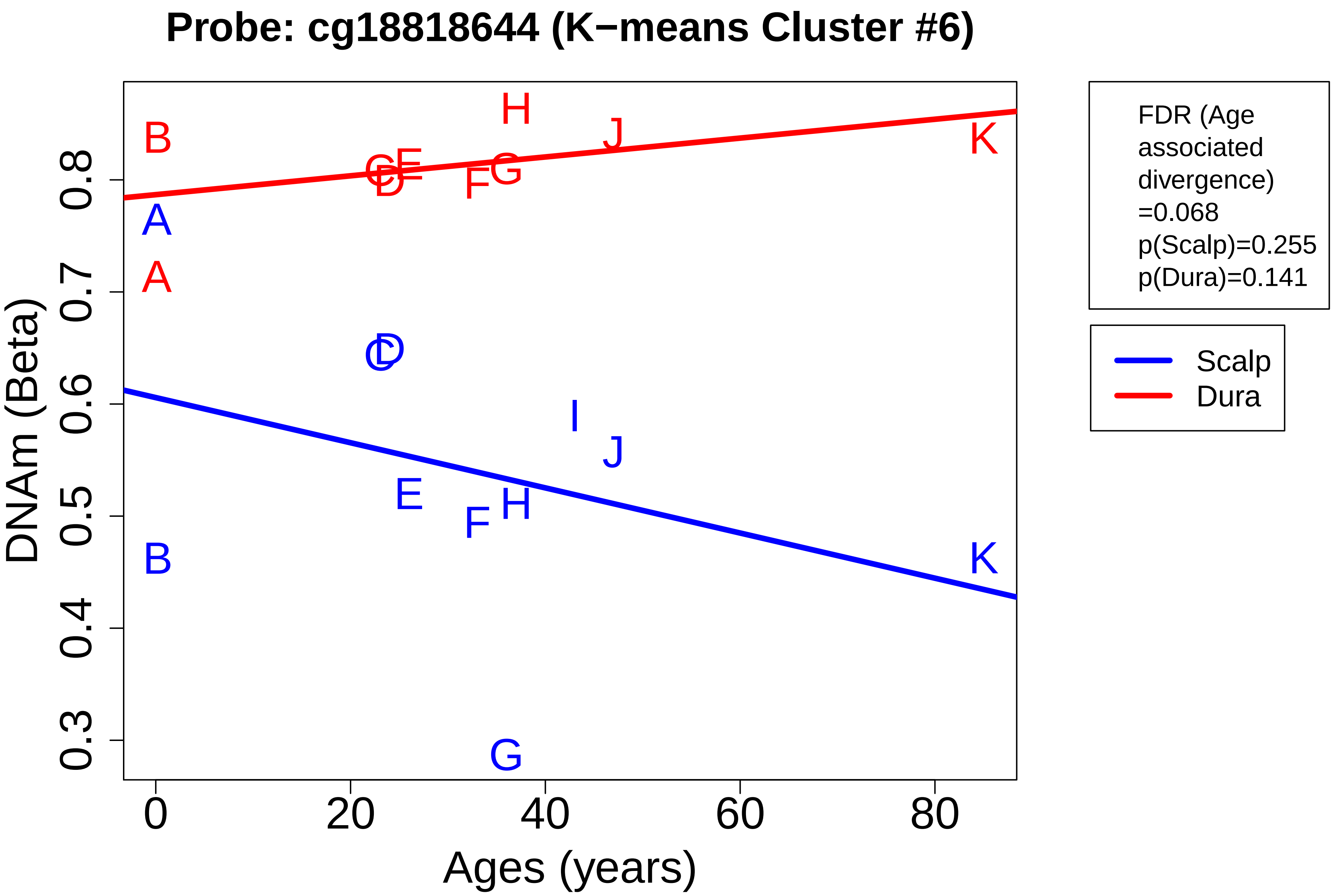

Probe: cg18818644 (K−means Cluster #6)
DNAm (Beta)
Ages (years)
0.3
0.4
0.5
0.6
0.7
0.8
0
20
40
60
80
FDR (Age associated divergence) =0.068
p(Scalp)=0.255
p(Dura)=0.141
Scalp
Dura

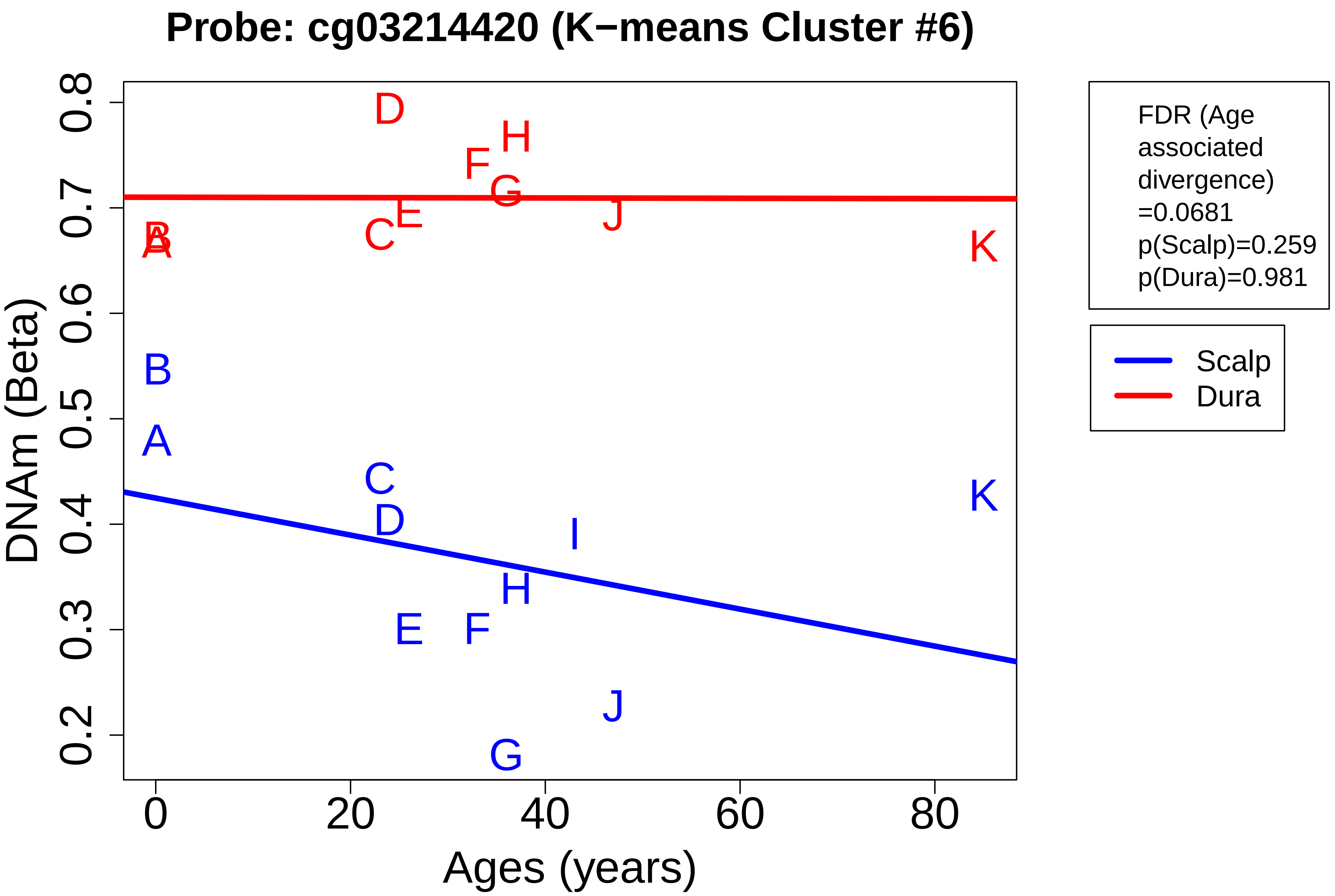
Probe: cg03214420 (K−means Cluster #6)
DNAm (Beta)
0.2
0.3
0.4
0.5
0.6
0.7
0.8
Ages (years)
0
20
40
60
80
FDR (Age associated divergence) =0.0681
p(Scalp)=0.259
p(Dura)=0.981
Scalp
Dura

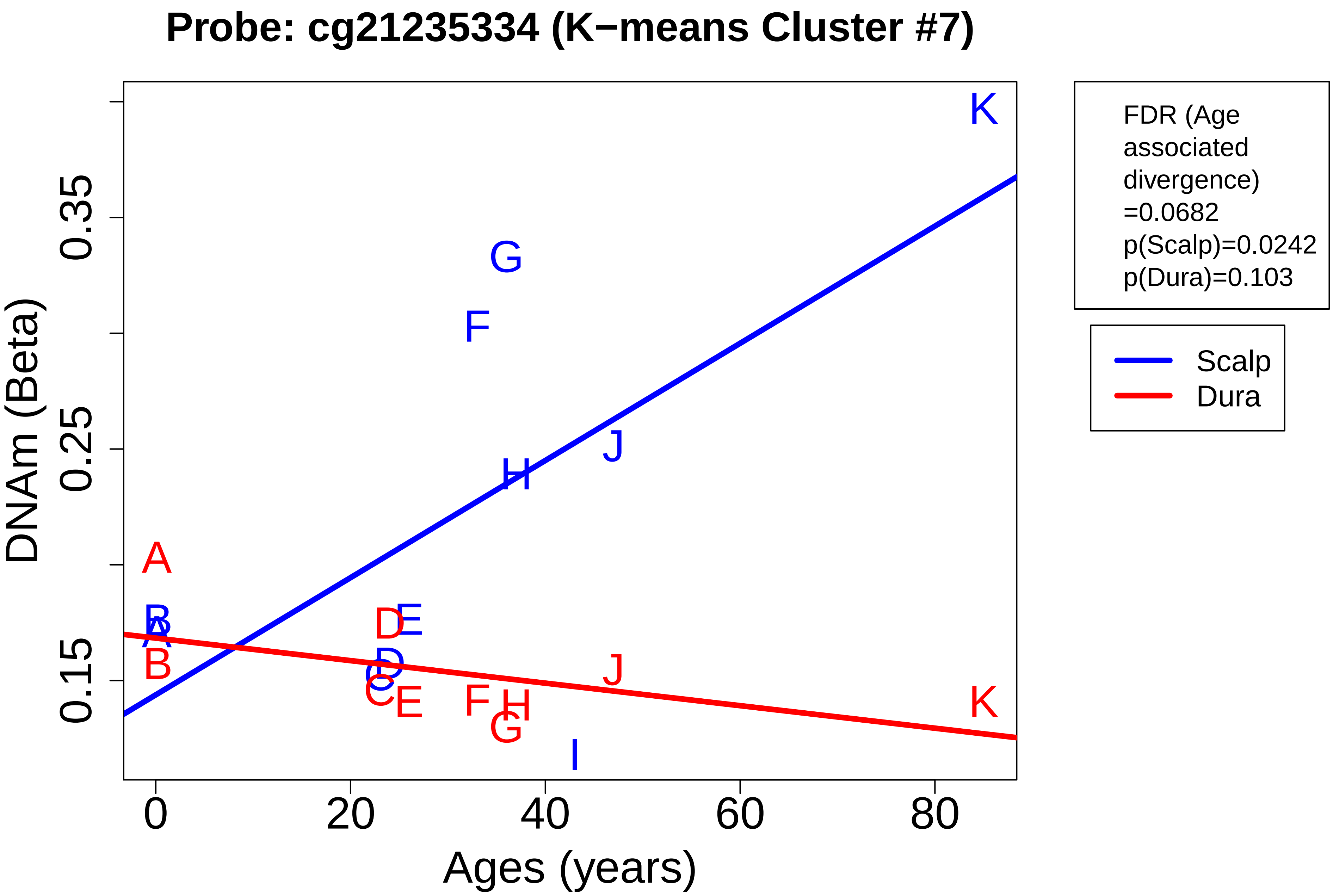

Probe: cg21235334 (K−means Cluster #7)
DNAm (Beta)
0.15
0.25
0.35
Ages (years)
0
20
40
60
80
FDR (Age associated divergence) =0.0682
p(Scalp)=0.0242
p(Dura)=0.103
Scalp
Dura

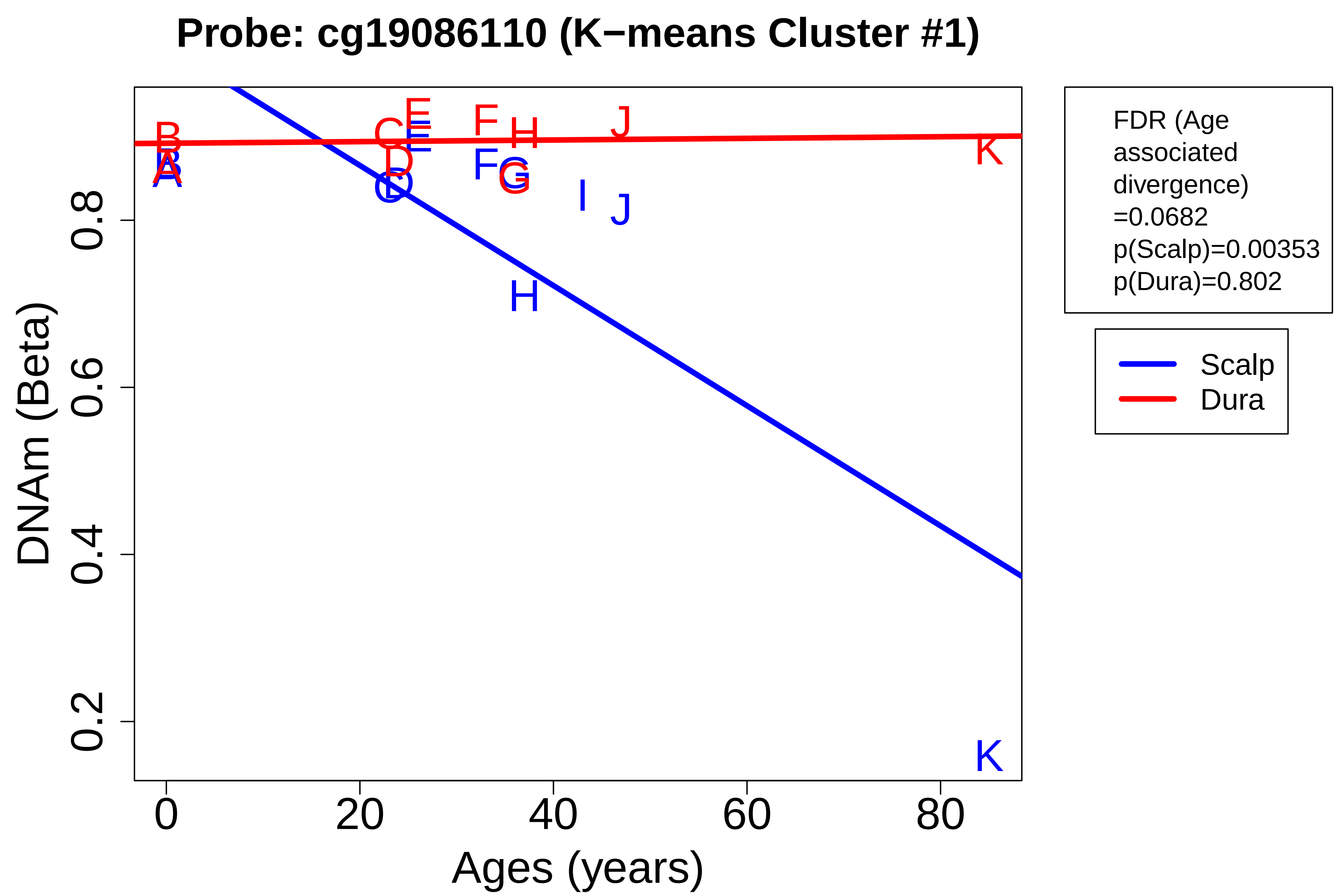

Probe: cg19086110 (K−means Cluster #1)
DNAm (Beta)
0.2
0.4
0.6
0.8
0
20
40
60
80
Ages (years)
A
B
C
D
E
F
G
H
I
J
K
FDR (Age associated divergence) =0.0682
p(Scalp)=0.00353
p(Dura)=0.802
Scalp
Dura

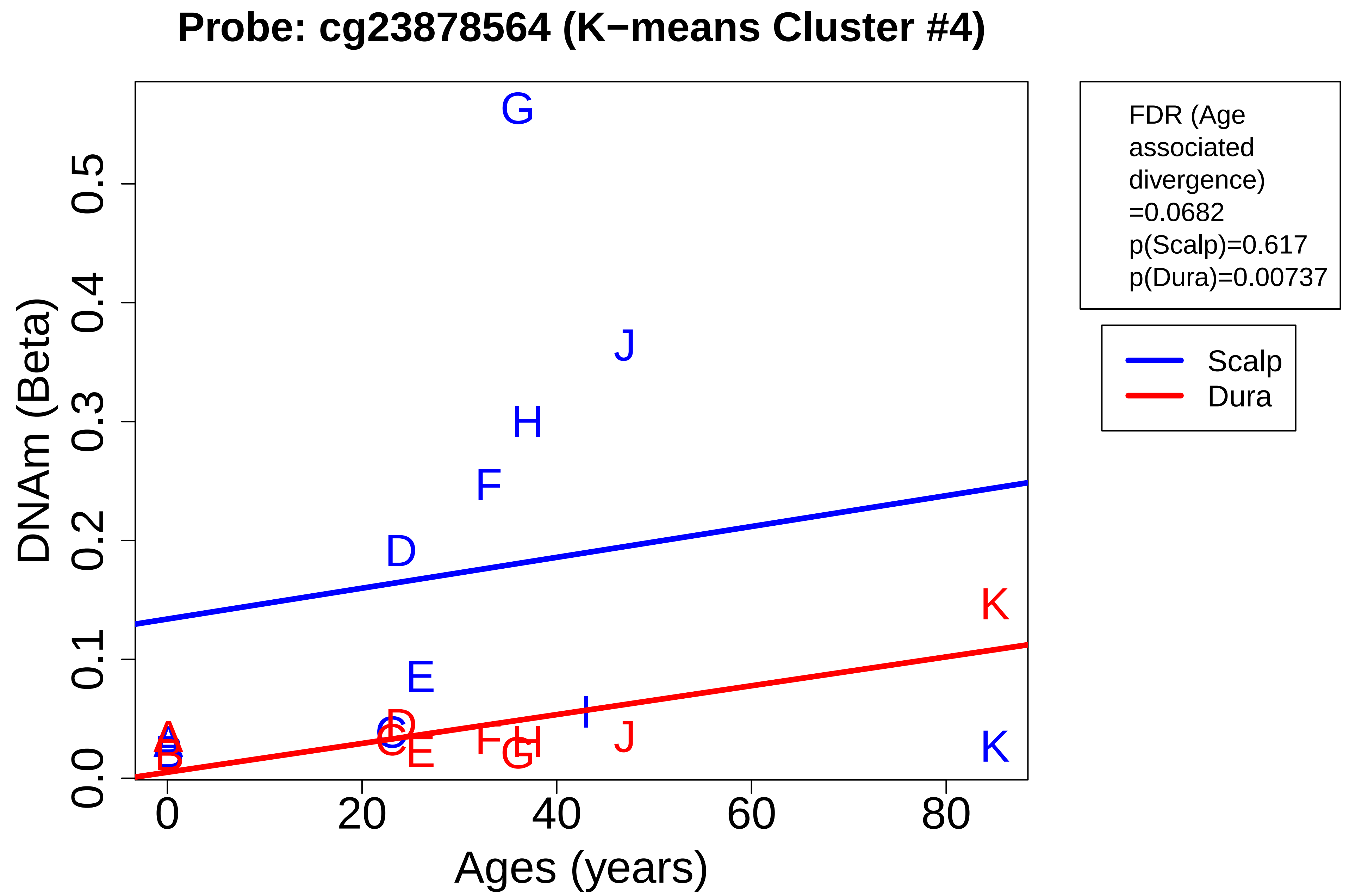

Probe: cg23878564 (K−means Cluster #4)
DNAm (Beta)
0.0
0.1
0.2
0.3
0.4
0.5
0
20
40
60
80
Ages (years)
A B C D E F G H I J K
FDR (Age associated divergence) =0.0682
p(Scalp)=0.617
p(Dura)=0.00737
Scalp
Dura

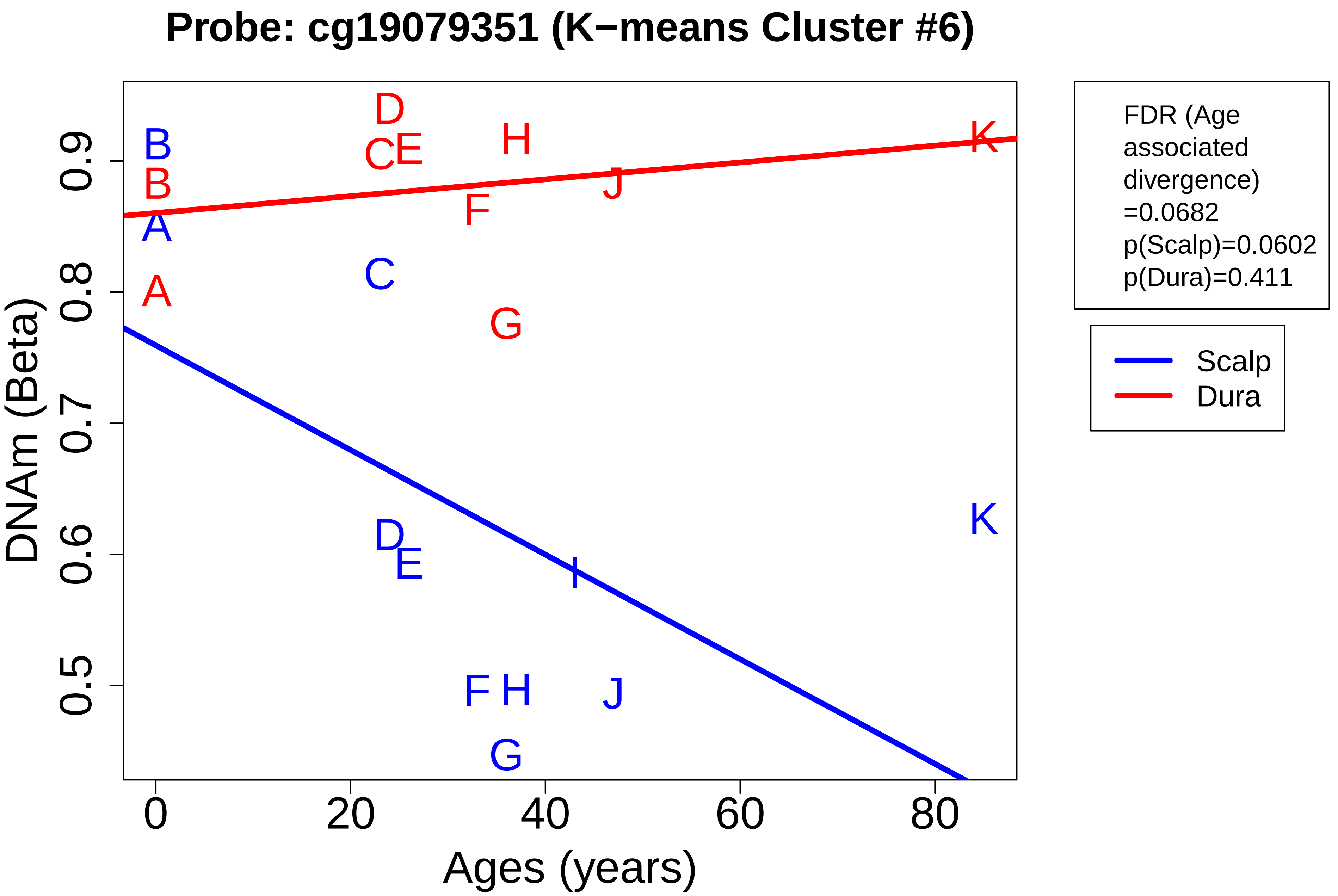

Probe: cg19079351 (K−means Cluster #6)
DNAm (Beta)
0.5
0.6
0.7
0.8
0.9
0
20
40
60
80
Ages (years)
FDR (Age associated divergence) =0.0682
p(Scalp)=0.0602
p(Dura)=0.411
Scalp
Dura

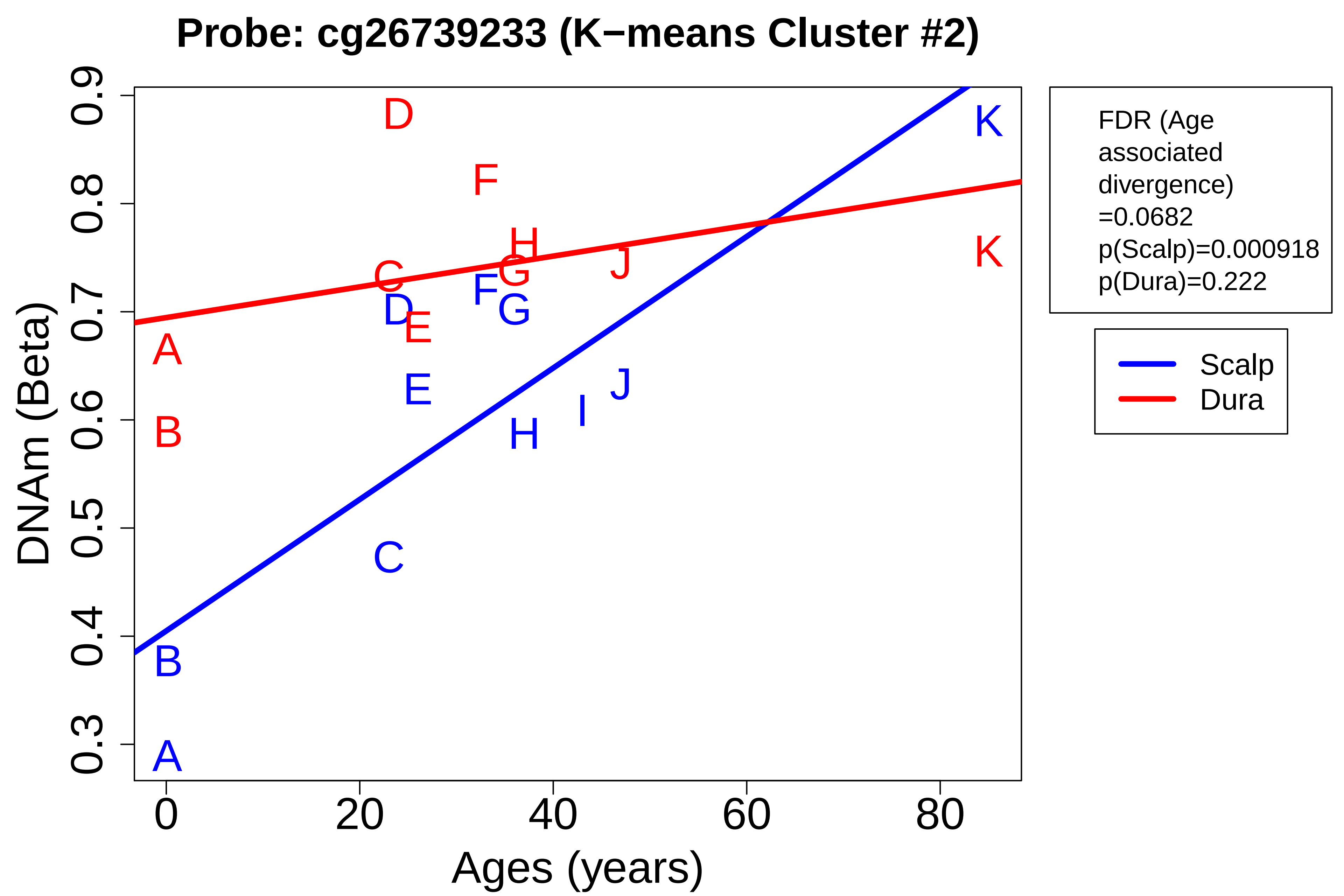
Probe: cg26739233 (K−means Cluster #2)
DNAm (Beta)
Ages (years)
0.3
0.4
0.5
0.6
0.7
0.8
0.9
0
20
40
60
80
FDR (Age associated divergence) =0.0682
p(Scalp)=0.000918
p(Dura)=0.222
Scalp
Dura

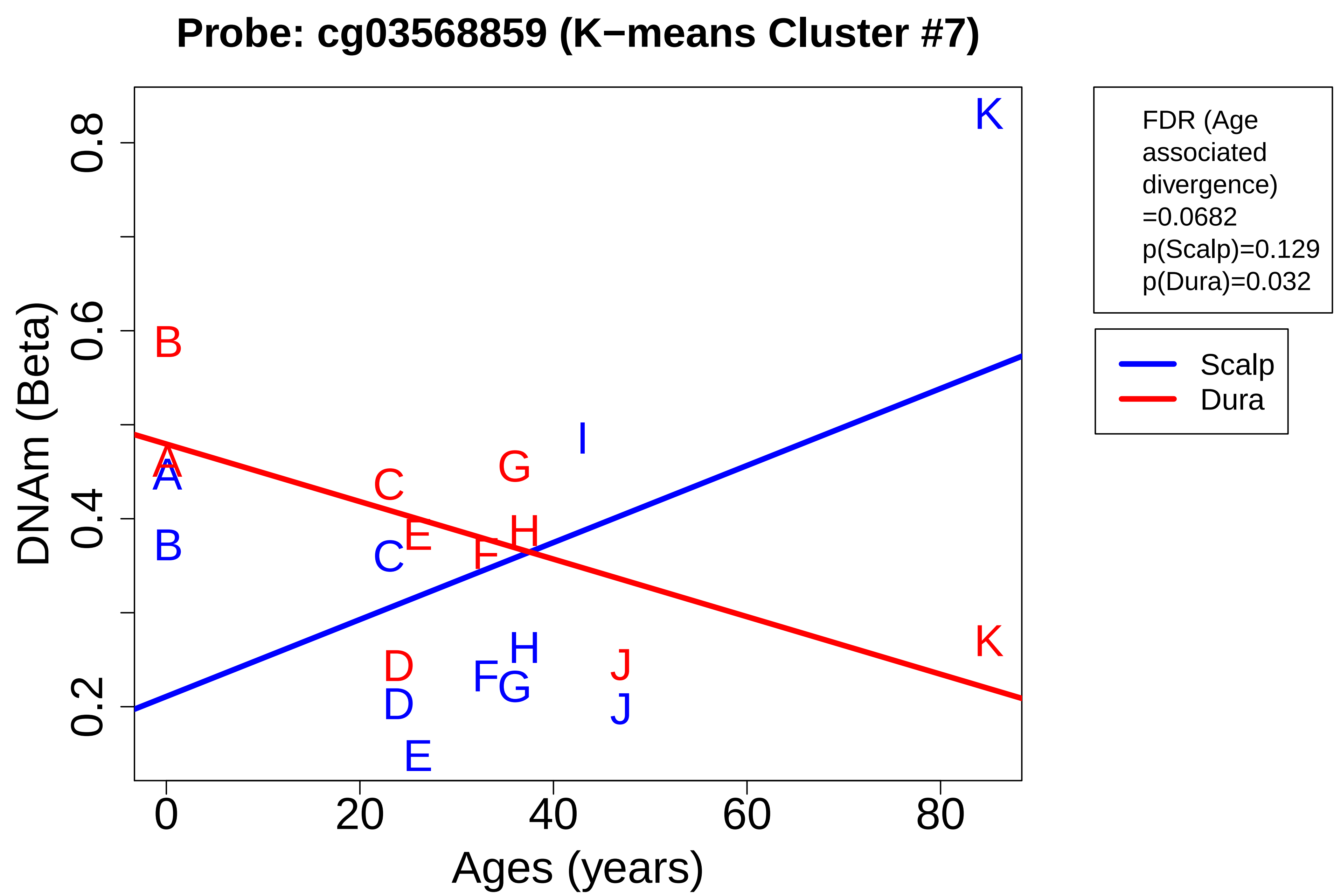

Probe: cg03568859 (K−means Cluster #7)
DNAm (Beta)
Ages (years)
0.2
0.4
0.6
0.8
0
20
40
60
80
FDR (Age associated divergence) =0.0682
p(Scalp)=0.129
p(Dura)=0.032
Scalp
Dura
A B C D E F G H I J K

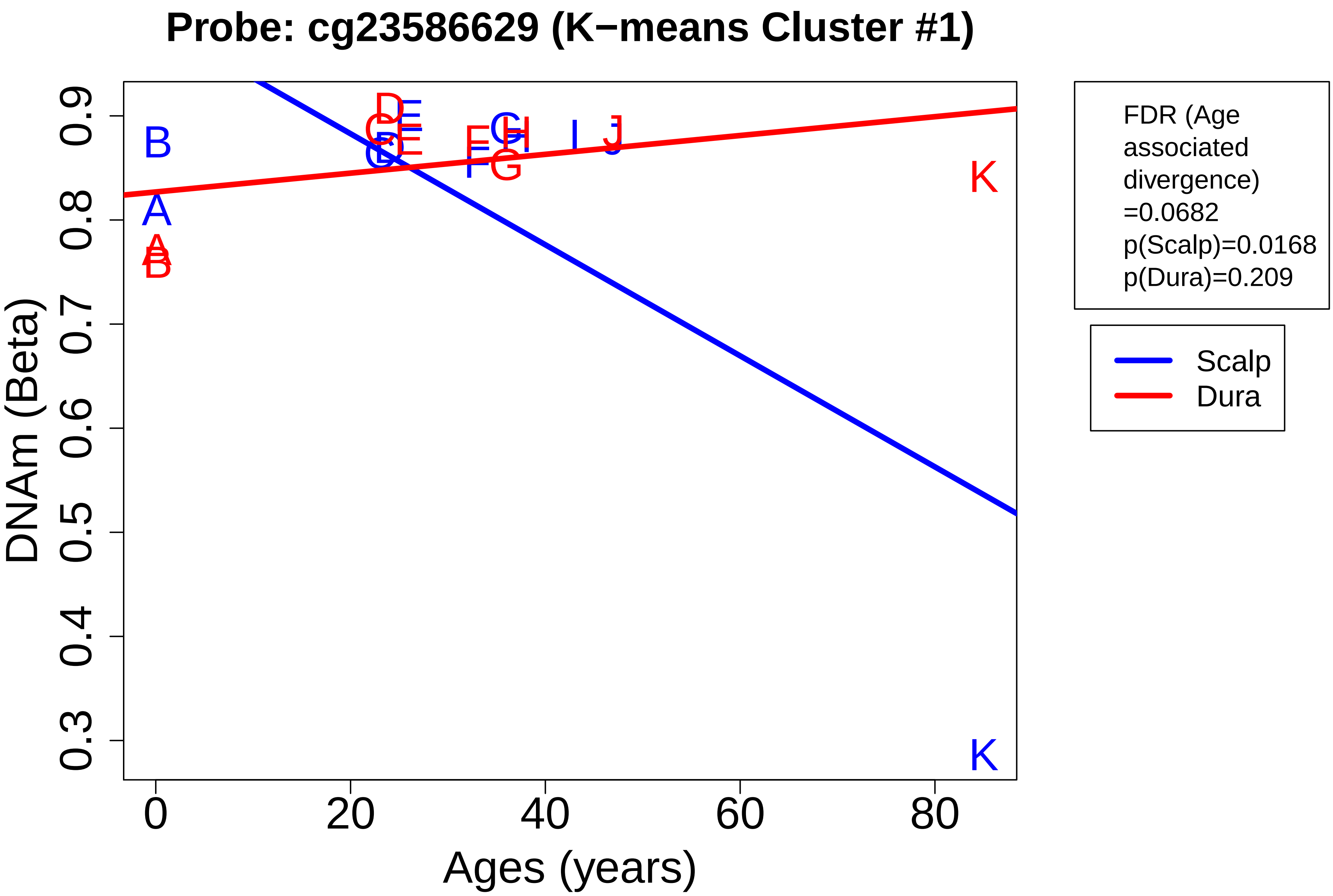
Probe: cg23586629 (K−means Cluster #1)
DNAm (Beta)
0.3
0.4
0.5
0.6
0.7
0.8
0.9
Ages (years)
0
20
40
60
80
FDR (Age associated divergence) =0.0682
p(Scalp)=0.0168
p(Dura)=0.209
Scalp
Dura

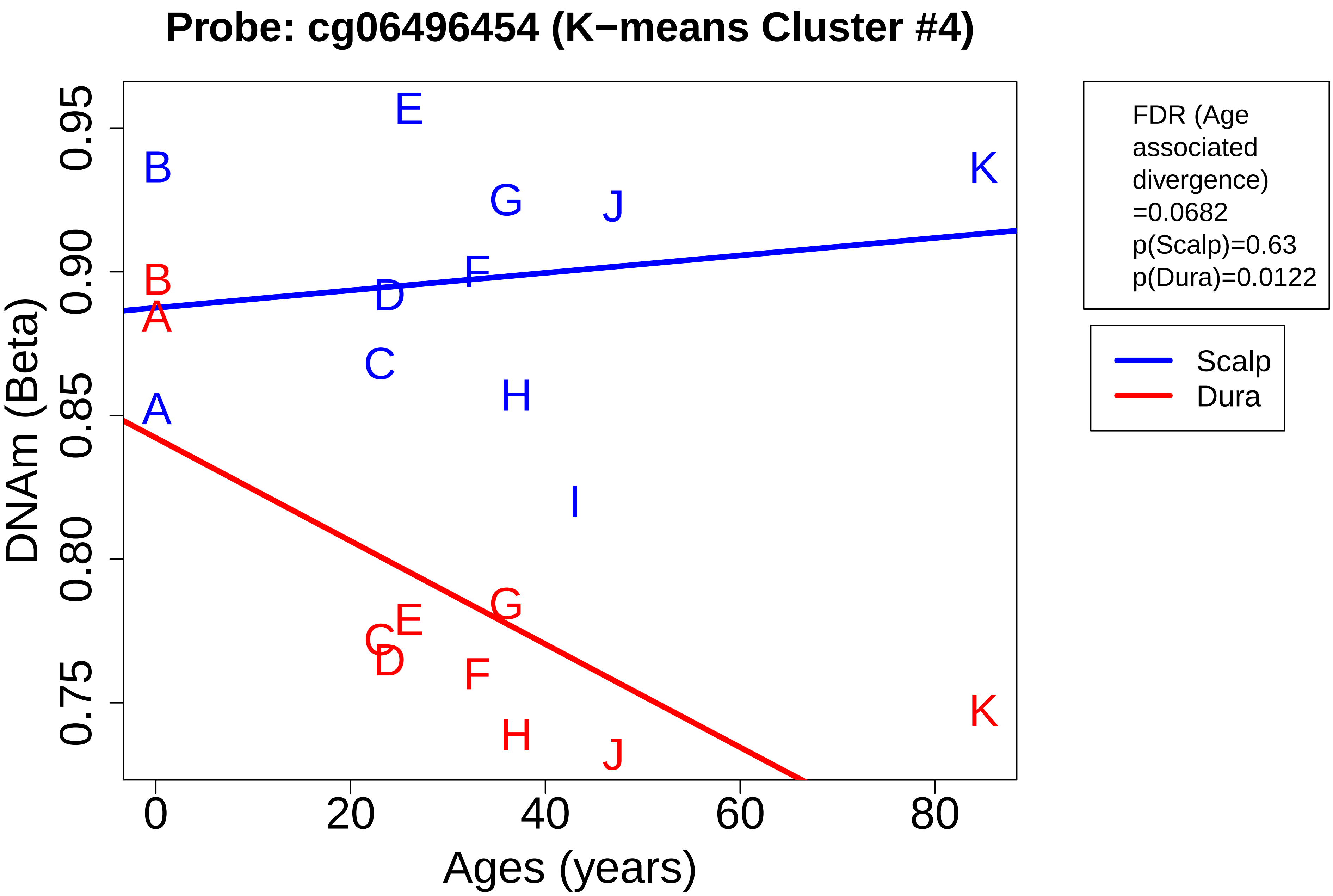

Probe: cg06496454 (K−means Cluster #4)
DNAm (Beta)
0.75
0.80
0.85
0.90
0.95
0
20
40
60
80
Ages (years)
FDR (Age associated divergence) =0.0682
p(Scalp)=0.63
p(Dura)=0.0122
Scalp
Dura

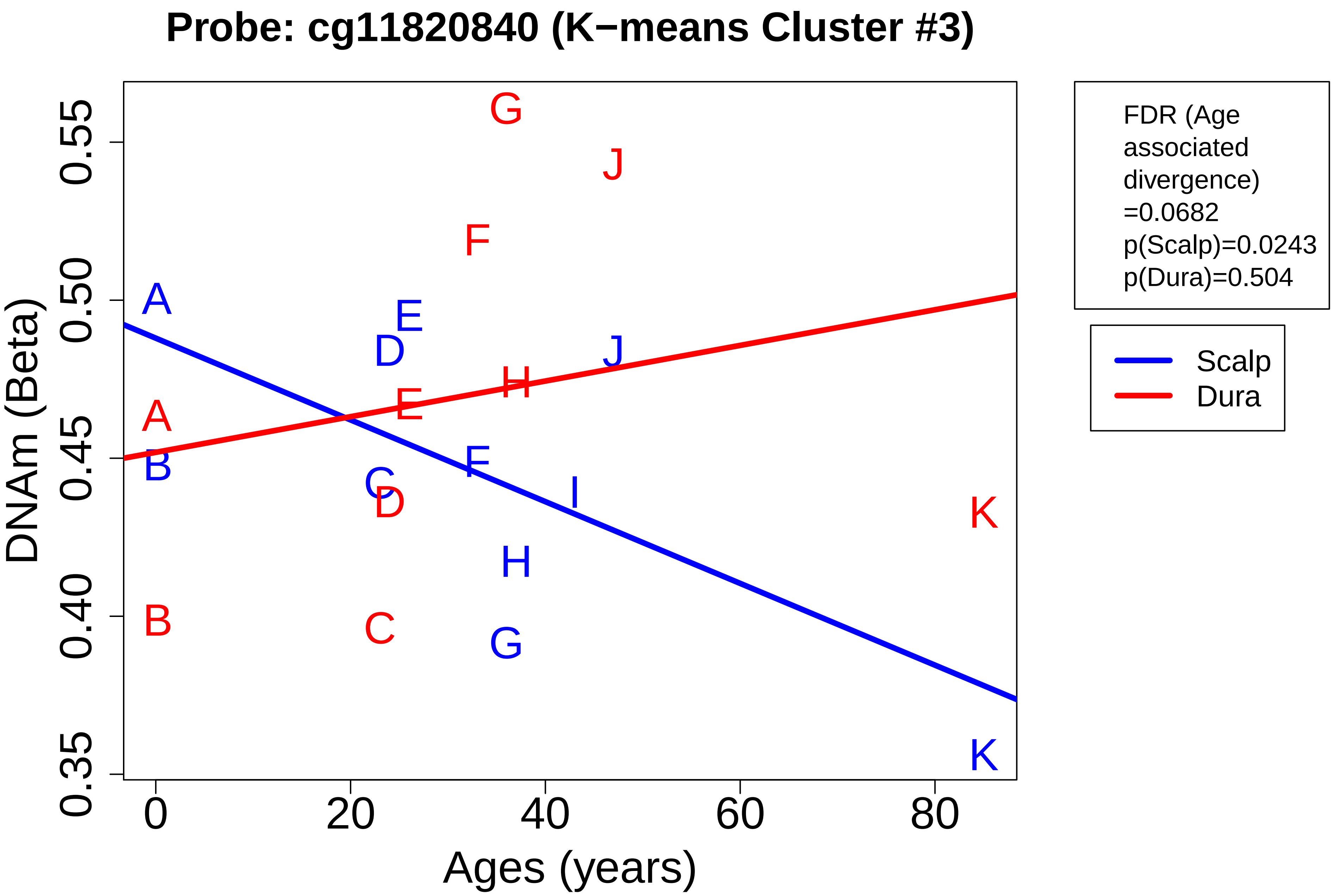

Probe: cg11820840 (K−means Cluster #3)
DNAm (Beta)
0.35
0.40
0.45
0.50
0.55
0
20
40
60
80
Ages (years)
FDR (Age associated divergence) =0.0682
p(Scalp)=0.0243
p(Dura)=0.504
Scalp
Dura
A
B
C
D
E
F
G
H
I
J
K

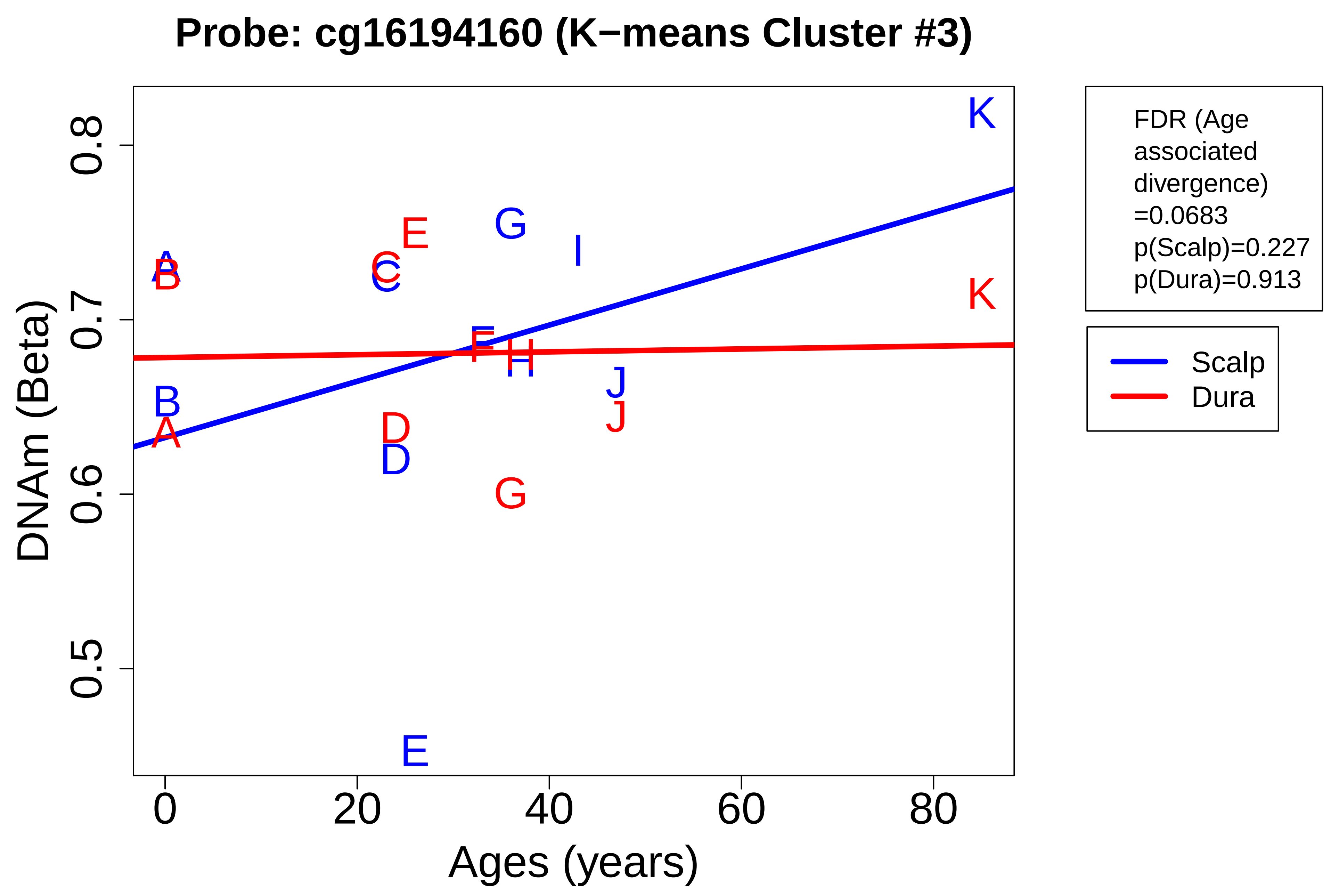

Probe: cg16194160 (K−means Cluster #3)
DNAm (Beta)
0.5
0.6
0.7
0.8
0
20
40
60
80
Ages (years)
FDR (Age associated divergence) =0.0683
p(Scalp)=0.227
p(Dura)=0.913
Scalp
Dura

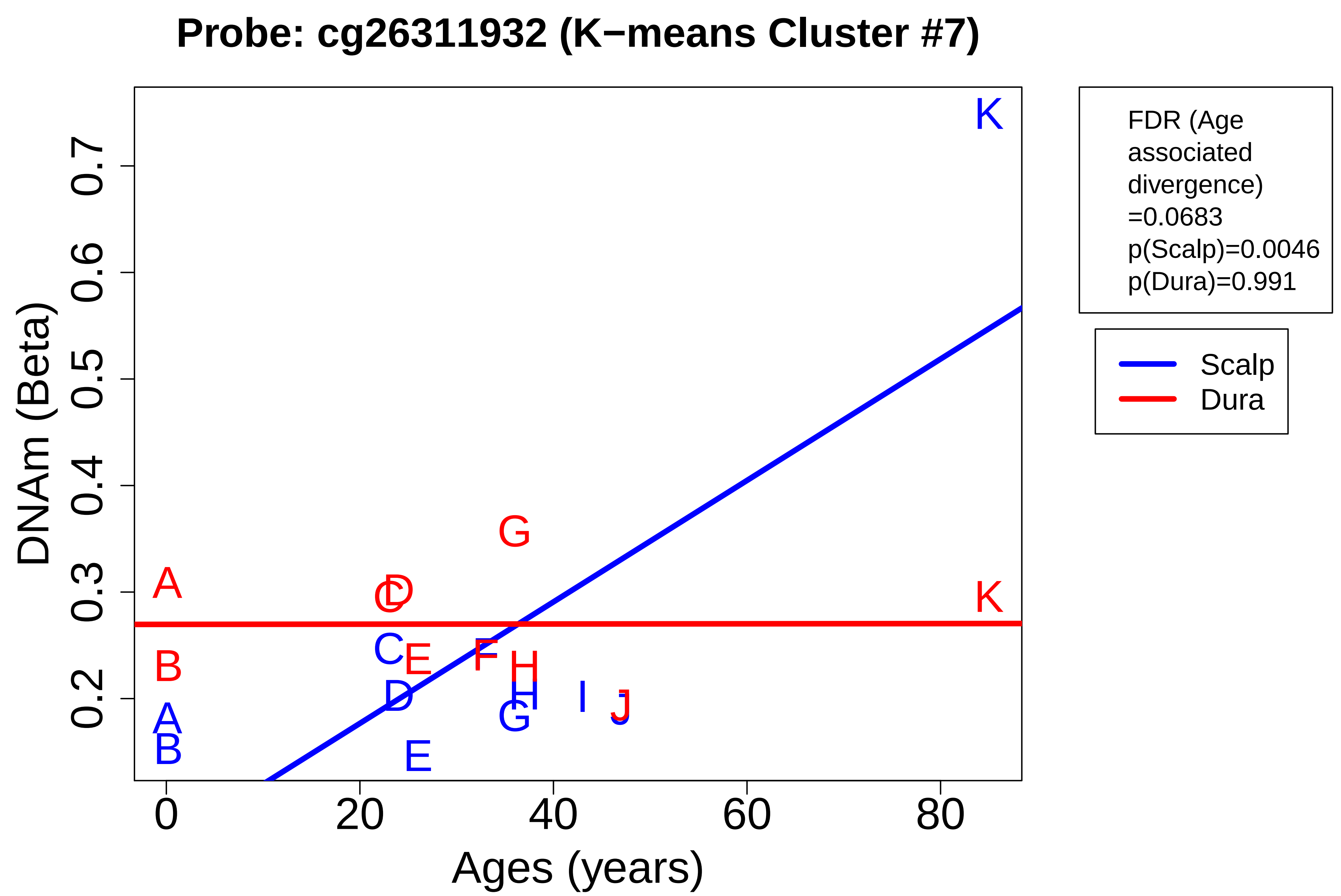

Probe: cg26311932 (K−means Cluster #7)
DNAm (Beta)
0.2
0.3
0.4
0.5
0.6
0.7
0
20
40
60
80
Ages (years)
A
B
C
D
E
F
G
H
I
J
K
FDR (Age associated divergence) =0.0683
p(Scalp)=0.0046
p(Dura)=0.991
Scalp
Dura

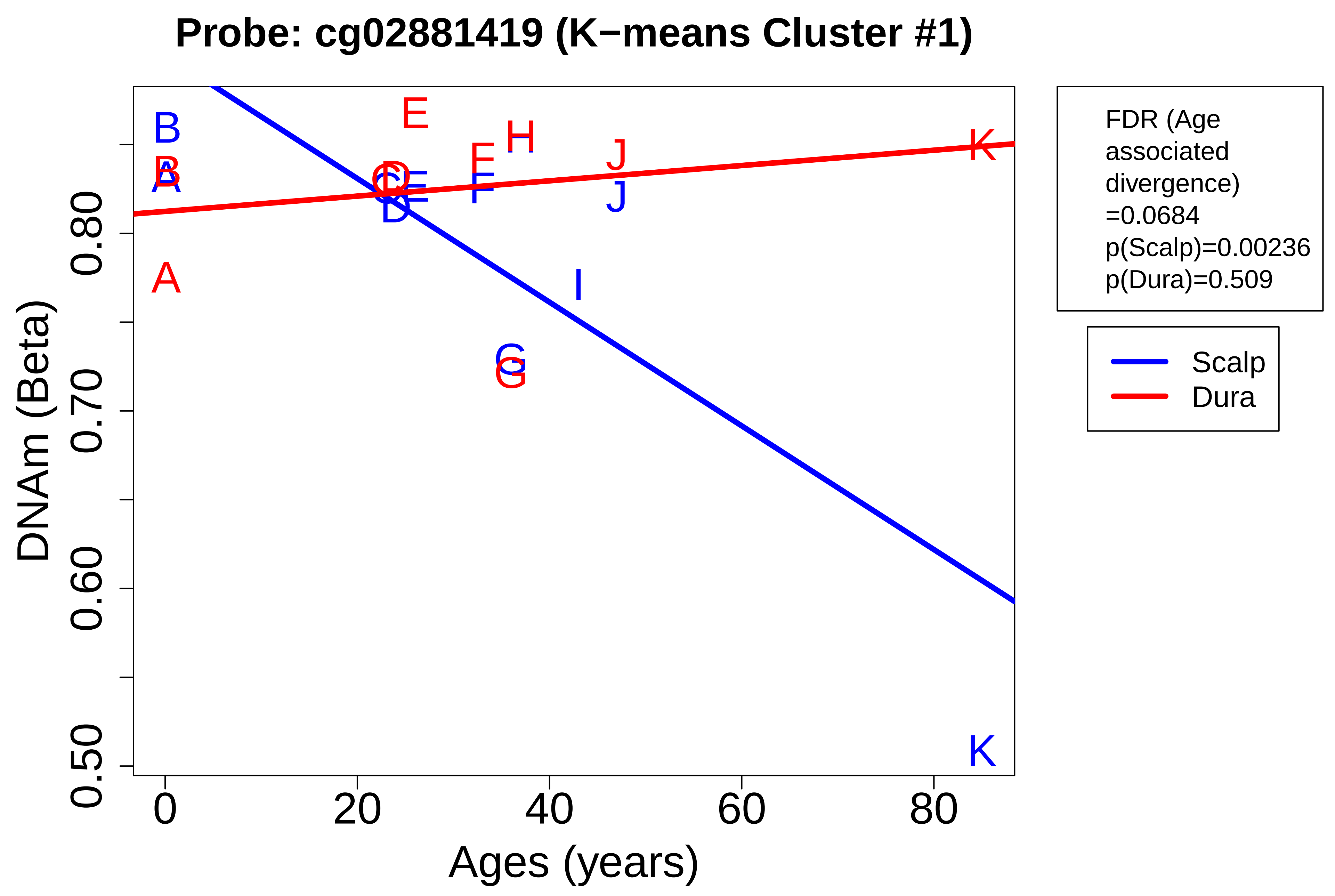

Probe: cg02881419 (K−means Cluster #1)
DNAm (Beta)
Ages (years)
0.50
0.60
0.70
0.80
0
20
40
60
80
FDR (Age associated divergence) =0.0684
p(Scalp)=0.00236
p(Dura)=0.509
Scalp
Dura

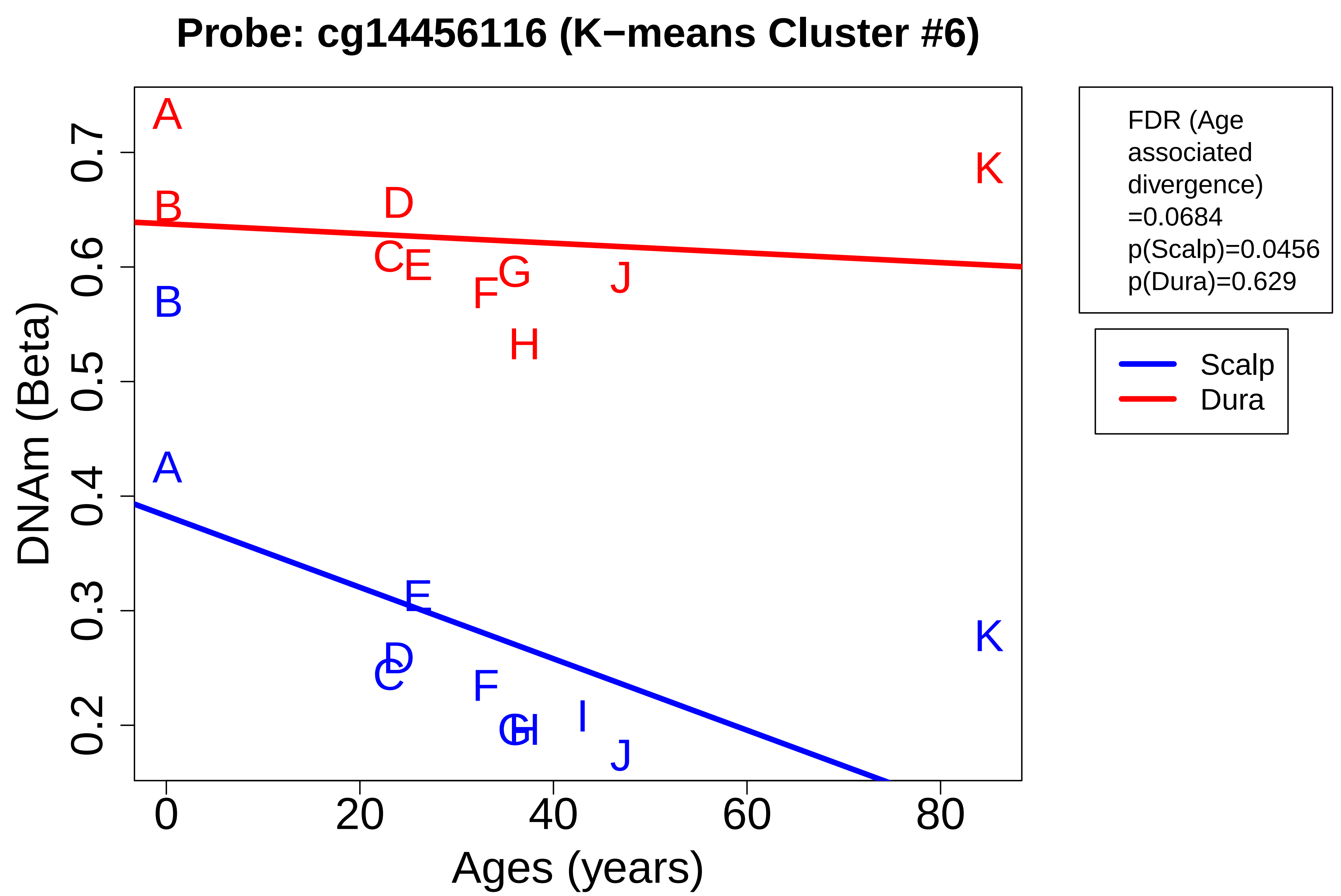

Probe: cg14456116 (K−means Cluster #6)
DNAm (Beta)
0.2
0.3
0.4
0.5
0.6
0.7
0
20
40
60
80
Ages (years)
A
B
D
K
C
E
F
G
J
H
B
A
E
D
C
F
G
H
I
J
K
FDR (Age associated divergence) =0.0684
p(Scalp)=0.0456
p(Dura)=0.629
Scalp
Dura

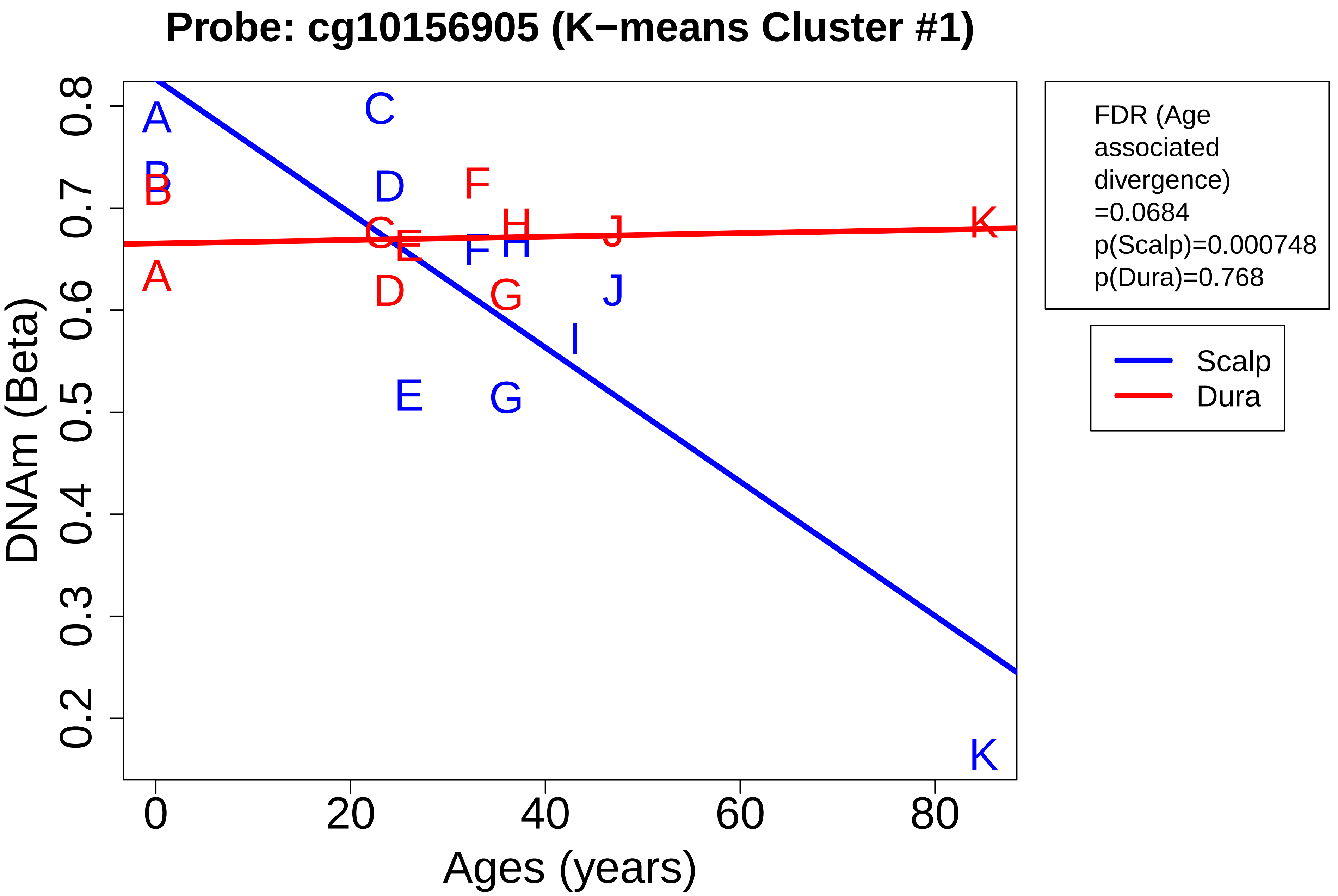

Probe: cg10156905 (K−means Cluster #1)
DNAm (Beta)
0.2
0.3
0.4
0.5
0.6
0.7
0.8
Ages (years)
0
20
40
60
80
FDR (Age associated divergence) =0.0684
p(Scalp)=0.000748
p(Dura)=0.768
Scalp
Dura

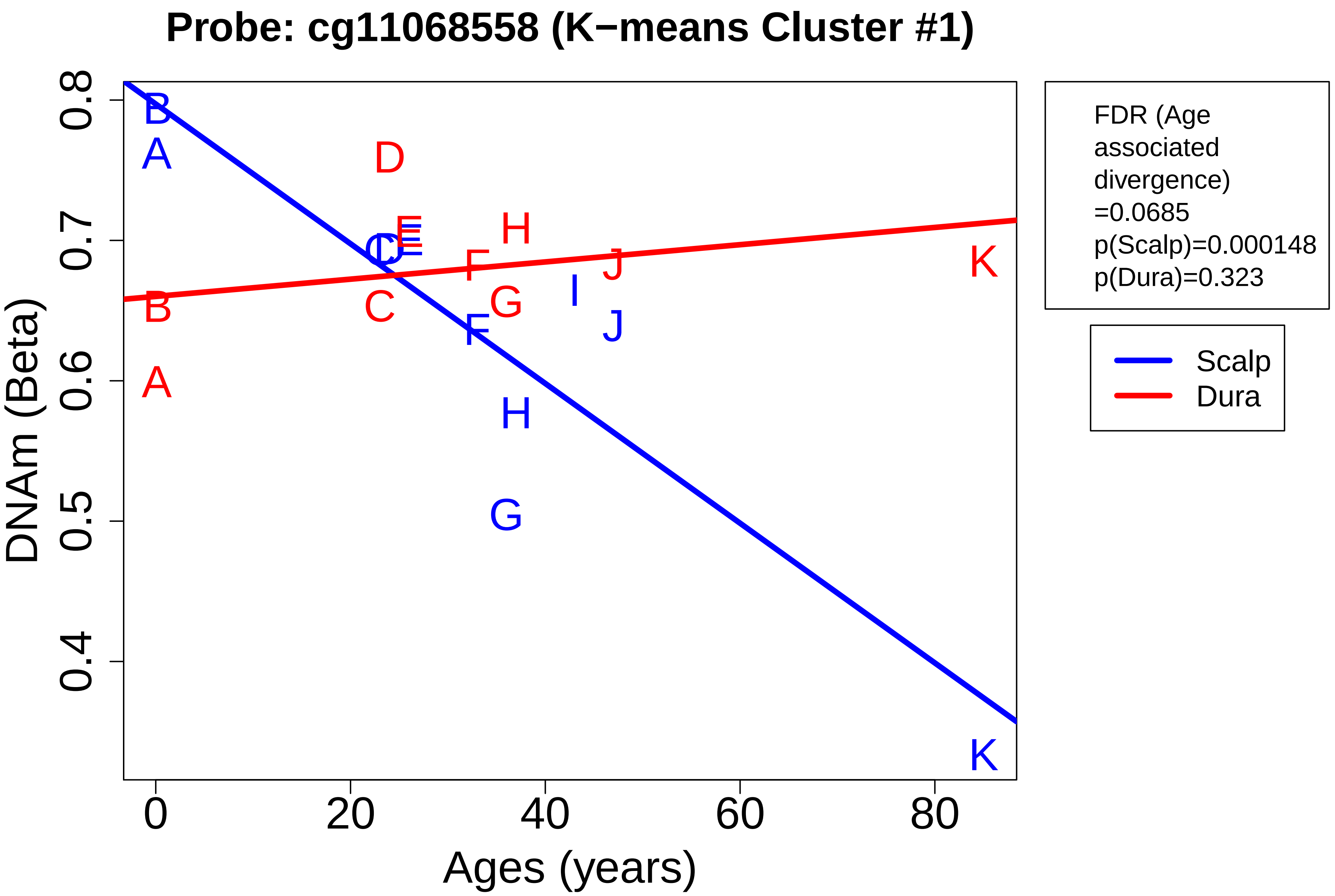

Probe: cg11068558 (K−means Cluster #1)
DNAm (Beta)
0.4
0.5
0.6
0.7
0.8
0
20
40
60
80
Ages (years)
FDR (Age associated divergence) =0.0685
p(Scalp)=0.000148
p(Dura)=0.323
Scalp
Dura

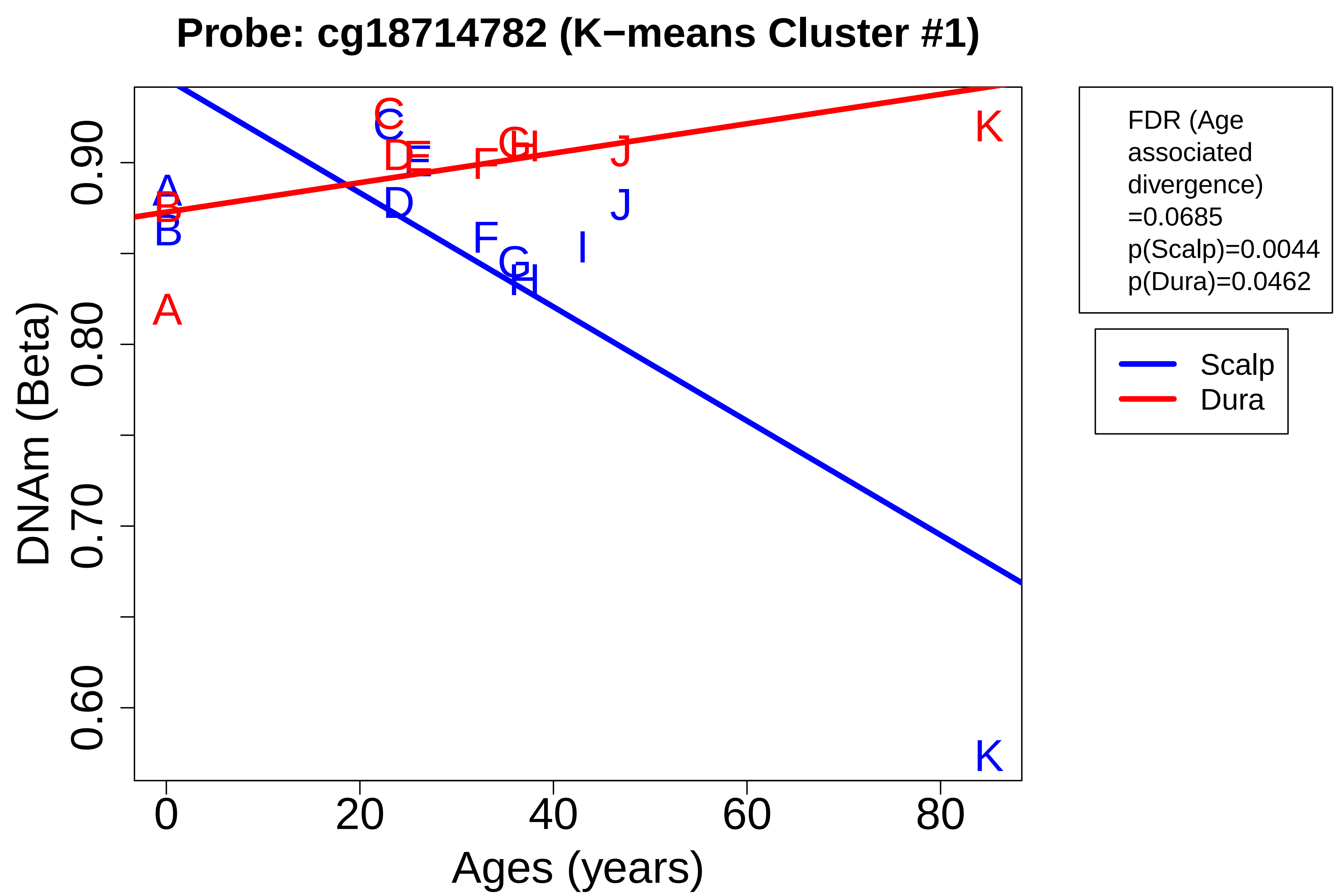
Probe: cg18714782 (K−means Cluster #1)
DNAm (Beta)
0.60
0.70
0.80
0.90
0
20
40
60
80
Ages (years)
FDR (Age associated divergence) =0.0685
p(Scalp)=0.0044
p(Dura)=0.0462
Scalp
Dura

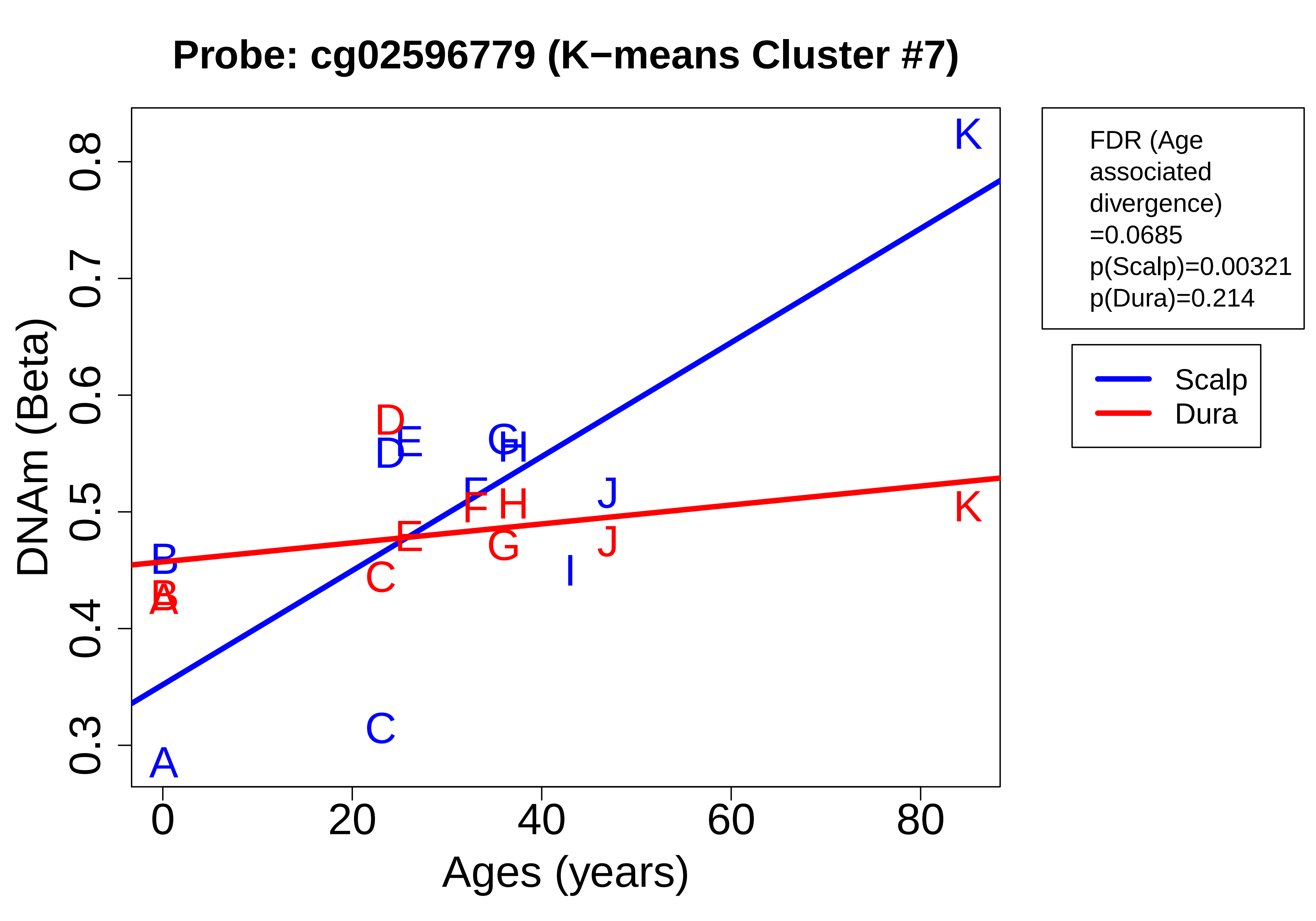
Probe: cg02596779 (K−means Cluster #7)
DNAm (Beta)
0.3
0.4
0.5
0.6
0.7
0.8
Ages (years)
0
20
40
60
80
FDR (Age associated divergence) =0.0685
p(Scalp)=0.00321
p(Dura)=0.214
Scalp
Dura
A B C D E F G H I J K

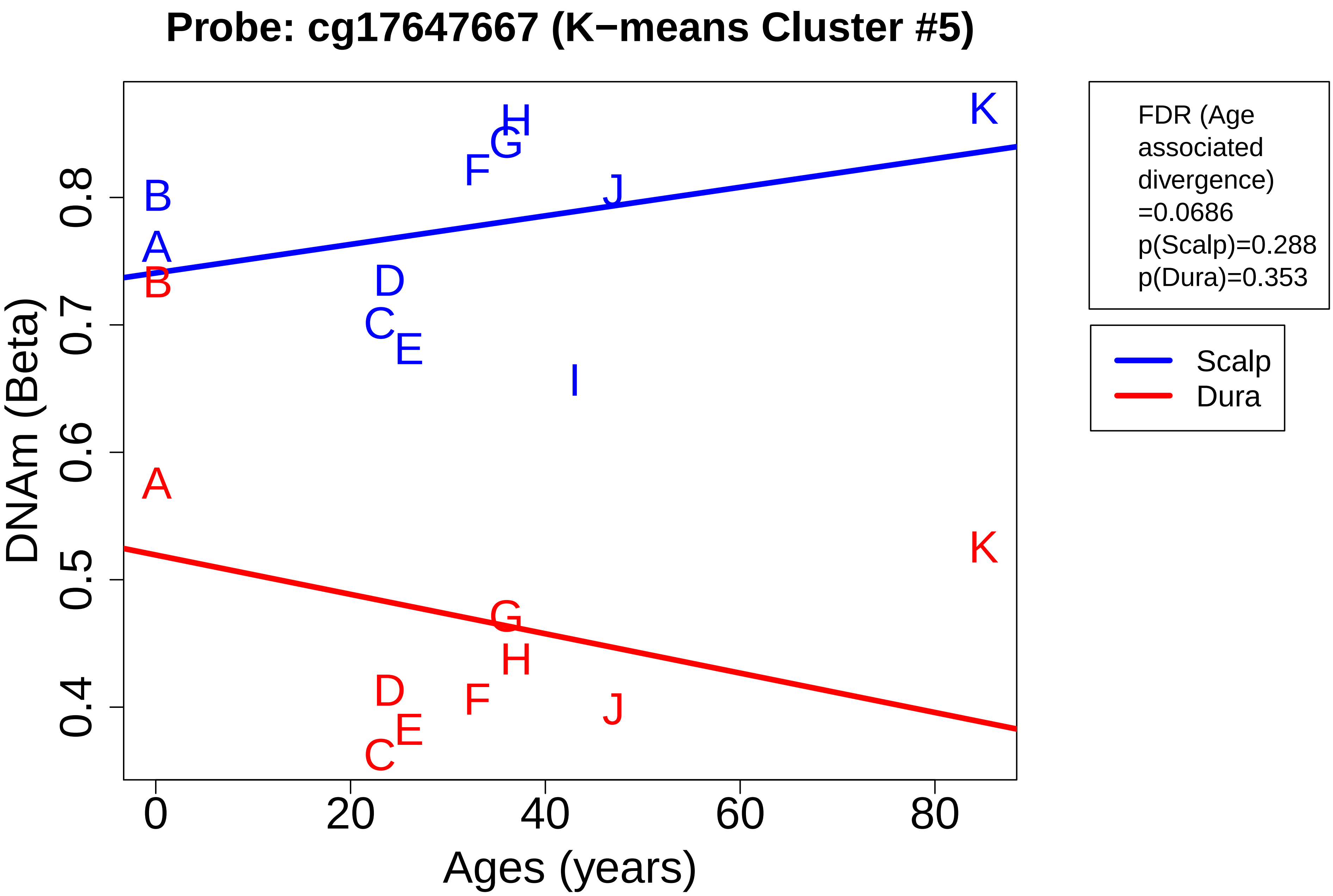

Probe: cg17647667 (K−means Cluster #5)
DNAm (Beta)
0.4
0.5
0.6
0.7
0.8
Ages (years)
0
20
40
60
80
FDR (Age associated divergence) =0.0686
p(Scalp)=0.288
p(Dura)=0.353
Scalp
Dura
A
B
C
D
E
F
G
H
I
J
K

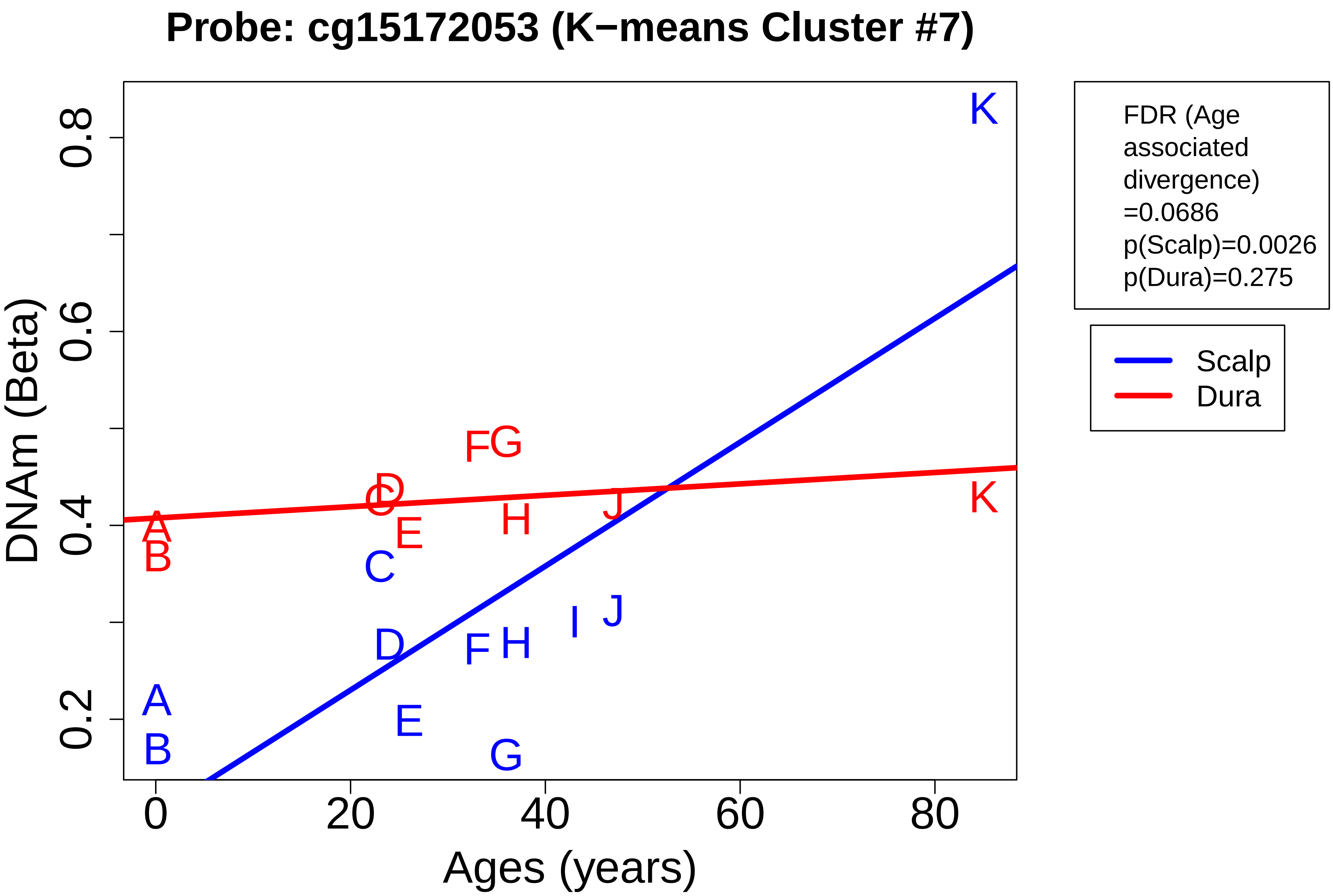

Probe: cg15172053 (K−means Cluster #7)
DNAm (Beta)
0.2
0.4
0.6
0.8
Ages (years)
0
20
40
60
80
FDR (Age associated divergence) =0.0686
p(Scalp)=0.0026
p(Dura)=0.275
Scalp
Dura
A
B
C
D
E
F
G
H
I
J
K

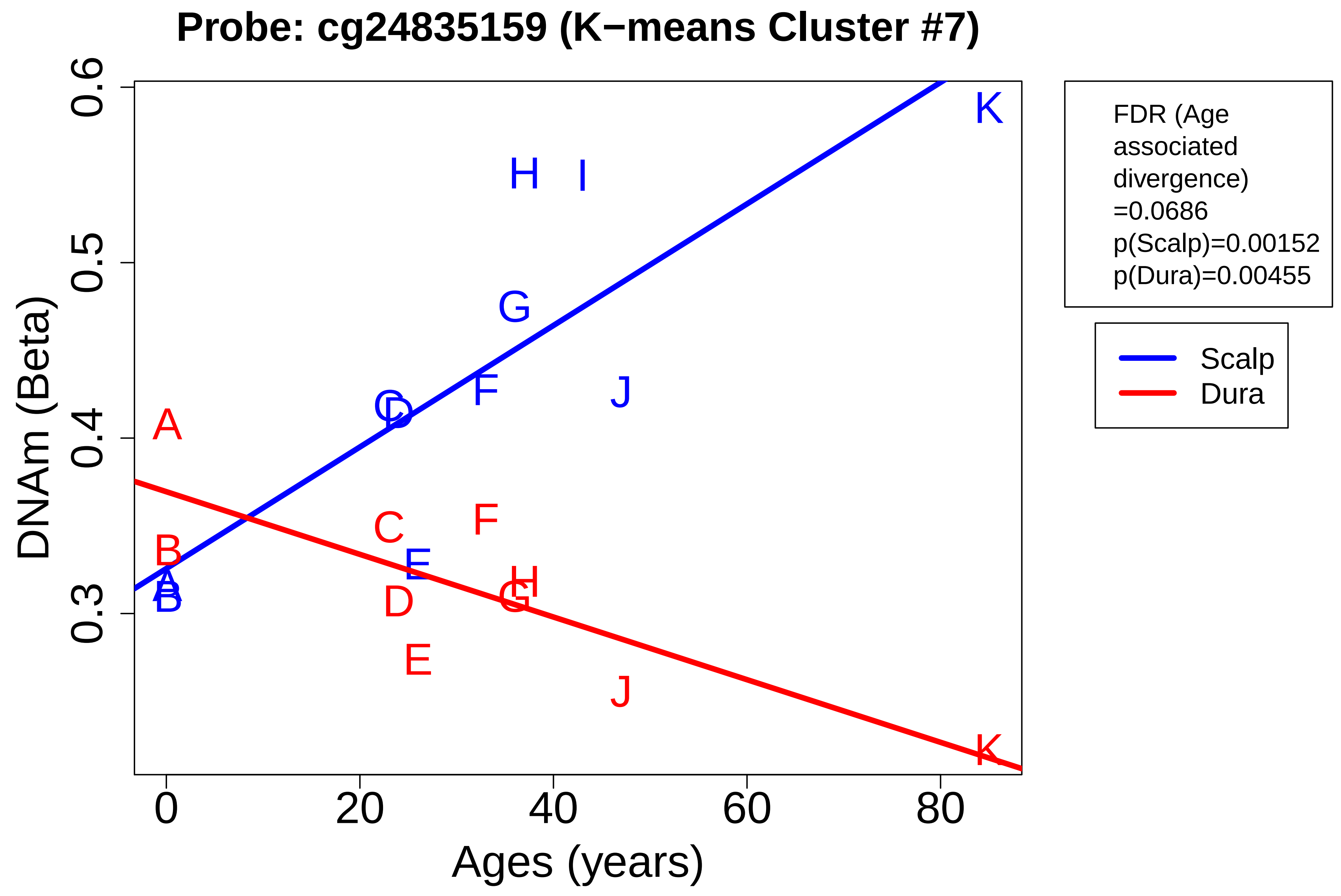

Probe: cg24835159 (K−means Cluster #7)
DNAm (Beta)
0.3
0.4
0.5
0.6
0
20
40
60
80
Ages (years)
FDR (Age associated divergence) =0.0686
p(Scalp)=0.00152
p(Dura)=0.00455
Scalp
Dura

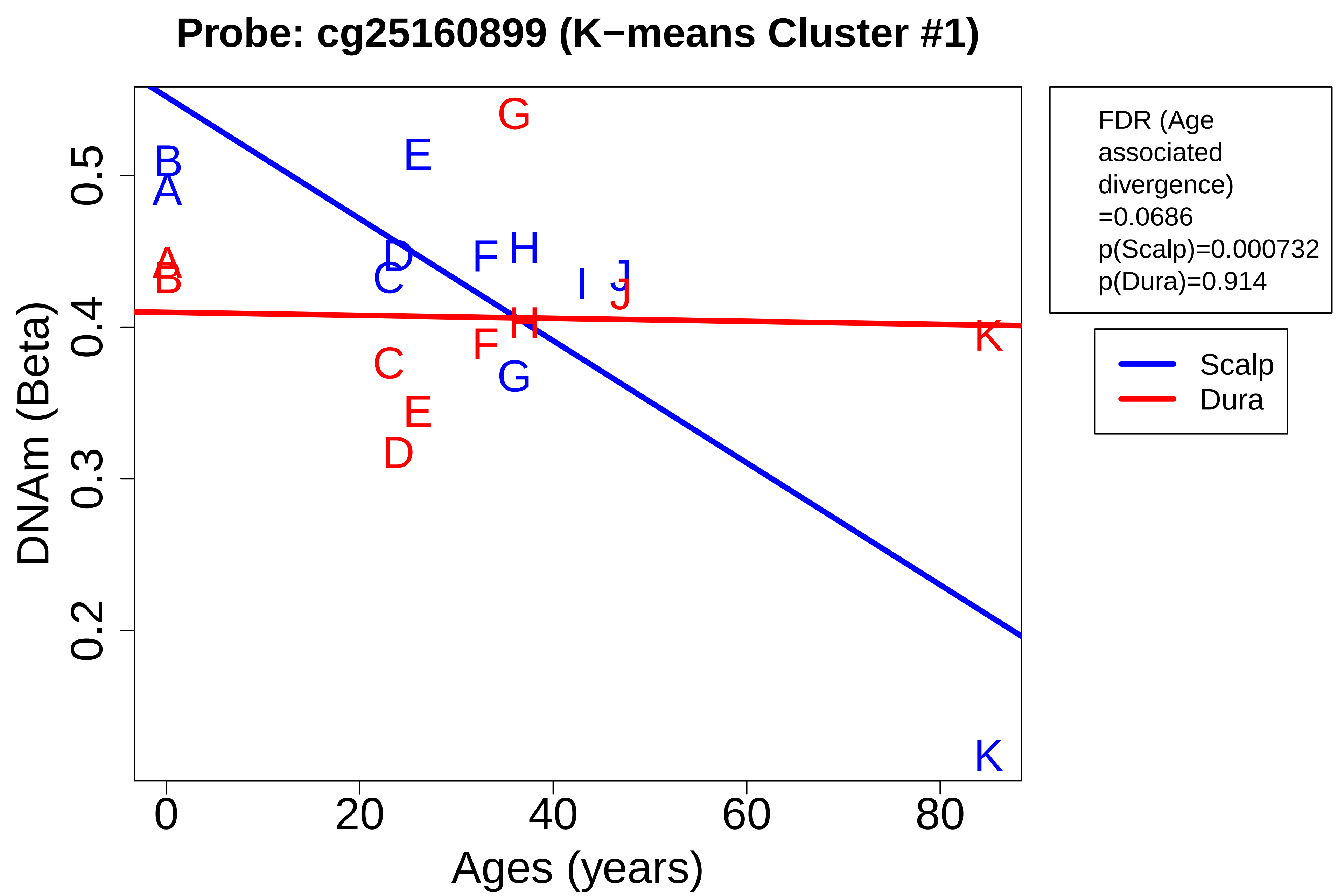

Probe: cg25160899 (K−means Cluster #1)
DNAm (Beta)
0.2
0.3
0.4
0.5
Ages (years)
0
20
40
60
80
FDR (Age associated divergence) =0.0686
p(Scalp)=0.000732
p(Dura)=0.914
Scalp
Dura

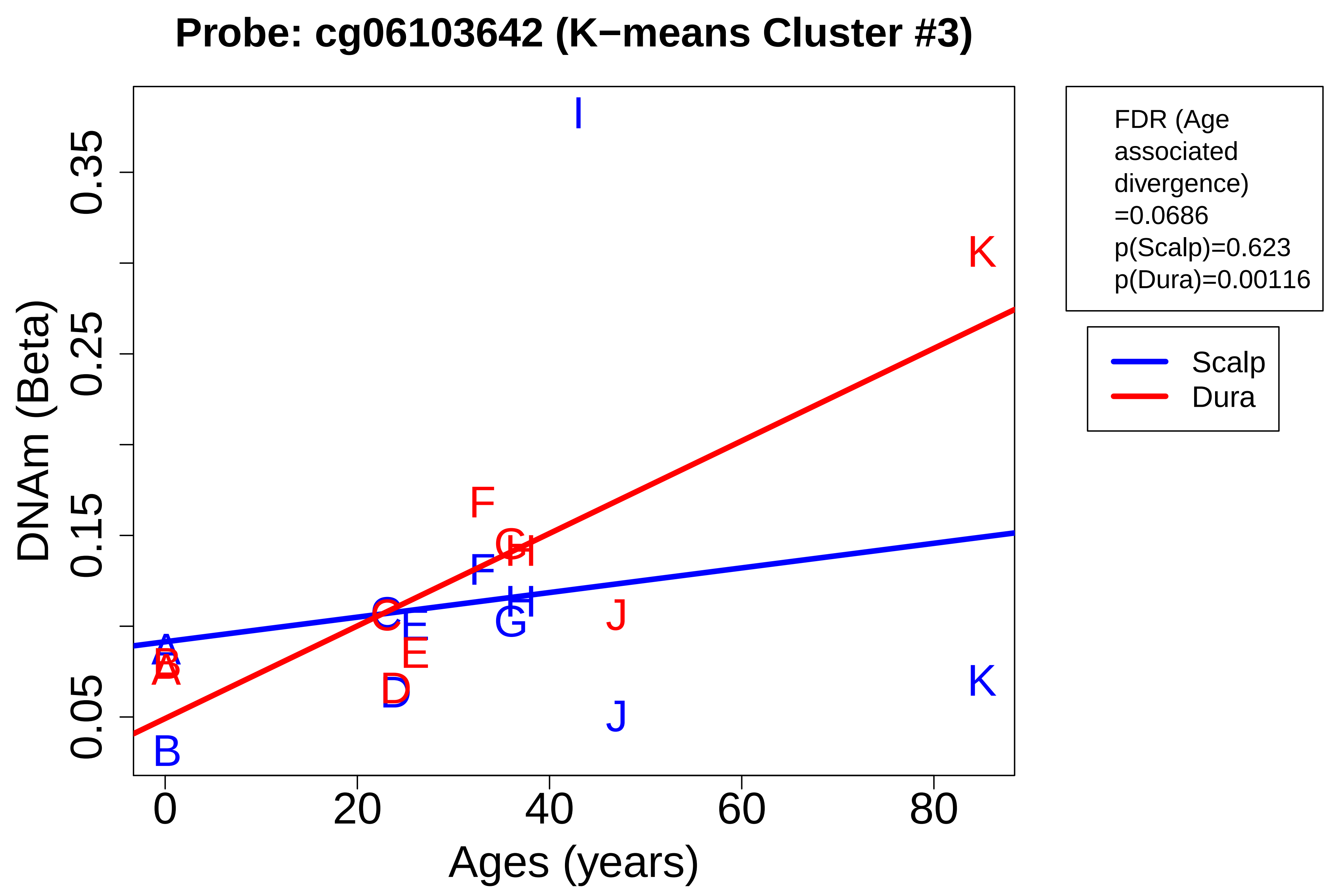

Probe: cg06103642 (K−means Cluster #3)
DNAm (Beta)
0.05
0.15
0.25
0.35
Ages (years)
0
20
40
60
80
FDR (Age associated divergence) =0.0686
p(Scalp)=0.623
p(Dura)=0.00116
Scalp
Dura
A
B
C
D
E
F
G
H
I
J
K

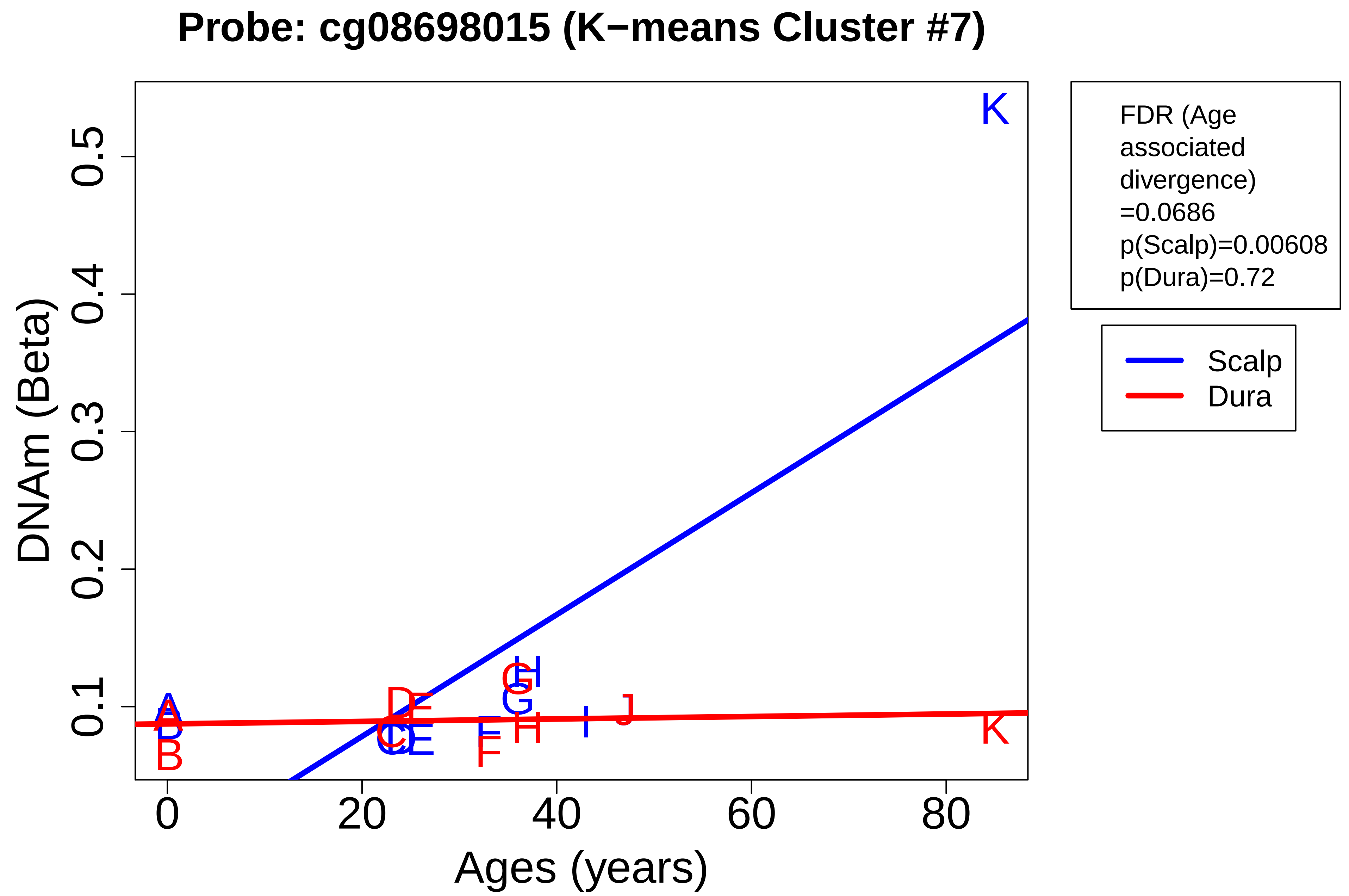

Probe: cg08698015 (K−means Cluster #7)
DNAm (Beta)
0.1
0.2
0.3
0.4
0.5
0
20
40
60
80
Ages (years)
FDR (Age associated divergence) =0.0686
p(Scalp)=0.00608
p(Dura)=0.72
Scalp
Dura

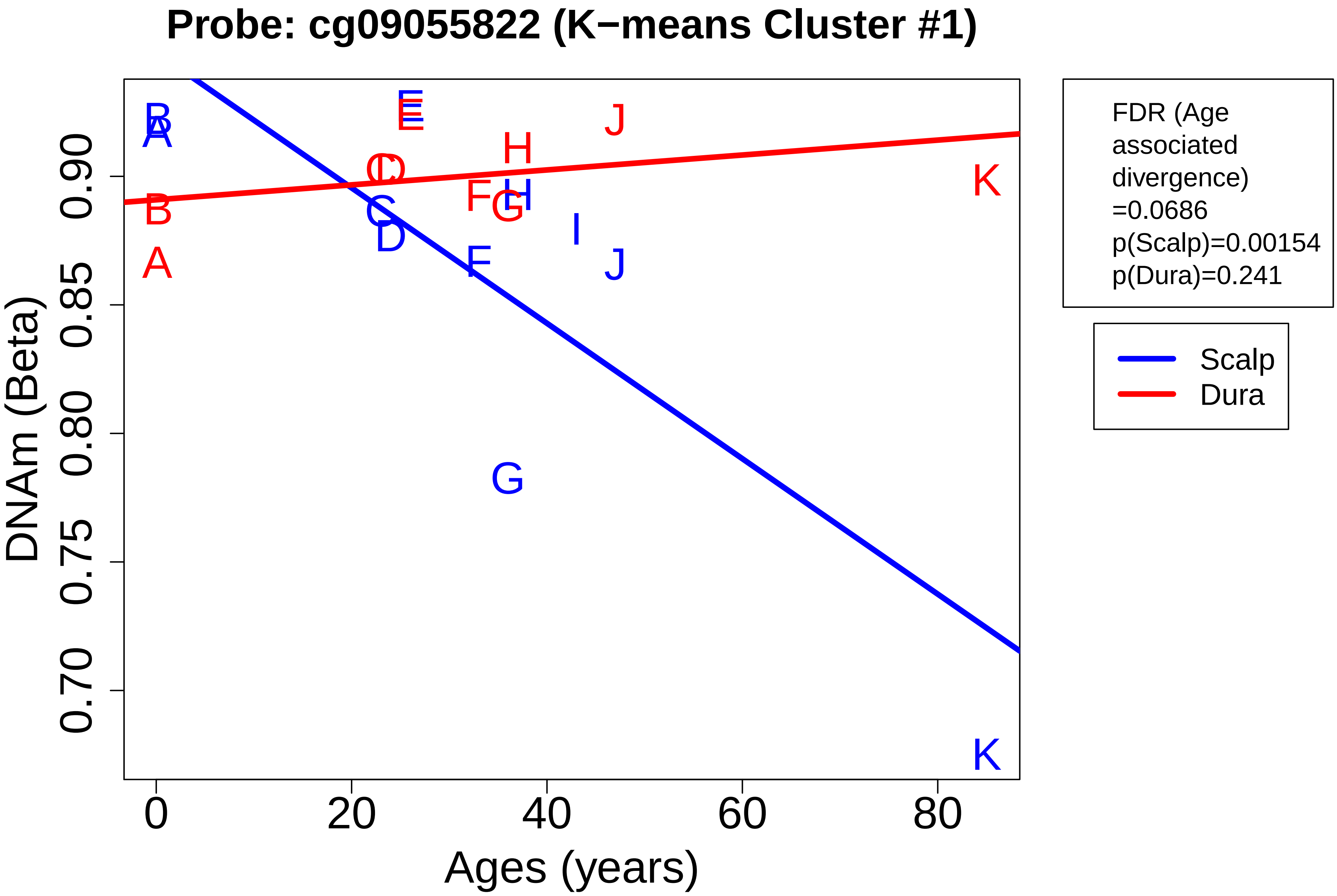

Probe: cg09055822 (K−means Cluster #1)
DNAm (Beta)
0.70
0.75
0.80
0.85
0.90
0
20
40
60
80
Ages (years)
FDR (Age associated divergence) =0.0686
p(Scalp)=0.00154
p(Dura)=0.241
Scalp
Dura

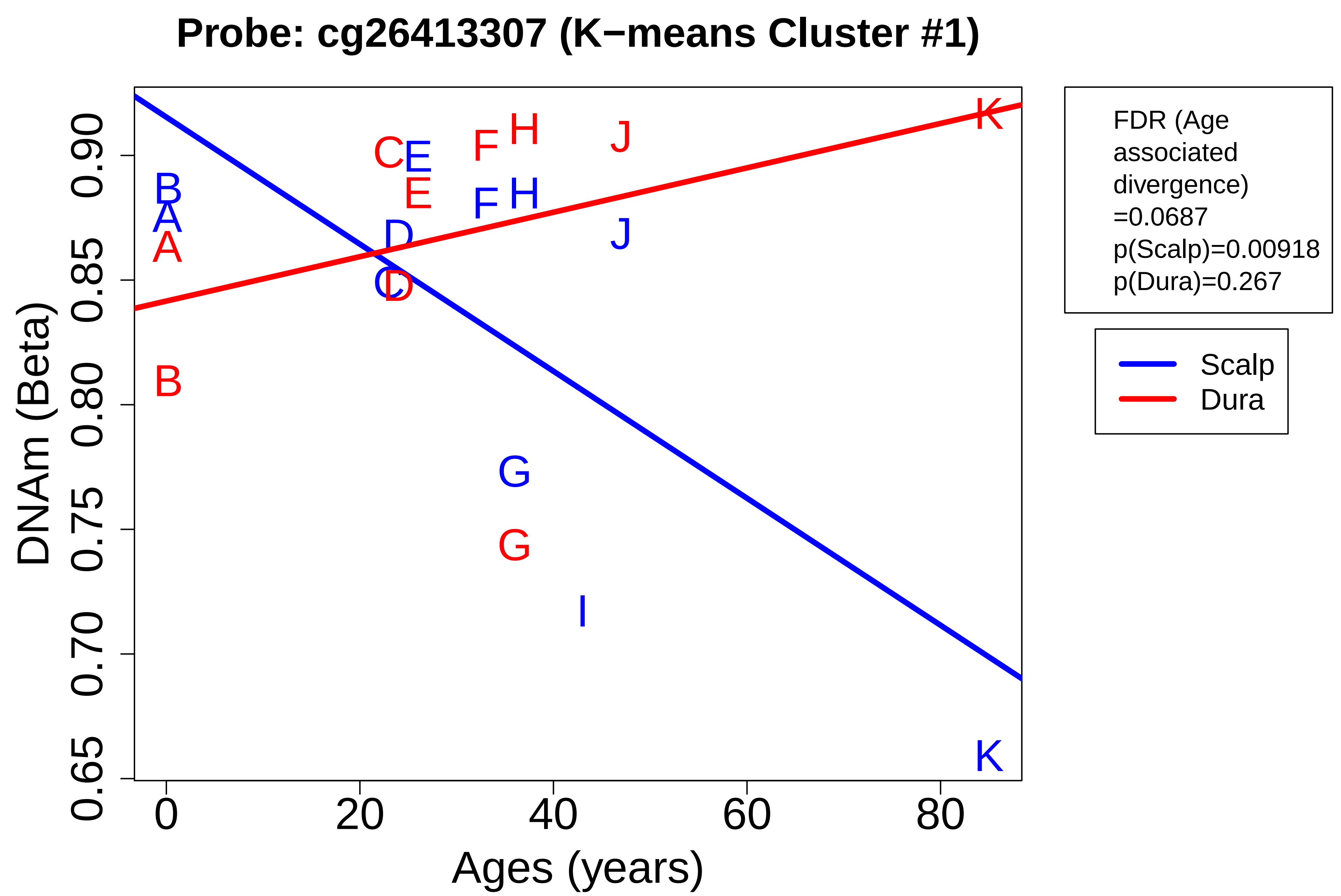

Probe: cg26413307 (K−means Cluster #1)
DNAm (Beta)
0.65
0.70
0.75
0.80
0.85
0.90
0
20
40
60
80
Ages (years)
A
B
C
D
E
F
G
H
I
J
K
FDR (Age associated divergence) =0.0687
p(Scalp)=0.00918
p(Dura)=0.267
Scalp
Dura

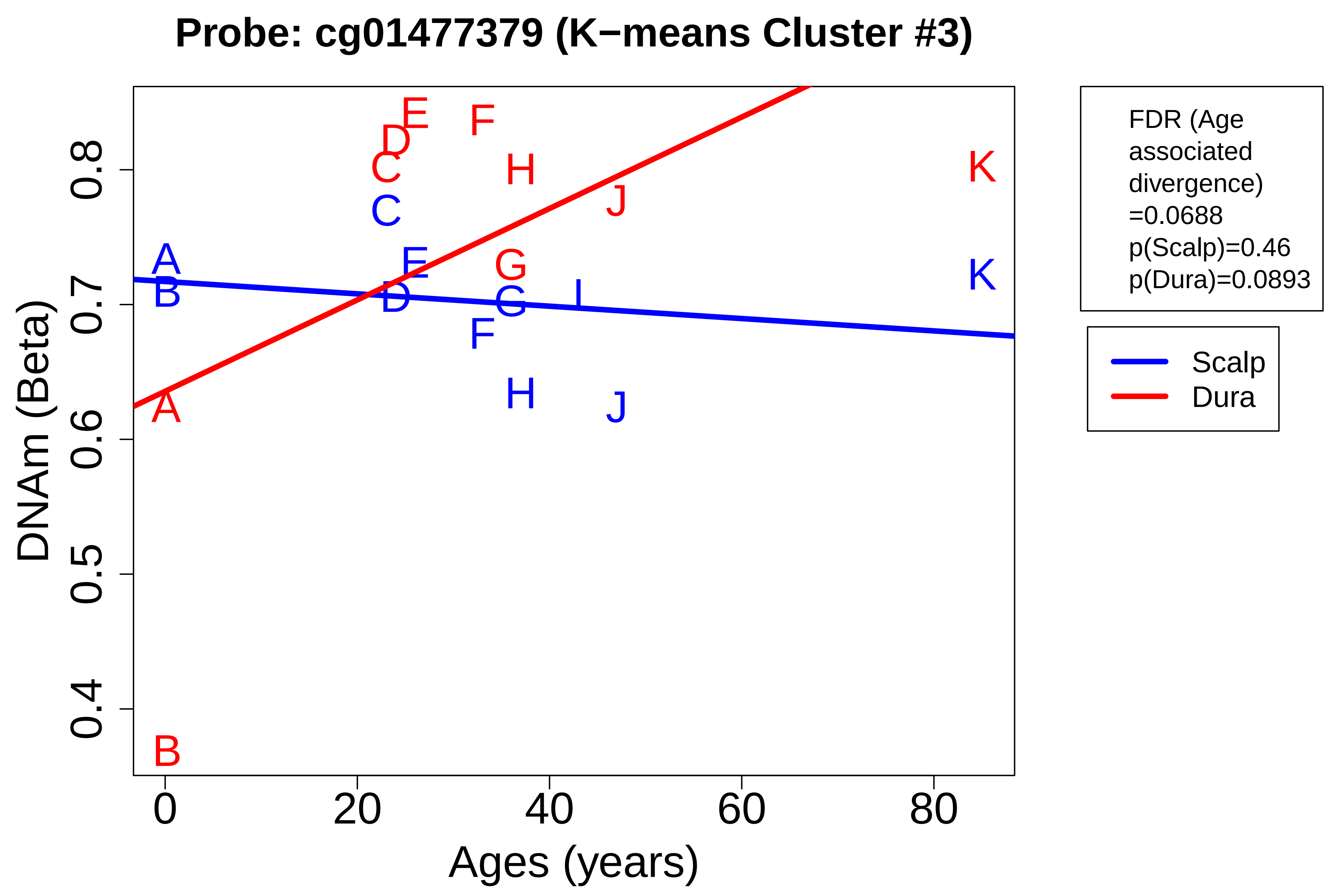
Probe: cg01477379 (K−means Cluster #3)
DNAm (Beta)
Ages (years)
0.4
0.5
0.6
0.7
0.8
0
20
40
60
80
FDR (Age associated divergence) =0.0688
p(Scalp)=0.46
p(Dura)=0.0893
Scalp
Dura

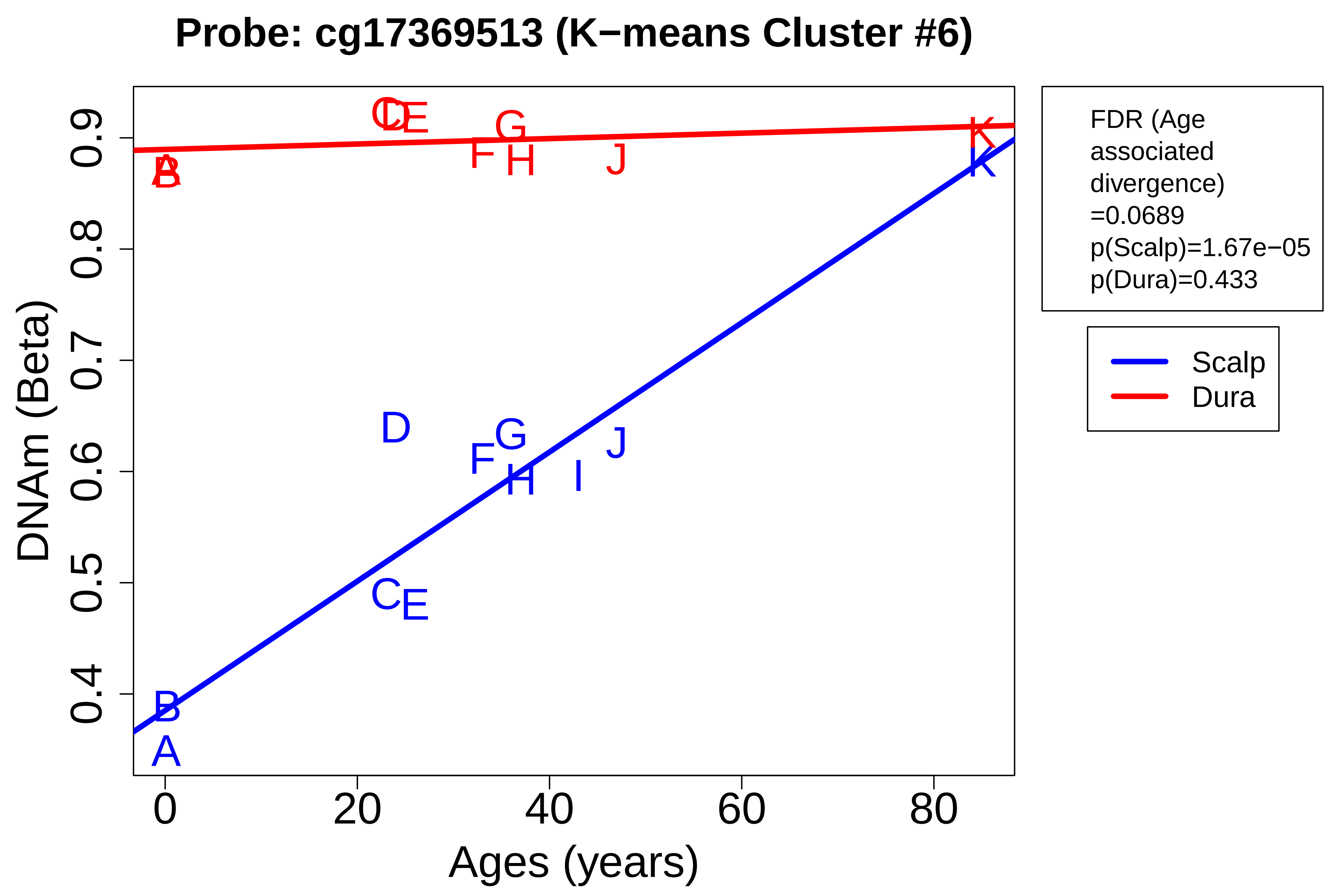

Probe: cg17369513 (K−means Cluster #6)
DNAm (Beta)
Ages (years)
0.4
0.5
0.6
0.7
0.8
0.9
0
20
40
60
80
FDR (Age associated divergence) =0.0689
p(Scalp)=1.67e−05
p(Dura)=0.433
Scalp
Dura

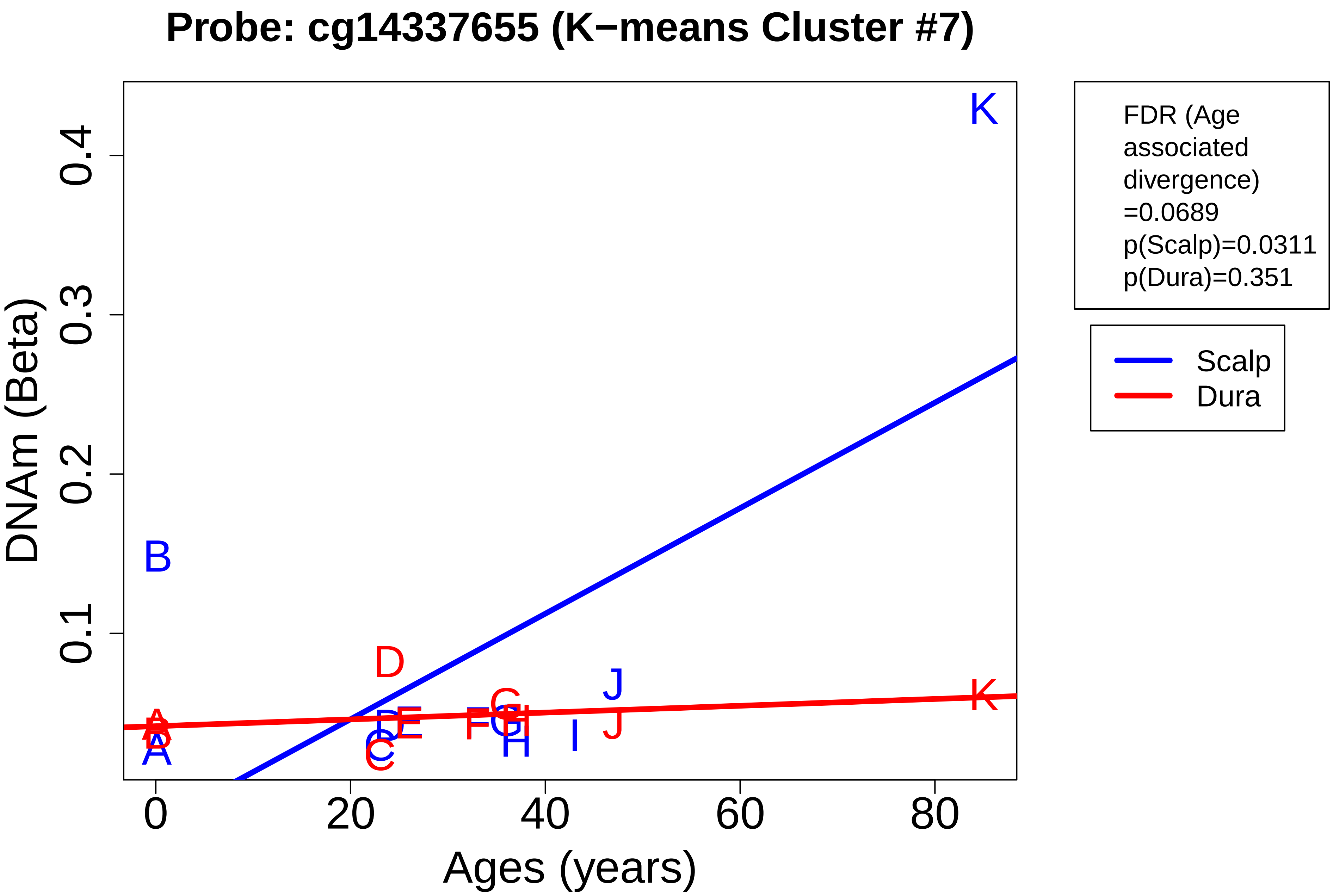

Probe: cg14337655 (K−means Cluster #7)
DNAm (Beta)
0.1
0.2
0.3
0.4
Ages (years)
0
20
40
60
80
FDR (Age associated divergence) =0.0689
p(Scalp)=0.0311
p(Dura)=0.351
Scalp
Dura
A
B
C
D
E
F
G
H
I
J
K

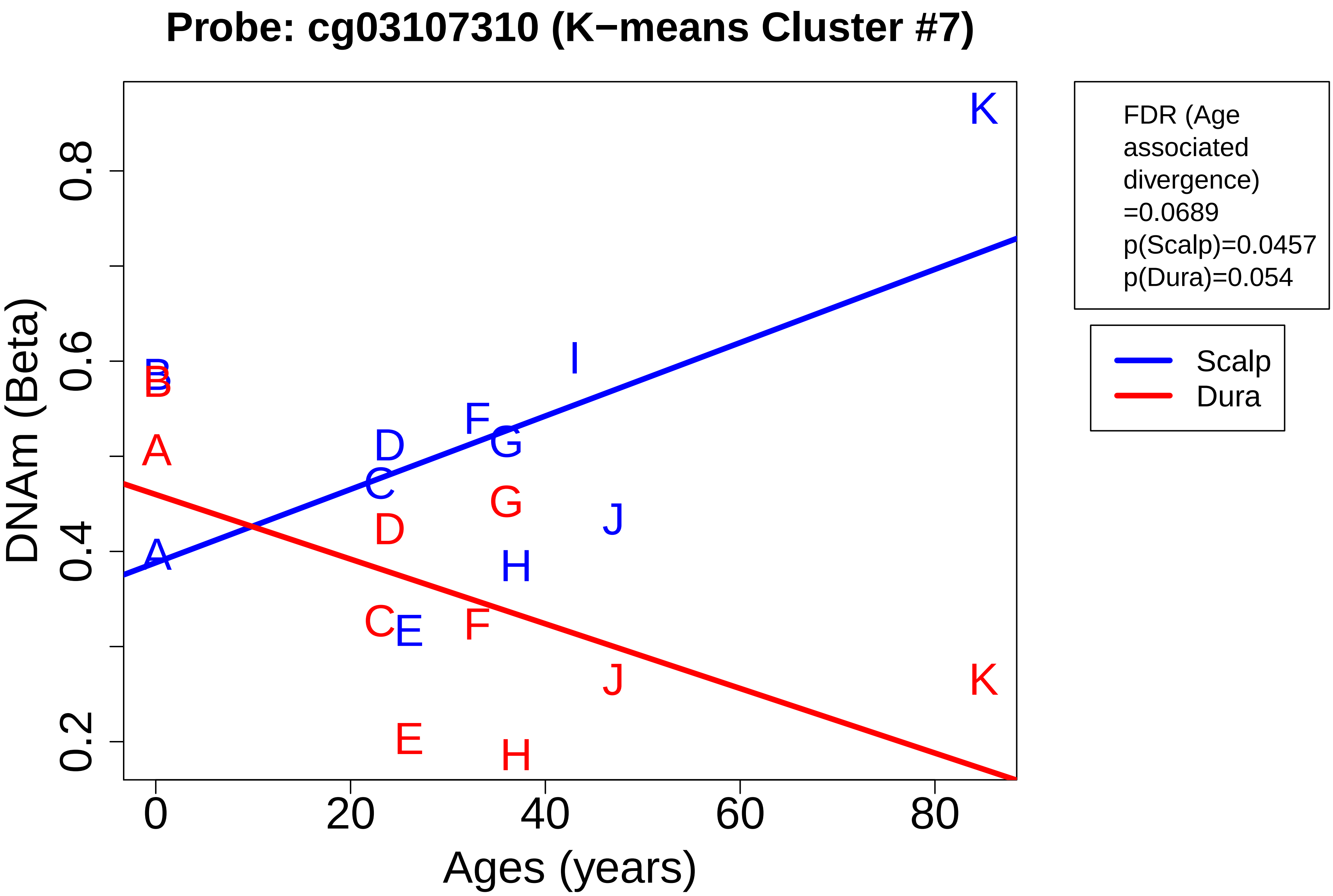

Probe: cg03107310 (K−means Cluster #7)
DNAm (Beta)
0.2
0.4
0.6
0.8
0
20
40
60
80
Ages (years)
FDR (Age associated divergence) =0.0689
p(Scalp)=0.0457
p(Dura)=0.054
Scalp
Dura
A
B
C
D
E
F
G
H
I
J
K

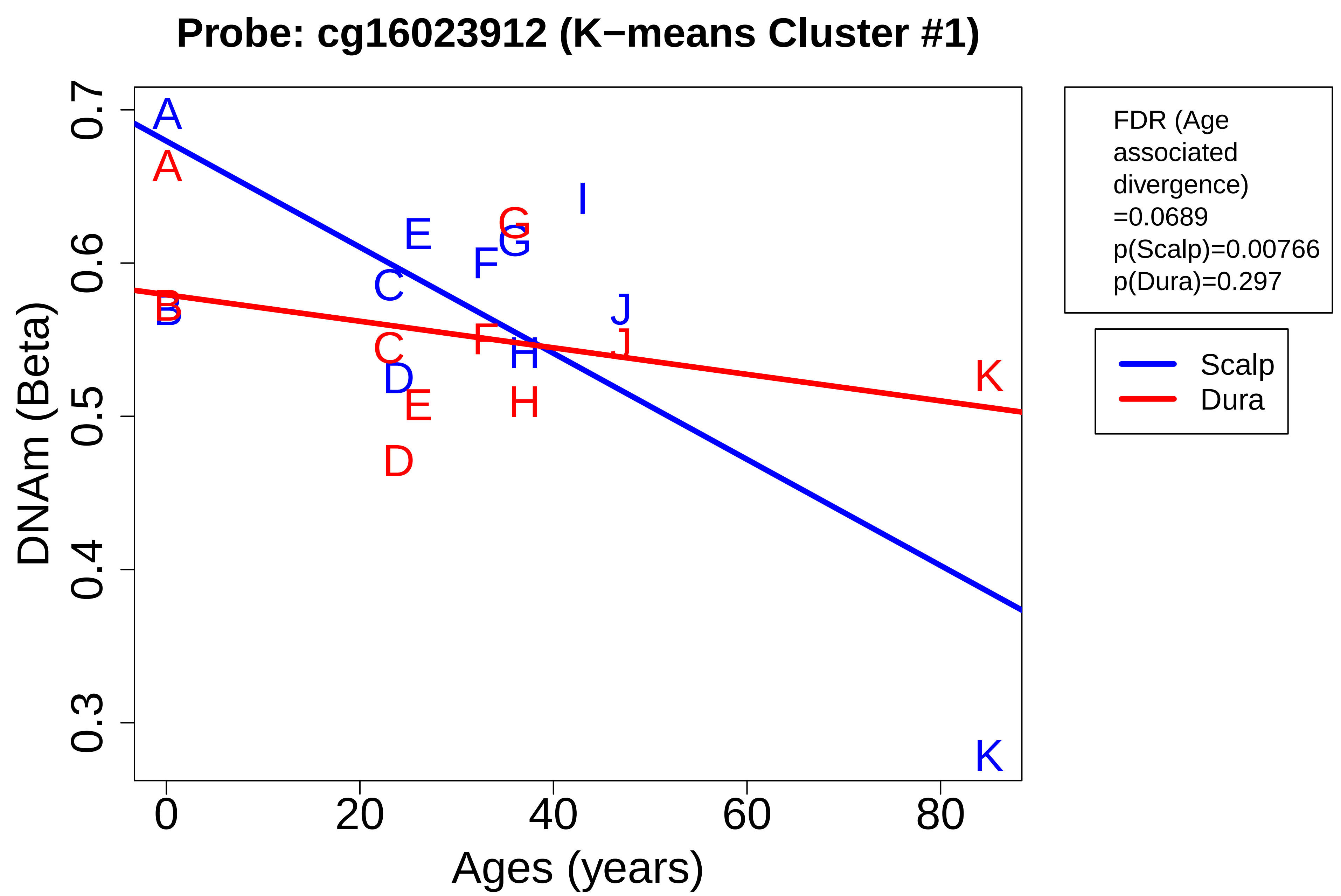

Probe: cg16023912 (K−means Cluster #1)
DNAm (Beta)
0.3
0.4
0.5
0.6
0.7
0
20
40
60
80
Ages (years)
A
B
C
D
E
F
G
H
I
J
K
FDR (Age associated divergence) =0.0689
p(Scalp)=0.00766
p(Dura)=0.297
Scalp
Dura

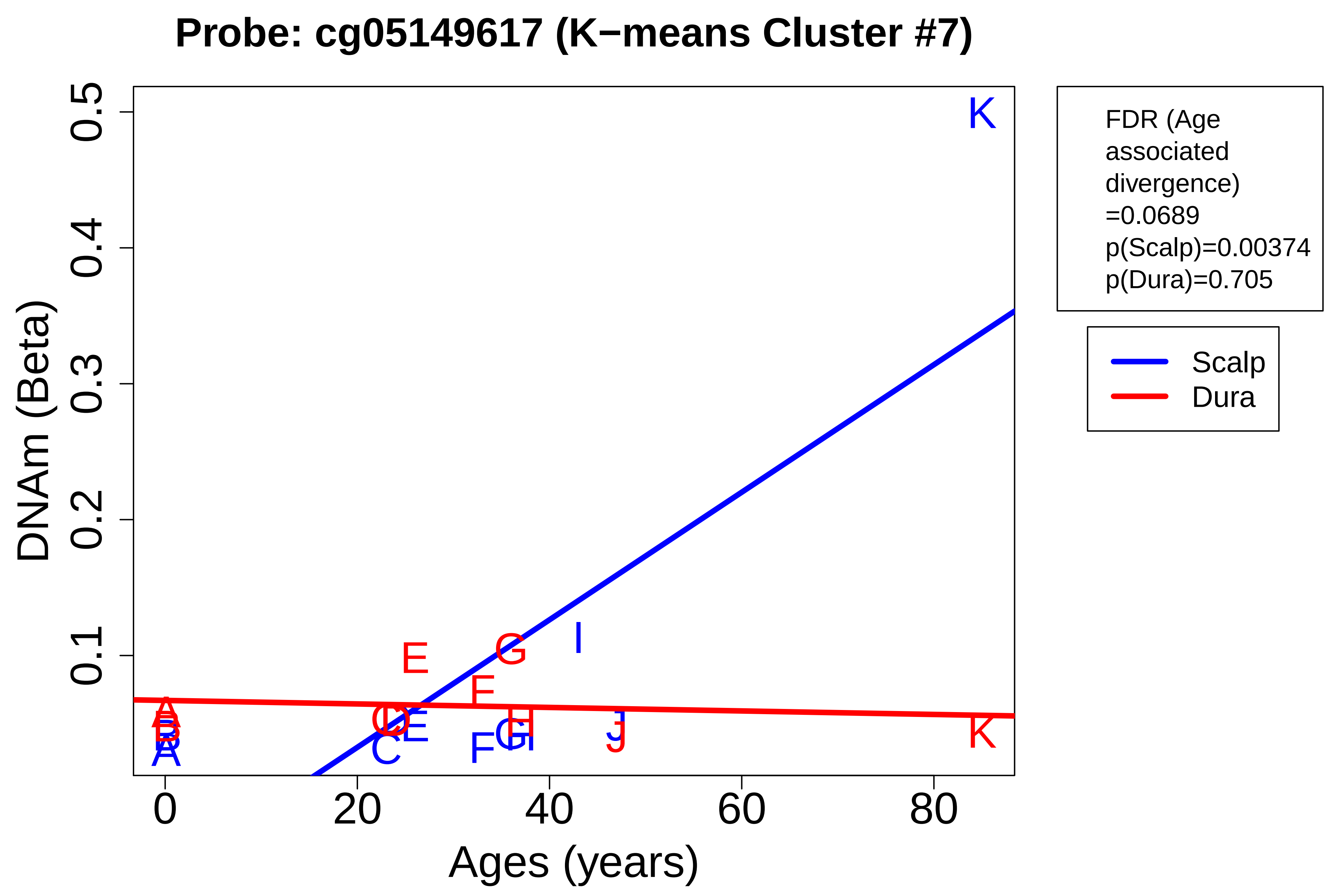

Probe: cg05149617 (K−means Cluster #7)
DNAm (Beta)
0.1
0.2
0.3
0.4
0.5
Ages (years)
0
20
40
60
80
FDR (Age associated divergence) =0.0689
p(Scalp)=0.00374
p(Dura)=0.705
Scalp
Dura

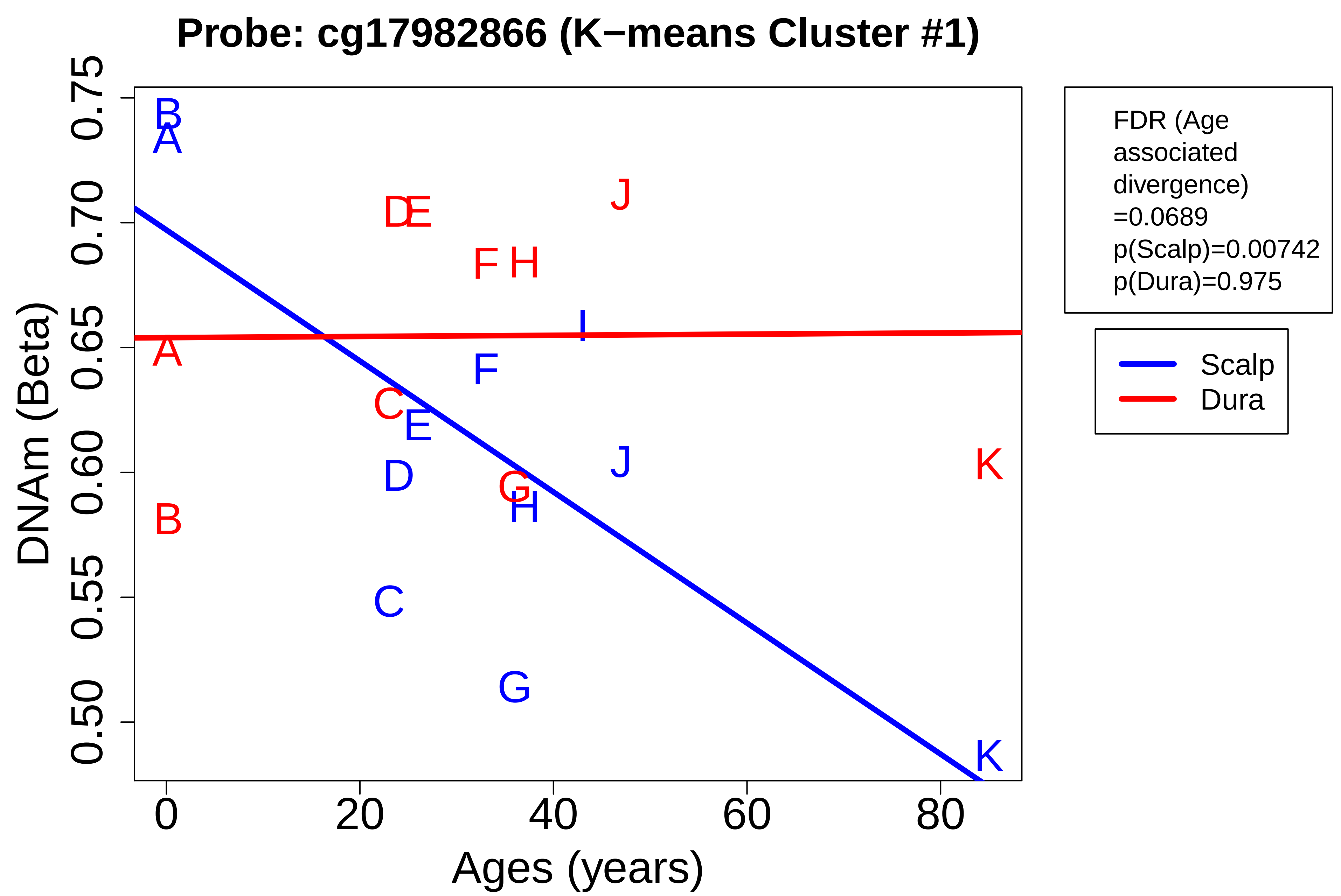

Probe: cg17982866 (K−means Cluster #1)
DNAm (Beta)
0.50
0.55
0.60
0.65
0.70
0.75
0
20
40
60
80
Ages (years)
A
B
C
D
E
F
G
H
I
J
K
FDR (Age associated divergence) =0.0689
p(Scalp)=0.00742
p(Dura)=0.975
Scalp
Dura

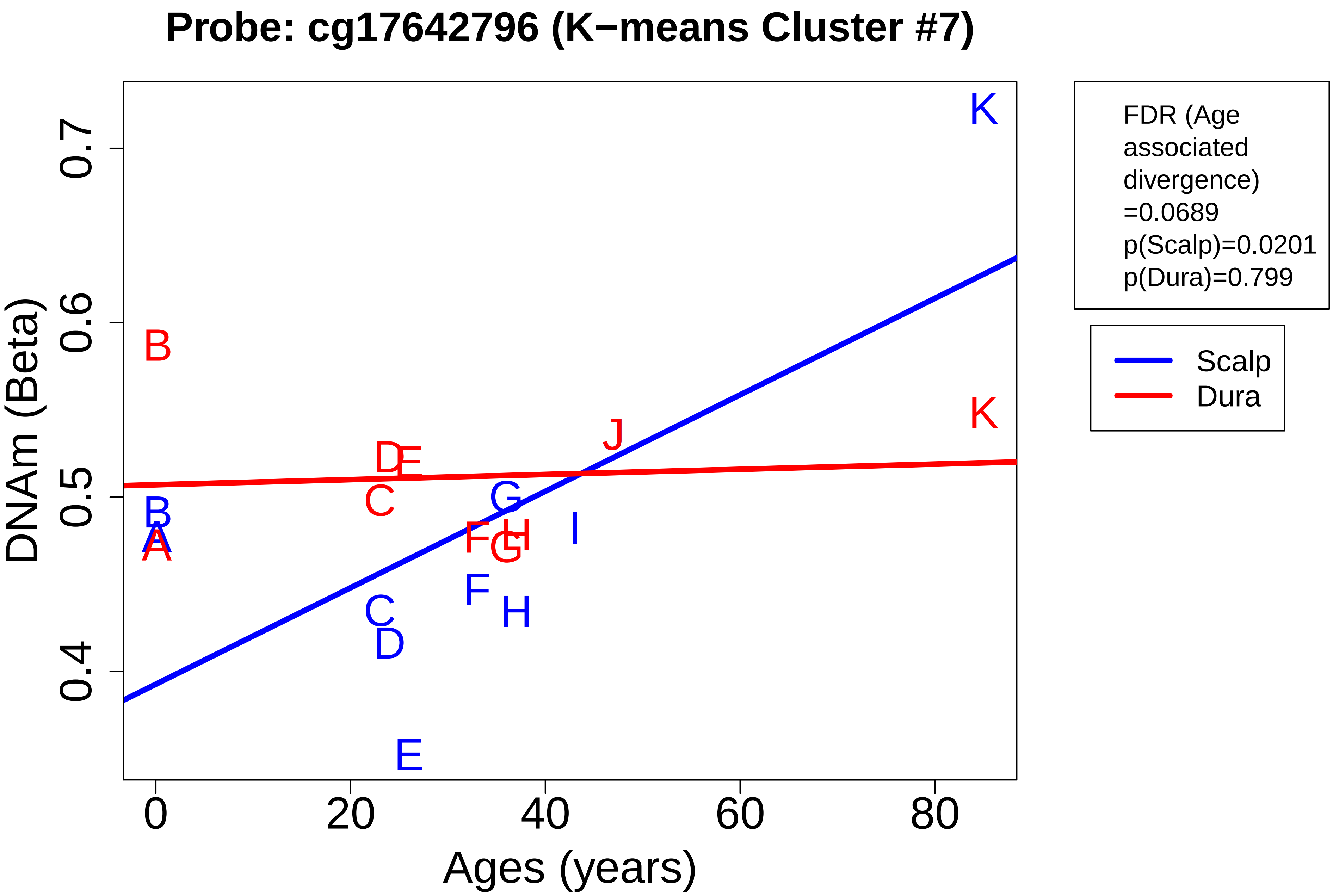

Probe: cg17642796 (K−means Cluster #7)
DNAm (Beta)
0.4
0.5
0.6
0.7
0
20
40
60
80
Ages (years)
FDR (Age associated divergence) =0.0689
p(Scalp)=0.0201
p(Dura)=0.799
Scalp
Dura
A
B
C
D
E
F
G
H
I
J
K

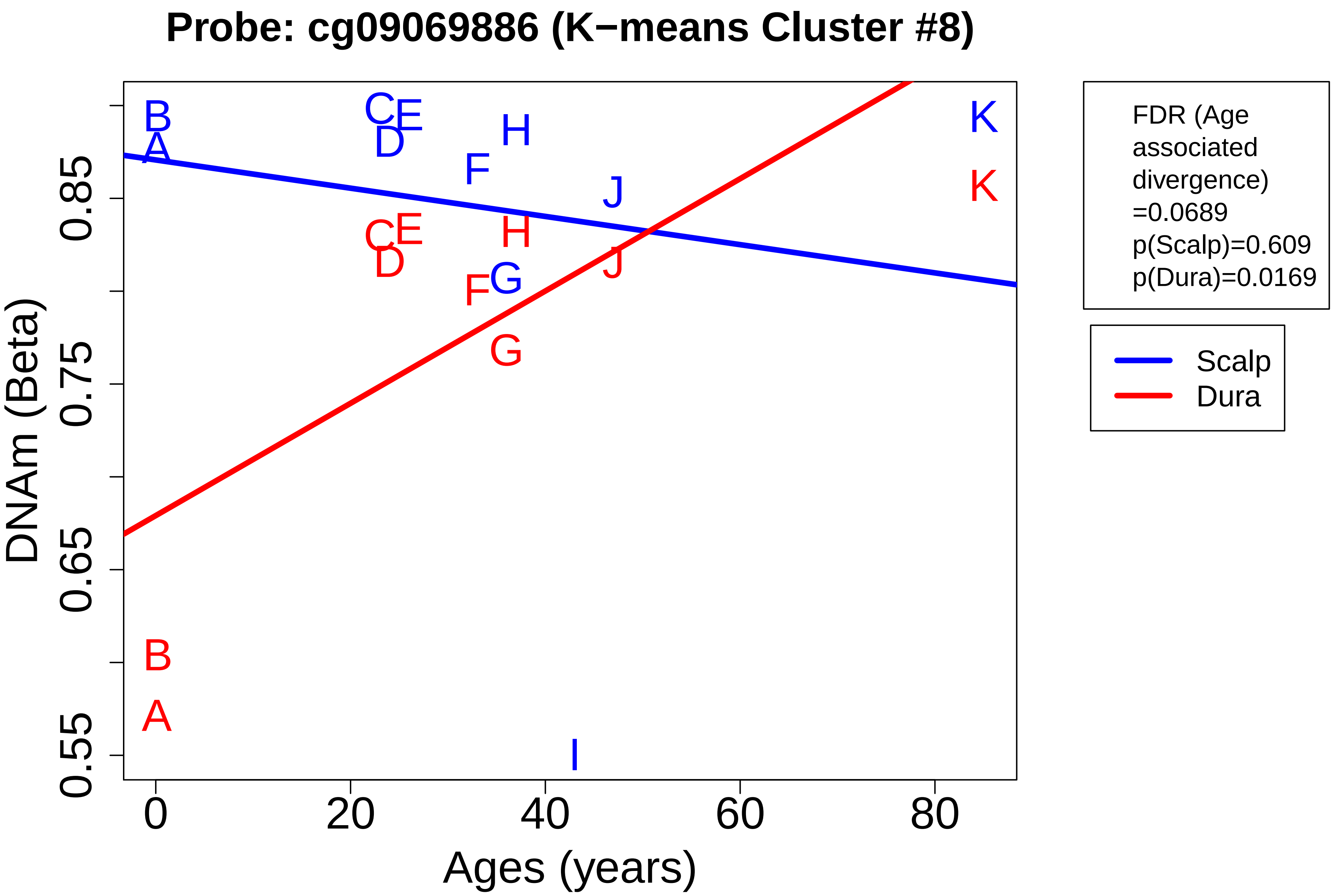

Probe: cg09069886 (K−means Cluster #8)
DNAm (Beta)
0.55
0.65
0.75
0.85
0
20
40
60
80
Ages (years)
FDR (Age associated divergence) =0.0689
p(Scalp)=0.609
p(Dura)=0.0169
Scalp
Dura

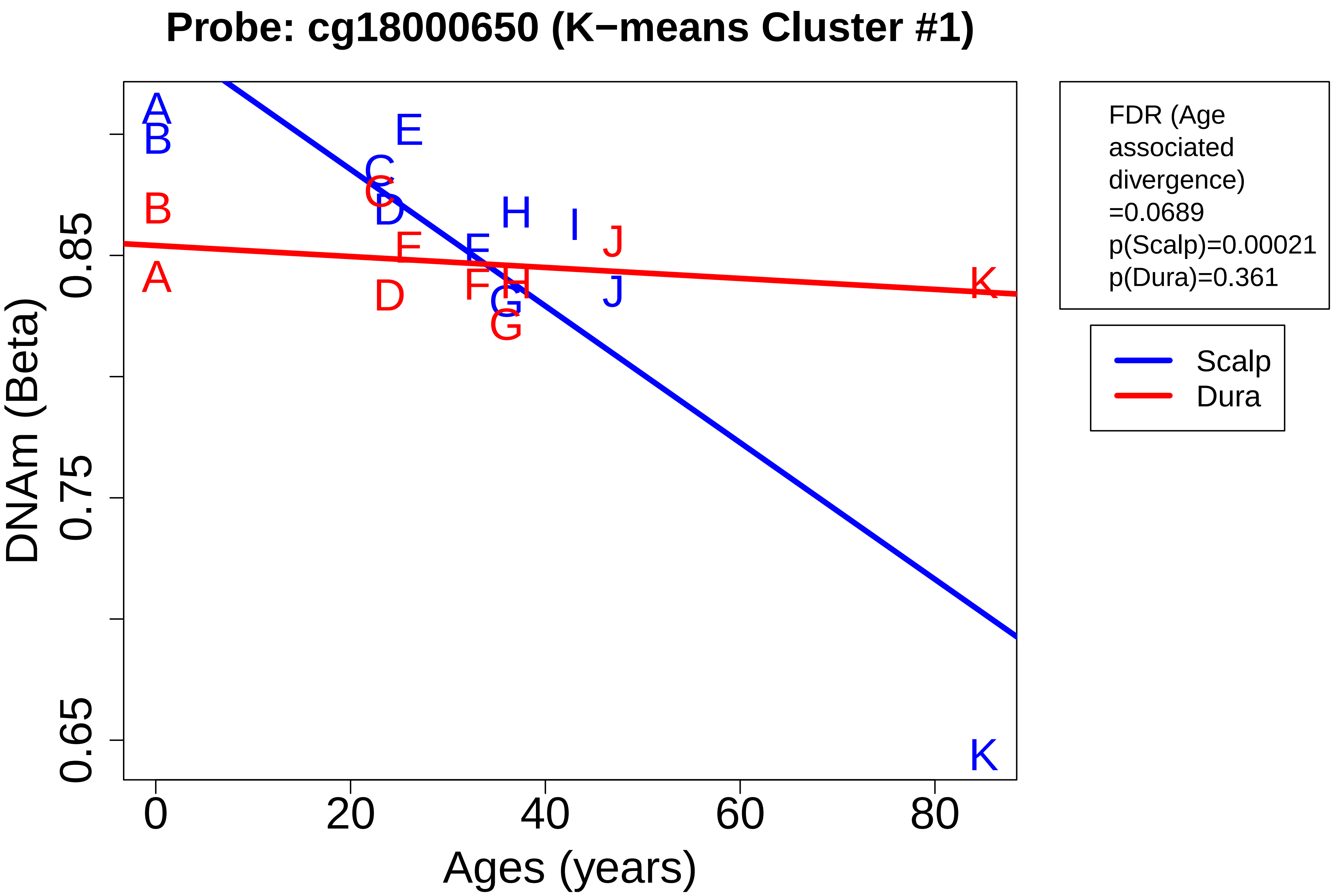

Probe: cg18000650 (K−means Cluster #1)
DNAm (Beta)
0.65
0.75
0.85
Ages (years)
0
20
40
60
80
FDR (Age
associated
divergence)
=0.0689
p(Scalp)=0.00021
p(Dura)=0.361
Scalp
Dura
A
B
C
D
E
F
G
H
I
J
K

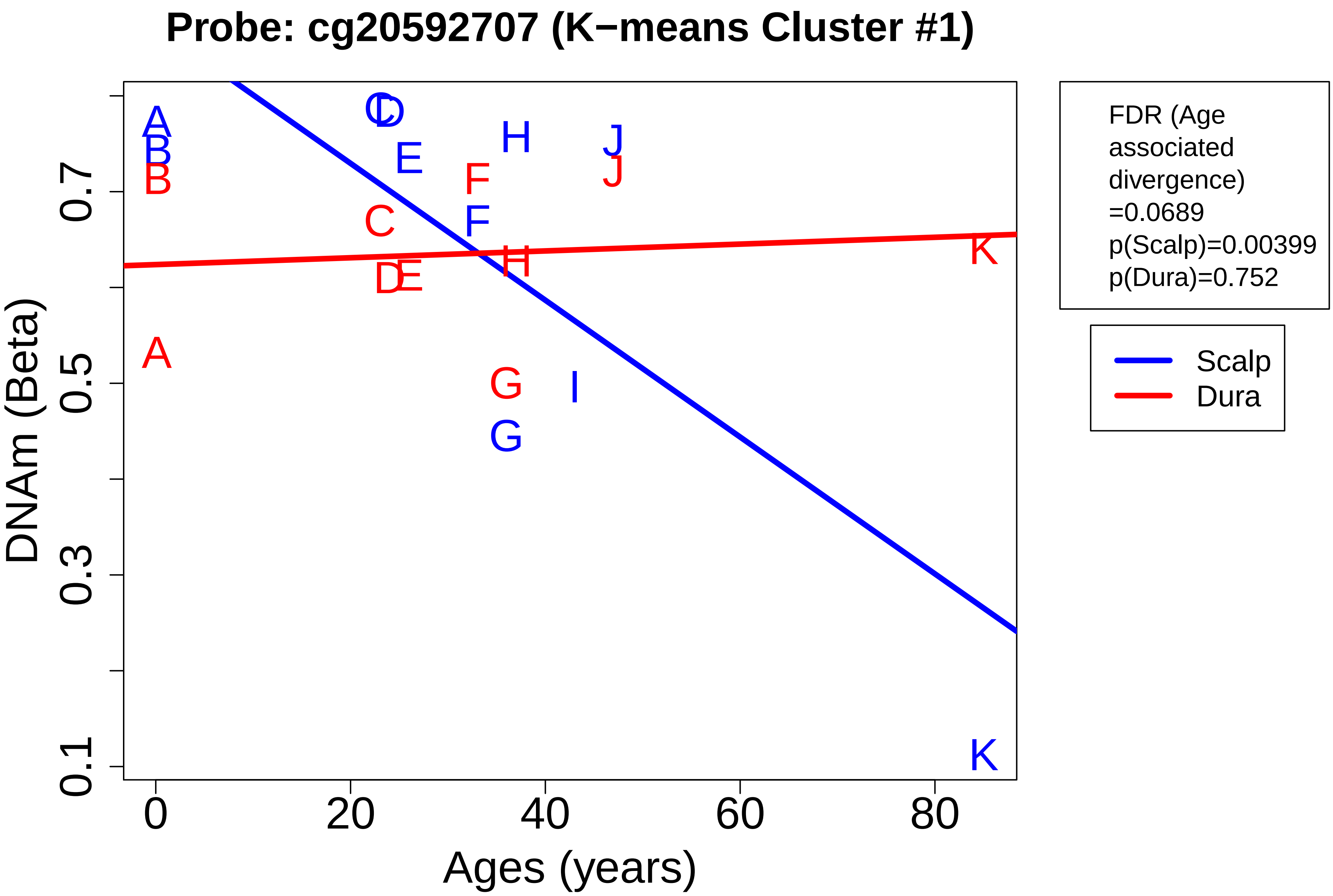

Probe: cg20592707 (K−means Cluster #1)
DNAm (Beta)
0.1
0.3
0.5
0.7
0
20
40
60
80
Ages (years)
A
B
B
C
D
E
F
C
F
H
H
J
J
D
E
A
G
I
G
K
K
FDR (Age associated divergence) =0.0689
p(Scalp)=0.00399
p(Dura)=0.752
Scalp
Dura

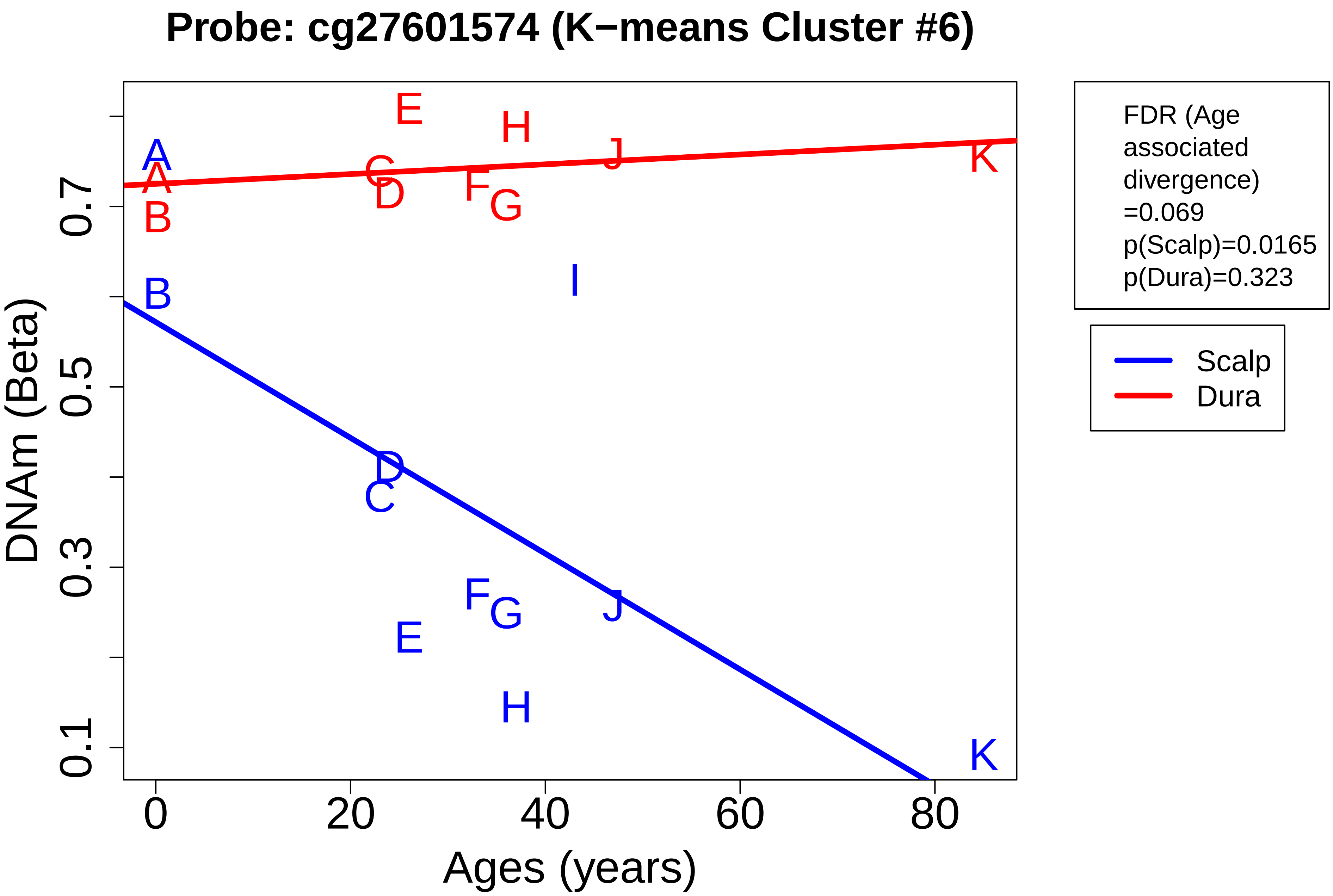

Probe: cg27601574 (K−means Cluster #6)
DNAm (Beta)
0.1
0.3
0.5
0.7
0
20
40
60
80
Ages (years)
FDR (Age associated divergence) =0.069
p(Scalp)=0.0165
p(Dura)=0.323
Scalp
Dura

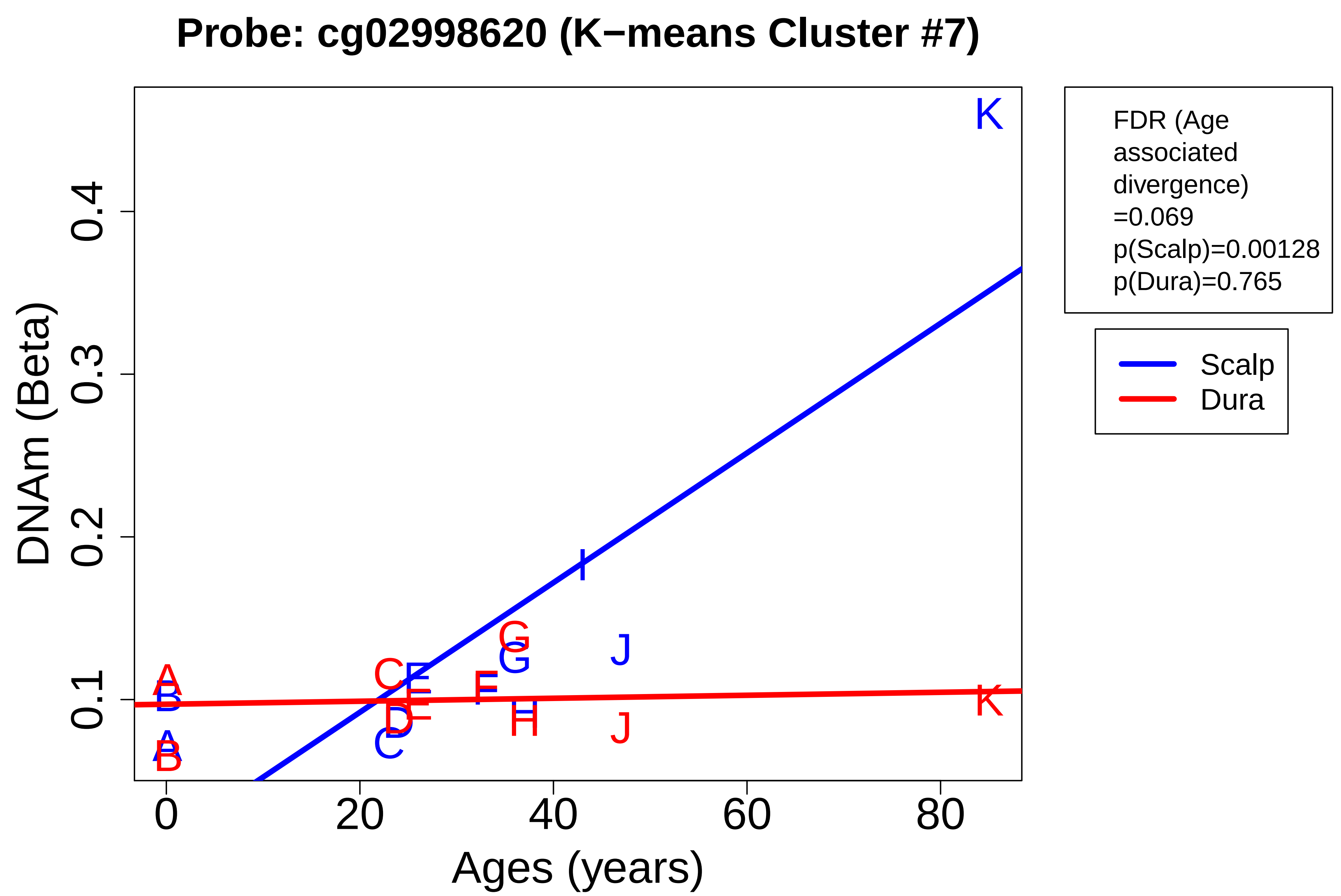

Probe: cg02998620 (K−means Cluster #7)
DNAm (Beta)
0.1
0.2
0.3
0.4
0
20
40
60
80
Ages (years)
FDR (Age associated divergence) =0.069
p(Scalp)=0.00128
p(Dura)=0.765
Scalp
Dura

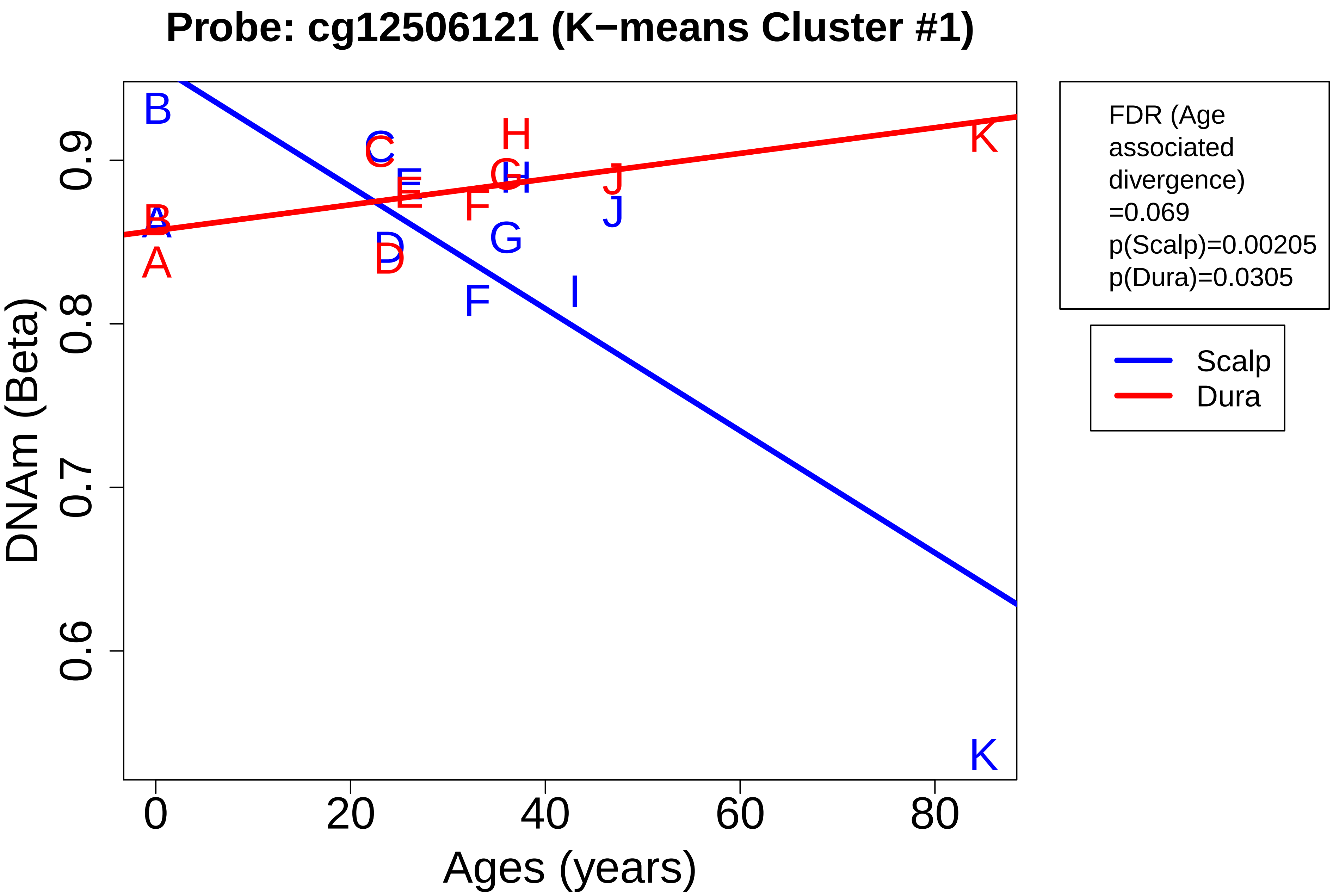

Probe: cg12506121 (K−means Cluster #1)
DNAm (Beta)
0.6
0.7
0.8
0.9
0
20
40
60
80
Ages (years)
FDR (Age associated divergence) =0.069
p(Scalp)=0.00205
p(Dura)=0.0305
Scalp
Dura
A
B
C
D
E
F
G
H
I
J
K

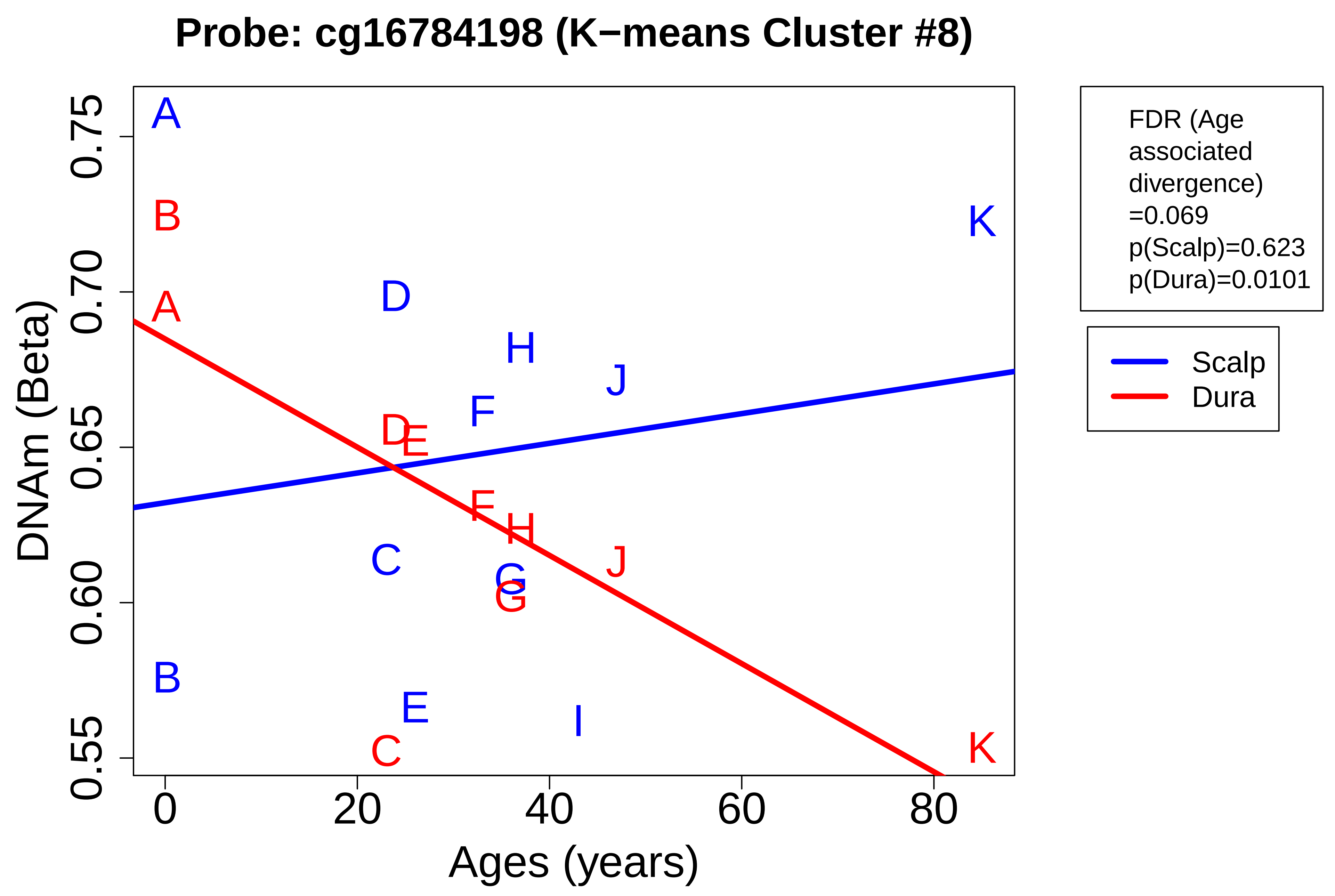

Probe: cg16784198 (K−means Cluster #8)
DNAm (Beta)
0.55
0.60
0.65
0.70
0.75
0
20
40
60
80
Ages (years)
A
B
A
D
H
J
F
D
E
F
H
C
J
G
G
B
E
I
C
K
K
FDR (Age associated divergence) =0.069
p(Scalp)=0.623
p(Dura)=0.0101
Scalp
Dura

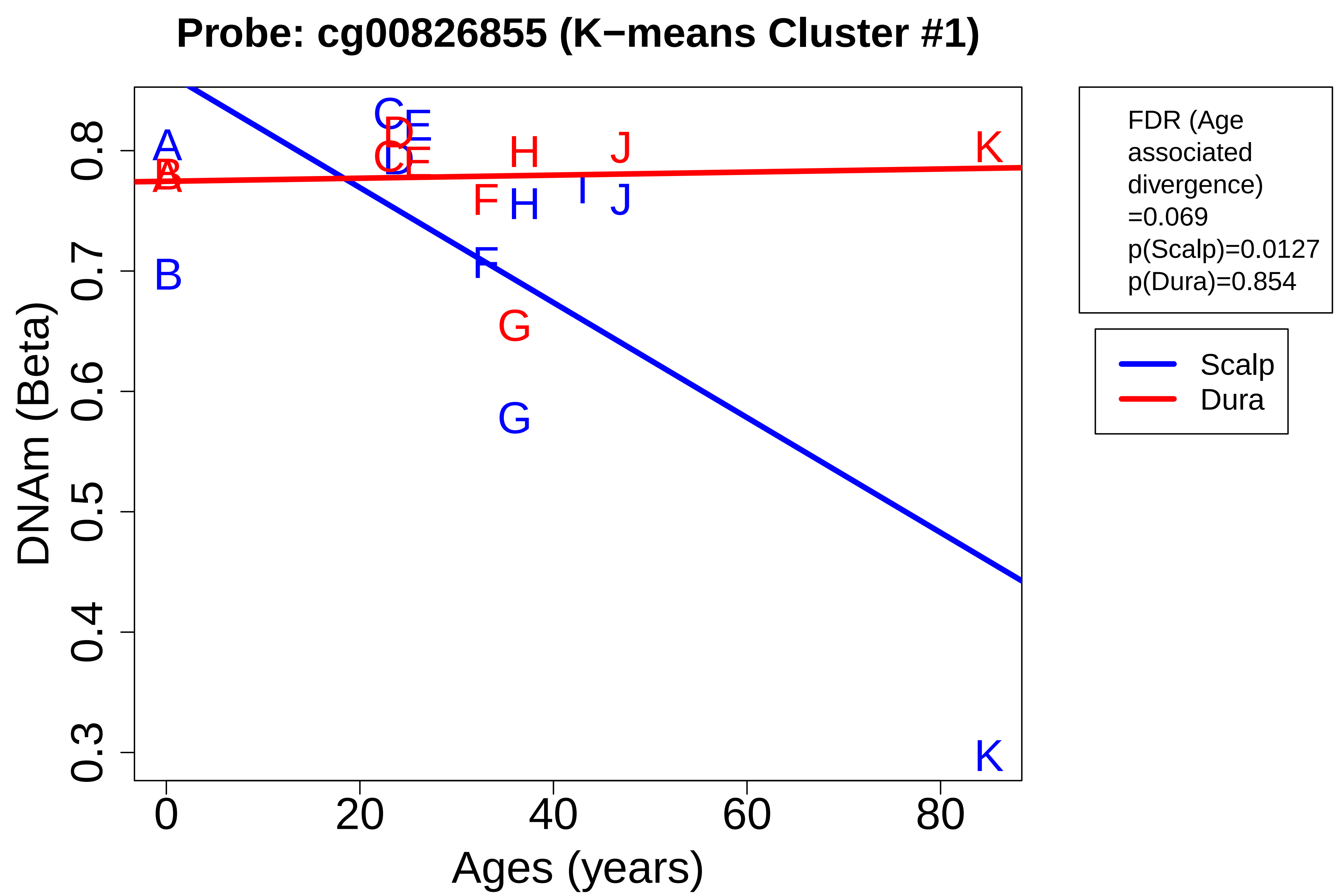

Probe: cg00826855 (K−means Cluster #1)
DNAm (Beta)
Ages (years)
0.3
0.4
0.5
0.6
0.7
0.8
0
20
40
60
80
FDR (Age associated divergence) =0.069
p(Scalp)=0.0127
p(Dura)=0.854
Scalp
Dura

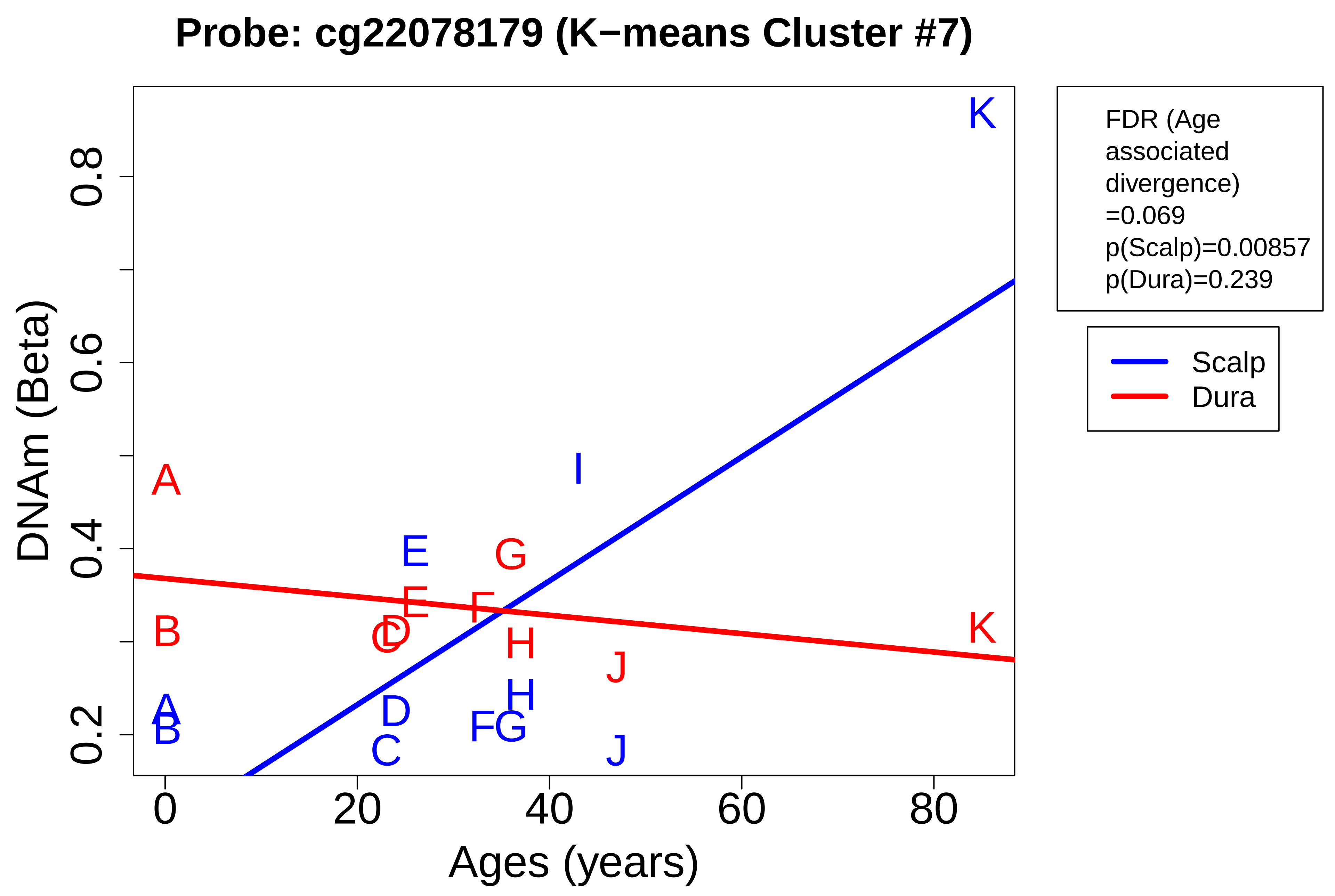
Probe: cg22078179 (K−means Cluster #7)
DNAm (Beta)
0.2
0.4
0.6
0.8
0
20
40
60
80
Ages (years)
A
B
C
D
E
F
G
H
I
J
K
FDR (Age associated divergence) =0.069
p(Scalp)=0.00857
p(Dura)=0.239
Scalp
Dura

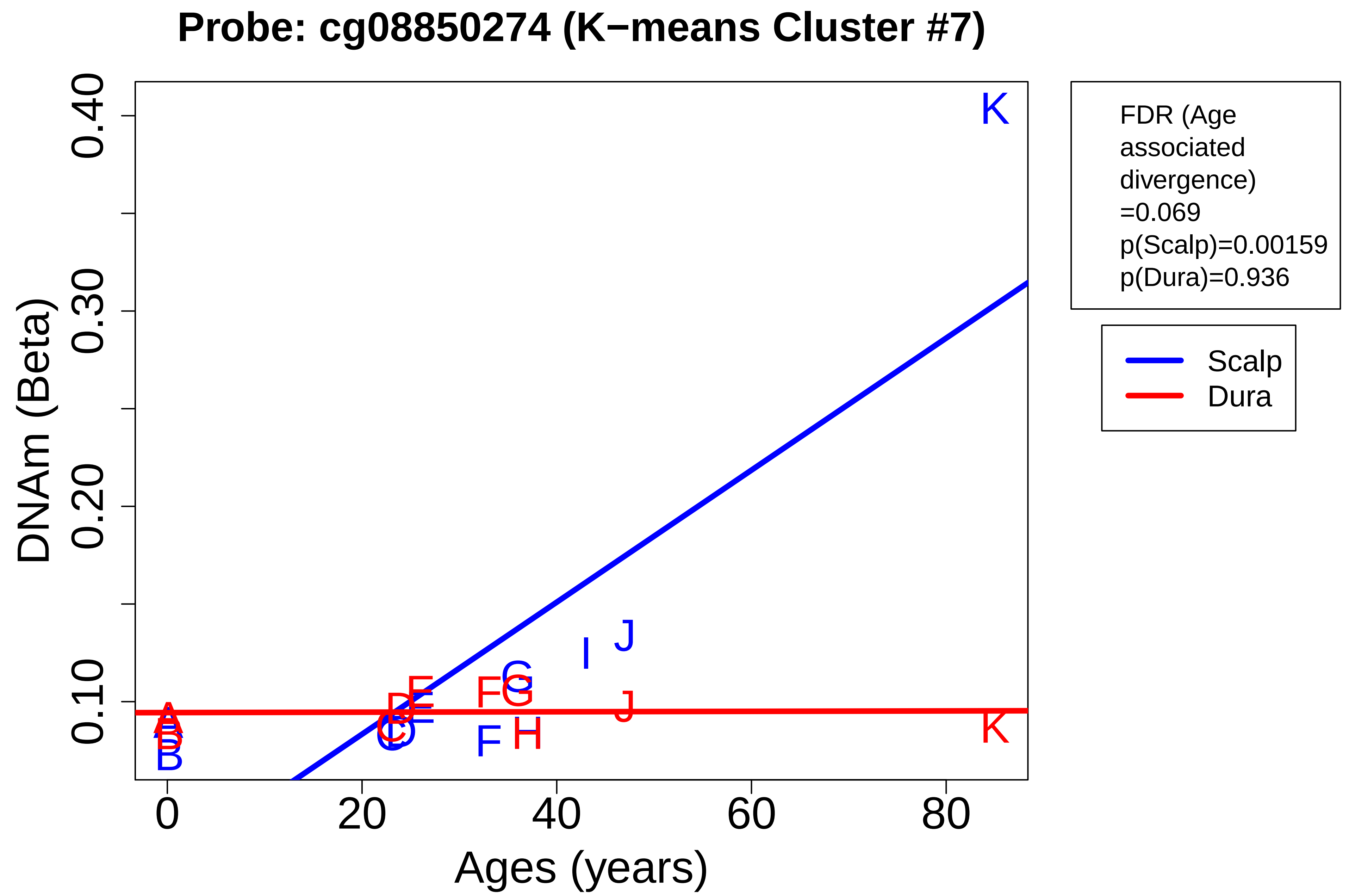

Probe: cg08850274 (K−means Cluster #7)
DNAm (Beta)
0.10
0.20
0.30
0.40
0
20
40
60
80
Ages (years)
FDR (Age associated divergence) =0.069
p(Scalp)=0.00159
p(Dura)=0.936
Scalp
Dura
A
B
C
D
E
F
G
H
I
J
K

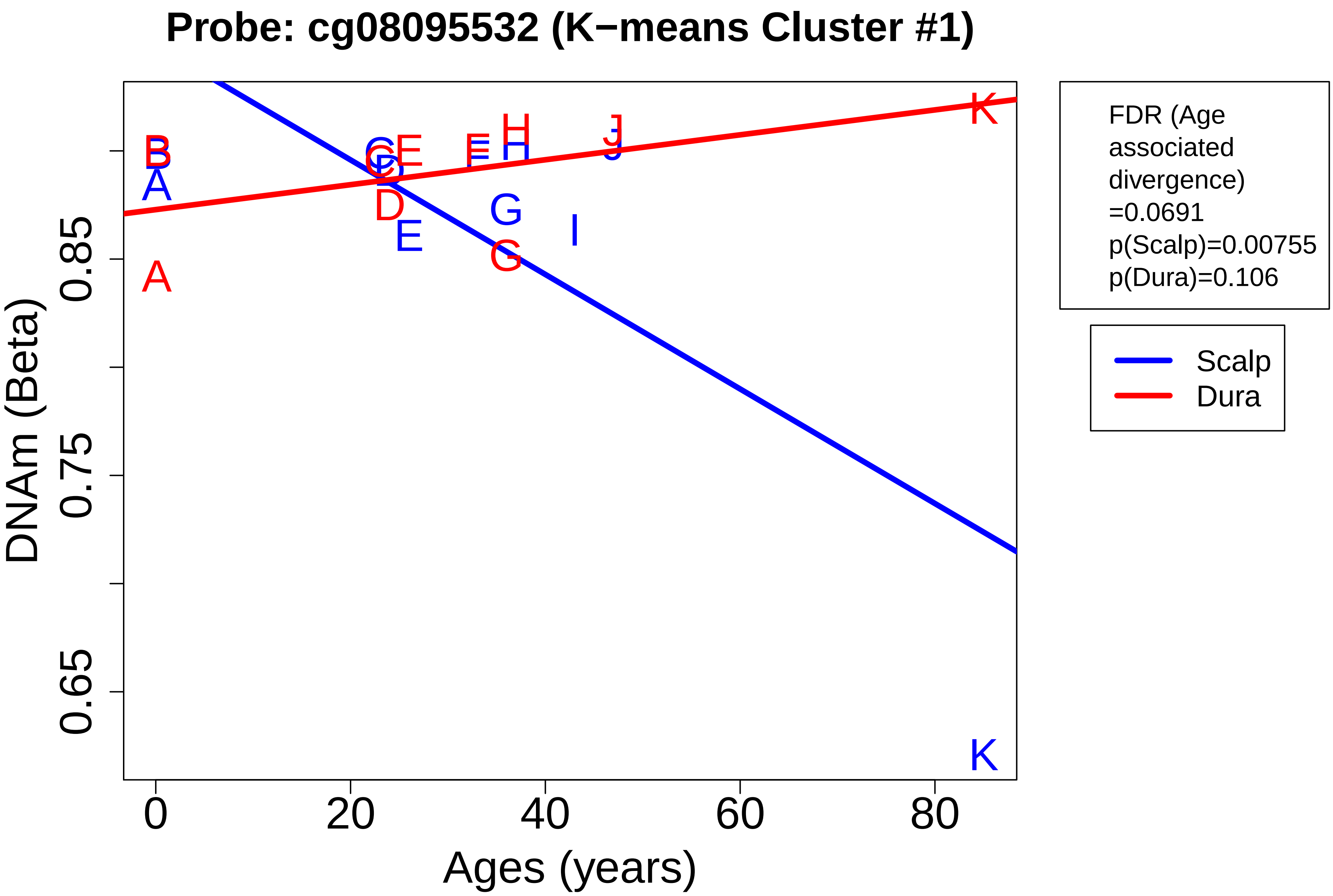

Probe: cg08095532 (K−means Cluster #1)
DNAm (Beta)
0.65
0.75
0.85
Ages (years)
0
20
40
60
80
FDR (Age associated divergence) =0.0691
p(Scalp)=0.00755
p(Dura)=0.106
Scalp
Dura
A
B
C
D
E
F
G
H
I
J
K

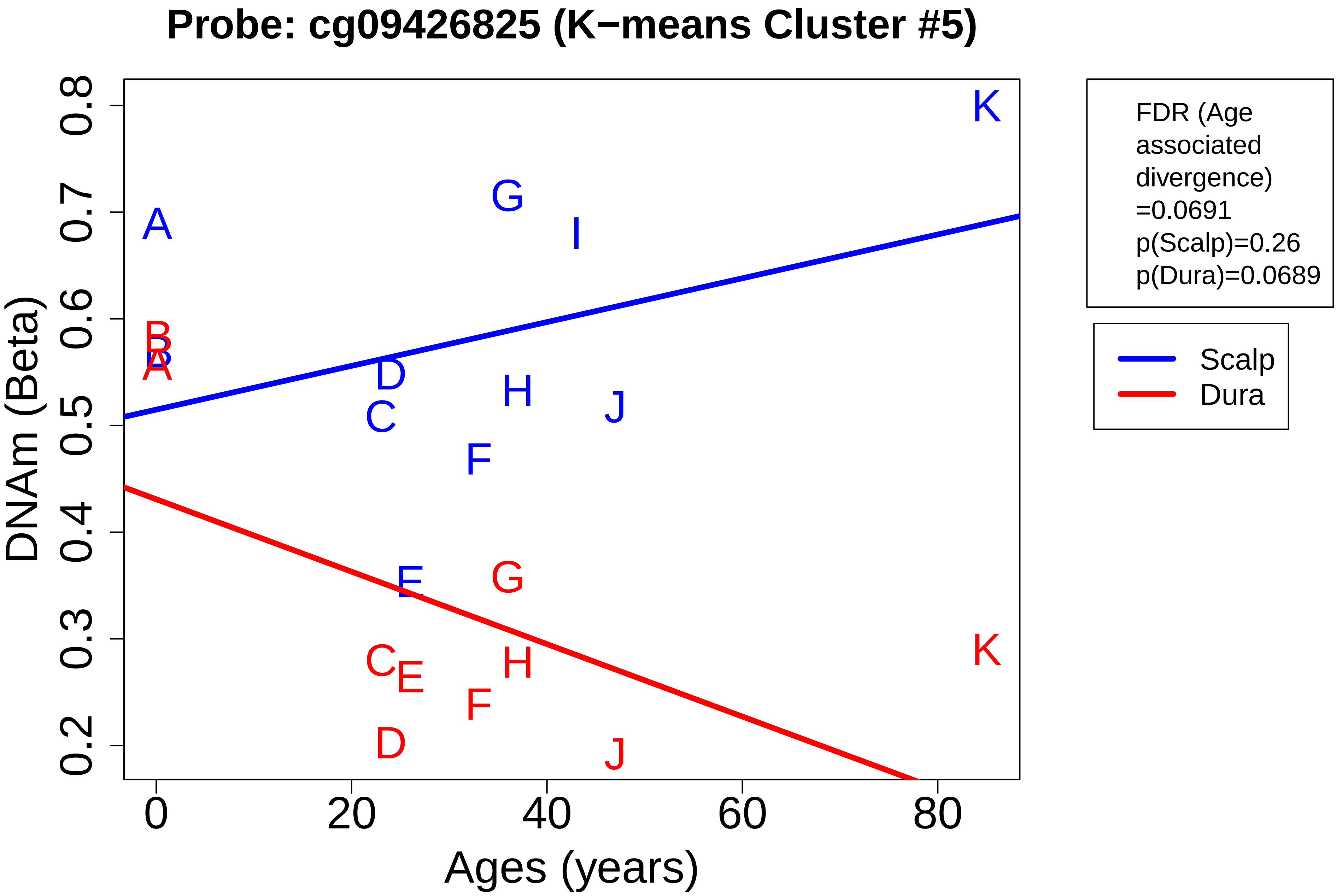

Probe: cg09426825 (K−means Cluster #5)
DNAm (Beta)
0.2
0.3
0.4
0.5
0.6
0.7
0.8
Ages (years)
0
20
40
60
80
A
B
C
D
E
F
G
H
I
J
K
FDR (Age associated divergence) =0.0691
p(Scalp)=0.26
p(Dura)=0.0689
Scalp
Dura

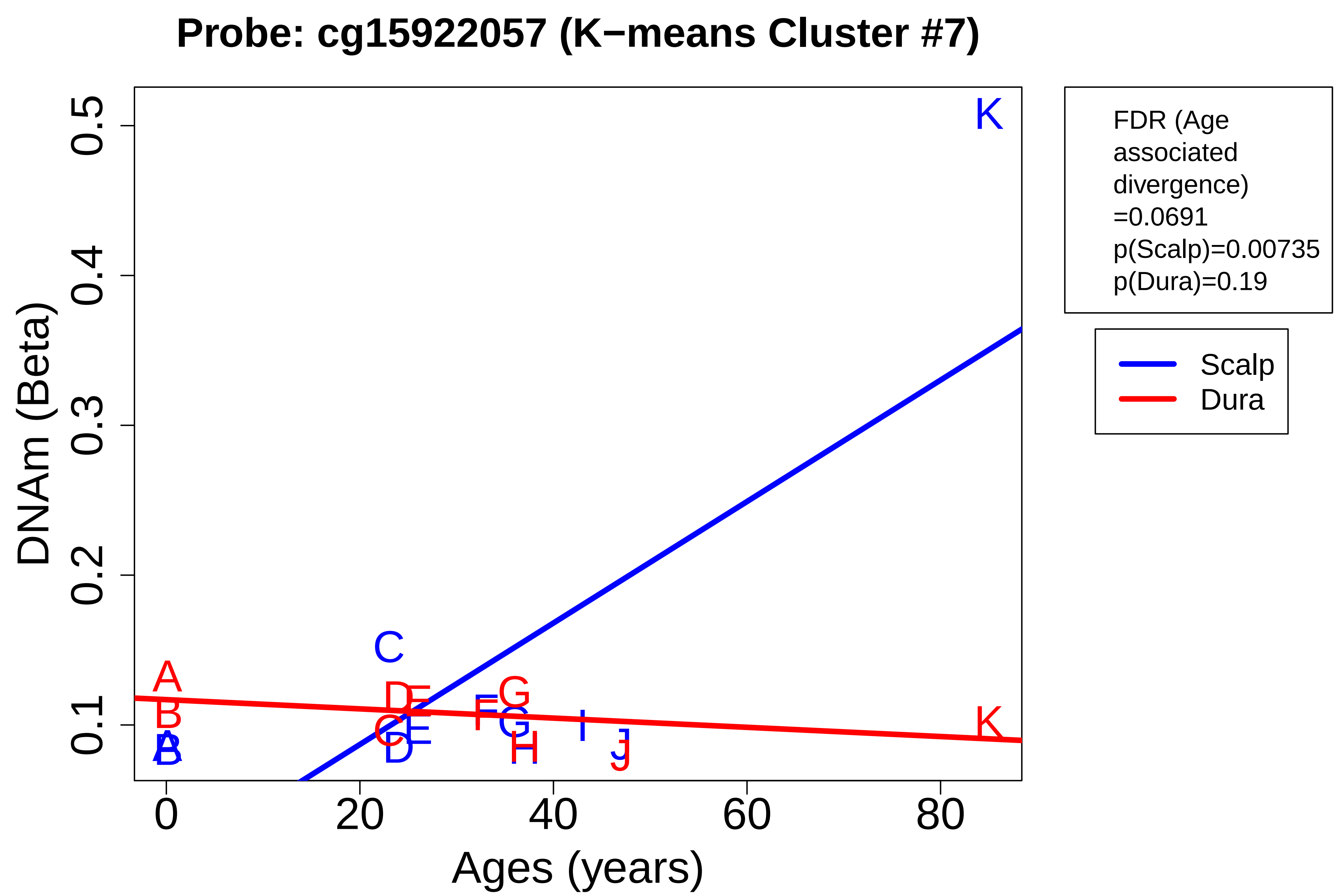

Probe: cg15922057 (K−means Cluster #7)
FDR (Age associated divergence) =0.0691
p(Scalp)=0.00735
p(Dura)=0.19
Scalp
Dura
DNAm (Beta)
Ages (years)
0.1
0.2
0.3
0.4
0.5
0
20
40
60
80

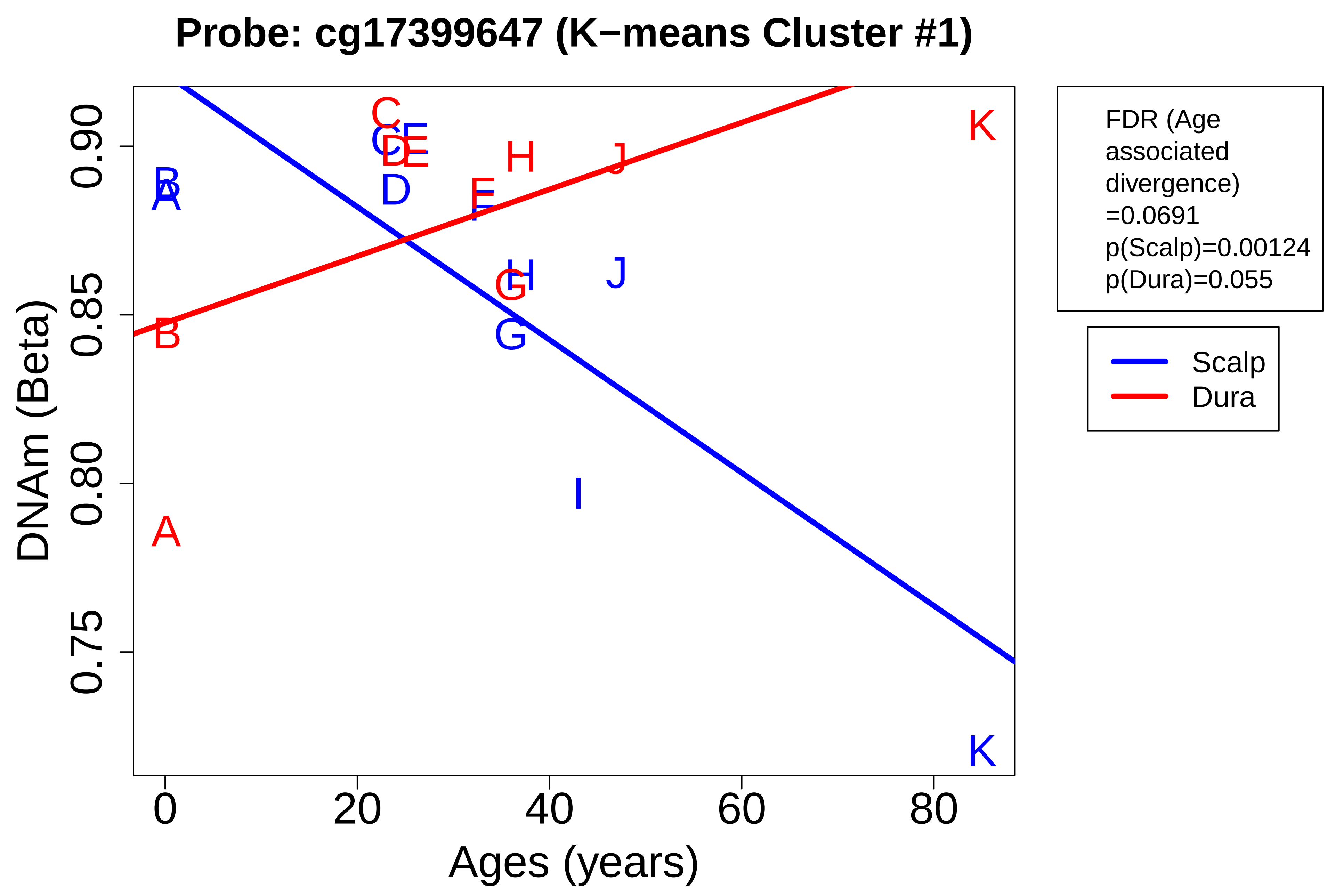
Probe: cg17399647 (K−means Cluster #1)
DNAm (Beta)
0.75
0.80
0.85
0.90
0
20
40
60
80
Ages (years)
FDR (Age associated divergence) =0.0691
p(Scalp)=0.00124
p(Dura)=0.055
Scalp
Dura

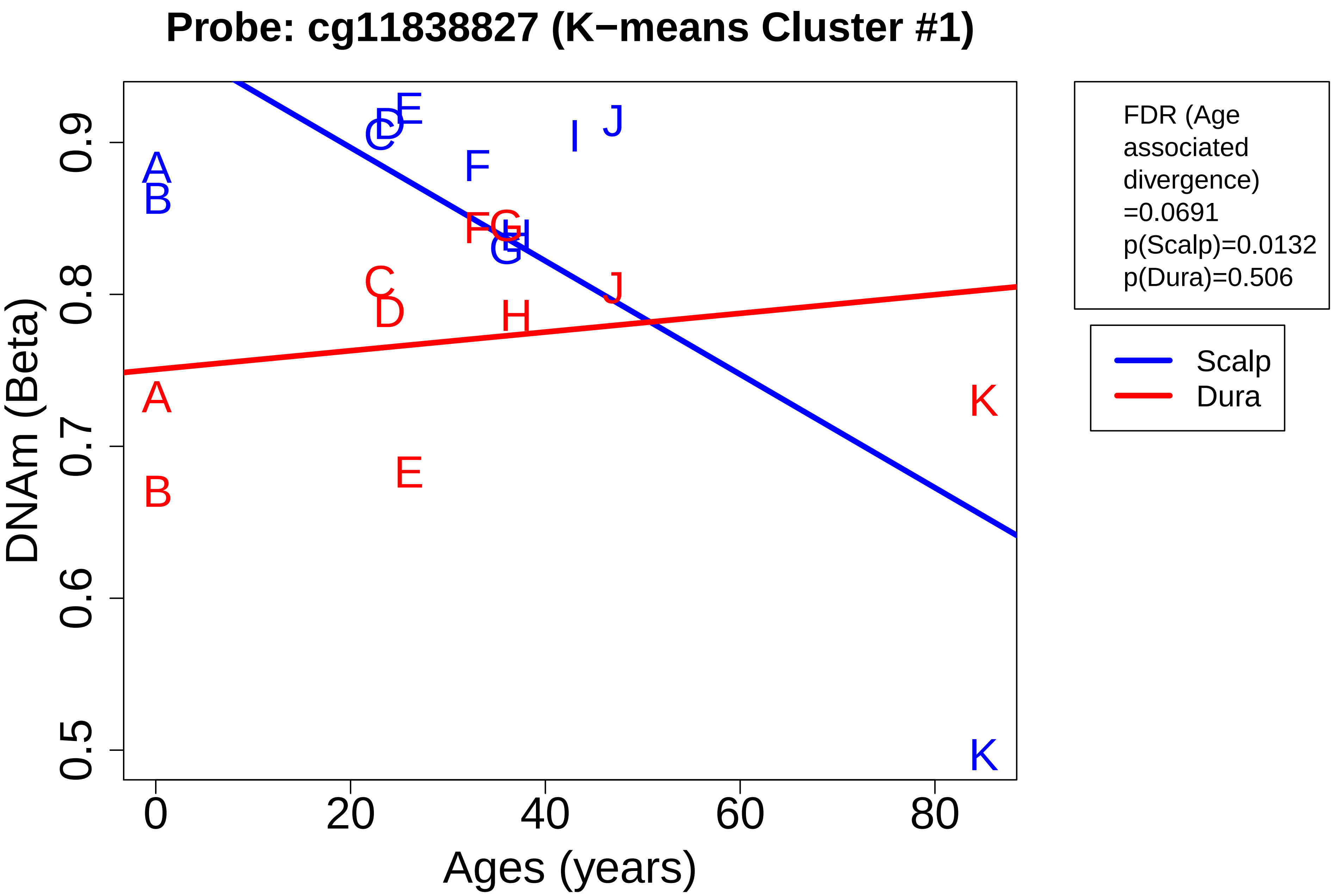

Probe: cg11838827 (K−means Cluster #1)
DNAm (Beta)
0.5
0.6
0.7
0.8
0.9
0
20
40
60
80
Ages (years)
FDR (Age associated divergence) =0.0691
p(Scalp)=0.0132
p(Dura)=0.506
Scalp
Dura

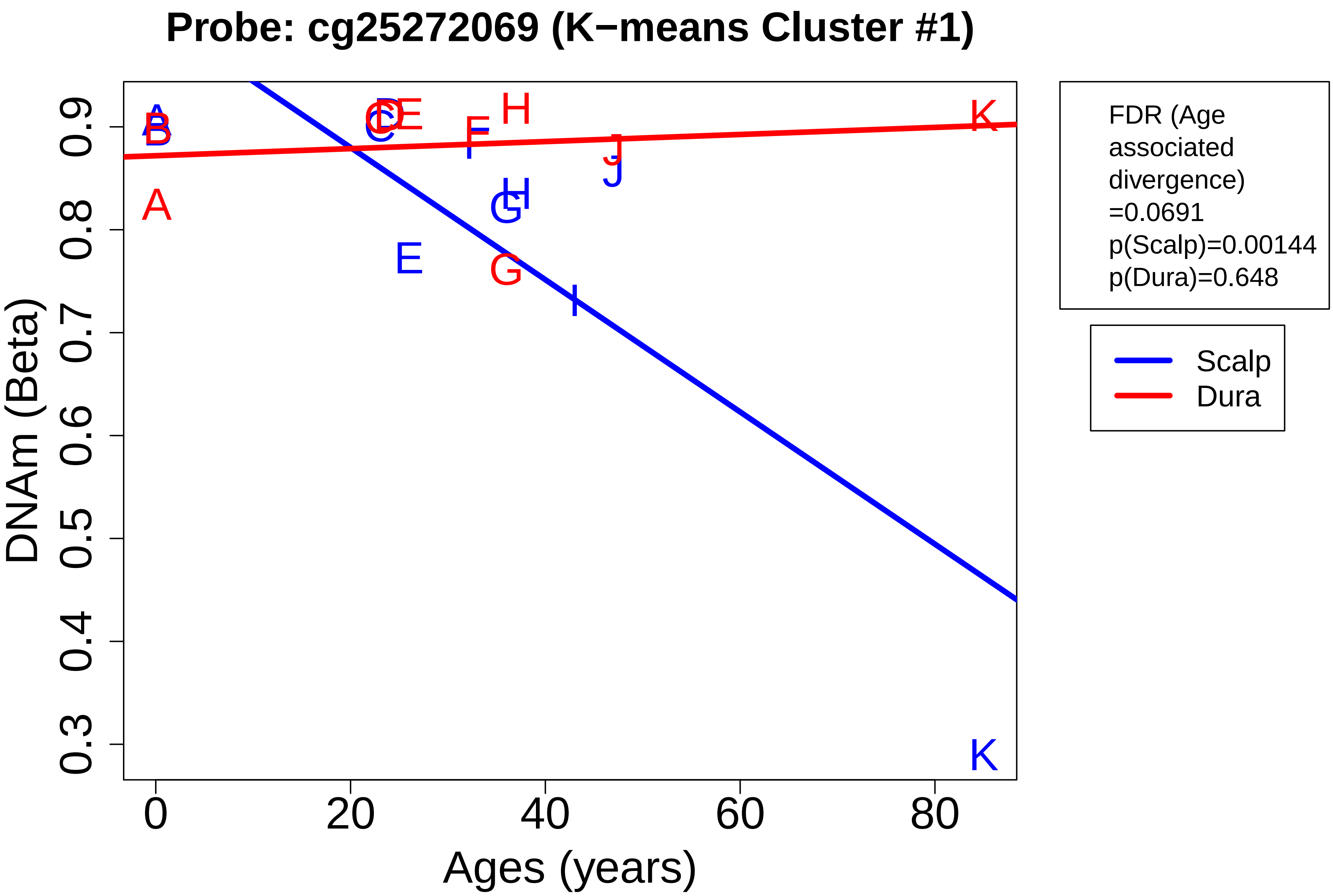

Probe: cg25272069 (K−means Cluster #1)
DNAm (Beta)
0.3
0.4
0.5
0.6
0.7
0.8
0.9
0
20
40
60
80
Ages (years)
FDR (Age associated divergence) =0.0691
p(Scalp)=0.00144
p(Dura)=0.648
Scalp
Dura

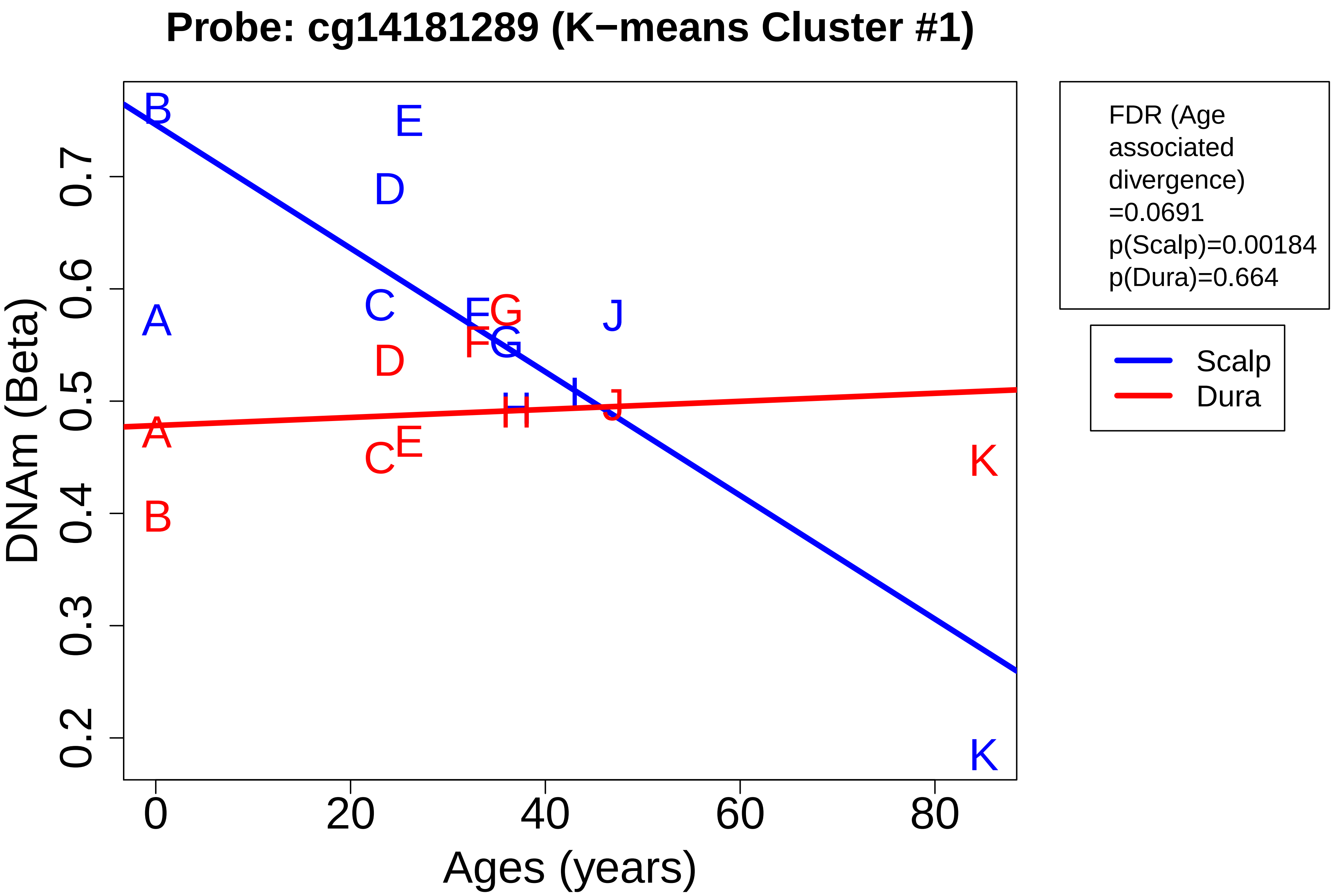

Probe: cg14181289 (K−means Cluster #1)
DNAm (Beta)
Ages (years)
0.2
0.3
0.4
0.5
0.6
0.7
0
20
40
60
80
FDR (Age associated divergence) =0.0691
p(Scalp)=0.00184
p(Dura)=0.664
Scalp
Dura

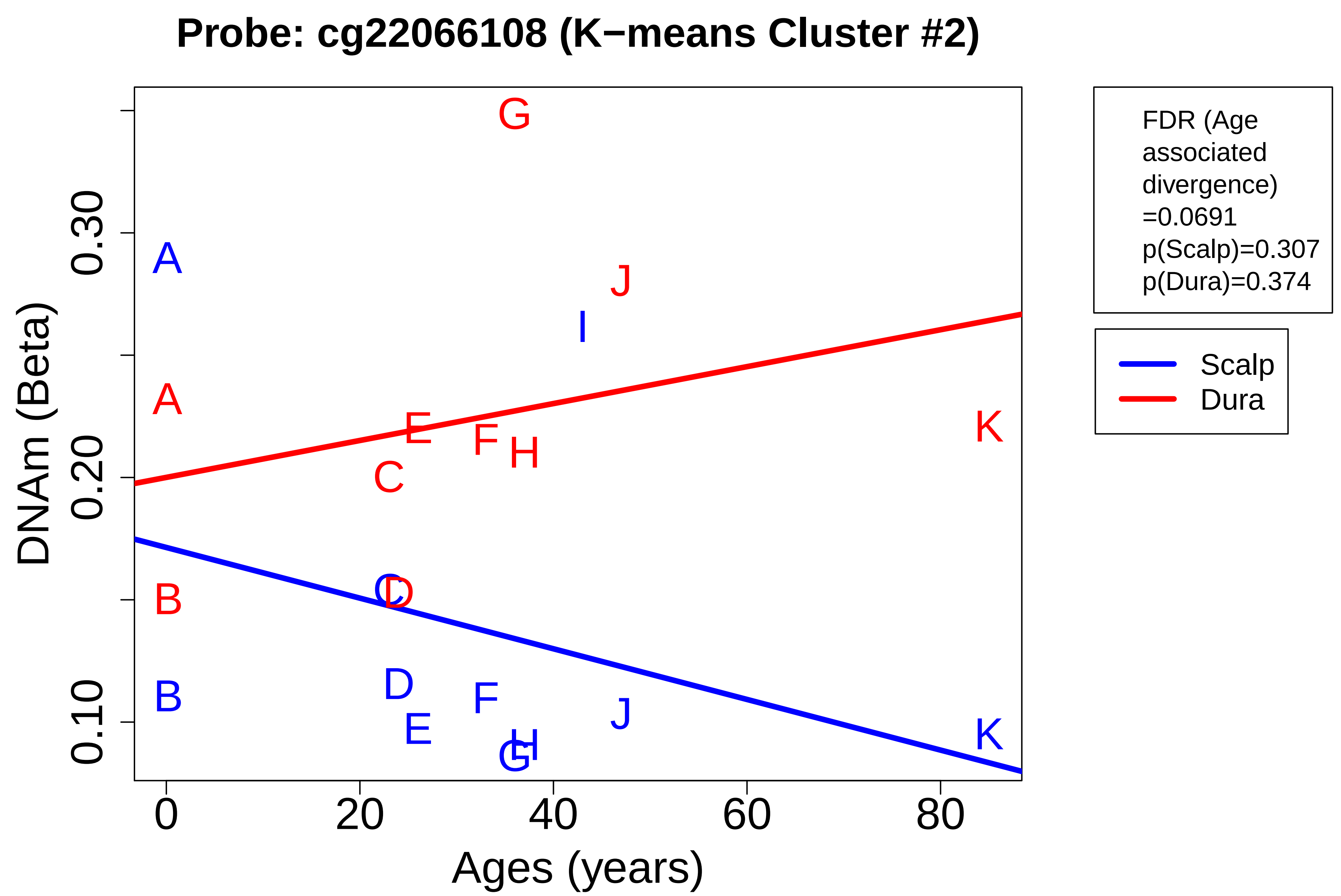

Probe: cg22066108 (K−means Cluster #2)
DNAm (Beta)
0.10
0.20
0.30
0
20
40
60
80
Ages (years)
FDR (Age associated divergence) =0.0691
p(Scalp)=0.307
p(Dura)=0.374
Scalp
Dura

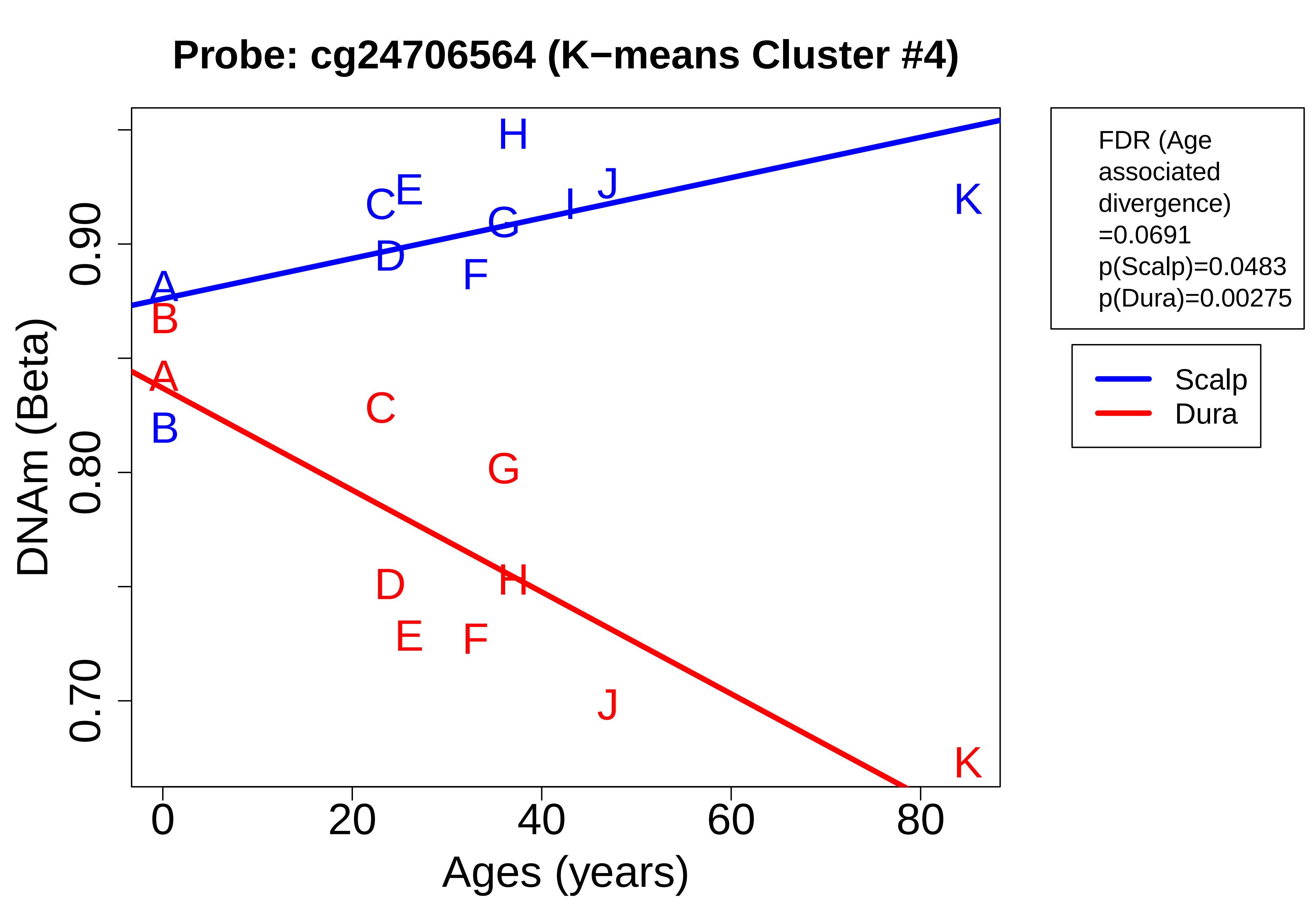

Probe: cg24706564 (K−means Cluster #4)
FDR (Age associated divergence) =0.0691
p(Scalp)=0.0483
p(Dura)=0.00275
Scalp
Dura
DNAm (Beta)
0.70
0.80
0.90
0
20
40
60
80
Ages (years)
A
B
C
D
E
F
G
H
I
J
K

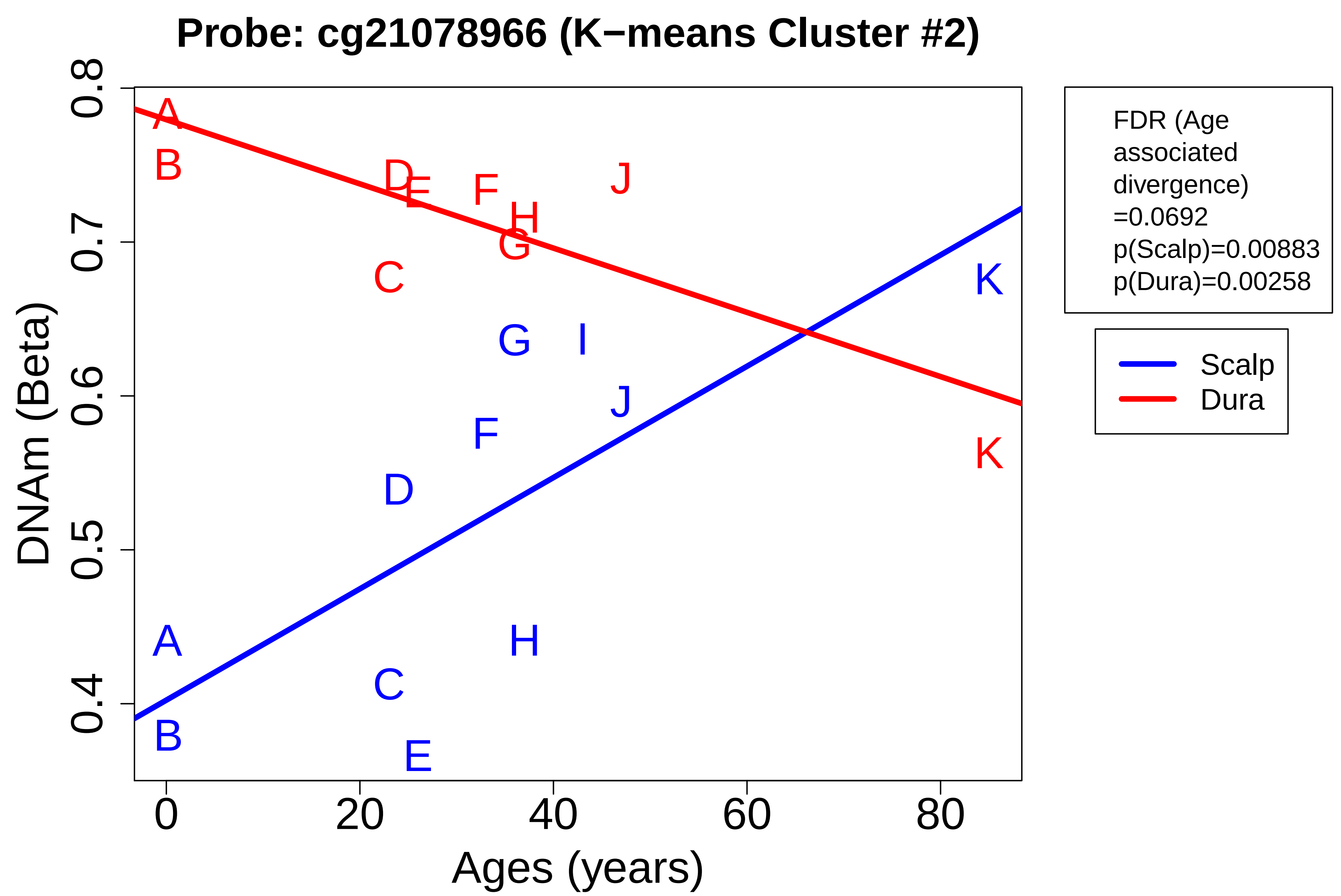

Probe: cg21078966 (K−means Cluster #2)
DNAm (Beta)
0.4
0.5
0.6
0.7
0.8
Ages (years)
0
20
40
60
80
FDR (Age associated divergence) =0.0692
p(Scalp)=0.00883
p(Dura)=0.00258
Scalp
Dura

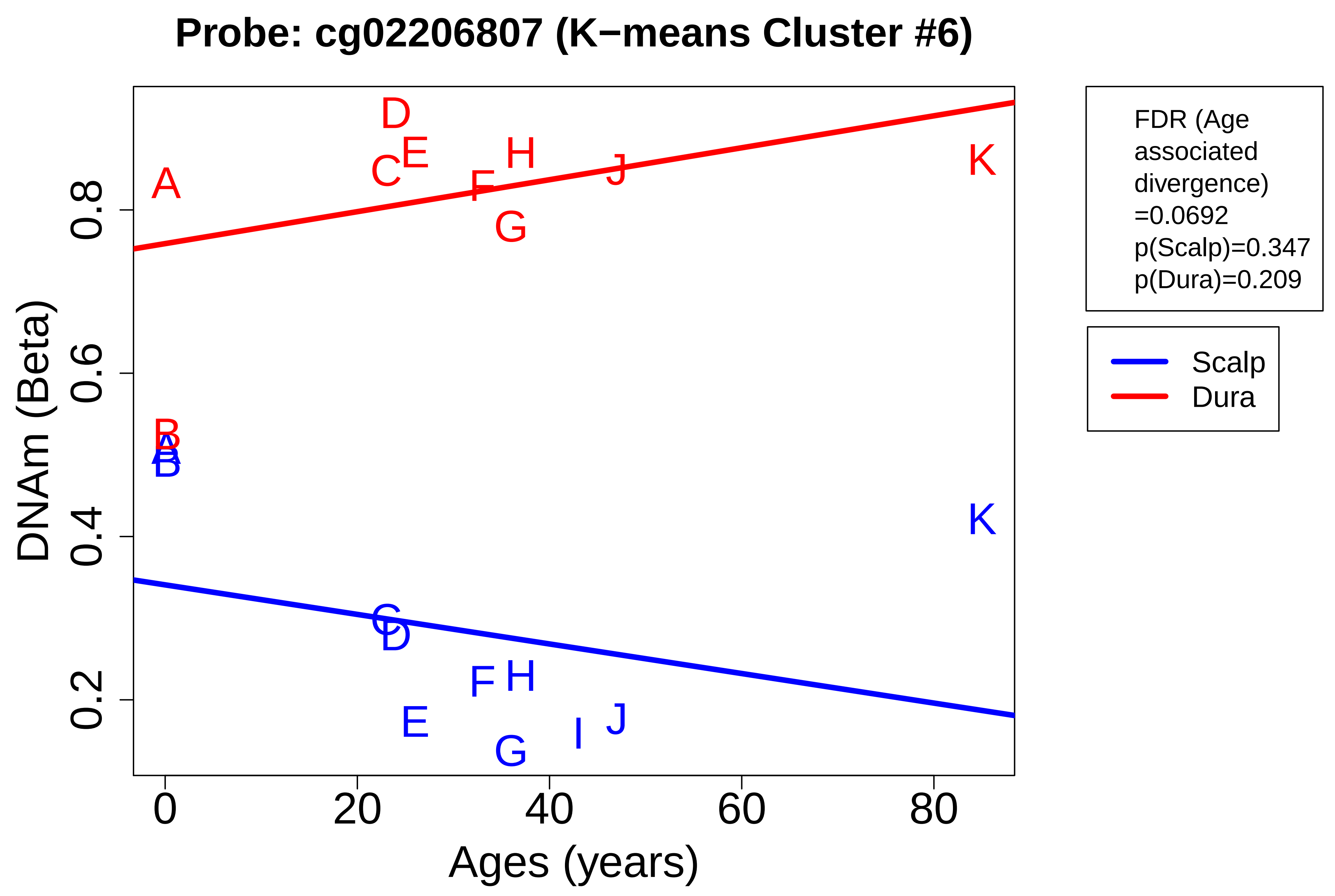

Probe: cg02206807 (K−means Cluster #6)
DNAm (Beta)
0.2
0.4
0.6
0.8
0
20
40
60
80
Ages (years)
FDR (Age associated divergence) =0.0692
p(Scalp)=0.347
p(Dura)=0.209
Scalp
Dura

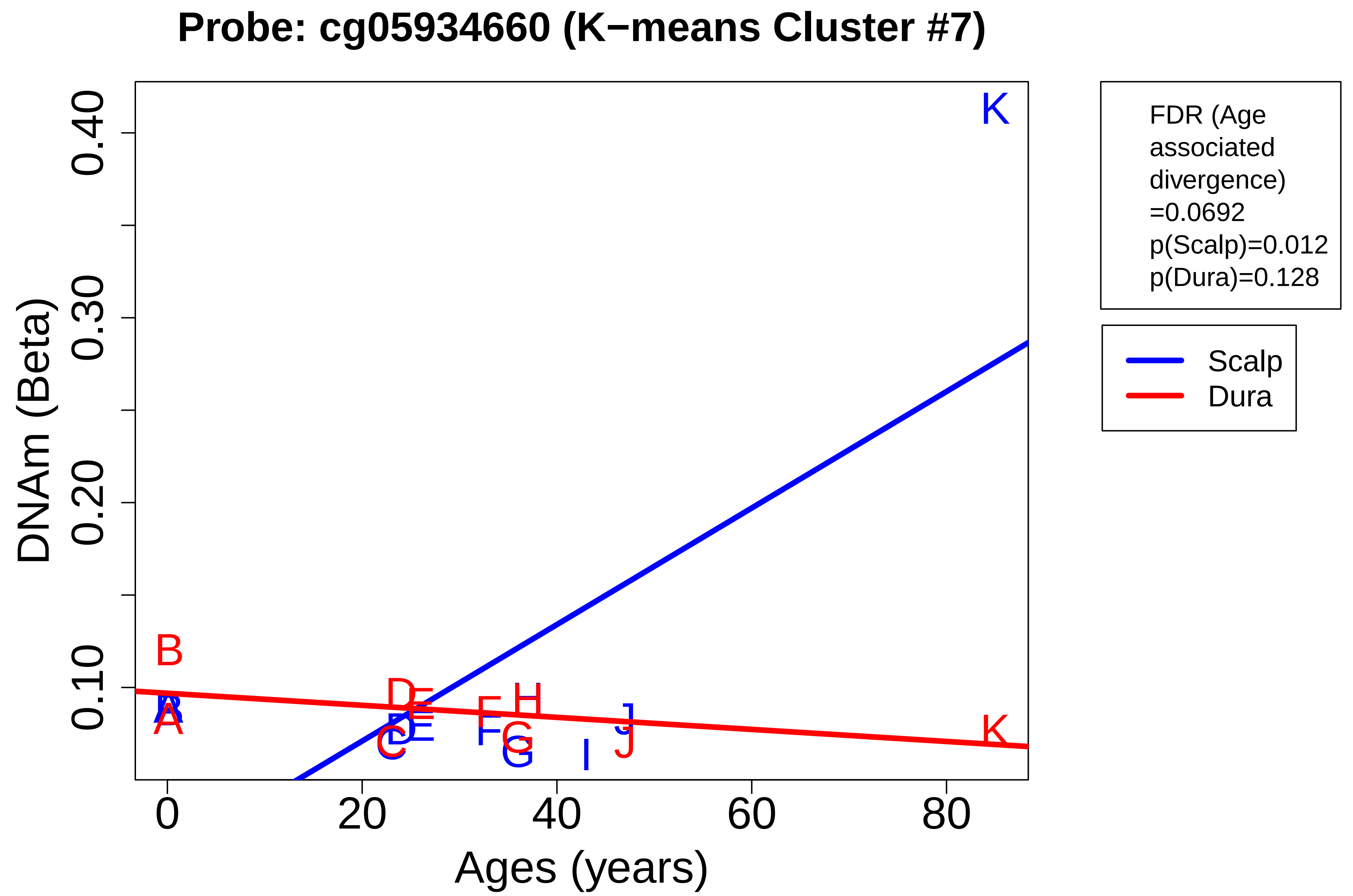
Probe: cg05934660 (K−means Cluster #7)
DNAm (Beta)
0.10
0.20
0.30
0.40
Ages (years)
0
20
40
60
80
FDR (Age associated divergence) =0.0692
p(Scalp)=0.012
p(Dura)=0.128
Scalp
Dura
A
B
C
D
E
F
G
H
I
J
K

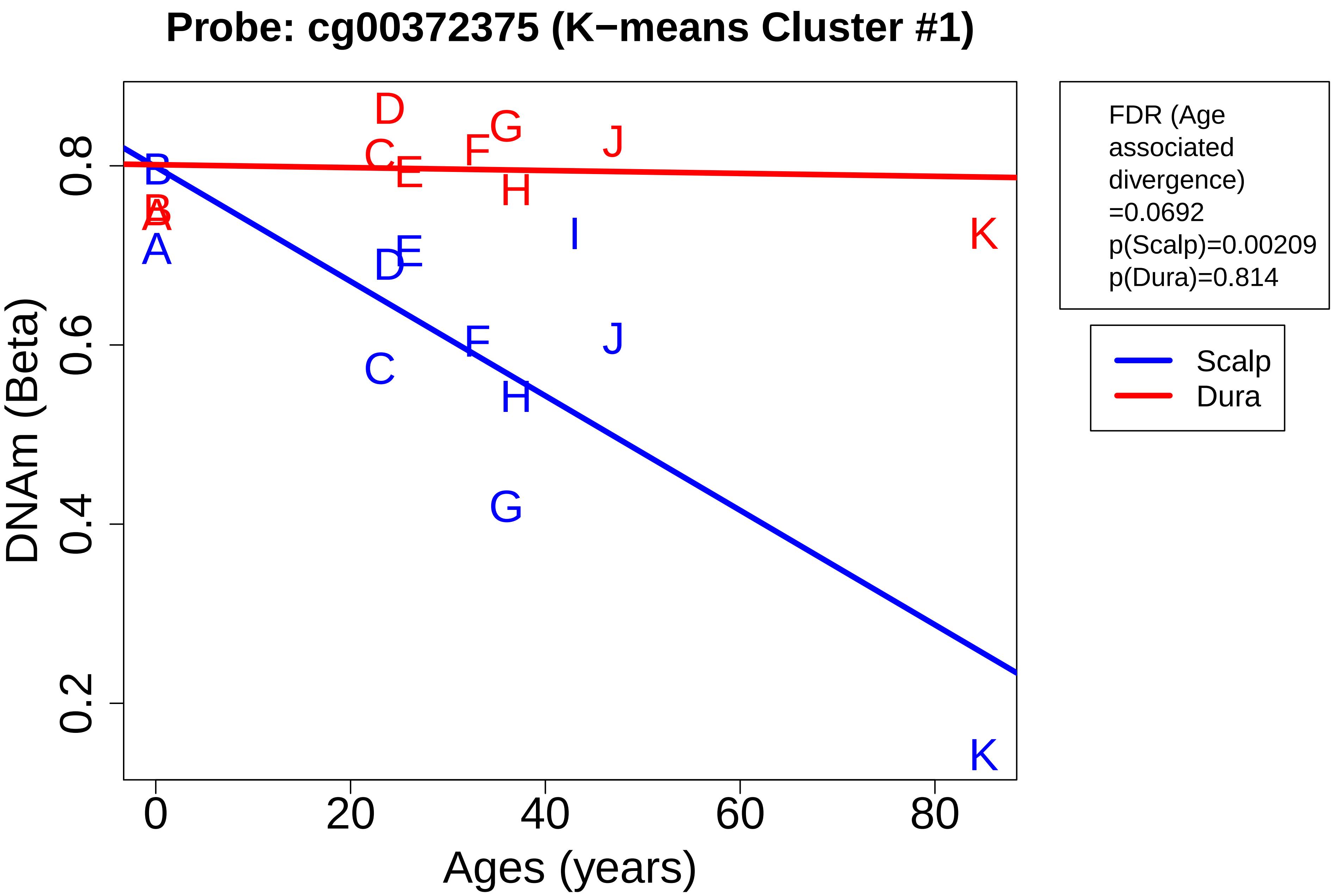

Probe: cg00372375 (K−means Cluster #1)
DNAm (Beta)
0.2
0.4
0.6
0.8
0
20
40
60
80
Ages (years)
FDR (Age associated divergence) =0.0692
p(Scalp)=0.00209
p(Dura)=0.814
Scalp
Dura

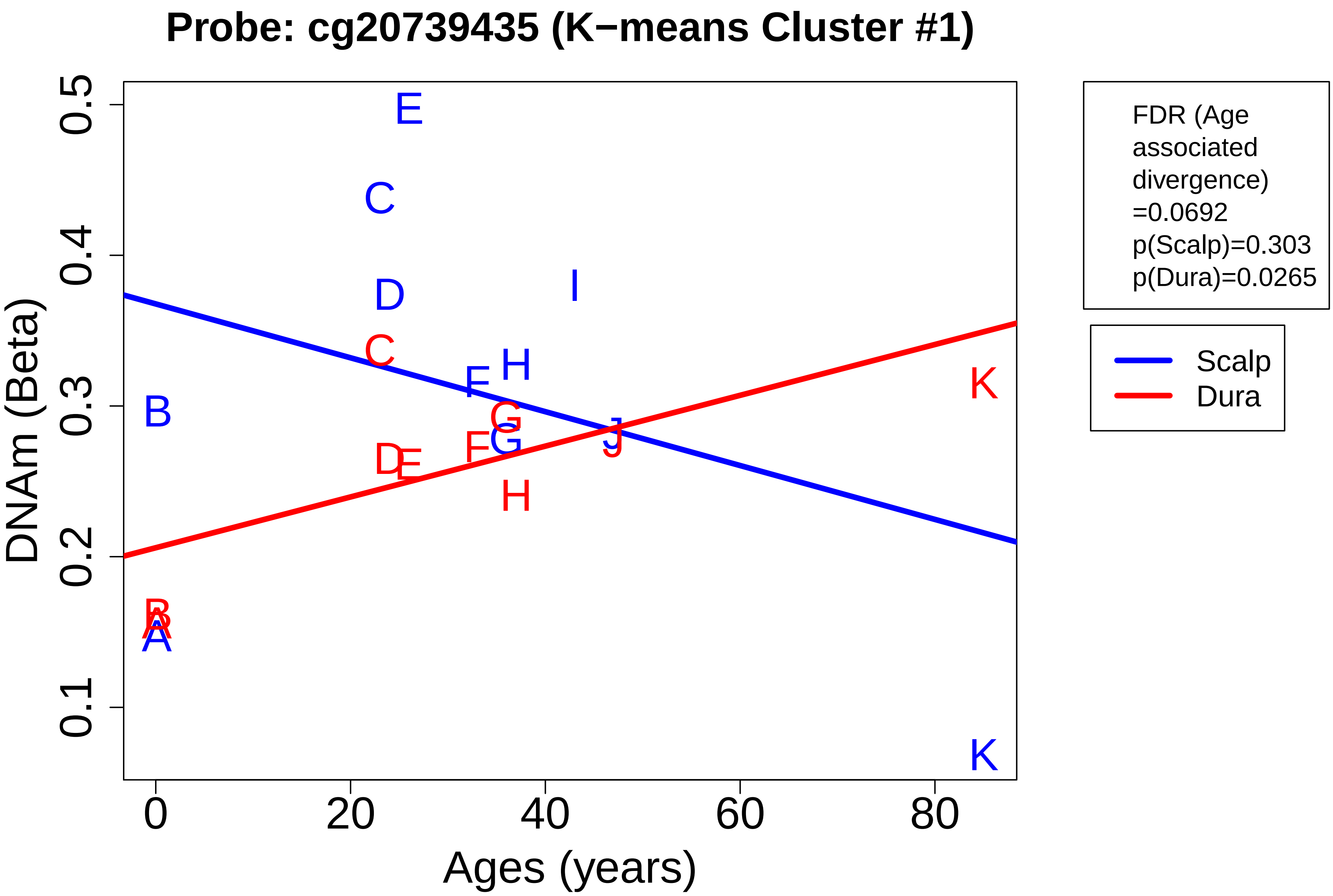

Probe: cg20739435 (K−means Cluster #1)
DNAm (Beta)
0.1
0.2
0.3
0.4
0.5
0
20
40
60
80
Ages (years)
FDR (Age associated divergence) =0.0692
p(Scalp)=0.303
p(Dura)=0.0265
Scalp
Dura

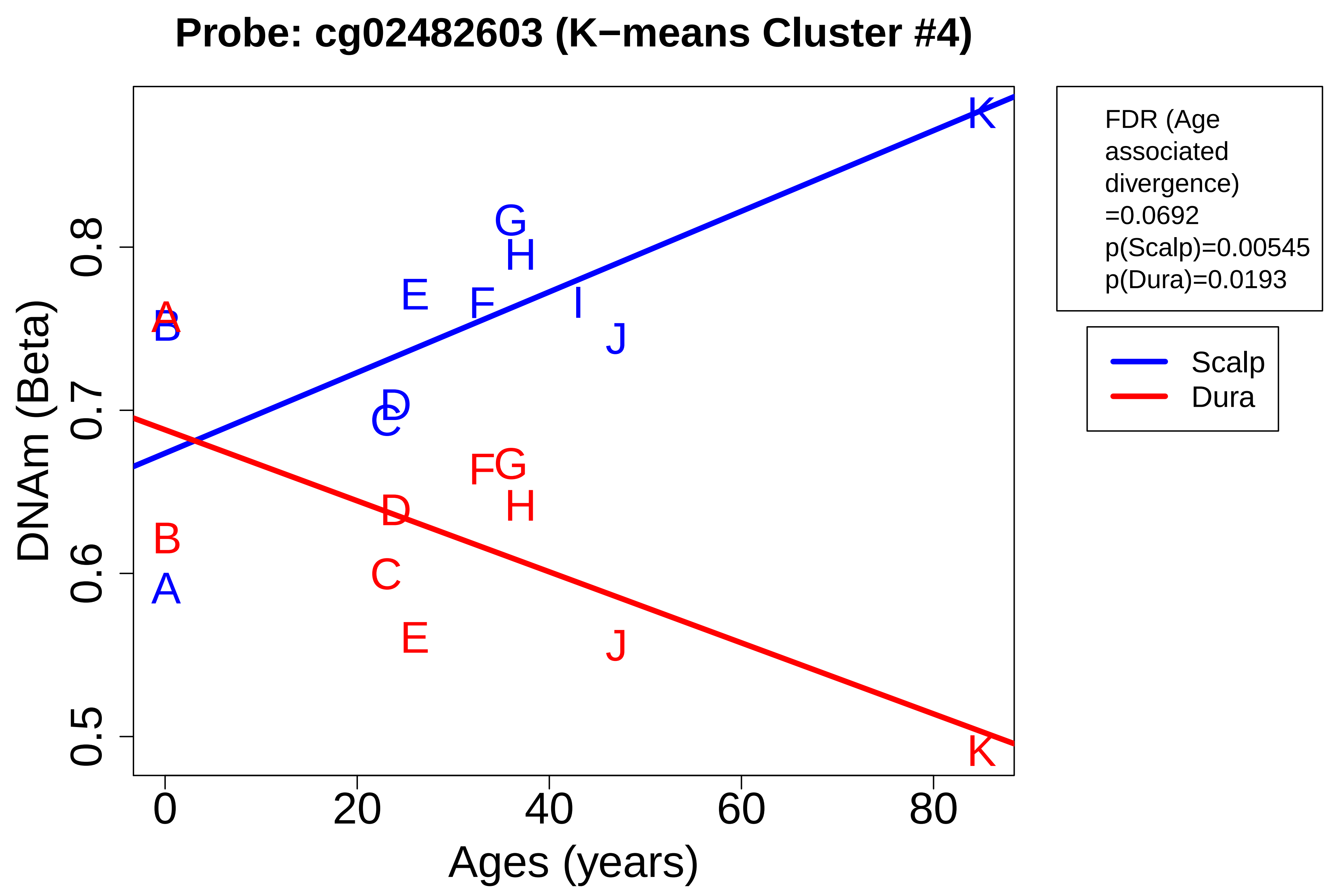

Probe: cg02482603 (K−means Cluster #4)
DNAm (Beta)
0.5
0.6
0.7
0.8
0
20
40
60
80
Ages (years)
A
B
C
D
E
F
G
H
I
J
K
FDR (Age associated divergence) =0.0692
p(Scalp)=0.00545
p(Dura)=0.0193
Scalp
Dura

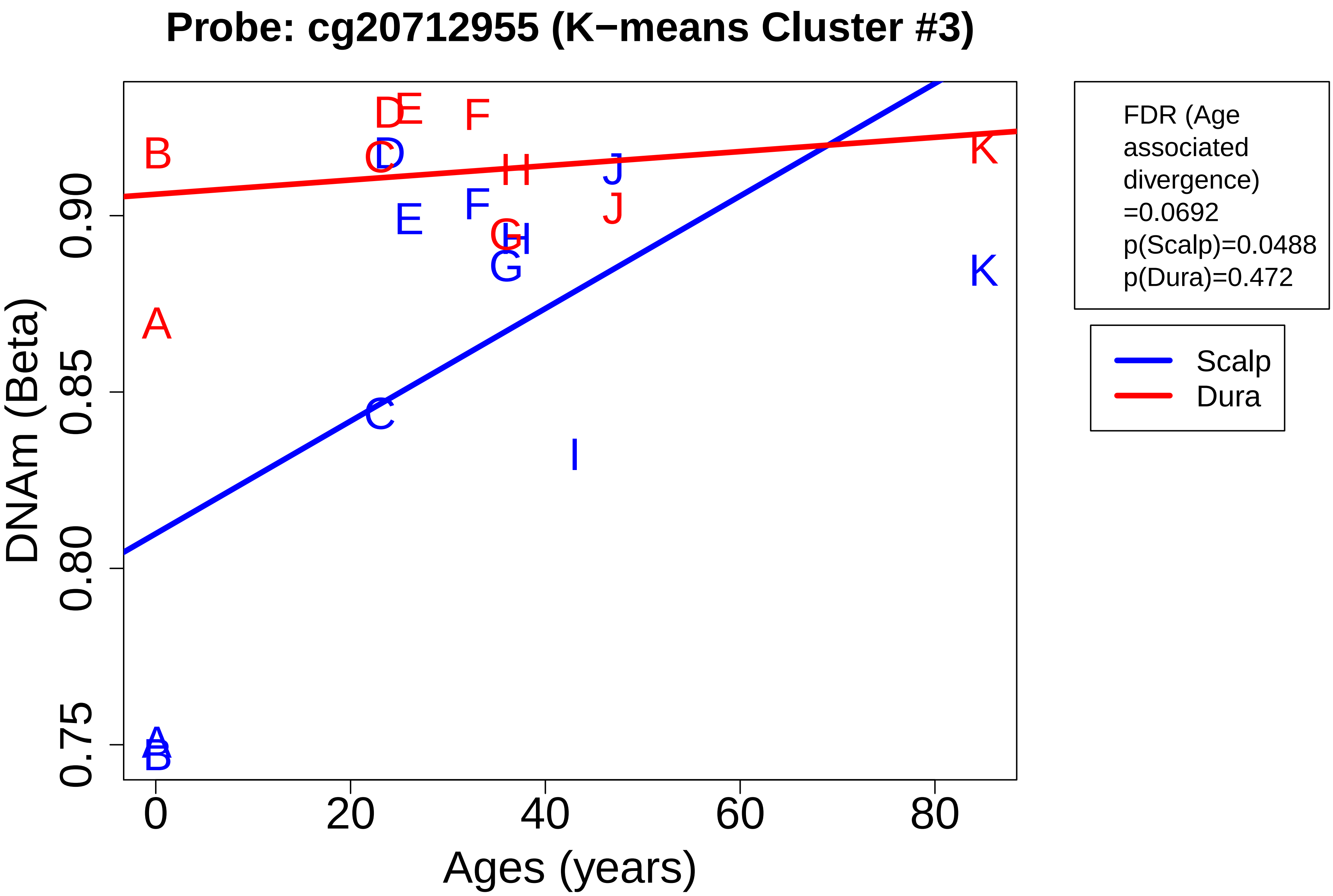
Probe: cg20712955 (K−means Cluster #3)
DNAm (Beta)
0.75
0.80
0.85
0.90
0
20
40
60
80
Ages (years)
FDR (Age associated divergence) =0.0692
p(Scalp)=0.0488
p(Dura)=0.472
Scalp
Dura

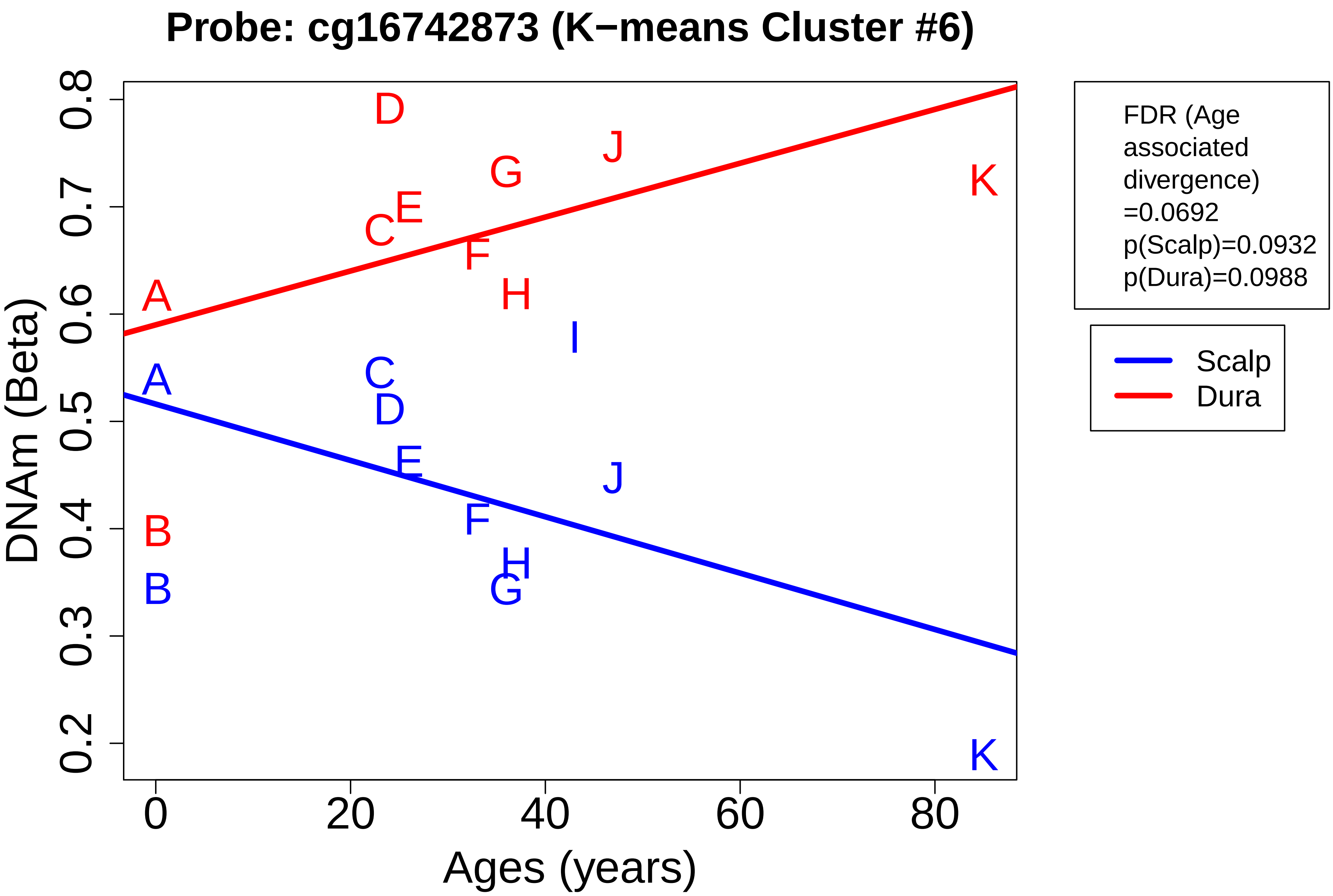

Probe: cg16742873 (K−means Cluster #6)
DNAm (Beta)
0.2
0.3
0.4
0.5
0.6
0.7
0.8
Ages (years)
0
20
40
60
80
A
B
C
D
E
F
G
H
I
J
K
FDR (Age associated divergence) =0.0692
p(Scalp)=0.0932
p(Dura)=0.0988
Scalp
Dura

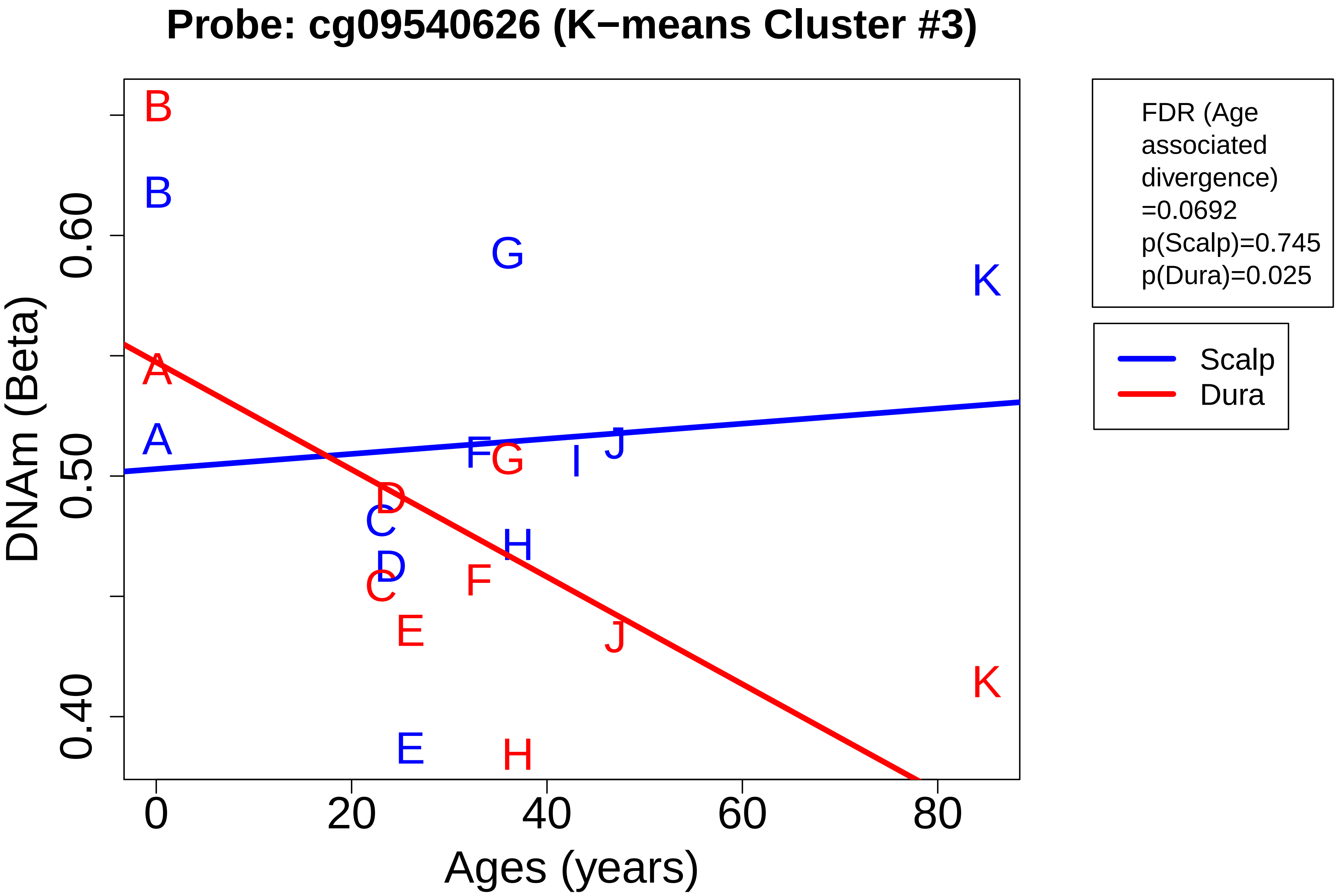
Probe: cg09540626 (K−means Cluster #3)
DNAm (Beta)
0.40
0.50
0.60
Ages (years)
0
20
40
60
80
B
B
G
K
A
A
F
G
I
J
D
C
H
D
C
F
E
J
K
E
H
FDR (Age associated divergence) =0.0692
p(Scalp)=0.745
p(Dura)=0.025
Scalp
Dura

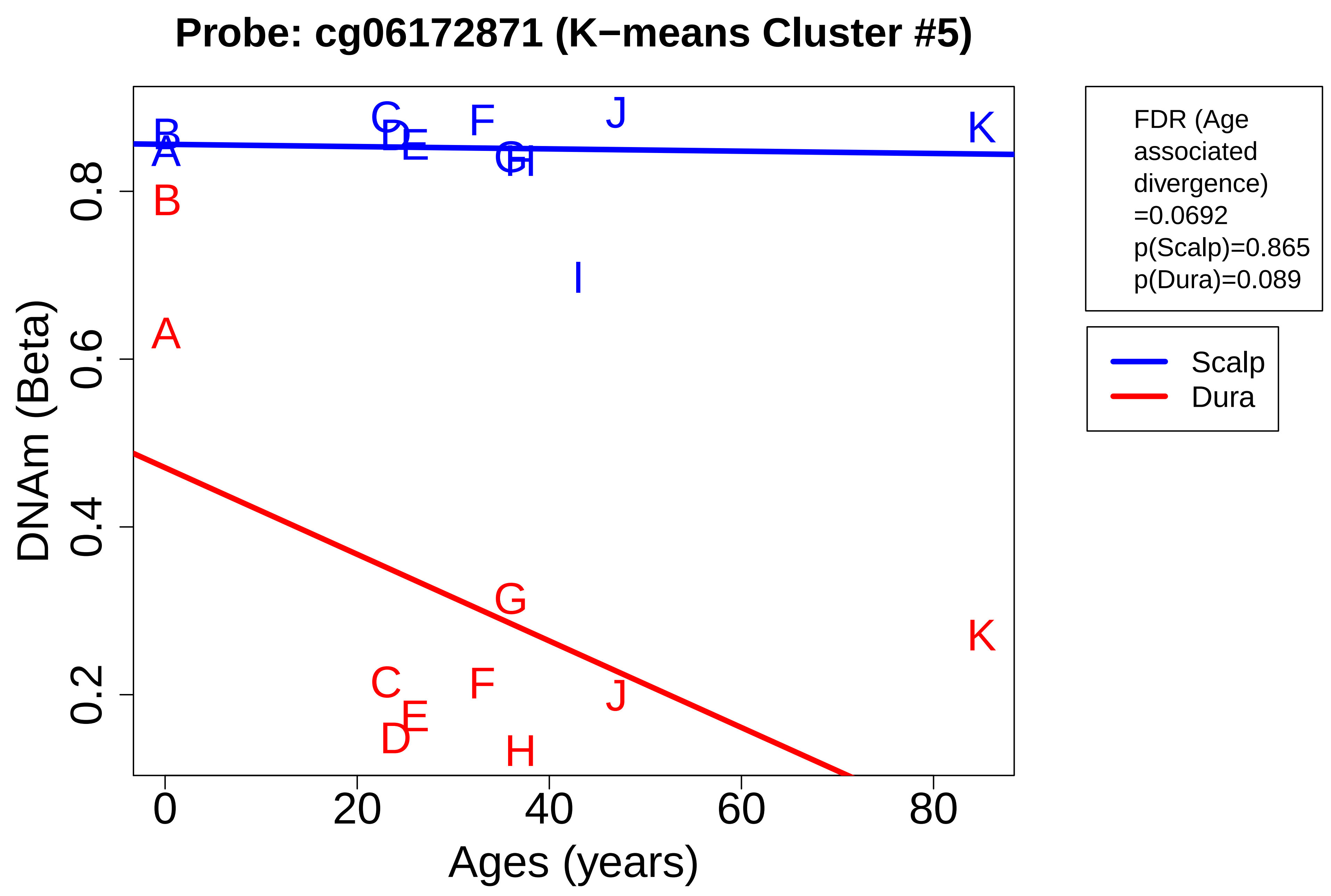

Probe: cg06172871 (K−means Cluster #5)
DNAm (Beta)
0.2
0.4
0.6
0.8
0
20
40
60
80
Ages (years)
A
B
C
D
E
F
G
H
I
J
K
FDR (Age associated divergence) =0.0692
p(Scalp)=0.865
p(Dura)=0.089
Scalp
Dura

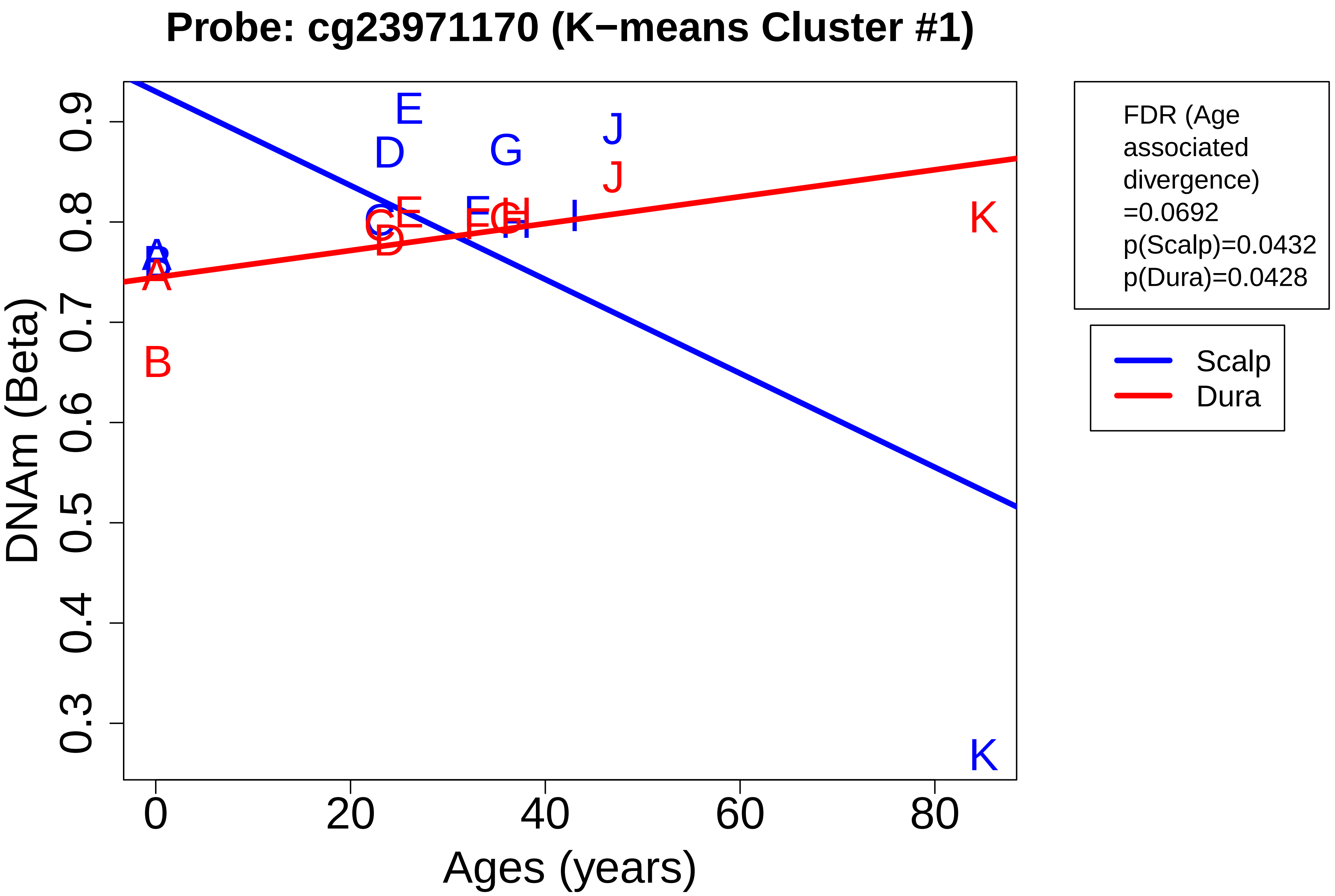

Probe: cg23971170 (K−means Cluster #1)
DNAm (Beta)
Ages (years)
0
20
40
60
80
0.3
0.4
0.5
0.6
0.7
0.8
0.9
A
B
C
D
E
F
G
H
I
J
K
FDR (Age associated divergence) =0.0692
p(Scalp)=0.0432
p(Dura)=0.0428
Scalp
Dura

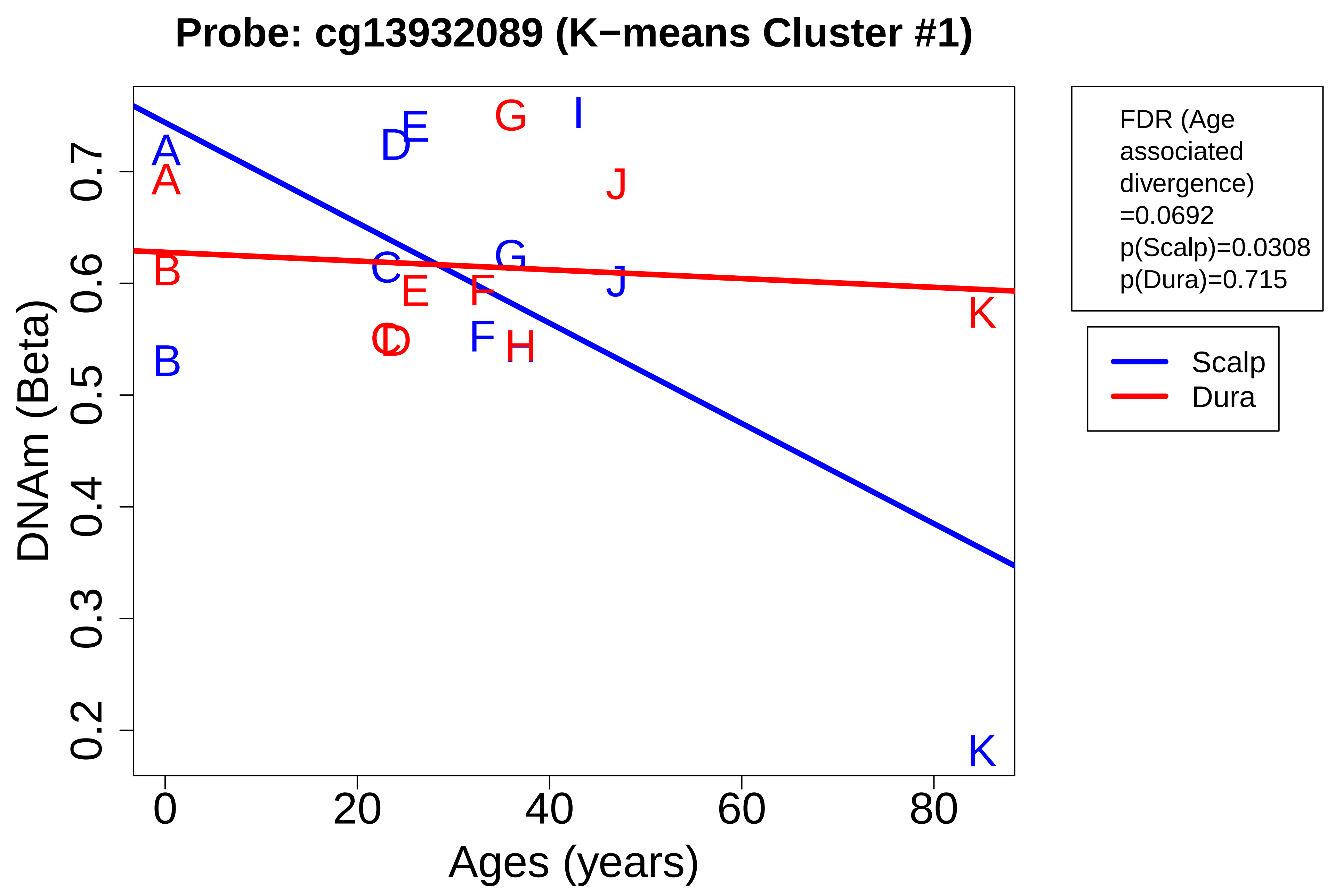

Probe: cg13932089 (K−means Cluster #1)
DNAm (Beta)
0.2
0.3
0.4
0.5
0.6
0.7
0
20
40
60
80
Ages (years)
A
B
C
D
E
F
G
H
I
J
K
FDR (Age associated divergence) =0.0692
p(Scalp)=0.0308
p(Dura)=0.715
Scalp
Dura

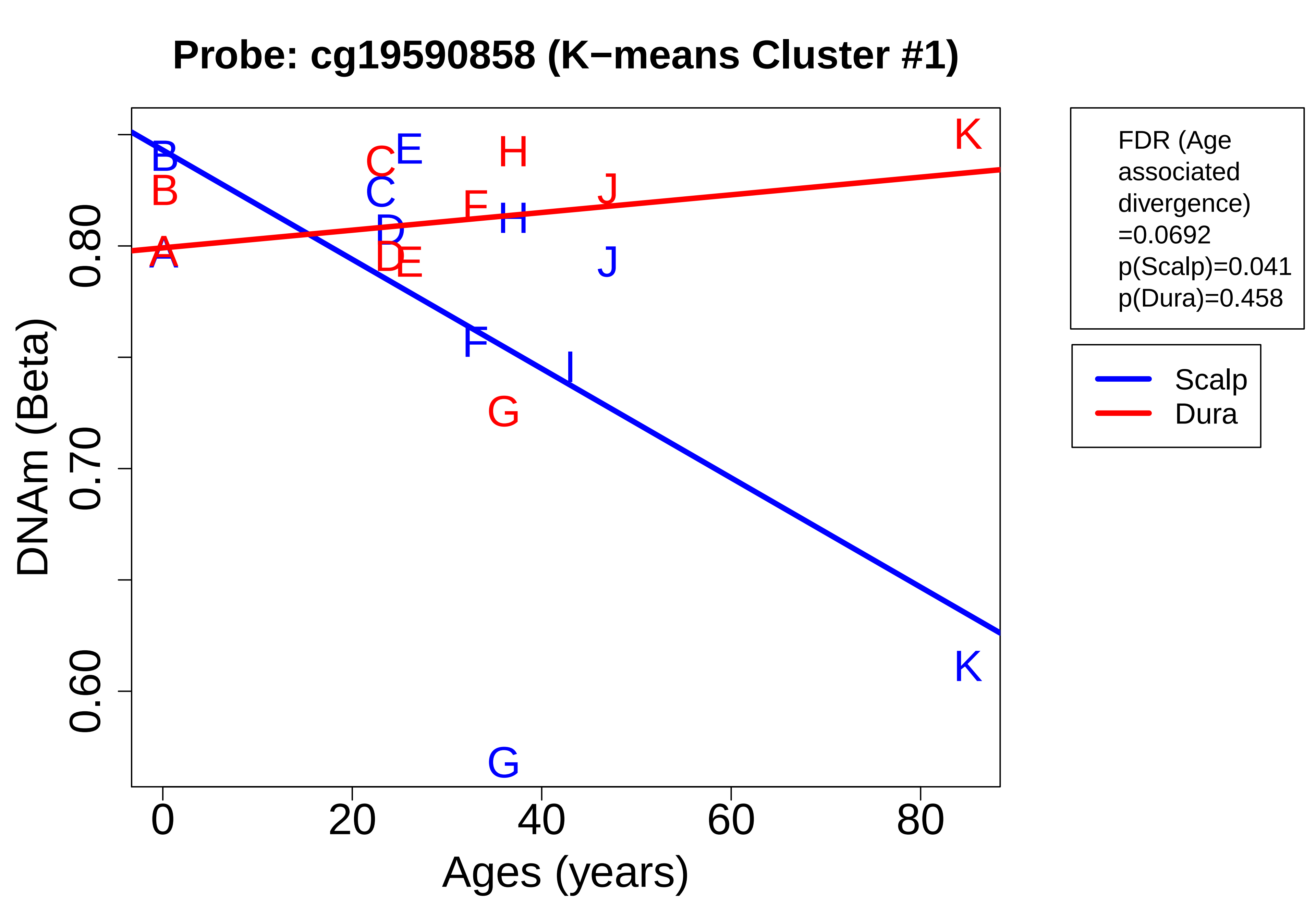

Probe: cg19590858 (K−means Cluster #1)
DNAm (Beta)
0.60
0.70
0.80
Ages (years)
0
20
40
60
80
FDR (Age associated divergence) =0.0692
p(Scalp)=0.041
p(Dura)=0.458
Scalp
Dura
A
B
C
D
E
F
G
H
I
J
K

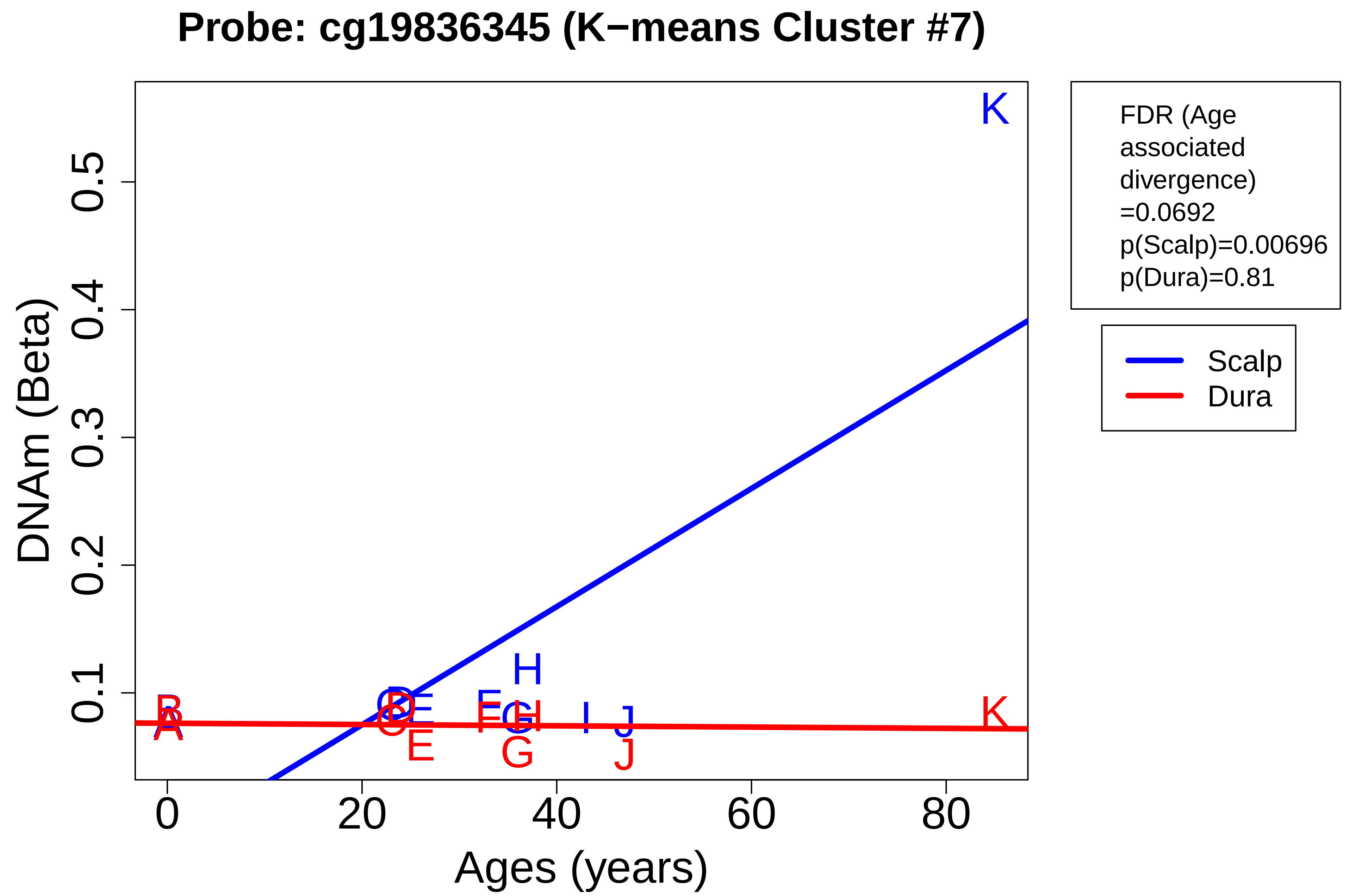

Probe: cg19836345 (K−means Cluster #7)
DNAm (Beta)
0.1
0.2
0.3
0.4
0.5
Ages (years)
0
20
40
60
80
FDR (Age associated divergence) =0.0692
p(Scalp)=0.00696
p(Dura)=0.81
Scalp
Dura

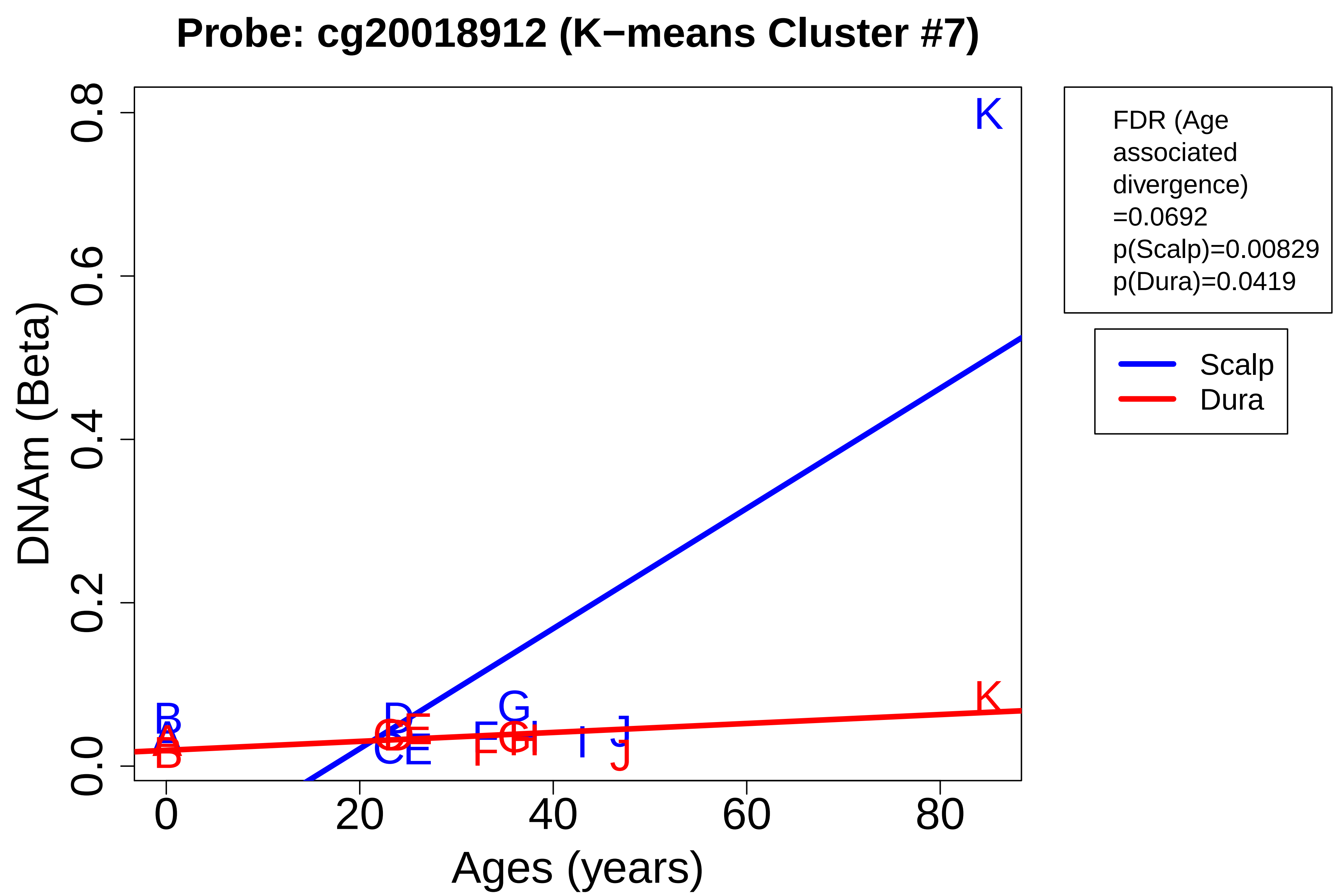

Probe: cg20018912 (K−means Cluster #7)
DNAm (Beta)
0.0
0.2
0.4
0.6
0.8
Ages (years)
0
20
40
60
80
FDR (Age associated divergence) =0.0692
p(Scalp)=0.00829
p(Dura)=0.0419
Scalp
Dura

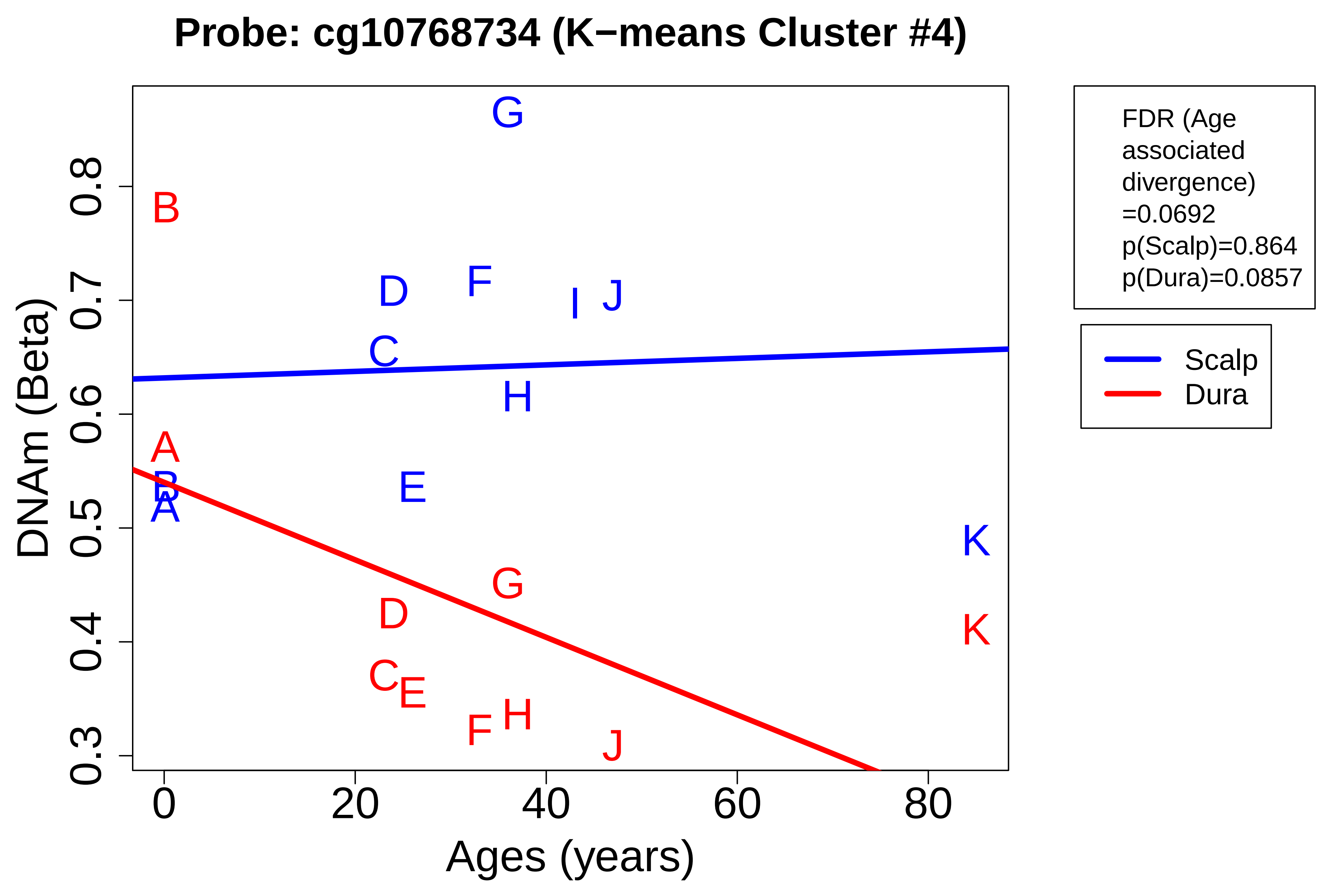

Probe: cg10768734 (K−means Cluster #4)
FDR (Age associated divergence) =0.0692
p(Scalp)=0.864
p(Dura)=0.0857
Scalp
Dura
DNAm (Beta)
Ages (years)
0.3
0.4
0.5
0.6
0.7
0.8
0
20
40
60
80

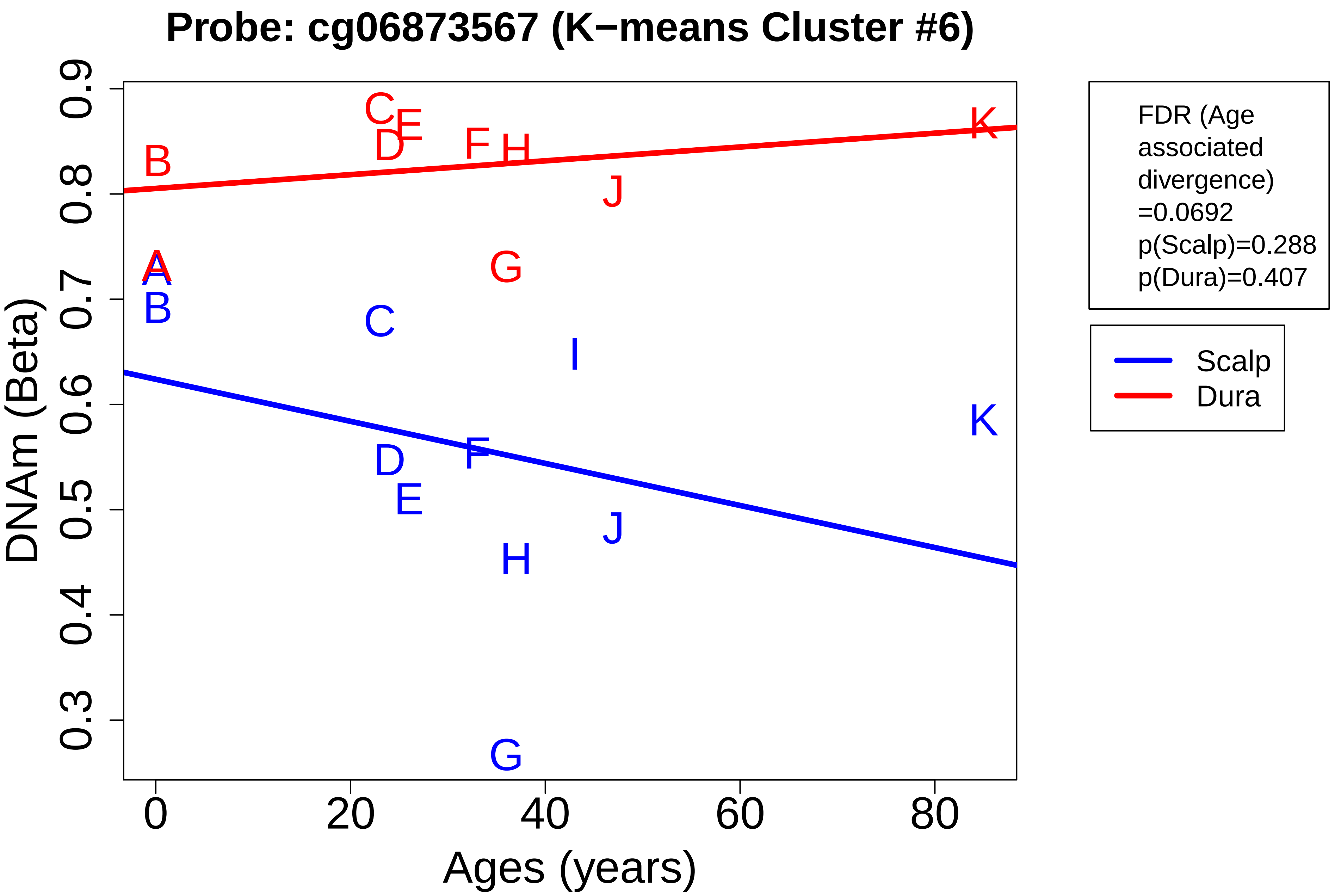

Probe: cg06873567 (K−means Cluster #6)
DNAm (Beta)
0.3
0.4
0.5
0.6
0.7
0.8
0.9
0
20
40
60
80
Ages (years)
FDR (Age associated divergence) =0.0692
p(Scalp)=0.288
p(Dura)=0.407
Scalp
Dura

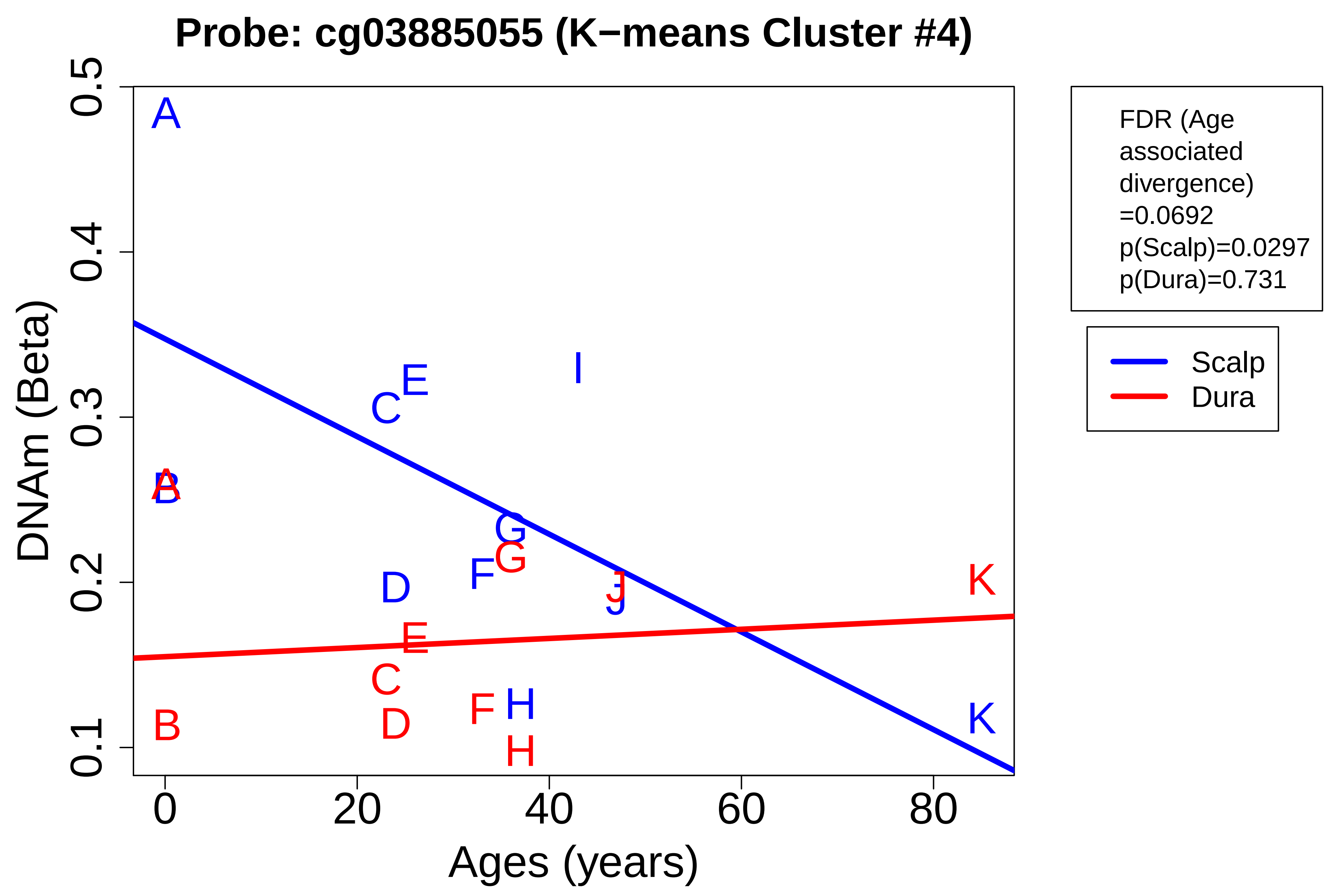

Probe: cg03885055 (K−means Cluster #4)
DNAm (Beta)
0.1
0.2
0.3
0.4
0.5
0
20
40
60
80
Ages (years)
A
B
C
D
E
F
G
H
I
J
K
FDR (Age associated divergence) =0.0692
p(Scalp)=0.0297
p(Dura)=0.731
Scalp
Dura

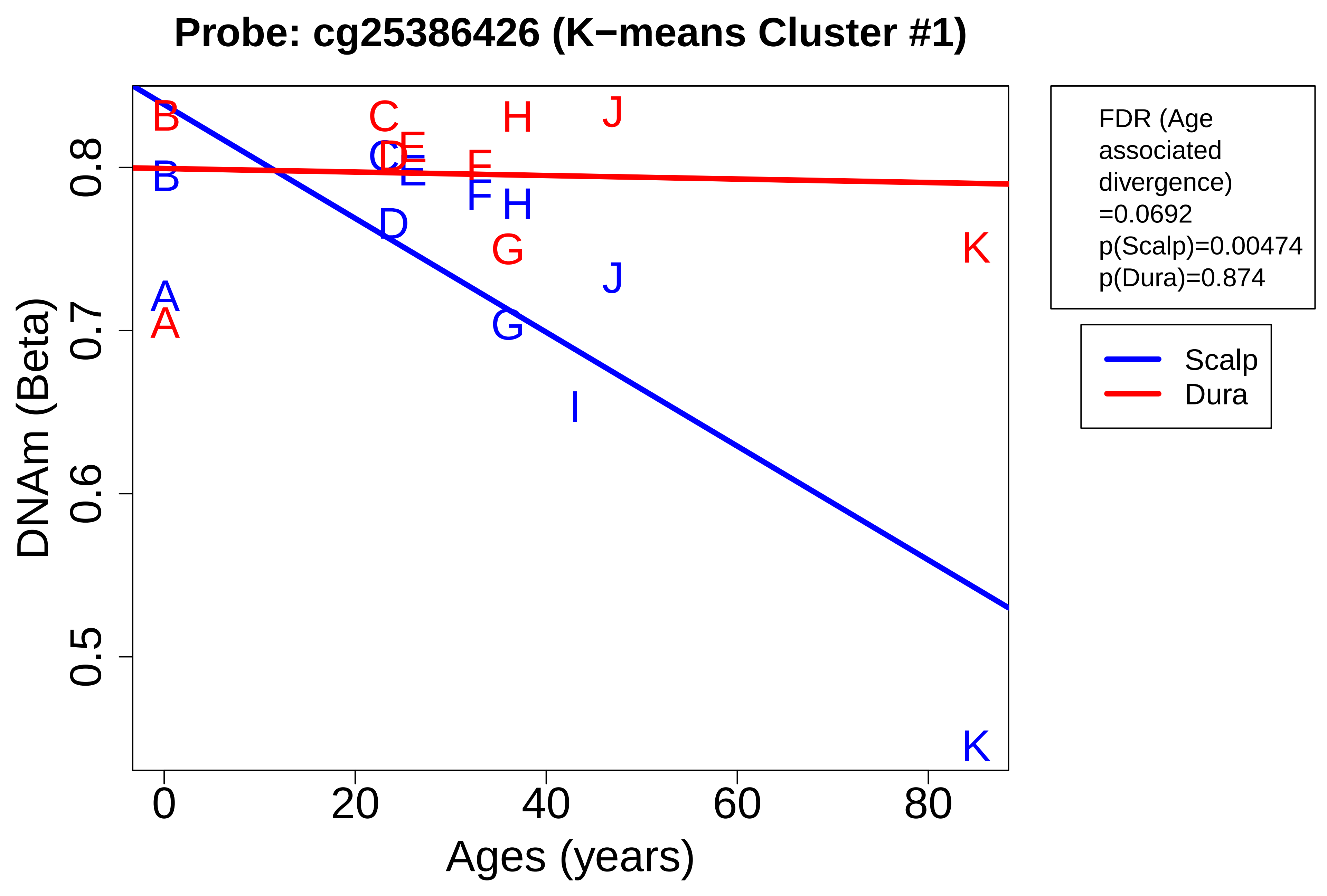

Probe: cg25386426 (K−means Cluster #1)
DNAm (Beta)
0.5
0.6
0.7
0.8
0
20
40
60
80
Ages (years)
FDR (Age associated divergence) =0.0692
p(Scalp)=0.00474
p(Dura)=0.874
Scalp
Dura

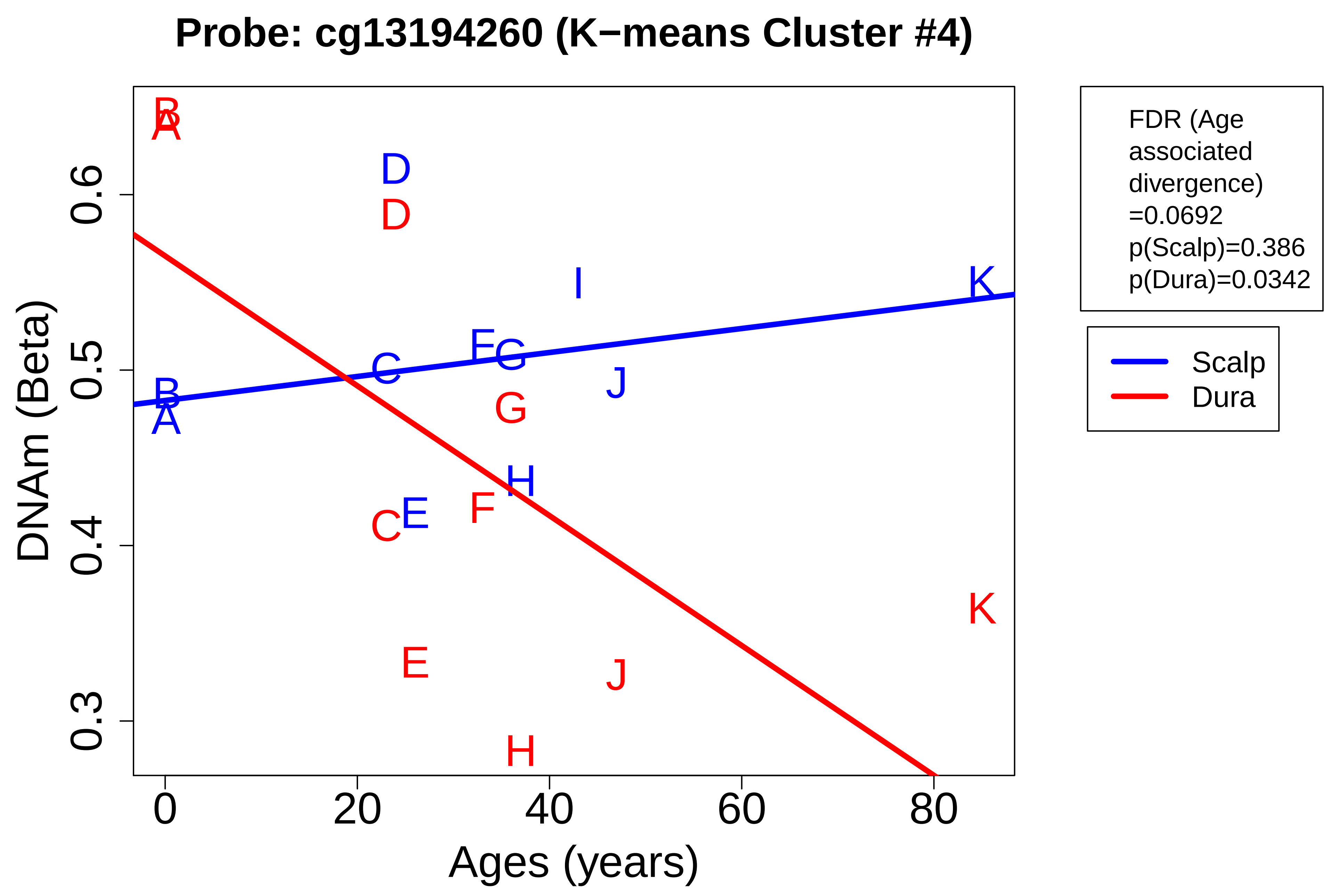
Probe: cg13194260 (K−means Cluster #4)
DNAm (Beta)
Ages (years)
0.3
0.4
0.5
0.6
0
20
40
60
80
FDR (Age associated divergence) =0.0692
p(Scalp)=0.386
p(Dura)=0.0342
Scalp
Dura
A
B
C
D
E
F
G
H
I
J
K
A
B
C
D
E
F
G
H
J
K

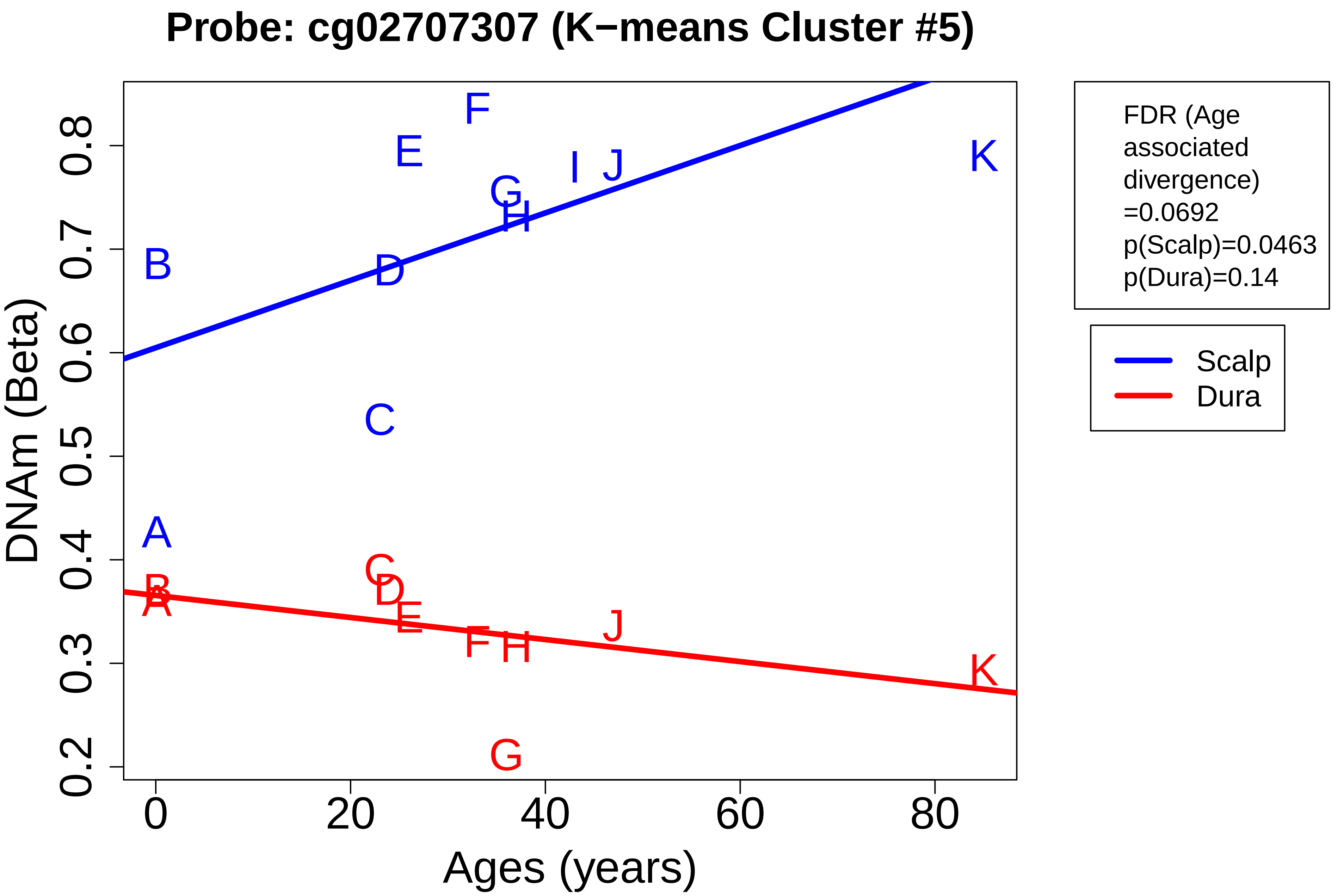
Probe: cg02707307 (K−means Cluster #5)
FDR (Age associated divergence) =0.0692
p(Scalp)=0.0463
p(Dura)=0.14
Scalp
Dura
DNAm (Beta)
Ages (years)
0.2
0.3
0.4
0.5
0.6
0.7
0.8
0
20
40
60
80
A
B
C
D
E
F
G
H
I
J
K

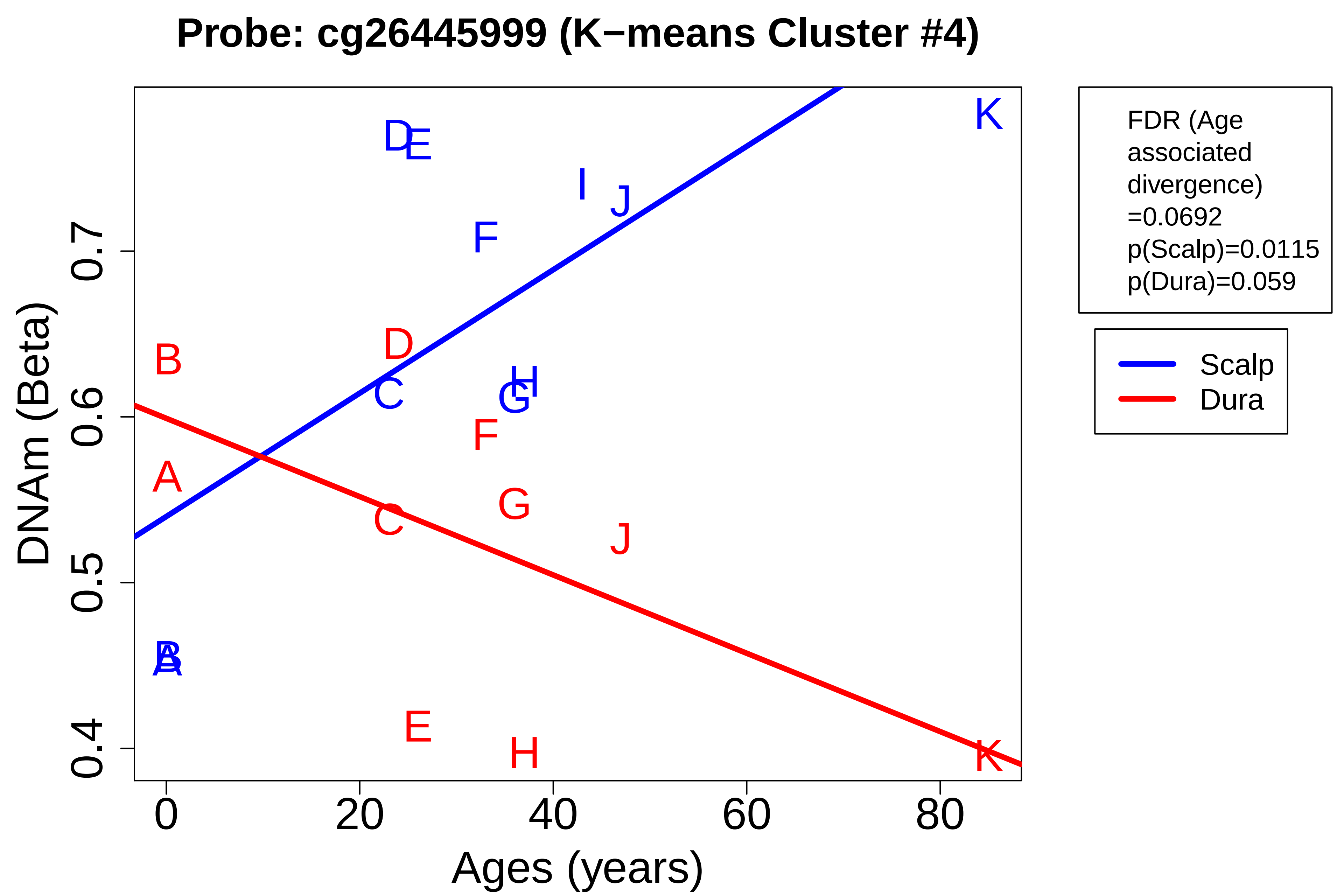

Probe: cg26445999 (K−means Cluster #4)
DNAm (Beta)
0.4
0.5
0.6
0.7
Ages (years)
0
20
40
60
80
FDR (Age associated divergence) =0.0692
p(Scalp)=0.0115
p(Dura)=0.059
Scalp
Dura

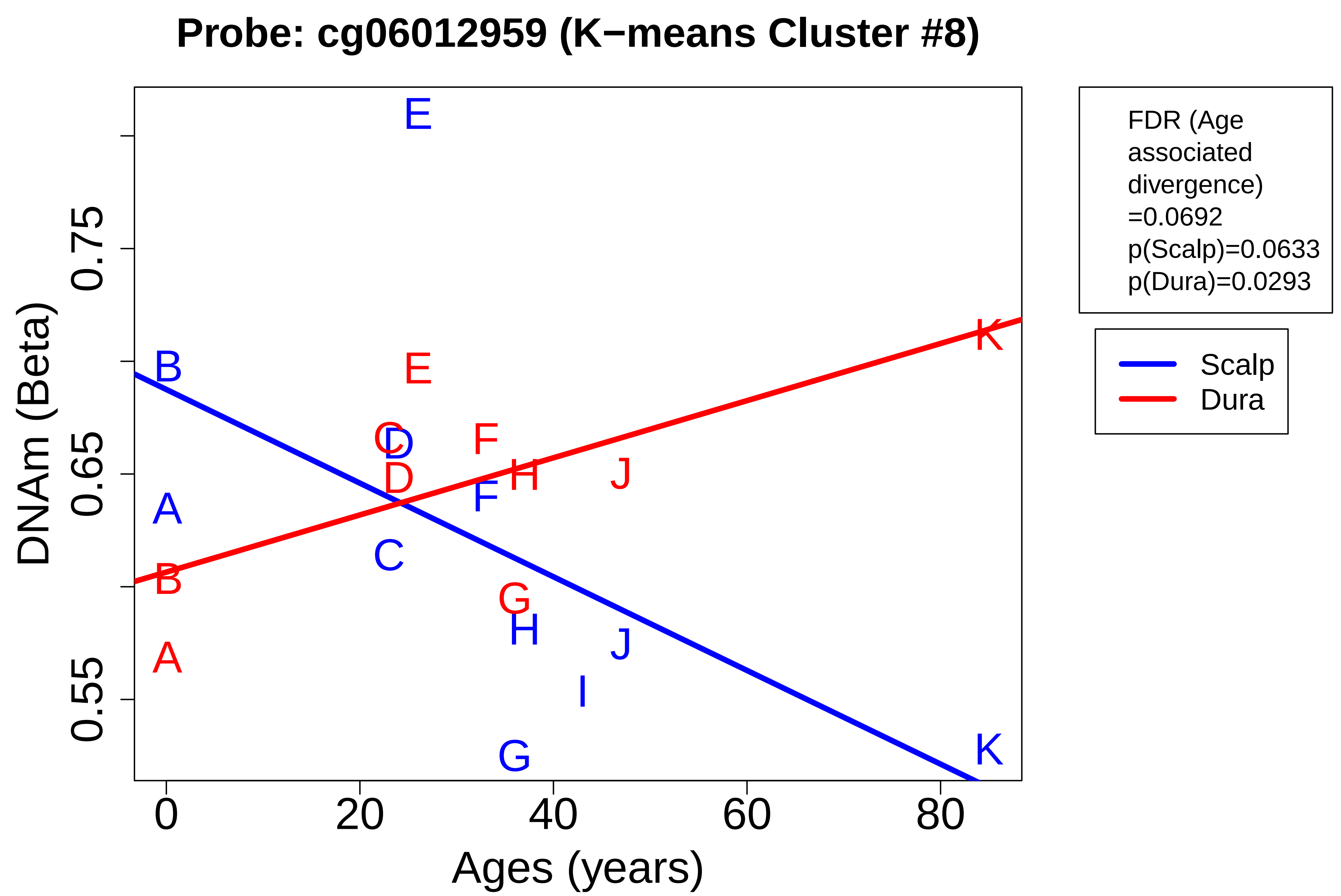

Probe: cg06012959 (K−means Cluster #8)
DNAm (Beta)
0.55
0.65
0.75
Ages (years)
0
20
40
60
80
FDR (Age associated divergence) =0.0692
p(Scalp)=0.0633
p(Dura)=0.0293
Scalp
Dura

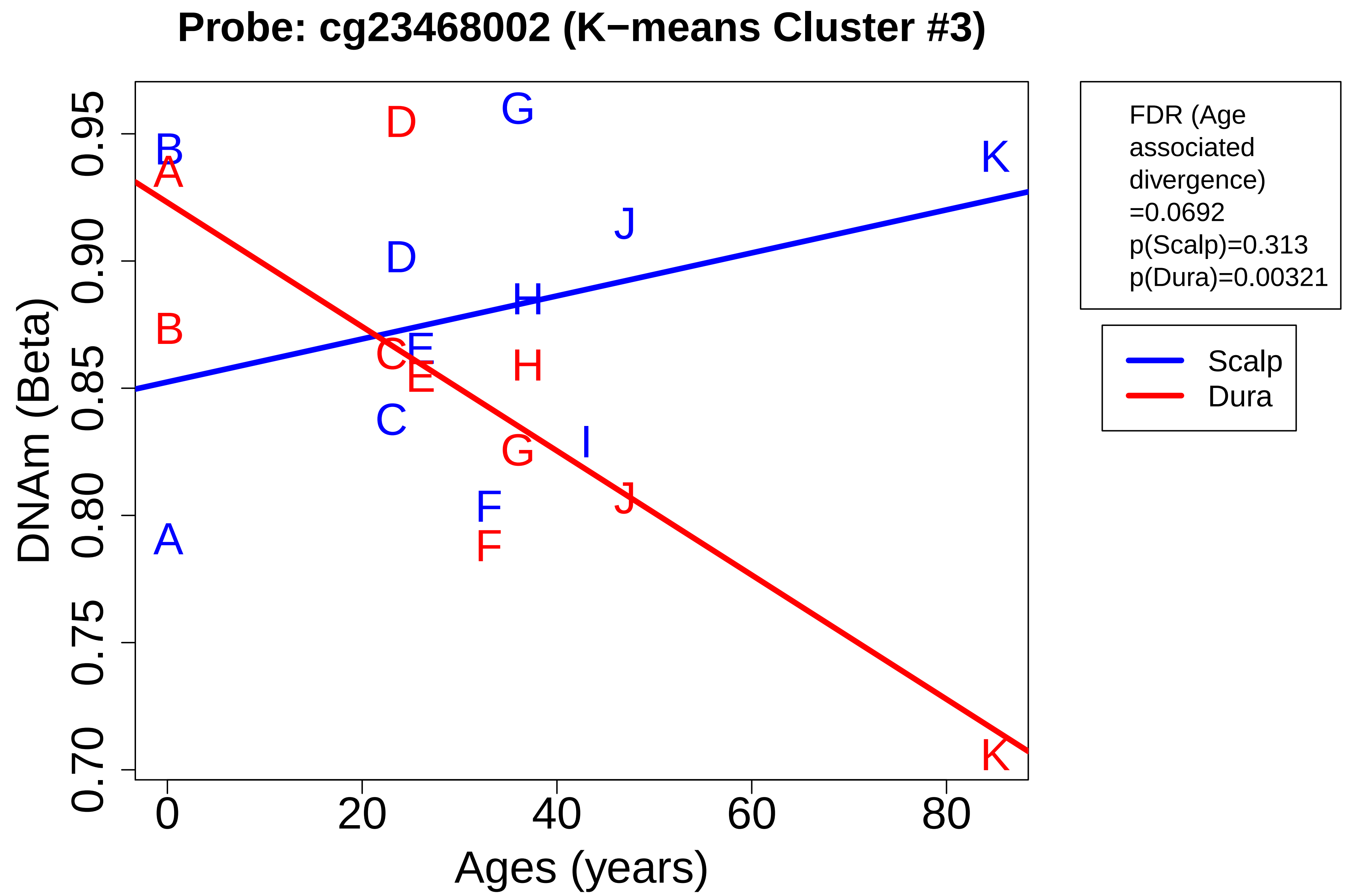

Probe: cg23468002 (K−means Cluster #3)
DNAm (Beta)
0.70
0.75
0.80
0.85
0.90
0.95
0
20
40
60
80
Ages (years)
FDR (Age associated divergence) =0.0692
p(Scalp)=0.313
p(Dura)=0.00321
Scalp
Dura

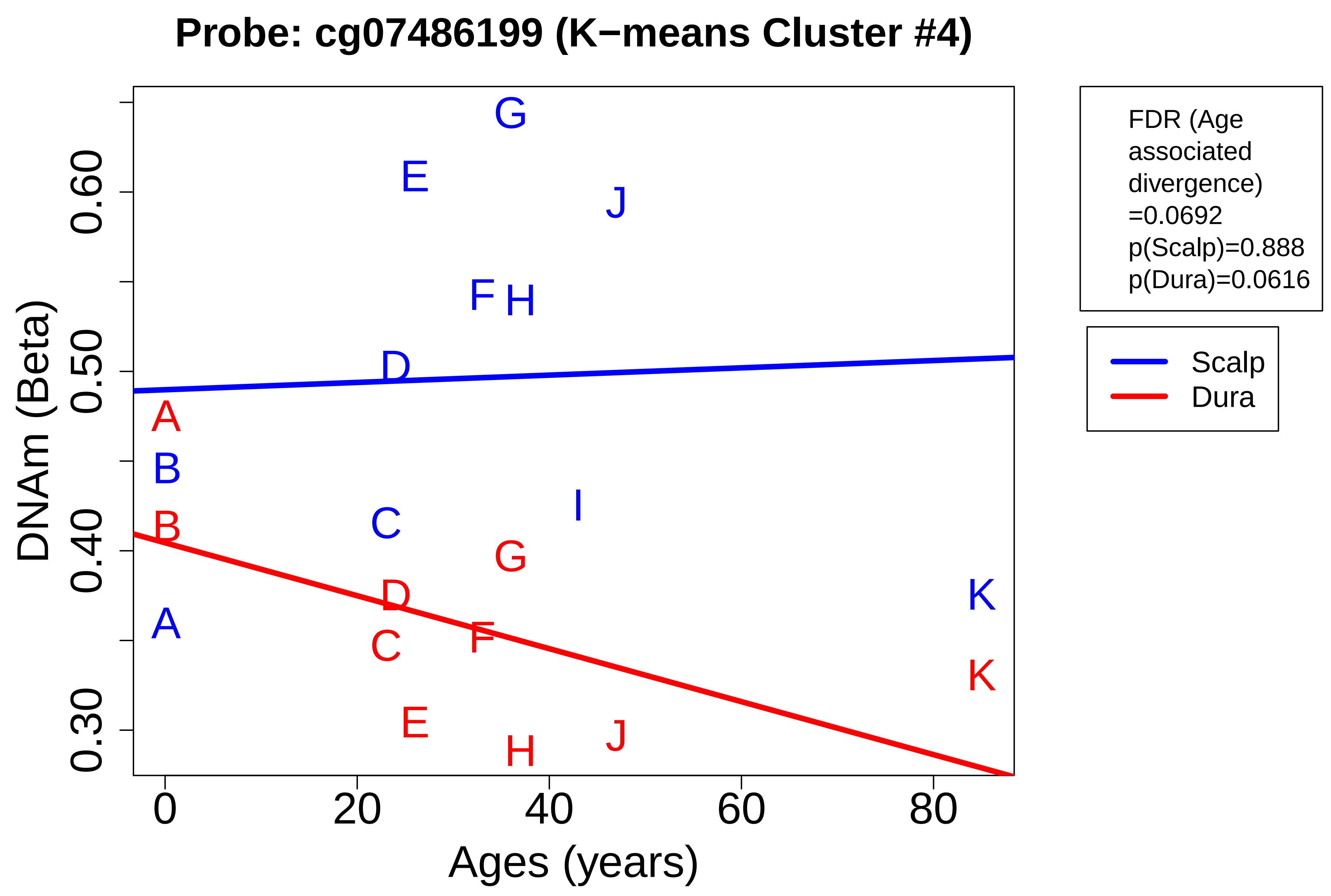

Probe: cg07486199 (K−means Cluster #4)
DNAm (Beta)
0.30
0.40
0.50
0.60
0
20
40
60
80
Ages (years)
FDR (Age associated divergence) =0.0692
p(Scalp)=0.888
p(Dura)=0.0616
Scalp
Dura

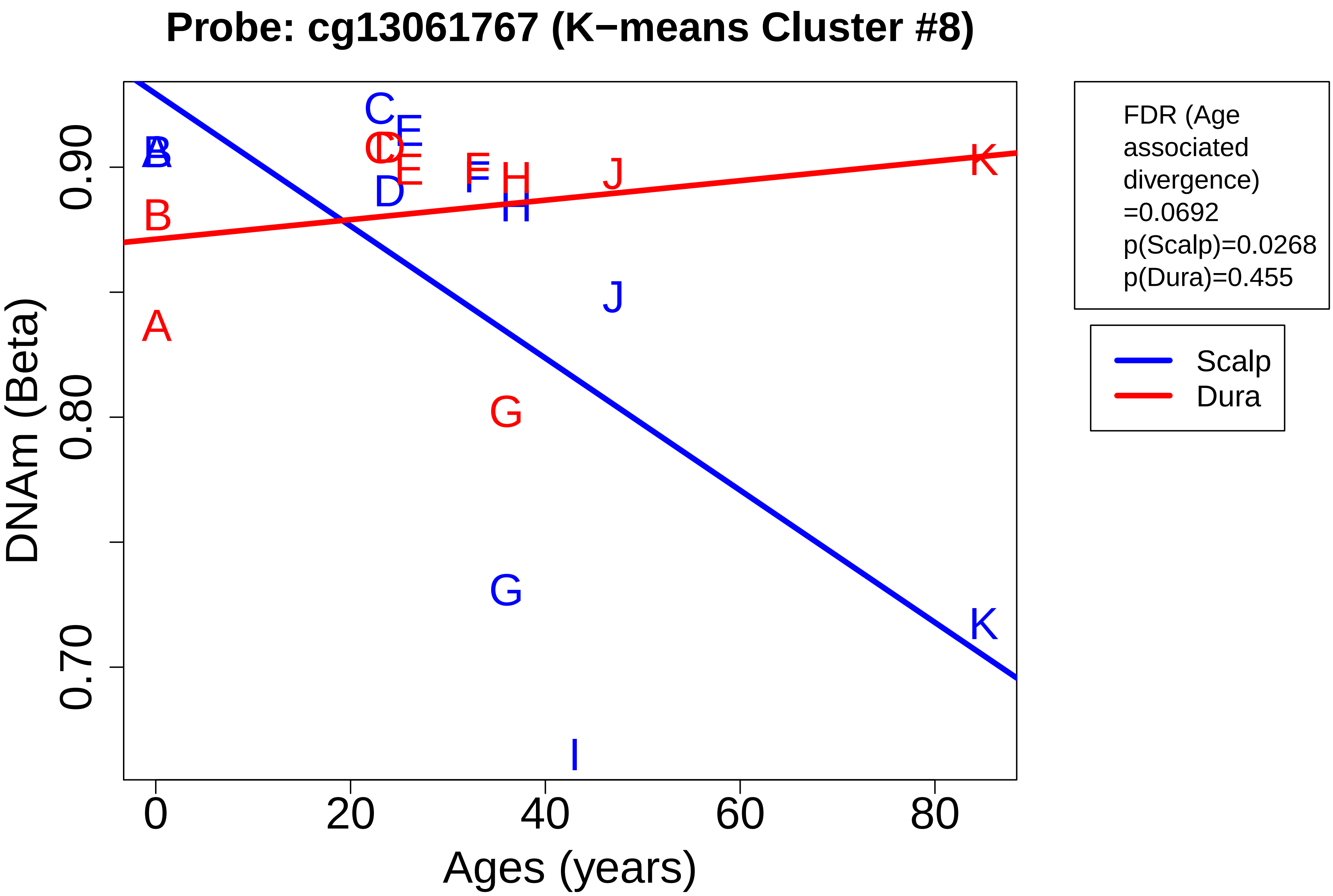

Probe: cg13061767 (K−means Cluster #8)
DNAm (Beta)
0.70
0.80
0.90
Ages (years)
0
20
40
60
80
FDR (Age associated divergence) =0.0692
p(Scalp)=0.0268
p(Dura)=0.455
Scalp
Dura

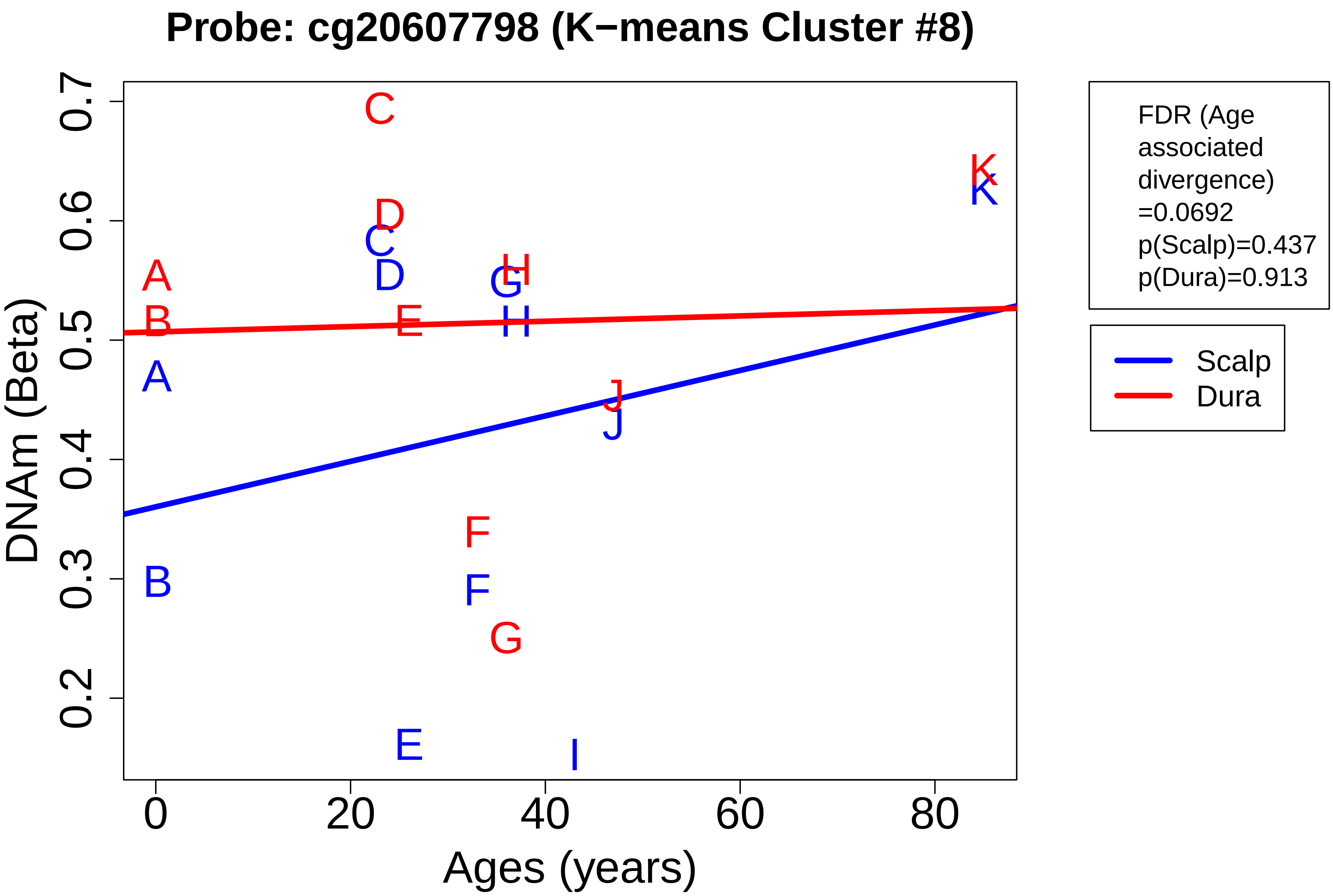

Probe: cg20607798 (K−means Cluster #8)
DNAm (Beta)
Ages (years)
0.2
0.3
0.4
0.5
0.6
0.7
0
20
40
60
80
FDR (Age associated divergence) =0.0692
p(Scalp)=0.437
p(Dura)=0.913
Scalp
Dura
A B C D E F G H I J K

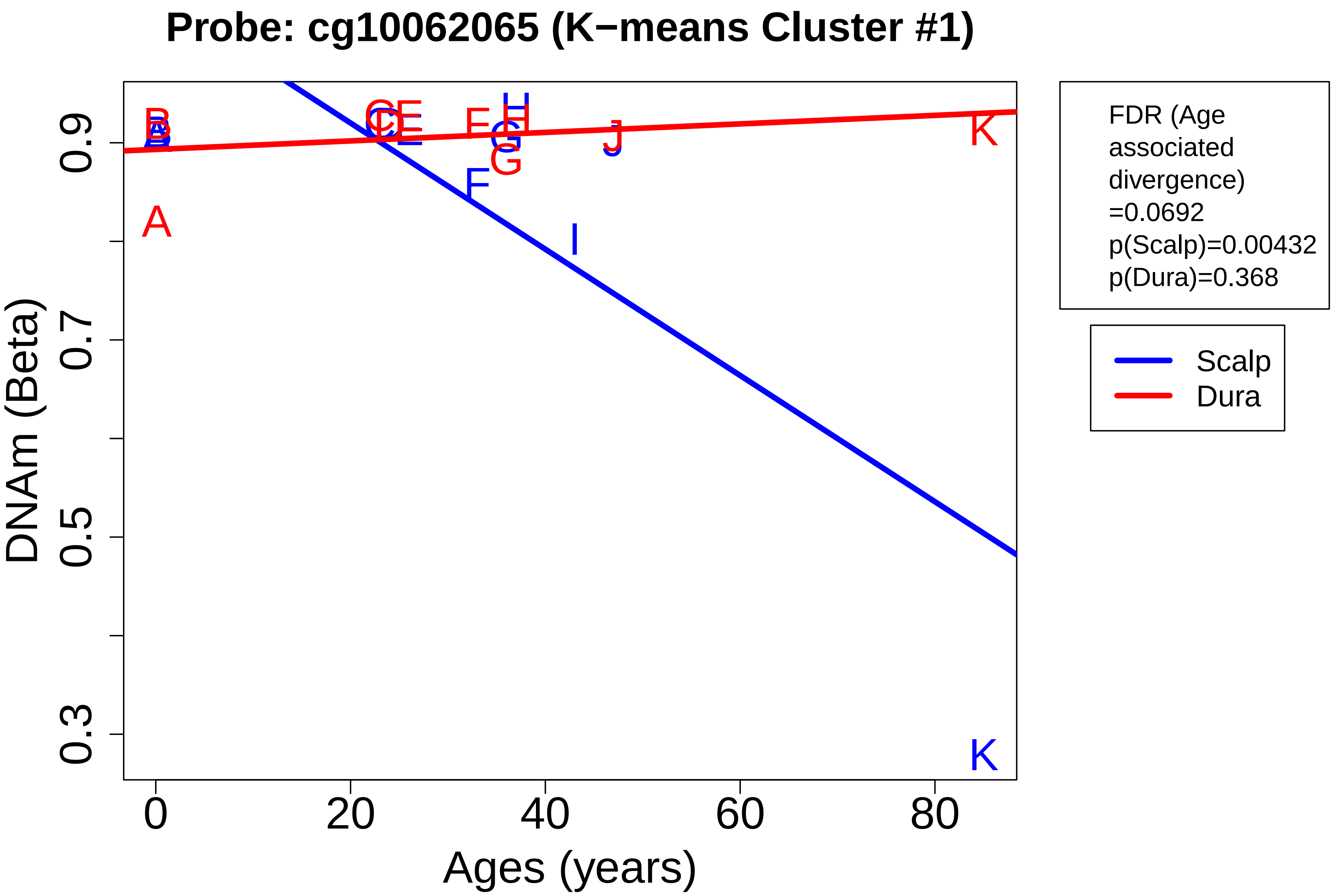

Probe: cg10062065 (K−means Cluster #1)
DNAm (Beta)
0.3
0.5
0.7
0.9
Ages (years)
0
20
40
60
80
FDR (Age associated divergence) =0.0692
p(Scalp)=0.00432
p(Dura)=0.368
Scalp
Dura

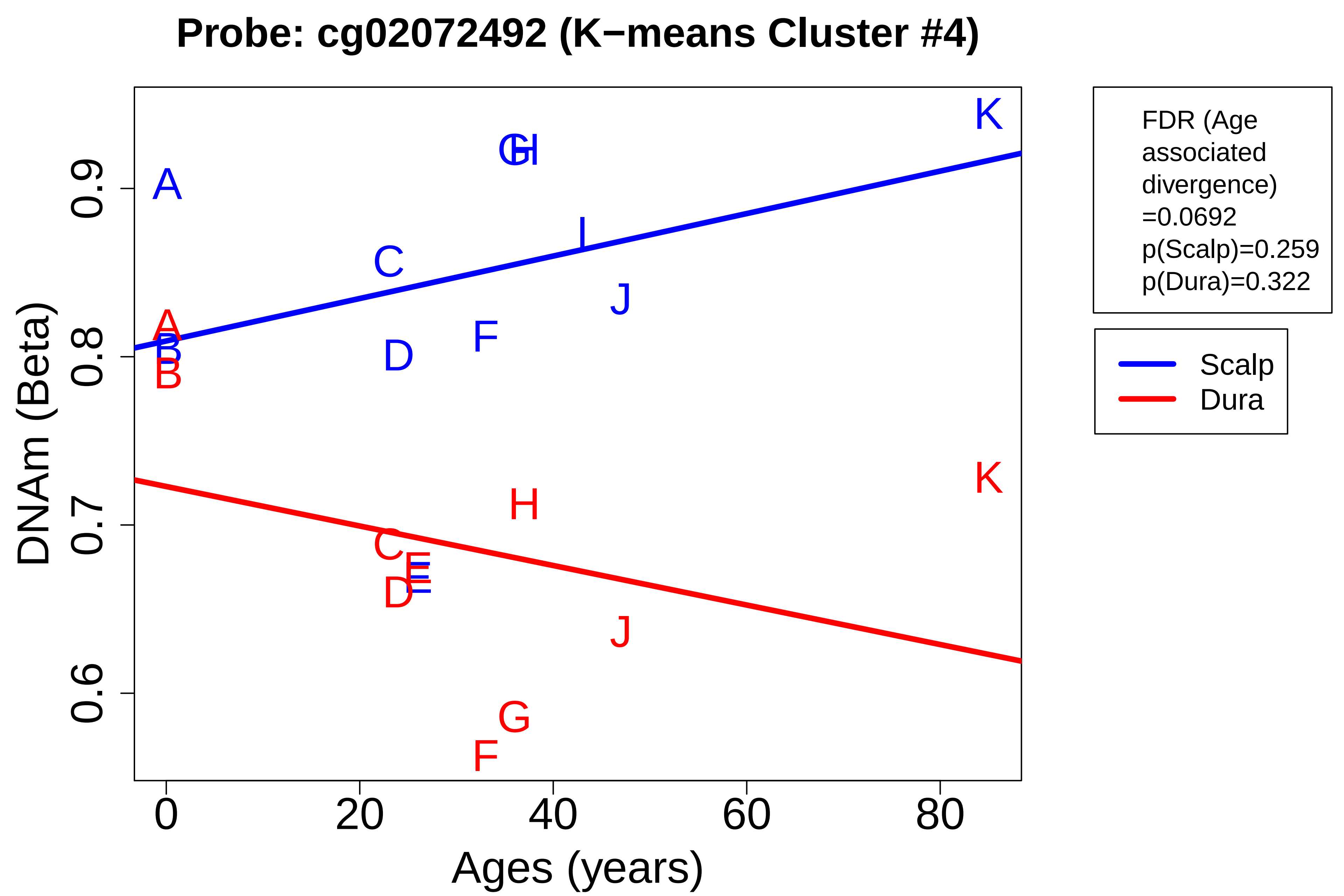

Probe: cg02072492 (K−means Cluster #4)
DNAm (Beta)
0.6
0.7
0.8
0.9
Ages (years)
0
20
40
60
80
A
B
C
D
E
F
G
H
I
J
K
FDR (Age associated divergence) =0.0692
p(Scalp)=0.259
p(Dura)=0.322
Scalp
Dura

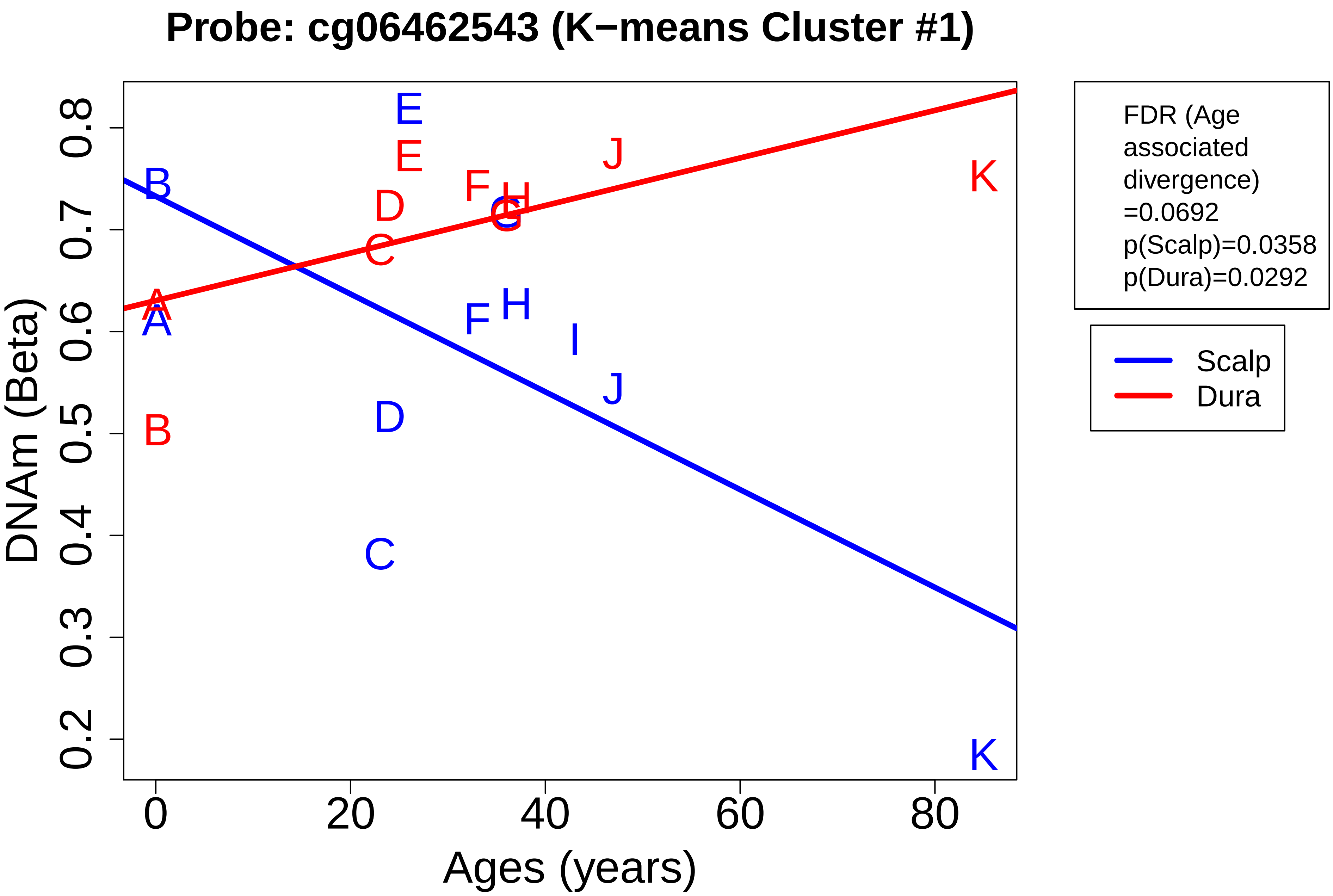

Probe: cg06462543 (K−means Cluster #1)
DNAm (Beta)
0.2
0.3
0.4
0.5
0.6
0.7
0.8
0
20
40
60
80
Ages (years)
FDR (Age associated divergence) =0.0692
p(Scalp)=0.0358
p(Dura)=0.0292
Scalp
Dura

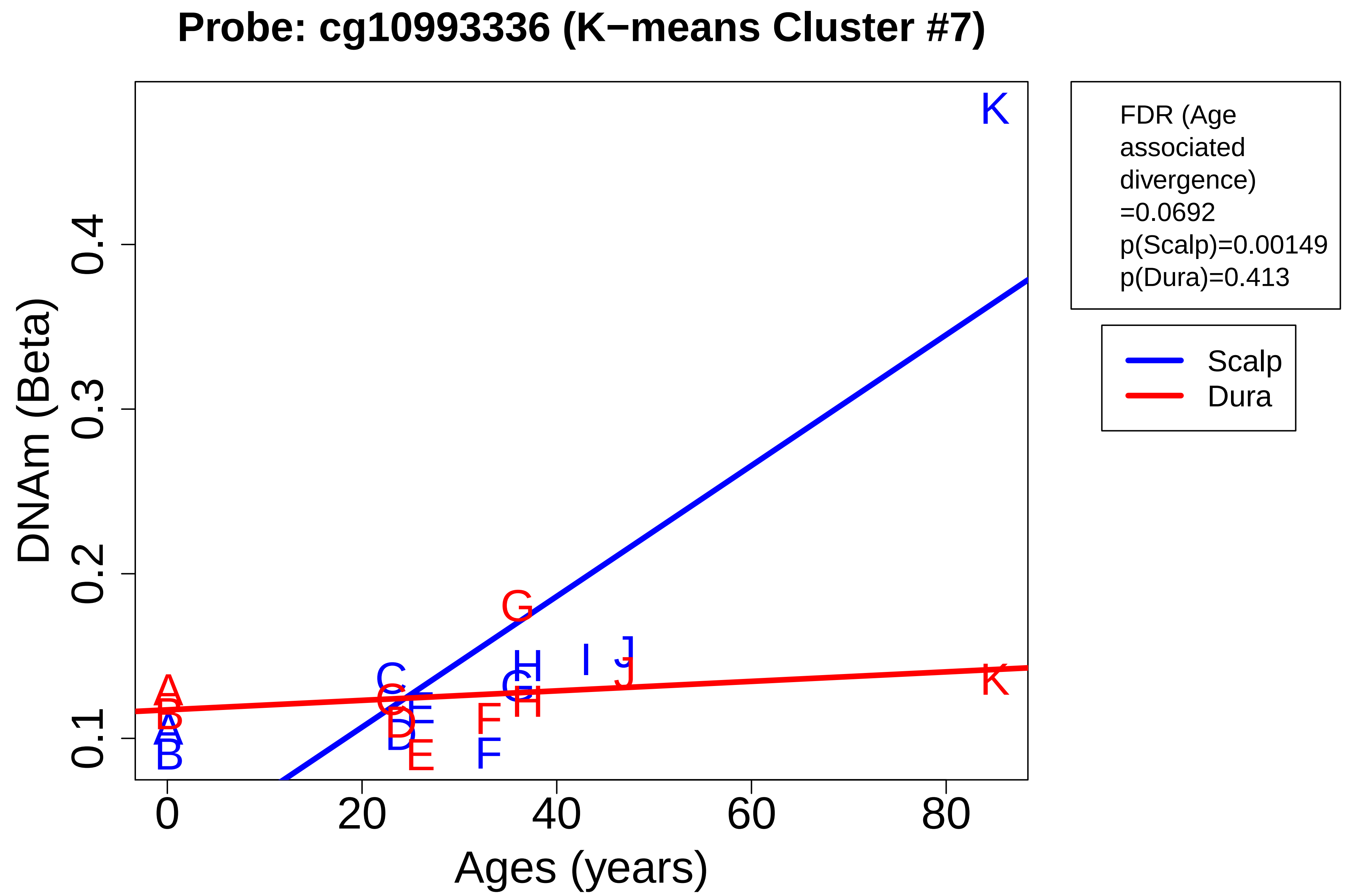

Probe: cg10993336 (K−means Cluster #7)
DNAm (Beta)
0.1
0.2
0.3
0.4
0
20
40
60
80
Ages (years)
A
B
C
D
E
F
G
H
I
J
K
FDR (Age associated divergence) =0.0692
p(Scalp)=0.00149
p(Dura)=0.413
Scalp
Dura

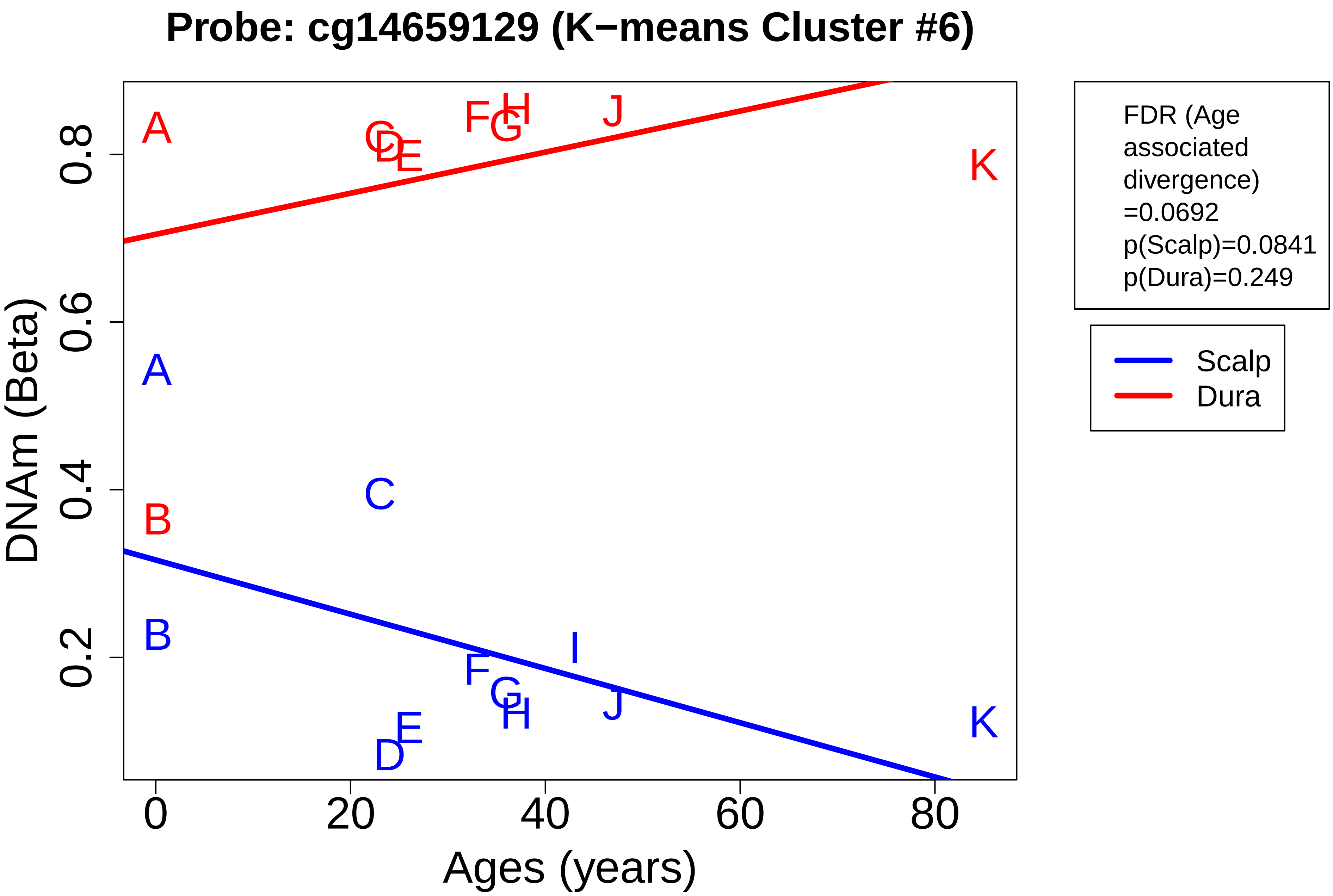

Probe: cg14659129 (K−means Cluster #6)
DNAm (Beta)
0.2
0.4
0.6
0.8
Ages (years)
0
20
40
60
80
FDR (Age associated divergence) =0.0692
p(Scalp)=0.0841
p(Dura)=0.249
Scalp
Dura

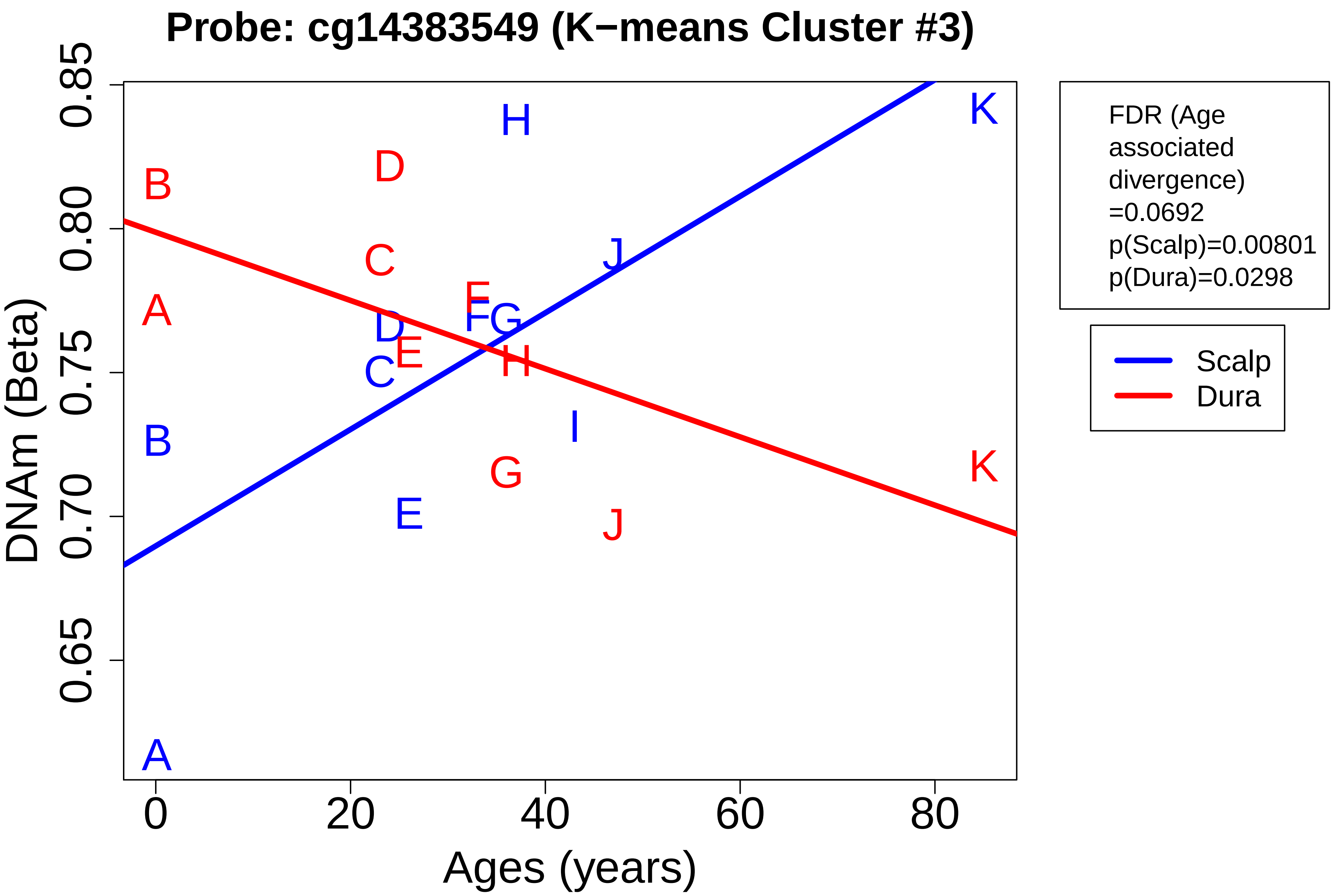

Probe: cg14383549 (K−means Cluster #3)
DNAm (Beta)
0.65
0.70
0.75
0.80
0.85
Ages (years)
0
20
40
60
80
A
B
C
D
E
F
G
H
I
J
K
FDR (Age associated divergence) =0.0692
p(Scalp)=0.00801
p(Dura)=0.0298
Scalp
Dura

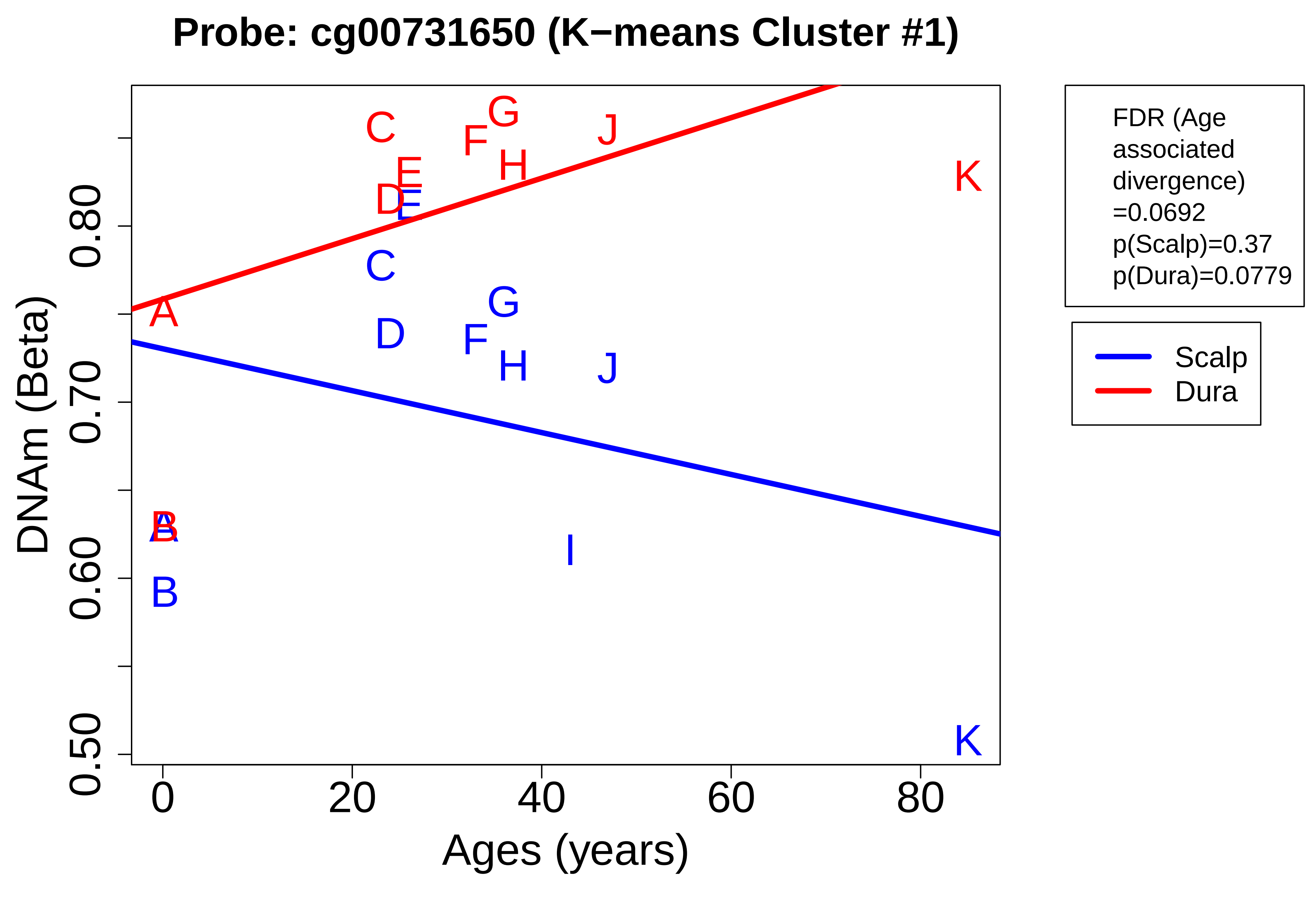

Probe: cg00731650 (K−means Cluster #1)
FDR (Age associated divergence) =0.0692
p(Scalp)=0.37
p(Dura)=0.0779
Scalp
Dura
DNAm (Beta)
0.50
0.60
0.70
0.80
0
20
40
60
80
Ages (years)
A
B
C
D
E
F
G
H
I
J
K

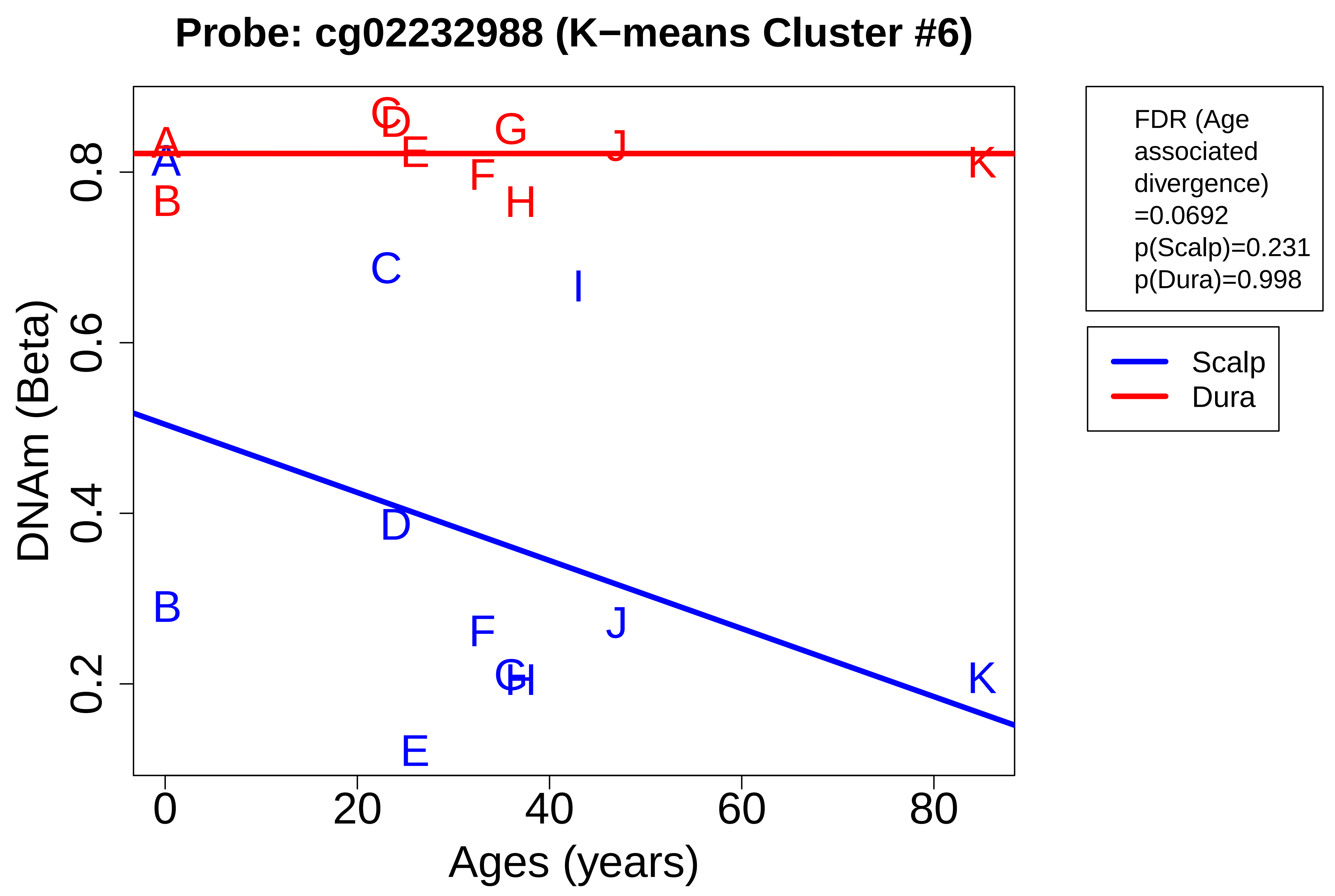

Probe: cg02232988 (K−means Cluster #6)
DNAm (Beta)
0.2
0.4
0.6
0.8
0
20
40
60
80
Ages (years)
A
B
C
D
E
F
G
H
I
J
K
FDR (Age associated divergence) =0.0692
p(Scalp)=0.231
p(Dura)=0.998
Scalp
Dura

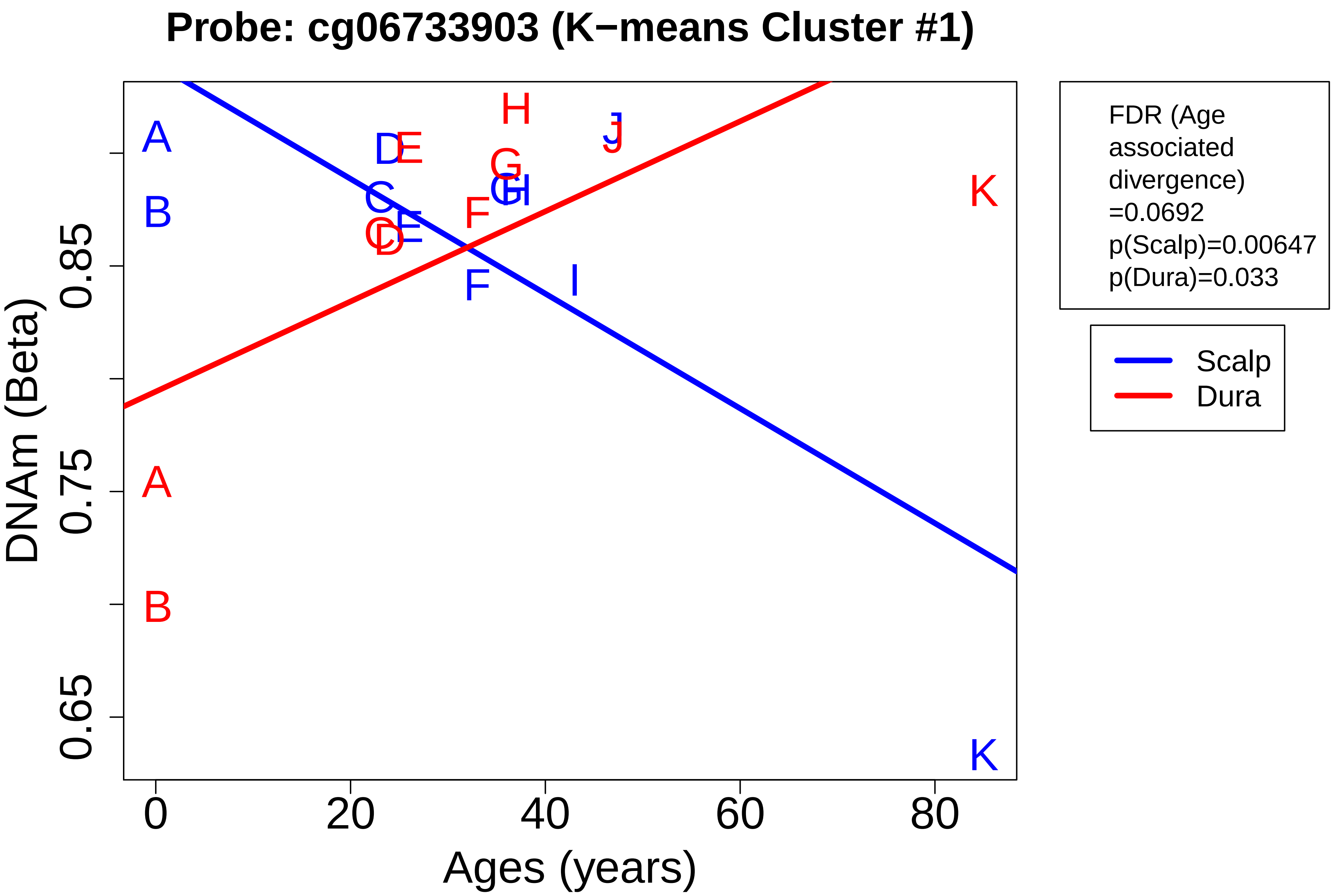

Probe: cg06733903 (K−means Cluster #1)
DNAm (Beta)
0.65
0.75
0.85
Ages (years)
0
20
40
60
80
FDR (Age associated divergence) =0.0692
p(Scalp)=0.00647
p(Dura)=0.033
Scalp
Dura
A
B
C
D
E
F
G
H
I
J
K

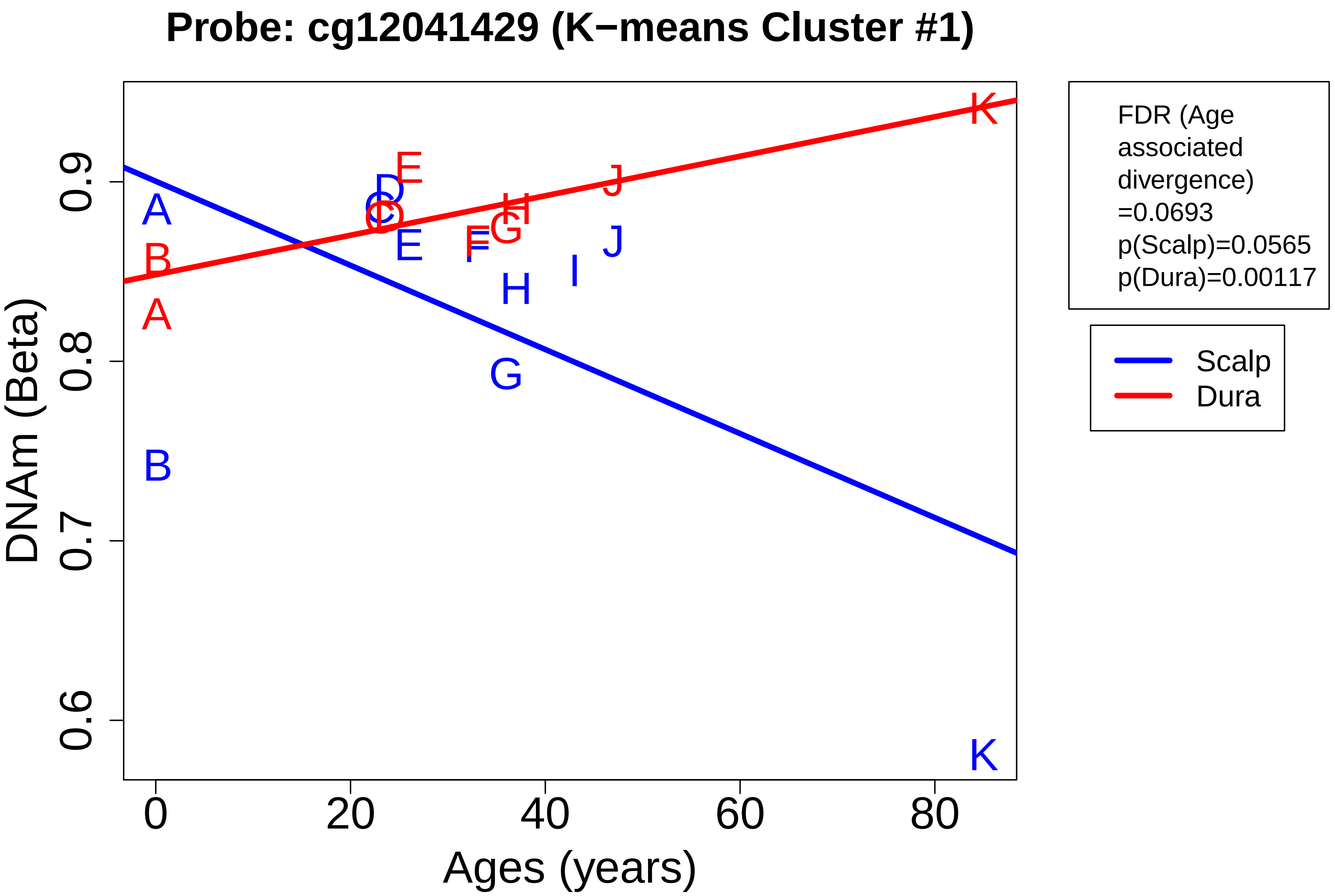

Probe: cg12041429 (K−means Cluster #1)
DNAm (Beta)
0.6
0.7
0.8
0.9
Ages (years)
0
20
40
60
80
A
B
C
D
E
F
G
H
I
J
K
FDR (Age associated divergence) =0.0693
p(Scalp)=0.0565
p(Dura)=0.00117
Scalp
Dura

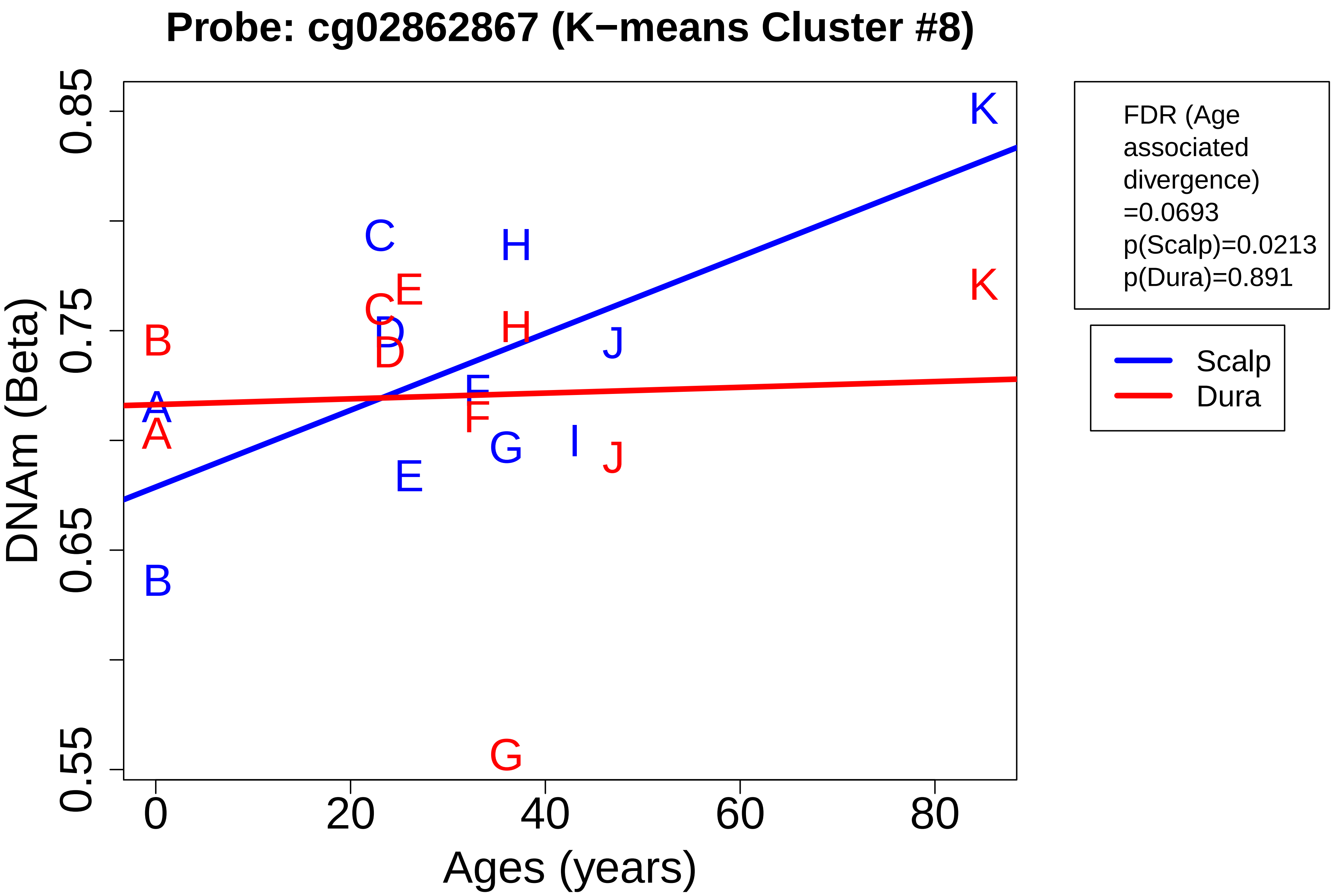
Probe: cg02862867 (K−means Cluster #8)
DNAm (Beta)
0.55
0.65
0.75
0.85
Ages (years)
0
20
40
60
80
FDR (Age associated divergence) =0.0693
p(Scalp)=0.0213
p(Dura)=0.891
Scalp
Dura

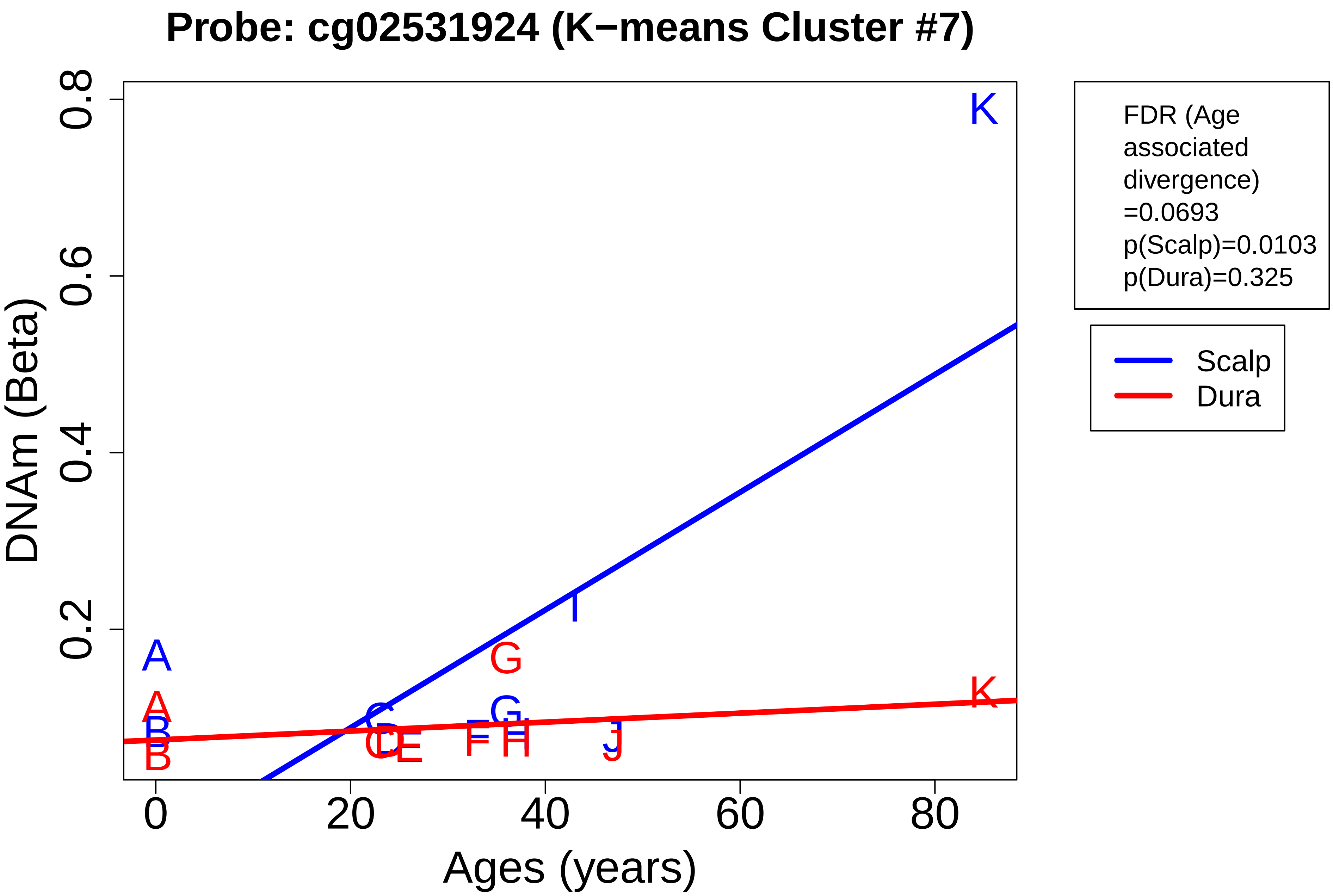

Probe: cg02531924 (K−means Cluster #7)
DNAm (Beta)
0.2
0.4
0.6
0.8
0
20
40
60
80
Ages (years)
FDR (Age associated divergence) =0.0693
p(Scalp)=0.0103
p(Dura)=0.325
Scalp
Dura

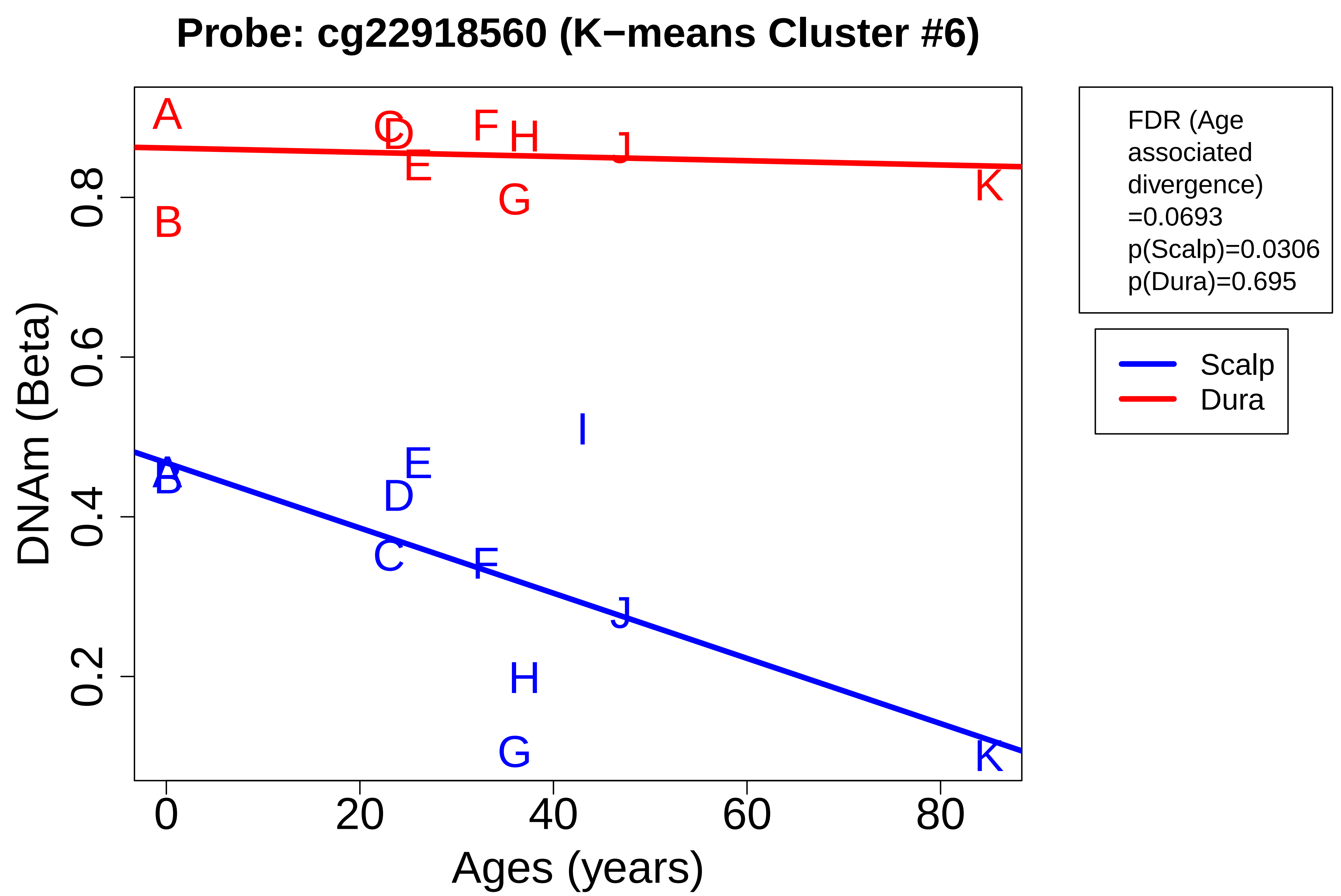

Probe: cg22918560 (K−means Cluster #6)
DNAm (Beta)
0.2
0.4
0.6
0.8
0
20
40
60
80
Ages (years)
FDR (Age associated divergence) =0.0693
p(Scalp)=0.0306
p(Dura)=0.695
Scalp
Dura
A
B
C
D
E
F
G
H
I
J
K

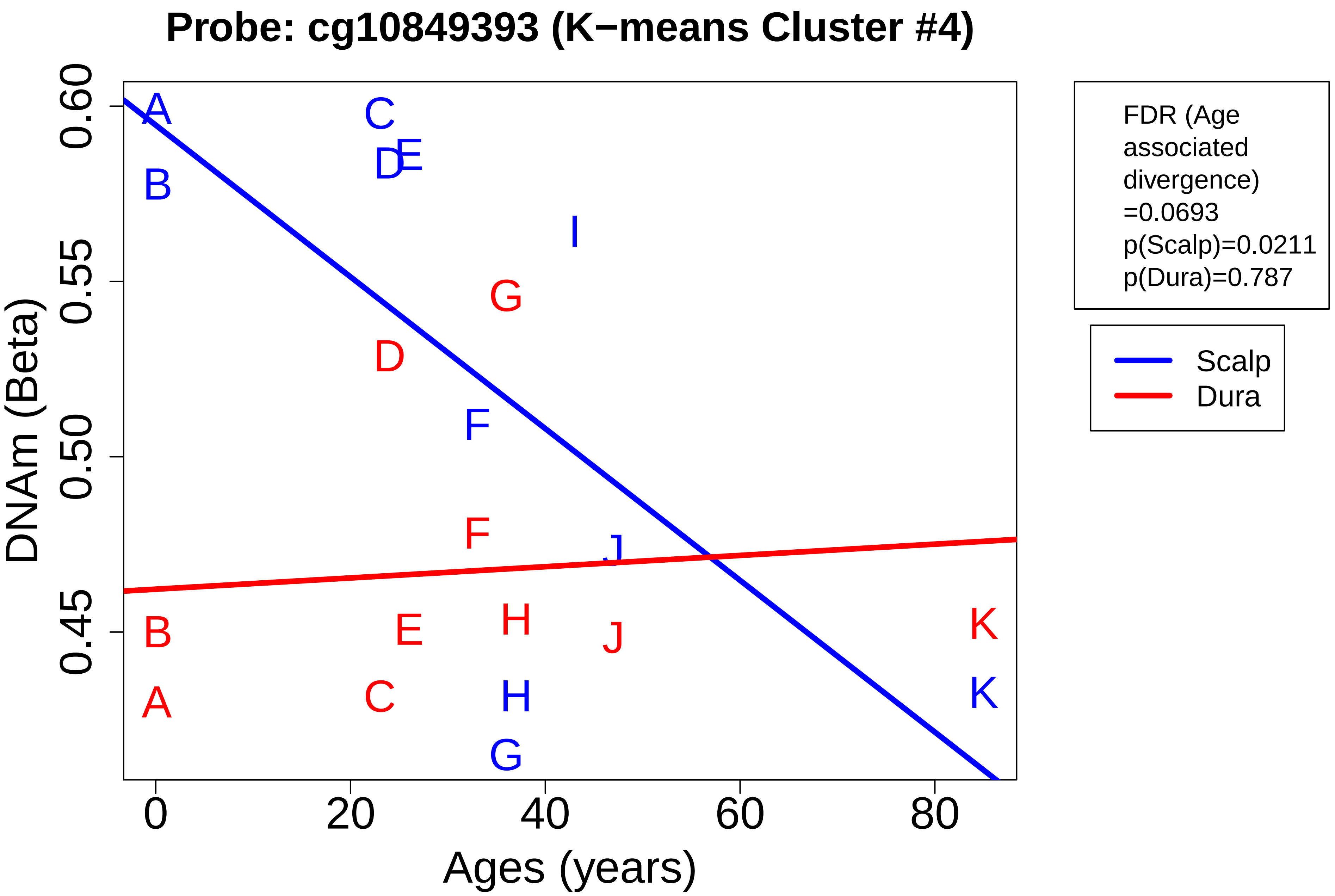
Probe: cg10849393 (K−means Cluster #4)
DNAm (Beta)
0.60
0.55
0.50
0.45
0
20
40
60
80
Ages (years)
A
B
C
D
E
F
G
H
I
J
K
FDR (Age associated divergence) =0.0693
p(Scalp)=0.0211
p(Dura)=0.787
Scalp
Dura

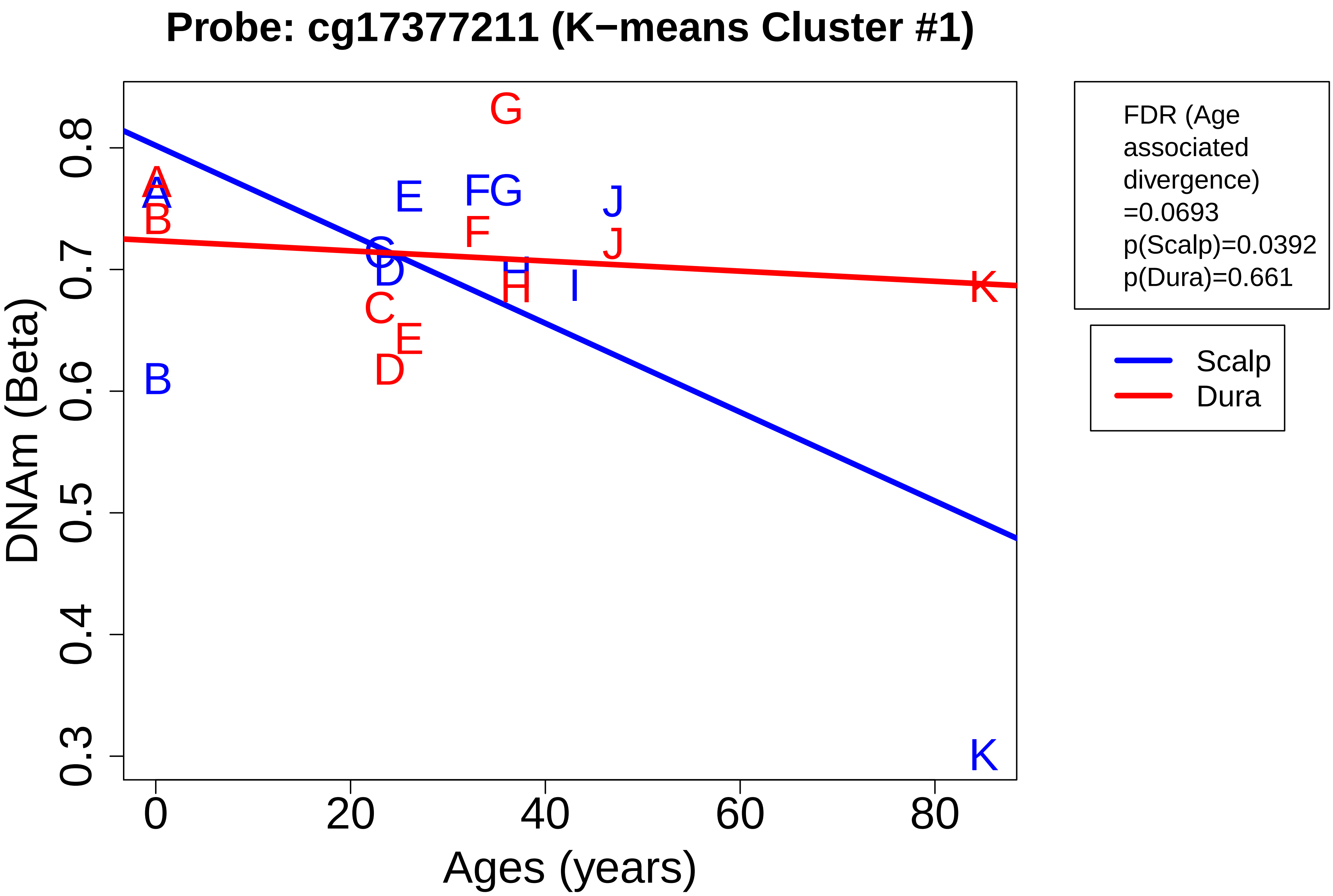

Probe: cg17377211 (K−means Cluster #1)
DNAm (Beta)
Ages (years)
0.3
0.4
0.5
0.6
0.7
0.8
0
20
40
60
80
FDR (Age associated divergence) =0.0693
p(Scalp)=0.0392
p(Dura)=0.661
Scalp
Dura

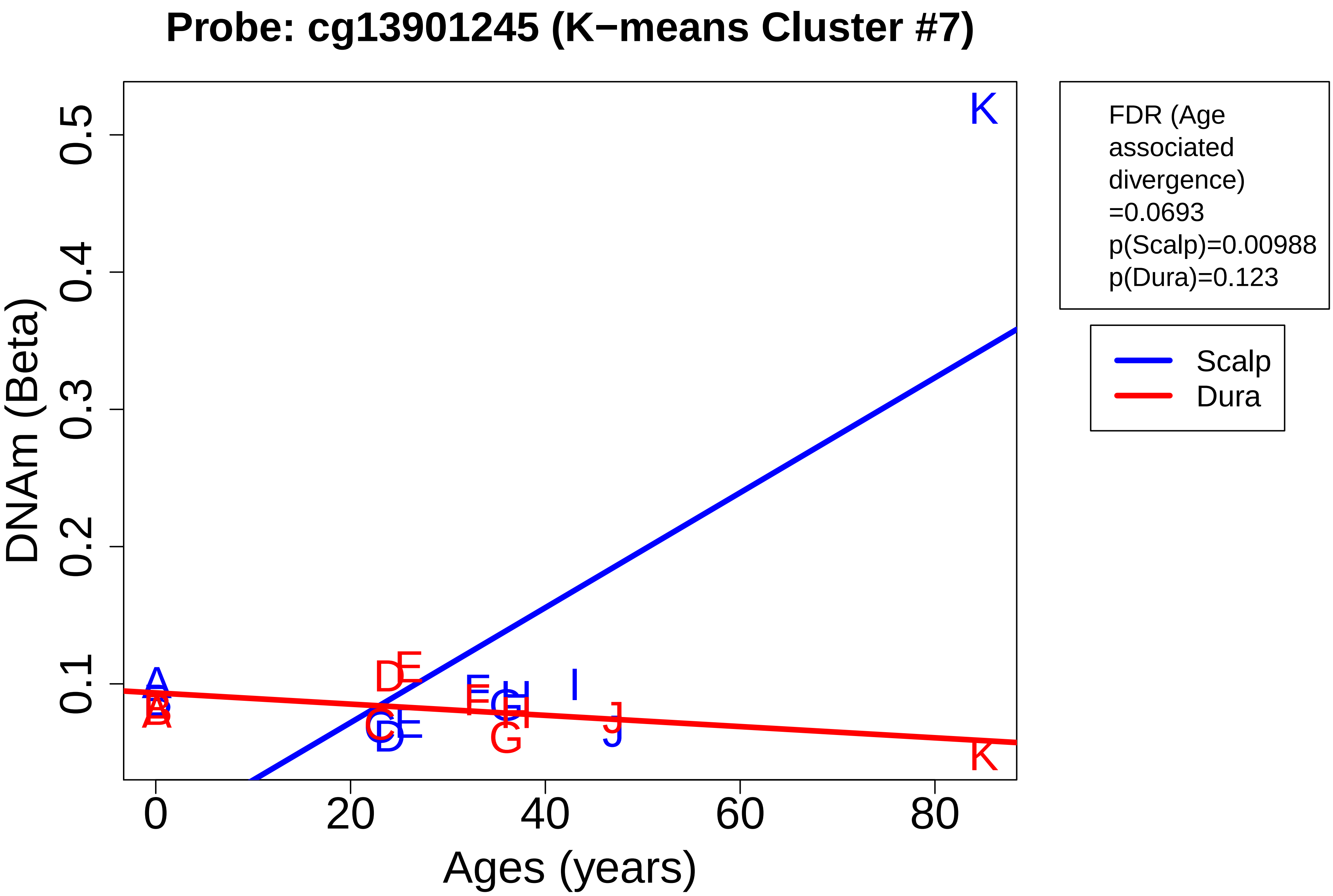

Probe: cg13901245 (K−means Cluster #7)
DNAm (Beta)
0.1
0.2
0.3
0.4
0.5
0
20
40
60
80
Ages (years)
FDR (Age associated divergence) =0.0693
p(Scalp)=0.00988
p(Dura)=0.123
Scalp
Dura

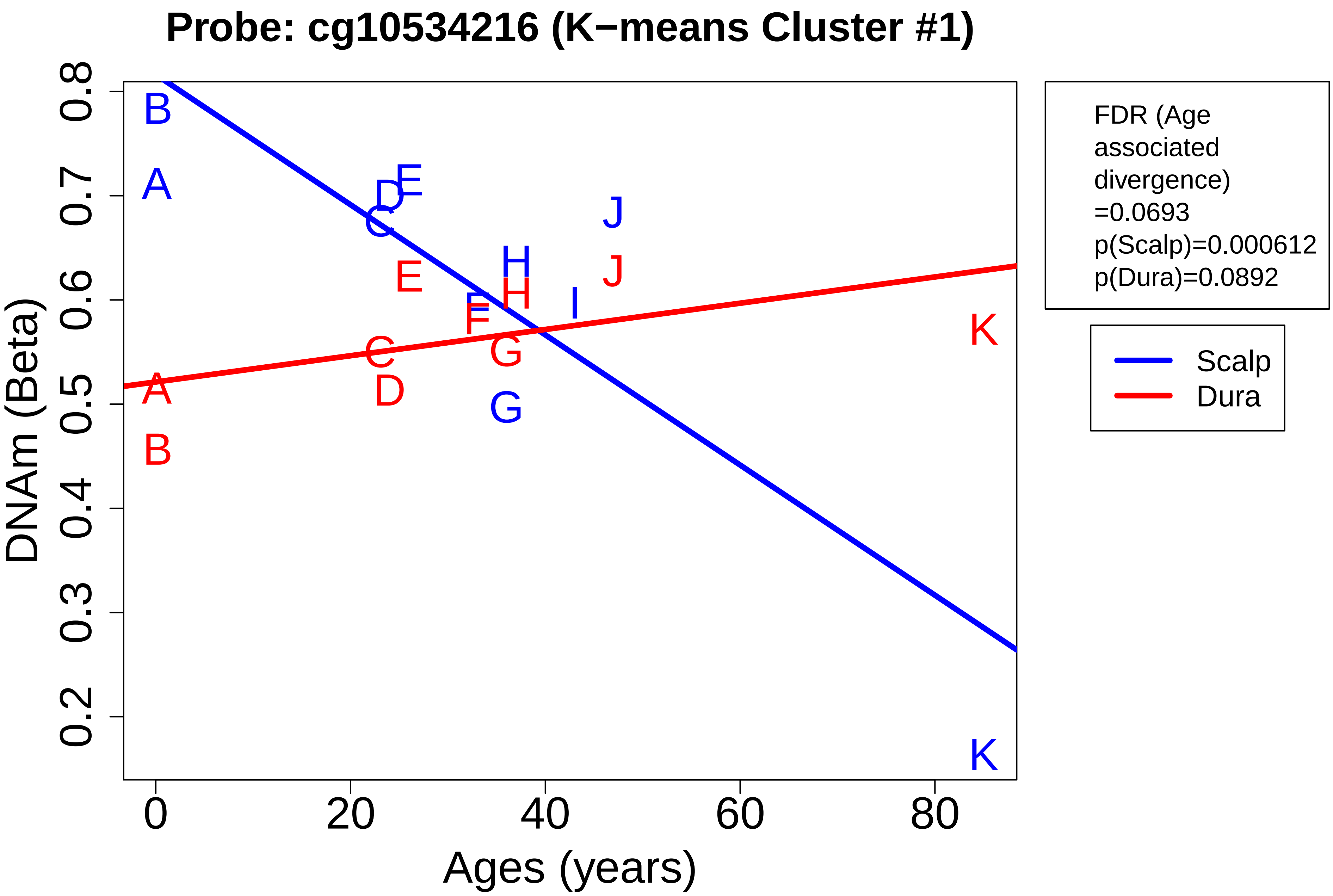

Probe: cg10534216 (K−means Cluster #1)
DNAm (Beta)
0.2
0.3
0.4
0.5
0.6
0.7
0.8
0
20
40
60
80
Ages (years)
FDR (Age associated divergence) =0.0693
p(Scalp)=0.000612
p(Dura)=0.0892
Scalp
Dura
A
B
C
D
E
F
G
H
I
J
K

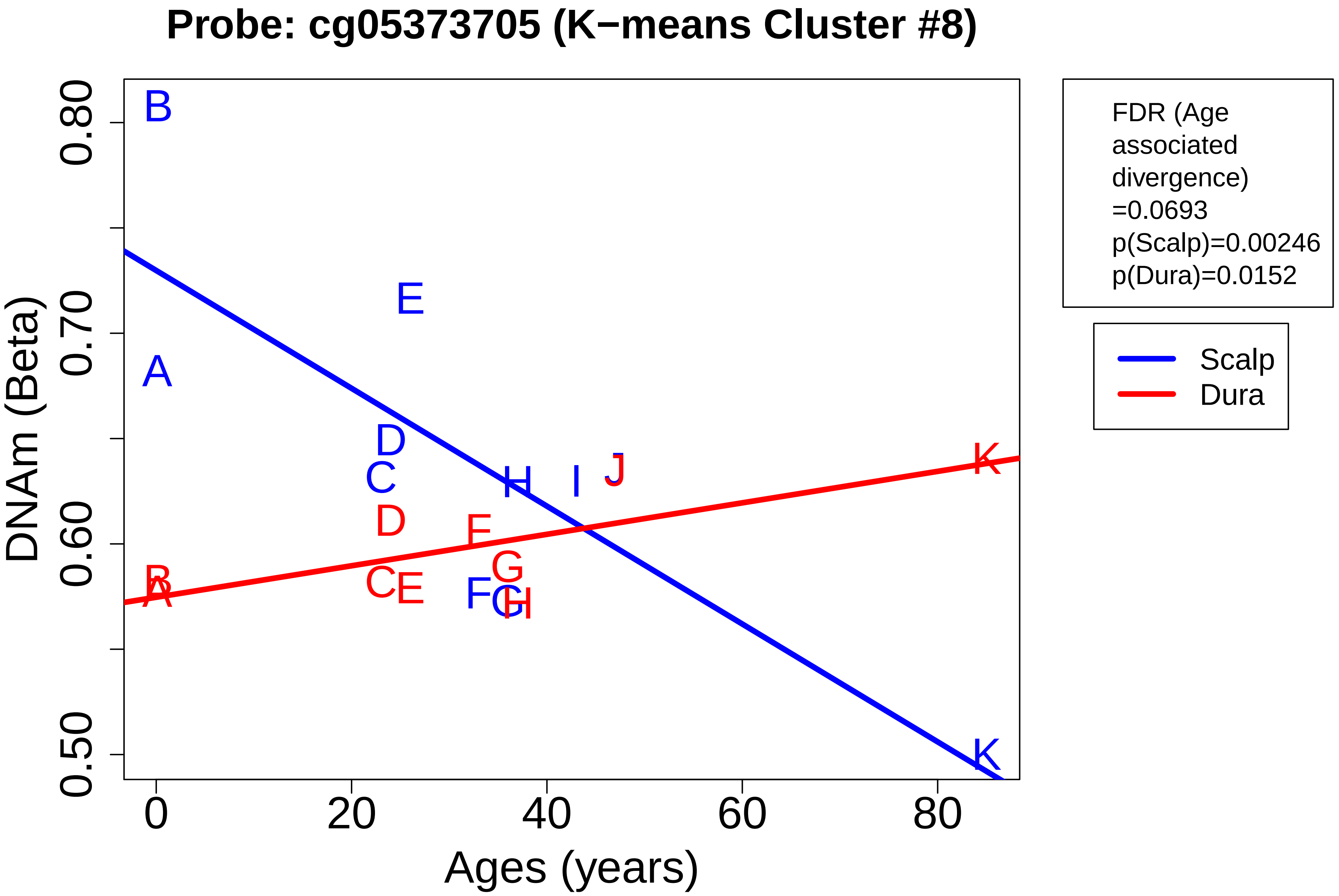

Probe: cg05373705 (K−means Cluster #8)
FDR (Age associated divergence) =0.0693
p(Scalp)=0.00246
p(Dura)=0.0152
Scalp
Dura
DNAm (Beta)
Ages (years)
0.50
0.60
0.70
0.80
0
20
40
60
80

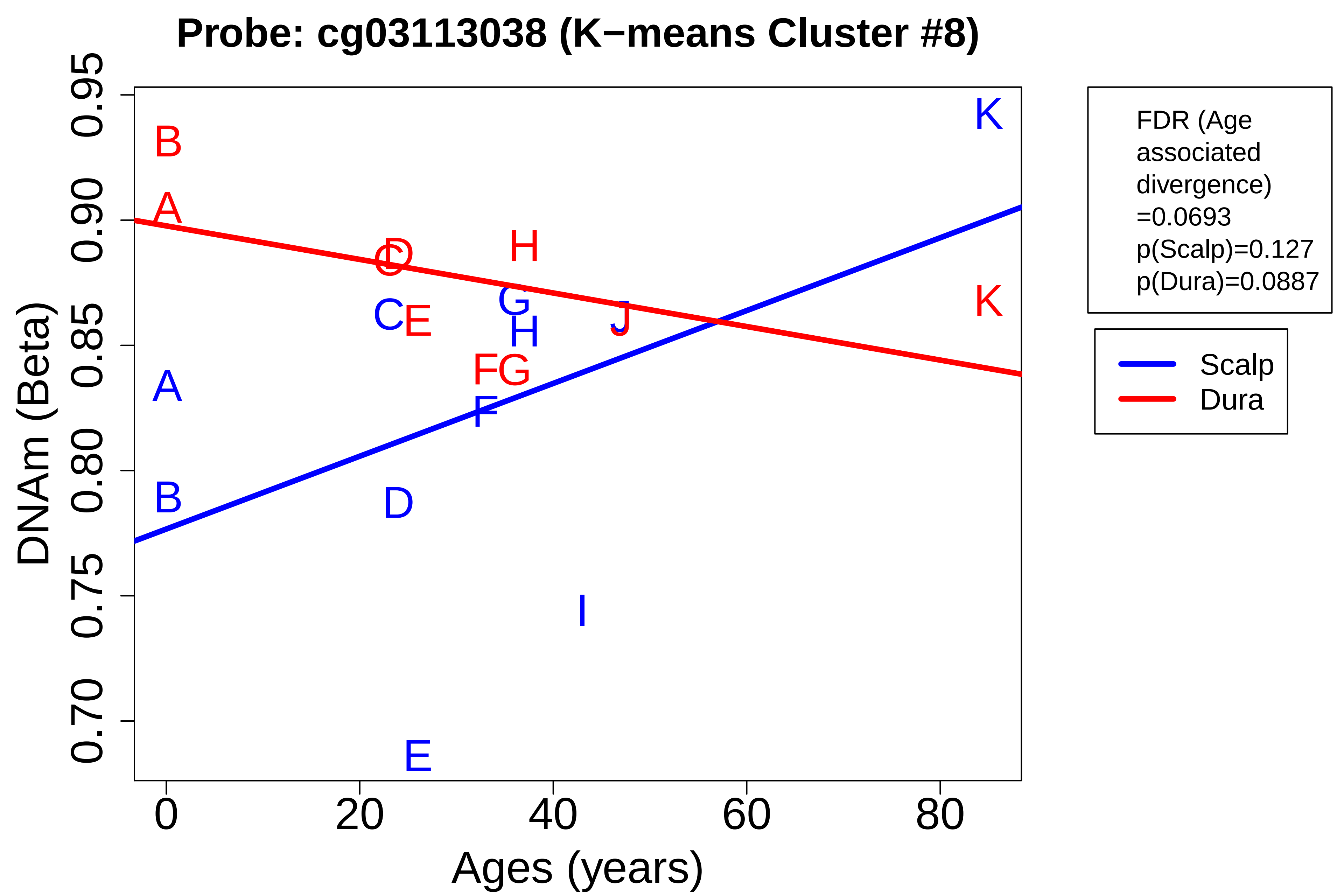

Probe: cg03113038 (K−means Cluster #8)
DNAm (Beta)
0.70
0.75
0.80
0.85
0.90
0.95
Ages (years)
0
20
40
60
80
FDR (Age associated divergence) =0.0693
p(Scalp)=0.127
p(Dura)=0.0887
Scalp
Dura

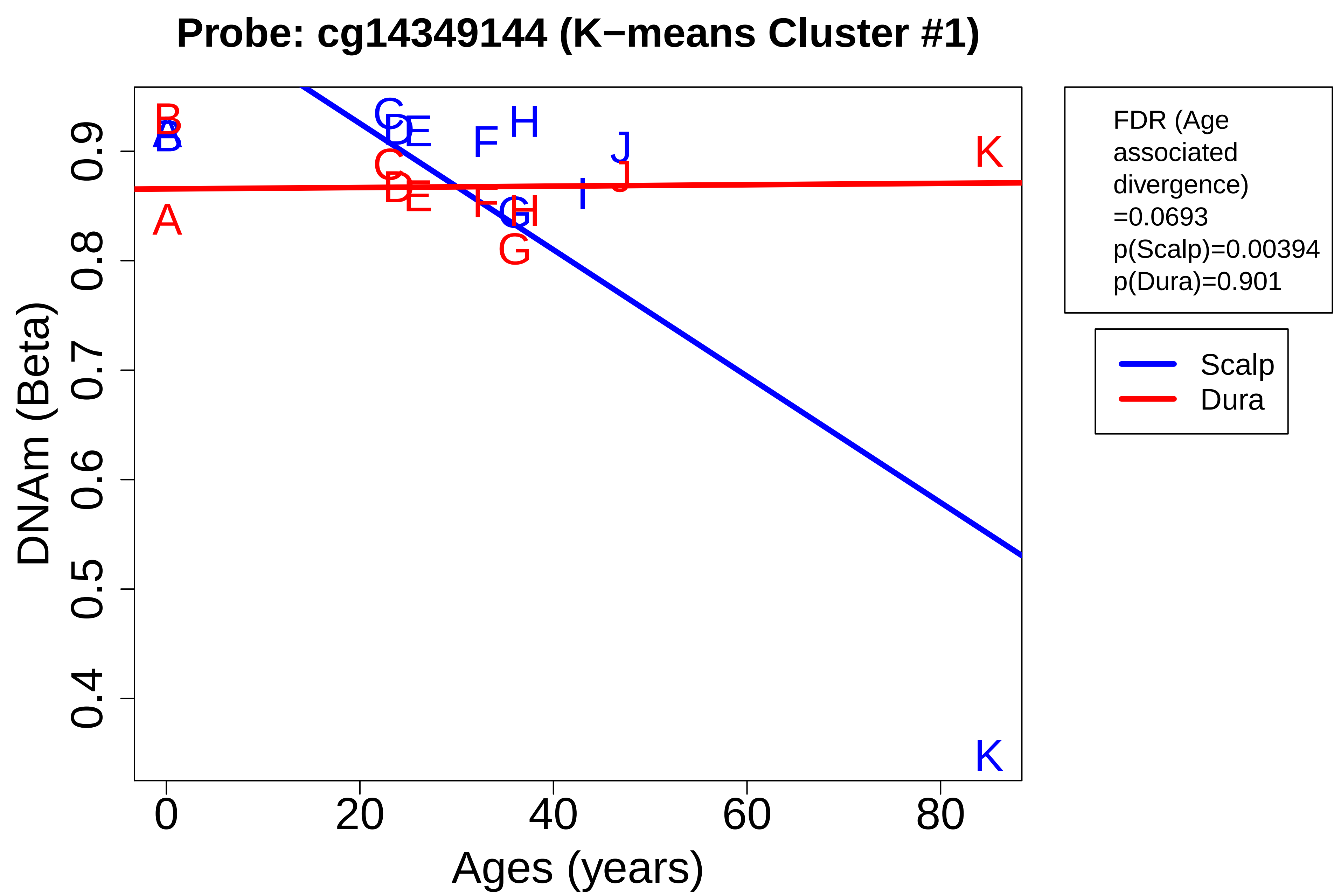

Probe: cg14349144 (K−means Cluster #1)
DNAm (Beta)
Ages (years)
0.4
0.5
0.6
0.7
0.8
0.9
0
20
40
60
80
FDR (Age associated divergence) =0.0693
p(Scalp)=0.00394
p(Dura)=0.901
Scalp
Dura

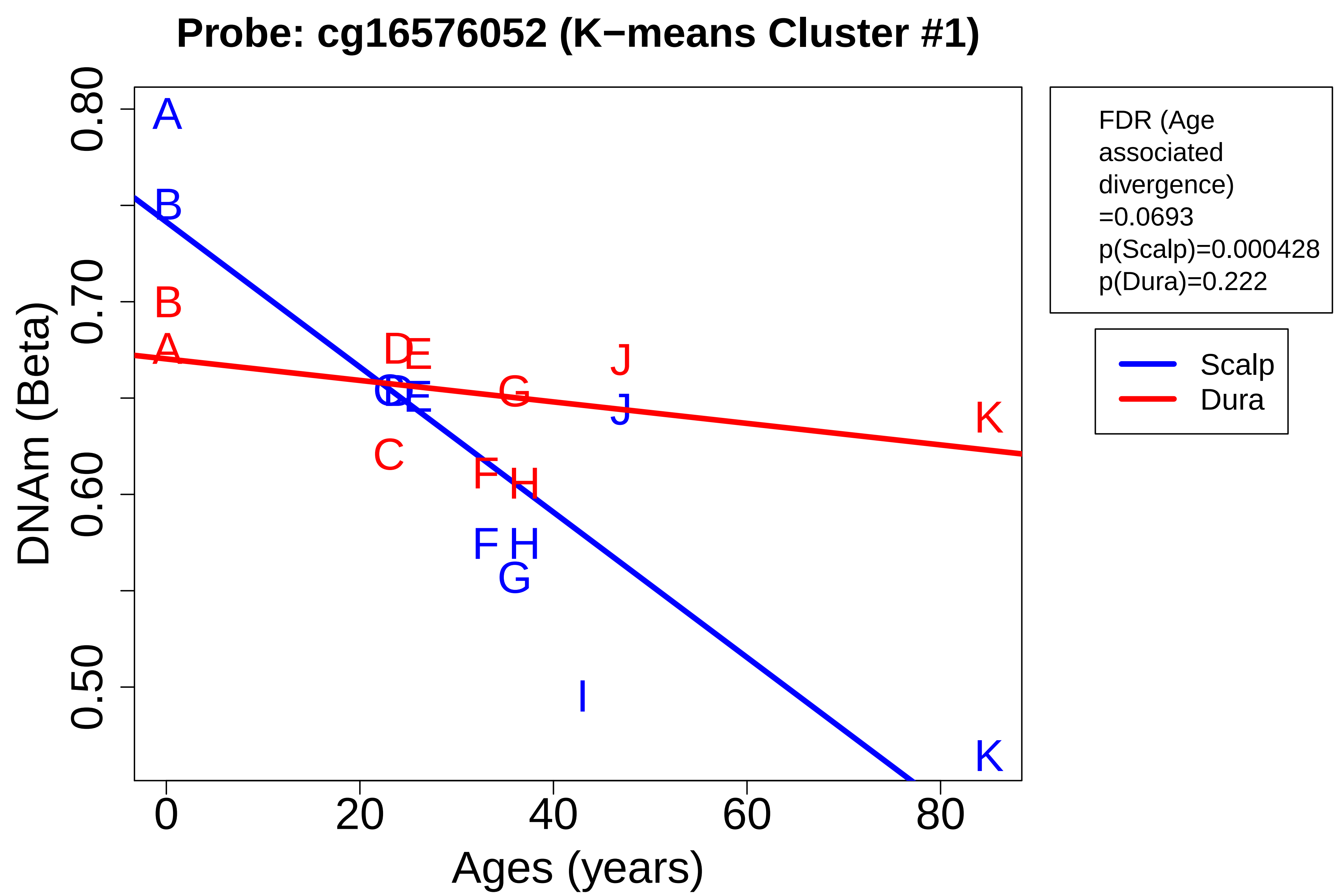

Probe: cg16576052 (K−means Cluster #1)
DNAm (Beta)
0.50
0.60
0.70
0.80
Ages (years)
0
20
40
60
80
A
B
C
D
E
F
G
H
I
J
K
FDR (Age associated divergence) =0.0693
p(Scalp)=0.000428
p(Dura)=0.222
Scalp
Dura

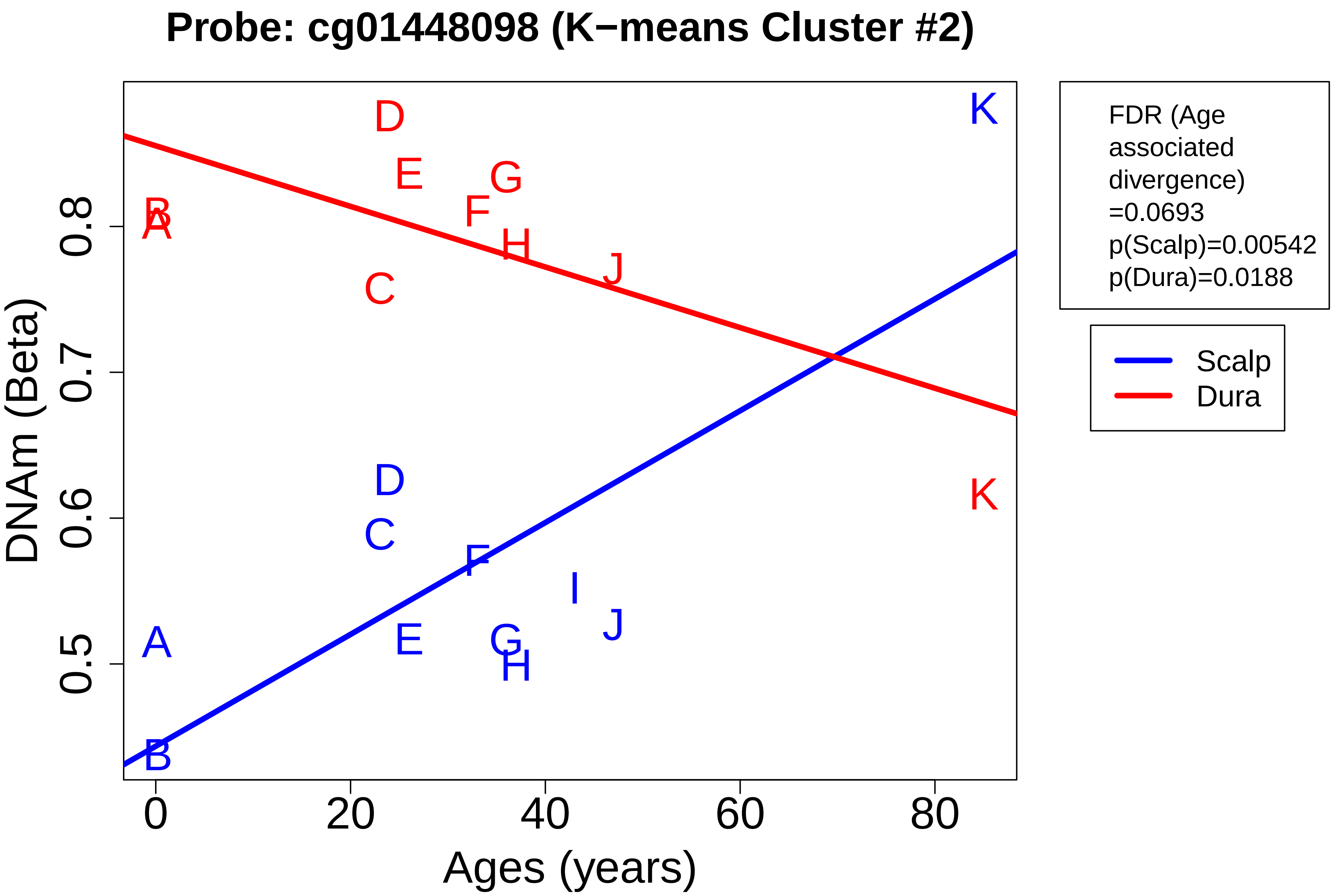

Probe: cg01448098 (K−means Cluster #2)
DNAm (Beta)
0.5
0.6
0.7
0.8
0
20
40
60
80
Ages (years)
FDR (Age associated divergence) =0.0693
p(Scalp)=0.00542
p(Dura)=0.0188
Scalp
Dura

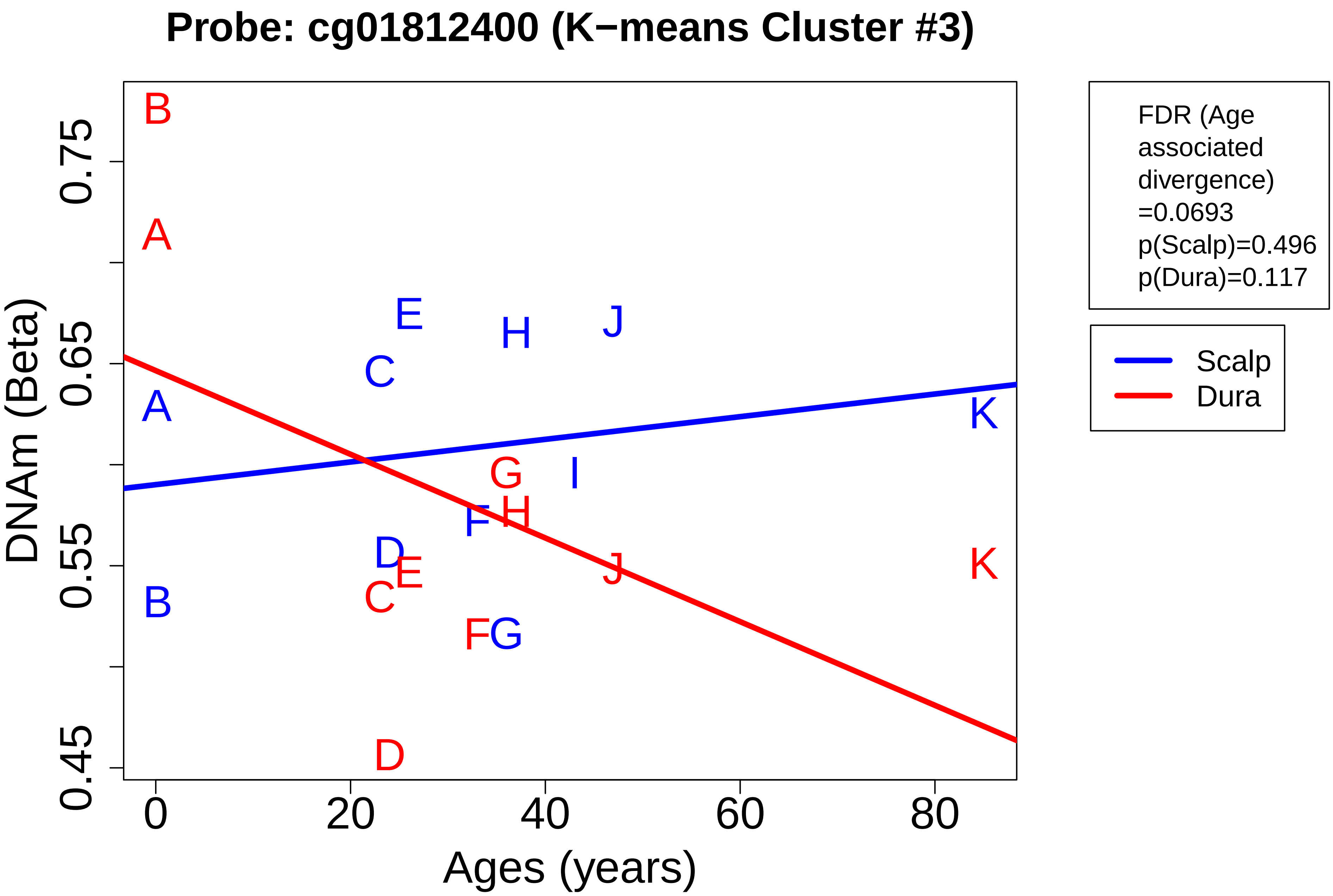
Probe: cg01812400 (K−means Cluster #3)
DNAm (Beta)
0.45
0.55
0.65
0.75
0
20
40
60
80
Ages (years)
FDR (Age associated divergence) =0.0693
p(Scalp)=0.496
p(Dura)=0.117
Scalp
Dura
A
B
C
D
E
F
G
H
I
J
K

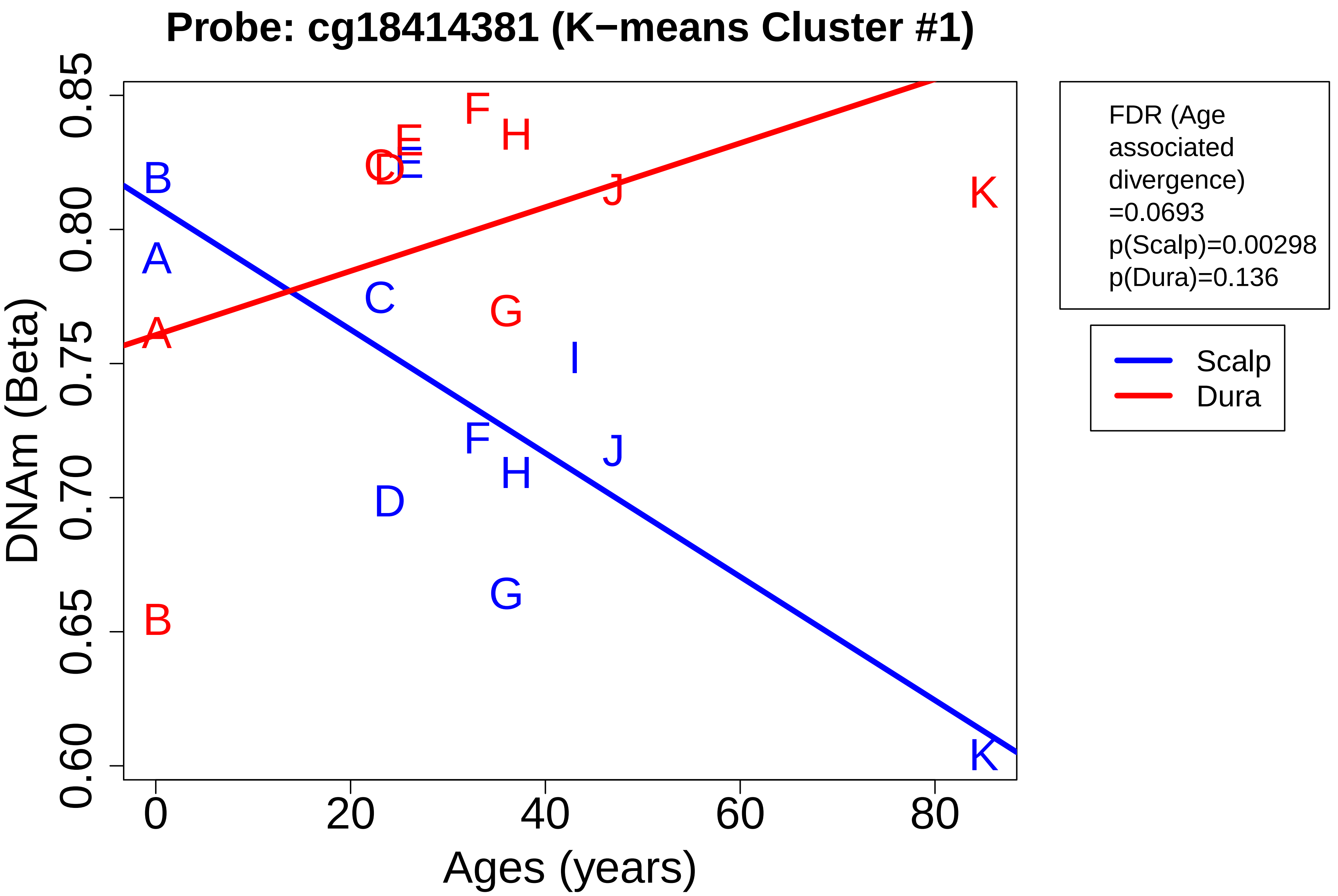

Probe: cg18414381 (K−means Cluster #1)
DNAm (Beta)
0.60
0.65
0.70
0.75
0.80
0.85
0
20
40
60
80
Ages (years)
FDR (Age associated divergence) =0.0693
p(Scalp)=0.00298
p(Dura)=0.136
Scalp
Dura

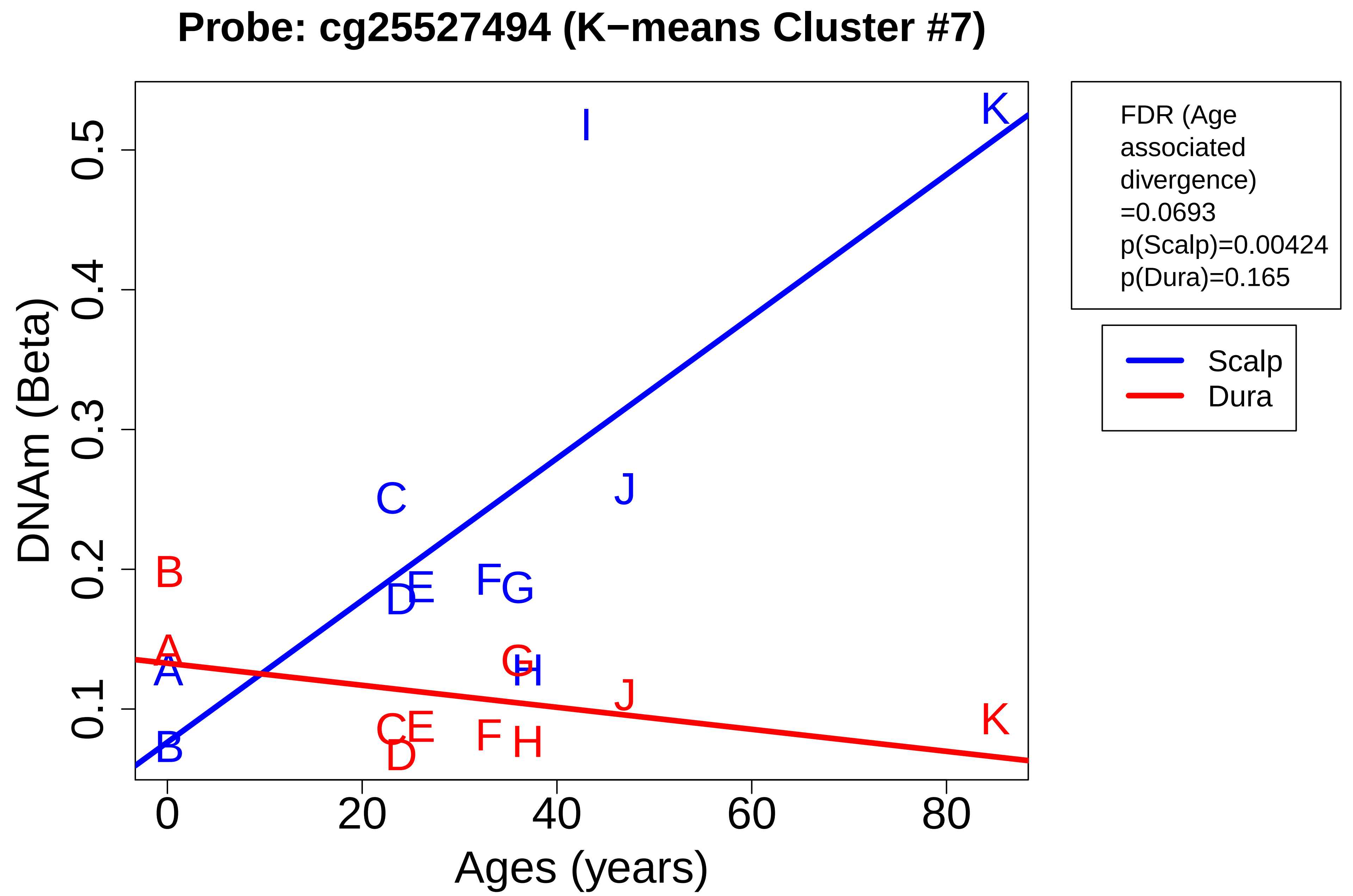

Probe: cg25527494 (K−means Cluster #7)
DNAm (Beta)
0.1
0.2
0.3
0.4
0.5
Ages (years)
0
20
40
60
80
FDR (Age associated divergence) =0.0693
p(Scalp)=0.00424
p(Dura)=0.165
Scalp
Dura

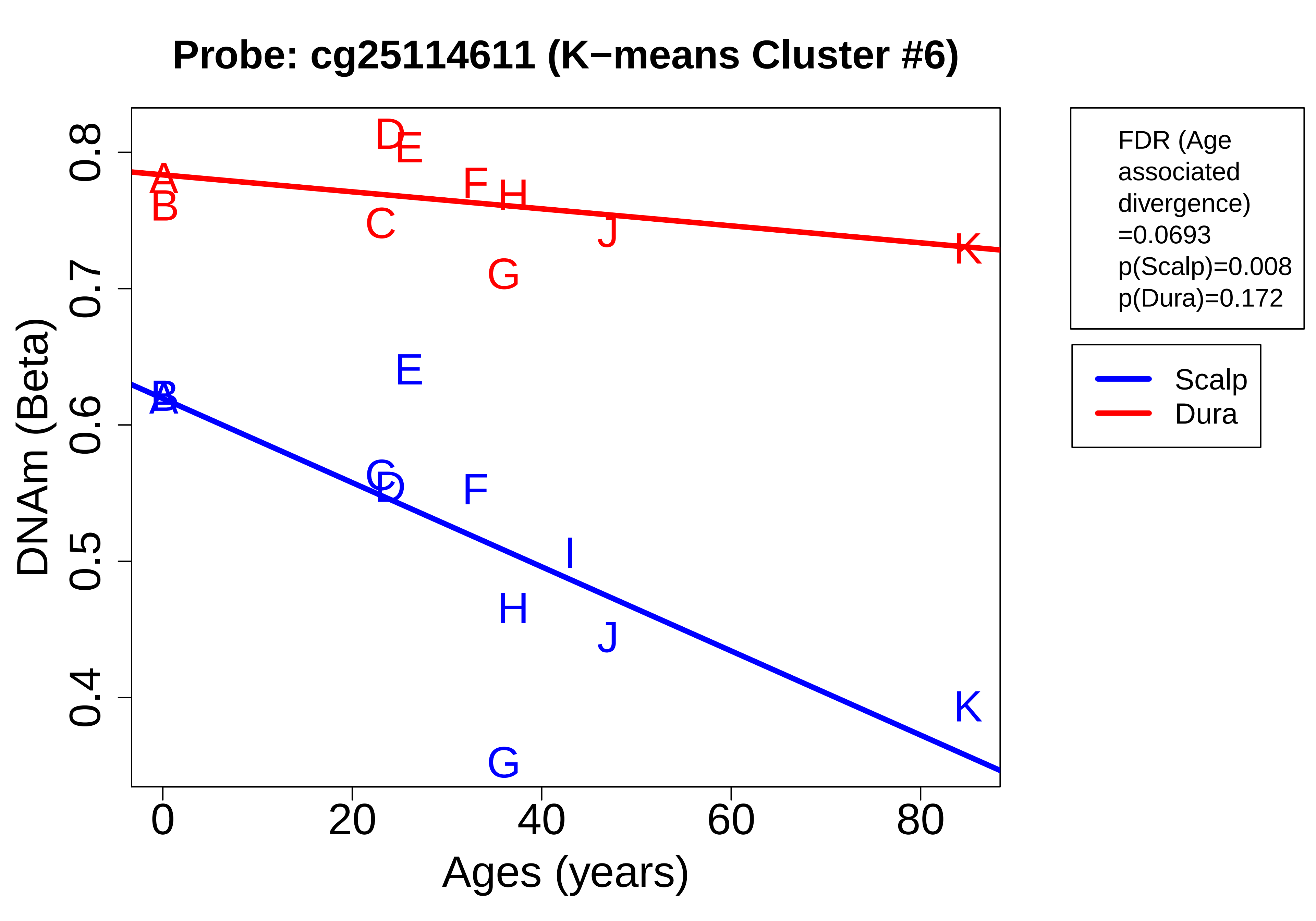
Probe: cg25114611 (K−means Cluster #6)
DNAm (Beta)
0.4
0.5
0.6
0.7
0.8
0
20
40
60
80
Ages (years)
FDR (Age associated divergence) =0.0693
p(Scalp)=0.008
p(Dura)=0.172
Scalp
Dura
A
B
C
D
E
F
G
H
I
J
K

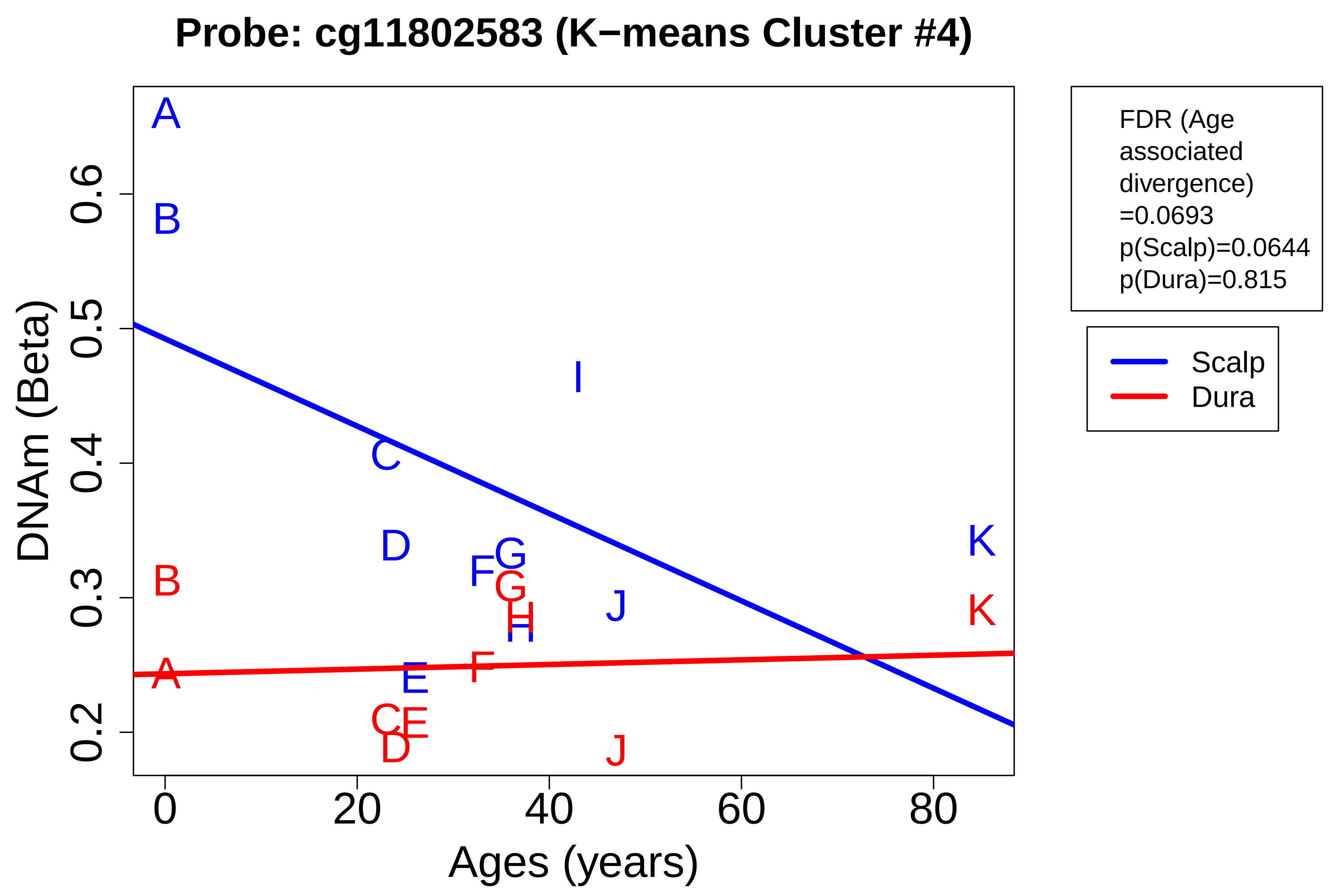

Probe: cg11802583 (K−means Cluster #4)
DNAm (Beta)
0.2
0.3
0.4
0.5
0.6
Ages (years)
0
20
40
60
80
FDR (Age associated divergence) =0.0693
p(Scalp)=0.0644
p(Dura)=0.815
Scalp
Dura
A
B
C
D
E
F
G
H
I
J
K

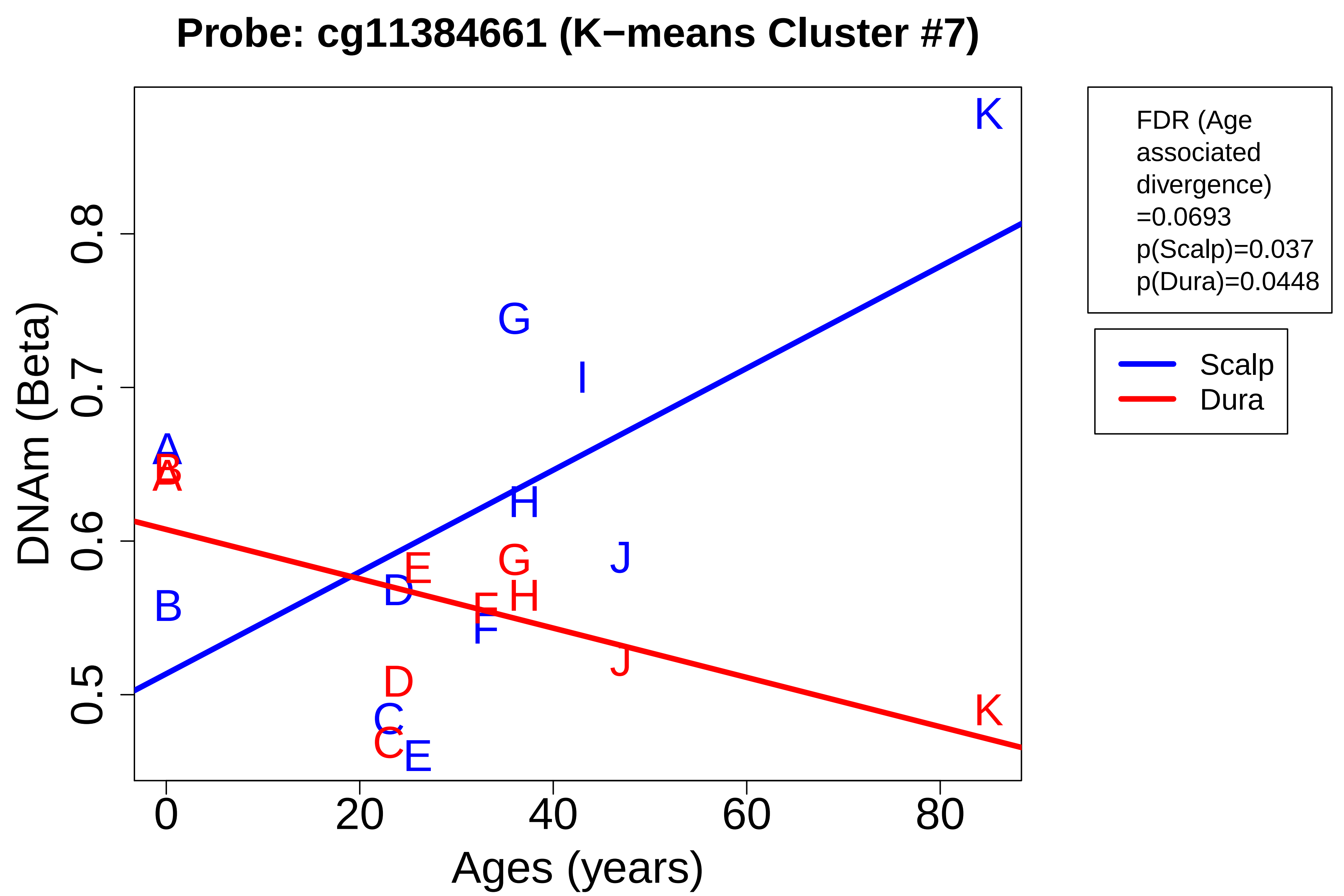

Probe: cg11384661 (K−means Cluster #7)
DNAm (Beta)
0.5
0.6
0.7
0.8
0
20
40
60
80
Ages (years)
FDR (Age associated divergence) =0.0693
p(Scalp)=0.037
p(Dura)=0.0448
Scalp
Dura

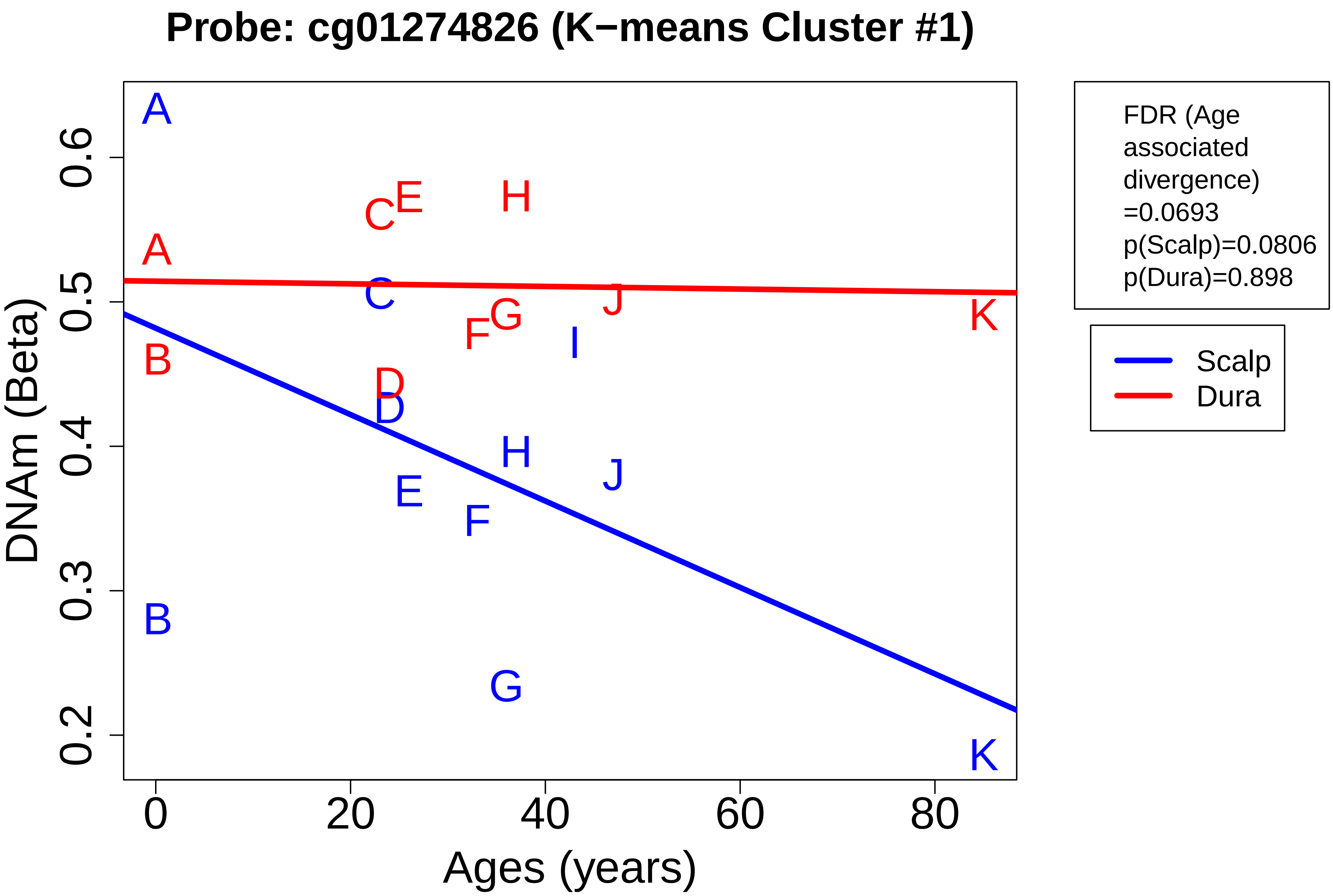
Probe: cg01274826 (K−means Cluster #1)
DNAm (Beta)
0.2
0.3
0.4
0.5
0.6
Ages (years)
0
20
40
60
80
A
B
C
D
E
F
G
H
I
J
K
A
B
C
D
E
F
G
H
J
K
FDR (Age associated divergence) =0.0693
p(Scalp)=0.0806
p(Dura)=0.898
Scalp
Dura

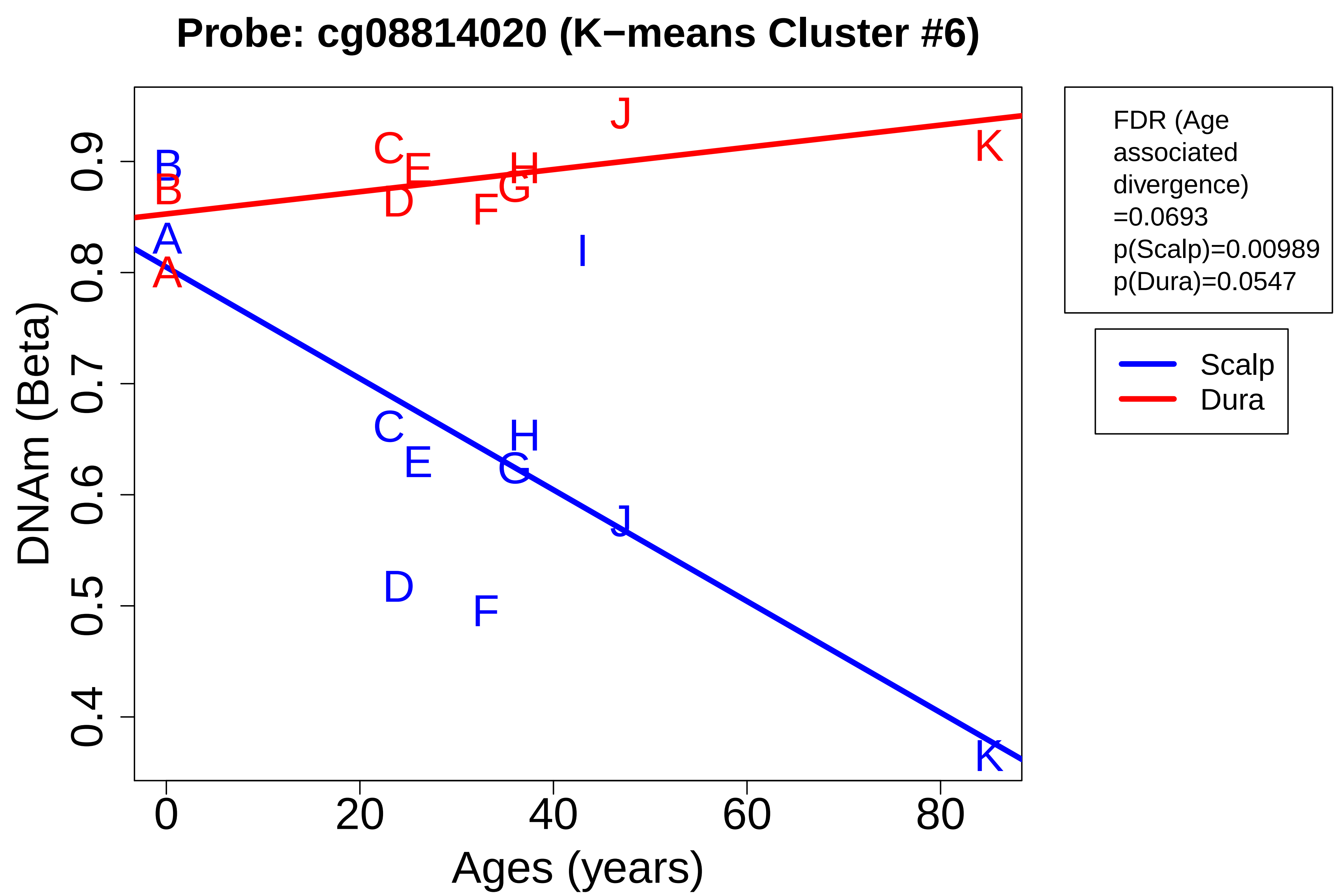

Probe: cg08814020 (K−means Cluster #6)
DNAm (Beta)
0.4
0.5
0.6
0.7
0.8
0.9
0
20
40
60
80
Ages (years)
A
B
C
D
E
F
G
H
I
J
K
FDR (Age associated divergence) =0.0693
p(Scalp)=0.00989
p(Dura)=0.0547
Scalp
Dura

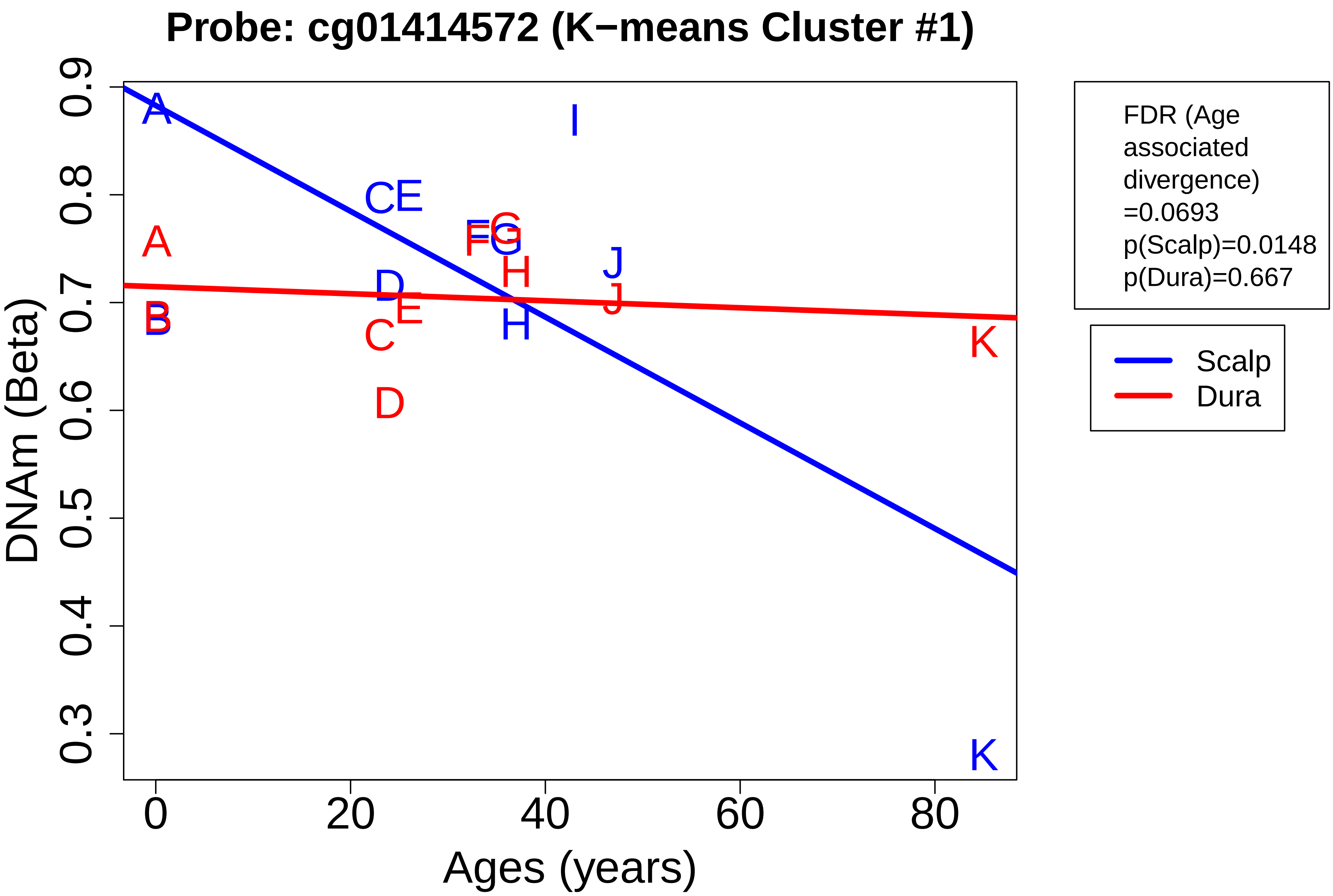

Probe: cg01414572 (K−means Cluster #1)
DNAm (Beta)
0.3
0.4
0.5
0.6
0.7
0.8
0.9
0
20
40
60
80
Ages (years)
FDR (Age associated divergence) =0.0693
p(Scalp)=0.0148
p(Dura)=0.667
Scalp
Dura

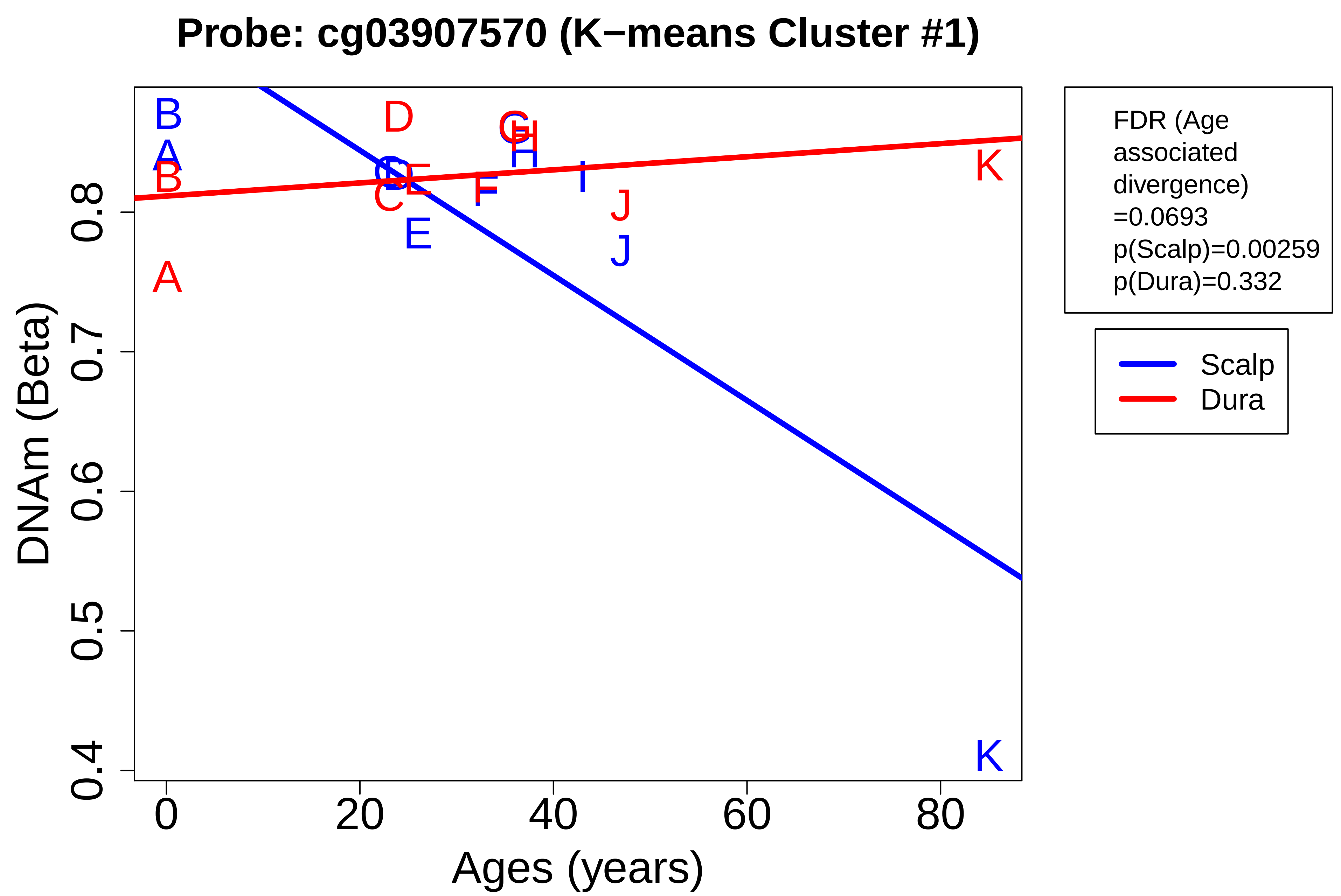

Probe: cg03907570 (K−means Cluster #1)
FDR (Age associated divergence) =0.0693
p(Scalp)=0.00259
p(Dura)=0.332
Scalp
Dura
DNAm (Beta)
Ages (years)
0.4
0.5
0.6
0.7
0.8
0
20
40
60
80

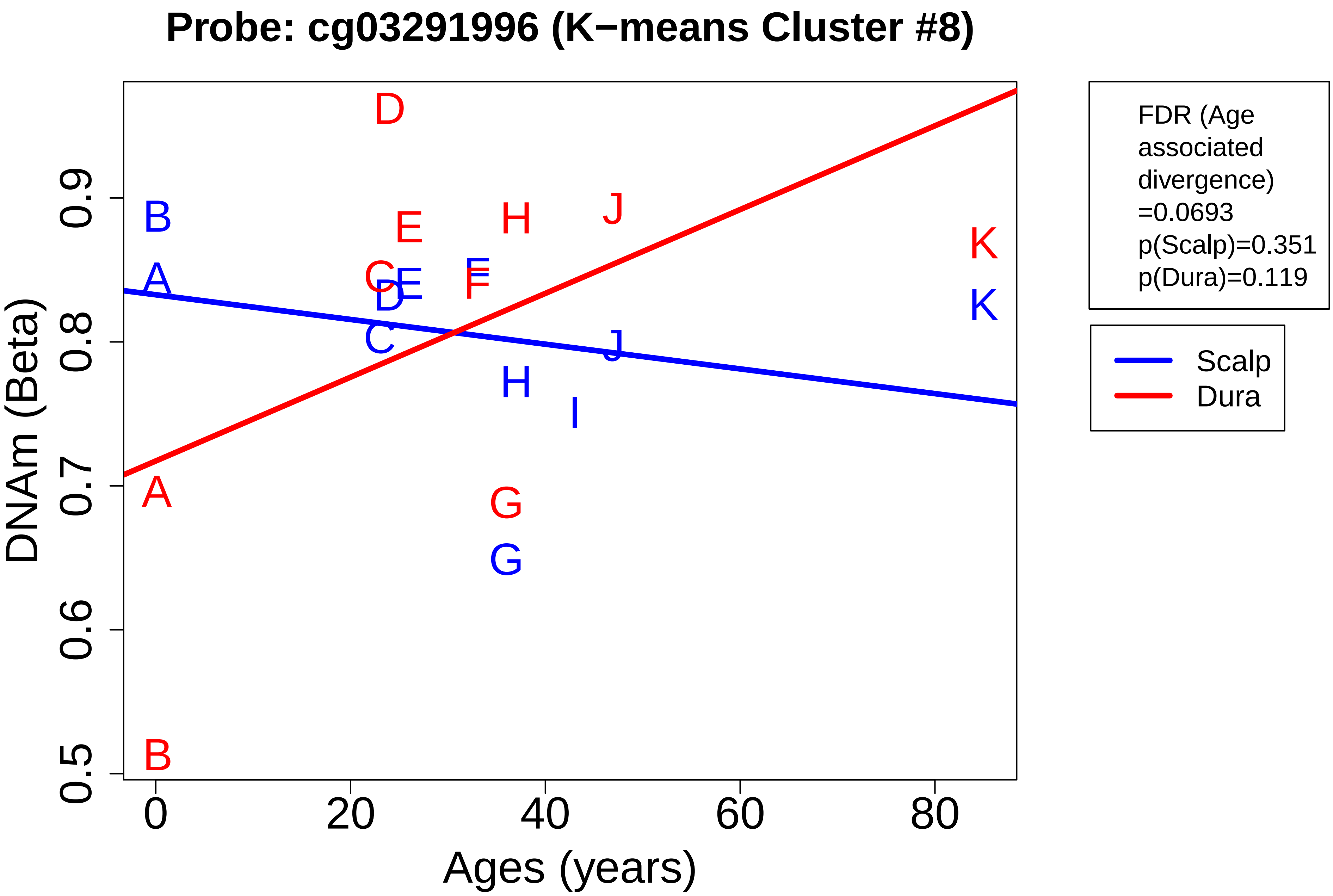

Probe: cg03291996 (K−means Cluster #8)
DNAm (Beta)
0.5
0.6
0.7
0.8
0.9
0
20
40
60
80
Ages (years)
A
B
C
D
E
F
G
H
I
J
K
FDR (Age associated divergence) =0.0693
p(Scalp)=0.351
p(Dura)=0.119
Scalp
Dura

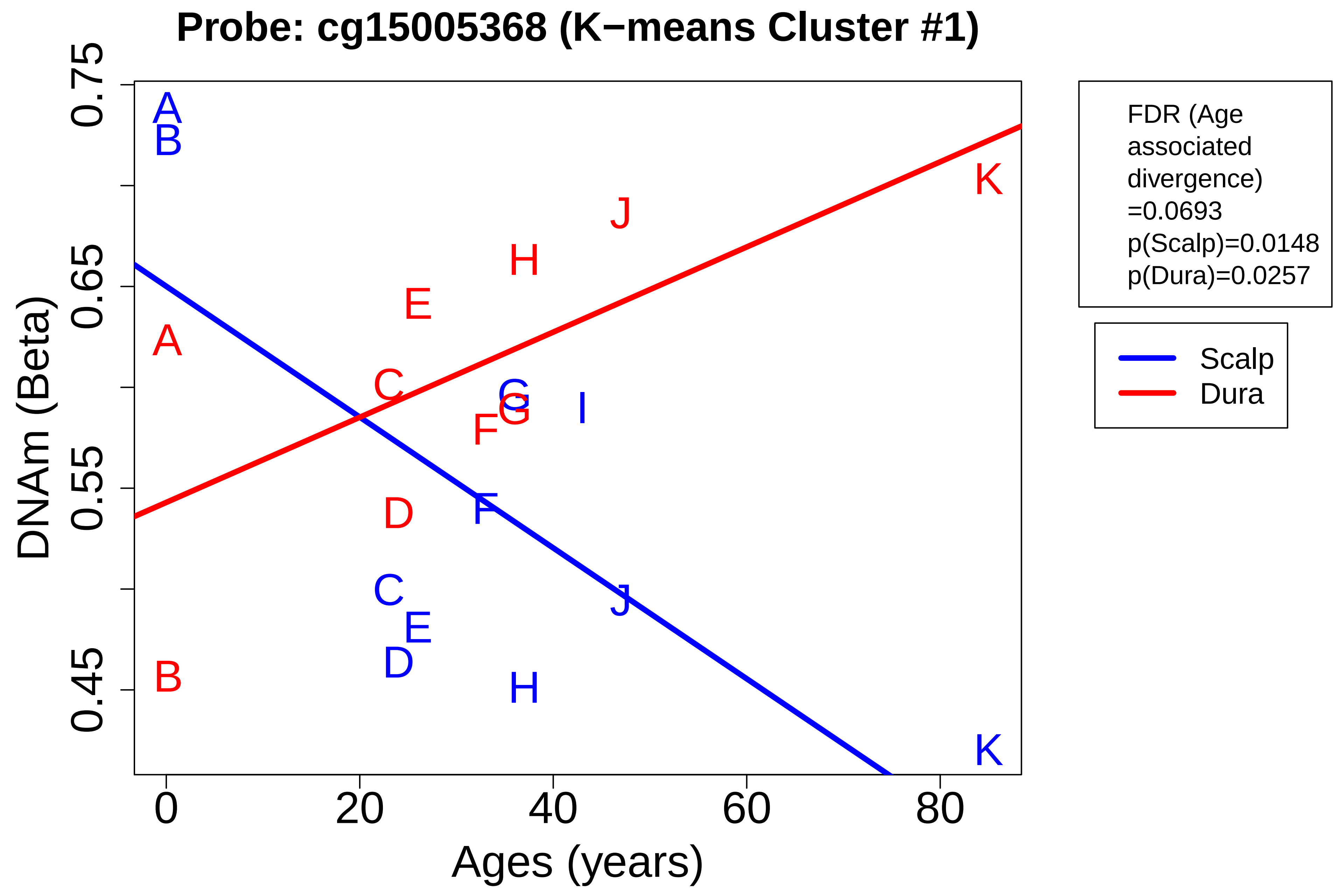

Probe: cg15005368 (K−means Cluster #1)
DNAm (Beta)
0.45
0.55
0.65
0.75
Ages (years)
0
20
40
60
80
A
B
C
D
E
F
G
H
I
J
K
FDR (Age associated divergence) =0.0693
p(Scalp)=0.0148
p(Dura)=0.0257
Scalp
Dura

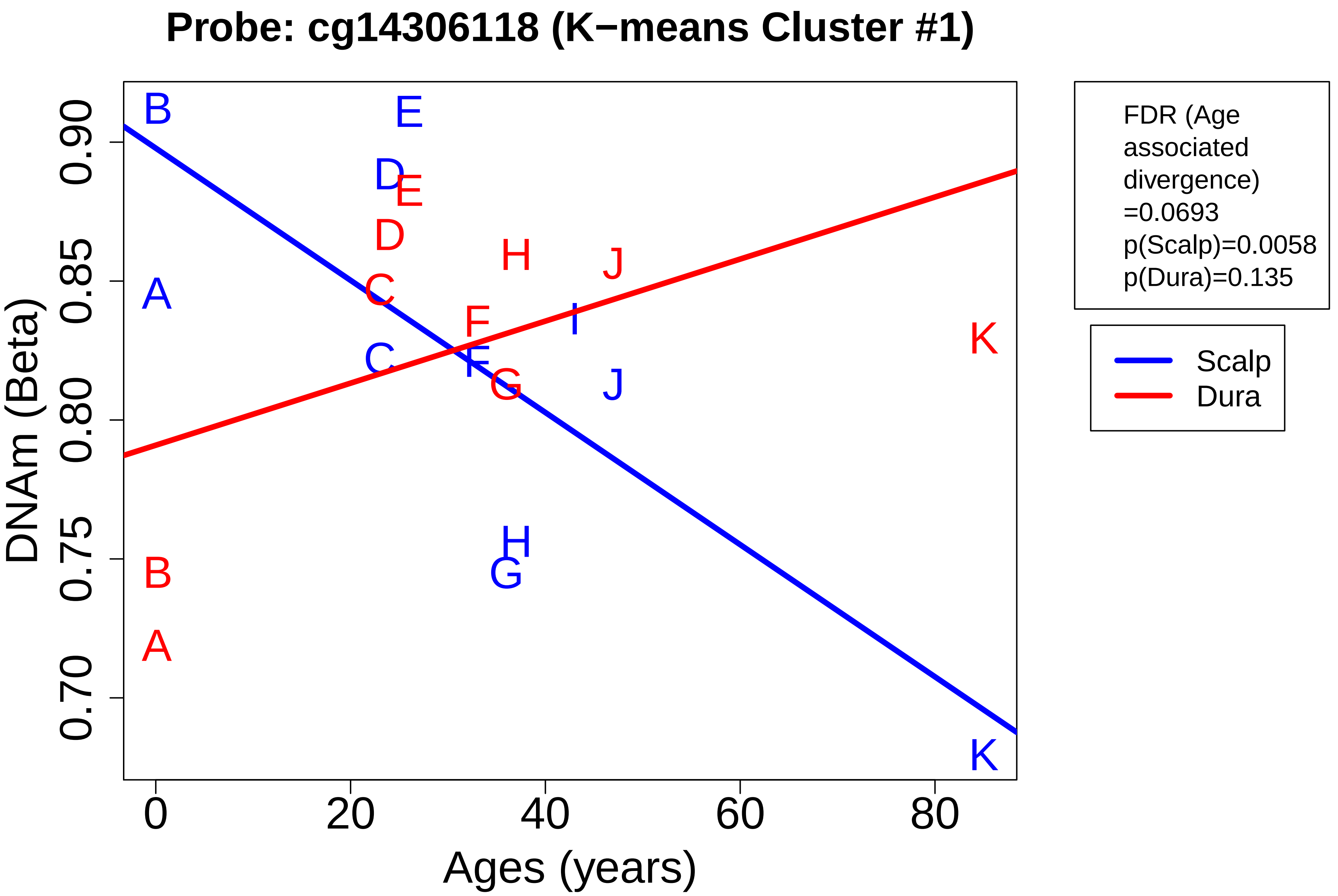
Probe: cg14306118 (K−means Cluster #1)
DNAm (Beta)
0.70
0.75
0.80
0.85
0.90
Ages (years)
0
20
40
60
80
FDR (Age associated divergence) =0.0693
p(Scalp)=0.0058
p(Dura)=0.135
Scalp
Dura

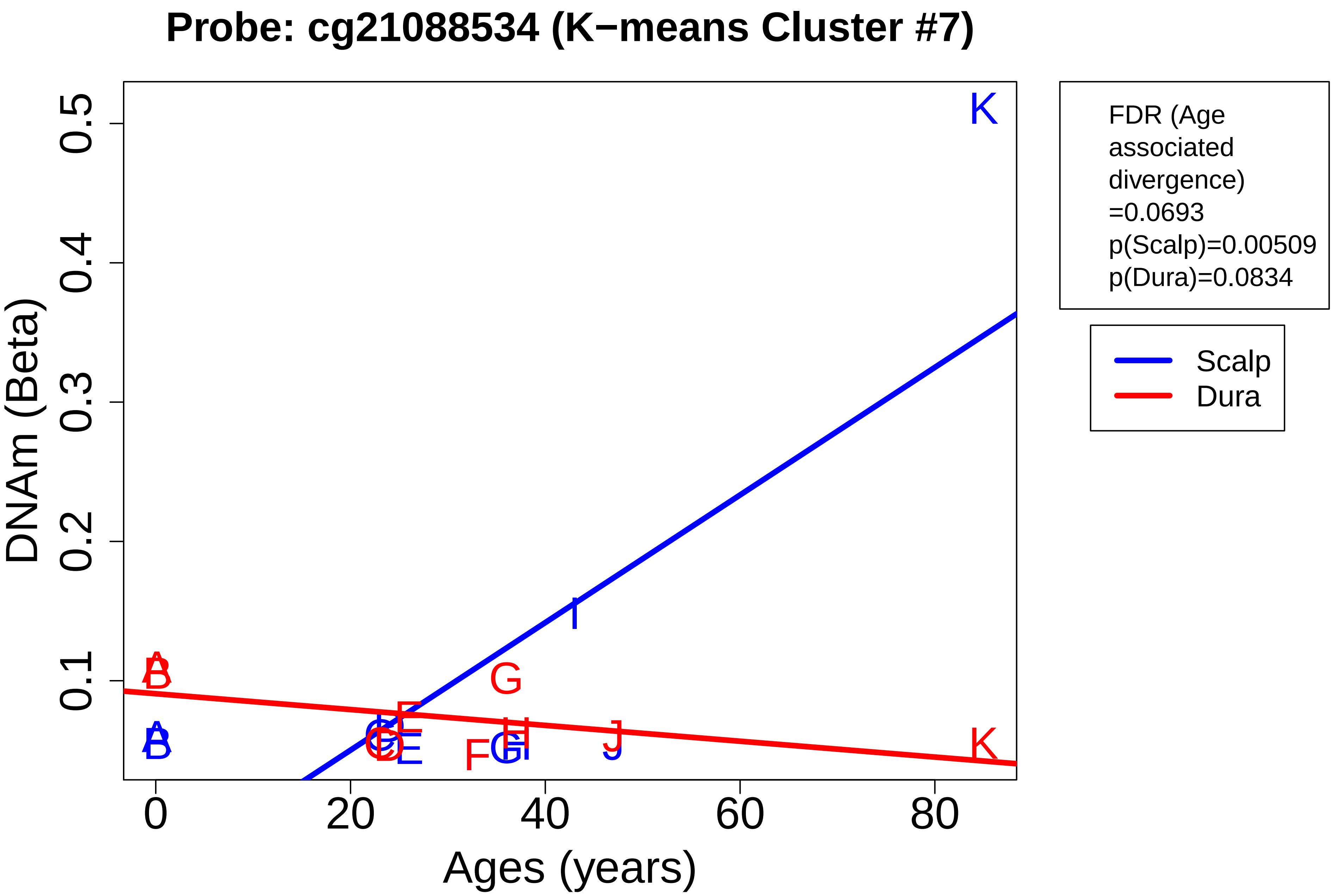

Probe: cg21088534 (K−means Cluster #7)
DNAm (Beta)
0.1
0.2
0.3
0.4
0.5
0
20
40
60
80
Ages (years)
FDR (Age associated divergence) =0.0693
p(Scalp)=0.00509
p(Dura)=0.0834
Scalp
Dura

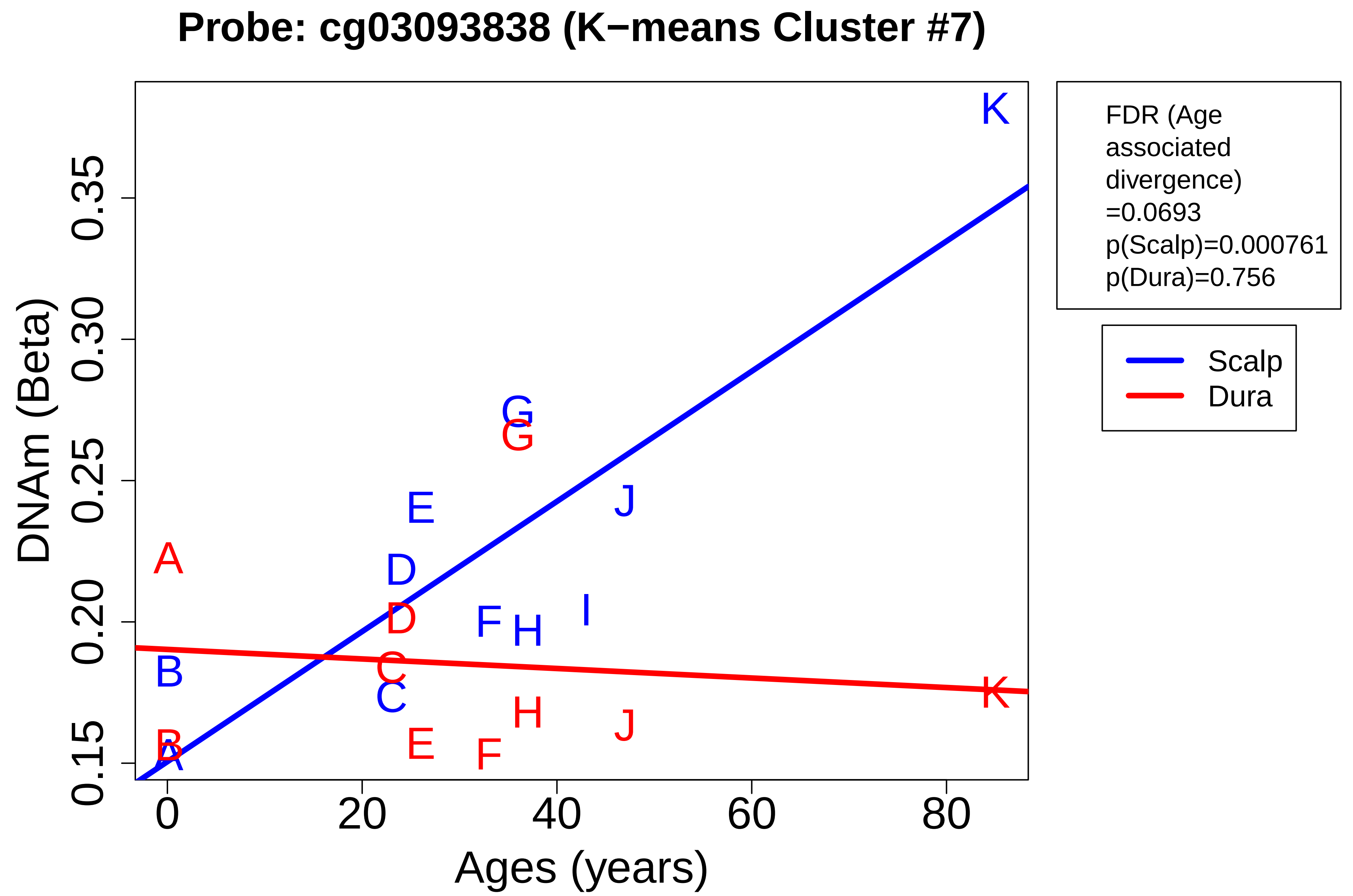

Probe: cg03093838 (K−means Cluster #7)
FDR (Age associated divergence) =0.0693
p(Scalp)=0.000761
p(Dura)=0.756
Scalp
Dura
DNAm (Beta)
0.15
0.20
0.25
0.30
0.35
Ages (years)
0
20
40
60
80

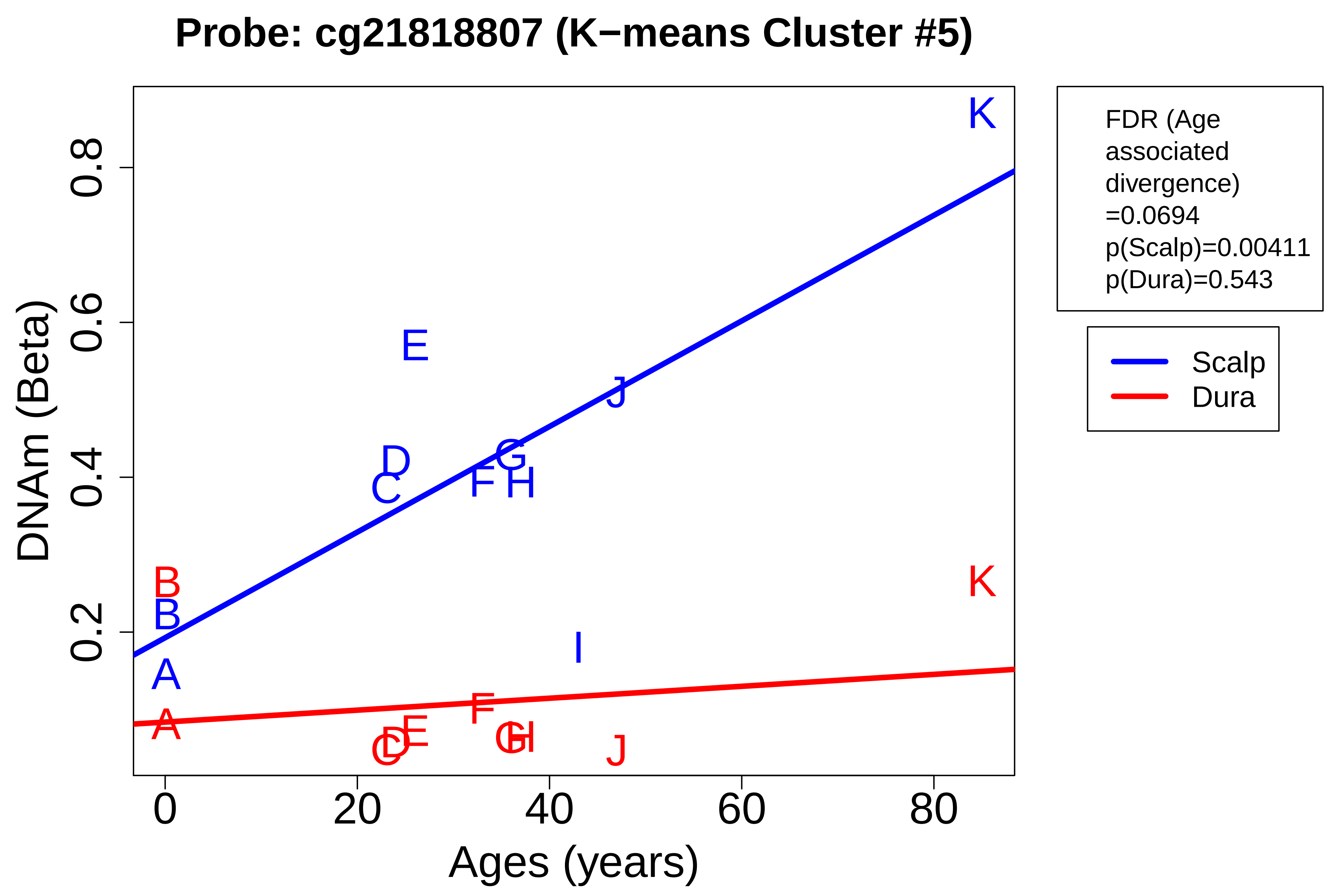
Probe: cg21818807 (K−means Cluster #5)
DNAm (Beta)
0.2
0.4
0.6
0.8
Ages (years)
0
20
40
60
80
FDR (Age associated divergence) =0.0694
p(Scalp)=0.00411
p(Dura)=0.543
Scalp
Dura
A
B
C
D
E
F
G
H
I
J
K

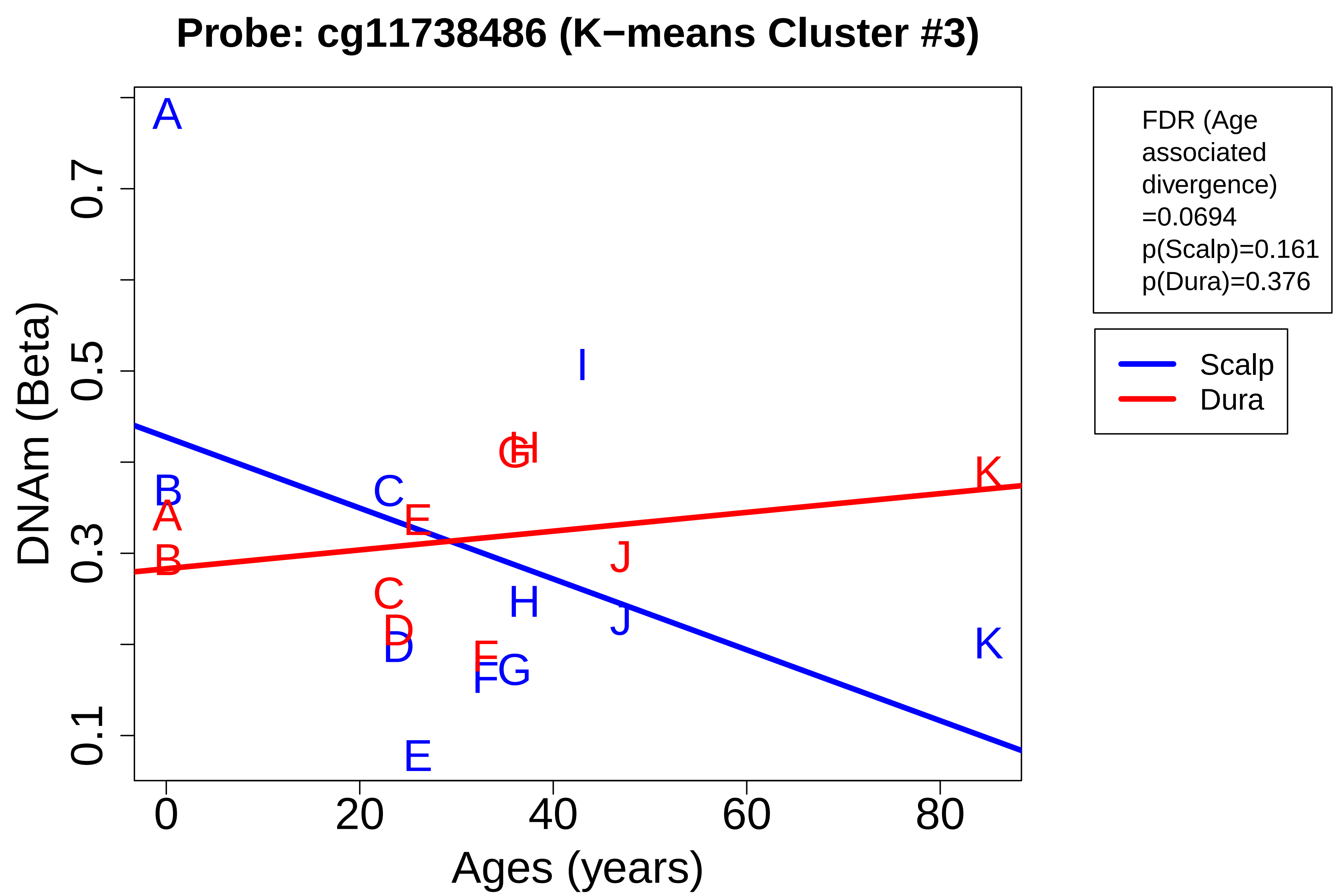
Probe: cg11738486 (K−means Cluster #3)
DNAm (Beta)
0.1
0.3
0.5
0.7
0
20
40
60
80
Ages (years)
A
B
C
D
E
F
G
H
I
J
K
FDR (Age associated divergence) =0.0694
p(Scalp)=0.161
p(Dura)=0.376
Scalp
Dura

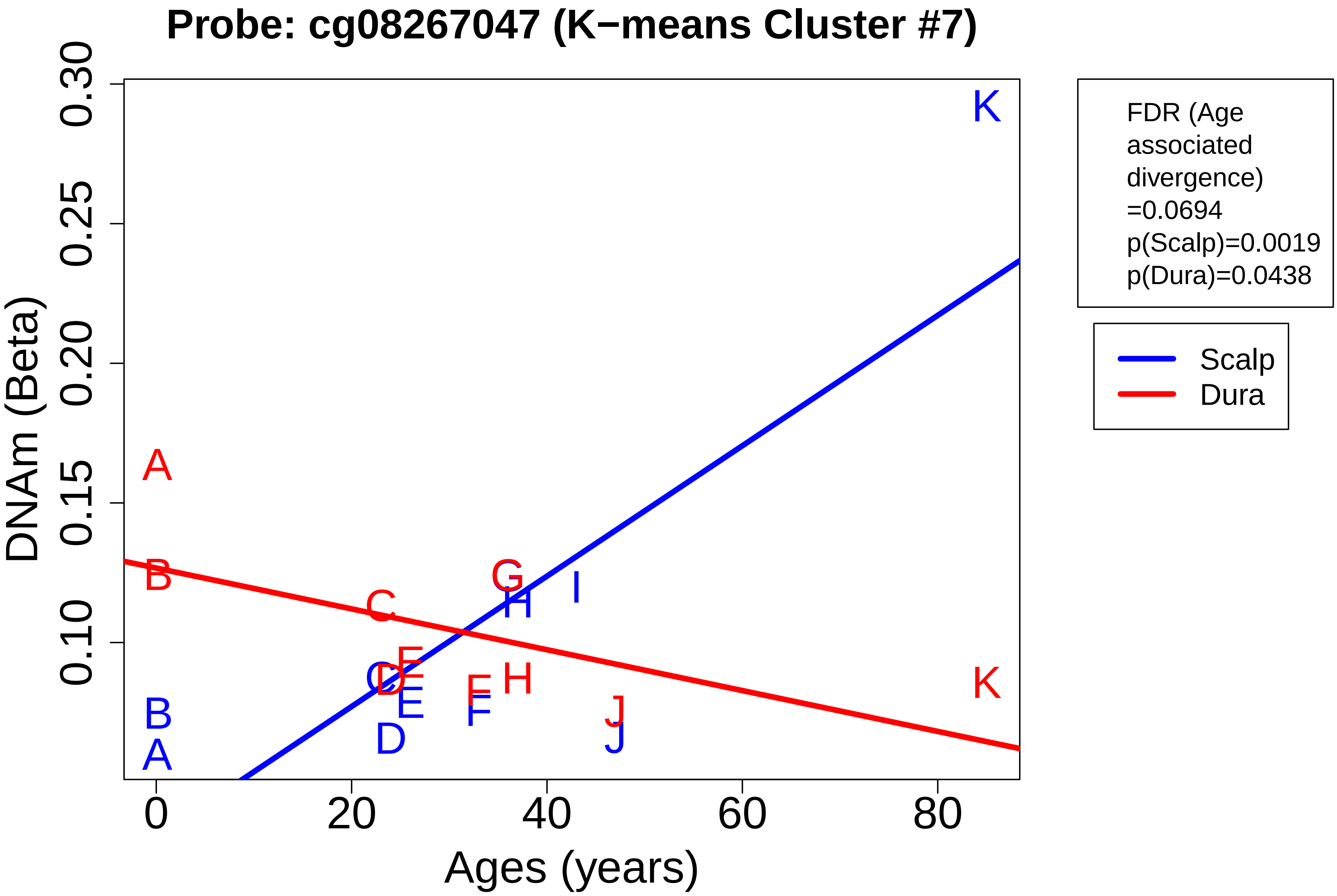

Probe: cg08267047 (K−means Cluster #7)
DNAm (Beta)
0.10
0.15
0.20
0.25
0.30
Ages (years)
0
20
40
60
80
FDR (Age associated divergence) =0.0694
p(Scalp)=0.0019
p(Dura)=0.0438
Scalp
Dura

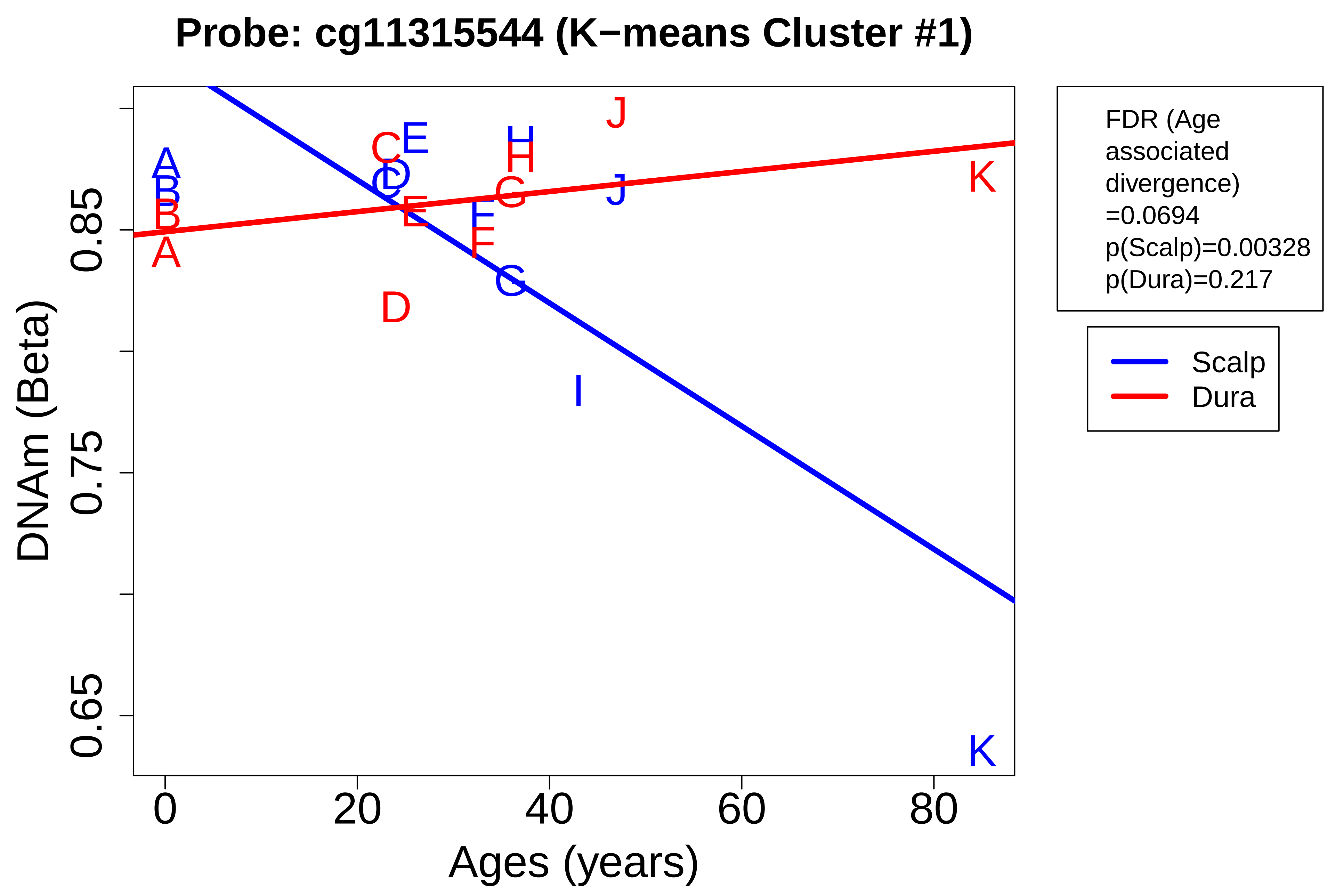

Probe: cg11315544 (K−means Cluster #1)
DNAm (Beta)
0.65
0.75
0.85
Ages (years)
0
20
40
60
80
A
B
C
D
E
F
G
H
I
J
K
FDR (Age associated divergence) =0.0694
p(Scalp)=0.00328
p(Dura)=0.217
Scalp
Dura

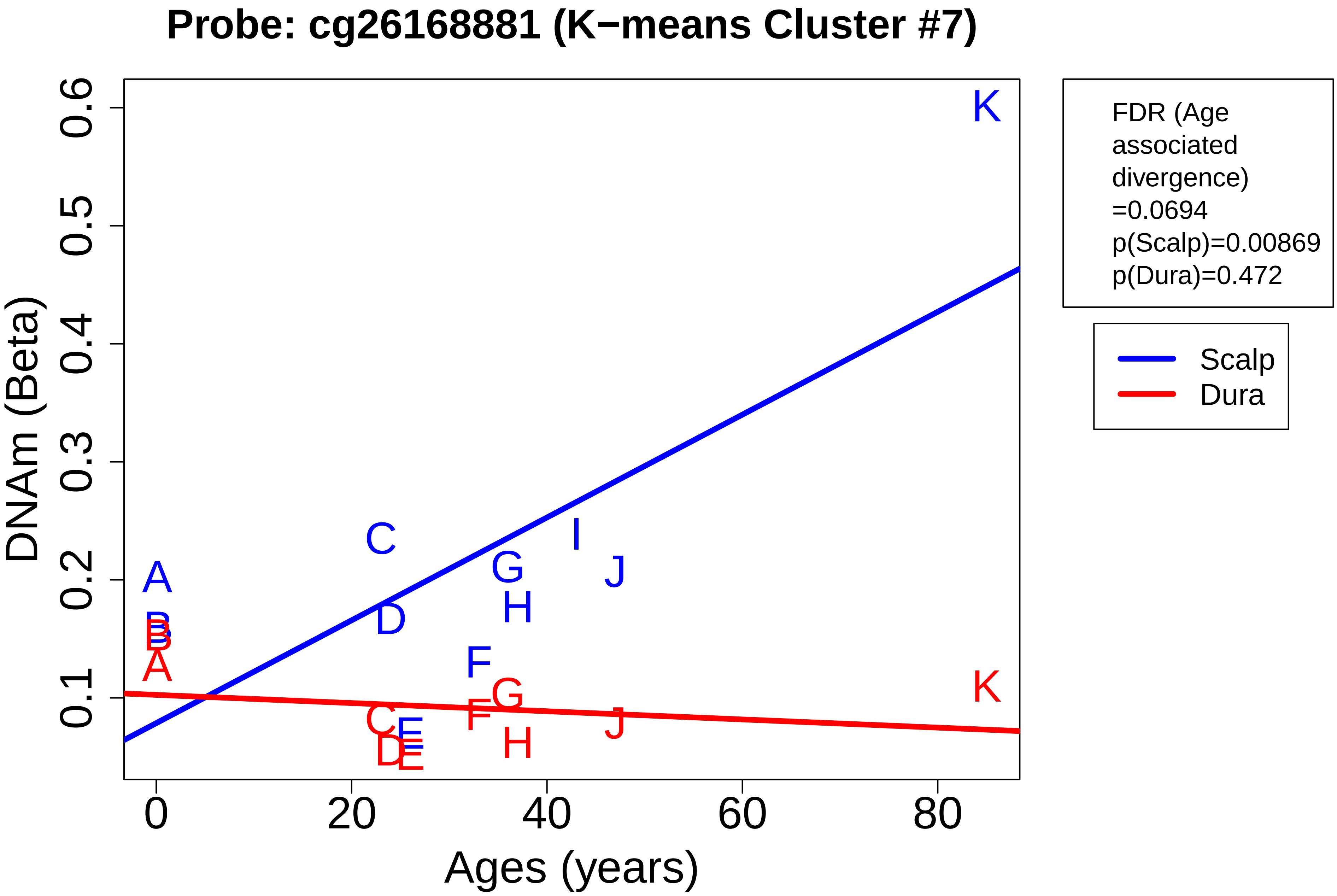

Probe: cg26168881 (K−means Cluster #7)
DNAm (Beta)
0.1
0.2
0.3
0.4
0.5
0.6
Ages (years)
0
20
40
60
80
A
B
C
D
E
F
G
H
I
J
K
FDR (Age associated divergence) =0.0694
p(Scalp)=0.00869
p(Dura)=0.472
Scalp
Dura

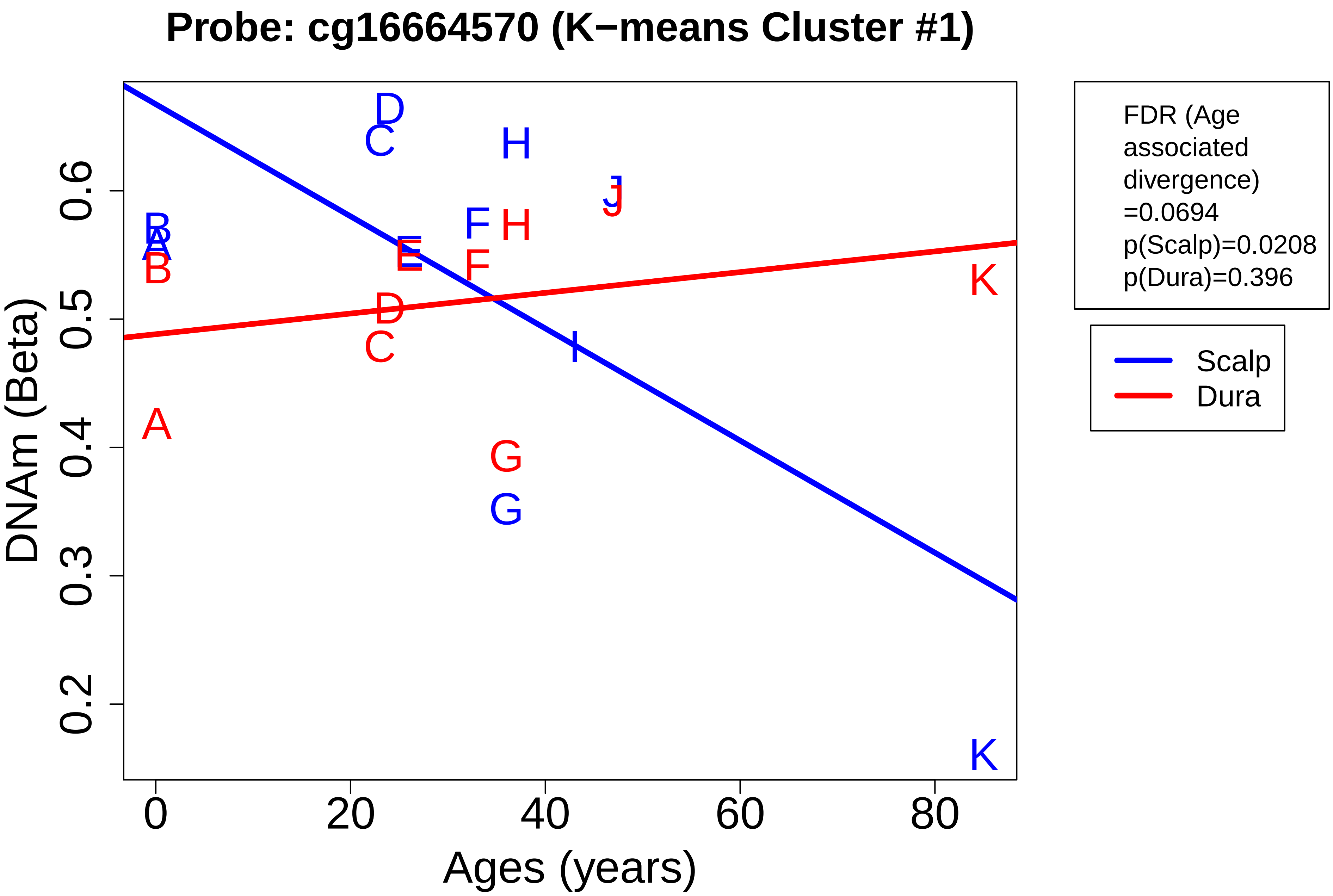

Probe: cg16664570 (K−means Cluster #1)
DNAm (Beta)
0.2
0.3
0.4
0.5
0.6
Ages (years)
0
20
40
60
80
A
B
C
D
E
F
G
H
I
J
K
FDR (Age
associated
divergence)
=0.0694
p(Scalp)=0.0208
p(Dura)=0.396
Scalp
Dura

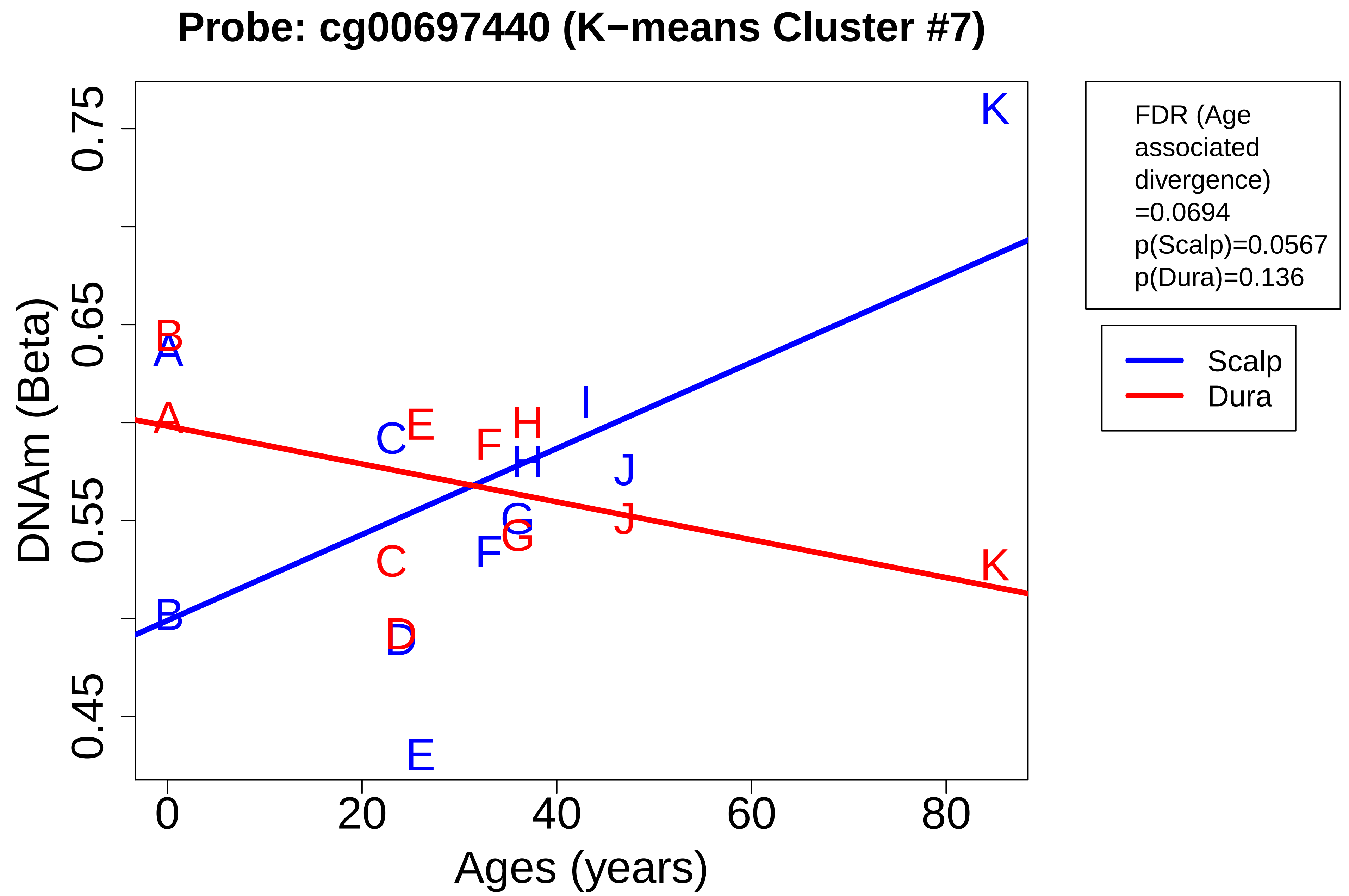

Probe: cg00697440 (K−means Cluster #7)
DNAm (Beta)
0.45
0.55
0.65
0.75
0
20
40
60
80
Ages (years)
FDR (Age associated divergence) =0.0694
p(Scalp)=0.0567
p(Dura)=0.136
Scalp
Dura

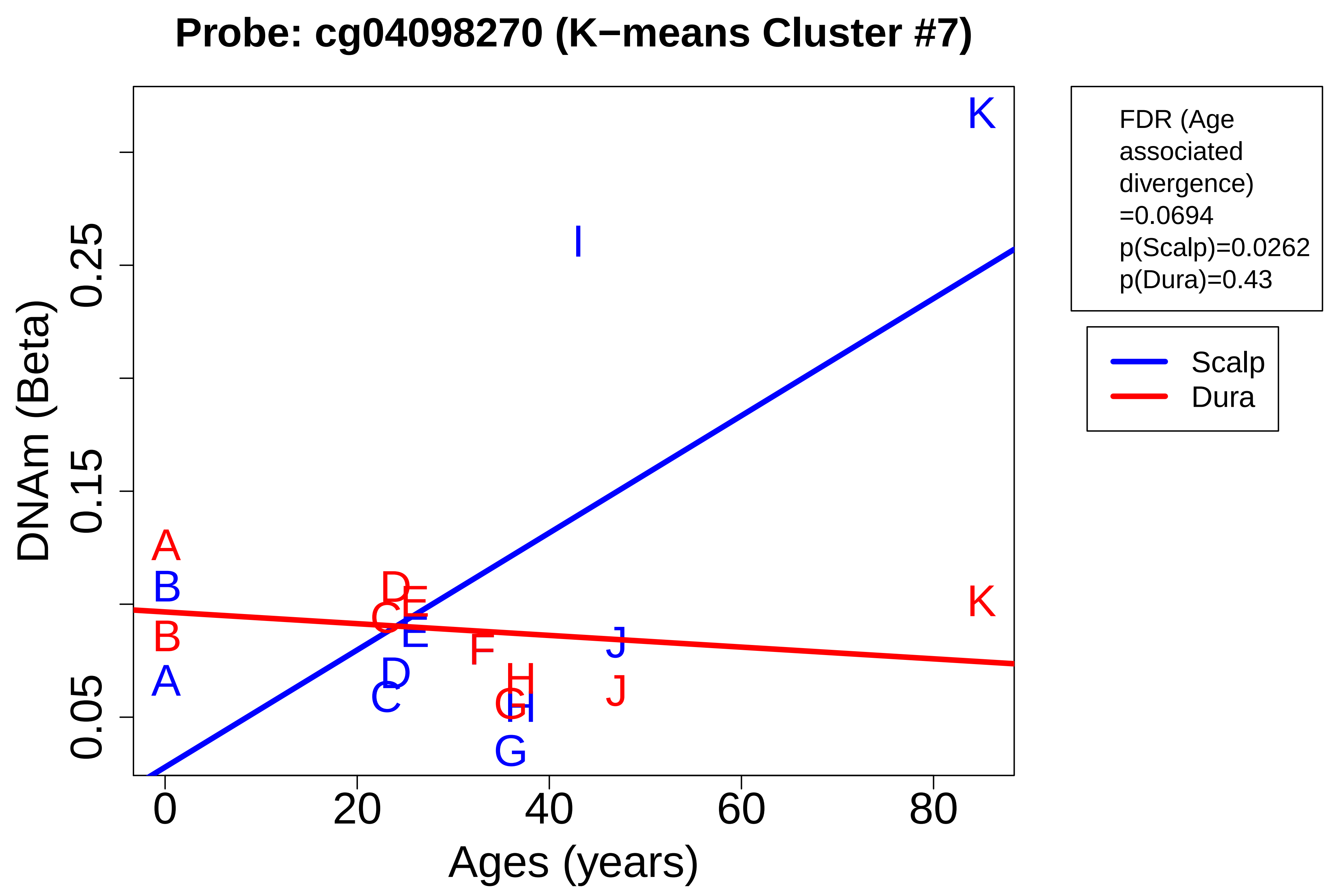

Probe: cg04098270 (K−means Cluster #7)
DNAm (Beta)
0.05
0.15
0.25
0
20
40
60
80
Ages (years)
A
B
C
D
E
F
G
H
I
J
K
FDR (Age associated divergence) =0.0694
p(Scalp)=0.0262
p(Dura)=0.43
Scalp
Dura

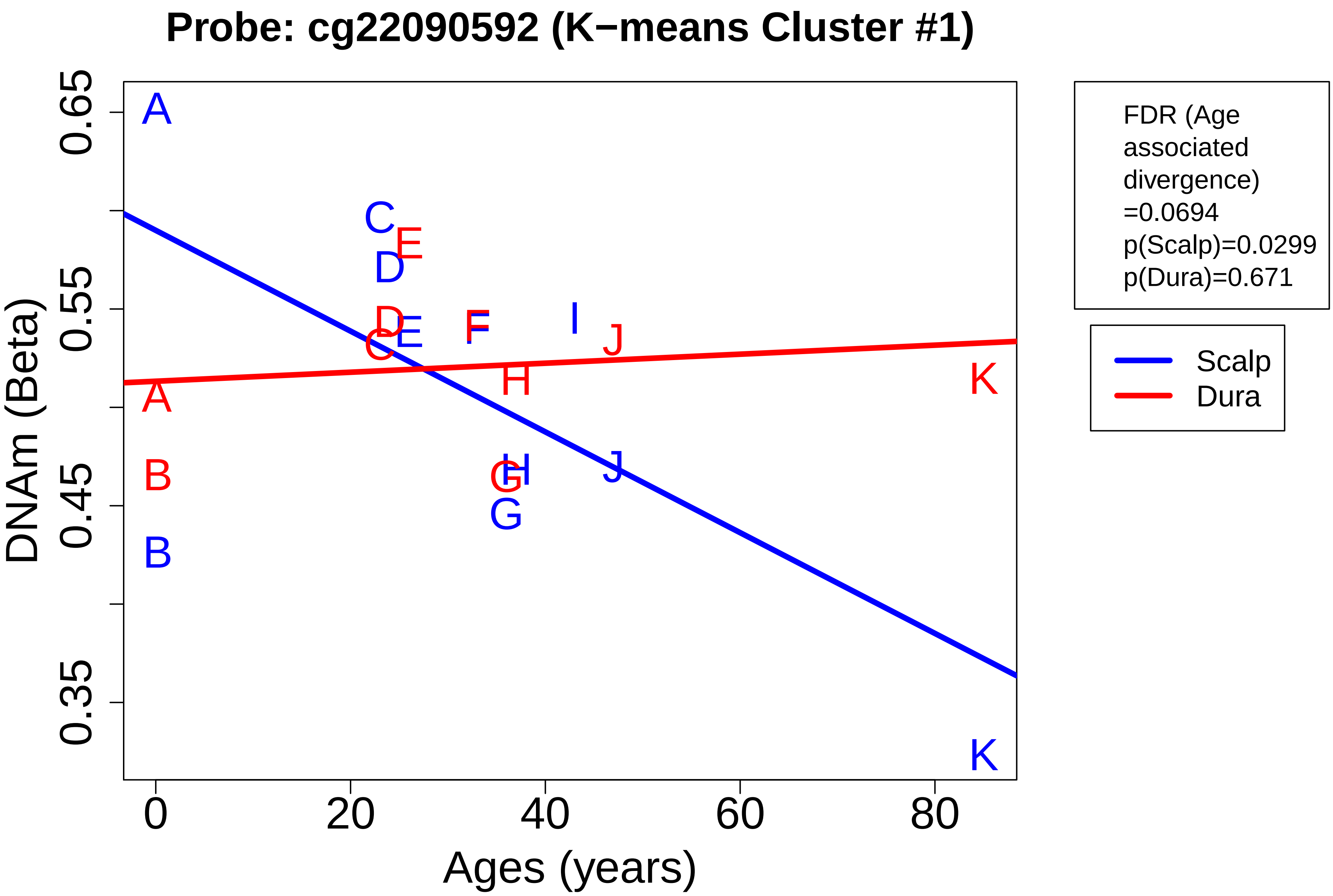
Probe: cg22090592 (K−means Cluster #1)
DNAm (Beta)
0.35
0.45
0.55
0.65
Ages (years)
0
20
40
60
80
FDR (Age associated divergence) =0.0694
p(Scalp)=0.0299
p(Dura)=0.671
Scalp
Dura
A
B
C
D
E
F
G
H
I
J
K

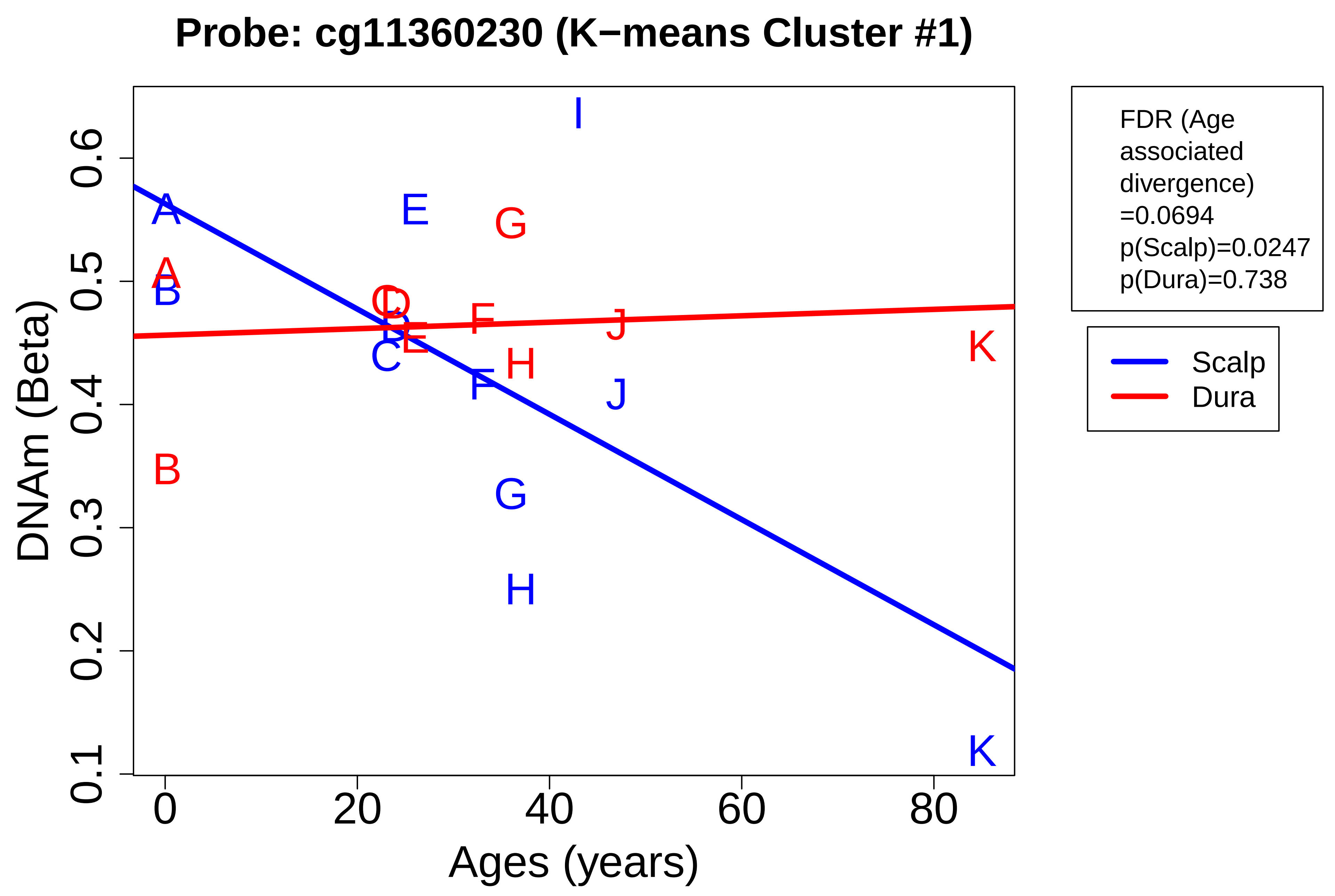

Probe: cg11360230 (K−means Cluster #1)
FDR (Age associated divergence) =0.0694
p(Scalp)=0.0247
p(Dura)=0.738
Scalp
Dura
DNAm (Beta)
0.1
0.2
0.3
0.4
0.5
0.6
Ages (years)
0
20
40
60
80

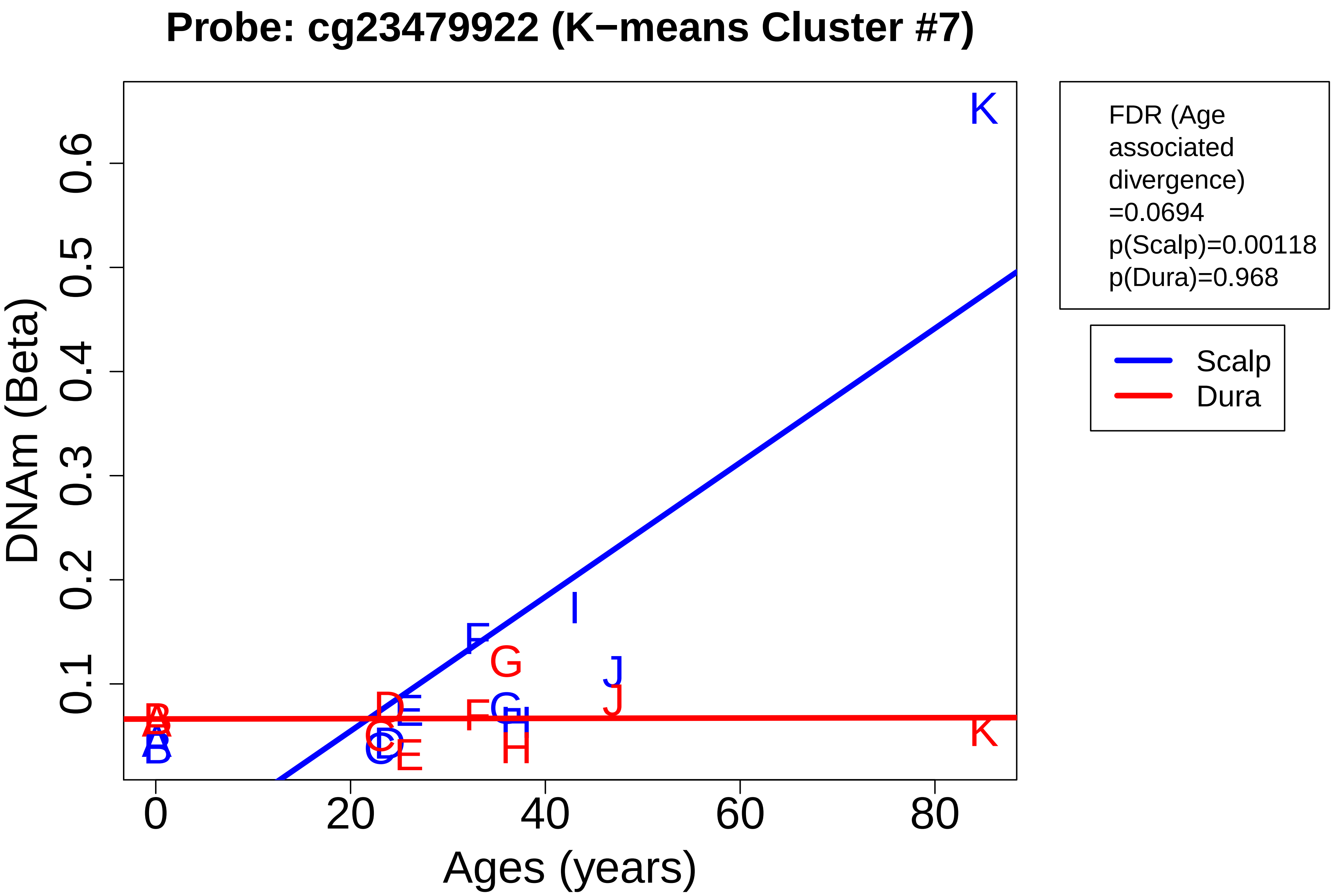
Probe: cg23479922 (K−means Cluster #7)
DNAm (Beta)
0.1
0.2
0.3
0.4
0.5
0.6
0
20
40
60
80
Ages (years)
FDR (Age associated divergence) =0.0694
p(Scalp)=0.00118
p(Dura)=0.968
Scalp
Dura

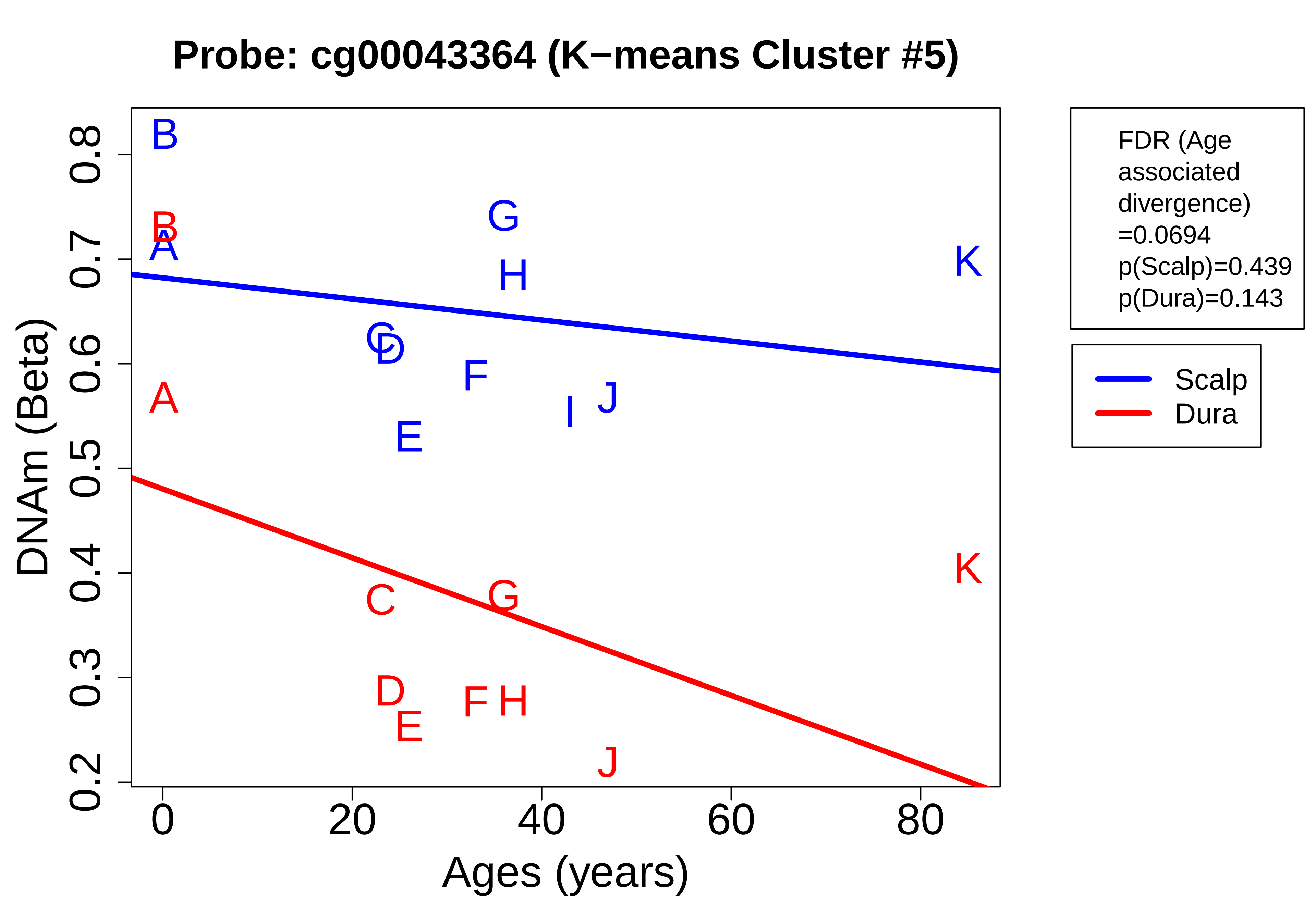

Probe: cg00043364 (K−means Cluster #5)
DNAm (Beta)
0.2
0.3
0.4
0.5
0.6
0.7
0.8
Ages (years)
0
20
40
60
80
FDR (Age associated divergence) =0.0694
p(Scalp)=0.439
p(Dura)=0.143
Scalp
Dura

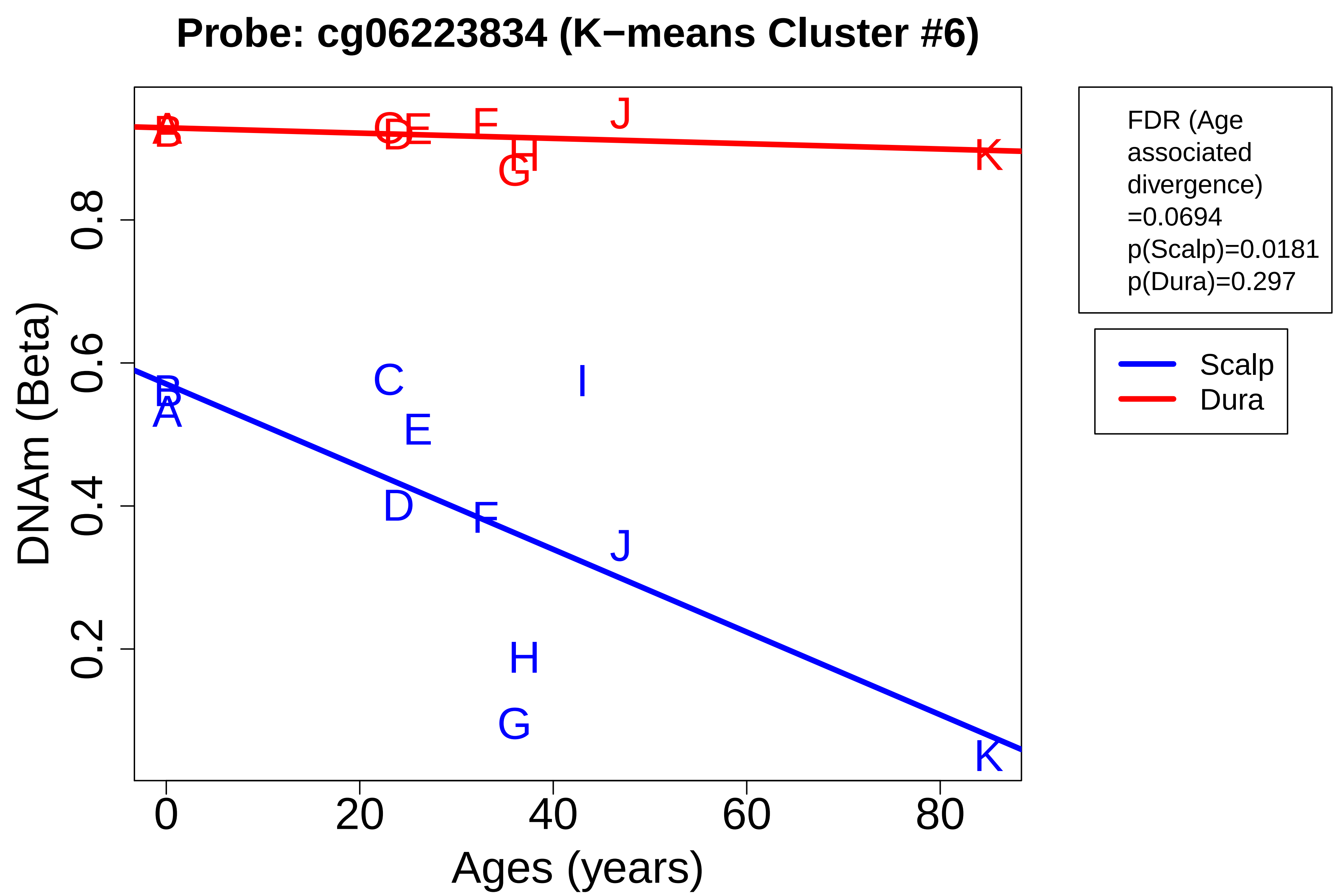
Probe: cg06223834 (K−means Cluster #6)
DNAm (Beta)
0.2
0.4
0.6
0.8
0
20
40
60
80
Ages (years)
FDR (Age associated divergence) =0.0694
p(Scalp)=0.0181
p(Dura)=0.297
Scalp
Dura
A
B
C
D
E
F
G
H
I
J
K

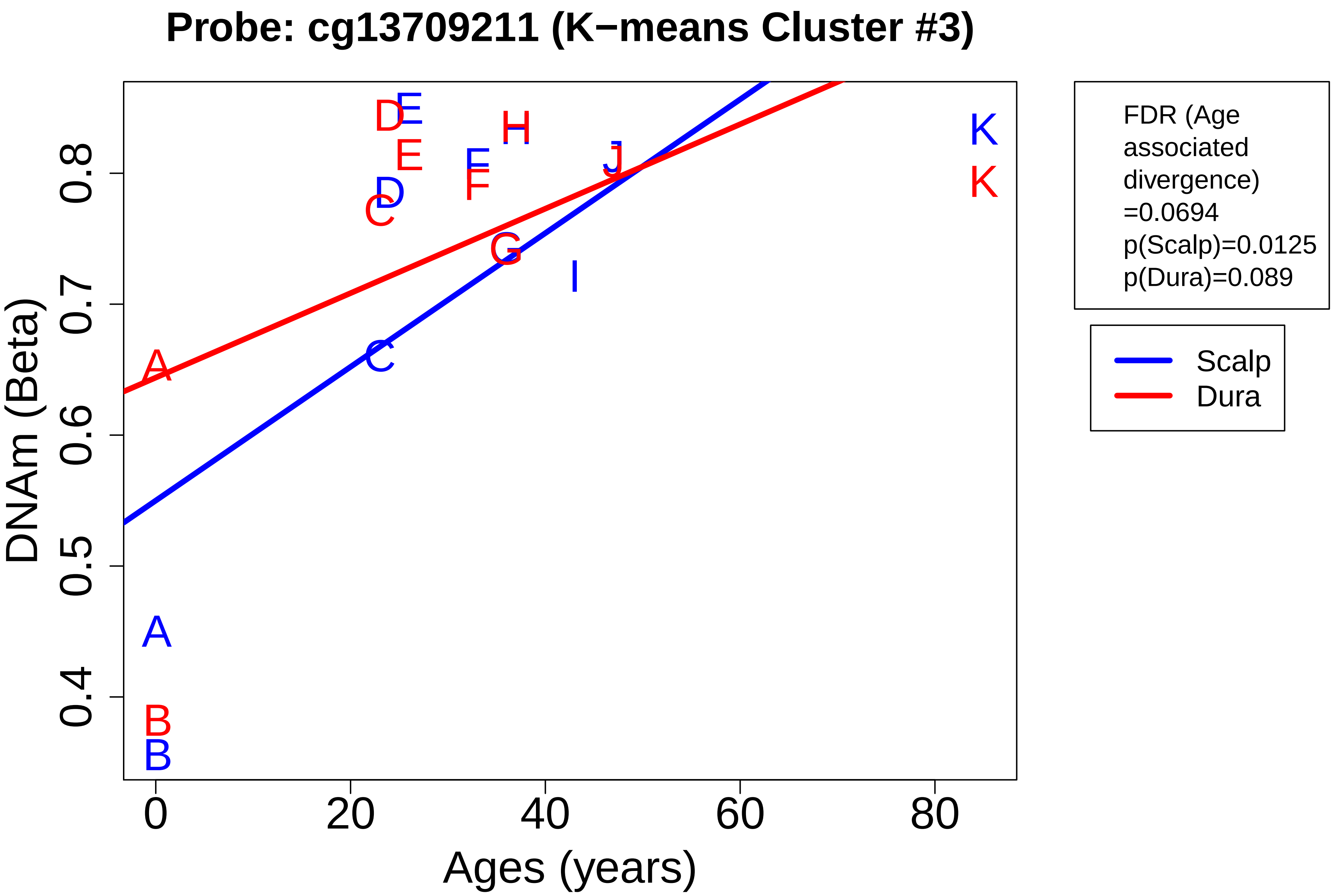

Probe: cg13709211 (K−means Cluster #3)
DNAm (Beta)
0.4
0.5
0.6
0.7
0.8
Ages (years)
0
20
40
60
80
FDR (Age associated divergence) =0.0694
p(Scalp)=0.0125
p(Dura)=0.089
Scalp
Dura
A
B
C
D
E
F
G
H
I
J
K

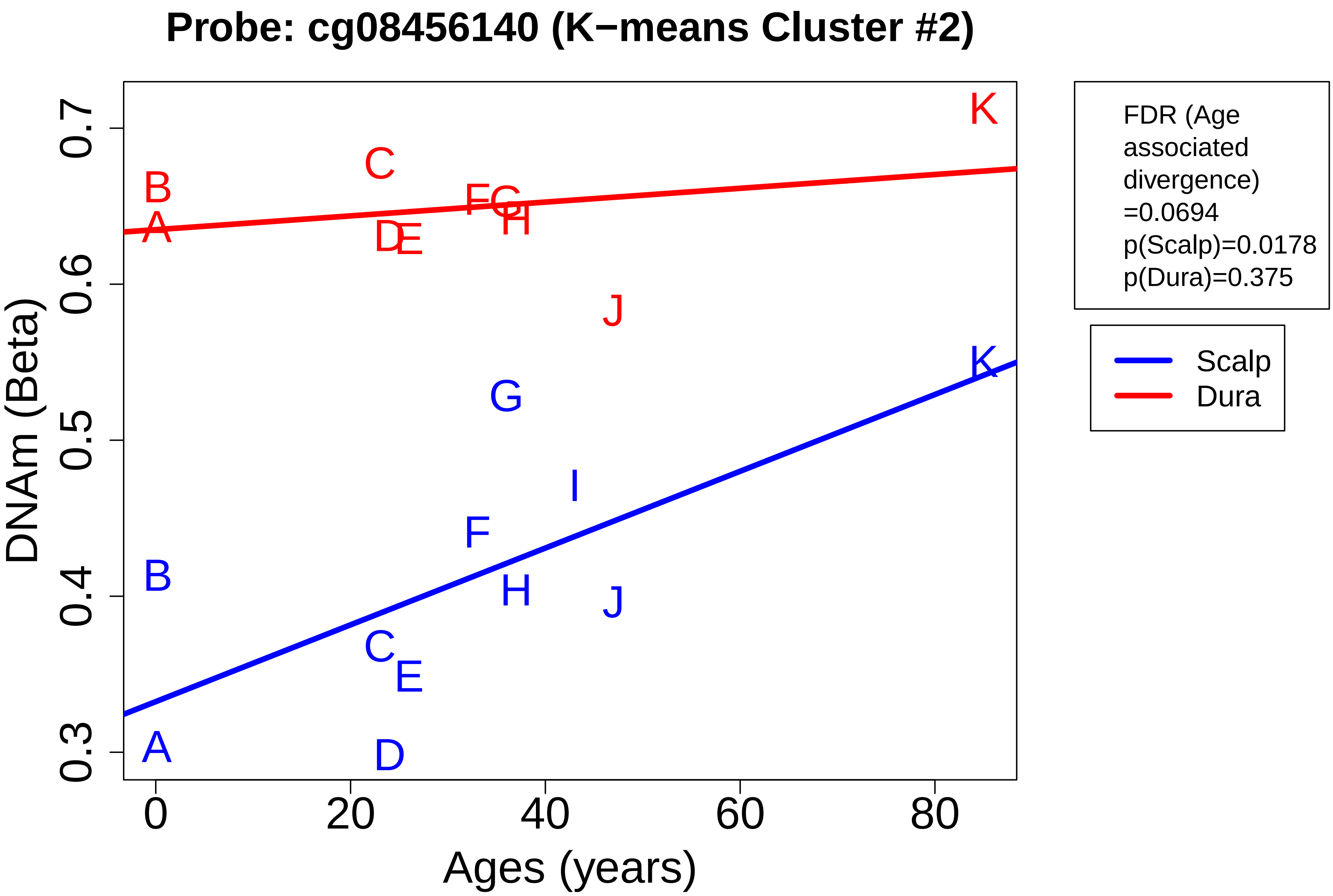

Probe: cg08456140 (K−means Cluster #2)
DNAm (Beta)
0.3
0.4
0.5
0.6
0.7
0
20
40
60
80
Ages (years)
FDR (Age associated divergence) =0.0694
p(Scalp)=0.0178
p(Dura)=0.375
Scalp
Dura
A
B
C
D
E
F
G
H
I
J
K

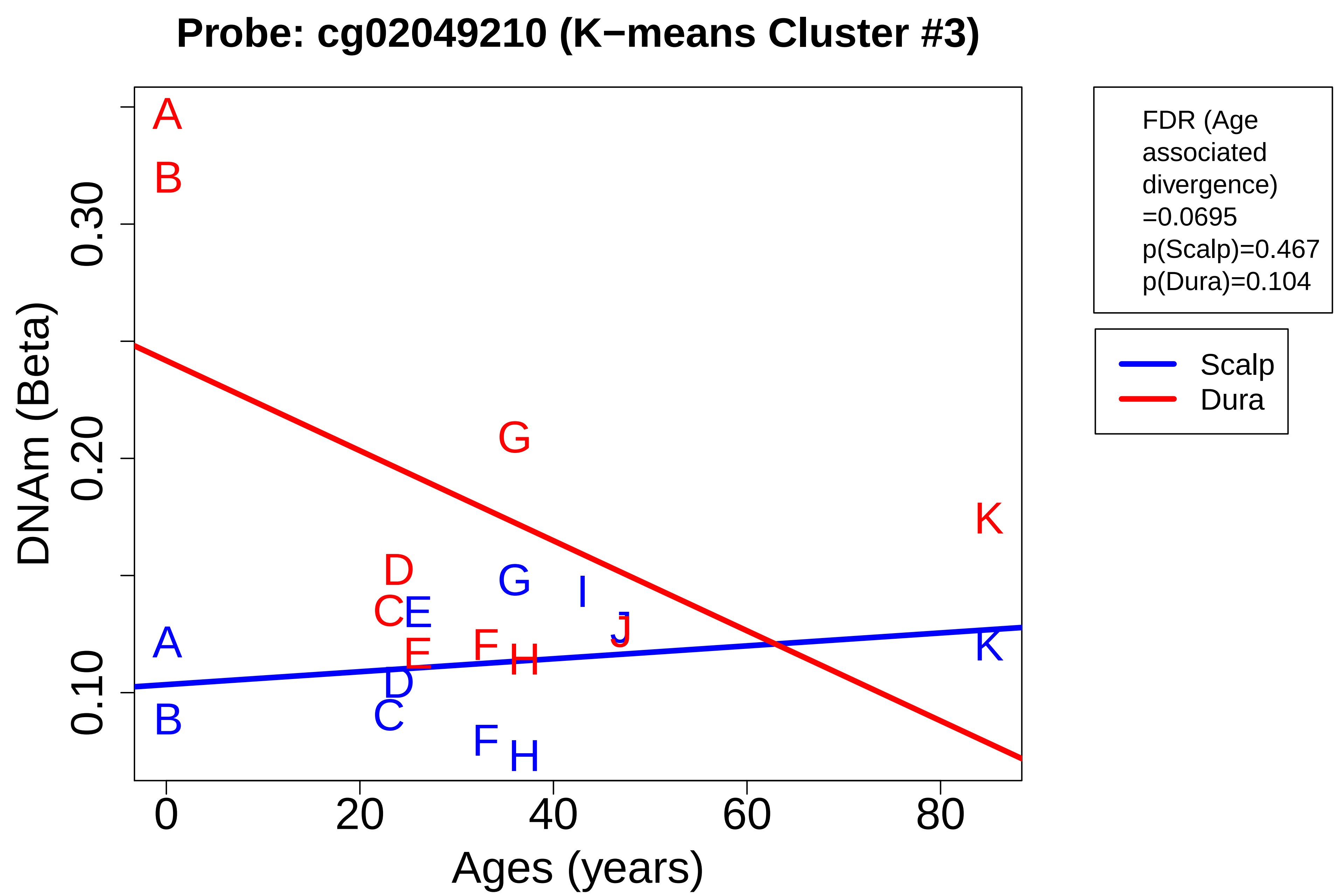

Probe: cg02049210 (K−means Cluster #3)
DNAm (Beta)
0.10
0.20
0.30
Ages (years)
0
20
40
60
80
A
B
C
D
E
F
G
H
I
J
K
FDR (Age associated divergence) =0.0695
p(Scalp)=0.467
p(Dura)=0.104
Scalp
Dura

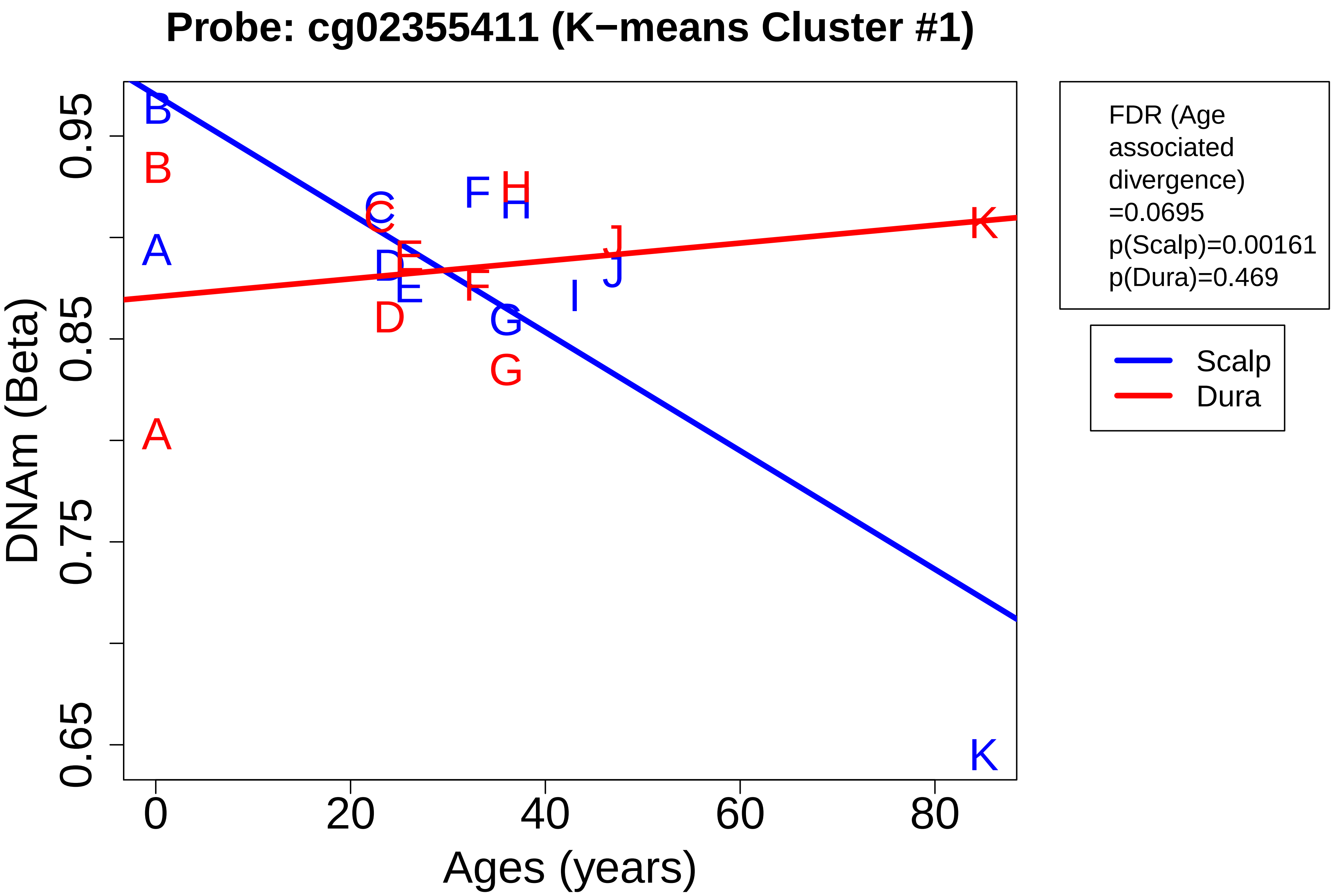

Probe: cg02355411 (K−means Cluster #1)
DNAm (Beta)
0.65
0.75
0.85
0.95
0
20
40
60
80
Ages (years)
FDR (Age associated divergence) =0.0695
p(Scalp)=0.00161
p(Dura)=0.469
Scalp
Dura

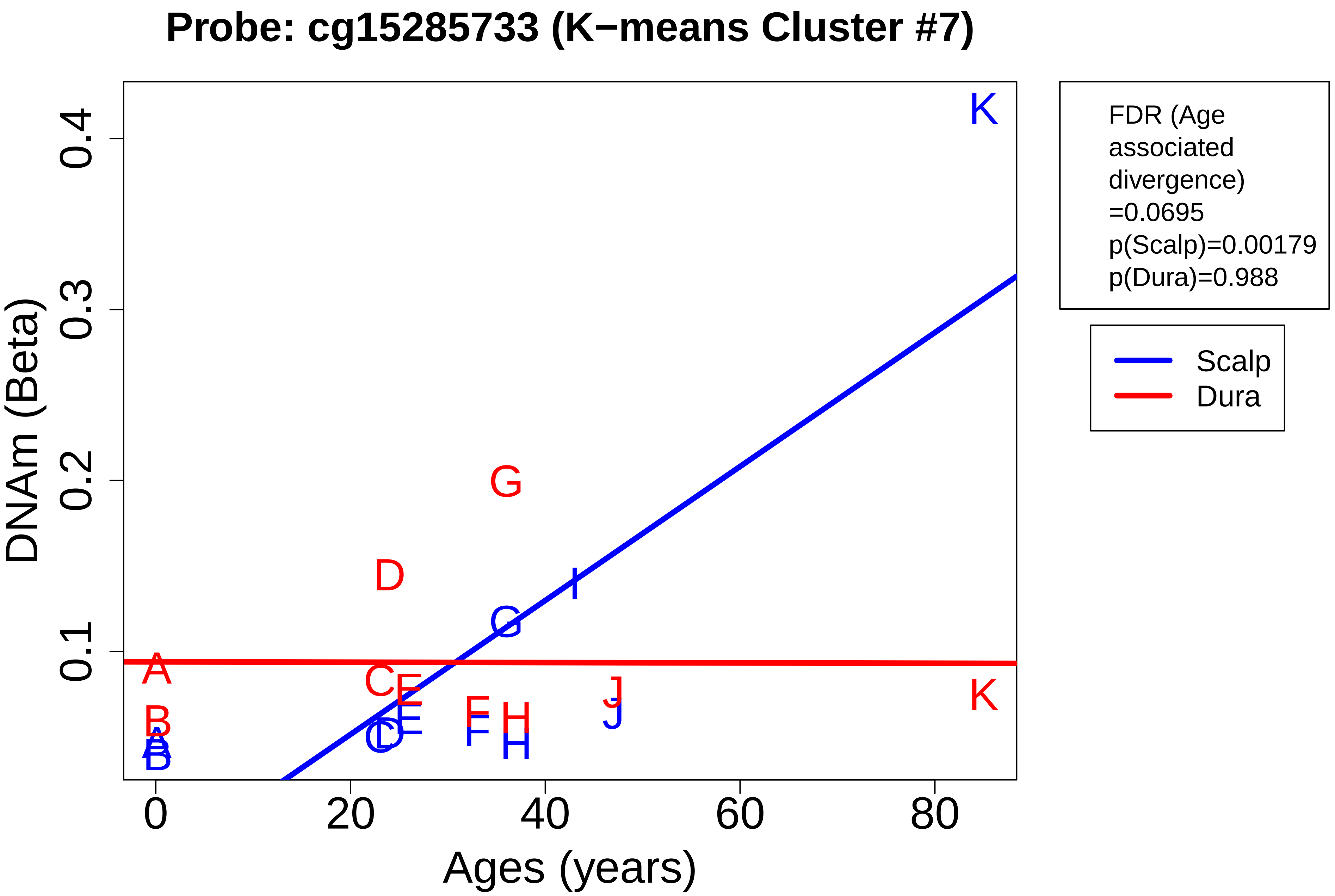

Probe: cg15285733 (K−means Cluster #7)
DNAm (Beta)
0.1
0.2
0.3
0.4
0
20
40
60
80
Ages (years)
FDR (Age associated divergence) =0.0695
p(Scalp)=0.00179
p(Dura)=0.988
Scalp
Dura

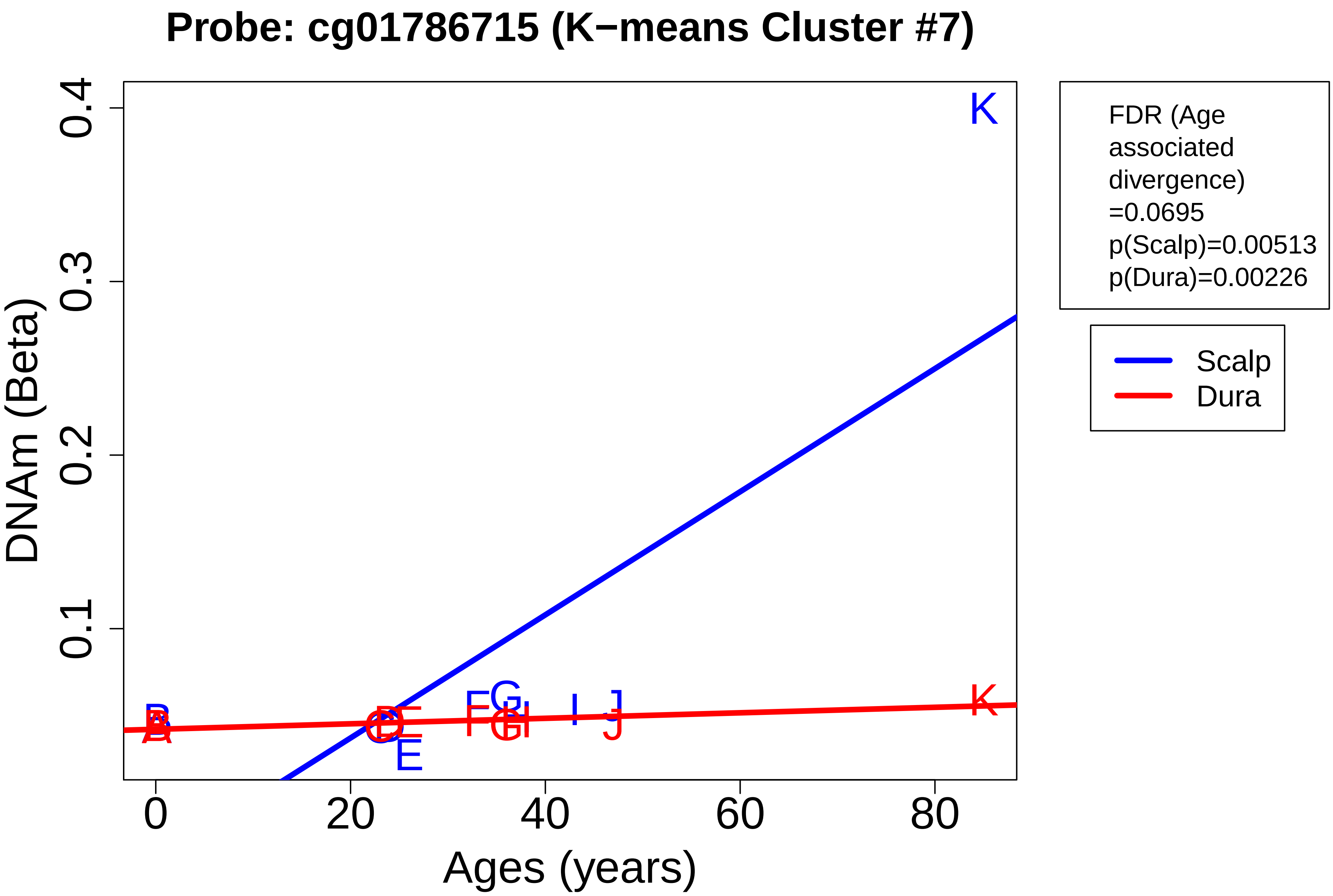

Probe: cg01786715 (K−means Cluster #7)
DNAm (Beta)
0.1
0.2
0.3
0.4
Ages (years)
0
20
40
60
80
K
FDR (Age associated divergence) =0.0695
p(Scalp)=0.00513
p(Dura)=0.00226
Scalp
Dura

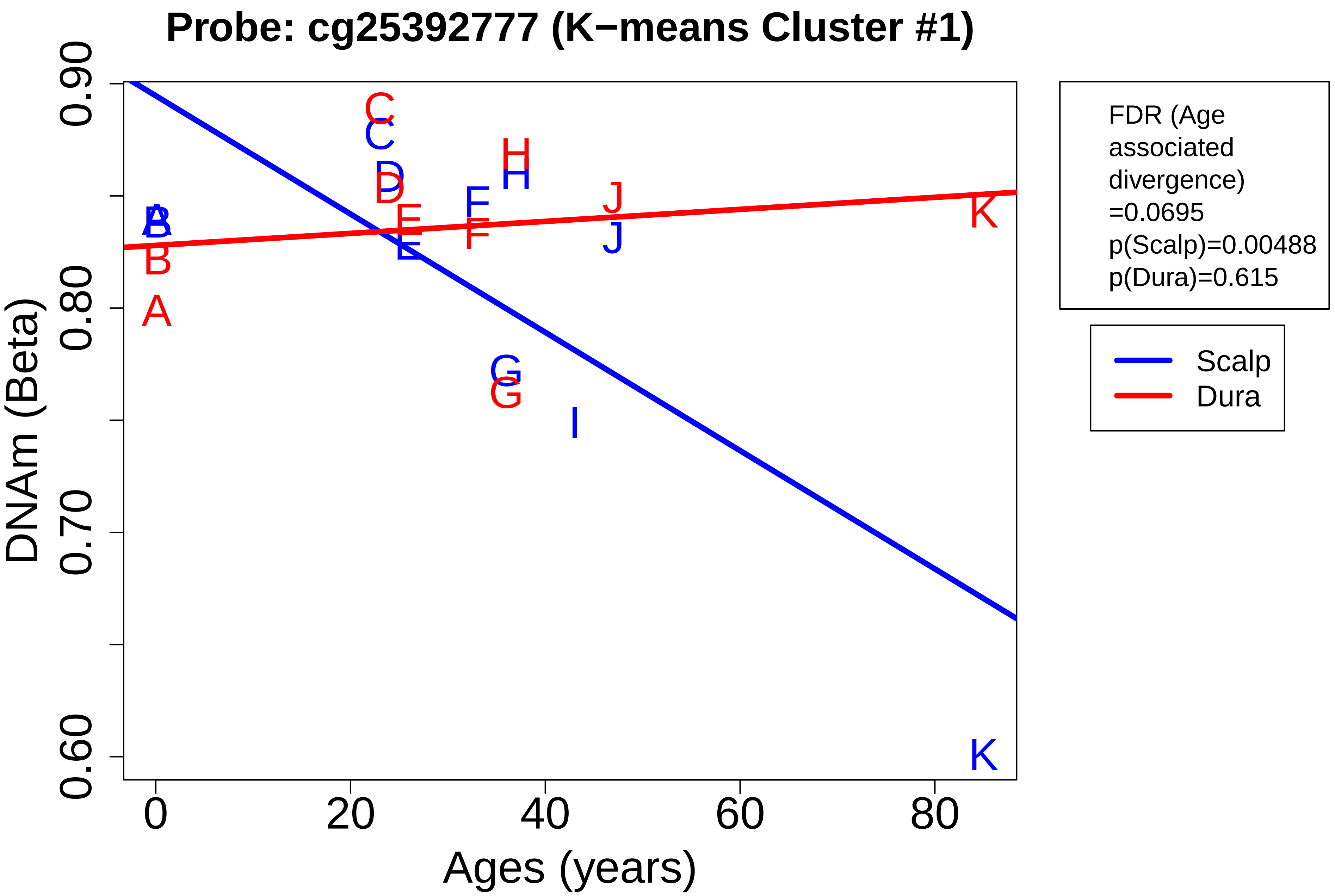

Probe: cg25392777 (K−means Cluster #1)
DNAm (Beta)
0.60
0.70
0.80
0.90
Ages (years)
0
20
40
60
80
A
B
C
D
E
F
G
H
I
J
K
FDR (Age associated divergence) =0.0695
p(Scalp)=0.00488
p(Dura)=0.615
Scalp
Dura

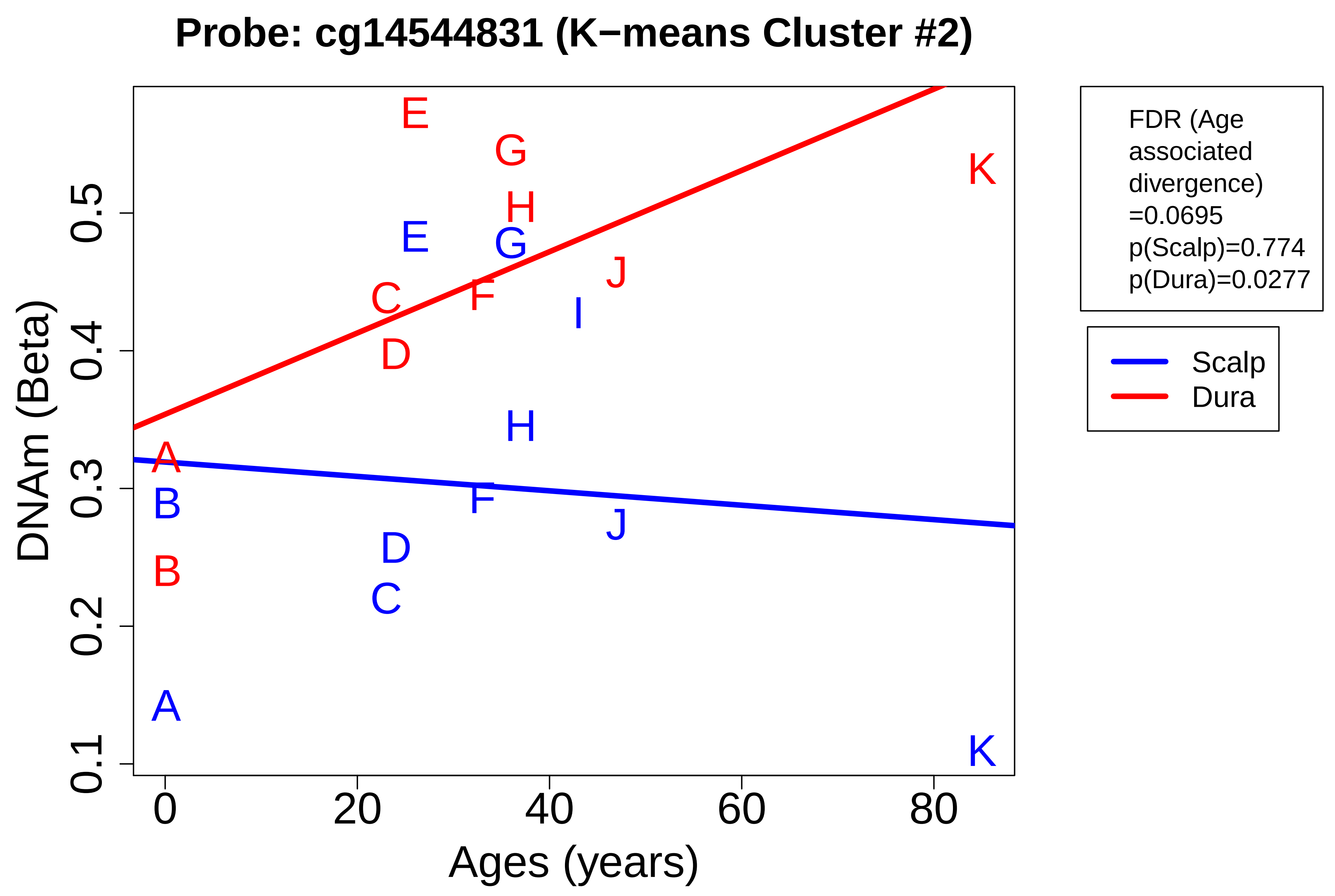
Probe: cg14544831 (K−means Cluster #2)
DNAm (Beta)
0.1
0.2
0.3
0.4
0.5
0
20
40
60
80
Ages (years)
FDR (Age associated divergence) =0.0695
p(Scalp)=0.774
p(Dura)=0.0277
Scalp
Dura
A
B
C
D
E
F
G
H
I
J
K

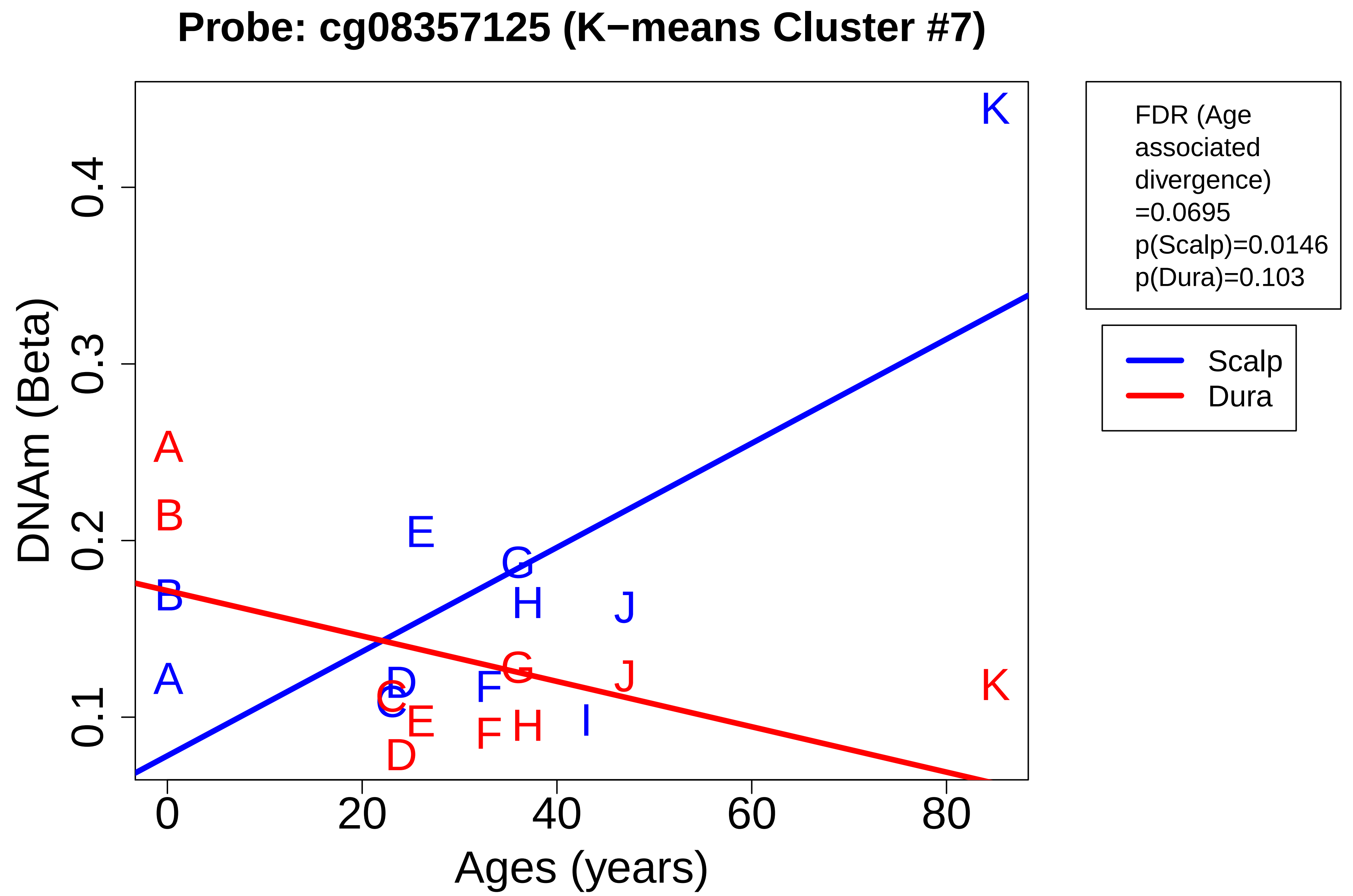

Probe: cg08357125 (K−means Cluster #7)
DNAm (Beta)
0.1
0.2
0.3
0.4
0
20
40
60
80
Ages (years)
A
B
C
D
E
F
G
H
I
J
K
FDR (Age associated divergence) =0.0695
p(Scalp)=0.0146
p(Dura)=0.103
Scalp
Dura

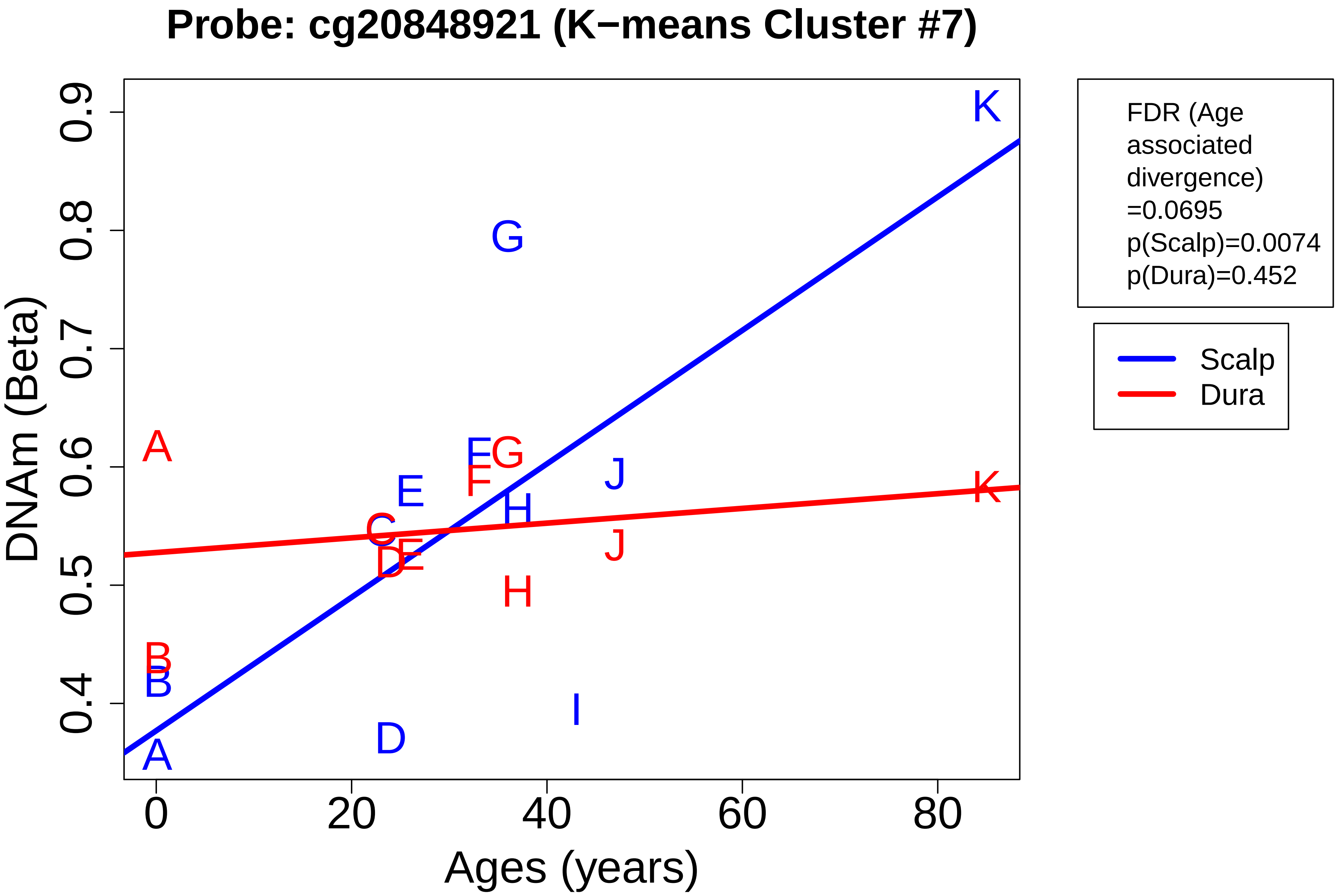

Probe: cg20848921 (K−means Cluster #7)
DNAm (Beta)
0.4
0.5
0.6
0.7
0.8
0.9
0
20
40
60
80
Ages (years)
A
B
C
D
E
F
G
H
I
J
K
FDR (Age associated divergence) =0.0695
p(Scalp)=0.0074
p(Dura)=0.452
Scalp
Dura

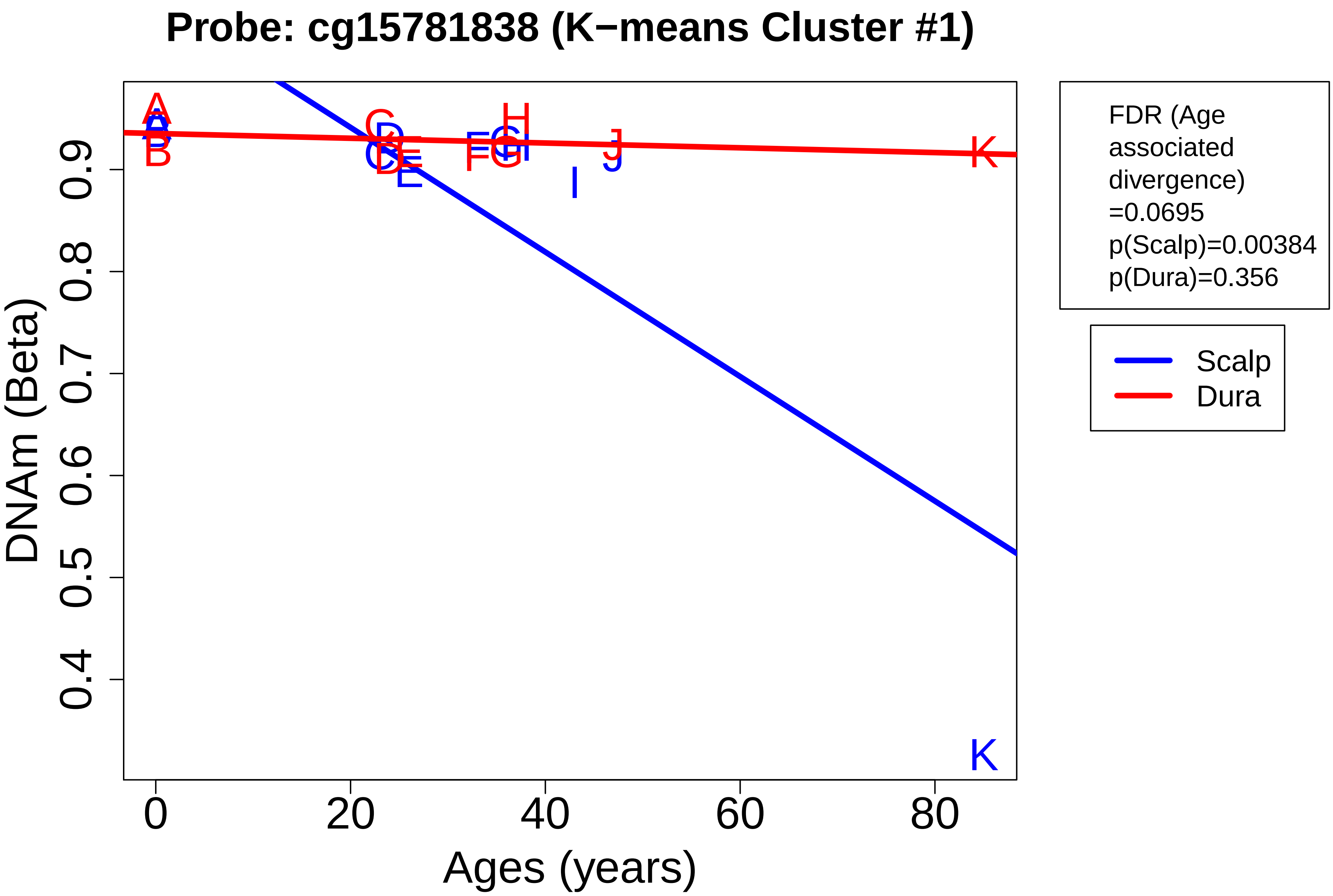

Probe: cg15781838 (K−means Cluster #1)
DNAm (Beta)
Ages (years)
0.4
0.5
0.6
0.7
0.8
0.9
0
20
40
60
80
FDR (Age associated divergence) =0.0695
p(Scalp)=0.00384
p(Dura)=0.356
Scalp
Dura

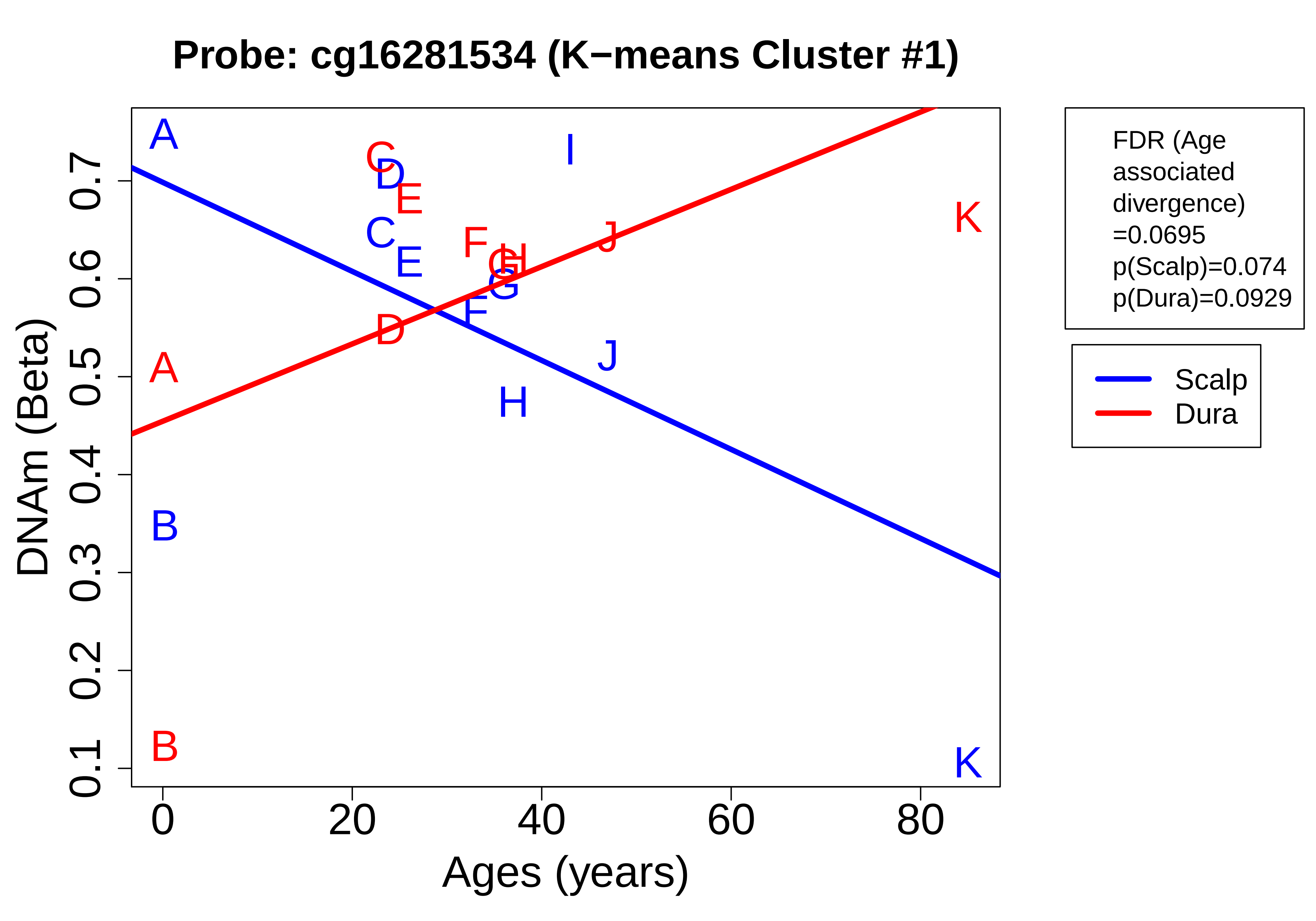
Probe: cg16281534 (K−means Cluster #1)
DNAm (Beta)
Ages (years)
0.1
0.2
0.3
0.4
0.5
0.6
0.7
0
20
40
60
80
FDR (Age associated divergence) =0.0695
p(Scalp)=0.074
p(Dura)=0.0929
Scalp
Dura

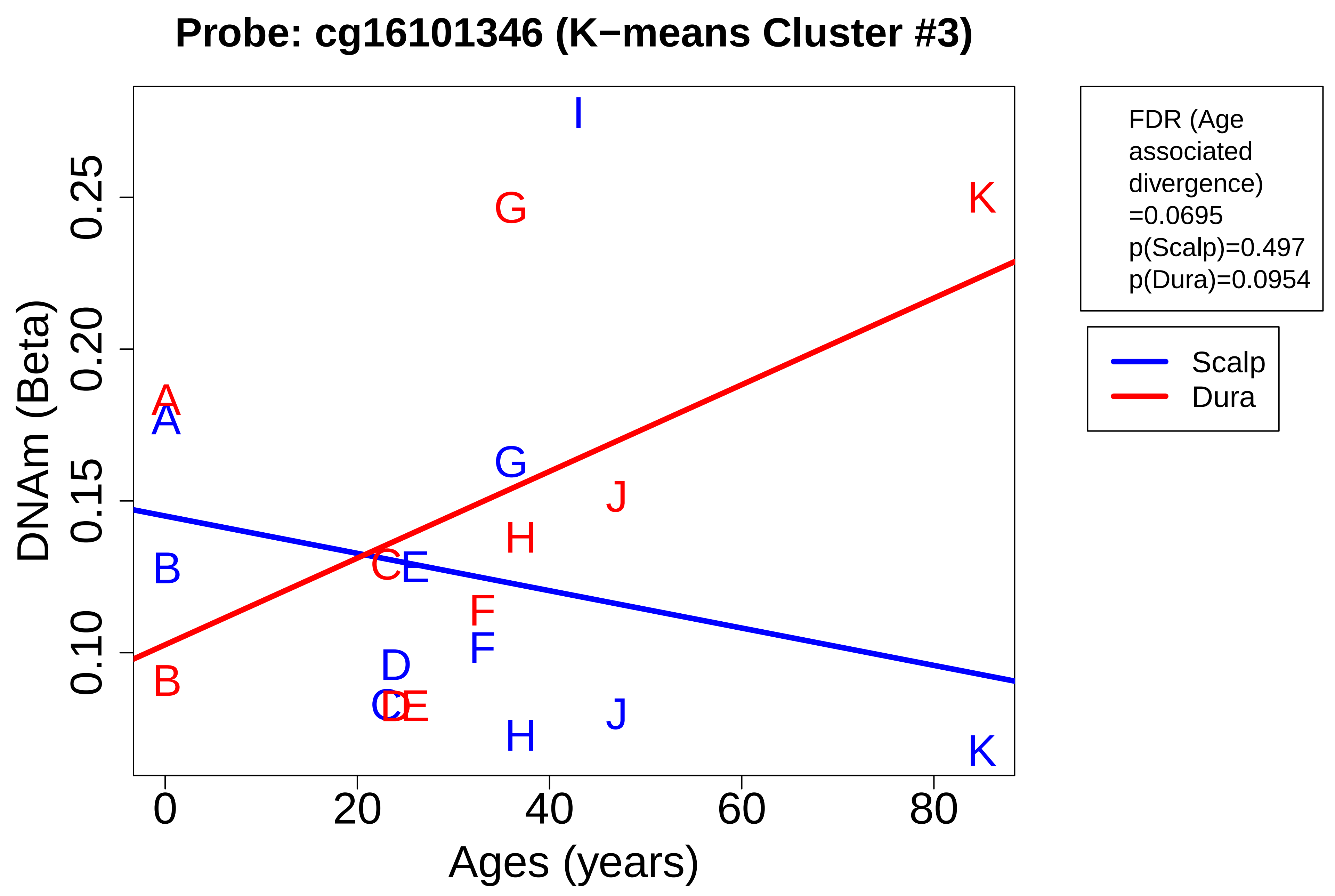

Probe: cg16101346 (K−means Cluster #3)
DNAm (Beta)
0.10
0.15
0.20
0.25
0
20
40
60
80
Ages (years)
FDR (Age associated divergence) =0.0695
p(Scalp)=0.497
p(Dura)=0.0954
Scalp
Dura

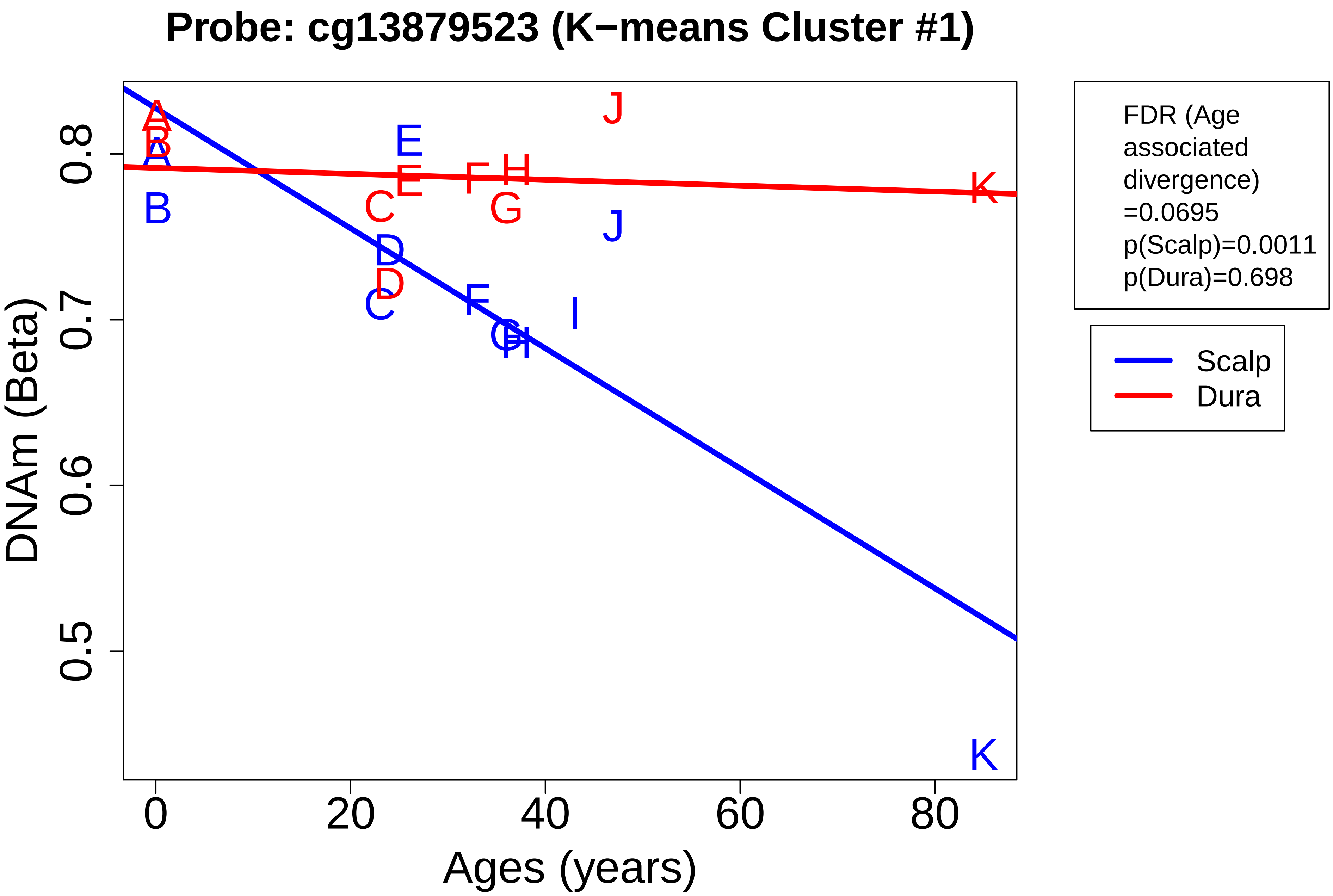

Probe: cg13879523 (K−means Cluster #1)
DNAm (Beta)
0.5
0.6
0.7
0.8
Ages (years)
0
20
40
60
80
FDR (Age associated divergence) =0.0695
p(Scalp)=0.0011
p(Dura)=0.698
Scalp
Dura

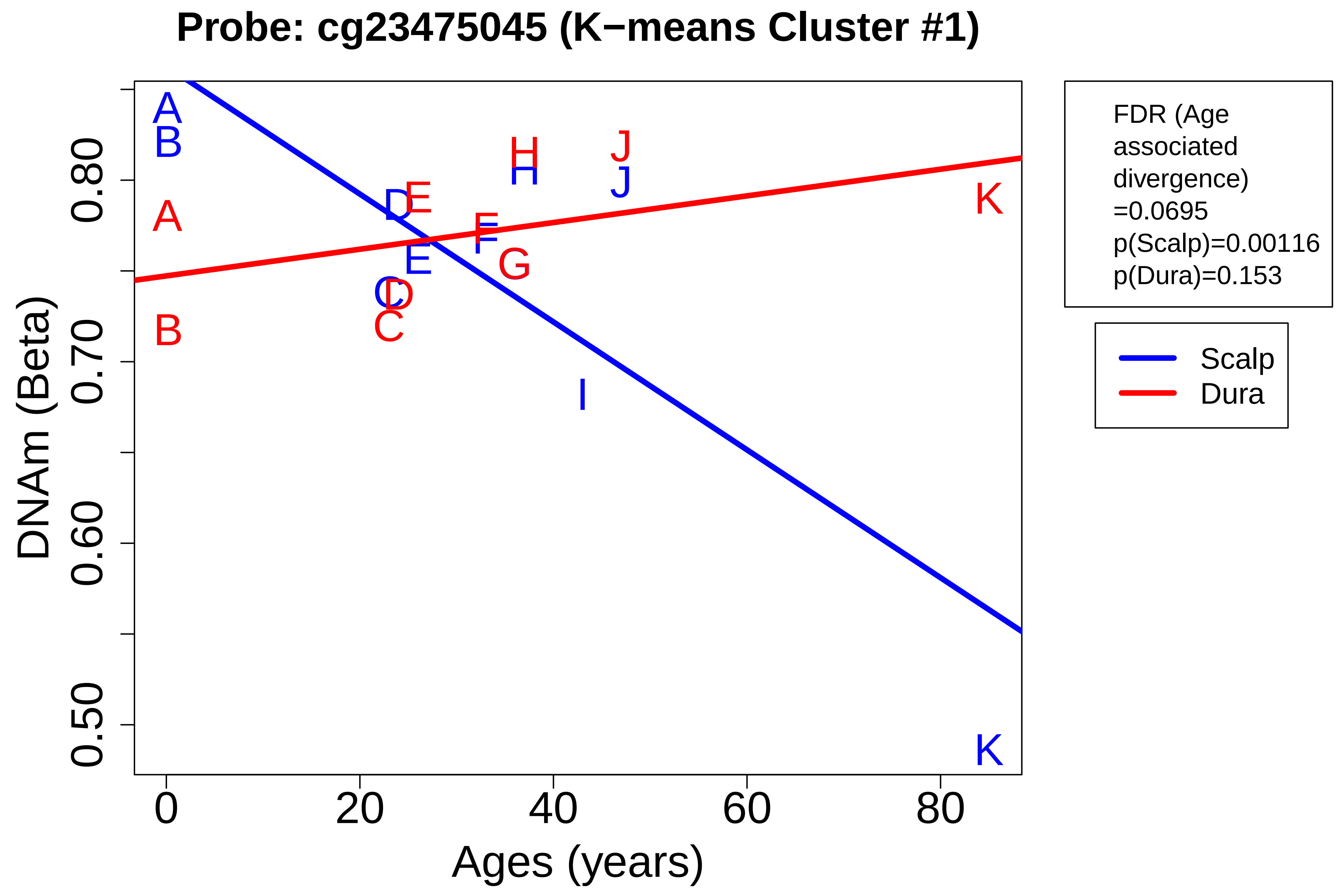

Probe: cg23475045 (K−means Cluster #1)
DNAm (Beta)
0.50
0.60
0.70
0.80
0
20
40
60
80
Ages (years)
A
B
C
D
E
F
G
H
I
J
K
FDR (Age associated divergence) =0.0695
p(Scalp)=0.00116
p(Dura)=0.153
Scalp
Dura

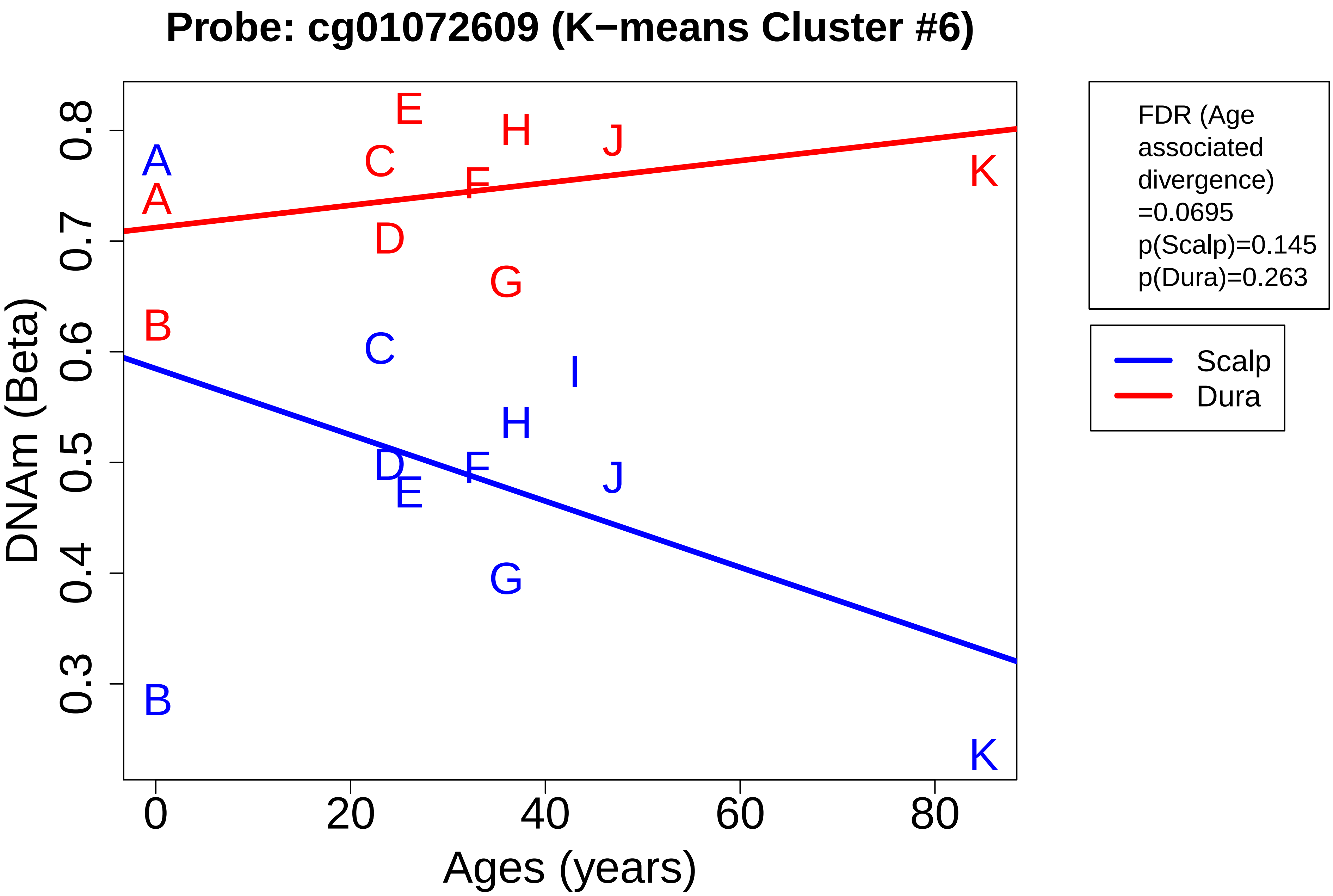

Probe: cg01072609 (K−means Cluster #6)
DNAm (Beta)
0.3
0.4
0.5
0.6
0.7
0.8
0
20
40
60
80
Ages (years)
A
B
C
D
E
F
G
H
I
J
K
FDR (Age associated divergence) =0.0695
p(Scalp)=0.145
p(Dura)=0.263
Scalp
Dura

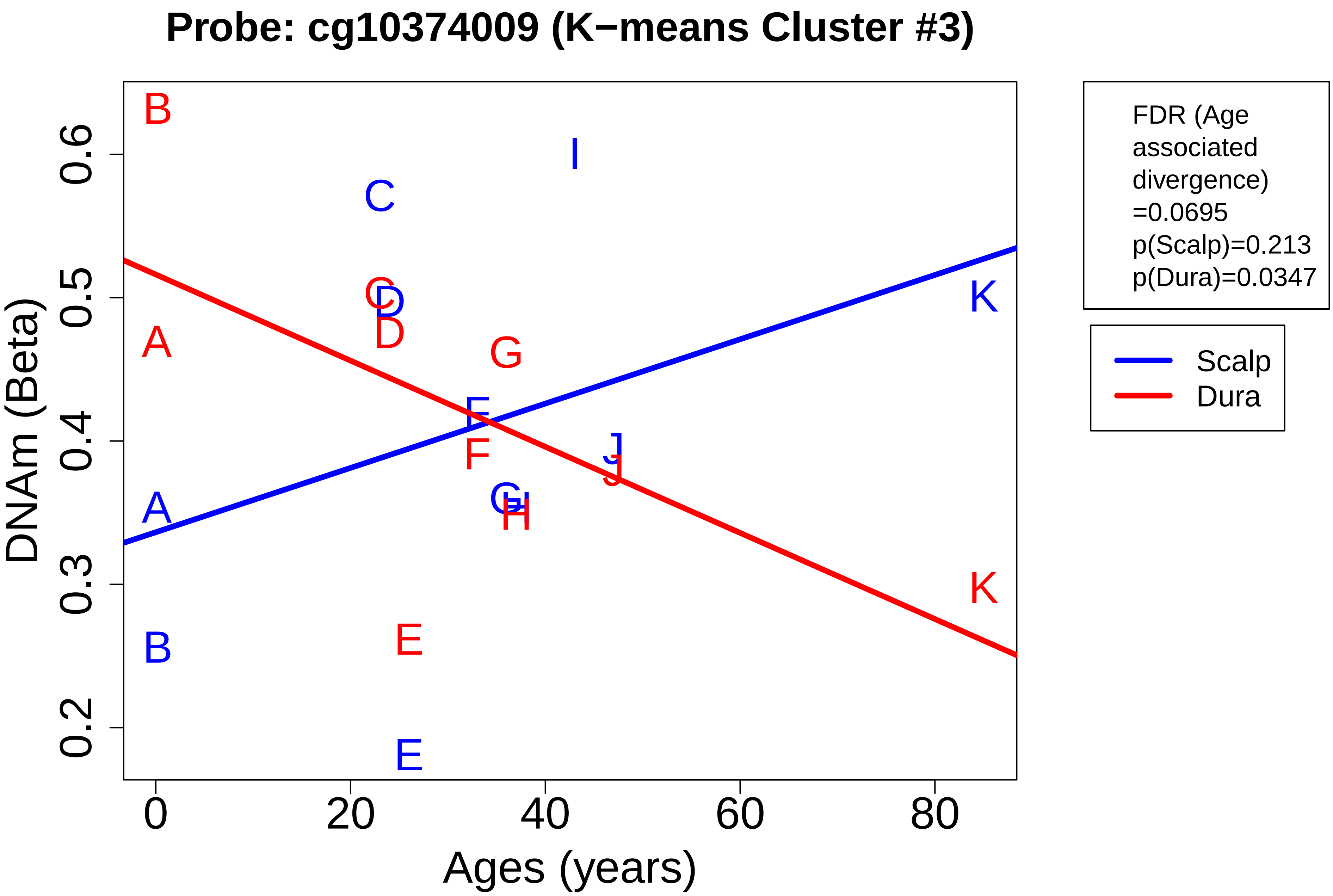

Probe: cg10374009 (K−means Cluster #3)
DNAm (Beta)
Ages (years)
0.2
0.3
0.4
0.5
0.6
0
20
40
60
80
FDR (Age associated divergence) =0.0695
p(Scalp)=0.213
p(Dura)=0.0347
Scalp
Dura

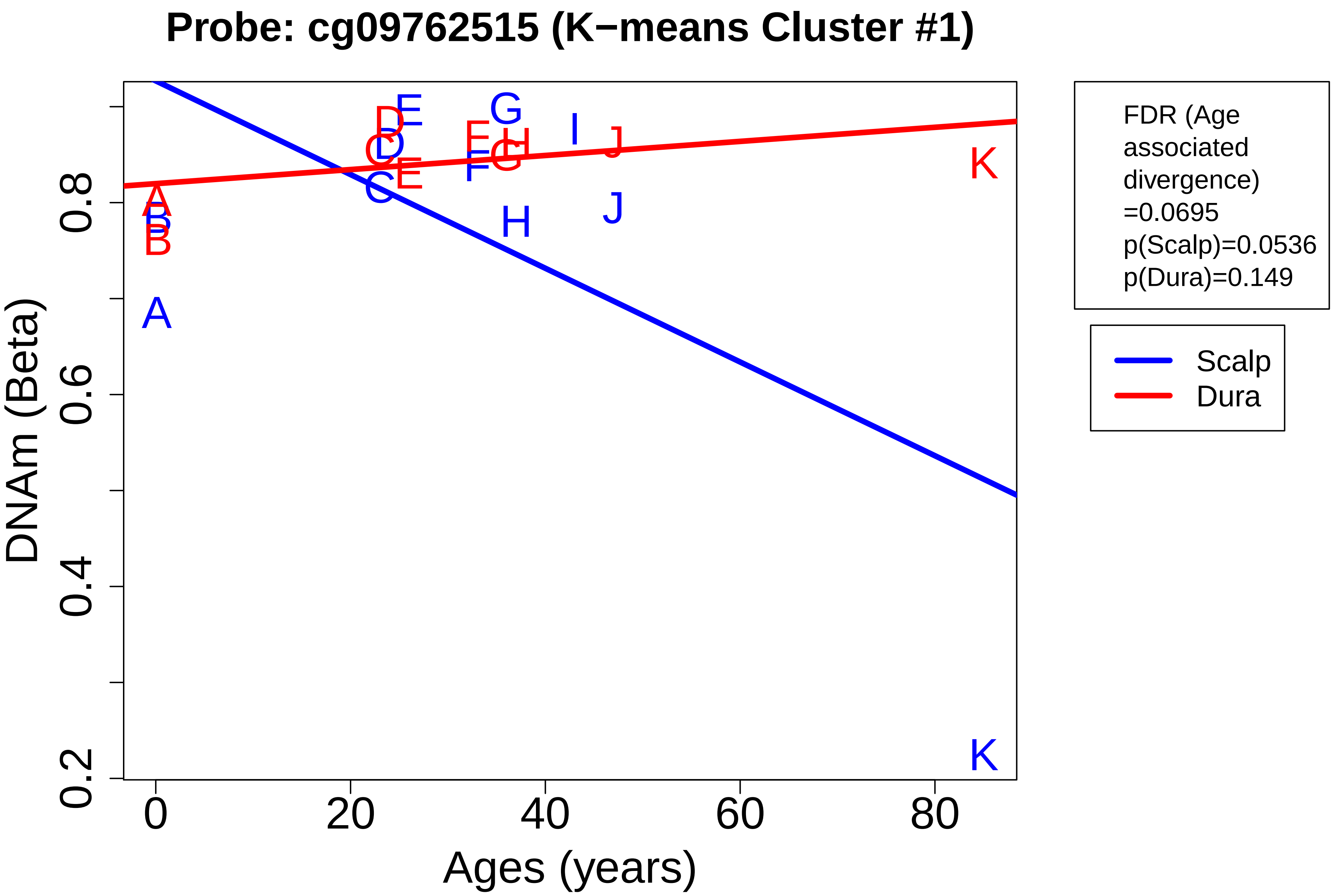

Probe: cg09762515 (K−means Cluster #1)
DNAm (Beta)
Ages (years)
0.2
0.4
0.6
0.8
0
20
40
60
80
FDR (Age associated divergence) =0.0695
p(Scalp)=0.0536
p(Dura)=0.149
Scalp
Dura

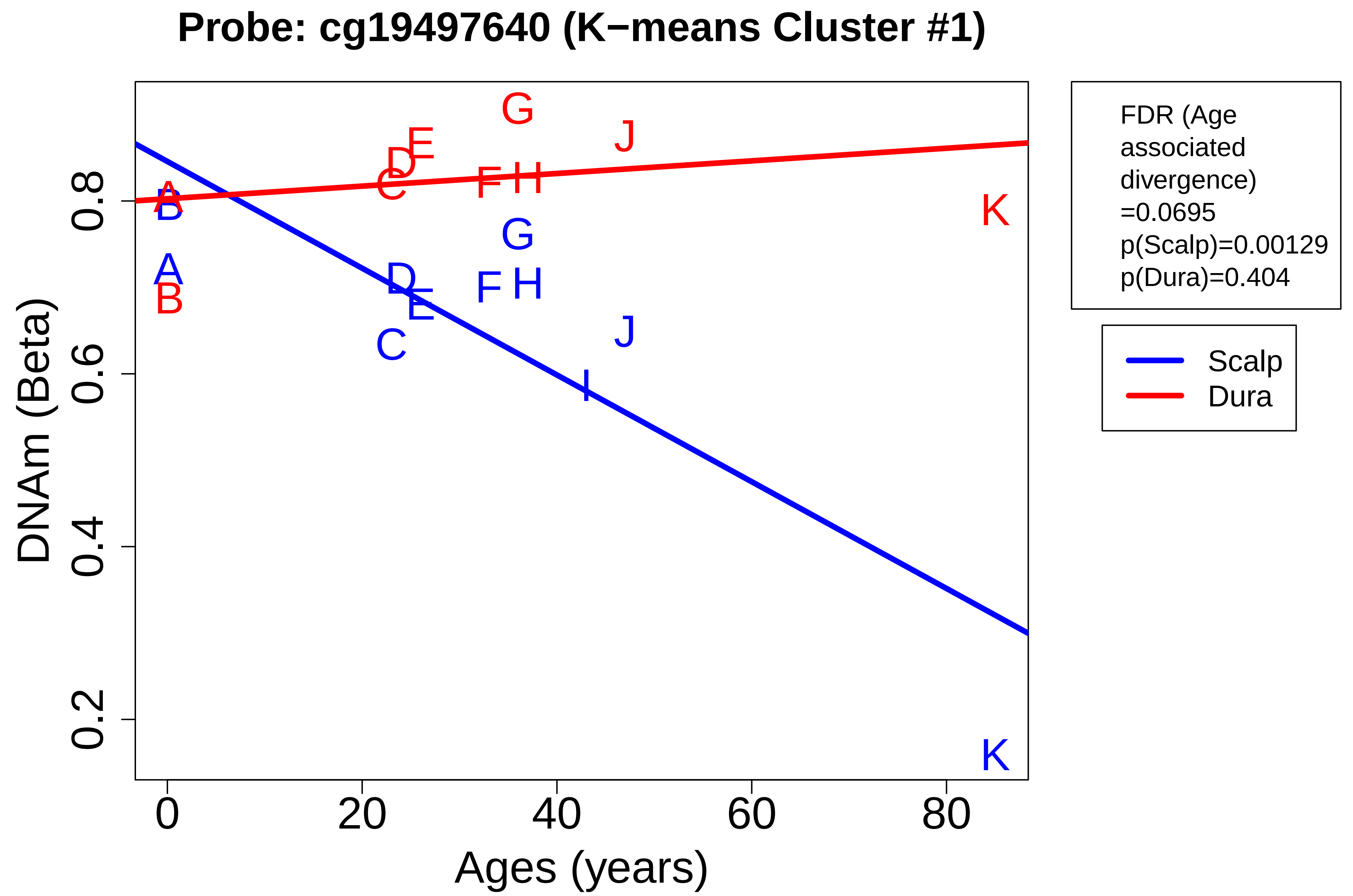

Probe: cg19497640 (K−means Cluster #1)
DNAm (Beta)
0.2
0.4
0.6
0.8
0
20
40
60
80
Ages (years)
FDR (Age associated divergence) =0.0695
p(Scalp)=0.00129
p(Dura)=0.404
Scalp
Dura

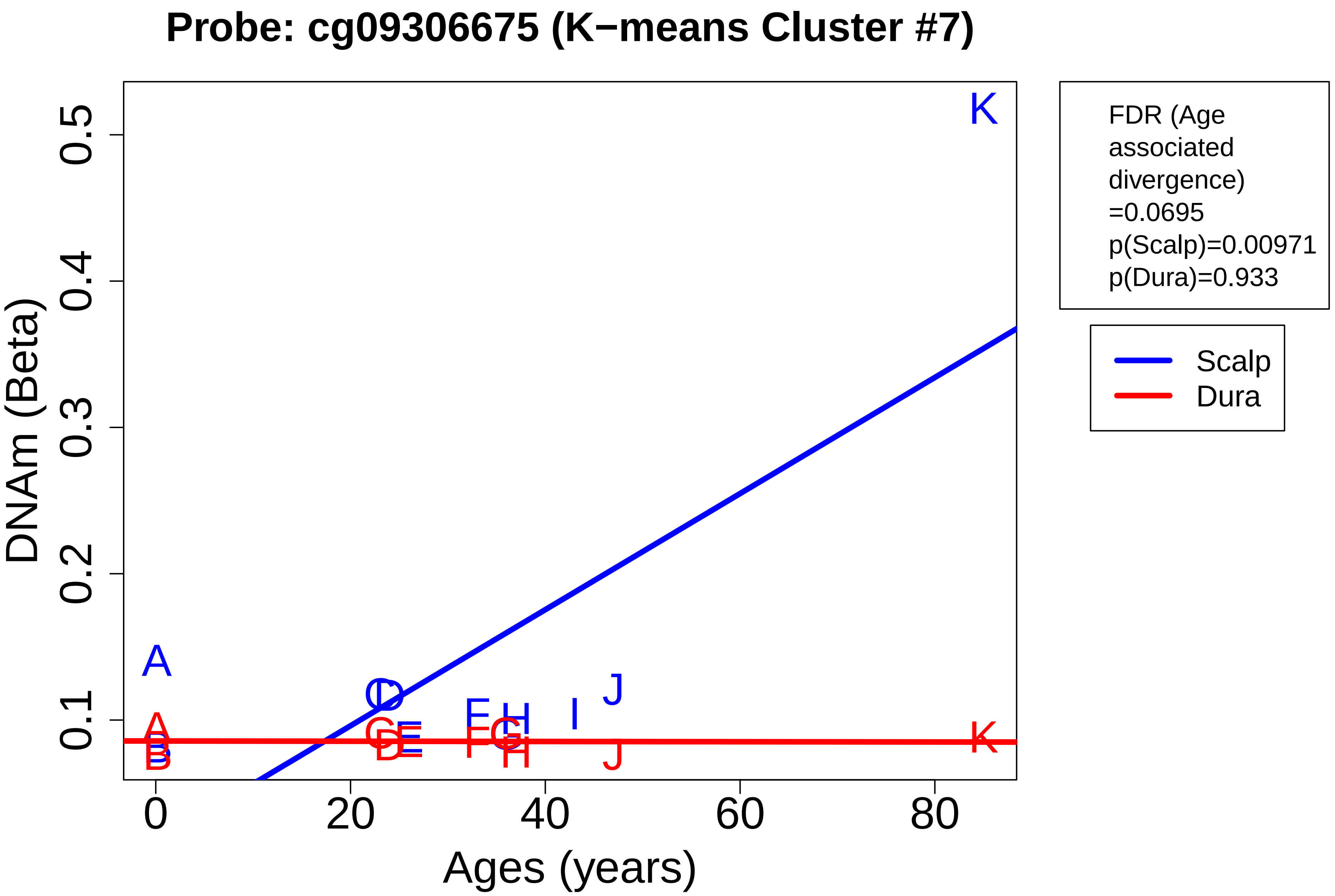

Probe: cg09306675 (K−means Cluster #7)
FDR (Age associated divergence) =0.0695
p(Scalp)=0.00971
p(Dura)=0.933
Scalp
Dura
DNAm (Beta)
Ages (years)
0.1
0.2
0.3
0.4
0.5
0
20
40
60
80

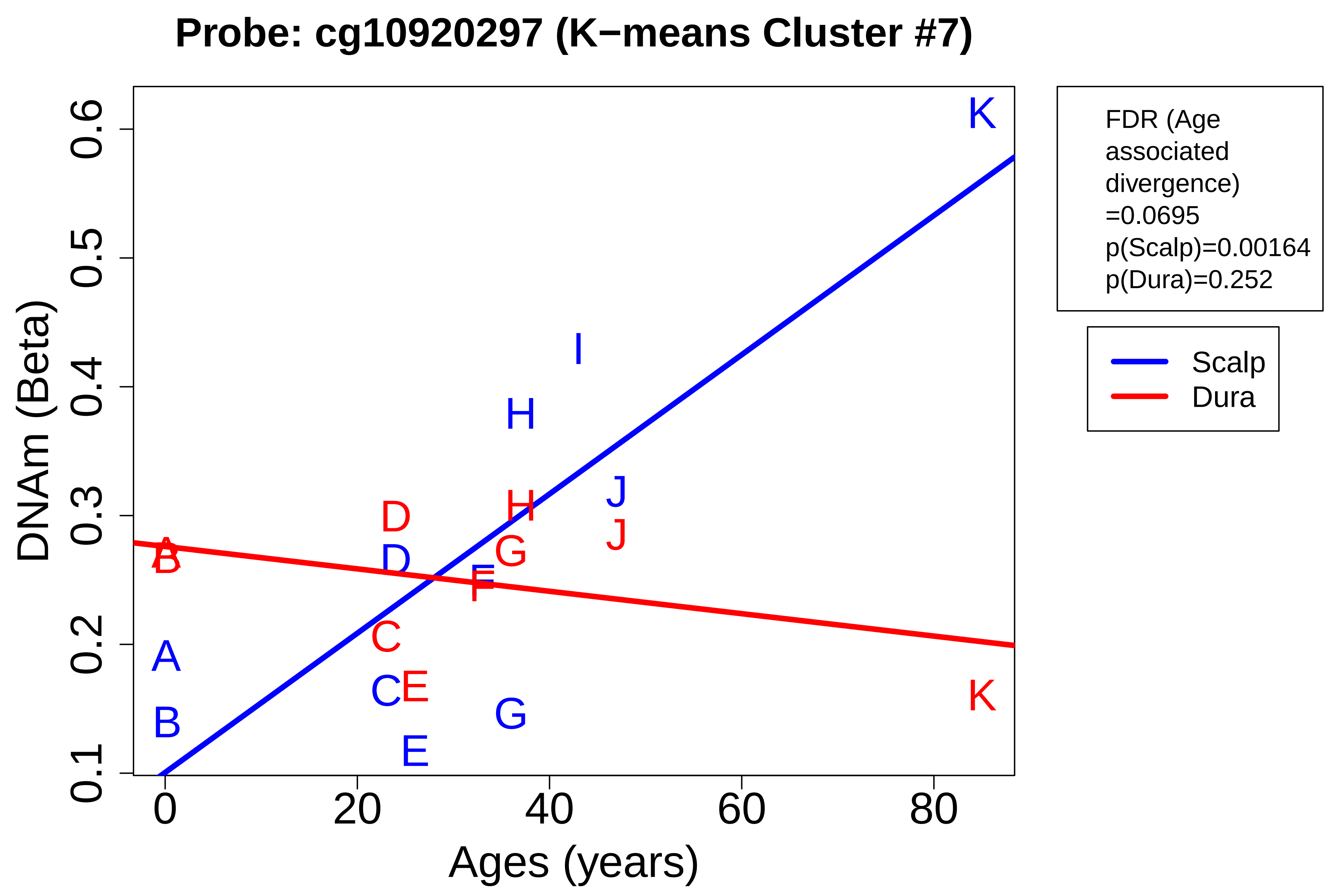

Probe: cg10920297 (K−means Cluster #7)
DNAm (Beta)
0.1
0.2
0.3
0.4
0.5
0.6
0
20
40
60
80
Ages (years)
A
B
C
D
E
F
G
H
I
J
K
FDR (Age associated divergence) =0.0695
p(Scalp)=0.00164
p(Dura)=0.252
Scalp
Dura

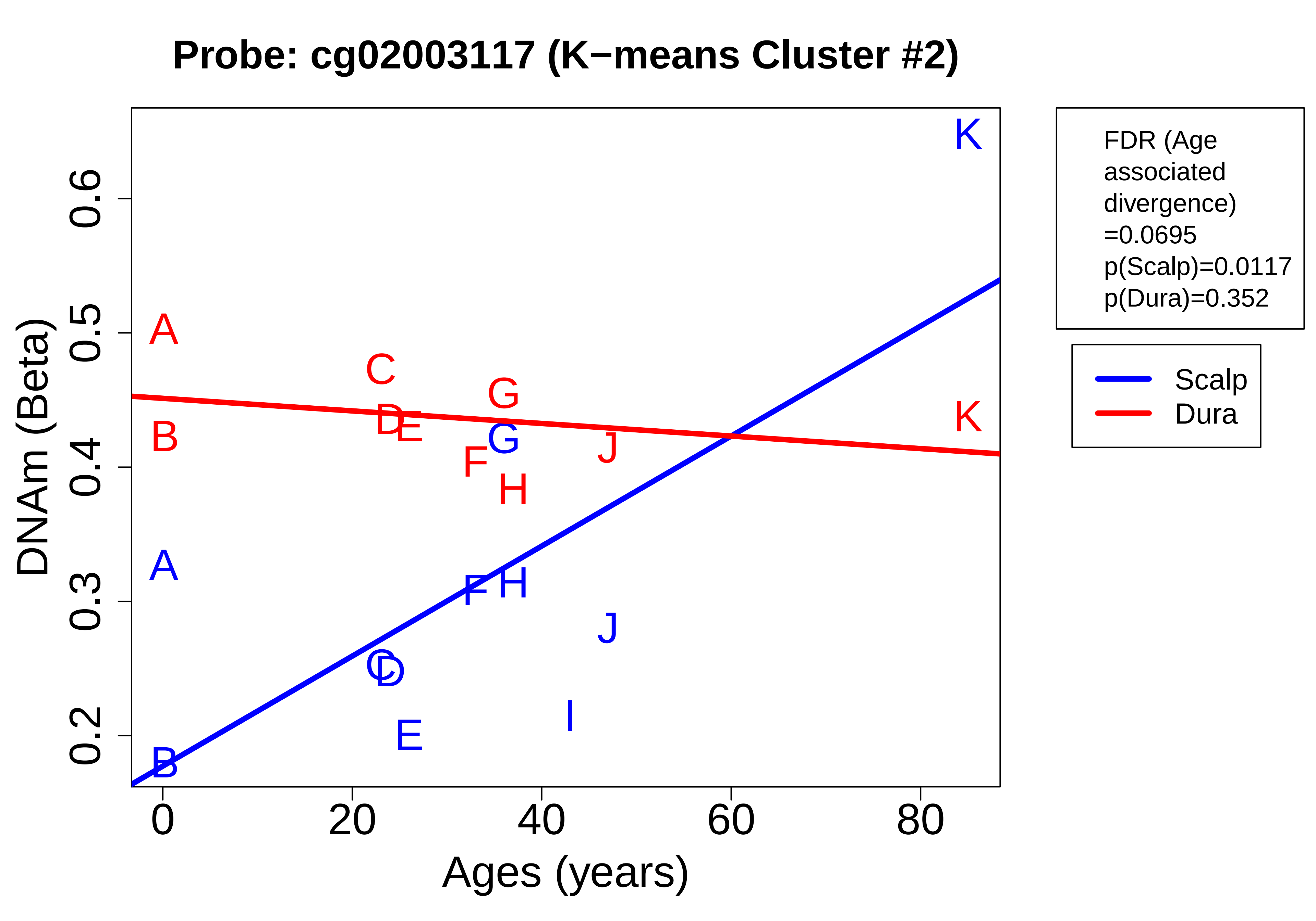
Probe: cg02003117 (K−means Cluster #2)
DNAm (Beta)
0.2
0.3
0.4
0.5
0.6
0
20
40
60
80
Ages (years)
FDR (Age associated divergence) =0.0695
p(Scalp)=0.0117
p(Dura)=0.352
Scalp
Dura
A
B
C
D
E
F
G
H
I
J
K

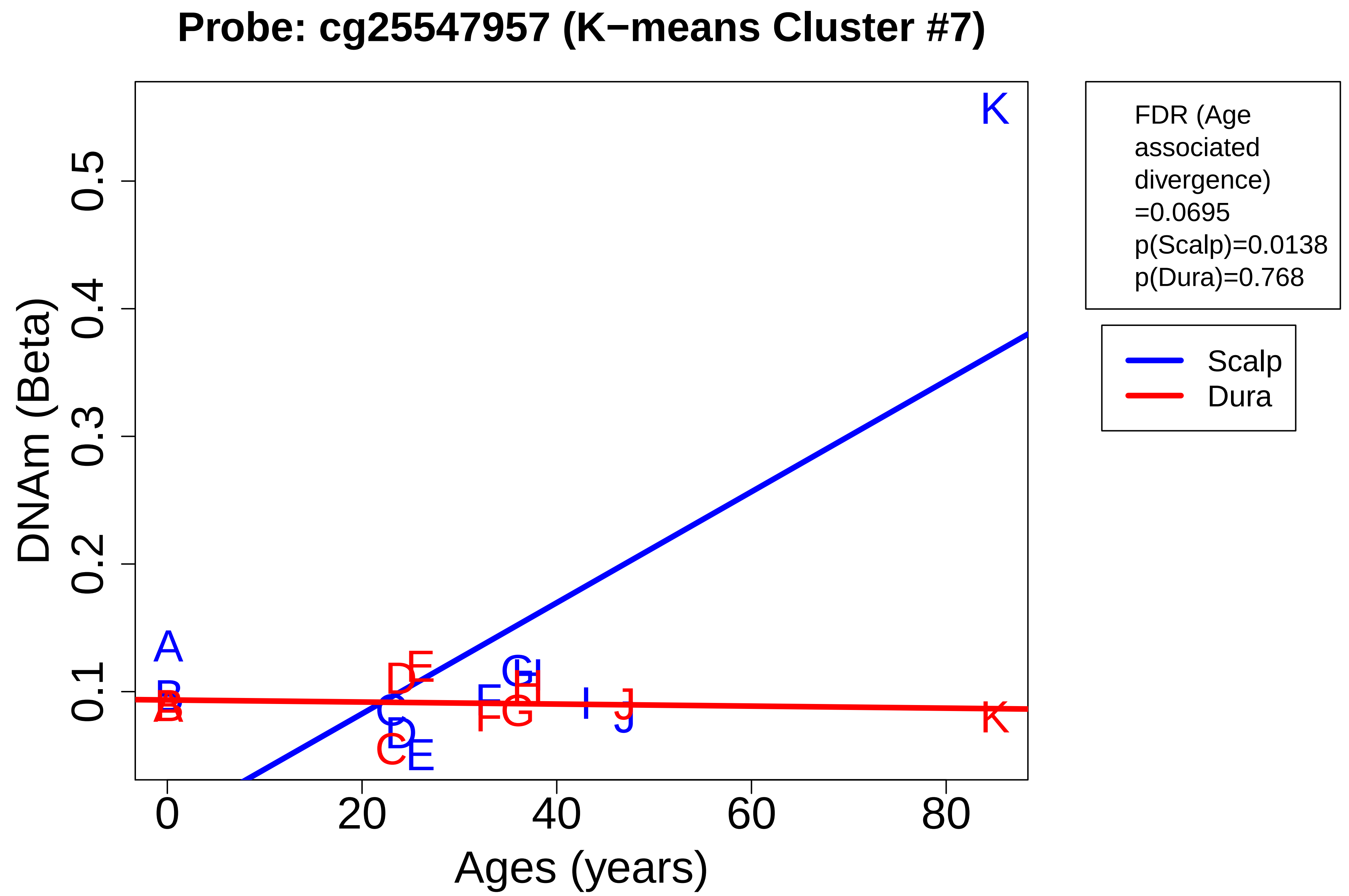
Probe: cg25547957 (K−means Cluster #7)
DNAm (Beta)
0.1
0.2
0.3
0.4
0.5
0
20
40
60
80
Ages (years)
FDR (Age associated divergence) =0.0695
p(Scalp)=0.0138
p(Dura)=0.768
Scalp
Dura

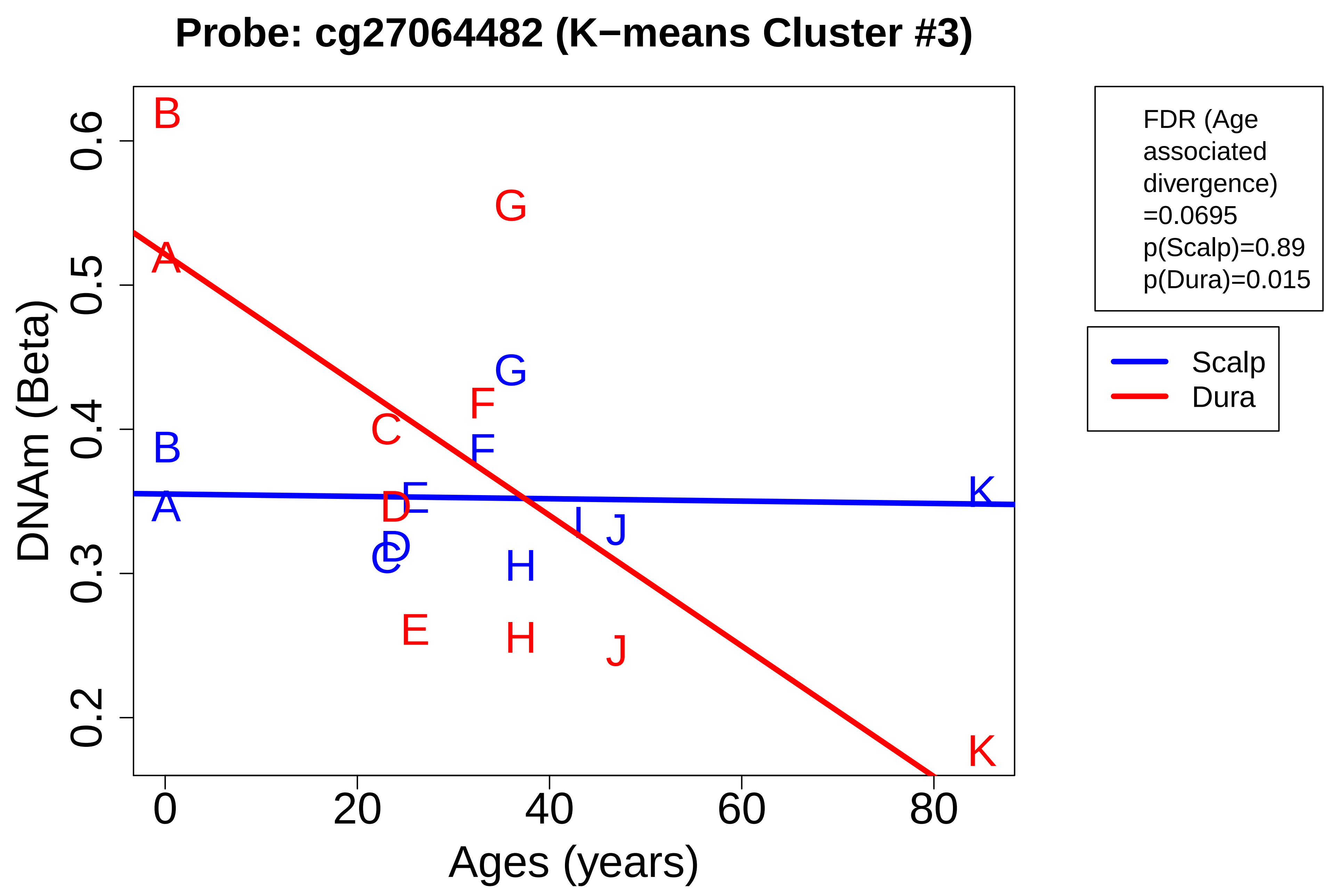

Probe: cg27064482 (K−means Cluster #3)
DNAm (Beta)
Ages (years)
0.2
0.3
0.4
0.5
0.6
0
20
40
60
80
FDR (Age associated divergence) =0.0695
p(Scalp)=0.89
p(Dura)=0.015
Scalp
Dura

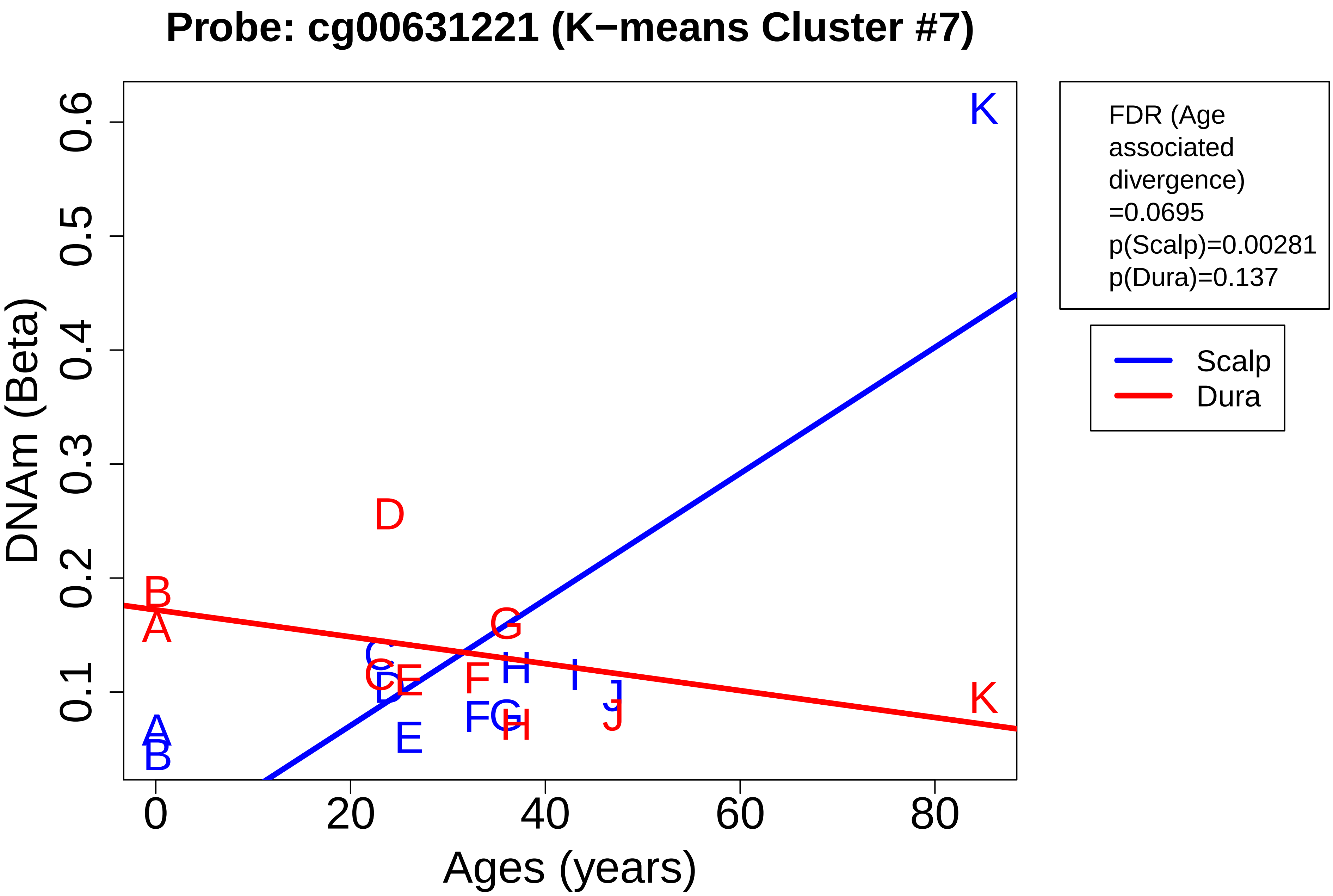

Probe: cg00631221 (K−means Cluster #7)
DNAm (Beta)
Ages (years)
FDR (Age associated divergence) =0.0695
p(Scalp)=0.00281
p(Dura)=0.137
Scalp
Dura

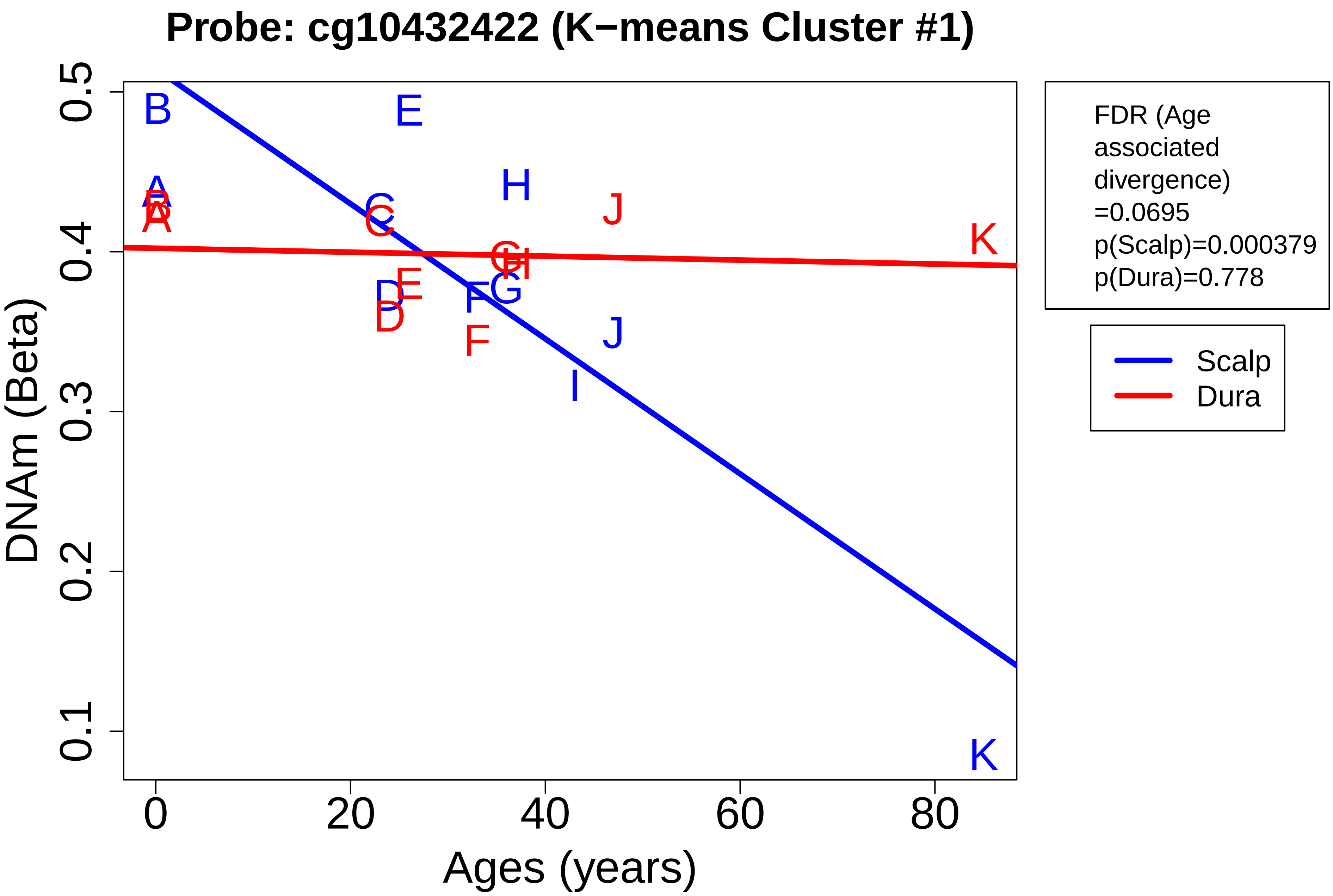
Probe: cg10432422 (K−means Cluster #1)
DNAm (Beta)
0.1
0.2
0.3
0.4
0.5
Ages (years)
0
20
40
60
80
FDR (Age associated divergence) =0.0695
p(Scalp)=0.000379
p(Dura)=0.778
Scalp
Dura
A
B
C
D
E
F
G
H
I
J
K

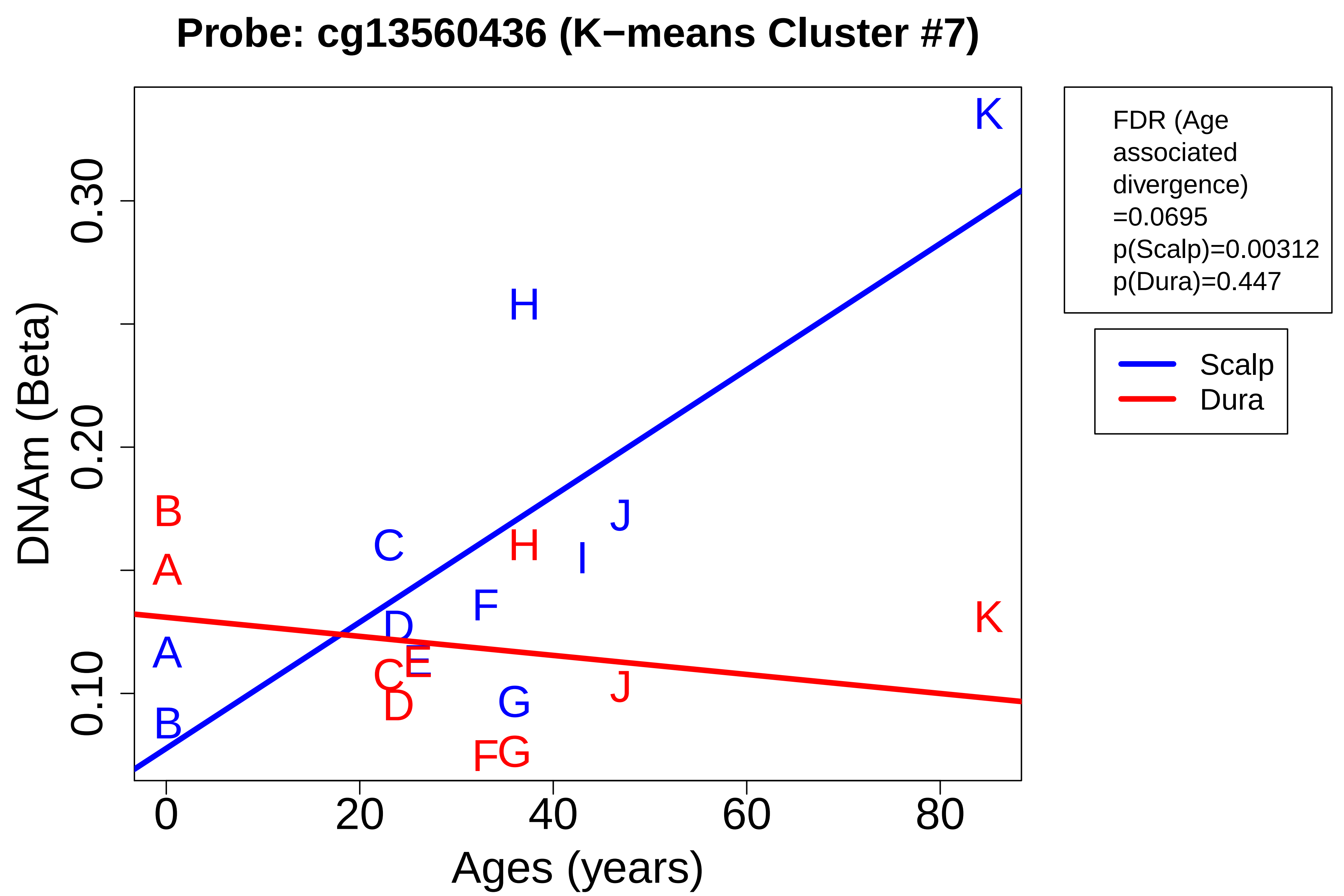
Probe: cg13560436 (K−means Cluster #7)
DNAm (Beta)
0.10
0.20
0.30
Ages (years)
0
20
40
60
80
FDR (Age associated divergence) =0.0695
p(Scalp)=0.00312
p(Dura)=0.447
Scalp
Dura
A
B
C
D
E
F
G
H
I
J
K

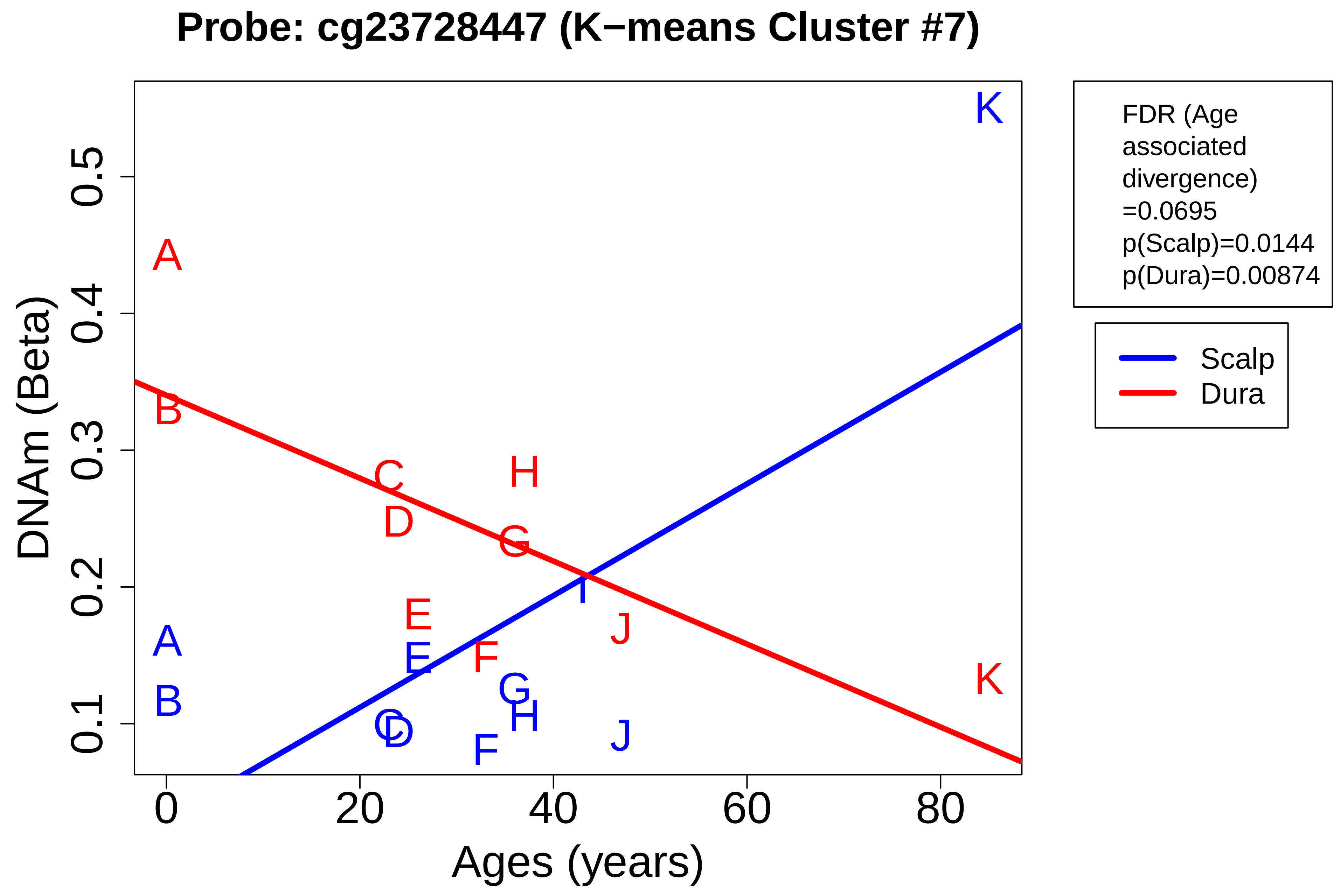

Probe: cg23728447 (K−means Cluster #7)
DNAm (Beta)
0.1
0.2
0.3
0.4
0.5
Ages (years)
0
20
40
60
80
A
B
C
D
E
F
G
H
I
J
K
FDR (Age associated divergence) =0.0695
p(Scalp)=0.0144
p(Dura)=0.00874
Scalp
Dura

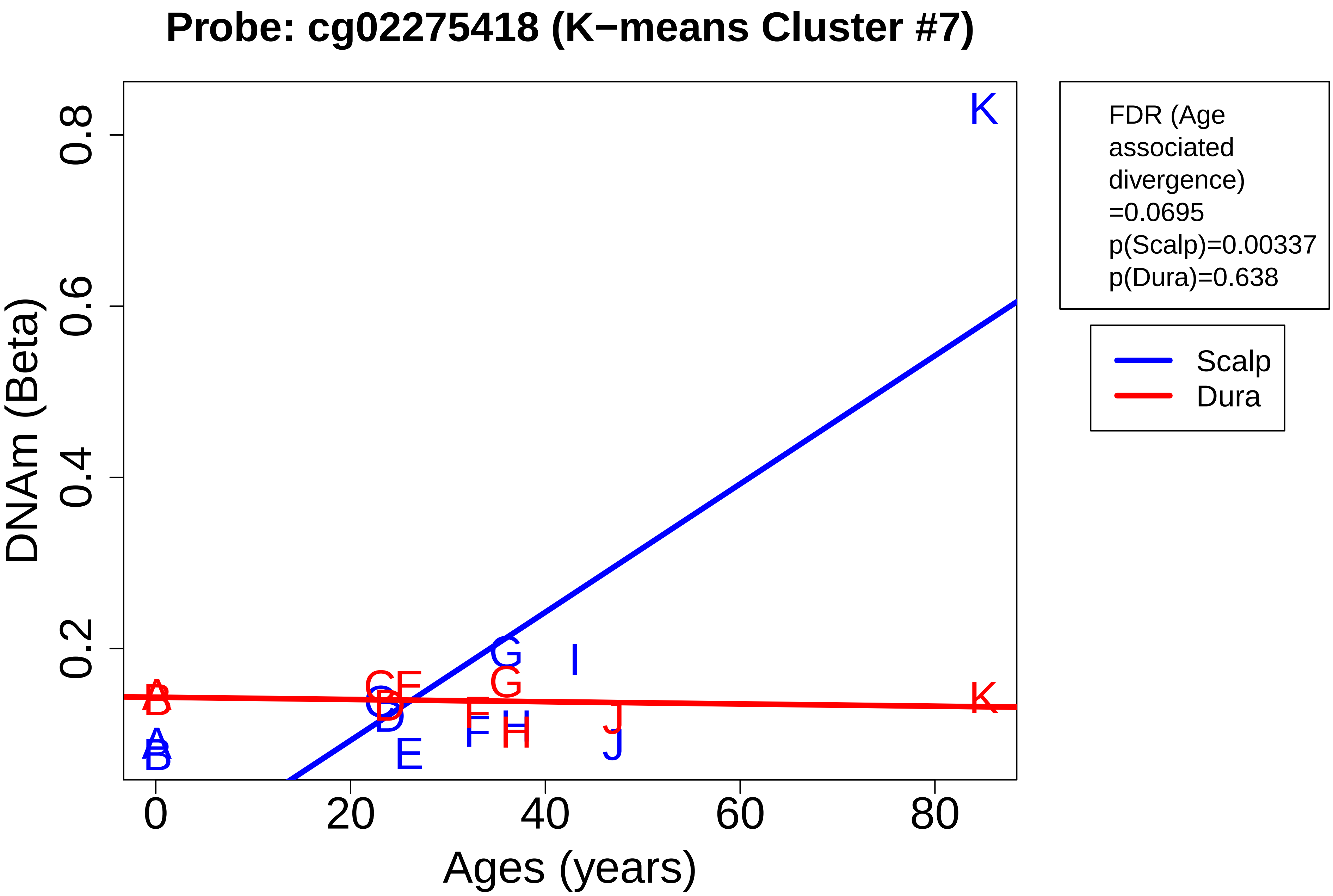
Probe: cg02275418 (K−means Cluster #7)
DNAm (Beta)
0.2
0.4
0.6
0.8
Ages (years)
0
20
40
60
80
K
A
B
C
D
E
F
G
H
I
J
FDR (Age associated divergence) =0.0695
p(Scalp)=0.00337
p(Dura)=0.638
Scalp
Dura

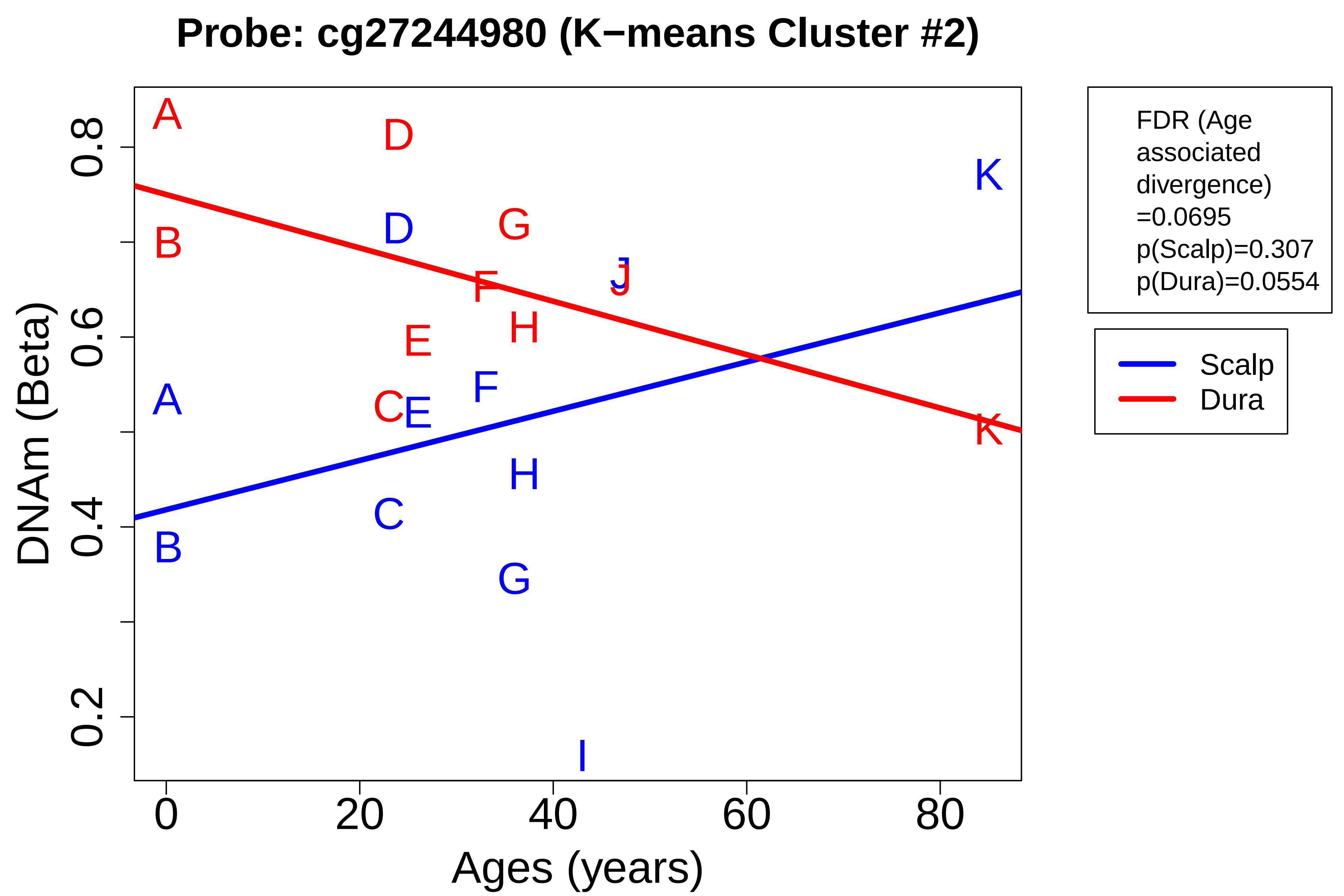

Probe: cg27244980 (K−means Cluster #2)
DNAm (Beta)
0.2
0.4
0.6
0.8
Ages (years)
0
20
40
60
80
A
B
C
D
E
F
G
H
I
J
K
FDR (Age associated divergence) =0.0695
p(Scalp)=0.307
p(Dura)=0.0554
Scalp
Dura

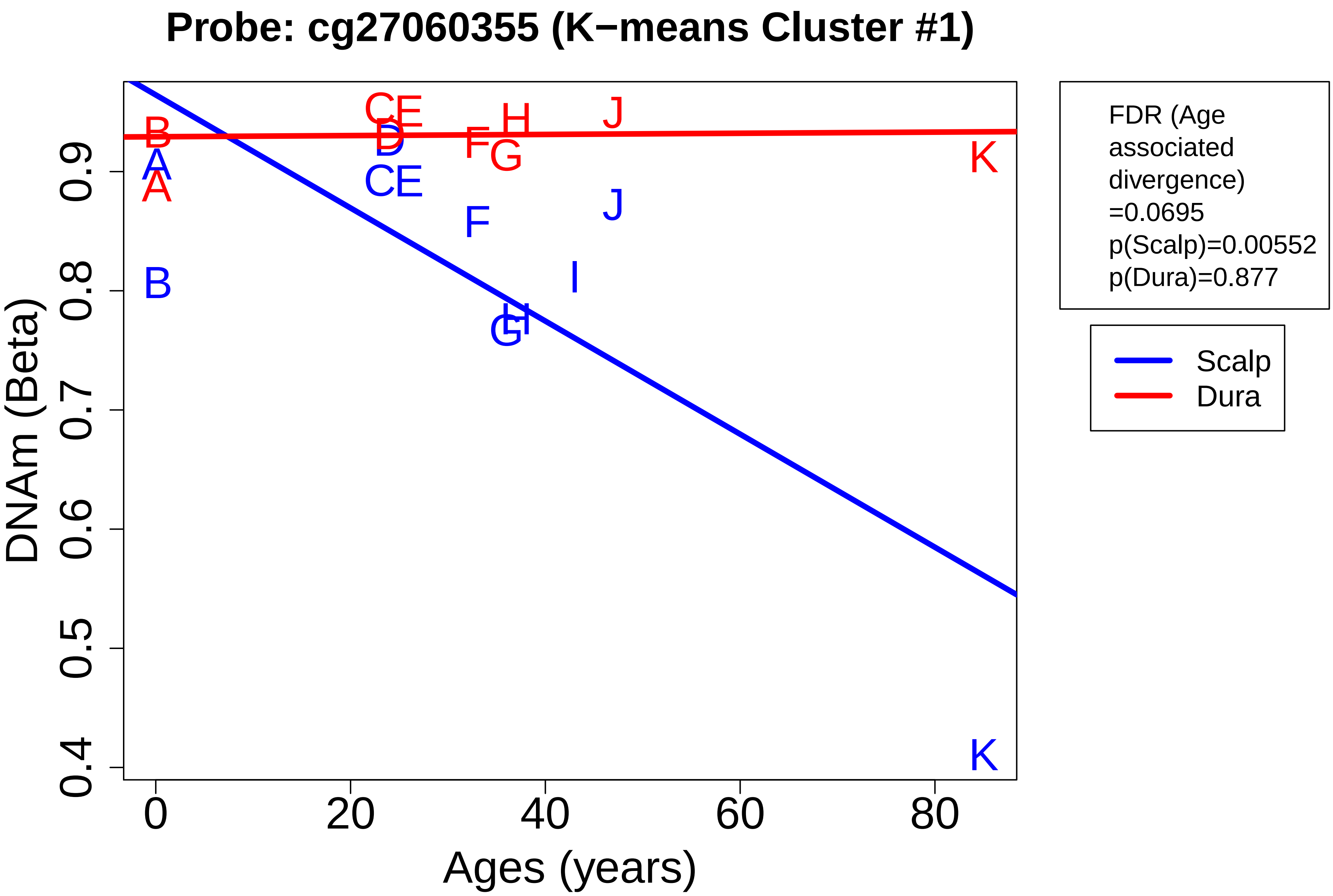

Probe: cg27060355 (K−means Cluster #1)
DNAm (Beta)
0.4
0.5
0.6
0.7
0.8
0.9
0
20
40
60
80
Ages (years)
FDR (Age associated divergence) =0.0695
p(Scalp)=0.00552
p(Dura)=0.877
Scalp
Dura

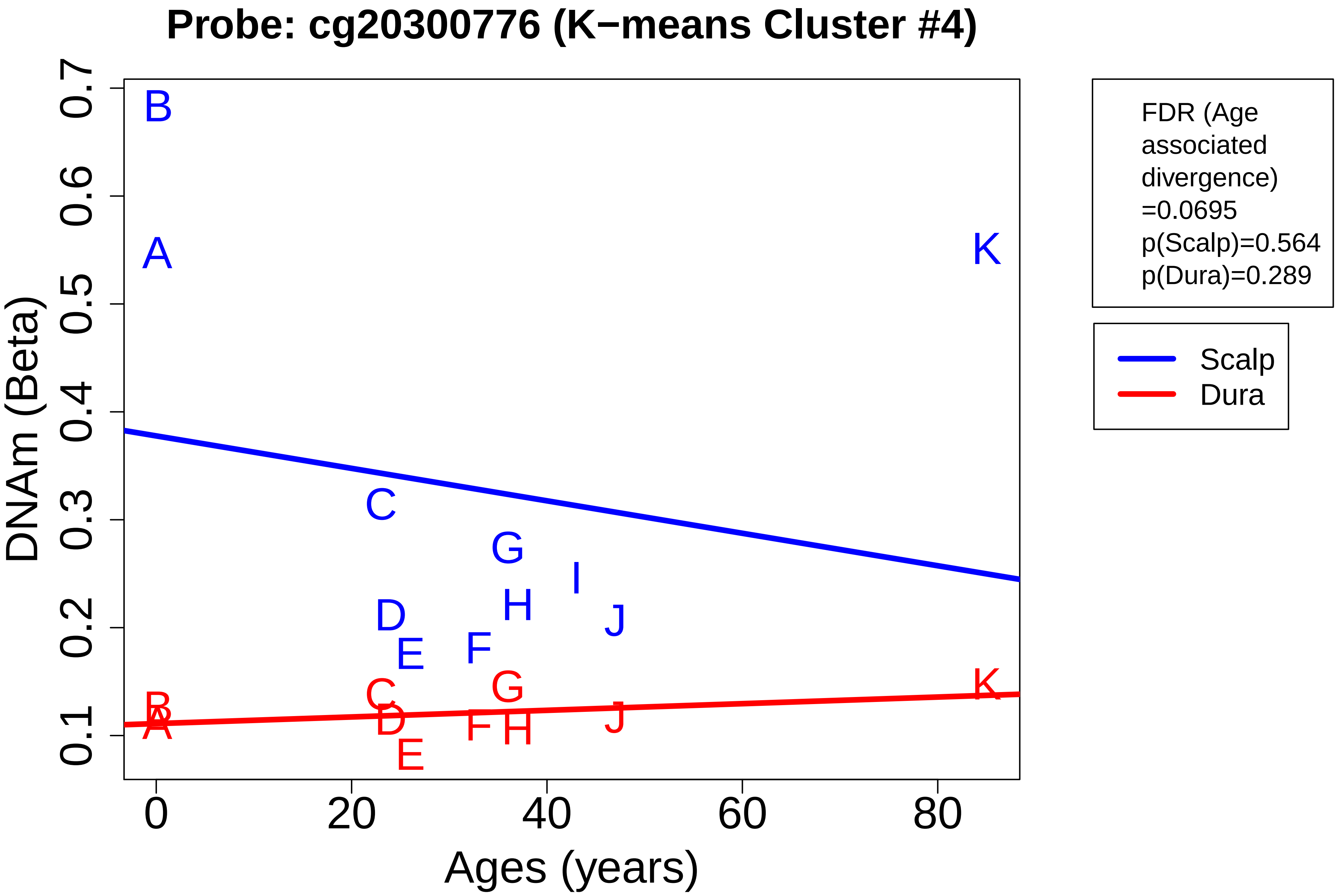
Probe: cg20300776 (K−means Cluster #4)
DNAm (Beta)
0.1
0.2
0.3
0.4
0.5
0.6
0.7
Ages (years)
0
20
40
60
80
A B C D E F G H I J K
A B C D E F G H J K
FDR (Age associated divergence) =0.0695
p(Scalp)=0.564
p(Dura)=0.289
Scalp
Dura

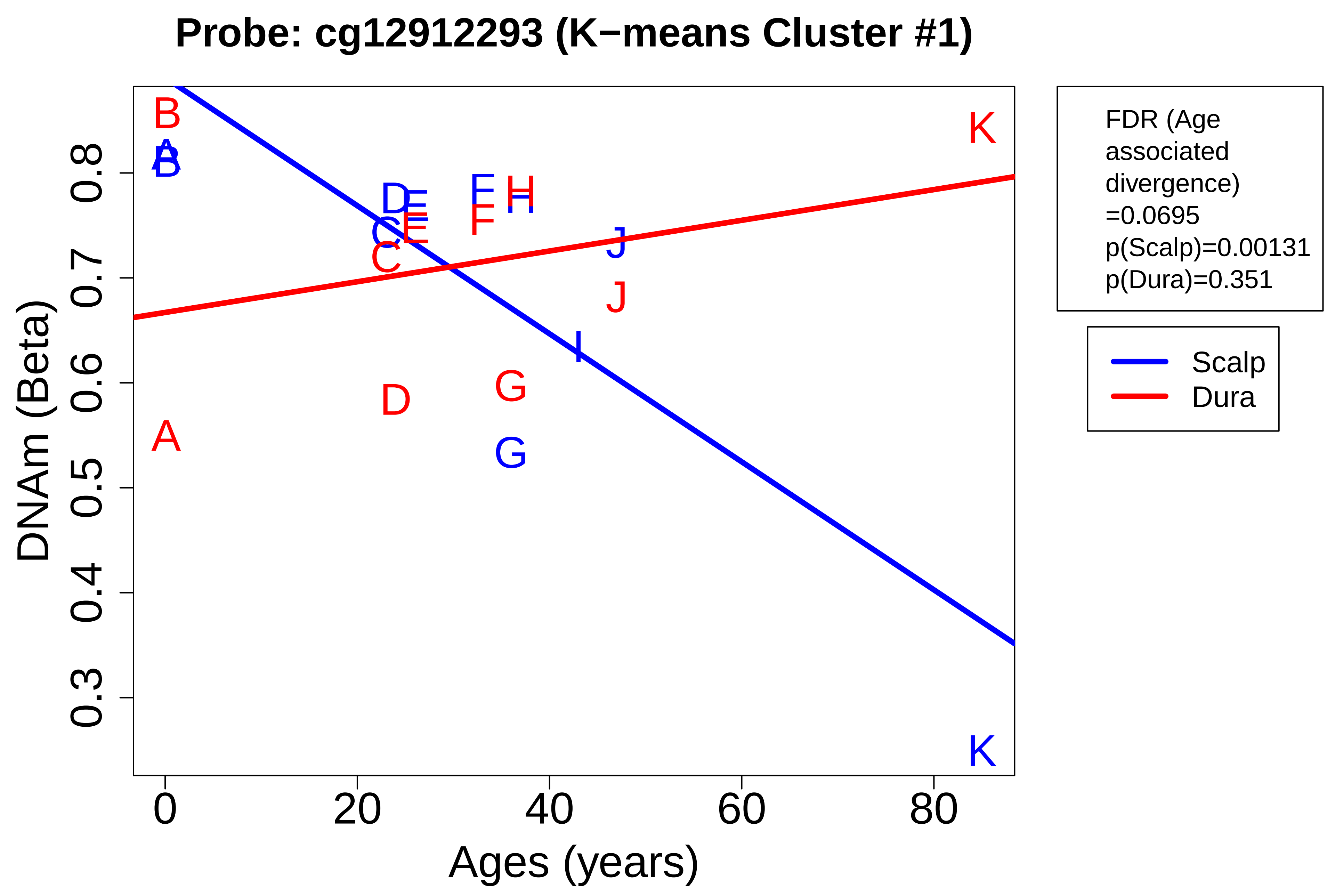

Probe: cg12912293 (K−means Cluster #1)
DNAm (Beta)
0.3
0.4
0.5
0.6
0.7
0.8
0
20
40
60
80
Ages (years)
A
B
C
D
E
F
G
H
I
J
K
FDR (Age associated divergence) =0.0695
p(Scalp)=0.00131
p(Dura)=0.351
Scalp
Dura

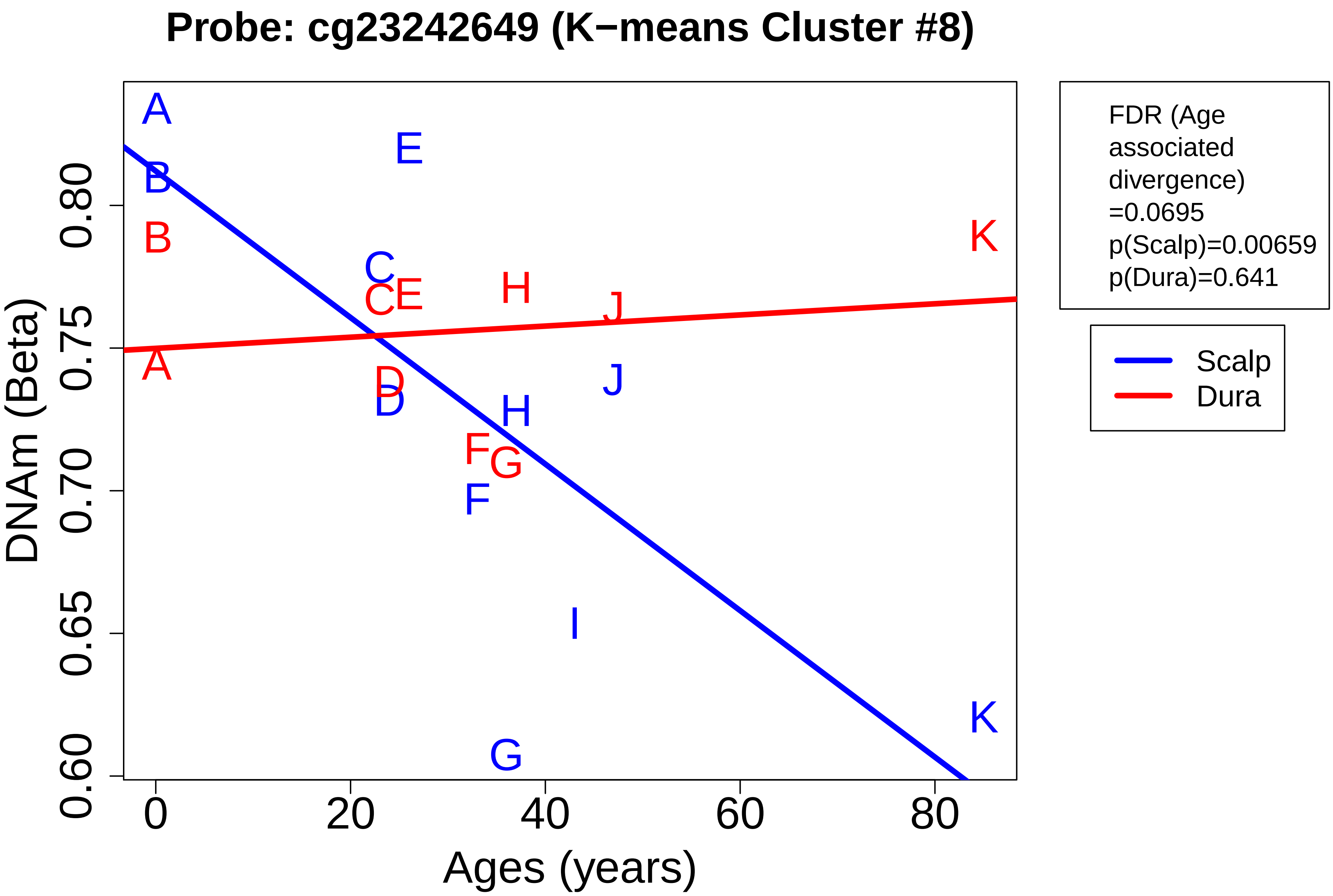

Probe: cg23242649 (K−means Cluster #8)
DNAm (Beta)
0.60
0.65
0.70
0.75
0.80
Ages (years)
0
20
40
60
80
FDR (Age associated divergence) =0.0695
p(Scalp)=0.00659
p(Dura)=0.641
Scalp
Dura
A
B
C
D
E
F
G
H
I
J
K

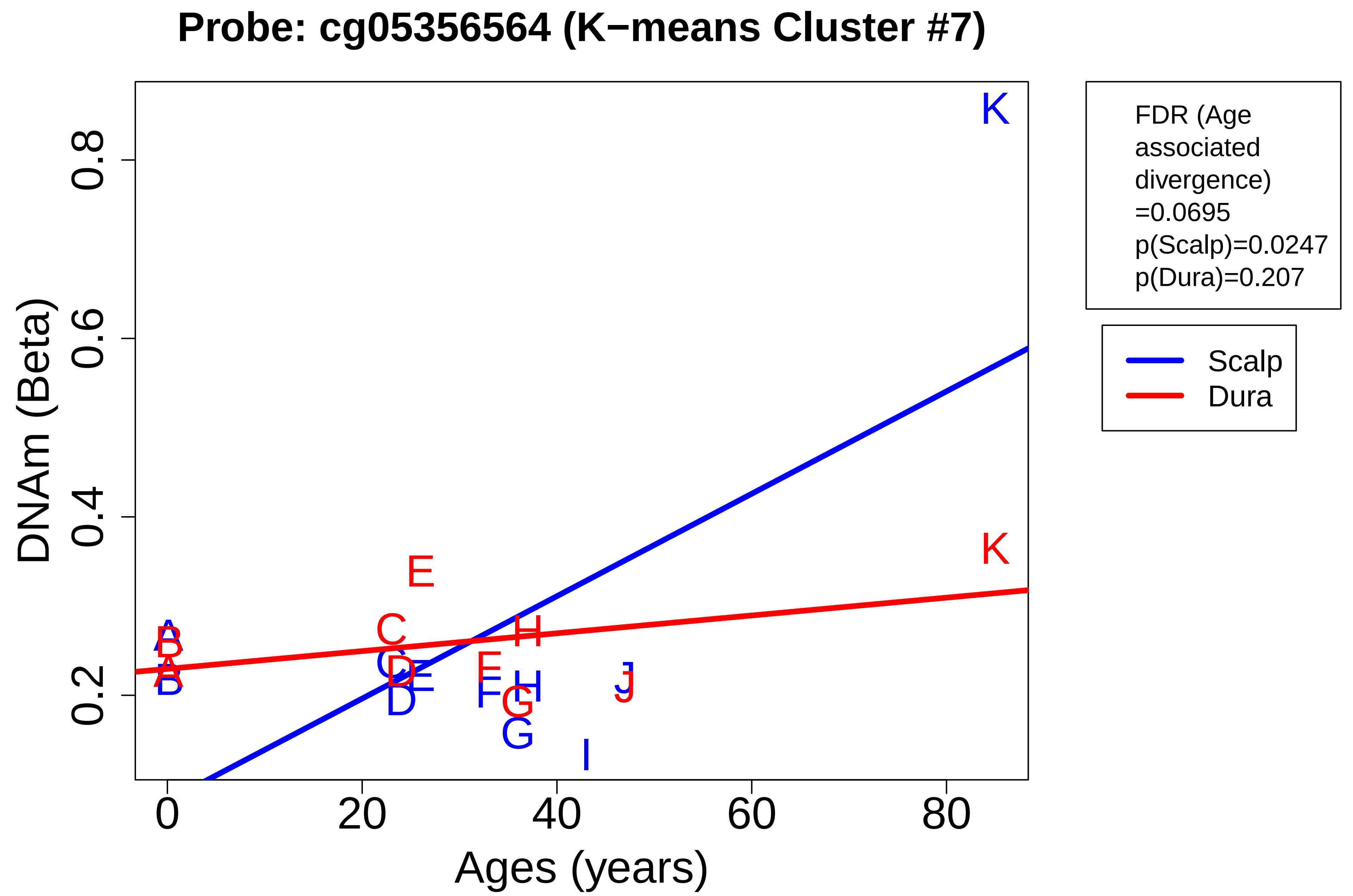

Probe: cg05356564 (K−means Cluster #7)
DNAm (Beta)
Ages (years)
0.2
0.4
0.6
0.8
0
20
40
60
80
FDR (Age associated divergence) =0.0695
p(Scalp)=0.0247
p(Dura)=0.207
Scalp
Dura

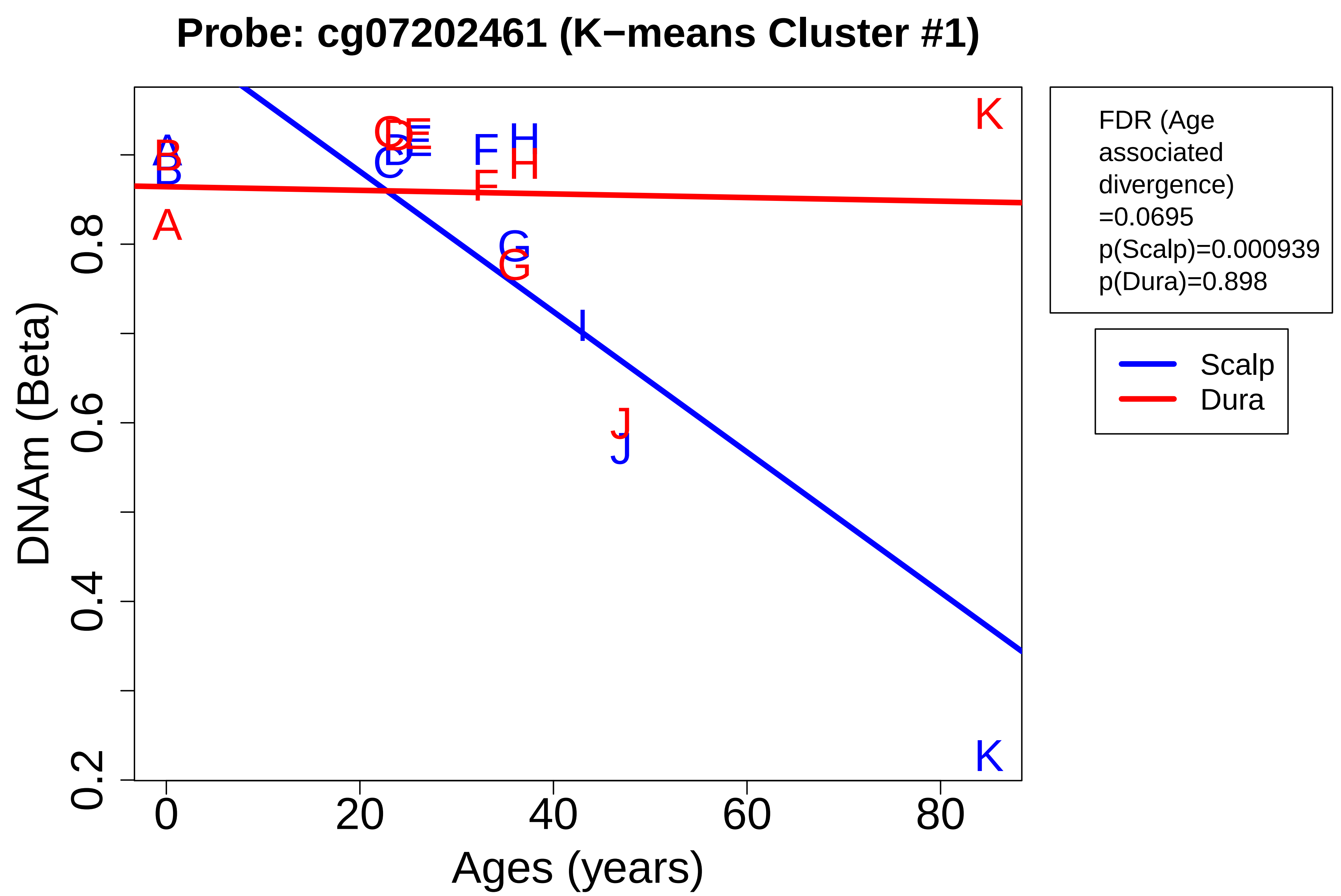

Probe: cg07202461 (K−means Cluster #1)
DNAm (Beta)
0.2
0.4
0.6
0.8
0
20
40
60
80
Ages (years)
A
B
C
D
E
F
G
H
I
J
K
FDR (Age associated divergence) =0.0695
p(Scalp)=0.000939
p(Dura)=0.898
Scalp
Dura

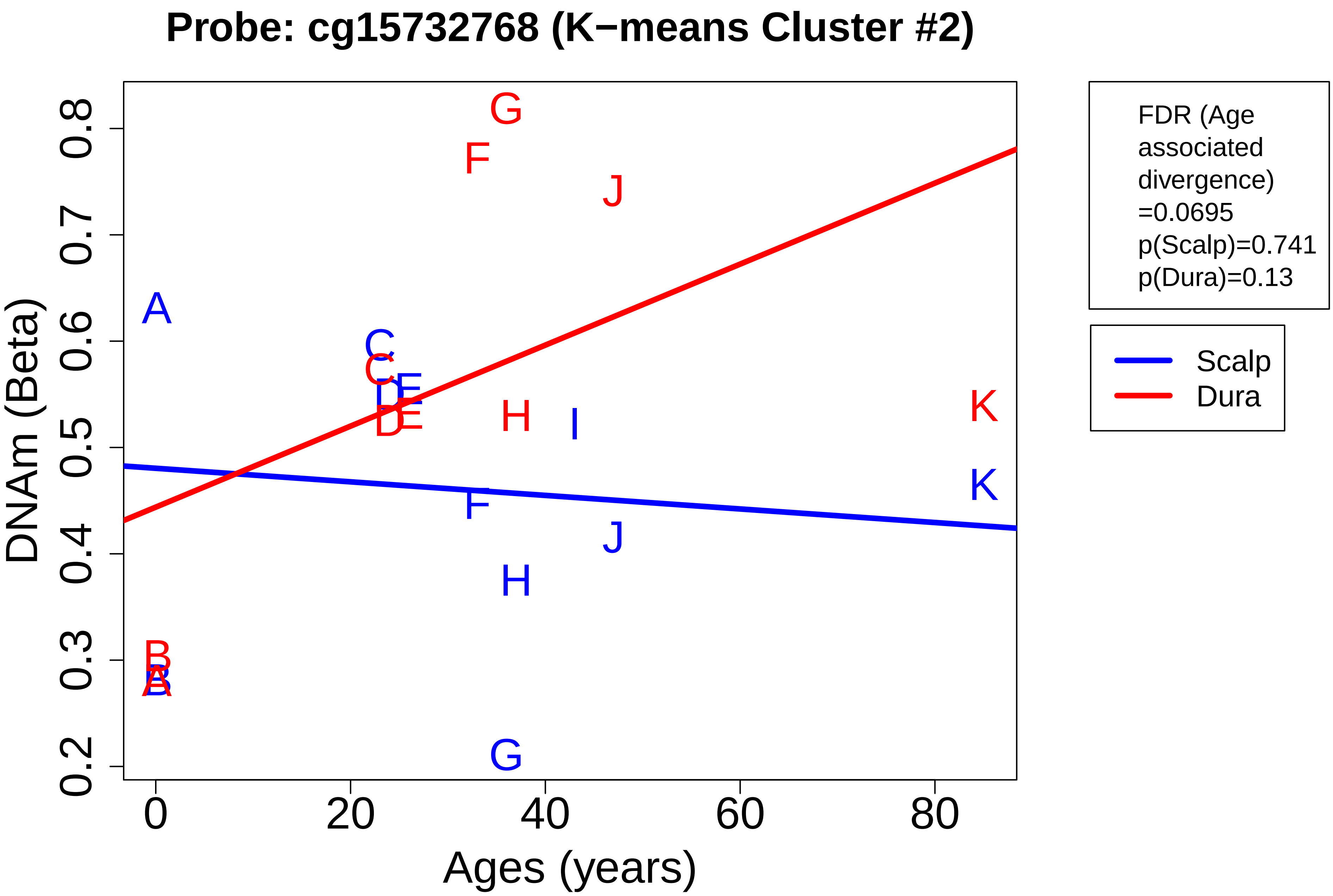

Probe: cg15732768 (K−means Cluster #2)
DNAm (Beta)
Ages (years)
0.2
0.3
0.4
0.5
0.6
0.7
0.8
0
20
40
60
80
FDR (Age associated divergence) =0.0695
p(Scalp)=0.741
p(Dura)=0.13
Scalp
Dura
A
B
C
D
E
F
G
H
I
J
K

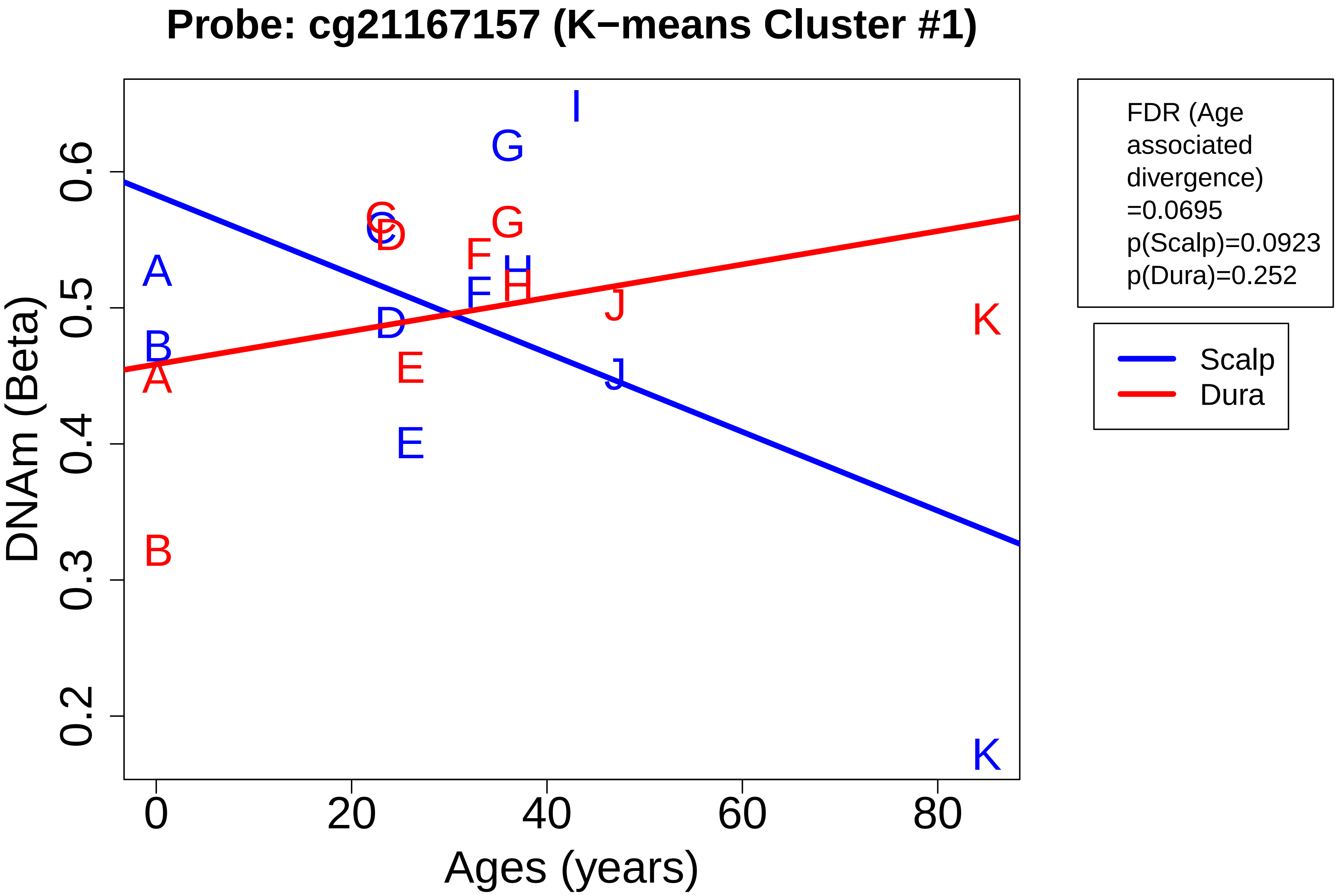

Probe: cg21167157 (K−means Cluster #1)
DNAm (Beta)
0.2
0.3
0.4
0.5
0.6
0
20
40
60
80
Ages (years)
A
B
C
D
E
F
G
H
I
J
K
FDR (Age associated divergence) =0.0695
p(Scalp)=0.0923
p(Dura)=0.252
Scalp
Dura

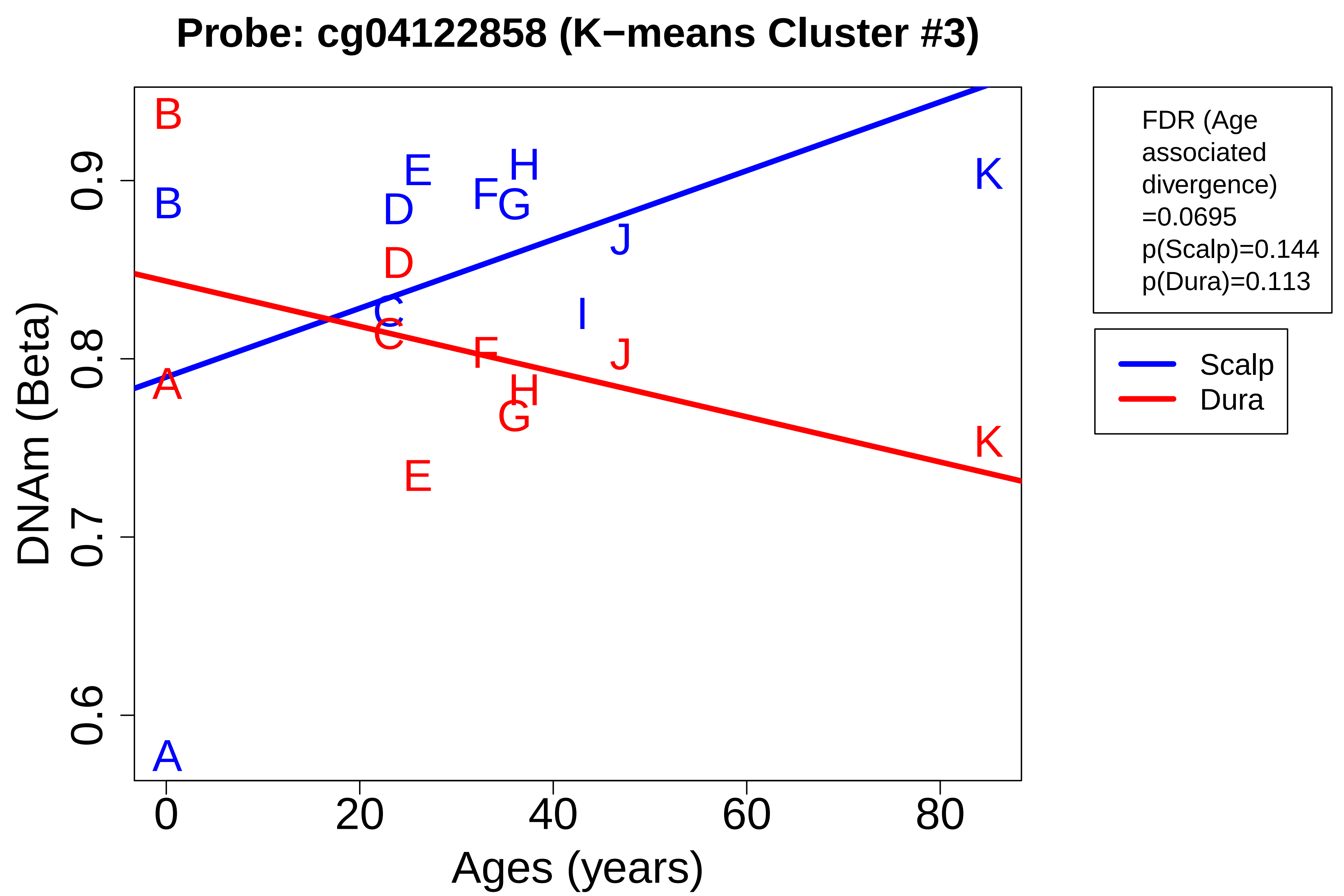

Probe: cg04122858 (K−means Cluster #3)
DNAm (Beta)
0.6
0.7
0.8
0.9
0
20
40
60
80
Ages (years)
FDR (Age associated divergence) =0.0695
p(Scalp)=0.144
p(Dura)=0.113
Scalp
Dura
A
B
C
D
E
F
G
H
I
J
K

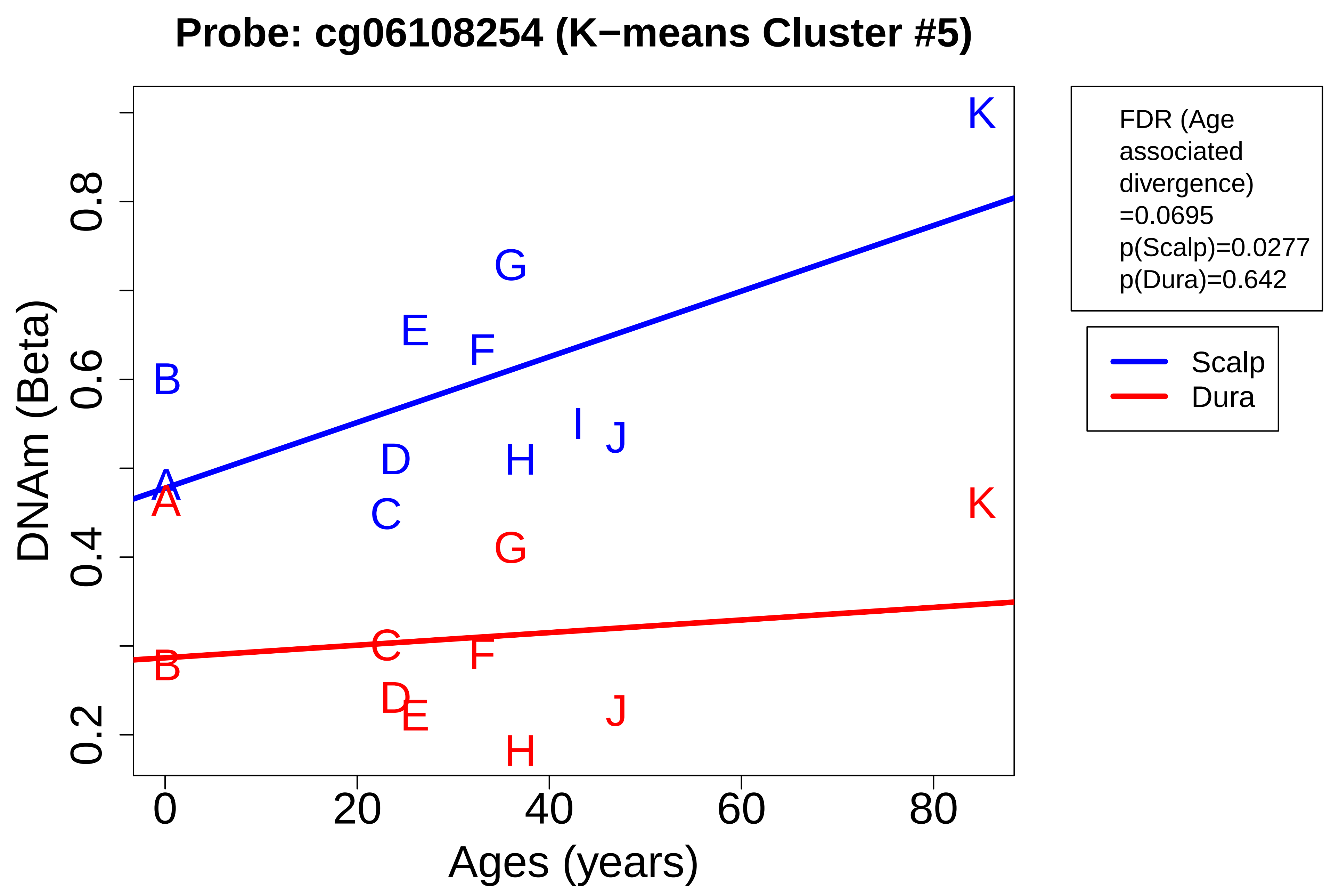

Probe: cg06108254 (K−means Cluster #5)
DNAm (Beta)
0.2
0.4
0.6
0.8
0
20
40
60
80
Ages (years)
FDR (Age associated divergence) =0.0695
p(Scalp)=0.0277
p(Dura)=0.642
Scalp
Dura
A B C D E F G H I J K
A B C D E F G H J K

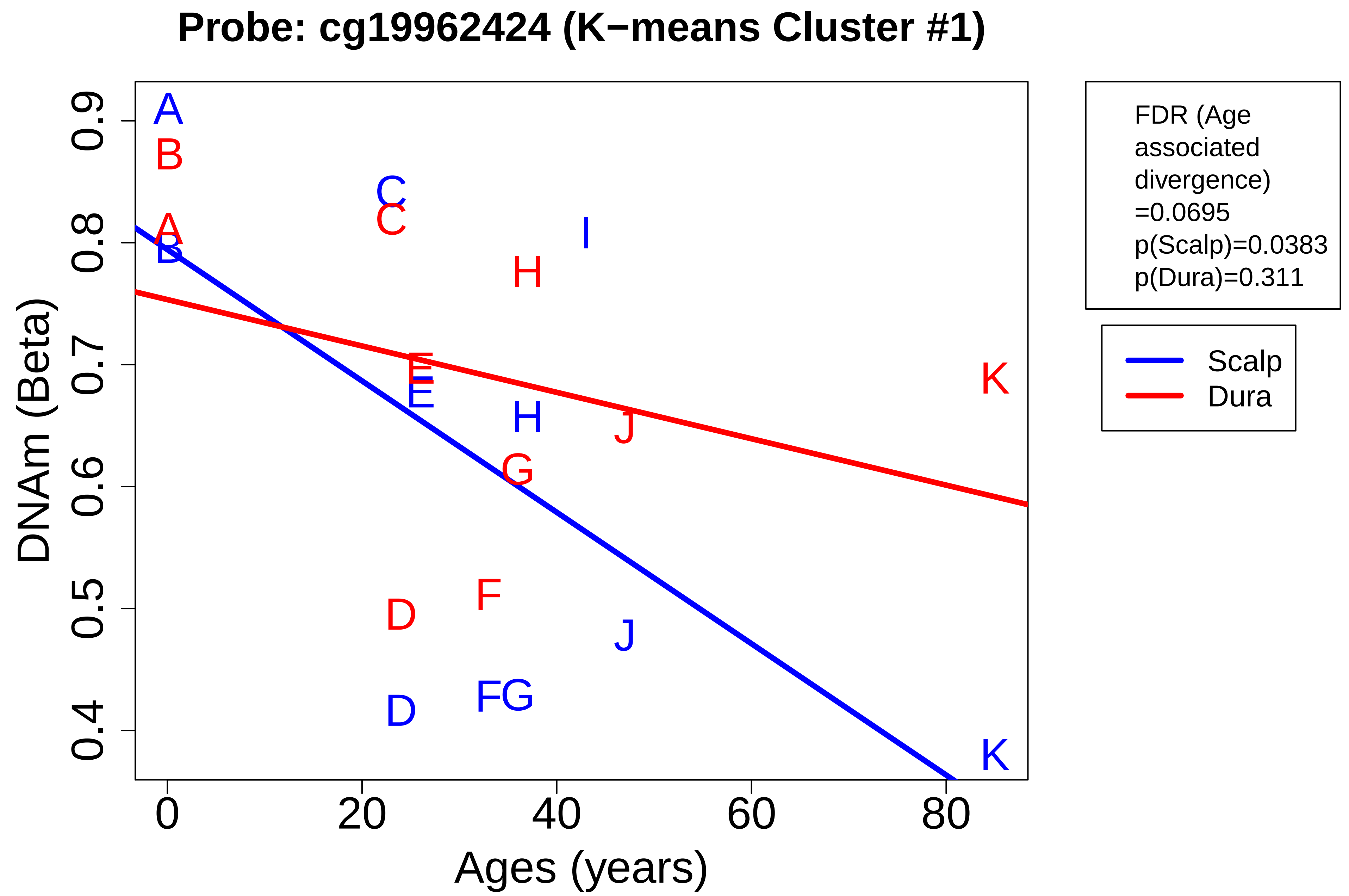

Probe: cg19962424 (K−means Cluster #1)
DNAm (Beta)
0.4
0.5
0.6
0.7
0.8
0.9
0
20
40
60
80
Ages (years)
A
B
C
C
A
B
I
H
E
E
H
J
G
F
D
J
FG
D
K
K
FDR (Age associated divergence) =0.0695
p(Scalp)=0.0383
p(Dura)=0.311
Scalp
Dura

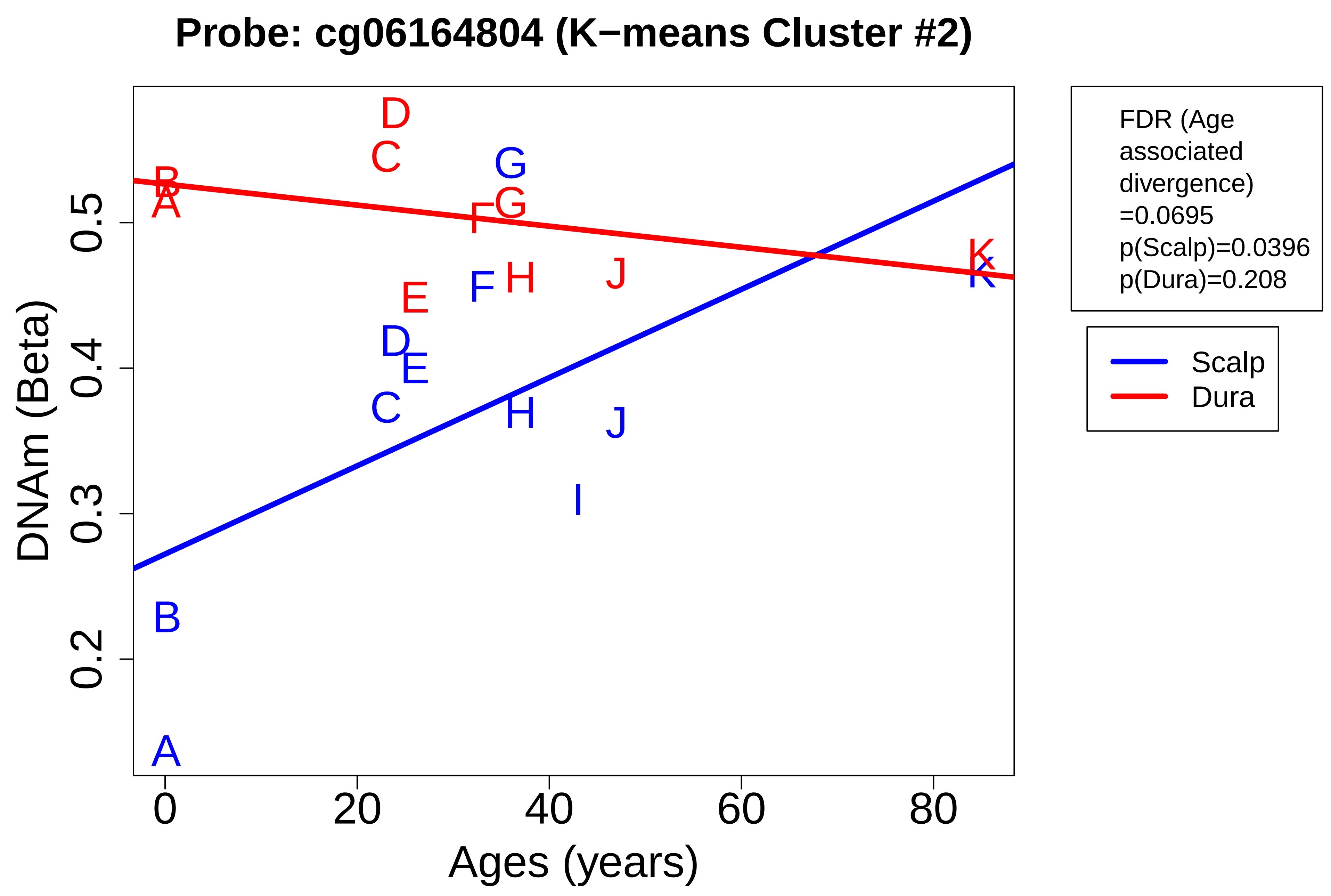

Probe: cg06164804 (K−means Cluster #2)
DNAm (Beta)
0.2
0.3
0.4
0.5
0
20
40
60
80
Ages (years)
A
B
C
D
E
F
G
H
I
J
K
FDR (Age associated divergence) =0.0695
p(Scalp)=0.0396
p(Dura)=0.208
Scalp
Dura

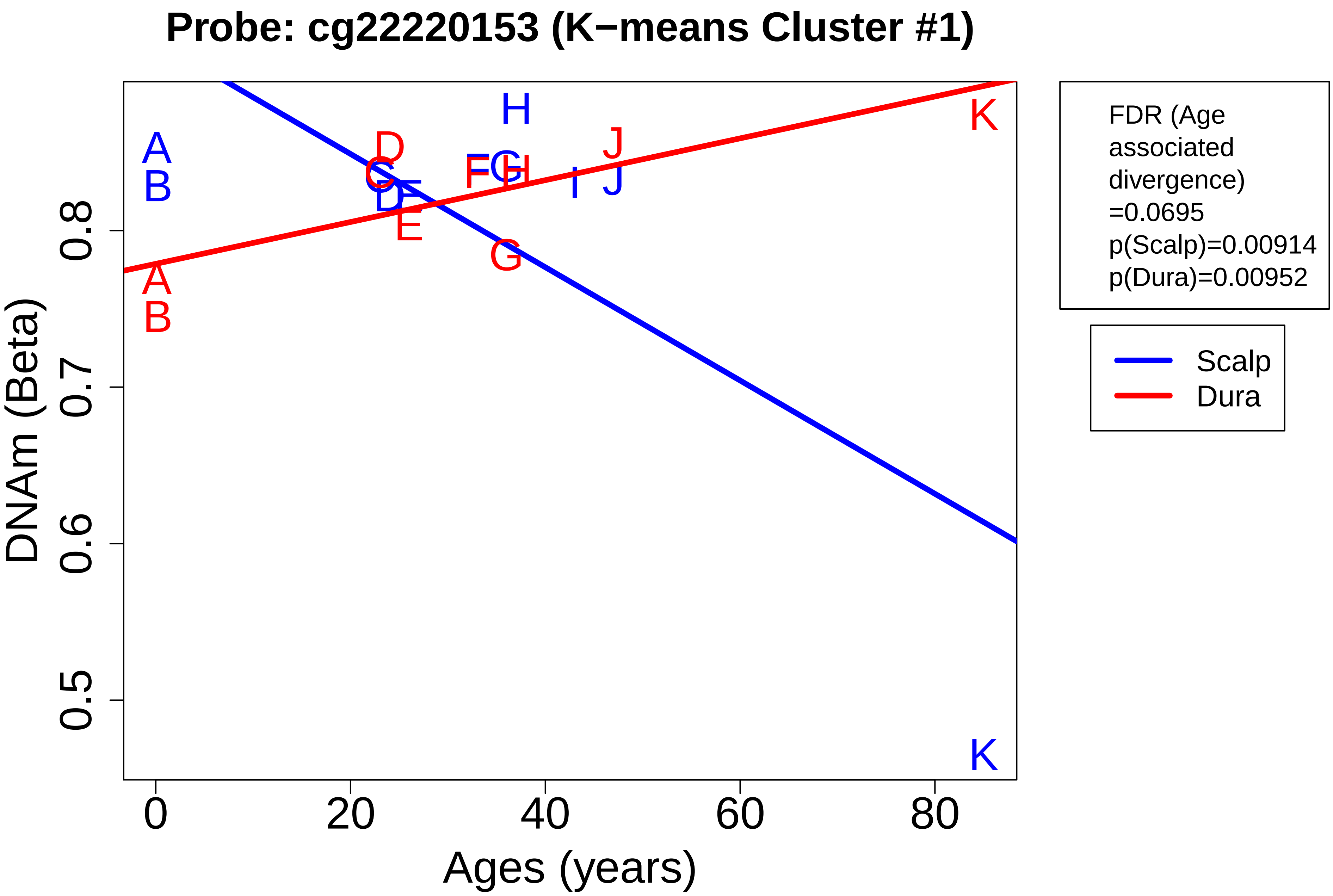
Probe: cg22220153 (K−means Cluster #1)
DNAm (Beta)
0.5
0.6
0.7
0.8
0
20
40
60
80
Ages (years)
A
B
C
D
E
F
G
H
I
J
K
FDR (Age associated divergence) =0.0695
p(Scalp)=0.00914
p(Dura)=0.00952
Scalp
Dura

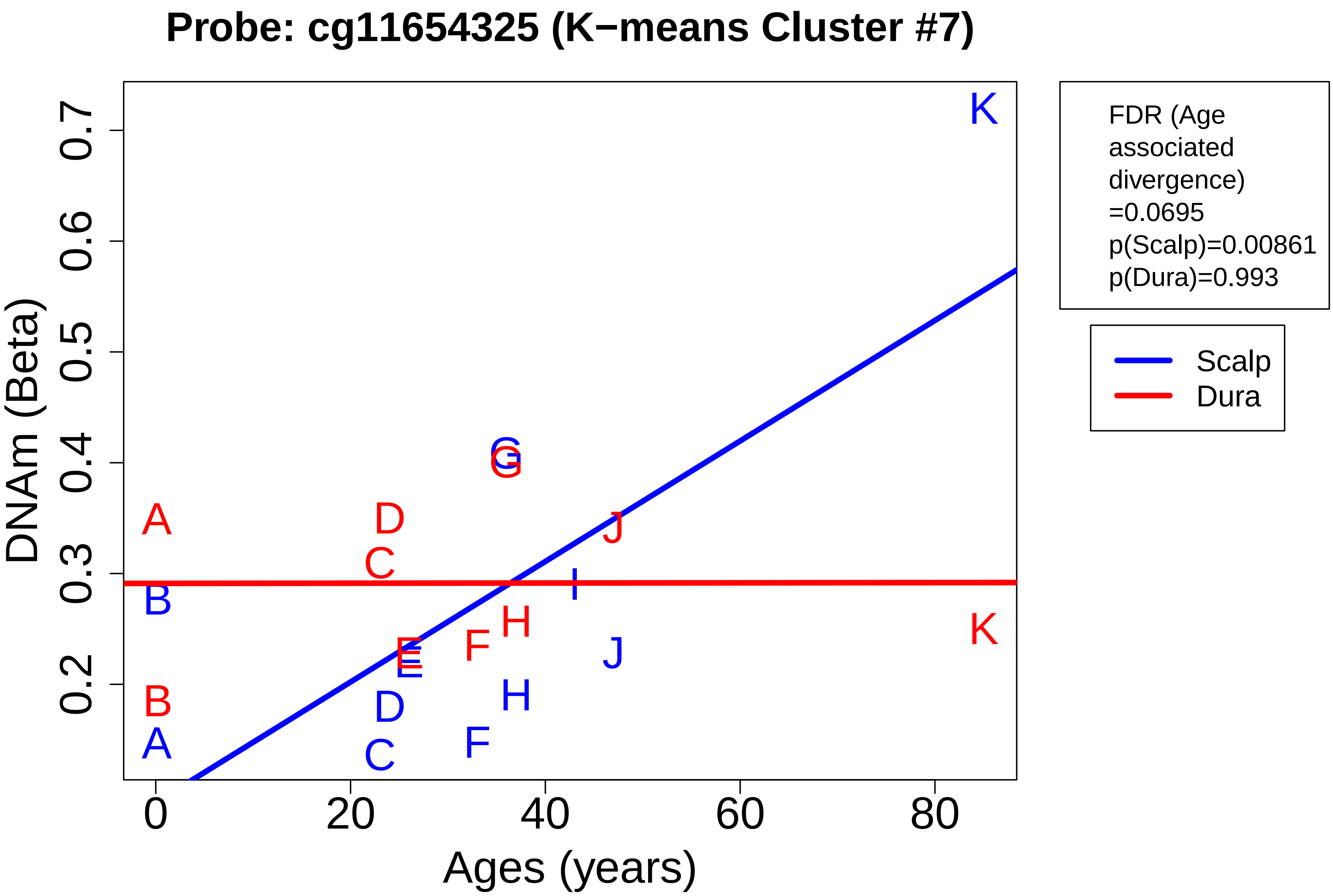

Probe: cg11654325 (K−means Cluster #7)
DNAm (Beta)
0.2
0.3
0.4
0.5
0.6
0.7
Ages (years)
0
20
40
60
80
FDR (Age associated divergence) =0.0695
p(Scalp)=0.00861
p(Dura)=0.993
Scalp
Dura
A
B
C
D
E
F
G
H
I
J
K

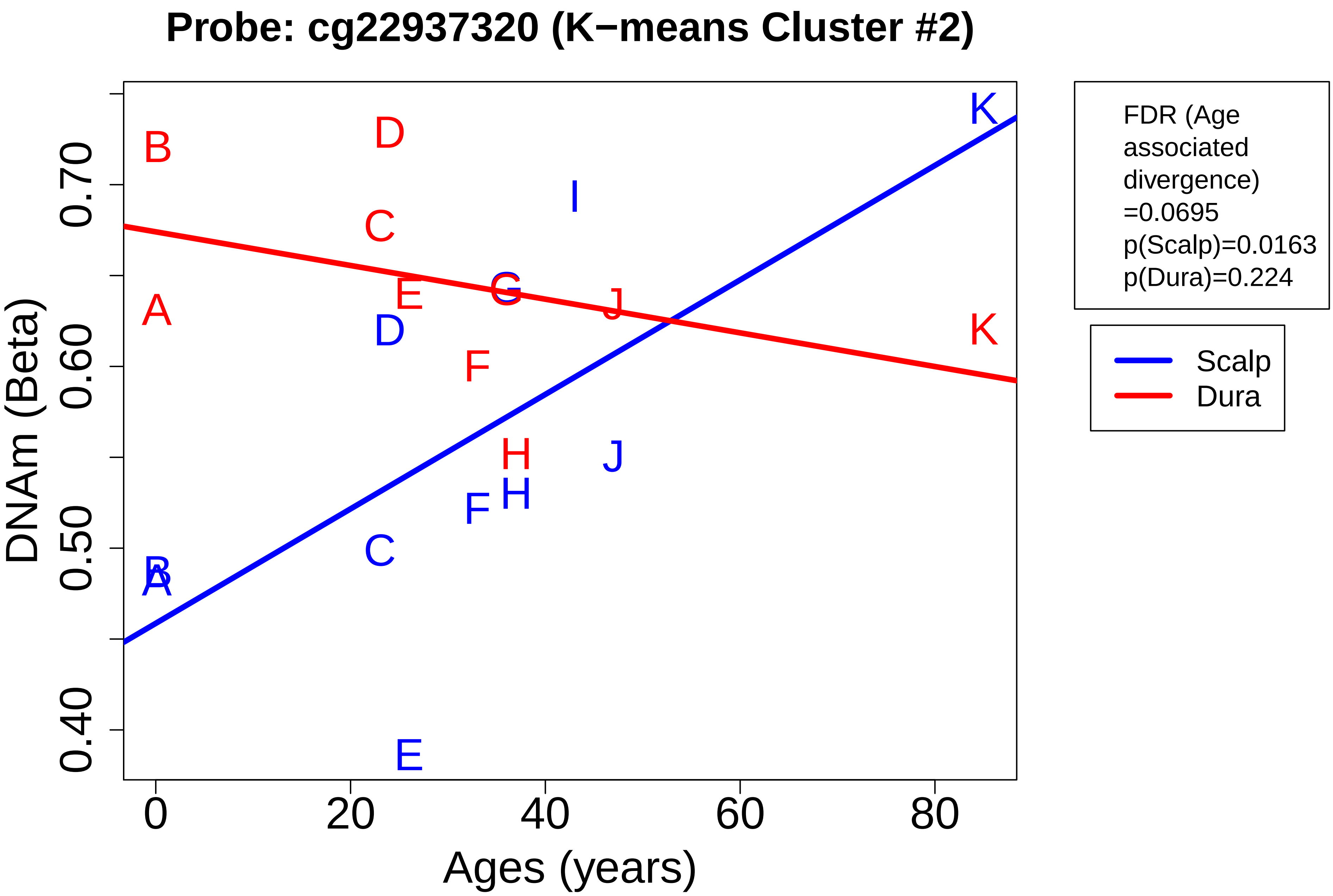

Probe: cg22937320 (K−means Cluster #2)
DNAm (Beta)
0.40
0.50
0.60
0.70
Ages (years)
0
20
40
60
80
FDR (Age associated divergence) =0.0695
p(Scalp)=0.0163
p(Dura)=0.224
Scalp
Dura
A
B
C
D
E
F
G
H
I
J
K

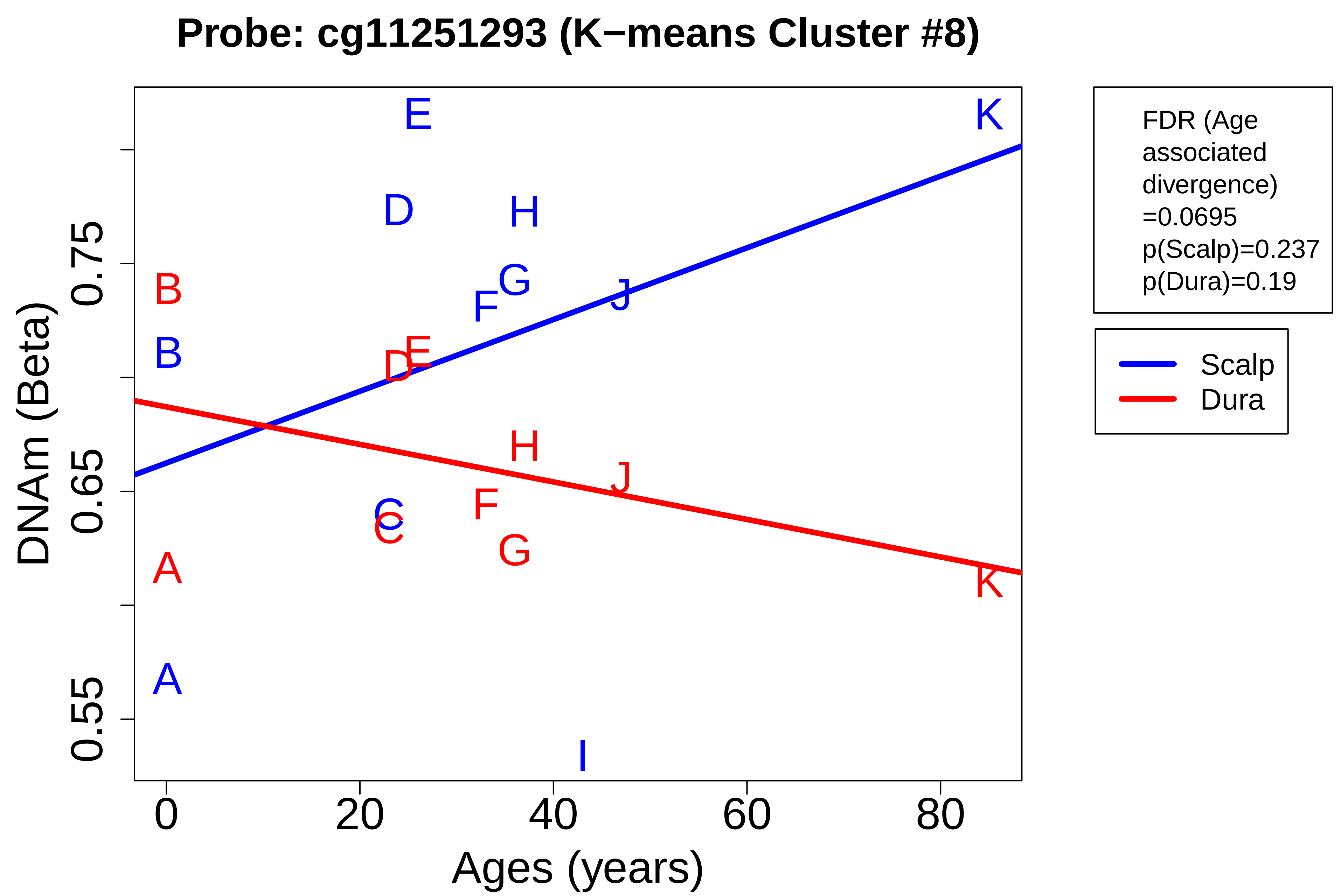
Probe: cg11251293 (K−means Cluster #8)
DNAm (Beta)
0.55
0.65
0.75
Ages (years)
0
20
40
60
80
FDR (Age associated divergence) =0.0695
p(Scalp)=0.237
p(Dura)=0.19
Scalp
Dura

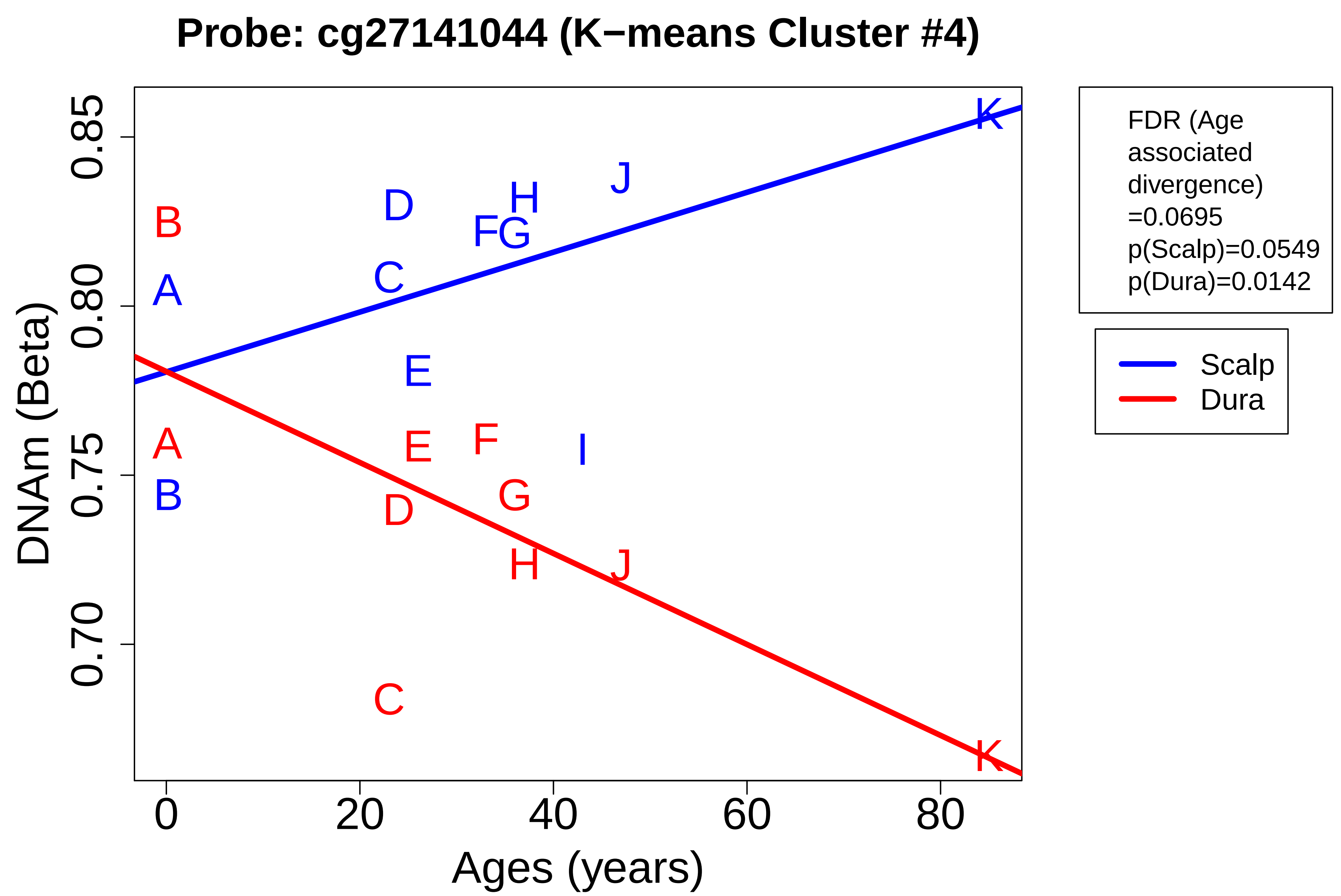

Probe: cg27141044 (K−means Cluster #4)
DNAm (Beta)
0.70
0.75
0.80
0.85
0
20
40
60
80
Ages (years)
FDR (Age associated divergence) =0.0695
p(Scalp)=0.0549
p(Dura)=0.0142
Scalp
Dura
A
B
C
D
E
F
G
H
I
J
K

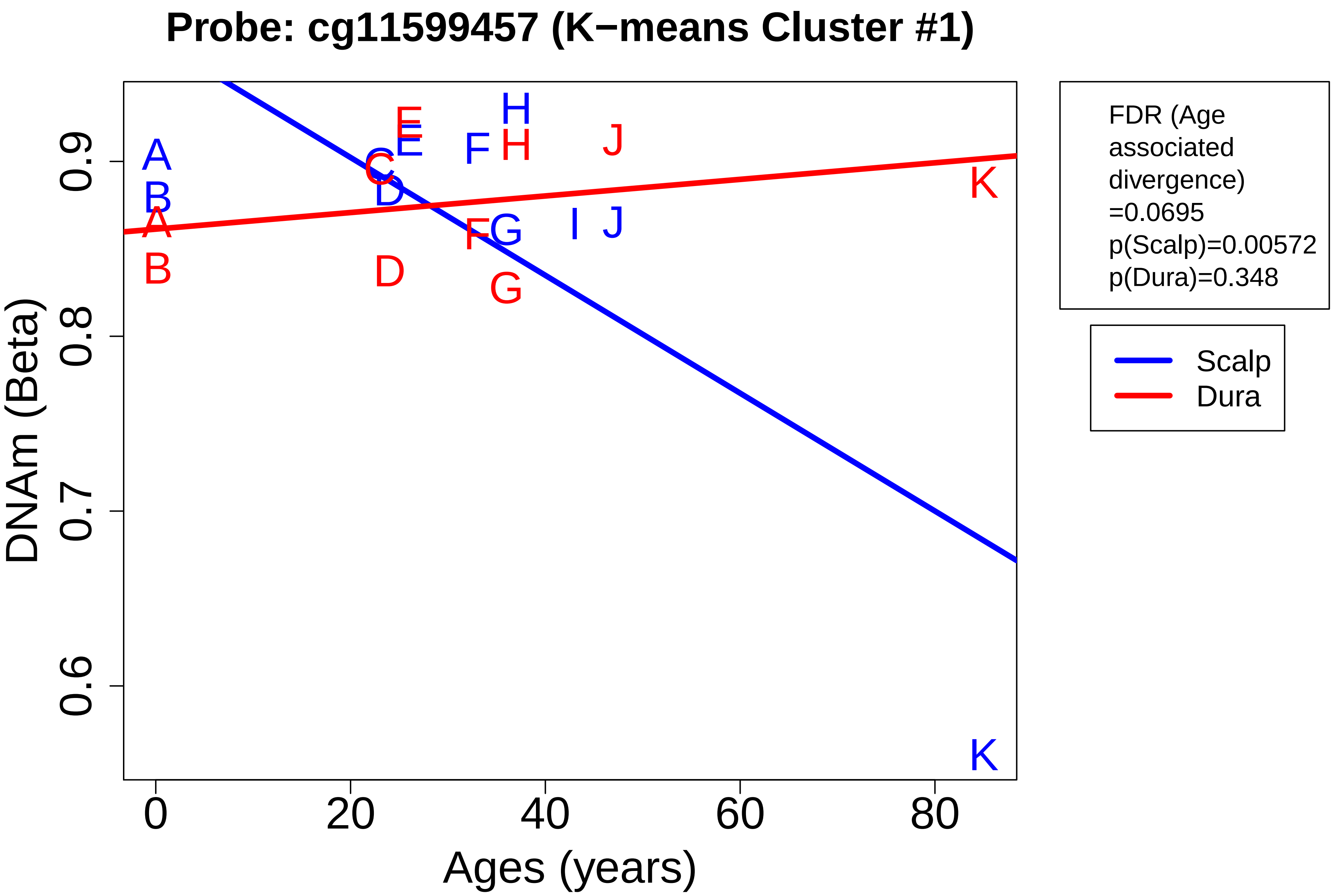

Probe: cg11599457 (K−means Cluster #1)
FDR (Age associated divergence) =0.0695
p(Scalp)=0.00572
p(Dura)=0.348
Scalp
Dura
DNAm (Beta)
0.6
0.7
0.8
0.9
Ages (years)
0
20
40
60
80
A
B
C
D
E
F
G
H
I
J
K

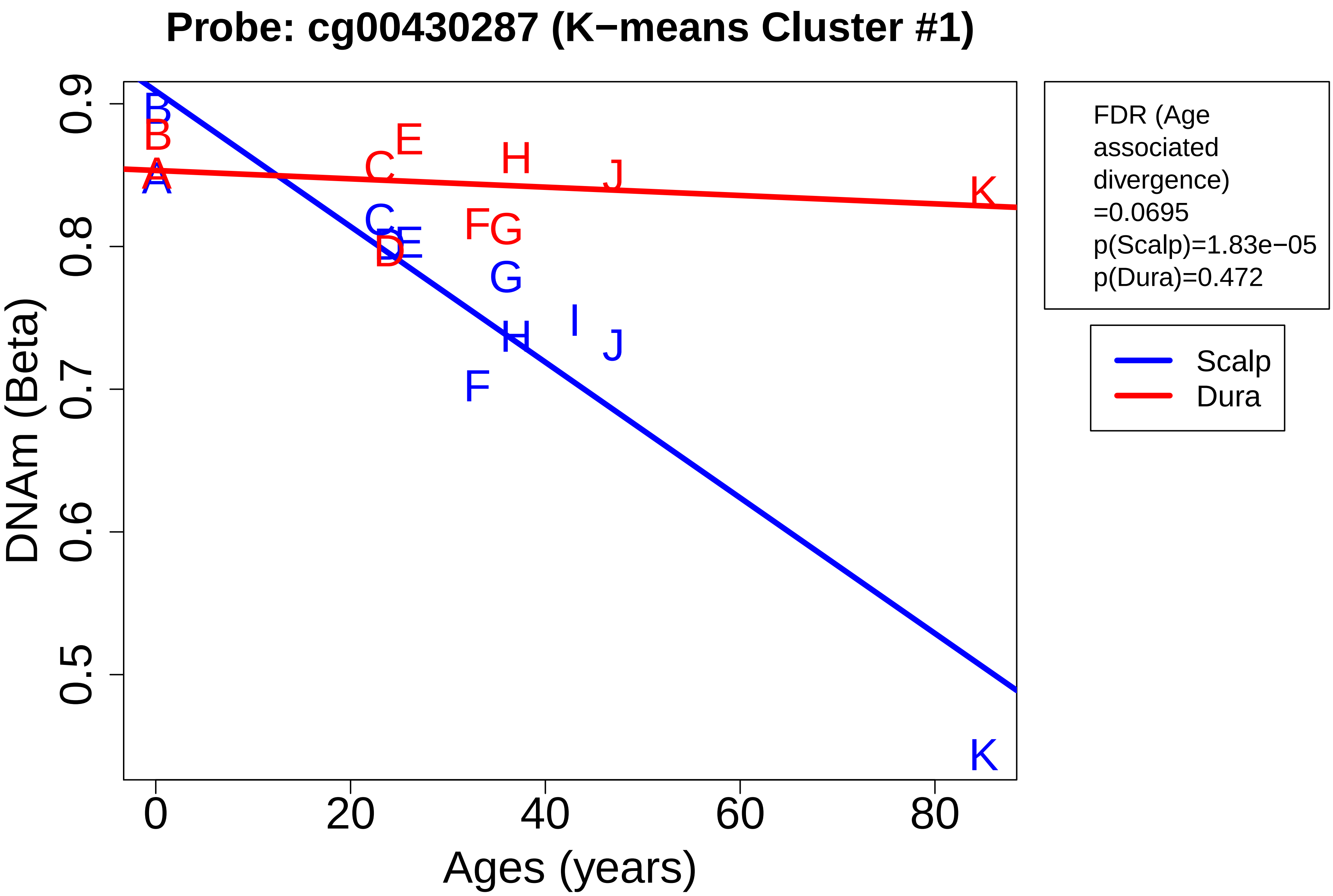

Probe: cg00430287 (K−means Cluster #1)
DNAm (Beta)
0.5
0.6
0.7
0.8
0.9
0
20
40
60
80
Ages (years)
FDR (Age associated divergence) =0.0695
p(Scalp)=1.83e−05
p(Dura)=0.472
Scalp
Dura

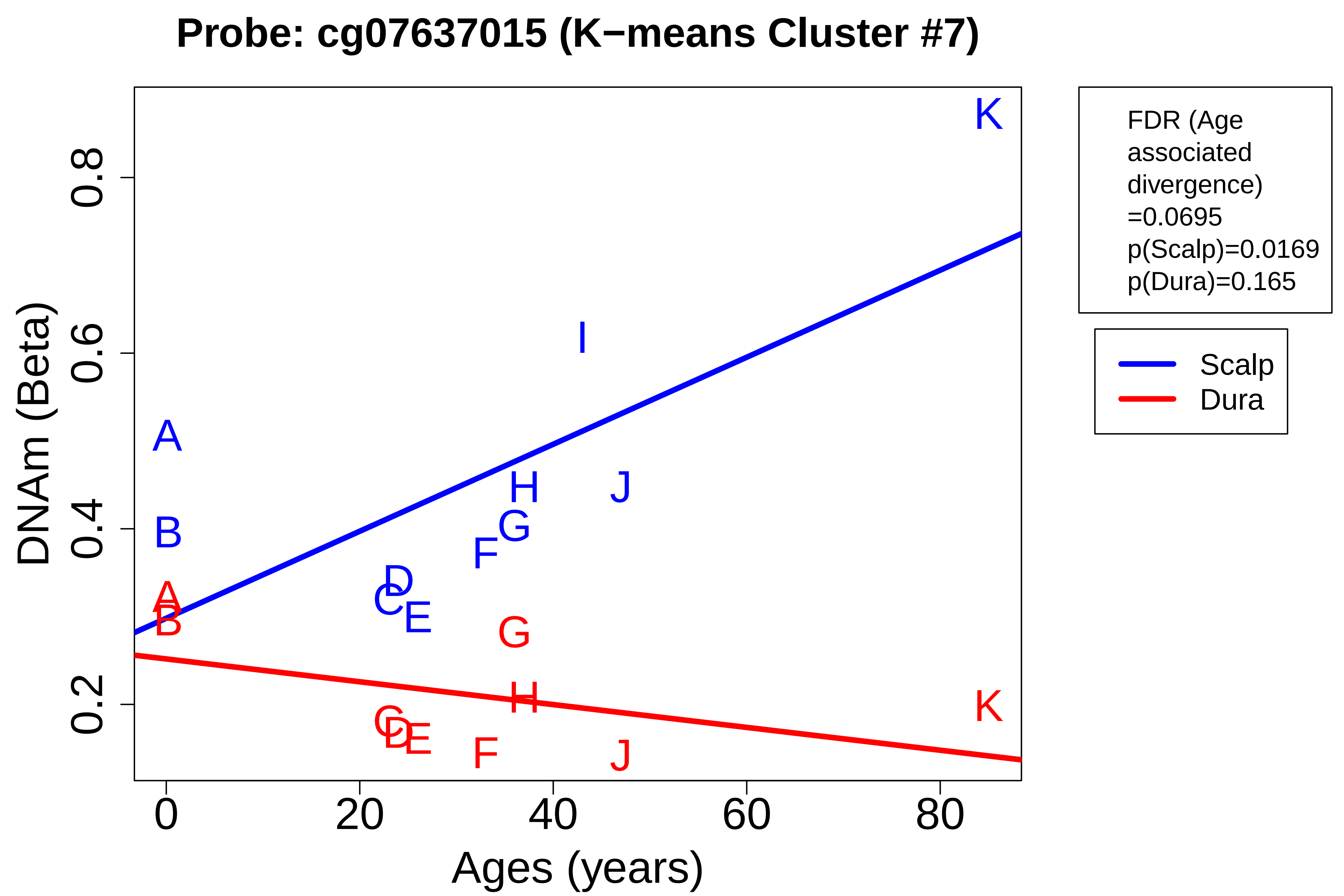

Probe: cg07637015 (K−means Cluster #7)
FDR (Age associated divergence) =0.0695
p(Scalp)=0.0169
p(Dura)=0.165
Scalp
Dura
DNAm (Beta)
0.2
0.4
0.6
0.8
0
20
40
60
80
Ages (years)
A
B
C
D
E
F
G
H
I
J
K

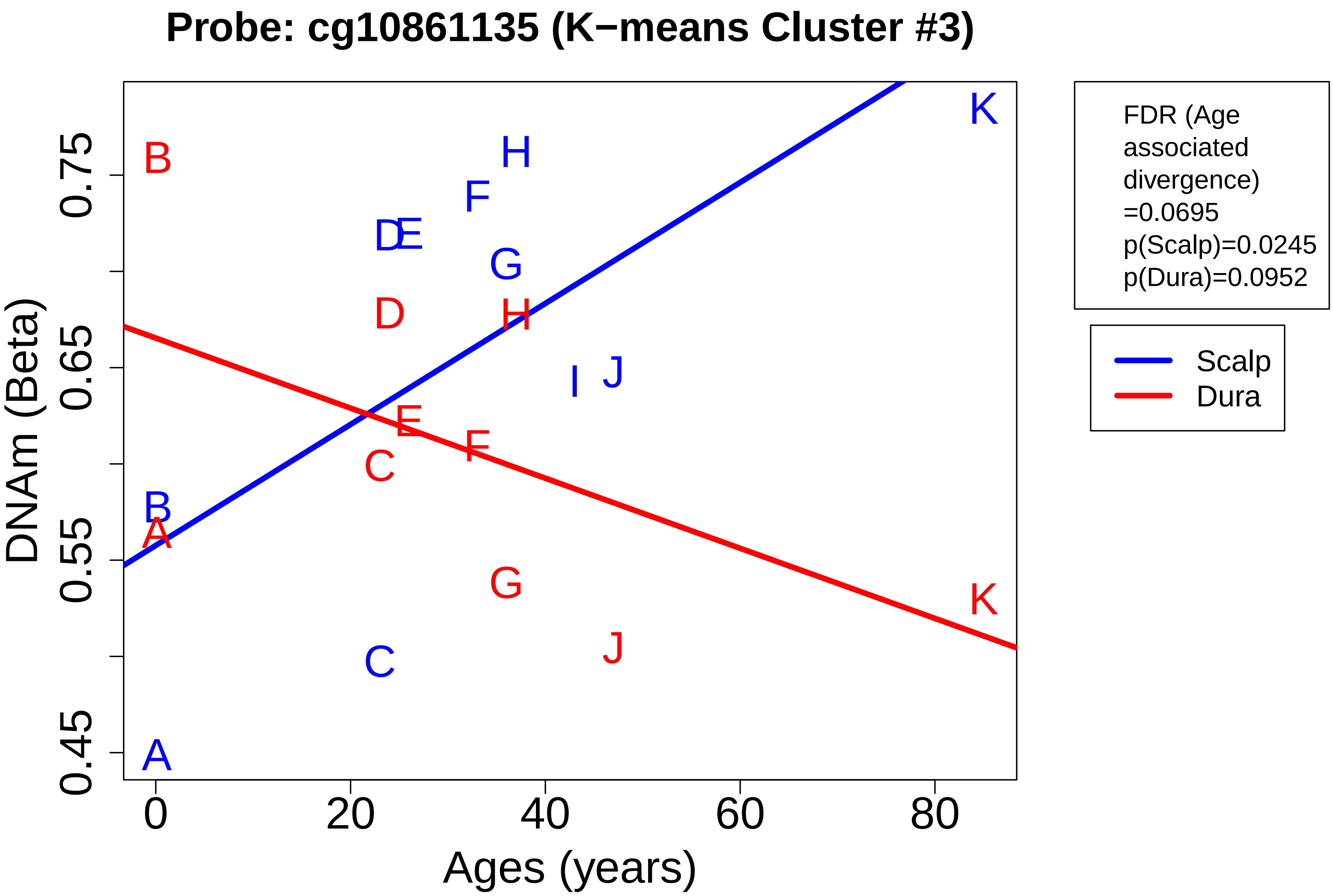

Probe: cg10861135 (K−means Cluster #3)
DNAm (Beta)
0.45
0.55
0.65
0.75
Ages (years)
0
20
40
60
80
A
B
C
D
E
F
G
H
I
J
K
FDR (Age associated divergence) =0.0695
p(Scalp)=0.0245
p(Dura)=0.0952
Scalp
Dura

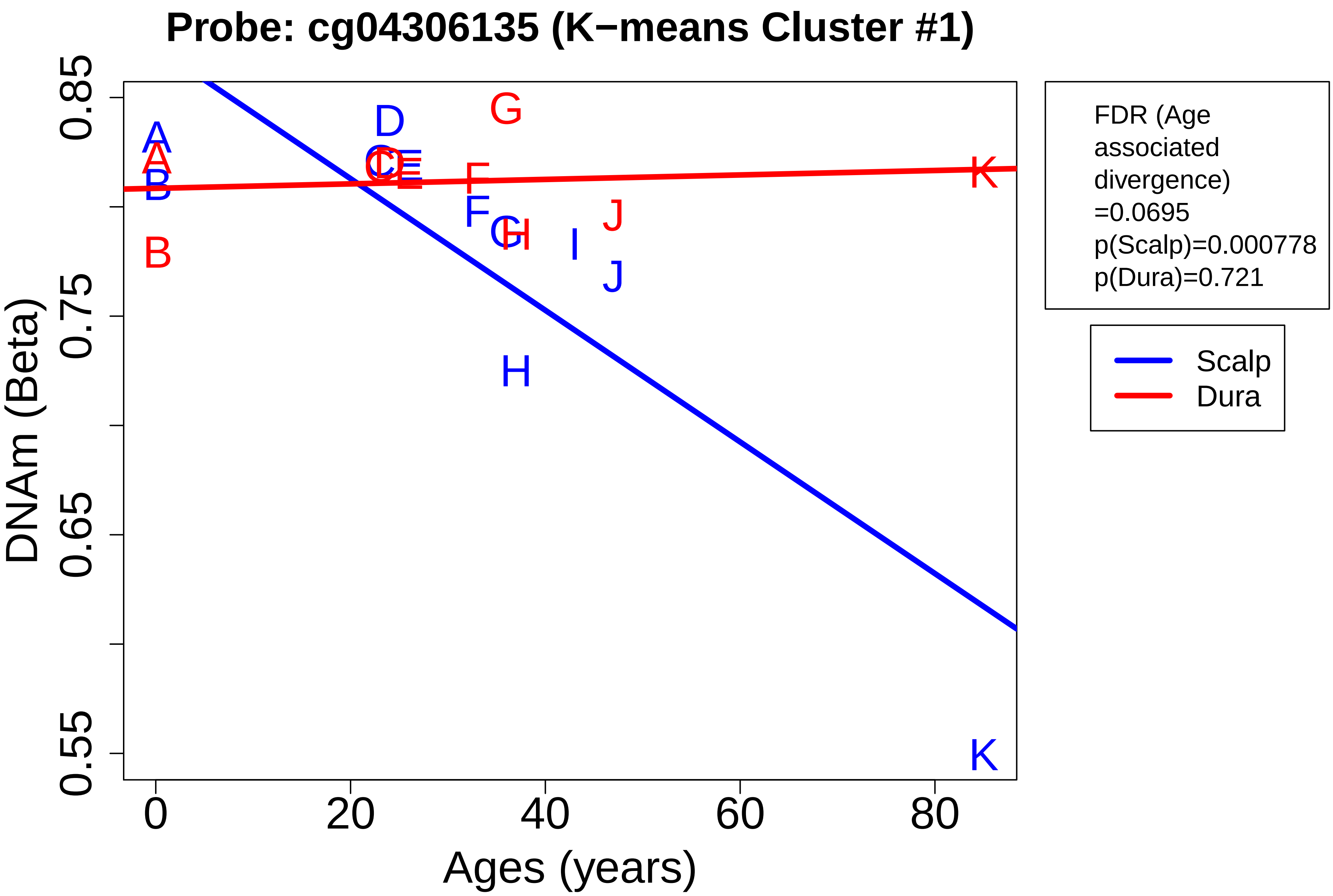

Probe: cg04306135 (K−means Cluster #1)
DNAm (Beta)
0.55
0.65
0.75
0.85
Ages (years)
0
20
40
60
80
A
B
C
D
E
F
G
H
I
J
K
FDR (Age associated divergence) =0.0695
p(Scalp)=0.000778
p(Dura)=0.721
Scalp
Dura

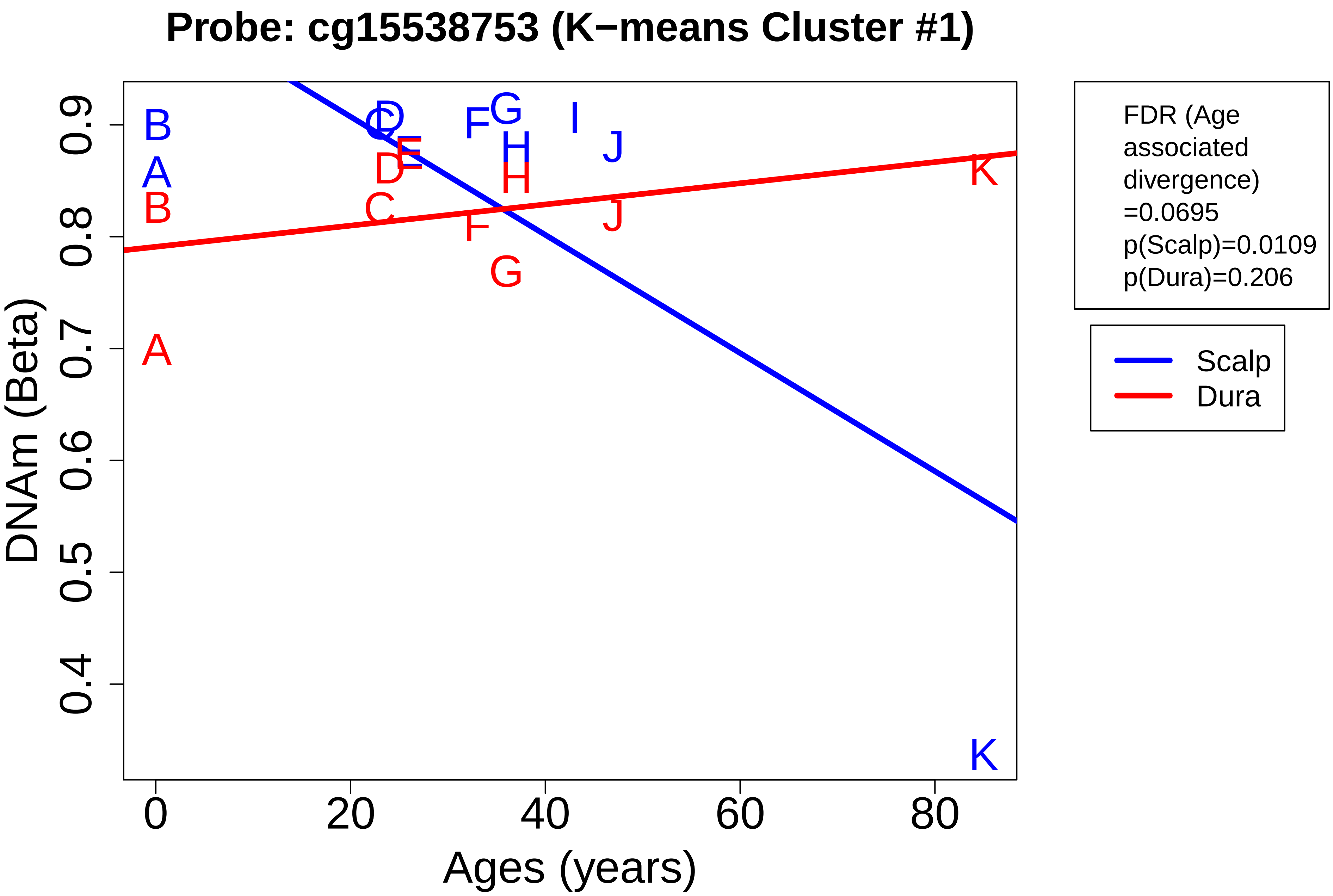

Probe: cg15538753 (K−means Cluster #1)
DNAm (Beta)
0.4
0.5
0.6
0.7
0.8
0.9
Ages (years)
0
20
40
60
80
B
A
B
A
C
D
E
D
C
F
G
H
H
F
G
I
J
J
K
K
FDR (Age associated divergence) =0.0695
p(Scalp)=0.0109
p(Dura)=0.206
Scalp
Dura

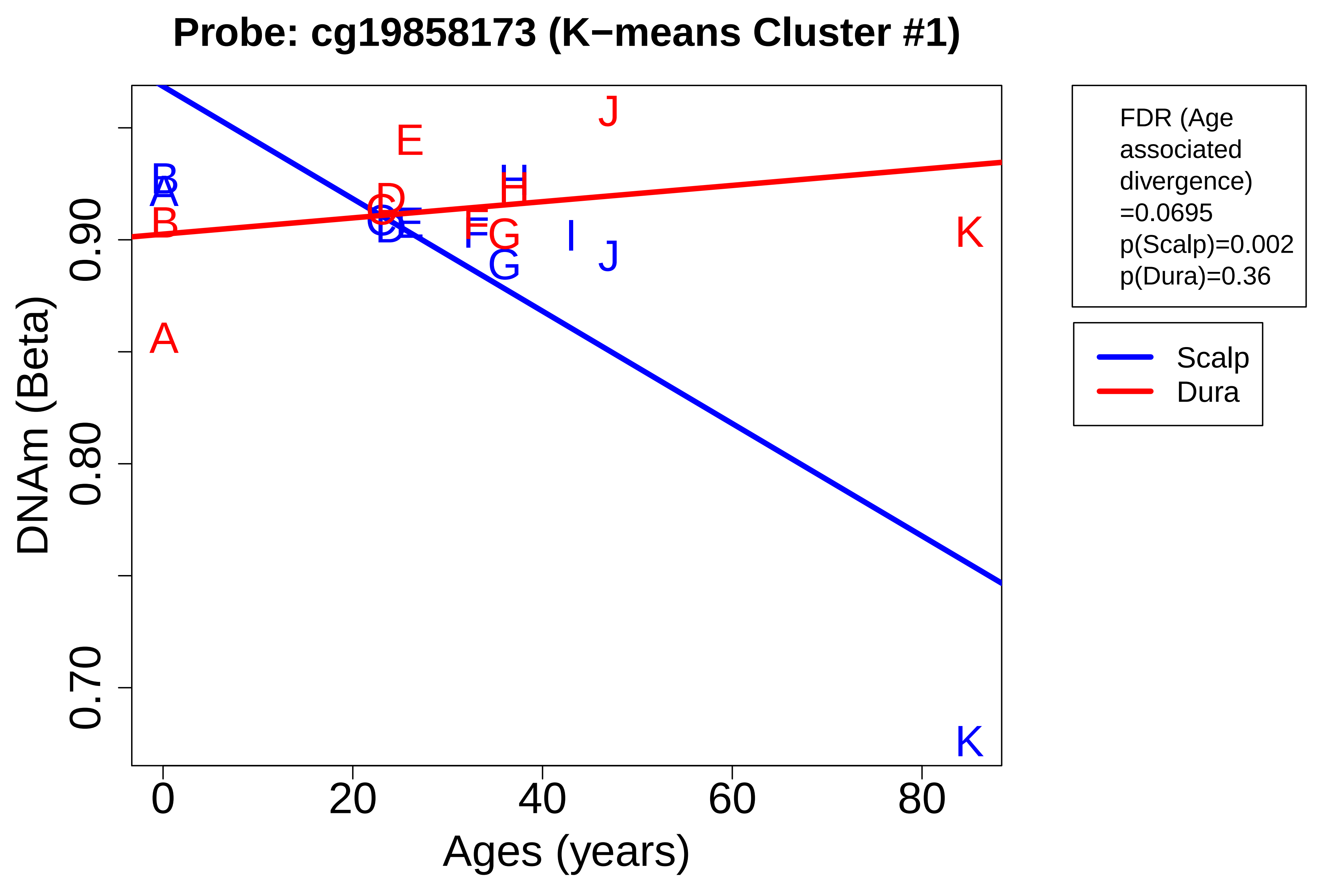
Probe: cg19858173 (K−means Cluster #1)
DNAm (Beta)
0.70
0.80
0.90
Ages (years)
0
20
40
60
80
A
B
C
D
E
F
G
H
I
J
K
FDR (Age associated divergence) =0.0695
p(Scalp)=0.002
p(Dura)=0.36
Scalp
Dura

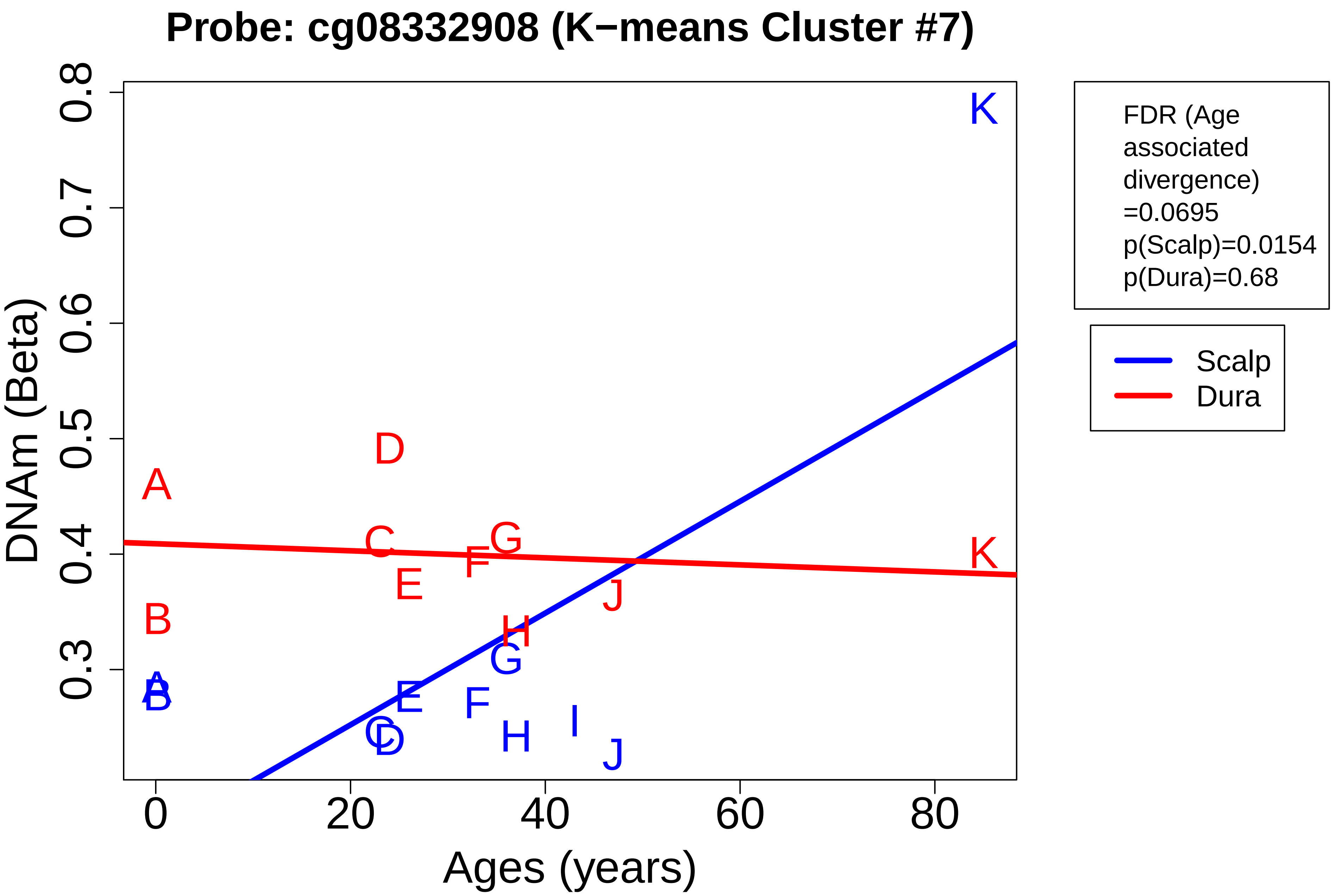
Probe: cg08332908 (K−means Cluster #7)
DNAm (Beta)
Ages (years)
0.3
0.4
0.5
0.6
0.7
0.8
0
20
40
60
80
FDR (Age associated divergence) =0.0695
p(Scalp)=0.0154
p(Dura)=0.68
Scalp
Dura
A
B
C
D
E
F
G
H
I
J
K

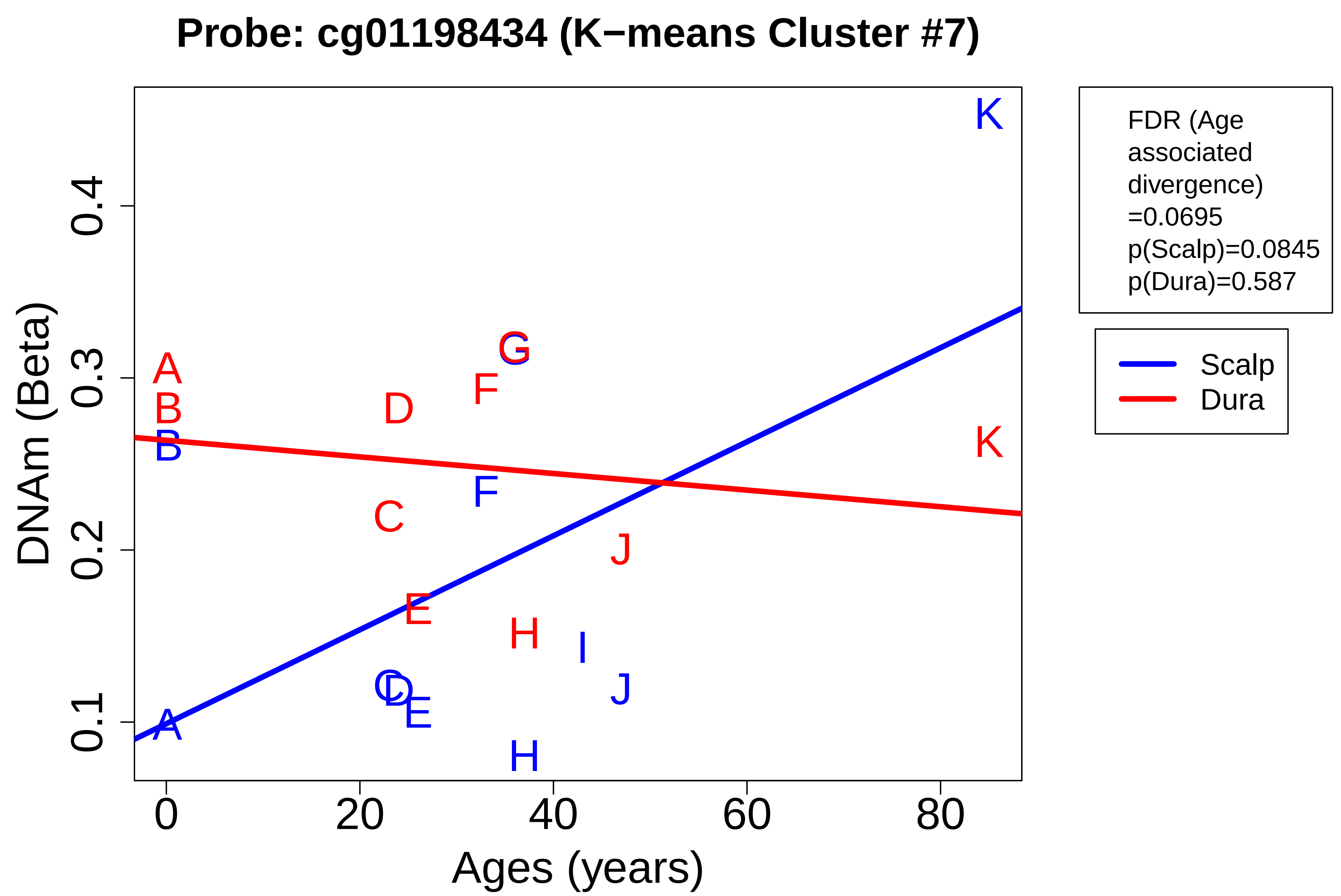

Probe: cg01198434 (K−means Cluster #7)
DNAm (Beta)
Ages (years)
0.1
0.2
0.3
0.4
0
20
40
60
80
FDR (Age associated divergence) =0.0695
p(Scalp)=0.0845
p(Dura)=0.587
Scalp
Dura
A
B
C
D
E
F
G
H
I
J
K

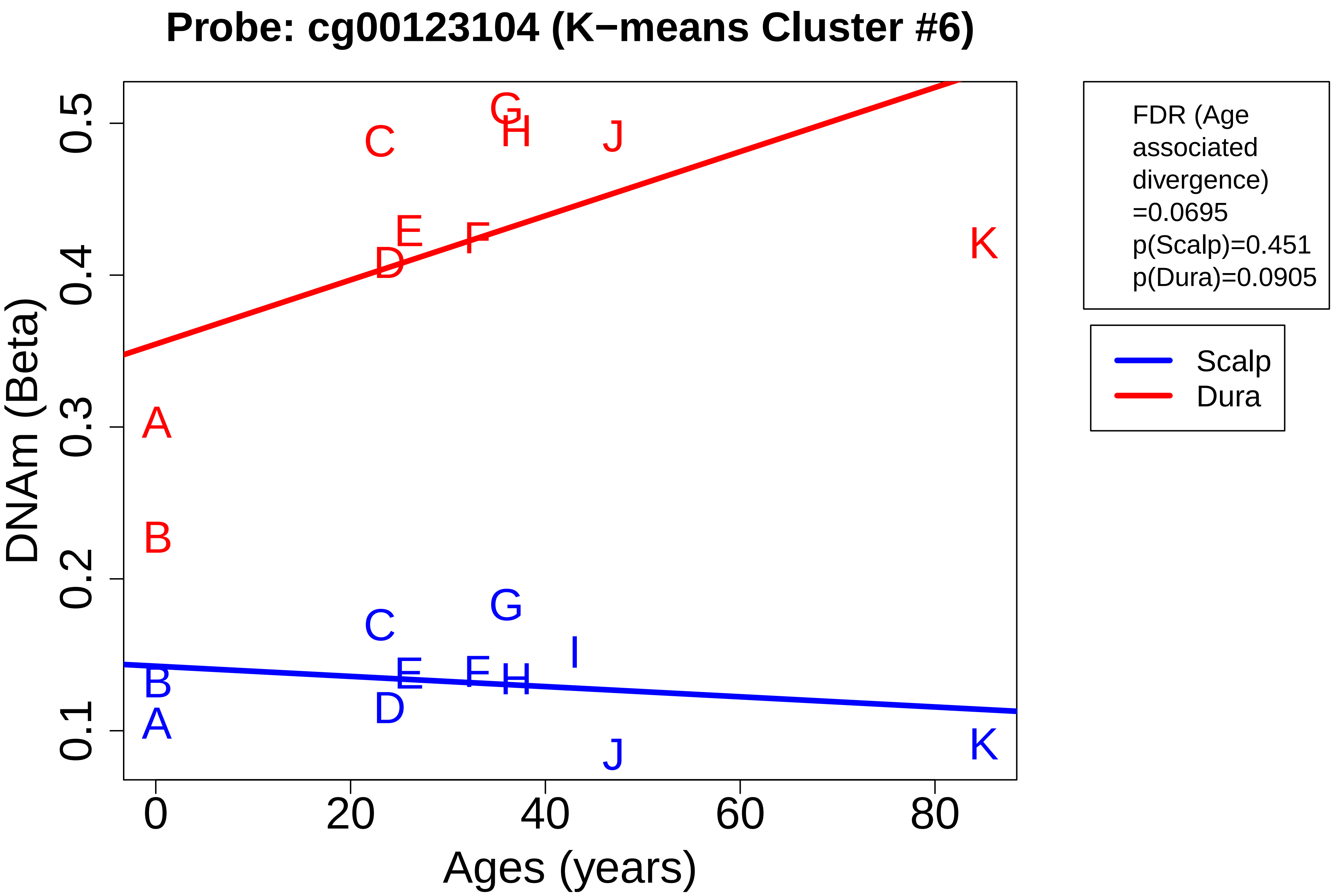
Probe: cg00123104 (K−means Cluster #6)
DNAm (Beta)
Ages (years)
0.1
0.2
0.3
0.4
0.5
0
20
40
60
80
FDR (Age associated divergence) =0.0695
p(Scalp)=0.451
p(Dura)=0.0905
Scalp
Dura
A
B
C
D
E
F
G
H
I
J
K

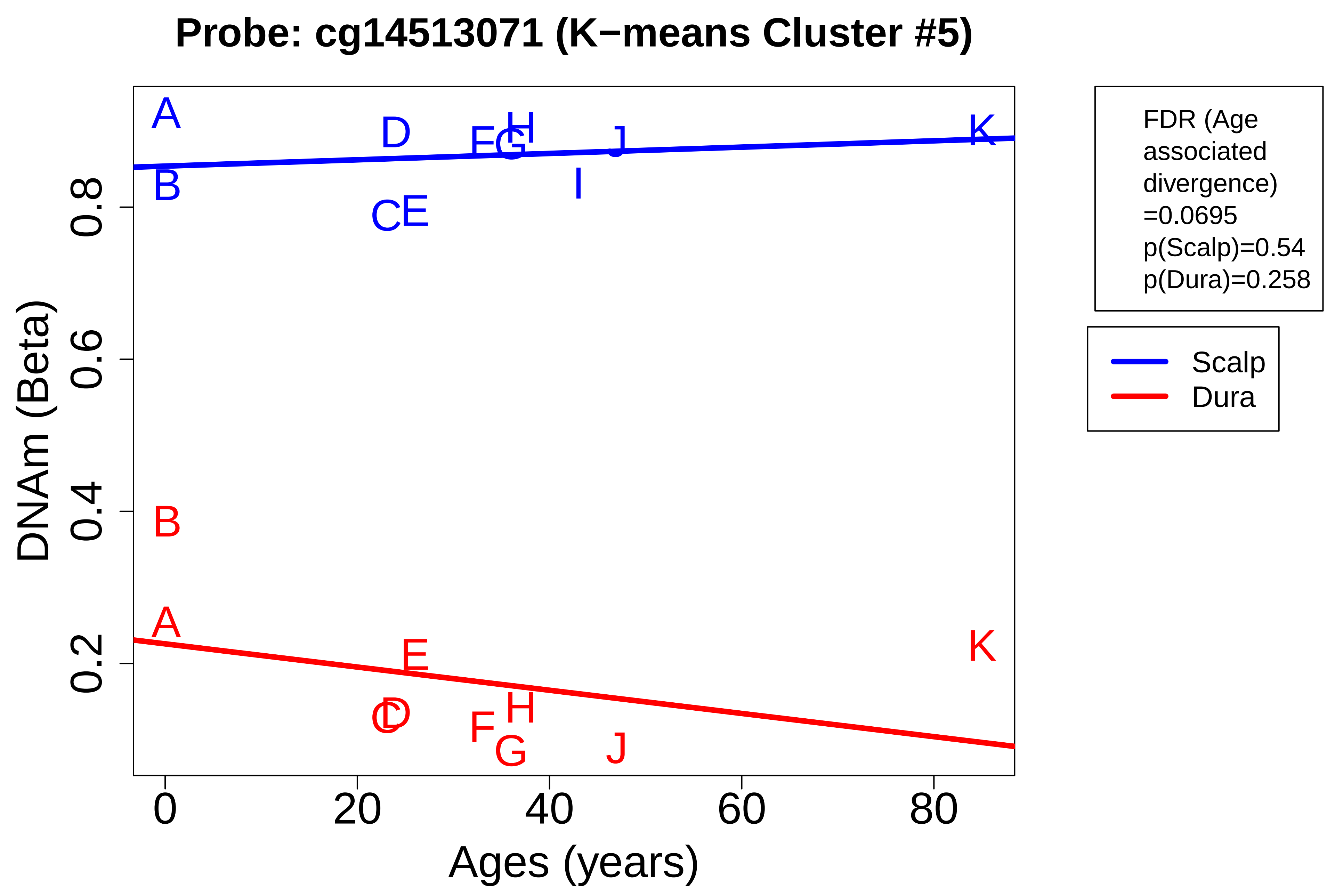

Probe: cg14513071 (K−means Cluster #5)
DNAm (Beta)
0.2
0.4
0.6
0.8
0
20
40
60
80
Ages (years)
A
B
C
D
E
F
G
H
I
J
K
FDR (Age associated divergence) =0.0695
p(Scalp)=0.54
p(Dura)=0.258
Scalp
Dura

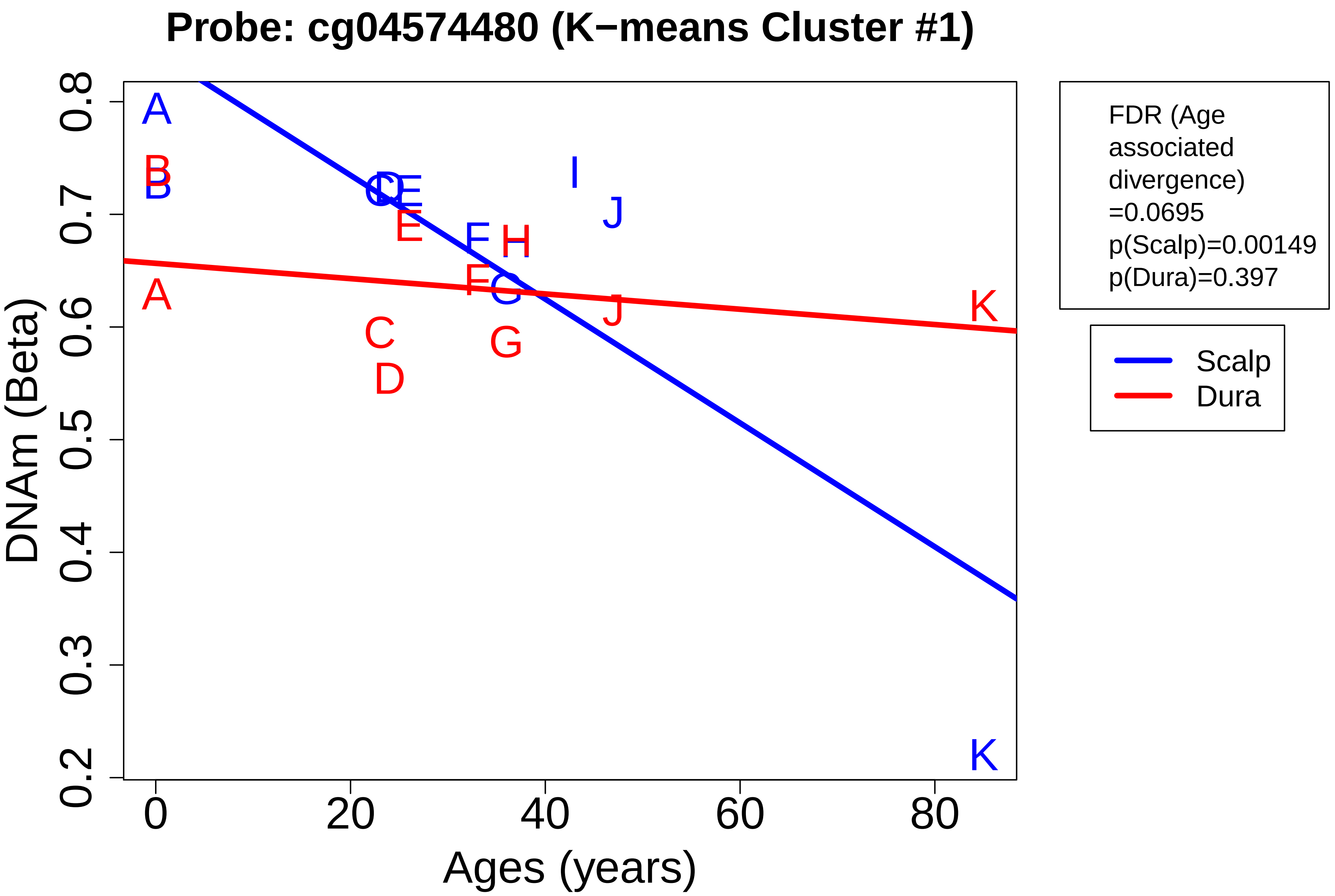

Probe: cg04574480 (K−means Cluster #1)
DNAm (Beta)
0.2
0.3
0.4
0.5
0.6
0.7
0.8
0
20
40
60
80
Ages (years)
FDR (Age associated divergence) =0.0695
p(Scalp)=0.00149
p(Dura)=0.397
Scalp
Dura

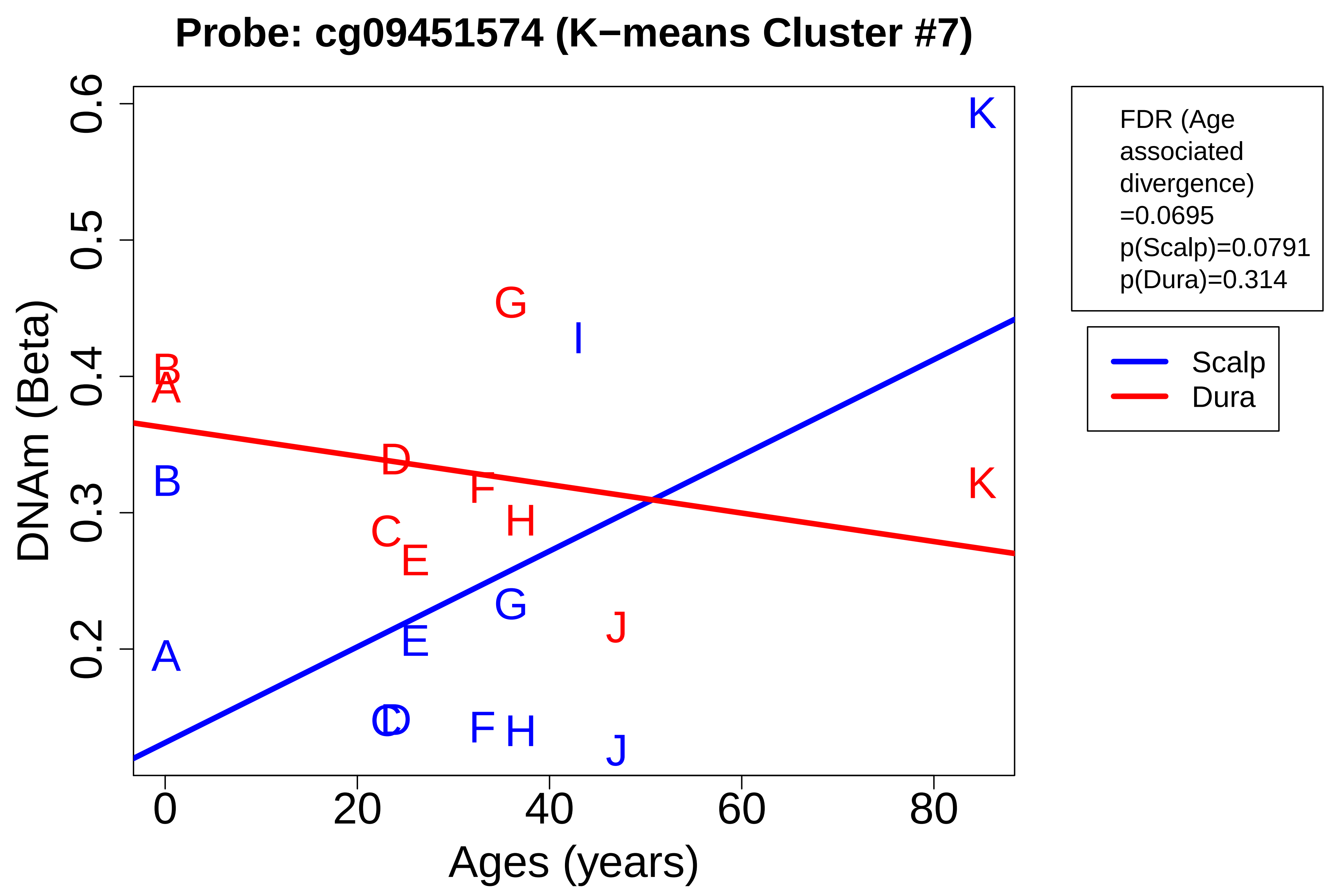

Probe: cg09451574 (K−means Cluster #7)
DNAm (Beta)
0.2
0.3
0.4
0.5
0.6
0
20
40
60
80
Ages (years)
FDR (Age associated divergence) =0.0695
p(Scalp)=0.0791
p(Dura)=0.314
Scalp
Dura

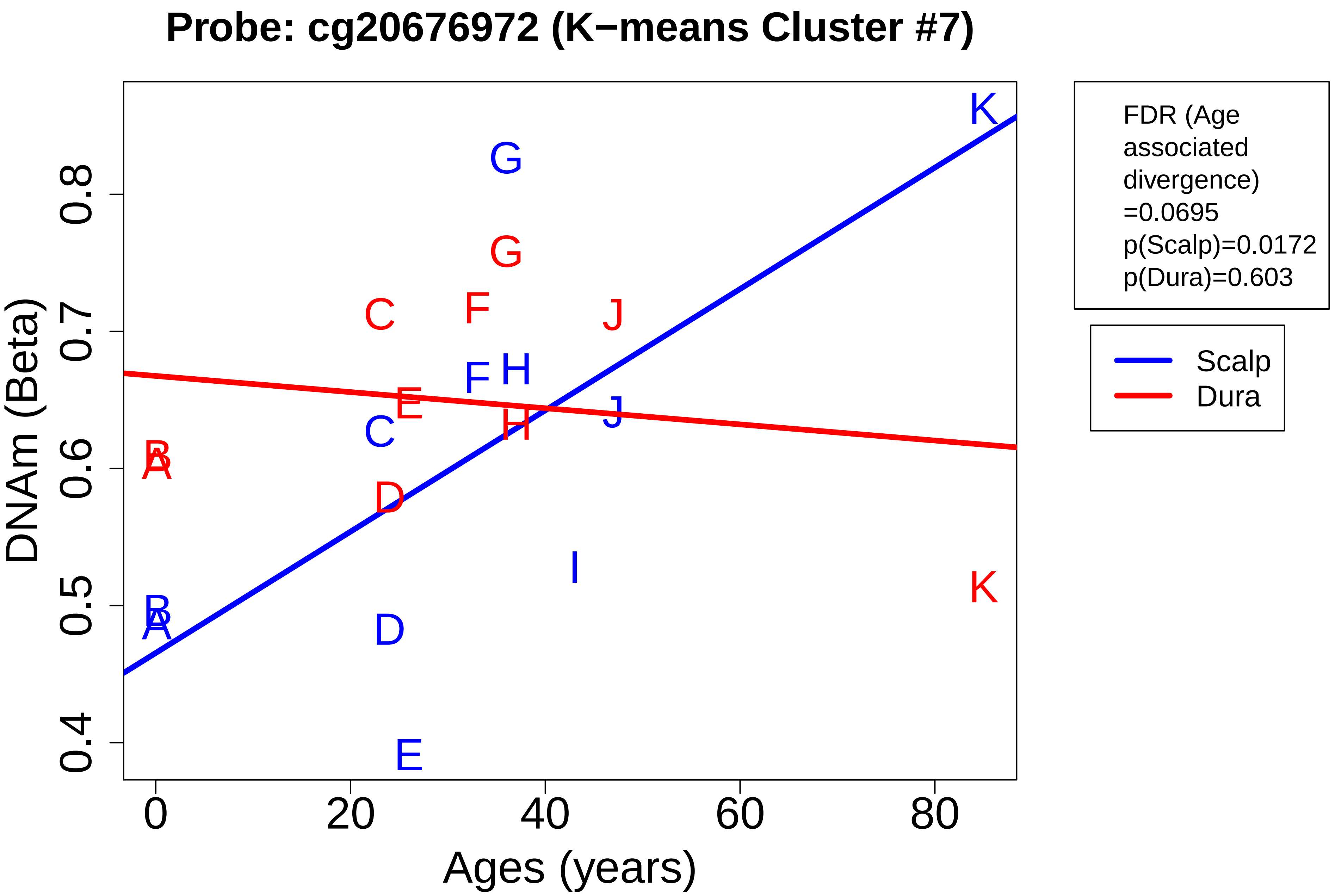

Probe: cg20676972 (K−means Cluster #7)
DNAm (Beta)
0.4
0.5
0.6
0.7
0.8
0
20
40
60
80
Ages (years)
FDR (Age associated divergence) =0.0695
p(Scalp)=0.0172
p(Dura)=0.603
Scalp
Dura

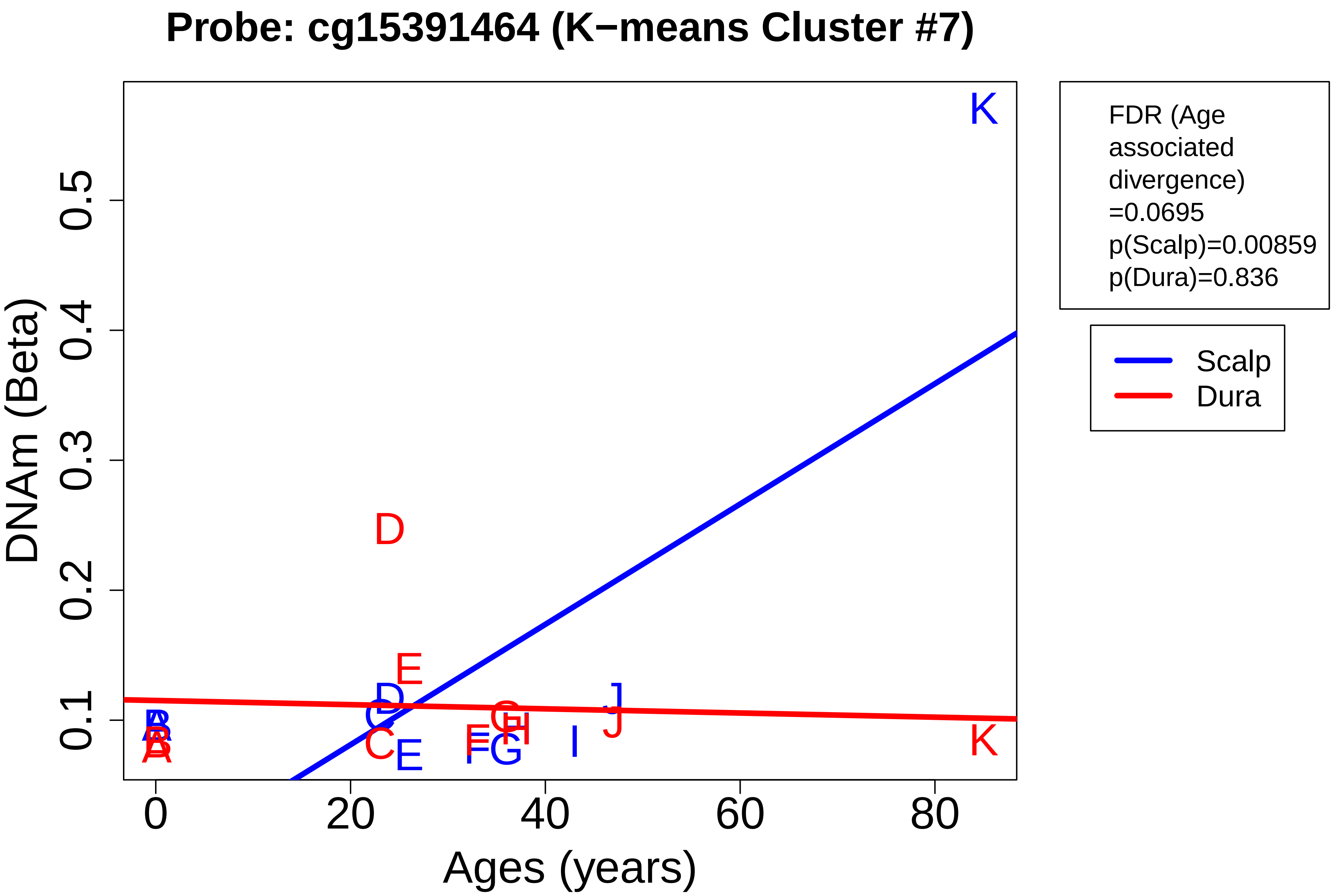

Probe: cg15391464 (K−means Cluster #7)
DNAm (Beta)
0.1
0.2
0.3
0.4
0.5
0
20
40
60
80
Ages (years)
FDR (Age associated divergence) =0.0695
p(Scalp)=0.00859
p(Dura)=0.836
Scalp
Dura

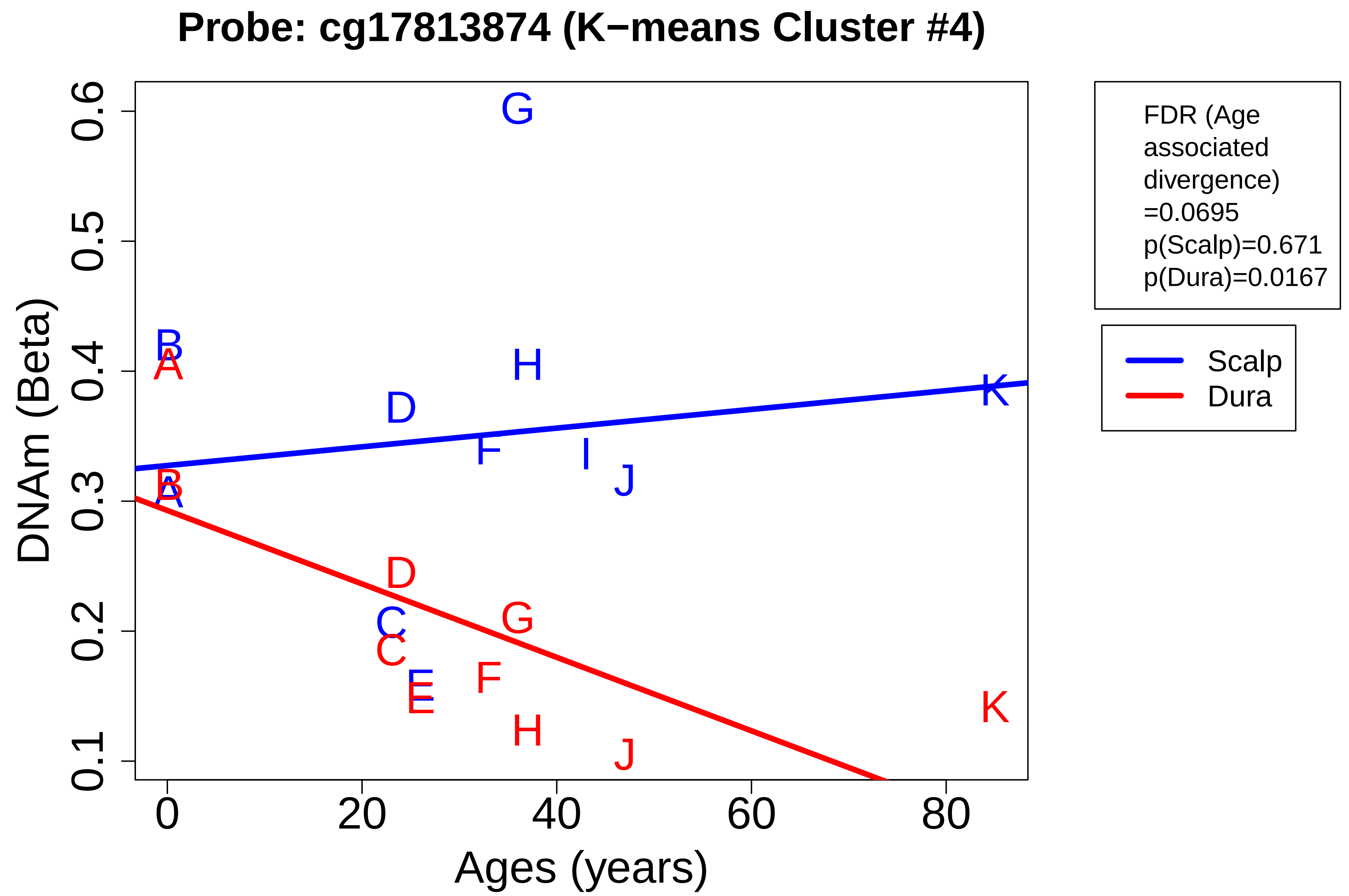
Probe: cg17813874 (K−means Cluster #4)
FDR (Age associated divergence) =0.0695
p(Scalp)=0.671
p(Dura)=0.0167
Scalp
Dura
DNAm (Beta)
Ages (years)
0.1
0.2
0.3
0.4
0.5
0.6
0
20
40
60
80

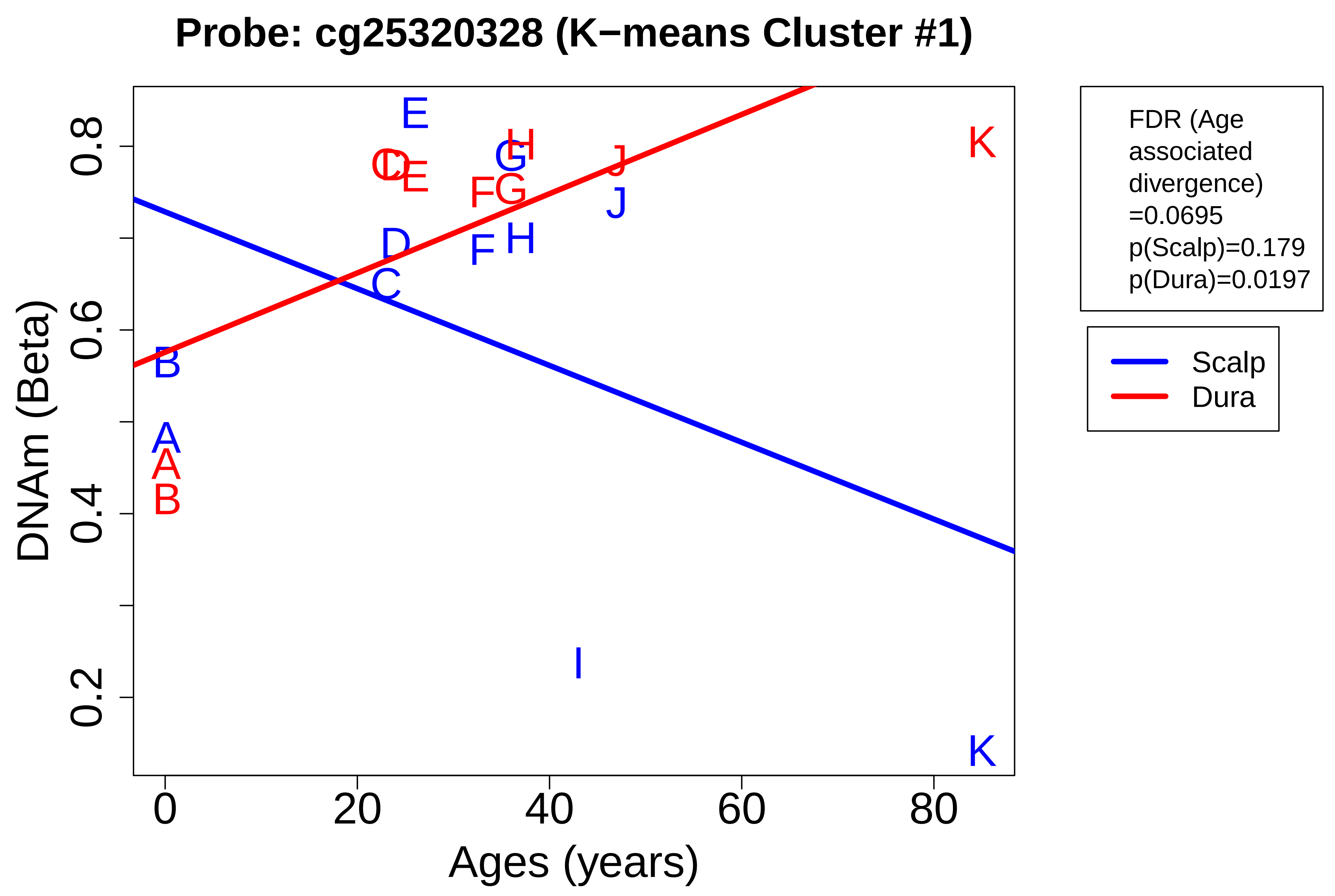

Probe: cg25320328 (K−means Cluster #1)
DNAm (Beta)
0.2
0.4
0.6
0.8
0
20
40
60
80
Ages (years)
FDR (Age associated divergence) =0.0695
p(Scalp)=0.179
p(Dura)=0.0197
Scalp
Dura

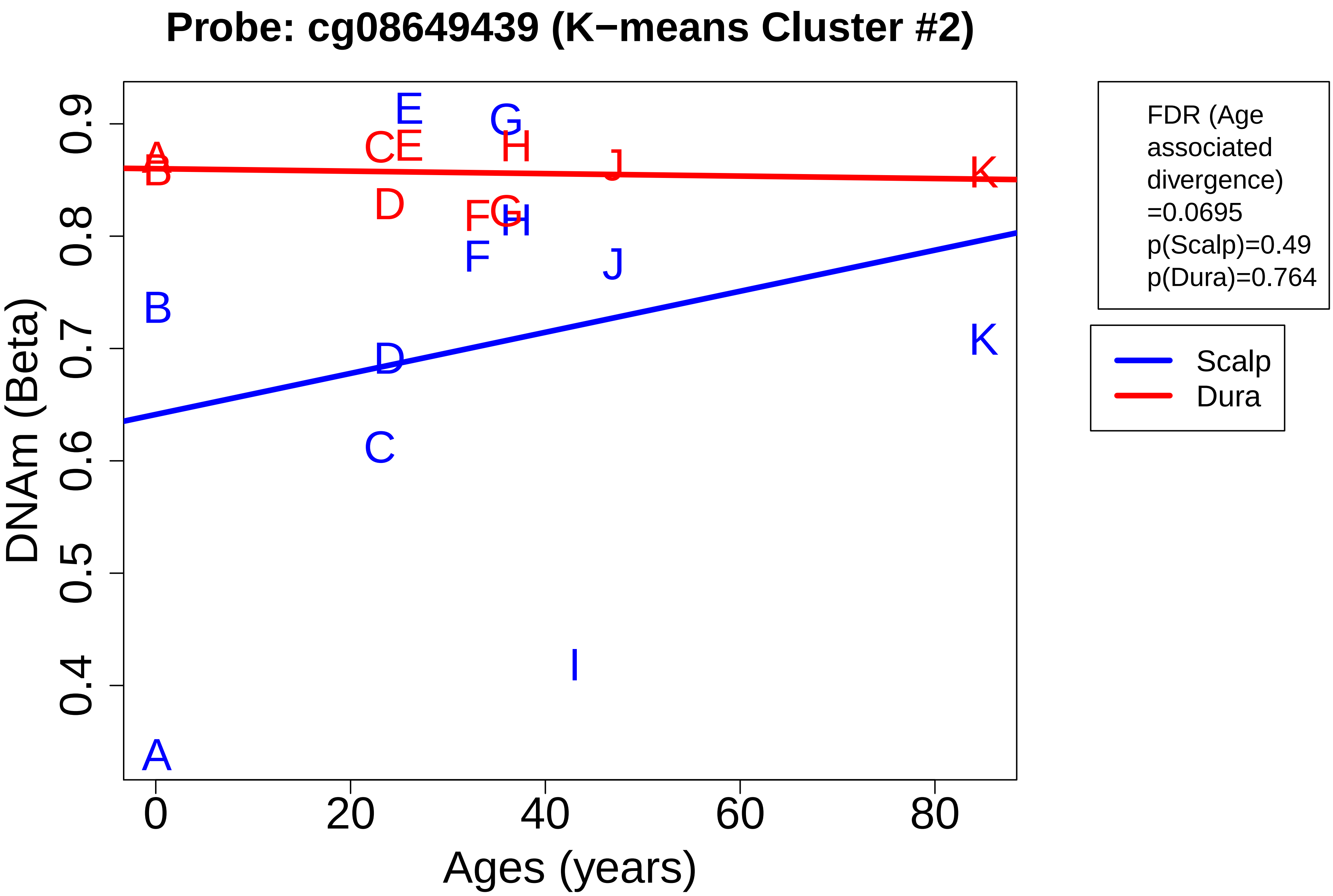

Probe: cg08649439 (K−means Cluster #2)
DNAm (Beta)
0.4
0.5
0.6
0.7
0.8
0.9
Ages (years)
0
20
40
60
80
FDR (Age associated divergence) =0.0695
p(Scalp)=0.49
p(Dura)=0.764
Scalp
Dura

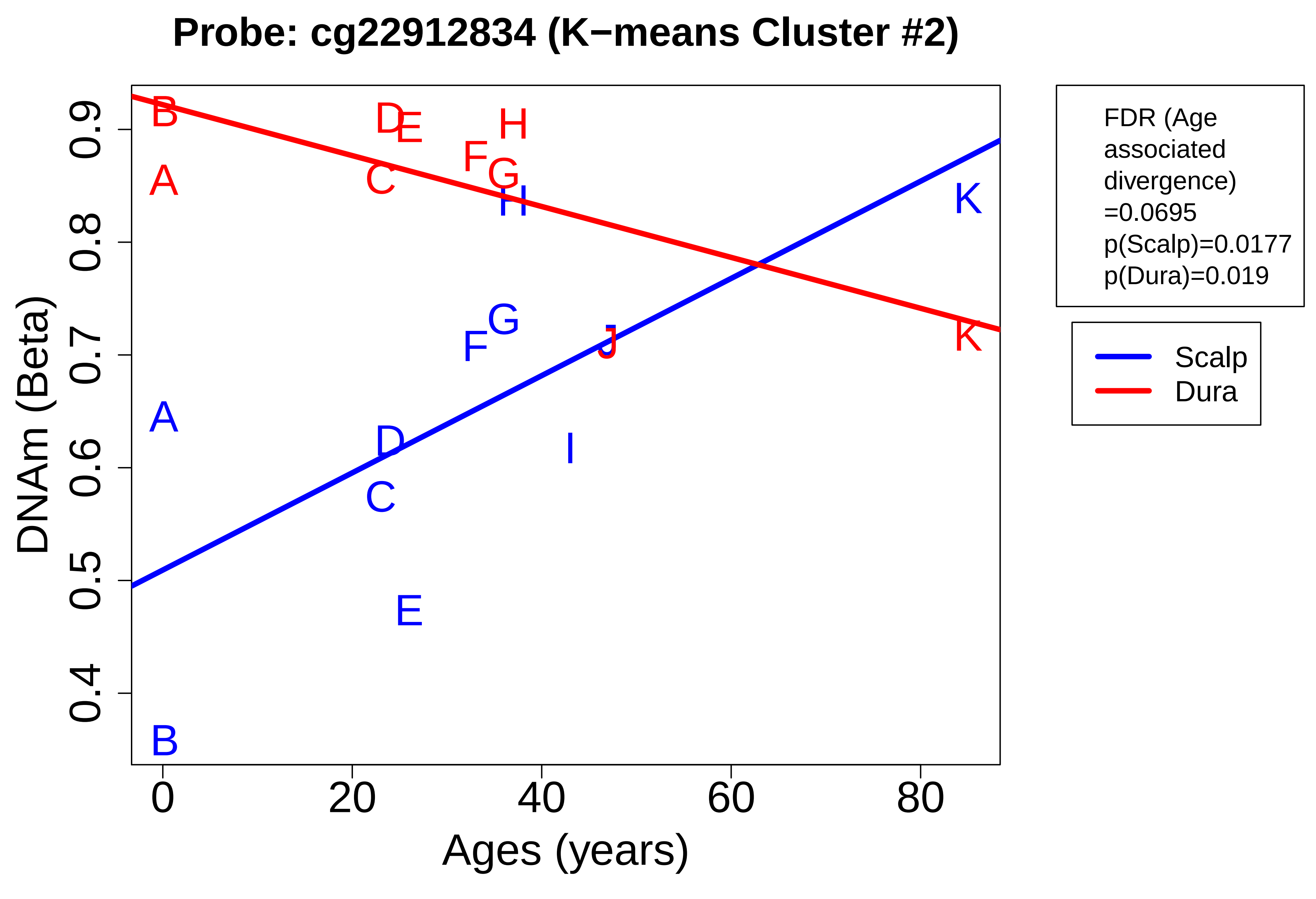

Probe: cg22912834 (K−means Cluster #2)
DNAm (Beta)
0.4
0.5
0.6
0.7
0.8
0.9
0
20
40
60
80
Ages (years)
FDR (Age associated divergence) =0.0695
p(Scalp)=0.0177
p(Dura)=0.019
Scalp
Dura

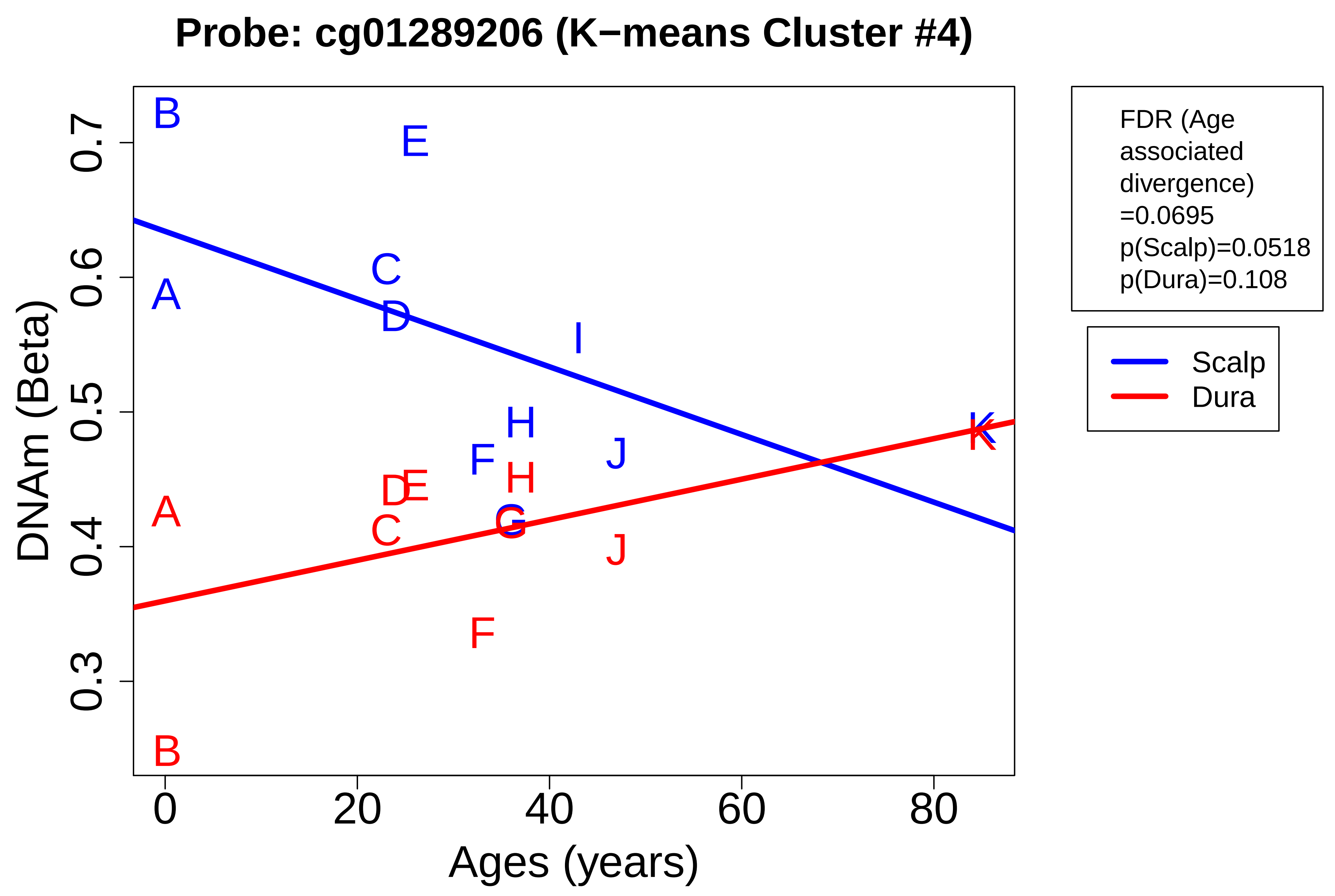

Probe: cg01289206 (K−means Cluster #4)
DNAm (Beta)
0.3
0.4
0.5
0.6
0.7
0
20
40
60
80
Ages (years)
FDR (Age associated divergence) =0.0695
p(Scalp)=0.0518
p(Dura)=0.108
Scalp
Dura

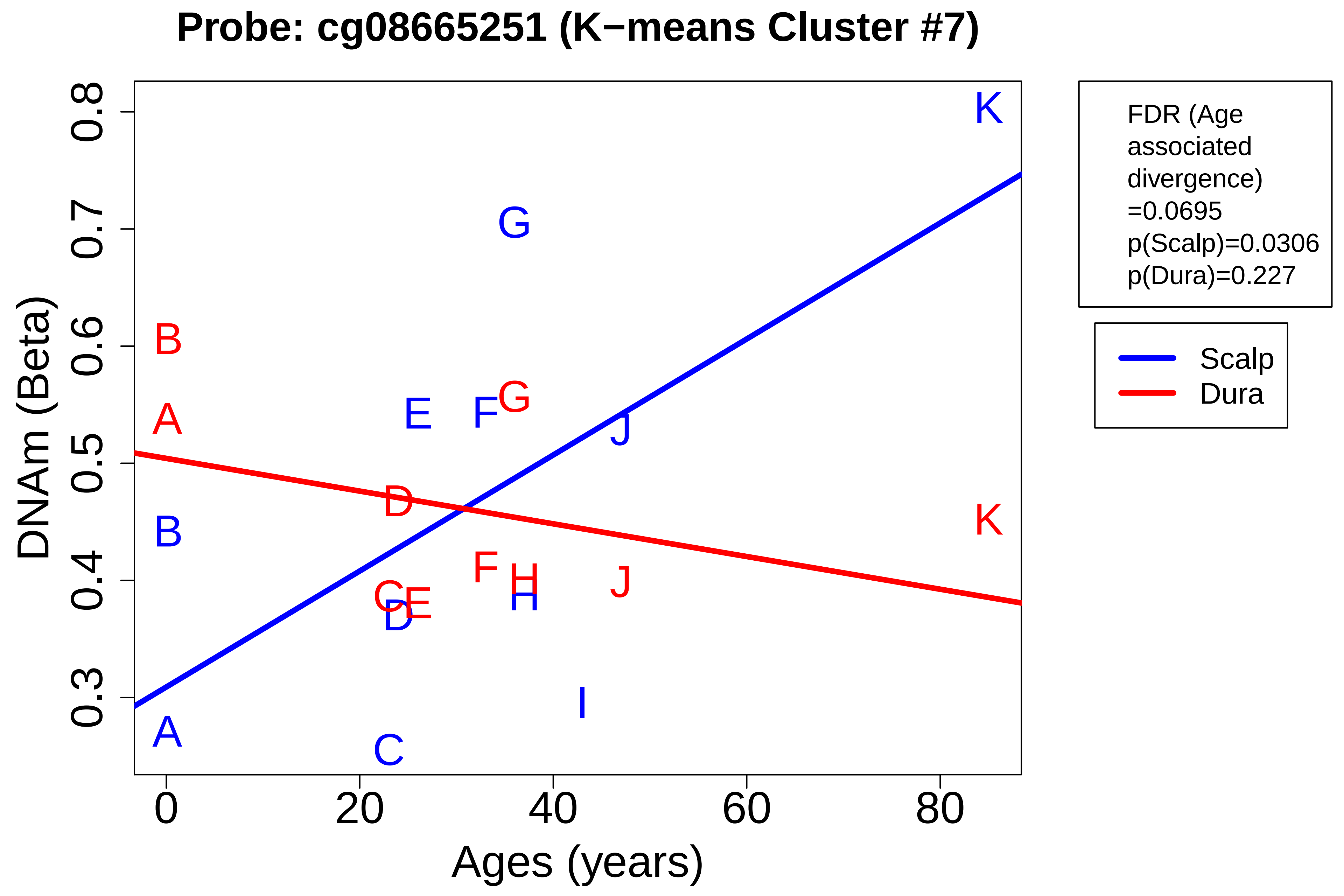
Probe: cg08665251 (K−means Cluster #7)
DNAm (Beta)
Ages (years)
0.3
0.4
0.5
0.6
0.7
0.8
0
20
40
60
80
FDR (Age associated divergence) =0.0695
p(Scalp)=0.0306
p(Dura)=0.227
Scalp
Dura

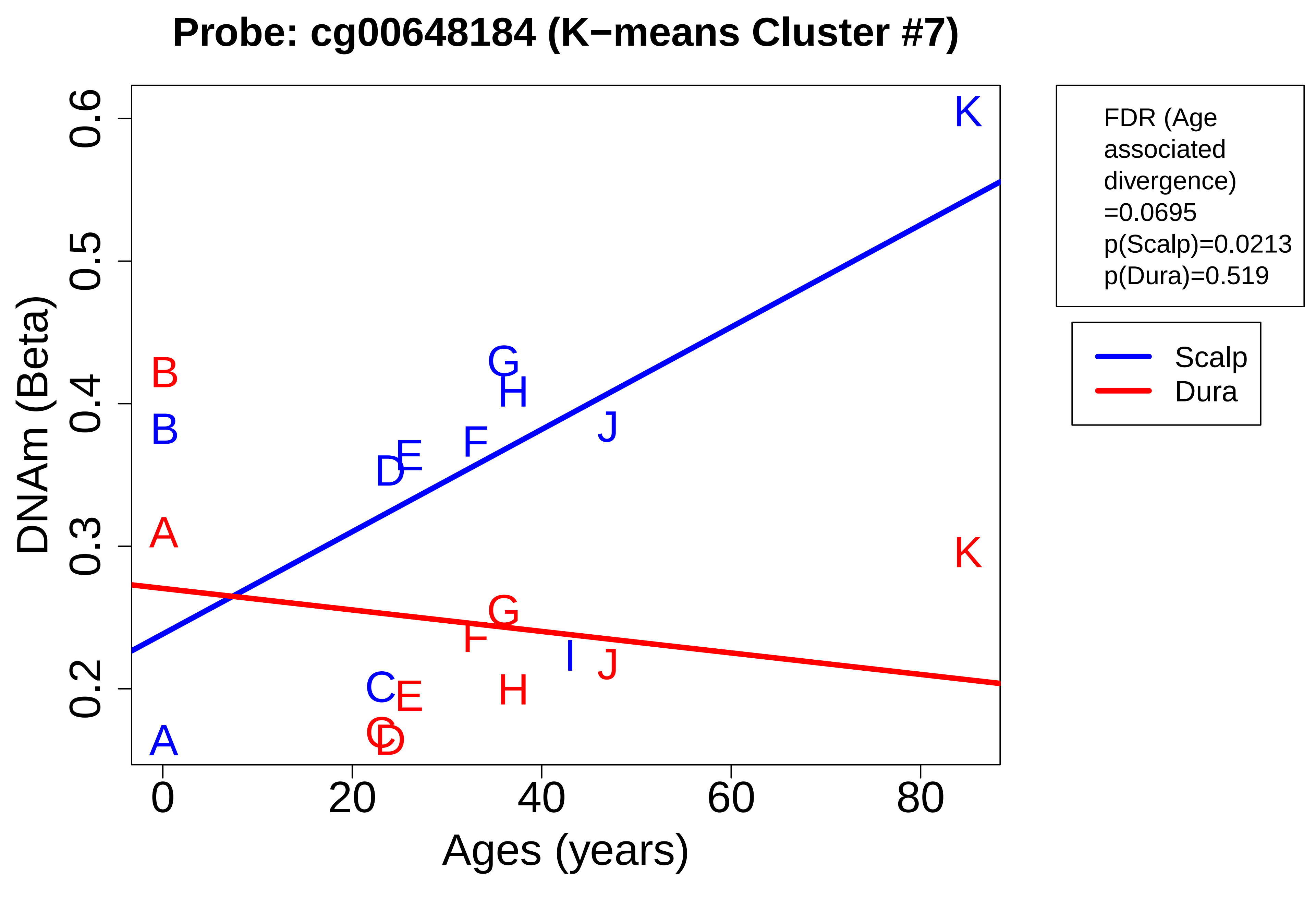

Probe: cg00648184 (K−means Cluster #7)
DNAm (Beta)
0.2
0.3
0.4
0.5
0.6
0
20
40
60
80
Ages (years)
A
B
C
D
E
F
G
H
I
J
K
FDR (Age associated divergence) =0.0695
p(Scalp)=0.0213
p(Dura)=0.519
Scalp
Dura

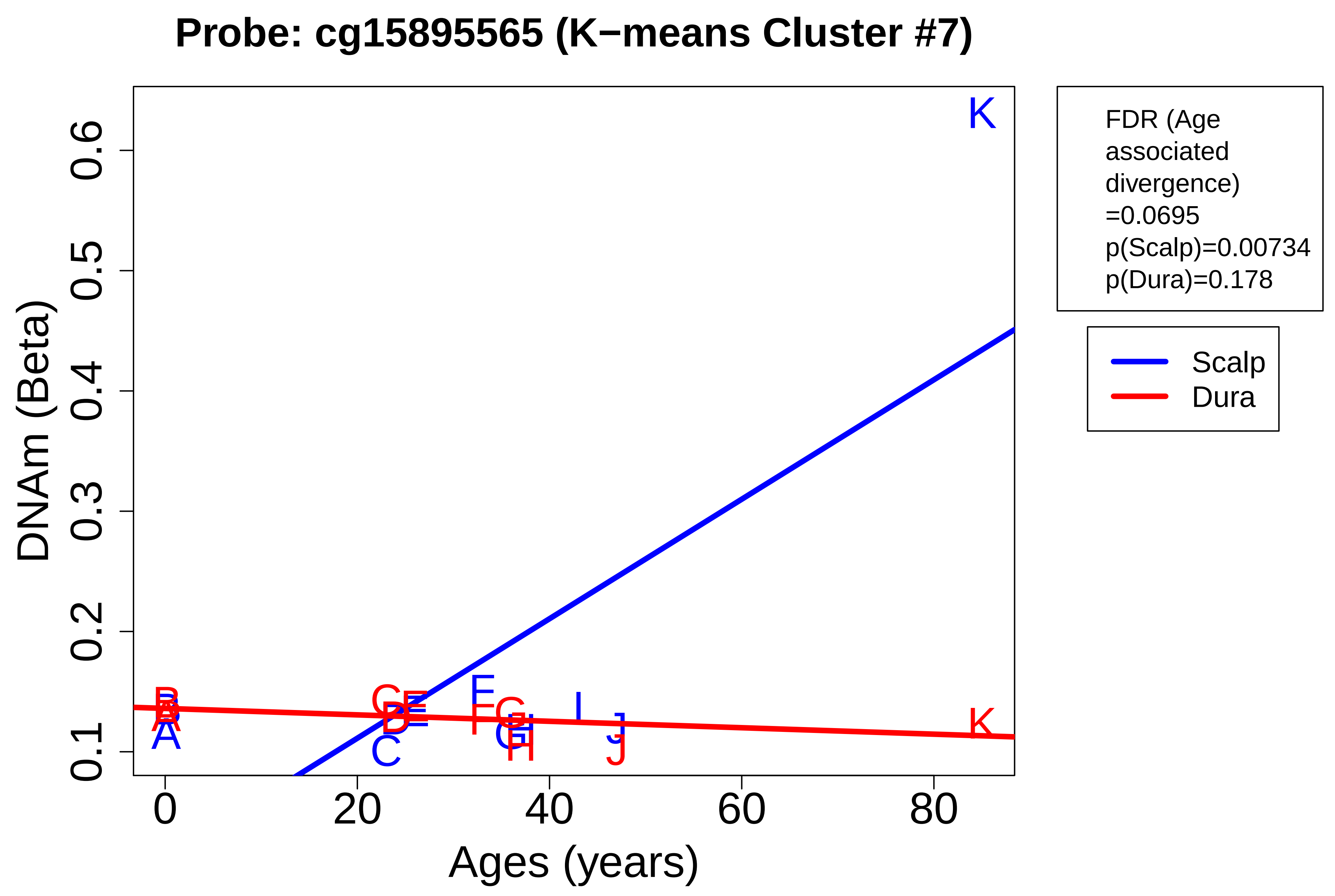
Probe: cg15895565 (K−means Cluster #7)
DNAm (Beta)
Ages (years)
0.1
0.2
0.3
0.4
0.5
0.6
0
20
40
60
80
FDR (Age associated divergence) =0.0695
p(Scalp)=0.00734
p(Dura)=0.178
Scalp
Dura

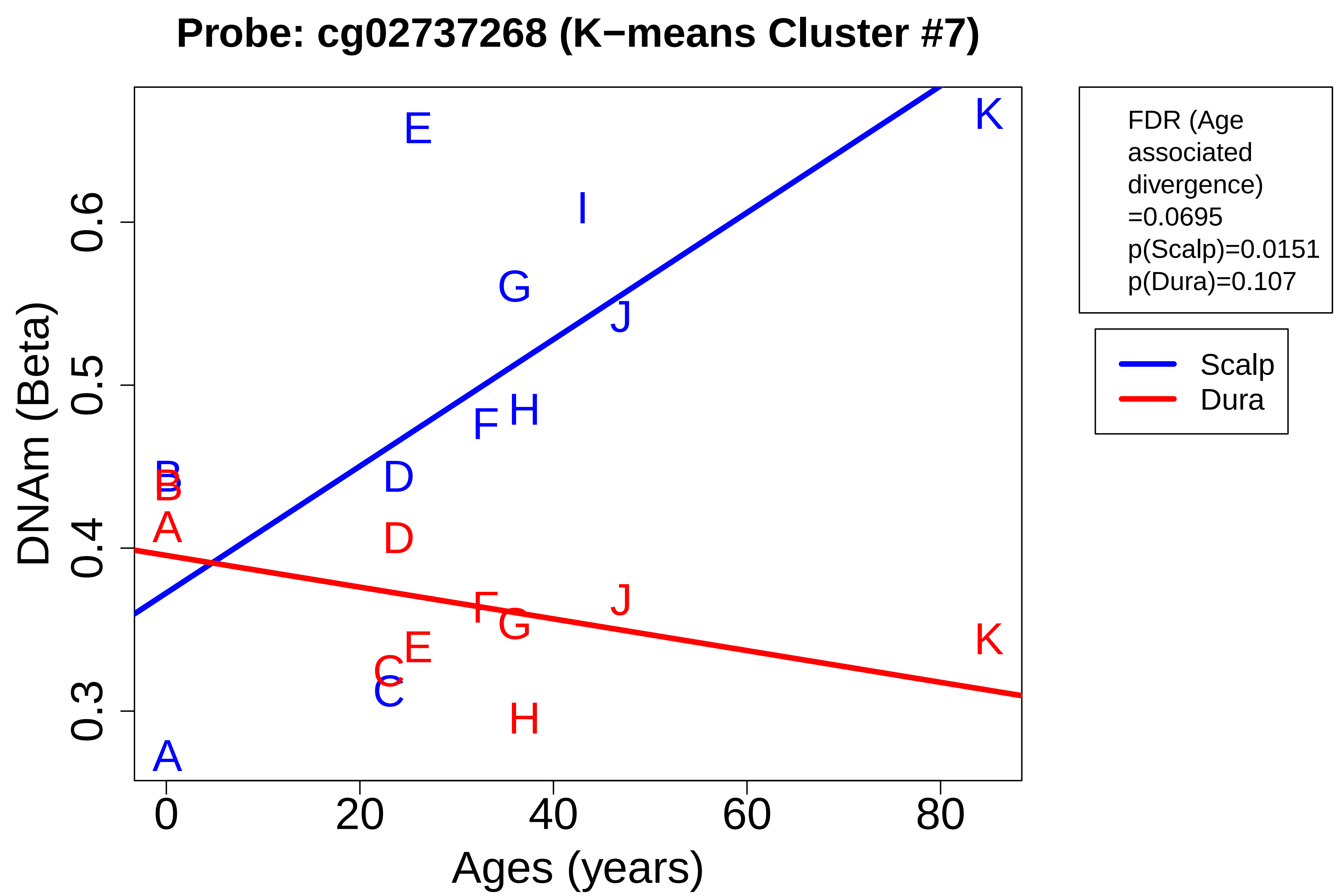

Probe: cg02737268 (K−means Cluster #7)
DNAm (Beta)
0.3
0.4
0.5
0.6
0
20
40
60
80
Ages (years)
E
I
G
J
K
H
F
B
D
A
C
B
A
D
F
G
J
E
C
K
H
FDR (Age associated divergence) =0.0695
p(Scalp)=0.0151
p(Dura)=0.107
Scalp
Dura

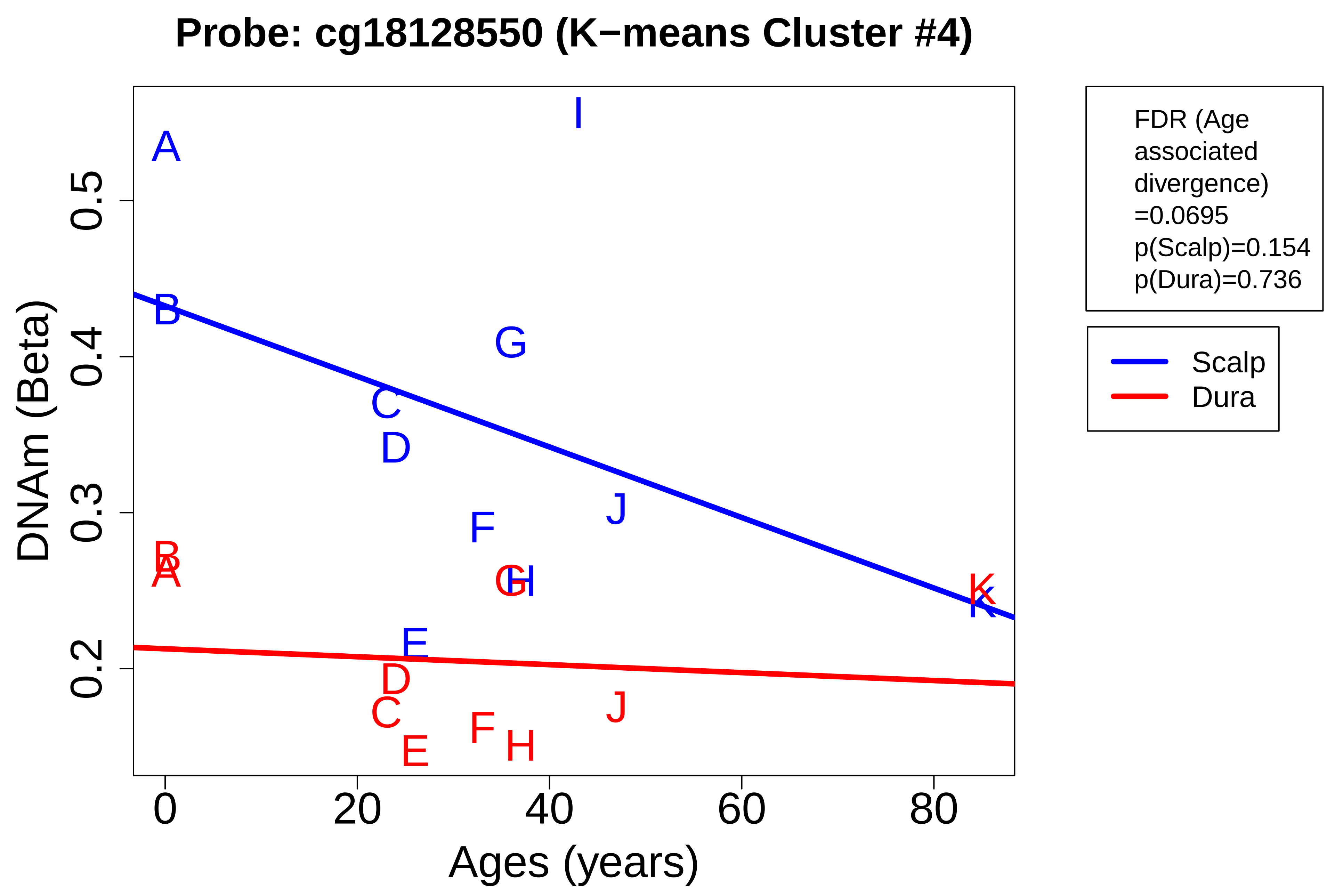

Probe: cg18128550 (K−means Cluster #4)
DNAm (Beta)
0.2
0.3
0.4
0.5
Ages (years)
0
20
40
60
80
A
B
C
D
E
F
G
H
I
J
K
FDR (Age associated divergence) =0.0695
p(Scalp)=0.154
p(Dura)=0.736
Scalp
Dura

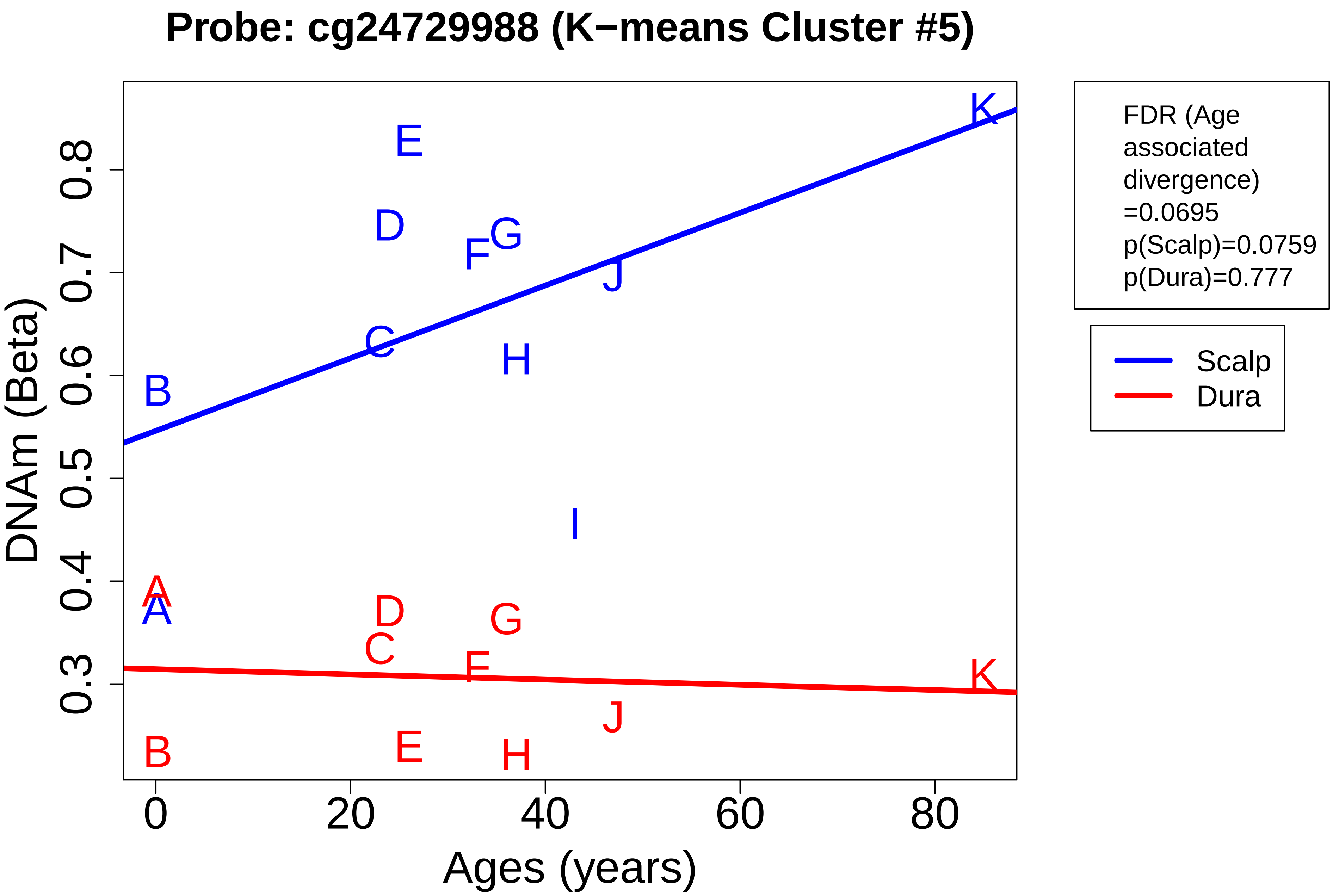

Probe: cg24729988 (K−means Cluster #5)
DNAm (Beta)
0.3
0.4
0.5
0.6
0.7
0.8
Ages (years)
0
20
40
60
80
A
B
C
D
E
F
G
H
I
J
K
FDR (Age associated divergence) =0.0695
p(Scalp)=0.0759
p(Dura)=0.777
Scalp
Dura

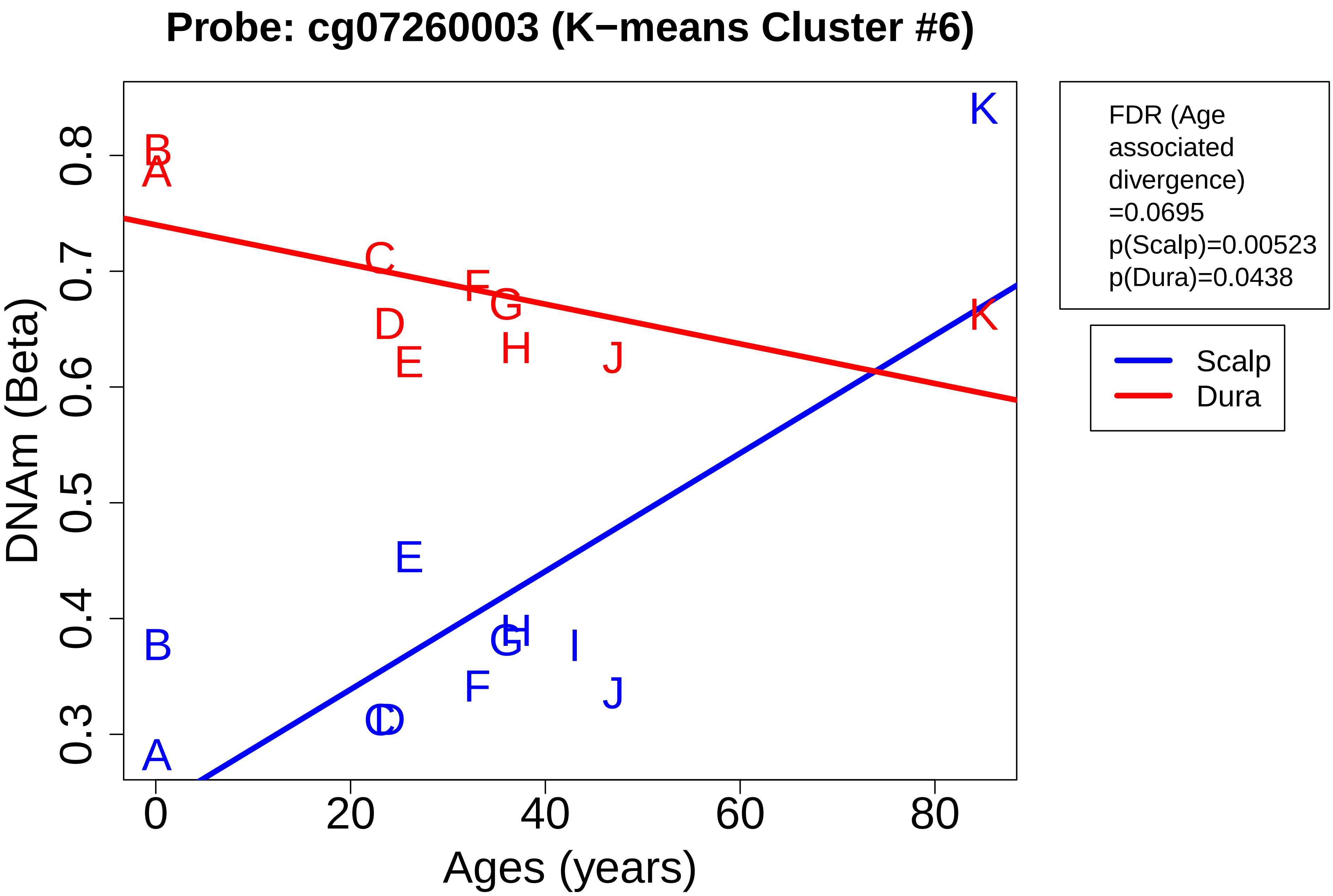

Probe: cg07260003 (K−means Cluster #6)
DNAm (Beta)
0.3
0.4
0.5
0.6
0.7
0.8
0
20
40
60
80
Ages (years)
FDR (Age associated divergence) =0.0695
p(Scalp)=0.00523
p(Dura)=0.0438
Scalp
Dura

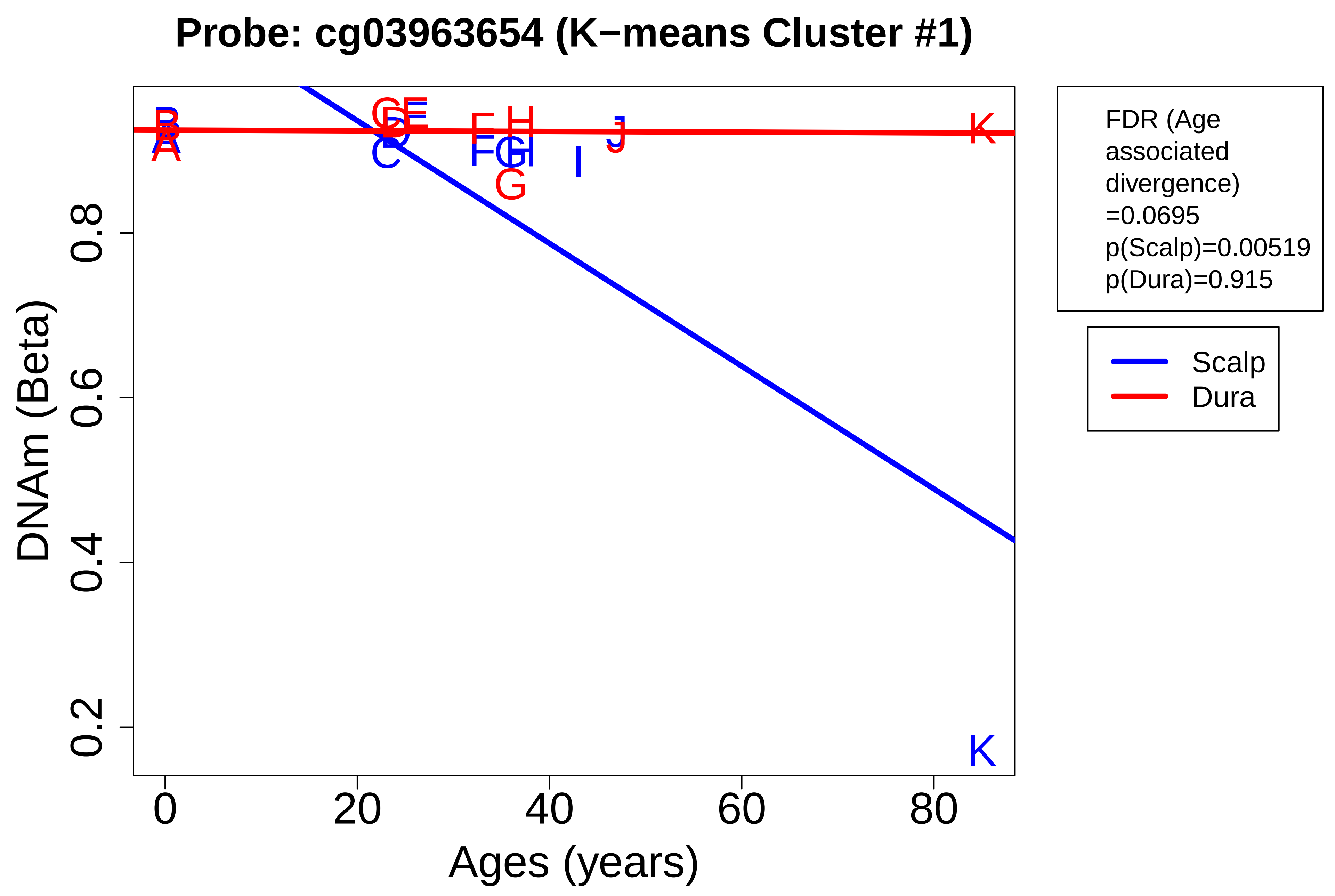

Probe: cg03963654 (K−means Cluster #1)
DNAm (Beta)
0.2
0.4
0.6
0.8
0
20
40
60
80
Ages (years)
A
B
C
D
E
F
G
H
I
J
K
FDR (Age associated divergence) =0.0695
p(Scalp)=0.00519
p(Dura)=0.915
Scalp
Dura

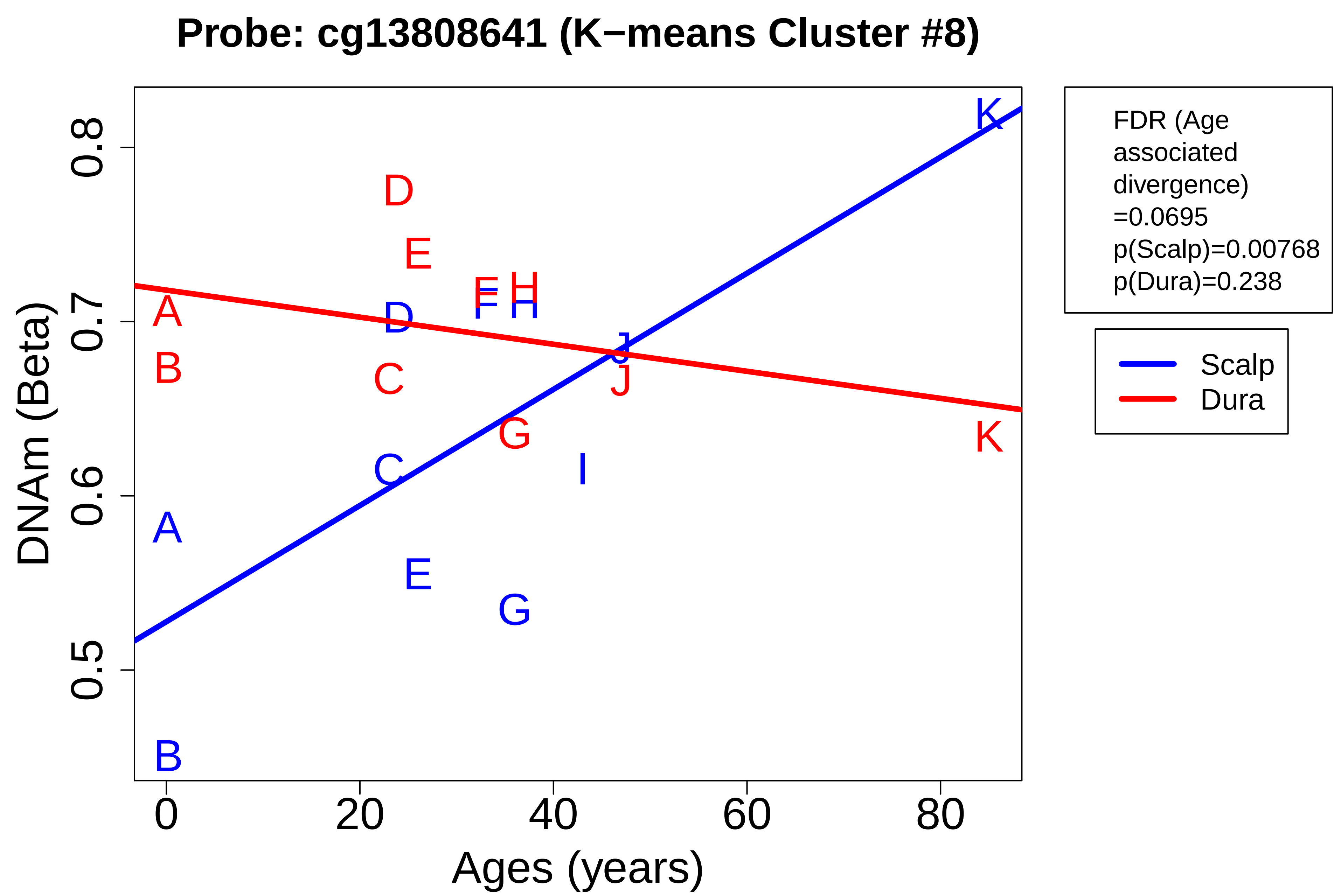

Probe: cg13808641 (K−means Cluster #8)
DNAm (Beta)
0.5
0.6
0.7
0.8
Ages (years)
0
20
40
60
80
FDR (Age associated divergence) =0.0695
p(Scalp)=0.00768
p(Dura)=0.238
Scalp
Dura
A
B
C
D
E
F
G
H
I
J
K

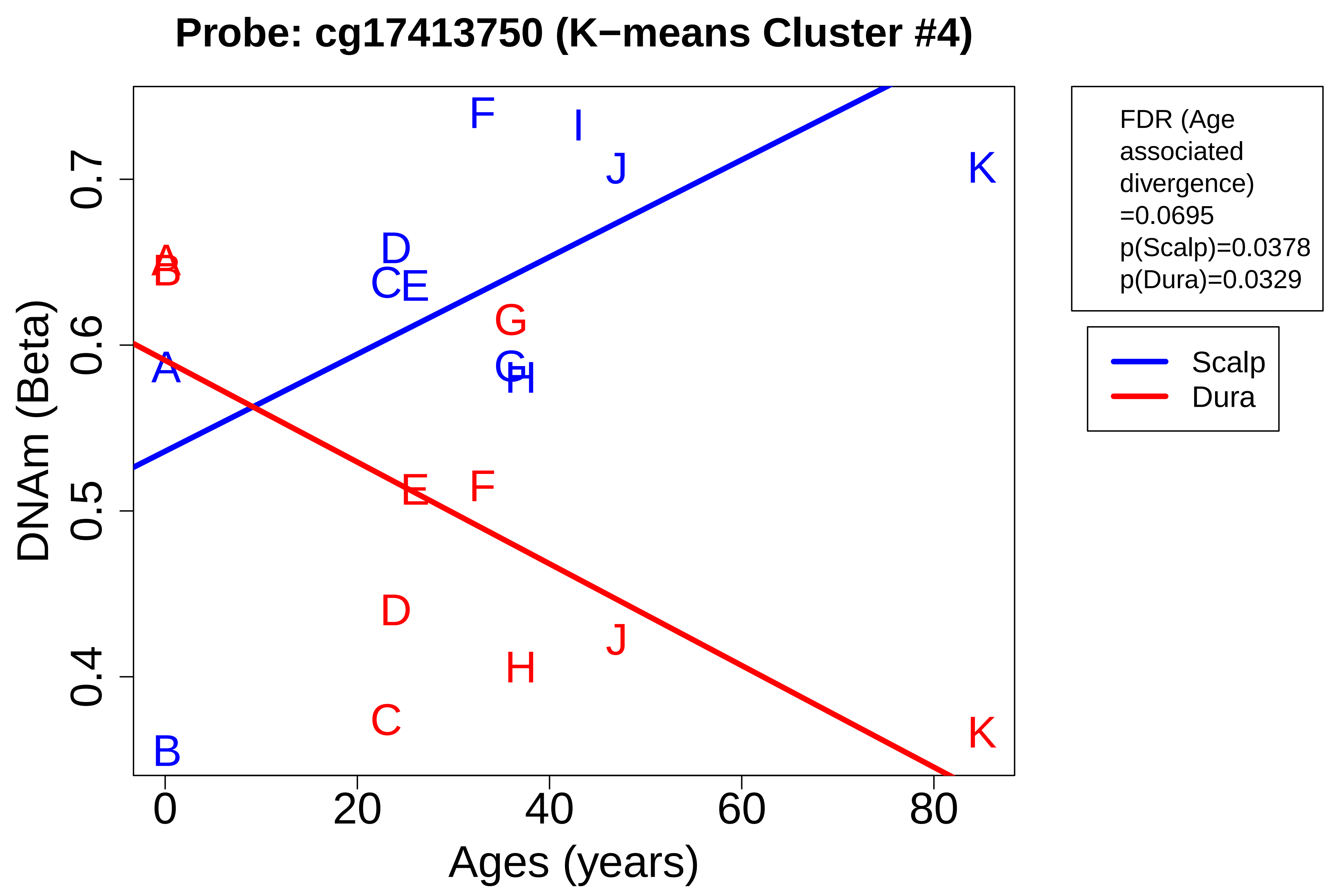

Probe: cg17413750 (K−means Cluster #4)
DNAm (Beta)
0.4
0.5
0.6
0.7
0
20
40
60
80
Ages (years)
FDR (Age associated divergence) =0.0695
p(Scalp)=0.0378
p(Dura)=0.0329
Scalp
Dura

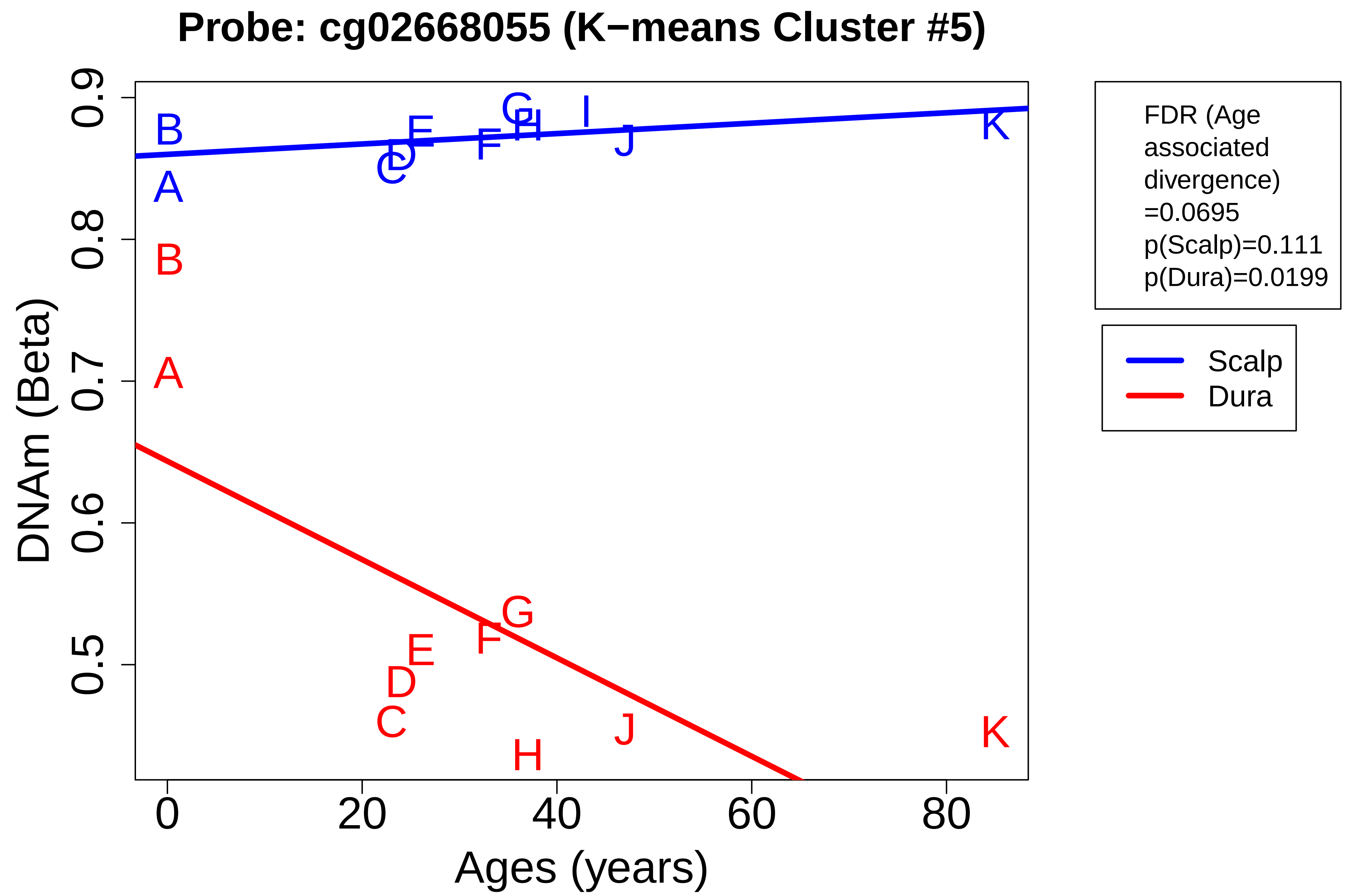

Probe: cg02668055 (K−means Cluster #5)
DNAm (Beta)
0.5
0.6
0.7
0.8
0.9
Ages (years)
0
20
40
60
80
FDR (Age associated divergence) =0.0695
p(Scalp)=0.111
p(Dura)=0.0199
Scalp
Dura

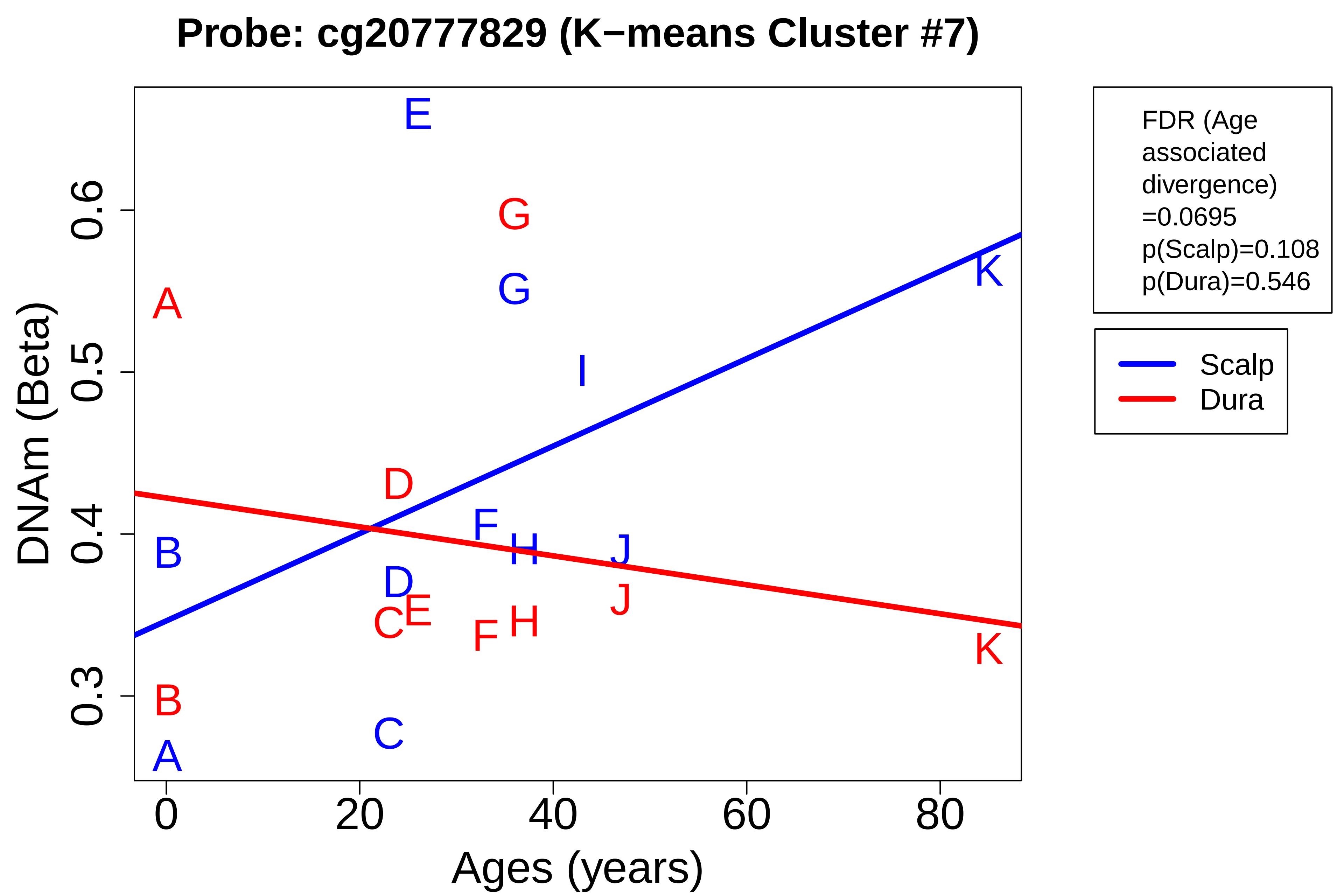
Probe: cg20777829 (K−means Cluster #7)
FDR (Age associated divergence) =0.0695
p(Scalp)=0.108
p(Dura)=0.546
Scalp
Dura
DNAm (Beta)
0.3
0.4
0.5
0.6
0
20
40
60
80
Ages (years)
A
B
C
D
E
F
G
H
I
J
K

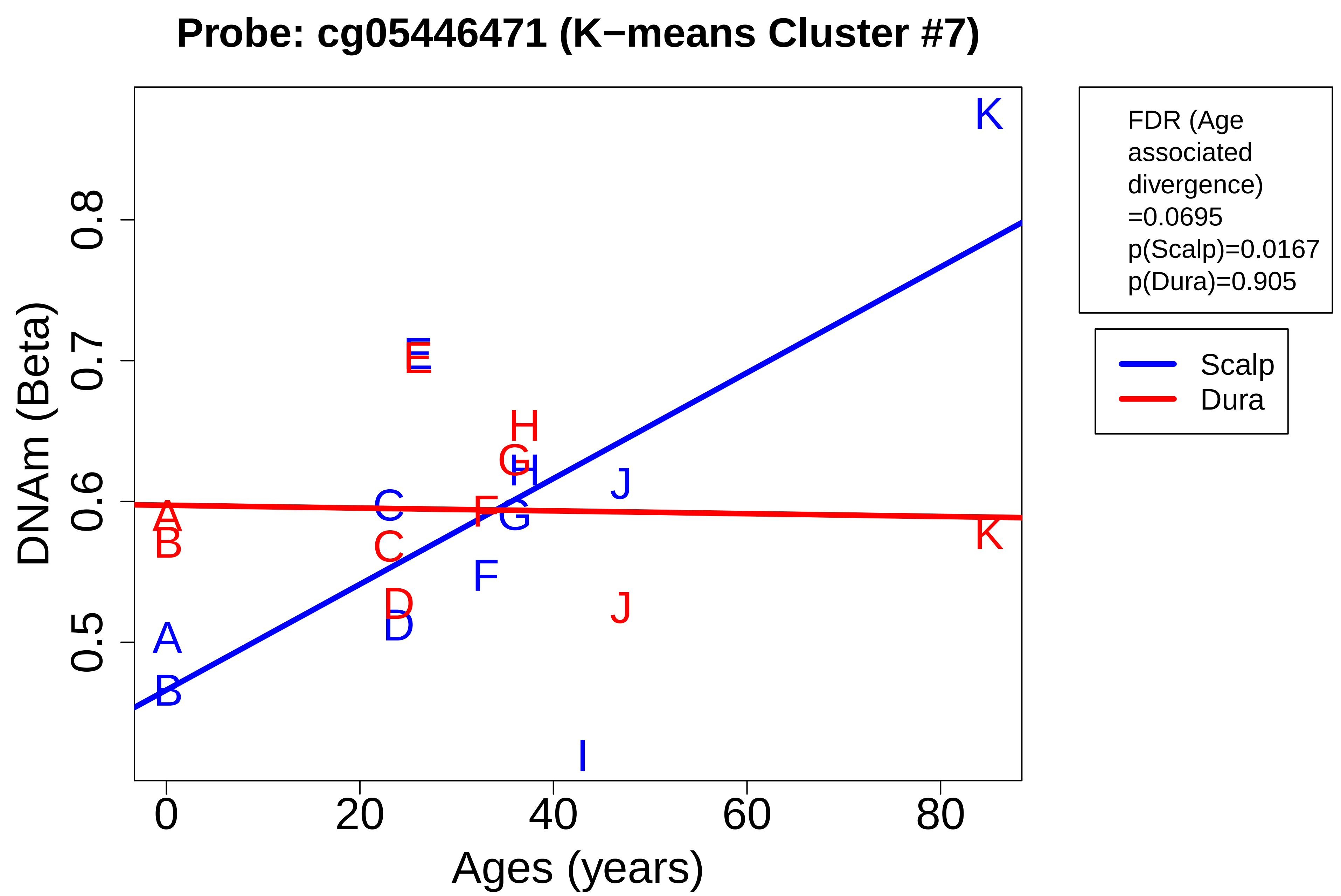

Probe: cg05446471 (K−means Cluster #7)
DNAm (Beta)
0.5
0.6
0.7
0.8
0
20
40
60
80
Ages (years)
A
B
C
D
E
F
G
H
I
J
K
FDR (Age associated divergence) =0.0695
p(Scalp)=0.0167
p(Dura)=0.905
Scalp
Dura

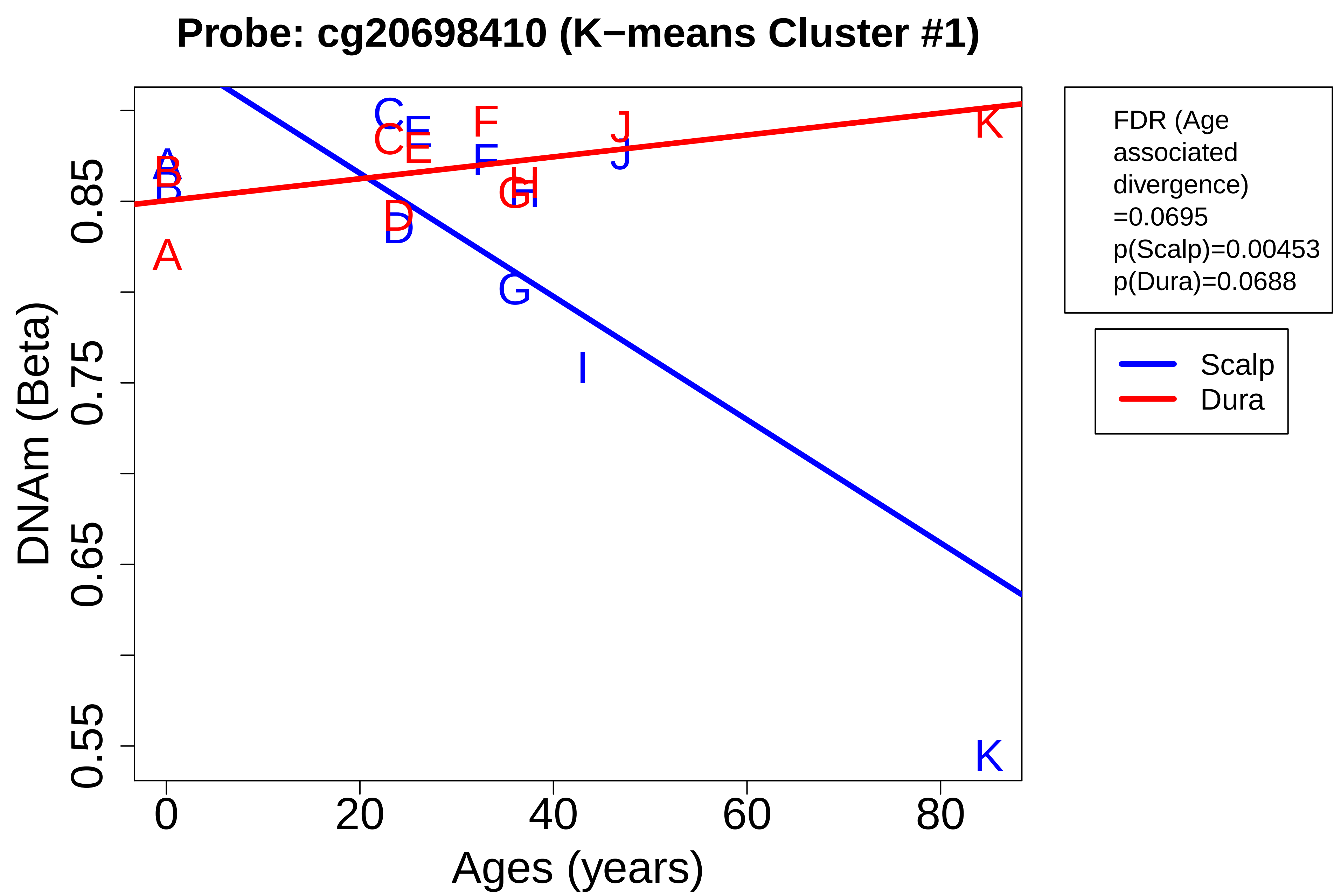

Probe: cg20698410 (K−means Cluster #1)
DNAm (Beta)
0.55
0.65
0.75
0.85
0
20
40
60
80
Ages (years)
FDR (Age associated divergence) =0.0695
p(Scalp)=0.00453
p(Dura)=0.0688
Scalp
Dura

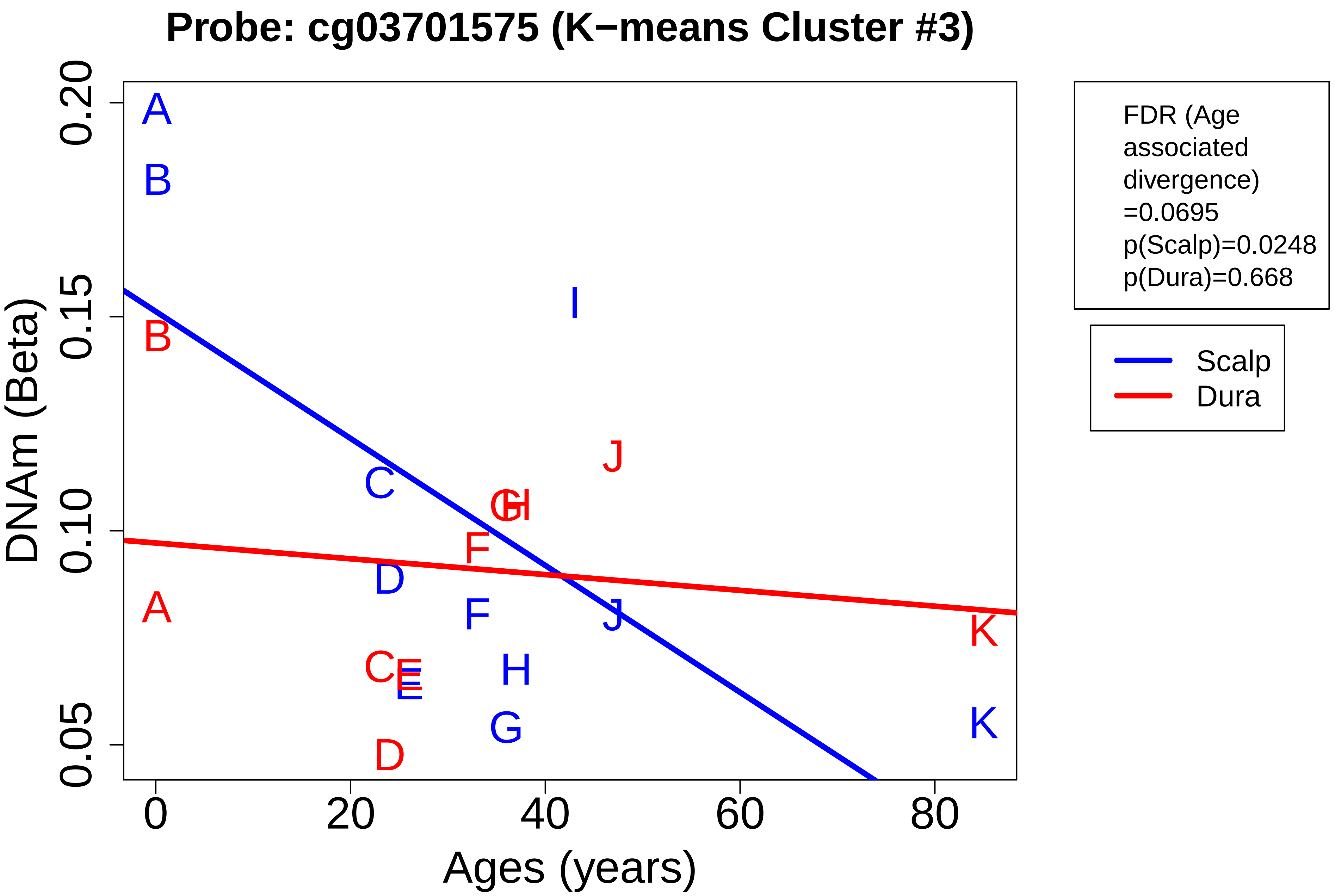

Probe: cg03701575 (K−means Cluster #3)
DNAm (Beta)
0.05
0.10
0.15
0.20
0
20
40
60
80
Ages (years)
FDR (Age associated divergence) =0.0695
p(Scalp)=0.0248
p(Dura)=0.668
Scalp
Dura

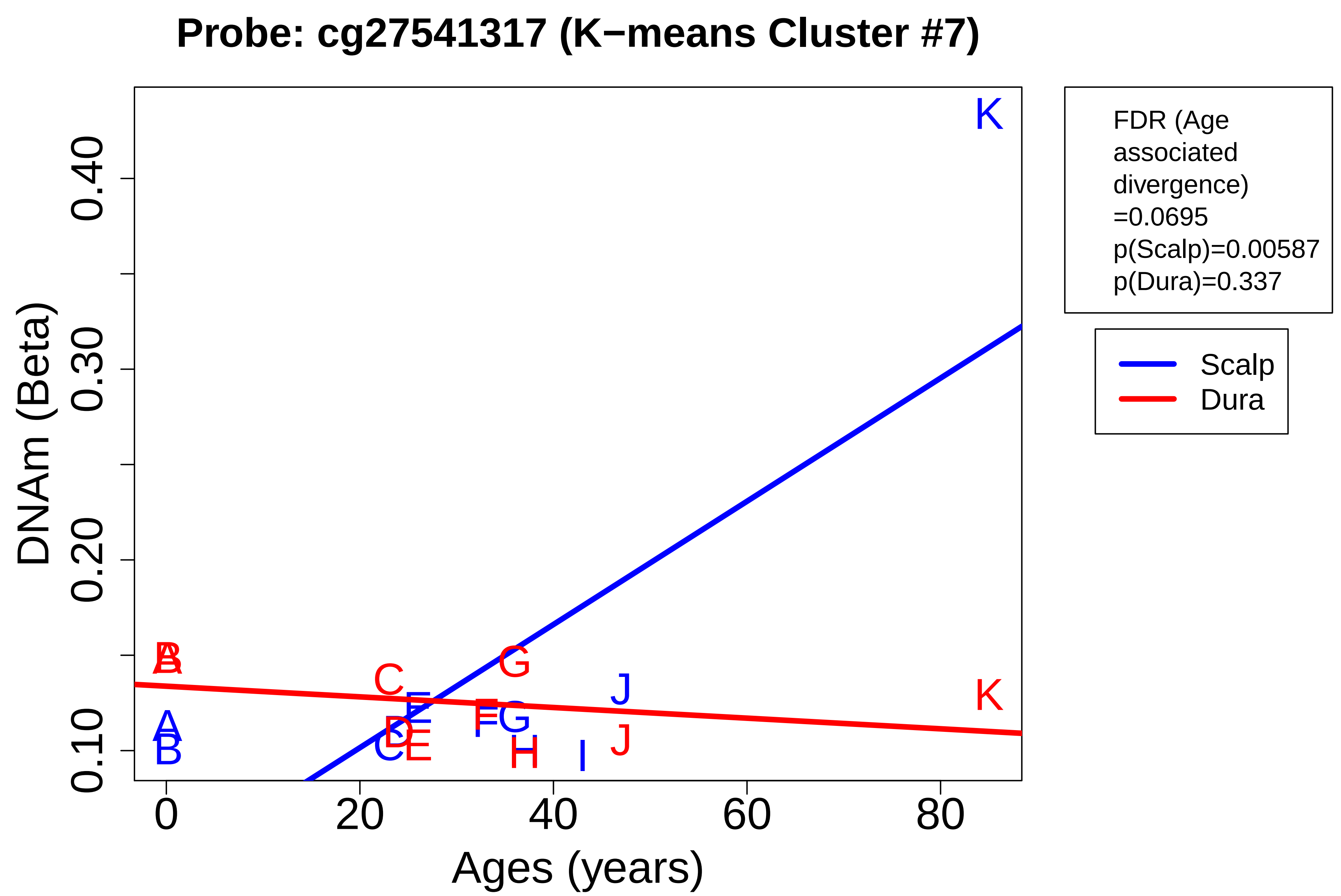

Probe: cg27541317 (K−means Cluster #7)
DNAm (Beta)
0.10
0.20
0.30
0.40
0
20
40
60
80
Ages (years)
FDR (Age associated divergence) =0.0695
p(Scalp)=0.00587
p(Dura)=0.337
Scalp
Dura
A
B
C
D
E
F
G
H
I
J
K

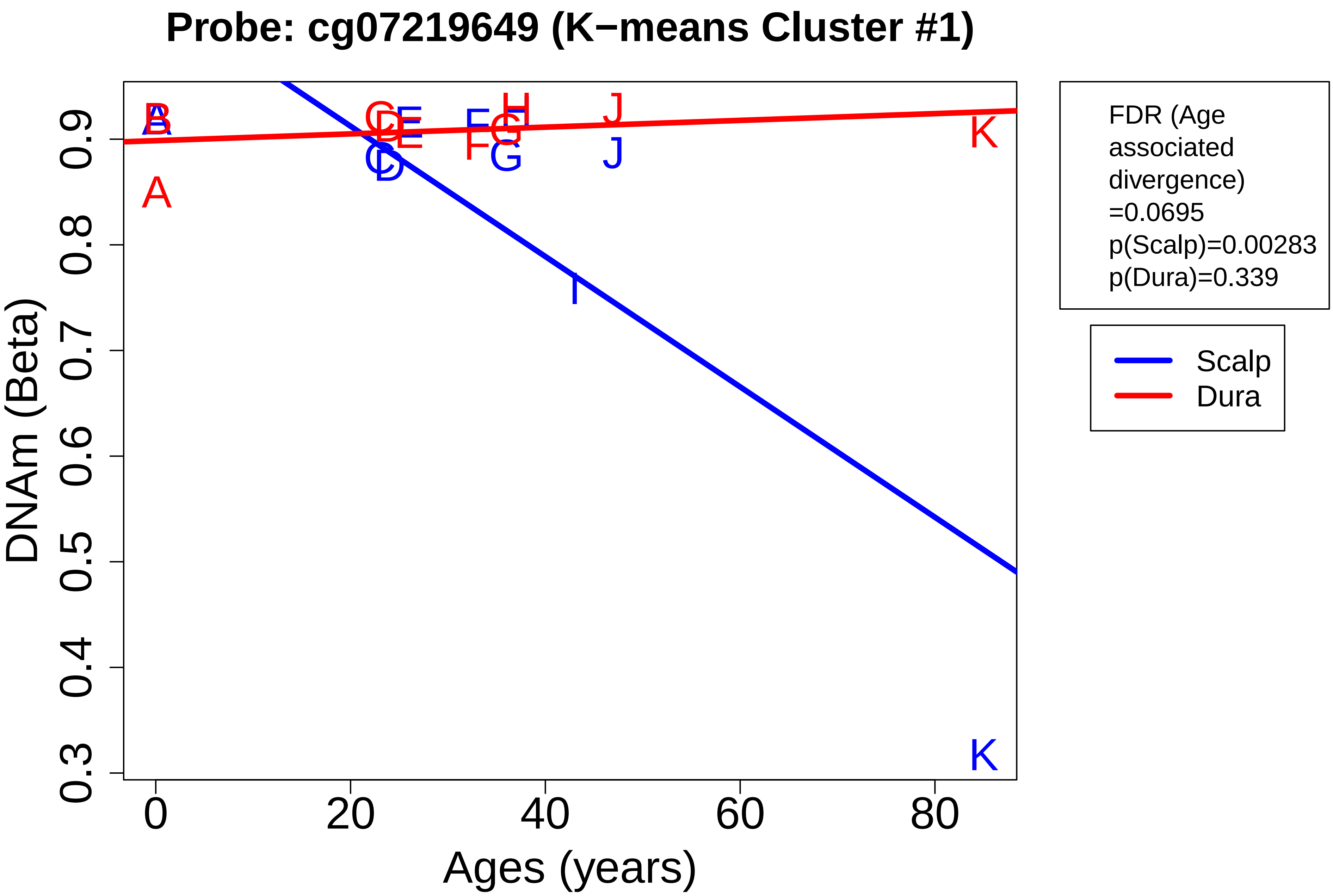

Probe: cg07219649 (K−means Cluster #1)
DNAm (Beta)
0.3
0.4
0.5
0.6
0.7
0.8
0.9
0
20
40
60
80
Ages (years)
A
B
C
D
E
F
G
H
I
J
K
FDR (Age associated divergence) =0.0695
p(Scalp)=0.00283
p(Dura)=0.339
Scalp
Dura

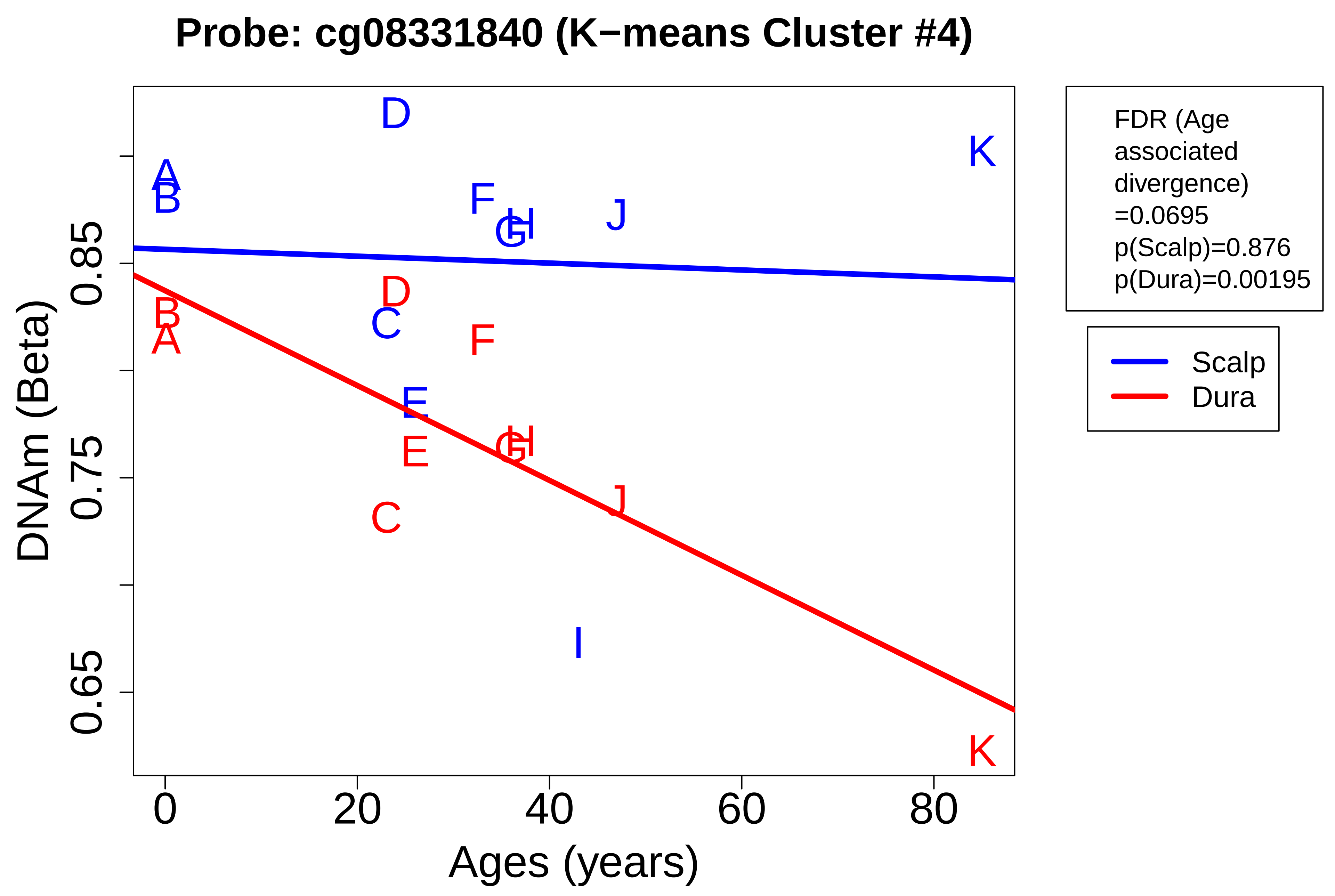
Probe: cg08331840 (K−means Cluster #4)
DNAm (Beta)
0.65
0.75
0.85
0
20
40
60
80
Ages (years)
A
B
C
D
E
F
G
H
I
J
K
FDR (Age associated divergence) =0.0695
p(Scalp)=0.876
p(Dura)=0.00195
Scalp
Dura

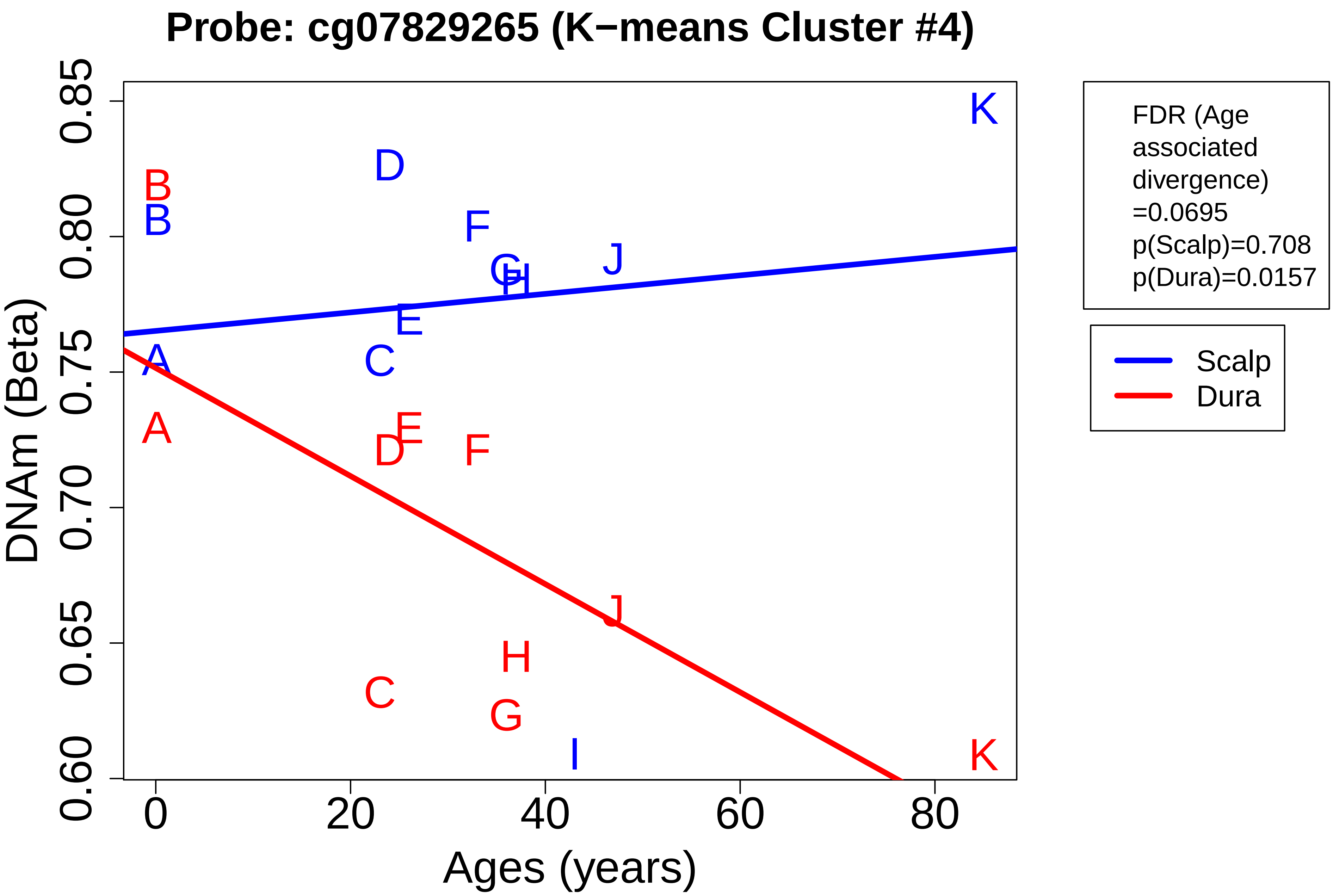

Probe: cg07829265 (K−means Cluster #4)
DNAm (Beta)
0.60
0.65
0.70
0.75
0.80
0.85
0
20
40
60
80
Ages (years)
FDR (Age associated divergence) =0.0695
p(Scalp)=0.708
p(Dura)=0.0157
Scalp
Dura
A
B
C
D
E
F
G
H
I
J
K

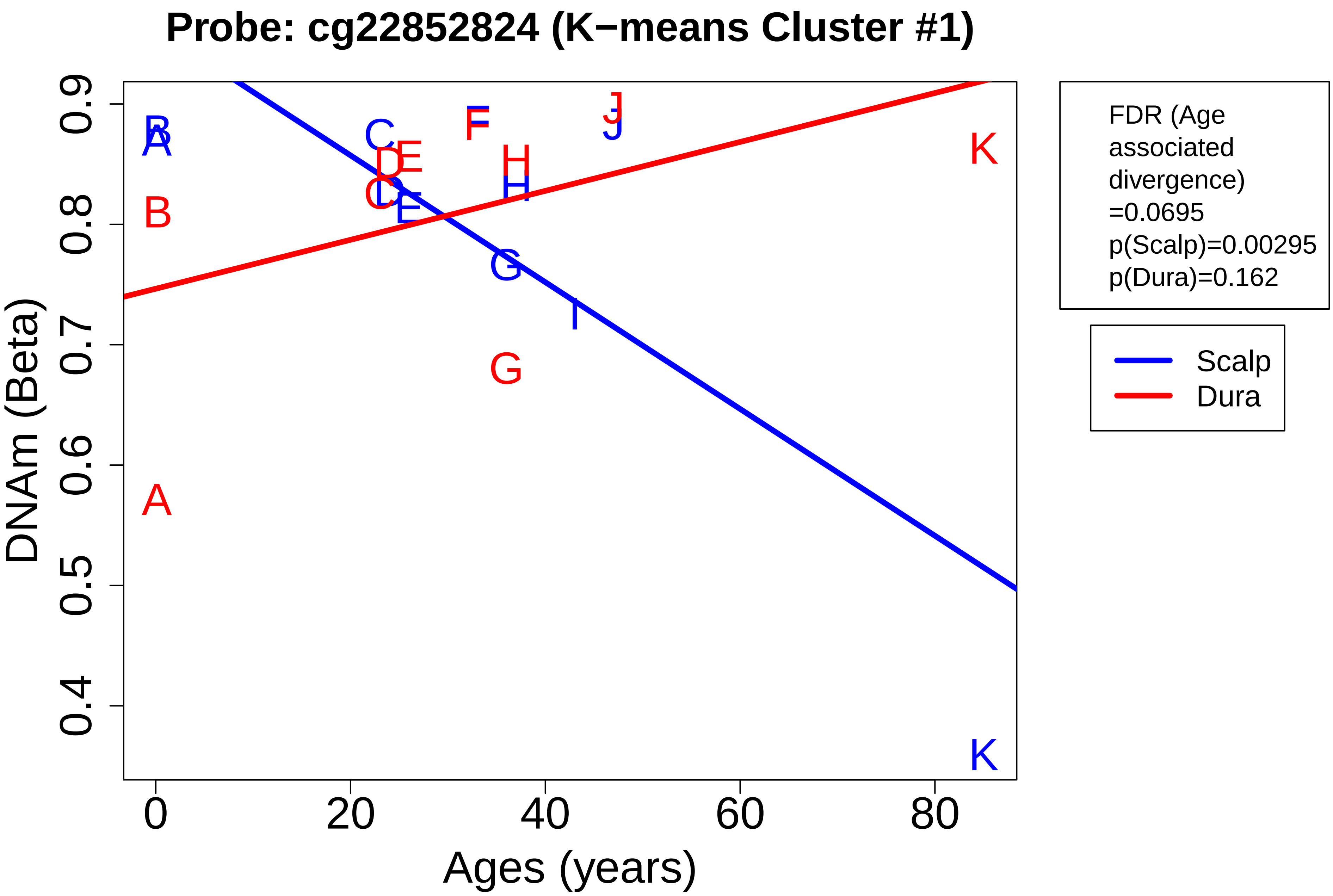

Probe: cg22852824 (K−means Cluster #1)
DNAm (Beta)
0.4
0.5
0.6
0.7
0.8
0.9
Ages (years)
0
20
40
60
80
FDR (Age associated divergence) =0.0695
p(Scalp)=0.00295
p(Dura)=0.162
Scalp
Dura

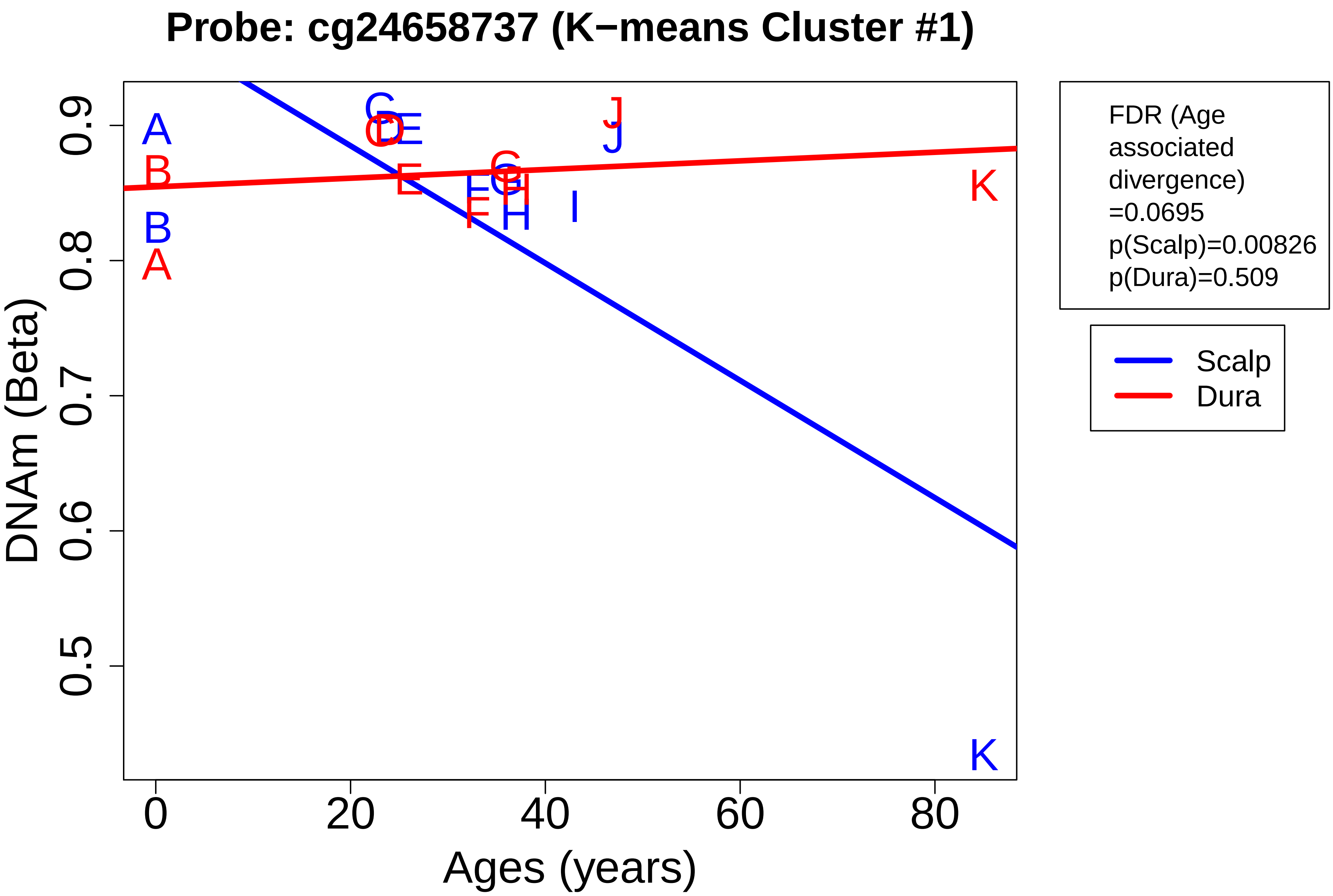
Probe: cg24658737 (K−means Cluster #1)
DNAm (Beta)
0.5
0.6
0.7
0.8
0.9
0
20
40
60
80
Ages (years)
FDR (Age associated divergence) =0.0695
p(Scalp)=0.00826
p(Dura)=0.509
Scalp
Dura

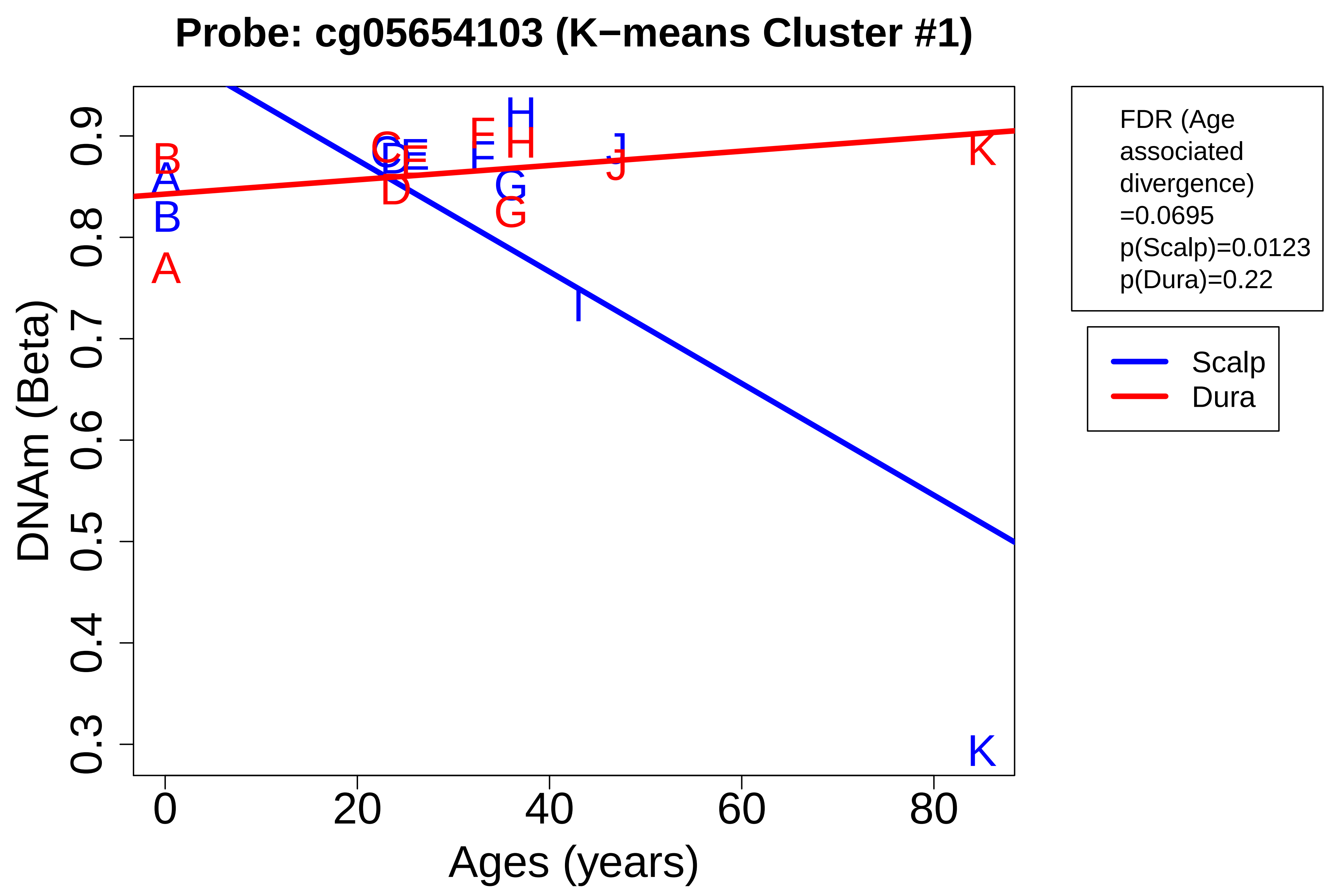

Probe: cg05654103 (K−means Cluster #1)
DNAm (Beta)
0.3
0.4
0.5
0.6
0.7
0.8
0.9
0
20
40
60
80
Ages (years)
FDR (Age associated divergence) =0.0695
p(Scalp)=0.0123
p(Dura)=0.22
Scalp
Dura

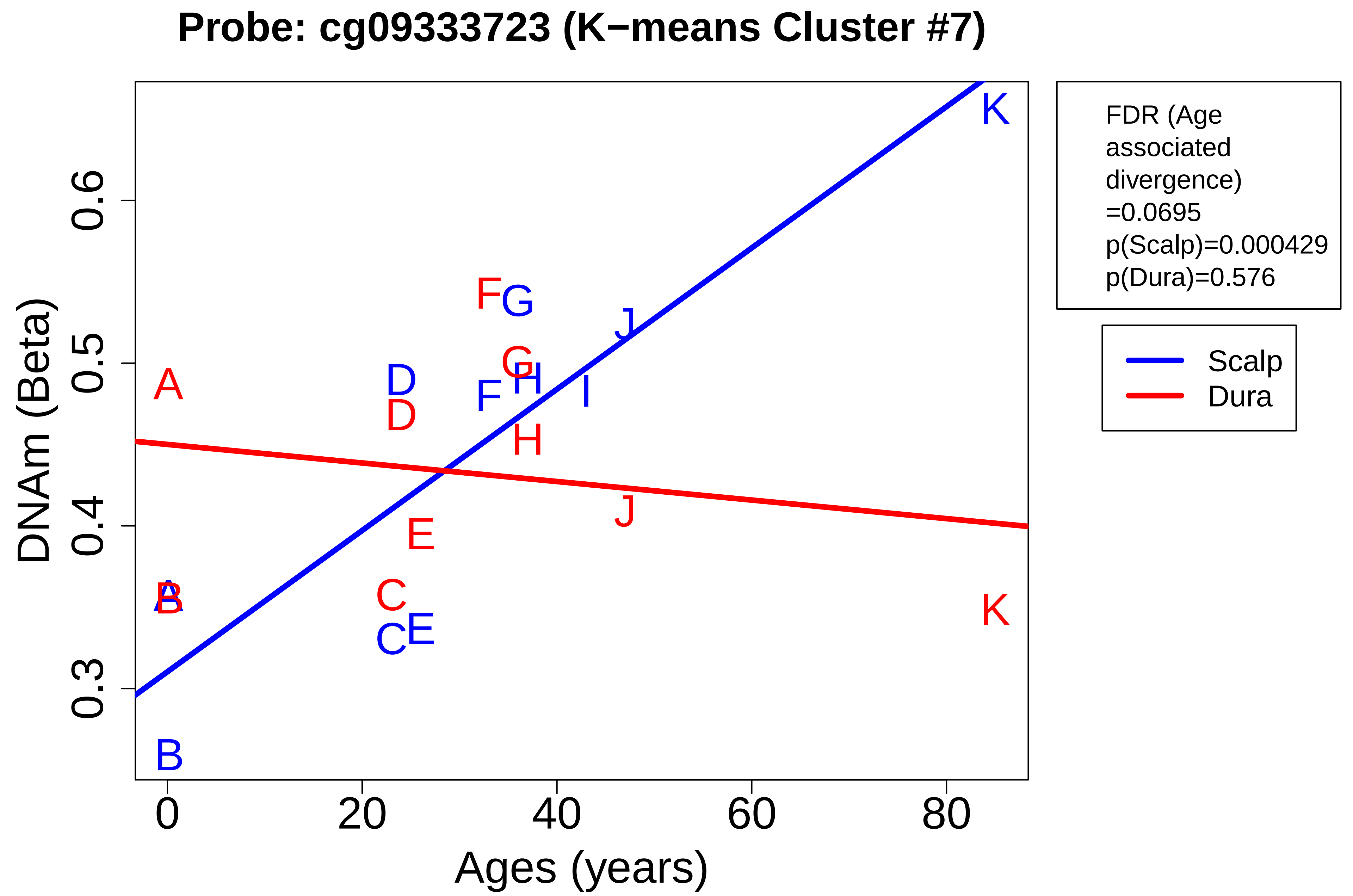

Probe: cg09333723 (K−means Cluster #7)
DNAm (Beta)
0.3
0.4
0.5
0.6
Ages (years)
0
20
40
60
80
FDR (Age associated divergence) =0.0695
p(Scalp)=0.000429
p(Dura)=0.576
Scalp
Dura

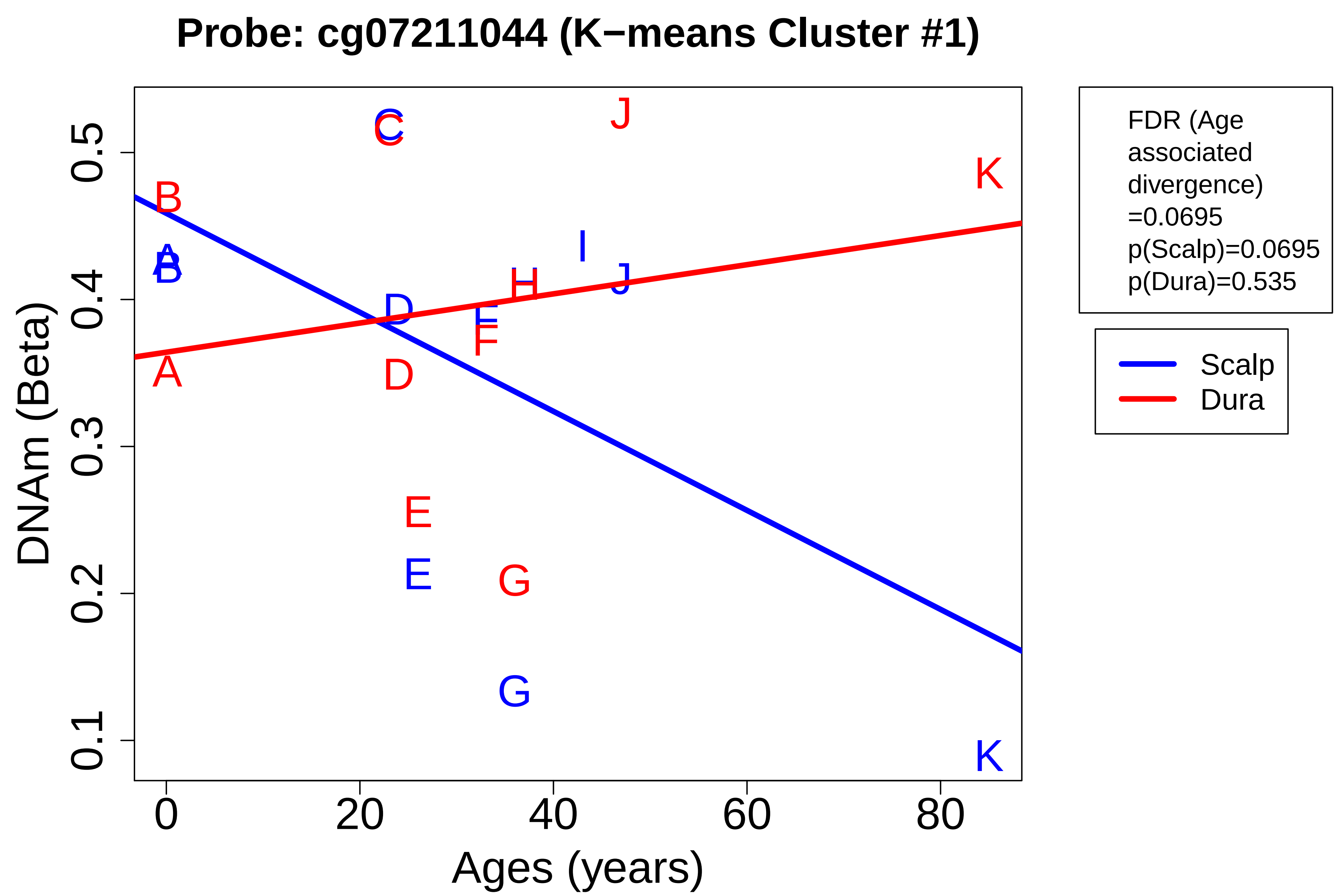

Probe: cg07211044 (K−means Cluster #1)
DNAm (Beta)
0.1
0.2
0.3
0.4
0.5
Ages (years)
0
20
40
60
80
A
B
C
D
E
F
G
H
I
J
K
FDR (Age associated divergence) =0.0695
p(Scalp)=0.0695
p(Dura)=0.535
Scalp
Dura

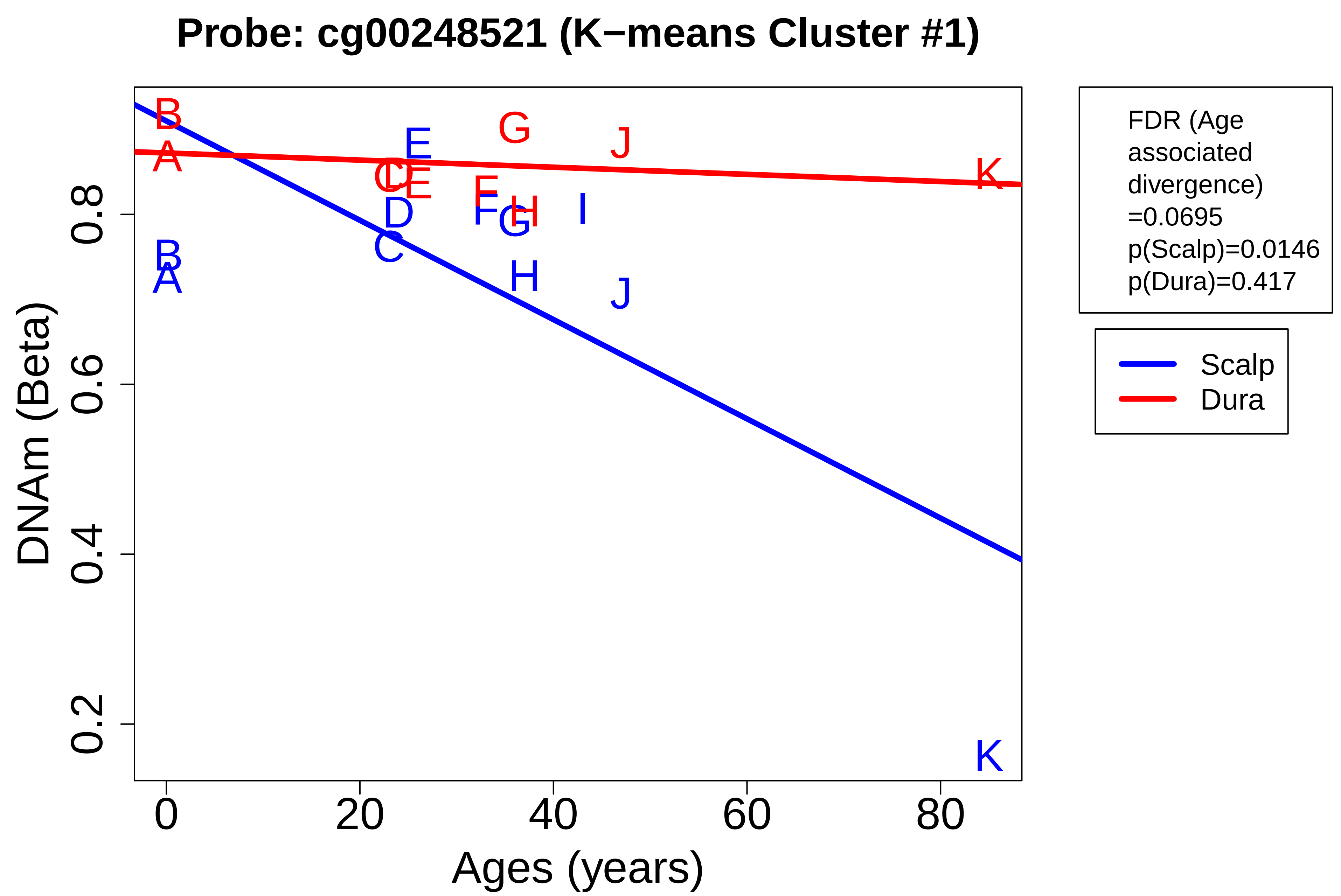
Probe: cg00248521 (K−means Cluster #1)
FDR (Age associated divergence) =0.0695
p(Scalp)=0.0146
p(Dura)=0.417
Scalp
Dura
DNAm (Beta)
0.2
0.4
0.6
0.8
0
20
40
60
80
Ages (years)

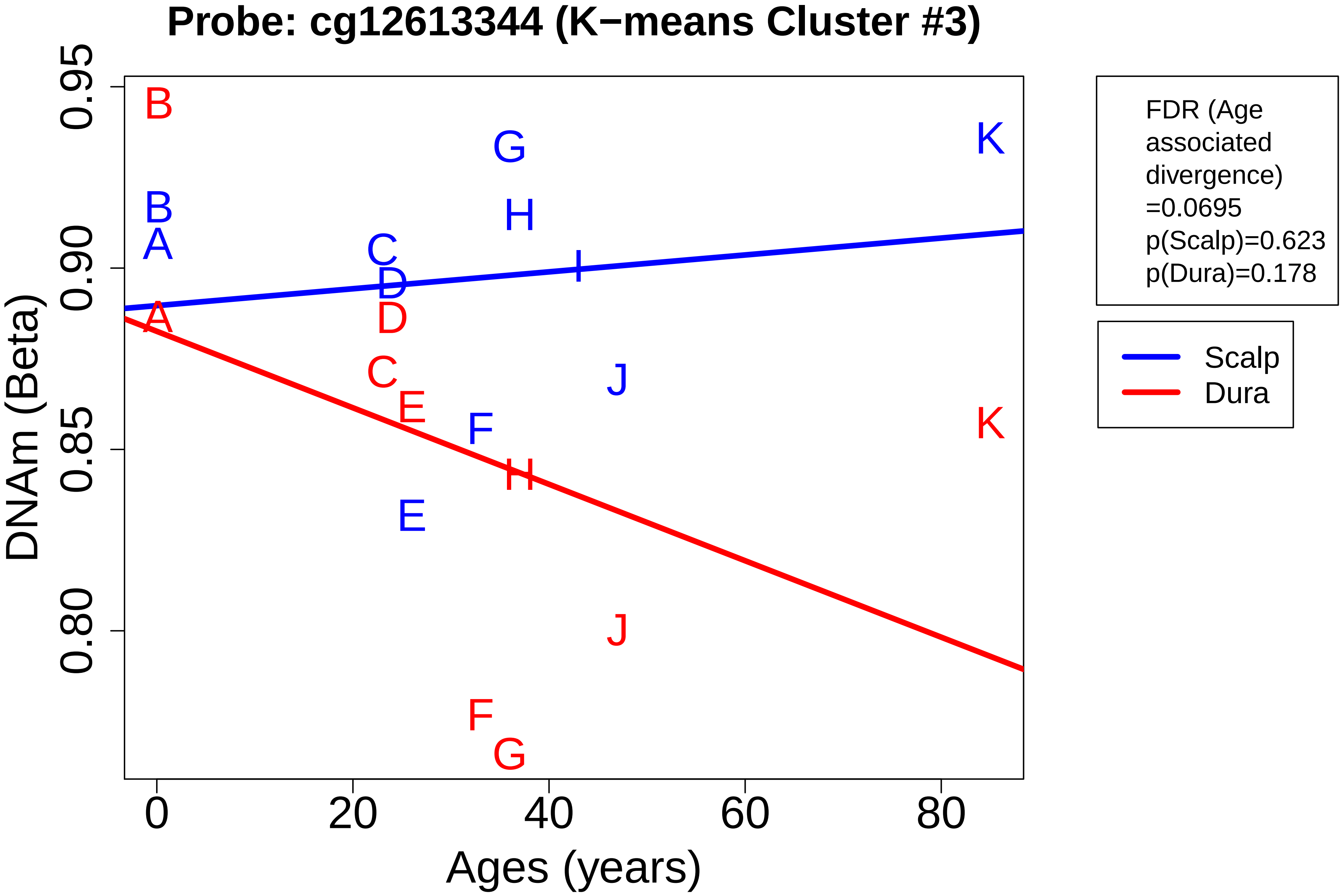

Probe: cg12613344 (K−means Cluster #3)
DNAm (Beta)
0.80
0.85
0.90
0.95
0
20
40
60
80
Ages (years)
A
B
C
D
E
F
G
H
I
J
K
FDR (Age associated divergence) =0.0695
p(Scalp)=0.623
p(Dura)=0.178
Scalp
Dura

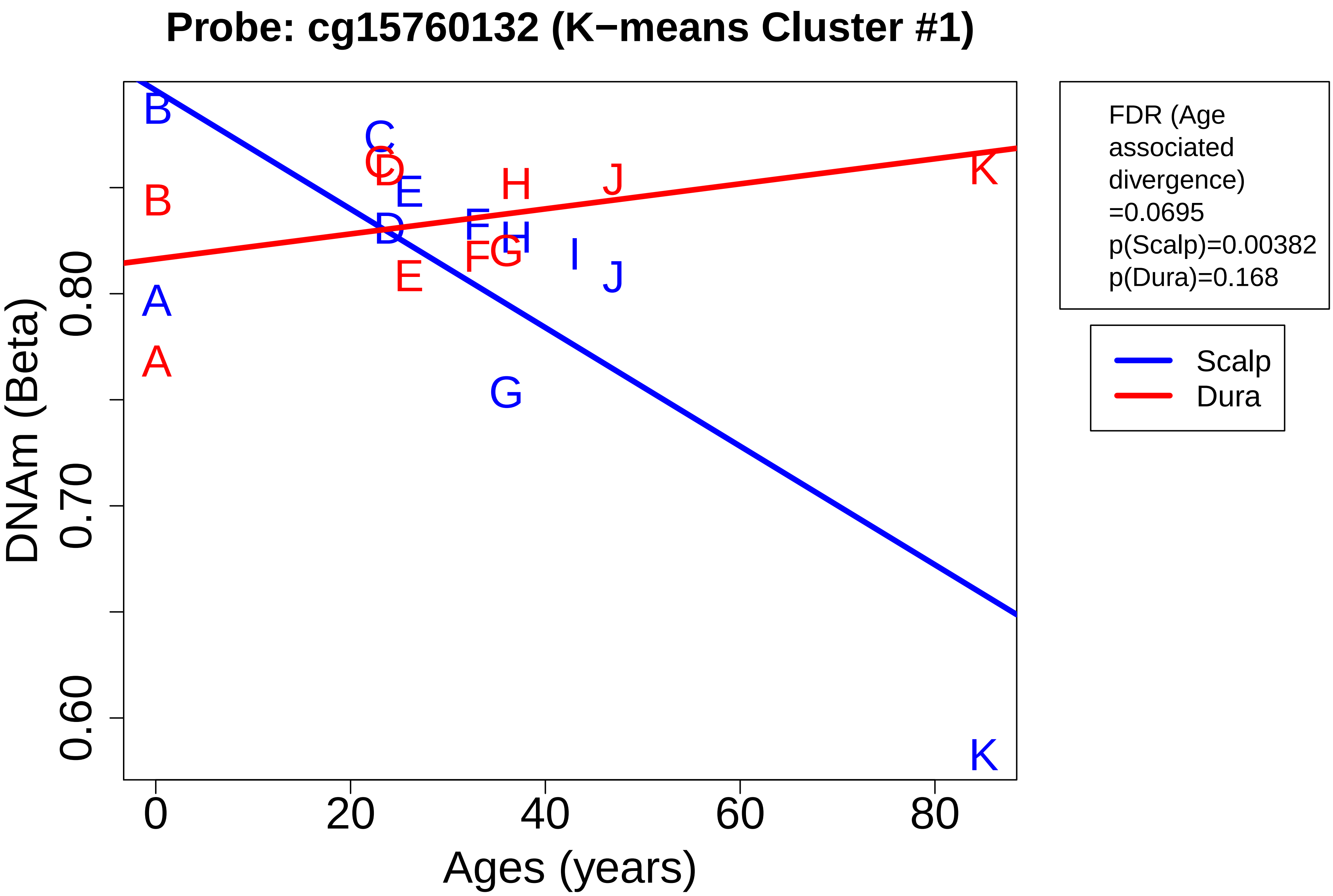
Probe: cg15760132 (K−means Cluster #1)
FDR (Age associated divergence) =0.0695
p(Scalp)=0.00382
p(Dura)=0.168
Scalp
Dura
DNAm (Beta)
0.60
0.70
0.80
0
20
40
60
80
Ages (years)

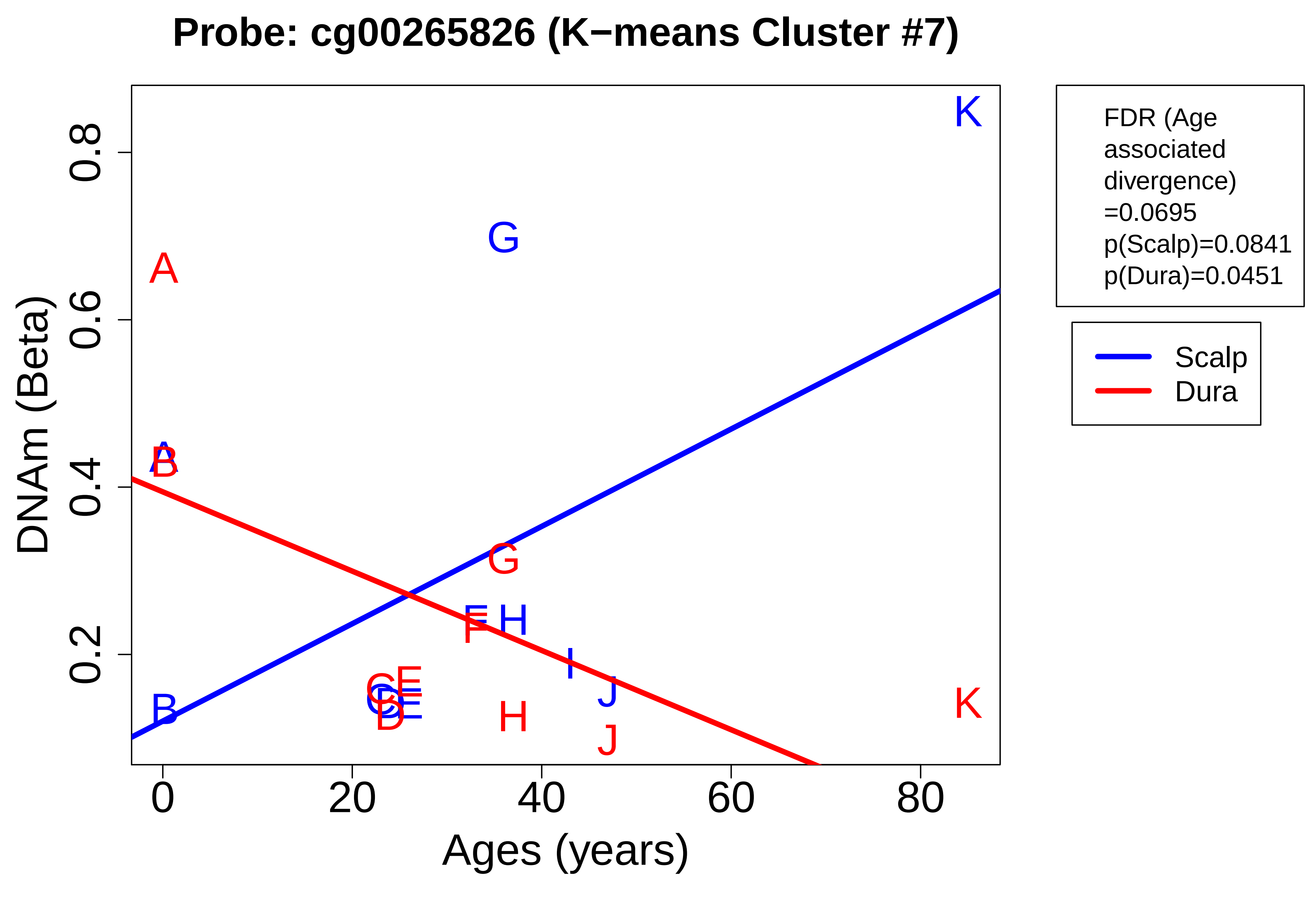

Probe: cg00265826 (K−means Cluster #7)
DNAm (Beta)
0.2
0.4
0.6
0.8
Ages (years)
0
20
40
60
80
FDR (Age associated divergence) =0.0695
p(Scalp)=0.0841
p(Dura)=0.0451
Scalp
Dura

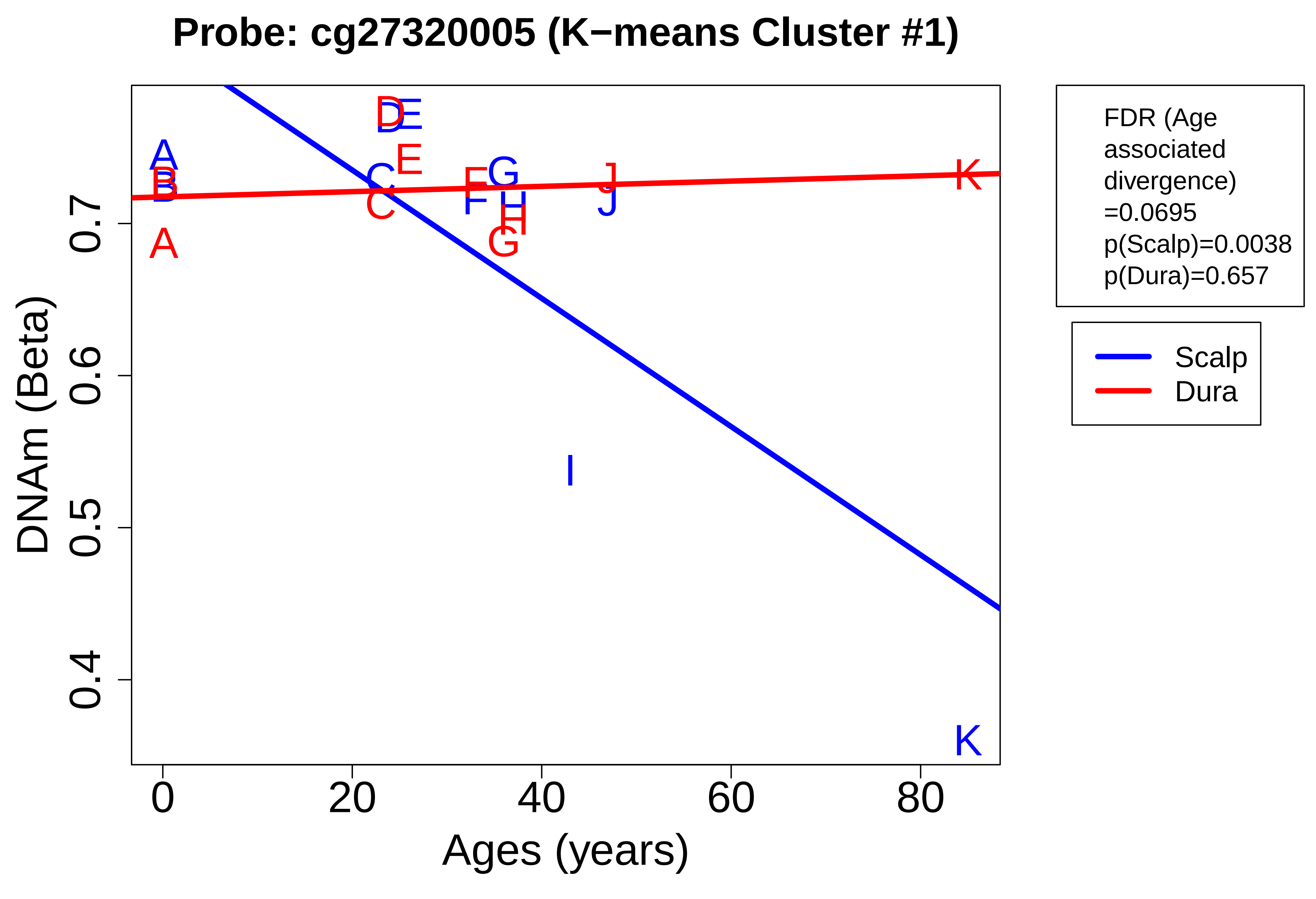

Probe: cg27320005 (K−means Cluster #1)
DNAm (Beta)
0.4
0.5
0.6
0.7
0
20
40
60
80
Ages (years)
A
B
C
D
E
F
G
H
I
J
K
FDR (Age associated divergence) =0.0695
p(Scalp)=0.0038
p(Dura)=0.657
Scalp
Dura

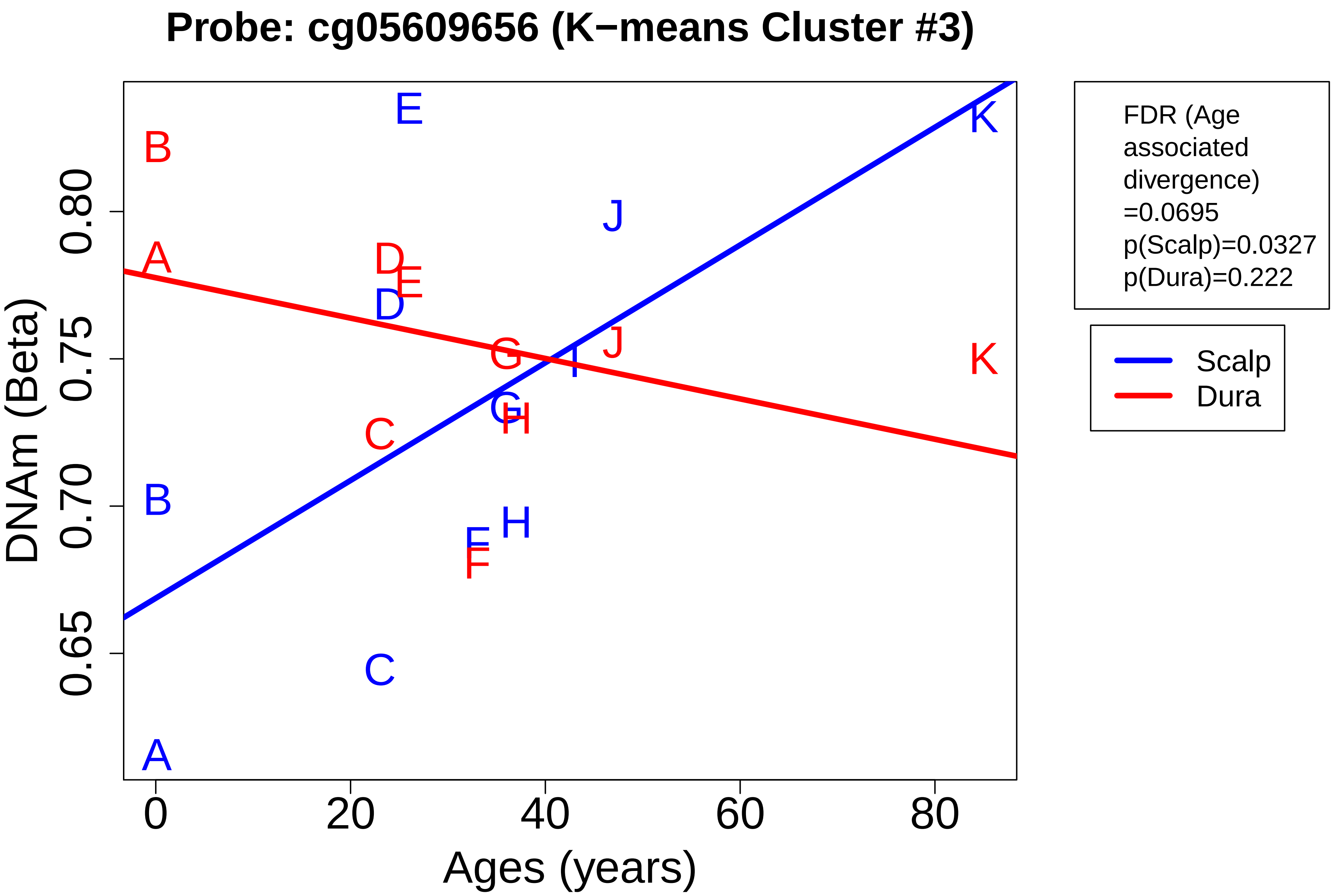

Probe: cg05609656 (K−means Cluster #3)
DNAm (Beta)
0.65
0.70
0.75
0.80
Ages (years)
0
20
40
60
80
A
B
C
D
E
F
G
H
I
J
K
FDR (Age associated divergence) =0.0695
p(Scalp)=0.0327
p(Dura)=0.222
Scalp
Dura

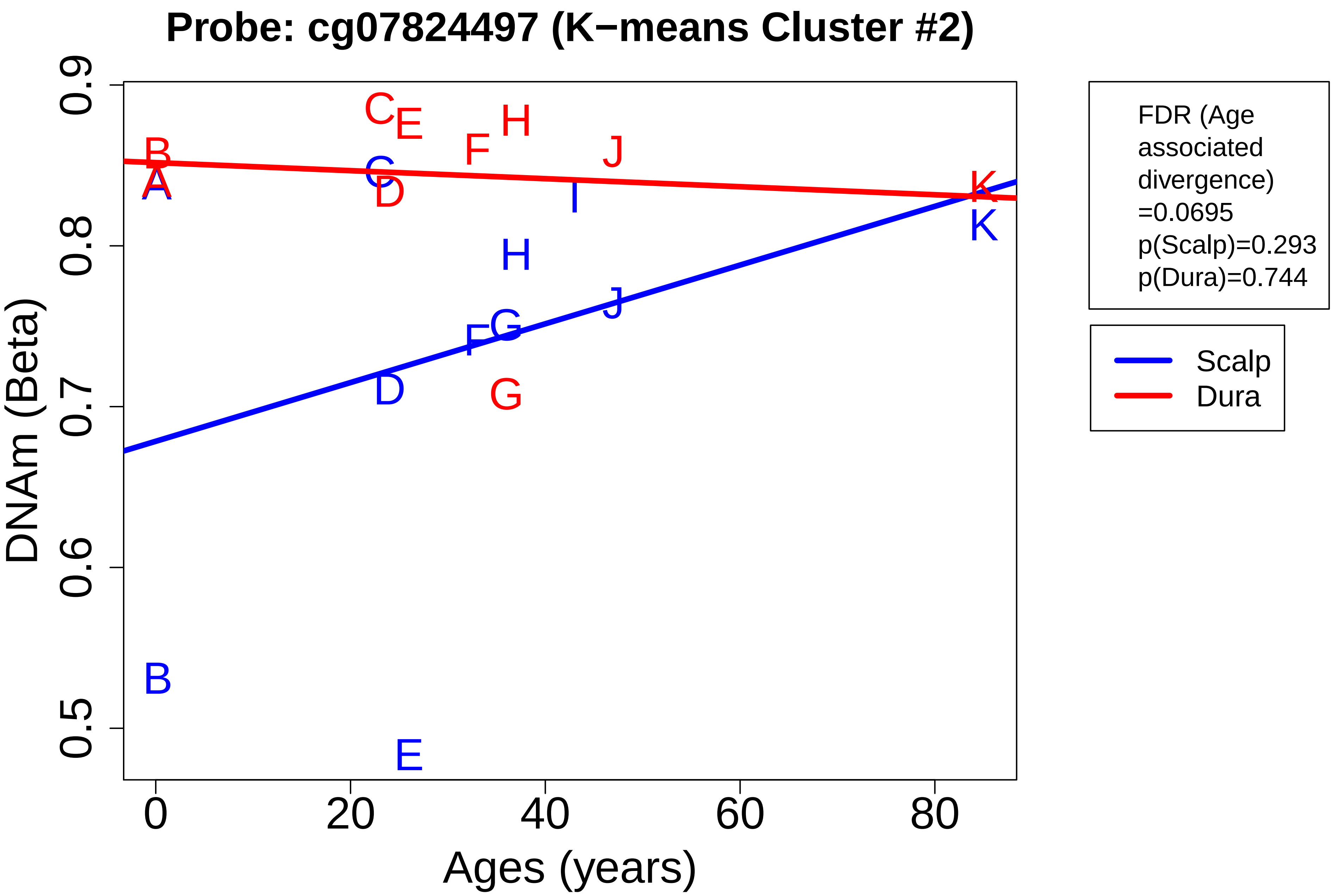

Probe: cg07824497 (K−means Cluster #2)
DNAm (Beta)
0.5
0.6
0.7
0.8
0.9
0
20
40
60
80
Ages (years)
FDR (Age associated divergence) =0.0695
p(Scalp)=0.293
p(Dura)=0.744
Scalp
Dura

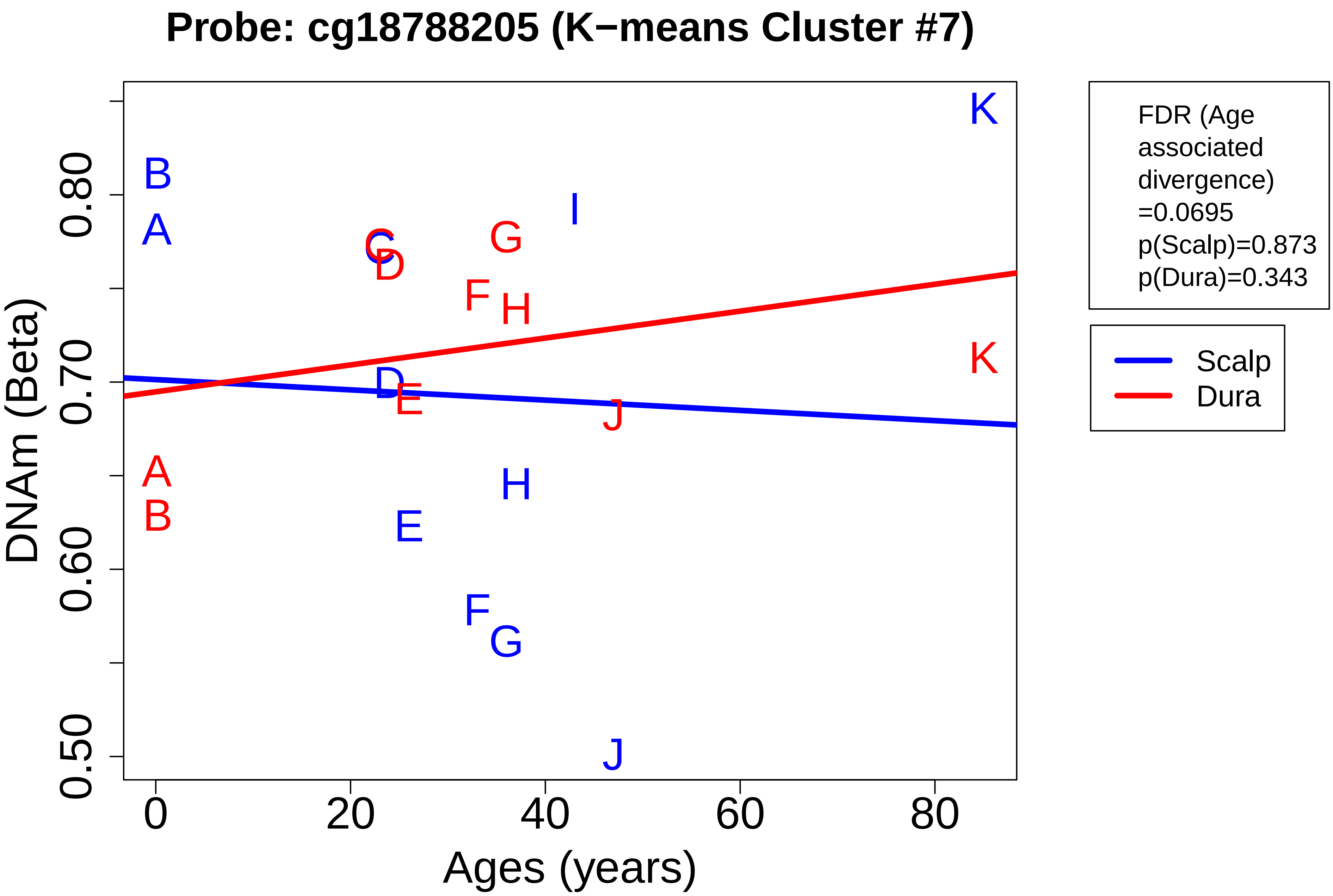

Probe: cg18788205 (K−means Cluster #7)
DNAm (Beta)
0.50
0.60
0.70
0.80
0
20
40
60
80
Ages (years)
A
B
C
D
E
F
G
H
I
J
K
FDR (Age associated divergence) =0.0695
p(Scalp)=0.873
p(Dura)=0.343
Scalp
Dura

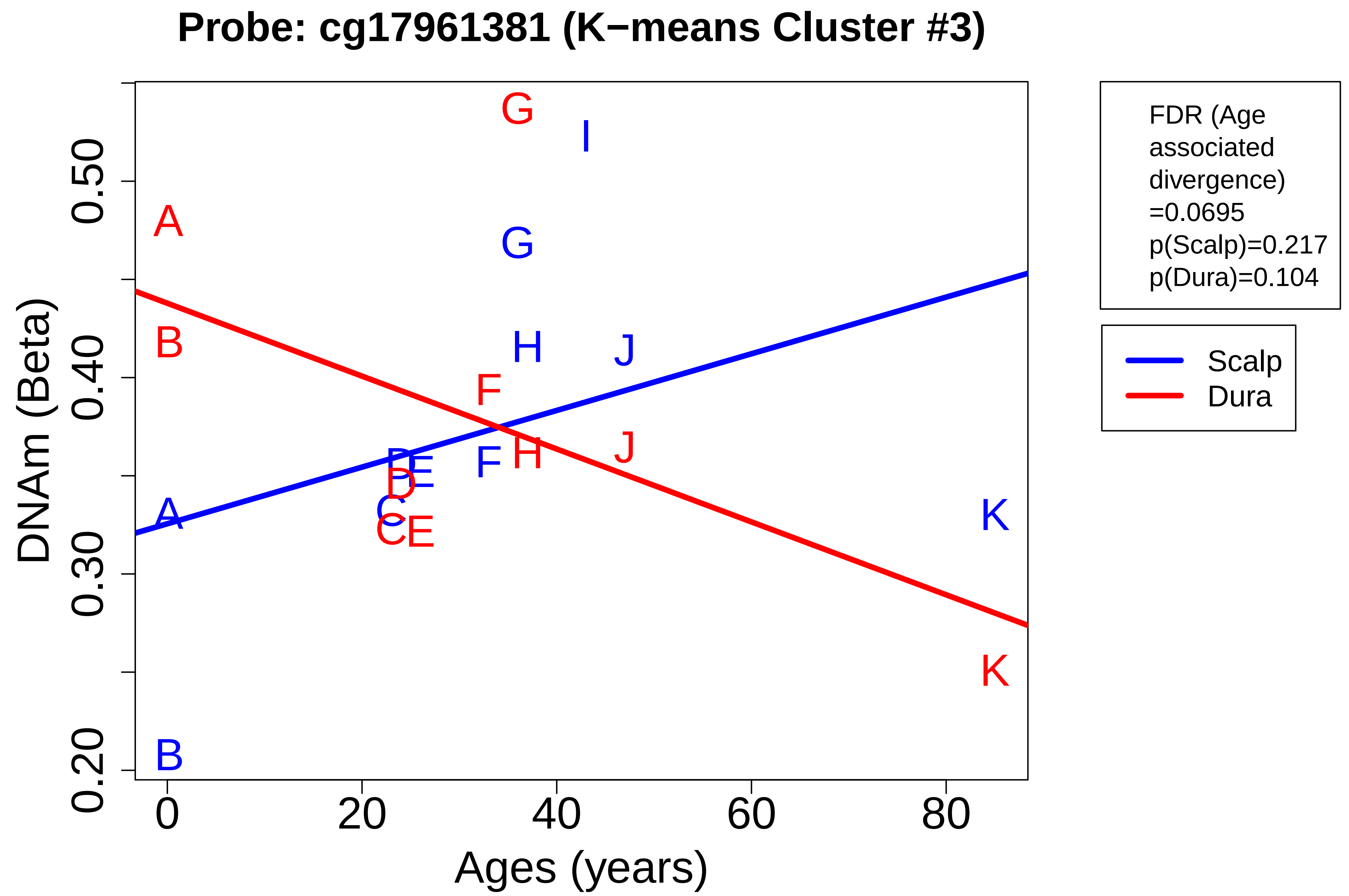

Probe: cg17961381 (K−means Cluster #3)
DNAm (Beta)
0.20
0.30
0.40
0.50
0
20
40
60
80
Ages (years)
FDR (Age associated divergence) =0.0695
p(Scalp)=0.217
p(Dura)=0.104
Scalp
Dura

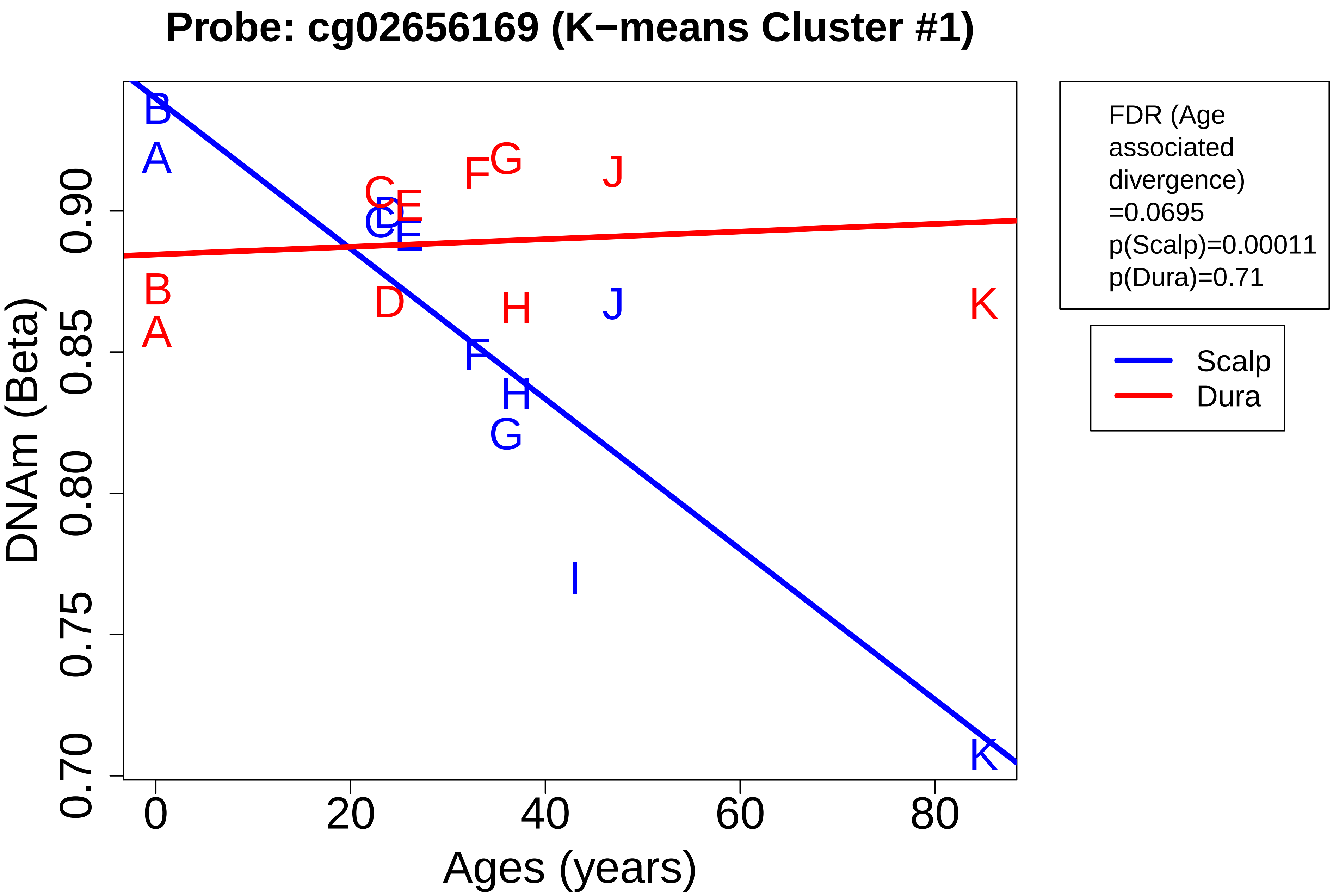

Probe: cg02656169 (K−means Cluster #1)
DNAm (Beta)
0.70
0.75
0.80
0.85
0.90
0
20
40
60
80
Ages (years)
FDR (Age associated divergence) =0.0695
p(Scalp)=0.00011
p(Dura)=0.71
Scalp
Dura

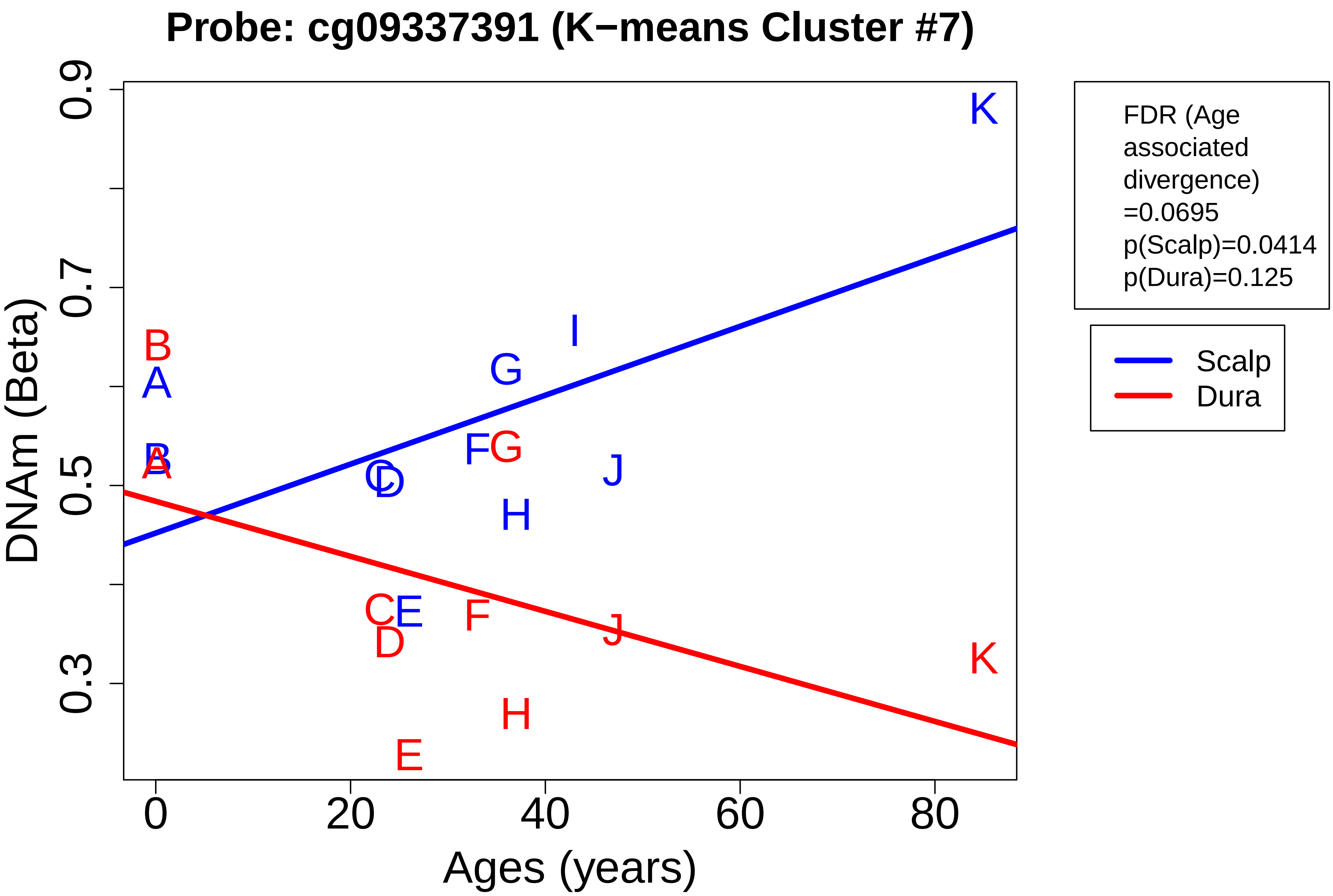

Probe: cg09337391 (K−means Cluster #7)
DNAm (Beta)
0.3
0.5
0.7
0.9
Ages (years)
0
20
40
60
80
FDR (Age associated divergence) =0.0695
p(Scalp)=0.0414
p(Dura)=0.125
Scalp
Dura

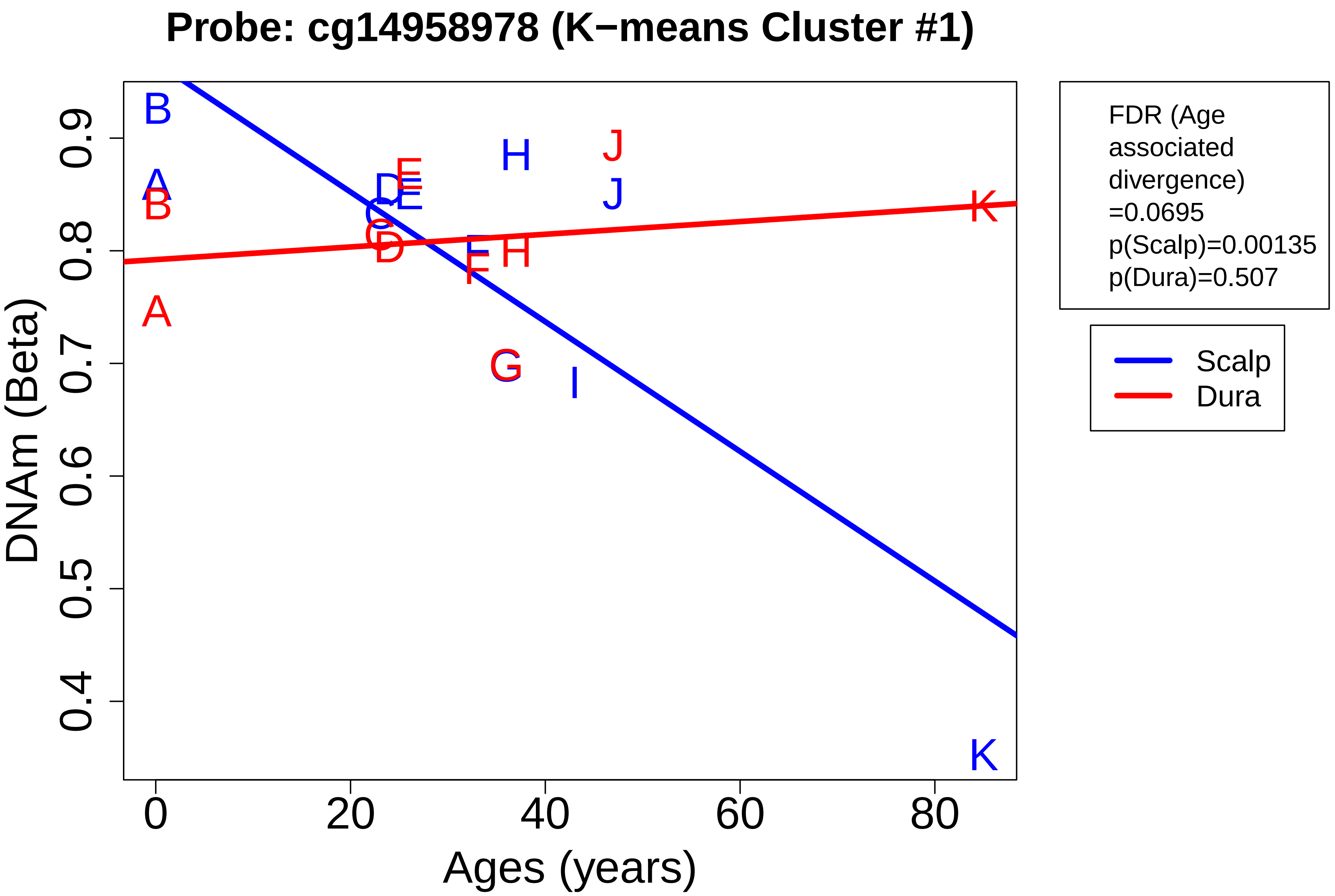

Probe: cg14958978 (K−means Cluster #1)
DNAm (Beta)
Ages (years)
0.4
0.5
0.6
0.7
0.8
0.9
0
20
40
60
80
FDR (Age associated divergence) =0.0695
p(Scalp)=0.00135
p(Dura)=0.507
Scalp
Dura

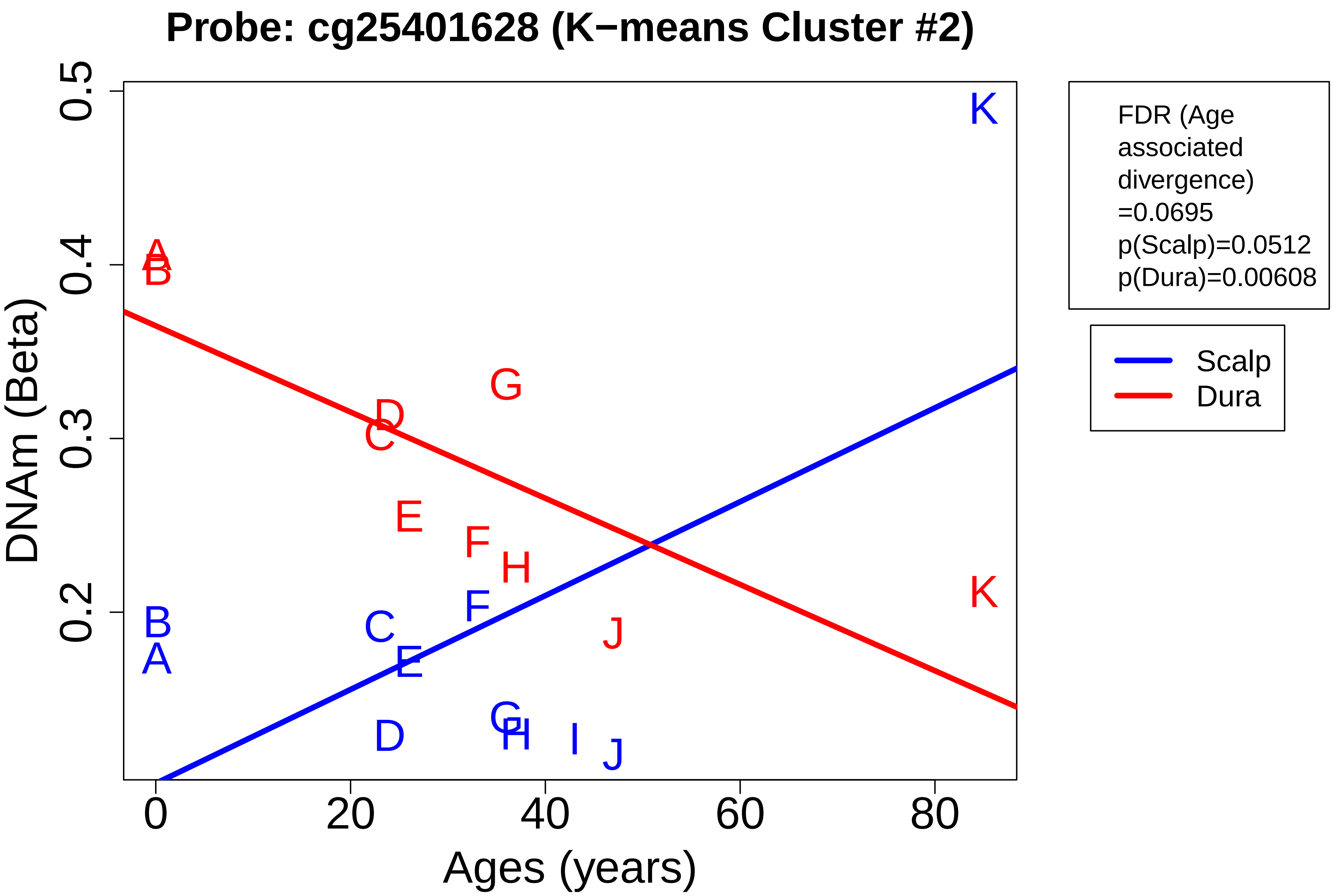
Probe: cg25401628 (K−means Cluster #2)
DNAm (Beta)
Ages (years)
0.2
0.3
0.4
0.5
0
20
40
60
80
FDR (Age associated divergence) =0.0695
p(Scalp)=0.0512
p(Dura)=0.00608
Scalp
Dura

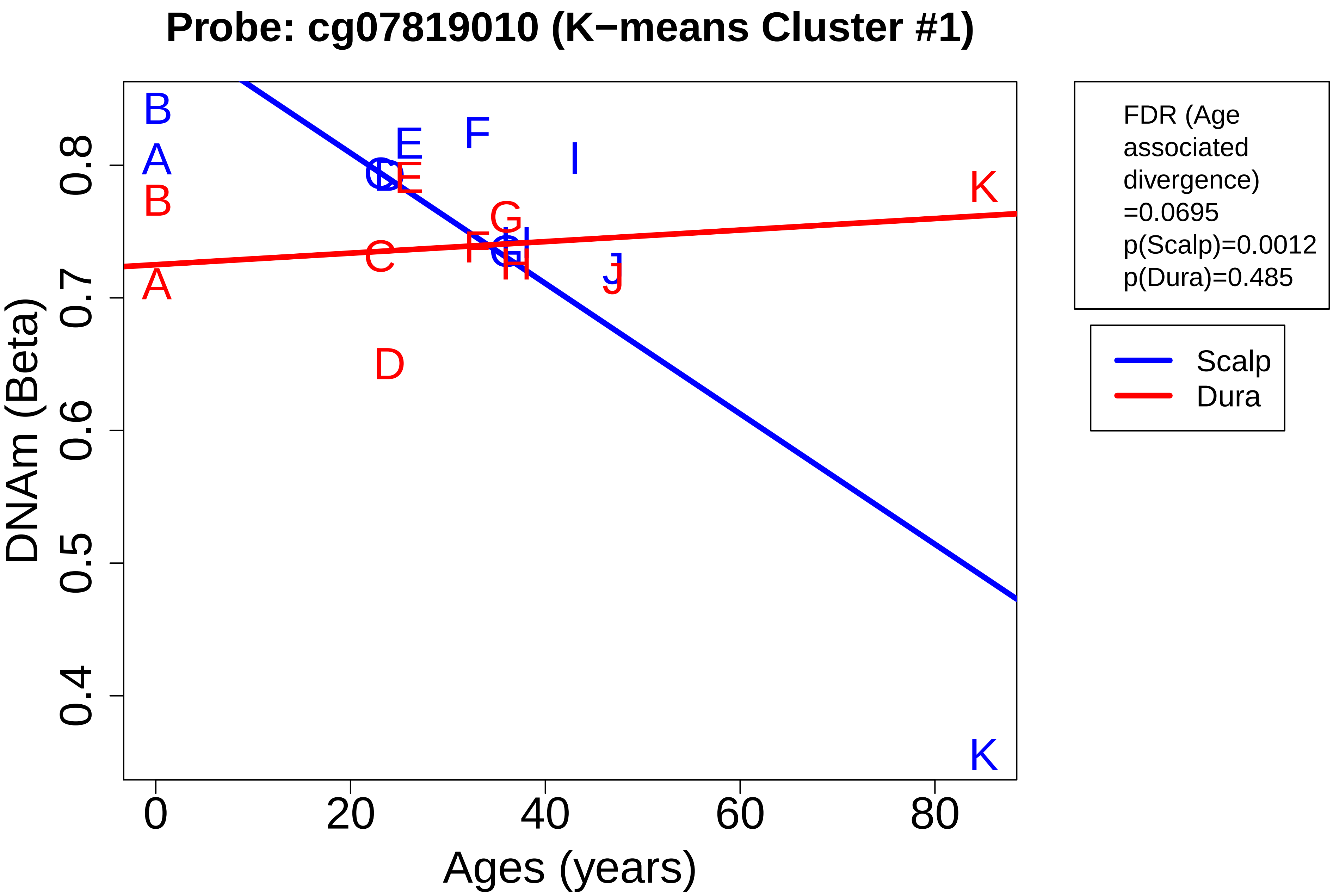
Probe: cg07819010 (K−means Cluster #1)
DNAm (Beta)
0.4
0.5
0.6
0.7
0.8
Ages (years)
0
20
40
60
80
FDR (Age associated divergence) =0.0695
p(Scalp)=0.0012
p(Dura)=0.485
Scalp
Dura
A
B
C
D
E
F
G
H
I
J
K

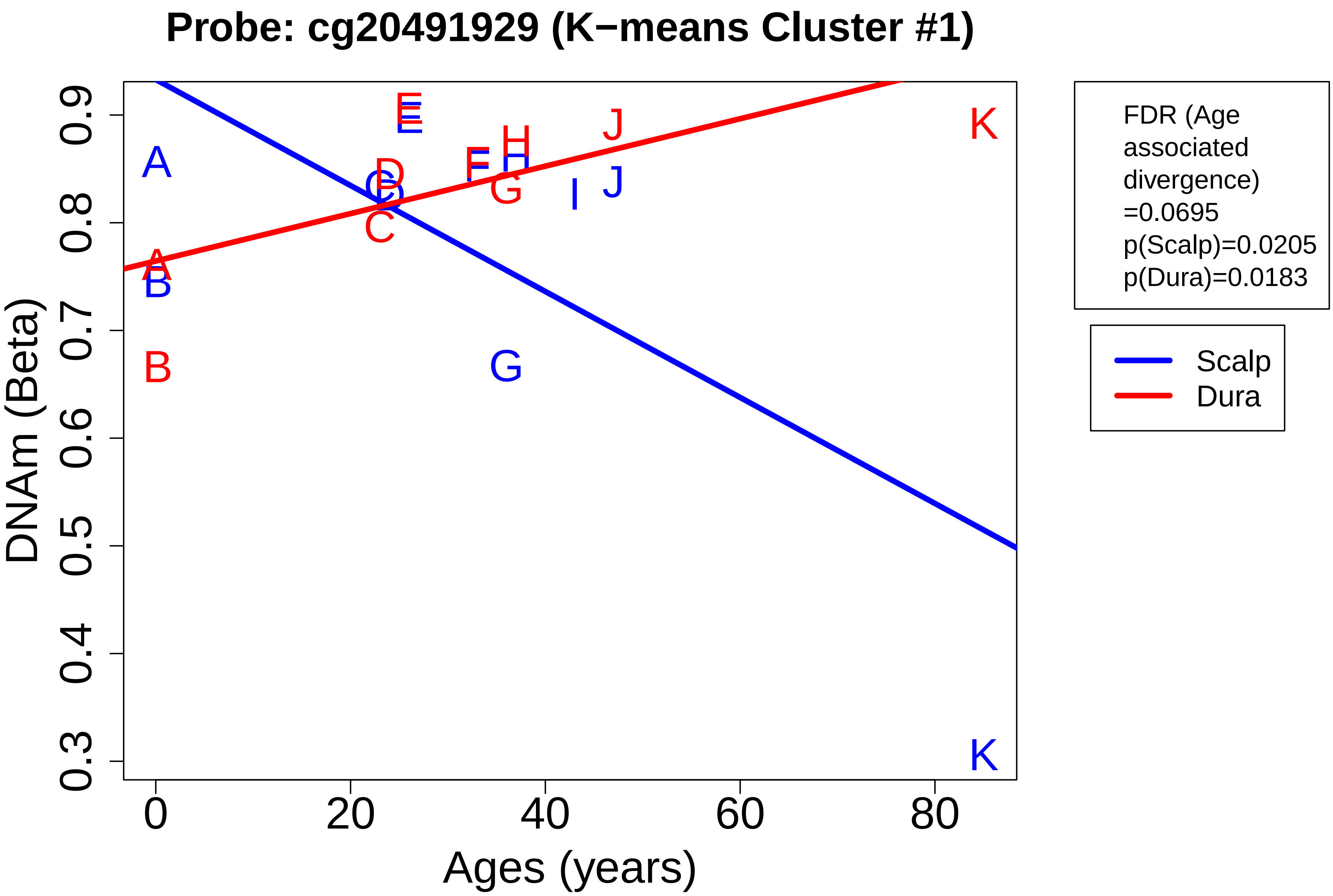

Probe: cg20491929 (K−means Cluster #1)
DNAm (Beta)
Ages (years)
0.3
0.4
0.5
0.6
0.7
0.8
0.9
0
20
40
60
80
FDR (Age associated divergence) =0.0695
p(Scalp)=0.0205
p(Dura)=0.0183
Scalp
Dura

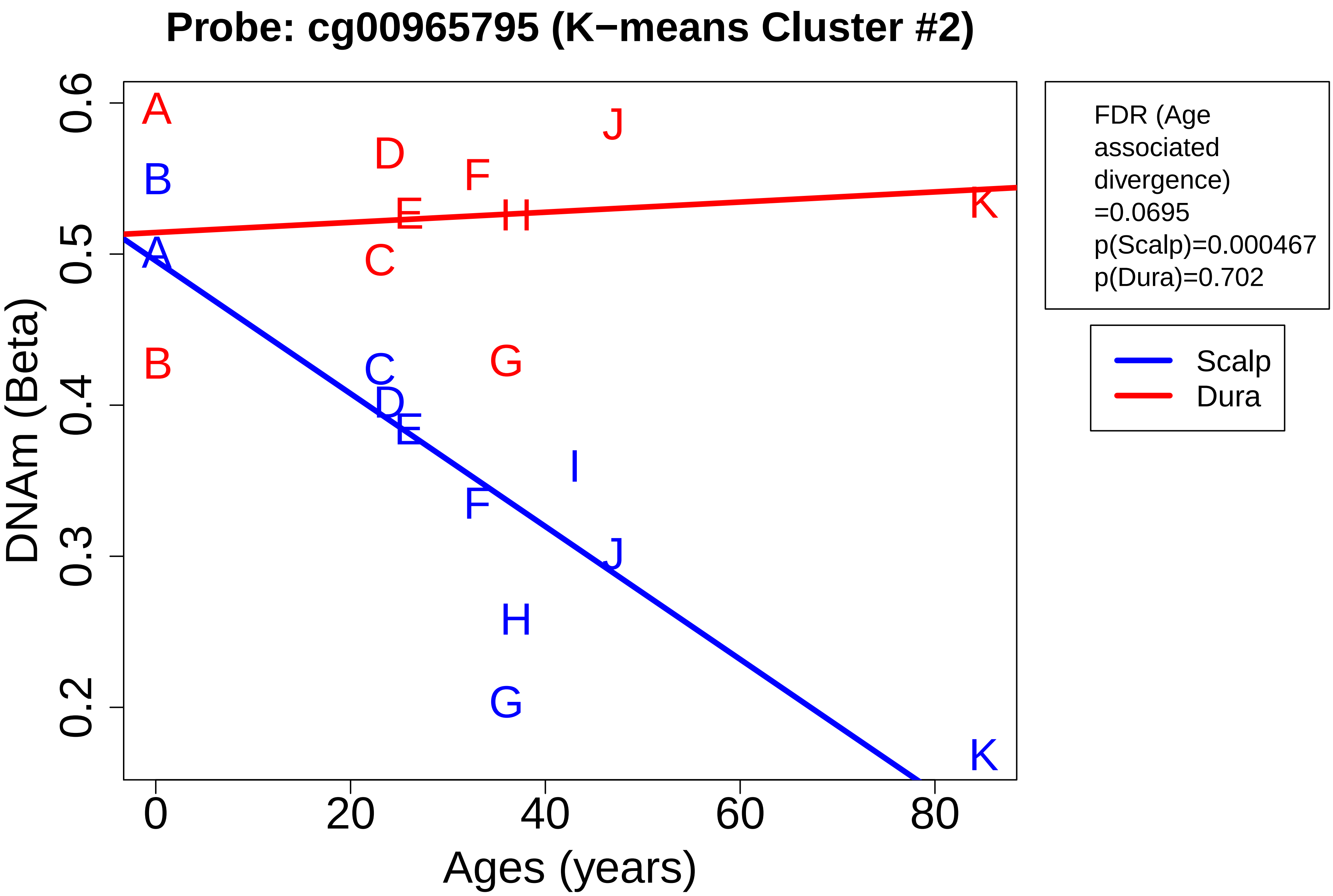

Probe: cg00965795 (K−means Cluster #2)
DNAm (Beta)
0.2
0.3
0.4
0.5
0.6
Ages (years)
0
20
40
60
80
FDR (Age associated divergence) =0.0695
p(Scalp)=0.000467
p(Dura)=0.702
Scalp
Dura
A
B
C
D
E
F
G
H
I
J
K

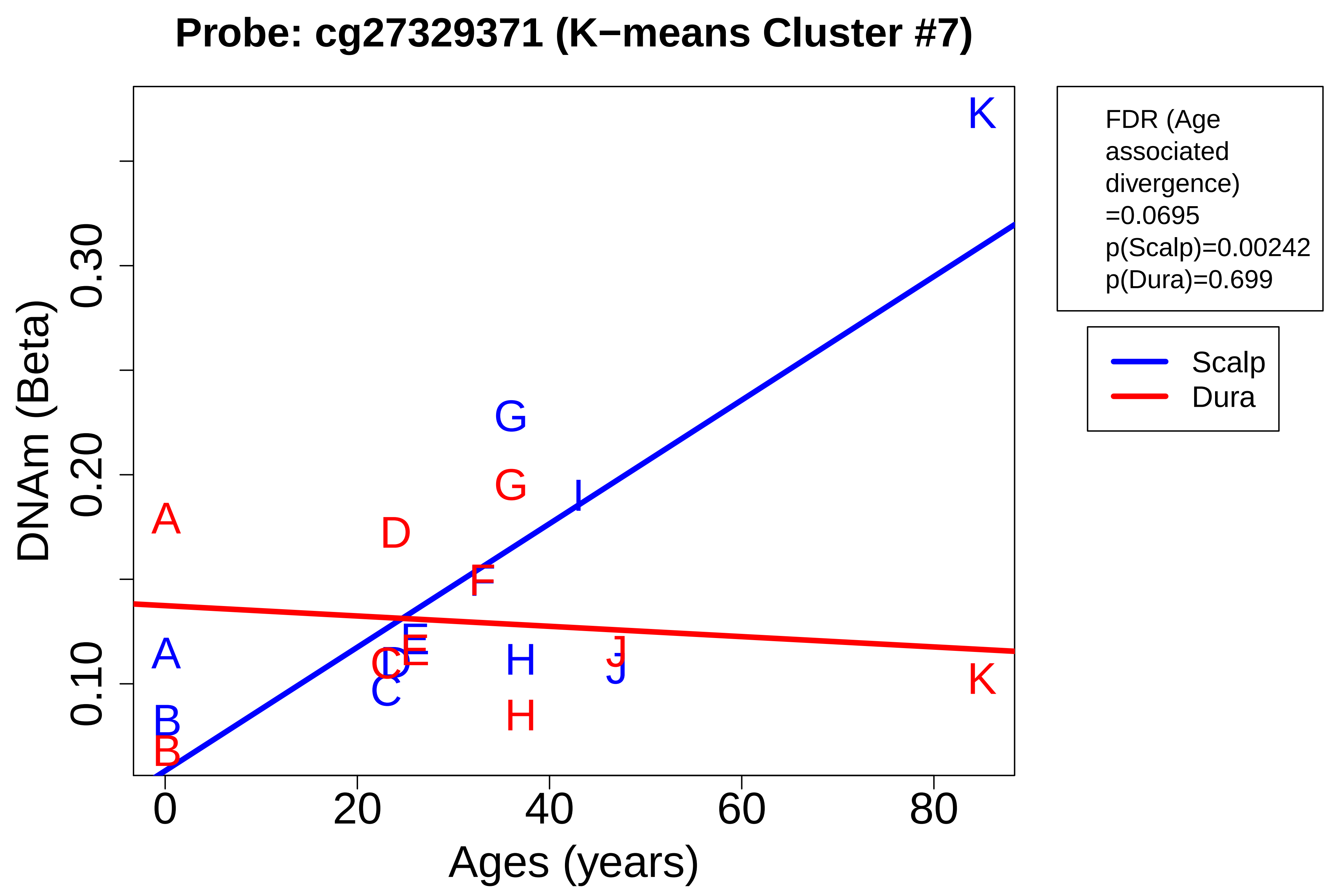

Probe: cg27329371 (K−means Cluster #7)
DNAm (Beta)
0.10
0.20
0.30
0
20
40
60
80
Ages (years)
FDR (Age associated divergence) =0.0695
p(Scalp)=0.00242
p(Dura)=0.699
Scalp
Dura
A
B
C
D
E
F
G
H
I
J
K

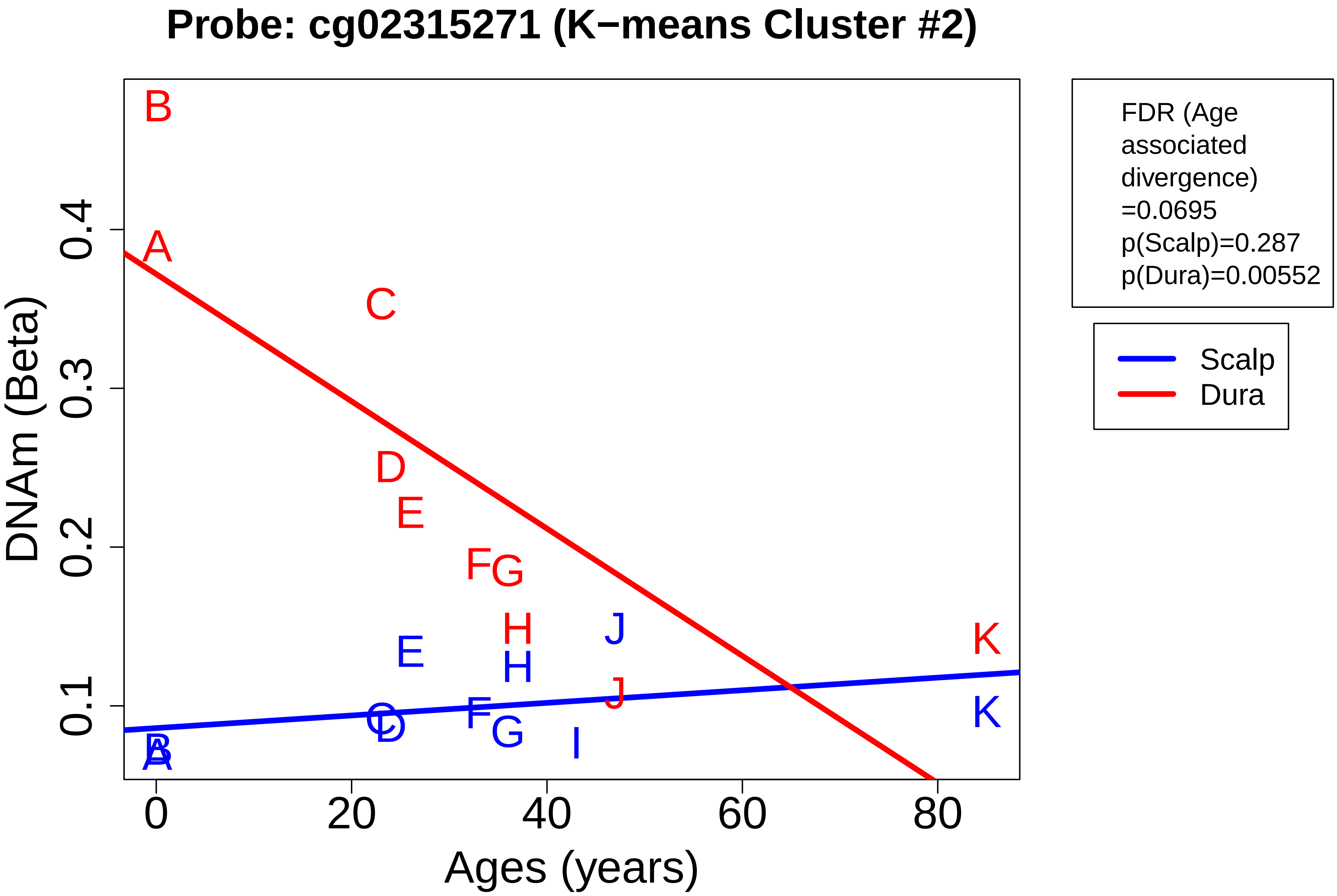

Probe: cg02315271 (K−means Cluster #2)
FDR (Age associated divergence) =0.0695
p(Scalp)=0.287
p(Dura)=0.00552
Scalp
Dura
DNAm (Beta)
0.1
0.2
0.3
0.4
Ages (years)
0
20
40
60
80
A
B
C
D
E
F
G
H
I
J
K

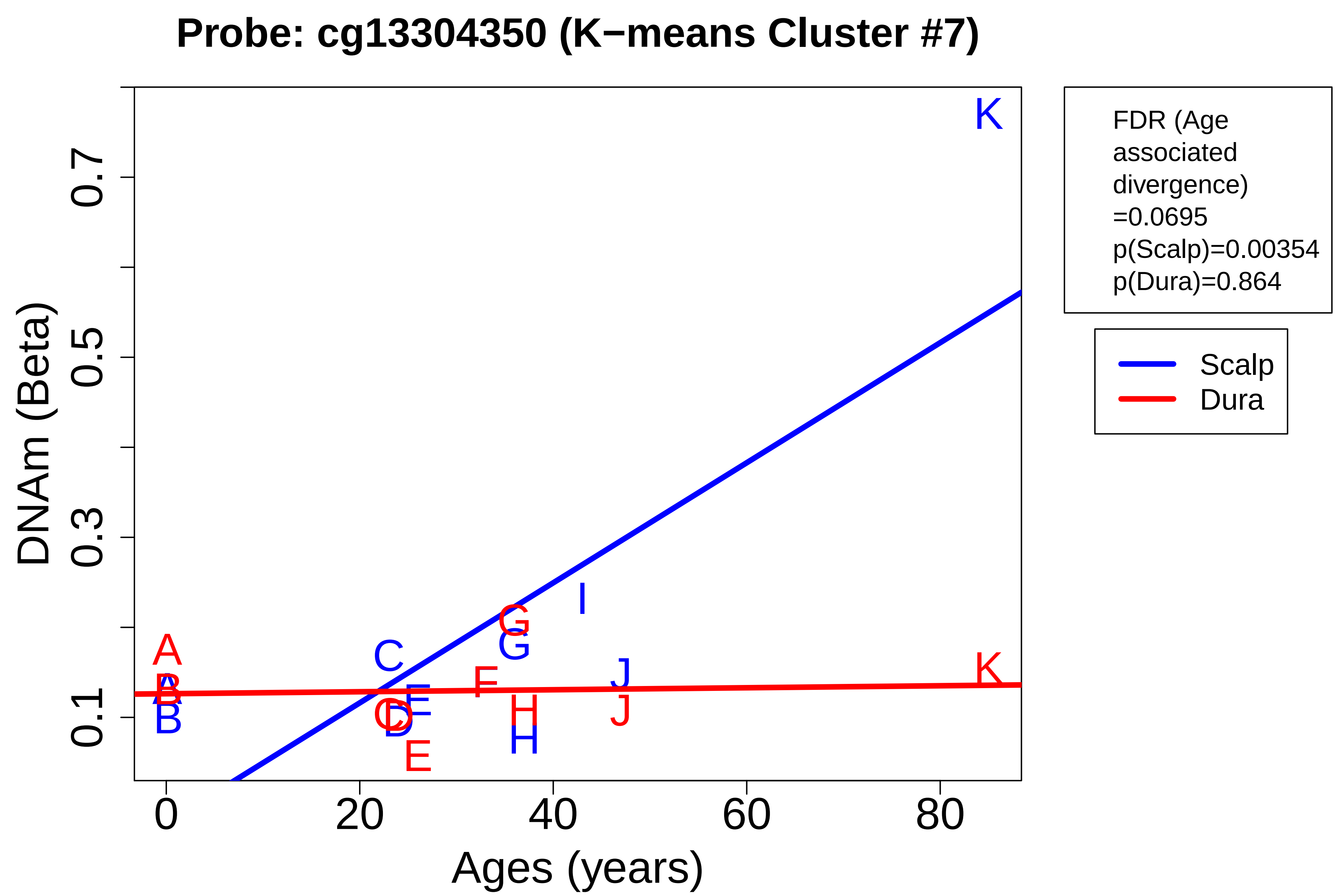

Probe: cg13304350 (K−means Cluster #7)
FDR (Age associated divergence) =0.0695
p(Scalp)=0.00354
p(Dura)=0.864
Scalp
Dura
DNAm (Beta)
0.1
0.3
0.5
0.7
Ages (years)
0
20
40
60
80
A
B
C
D
E
F
G
H
I
J
K

Probe: cg13705830 (K−means Cluster #2)

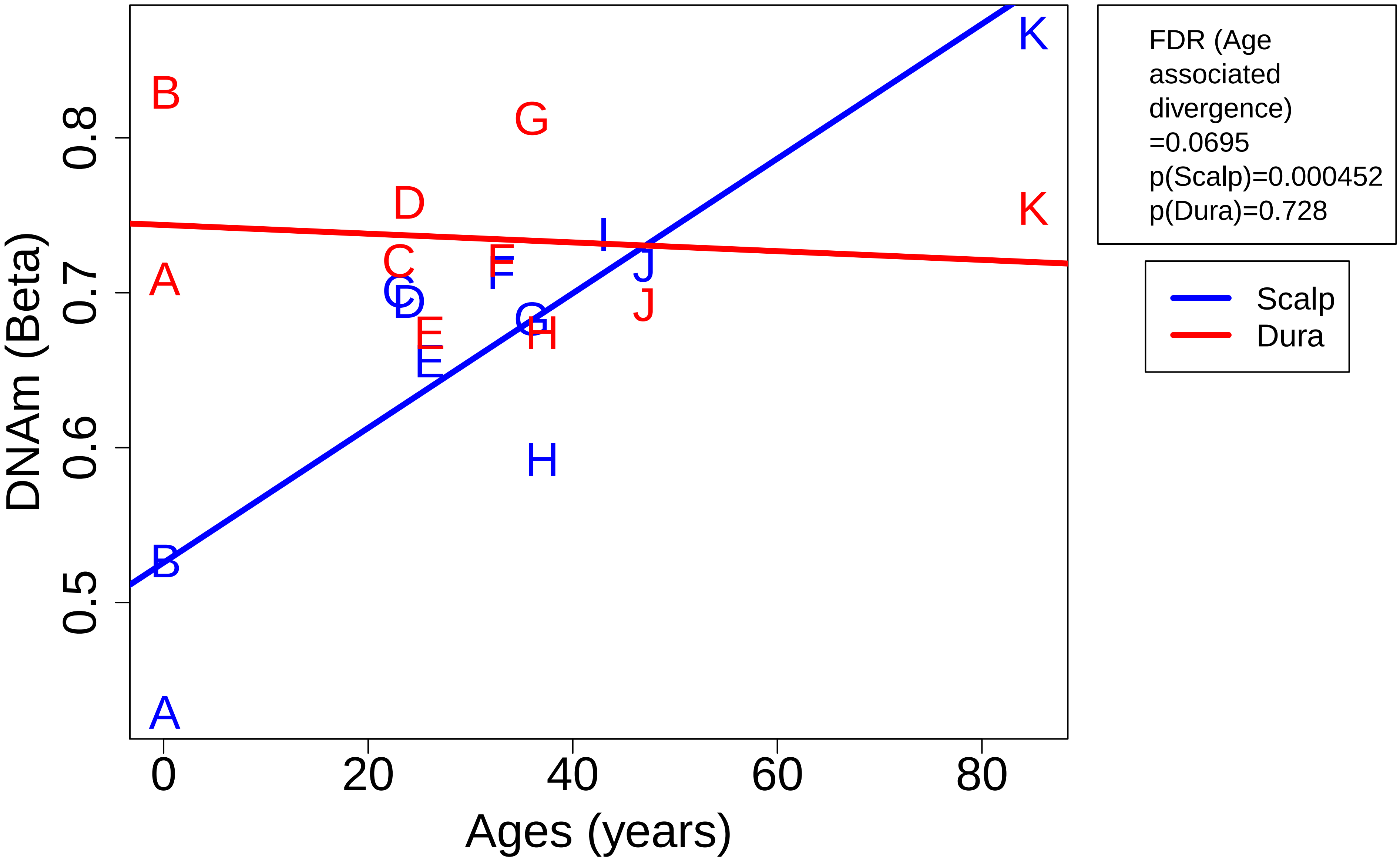

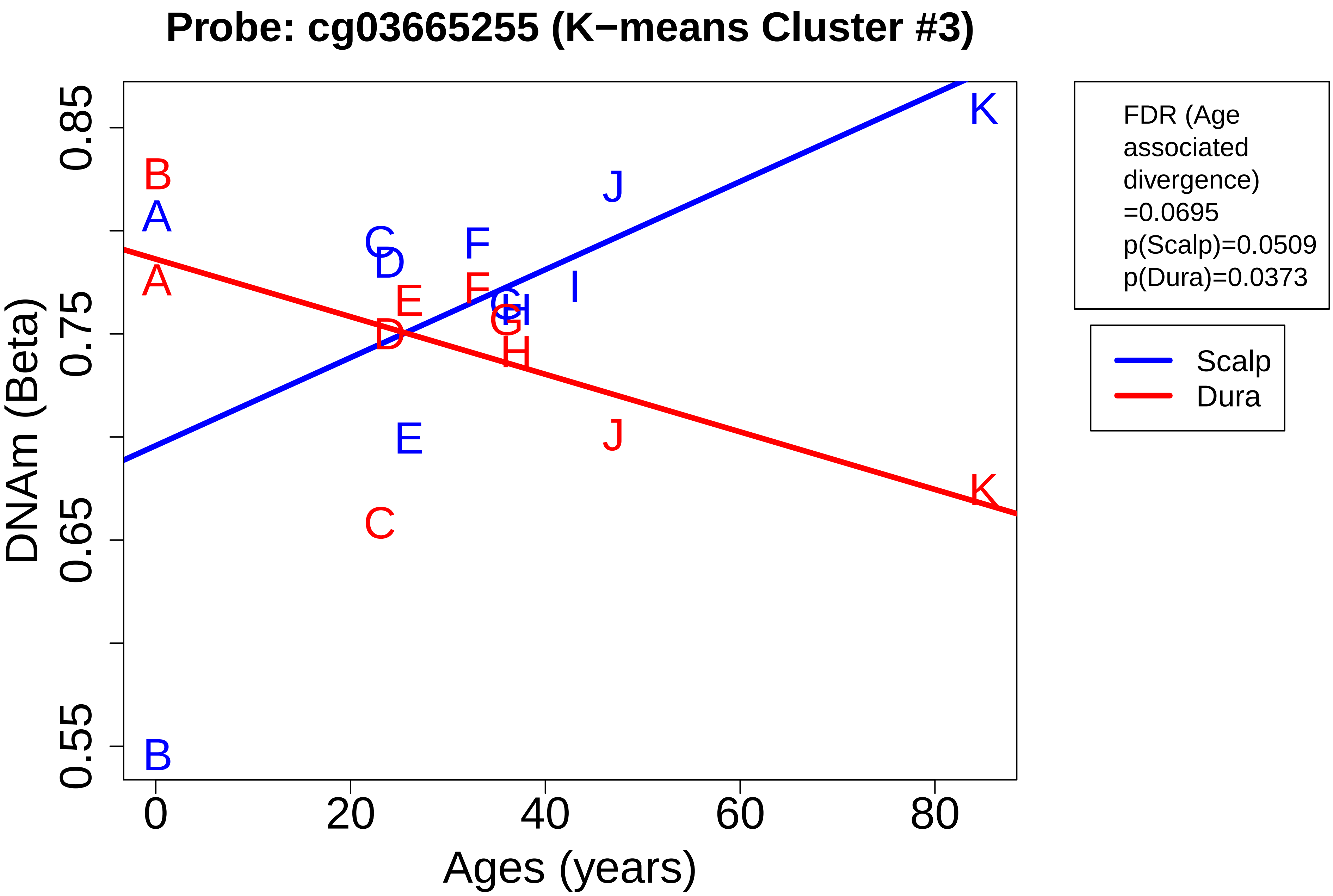

Probe: cg03665255 (K−means Cluster #3)
DNAm (Beta)
0.55
0.65
0.75
0.85
Ages (years)
0
20
40
60
80
A
B
C
D
E
F
G
H
I
J
K
FDR (Age associated divergence) =0.0695
p(Scalp)=0.0509
p(Dura)=0.0373
Scalp
Dura

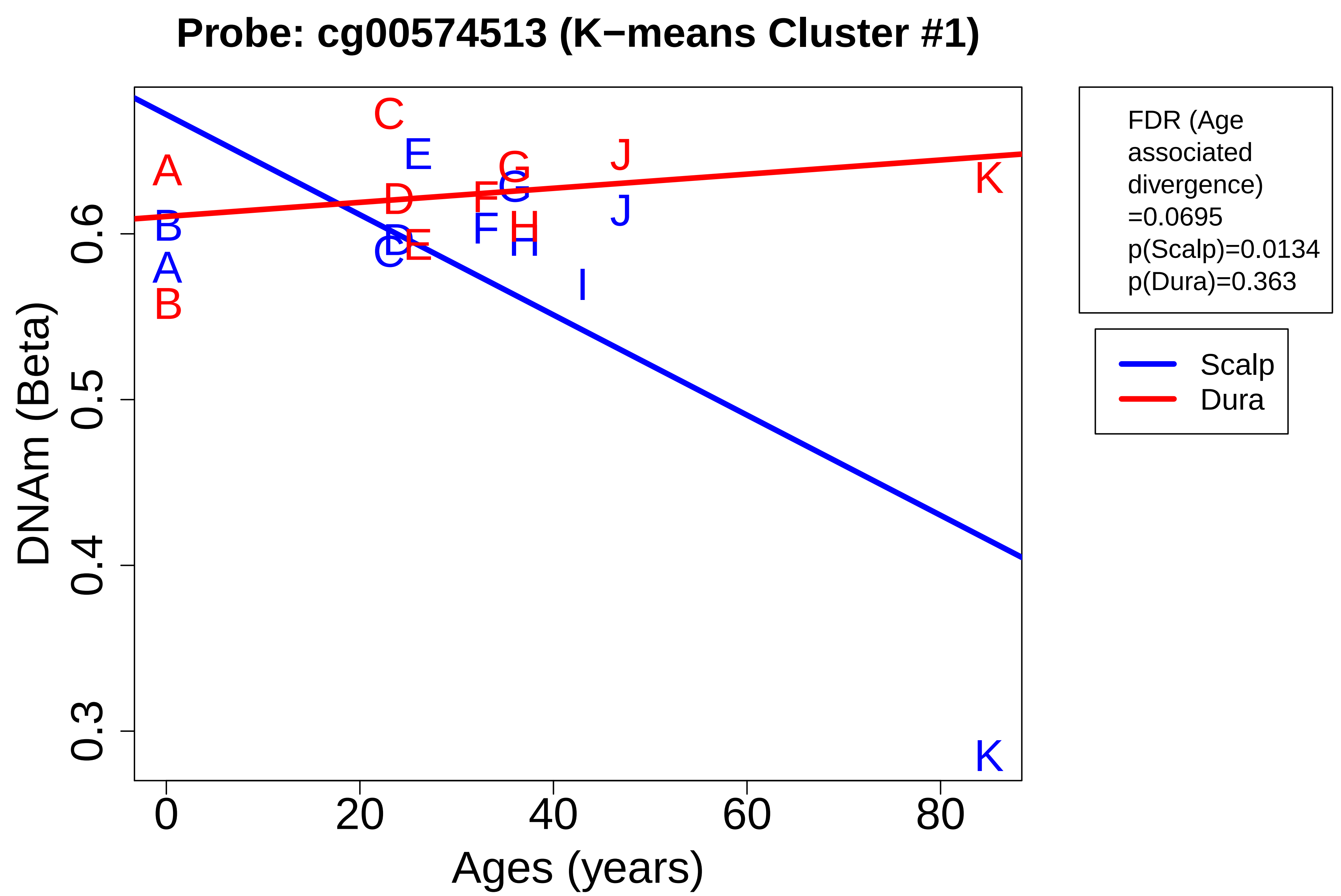

Probe: cg00574513 (K−means Cluster #1)
DNAm (Beta)
Ages (years)
0.3
0.4
0.5
0.6
0
20
40
60
80
A
B
C
D
E
F
G
H
I
J
K
FDR (Age associated divergence) =0.0695
p(Scalp)=0.0134
p(Dura)=0.363
Scalp
Dura

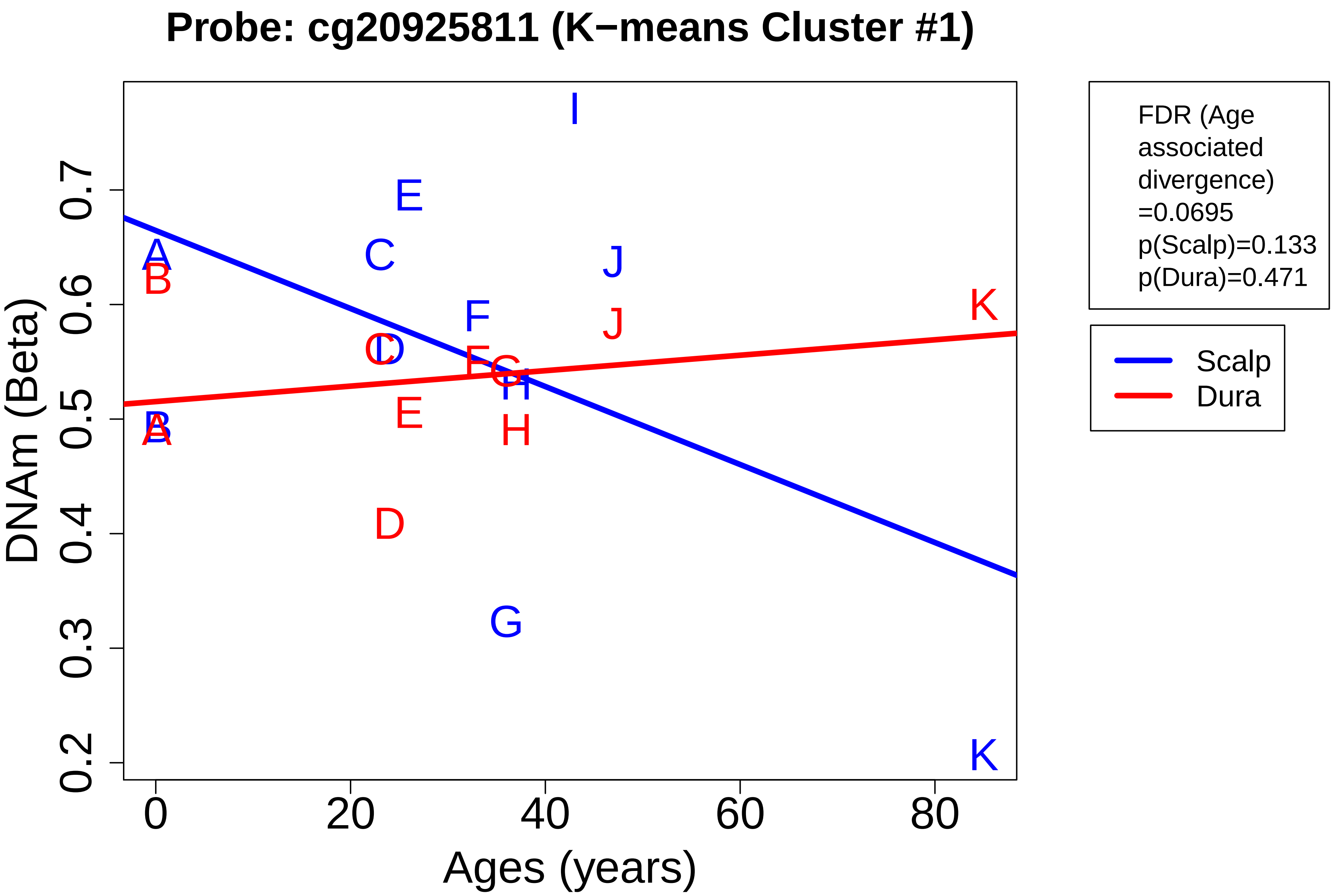

Probe: cg20925811 (K−means Cluster #1)
FDR (Age associated divergence) =0.0695
p(Scalp)=0.133
p(Dura)=0.471
Scalp
Dura
DNAm (Beta)
Ages (years)
0.2
0.3
0.4
0.5
0.6
0.7
0
20
40
60
80

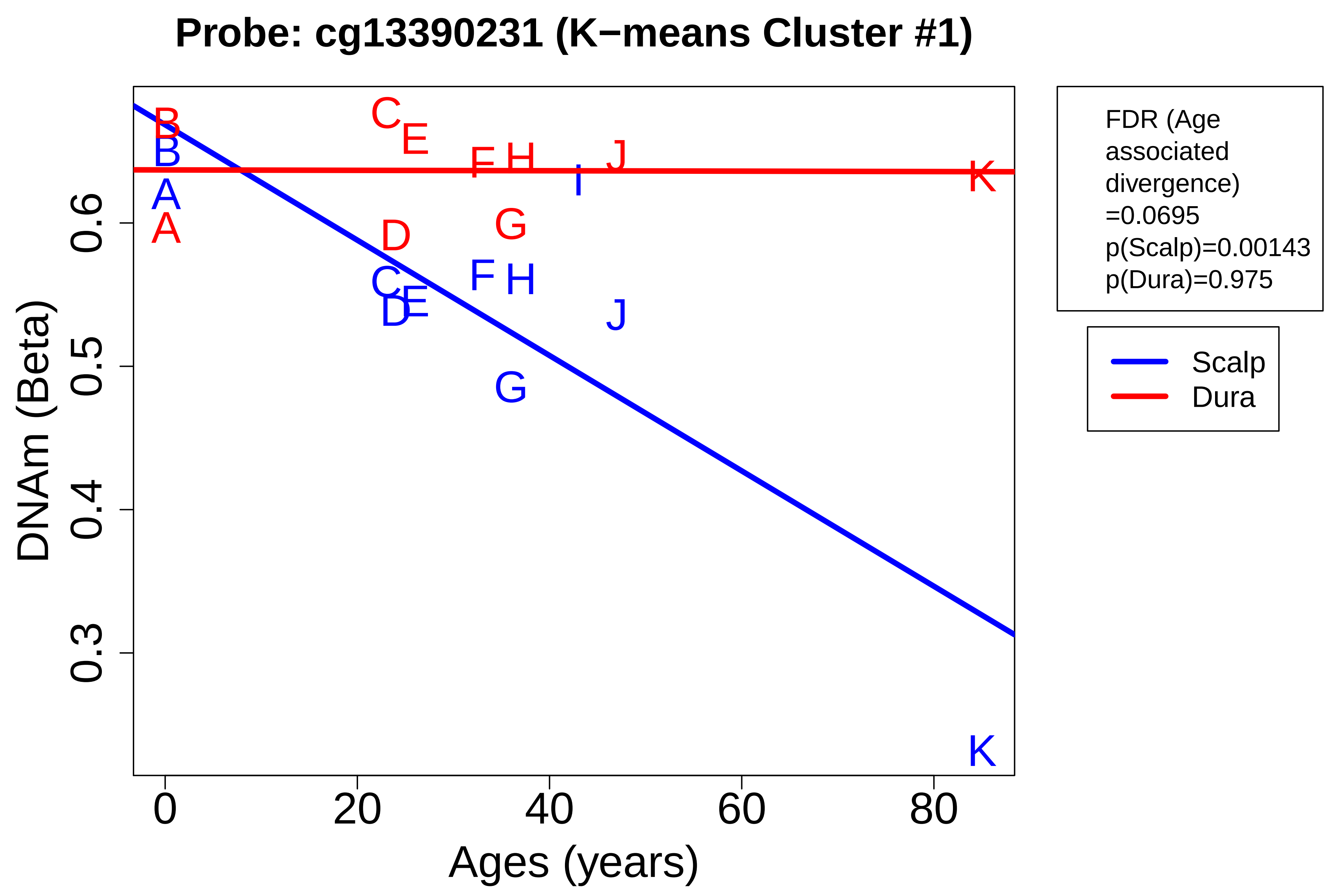

Probe: cg13390231 (K−means Cluster #1)
DNAm (Beta)
0.3
0.4
0.5
0.6
0
20
40
60
80
Ages (years)
FDR (Age associated divergence) =0.0695
p(Scalp)=0.00143
p(Dura)=0.975
Scalp
Dura

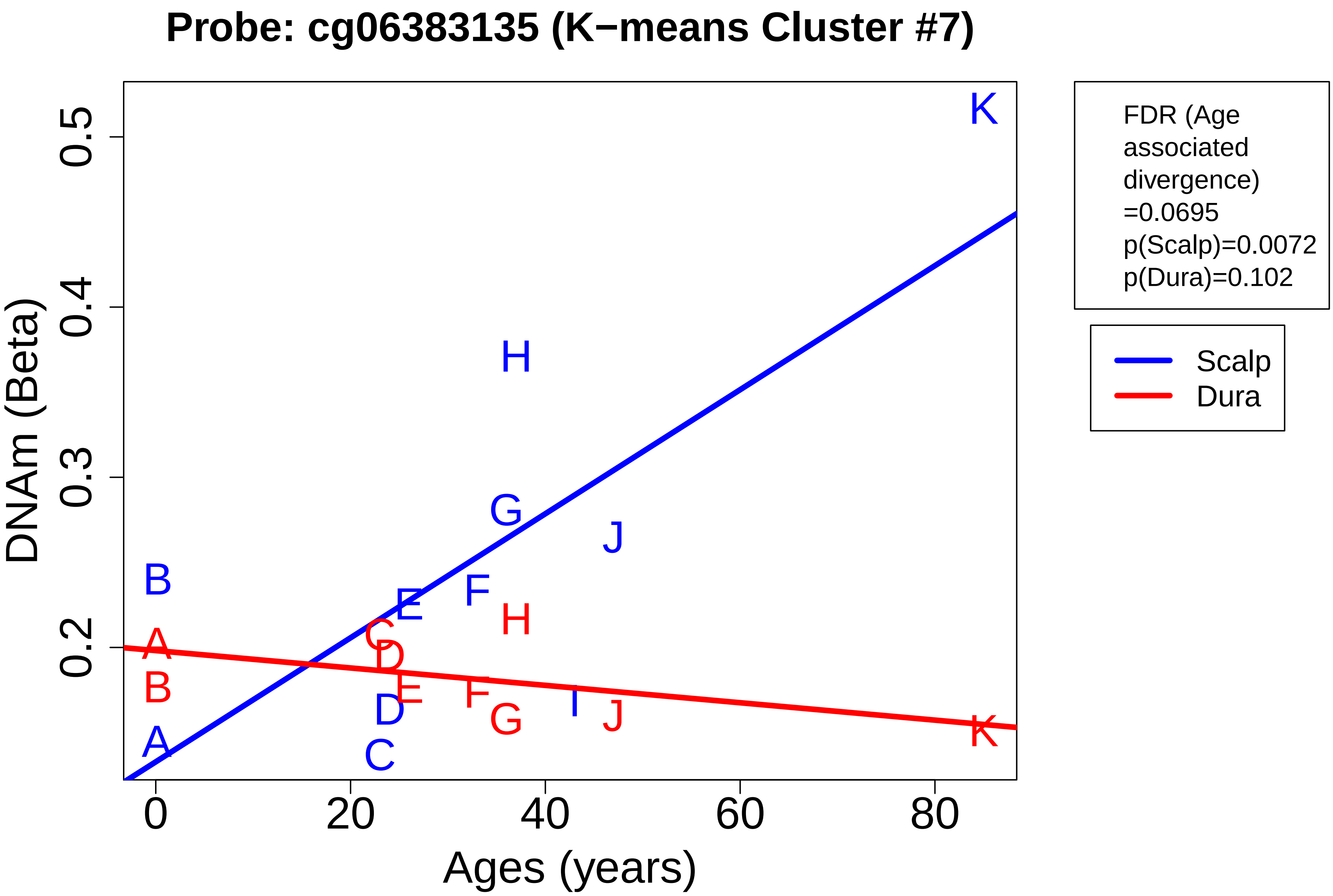

Probe: cg06383135 (K−means Cluster #7)
DNAm (Beta)
0.2
0.3
0.4
0.5
0
20
40
60
80
Ages (years)
FDR (Age associated divergence) =0.0695
p(Scalp)=0.0072
p(Dura)=0.102
Scalp
Dura

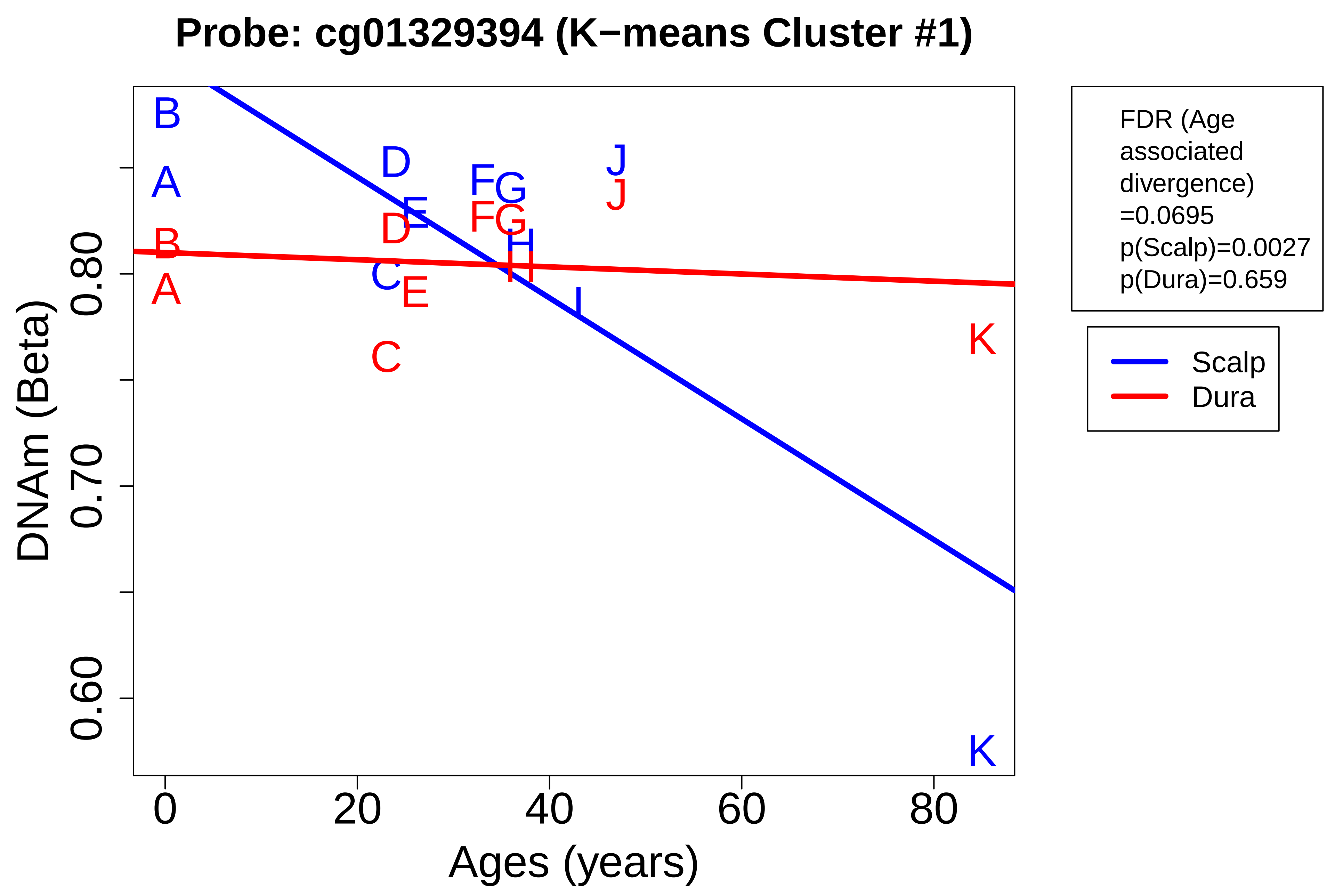

Probe: cg01329394 (K−means Cluster #1)
DNAm (Beta)
0.60
0.70
0.80
0
20
40
60
80
Ages (years)
FDR (Age associated divergence) =0.0695
p(Scalp)=0.0027
p(Dura)=0.659
Scalp
Dura
A B C D E F G H I J K

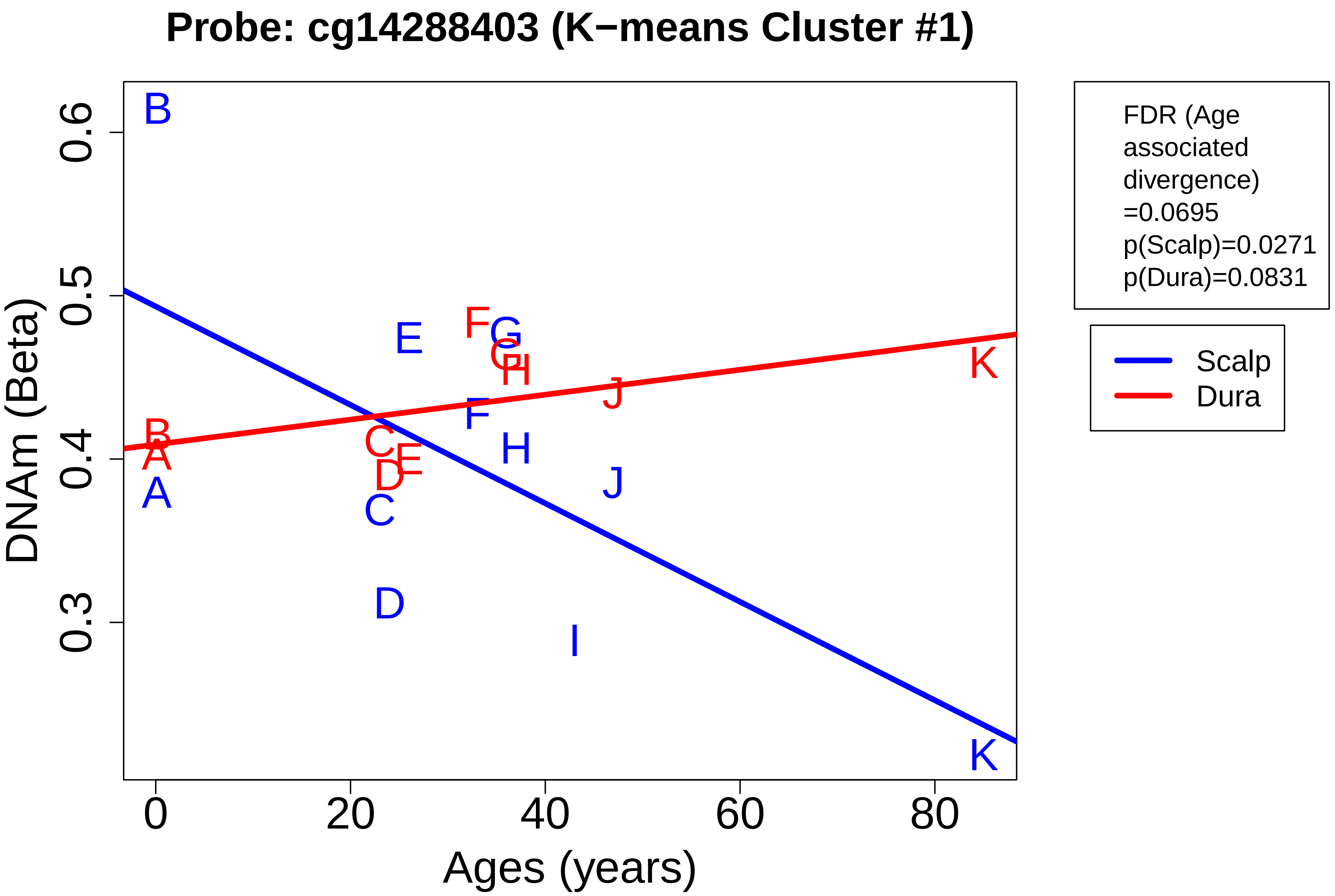

Probe: cg14288403 (K−means Cluster #1)
DNAm (Beta)
0.3
0.4
0.5
0.6
Ages (years)
0
20
40
60
80
FDR (Age associated divergence) =0.0695
p(Scalp)=0.0271
p(Dura)=0.0831
Scalp
Dura

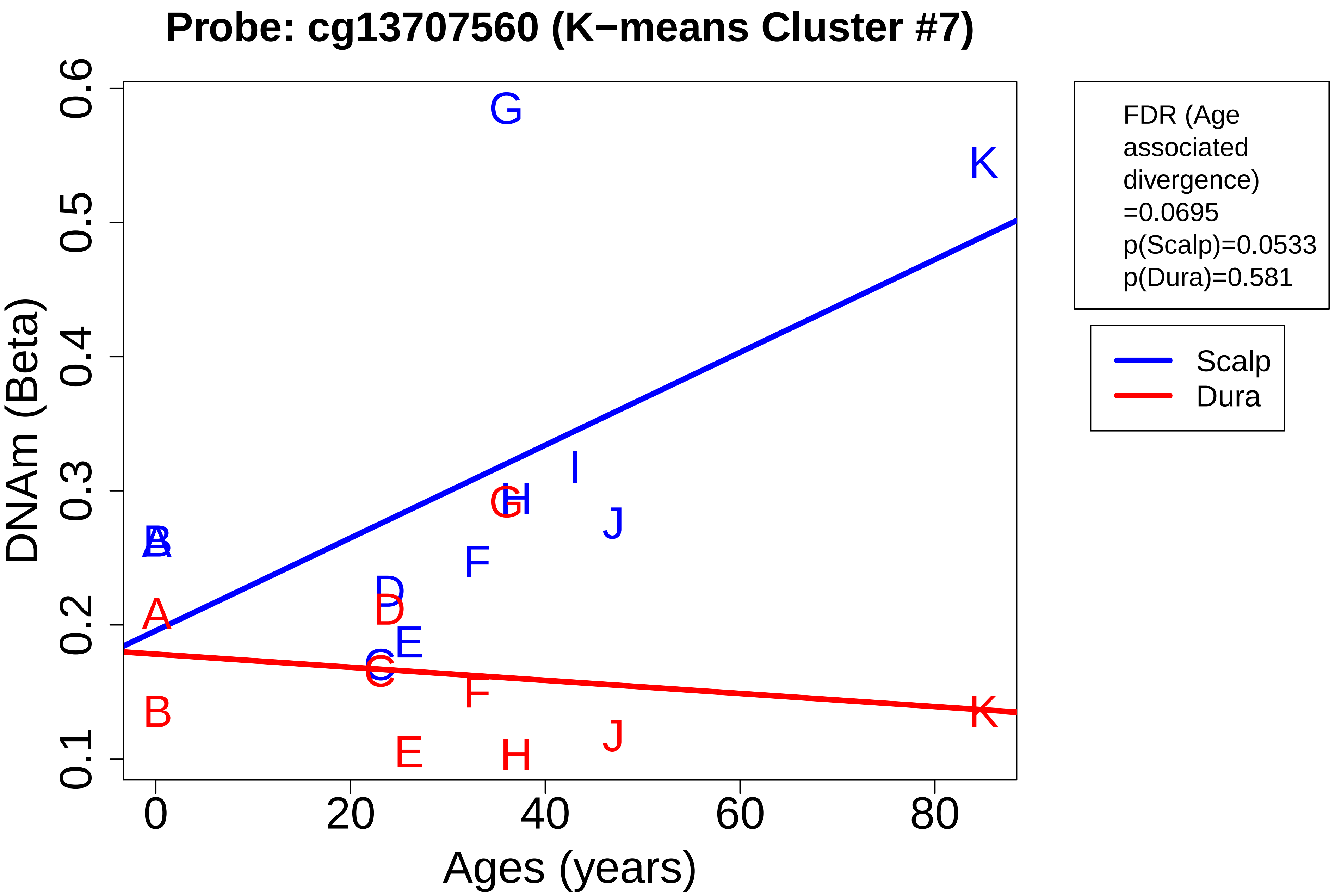

Probe: cg13707560 (K−means Cluster #7)
DNAm (Beta)
0.1
0.2
0.3
0.4
0.5
0.6
Ages (years)
0
20
40
60
80
FDR (Age associated divergence) =0.0695
p(Scalp)=0.0533
p(Dura)=0.581
Scalp
Dura

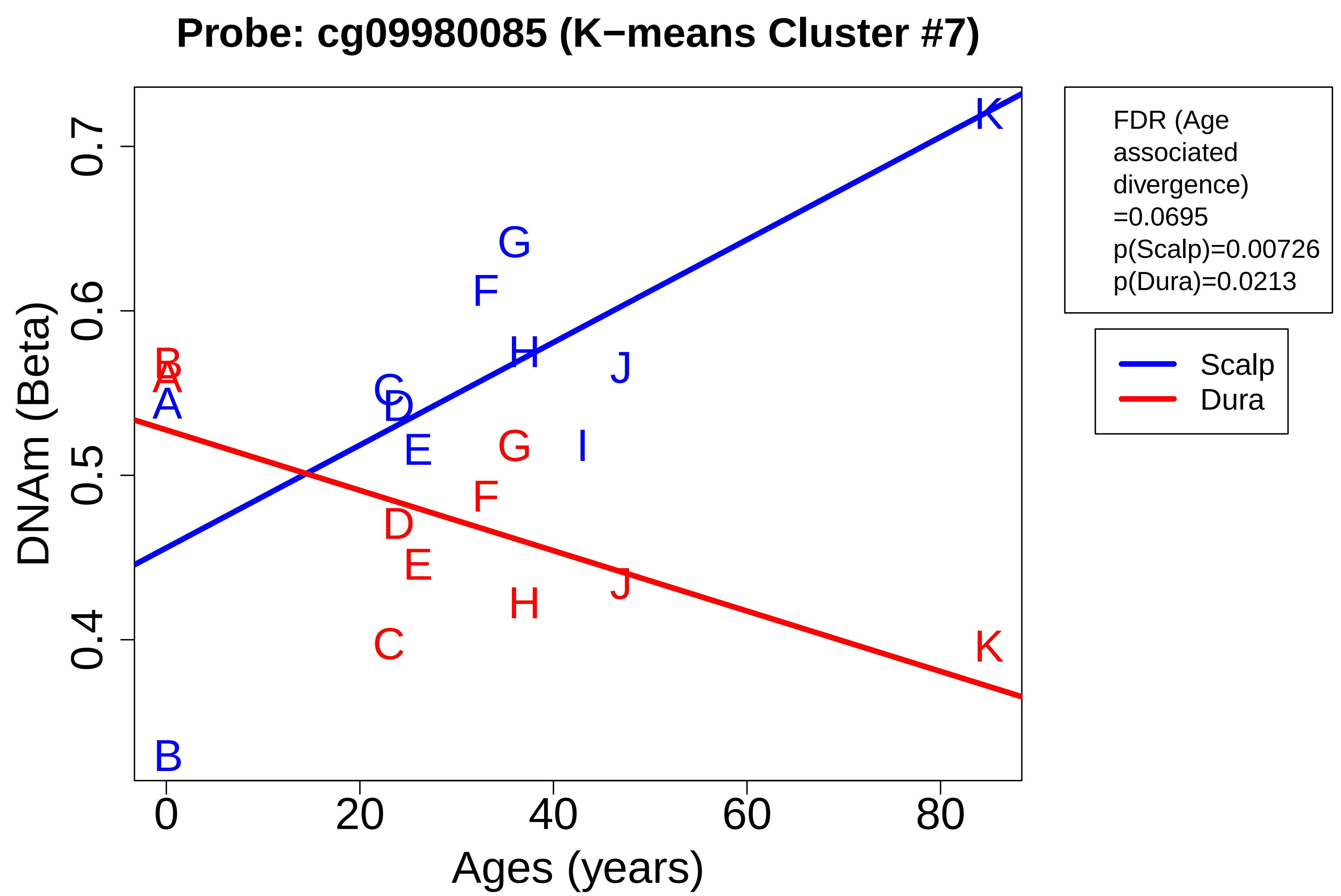

Probe: cg09980085 (K−means Cluster #7)
FDR (Age associated divergence) =0.0695
p(Scalp)=0.00726
p(Dura)=0.0213
Scalp
Dura
DNAm (Beta)
0.4
0.5
0.6
0.7
0
20
40
60
80
Ages (years)
A
B
C
D
E
F
G
H
I
J
K

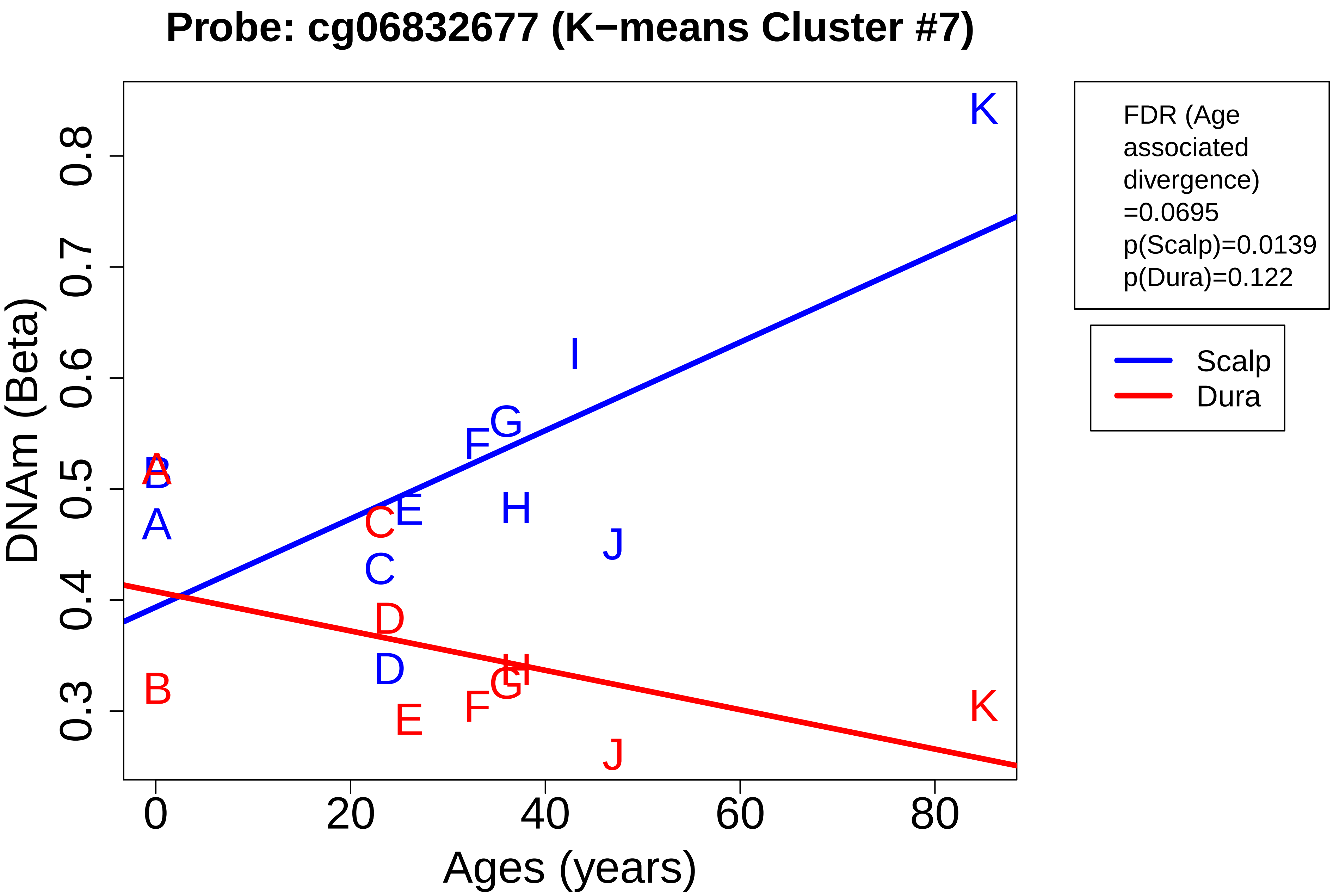

Probe: cg06832677 (K−means Cluster #7)
DNAm (Beta)
0.3
0.4
0.5
0.6
0.7
0.8
0
20
40
60
80
Ages (years)
FDR (Age associated divergence) =0.0695
p(Scalp)=0.0139
p(Dura)=0.122
Scalp
Dura
A
B
C
D
E
F
G
H
I
J
K

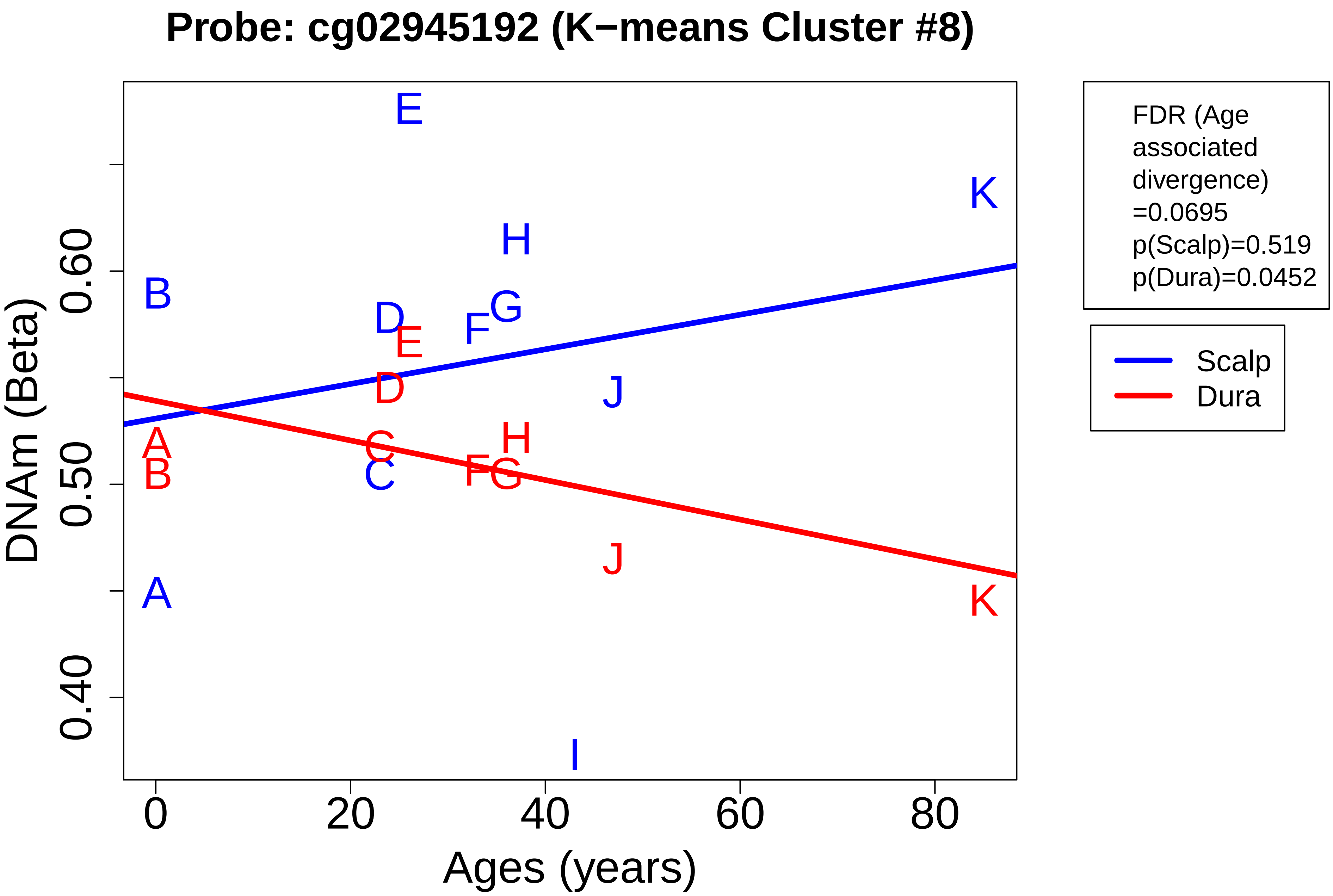

Probe: cg02945192 (K−means Cluster #8)
FDR (Age associated divergence) =0.0695
p(Scalp)=0.519
p(Dura)=0.0452
Scalp
Dura
DNAm (Beta)
0.40
0.50
0.60
Ages (years)
0
20
40
60
80
A
B
C
D
E
F
G
H
I
J
K

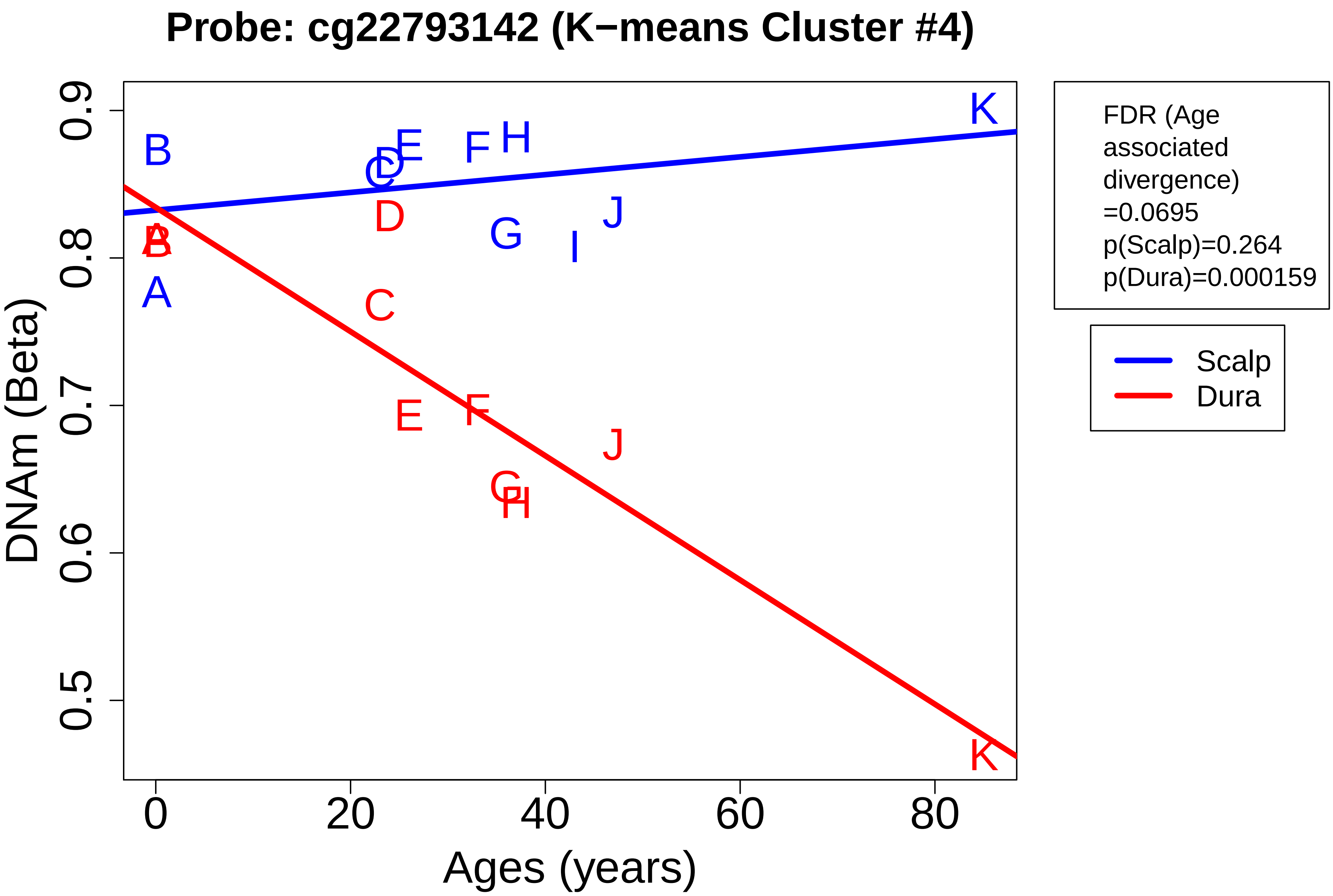
Probe: cg22793142 (K−means Cluster #4)
DNAm (Beta)
Ages (years)
0.5
0.6
0.7
0.8
0.9
0
20
40
60
80
FDR (Age
associated
divergence)
=0.0695
p(Scalp)=0.264
p(Dura)=0.000159
Scalp
Dura

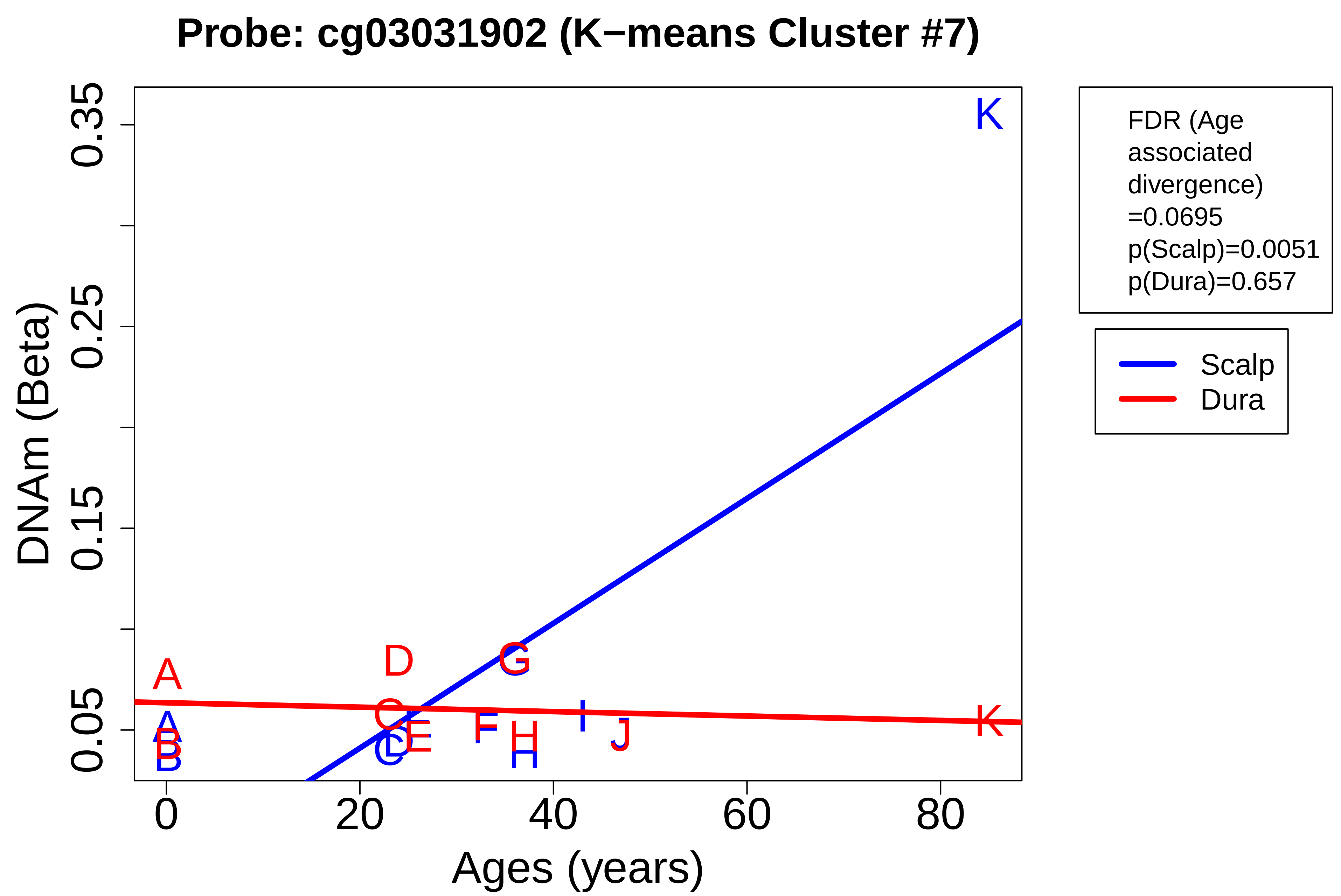

Probe: cg03031902 (K−means Cluster #7)
DNAm (Beta)
0.05
0.15
0.25
0.35
0
20
40
60
80
Ages (years)
FDR (Age associated divergence) =0.0695
p(Scalp)=0.0051
p(Dura)=0.657
Scalp
Dura
A
B
C
D
E
F
G
H
I
J
K

Probe: cg21200067 (K−means Cluster #1)

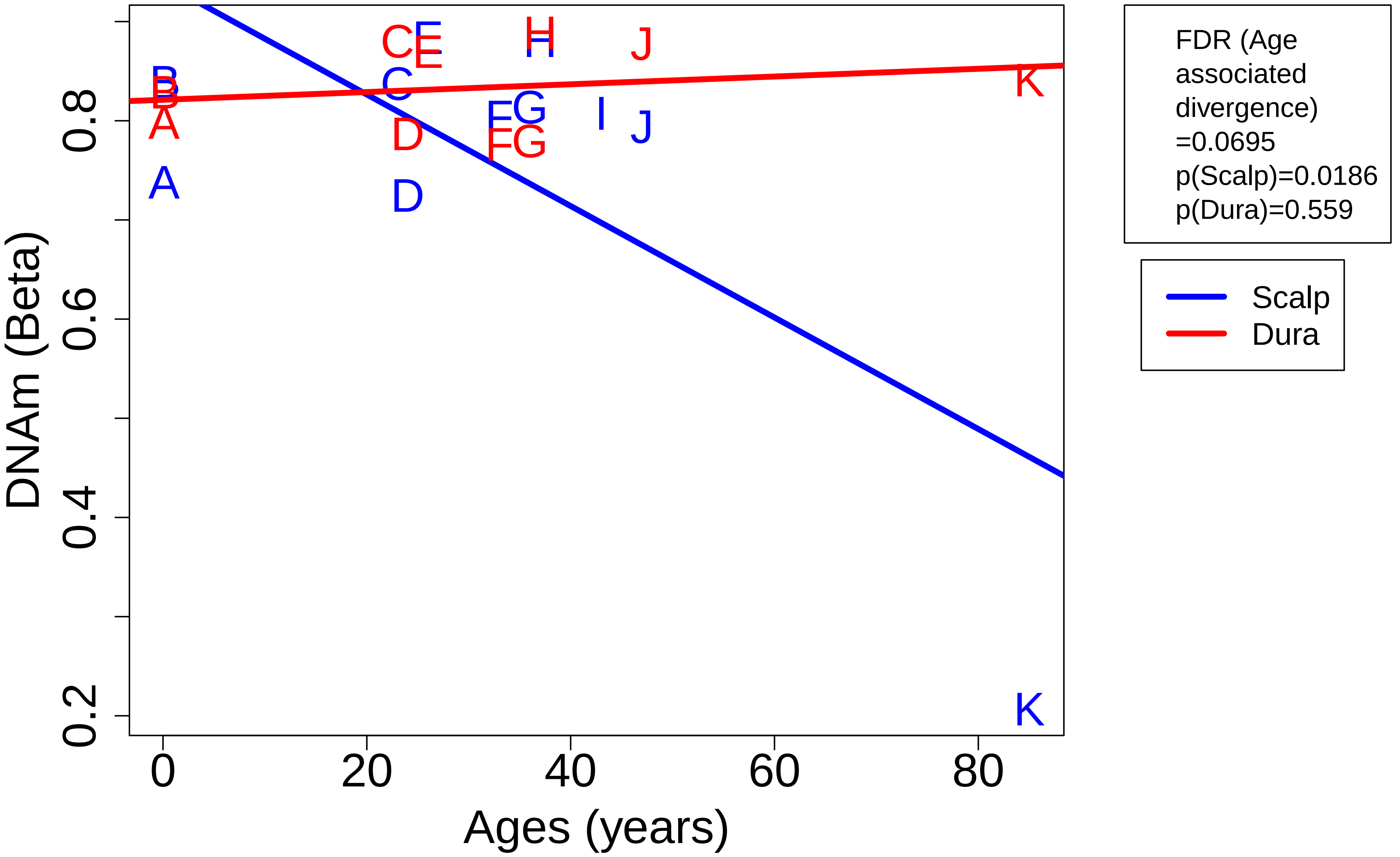

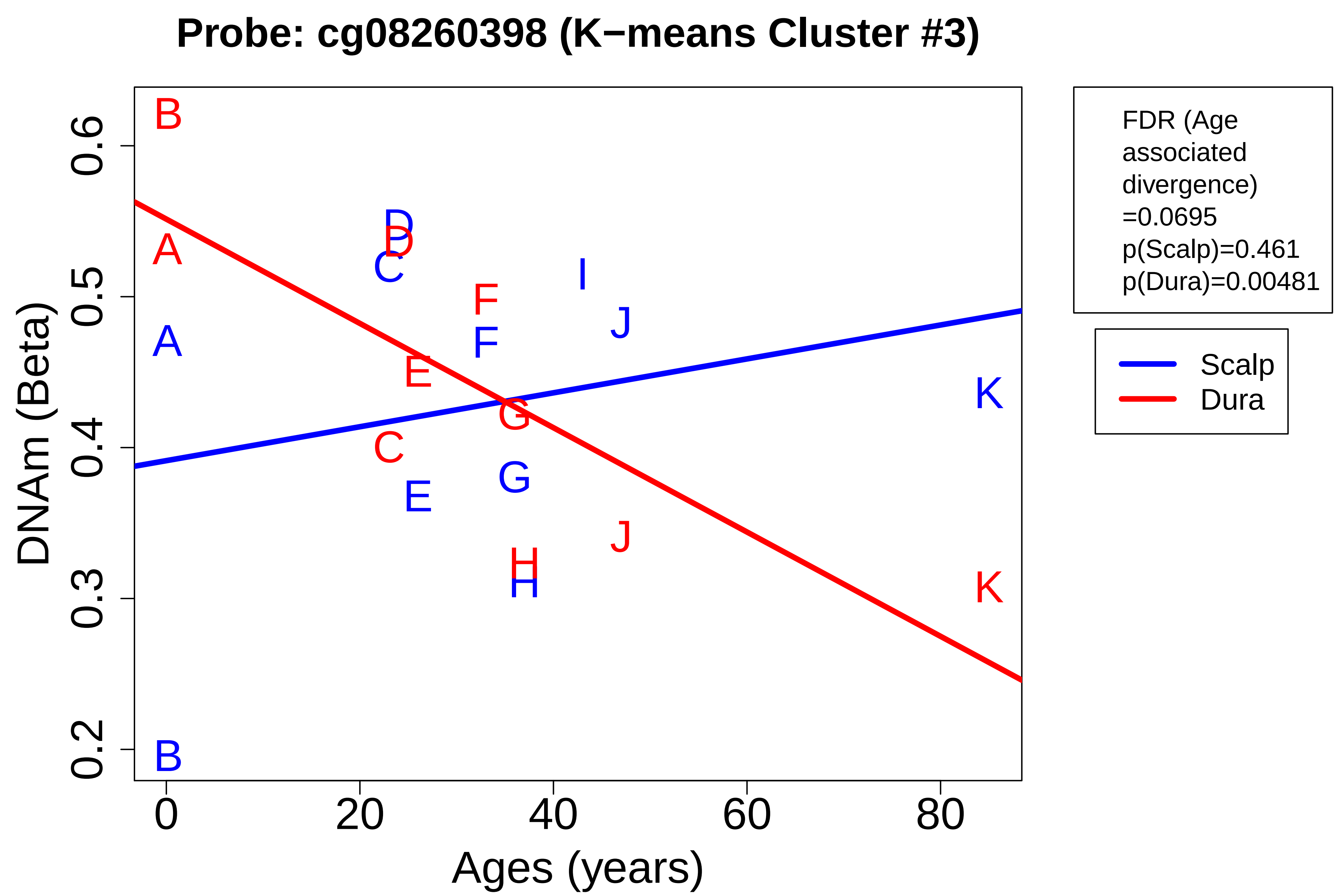

Probe: cg08260398 (K−means Cluster #3)
FDR (Age associated divergence) =0.0695
p(Scalp)=0.461
p(Dura)=0.00481
Scalp
Dura
DNAm (Beta)
Ages (years)
0.2
0.3
0.4
0.5
0.6
0
20
40
60
80

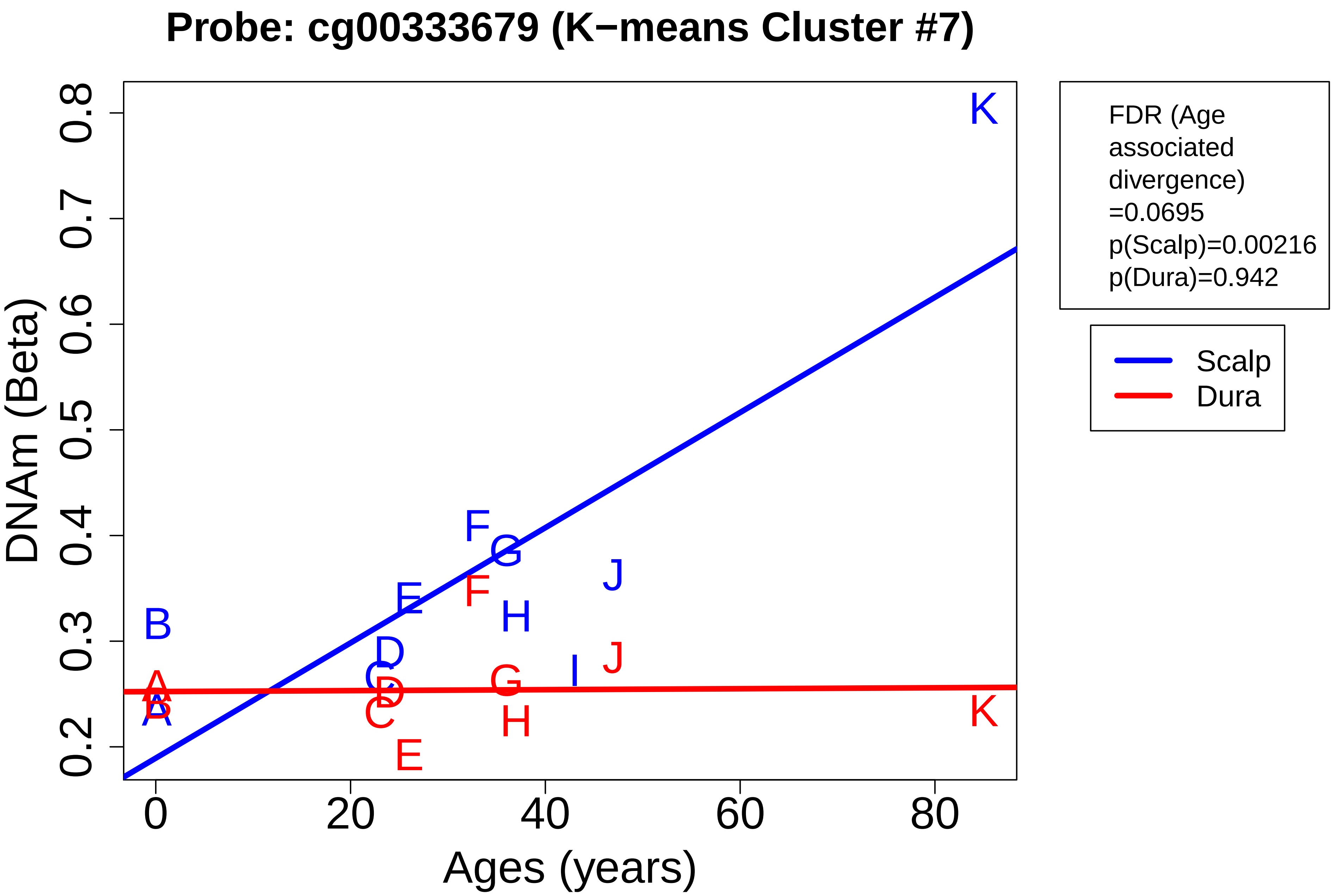

Probe: cg00333679 (K−means Cluster #7)
DNAm (Beta)
0.2
0.3
0.4
0.5
0.6
0.7
0.8
Ages (years)
0
20
40
60
80
FDR (Age associated divergence) =0.0695
p(Scalp)=0.00216
p(Dura)=0.942
Scalp
Dura

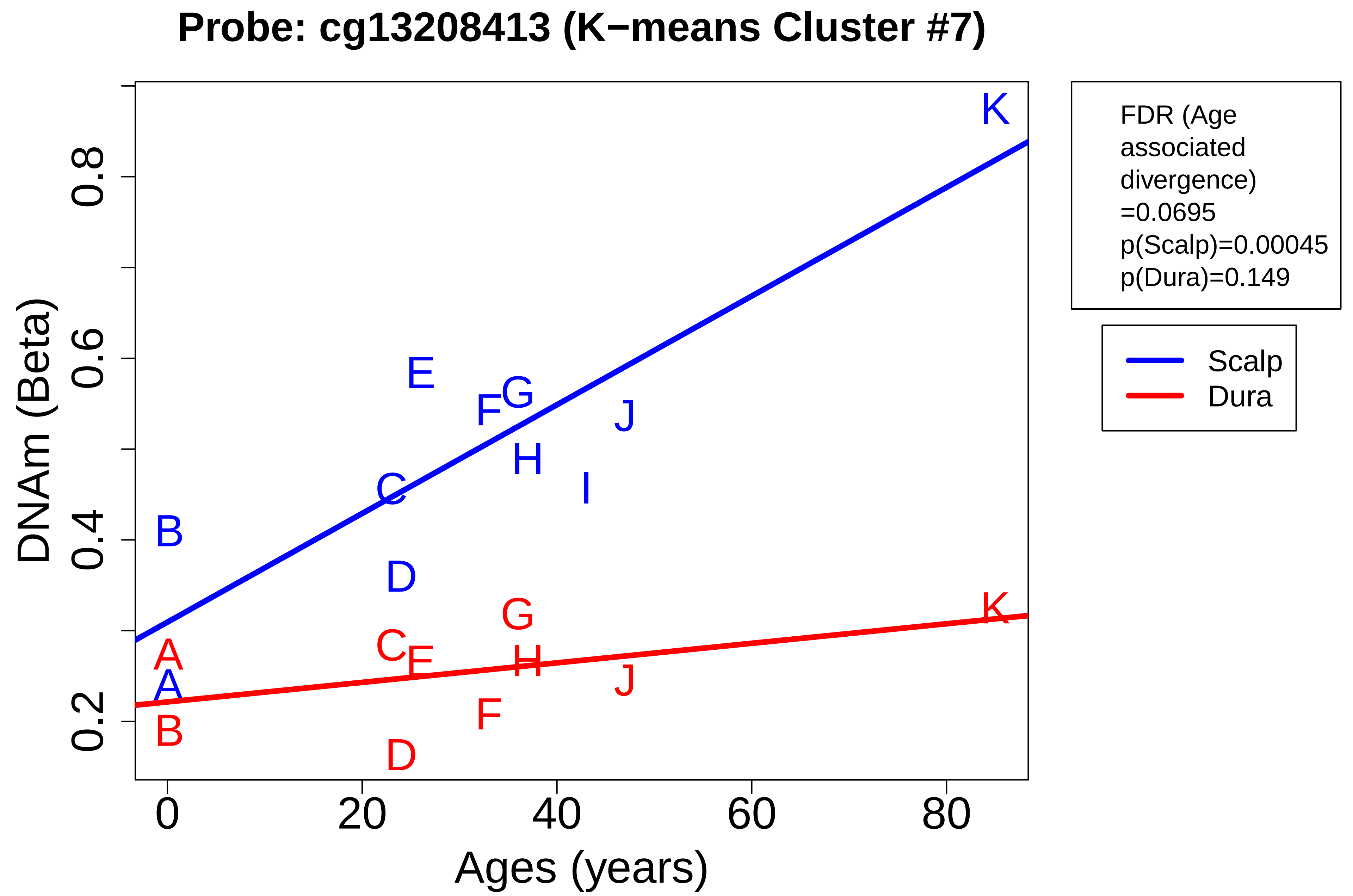

Probe: cg13208413 (K−means Cluster #7)
DNAm (Beta)
0.2
0.4
0.6
0.8
0
20
40
60
80
Ages (years)
FDR (Age associated divergence) =0.0695
p(Scalp)=0.00045
p(Dura)=0.149
Scalp
Dura
A
B
C
D
E
F
G
H
I
J
K

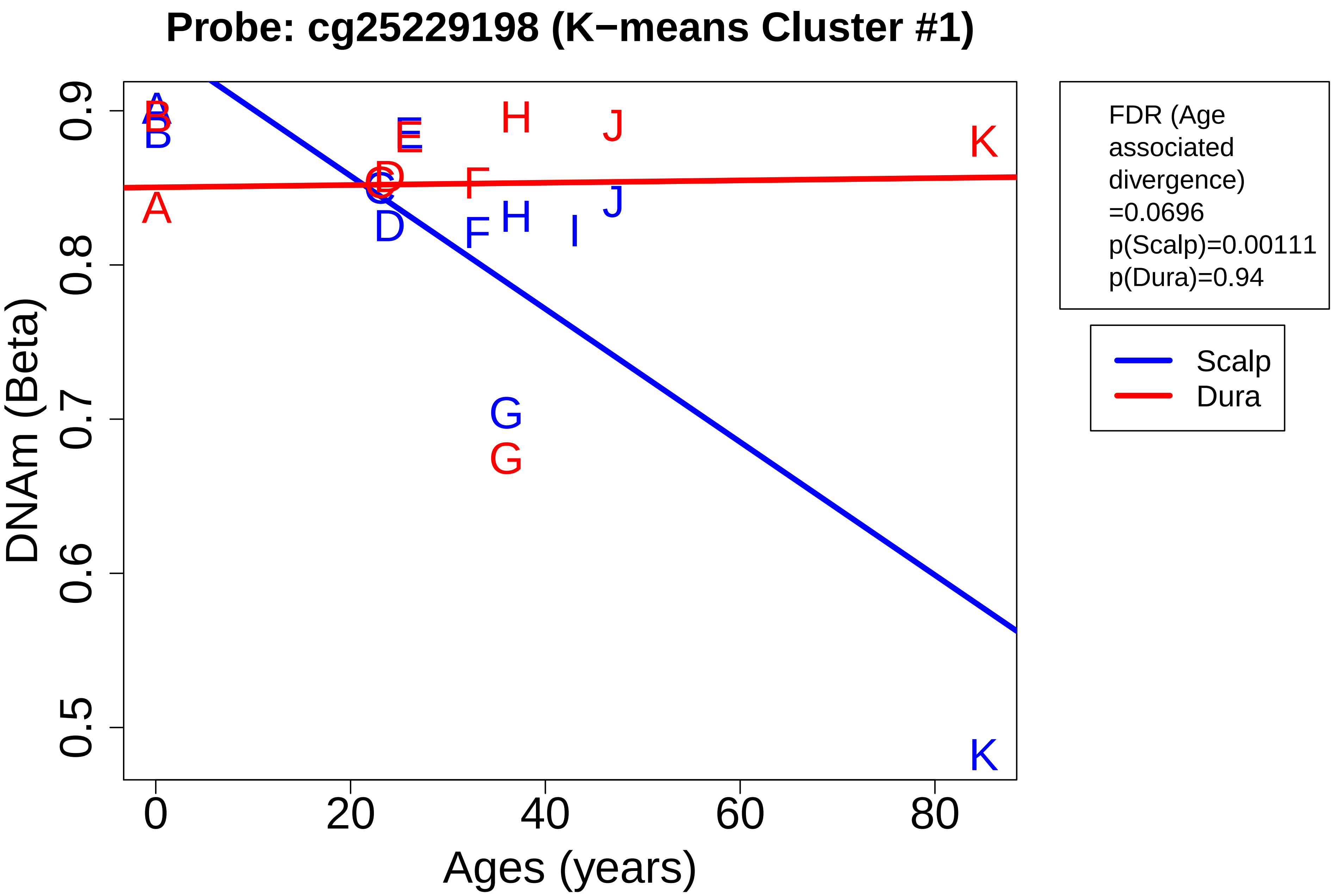

Probe: cg25229198 (K−means Cluster #1)
DNAm (Beta)
Ages (years)
0.5
0.6
0.7
0.8
0.9
0
20
40
60
80
A
B
C
D
E
F
G
H
I
J
K
FDR (Age associated divergence) =0.0696
p(Scalp)=0.00111
p(Dura)=0.94
Scalp
Dura

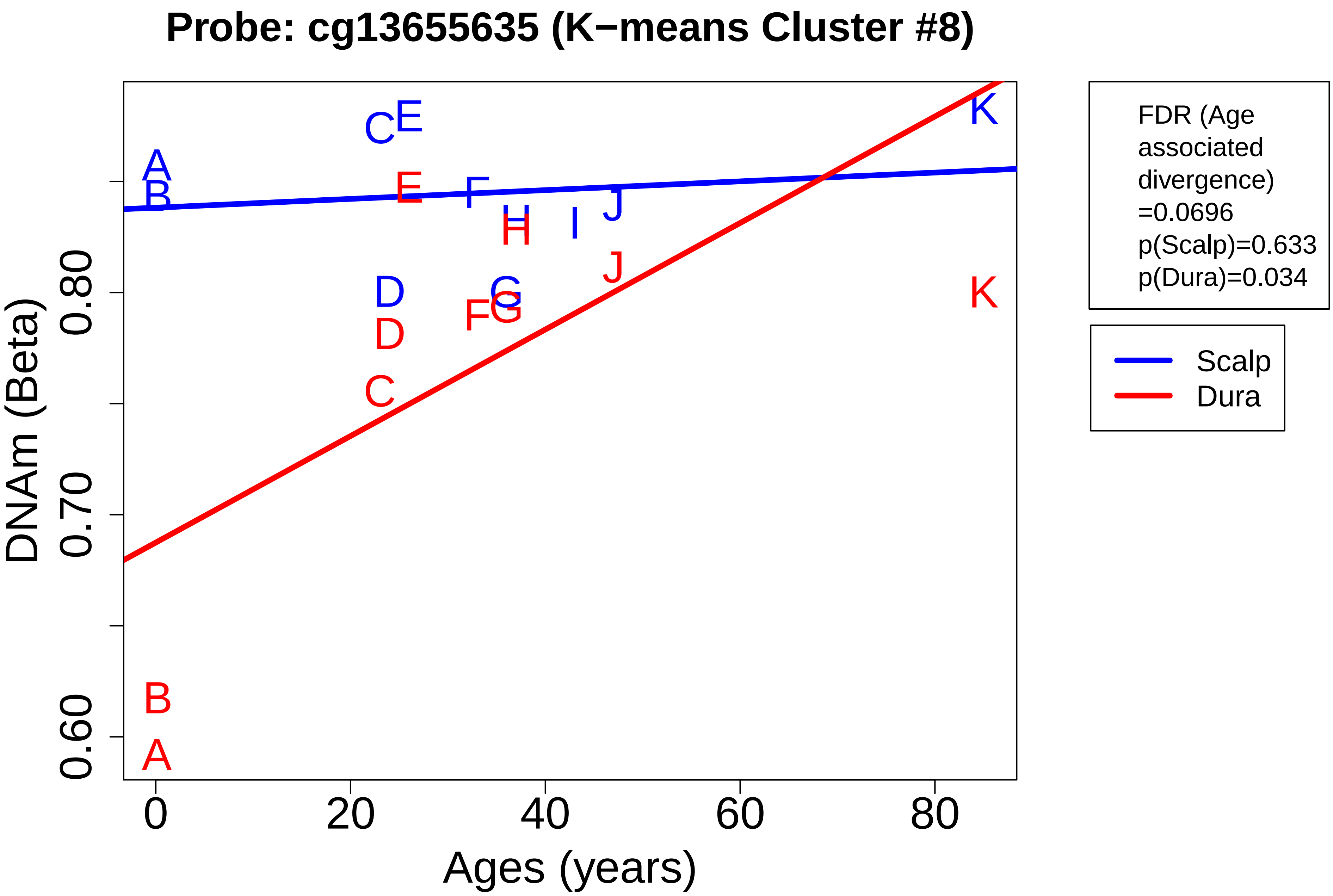

Probe: cg13655635 (K−means Cluster #8)
DNAm (Beta)
0.60
0.70
0.80
0
20
40
60
80
Ages (years)
FDR (Age associated divergence) =0.0696
p(Scalp)=0.633
p(Dura)=0.034
Scalp
Dura

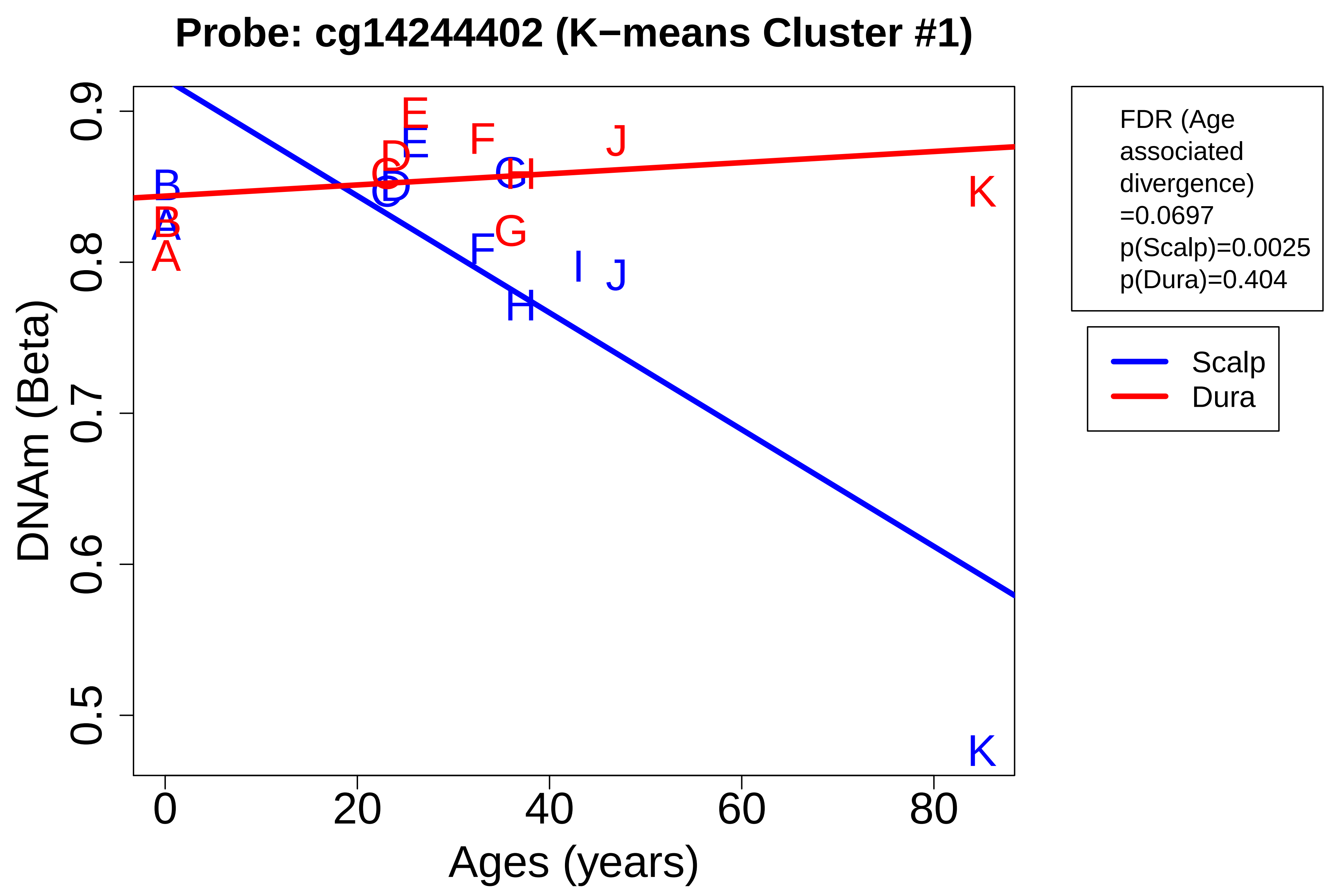

Probe: cg14244402 (K−means Cluster #1)
DNAm (Beta)
0.5
0.6
0.7
0.8
0.9
Ages (years)
0
20
40
60
80
FDR (Age associated divergence) =0.0697
p(Scalp)=0.0025
p(Dura)=0.404
Scalp
Dura
A
B
C
D
E
F
G
H
I
J
K

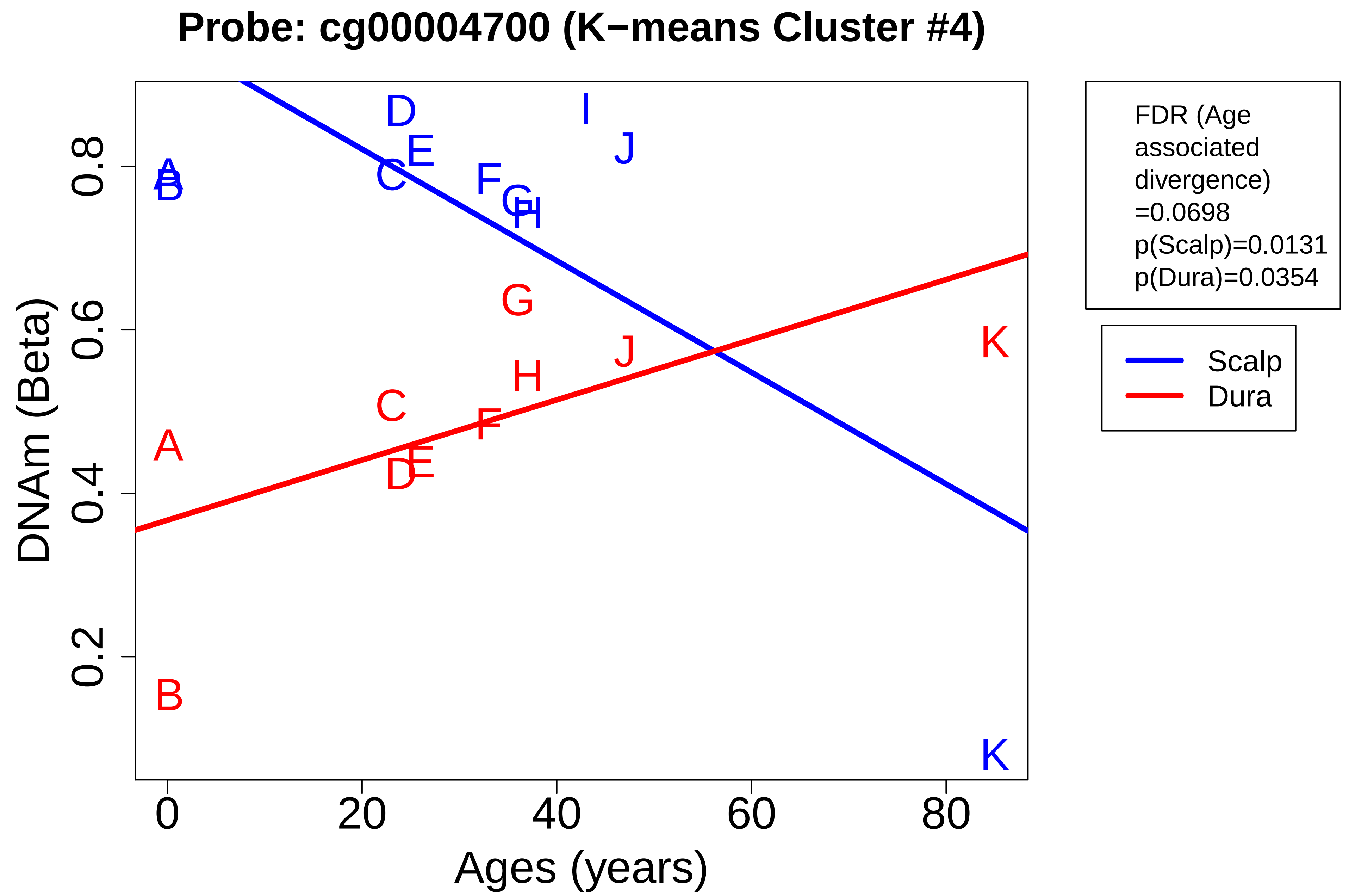

Probe: cg00004700 (K−means Cluster #4)
DNAm (Beta)
Ages (years)
0.2
0.4
0.6
0.8
0
20
40
60
80
FDR (Age associated divergence) =0.0698
p(Scalp)=0.0131
p(Dura)=0.0354
Scalp
Dura
A
B
C
D
E
F
G
H
I
J
K

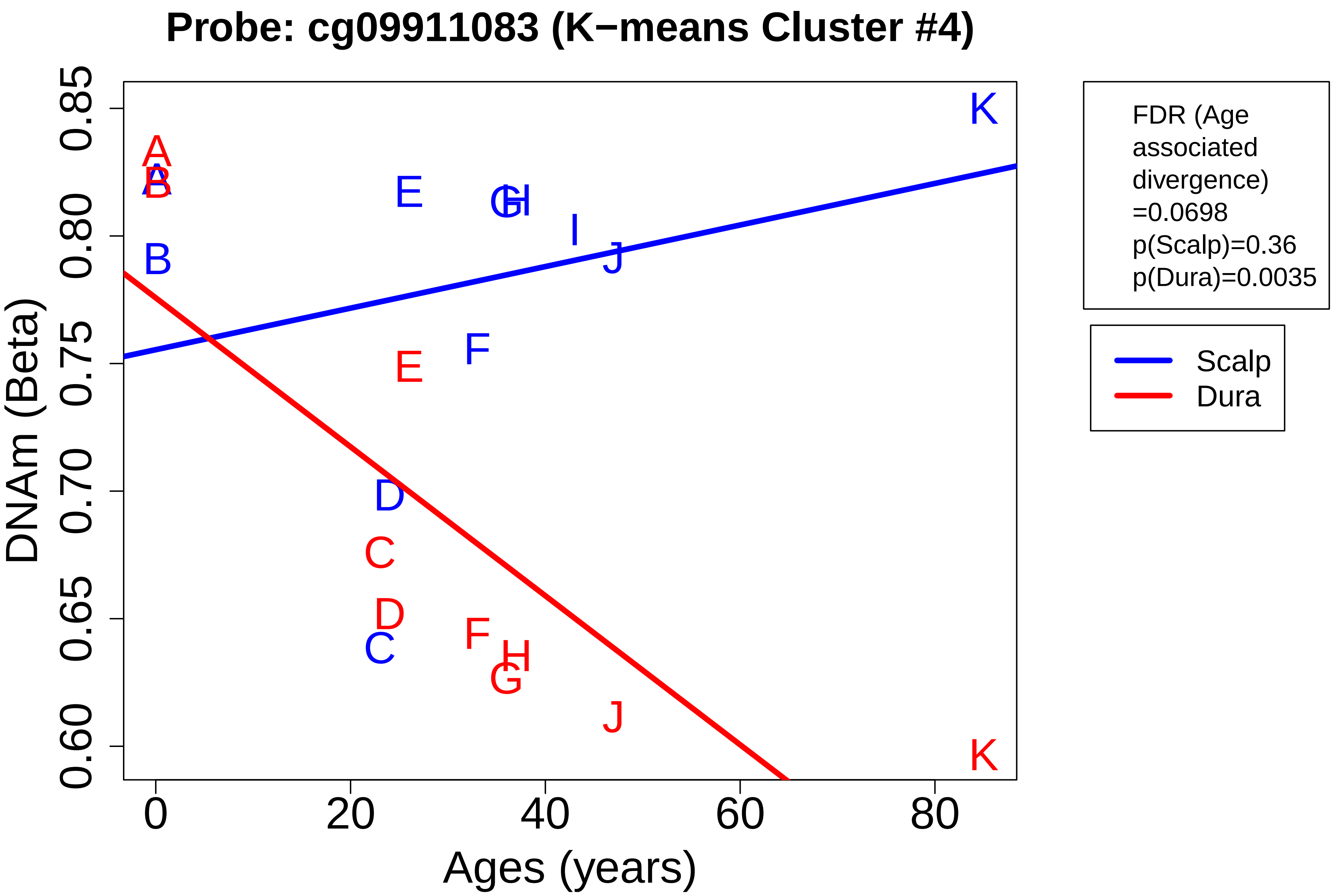

Probe: cg09911083 (K−means Cluster #4)
DNAm (Beta)
0.60
0.65
0.70
0.75
0.80
0.85
Ages (years)
0
20
40
60
80
A
B
C
D
E
F
G
H
I
J
K
FDR (Age associated divergence) =0.0698
p(Scalp)=0.36
p(Dura)=0.0035
Scalp
Dura

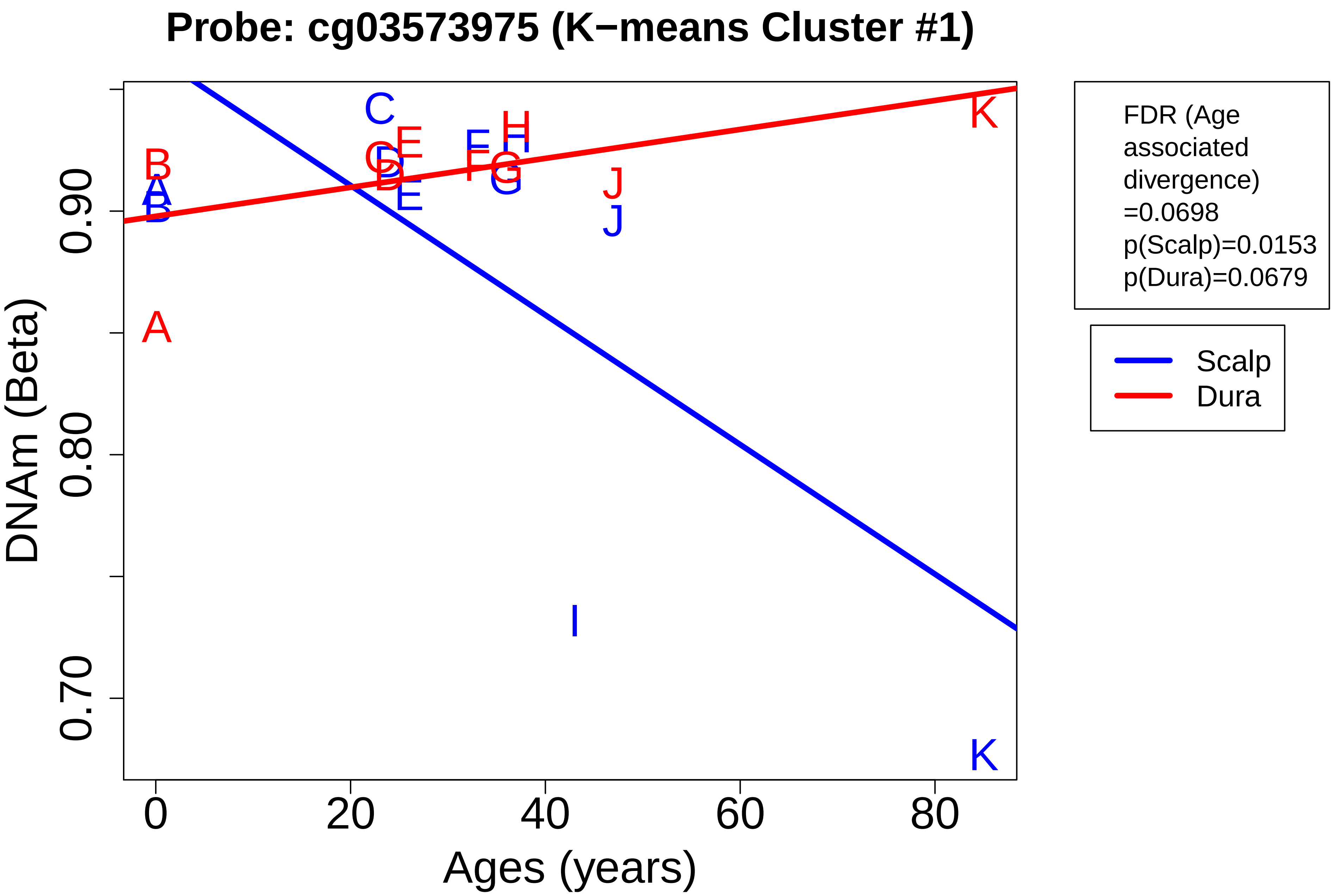

Probe: cg03573975 (K−means Cluster #1)
DNAm (Beta)
0.70
0.80
0.90
0
20
40
60
80
Ages (years)
FDR (Age associated divergence) =0.0698
p(Scalp)=0.0153
p(Dura)=0.0679
Scalp
Dura

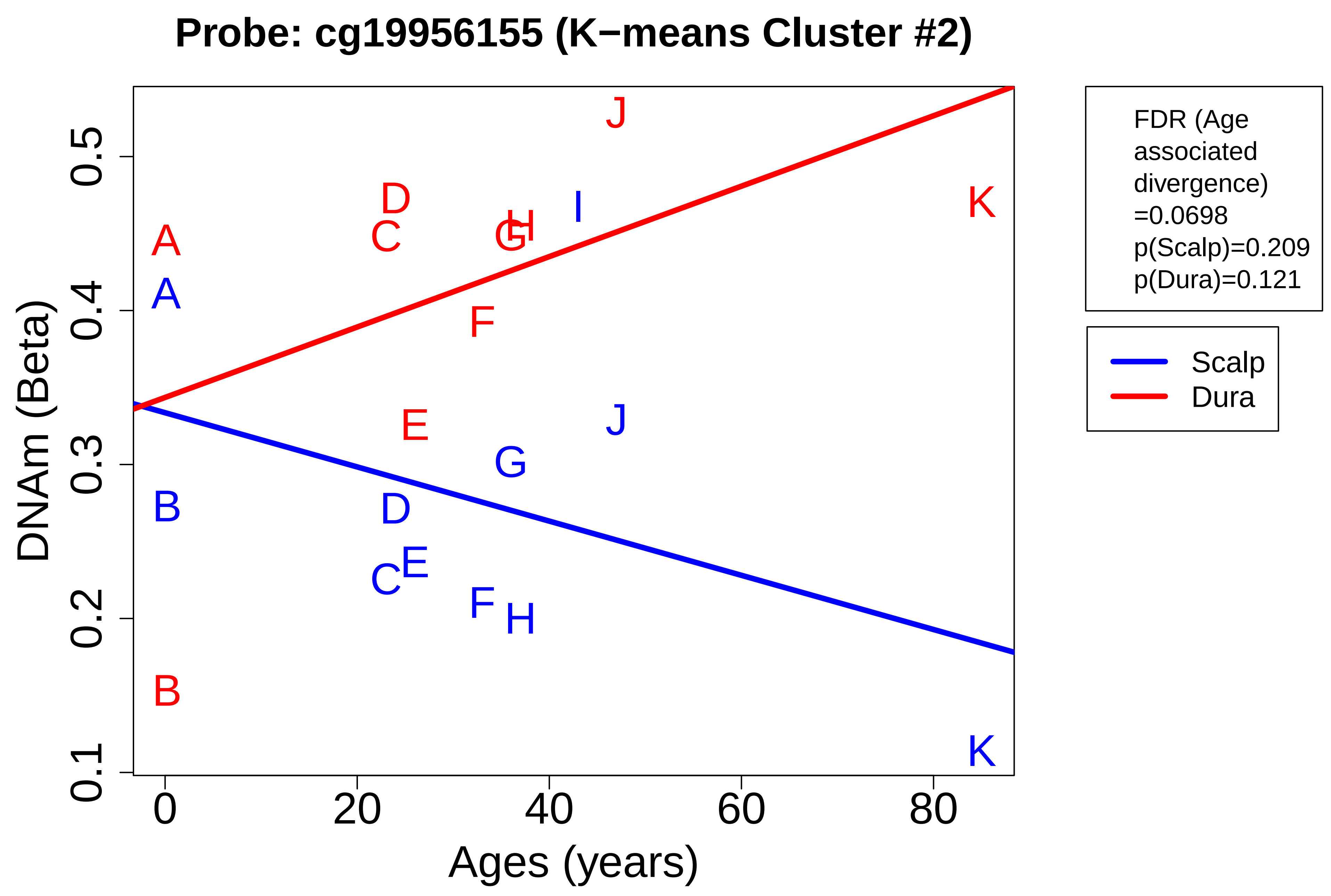

Probe: cg19956155 (K−means Cluster #2)
DNAm (Beta)
Ages (years)
0.1
0.2
0.3
0.4
0.5
0
20
40
60
80
A
B
C
D
E
F
G
H
I
J
K
A
B
C
D
E
F
G
H
J
K
FDR (Age associated divergence) =0.0698
p(Scalp)=0.209
p(Dura)=0.121
Scalp
Dura

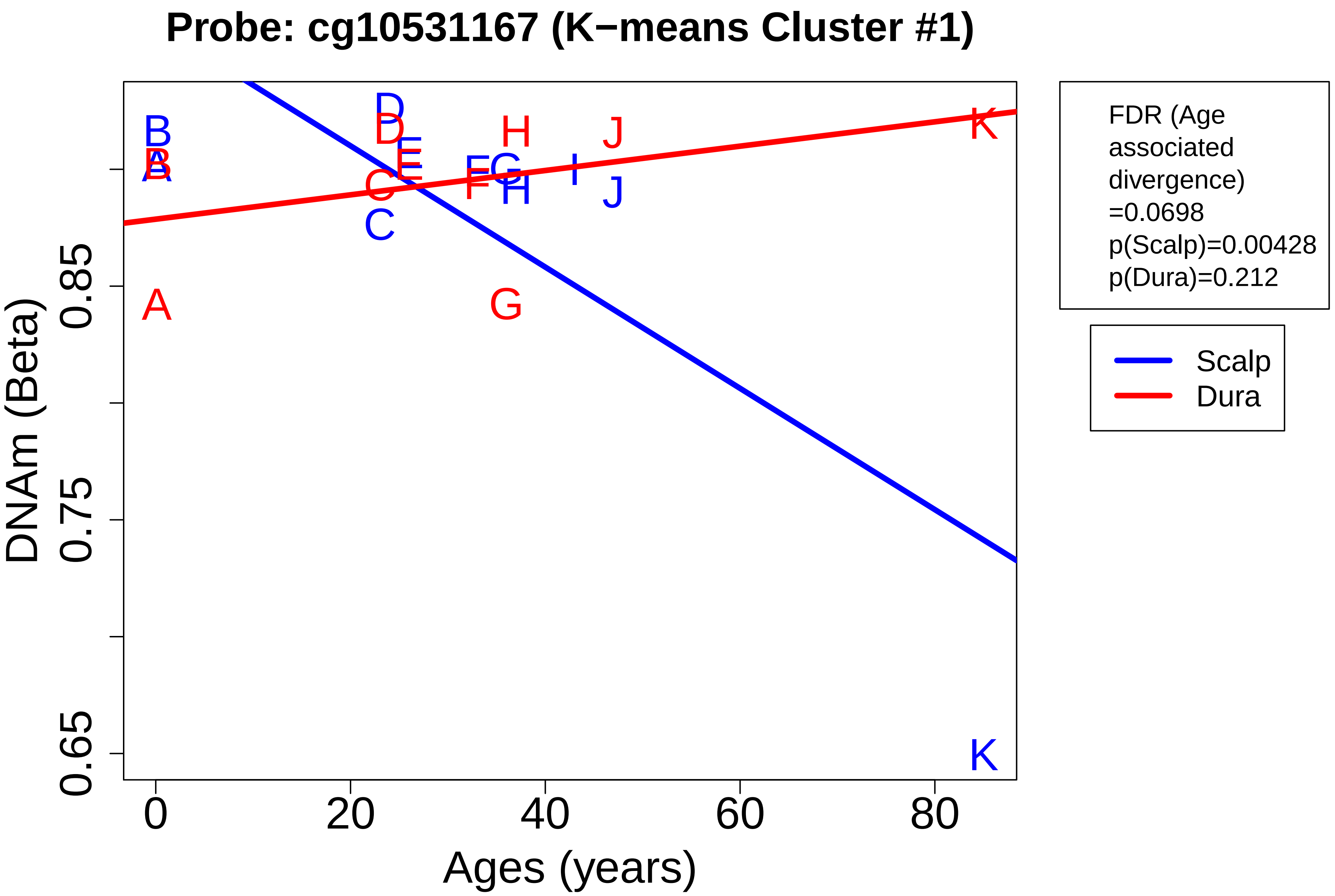

Probe: cg10531167 (K−means Cluster #1)
DNAm (Beta)
0.65
0.75
0.85
Ages (years)
0
20
40
60
80
FDR (Age associated divergence) =0.0698
p(Scalp)=0.00428
p(Dura)=0.212
Scalp
Dura

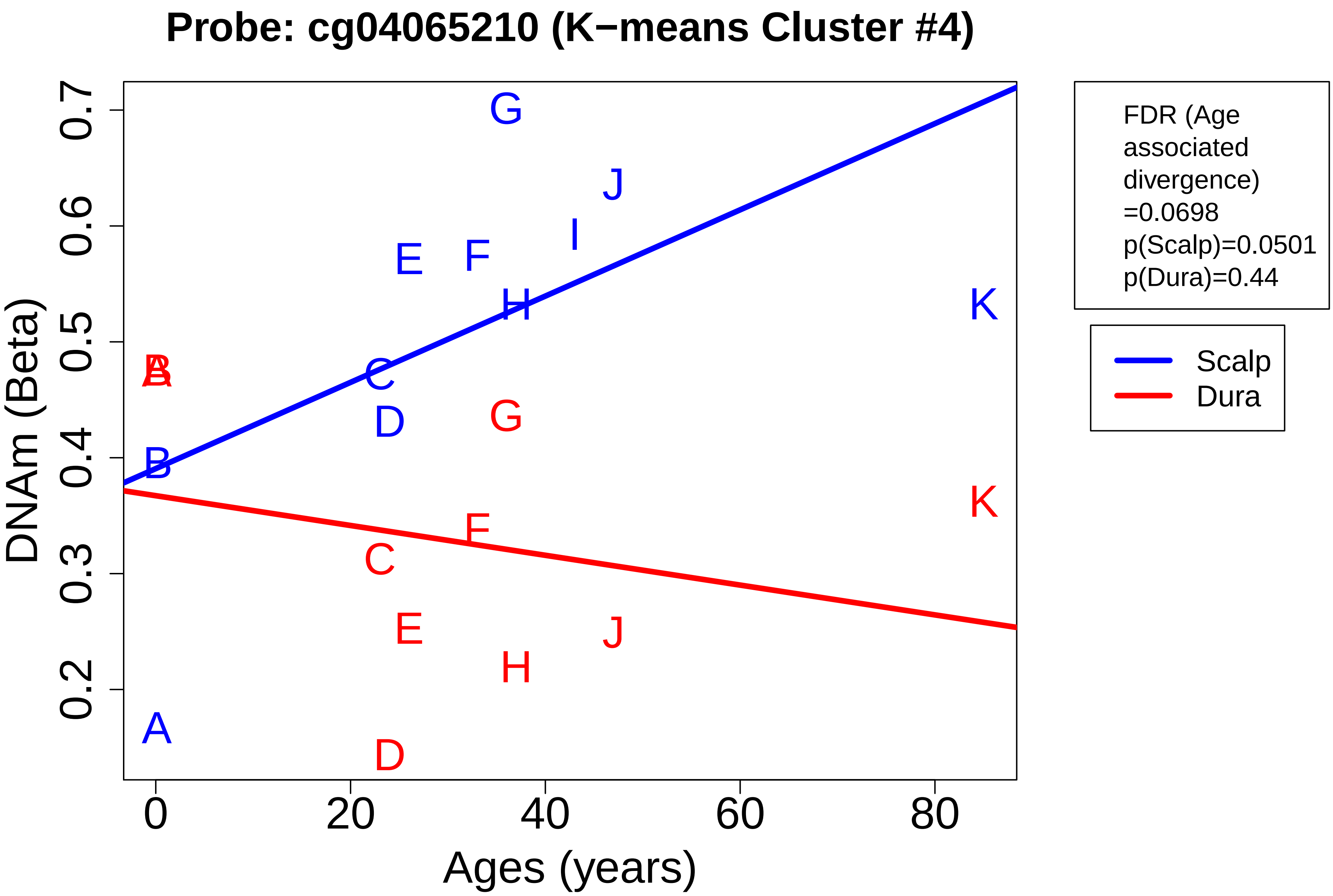
Probe: cg04065210 (K−means Cluster #4)
DNAm (Beta)
0.2
0.3
0.4
0.5
0.6
0.7
0
20
40
60
80
Ages (years)
FDR (Age associated divergence) =0.0698
p(Scalp)=0.0501
p(Dura)=0.44
Scalp
Dura
A
B
C
D
E
F
G
H
I
J
K

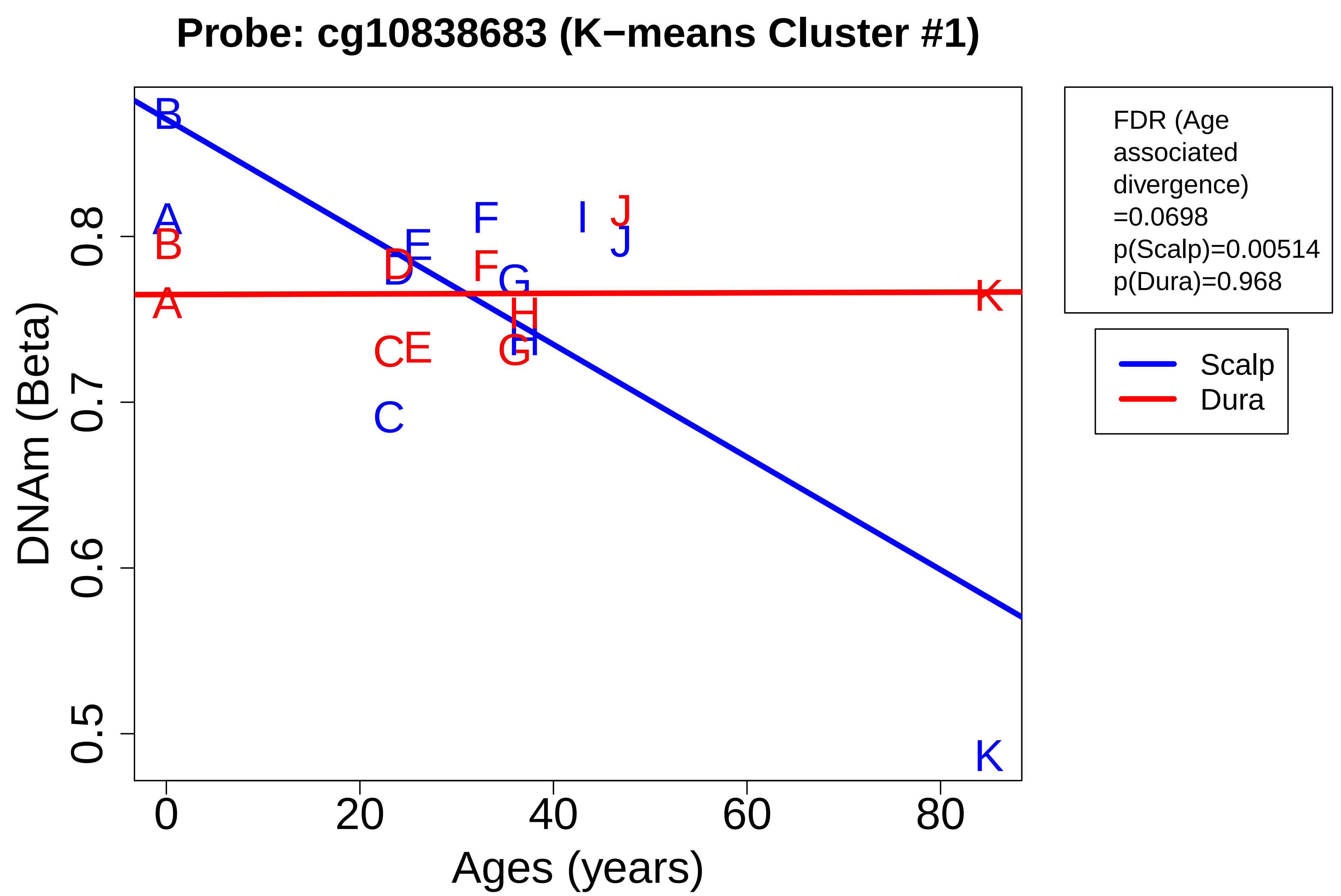

Probe: cg10838683 (K−means Cluster #1)
DNAm (Beta)
0.5
0.6
0.7
0.8
0
20
40
60
80
Ages (years)
FDR (Age associated divergence) =0.0698
p(Scalp)=0.00514
p(Dura)=0.968
Scalp
Dura

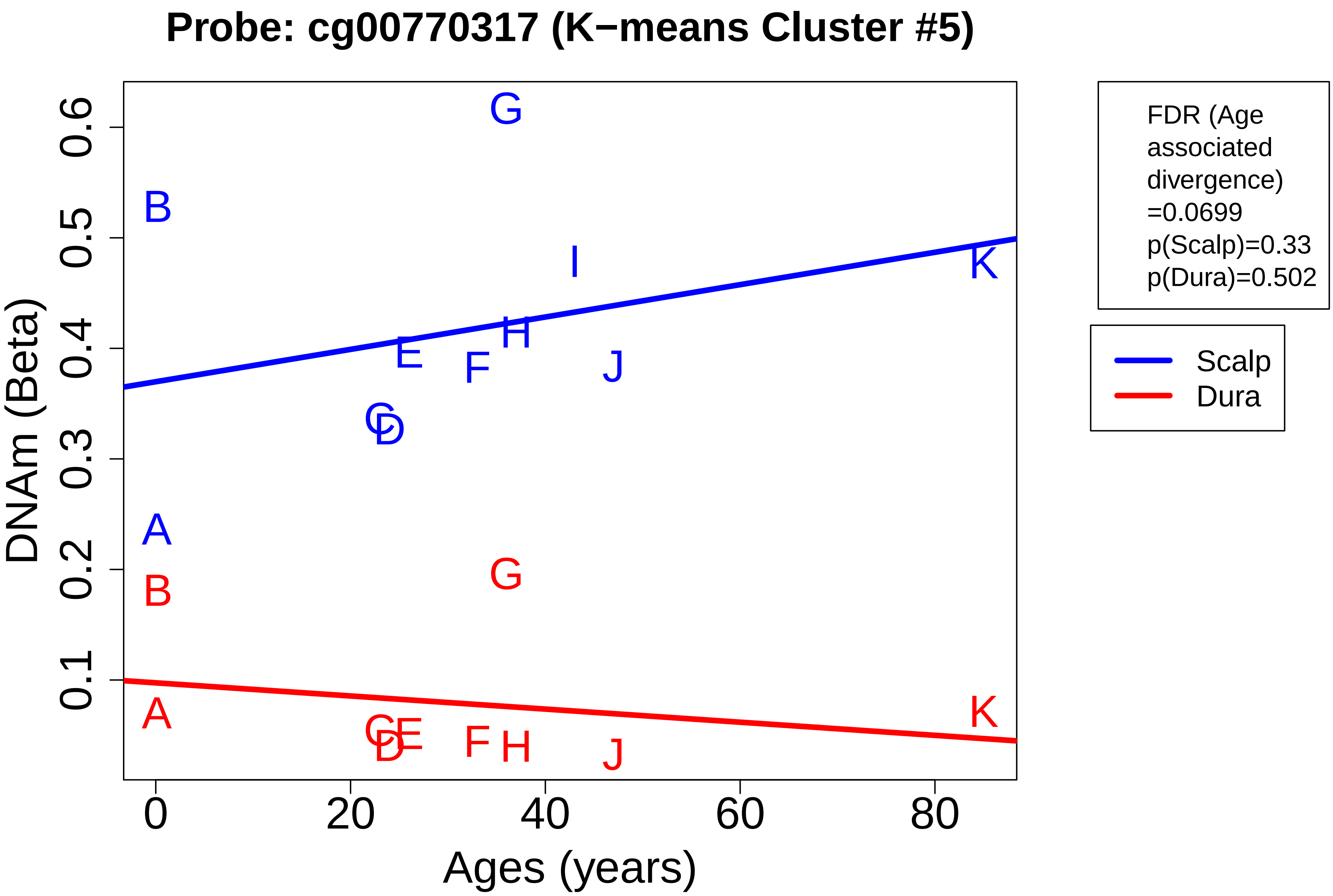

Probe: cg00770317 (K−means Cluster #5)
DNAm (Beta)
0.1
0.2
0.3
0.4
0.5
0.6
0
20
40
60
80
Ages (years)
FDR (Age associated divergence) =0.0699
p(Scalp)=0.33
p(Dura)=0.502
Scalp
Dura
A
B
C
D
E
F
G
H
I
J
K

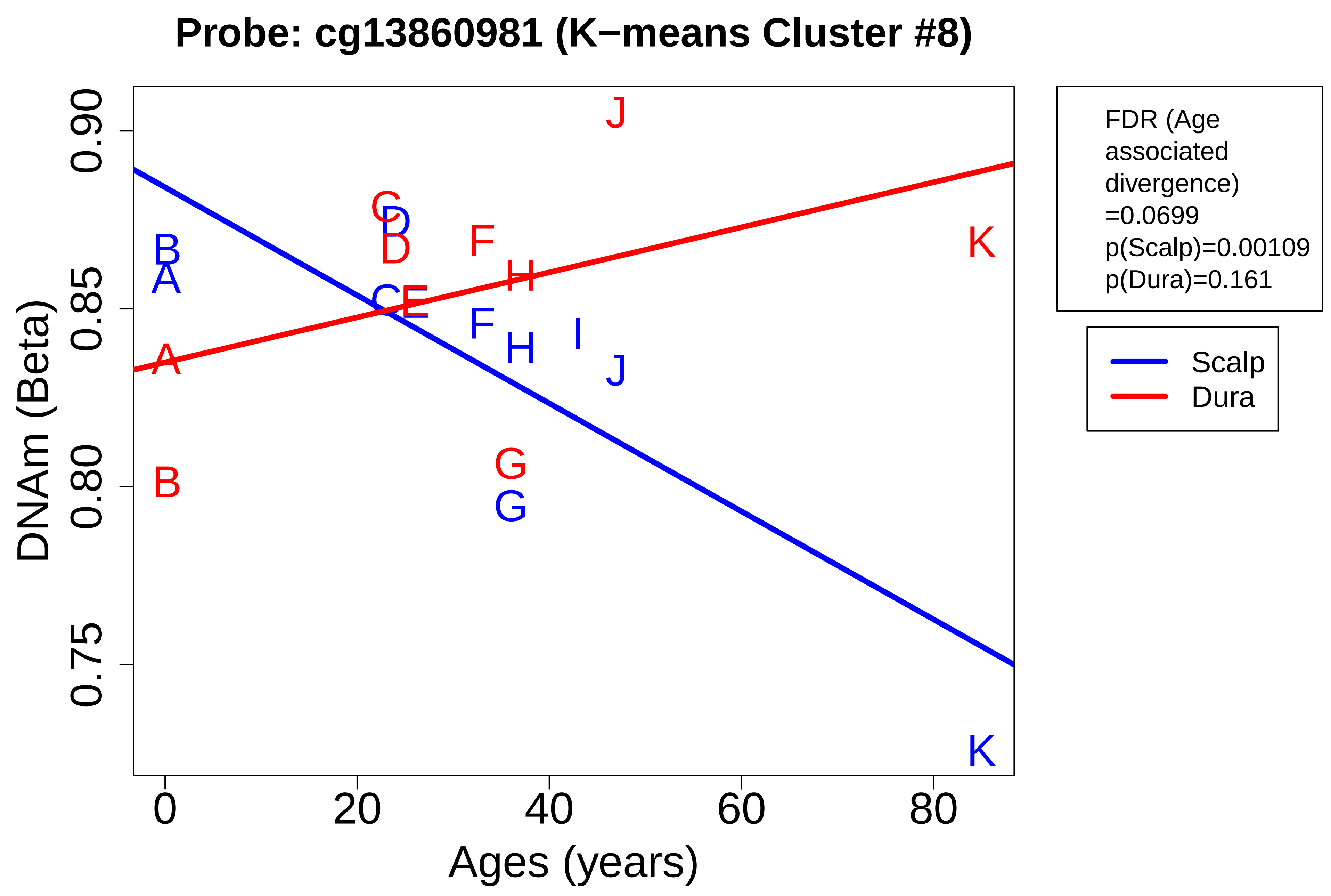

Probe: cg13860981 (K−means Cluster #8)
DNAm (Beta)
0.75
0.80
0.85
0.90
0
20
40
60
80
Ages (years)
FDR (Age associated divergence) =0.0699
p(Scalp)=0.00109
p(Dura)=0.161
Scalp
Dura

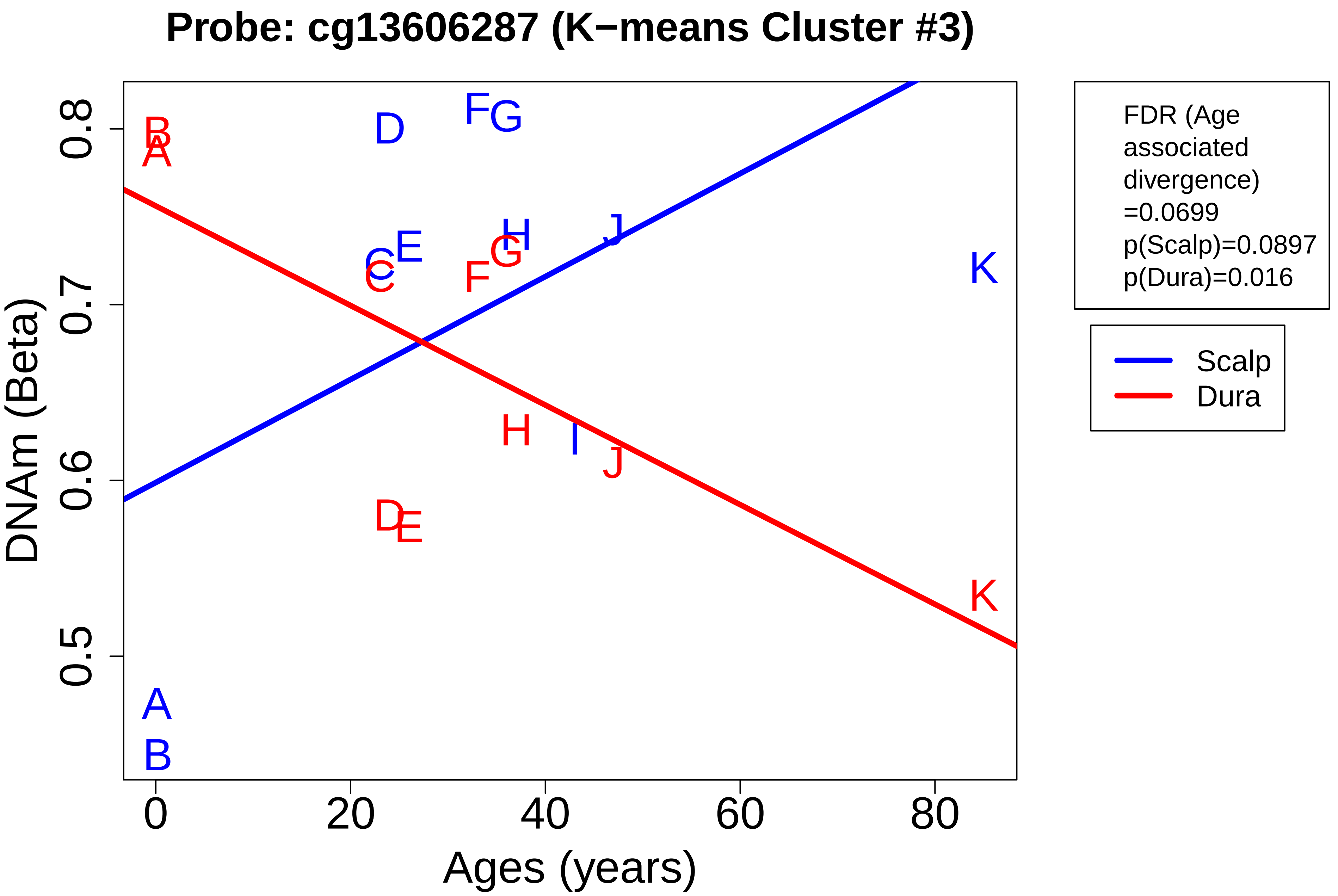

Probe: cg13606287 (K−means Cluster #3)
DNAm (Beta)
0.5
0.6
0.7
0.8
0
20
40
60
80
Ages (years)
FDR (Age associated divergence) =0.0699
p(Scalp)=0.0897
p(Dura)=0.016
Scalp
Dura

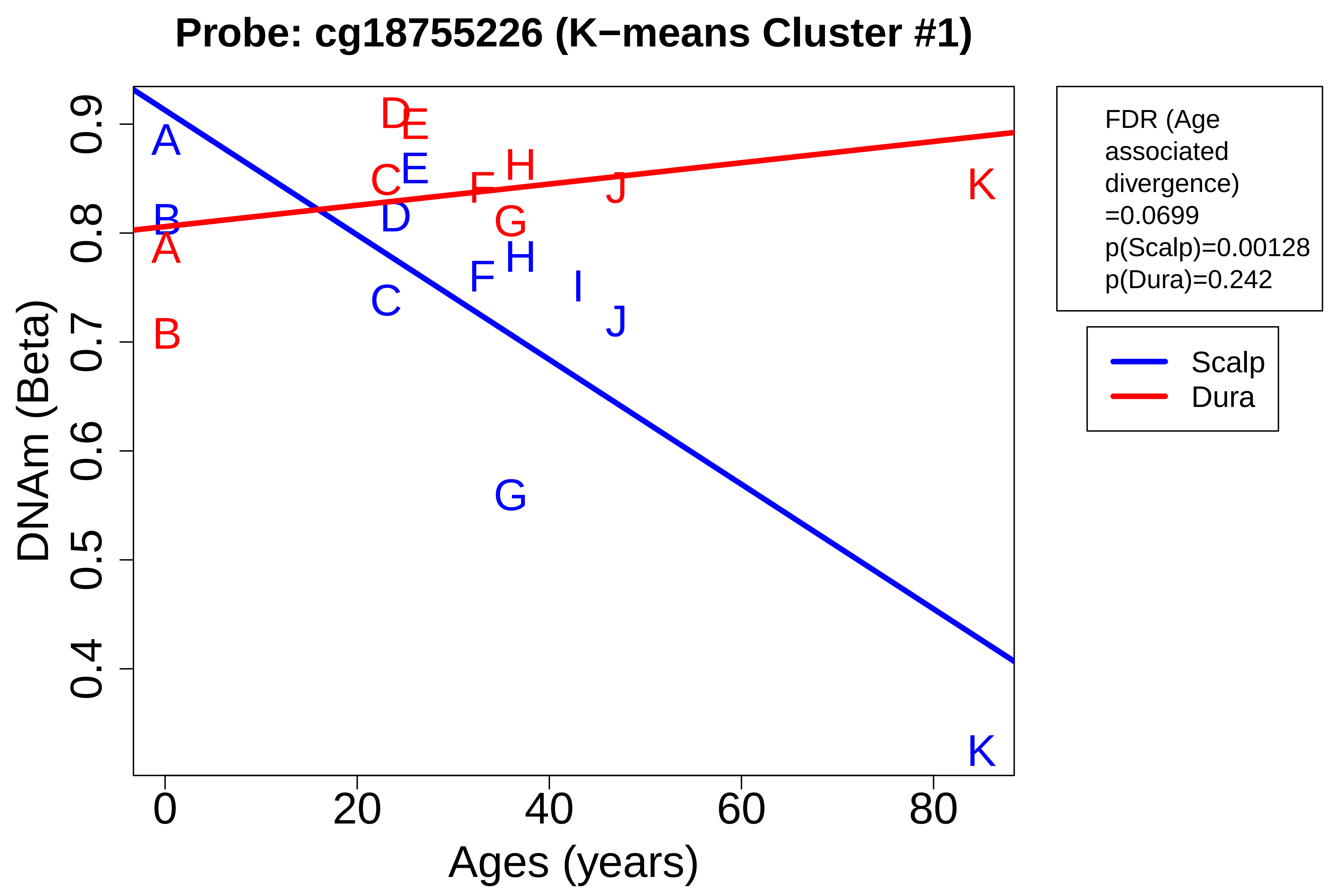
Probe: cg18755226 (K−means Cluster #1)
DNAm (Beta)
0.4
0.5
0.6
0.7
0.8
0.9
0
20
40
60
80
Ages (years)
A
B
C
D
E
F
G
H
I
J
K
A
B
C
D
E
F
G
H
J
K
FDR (Age associated divergence) =0.0699
p(Scalp)=0.00128
p(Dura)=0.242
Scalp
Dura

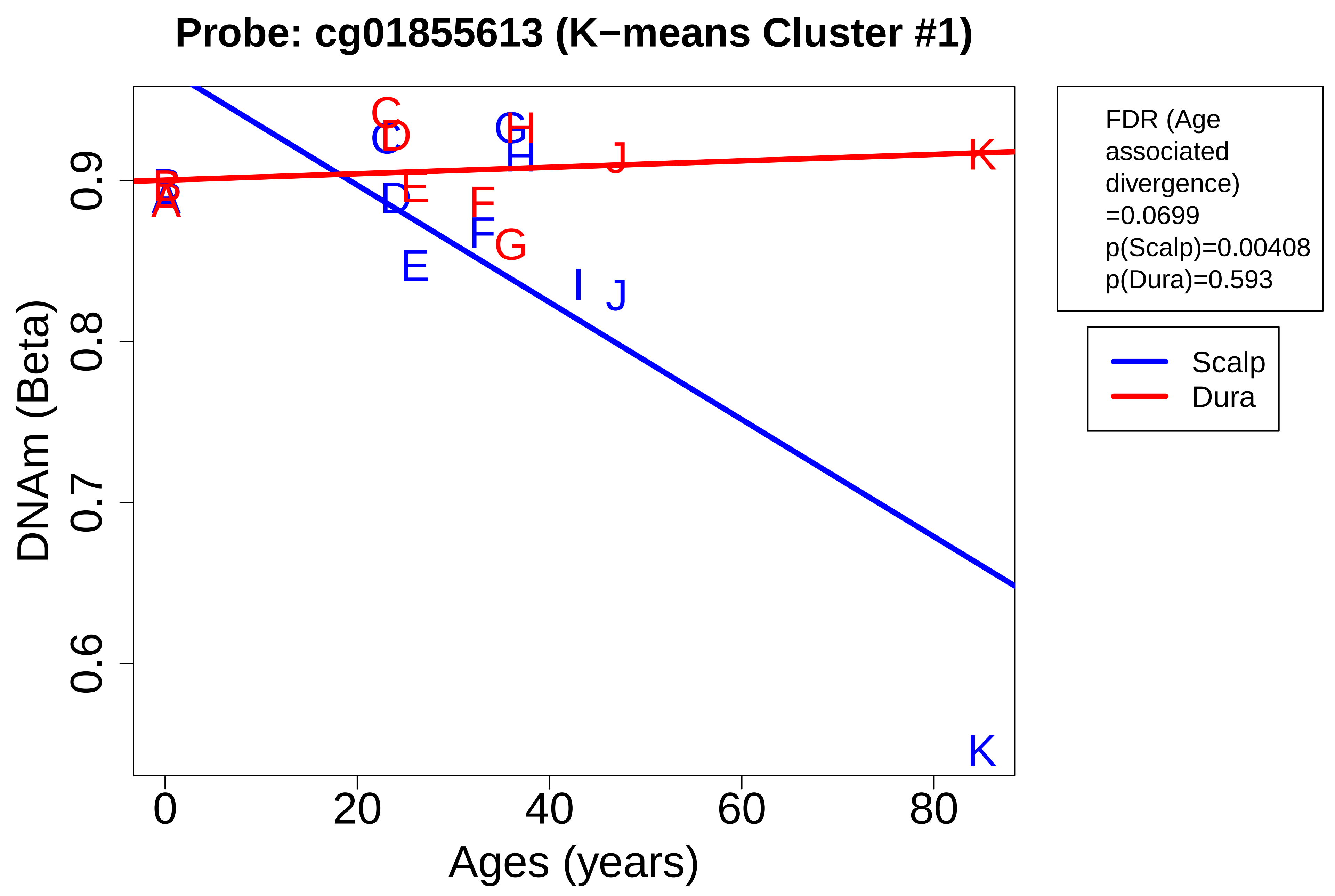

Probe: cg01855613 (K−means Cluster #1)
DNAm (Beta)
0.6
0.7
0.8
0.9
Ages (years)
0
20
40
60
80
A
B
C
D
E
F
G
H
I
J
K
FDR (Age associated divergence) =0.0699
p(Scalp)=0.00408
p(Dura)=0.593
Scalp
Dura

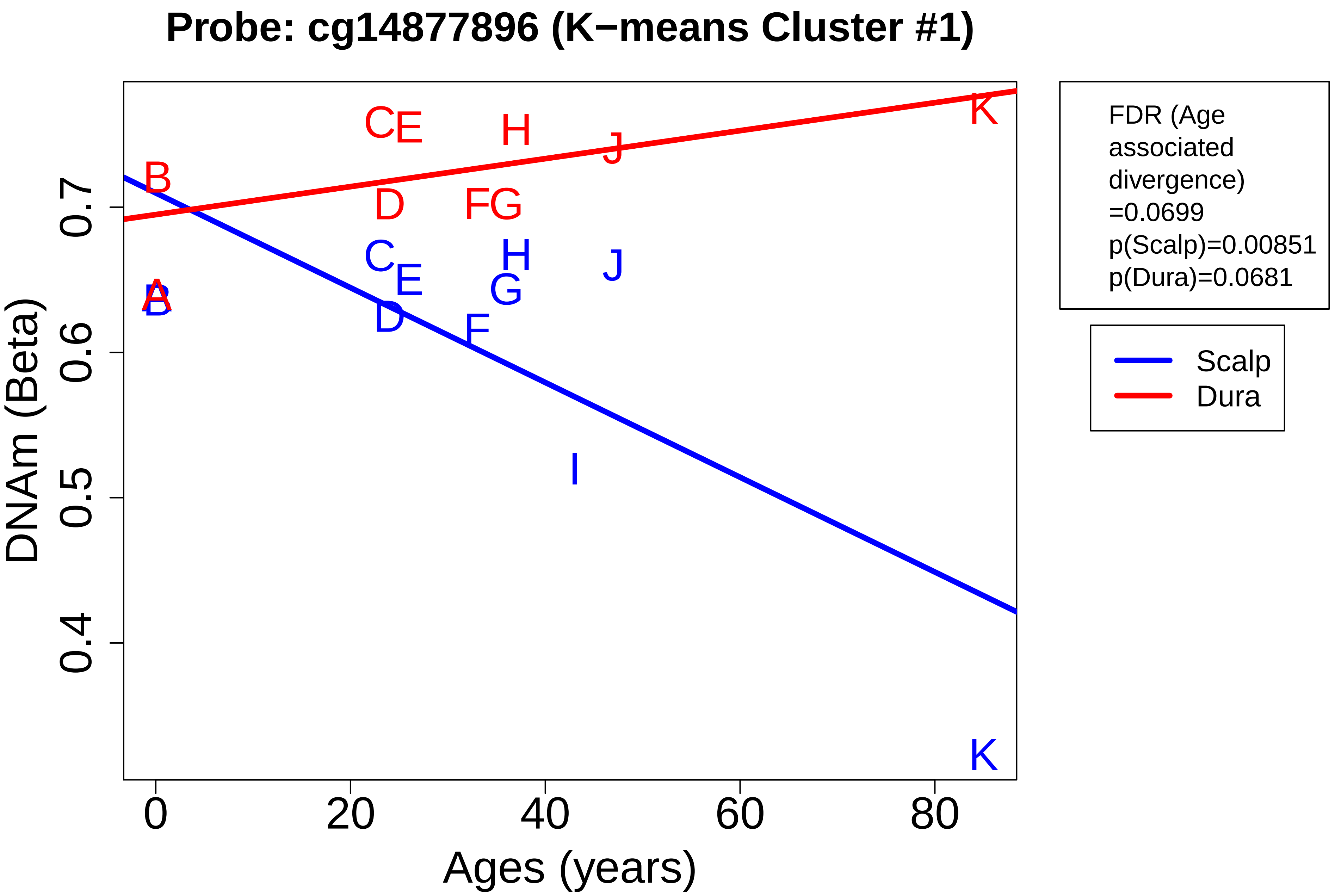

Probe: cg14877896 (K−means Cluster #1)
DNAm (Beta)
0.4
0.5
0.6
0.7
Ages (years)
0
20
40
60
80
A
B
C
D
E
F
G
H
I
J
K
FDR (Age associated divergence) =0.0699
p(Scalp)=0.00851
p(Dura)=0.0681
Scalp
Dura

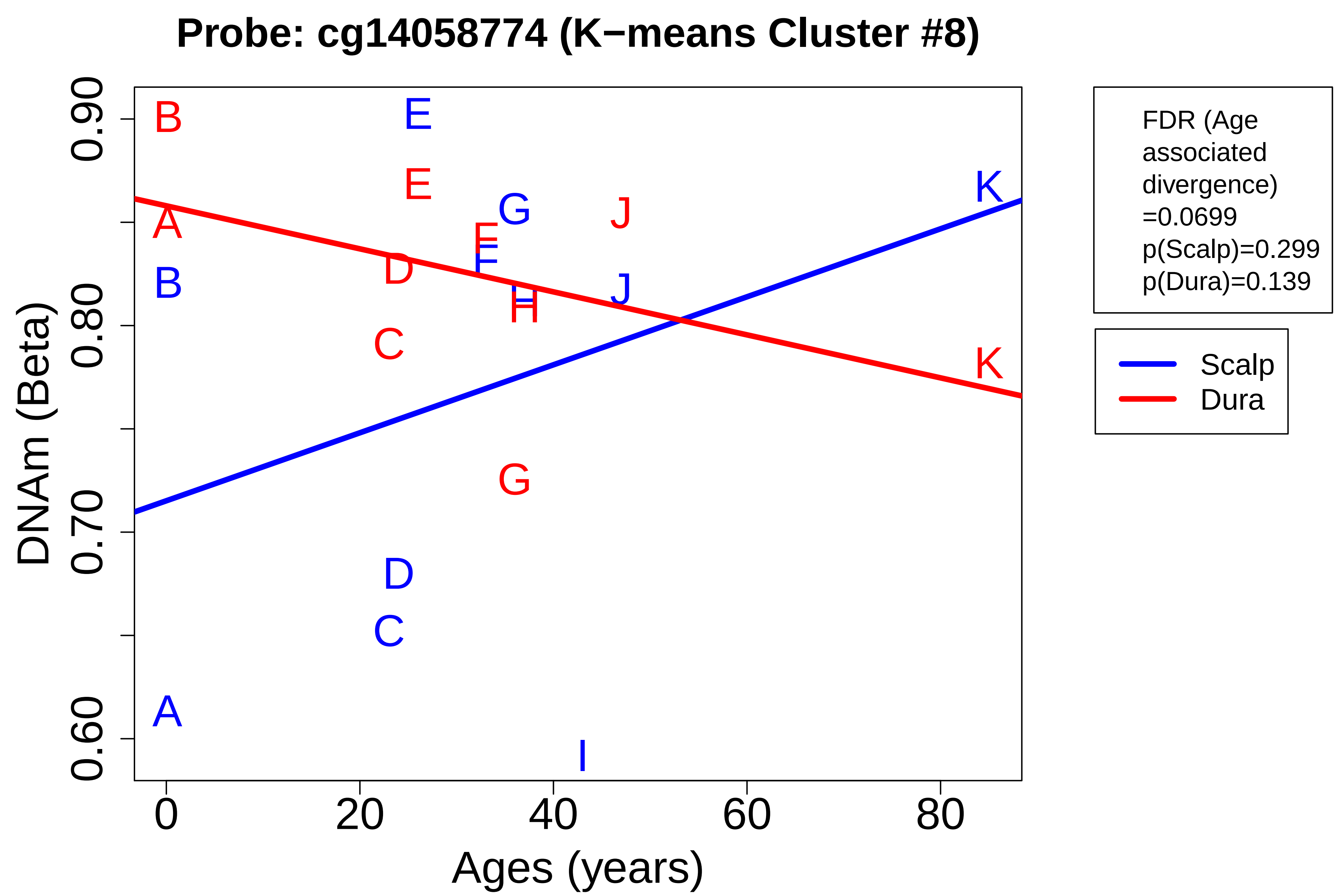
Probe: cg14058774 (K−means Cluster #8)
DNAm (Beta)
0.60
0.70
0.80
0.90
0
20
40
60
80
Ages (years)
FDR (Age associated divergence) =0.0699
p(Scalp)=0.299
p(Dura)=0.139
Scalp
Dura
A
B
C
D
E
F
G
H
I
J
K

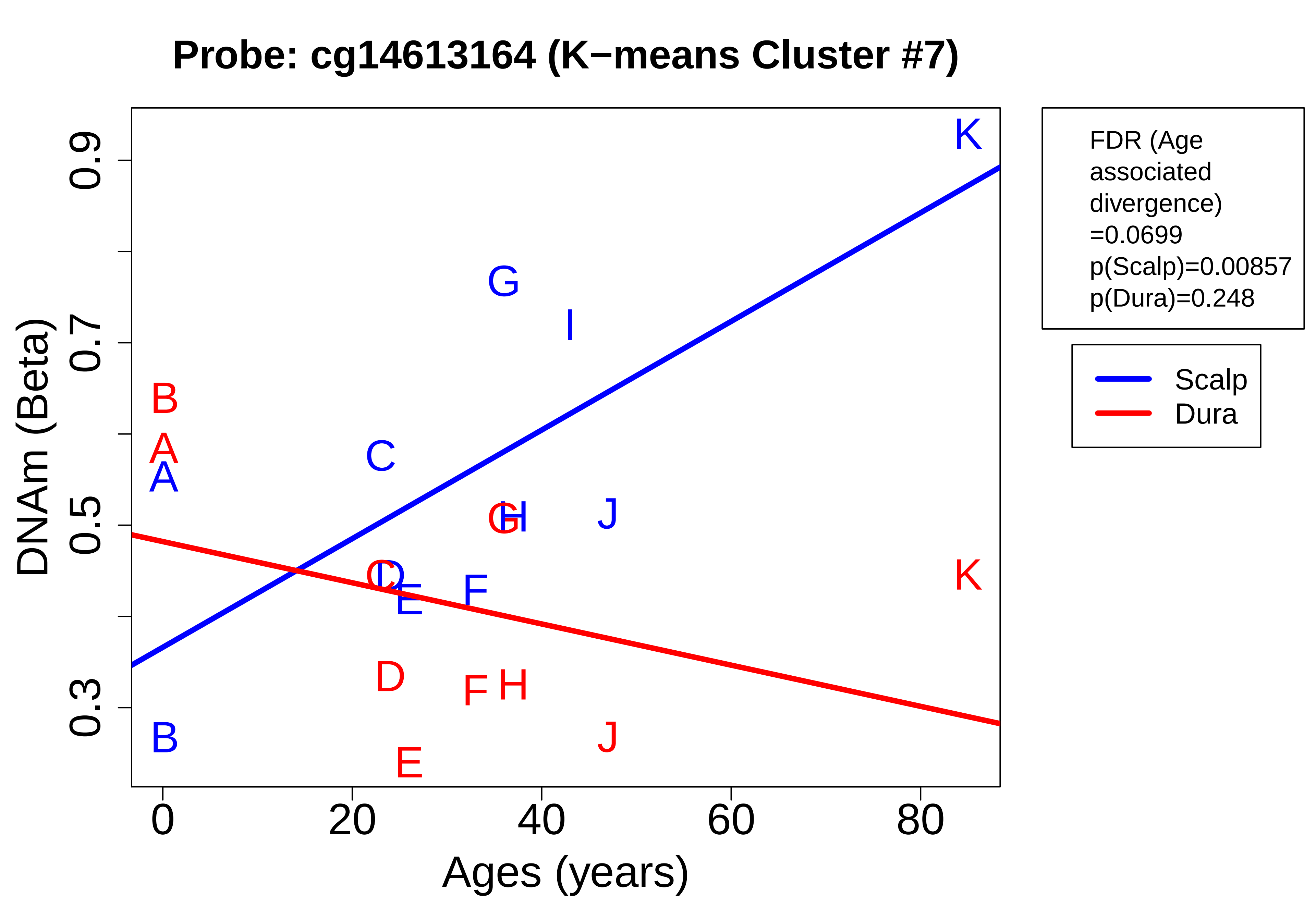

Probe: cg14613164 (K−means Cluster #7)
DNAm (Beta)
Ages (years)
0.3
0.5
0.7
0.9
0
20
40
60
80
FDR (Age associated divergence) =0.0699
p(Scalp)=0.00857
p(Dura)=0.248
Scalp
Dura
A
B
C
D
E
F
G
H
I
J
K

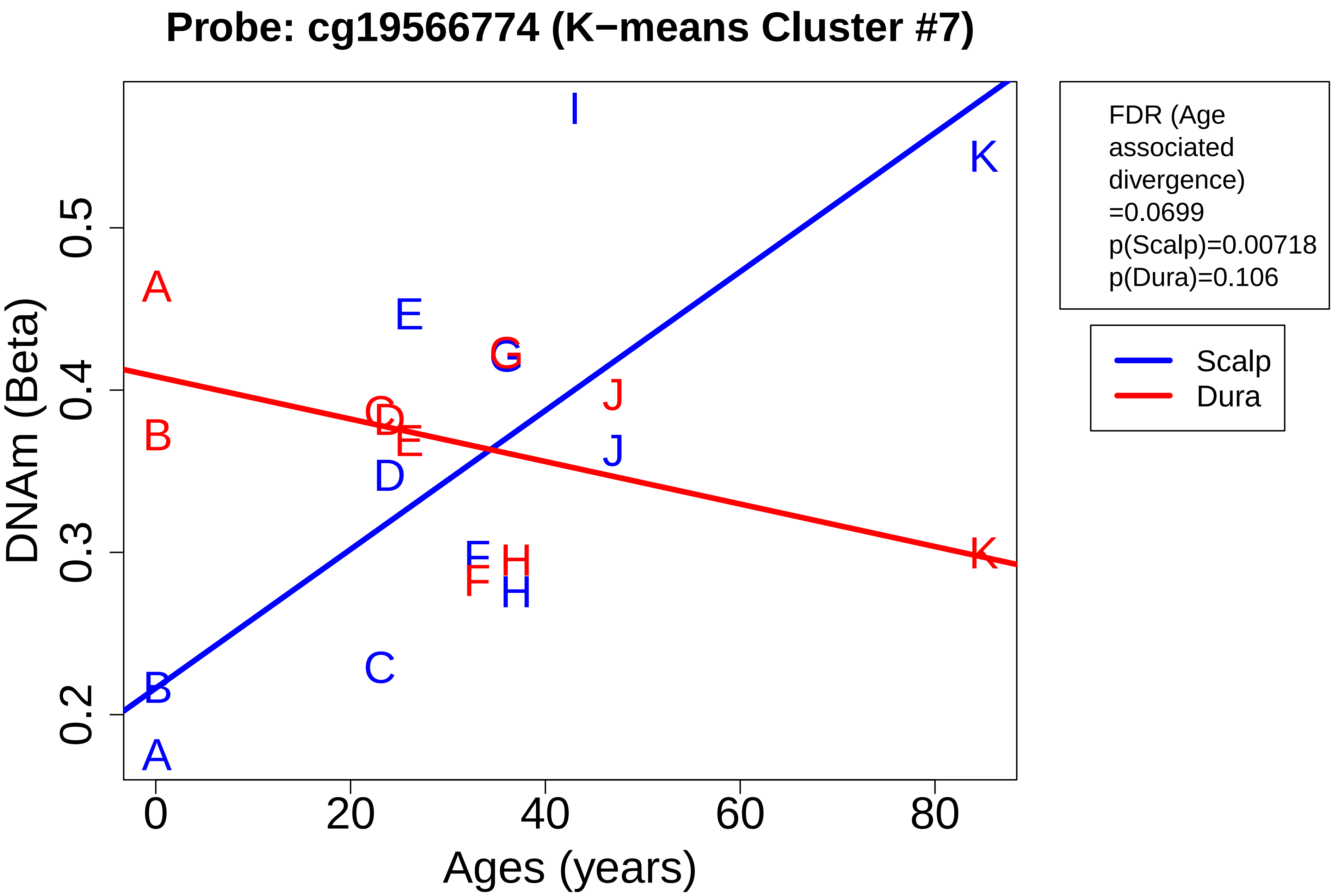
Probe: cg19566774 (K−means Cluster #7)
DNAm (Beta)
0.2
0.3
0.4
0.5
0
20
40
60
80
Ages (years)
FDR (Age associated divergence) =0.0699
p(Scalp)=0.00718
p(Dura)=0.106
Scalp
Dura
A
B
C
D
E
F
G
H
I
J
K

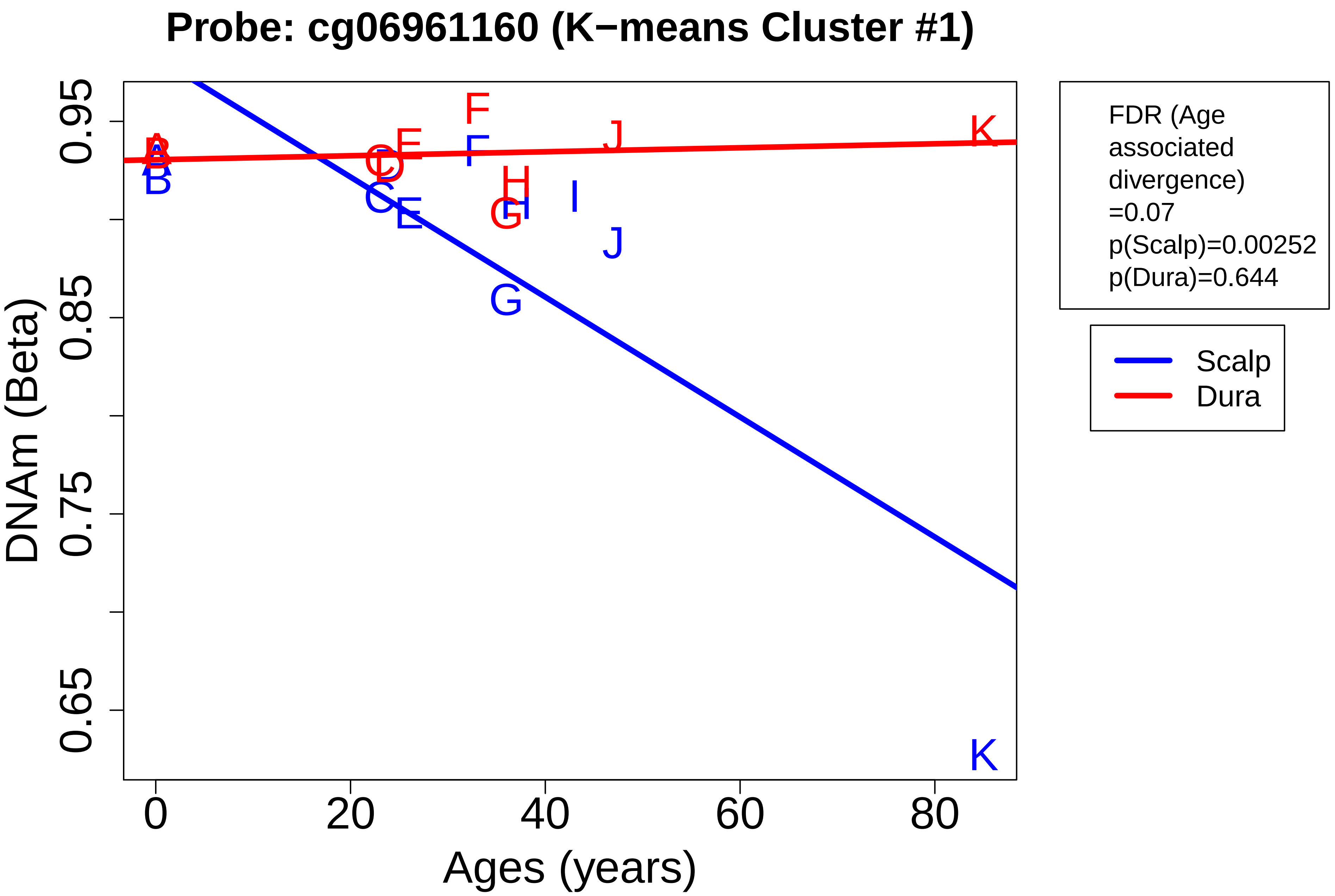
Probe: cg06961160 (K−means Cluster #1)
DNAm (Beta)
0.65
0.75
0.85
0.95
Ages (years)
0
20
40
60
80
FDR (Age associated divergence) =0.07
p(Scalp)=0.00252
p(Dura)=0.644
Scalp
Dura

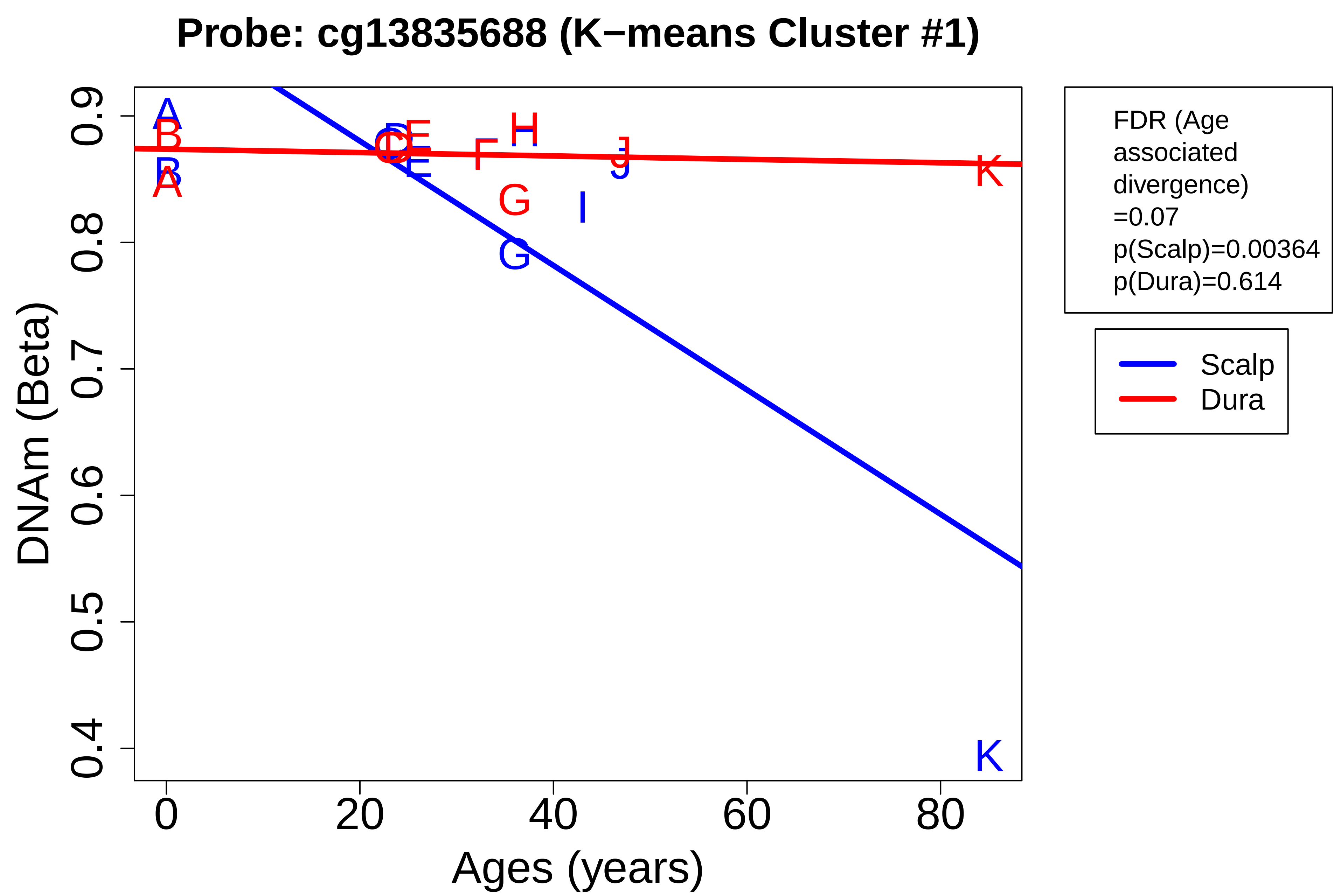

Probe: cg13835688 (K−means Cluster #1)
DNAm (Beta)
0.4
0.5
0.6
0.7
0.8
0.9
0
20
40
60
80
Ages (years)
FDR (Age associated divergence) =0.07
p(Scalp)=0.00364
p(Dura)=0.614
Scalp
Dura

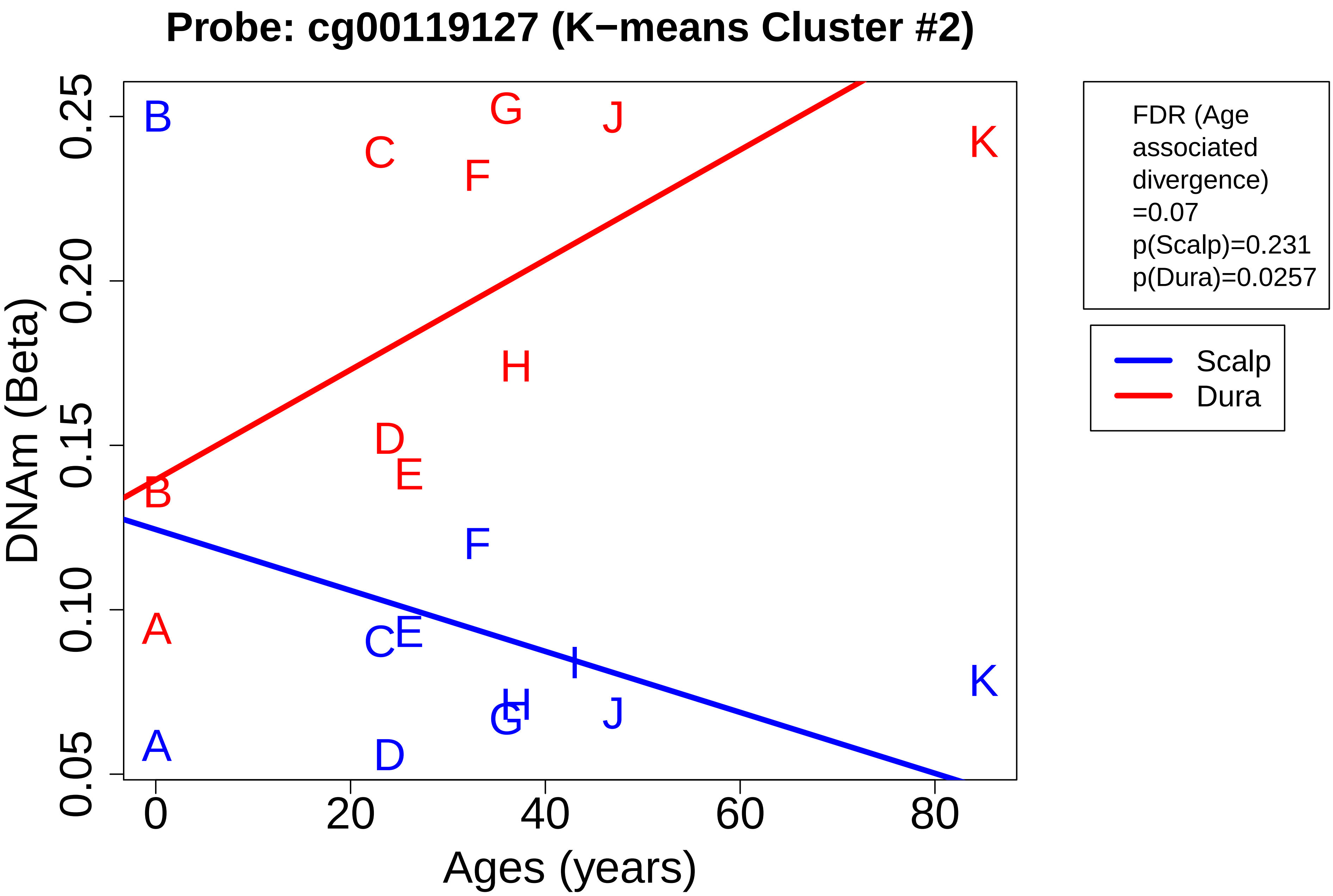

Probe: cg00119127 (K−means Cluster #2)
FDR (Age associated divergence) =0.07
p(Scalp)=0.231
p(Dura)=0.0257
Scalp
Dura
DNAm (Beta)
0.05
0.10
0.15
0.20
0.25
0
20
40
60
80
Ages (years)

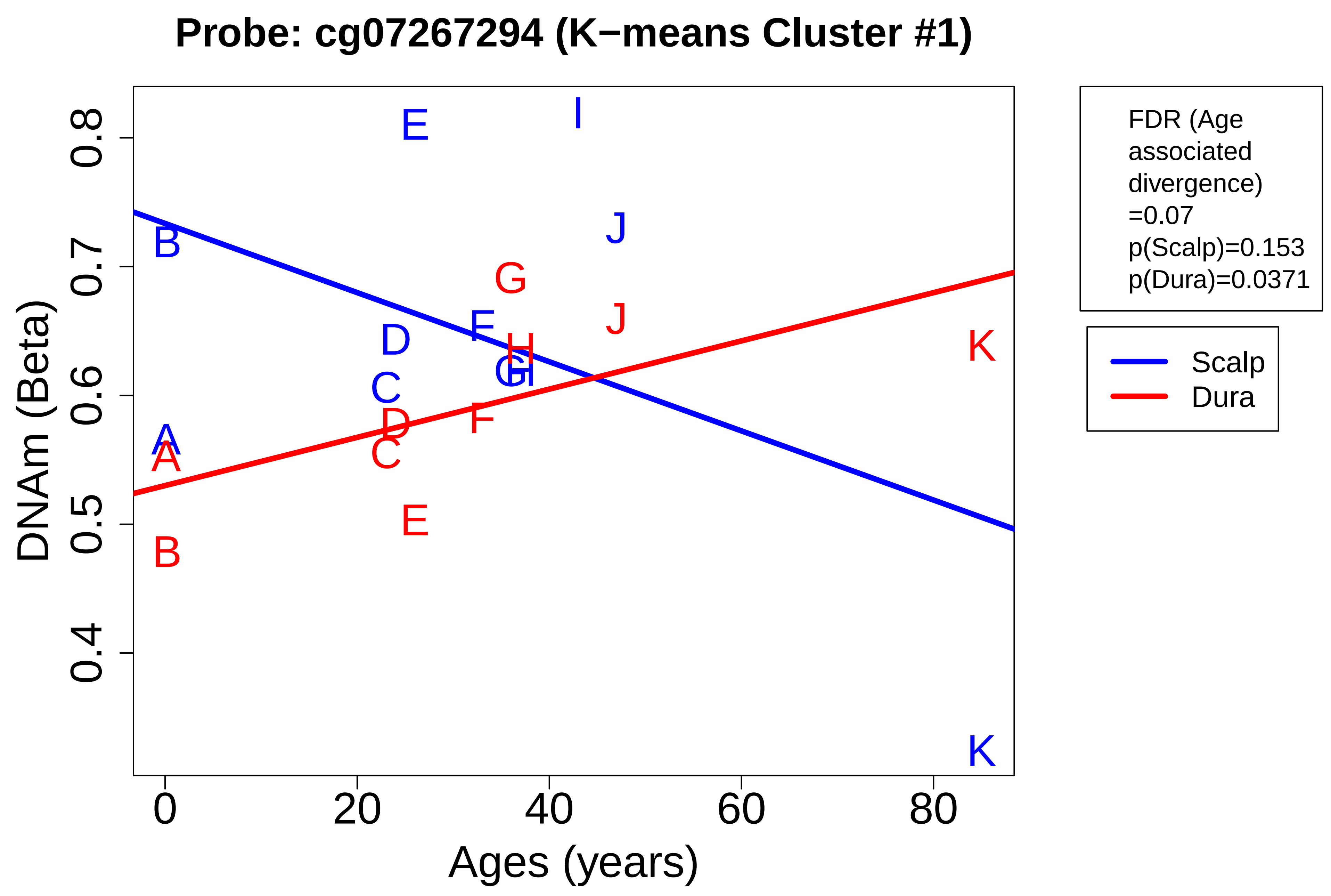

Probe: cg07267294 (K−means Cluster #1)
DNAm (Beta)
0.4
0.5
0.6
0.7
0.8
0
20
40
60
80
Ages (years)
FDR (Age associated divergence) =0.07
p(Scalp)=0.153
p(Dura)=0.0371
Scalp
Dura
A
B
C
D
E
F
G
H
I
J
K

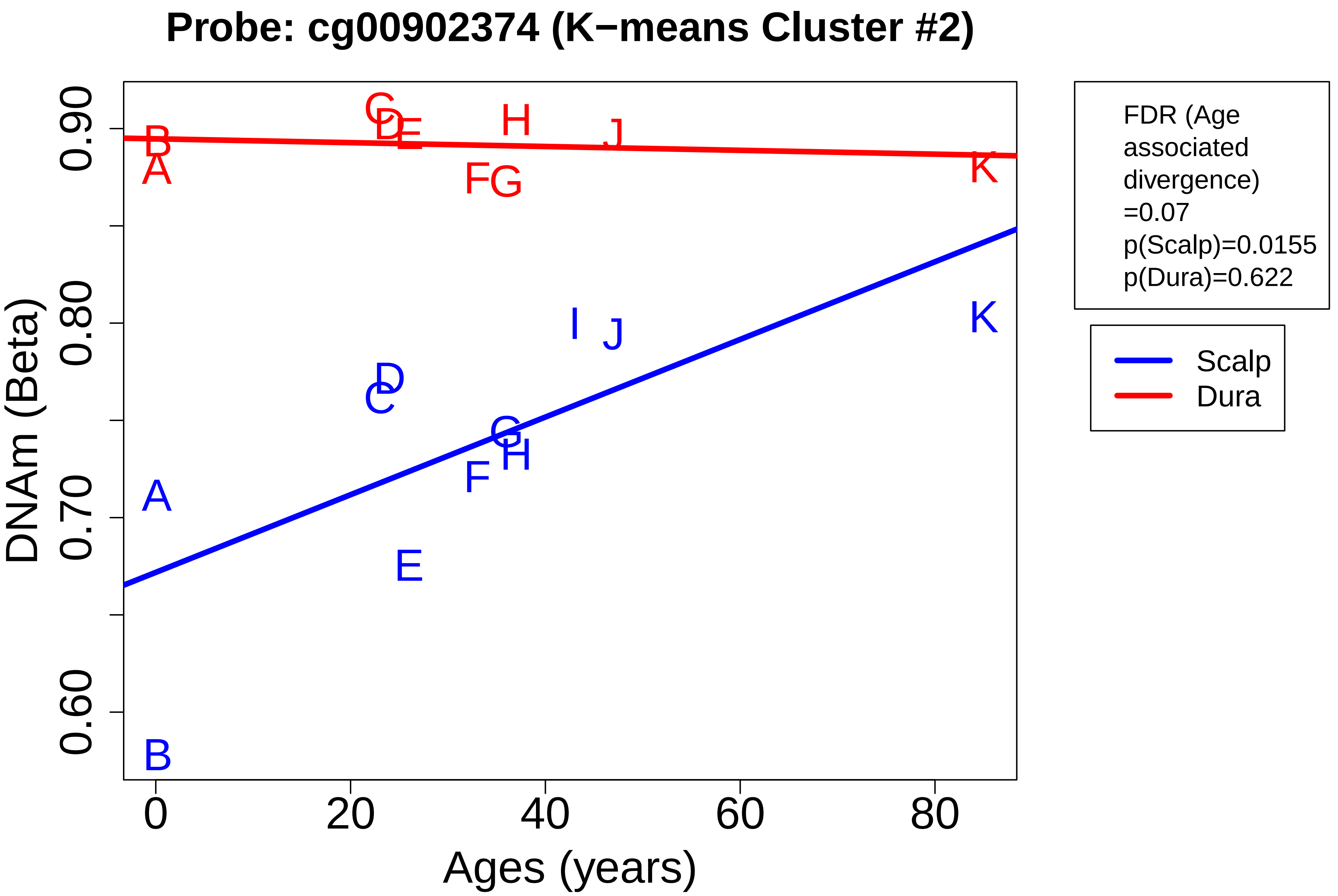

Probe: cg00902374 (K−means Cluster #2)
DNAm (Beta)
0.60
0.70
0.80
0.90
0
20
40
60
80
Ages (years)
FDR (Age associated divergence) =0.07
p(Scalp)=0.0155
p(Dura)=0.622
Scalp
Dura
A
B
C
D
E
F
G
H
I
J
K

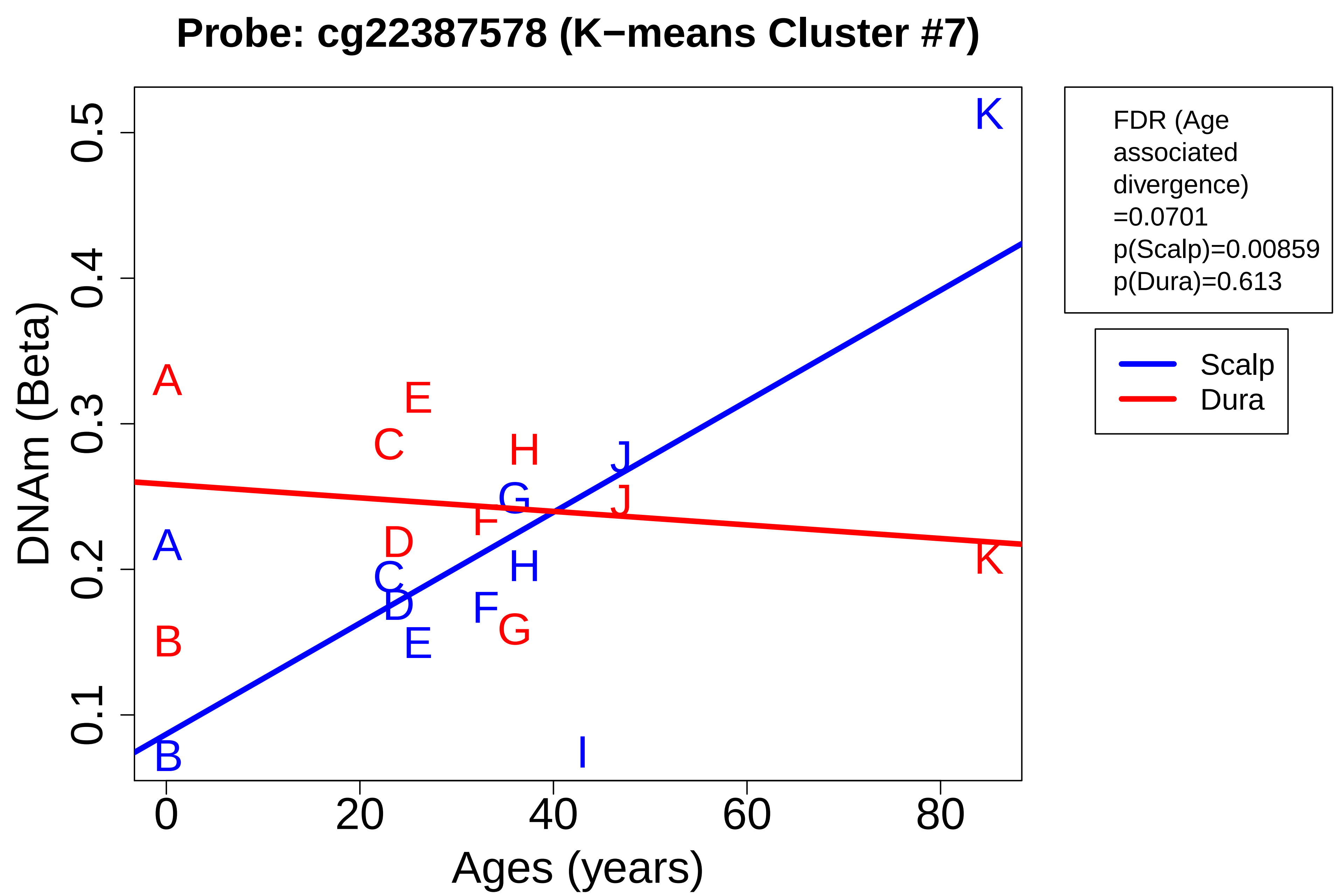

Probe: cg22387578 (K−means Cluster #7)
DNAm (Beta)
0.1
0.2
0.3
0.4
0.5
0
20
40
60
80
Ages (years)
A
B
C
D
E
F
G
H
I
J
K
A
B
C
D
E
F
G
H
J
K
FDR (Age associated divergence) =0.0701
p(Scalp)=0.00859
p(Dura)=0.613
Scalp
Dura

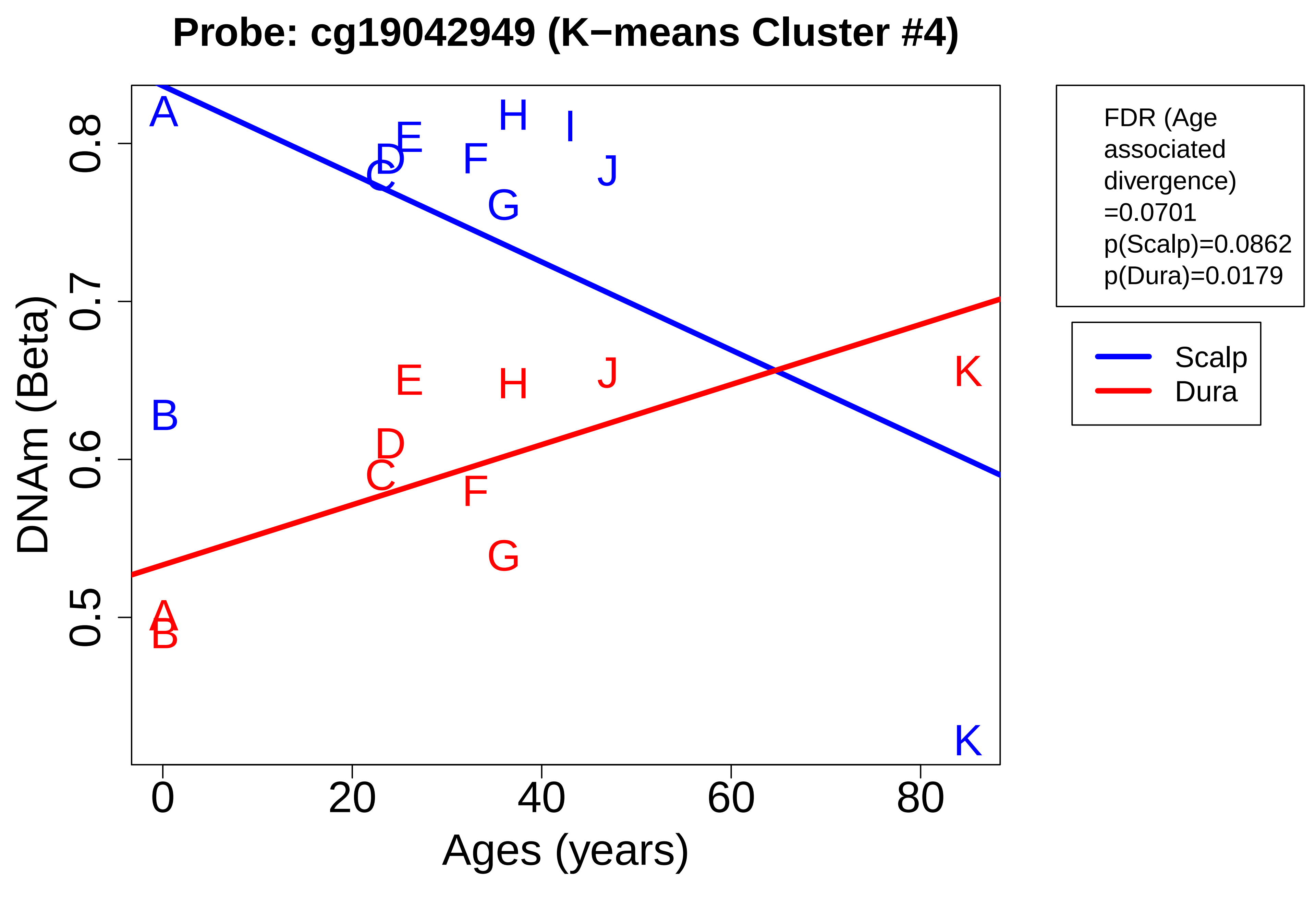

Probe: cg19042949 (K−means Cluster #4)
DNAm (Beta)
0.5
0.6
0.7
0.8
0
20
40
60
80
Ages (years)
A
B
C
D
E
F
G
H
I
J
K
FDR (Age associated divergence) =0.0701
p(Scalp)=0.0862
p(Dura)=0.0179
Scalp
Dura

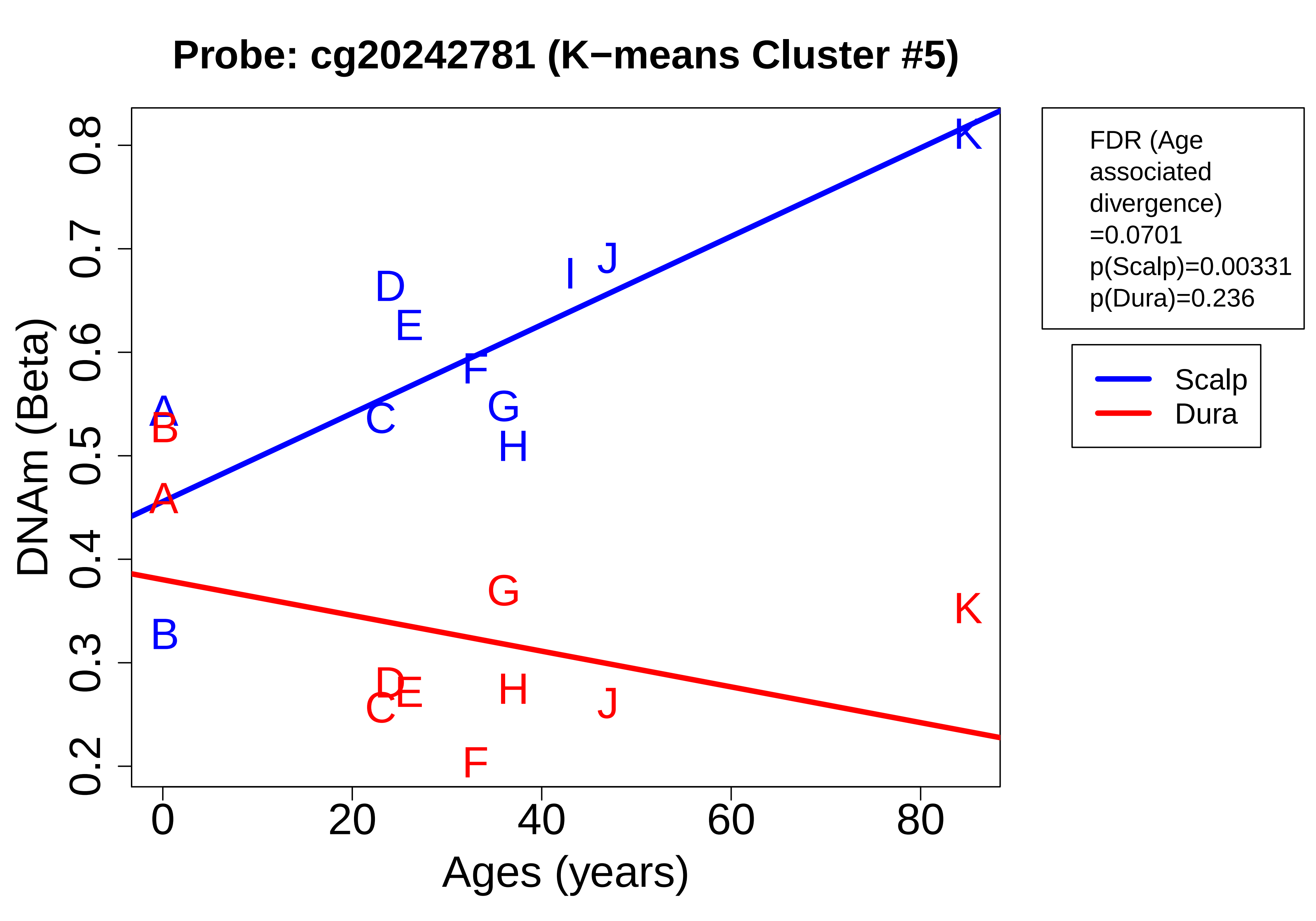

Probe: cg20242781 (K−means Cluster #5)
DNAm (Beta)
0.2
0.3
0.4
0.5
0.6
0.7
0.8
0
20
40
60
80
Ages (years)
A
B
C
D
E
F
G
H
I
J
K
A
B
C
D
E
F
G
H
J
K
FDR (Age associated divergence) =0.0701
p(Scalp)=0.00331
p(Dura)=0.236
Scalp
Dura

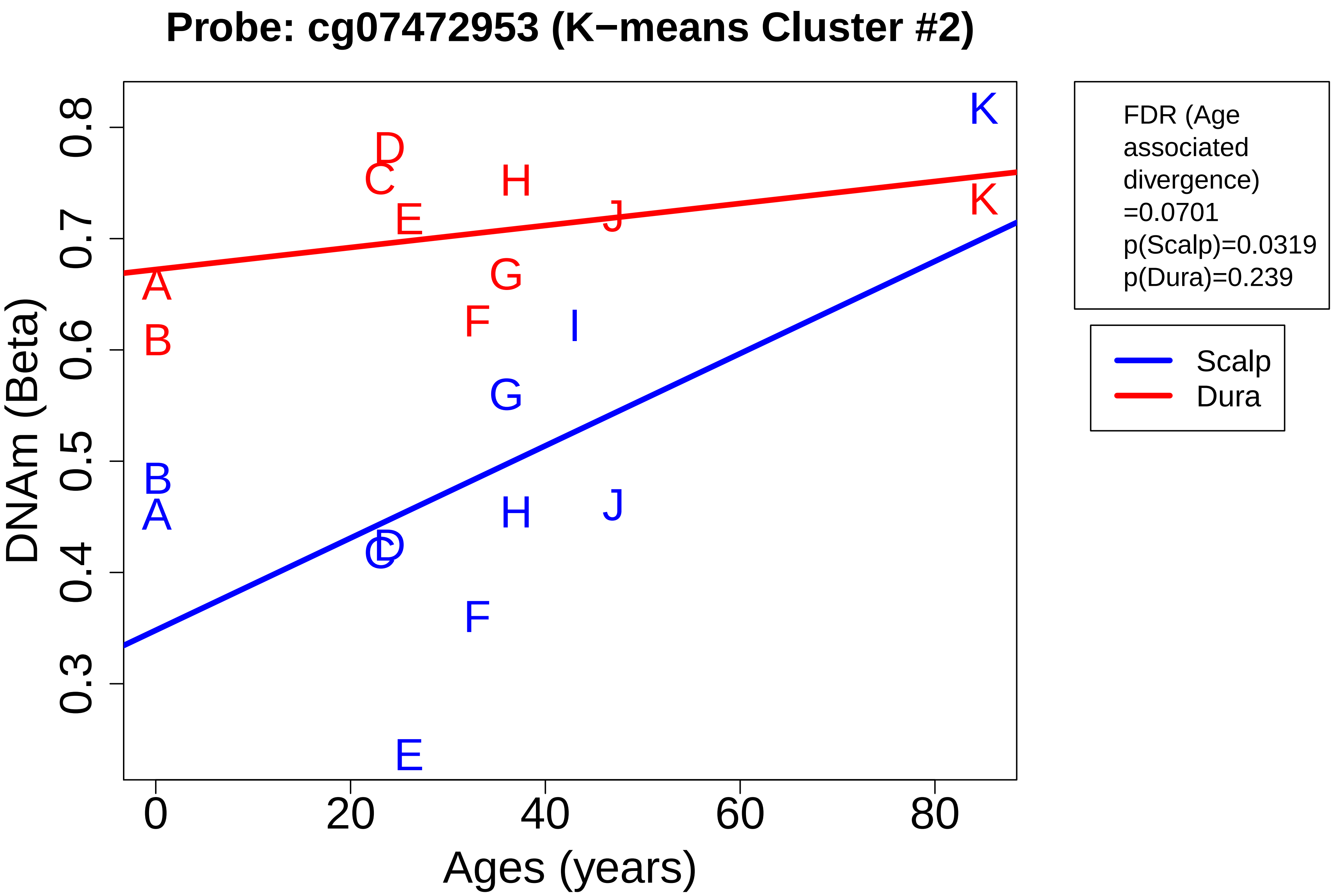

Probe: cg07472953 (K−means Cluster #2)
DNAm (Beta)
0.3
0.4
0.5
0.6
0.7
0.8
0
20
40
60
80
Ages (years)
A
B
C
D
E
F
G
H
I
J
K
FDR (Age associated divergence) =0.0701
p(Scalp)=0.0319
p(Dura)=0.239
Scalp
Dura

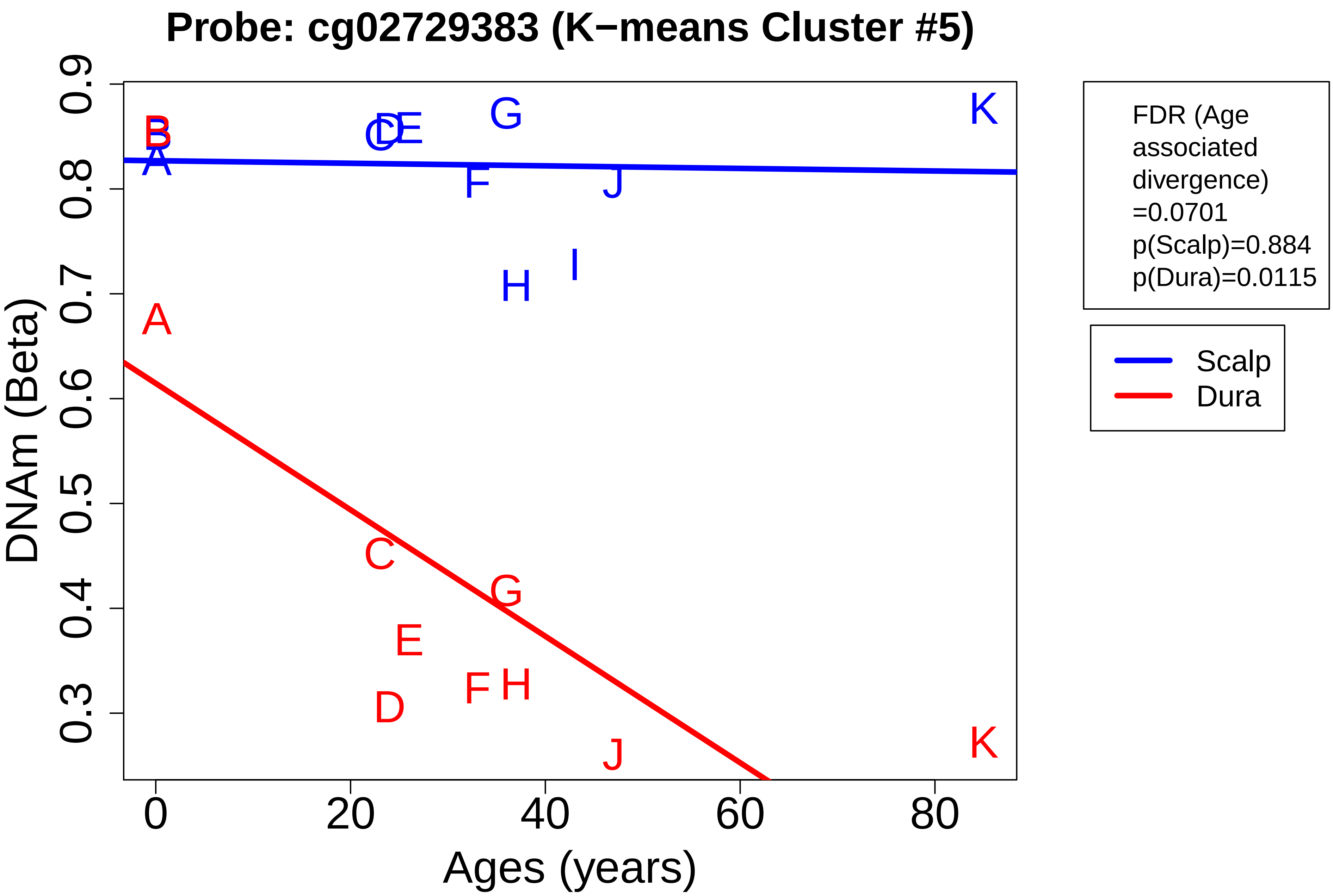
Probe: cg02729383 (K−means Cluster #5)
DNAm (Beta)
Ages (years)
0.3
0.4
0.5
0.6
0.7
0.8
0.9
0
20
40
60
80
FDR (Age associated divergence) =0.0701
p(Scalp)=0.884
p(Dura)=0.0115
Scalp
Dura

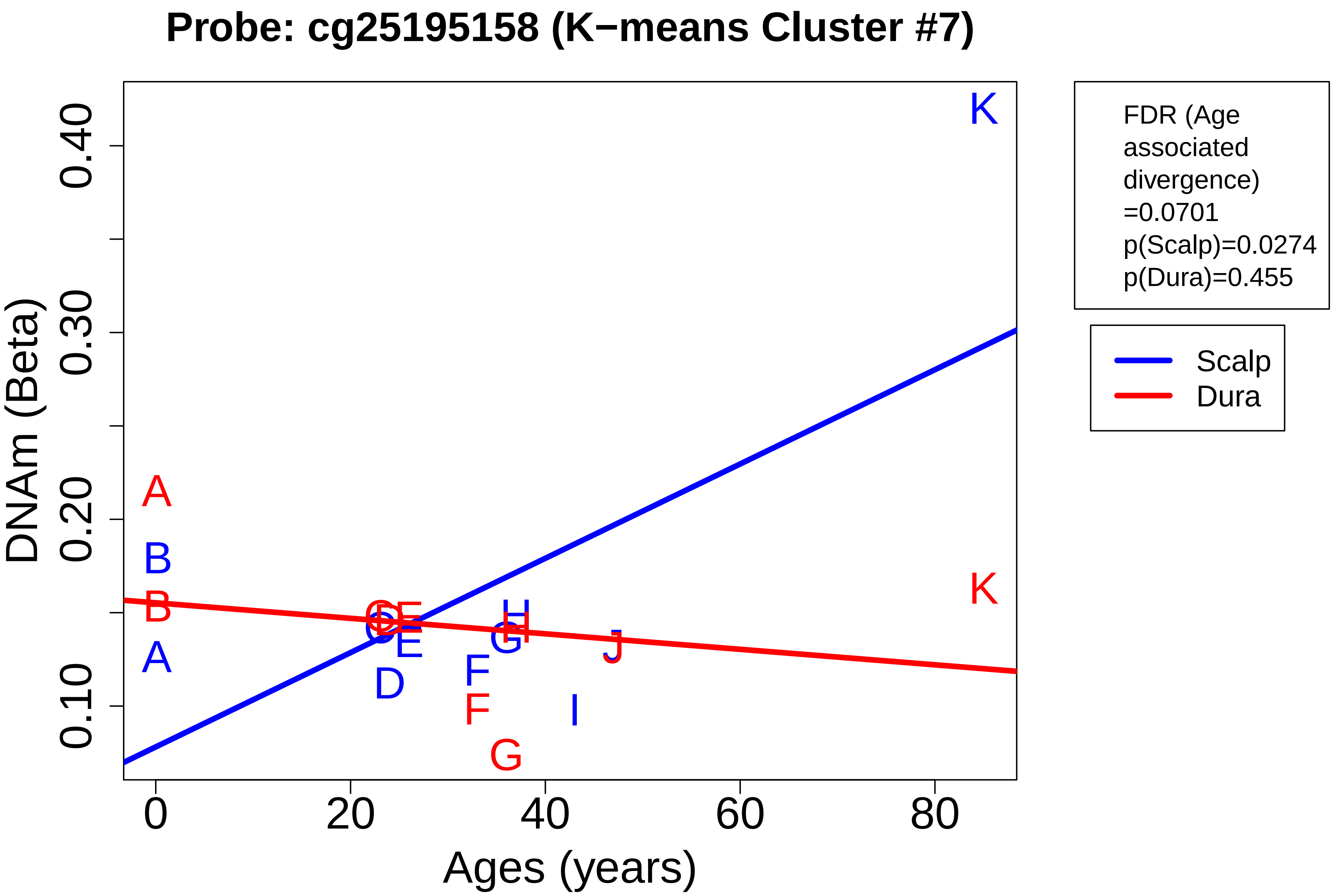

Probe: cg25195158 (K−means Cluster #7)
DNAm (Beta)
0.10
0.20
0.30
0.40
Ages (years)
0
20
40
60
80
A
B
C
D
E
F
G
H
I
J
K
FDR (Age associated divergence) =0.0701
p(Scalp)=0.0274
p(Dura)=0.455
Scalp
Dura

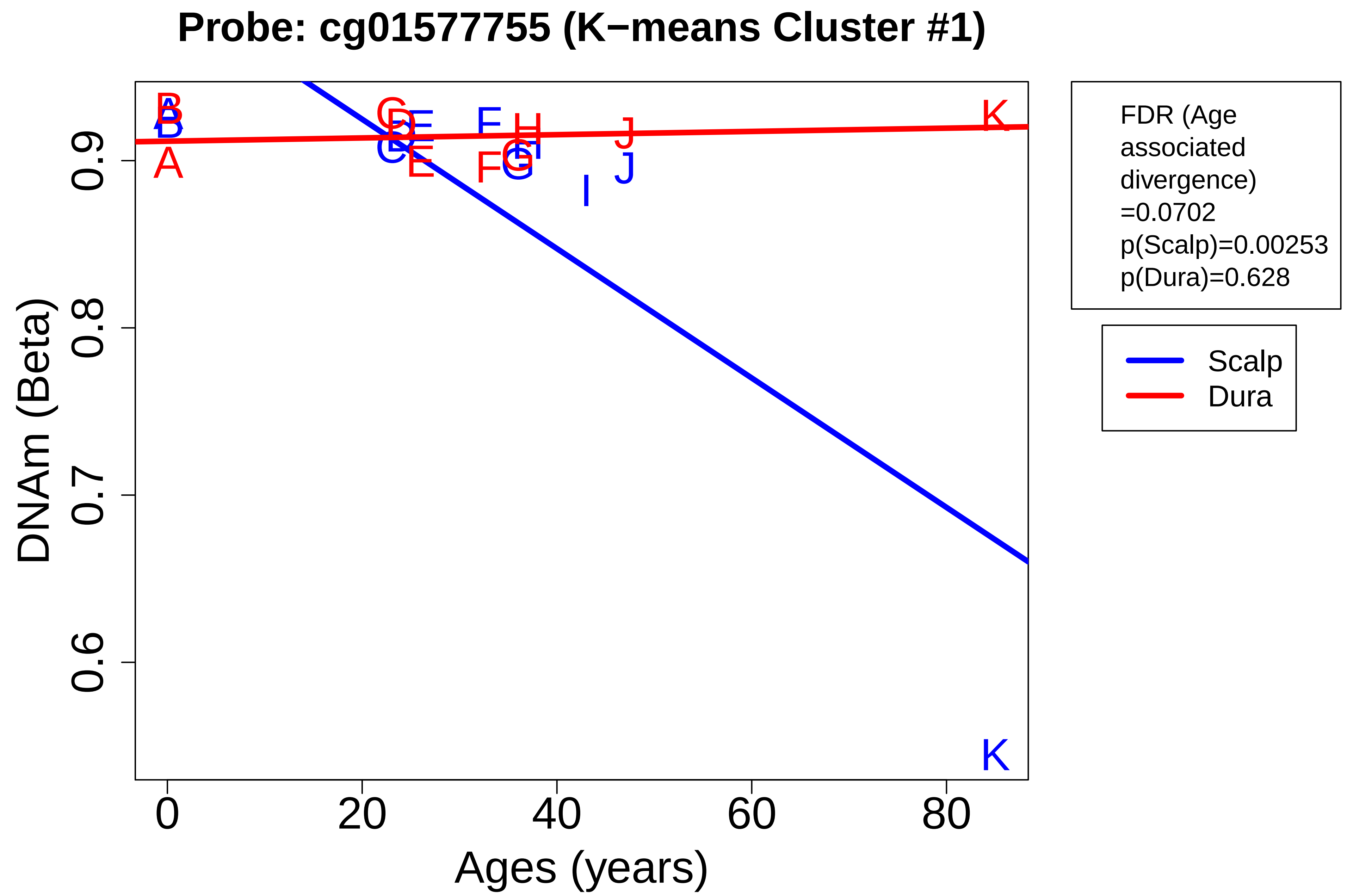

Probe: cg01577755 (K−means Cluster #1)
DNAm (Beta)
0.6
0.7
0.8
0.9
Ages (years)
0
20
40
60
80
FDR (Age associated divergence) =0.0702
p(Scalp)=0.00253
p(Dura)=0.628
Scalp
Dura
A
B
C
D
E
F
G
H
I
J
K

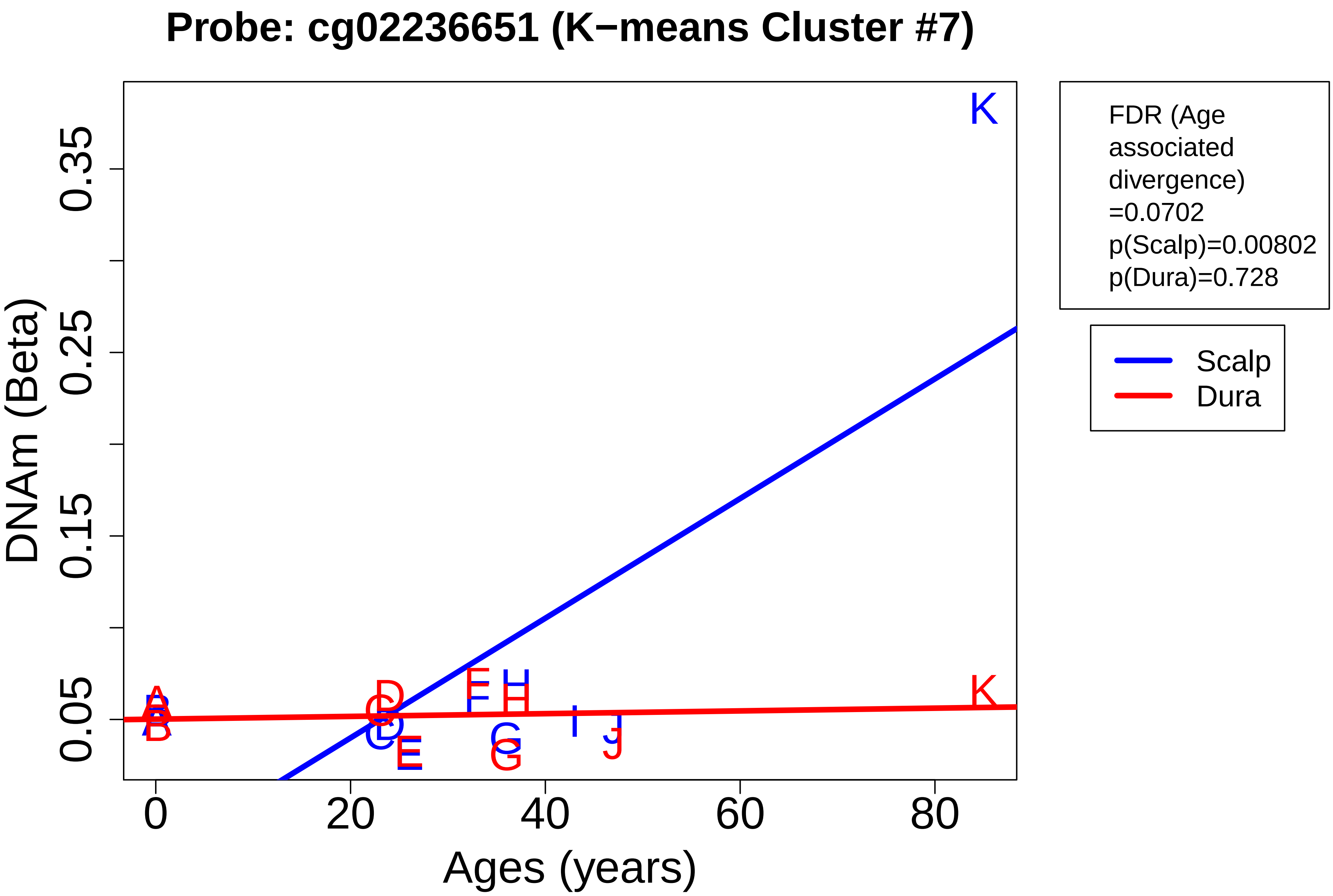

Probe: cg02236651 (K−means Cluster #7)
DNAm (Beta)
0.05
0.15
0.25
0.35
Ages (years)
0
20
40
60
80
FDR (Age associated divergence) =0.0702
p(Scalp)=0.00802
p(Dura)=0.728
Scalp
Dura

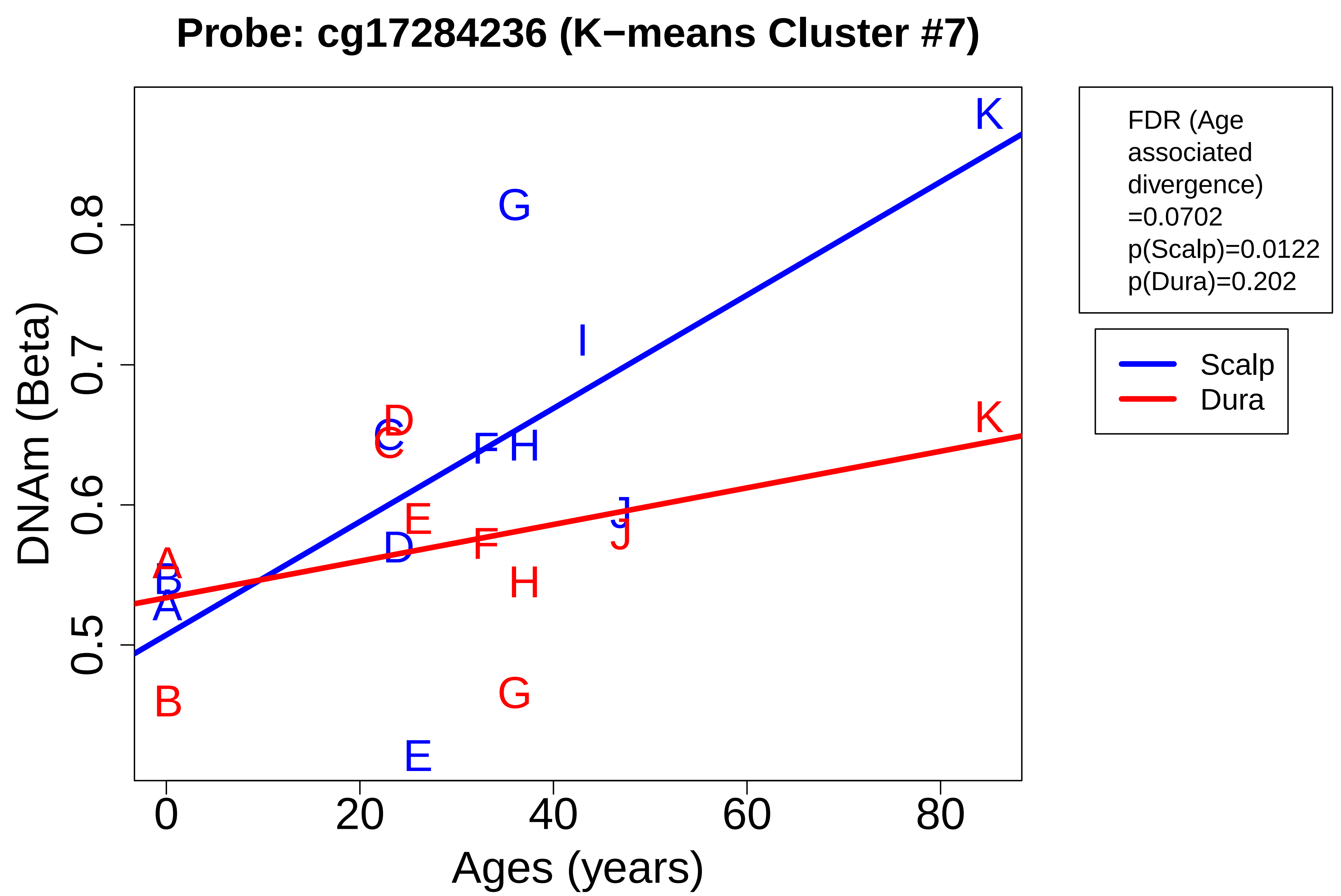

Probe: cg17284236 (K−means Cluster #7)
DNAm (Beta)
Ages (years)
0.5
0.6
0.7
0.8
0
20
40
60
80
FDR (Age associated divergence) =0.0702
p(Scalp)=0.0122
p(Dura)=0.202
Scalp
Dura

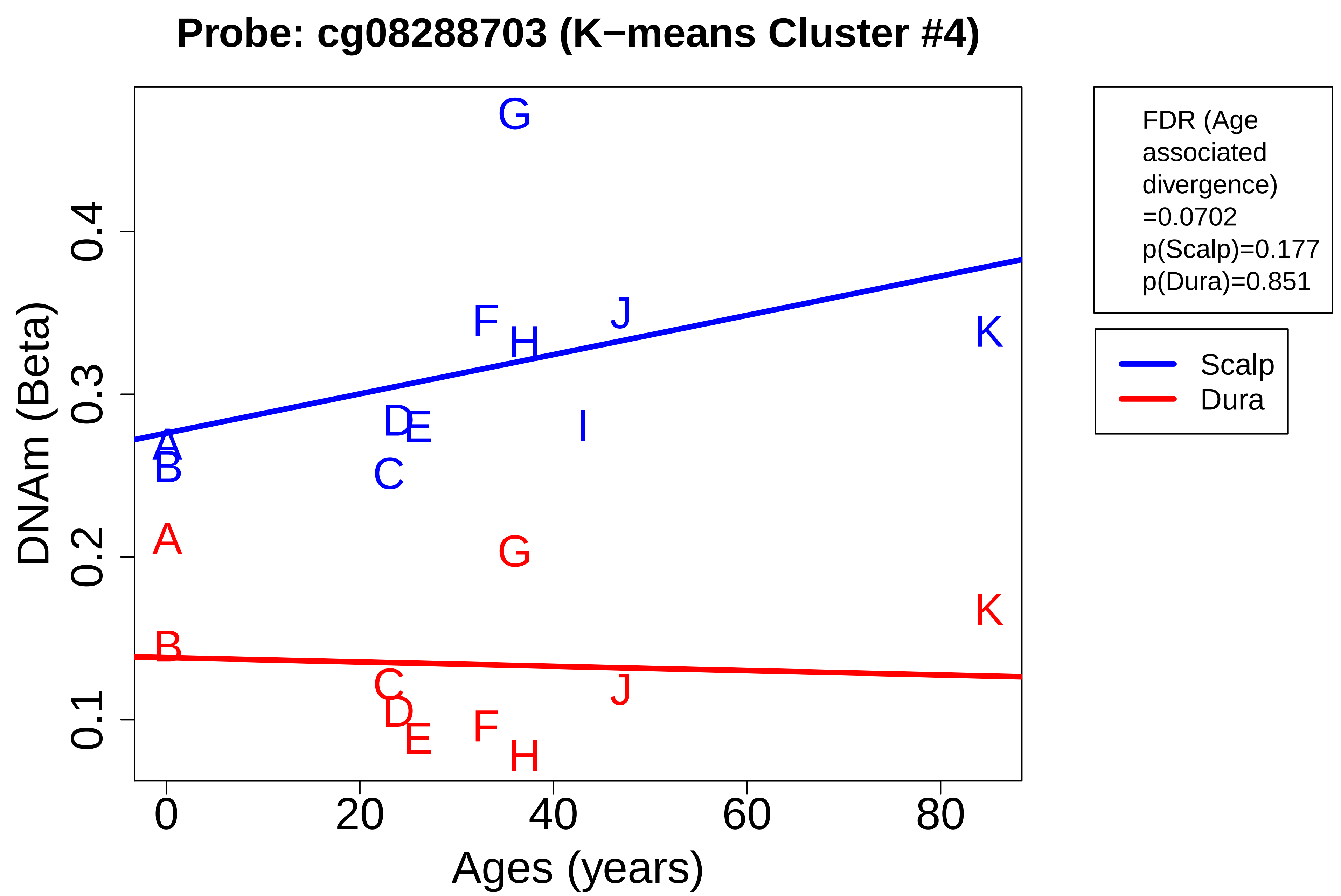

Probe: cg08288703 (K−means Cluster #4)
DNAm (Beta)
0.1
0.2
0.3
0.4
0
20
40
60
80
Ages (years)
FDR (Age associated divergence) =0.0702
p(Scalp)=0.177
p(Dura)=0.851
Scalp
Dura
A
B
C
D
E
F
G
H
I
J
K

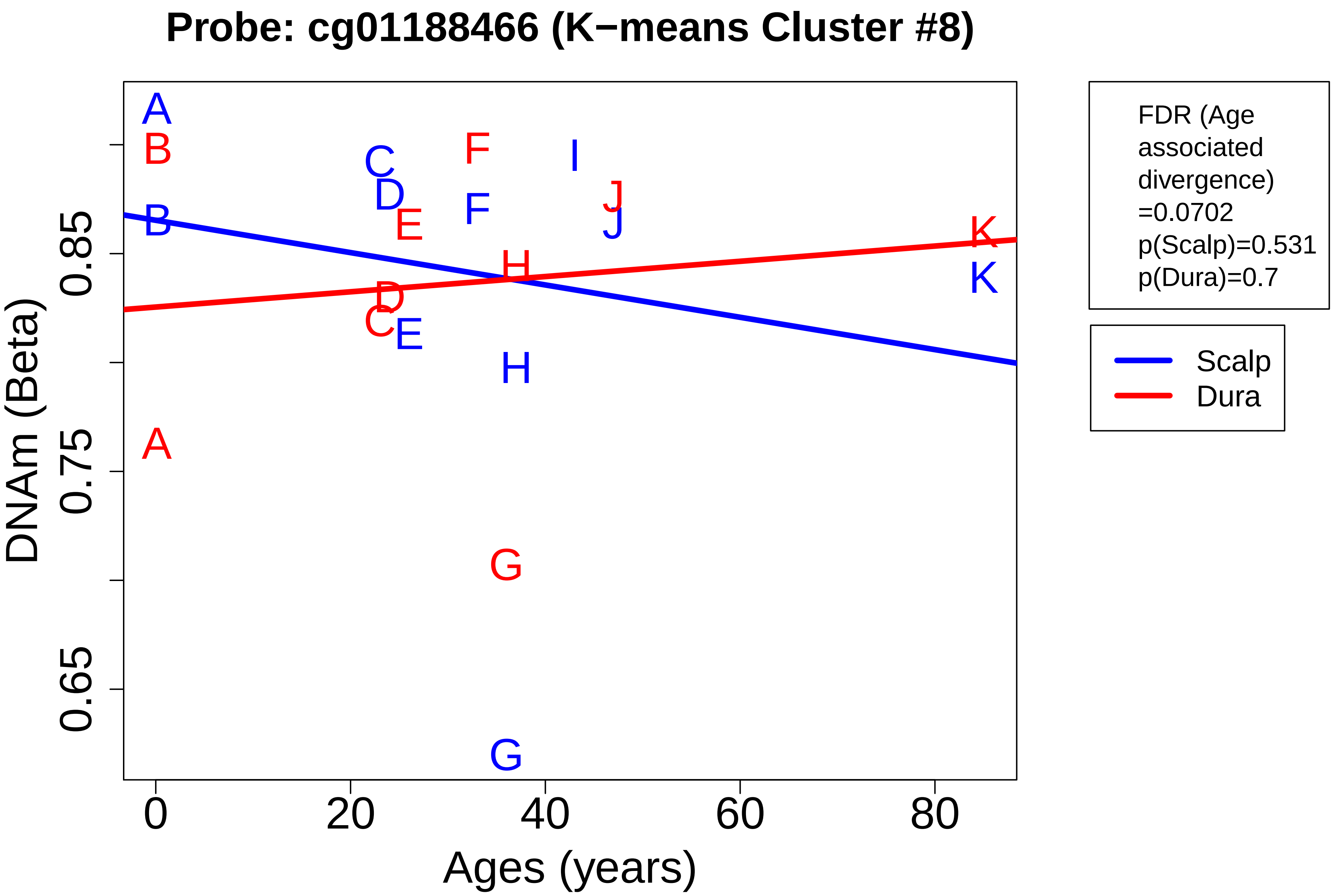

Probe: cg01188466 (K−means Cluster #8)
DNAm (Beta)
0.65
0.75
0.85
0
20
40
60
80
Ages (years)
A
B
C
D
E
F
G
H
I
J
K
FDR (Age associated divergence) =0.0702
p(Scalp)=0.531
p(Dura)=0.7
Scalp
Dura

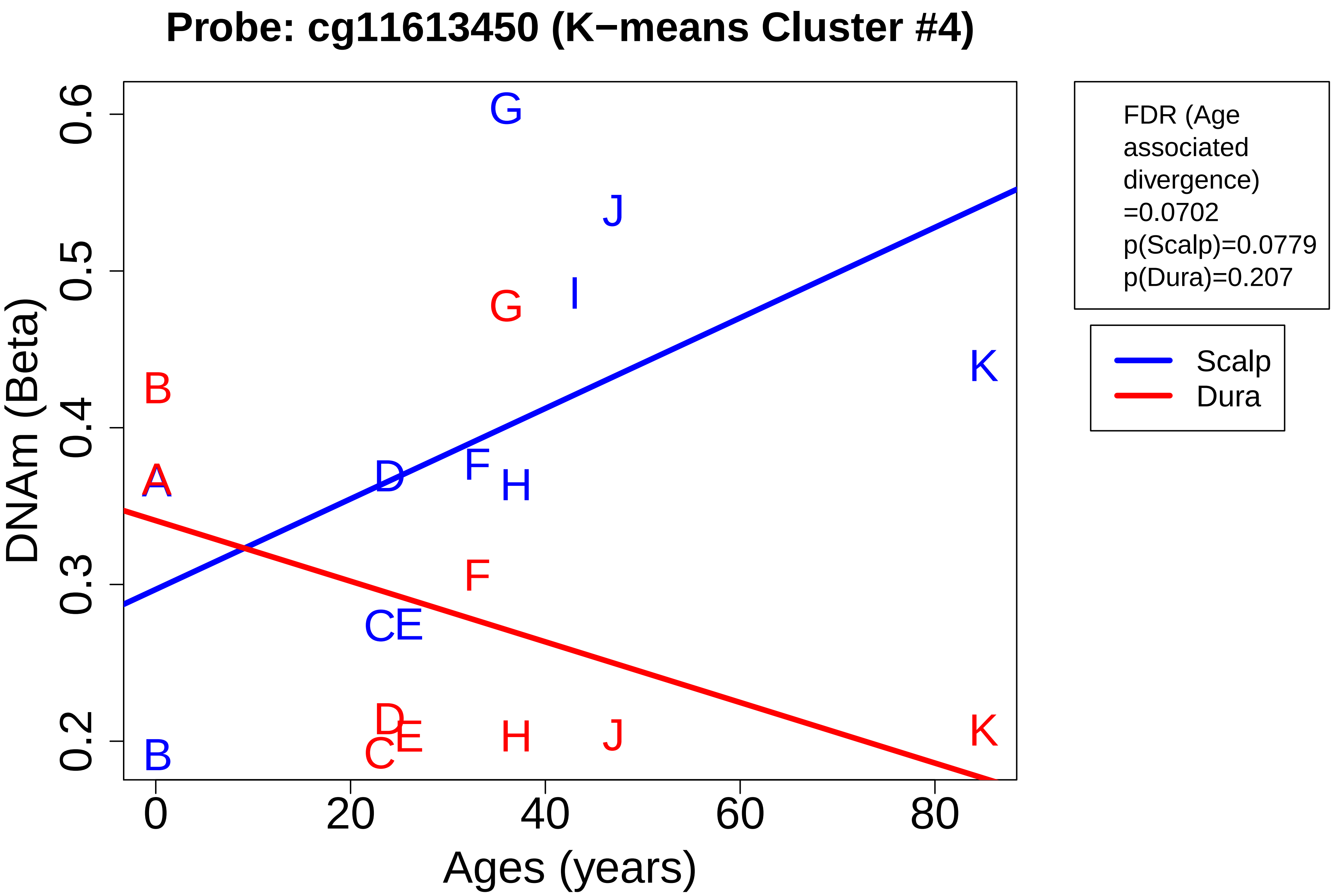

Probe: cg11613450 (K−means Cluster #4)
DNAm (Beta)
Ages (years)
0.2
0.3
0.4
0.5
0.6
0
20
40
60
80
FDR (Age associated divergence) =0.0702
p(Scalp)=0.0779
p(Dura)=0.207
Scalp
Dura
A
B
C
D
E
F
G
H
I
J
K

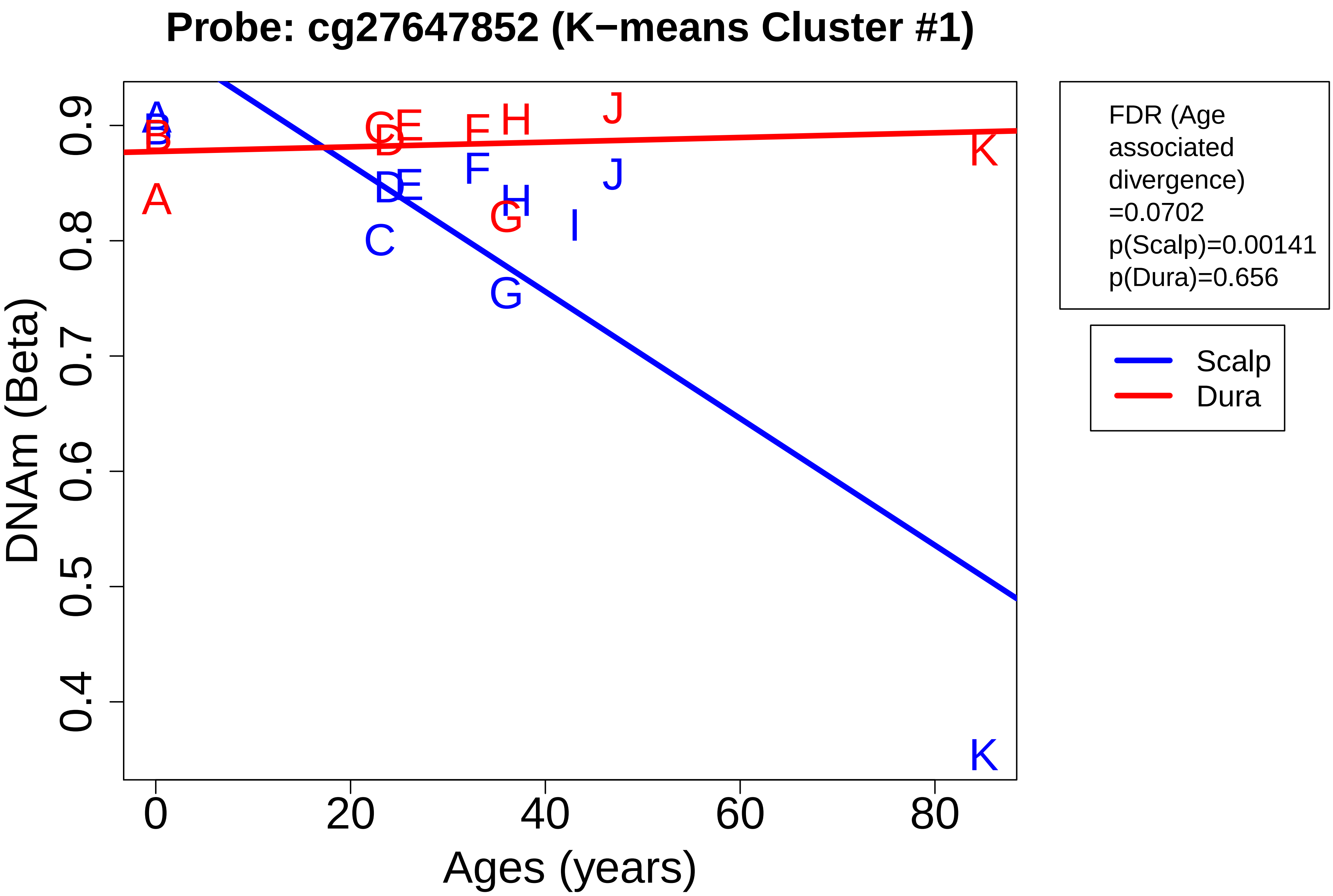

Probe: cg27647852 (K−means Cluster #1)
DNAm (Beta)
0.4
0.5
0.6
0.7
0.8
0.9
0
20
40
60
80
Ages (years)
A
B
C
D
E
F
G
H
I
J
K
FDR (Age associated divergence) =0.0702
p(Scalp)=0.00141
p(Dura)=0.656
Scalp
Dura

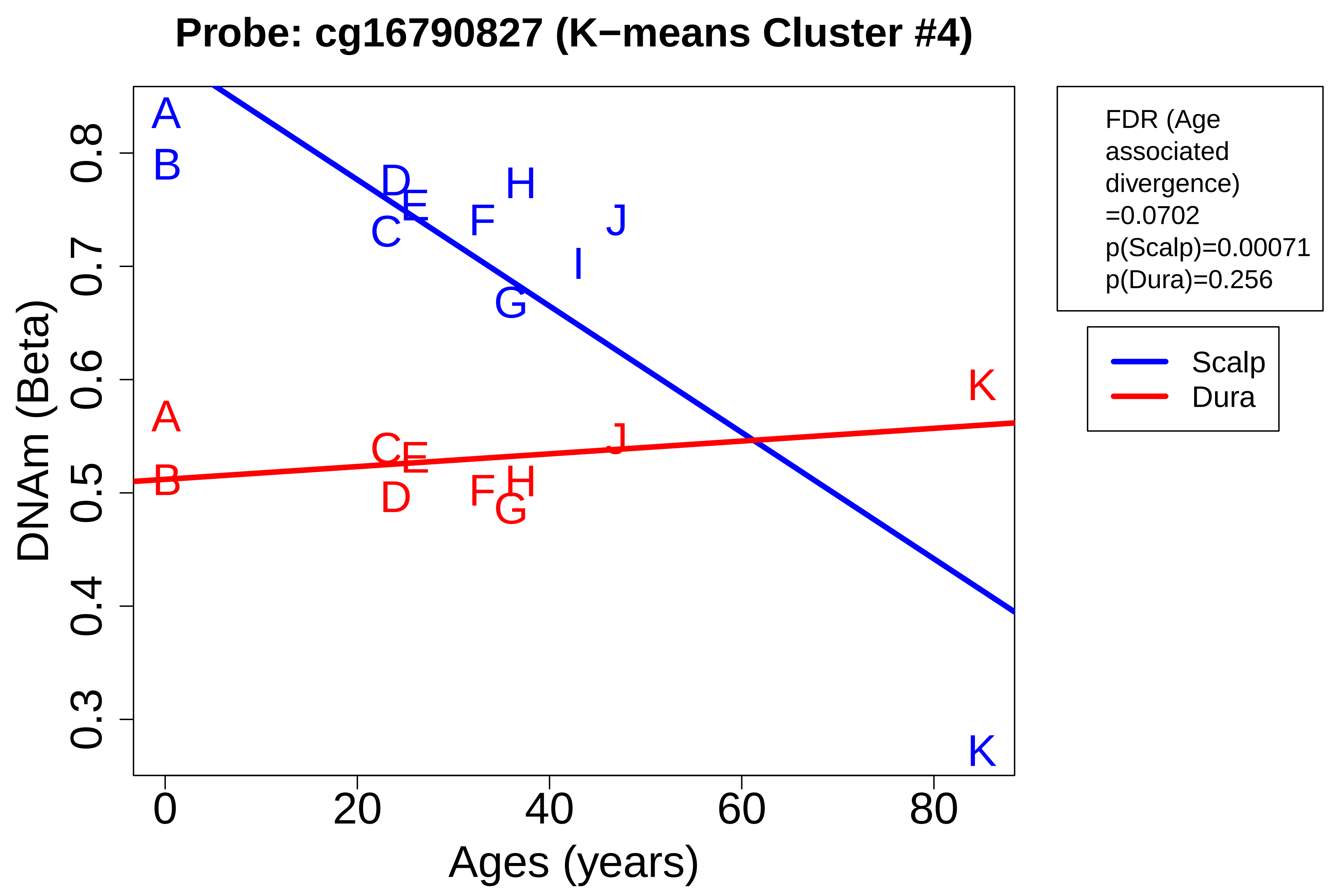

Probe: cg16790827 (K−means Cluster #4)
FDR (Age associated divergence) =0.0702
p(Scalp)=0.00071
p(Dura)=0.256
Scalp
Dura
DNAm (Beta)
0.3
0.4
0.5
0.6
0.7
0.8
0
20
40
60
80
Ages (years)
A
B
C
D
E
F
G
H
I
J
K

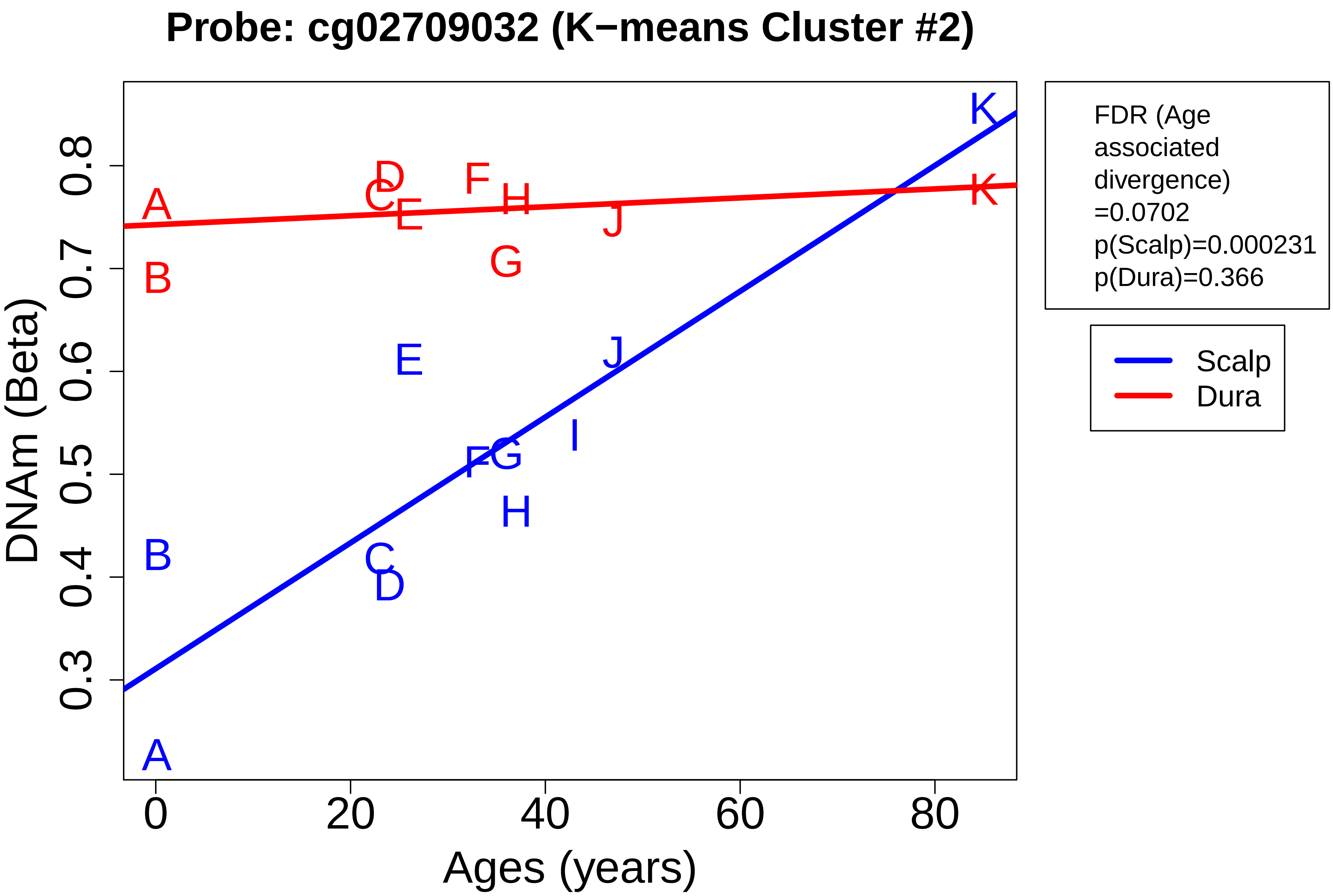
Probe: cg02709032 (K−means Cluster #2)
DNAm (Beta)
0.3
0.4
0.5
0.6
0.7
0.8
Ages (years)
0
20
40
60
80
FDR (Age associated divergence) =0.0702
p(Scalp)=0.000231
p(Dura)=0.366
Scalp
Dura
A
B
C
D
E
F
G
H
I
J
K

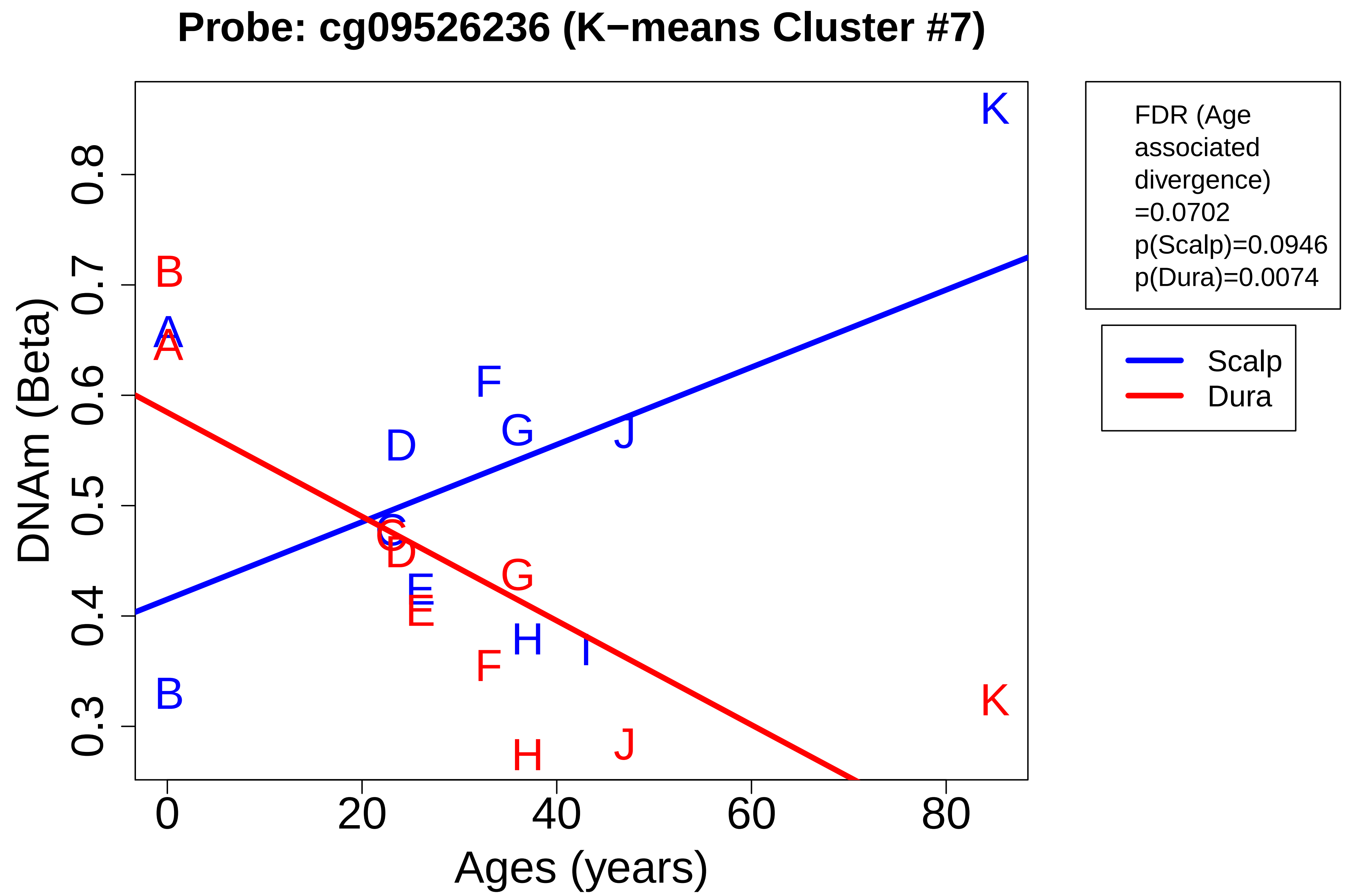

Probe: cg09526236 (K−means Cluster #7)
FDR (Age associated divergence) =0.0702
p(Scalp)=0.0946
p(Dura)=0.0074
Scalp
Dura
DNAm (Beta)
Ages (years)
0.3
0.4
0.5
0.6
0.7
0.8
0
20
40
60
80

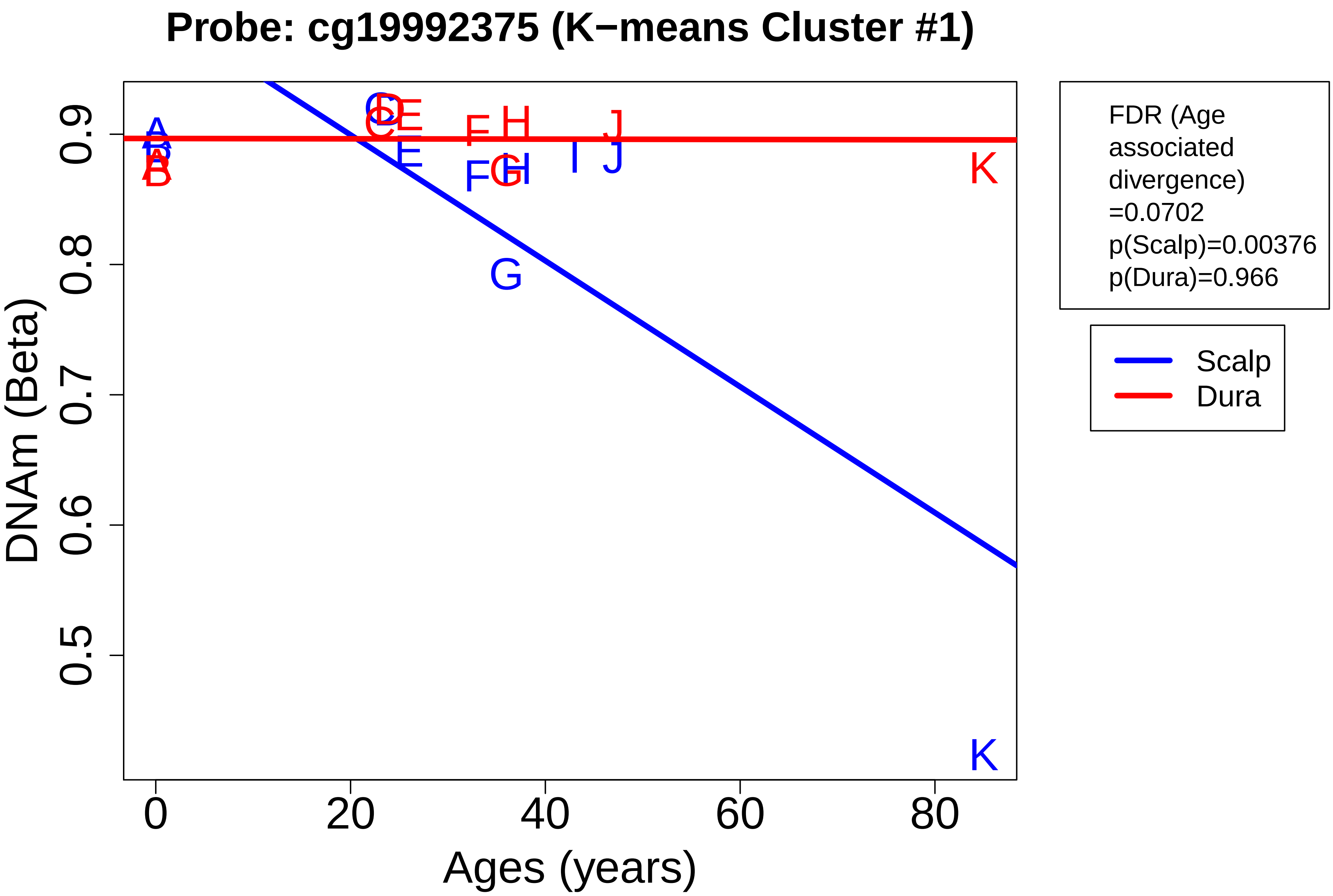

Probe: cg19992375 (K−means Cluster #1)
FDR (Age associated divergence) =0.0702
p(Scalp)=0.00376
p(Dura)=0.966
Scalp
Dura
DNAm (Beta)
0.5
0.6
0.7
0.8
0.9
Ages (years)
0
20
40
60
80
A
B
C
D
E
F
G
H
I
J
K

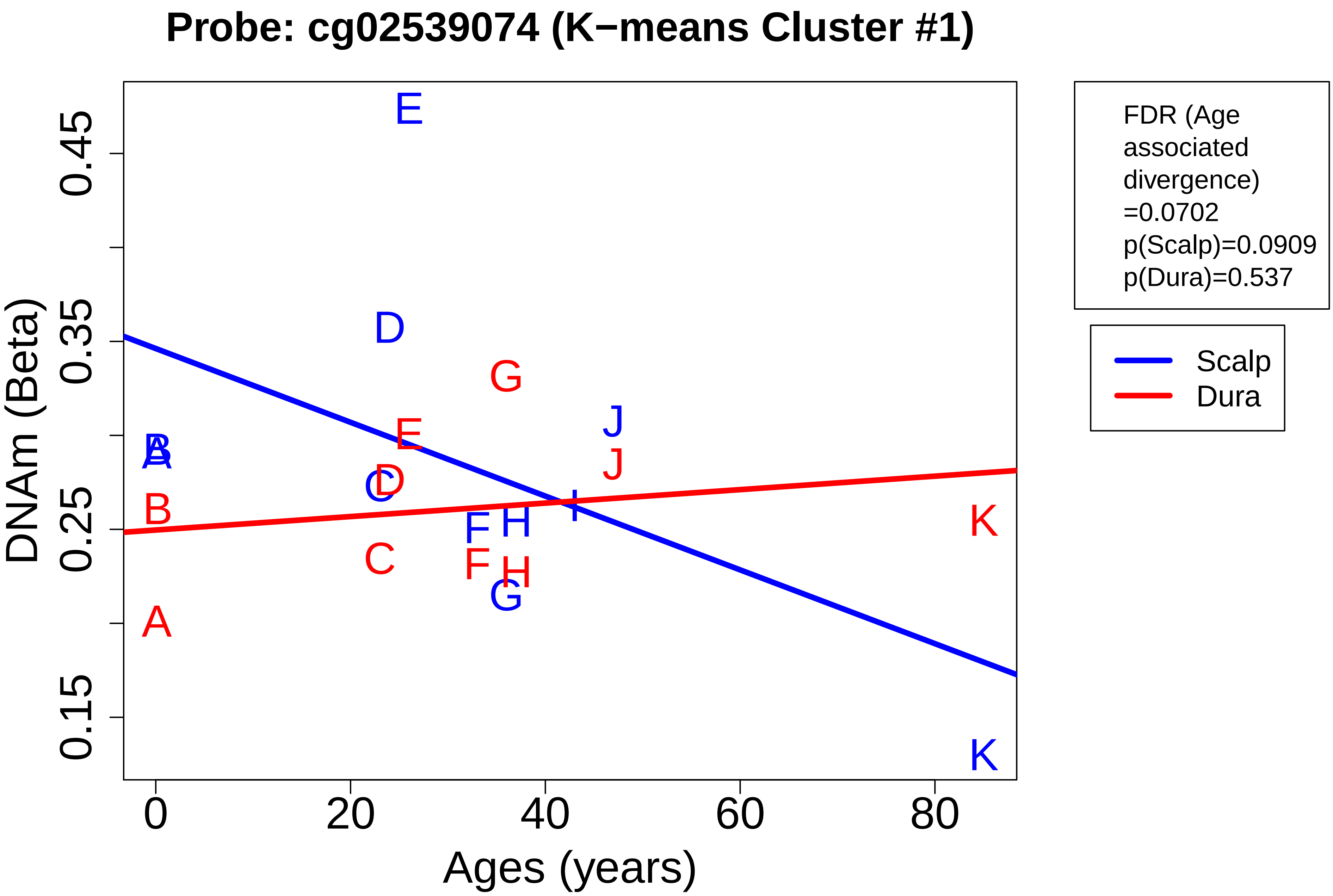

Probe: cg02539074 (K−means Cluster #1)
DNAm (Beta)
Ages (years)
0.15
0.25
0.35
0.45
0
20
40
60
80
FDR (Age associated divergence) =0.0702
p(Scalp)=0.0909
p(Dura)=0.537
Scalp
Dura

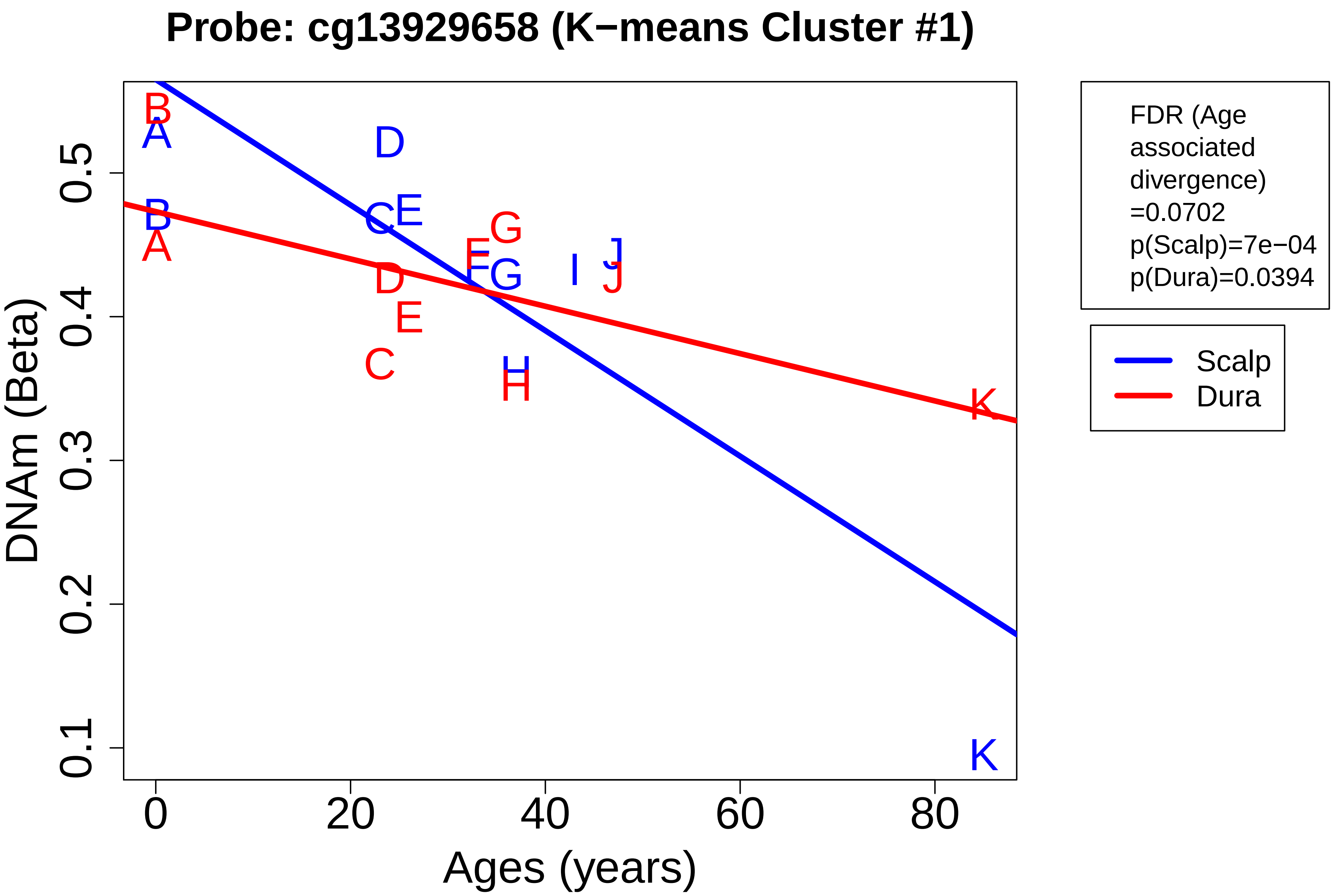
Probe: cg13929658 (K−means Cluster #1)
DNAm (Beta)
0.1
0.2
0.3
0.4
0.5
0
20
40
60
80
Ages (years)
A
B
C
D
E
F
G
H
I
J
K
FDR (Age associated divergence) =0.0702
p(Scalp)=7e−04
p(Dura)=0.0394
Scalp
Dura

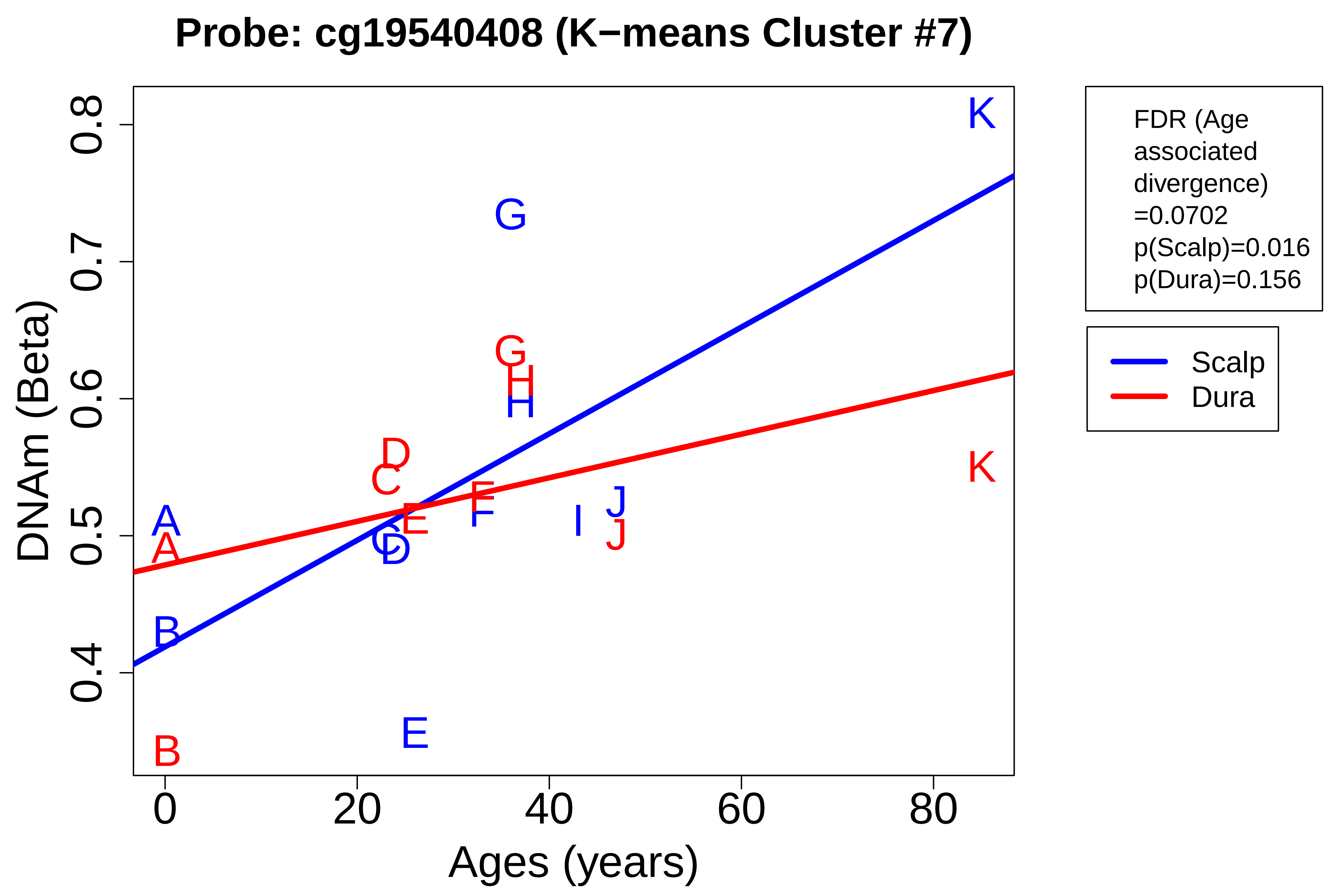

Probe: cg19540408 (K−means Cluster #7)
DNAm (Beta)
0.4
0.5
0.6
0.7
0.8
0
20
40
60
80
Ages (years)
A
B
C
D
E
F
G
H
I
J
K
FDR (Age associated divergence) =0.0702
p(Scalp)=0.016
p(Dura)=0.156
Scalp
Dura

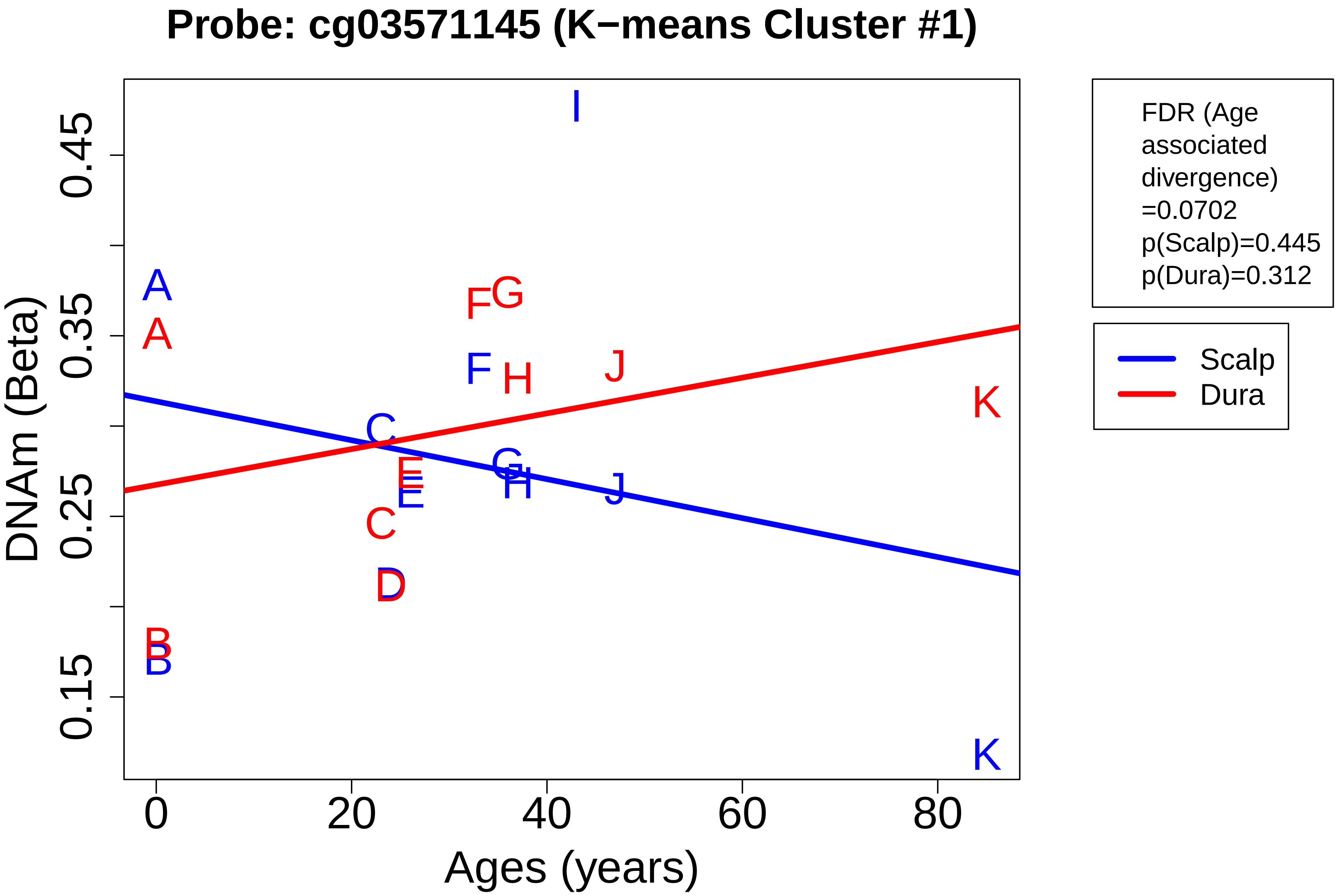
Probe: cg03571145 (K−means Cluster #1)
DNAm (Beta)
0.15
0.25
0.35
0.45
Ages (years)
0
20
40
60
80
A
B
C
D
E
F
G
H
I
J
K
FDR (Age associated divergence) =0.0702
p(Scalp)=0.445
p(Dura)=0.312
Scalp
Dura

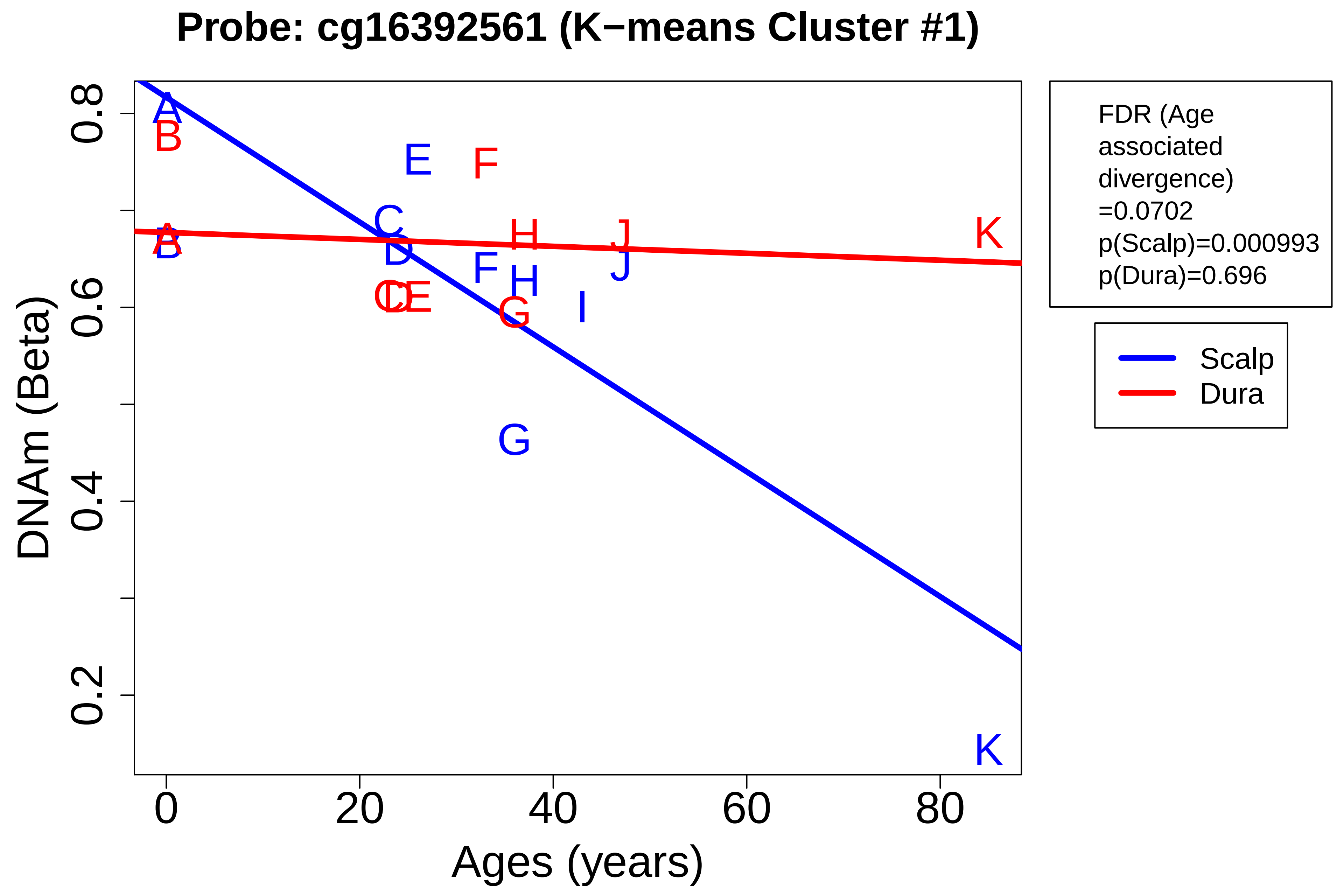

Probe: cg16392561 (K−means Cluster #1)
DNAm (Beta)
0.2
0.4
0.6
0.8
Ages (years)
0
20
40
60
80
FDR (Age associated divergence) =0.0702
p(Scalp)=0.000993
p(Dura)=0.696
Scalp
Dura
A
B
C
D
E
F
G
H
I
J
K

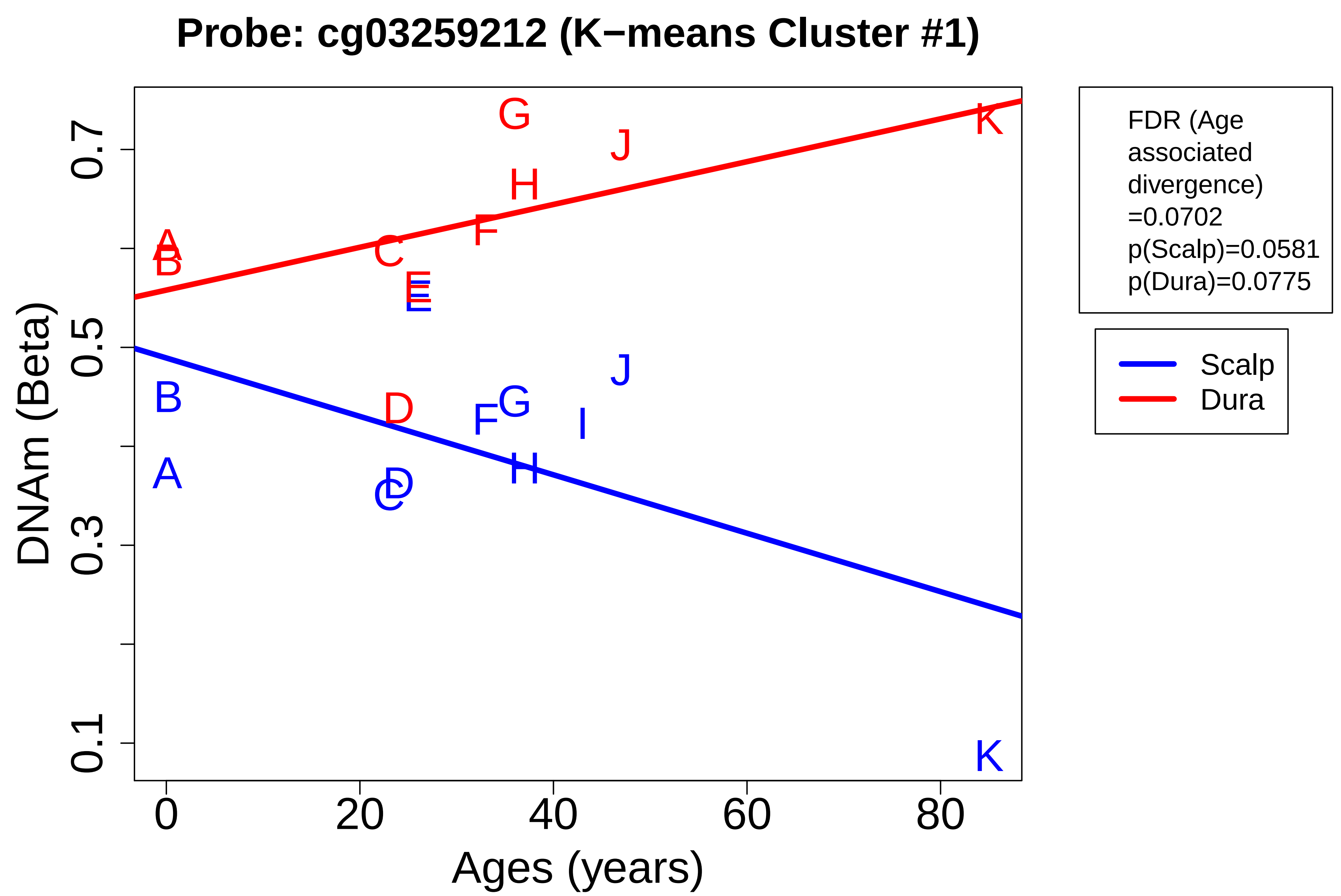

Probe: cg03259212 (K−means Cluster #1)
DNAm (Beta)
0.1
0.3
0.5
0.7
0
20
40
60
80
Ages (years)
FDR (Age associated divergence) =0.0702
p(Scalp)=0.0581
p(Dura)=0.0775
Scalp
Dura

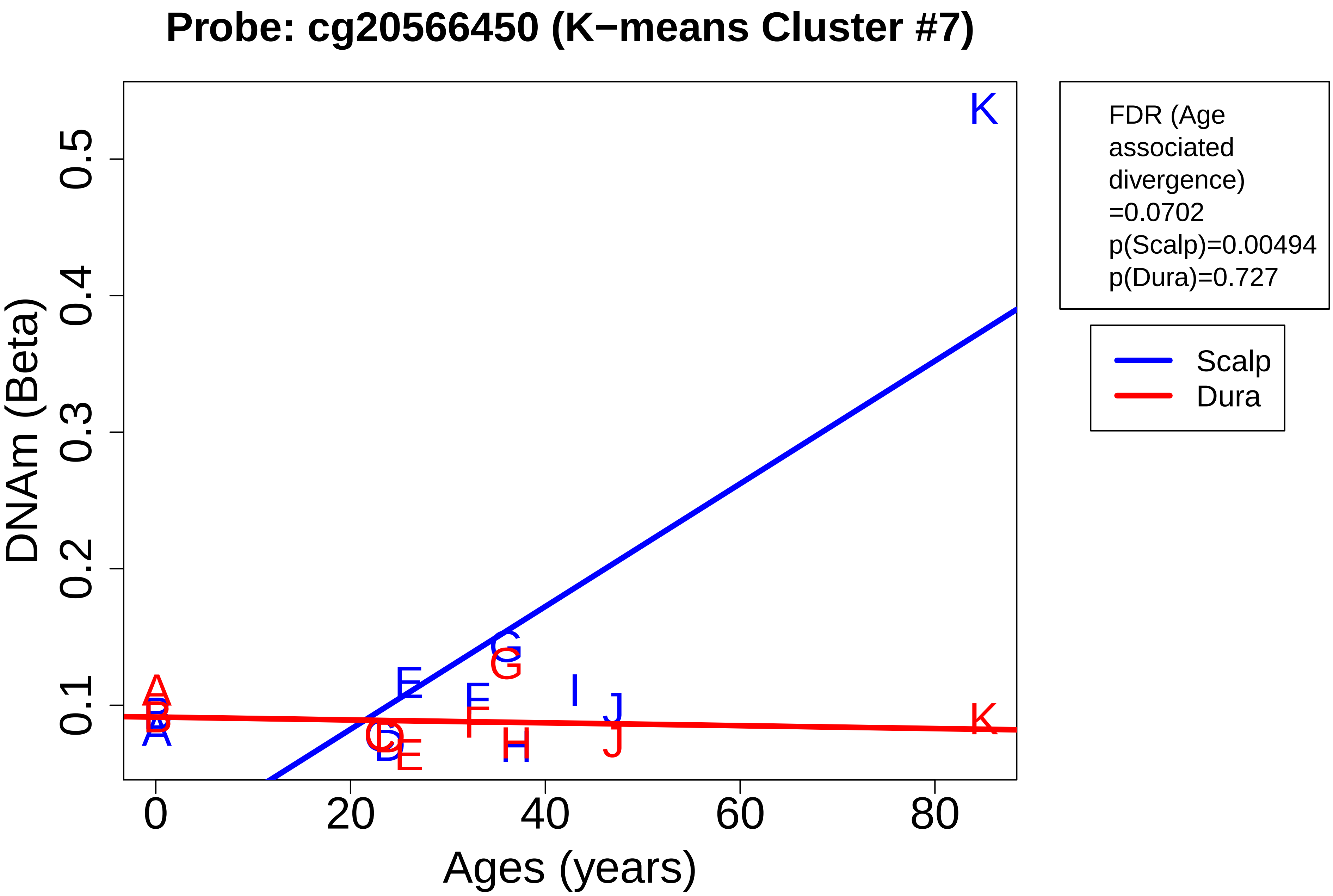

Probe: cg20566450 (K−means Cluster #7)
DNAm (Beta)
0.1
0.2
0.3
0.4
0.5
0
20
40
60
80
Ages (years)
FDR (Age associated divergence) =0.0702
p(Scalp)=0.00494
p(Dura)=0.727
Scalp
Dura

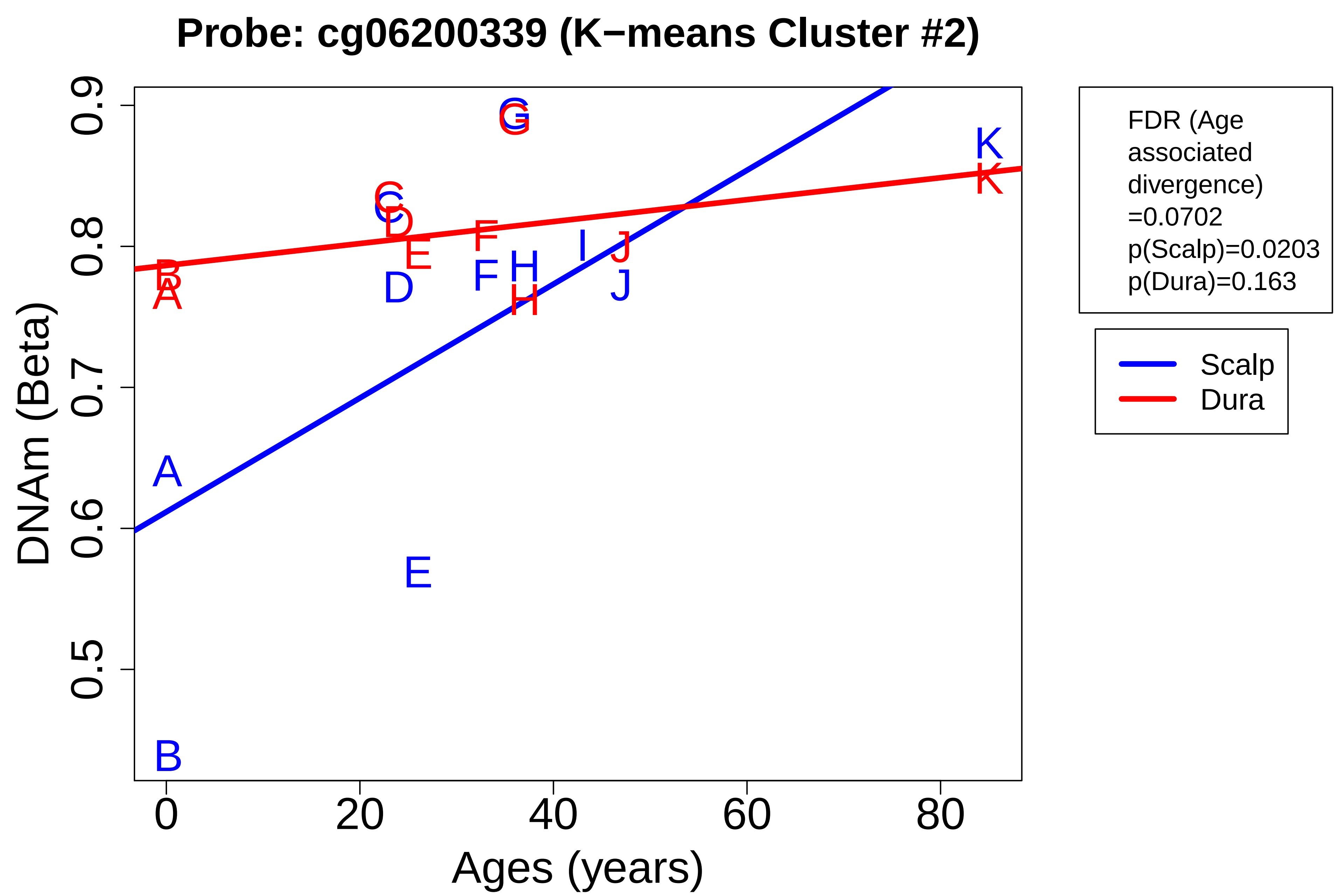

Probe: cg06200339 (K−means Cluster #2)
DNAm (Beta)
0.5
0.6
0.7
0.8
0.9
0
20
40
60
80
Ages (years)
A
B
C
D
E
F
G
H
I
J
K
FDR (Age associated divergence) =0.0702
p(Scalp)=0.0203
p(Dura)=0.163
Scalp
Dura

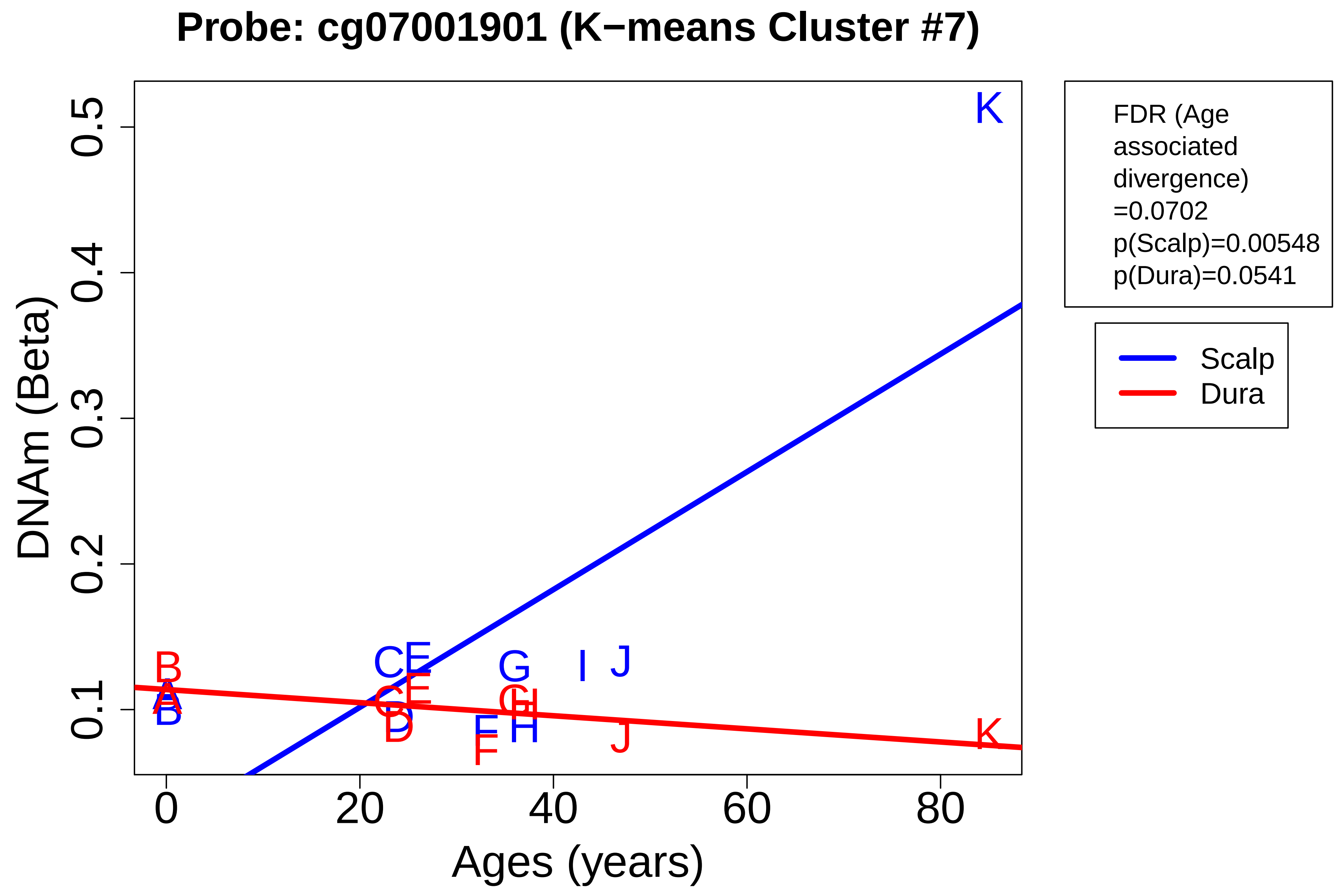

Probe: cg07001901 (K−means Cluster #7)
DNAm (Beta)
0.1
0.2
0.3
0.4
0.5
0
20
40
60
80
Ages (years)
FDR (Age associated divergence) =0.0702
p(Scalp)=0.00548
p(Dura)=0.0541
Scalp
Dura

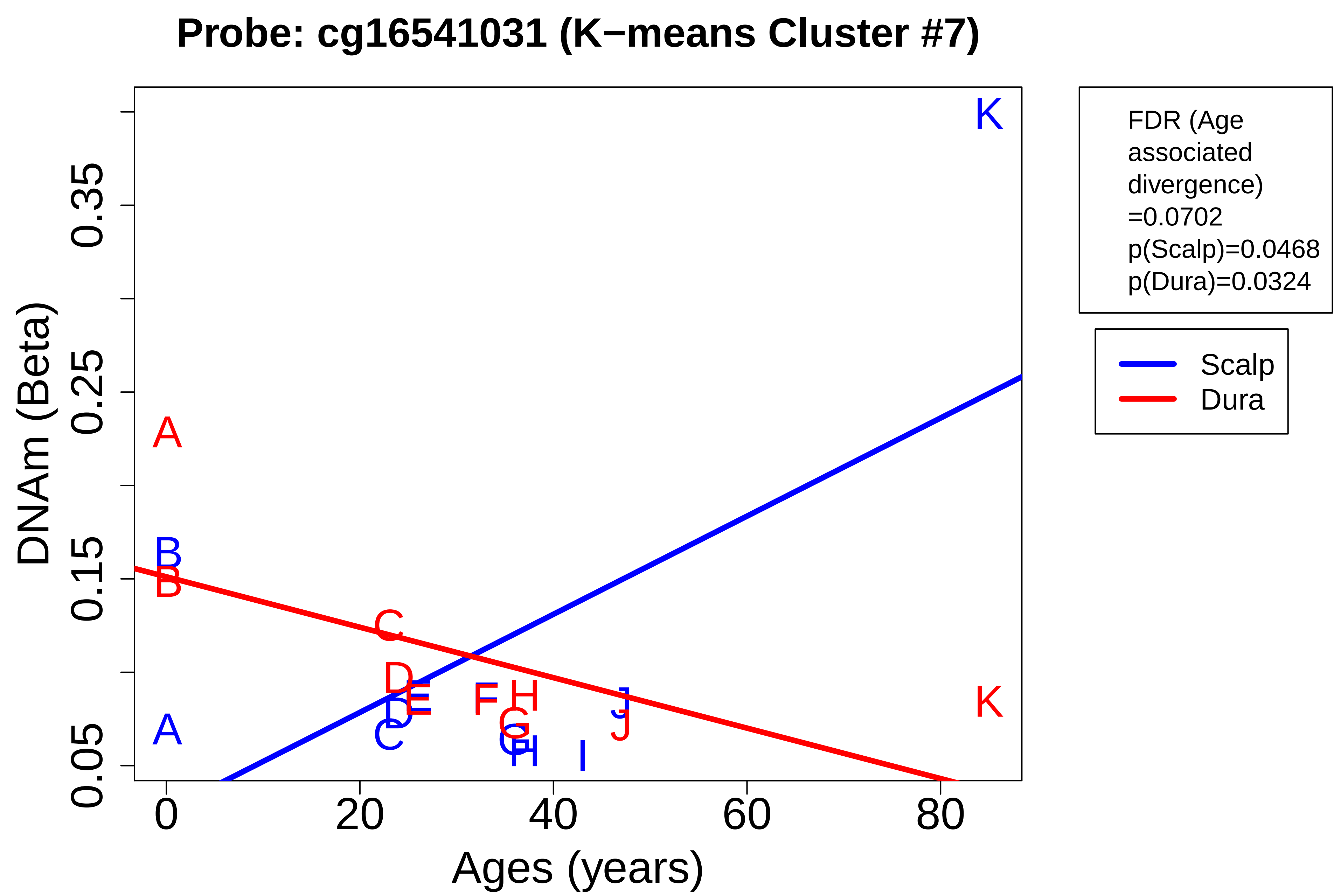

Probe: cg16541031 (K−means Cluster #7)
DNAm (Beta)
0.05
0.15
0.25
0.35
Ages (years)
0
20
40
60
80
A
B
C
D
E
F
G
H
I
J
K
FDR (Age associated divergence) =0.0702
p(Scalp)=0.0468
p(Dura)=0.0324
Scalp
Dura

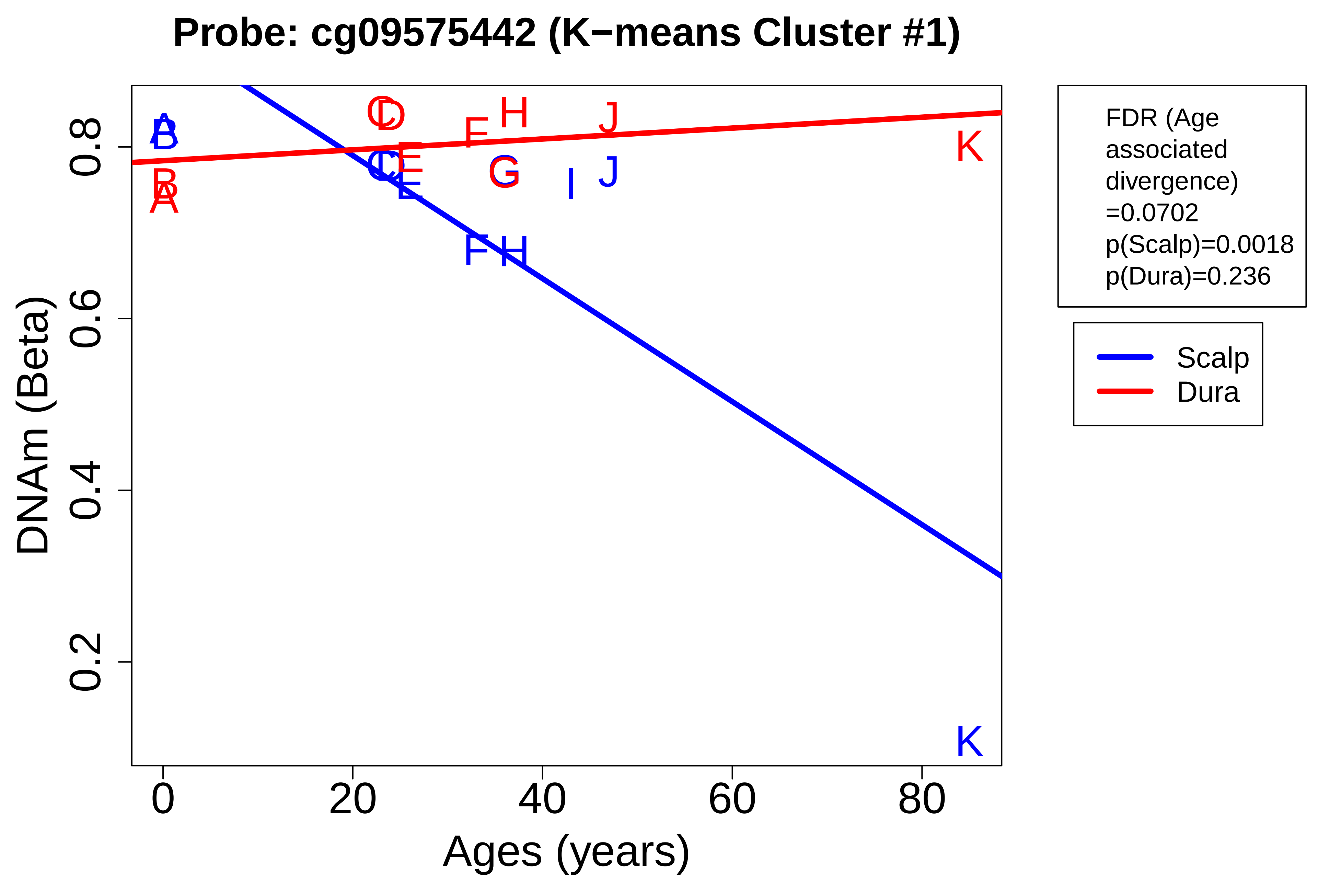

Probe: cg09575442 (K−means Cluster #1)
DNAm (Beta)
0.2
0.4
0.6
0.8
Ages (years)
0
20
40
60
80
A
B
C
D
E
F
G
H
I
J
K
FDR (Age associated divergence) =0.0702
p(Scalp)=0.0018
p(Dura)=0.236
Scalp
Dura

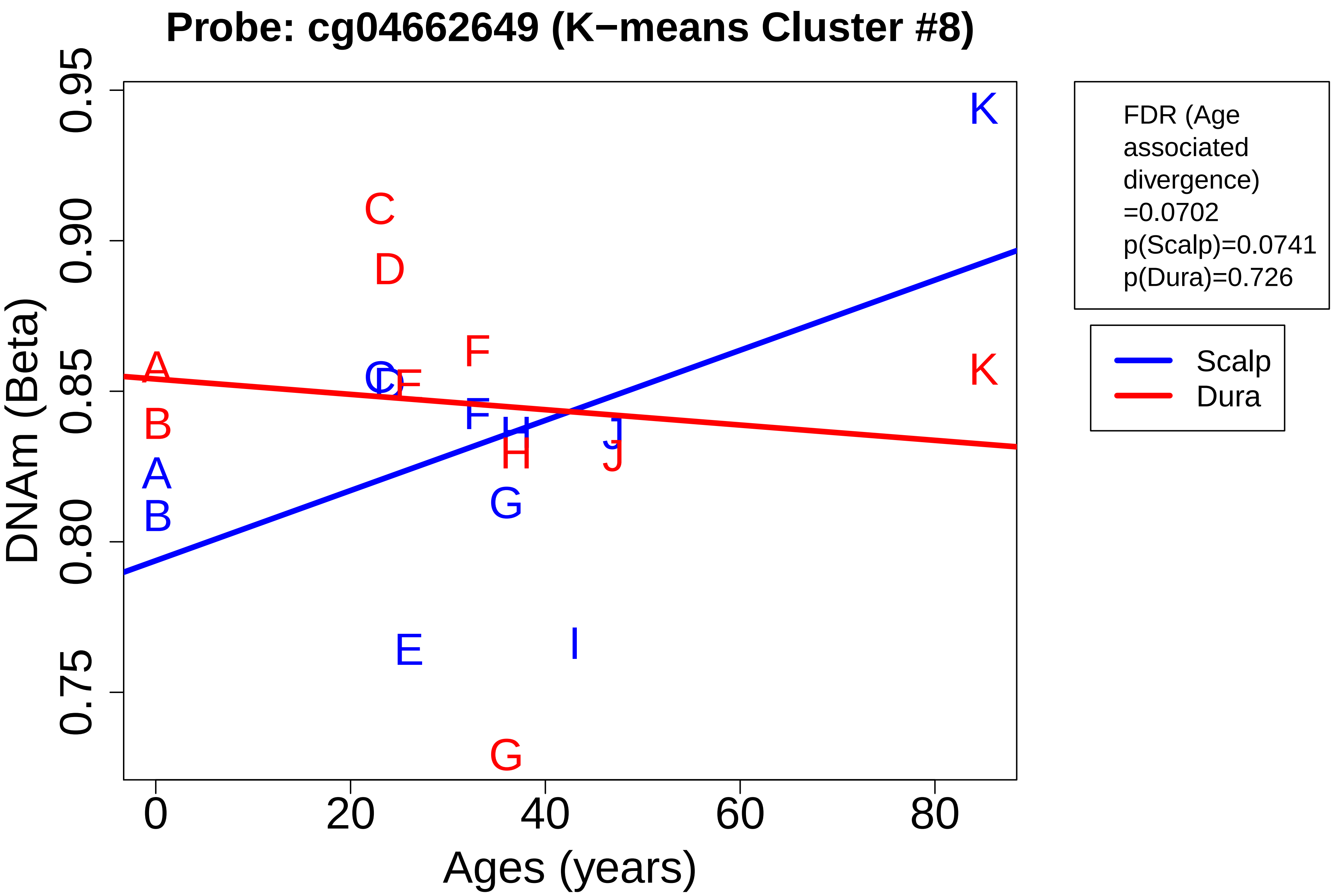

Probe: cg04662649 (K−means Cluster #8)
DNAm (Beta)
0.75
0.80
0.85
0.90
0.95
0
20
40
60
80
Ages (years)
FDR (Age associated divergence) =0.0702
p(Scalp)=0.0741
p(Dura)=0.726
Scalp
Dura

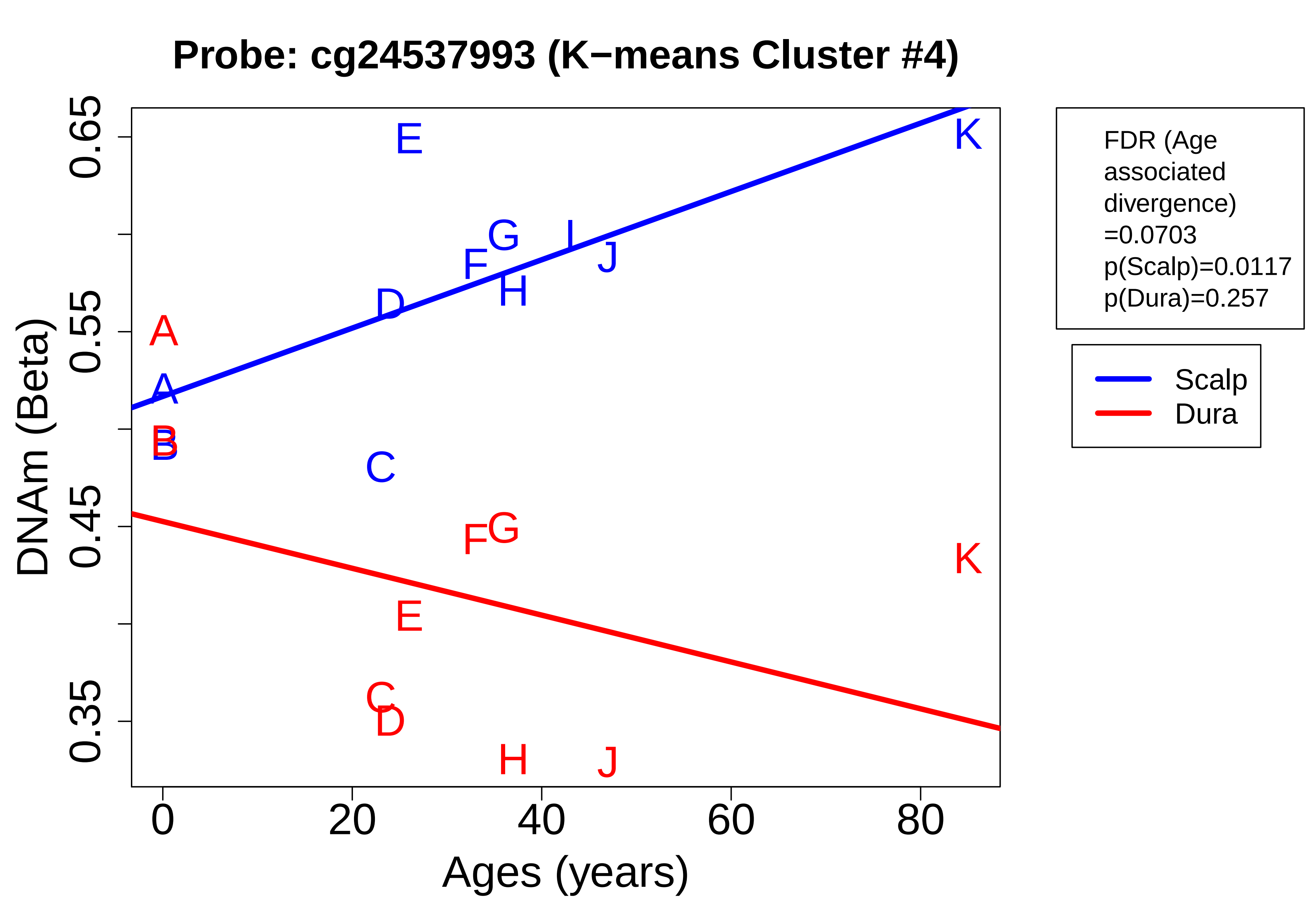

Probe: cg24537993 (K−means Cluster #4)
DNAm (Beta)
0.35
0.45
0.55
0.65
Ages (years)
0
20
40
60
80
A
B
C
D
E
F
G
H
I
J
K
FDR (Age associated divergence) =0.0703
p(Scalp)=0.0117
p(Dura)=0.257
Scalp
Dura

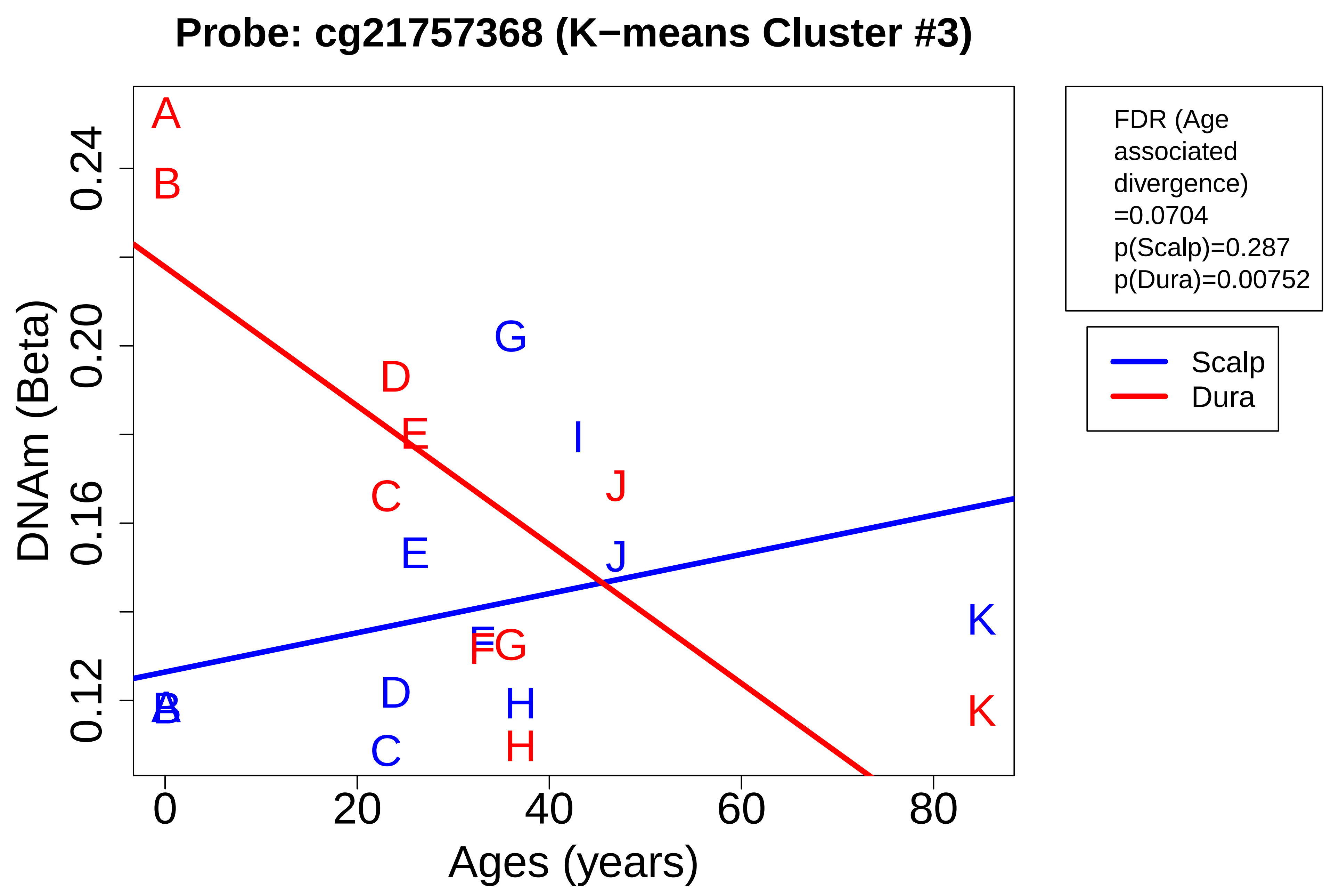
Probe: cg21757368 (K−means Cluster #3)
DNAm (Beta)
Ages (years)
0.12
0.16
0.20
0.24
0
20
40
60
80
FDR (Age associated divergence) =0.0704
p(Scalp)=0.287
p(Dura)=0.00752
Scalp
Dura
A
B
C
D
E
F
G
H
I
J
K

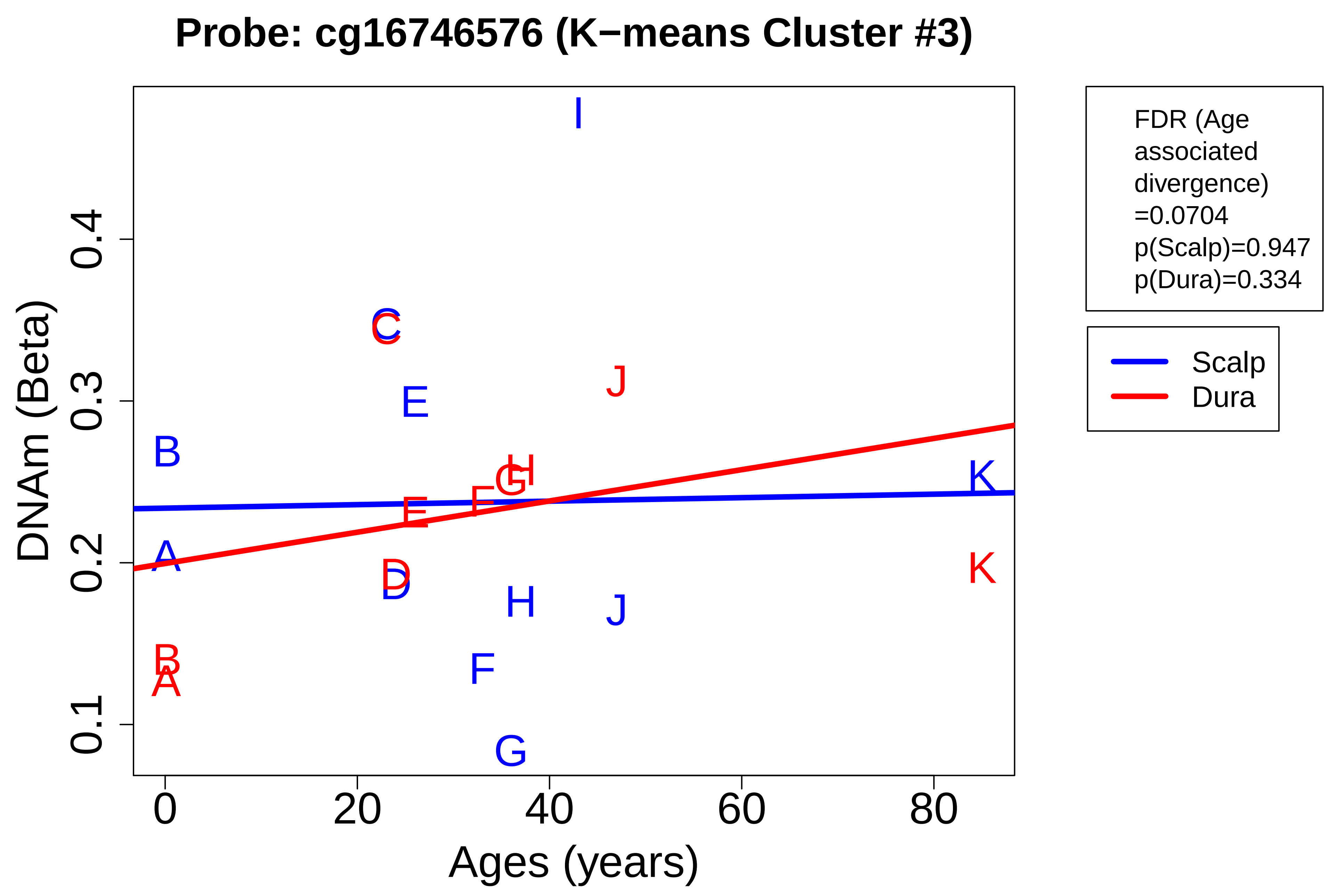

Probe: cg16746576 (K−means Cluster #3)
DNAm (Beta)
0.1
0.2
0.3
0.4
Ages (years)
0
20
40
60
80
FDR (Age associated divergence) =0.0704
p(Scalp)=0.947
p(Dura)=0.334
Scalp
Dura
A
B
C
D
E
F
G
H
I
J
K

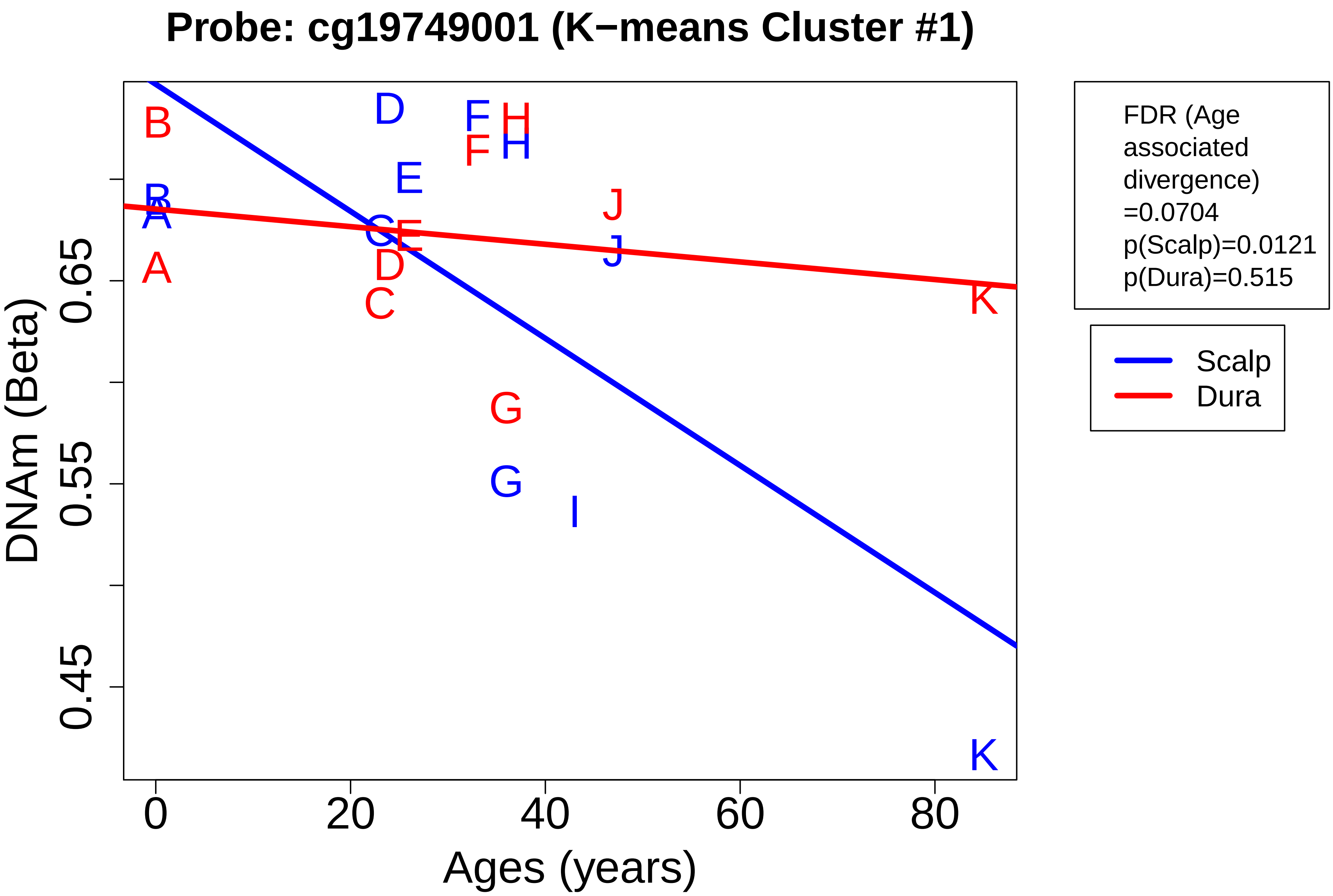

Probe: cg19749001 (K−means Cluster #1)
DNAm (Beta)
0.45
0.55
0.65
0
20
40
60
80
Ages (years)
FDR (Age associated divergence) =0.0704
p(Scalp)=0.0121
p(Dura)=0.515
Scalp
Dura

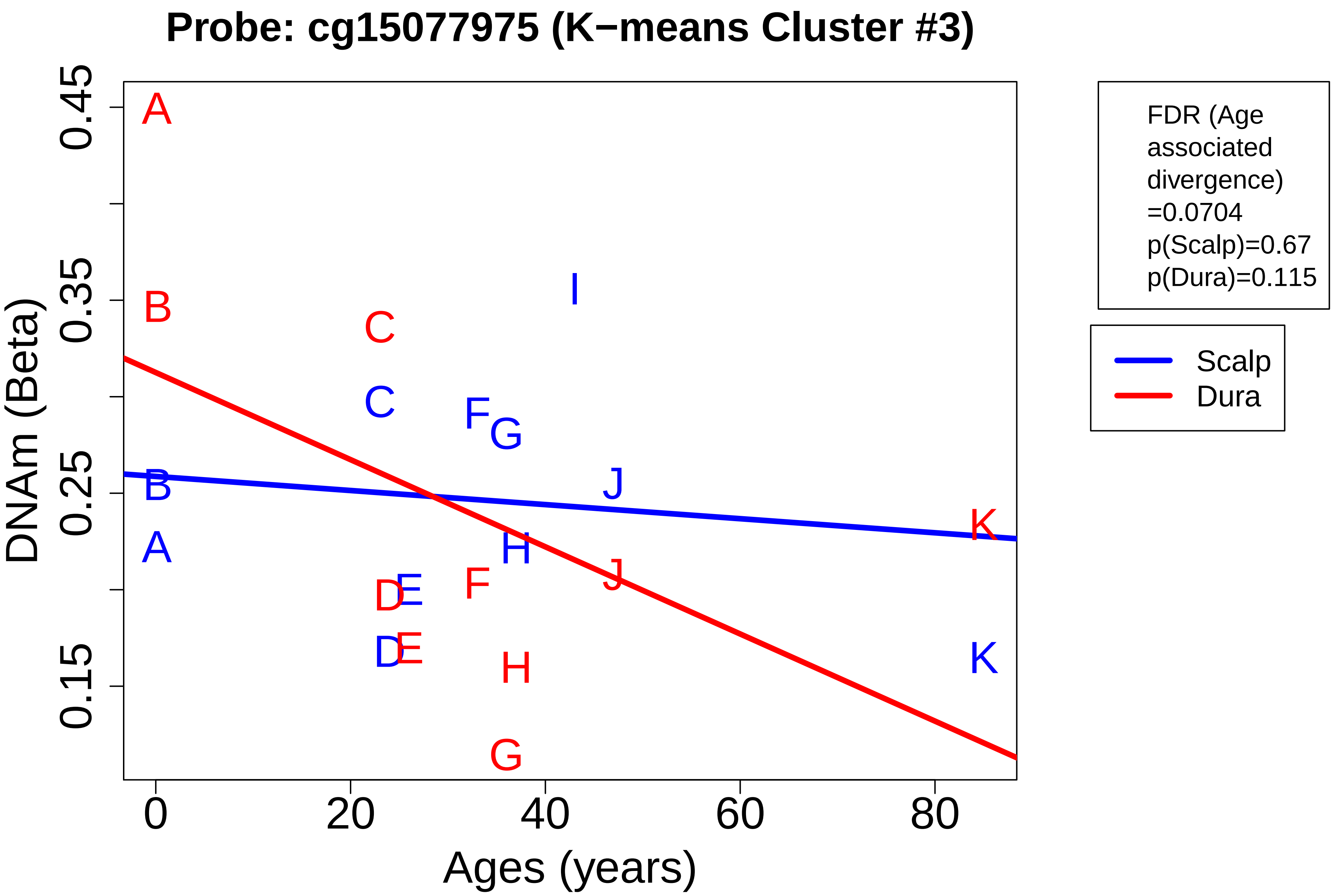
Probe: cg15077975 (K−means Cluster #3)
DNAm (Beta)
0.15
0.25
0.35
0.45
Ages (years)
0
20
40
60
80
A
B
C
D
E
F
G
H
I
J
K
FDR (Age associated divergence) =0.0704
p(Scalp)=0.67
p(Dura)=0.115
Scalp
Dura

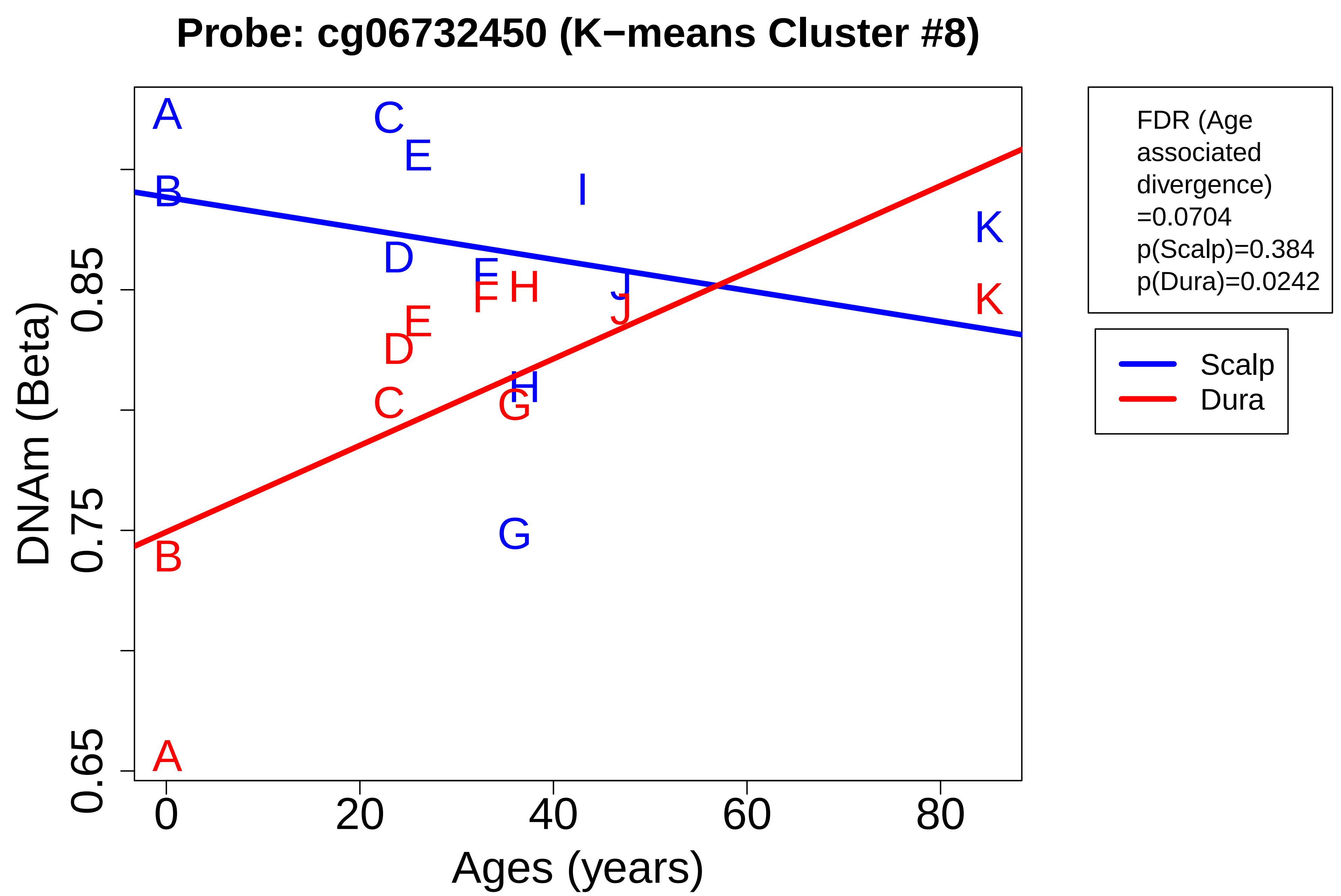

Probe: cg06732450 (K−means Cluster #8)
DNAm (Beta)
0.65
0.75
0.85
Ages (years)
0
20
40
60
80
FDR (Age associated divergence) =0.0704
p(Scalp)=0.384
p(Dura)=0.0242
Scalp
Dura
A
B
C
D
E
F
G
H
I
J
K

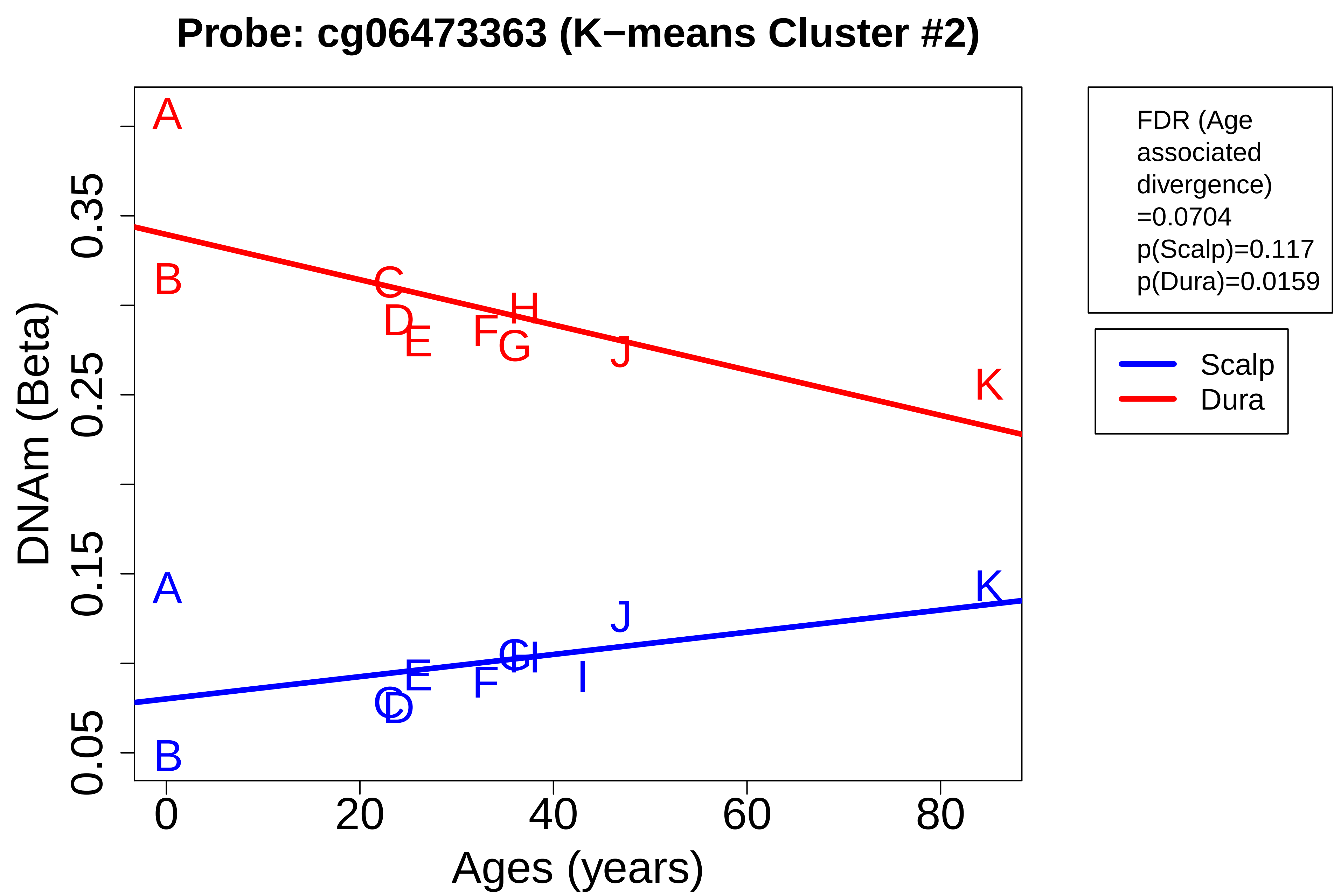

Probe: cg06473363 (K−means Cluster #2)
DNAm (Beta)
0.05
0.15
0.25
0.35
Ages (years)
0
20
40
60
80
FDR (Age associated divergence) =0.0704
p(Scalp)=0.117
p(Dura)=0.0159
Scalp
Dura

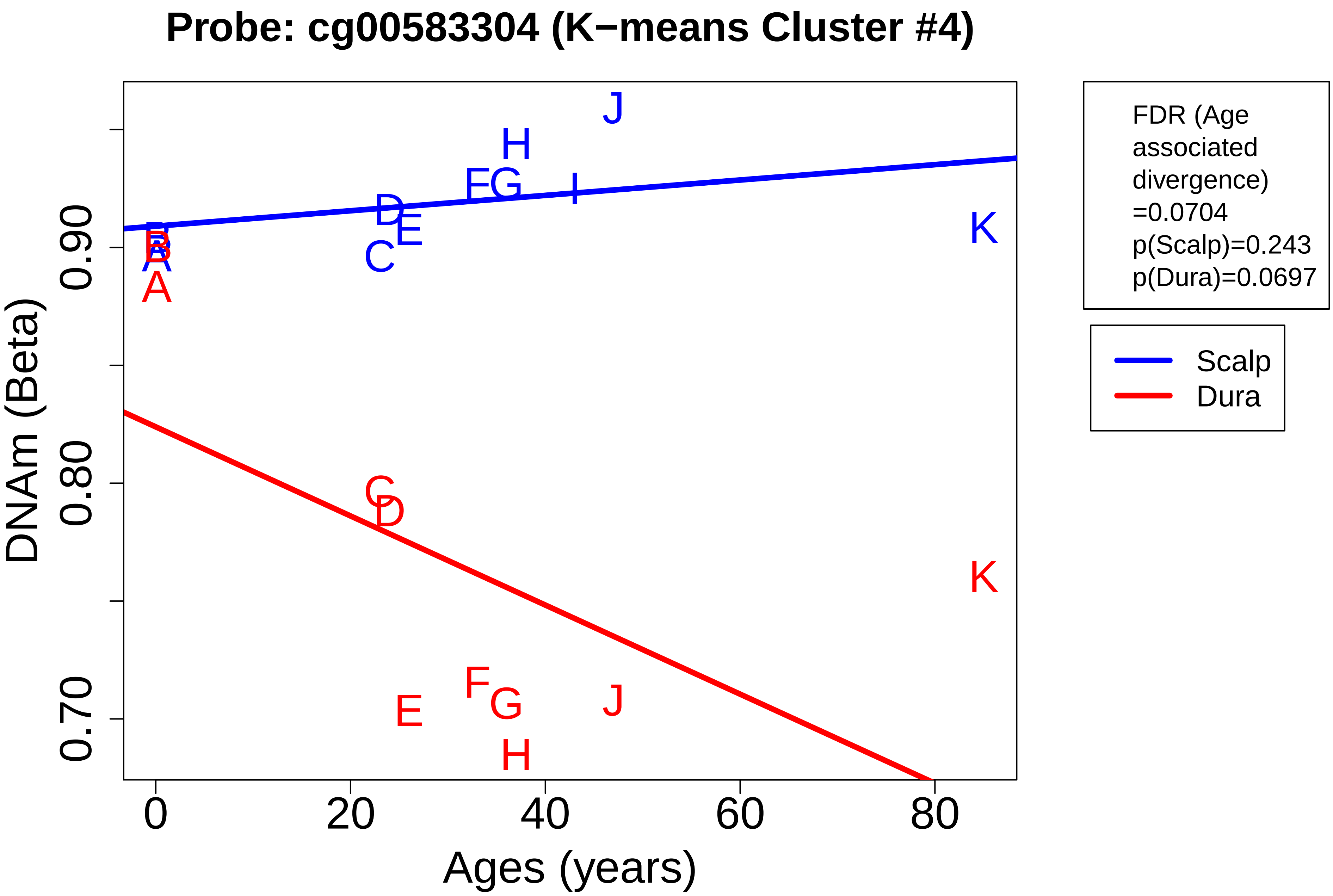
Probe: cg00583304 (K−means Cluster #4)
DNAm (Beta)
0.70
0.80
0.90
Ages (years)
0
20
40
60
80
A
B
C
D
E
F
G
H
I
J
K
FDR (Age associated divergence) =0.0704
p(Scalp)=0.243
p(Dura)=0.0697
Scalp
Dura

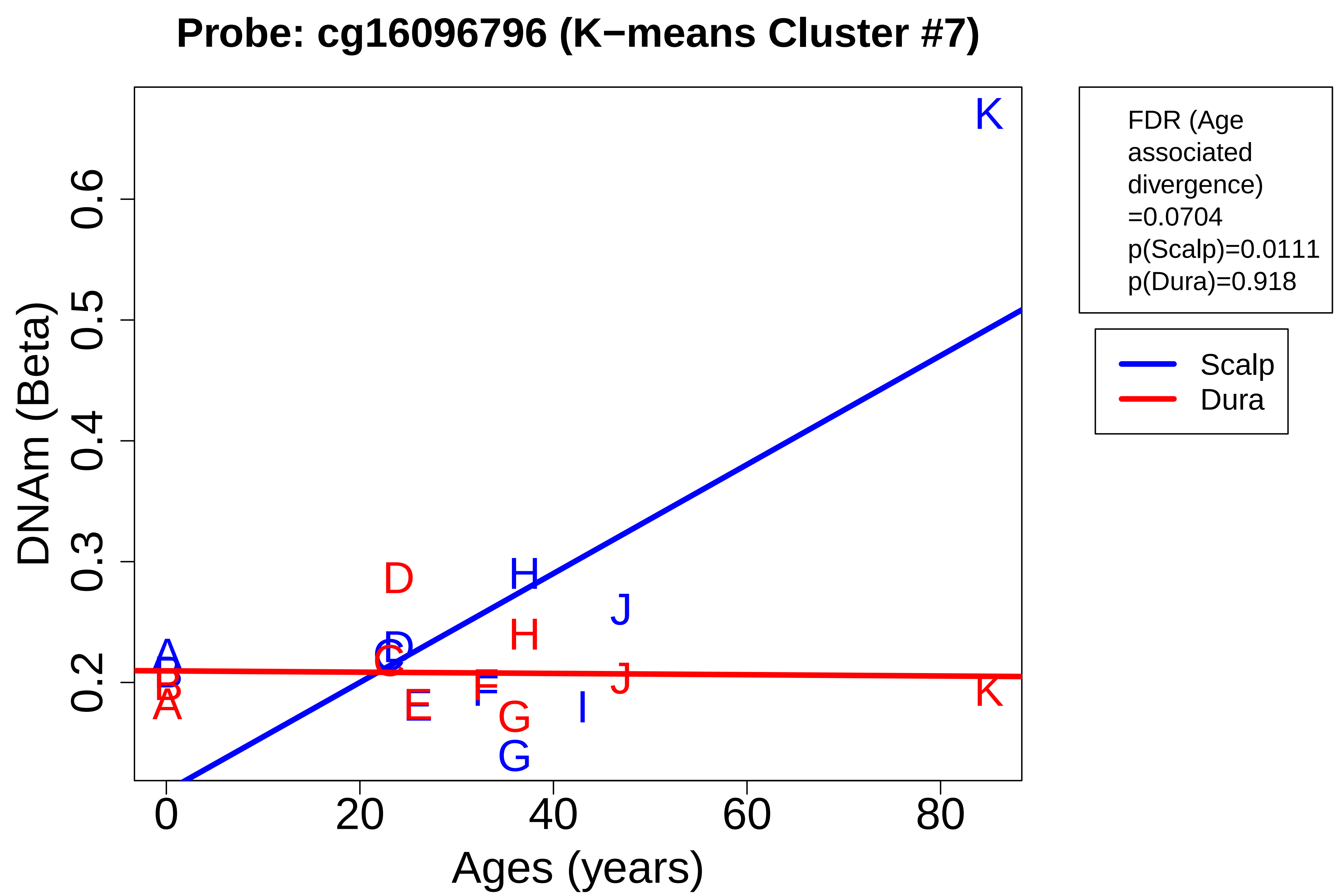

Probe: cg16096796 (K−means Cluster #7)
DNAm (Beta)
0.2
0.3
0.4
0.5
0.6
0
20
40
60
80
Ages (years)
FDR (Age associated divergence) =0.0704
p(Scalp)=0.0111
p(Dura)=0.918
Scalp
Dura

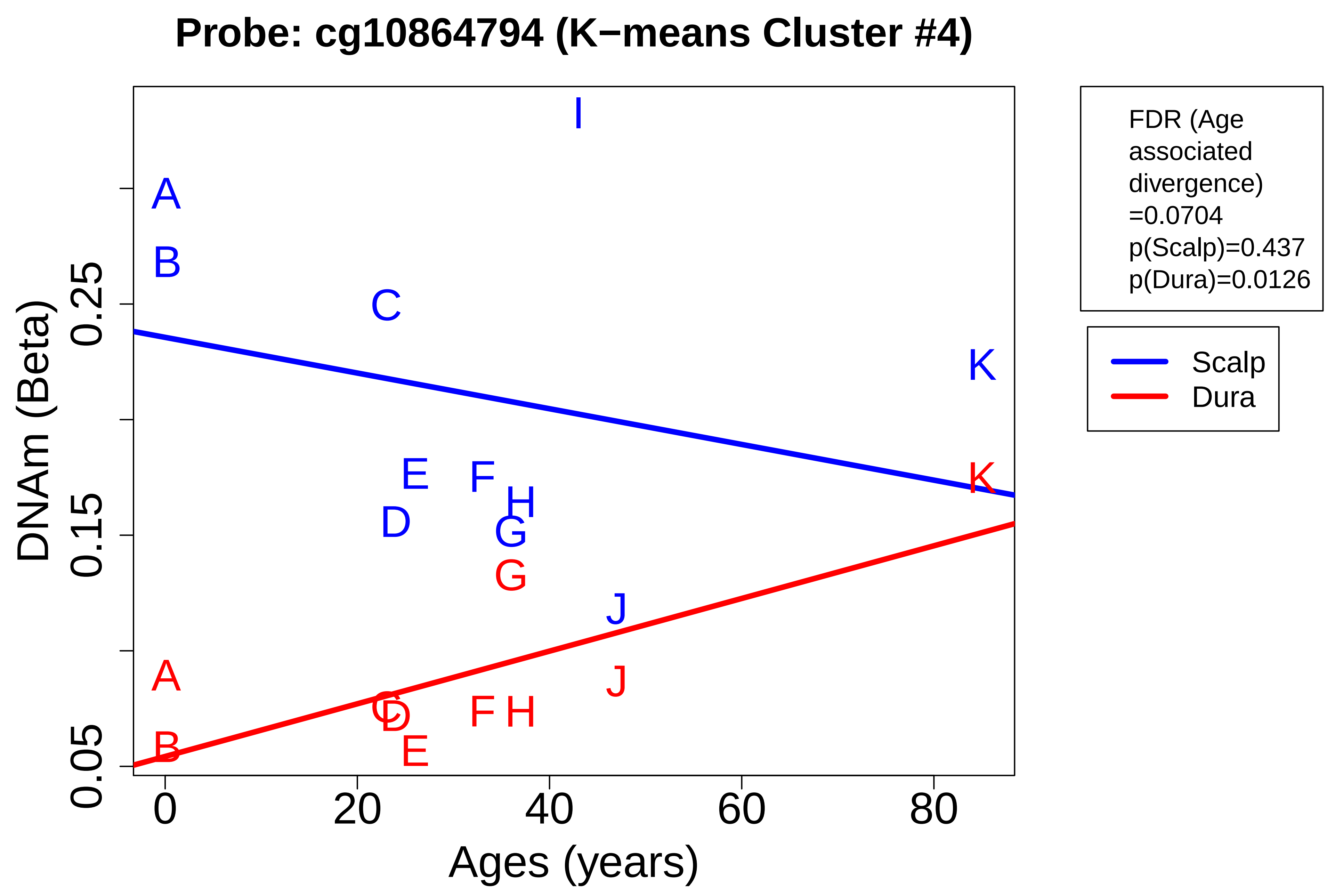
Probe: cg10864794 (K−means Cluster #4)
DNAm (Beta)
0.05
0.15
0.25
0
20
40
60
80
Ages (years)
A
B
C
D
E
F
G
H
I
J
K
FDR (Age associated divergence) =0.0704
p(Scalp)=0.437
p(Dura)=0.0126
Scalp
Dura

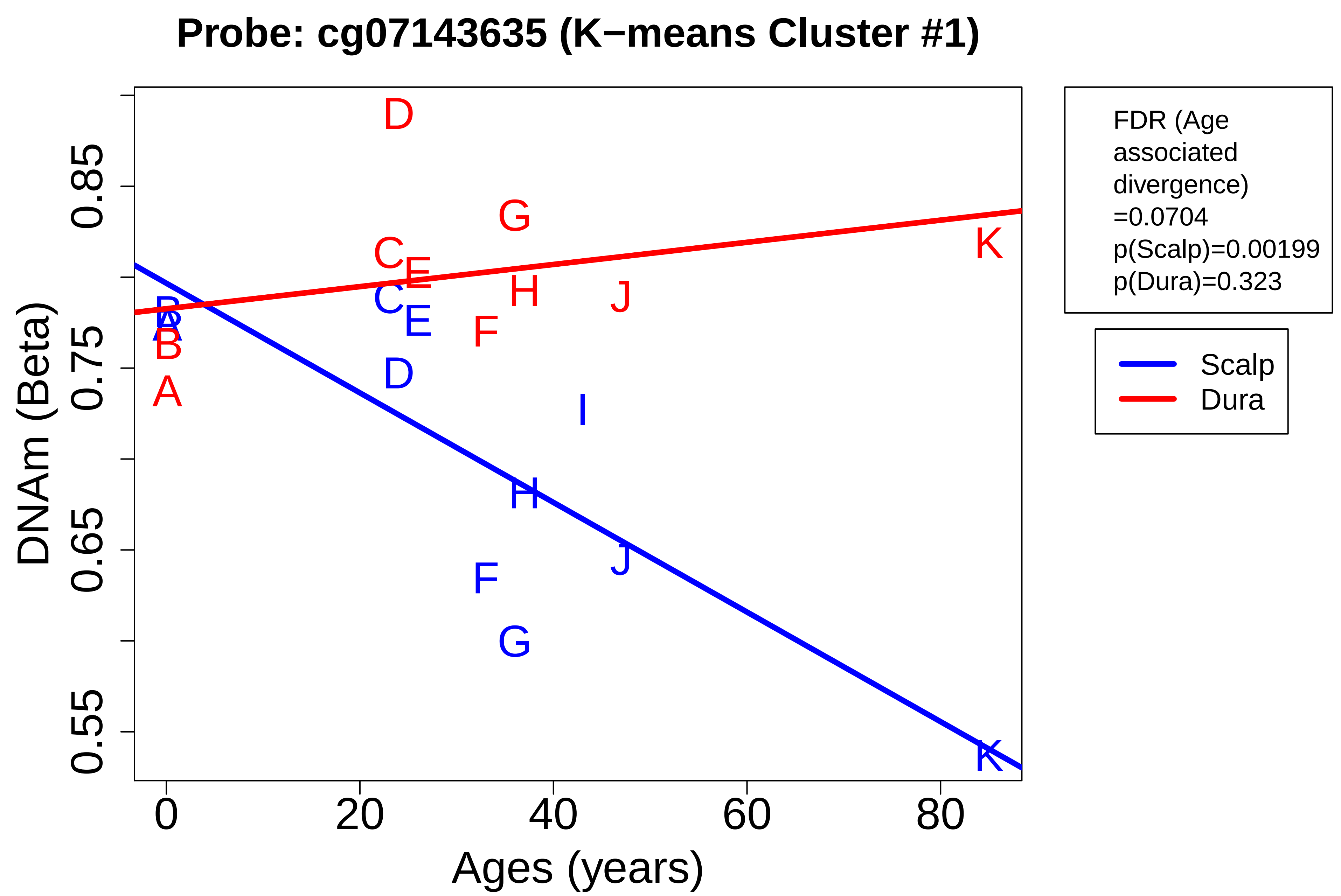

Probe: cg07143635 (K−means Cluster #1)
DNAm (Beta)
0.55
0.65
0.75
0.85
0
20
40
60
80
Ages (years)
FDR (Age associated divergence) =0.0704
p(Scalp)=0.00199
p(Dura)=0.323
Scalp
Dura

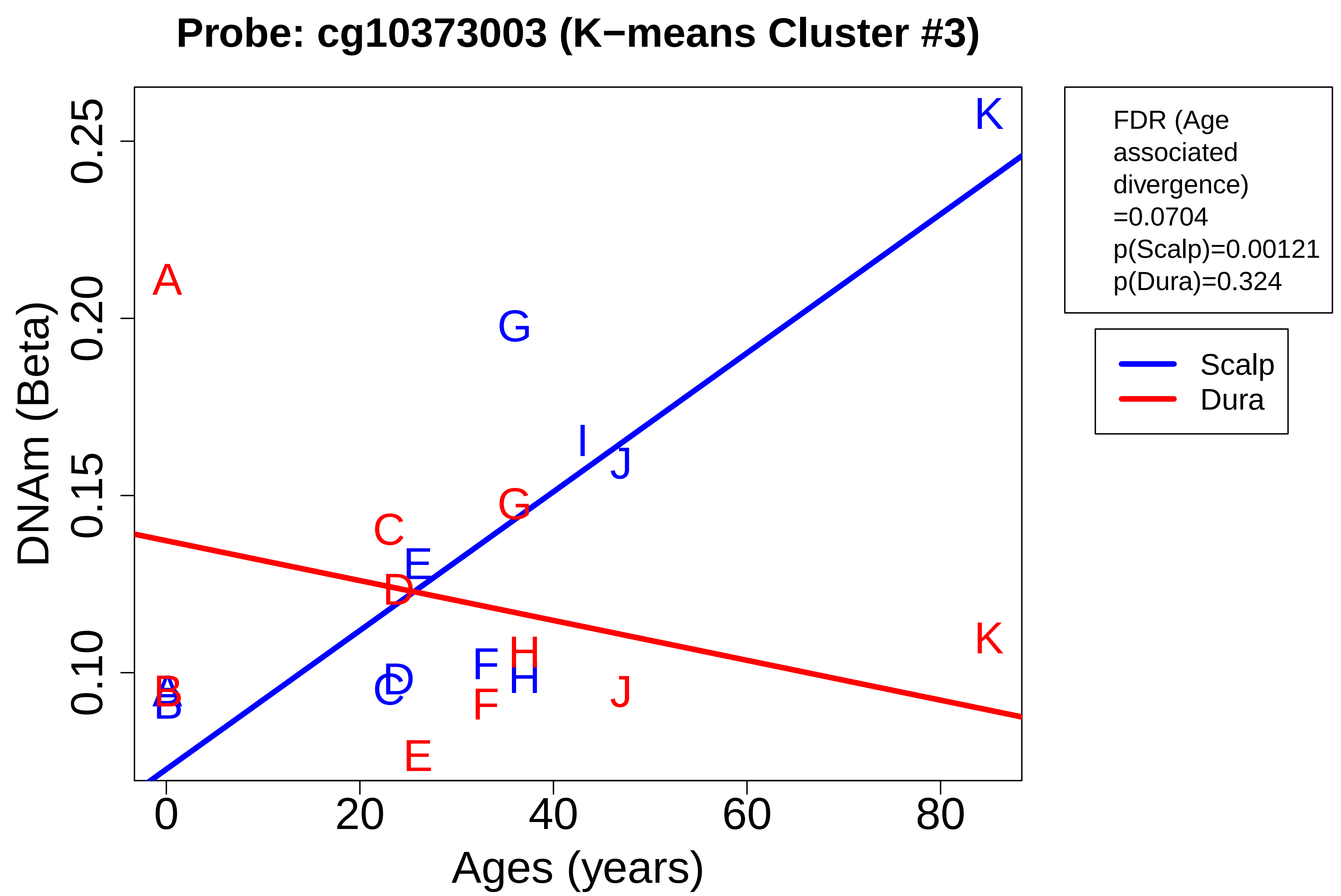
Probe: cg10373003 (K−means Cluster #3)
DNAm (Beta)
0.10
0.15
0.20
0.25
0
20
40
60
80
Ages (years)
A B C D E F G H I J K
FDR (Age associated divergence) =0.0704
p(Scalp)=0.00121
p(Dura)=0.324
Scalp
Dura

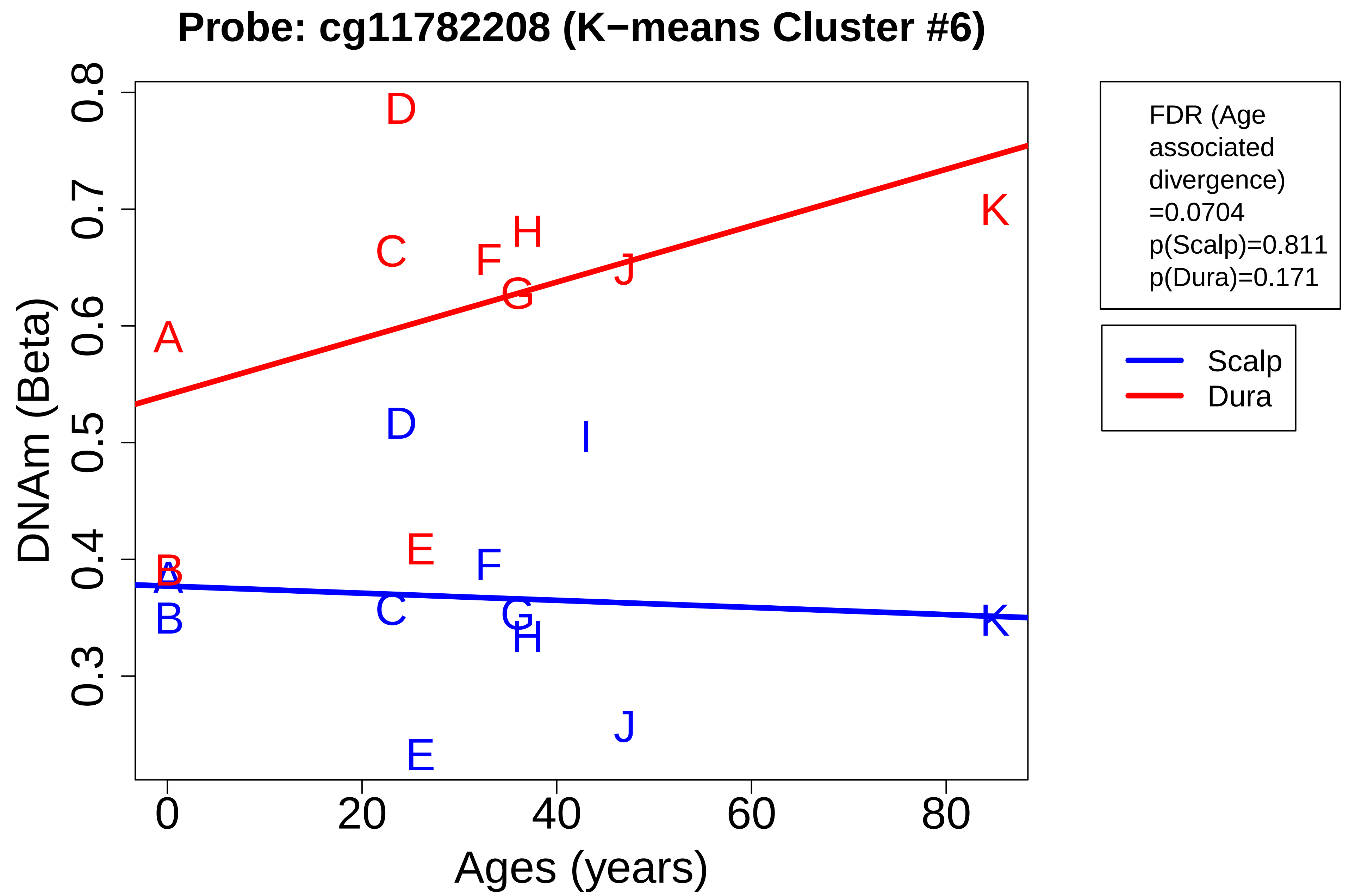

Probe: cg11782208 (K−means Cluster #6)
DNAm (Beta)
0.3
0.4
0.5
0.6
0.7
0.8
0
20
40
60
80
Ages (years)
FDR (Age associated divergence) =0.0704
p(Scalp)=0.811
p(Dura)=0.171
Scalp
Dura

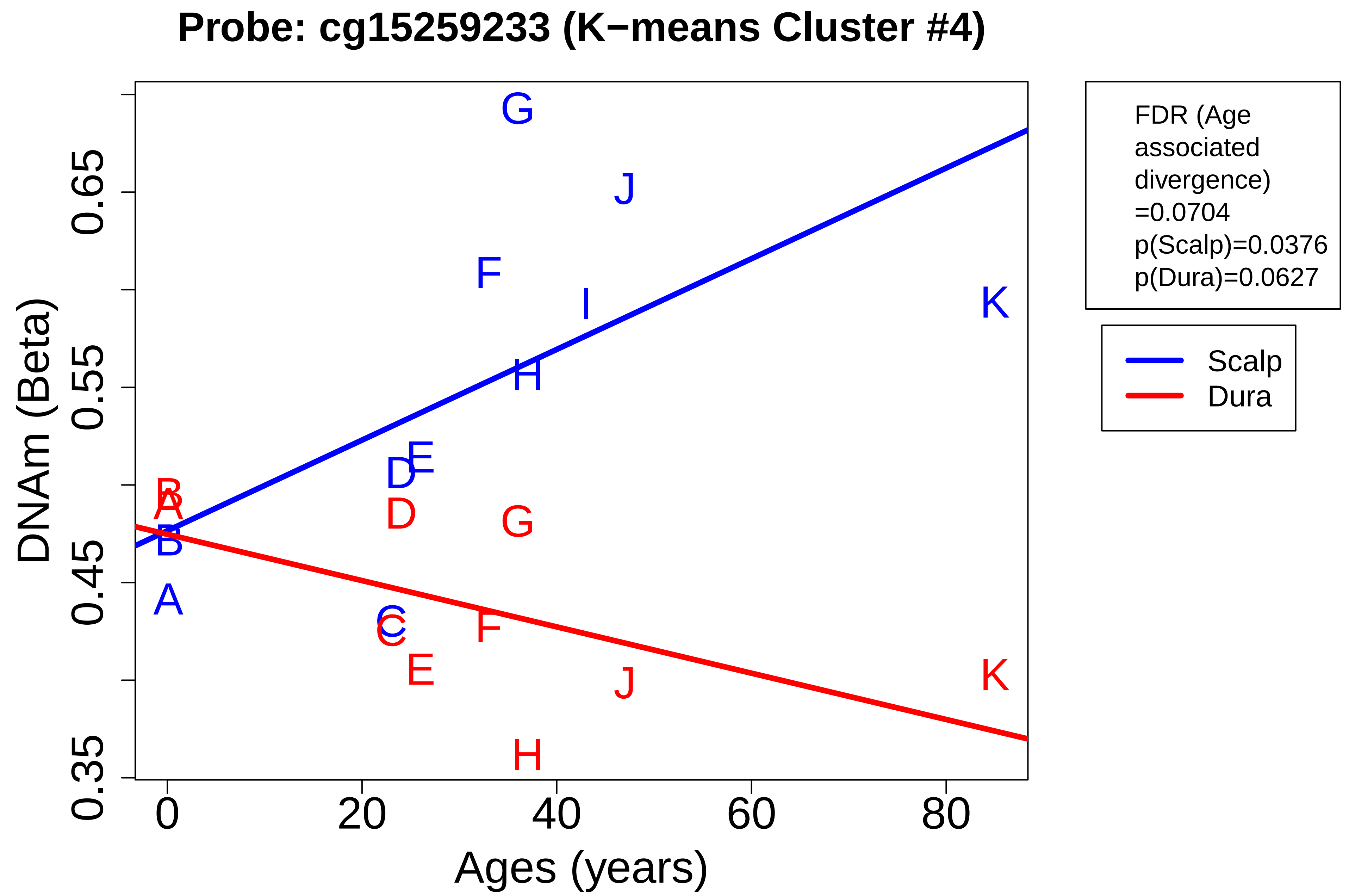

Probe: cg15259233 (K−means Cluster #4)
FDR (Age associated divergence) =0.0704
p(Scalp)=0.0376
p(Dura)=0.0627
Scalp
Dura
DNAm (Beta)
0.35
0.45
0.55
0.65
0
20
40
60
80
Ages (years)

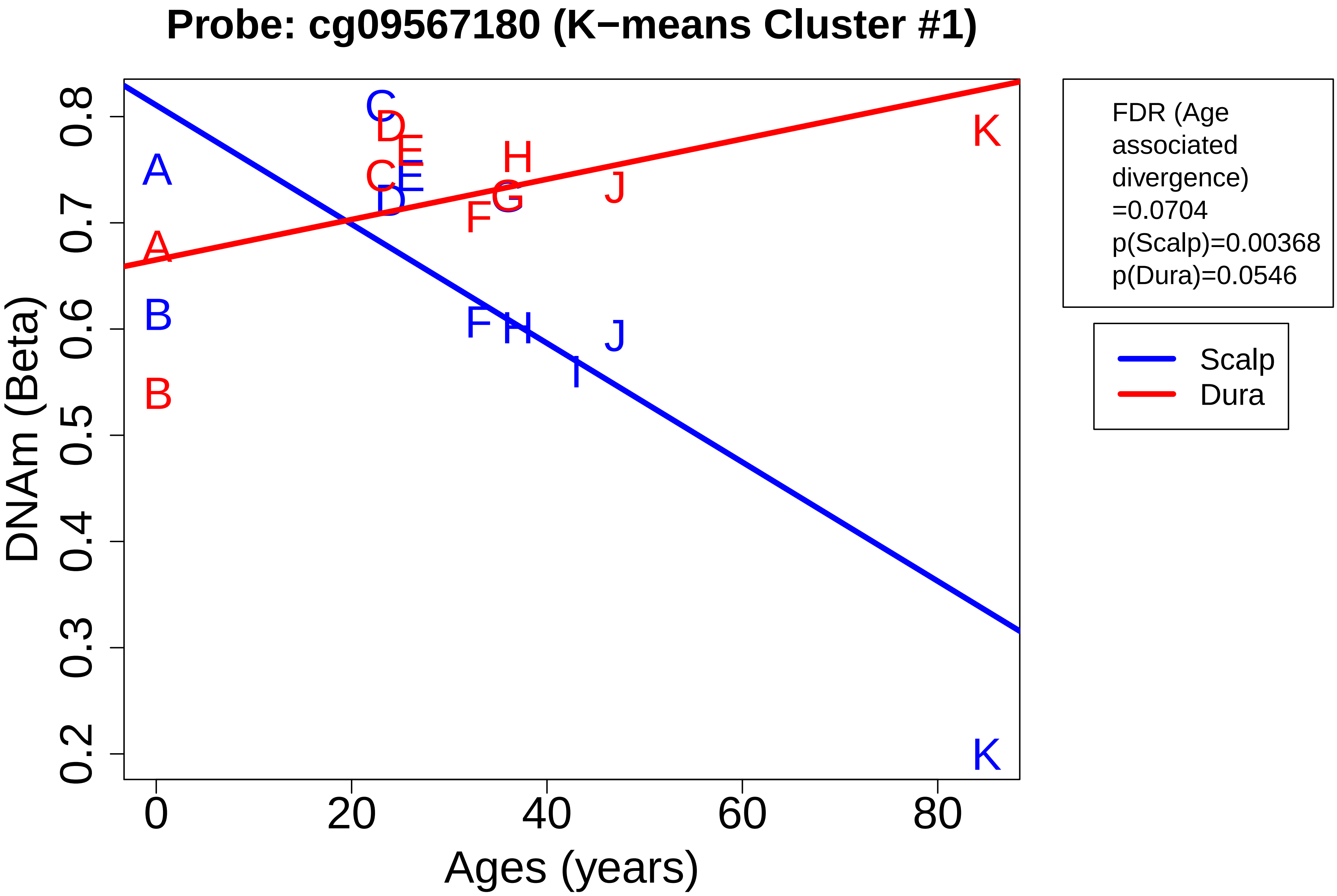

Probe: cg09567180 (K−means Cluster #1)
DNAm (Beta)
0.2
0.3
0.4
0.5
0.6
0.7
0.8
Ages (years)
0
20
40
60
80
A
B
C
D
E
F
G
H
I
J
K
FDR (Age associated divergence) =0.0704
p(Scalp)=0.00368
p(Dura)=0.0546
Scalp
Dura

Probe: cg00155397 (K−means Cluster #7)

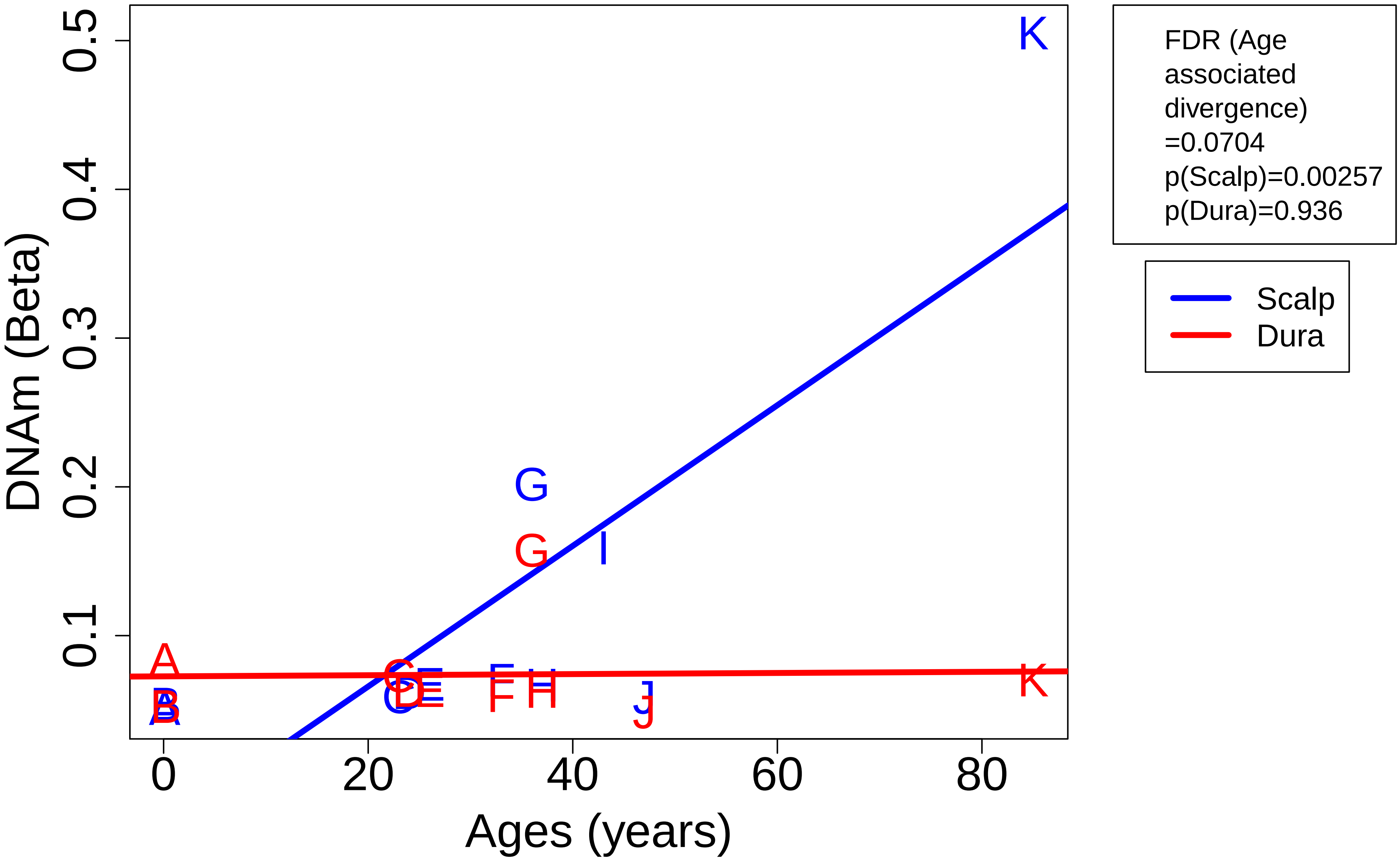

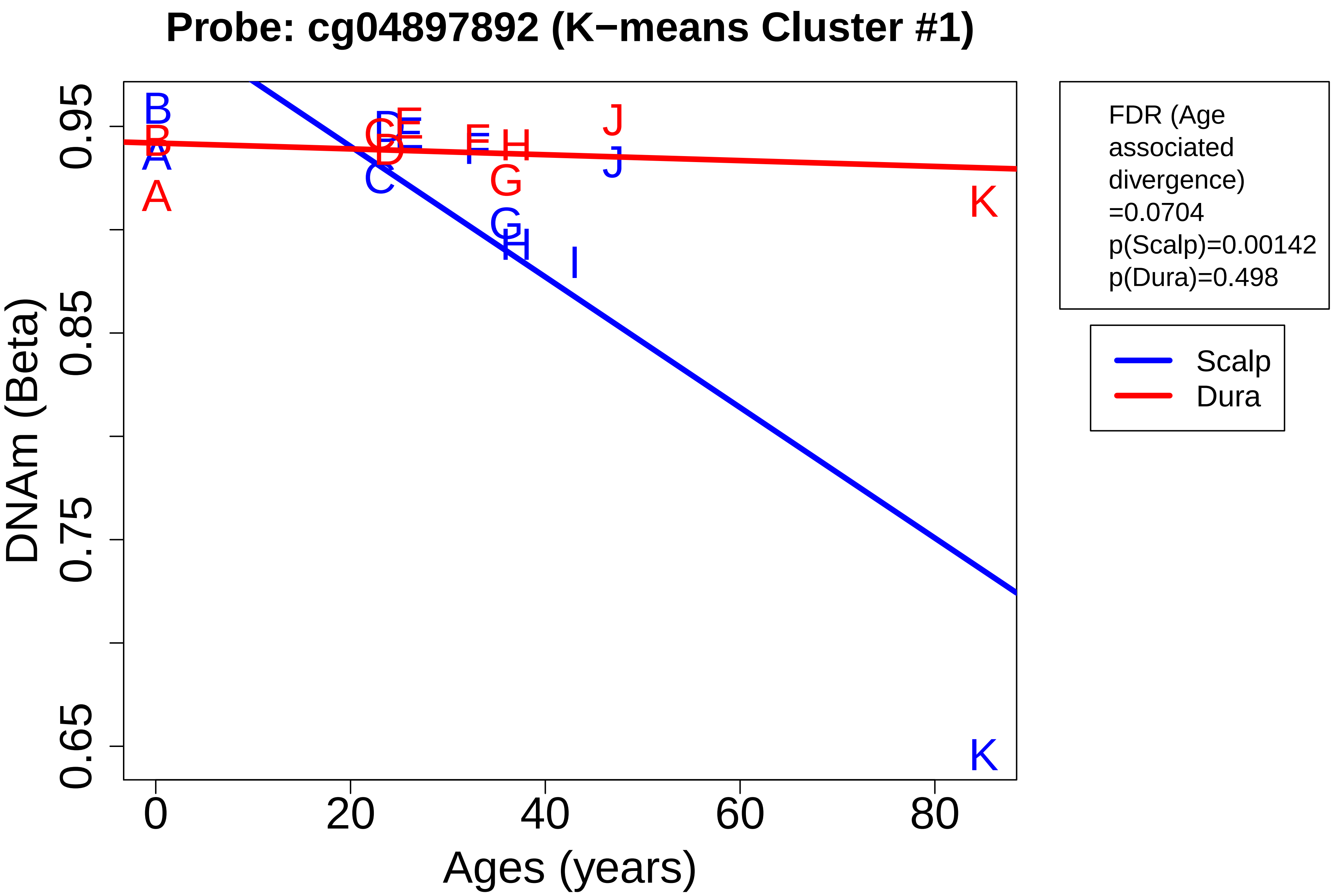

Probe: cg04897892 (K−means Cluster #1)
DNAm (Beta)
Ages (years)
0.65
0.75
0.85
0.95
0
20
40
60
80
FDR (Age associated divergence) =0.0704
p(Scalp)=0.00142
p(Dura)=0.498
Scalp
Dura

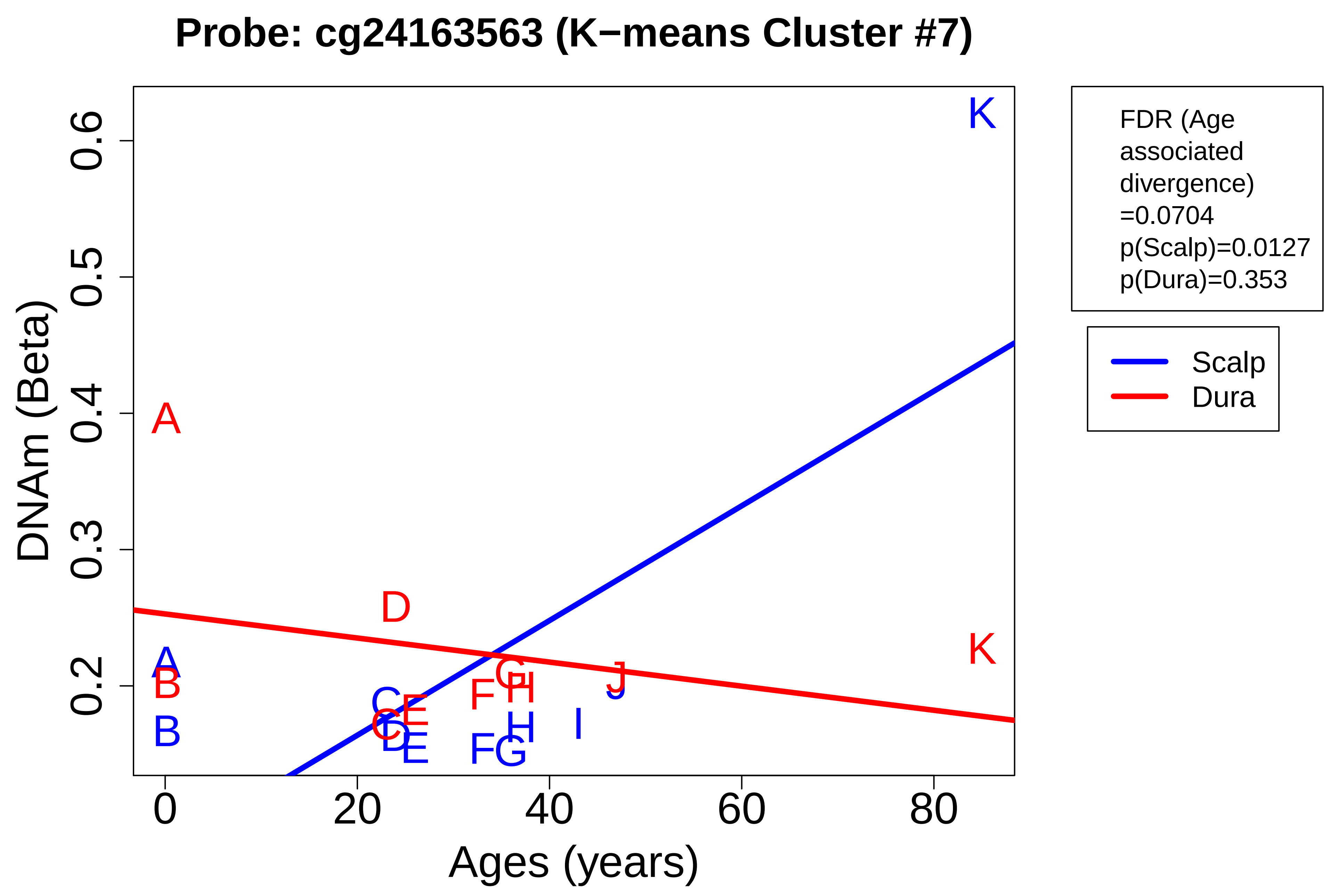

Probe: cg24163563 (K−means Cluster #7)
DNAm (Beta)
0.2
0.3
0.4
0.5
0.6
0
20
40
60
80
Ages (years)
A
B
C
D
E
F
G
H
I
J
K
FDR (Age associated divergence) =0.0704
p(Scalp)=0.0127
p(Dura)=0.353
Scalp
Dura

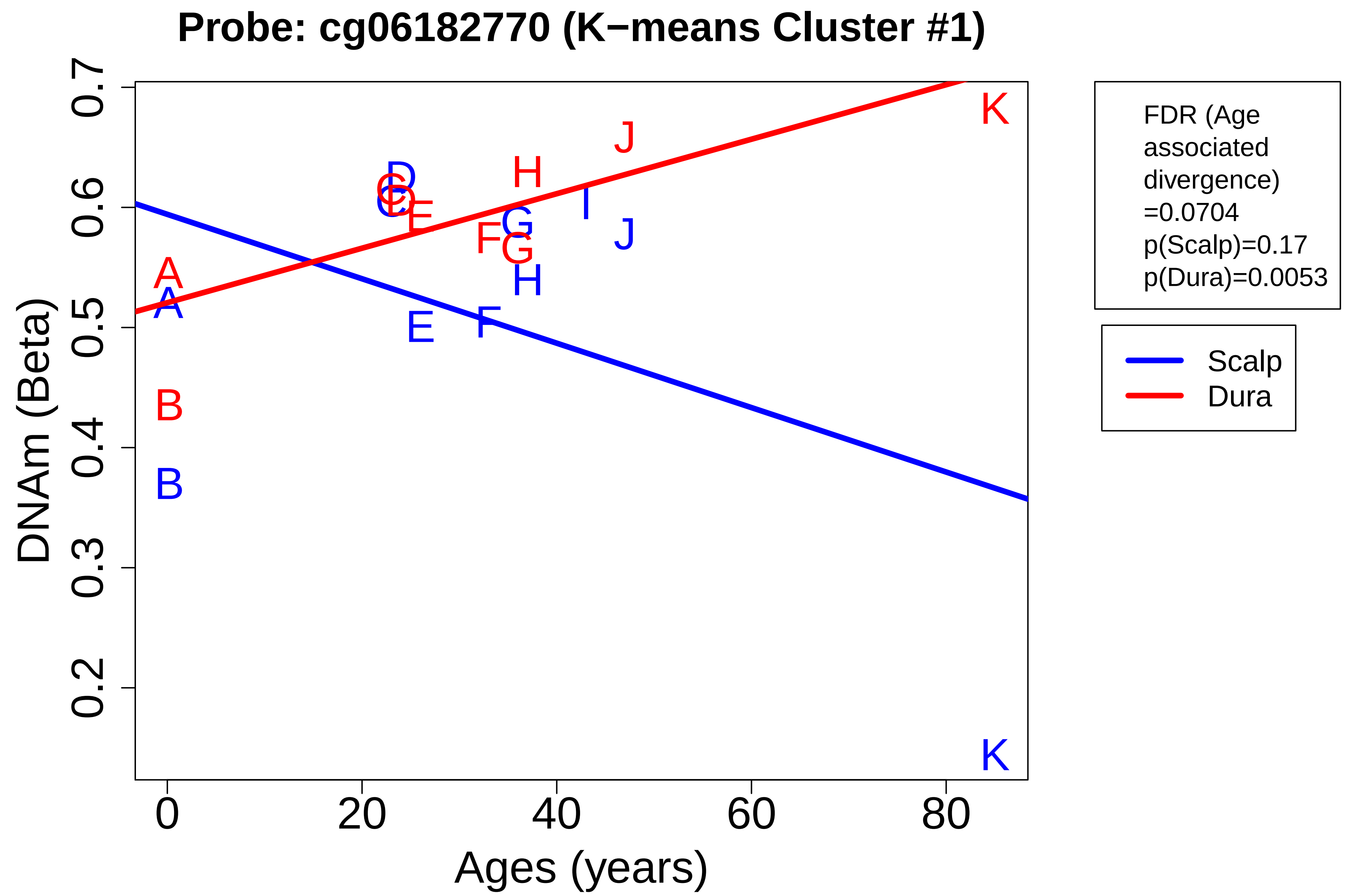

Probe: cg06182770 (K−means Cluster #1)
DNAm (Beta)
0.2
0.3
0.4
0.5
0.6
0.7
0
20
40
60
80
Ages (years)
FDR (Age associated divergence) =0.0704
p(Scalp)=0.17
p(Dura)=0.0053
Scalp
Dura

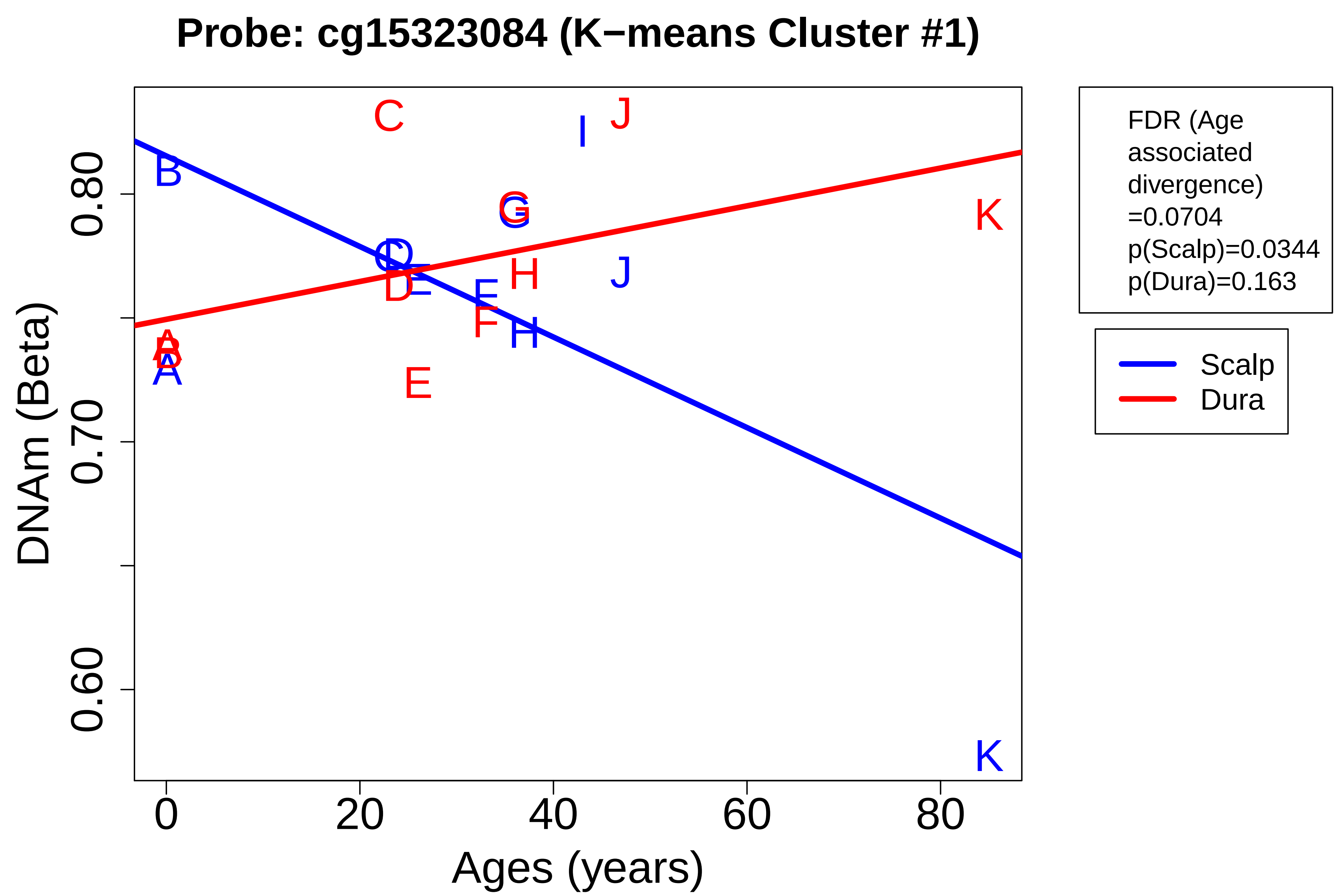

Probe: cg15323084 (K−means Cluster #1)
DNAm (Beta)
0.60
0.70
0.80
0
20
40
60
80
Ages (years)
FDR (Age associated divergence) =0.0704
p(Scalp)=0.0344
p(Dura)=0.163
Scalp
Dura

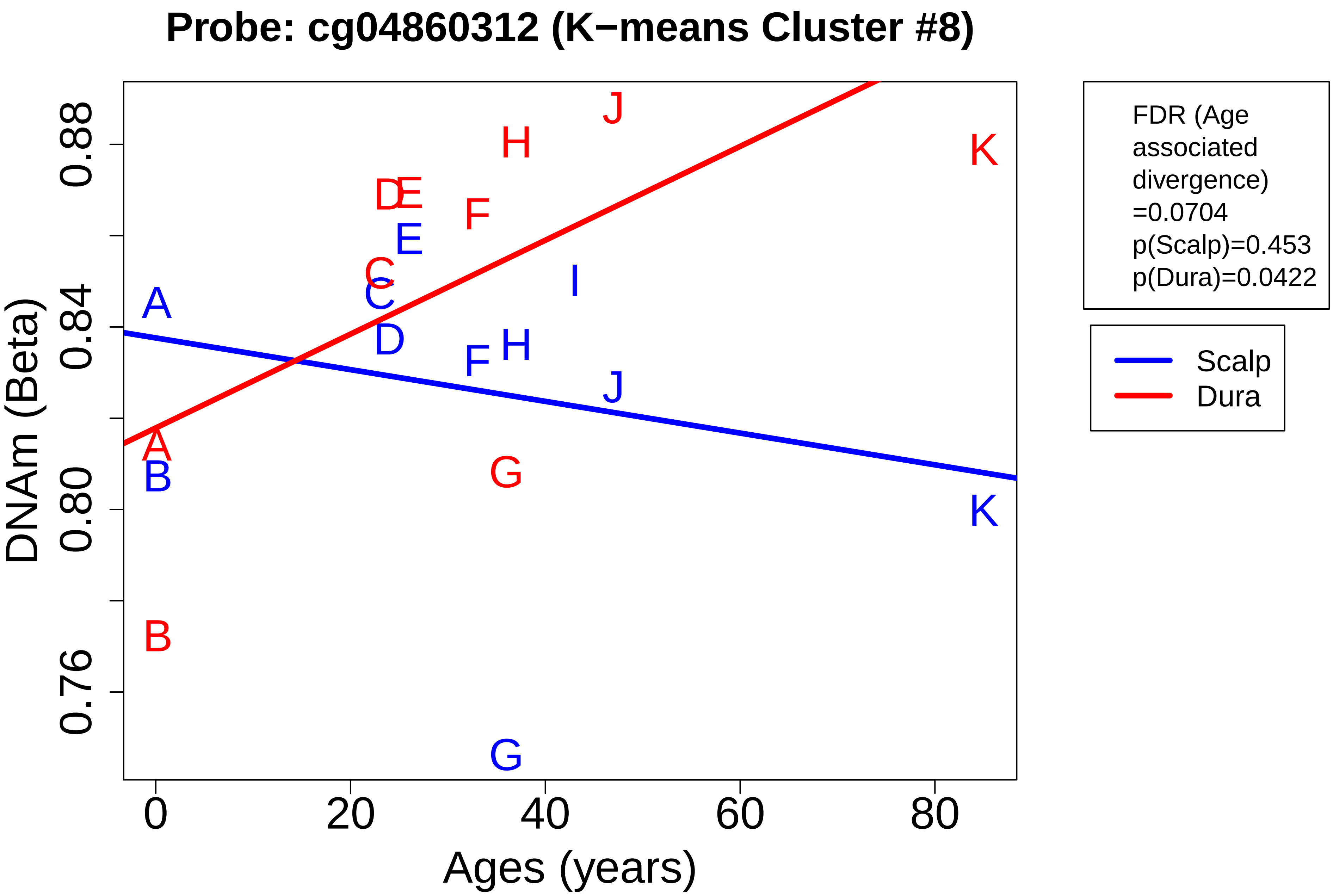

Probe: cg04860312 (K−means Cluster #8)
DNAm (Beta)
0.76
0.80
0.84
0.88
Ages (years)
0
20
40
60
80
A
B
C
D
E
F
G
H
I
J
K
A
B
C
D
E
F
G
H
J
K
FDR (Age associated divergence) =0.0704
p(Scalp)=0.453
p(Dura)=0.0422
Scalp
Dura

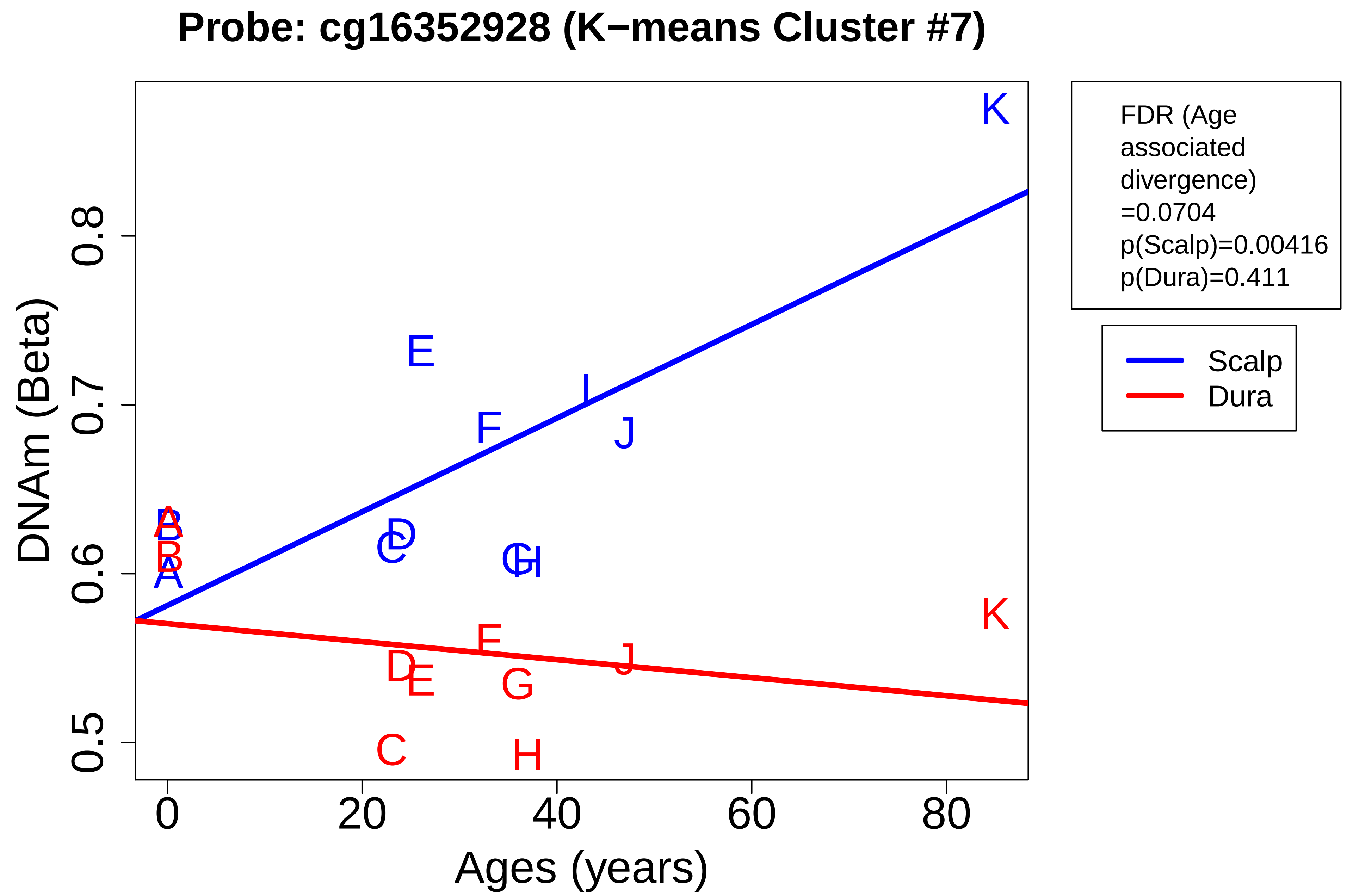

Probe: cg16352928 (K−means Cluster #7)
FDR (Age associated divergence) =0.0704
p(Scalp)=0.00416
p(Dura)=0.411
Scalp
Dura
DNAm (Beta)
0.5
0.6
0.7
0.8
0
20
40
60
80
Ages (years)
A
B
C
D
E
F
G
H
I
J
K

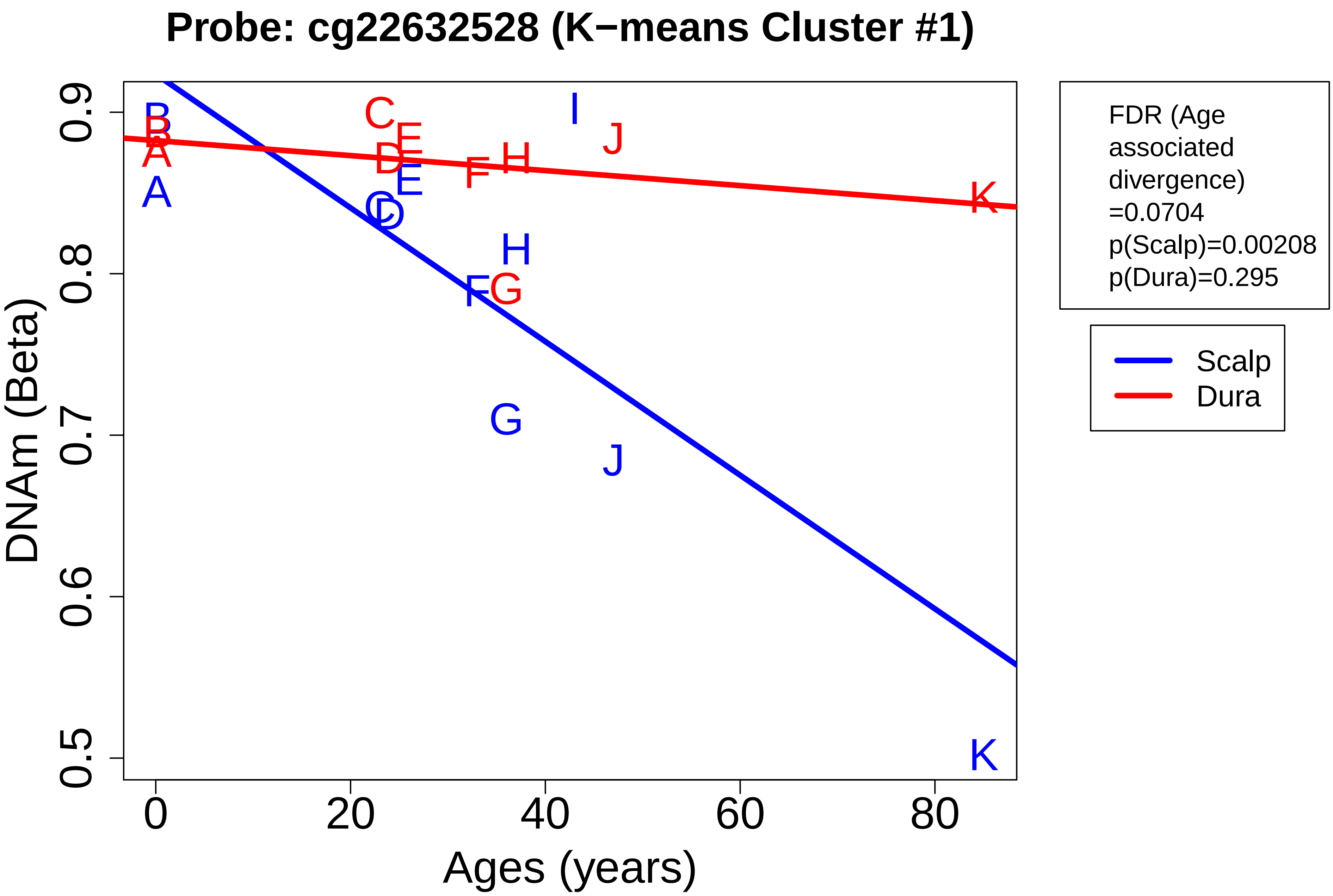

Probe: cg22632528 (K−means Cluster #1)
DNAm (Beta)
0.9
0.8
0.7
0.6
0.5
0
20
40
60
80
Ages (years)
A
B
C
D
E
F
G
H
I
J
K
FDR (Age associated divergence) =0.0704
p(Scalp)=0.00208
p(Dura)=0.295
Scalp
Dura

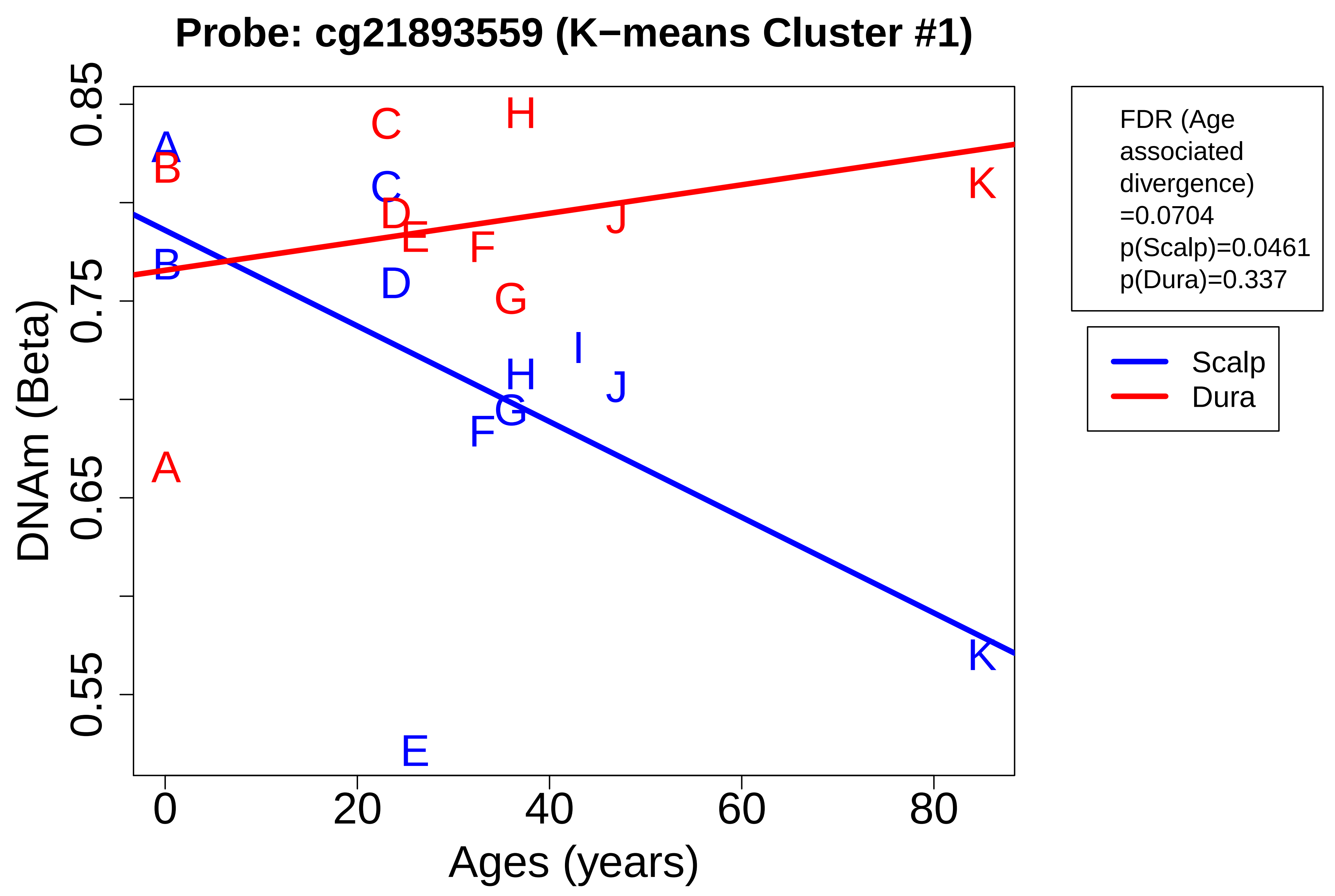

Probe: cg21893559 (K−means Cluster #1)
DNAm (Beta)
0.85
0.75
0.65
0.55
0
20
40
60
80
Ages (years)
FDR (Age associated divergence) =0.0704
p(Scalp)=0.0461
p(Dura)=0.337
Scalp
Dura

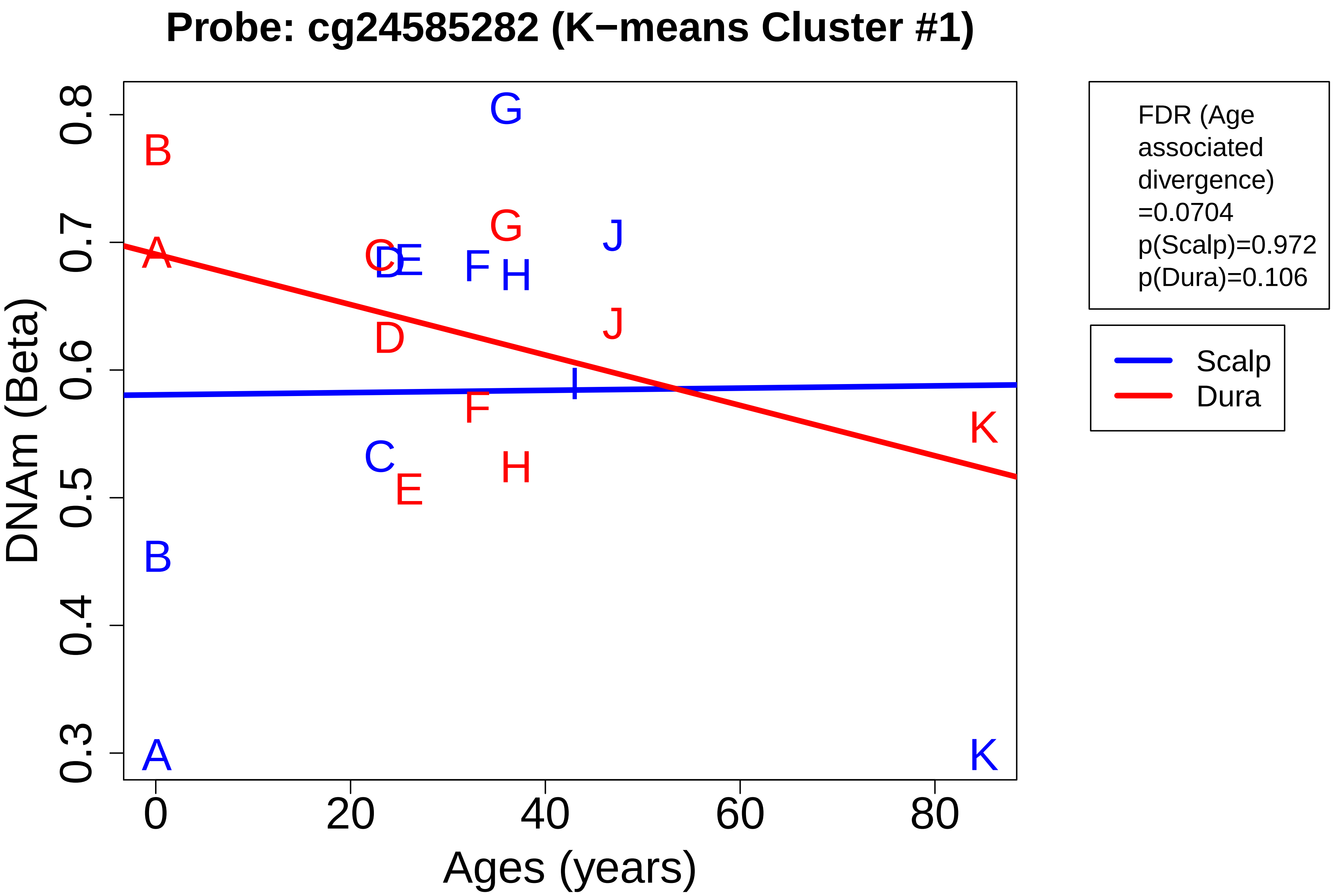

Probe: cg24585282 (K−means Cluster #1)
DNAm (Beta)
0.3
0.4
0.5
0.6
0.7
0.8
0
20
40
60
80
Ages (years)
FDR (Age associated divergence) =0.0704
p(Scalp)=0.972
p(Dura)=0.106
Scalp
Dura

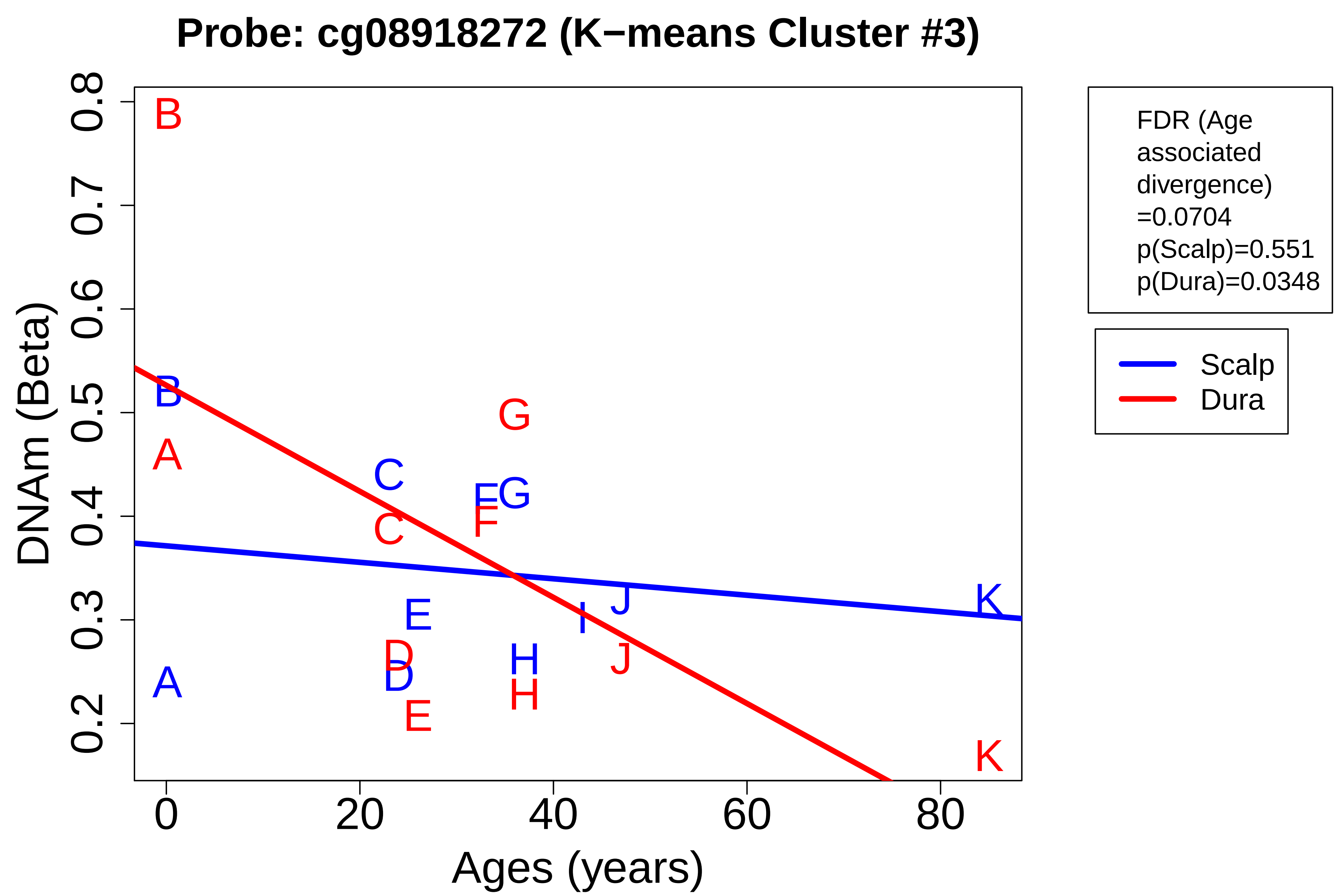
Probe: cg08918272 (K−means Cluster #3)
DNAm (Beta)
0.2
0.3
0.4
0.5
0.6
0.7
0.8
Ages (years)
0
20
40
60
80
FDR (Age associated divergence) =0.0704
p(Scalp)=0.551
p(Dura)=0.0348
Scalp
Dura
A
B
C
D
E
F
G
H
I
J
K

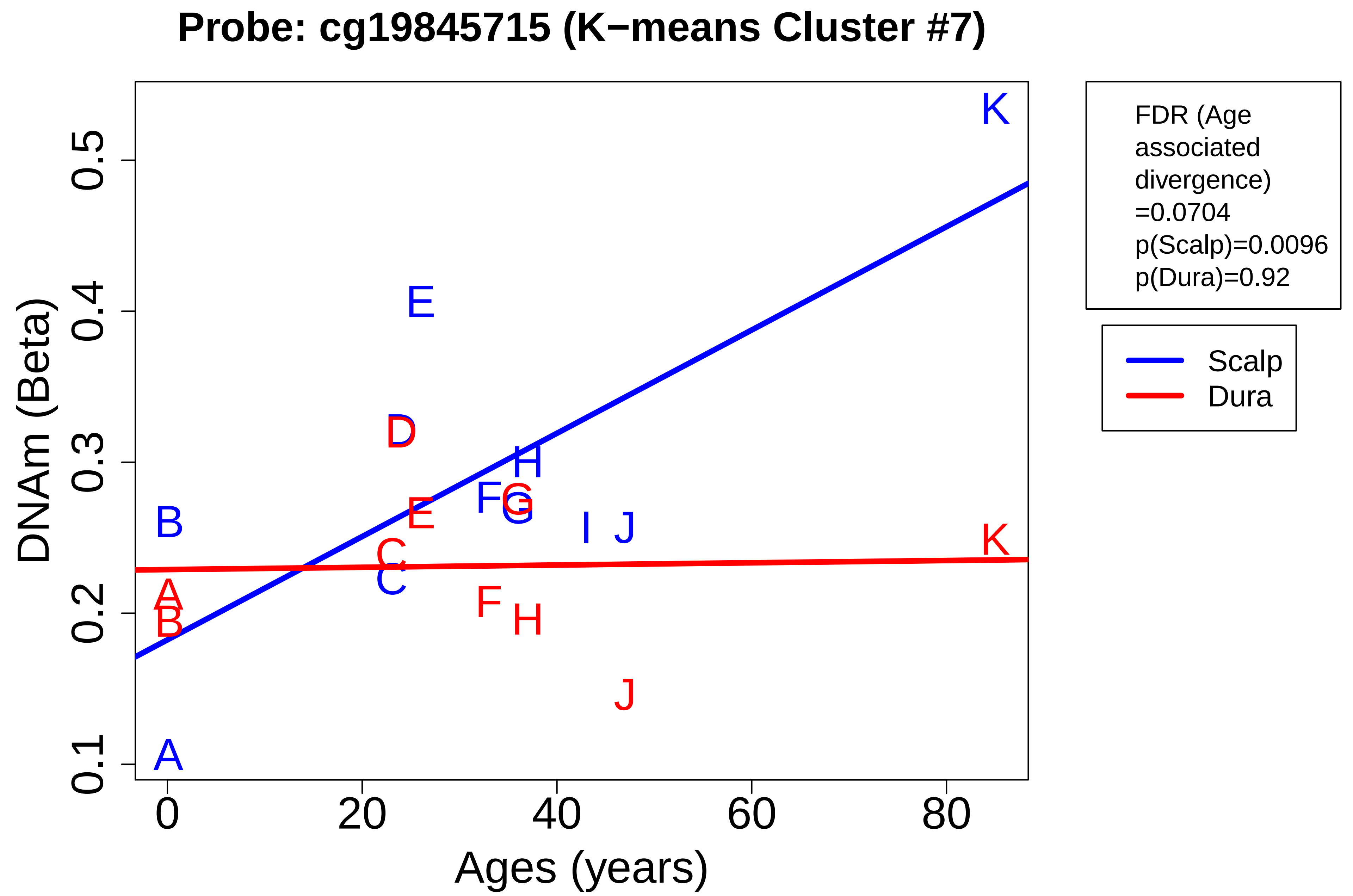

Probe: cg19845715 (K−means Cluster #7)
DNAm (Beta)
0.1
0.2
0.3
0.4
0.5
0
20
40
60
80
Ages (years)
A
B
C
D
E
F
G
H
I
J
K
FDR (Age associated divergence) =0.0704
p(Scalp)=0.0096
p(Dura)=0.92
Scalp
Dura

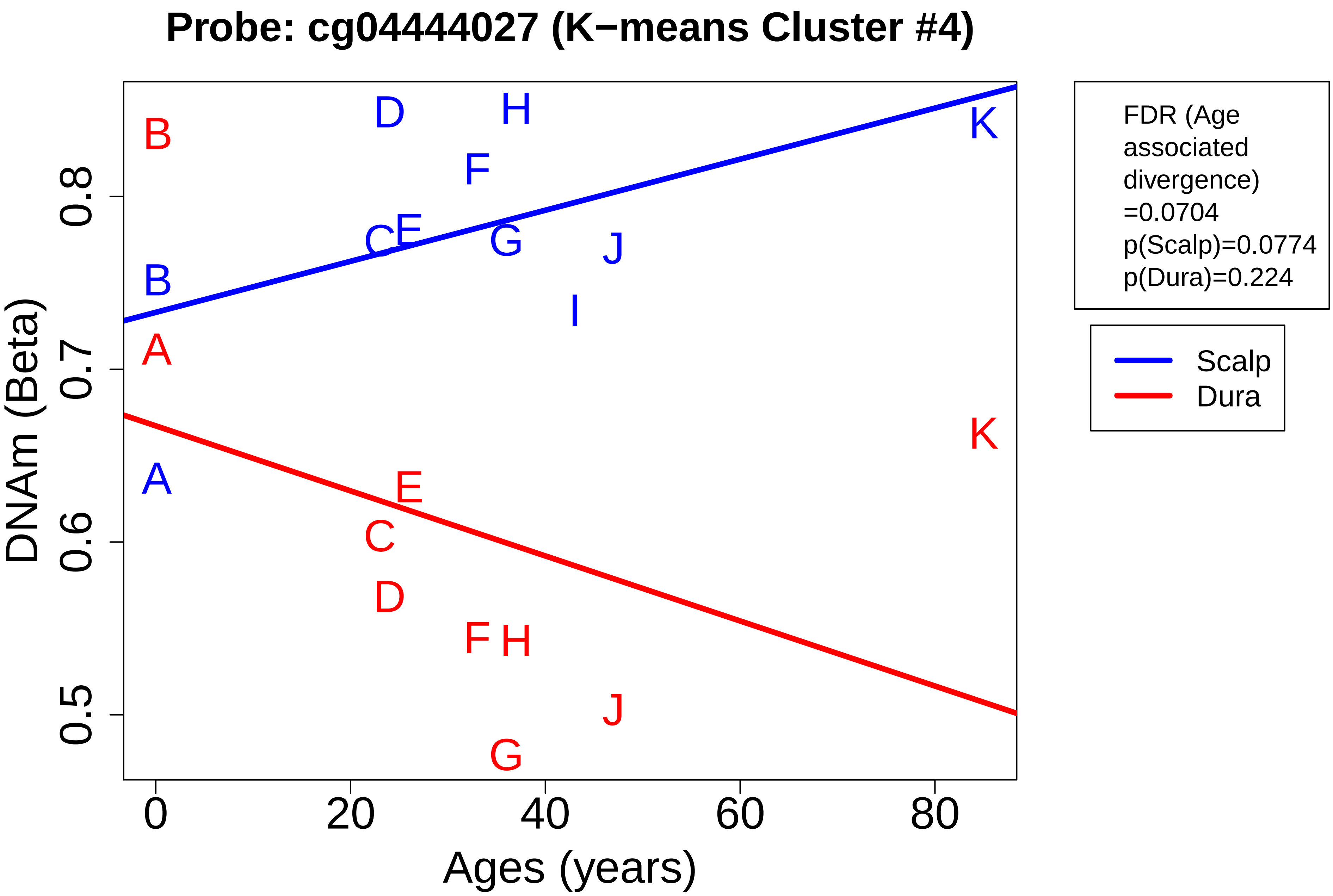

Probe: cg04444027 (K−means Cluster #4)
DNAm (Beta)
0.5
0.6
0.7
0.8
0
20
40
60
80
Ages (years)
A
B
C
D
E
F
G
H
I
J
K
FDR (Age associated divergence) =0.0704
p(Scalp)=0.0774
p(Dura)=0.224
Scalp
Dura

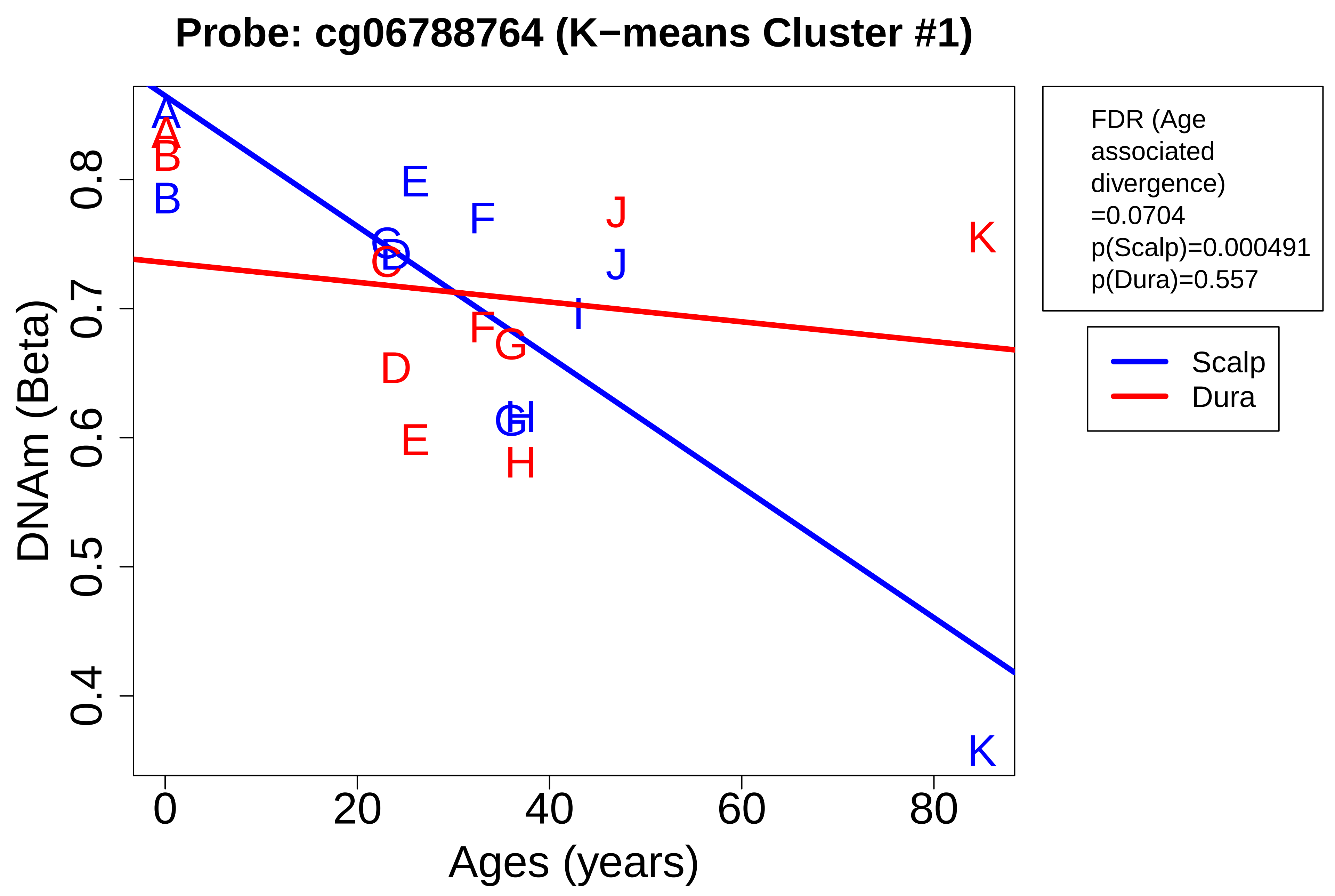

Probe: cg06788764 (K−means Cluster #1)
DNAm (Beta)
0.4
0.5
0.6
0.7
0.8
Ages (years)
0
20
40
60
80
FDR (Age associated divergence) =0.0704
p(Scalp)=0.000491
p(Dura)=0.557
Scalp
Dura

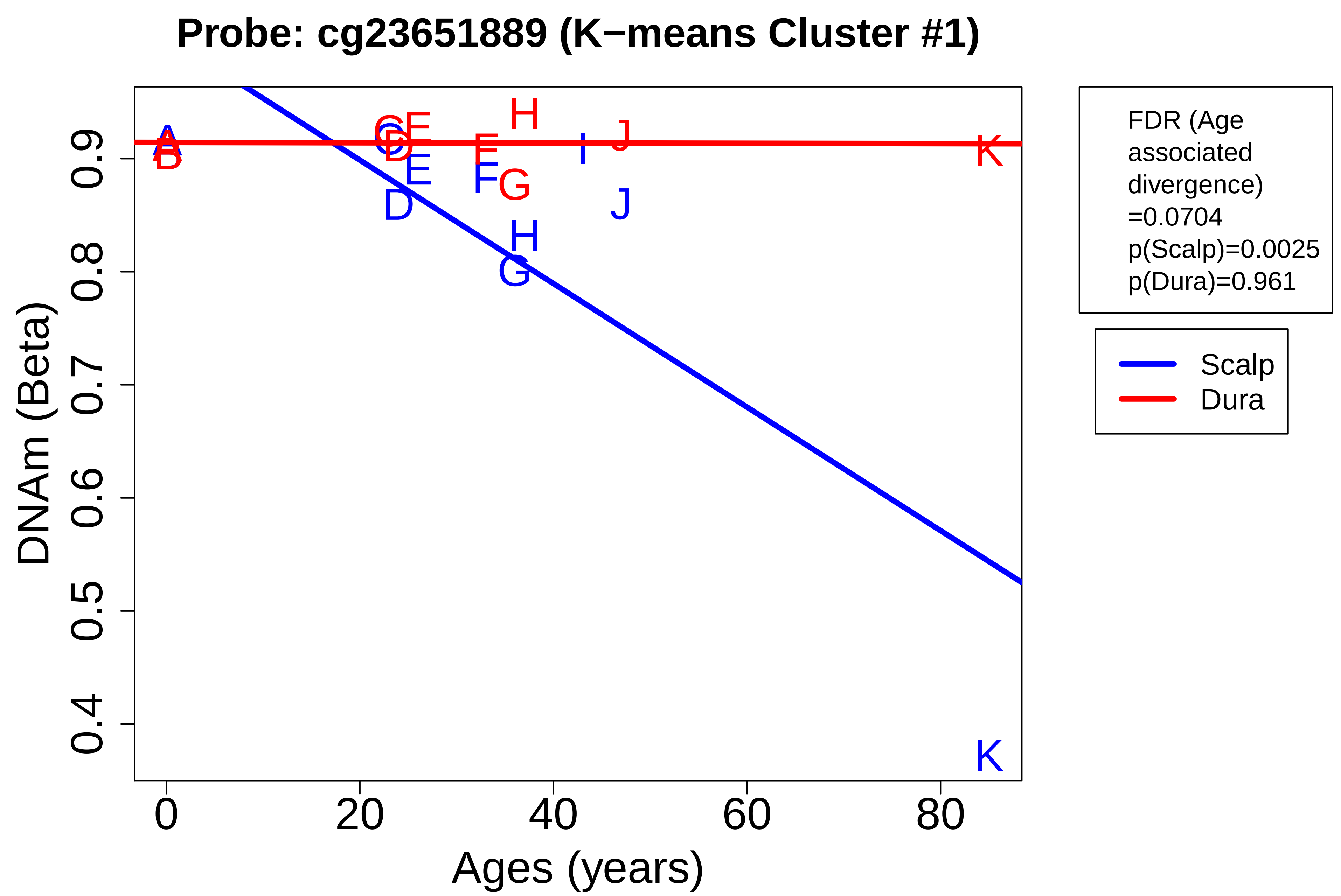
Probe: cg23651889 (K−means Cluster #1)
DNAm (Beta)
Ages (years)
0
20
40
60
80
0.4
0.5
0.6
0.7
0.8
0.9
FDR (Age associated divergence) =0.0704
p(Scalp)=0.0025
p(Dura)=0.961
Scalp
Dura

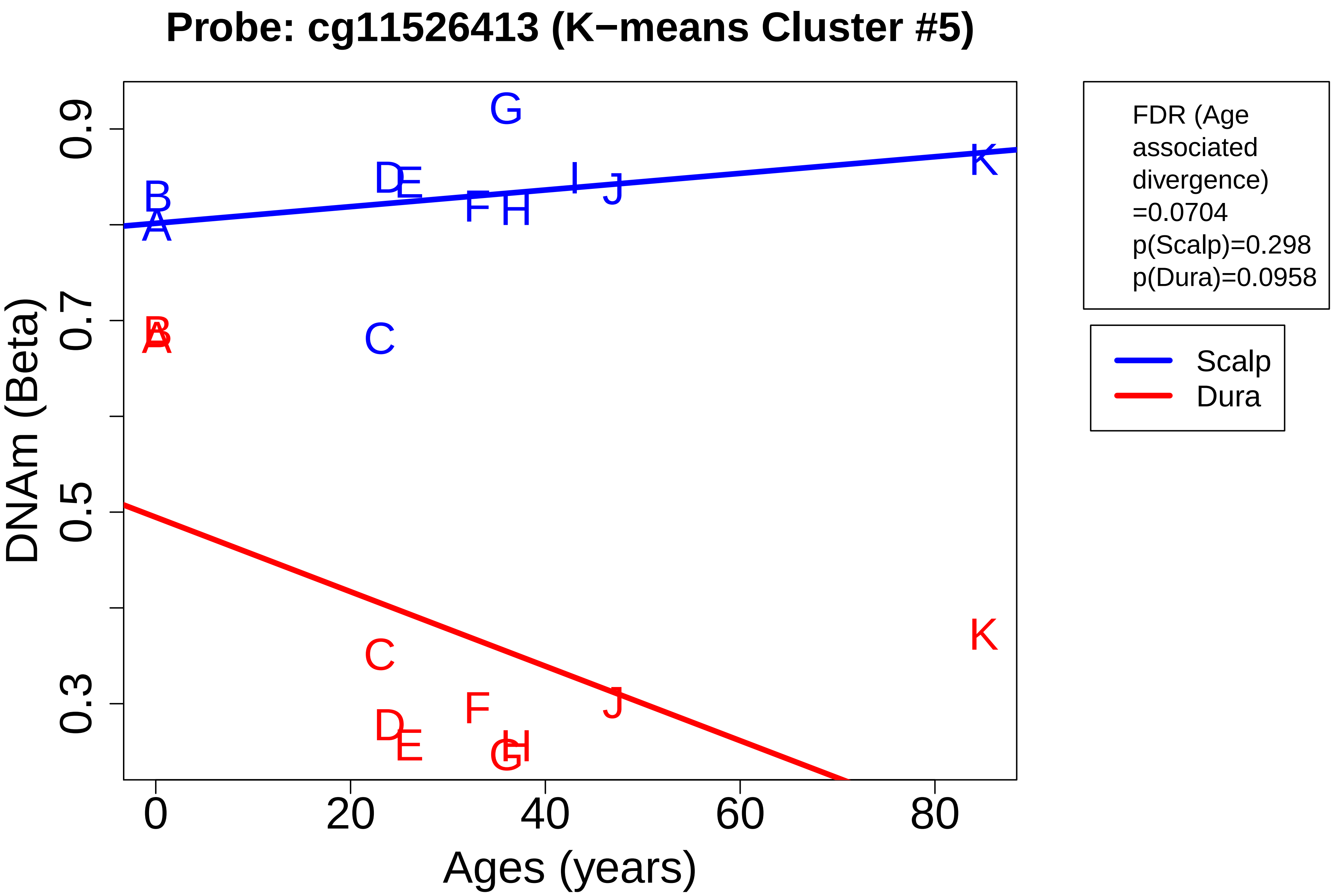

Probe: cg11526413 (K−means Cluster #5)
DNAm (Beta)
0.3
0.5
0.7
0.9
Ages (years)
0
20
40
60
80
A
B
C
D
E
F
G
H
I
J
K
FDR (Age associated divergence) =0.0704
p(Scalp)=0.298
p(Dura)=0.0958
Scalp
Dura

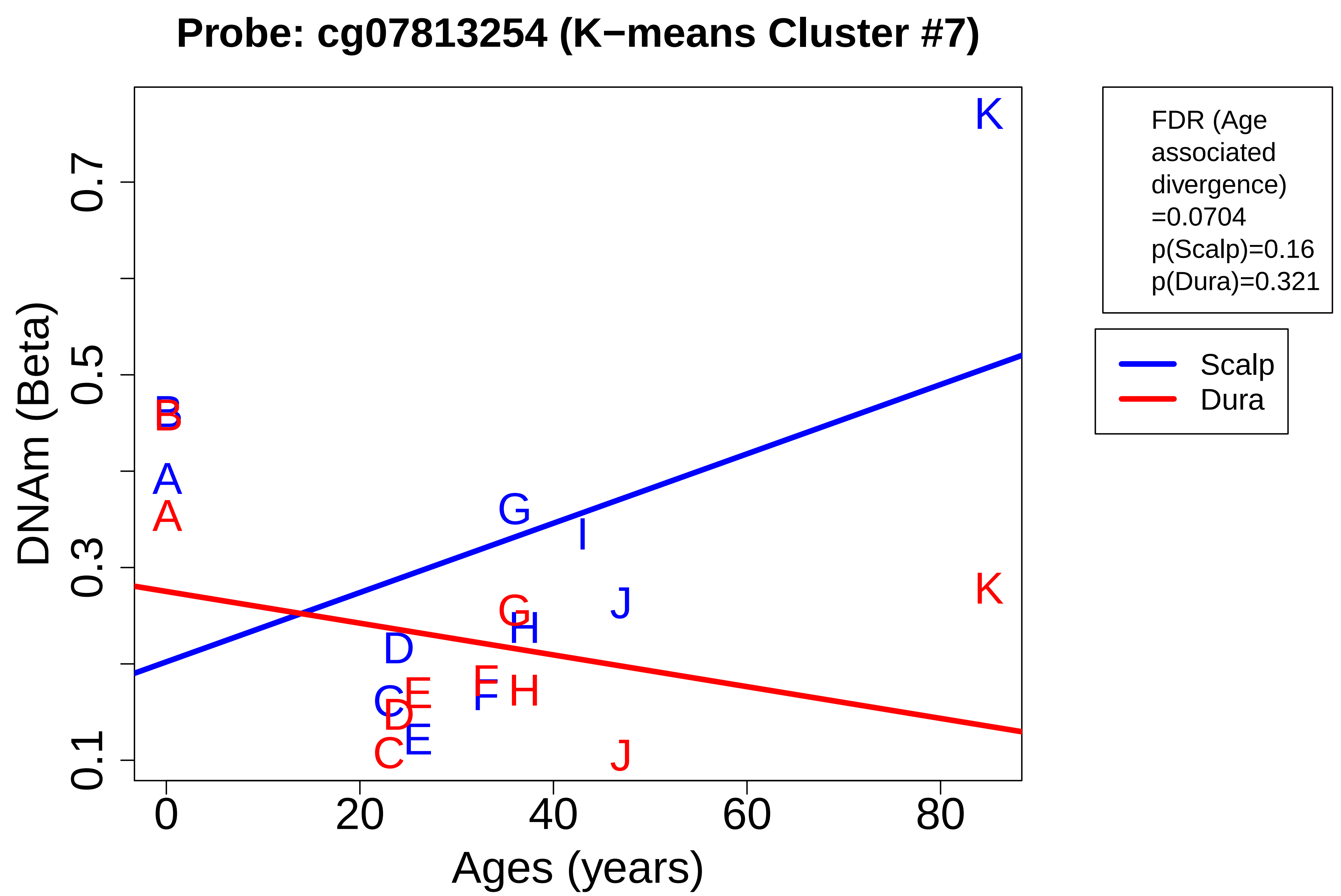

Probe: cg07813254 (K−means Cluster #7)
DNAm (Beta)
0.1
0.3
0.5
0.7
0
20
40
60
80
Ages (years)
FDR (Age associated divergence) =0.0704
p(Scalp)=0.16
p(Dura)=0.321
Scalp
Dura

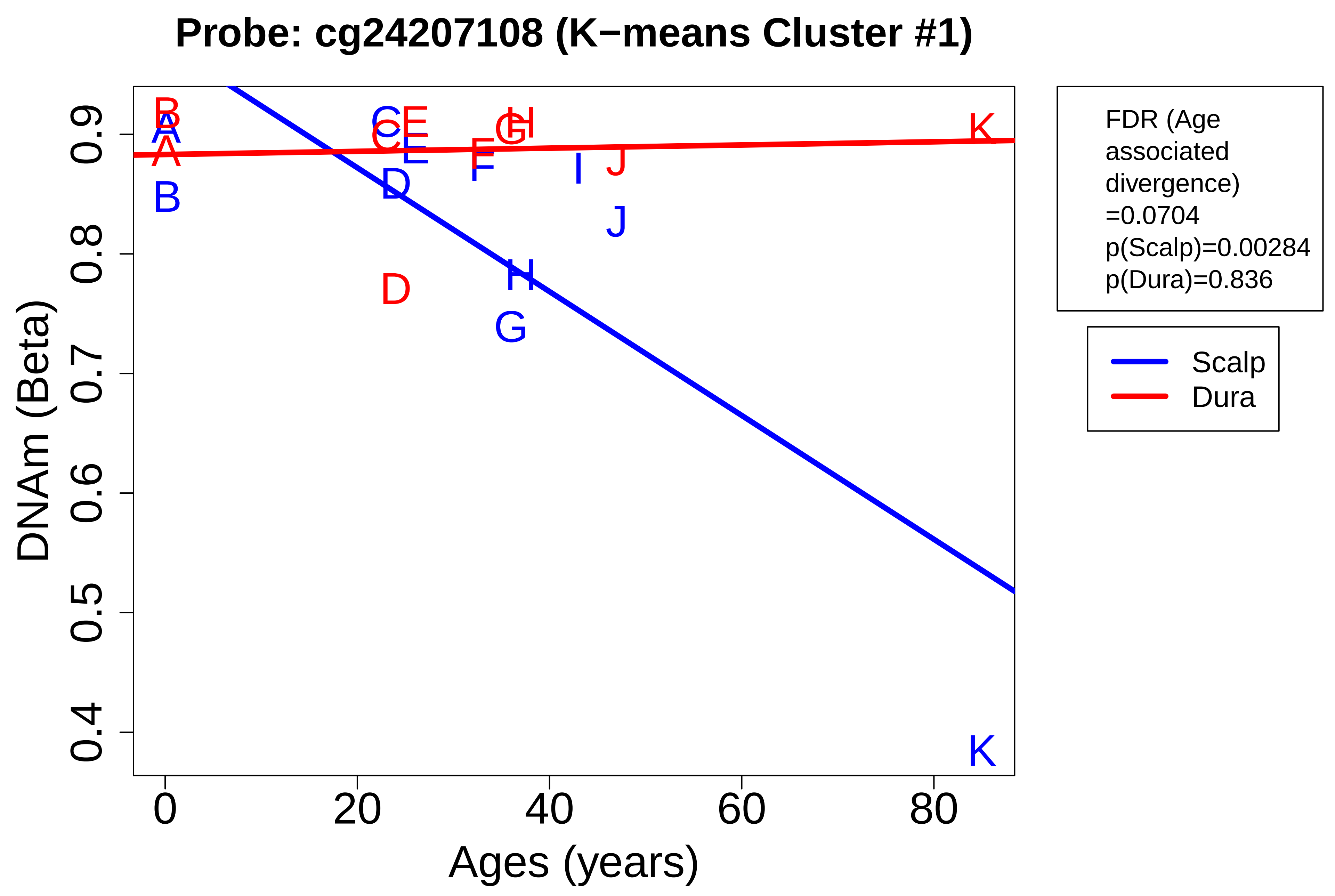

Probe: cg24207108 (K−means Cluster #1)
DNAm (Beta)
0.4
0.5
0.6
0.7
0.8
0.9
0
20
40
60
80
Ages (years)
FDR (Age associated divergence) =0.0704
p(Scalp)=0.00284
p(Dura)=0.836
Scalp
Dura

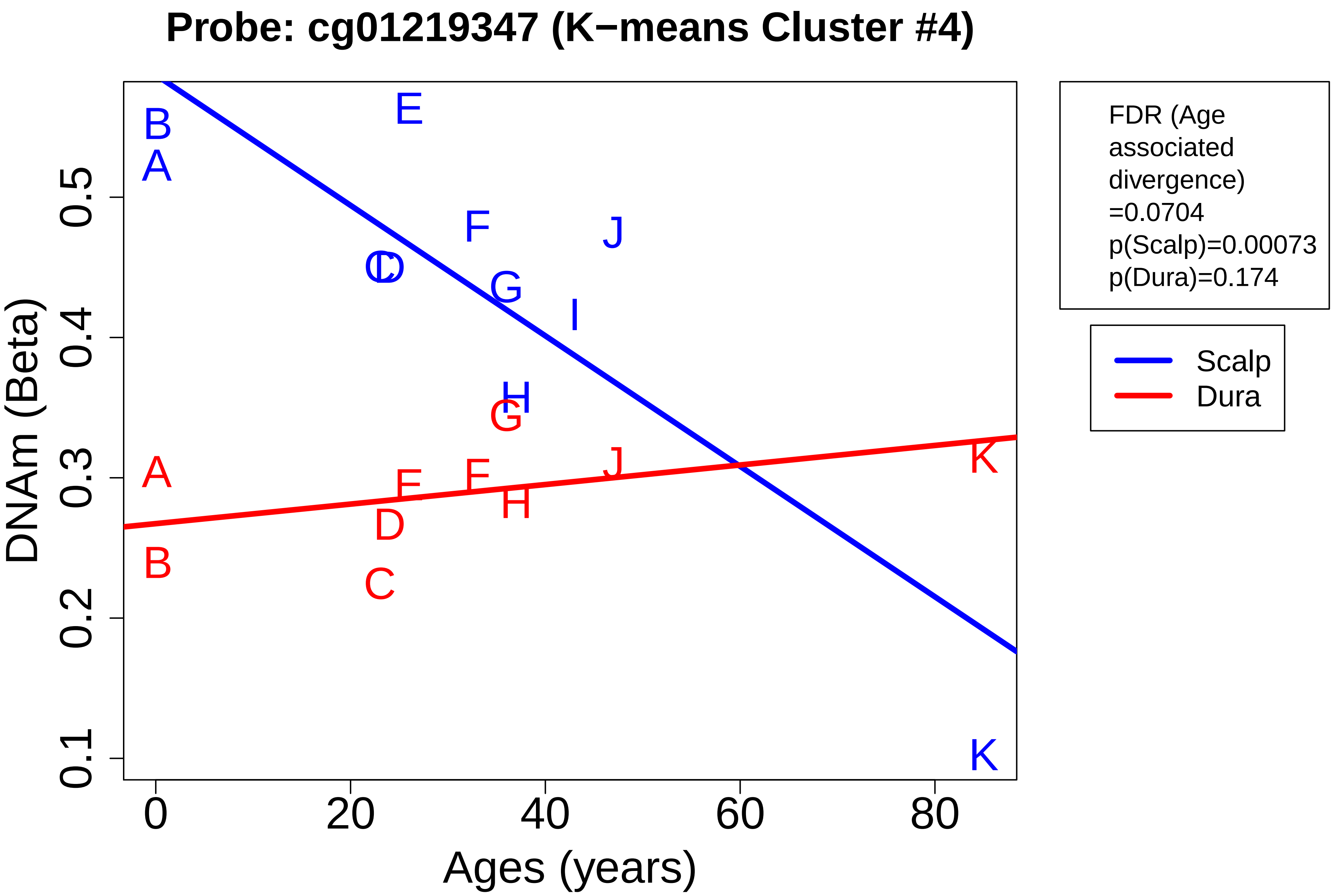

Probe: cg01219347 (K−means Cluster #4)
DNAm (Beta)
Ages (years)
0.1
0.2
0.3
0.4
0.5
0
20
40
60
80
FDR (Age associated divergence) =0.0704
p(Scalp)=0.00073
p(Dura)=0.174
Scalp
Dura

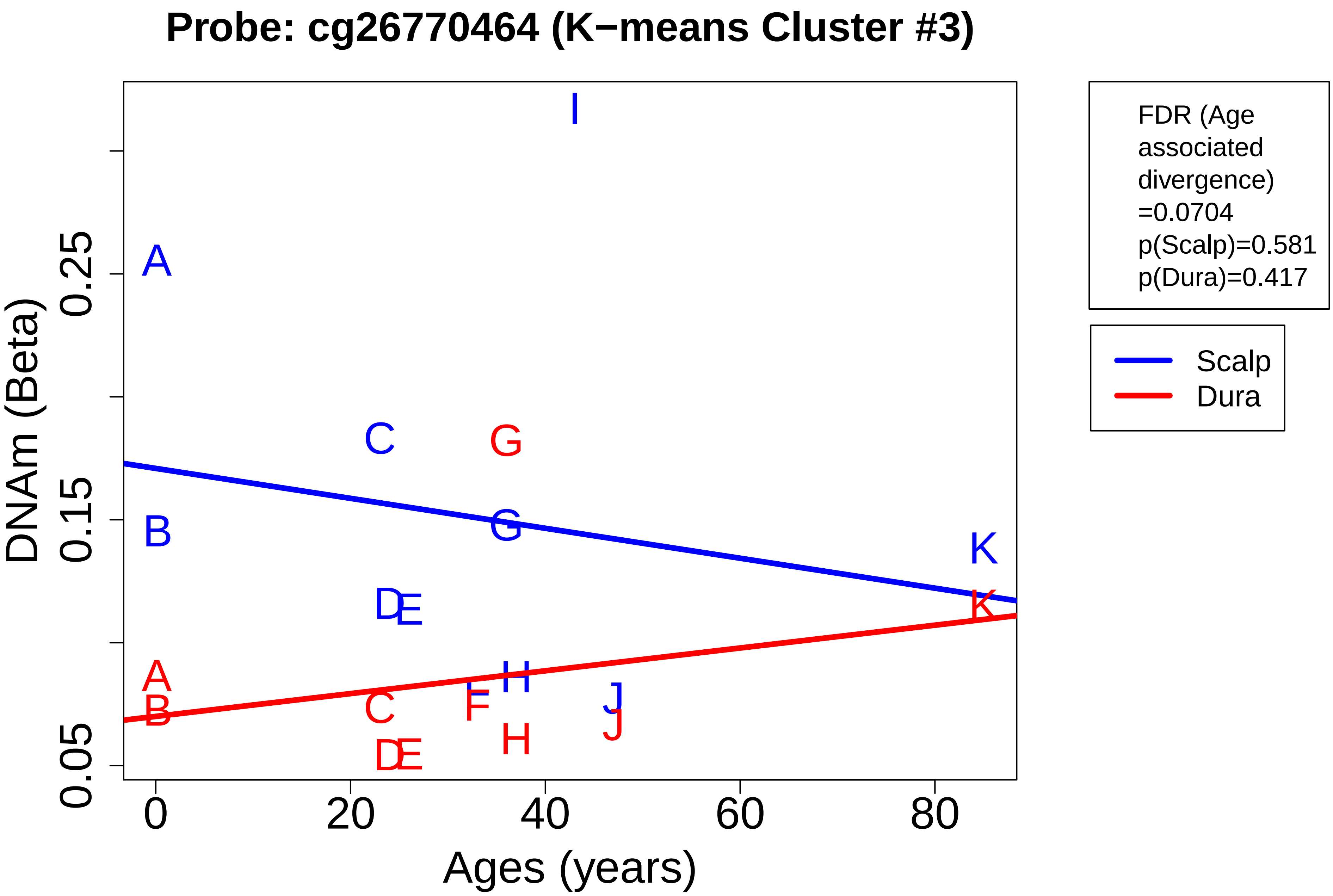

Probe: cg26770464 (K−means Cluster #3)
DNAm (Beta)
0.05
0.15
0.25
0
20
40
60
80
Ages (years)
FDR (Age associated divergence) =0.0704
p(Scalp)=0.581
p(Dura)=0.417
Scalp
Dura

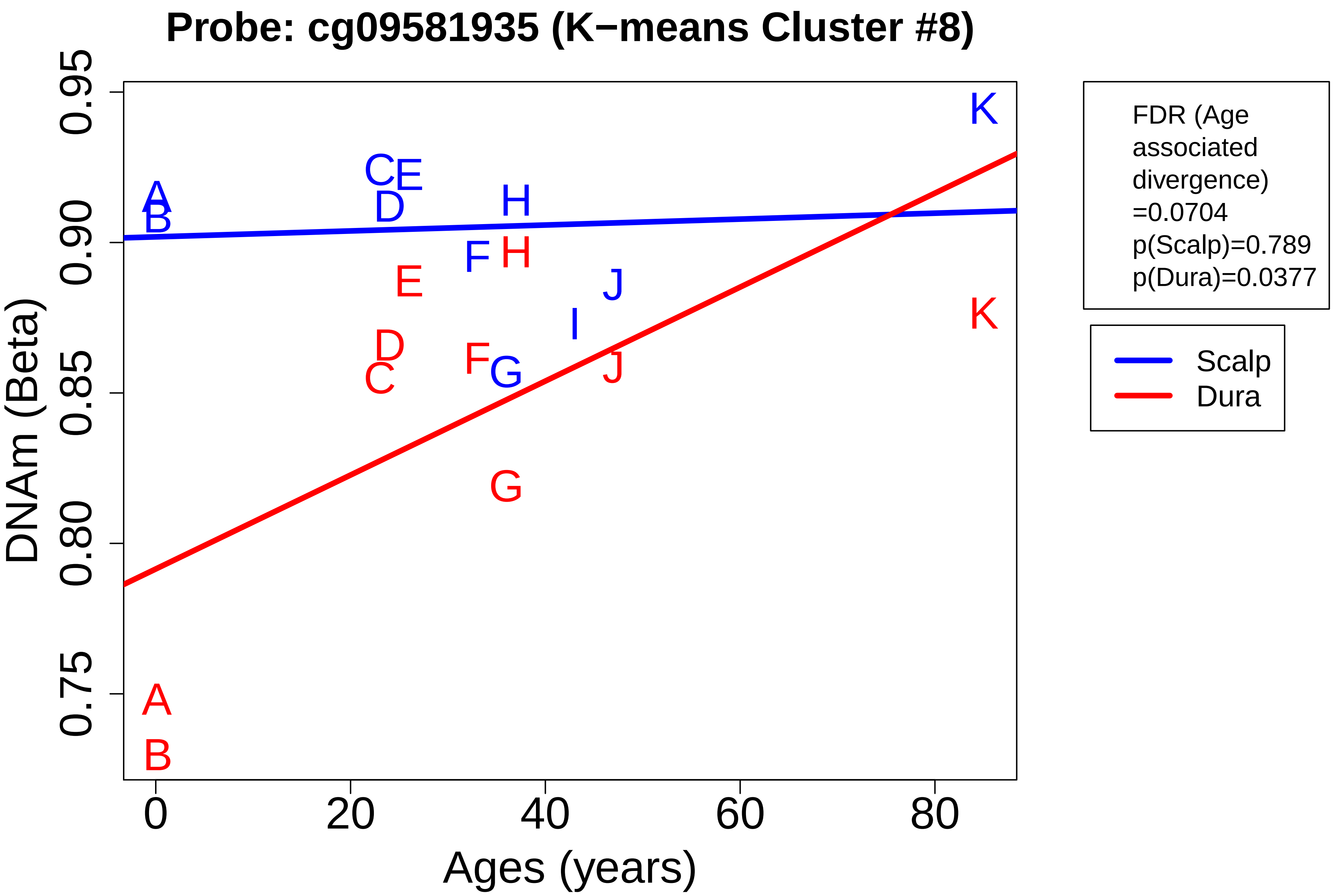

Probe: cg09581935 (K−means Cluster #8)
DNAm (Beta)
0.75
0.80
0.85
0.90
0.95
0
20
40
60
80
Ages (years)
A
B
C
D
E
F
G
H
I
J
K
FDR (Age associated divergence) =0.0704
p(Scalp)=0.789
p(Dura)=0.0377
Scalp
Dura

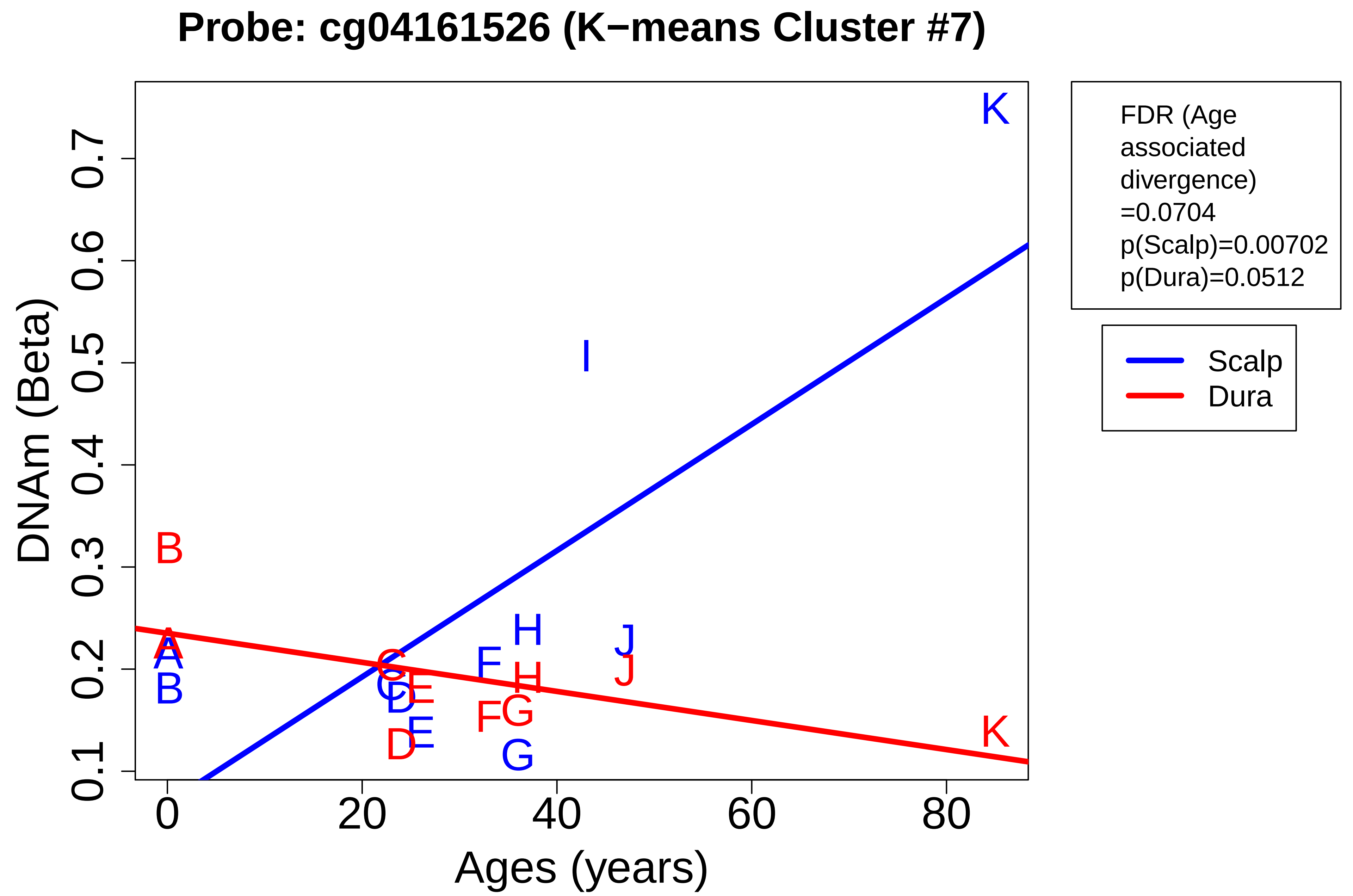
Probe: cg04161526 (K−means Cluster #7)
DNAm (Beta)
0.1
0.2
0.3
0.4
0.5
0.6
0.7
0
20
40
60
80
Ages (years)
FDR (Age associated divergence) =0.0704
p(Scalp)=0.00702
p(Dura)=0.0512
Scalp
Dura
A
B
C
D
E
F
G
H
I
J
K

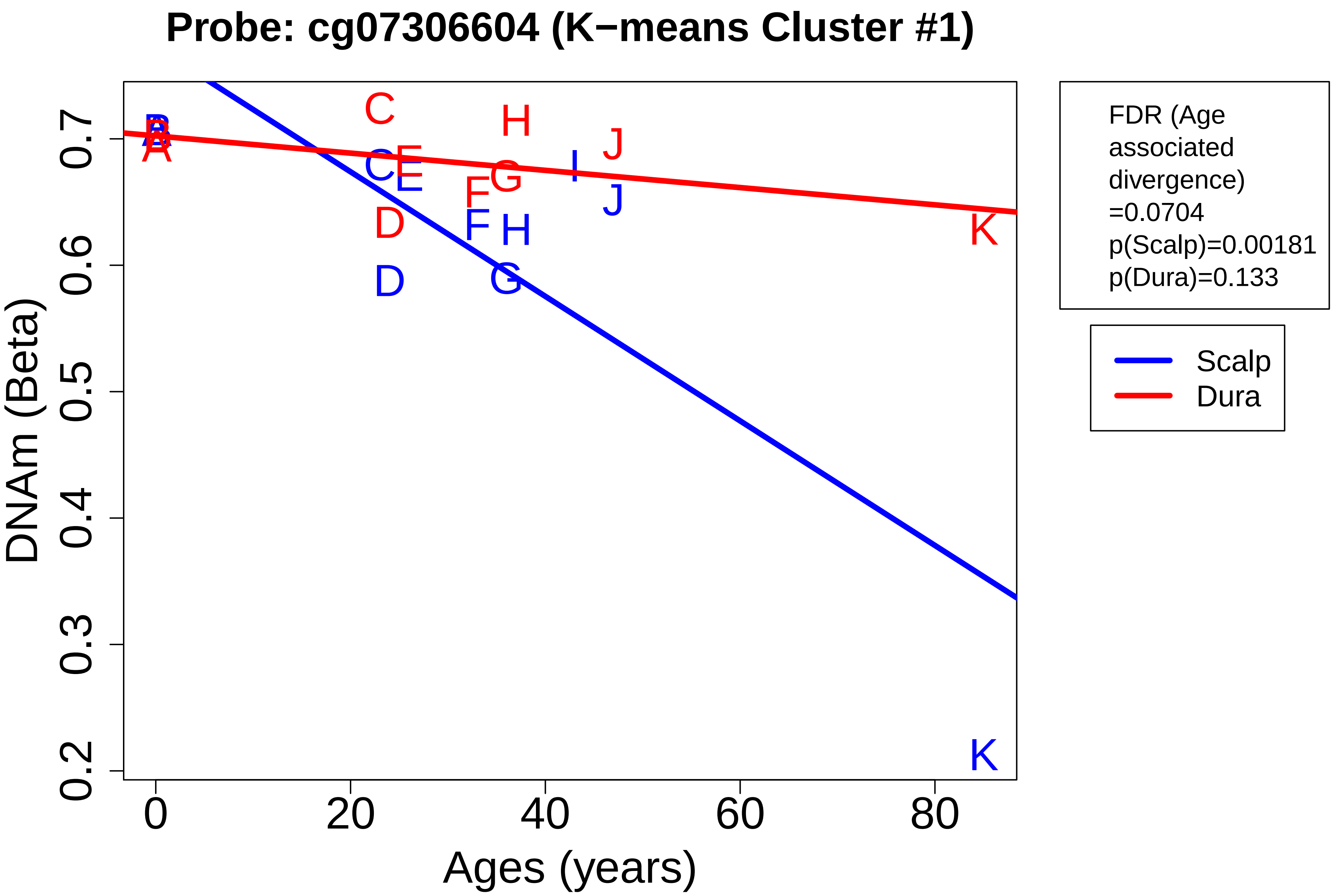

Probe: cg07306604 (K−means Cluster #1)
FDR (Age associated divergence) =0.0704
p(Scalp)=0.00181
p(Dura)=0.133
Scalp
Dura
DNAm (Beta)
0.2
0.3
0.4
0.5
0.6
0.7
0
20
40
60
80
Ages (years)

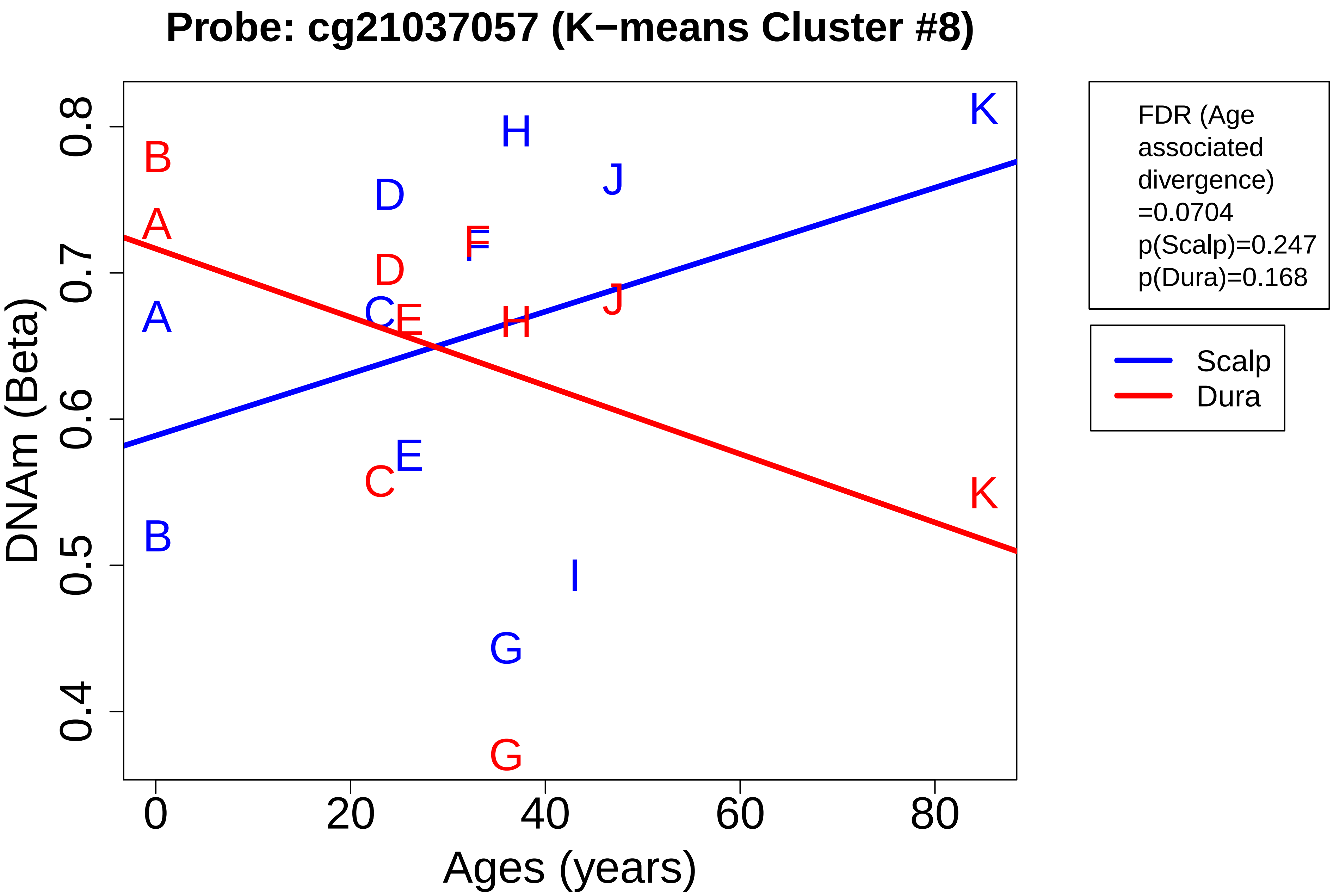

Probe: cg21037057 (K−means Cluster #8)
DNAm (Beta)
0.4
0.5
0.6
0.7
0.8
0
20
40
60
80
Ages (years)
FDR (Age associated divergence) =0.0704
p(Scalp)=0.247
p(Dura)=0.168
Scalp
Dura

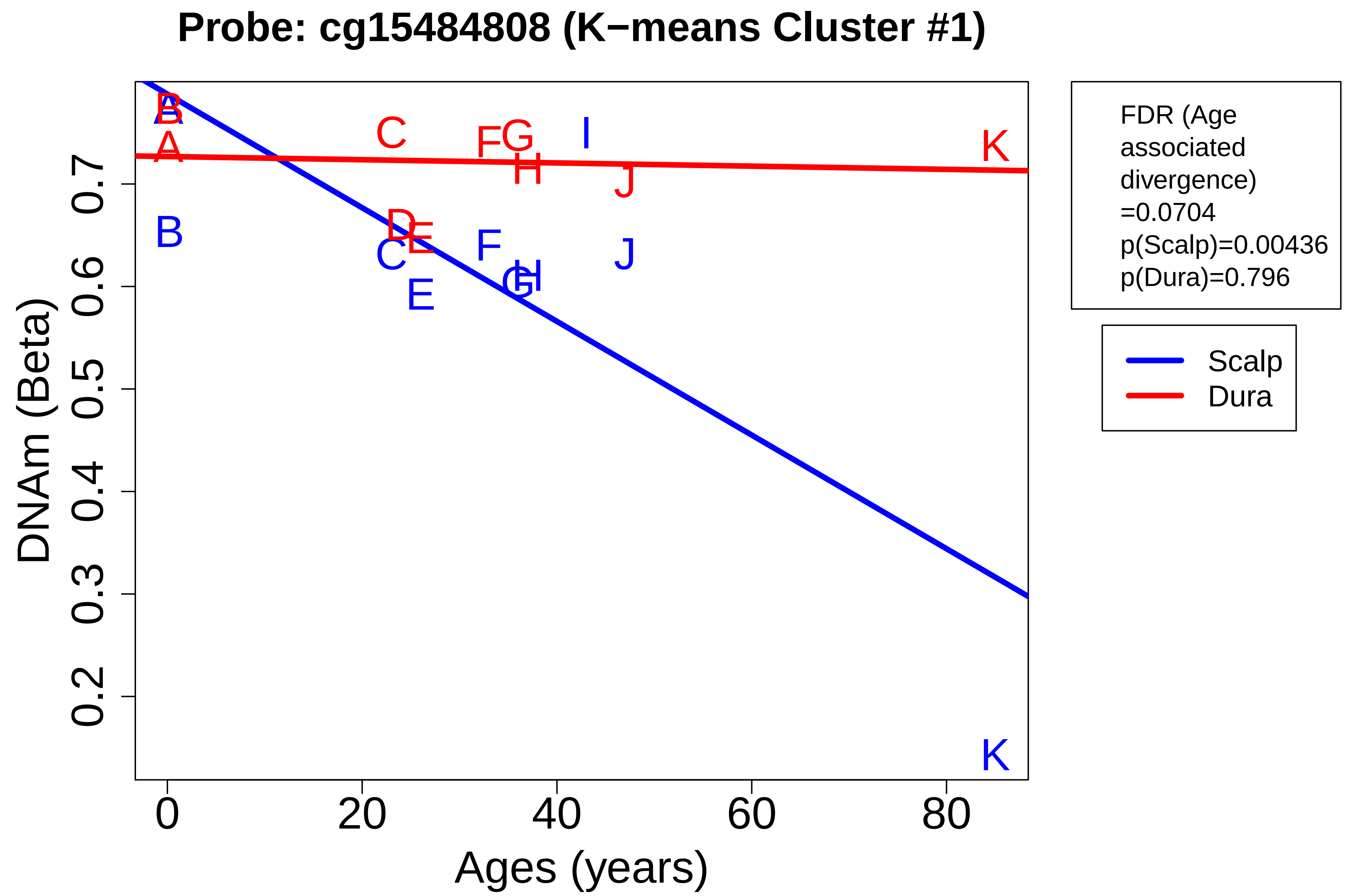

Probe: cg15484808 (K−means Cluster #1)
DNAm (Beta)
0.2
0.3
0.4
0.5
0.6
0.7
Ages (years)
0
20
40
60
80
FDR (Age associated divergence) =0.0704
p(Scalp)=0.00436
p(Dura)=0.796
Scalp
Dura

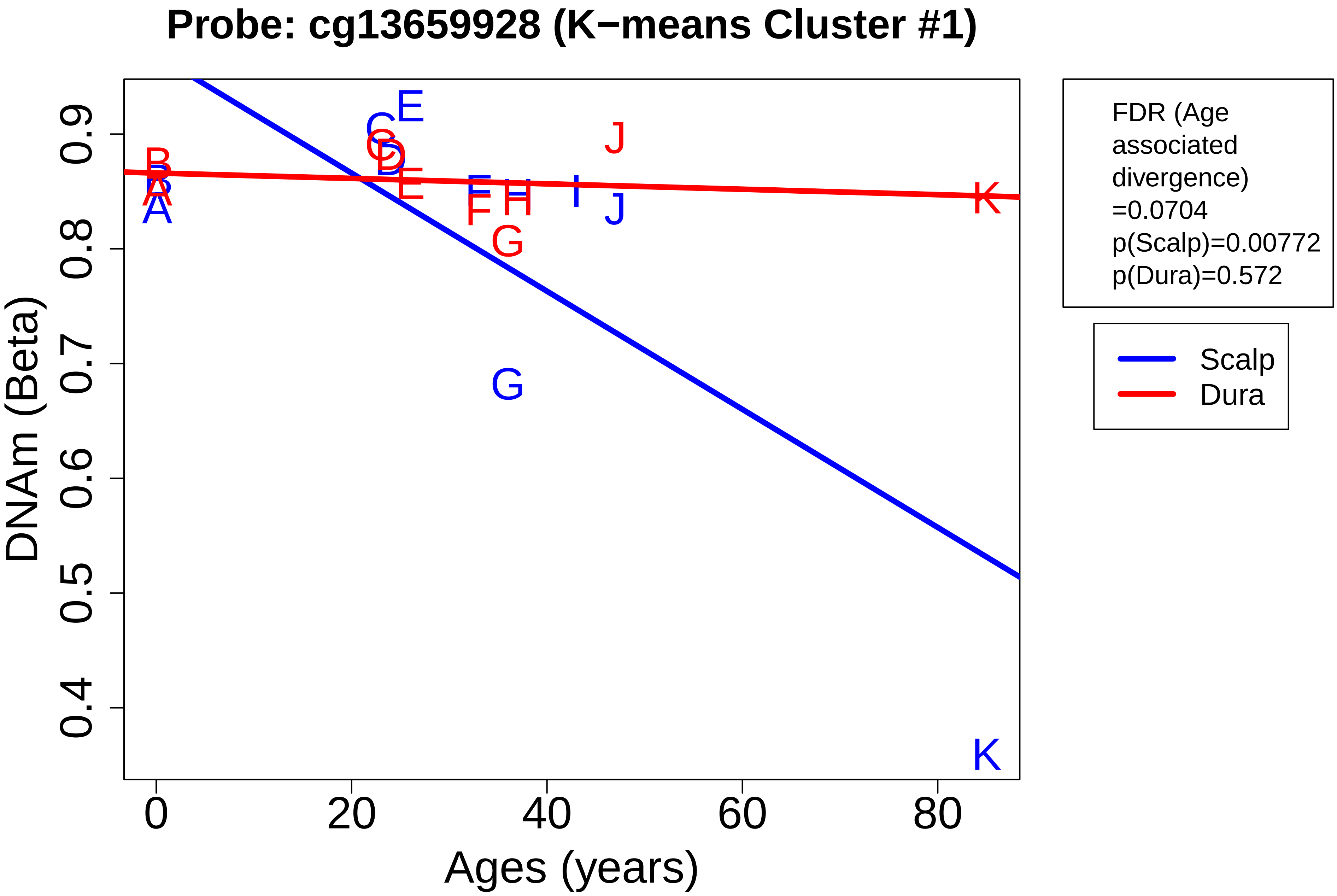

Probe: cg13659928 (K−means Cluster #1)
DNAm (Beta)
0.4
0.5
0.6
0.7
0.8
0.9
0
20
40
60
80
Ages (years)
FDR (Age associated divergence) =0.0704
p(Scalp)=0.00772
p(Dura)=0.572
Scalp
Dura

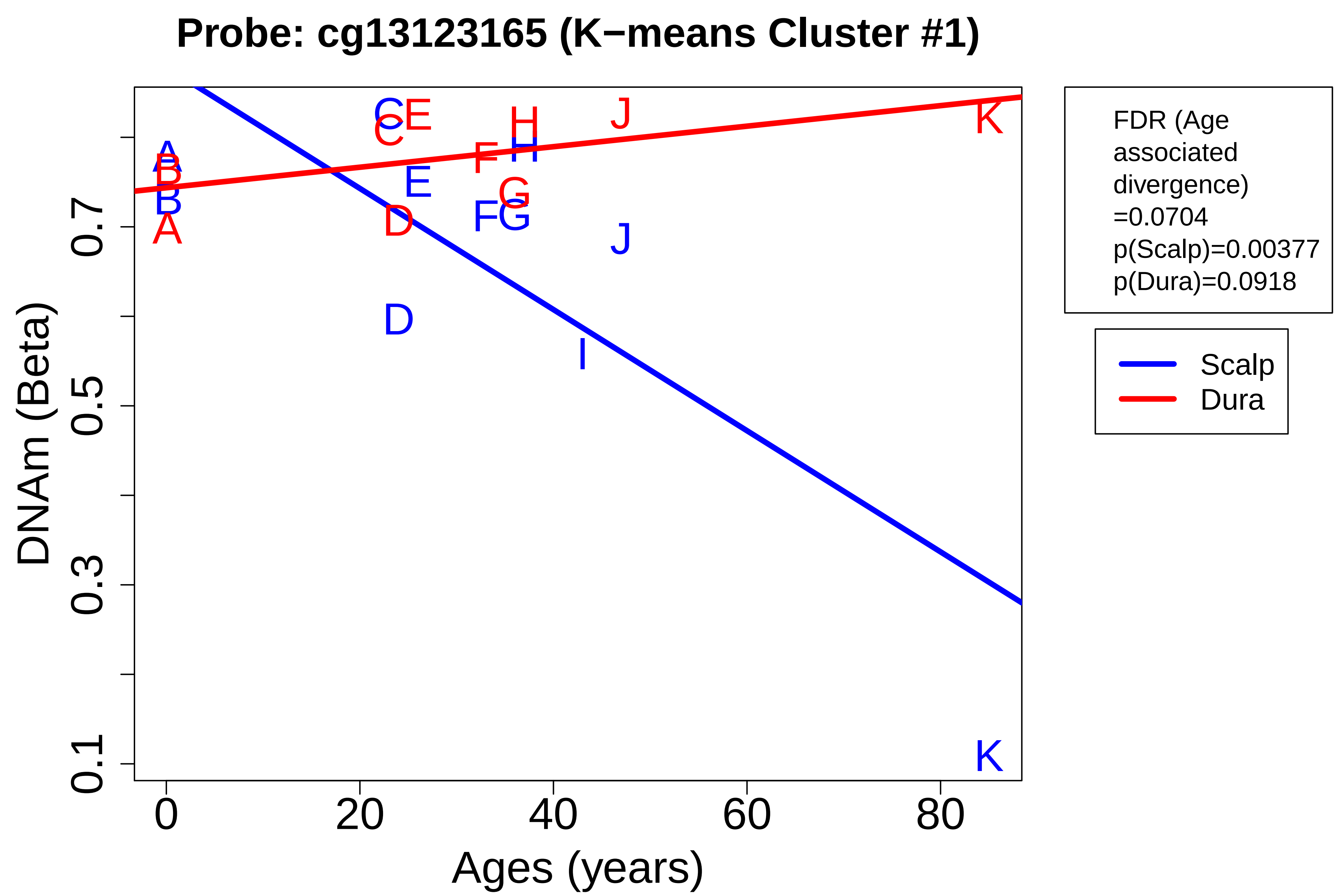

Probe: cg13123165 (K−means Cluster #1)
DNAm (Beta)
0.1
0.3
0.5
0.7
0
20
40
60
80
Ages (years)
FDR (Age associated divergence) =0.0704
p(Scalp)=0.00377
p(Dura)=0.0918
Scalp
Dura

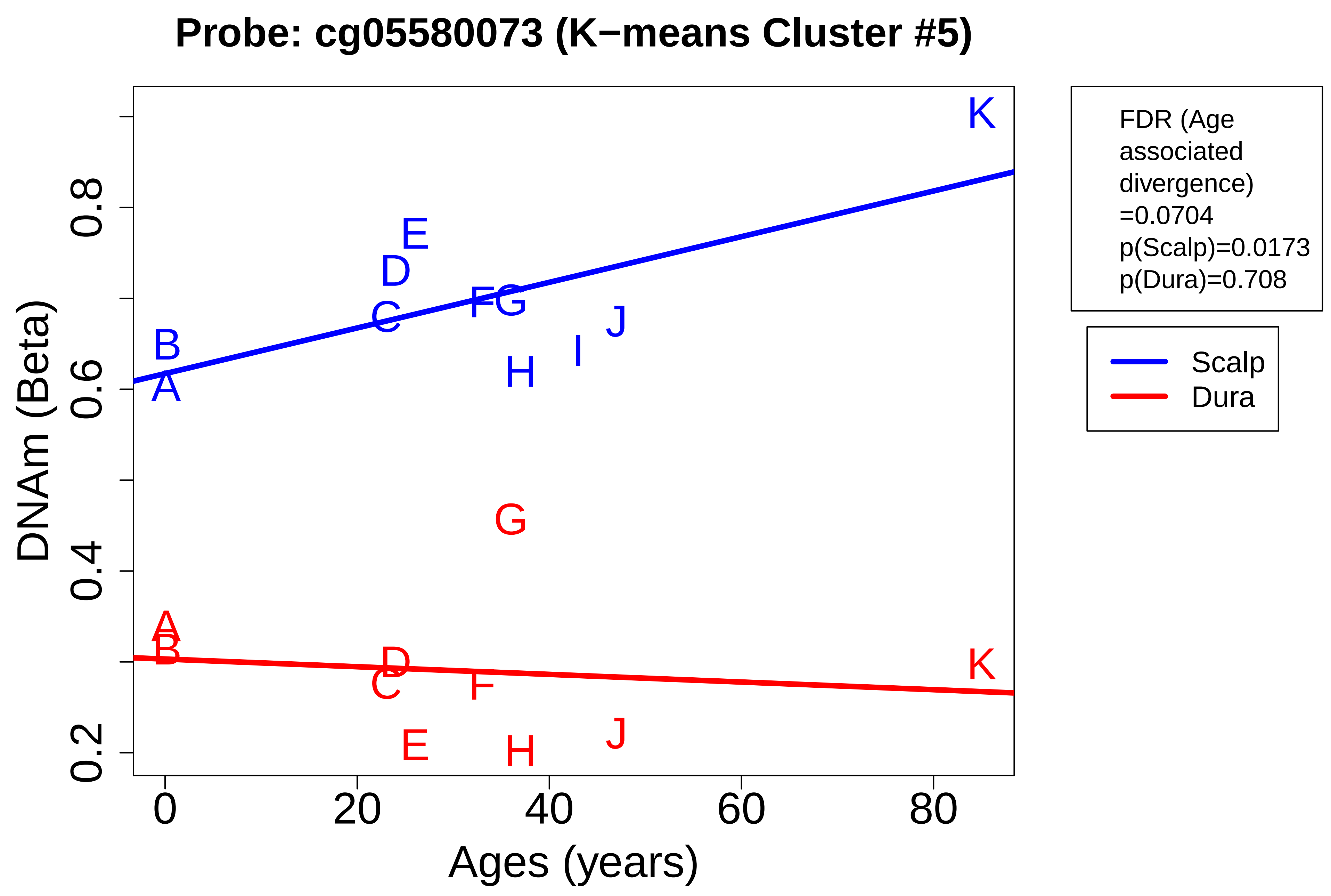

Probe: cg05580073 (K−means Cluster #5)
DNAm (Beta)
0.2
0.4
0.6
0.8
0
20
40
60
80
Ages (years)
FDR (Age associated divergence) =0.0704
p(Scalp)=0.0173
p(Dura)=0.708
Scalp
Dura

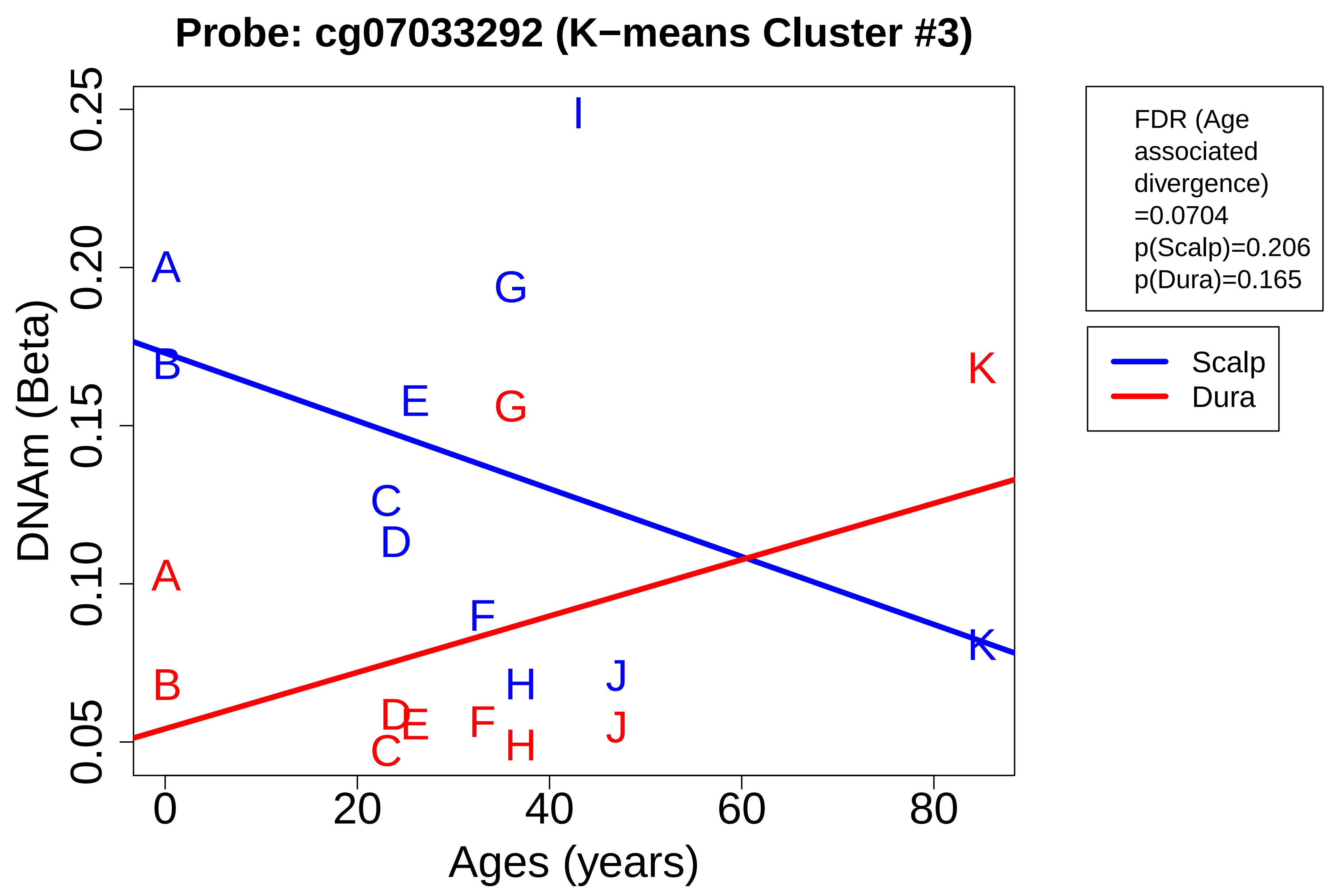
Probe: cg07033292 (K−means Cluster #3)
DNAm (Beta)
0.05
0.10
0.15
0.20
0.25
0
20
40
60
80
Ages (years)
A
B
C
D
E
F
G
H
I
J
K
A
B
C
D
E
F
G
H
J
K
FDR (Age associated divergence) =0.0704
p(Scalp)=0.206
p(Dura)=0.165
Scalp
Dura

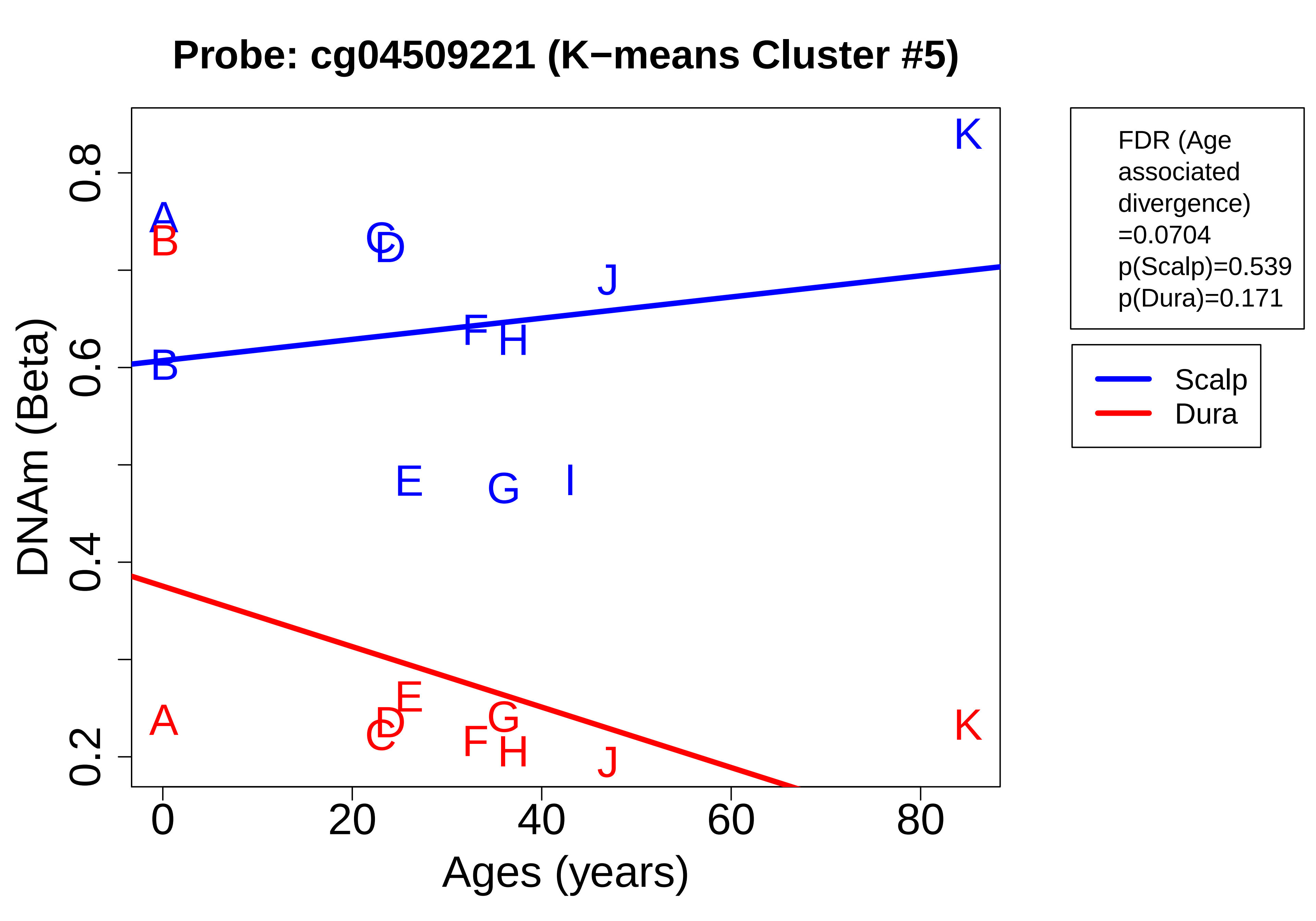
Probe: cg04509221 (K−means Cluster #5)
DNAm (Beta)
0.2
0.4
0.6
0.8
0
20
40
60
80
Ages (years)
A
B
C
D
E
F
G
H
I
J
K
FDR (Age associated divergence) =0.0704
p(Scalp)=0.539
p(Dura)=0.171
Scalp
Dura

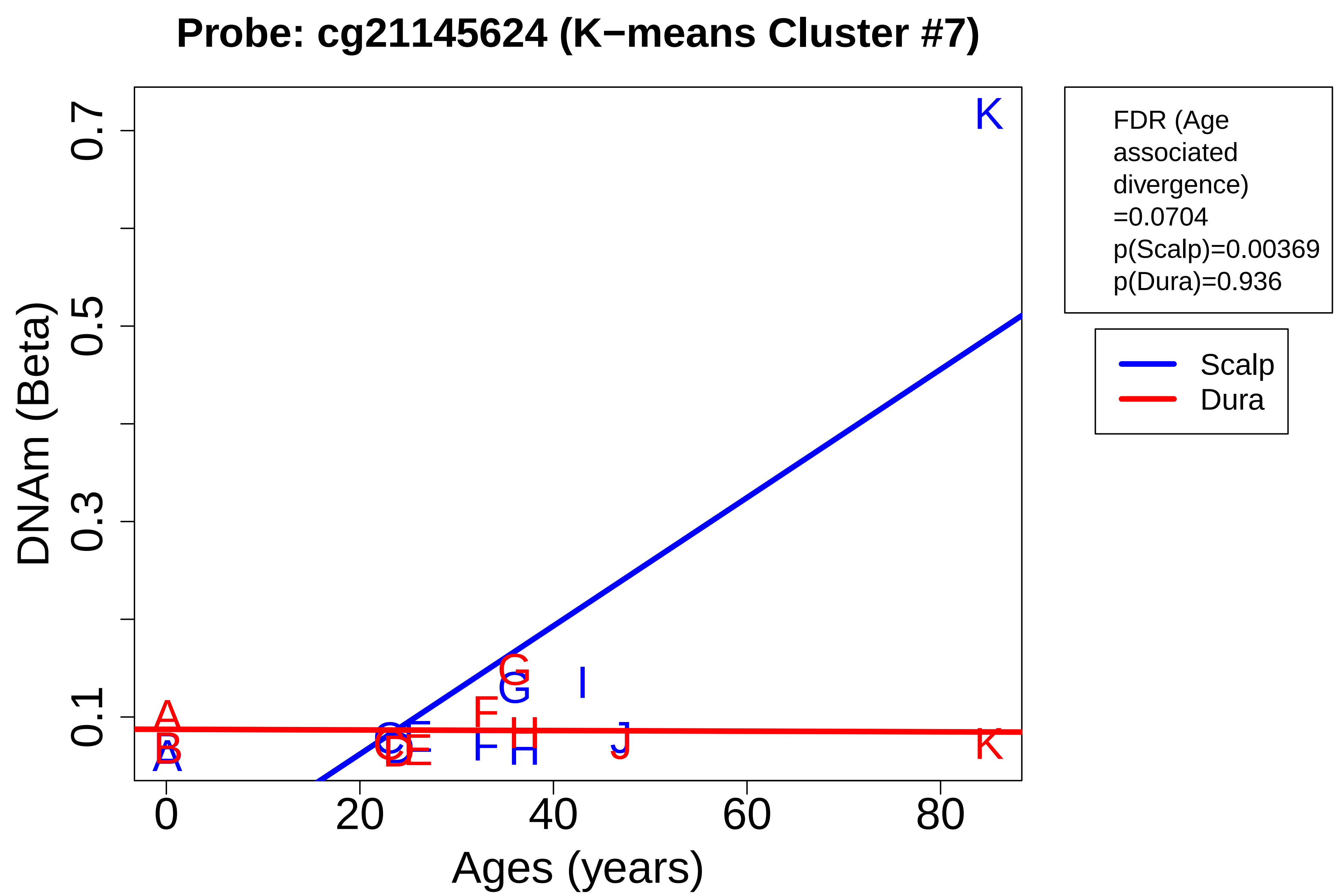
Probe: cg21145624 (K−means Cluster #7)
DNAm (Beta)
0.1
0.3
0.5
0.7
0
20
40
60
80
Ages (years)
A
B
C
D
E
F
G
H
I
J
K
FDR (Age associated divergence) =0.0704
p(Scalp)=0.00369
p(Dura)=0.936
Scalp
Dura

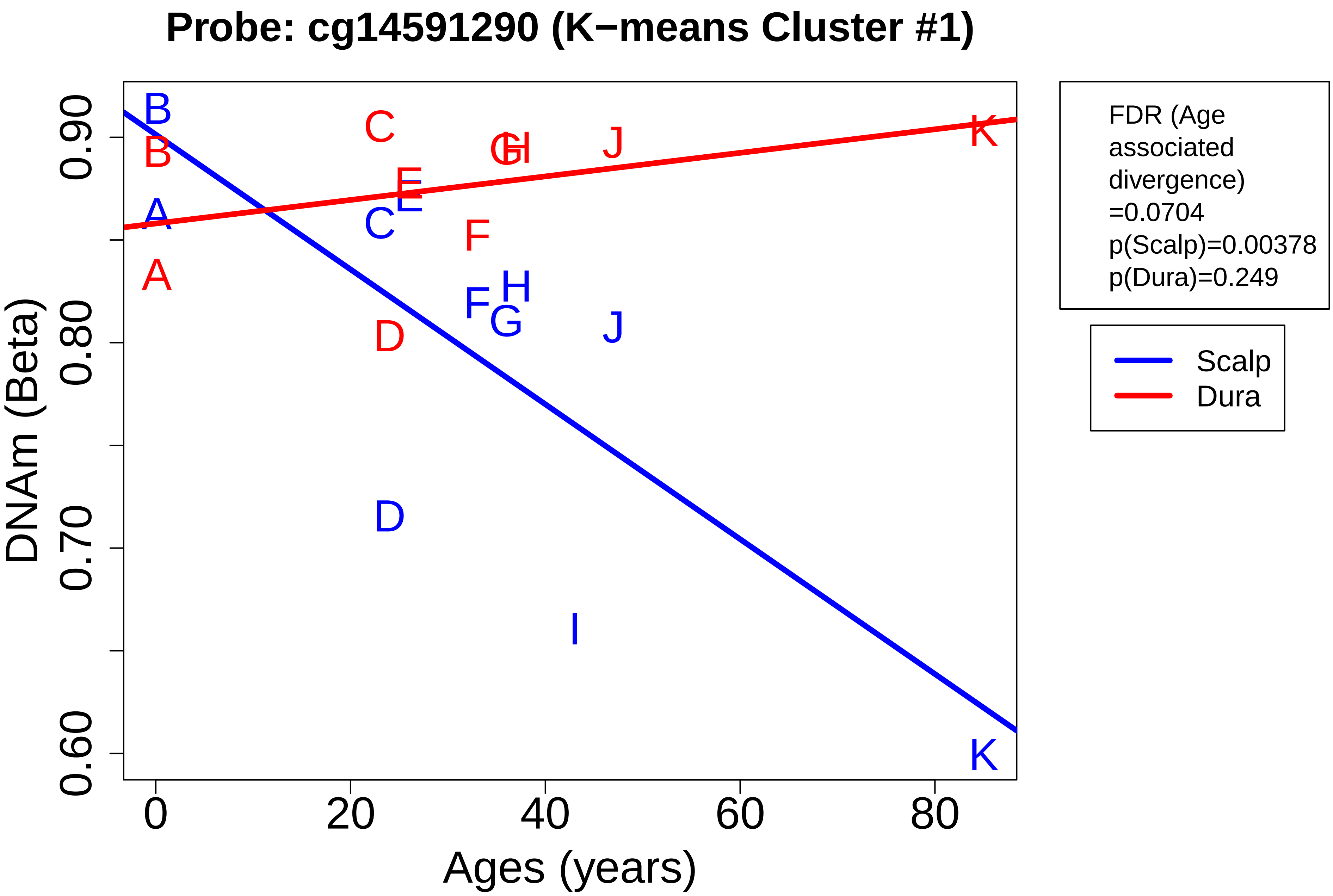

Probe: cg14591290 (K−means Cluster #1)
DNAm (Beta)
0.60
0.70
0.80
0.90
0
20
40
60
80
Ages (years)
FDR (Age associated divergence) =0.0704
p(Scalp)=0.00378
p(Dura)=0.249
Scalp
Dura

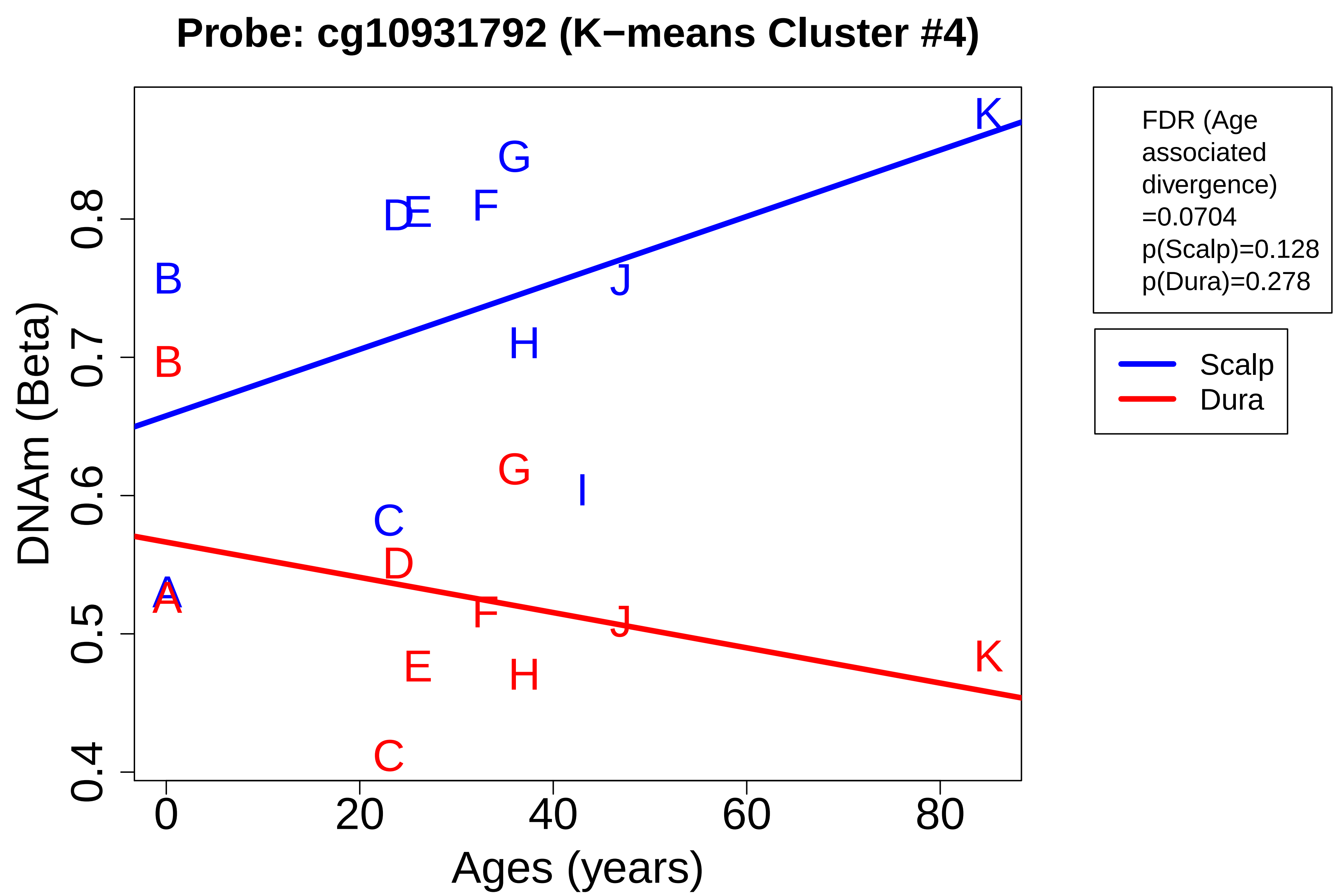

Probe: cg10931792 (K−means Cluster #4)
DNAm (Beta)
0.4
0.5
0.6
0.7
0.8
0
20
40
60
80
Ages (years)
A
B
C
D
E
F
G
H
I
J
K
FDR (Age associated divergence) =0.0704
p(Scalp)=0.128
p(Dura)=0.278
Scalp
Dura

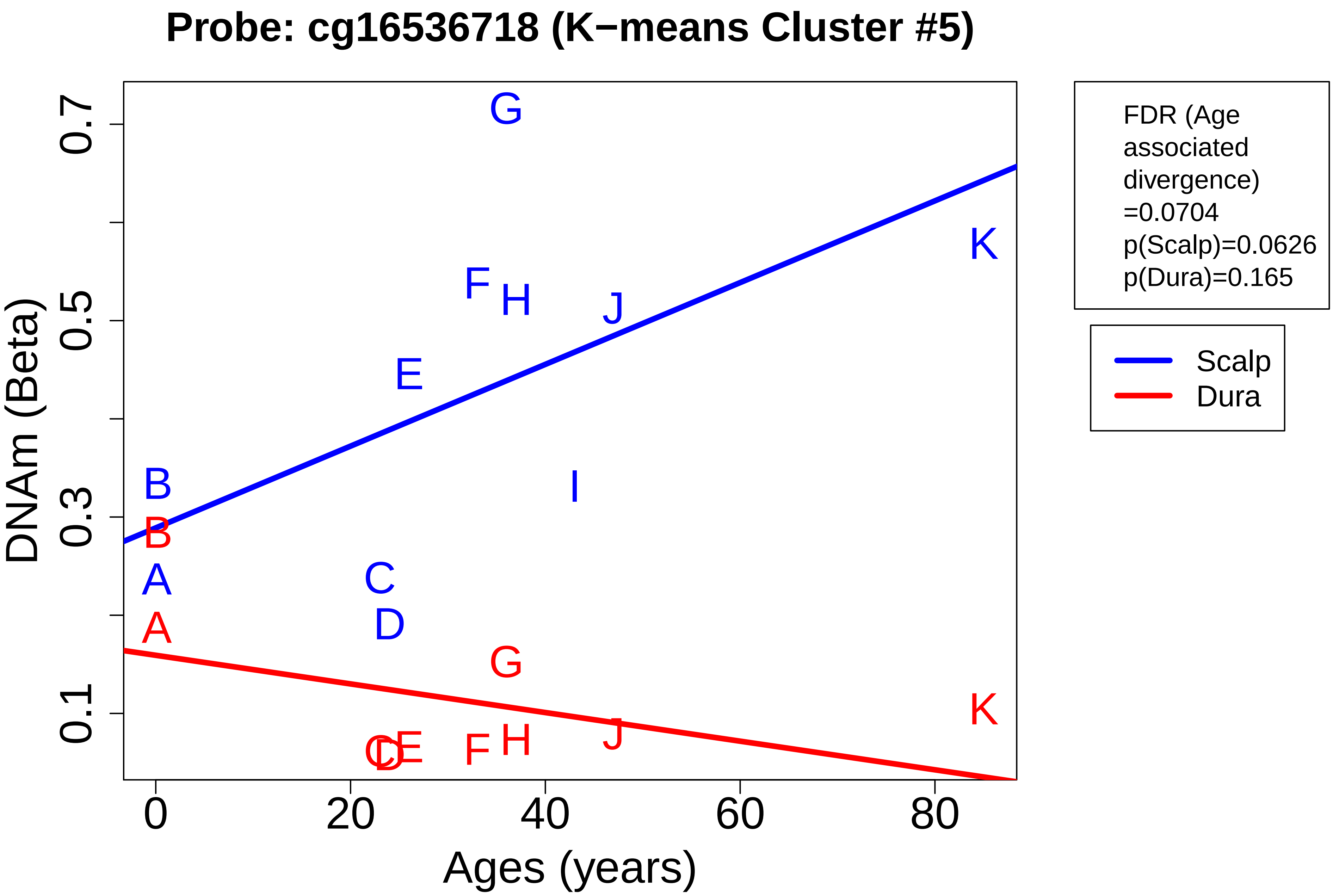
Probe: cg16536718 (K−means Cluster #5)
DNAm (Beta)
0.1
0.3
0.5
0.7
Ages (years)
0
20
40
60
80
FDR (Age associated divergence) =0.0704
p(Scalp)=0.0626
p(Dura)=0.165
Scalp
Dura
A
B
C
D
E
F
G
H
I
J
K

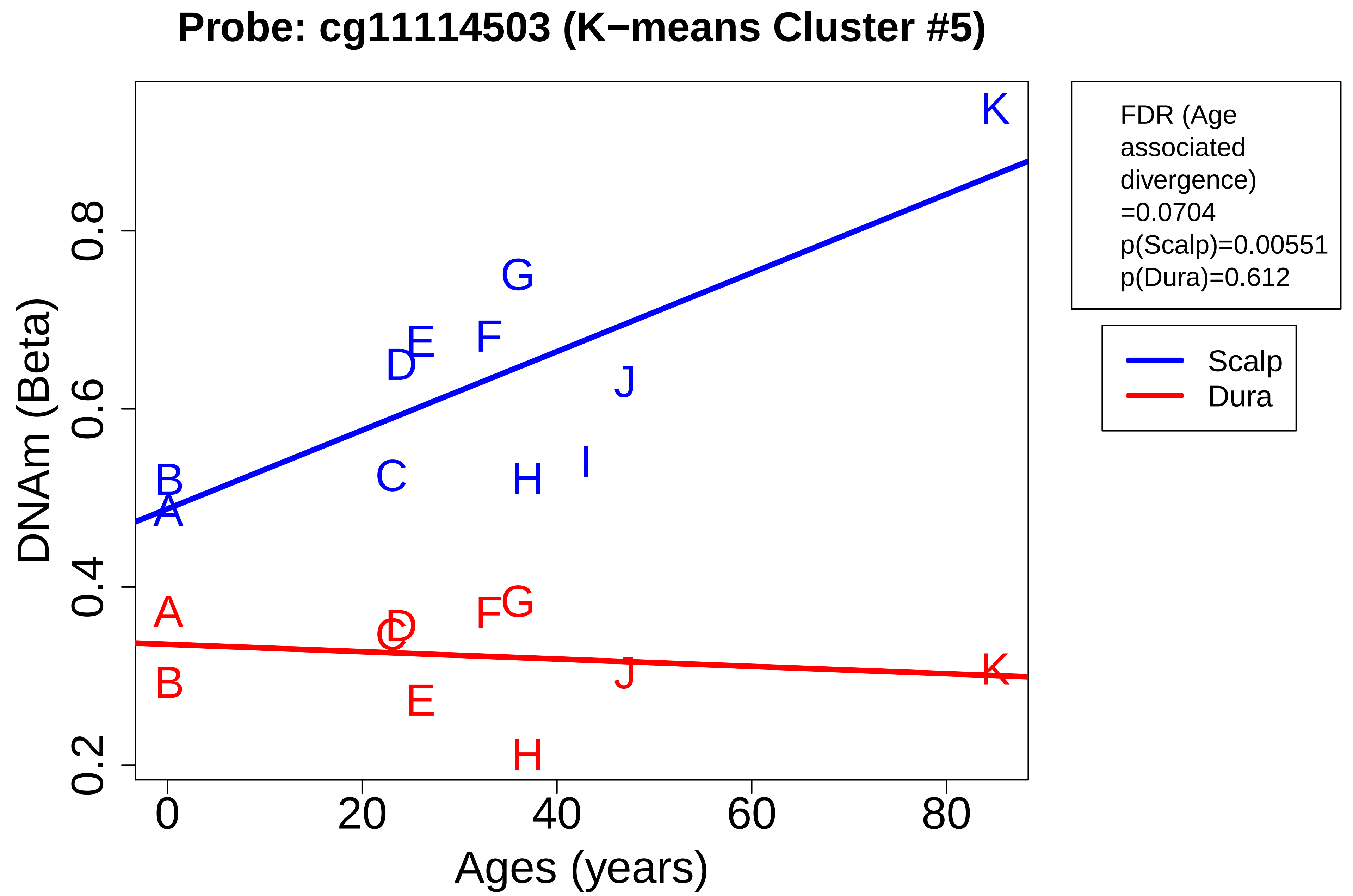

Probe: cg11114503 (K−means Cluster #5)
FDR (Age associated divergence) =0.0704
p(Scalp)=0.00551
p(Dura)=0.612
Scalp
Dura
DNAm (Beta)
0.2
0.4
0.6
0.8
0
20
40
60
80
Ages (years)
A
B
C
D
E
F
G
H
I
J
K

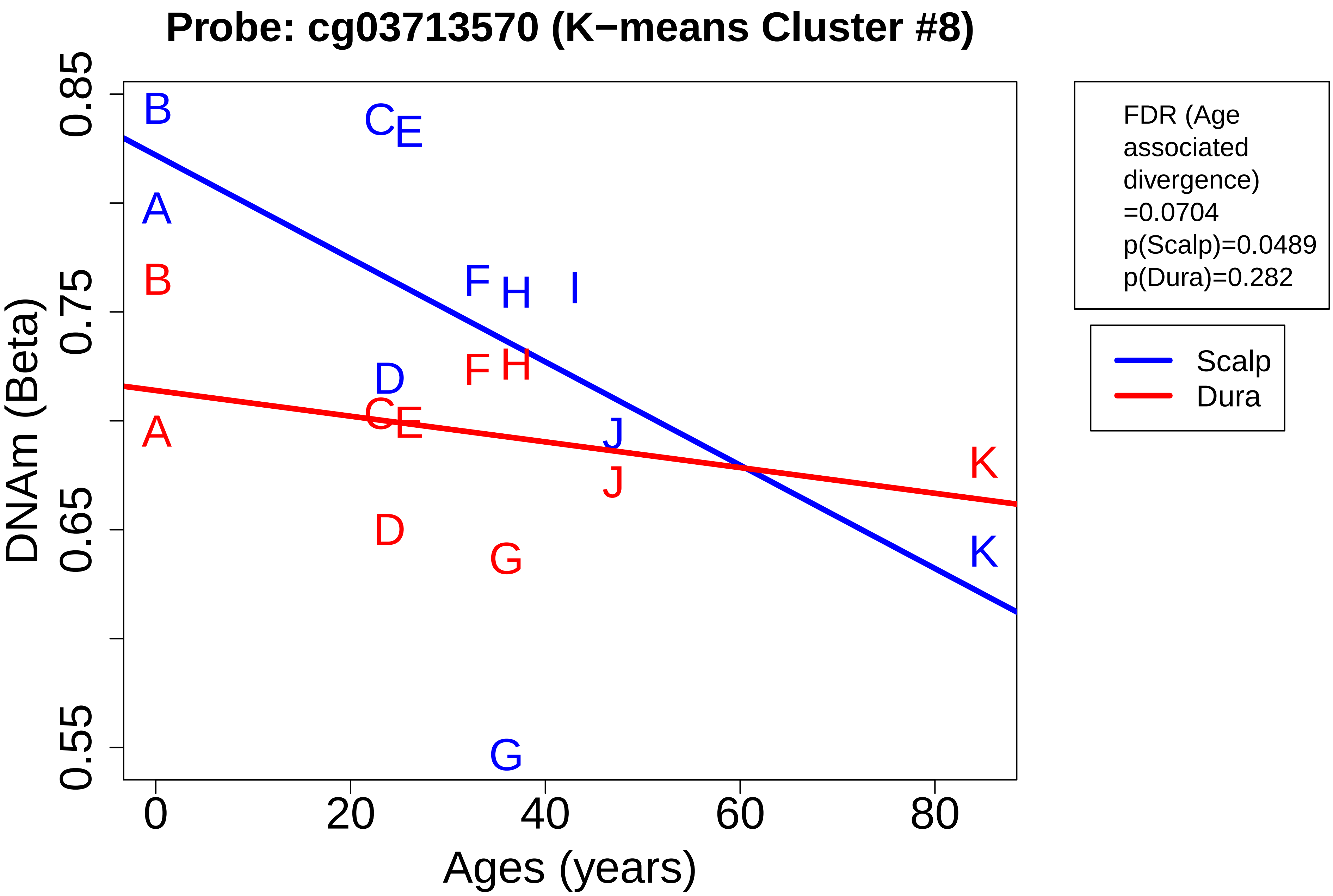
Probe: cg03713570 (K−means Cluster #8)
DNAm (Beta)
0.55
0.65
0.75
0.85
0
20
40
60
80
Ages (years)
B
C
E
A
B
F
H
I
D
F
H
C
E
A
J
K
J
D
G
K
G
FDR (Age associated divergence) =0.0704
p(Scalp)=0.0489
p(Dura)=0.282
Scalp
Dura

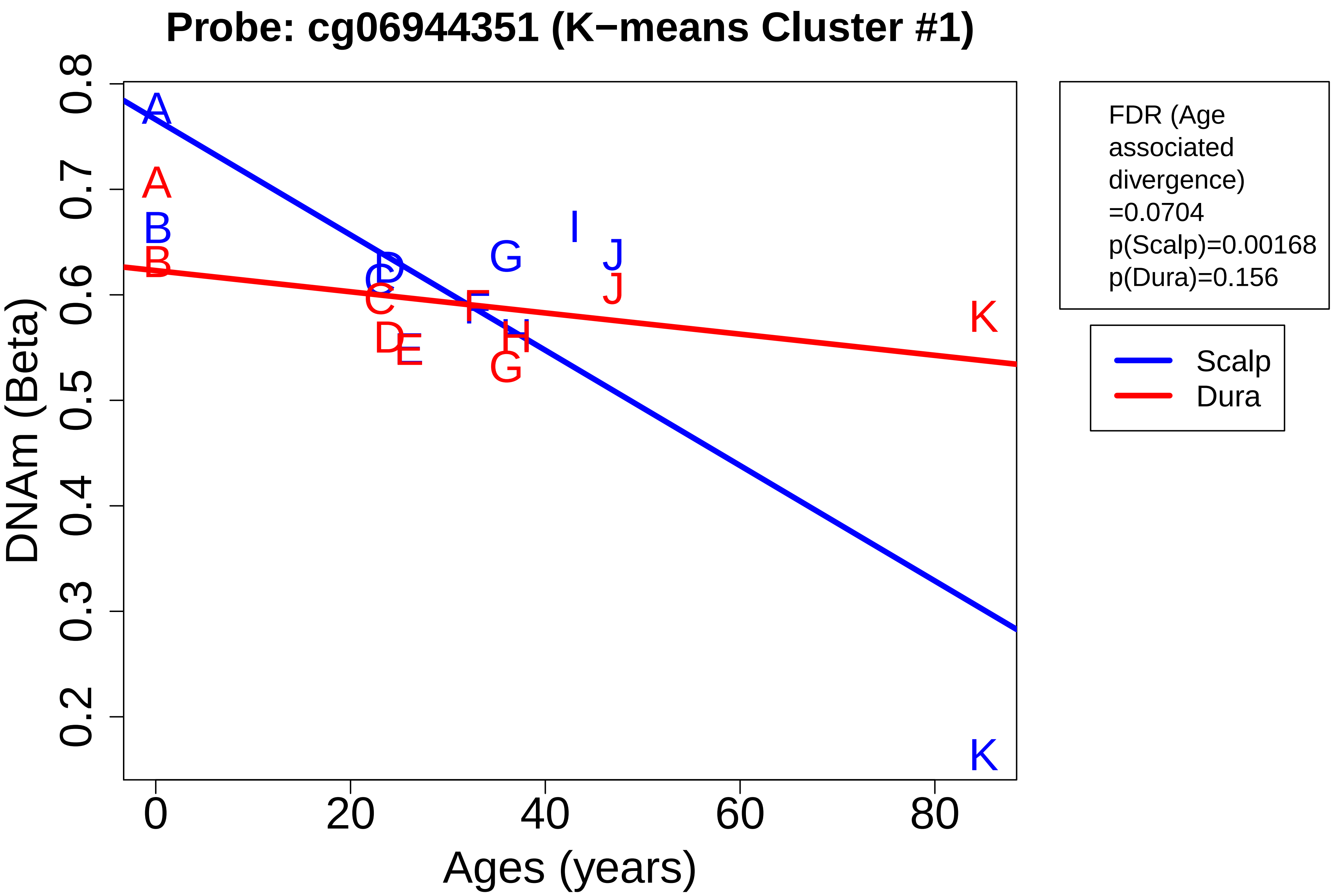

Probe: cg06944351 (K−means Cluster #1)
FDR (Age associated divergence) =0.0704
p(Scalp)=0.00168
p(Dura)=0.156
Scalp
Dura
DNAm (Beta)
0.2
0.3
0.4
0.5
0.6
0.7
0.8
Ages (years)
0
20
40
60
80
A
B
C
D
E
F
G
H
I
J
K

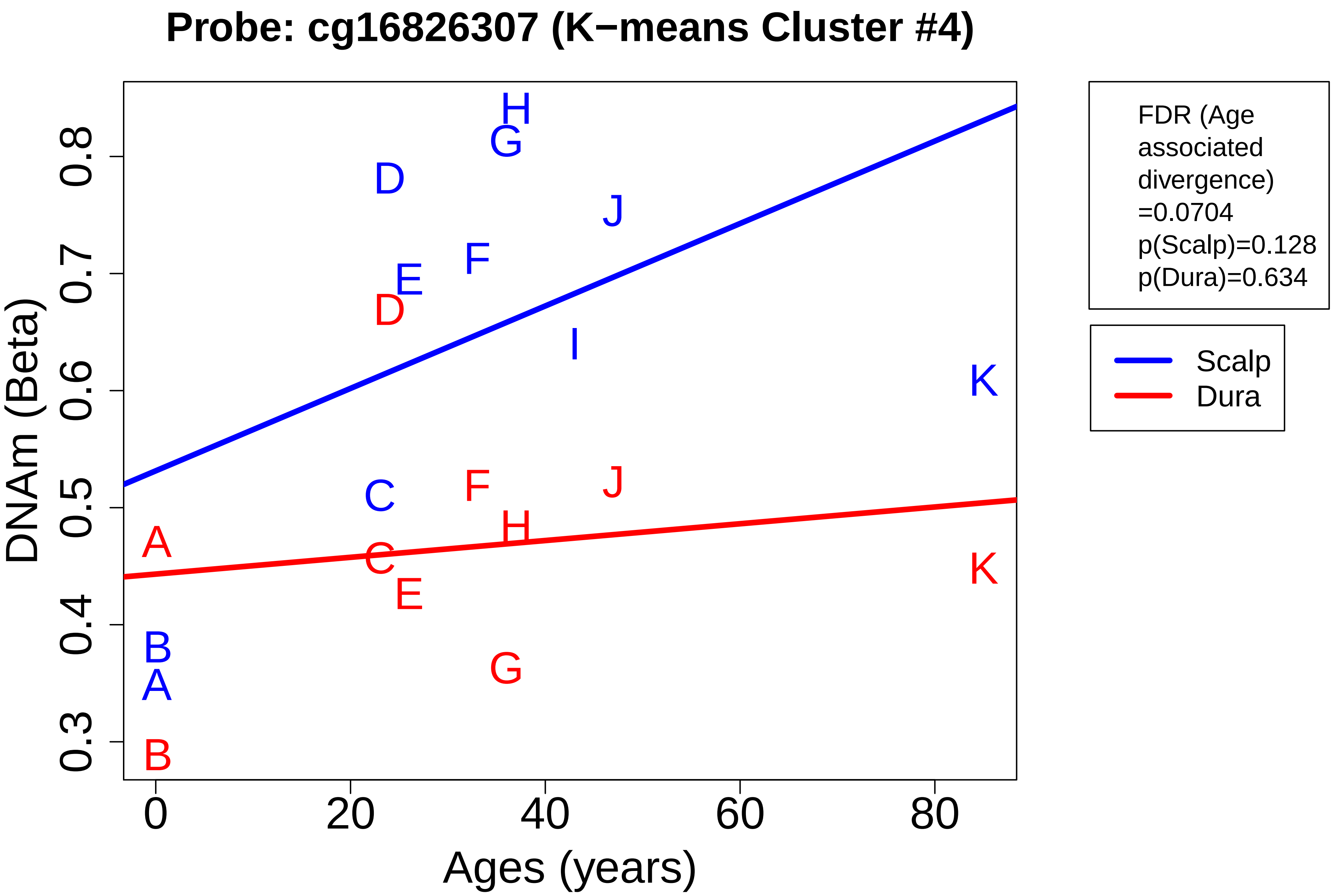
Probe: cg16826307 (K−means Cluster #4)
DNAm (Beta)
0.3
0.4
0.5
0.6
0.7
0.8
0
20
40
60
80
Ages (years)
FDR (Age associated divergence) =0.0704
p(Scalp)=0.128
p(Dura)=0.634
Scalp
Dura
A
B
C
D
E
F
G
H
I
J
K

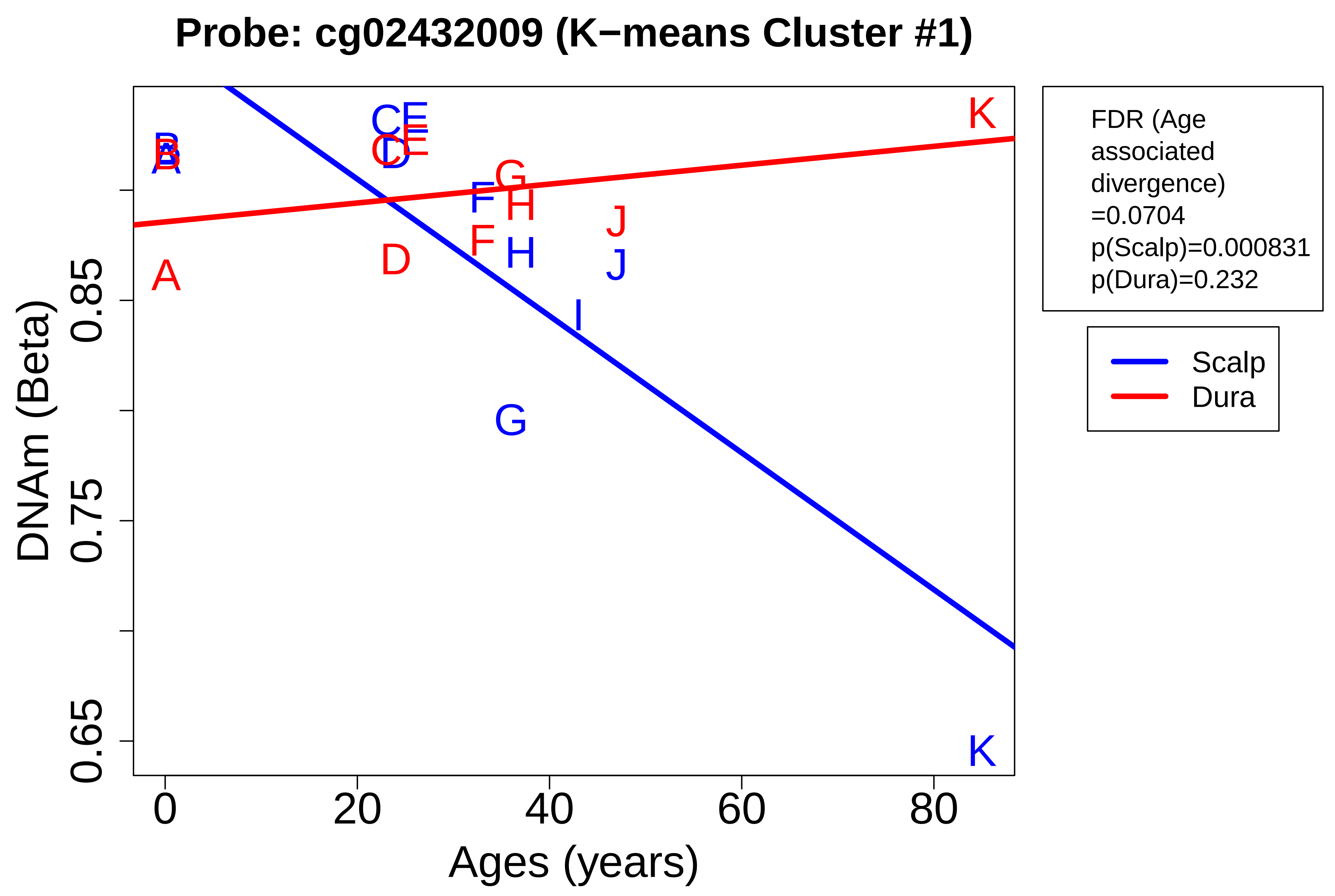

Probe: cg02432009 (K−means Cluster #1)
DNAm (Beta)
0.65
0.75
0.85
0
20
40
60
80
Ages (years)
A B C D E F G H I J K
FDR (Age associated divergence) =0.0704
p(Scalp)=0.000831
p(Dura)=0.232
Scalp
Dura

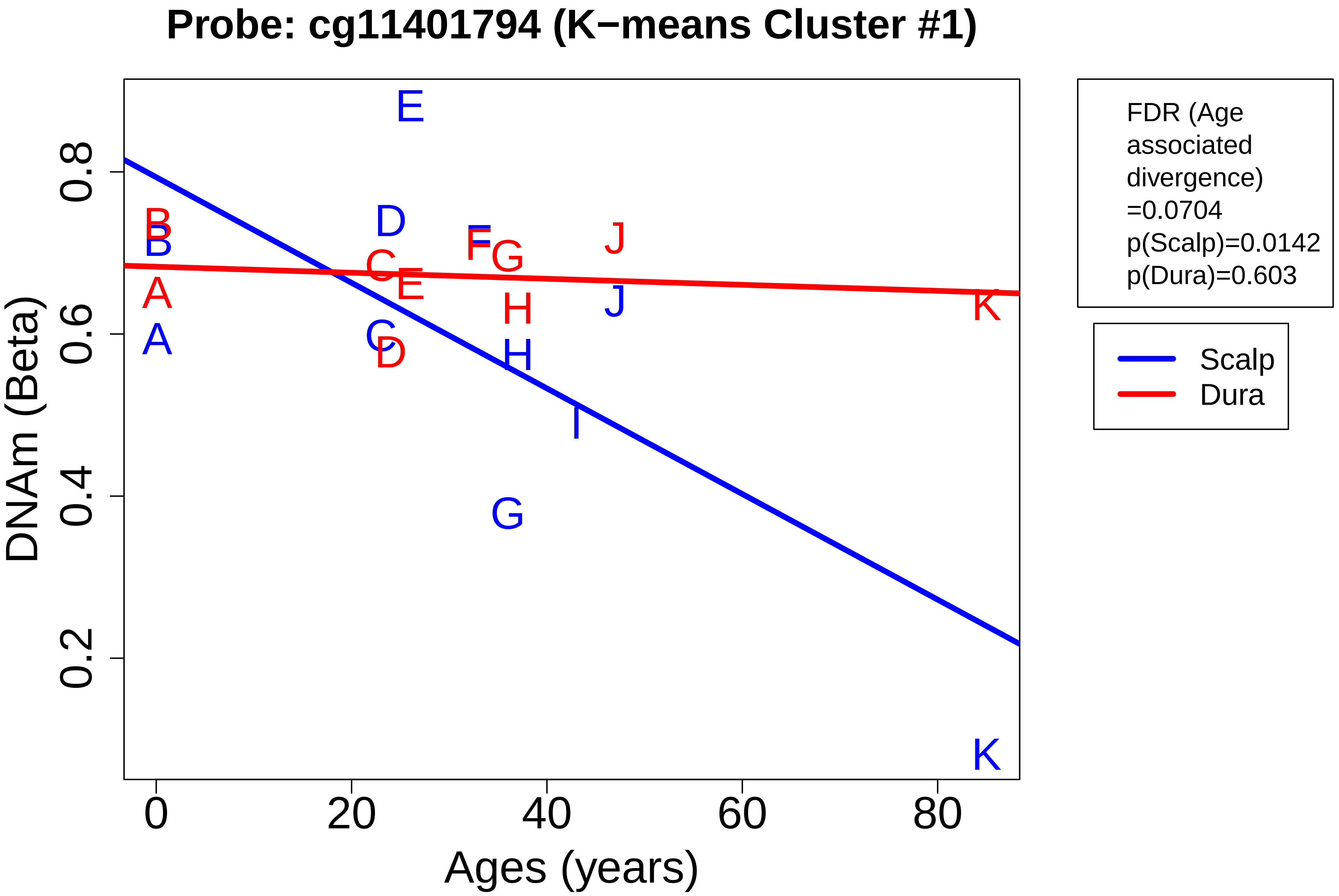

Probe: cg11401794 (K−means Cluster #1)
DNAm (Beta)
0.2
0.4
0.6
0.8
0
20
40
60
80
Ages (years)
FDR (Age associated divergence) =0.0704
p(Scalp)=0.0142
p(Dura)=0.603
Scalp
Dura

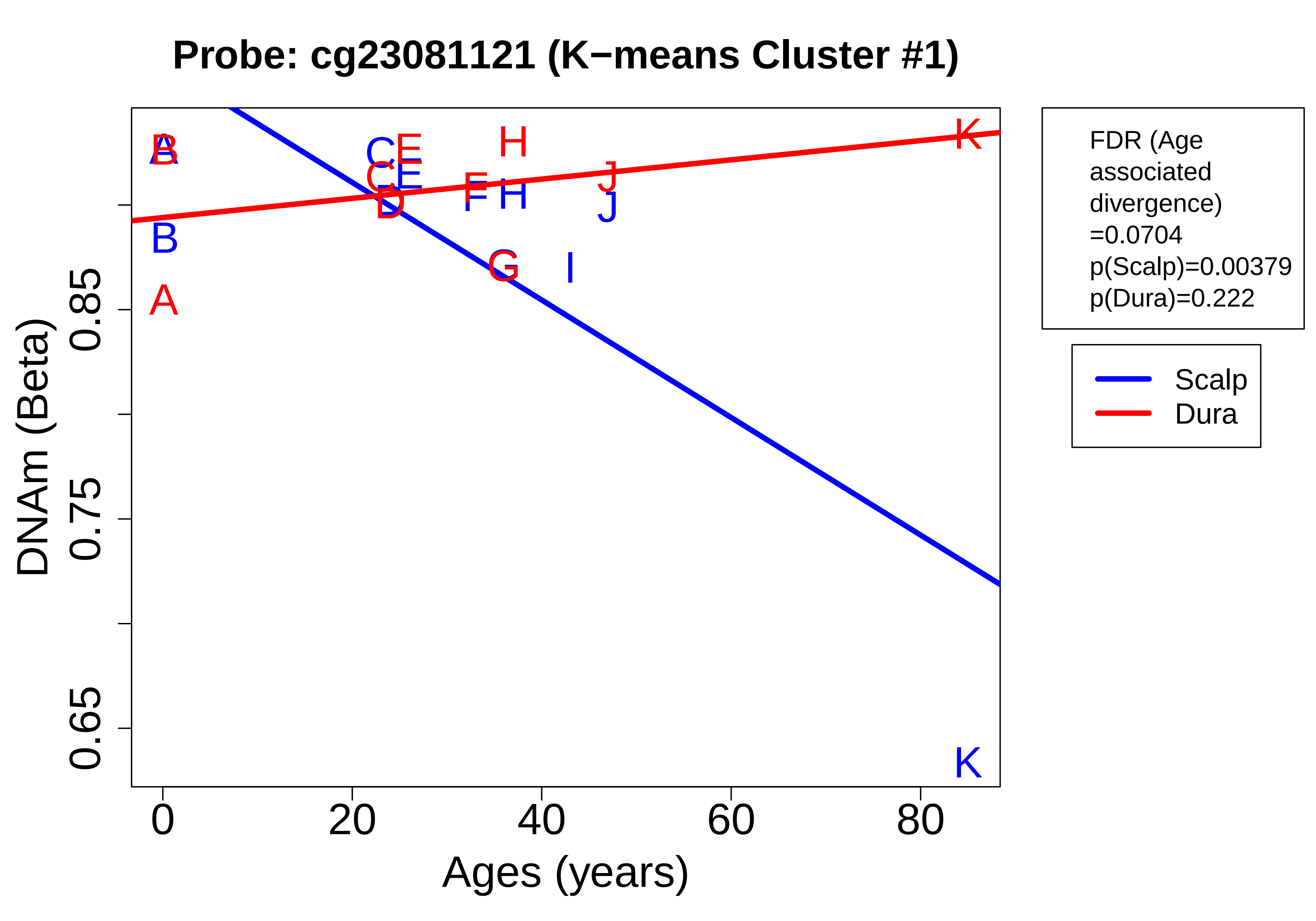

Probe: cg23081121 (K−means Cluster #1)
DNAm (Beta)
0.65
0.75
0.85
Ages (years)
0
20
40
60
80
FDR (Age associated divergence) =0.0704
p(Scalp)=0.00379
p(Dura)=0.222
Scalp
Dura

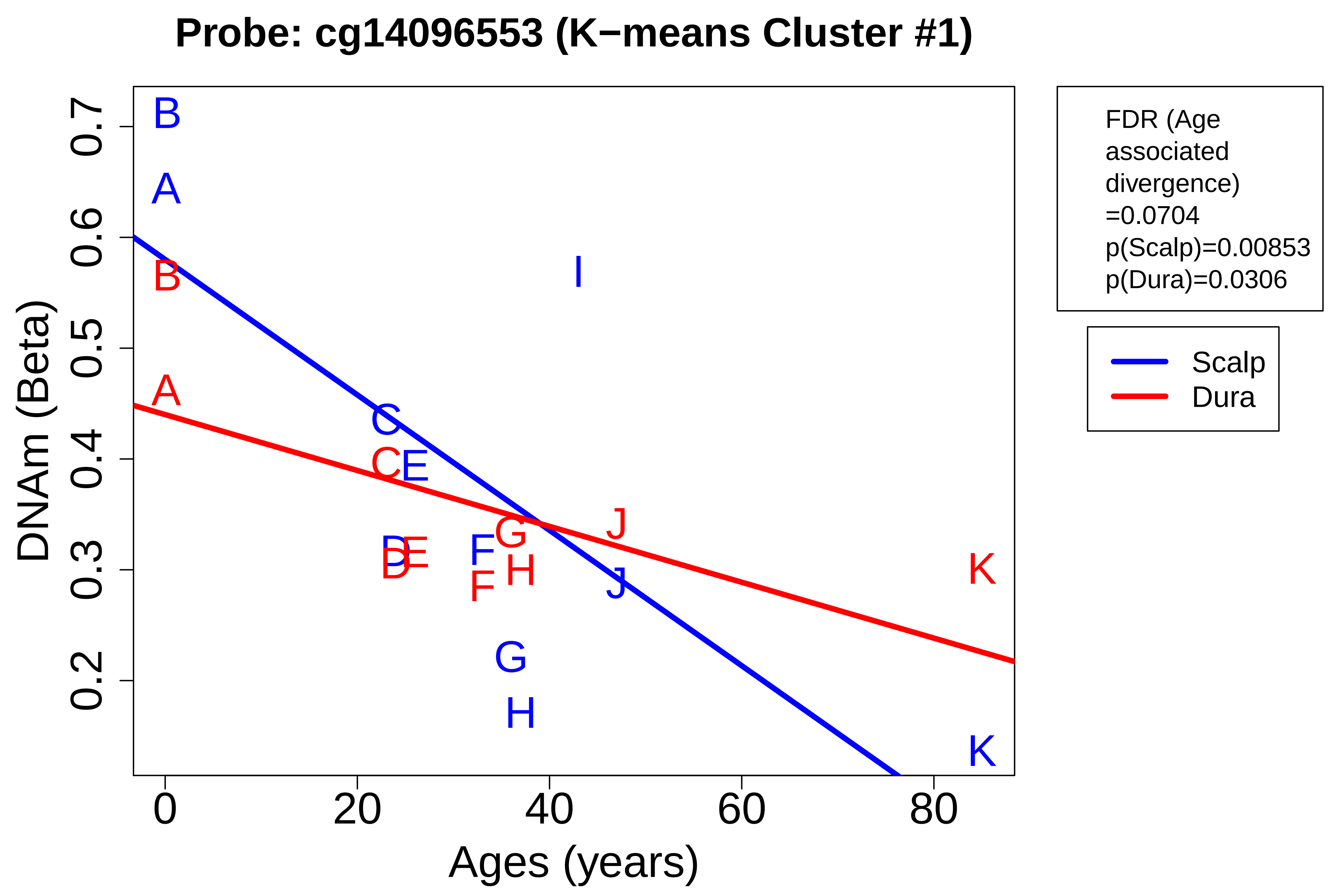

Probe: cg14096553 (K−means Cluster #1)
DNAm (Beta)
0.2
0.3
0.4
0.5
0.6
0.7
Ages (years)
0
20
40
60
80
FDR (Age associated divergence) =0.0704
p(Scalp)=0.00853
p(Dura)=0.0306
Scalp
Dura
A
B
C
D
E
F
G
H
I
J
K

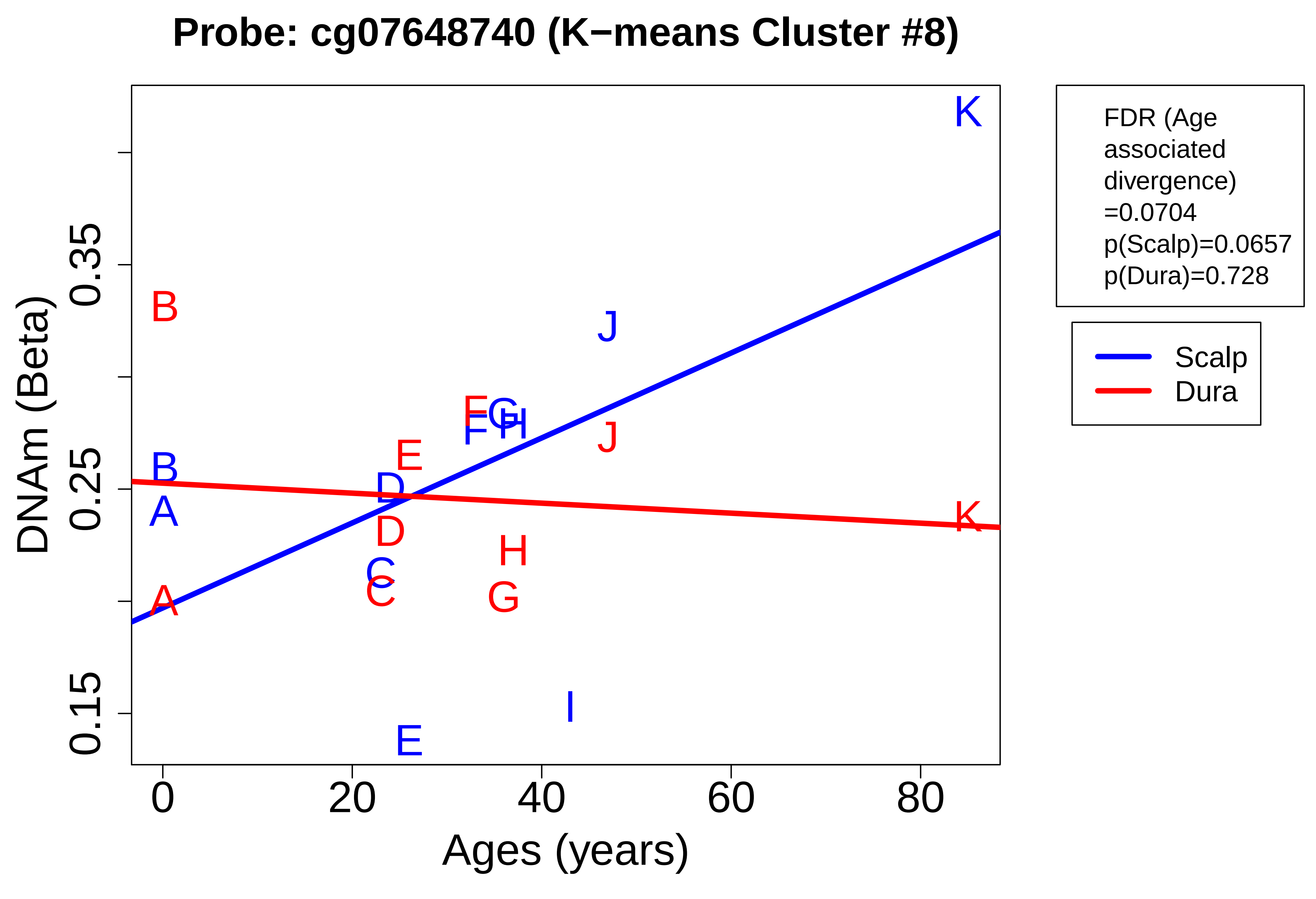

Probe: cg07648740 (K−means Cluster #8)
FDR (Age associated divergence) =0.0704
p(Scalp)=0.0657
p(Dura)=0.728
Scalp
Dura
DNAm (Beta)
0.15
0.25
0.35
Ages (years)
0
20
40
60
80
A
B
C
D
E
F
G
H
I
J
K

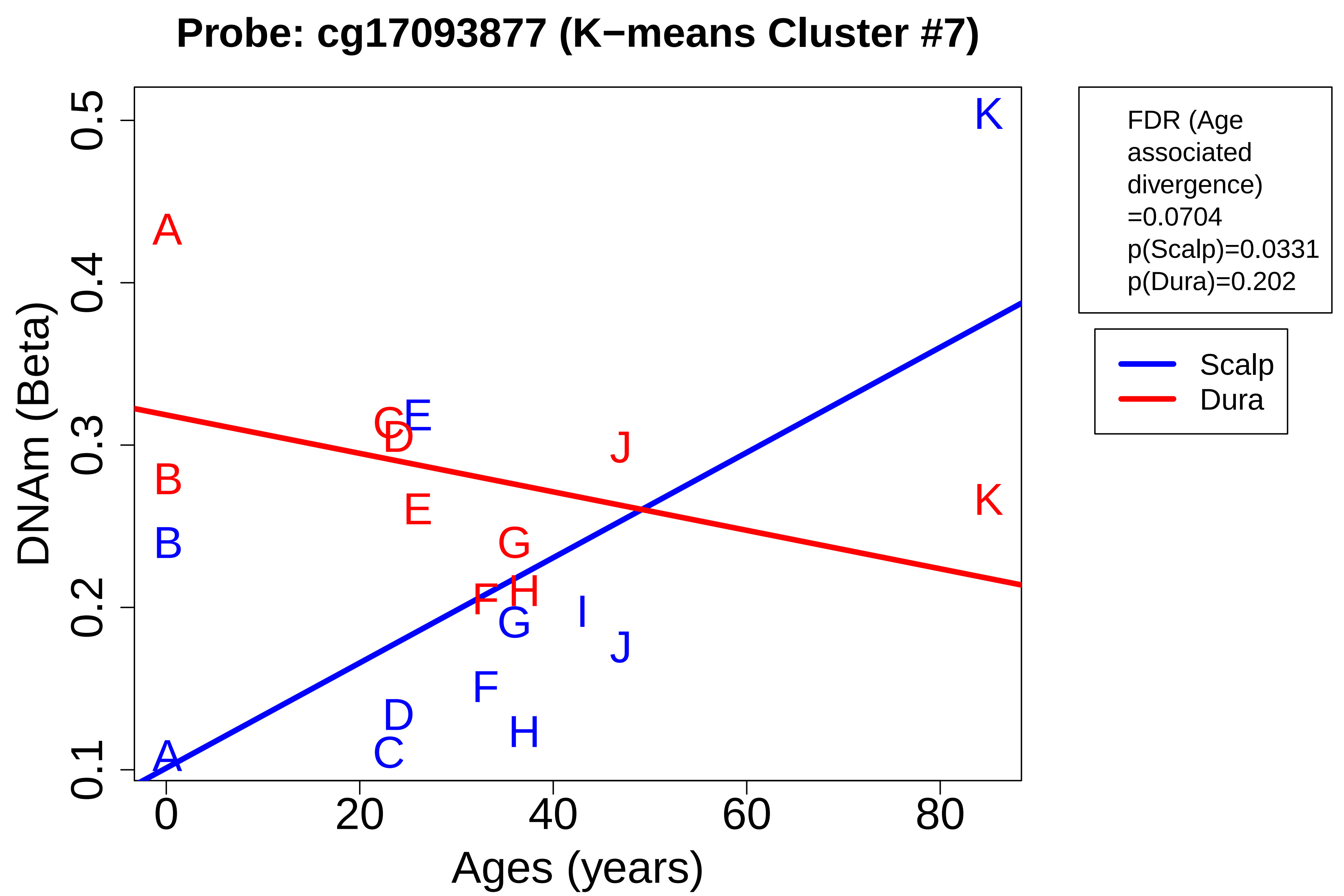

Probe: cg17093877 (K−means Cluster #7)
DNAm (Beta)
0.1
0.2
0.3
0.4
0.5
0
20
40
60
80
Ages (years)
A
B
C
D
E
F
G
H
I
J
K
FDR (Age associated divergence) =0.0704
p(Scalp)=0.0331
p(Dura)=0.202
Scalp
Dura

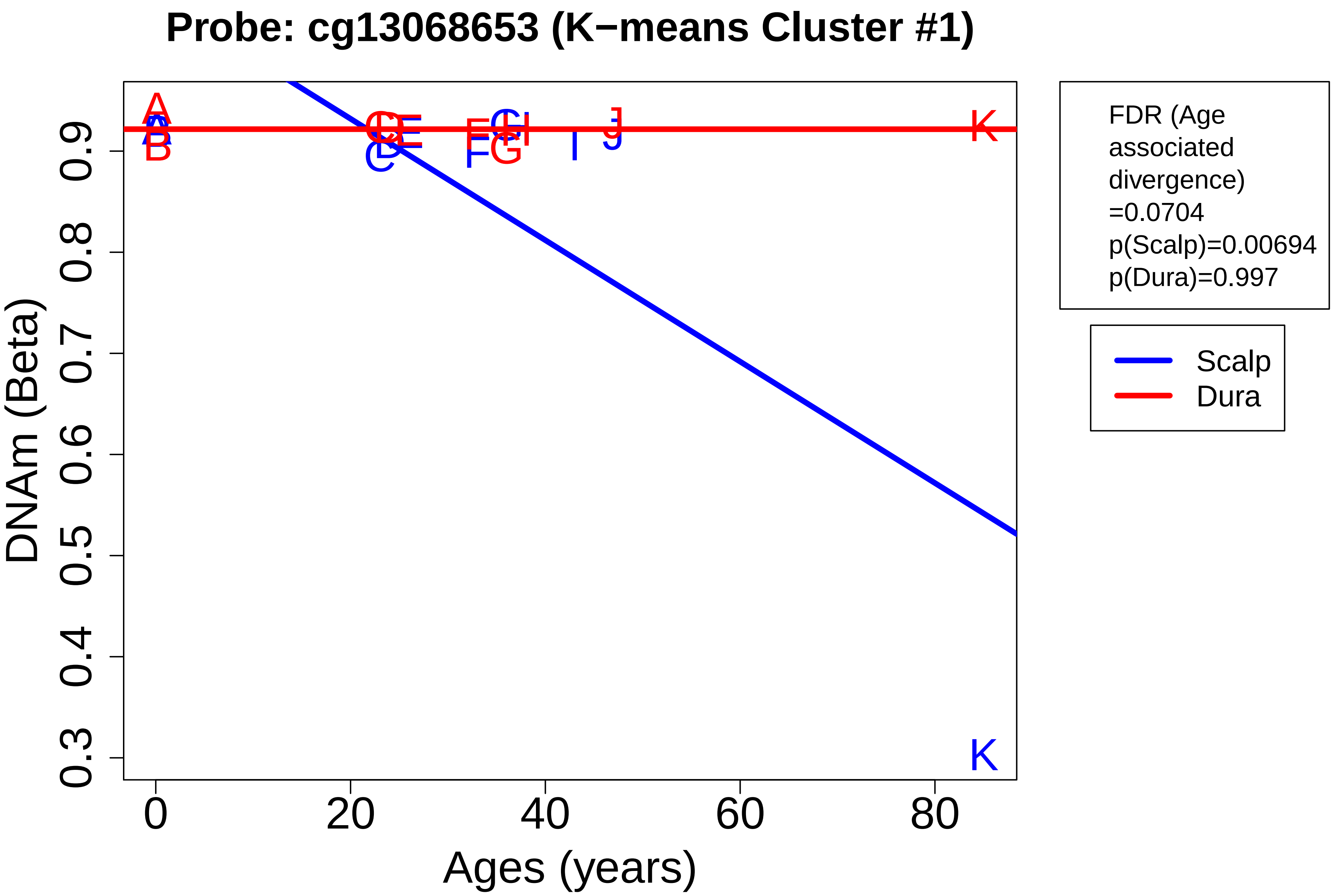

Probe: cg13068653 (K−means Cluster #1)
DNAm (Beta)
0.3
0.4
0.5
0.6
0.7
0.8
0.9
0
20
40
60
80
Ages (years)
FDR (Age associated divergence) =0.0704
p(Scalp)=0.00694
p(Dura)=0.997
Scalp
Dura

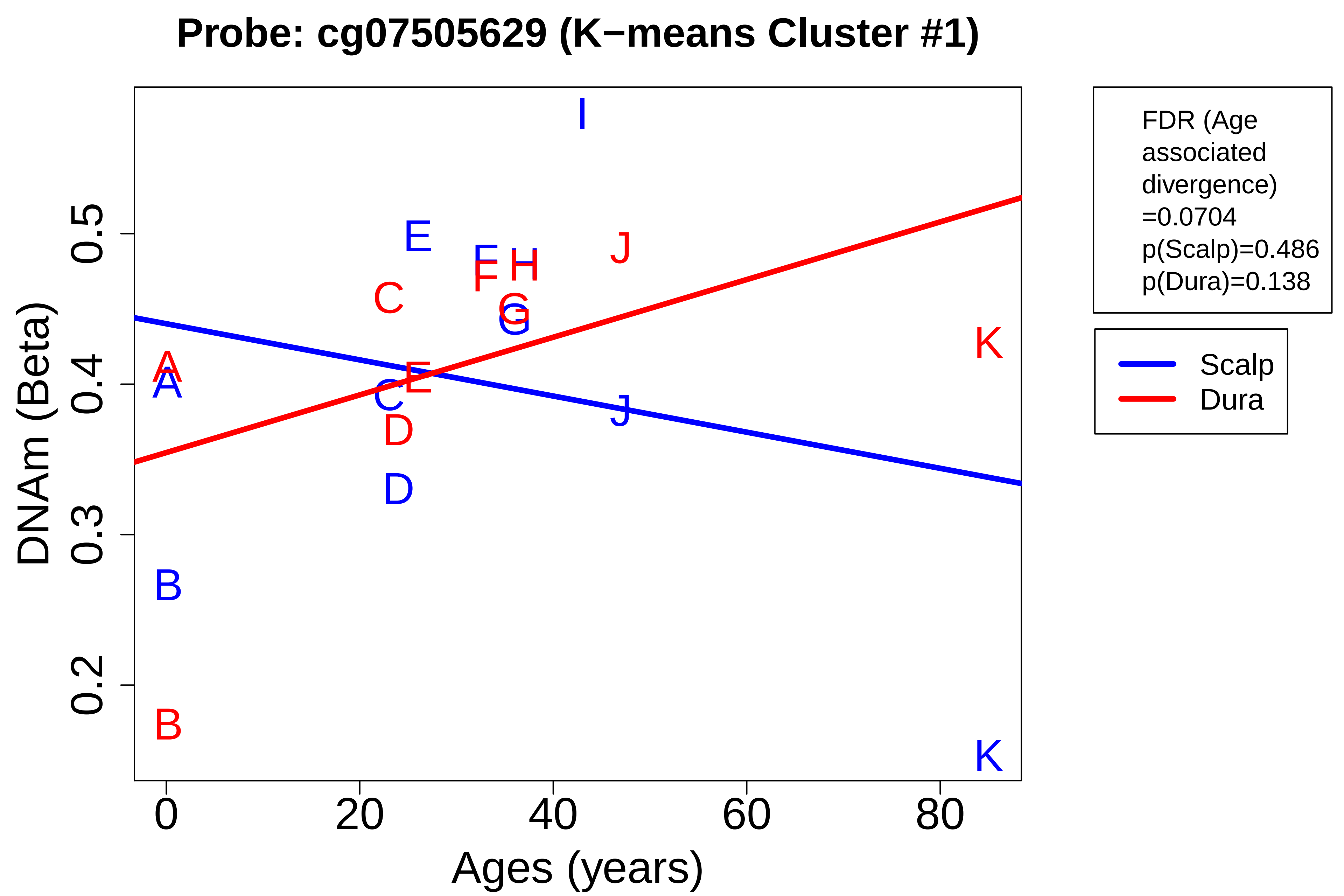

Probe: cg07505629 (K−means Cluster #1)
DNAm (Beta)
0.2
0.3
0.4
0.5
Ages (years)
0
20
40
60
80
FDR (Age associated divergence) =0.0704
p(Scalp)=0.486
p(Dura)=0.138
Scalp
Dura
A
B
C
D
E
F
G
H
I
J
K

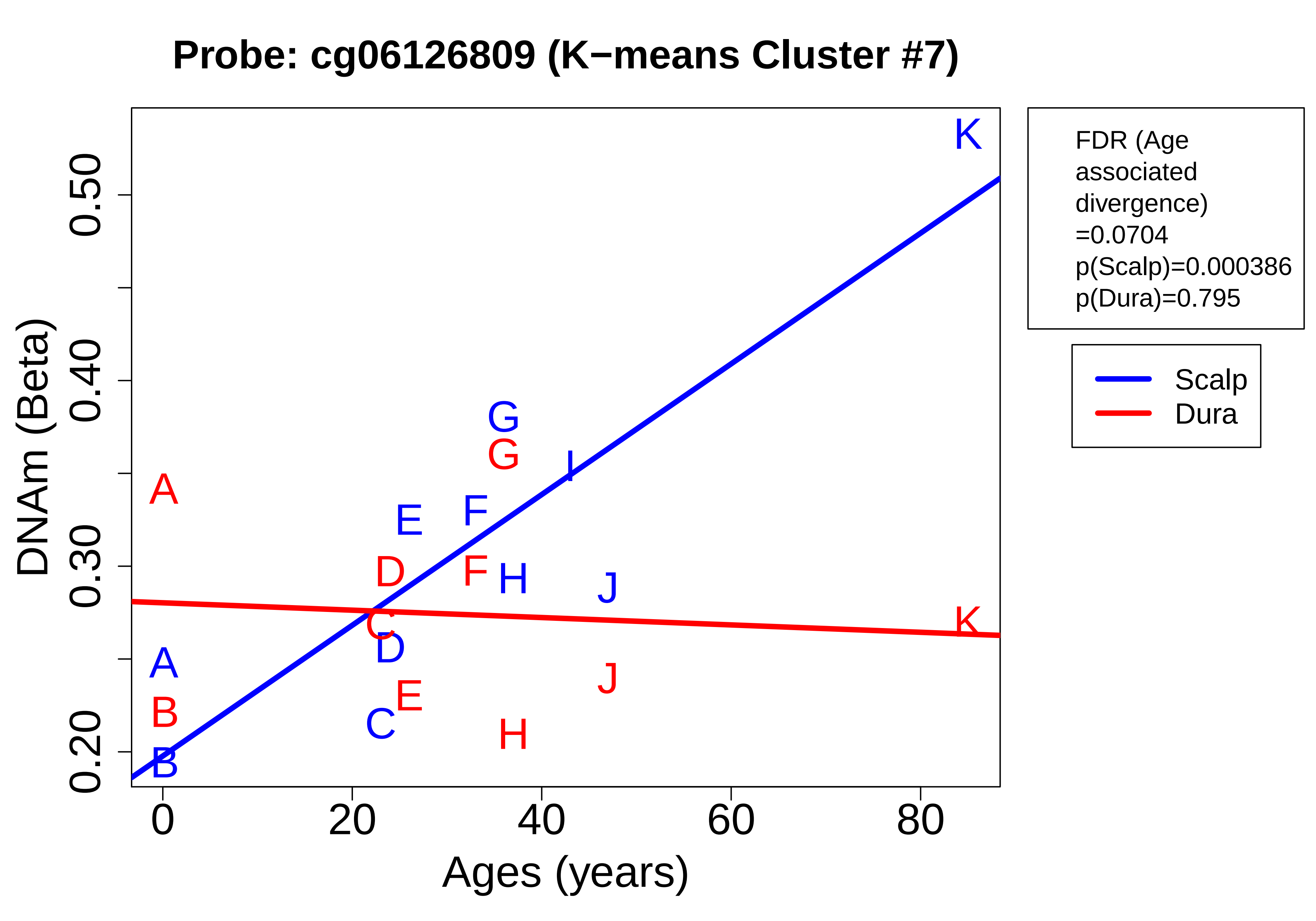
Probe: cg06126809 (K−means Cluster #7)
DNAm (Beta)
0.20
0.30
0.40
0.50
0
20
40
60
80
Ages (years)
FDR (Age associated divergence) =0.0704
p(Scalp)=0.000386
p(Dura)=0.795
Scalp
Dura
A
B
C
D
E
F
G
H
I
J
K
A
B
C
D
E
F
G
H
J
K

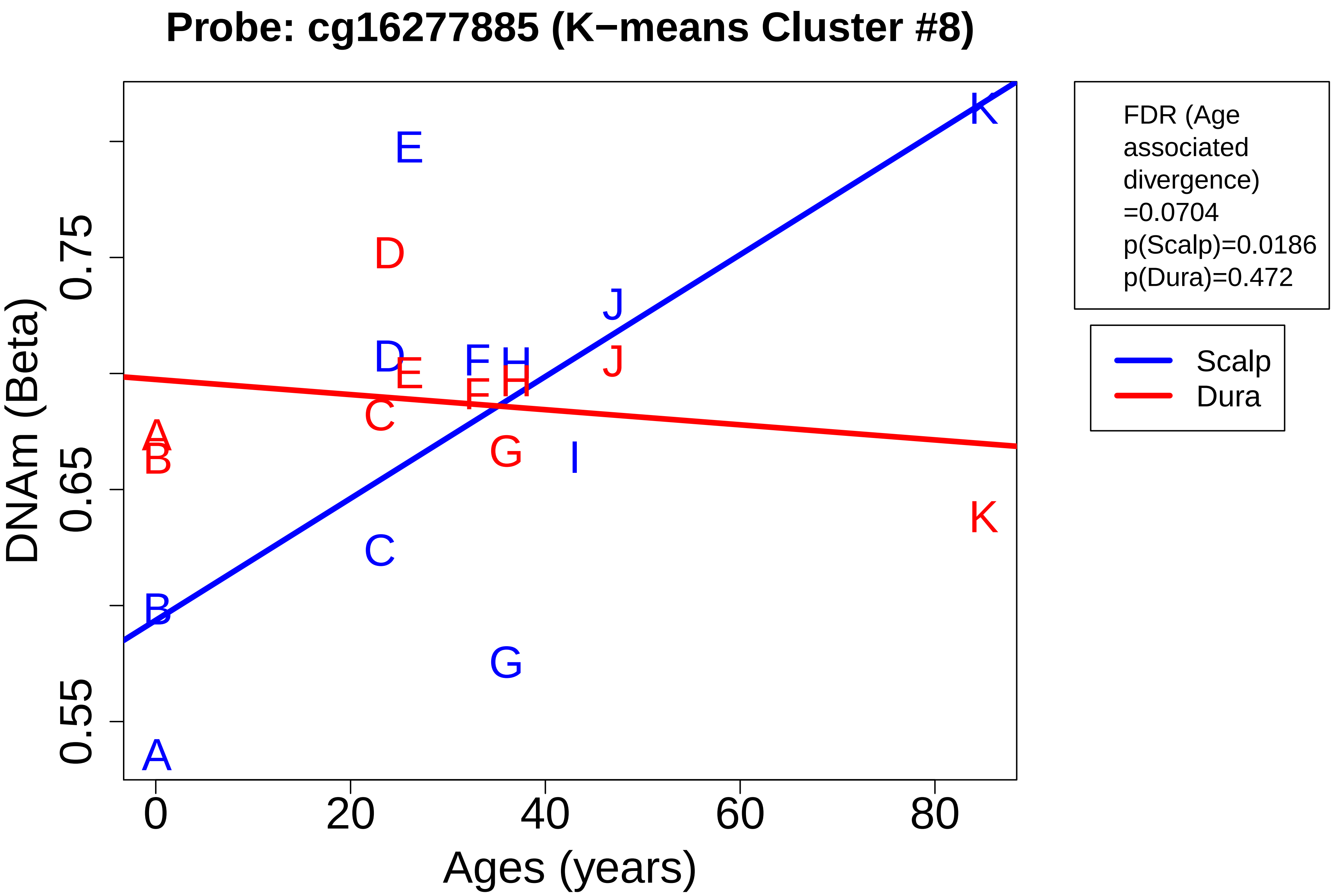

Probe: cg16277885 (K−means Cluster #8)
DNAm (Beta)
0.55
0.65
0.75
Ages (years)
0
20
40
60
80
FDR (Age associated divergence) =0.0704
p(Scalp)=0.0186
p(Dura)=0.472
Scalp
Dura
A B C D E F G H I J K
A B C D E F G H J K

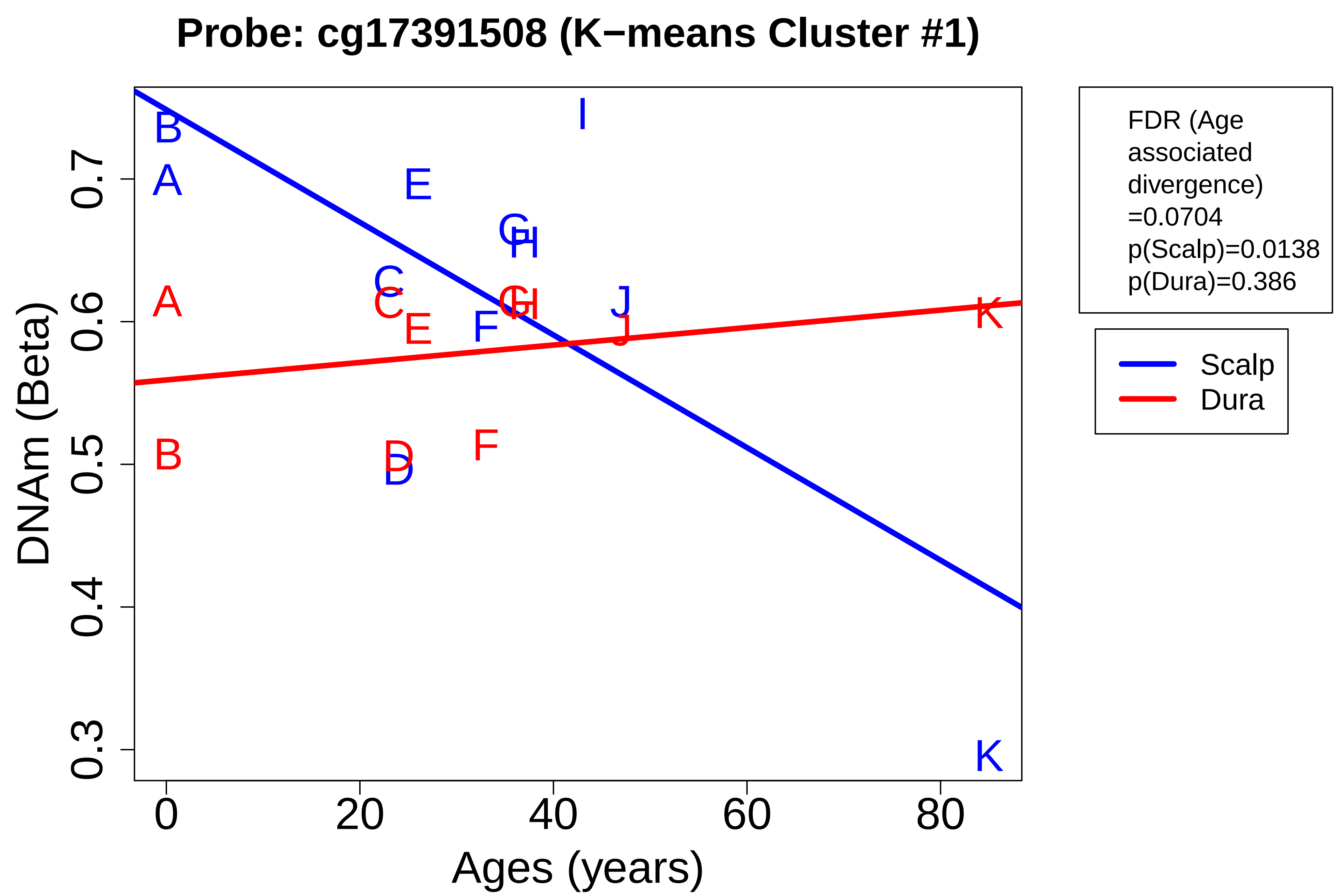

Probe: cg17391508 (K−means Cluster #1)
FDR (Age associated divergence) =0.0704
p(Scalp)=0.0138
p(Dura)=0.386
Scalp
Dura
DNAm (Beta)
0.3
0.4
0.5
0.6
0.7
0
20
40
60
80
Ages (years)
A
B
C
D
E
F
G
H
I
J
K

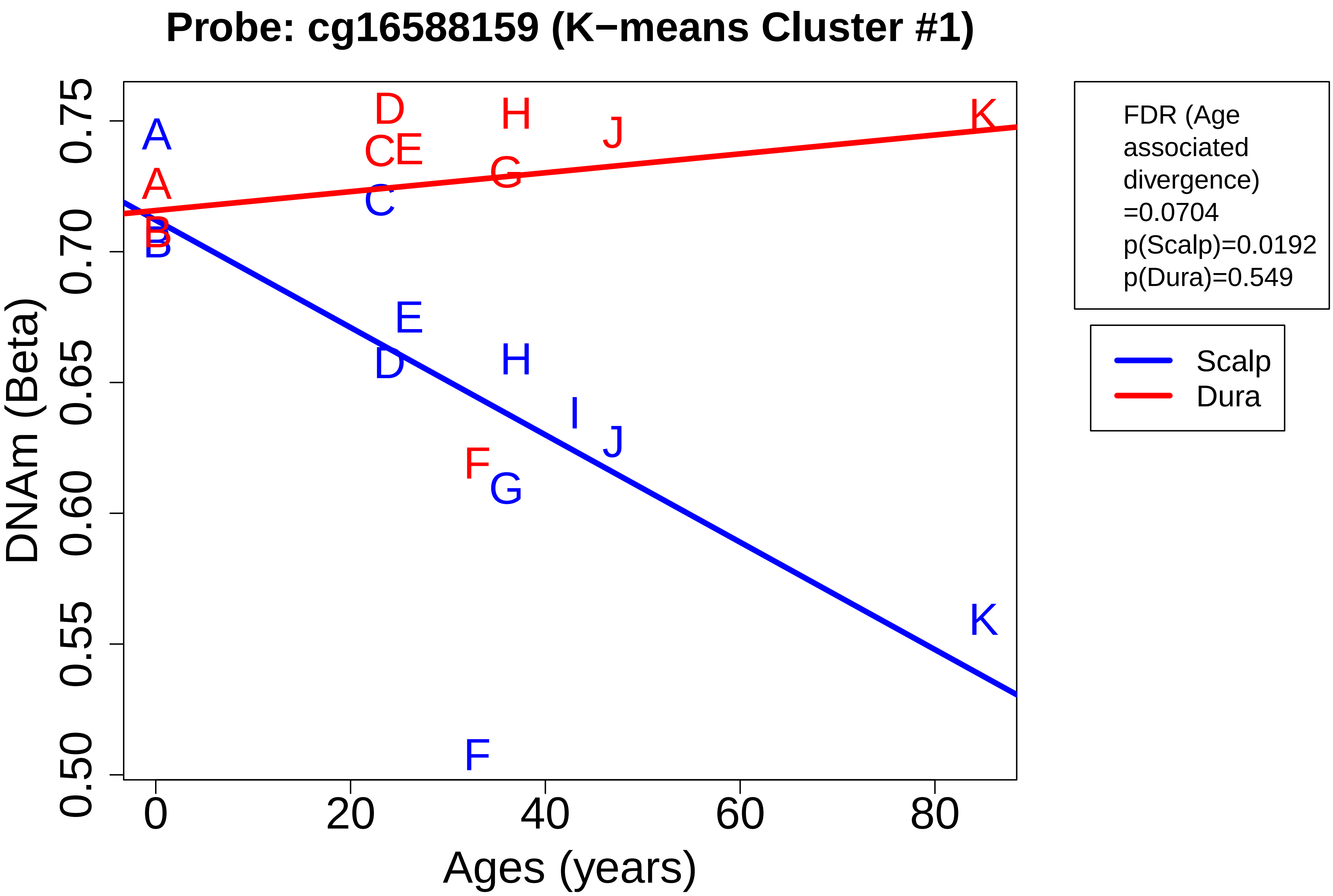

Probe: cg16588159 (K−means Cluster #1)
DNAm (Beta)
0.50
0.55
0.60
0.65
0.70
0.75
0
20
40
60
80
Ages (years)
FDR (Age associated divergence) =0.0704
p(Scalp)=0.0192
p(Dura)=0.549
Scalp
Dura
A
B
C
D
E
F
G
H
I
J
K

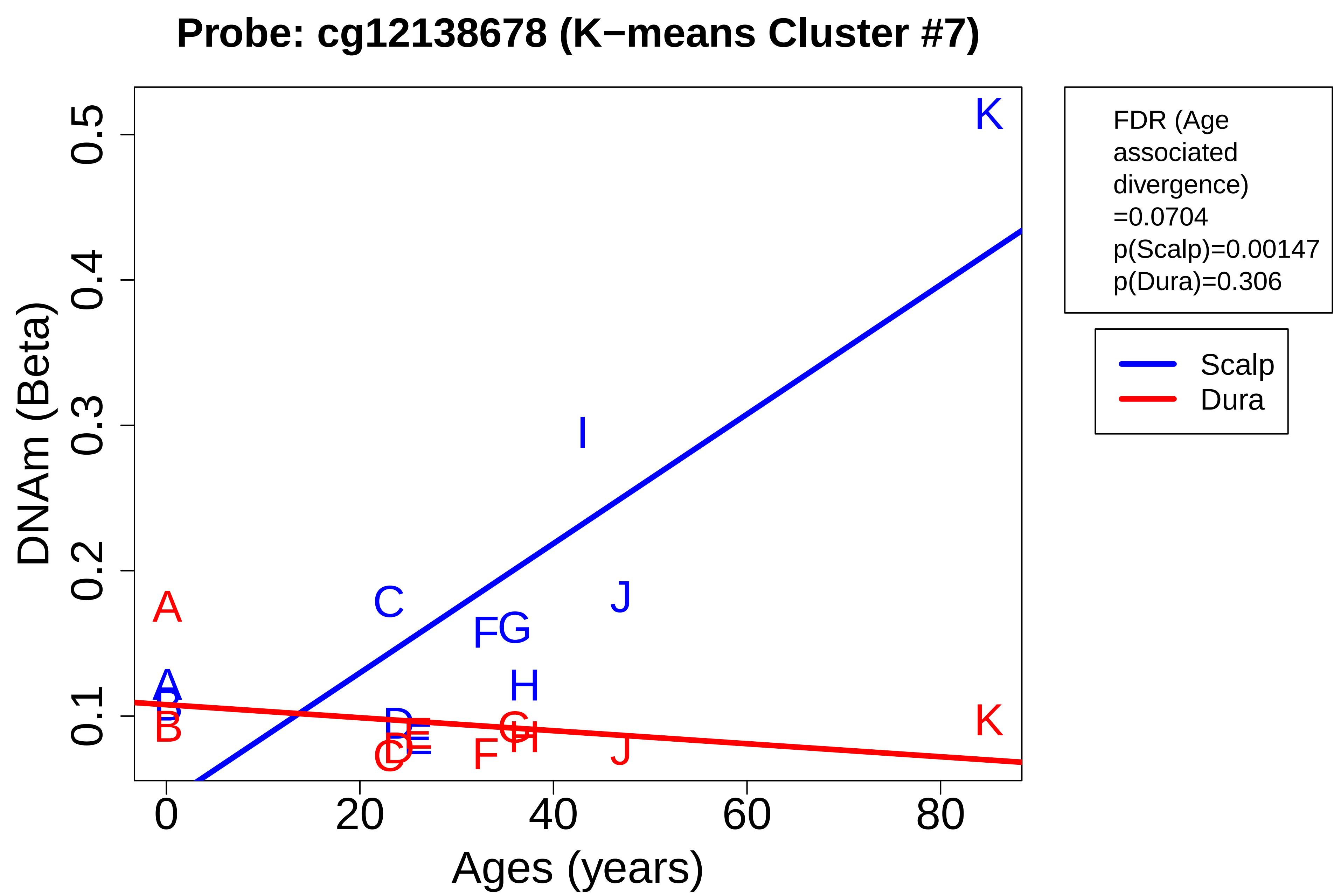

Probe: cg12138678 (K−means Cluster #7)
DNAm (Beta)
0.1
0.2
0.3
0.4
0.5
0
20
40
60
80
Ages (years)
A
B
C
D
E
F
G
H
I
J
K
FDR (Age associated divergence) =0.0704
p(Scalp)=0.00147
p(Dura)=0.306
Scalp
Dura

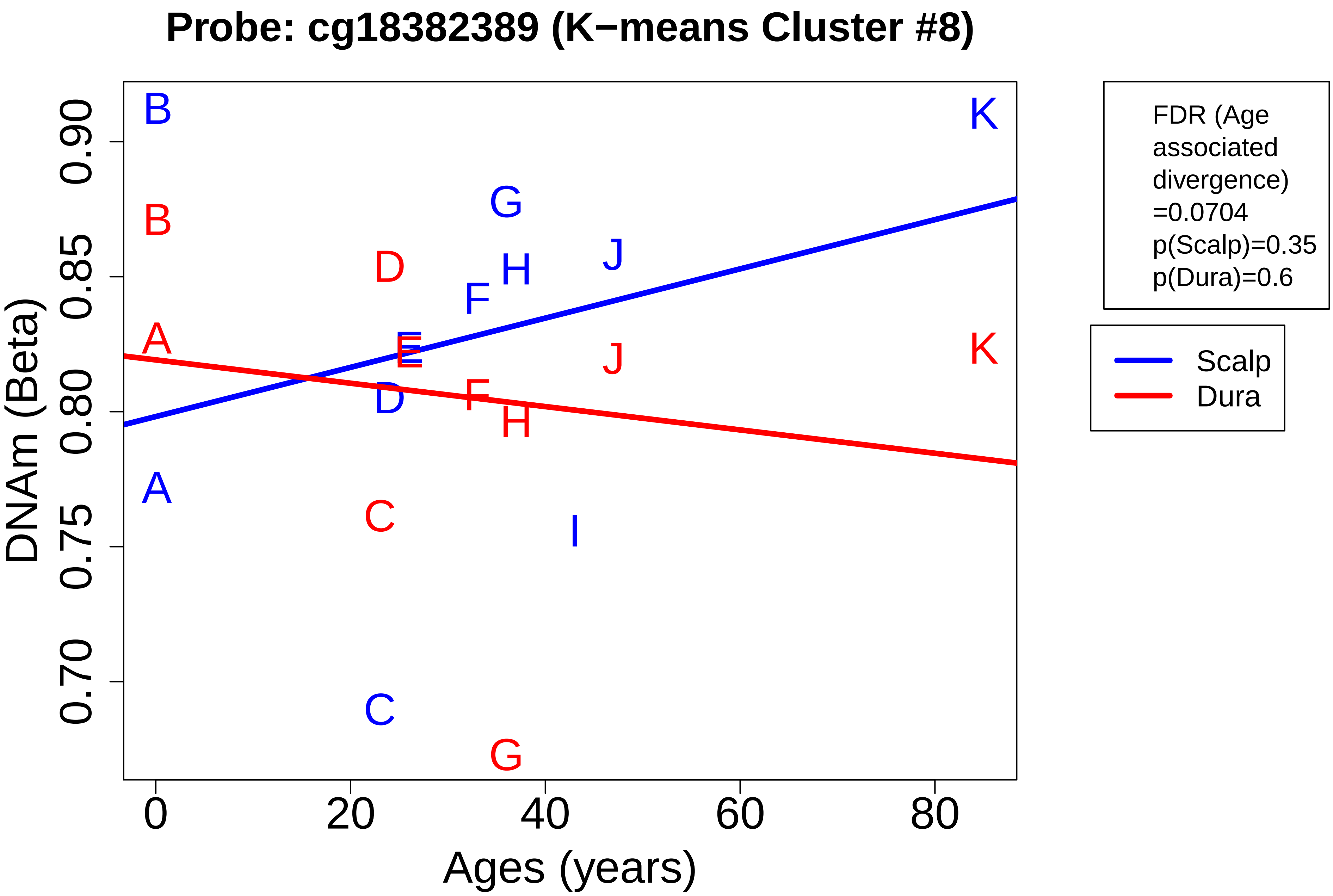

Probe: cg18382389 (K−means Cluster #8)
DNAm (Beta)
0.70
0.75
0.80
0.85
0.90
Ages (years)
0
20
40
60
80
A
B
C
D
E
F
G
H
I
J
K
FDR (Age associated divergence) =0.0704
p(Scalp)=0.35
p(Dura)=0.6
Scalp
Dura

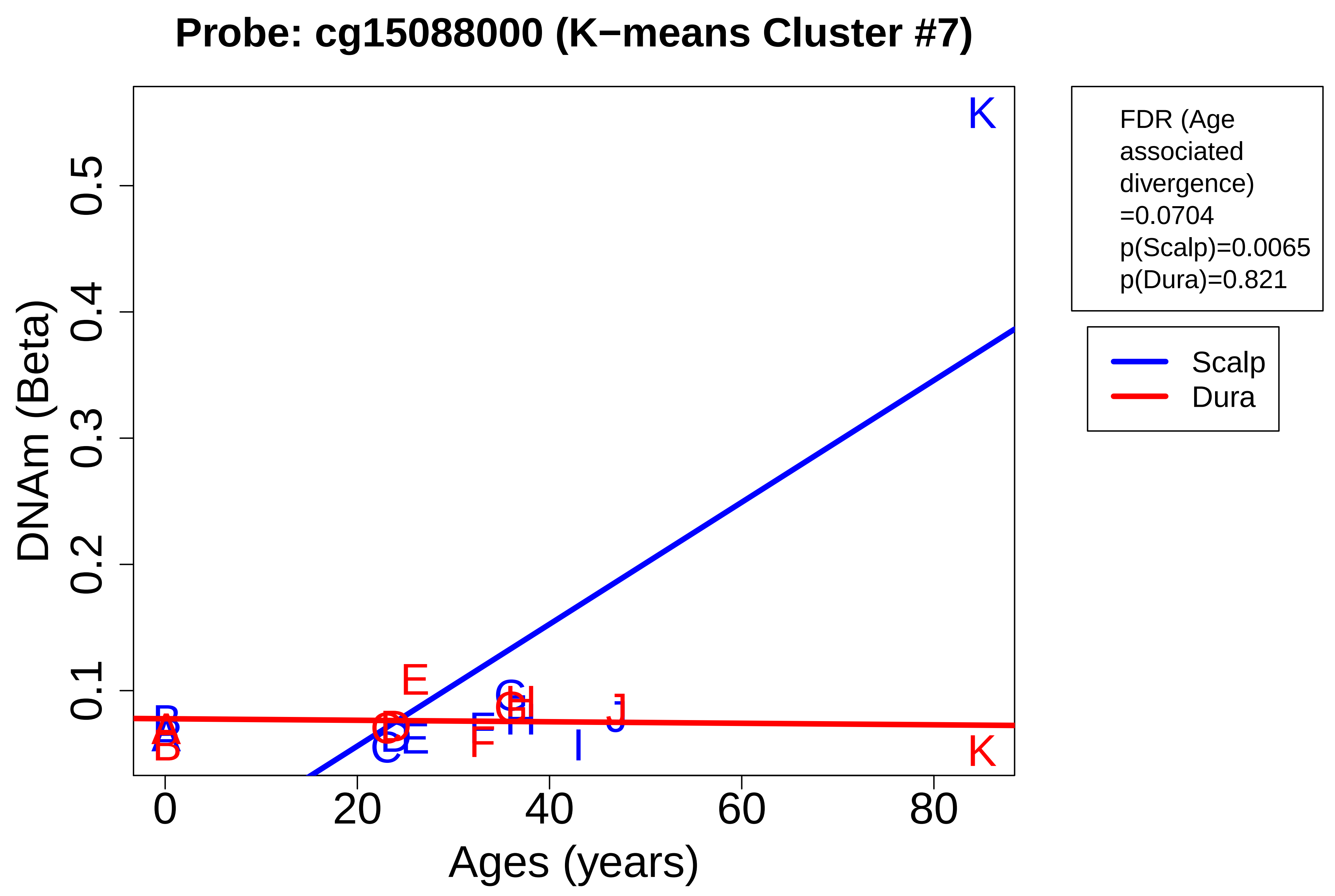
Probe: cg15088000 (K−means Cluster #7)
DNAm (Beta)
0.1
0.2
0.3
0.4
0.5
0
20
40
60
80
Ages (years)
FDR (Age associated divergence) =0.0704
p(Scalp)=0.0065
p(Dura)=0.821
Scalp
Dura

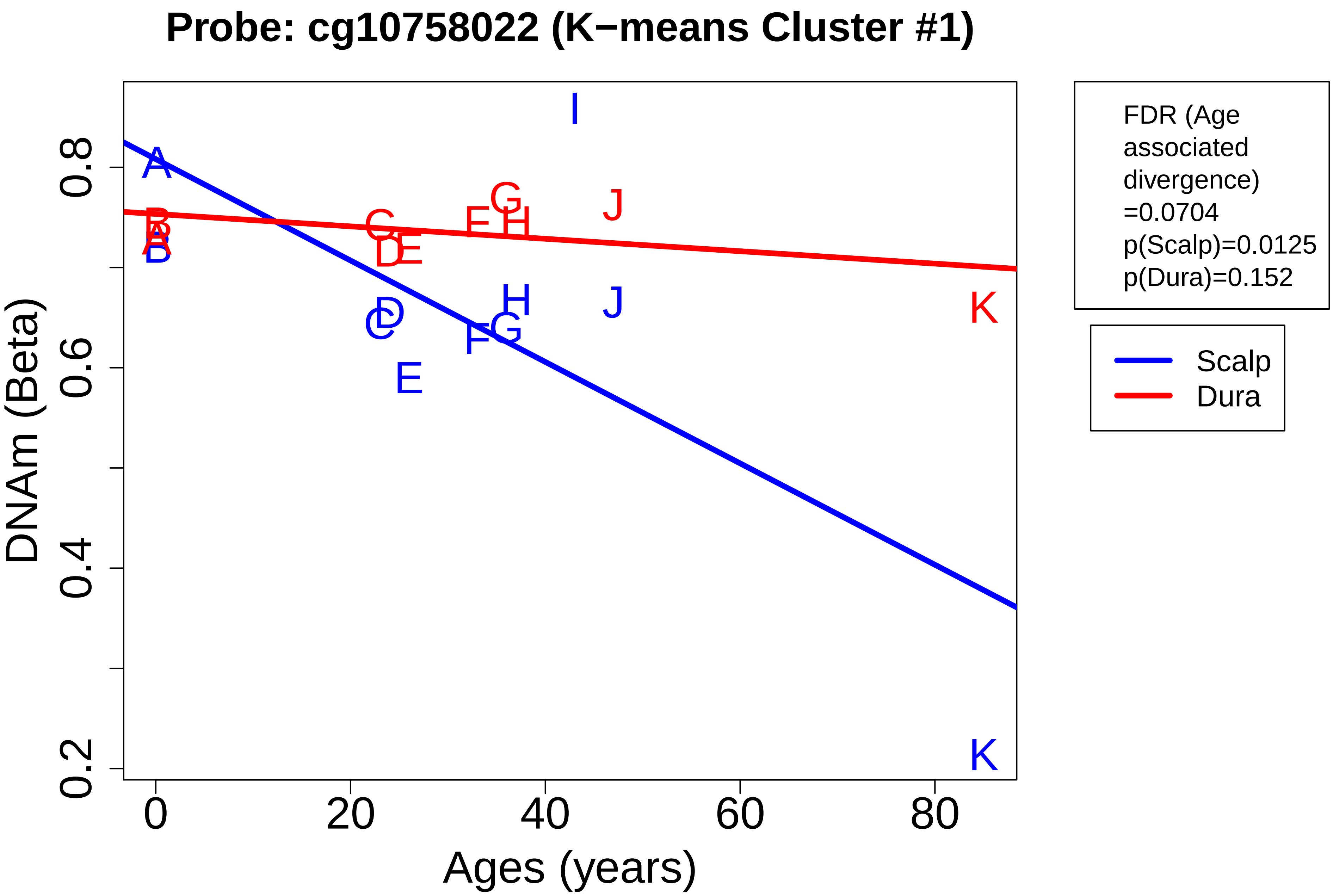

Probe: cg10758022 (K−means Cluster #1)
DNAm (Beta)
0.2
0.4
0.6
0.8
0
20
40
60
80
Ages (years)
FDR (Age associated divergence) =0.0704
p(Scalp)=0.0125
p(Dura)=0.152
Scalp
Dura

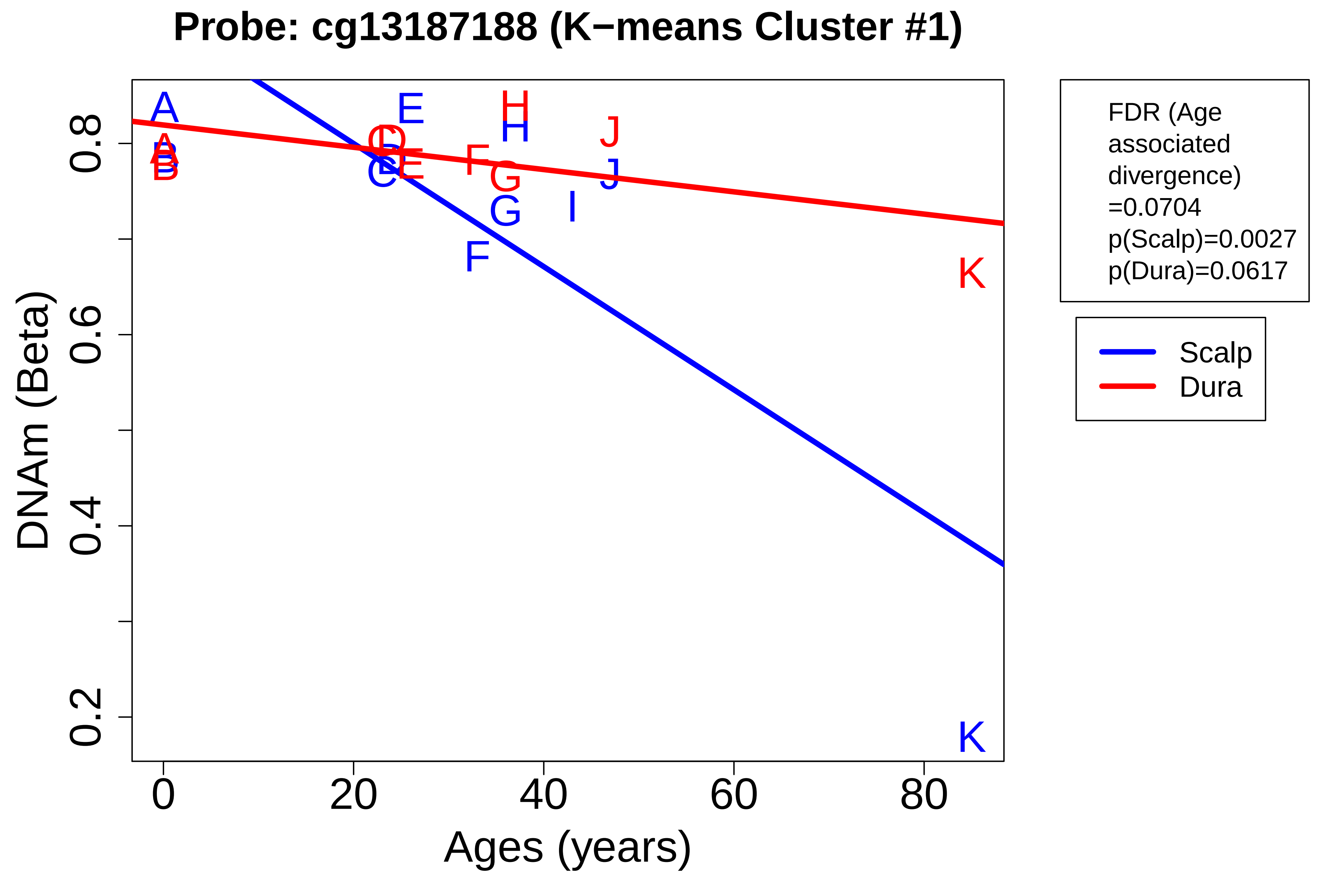
Probe: cg13187188 (K−means Cluster #1)
DNAm (Beta)
0.2
0.4
0.6
0.8
0
20
40
60
80
Ages (years)
A
B
C
D
E
F
G
H
I
J
K
FDR (Age associated divergence) =0.0704
p(Scalp)=0.0027
p(Dura)=0.0617
Scalp
Dura

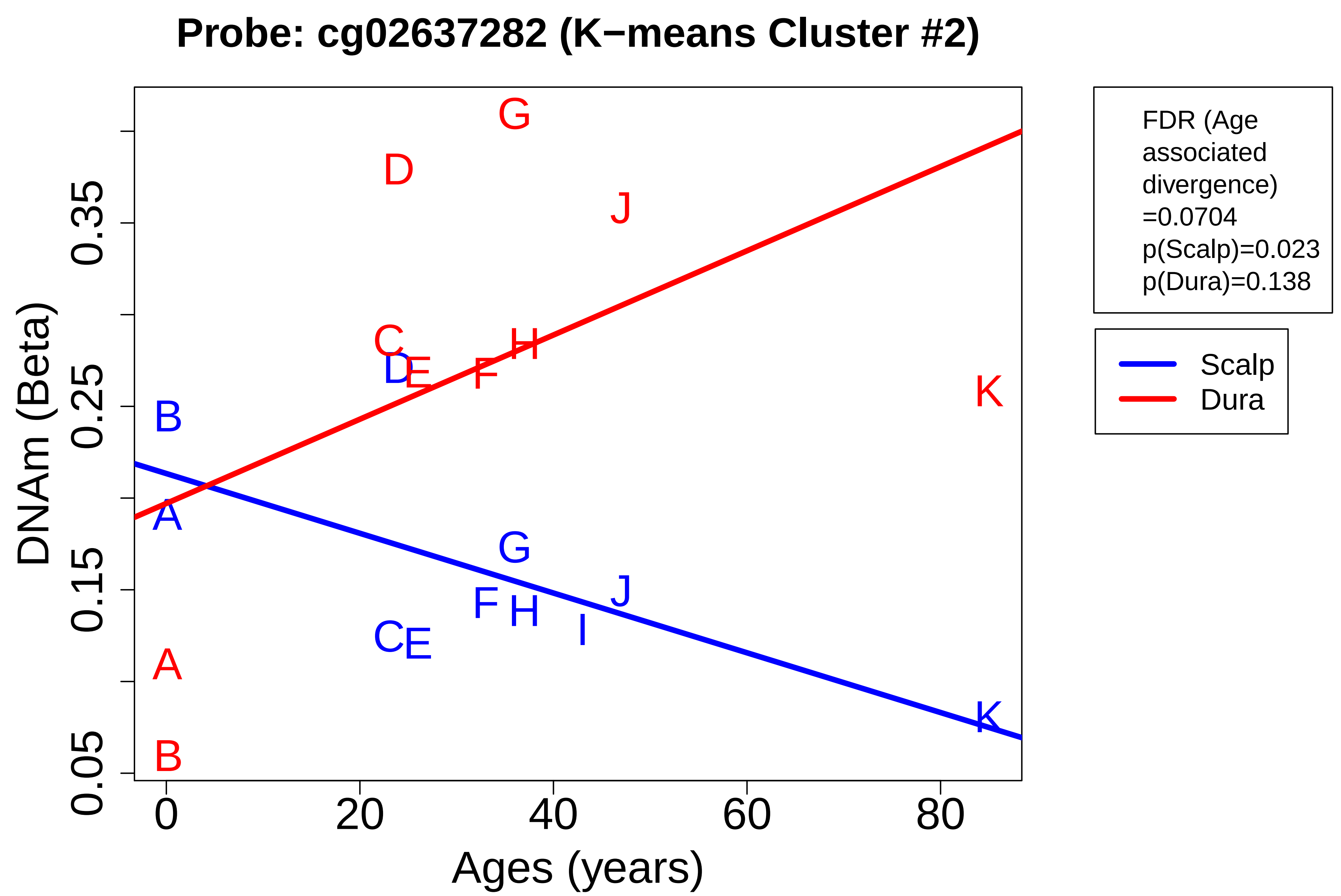

Probe: cg02637282 (K−means Cluster #2)
DNAm (Beta)
0.05
0.15
0.25
0.35
Ages (years)
0
20
40
60
80
A
B
C
D
E
F
G
H
I
J
K
FDR (Age associated divergence) =0.0704
p(Scalp)=0.023
p(Dura)=0.138
Scalp
Dura

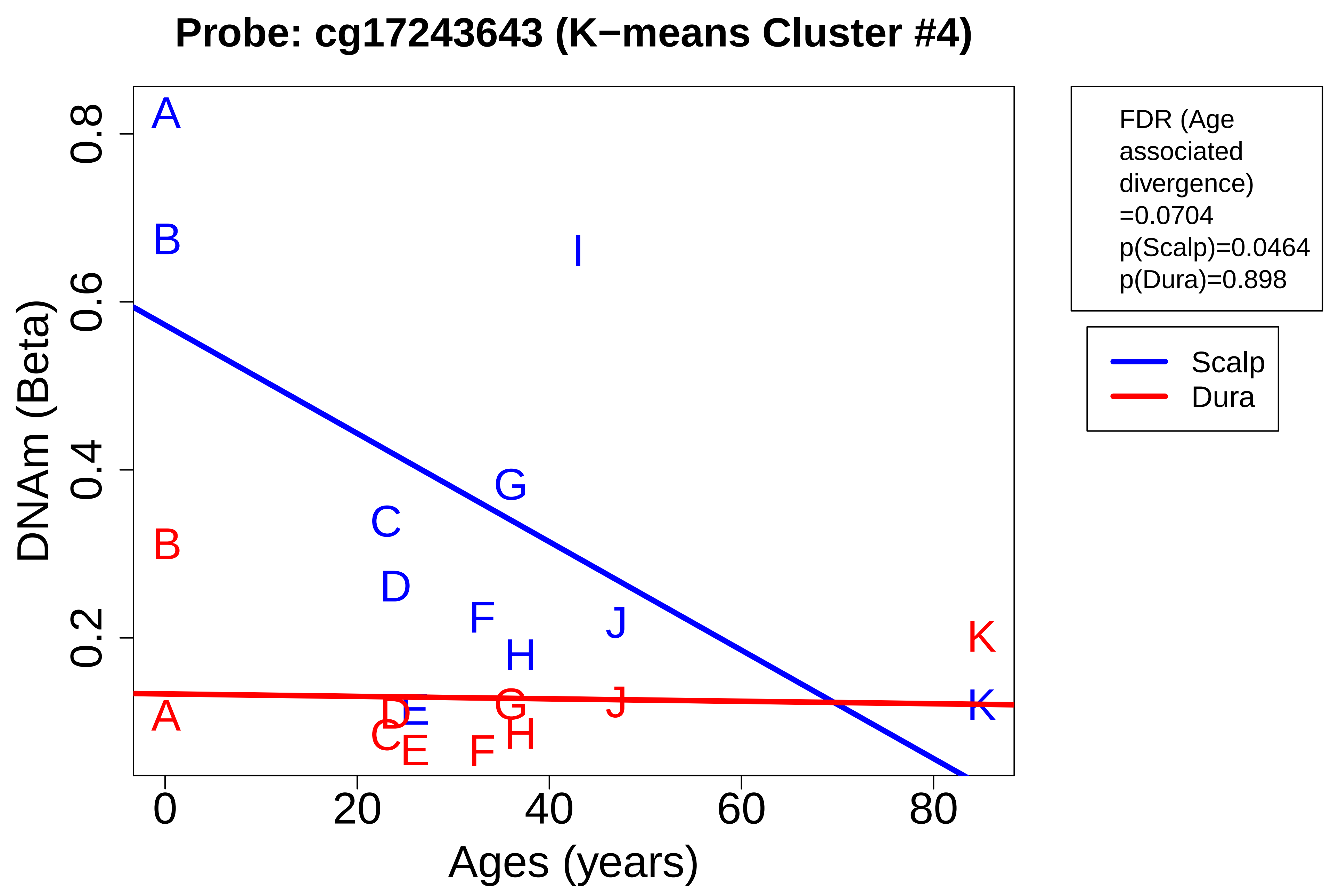

Probe: cg17243643 (K−means Cluster #4)
DNAm (Beta)
0.2
0.4
0.6
0.8
Ages (years)
0
20
40
60
80
A
B
C
D
E
F
G
H
I
J
K
FDR (Age associated divergence) =0.0704
p(Scalp)=0.0464
p(Dura)=0.898
Scalp
Dura

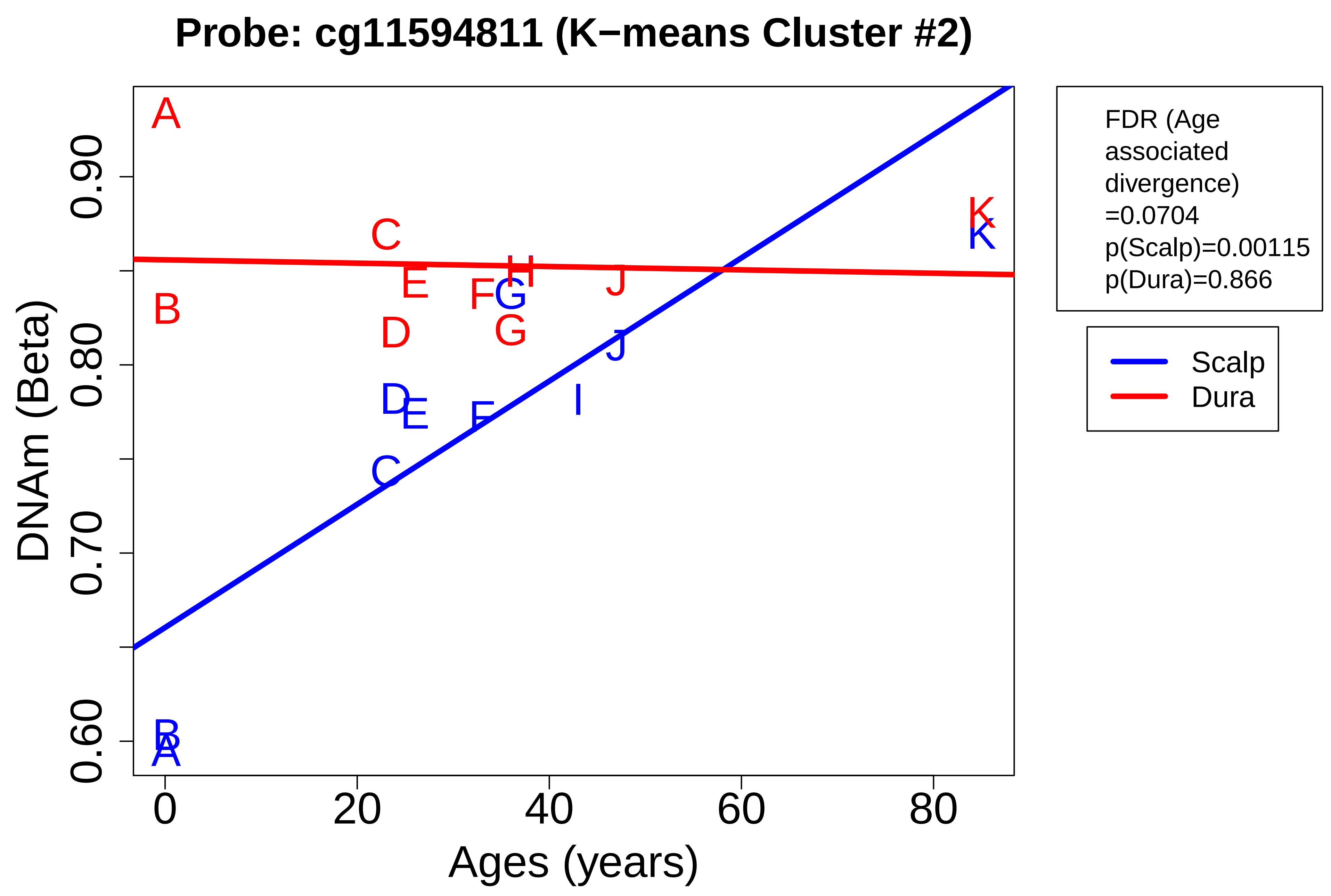
Probe: cg11594811 (K−means Cluster #2)
DNAm (Beta)
0.60
0.70
0.80
0.90
0
20
40
60
80
Ages (years)
FDR (Age associated divergence) =0.0704
p(Scalp)=0.00115
p(Dura)=0.866
Scalp
Dura

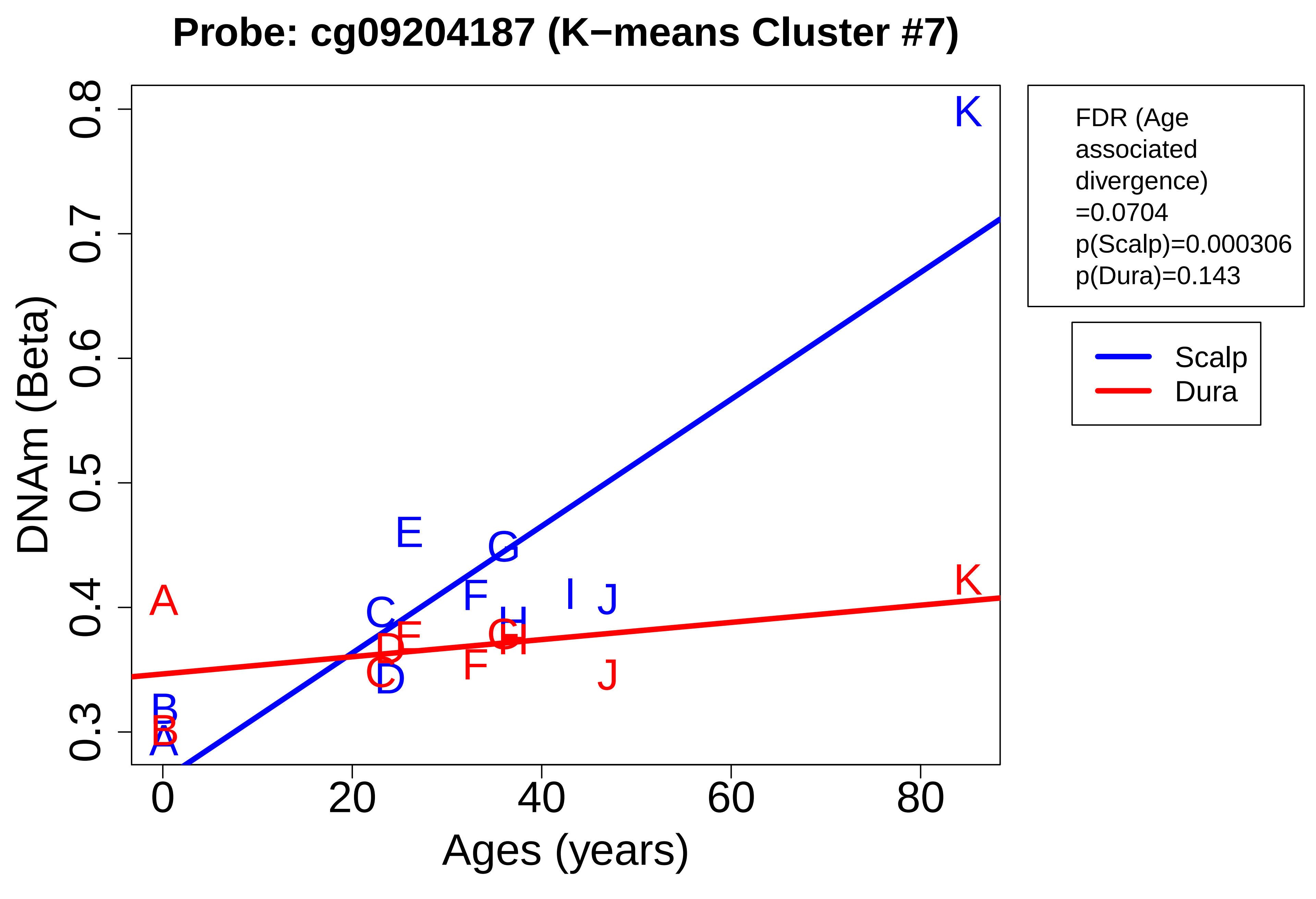

Probe: cg09204187 (K−means Cluster #7)
DNAm (Beta)
0.3
0.4
0.5
0.6
0.7
0.8
Ages (years)
0
20
40
60
80
FDR (Age associated divergence) =0.0704
p(Scalp)=0.000306
p(Dura)=0.143
Scalp
Dura
A
B
C
D
E
F
G
H
I
J
K

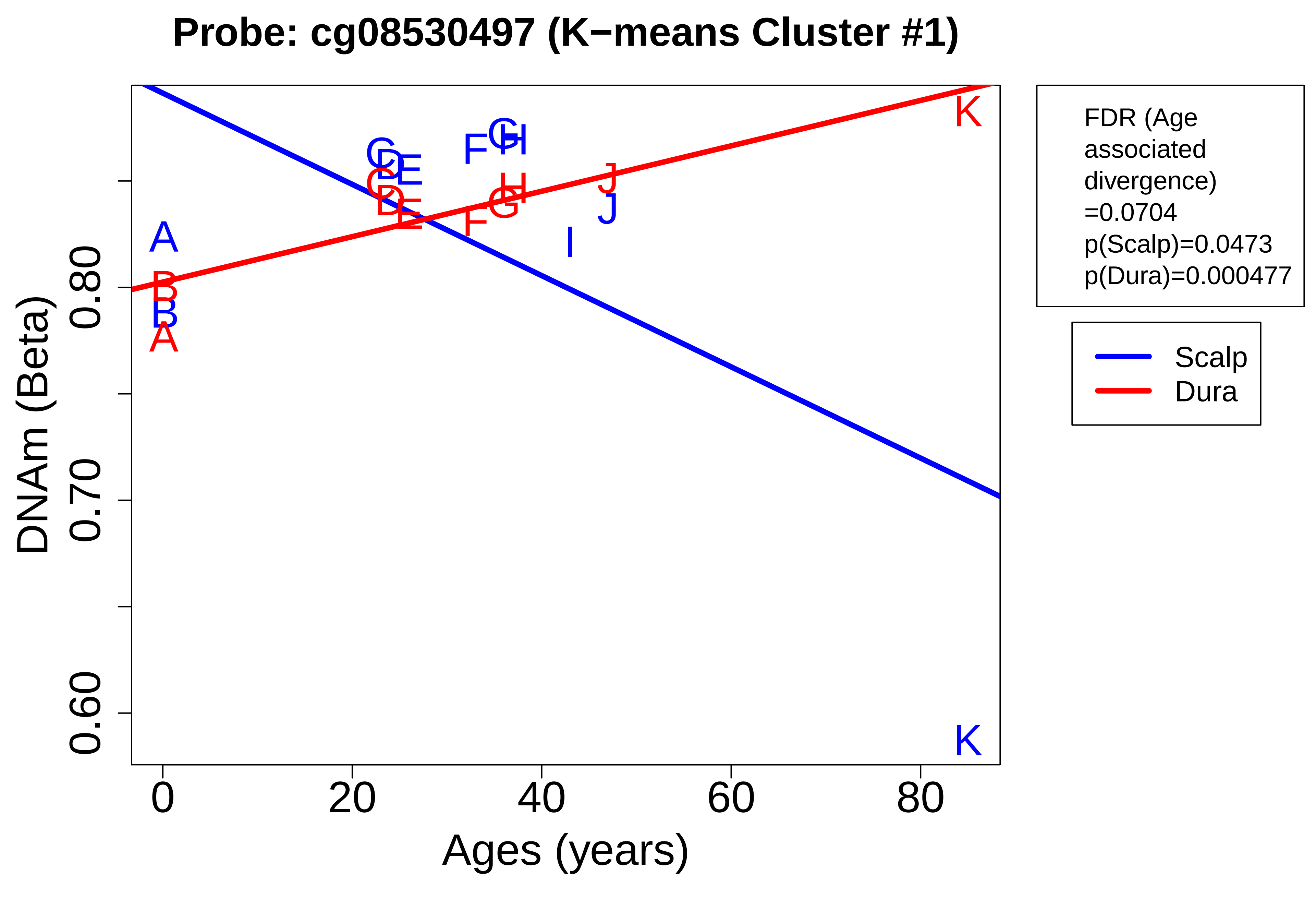

Probe: cg08530497 (K−means Cluster #1)
DNAm (Beta)
0.60
0.70
0.80
0
20
40
60
80
Ages (years)
A
B
C
D
E
F
G
H
I
J
K
FDR (Age associated divergence) =0.0704
p(Scalp)=0.0473
p(Dura)=0.000477
Scalp
Dura

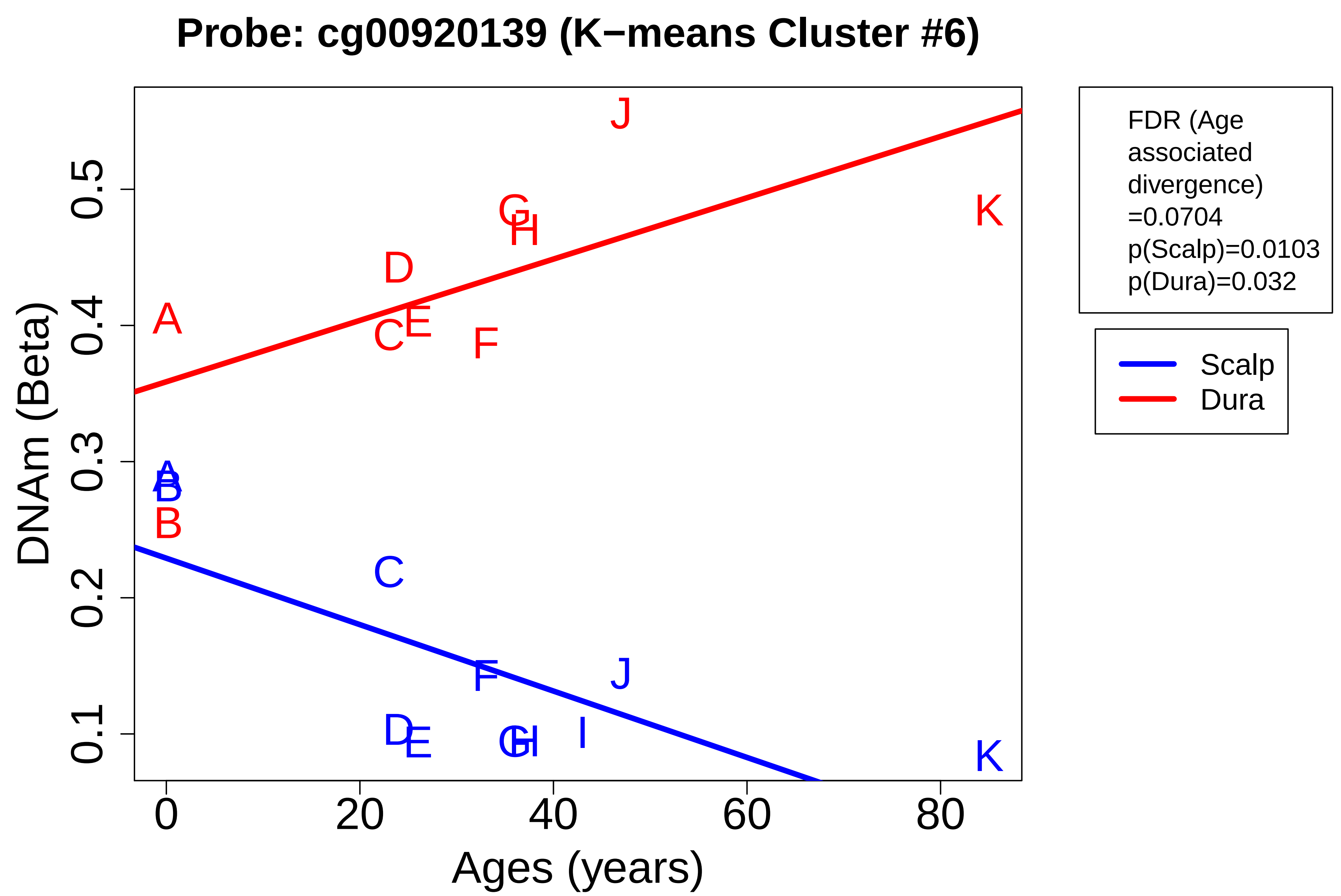

Probe: cg00920139 (K−means Cluster #6)
DNAm (Beta)
0.1
0.2
0.3
0.4
0.5
0
20
40
60
80
Ages (years)
A
B
C
D
E
F
G
H
J
K
A
B
C
D
E
F
G
H
I
J
K
FDR (Age associated divergence) =0.0704
p(Scalp)=0.0103
p(Dura)=0.032
Scalp
Dura

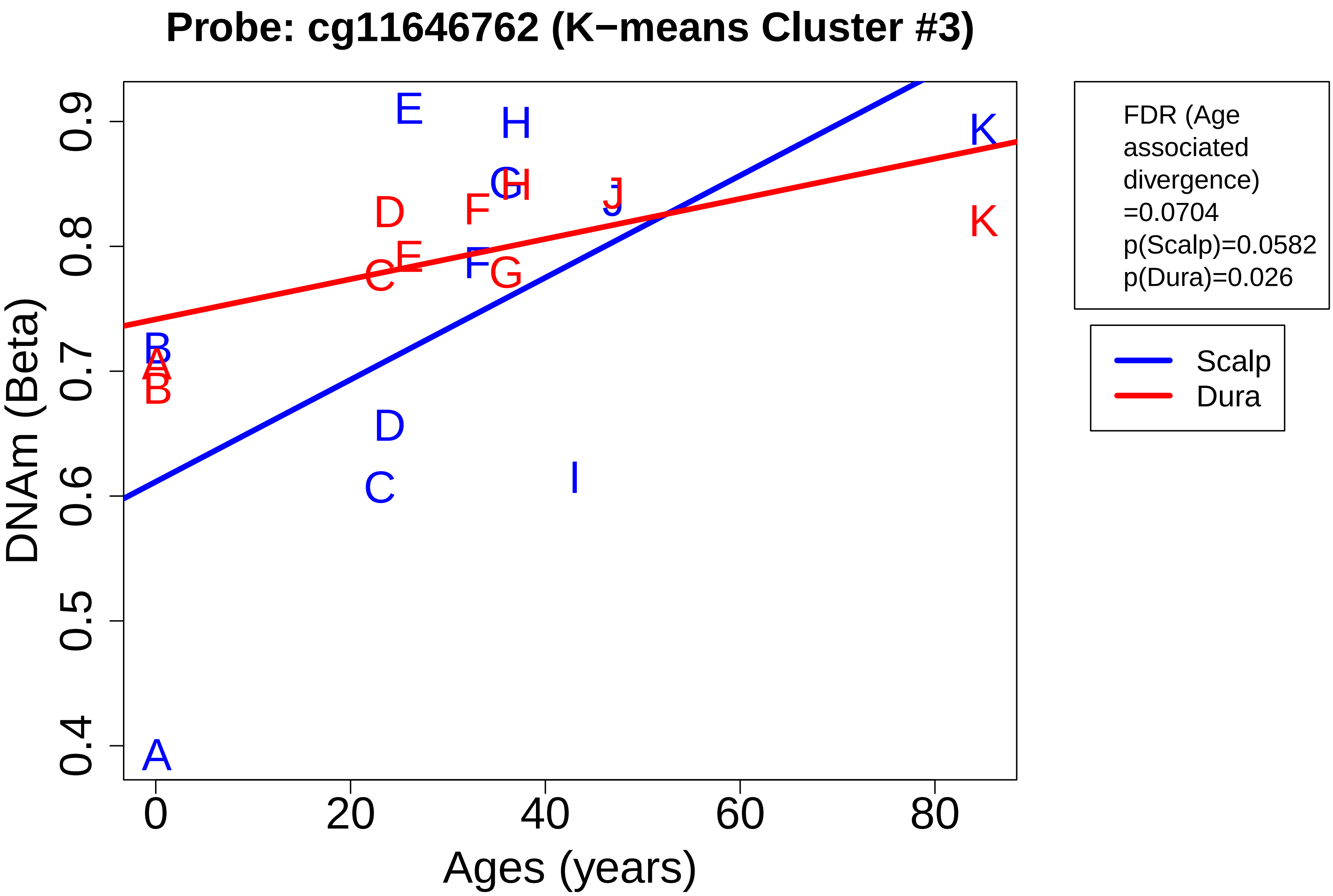

Probe: cg11646762 (K−means Cluster #3)
DNAm (Beta)
Ages (years)
0.4
0.5
0.6
0.7
0.8
0.9
0
20
40
60
80
FDR (Age associated divergence) =0.0704
p(Scalp)=0.0582
p(Dura)=0.026
Scalp
Dura

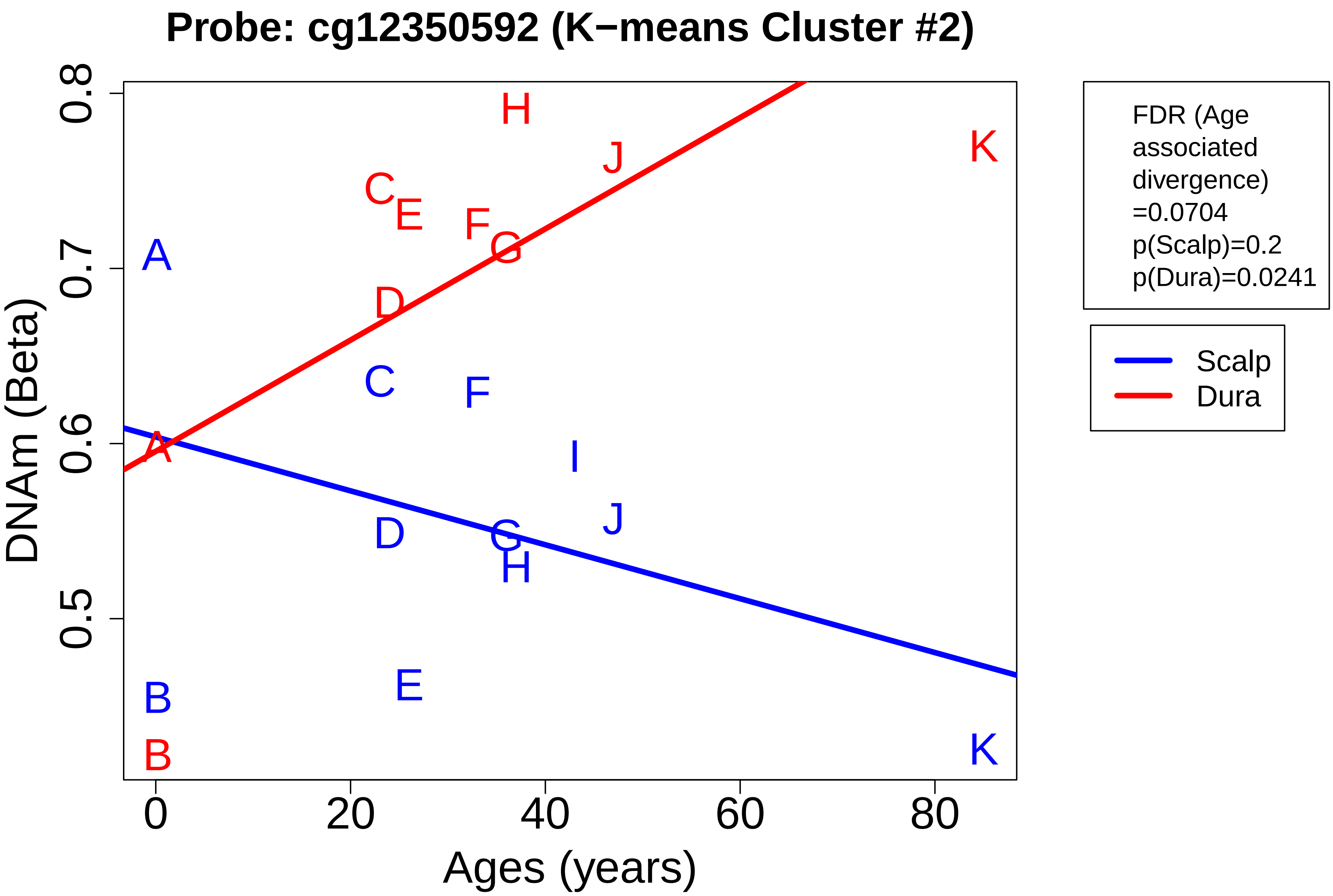
Probe: cg12350592 (K−means Cluster #2)
DNAm (Beta)
0.5
0.6
0.7
0.8
0
20
40
60
80
Ages (years)
A
B
C
D
E
F
G
H
I
J
K
FDR (Age associated divergence) =0.0704
p(Scalp)=0.2
p(Dura)=0.0241
Scalp
Dura

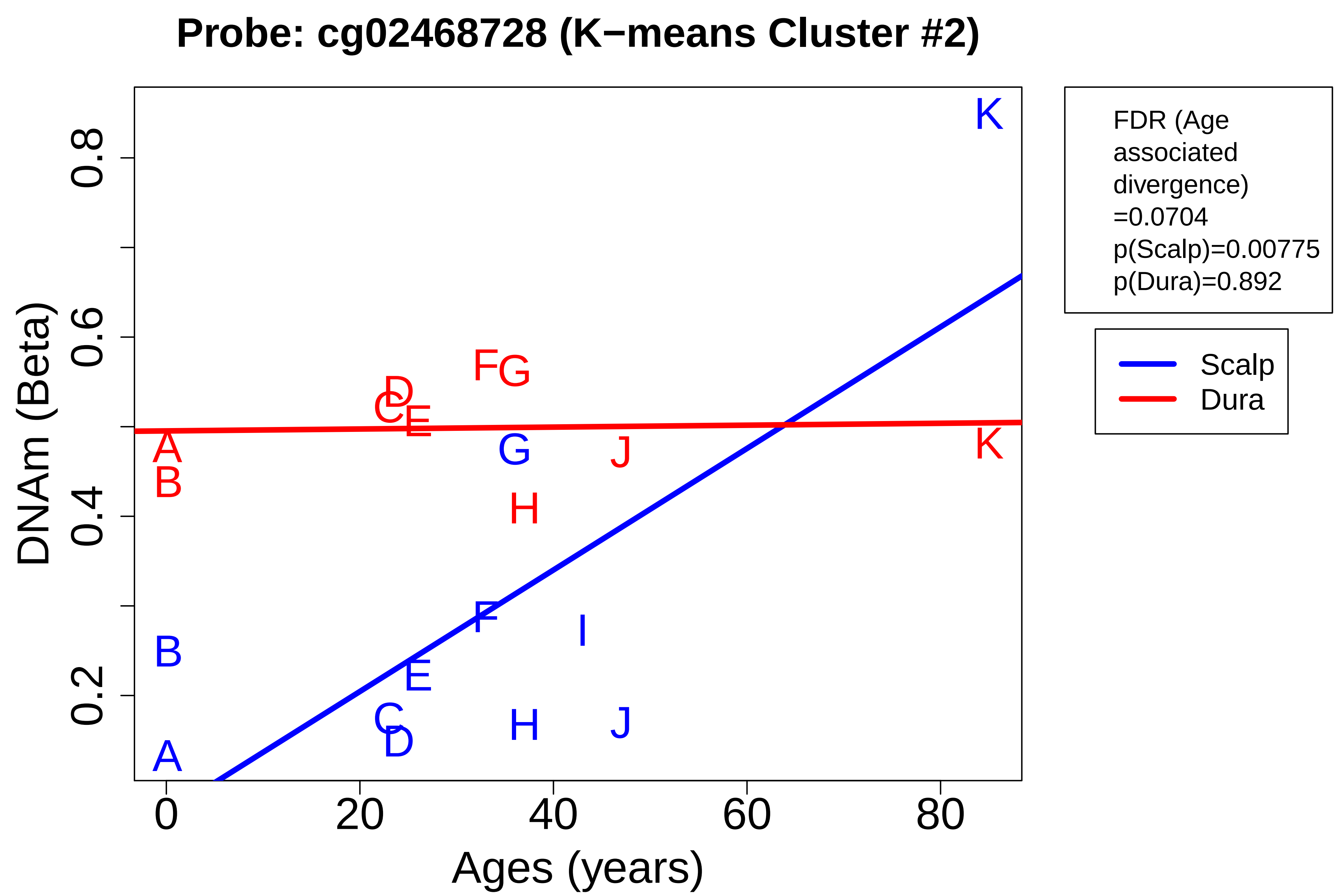

Probe: cg02468728 (K−means Cluster #2)
FDR (Age associated divergence) =0.0704
p(Scalp)=0.00775
p(Dura)=0.892
Scalp
Dura
DNAm (Beta)
0.2
0.4
0.6
0.8
0
20
40
60
80
Ages (years)
A
B
C
D
E
F
G
H
I
J
K

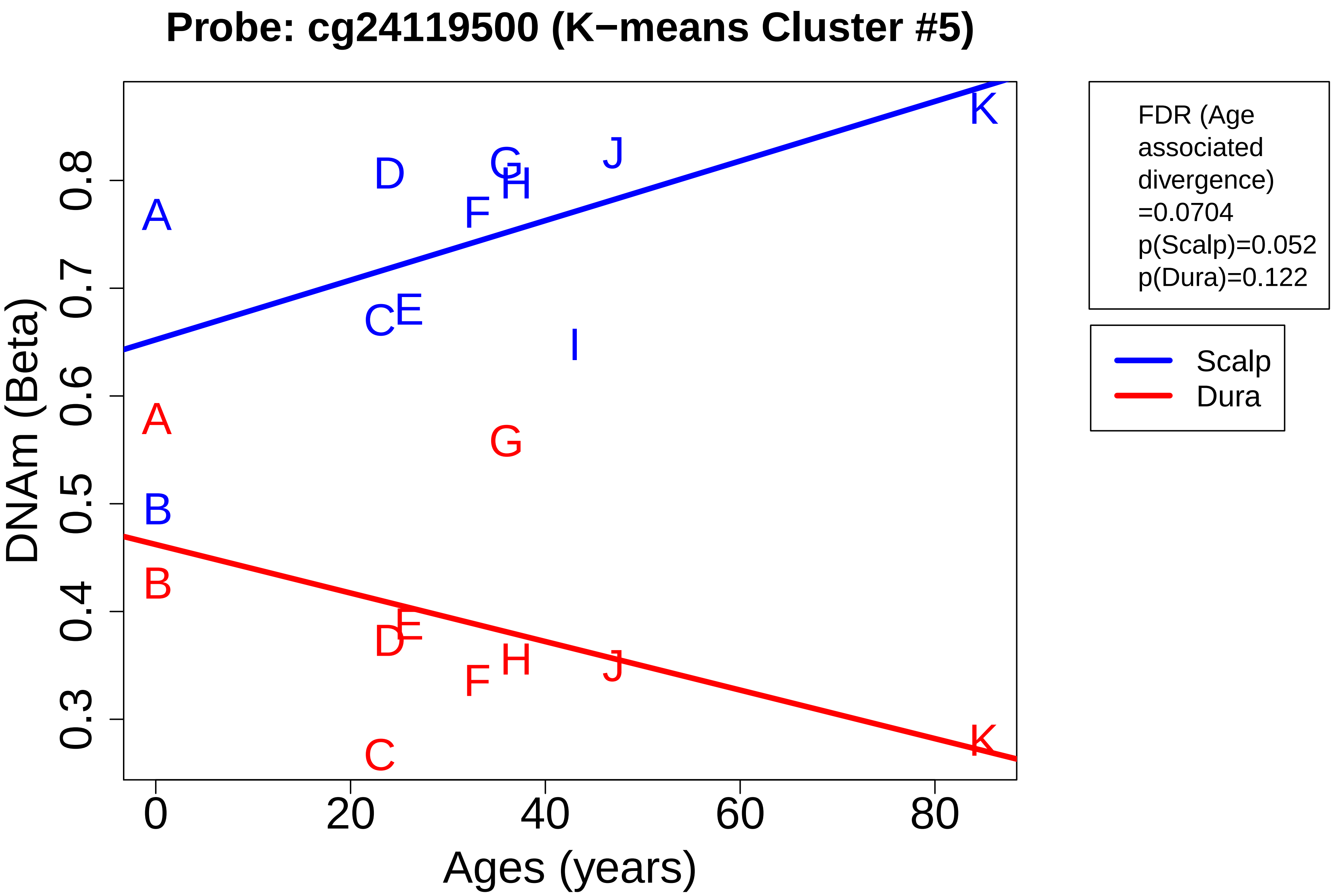

Probe: cg24119500 (K−means Cluster #5)
DNAm (Beta)
0.3
0.4
0.5
0.6
0.7
0.8
Ages (years)
0
20
40
60
80
A
B
C
D
E
F
G
H
I
J
K
FDR (Age associated divergence) =0.0704
p(Scalp)=0.052
p(Dura)=0.122
Scalp
Dura

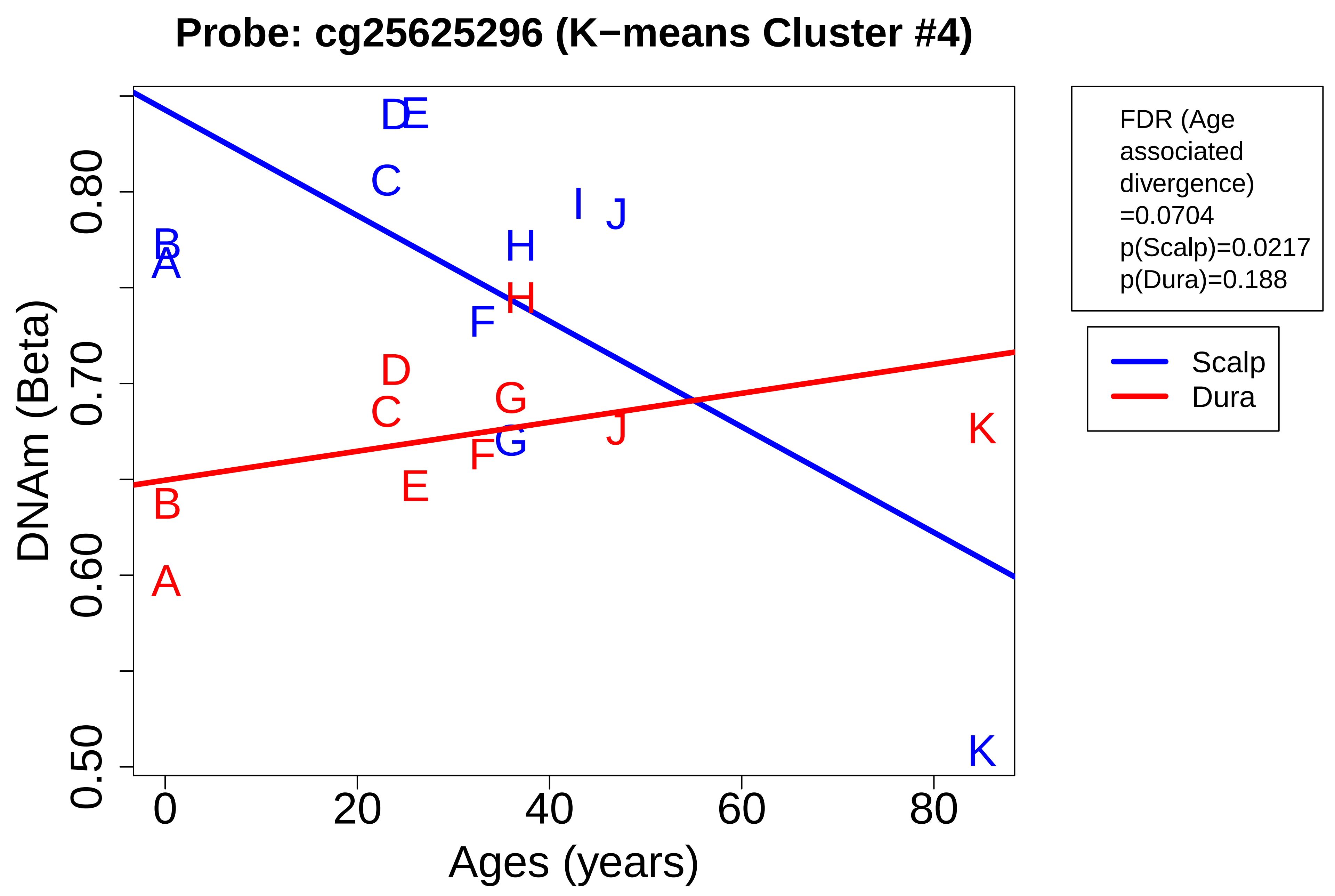

Probe: cg25625296 (K−means Cluster #4)
DNAm (Beta)
0.50
0.60
0.70
0.80
0
20
40
60
80
Ages (years)
FDR (Age associated divergence) =0.0704
p(Scalp)=0.0217
p(Dura)=0.188
Scalp
Dura

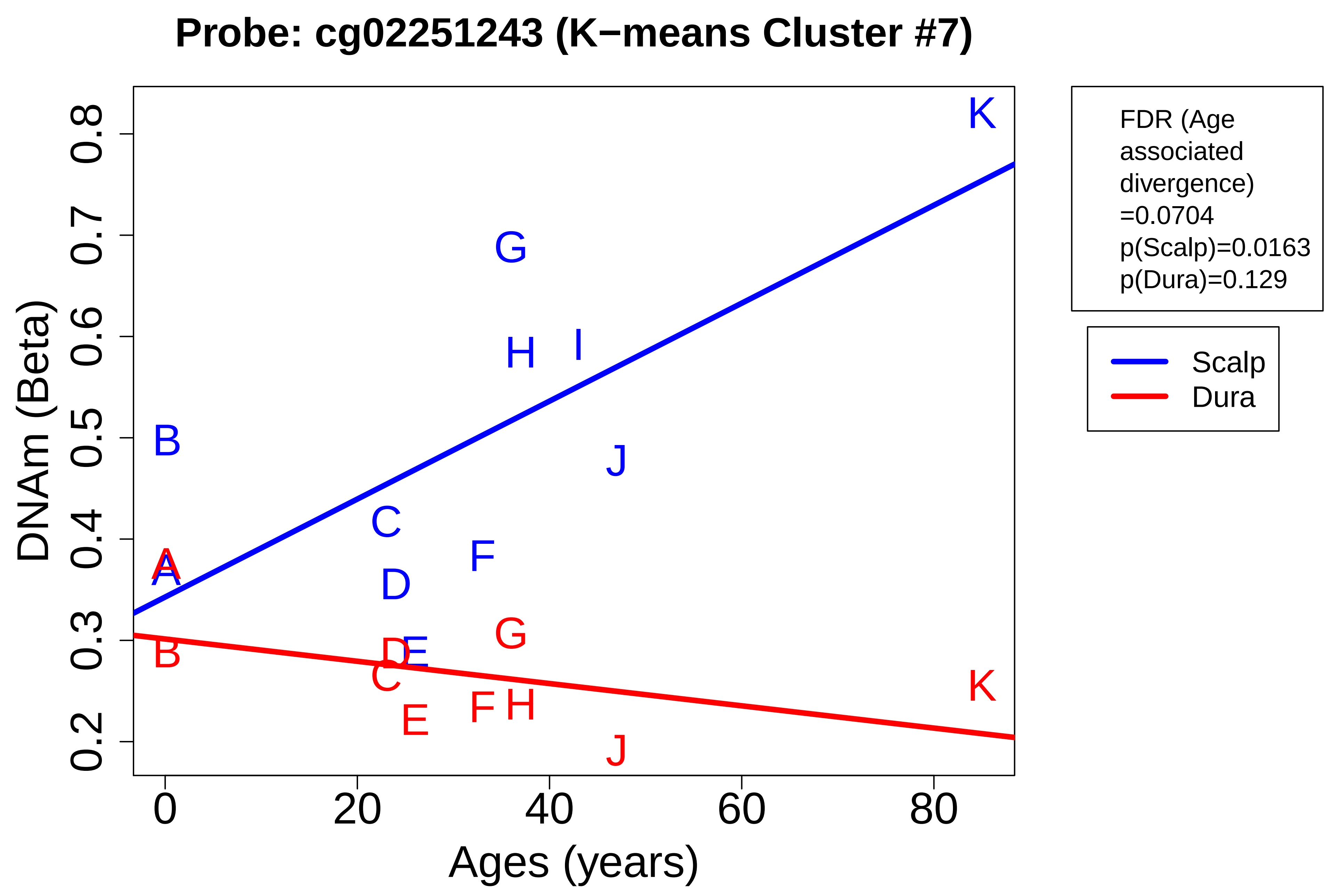

Probe: cg02251243 (K−means Cluster #7)
DNAm (Beta)
Ages (years)
0.2
0.3
0.4
0.5
0.6
0.7
0.8
0
20
40
60
80
FDR (Age associated divergence) =0.0704
p(Scalp)=0.0163
p(Dura)=0.129
Scalp
Dura

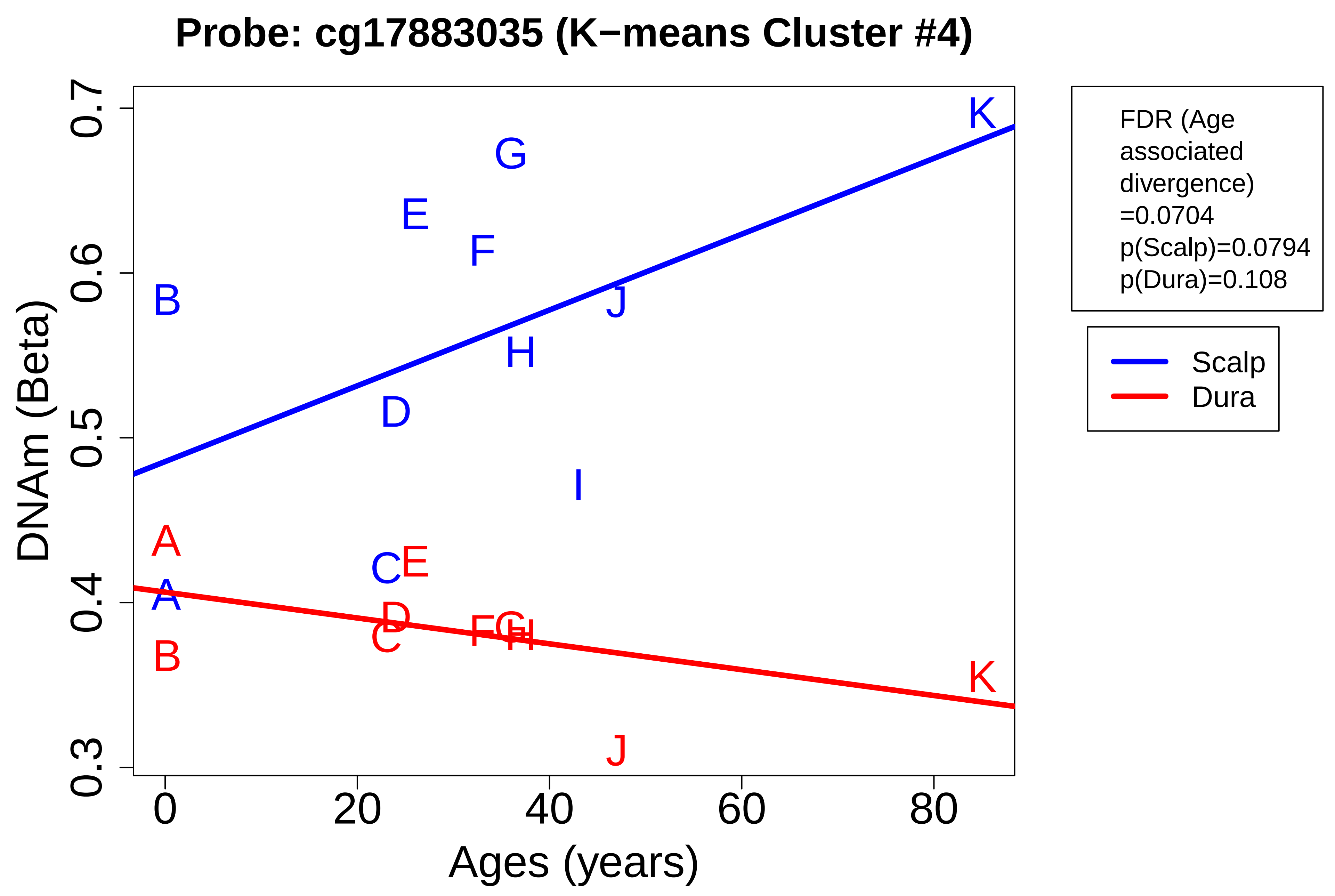

Probe: cg17883035 (K−means Cluster #4)
DNAm (Beta)
0.3
0.4
0.5
0.6
0.7
Ages (years)
0
20
40
60
80
FDR (Age associated divergence) =0.0704
p(Scalp)=0.0794
p(Dura)=0.108
Scalp
Dura
A
B
C
D
E
F
G
H
I
J
K

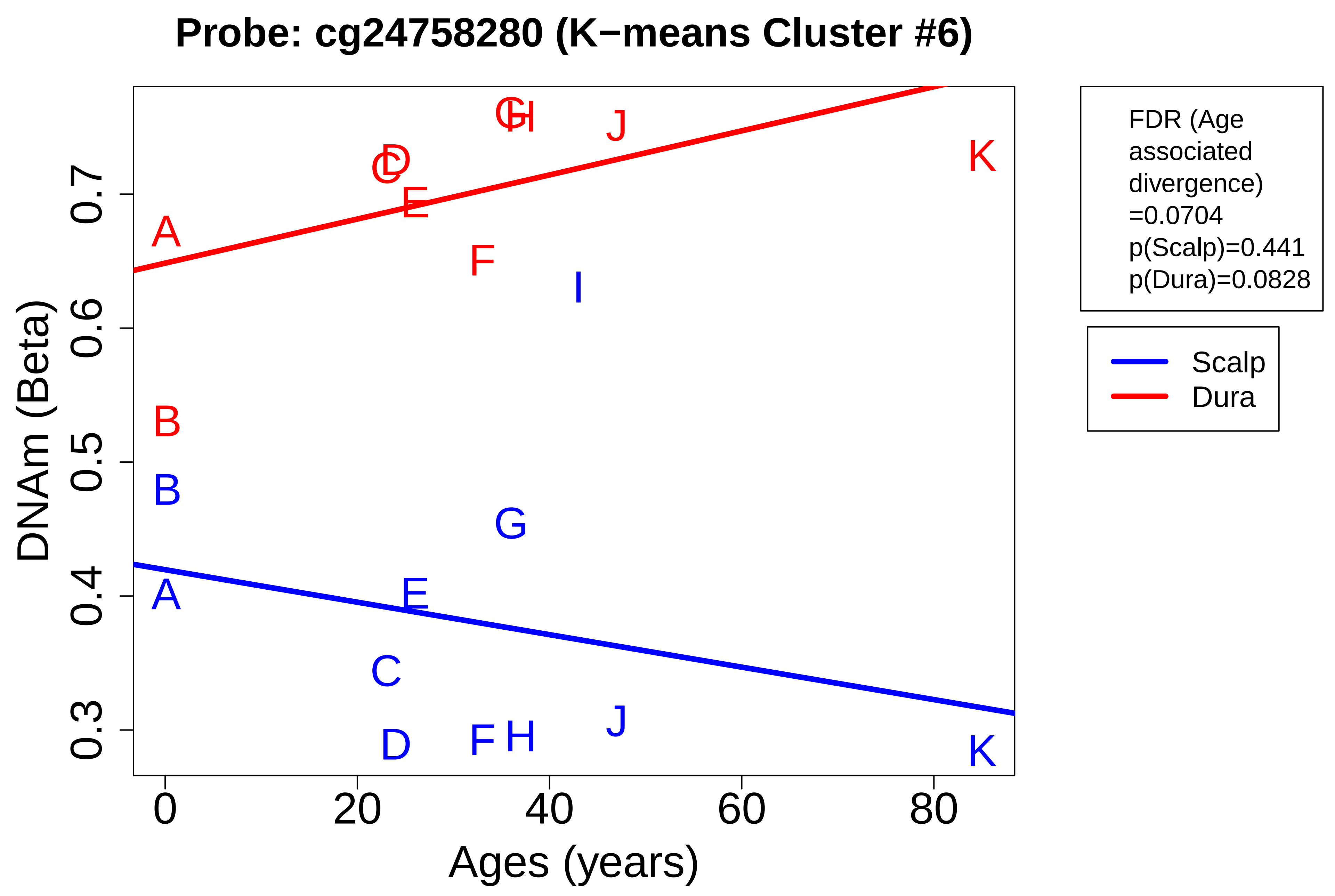

Probe: cg24758280 (K−means Cluster #6)
DNAm (Beta)
0.3
0.4
0.5
0.6
0.7
0
20
40
60
80
Ages (years)
FDR (Age associated divergence) =0.0704
p(Scalp)=0.441
p(Dura)=0.0828
Scalp
Dura

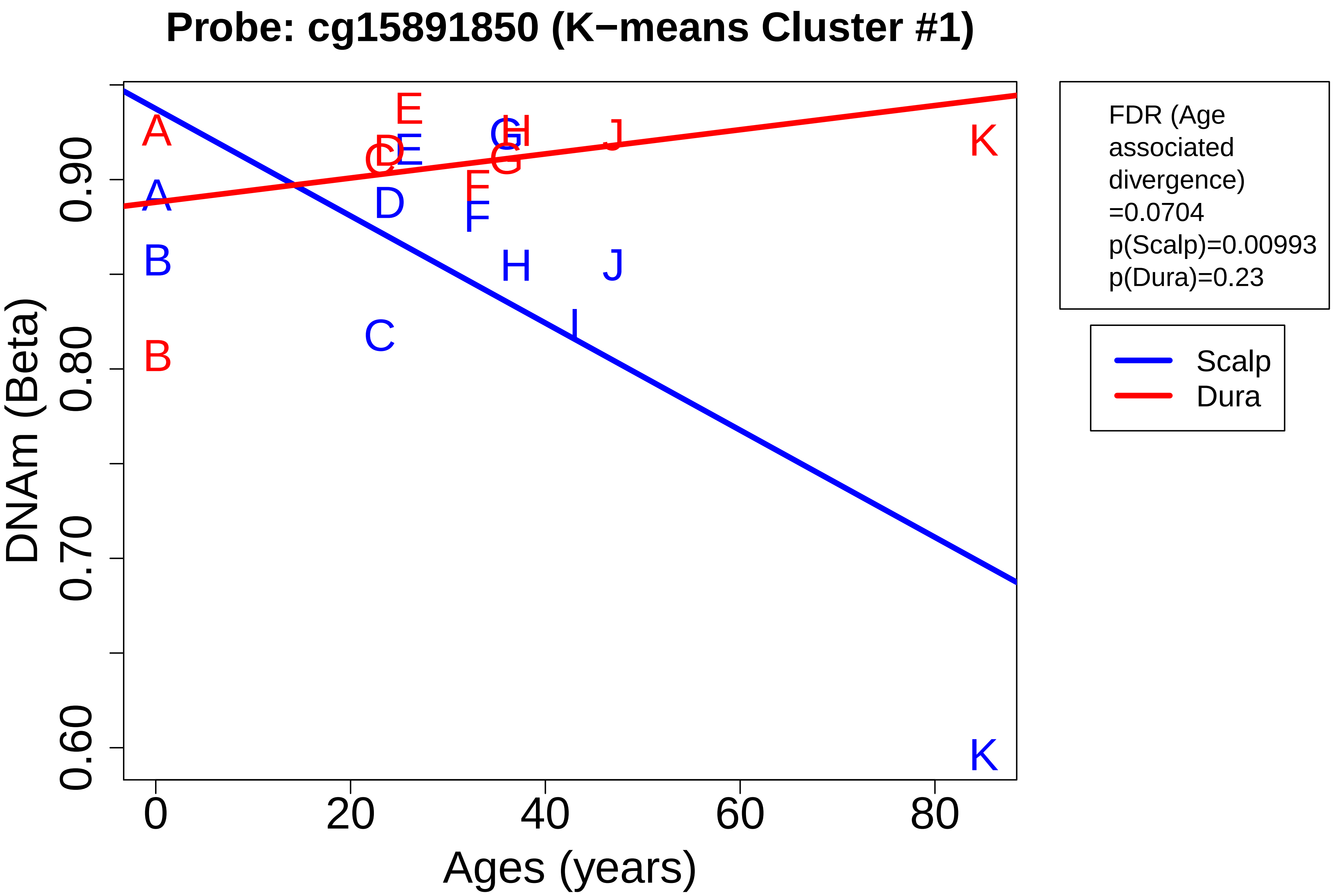

Probe: cg15891850 (K−means Cluster #1)
DNAm (Beta)
0.60
0.70
0.80
0.90
0
20
40
60
80
Ages (years)
FDR (Age associated divergence) =0.0704
p(Scalp)=0.00993
p(Dura)=0.23
Scalp
Dura
A
B
C
D
E
F
G
H
I
J
K

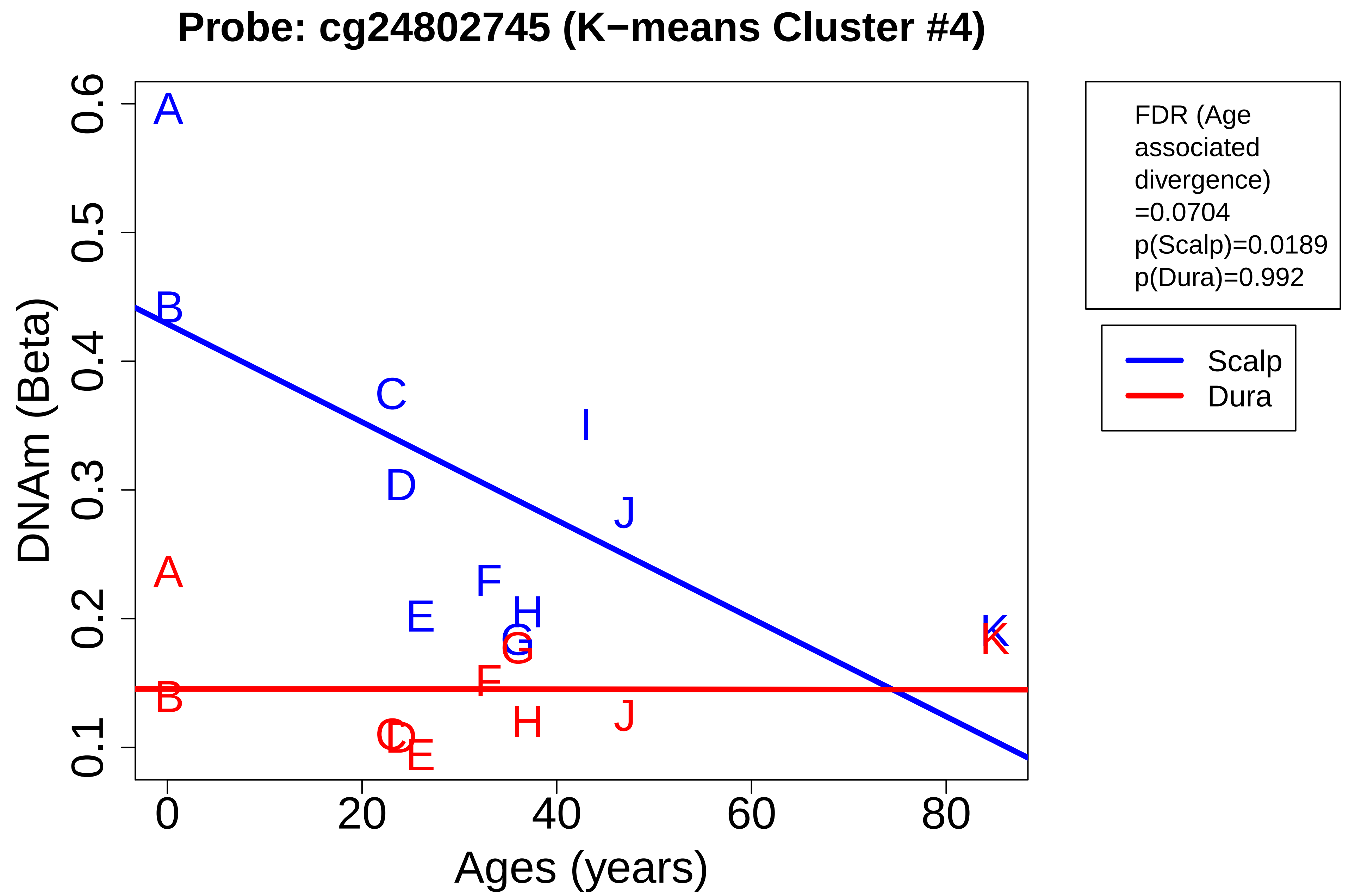

Probe: cg24802745 (K−means Cluster #4)
DNAm (Beta)
0.1
0.2
0.3
0.4
0.5
0.6
0
20
40
60
80
Ages (years)
FDR (Age associated divergence) =0.0704
p(Scalp)=0.0189
p(Dura)=0.992
Scalp
Dura
A
B
C
D
E
F
G
H
I
J
K

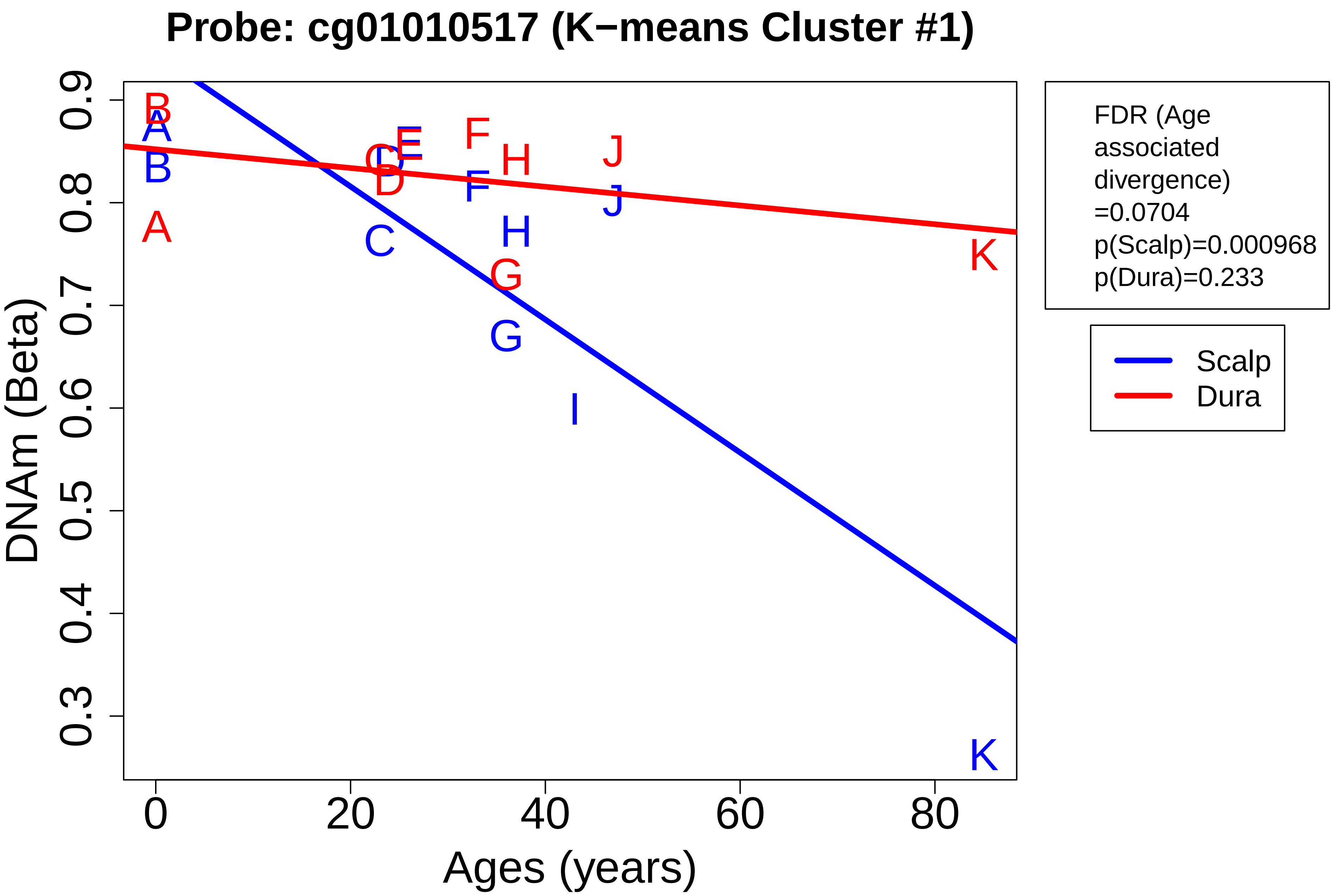

Probe: cg01010517 (K−means Cluster #1)
DNAm (Beta)
Ages (years)
0.3
0.4
0.5
0.6
0.7
0.8
0.9
0
20
40
60
80
FDR (Age associated divergence) =0.0704
p(Scalp)=0.000968
p(Dura)=0.233
Scalp
Dura

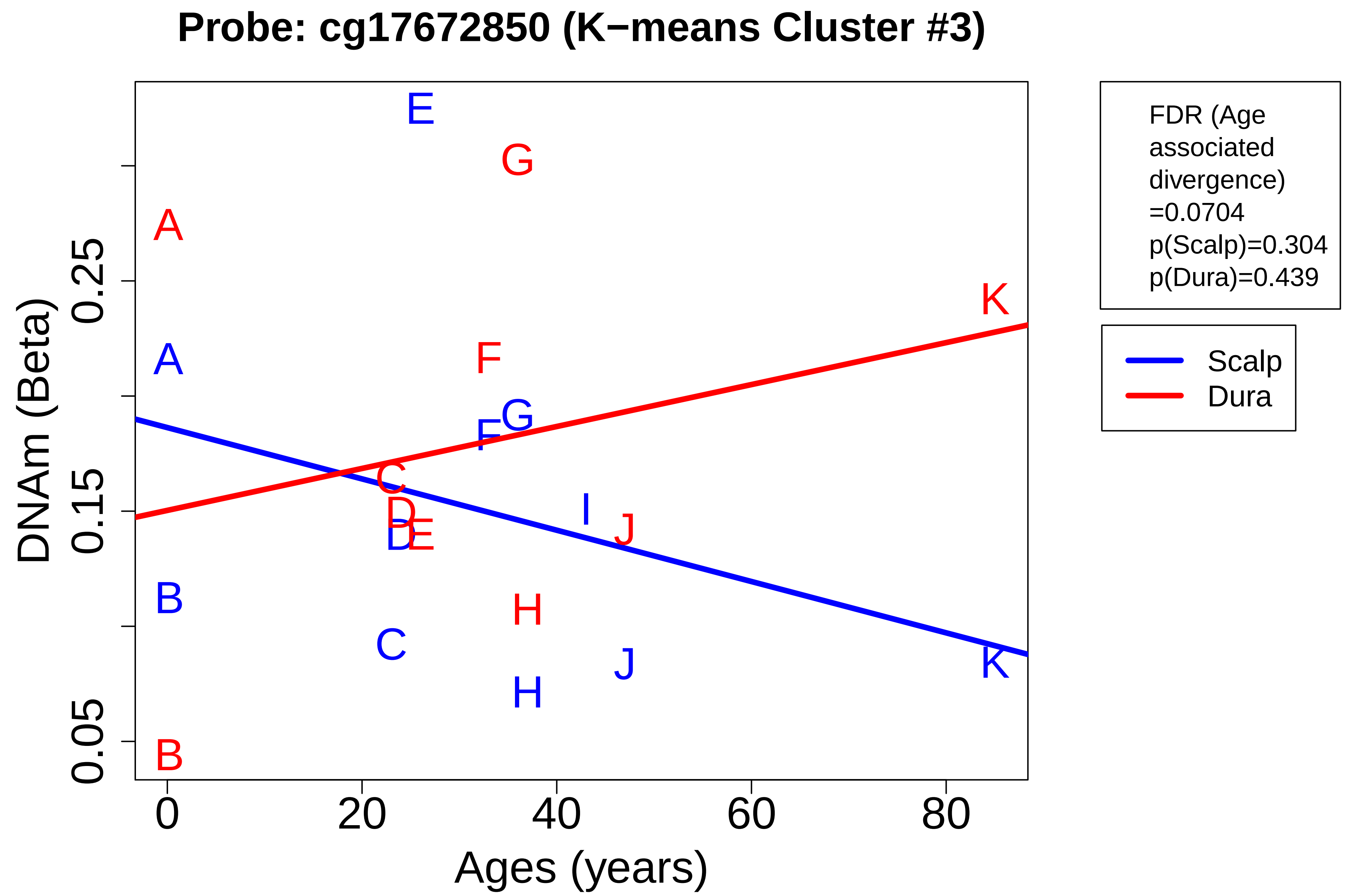

Probe: cg17672850 (K−means Cluster #3)
DNAm (Beta)
0.05
0.15
0.25
Ages (years)
0
20
40
60
80
FDR (Age associated divergence) =0.0704
p(Scalp)=0.304
p(Dura)=0.439
Scalp
Dura

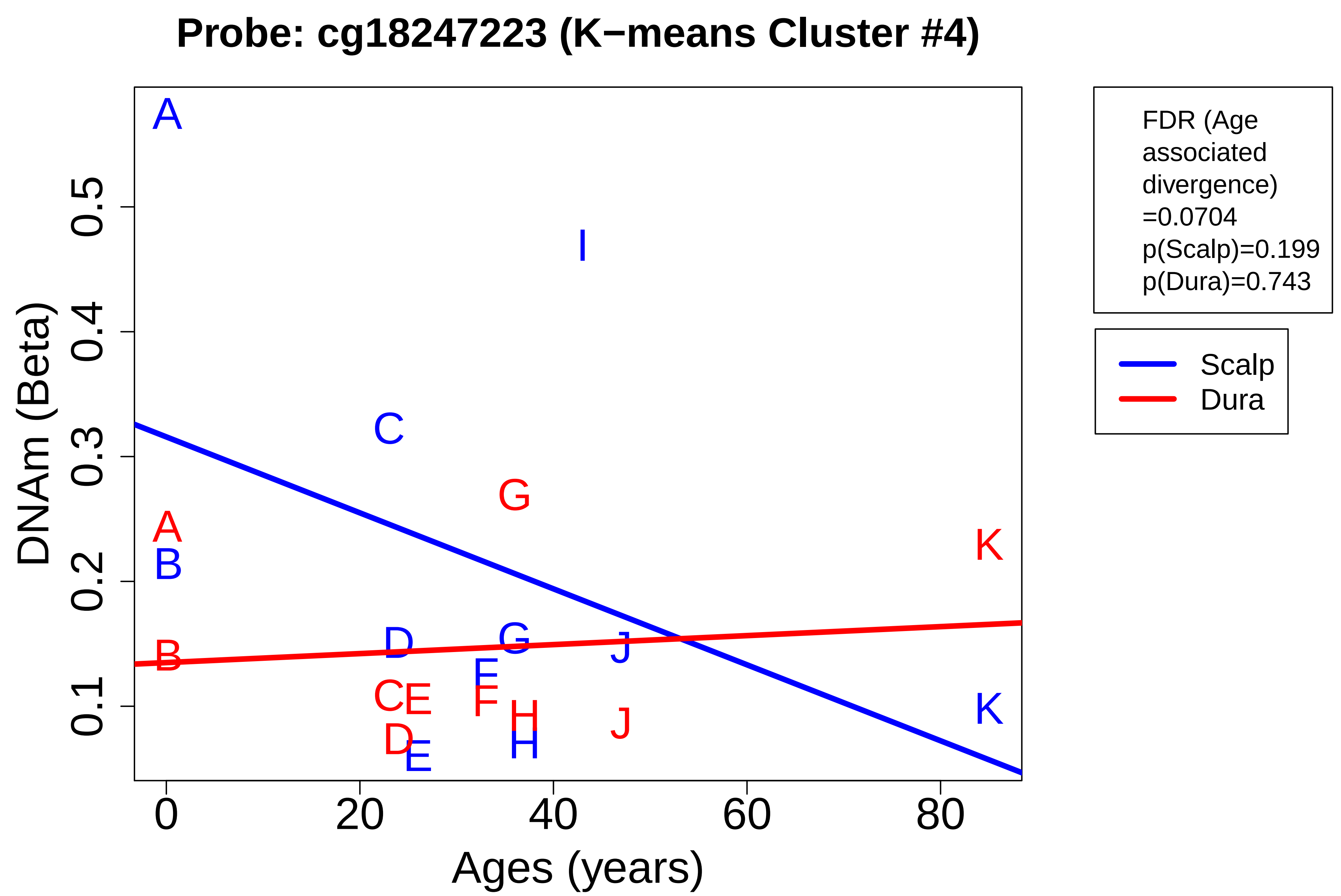

Probe: cg18247223 (K−means Cluster #4)
DNAm (Beta)
0.1
0.2
0.3
0.4
0.5
Ages (years)
0
20
40
60
80
FDR (Age associated divergence) =0.0704
p(Scalp)=0.199
p(Dura)=0.743
Scalp
Dura

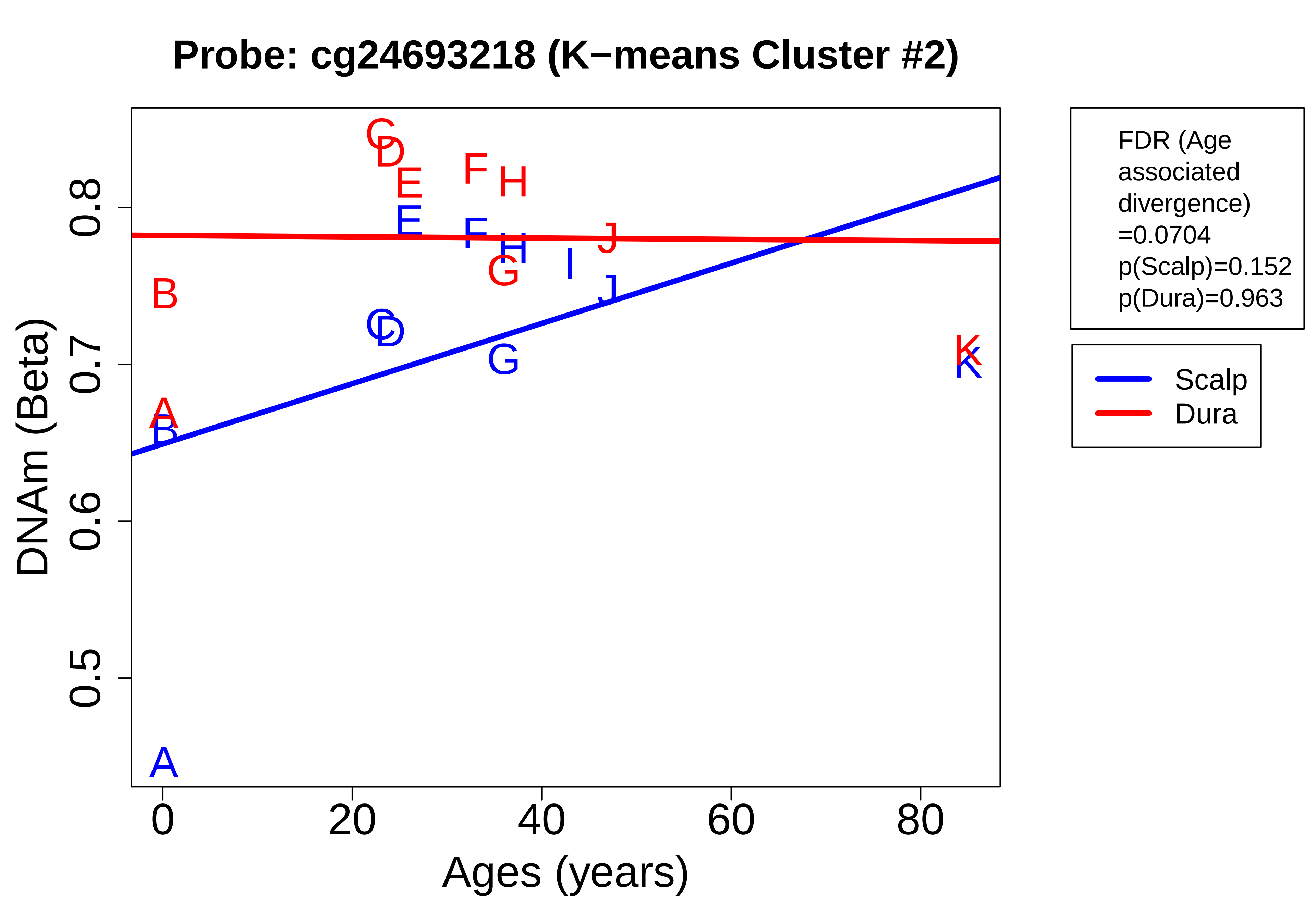

Probe: cg24693218 (K−means Cluster #2)
DNAm (Beta)
0.5
0.6
0.7
0.8
0
20
40
60
80
Ages (years)
FDR (Age associated divergence) =0.0704
p(Scalp)=0.152
p(Dura)=0.963
Scalp
Dura

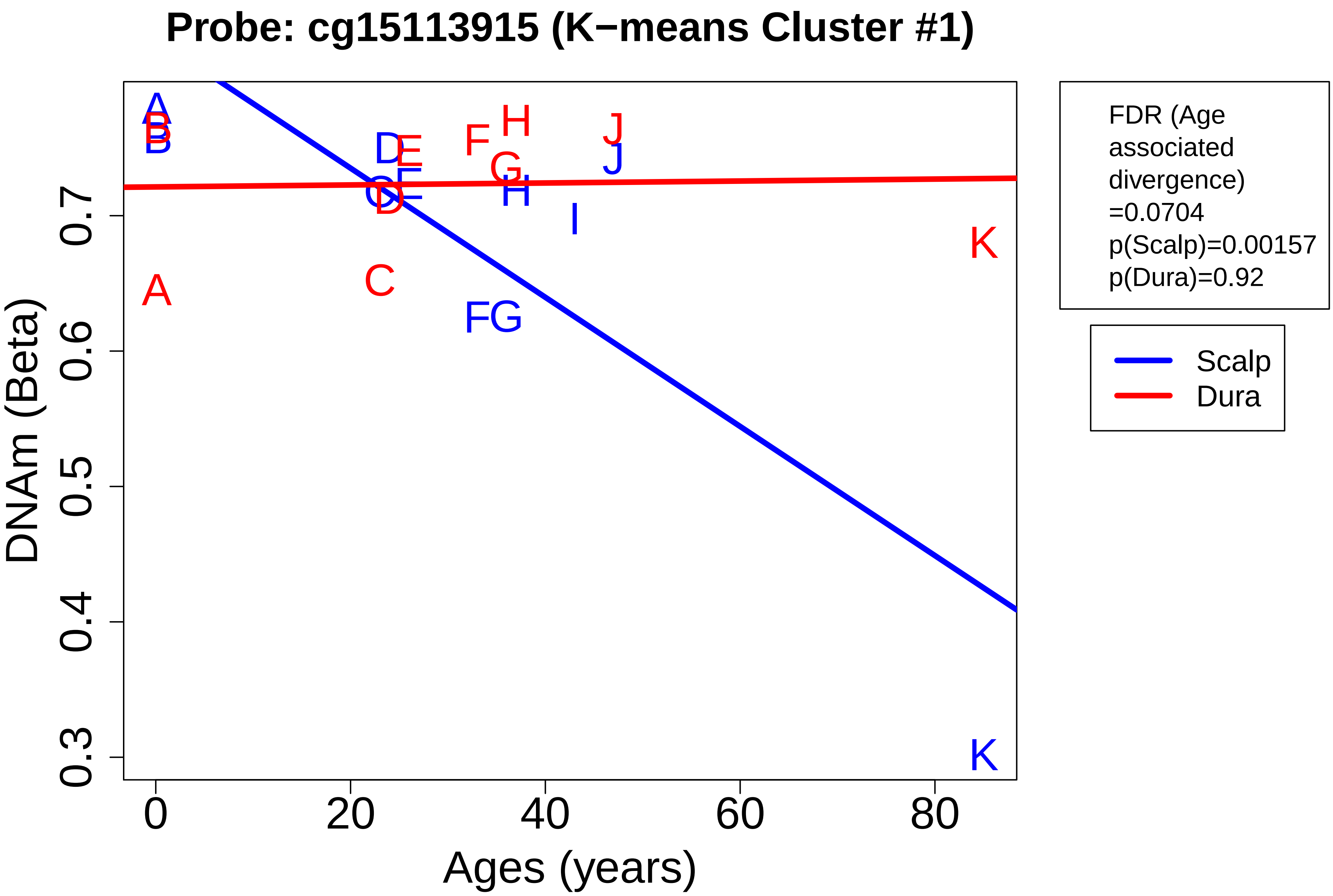

Probe: cg15113915 (K−means Cluster #1)
FDR (Age associated divergence) =0.0704
p(Scalp)=0.00157
p(Dura)=0.92
Scalp
Dura
DNAm (Beta)
0.3
0.4
0.5
0.6
0.7
0
20
40
60
80
Ages (years)

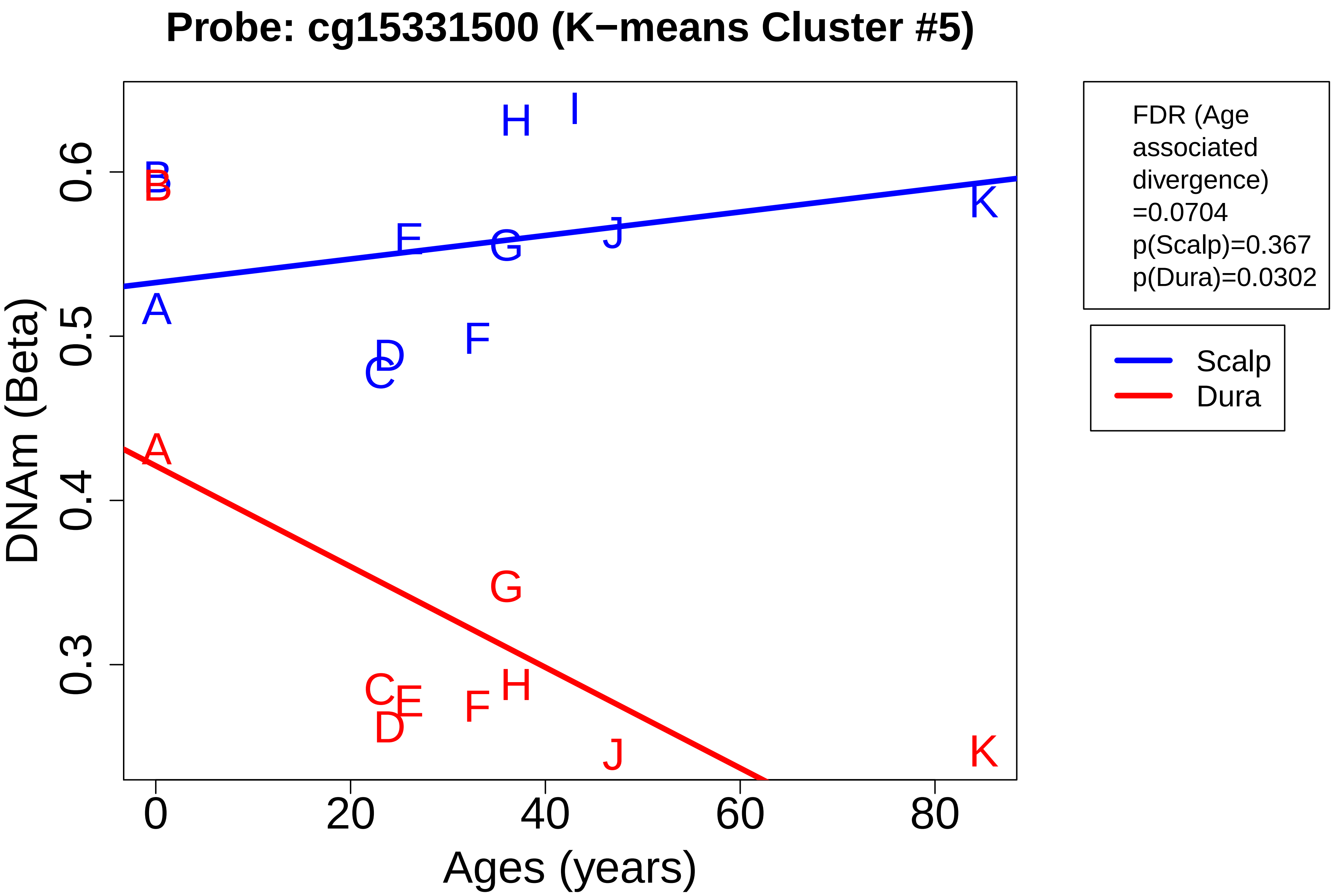

Probe: cg15331500 (K−means Cluster #5)
FDR (Age associated divergence) =0.0704
p(Scalp)=0.367
p(Dura)=0.0302
Scalp
Dura
DNAm (Beta)
0.3
0.4
0.5
0.6
0
20
40
60
80
Ages (years)
A
B
C
D
E
F
G
H
I
J
K

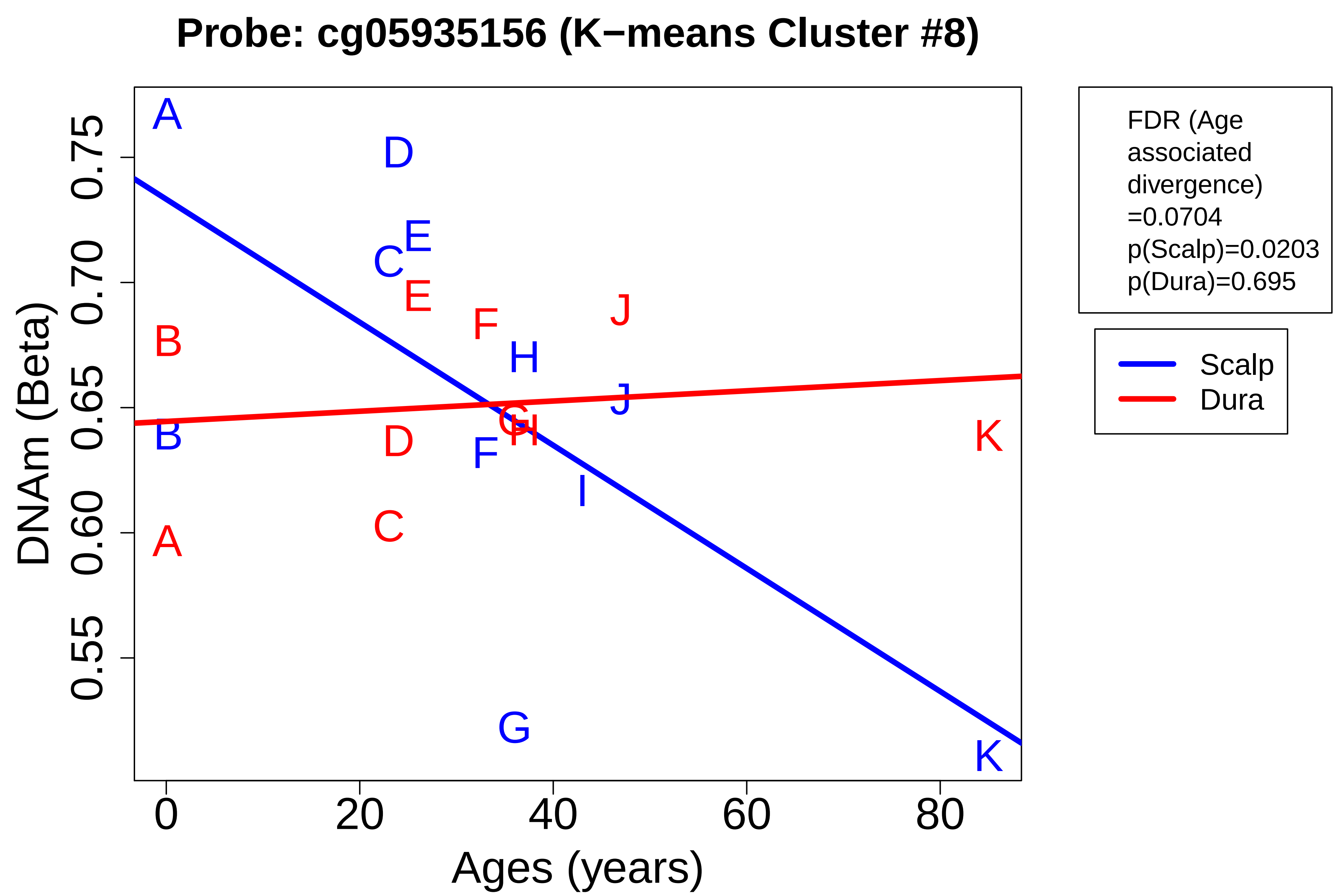

Probe: cg05935156 (K−means Cluster #8)
DNAm (Beta)
0.55
0.60
0.65
0.70
0.75
0
20
40
60
80
Ages (years)
FDR (Age associated divergence) =0.0704
p(Scalp)=0.0203
p(Dura)=0.695
Scalp
Dura

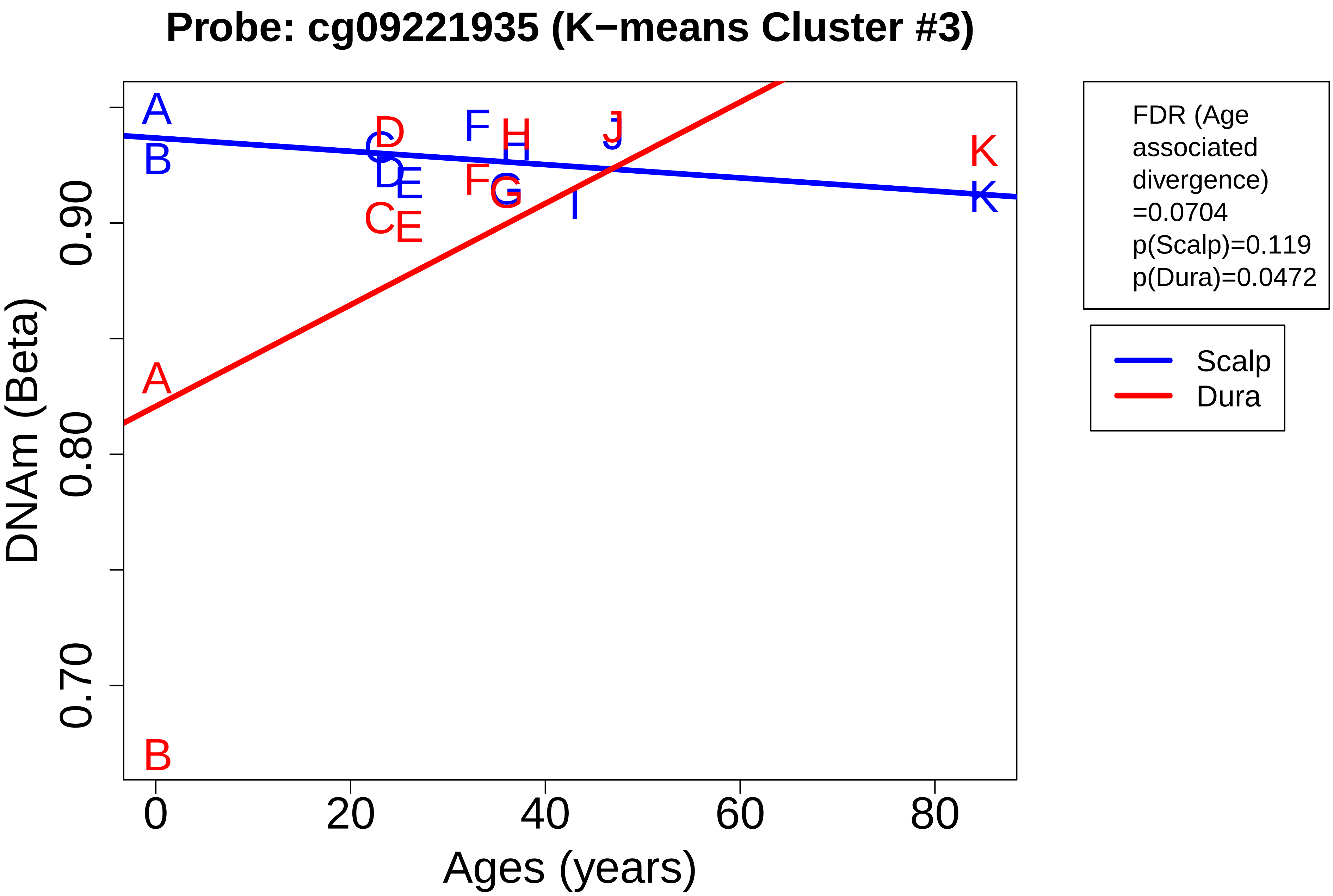

Probe: cg09221935 (K−means Cluster #3)
DNAm (Beta)
0.70
0.80
0.90
0
20
40
60
80
Ages (years)
FDR (Age associated divergence) =0.0704
p(Scalp)=0.119
p(Dura)=0.0472
Scalp
Dura

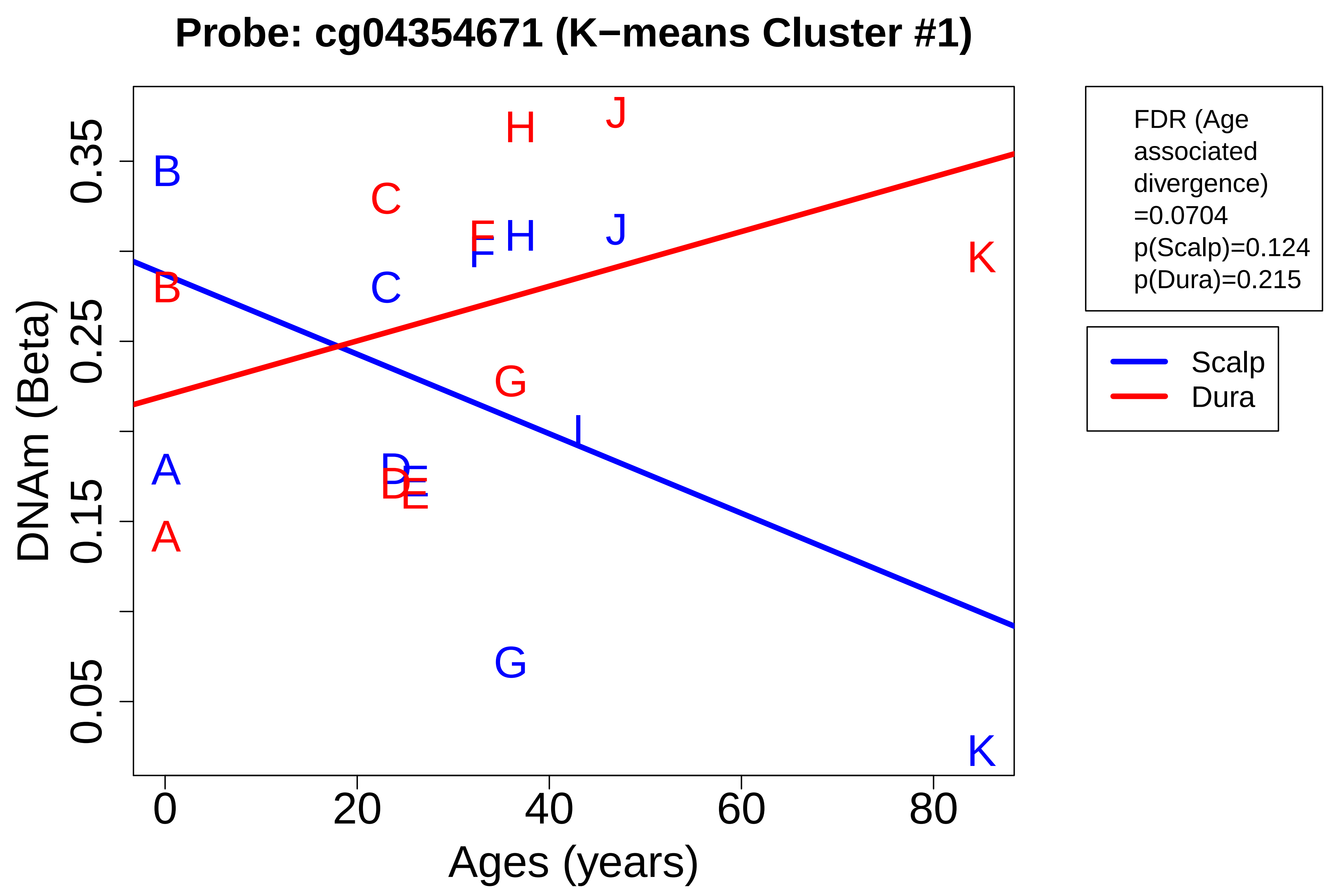

Probe: cg04354671 (K−means Cluster #1)
DNAm (Beta)
0.05
0.15
0.25
0.35
0
20
40
60
80
Ages (years)
A
B
C
D
E
F
G
H
I
J
K
A
B
C
D
E
F
G
H
J
K
FDR (Age associated divergence) =0.0704
p(Scalp)=0.124
p(Dura)=0.215
Scalp
Dura

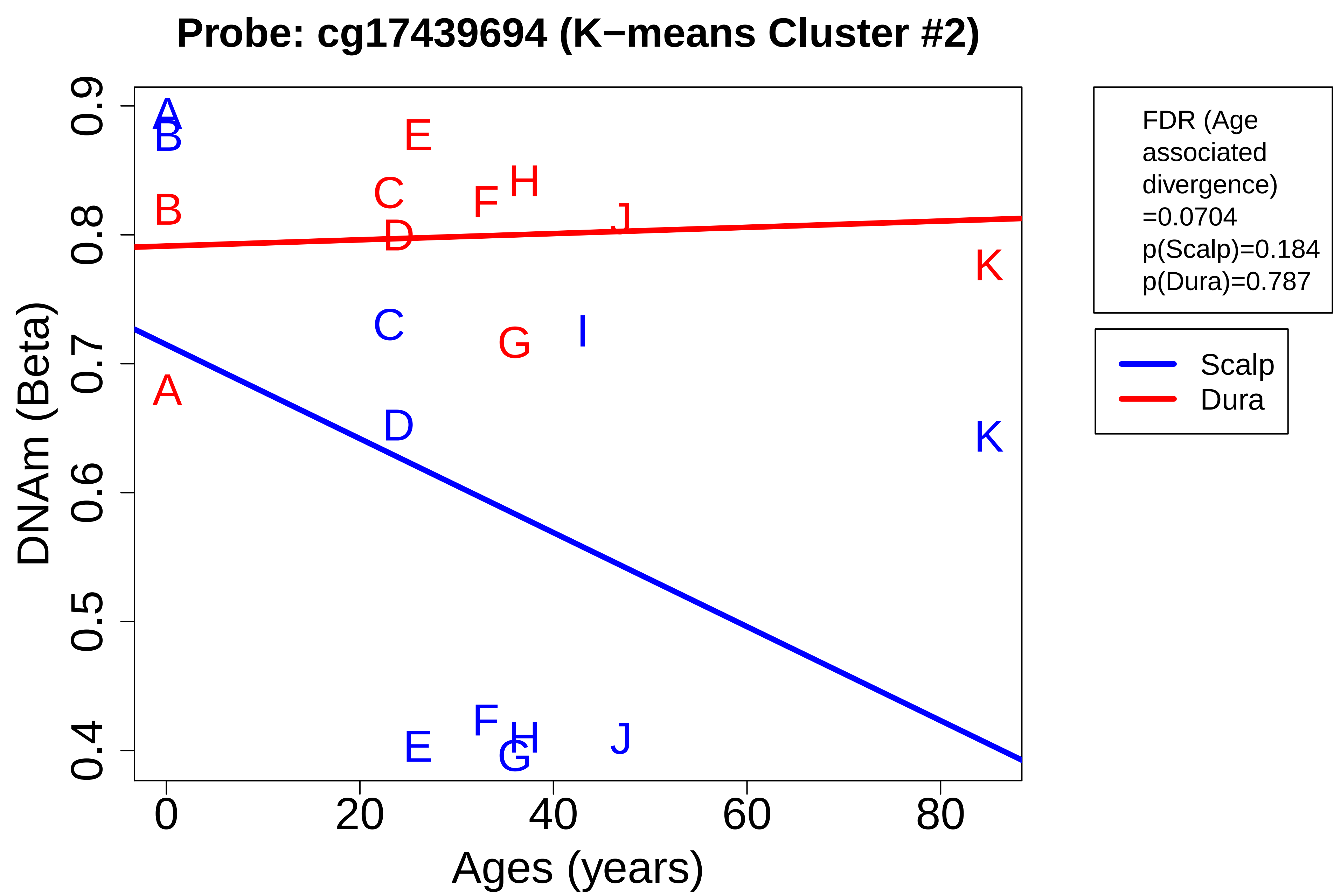

Probe: cg17439694 (K−means Cluster #2)
DNAm (Beta)
0.4
0.5
0.6
0.7
0.8
0.9
0
20
40
60
80
Ages (years)
FDR (Age associated divergence) =0.0704
p(Scalp)=0.184
p(Dura)=0.787
Scalp
Dura

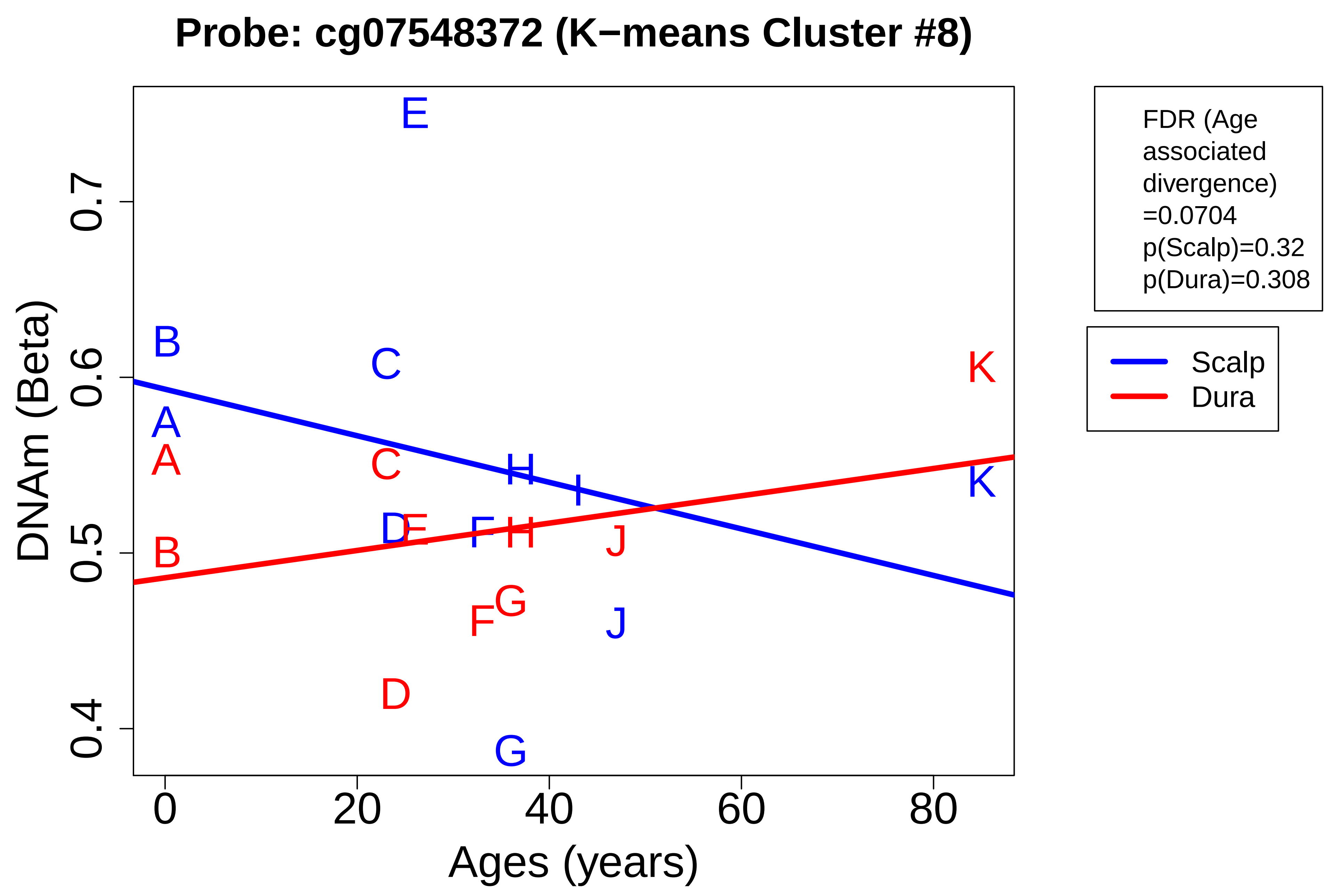
Probe: cg07548372 (K−means Cluster #8)
DNAm (Beta)
0.4
0.5
0.6
0.7
0
20
40
60
80
Ages (years)
FDR (Age associated divergence) =0.0704
p(Scalp)=0.32
p(Dura)=0.308
Scalp
Dura
A
B
C
D
E
F
G
H
I
J
K

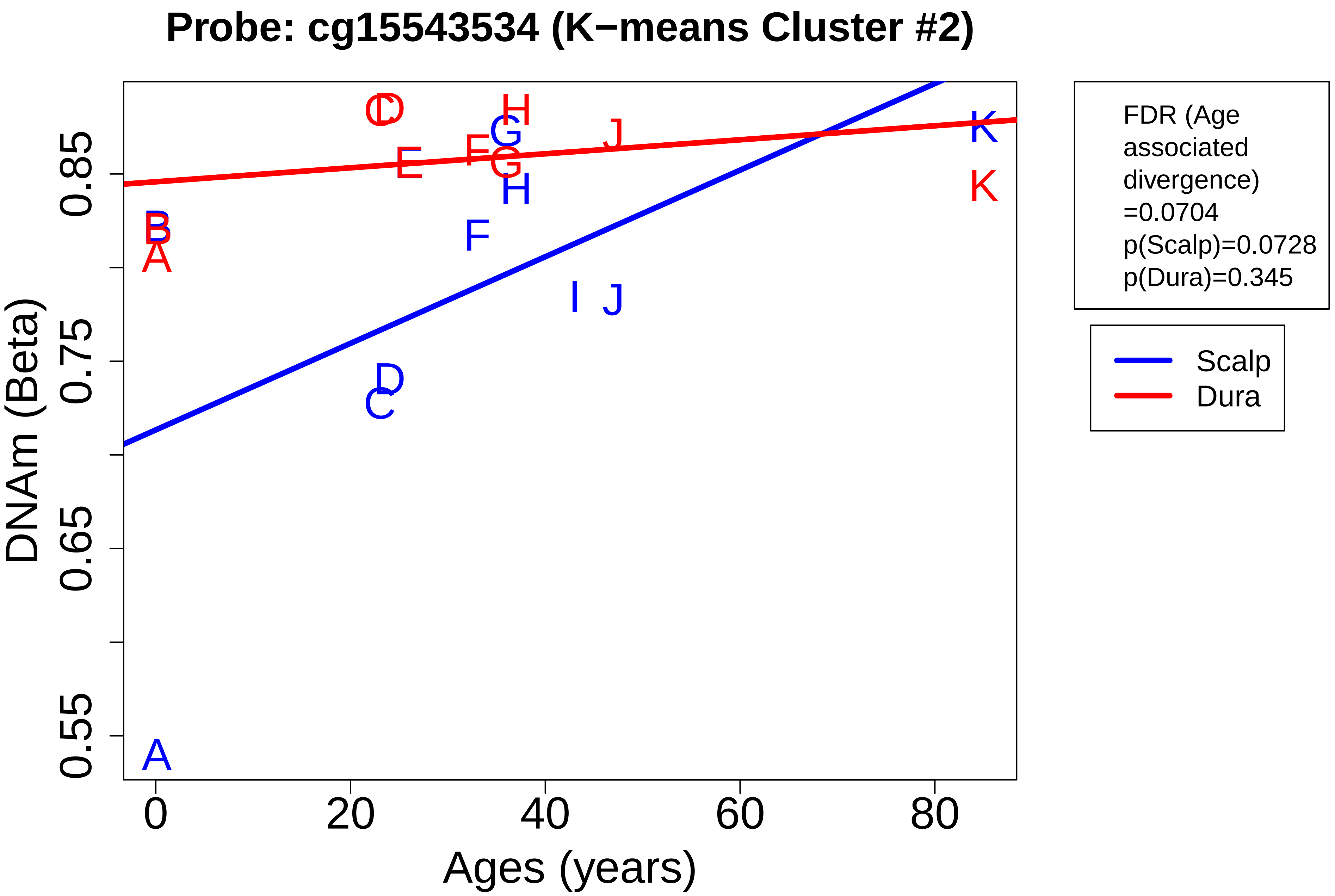
Probe: cg15543534 (K−means Cluster #2)
DNAm (Beta)
0.55
0.65
0.75
0.85
0
20
40
60
80
Ages (years)
FDR (Age associated divergence) =0.0704
p(Scalp)=0.0728
p(Dura)=0.345
Scalp
Dura
A
B
C
D
E
F
G
H
I
J
K

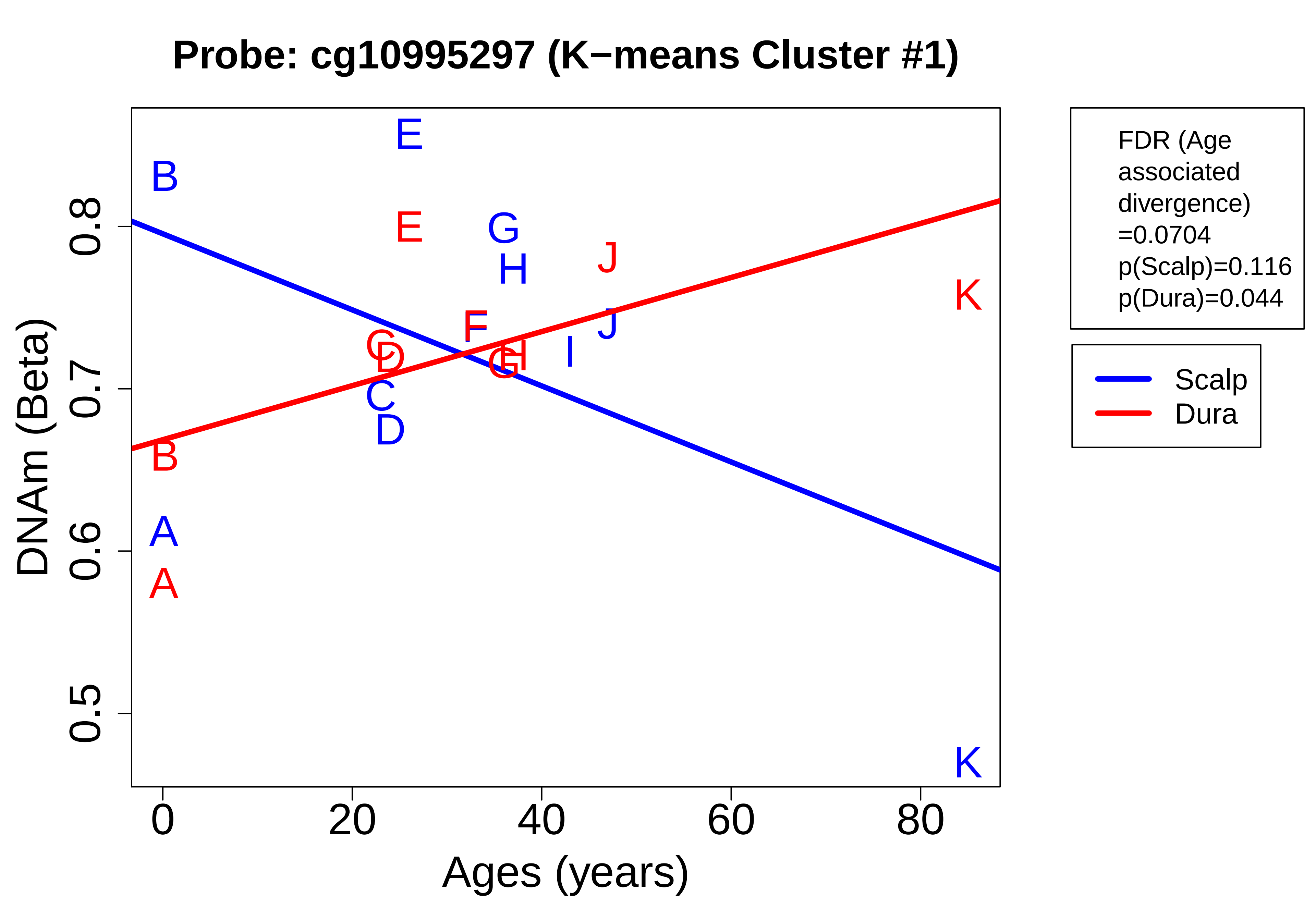

Probe: cg10995297 (K−means Cluster #1)
DNAm (Beta)
0.5
0.6
0.7
0.8
0
20
40
60
80
Ages (years)
FDR (Age associated divergence) =0.0704
p(Scalp)=0.116
p(Dura)=0.044
Scalp
Dura

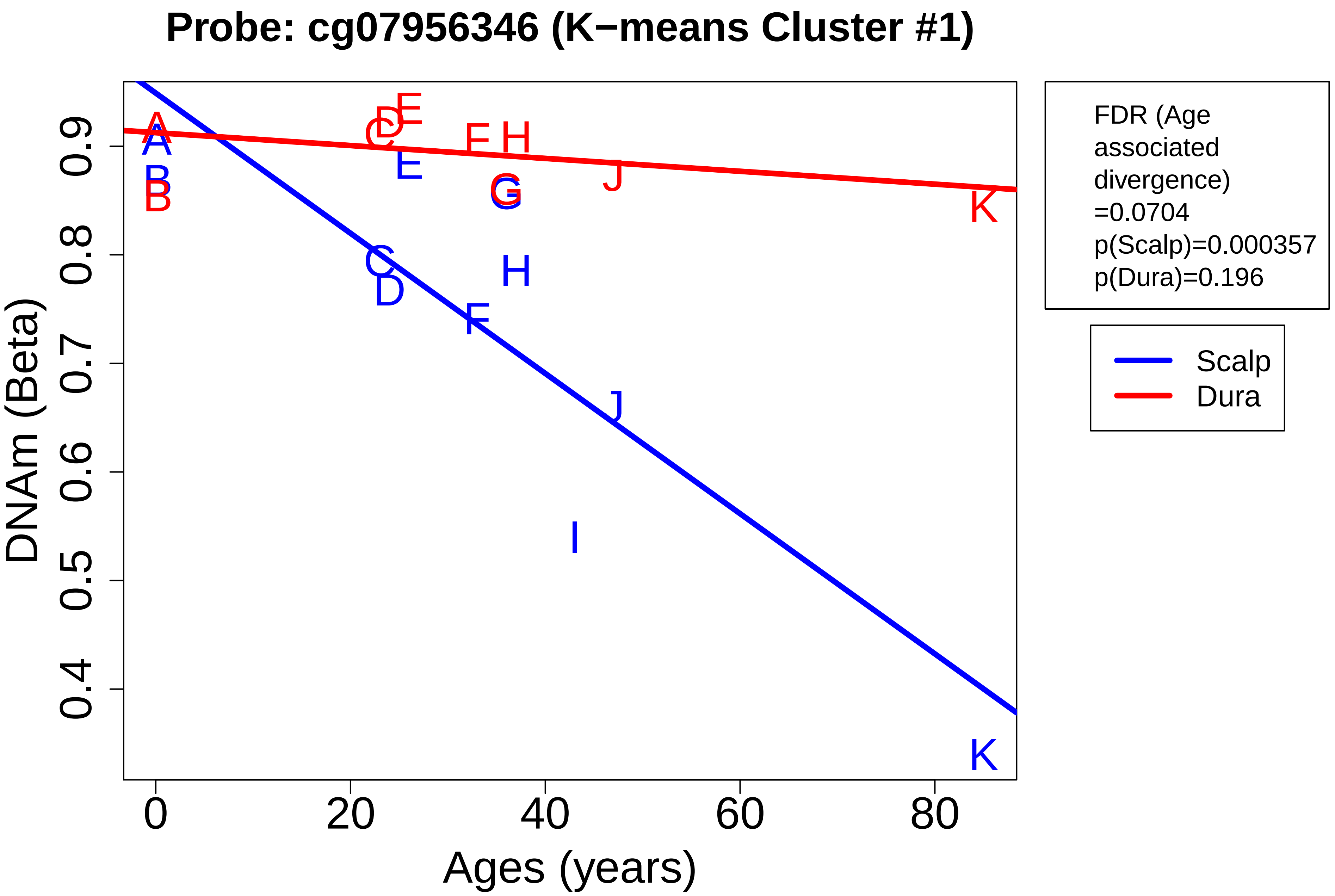
Probe: cg07956346 (K−means Cluster #1)
DNAm (Beta)
Ages (years)
0.4
0.5
0.6
0.7
0.8
0.9
0
20
40
60
80
FDR (Age associated divergence) =0.0704
p(Scalp)=0.000357
p(Dura)=0.196
Scalp
Dura
A
B
C
D
E
F
G
H
I
J
K

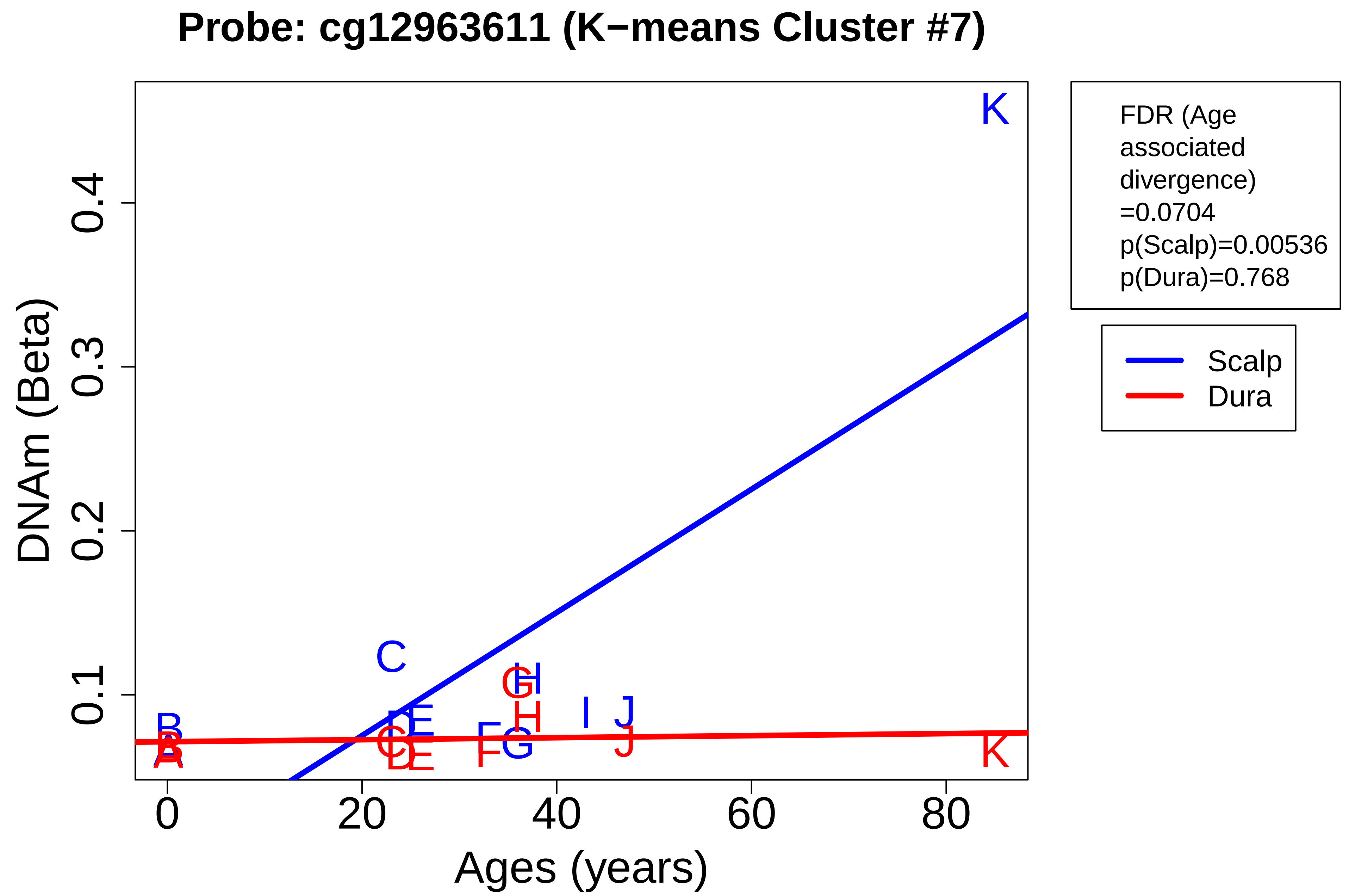

Probe: cg12963611 (K−means Cluster #7)
DNAm (Beta)
0.1
0.2
0.3
0.4
0
20
40
60
80
Ages (years)
FDR (Age associated divergence) =0.0704
p(Scalp)=0.00536
p(Dura)=0.768
Scalp
Dura

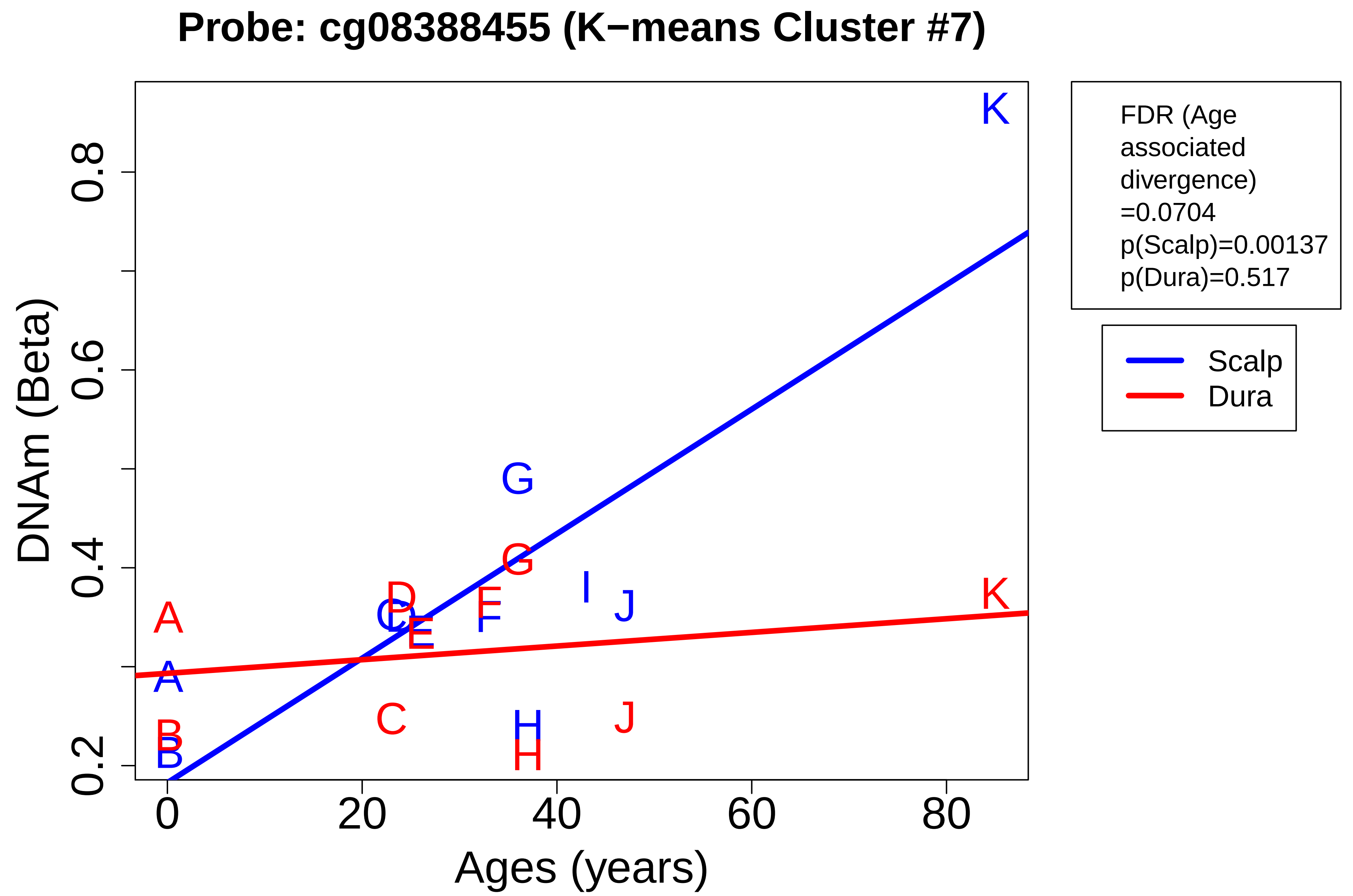

Probe: cg08388455 (K−means Cluster #7)
DNAm (Beta)
0.2
0.4
0.6
0.8
0
20
40
60
80
Ages (years)
A
B
C
D
E
F
G
H
I
J
K
A
B
C
D
E
F
G
H
J
K
FDR (Age associated divergence) =0.0704
p(Scalp)=0.00137
p(Dura)=0.517
Scalp
Dura

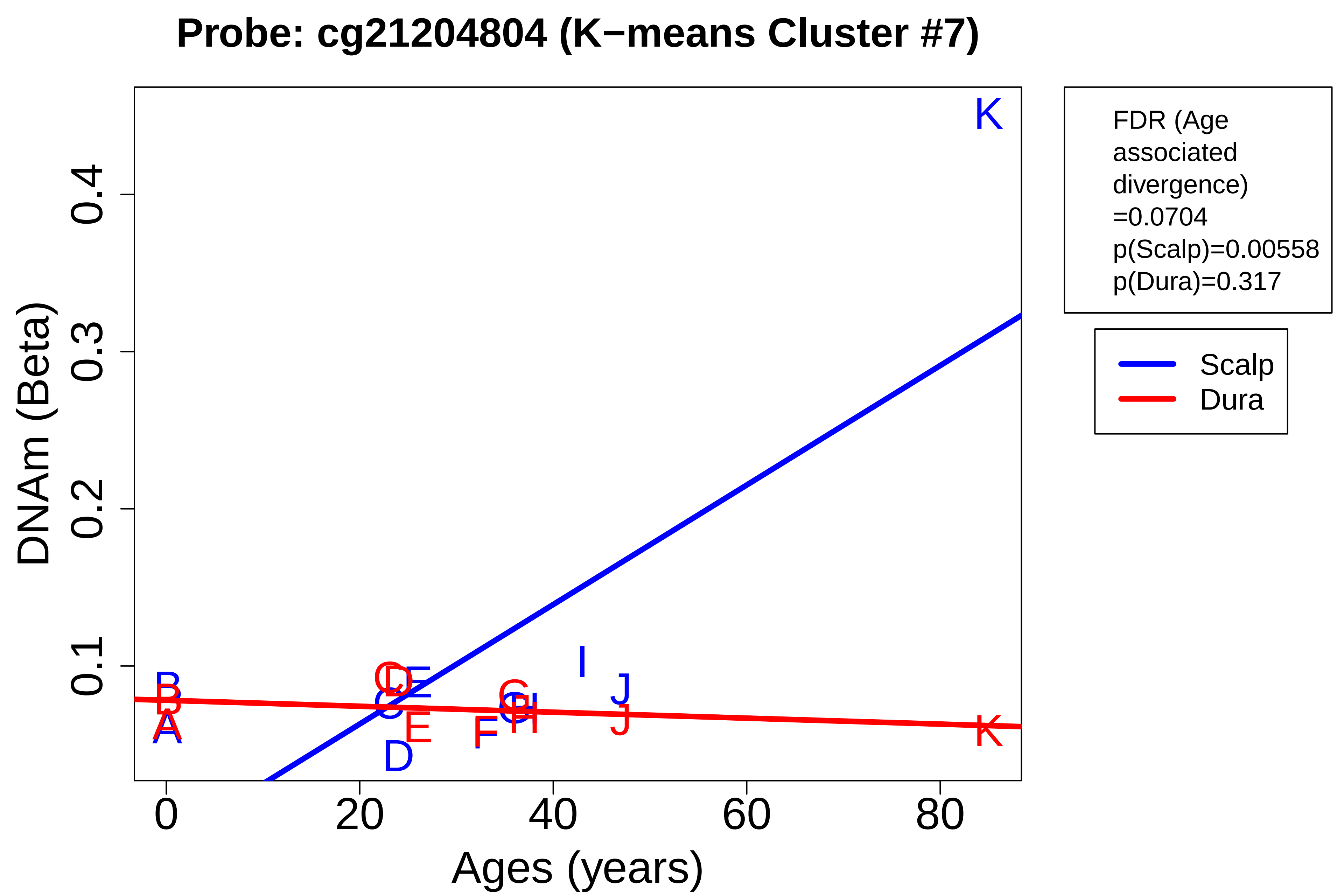

Probe: cg21204804 (K−means Cluster #7)
DNAm (Beta)
0.1
0.2
0.3
0.4
Ages (years)
0
20
40
60
80
FDR (Age associated divergence) =0.0704
p(Scalp)=0.00558
p(Dura)=0.317
Scalp
Dura
A
B
C
D
E
F
G
H
I
J
K

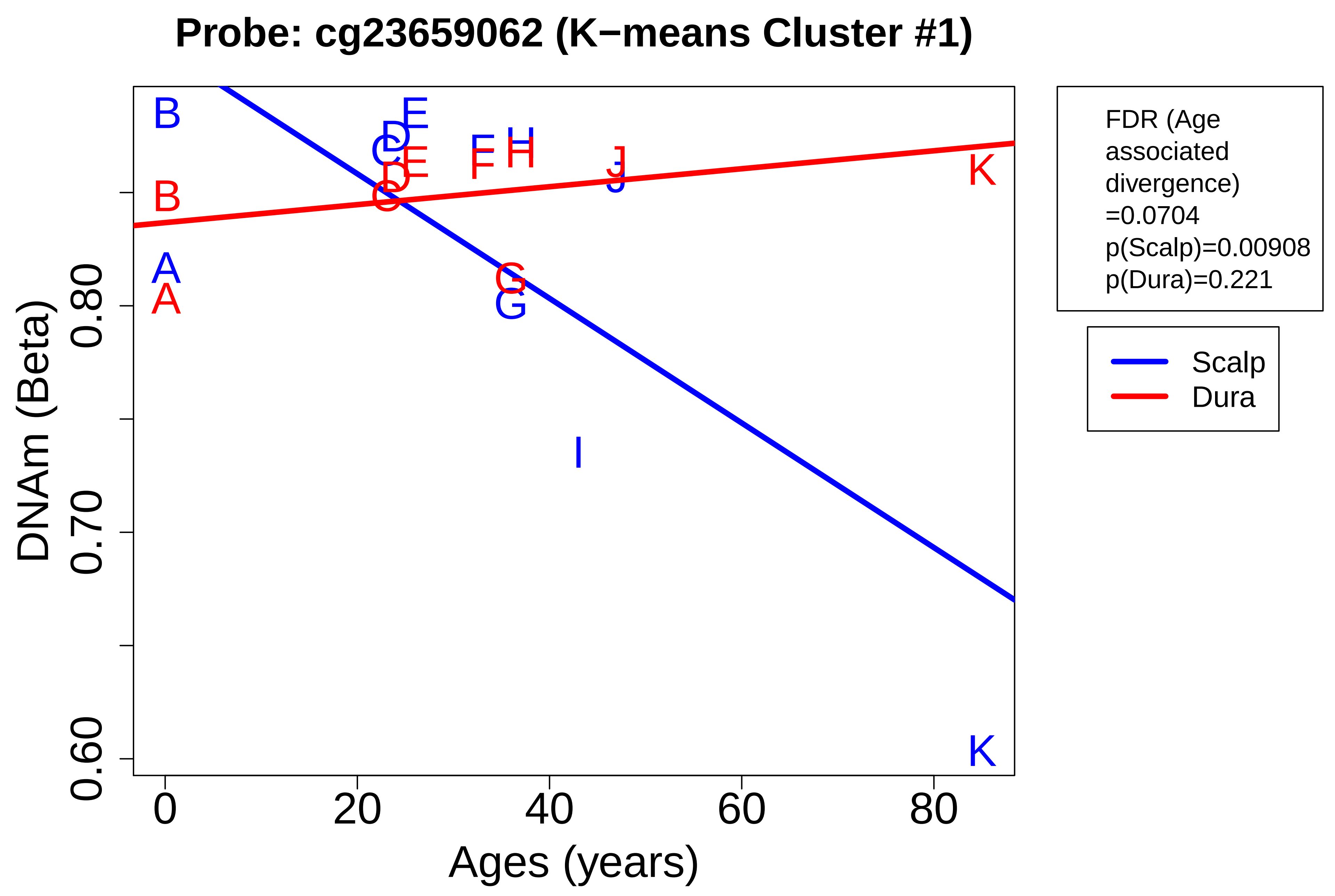
Probe: cg23659062 (K−means Cluster #1)
DNAm (Beta)
0.60
0.70
0.80
Ages (years)
0
20
40
60
80
FDR (Age associated divergence) =0.0704
p(Scalp)=0.00908
p(Dura)=0.221
Scalp
Dura
A
B
C
D
E
F
G
H
I
J
K

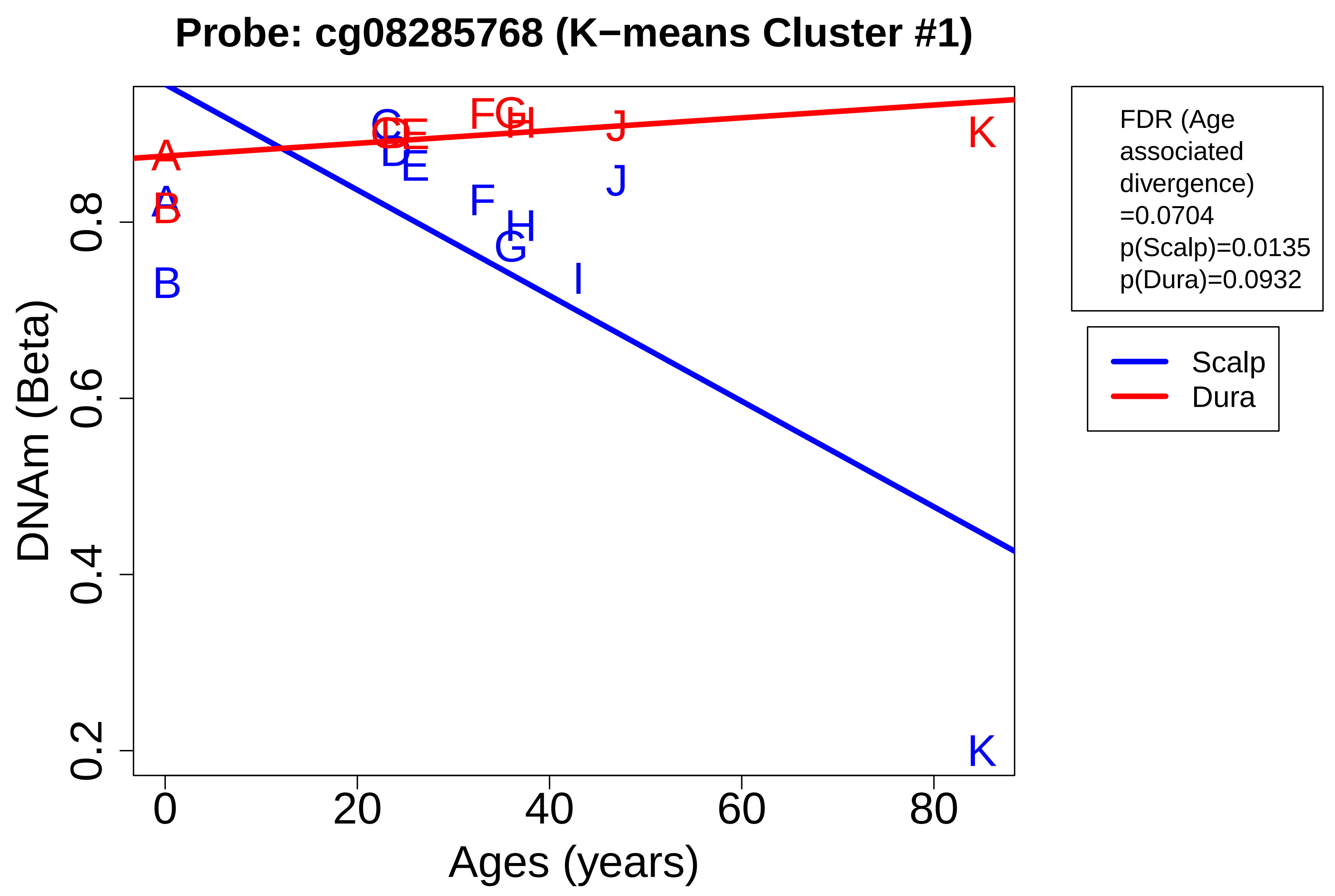
Probe: cg08285768 (K−means Cluster #1)
DNAm (Beta)
0.2
0.4
0.6
0.8
0
20
40
60
80
Ages (years)
A
B
C
D
E
F
G
H
I
J
K
FDR (Age associated divergence) =0.0704
p(Scalp)=0.0135
p(Dura)=0.0932
Scalp
Dura

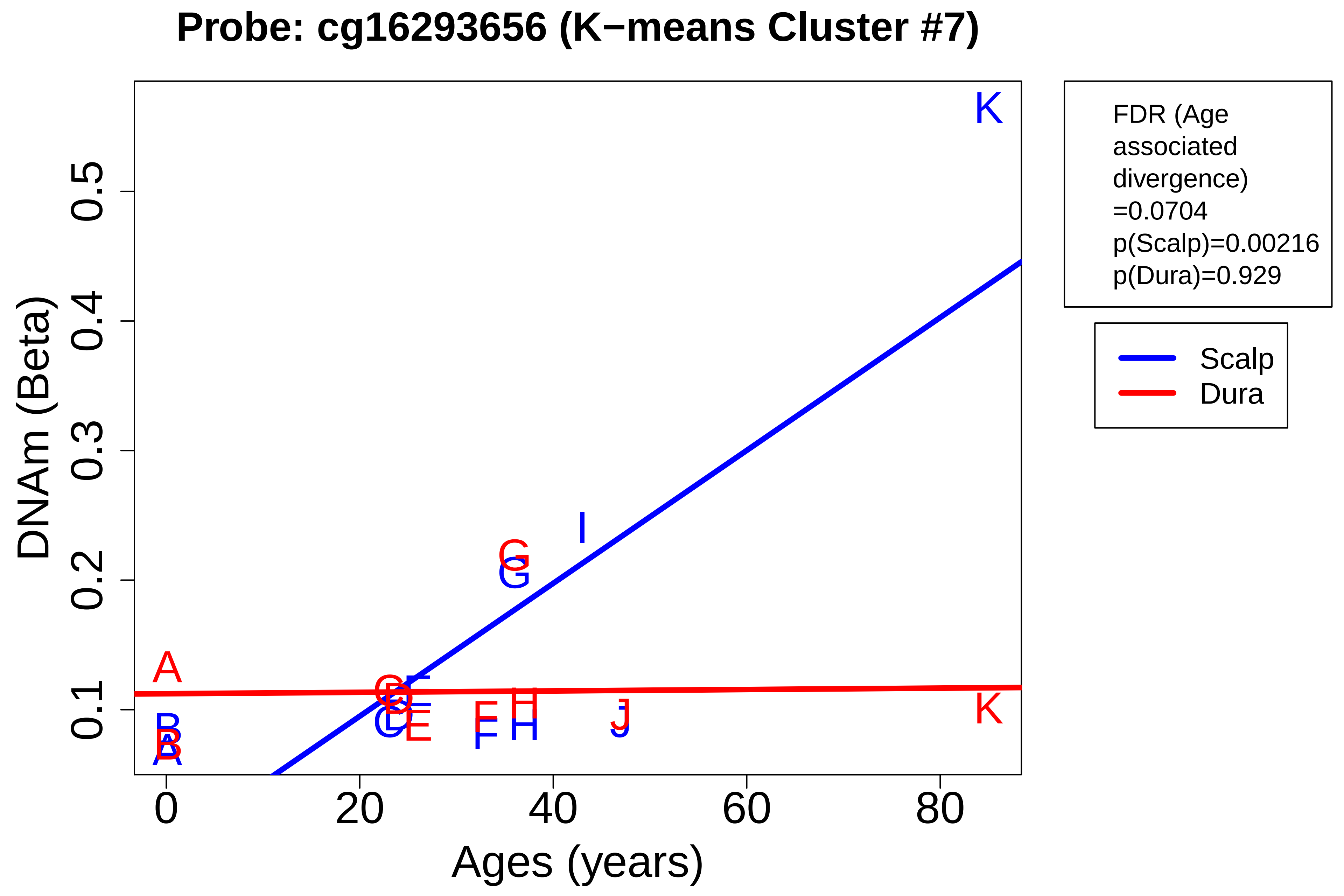

Probe: cg16293656 (K−means Cluster #7)
DNAm (Beta)
0.1
0.2
0.3
0.4
0.5
0
20
40
60
80
Ages (years)
FDR (Age associated divergence) =0.0704
p(Scalp)=0.00216
p(Dura)=0.929
Scalp
Dura
A
B
C
D
E
F
G
H
I
J
K

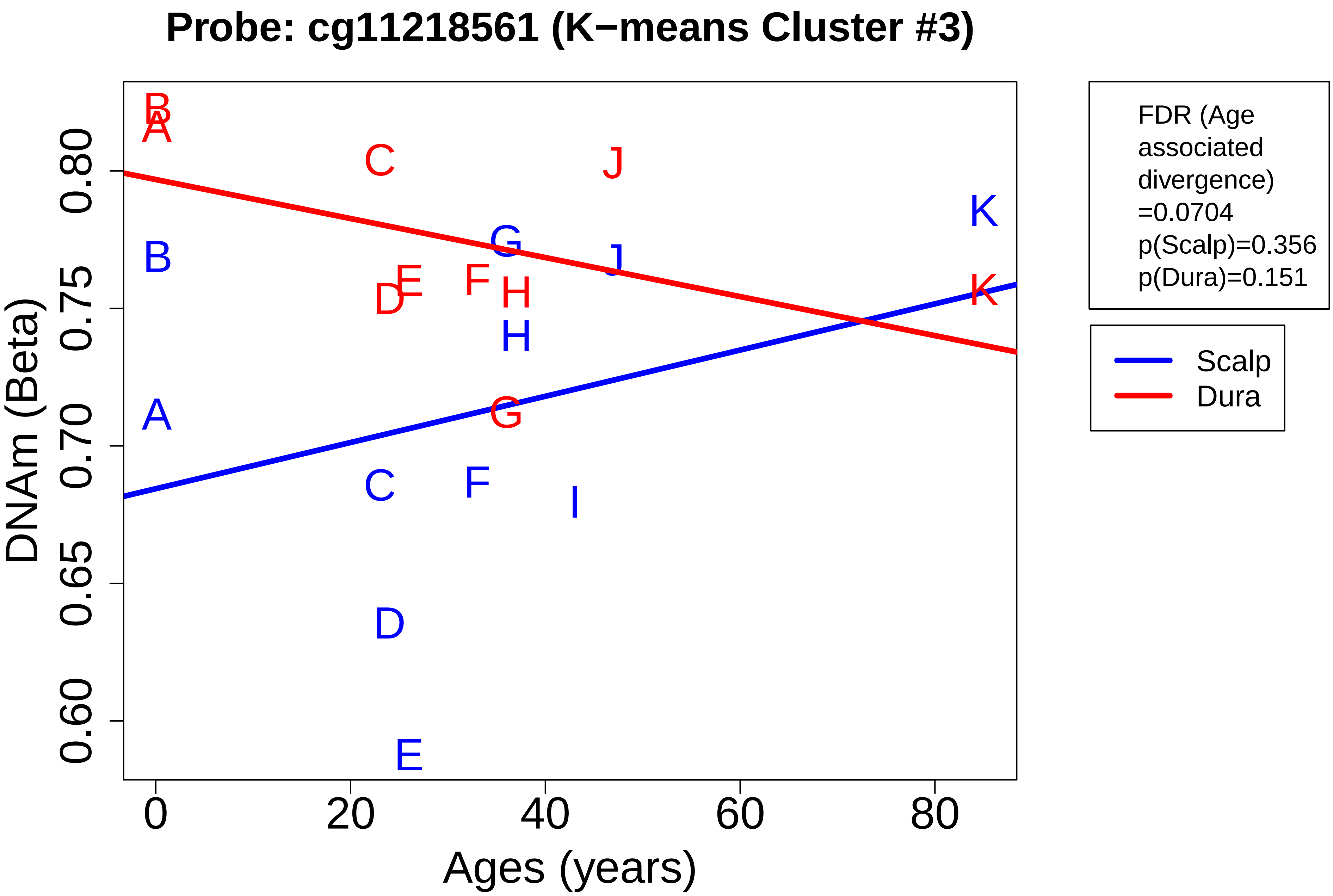

Probe: cg11218561 (K−means Cluster #3)
DNAm (Beta)
0.60
0.65
0.70
0.75
0.80
0
20
40
60
80
Ages (years)
FDR (Age associated divergence) =0.0704
p(Scalp)=0.356
p(Dura)=0.151
Scalp
Dura

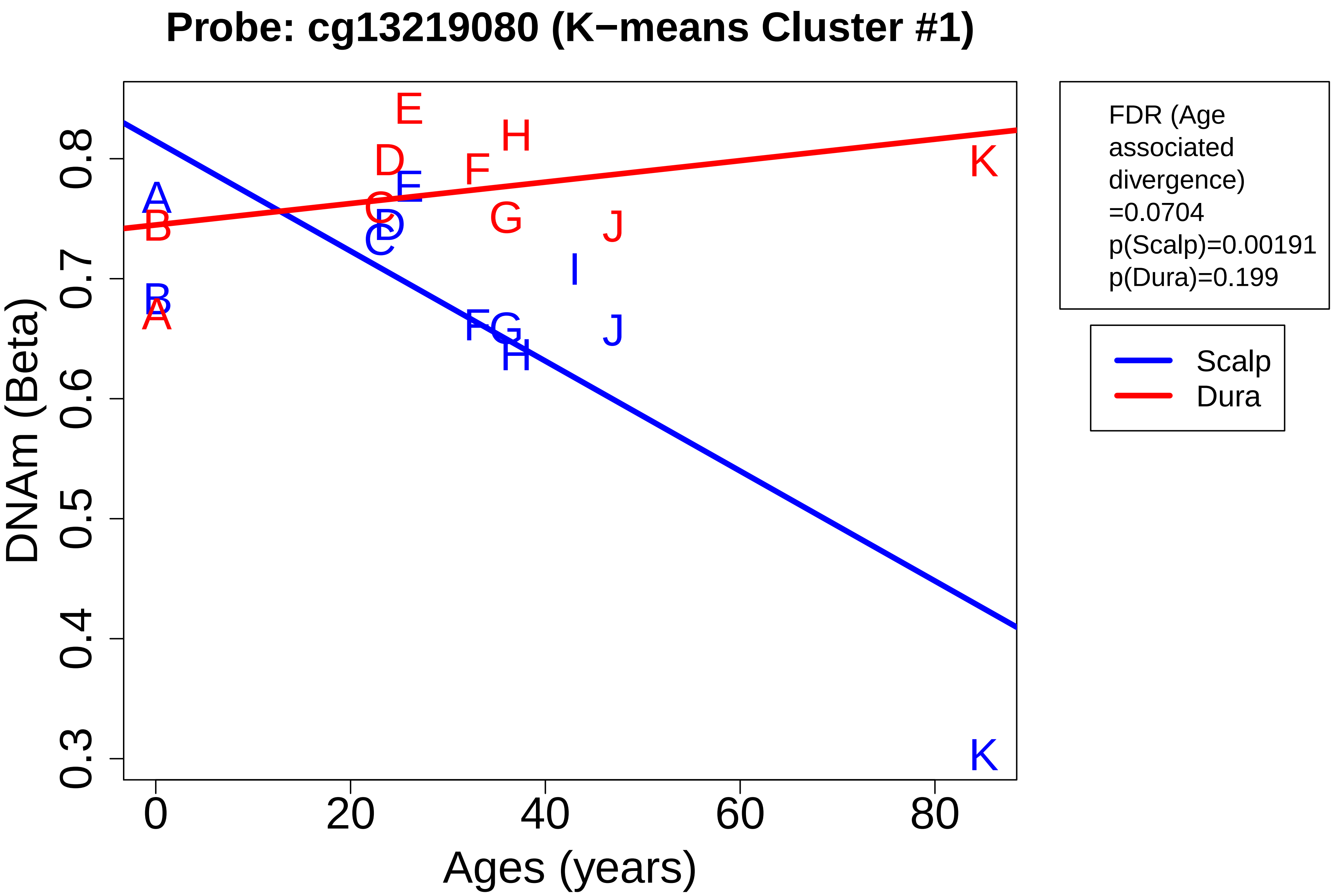

Probe: cg13219080 (K−means Cluster #1)
DNAm (Beta)
0.3
0.4
0.5
0.6
0.7
0.8
0
20
40
60
80
Ages (years)
FDR (Age associated divergence) =0.0704
p(Scalp)=0.00191
p(Dura)=0.199
Scalp
Dura

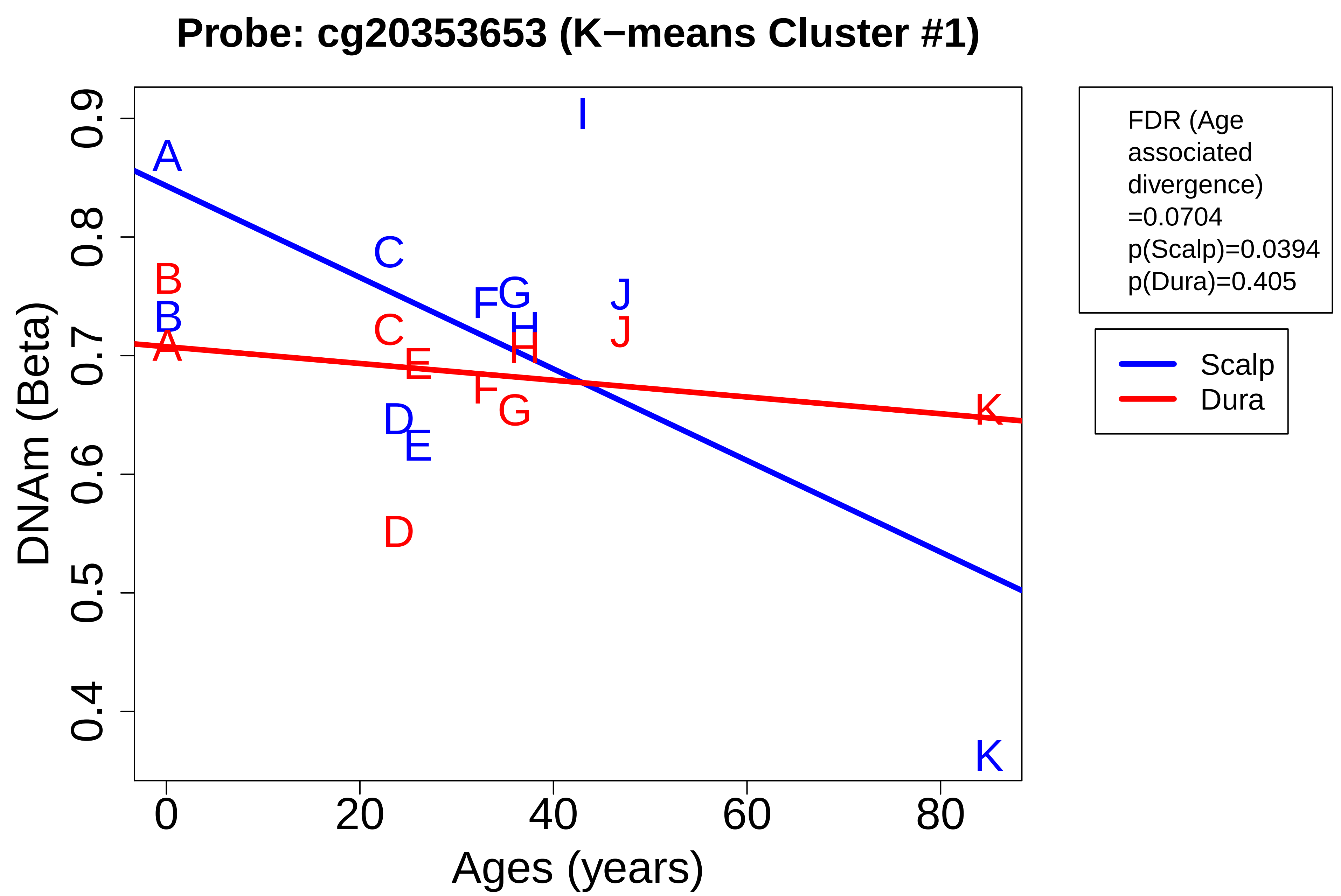
Probe: cg20353653 (K−means Cluster #1)
DNAm (Beta)
0.4
0.5
0.6
0.7
0.8
0.9
0
20
40
60
80
Ages (years)
A
B
C
D
E
F
G
H
I
J
K
FDR (Age associated divergence) =0.0704
p(Scalp)=0.0394
p(Dura)=0.405
Scalp
Dura

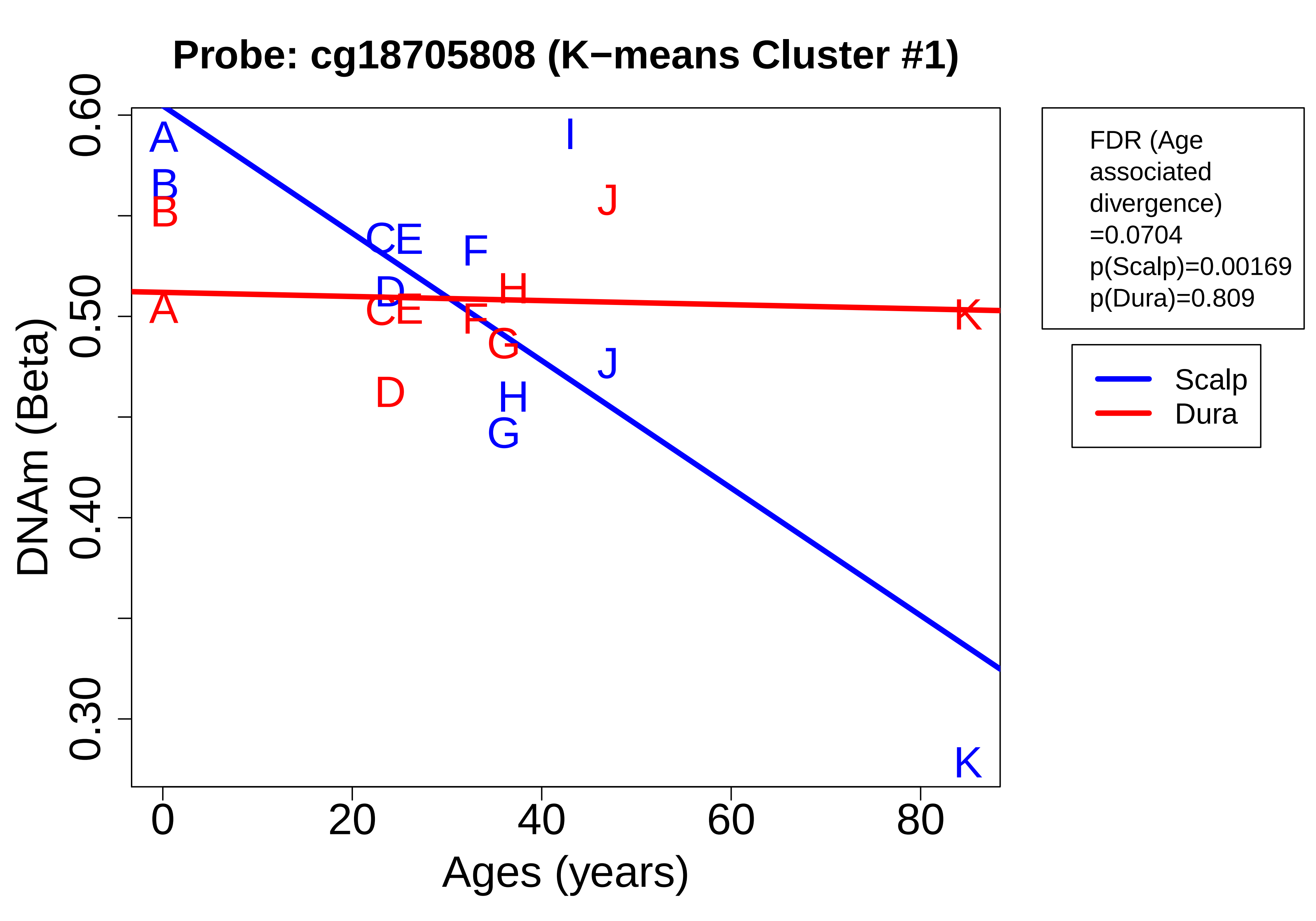

Probe: cg18705808 (K−means Cluster #1)
DNAm (Beta)
0.30
0.40
0.50
0.60
0
20
40
60
80
Ages (years)
A
B
B
A
CE
D
CE
F
F
H
G
D
H
G
I
J
J
K
K
FDR (Age associated divergence) =0.0704
p(Scalp)=0.00169
p(Dura)=0.809
Scalp
Dura

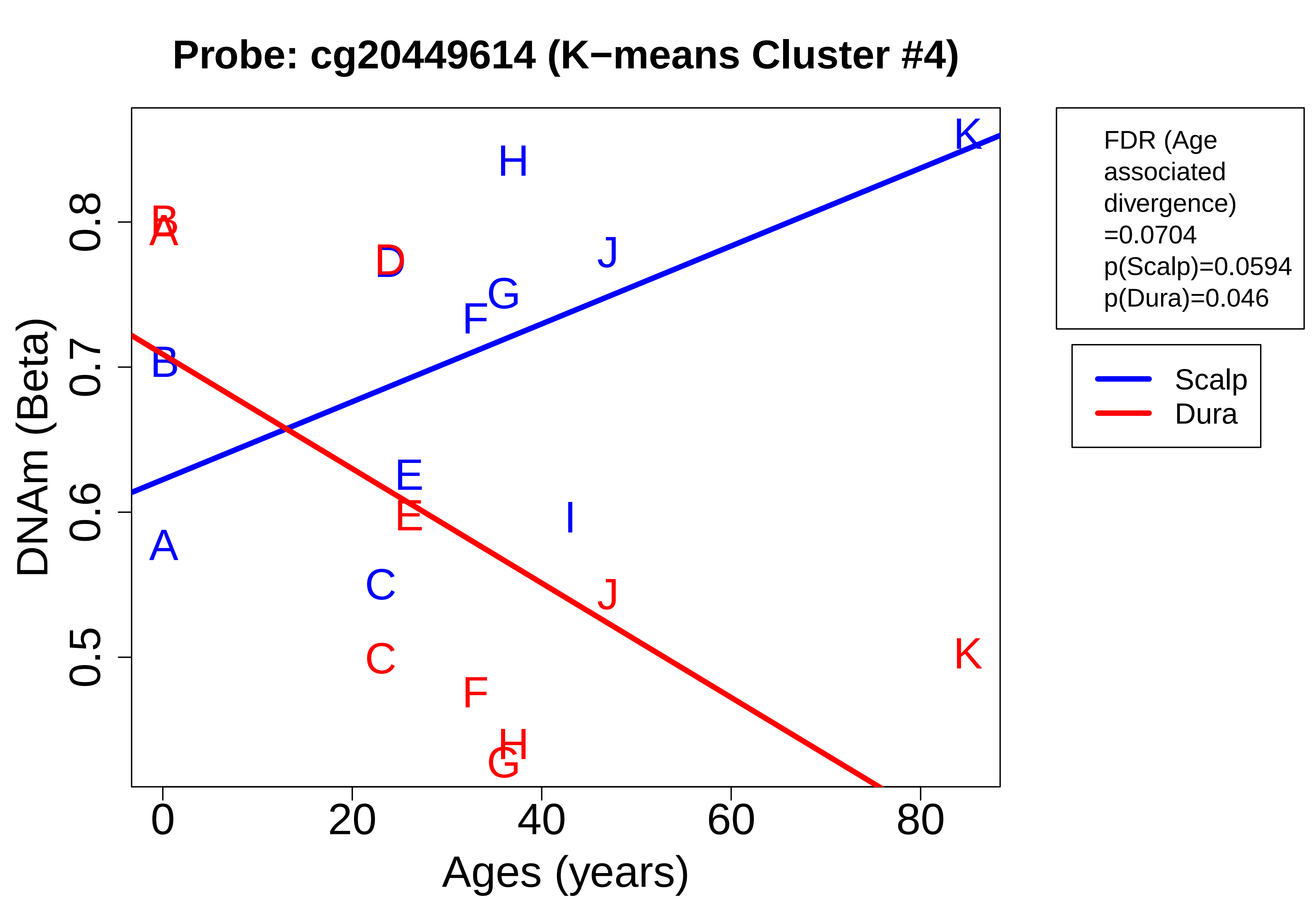

Probe: cg20449614 (K−means Cluster #4)
DNAm (Beta)
0.5
0.6
0.7
0.8
0
20
40
60
80
Ages (years)
FDR (Age associated divergence) =0.0704
p(Scalp)=0.0594
p(Dura)=0.046
Scalp
Dura

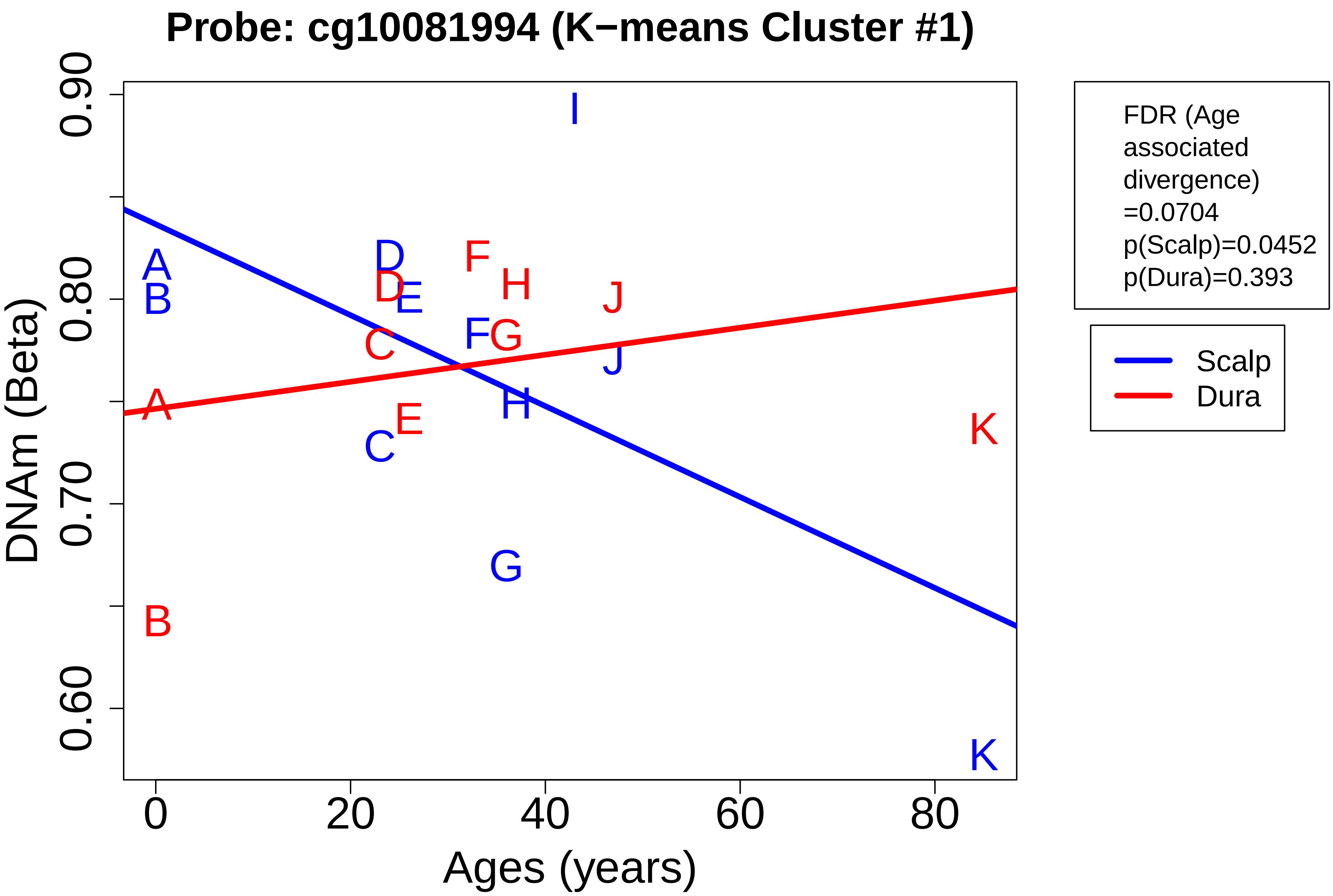

Probe: cg10081994 (K−means Cluster #1)
DNAm (Beta)
0.60
0.70
0.80
0.90
0
20
40
60
80
Ages (years)
A
B
C
D
E
F
G
H
I
J
K
FDR (Age associated divergence) =0.0704
p(Scalp)=0.0452
p(Dura)=0.393
Scalp
Dura

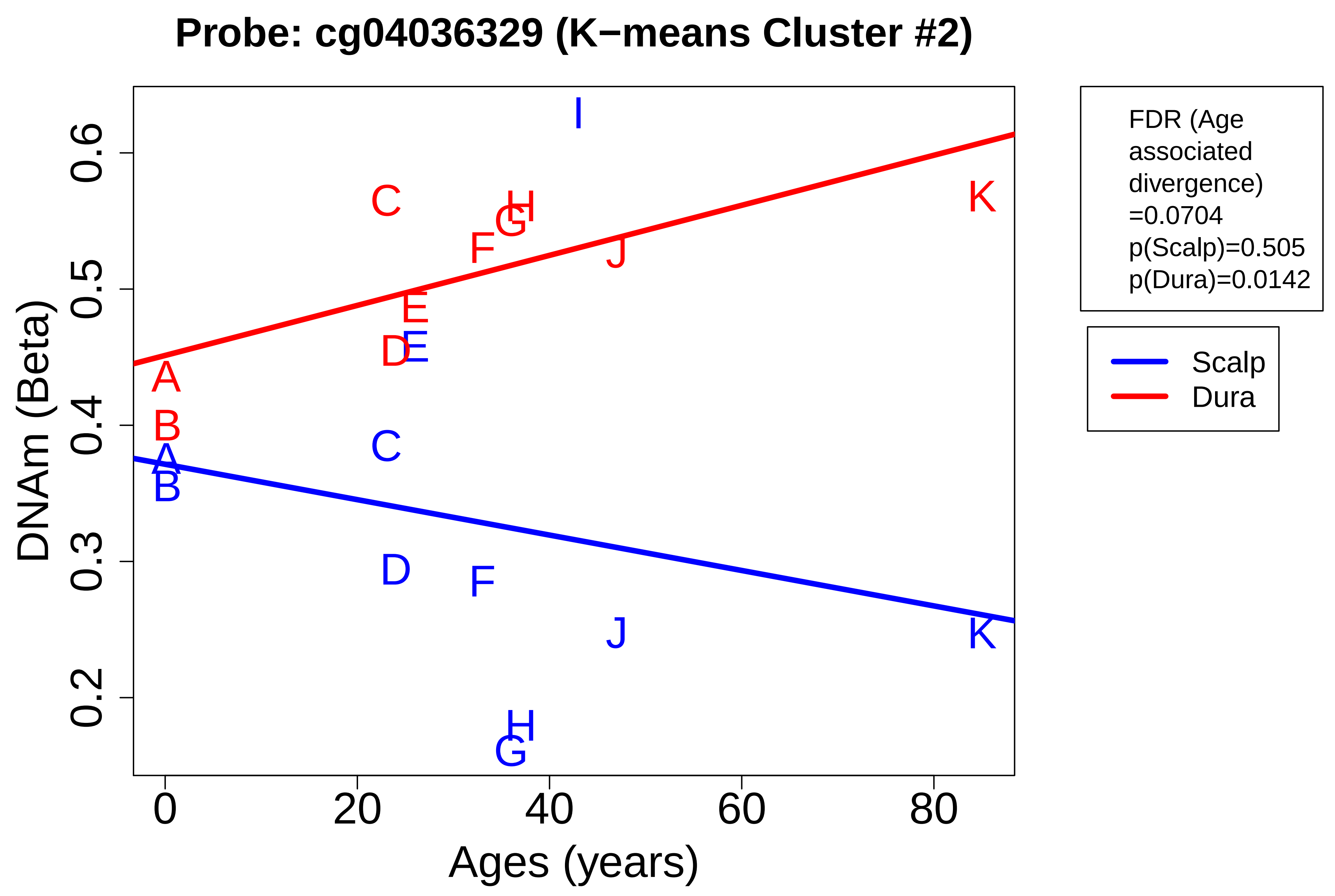

Probe: cg04036329 (K−means Cluster #2)
DNAm (Beta)
0.2
0.3
0.4
0.5
0.6
Ages (years)
0
20
40
60
80
A
B
C
D
E
F
G
H
I
J
K
FDR (Age associated divergence) =0.0704
p(Scalp)=0.505
p(Dura)=0.0142
Scalp
Dura

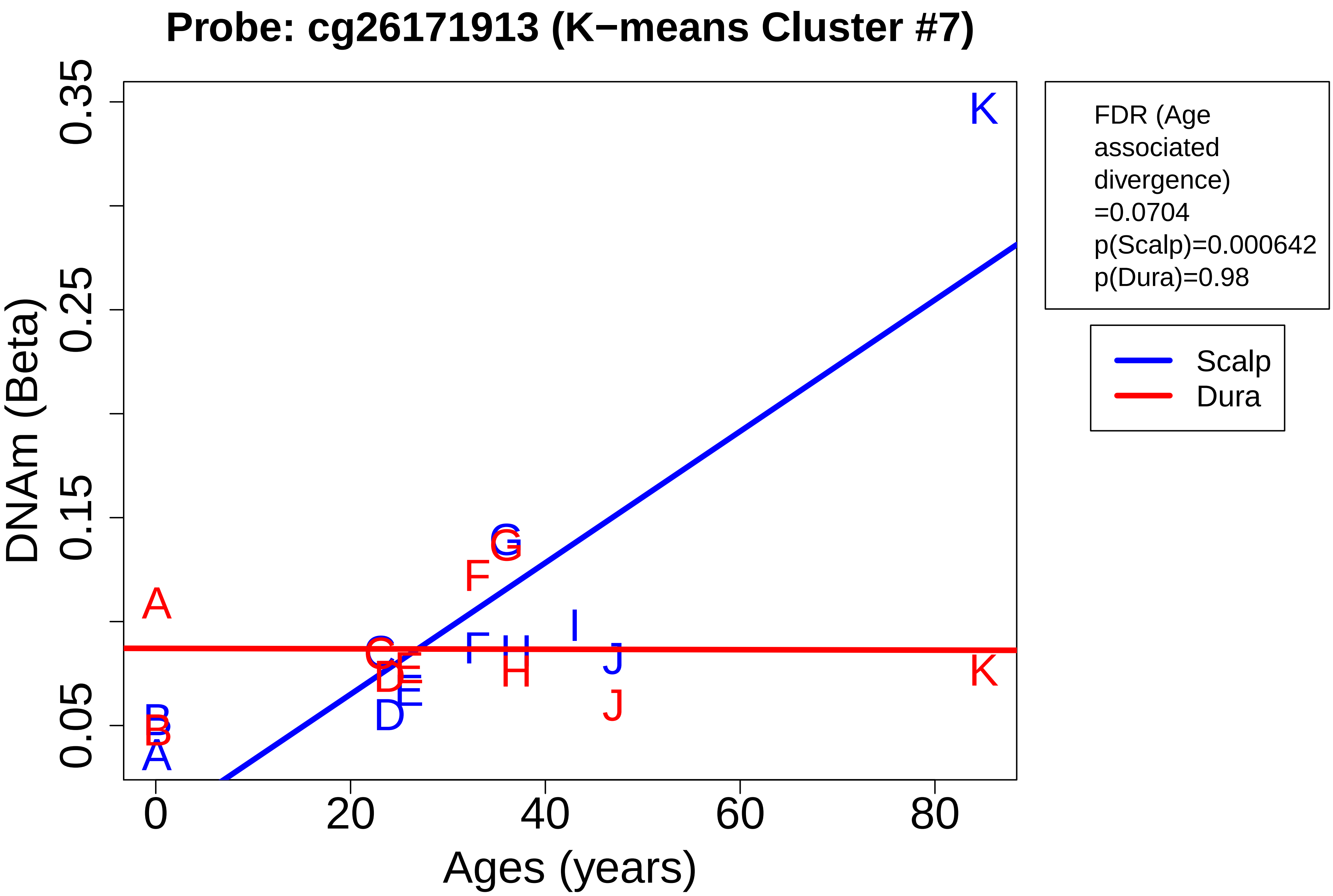

Probe: cg26171913 (K−means Cluster #7)
DNAm (Beta)
0.05
0.15
0.25
0.35
0
20
40
60
80
Ages (years)
FDR (Age associated divergence) =0.0704
p(Scalp)=0.000642
p(Dura)=0.98
Scalp
Dura

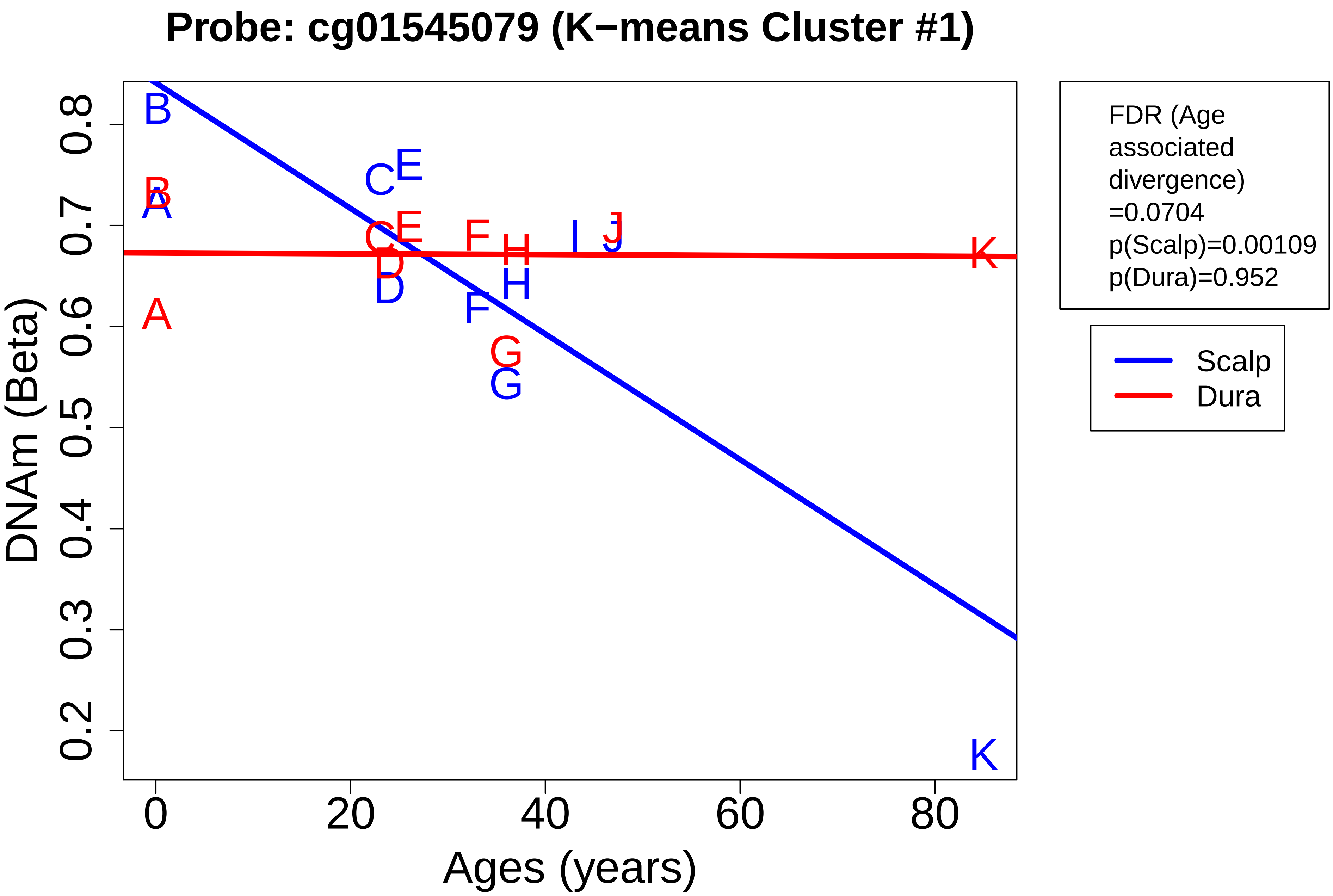

Probe: cg01545079 (K−means Cluster #1)
DNAm (Beta)
0.2
0.3
0.4
0.5
0.6
0.7
0.8
Ages (years)
0
20
40
60
80
A
B
C
D
E
F
G
H
I
J
K
FDR (Age associated divergence) =0.0704
p(Scalp)=0.00109
p(Dura)=0.952
Scalp
Dura

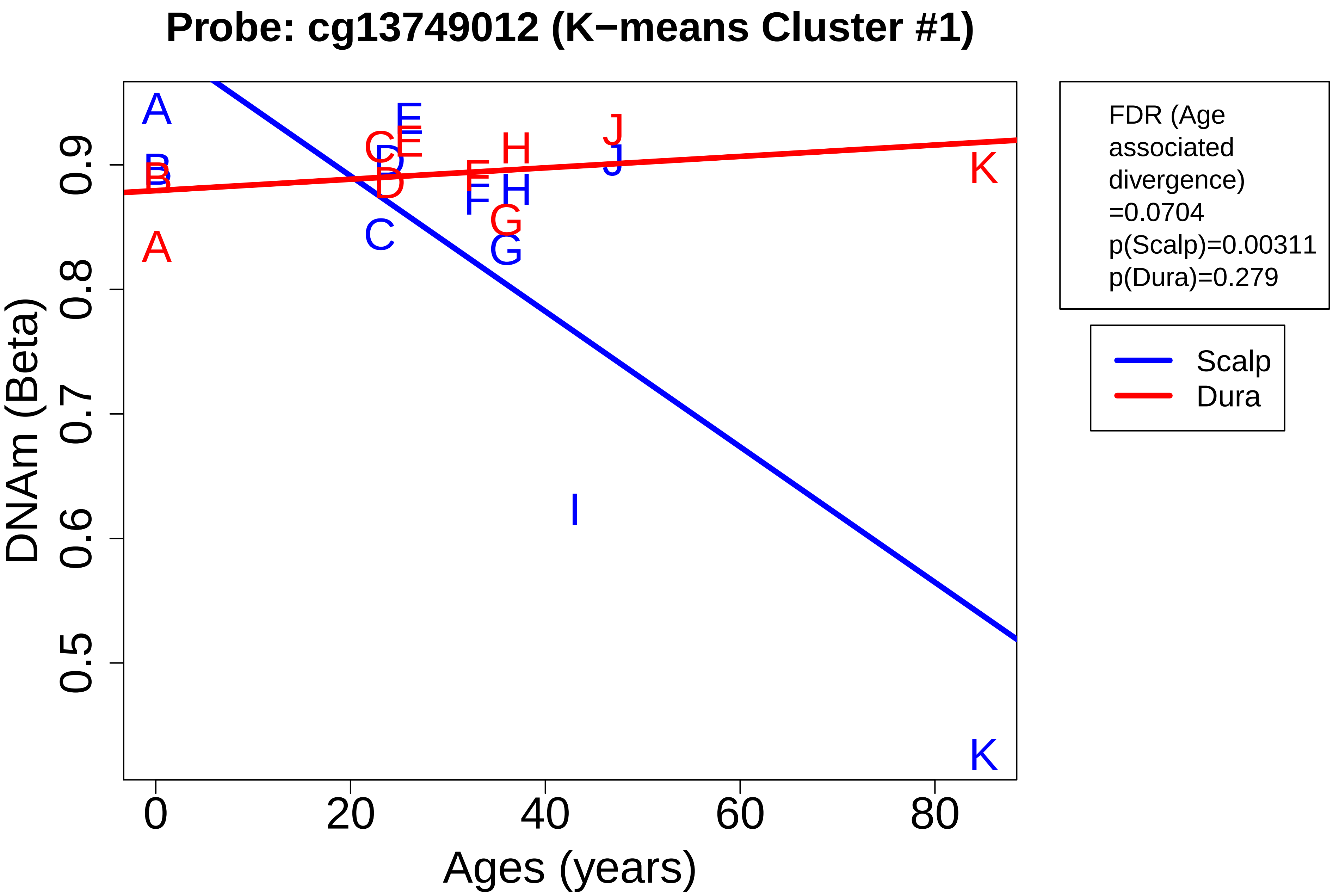

Probe: cg13749012 (K−means Cluster #1)
DNAm (Beta)
0.5
0.6
0.7
0.8
0.9
Ages (years)
0
20
40
60
80
A
B
C
D
E
F
G
H
I
J
K
FDR (Age associated divergence) =0.0704
p(Scalp)=0.00311
p(Dura)=0.279
Scalp
Dura

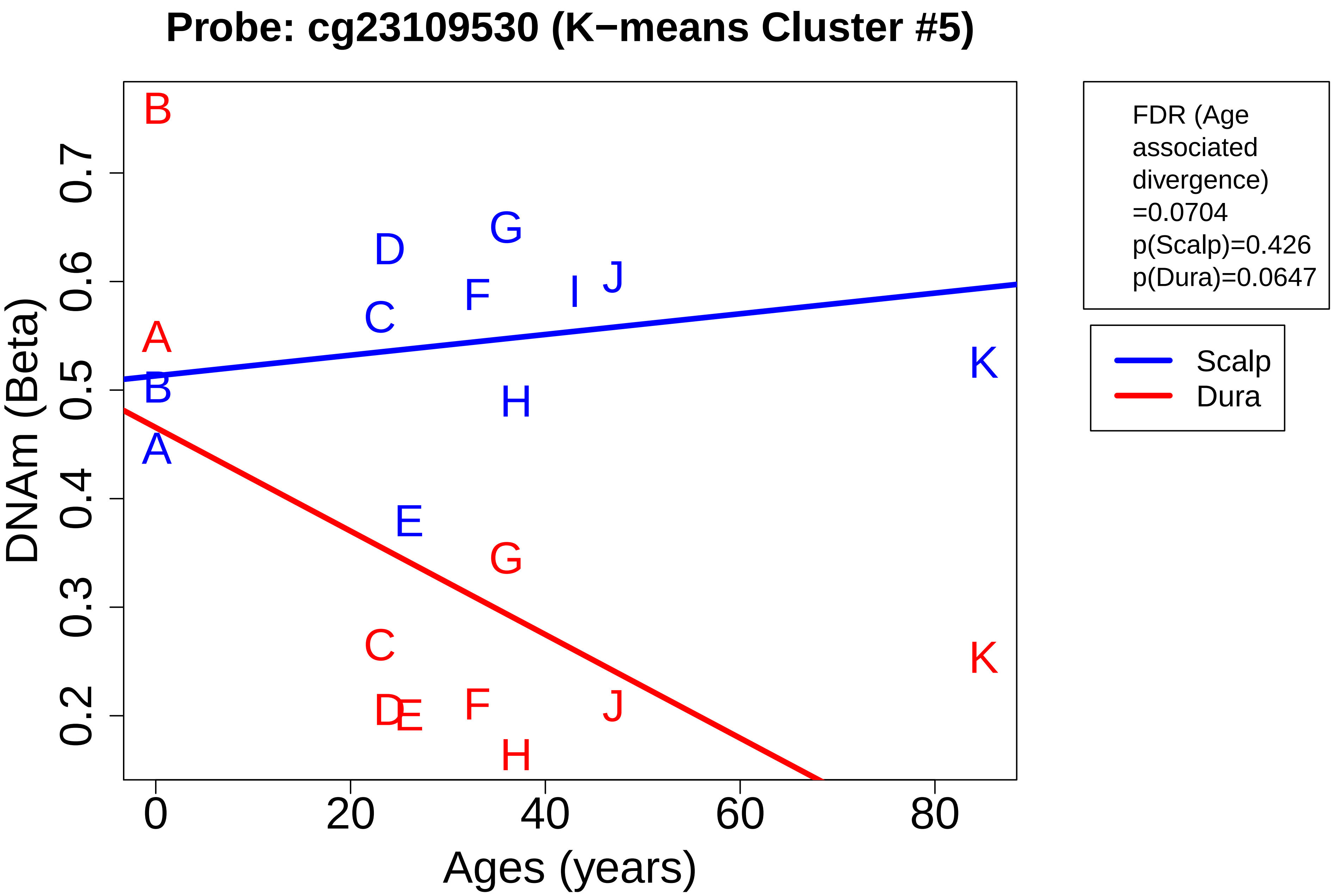

Probe: cg23109530 (K−means Cluster #5)
DNAm (Beta)
Ages (years)
0.2
0.3
0.4
0.5
0.6
0.7
0
20
40
60
80
B
A
B
A
C
D
G
F
I
J
H
K
E
G
C
D
E
F
J
H
K
FDR (Age associated divergence) =0.0704
p(Scalp)=0.426
p(Dura)=0.0647
Scalp
Dura

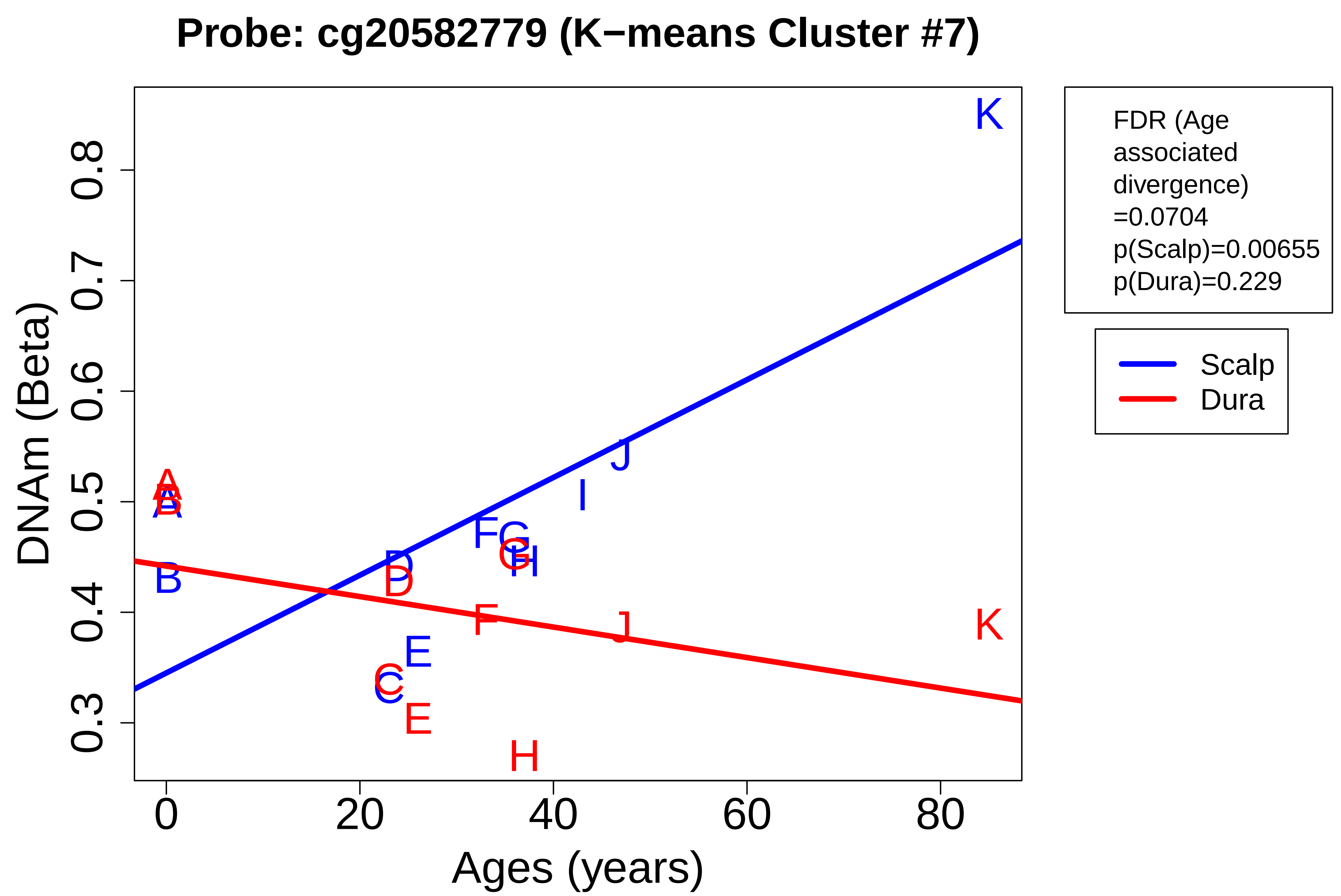

Probe: cg20582779 (K−means Cluster #7)
DNAm (Beta)
0.3
0.4
0.5
0.6
0.7
0.8
Ages (years)
0
20
40
60
80
FDR (Age associated divergence) =0.0704
p(Scalp)=0.00655
p(Dura)=0.229
Scalp
Dura

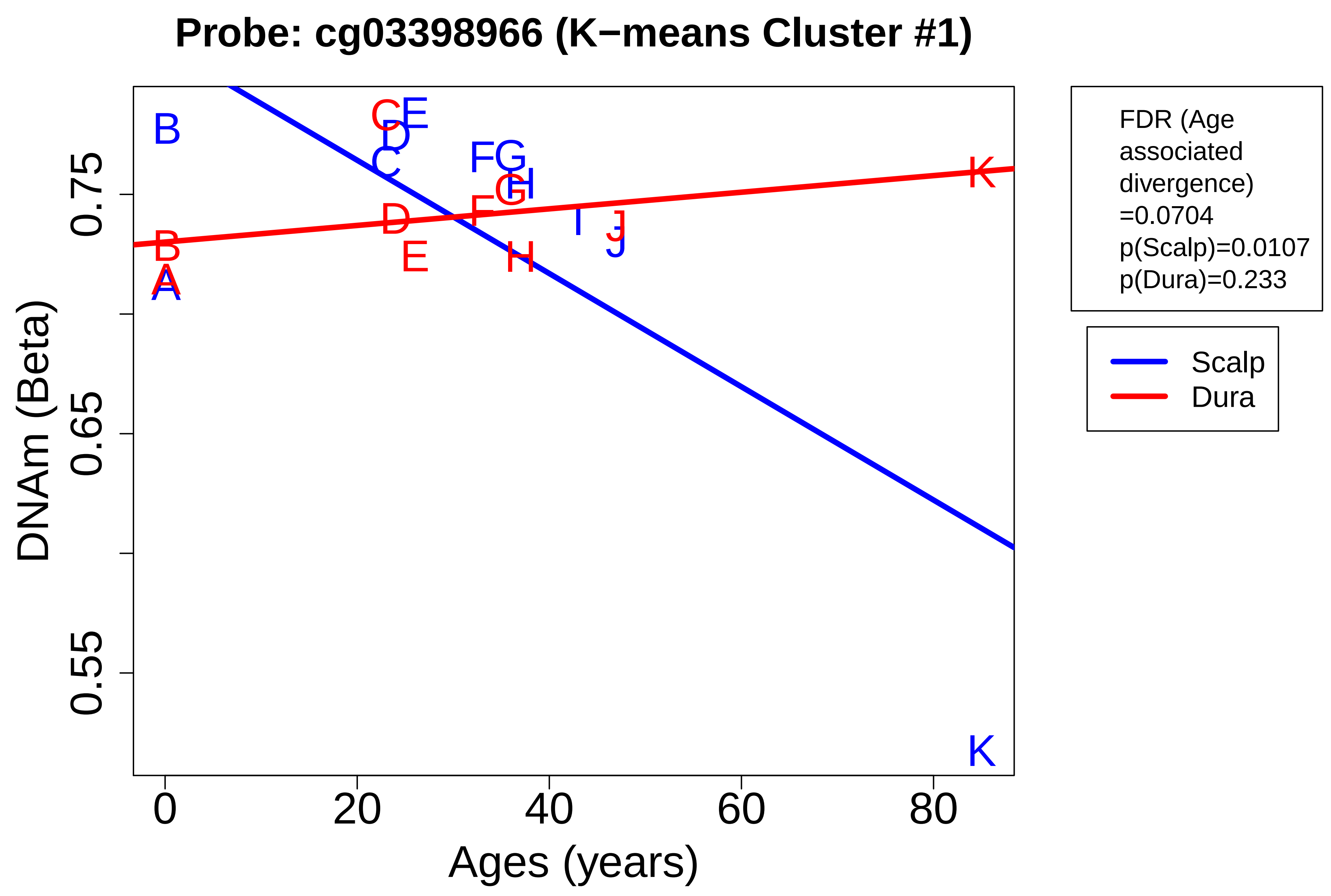

Probe: cg03398966 (K−means Cluster #1)
DNAm (Beta)
0.55
0.65
0.75
Ages (years)
0
20
40
60
80
B
C
E
D
C
F
G
H
G
D
F
I
J
J
B
A
A
E
H
K
K
FDR (Age associated divergence) =0.0704
p(Scalp)=0.0107
p(Dura)=0.233
Scalp
Dura

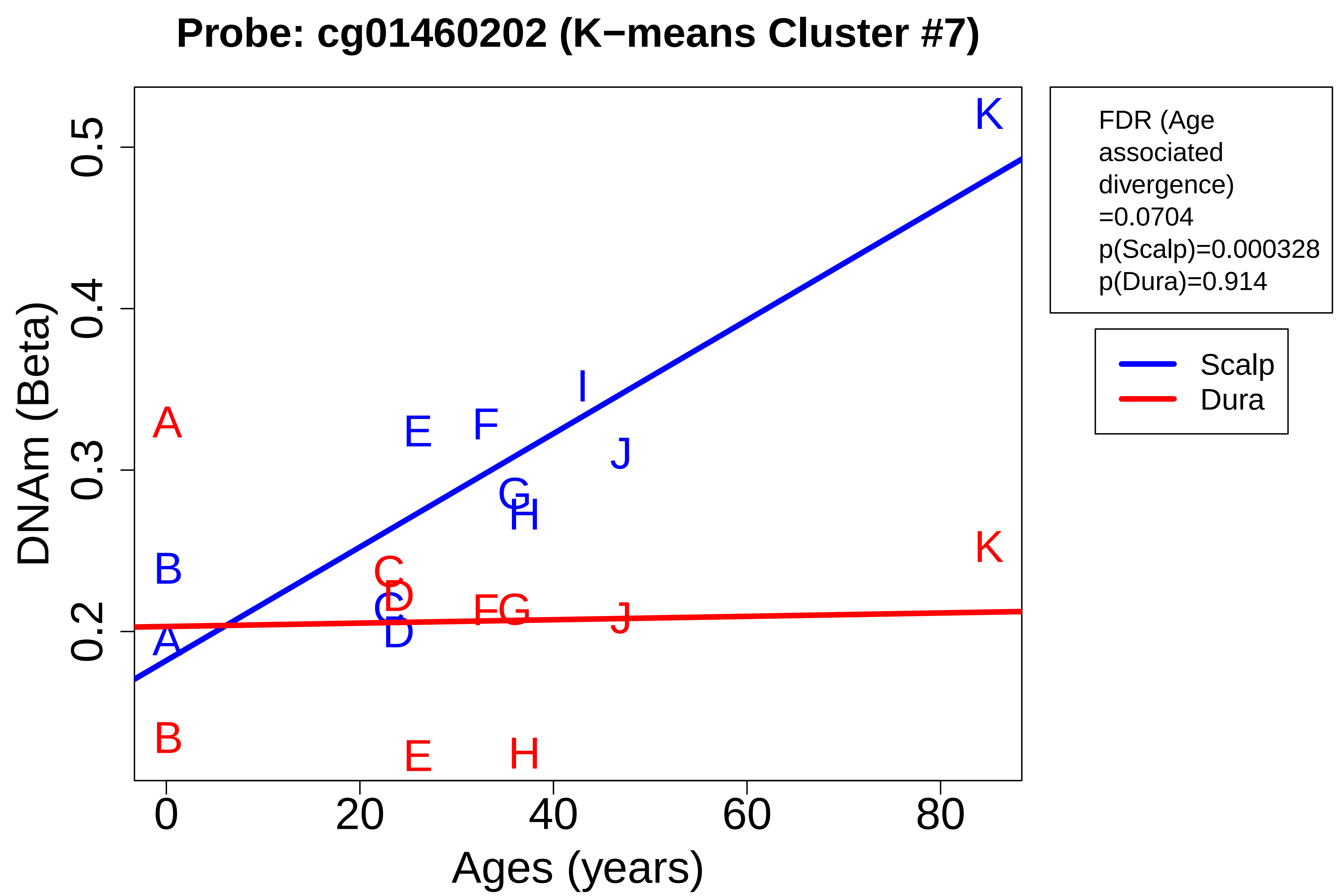
Probe: cg01460202 (K−means Cluster #7)
DNAm (Beta)
0.2
0.3
0.4
0.5
0
20
40
60
80
Ages (years)
FDR (Age associated divergence) =0.0704
p(Scalp)=0.000328
p(Dura)=0.914
Scalp
Dura

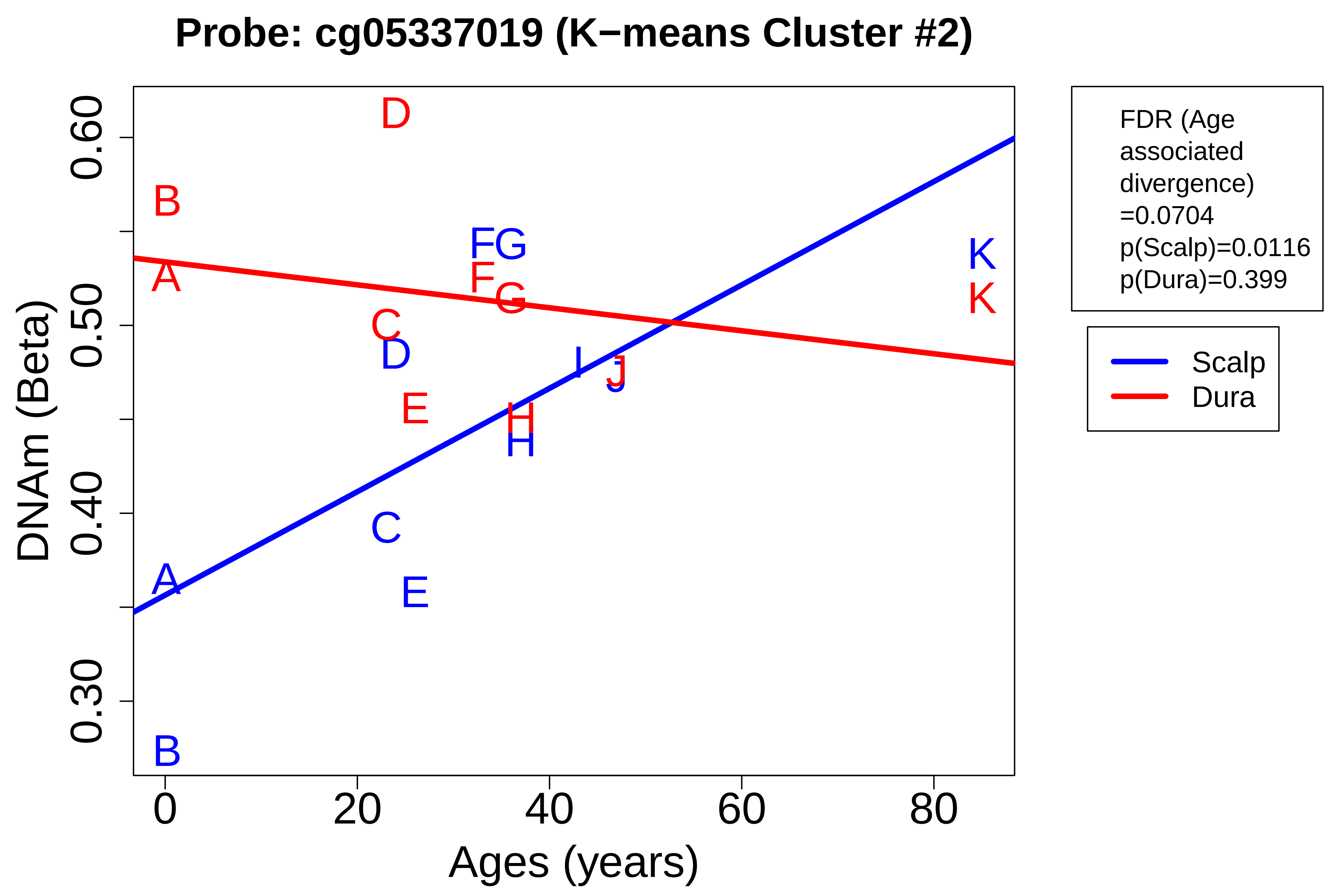

Probe: cg05337019 (K−means Cluster #2)
DNAm (Beta)
0.30
0.40
0.50
0.60
Ages (years)
0
20
40
60
80
FDR (Age associated divergence) =0.0704
p(Scalp)=0.0116
p(Dura)=0.399
Scalp
Dura
A
B
C
D
E
F
G
H
I
J
K

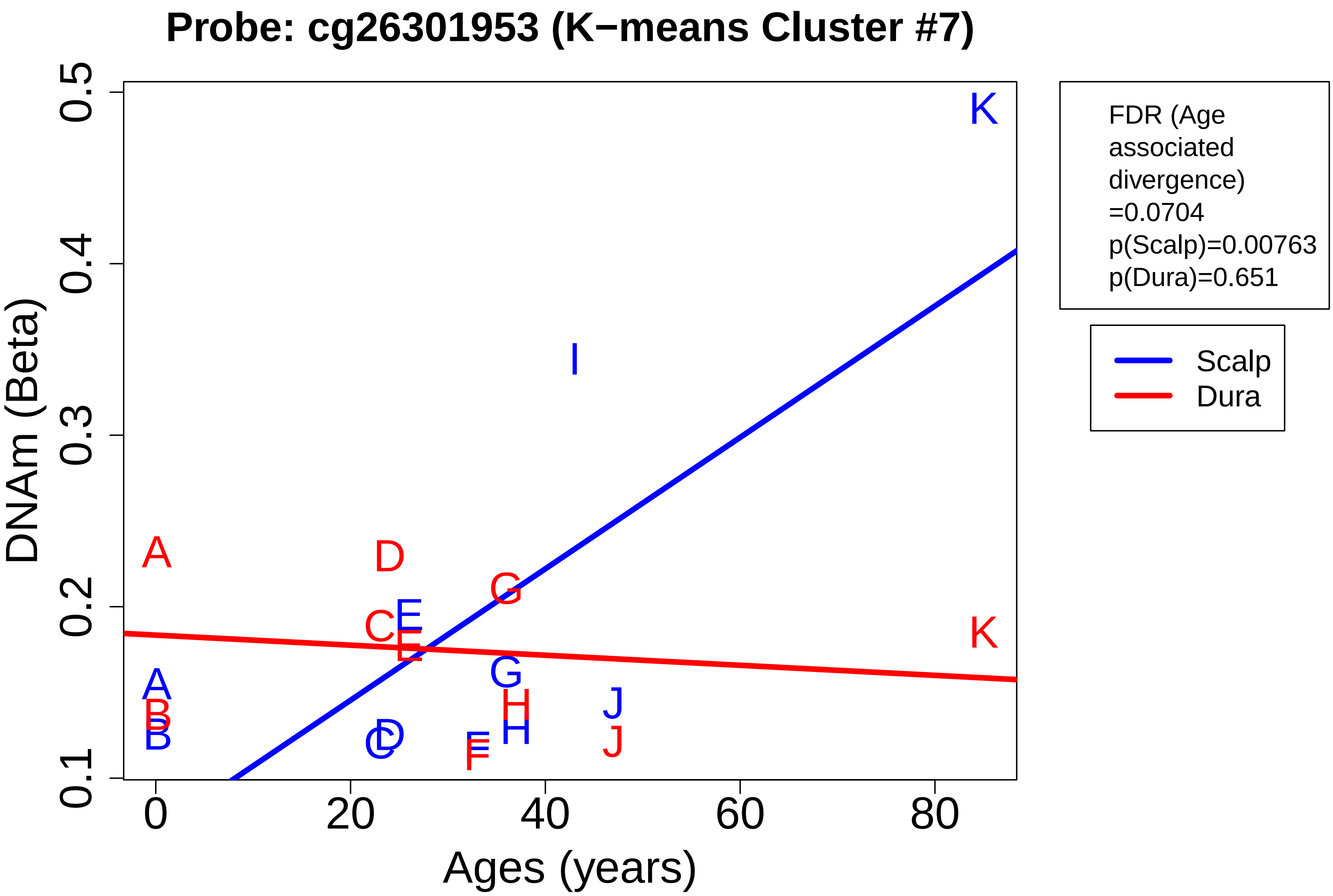

Probe: cg26301953 (K−means Cluster #7)
DNAm (Beta)
0.1
0.2
0.3
0.4
0.5
0
20
40
60
80
Ages (years)
A
B
C
D
E
F
G
H
I
J
K
FDR (Age associated divergence) =0.0704
p(Scalp)=0.00763
p(Dura)=0.651
Scalp
Dura

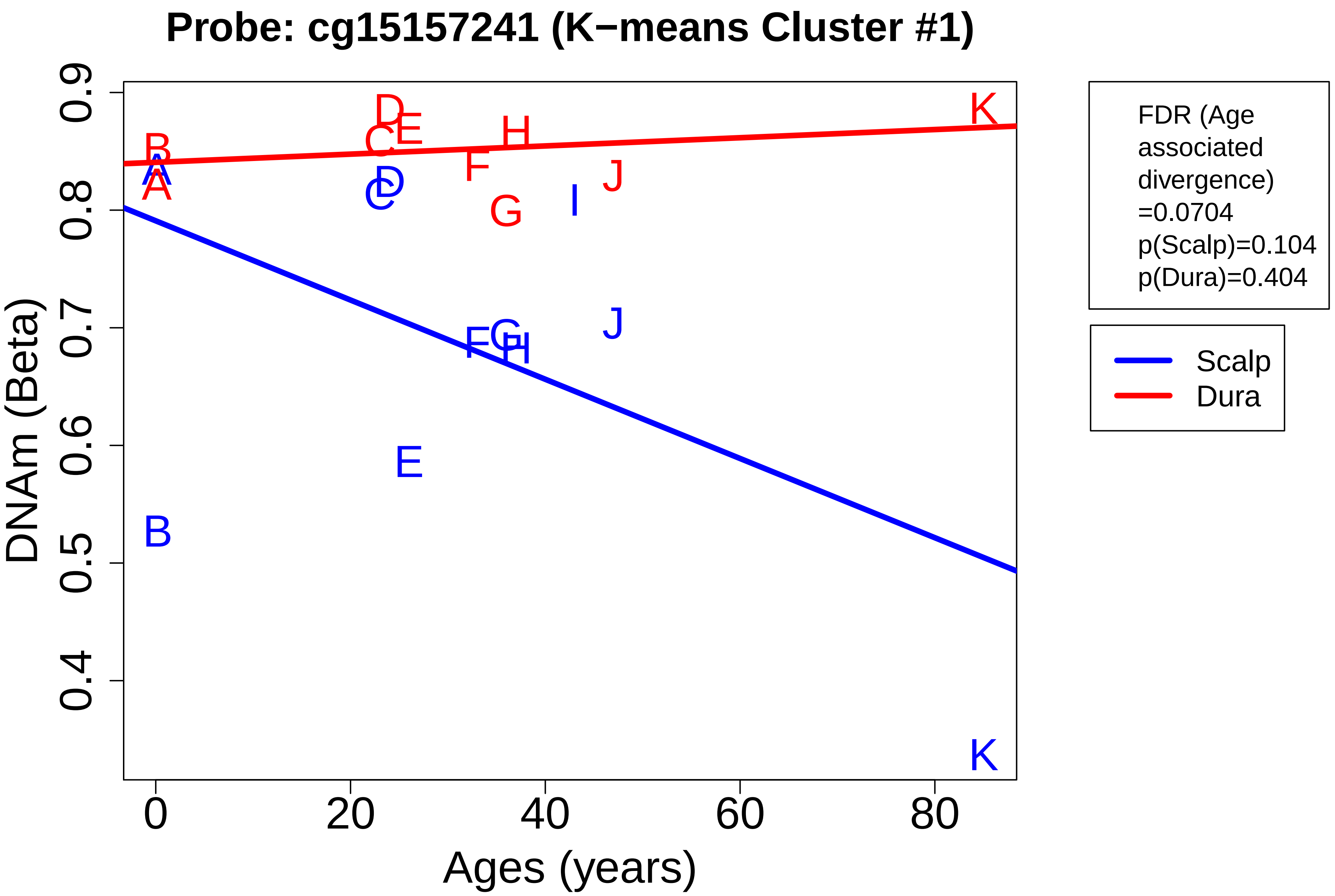

Probe: cg15157241 (K−means Cluster #1)
DNAm (Beta)
0.4
0.5
0.6
0.7
0.8
0.9
0
20
40
60
80
Ages (years)
FDR (Age associated divergence) =0.0704
p(Scalp)=0.104
p(Dura)=0.404
Scalp
Dura
A
B
C
D
E
F
G
H
I
J
K

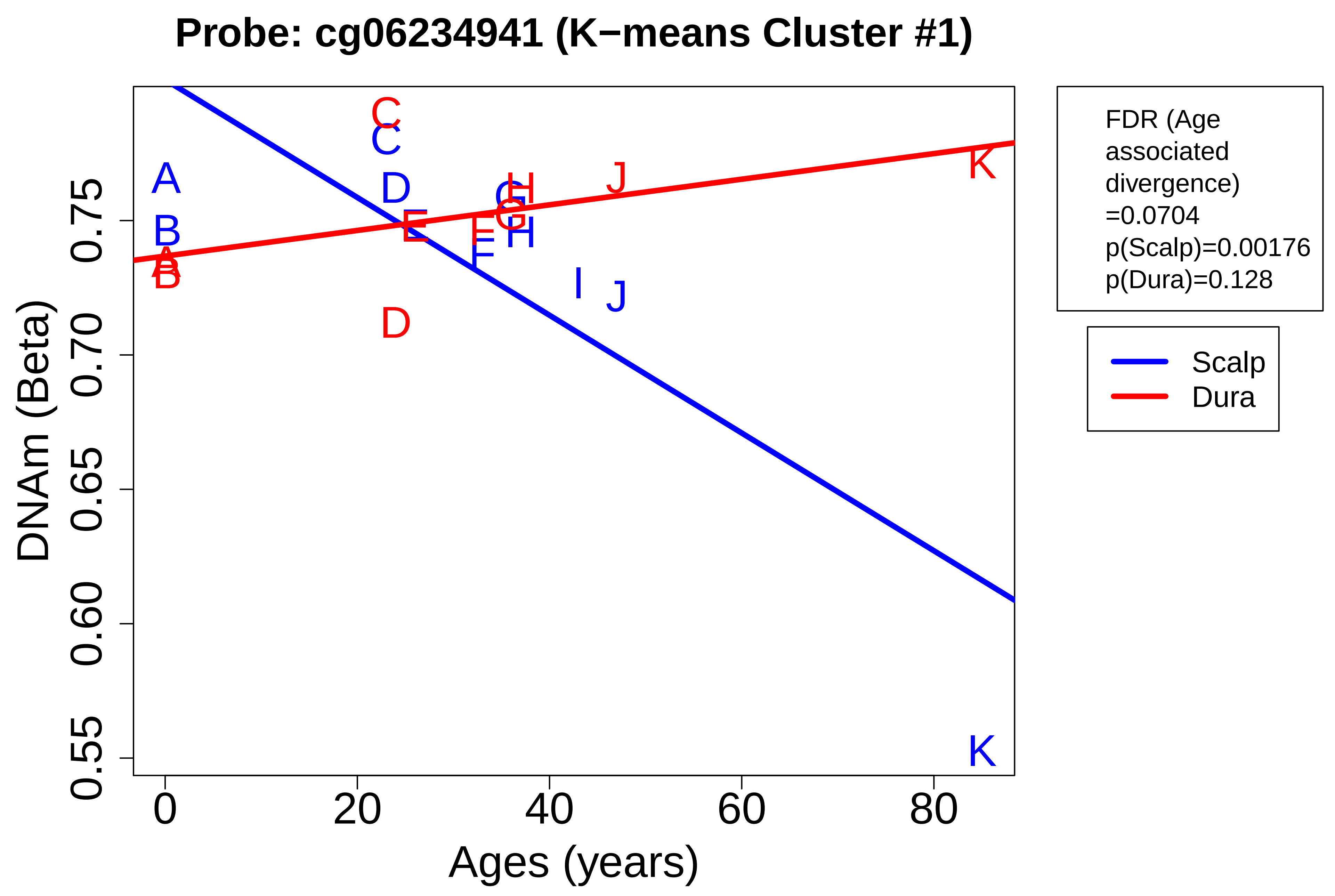
Probe: cg06234941 (K−means Cluster #1)
DNAm (Beta)
0.55
0.60
0.65
0.70
0.75
0
20
40
60
80
Ages (years)
A
B
C
D
E
F
G
H
I
J
K
FDR (Age associated divergence) =0.0704
p(Scalp)=0.00176
p(Dura)=0.128
Scalp
Dura

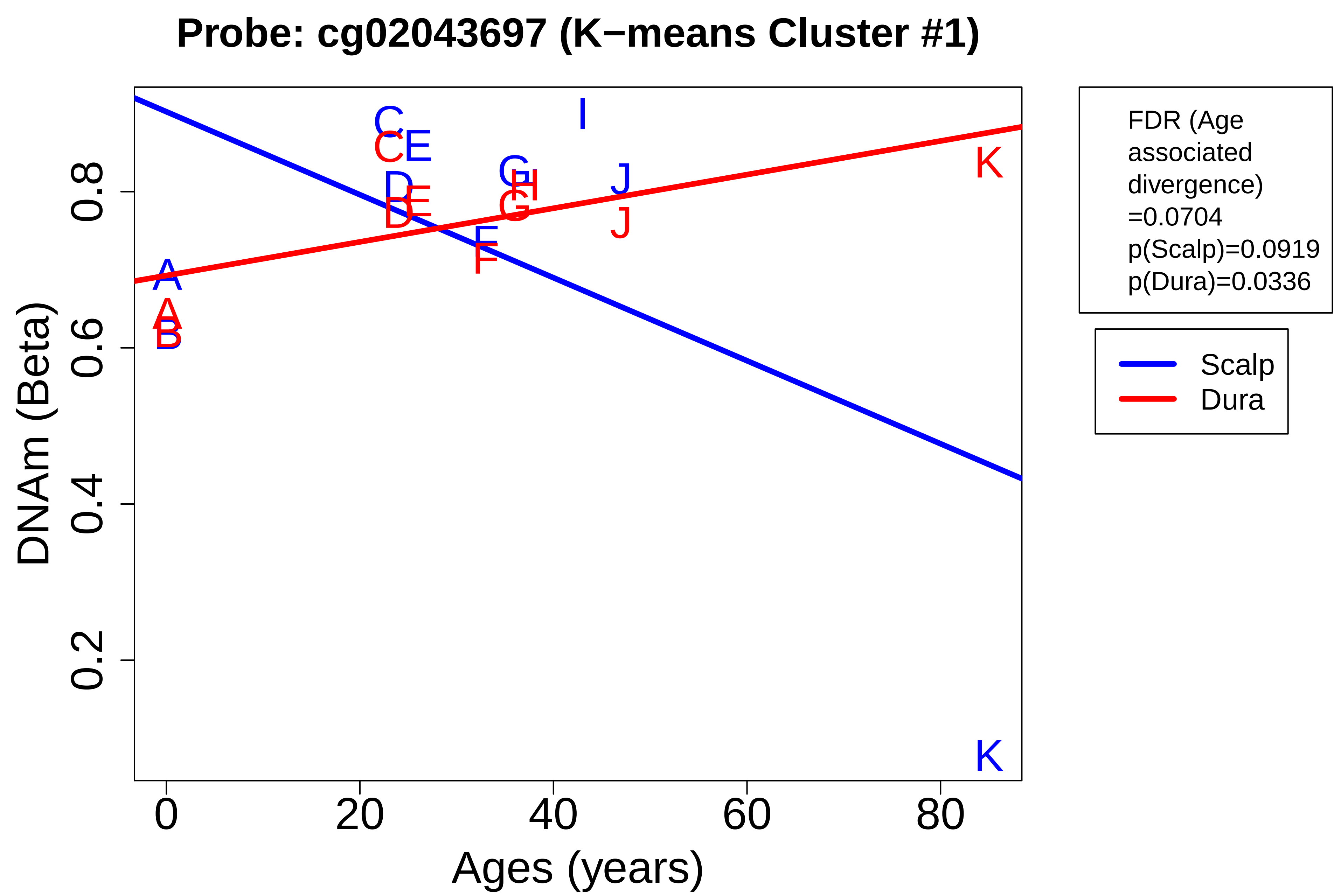
Probe: cg02043697 (K−means Cluster #1)
FDR (Age associated divergence) =0.0704
p(Scalp)=0.0919
p(Dura)=0.0336
Scalp
Dura
DNAm (Beta)
0.2
0.4
0.6
0.8
0
20
40
60
80
Ages (years)
A
B
C
D
E
F
G
H
I
J
K

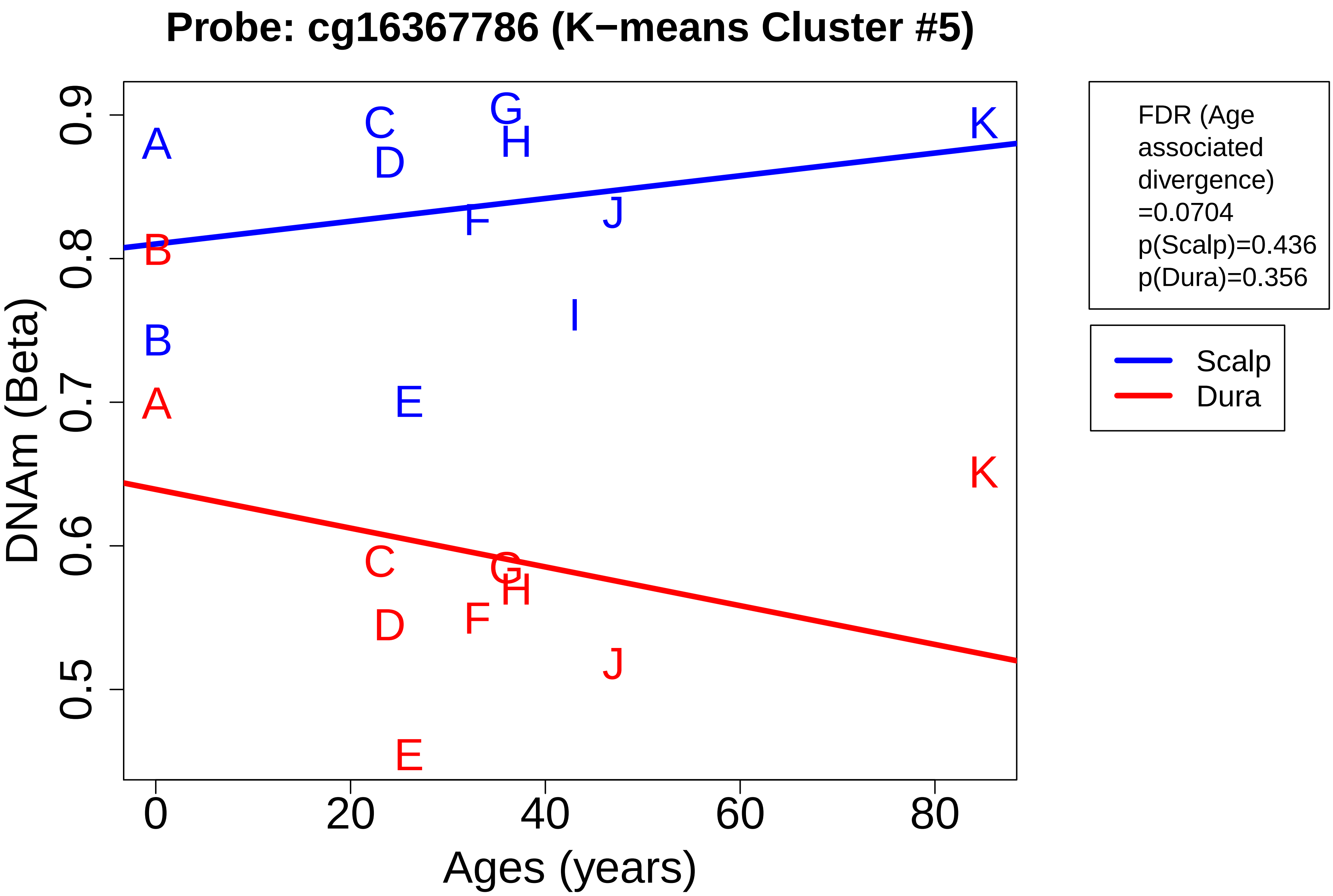

Probe: cg16367786 (K−means Cluster #5)
DNAm (Beta)
0.9
0.8
0.7
0.6
0.5
Ages (years)
0
20
40
60
80
A
B
C
D
E
F
G
H
I
J
K
FDR (Age associated divergence) =0.0704
p(Scalp)=0.436
p(Dura)=0.356
Scalp
Dura

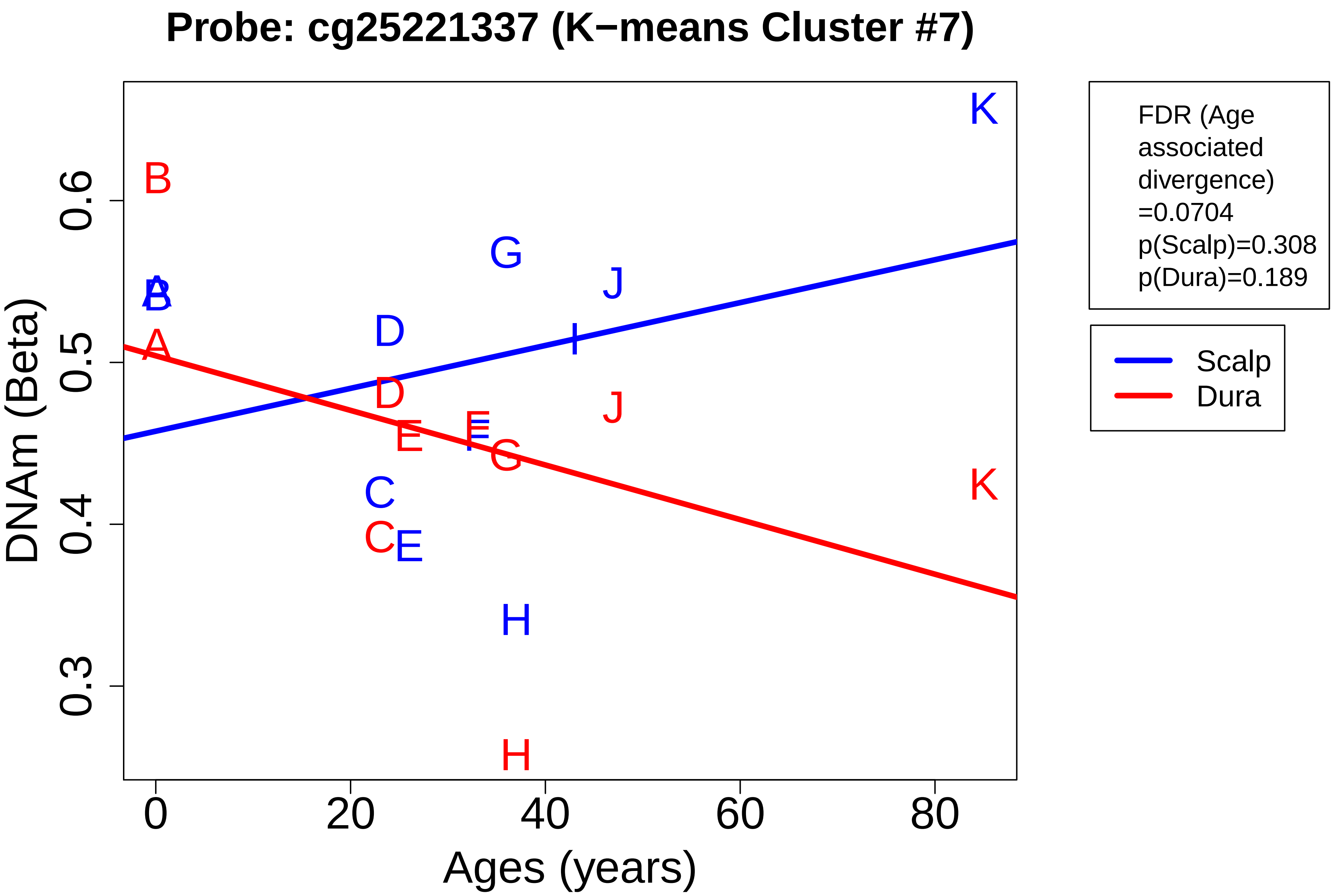

Probe: cg25221337 (K−means Cluster #7)
DNAm (Beta)
0.3
0.4
0.5
0.6
Ages (years)
0
20
40
60
80
K
B
G
J
A
B
D
I
A
D
J
E
E
G
C
K
C
E
H
H
FDR (Age associated divergence) =0.0704
p(Scalp)=0.308
p(Dura)=0.189
Scalp
Dura

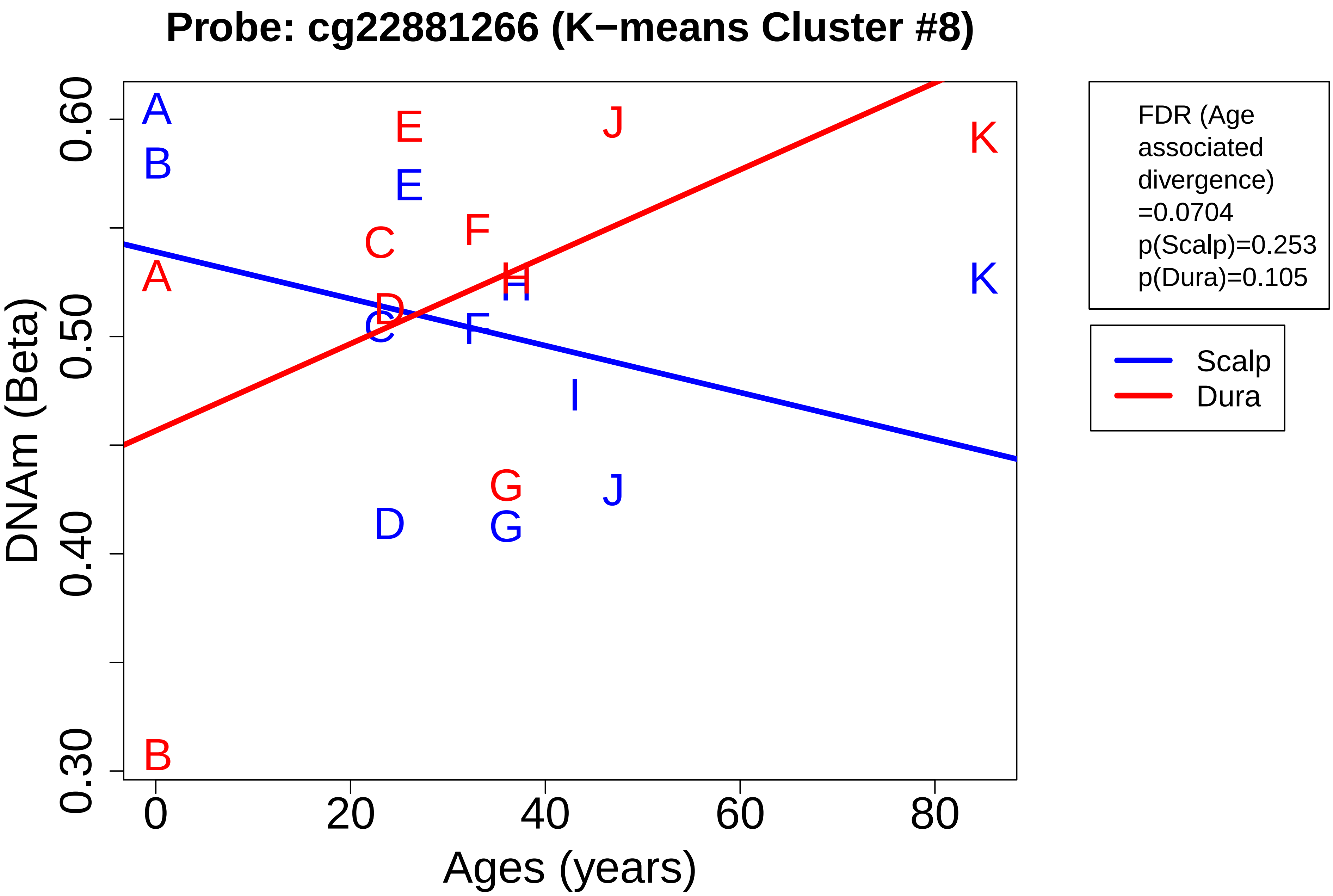

Probe: cg22881266 (K−means Cluster #8)
DNAm (Beta)
0.30
0.40
0.50
0.60
Ages (years)
0
20
40
60
80
A
B
C
D
E
F
G
H
I
J
K
FDR (Age associated divergence) =0.0704
p(Scalp)=0.253
p(Dura)=0.105
Scalp
Dura

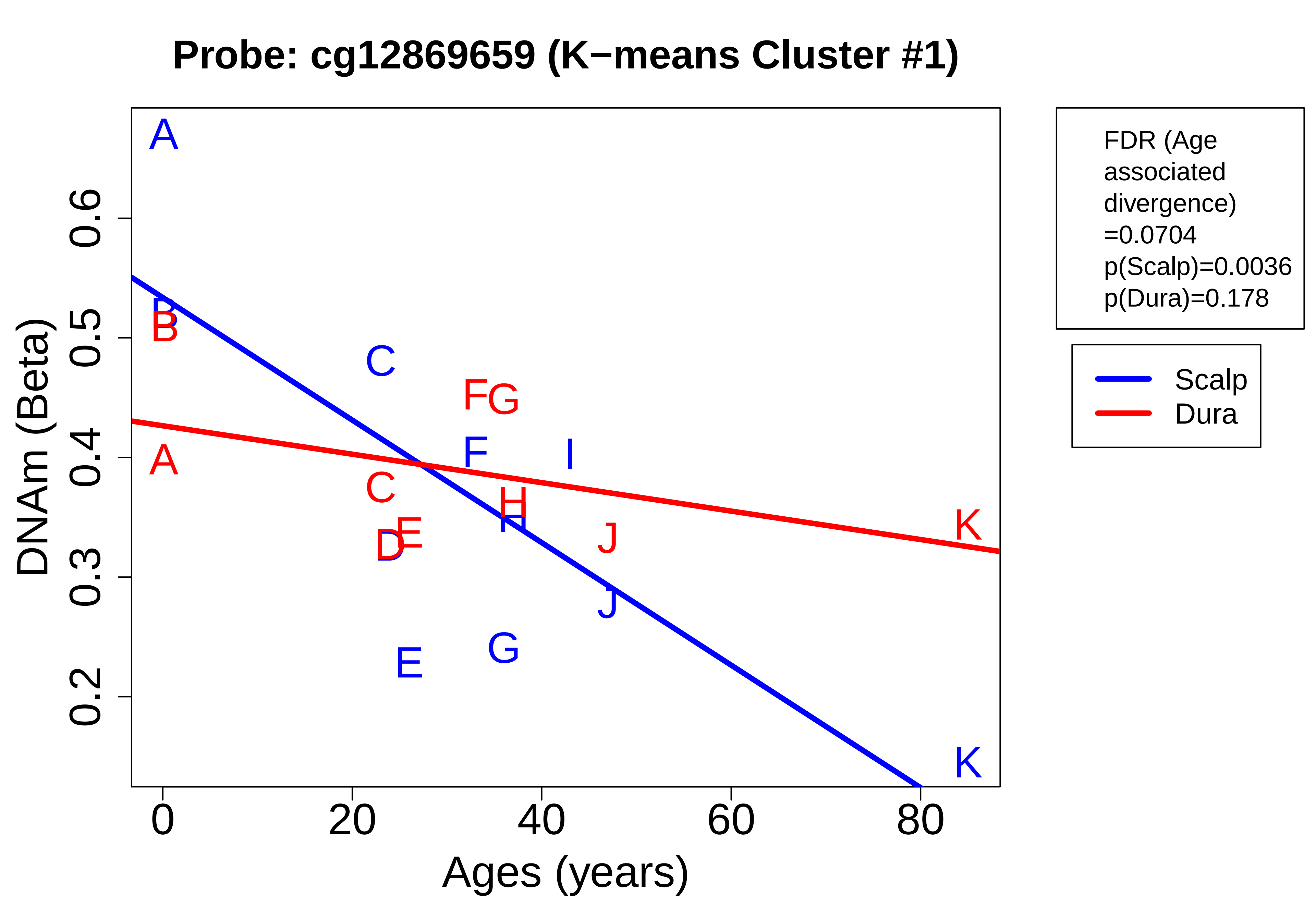

Probe: cg12869659 (K−means Cluster #1)
DNAm (Beta)
0.2
0.3
0.4
0.5
0.6
0
20
40
60
80
Ages (years)
A
B
C
D
E
F
G
H
I
J
K
FDR (Age associated divergence) =0.0704
p(Scalp)=0.0036
p(Dura)=0.178
Scalp
Dura

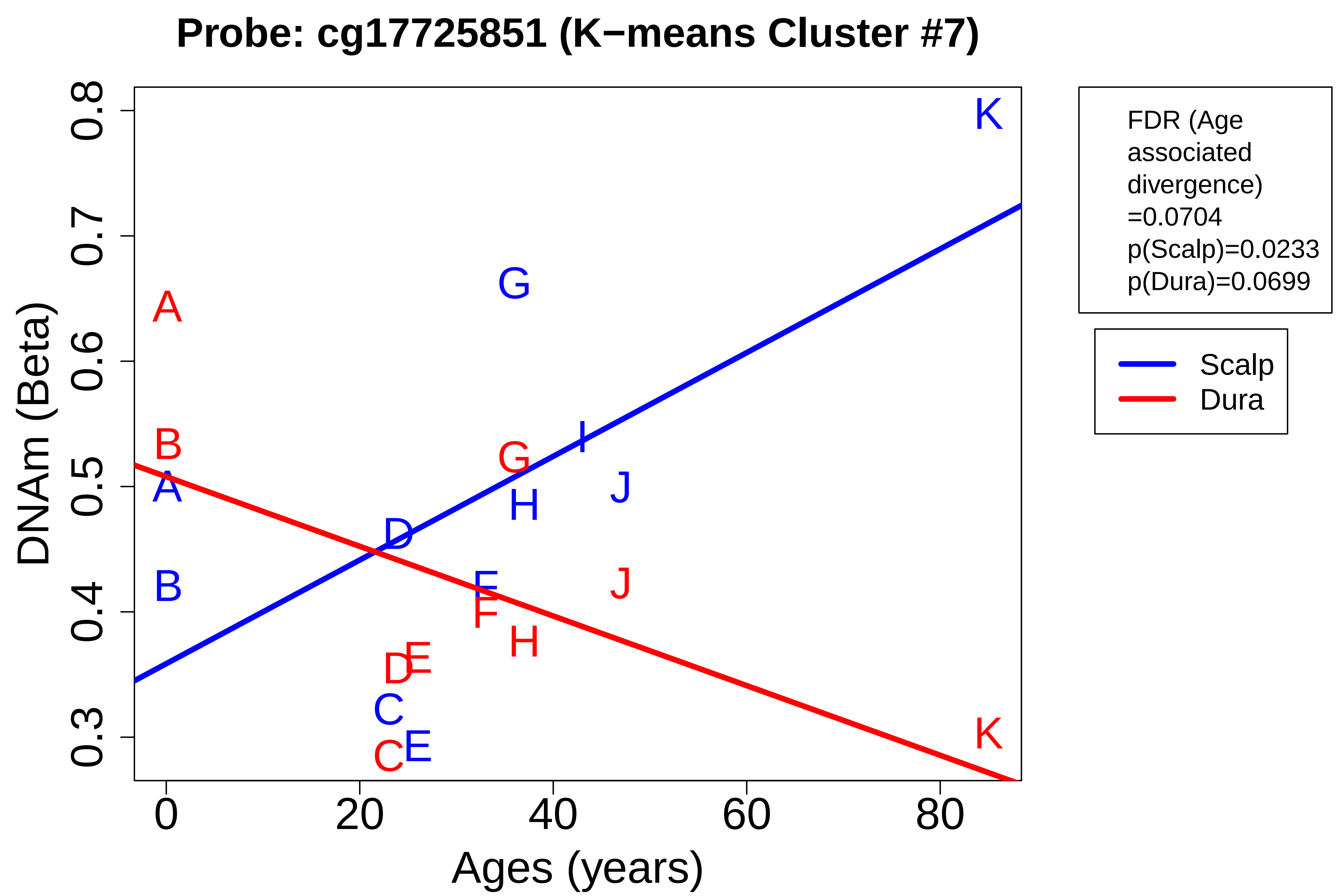
Probe: cg17725851 (K−means Cluster #7)
DNAm (Beta)
Ages (years)
0.3
0.4
0.5
0.6
0.7
0.8
0
20
40
60
80
FDR (Age associated divergence) =0.0704
p(Scalp)=0.0233
p(Dura)=0.0699
Scalp
Dura

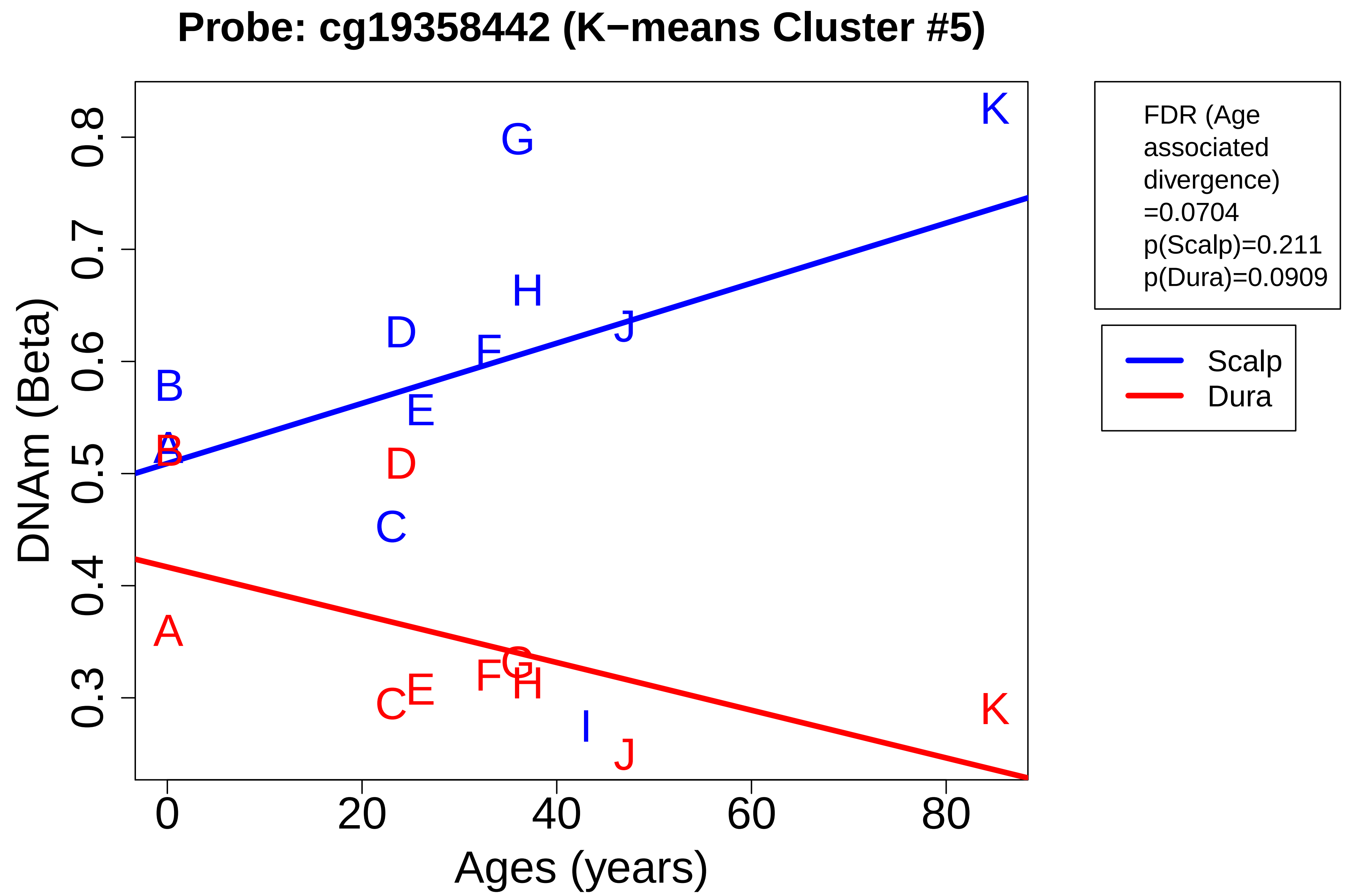

Probe: cg19358442 (K−means Cluster #5)
DNAm (Beta)
0.3
0.4
0.5
0.6
0.7
0.8
0
20
40
60
80
Ages (years)
FDR (Age associated divergence) =0.0704
p(Scalp)=0.211
p(Dura)=0.0909
Scalp
Dura

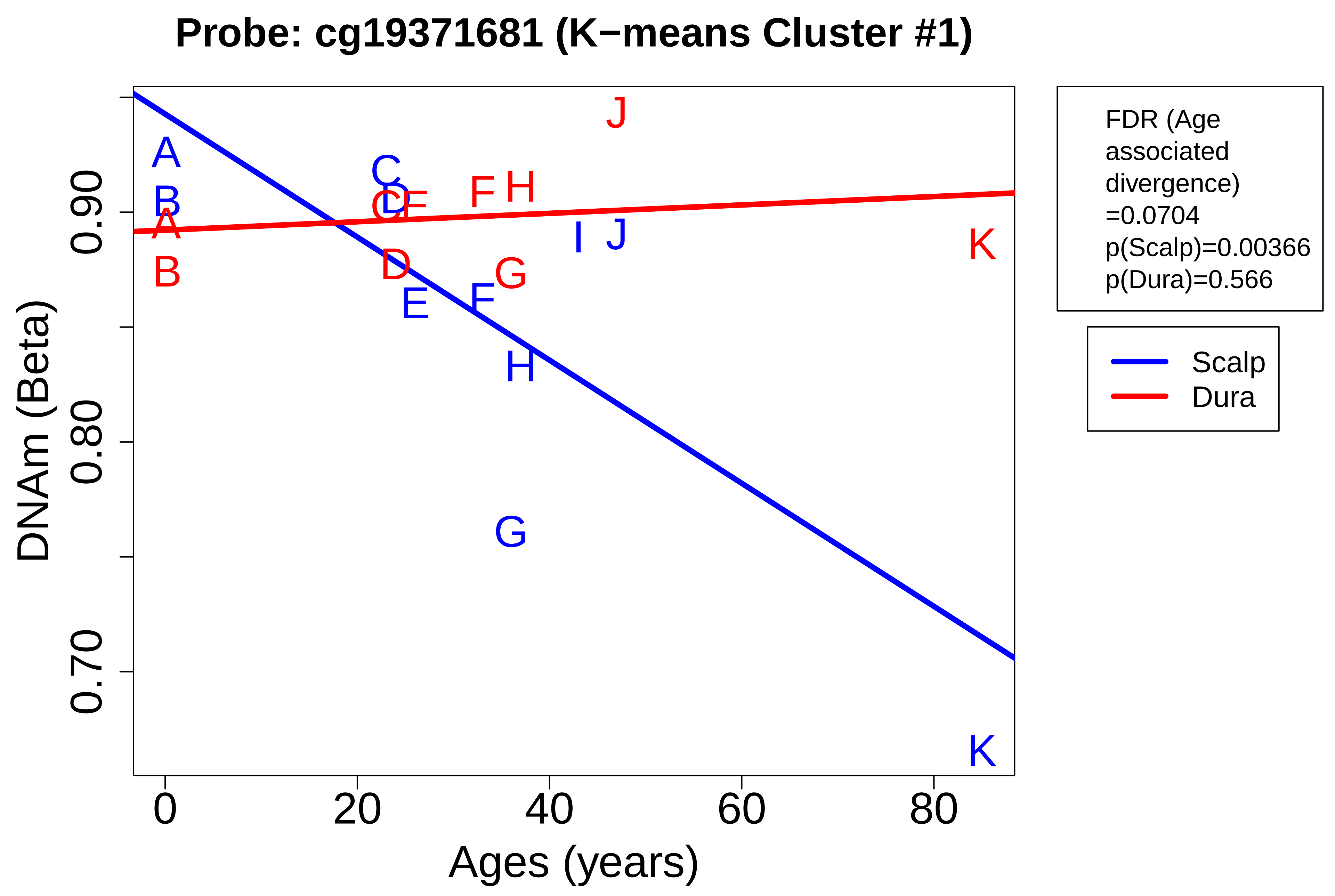

Probe: cg19371681 (K−means Cluster #1)
DNAm (Beta)
0.70
0.80
0.90
0
20
40
60
80
Ages (years)
A
B
C
D
E
F
G
H
I
J
K
FDR (Age associated divergence) =0.0704
p(Scalp)=0.00366
p(Dura)=0.566
Scalp
Dura

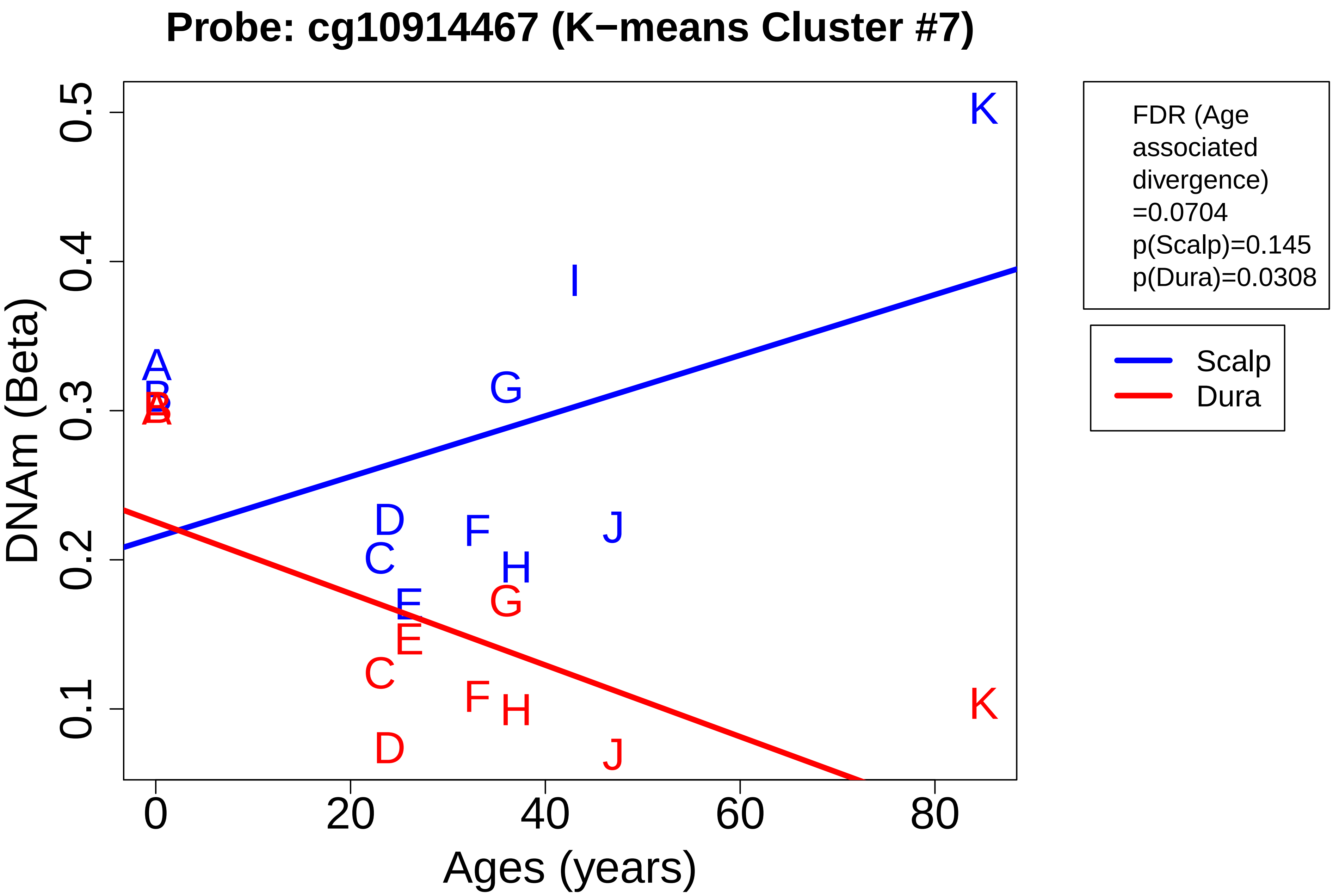

Probe: cg10914467 (K−means Cluster #7)
DNAm (Beta)
0.1
0.2
0.3
0.4
0.5
Ages (years)
0
20
40
60
80
FDR (Age associated divergence) =0.0704
p(Scalp)=0.145
p(Dura)=0.0308
Scalp
Dura
A
B
C
D
E
F
G
H
I
J
K

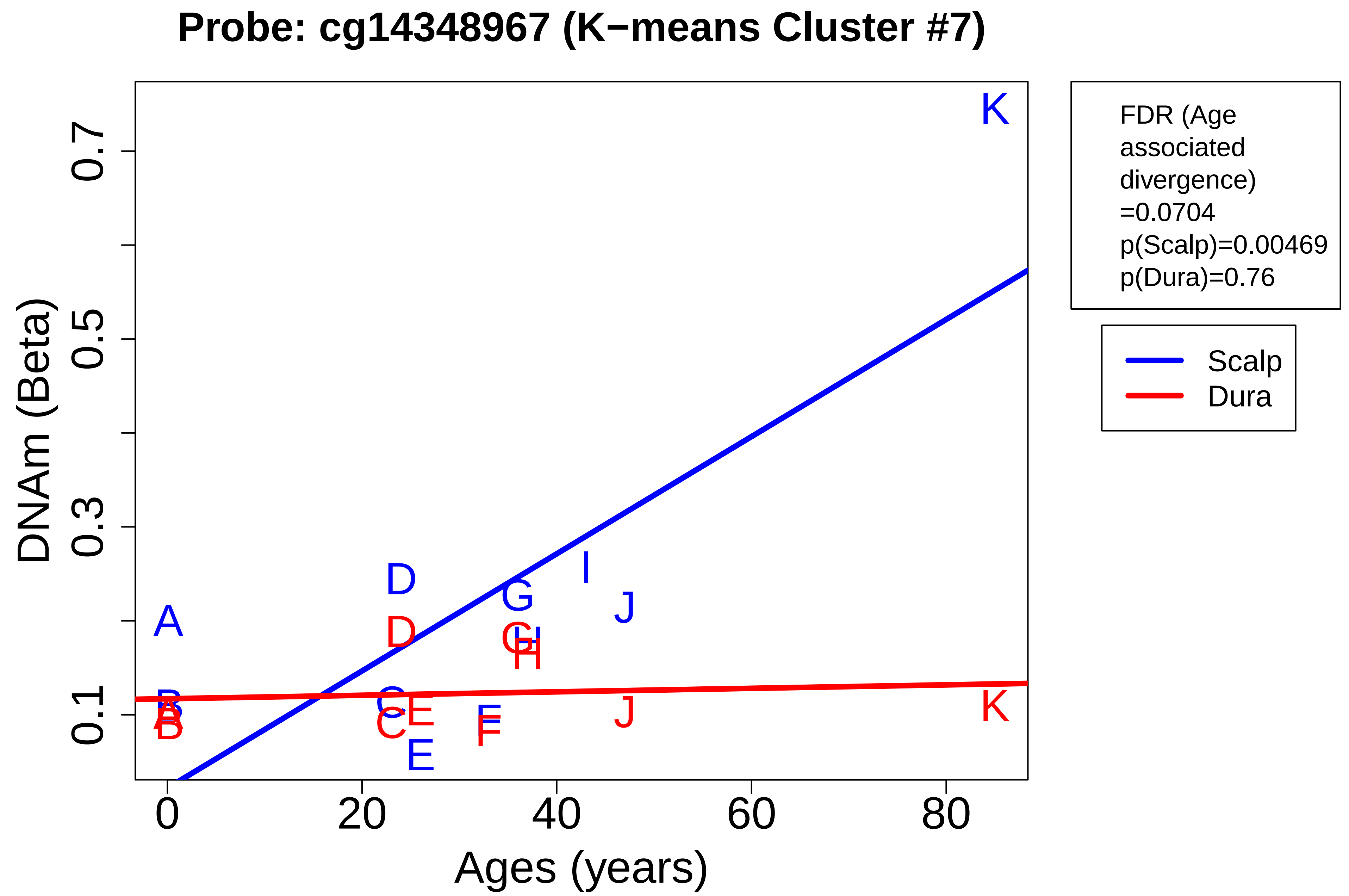

Probe: cg14348967 (K−means Cluster #7)
DNAm (Beta)
0.1
0.3
0.5
0.7
Ages (years)
0
20
40
60
80
A
B
C
D
E
F
G
H
I
J
K
FDR (Age associated divergence) =0.0704
p(Scalp)=0.00469
p(Dura)=0.76
Scalp
Dura

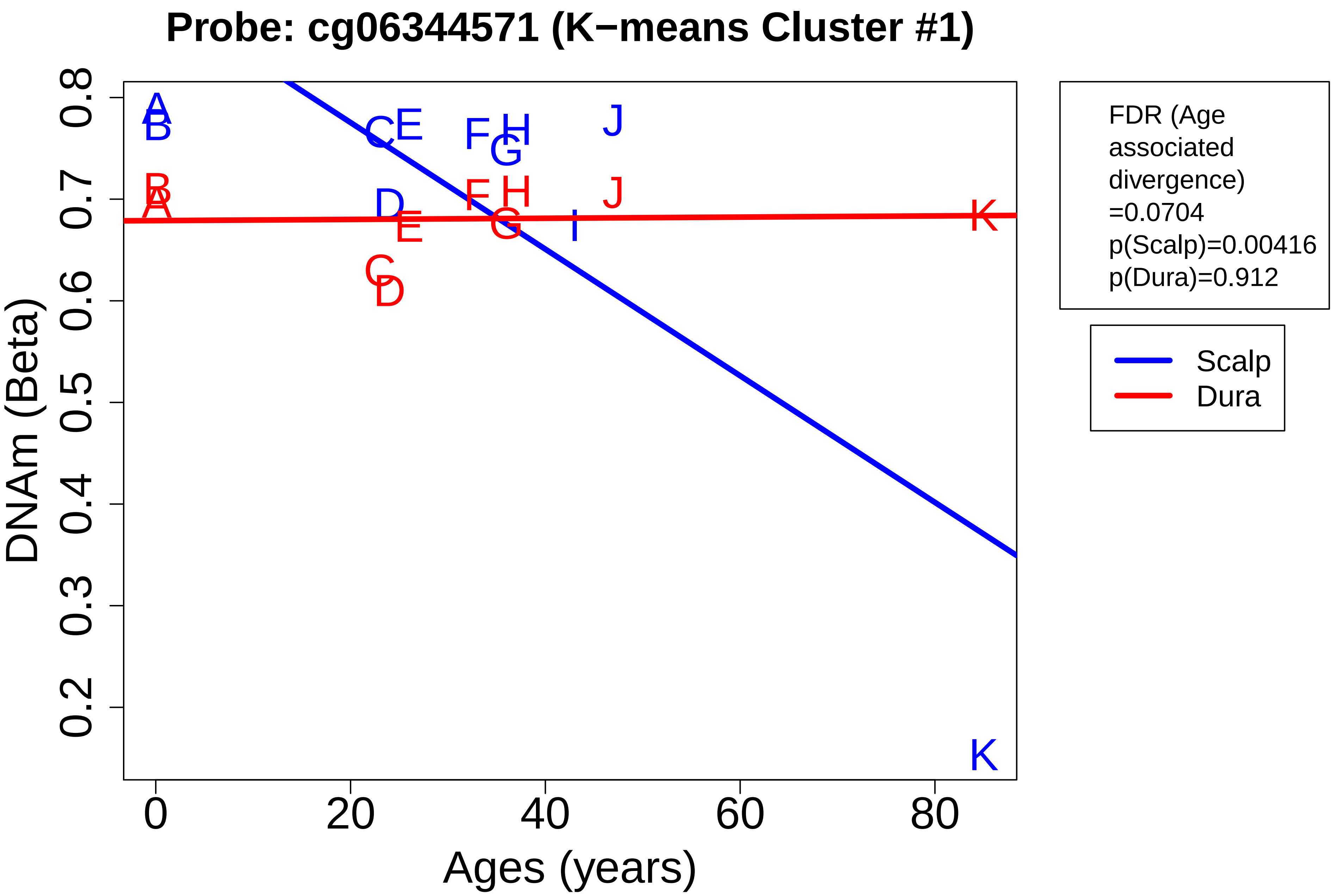

Probe: cg06344571 (K−means Cluster #1)
DNAm (Beta)
0.2
0.3
0.4
0.5
0.6
0.7
0.8
Ages (years)
0
20
40
60
80
A
B
C
D
E
F
G
H
I
J
K
FDR (Age associated divergence) =0.0704
p(Scalp)=0.00416
p(Dura)=0.912
Scalp
Dura

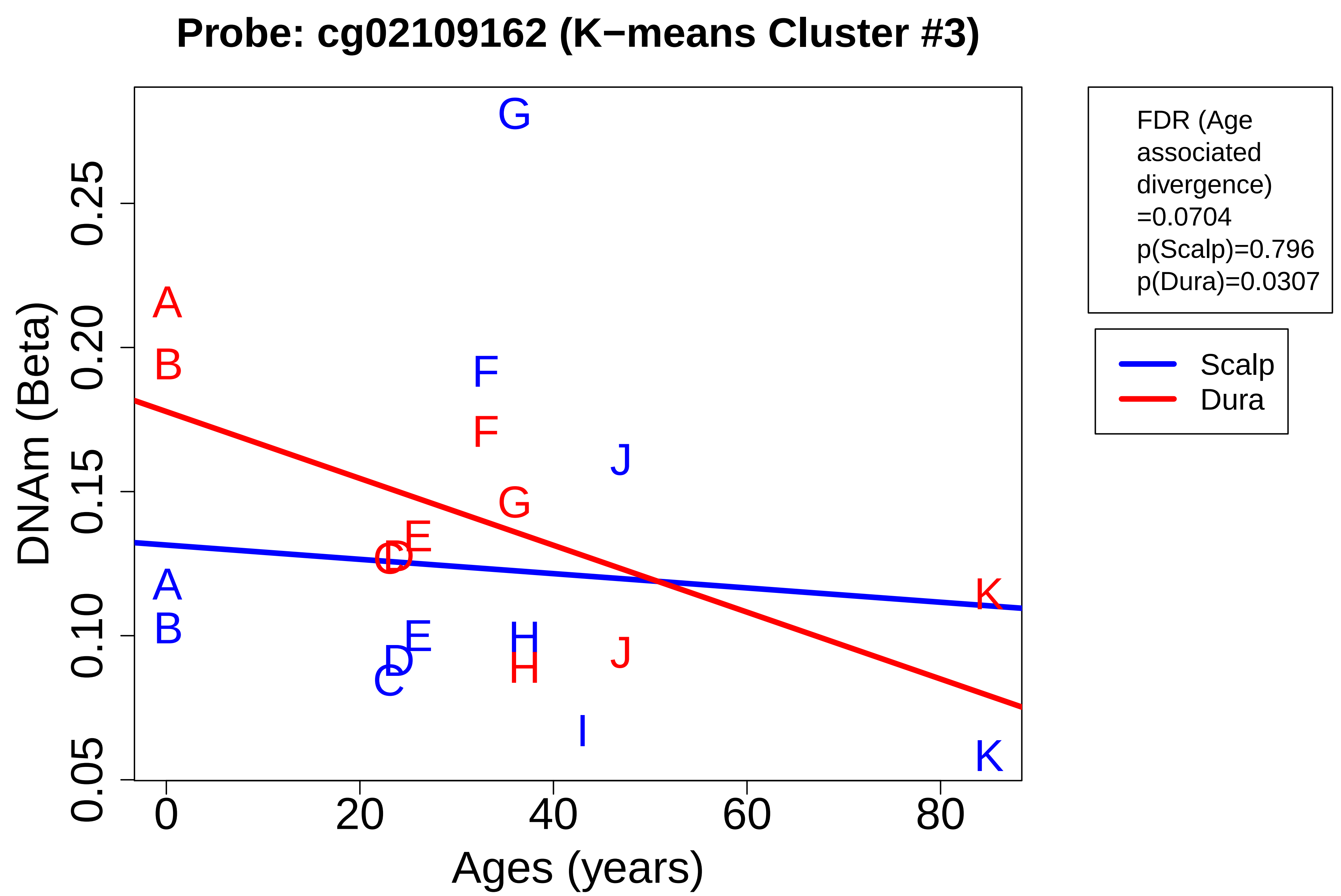
Probe: cg02109162 (K−means Cluster #3)
DNAm (Beta)
0.05
0.10
0.15
0.20
0.25
Ages (years)
0
20
40
60
80
G
A
B
F
F
J
G
E
C
D
A
B
E
D
C
H
H
J
I
K
K
FDR (Age associated divergence) =0.0704
p(Scalp)=0.796
p(Dura)=0.0307
Scalp
Dura

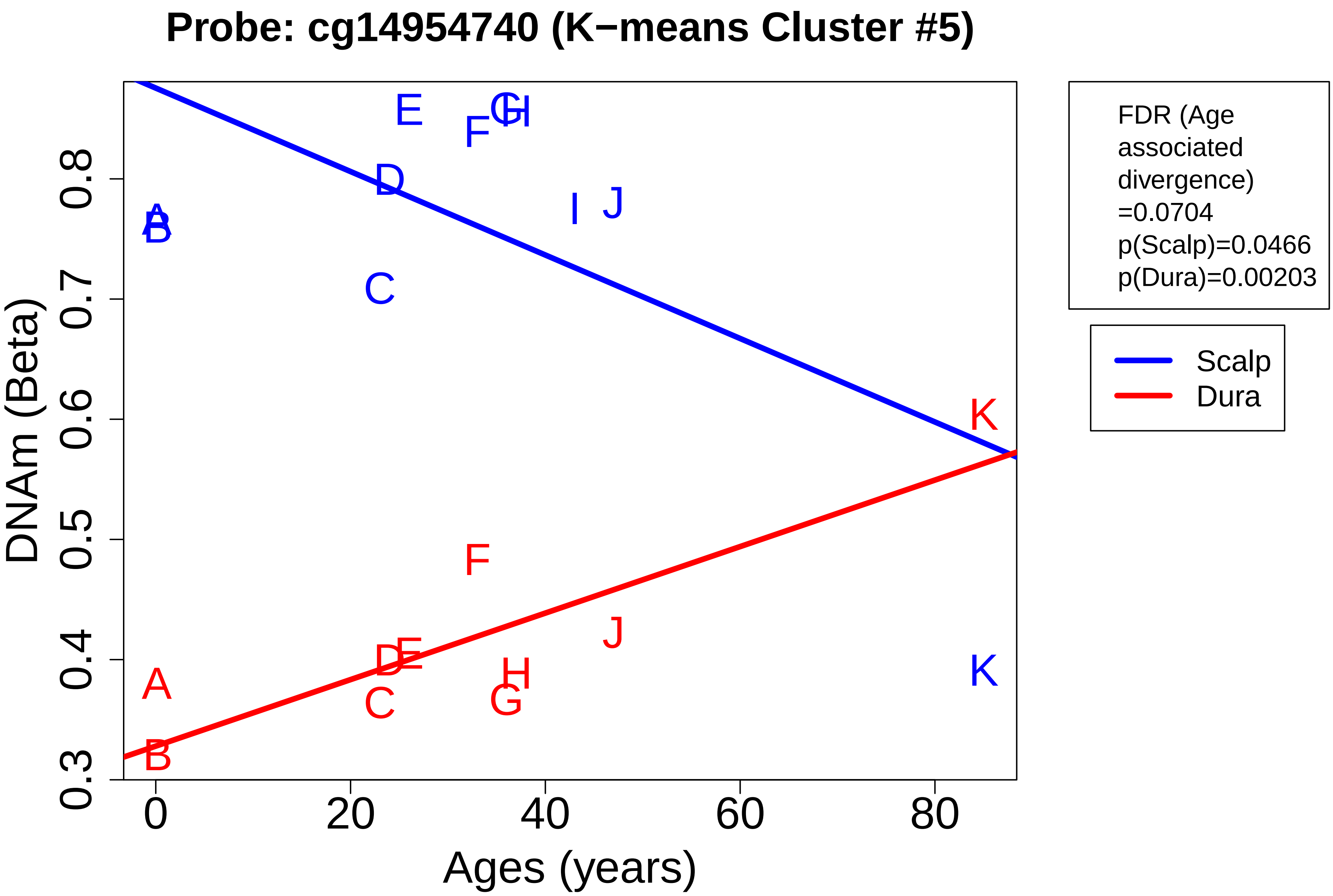

Probe: cg14954740 (K−means Cluster #5)
DNAm (Beta)
0.3
0.4
0.5
0.6
0.7
0.8
0
20
40
60
80
Ages (years)
A
B
C
D
E
F
G
H
I
J
K
FDR (Age associated divergence) =0.0704
p(Scalp)=0.0466
p(Dura)=0.00203
Scalp
Dura

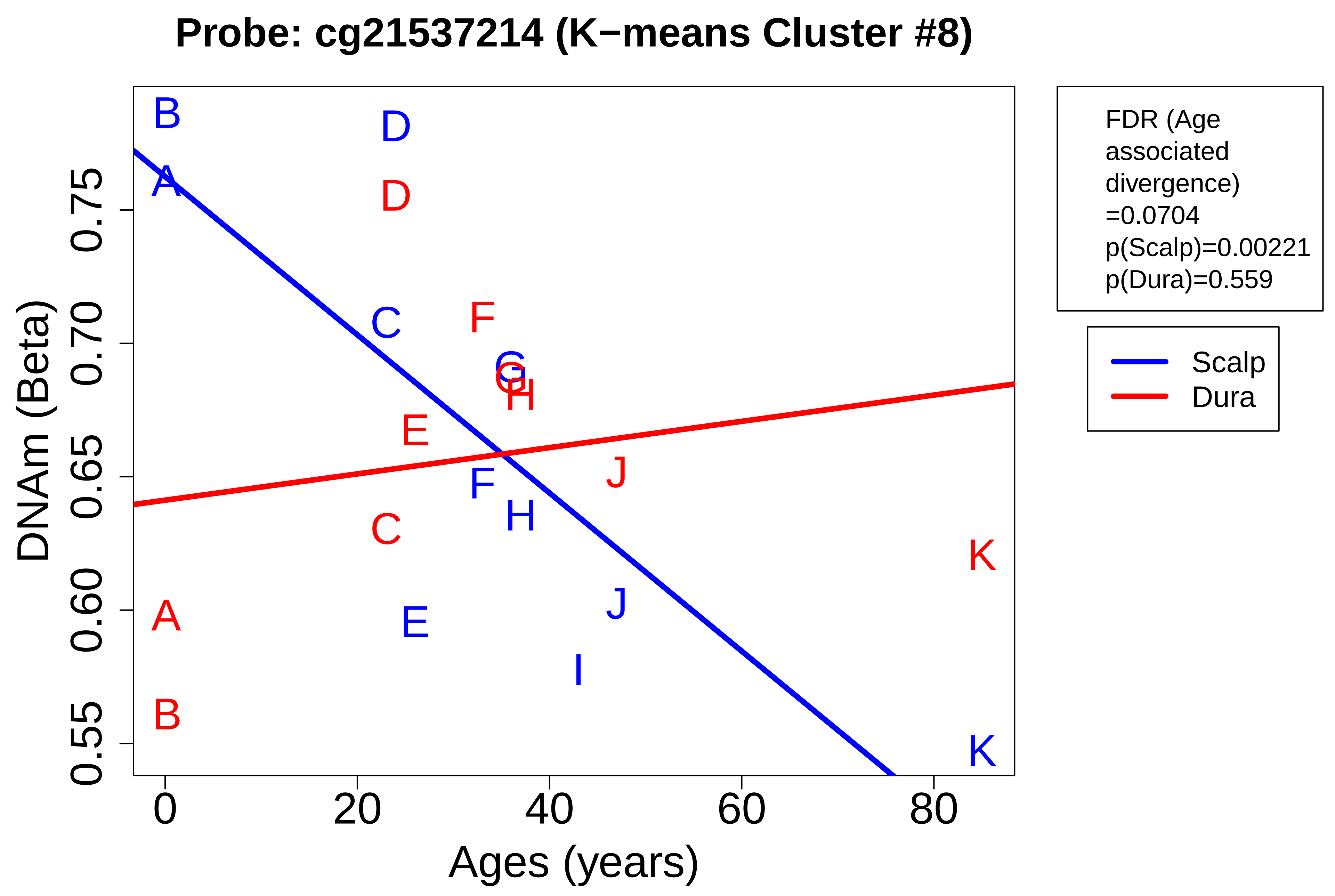

Probe: cg21537214 (K−means Cluster #8)
DNAm (Beta)
0.55
0.60
0.65
0.70
0.75
Ages (years)
0
20
40
60
80
FDR (Age associated divergence) =0.0704
p(Scalp)=0.00221
p(Dura)=0.559
Scalp
Dura

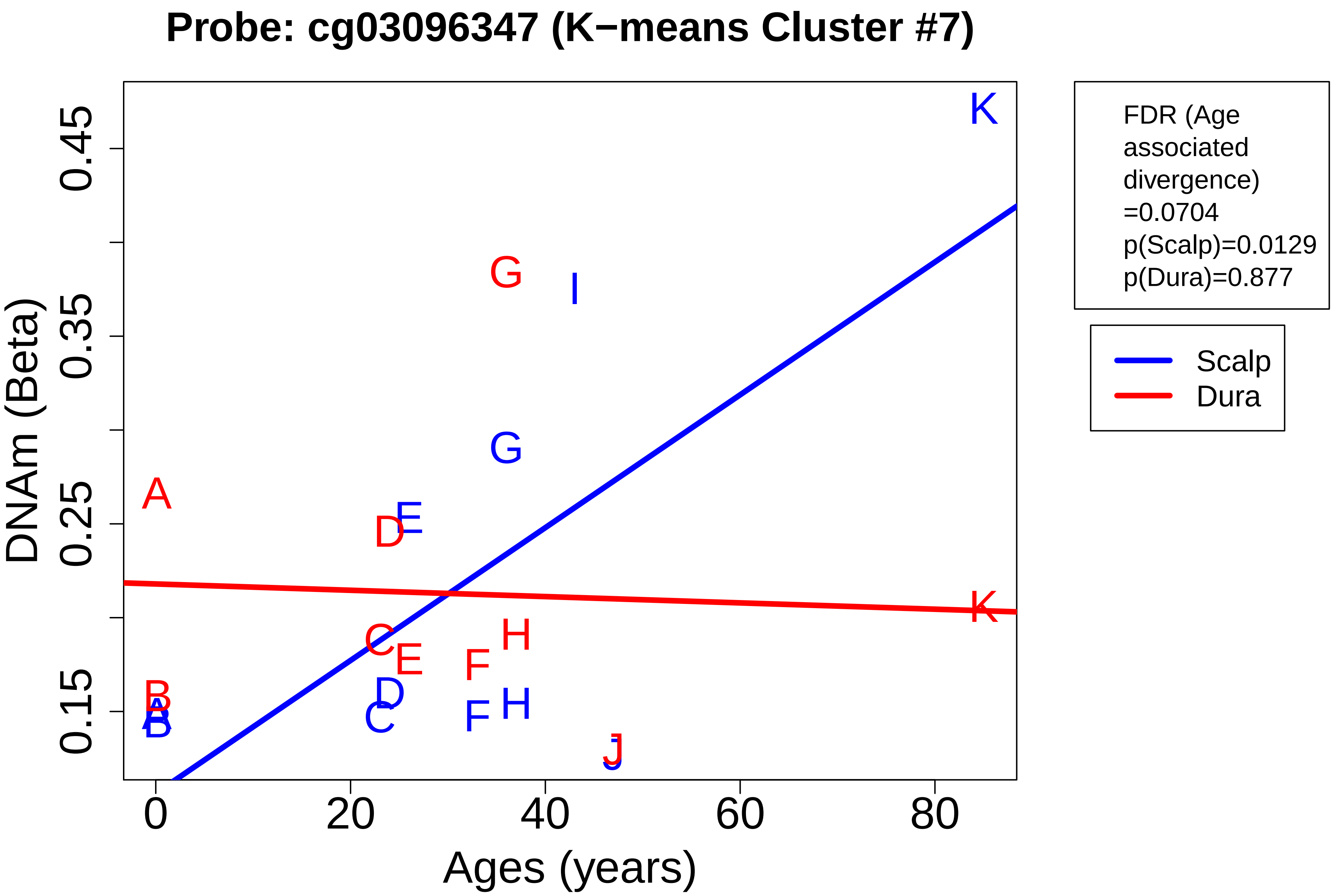

Probe: cg03096347 (K−means Cluster #7)
DNAm (Beta)
0.15
0.25
0.35
0.45
Ages (years)
0
20
40
60
80
FDR (Age associated divergence) =0.0704
p(Scalp)=0.0129
p(Dura)=0.877
Scalp
Dura

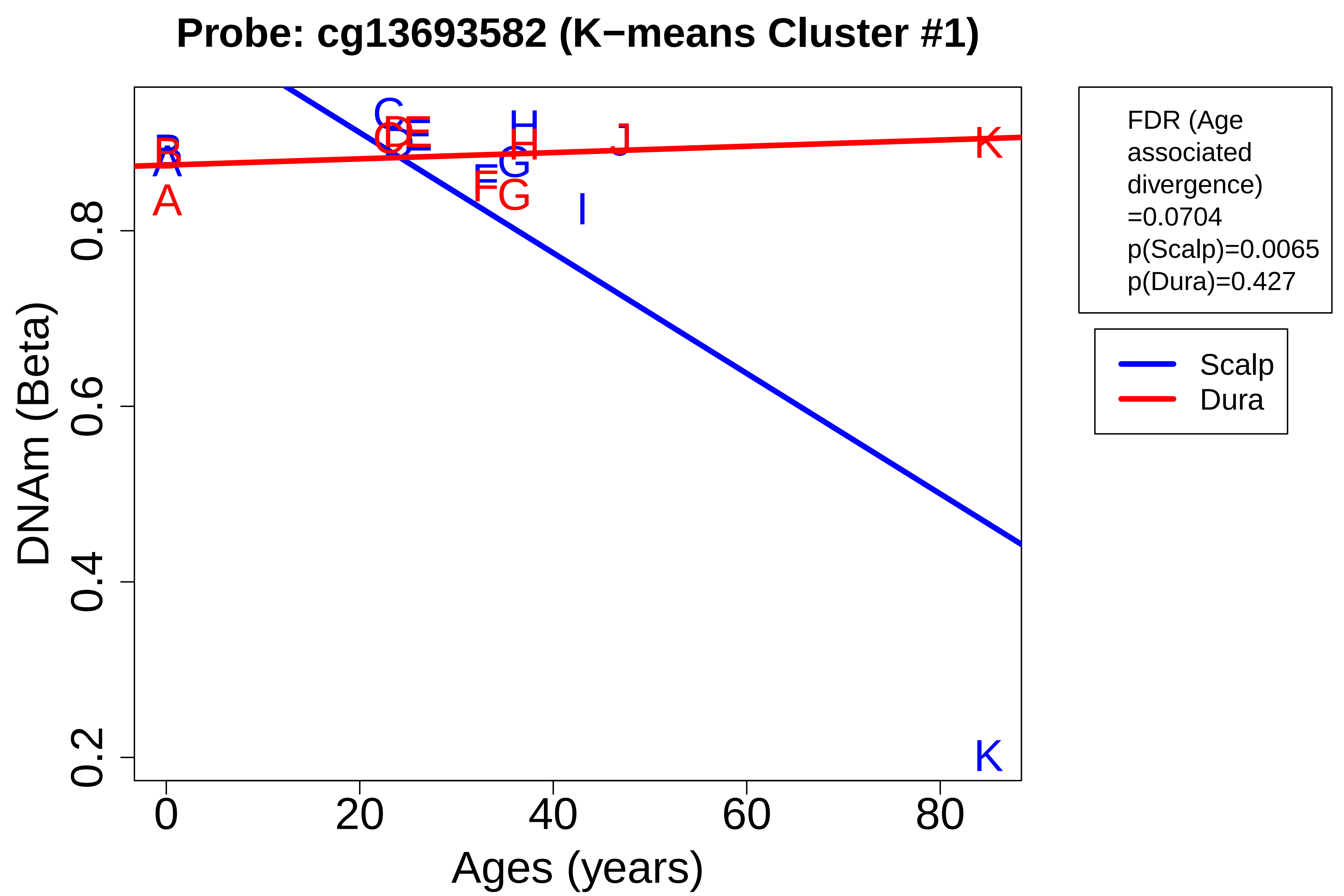

Probe: cg13693582 (K−means Cluster #1)
DNAm (Beta)
0.2
0.4
0.6
0.8
0
20
40
60
80
Ages (years)
FDR (Age associated divergence) =0.0704
p(Scalp)=0.0065
p(Dura)=0.427
Scalp
Dura

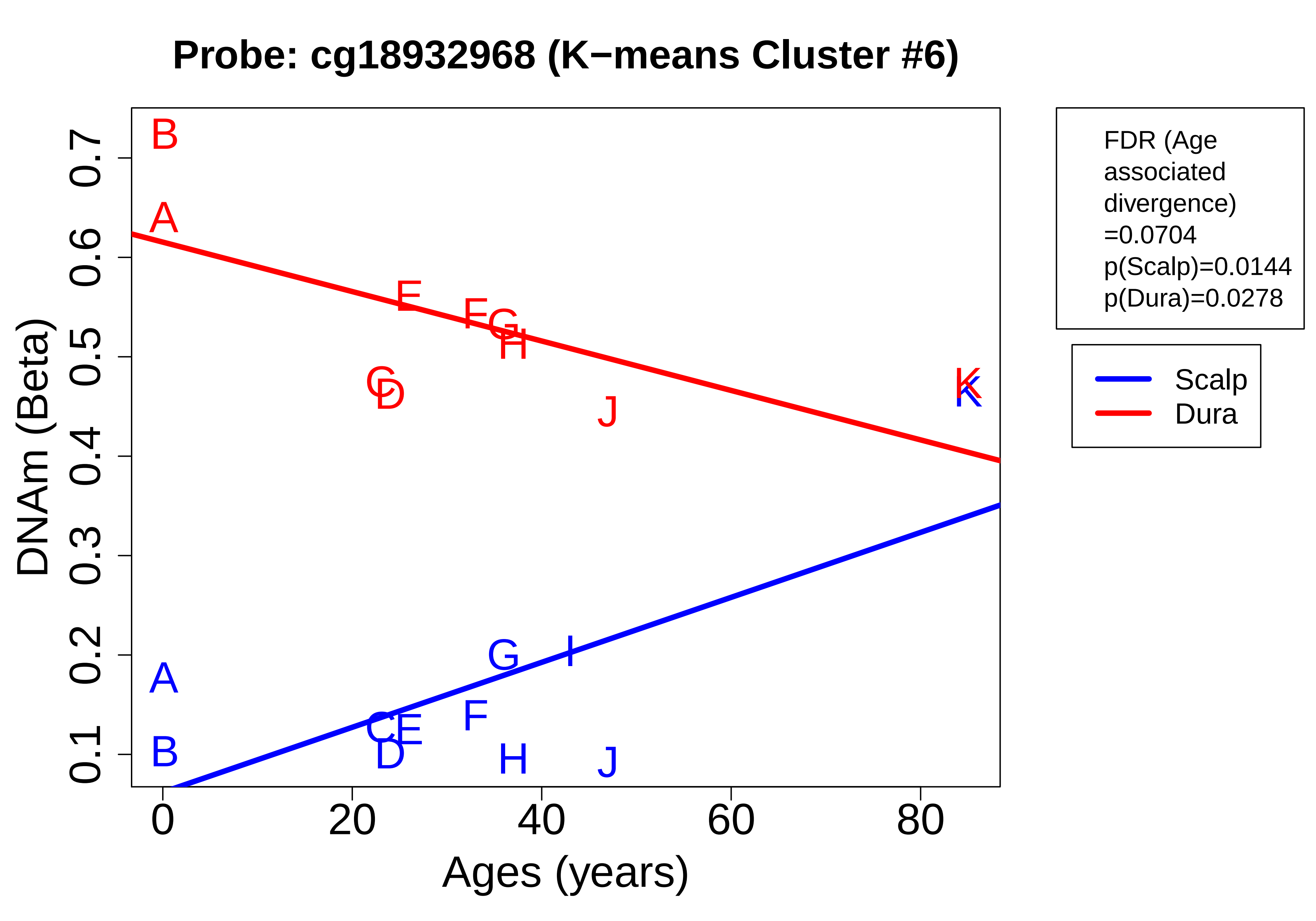

Probe: cg18932968 (K−means Cluster #6)
DNAm (Beta)
0.1
0.2
0.3
0.4
0.5
0.6
0.7
0
20
40
60
80
Ages (years)
FDR (Age associated divergence) =0.0704
p(Scalp)=0.0144
p(Dura)=0.0278
Scalp
Dura

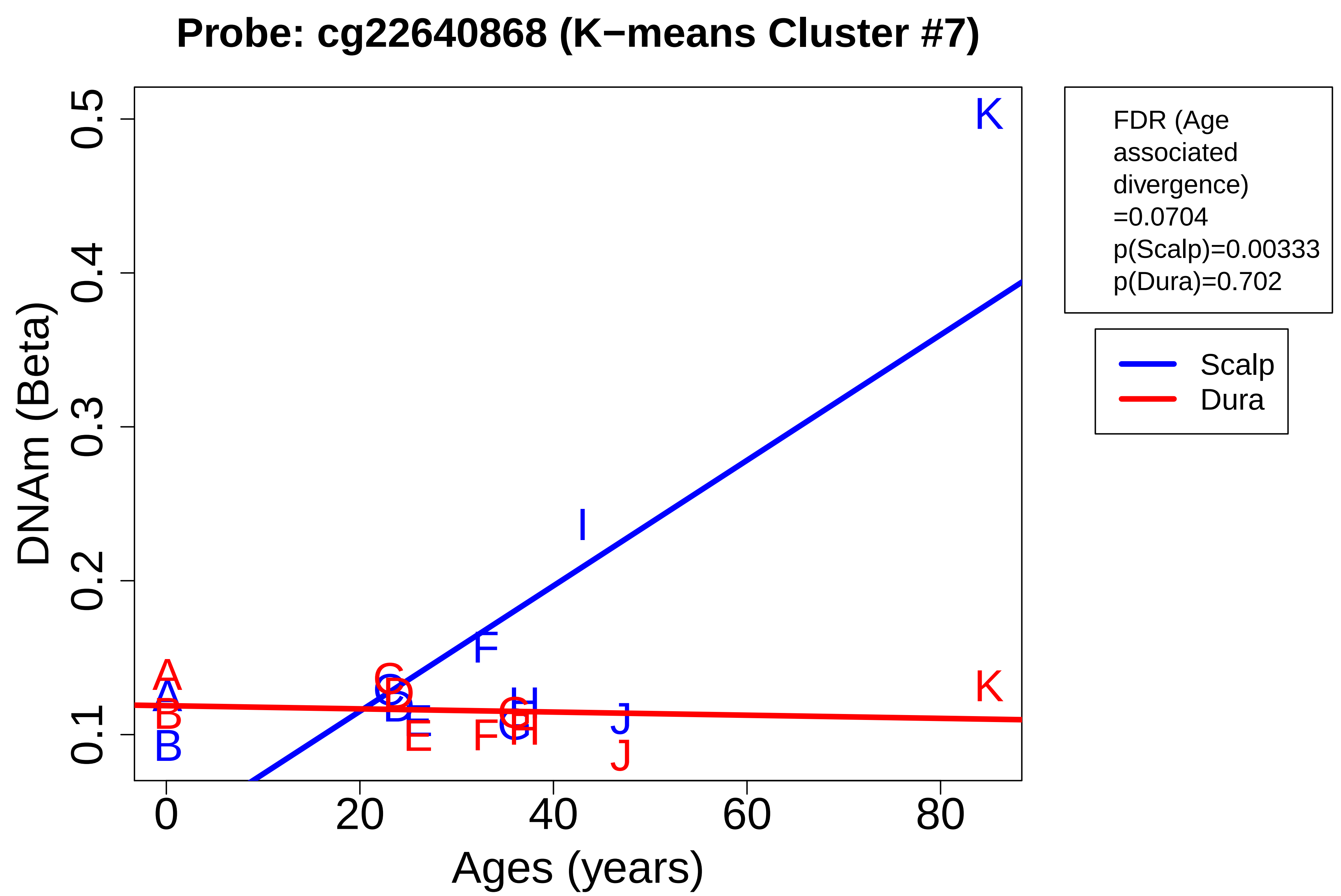

Probe: cg22640868 (K−means Cluster #7)
DNAm (Beta)
0.1
0.2
0.3
0.4
0.5
Ages (years)
0
20
40
60
80
A
B
C
D
E
F
G
H
I
J
K
FDR (Age associated divergence) =0.0704
p(Scalp)=0.00333
p(Dura)=0.702
Scalp
Dura

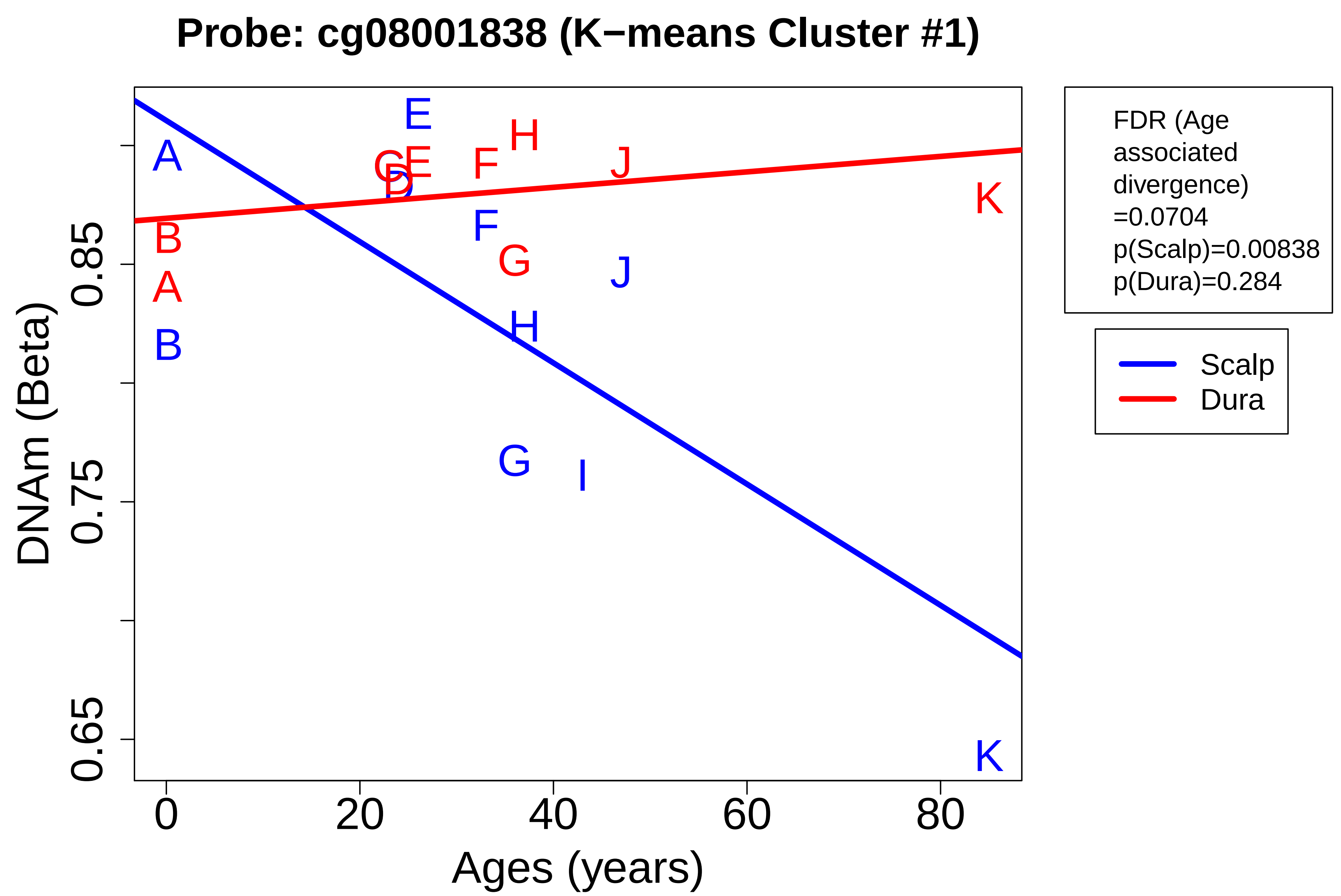

Probe: cg08001838 (K−means Cluster #1)
DNAm (Beta)
0.65
0.75
0.85
0
20
40
60
80
Ages (years)
FDR (Age associated divergence) =0.0704
p(Scalp)=0.00838
p(Dura)=0.284
Scalp
Dura

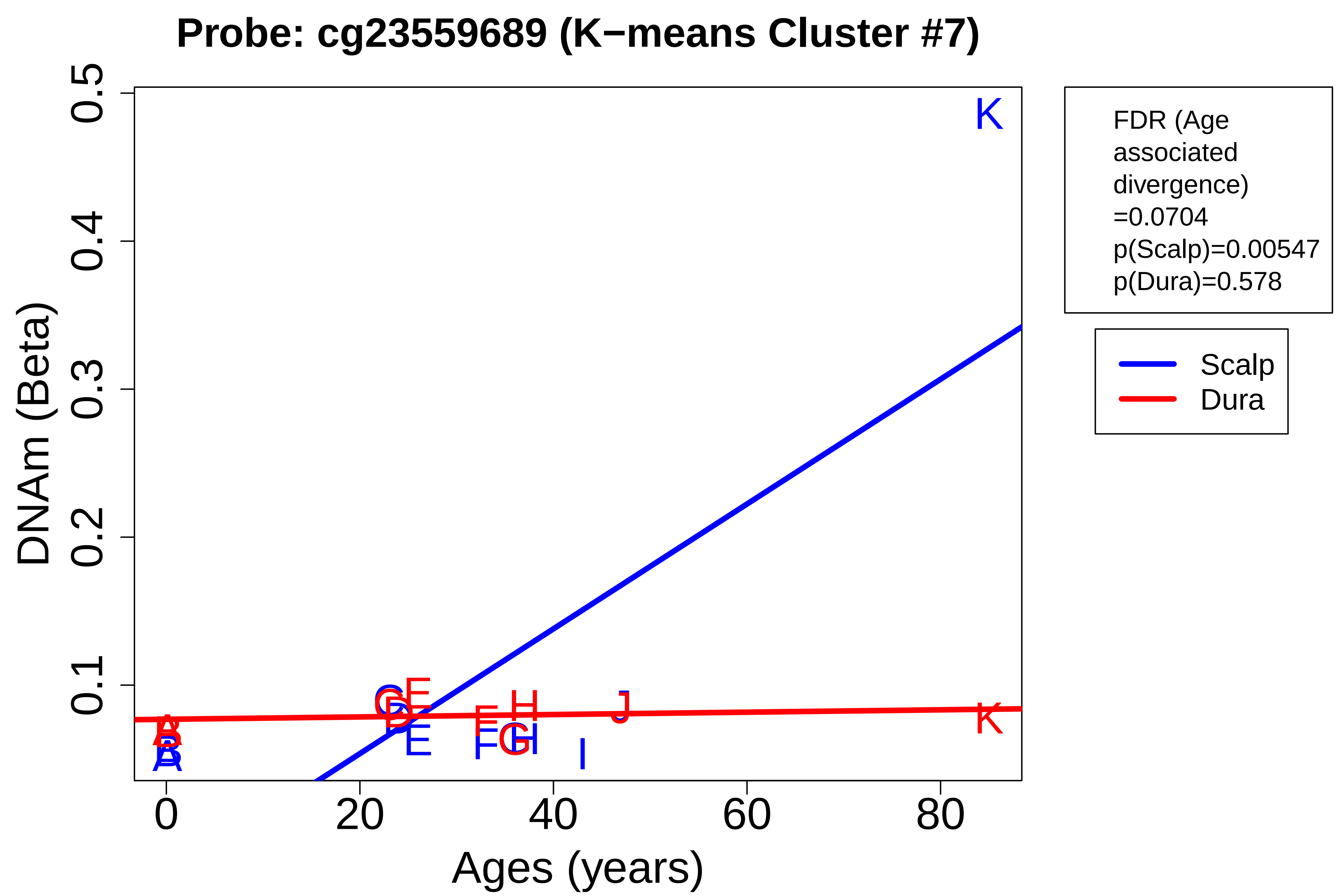
Probe: cg23559689 (K−means Cluster #7)
DNAm (Beta)
Ages (years)
0.1
0.2
0.3
0.4
0.5
0
20
40
60
80
FDR (Age associated divergence) =0.0704
p(Scalp)=0.00547
p(Dura)=0.578
Scalp
Dura

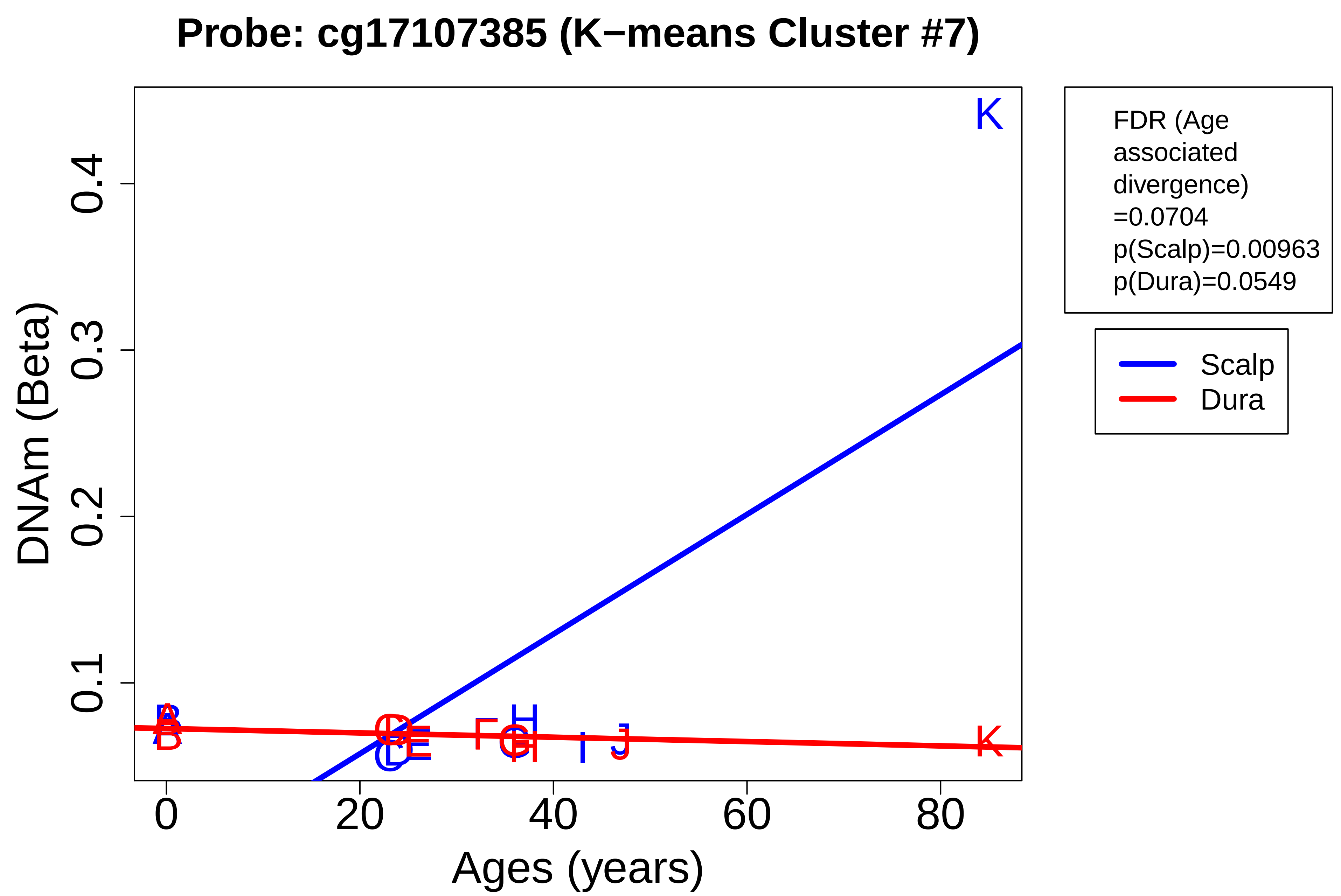

Probe: cg17107385 (K−means Cluster #7)
DNAm (Beta)
0.1
0.2
0.3
0.4
Ages (years)
0
20
40
60
80
FDR (Age associated divergence) =0.0704
p(Scalp)=0.00963
p(Dura)=0.0549
Scalp
Dura

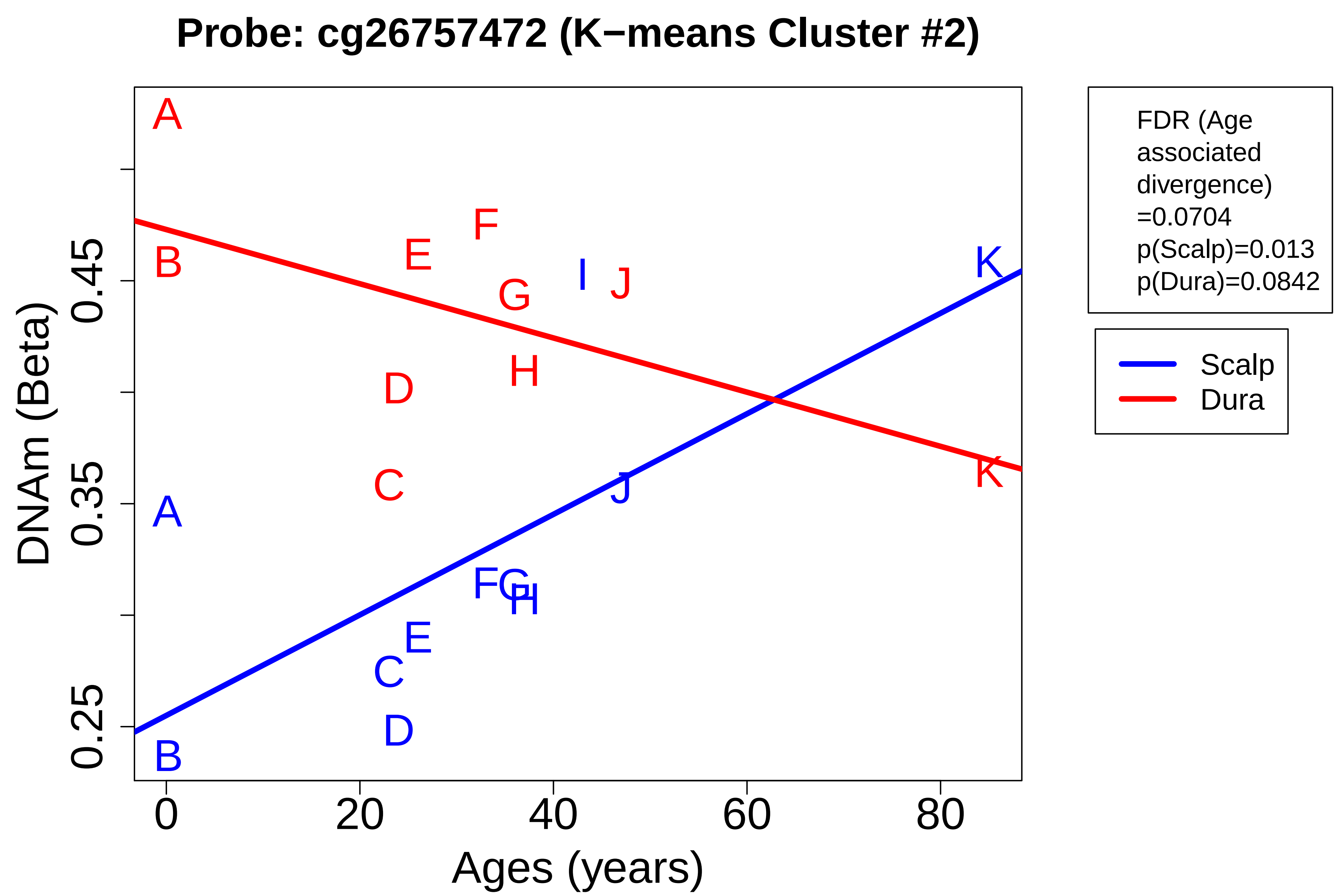
Probe: cg26757472 (K−means Cluster #2)
DNAm (Beta)
0.25
0.35
0.45
0
20
40
60
80
Ages (years)
A
B
C
D
E
F
G
H
I
J
K
FDR (Age associated divergence) =0.0704
p(Scalp)=0.013
p(Dura)=0.0842
Scalp
Dura

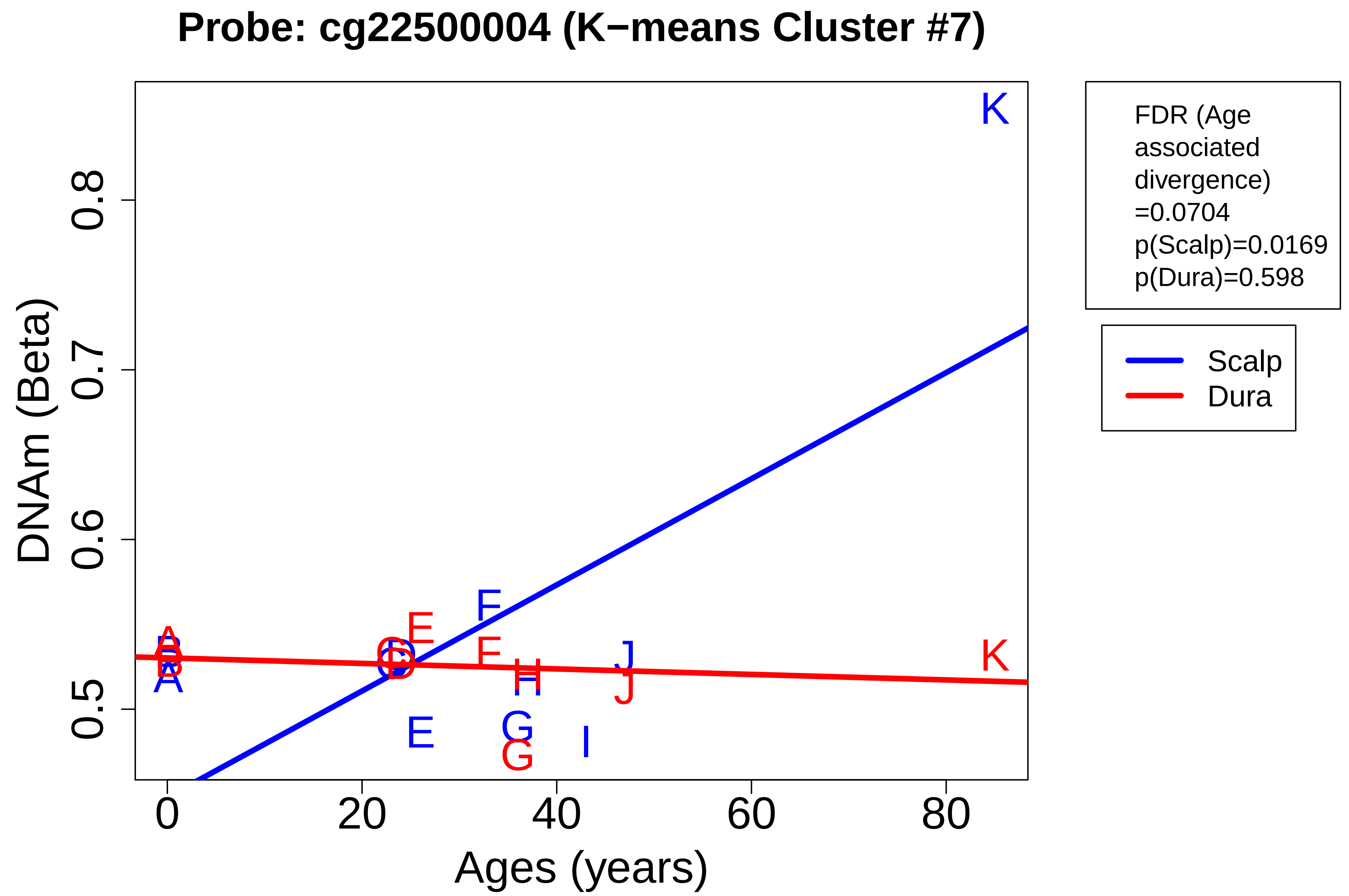

Probe: cg22500004 (K−means Cluster #7)
DNAm (Beta)
0.5
0.6
0.7
0.8
0
20
40
60
80
Ages (years)
FDR (Age associated divergence) =0.0704
p(Scalp)=0.0169
p(Dura)=0.598
Scalp
Dura

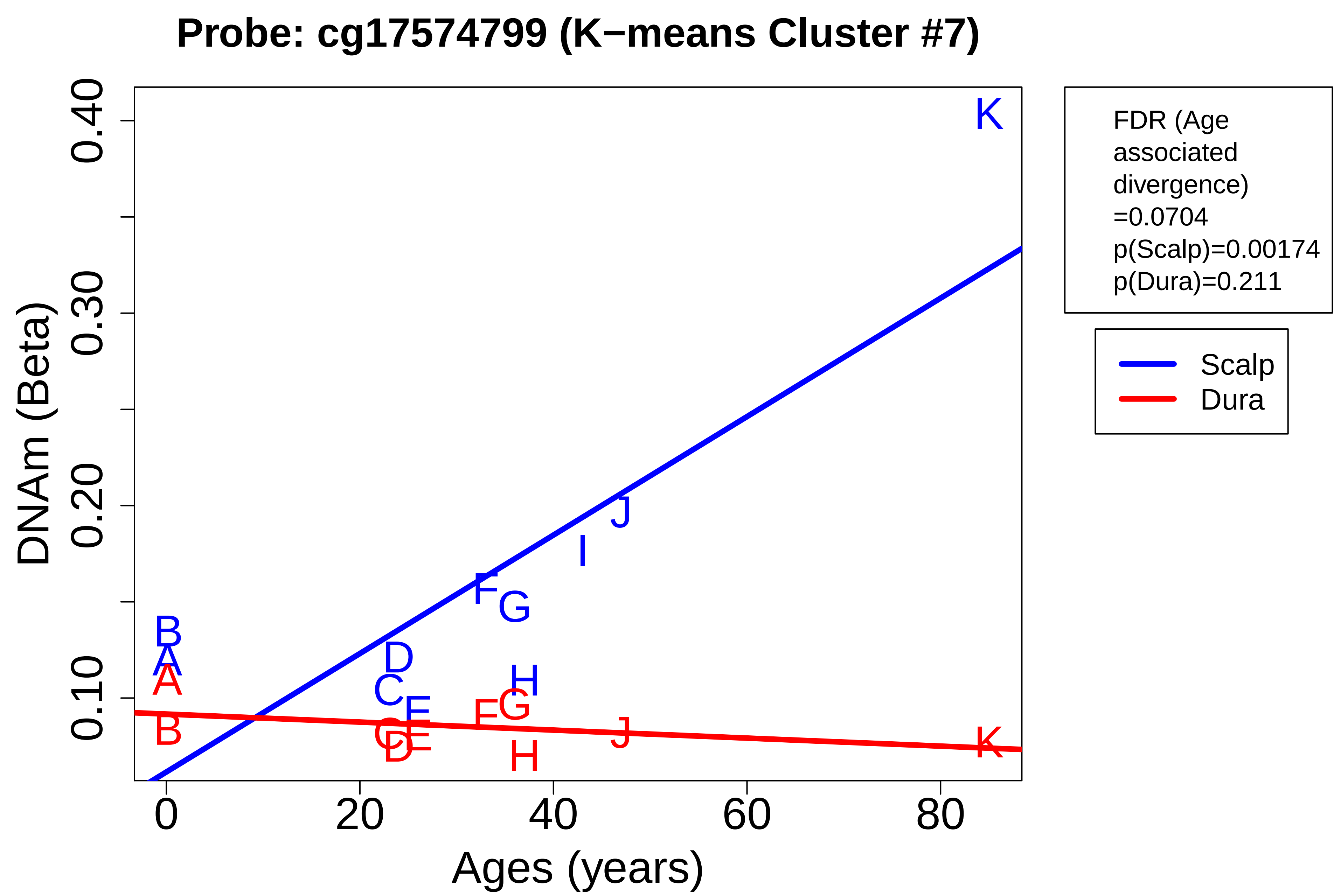

Probe: cg17574799 (K−means Cluster #7)
DNAm (Beta)
0.10
0.20
0.30
0.40
0
20
40
60
80
Ages (years)
FDR (Age associated divergence) =0.0704
p(Scalp)=0.00174
p(Dura)=0.211
Scalp
Dura

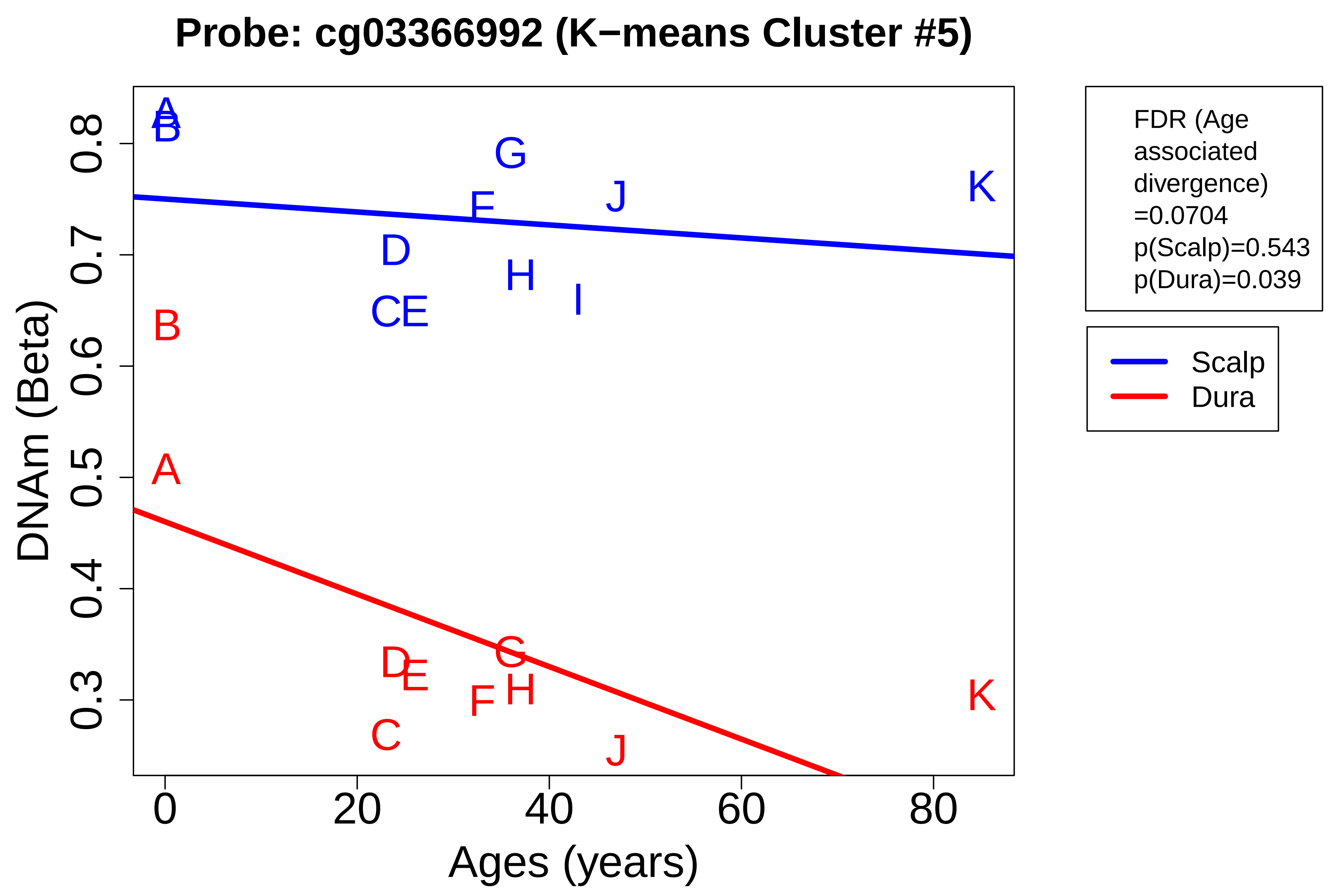

Probe: cg03366992 (K−means Cluster #5)
DNAm (Beta)
0.3
0.4
0.5
0.6
0.7
0.8
0
20
40
60
80
Ages (years)
FDR (Age associated divergence) =0.0704
p(Scalp)=0.543
p(Dura)=0.039
Scalp
Dura

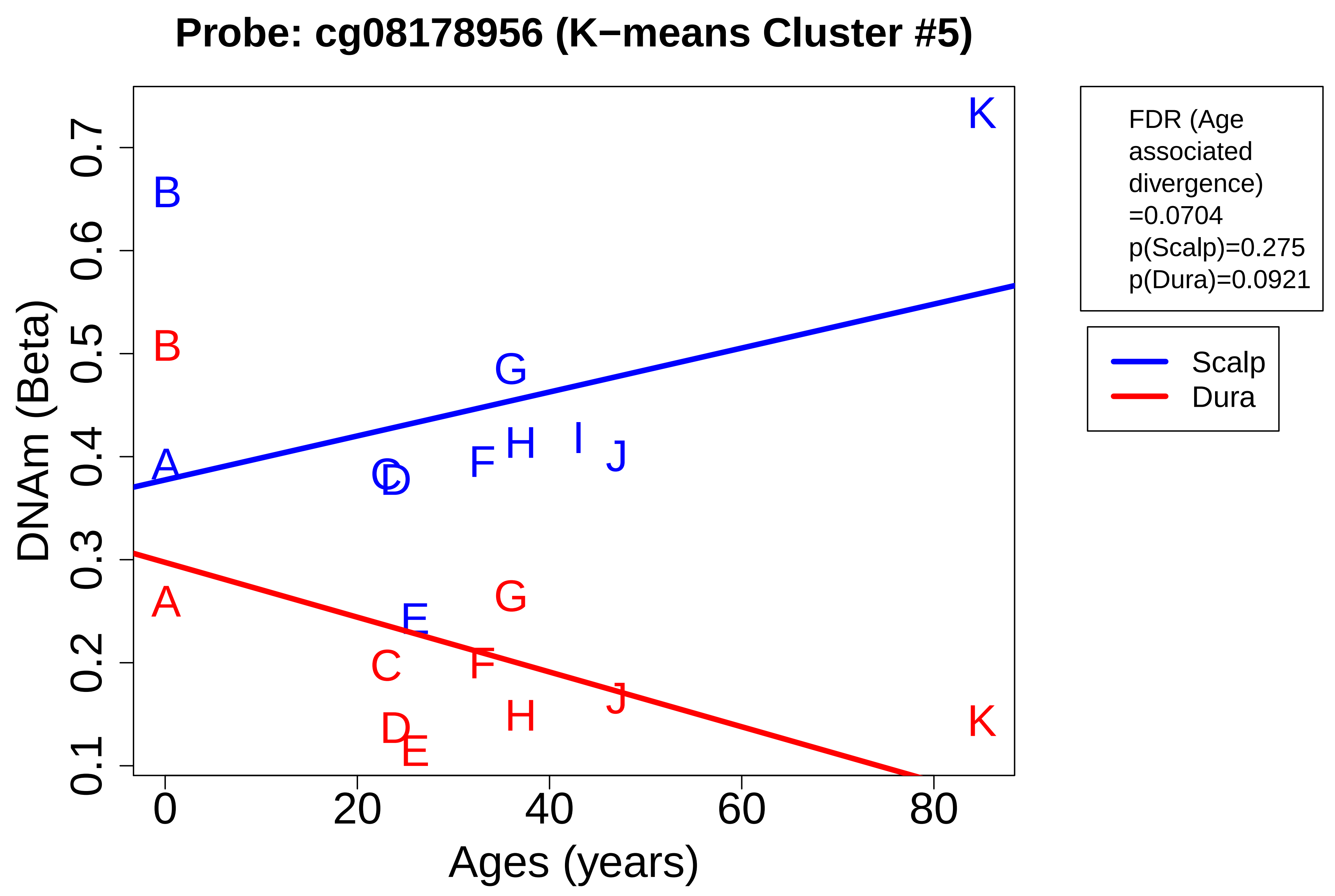

Probe: cg08178956 (K−means Cluster #5)
FDR (Age associated divergence) =0.0704
p(Scalp)=0.275
p(Dura)=0.0921
Scalp
Dura
DNAm (Beta)
0.1
0.2
0.3
0.4
0.5
0.6
0.7
0
20
40
60
80
Ages (years)

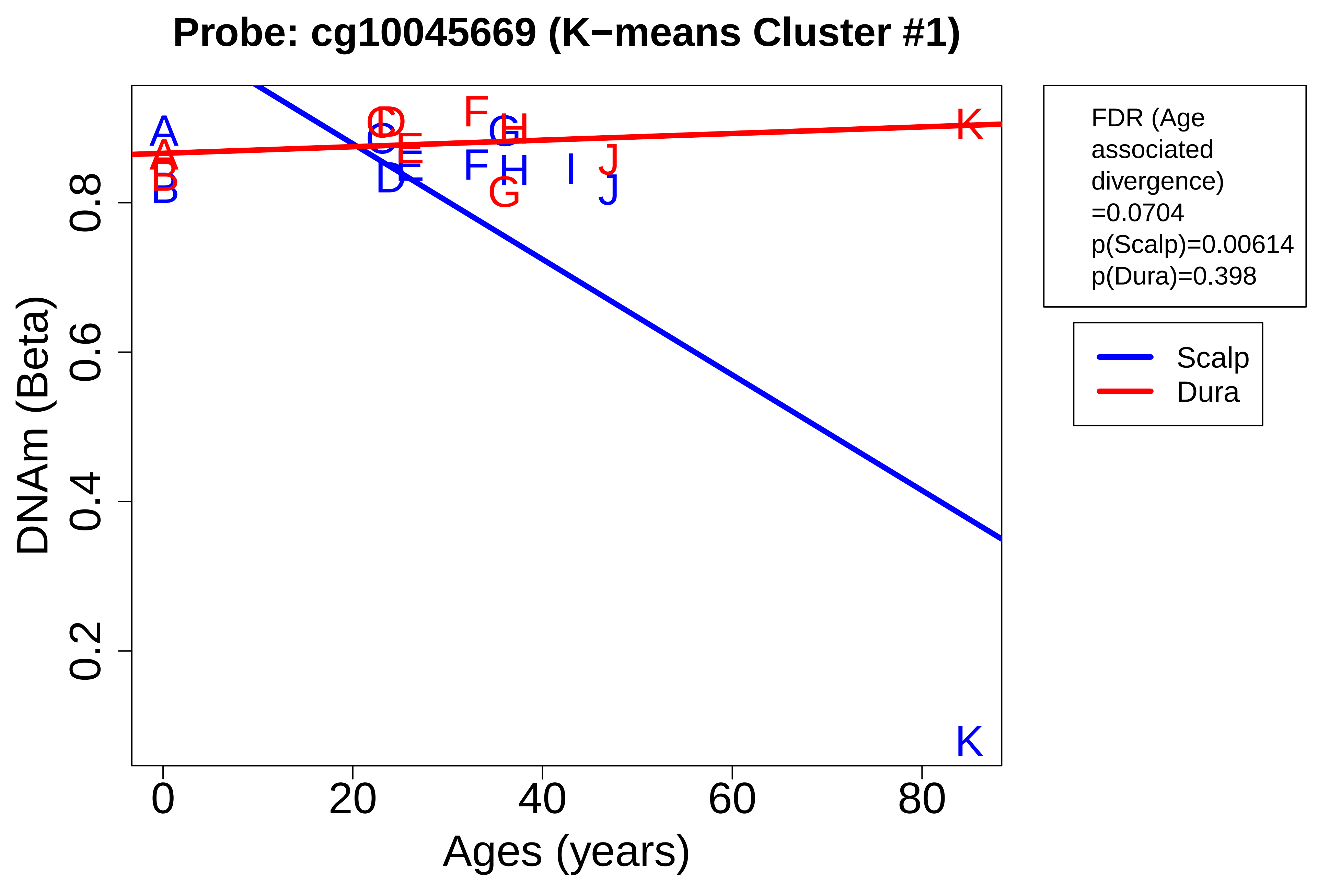
Probe: cg10045669 (K−means Cluster #1)
DNAm (Beta)
0.2
0.4
0.6
0.8
0
20
40
60
80
Ages (years)
FDR (Age associated divergence) =0.0704
p(Scalp)=0.00614
p(Dura)=0.398
Scalp
Dura

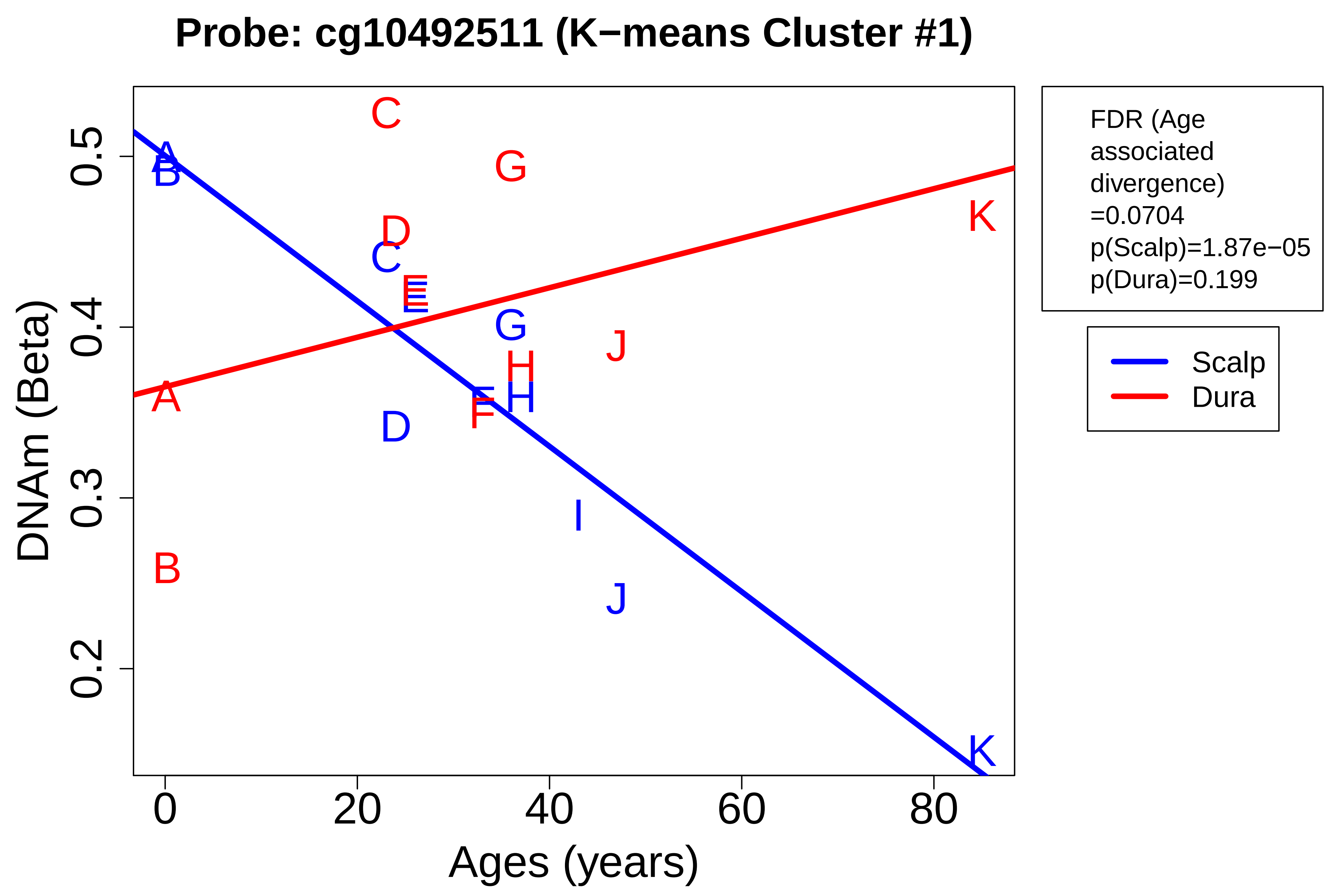
Probe: cg10492511 (K−means Cluster #1)
DNAm (Beta)
0.2
0.3
0.4
0.5
0
20
40
60
80
Ages (years)
FDR (Age associated divergence) =0.0704
p(Scalp)=1.87e−05
p(Dura)=0.199
Scalp
Dura
A
B
C
D
E
F
G
H
I
J
K

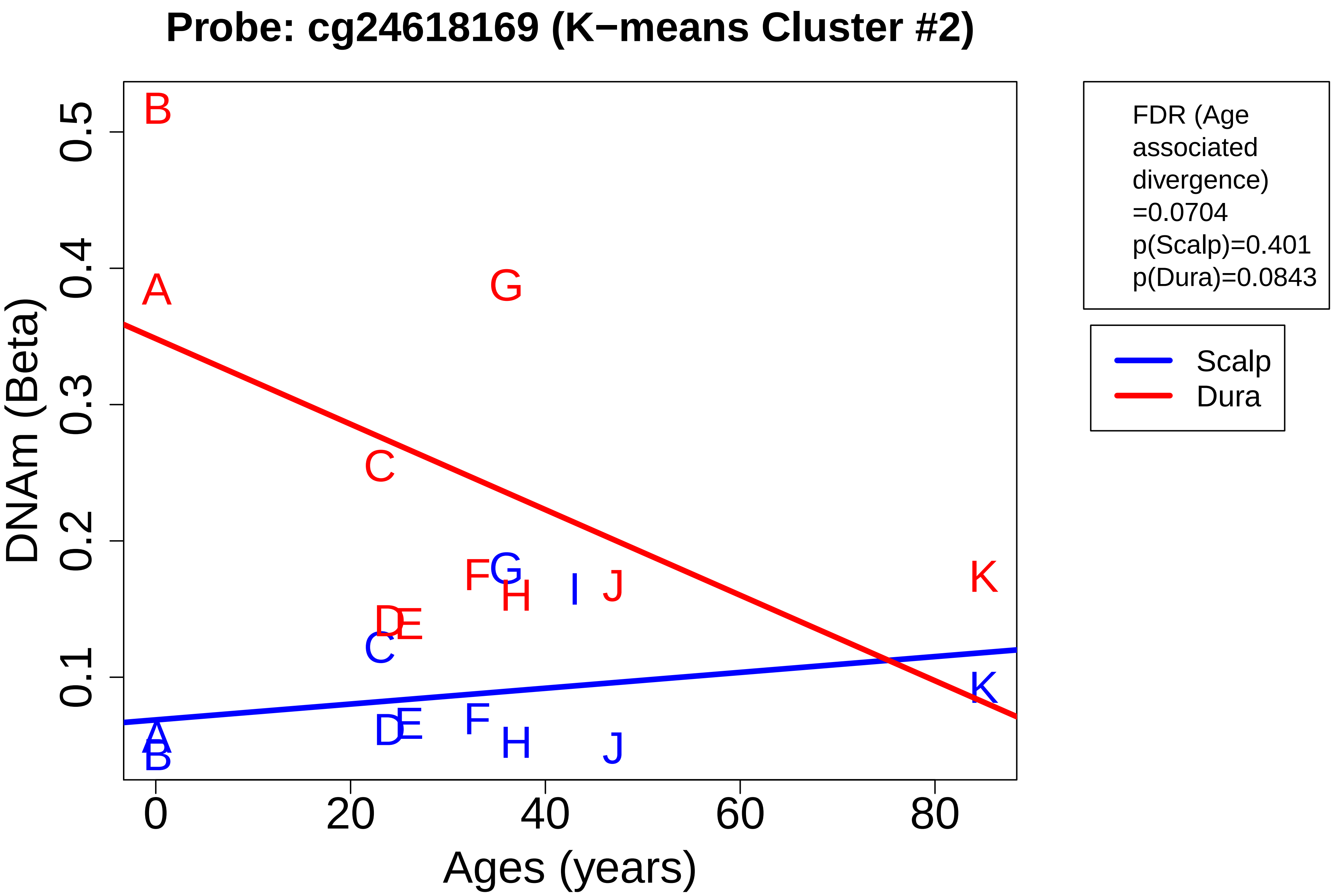
Probe: cg24618169 (K−means Cluster #2)
DNAm (Beta)
0.1
0.2
0.3
0.4
0.5
Ages (years)
0
20
40
60
80
FDR (Age associated divergence) =0.0704
p(Scalp)=0.401
p(Dura)=0.0843
Scalp
Dura
A
B
C
D
E
F
G
H
I
J
K

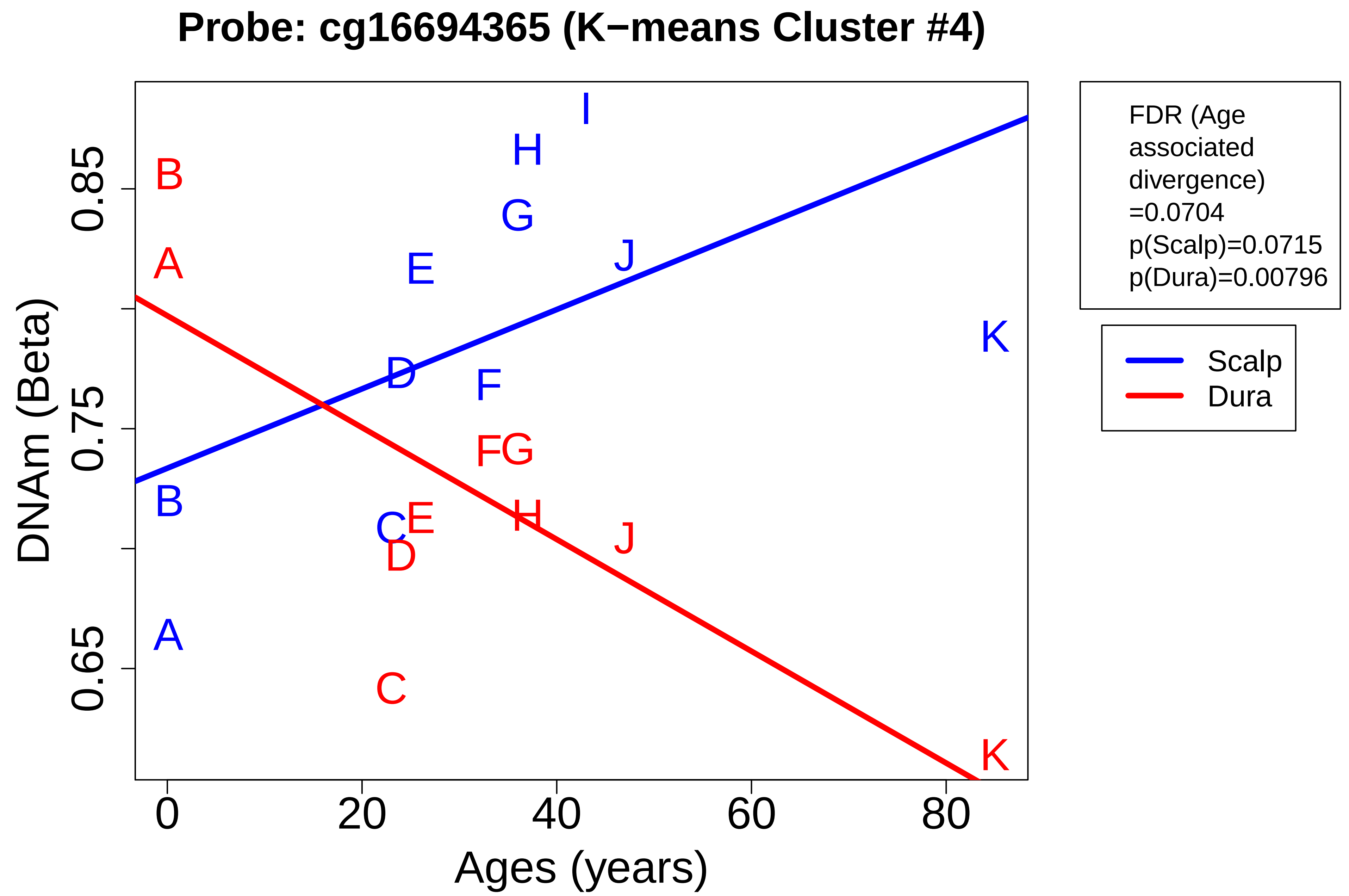
Probe: cg16694365 (K−means Cluster #4)
DNAm (Beta)
0.65
0.75
0.85
0
20
40
60
80
Ages (years)
FDR (Age associated divergence) =0.0704
p(Scalp)=0.0715
p(Dura)=0.00796
Scalp
Dura
A
B
C
D
E
F
G
H
I
J
K
A
B
C
D
E
FG
H
J
K

Probe: cg12031962 (K−means Cluster #1)

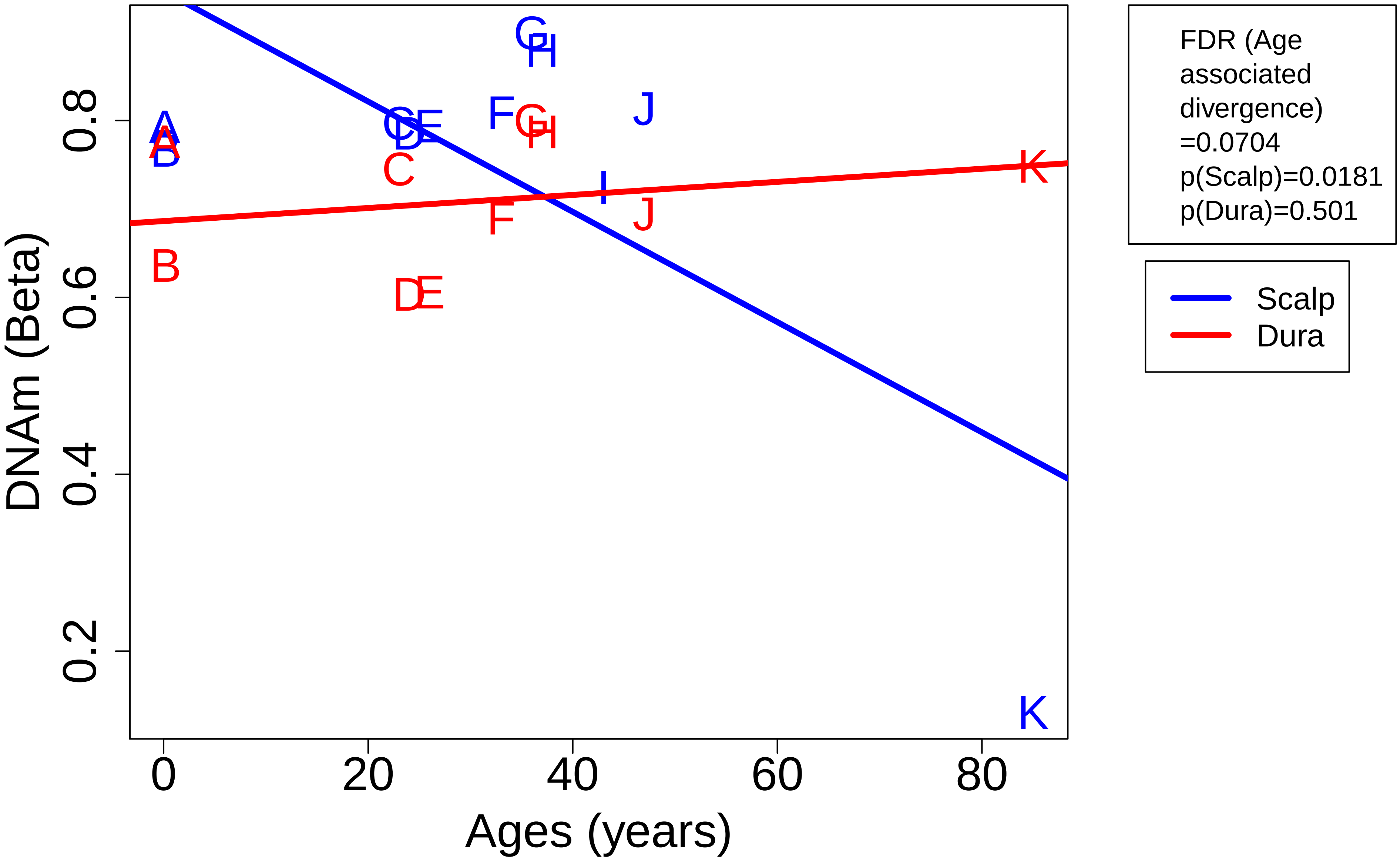

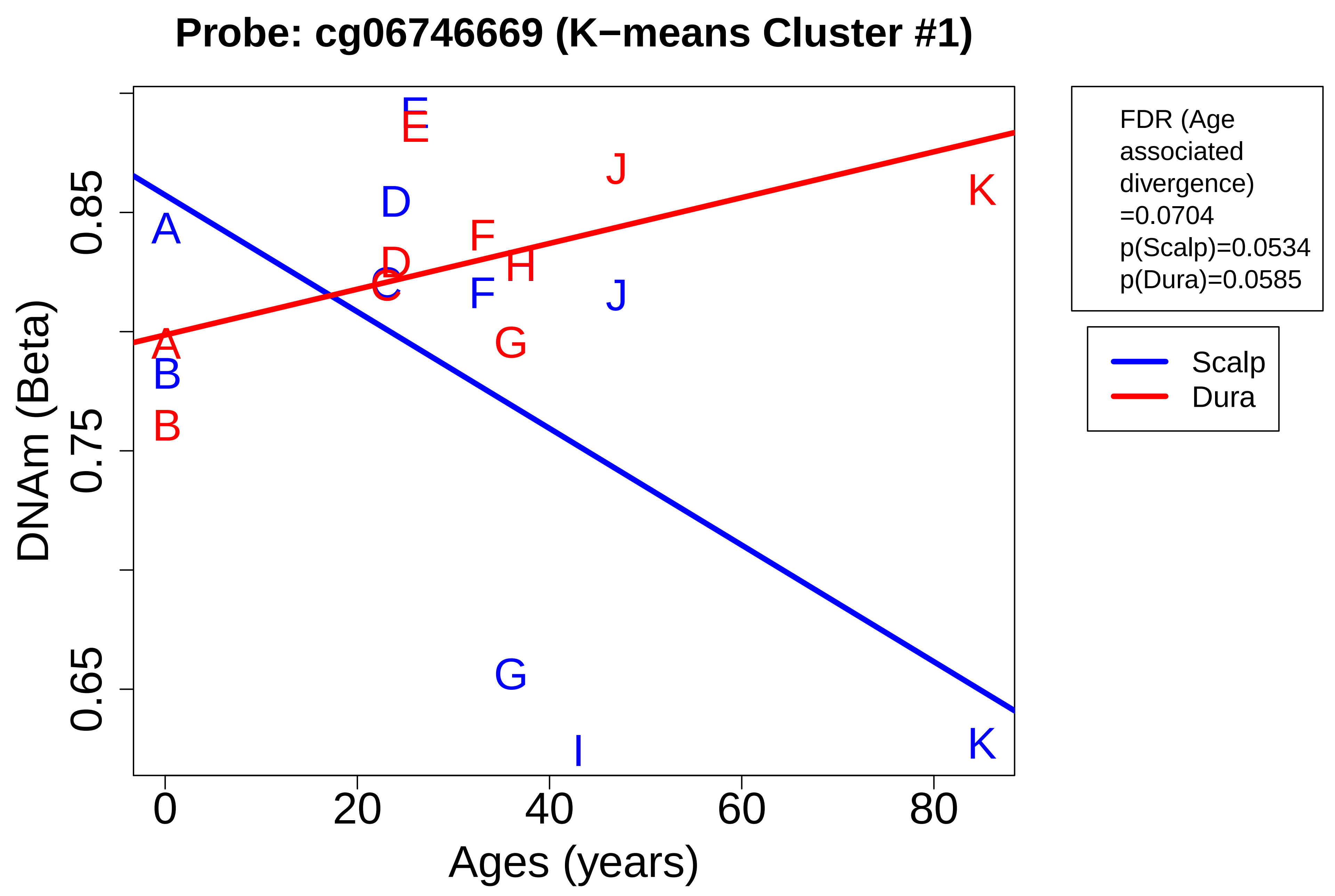

Probe: cg06746669 (K−means Cluster #1)
DNAm (Beta)
0.65
0.75
0.85
Ages (years)
0
20
40
60
80
A
B
C
D
E
F
G
H
I
J
K
FDR (Age associated divergence) =0.0704
p(Scalp)=0.0534
p(Dura)=0.0585
Scalp
Dura

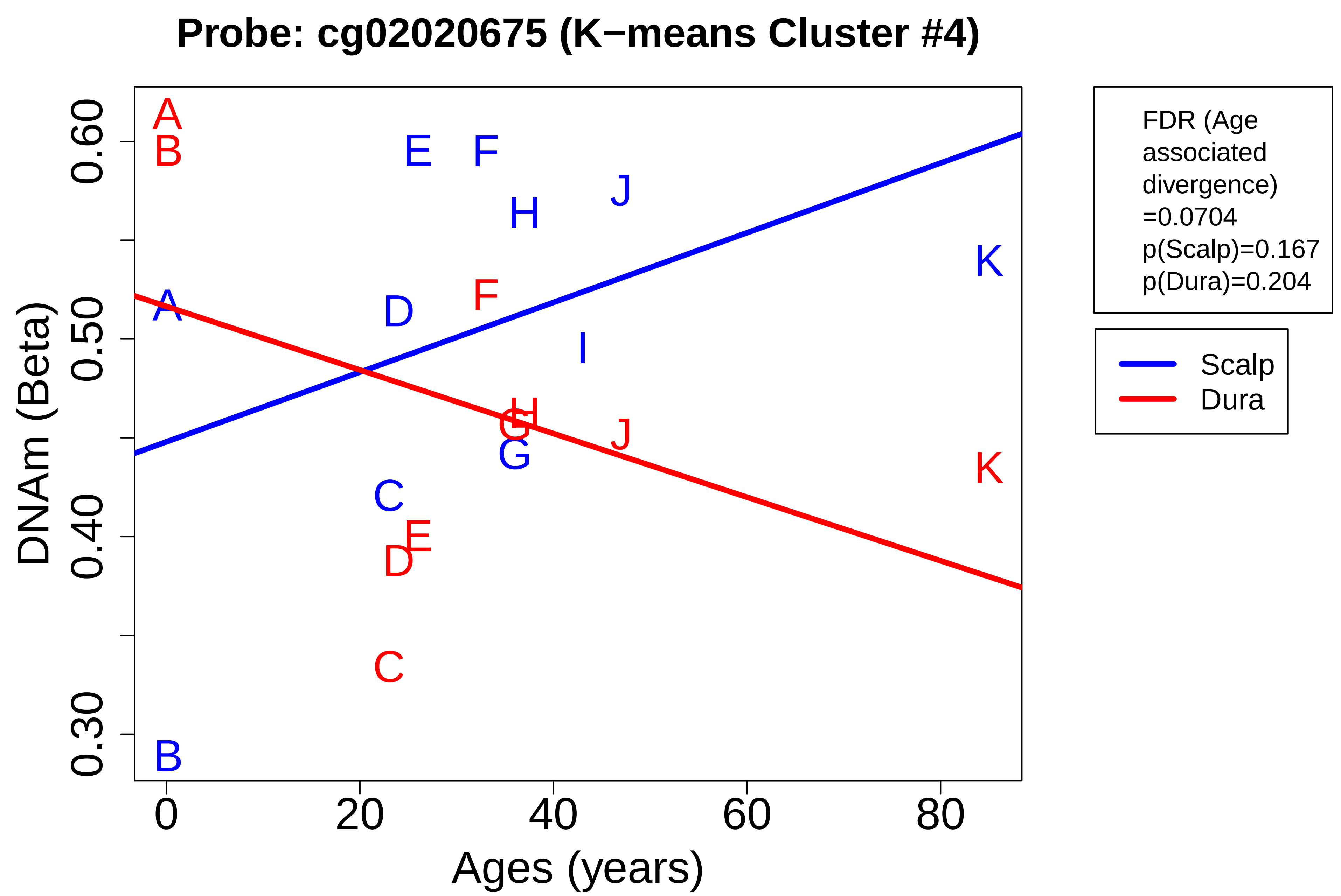

Probe: cg02020675 (K−means Cluster #4)
DNAm (Beta)
0.30
0.40
0.50
0.60
Ages (years)
0
20
40
60
80
A
B
E
F
J
H
K
A
D
F
I
H
G
J
G
K
C
E
D
C
B
FDR (Age associated divergence) =0.0704
p(Scalp)=0.167
p(Dura)=0.204
Scalp
Dura

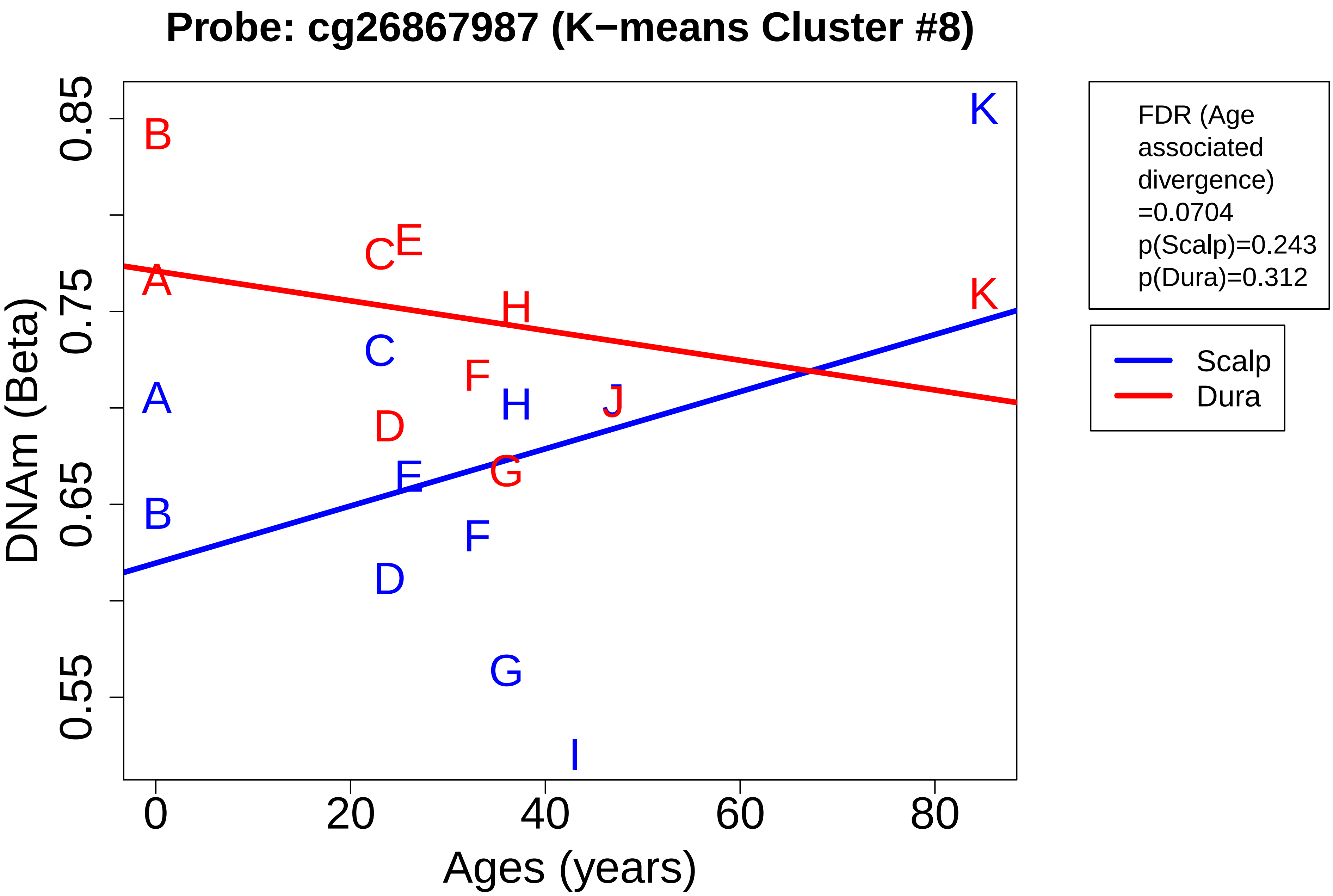

Probe: cg26867987 (K−means Cluster #8)
DNAm (Beta)
0.85
0.75
0.65
0.55
0
20
40
60
80
Ages (years)
FDR (Age associated divergence) =0.0704
p(Scalp)=0.243
p(Dura)=0.312
Scalp
Dura

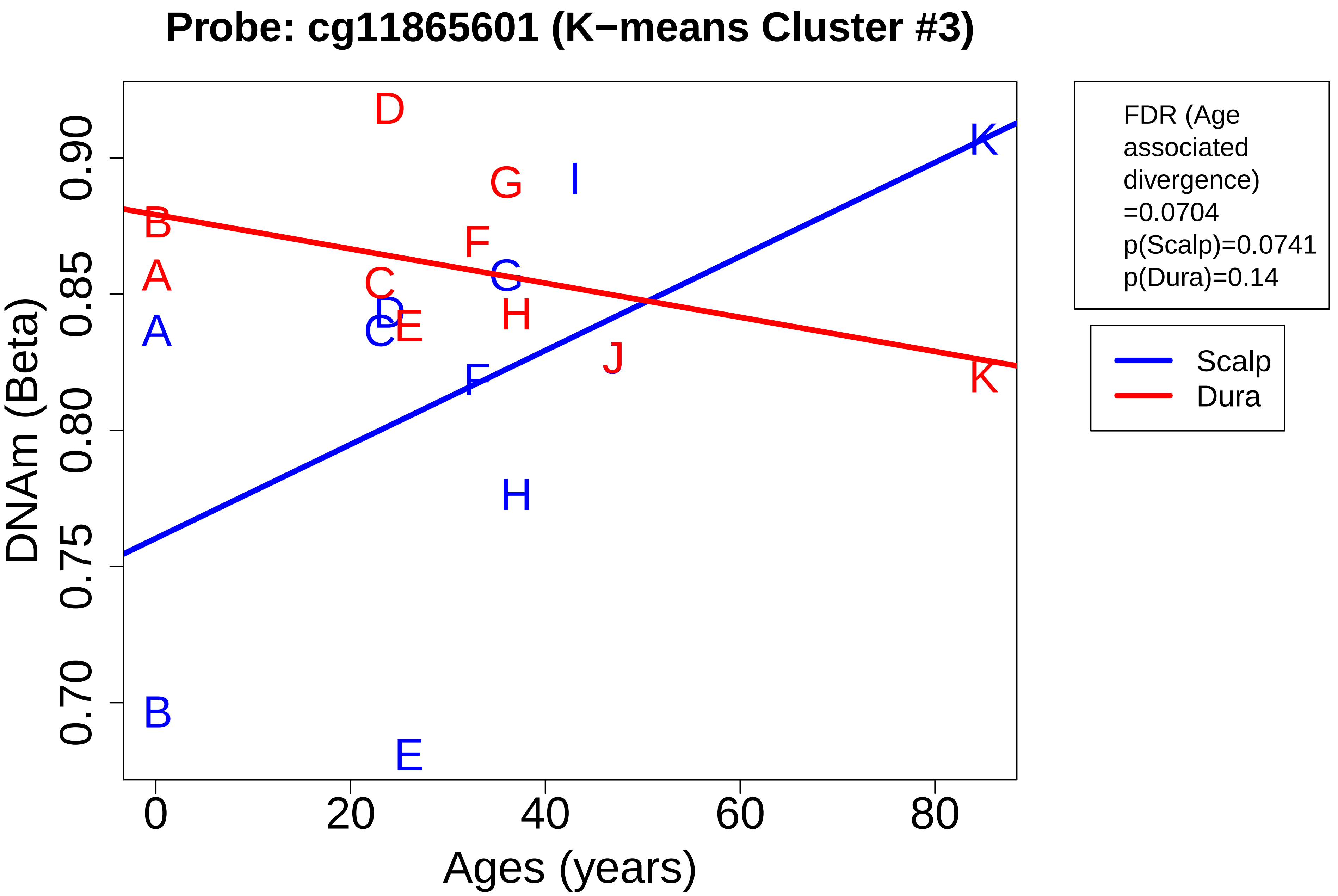
Probe: cg11865601 (K−means Cluster #3)
FDR (Age associated divergence) =0.0704
p(Scalp)=0.0741
p(Dura)=0.14
Scalp
Dura
DNAm (Beta)
0.70
0.75
0.80
0.85
0.90
0
20
40
60
80
Ages (years)
A
B
C
D
E
F
G
H
I
J
K

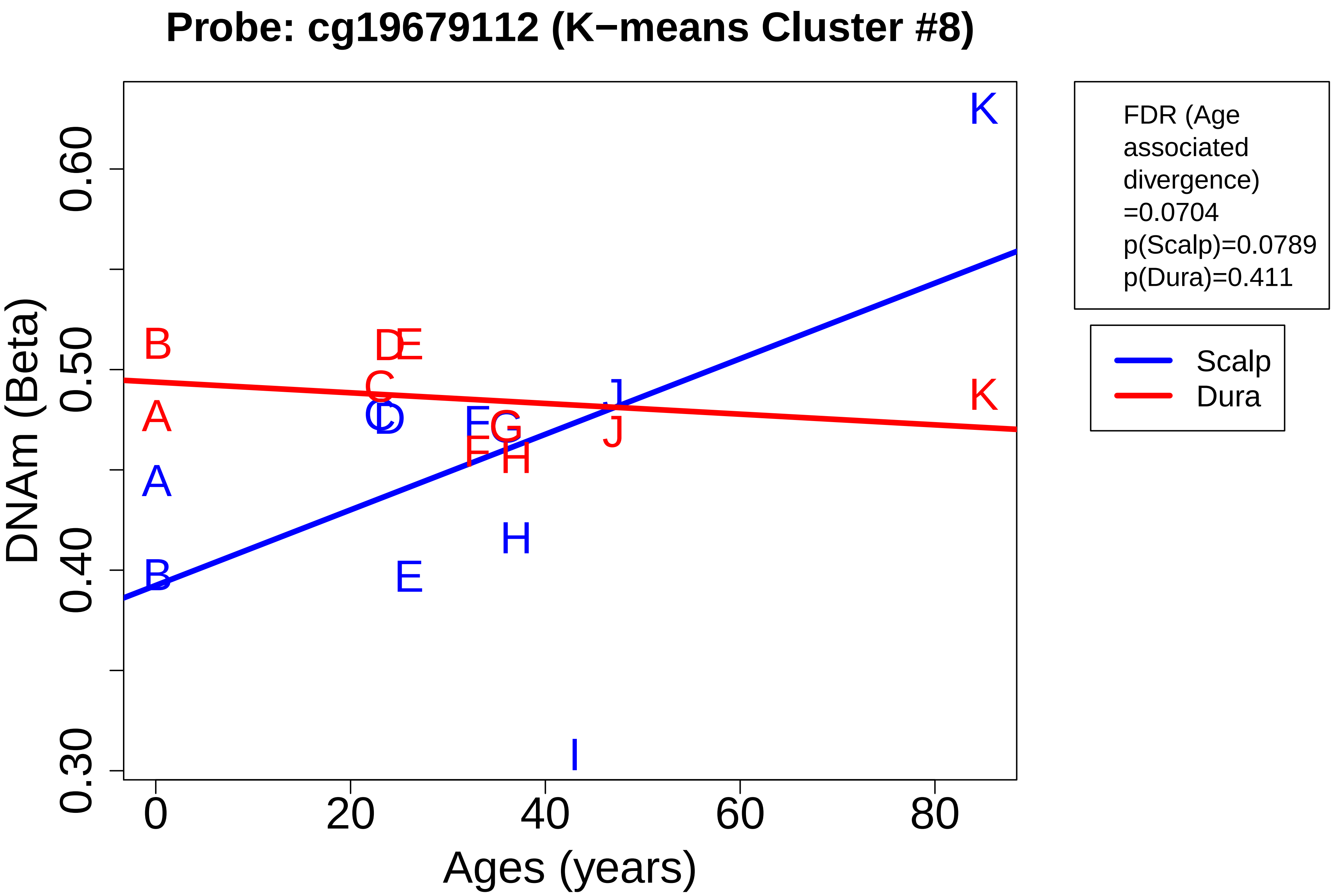

Probe: cg19679112 (K−means Cluster #8)
DNAm (Beta)
0.30
0.40
0.50
0.60
Ages (years)
0
20
40
60
80
FDR (Age associated divergence) =0.0704
p(Scalp)=0.0789
p(Dura)=0.411
Scalp
Dura

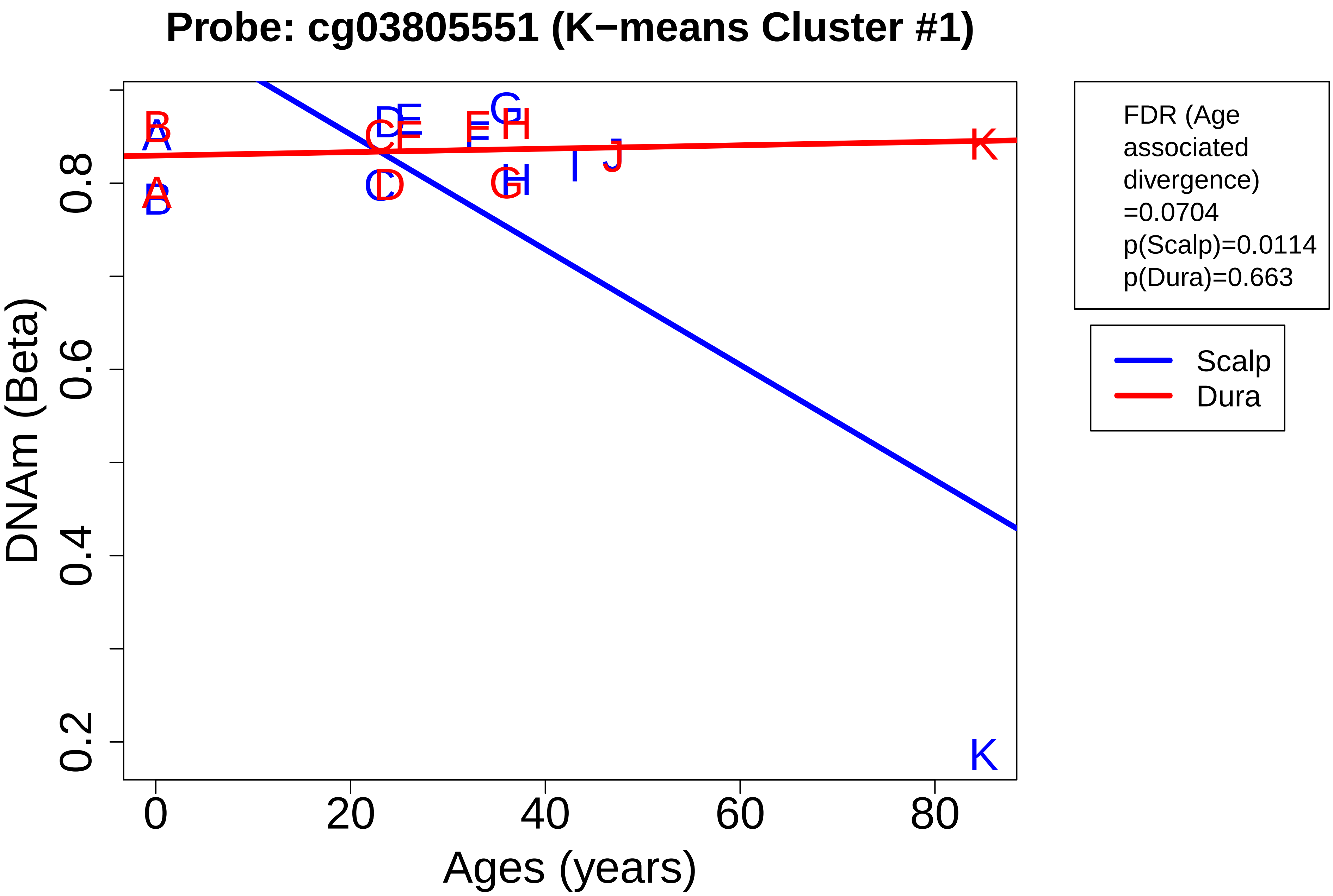

Probe: cg03805551 (K−means Cluster #1)
DNAm (Beta)
0.2
0.4
0.6
0.8
Ages (years)
0
20
40
60
80
FDR (Age associated divergence) =0.0704
p(Scalp)=0.0114
p(Dura)=0.663
Scalp
Dura

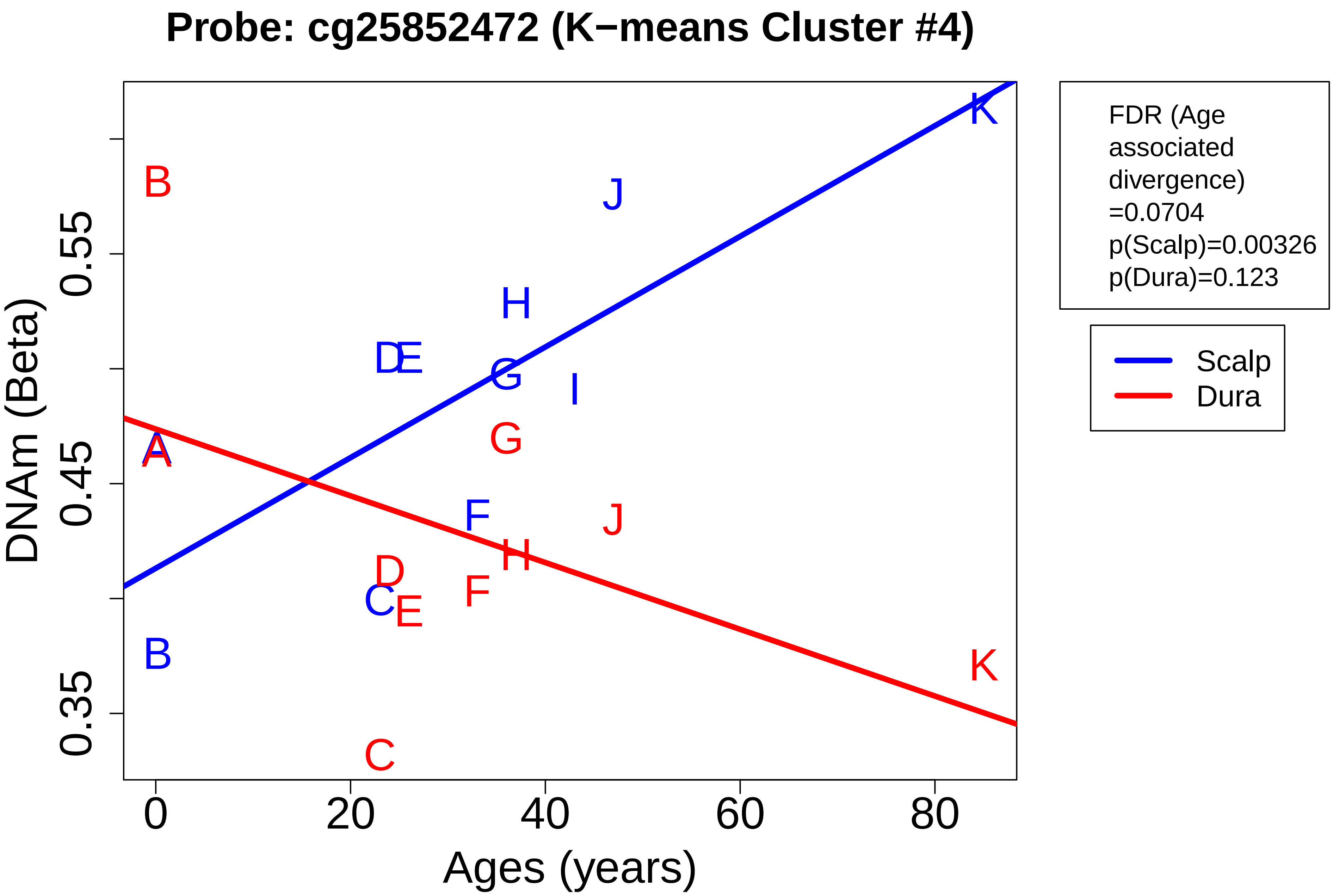
Probe: cg25852472 (K−means Cluster #4)
DNAm (Beta)
0.35
0.45
0.55
0
20
40
60
80
Ages (years)
A
B
C
D
E
F
G
H
I
J
K
FDR (Age associated divergence) =0.0704
p(Scalp)=0.00326
p(Dura)=0.123
Scalp
Dura

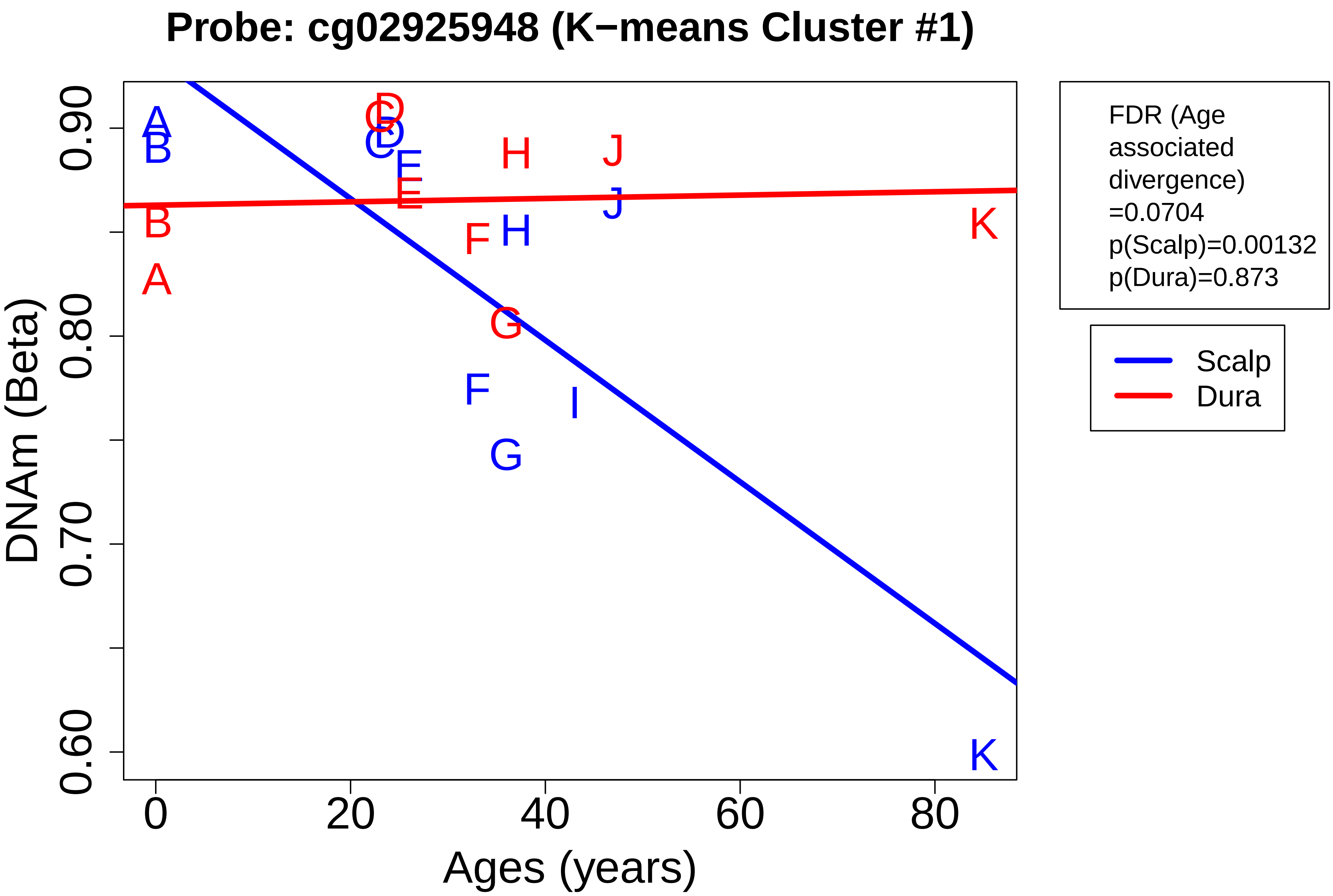
Probe: cg02925948 (K−means Cluster #1)
DNAm (Beta)
0.60
0.70
0.80
0.90
0
20
40
60
80
Ages (years)
A
B
C
D
E
F
G
H
I
J
K
FDR (Age associated divergence) =0.0704
p(Scalp)=0.00132
p(Dura)=0.873
Scalp
Dura

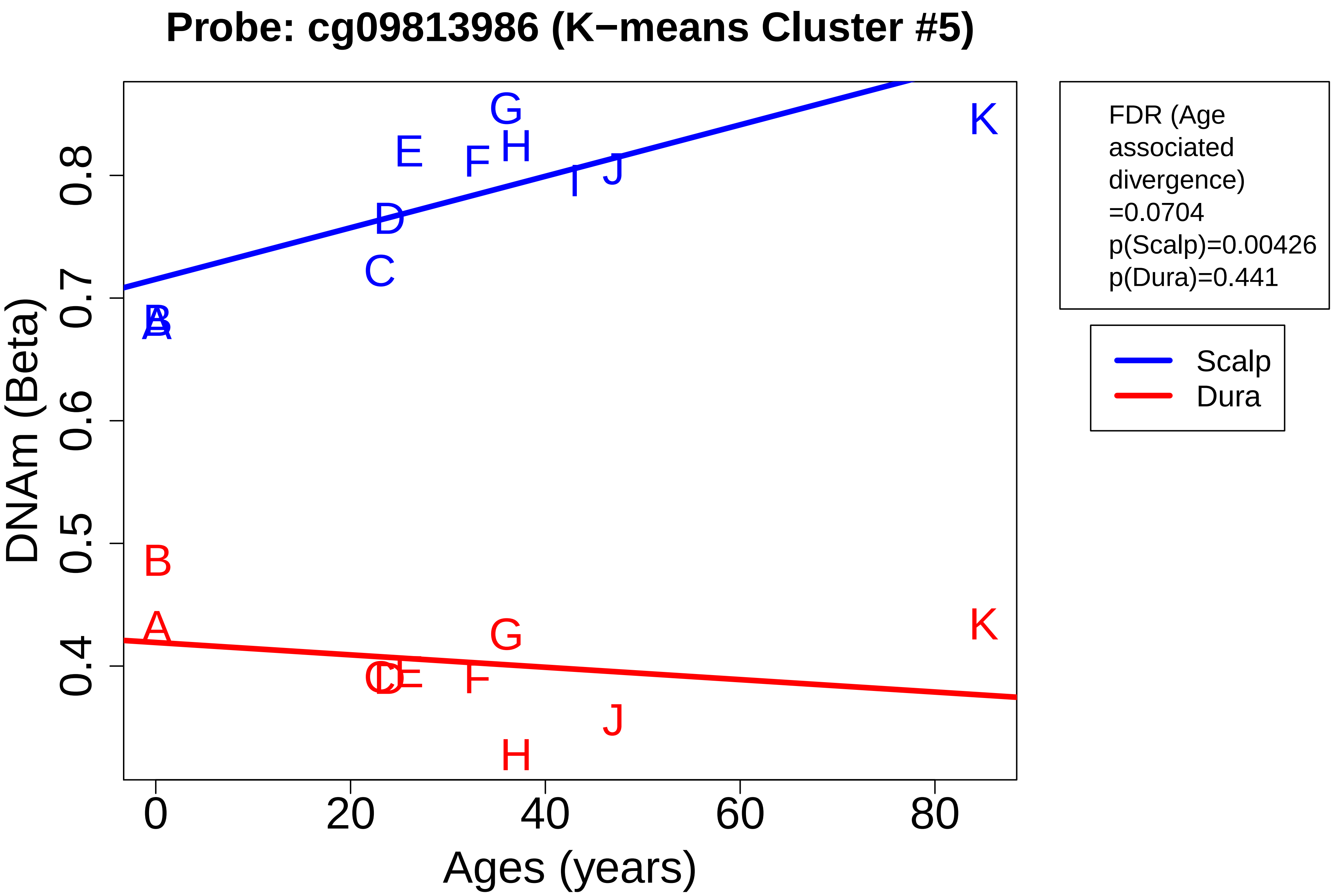

Probe: cg09813986 (K−means Cluster #5)
DNAm (Beta)
0.4
0.5
0.6
0.7
0.8
0
20
40
60
80
Ages (years)
A
B
C
D
E
F
G
H
I
J
K
FDR (Age associated divergence) =0.0704
p(Scalp)=0.00426
p(Dura)=0.441
Scalp
Dura

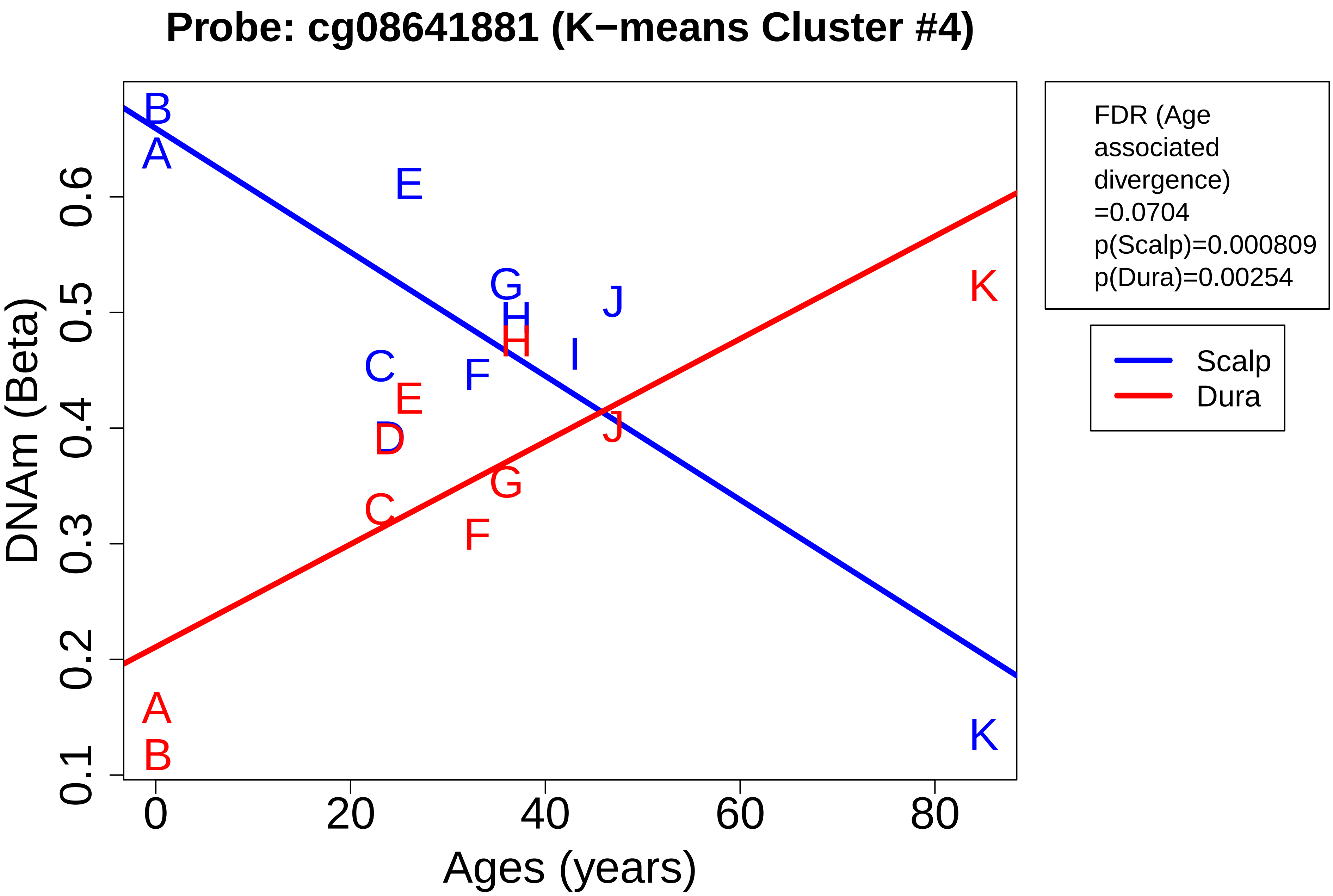

Probe: cg08641881 (K−means Cluster #4)
FDR (Age associated divergence) =0.0704
p(Scalp)=0.000809
p(Dura)=0.00254
Scalp
Dura
DNAm (Beta)
Ages (years)
0.1
0.2
0.3
0.4
0.5
0.6
0
20
40
60
80

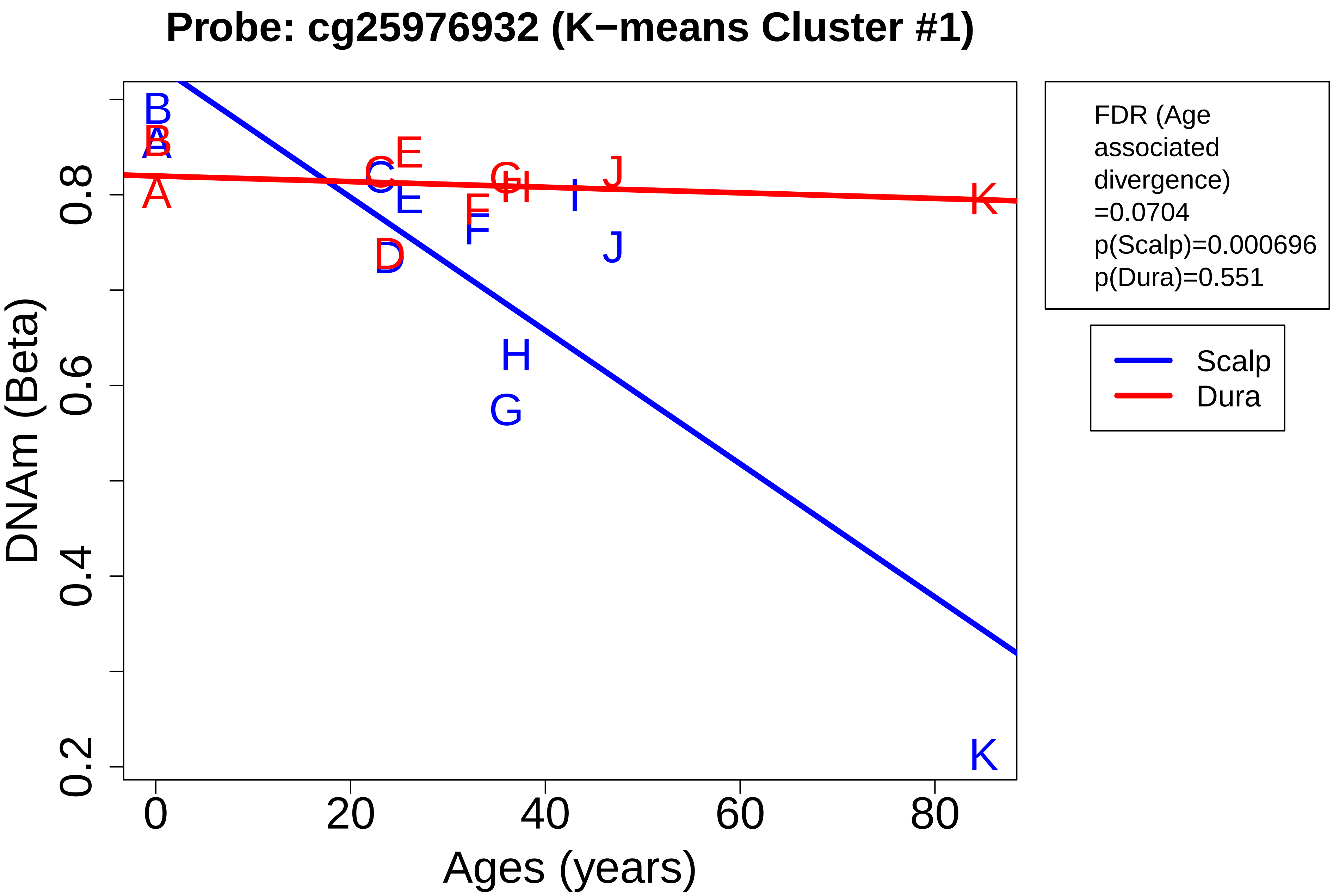

Probe: cg25976932 (K−means Cluster #1)
DNAm (Beta)
0.2
0.4
0.6
0.8
0
20
40
60
80
Ages (years)
FDR (Age associated divergence) =0.0704
p(Scalp)=0.000696
p(Dura)=0.551
Scalp
Dura

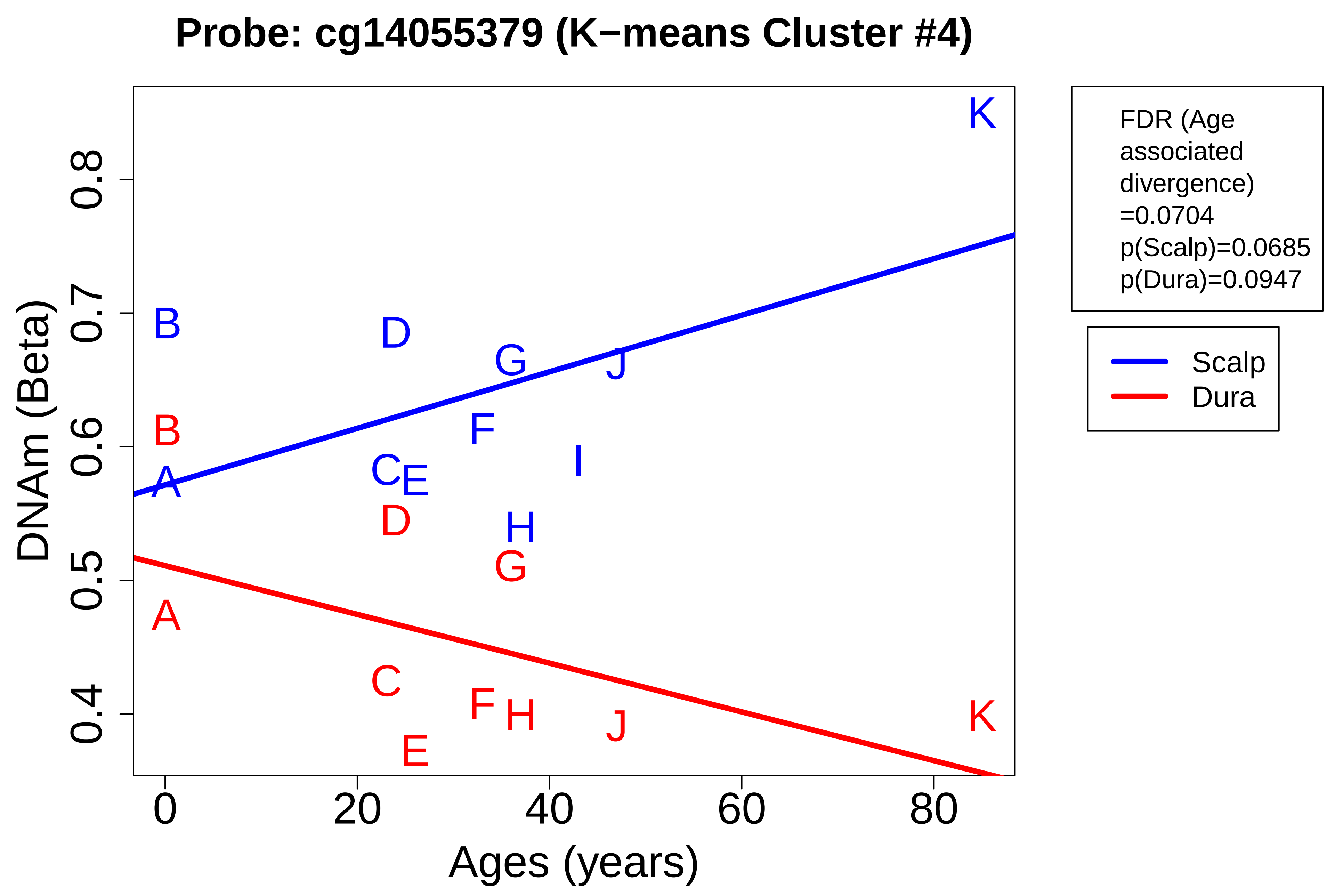

Probe: cg14055379 (K−means Cluster #4)
FDR (Age associated divergence) =0.0704
p(Scalp)=0.0685
p(Dura)=0.0947
Scalp
Dura
DNAm (Beta)
0.4
0.5
0.6
0.7
0.8
0
20
40
60
80
Ages (years)
A
B
C
D
E
F
G
H
I
J
K

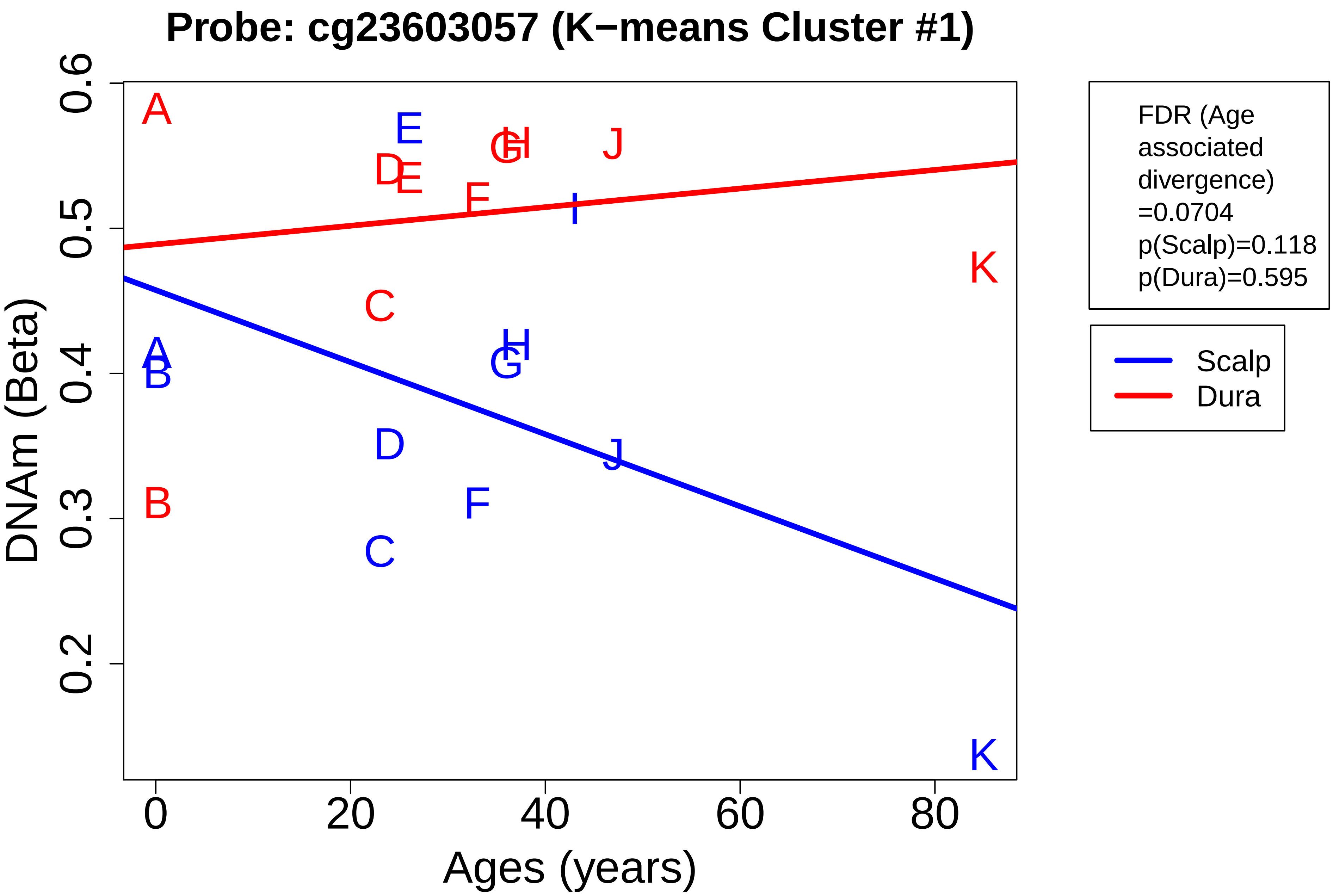
Probe: cg23603057 (K−means Cluster #1)
DNAm (Beta)
Ages (years)
0.2
0.3
0.4
0.5
0.6
0
20
40
60
80
FDR (Age associated divergence) =0.0704
p(Scalp)=0.118
p(Dura)=0.595
Scalp
Dura
A
B
C
D
E
F
G
H
I
J
K

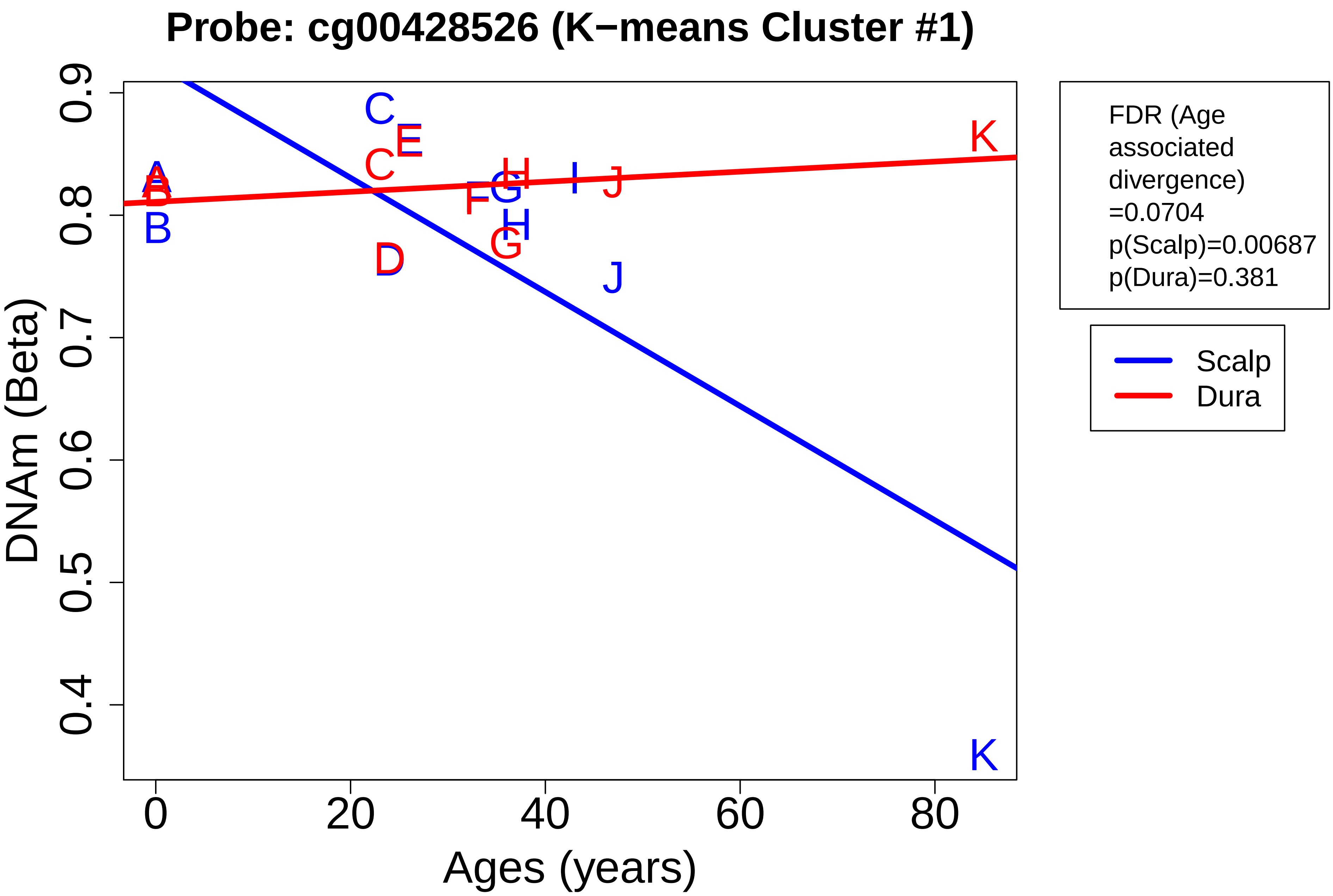

Probe: cg00428526 (K−means Cluster #1)
DNAm (Beta)
0.4
0.5
0.6
0.7
0.8
0.9
0
20
40
60
80
Ages (years)
A
B
C
D
E
F
G
H
I
J
K
FDR (Age associated divergence) =0.0704
p(Scalp)=0.00687
p(Dura)=0.381
Scalp
Dura

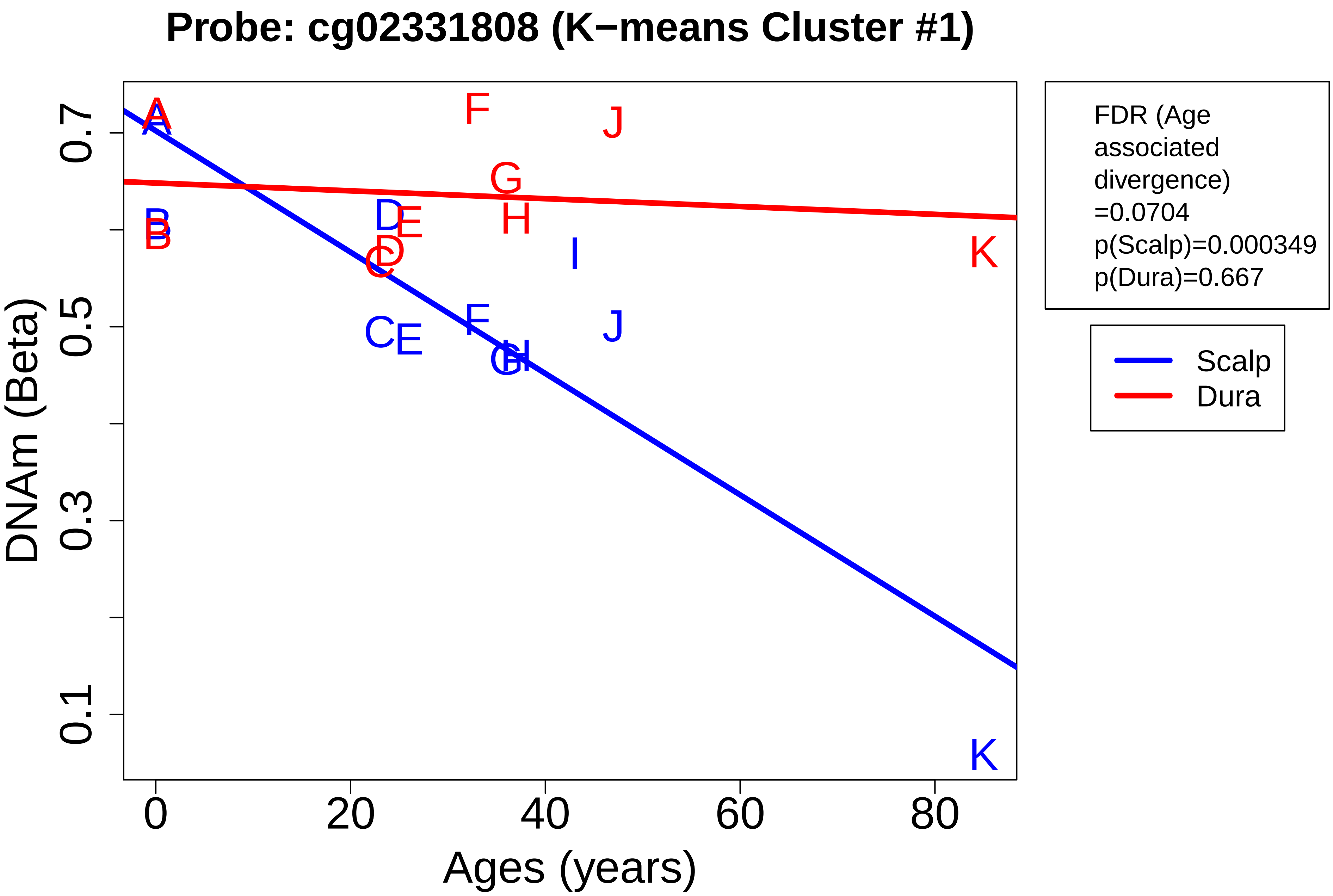

Probe: cg02331808 (K−means Cluster #1)
FDR (Age associated divergence) =0.0704
p(Scalp)=0.000349
p(Dura)=0.667
Scalp
Dura
DNAm (Beta)
0.1
0.3
0.5
0.7
Ages (years)
0
20
40
60
80

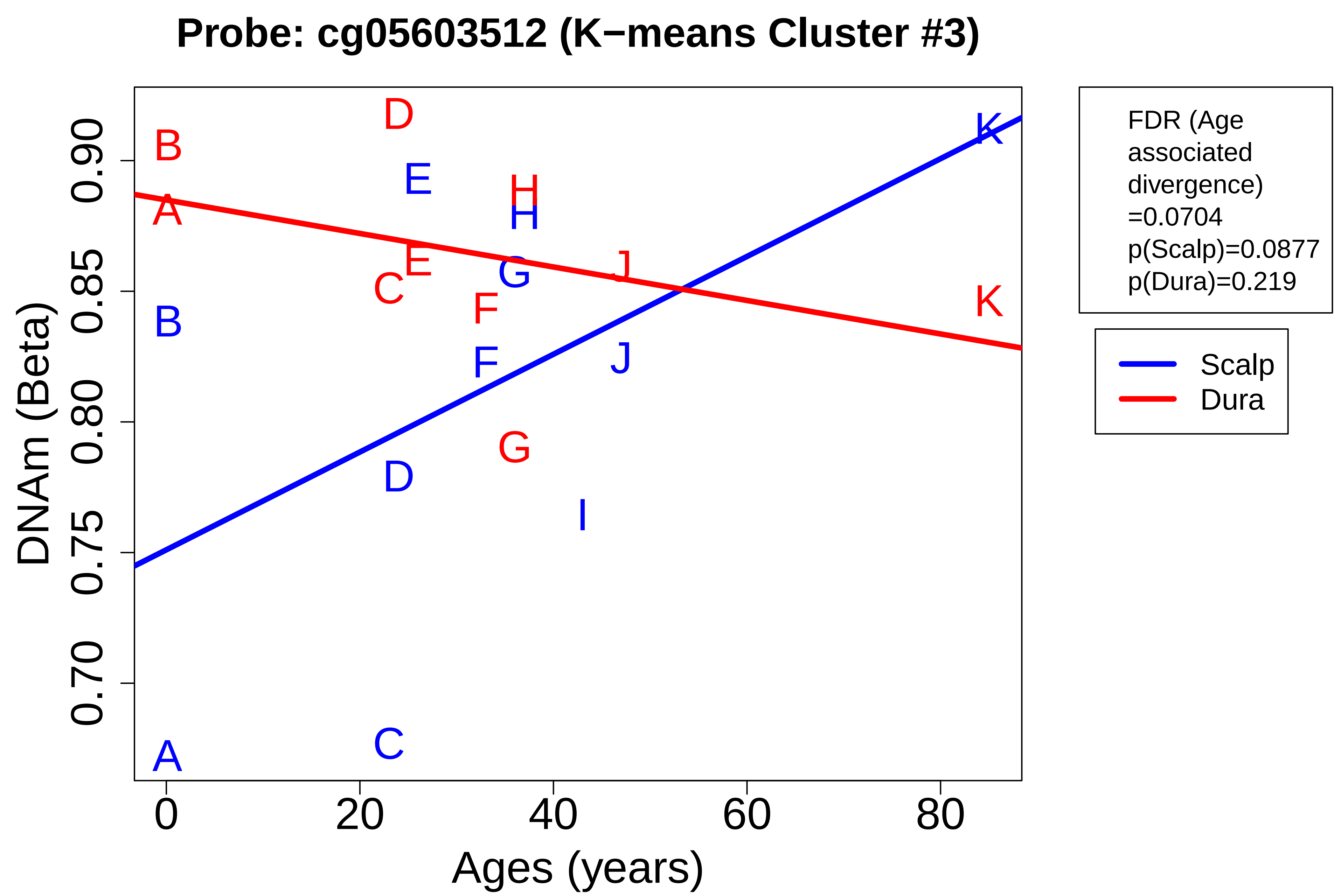
Probe: cg05603512 (K−means Cluster #3)
FDR (Age associated divergence) =0.0704
p(Scalp)=0.0877
p(Dura)=0.219
Scalp
Dura
DNAm (Beta)
0.70
0.75
0.80
0.85
0.90
0
20
40
60
80
Ages (years)

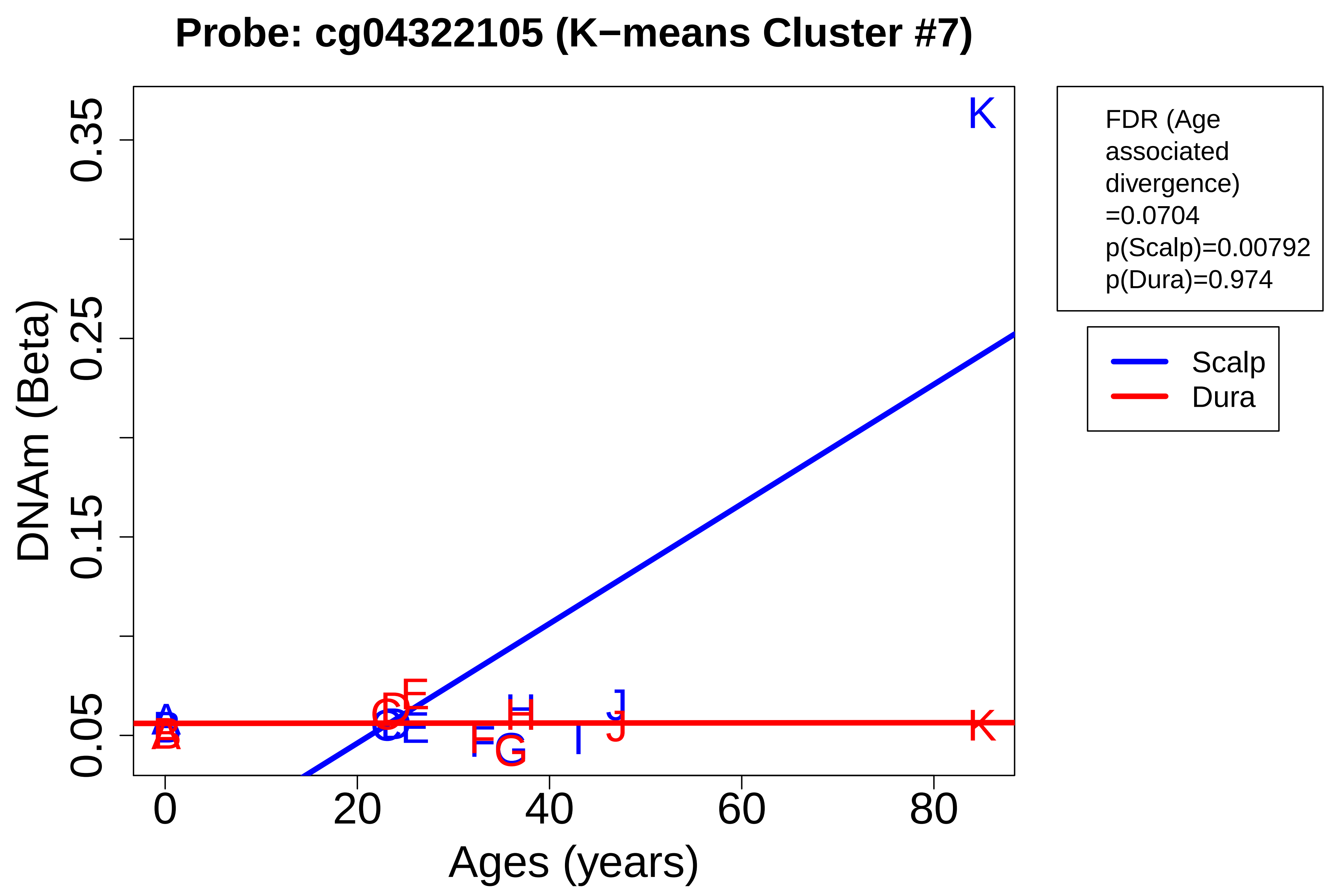

Probe: cg04322105 (K−means Cluster #7)
DNAm (Beta)
0.05
0.15
0.25
0.35
Ages (years)
0
20
40
60
80
FDR (Age associated divergence) =0.0704
p(Scalp)=0.00792
p(Dura)=0.974
Scalp
Dura

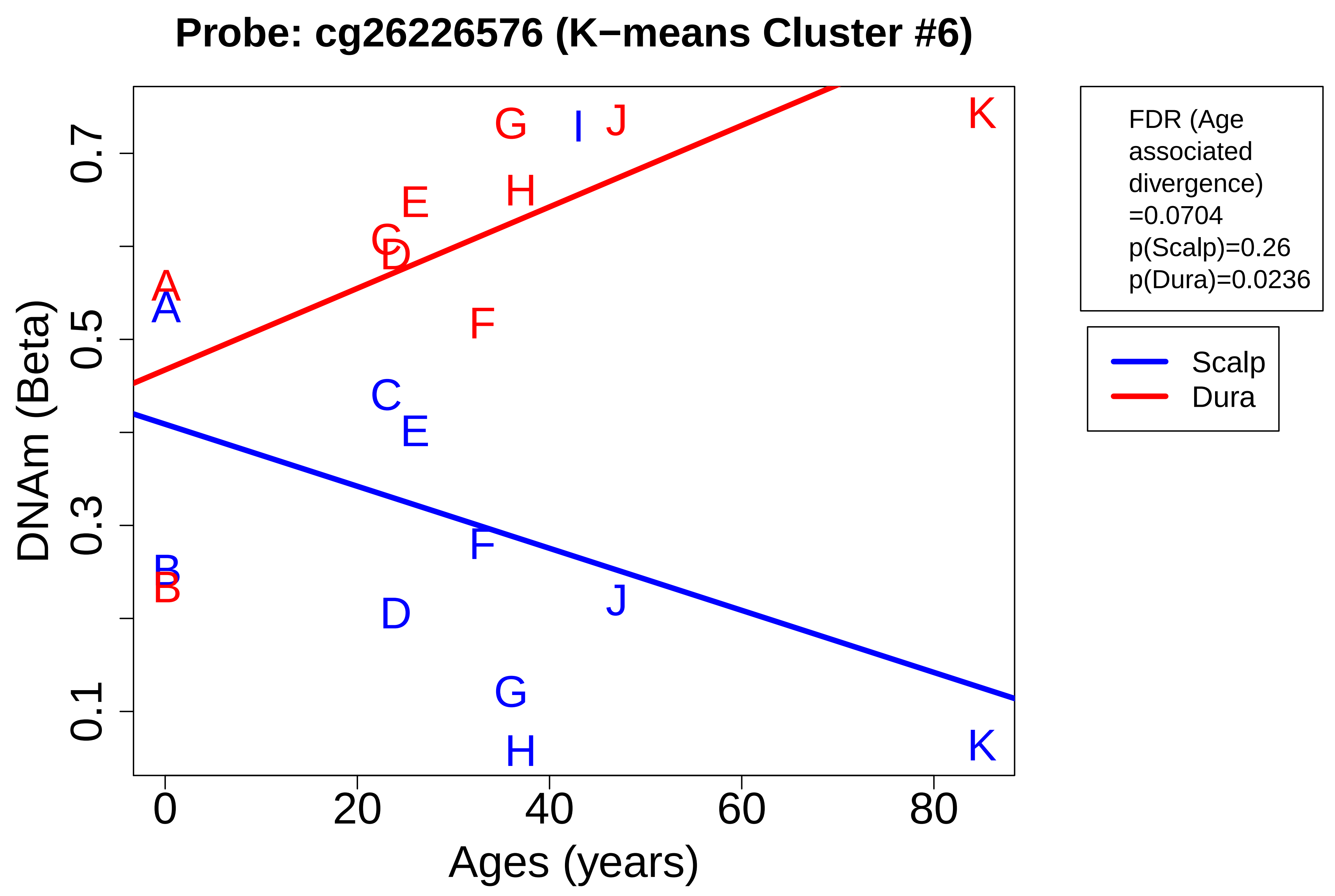

Probe: cg26226576 (K−means Cluster #6)
DNAm (Beta)
0.1
0.3
0.5
0.7
Ages (years)
0
20
40
60
80
FDR (Age associated divergence) =0.0704
p(Scalp)=0.26
p(Dura)=0.0236
Scalp
Dura
A
B
C
D
E
F
G
H
I
J
K

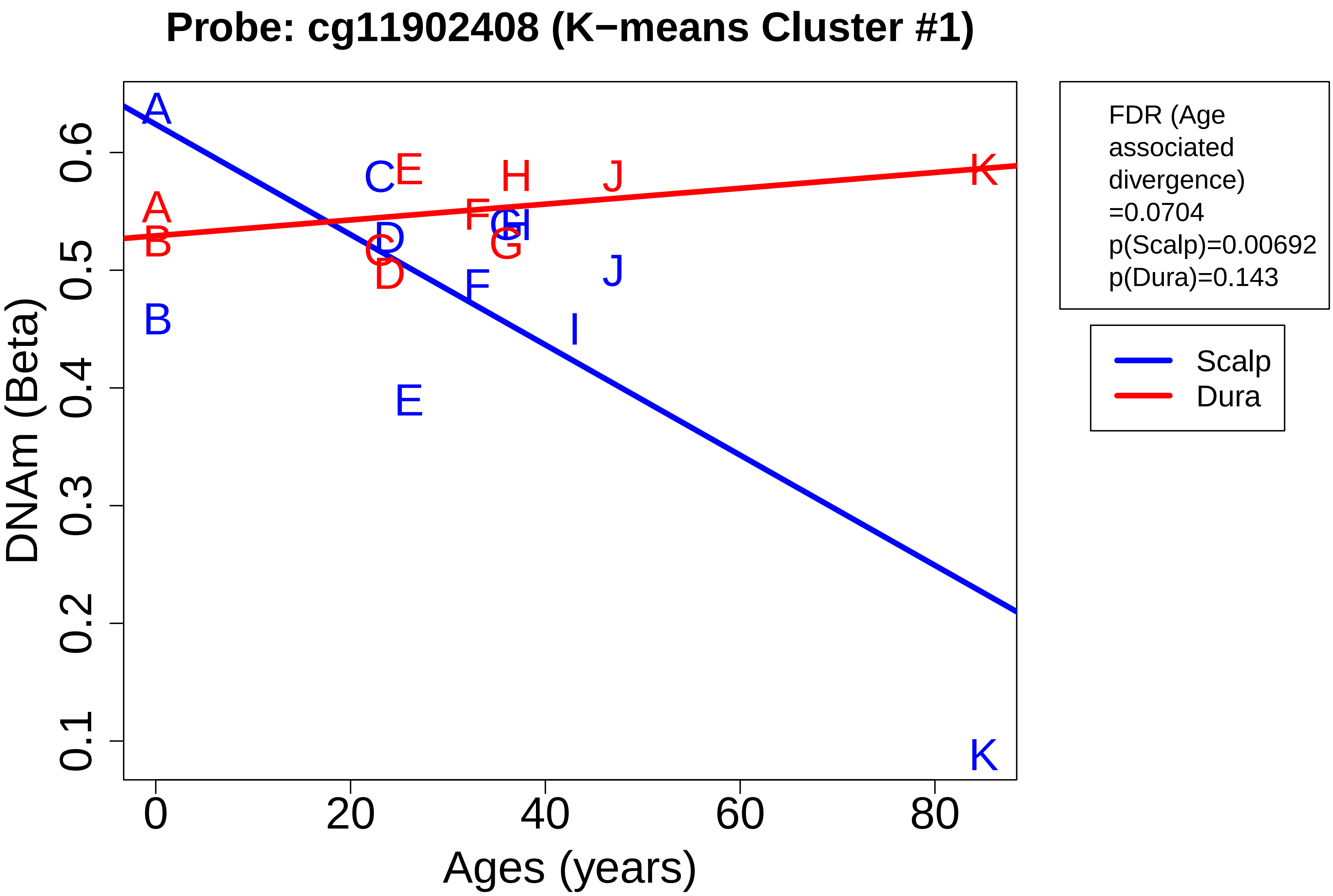

Probe: cg11902408 (K−means Cluster #1)
DNAm (Beta)
Ages (years)
0.1
0.2
0.3
0.4
0.5
0.6
0
20
40
60
80
FDR (Age associated divergence) =0.0704
p(Scalp)=0.00692
p(Dura)=0.143
Scalp
Dura

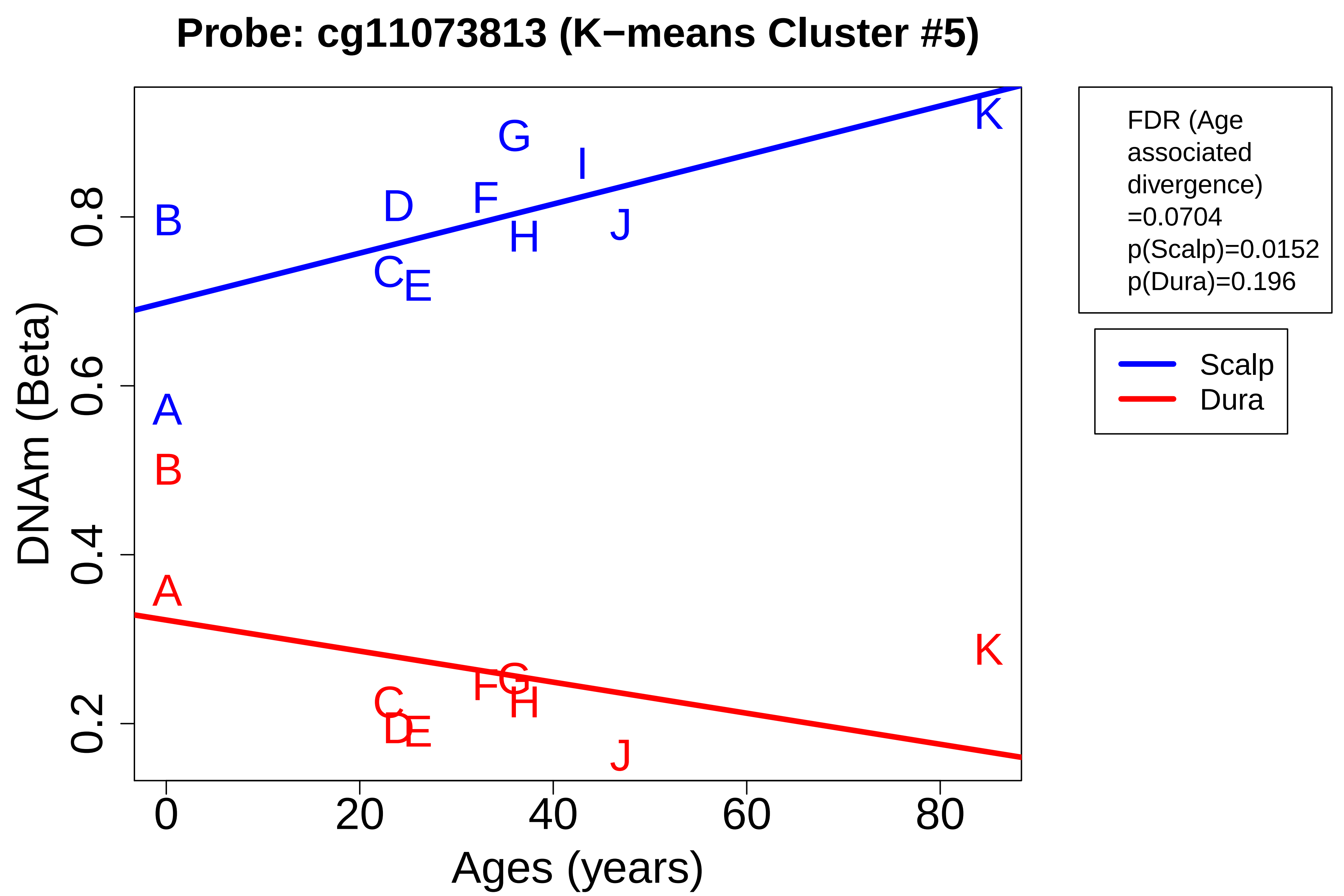

Probe: cg11073813 (K−means Cluster #5)
DNAm (Beta)
0.2
0.4
0.6
0.8
Ages (years)
0
20
40
60
80
A
B
C
D
E
F
G
H
I
J
K
FDR (Age associated divergence) =0.0704
p(Scalp)=0.0152
p(Dura)=0.196
Scalp
Dura

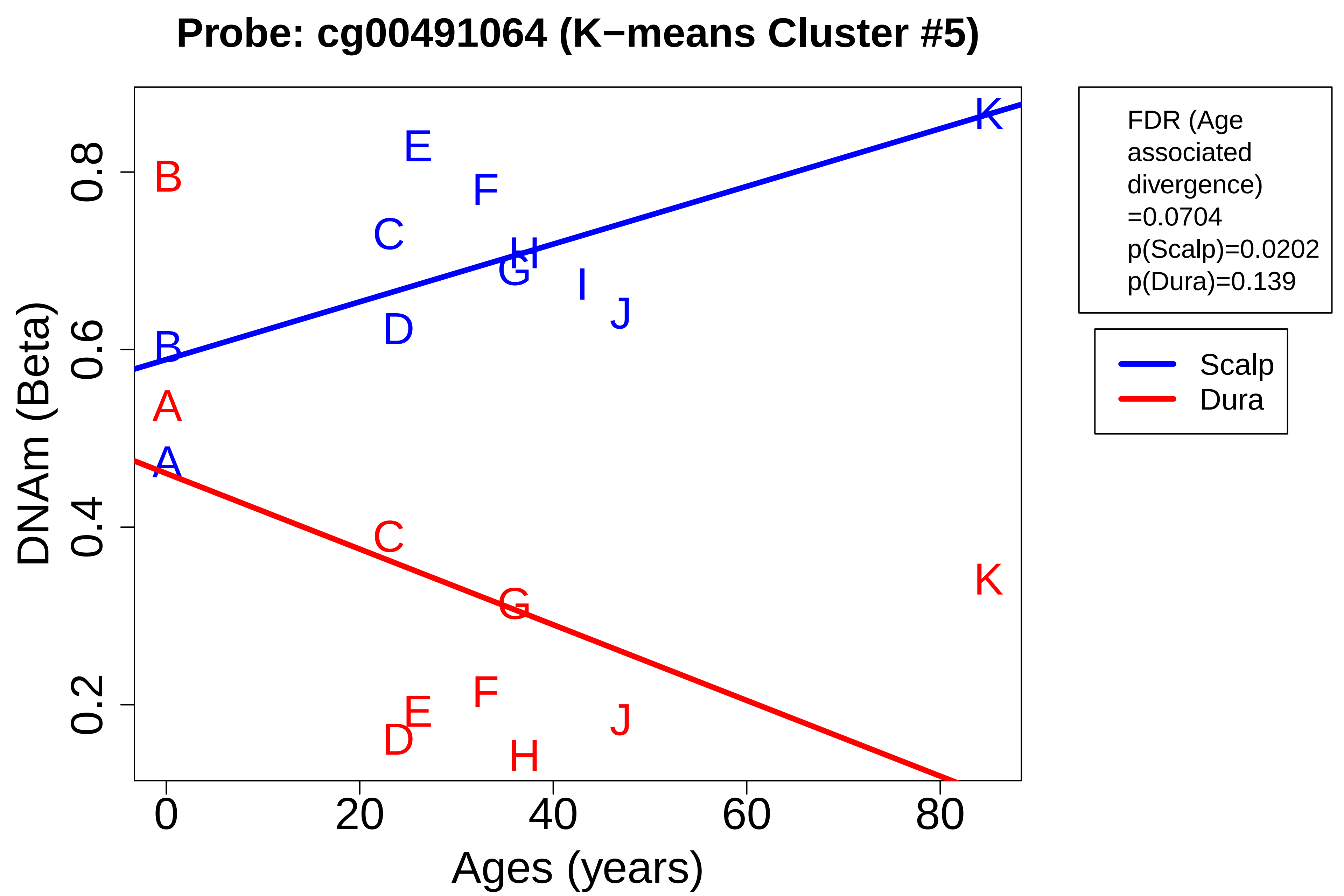
Probe: cg00491064 (K−means Cluster #5)
DNAm (Beta)
0.2
0.4
0.6
0.8
Ages (years)
0
20
40
60
80
A
B
C
D
E
F
G
H
I
J
K
FDR (Age associated divergence) =0.0704
p(Scalp)=0.0202
p(Dura)=0.139
Scalp
Dura

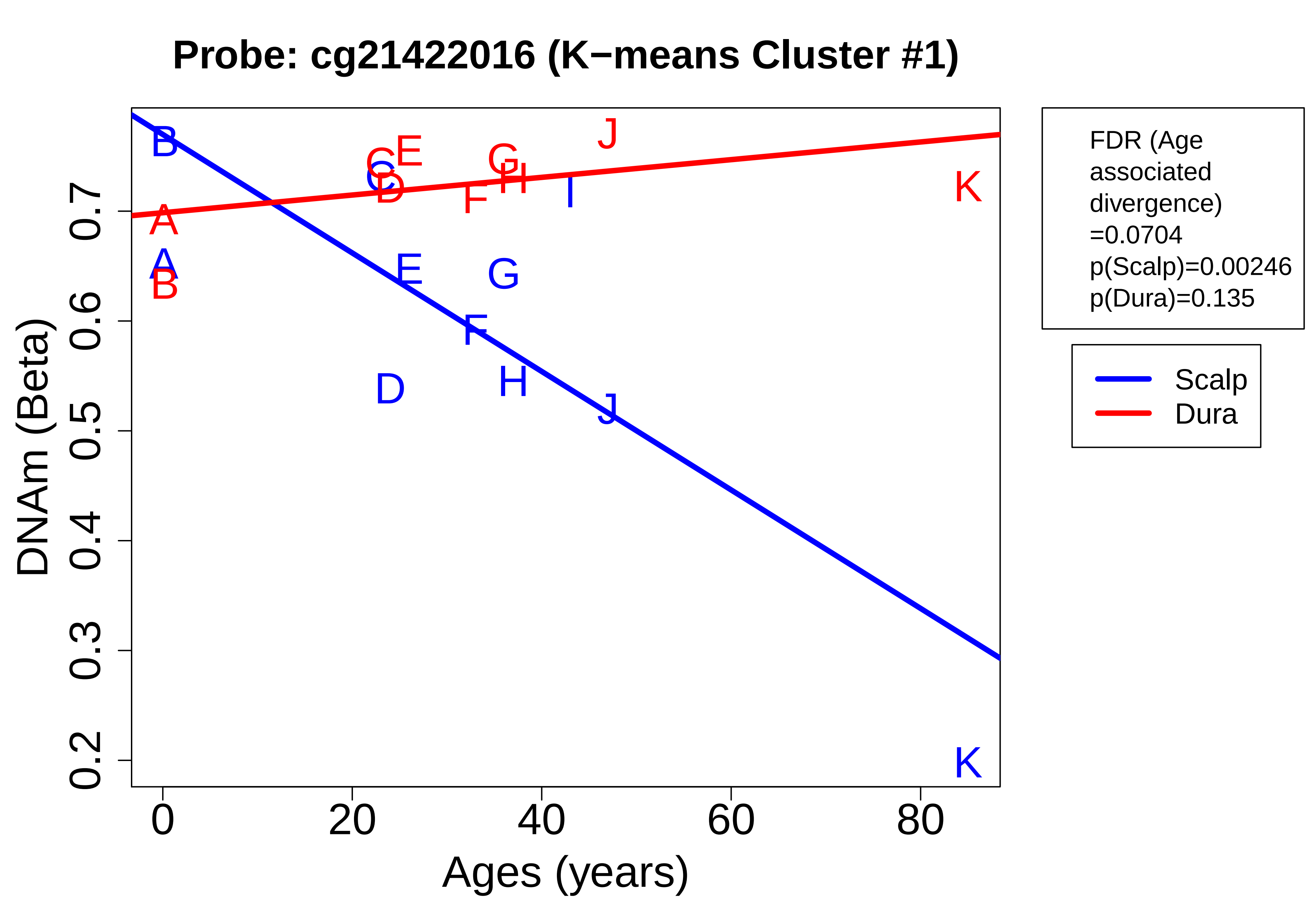

Probe: cg21422016 (K−means Cluster #1)
DNAm (Beta)
Ages (years)
0.2
0.3
0.4
0.5
0.6
0.7
0
20
40
60
80
FDR (Age associated divergence) =0.0704
p(Scalp)=0.00246
p(Dura)=0.135
Scalp
Dura

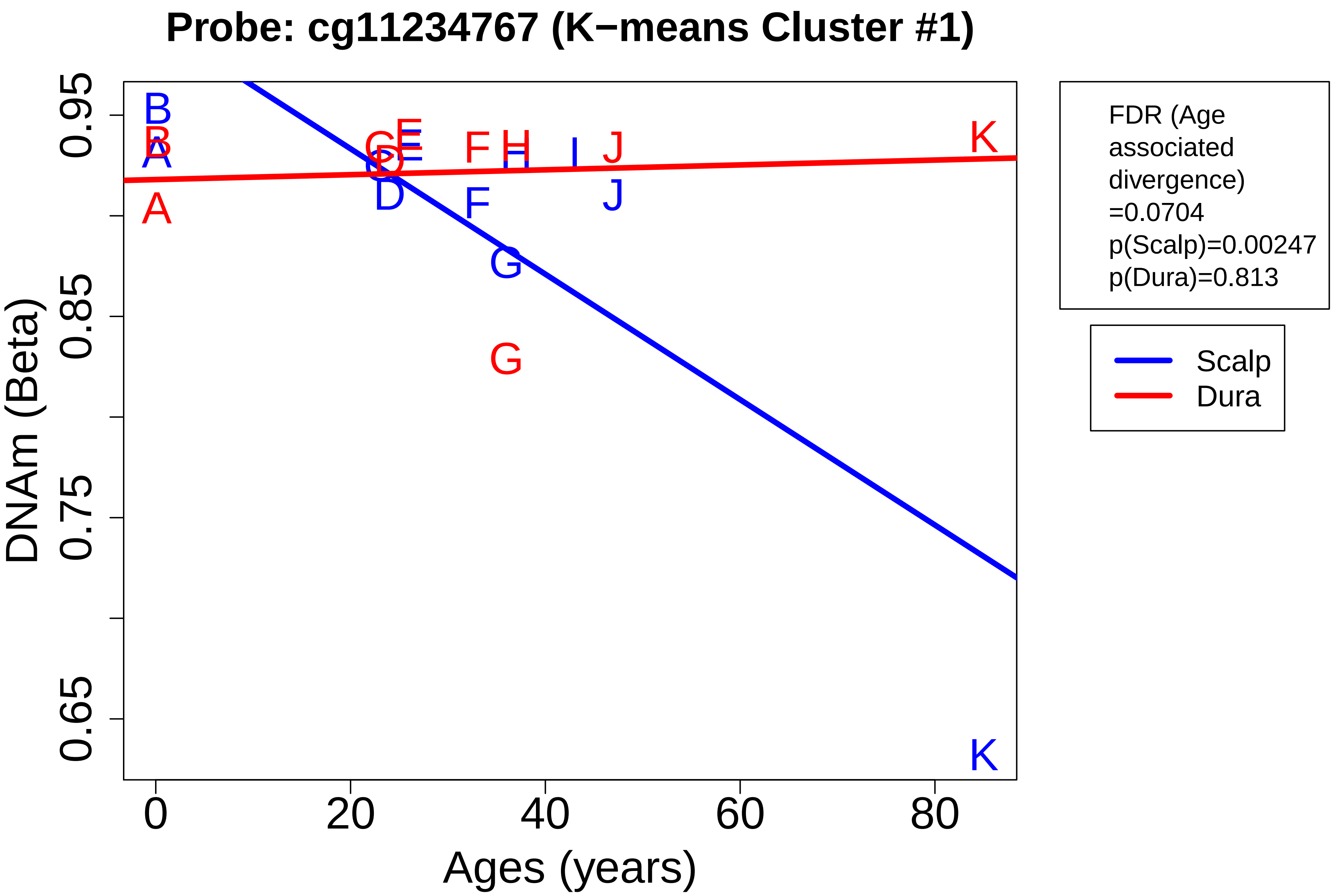

Probe: cg11234767 (K−means Cluster #1)
DNAm (Beta)
0.65
0.75
0.85
0.95
Ages (years)
0
20
40
60
80
FDR (Age associated divergence) =0.0704
p(Scalp)=0.00247
p(Dura)=0.813
Scalp
Dura

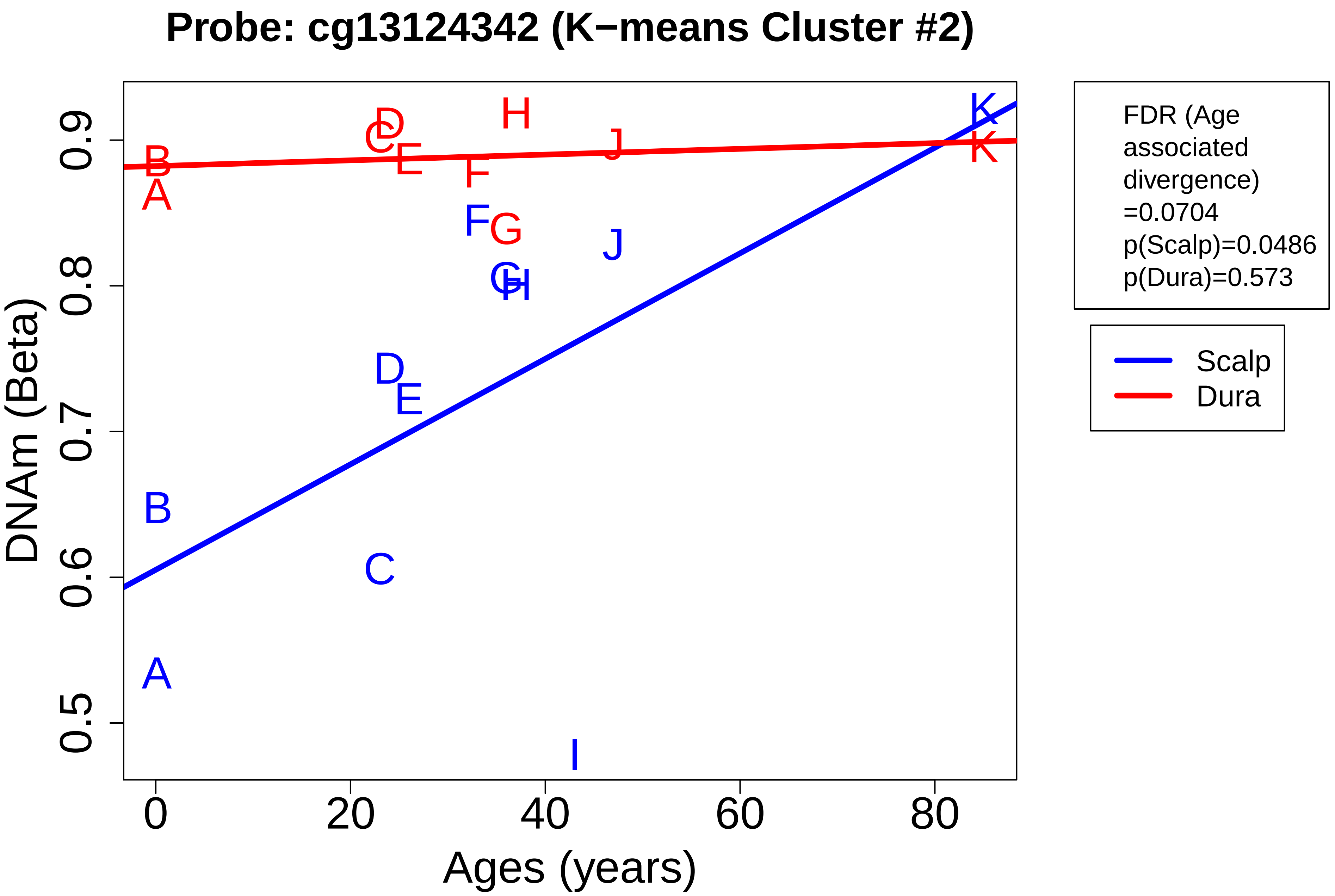

Probe: cg13124342 (K−means Cluster #2)
DNAm (Beta)
0.5
0.6
0.7
0.8
0.9
0
20
40
60
80
Ages (years)
A
B
C
D
E
F
G
H
I
J
K
FDR (Age associated divergence) =0.0704
p(Scalp)=0.0486
p(Dura)=0.573
Scalp
Dura

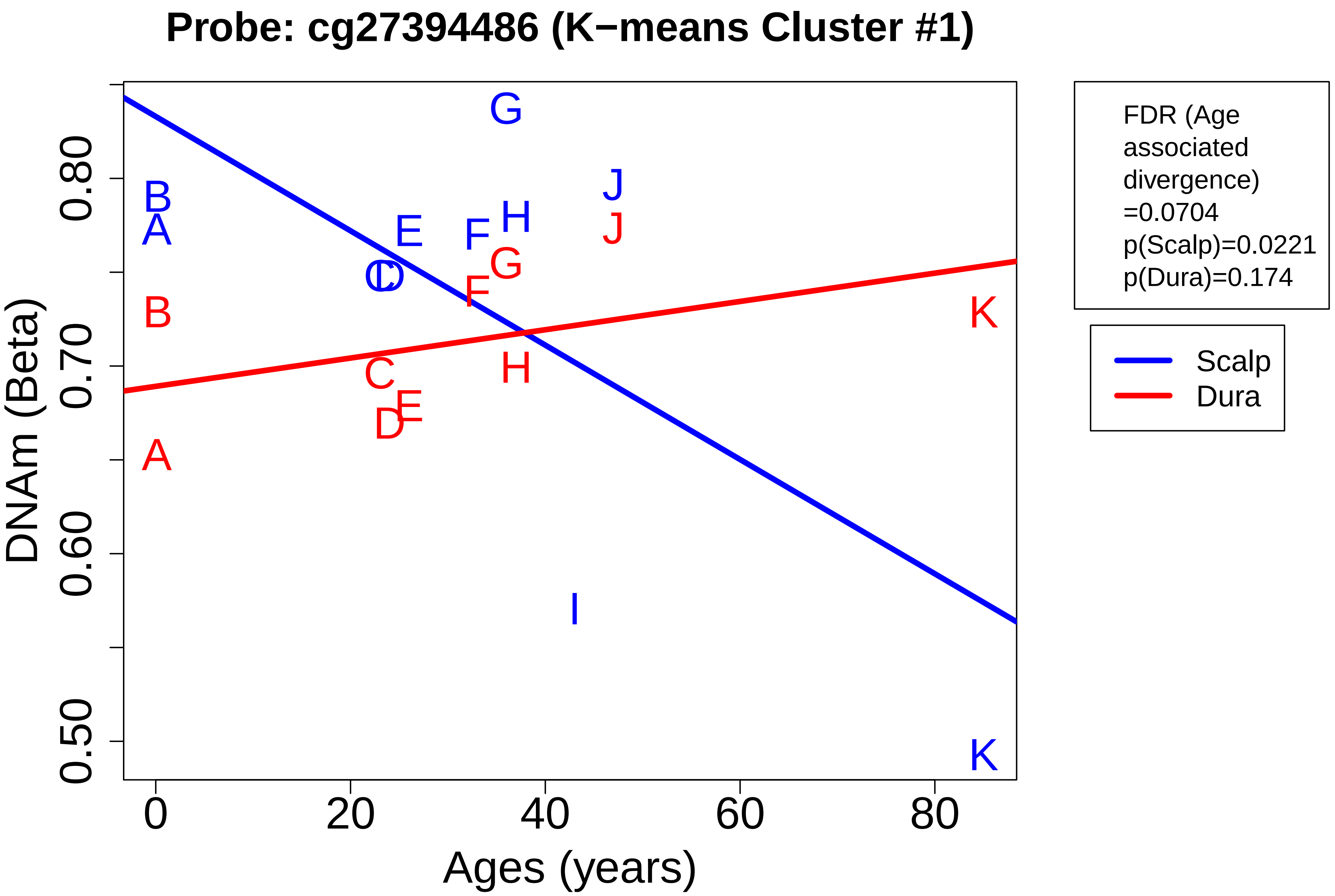

Probe: cg27394486 (K−means Cluster #1)
DNAm (Beta)
0.50
0.60
0.70
0.80
0
20
40
60
80
Ages (years)
FDR (Age associated divergence) =0.0704
p(Scalp)=0.0221
p(Dura)=0.174
Scalp
Dura

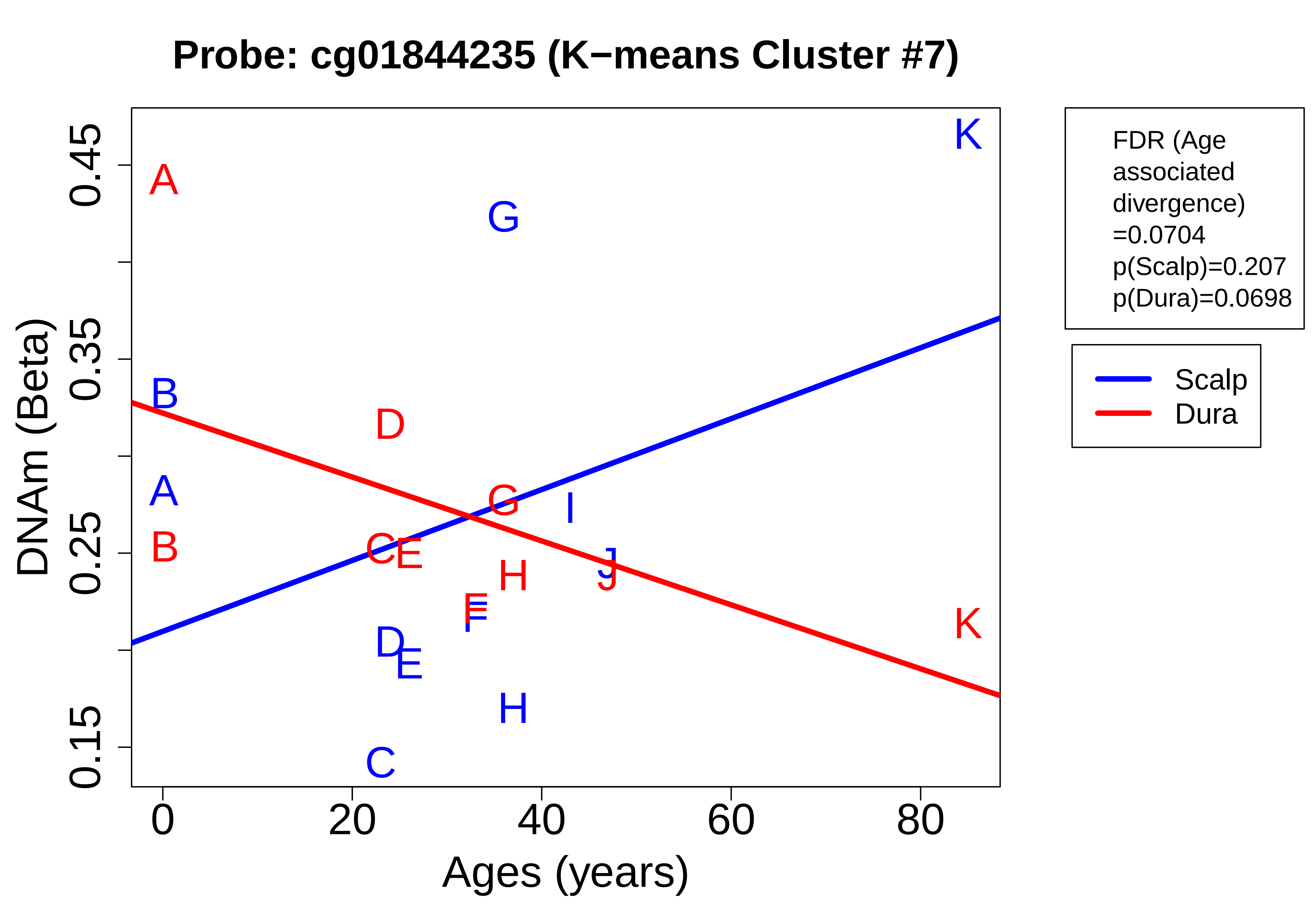
Probe: cg01844235 (K−means Cluster #7)
FDR (Age associated divergence) =0.0704
p(Scalp)=0.207
p(Dura)=0.0698
Scalp
Dura
DNAm (Beta)
0.15
0.25
0.35
0.45
0
20
40
60
80
Ages (years)
A
B
C
D
E
F
G
H
I
J
K

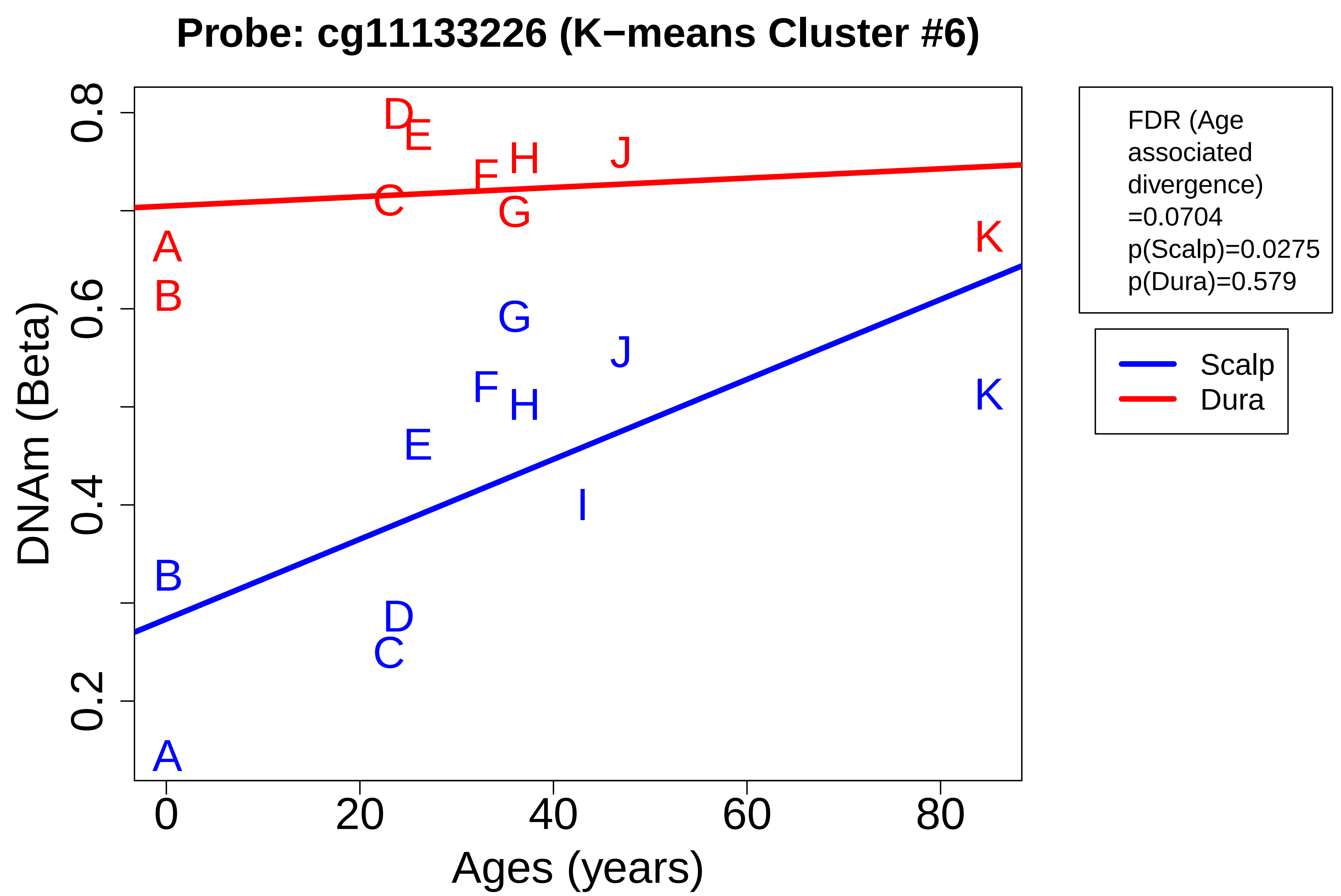

Probe: cg11133226 (K−means Cluster #6)
DNAm (Beta)
0.2
0.4
0.6
0.8
Ages (years)
0
20
40
60
80
A
B
C
D
E
F
G
H
I
J
K
FDR (Age associated divergence) =0.0704
p(Scalp)=0.0275
p(Dura)=0.579
Scalp
Dura

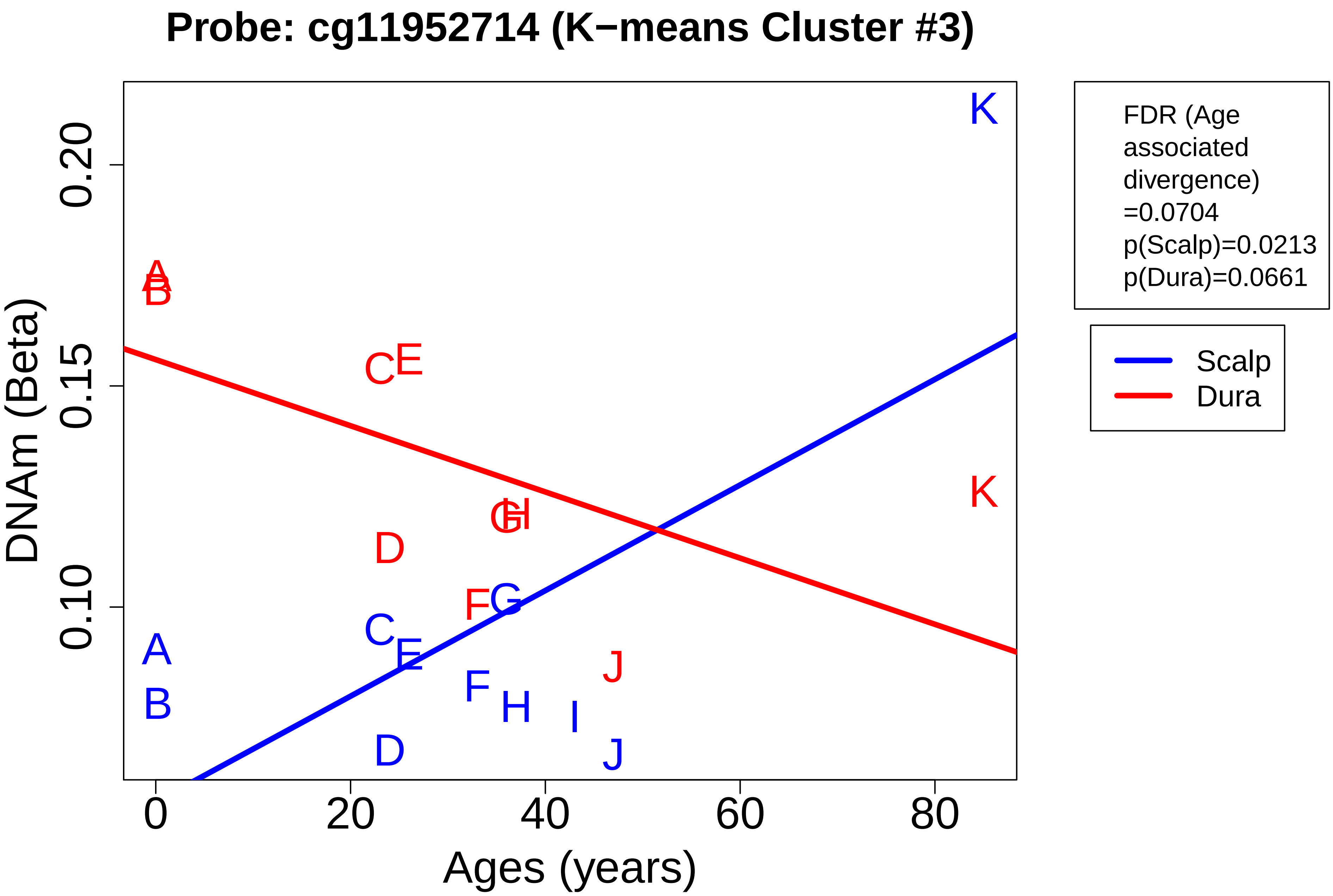

Probe: cg11952714 (K−means Cluster #3)
DNAm (Beta)
0.10
0.15
0.20
Ages (years)
0
20
40
60
80
FDR (Age associated divergence) =0.0704
p(Scalp)=0.0213
p(Dura)=0.0661
Scalp
Dura

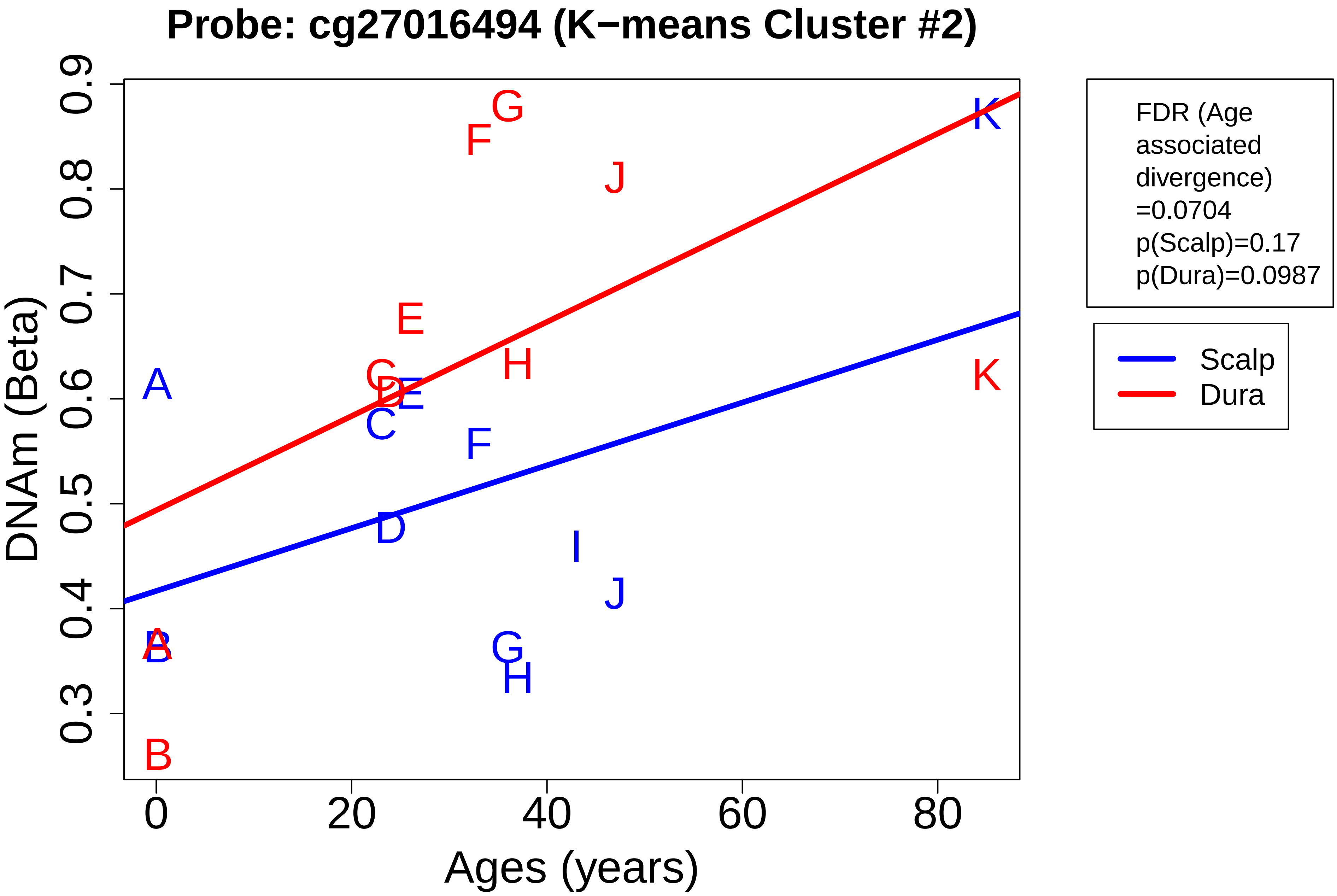

Probe: cg27016494 (K−means Cluster #2)
DNAm (Beta)
0.3
0.4
0.5
0.6
0.7
0.8
0.9
0
20
40
60
80
Ages (years)
A
B
C
D
E
F
G
H
I
J
K
FDR (Age associated divergence) =0.0704
p(Scalp)=0.17
p(Dura)=0.0987
Scalp
Dura

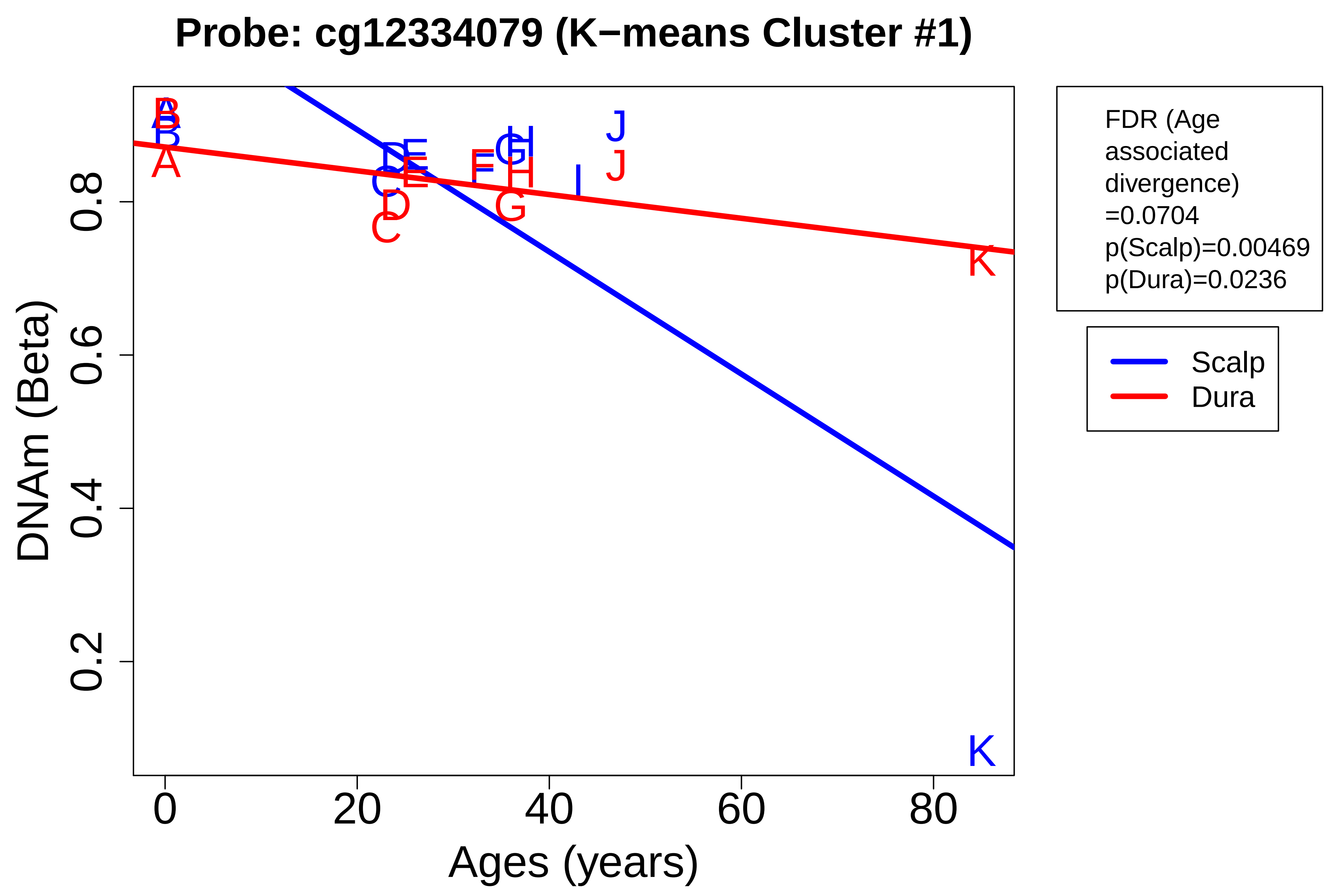

Probe: cg12334079 (K−means Cluster #1)
DNAm (Beta)
0.2
0.4
0.6
0.8
Ages (years)
0
20
40
60
80
FDR (Age associated divergence) =0.0704
p(Scalp)=0.00469
p(Dura)=0.0236
Scalp
Dura
A
B
C
D
E
F
G
H
I
J
K

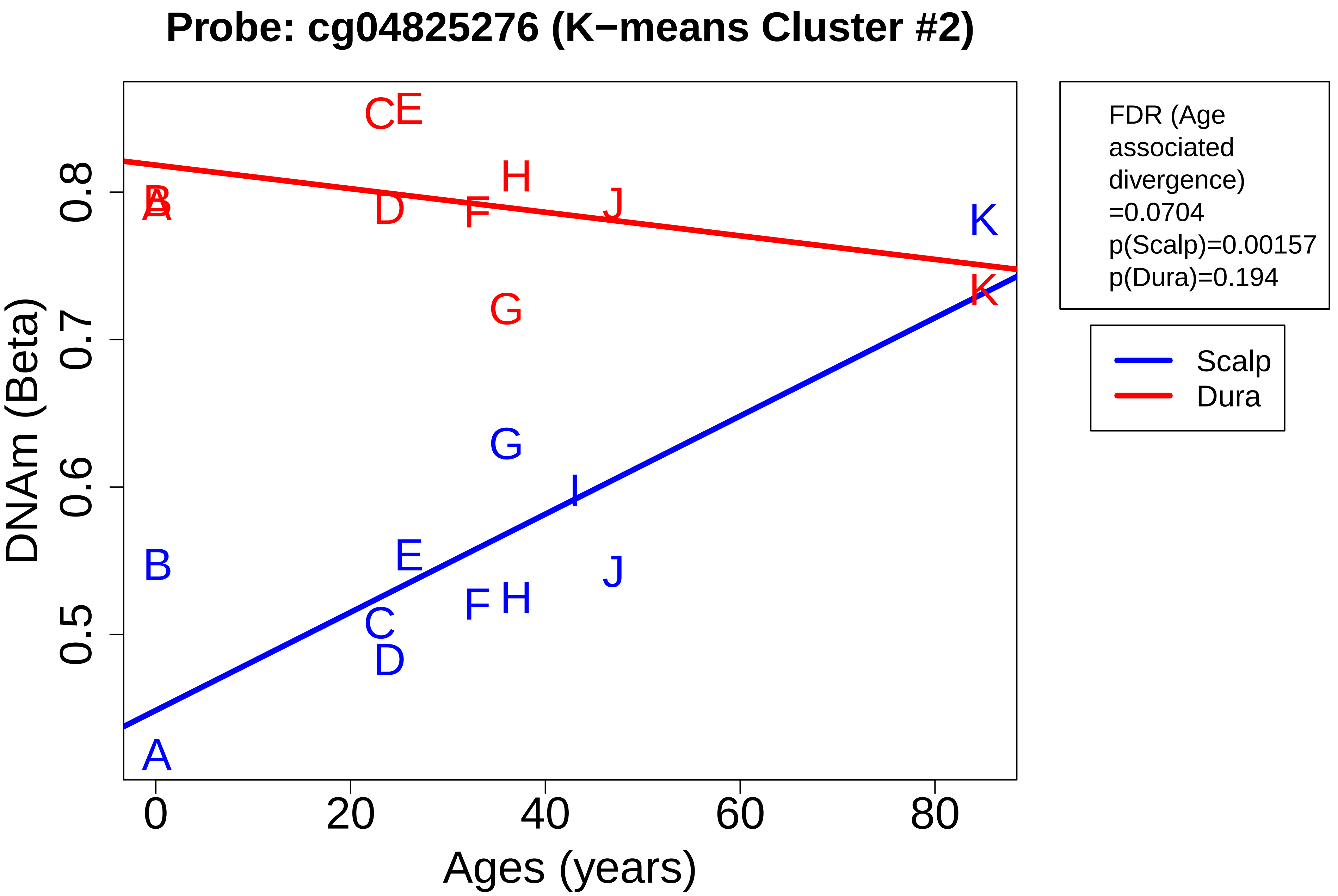

Probe: cg04825276 (K−means Cluster #2)
DNAm (Beta)
0.5
0.6
0.7
0.8
0
20
40
60
80
Ages (years)
FDR (Age associated divergence) =0.0704
p(Scalp)=0.00157
p(Dura)=0.194
Scalp
Dura

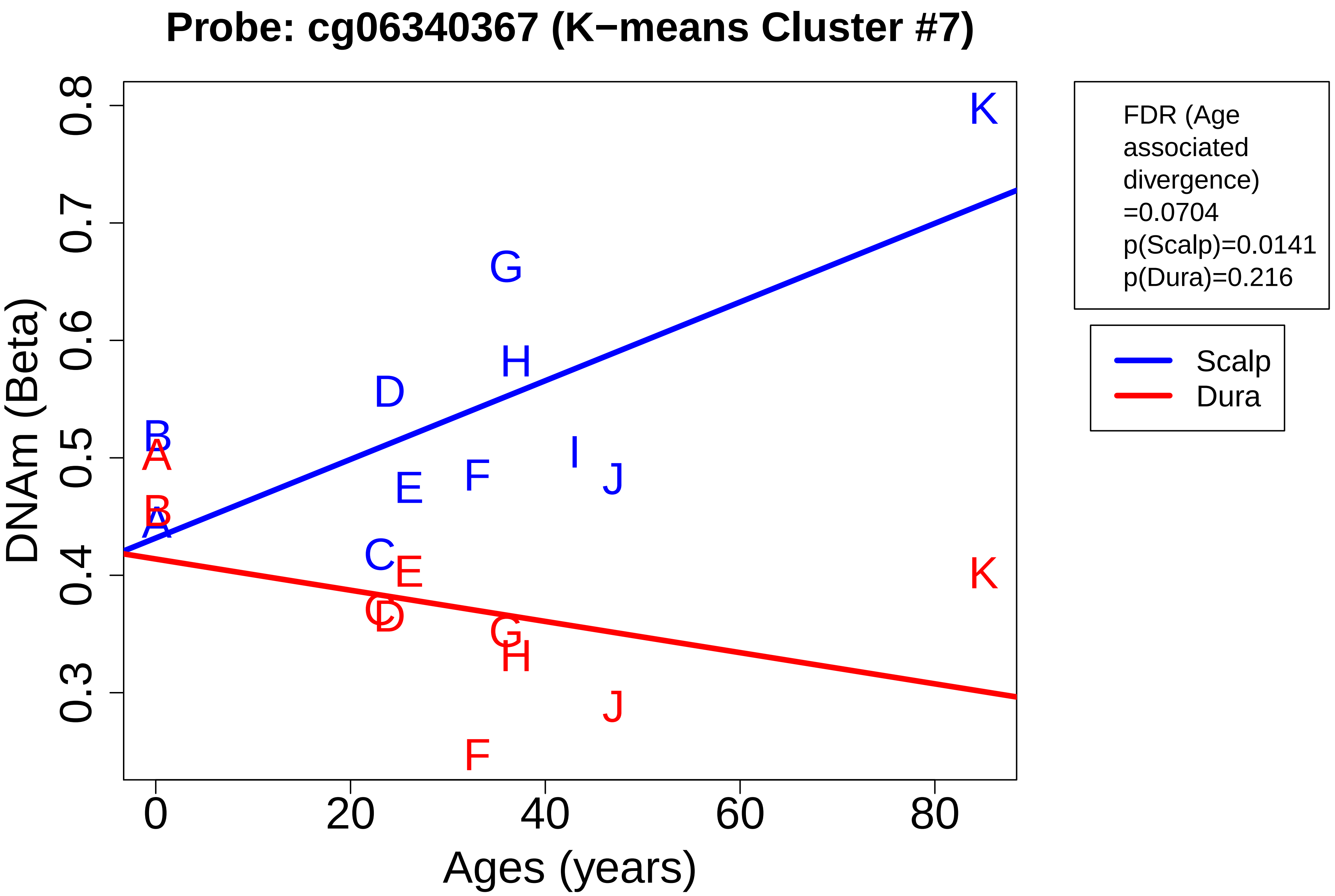
Probe: cg06340367 (K−means Cluster #7)
DNAm (Beta)
0.3
0.4
0.5
0.6
0.7
0.8
0
20
40
60
80
Ages (years)
FDR (Age associated divergence) =0.0704
p(Scalp)=0.0141
p(Dura)=0.216
Scalp
Dura
A
B
C
D
E
F
G
H
I
J
K

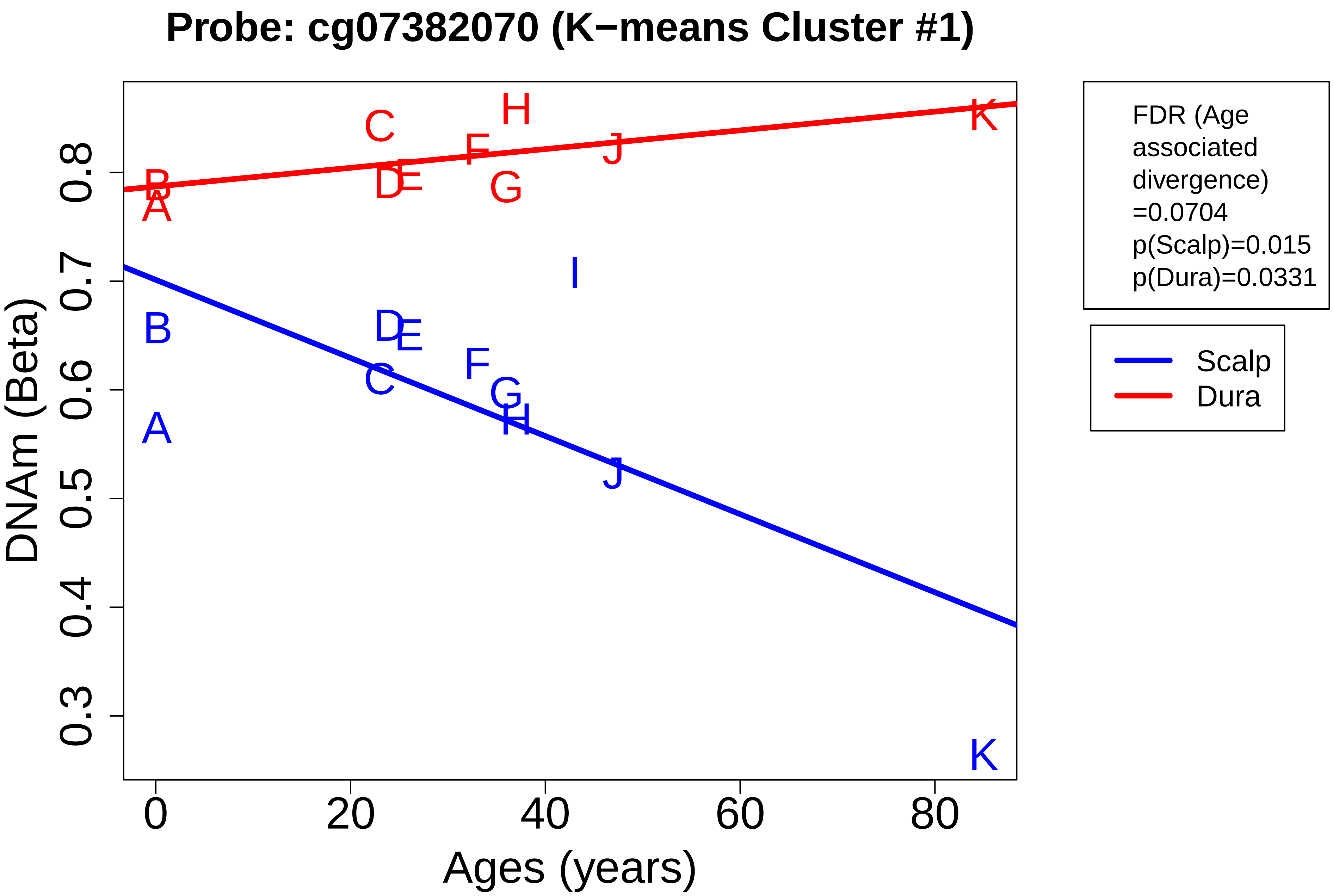

Probe: cg07382070 (K−means Cluster #1)
DNAm (Beta)
0.3
0.4
0.5
0.6
0.7
0.8
0
20
40
60
80
Ages (years)
FDR (Age associated divergence) =0.0704
p(Scalp)=0.015
p(Dura)=0.0331
Scalp
Dura

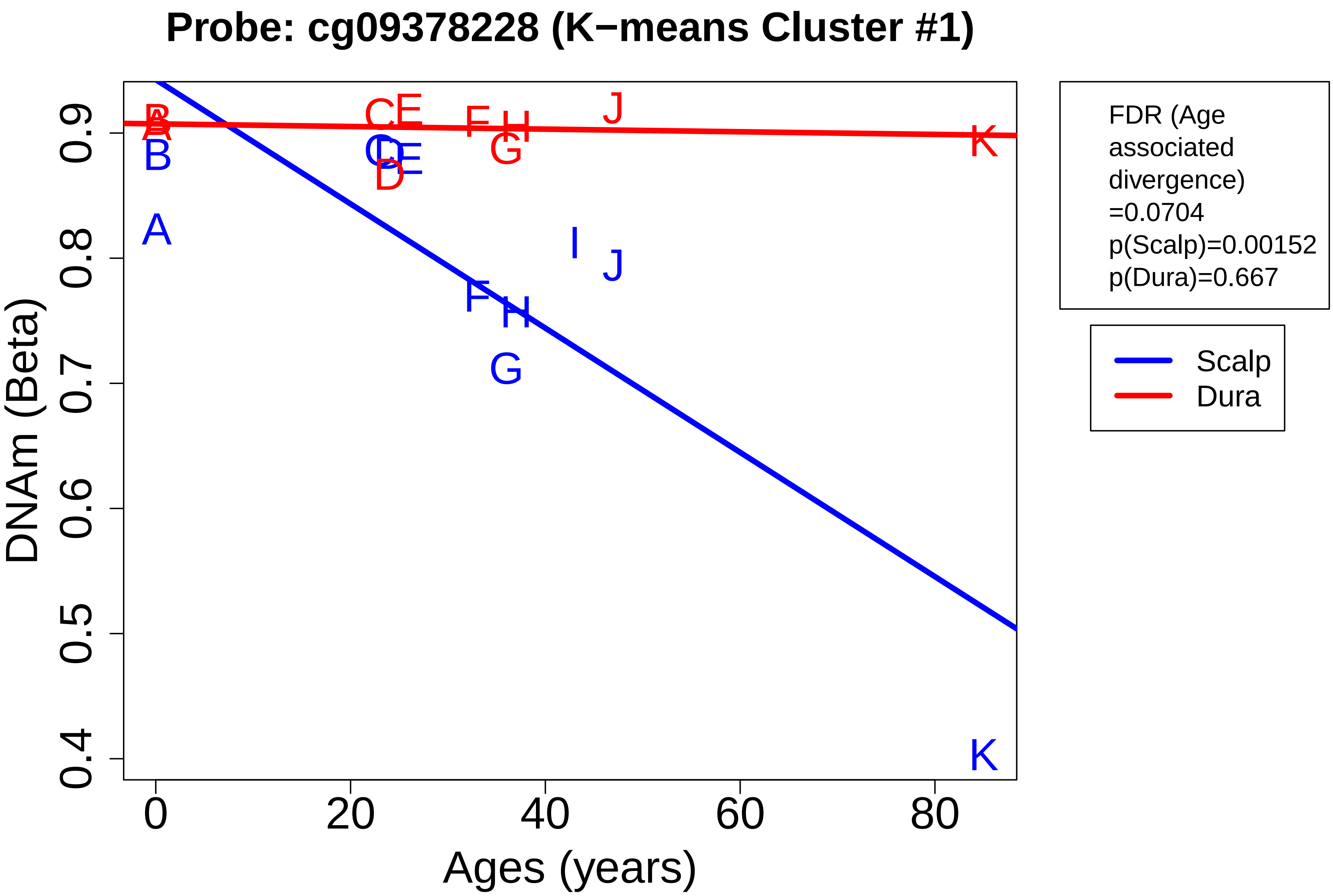

Probe: cg09378228 (K−means Cluster #1)
DNAm (Beta)
0.4
0.5
0.6
0.7
0.8
0.9
0
20
40
60
80
Ages (years)
A
B
C
D
E
F
G
H
I
J
K
FDR (Age associated divergence) =0.0704
p(Scalp)=0.00152
p(Dura)=0.667
Scalp
Dura

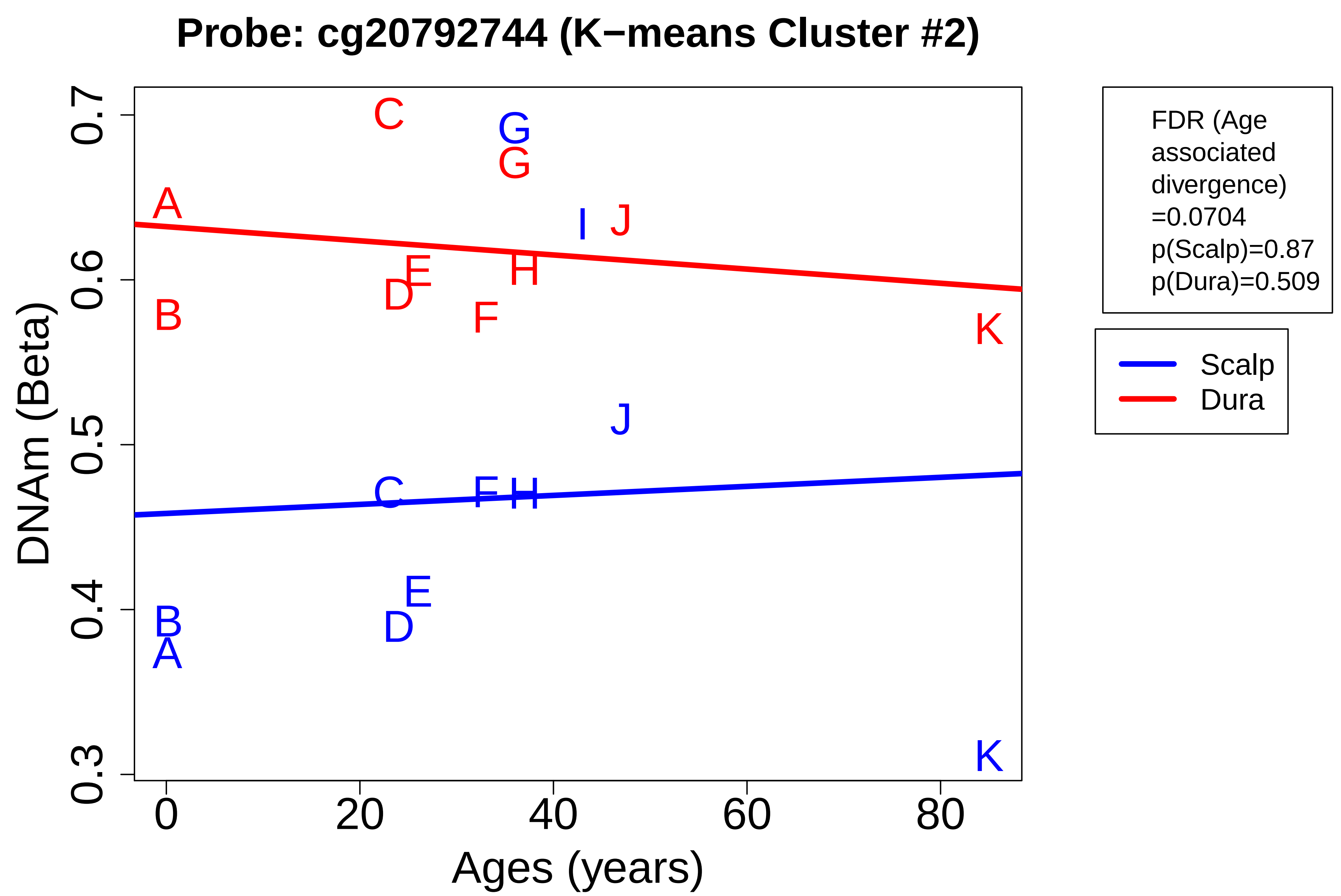

Probe: cg20792744 (K−means Cluster #2)
DNAm (Beta)
0.3
0.4
0.5
0.6
0.7
Ages (years)
0
20
40
60
80
FDR (Age associated divergence) =0.0704
p(Scalp)=0.87
p(Dura)=0.509
Scalp
Dura
A
B
C
D
E
F
G
H
I
J
K

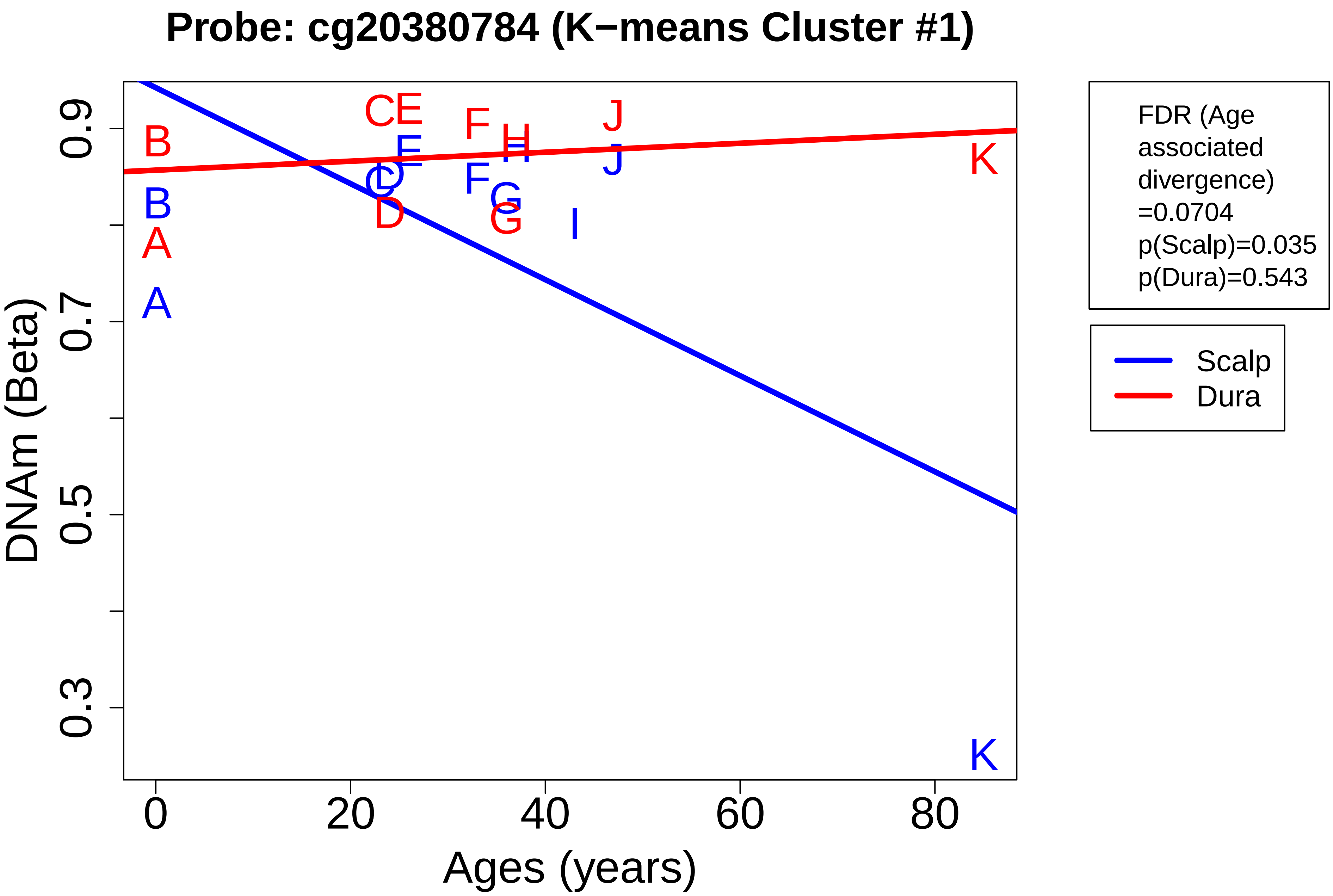

Probe: cg20380784 (K−means Cluster #1)
DNAm (Beta)
0.3
0.5
0.7
0.9
Ages (years)
0
20
40
60
80
FDR (Age associated divergence) =0.0704
p(Scalp)=0.035
p(Dura)=0.543
Scalp
Dura

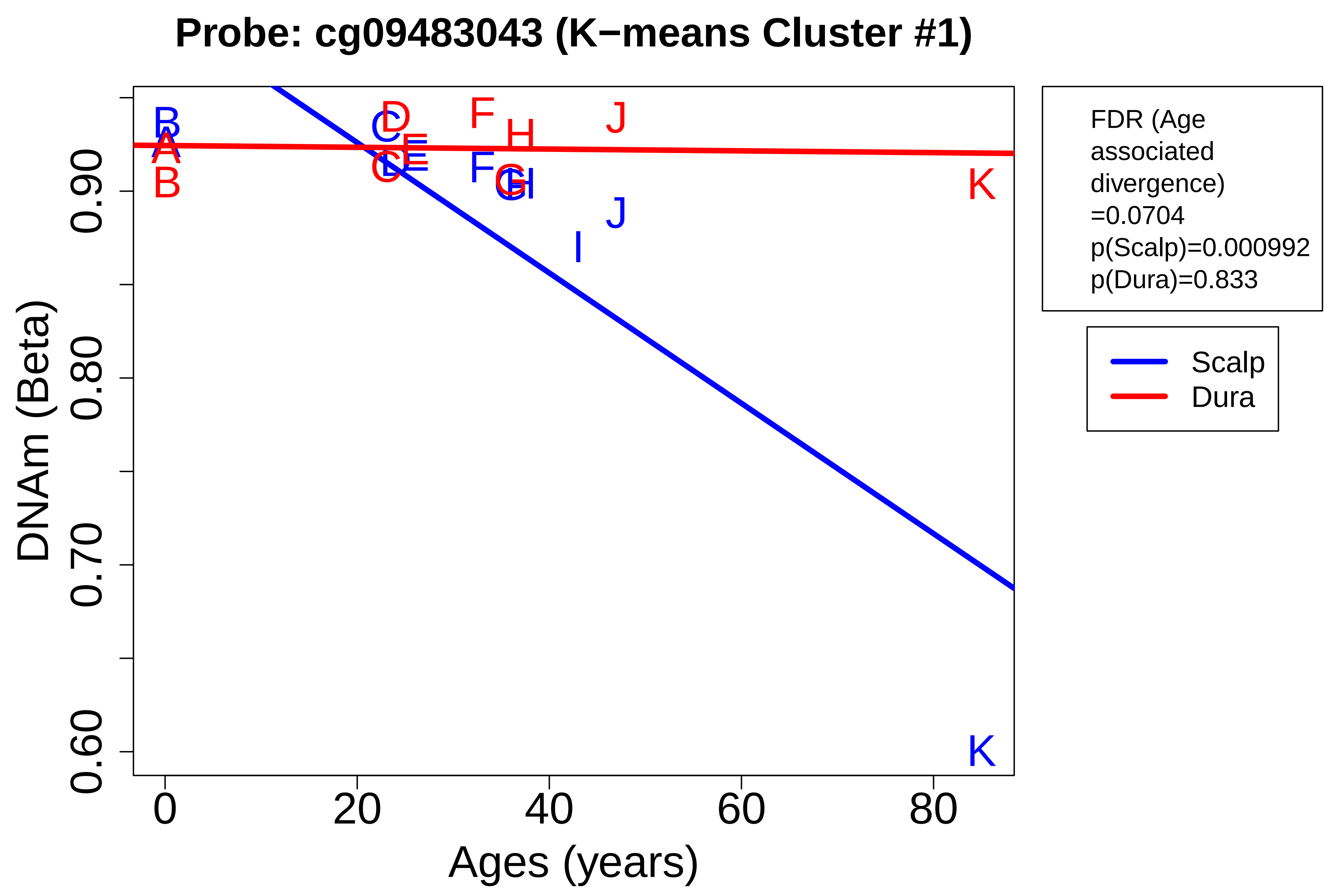

Probe: cg09483043 (K−means Cluster #1)
DNAm (Beta)
0.60
0.70
0.80
0.90
0
20
40
60
80
Ages (years)
FDR (Age associated divergence) =0.0704
p(Scalp)=0.000992
p(Dura)=0.833
Scalp
Dura

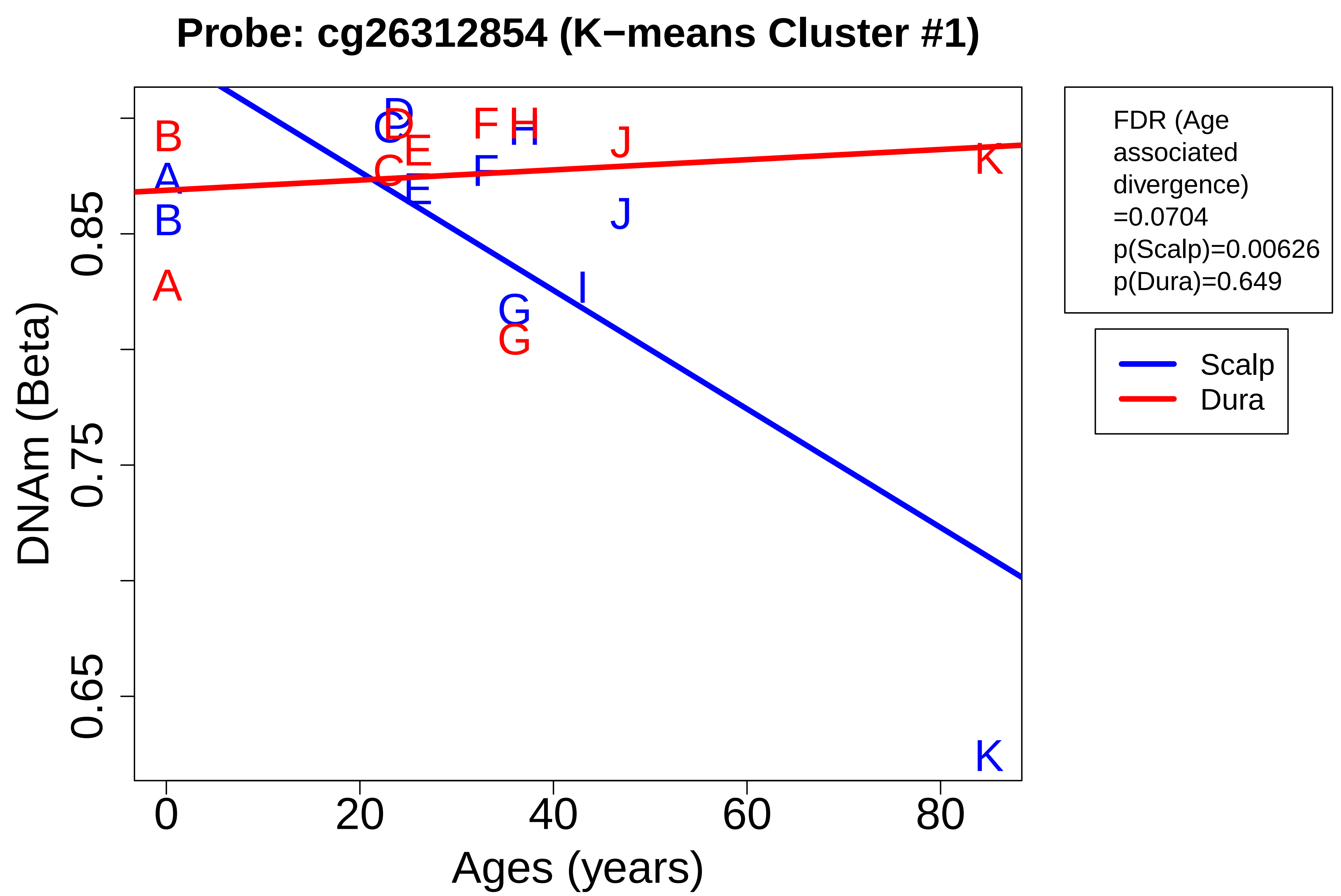
Probe: cg26312854 (K−means Cluster #1)
DNAm (Beta)
0.65
0.75
0.85
Ages (years)
0
20
40
60
80
FDR (Age associated divergence) =0.0704
p(Scalp)=0.00626
p(Dura)=0.649
Scalp
Dura

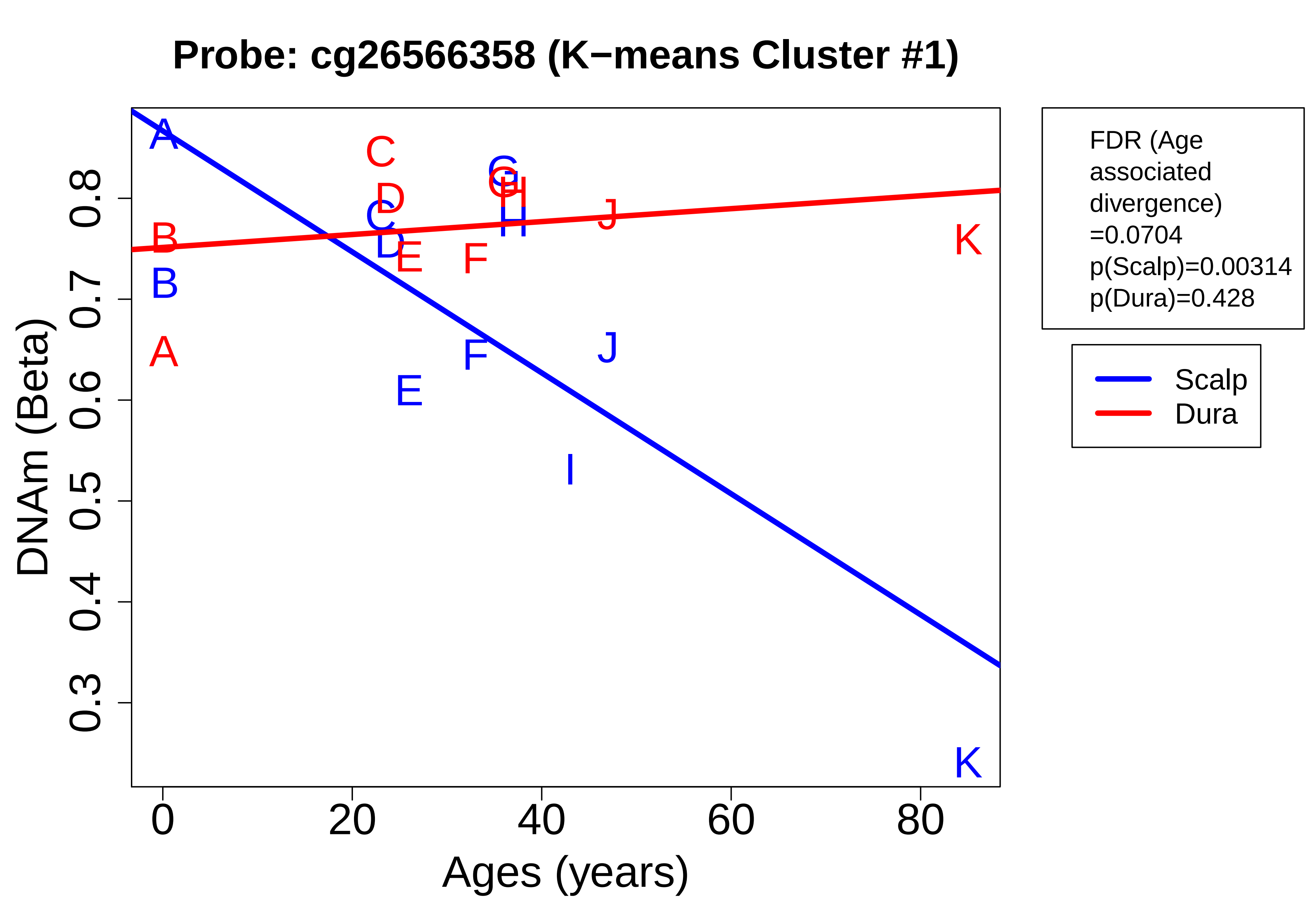
Probe: cg26566358 (K−means Cluster #1)
FDR (Age associated divergence) =0.0704
p(Scalp)=0.00314
p(Dura)=0.428
Scalp
Dura
DNAm (Beta)
Ages (years)
0.3
0.4
0.5
0.6
0.7
0.8
0
20
40
60
80
A
B
C
D
E
F
G
H
I
J
K

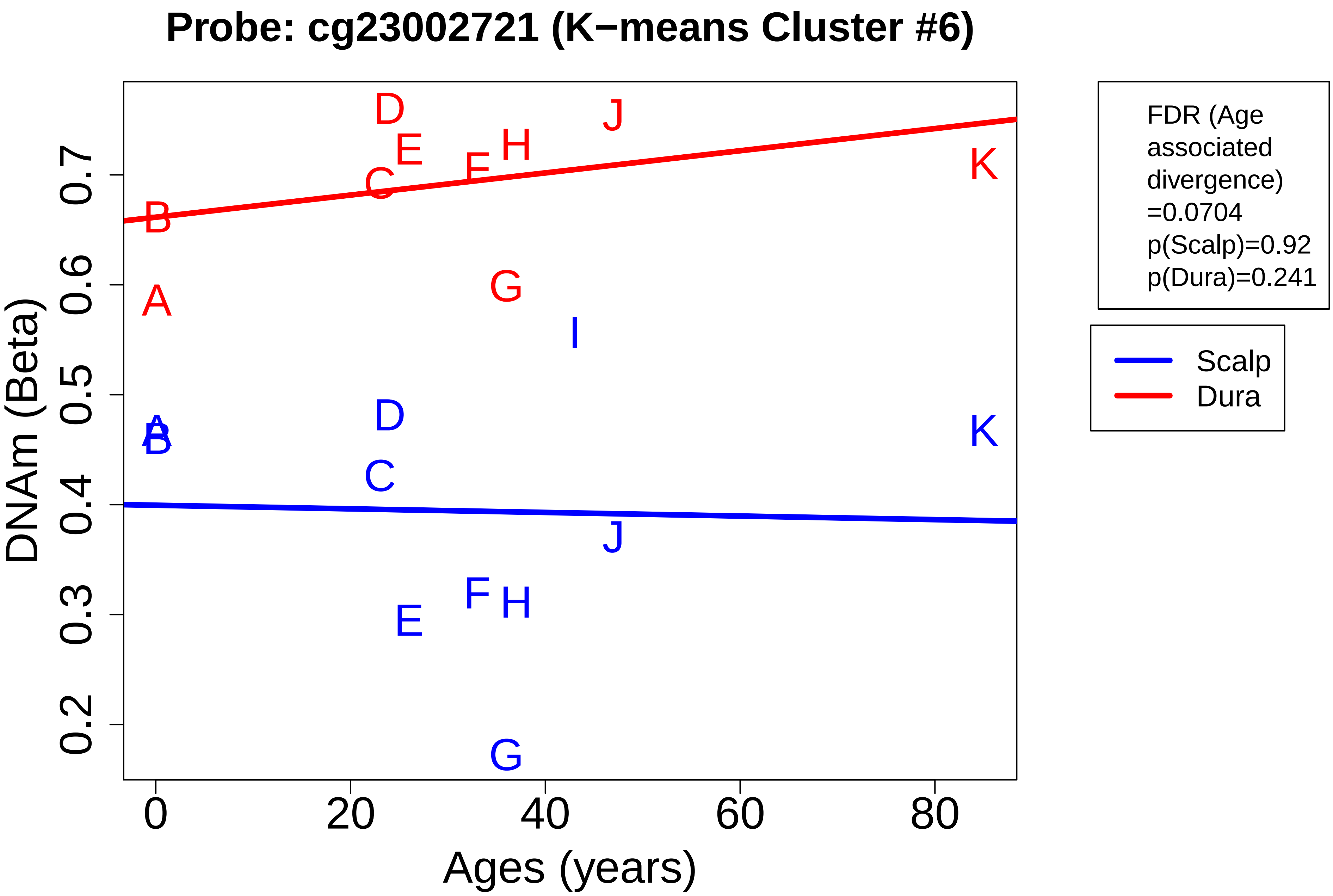

Probe: cg23002721 (K−means Cluster #6)
DNAm (Beta)
0.2
0.3
0.4
0.5
0.6
0.7
0
20
40
60
80
Ages (years)
FDR (Age associated divergence) =0.0704
p(Scalp)=0.92
p(Dura)=0.241
Scalp
Dura

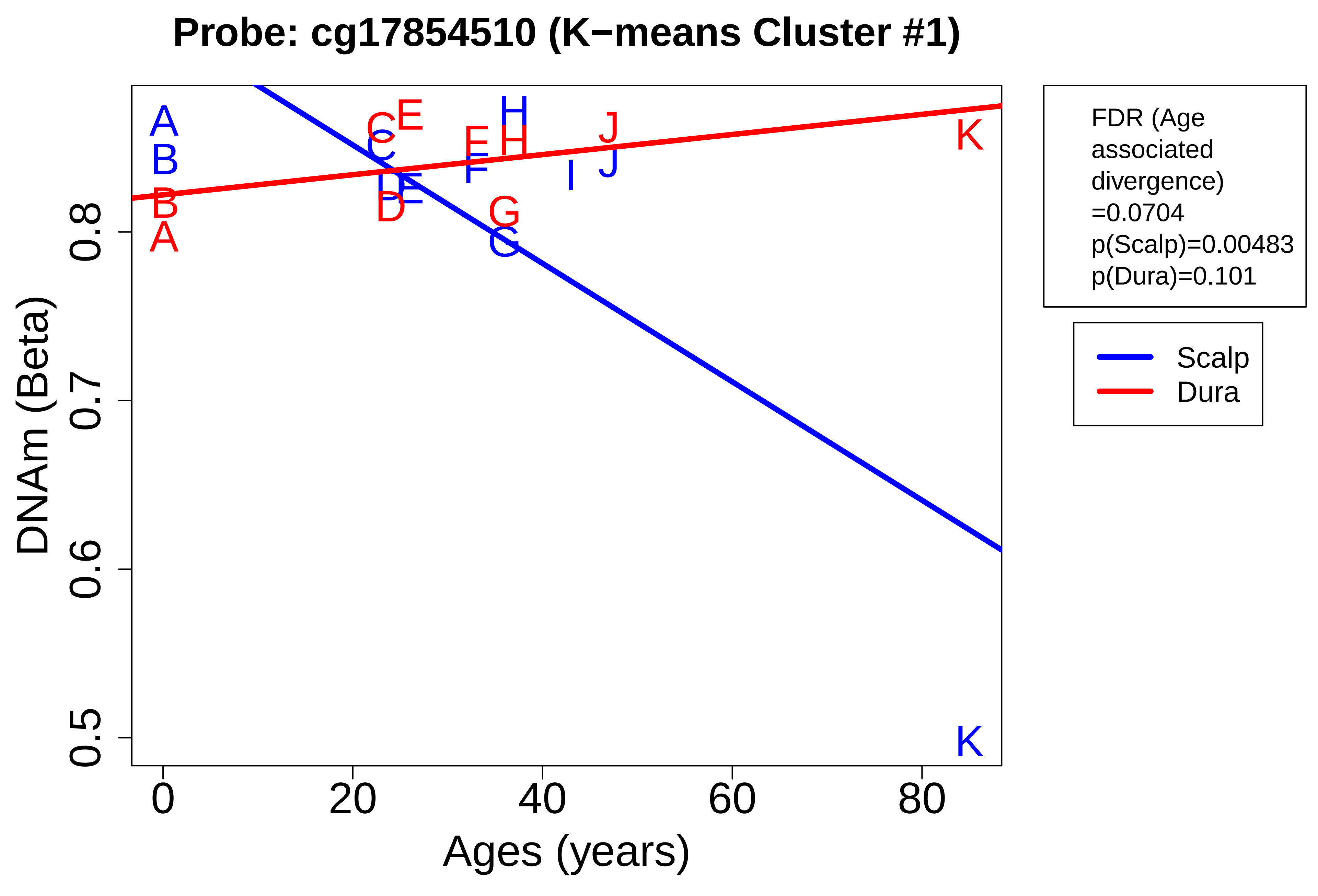
Probe: cg17854510 (K−means Cluster #1)
DNAm (Beta)
0.5
0.6
0.7
0.8
Ages (years)
0
20
40
60
80
A
B
C
D
E
F
G
H
I
J
K
FDR (Age
associated
divergence)
=0.0704
p(Scalp)=0.00483
p(Dura)=0.101
Scalp
Dura

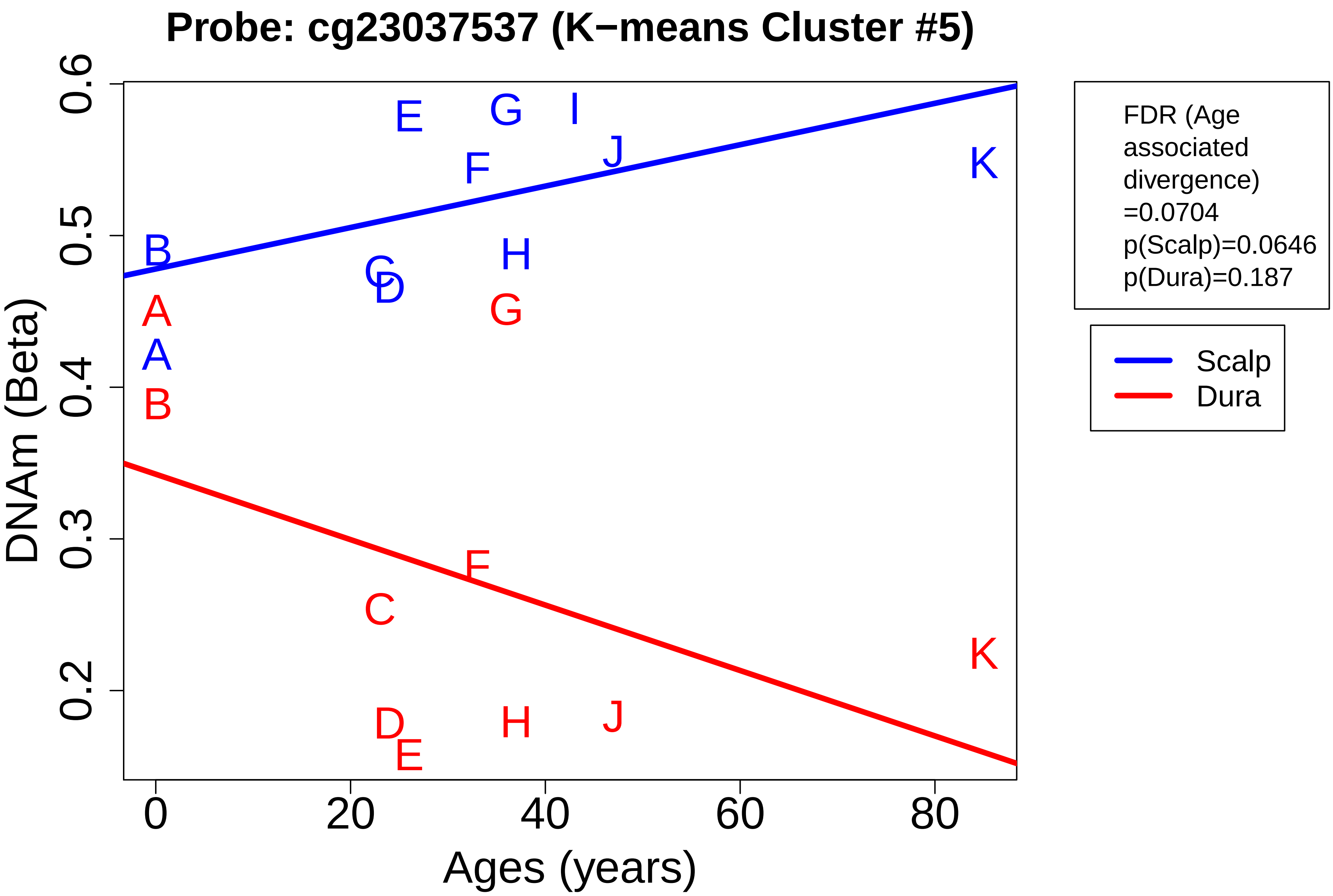

Probe: cg23037537 (K−means Cluster #5)
DNAm (Beta)
0.2
0.3
0.4
0.5
0.6
Ages (years)
0
20
40
60
80
A B C D E F G H I J K
A B C D E F G H J K
FDR (Age associated divergence) =0.0704
p(Scalp)=0.0646
p(Dura)=0.187
Scalp
Dura

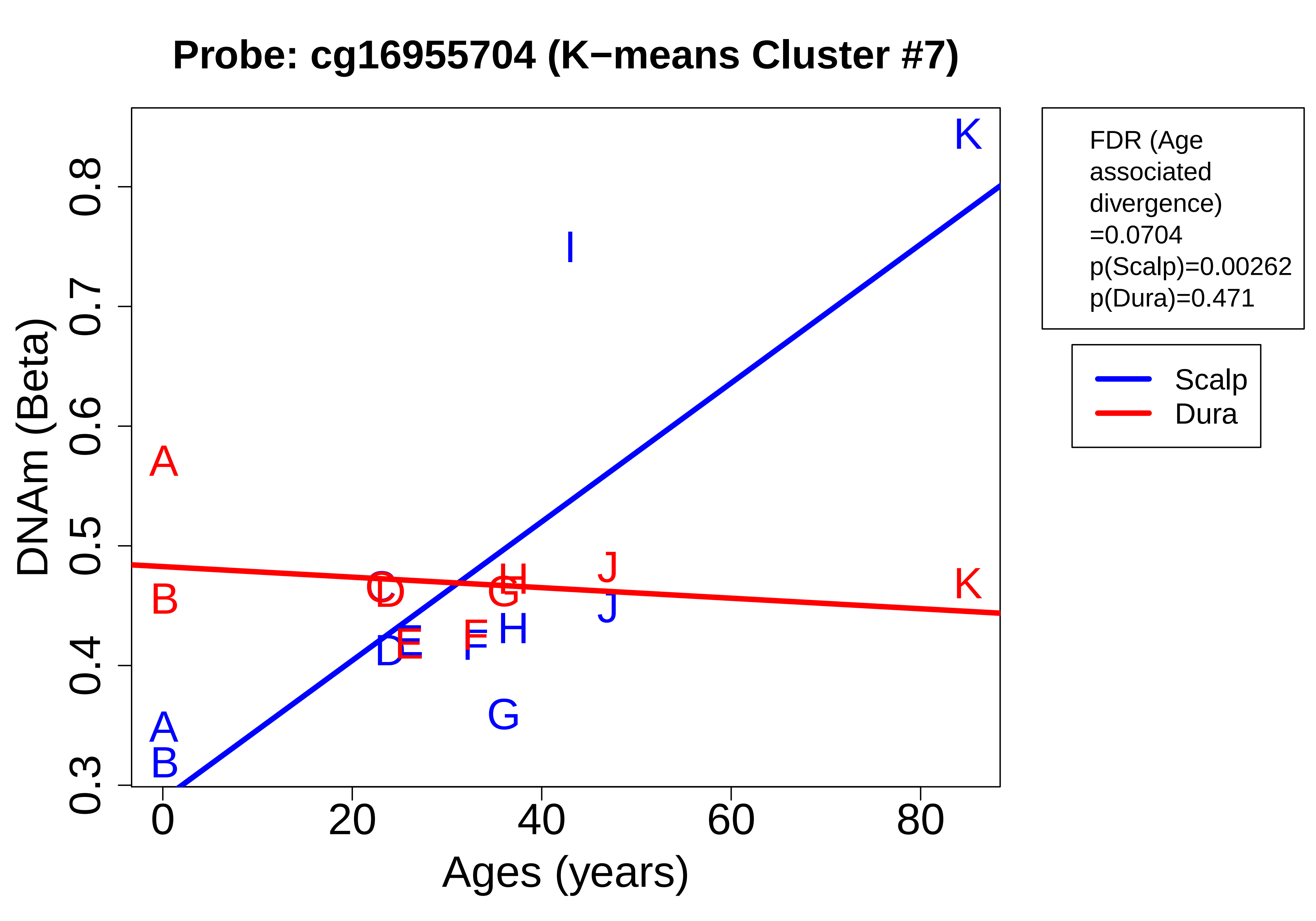

Probe: cg16955704 (K−means Cluster #7)
DNAm (Beta)
0.3
0.4
0.5
0.6
0.7
0.8
0
20
40
60
80
Ages (years)
FDR (Age associated divergence) =0.0704
p(Scalp)=0.00262
p(Dura)=0.471
Scalp
Dura

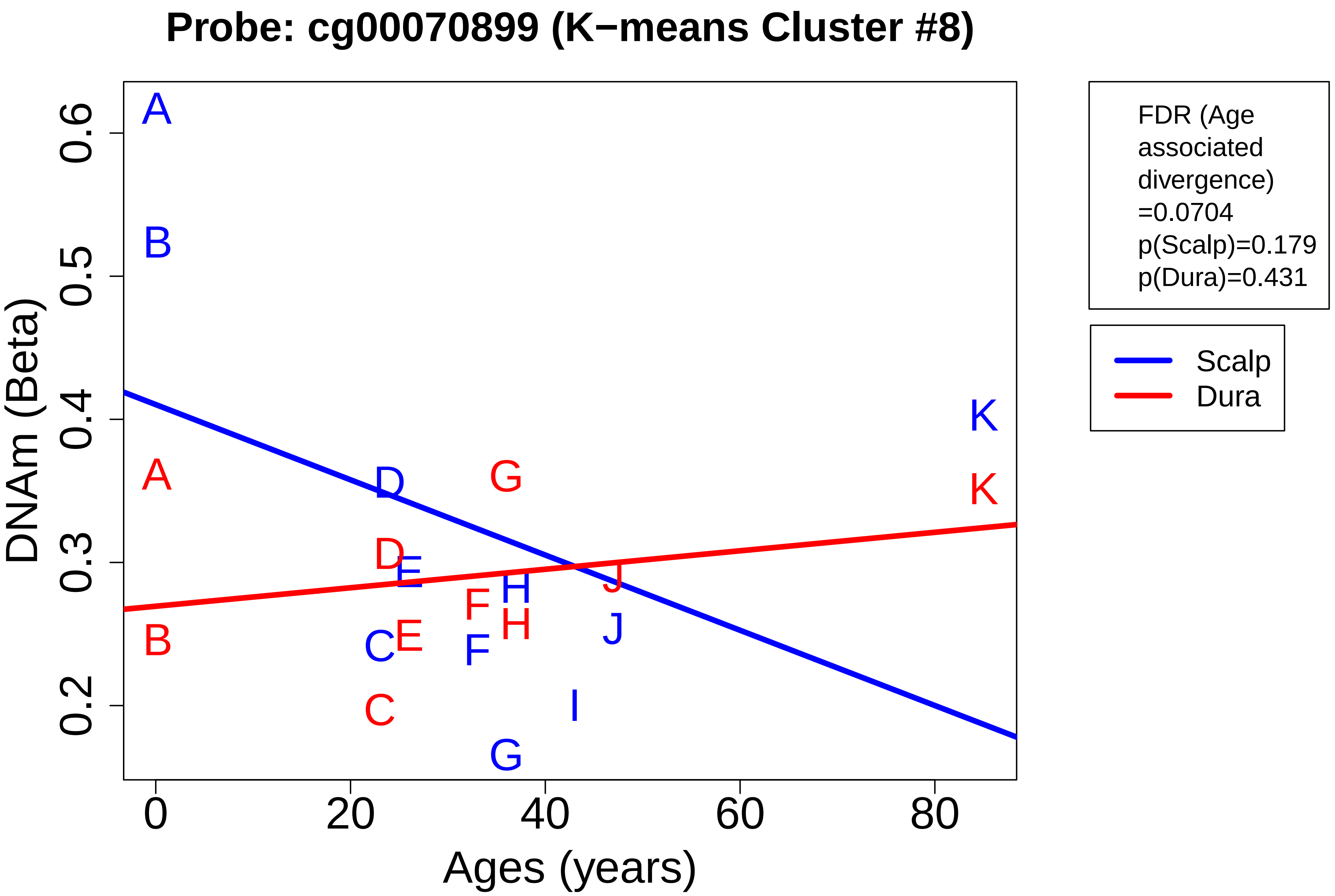

Probe: cg00070899 (K−means Cluster #8)
DNAm (Beta)
0.2
0.3
0.4
0.5
0.6
Ages (years)
0
20
40
60
80
A
B
C
D
E
F
G
H
I
J
K
FDR (Age associated divergence) =0.0704
p(Scalp)=0.179
p(Dura)=0.431
Scalp
Dura

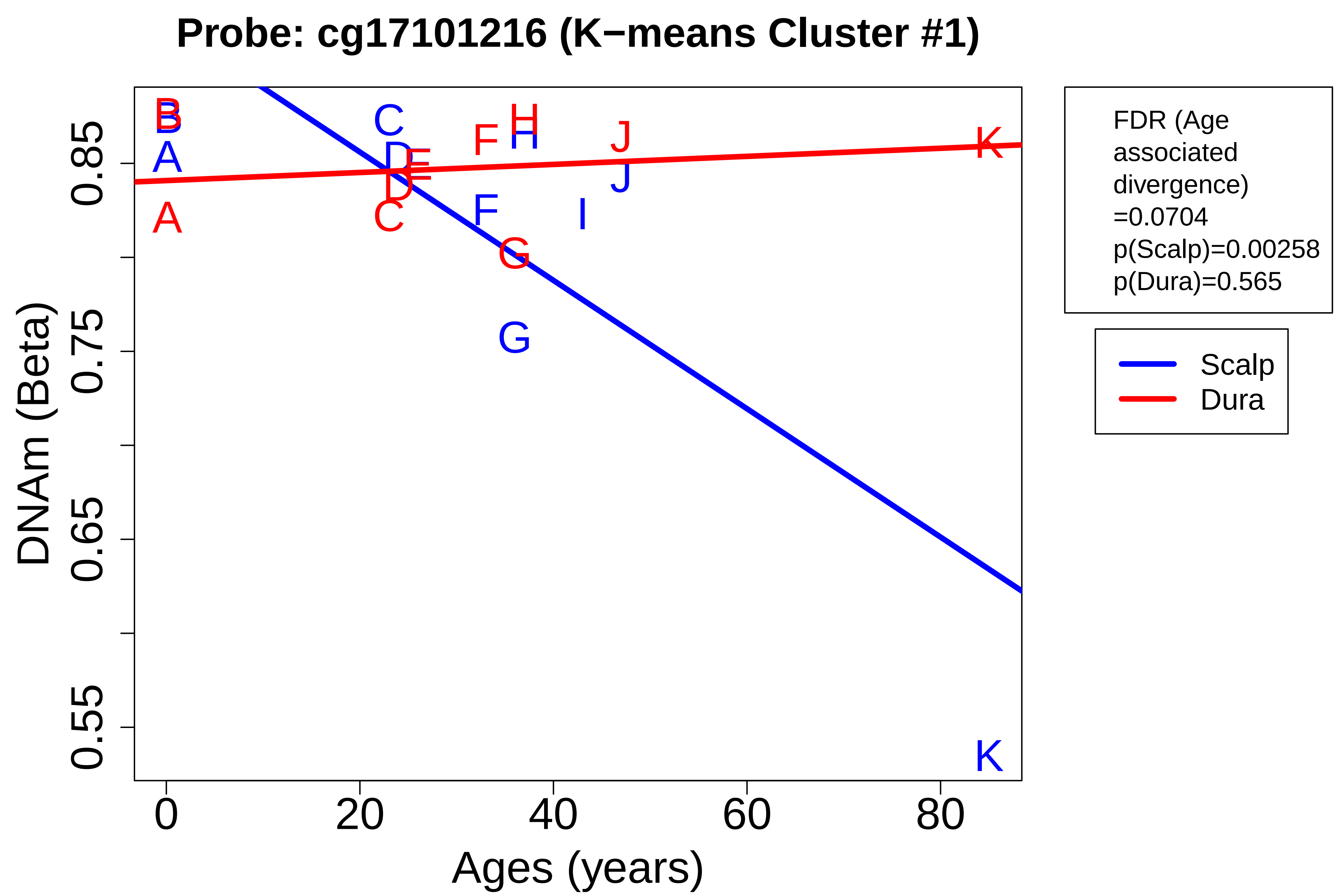

Probe: cg17101216 (K−means Cluster #1)
DNAm (Beta)
0.55
0.65
0.75
0.85
0
20
40
60
80
Ages (years)
A
B
C
D
E
F
G
H
I
J
K
FDR (Age associated divergence) =0.0704
p(Scalp)=0.00258
p(Dura)=0.565
Scalp
Dura

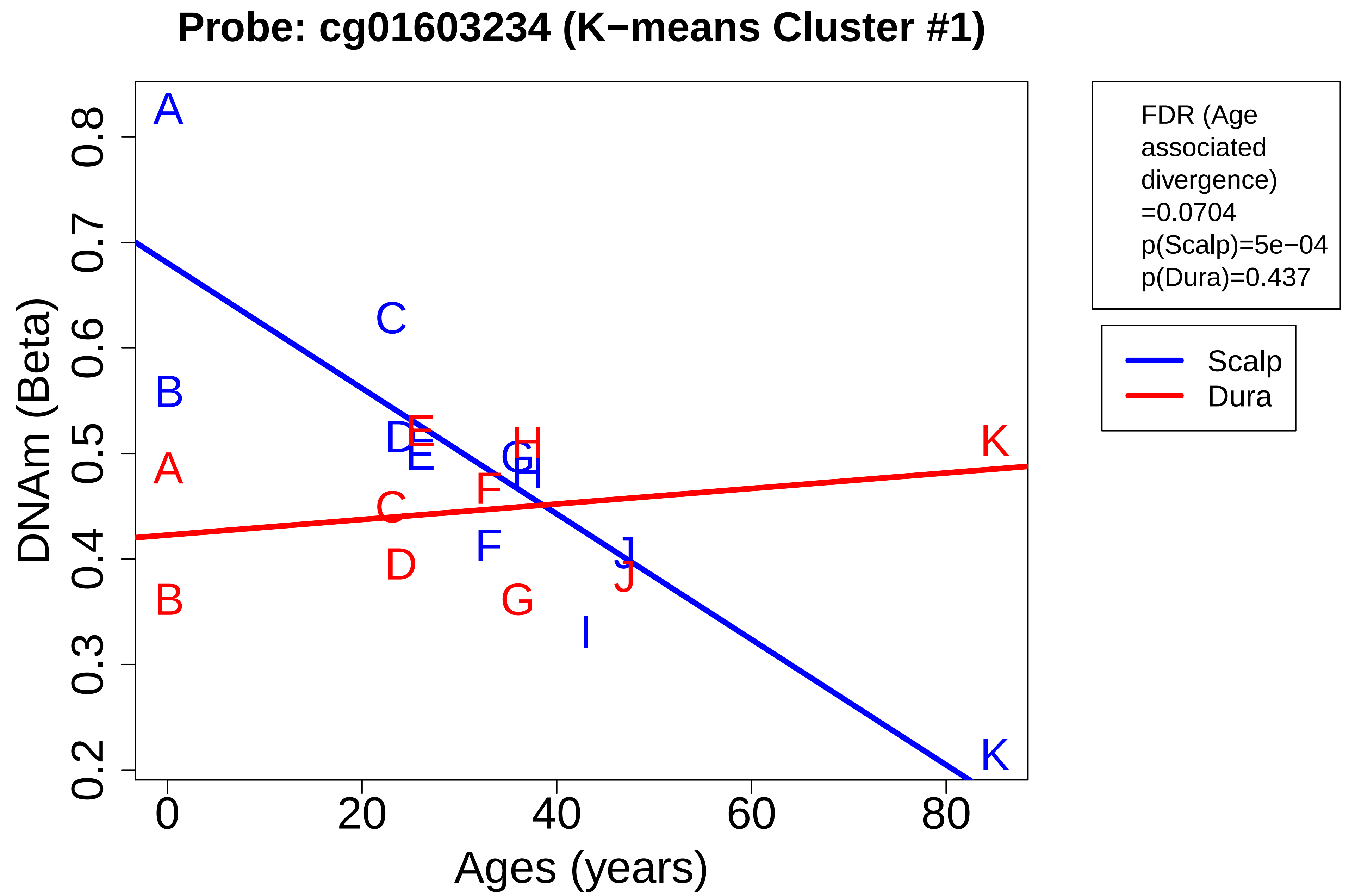

Probe: cg01603234 (K−means Cluster #1)
DNAm (Beta)
0.2
0.3
0.4
0.5
0.6
0.7
0.8
0
20
40
60
80
Ages (years)
FDR (Age associated divergence) =0.0704
p(Scalp)=5e−04
p(Dura)=0.437
Scalp
Dura

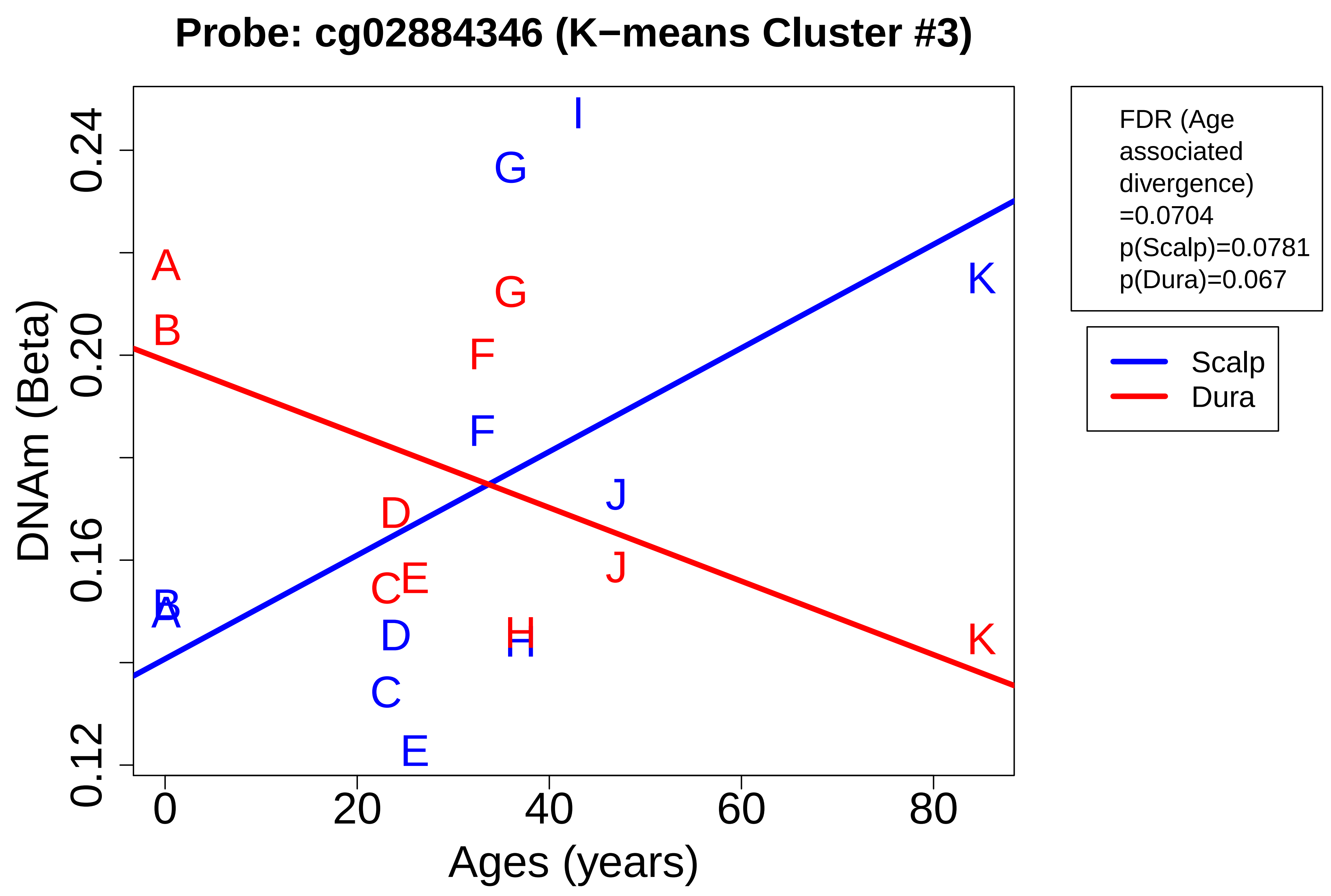

Probe: cg02884346 (K−means Cluster #3)
DNAm (Beta)
Ages (years)
0.12
0.16
0.20
0.24
0
20
40
60
80
FDR (Age associated divergence) =0.0704
p(Scalp)=0.0781
p(Dura)=0.067
Scalp
Dura
A
B
C
D
E
F
G
H
I
J
K

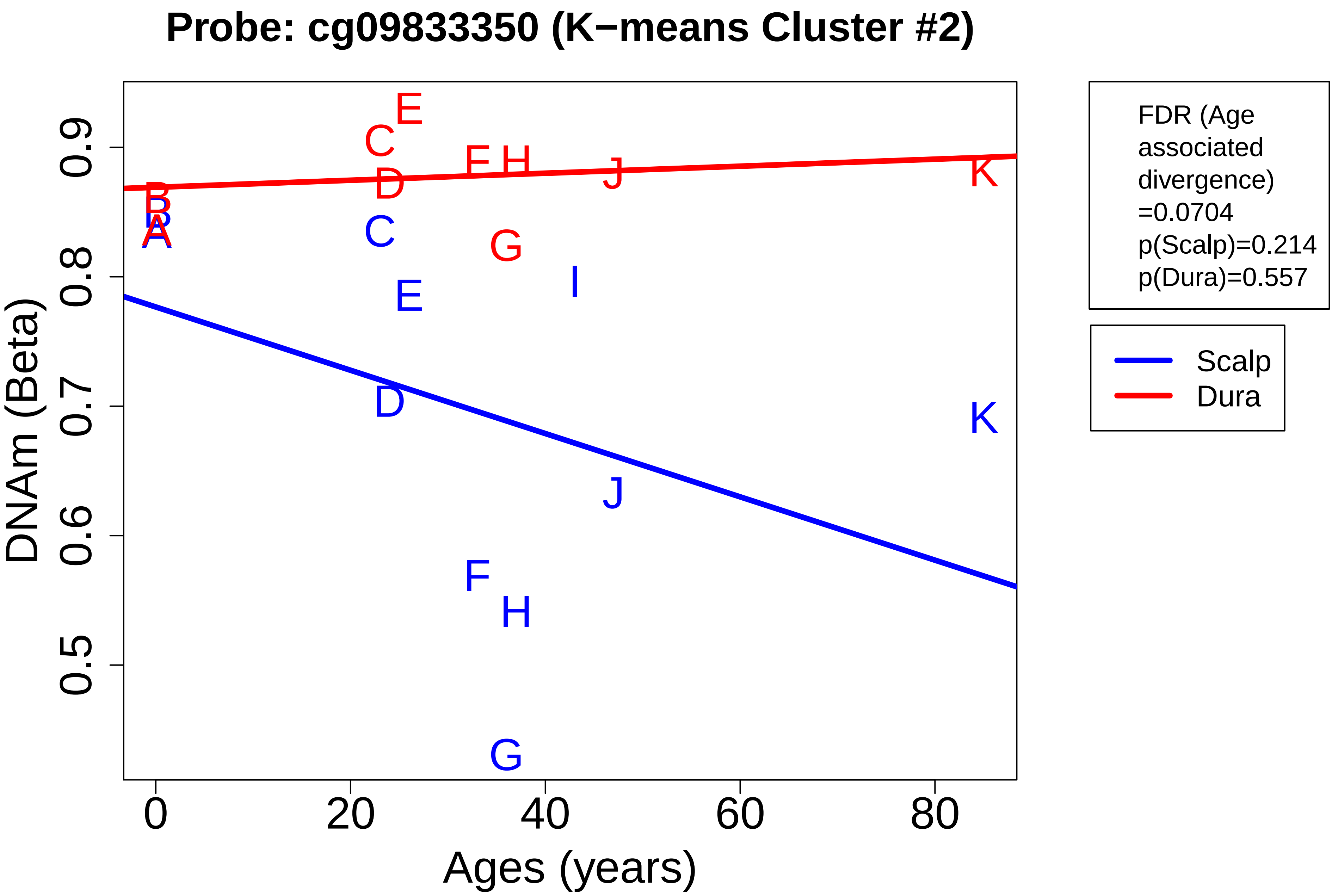

Probe: cg09833350 (K−means Cluster #2)
DNAm (Beta)
0.5
0.6
0.7
0.8
0.9
0
20
40
60
80
Ages (years)
FDR (Age associated divergence) =0.0704
p(Scalp)=0.214
p(Dura)=0.557
Scalp
Dura

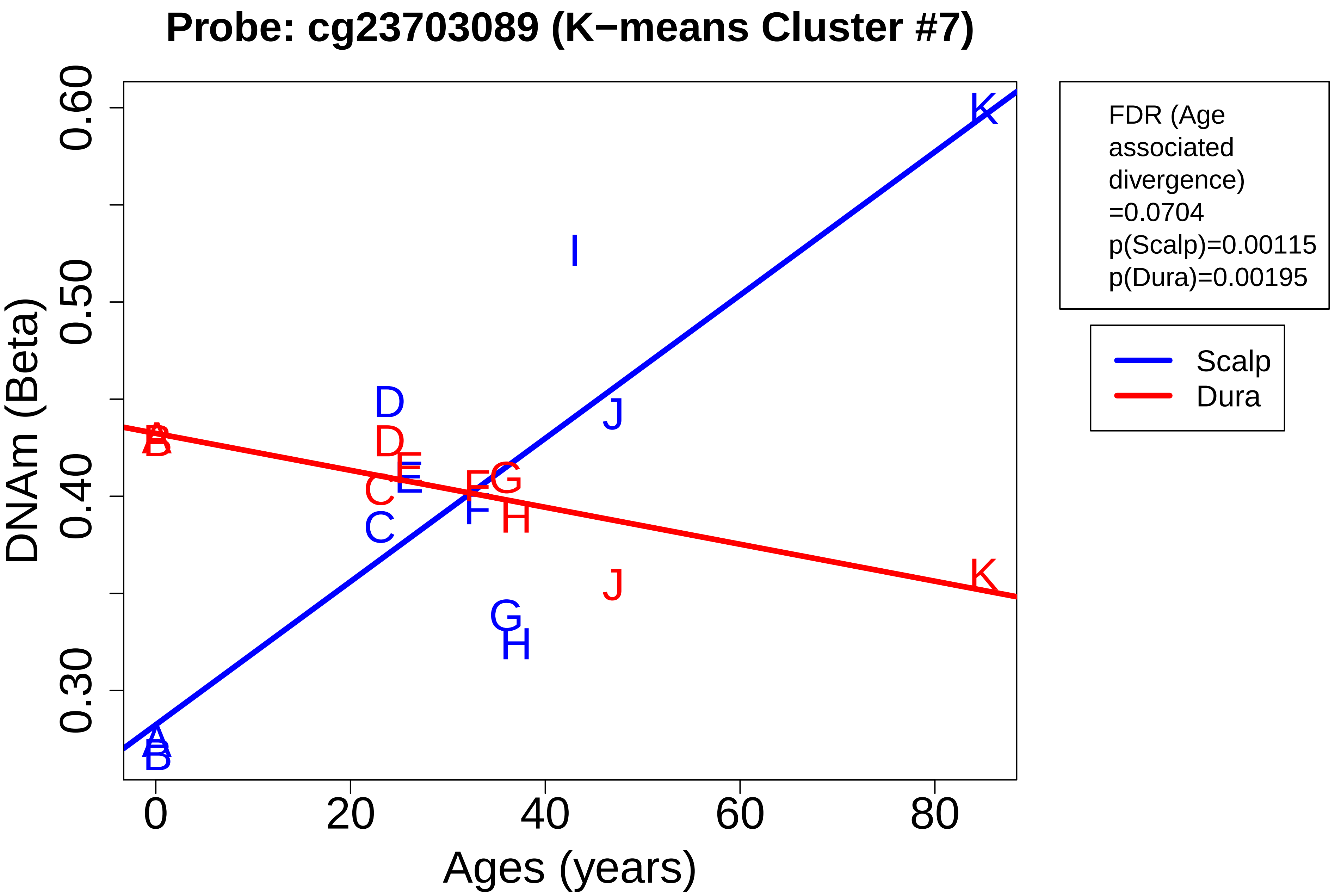

Probe: cg23703089 (K−means Cluster #7)
DNAm (Beta)
0.30
0.40
0.50
0.60
0
20
40
60
80
Ages (years)
FDR (Age associated divergence) =0.0704
p(Scalp)=0.00115
p(Dura)=0.00195
Scalp
Dura

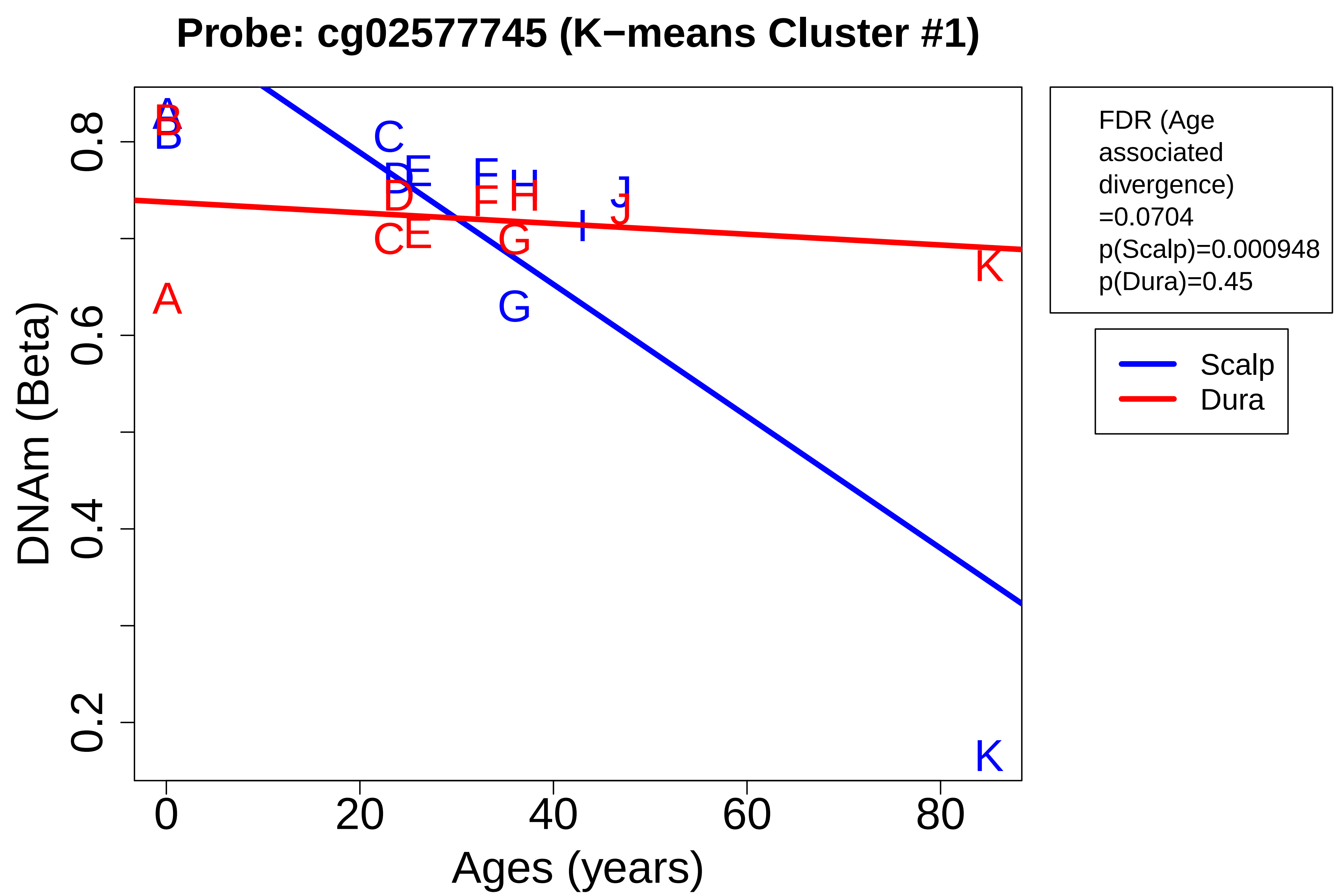

Probe: cg02577745 (K−means Cluster #1)
DNAm (Beta)
0.2
0.4
0.6
0.8
Ages (years)
0
20
40
60
80
A
B
C
D
E
F
G
H
I
J
K
FDR (Age associated divergence) =0.0704
p(Scalp)=0.000948
p(Dura)=0.45
Scalp
Dura

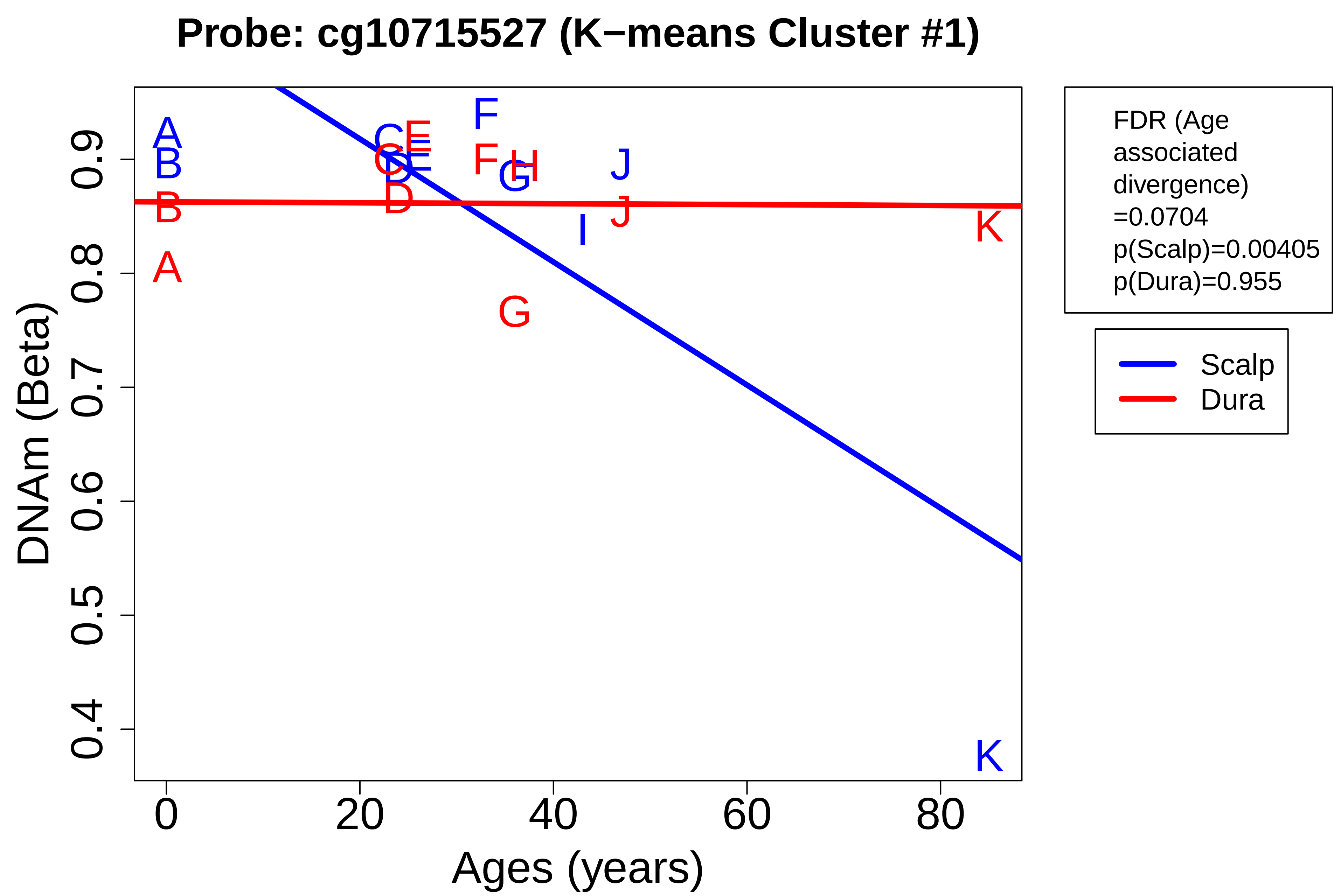
Probe: cg10715527 (K−means Cluster #1)
DNAm (Beta)
0.4
0.5
0.6
0.7
0.8
0.9
0
20
40
60
80
Ages (years)
FDR (Age associated divergence) =0.0704
p(Scalp)=0.00405
p(Dura)=0.955
Scalp
Dura
A
B
C
D
E
F
G
H
I
J
K

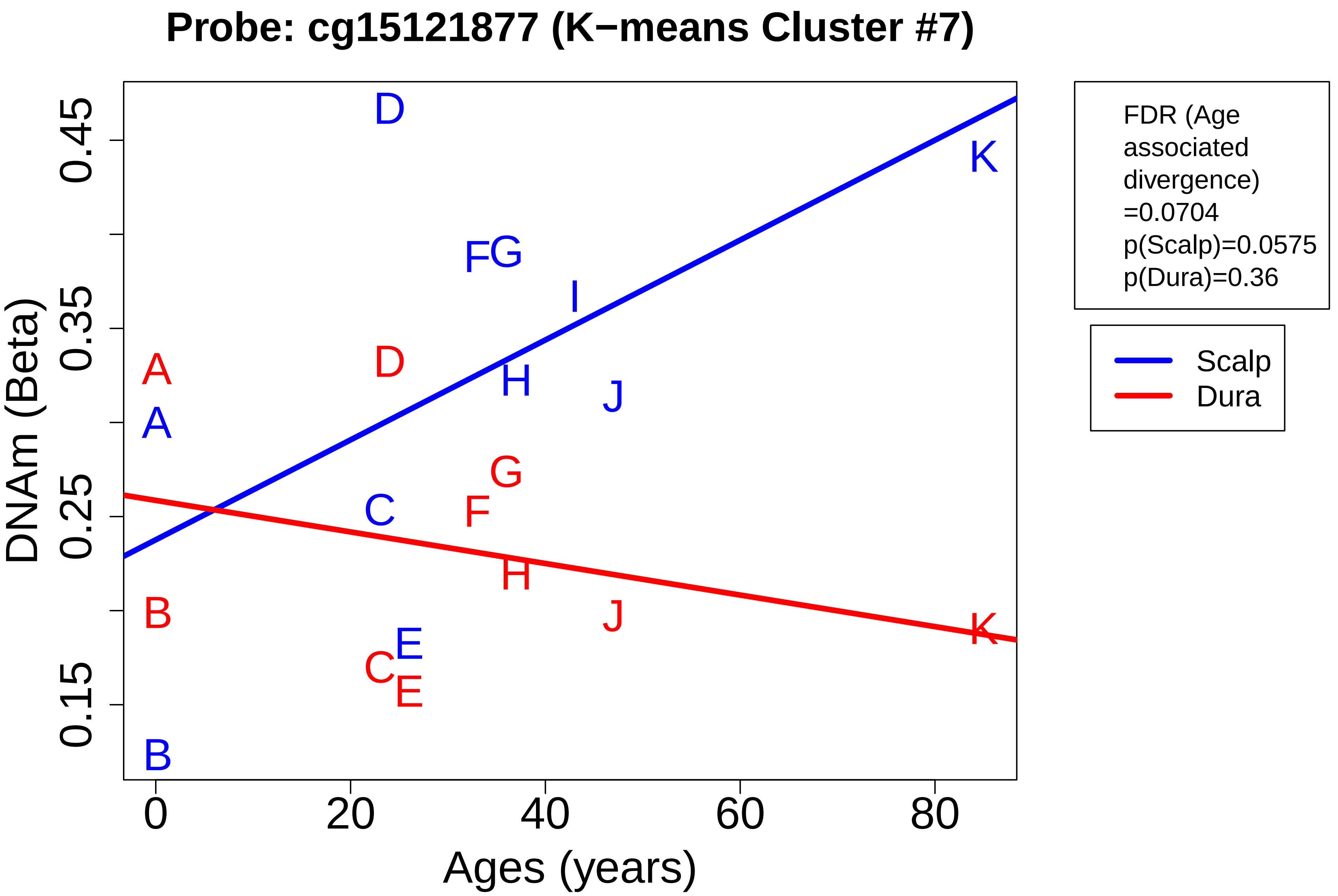

Probe: cg15121877 (K−means Cluster #7)
DNAm (Beta)
0.45
0.35
0.25
0.15
0
20
40
60
80
Ages (years)
FDR (Age associated divergence) =0.0704
p(Scalp)=0.0575
p(Dura)=0.36
Scalp
Dura
A
B
C
D
E
F
G
H
I
J
K

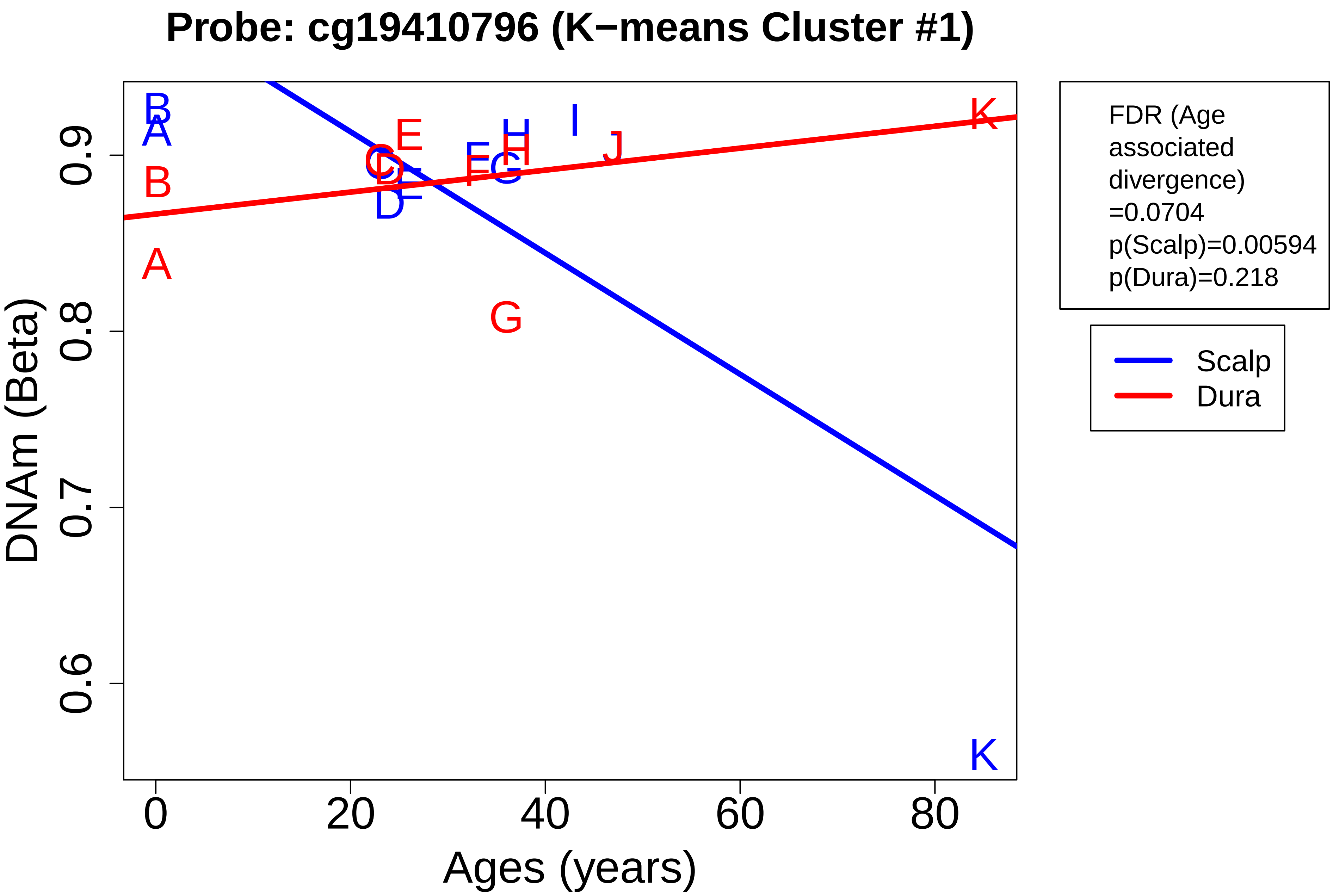
Probe: cg19410796 (K−means Cluster #1)
DNAm (Beta)
0.6
0.7
0.8
0.9
0
20
40
60
80
Ages (years)
FDR (Age associated divergence) =0.0704
p(Scalp)=0.00594
p(Dura)=0.218
Scalp
Dura

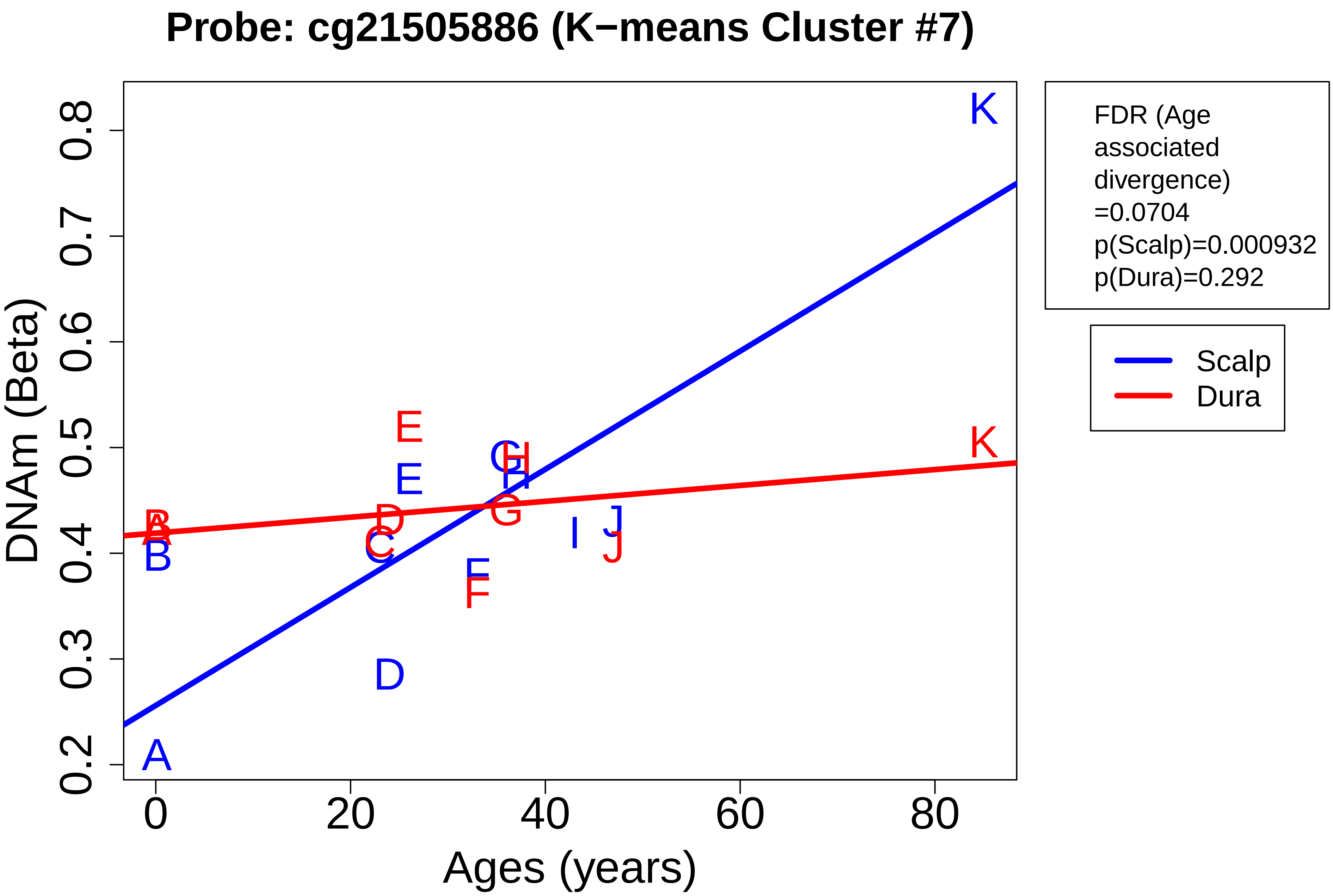

Probe: cg21505886 (K−means Cluster #7)
DNAm (Beta)
Ages (years)
0.2
0.3
0.4
0.5
0.6
0.7
0.8
0
20
40
60
80
FDR (Age associated divergence) =0.0704
p(Scalp)=0.000932
p(Dura)=0.292
Scalp
Dura

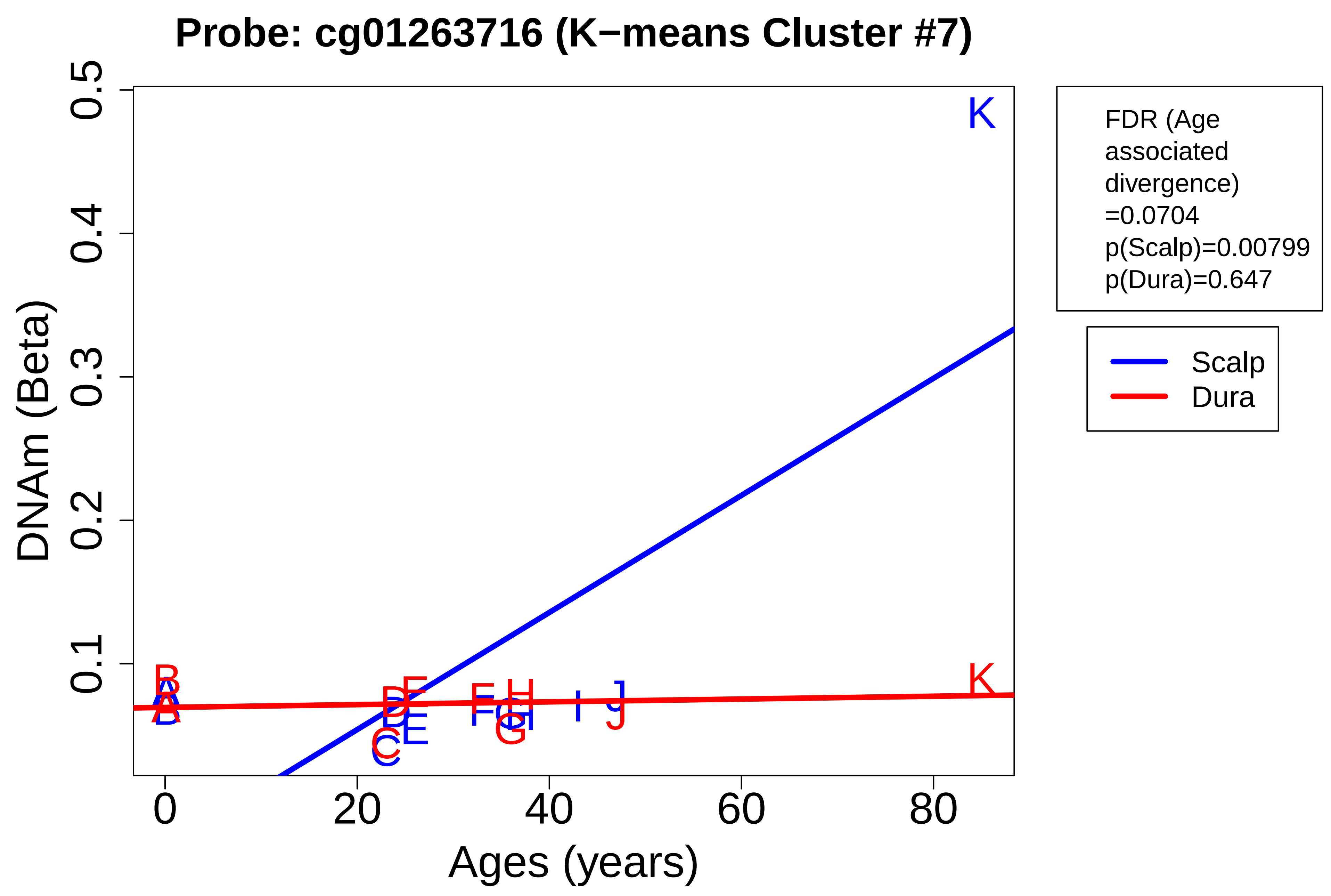
Probe: cg01263716 (K−means Cluster #7)
DNAm (Beta)
0.1
0.2
0.3
0.4
0.5
0
20
40
60
80
Ages (years)
FDR (Age associated divergence) =0.0704
p(Scalp)=0.00799
p(Dura)=0.647
Scalp
Dura
A
B
C
D
E
F
G
H
I
J
K

Probe: cg21821388 (K−means Cluster #7)

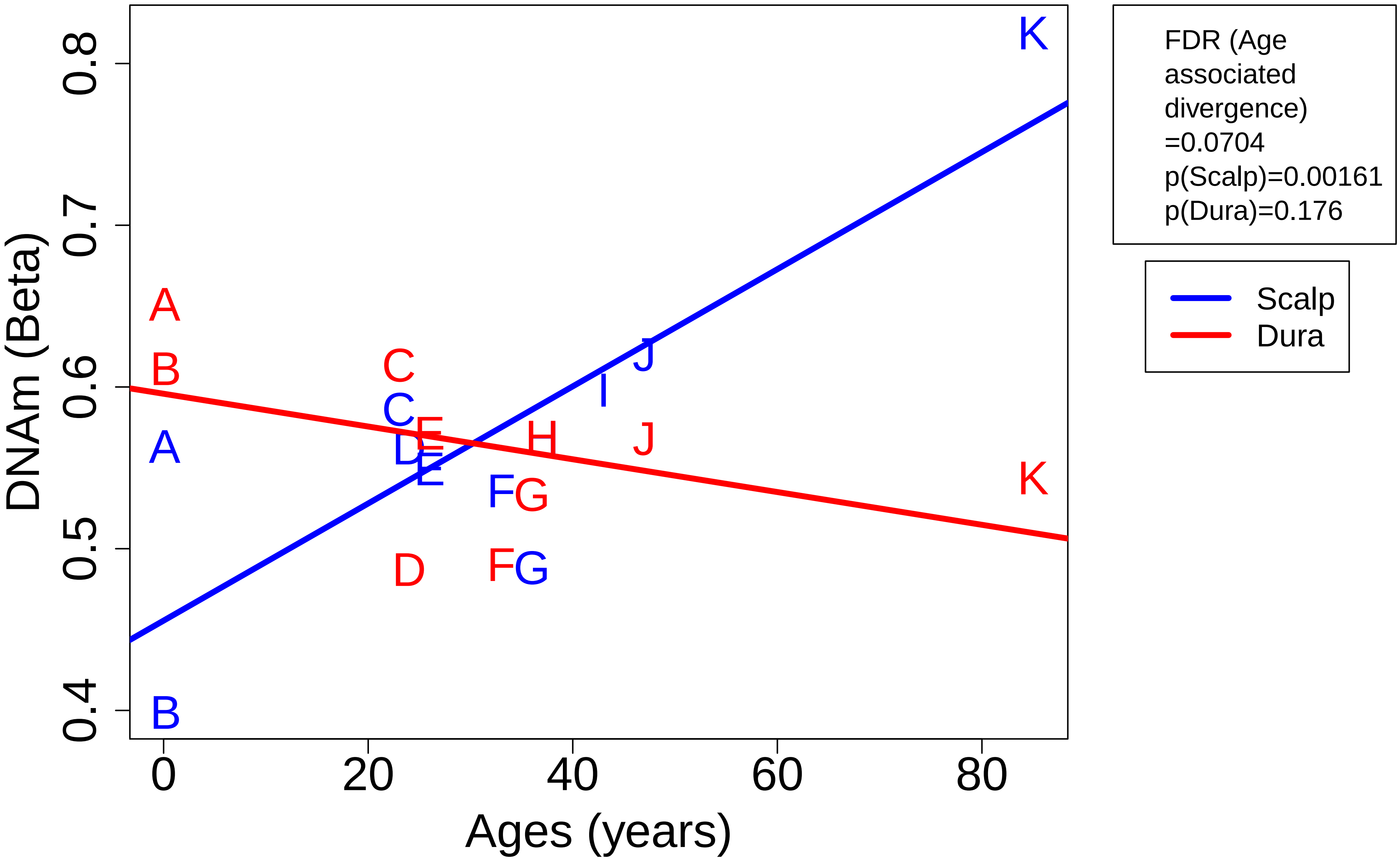

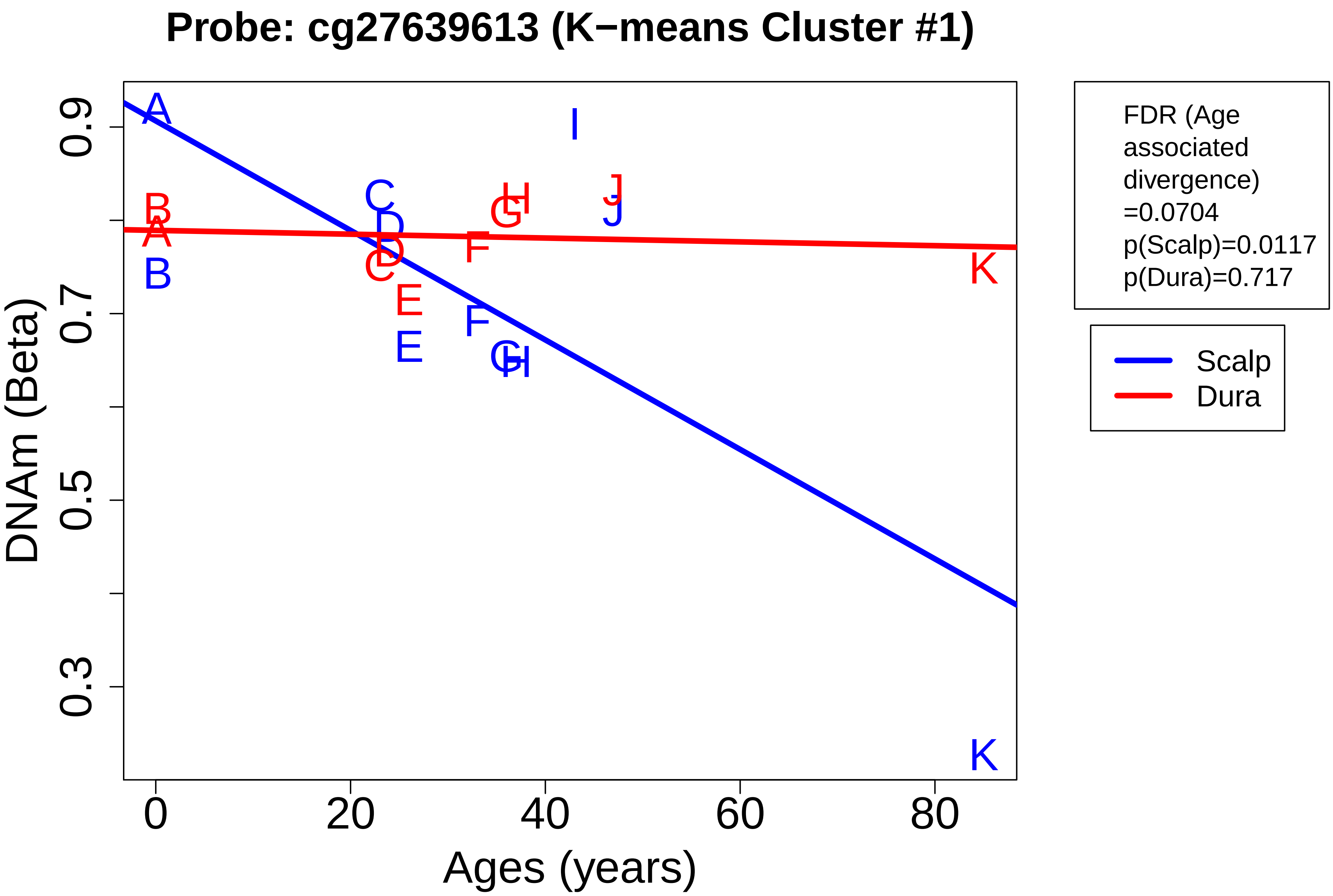

Probe: cg27639613 (K−means Cluster #1)
FDR (Age associated divergence) =0.0704
p(Scalp)=0.0117
p(Dura)=0.717
Scalp
Dura
DNAm (Beta)
0.3
0.5
0.7
0.9
0
20
40
60
80
Ages (years)

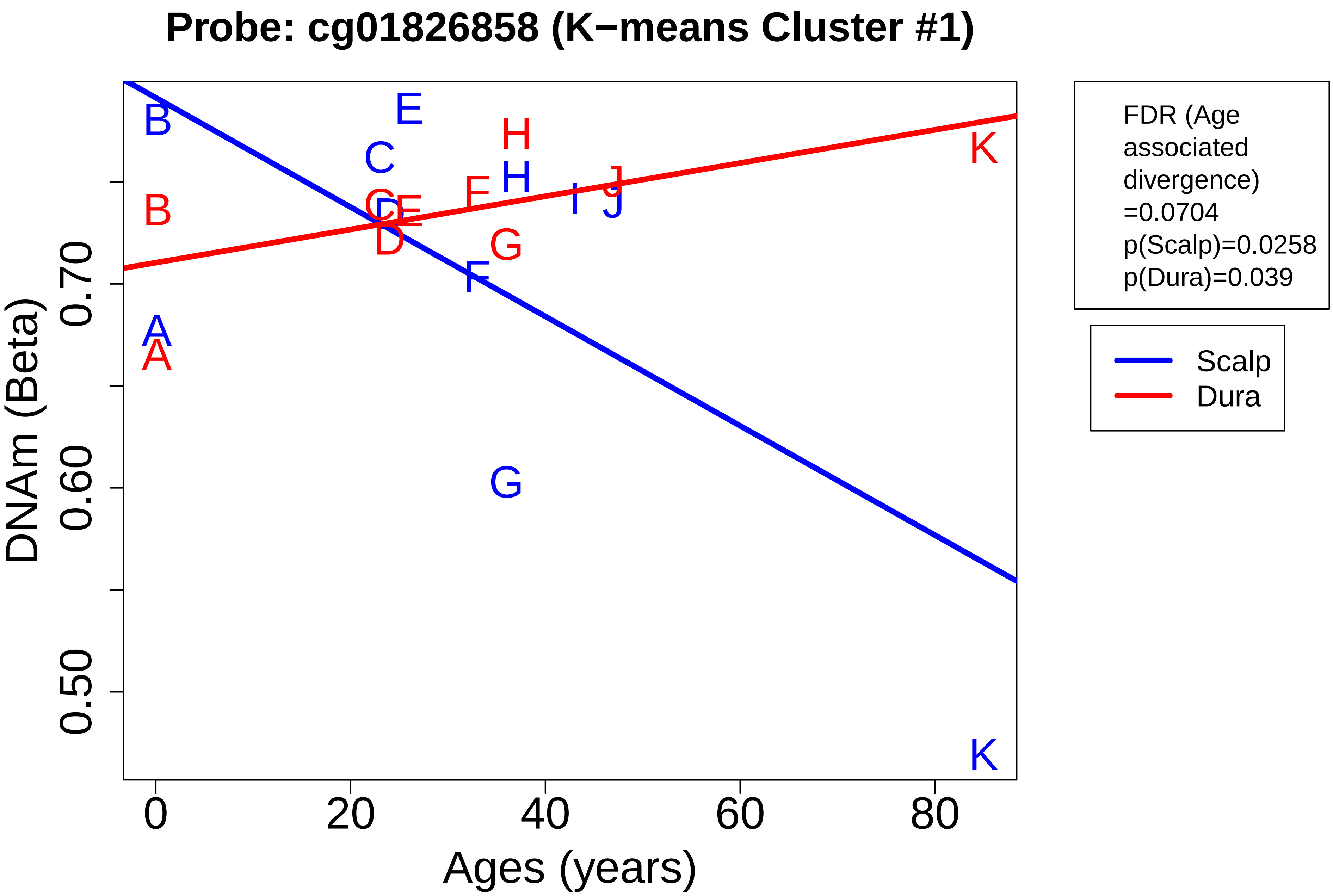

Probe: cg01826858 (K−means Cluster #1)
FDR (Age associated divergence) =0.0704
p(Scalp)=0.0258
p(Dura)=0.039
Scalp
Dura
DNAm (Beta)
0.50
0.60
0.70
0
20
40
60
80
Ages (years)
A
B
C
D
E
F
G
H
I
J
K

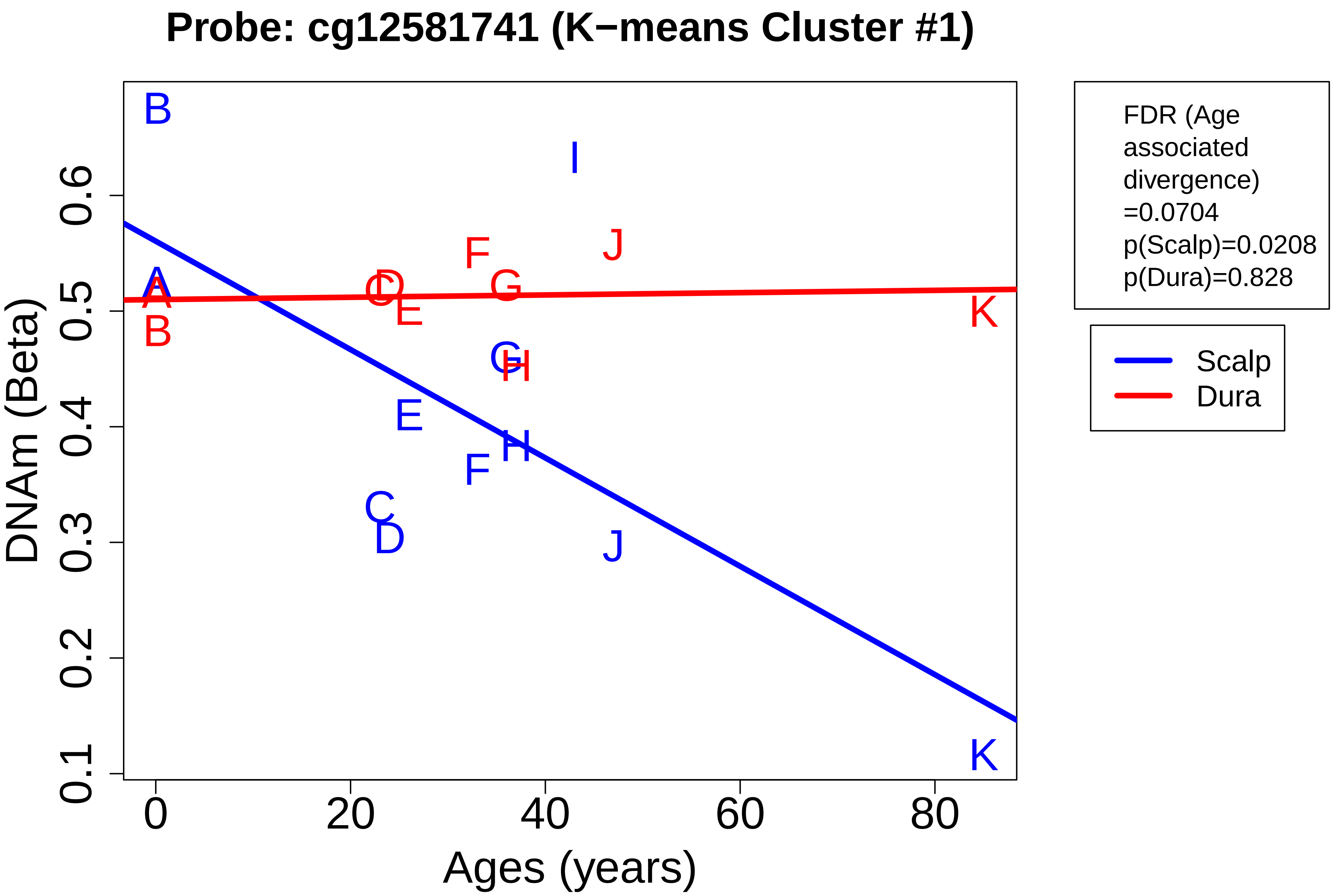
Probe: cg12581741 (K−means Cluster #1)
DNAm (Beta)
0.1
0.2
0.3
0.4
0.5
0.6
0
20
40
60
80
Ages (years)
FDR (Age associated divergence) =0.0704
p(Scalp)=0.0208
p(Dura)=0.828
Scalp
Dura
A
B
C
D
E
F
G
H
I
J
K

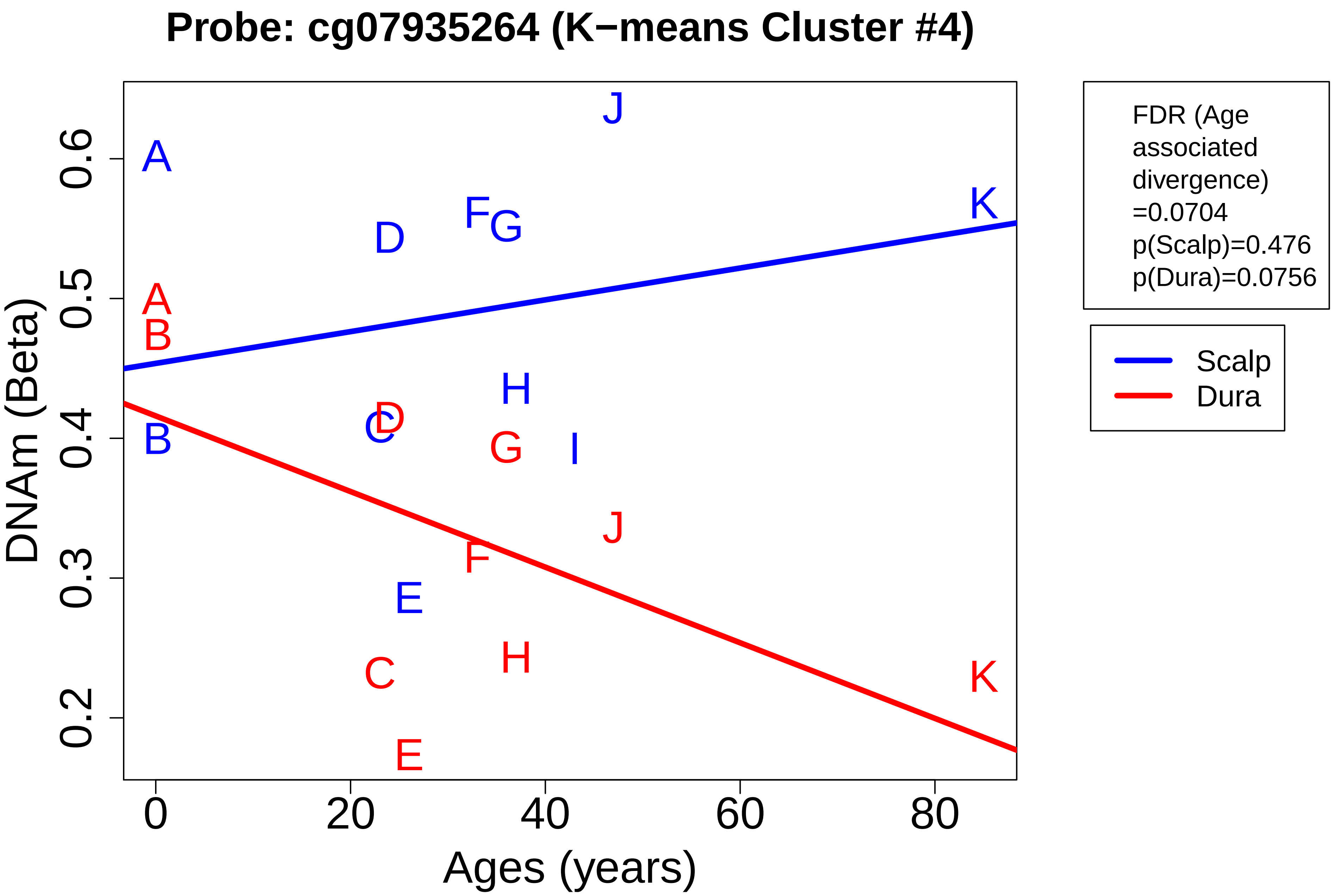
Probe: cg07935264 (K−means Cluster #4)
DNAm (Beta)
0.2
0.3
0.4
0.5
0.6
0
20
40
60
80
Ages (years)
A
B
C
D
E
F
G
H
I
J
K
FDR (Age associated divergence) =0.0704
p(Scalp)=0.476
p(Dura)=0.0756
Scalp
Dura

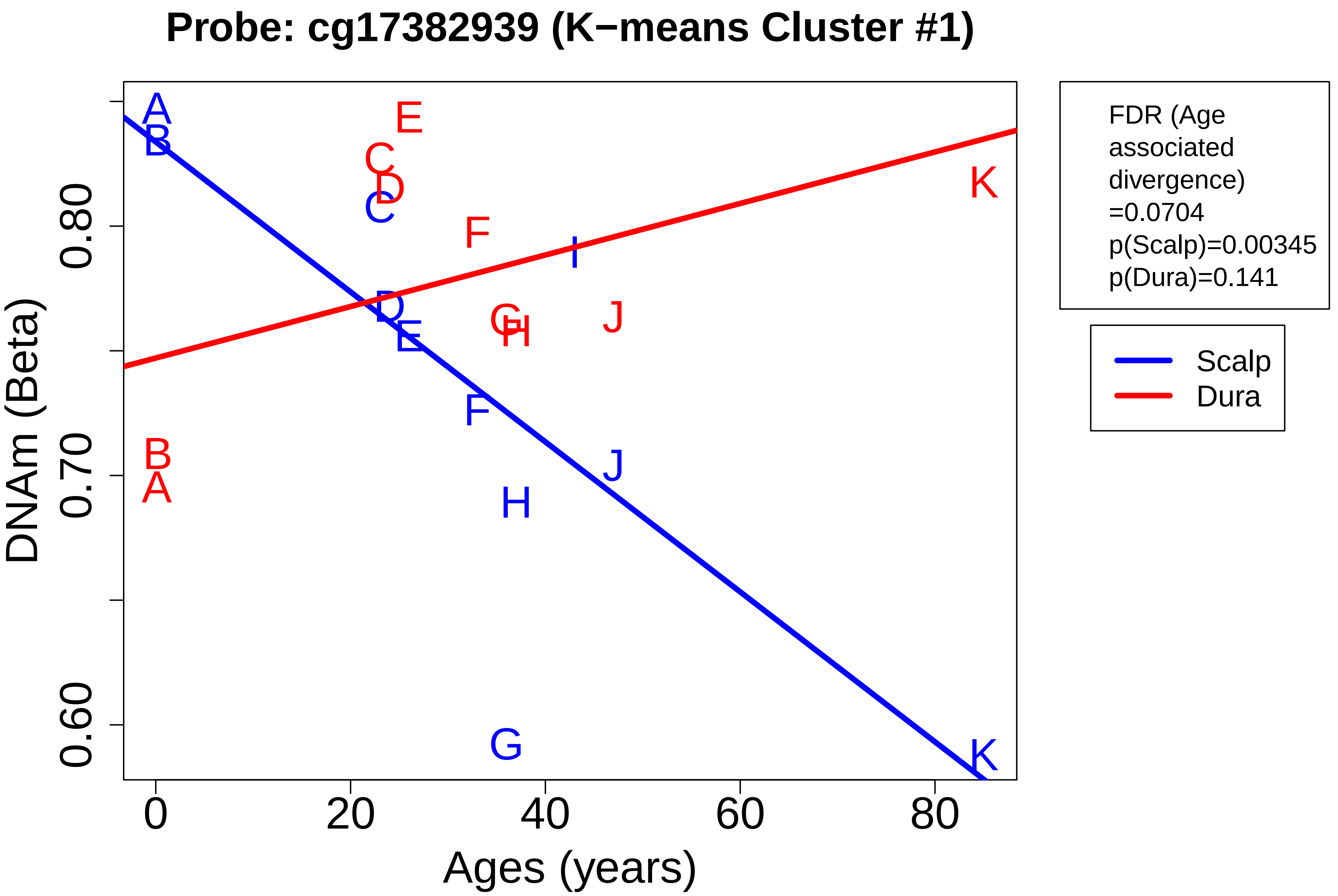

Probe: cg17382939 (K−means Cluster #1)
DNAm (Beta)
0.60
0.70
0.80
Ages (years)
0
20
40
60
80
FDR (Age associated divergence) =0.0704
p(Scalp)=0.00345
p(Dura)=0.141
Scalp
Dura

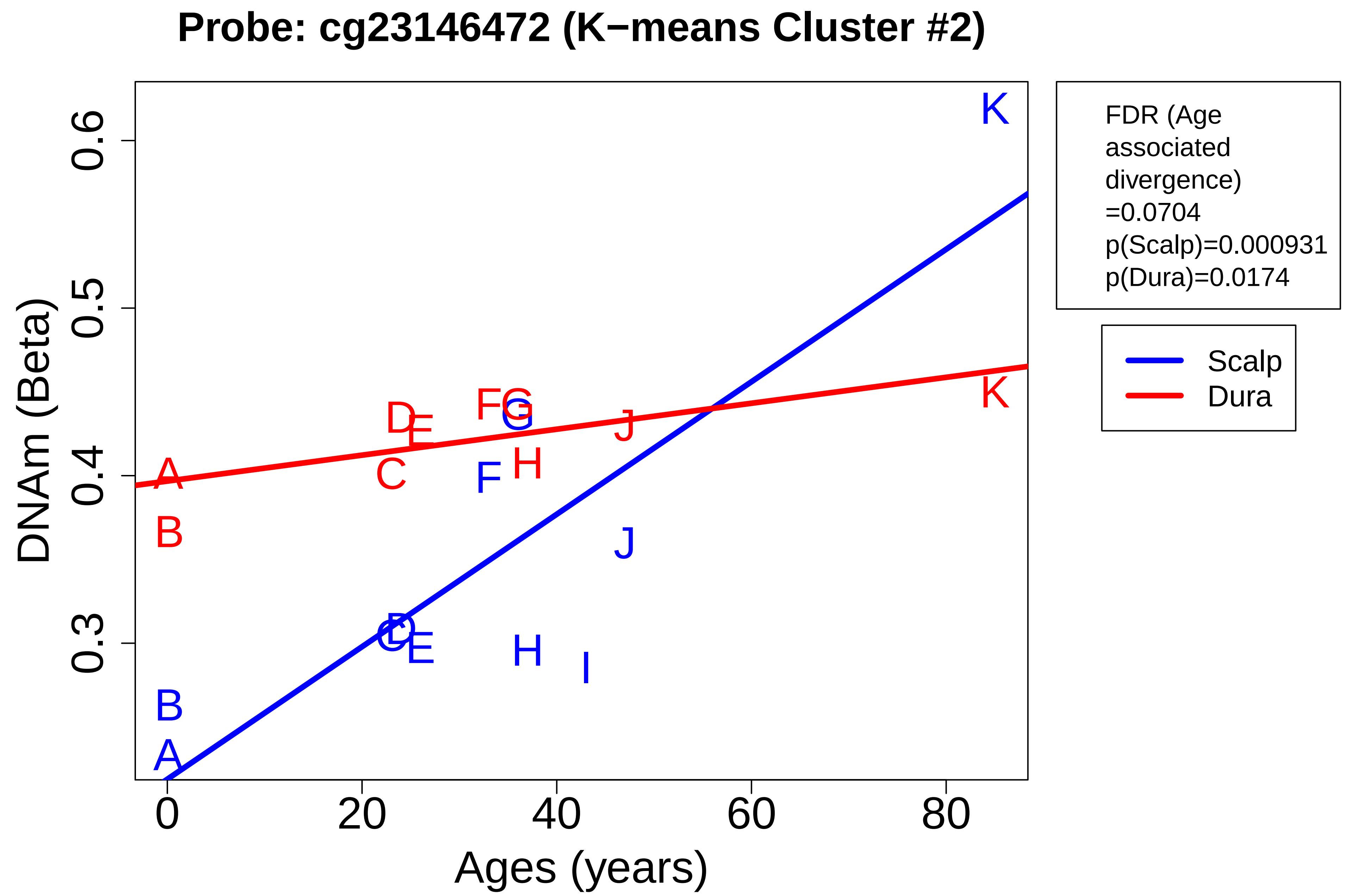

Probe: cg23146472 (K−means Cluster #2)
FDR (Age associated divergence) =0.0704
p(Scalp)=0.000931
p(Dura)=0.0174
Scalp
Dura
DNAm (Beta)
Ages (years)
0.3
0.4
0.5
0.6
0
20
40
60
80

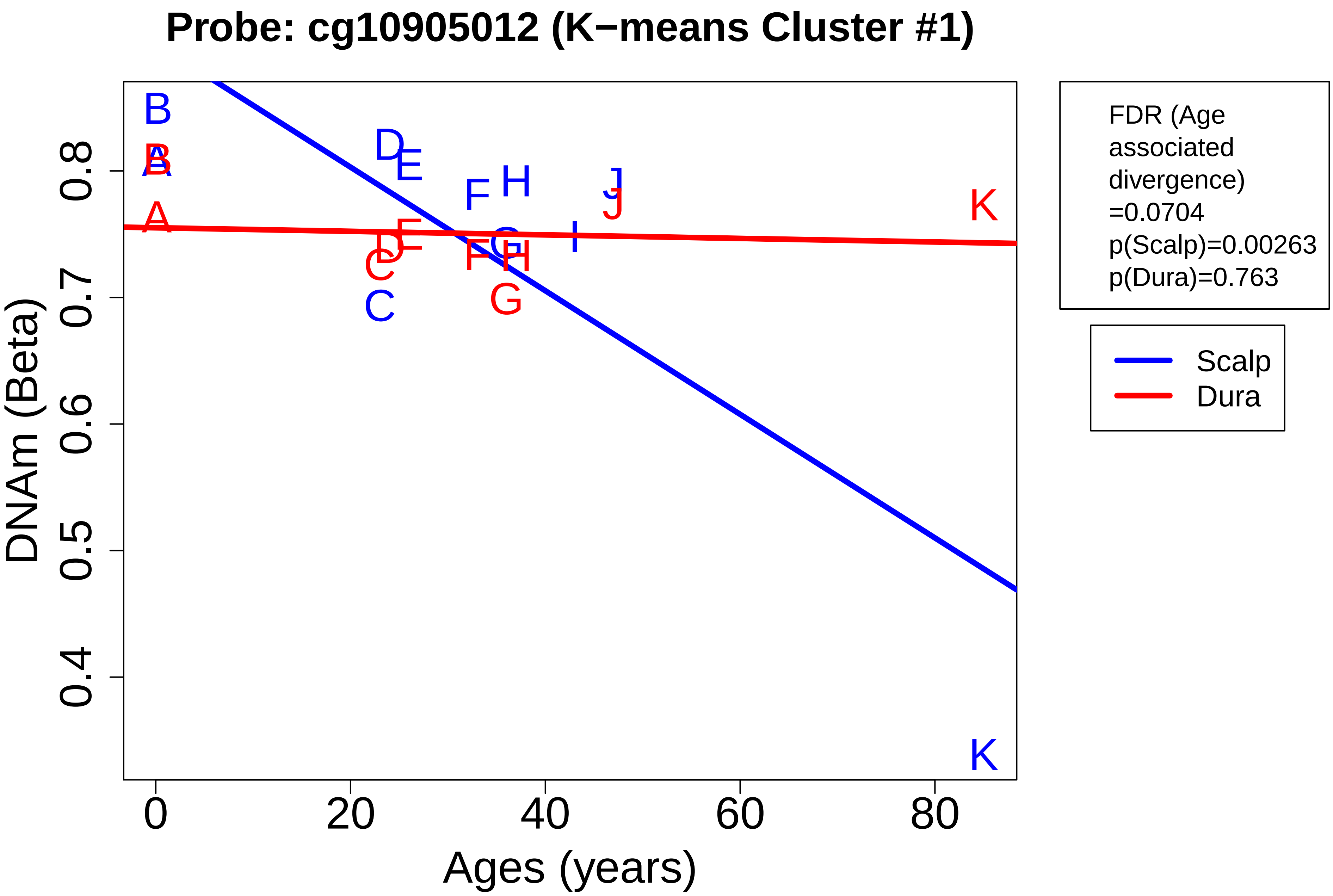

Probe: cg10905012 (K−means Cluster #1)
DNAm (Beta)
0.4
0.5
0.6
0.7
0.8
Ages (years)
0
20
40
60
80
FDR (Age associated divergence) =0.0704
p(Scalp)=0.00263
p(Dura)=0.763
Scalp
Dura

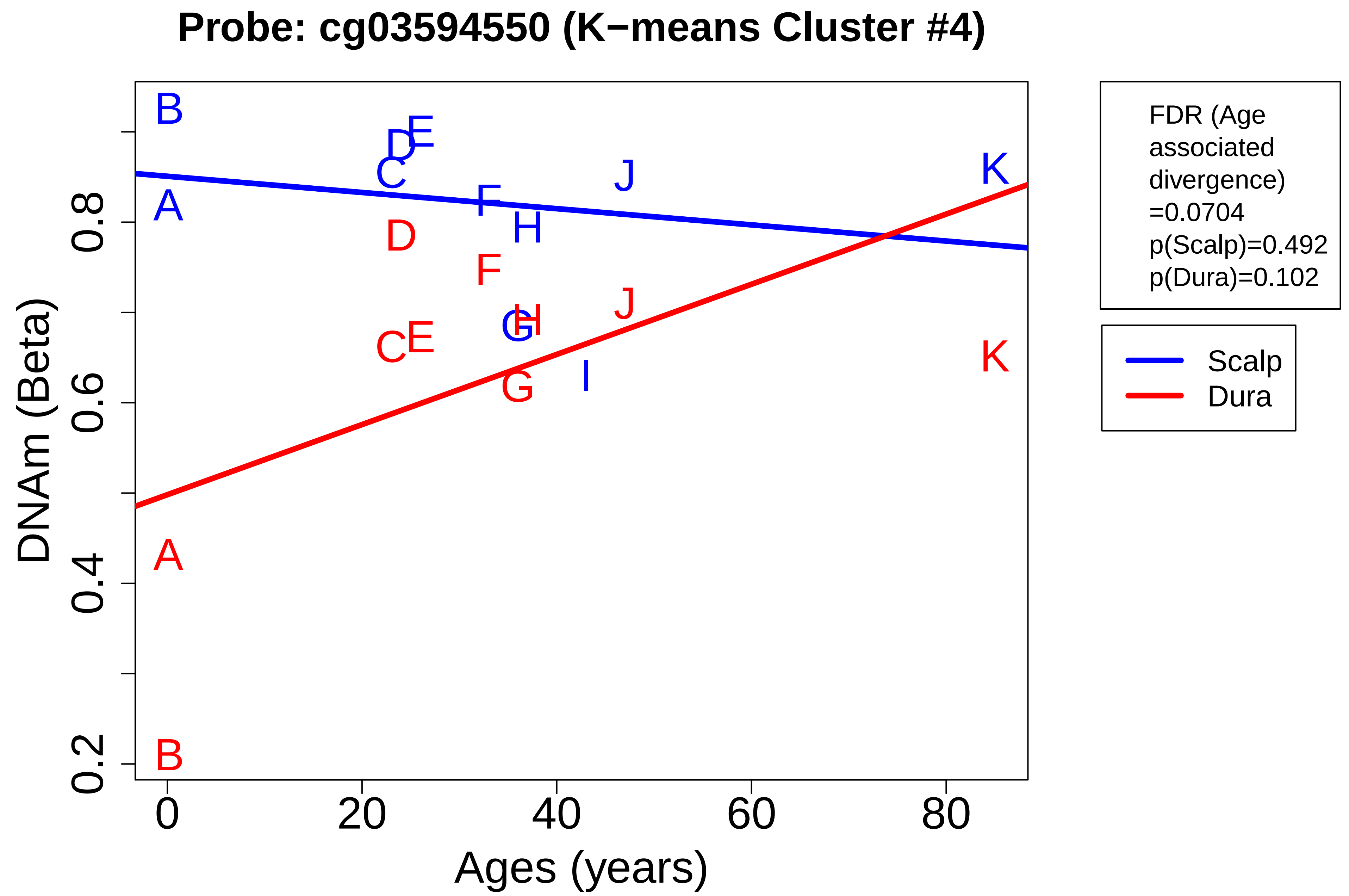
Probe: cg03594550 (K−means Cluster #4)
DNAm (Beta)
0.2
0.4
0.6
0.8
0
20
40
60
80
Ages (years)
A
B
C
D
E
F
G
H
I
J
K
FDR (Age associated divergence) =0.0704
p(Scalp)=0.492
p(Dura)=0.102
Scalp
Dura

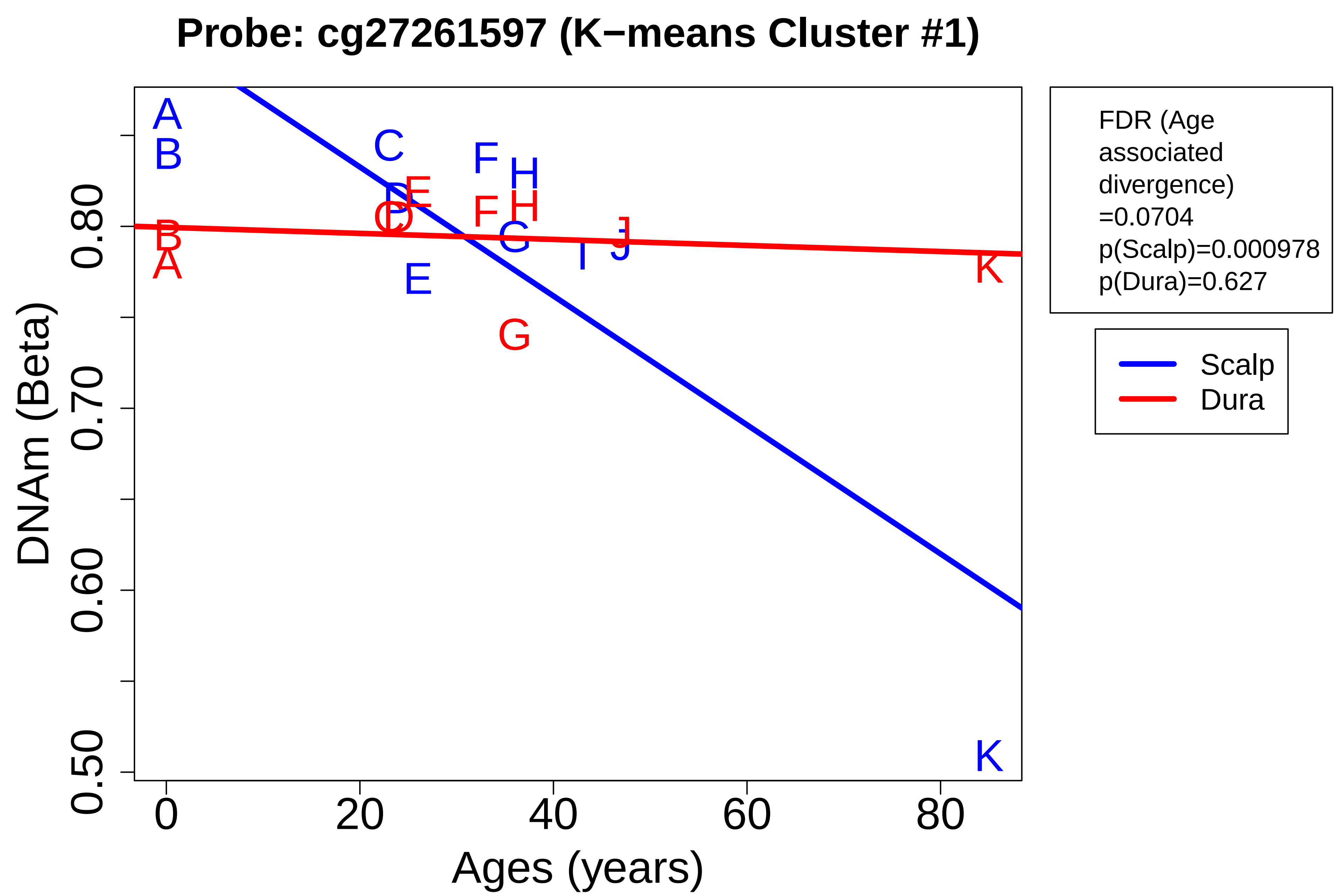

Probe: cg27261597 (K−means Cluster #1)
DNAm (Beta)
0.50
0.60
0.70
0.80
0
20
40
60
80
Ages (years)
A
B
C
D
E
F
G
H
I
J
K
FDR (Age associated divergence) =0.0704
p(Scalp)=0.000978
p(Dura)=0.627
Scalp
Dura

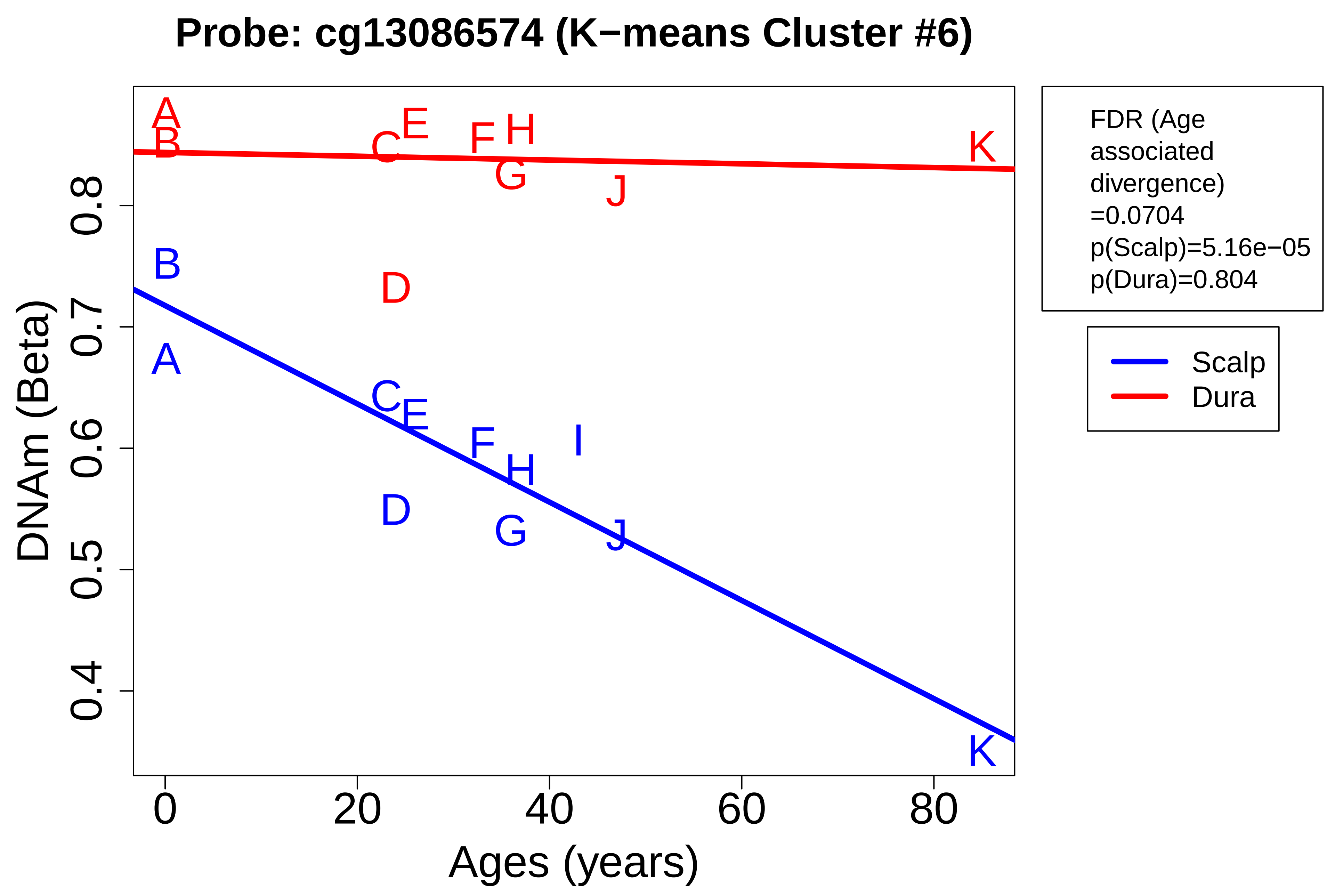
Probe: cg13086574 (K−means Cluster #6)
DNAm (Beta)
0.4
0.5
0.6
0.7
0.8
Ages (years)
0
20
40
60
80
FDR (Age associated divergence) =0.0704
p(Scalp)=5.16e−05
p(Dura)=0.804
Scalp
Dura
A
B
C
D
E
F
G
H
I
J
K

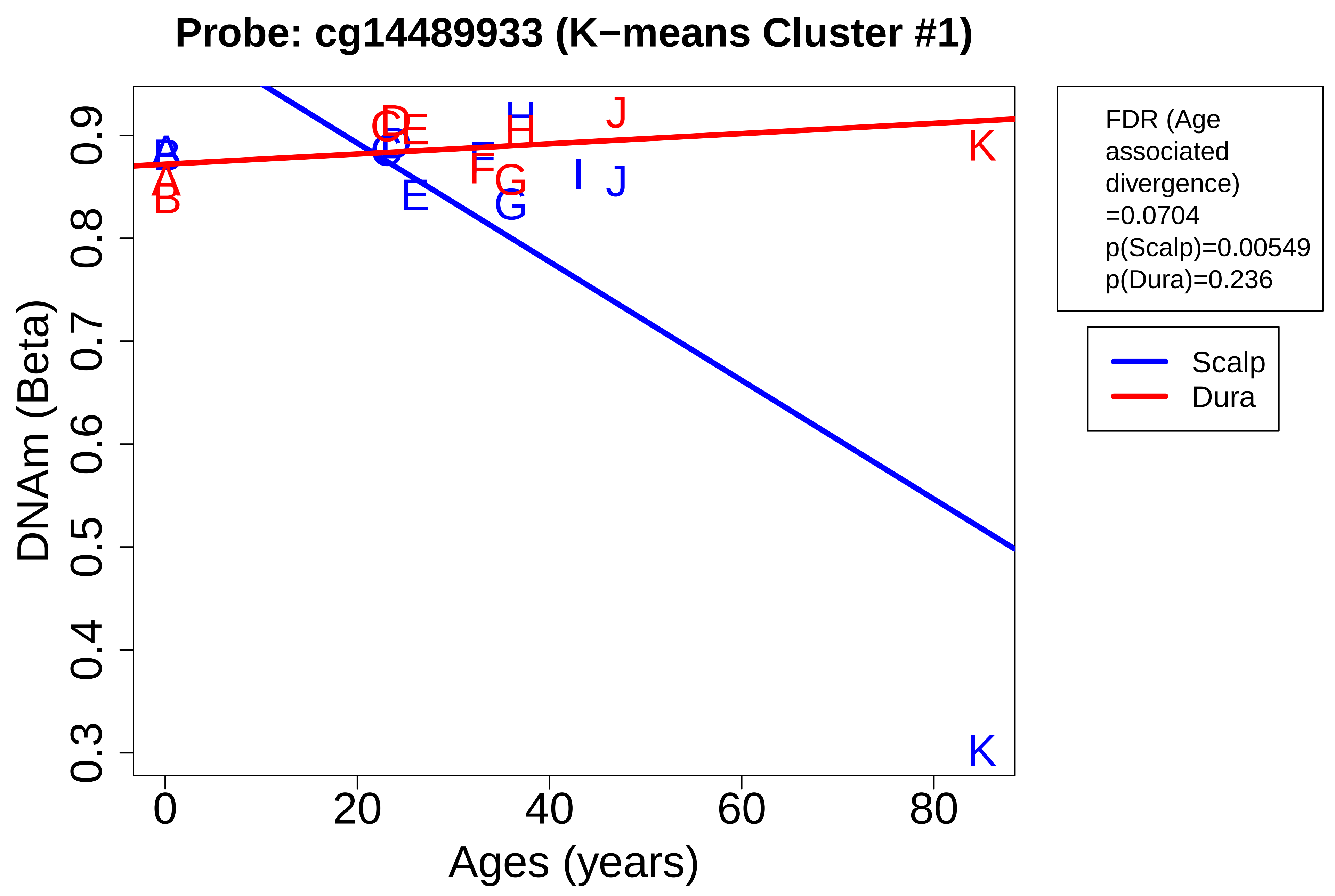

Probe: cg14489933 (K−means Cluster #1)
DNAm (Beta)
Ages (years)
0.3
0.4
0.5
0.6
0.7
0.8
0.9
0
20
40
60
80
FDR (Age associated divergence) =0.0704
p(Scalp)=0.00549
p(Dura)=0.236
Scalp
Dura

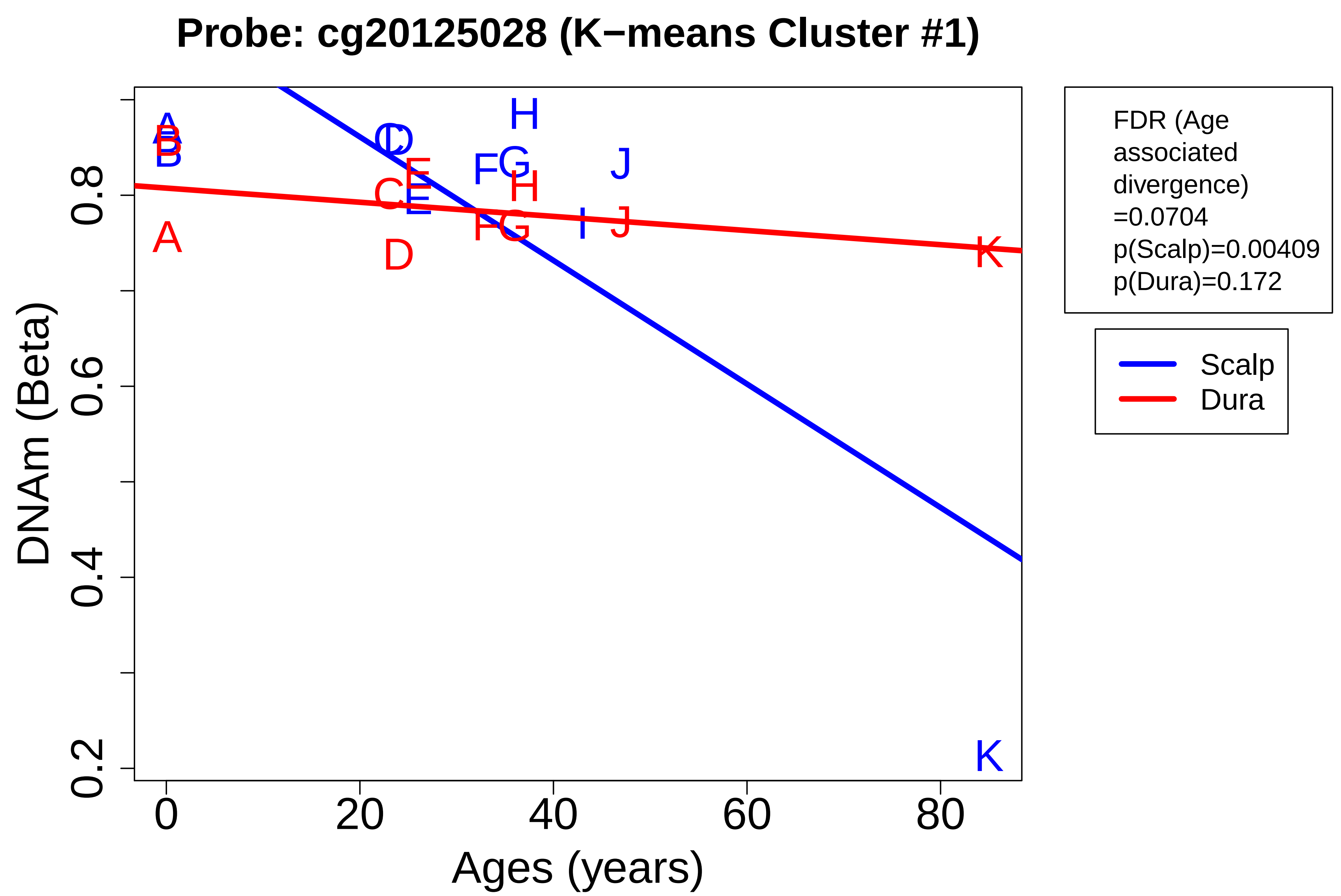

Probe: cg20125028 (K−means Cluster #1)
DNAm (Beta)
0.2
0.4
0.6
0.8
0
20
40
60
80
Ages (years)
FDR (Age associated divergence) =0.0704
p(Scalp)=0.00409
p(Dura)=0.172
Scalp
Dura

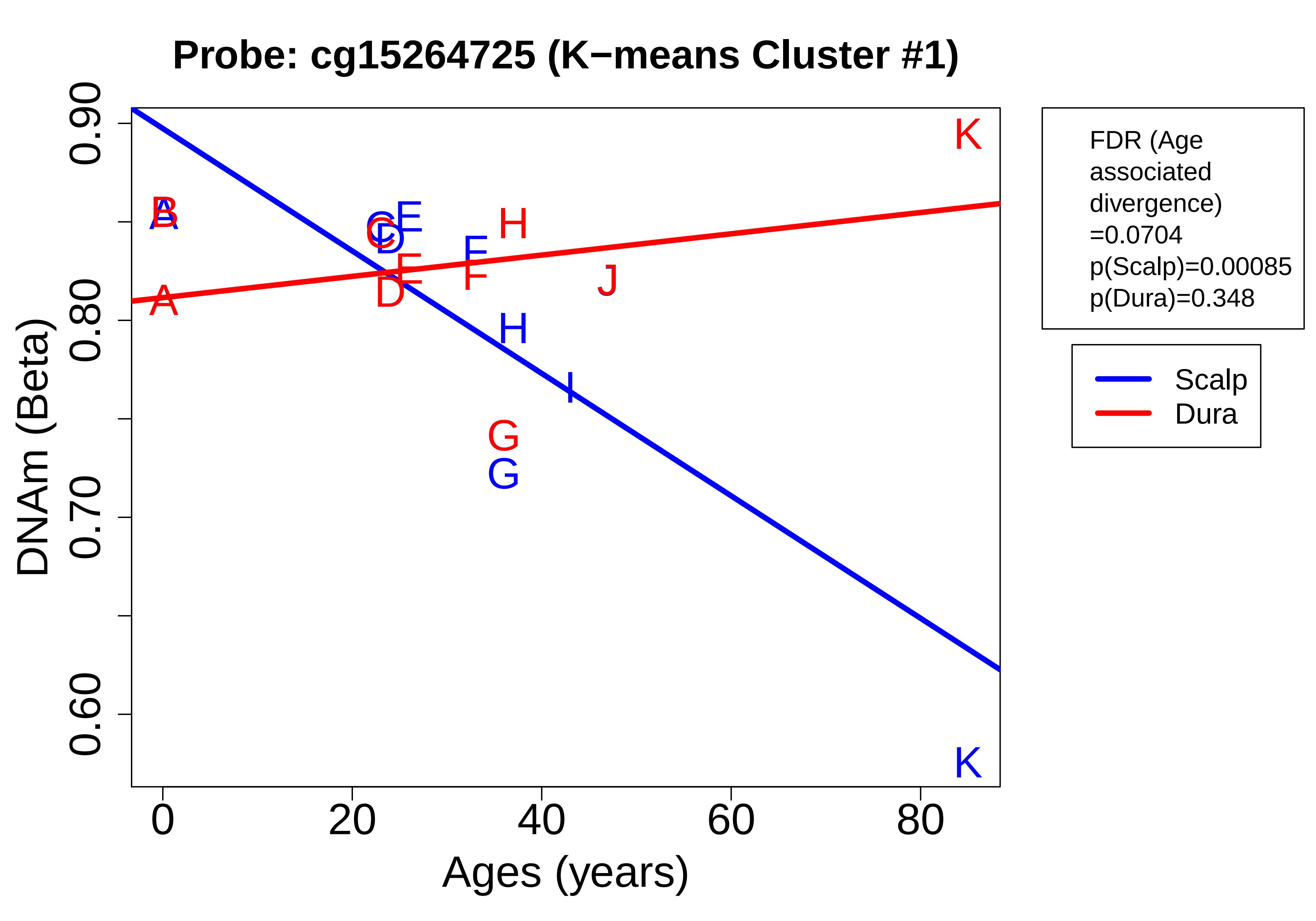
Probe: cg15264725 (K−means Cluster #1)
DNAm (Beta)
0.60
0.70
0.80
0.90
0
20
40
60
80
Ages (years)
A
B
C
D
E
F
G
H
I
J
K
FDR (Age associated divergence) =0.0704
p(Scalp)=0.00085
p(Dura)=0.348
Scalp
Dura

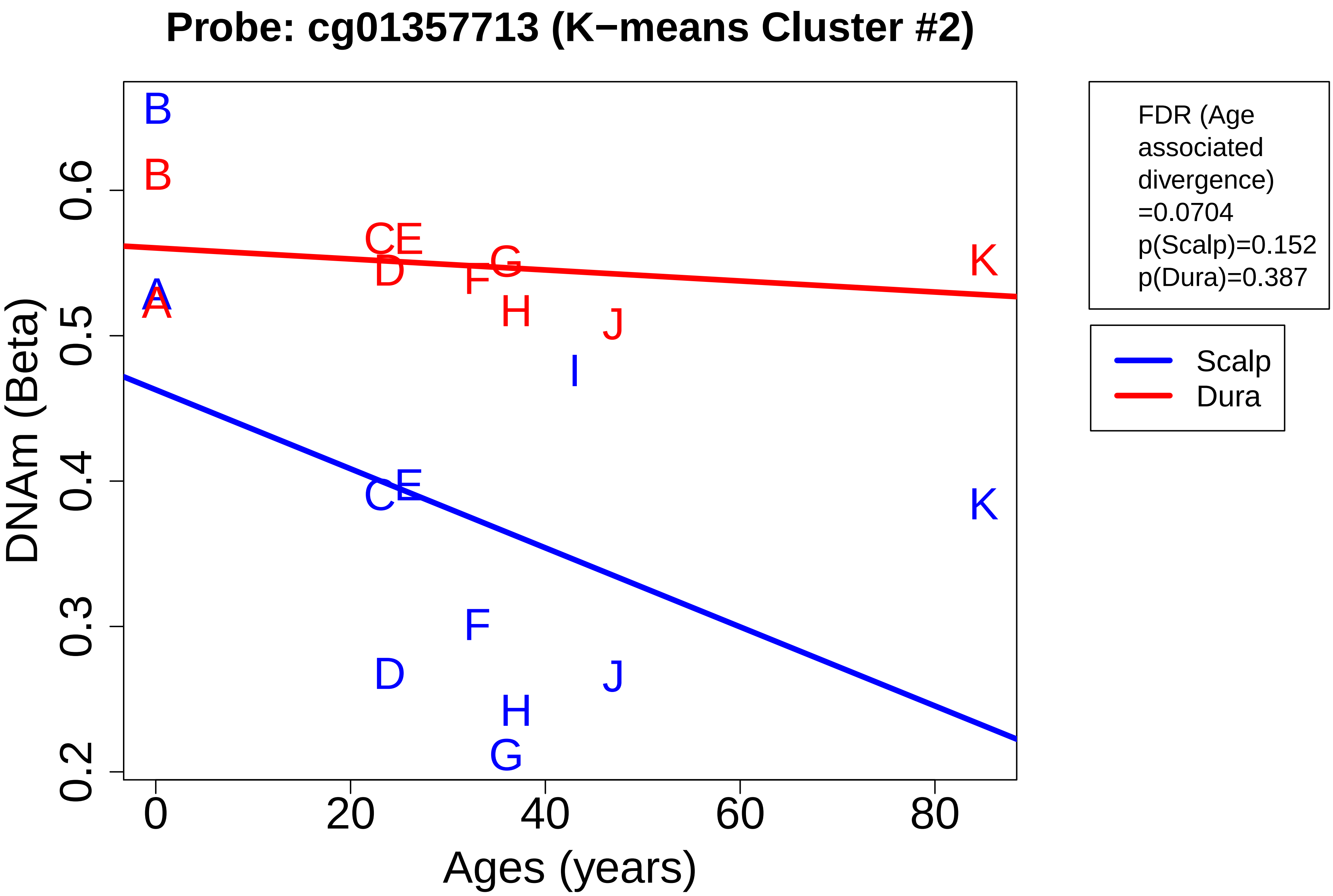
Probe: cg01357713 (K−means Cluster #2)
DNAm (Beta)
Ages (years)
0.2
0.3
0.4
0.5
0.6
0
20
40
60
80
FDR (Age associated divergence) =0.0704
p(Scalp)=0.152
p(Dura)=0.387
Scalp
Dura

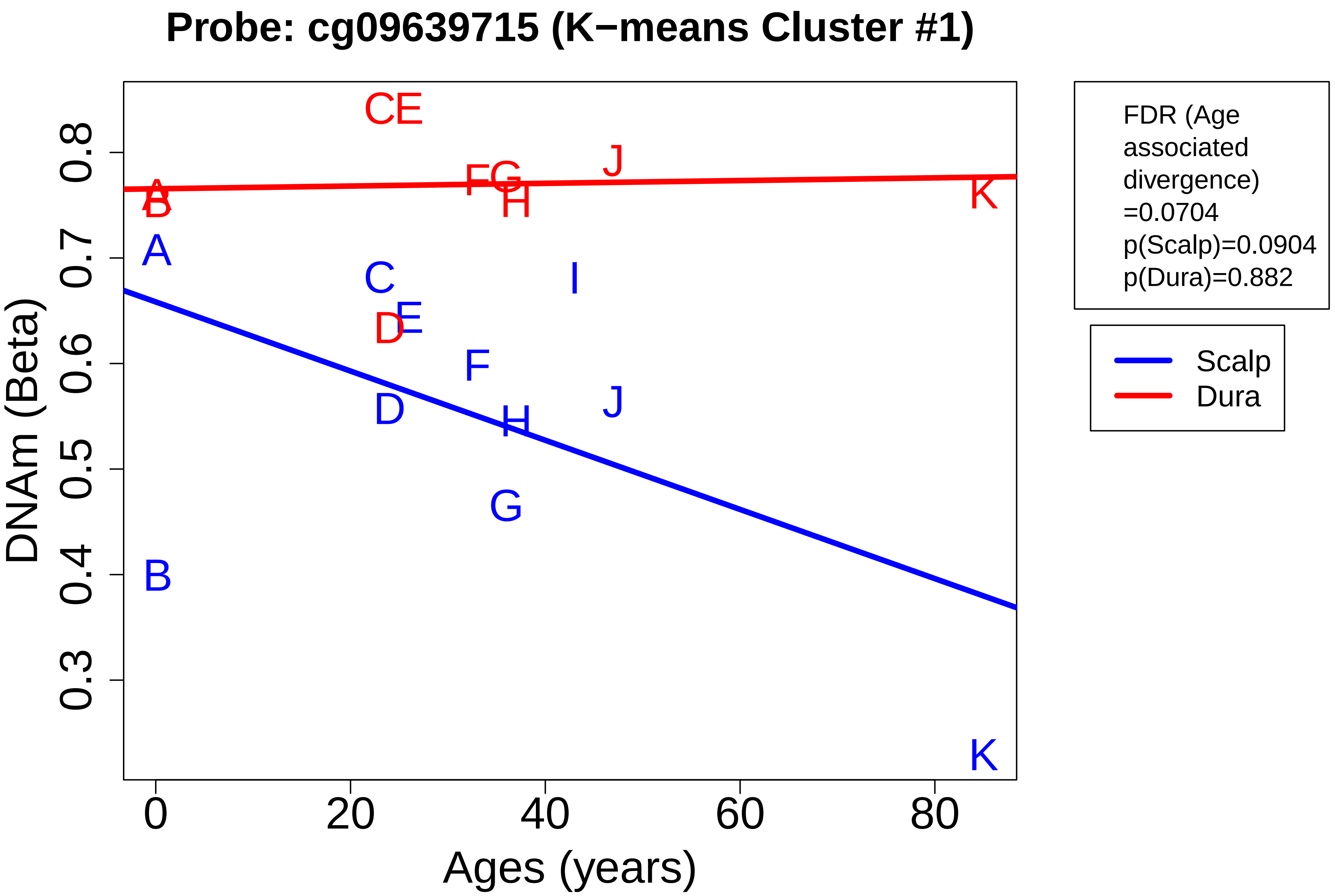

Probe: cg09639715 (K−means Cluster #1)
DNAm (Beta)
0.3
0.4
0.5
0.6
0.7
0.8
0
20
40
60
80
Ages (years)
FDR (Age associated divergence) =0.0704
p(Scalp)=0.0904
p(Dura)=0.882
Scalp
Dura

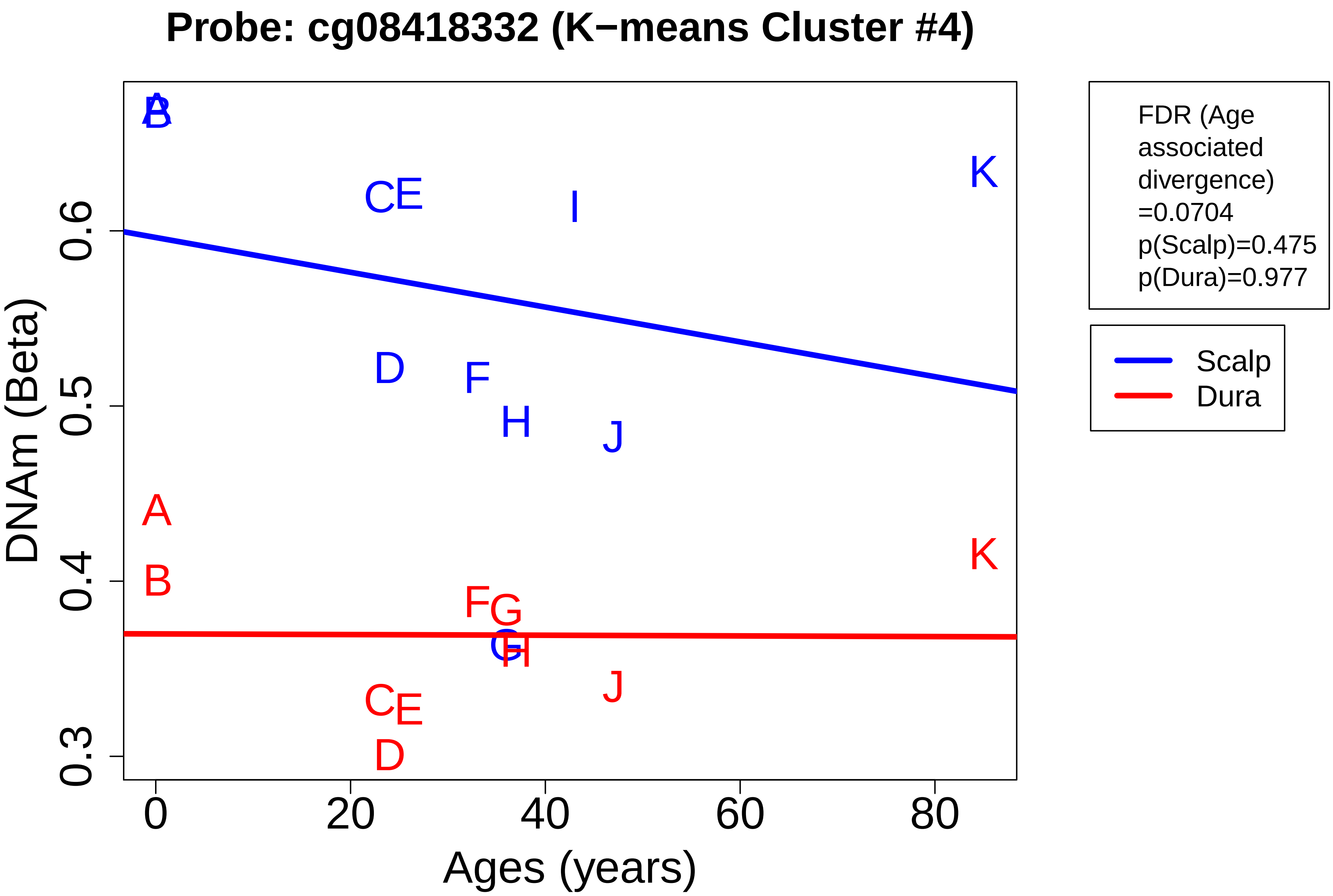

Probe: cg08418332 (K−means Cluster #4)
DNAm (Beta)
0.3
0.4
0.5
0.6
0
20
40
60
80
Ages (years)
FDR (Age associated divergence) =0.0704
p(Scalp)=0.475
p(Dura)=0.977
Scalp
Dura

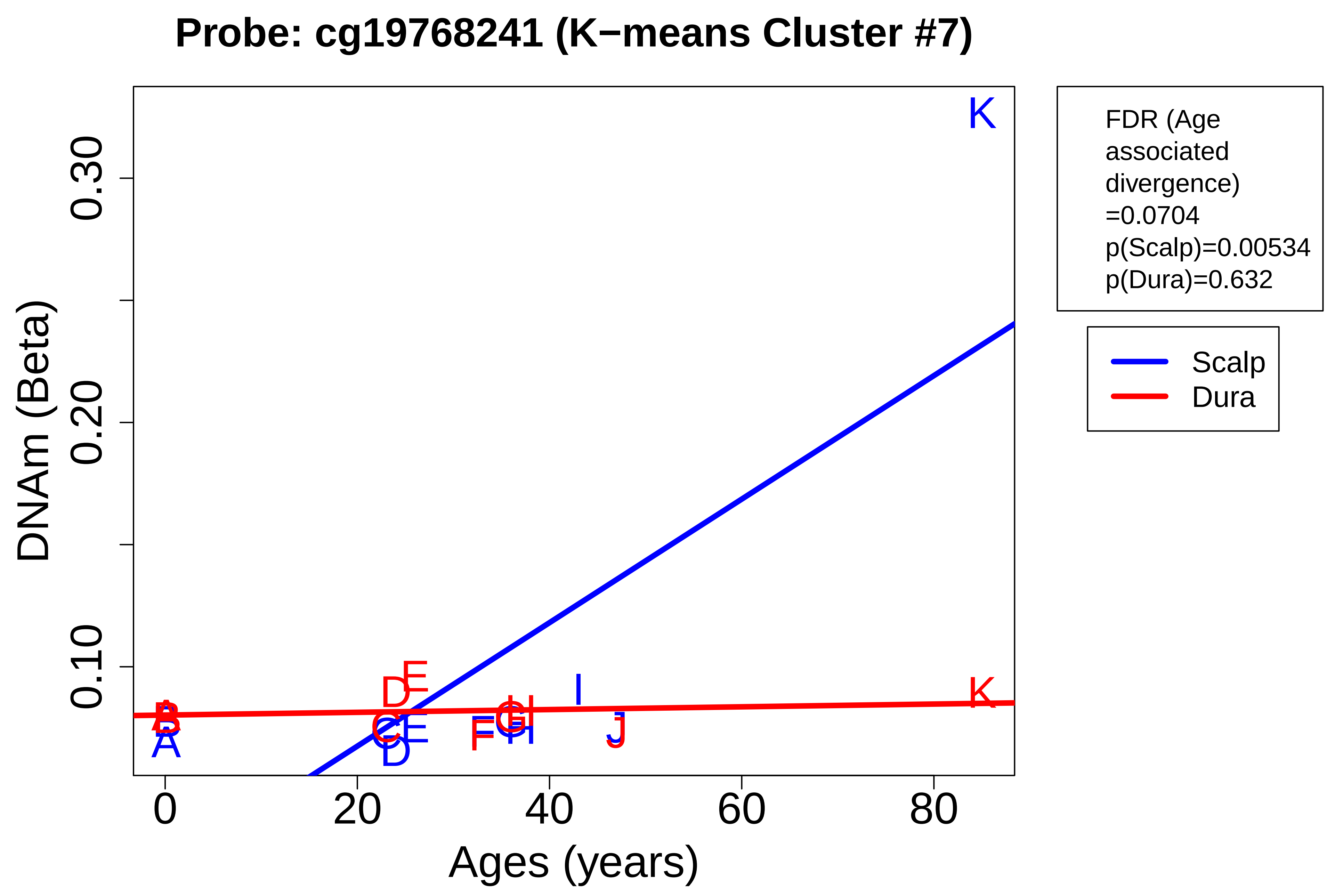

Probe: cg19768241 (K−means Cluster #7)
DNAm (Beta)
0.10
0.20
0.30
Ages (years)
0
20
40
60
80
FDR (Age associated divergence) =0.0704
p(Scalp)=0.00534
p(Dura)=0.632
Scalp
Dura

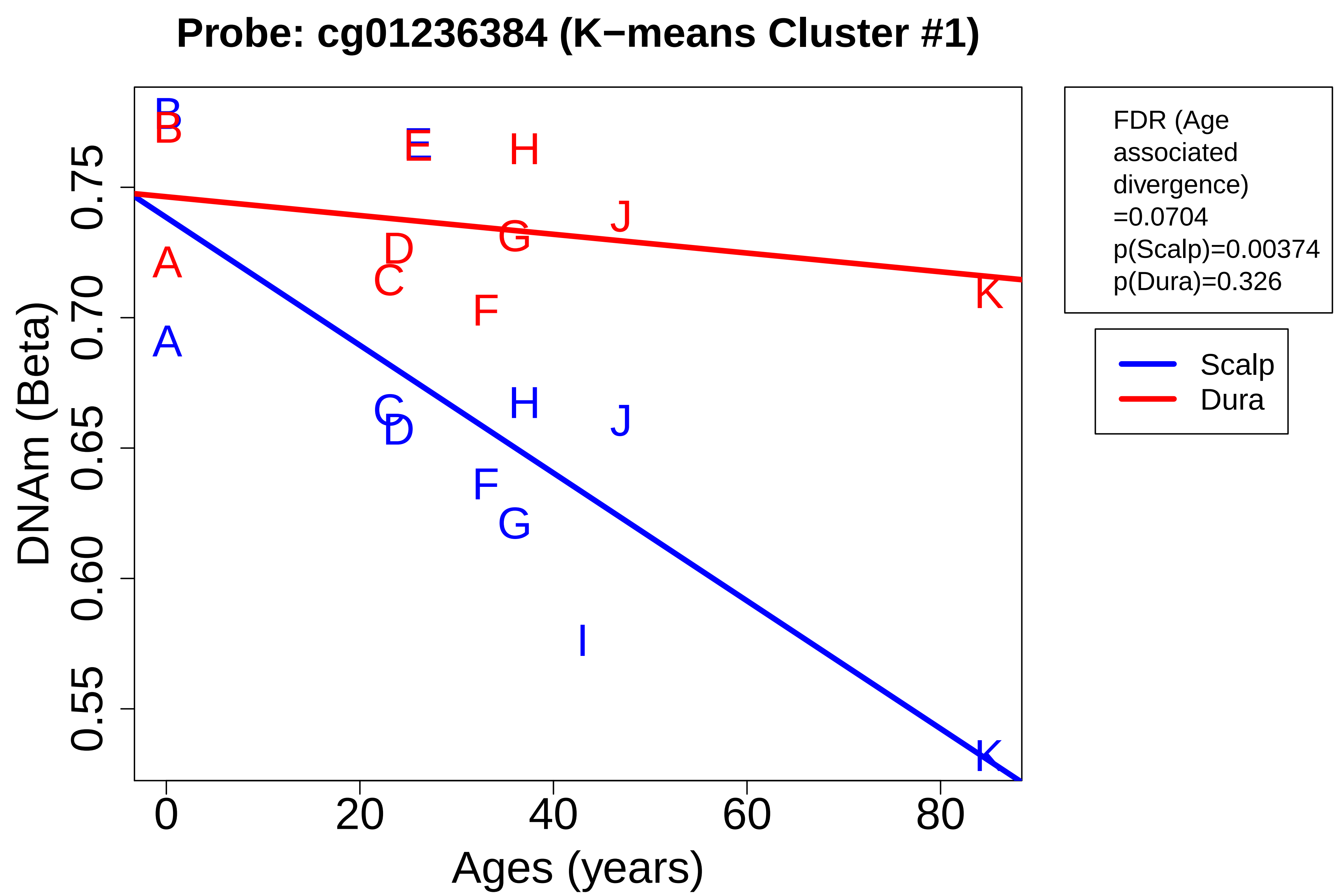

Probe: cg01236384 (K−means Cluster #1)
FDR (Age associated divergence) =0.0704
p(Scalp)=0.00374
p(Dura)=0.326
Scalp
Dura
DNAm (Beta)
0.55
0.60
0.65
0.70
0.75
0
20
40
60
80
Ages (years)
A
B
C
D
E
F
G
H
I
J
K

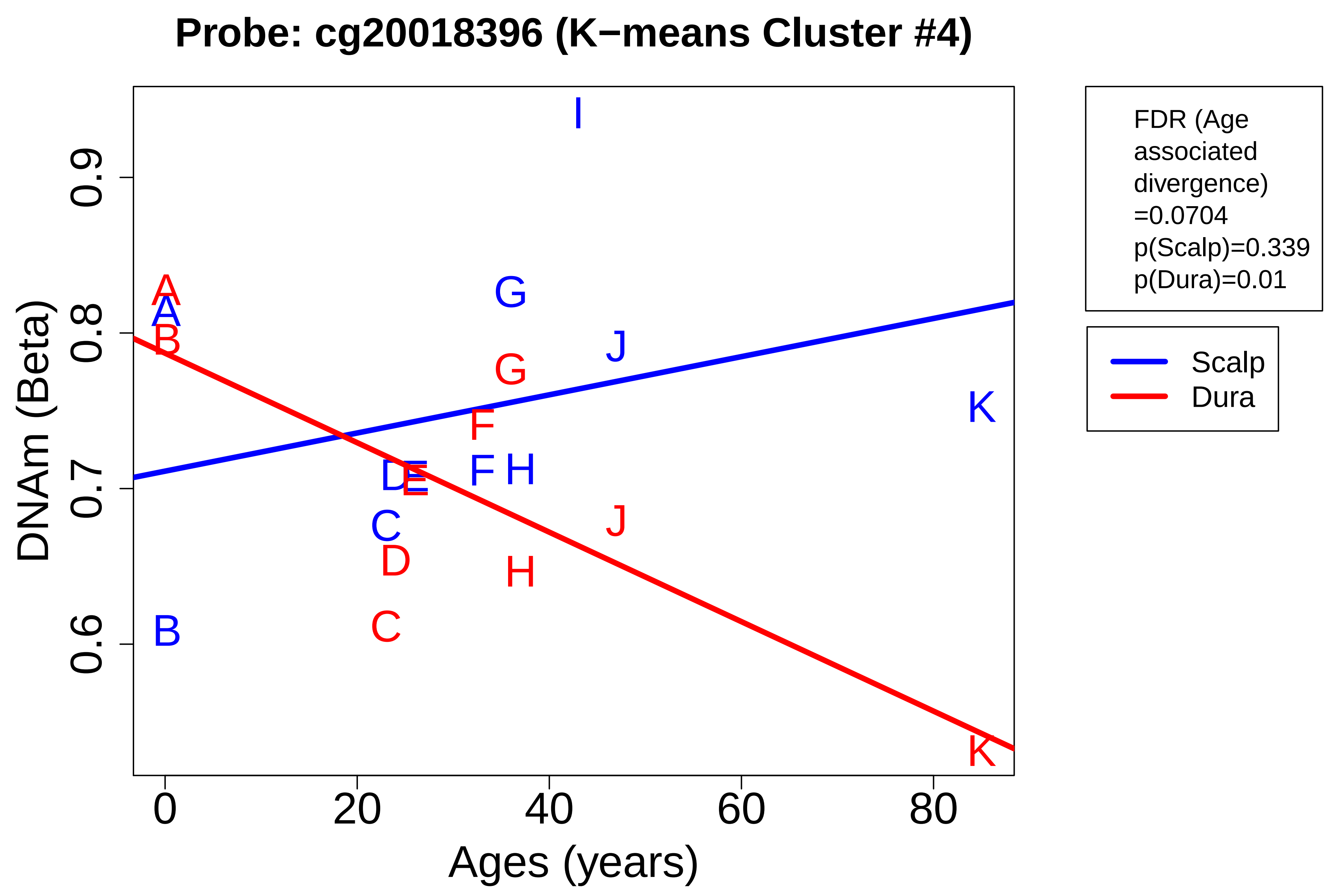
Probe: cg20018396 (K−means Cluster #4)
DNAm (Beta)
Ages (years)
0.6
0.7
0.8
0.9
0
20
40
60
80
FDR (Age associated divergence) =0.0704
p(Scalp)=0.339
p(Dura)=0.01
Scalp
Dura
A
B
C
D
E
F
G
H
I
J
K

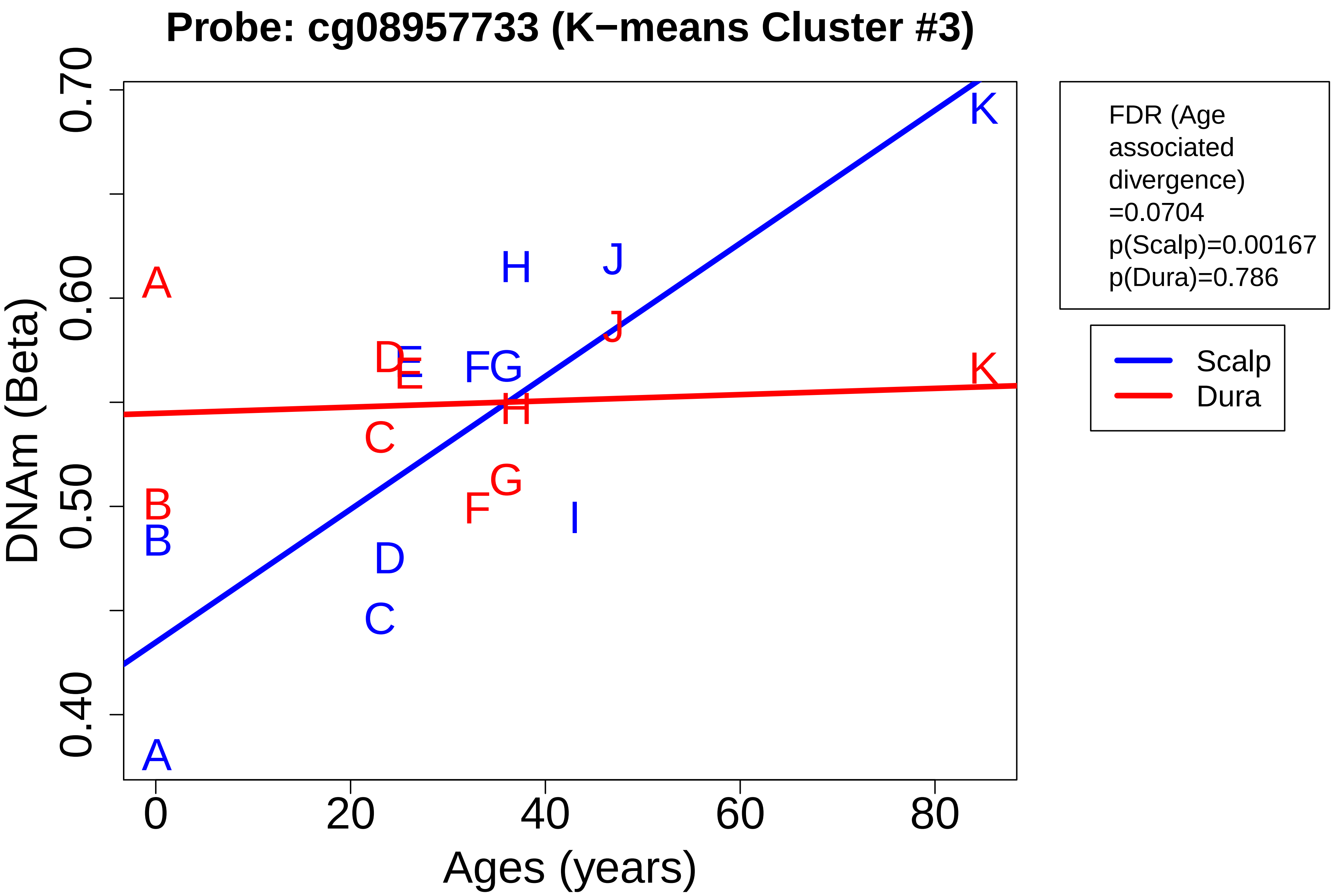

Probe: cg08957733 (K−means Cluster #3)
DNAm (Beta)
0.40
0.50
0.60
0.70
Ages (years)
0
20
40
60
80
A
B
C
D
E
F
G
H
I
J
K
FDR (Age associated divergence) =0.0704
p(Scalp)=0.00167
p(Dura)=0.786
Scalp
Dura

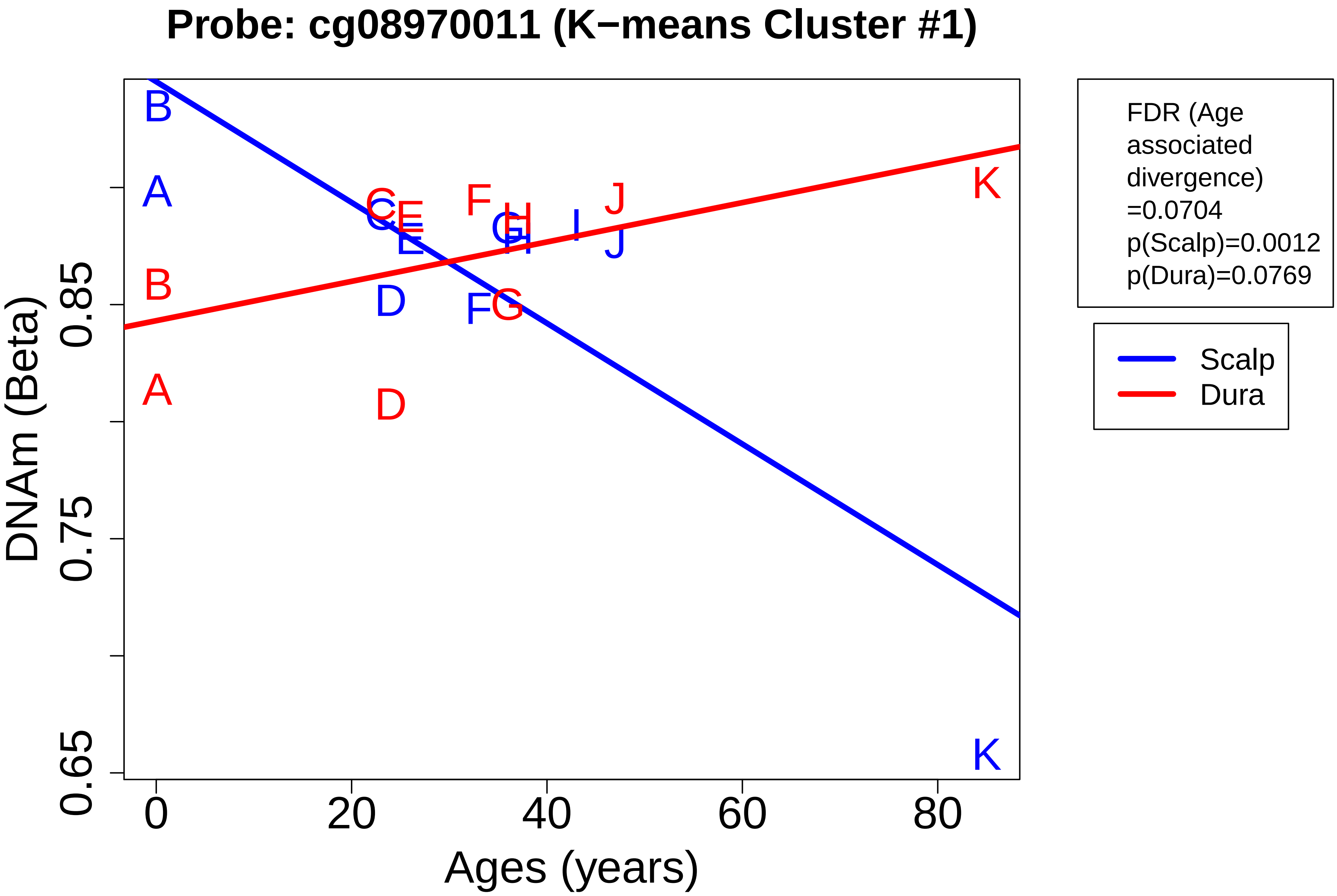

Probe: cg08970011 (K−means Cluster #1)
DNAm (Beta)
0.65
0.75
0.85
Ages (years)
0
20
40
60
80
FDR (Age associated divergence) =0.0704
p(Scalp)=0.0012
p(Dura)=0.0769
Scalp
Dura

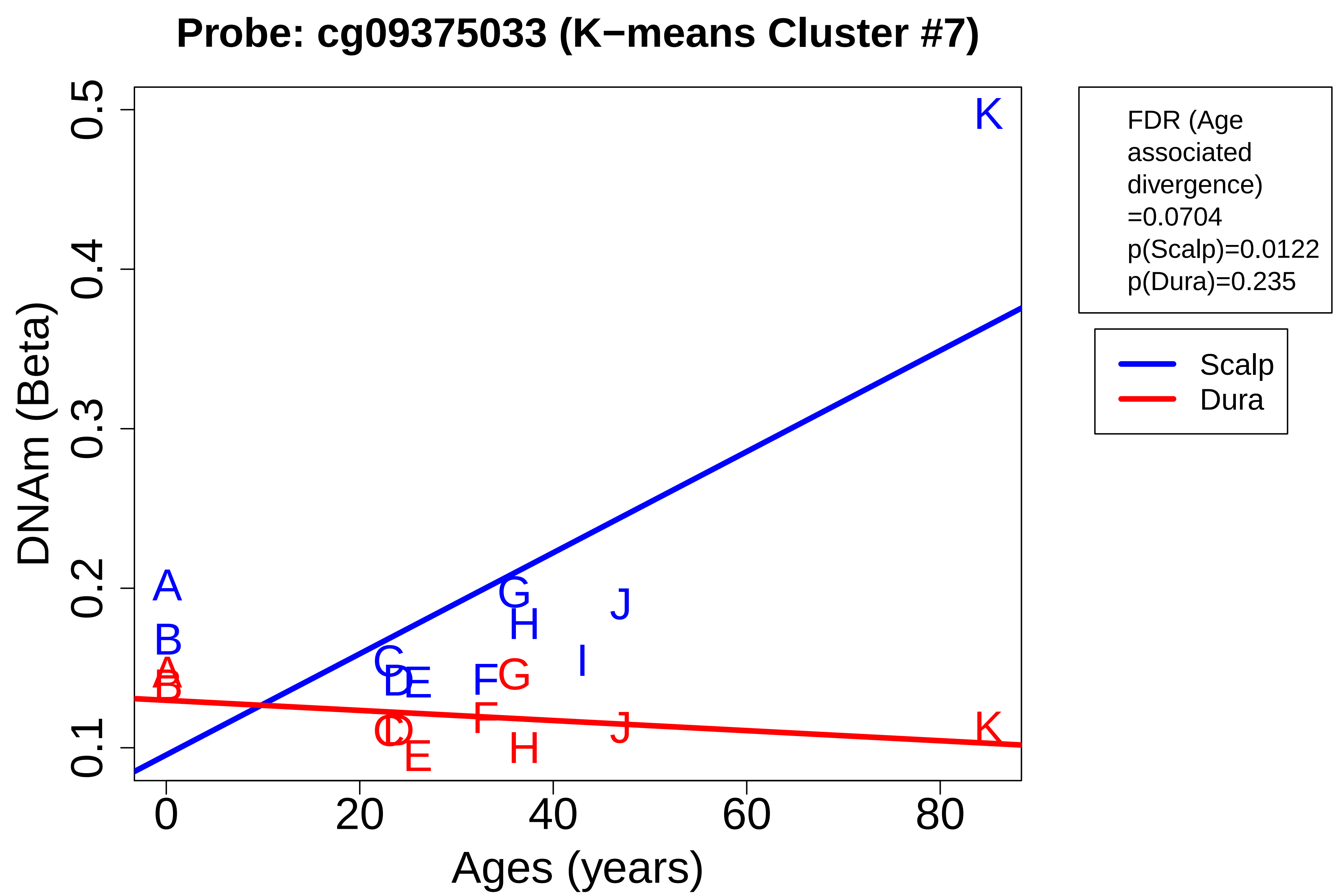

Probe: cg09375033 (K−means Cluster #7)
DNAm (Beta)
0.1
0.2
0.3
0.4
0.5
Ages (years)
0
20
40
60
80
A
B
C
D
E
F
G
H
I
J
K
FDR (Age associated divergence) =0.0704
p(Scalp)=0.0122
p(Dura)=0.235
Scalp
Dura

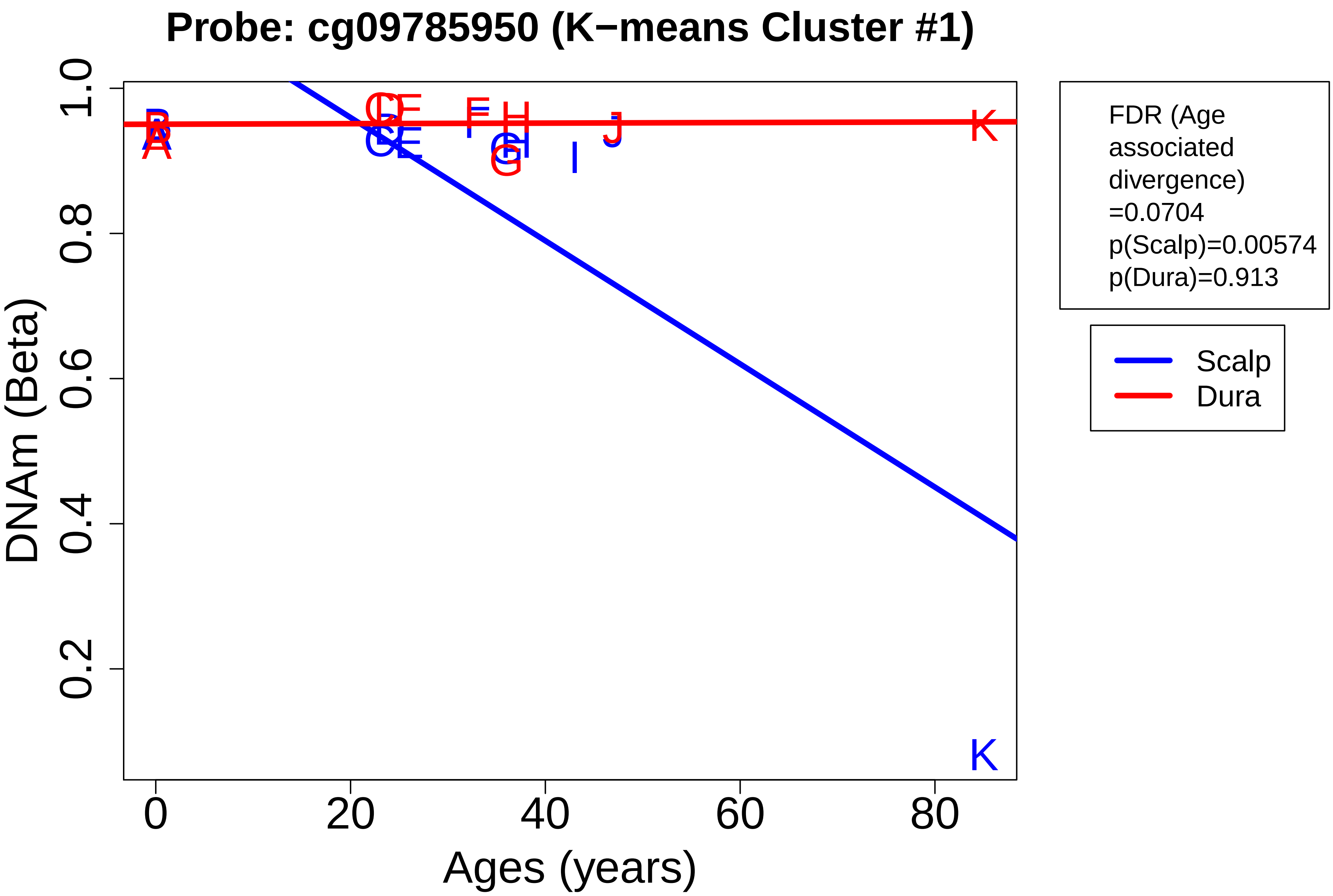

Probe: cg09785950 (K−means Cluster #1)
DNAm (Beta)
Ages (years)
FDR (Age associated divergence) =0.0704
p(Scalp)=0.00574
p(Dura)=0.913
Scalp
Dura
0.2
0.4
0.6
0.8
1.0
0
20
40
60
80

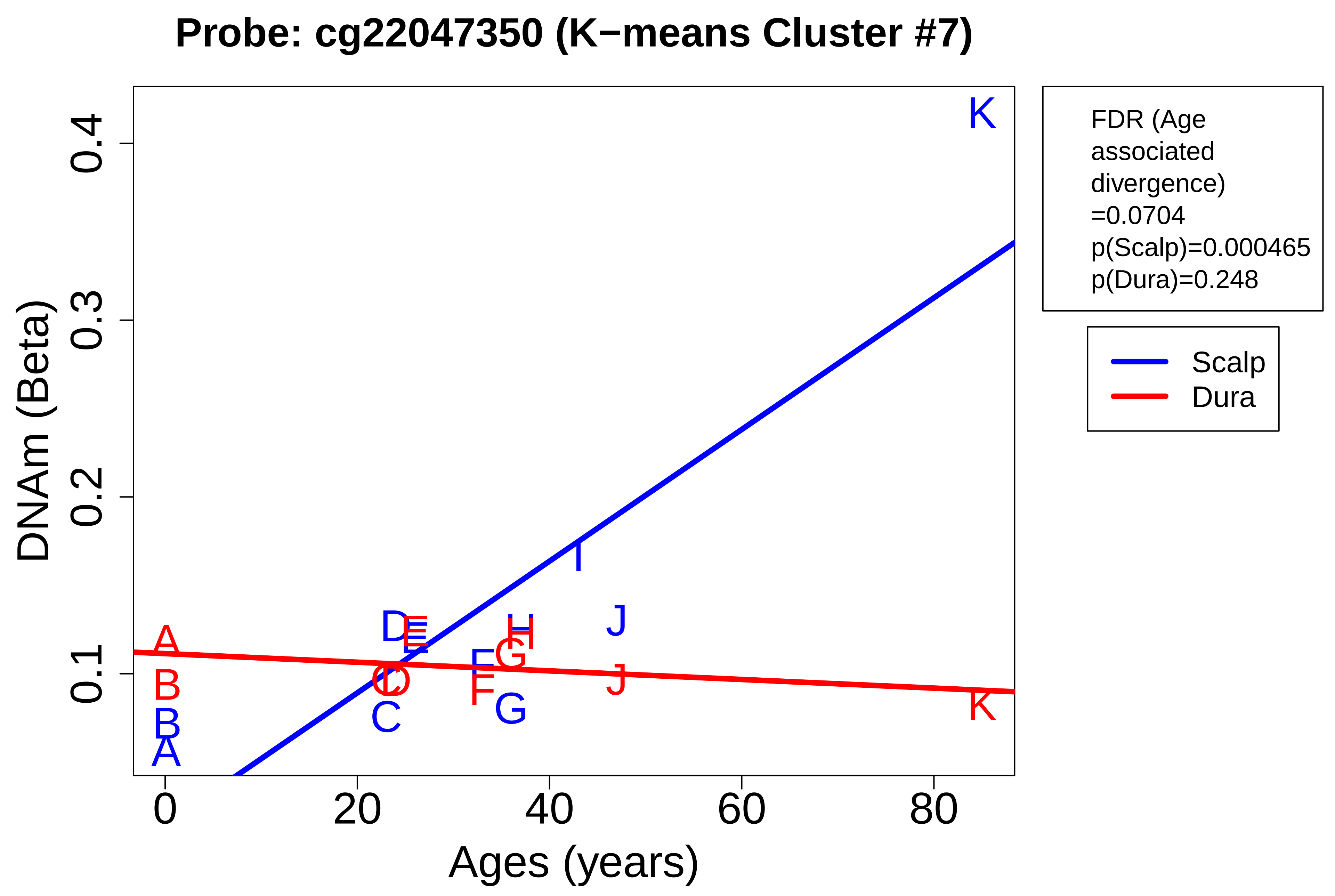

Probe: cg22047350 (K−means Cluster #7)
FDR (Age associated divergence) =0.0704
p(Scalp)=0.000465
p(Dura)=0.248
Scalp
Dura
DNAm (Beta)
Ages (years)
0.1
0.2
0.3
0.4
0
20
40
60
80

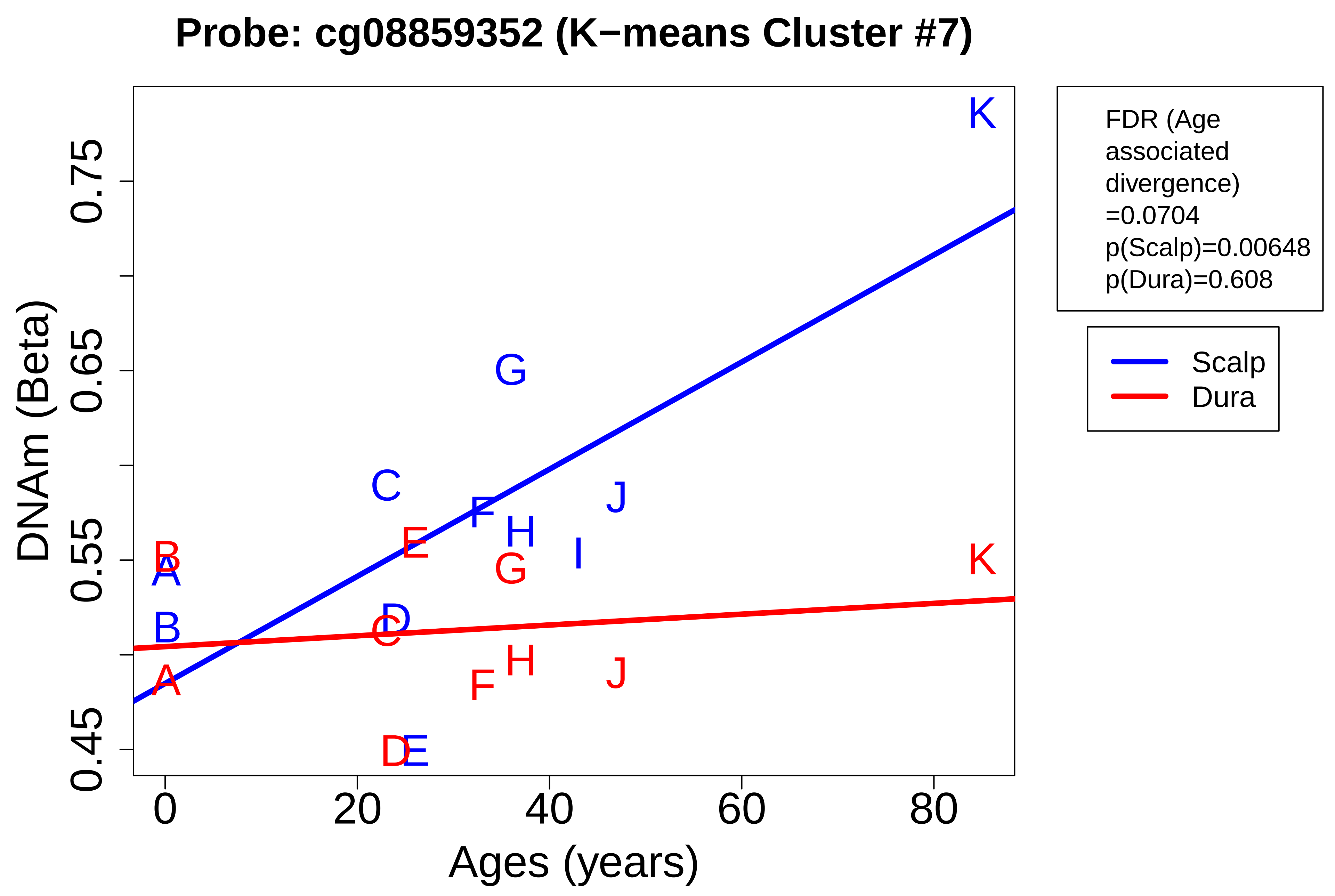

Probe: cg08859352 (K−means Cluster #7)
DNAm (Beta)
0.45
0.55
0.65
0.75
0
20
40
60
80
Ages (years)
K
G
C
F
J
H
I
E
B
A
G
K
B
D
C
A
H
F
J
D
E
FDR (Age associated divergence) =0.0704
p(Scalp)=0.00648
p(Dura)=0.608
Scalp
Dura

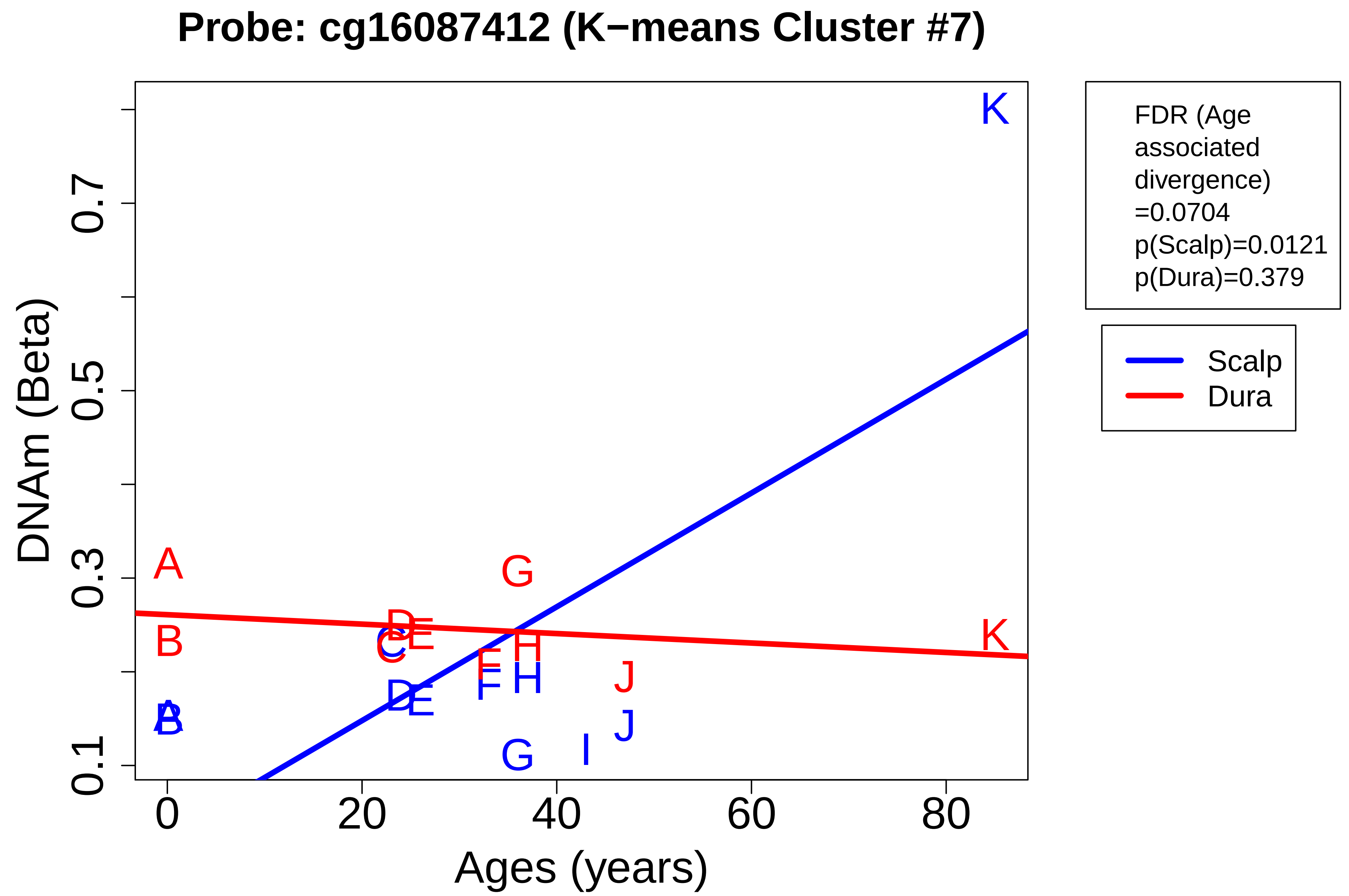

Probe: cg16087412 (K−means Cluster #7)
DNAm (Beta)
0.1
0.3
0.5
0.7
0
20
40
60
80
Ages (years)
FDR (Age associated divergence) =0.0704
p(Scalp)=0.0121
p(Dura)=0.379
Scalp
Dura

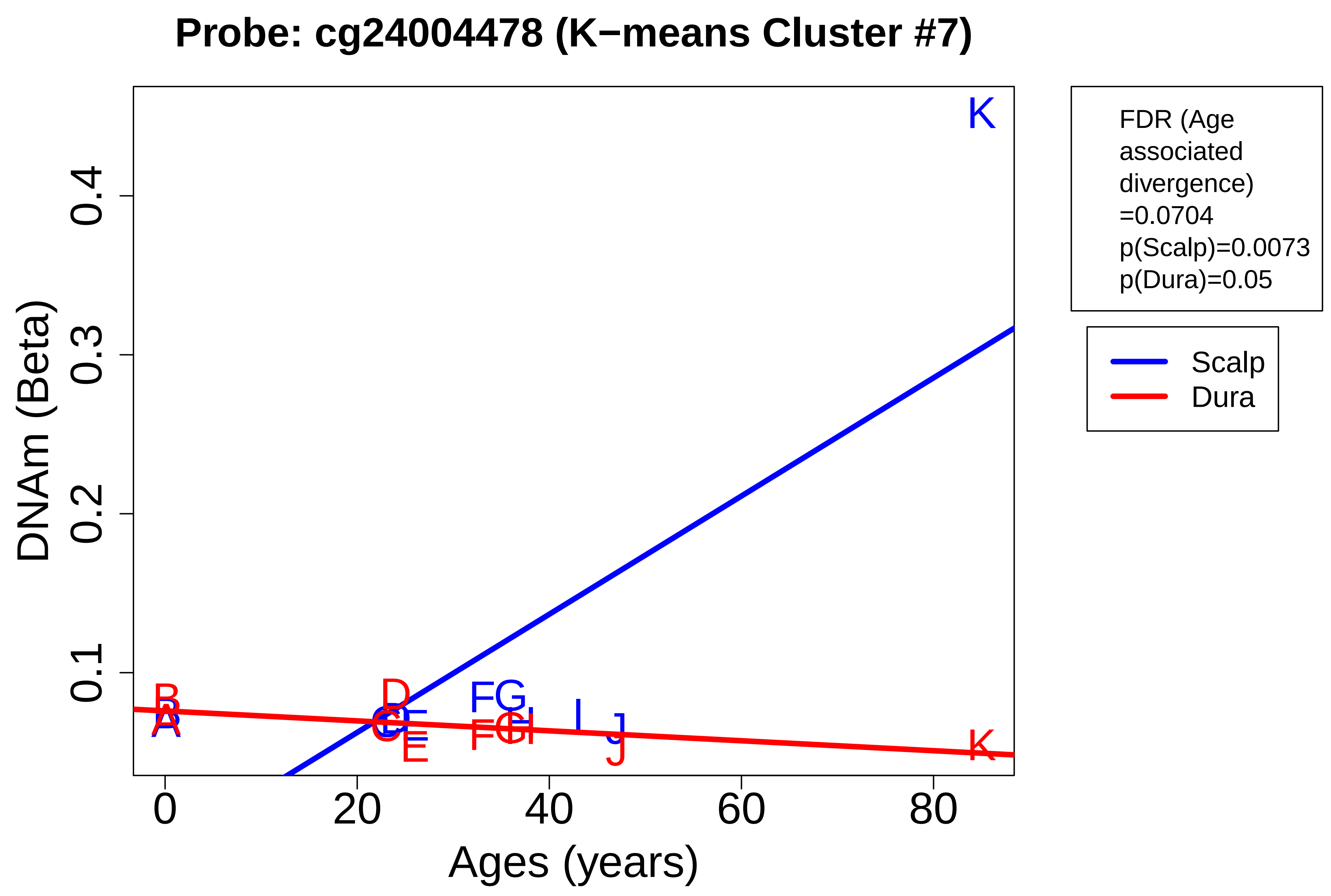

Probe: cg24004478 (K−means Cluster #7)
FDR (Age associated divergence) =0.0704
p(Scalp)=0.0073
p(Dura)=0.05
Scalp
Dura
DNAm (Beta)
Ages (years)
0.1
0.2
0.3
0.4
0
20
40
60
80

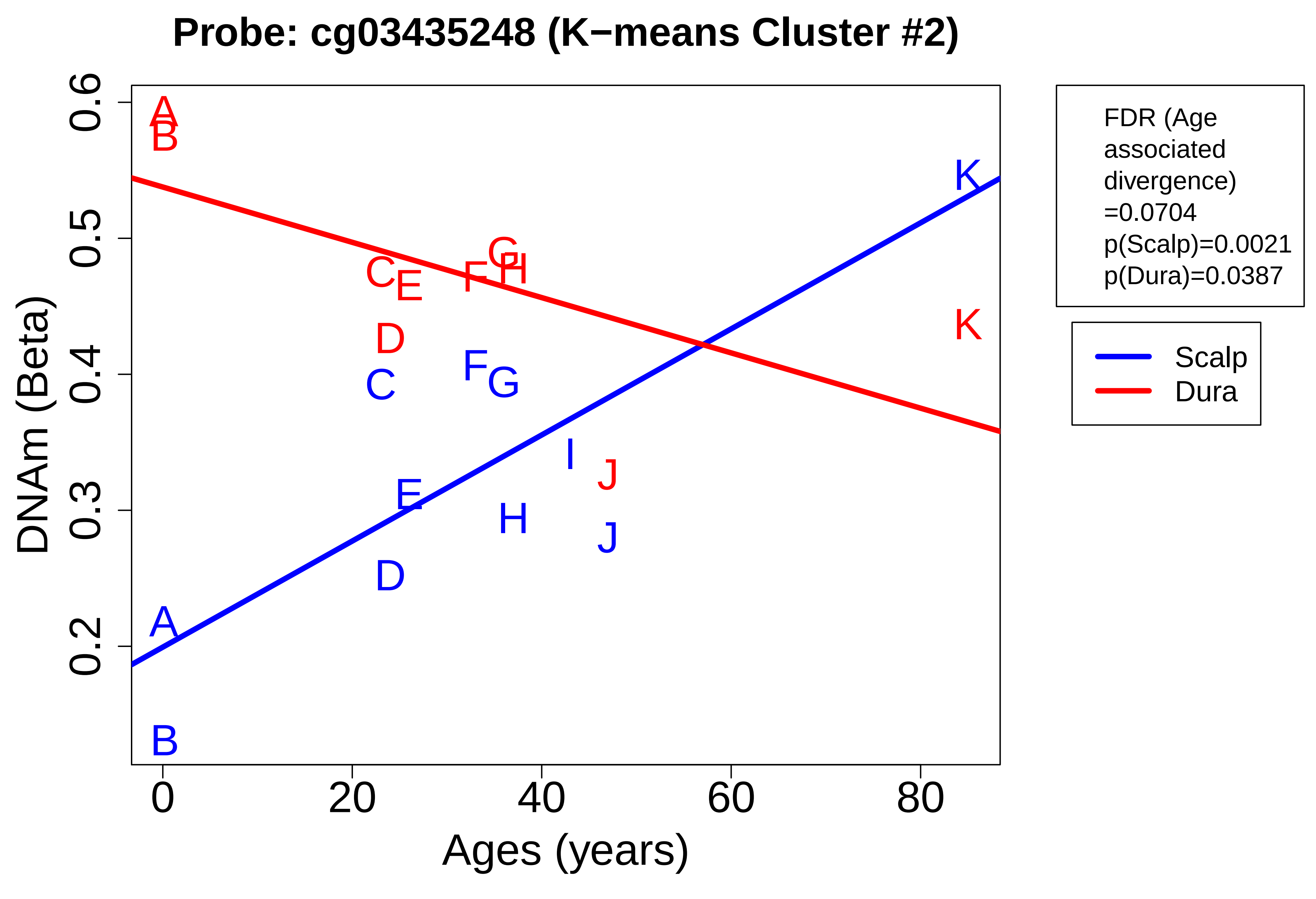

Probe: cg03435248 (K−means Cluster #2)
DNAm (Beta)
0.2
0.3
0.4
0.5
0.6
Ages (years)
0
20
40
60
80
A
B
C
D
E
F
G
H
I
J
K
FDR (Age associated divergence) =0.0704
p(Scalp)=0.0021
p(Dura)=0.0387
Scalp
Dura

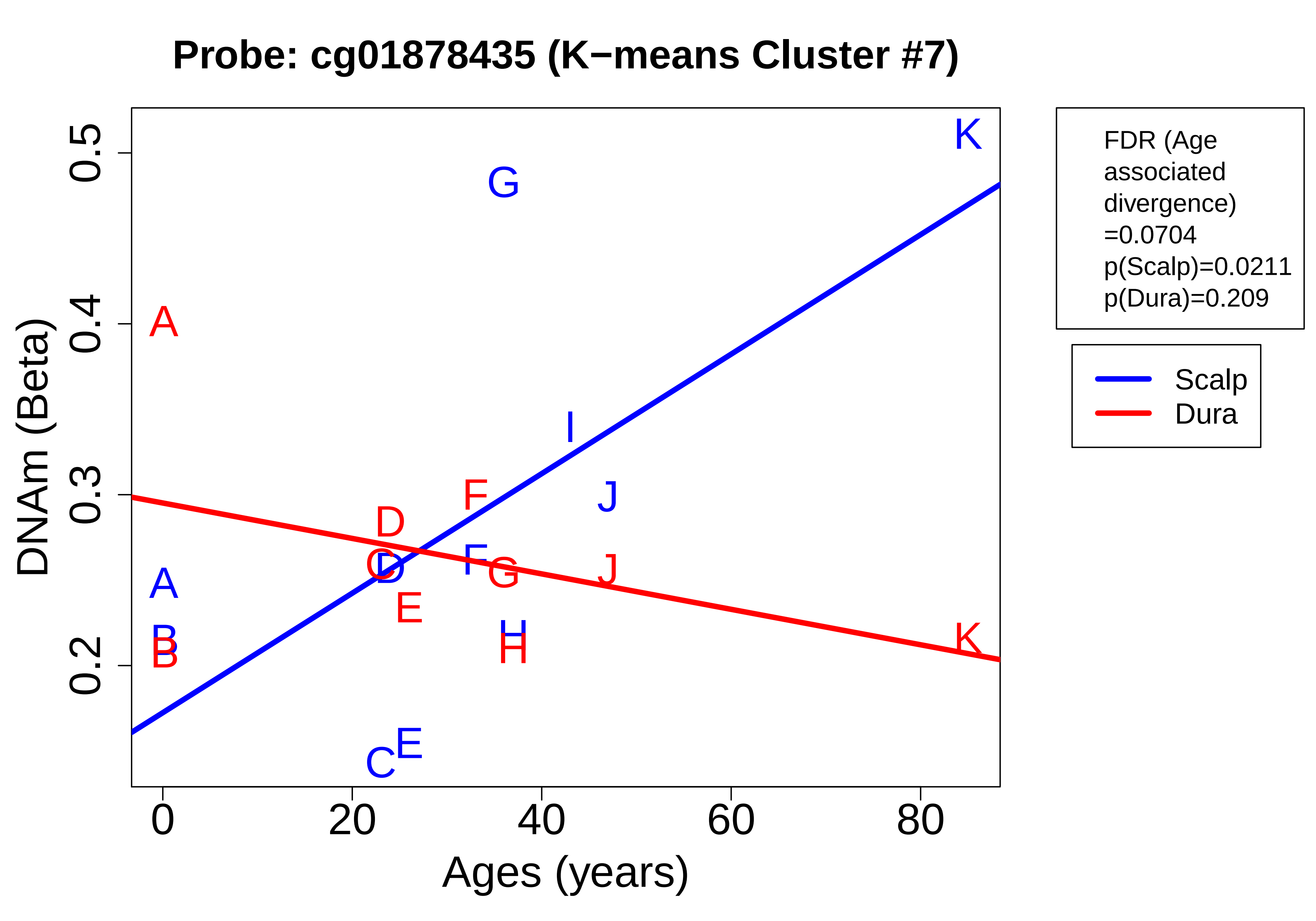
Probe: cg01878435 (K−means Cluster #7)
DNAm (Beta)
0.2
0.3
0.4
0.5
Ages (years)
0
20
40
60
80
FDR (Age associated divergence) =0.0704
p(Scalp)=0.0211
p(Dura)=0.209
Scalp
Dura
A
B
C
D
E
F
G
H
I
J
K

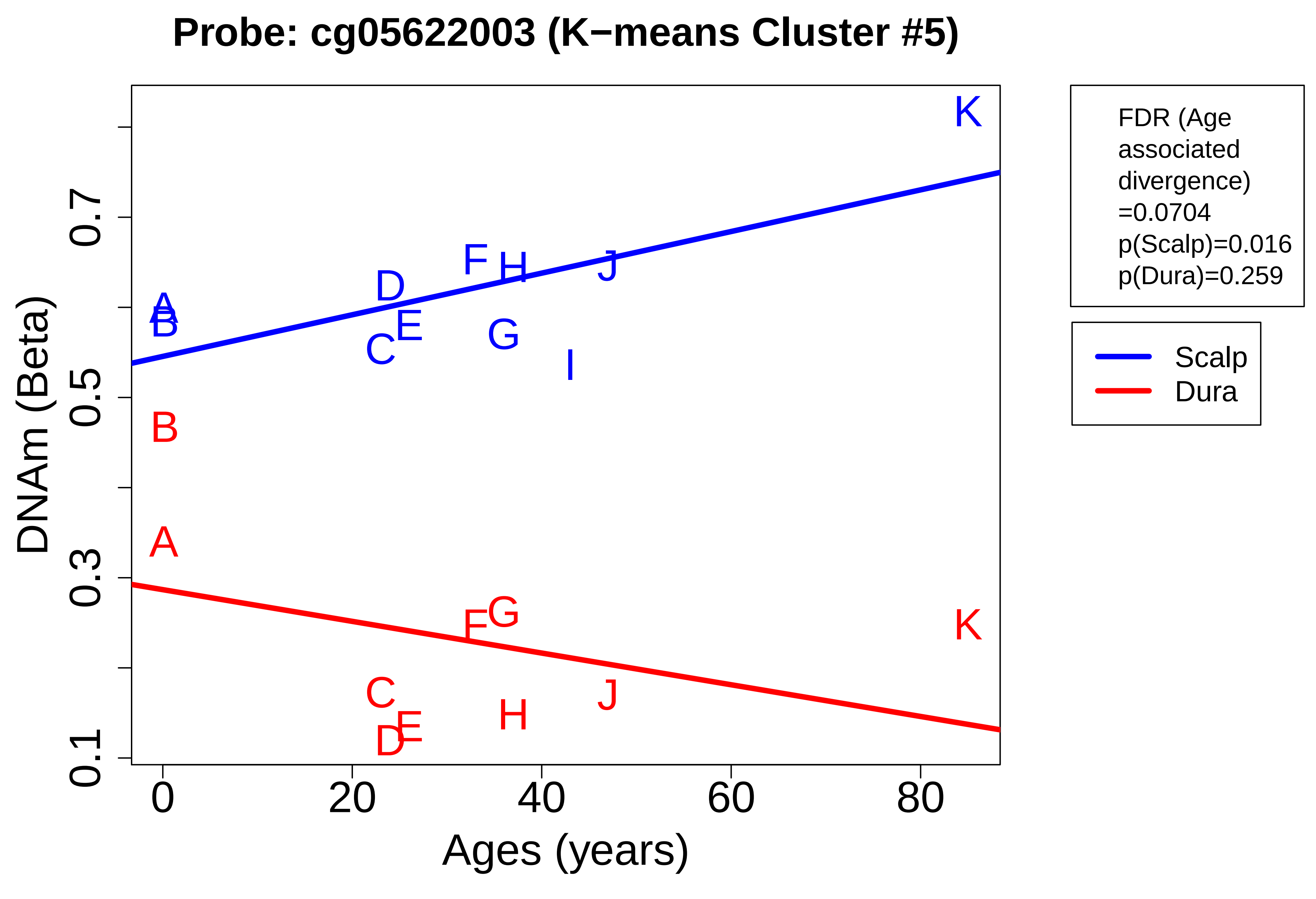

Probe: cg05622003 (K−means Cluster #5)
DNAm (Beta)
0.1
0.3
0.5
0.7
Ages (years)
0
20
40
60
80
A
B
C
D
E
F
G
H
I
J
K
A
B
C
D
E
F
G
H
J
K
FDR (Age associated divergence) =0.0704
p(Scalp)=0.016
p(Dura)=0.259
Scalp
Dura

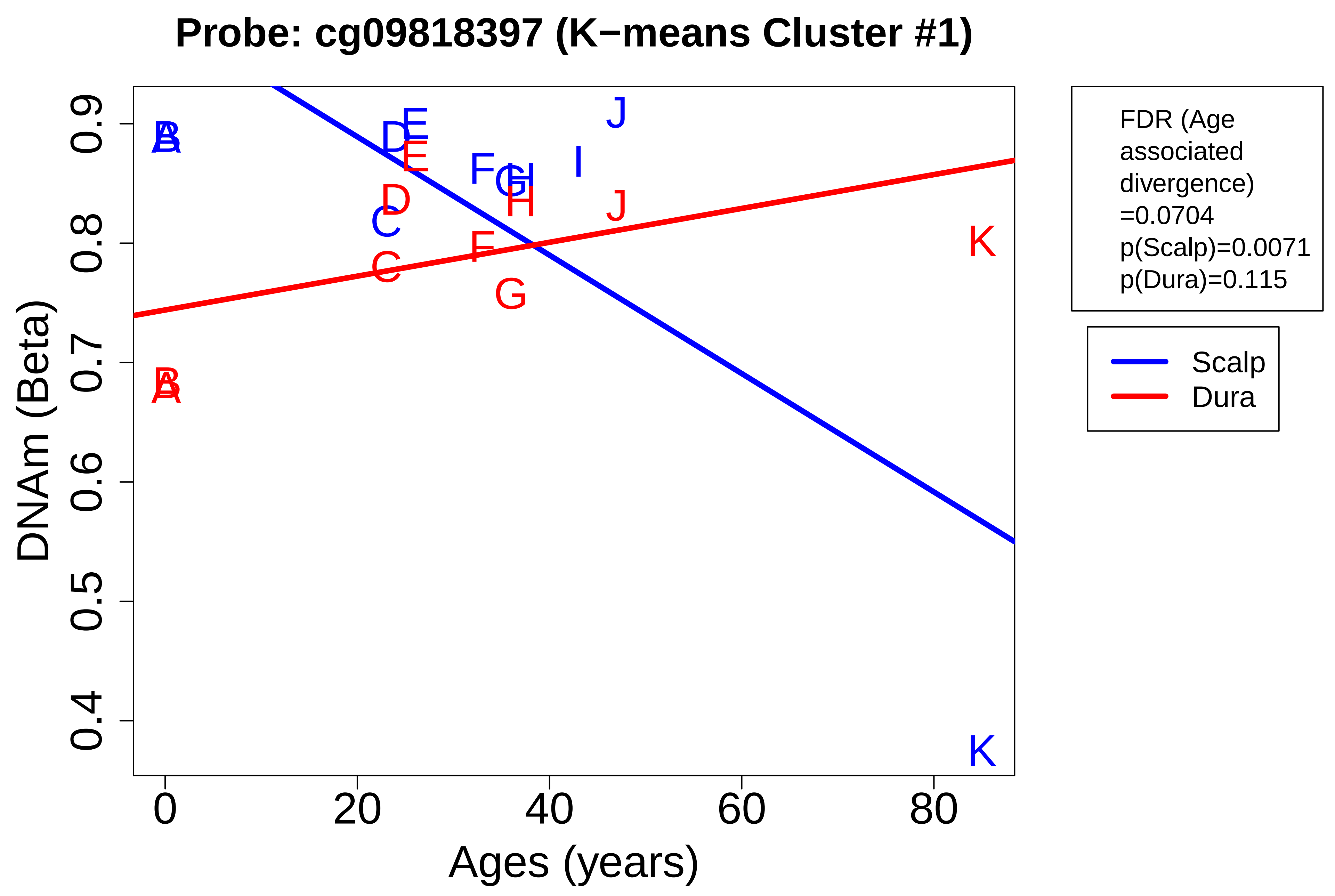
Probe: cg09818397 (K−means Cluster #1)
DNAm (Beta)
0.4
0.5
0.6
0.7
0.8
0.9
0
20
40
60
80
Ages (years)
A
B
C
D
E
F
G
H
I
J
K
FDR (Age associated divergence) =0.0704
p(Scalp)=0.0071
p(Dura)=0.115
Scalp
Dura

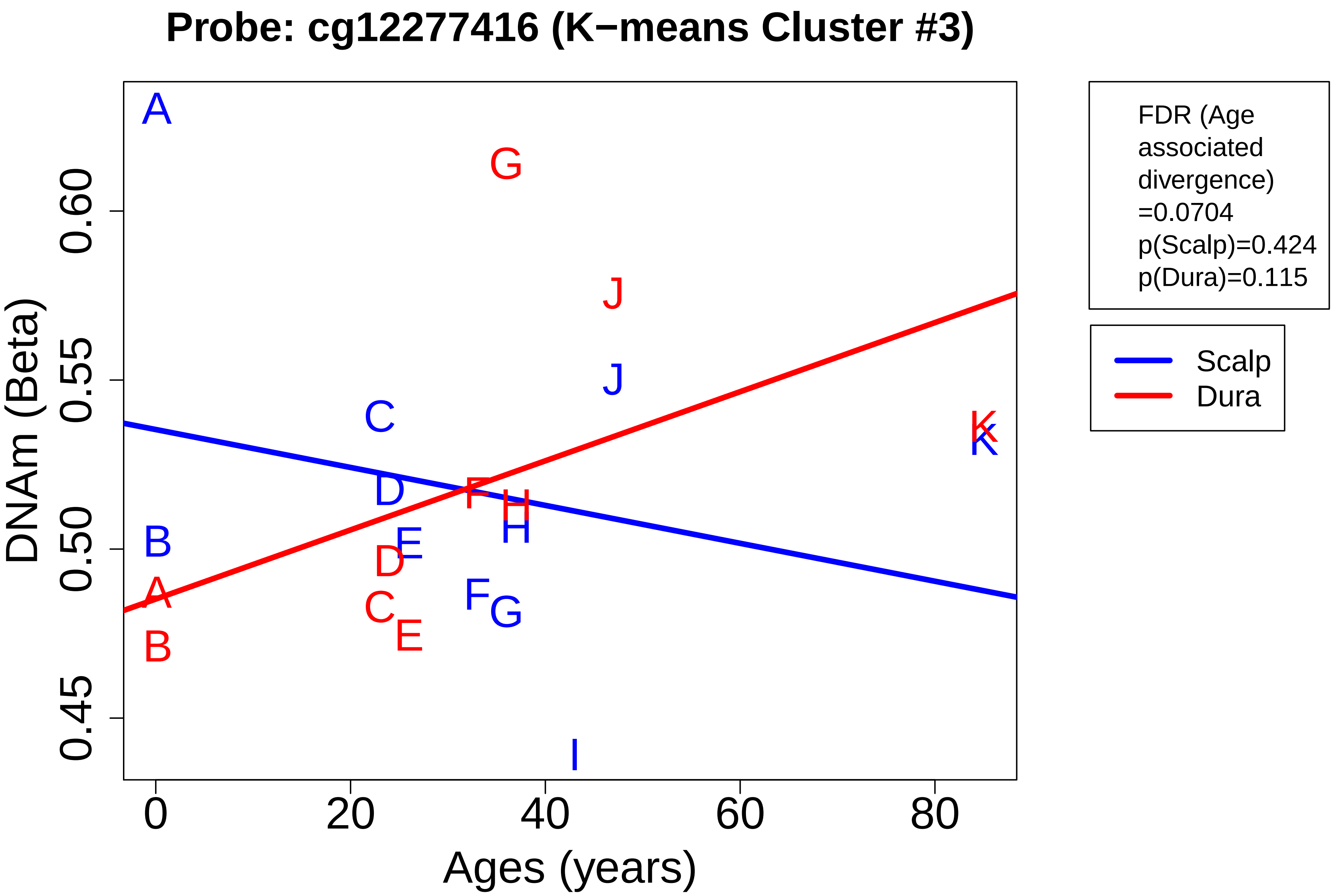

Probe: cg12277416 (K−means Cluster #3)
FDR (Age associated divergence) =0.0704
p(Scalp)=0.424
p(Dura)=0.115
Scalp
Dura
DNAm (Beta)
0.45
0.50
0.55
0.60
0
20
40
60
80
Ages (years)

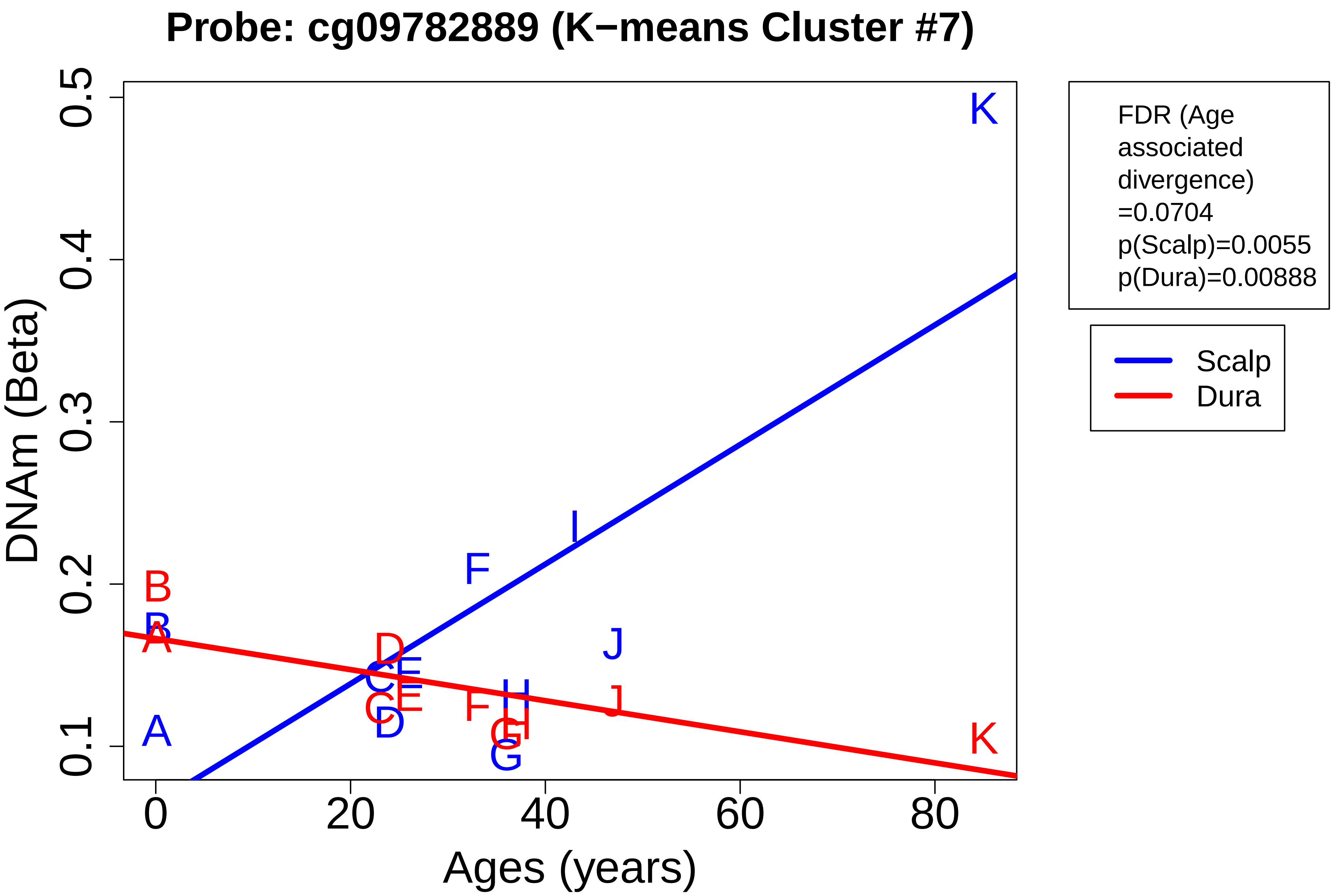

Probe: cg09782889 (K−means Cluster #7)
DNAm (Beta)
Ages (years)
0.1
0.2
0.3
0.4
0.5
0
20
40
60
80
FDR (Age associated divergence) =0.0704
p(Scalp)=0.0055
p(Dura)=0.00888
Scalp
Dura

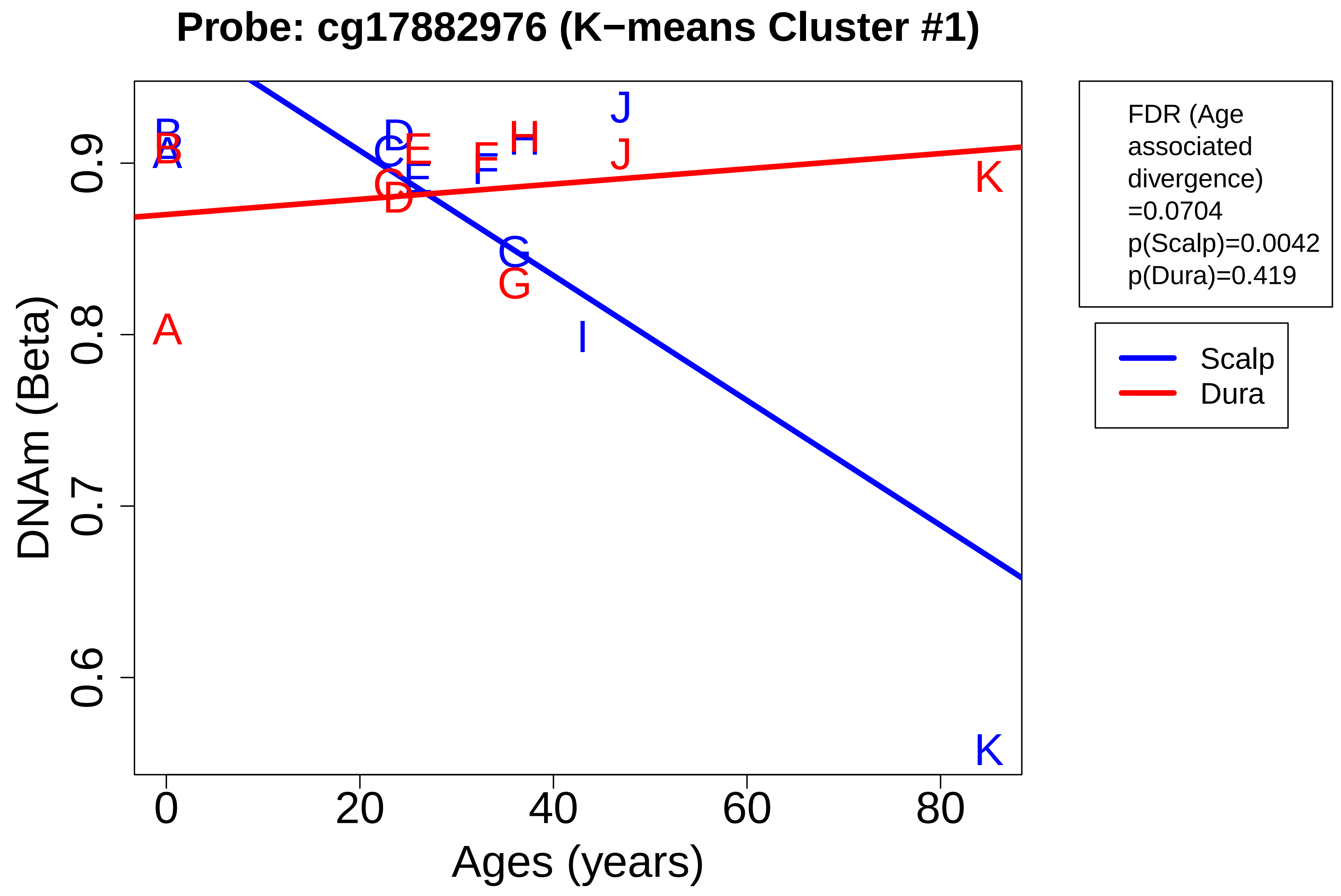

Probe: cg17882976 (K−means Cluster #1)
DNAm (Beta)
0.6
0.7
0.8
0.9
0
20
40
60
80
Ages (years)
A
B
C
D
E
F
G
H
I
J
K
FDR (Age associated divergence) =0.0704
p(Scalp)=0.0042
p(Dura)=0.419
Scalp
Dura

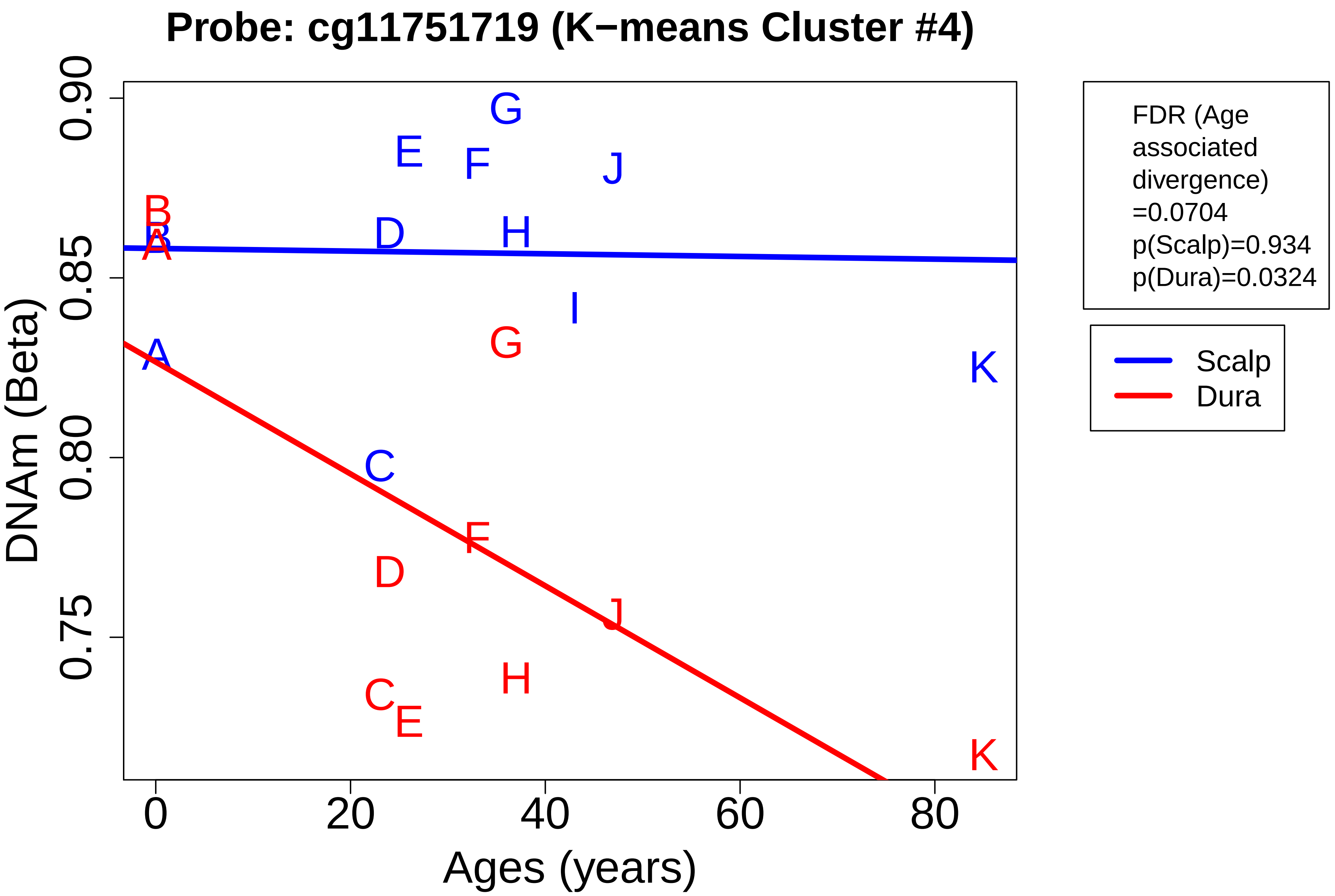
Probe: cg11751719 (K−means Cluster #4)
DNAm (Beta)
0.90
0.85
0.80
0.75
0
20
40
60
80
Ages (years)
FDR (Age associated divergence) =0.0704
p(Scalp)=0.934
p(Dura)=0.0324
Scalp
Dura

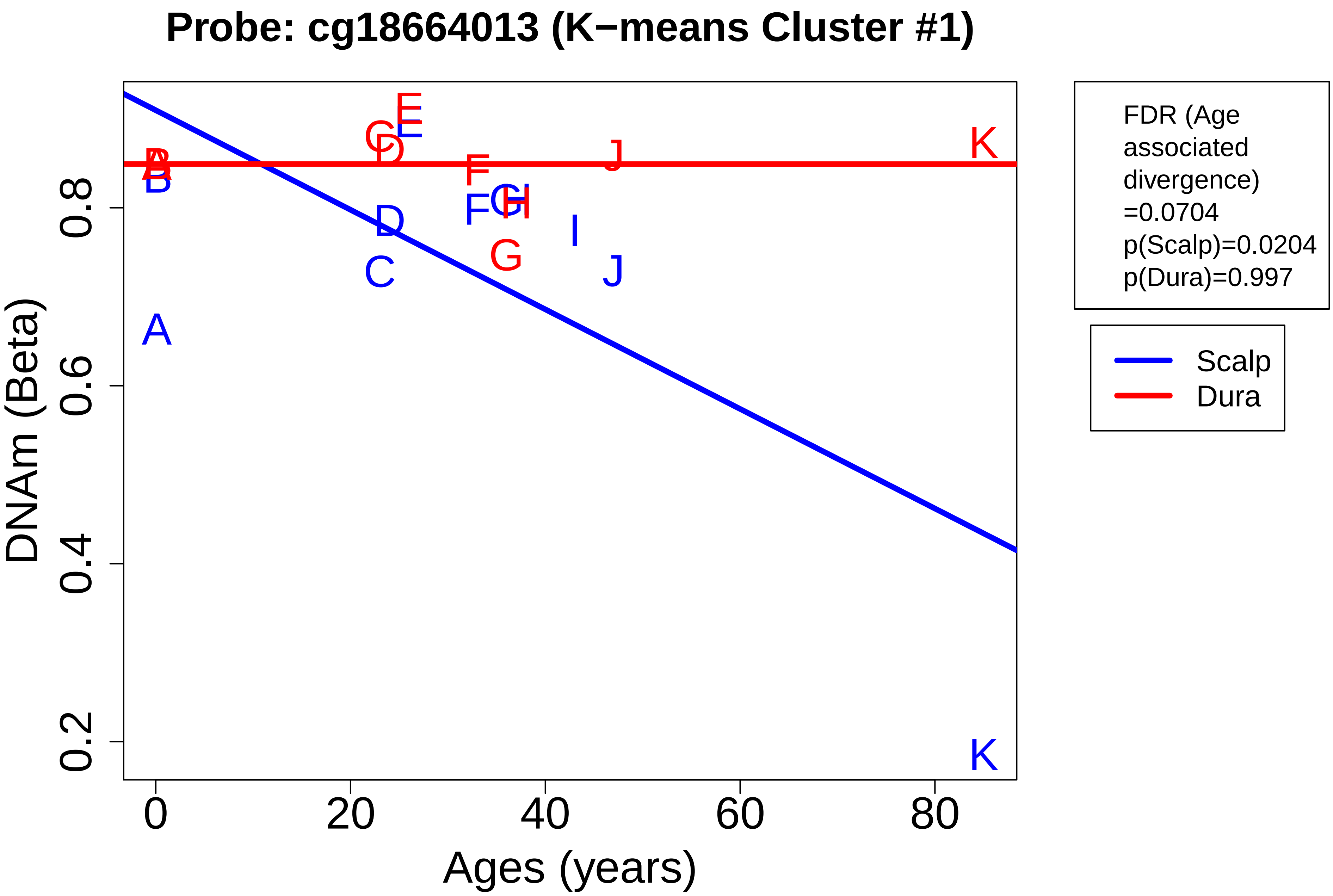

Probe: cg18664013 (K−means Cluster #1)
DNAm (Beta)
0.2
0.4
0.6
0.8
0
20
40
60
80
Ages (years)
FDR (Age associated divergence) =0.0704
p(Scalp)=0.0204
p(Dura)=0.997
Scalp
Dura

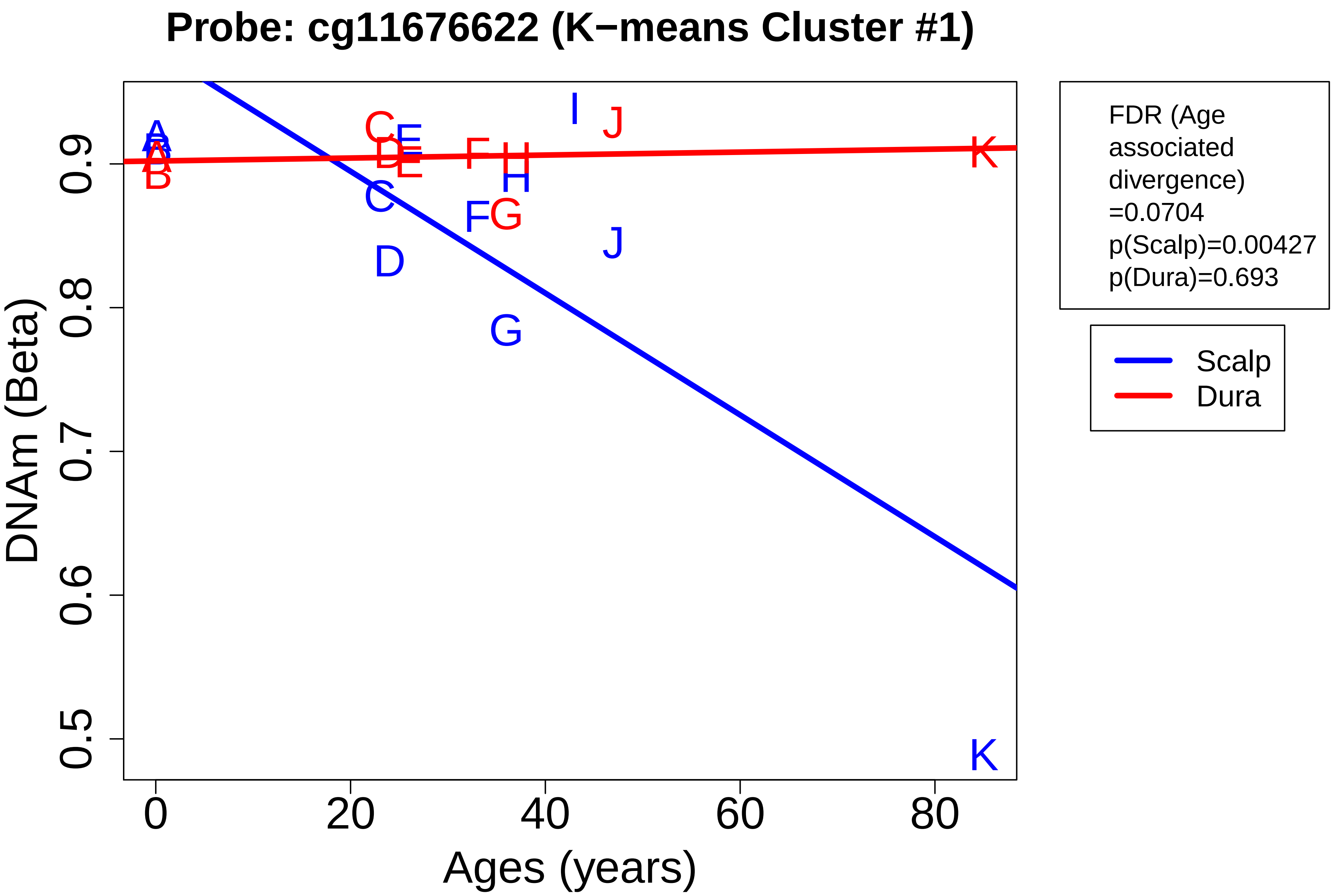
Probe: cg11676622 (K−means Cluster #1)
DNAm (Beta)
0.5
0.6
0.7
0.8
0.9
0
20
40
60
80
Ages (years)
A
B
C
D
E
F
G
H
I
J
K
FDR (Age associated divergence) =0.0704
p(Scalp)=0.00427
p(Dura)=0.693
Scalp
Dura

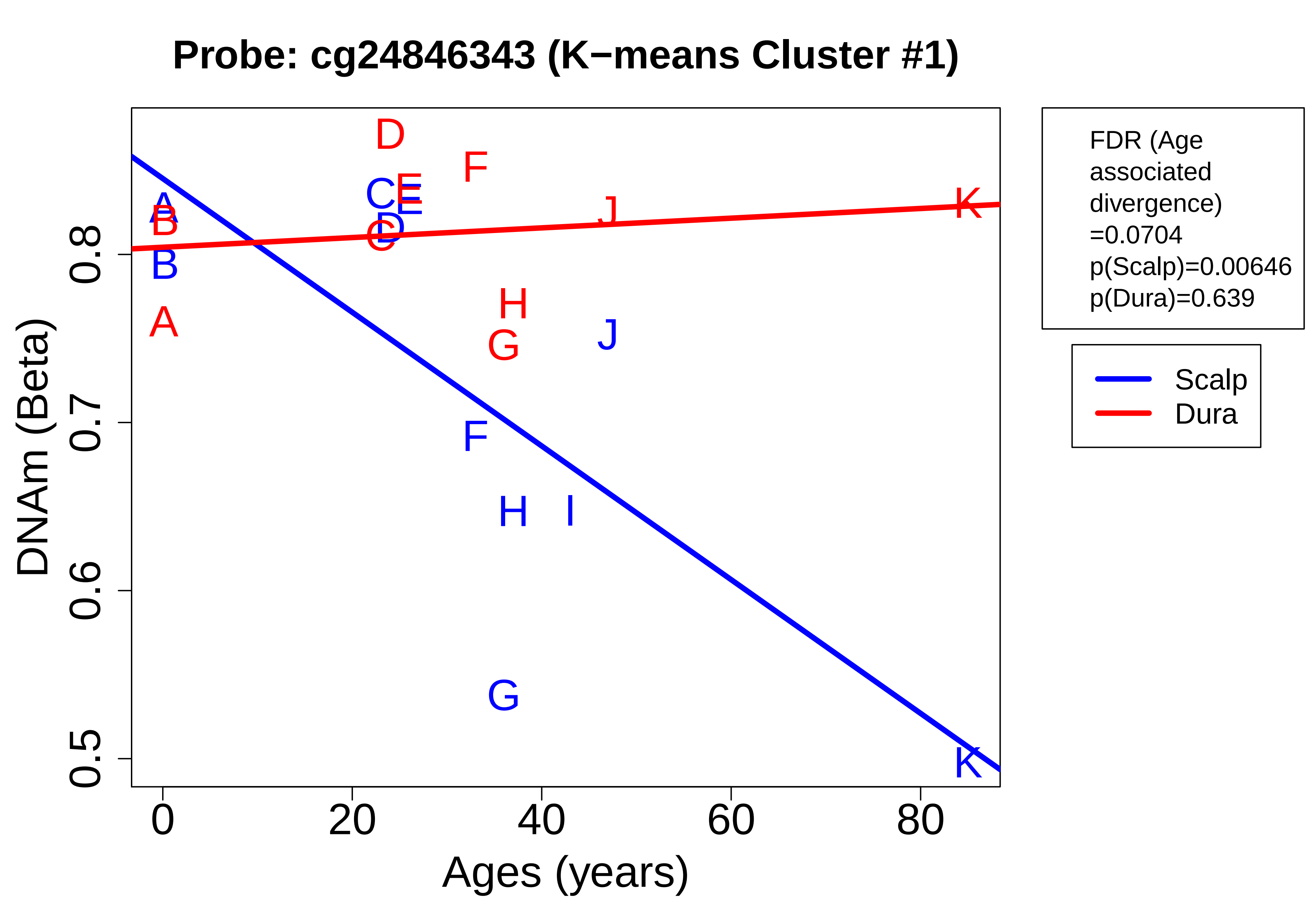

Probe: cg24846343 (K−means Cluster #1)
DNAm (Beta)
0.5
0.6
0.7
0.8
Ages (years)
0
20
40
60
80
FDR (Age associated divergence) =0.0704
p(Scalp)=0.00646
p(Dura)=0.639
Scalp
Dura

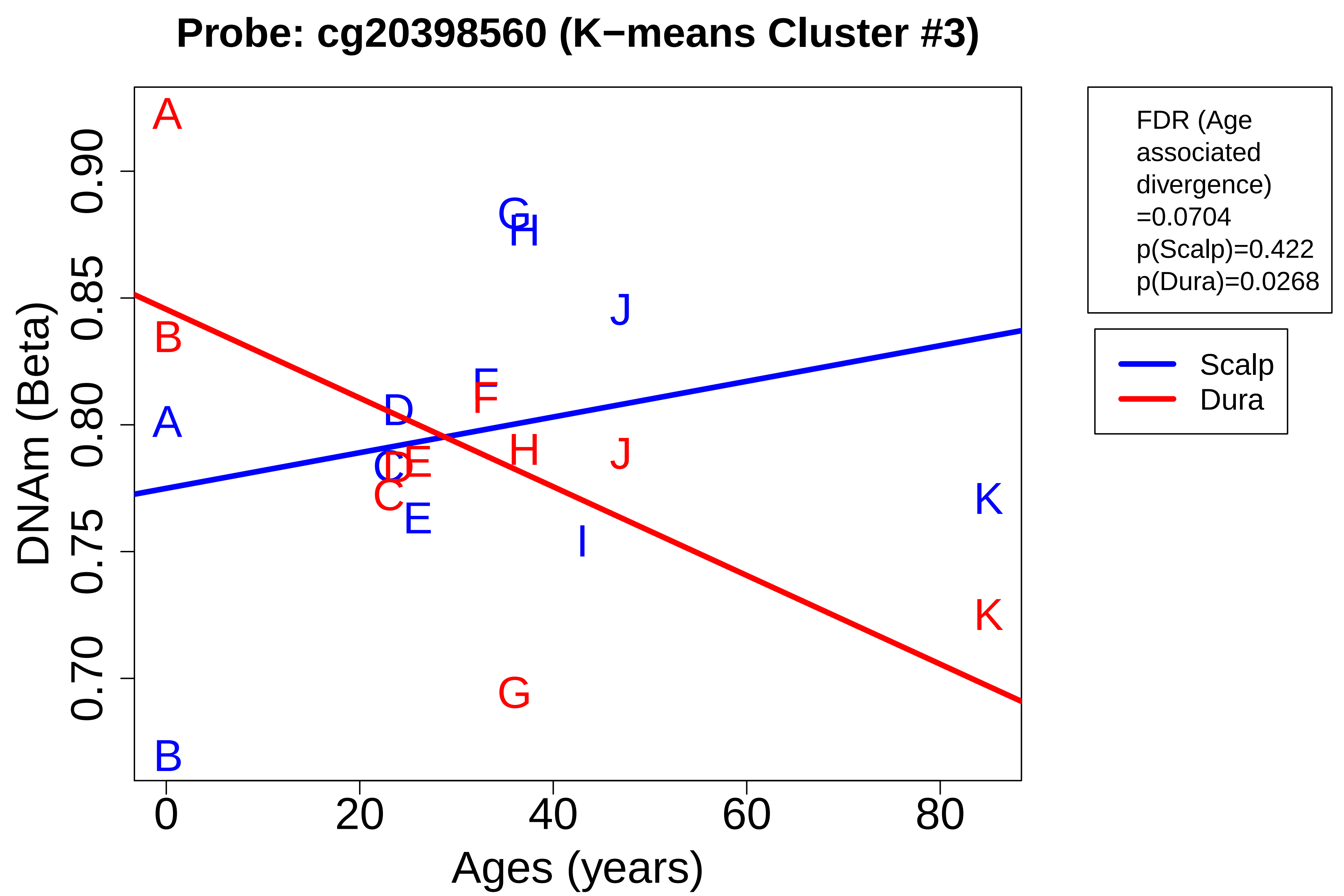

Probe: cg20398560 (K−means Cluster #3)
DNAm (Beta)
0.70
0.75
0.80
0.85
0.90
0
20
40
60
80
Ages (years)
FDR (Age associated divergence) =0.0704
p(Scalp)=0.422
p(Dura)=0.0268
Scalp
Dura
A
B
C
D
E
F
G
H
I
J
K

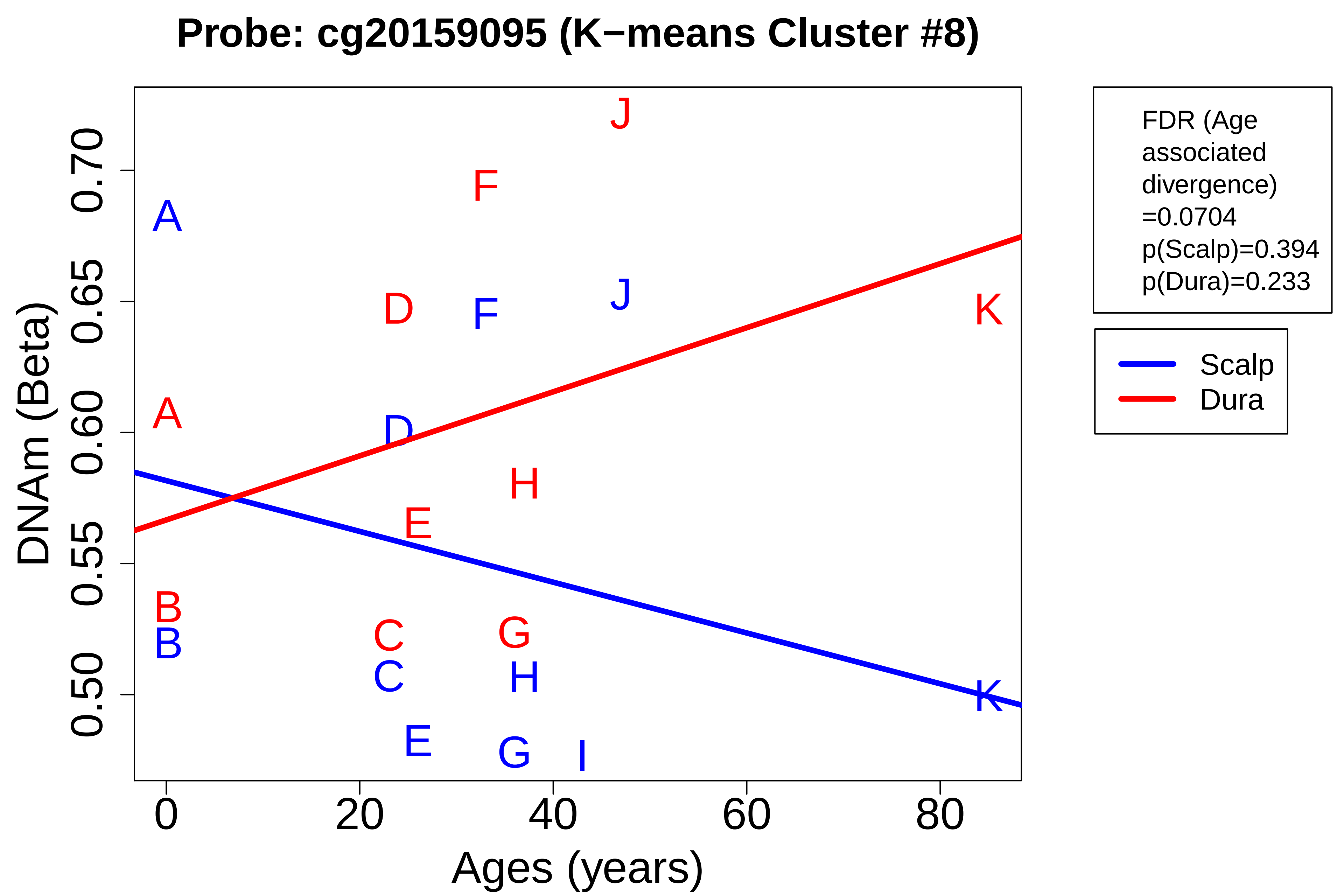

Probe: cg20159095 (K−means Cluster #8)
DNAm (Beta)
0.50
0.55
0.60
0.65
0.70
0
20
40
60
80
Ages (years)
FDR (Age associated divergence) =0.0704
p(Scalp)=0.394
p(Dura)=0.233
Scalp
Dura
A
B
C
D
E
F
G
H
I
J
K

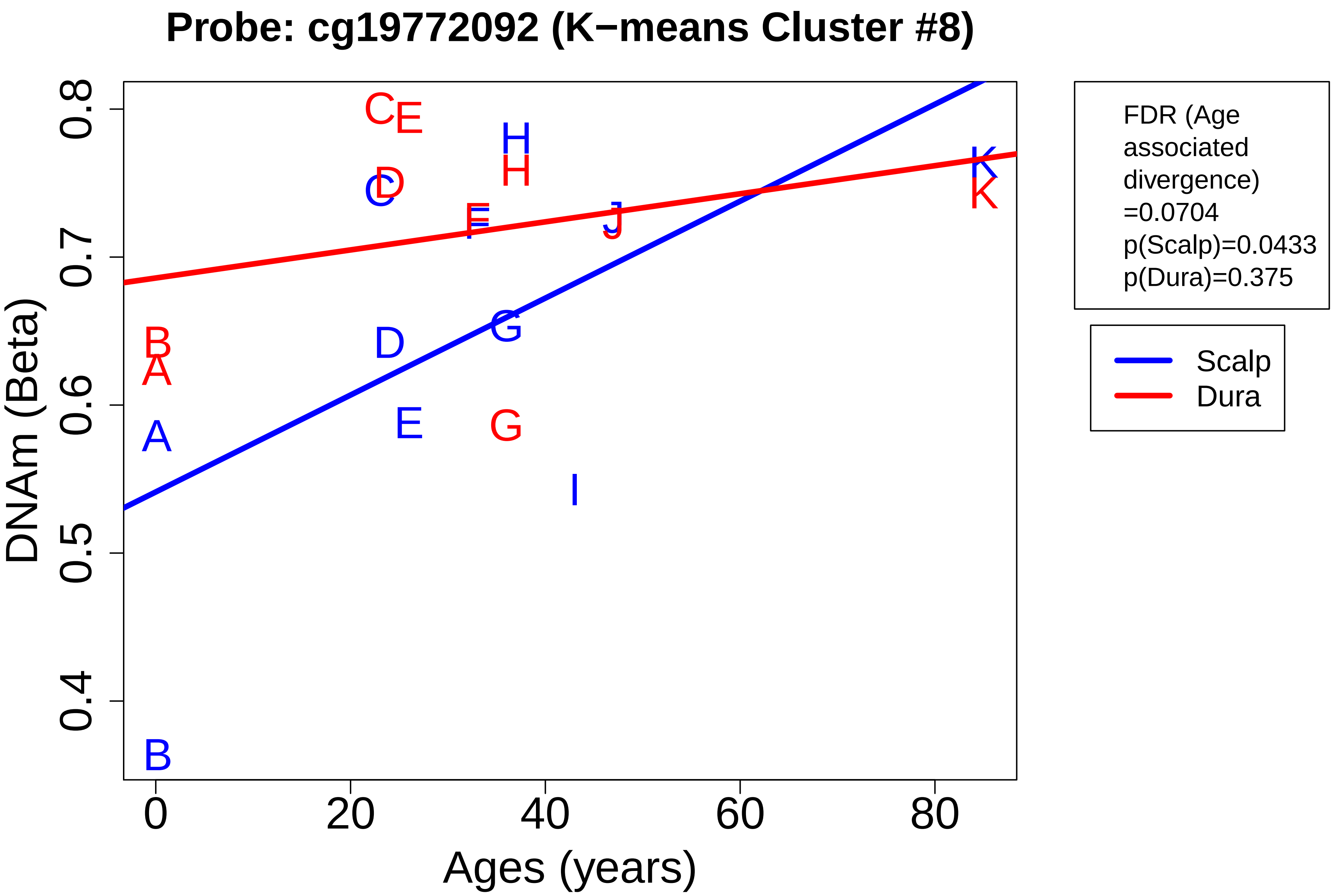

Probe: cg19772092 (K−means Cluster #8)
DNAm (Beta)
0.4
0.5
0.6
0.7
0.8
0
20
40
60
80
Ages (years)
C
E
H
K
D
C
H
K
F
J
B
D
G
A
A
E
G
I
B
FDR (Age associated divergence) =0.0704
p(Scalp)=0.0433
p(Dura)=0.375
Scalp
Dura

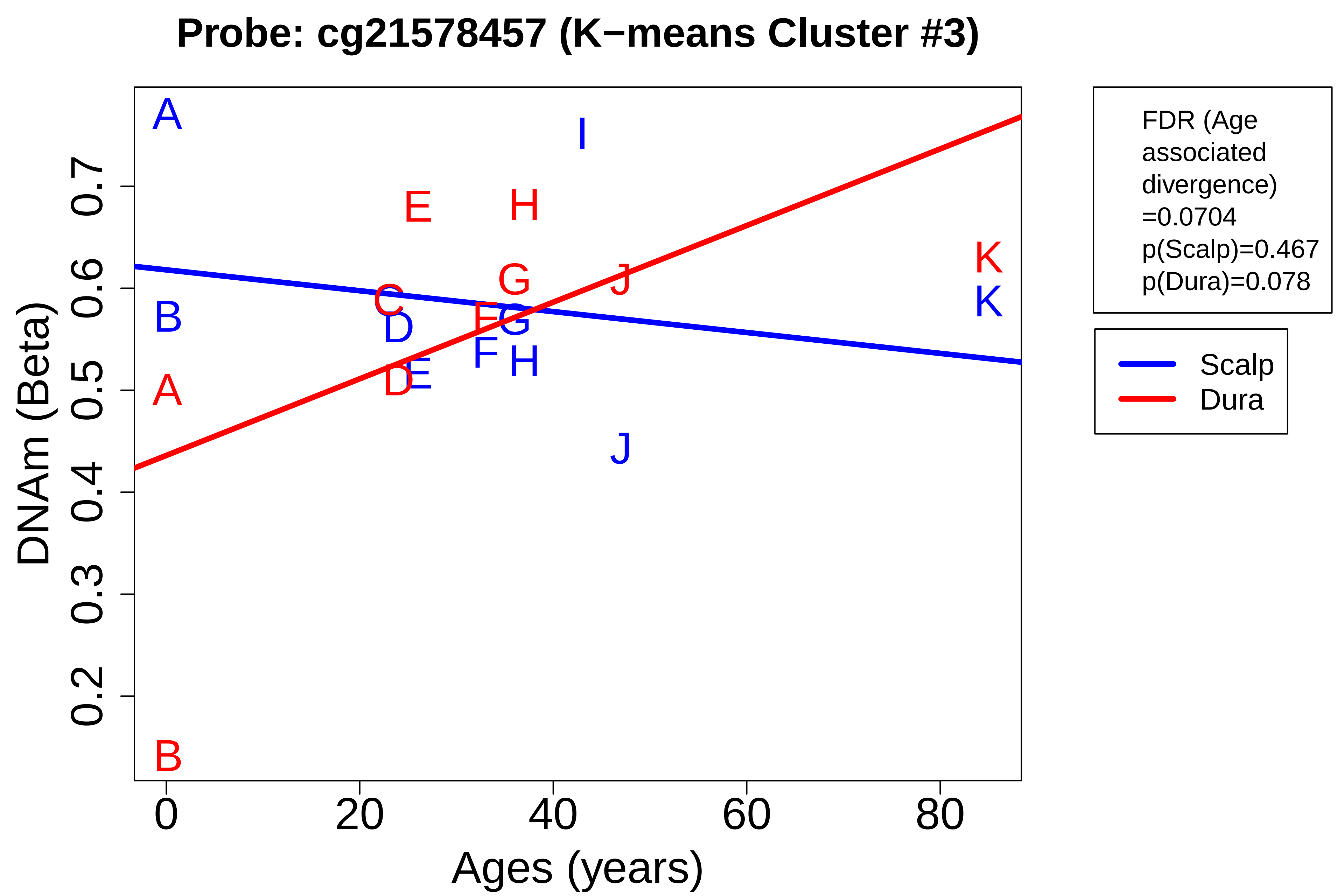

Probe: cg21578457 (K−means Cluster #3)
DNAm (Beta)
0.2
0.3
0.4
0.5
0.6
0.7
Ages (years)
0
20
40
60
80
FDR (Age associated divergence) =0.0704
p(Scalp)=0.467
p(Dura)=0.078
Scalp
Dura
A
B
C
D
E
F
G
H
I
J
K

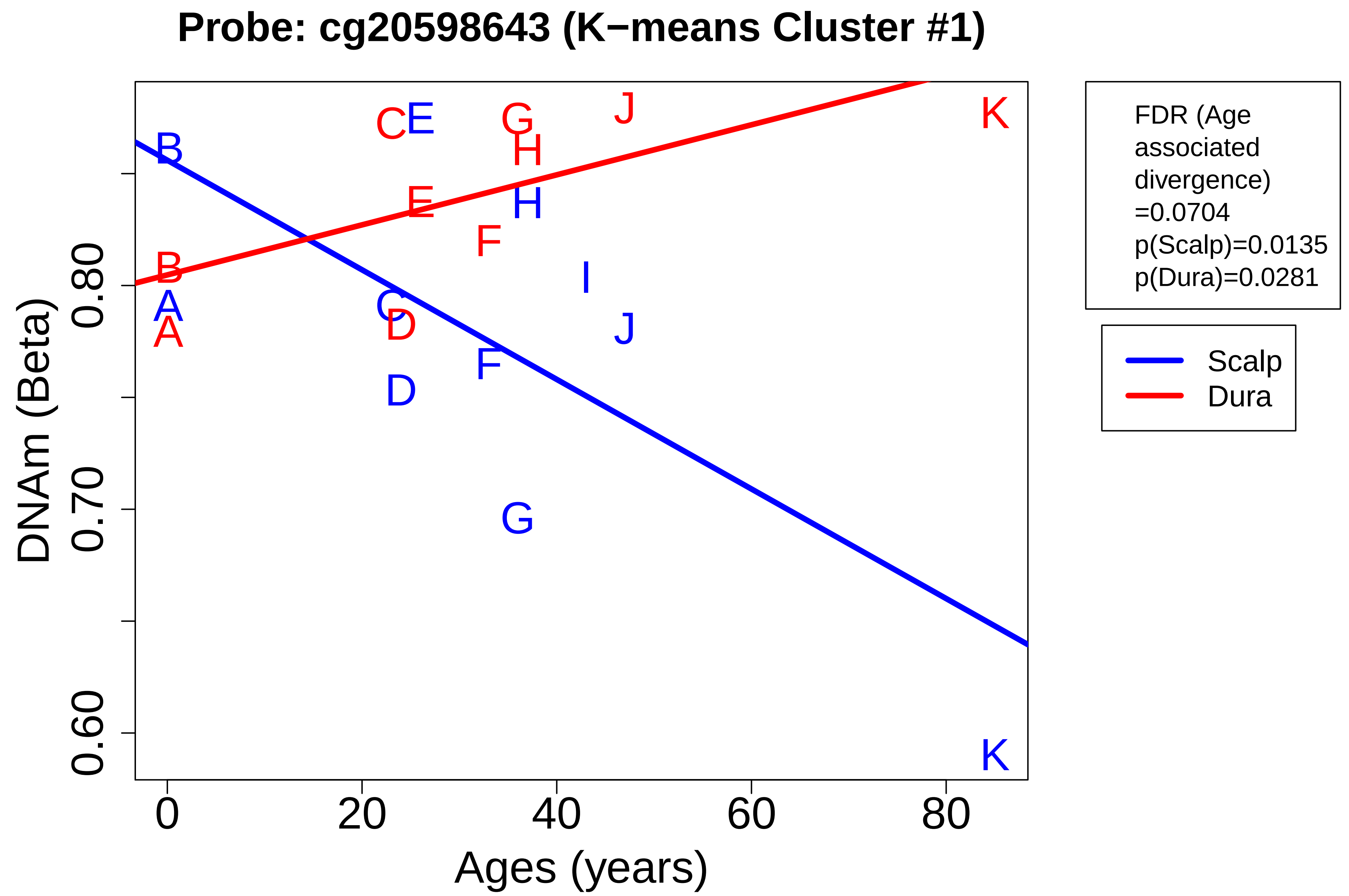

Probe: cg20598643 (K−means Cluster #1)
DNAm (Beta)
0.60
0.70
0.80
Ages (years)
0
20
40
60
80
FDR (Age associated divergence) =0.0704
p(Scalp)=0.0135
p(Dura)=0.0281
Scalp
Dura

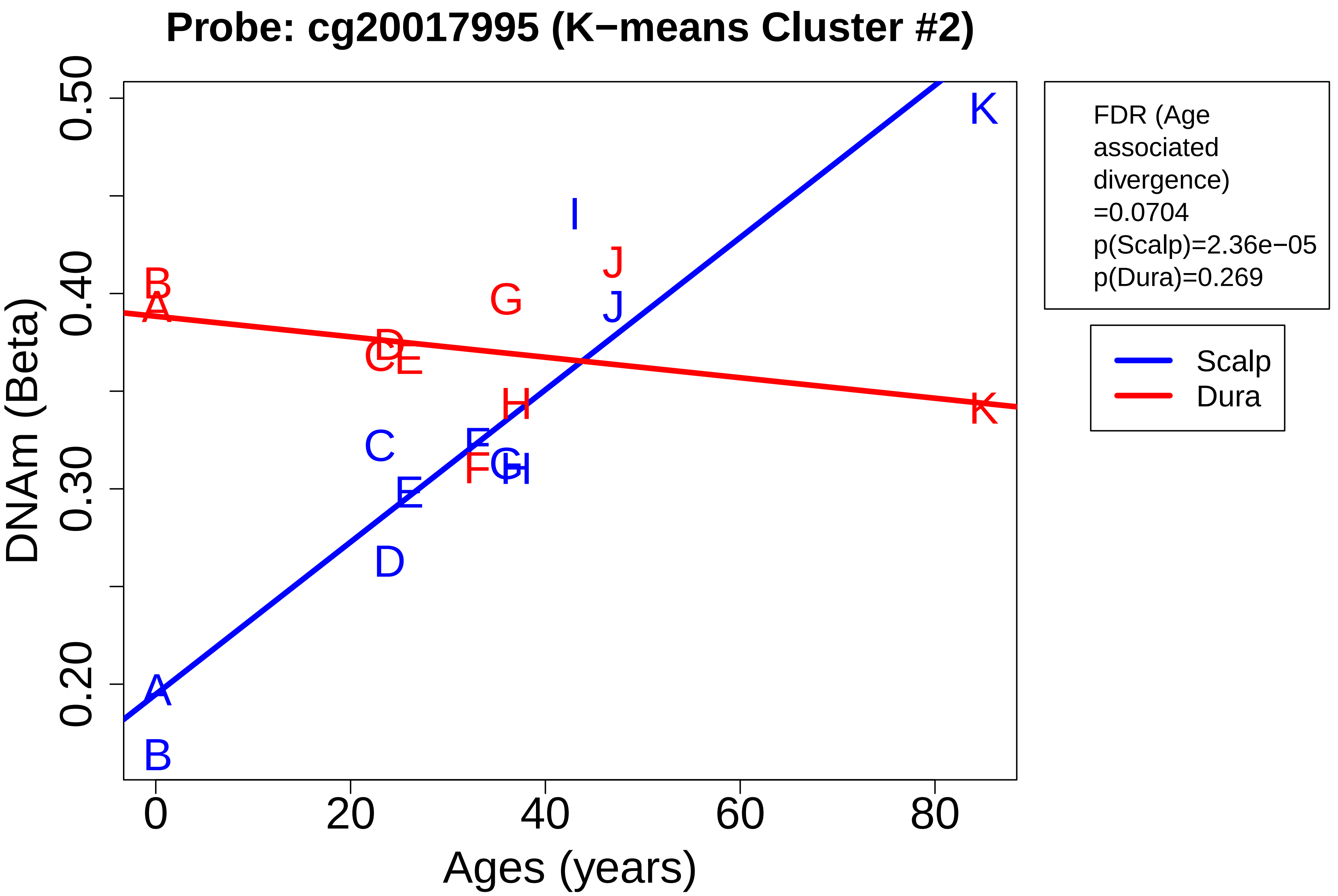

Probe: cg20017995 (K−means Cluster #2)
DNAm (Beta)
0.20
0.30
0.40
0.50
Ages (years)
0
20
40
60
80
FDR (Age
associated
divergence)
=0.0704
p(Scalp)=2.36e−05
p(Dura)=0.269
Scalp
Dura

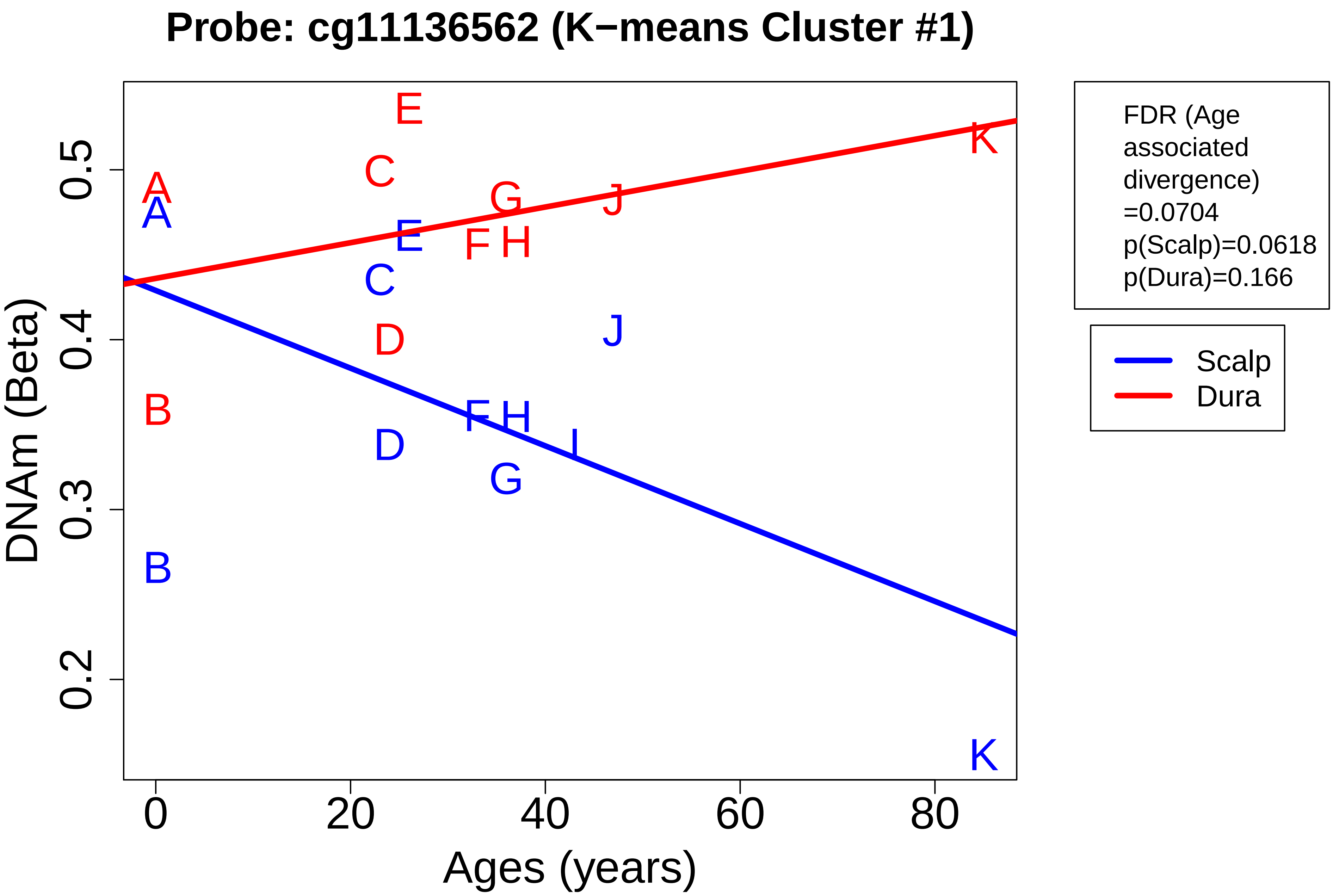

Probe: cg11136562 (K−means Cluster #1)
DNAm (Beta)
0.2
0.3
0.4
0.5
Ages (years)
0
20
40
60
80
FDR (Age associated divergence) =0.0704
p(Scalp)=0.0618
p(Dura)=0.166
Scalp
Dura

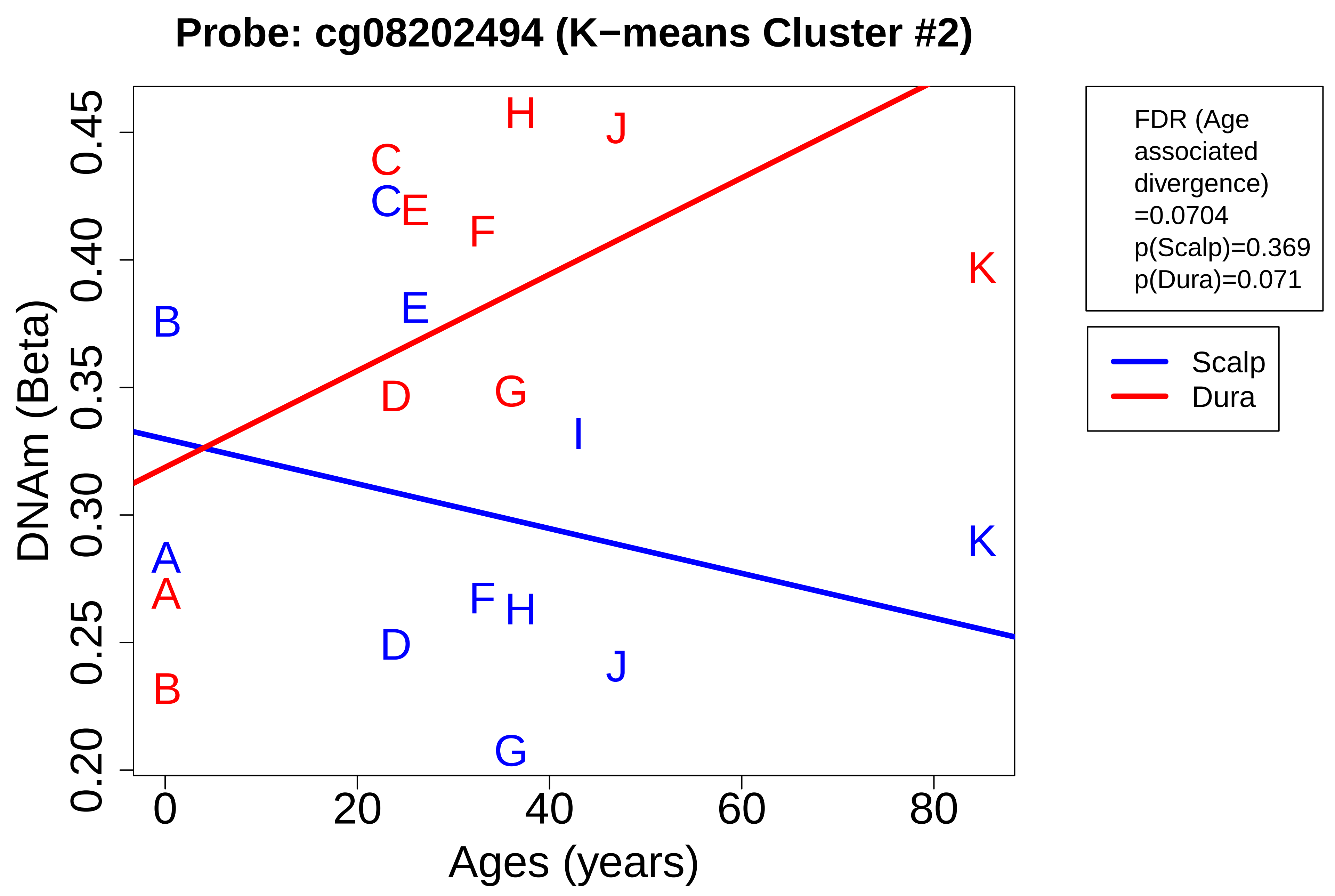
Probe: cg08202494 (K−means Cluster #2)
DNAm (Beta)
0.20
0.25
0.30
0.35
0.40
0.45
0
20
40
60
80
Ages (years)
FDR (Age associated divergence) =0.0704
p(Scalp)=0.369
p(Dura)=0.071
Scalp
Dura
A
B
C
D
E
F
G
H
I
J
K

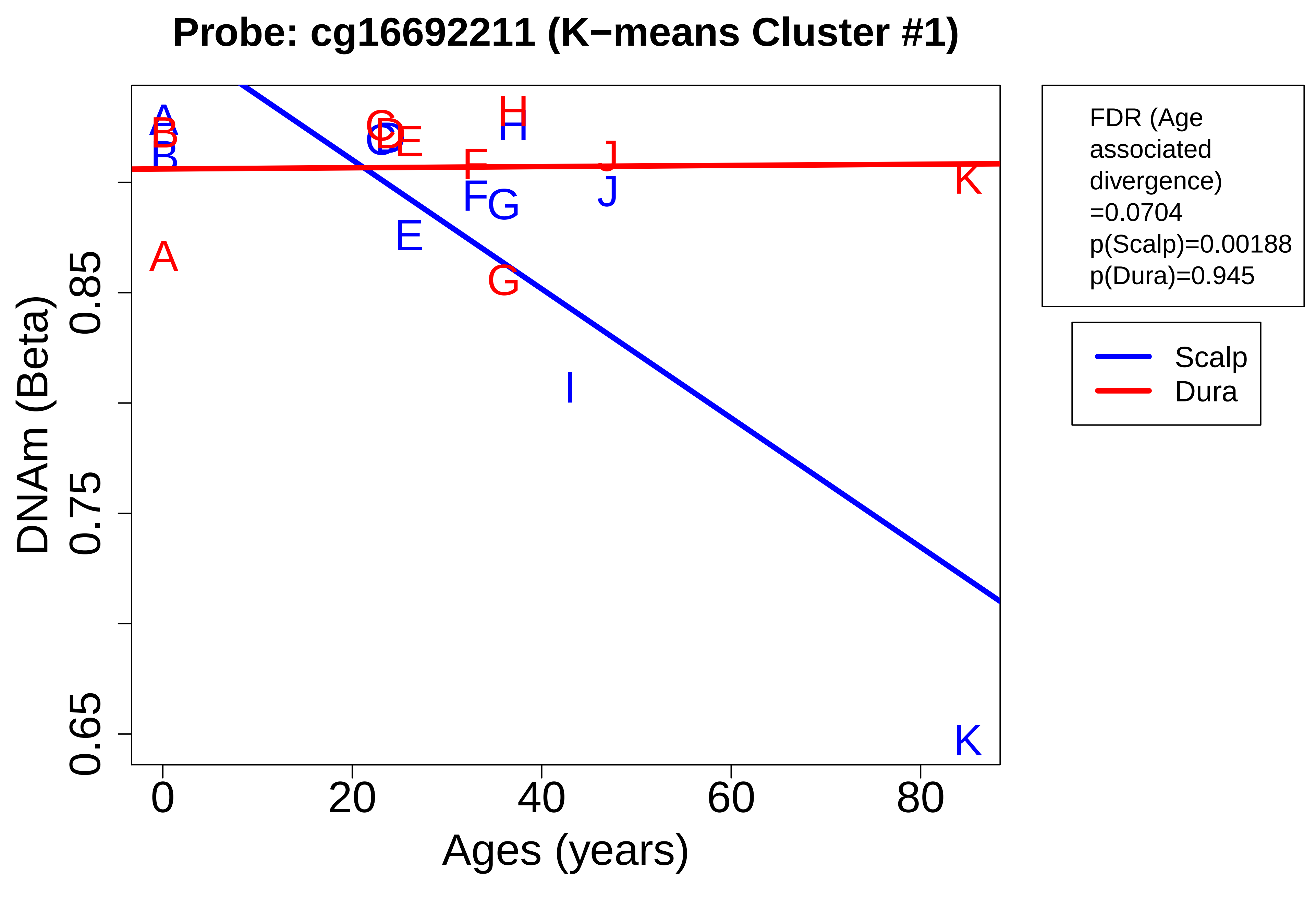

Probe: cg16692211 (K−means Cluster #1)
DNAm (Beta)
0.65
0.75
0.85
0
20
40
60
80
Ages (years)
FDR (Age associated divergence) =0.0704
p(Scalp)=0.00188
p(Dura)=0.945
Scalp
Dura

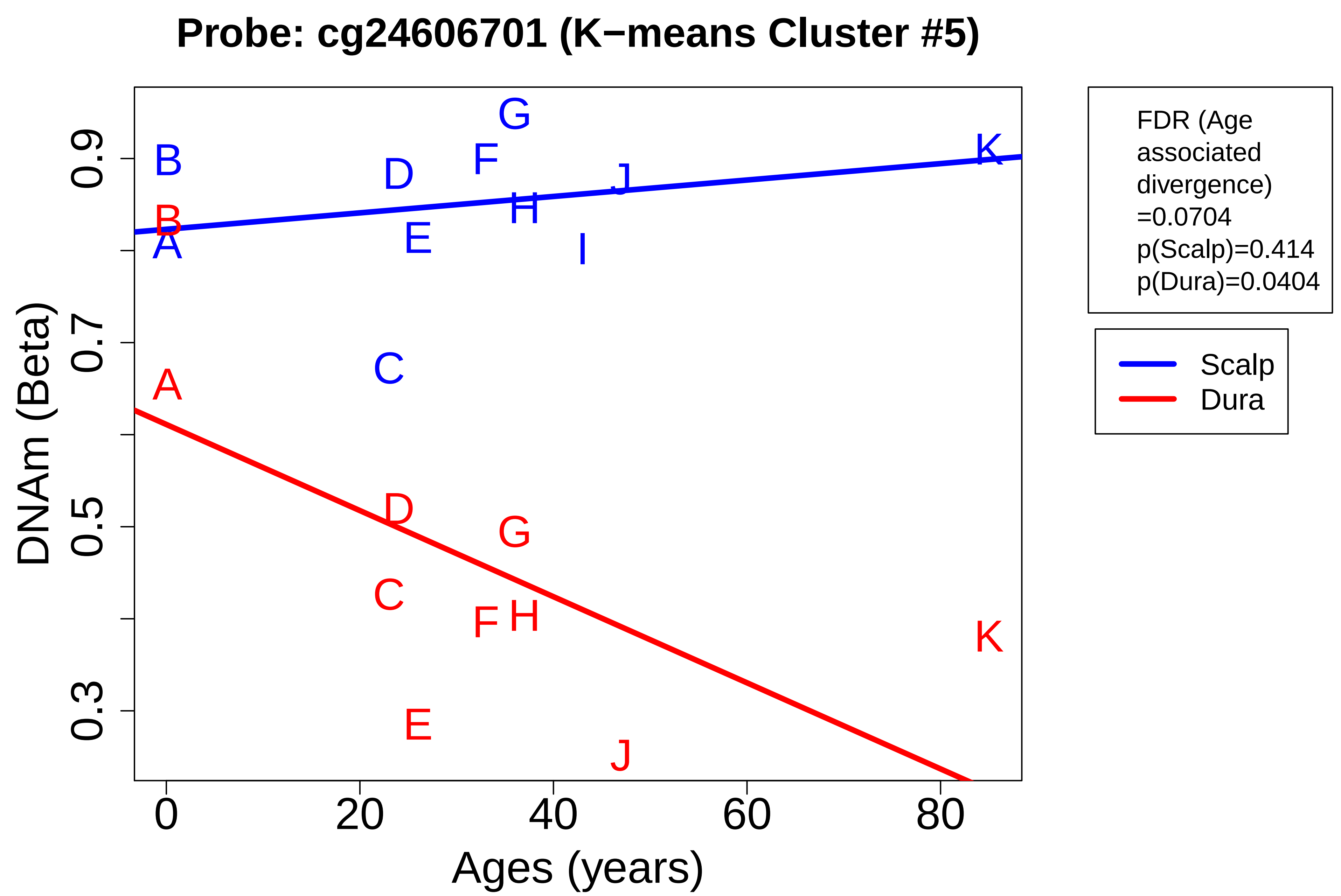

Probe: cg24606701 (K−means Cluster #5)
DNAm (Beta)
0.9
0.7
0.5
0.3
0
20
40
60
80
Ages (years)
FDR (Age associated divergence) =0.0704
p(Scalp)=0.414
p(Dura)=0.0404
Scalp
Dura

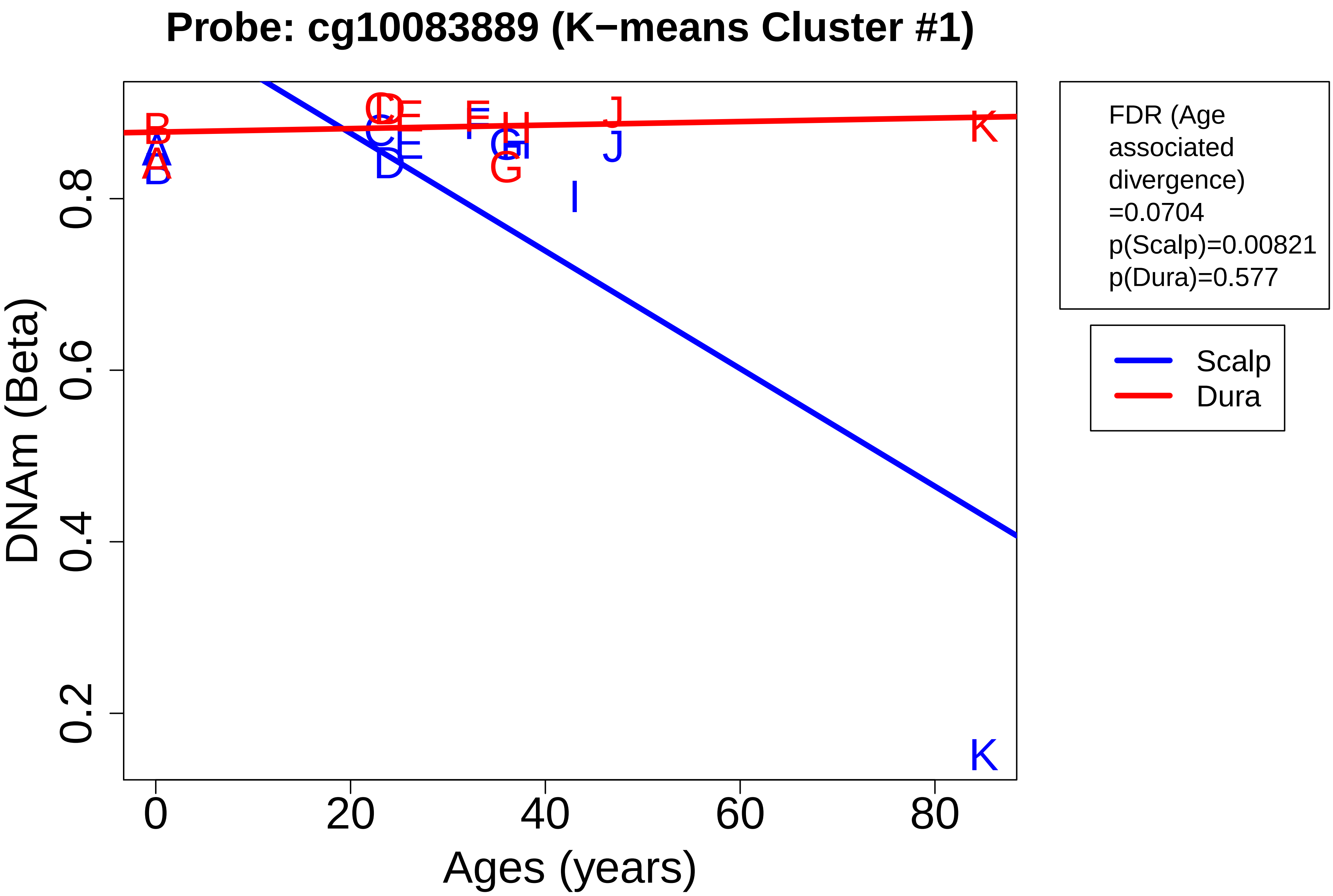

Probe: cg10083889 (K−means Cluster #1)
DNAm (Beta)
0.2
0.4
0.6
0.8
0
20
40
60
80
Ages (years)
FDR (Age associated divergence) =0.0704
p(Scalp)=0.00821
p(Dura)=0.577
Scalp
Dura

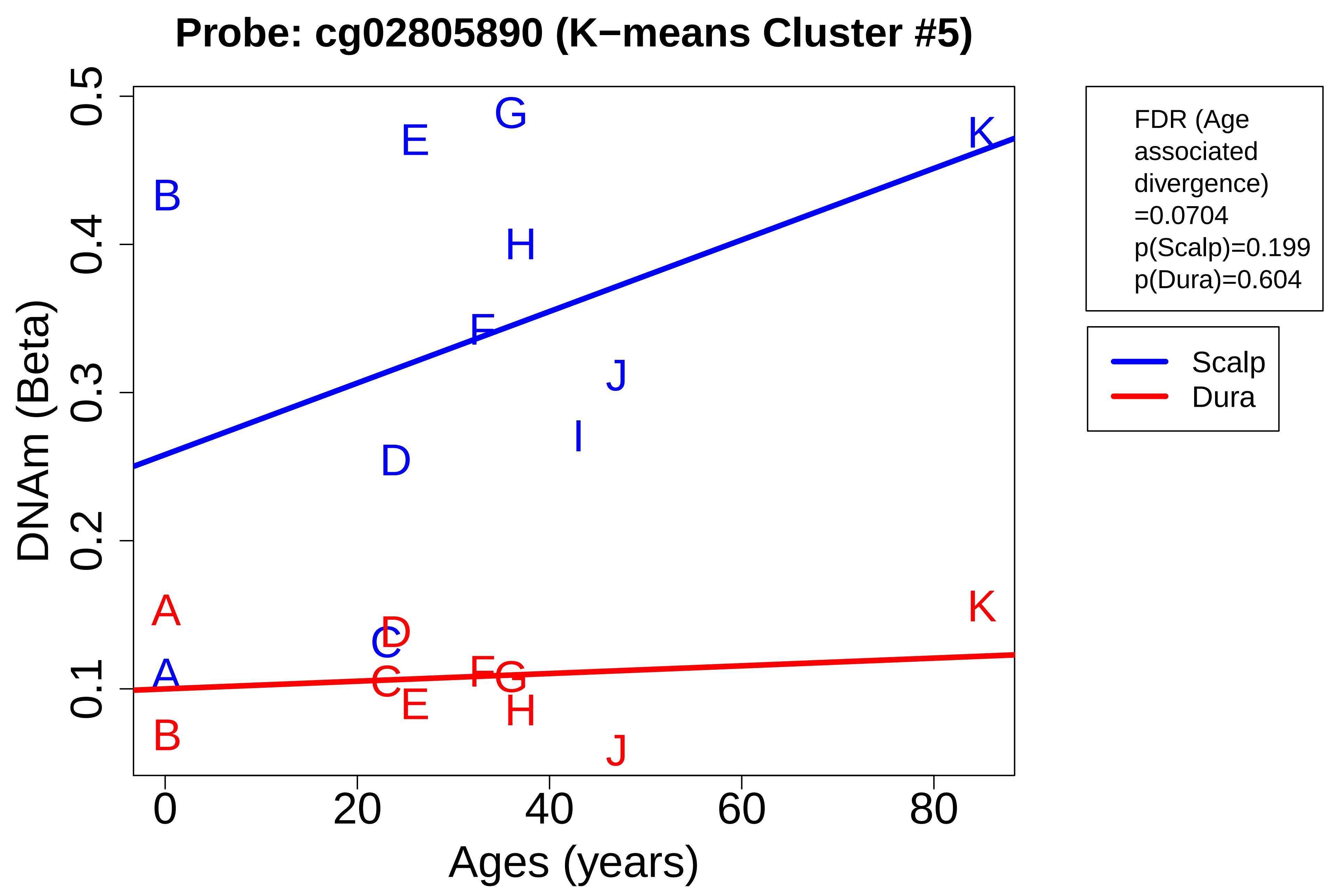

Probe: cg02805890 (K−means Cluster #5)
DNAm (Beta)
0.1
0.2
0.3
0.4
0.5
Ages (years)
0
20
40
60
80
A
B
C
D
E
F
G
H
I
J
K
FDR (Age associated divergence) =0.0704
p(Scalp)=0.199
p(Dura)=0.604
Scalp
Dura

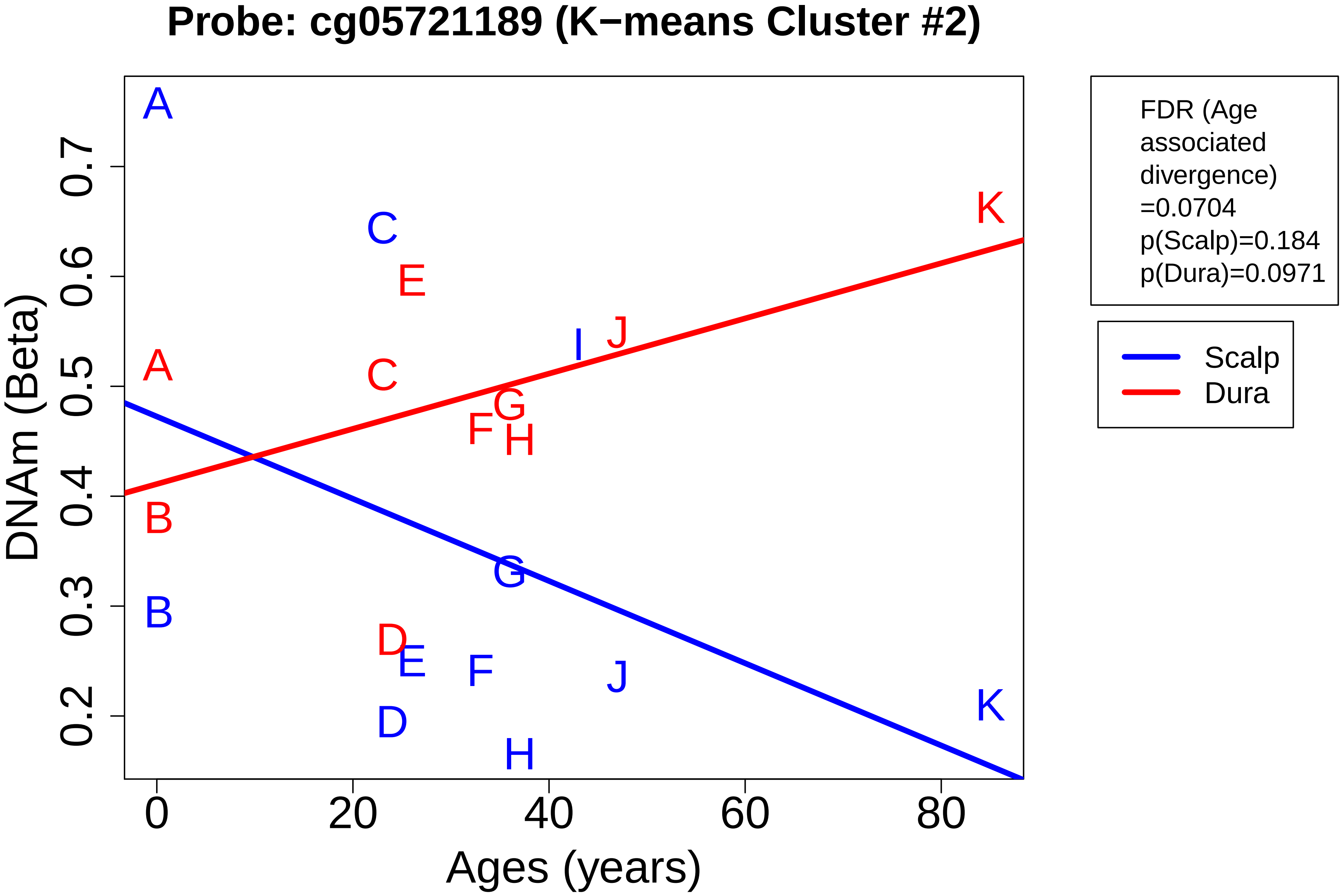
Probe: cg05721189 (K−means Cluster #2)
DNAm (Beta)
Ages (years)
0.2
0.3
0.4
0.5
0.6
0.7
0
20
40
60
80
A
B
C
D
E
F
G
H
I
J
K
FDR (Age associated divergence) =0.0704
p(Scalp)=0.184
p(Dura)=0.0971
Scalp
Dura

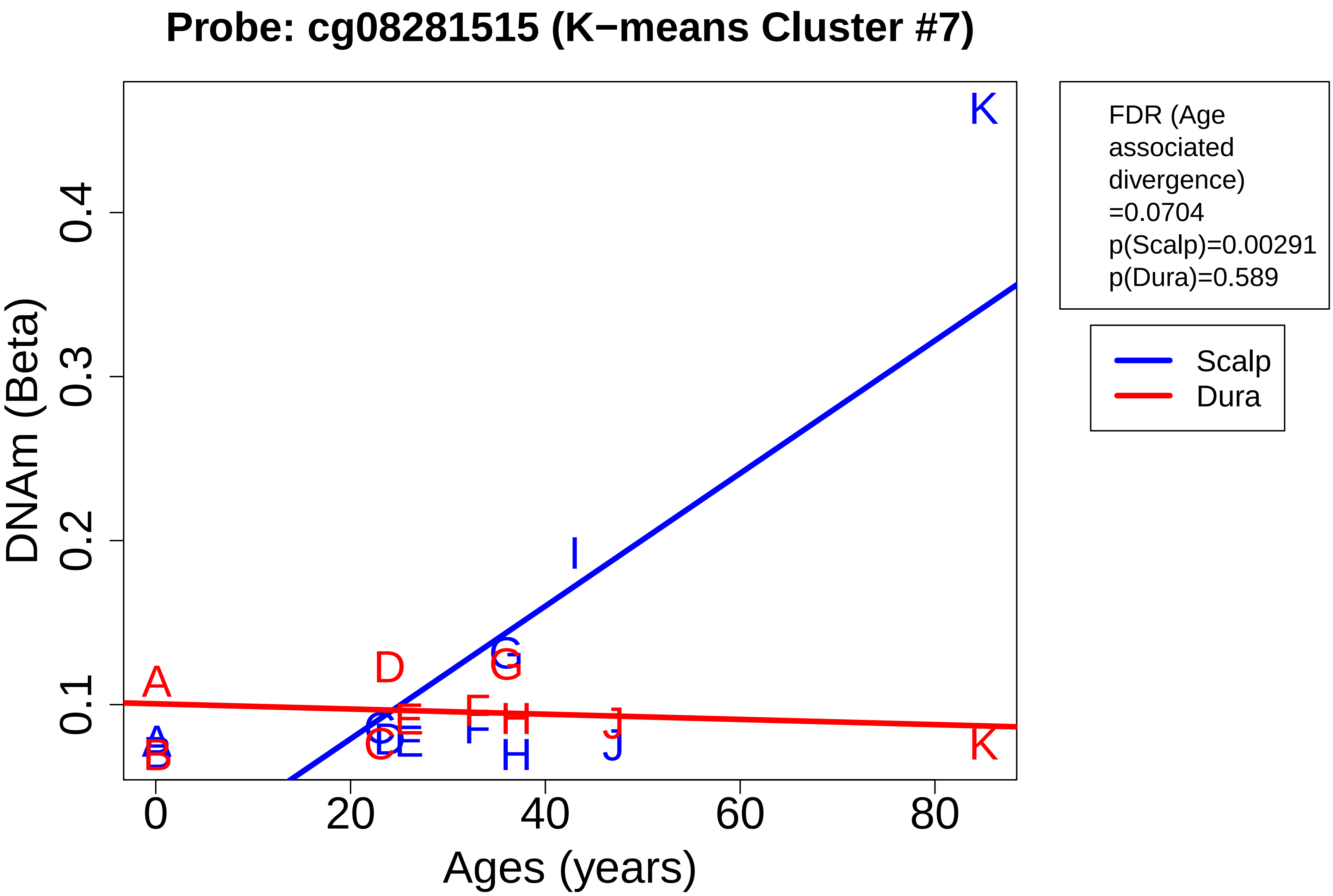
Probe: cg08281515 (K−means Cluster #7)
DNAm (Beta)
Ages (years)
0.1
0.2
0.3
0.4
0
20
40
60
80
FDR (Age associated divergence) =0.0704
p(Scalp)=0.00291
p(Dura)=0.589
Scalp
Dura

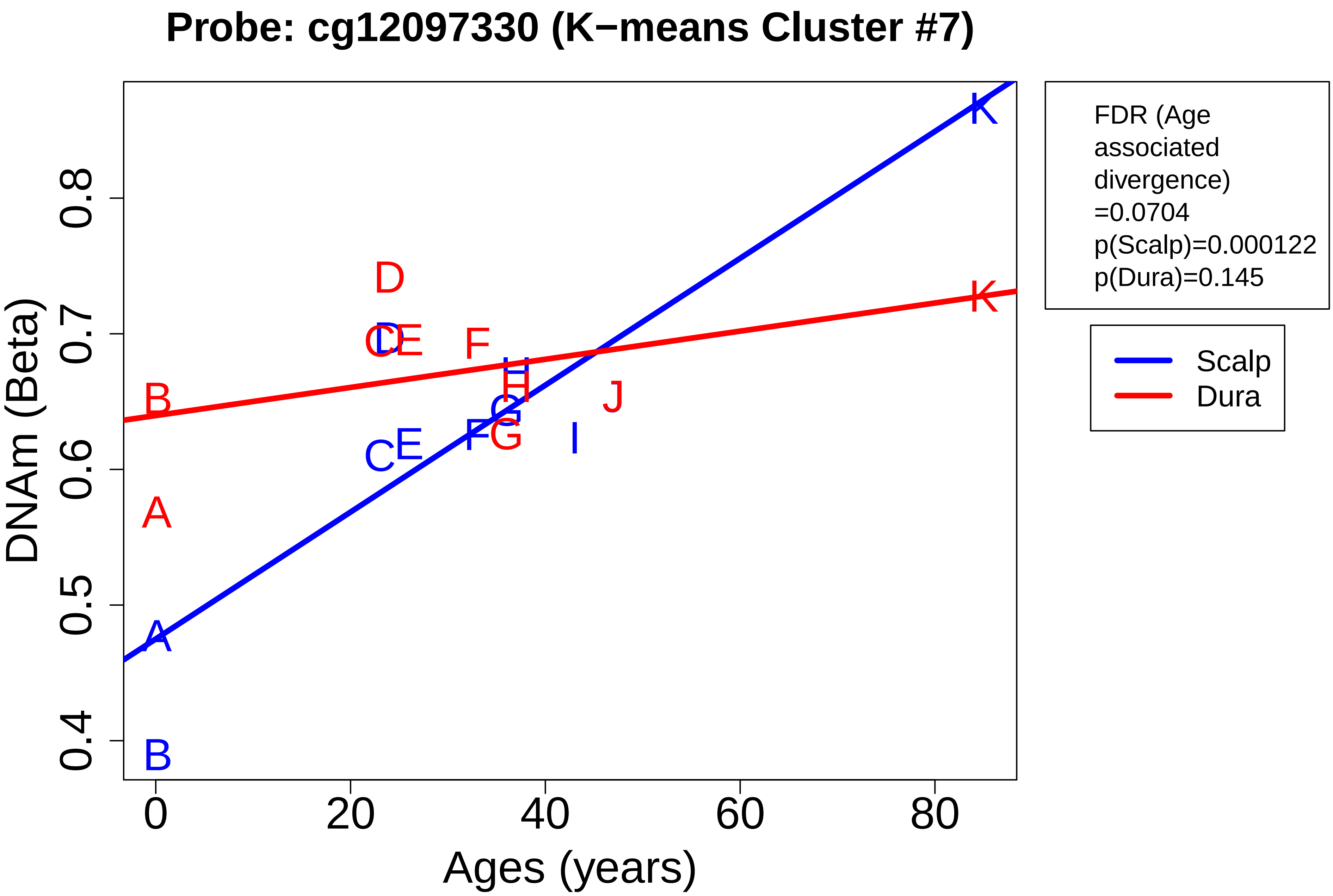

Probe: cg12097330 (K−means Cluster #7)
FDR (Age associated divergence) =0.0704
p(Scalp)=0.000122
p(Dura)=0.145
Scalp
Dura
DNAm (Beta)
0.4
0.5
0.6
0.7
0.8
Ages (years)
0
20
40
60
80
A
B
C
D
E
F
G
H
I
J
K

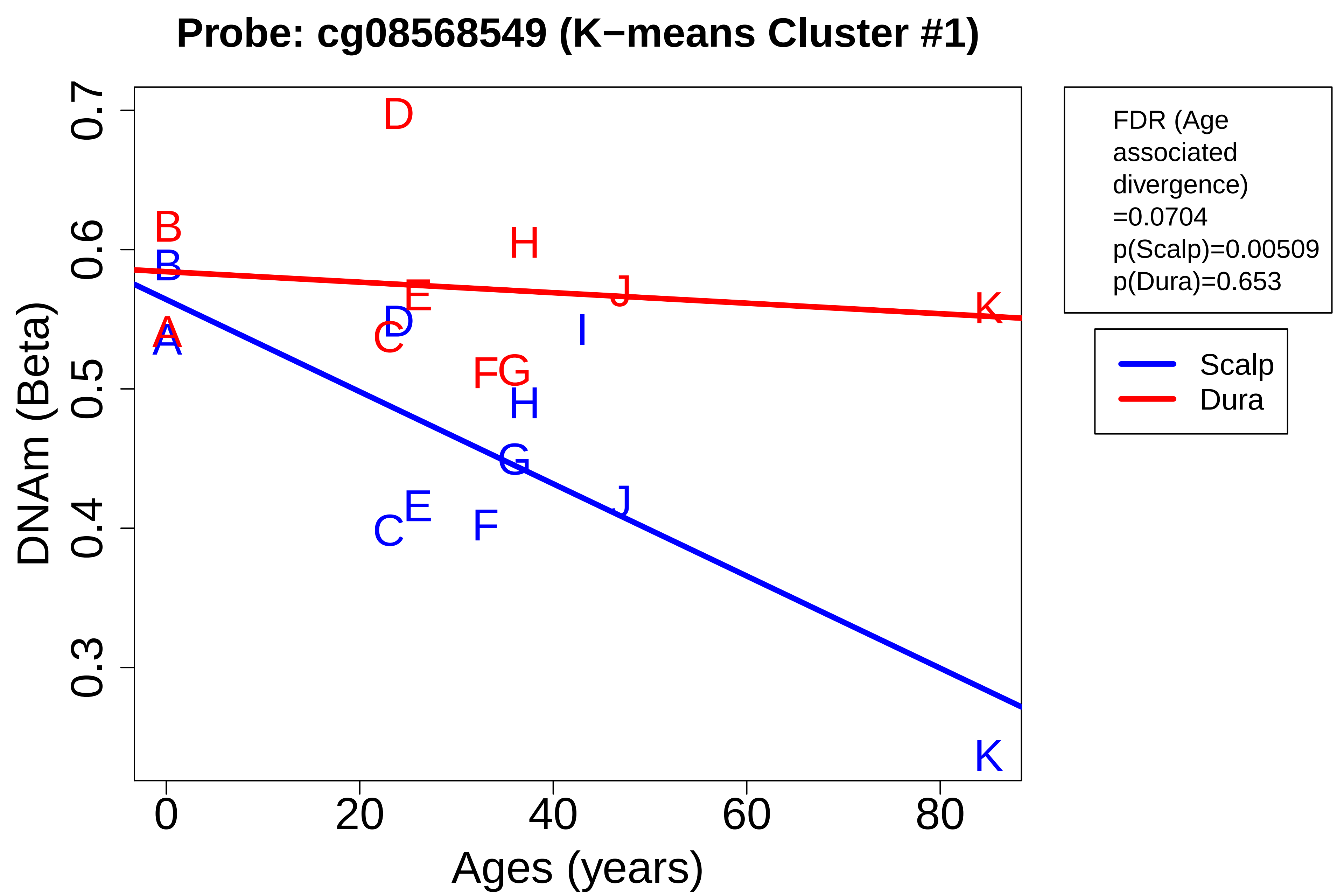

Probe: cg08568549 (K−means Cluster #1)
DNAm (Beta)
0.3
0.4
0.5
0.6
0.7
Ages (years)
0
20
40
60
80
FDR (Age associated divergence) =0.0704
p(Scalp)=0.00509
p(Dura)=0.653
Scalp
Dura

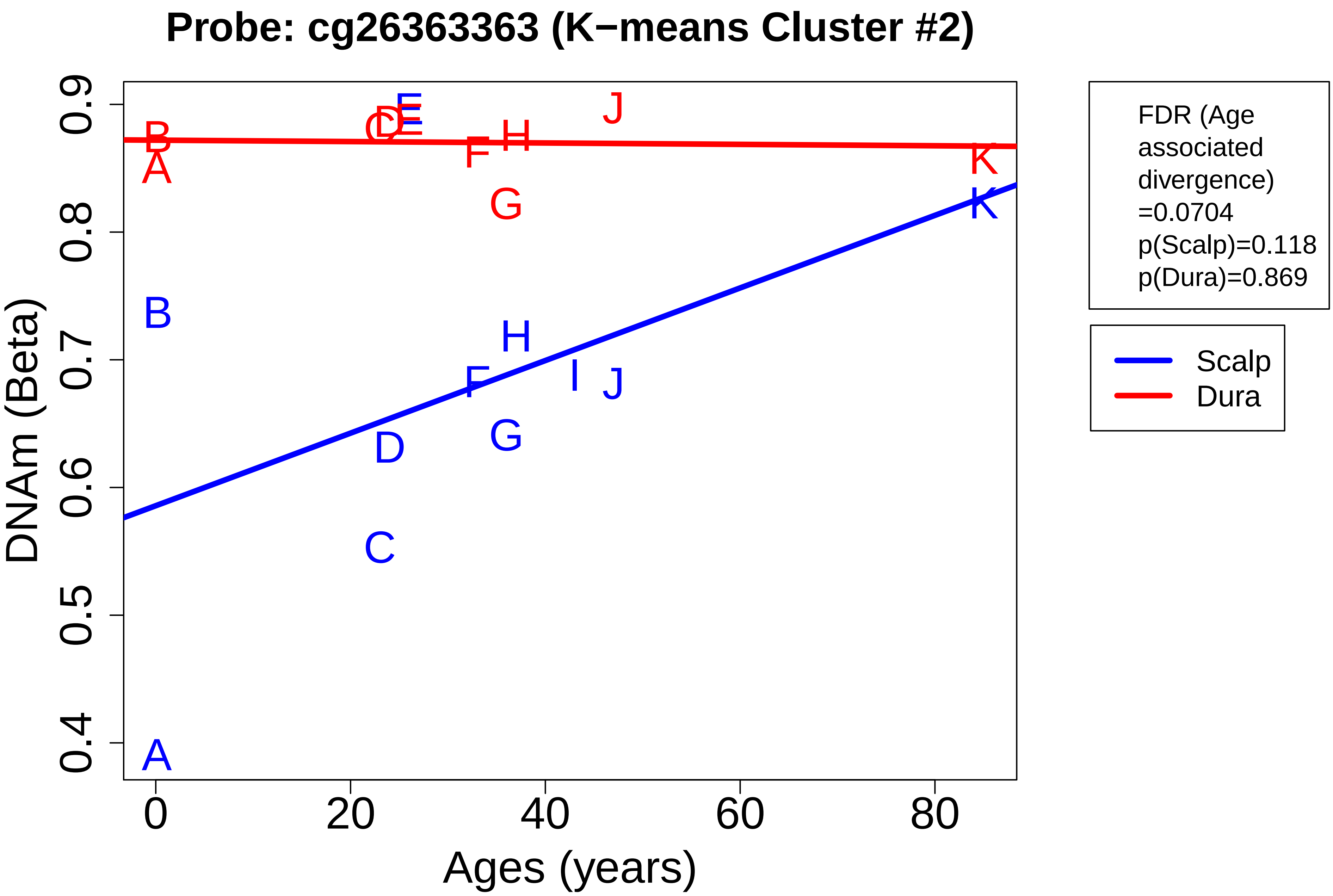

Probe: cg26363363 (K−means Cluster #2)
DNAm (Beta)
0.4
0.5
0.6
0.7
0.8
0.9
Ages (years)
0
20
40
60
80
FDR (Age associated divergence) =0.0704
p(Scalp)=0.118
p(Dura)=0.869
Scalp
Dura

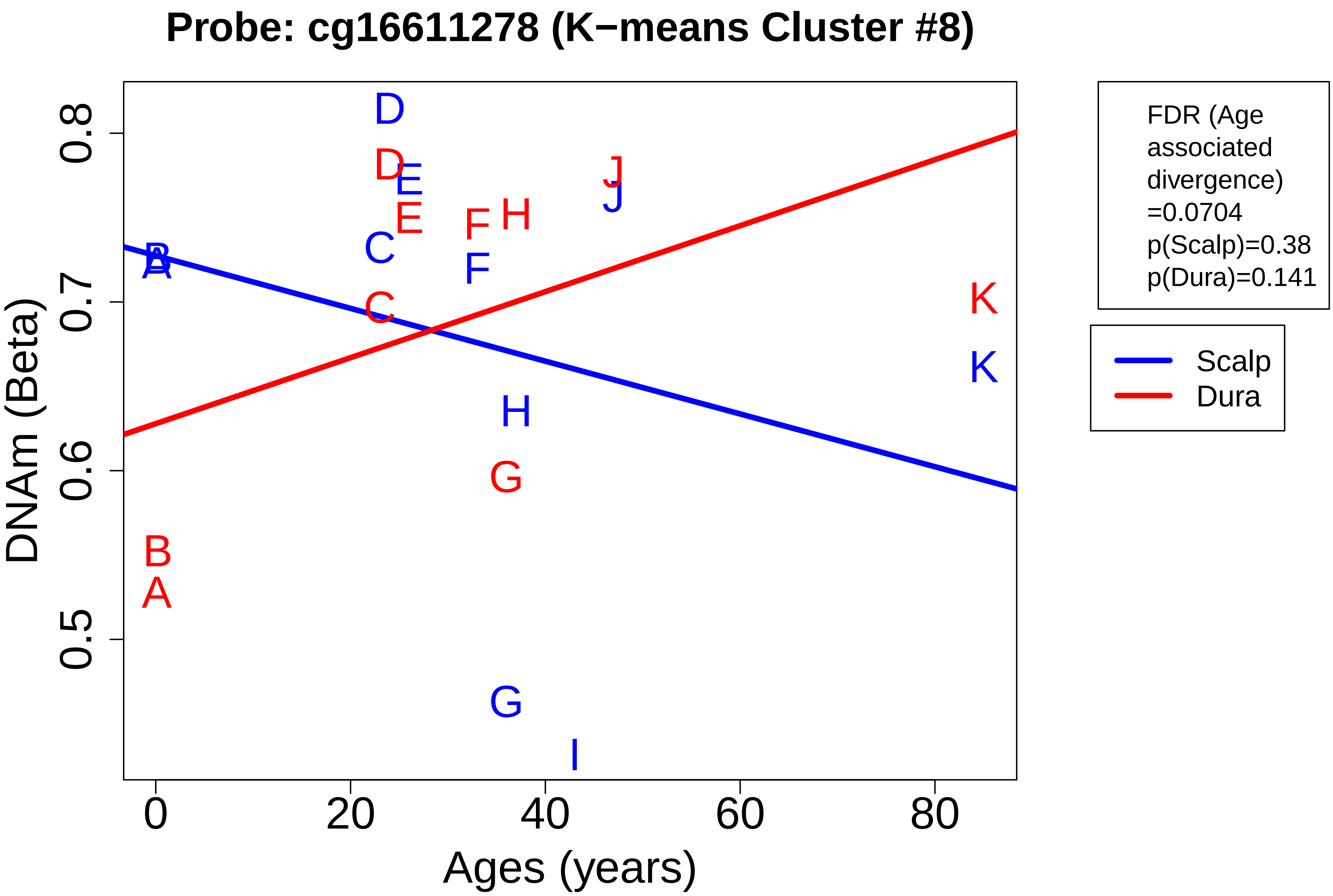
Probe: cg16611278 (K−means Cluster #8)
DNAm (Beta)
0.5
0.6
0.7
0.8
Ages (years)
0
20
40
60
80
FDR (Age associated divergence) =0.0704
p(Scalp)=0.38
p(Dura)=0.141
Scalp
Dura

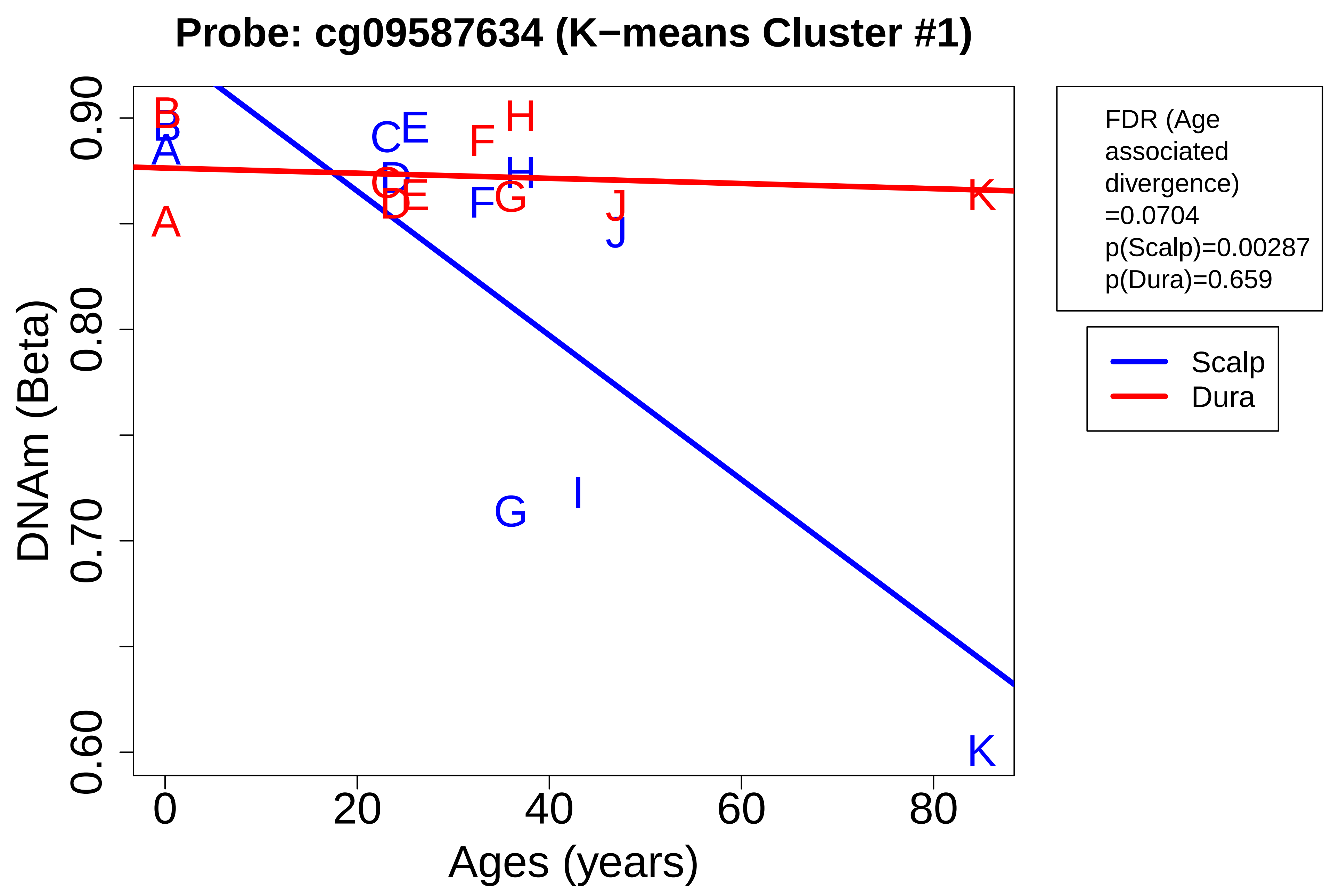

Probe: cg09587634 (K−means Cluster #1)
DNAm (Beta)
0.60
0.70
0.80
0.90
Ages (years)
0
20
40
60
80
FDR (Age associated divergence) =0.0704
p(Scalp)=0.00287
p(Dura)=0.659
Scalp
Dura

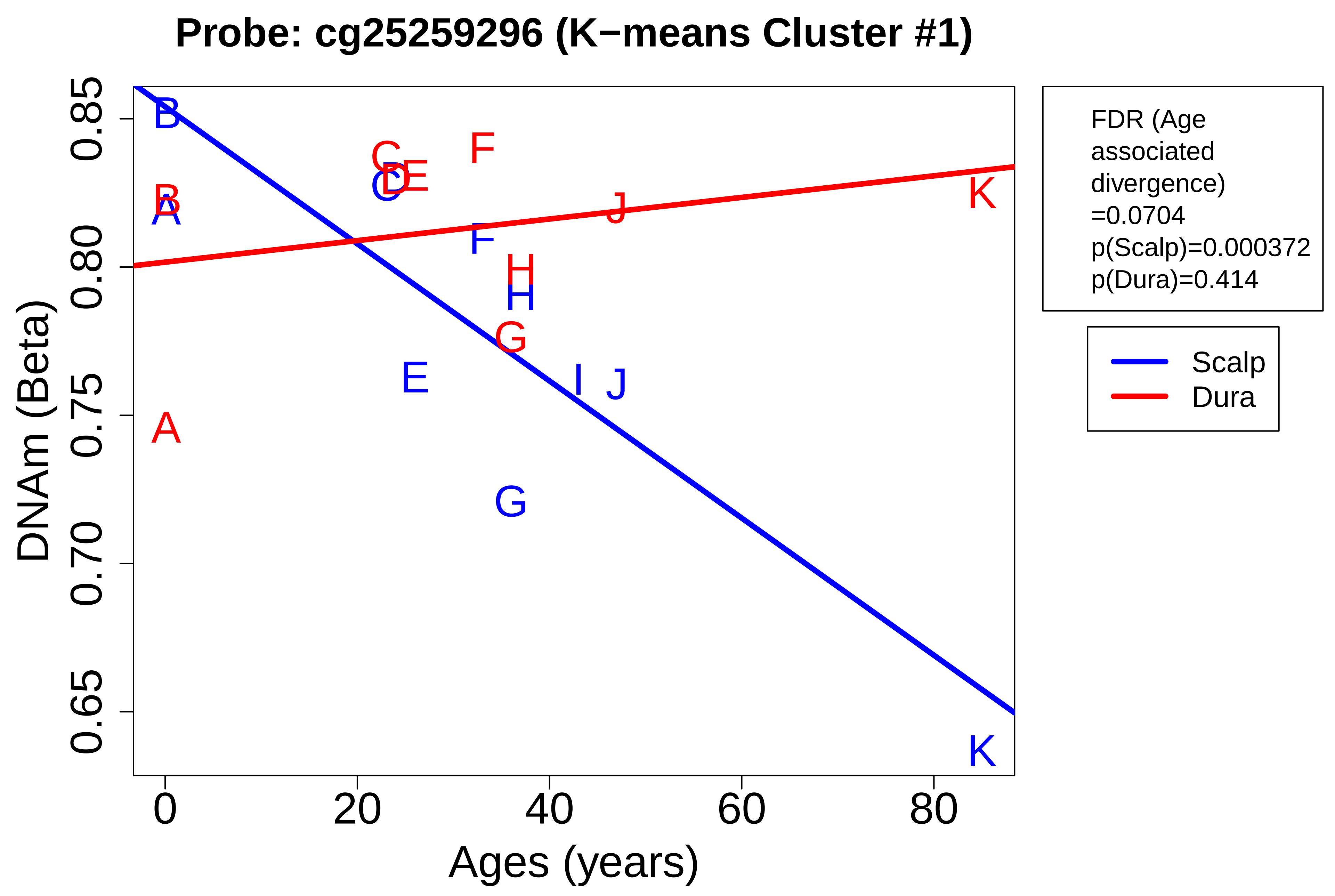

Probe: cg25259296 (K−means Cluster #1)
FDR (Age associated divergence) =0.0704
p(Scalp)=0.000372
p(Dura)=0.414
Scalp
Dura
DNAm (Beta)
0.65
0.70
0.75
0.80
0.85
0
20
40
60
80
Ages (years)

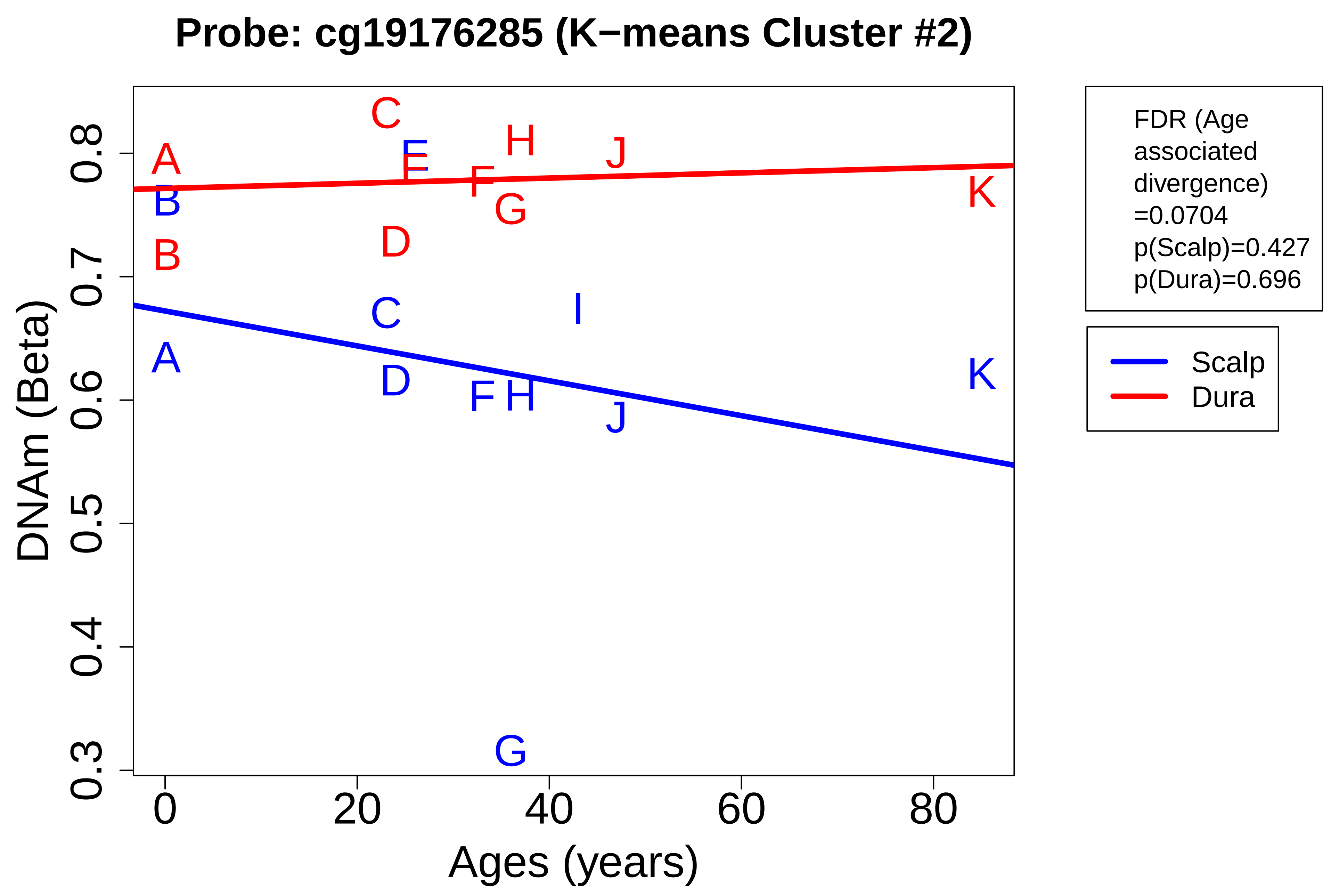

Probe: cg19176285 (K−means Cluster #2)
DNAm (Beta)
0.3
0.4
0.5
0.6
0.7
0.8
0
20
40
60
80
Ages (years)
A
B
C
D
E
F
G
H
I
J
K
FDR (Age associated divergence) =0.0704
p(Scalp)=0.427
p(Dura)=0.696
Scalp
Dura

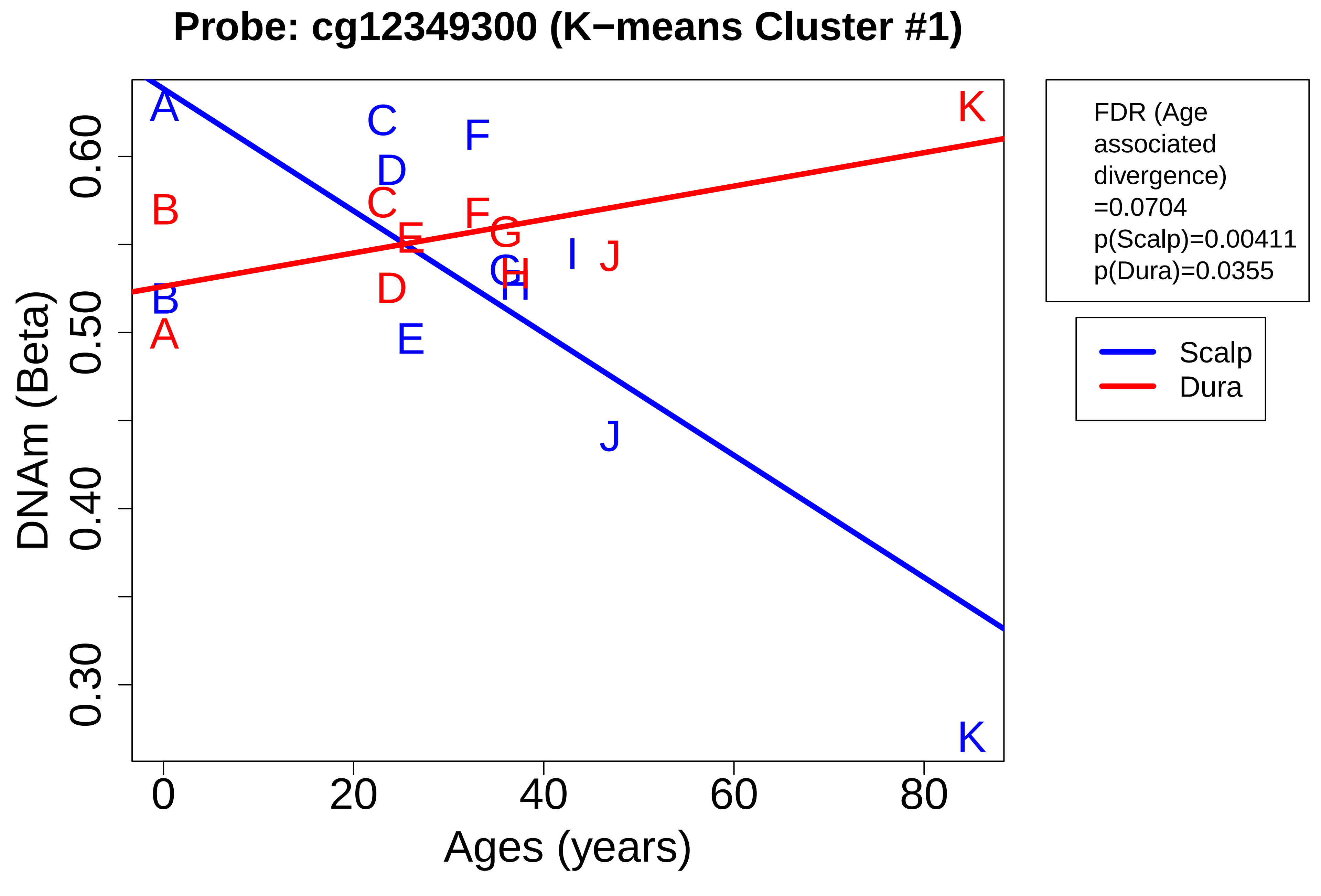

Probe: cg12349300 (K−means Cluster #1)
DNAm (Beta)
0.30
0.40
0.50
0.60
0
20
40
60
80
Ages (years)
A
B
C
D
E
F
G
H
I
J
K
FDR (Age associated divergence) =0.0704
p(Scalp)=0.00411
p(Dura)=0.0355
Scalp
Dura

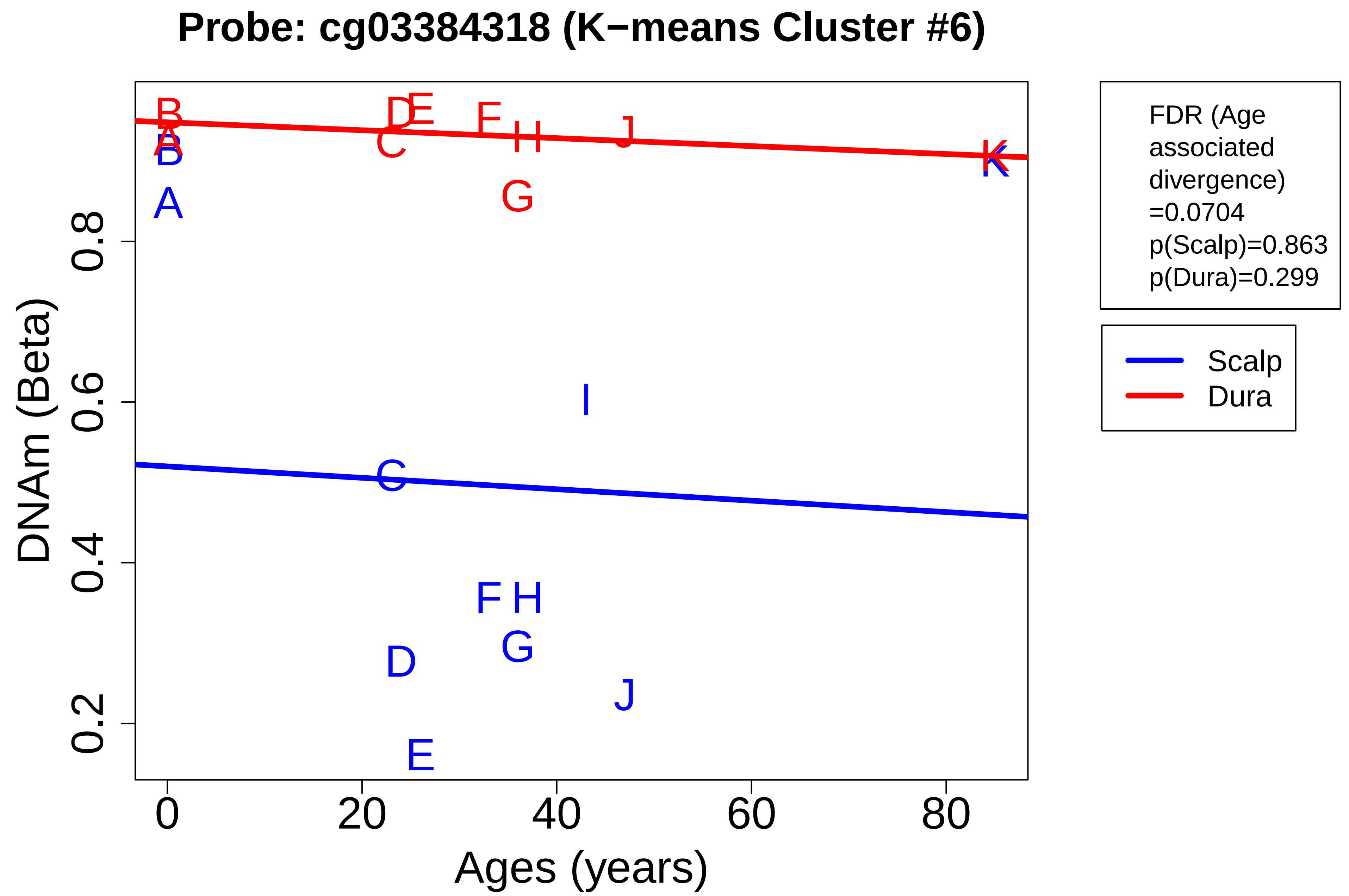
Probe: cg03384318 (K−means Cluster #6)
DNAm (Beta)
0.2
0.4
0.6
0.8
0
20
40
60
80
Ages (years)
FDR (Age associated divergence) =0.0704
p(Scalp)=0.863
p(Dura)=0.299
Scalp
Dura
A
B
C
D
E
F
G
H
I
J
K

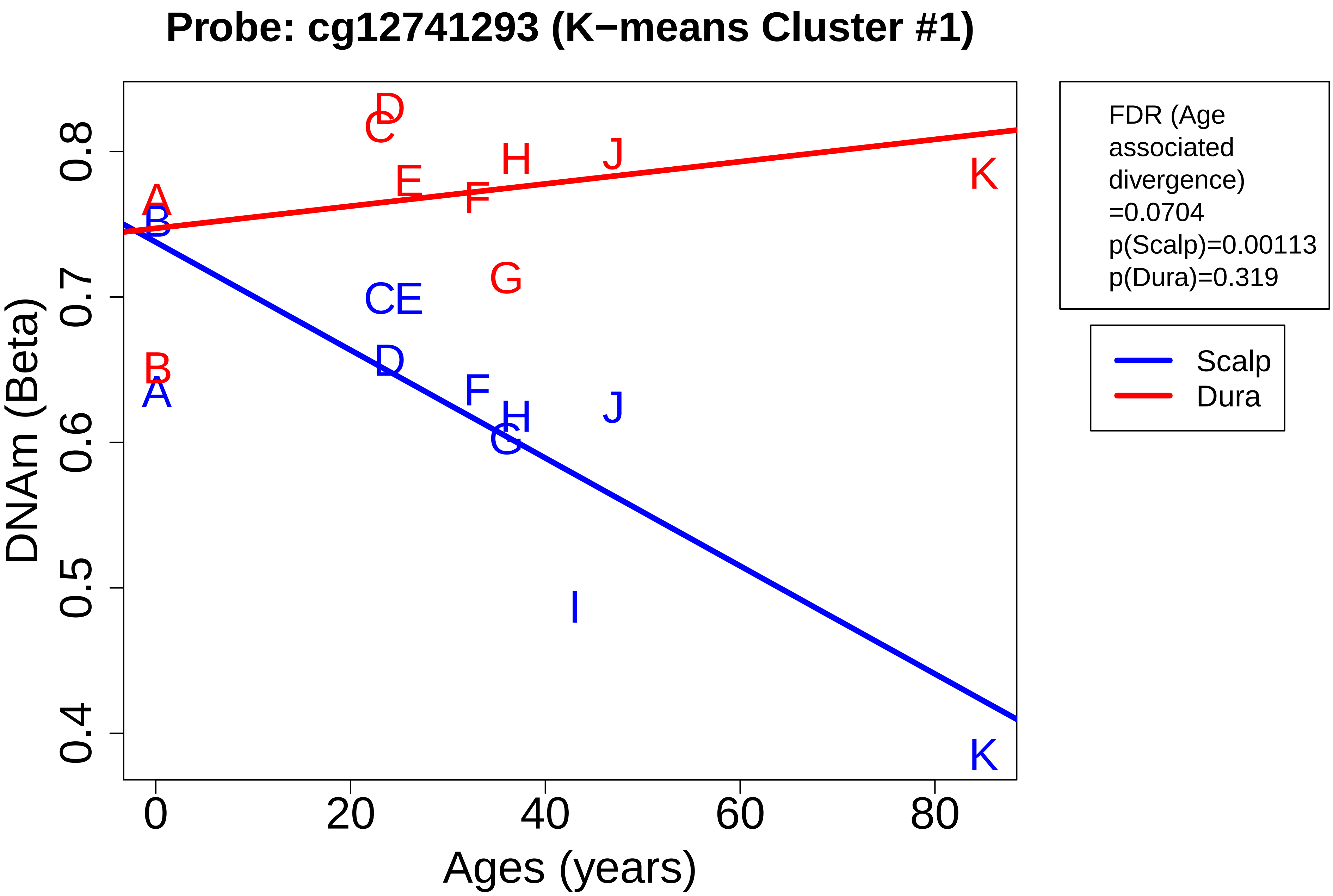

Probe: cg12741293 (K−means Cluster #1)
DNAm (Beta)
0.4
0.5
0.6
0.7
0.8
0
20
40
60
80
Ages (years)
FDR (Age associated divergence) =0.0704
p(Scalp)=0.00113
p(Dura)=0.319
Scalp
Dura

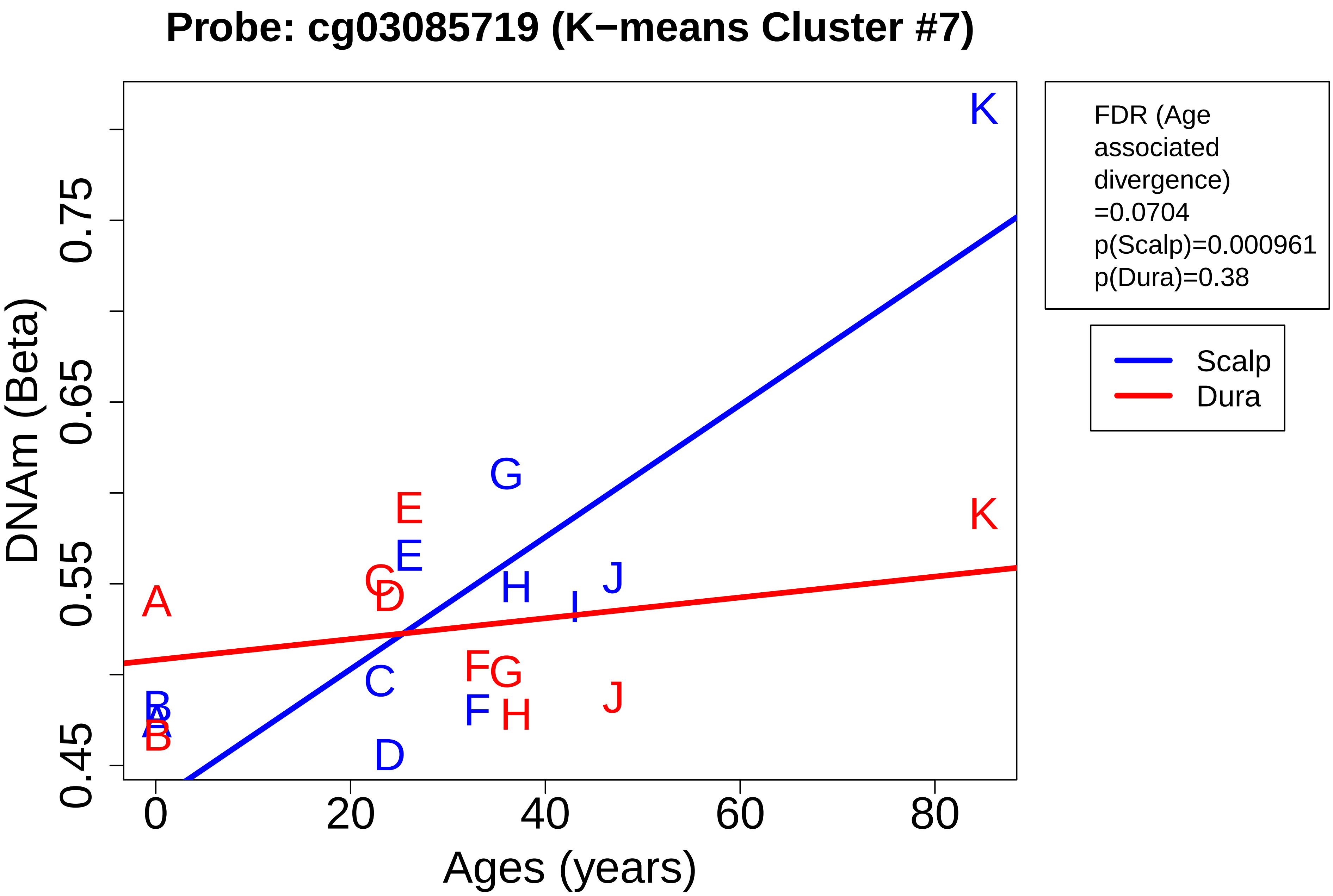
Probe: cg03085719 (K−means Cluster #7)
DNAm (Beta)
0.45
0.55
0.65
0.75
0
20
40
60
80
Ages (years)
FDR (Age associated divergence) =0.0704
p(Scalp)=0.000961
p(Dura)=0.38
Scalp
Dura
A
B
C
D
E
F
G
H
I
J
K

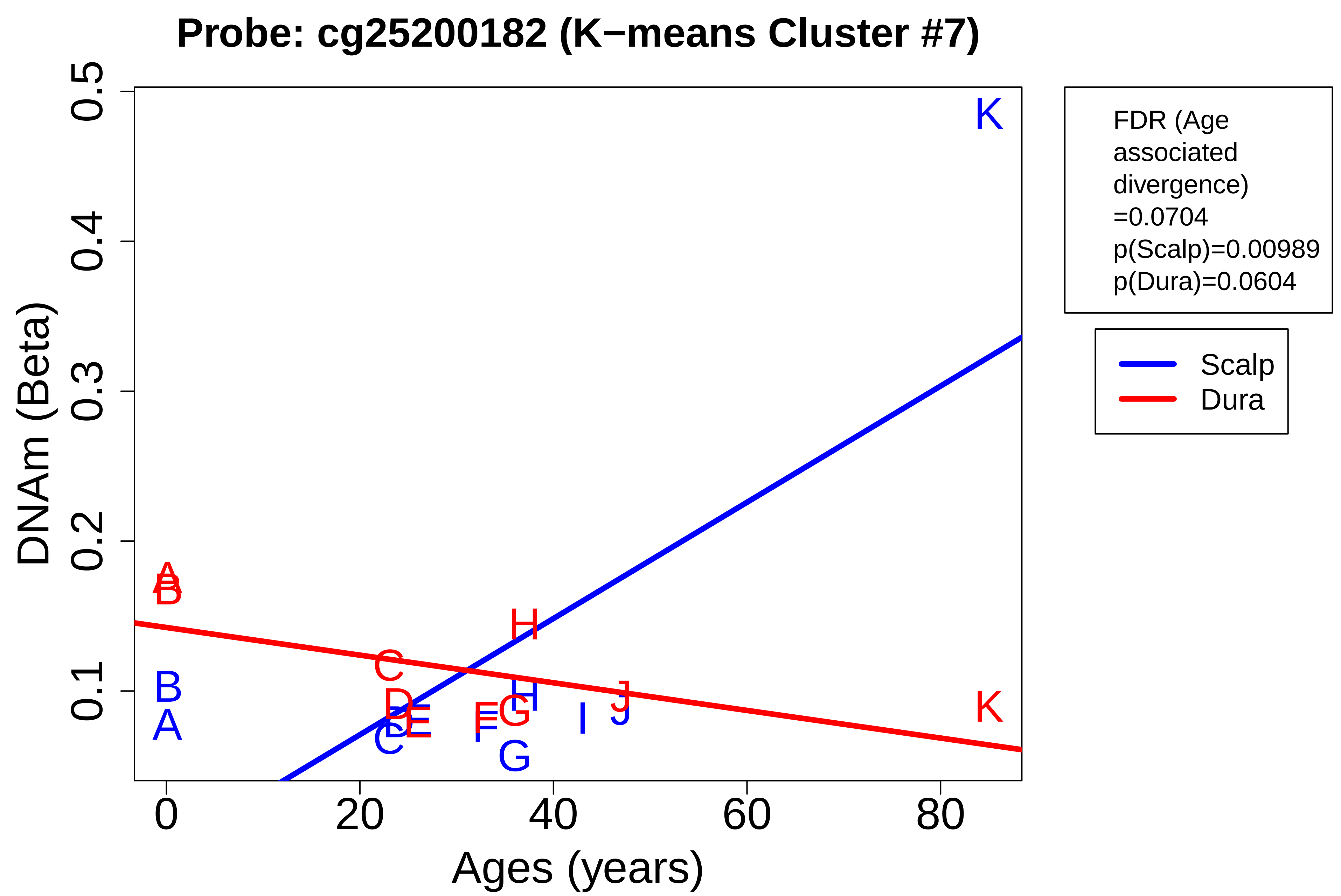
Probe: cg25200182 (K−means Cluster #7)
DNAm (Beta)
0.1
0.2
0.3
0.4
0.5
0
20
40
60
80
Ages (years)
FDR (Age associated divergence) =0.0704
p(Scalp)=0.00989
p(Dura)=0.0604
Scalp
Dura

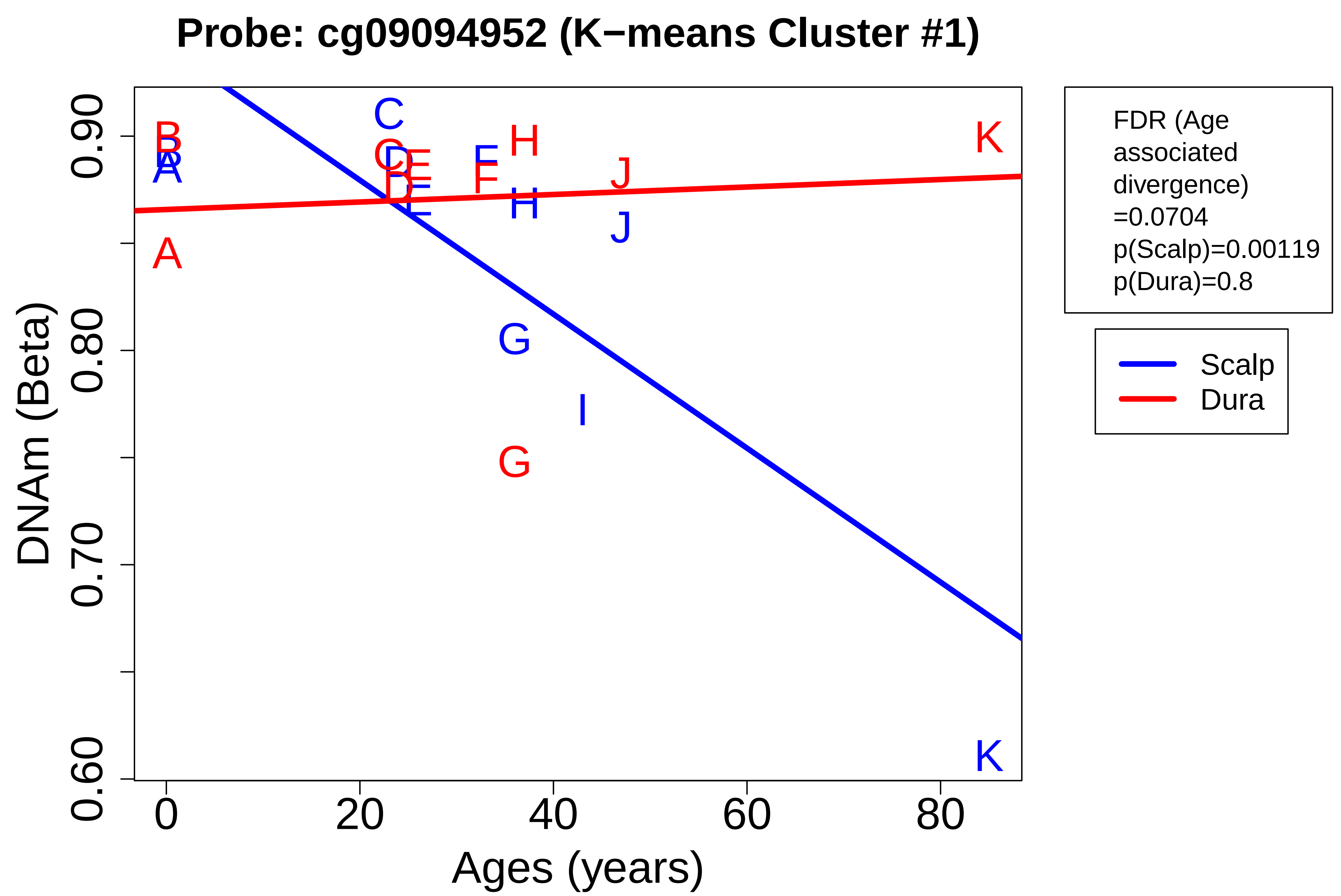

Probe: cg09094952 (K−means Cluster #1)
DNAm (Beta)
0.60
0.70
0.80
0.90
Ages (years)
0
20
40
60
80
A
B
C
D
E
F
G
H
I
J
K
FDR (Age associated divergence) =0.0704
p(Scalp)=0.00119
p(Dura)=0.8
Scalp
Dura

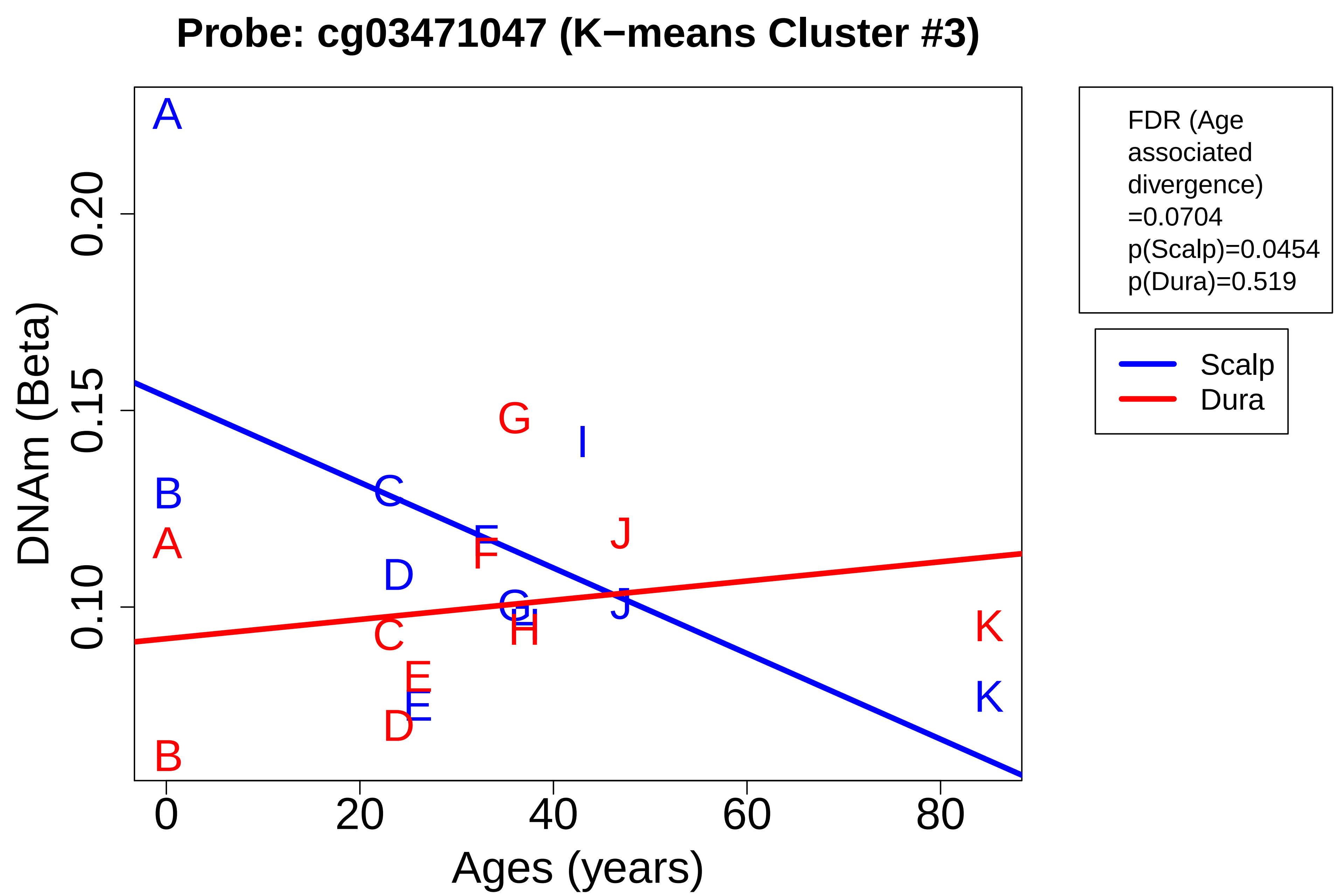

Probe: cg03471047 (K−means Cluster #3)
DNAm (Beta)
0.10
0.15
0.20
Ages (years)
0
20
40
60
80
A
B
C
D
E
F
G
H
I
J
K
FDR (Age associated divergence) =0.0704
p(Scalp)=0.0454
p(Dura)=0.519
Scalp
Dura

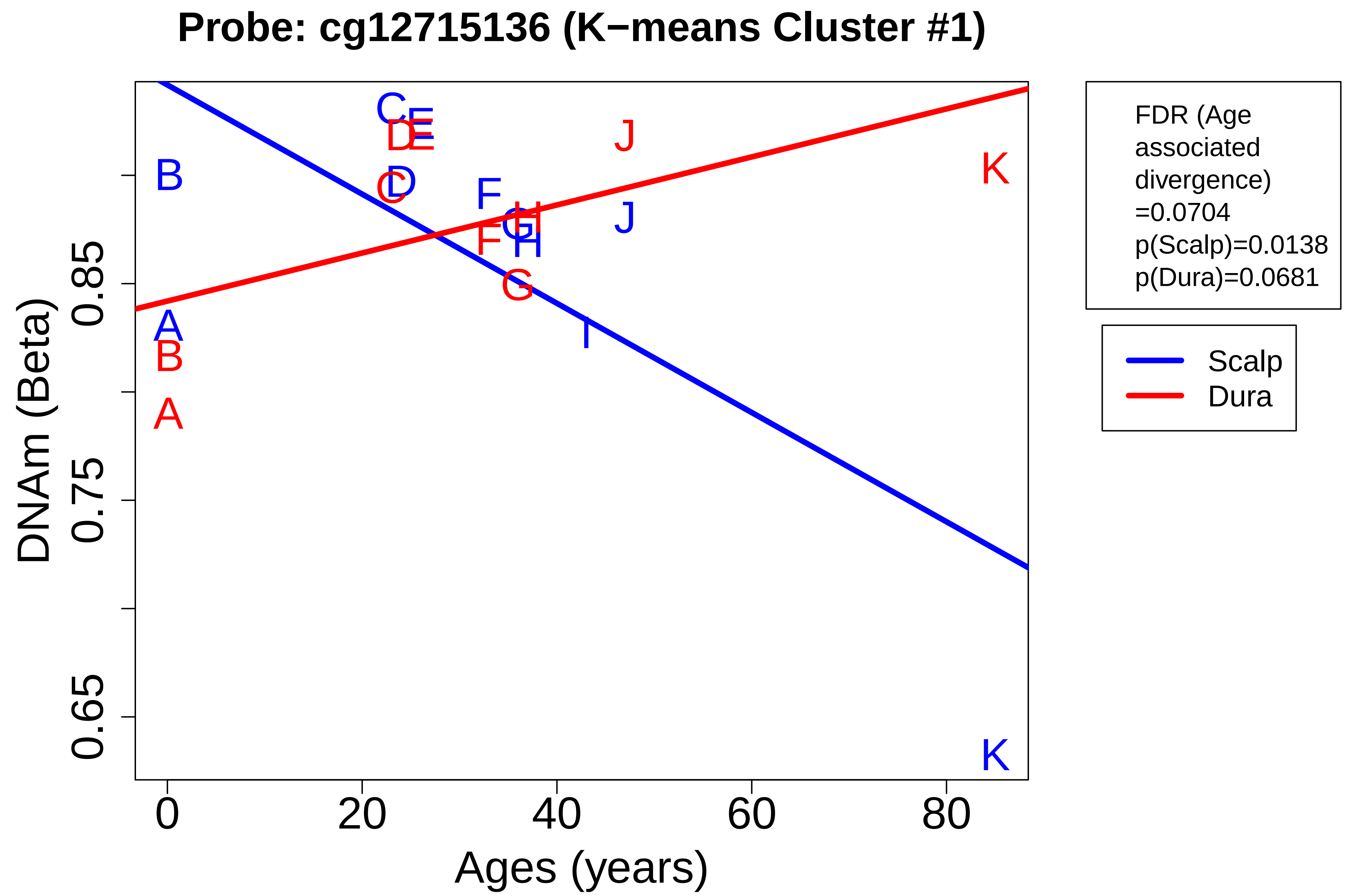

Probe: cg12715136 (K−means Cluster #1)
DNAm (Beta)
0.65
0.75
0.85
Ages (years)
0
20
40
60
80
FDR (Age associated divergence) =0.0704
p(Scalp)=0.0138
p(Dura)=0.0681
Scalp
Dura
A
B
C
D
E
F
G
H
I
J
K

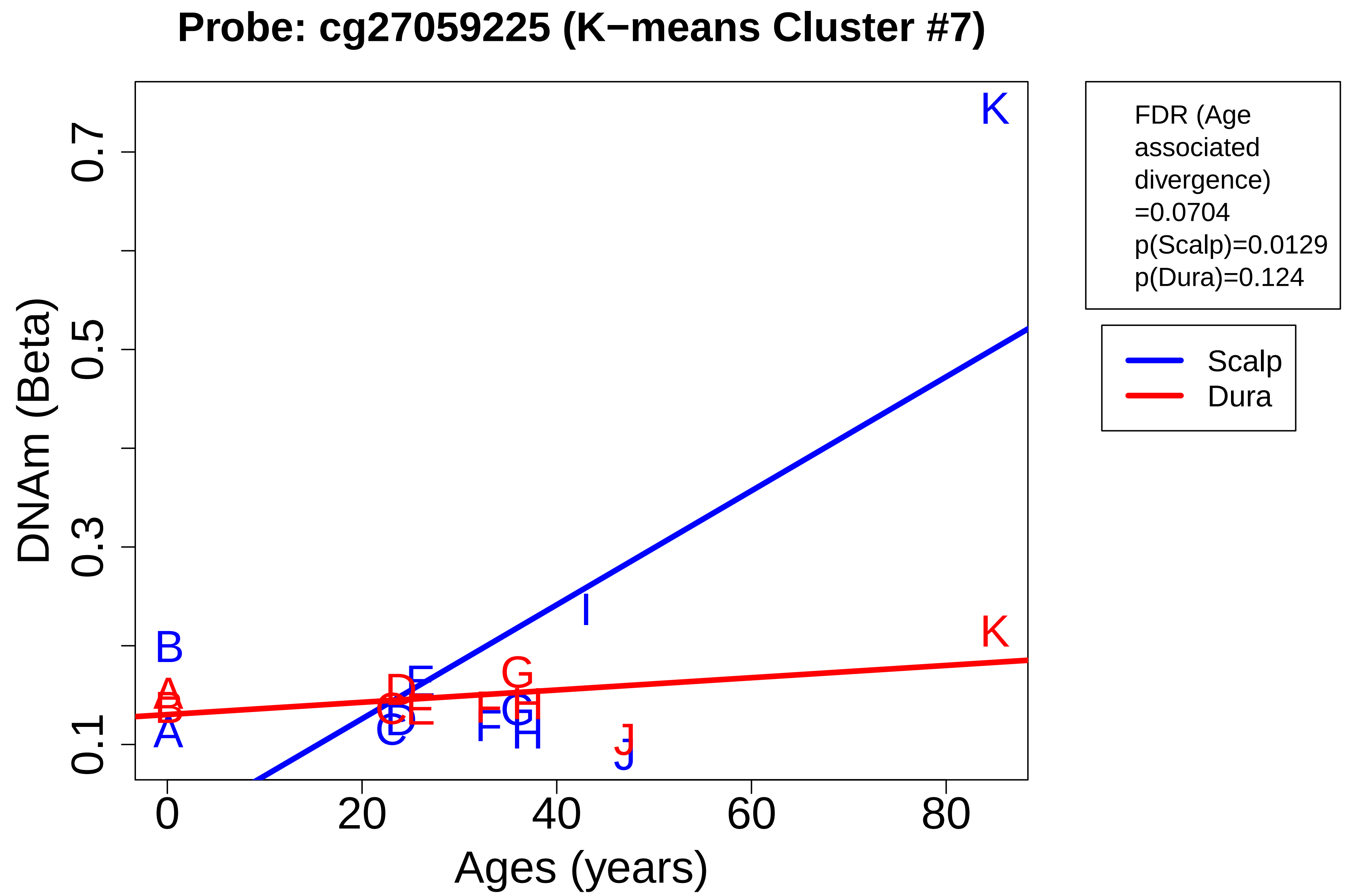

Probe: cg27059225 (K−means Cluster #7)
FDR (Age associated divergence) =0.0704
p(Scalp)=0.0129
p(Dura)=0.124
Scalp
Dura
DNAm (Beta)
Ages (years)
0.1
0.3
0.5
0.7
0
20
40
60
80

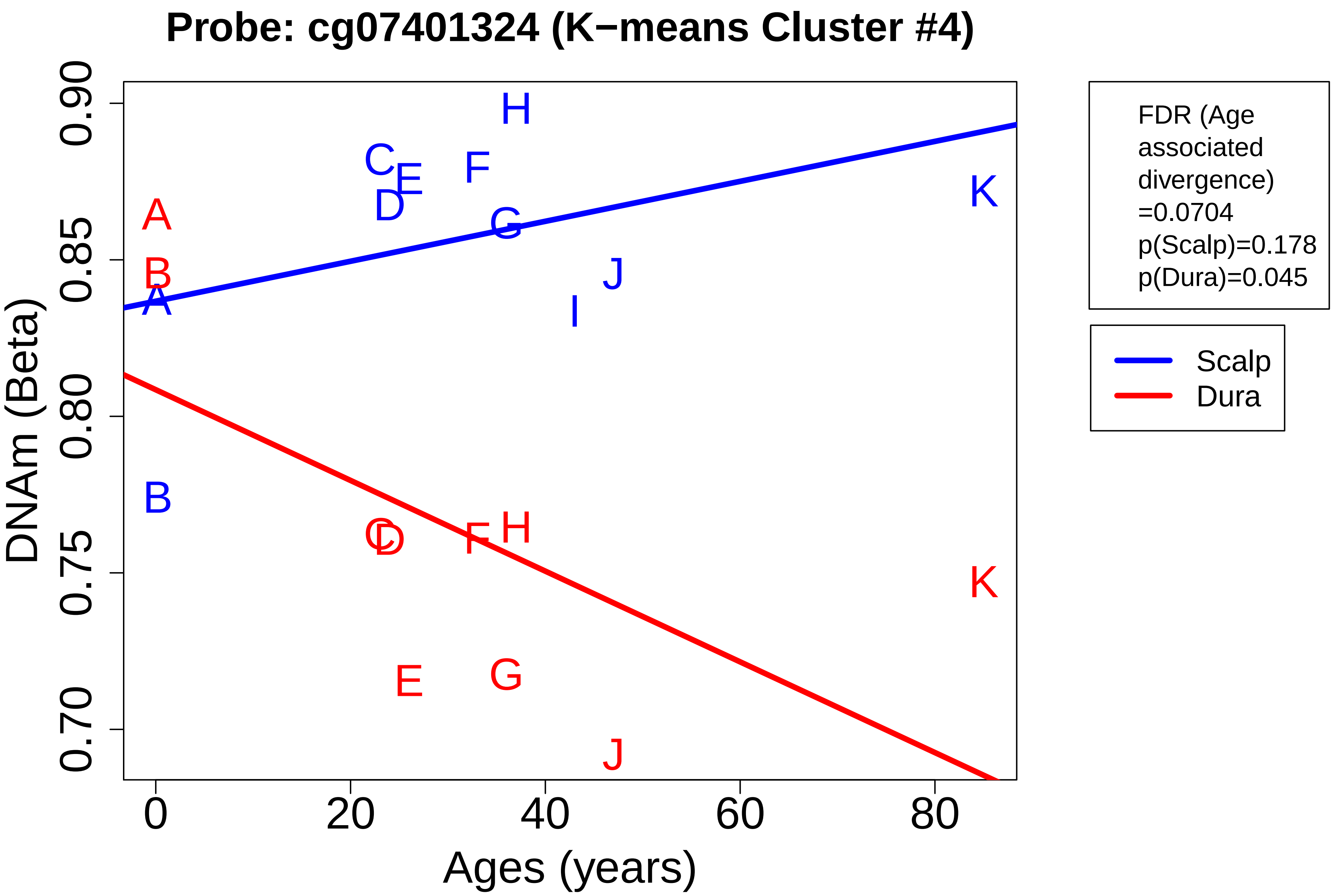

Probe: cg07401324 (K−means Cluster #4)
DNAm (Beta)
0.70
0.75
0.80
0.85
0.90
Ages (years)
0
20
40
60
80
A
B
C
D
E
F
G
H
I
J
K
FDR (Age associated divergence) =0.0704
p(Scalp)=0.178
p(Dura)=0.045
Scalp
Dura

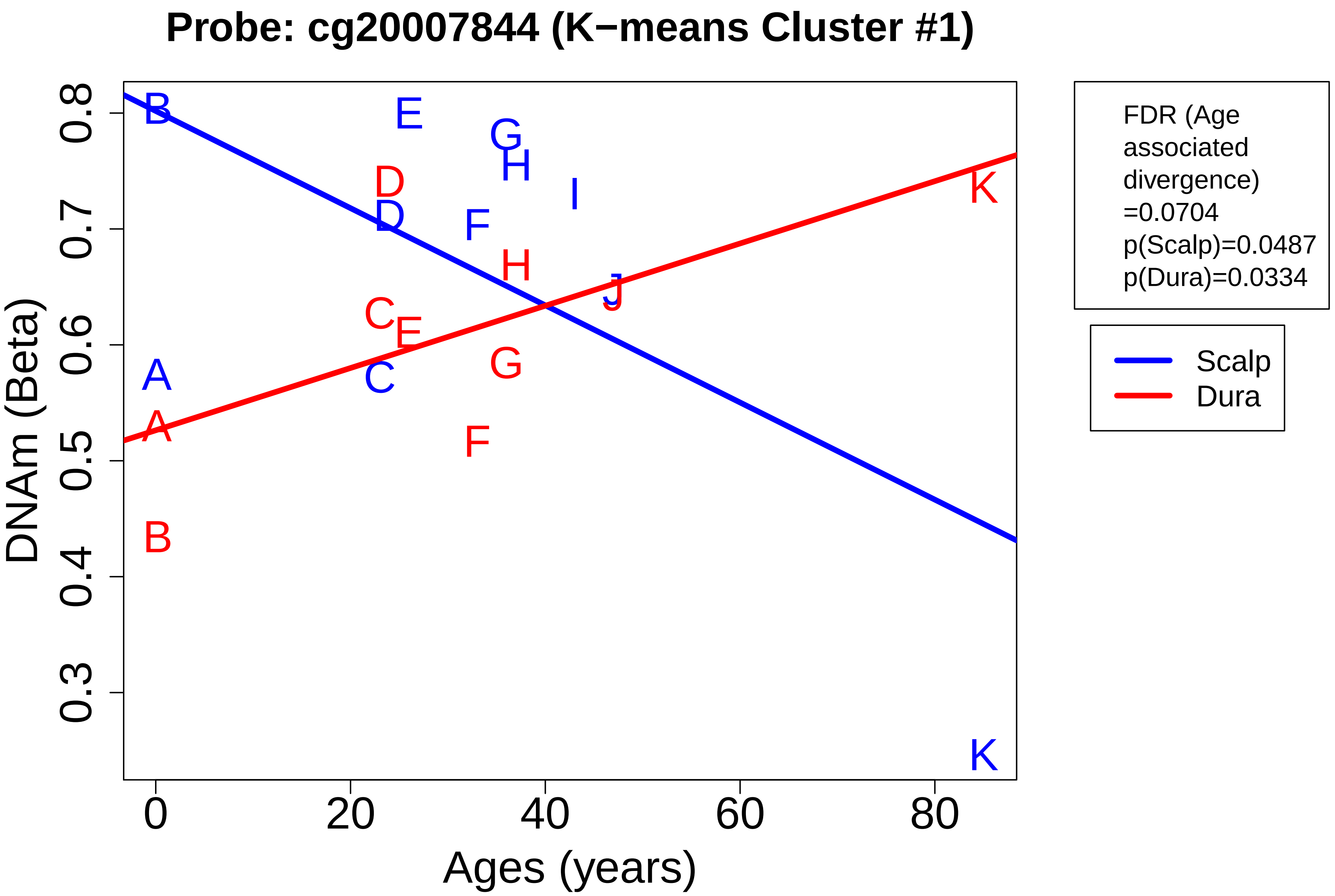

Probe: cg20007844 (K−means Cluster #1)
DNAm (Beta)
0.3
0.4
0.5
0.6
0.7
0.8
Ages (years)
0
20
40
60
80
A
B
C
D
E
F
G
H
I
J
K
A
B
C
D
E
F
G
H
J
K
FDR (Age associated divergence) =0.0704
p(Scalp)=0.0487
p(Dura)=0.0334
Scalp
Dura

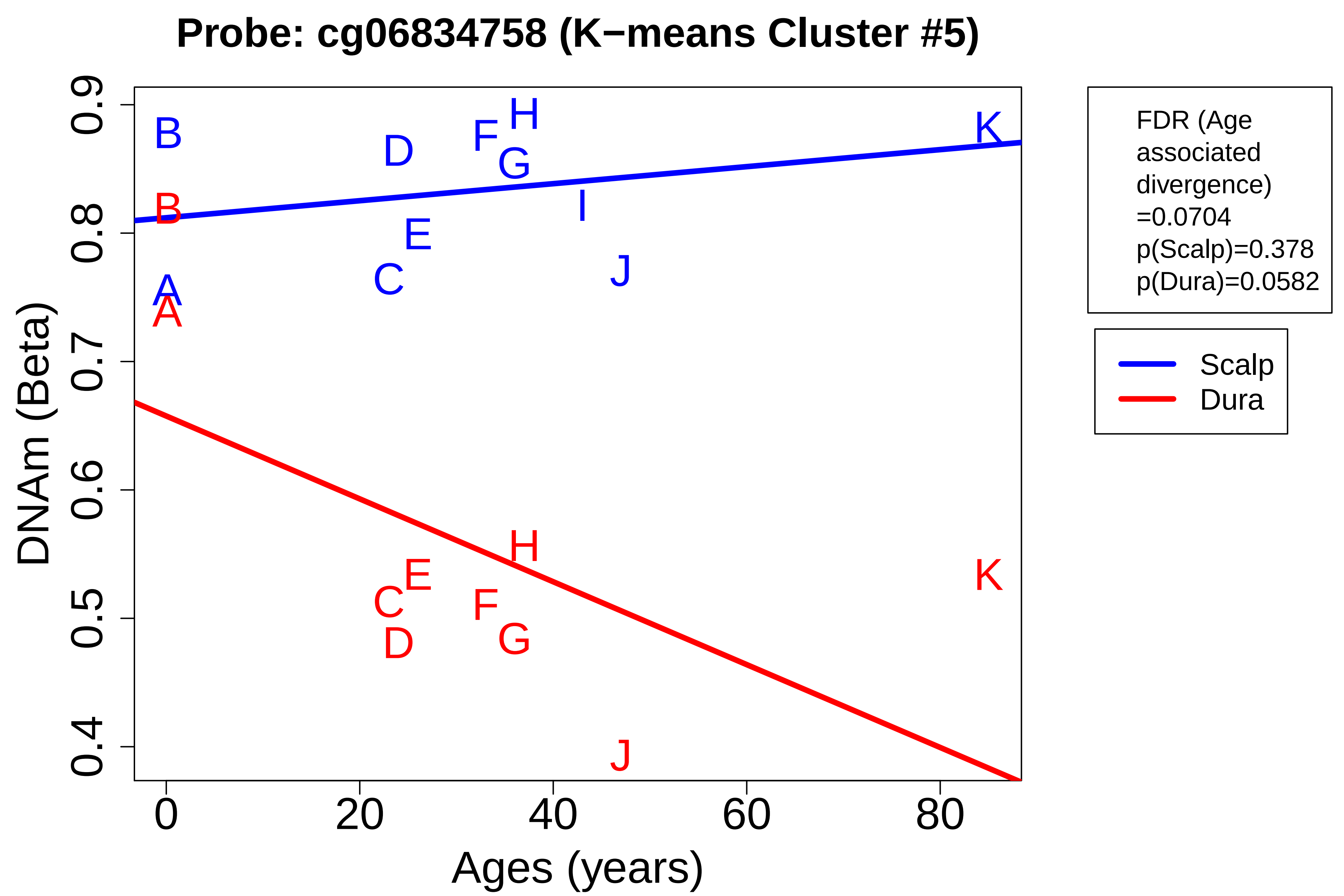
Probe: cg06834758 (K−means Cluster #5)
DNAm (Beta)
Ages (years)
0.4
0.5
0.6
0.7
0.8
0.9
0
20
40
60
80
A B C D E F G H I J K
A B C D E F G H J K
FDR (Age associated divergence) =0.0704
p(Scalp)=0.378
p(Dura)=0.0582
Scalp
Dura

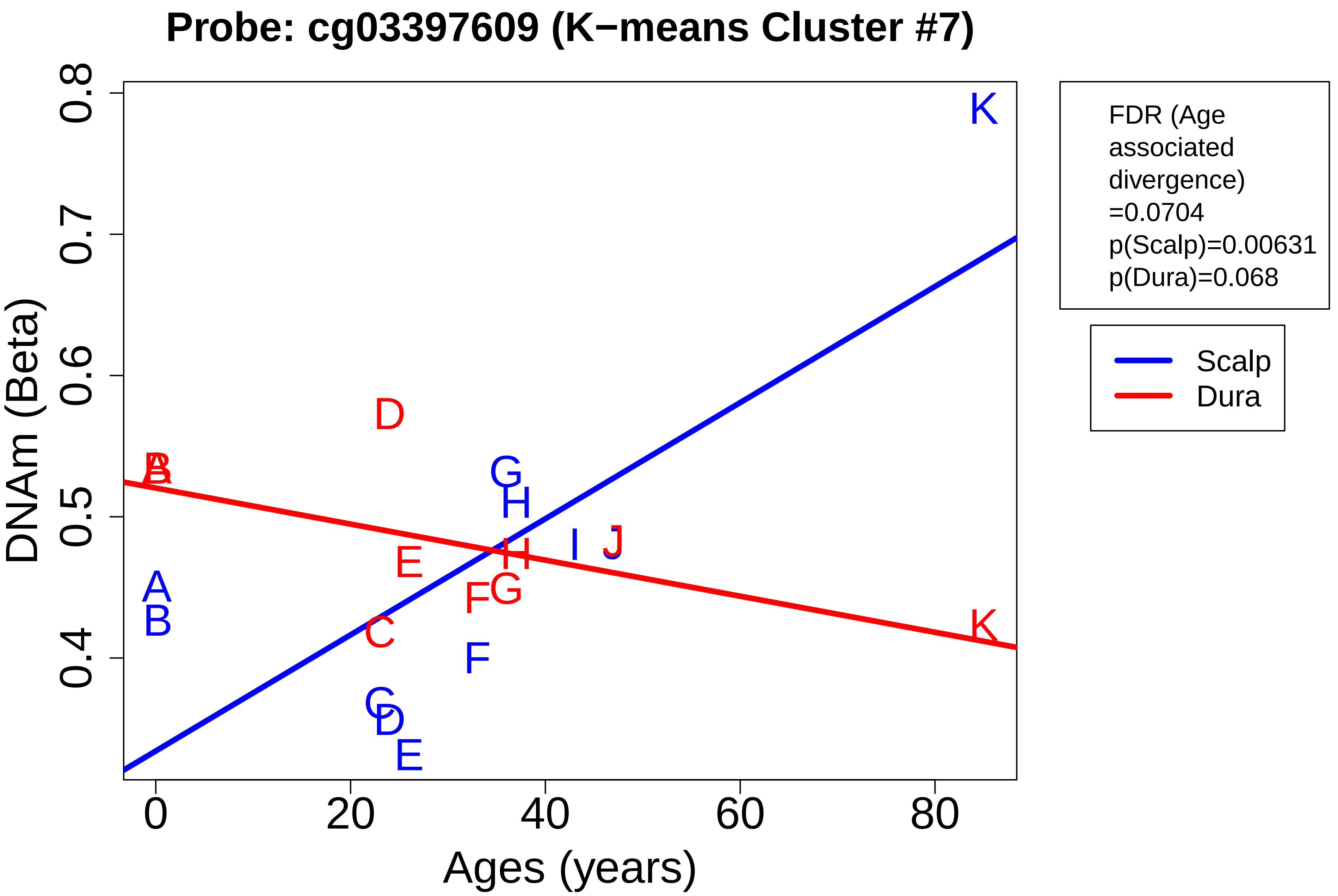

Probe: cg03397609 (K−means Cluster #7)
DNAm (Beta)
0.4
0.5
0.6
0.7
0.8
Ages (years)
0
20
40
60
80
FDR (Age associated divergence) =0.0704
p(Scalp)=0.00631
p(Dura)=0.068
Scalp
Dura
A
B
C
D
E
F
G
H
I
J
K

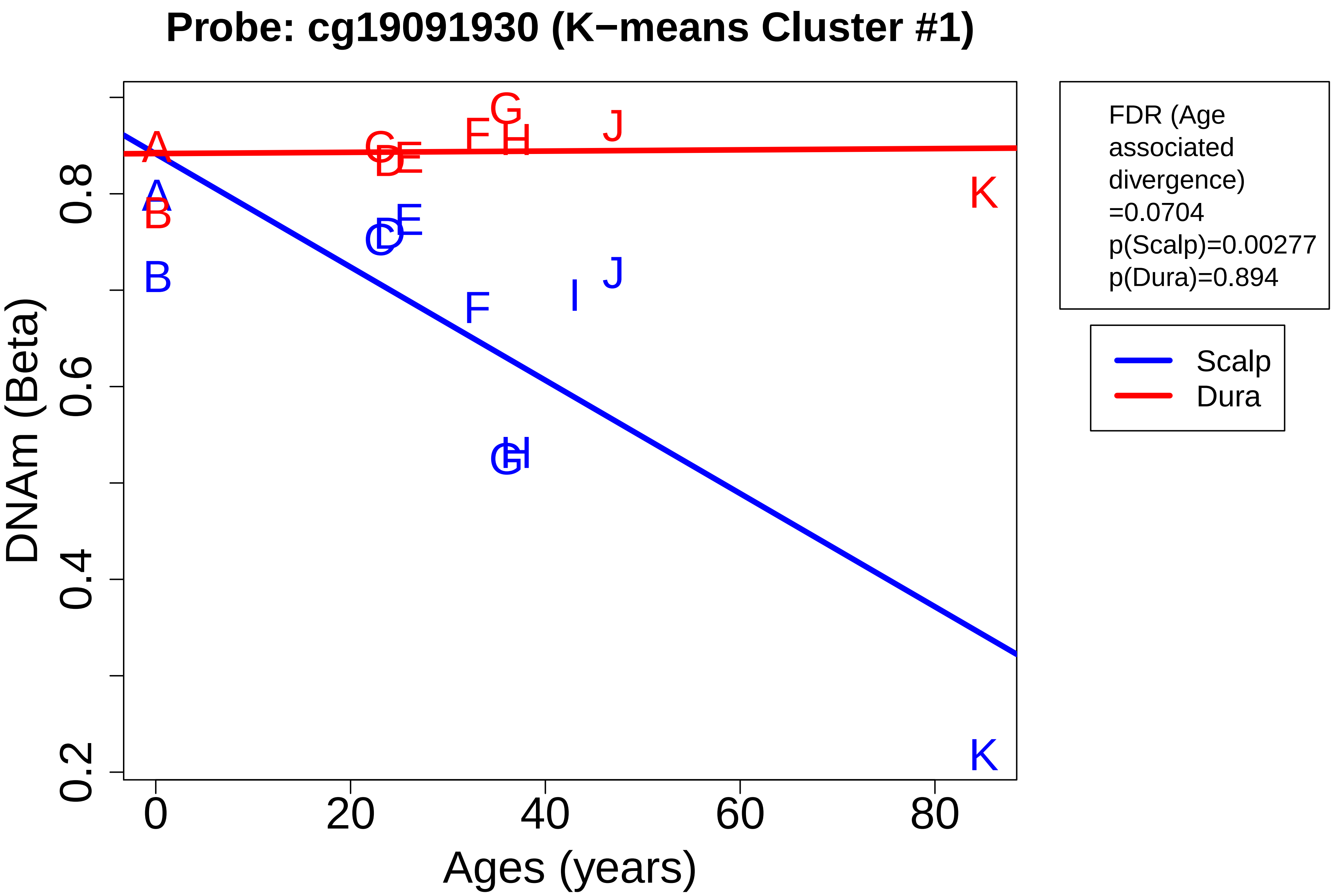

Probe: cg19091930 (K−means Cluster #1)
FDR (Age associated divergence) =0.0704
p(Scalp)=0.00277
p(Dura)=0.894
Scalp
Dura
DNAm (Beta)
0.2
0.4
0.6
0.8
Ages (years)
0
20
40
60
80

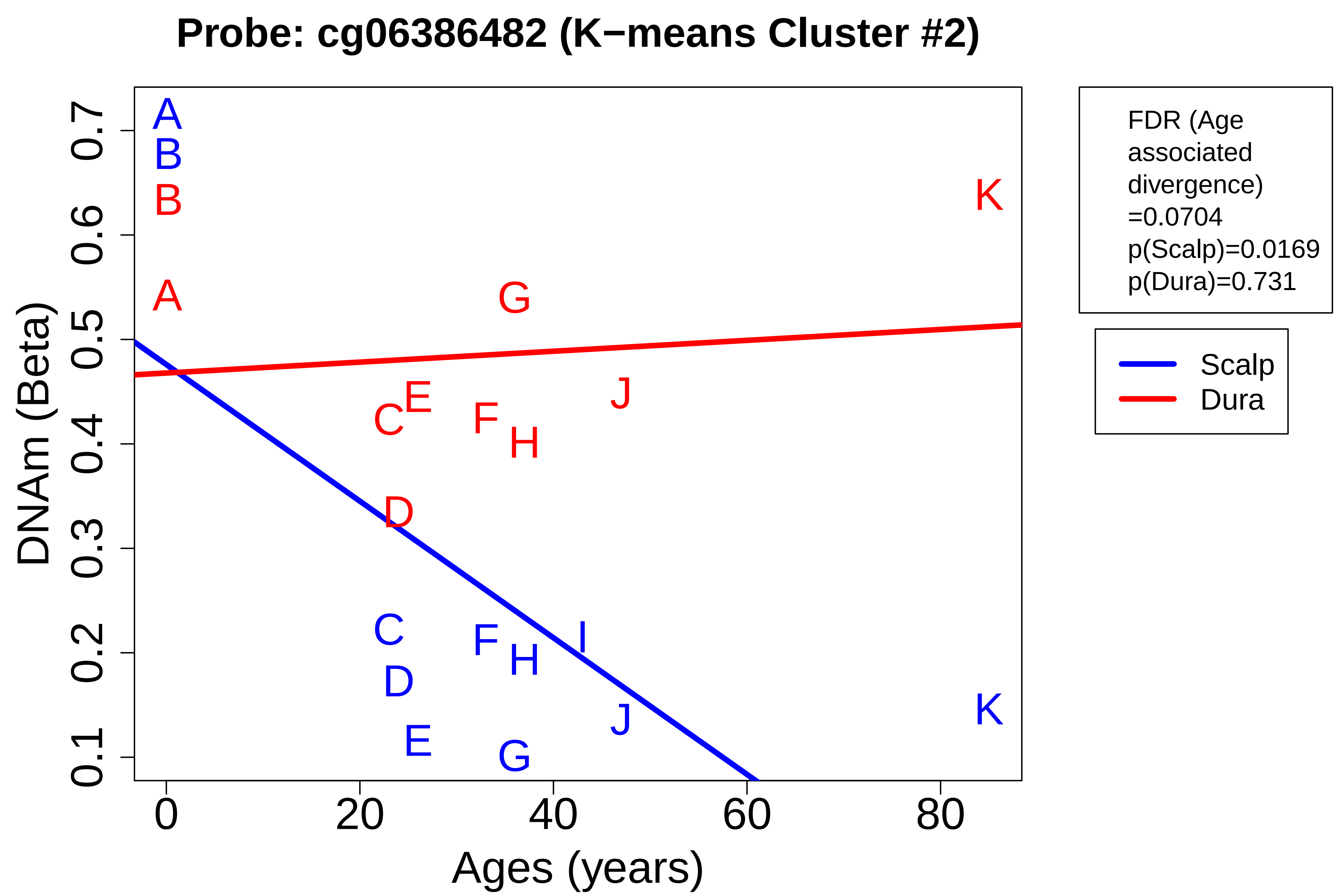

Probe: cg06386482 (K−means Cluster #2)
DNAm (Beta)
0.1
0.2
0.3
0.4
0.5
0.6
0.7
Ages (years)
0
20
40
60
80
A
B
B
A
G
K
C
E
F
H
J
D
C
F
H
I
D
J
E
G
K
FDR (Age associated divergence) =0.0704
p(Scalp)=0.0169
p(Dura)=0.731
Scalp
Dura

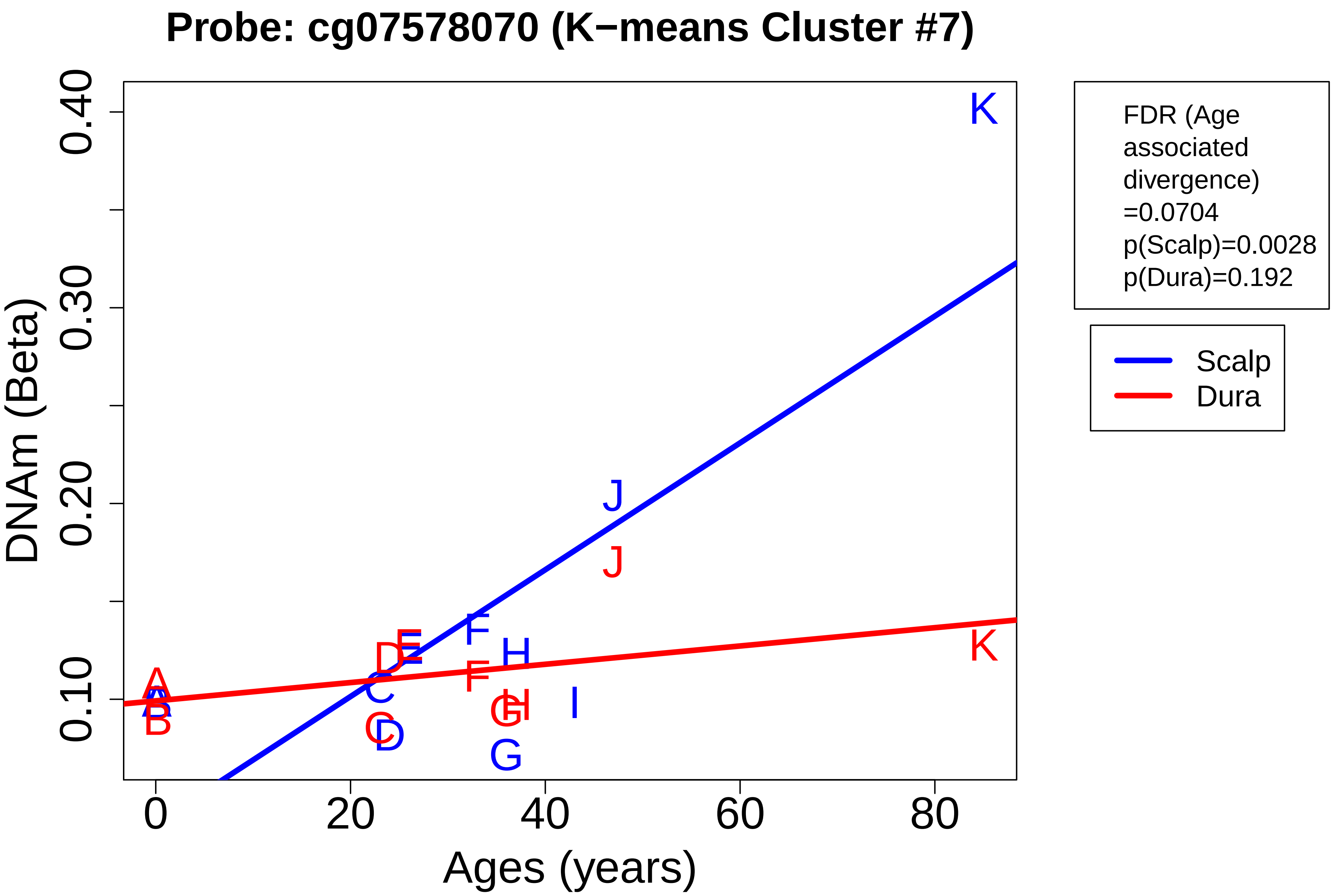

Probe: cg07578070 (K−means Cluster #7)
DNAm (Beta)
0.10
0.20
0.30
0.40
0
20
40
60
80
Ages (years)
FDR (Age associated divergence) =0.0704
p(Scalp)=0.0028
p(Dura)=0.192
Scalp
Dura

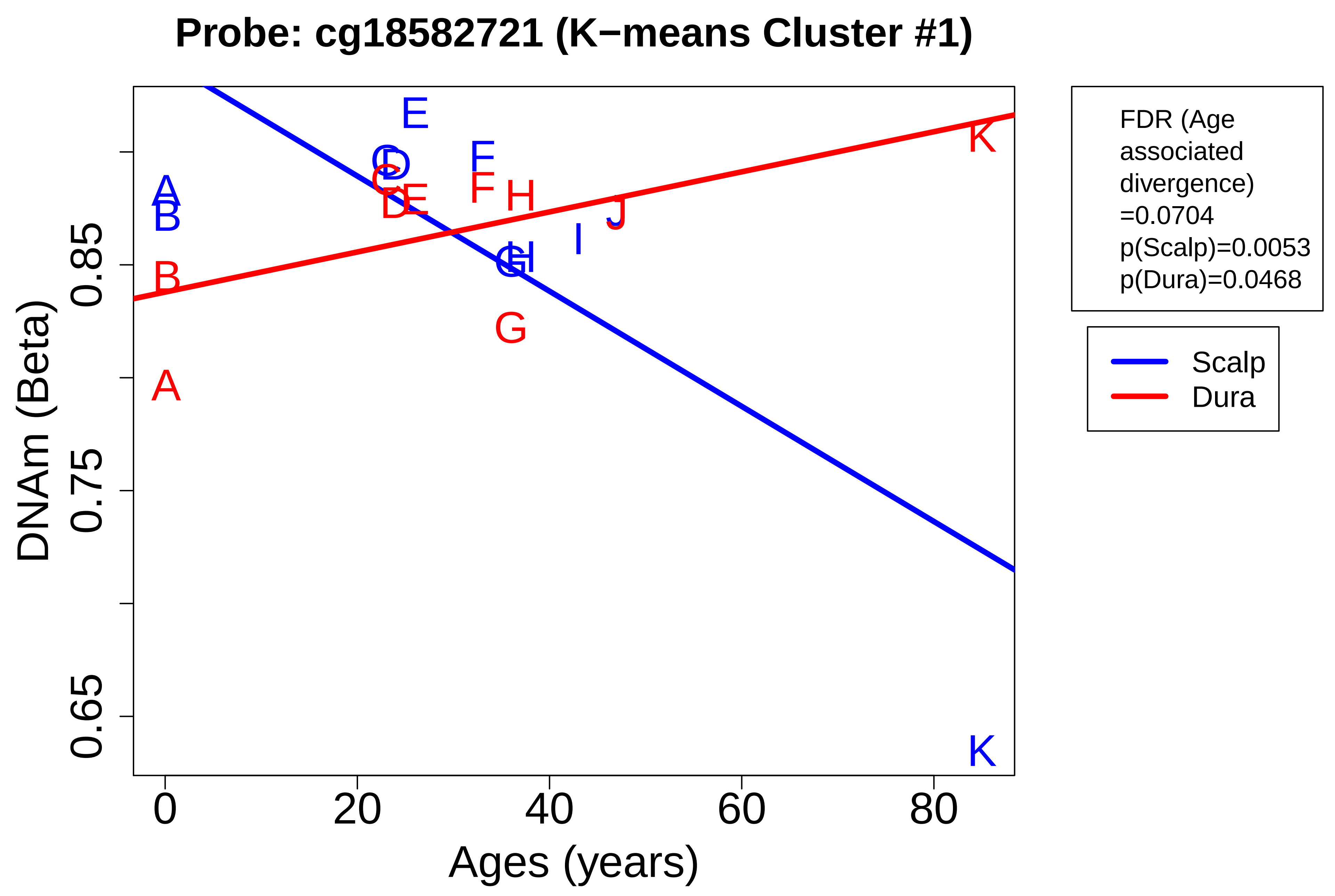

Probe: cg18582721 (K−means Cluster #1)
FDR (Age associated divergence) =0.0704
p(Scalp)=0.0053
p(Dura)=0.0468
Scalp
Dura
DNAm (Beta)
0.65
0.75
0.85
Ages (years)
0
20
40
60
80
A
B
C
D
E
F
G
H
I
J
K

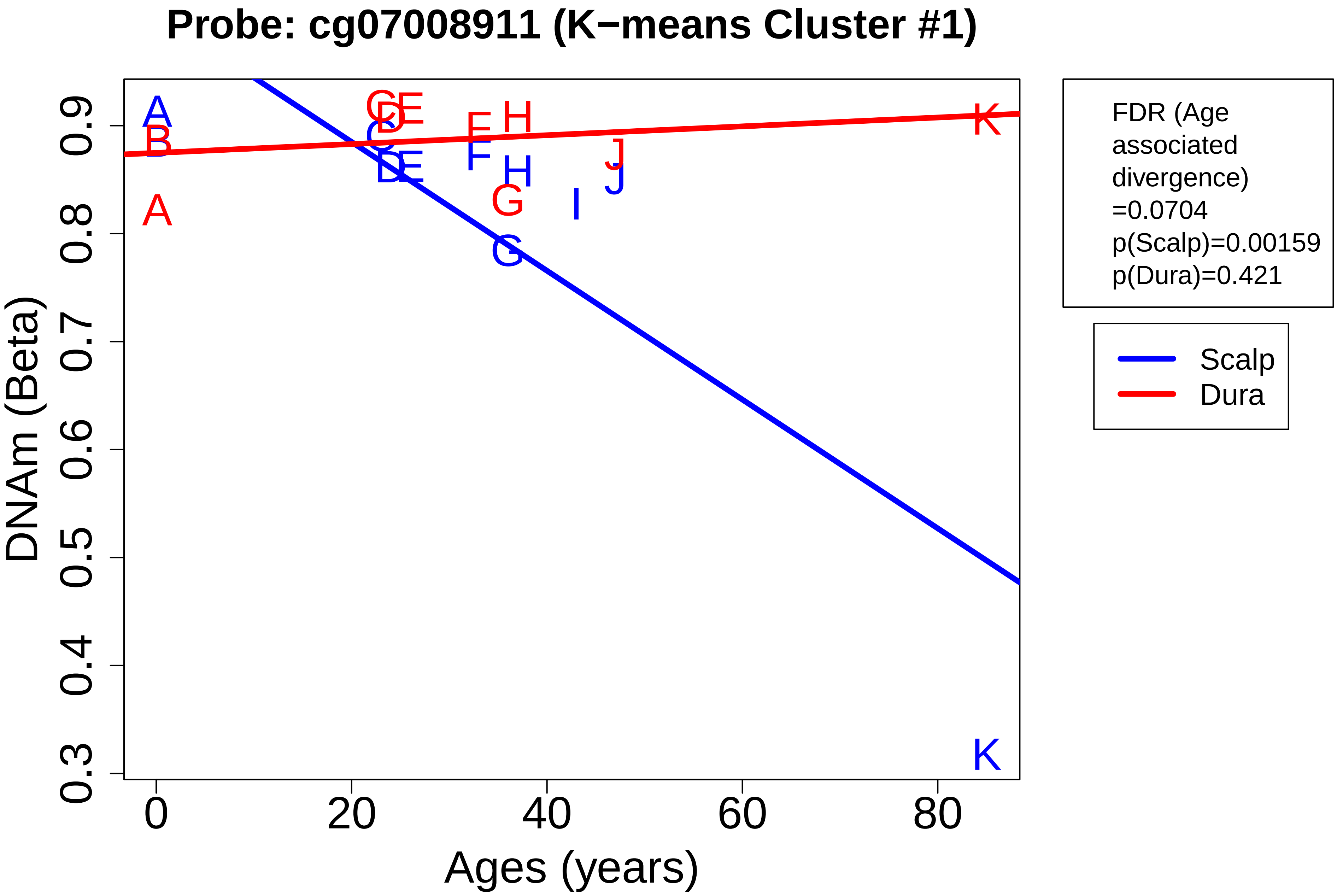

Probe: cg07008911 (K−means Cluster #1)
DNAm (Beta)
0.3
0.4
0.5
0.6
0.7
0.8
0.9
0
20
40
60
80
Ages (years)
A
B
C
D
E
F
G
H
I
J
K
FDR (Age associated divergence) =0.0704
p(Scalp)=0.00159
p(Dura)=0.421
Scalp
Dura

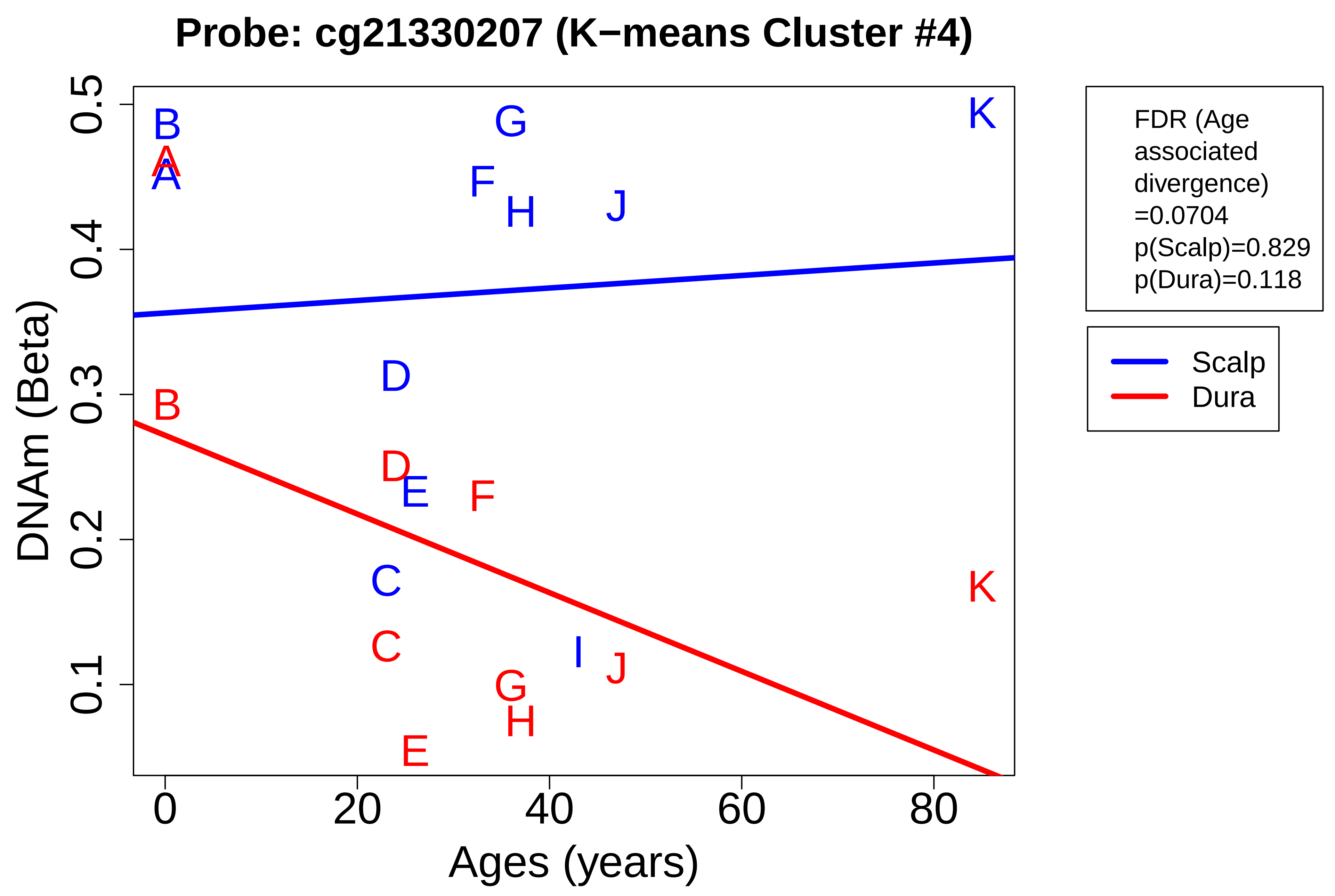

Probe: cg21330207 (K−means Cluster #4)
DNAm (Beta)
Ages (years)
0.1
0.2
0.3
0.4
0.5
0
20
40
60
80
FDR (Age associated divergence) =0.0704
p(Scalp)=0.829
p(Dura)=0.118
Scalp
Dura

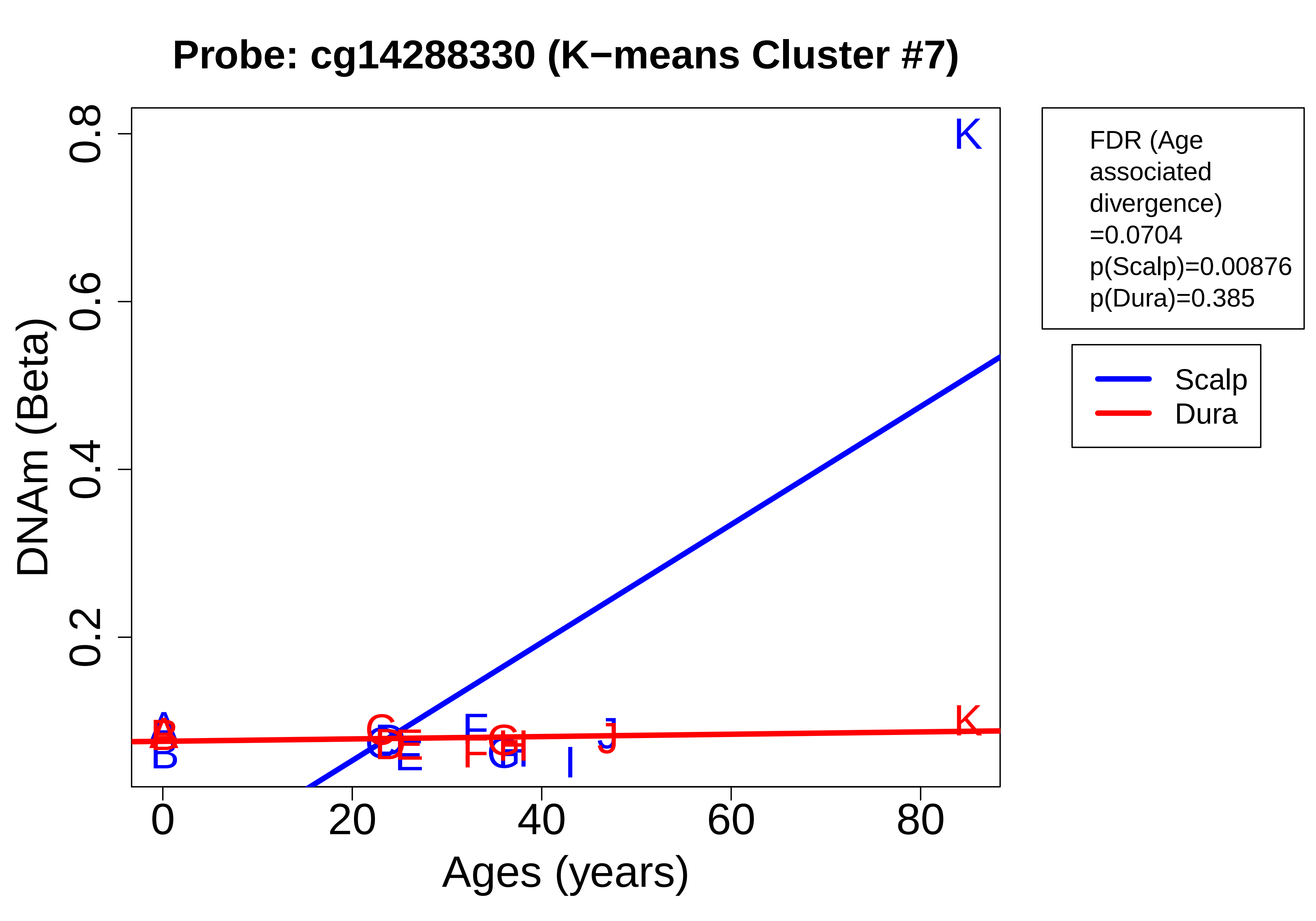

Probe: cg14288330 (K−means Cluster #7)
DNAm (Beta)
0.2
0.4
0.6
0.8
0
20
40
60
80
Ages (years)
A
B
C
D
E
F
G
H
I
J
K
FDR (Age associated divergence) =0.0704
p(Scalp)=0.00876
p(Dura)=0.385
Scalp
Dura

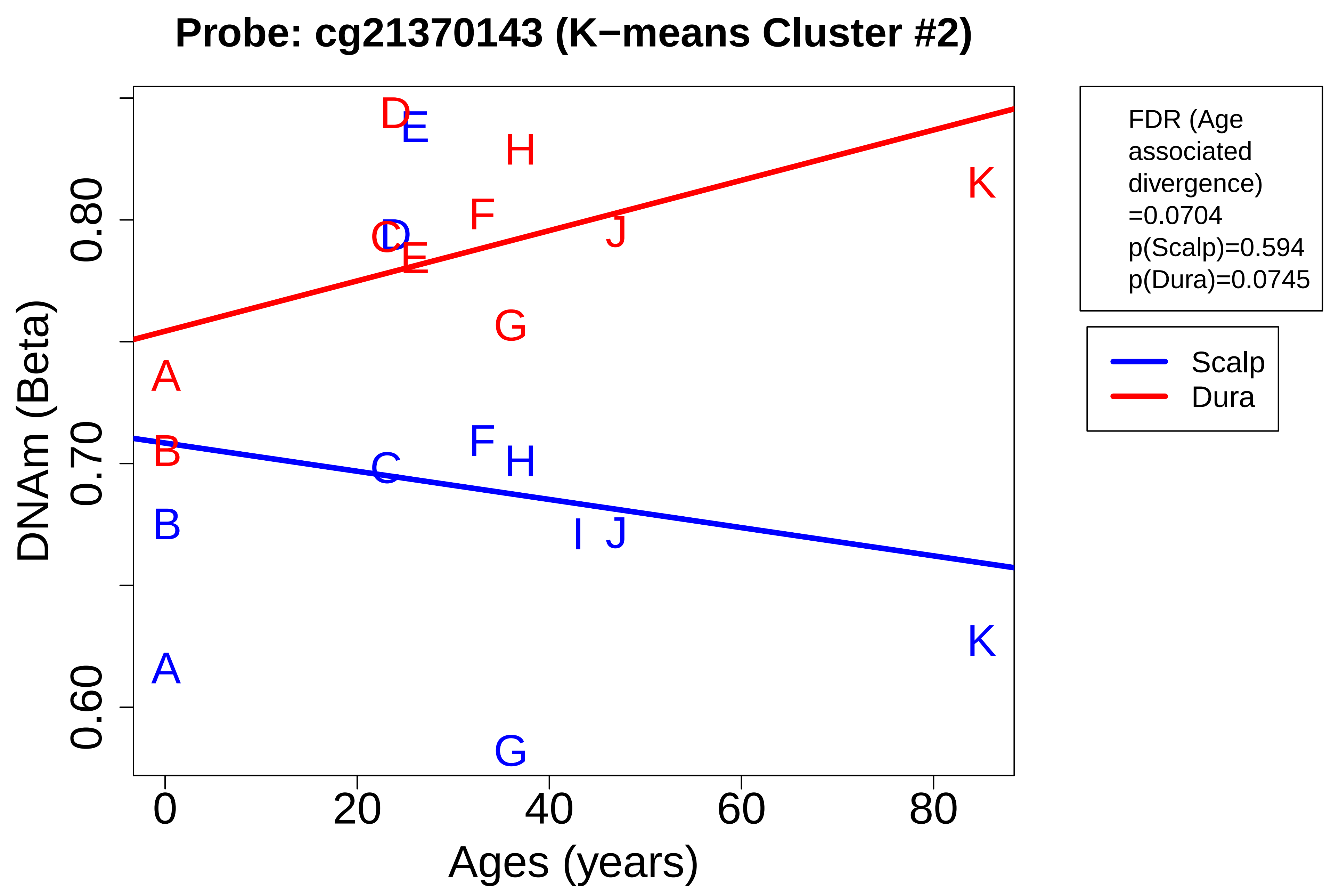

Probe: cg21370143 (K−means Cluster #2)
FDR (Age associated divergence) =0.0704
p(Scalp)=0.594
p(Dura)=0.0745
Scalp
Dura
DNAm (Beta)
0.60
0.70
0.80
0
20
40
60
80
Ages (years)
A
B
C
D
E
F
G
H
I
J
K

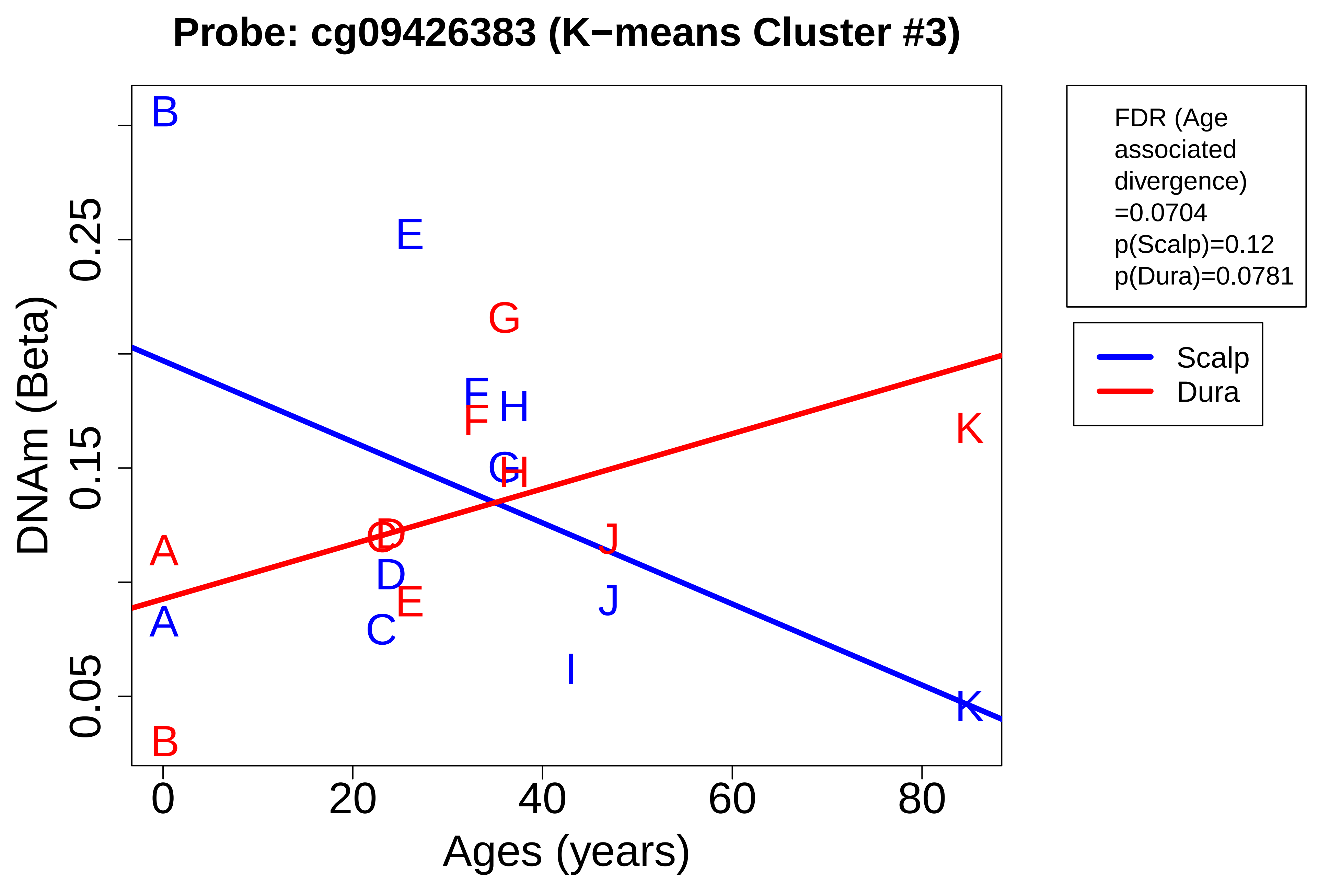

Probe: cg09426383 (K−means Cluster #3)
DNAm (Beta)
0.05
0.15
0.25
Ages (years)
0
20
40
60
80
FDR (Age associated divergence) =0.0704
p(Scalp)=0.12
p(Dura)=0.0781
Scalp
Dura
A
B
C
D
E
F
G
H
I
J
K

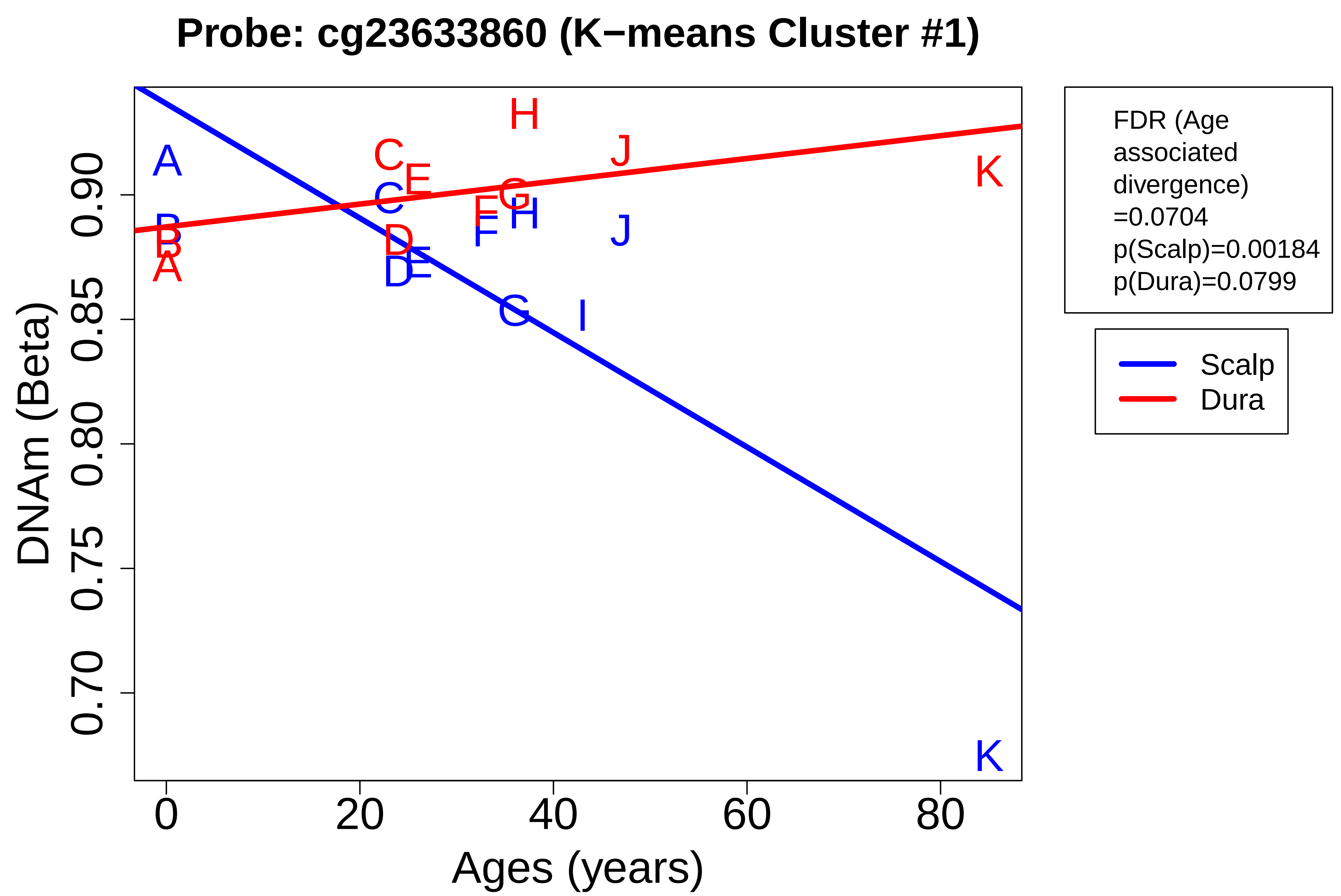

Probe: cg23633860 (K−means Cluster #1)
DNAm (Beta)
0.70
0.75
0.80
0.85
0.90
0
20
40
60
80
Ages (years)
A
B
C
D
E
F
G
H
I
J
K
FDR (Age associated divergence) =0.0704
p(Scalp)=0.00184
p(Dura)=0.0799
Scalp
Dura

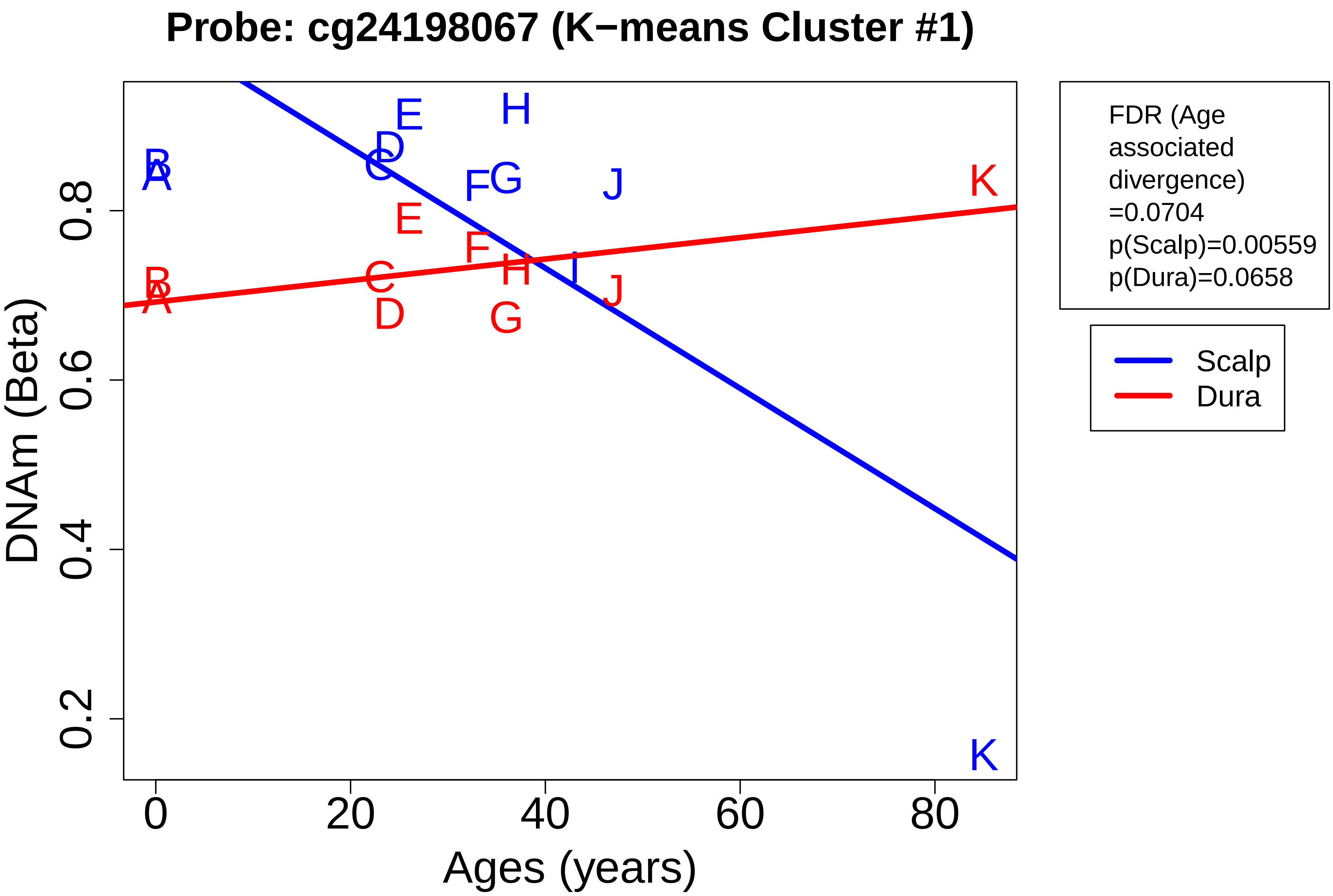
Probe: cg24198067 (K−means Cluster #1)
DNAm (Beta)
0.2
0.4
0.6
0.8
Ages (years)
0
20
40
60
80
FDR (Age associated divergence) =0.0704
p(Scalp)=0.00559
p(Dura)=0.0658
Scalp
Dura

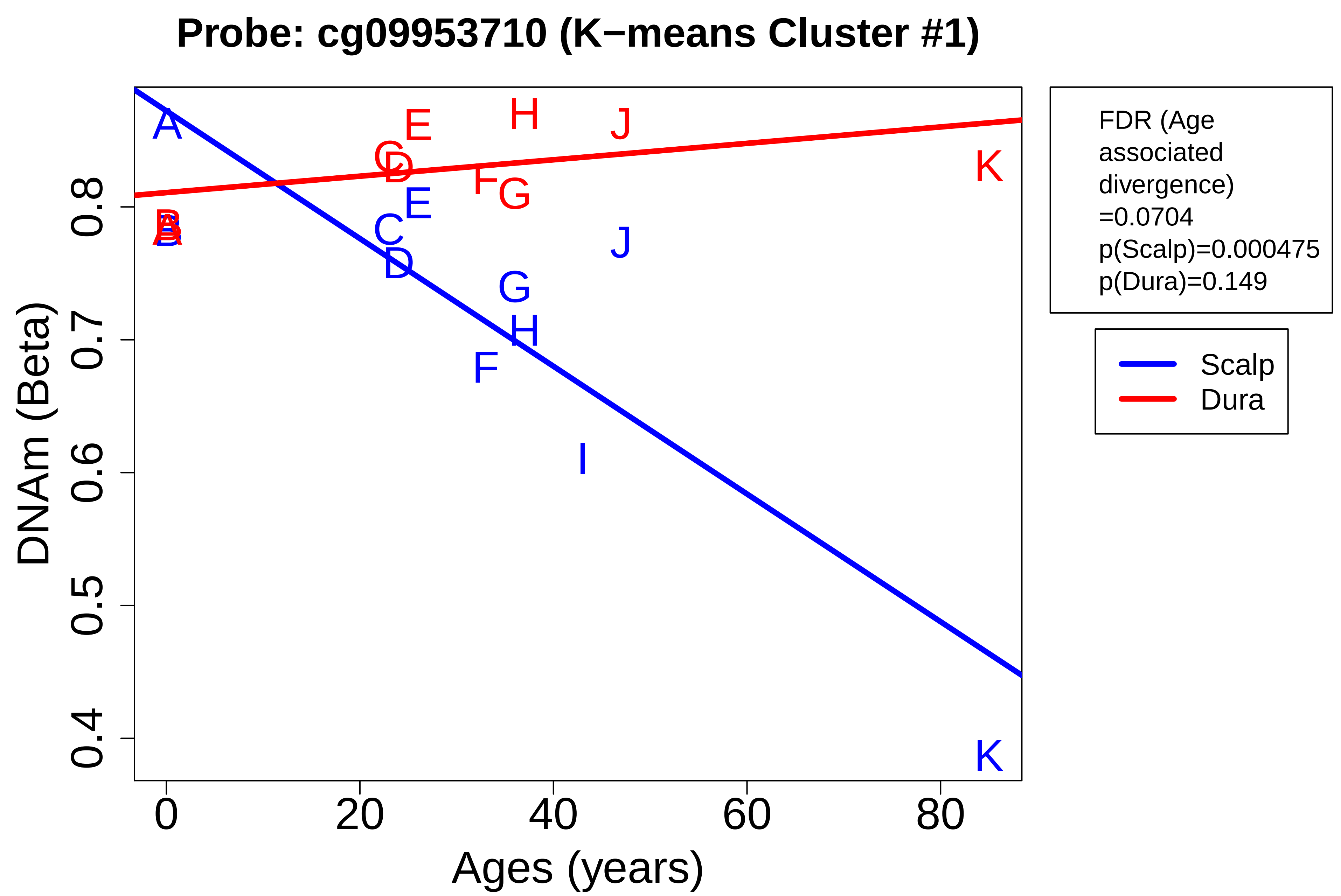

Probe: cg09953710 (K−means Cluster #1)
DNAm (Beta)
0.4
0.5
0.6
0.7
0.8
Ages (years)
0
20
40
60
80
FDR (Age associated divergence) =0.0704
p(Scalp)=0.000475
p(Dura)=0.149
Scalp
Dura

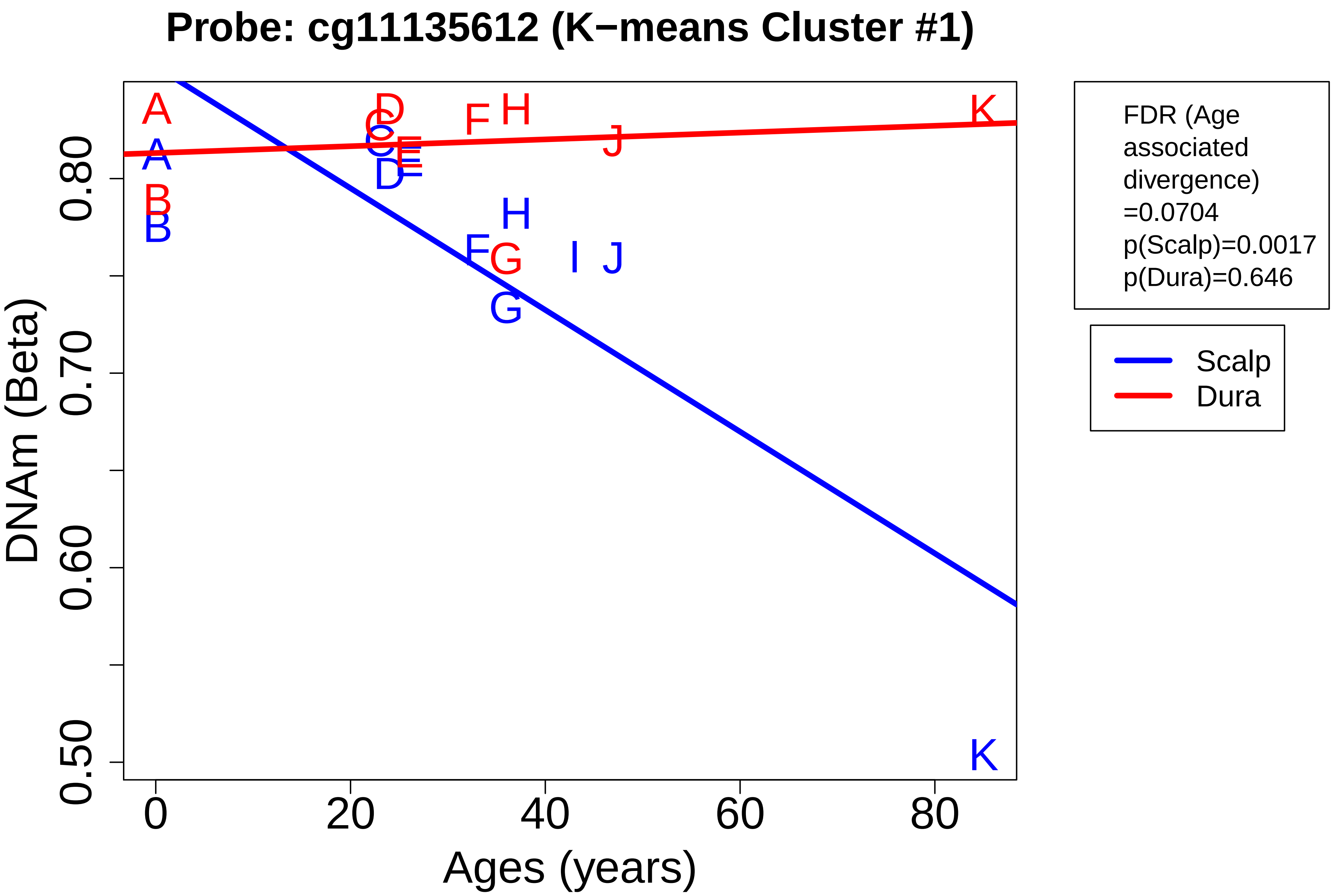

Probe: cg11135612 (K−means Cluster #1)
DNAm (Beta)
0.50
0.60
0.70
0.80
0
20
40
60
80
Ages (years)
A
B
C
D
E
F
G
H
I
J
K
FDR (Age associated divergence) =0.0704
p(Scalp)=0.0017
p(Dura)=0.646
Scalp
Dura

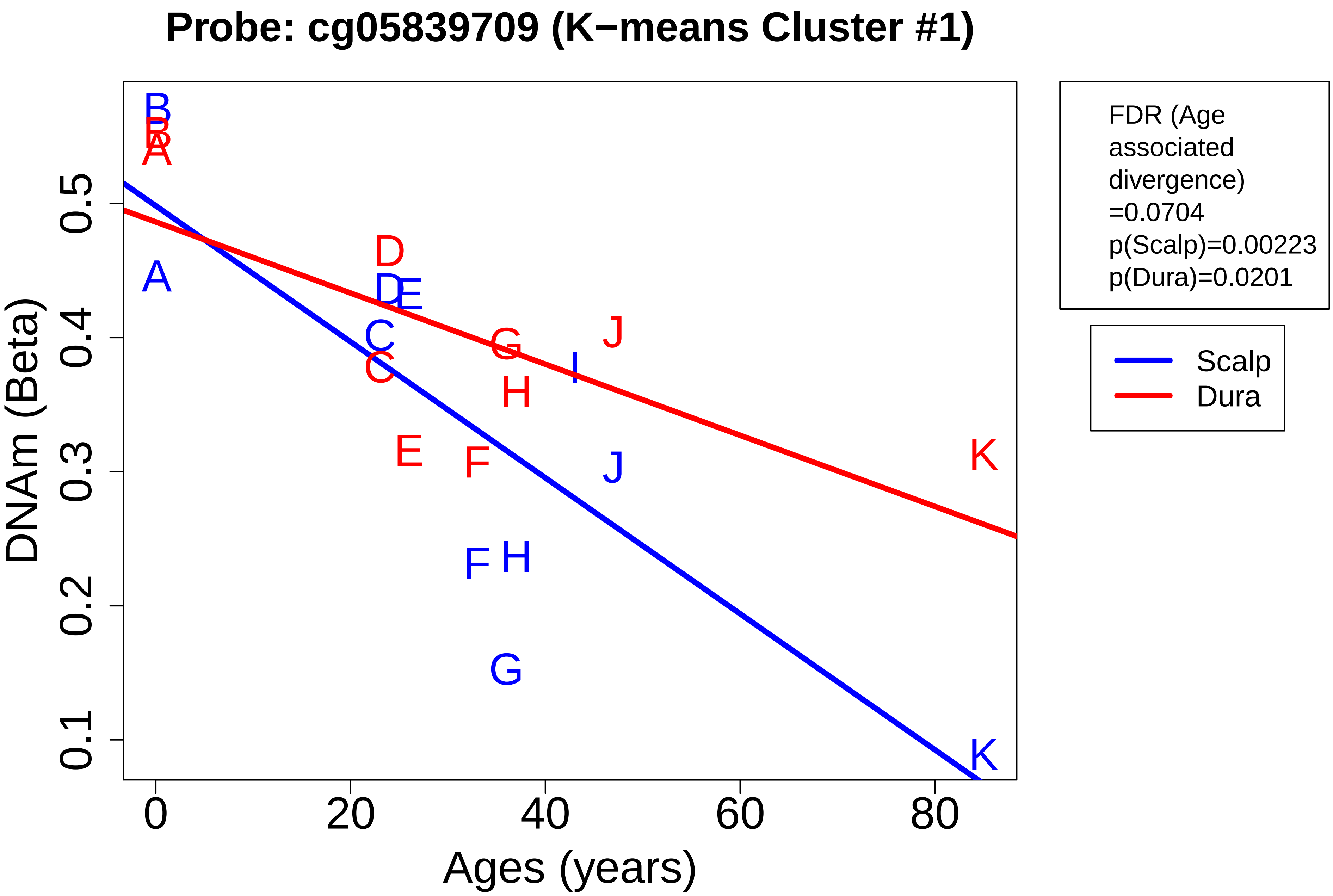

Probe: cg05839709 (K−means Cluster #1)
DNAm (Beta)
0.1
0.2
0.3
0.4
0.5
0
20
40
60
80
Ages (years)
FDR (Age associated divergence) =0.0704
p(Scalp)=0.00223
p(Dura)=0.0201
Scalp
Dura

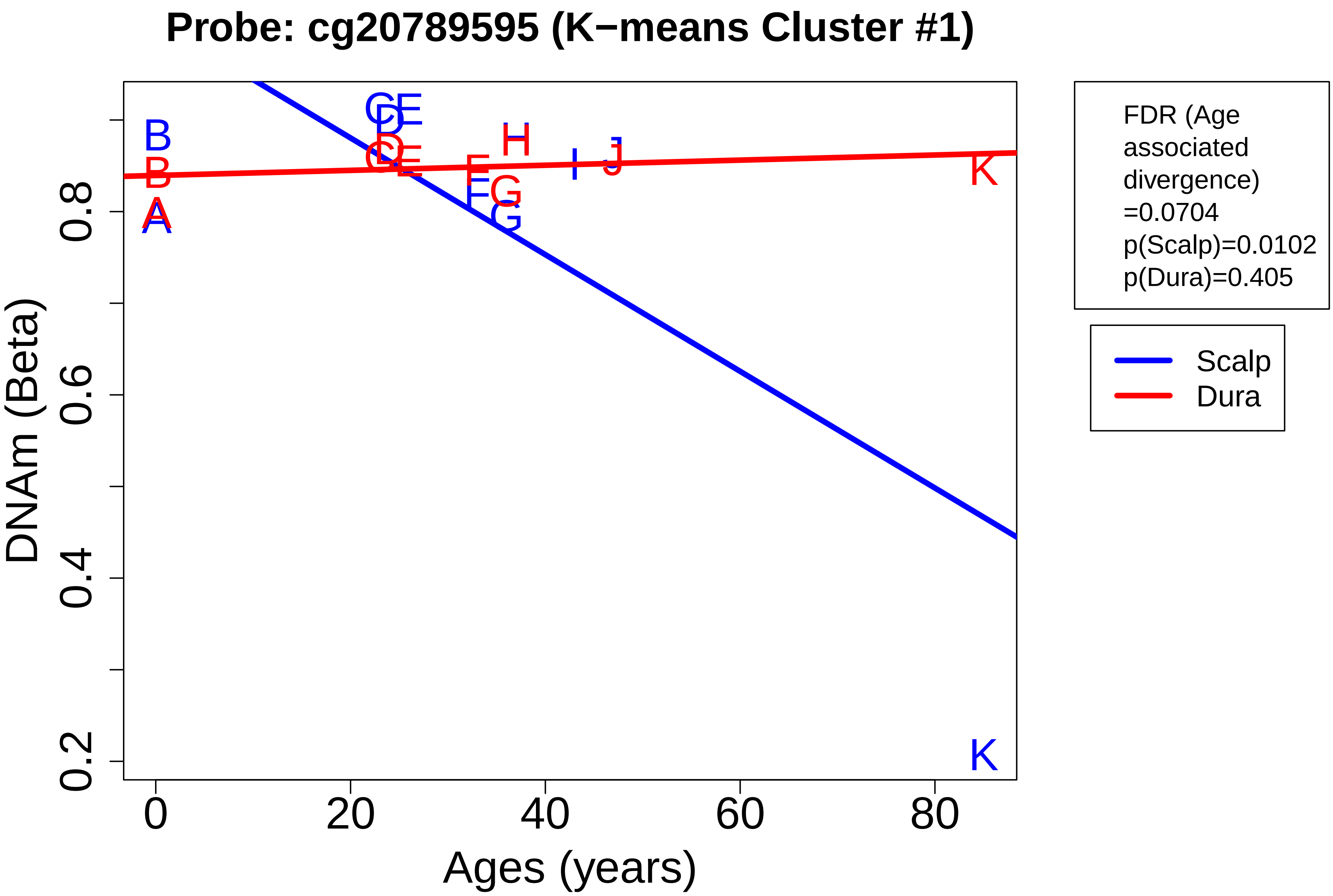

Probe: cg20789595 (K−means Cluster #1)
DNAm (Beta)
Ages (years)
0.2
0.4
0.6
0.8
0
20
40
60
80
FDR (Age associated divergence) =0.0704
p(Scalp)=0.0102
p(Dura)=0.405
Scalp
Dura

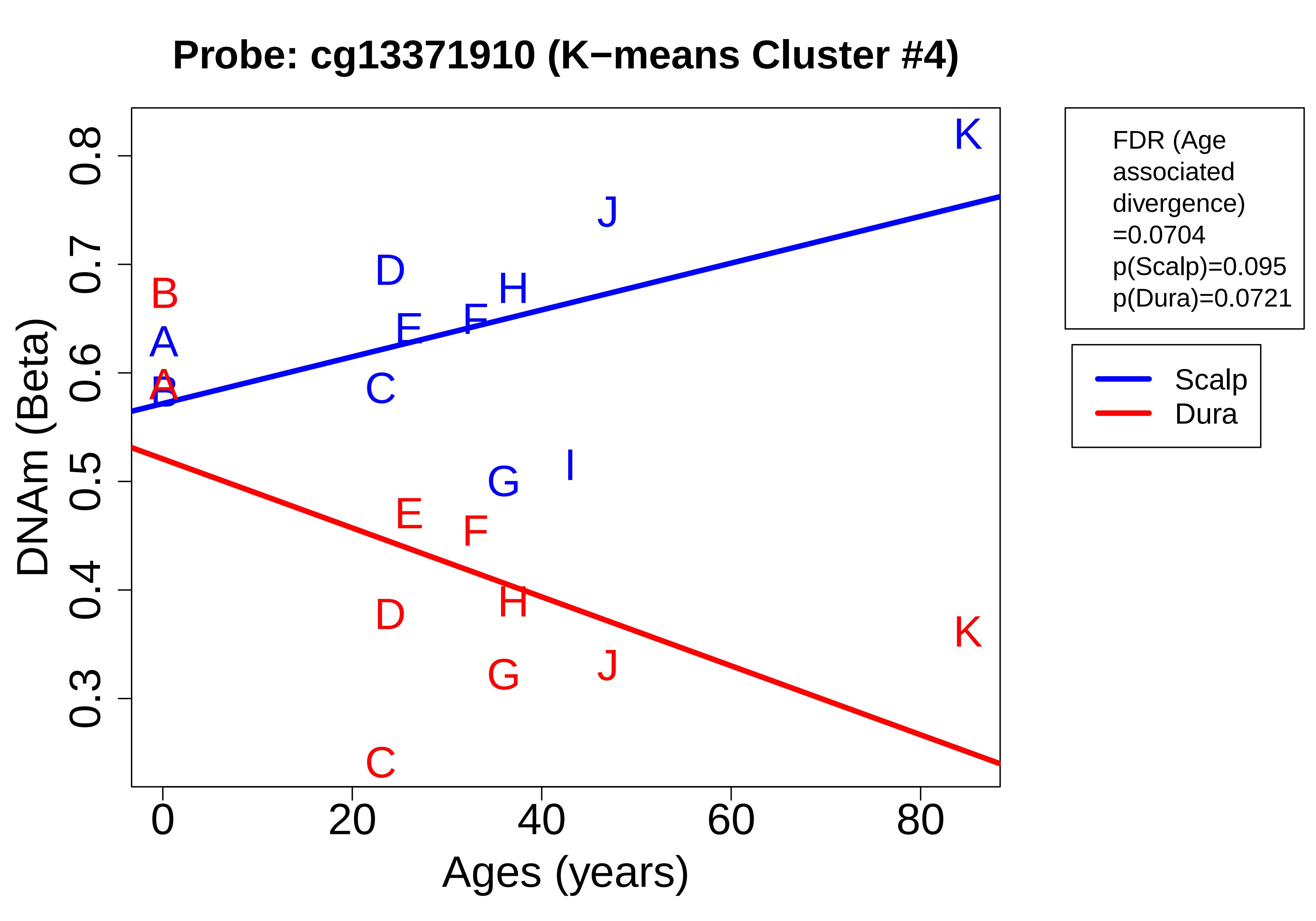
Probe: cg13371910 (K−means Cluster #4)
DNAm (Beta)
Ages (years)
0.3
0.4
0.5
0.6
0.7
0.8
0
20
40
60
80
FDR (Age associated divergence) =0.0704
p(Scalp)=0.095
p(Dura)=0.0721
Scalp
Dura
A
B
C
D
E
F
G
H
I
J
K

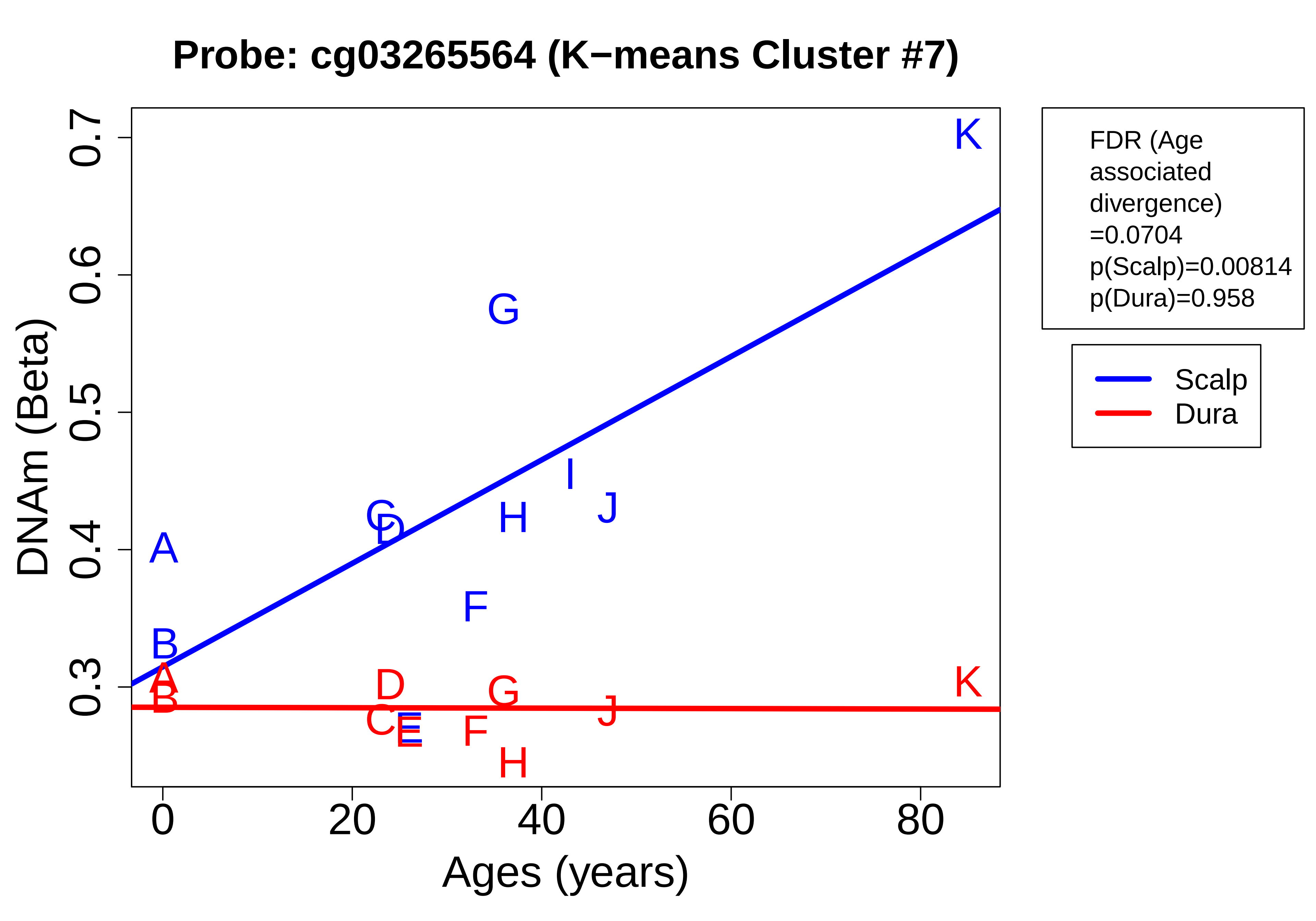
Probe: cg03265564 (K−means Cluster #7)
DNAm (Beta)
0.3
0.4
0.5
0.6
0.7
0
20
40
60
80
Ages (years)
A
B
C
D
E
F
G
H
I
J
K
FDR (Age associated divergence) =0.0704
p(Scalp)=0.00814
p(Dura)=0.958
Scalp
Dura

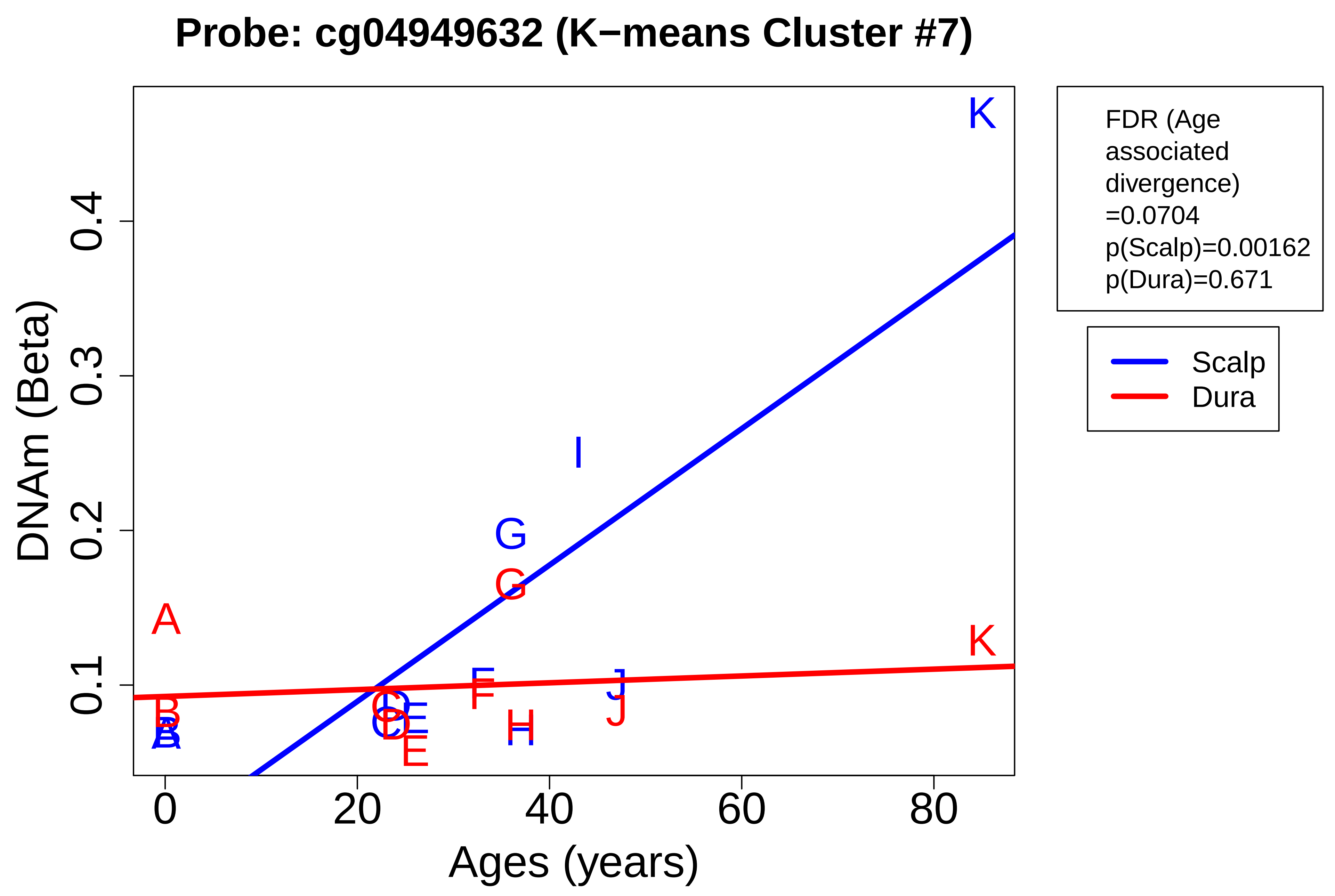

Probe: cg04949632 (K−means Cluster #7)
DNAm (Beta)
0.1
0.2
0.3
0.4
0
20
40
60
80
Ages (years)
FDR (Age associated divergence) =0.0704
p(Scalp)=0.00162
p(Dura)=0.671
Scalp
Dura

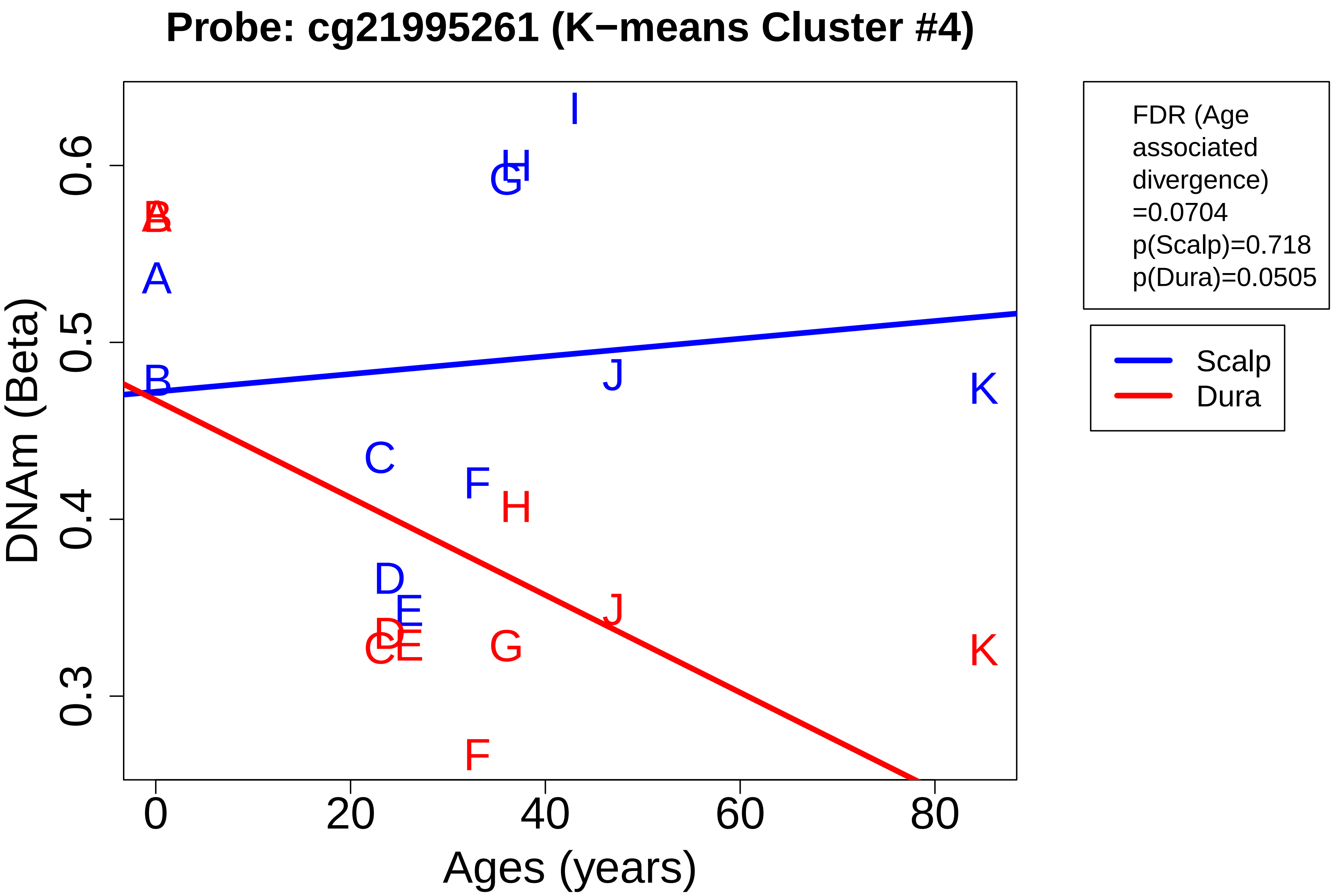

Probe: cg21995261 (K−means Cluster #4)
DNAm (Beta)
0.3
0.4
0.5
0.6
0
20
40
60
80
Ages (years)
FDR (Age associated divergence) =0.0704
p(Scalp)=0.718
p(Dura)=0.0505
Scalp
Dura

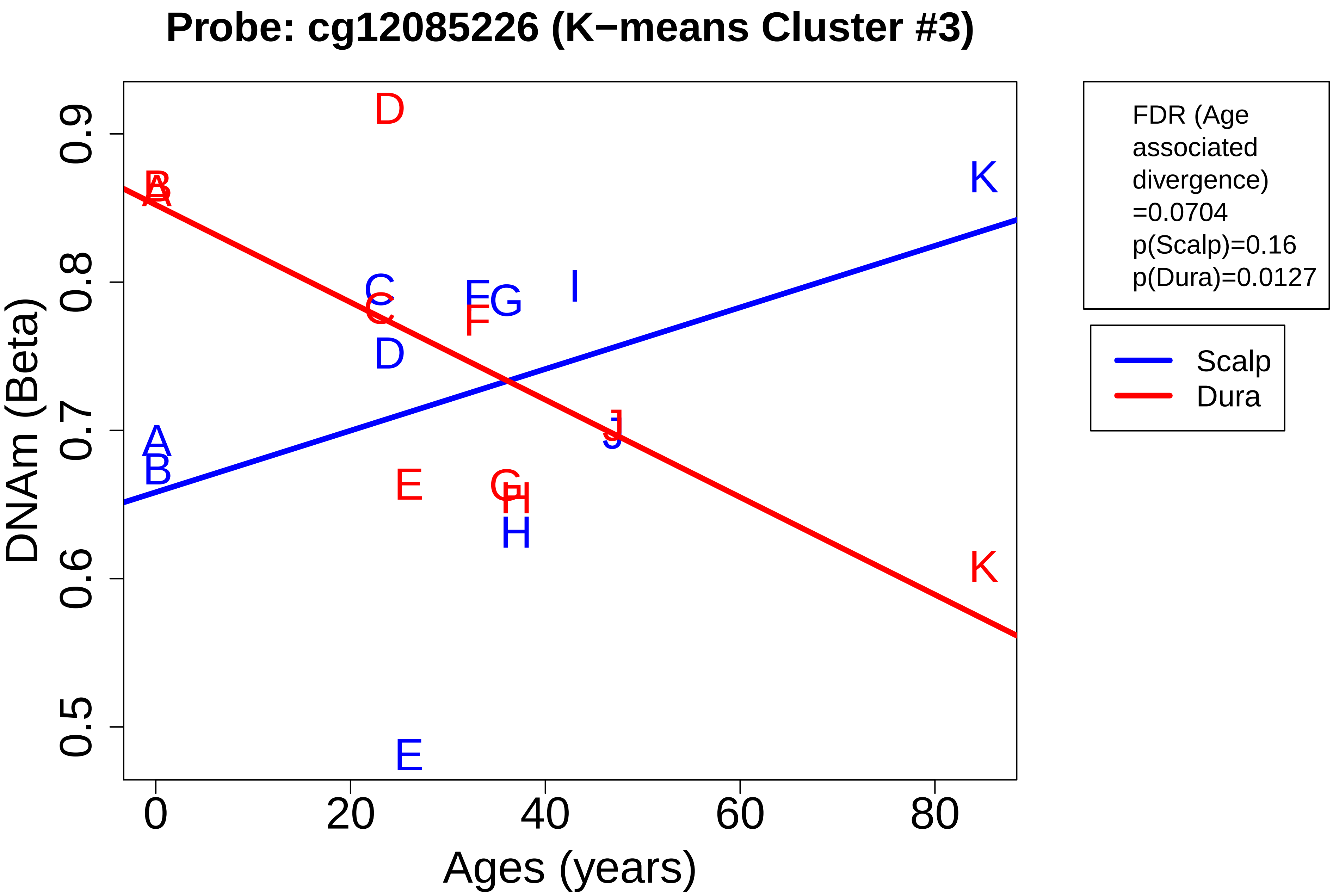

Probe: cg12085226 (K−means Cluster #3)
DNAm (Beta)
Ages (years)
0.5
0.6
0.7
0.8
0.9
0
20
40
60
80
FDR (Age associated divergence) =0.0704
p(Scalp)=0.16
p(Dura)=0.0127
Scalp
Dura

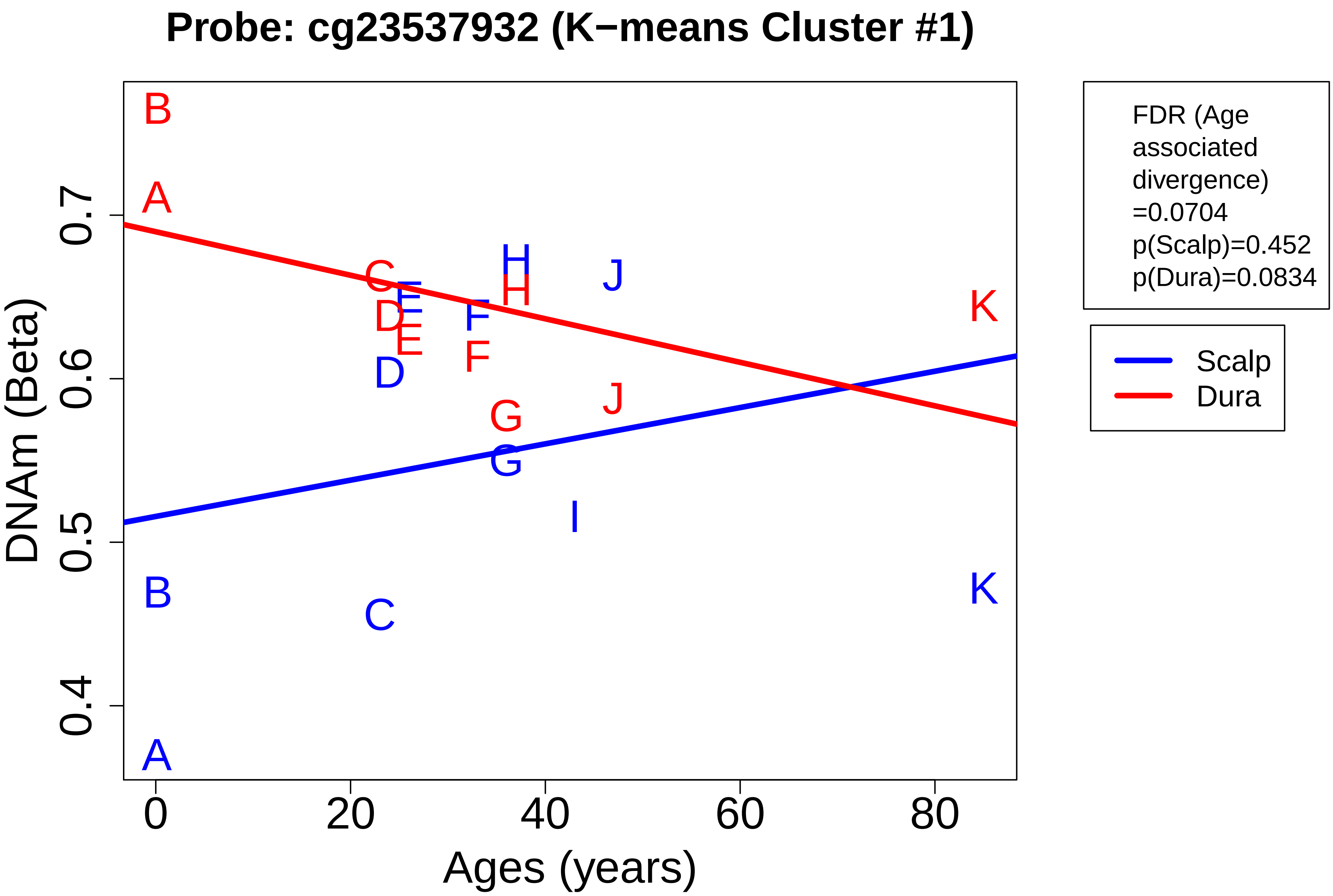

Probe: cg23537932 (K−means Cluster #1)
DNAm (Beta)
0.4
0.5
0.6
0.7
0
20
40
60
80
Ages (years)
FDR (Age associated divergence) =0.0704
p(Scalp)=0.452
p(Dura)=0.0834
Scalp
Dura

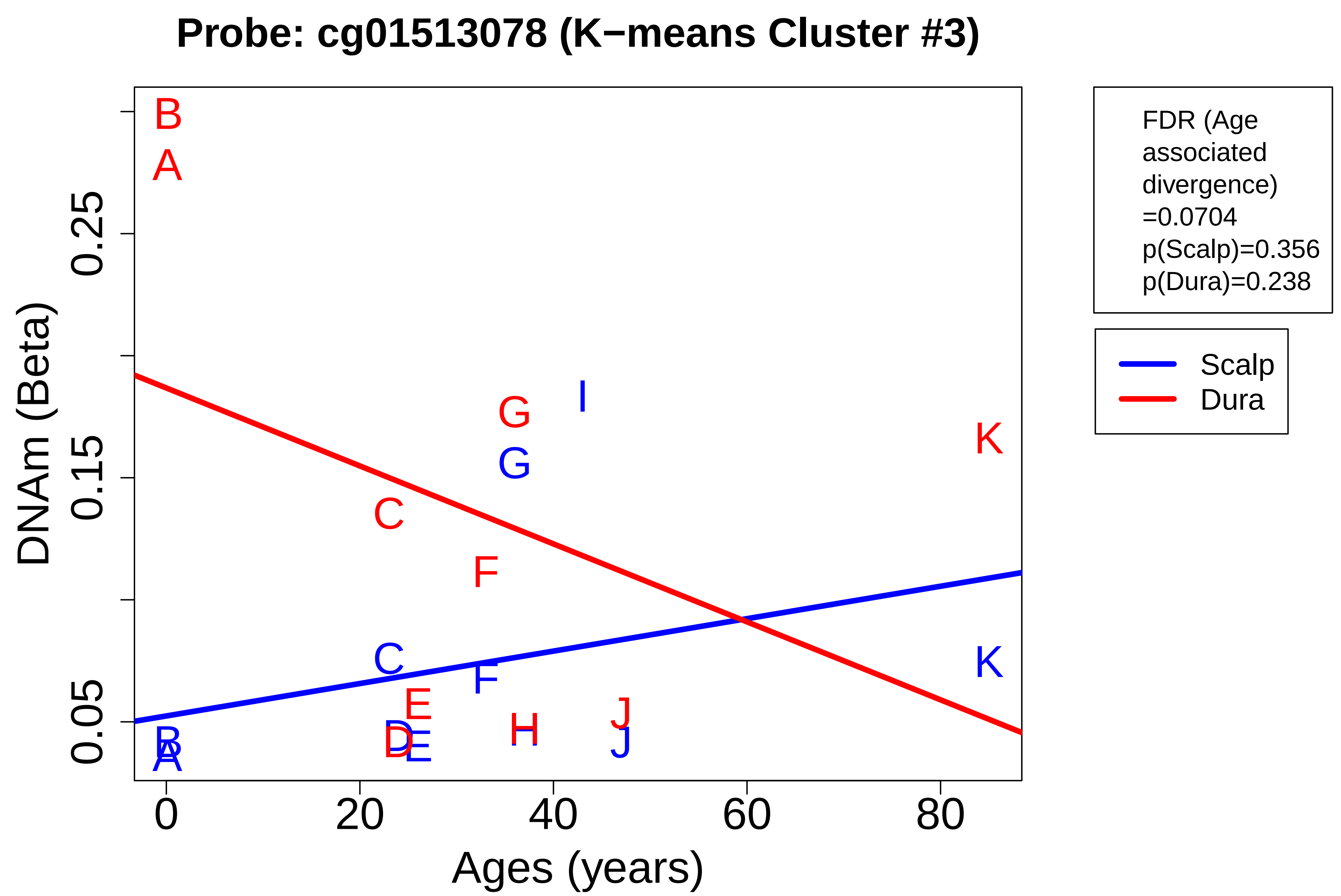

Probe: cg01513078 (K−means Cluster #3)
DNAm (Beta)
0.05
0.15
0.25
0
20
40
60
80
Ages (years)
FDR (Age associated divergence) =0.0704
p(Scalp)=0.356
p(Dura)=0.238
Scalp
Dura

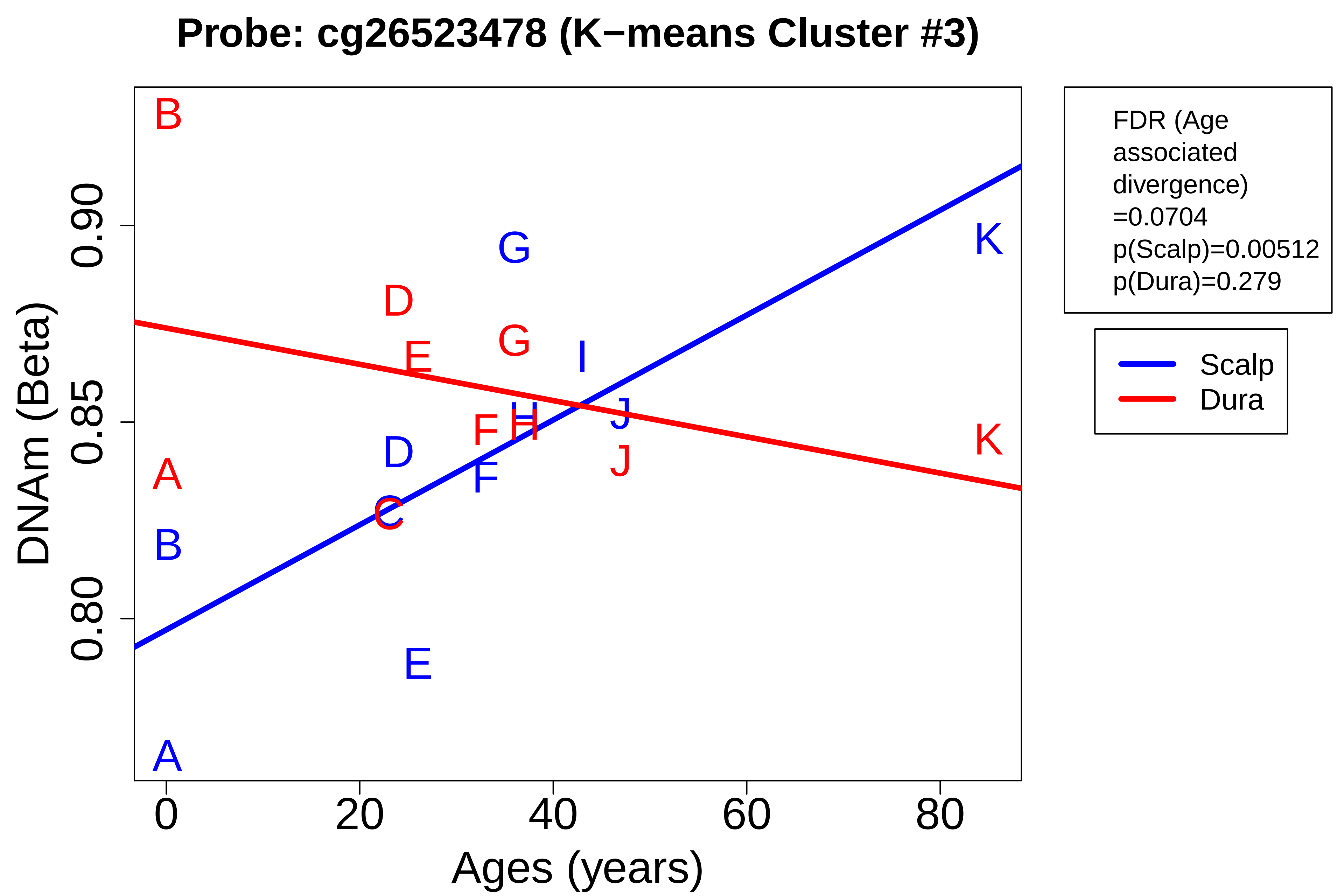

Probe: cg26523478 (K−means Cluster #3)
FDR (Age associated divergence) =0.0704
p(Scalp)=0.00512
p(Dura)=0.279
Scalp
Dura
DNAm (Beta)
0.80
0.85
0.90
0
20
40
60
80
Ages (years)
A
B
C
D
E
F
G
H
I
J
K

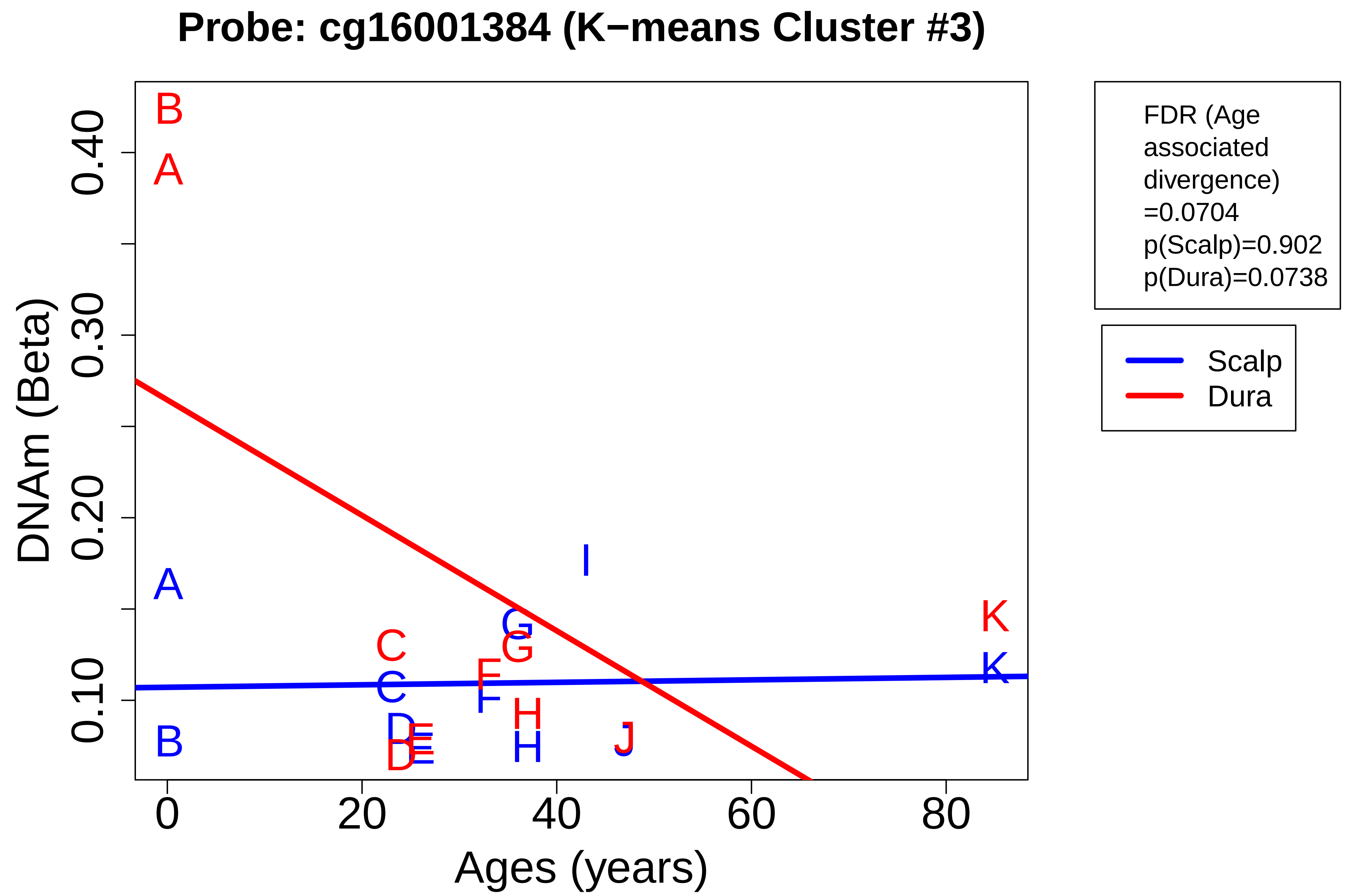

Probe: cg16001384 (K−means Cluster #3)
DNAm (Beta)
0.10
0.20
0.30
0.40
Ages (years)
0
20
40
60
80
FDR (Age associated divergence) =0.0704
p(Scalp)=0.902
p(Dura)=0.0738
Scalp
Dura

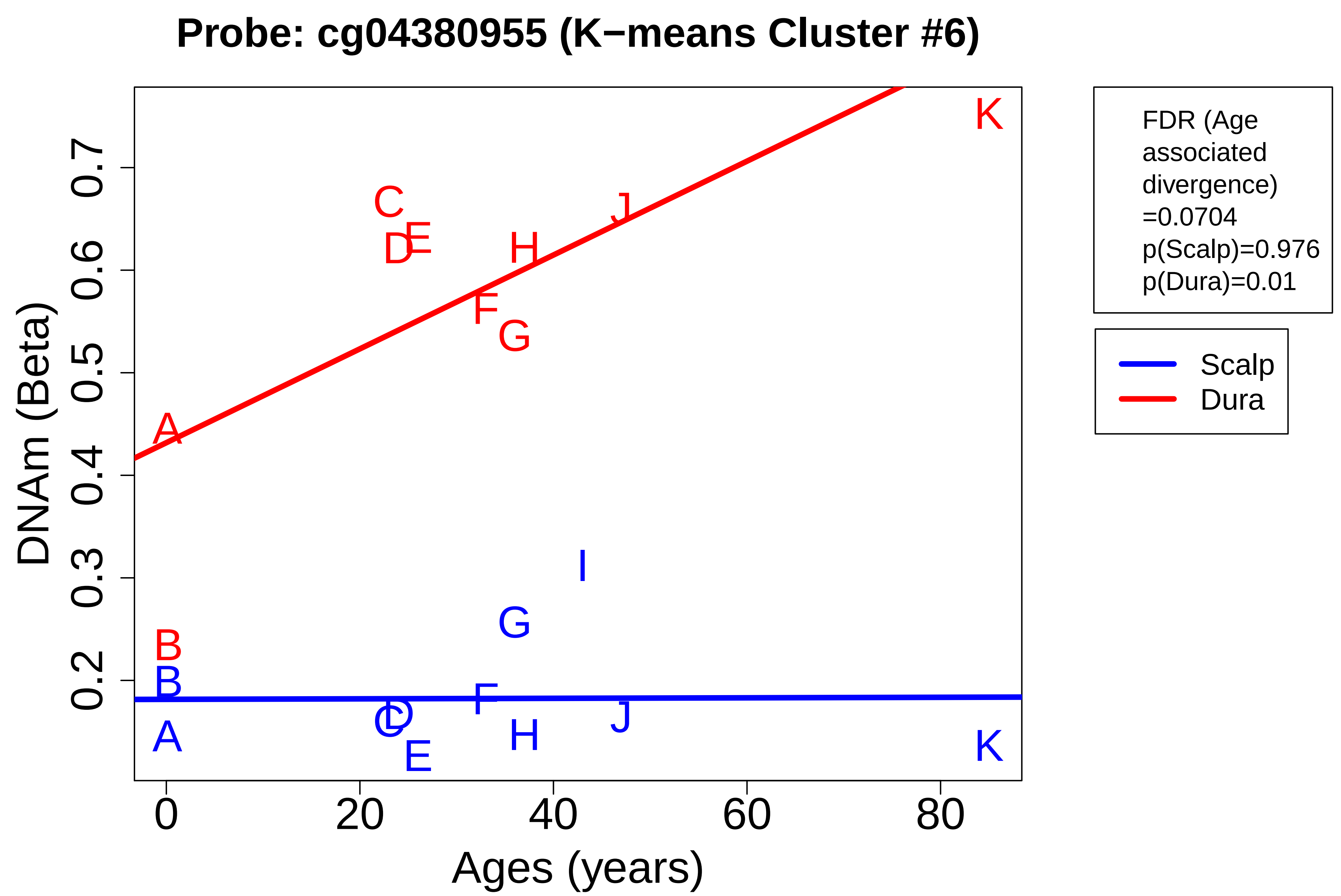

Probe: cg04380955 (K−means Cluster #6)
DNAm (Beta)
Ages (years)
0.2
0.3
0.4
0.5
0.6
0.7
0
20
40
60
80
FDR (Age associated divergence) =0.0704
p(Scalp)=0.976
p(Dura)=0.01
Scalp
Dura

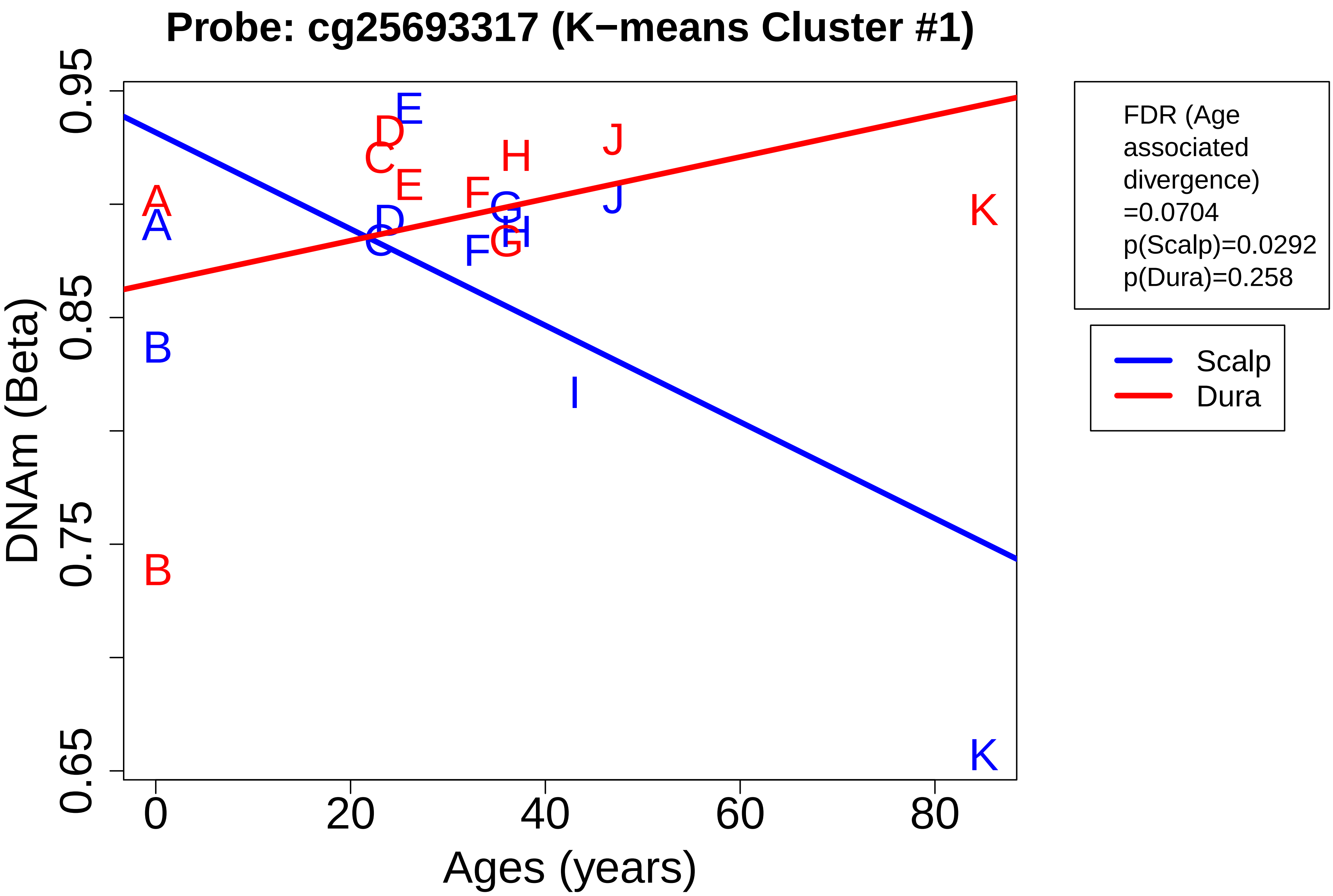

Probe: cg25693317 (K−means Cluster #1)
DNAm (Beta)
0.65
0.75
0.85
0.95
0
20
40
60
80
Ages (years)
A
B
C
D
E
F
G
H
I
J
K
FDR (Age associated divergence) =0.0704
p(Scalp)=0.0292
p(Dura)=0.258
Scalp
Dura

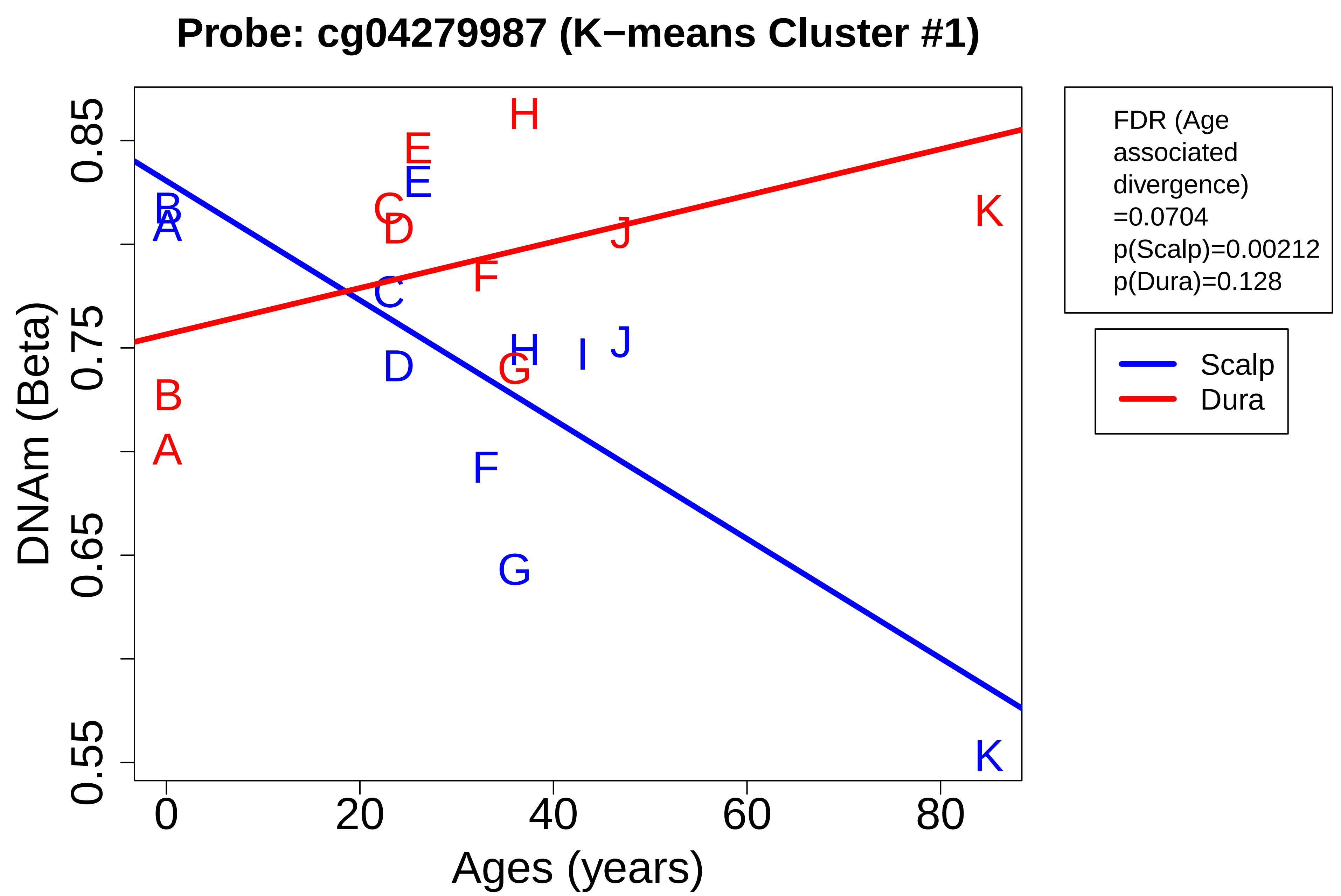
Probe: cg04279987 (K−means Cluster #1)
DNAm (Beta)
0.55
0.65
0.75
0.85
Ages (years)
0
20
40
60
80
FDR (Age associated divergence) =0.0704
p(Scalp)=0.00212
p(Dura)=0.128
Scalp
Dura
A B C D E F G H I J K

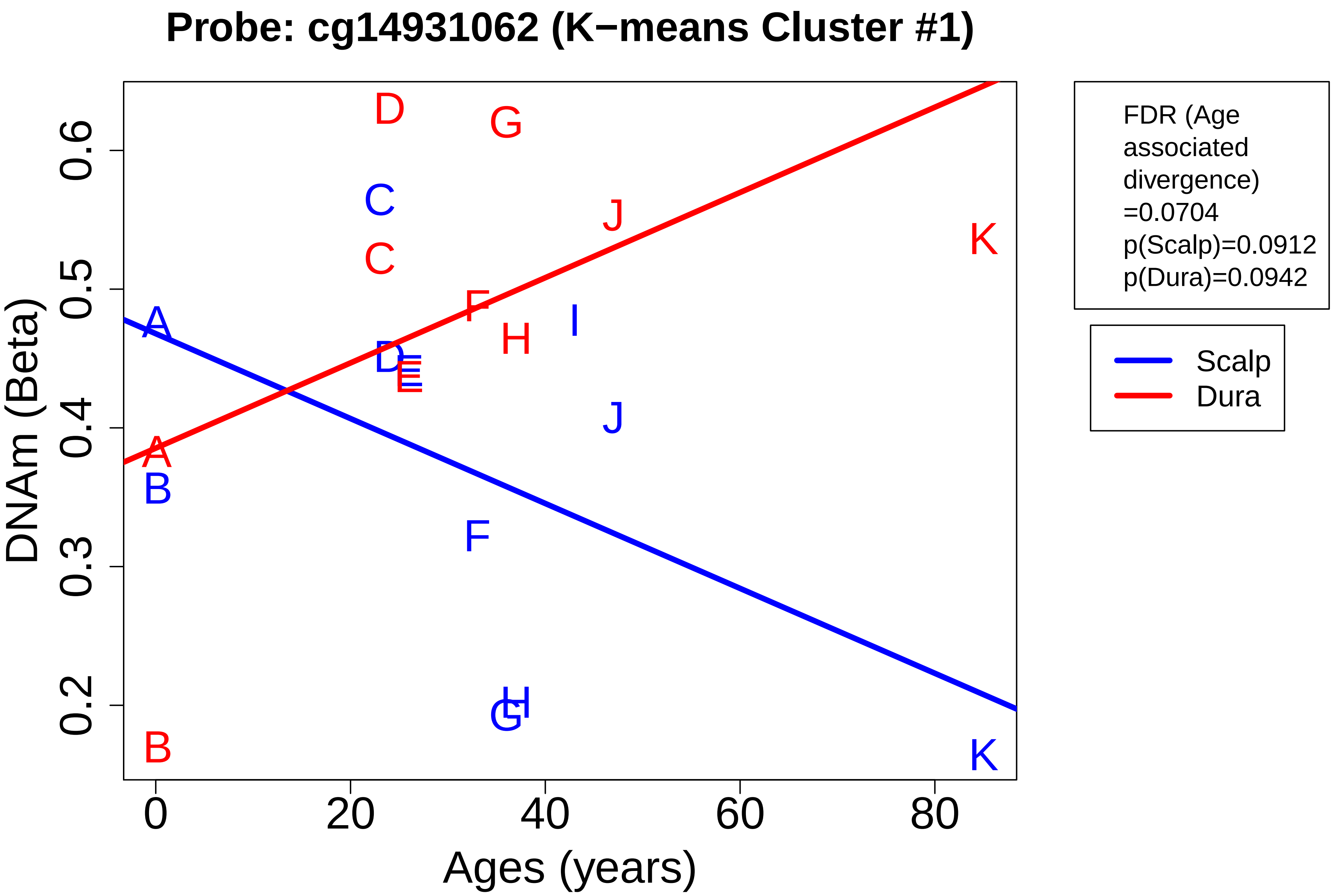
Probe: cg14931062 (K−means Cluster #1)
DNAm (Beta)
0.2
0.3
0.4
0.5
0.6
Ages (years)
0
20
40
60
80
FDR (Age associated divergence) =0.0704
p(Scalp)=0.0912
p(Dura)=0.0942
Scalp
Dura
A
B
C
D
E
F
G
H
I
J
K

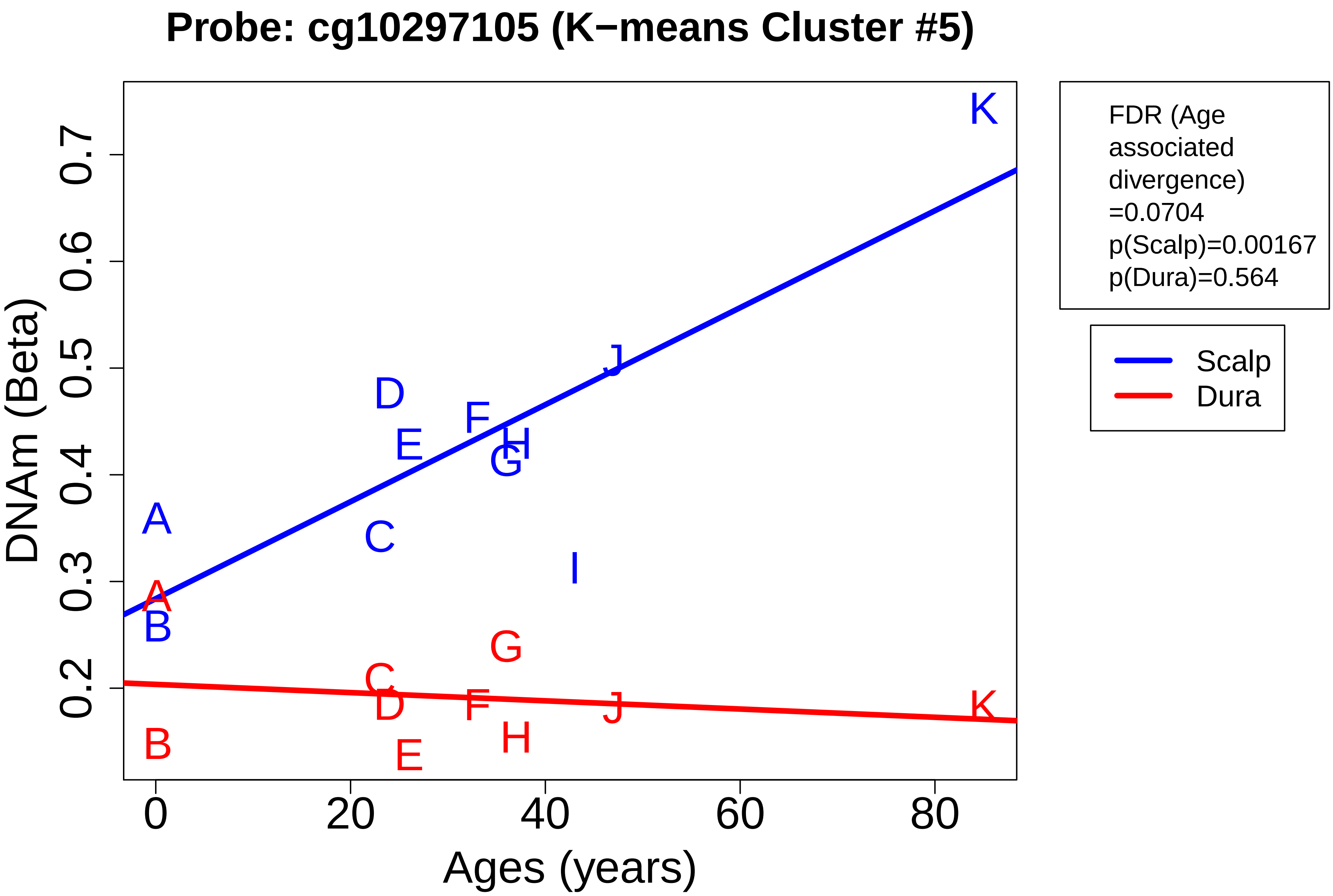
Probe: cg10297105 (K−means Cluster #5)
DNAm (Beta)
0.2
0.3
0.4
0.5
0.6
0.7
Ages (years)
0
20
40
60
80
FDR (Age associated divergence) =0.0704
p(Scalp)=0.00167
p(Dura)=0.564
Scalp
Dura
A
B
C
D
E
F
G
H
I
J
K

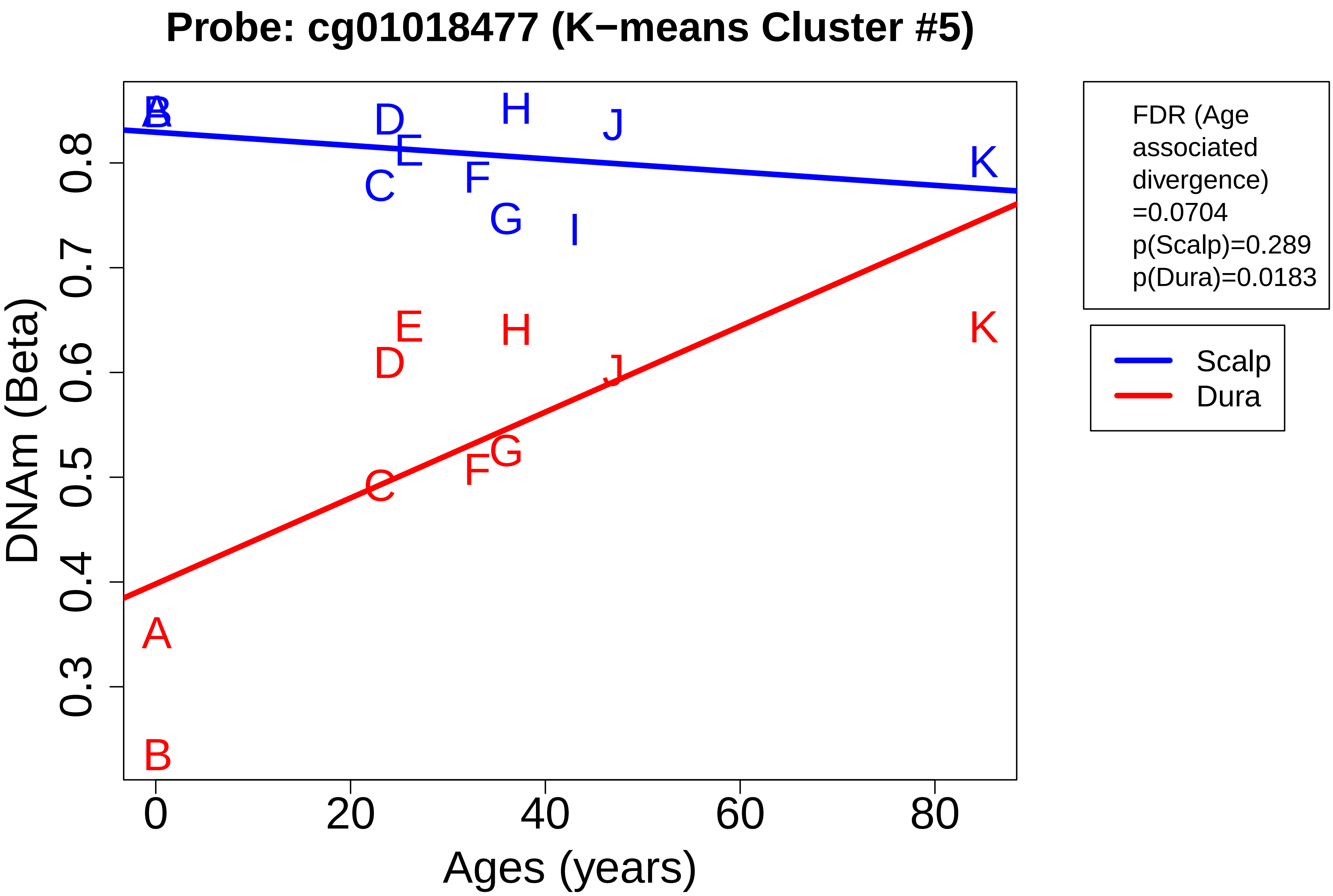

Probe: cg01018477 (K−means Cluster #5)
DNAm (Beta)
0.3
0.4
0.5
0.6
0.7
0.8
0
20
40
60
80
Ages (years)
FDR (Age associated divergence) =0.0704
p(Scalp)=0.289
p(Dura)=0.0183
Scalp
Dura

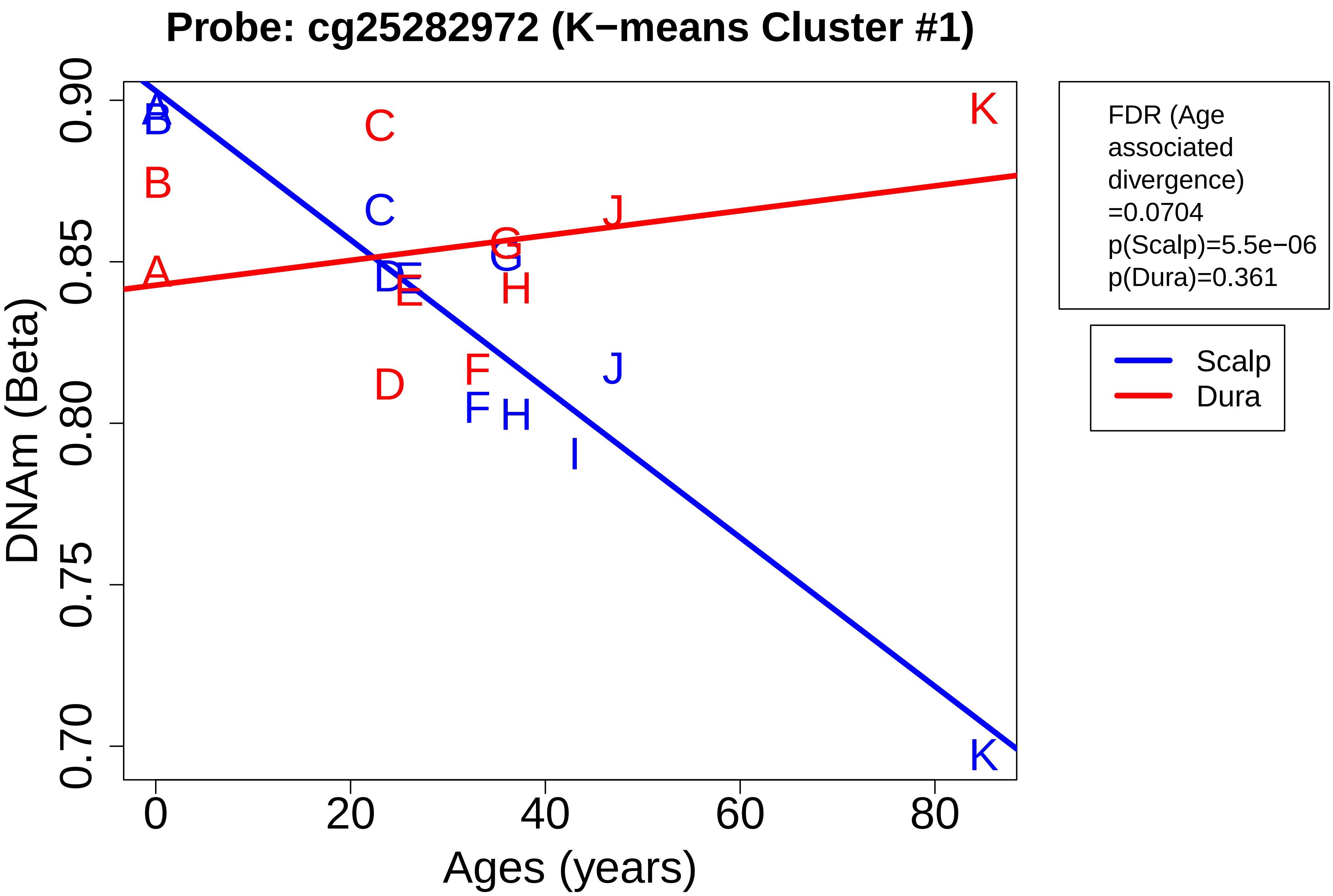

Probe: cg25282972 (K−means Cluster #1)
DNAm (Beta)
0.70
0.75
0.80
0.85
0.90
0
20
40
60
80
Ages (years)
FDR (Age associated divergence) =0.0704
p(Scalp)=5.5e−06
p(Dura)=0.361
Scalp
Dura

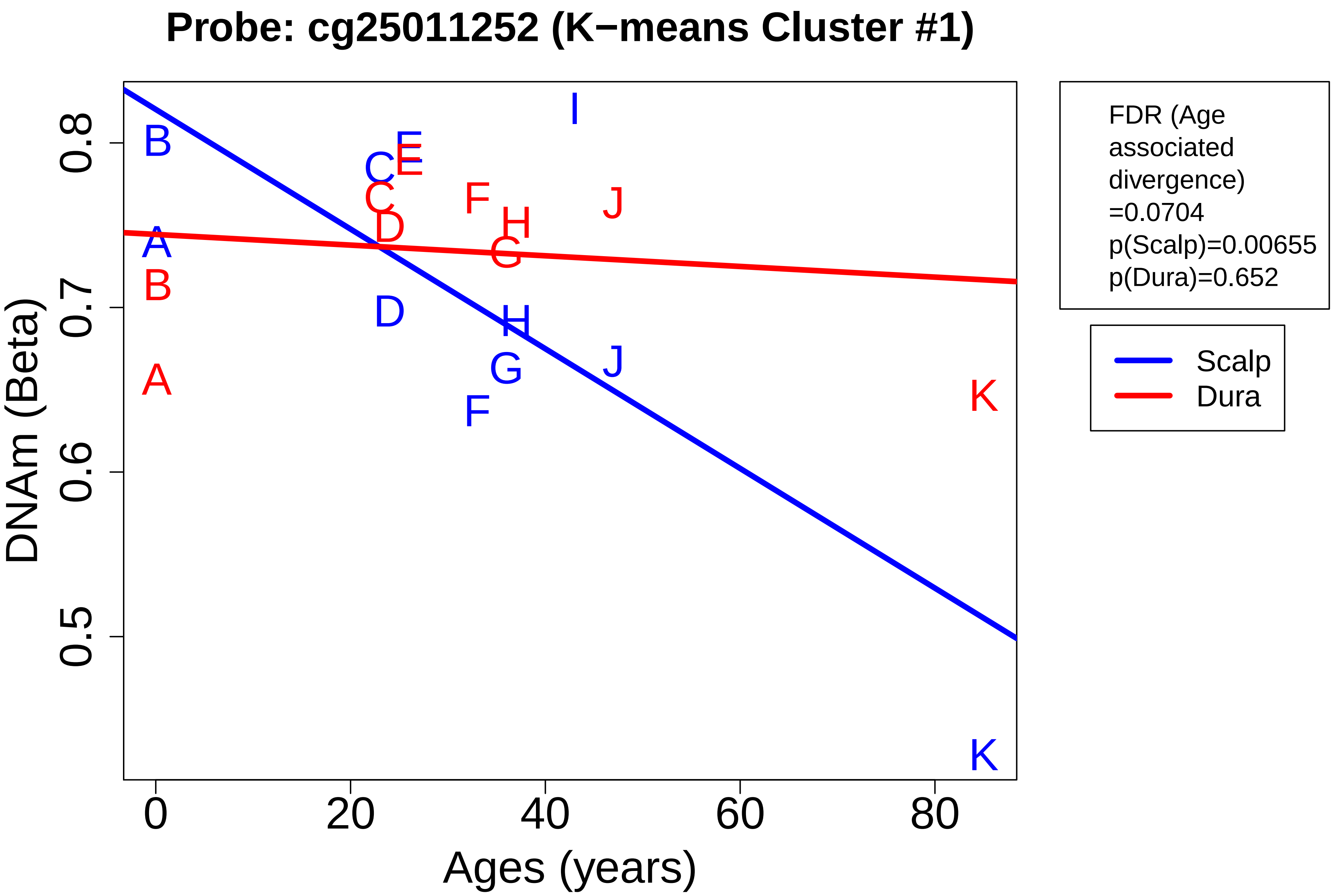

Probe: cg25011252 (K−means Cluster #1)
DNAm (Beta)
0.5
0.6
0.7
0.8
Ages (years)
0
20
40
60
80
A B C D E F G H I J K
A B C D E F G H J K
FDR (Age associated divergence) =0.0704
p(Scalp)=0.00655
p(Dura)=0.652
Scalp
Dura

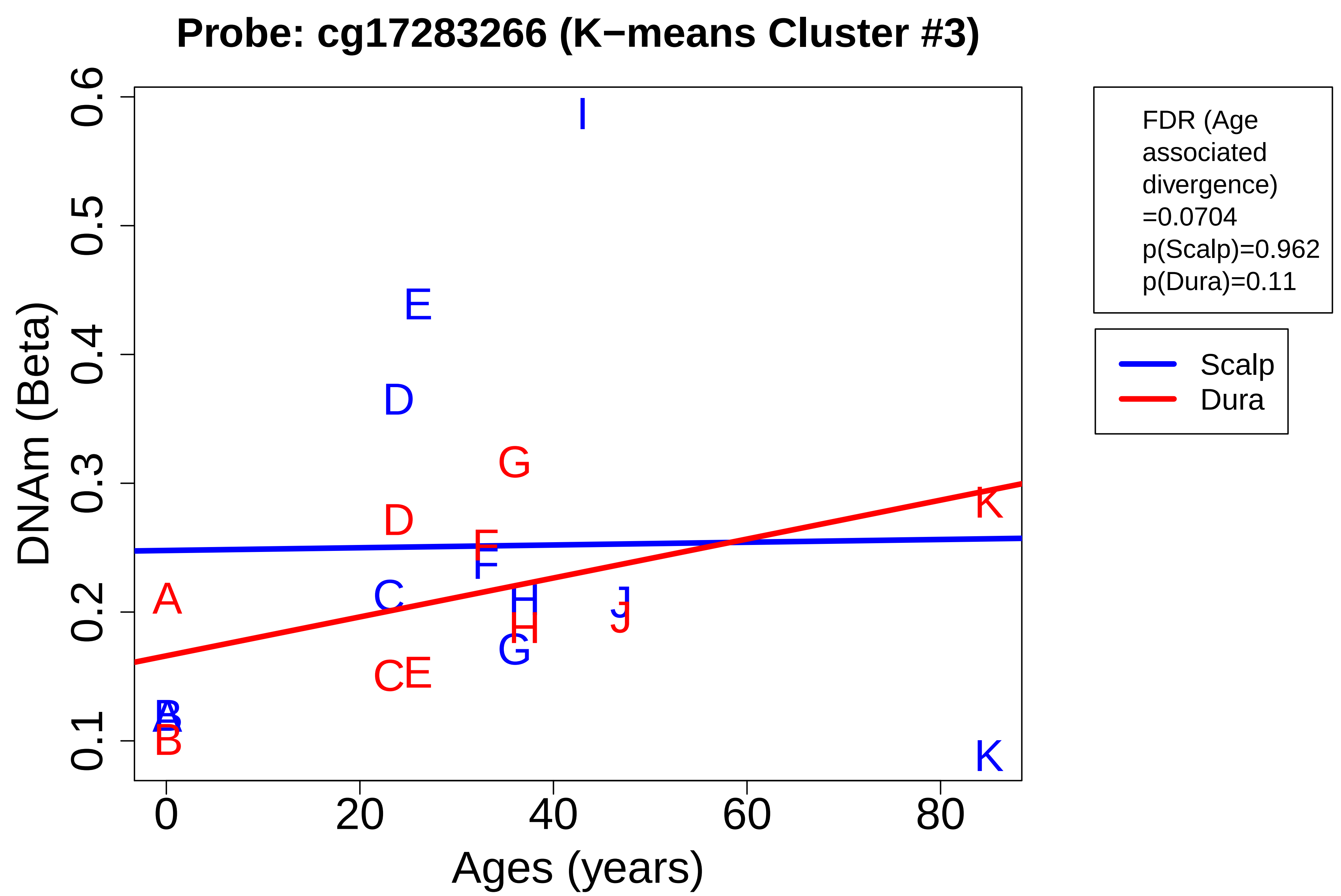

Probe: cg17283266 (K−means Cluster #3)
DNAm (Beta)
Ages (years)
0.1
0.2
0.3
0.4
0.5
0.6
0
20
40
60
80
FDR (Age associated divergence) =0.0704
p(Scalp)=0.962
p(Dura)=0.11
Scalp
Dura

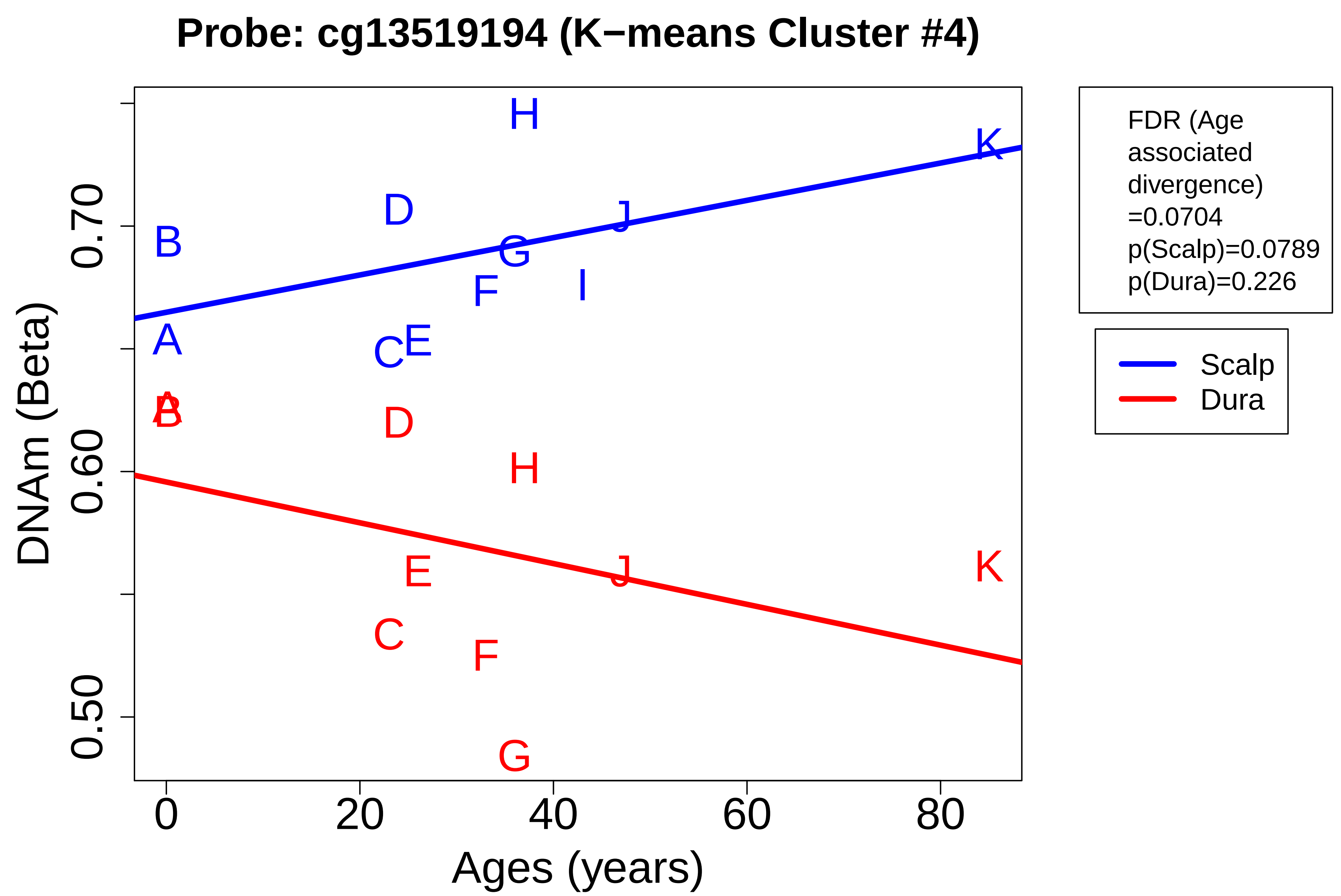

Probe: cg13519194 (K−means Cluster #4)
FDR (Age associated divergence) =0.0704
p(Scalp)=0.0789
p(Dura)=0.226
Scalp
Dura
DNAm (Beta)
0.50
0.60
0.70
0
20
40
60
80
Ages (years)

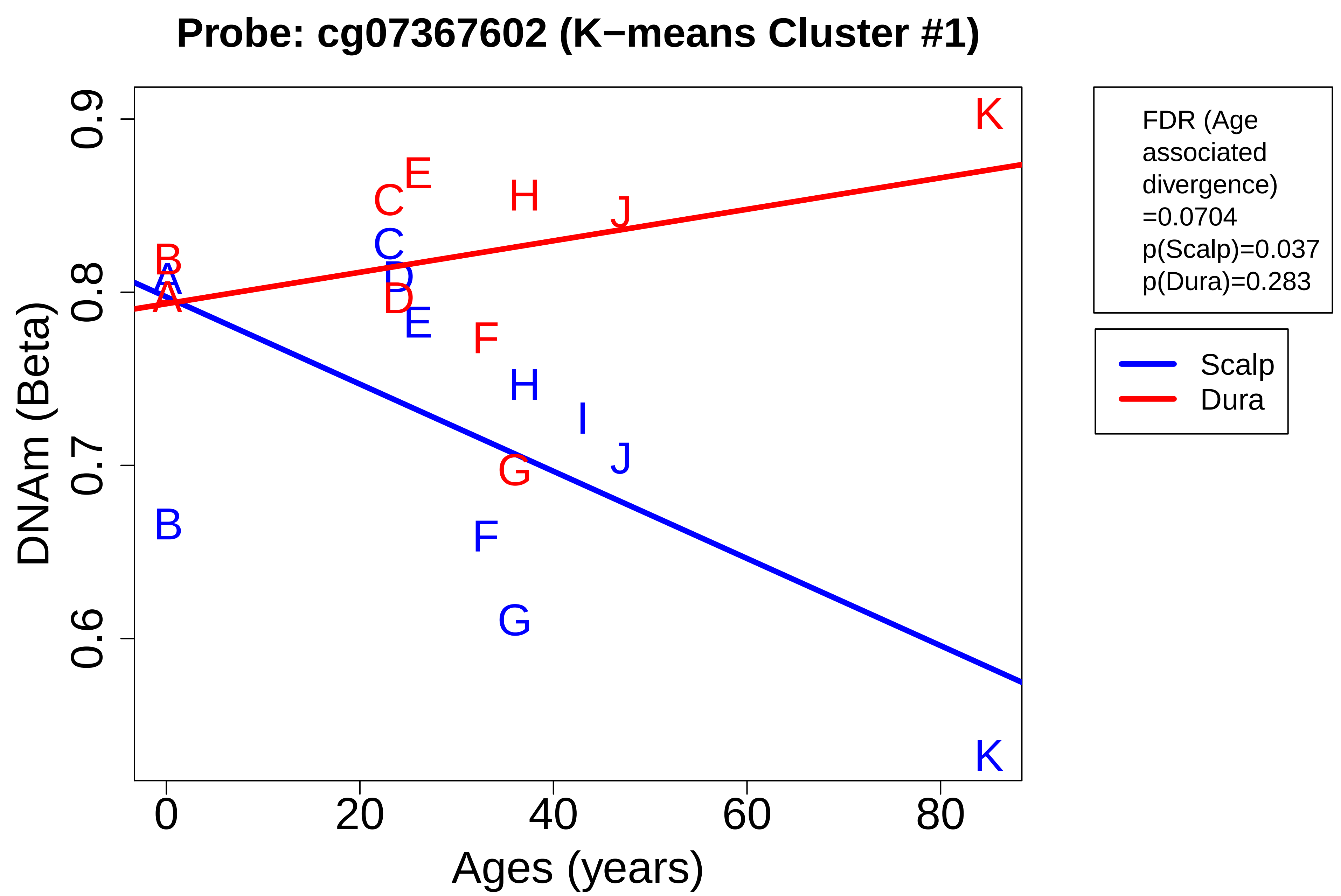

Probe: cg07367602 (K−means Cluster #1)
DNAm (Beta)
0.6
0.7
0.8
0.9
0
20
40
60
80
Ages (years)
A
B
C
D
E
F
G
H
I
J
K
FDR (Age associated divergence) =0.0704
p(Scalp)=0.037
p(Dura)=0.283
Scalp
Dura

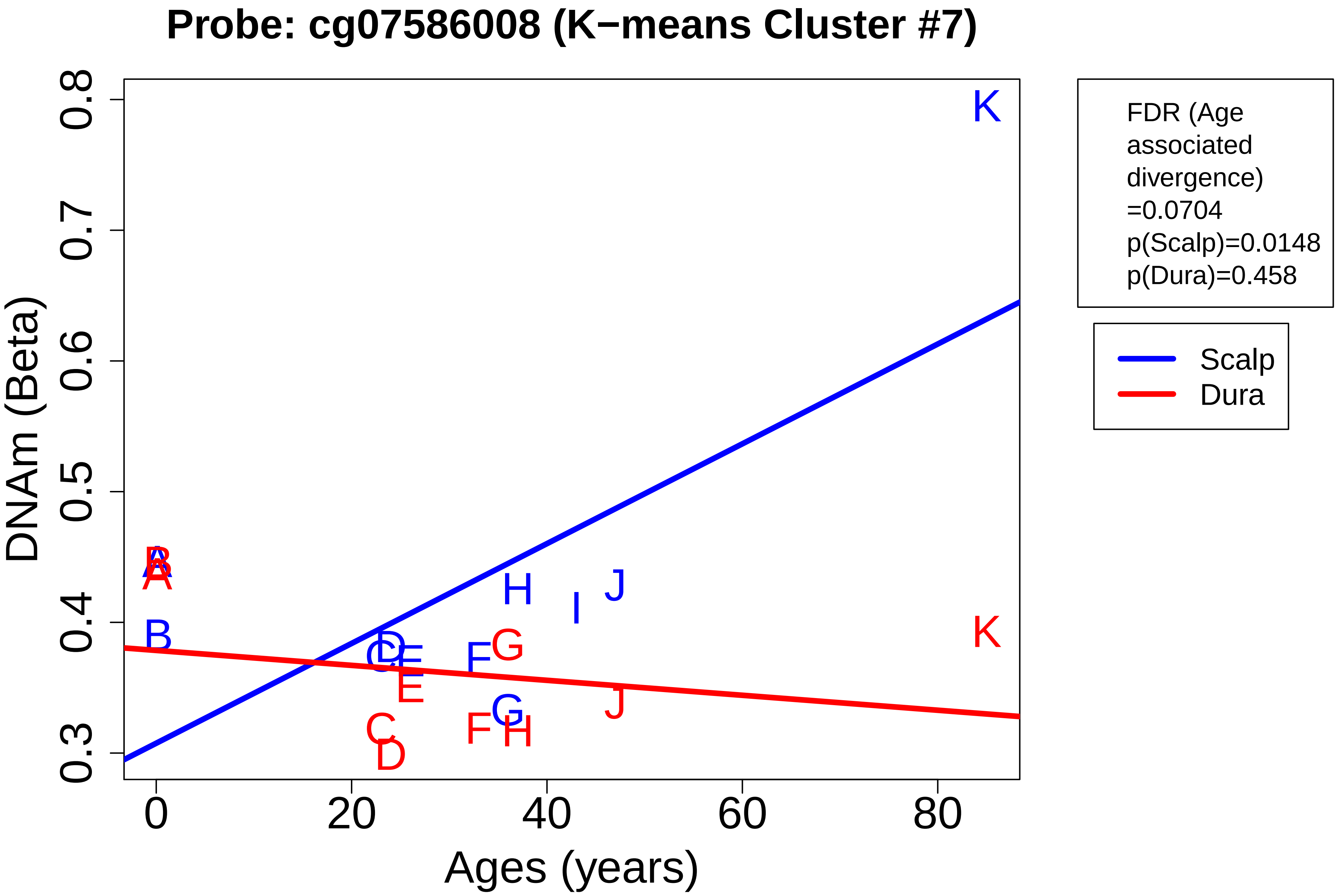
Probe: cg07586008 (K−means Cluster #7)
DNAm (Beta)
Ages (years)
0.3
0.4
0.5
0.6
0.7
0.8
0
20
40
60
80
FDR (Age associated divergence) =0.0704
p(Scalp)=0.0148
p(Dura)=0.458
Scalp
Dura
A
B
C
D
E
F
G
H
I
J
K

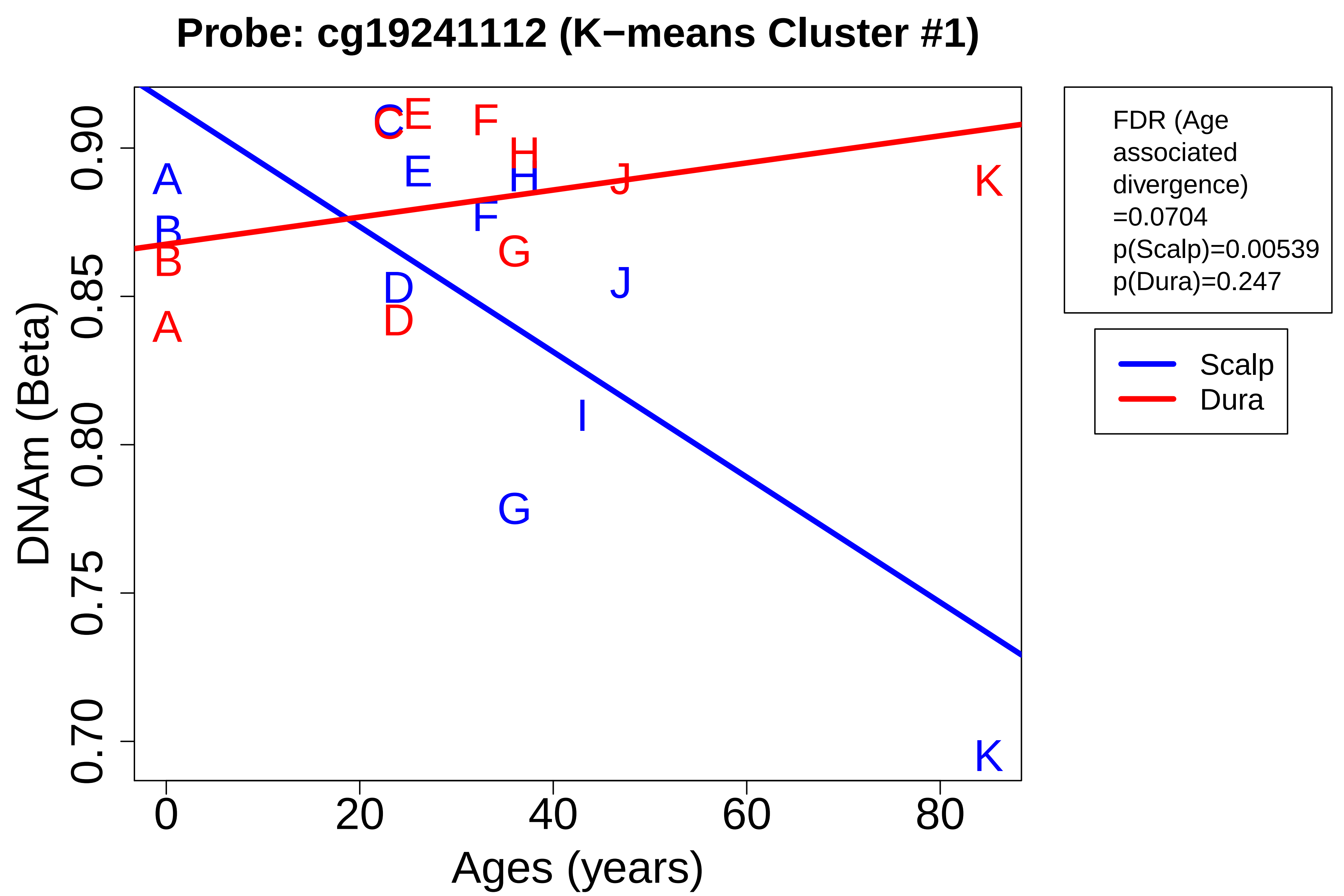

Probe: cg19241112 (K−means Cluster #1)
DNAm (Beta)
0.70
0.75
0.80
0.85
0.90
0
20
40
60
80
Ages (years)
A
B
C
D
E
F
G
H
I
J
K
FDR (Age associated divergence) =0.0704
p(Scalp)=0.00539
p(Dura)=0.247
Scalp
Dura

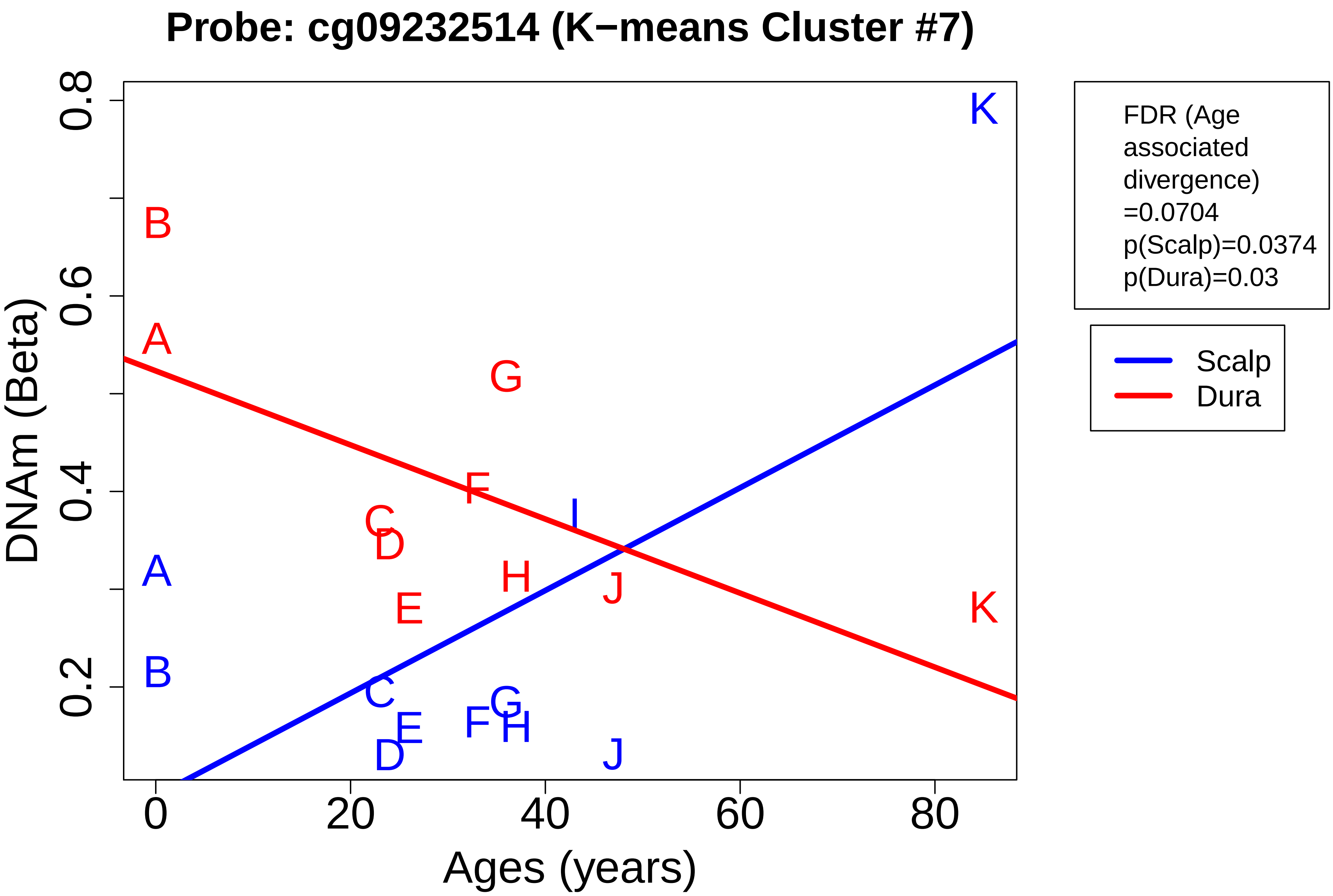

Probe: cg09232514 (K−means Cluster #7)
DNAm (Beta)
0.2
0.4
0.6
0.8
Ages (years)
0
20
40
60
80
FDR (Age associated divergence) =0.0704
p(Scalp)=0.0374
p(Dura)=0.03
Scalp
Dura
A
B
C
D
E
F
G
H
I
J
K

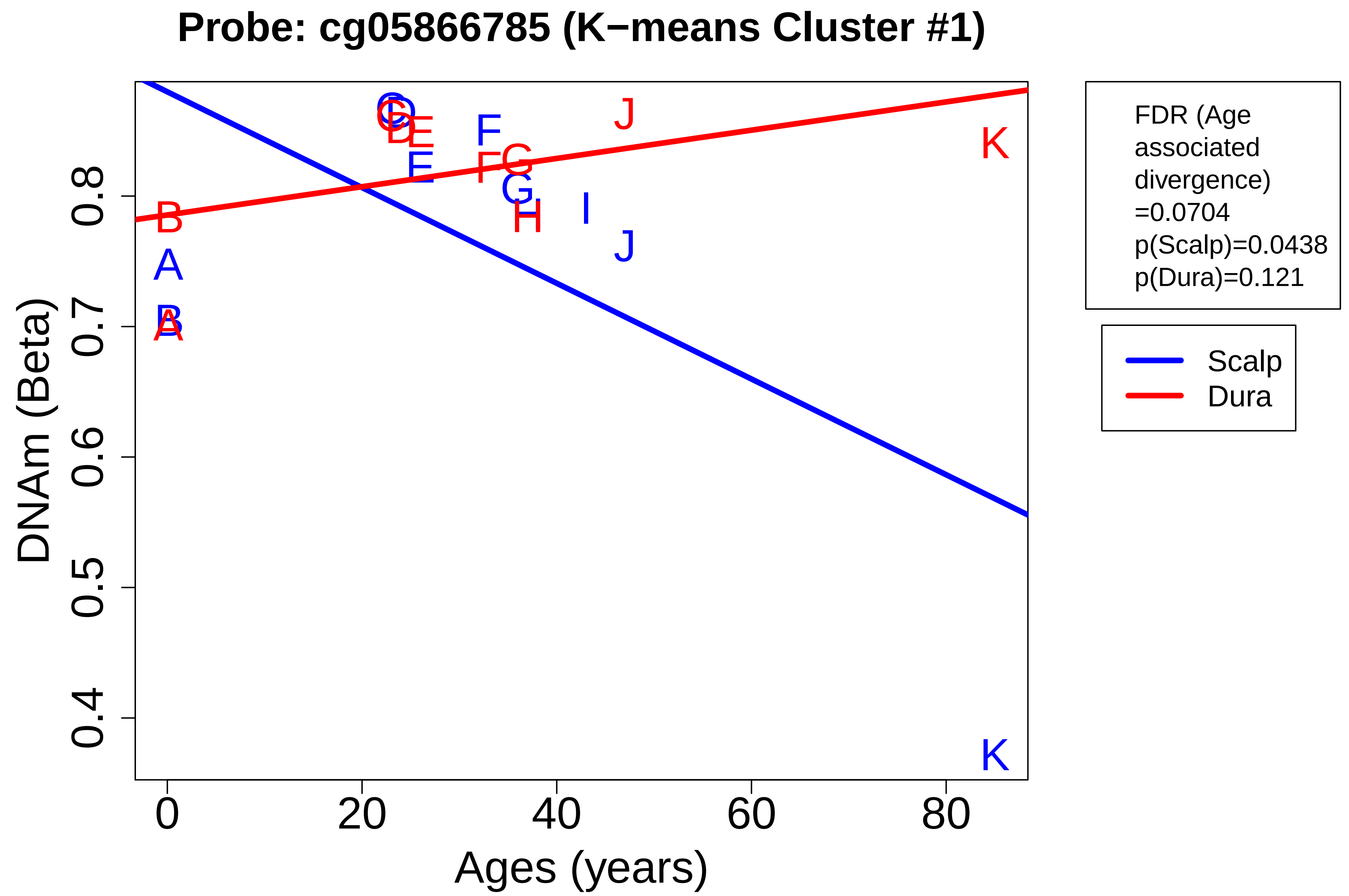

Probe: cg05866785 (K−means Cluster #1)
FDR (Age associated divergence) =0.0704
p(Scalp)=0.0438
p(Dura)=0.121
Scalp
Dura
DNAm (Beta)
0.4
0.5
0.6
0.7
0.8
0
20
40
60
80
Ages (years)
A
B
C
D
E
F
G
H
I
J
K

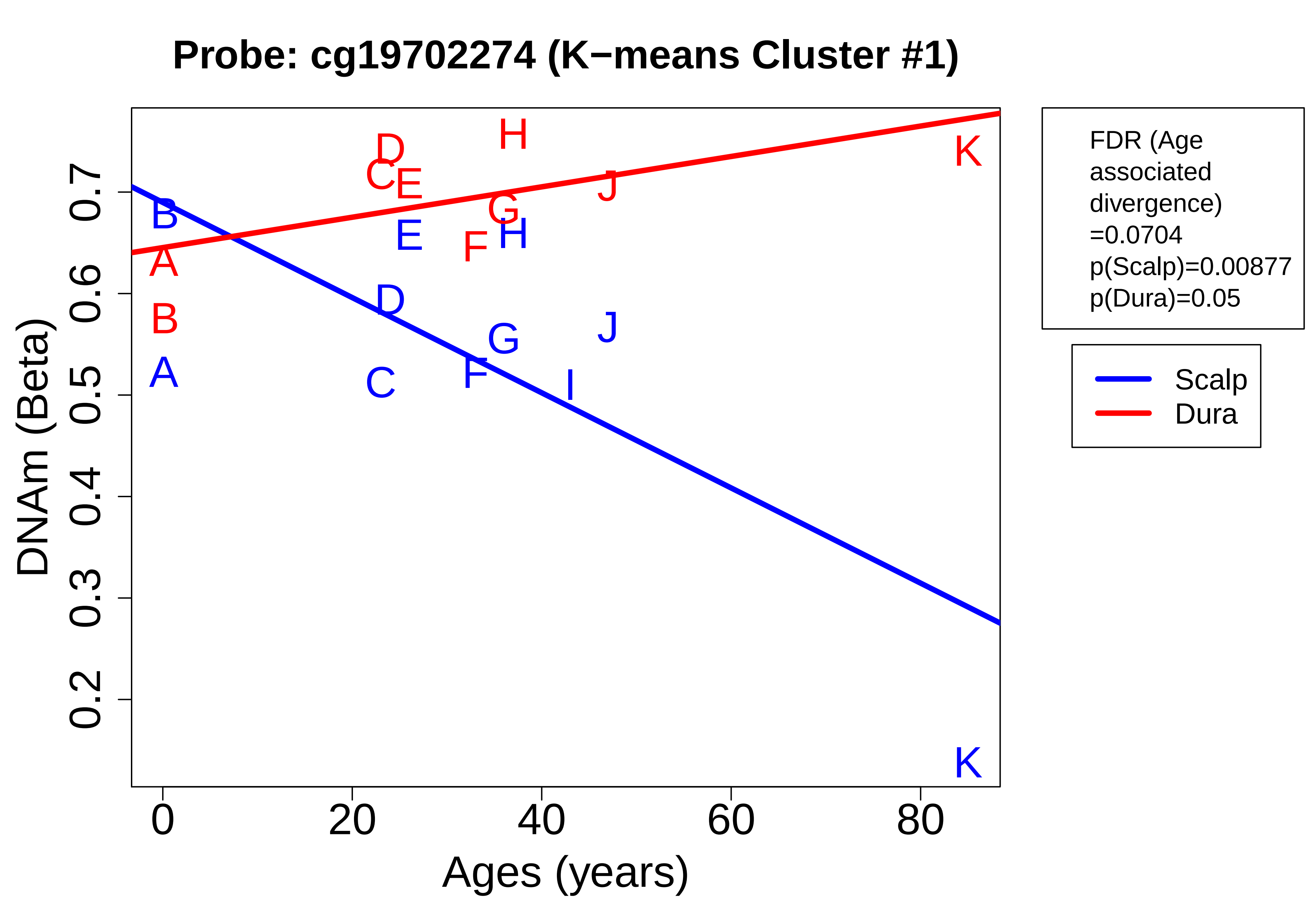

Probe: cg19702274 (K−means Cluster #1)
DNAm (Beta)
Ages (years)
0.2
0.3
0.4
0.5
0.6
0.7
0
20
40
60
80
FDR (Age associated divergence) =0.0704
p(Scalp)=0.00877
p(Dura)=0.05
Scalp
Dura
A
B
C
D
E
F
G
H
I
J
K

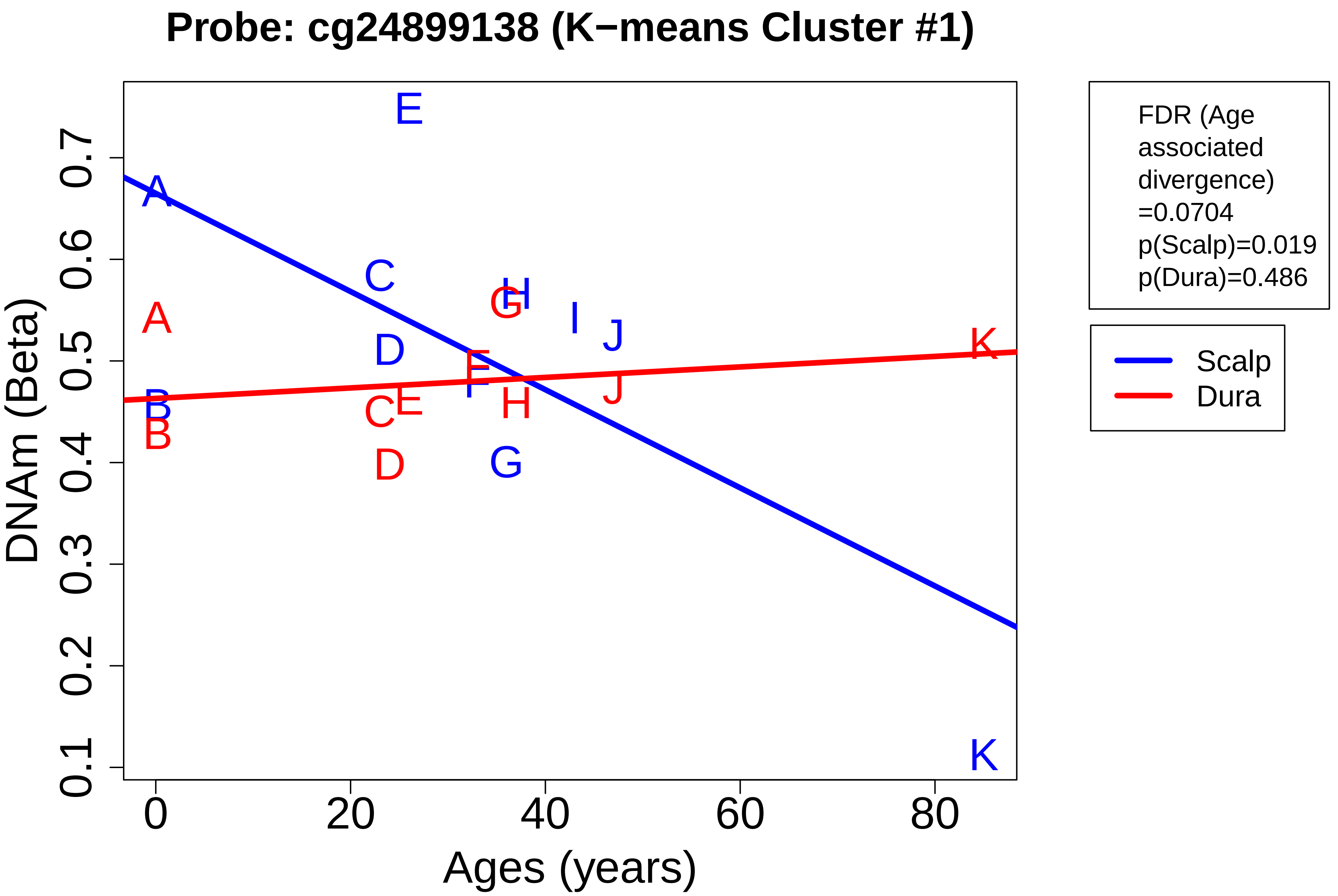

Probe: cg24899138 (K−means Cluster #1)
DNAm (Beta)
0.1
0.2
0.3
0.4
0.5
0.6
0.7
0
20
40
60
80
Ages (years)
A
B
C
D
E
F
G
H
I
J
K
FDR (Age associated divergence) =0.0704
p(Scalp)=0.019
p(Dura)=0.486
Scalp
Dura

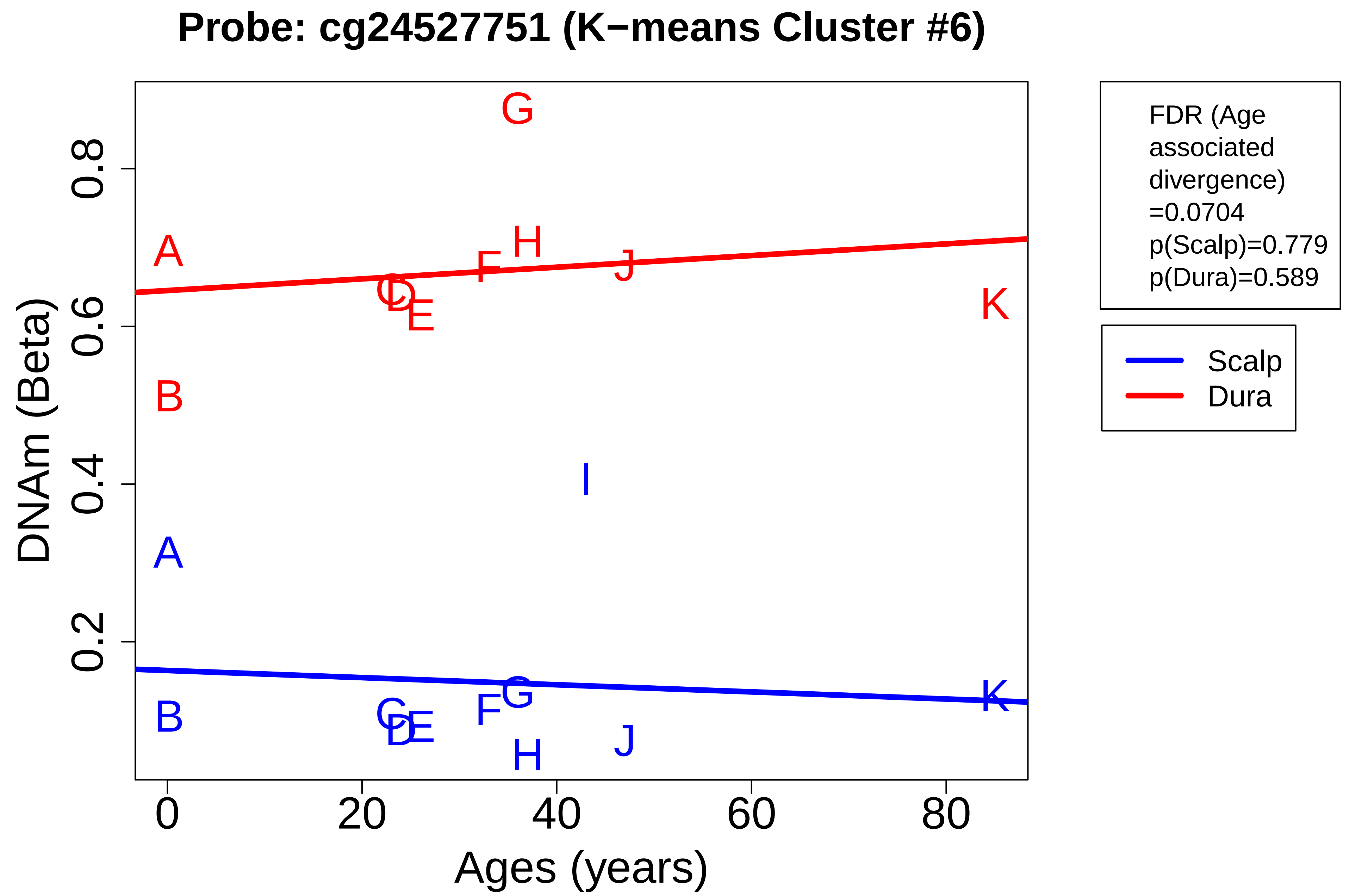

Probe: cg24527751 (K−means Cluster #6)
DNAm (Beta)
0.2
0.4
0.6
0.8
Ages (years)
0
20
40
60
80
A
B
C
D
E
F
G
H
I
J
K
FDR (Age associated divergence) =0.0704
p(Scalp)=0.779
p(Dura)=0.589
Scalp
Dura

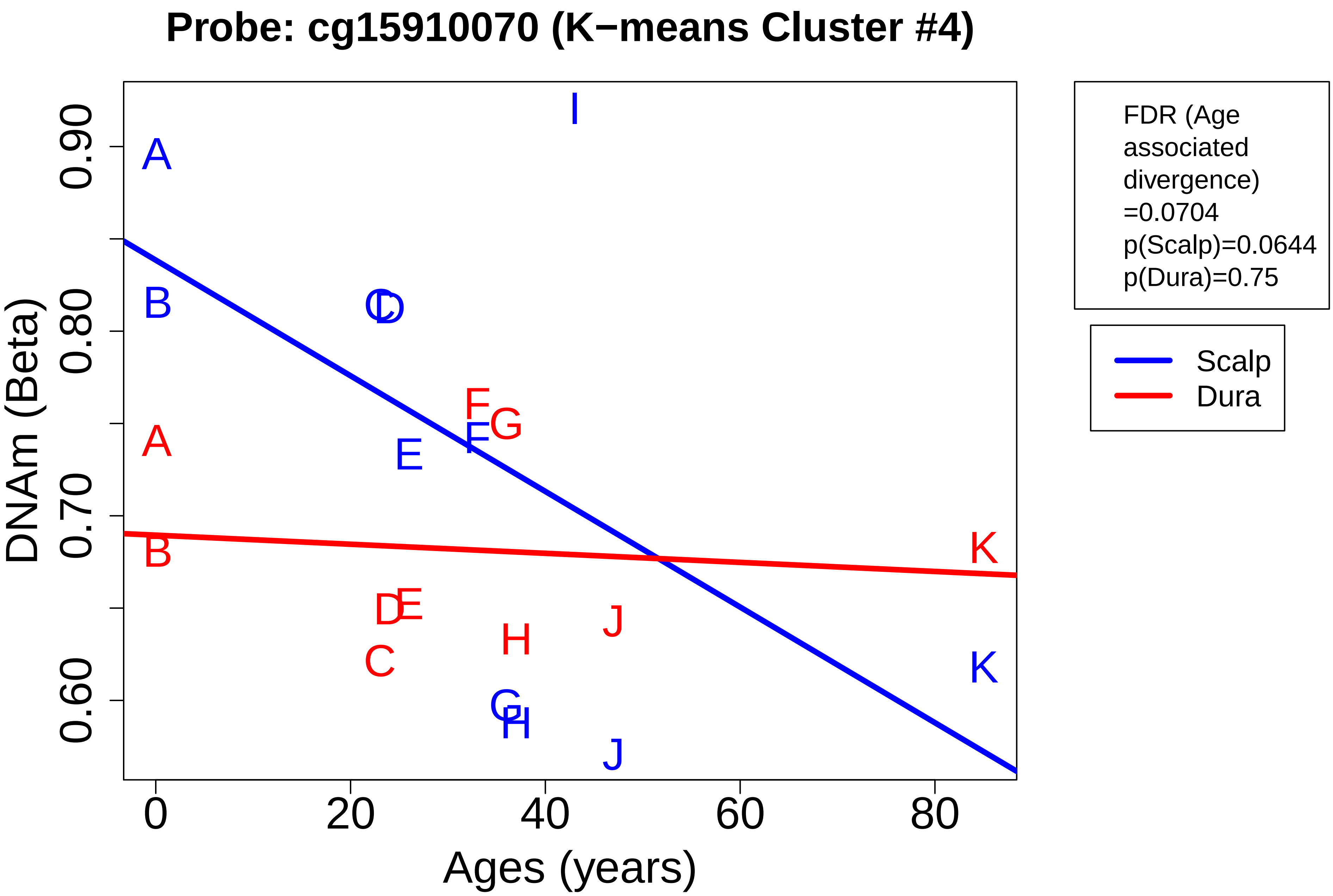

Probe: cg15910070 (K−means Cluster #4)
DNAm (Beta)
0.60
0.70
0.80
0.90
Ages (years)
0
20
40
60
80
A
B
C
D
E
F
G
H
I
J
K
FDR (Age associated divergence) =0.0704
p(Scalp)=0.0644
p(Dura)=0.75
Scalp
Dura

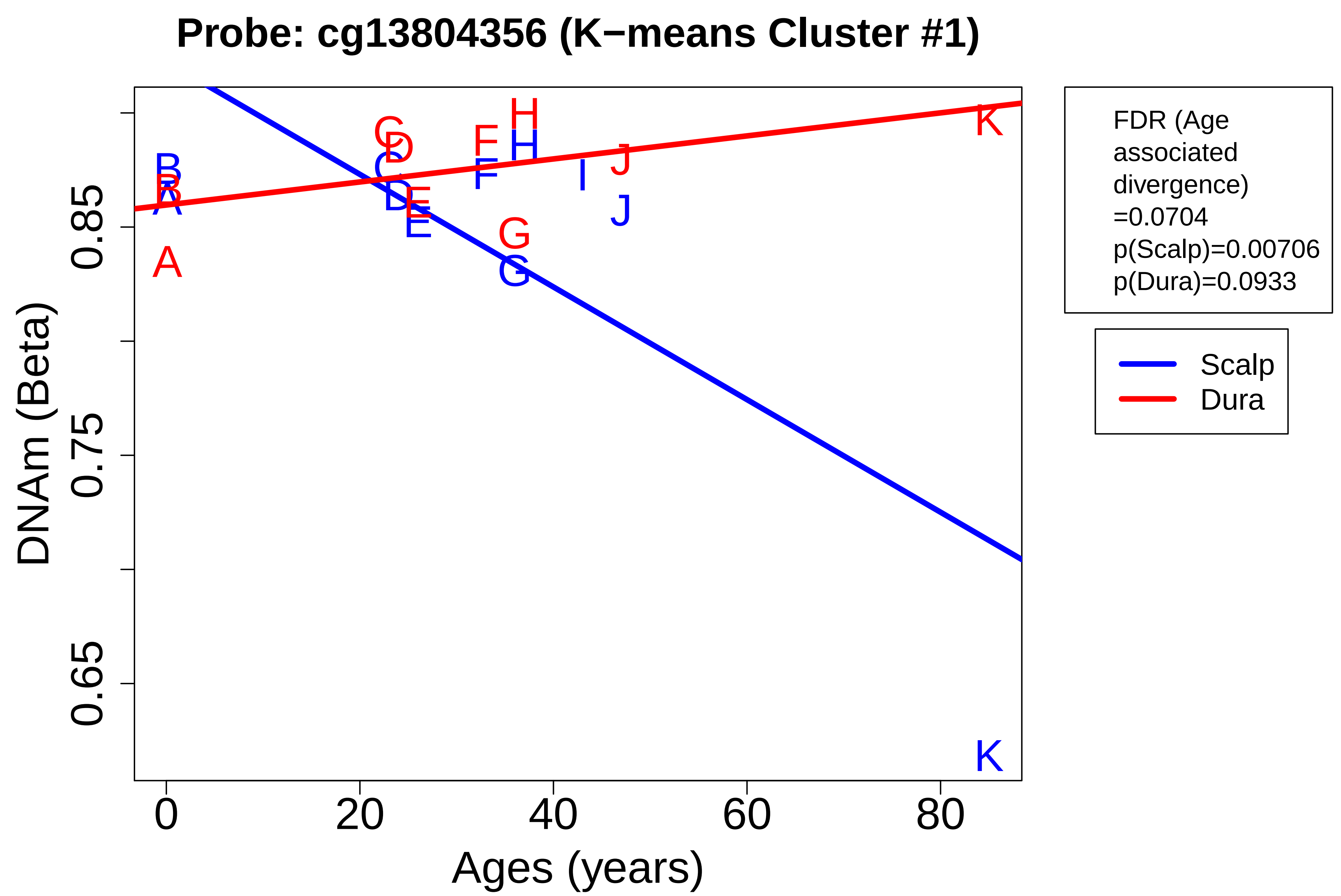

Probe: cg13804356 (K−means Cluster #1)
DNAm (Beta)
0.65
0.75
0.85
0
20
40
60
80
Ages (years)
A
B
C
D
E
F
G
H
I
J
K
FDR (Age
associated
divergence)
=0.0704
p(Scalp)=0.00706
p(Dura)=0.0933
Scalp
Dura

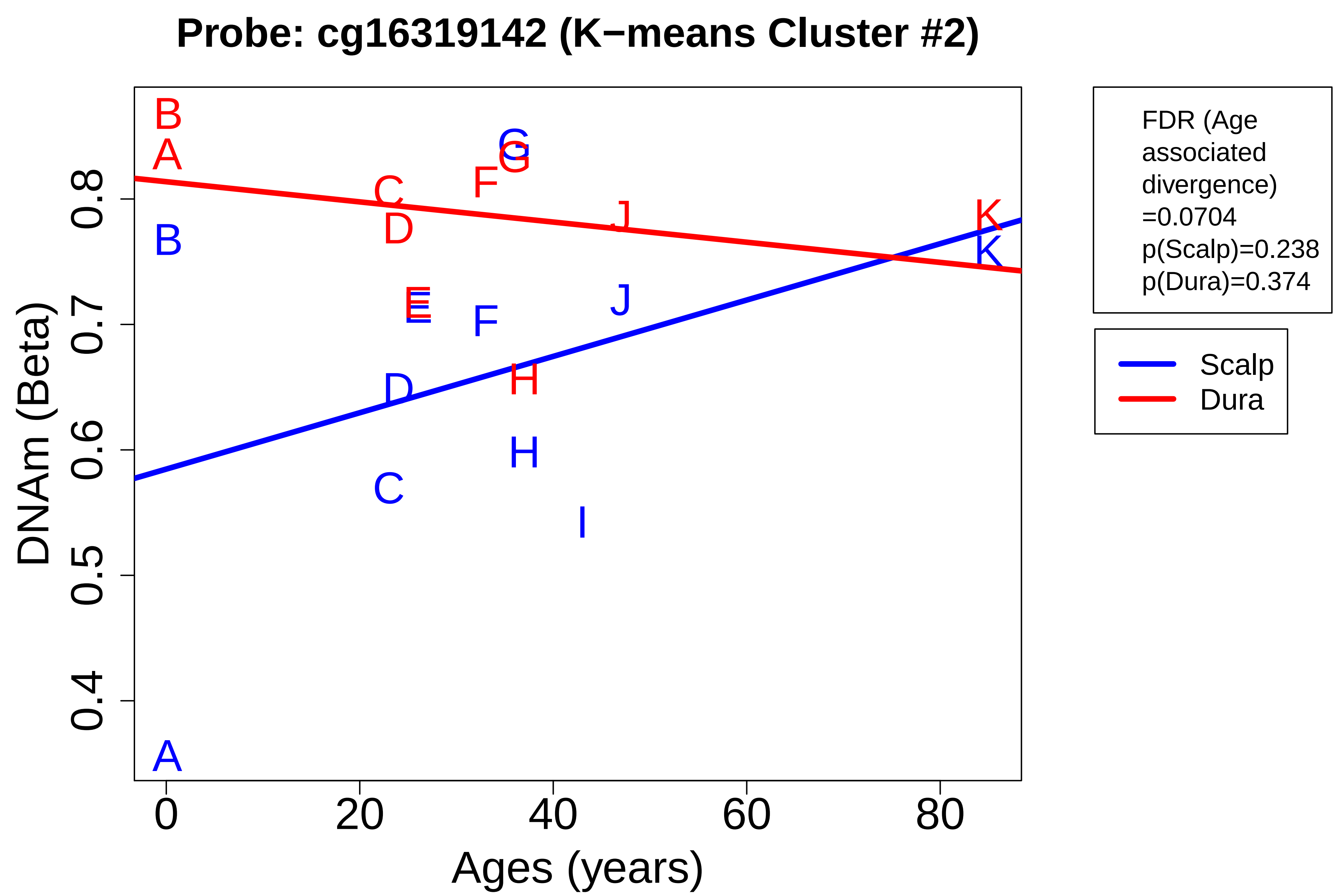

Probe: cg16319142 (K−means Cluster #2)
DNAm (Beta)
0.4
0.5
0.6
0.7
0.8
0
20
40
60
80
Ages (years)
FDR (Age associated divergence) =0.0704
p(Scalp)=0.238
p(Dura)=0.374
Scalp
Dura

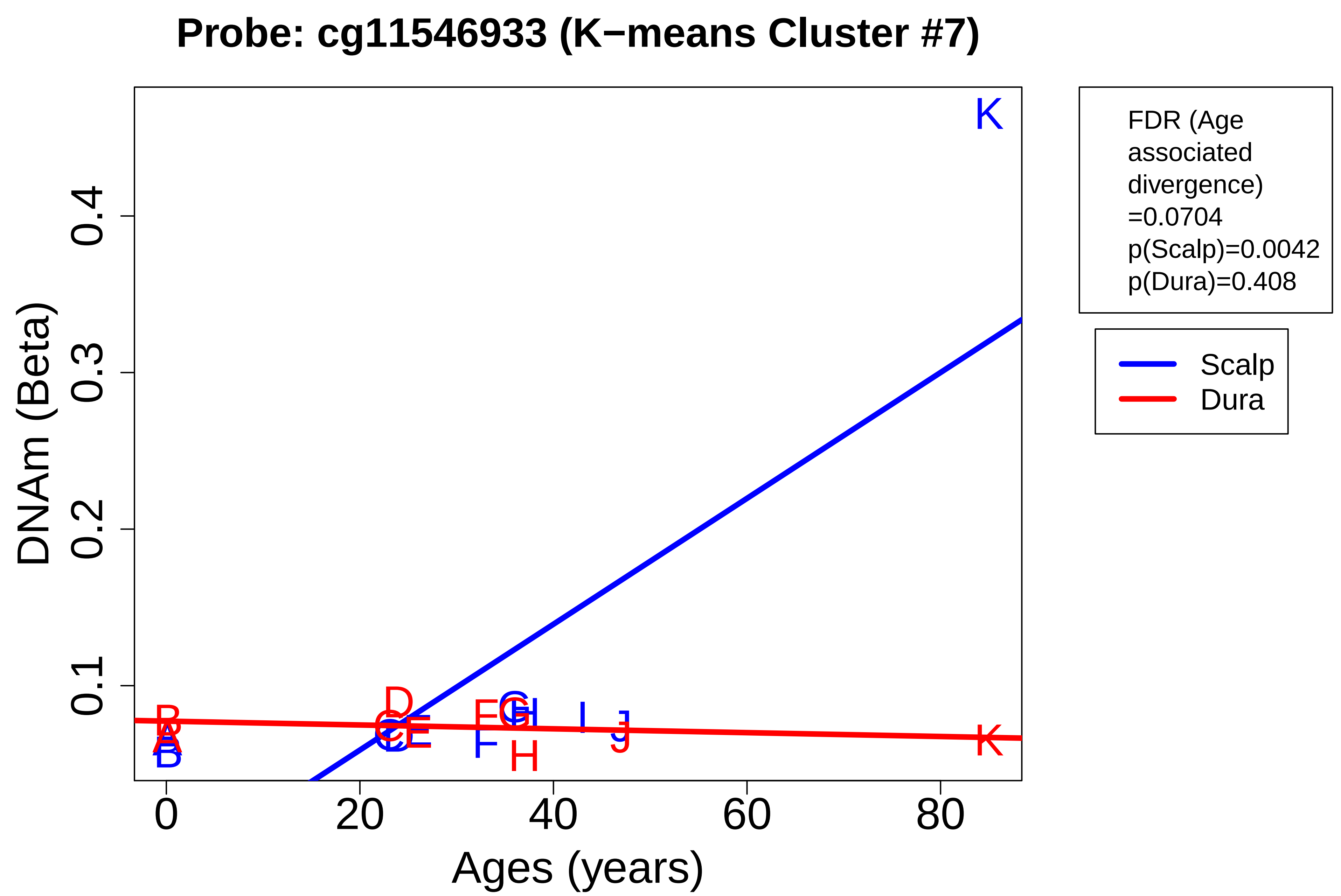

Probe: cg11546933 (K−means Cluster #7)
DNAm (Beta)
0.1
0.2
0.3
0.4
0
20
40
60
80
Ages (years)
FDR (Age associated divergence) =0.0704
p(Scalp)=0.0042
p(Dura)=0.408
Scalp
Dura

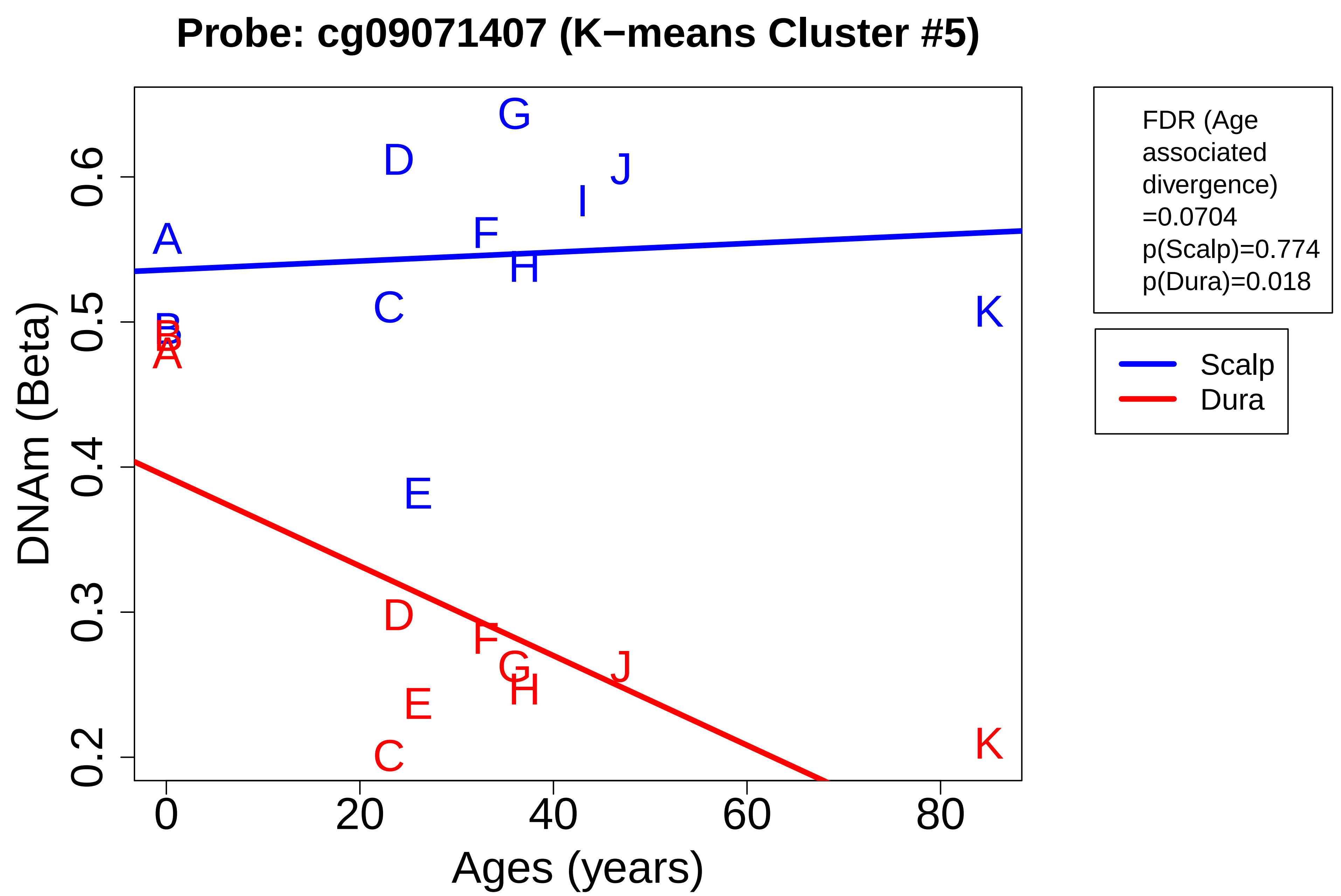

Probe: cg09071407 (K−means Cluster #5)
DNAm (Beta)
0.2
0.3
0.4
0.5
0.6
Ages (years)
0
20
40
60
80
A
B
C
D
E
F
G
H
I
J
K
FDR (Age associated divergence) =0.0704
p(Scalp)=0.774
p(Dura)=0.018
Scalp
Dura

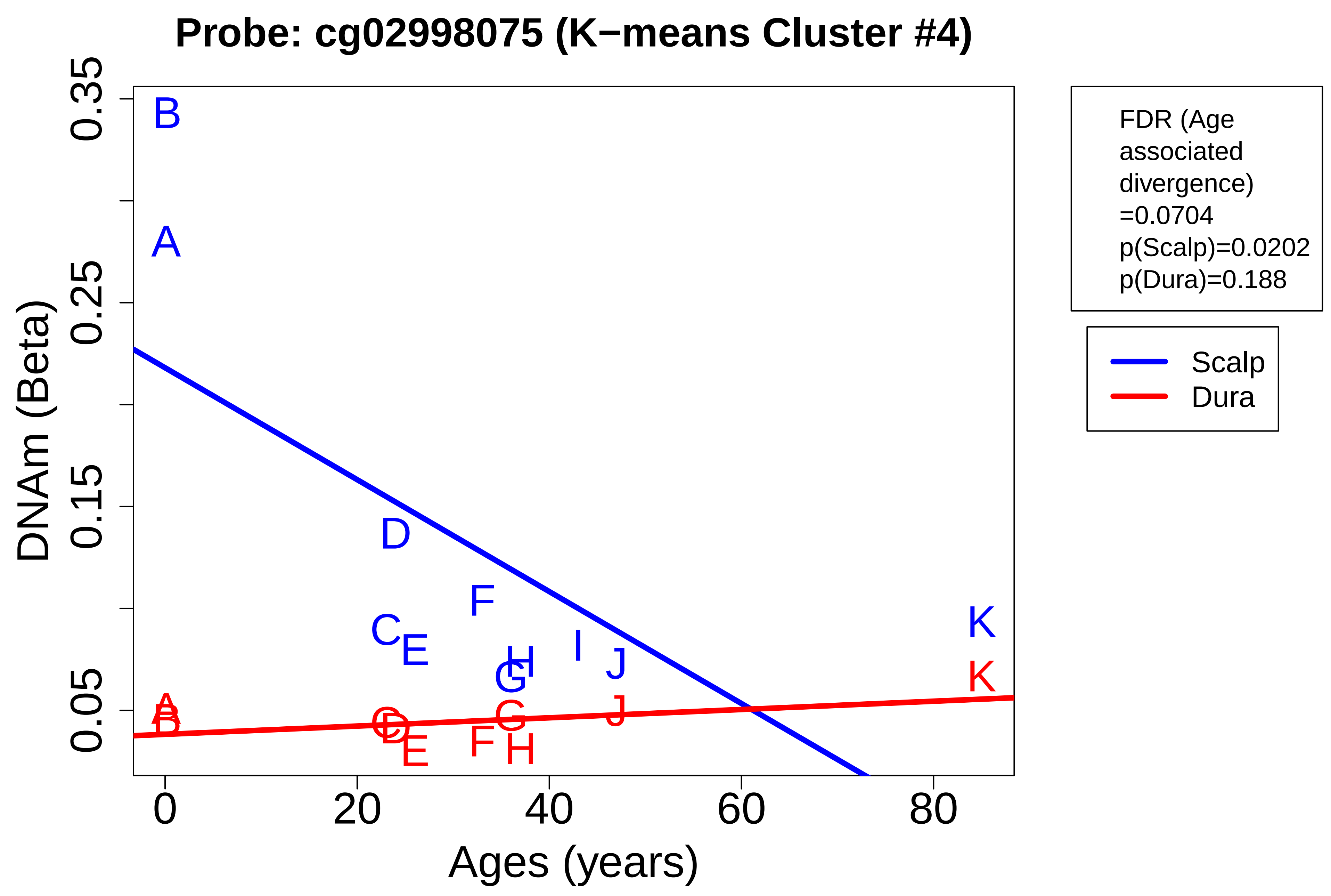

Probe: cg02998075 (K−means Cluster #4)
DNAm (Beta)
0.05
0.15
0.25
0.35
Ages (years)
0
20
40
60
80
A
B
C
D
E
F
G
H
I
J
K
FDR (Age associated divergence) =0.0704
p(Scalp)=0.0202
p(Dura)=0.188
Scalp
Dura

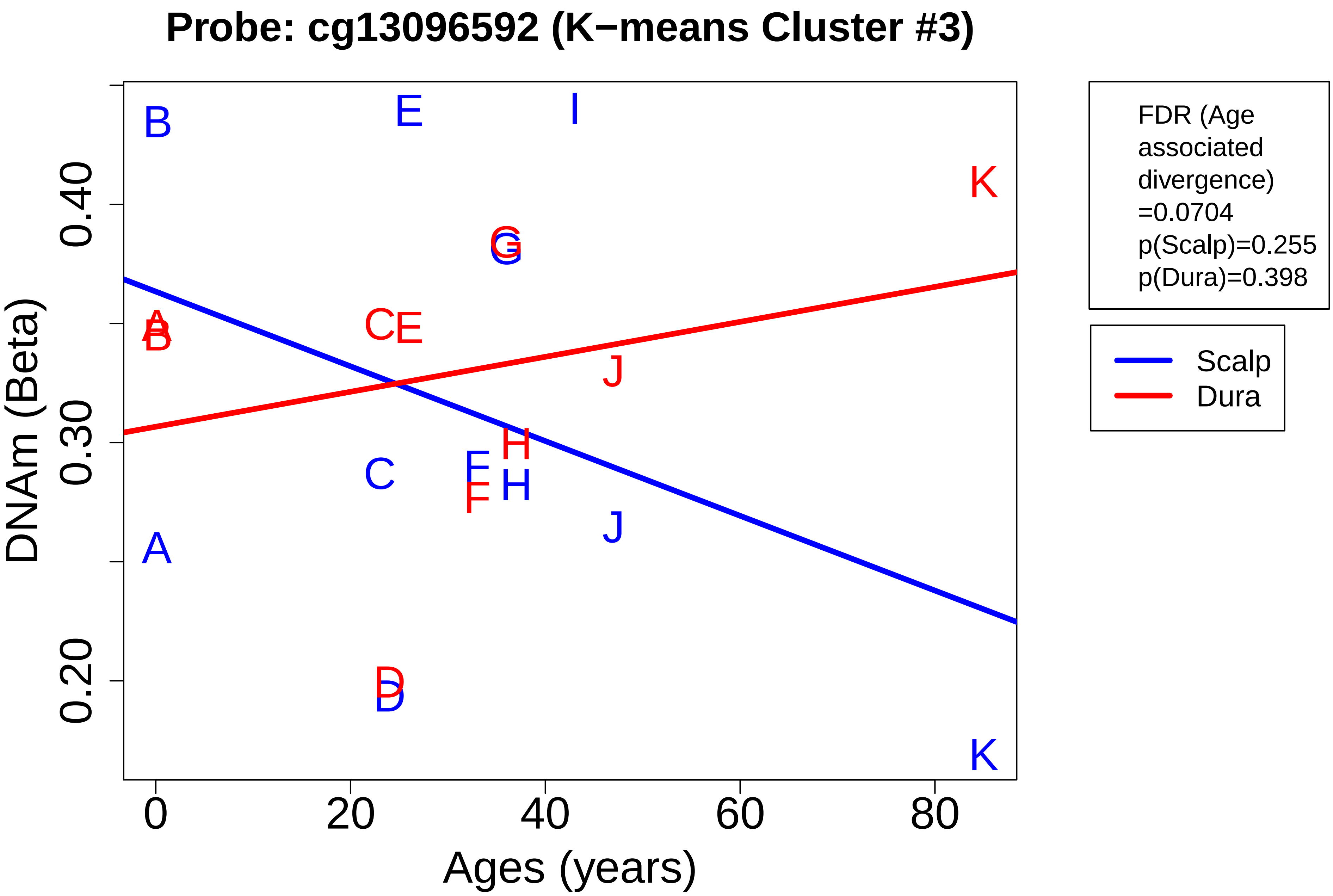

Probe: cg13096592 (K−means Cluster #3)
DNAm (Beta)
0.20
0.30
0.40
0
20
40
60
80
Ages (years)
FDR (Age associated divergence) =0.0704
p(Scalp)=0.255
p(Dura)=0.398
Scalp
Dura

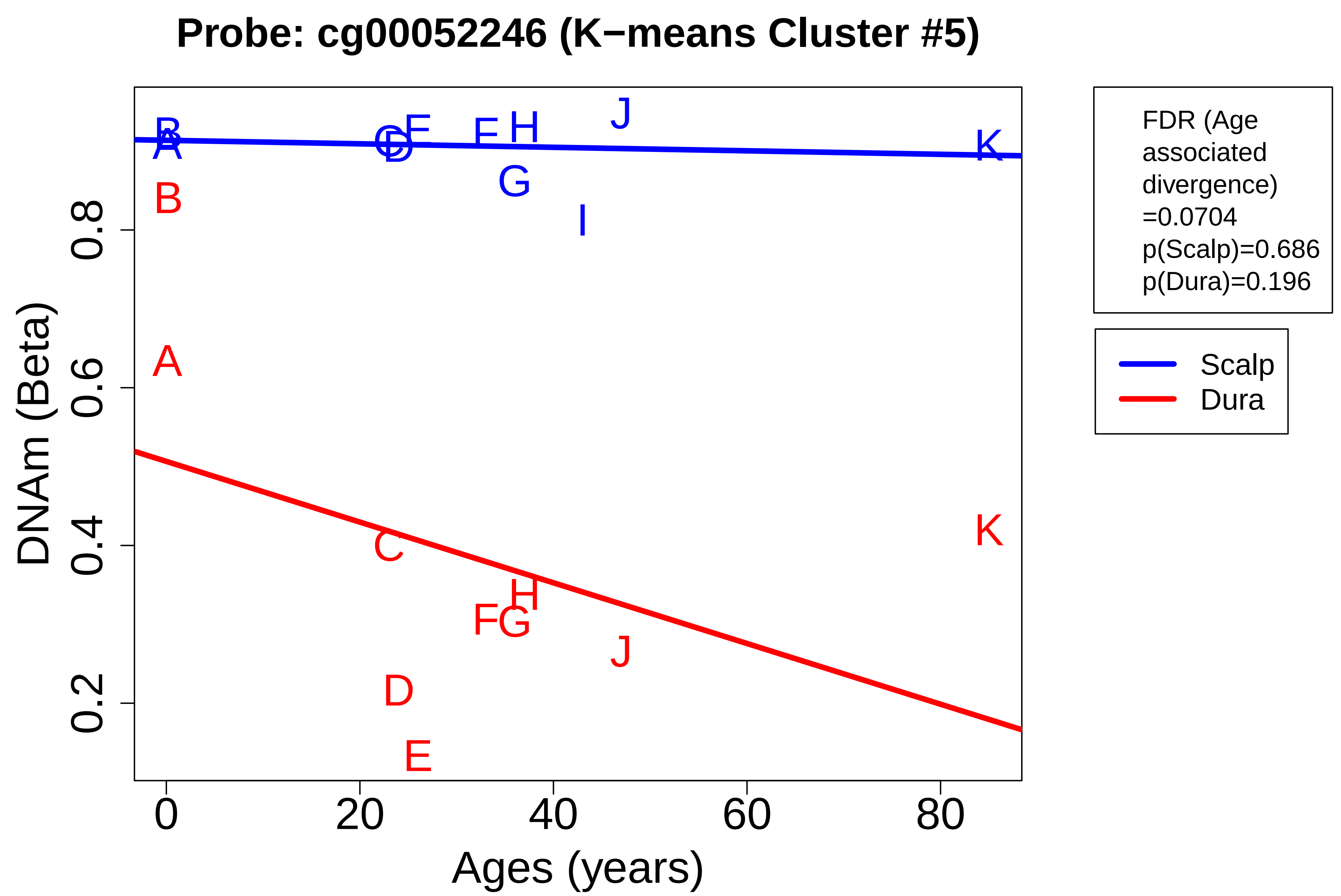

Probe: cg00052246 (K−means Cluster #5)
DNAm (Beta)
0.2
0.4
0.6
0.8
Ages (years)
0
20
40
60
80
FDR (Age associated divergence) =0.0704
p(Scalp)=0.686
p(Dura)=0.196
Scalp
Dura

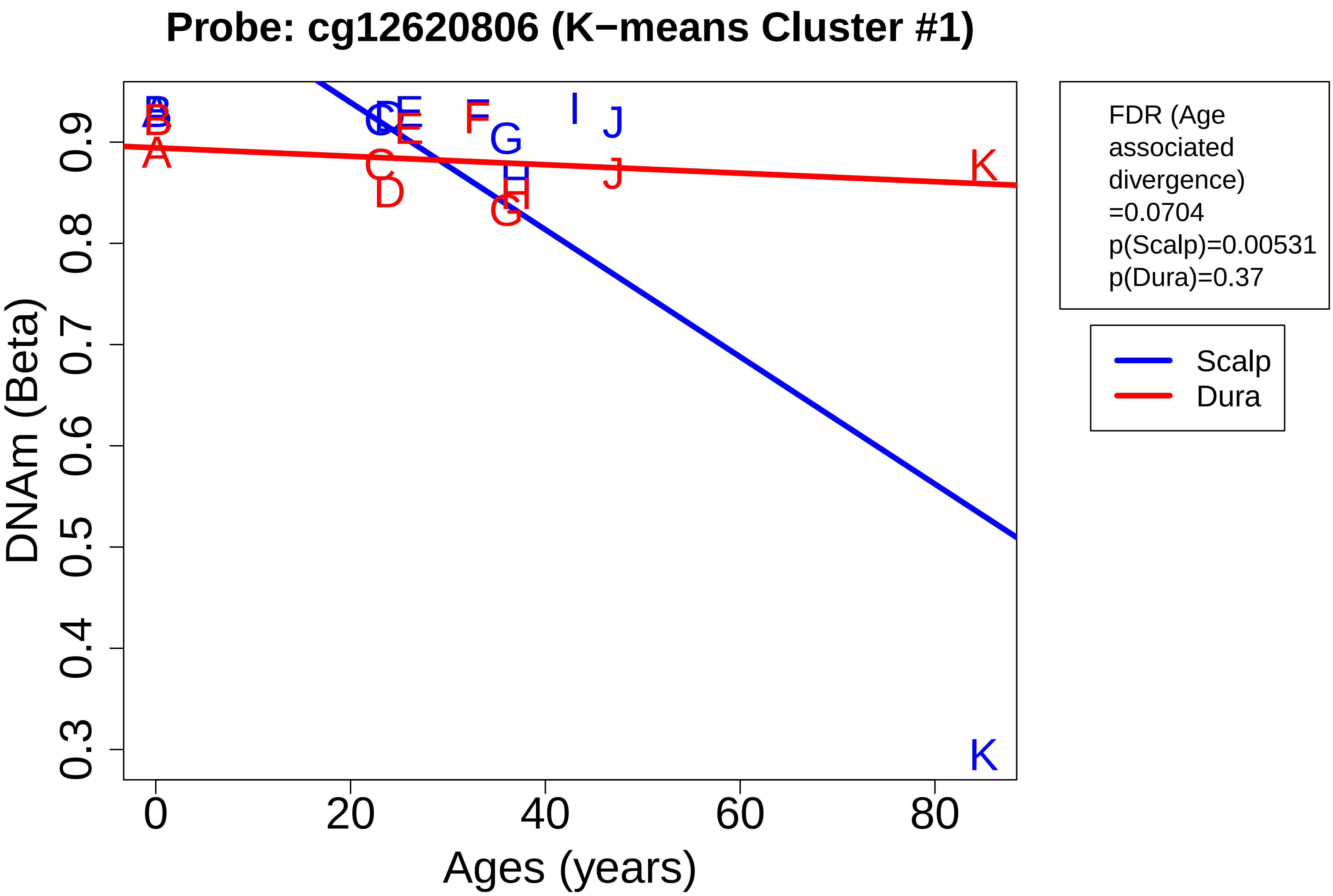

Probe: cg12620806 (K−means Cluster #1)
DNAm (Beta)
0.3
0.4
0.5
0.6
0.7
0.8
0.9
0
20
40
60
80
Ages (years)
A
B
C
D
E
F
G
H
I
J
K
FDR (Age associated divergence) =0.0704
p(Scalp)=0.00531
p(Dura)=0.37
Scalp
Dura

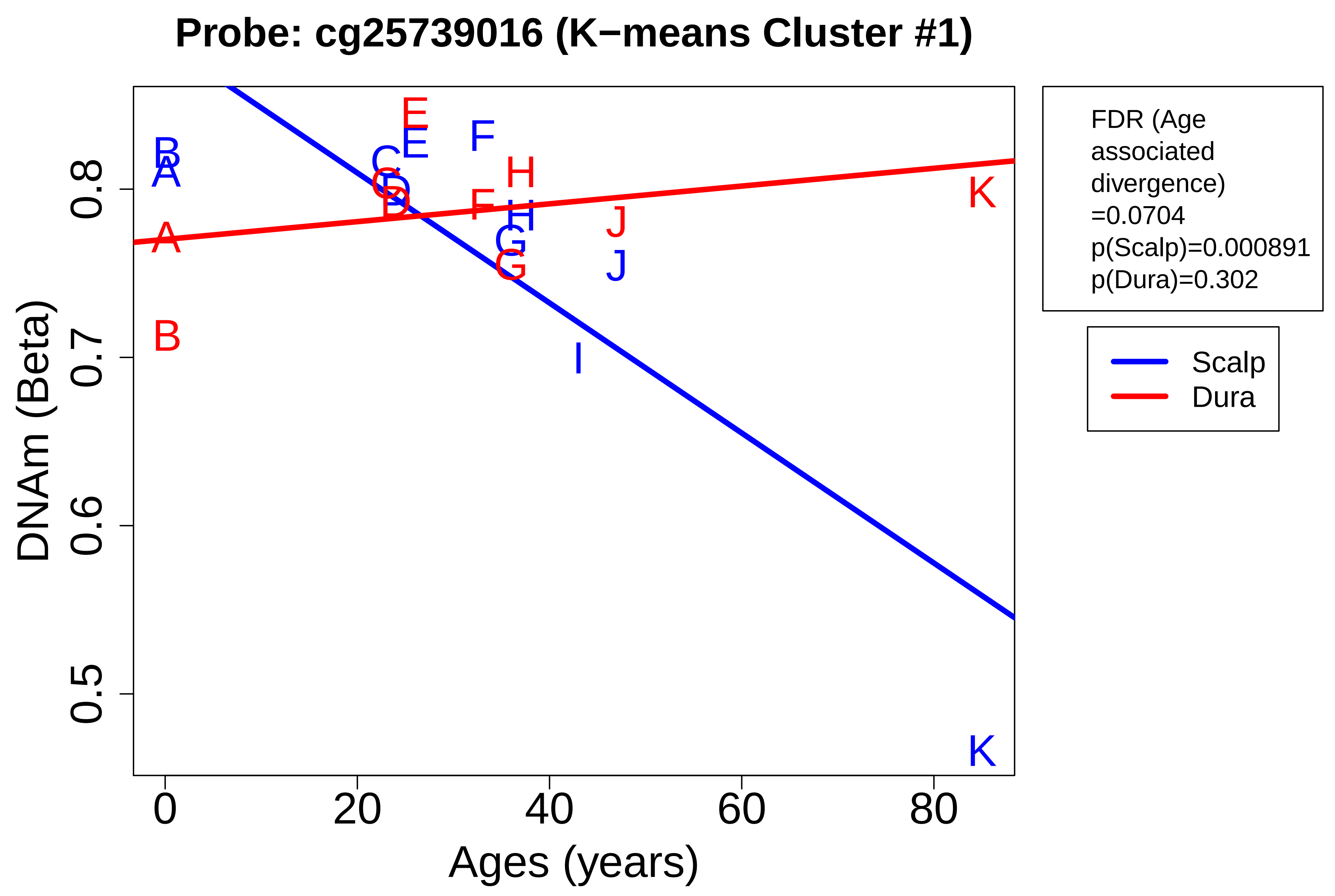

Probe: cg25739016 (K−means Cluster #1)
DNAm (Beta)
0.5
0.6
0.7
0.8
Ages (years)
0
20
40
60
80
FDR (Age associated divergence) =0.0704
p(Scalp)=0.000891
p(Dura)=0.302
Scalp
Dura

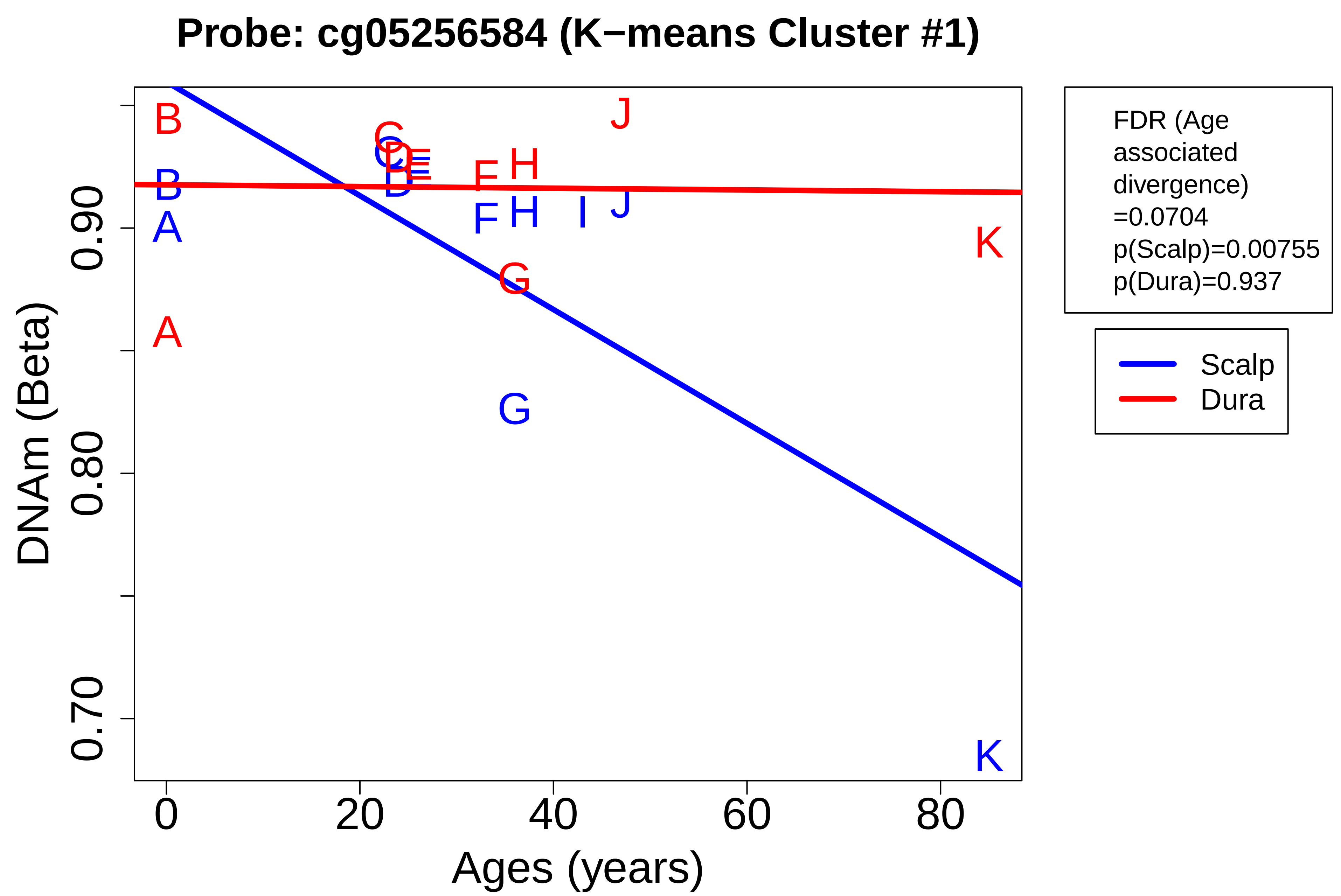

Probe: cg05256584 (K−means Cluster #1)
FDR (Age associated divergence) =0.0704
p(Scalp)=0.00755
p(Dura)=0.937
Scalp
Dura
DNAm (Beta)
0.70
0.80
0.90
Ages (years)
0
20
40
60
80
A
B
C
D
E
F
G
H
I
J
K

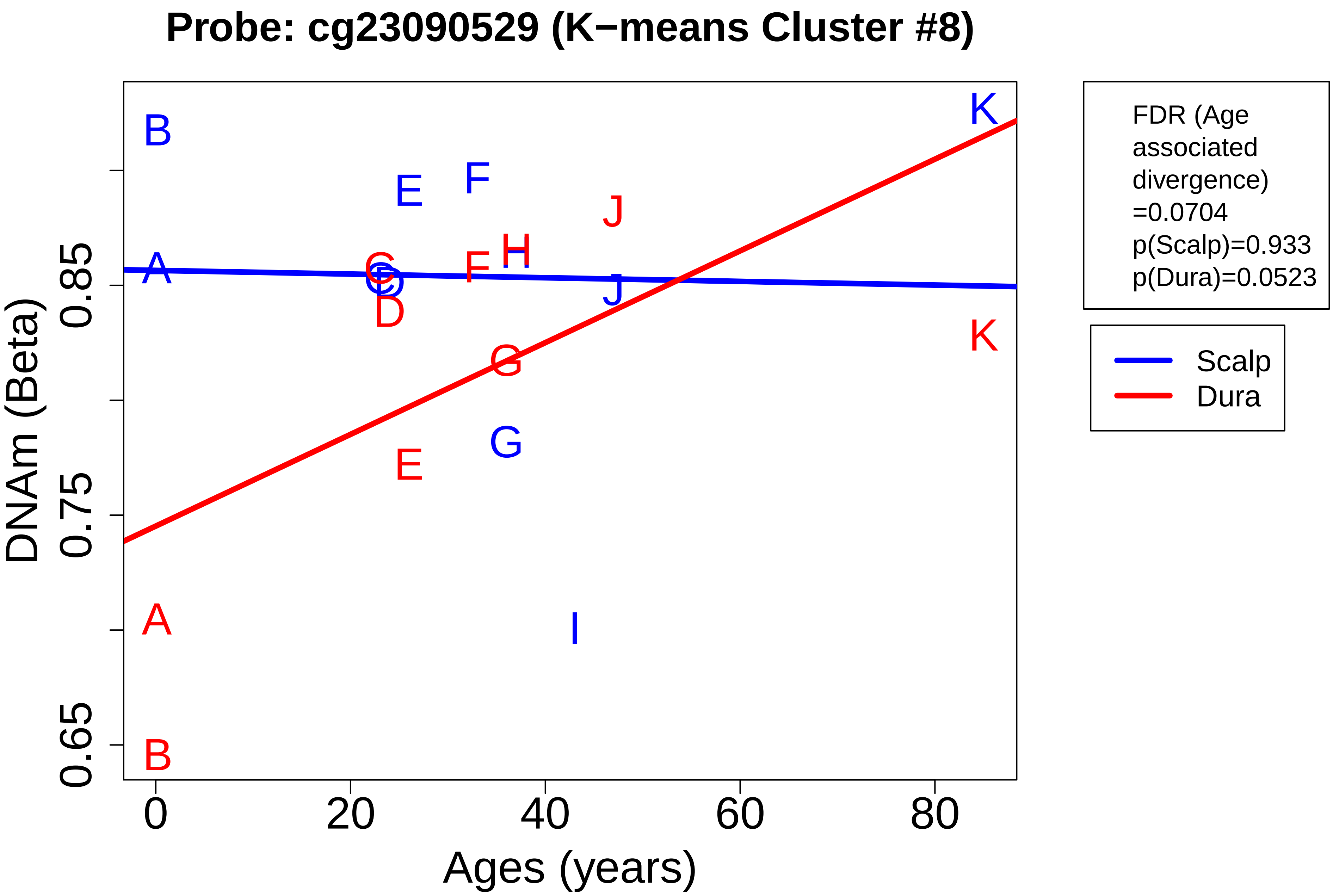

Probe: cg23090529 (K−means Cluster #8)
FDR (Age associated divergence) =0.0704
p(Scalp)=0.933
p(Dura)=0.0523
Scalp
Dura
DNAm (Beta)
0.65
0.75
0.85
Ages (years)
0
20
40
60
80

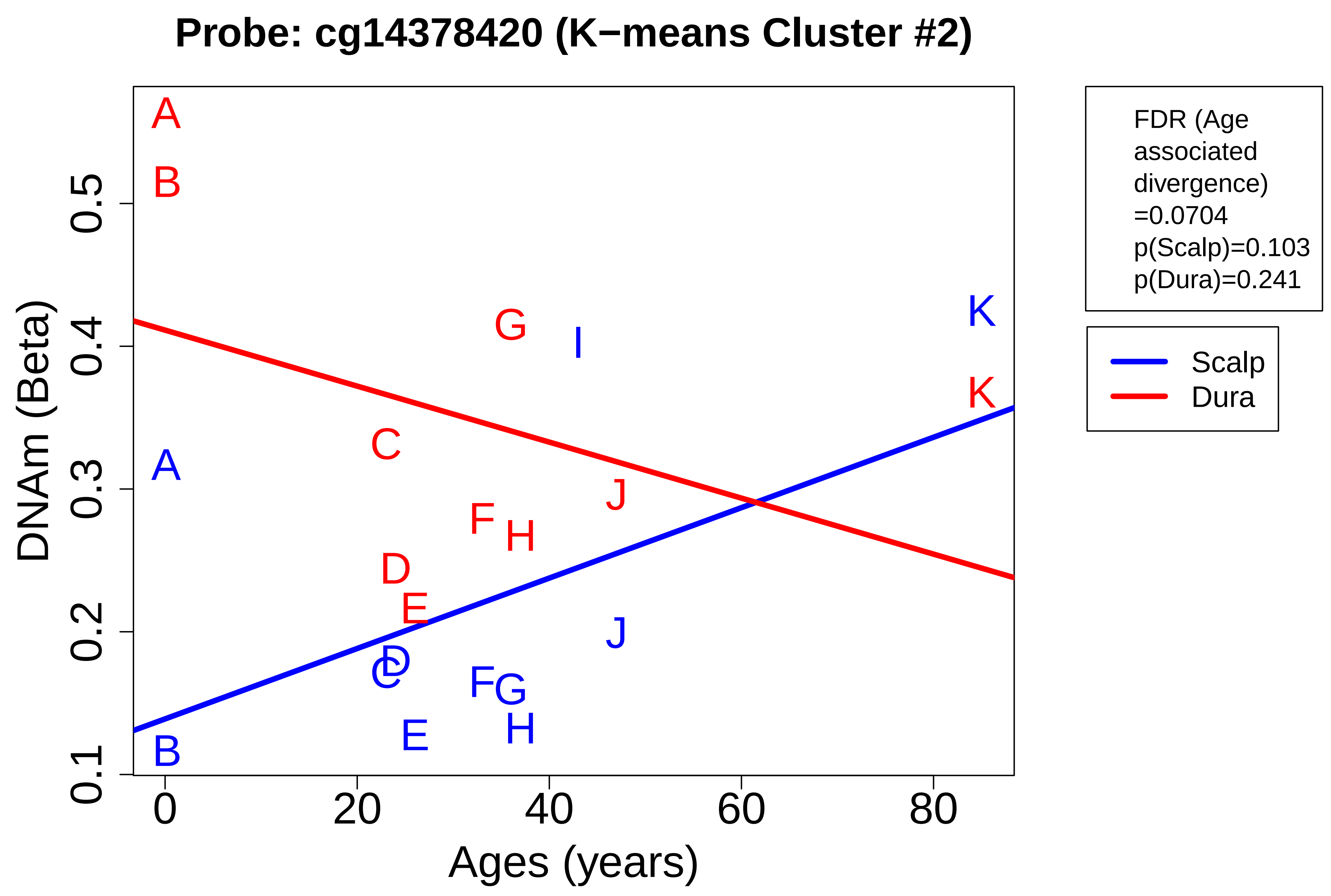
Probe: cg14378420 (K−means Cluster #2)
DNAm (Beta)
0.1
0.2
0.3
0.4
0.5
Ages (years)
0
20
40
60
80
A
B
C
D
E
F
G
H
I
J
K
FDR (Age associated divergence) =0.0704
p(Scalp)=0.103
p(Dura)=0.241
Scalp
Dura

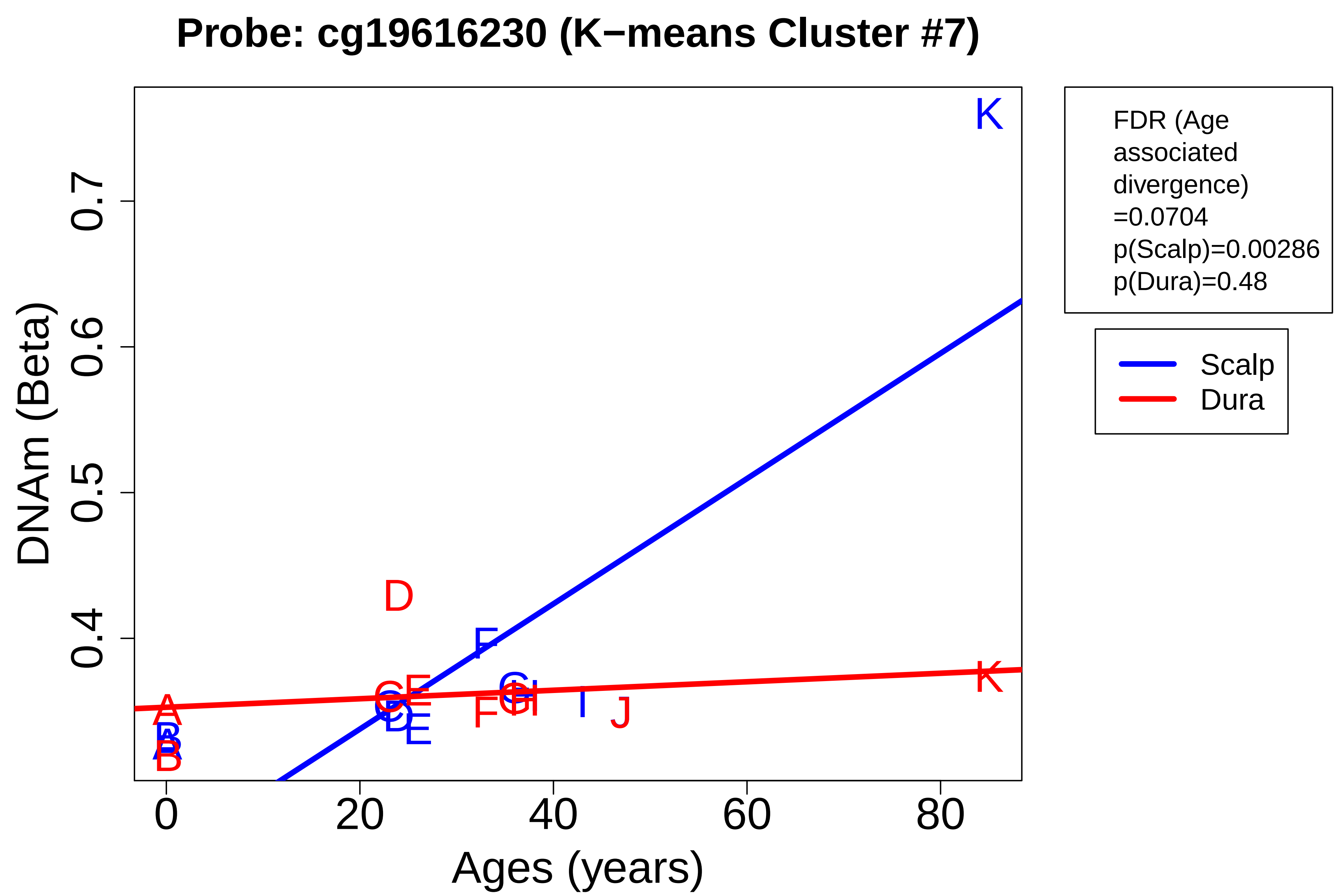

Probe: cg19616230 (K−means Cluster #7)
DNAm (Beta)
0.4
0.5
0.6
0.7
Ages (years)
0
20
40
60
80
FDR (Age associated divergence) =0.0704
p(Scalp)=0.00286
p(Dura)=0.48
Scalp
Dura

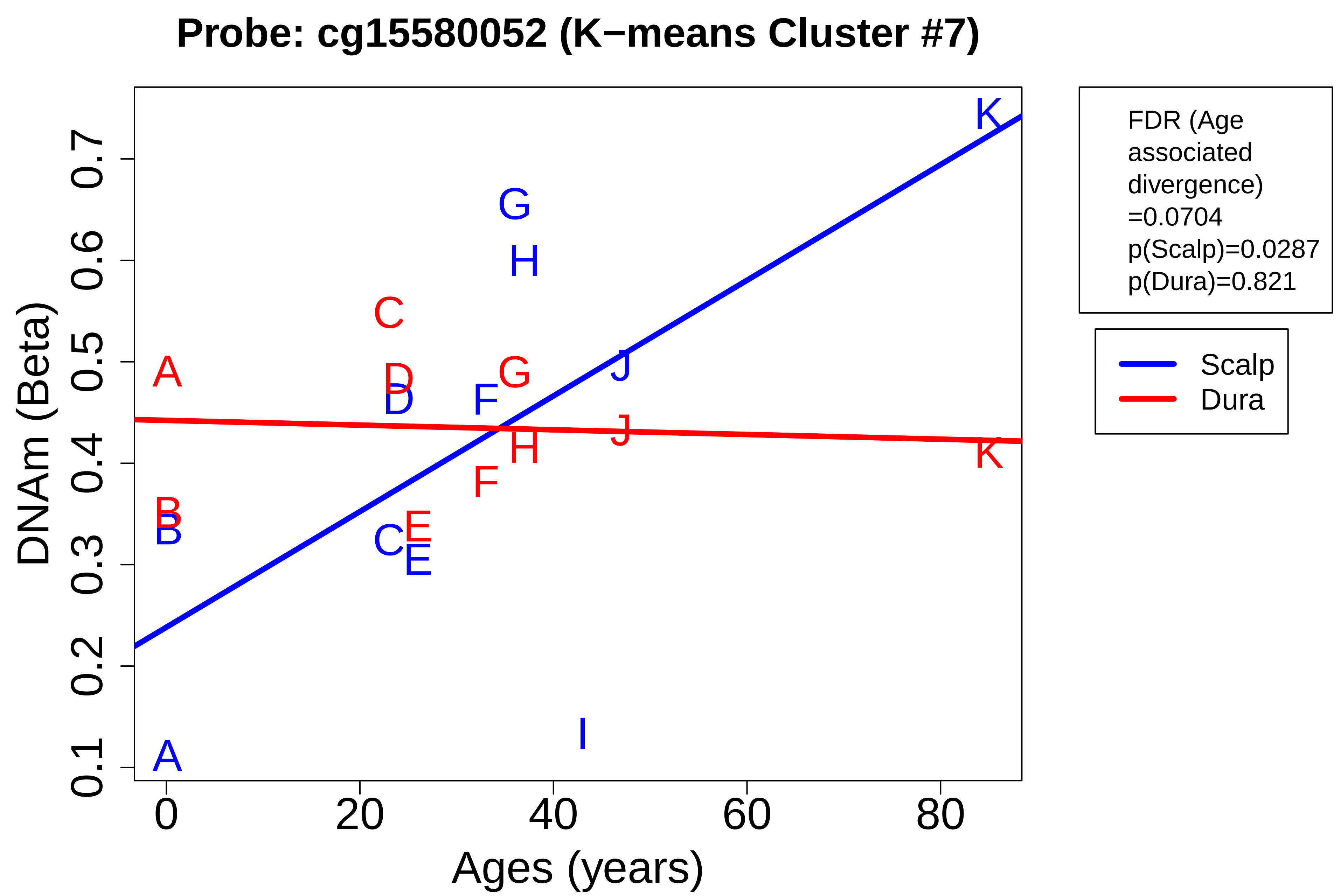

Probe: cg15580052 (K−means Cluster #7)
DNAm (Beta)
0.1
0.2
0.3
0.4
0.5
0.6
0.7
Ages (years)
0
20
40
60
80
A
B
C
D
E
F
G
H
I
J
K
FDR (Age associated divergence) =0.0704
p(Scalp)=0.0287
p(Dura)=0.821
Scalp
Dura

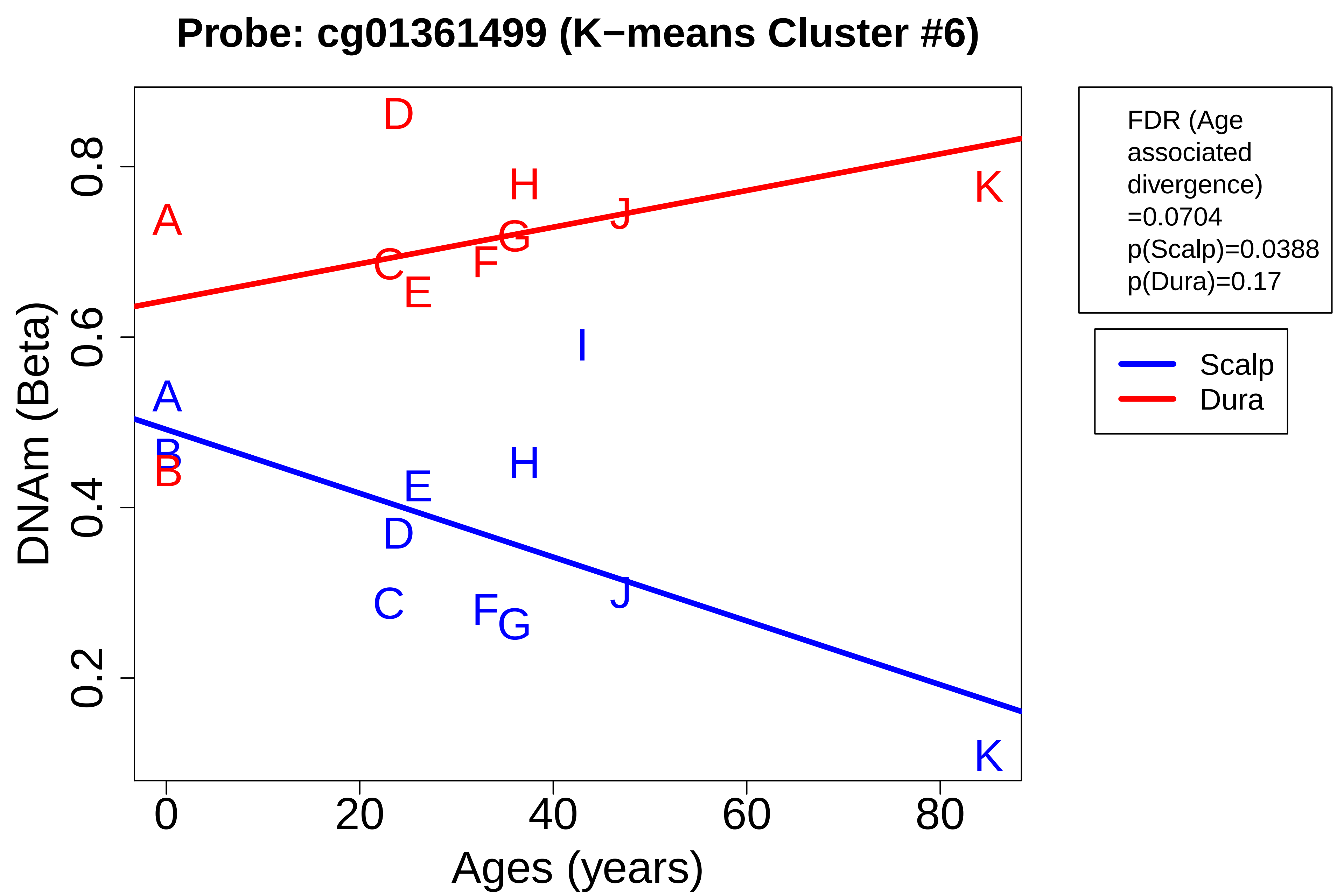

Probe: cg01361499 (K−means Cluster #6)
DNAm (Beta)
0.2
0.4
0.6
0.8
0
20
40
60
80
Ages (years)
FDR (Age associated divergence) =0.0704
p(Scalp)=0.0388
p(Dura)=0.17
Scalp
Dura
A
B
C
D
E
F
G
H
I
J
K

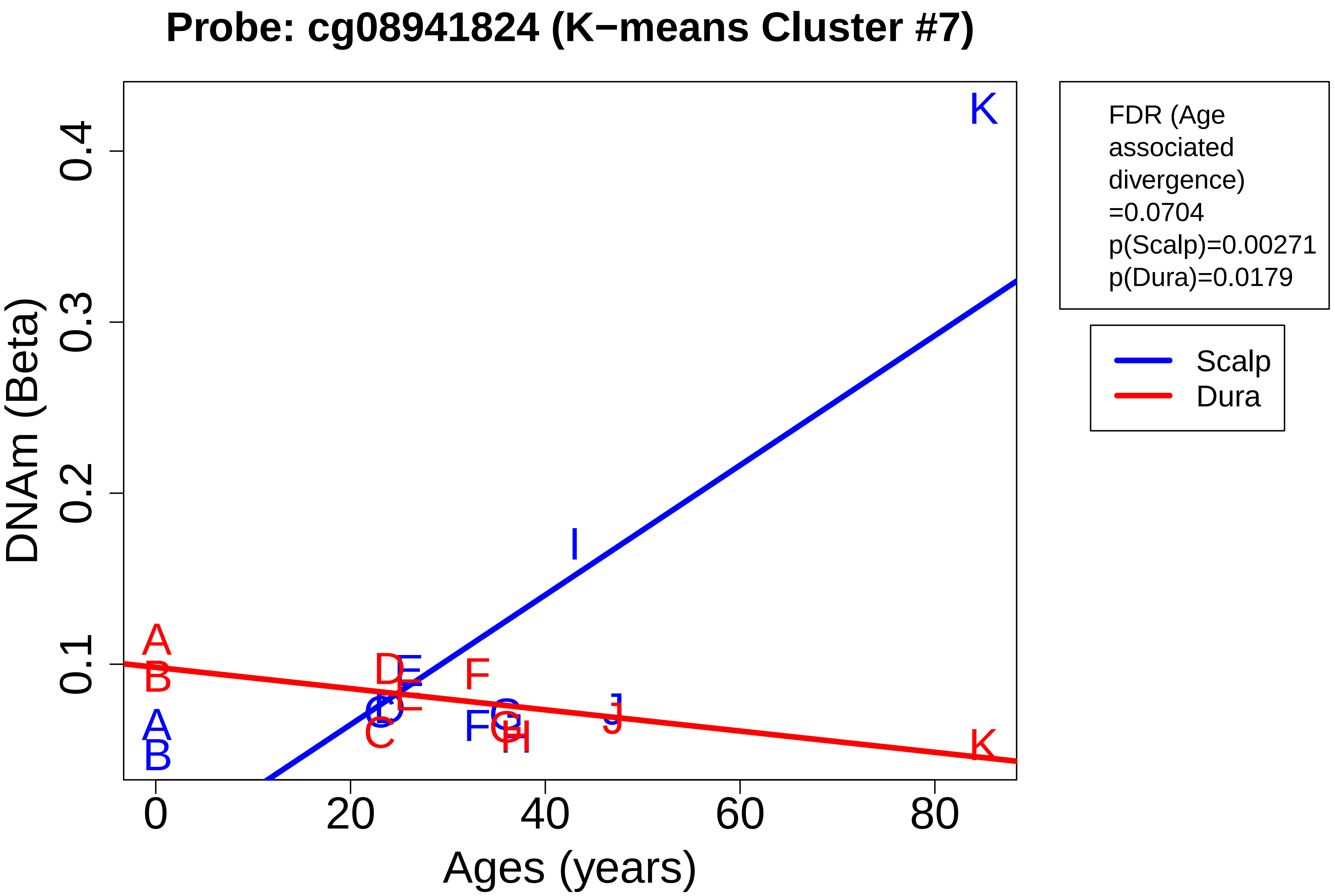
Probe: cg08941824 (K−means Cluster #7)
DNAm (Beta)
0.1
0.2
0.3
0.4
0
20
40
60
80
Ages (years)
FDR (Age associated divergence) =0.0704
p(Scalp)=0.00271
p(Dura)=0.0179
Scalp
Dura

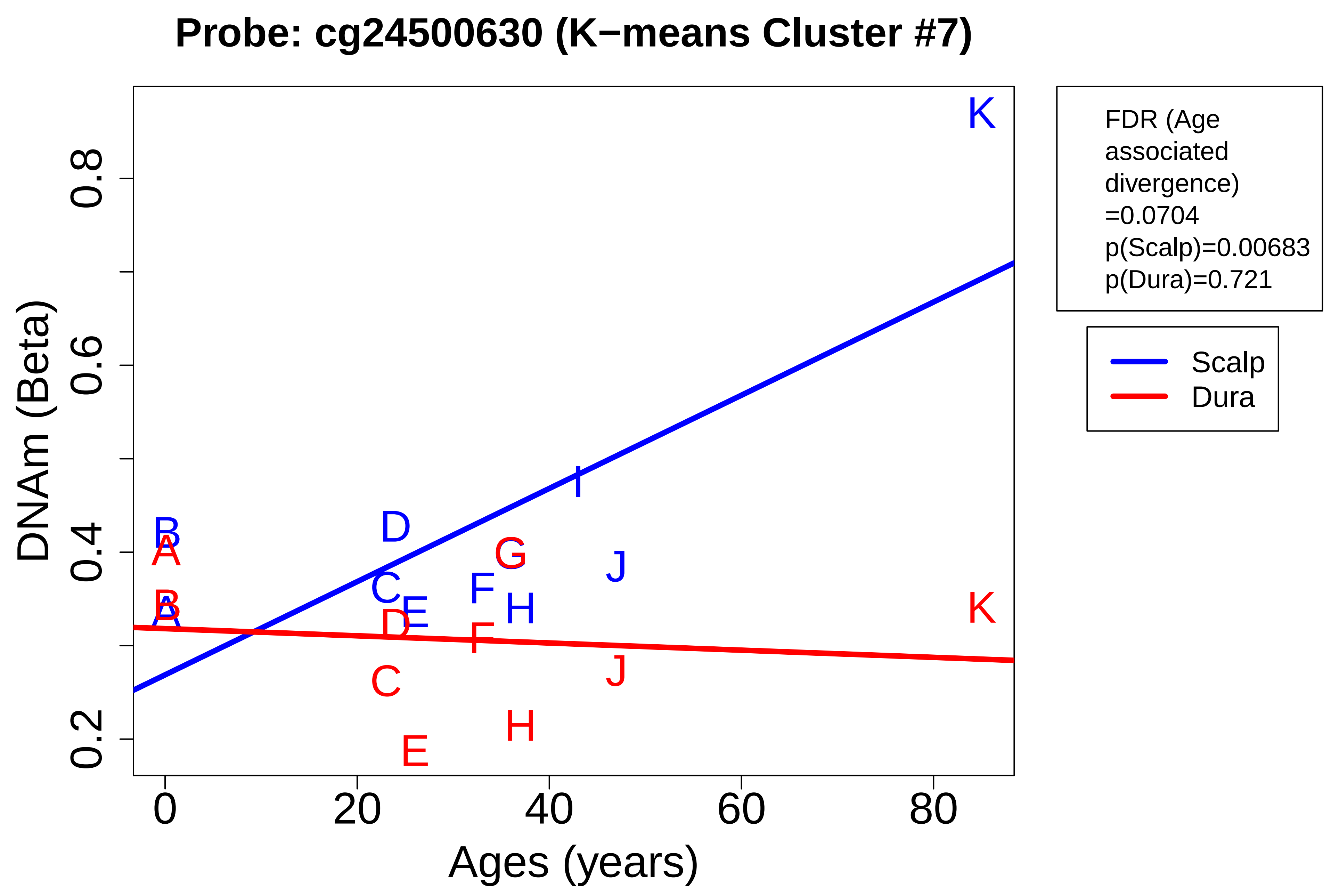

Probe: cg24500630 (K−means Cluster #7)
DNAm (Beta)
0.2
0.4
0.6
0.8
0
20
40
60
80
Ages (years)
A
B
C
D
E
F
G
H
I
J
K
FDR (Age associated divergence) =0.0704
p(Scalp)=0.00683
p(Dura)=0.721
Scalp
Dura

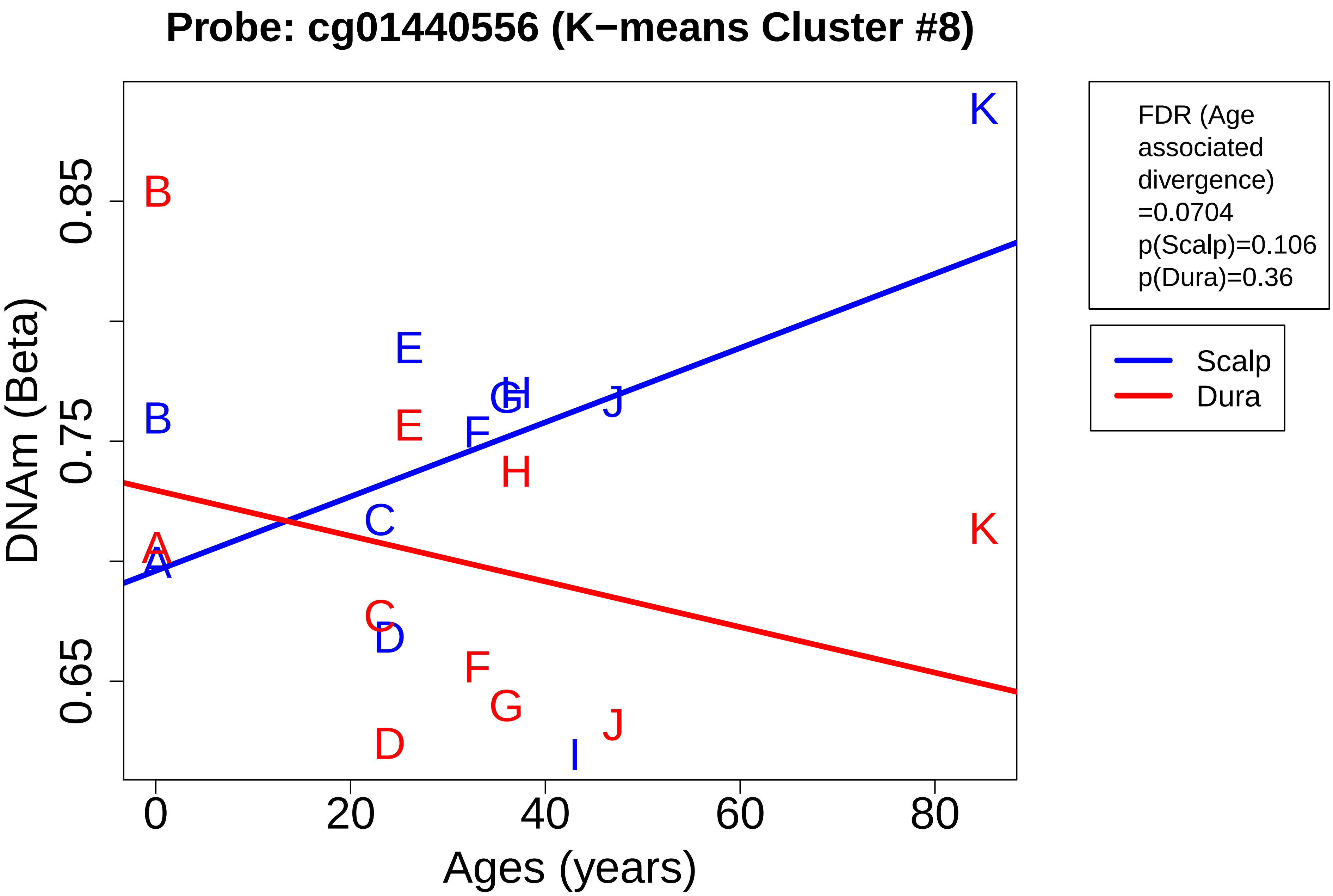

Probe: cg01440556 (K−means Cluster #8)
FDR (Age associated divergence) =0.0704
p(Scalp)=0.106
p(Dura)=0.36
Scalp
Dura
DNAm (Beta)
0.65
0.75
0.85
Ages (years)
0
20
40
60
80

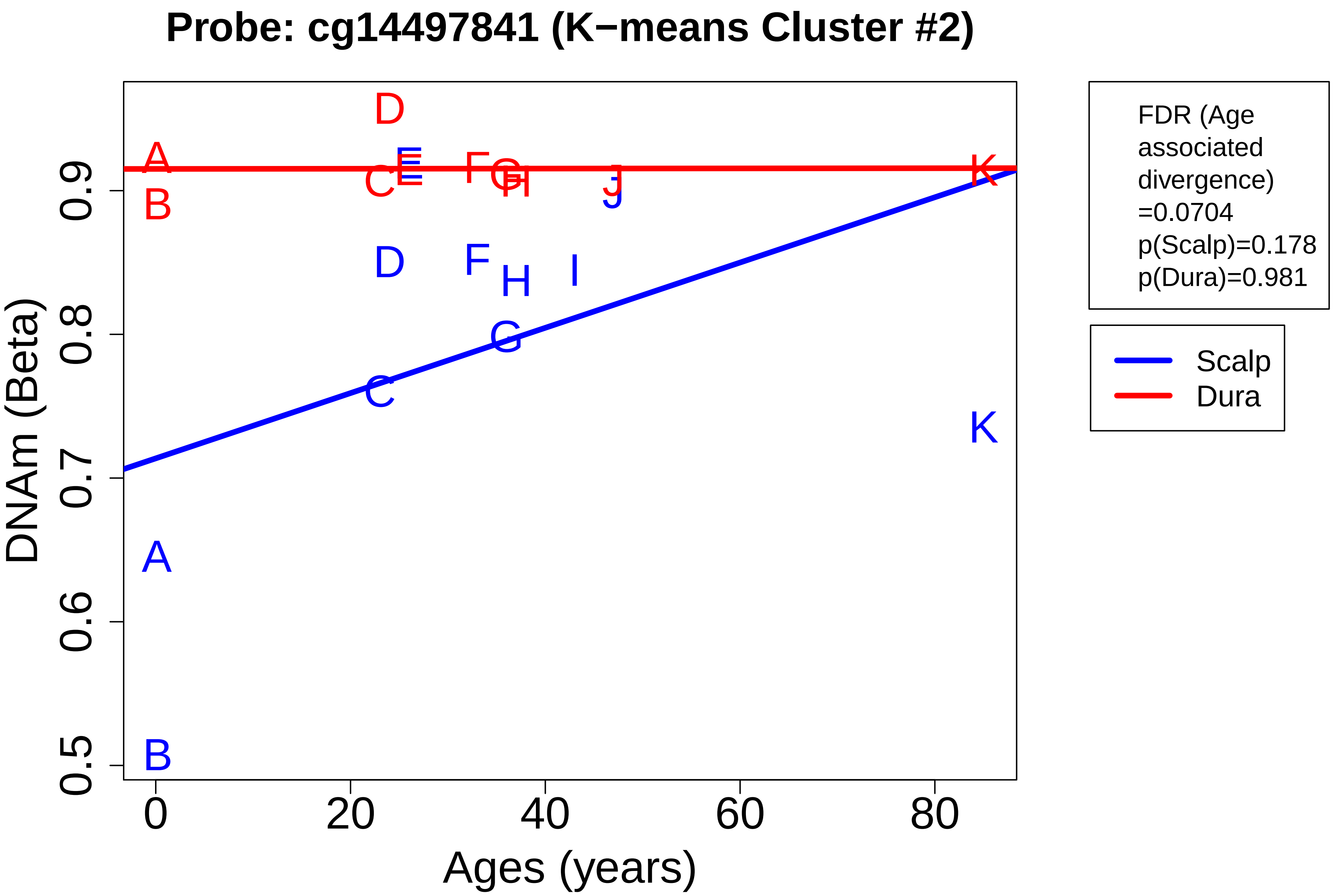

Probe: cg14497841 (K−means Cluster #2)
DNAm (Beta)
0.5
0.6
0.7
0.8
0.9
0
20
40
60
80
Ages (years)
FDR (Age associated divergence) =0.0704
p(Scalp)=0.178
p(Dura)=0.981
Scalp
Dura
A
B
C
D
E
F
G
H
I
J
K

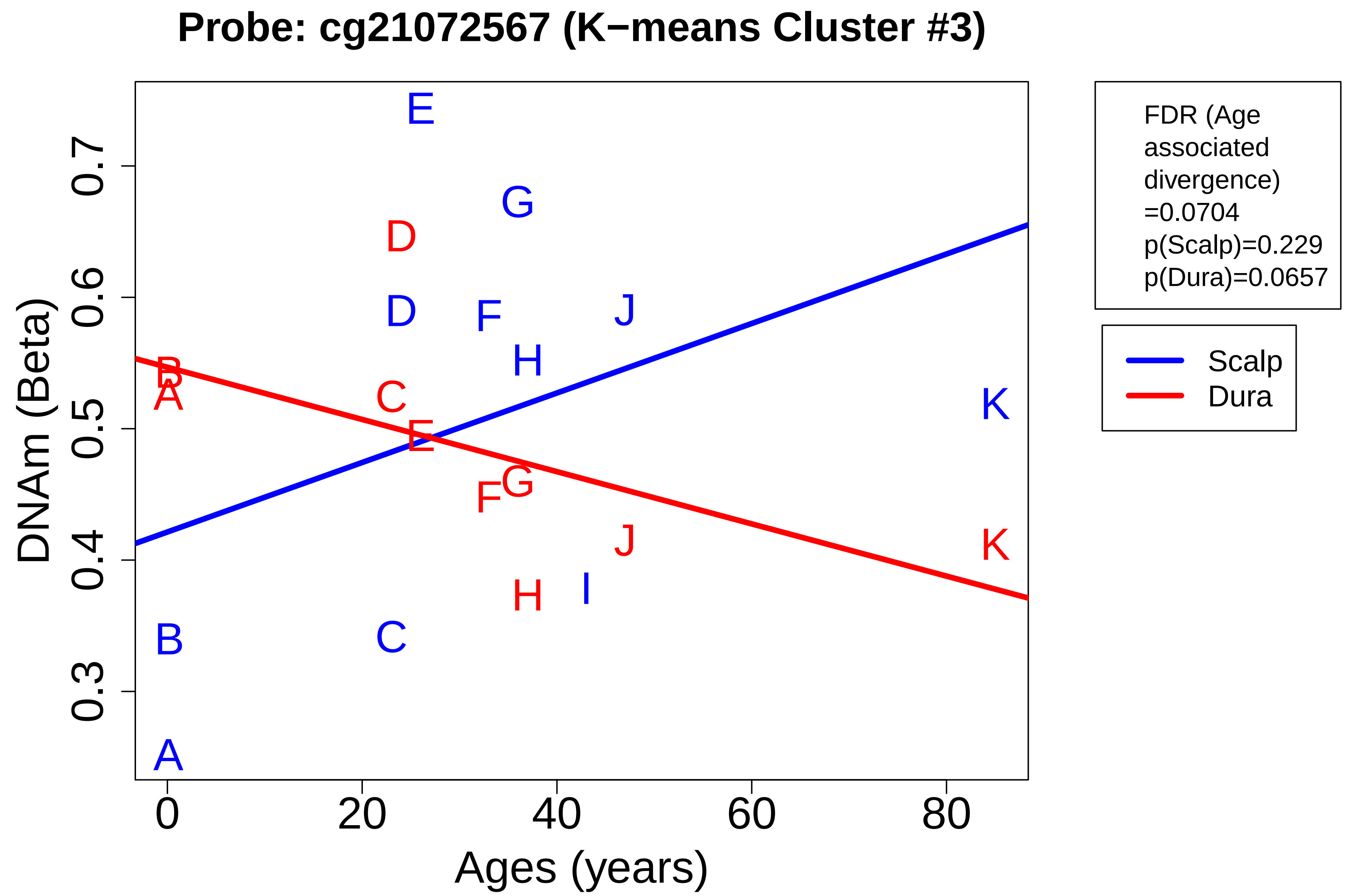

Probe: cg21072567 (K−means Cluster #3)
DNAm (Beta)
0.3
0.4
0.5
0.6
0.7
0
20
40
60
80
Ages (years)
FDR (Age associated divergence) =0.0704
p(Scalp)=0.229
p(Dura)=0.0657
Scalp
Dura

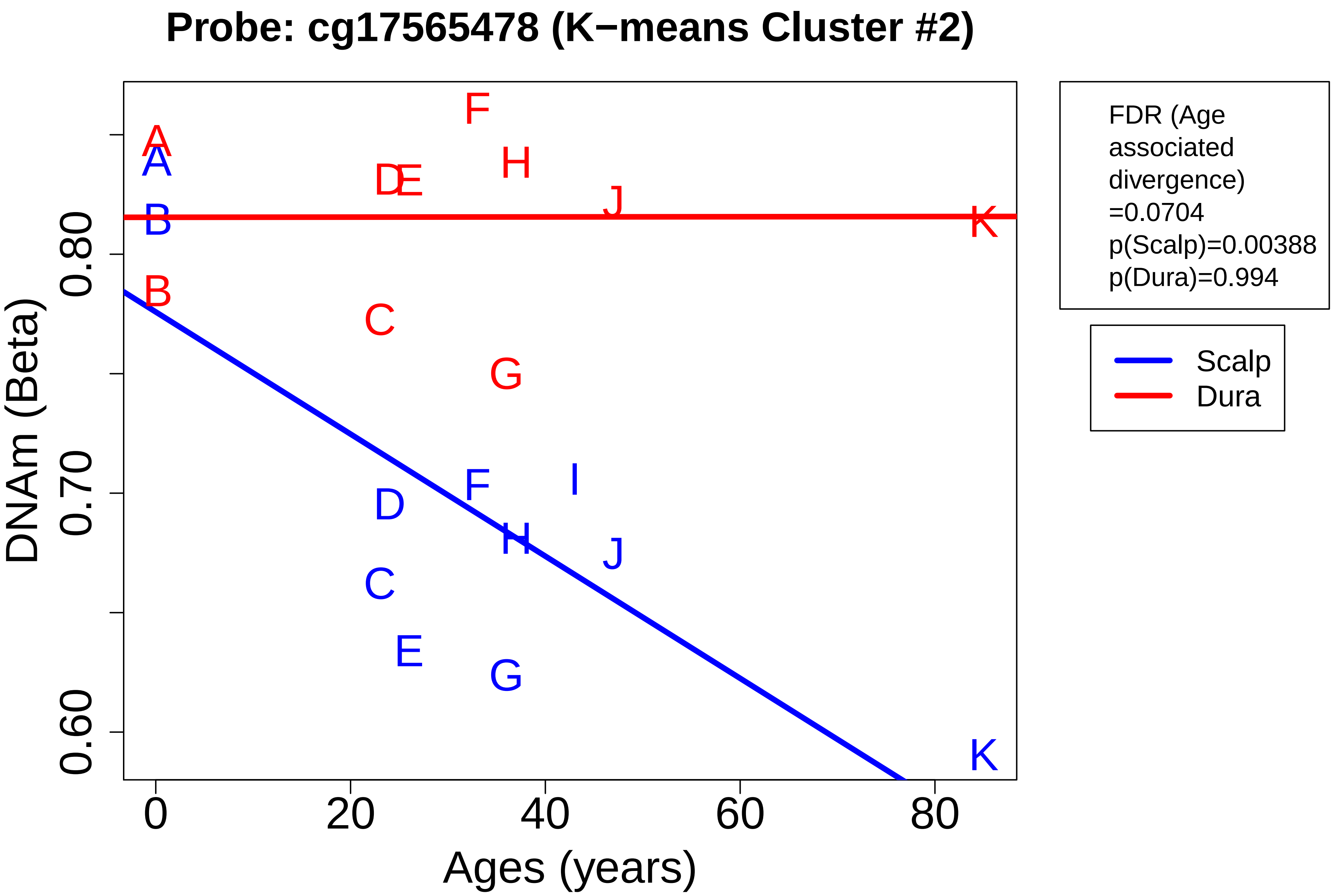

Probe: cg17565478 (K−means Cluster #2)
DNAm (Beta)
0.60
0.70
0.80
0
20
40
60
80
Ages (years)
A
B
C
D
E
F
G
H
I
J
K
FDR (Age associated divergence) =0.0704
p(Scalp)=0.00388
p(Dura)=0.994
Scalp
Dura

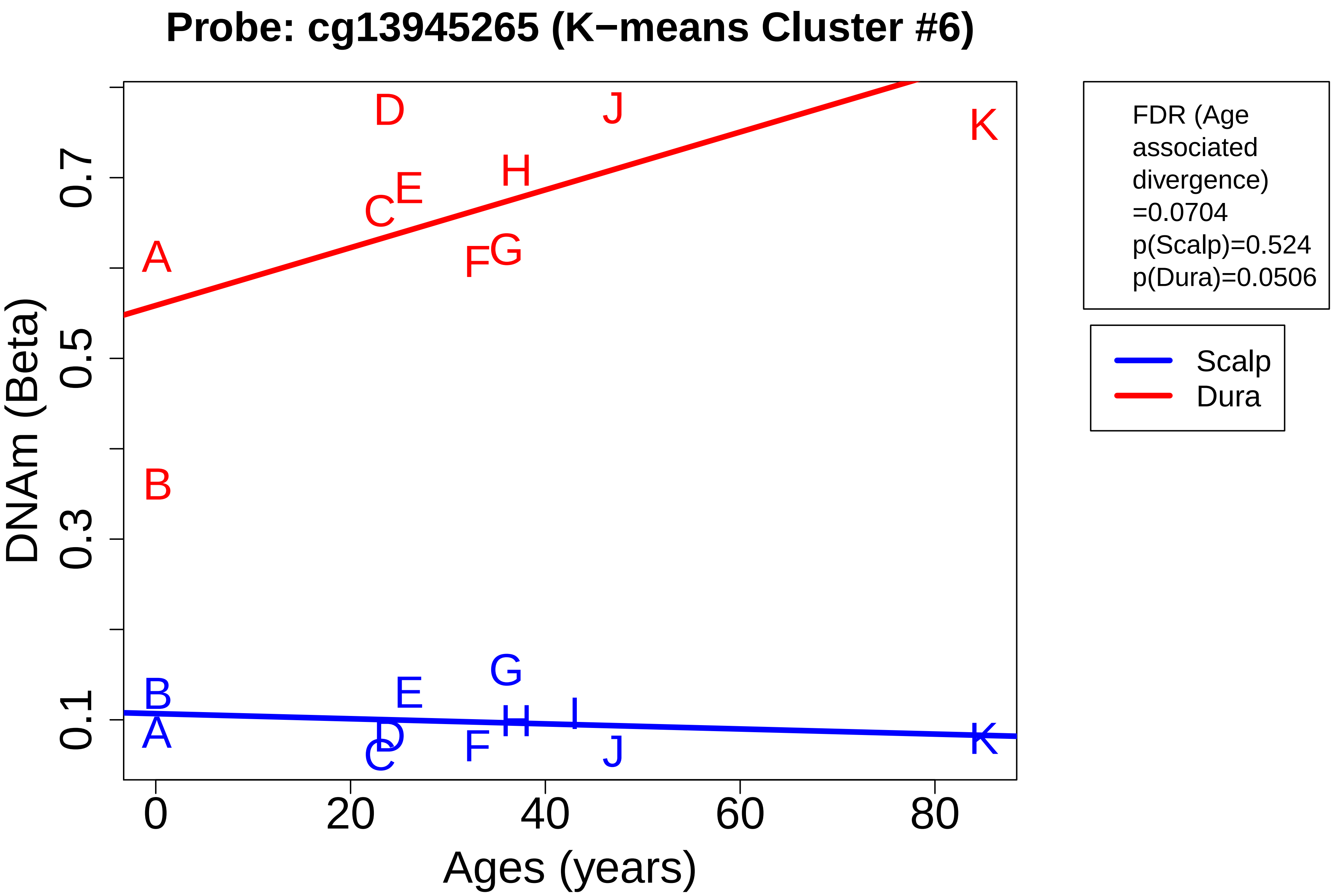

Probe: cg13945265 (K−means Cluster #6)
DNAm (Beta)
0.1
0.3
0.5
0.7
0
20
40
60
80
Ages (years)
A
B
C
D
E
F
G
H
I
J
K
FDR (Age associated divergence) =0.0704
p(Scalp)=0.524
p(Dura)=0.0506
Scalp
Dura

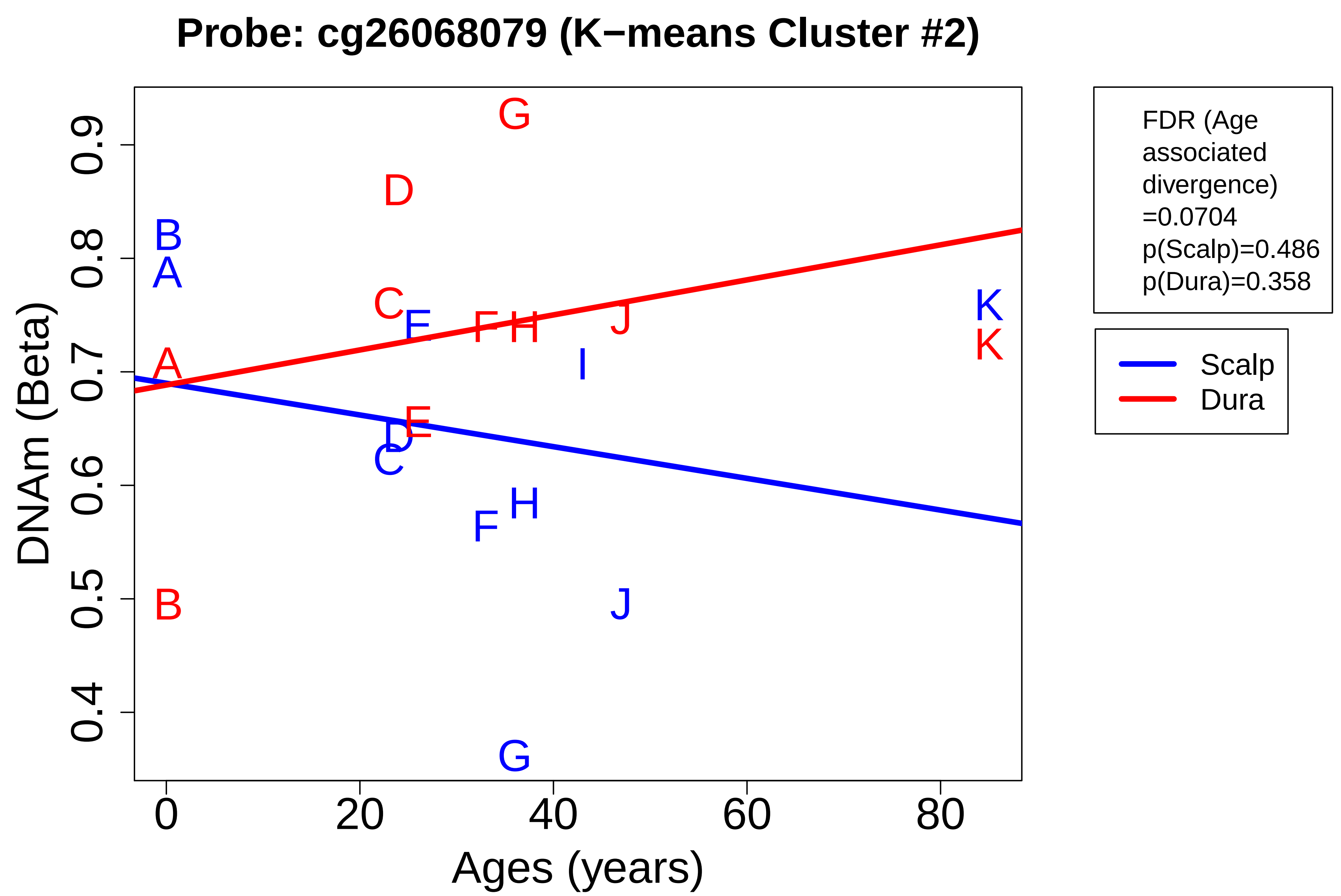

Probe: cg26068079 (K−means Cluster #2)
DNAm (Beta)
0.4
0.5
0.6
0.7
0.8
0.9
0
20
40
60
80
Ages (years)
FDR (Age associated divergence) =0.0704
p(Scalp)=0.486
p(Dura)=0.358
Scalp
Dura

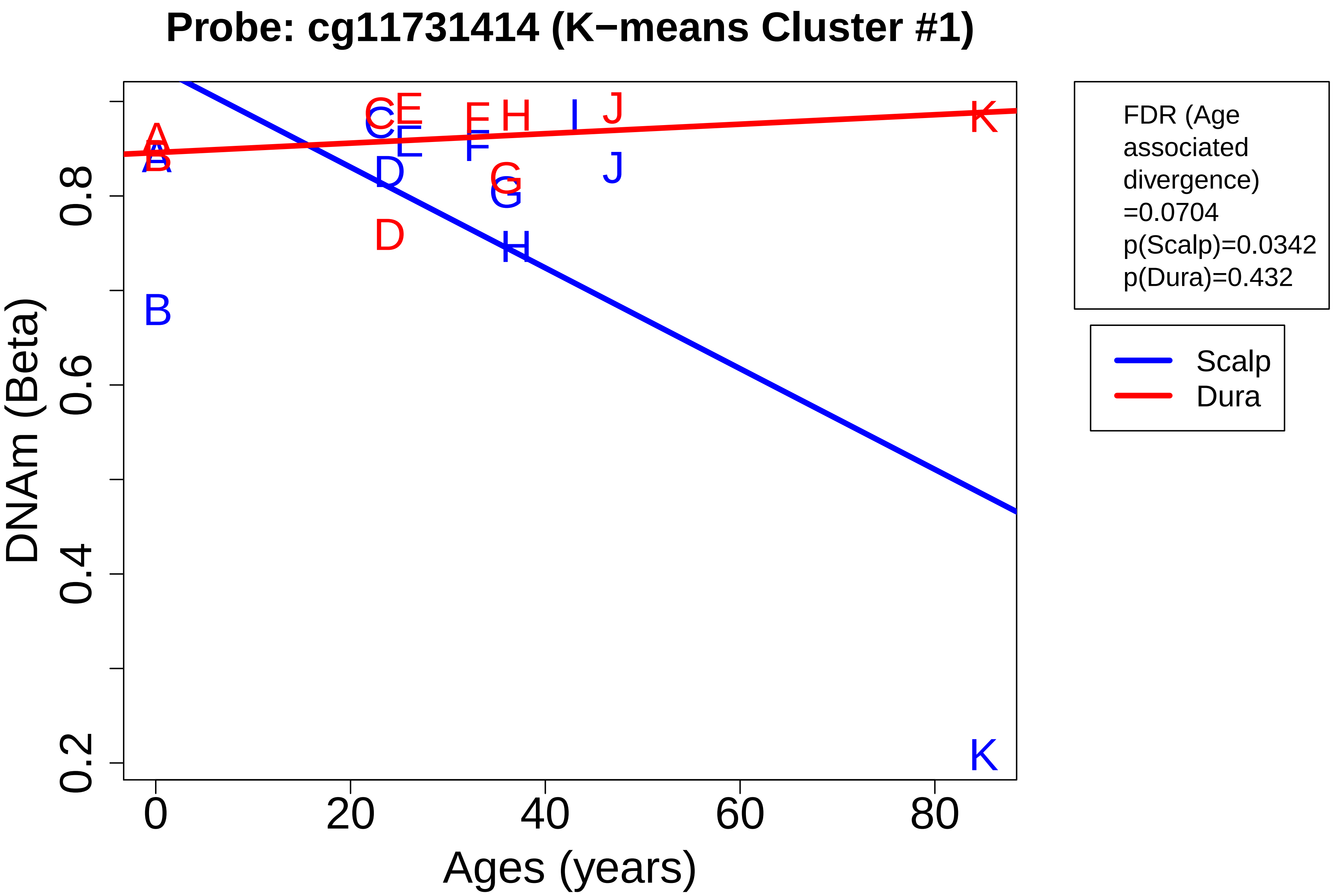

Probe: cg11731414 (K−means Cluster #1)
DNAm (Beta)
0.2
0.4
0.6
0.8
0
20
40
60
80
Ages (years)
FDR (Age associated divergence) =0.0704
p(Scalp)=0.0342
p(Dura)=0.432
Scalp
Dura

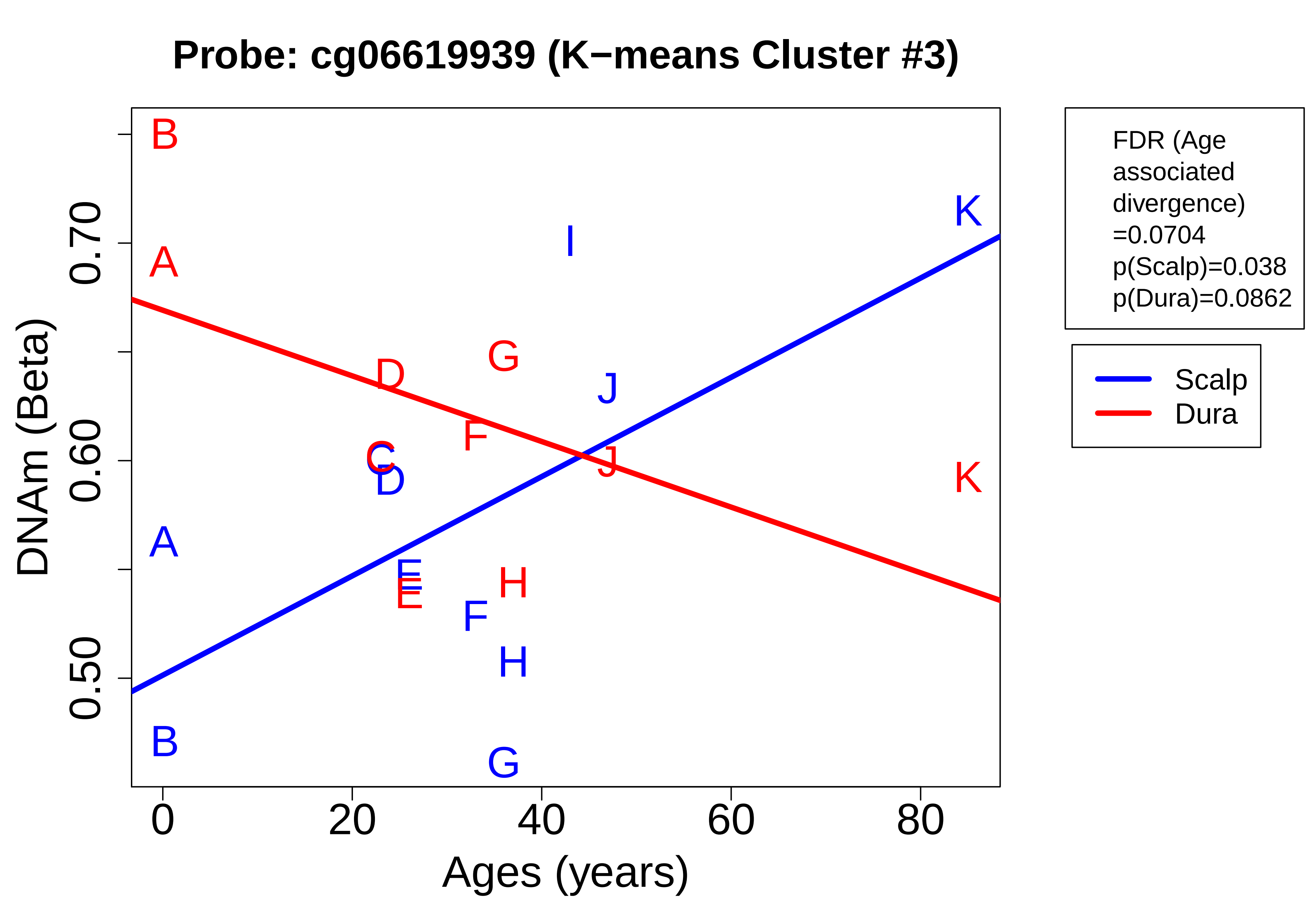

Probe: cg06619939 (K−means Cluster #3)
DNAm (Beta)
0.50
0.60
0.70
Ages (years)
0
20
40
60
80
FDR (Age associated divergence) =0.0704
p(Scalp)=0.038
p(Dura)=0.0862
Scalp
Dura
A
B
C
D
E
F
G
H
I
J
K

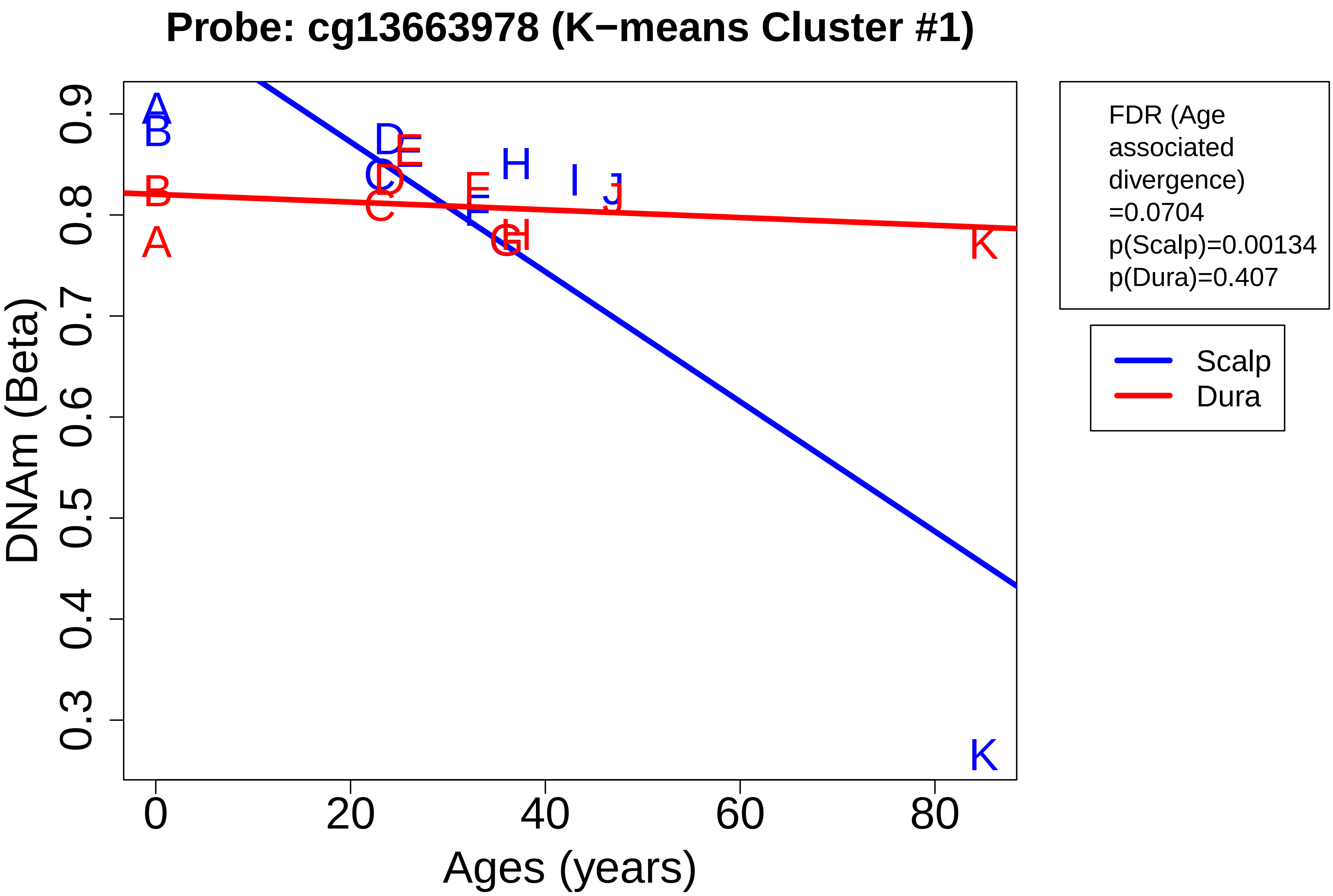

Probe: cg13663978 (K−means Cluster #1)
DNAm (Beta)
0.3
0.4
0.5
0.6
0.7
0.8
0.9
0
20
40
60
80
Ages (years)
A
B
C
D
E
F
G
H
I
J
K
FDR (Age associated divergence) =0.0704
p(Scalp)=0.00134
p(Dura)=0.407
Scalp
Dura

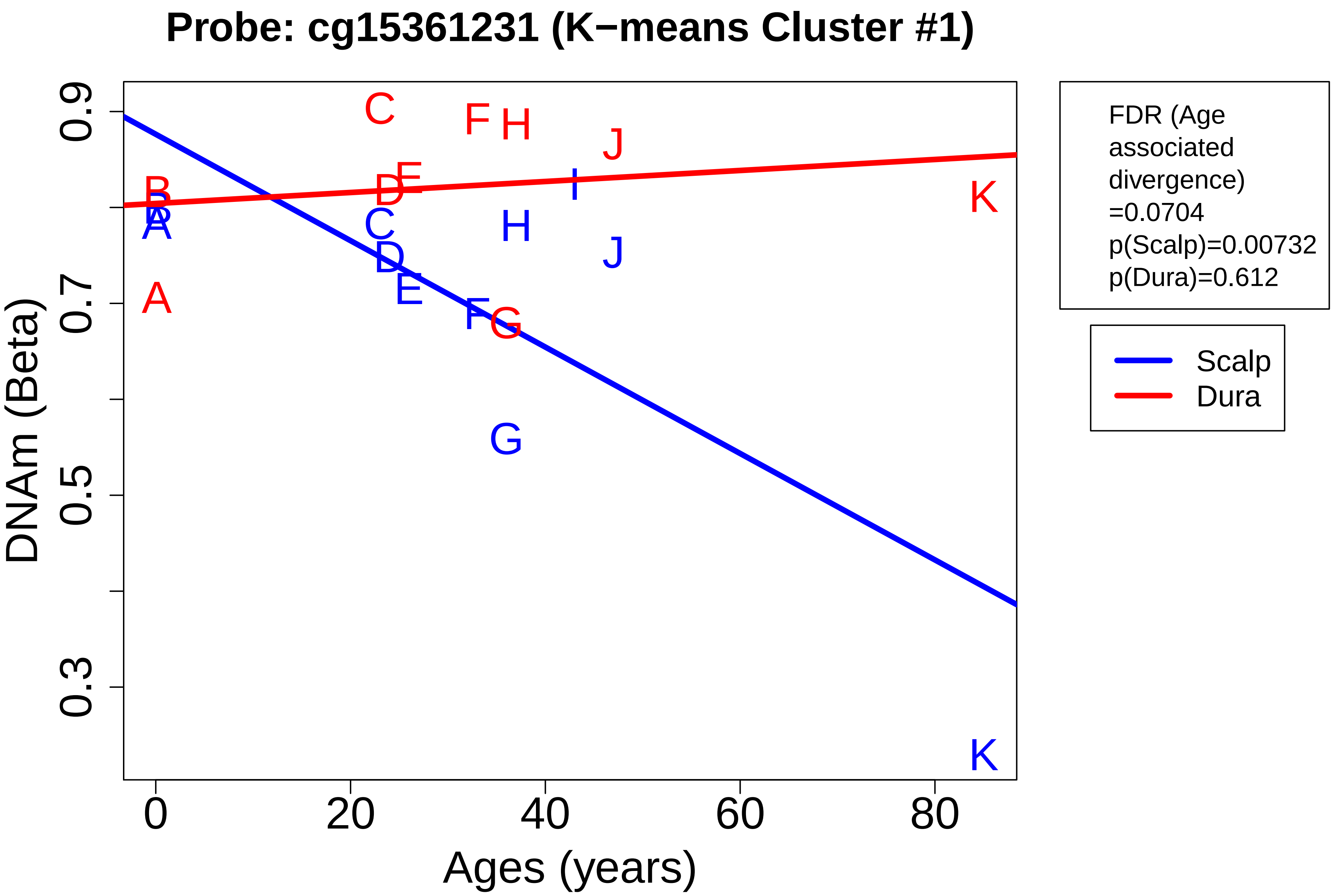

Probe: cg15361231 (K−means Cluster #1)
DNAm (Beta)
0.3
0.5
0.7
0.9
0
20
40
60
80
Ages (years)
FDR (Age associated divergence) =0.0704
p(Scalp)=0.00732
p(Dura)=0.612
Scalp
Dura

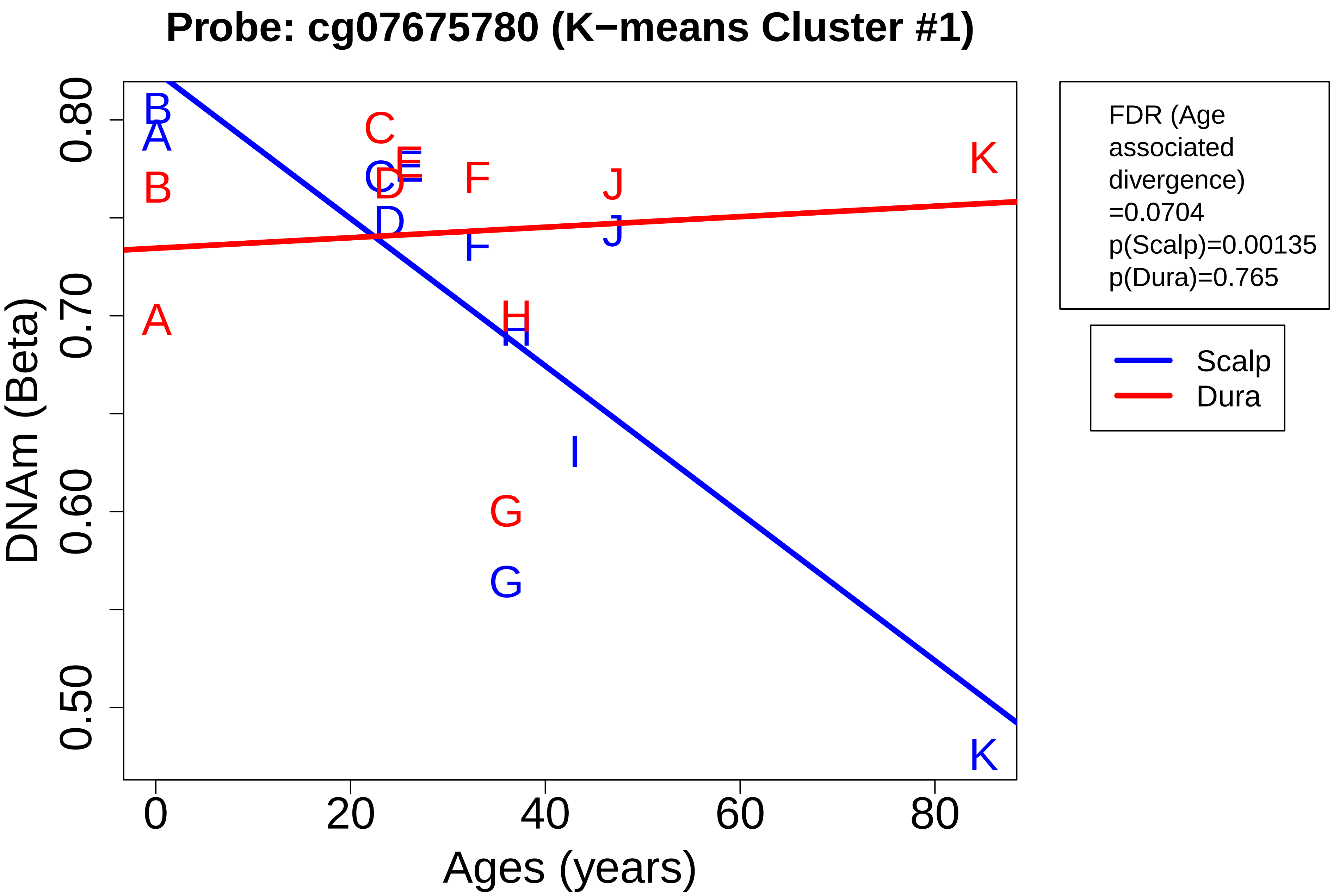

Probe: cg07675780 (K−means Cluster #1)
DNAm (Beta)
0.50
0.60
0.70
0.80
Ages (years)
0
20
40
60
80
A
B
C
D
E
F
G
H
I
J
K
FDR (Age associated divergence) =0.0704
p(Scalp)=0.00135
p(Dura)=0.765
Scalp
Dura

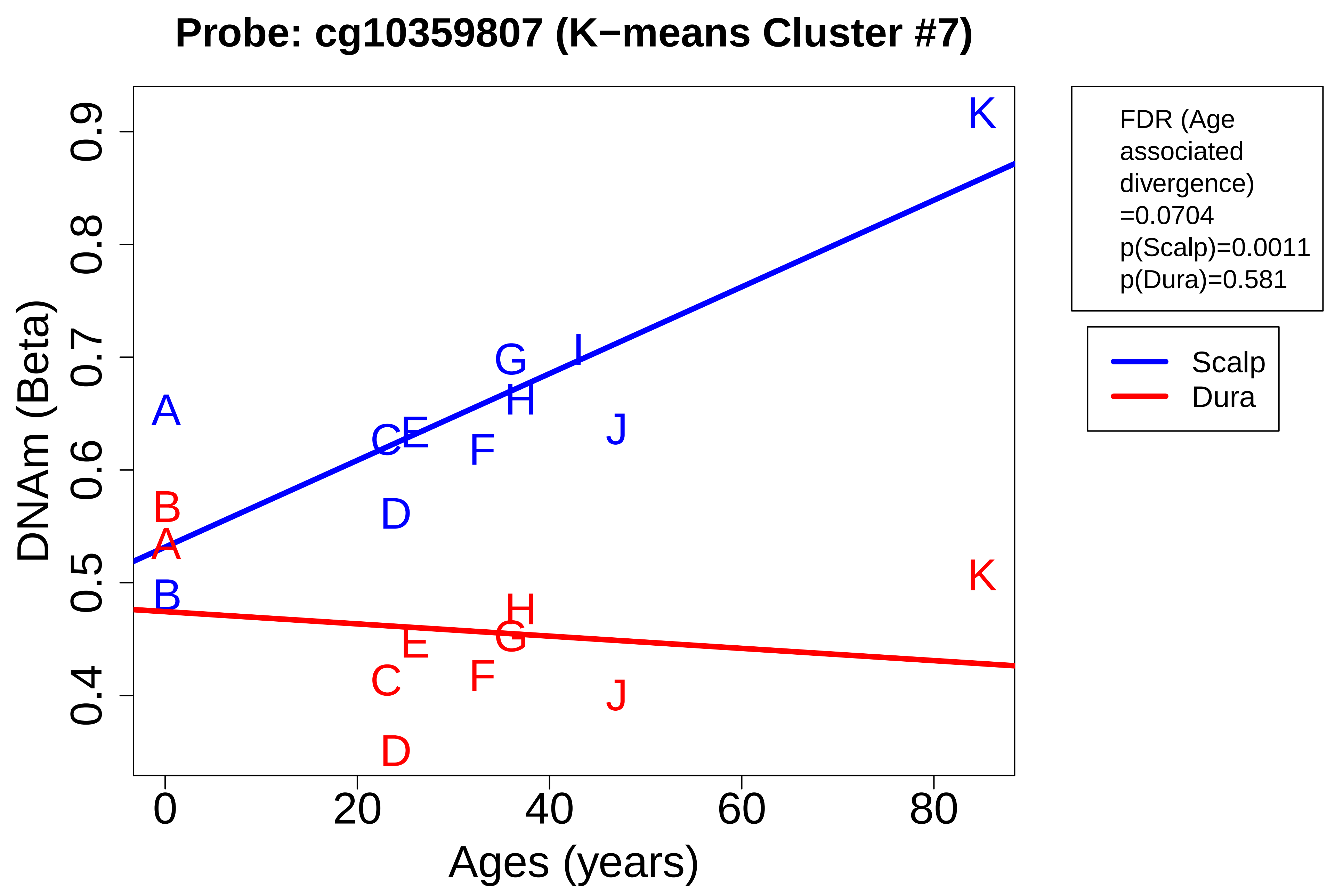

Probe: cg10359807 (K−means Cluster #7)
DNAm (Beta)
0.4
0.5
0.6
0.7
0.8
0.9
Ages (years)
0
20
40
60
80
FDR (Age associated divergence) =0.0704
p(Scalp)=0.0011
p(Dura)=0.581
Scalp
Dura
A
B
C
D
E
F
G
H
I
J
K

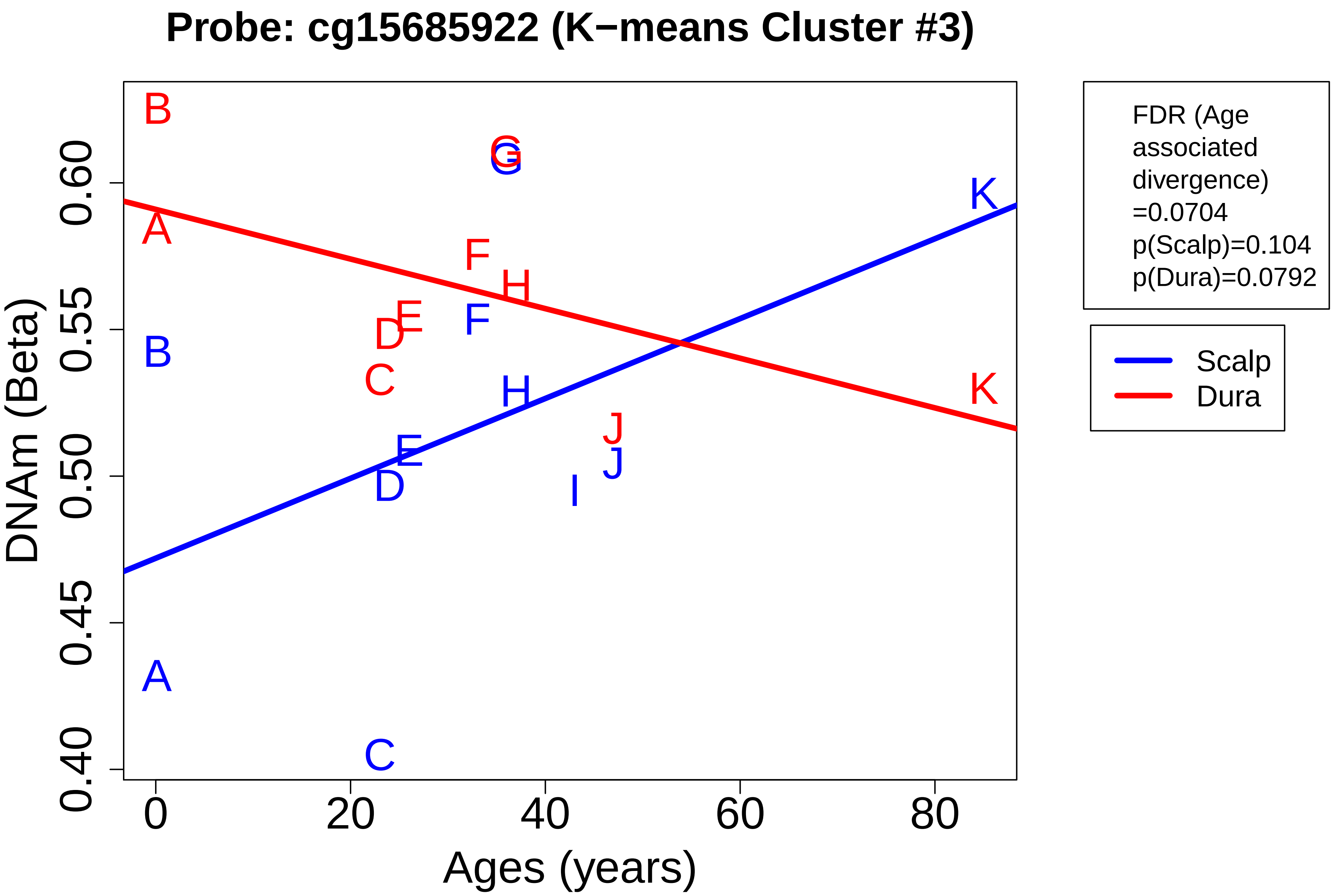
Probe: cg15685922 (K−means Cluster #3)
FDR (Age associated divergence) =0.0704
p(Scalp)=0.104
p(Dura)=0.0792
Scalp
Dura
DNAm (Beta)
0.40
0.45
0.50
0.55
0.60
0
20
40
60
80
Ages (years)
A
B
C
D
E
F
G
H
I
J
K

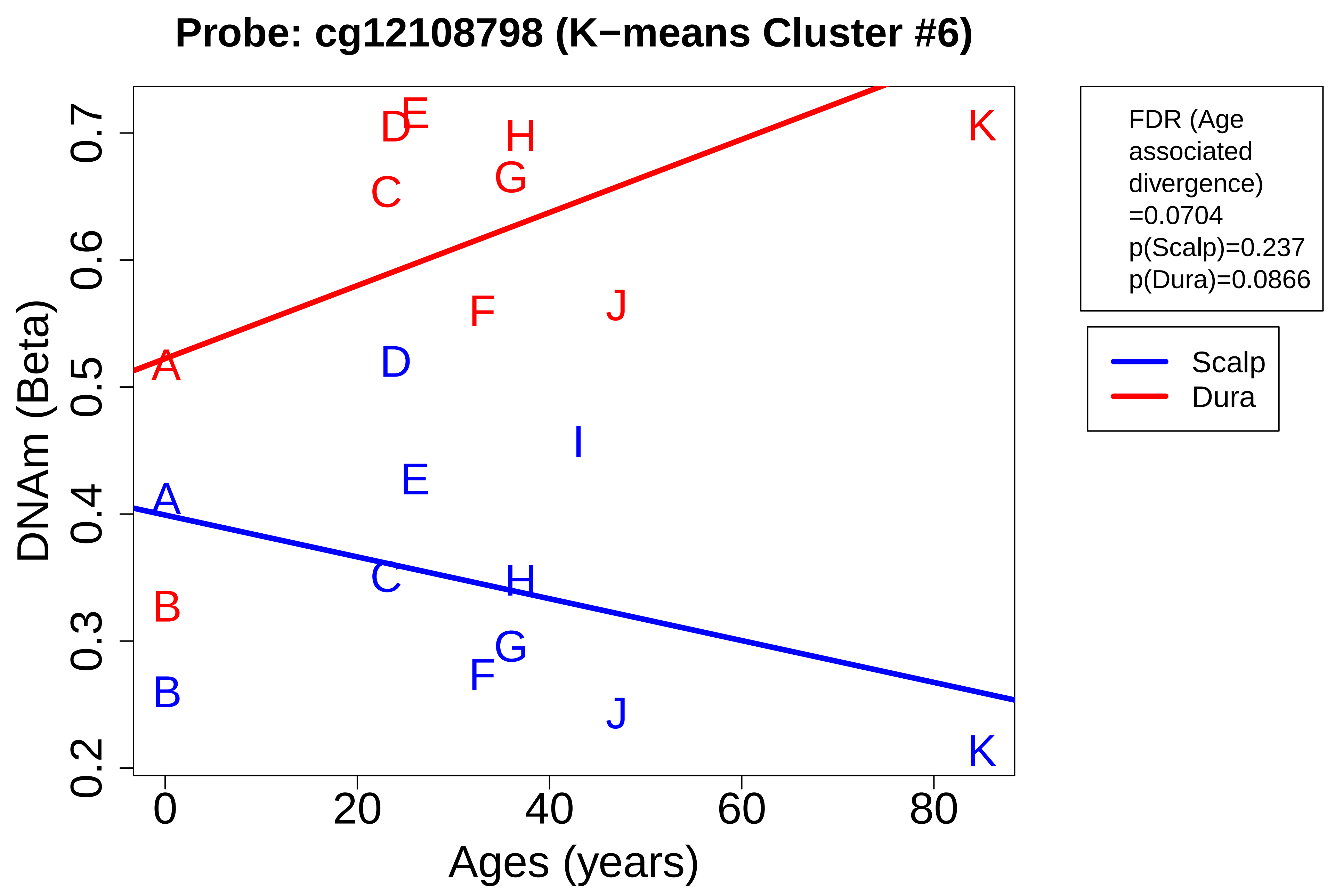

Probe: cg12108798 (K−means Cluster #6)
DNAm (Beta)
0.2
0.3
0.4
0.5
0.6
0.7
0
20
40
60
80
Ages (years)
FDR (Age associated divergence) =0.0704
p(Scalp)=0.237
p(Dura)=0.0866
Scalp
Dura
A
B
C
D
E
F
G
H
I
J
K

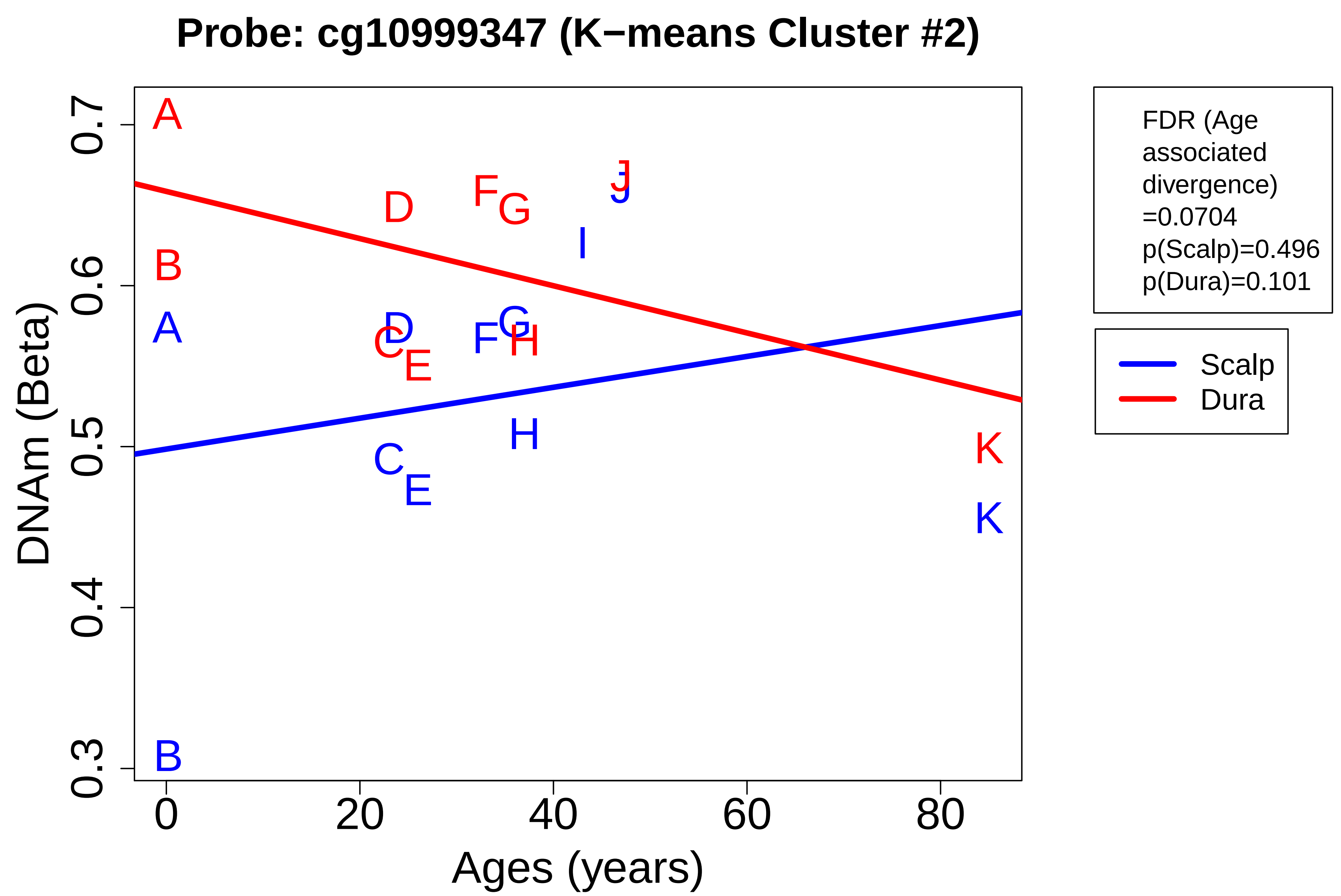

Probe: cg10999347 (K−means Cluster #2)
DNAm (Beta)
0.3
0.4
0.5
0.6
0.7
0
20
40
60
80
Ages (years)
A
B
C
D
E
F
G
H
I
J
K
FDR (Age associated divergence) =0.0704
p(Scalp)=0.496
p(Dura)=0.101
Scalp
Dura

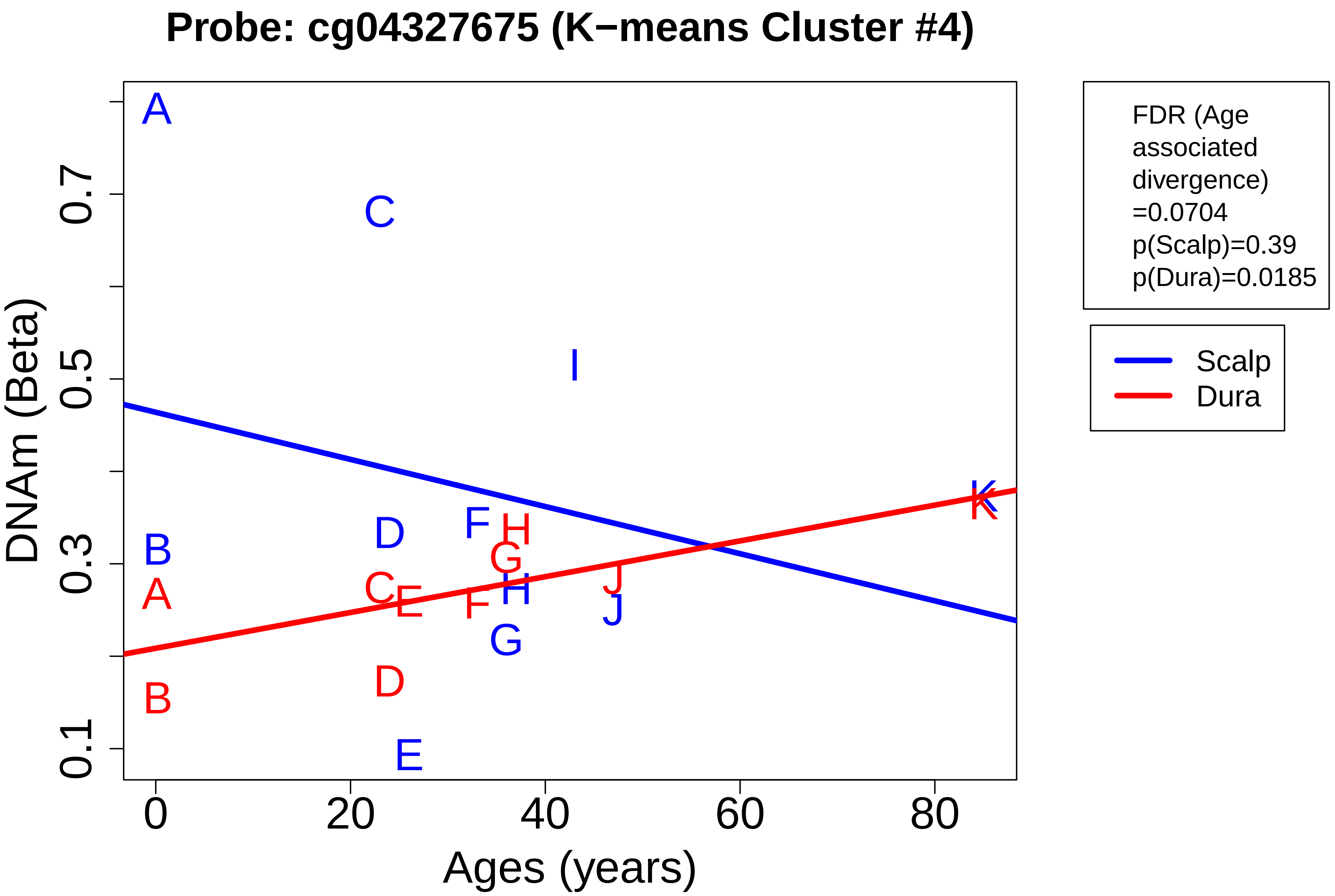
Probe: cg04327675 (K−means Cluster #4)
DNAm (Beta)
0.1
0.3
0.5
0.7
0
20
40
60
80
Ages (years)
A
B
C
D
E
F
G
H
I
J
K
FDR (Age associated divergence) =0.0704
p(Scalp)=0.39
p(Dura)=0.0185
Scalp
Dura

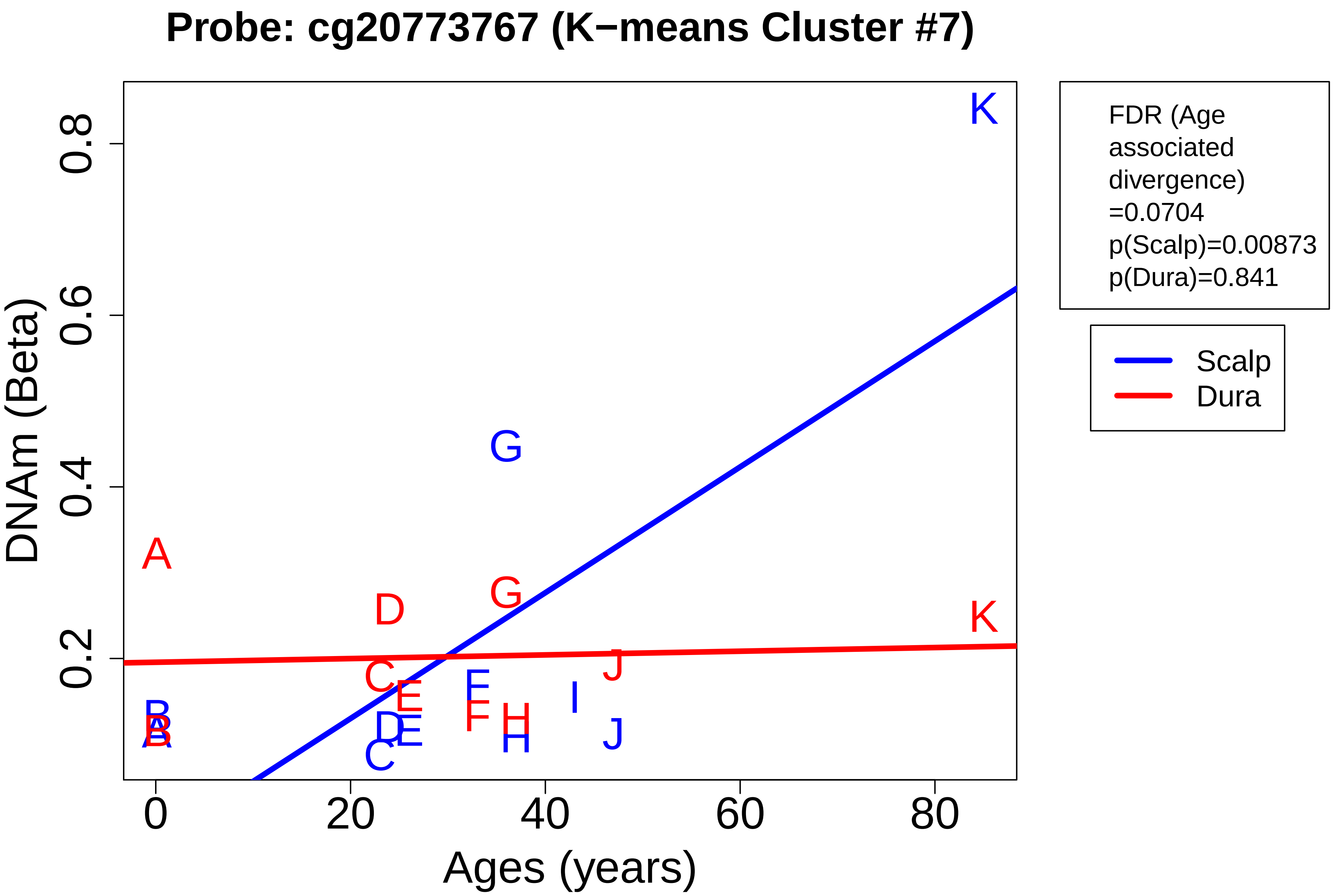
Probe: cg20773767 (K−means Cluster #7)
DNAm (Beta)
Ages (years)
0.2
0.4
0.6
0.8
0
20
40
60
80
FDR (Age associated divergence) =0.0704
p(Scalp)=0.00873
p(Dura)=0.841
Scalp
Dura

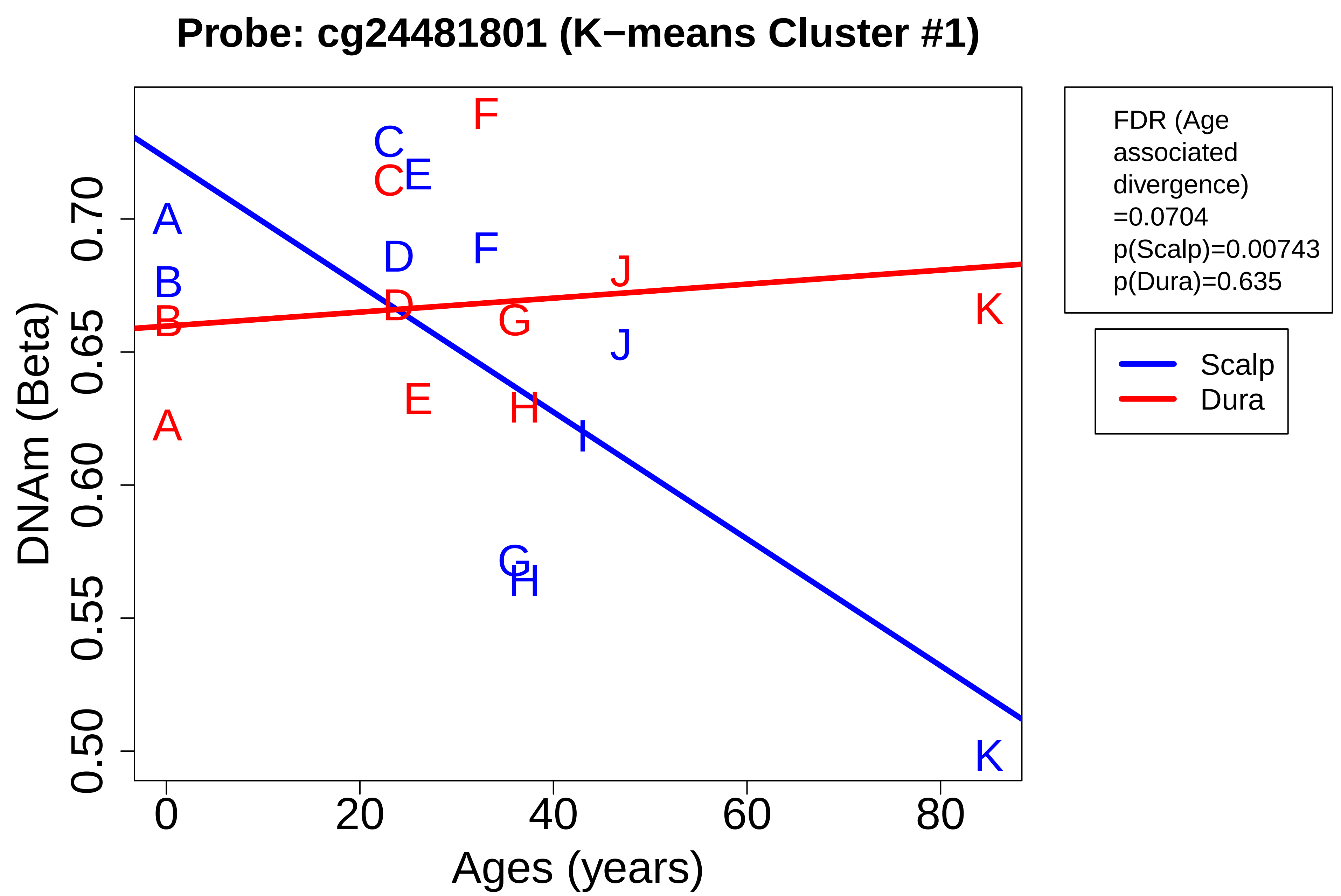
Probe: cg24481801 (K−means Cluster #1)
DNAm (Beta)
0.50
0.55
0.60
0.65
0.70
Ages (years)
0
20
40
60
80
FDR (Age associated divergence) =0.0704
p(Scalp)=0.00743
p(Dura)=0.635
Scalp
Dura
A
B
C
D
E
F
G
H
I
J
K

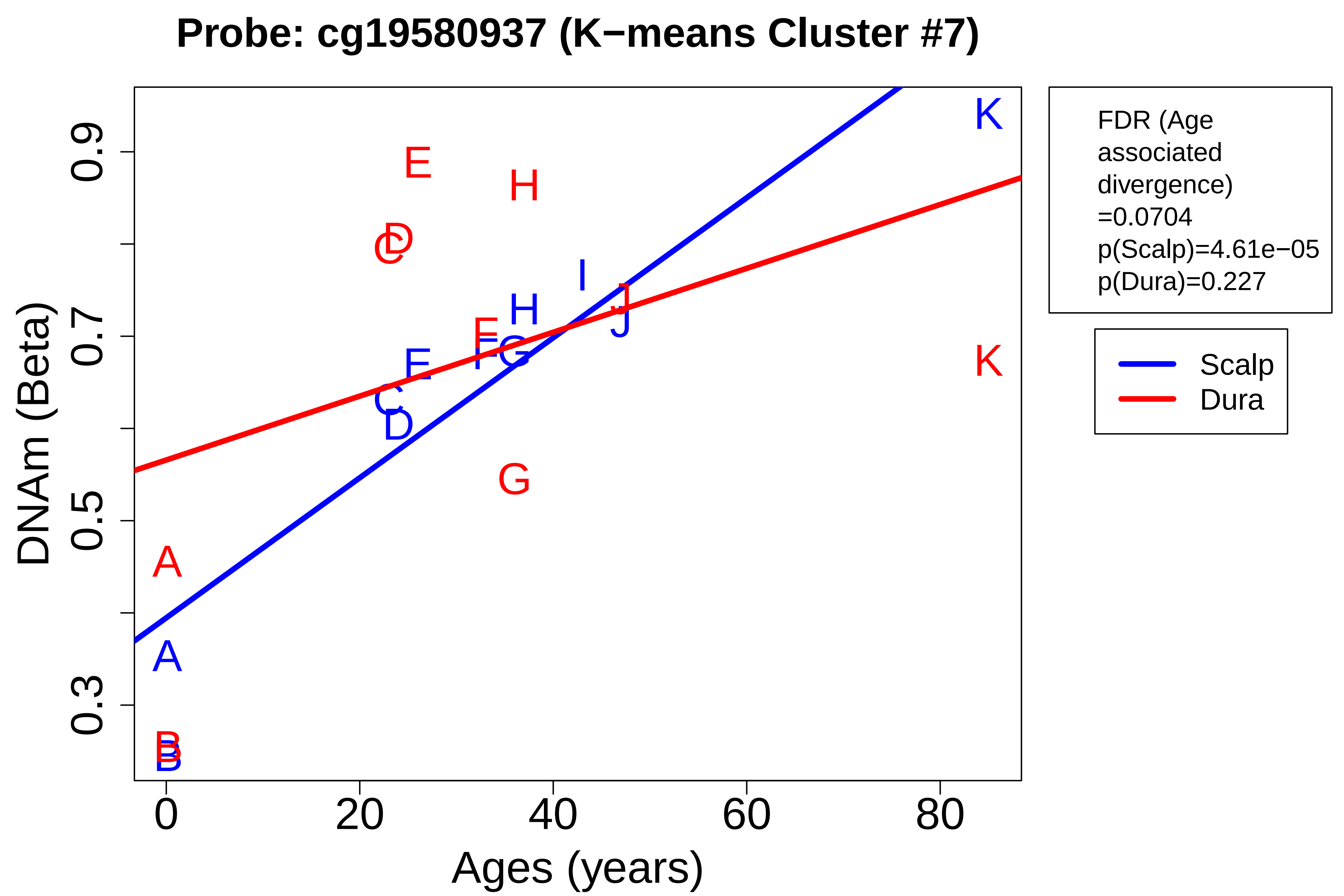

Probe: cg19580937 (K−means Cluster #7)
DNAm (Beta)
0.3
0.5
0.7
0.9
Ages (years)
0
20
40
60
80
A
B
C
D
E
F
G
H
I
J
K
FDR (Age associated divergence) =0.0704
p(Scalp)=4.61e−05
p(Dura)=0.227
Scalp
Dura

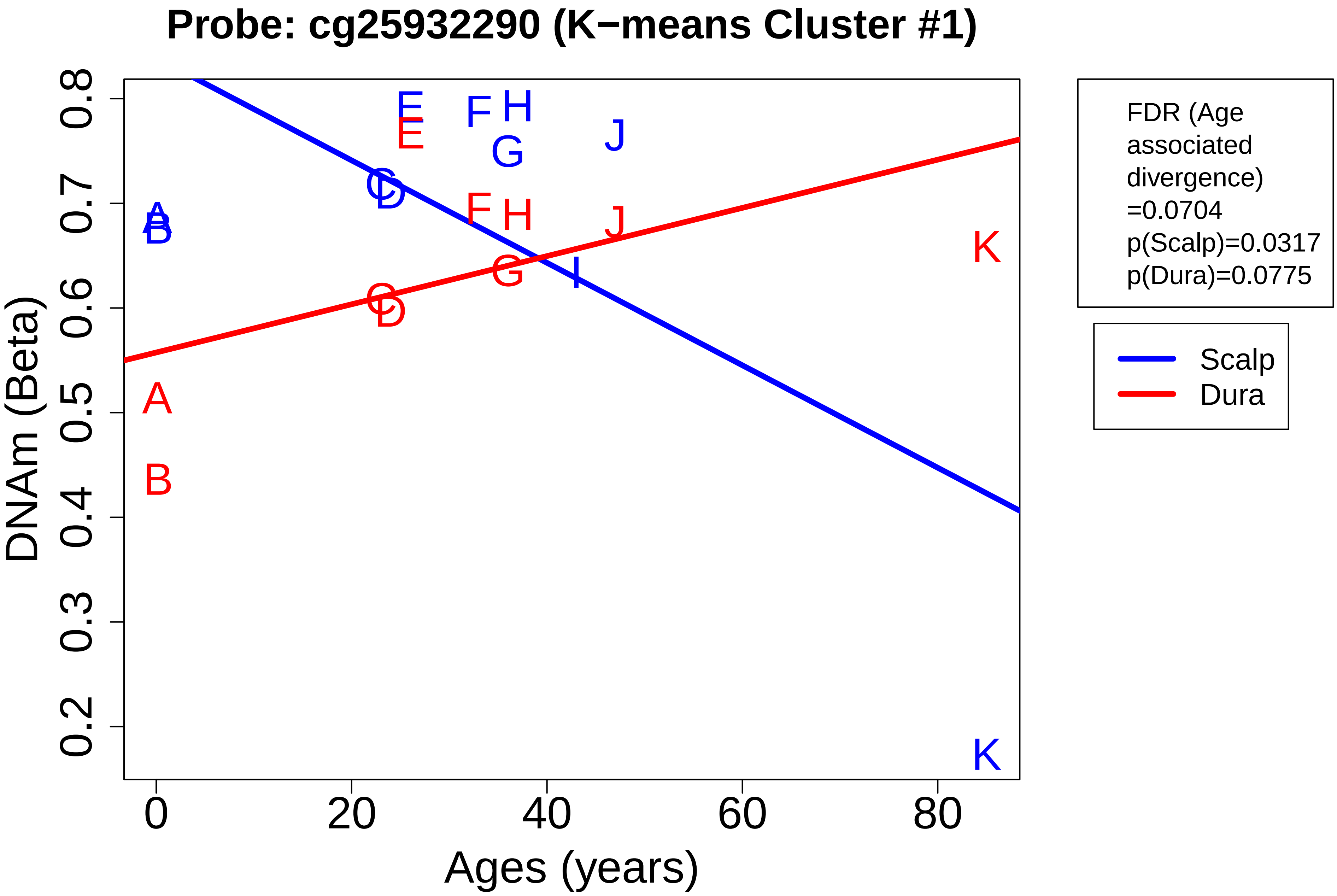
Probe: cg25932290 (K−means Cluster #1)
DNAm (Beta)
0.2
0.3
0.4
0.5
0.6
0.7
0.8
0
20
40
60
80
Ages (years)
A
B
C
D
E
F
G
H
I
J
K
FDR (Age associated divergence) =0.0704
p(Scalp)=0.0317
p(Dura)=0.0775
Scalp
Dura

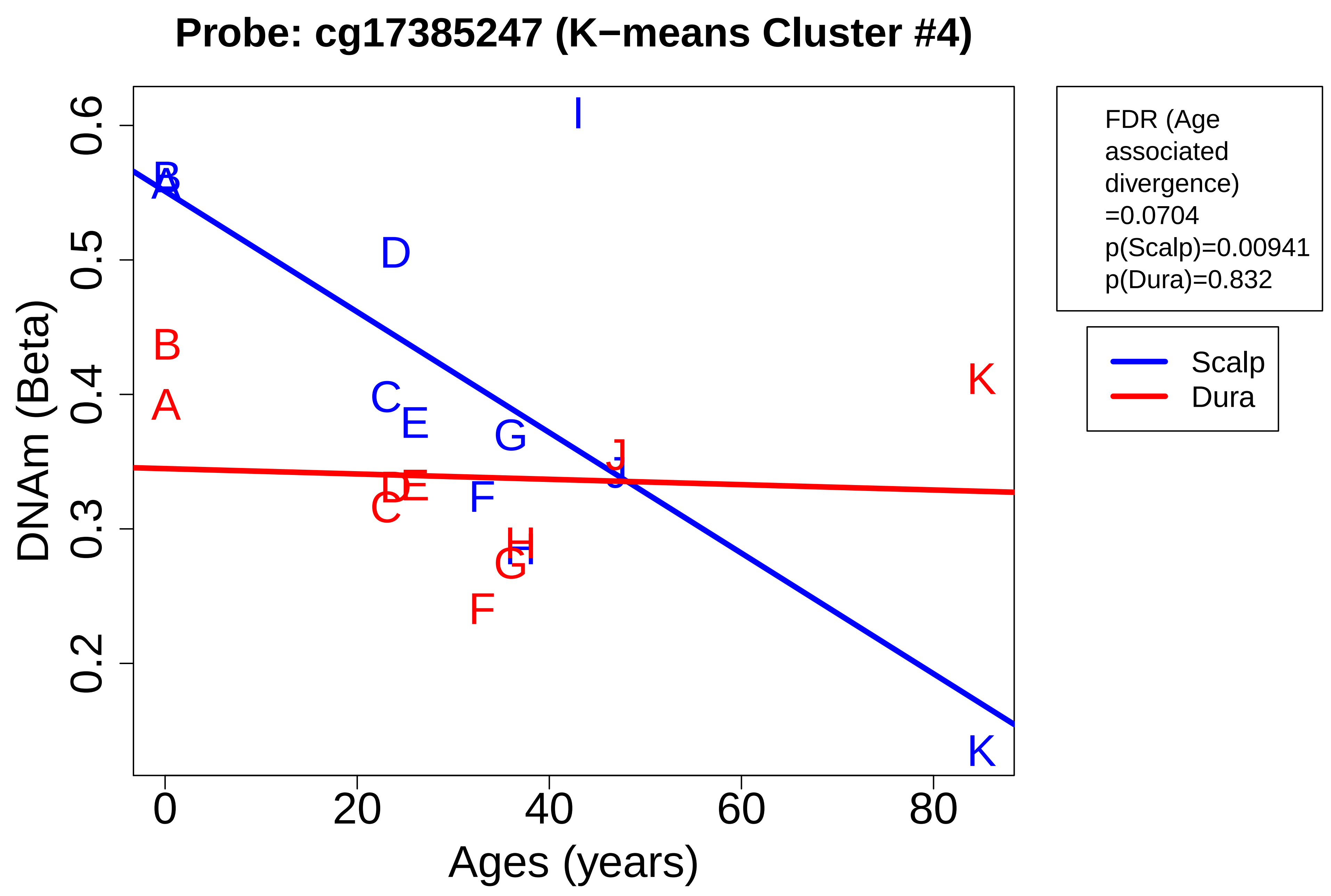

Probe: cg17385247 (K−means Cluster #4)
DNAm (Beta)
0.2
0.3
0.4
0.5
0.6
0
20
40
60
80
Ages (years)
FDR (Age associated divergence) =0.0704
p(Scalp)=0.00941
p(Dura)=0.832
Scalp
Dura

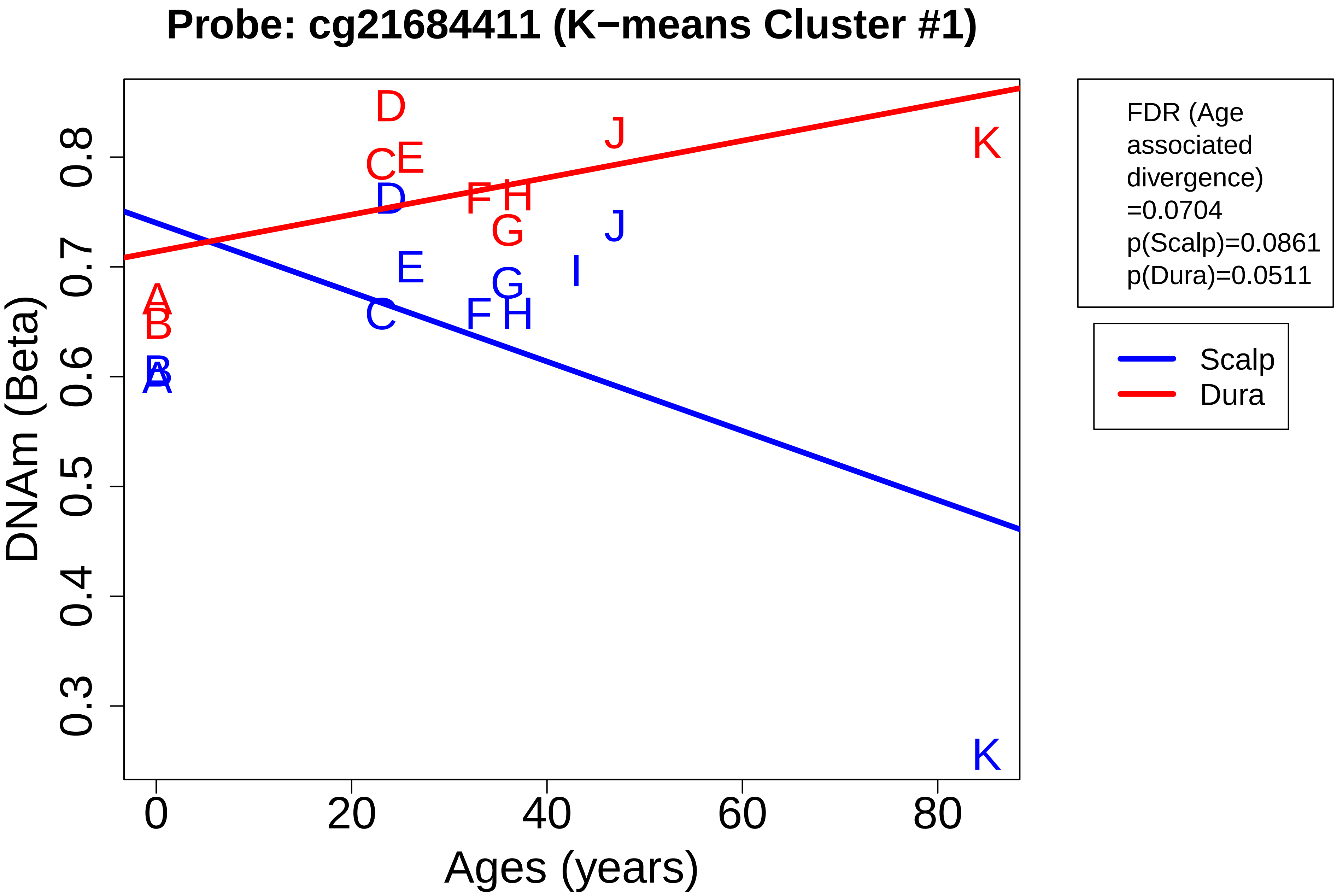

Probe: cg21684411 (K−means Cluster #1)
DNAm (Beta)
0.3
0.4
0.5
0.6
0.7
0.8
Ages (years)
0
20
40
60
80
FDR (Age associated divergence) =0.0704
p(Scalp)=0.0861
p(Dura)=0.0511
Scalp
Dura
A B C D E F G H I J K

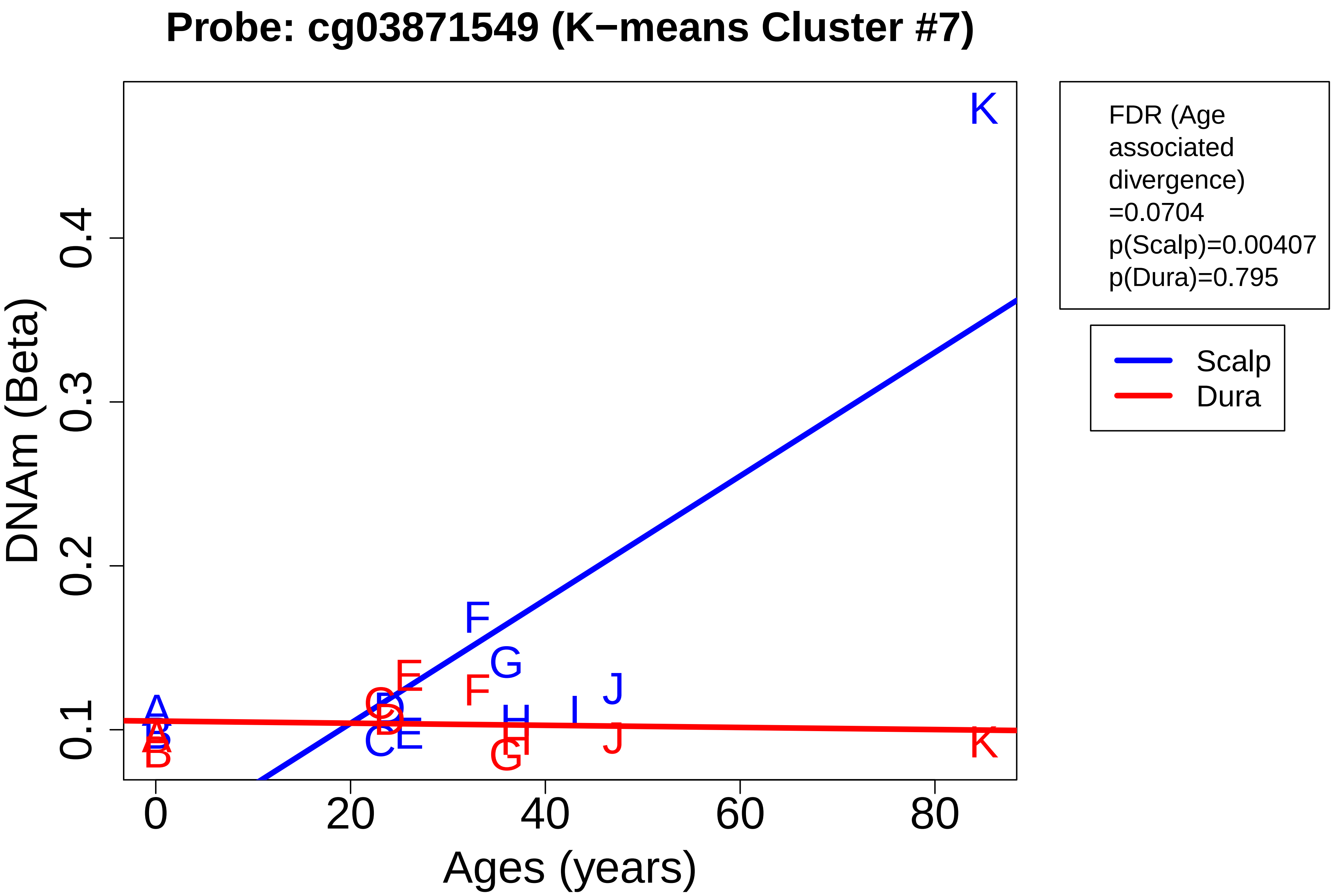

Probe: cg03871549 (K−means Cluster #7)
FDR (Age associated divergence) =0.0704
p(Scalp)=0.00407
p(Dura)=0.795
Scalp
Dura
DNAm (Beta)
0.1
0.2
0.3
0.4
0
20
40
60
80
Ages (years)

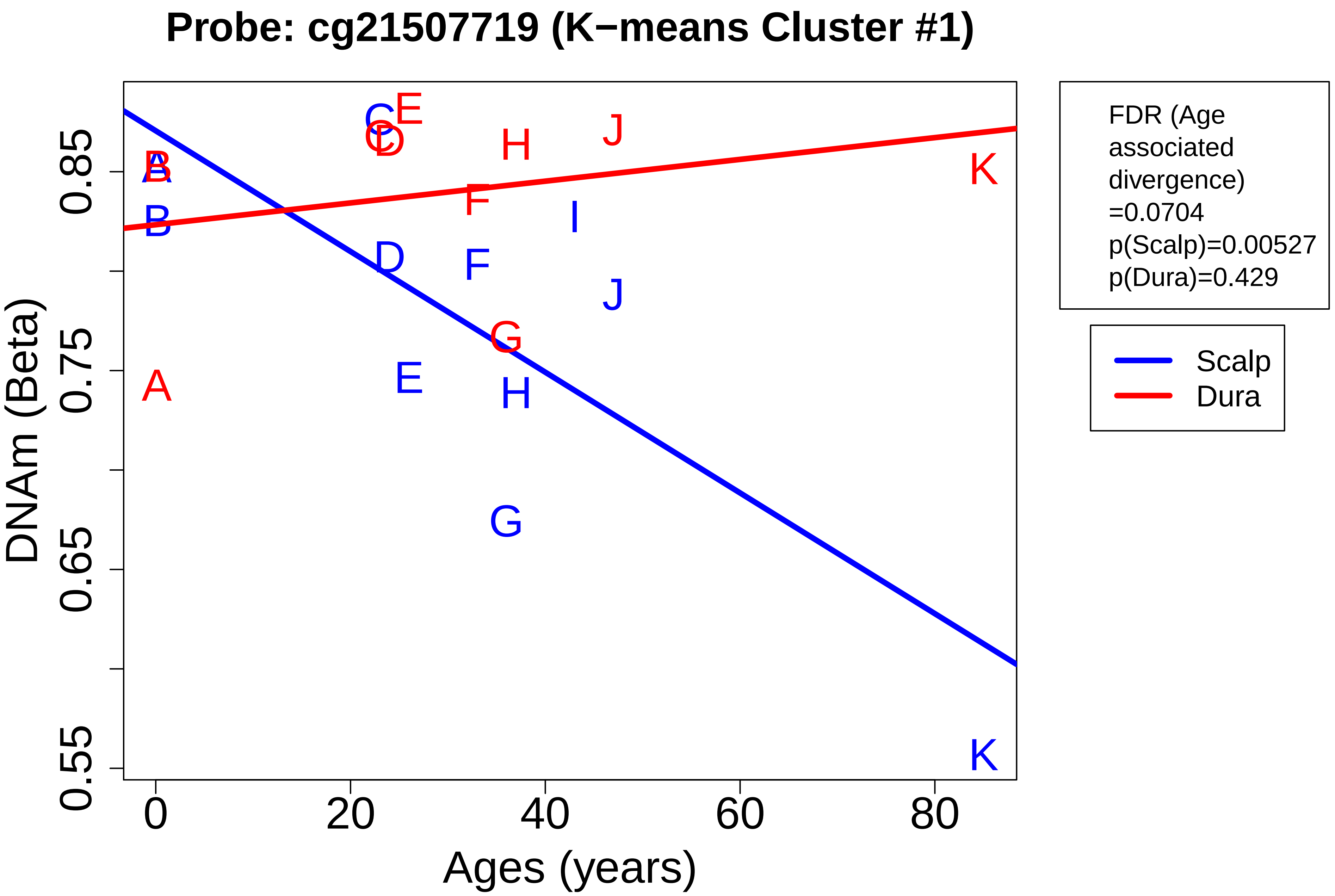
Probe: cg21507719 (K−means Cluster #1)
DNAm (Beta)
0.55
0.65
0.75
0.85
0
20
40
60
80
Ages (years)
FDR (Age associated divergence) =0.0704
p(Scalp)=0.00527
p(Dura)=0.429
Scalp
Dura

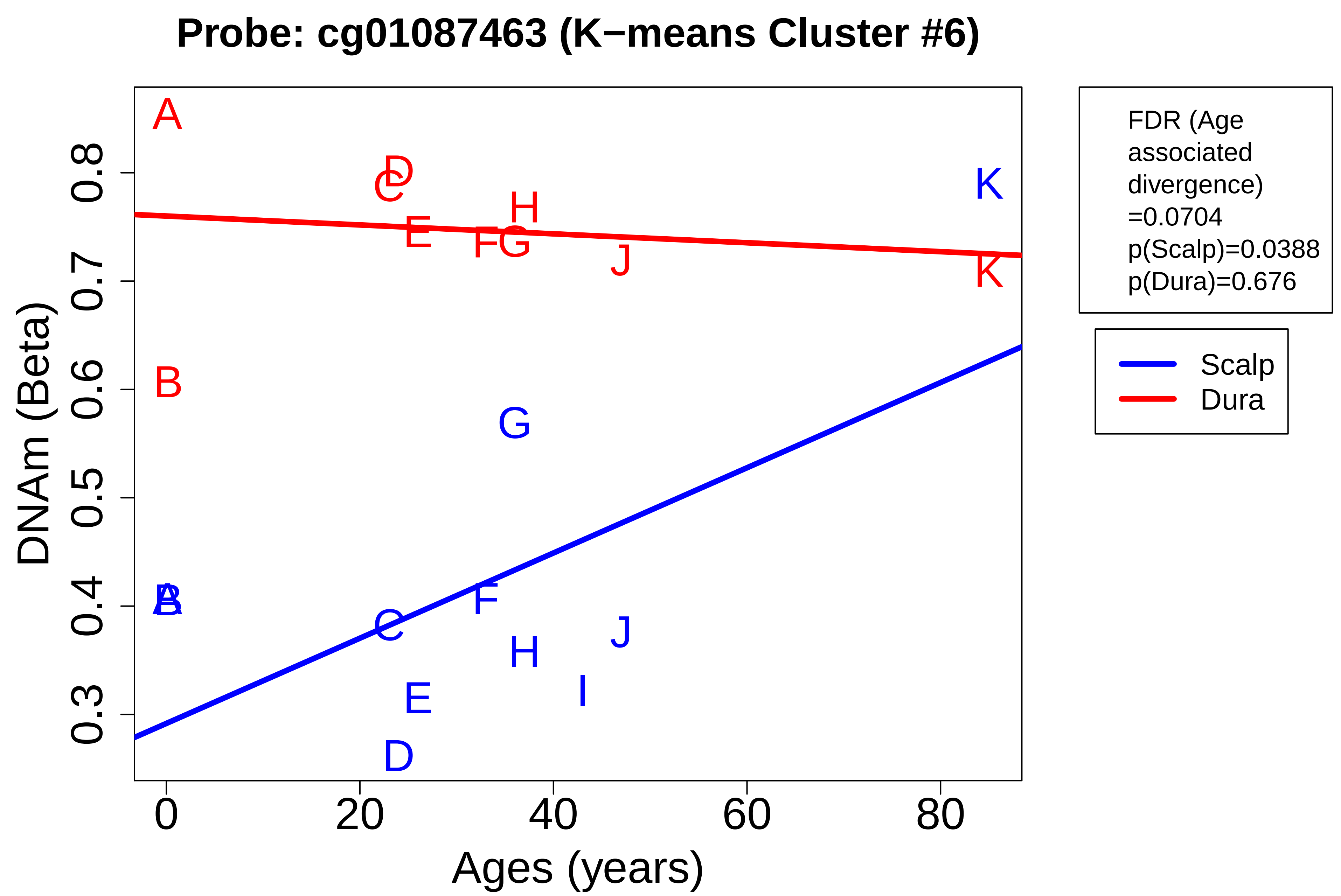

Probe: cg01087463 (K−means Cluster #6)
DNAm (Beta)
0.3
0.4
0.5
0.6
0.7
0.8
0
20
40
60
80
Ages (years)
FDR (Age associated divergence) =0.0704
p(Scalp)=0.0388
p(Dura)=0.676
Scalp
Dura

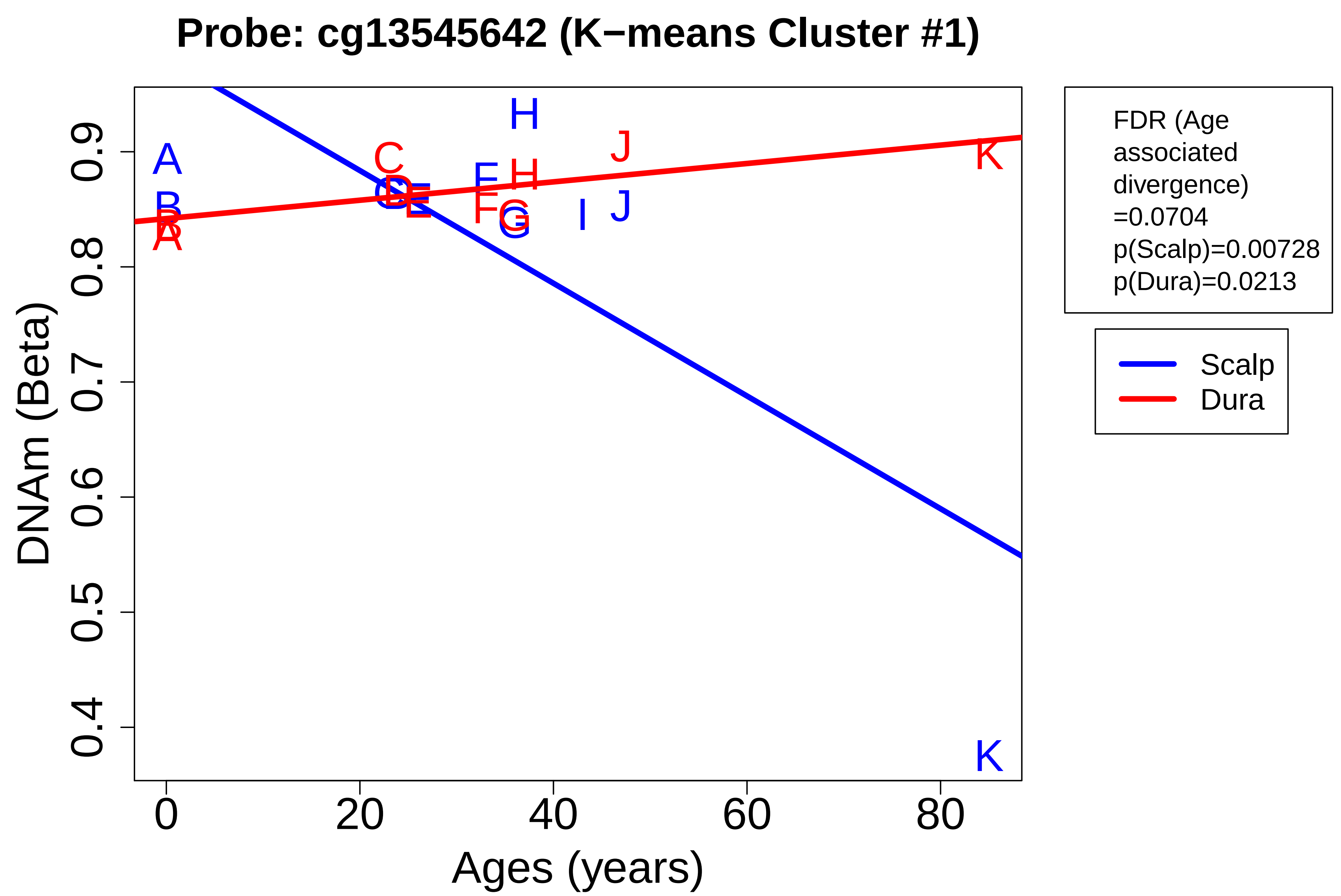

Probe: cg13545642 (K−means Cluster #1)
DNAm (Beta)
0.4
0.5
0.6
0.7
0.8
0.9
0
20
40
60
80
Ages (years)
FDR (Age associated divergence) =0.0704
p(Scalp)=0.00728
p(Dura)=0.0213
Scalp
Dura

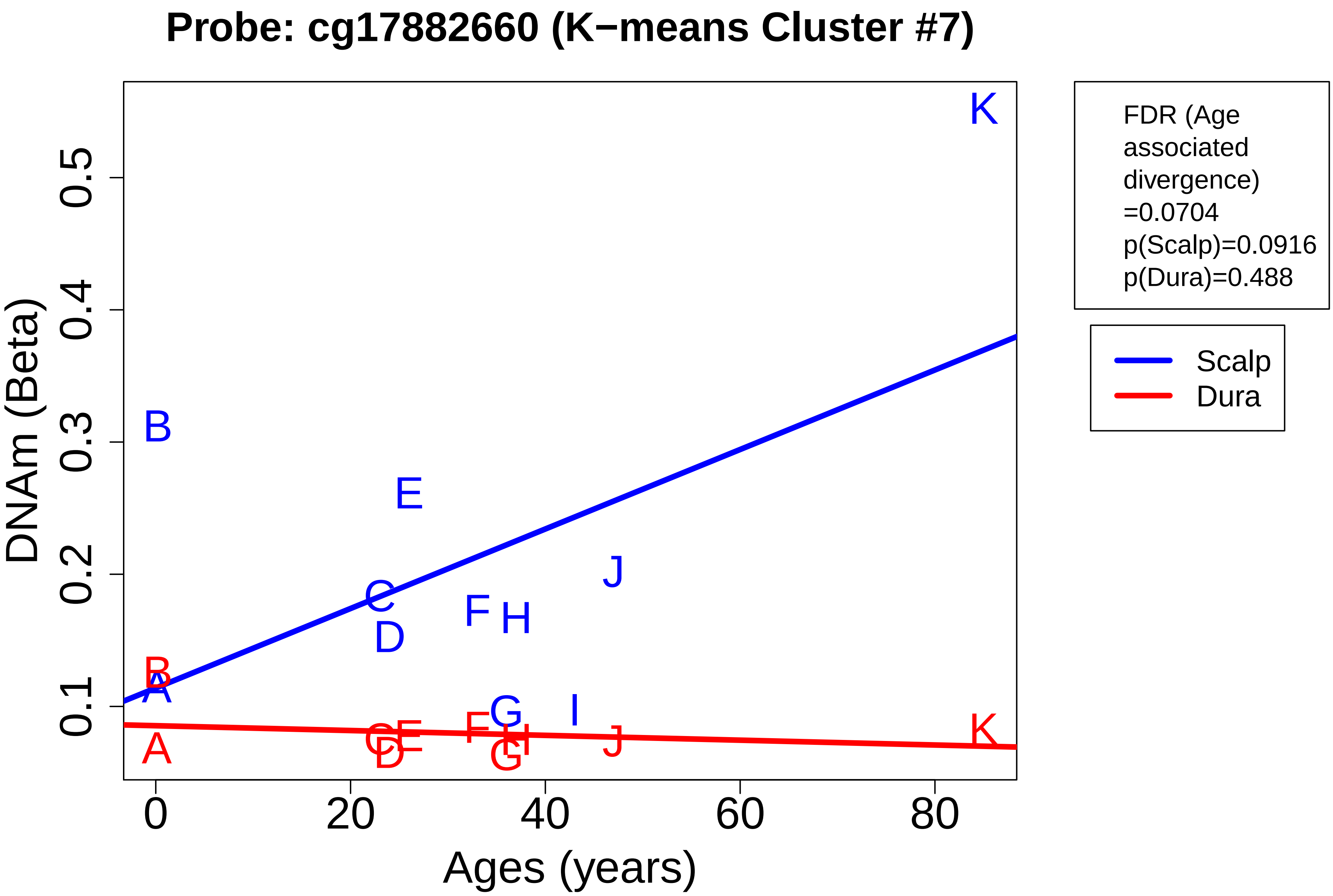

Probe: cg17882660 (K−means Cluster #7)
DNAm (Beta)
0.1
0.2
0.3
0.4
0.5
0
20
40
60
80
Ages (years)
FDR (Age associated divergence) =0.0704
p(Scalp)=0.0916
p(Dura)=0.488
Scalp
Dura

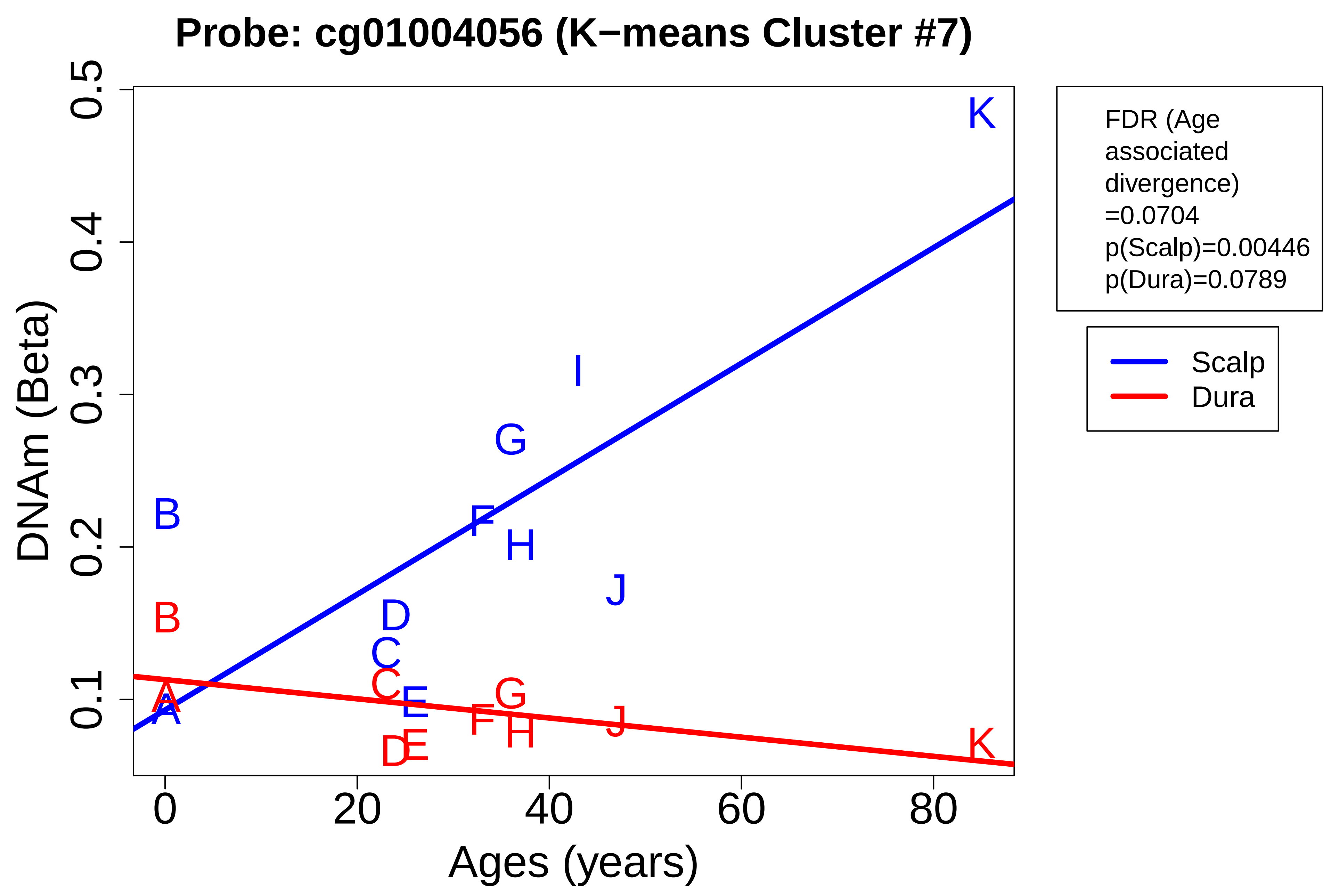

Probe: cg01004056 (K−means Cluster #7)
DNAm (Beta)
0.1
0.2
0.3
0.4
0.5
0
20
40
60
80
Ages (years)
FDR (Age associated divergence) =0.0704
p(Scalp)=0.00446
p(Dura)=0.0789
Scalp
Dura
A
B
C
D
E
F
G
H
I
J
K

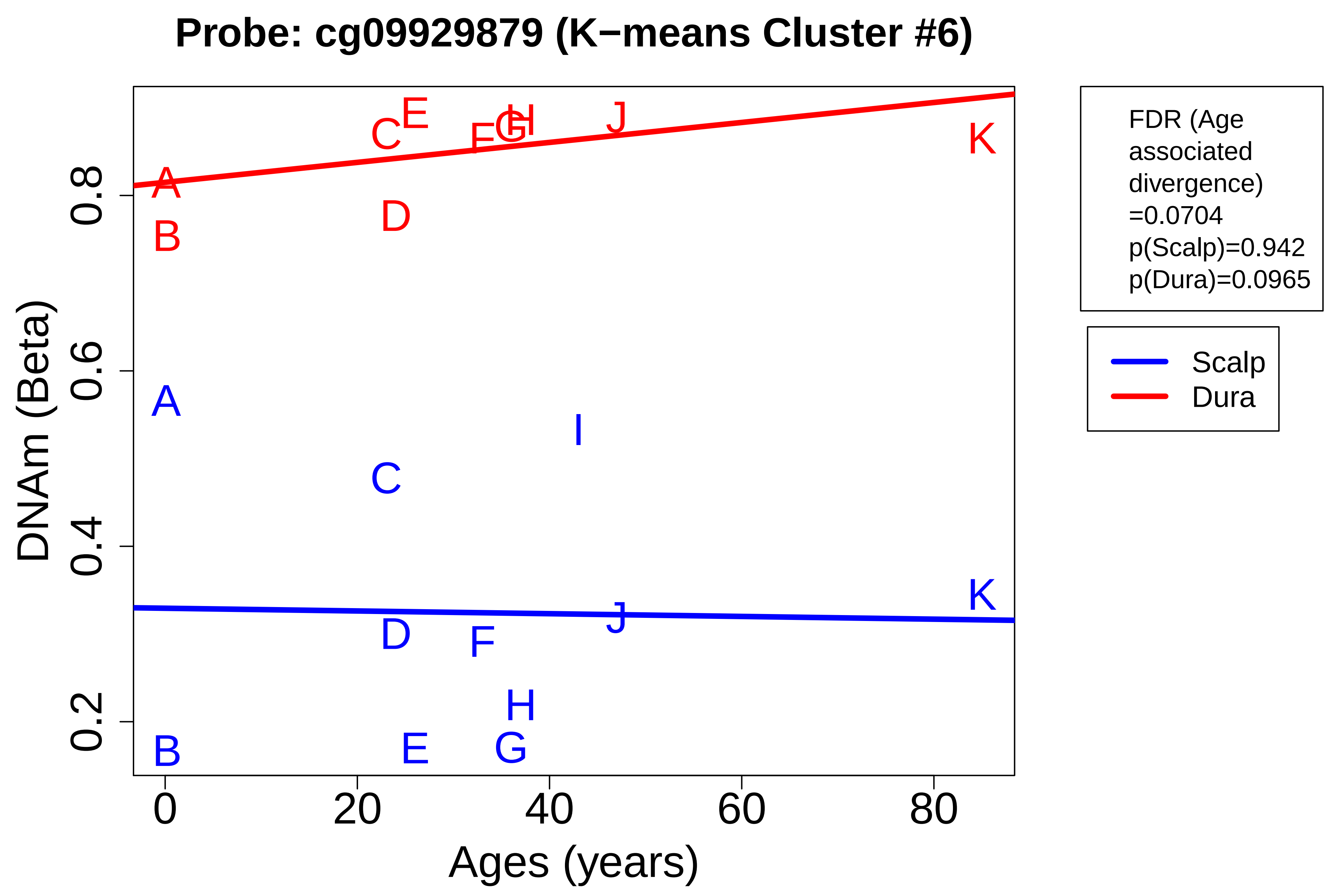

Probe: cg09929879 (K−means Cluster #6)
FDR (Age associated divergence) =0.0704
p(Scalp)=0.942
p(Dura)=0.0965
Scalp
Dura
DNAm (Beta)
0.2
0.4
0.6
0.8
Ages (years)
0
20
40
60
80
A
B
C
D
E
F
G
H
I
J
K

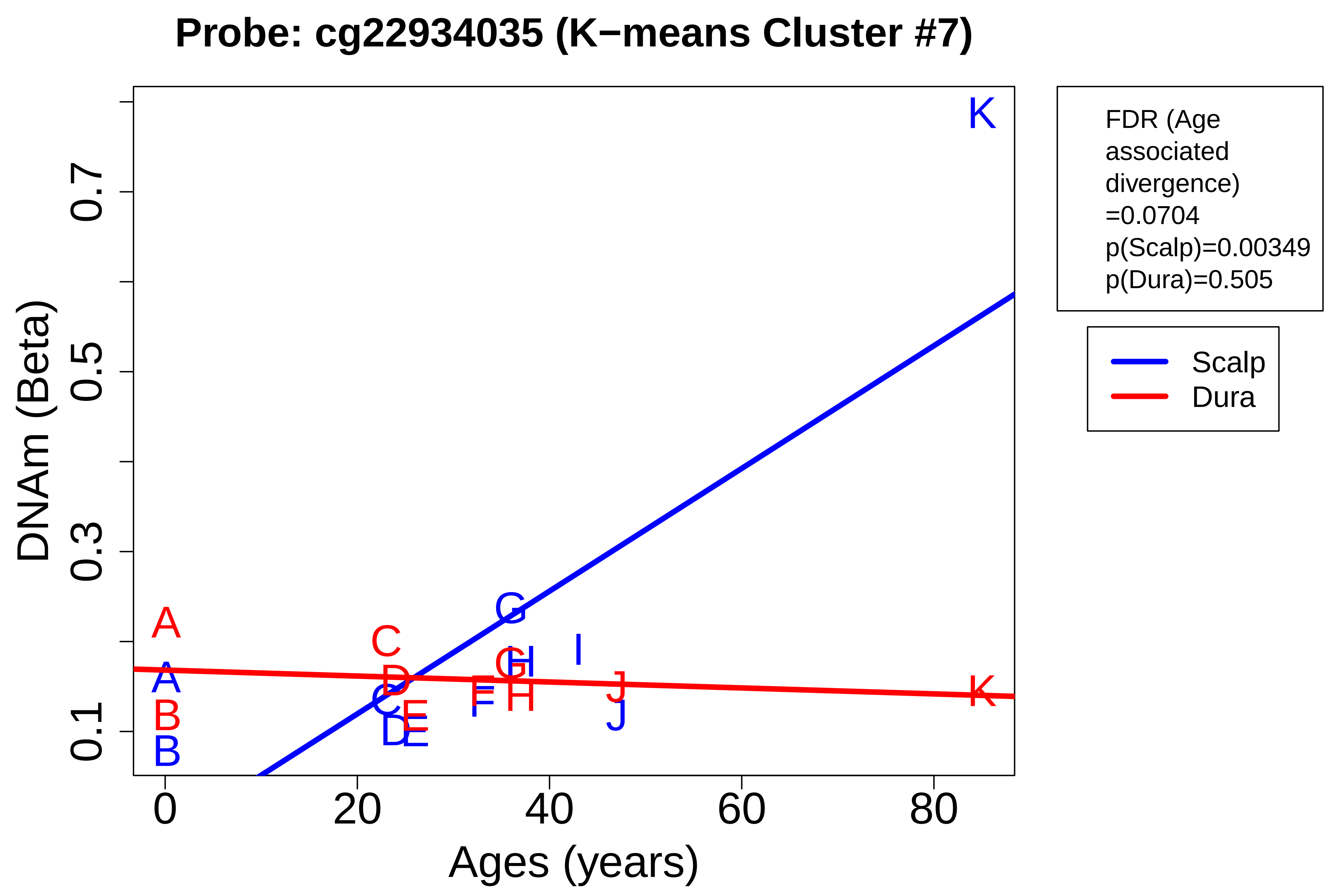

Probe: cg22934035 (K−means Cluster #7)
DNAm (Beta)
0.1
0.3
0.5
0.7
0
20
40
60
80
Ages (years)
FDR (Age associated divergence) =0.0704
p(Scalp)=0.00349
p(Dura)=0.505
Scalp
Dura
A
B
C
D
E
F
G
H
I
J
K

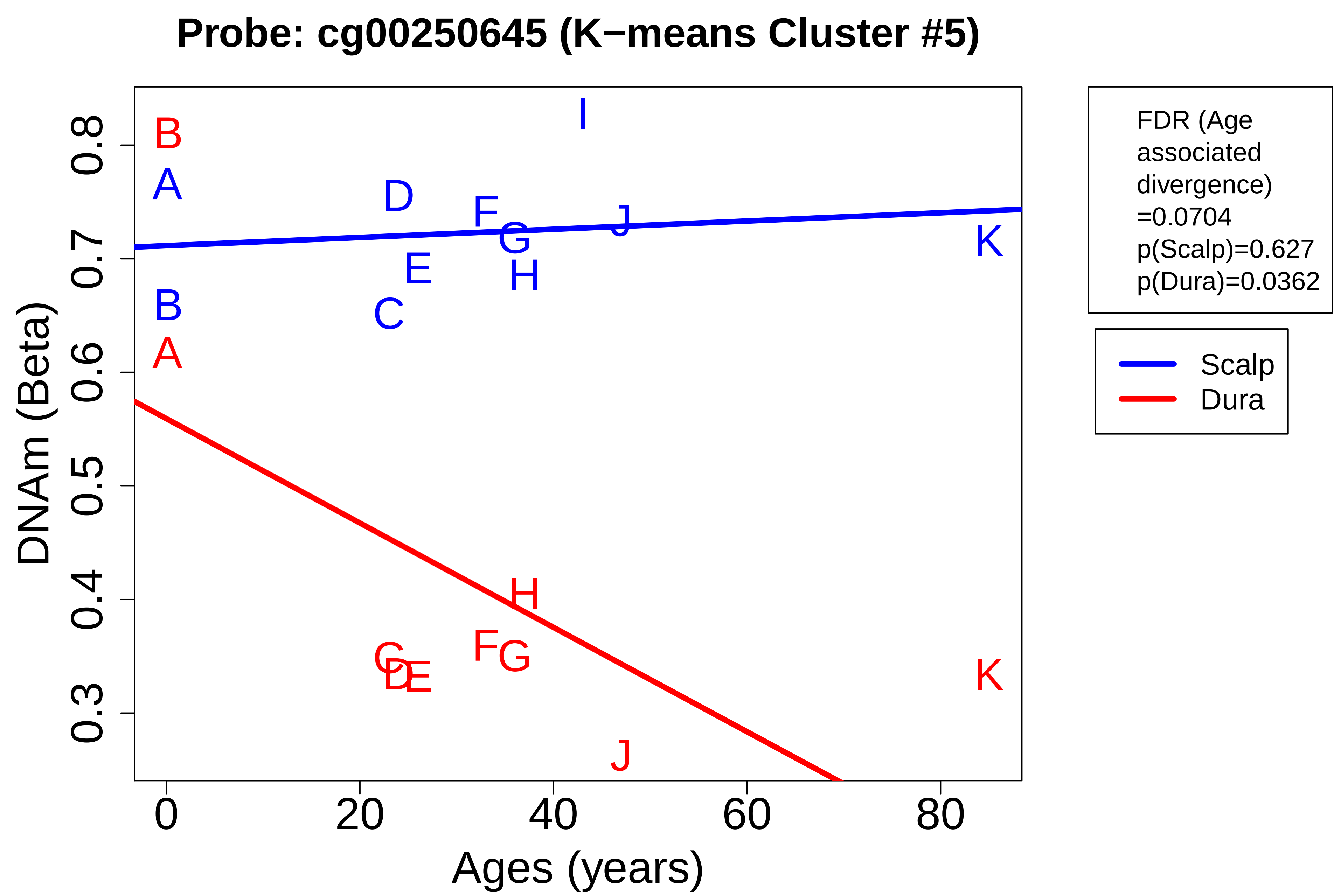

Probe: cg00250645 (K−means Cluster #5)
DNAm (Beta)
0.3
0.4
0.5
0.6
0.7
0.8
Ages (years)
0
20
40
60
80
B
A
D
F
G
J
I
K
E
H
B
C
A
H
C
D
E
F
G
K
J
FDR (Age associated divergence) =0.0704
p(Scalp)=0.627
p(Dura)=0.0362
Scalp
Dura

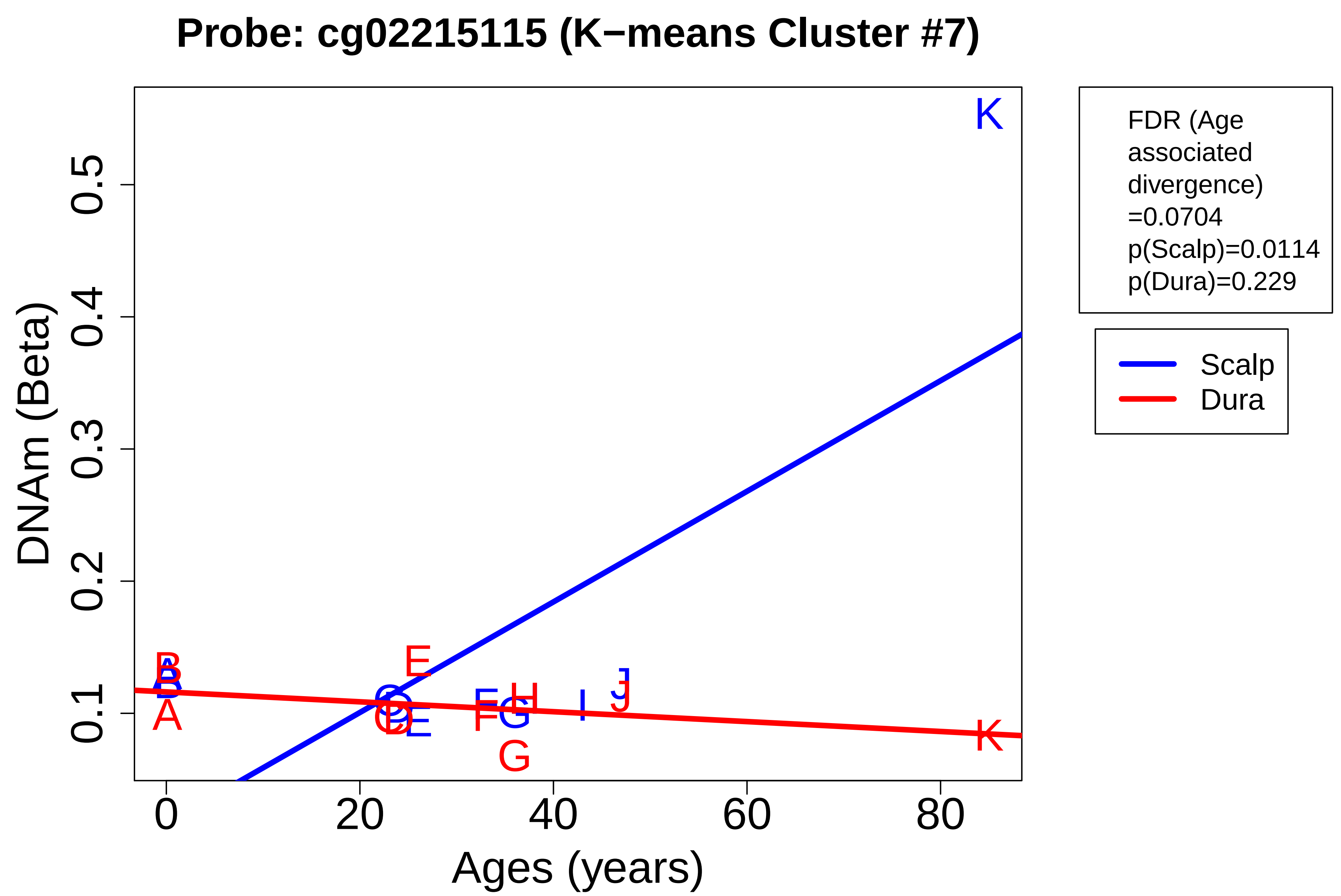
Probe: cg02215115 (K−means Cluster #7)
DNAm (Beta)
0.1
0.2
0.3
0.4
0.5
Ages (years)
0
20
40
60
80
FDR (Age associated divergence) =0.0704
p(Scalp)=0.0114
p(Dura)=0.229
Scalp
Dura

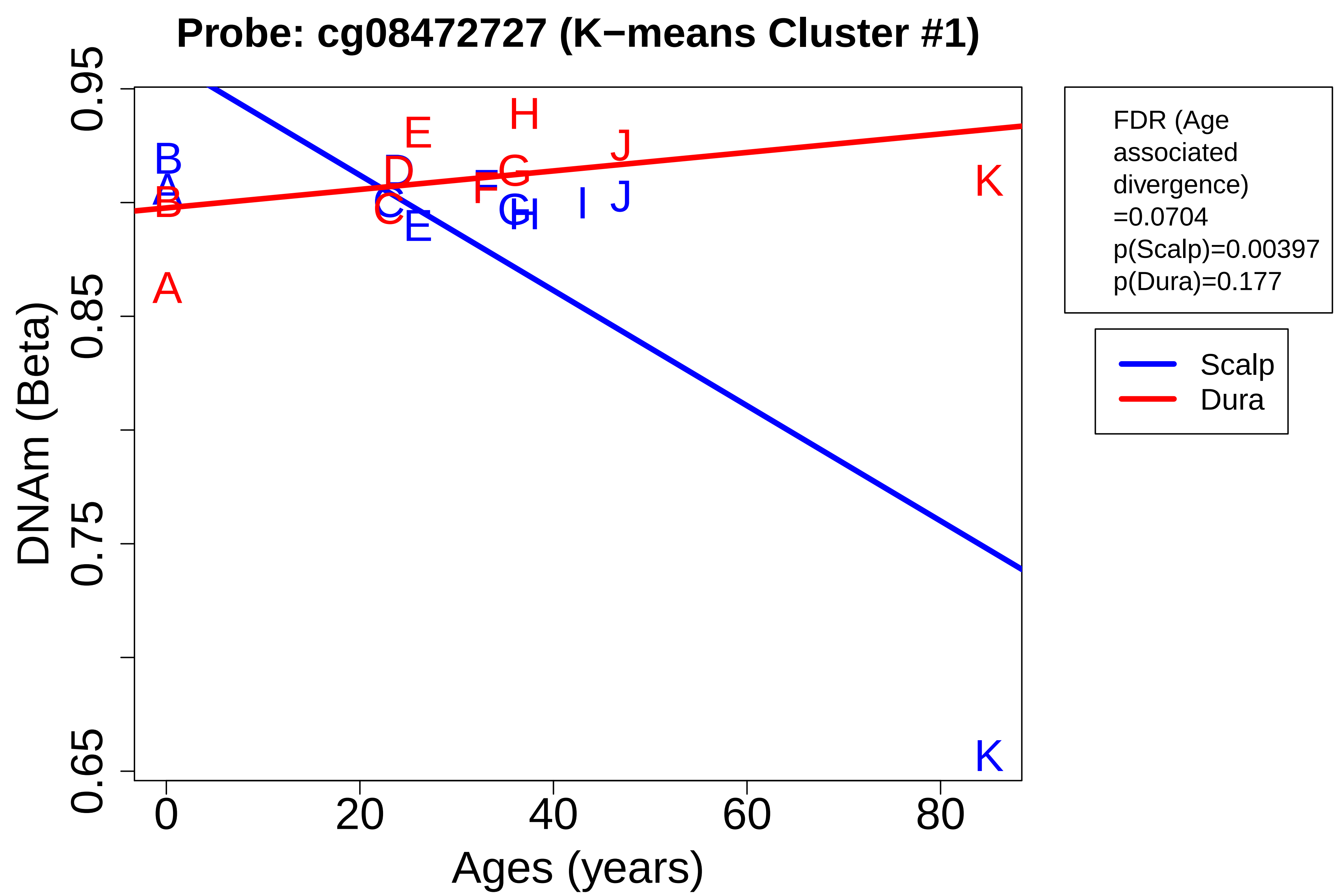

Probe: cg08472727 (K−means Cluster #1)
DNAm (Beta)
0.65
0.75
0.85
0.95
0
20
40
60
80
Ages (years)
A
B
C
D
E
F
G
H
I
J
K
FDR (Age associated divergence) =0.0704
p(Scalp)=0.00397
p(Dura)=0.177
Scalp
Dura

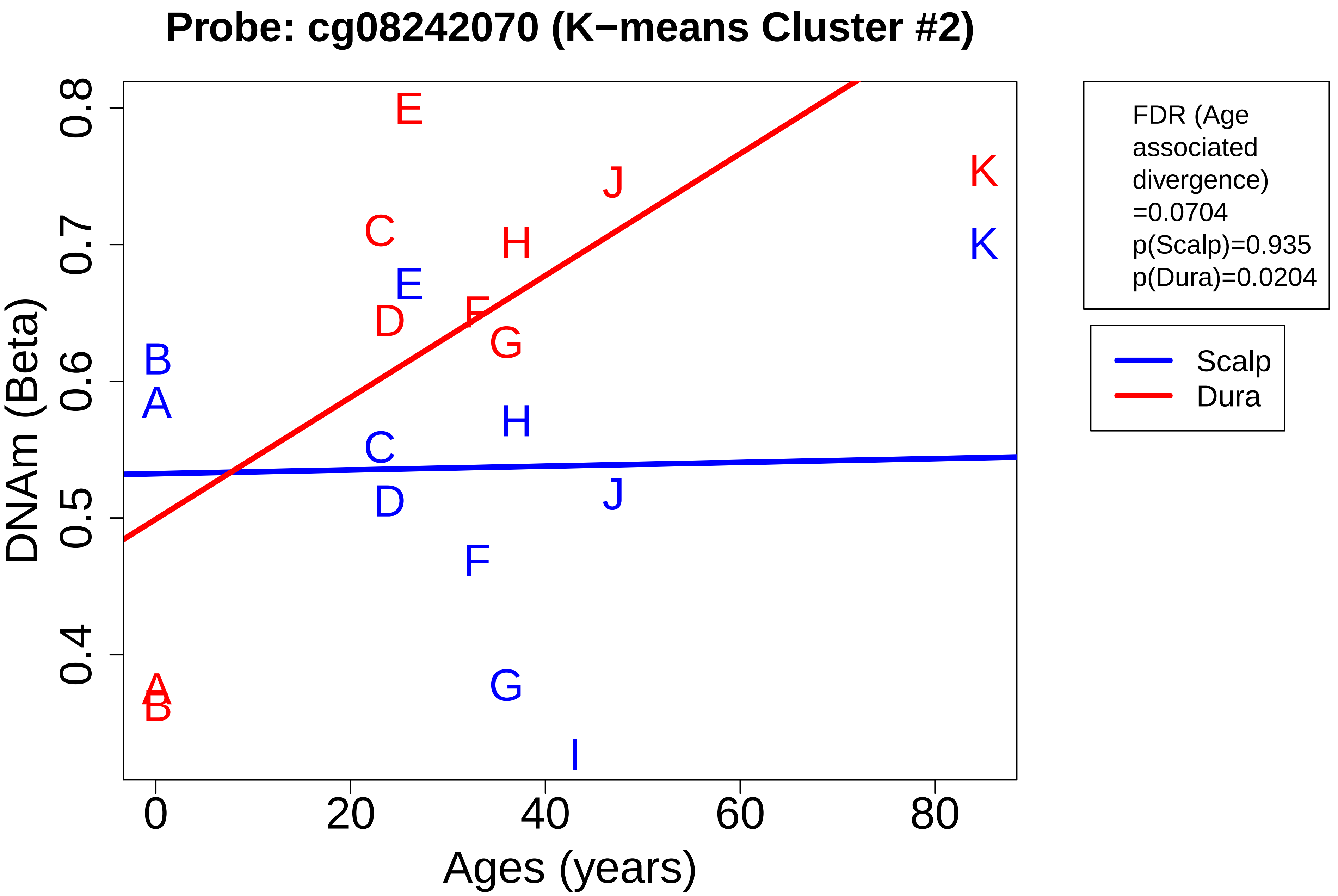

Probe: cg08242070 (K−means Cluster #2)
DNAm (Beta)
0.4
0.5
0.6
0.7
0.8
0
20
40
60
80
Ages (years)
FDR (Age associated divergence) =0.0704
p(Scalp)=0.935
p(Dura)=0.0204
Scalp
Dura

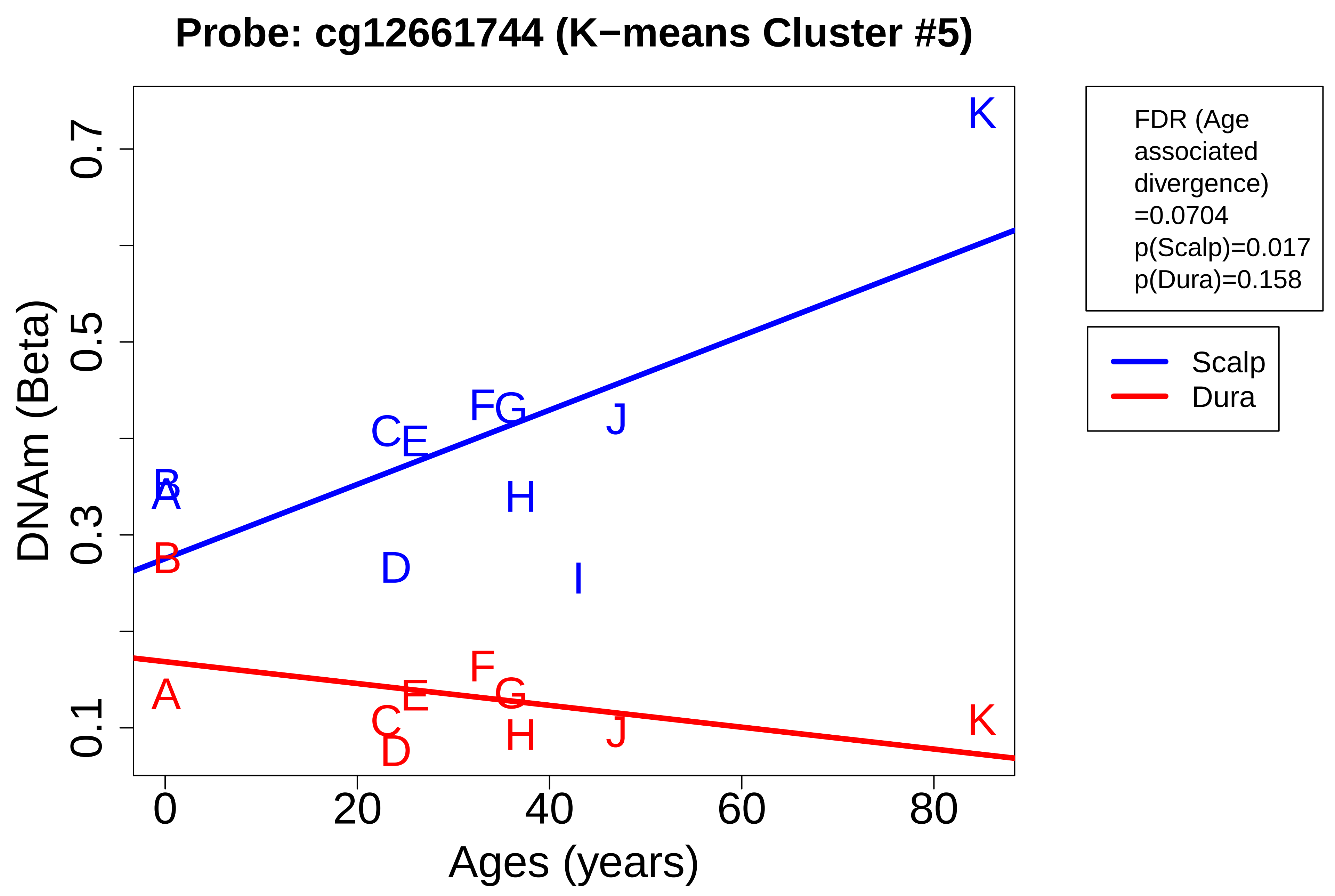
Probe: cg12661744 (K−means Cluster #5)
DNAm (Beta)
0.1
0.3
0.5
0.7
Ages (years)
0
20
40
60
80
FDR (Age associated divergence) =0.0704
p(Scalp)=0.017
p(Dura)=0.158
Scalp
Dura

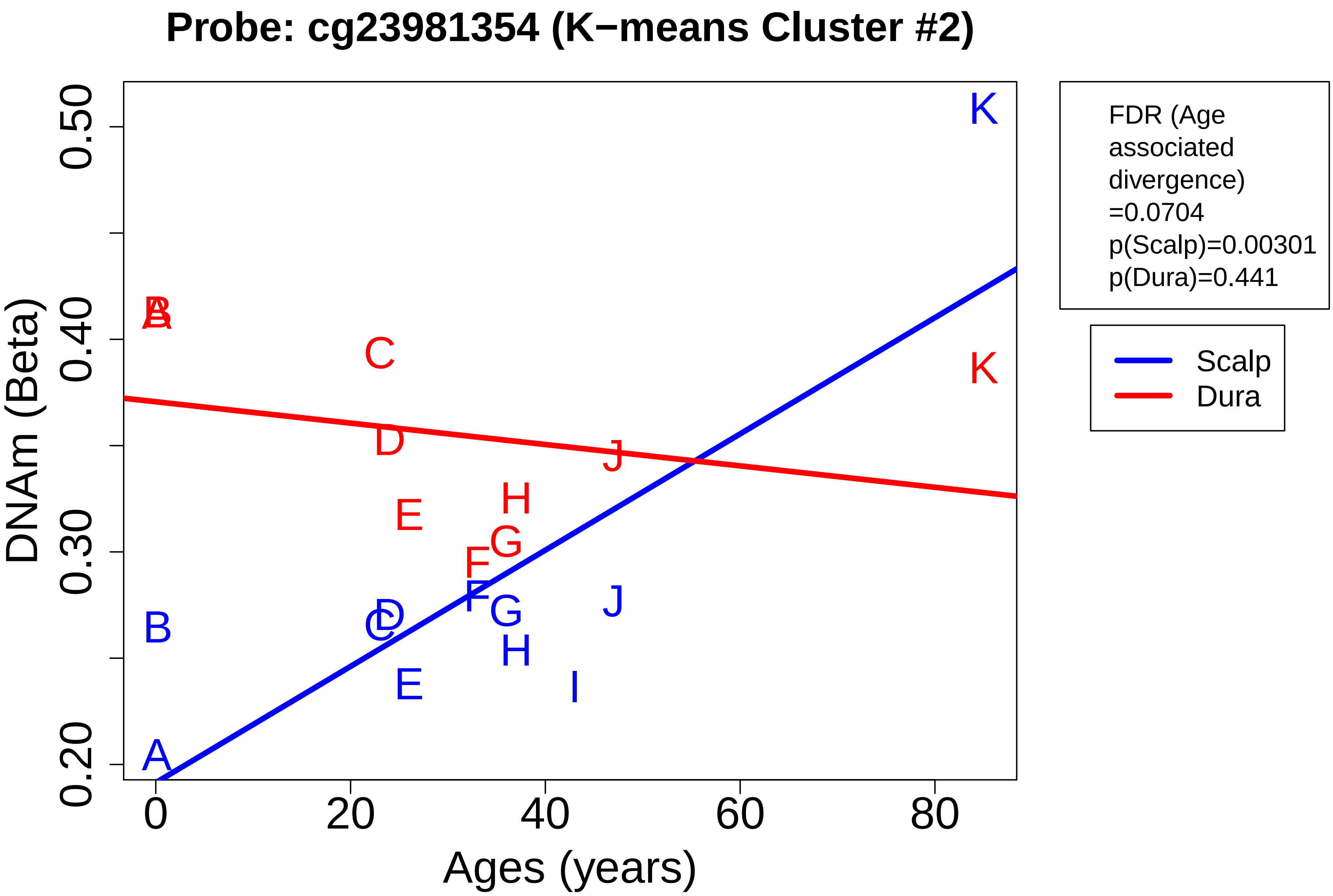

Probe: cg23981354 (K−means Cluster #2)
DNAm (Beta)
0.20
0.30
0.40
0.50
0
20
40
60
80
Ages (years)
FDR (Age associated divergence) =0.0704
p(Scalp)=0.00301
p(Dura)=0.441
Scalp
Dura

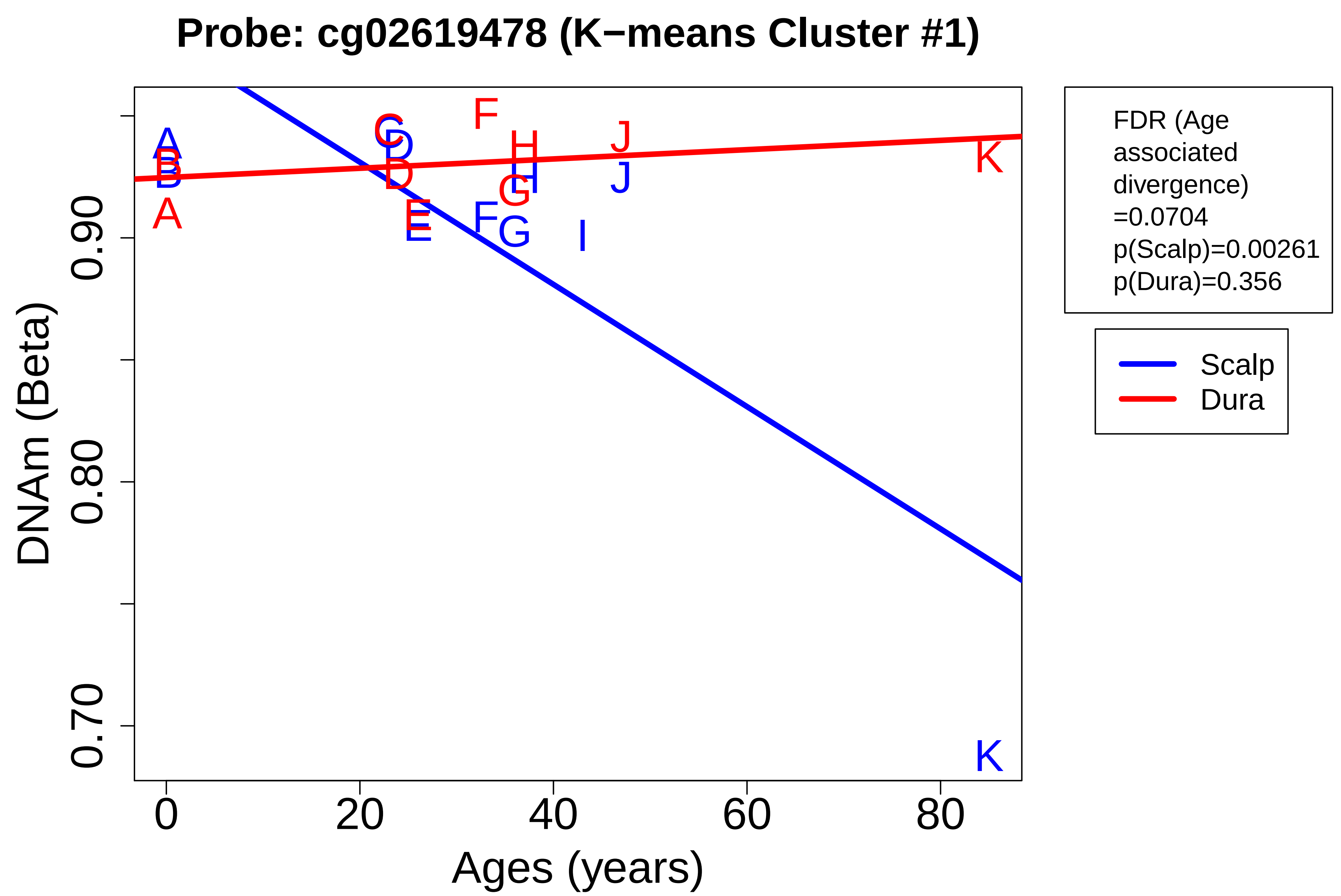

Probe: cg02619478 (K−means Cluster #1)
DNAm (Beta)
0.70
0.80
0.90
0
20
40
60
80
Ages (years)
A
B
C
D
E
F
G
H
I
J
K
FDR (Age associated divergence) =0.0704
p(Scalp)=0.00261
p(Dura)=0.356
Scalp
Dura

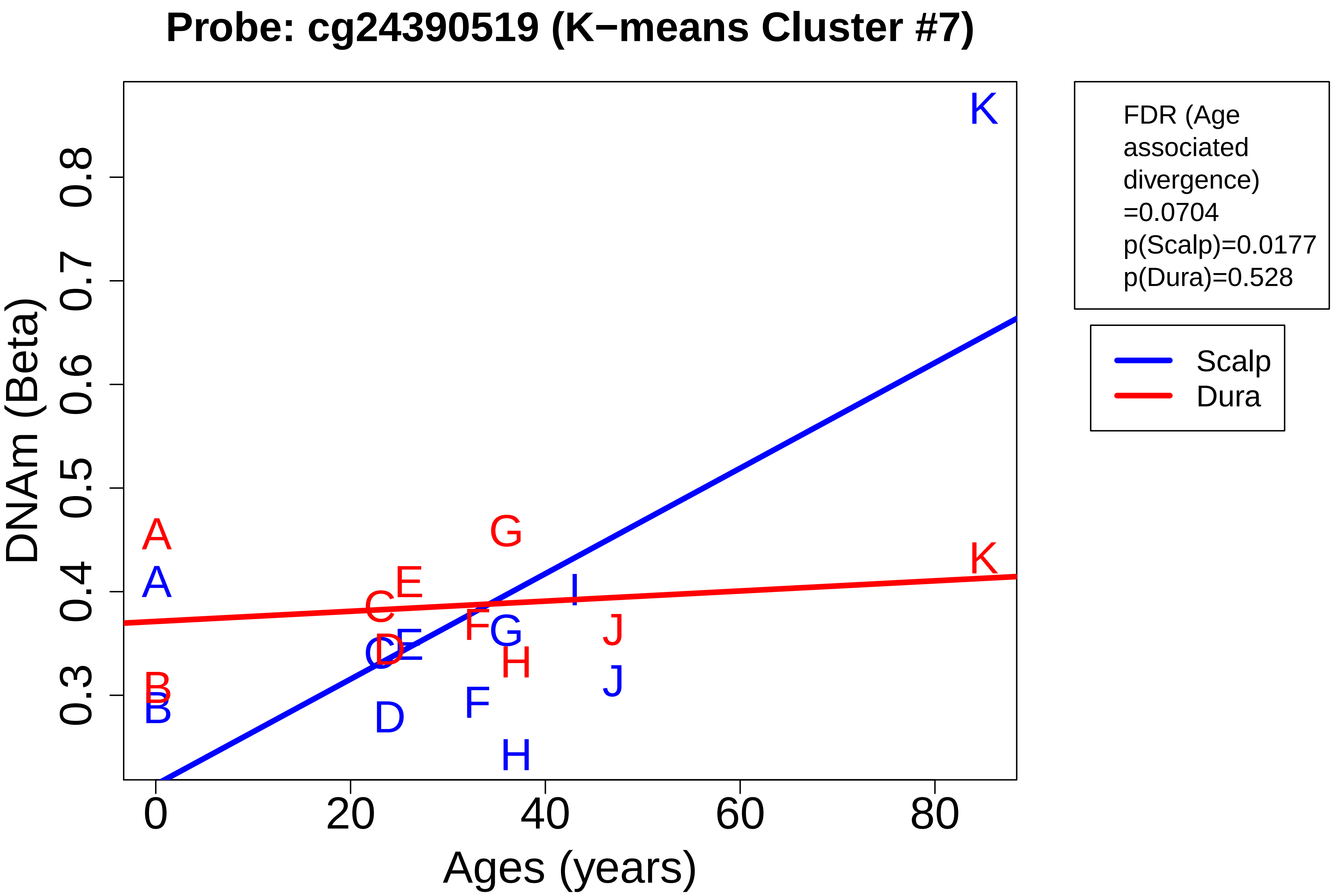

Probe: cg24390519 (K−means Cluster #7)
DNAm (Beta)
0.3
0.4
0.5
0.6
0.7
0.8
0
20
40
60
80
Ages (years)
A
B
C
D
E
F
G
H
I
J
K
FDR (Age associated divergence) =0.0704
p(Scalp)=0.0177
p(Dura)=0.528
Scalp
Dura

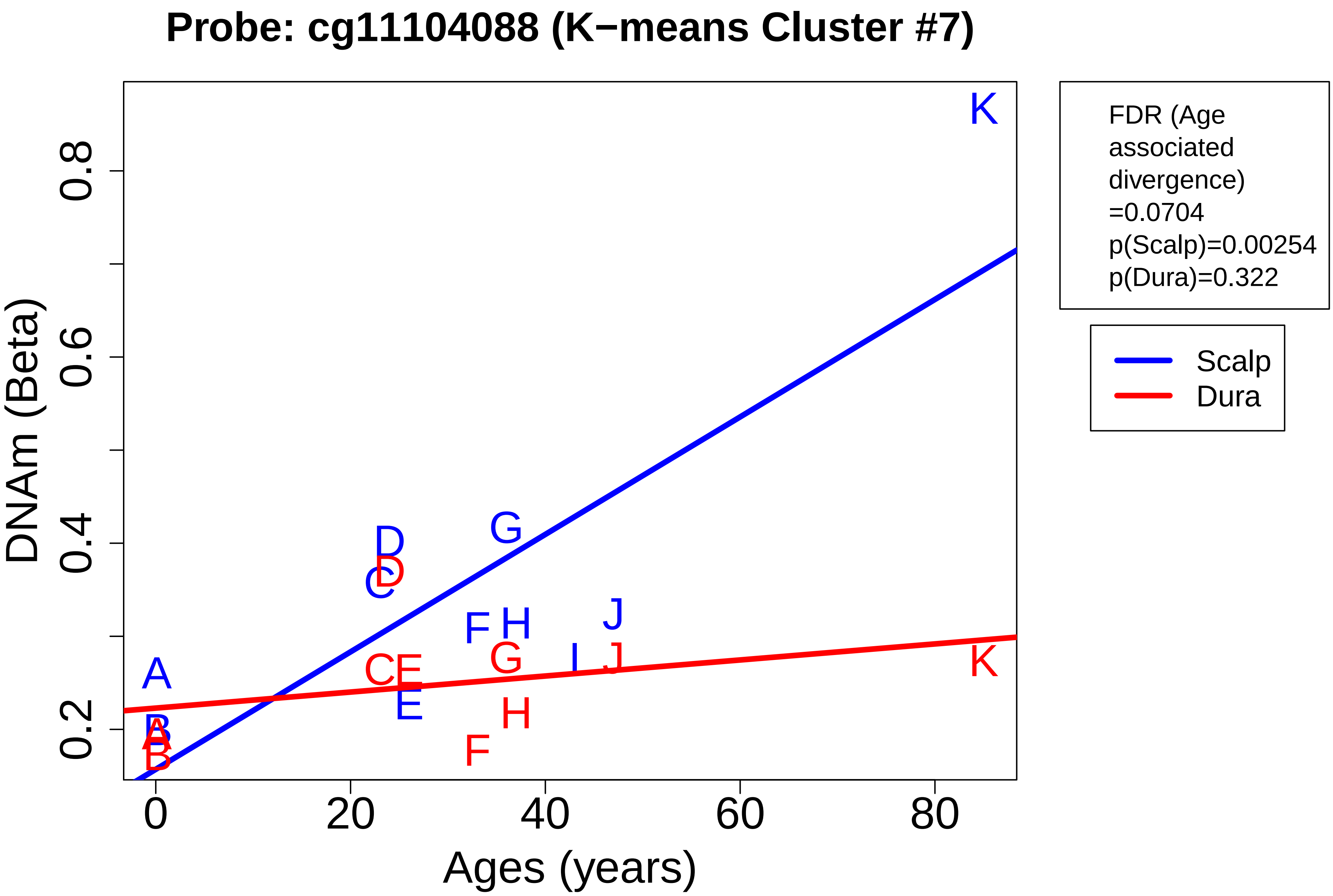

Probe: cg11104088 (K−means Cluster #7)
DNAm (Beta)
0.2
0.4
0.6
0.8
0
20
40
60
80
Ages (years)
FDR (Age associated divergence) =0.0704
p(Scalp)=0.00254
p(Dura)=0.322
Scalp
Dura

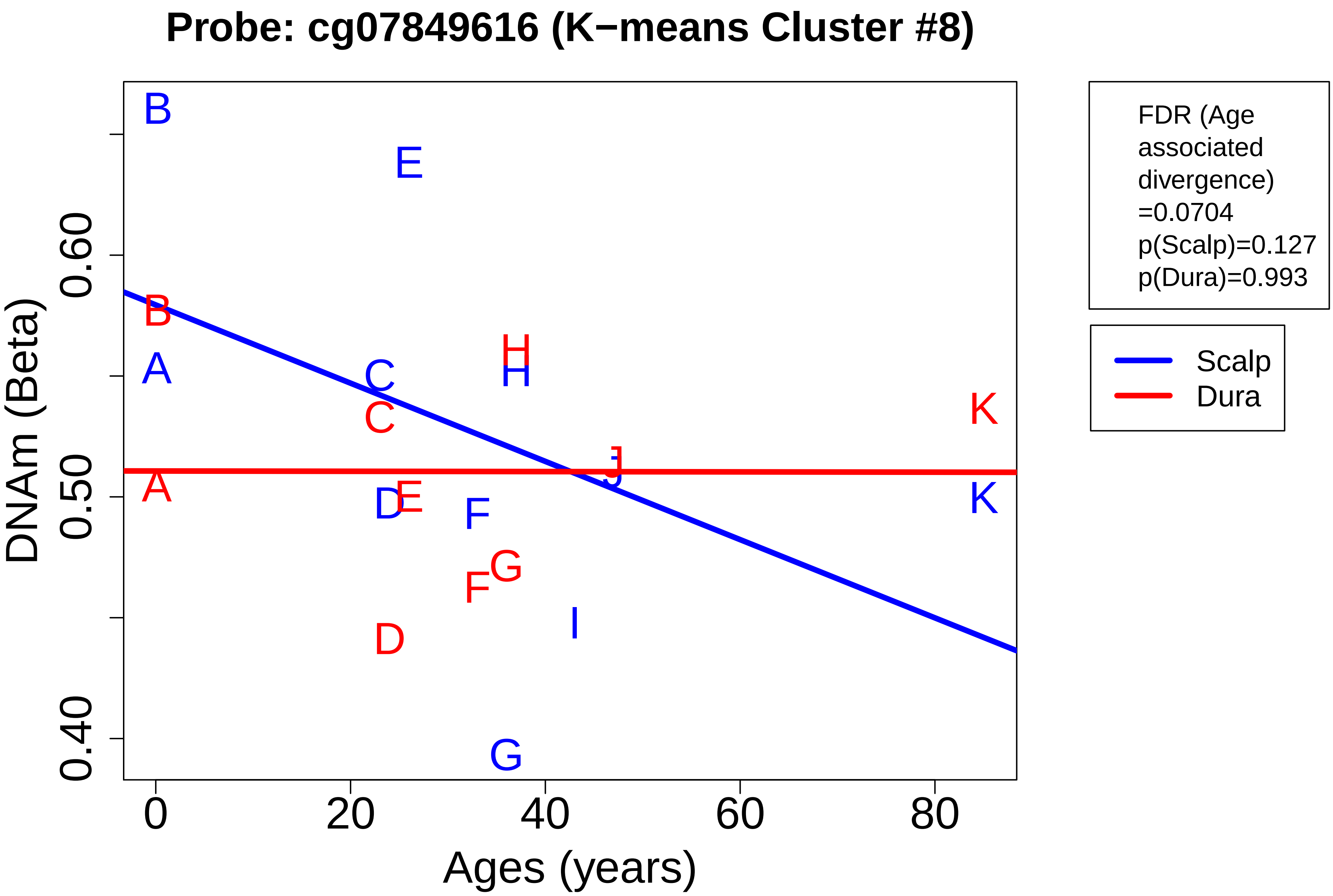

Probe: cg07849616 (K−means Cluster #8)
DNAm (Beta)
0.40
0.50
0.60
0
20
40
60
80
Ages (years)
FDR (Age associated divergence) =0.0704
p(Scalp)=0.127
p(Dura)=0.993
Scalp
Dura

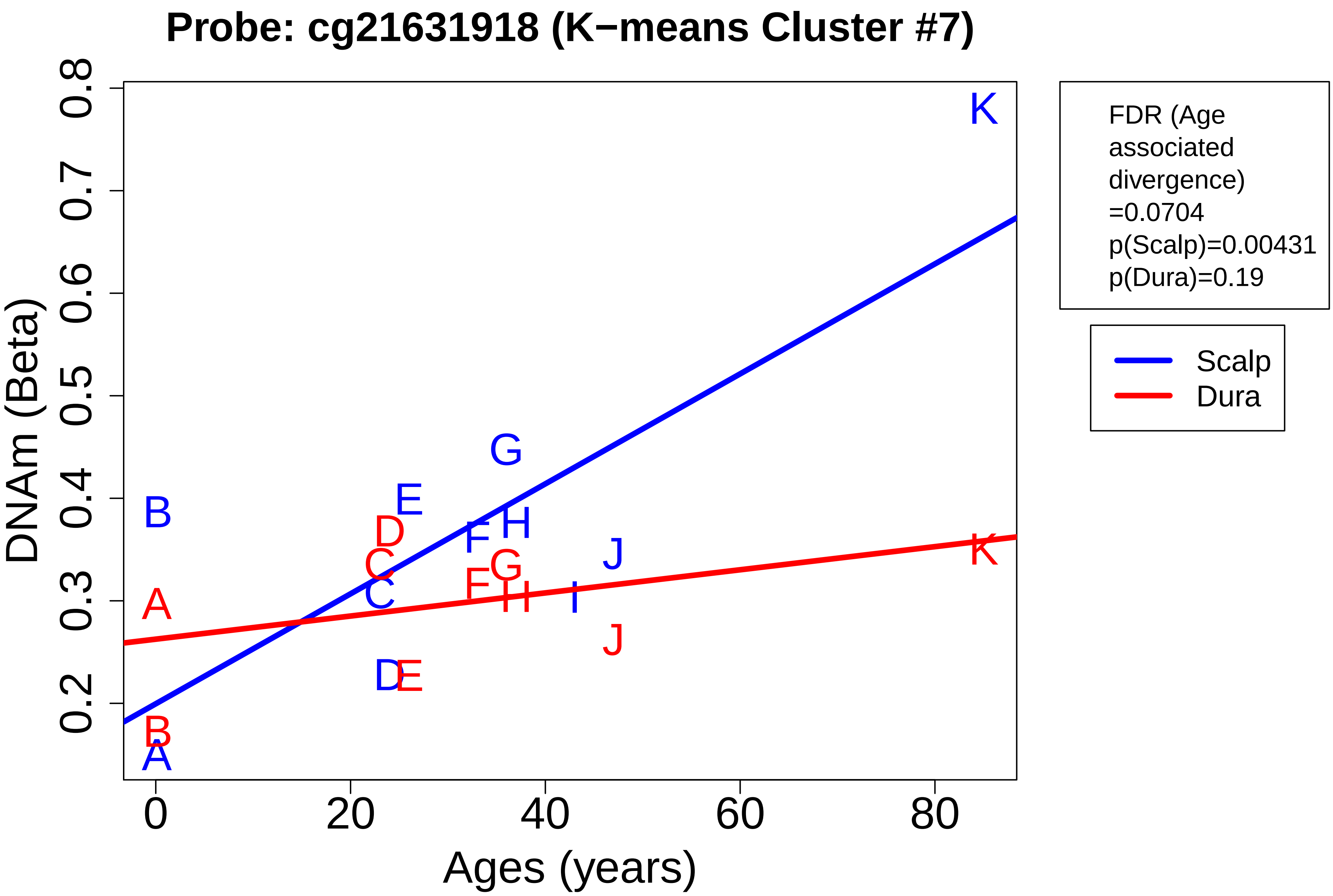

Probe: cg21631918 (K−means Cluster #7)
DNAm (Beta)
0.2
0.3
0.4
0.5
0.6
0.7
0.8
0
20
40
60
80
Ages (years)
FDR (Age associated divergence) =0.0704
p(Scalp)=0.00431
p(Dura)=0.19
Scalp
Dura

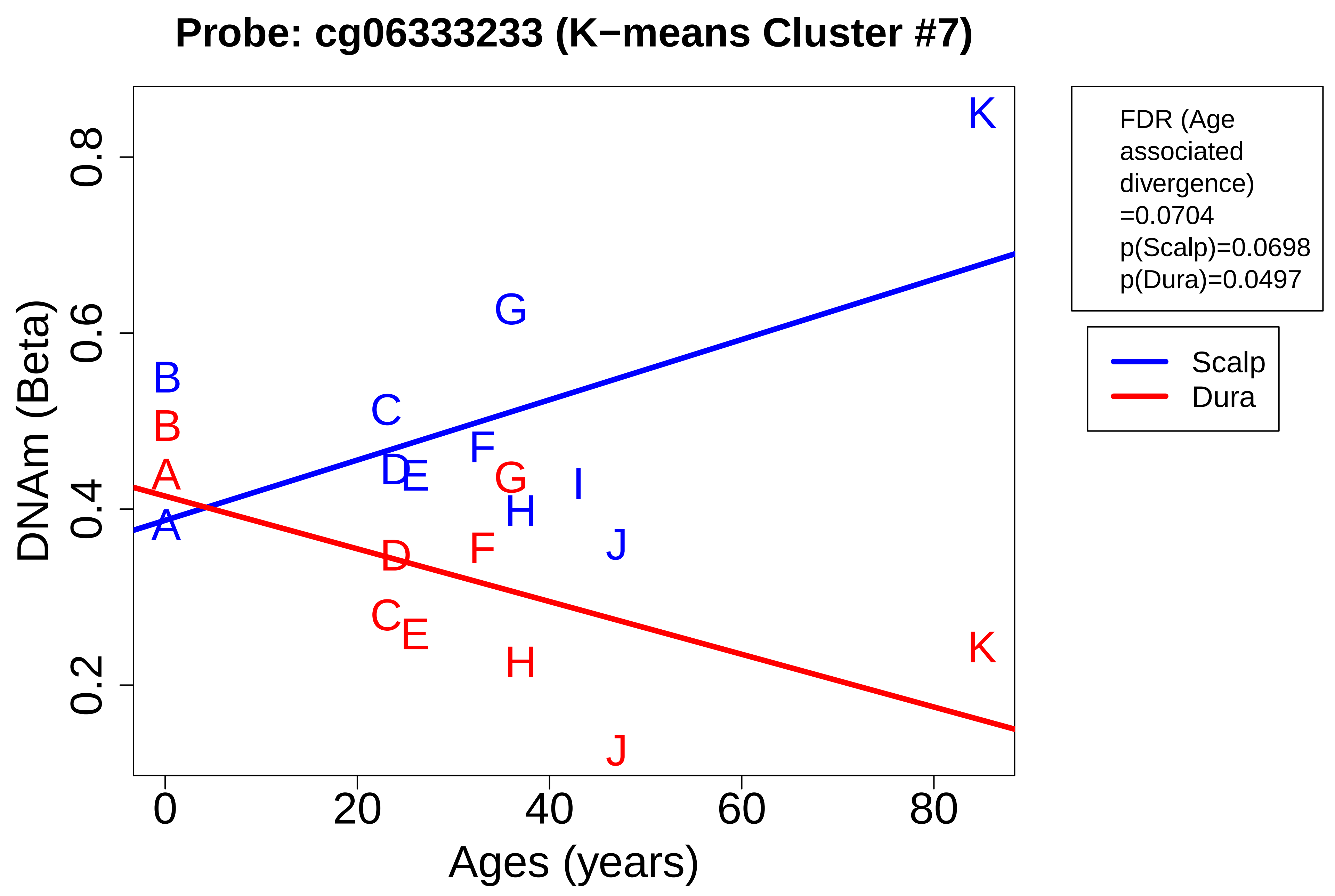

Probe: cg06333233 (K−means Cluster #7)
DNAm (Beta)
0.2
0.4
0.6
0.8
0
20
40
60
80
Ages (years)
FDR (Age associated divergence) =0.0704
p(Scalp)=0.0698
p(Dura)=0.0497
Scalp
Dura
A
B
C
D
E
F
G
H
I
J
K

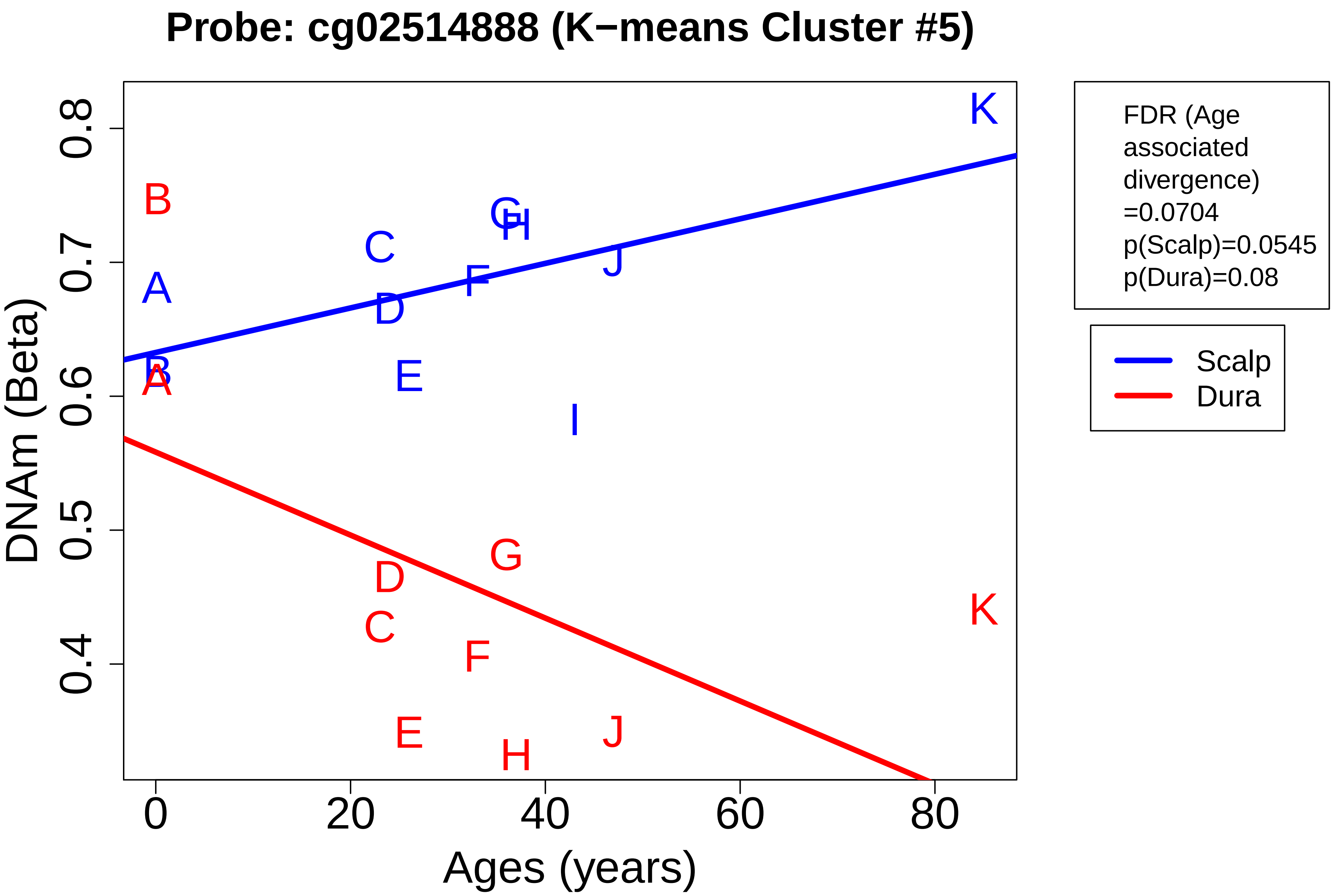

Probe: cg02514888 (K−means Cluster #5)
DNAm (Beta)
Ages (years)
0.4
0.5
0.6
0.7
0.8
0
20
40
60
80
FDR (Age associated divergence) =0.0704
p(Scalp)=0.0545
p(Dura)=0.08
Scalp
Dura

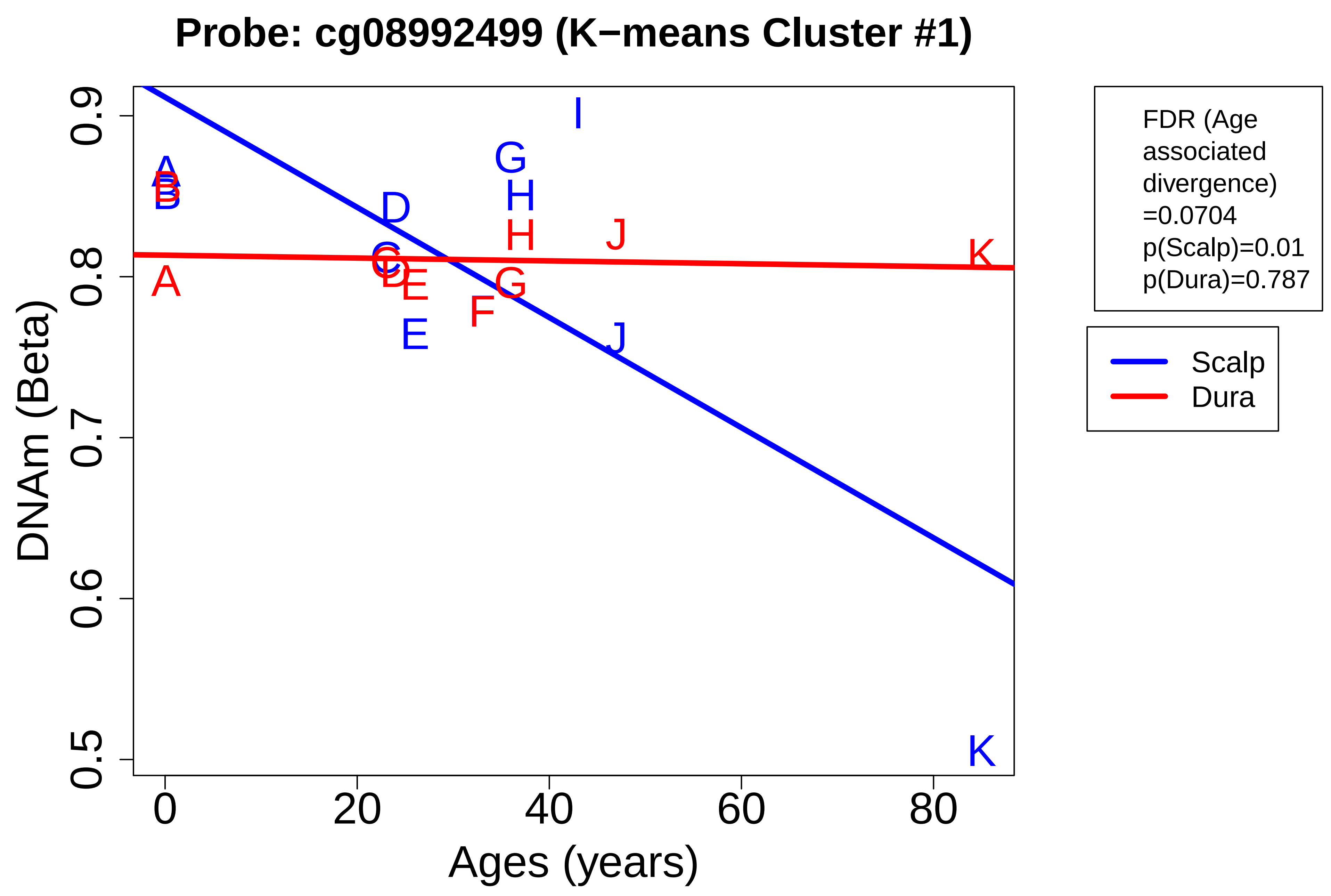

Probe: cg08992499 (K−means Cluster #1)
DNAm (Beta)
Ages (years)
0.5
0.6
0.7
0.8
0.9
0
20
40
60
80
FDR (Age associated divergence) =0.0704
p(Scalp)=0.01
p(Dura)=0.787
Scalp
Dura

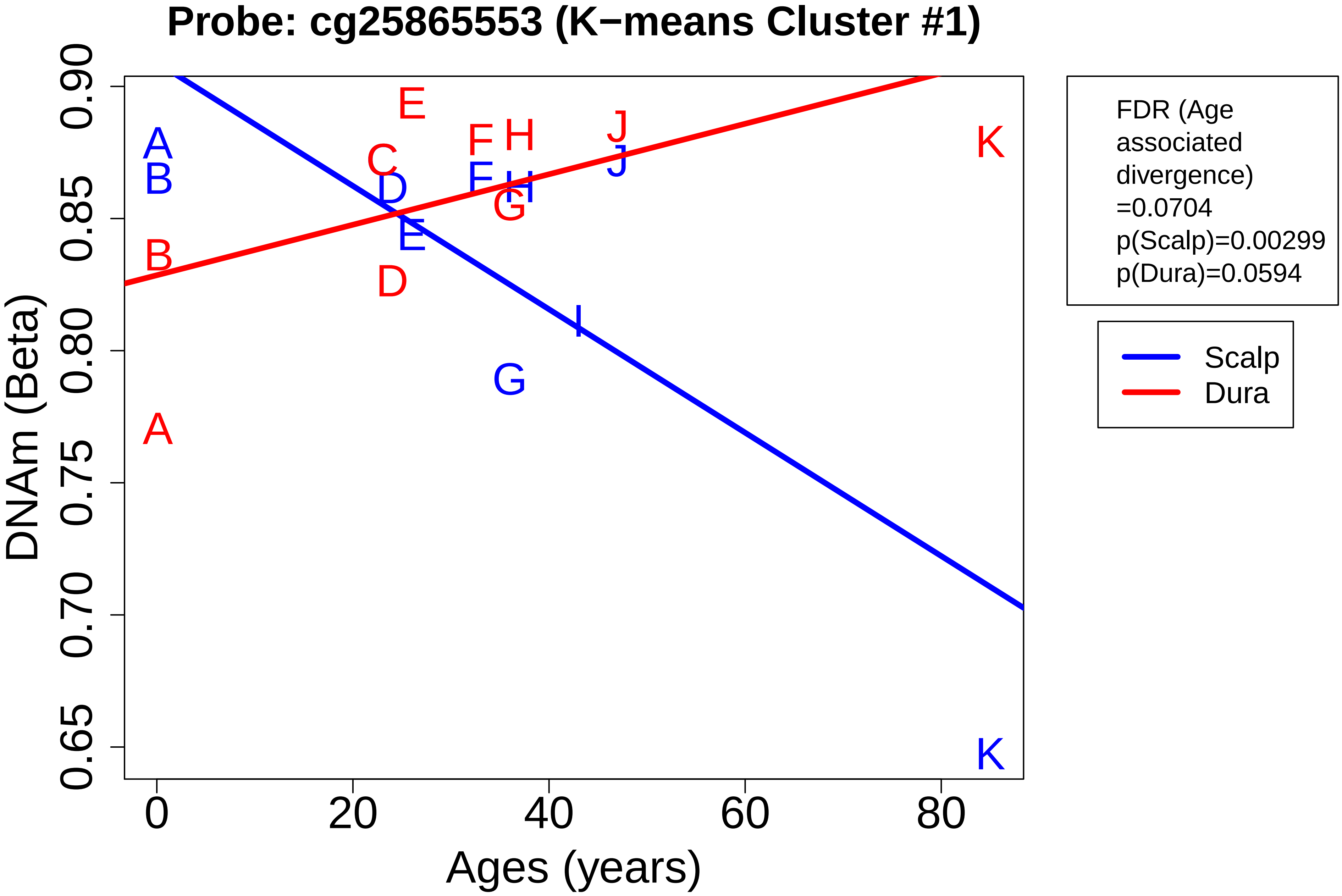
Probe: cg25865553 (K−means Cluster #1)
DNAm (Beta)
0.65
0.70
0.75
0.80
0.85
0.90
0
20
40
60
80
Ages (years)
A
B
C
D
E
F
G
H
I
J
K
FDR (Age associated divergence) =0.0704
p(Scalp)=0.00299
p(Dura)=0.0594
Scalp
Dura

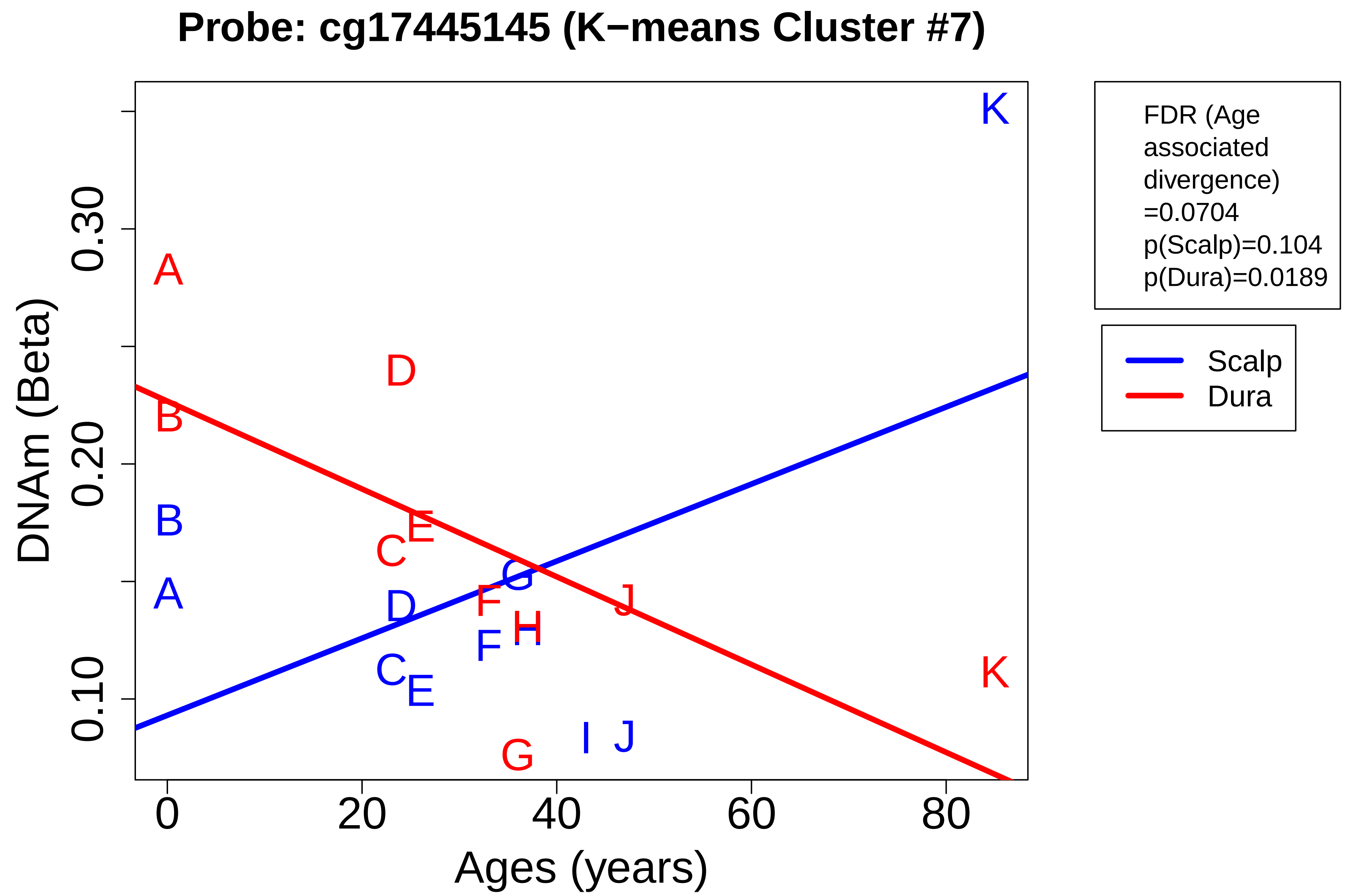

Probe: cg17445145 (K−means Cluster #7)
DNAm (Beta)
0.10
0.20
0.30
Ages (years)
0
20
40
60
80
A
B
C
D
E
F
G
H
I
J
K
FDR (Age associated divergence) =0.0704
p(Scalp)=0.104
p(Dura)=0.0189
Scalp
Dura

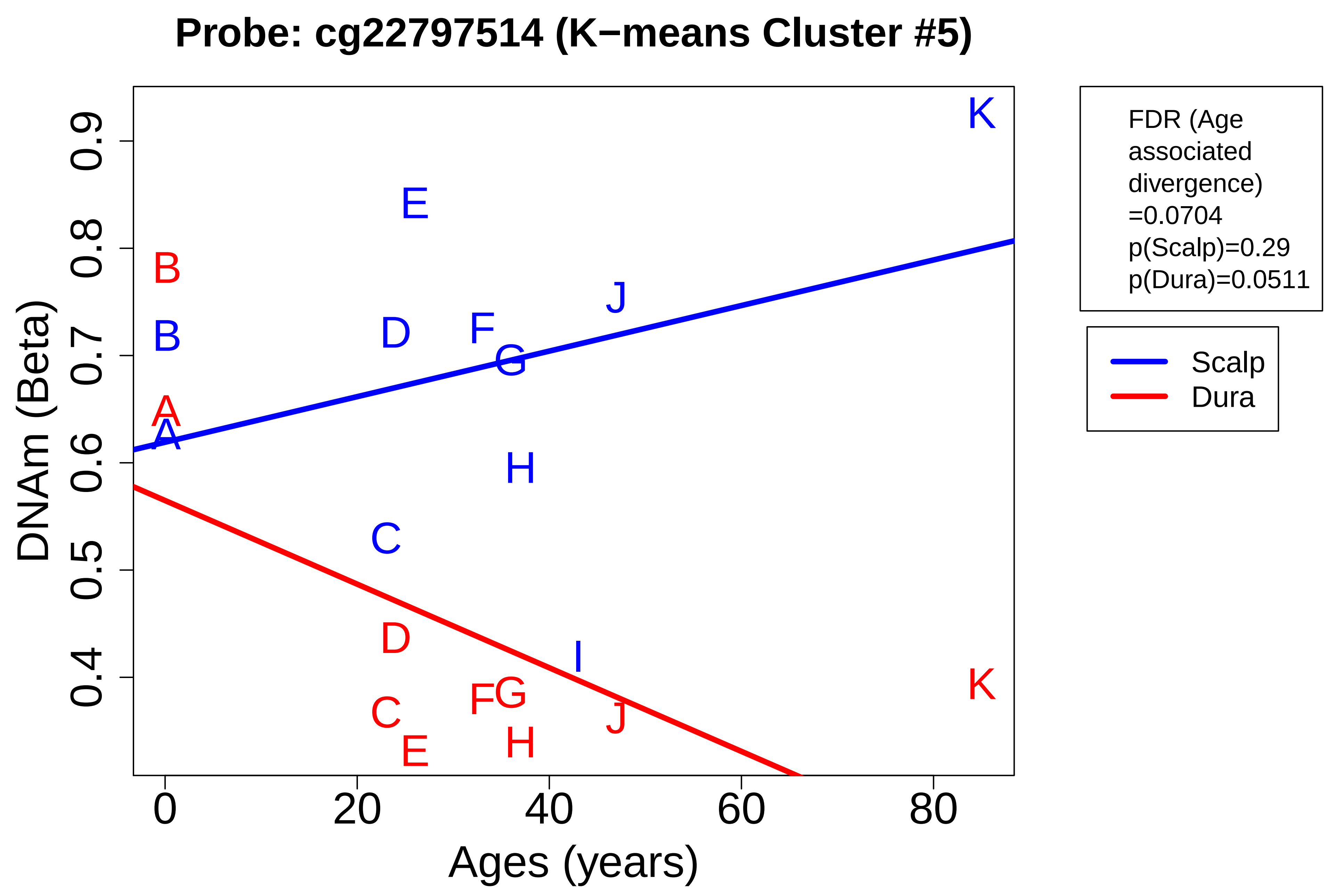

Probe: cg22797514 (K−means Cluster #5)
DNAm (Beta)
0.4
0.5
0.6
0.7
0.8
0.9
0
20
40
60
80
Ages (years)
FDR (Age associated divergence) =0.0704
p(Scalp)=0.29
p(Dura)=0.0511
Scalp
Dura

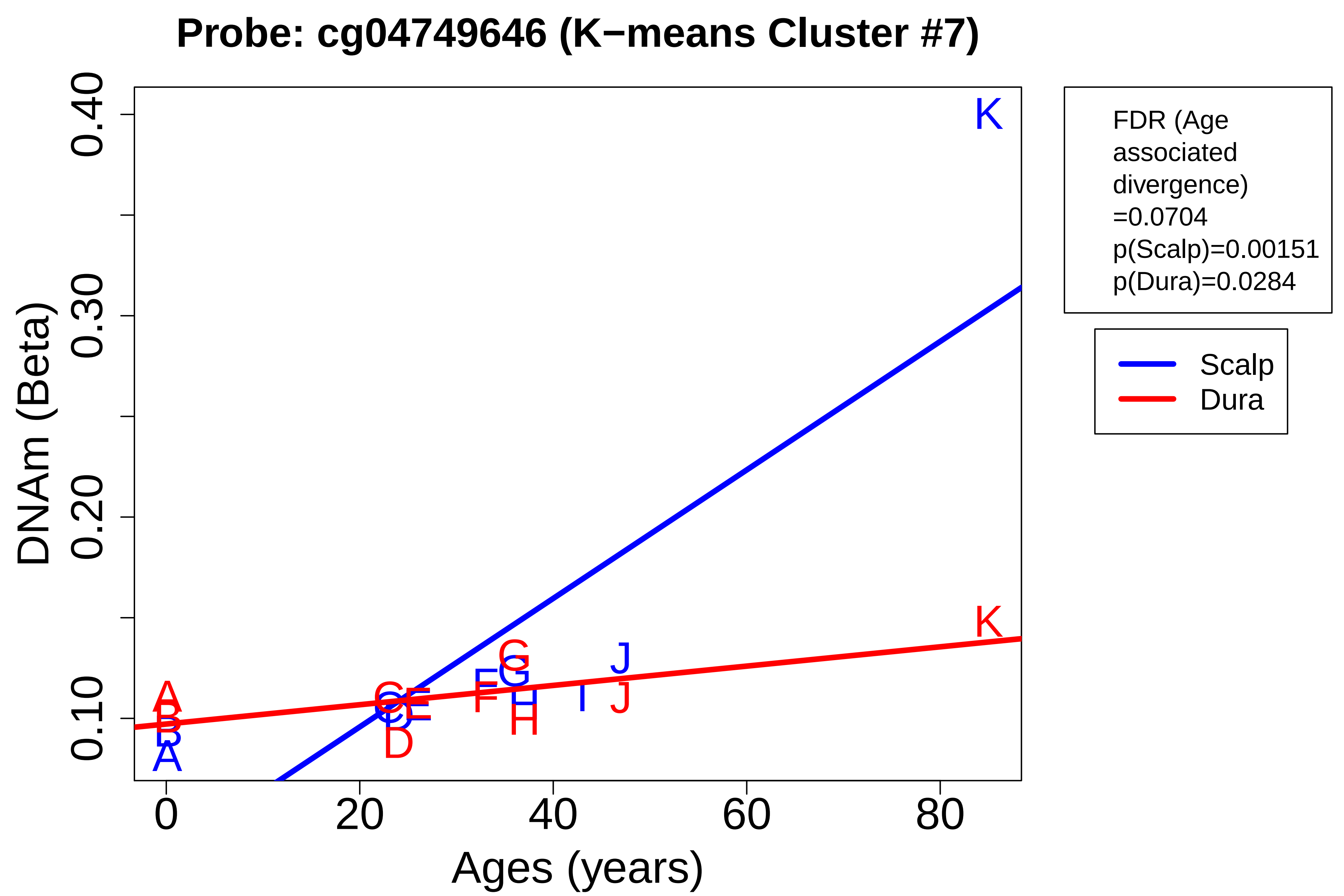

Probe: cg04749646 (K−means Cluster #7)
DNAm (Beta)
0.10
0.20
0.30
0.40
Ages (years)
0
20
40
60
80
A
B
C
D
E
F
G
H
I
J
K
FDR (Age associated divergence) =0.0704
p(Scalp)=0.00151
p(Dura)=0.0284
Scalp
Dura

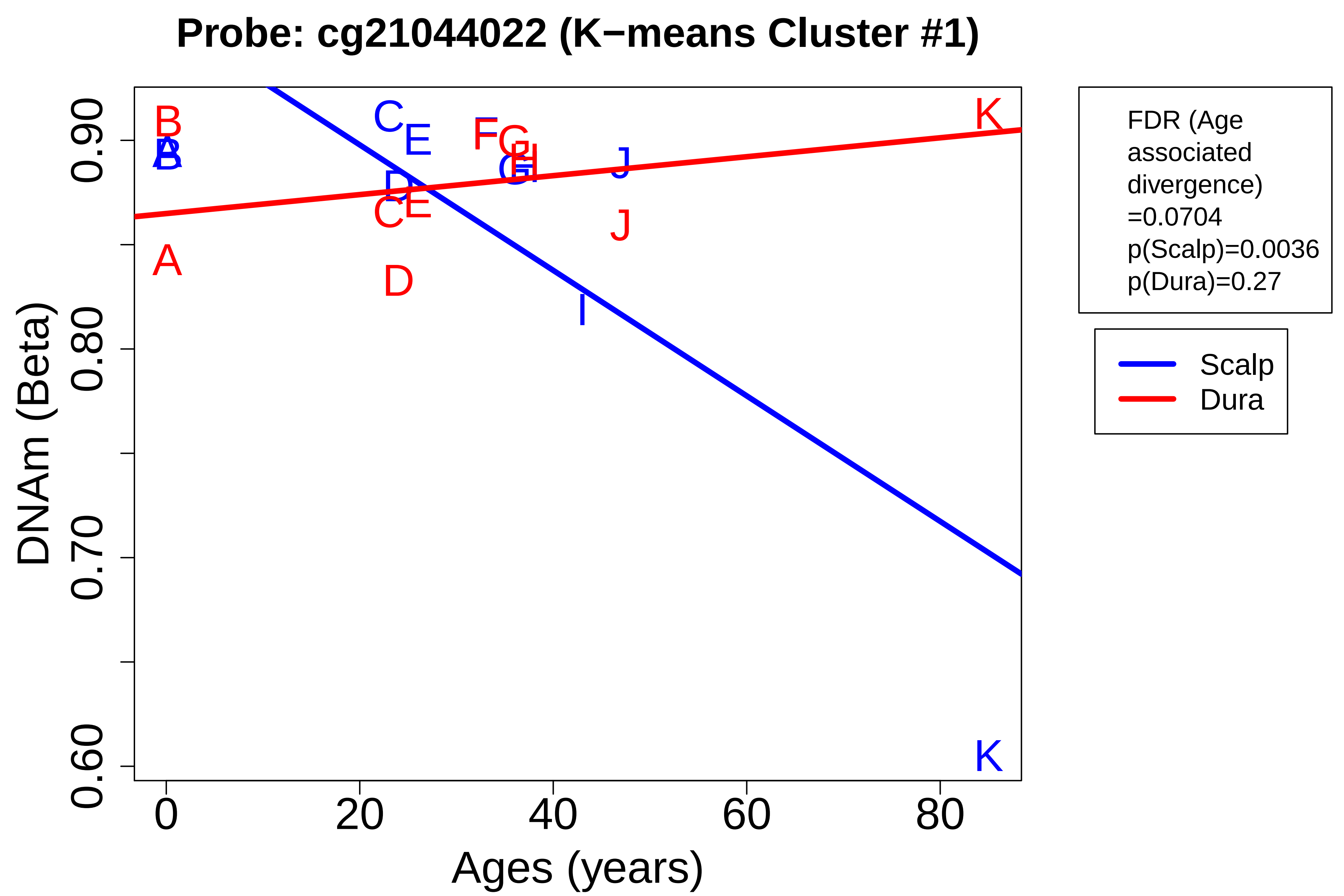

Probe: cg21044022 (K−means Cluster #1)
DNAm (Beta)
0.60
0.70
0.80
0.90
0
20
40
60
80
Ages (years)
FDR (Age associated divergence) =0.0704
p(Scalp)=0.0036
p(Dura)=0.27
Scalp
Dura
A
B
C
D
E
F
G
H
I
J
K

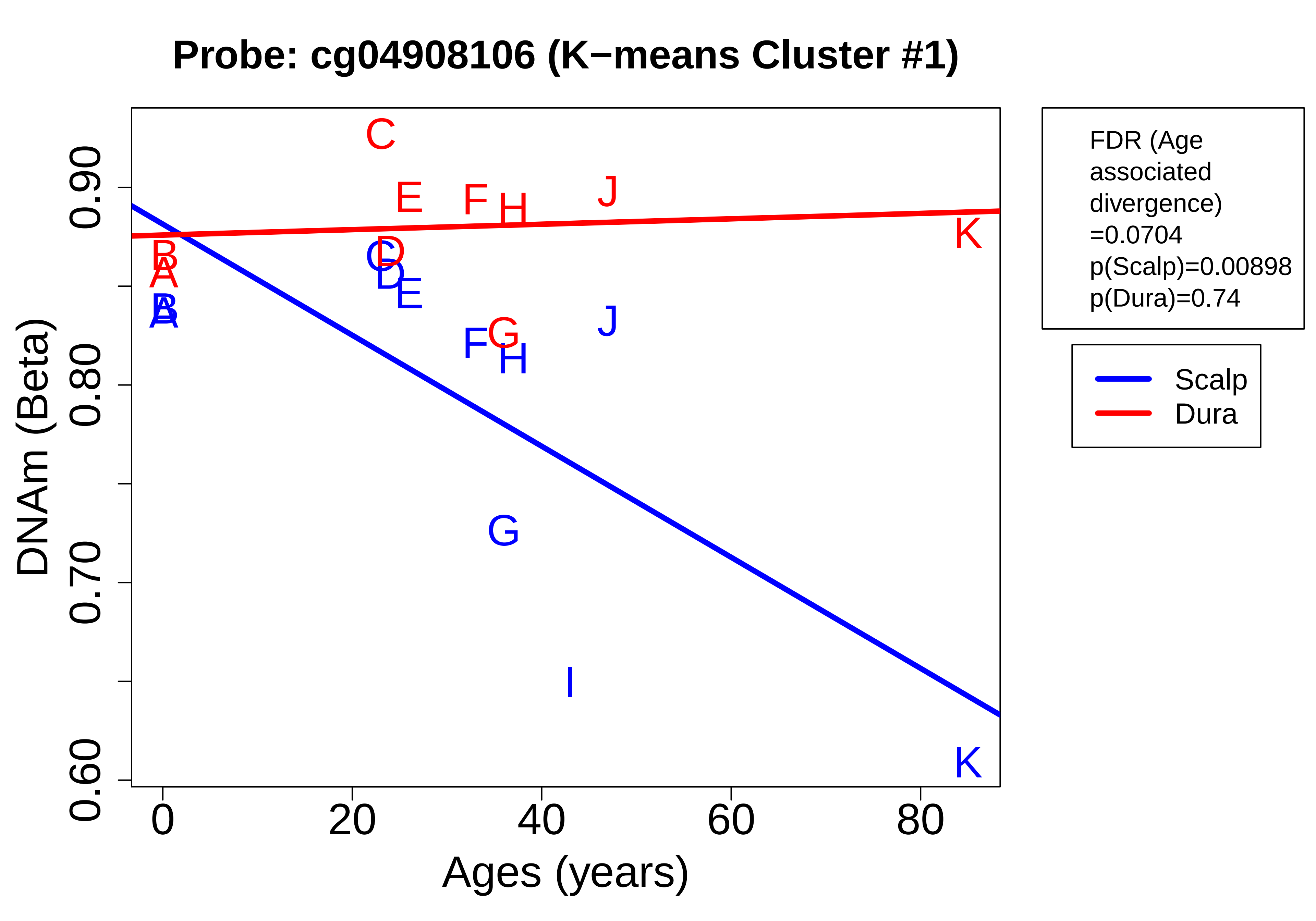
Probe: cg04908106 (K−means Cluster #1)
DNAm (Beta)
0.60
0.70
0.80
0.90
0
20
40
60
80
Ages (years)
A
B
C
D
E
F
G
H
I
J
K
FDR (Age associated divergence) =0.0704
p(Scalp)=0.00898
p(Dura)=0.74
Scalp
Dura

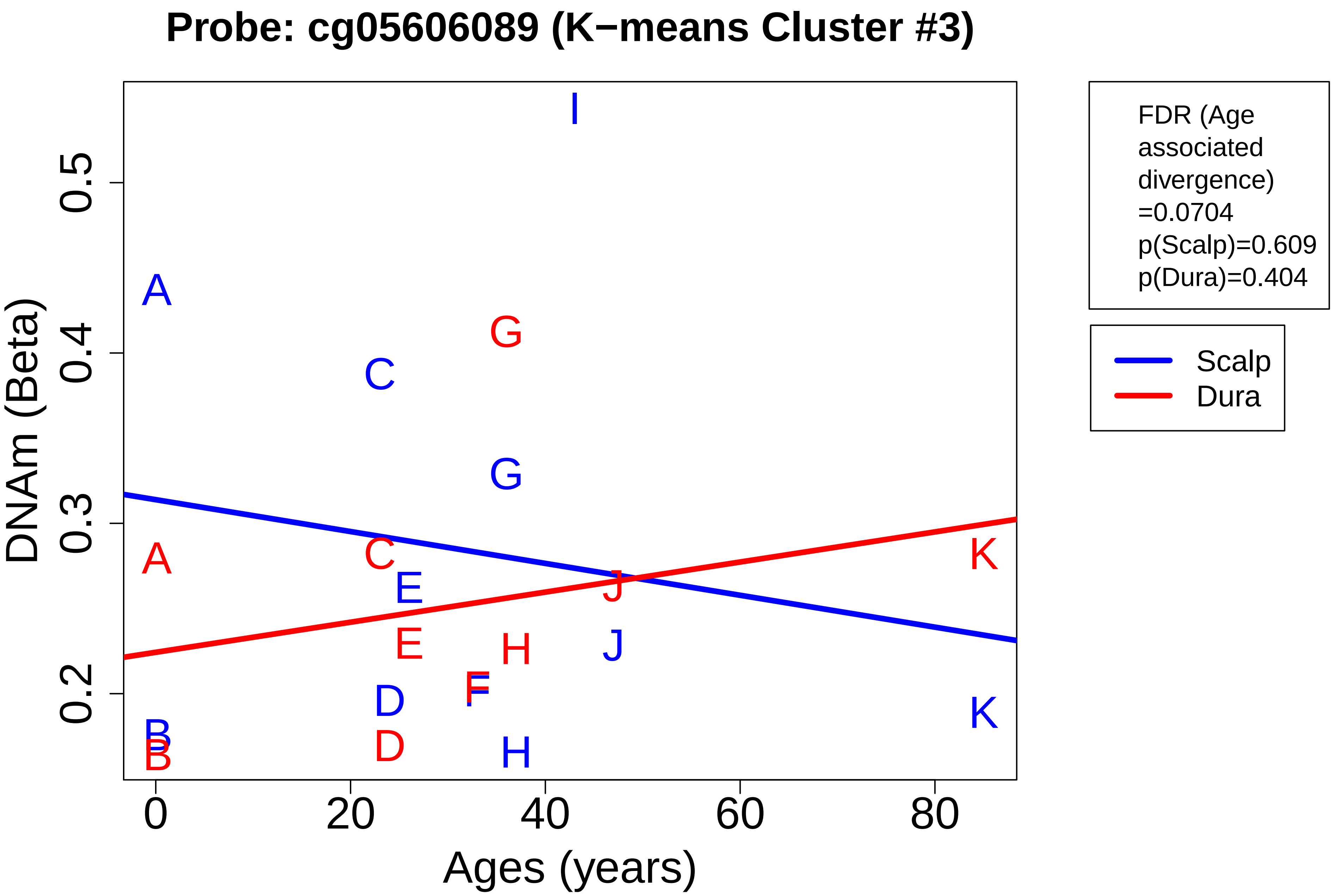

Probe: cg05606089 (K−means Cluster #3)
DNAm (Beta)
0.2
0.3
0.4
0.5
0
20
40
60
80
Ages (years)
A
B
C
D
E
F
G
H
I
J
K
FDR (Age associated divergence) =0.0704
p(Scalp)=0.609
p(Dura)=0.404
Scalp
Dura

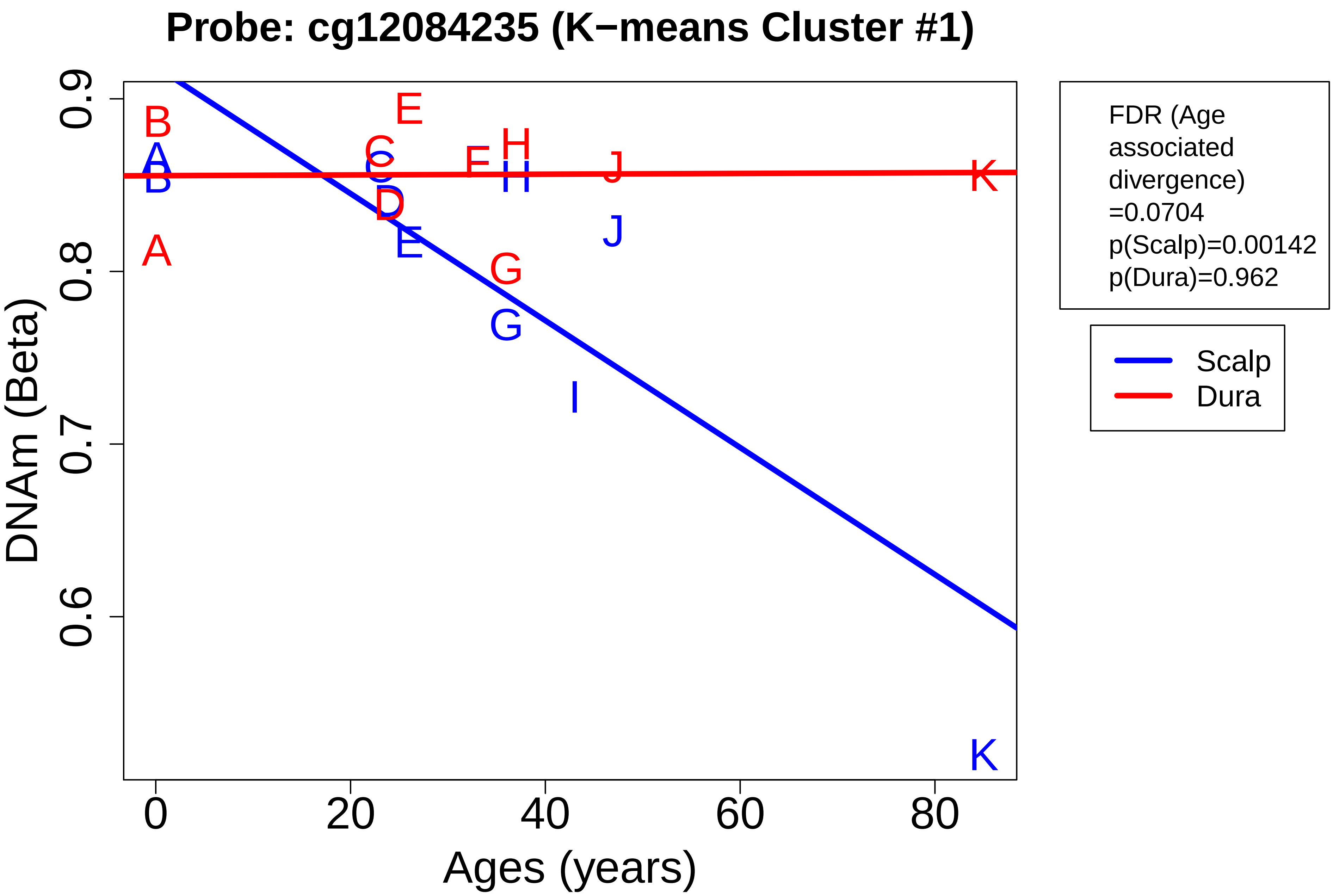

Probe: cg12084235 (K−means Cluster #1)
DNAm (Beta)
0.6
0.7
0.8
0.9
Ages (years)
0
20
40
60
80
A
B
C
D
E
F
G
H
I
J
K
FDR (Age
associated
divergence)
=0.0704
p(Scalp)=0.00142
p(Dura)=0.962
Scalp
Dura

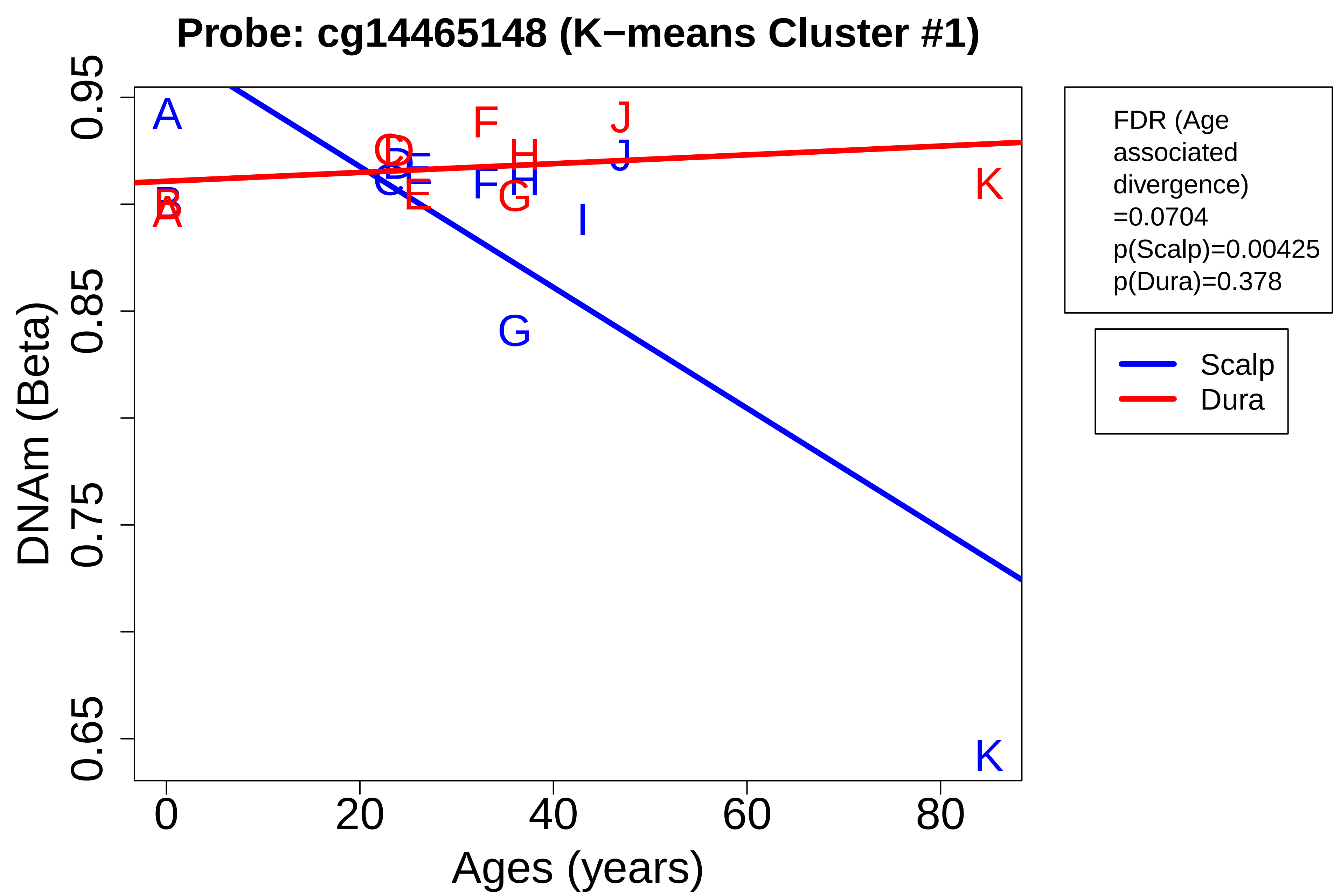
Probe: cg14465148 (K−means Cluster #1)
DNAm (Beta)
0.65
0.75
0.85
0.95
Ages (years)
0
20
40
60
80
A
B
C
D
E
F
G
H
I
J
K
FDR (Age associated divergence) =0.0704
p(Scalp)=0.00425
p(Dura)=0.378
Scalp
Dura

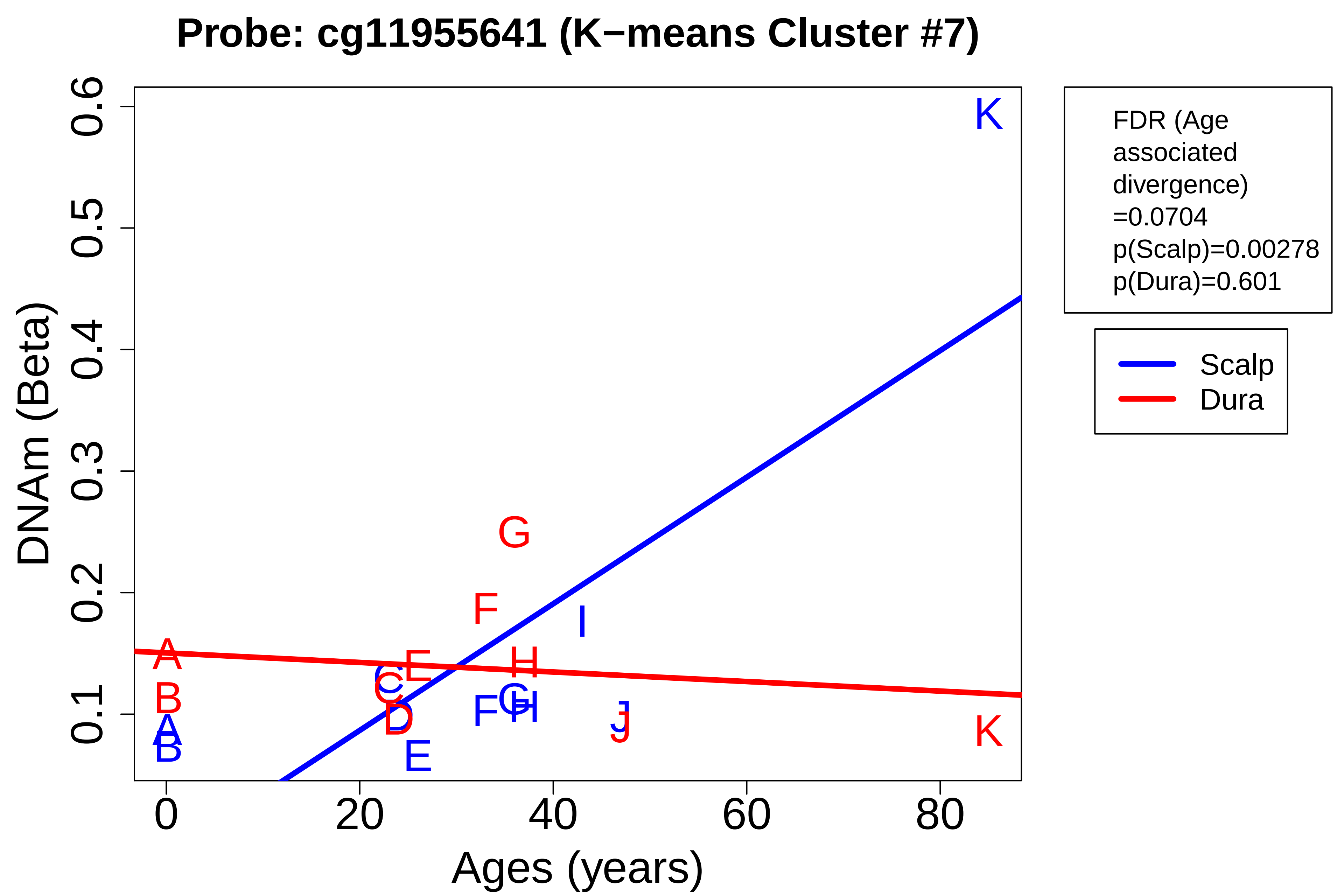

Probe: cg11955641 (K−means Cluster #7)
DNAm (Beta)
0.1
0.2
0.3
0.4
0.5
0.6
0
20
40
60
80
Ages (years)
FDR (Age associated divergence) =0.0704
p(Scalp)=0.00278
p(Dura)=0.601
Scalp
Dura

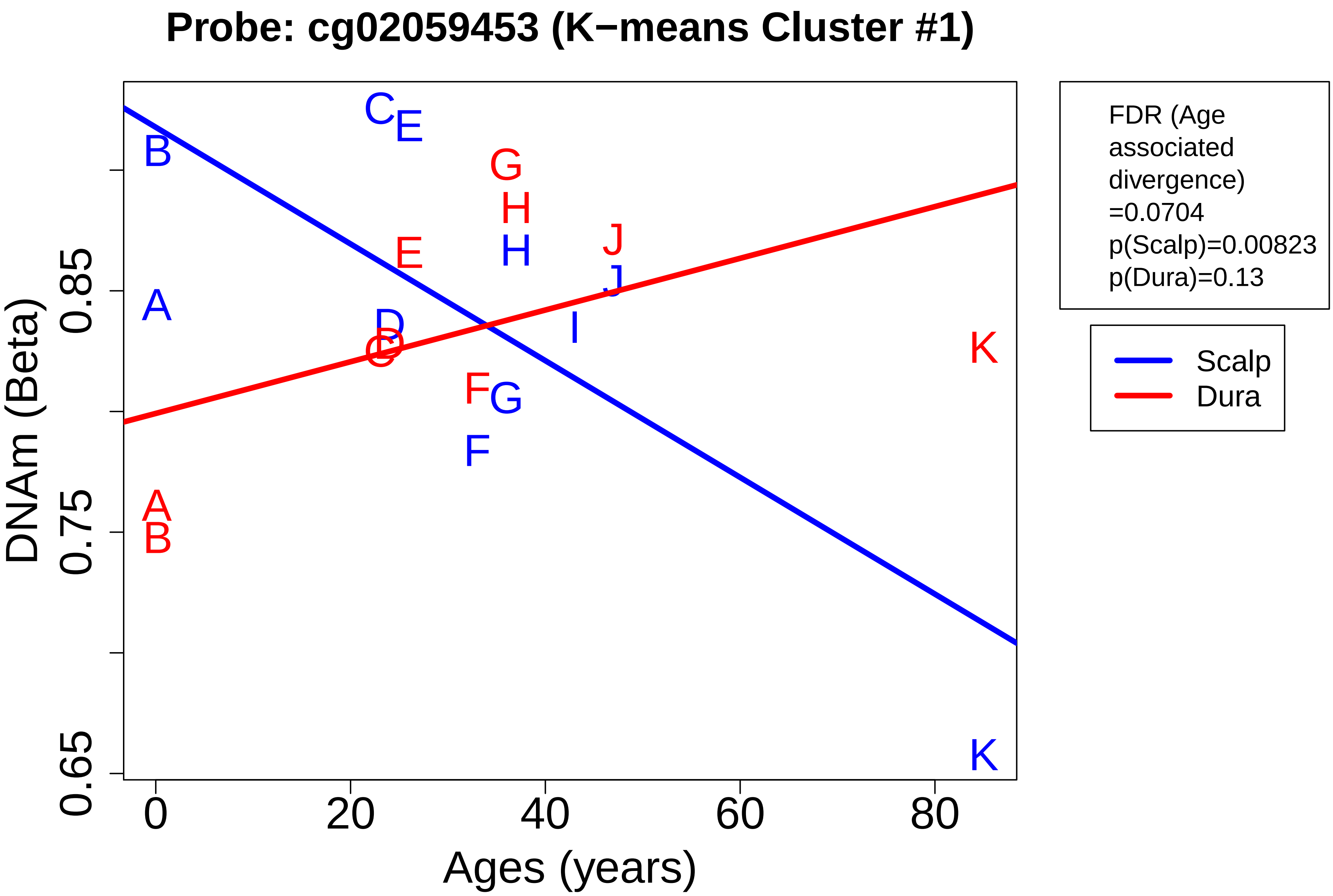
Probe: cg02059453 (K−means Cluster #1)
DNAm (Beta)
0.65
0.75
0.85
0
20
40
60
80
Ages (years)
FDR (Age associated divergence) =0.0704
p(Scalp)=0.00823
p(Dura)=0.13
Scalp
Dura

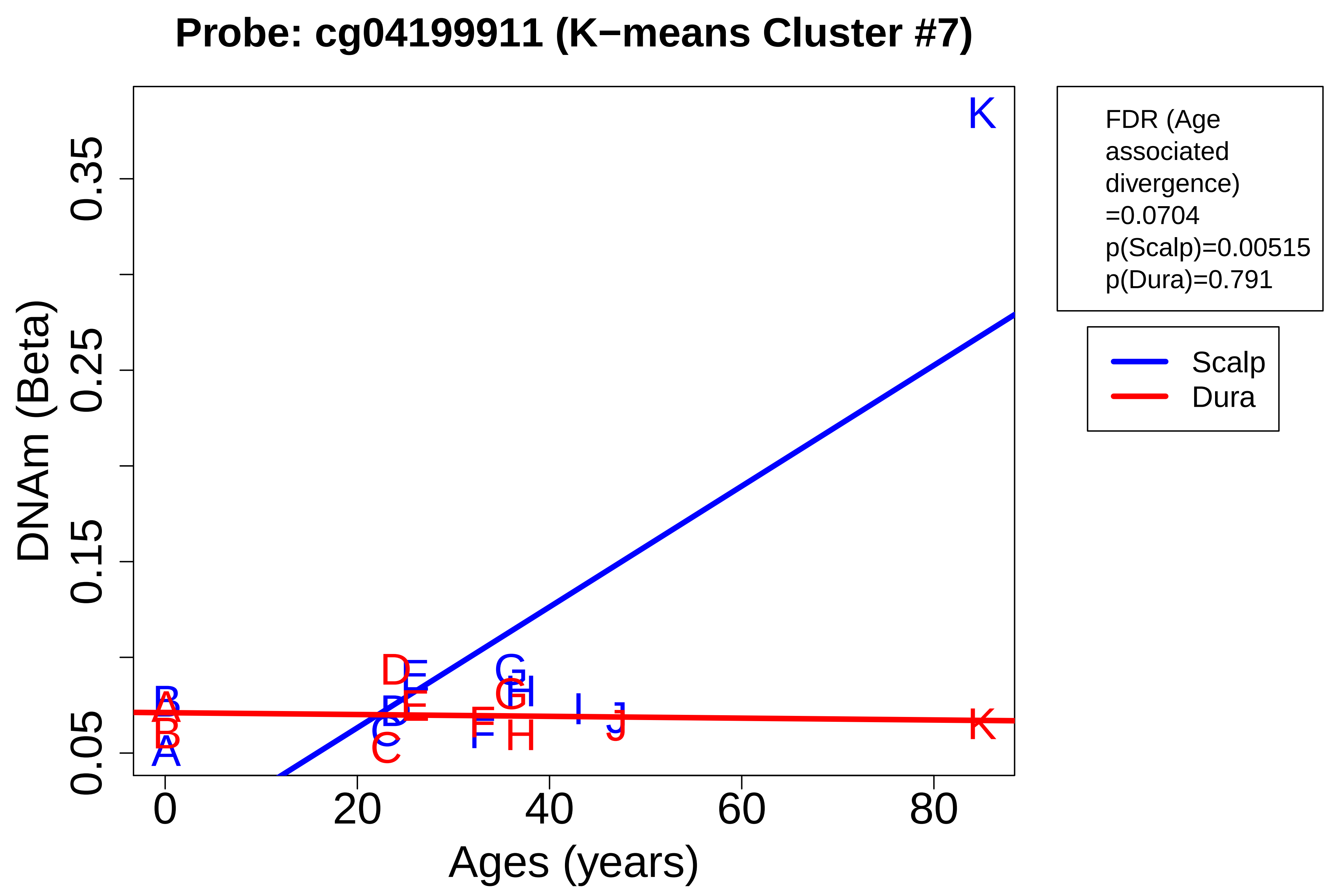

Probe: cg04199911 (K−means Cluster #7)
DNAm (Beta)
0.05
0.15
0.25
0.35
0
20
40
60
80
Ages (years)
FDR (Age associated divergence) =0.0704
p(Scalp)=0.00515
p(Dura)=0.791
Scalp
Dura

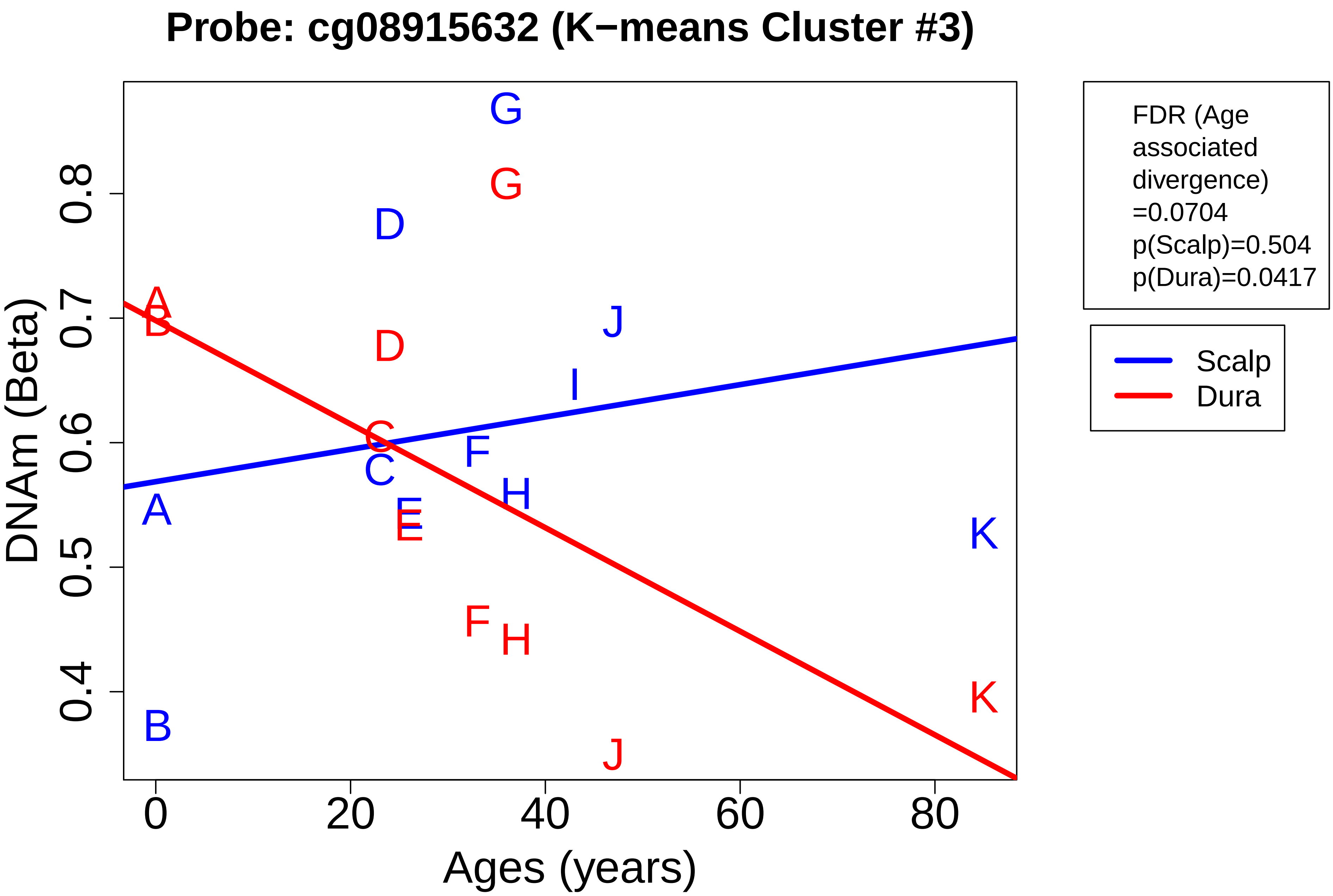

Probe: cg08915632 (K−means Cluster #3)
FDR (Age associated divergence) =0.0704
p(Scalp)=0.504
p(Dura)=0.0417
Scalp
Dura
DNAm (Beta)
0.4
0.5
0.6
0.7
0.8
0
20
40
60
80
Ages (years)

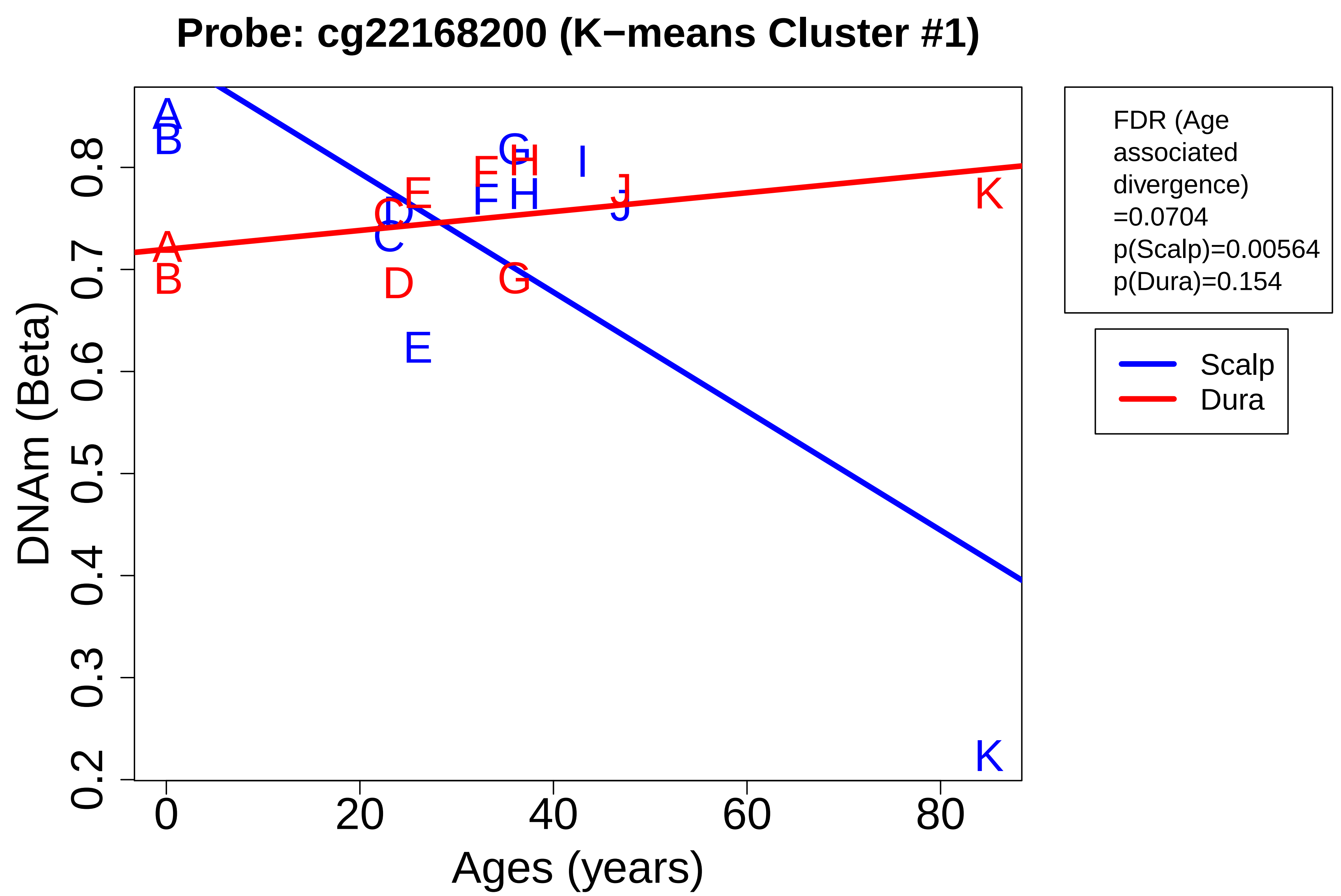

Probe: cg22168200 (K−means Cluster #1)
DNAm (Beta)
Ages (years)
0.2
0.3
0.4
0.5
0.6
0.7
0.8
0
20
40
60
80
FDR (Age associated divergence) =0.0704
p(Scalp)=0.00564
p(Dura)=0.154
Scalp
Dura

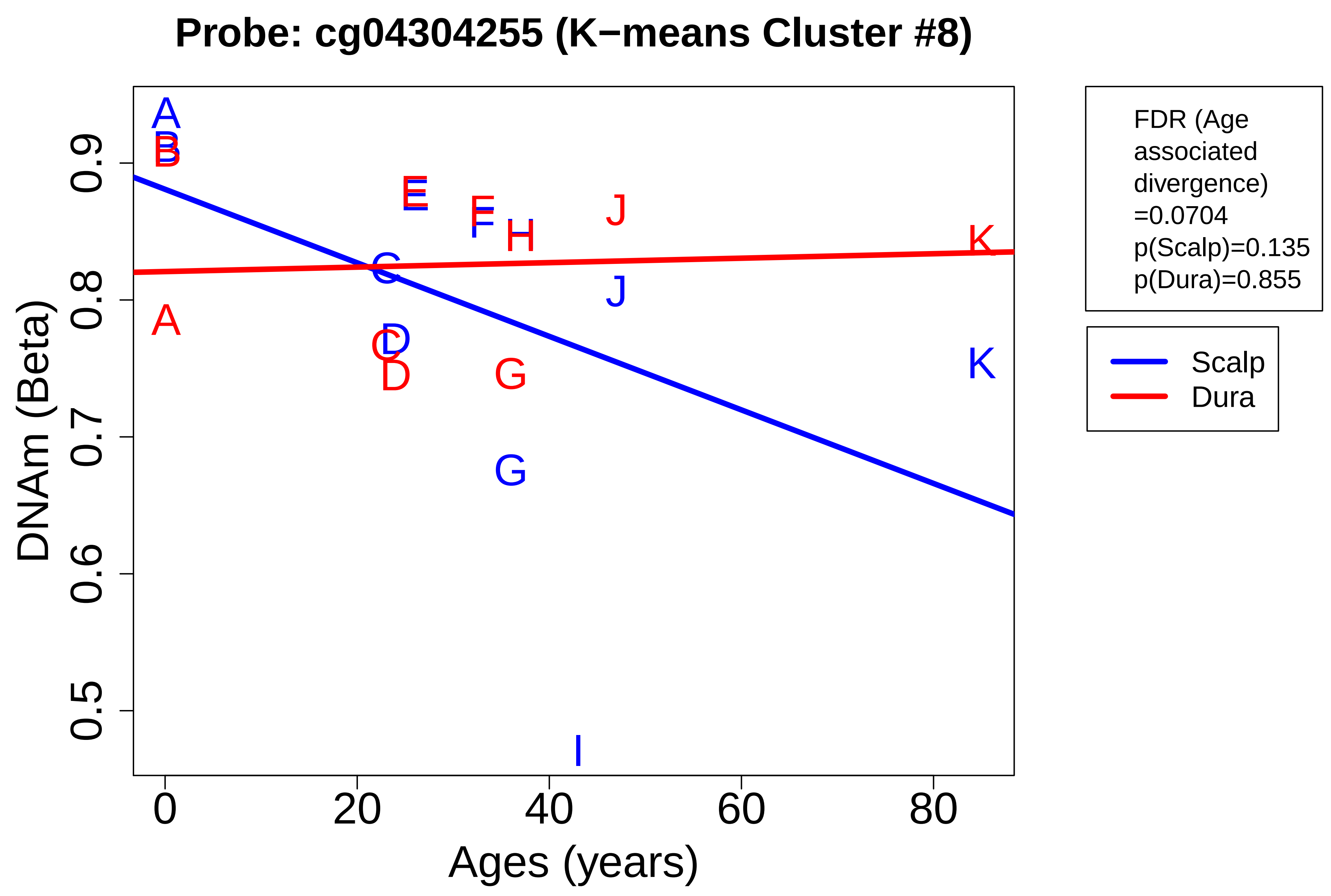

Probe: cg04304255 (K−means Cluster #8)
DNAm (Beta)
0.5
0.6
0.7
0.8
0.9
Ages (years)
0
20
40
60
80
FDR (Age associated divergence) =0.0704
p(Scalp)=0.135
p(Dura)=0.855
Scalp
Dura

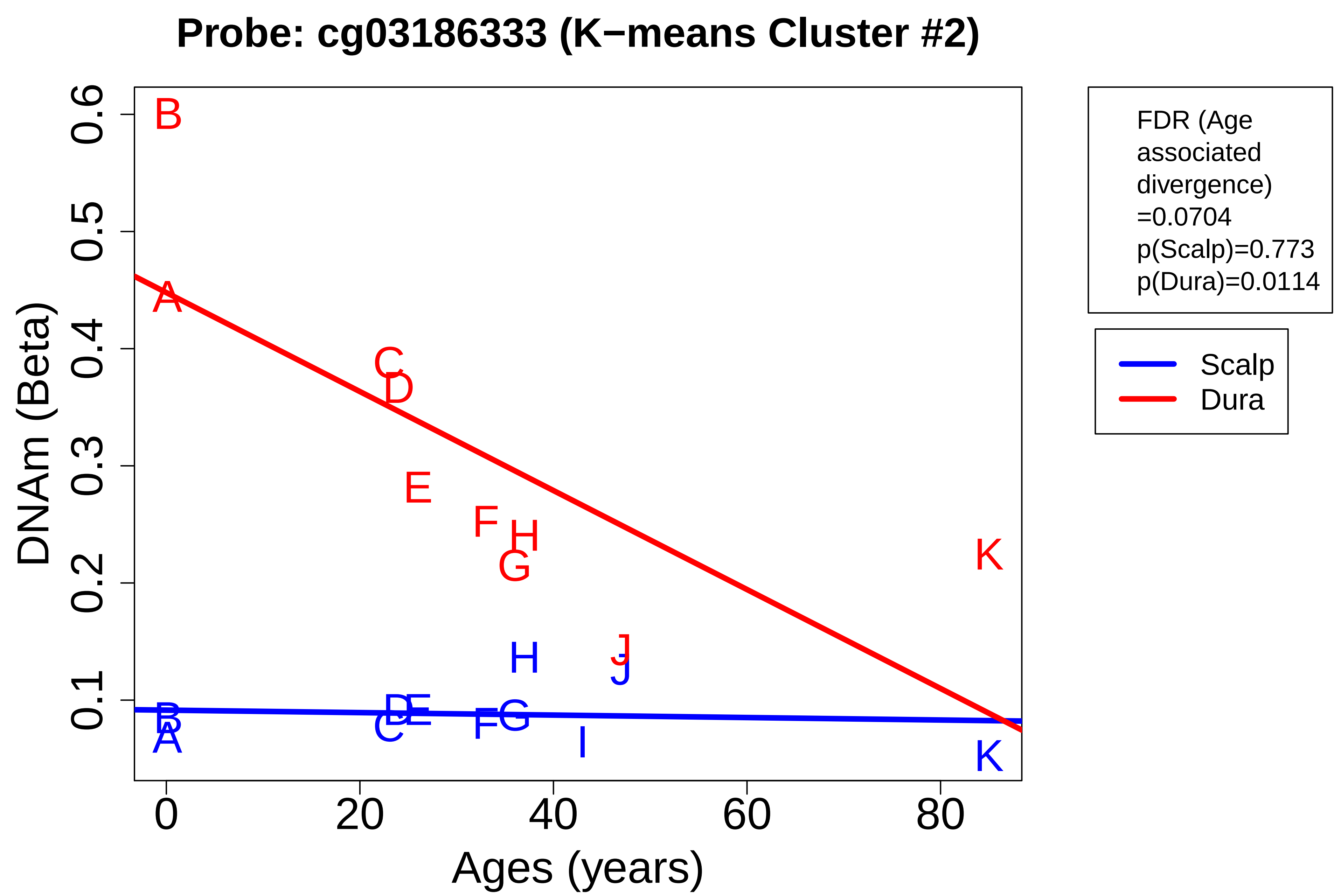

Probe: cg03186333 (K−means Cluster #2)
DNAm (Beta)
0.1
0.2
0.3
0.4
0.5
0.6
Ages (years)
0
20
40
60
80
B
A
C
D
E
F
H
G
K
J
FDR (Age
associated
divergence)
=0.0704
p(Scalp)=0.773
p(Dura)=0.0114
Scalp
Dura

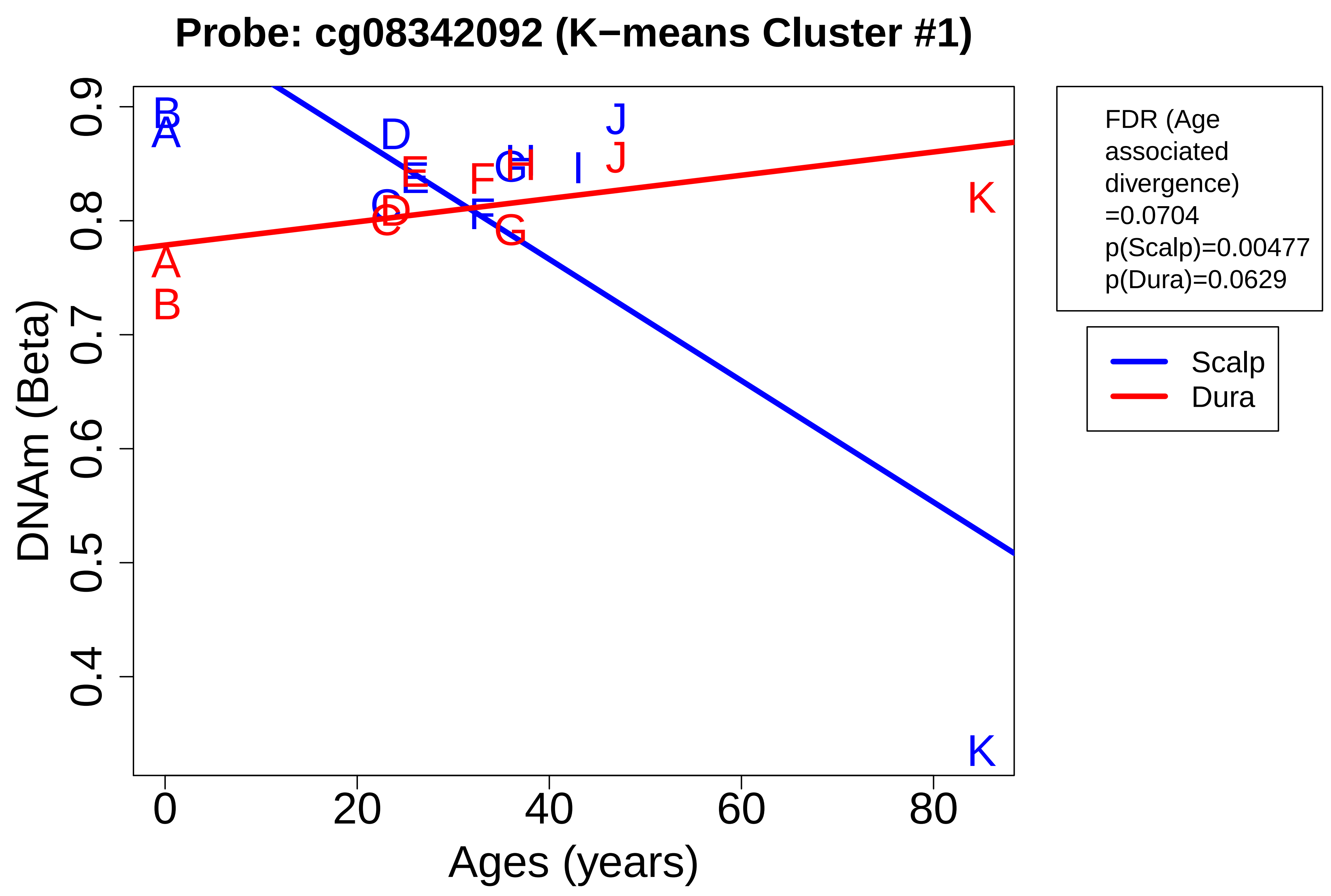
Probe: cg08342092 (K−means Cluster #1)
DNAm (Beta)
0.4
0.5
0.6
0.7
0.8
0.9
Ages (years)
0
20
40
60
80
A
B
C
D
E
F
G
H
I
J
K
FDR (Age associated divergence) =0.0704
p(Scalp)=0.00477
p(Dura)=0.0629
Scalp
Dura

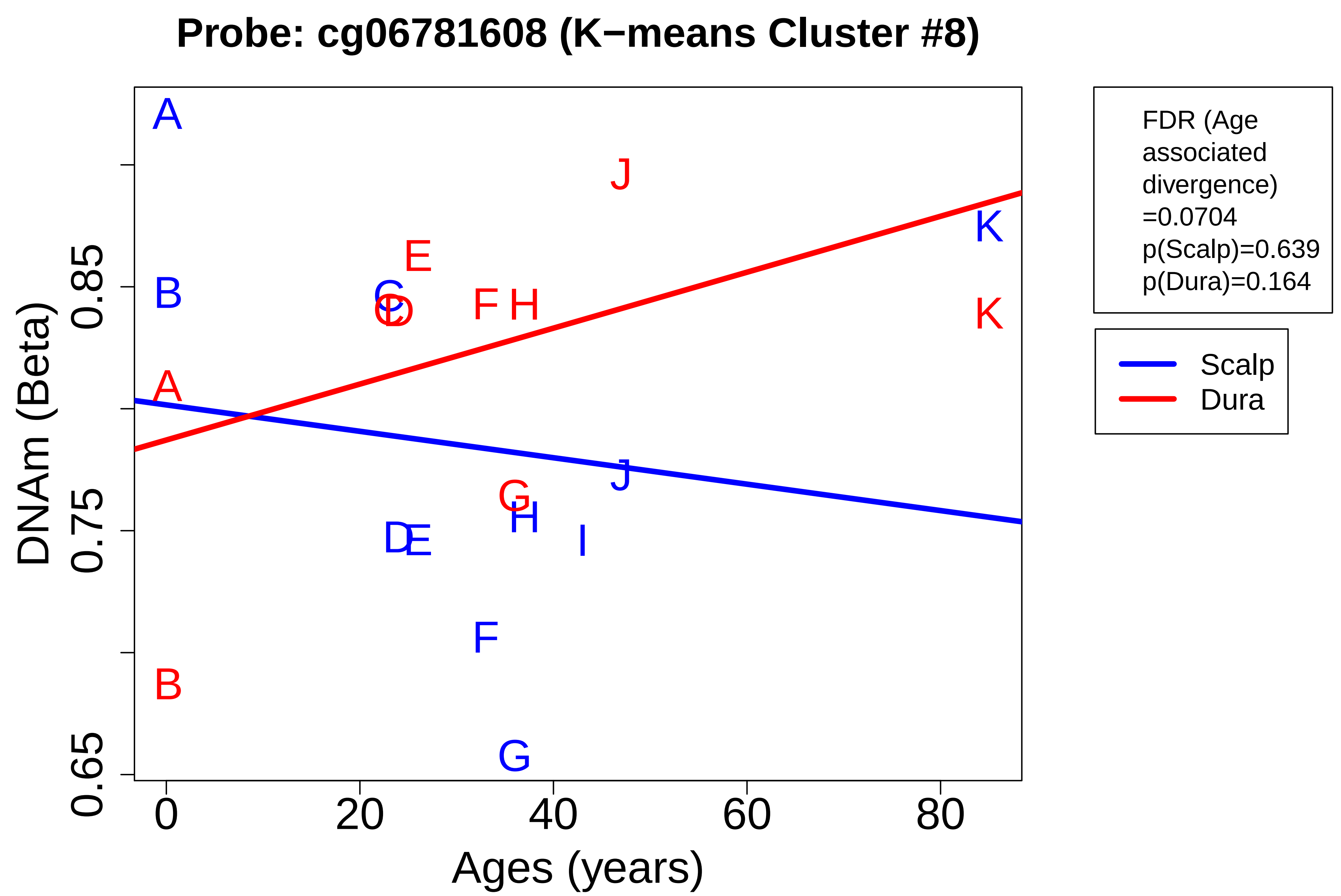
Probe: cg06781608 (K−means Cluster #8)
DNAm (Beta)
0.65
0.75
0.85
0
20
40
60
80
Ages (years)
A
B
C
D
E
F
G
H
I
J
K
FDR (Age associated divergence) =0.0704
p(Scalp)=0.639
p(Dura)=0.164
Scalp
Dura

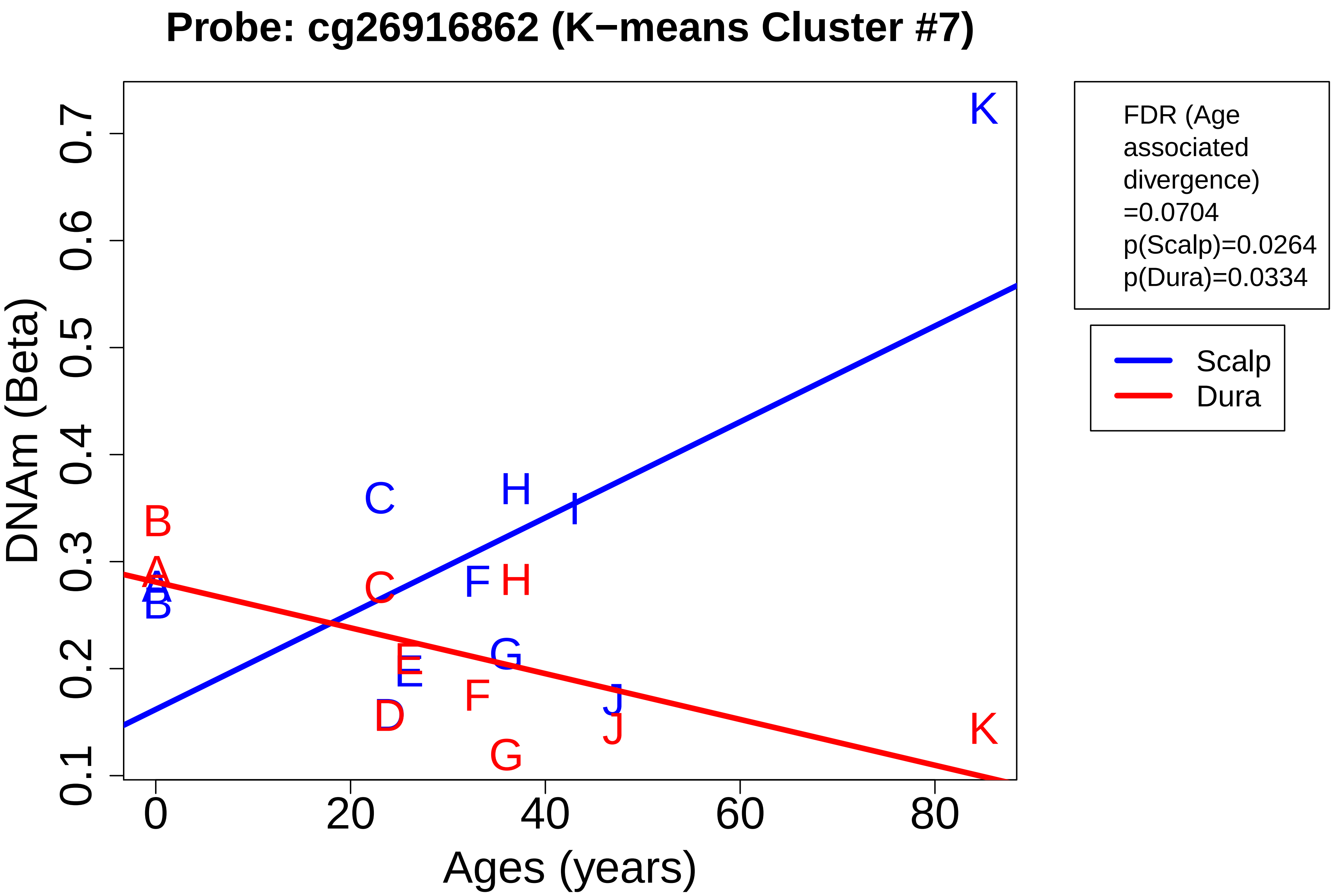

Probe: cg26916862 (K−means Cluster #7)
DNAm (Beta)
0.1
0.2
0.3
0.4
0.5
0.6
0.7
Ages (years)
0
20
40
60
80
A
B
C
D
E
F
G
H
I
J
K
FDR (Age associated divergence) =0.0704
p(Scalp)=0.0264
p(Dura)=0.0334
Scalp
Dura

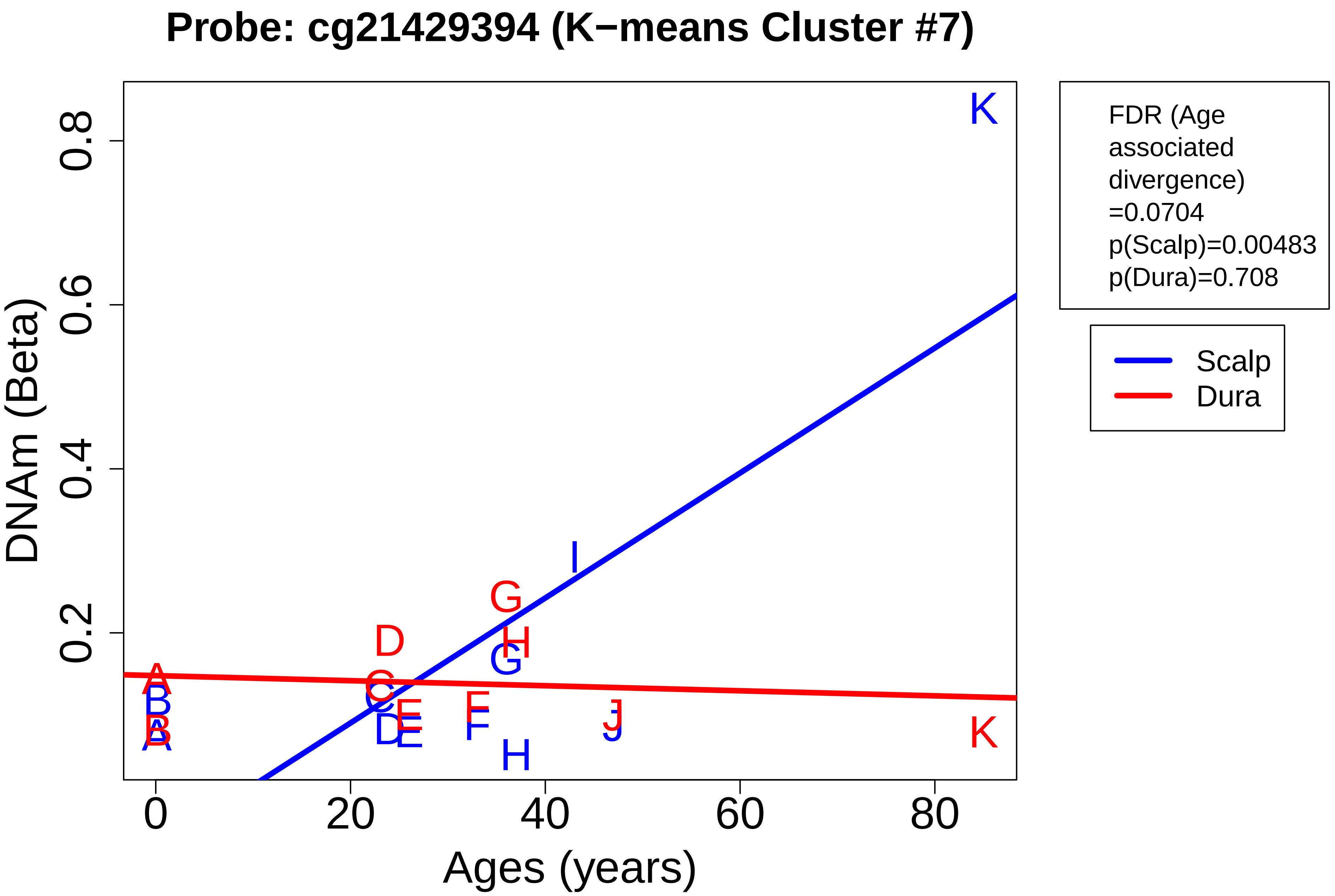

Probe: cg21429394 (K−means Cluster #7)
DNAm (Beta)
0.2
0.4
0.6
0.8
Ages (years)
0
20
40
60
80
FDR (Age associated divergence) =0.0704
p(Scalp)=0.00483
p(Dura)=0.708
Scalp
Dura

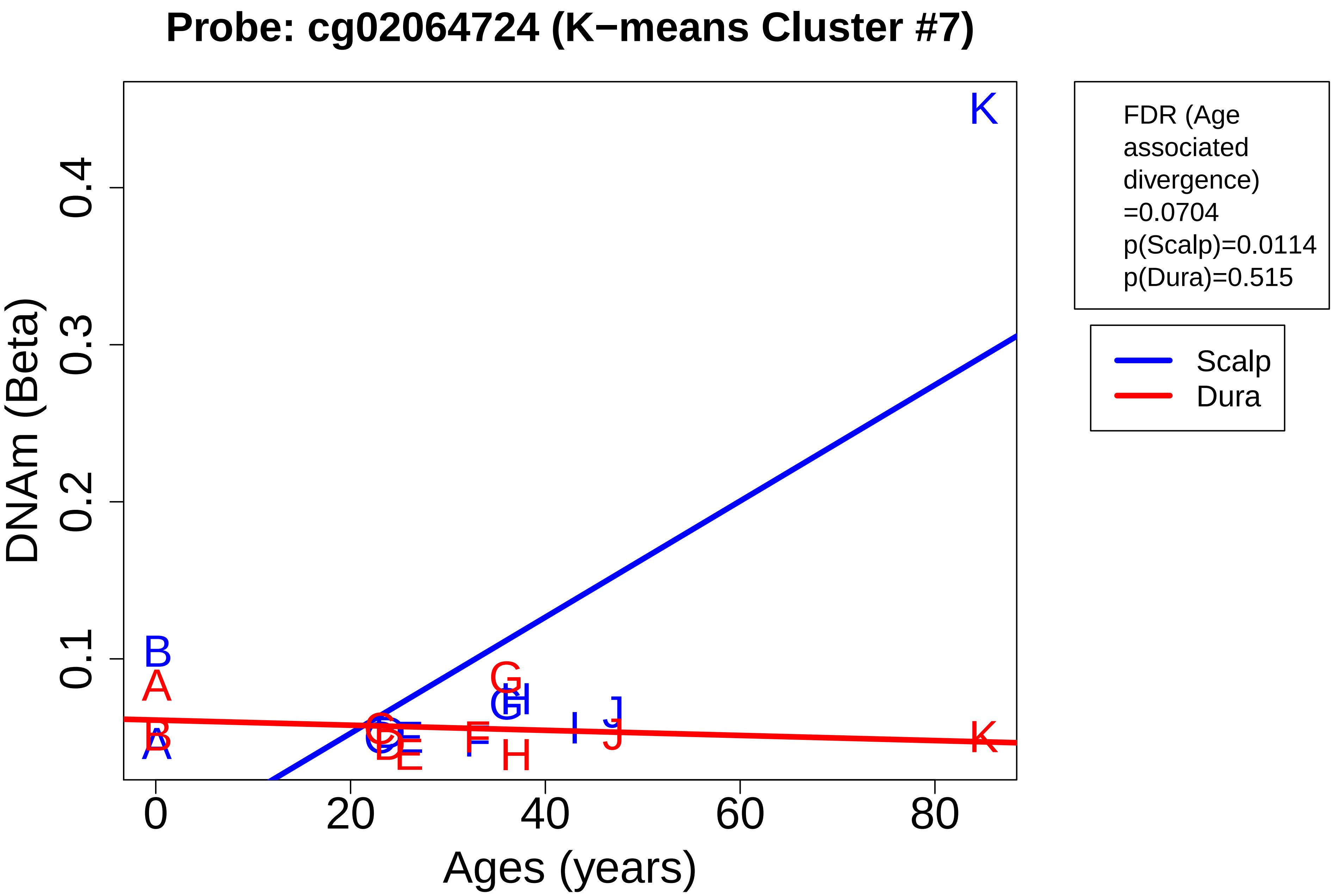

Probe: cg02064724 (K−means Cluster #7)
DNAm (Beta)
0.1
0.2
0.3
0.4
Ages (years)
0
20
40
60
80
FDR (Age associated divergence) =0.0704
p(Scalp)=0.0114
p(Dura)=0.515
Scalp
Dura

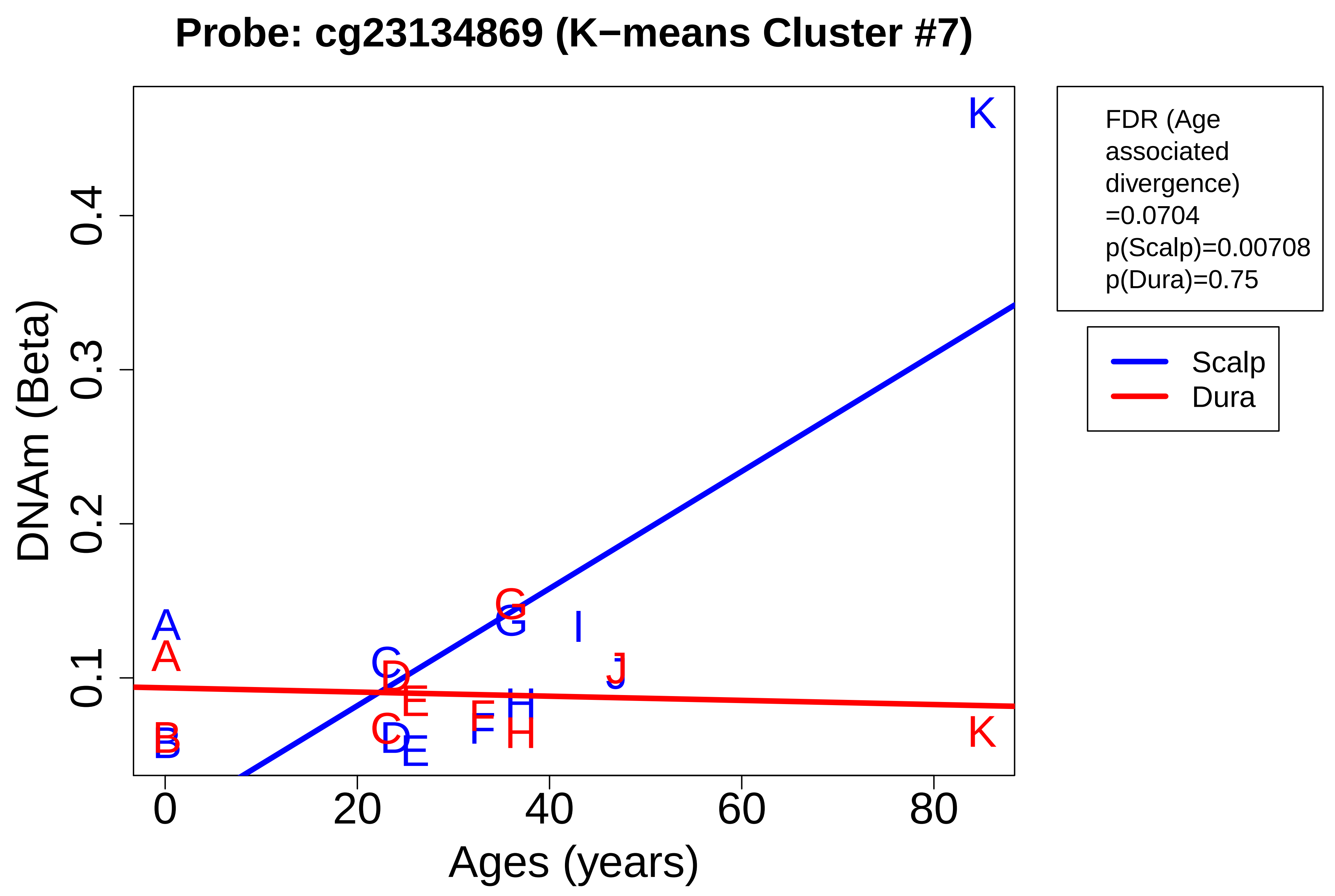

Probe: cg23134869 (K−means Cluster #7)
DNAm (Beta)
0.1
0.2
0.3
0.4
0
20
40
60
80
Ages (years)
A
B
C
D
E
F
G
H
I
J
K
FDR (Age associated divergence) =0.0704
p(Scalp)=0.00708
p(Dura)=0.75
Scalp
Dura

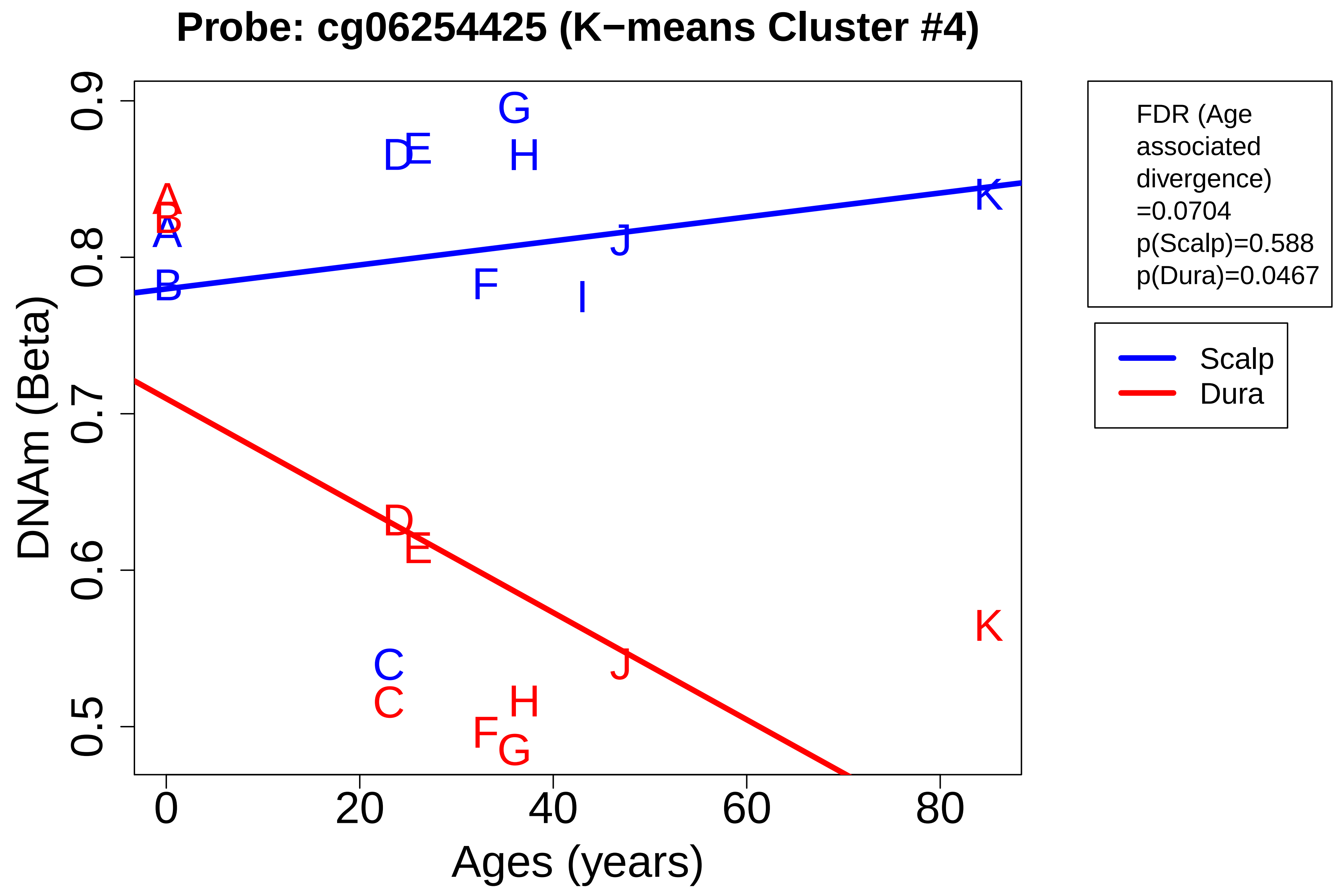

Probe: cg06254425 (K−means Cluster #4)
DNAm (Beta)
0.5
0.6
0.7
0.8
0.9
0
20
40
60
80
Ages (years)
A
B
C
D
E
F
G
H
I
J
K
FDR (Age associated divergence) =0.0704
p(Scalp)=0.588
p(Dura)=0.0467
Scalp
Dura

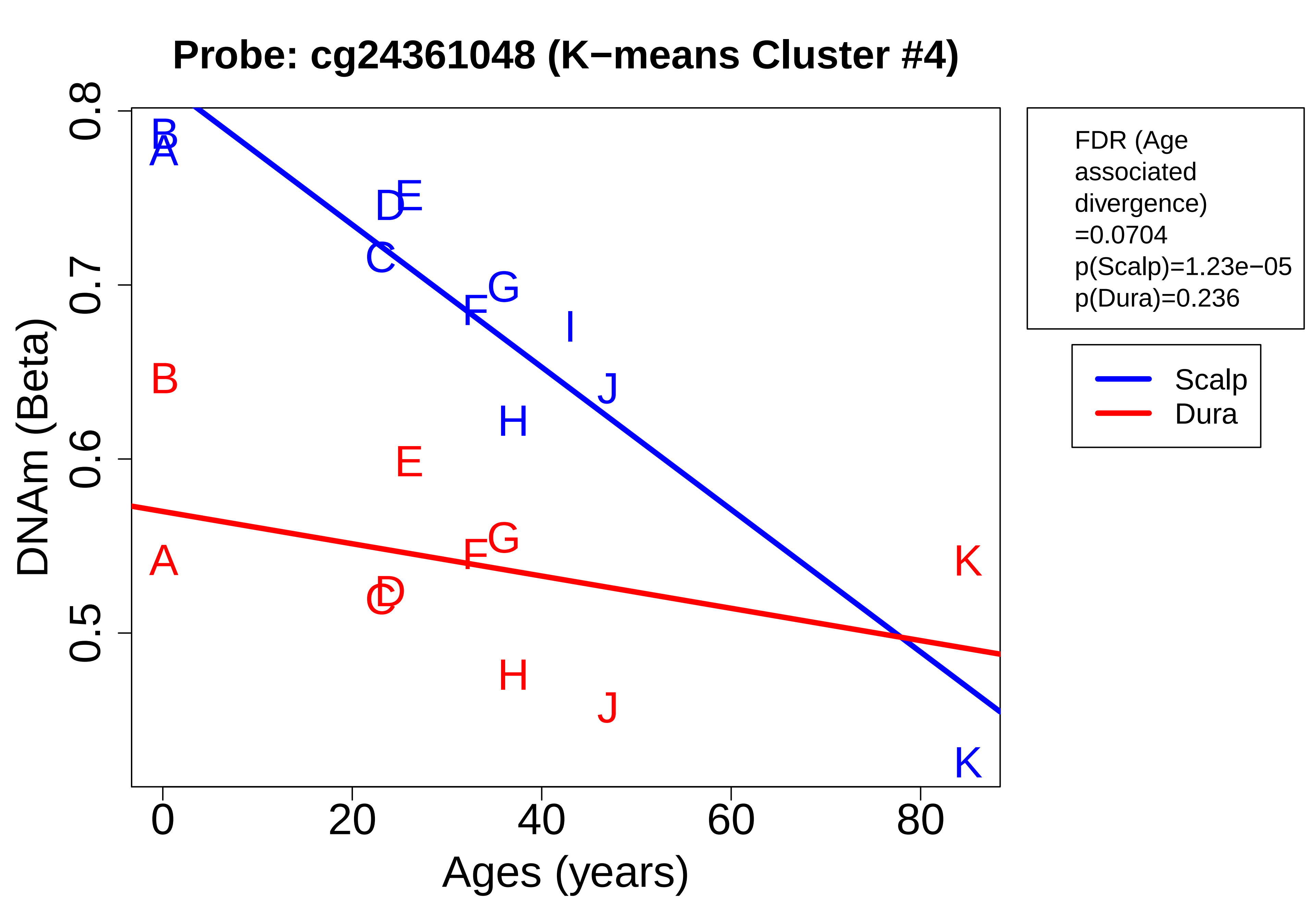

Probe: cg24361048 (K−means Cluster #4)
DNAm (Beta)
0.8
0.7
0.6
0.5
Ages (years)
0
20
40
60
80
FDR (Age associated divergence) =0.0704
p(Scalp)=1.23e−05
p(Dura)=0.236
Scalp
Dura
B
A
D
E
C
G
F
I
J
H
K

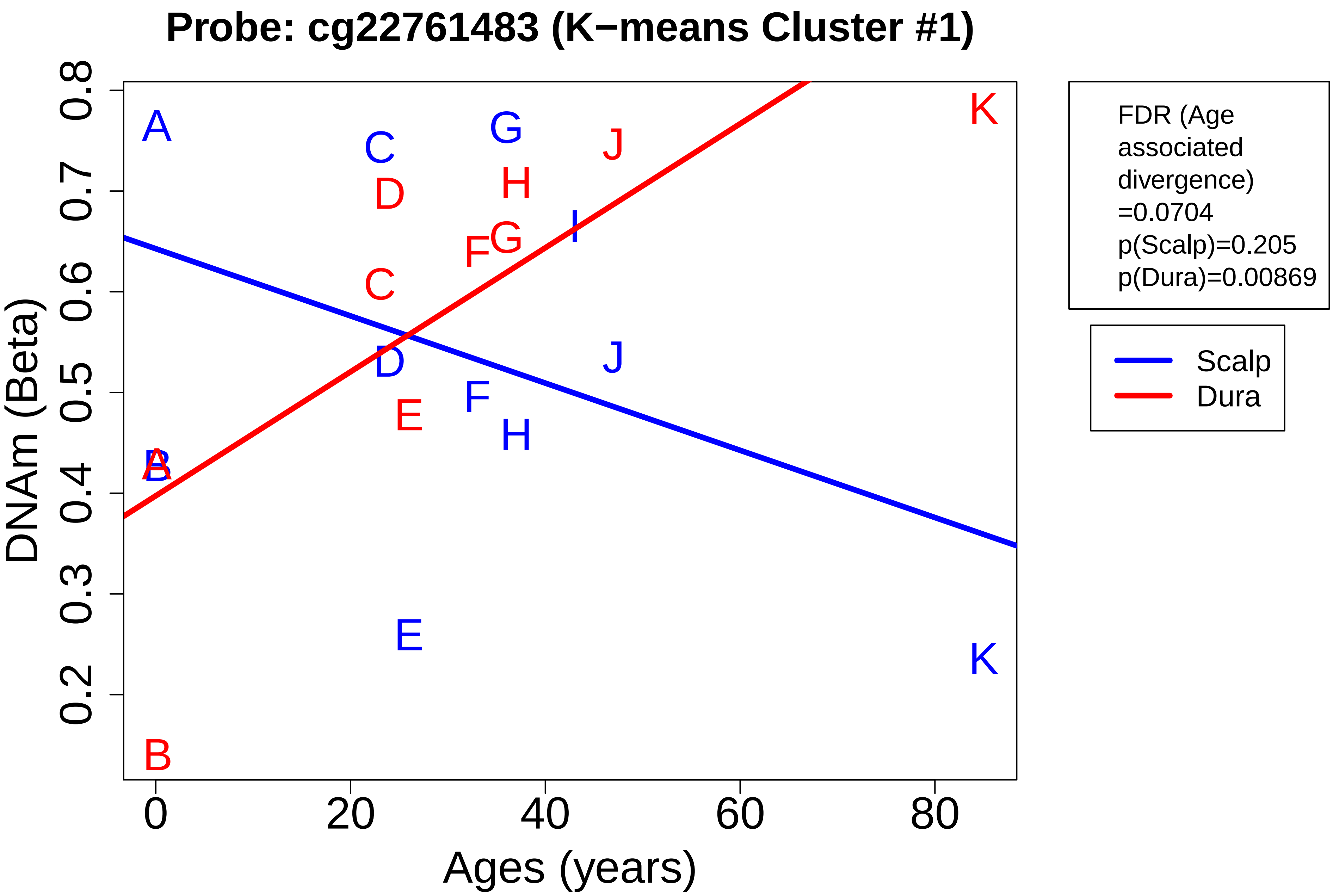
Probe: cg22761483 (K−means Cluster #1)
DNAm (Beta)
0.2
0.3
0.4
0.5
0.6
0.7
0.8
Ages (years)
0
20
40
60
80
A
B
C
D
E
F
G
H
I
J
K
FDR (Age associated divergence) =0.0704
p(Scalp)=0.205
p(Dura)=0.00869
Scalp
Dura

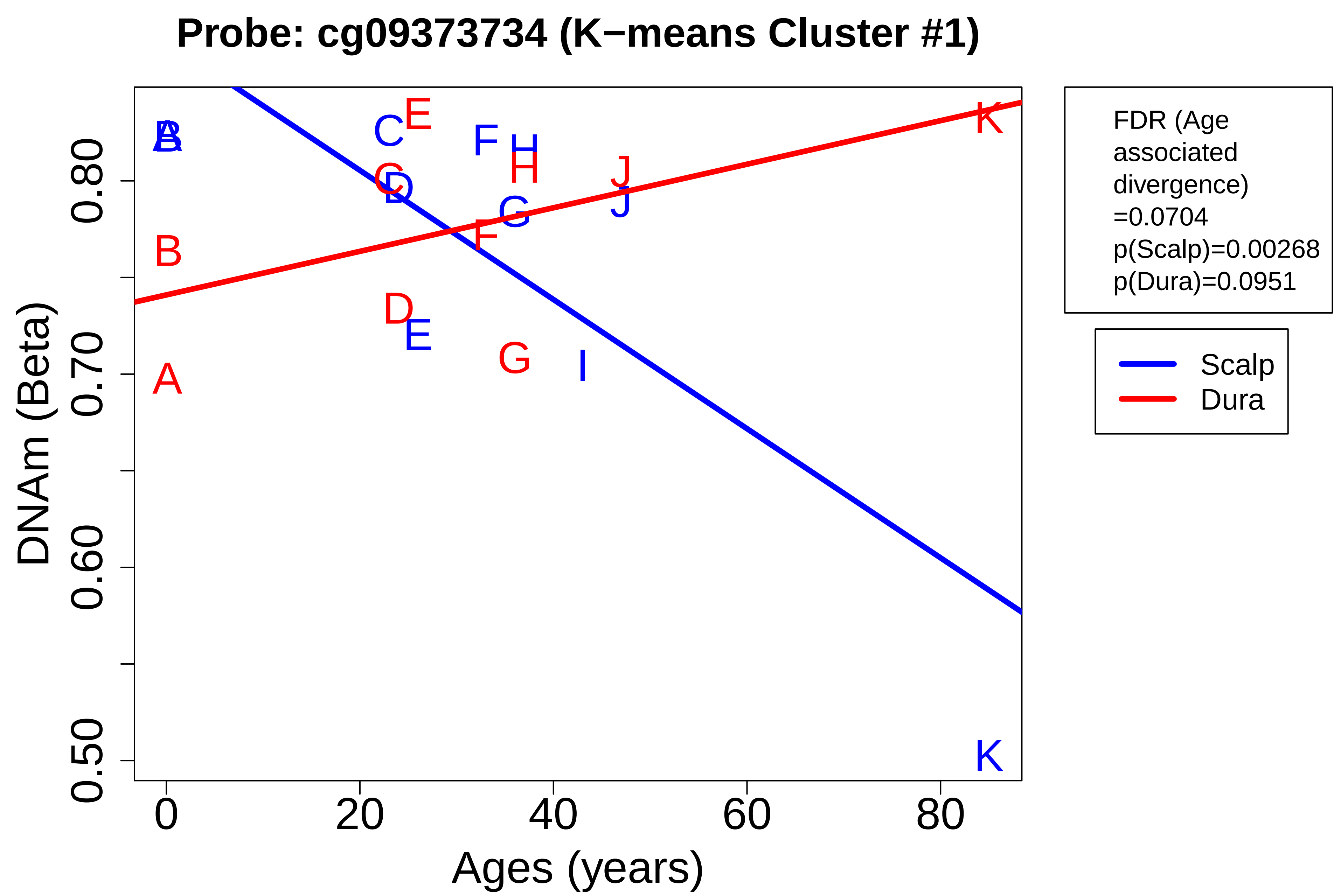

Probe: cg09373734 (K−means Cluster #1)
DNAm (Beta)
0.50
0.60
0.70
0.80
0
20
40
60
80
Ages (years)
FDR (Age associated divergence) =0.0704
p(Scalp)=0.00268
p(Dura)=0.0951
Scalp
Dura

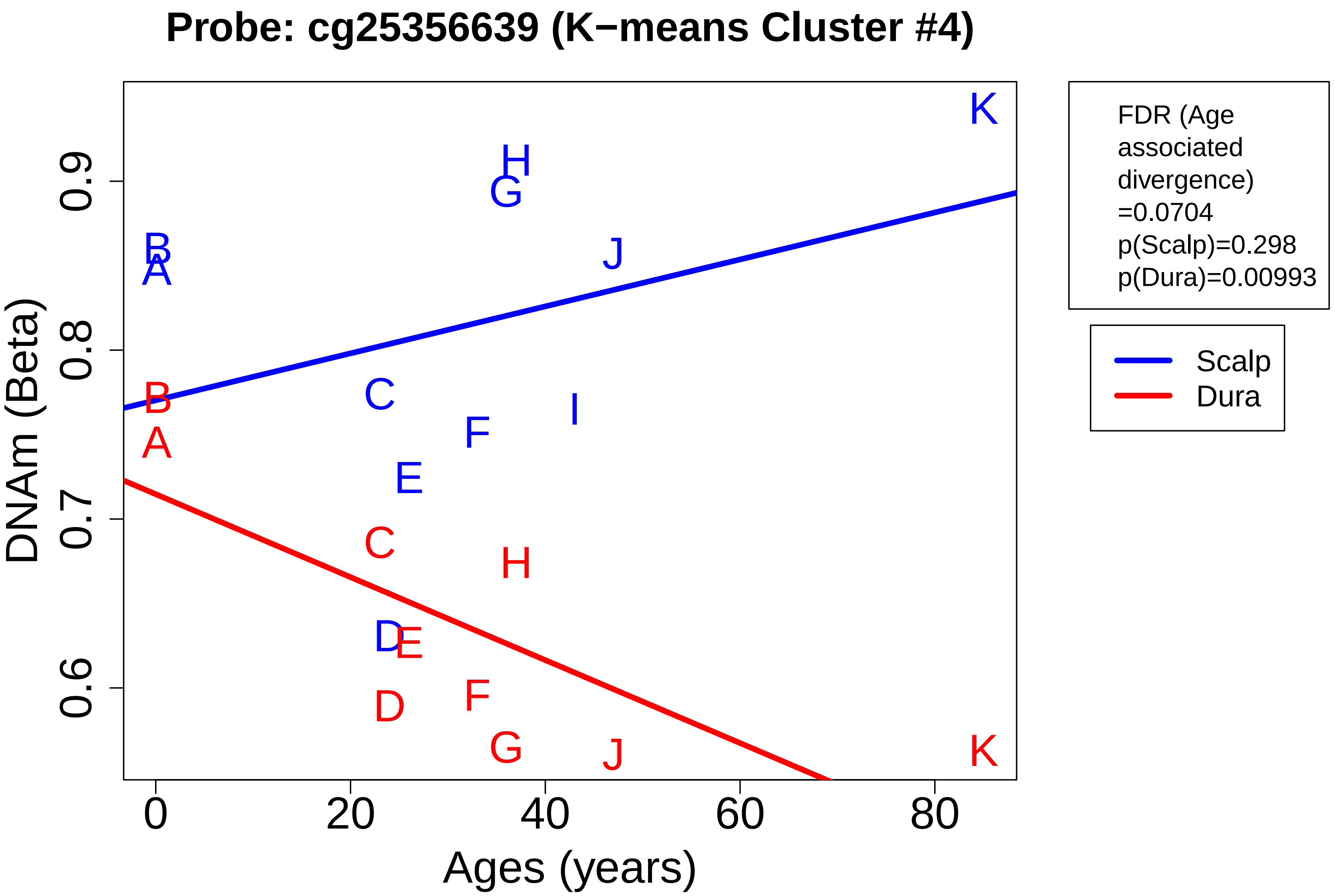

Probe: cg25356639 (K−means Cluster #4)
DNAm (Beta)
0.6
0.7
0.8
0.9
0
20
40
60
80
Ages (years)
FDR (Age associated divergence) =0.0704
p(Scalp)=0.298
p(Dura)=0.00993
Scalp
Dura
A
B
C
D
E
F
G
H
I
J
K

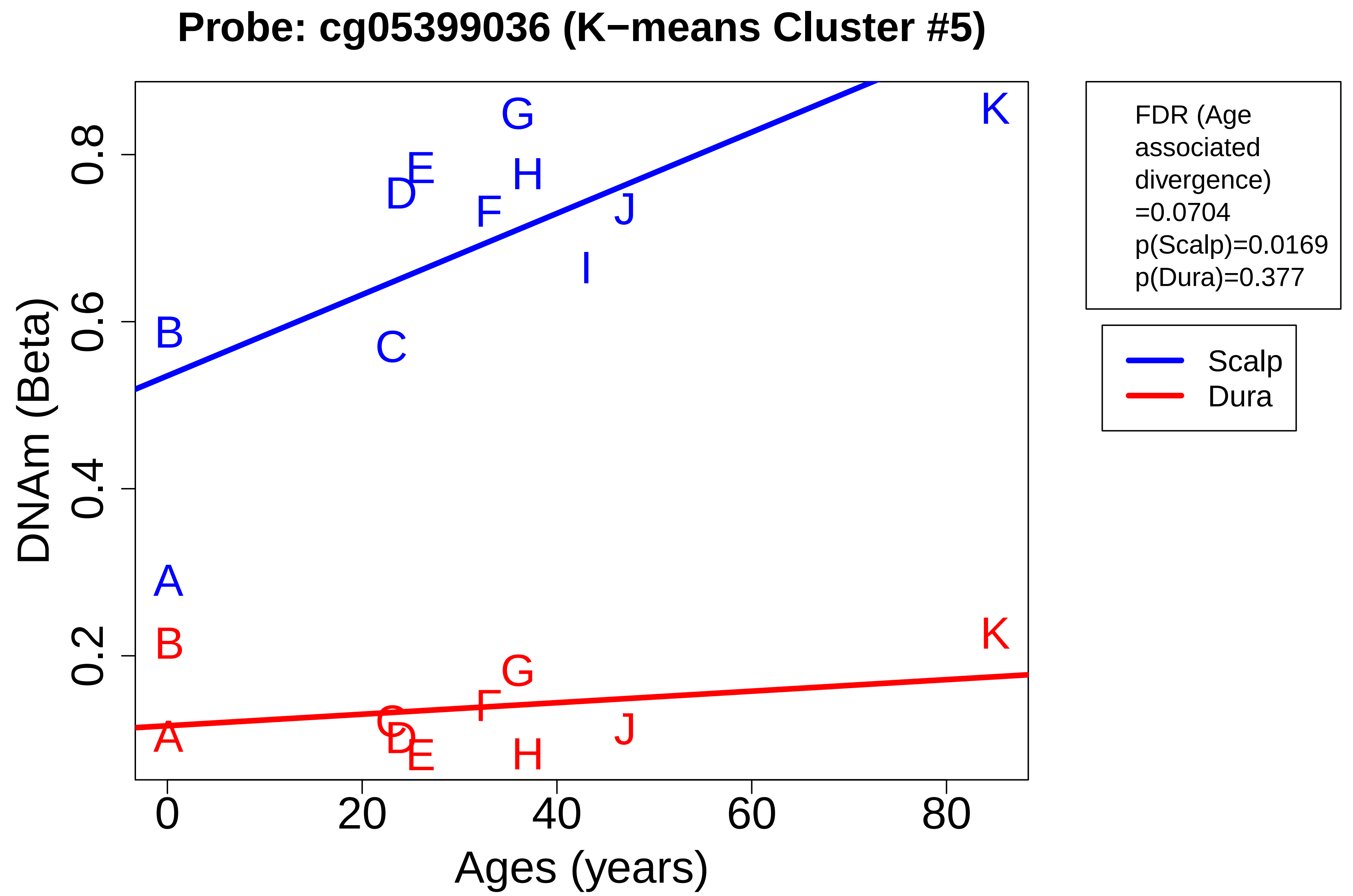

Probe: cg05399036 (K−means Cluster #5)
DNAm (Beta)
0.2
0.4
0.6
0.8
Ages (years)
0
20
40
60
80
A
B
C
D
E
F
G
H
I
J
K
FDR (Age associated divergence) =0.0704
p(Scalp)=0.0169
p(Dura)=0.377
Scalp
Dura

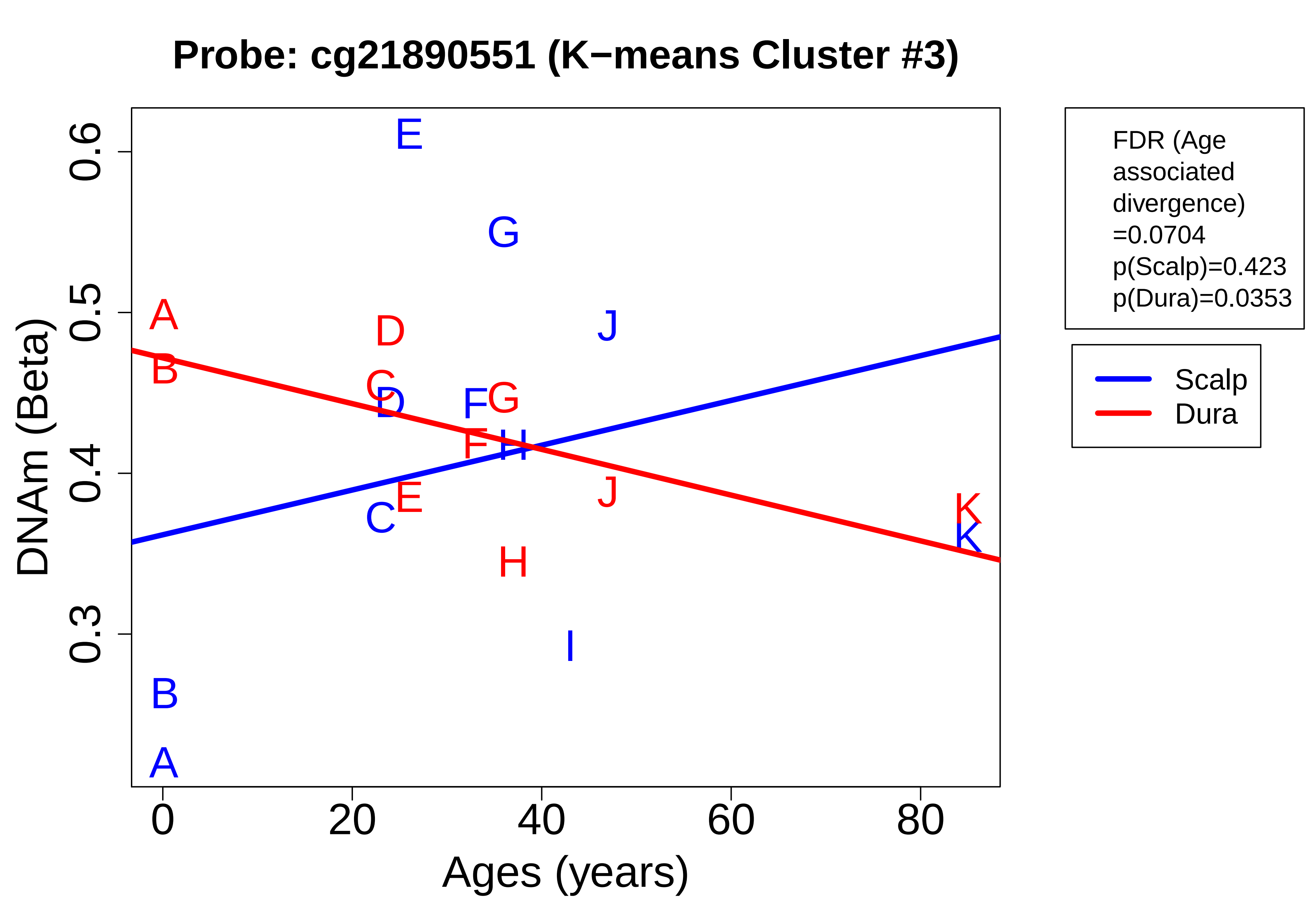
Probe: cg21890551 (K−means Cluster #3)
DNAm (Beta)
Ages (years)
0.3
0.4
0.5
0.6
0
20
40
60
80
FDR (Age associated divergence) =0.0704
p(Scalp)=0.423
p(Dura)=0.0353
Scalp
Dura

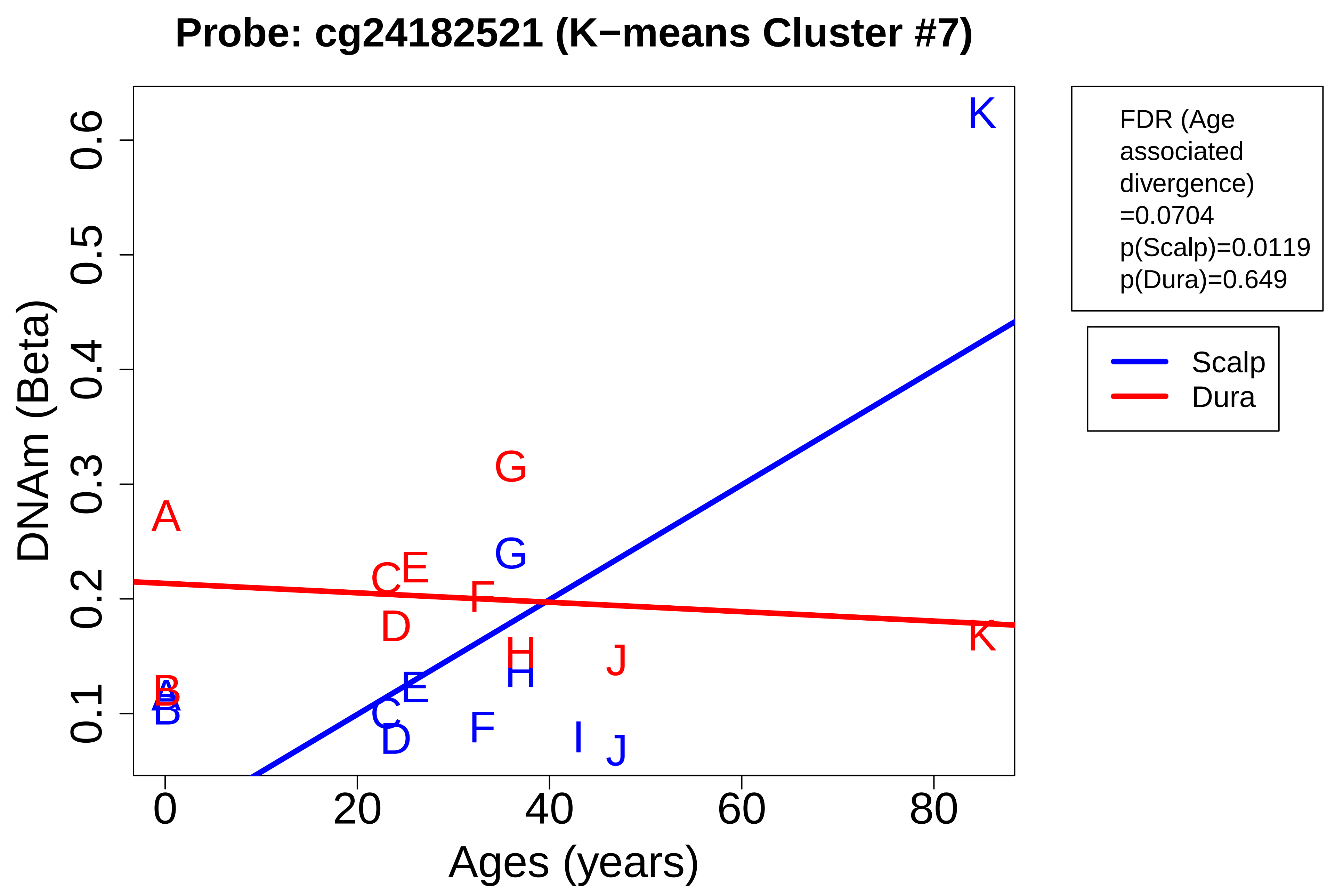

Probe: cg24182521 (K−means Cluster #7)
FDR (Age associated divergence) =0.0704
p(Scalp)=0.0119
p(Dura)=0.649
Scalp
Dura
DNAm (Beta)
Ages (years)
0
20
40
60
80
0.1
0.2
0.3
0.4
0.5
0.6

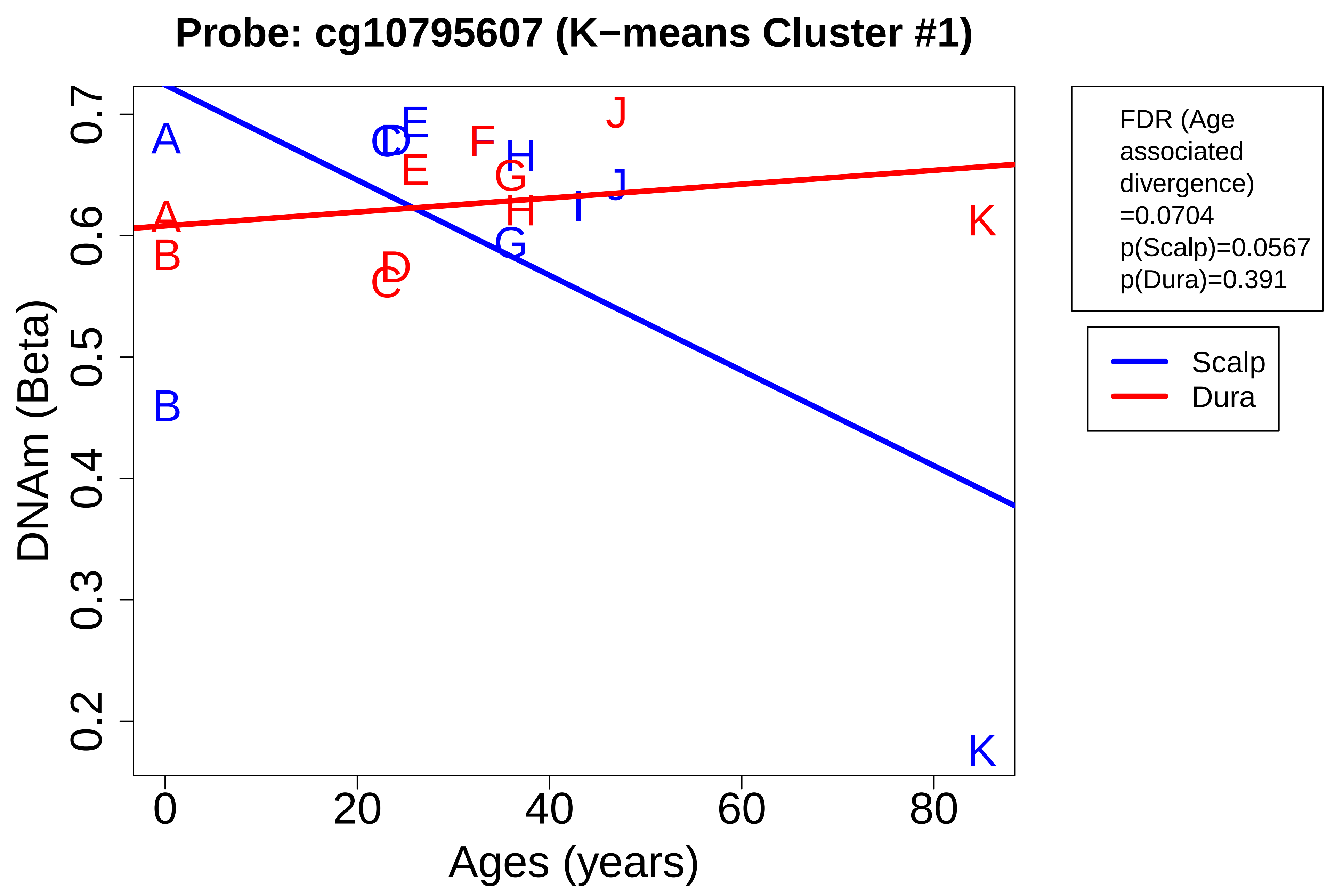
Probe: cg10795607 (K−means Cluster #1)
DNAm (Beta)
0.2
0.3
0.4
0.5
0.6
0.7
0
20
40
60
80
Ages (years)
A
B
C
D
E
F
G
H
I
J
K
FDR (Age associated divergence) =0.0704
p(Scalp)=0.0567
p(Dura)=0.391
Scalp
Dura

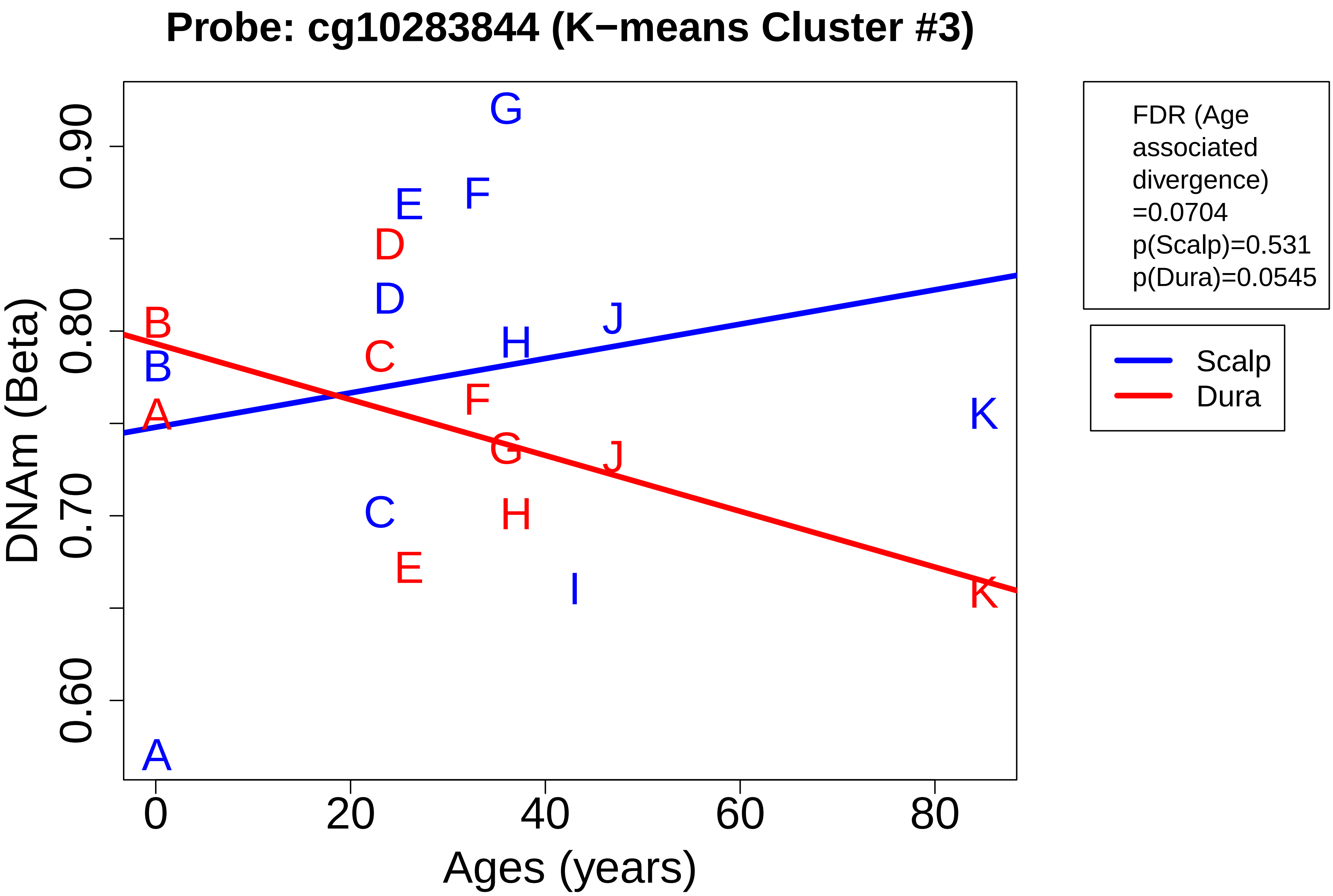

Probe: cg10283844 (K−means Cluster #3)
DNAm (Beta)
0.60
0.70
0.80
0.90
0
20
40
60
80
Ages (years)
FDR (Age associated divergence) =0.0704
p(Scalp)=0.531
p(Dura)=0.0545
Scalp
Dura

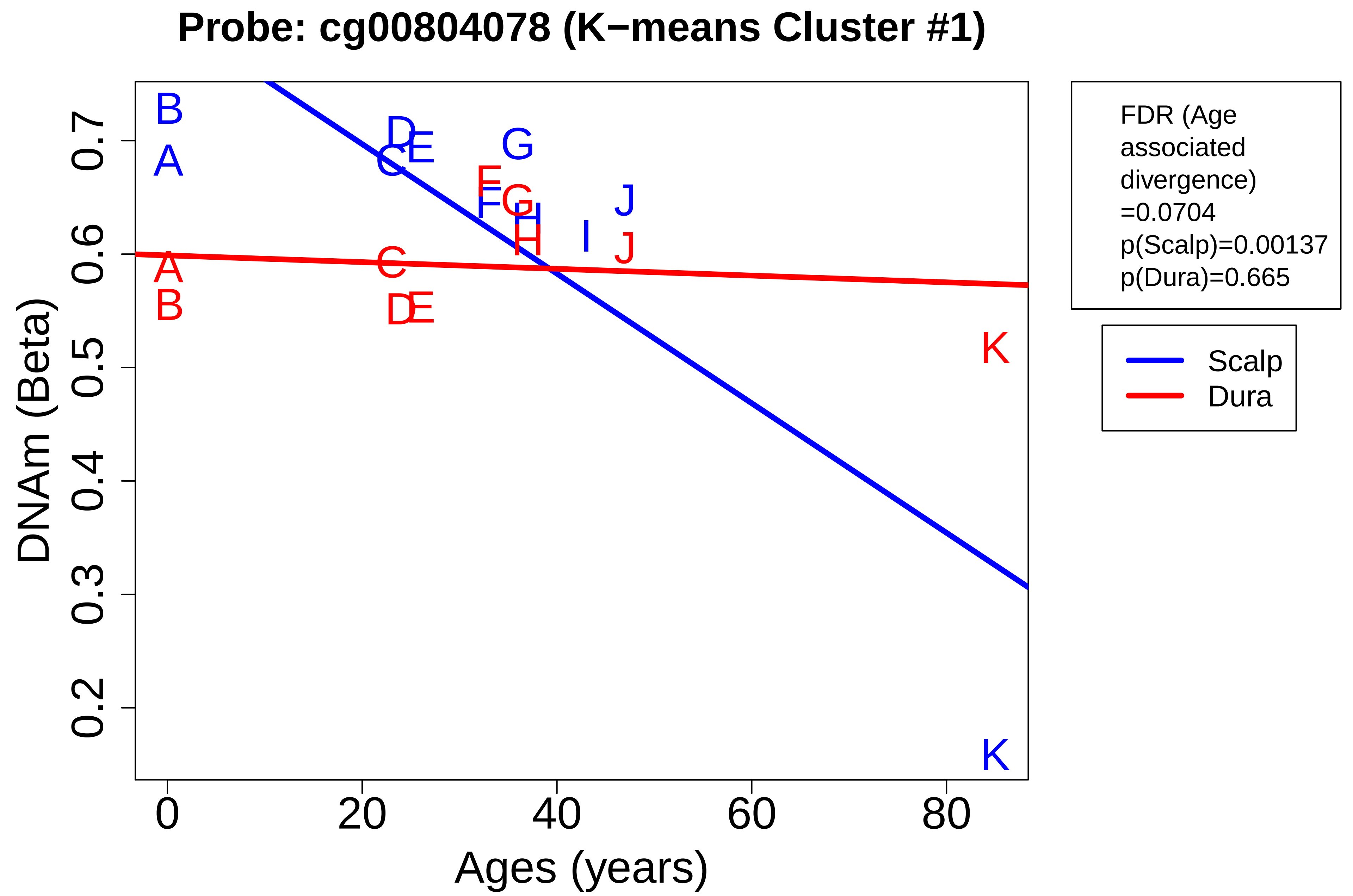

Probe: cg00804078 (K−means Cluster #1)
DNAm (Beta)
0.2
0.3
0.4
0.5
0.6
0.7
0
20
40
60
80
Ages (years)
A
B
C
D
E
F
G
H
I
J
K
FDR (Age associated divergence) =0.0704
p(Scalp)=0.00137
p(Dura)=0.665
Scalp
Dura

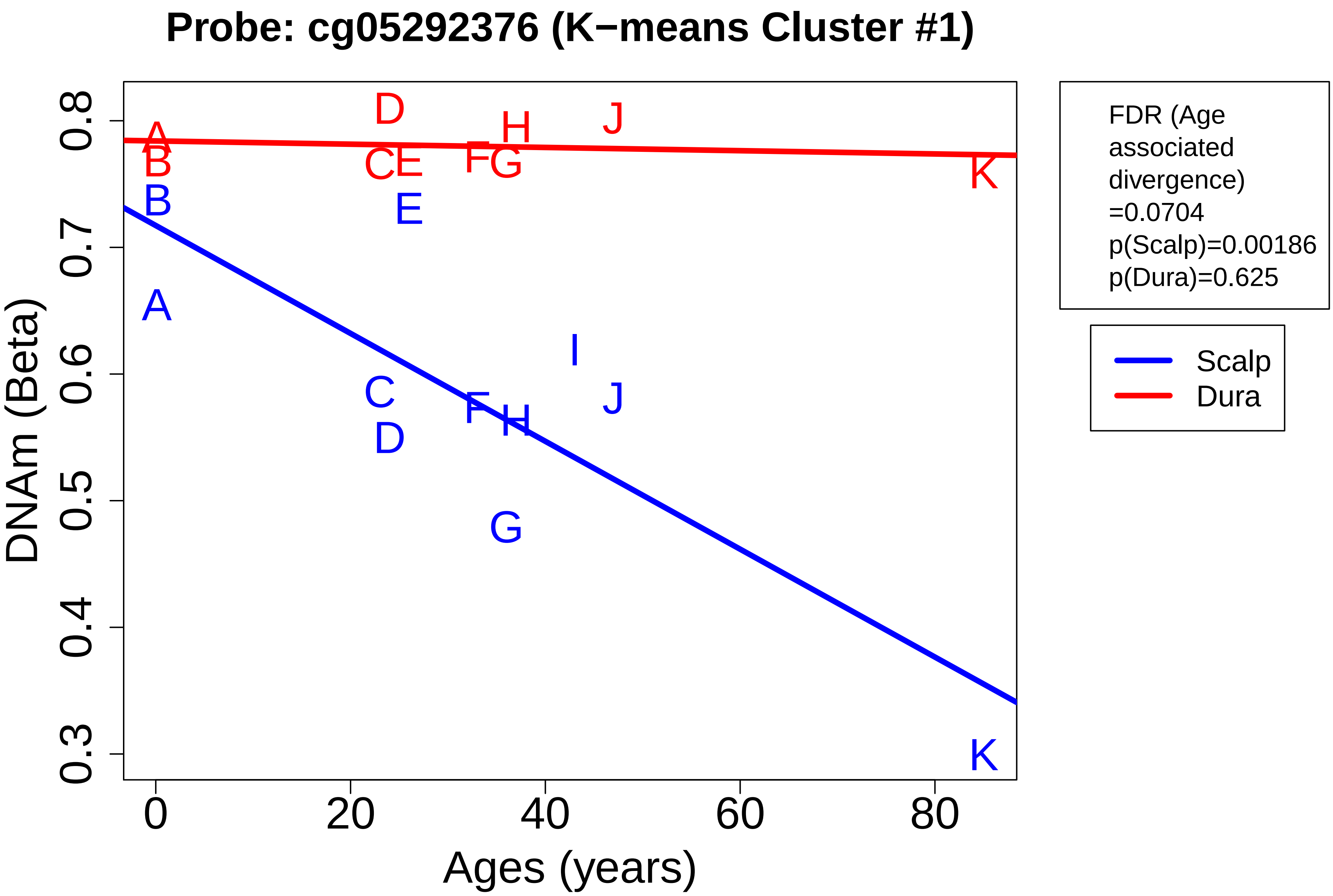

Probe: cg05292376 (K−means Cluster #1)
DNAm (Beta)
Ages (years)
0.3
0.4
0.5
0.6
0.7
0.8
0
20
40
60
80
A
B
C
D
E
F
G
H
I
J
K
FDR (Age associated divergence) =0.0704
p(Scalp)=0.00186
p(Dura)=0.625
Scalp
Dura

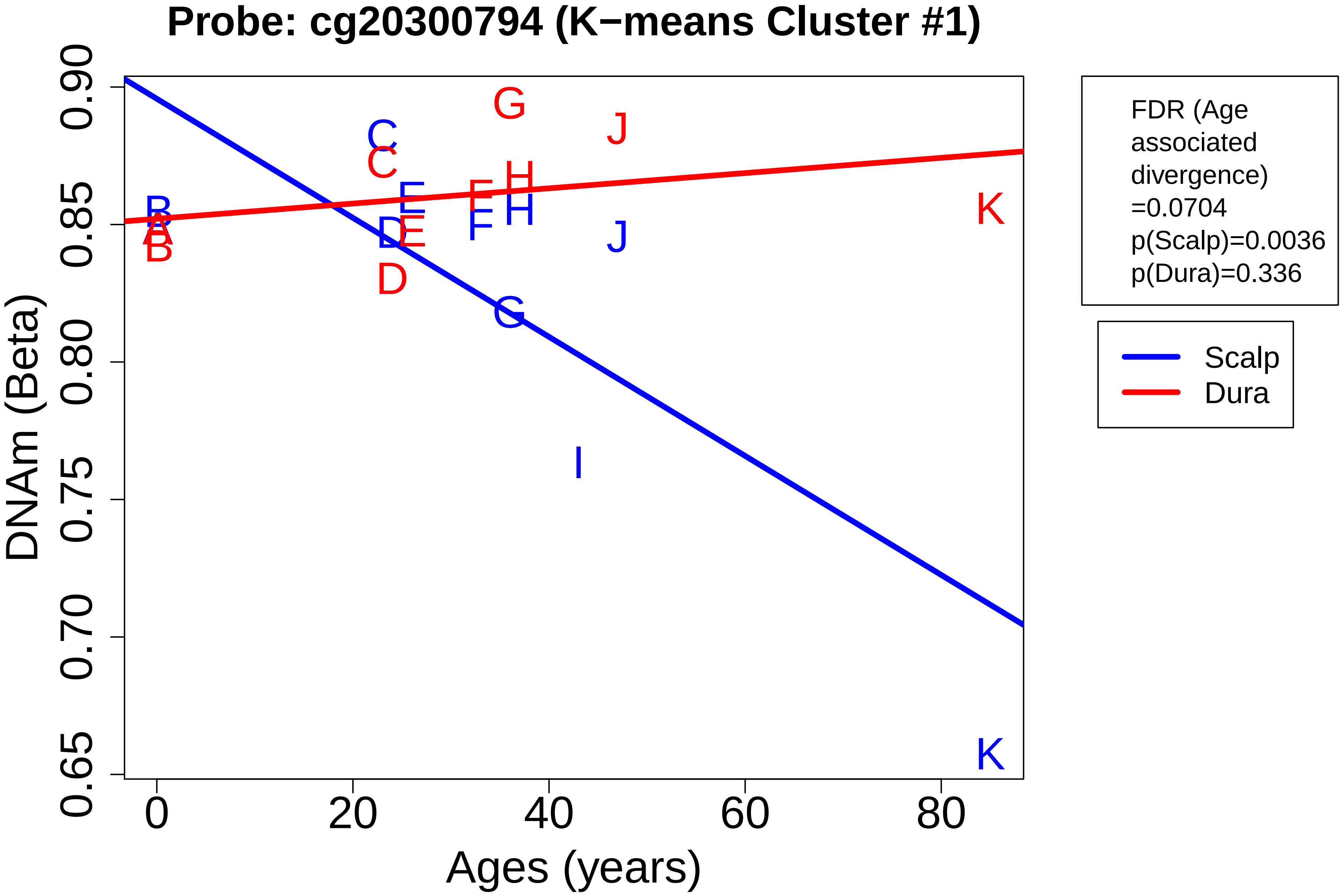

Probe: cg20300794 (K−means Cluster #1)
DNAm (Beta)
0.65
0.70
0.75
0.80
0.85
0.90
0
20
40
60
80
Ages (years)
A
B
C
D
E
F
G
H
I
J
K
FDR (Age associated divergence) =0.0704
p(Scalp)=0.0036
p(Dura)=0.336
Scalp
Dura

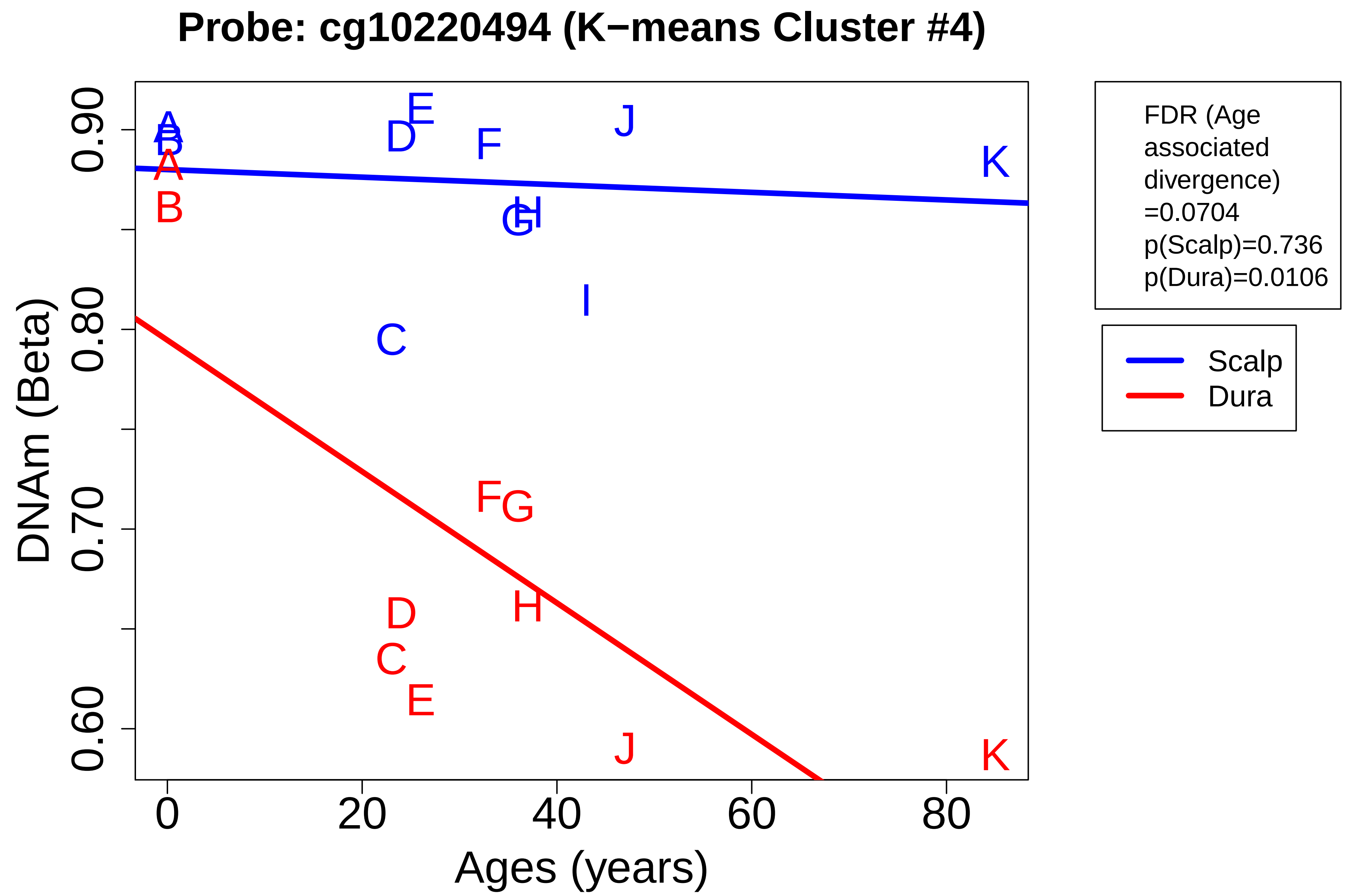

Probe: cg10220494 (K−means Cluster #4)
DNAm (Beta)
0.60
0.70
0.80
0.90
0
20
40
60
80
Ages (years)
A
B
C
D
E
F
G
H
I
J
K
FDR (Age associated divergence) =0.0704
p(Scalp)=0.736
p(Dura)=0.0106
Scalp
Dura

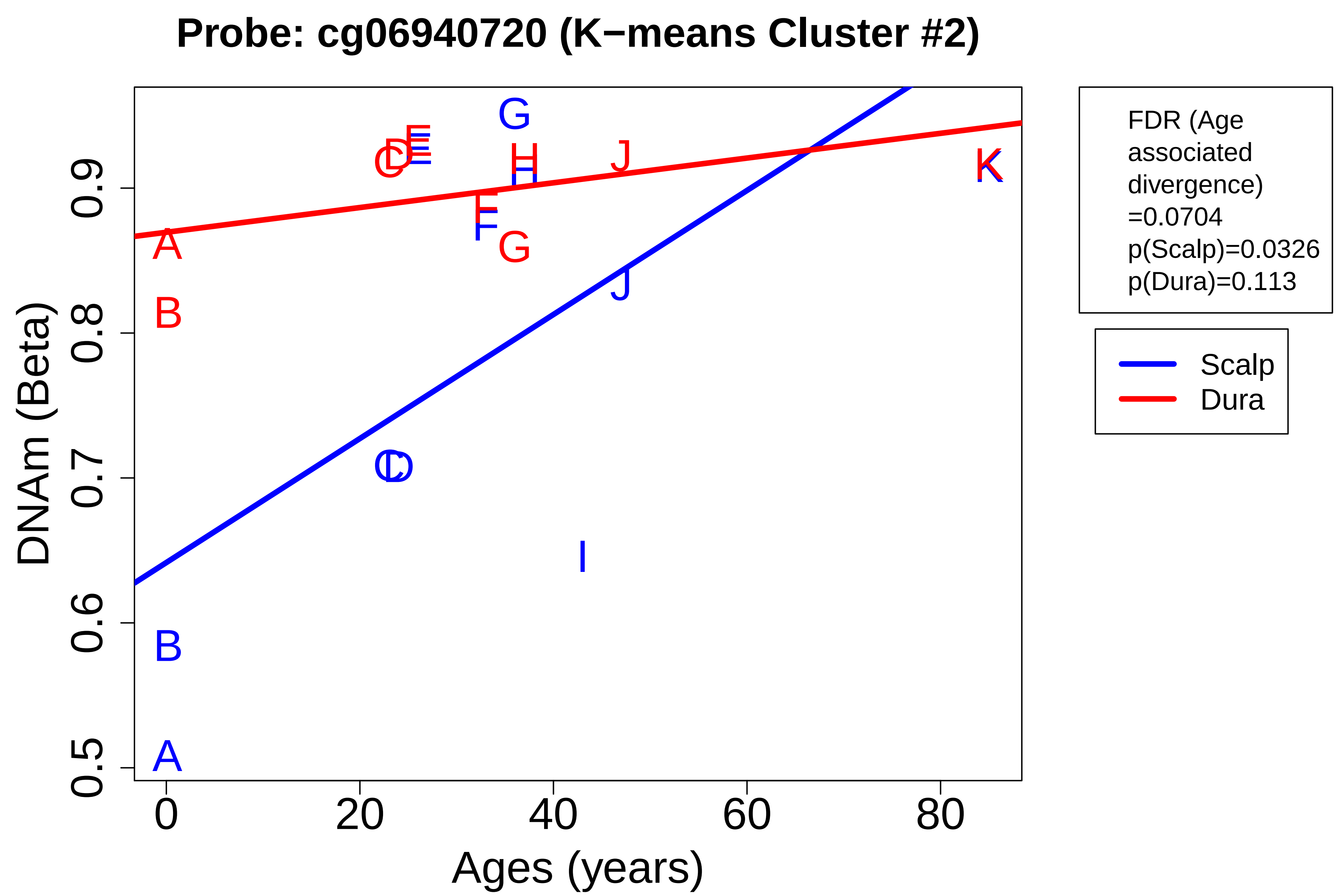

Probe: cg06940720 (K−means Cluster #2)
DNAm (Beta)
0.5
0.6
0.7
0.8
0.9
Ages (years)
0
20
40
60
80
A
B
C
D
E
F
G
H
I
J
K
FDR (Age associated divergence) =0.0704
p(Scalp)=0.0326
p(Dura)=0.113
Scalp
Dura

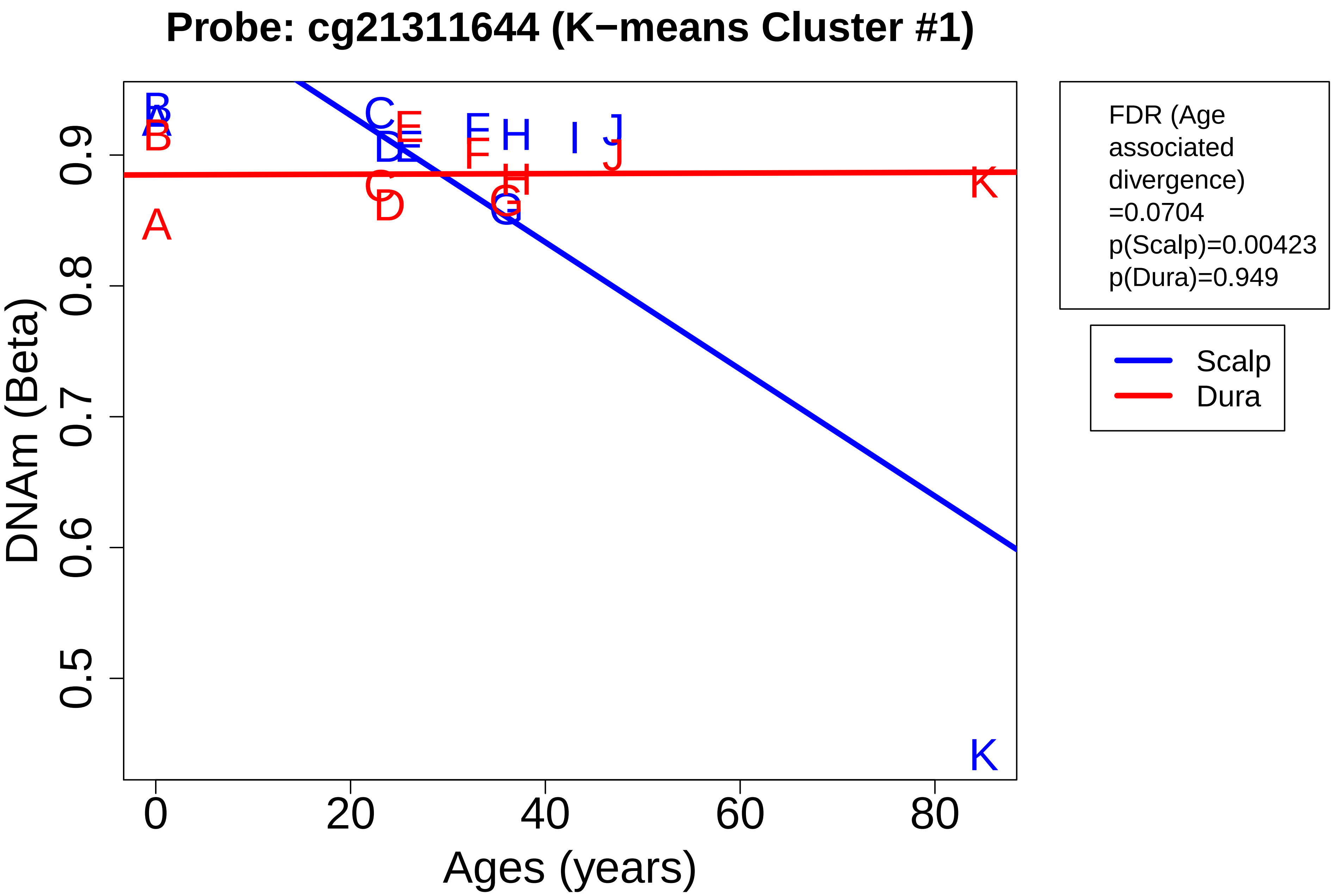

Probe: cg21311644 (K−means Cluster #1)
DNAm (Beta)
0.5
0.6
0.7
0.8
0.9
0
20
40
60
80
Ages (years)
A
B
C
D
E
F
G
H
I
J
K
FDR (Age
associated
divergence)
=0.0704
p(Scalp)=0.00423
p(Dura)=0.949
Scalp
Dura

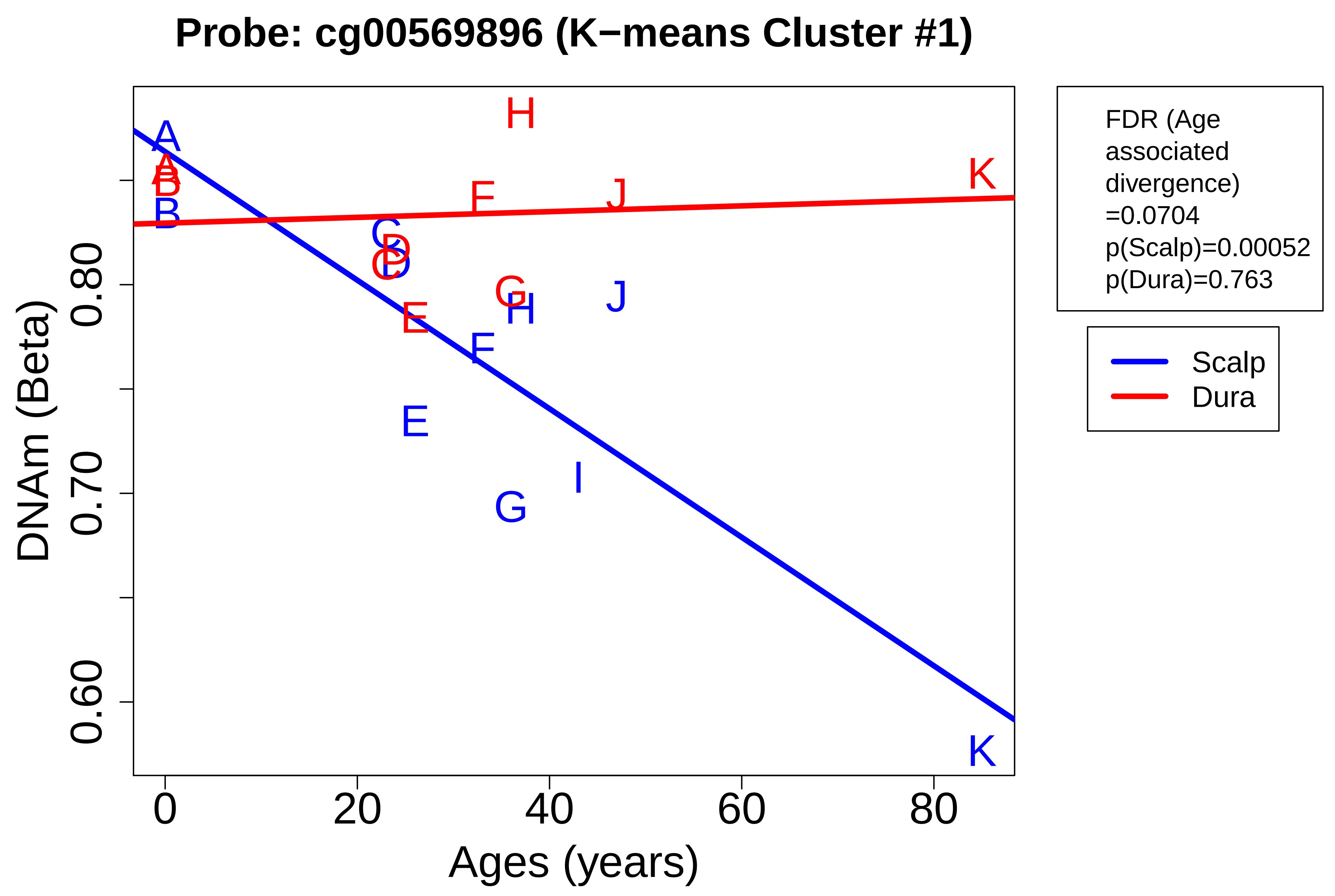

Probe: cg00569896 (K−means Cluster #1)
DNAm (Beta)
0.60
0.70
0.80
Ages (years)
0
20
40
60
80
FDR (Age associated divergence) =0.0704
p(Scalp)=0.00052
p(Dura)=0.763
Scalp
Dura

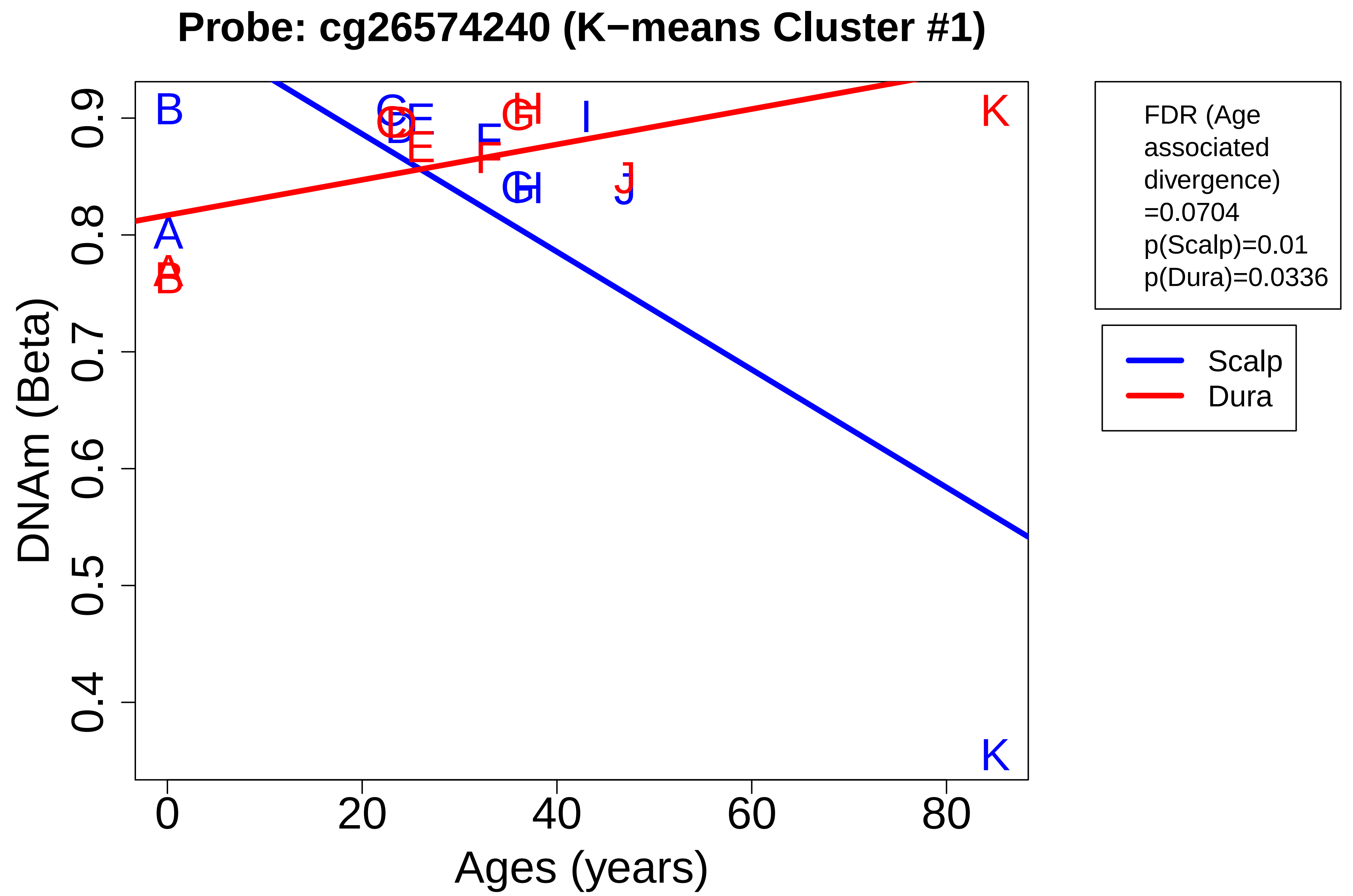

Probe: cg26574240 (K−means Cluster #1)
DNAm (Beta)
0.4
0.5
0.6
0.7
0.8
0.9
Ages (years)
0
20
40
60
80
FDR (Age associated divergence) =0.0704
p(Scalp)=0.01
p(Dura)=0.0336
Scalp
Dura

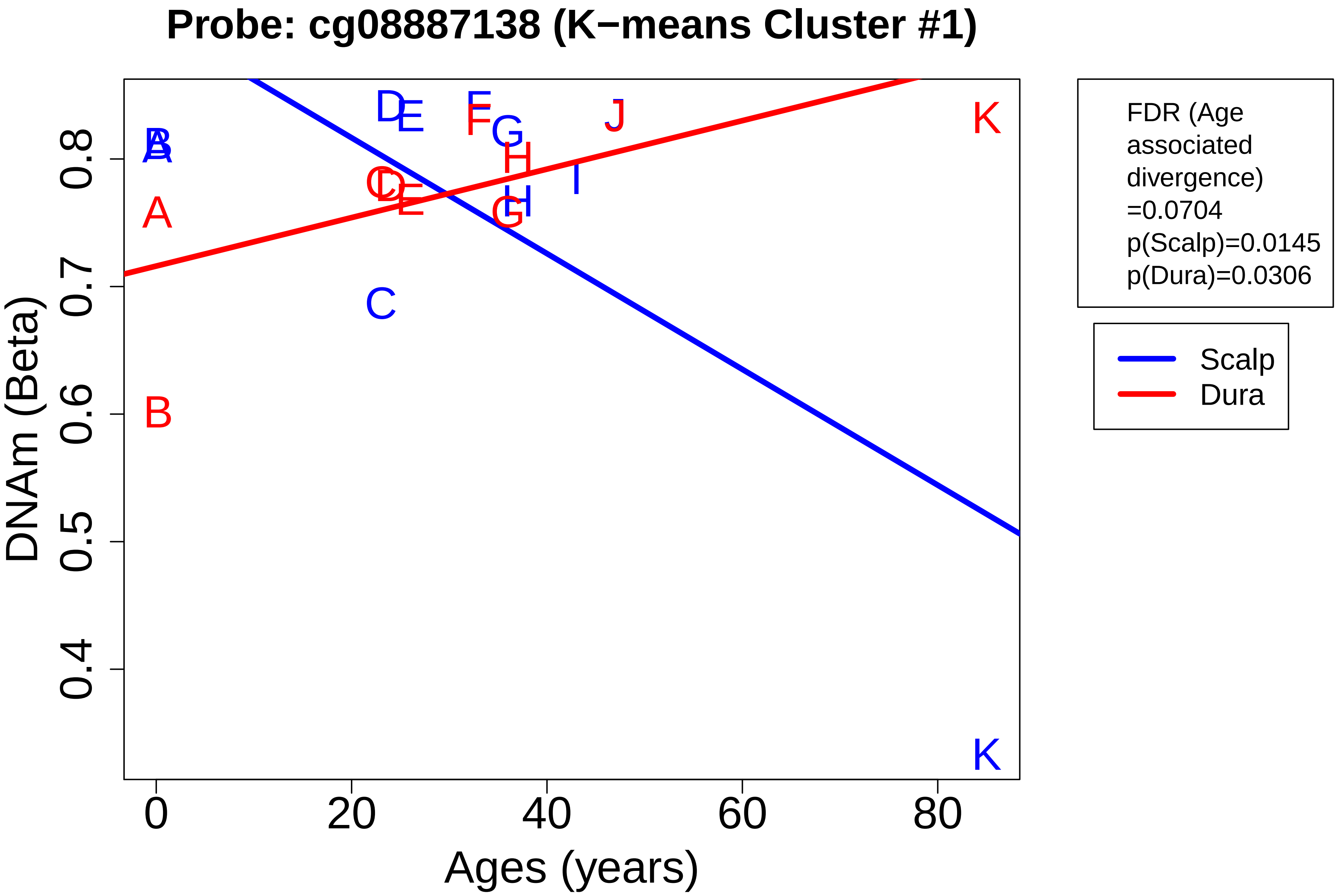
Probe: cg08887138 (K−means Cluster #1)
DNAm (Beta)
0.4
0.5
0.6
0.7
0.8
0
20
40
60
80
Ages (years)
A B C D E F G H I J K
FDR (Age associated divergence) =0.0704
p(Scalp)=0.0145
p(Dura)=0.0306
Scalp
Dura

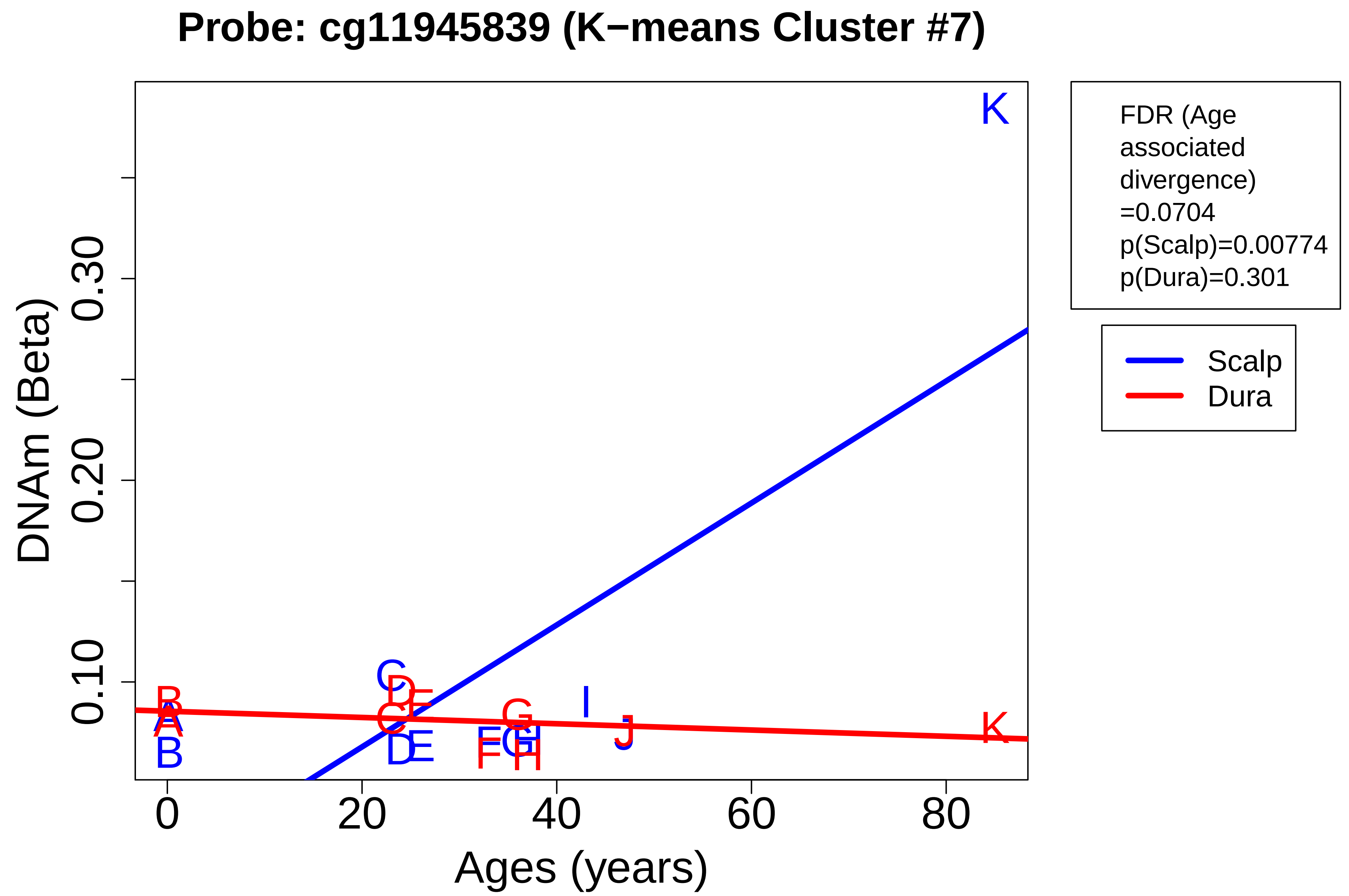

Probe: cg11945839 (K−means Cluster #7)
DNAm (Beta)
0.10
0.20
0.30
0
20
40
60
80
Ages (years)
FDR (Age associated divergence) =0.0704
p(Scalp)=0.00774
p(Dura)=0.301
Scalp
Dura

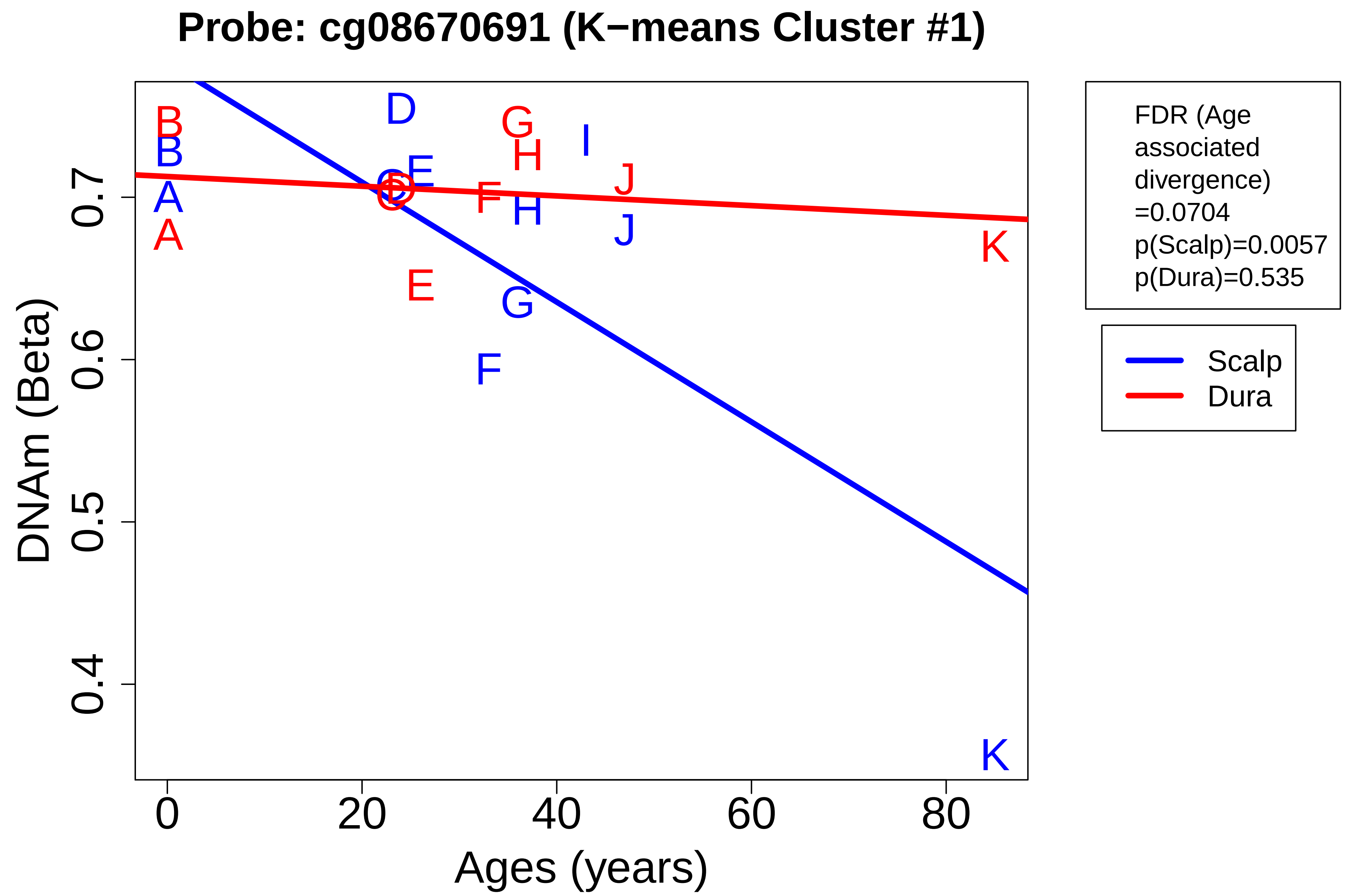

Probe: cg08670691 (K−means Cluster #1)
DNAm (Beta)
0.4
0.5
0.6
0.7
0
20
40
60
80
Ages (years)
FDR (Age associated divergence) =0.0704
p(Scalp)=0.0057
p(Dura)=0.535
Scalp
Dura

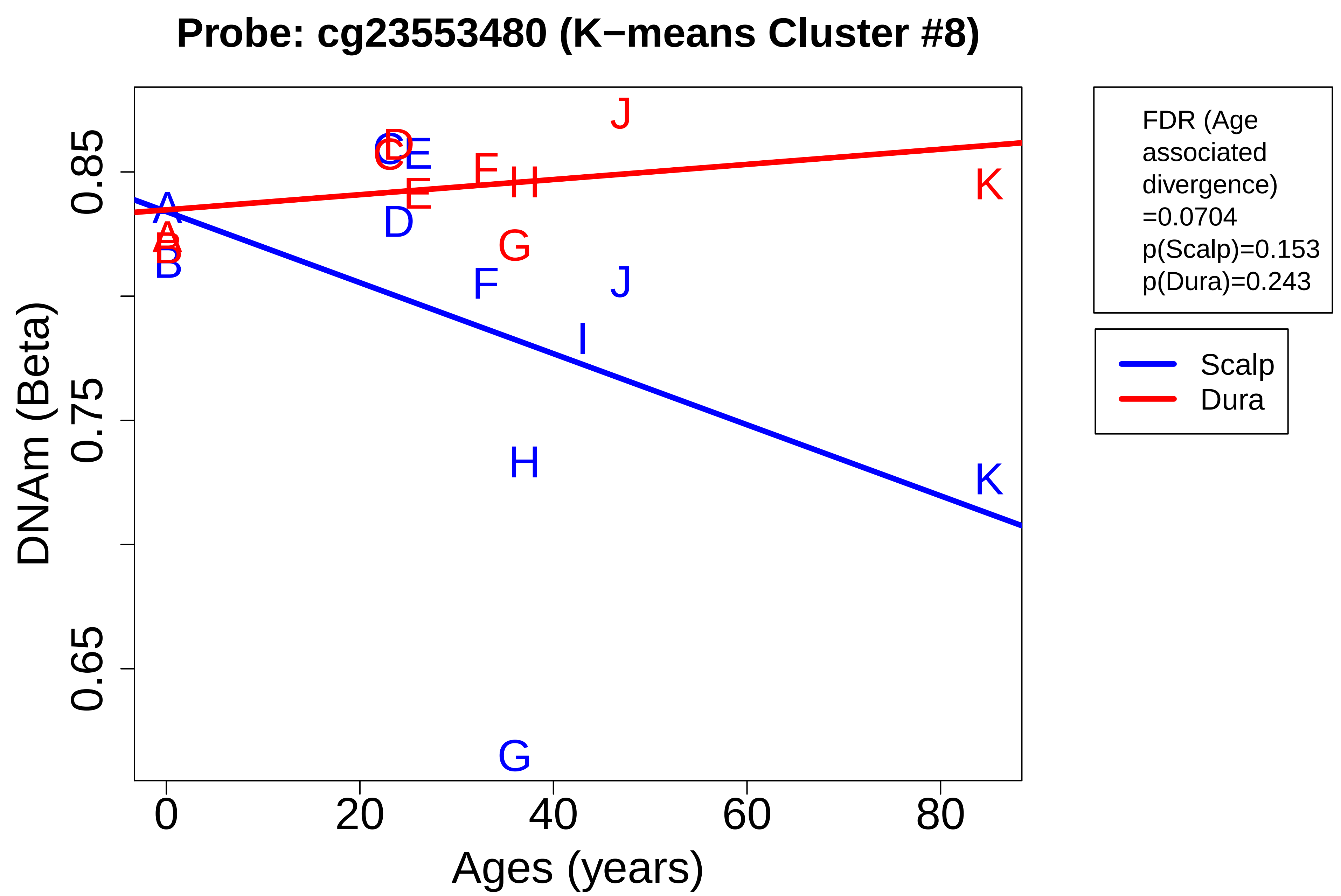

Probe: cg23553480 (K−means Cluster #8)
DNAm (Beta)
0.65
0.75
0.85
Ages (years)
0
20
40
60
80
FDR (Age associated divergence) =0.0704
p(Scalp)=0.153
p(Dura)=0.243
Scalp
Dura

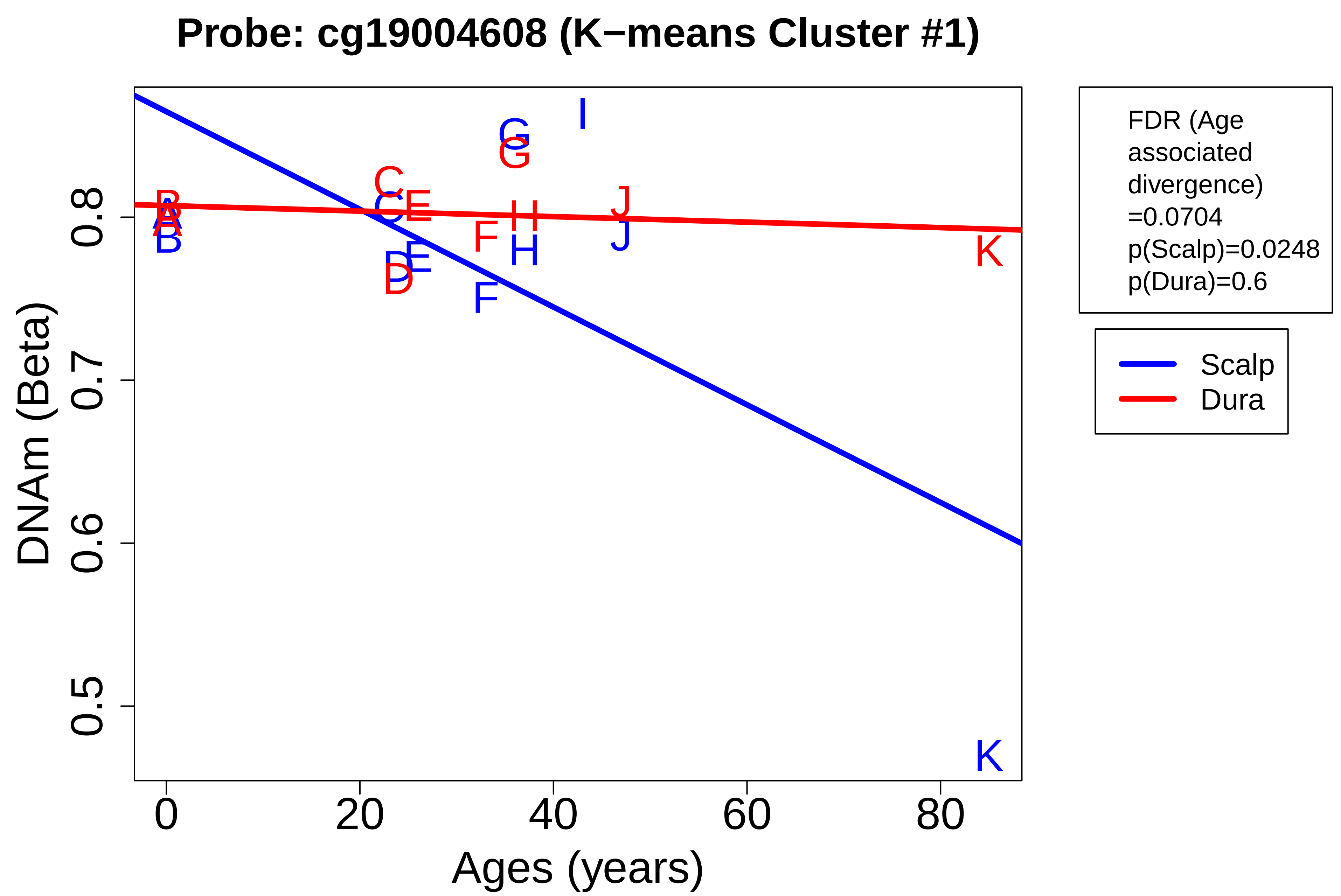
Probe: cg19004608 (K−means Cluster #1)
DNAm (Beta)
0.5
0.6
0.7
0.8
Ages (years)
0
20
40
60
80
A
B
C
D
E
F
G
H
I
J
K
FDR (Age associated divergence) =0.0704
p(Scalp)=0.0248
p(Dura)=0.6
Scalp
Dura

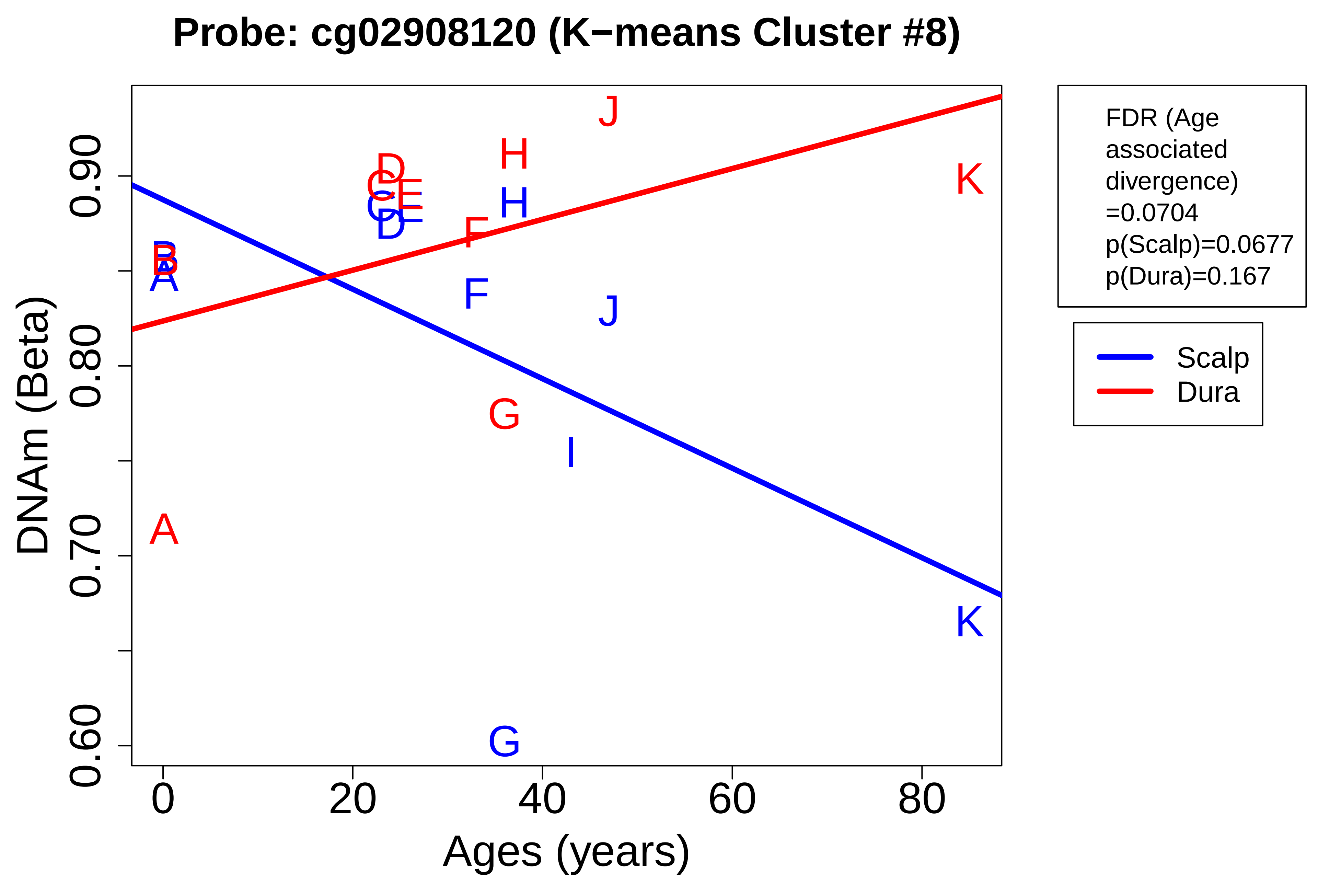

Probe: cg02908120 (K−means Cluster #8)
DNAm (Beta)
0.60
0.70
0.80
0.90
Ages (years)
0
20
40
60
80
FDR (Age associated divergence) =0.0704
p(Scalp)=0.0677
p(Dura)=0.167
Scalp
Dura

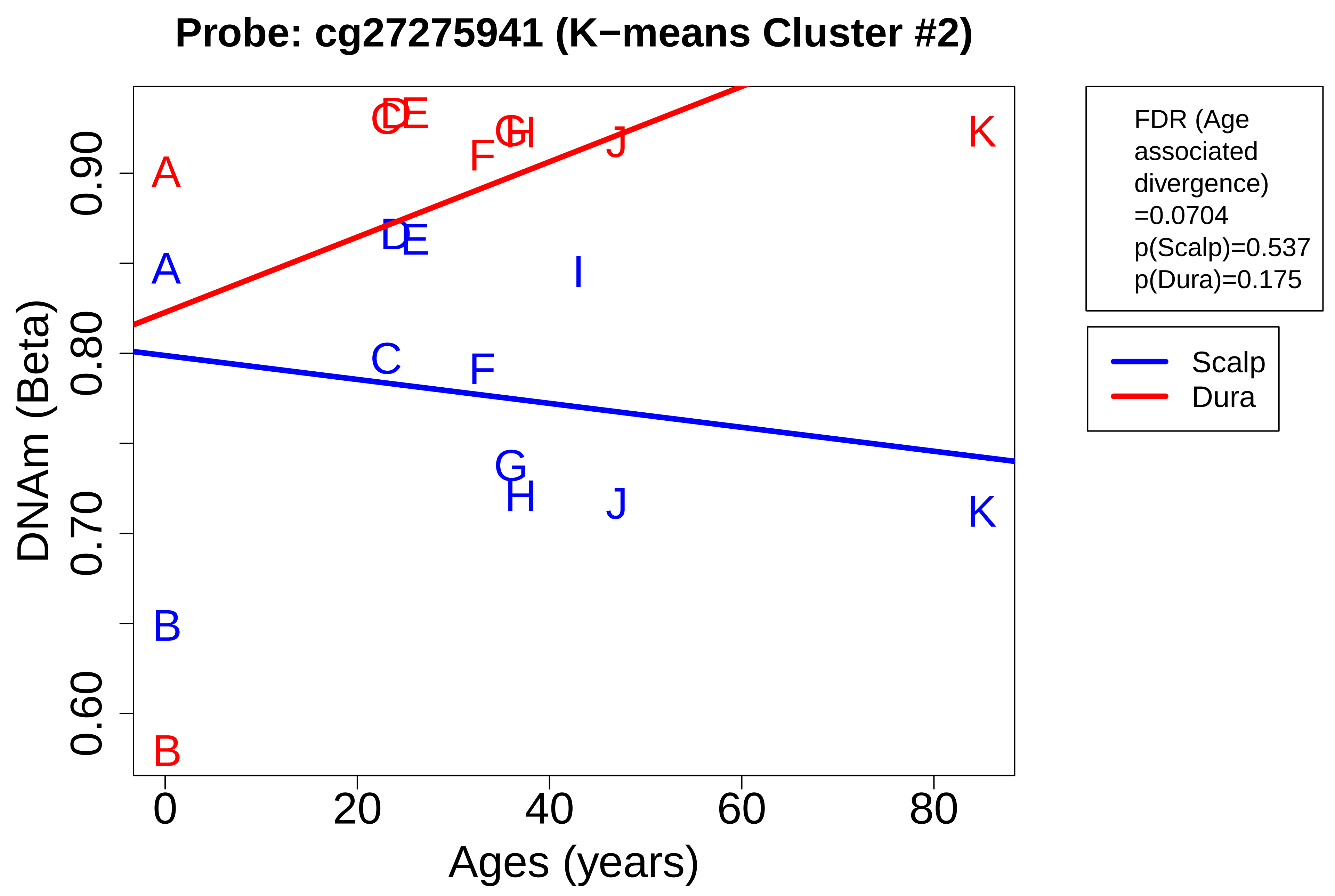

Probe: cg27275941 (K−means Cluster #2)
FDR (Age associated divergence) =0.0704
p(Scalp)=0.537
p(Dura)=0.175
Scalp
Dura
DNAm (Beta)
0.60
0.70
0.80
0.90
0
20
40
60
80
Ages (years)
A
B
C
D
E
F
G
H
I
J
K

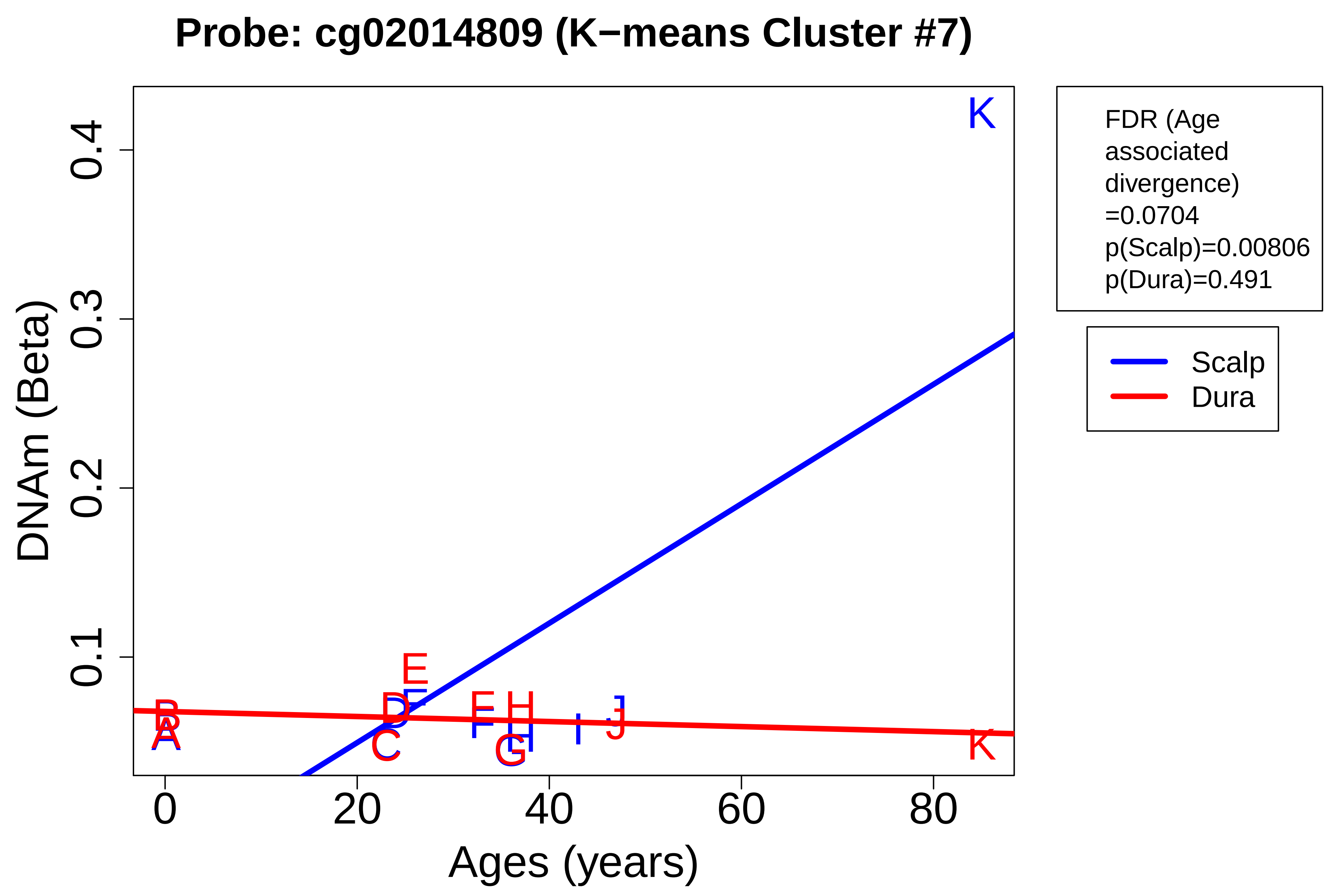

Probe: cg02014809 (K−means Cluster #7)
DNAm (Beta)
0.1
0.2
0.3
0.4
0
20
40
60
80
Ages (years)
A
B
C
D
E
F
G
H
I
J
K
FDR (Age associated divergence) =0.0704
p(Scalp)=0.00806
p(Dura)=0.491
Scalp
Dura

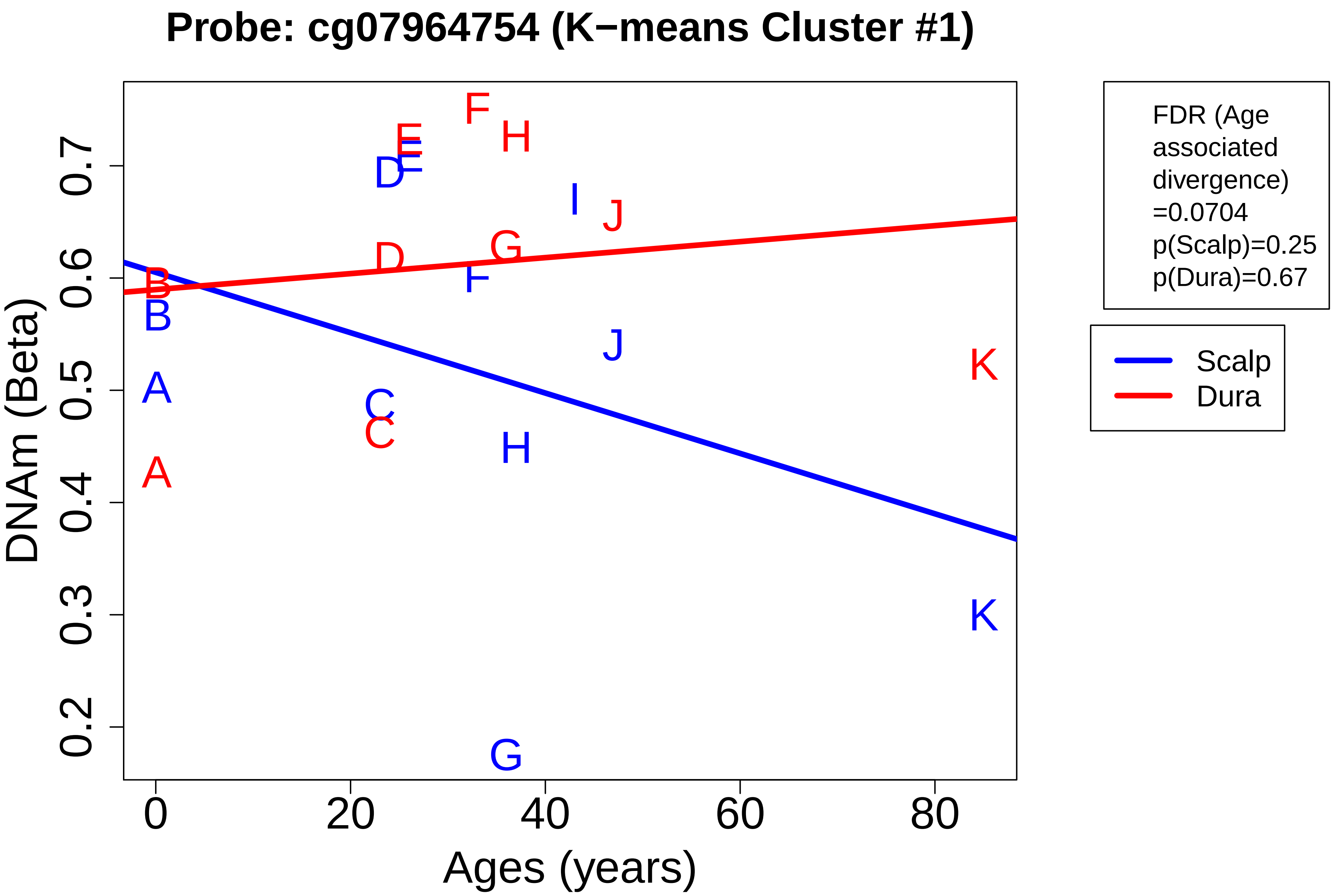
Probe: cg07964754 (K−means Cluster #1)
DNAm (Beta)
Ages (years)
0.2
0.3
0.4
0.5
0.6
0.7
0
20
40
60
80
A
B
C
D
E
F
G
H
I
J
K
FDR (Age associated divergence) =0.0704
p(Scalp)=0.25
p(Dura)=0.67
Scalp
Dura

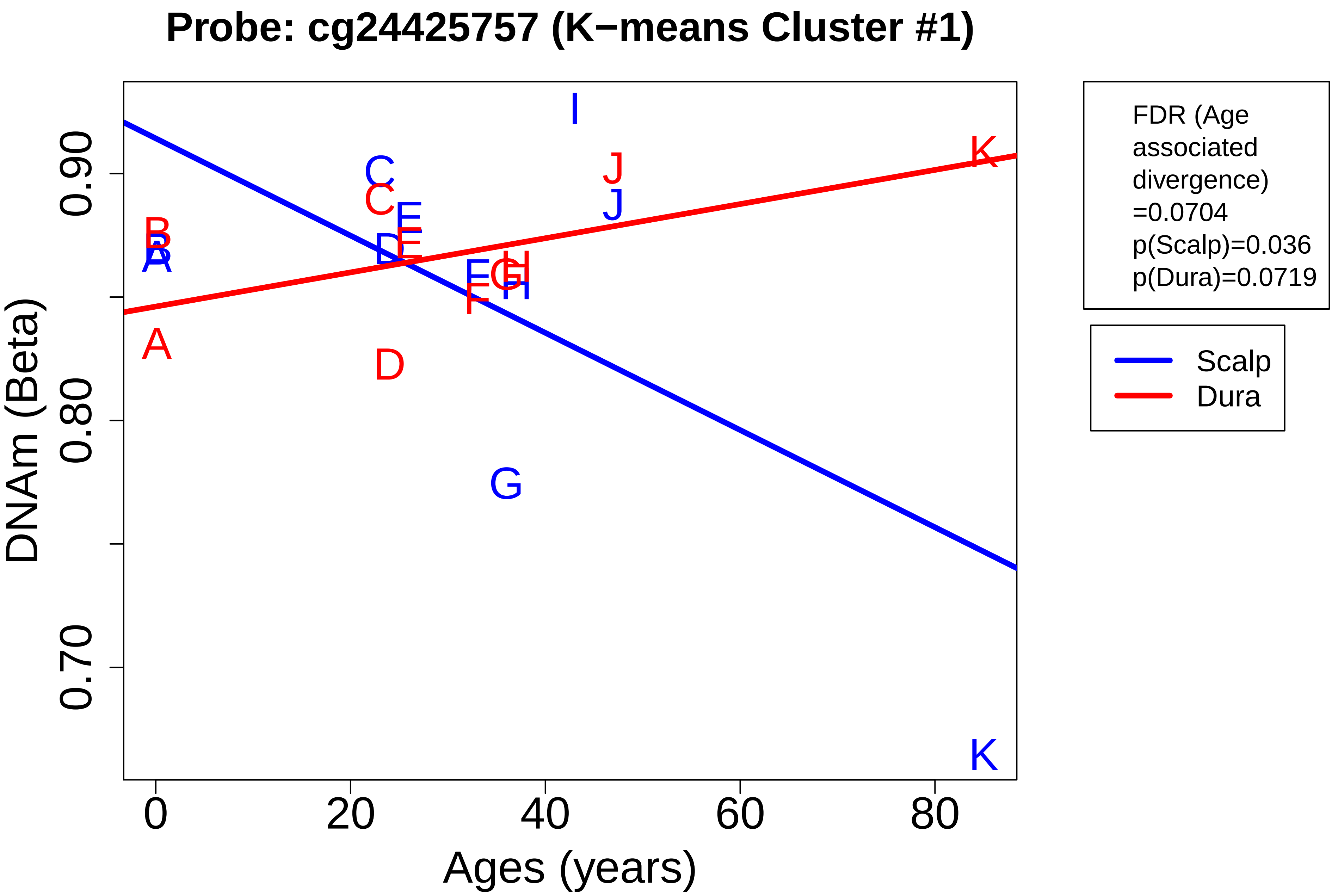

Probe: cg24425757 (K−means Cluster #1)
DNAm (Beta)
0.70
0.80
0.90
0
20
40
60
80
Ages (years)
FDR (Age associated divergence) =0.0704
p(Scalp)=0.036
p(Dura)=0.0719
Scalp
Dura

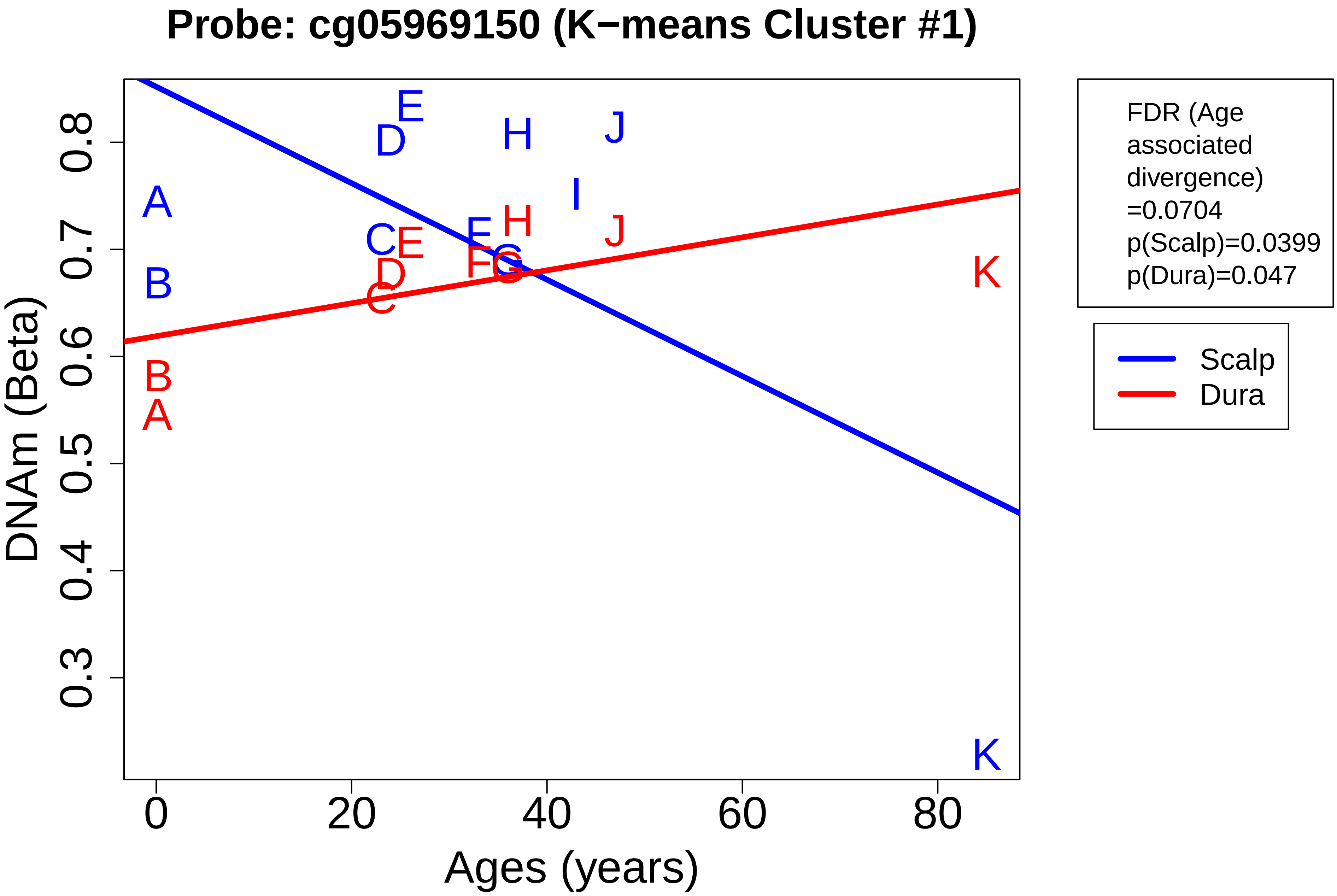

Probe: cg05969150 (K−means Cluster #1)
DNAm (Beta)
0.3
0.4
0.5
0.6
0.7
0.8
0
20
40
60
80
Ages (years)
A
B
C
D
E
F
G
H
I
J
K
FDR (Age associated divergence) =0.0704
p(Scalp)=0.0399
p(Dura)=0.047
Scalp
Dura

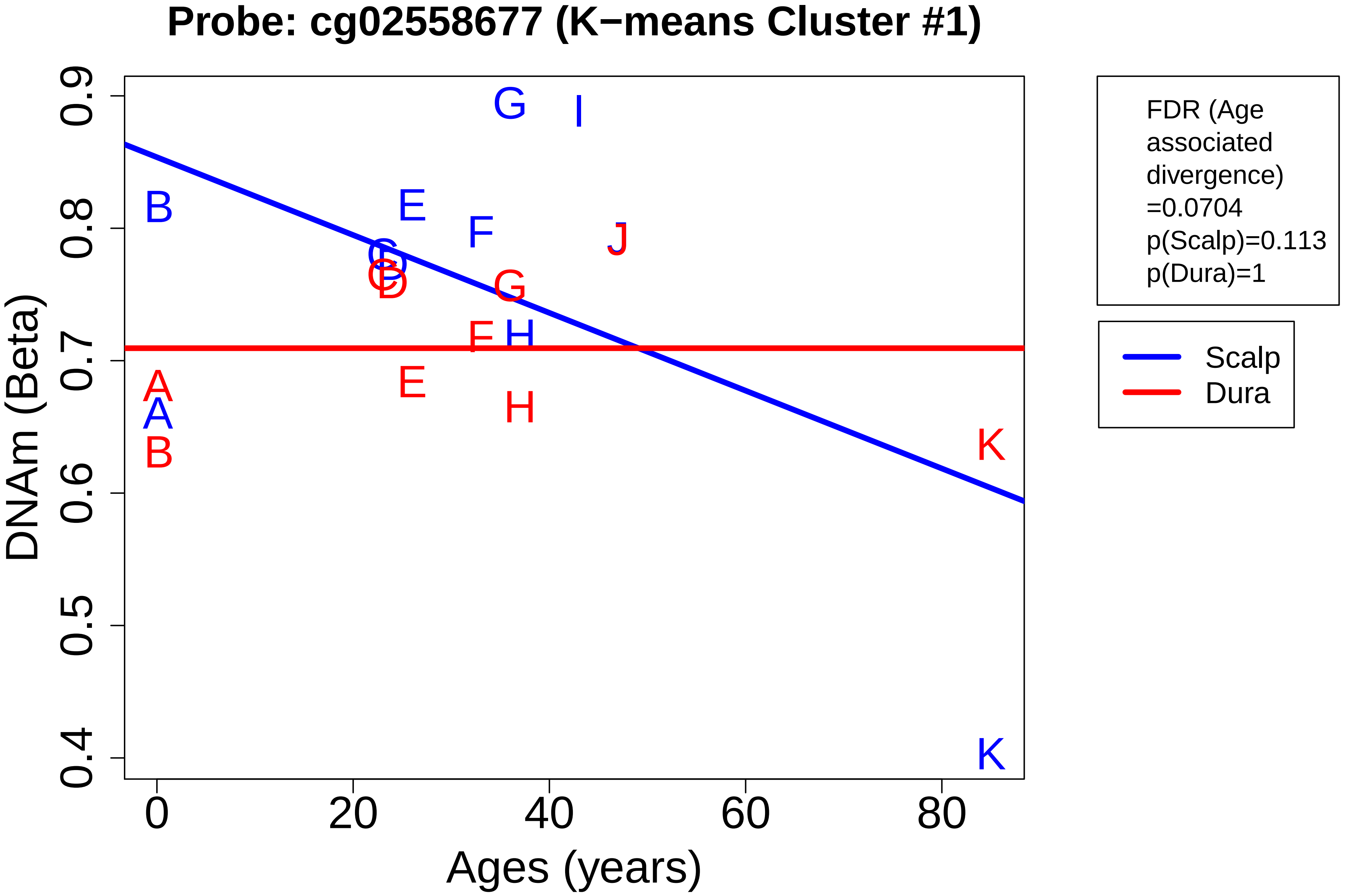

Probe: cg02558677 (K−means Cluster #1)
DNAm (Beta)
0.4
0.5
0.6
0.7
0.8
0.9
0
20
40
60
80
Ages (years)
FDR (Age associated divergence) =0.0704
p(Scalp)=0.113
p(Dura)=1
Scalp
Dura

Probe: cg19478638 (K−means Cluster #1)

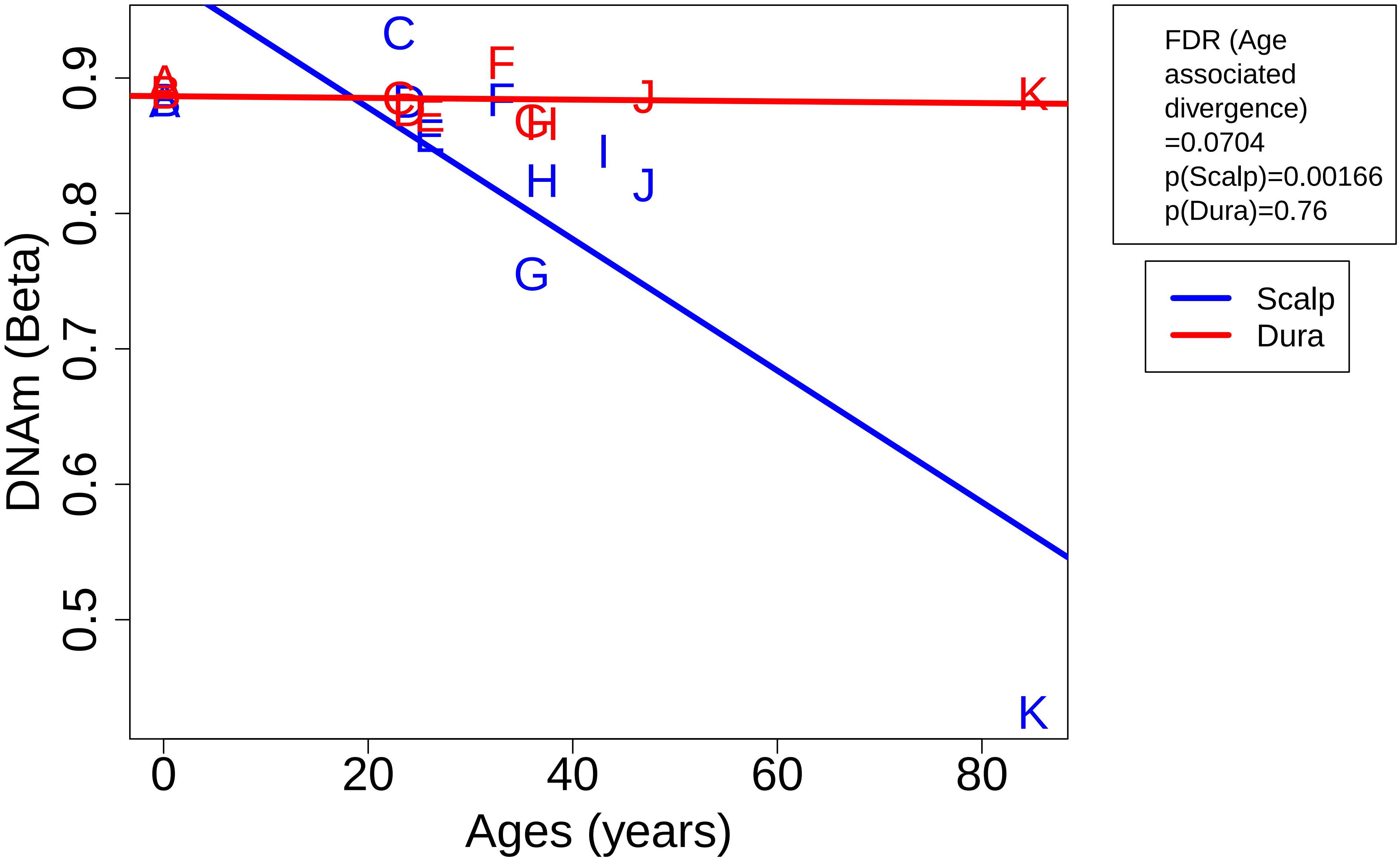

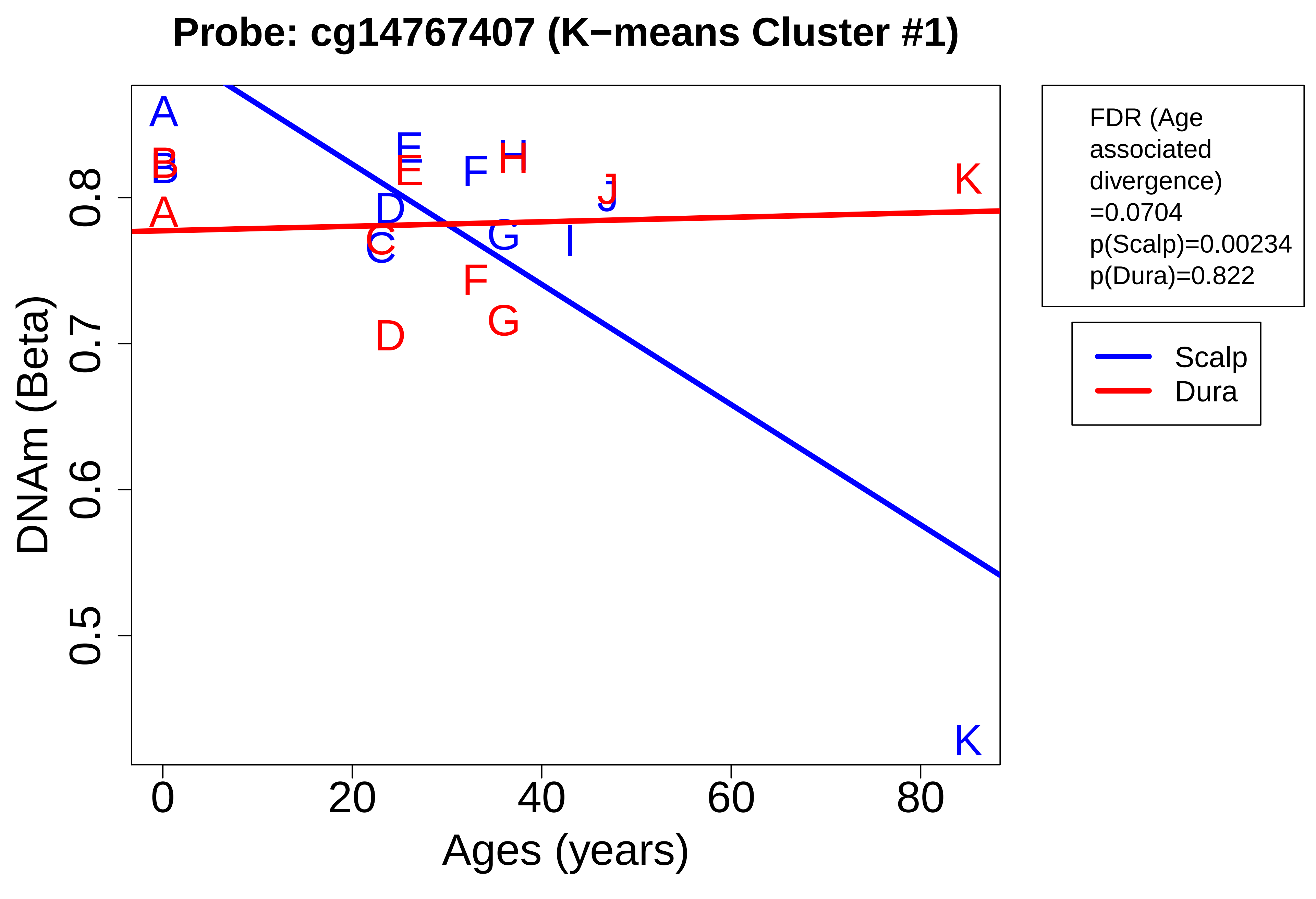

Probe: cg14767407 (K−means Cluster #1)
DNAm (Beta)
0.5
0.6
0.7
0.8
Ages (years)
0
20
40
60
80
A
B
C
D
E
F
G
H
I
J
K
FDR (Age associated divergence) =0.0704
p(Scalp)=0.00234
p(Dura)=0.822
Scalp
Dura

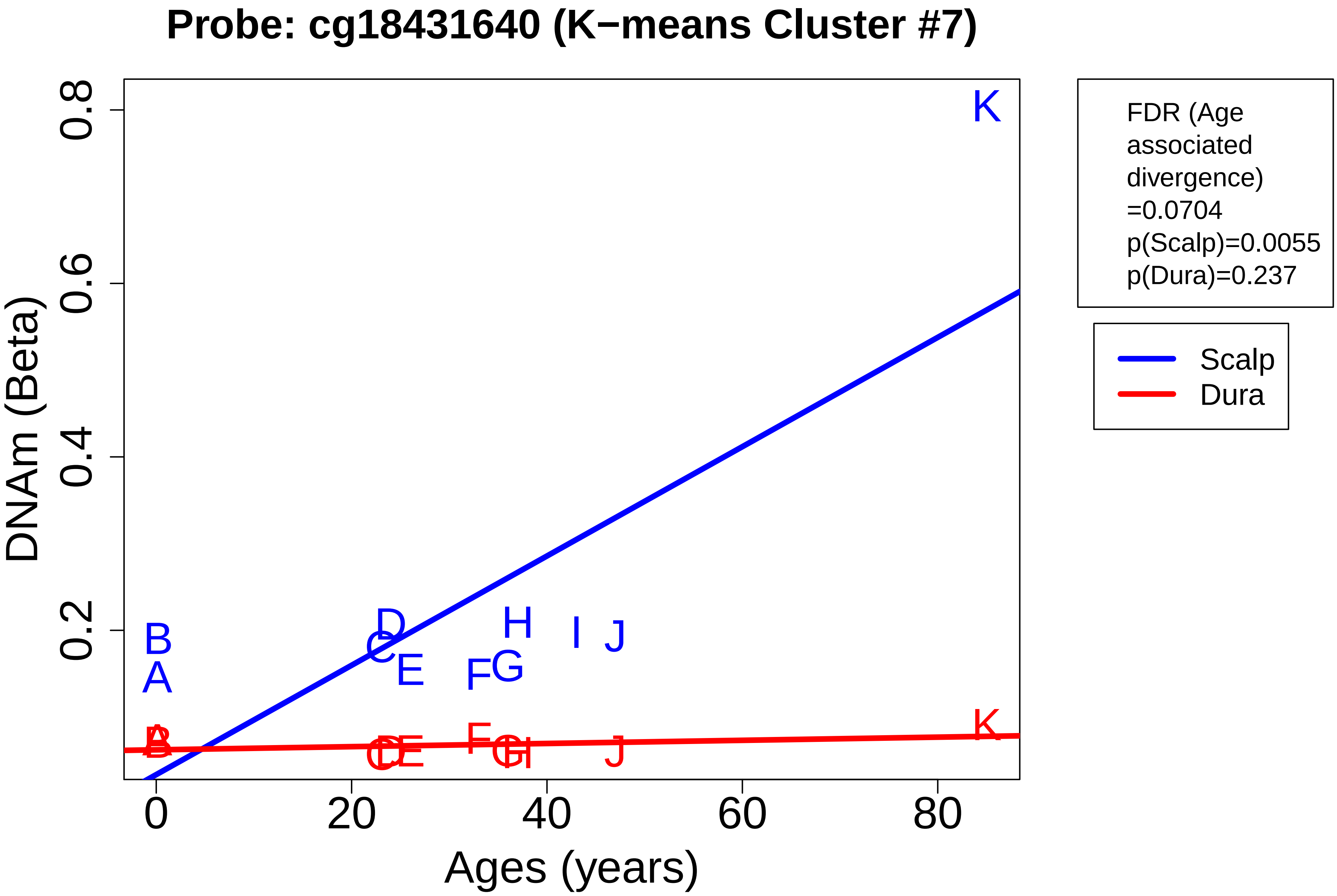

Probe: cg18431640 (K−means Cluster #7)
DNAm (Beta)
0.2
0.4
0.6
0.8
Ages (years)
0
20
40
60
80
FDR (Age associated divergence) =0.0704
p(Scalp)=0.0055
p(Dura)=0.237
Scalp
Dura

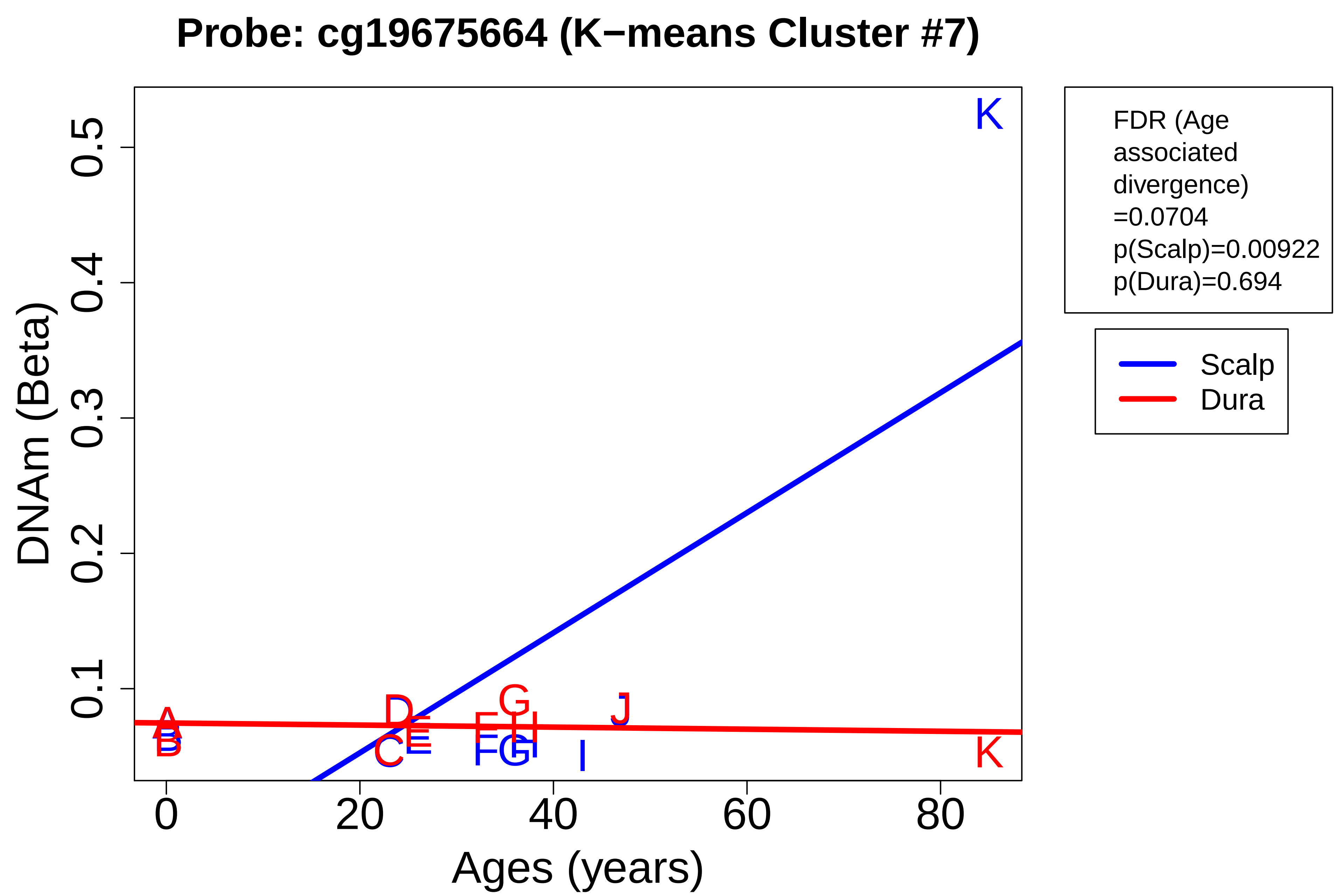

Probe: cg19675664 (K−means Cluster #7)
DNAm (Beta)
0.1
0.2
0.3
0.4
0.5
0
20
40
60
80
Ages (years)
A
B
C
D
E
F
G
H
I
J
K
FDR (Age associated divergence) =0.0704
p(Scalp)=0.00922
p(Dura)=0.694
Scalp
Dura

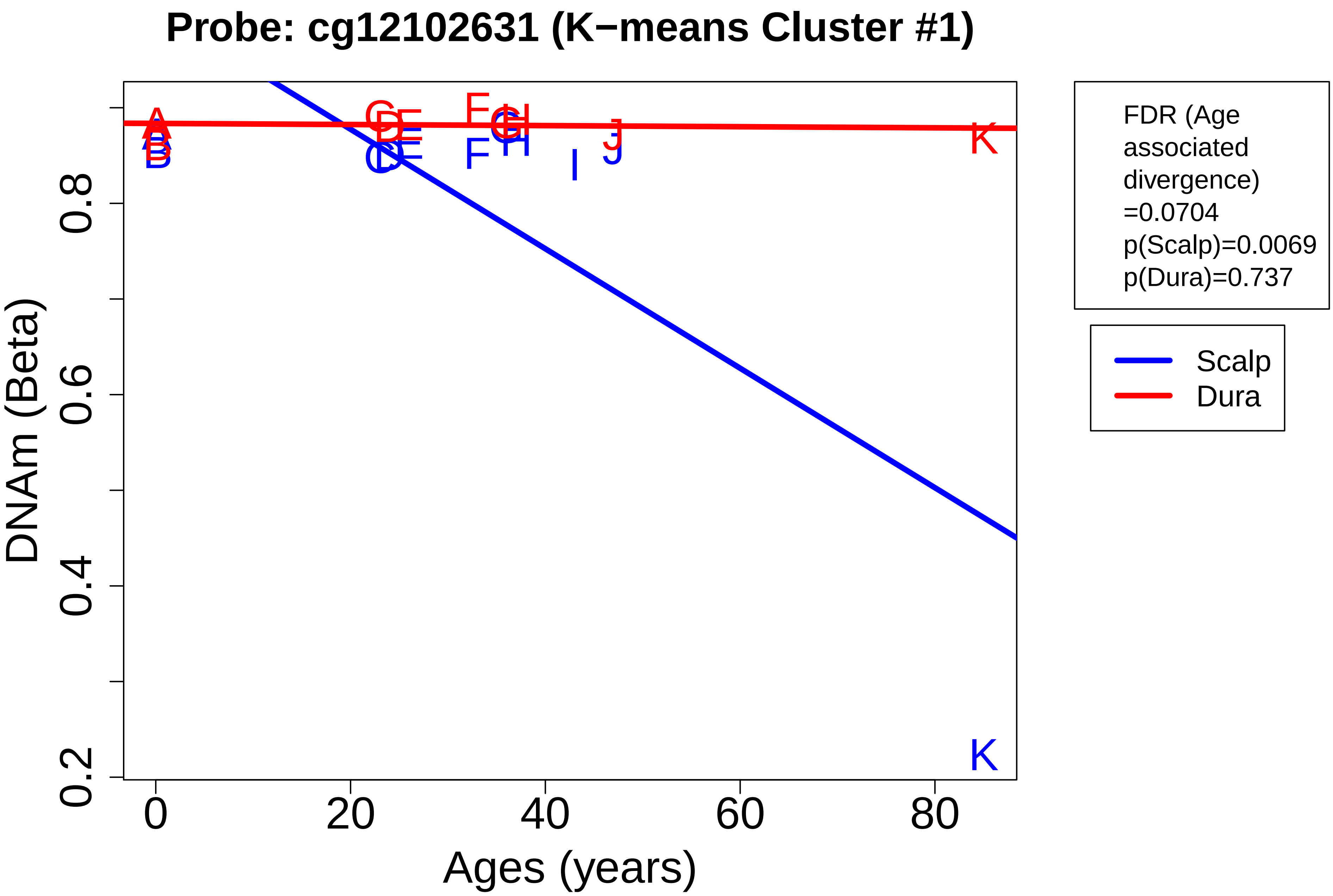

Probe: cg12102631 (K−means Cluster #1)
DNAm (Beta)
Ages (years)
0.2
0.4
0.6
0.8
0
20
40
60
80
FDR (Age associated divergence) =0.0704
p(Scalp)=0.0069
p(Dura)=0.737
Scalp
Dura

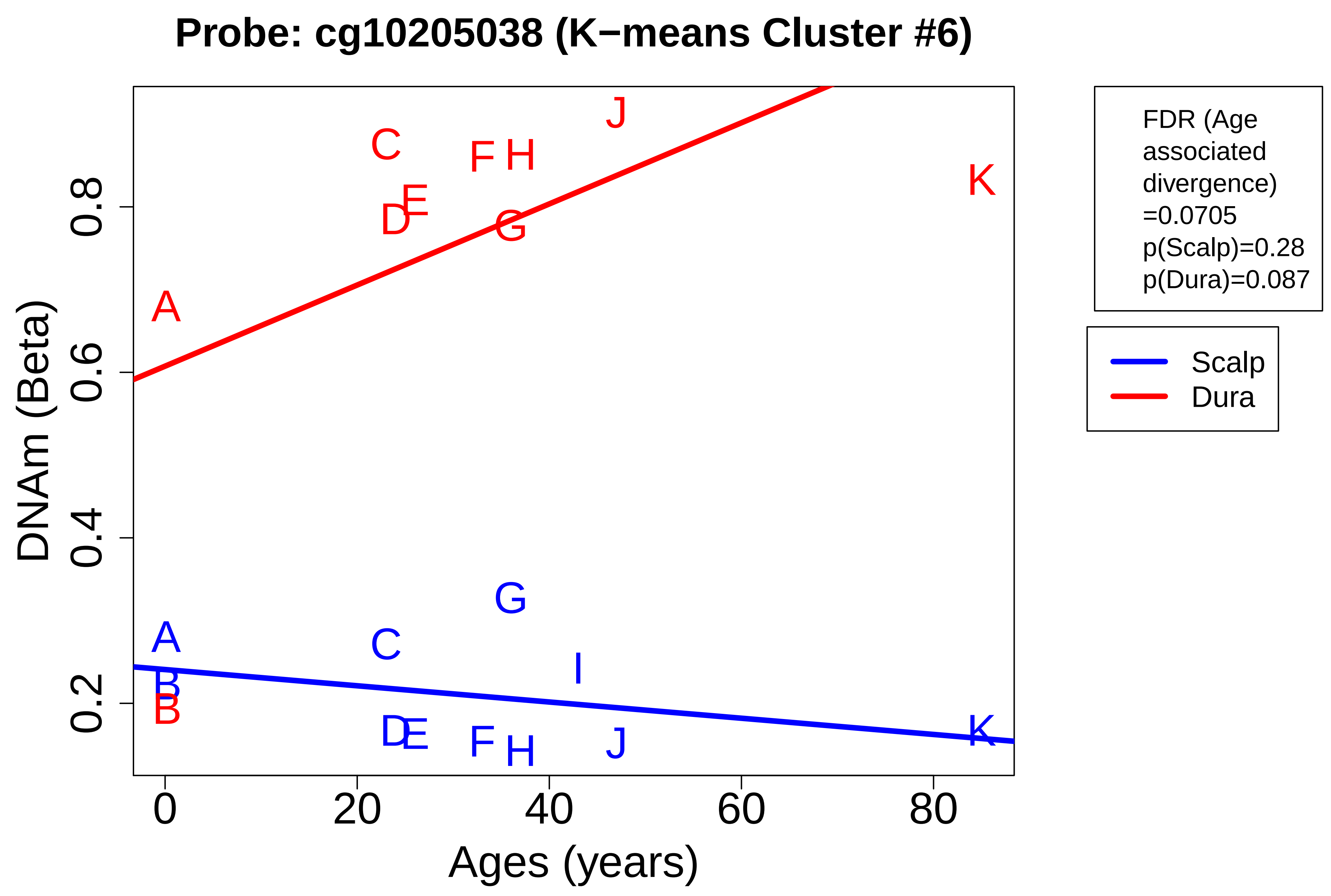
Probe: cg10205038 (K−means Cluster #6)
DNAm (Beta)
0.2
0.4
0.6
0.8
0
20
40
60
80
Ages (years)
A
B
C
D
E
F
G
H
I
J
K
FDR (Age associated divergence) =0.0705
p(Scalp)=0.28
p(Dura)=0.087
Scalp
Dura

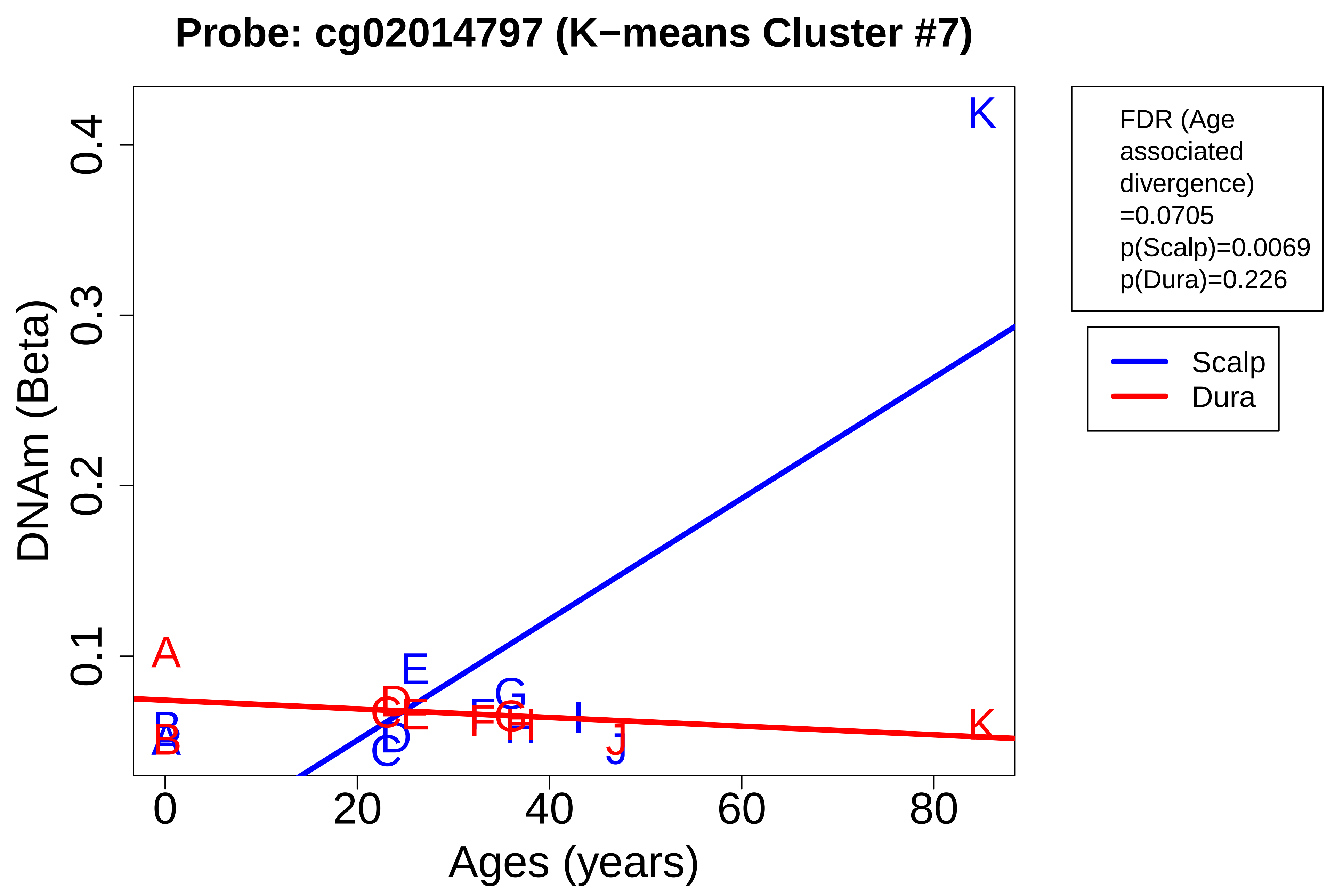

Probe: cg02014797 (K−means Cluster #7)
DNAm (Beta)
0.1
0.2
0.3
0.4
Ages (years)
0
20
40
60
80
A
B
C
D
E
F
G
H
I
J
K
FDR (Age associated divergence) =0.0705
p(Scalp)=0.0069
p(Dura)=0.226
Scalp
Dura

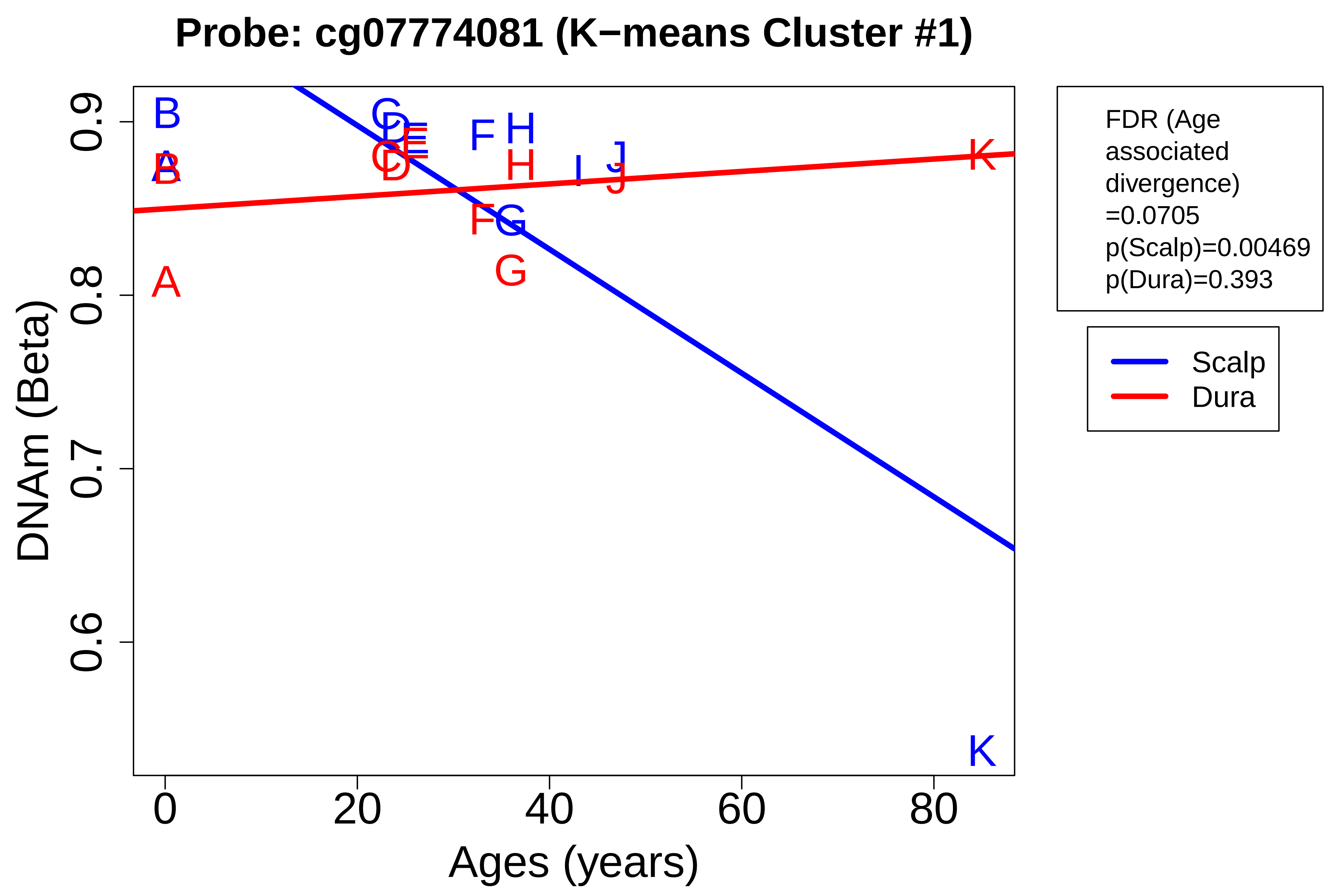

Probe: cg07774081 (K−means Cluster #1)
DNAm (Beta)
0.6
0.7
0.8
0.9
0
20
40
60
80
Ages (years)
FDR (Age associated divergence) =0.0705
p(Scalp)=0.00469
p(Dura)=0.393
Scalp
Dura

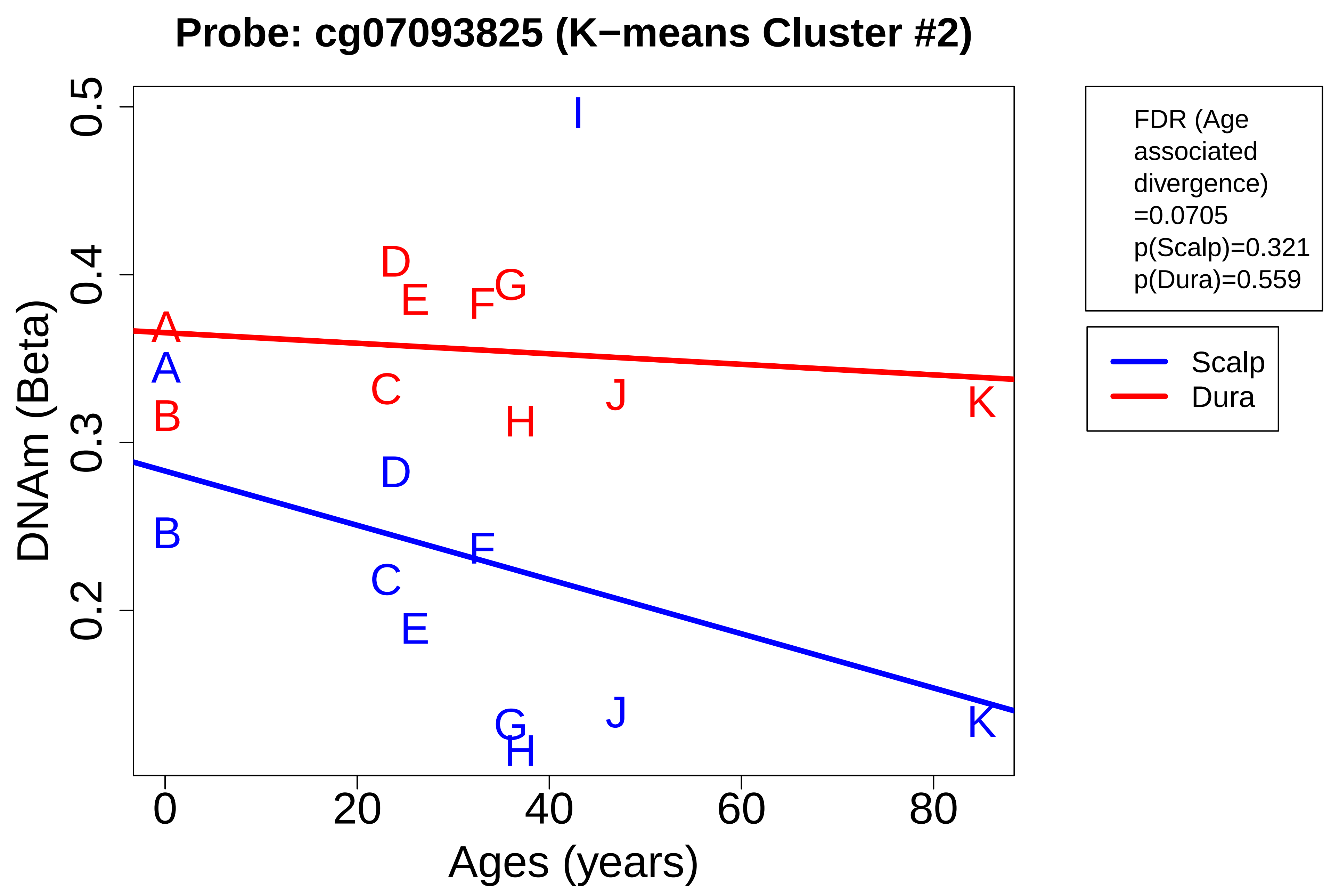

Probe: cg07093825 (K−means Cluster #2)
DNAm (Beta)
0.2
0.3
0.4
0.5
Ages (years)
0
20
40
60
80
I
D
E
F
G
A
A
B
C
J
K
H
D
B
E
C
E
G
H
J
K
FDR (Age associated divergence) =0.0705
p(Scalp)=0.321
p(Dura)=0.559
Scalp
Dura

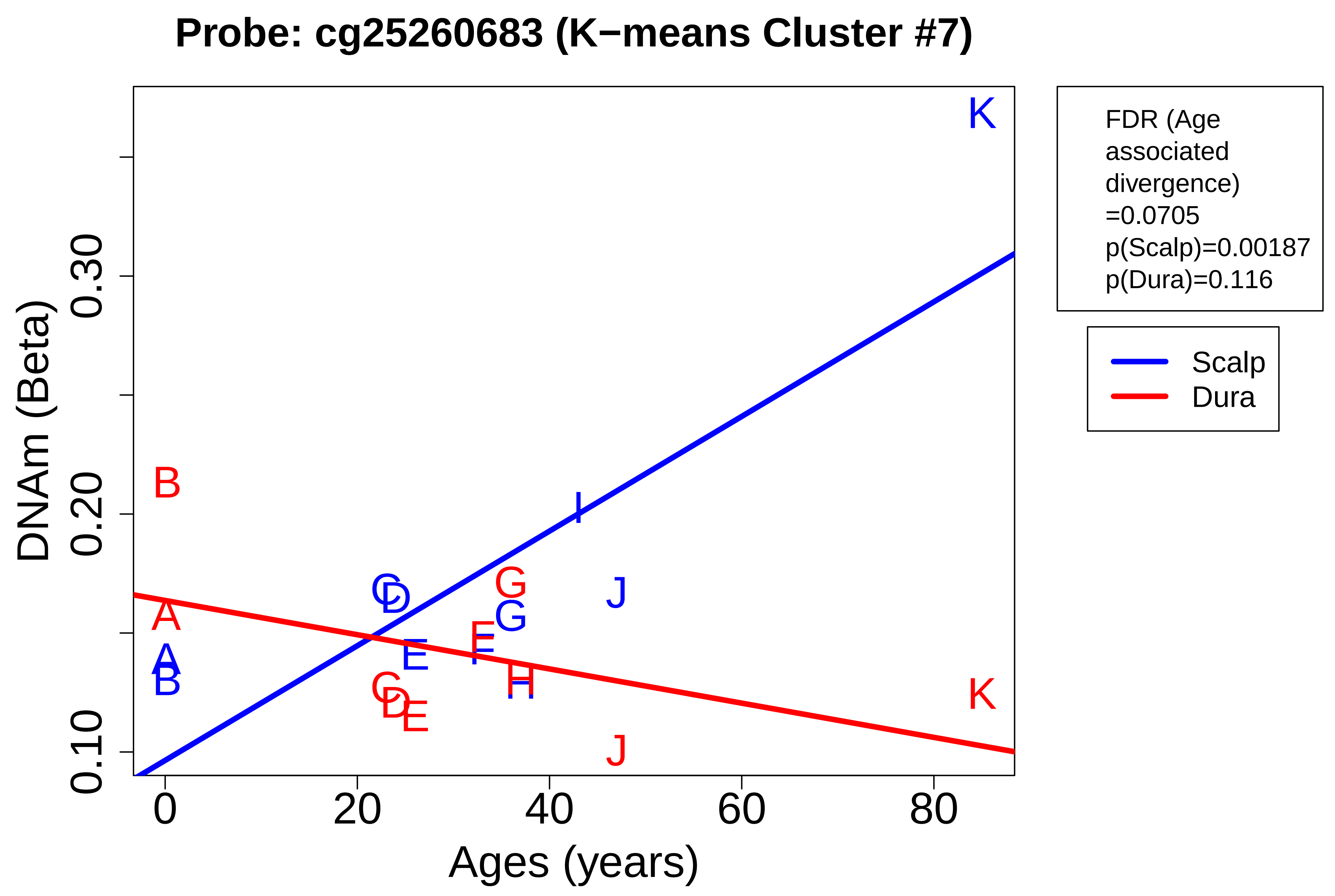

Probe: cg25260683 (K−means Cluster #7)
DNAm (Beta)
0.10
0.20
0.30
0
20
40
60
80
Ages (years)
FDR (Age associated divergence) =0.0705
p(Scalp)=0.00187
p(Dura)=0.116
Scalp
Dura
A
B
C
D
E
F
G
H
I
J
K

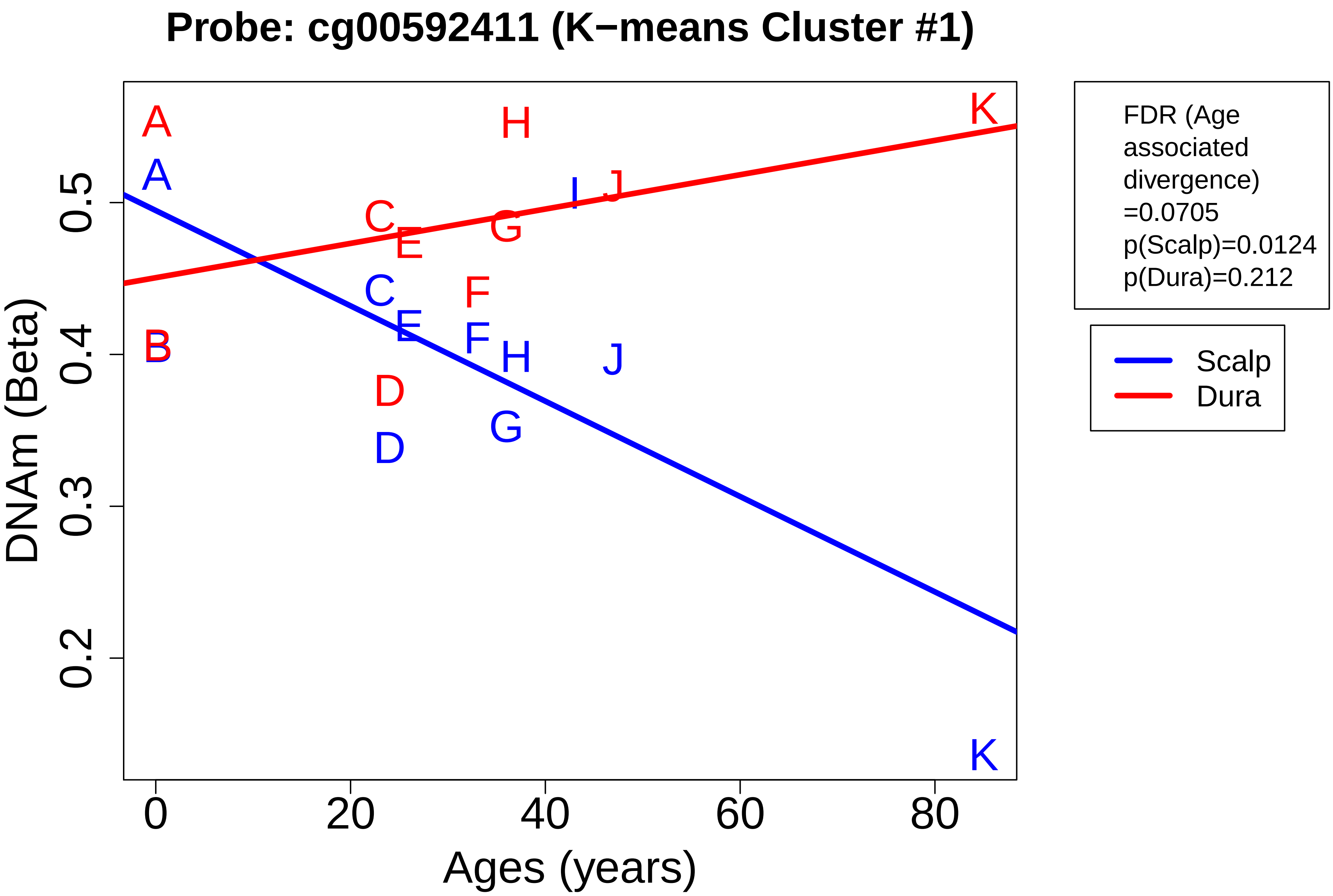
Probe: cg00592411 (K−means Cluster #1)
DNAm (Beta)
0.2
0.3
0.4
0.5
Ages (years)
0
20
40
60
80
A
B
C
D
E
F
G
H
I
J
K
FDR (Age associated divergence) =0.0705
p(Scalp)=0.0124
p(Dura)=0.212
Scalp
Dura

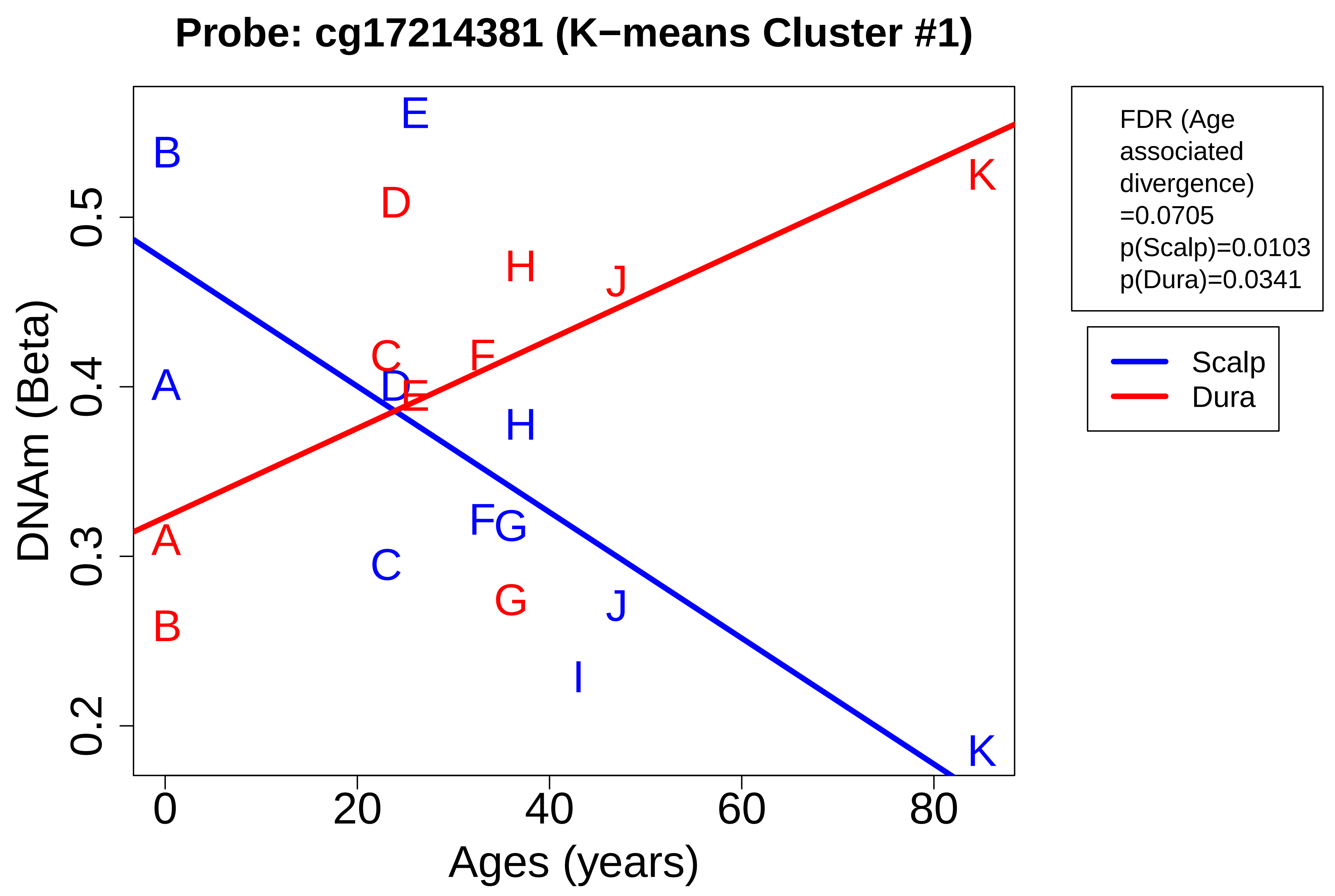

Probe: cg17214381 (K−means Cluster #1)
DNAm (Beta)
0.2
0.3
0.4
0.5
0
20
40
60
80
Ages (years)
FDR (Age associated divergence) =0.0705
p(Scalp)=0.0103
p(Dura)=0.0341
Scalp
Dura
A
B
C
D
E
F
G
H
I
J
K

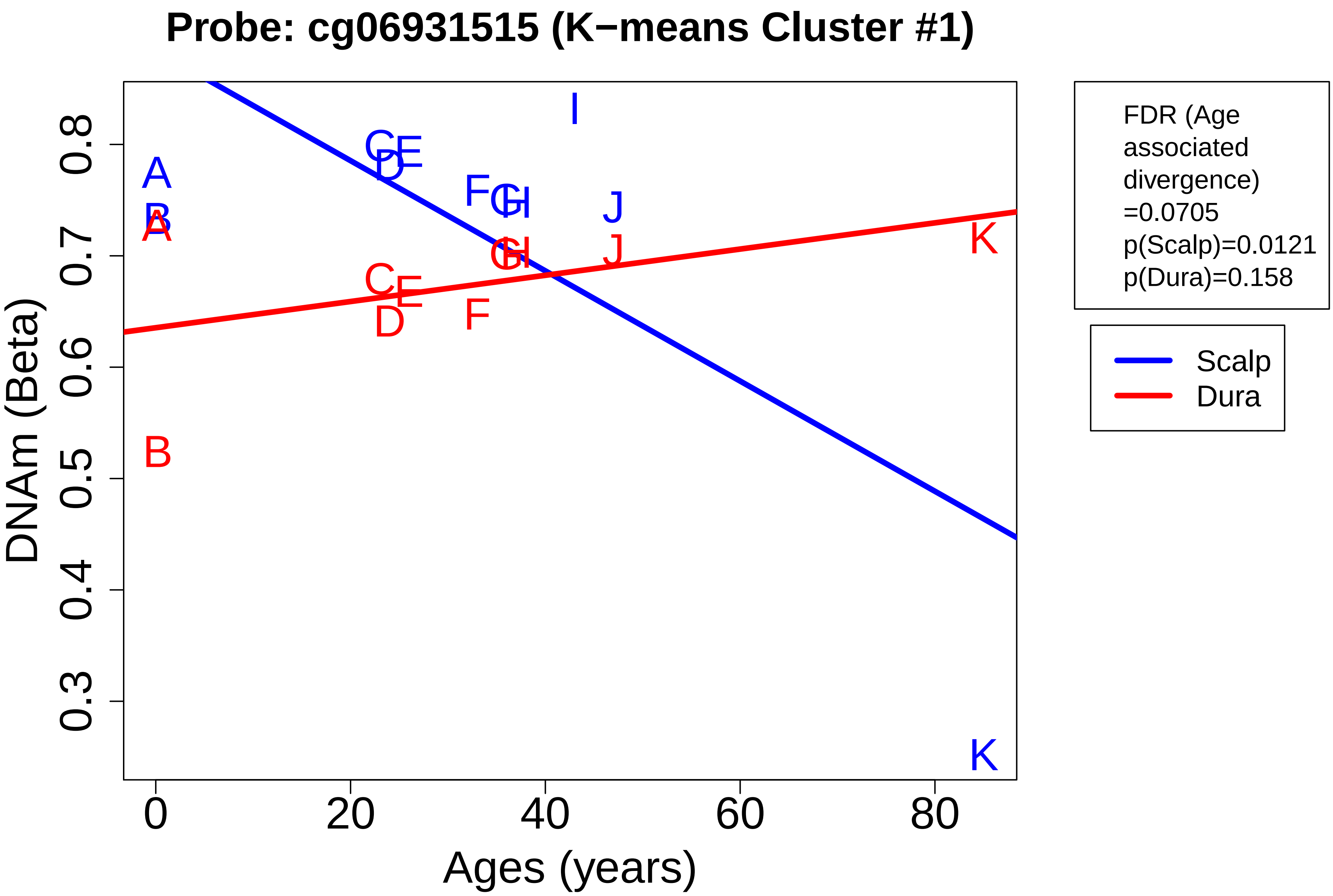

Probe: cg06931515 (K−means Cluster #1)
DNAm (Beta)
0.3
0.4
0.5
0.6
0.7
0.8
0
20
40
60
80
Ages (years)
A
B
C
D
E
F
G
H
I
J
K
FDR (Age associated divergence) =0.0705
p(Scalp)=0.0121
p(Dura)=0.158
Scalp
Dura

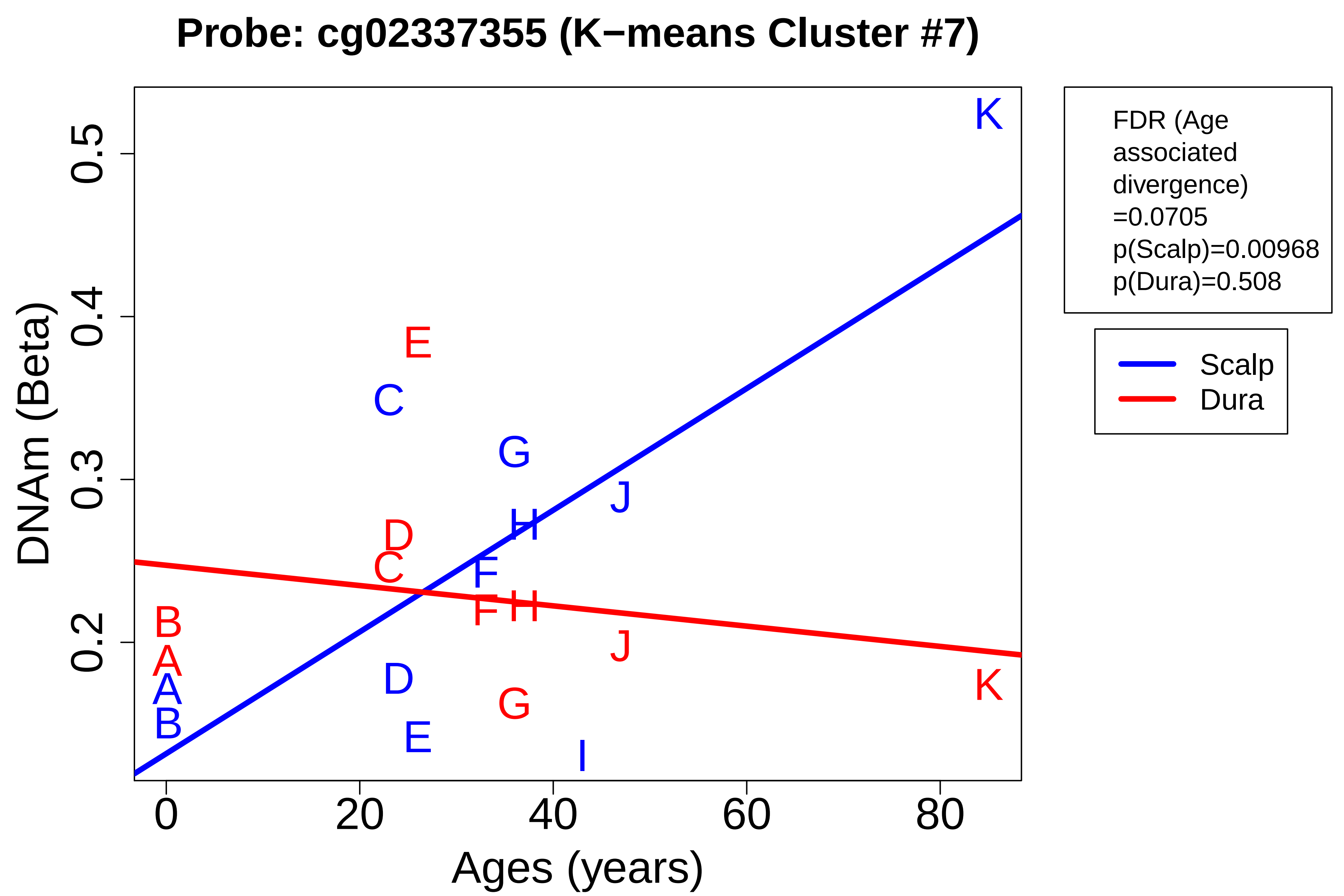

Probe: cg02337355 (K−means Cluster #7)
DNAm (Beta)
0.2
0.3
0.4
0.5
0
20
40
60
80
Ages (years)
FDR (Age associated divergence) =0.0705
p(Scalp)=0.00968
p(Dura)=0.508
Scalp
Dura

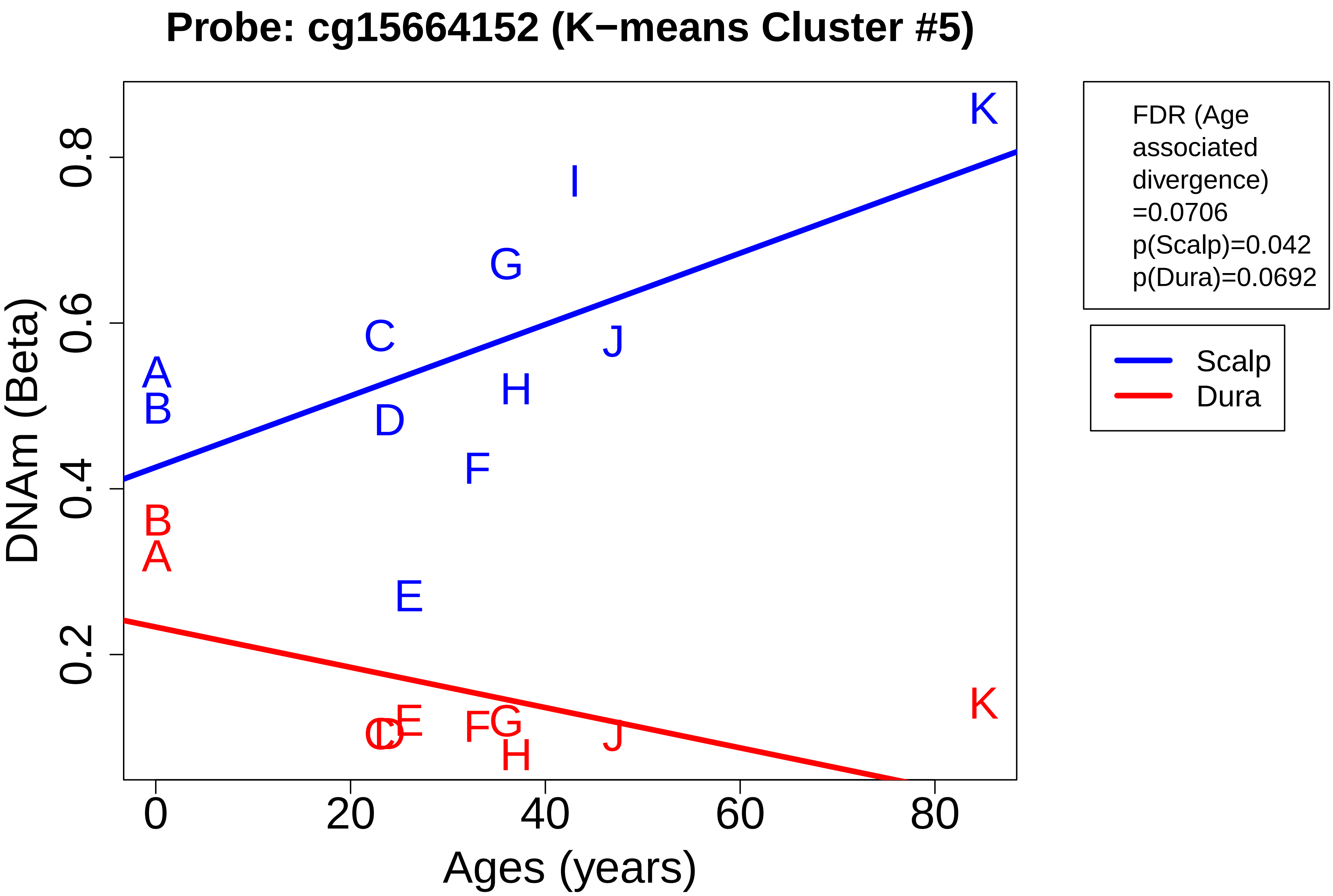

Probe: cg15664152 (K−means Cluster #5)
DNAm (Beta)
0.2
0.4
0.6
0.8
0
20
40
60
80
Ages (years)
FDR (Age associated divergence) =0.0706
p(Scalp)=0.042
p(Dura)=0.0692
Scalp
Dura
A
B
C
D
E
F
G
H
I
J
K

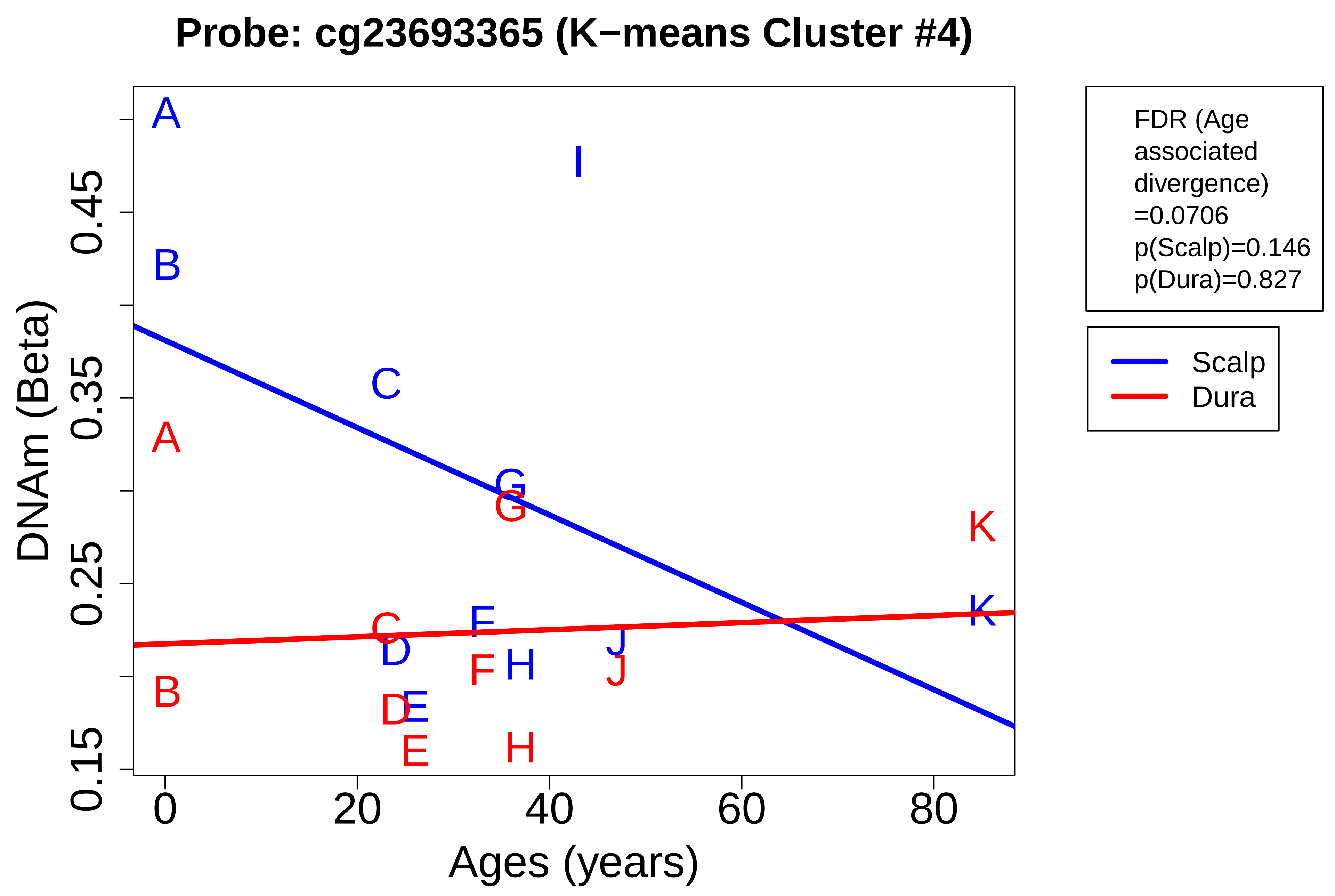

Probe: cg23693365 (K−means Cluster #4)
DNAm (Beta)
0.15
0.25
0.35
0.45
Ages (years)
0
20
40
60
80
FDR (Age associated divergence) =0.0706
p(Scalp)=0.146
p(Dura)=0.827
Scalp
Dura
A
B
C
D
E
F
G
H
I
J
K

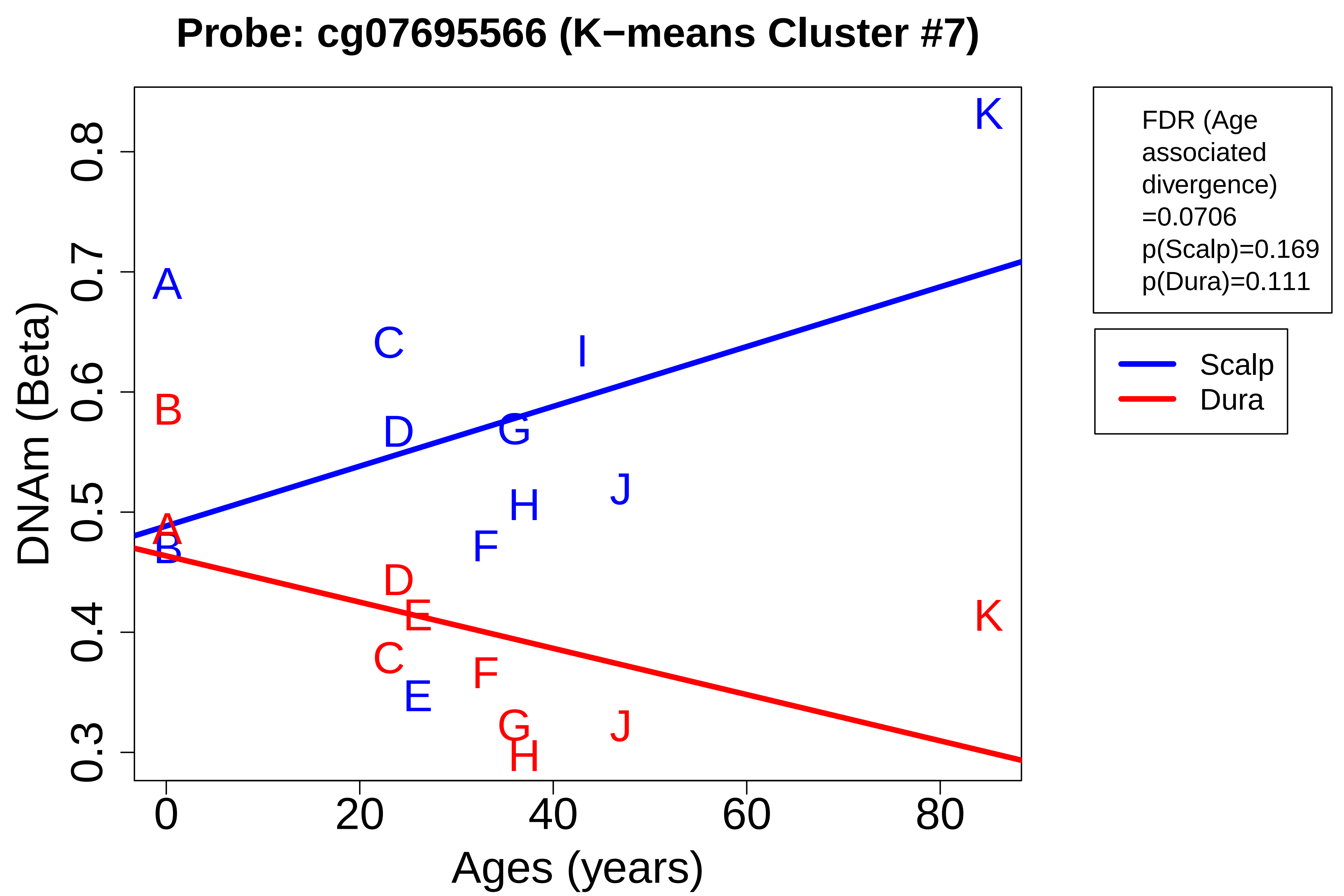

Probe: cg07695566 (K−means Cluster #7)
DNAm (Beta)
Ages (years)
0.3
0.4
0.5
0.6
0.7
0.8
0
20
40
60
80
A
B
C
D
E
F
G
H
I
J
K
FDR (Age associated divergence) =0.0706
p(Scalp)=0.169
p(Dura)=0.111
Scalp
Dura

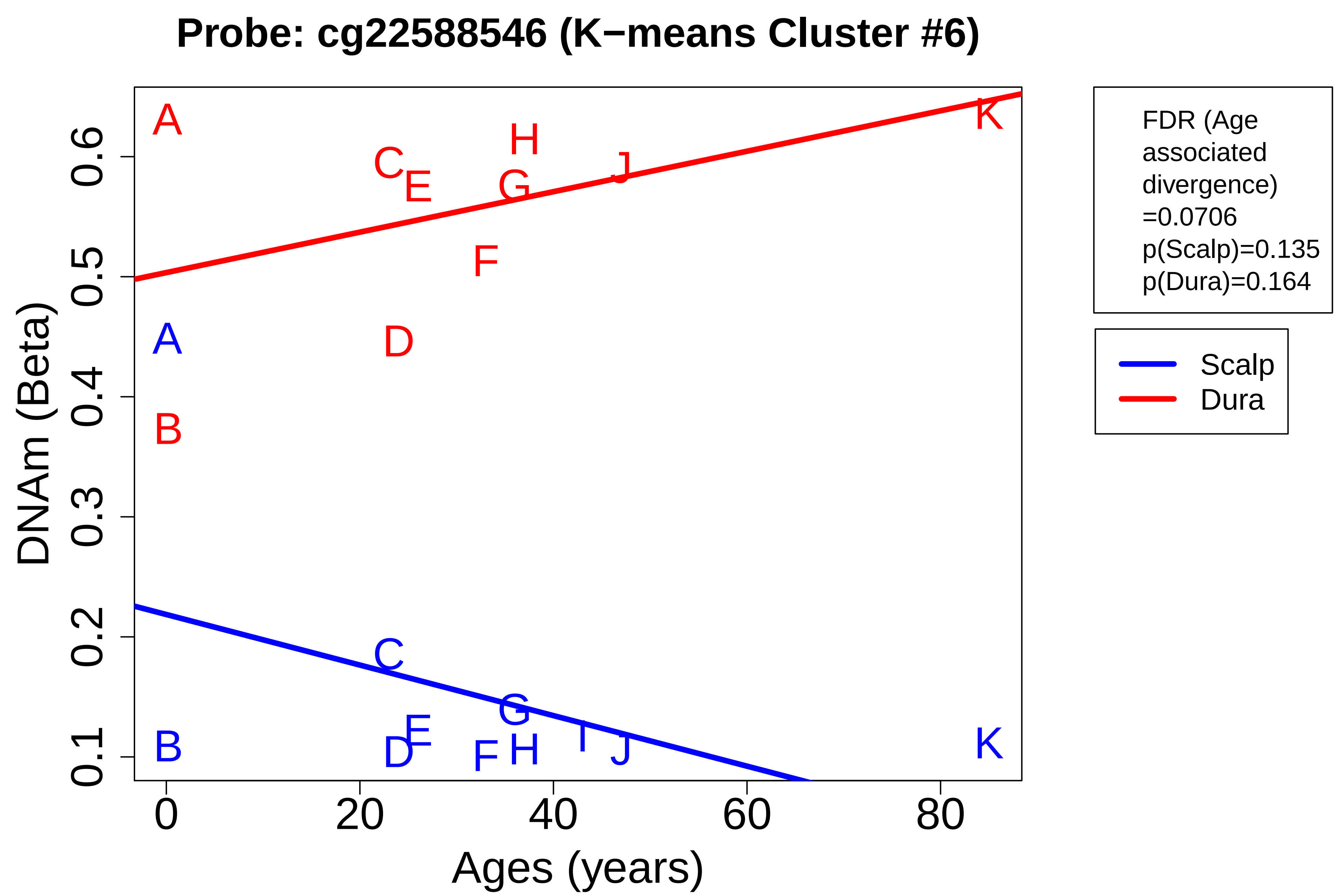

Probe: cg22588546 (K−means Cluster #6)
DNAm (Beta)
0.1
0.2
0.3
0.4
0.5
0.6
0
20
40
60
80
Ages (years)
A
B
C
D
E
F
G
H
I
J
K
FDR (Age associated divergence) =0.0706
p(Scalp)=0.135
p(Dura)=0.164
Scalp
Dura

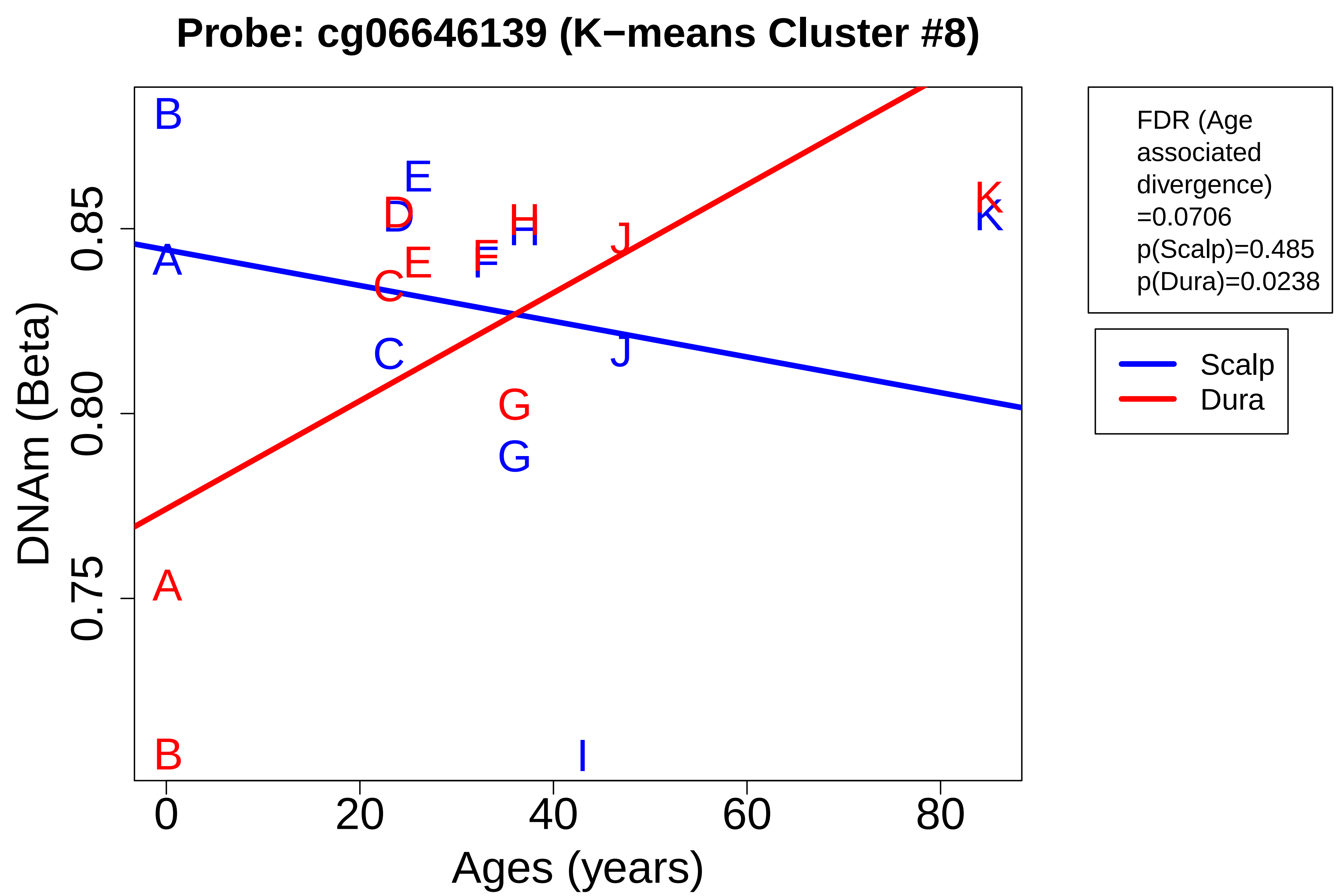
Probe: cg06646139 (K−means Cluster #8)
DNAm (Beta)
0.75
0.80
0.85
0
20
40
60
80
Ages (years)
FDR (Age associated divergence) =0.0706
p(Scalp)=0.485
p(Dura)=0.0238
Scalp
Dura

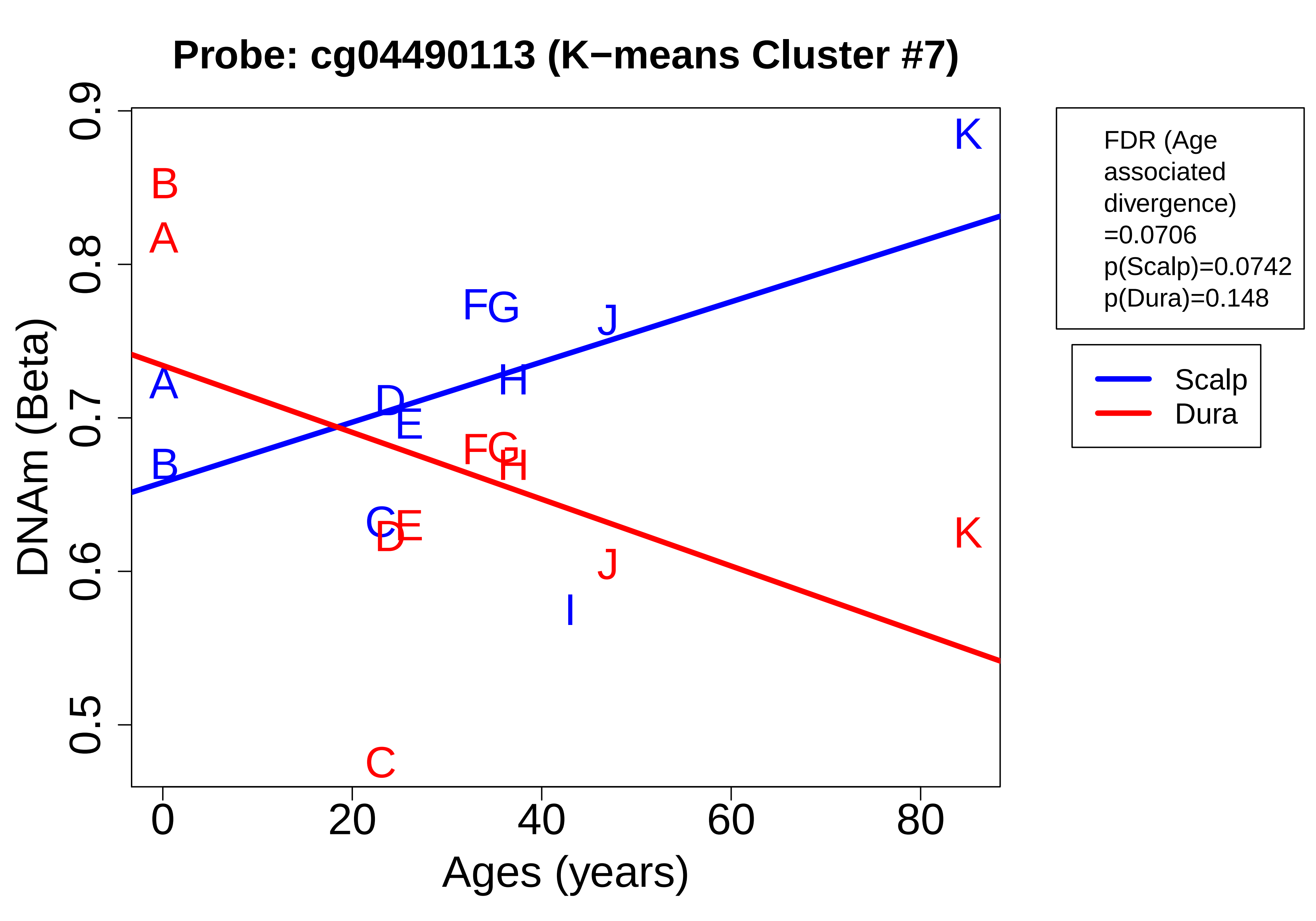
Probe: cg04490113 (K−means Cluster #7)
DNAm (Beta)
0.5
0.6
0.7
0.8
0.9
0
20
40
60
80
Ages (years)
A
B
C
D
E
F
G
H
I
J
K
FDR (Age associated divergence) =0.0706
p(Scalp)=0.0742
p(Dura)=0.148
Scalp
Dura

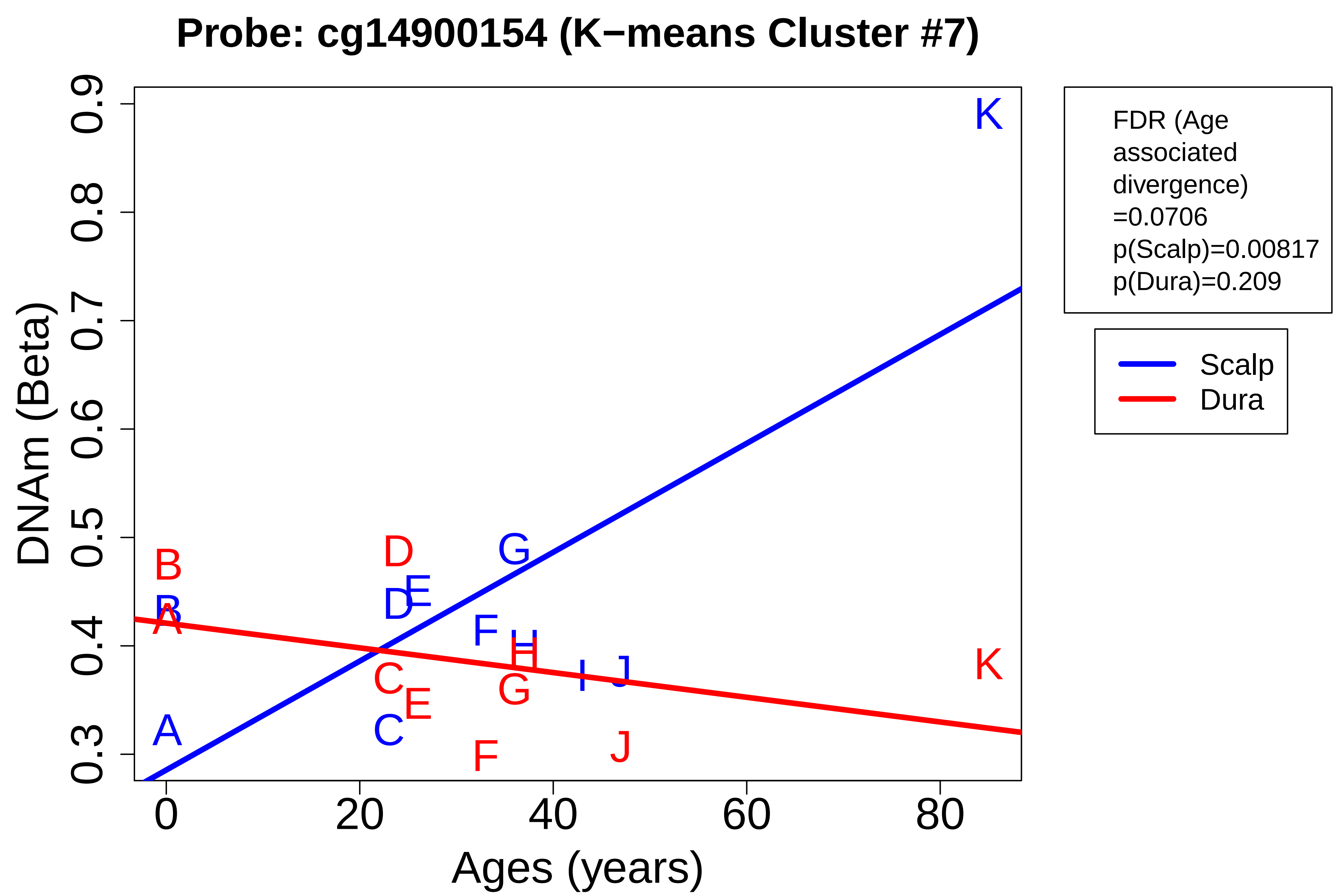

Probe: cg14900154 (K−means Cluster #7)
DNAm (Beta)
0.3
0.4
0.5
0.6
0.7
0.8
0.9
0
20
40
60
80
Ages (years)
FDR (Age associated divergence) =0.0706
p(Scalp)=0.00817
p(Dura)=0.209
Scalp
Dura

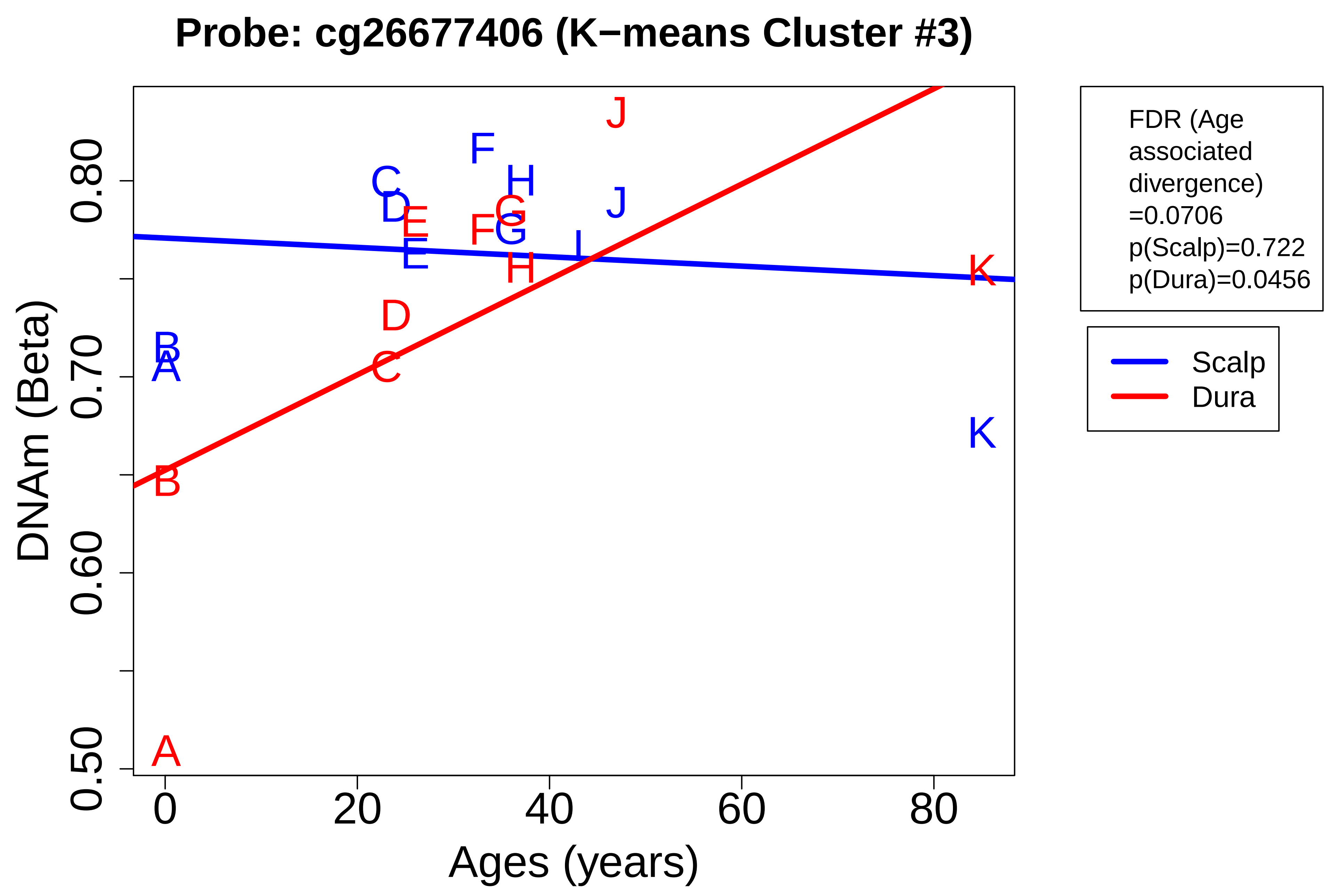
Probe: cg26677406 (K−means Cluster #3)
DNAm (Beta)
0.50
0.60
0.70
0.80
0
20
40
60
80
Ages (years)
FDR (Age associated divergence) =0.0706
p(Scalp)=0.722
p(Dura)=0.0456
Scalp
Dura
A
B
C
D
E
F
G
H
I
J
K

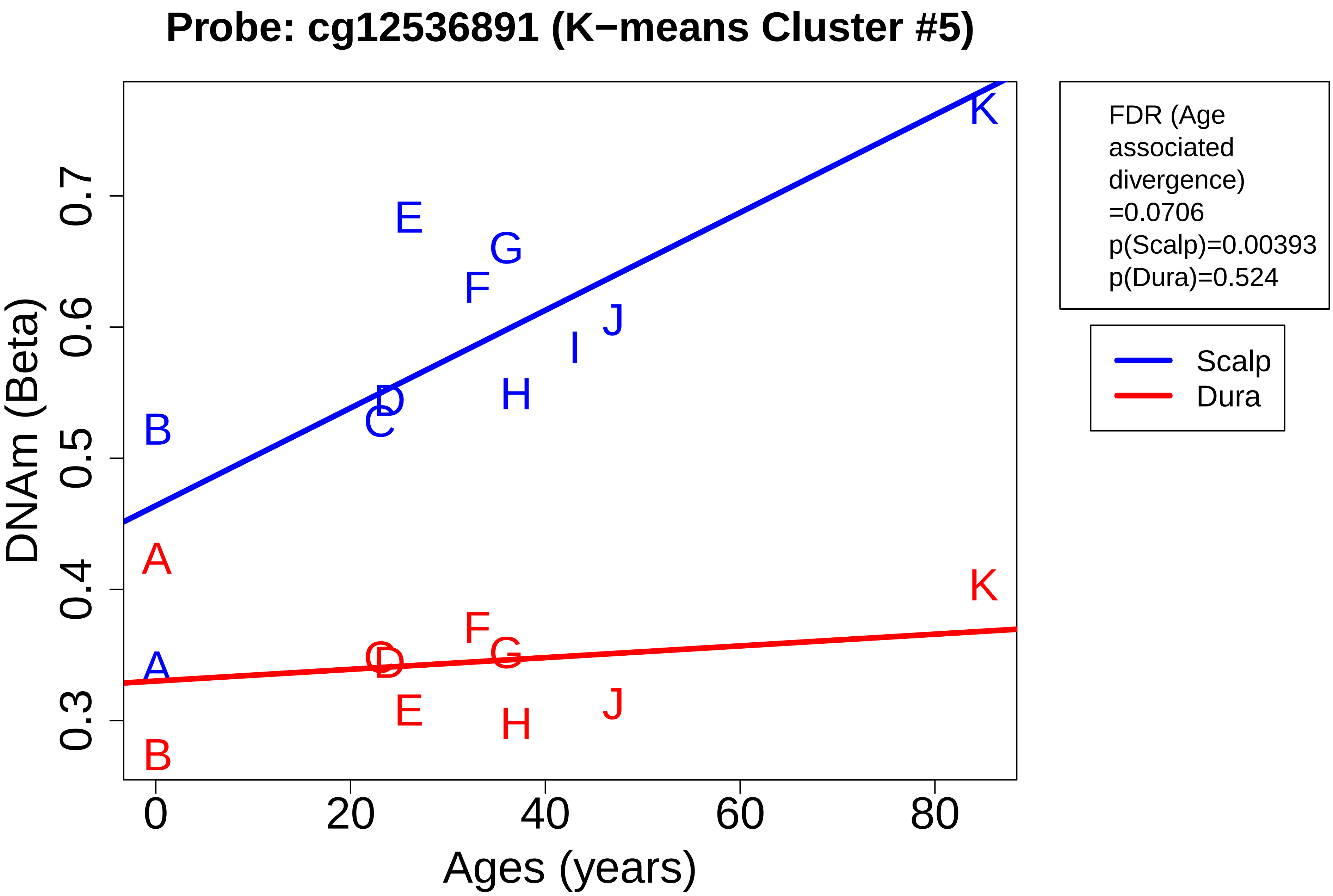

Probe: cg12536891 (K−means Cluster #5)
DNAm (Beta)
0.3
0.4
0.5
0.6
0.7
Ages (years)
0
20
40
60
80
A
B
C
D
E
F
G
H
I
J
K
FDR (Age associated divergence) =0.0706
p(Scalp)=0.00393
p(Dura)=0.524
Scalp
Dura

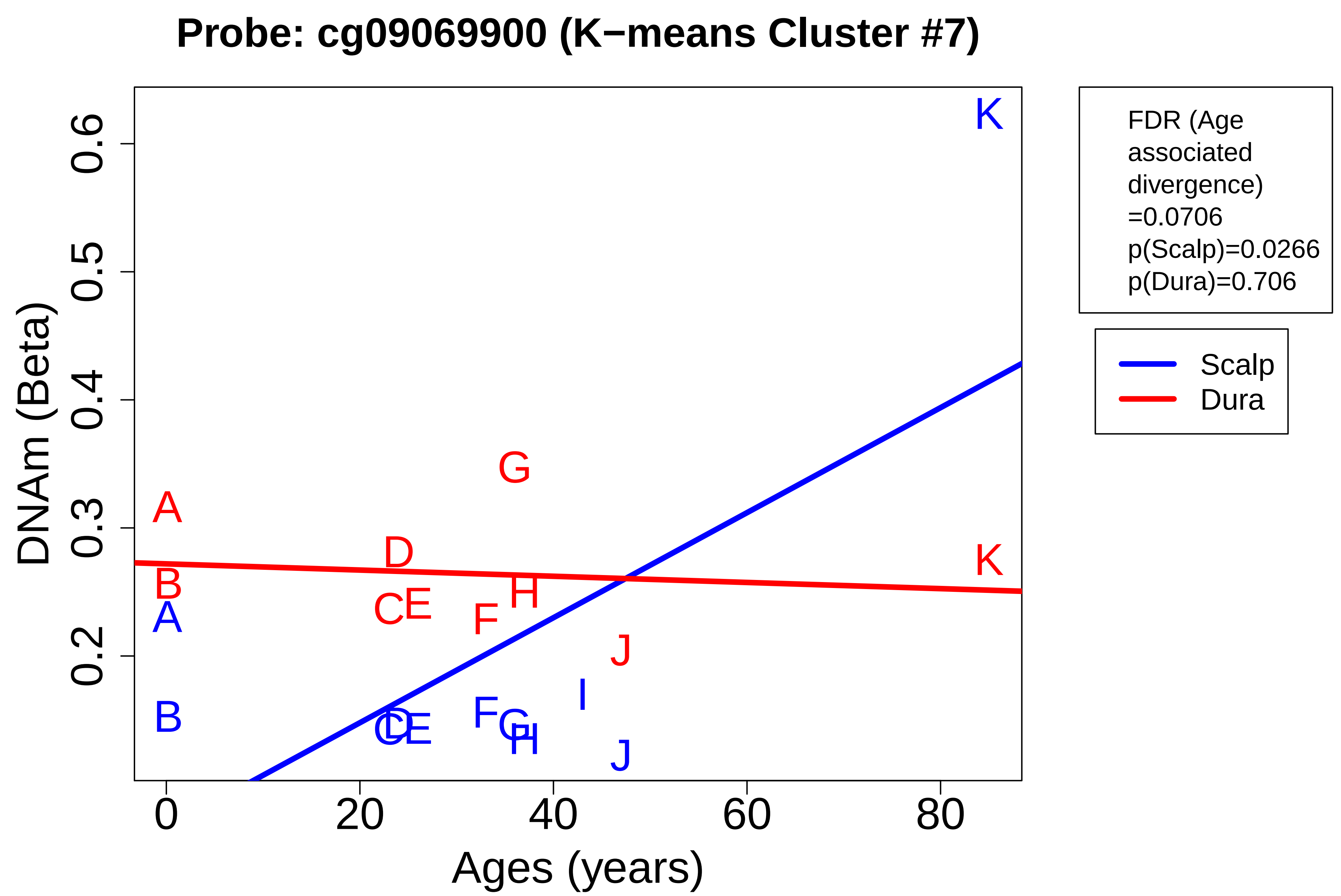
Probe: cg09069900 (K−means Cluster #7)
DNAm (Beta)
0.2
0.3
0.4
0.5
0.6
Ages (years)
0
20
40
60
80
FDR (Age associated divergence) =0.0706
p(Scalp)=0.0266
p(Dura)=0.706
Scalp
Dura
A
B
C
D
E
F
G
H
I
J
K

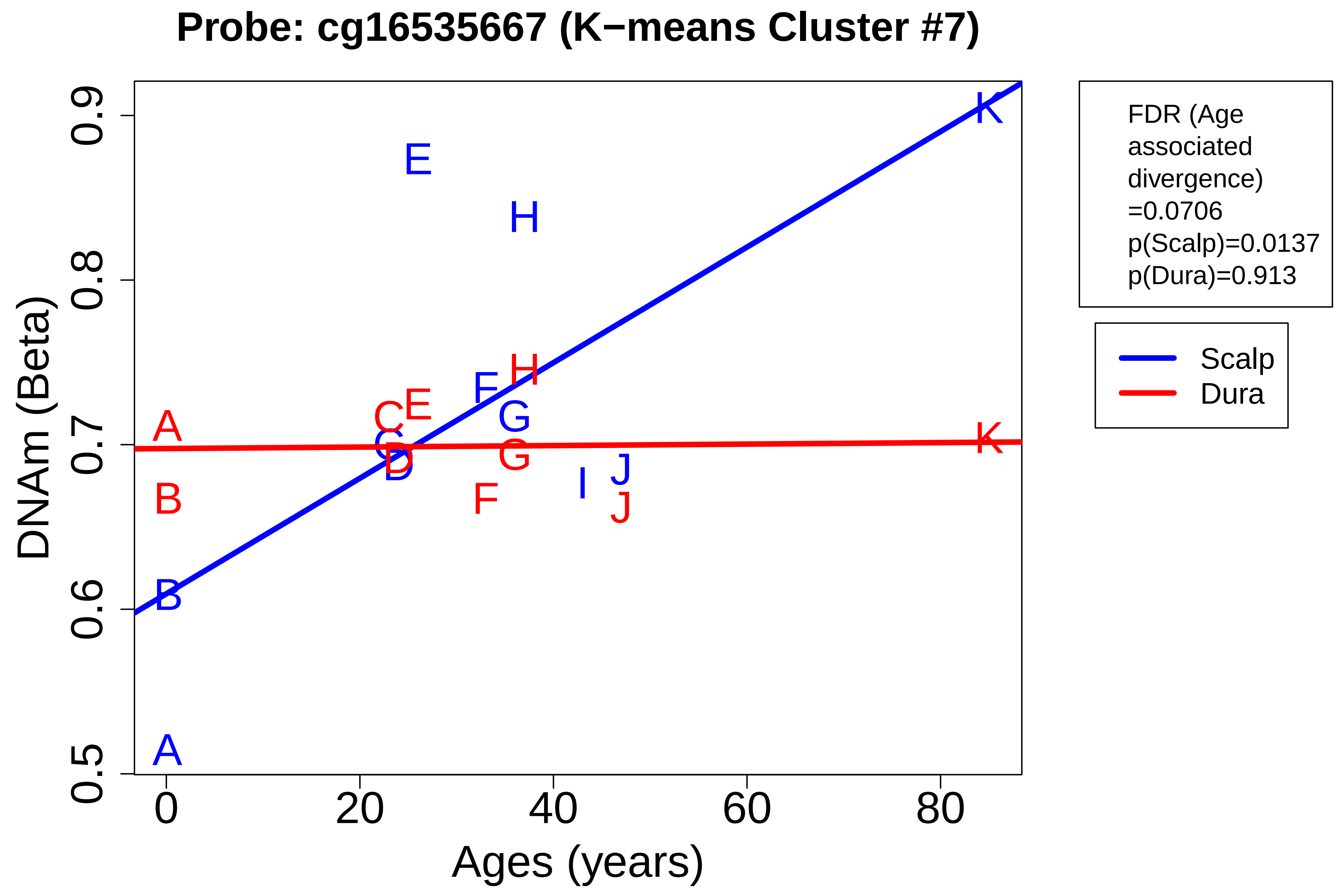

Probe: cg16535667 (K−means Cluster #7)
DNAm (Beta)
0.5
0.6
0.7
0.8
0.9
0
20
40
60
80
Ages (years)
A
B
C
D
E
F
G
H
I
J
K
FDR (Age associated divergence) =0.0706
p(Scalp)=0.0137
p(Dura)=0.913
Scalp
Dura

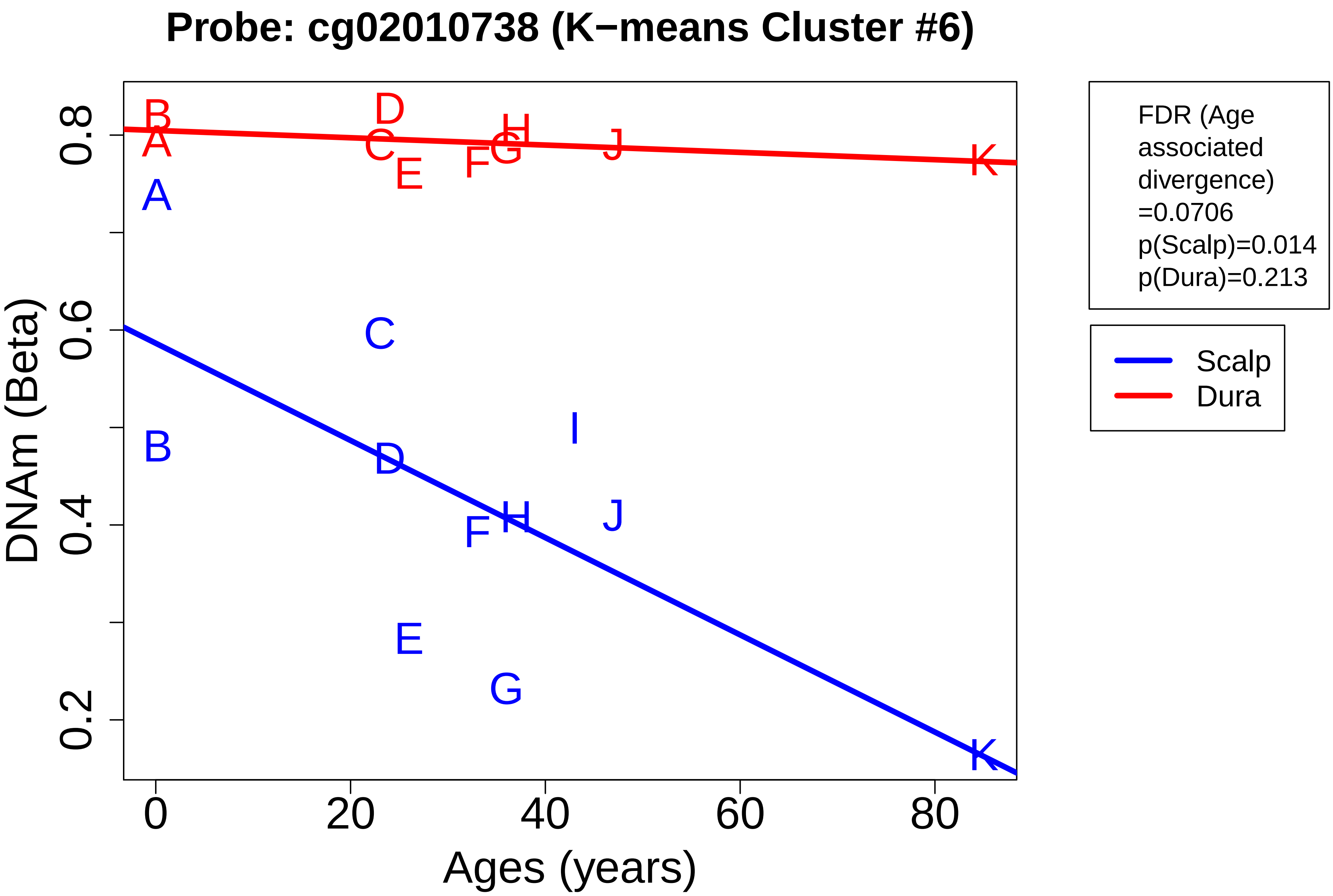

Probe: cg02010738 (K−means Cluster #6)
DNAm (Beta)
0.2
0.4
0.6
0.8
0
20
40
60
80
Ages (years)
A
B
C
D
E
F
G
H
I
J
K
FDR (Age associated divergence) =0.0706
p(Scalp)=0.014
p(Dura)=0.213
Scalp
Dura

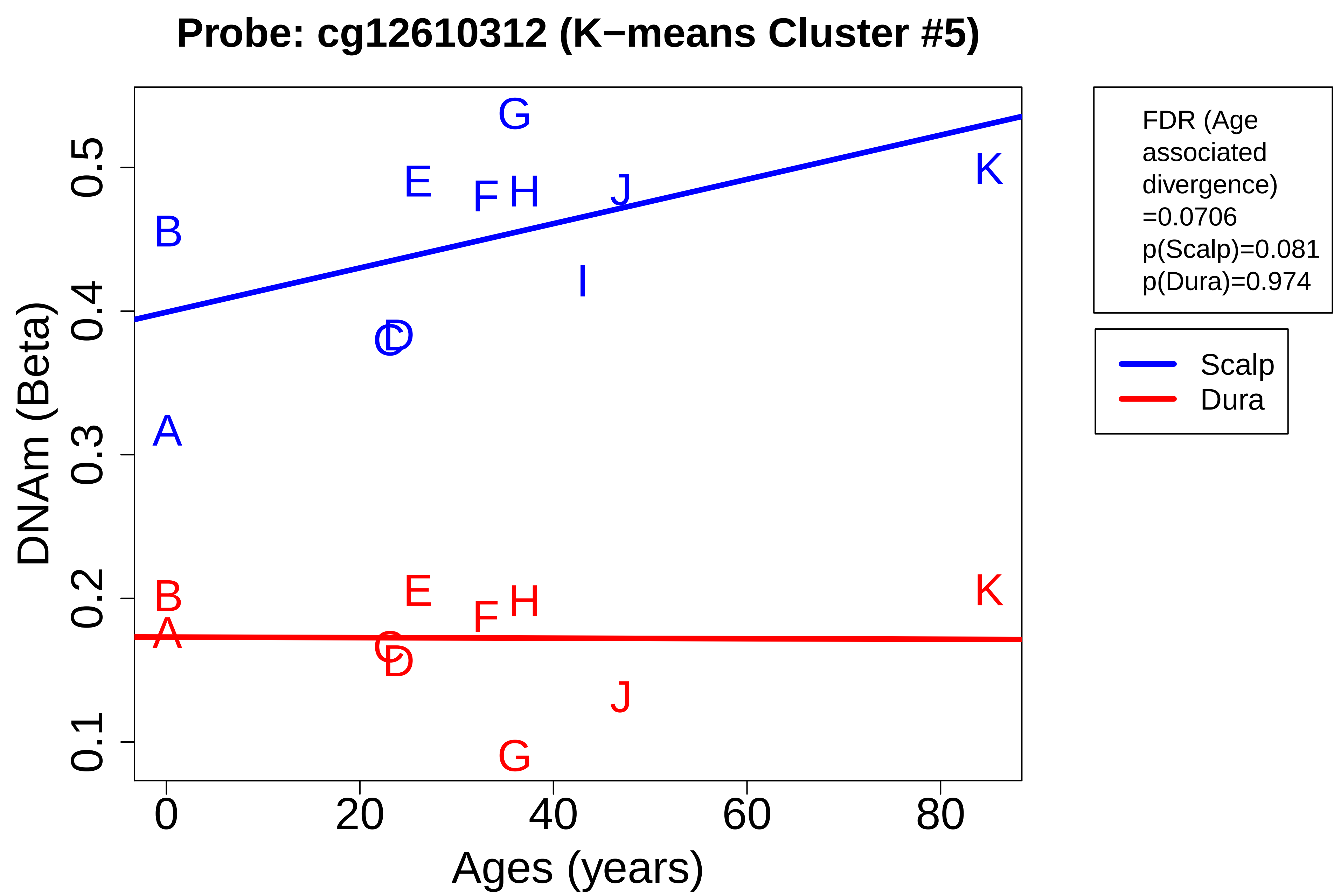

Probe: cg12610312 (K−means Cluster #5)
DNAm (Beta)
0.1
0.2
0.3
0.4
0.5
Ages (years)
0
20
40
60
80
FDR (Age associated divergence) =0.0706
p(Scalp)=0.081
p(Dura)=0.974
Scalp
Dura
A
B
C
D
E
F
G
H
I
J
K

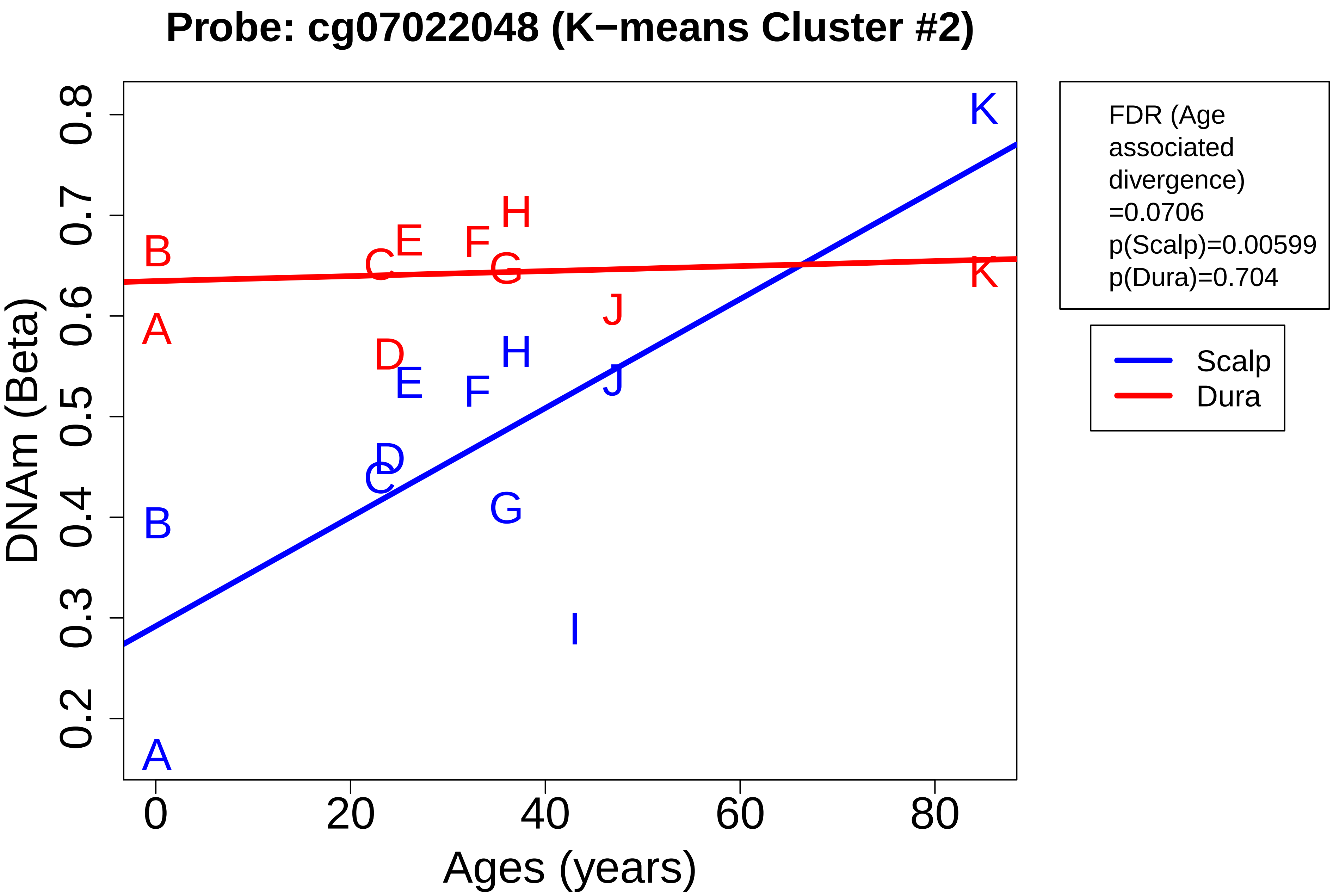

Probe: cg07022048 (K−means Cluster #2)
DNAm (Beta)
0.2
0.3
0.4
0.5
0.6
0.7
0.8
0
20
40
60
80
Ages (years)
A
B
C
D
E
F
G
H
I
J
K
FDR (Age associated divergence) =0.0706
p(Scalp)=0.00599
p(Dura)=0.704
Scalp
Dura

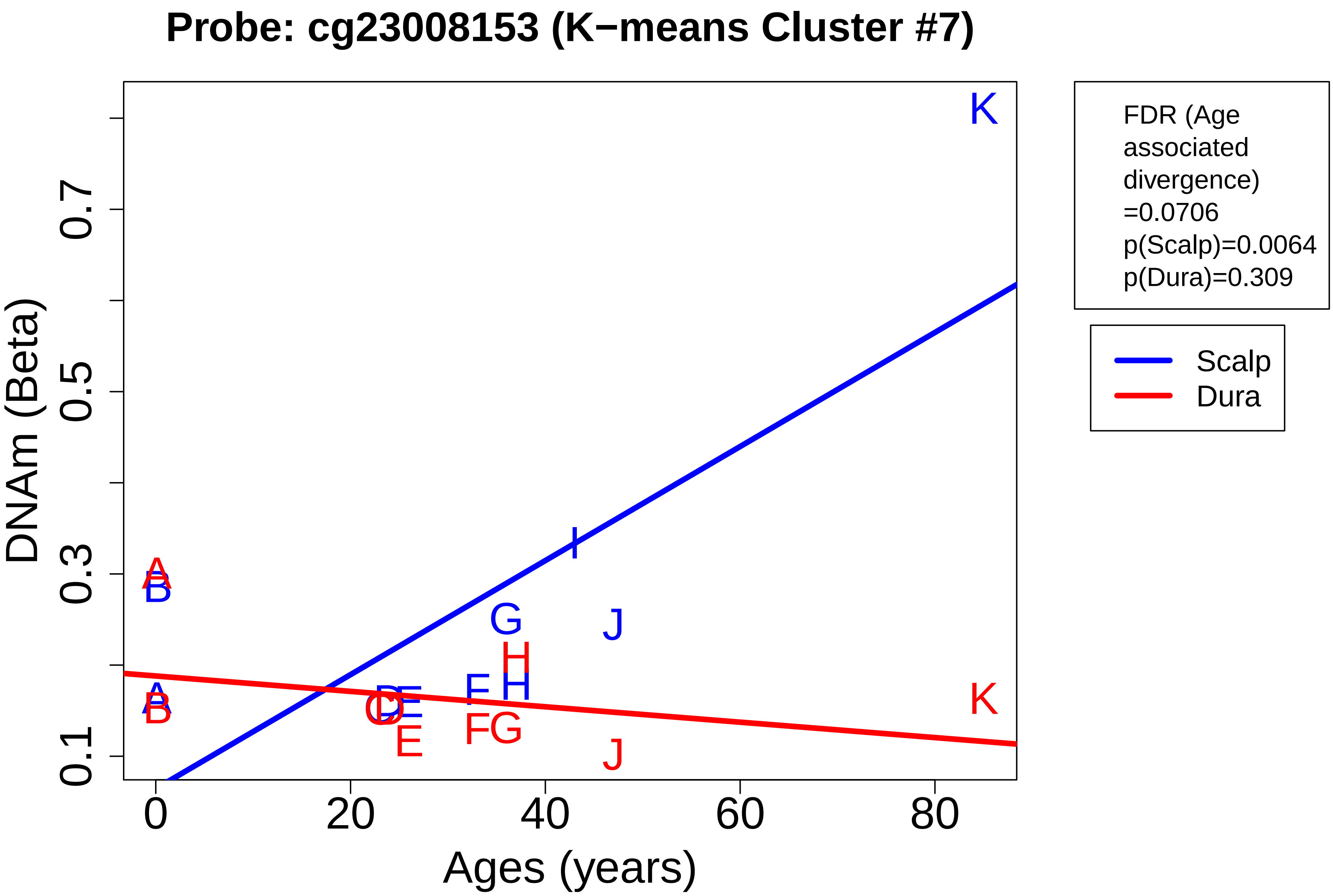
Probe: cg23008153 (K−means Cluster #7)
DNAm (Beta)
0.1
0.3
0.5
0.7
0
20
40
60
80
Ages (years)
FDR (Age associated divergence) =0.0706
p(Scalp)=0.0064
p(Dura)=0.309
Scalp
Dura
A
B
C
D
E
F
G
H
I
J
K

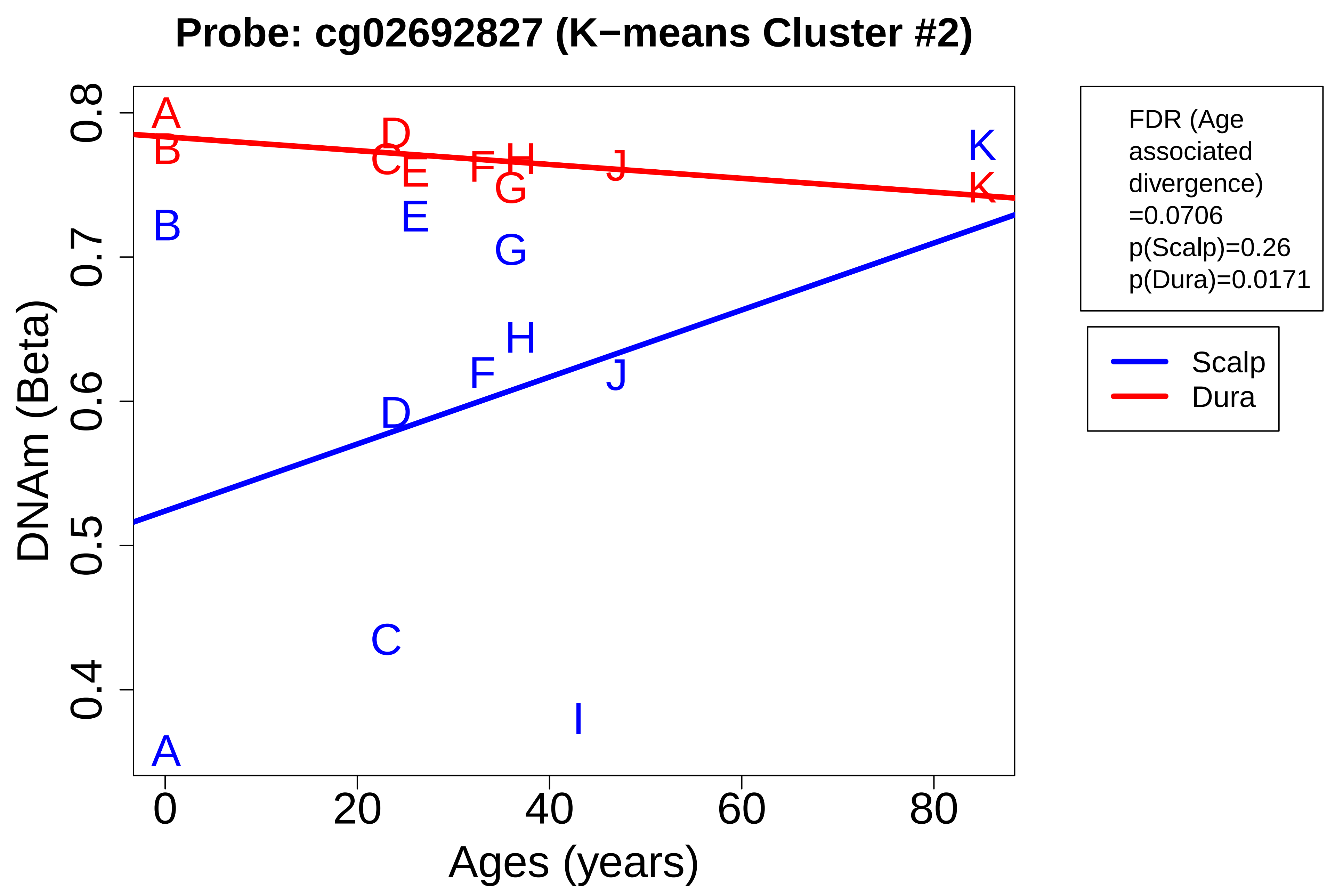
Probe: cg02692827 (K−means Cluster #2)
DNAm (Beta)
0.4
0.5
0.6
0.7
0.8
Ages (years)
0
20
40
60
80
FDR (Age associated divergence) =0.0706
p(Scalp)=0.26
p(Dura)=0.0171
Scalp
Dura
A
B
C
D
E
F
G
H
I
J
K

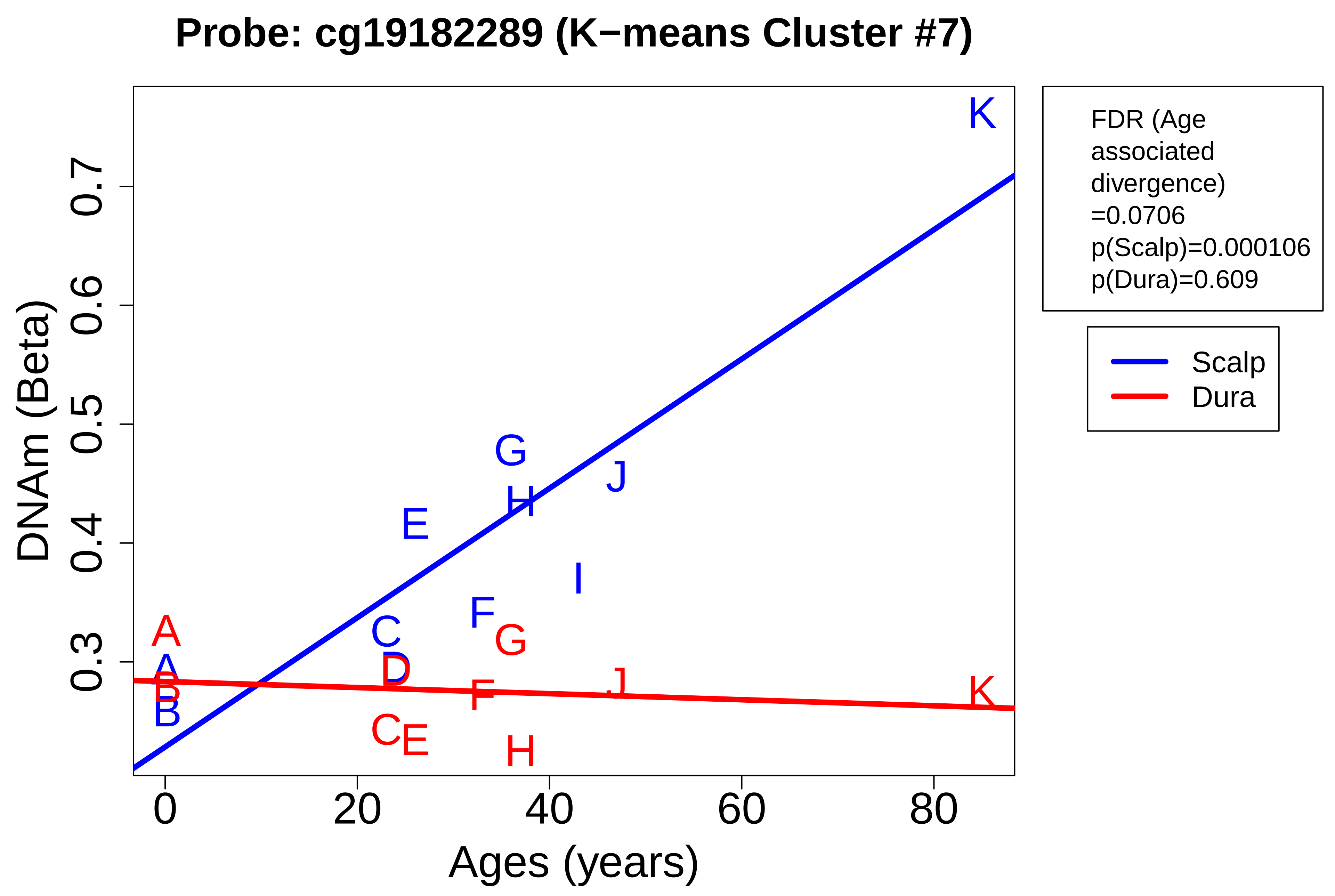

Probe: cg19182289 (K−means Cluster #7)
DNAm (Beta)
0.3
0.4
0.5
0.6
0.7
0
20
40
60
80
Ages (years)
A B C D E F G H I J K
A B C D E F G H J K
FDR (Age associated divergence) =0.0706
p(Scalp)=0.000106
p(Dura)=0.609
Scalp
Dura

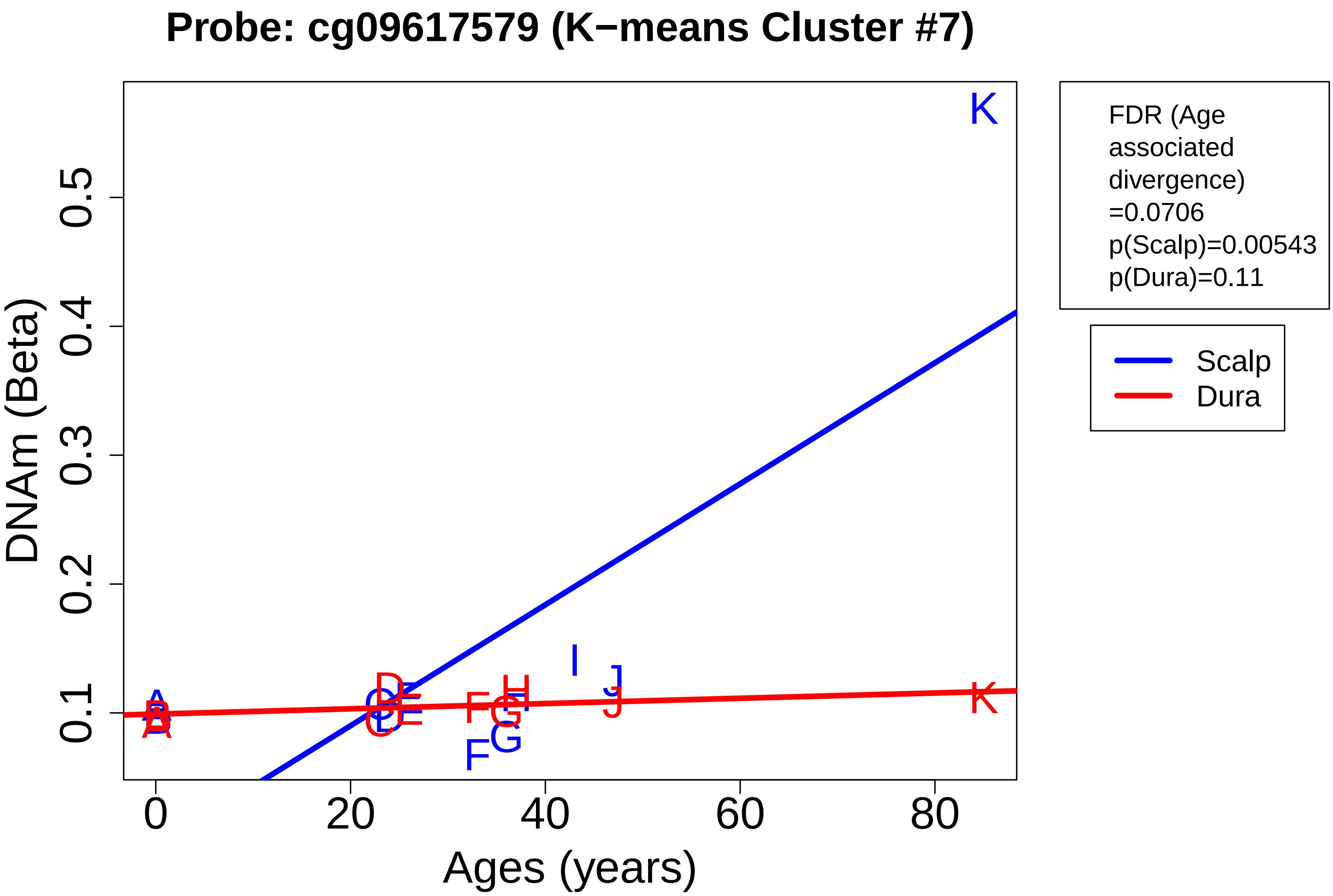

Probe: cg09617579 (K−means Cluster #7)
DNAm (Beta)
0.1
0.2
0.3
0.4
0.5
0
20
40
60
80
Ages (years)
FDR (Age associated divergence) =0.0706
p(Scalp)=0.00543
p(Dura)=0.11
Scalp
Dura

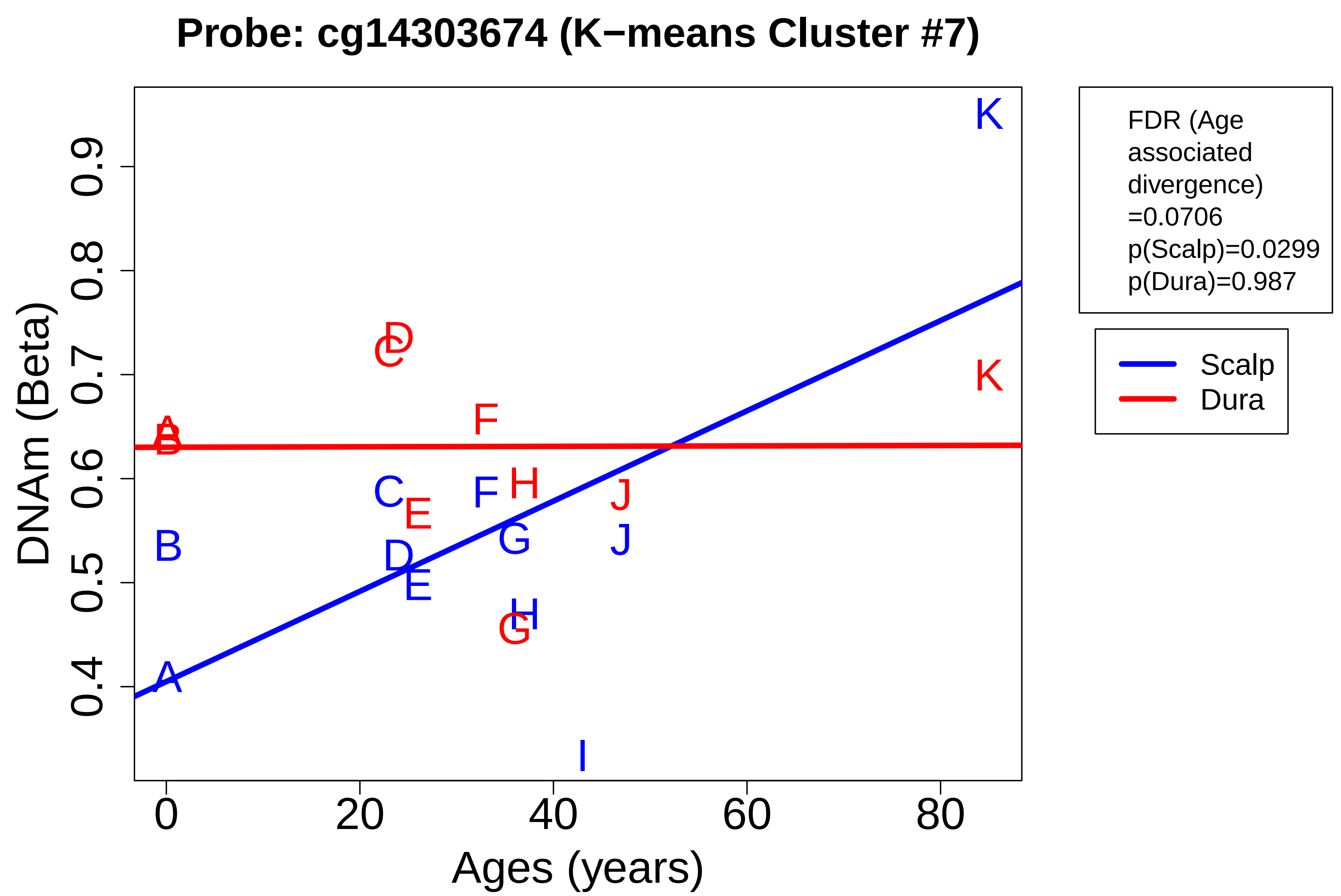

Probe: cg14303674 (K−means Cluster #7)
DNAm (Beta)
0.4
0.5
0.6
0.7
0.8
0.9
0
20
40
60
80
Ages (years)
A
B
C
D
E
F
G
H
I
J
K
FDR (Age associated divergence) =0.0706
p(Scalp)=0.0299
p(Dura)=0.987
Scalp
Dura

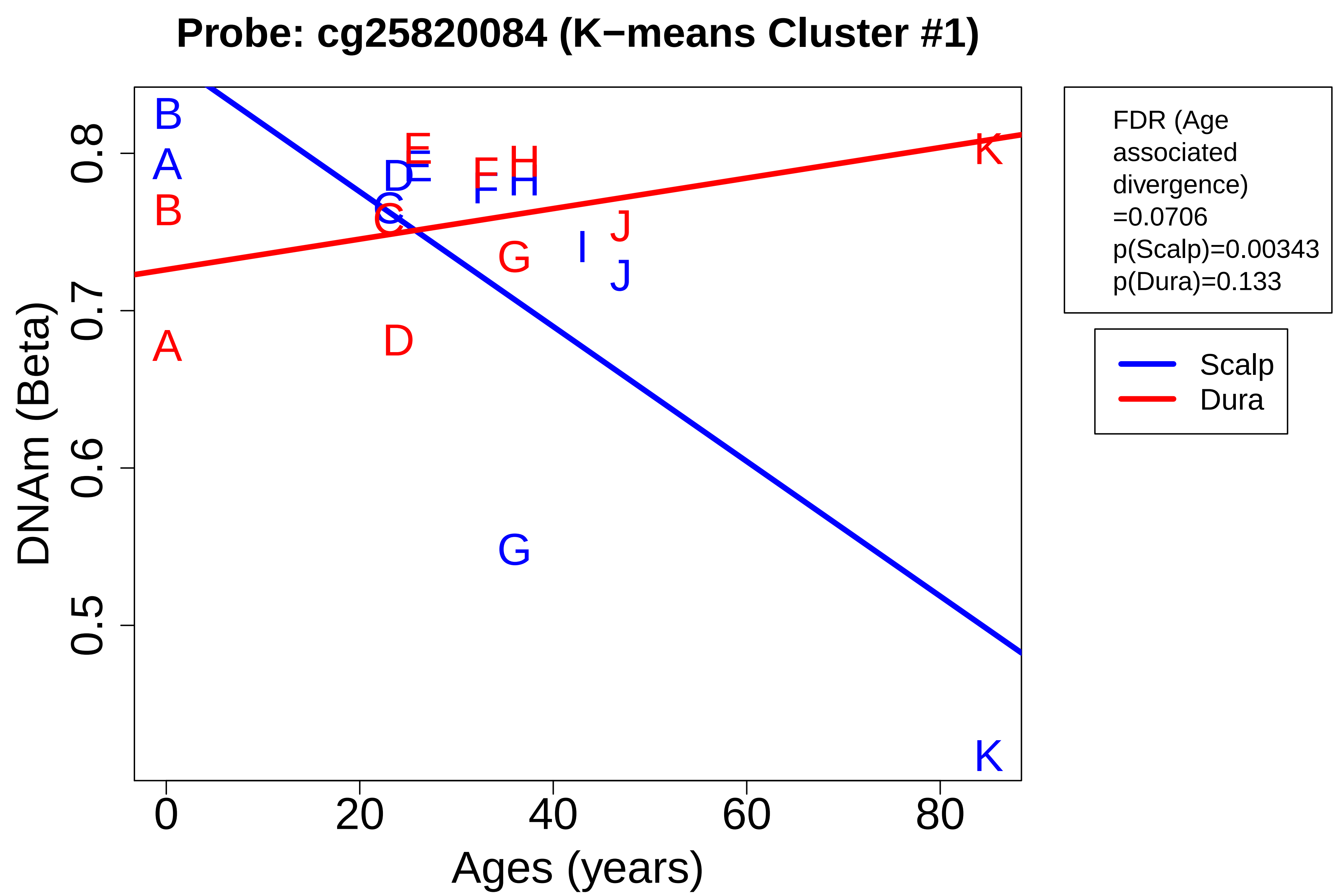

Probe: cg25820084 (K−means Cluster #1)
DNAm (Beta)
0.5
0.6
0.7
0.8
0
20
40
60
80
Ages (years)
B
A
B
A
C
C
D
E
E
D
F
F
H
H
G
G
I
J
J
K
K
FDR (Age associated divergence) =0.0706
p(Scalp)=0.00343
p(Dura)=0.133
Scalp
Dura

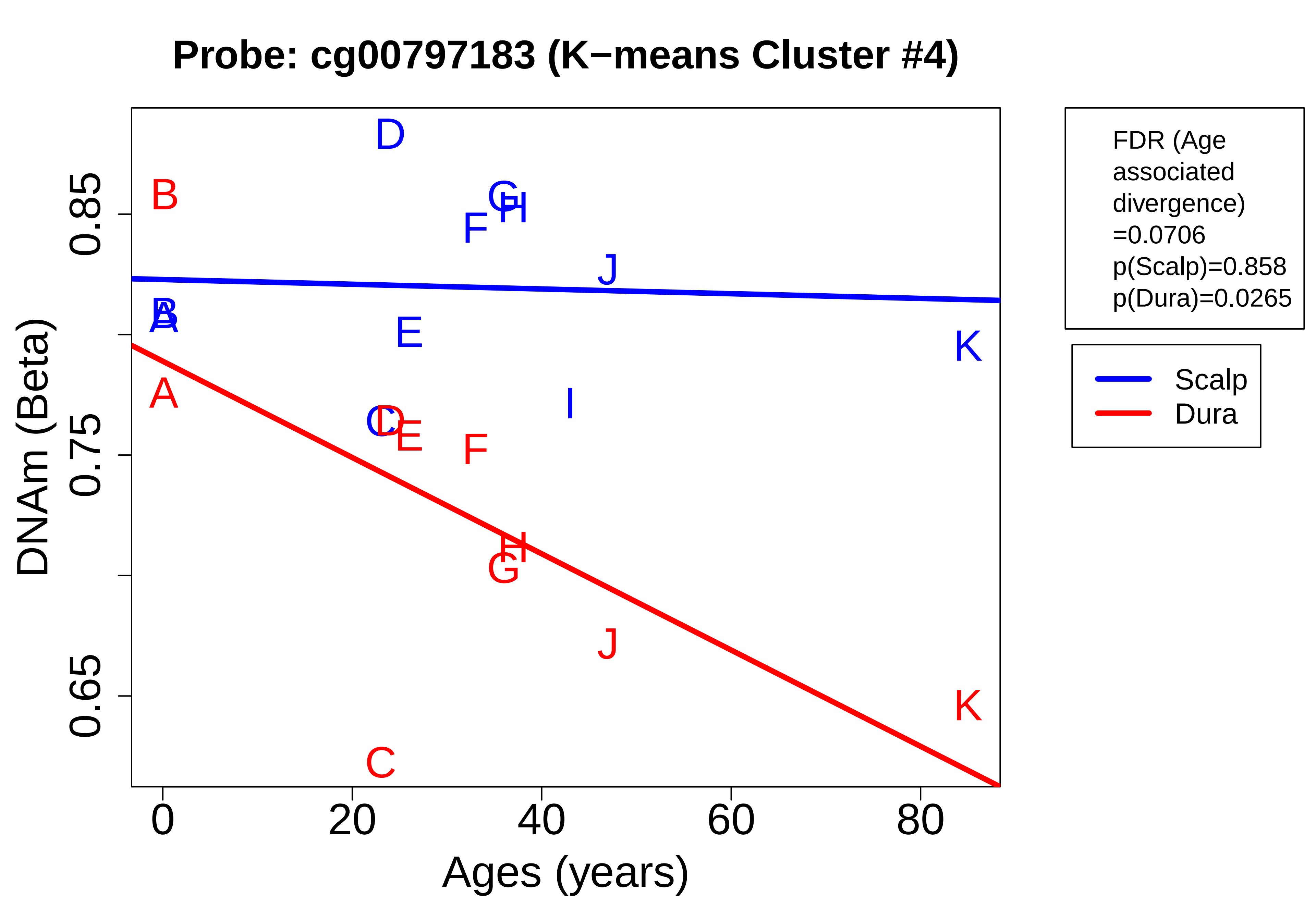

Probe: cg00797183 (K−means Cluster #4)
DNAm (Beta)
0.65
0.75
0.85
0
20
40
60
80
Ages (years)
FDR (Age associated divergence) =0.0706
p(Scalp)=0.858
p(Dura)=0.0265
Scalp
Dura

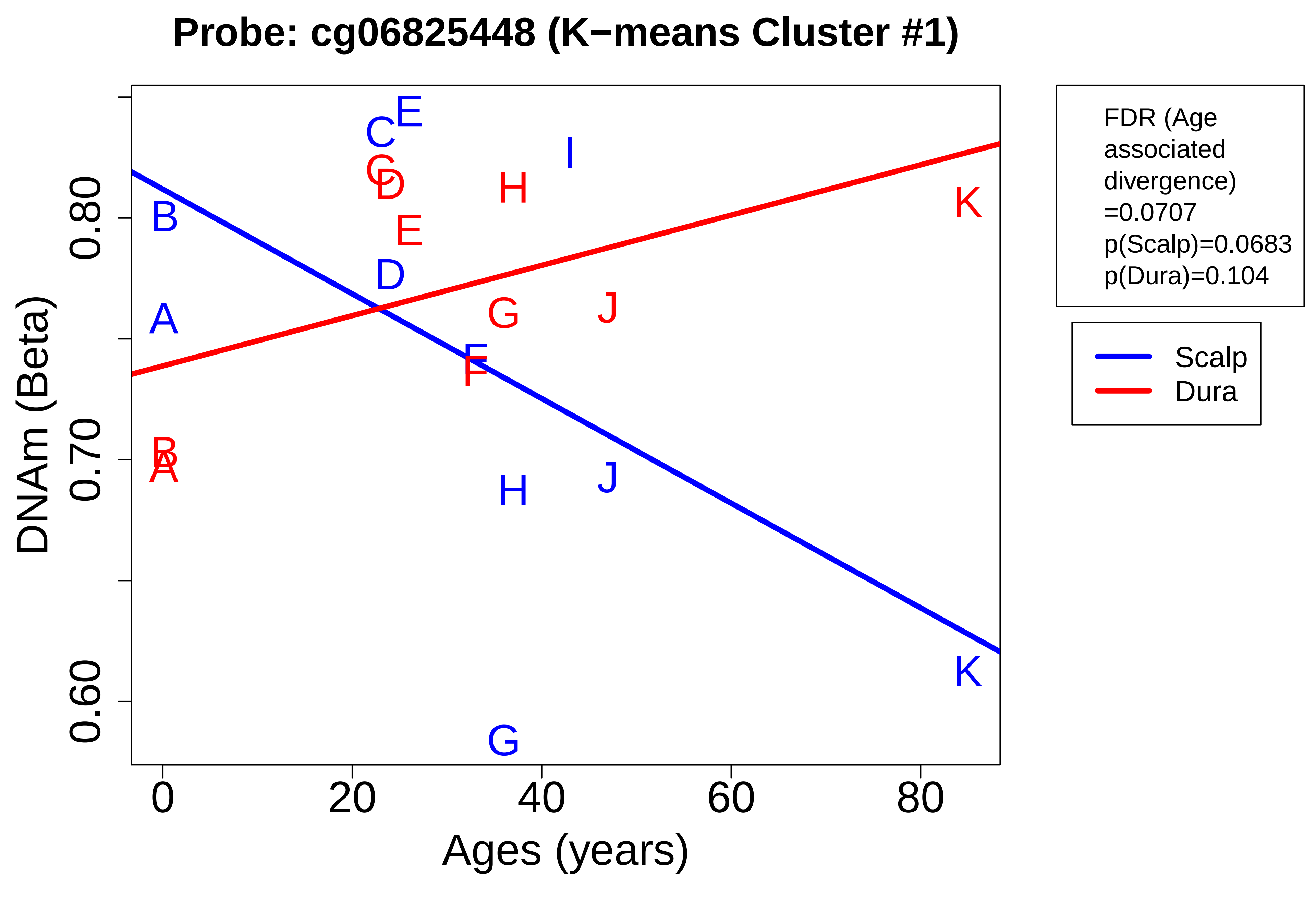

Probe: cg06825448 (K−means Cluster #1)
DNAm (Beta)
0.60
0.70
0.80
0
20
40
60
80
Ages (years)
FDR (Age associated divergence) =0.0707
p(Scalp)=0.0683
p(Dura)=0.104
Scalp
Dura

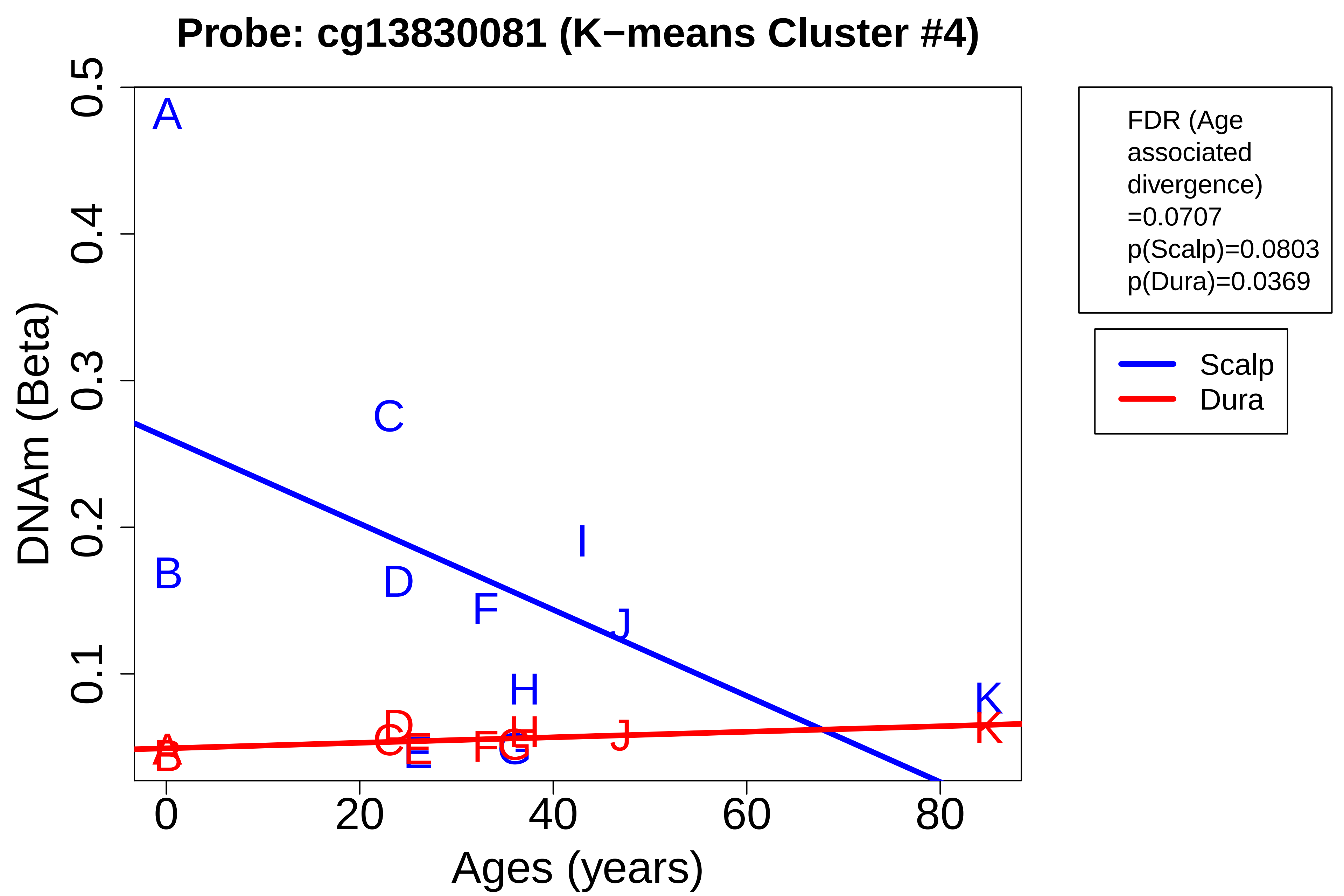

Probe: cg13830081 (K−means Cluster #4)
DNAm (Beta)
0.1
0.2
0.3
0.4
0.5
Ages (years)
0
20
40
60
80
FDR (Age associated divergence) =0.0707
p(Scalp)=0.0803
p(Dura)=0.0369
Scalp
Dura

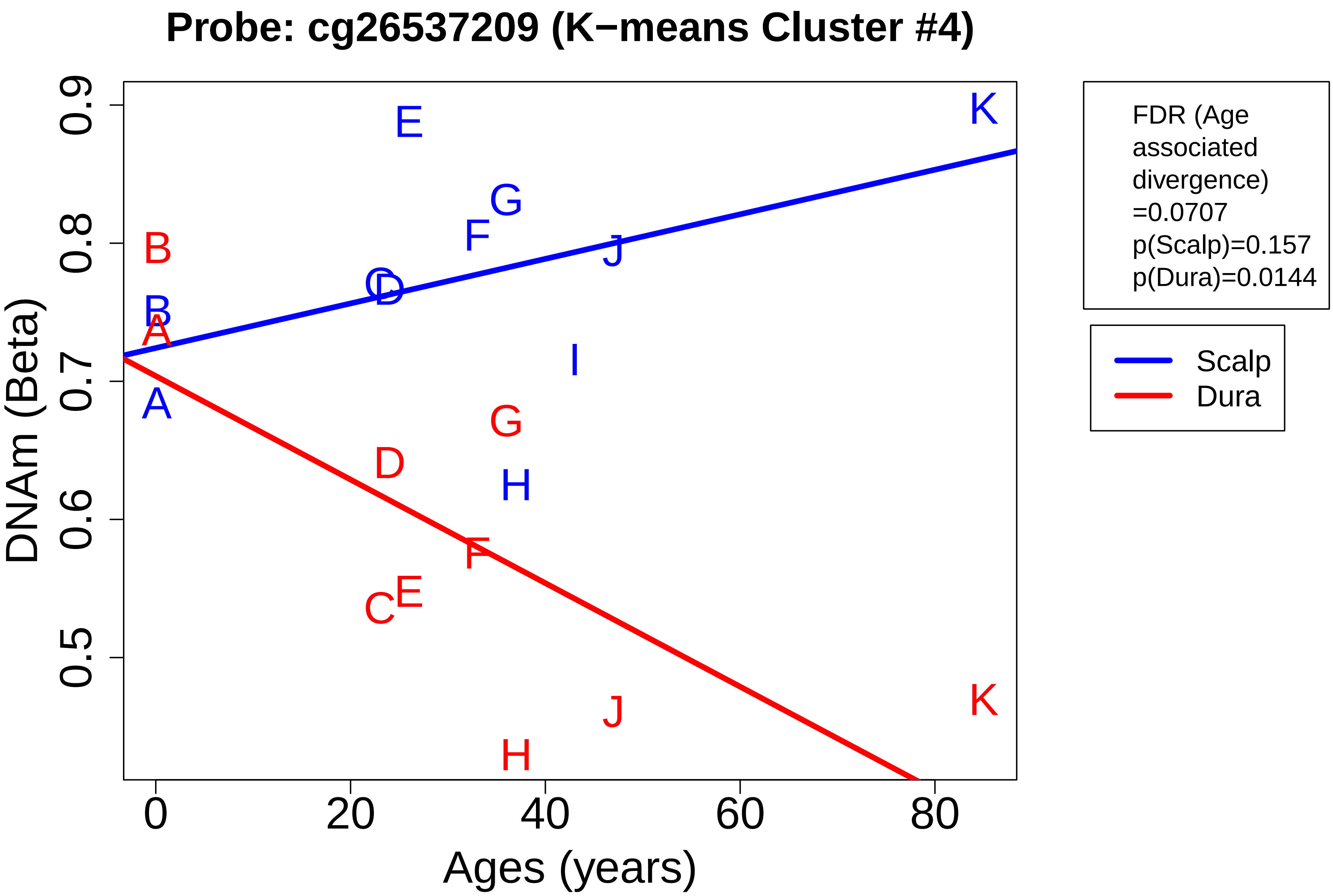
Probe: cg26537209 (K−means Cluster #4)
DNAm (Beta)
0.5
0.6
0.7
0.8
0.9
0
20
40
60
80
Ages (years)
FDR (Age associated divergence) =0.0707
p(Scalp)=0.157
p(Dura)=0.0144
Scalp
Dura

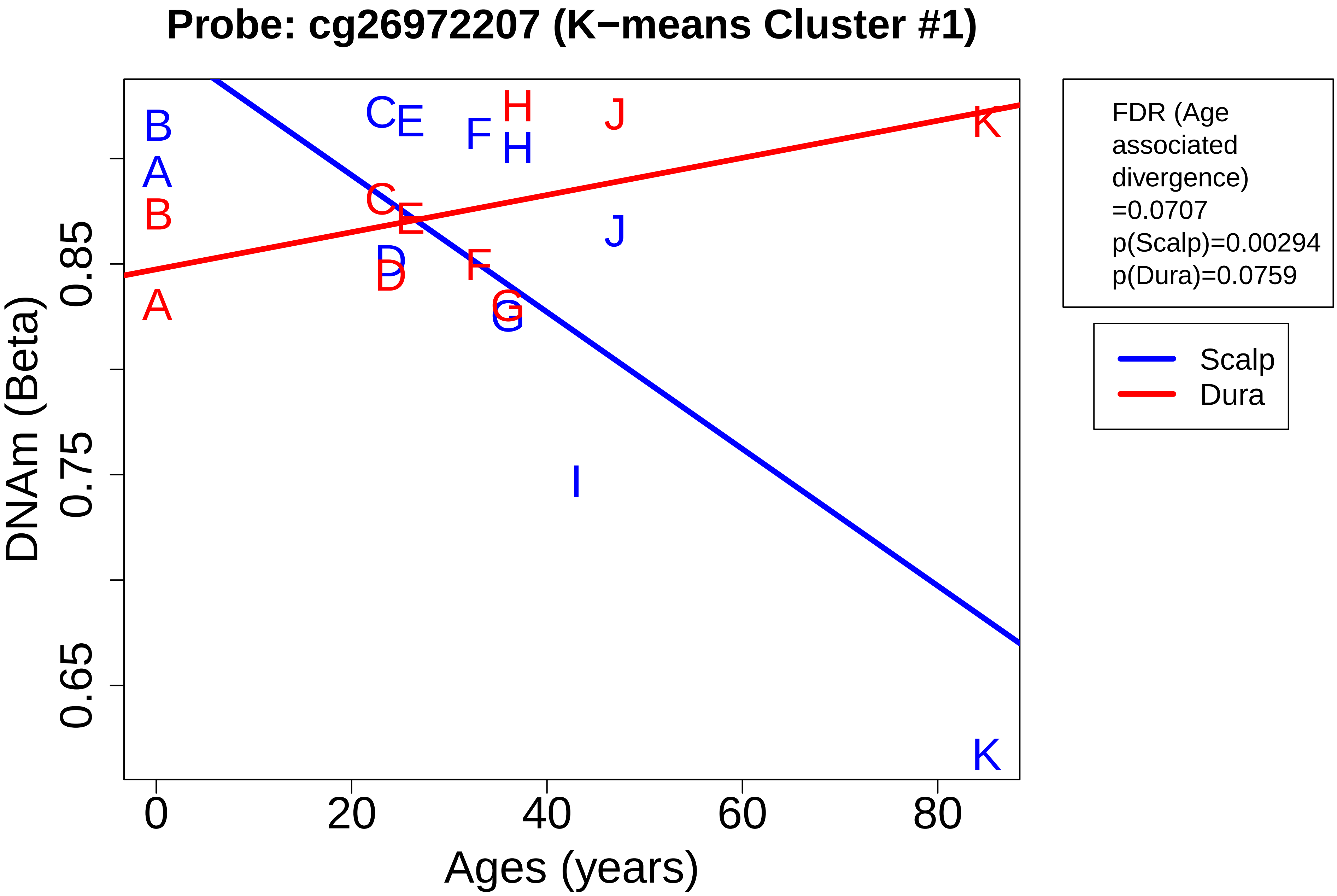
Probe: cg26972207 (K−means Cluster #1)
DNAm (Beta)
0.65
0.75
0.85
Ages (years)
0
20
40
60
80
FDR (Age associated divergence) =0.0707
p(Scalp)=0.00294
p(Dura)=0.0759
Scalp
Dura

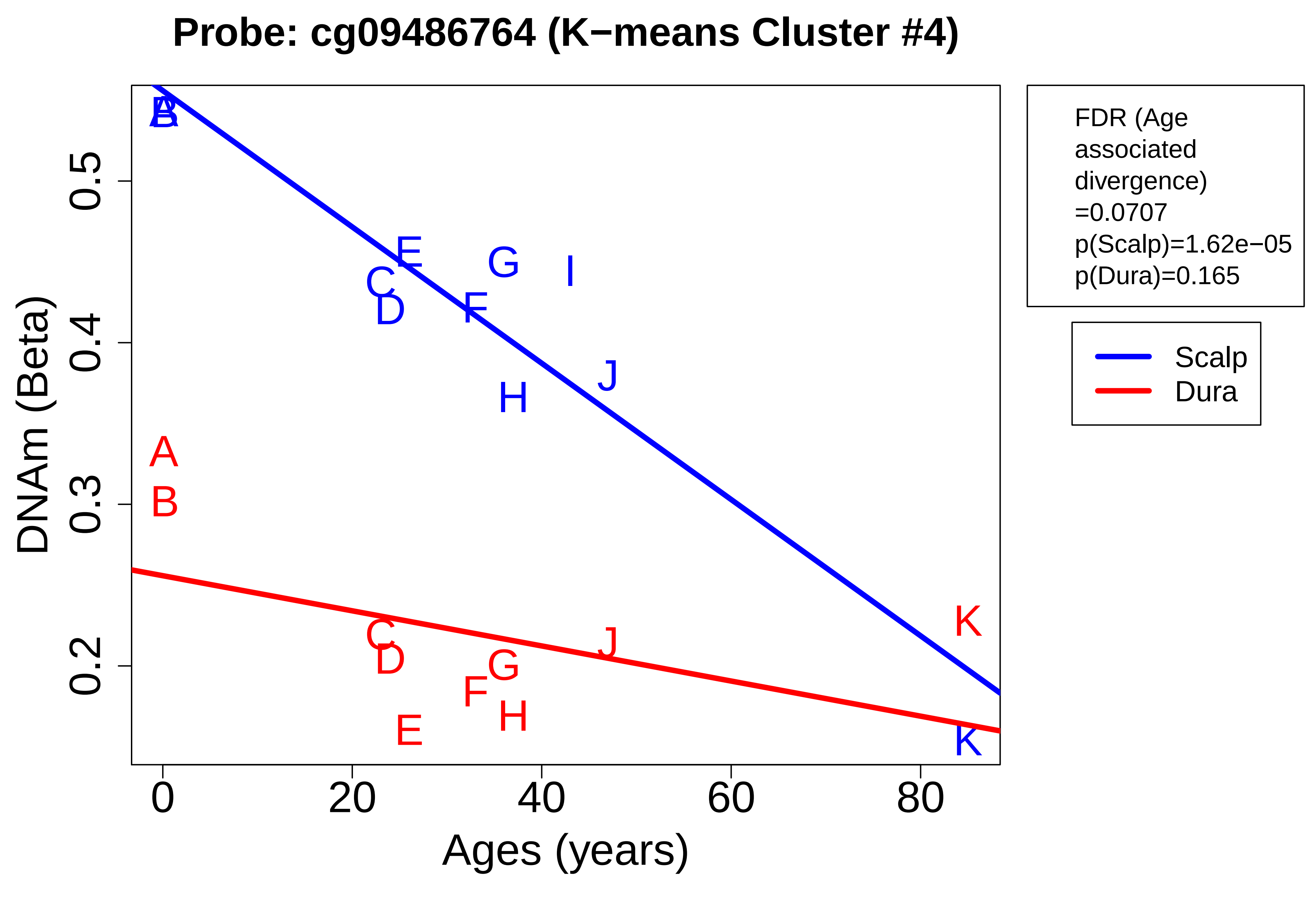

Probe: cg09486764 (K−means Cluster #4)
FDR (Age associated divergence) =0.0707
p(Scalp)=1.62e−05
p(Dura)=0.165
Scalp
Dura
DNAm (Beta)
0.2
0.3
0.4
0.5
0
20
40
60
80
Ages (years)

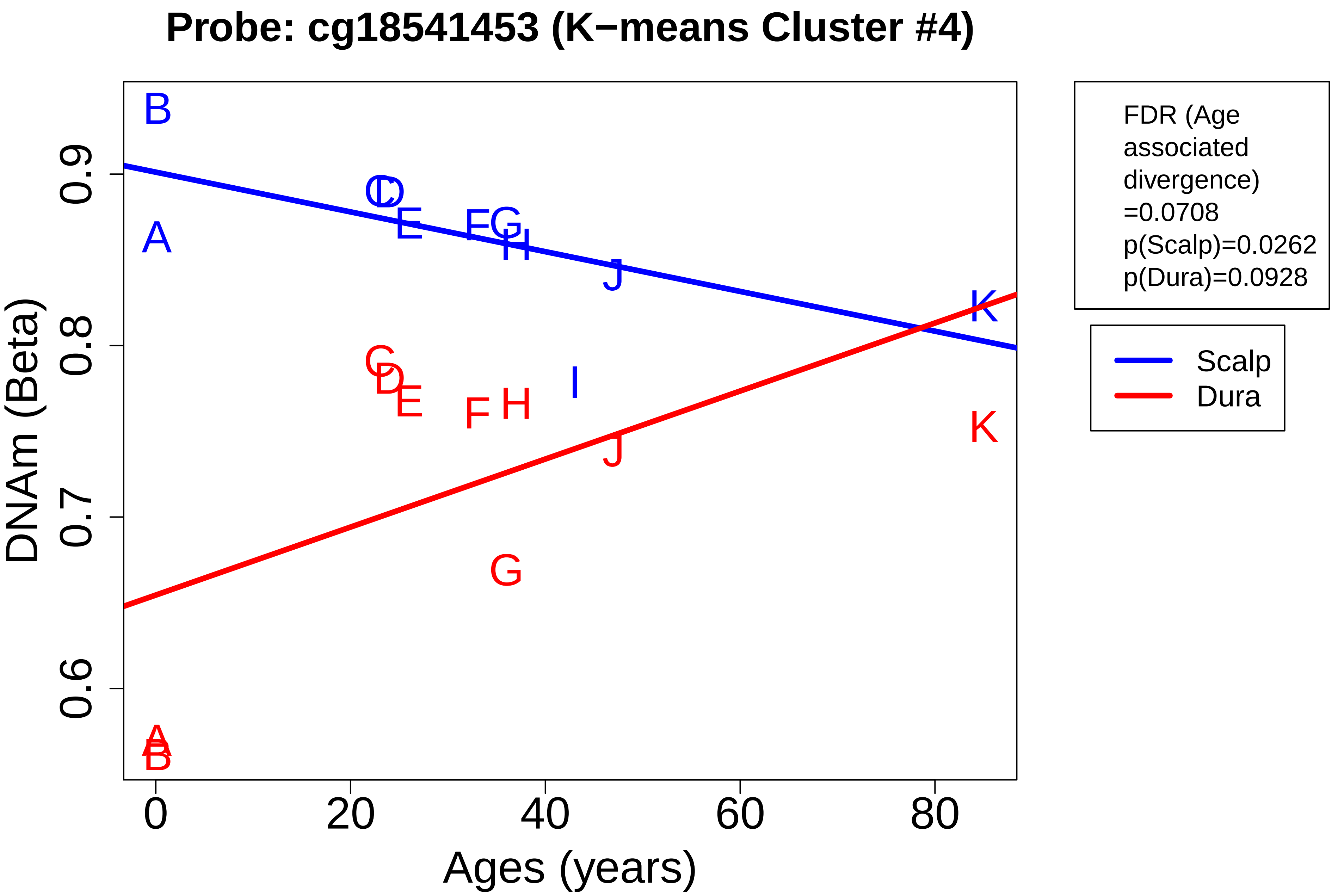

Probe: cg18541453 (K−means Cluster #4)
DNAm (Beta)
0.6
0.7
0.8
0.9
0
20
40
60
80
Ages (years)
A
B
C
D
E
F
G
H
I
J
K
FDR (Age associated divergence) =0.0708
p(Scalp)=0.0262
p(Dura)=0.0928
Scalp
Dura

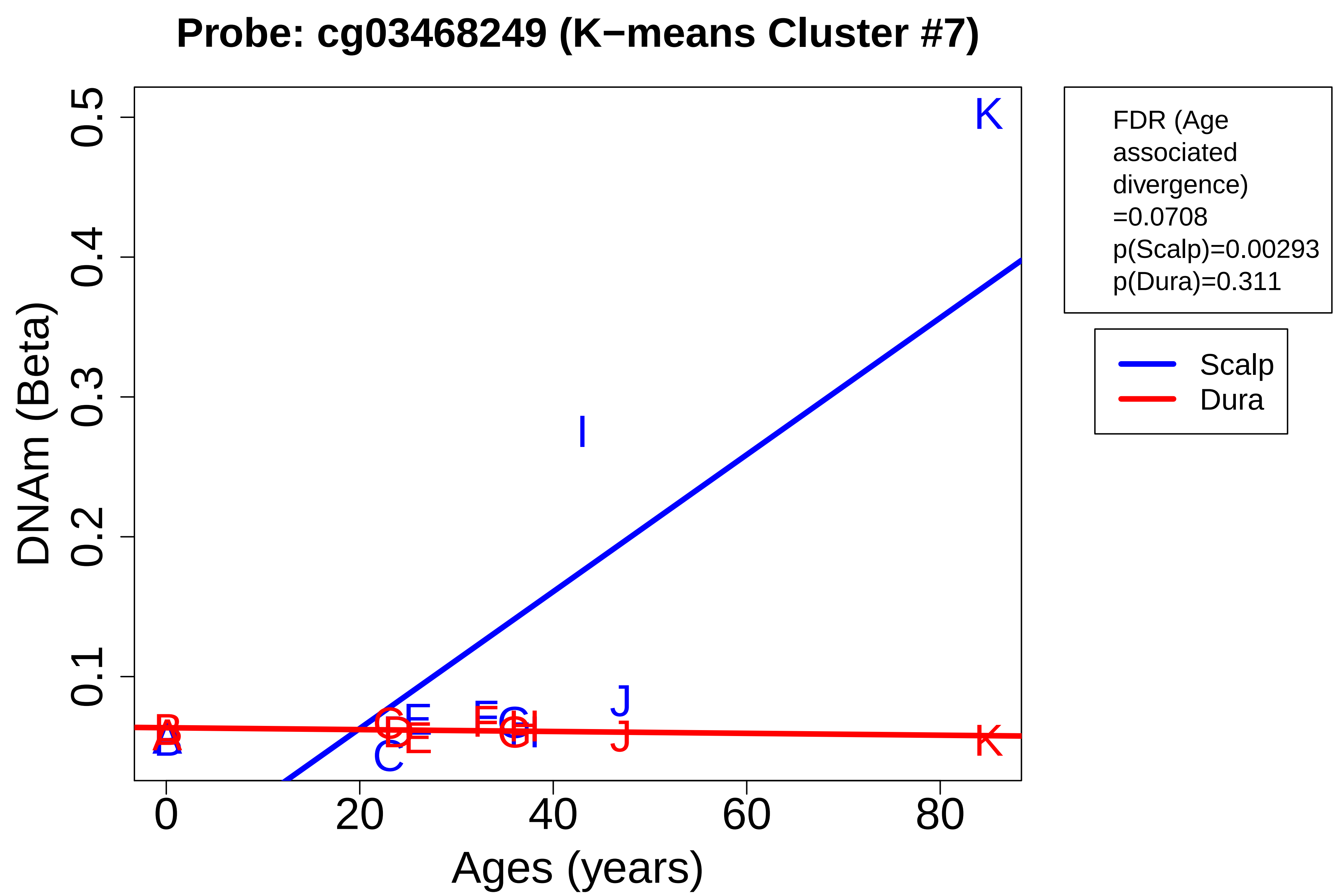

Probe: cg03468249 (K−means Cluster #7)
DNAm (Beta)
0.1
0.2
0.3
0.4
0.5
Ages (years)
0
20
40
60
80
FDR (Age associated divergence) =0.0708
p(Scalp)=0.00293
p(Dura)=0.311
Scalp
Dura

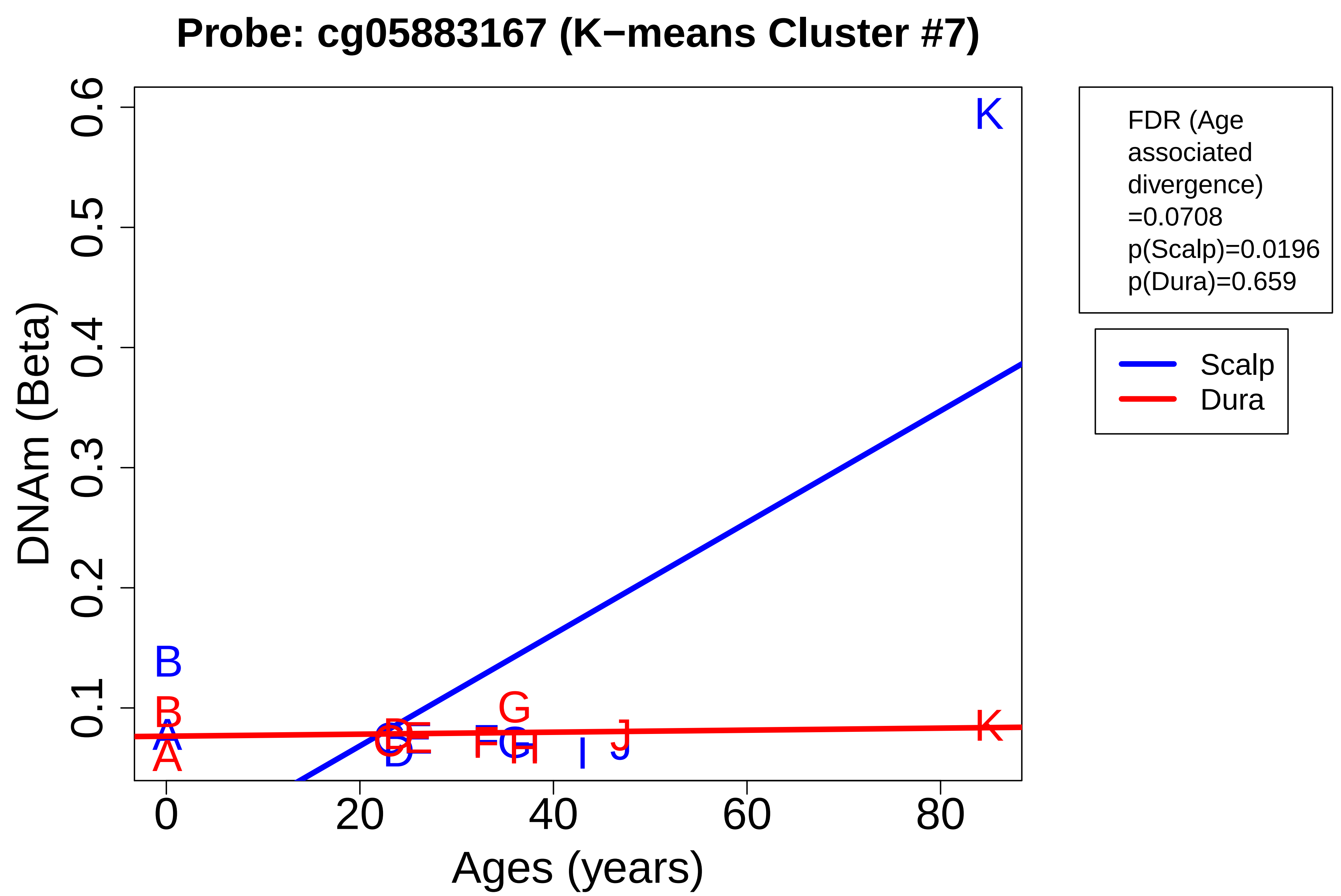

Probe: cg05883167 (K−means Cluster #7)
DNAm (Beta)
0.1
0.2
0.3
0.4
0.5
0.6
0
20
40
60
80
Ages (years)
FDR (Age associated divergence) =0.0708
p(Scalp)=0.0196
p(Dura)=0.659
Scalp
Dura

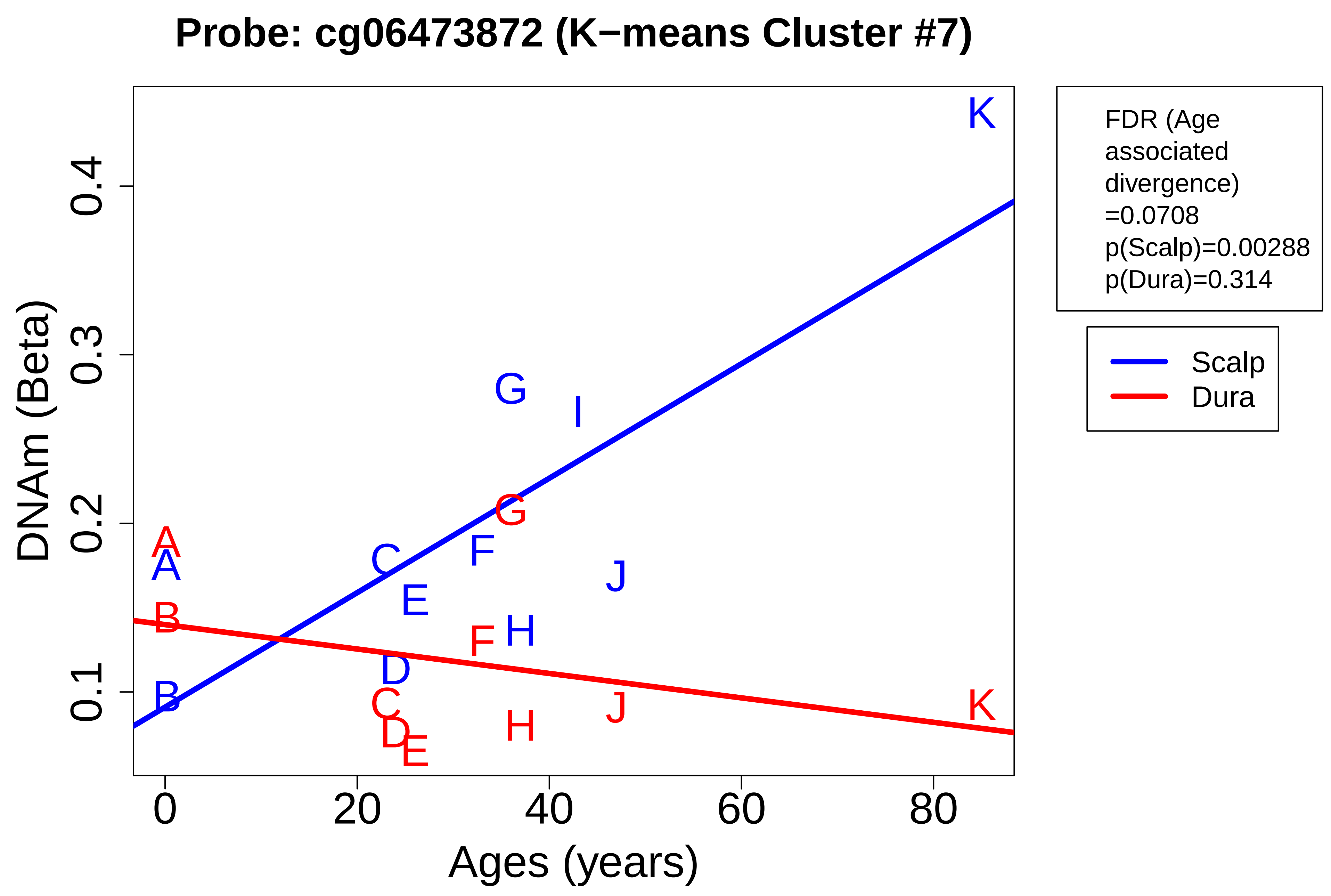

Probe: cg06473872 (K−means Cluster #7)
DNAm (Beta)
0.1
0.2
0.3
0.4
Ages (years)
0
20
40
60
80
A
B
C
D
E
F
G
H
I
J
K
FDR (Age associated divergence) =0.0708
p(Scalp)=0.00288
p(Dura)=0.314
Scalp
Dura

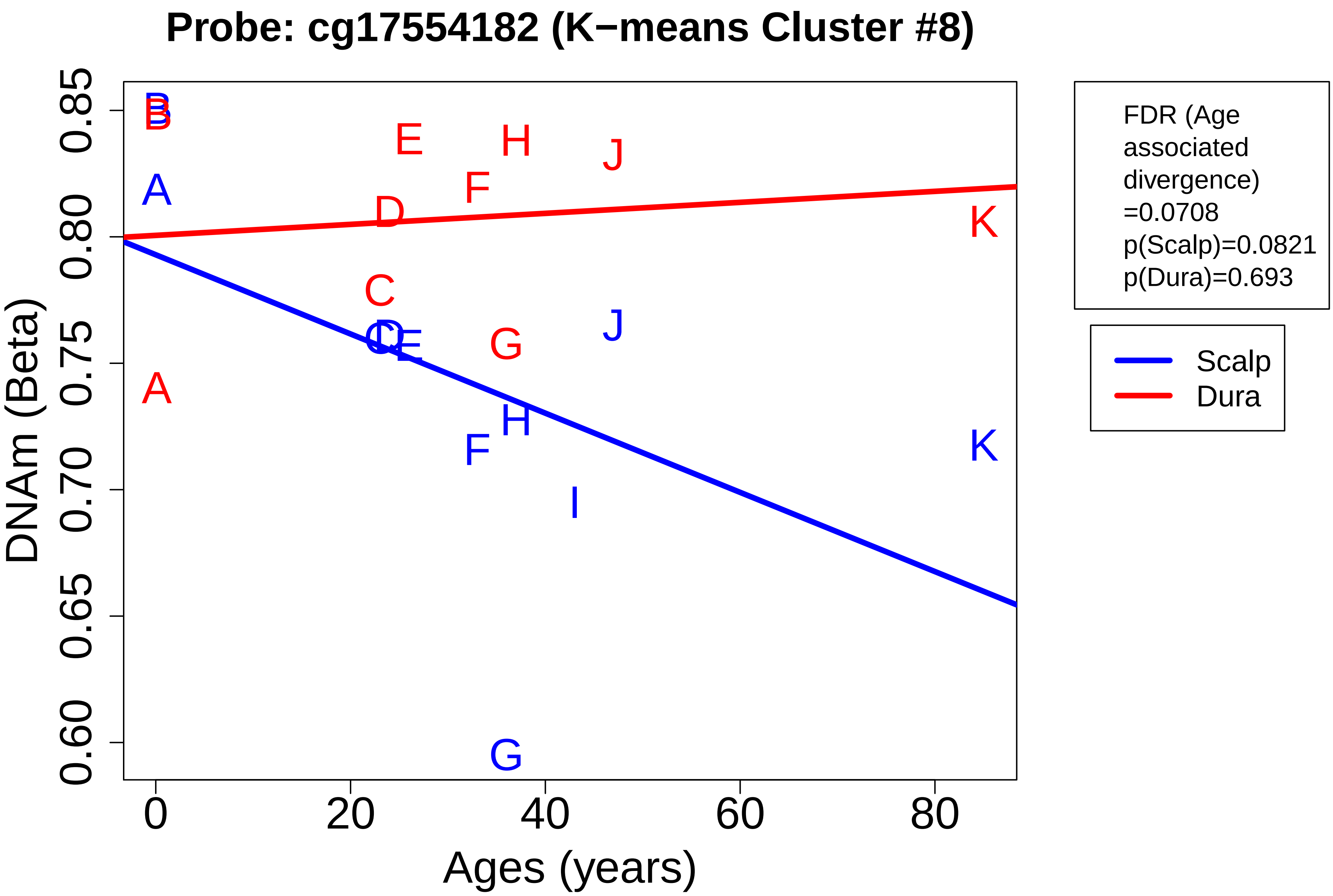

Probe: cg17554182 (K−means Cluster #8)
DNAm (Beta)
Ages (years)
0.60
0.65
0.70
0.75
0.80
0.85
0
20
40
60
80
FDR (Age associated divergence) =0.0708
p(Scalp)=0.0821
p(Dura)=0.693
Scalp
Dura

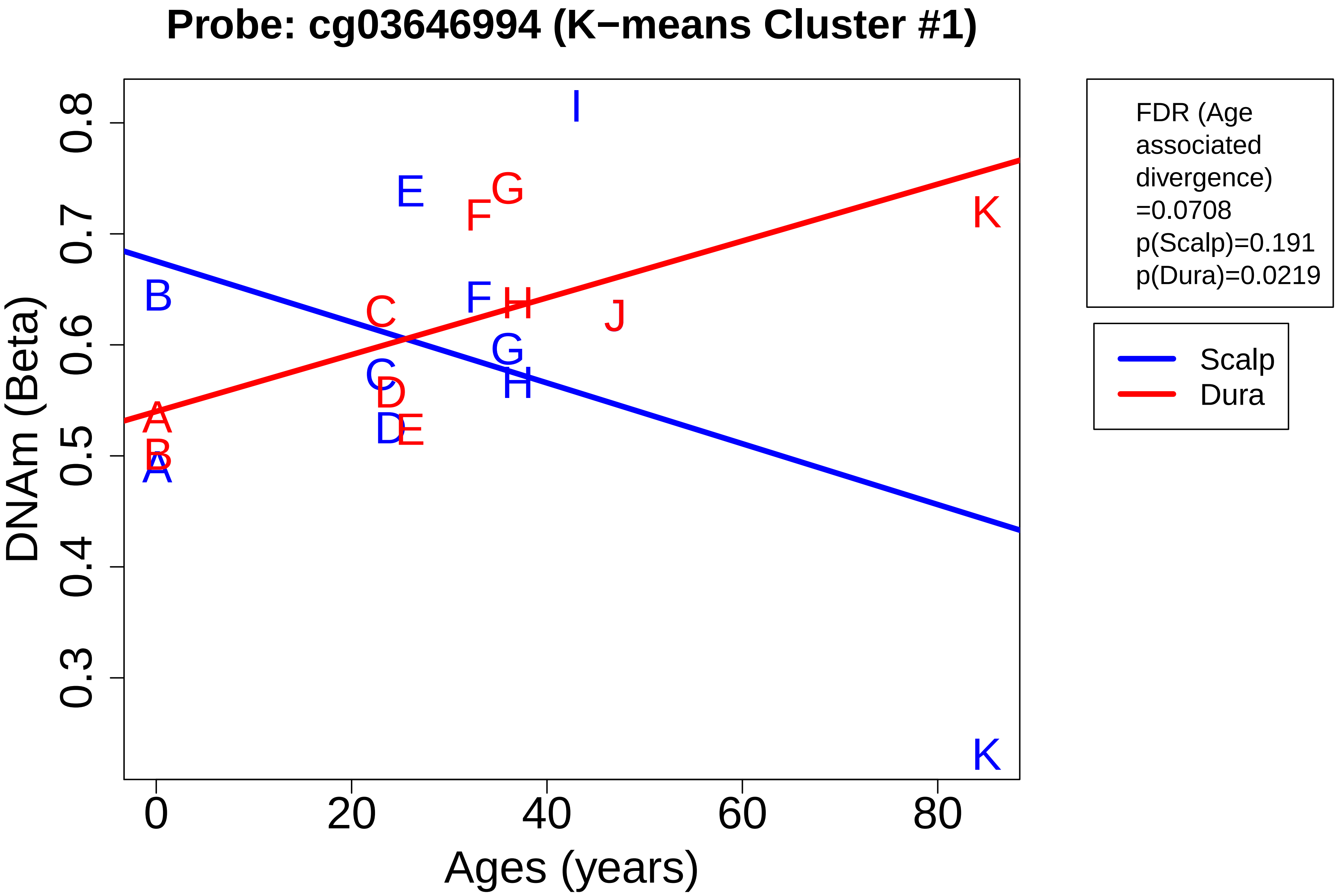

Probe: cg03646994 (K−means Cluster #1)
DNAm (Beta)
0.3
0.4
0.5
0.6
0.7
0.8
0
20
40
60
80
Ages (years)
FDR (Age associated divergence) =0.0708
p(Scalp)=0.191
p(Dura)=0.0219
Scalp
Dura
A
B
C
D
E
F
G
H
I
J
K

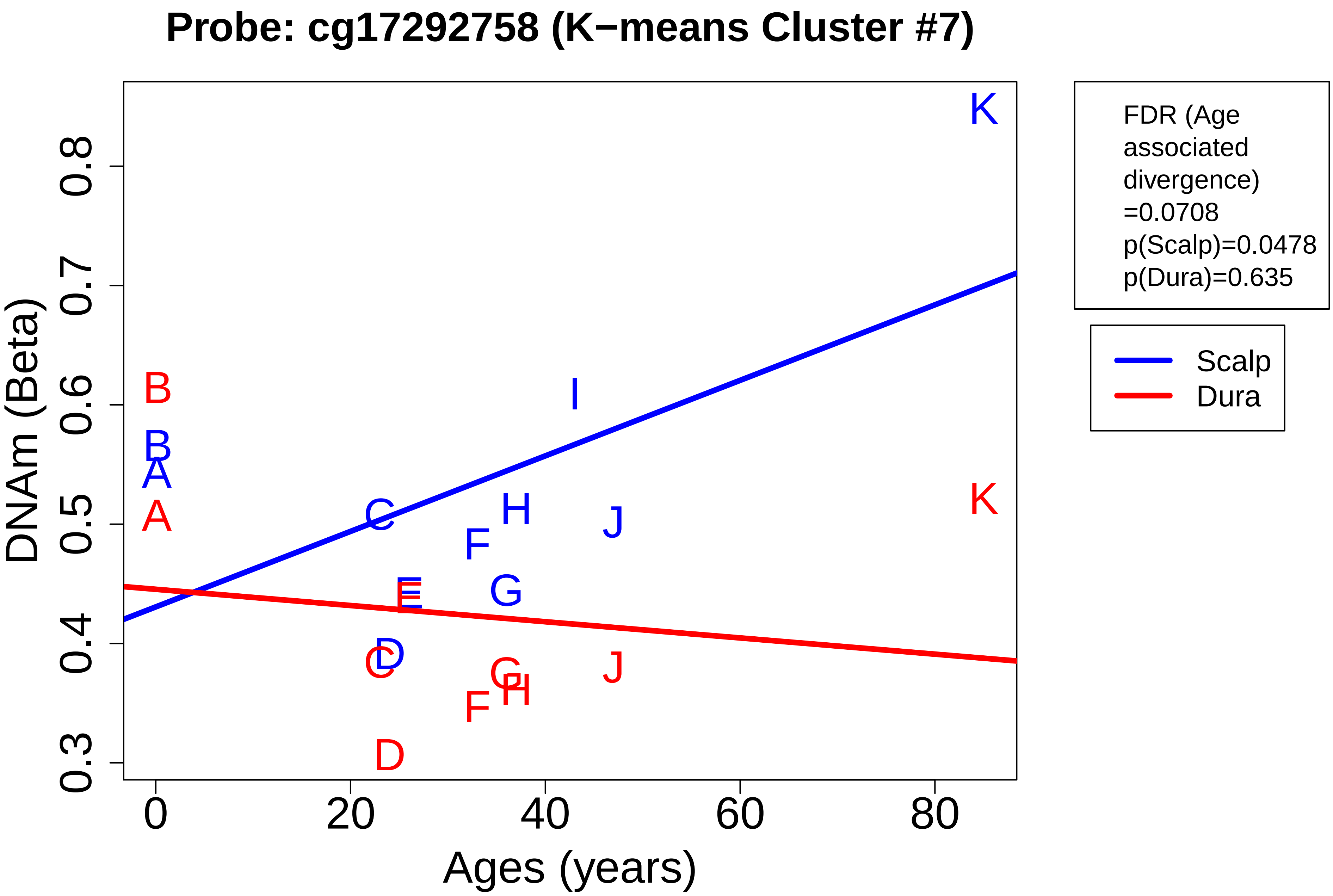

Probe: cg17292758 (K−means Cluster #7)
DNAm (Beta)
0.3
0.4
0.5
0.6
0.7
0.8
0
20
40
60
80
Ages (years)
FDR (Age associated divergence) =0.0708
p(Scalp)=0.0478
p(Dura)=0.635
Scalp
Dura
A
B
C
D
E
F
G
H
I
J
K

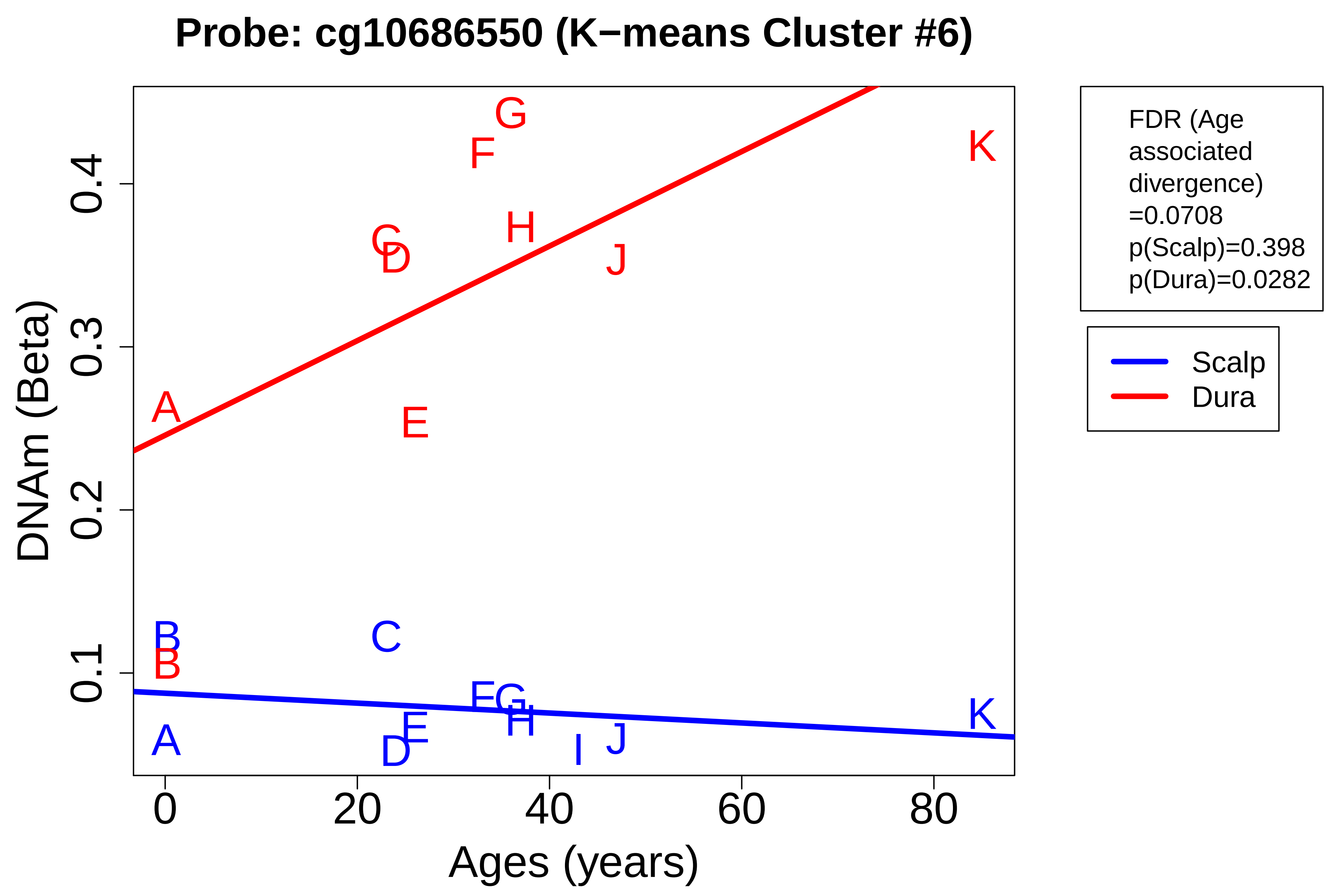

Probe: cg10686550 (K−means Cluster #6)
DNAm (Beta)
0.1
0.2
0.3
0.4
0
20
40
60
80
Ages (years)
A
B
C
D
E
F
G
H
I
J
K
FDR (Age associated divergence) =0.0708
p(Scalp)=0.398
p(Dura)=0.0282
Scalp
Dura

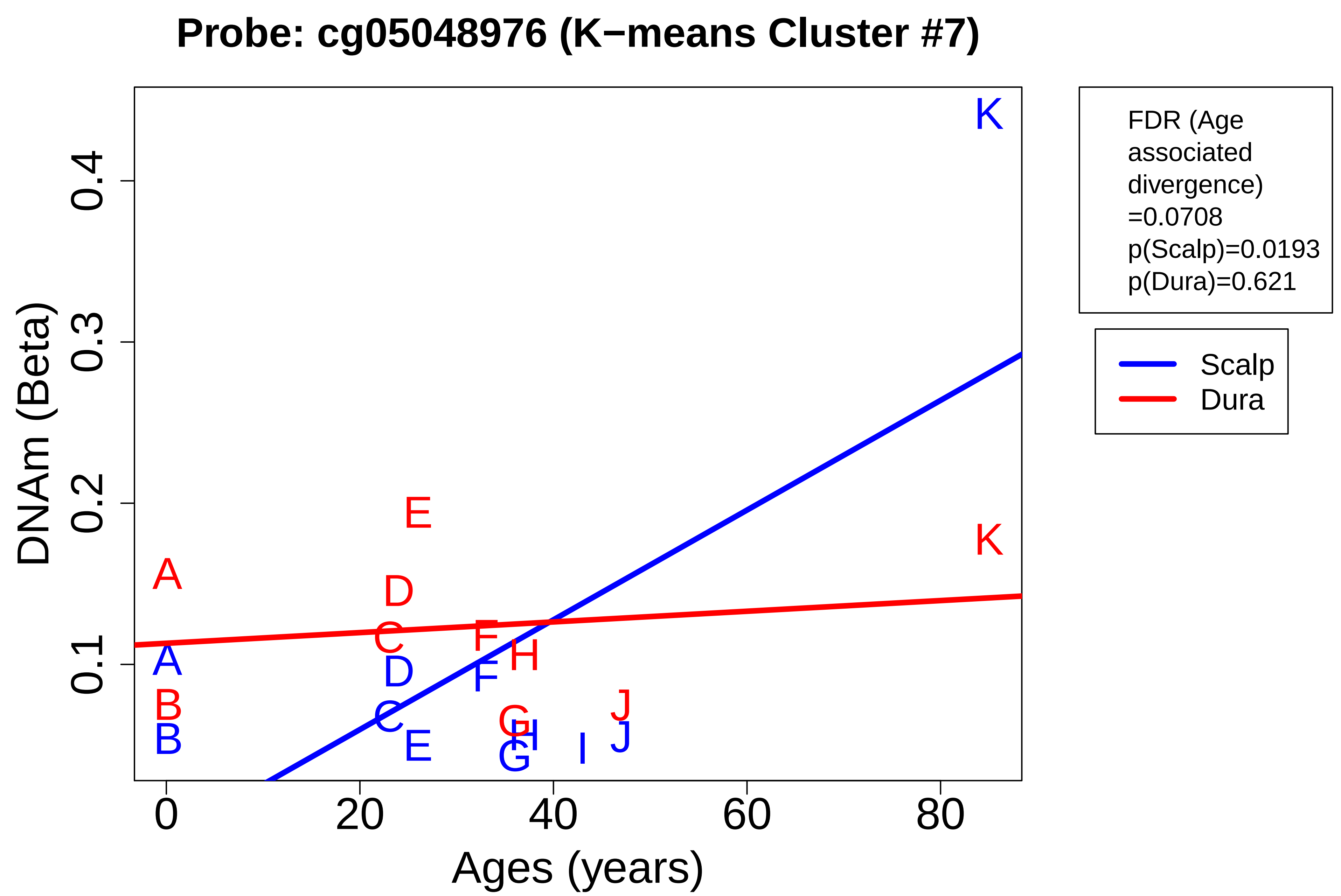

Probe: cg05048976 (K−means Cluster #7)
DNAm (Beta)
0.1
0.2
0.3
0.4
0
20
40
60
80
Ages (years)
FDR (Age associated divergence) =0.0708
p(Scalp)=0.0193
p(Dura)=0.621
Scalp
Dura
A
B
C
D
E
F
G
H
I
J
K

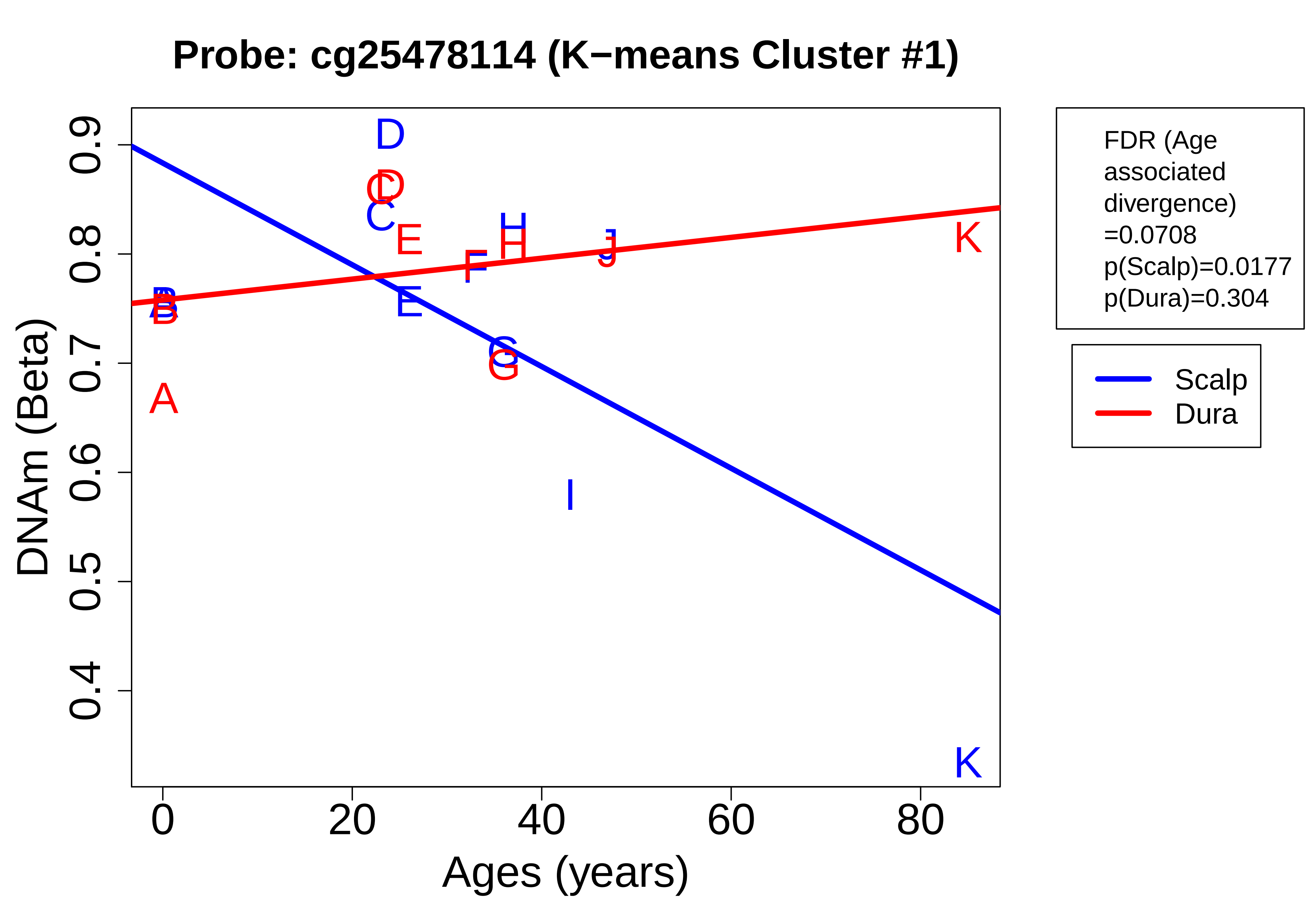

Probe: cg25478114 (K−means Cluster #1)
DNAm (Beta)
Ages (years)
0.4
0.5
0.6
0.7
0.8
0.9
0
20
40
60
80
FDR (Age associated divergence) =0.0708
p(Scalp)=0.0177
p(Dura)=0.304
Scalp
Dura

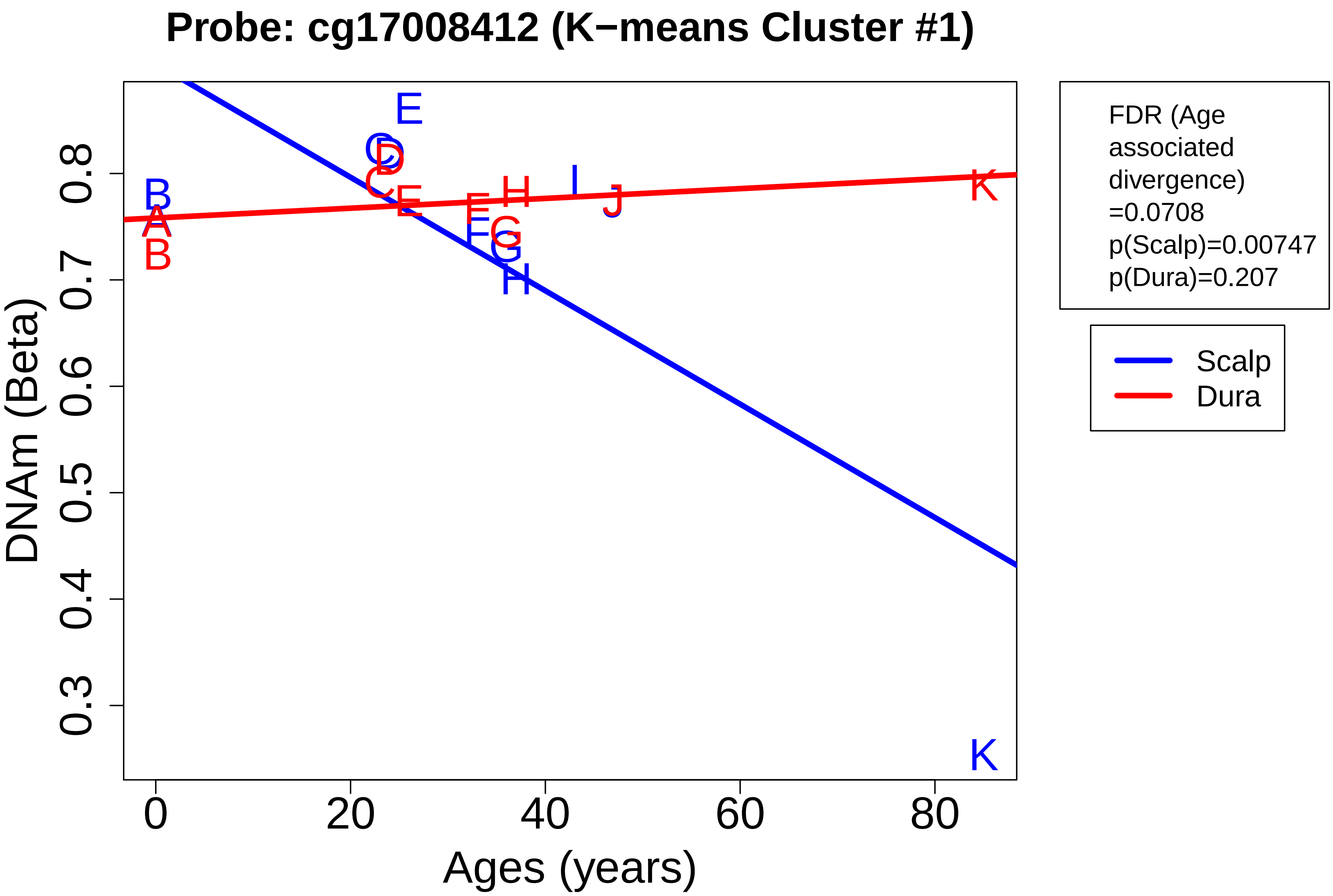

Probe: cg17008412 (K−means Cluster #1)
DNAm (Beta)
0.3
0.4
0.5
0.6
0.7
0.8
0
20
40
60
80
Ages (years)
FDR (Age associated divergence) =0.0708
p(Scalp)=0.00747
p(Dura)=0.207
Scalp
Dura

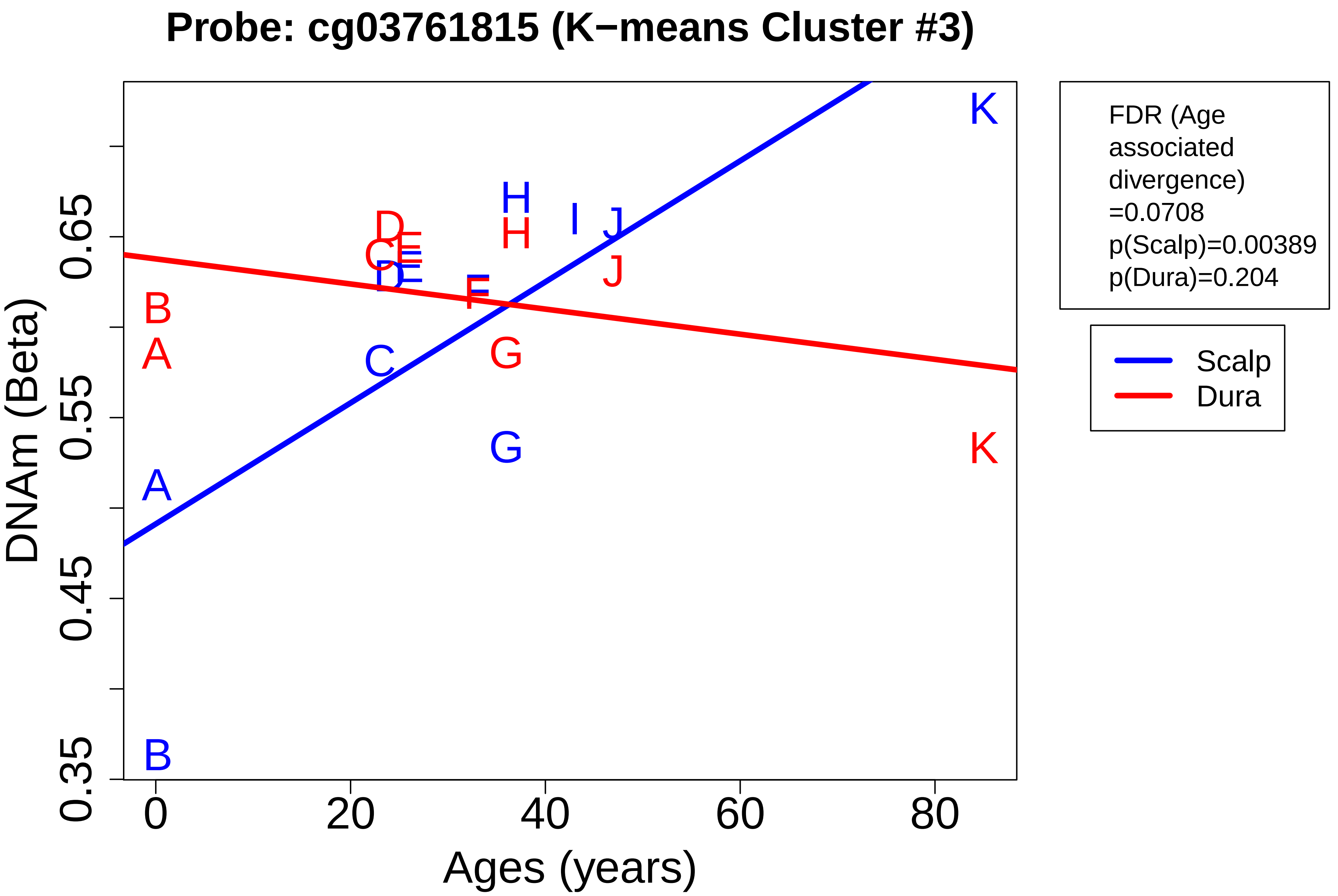

Probe: cg03761815 (K−means Cluster #3)
DNAm (Beta)
0.35
0.45
0.55
0.65
0
20
40
60
80
Ages (years)
FDR (Age associated divergence) =0.0708
p(Scalp)=0.00389
p(Dura)=0.204
Scalp
Dura

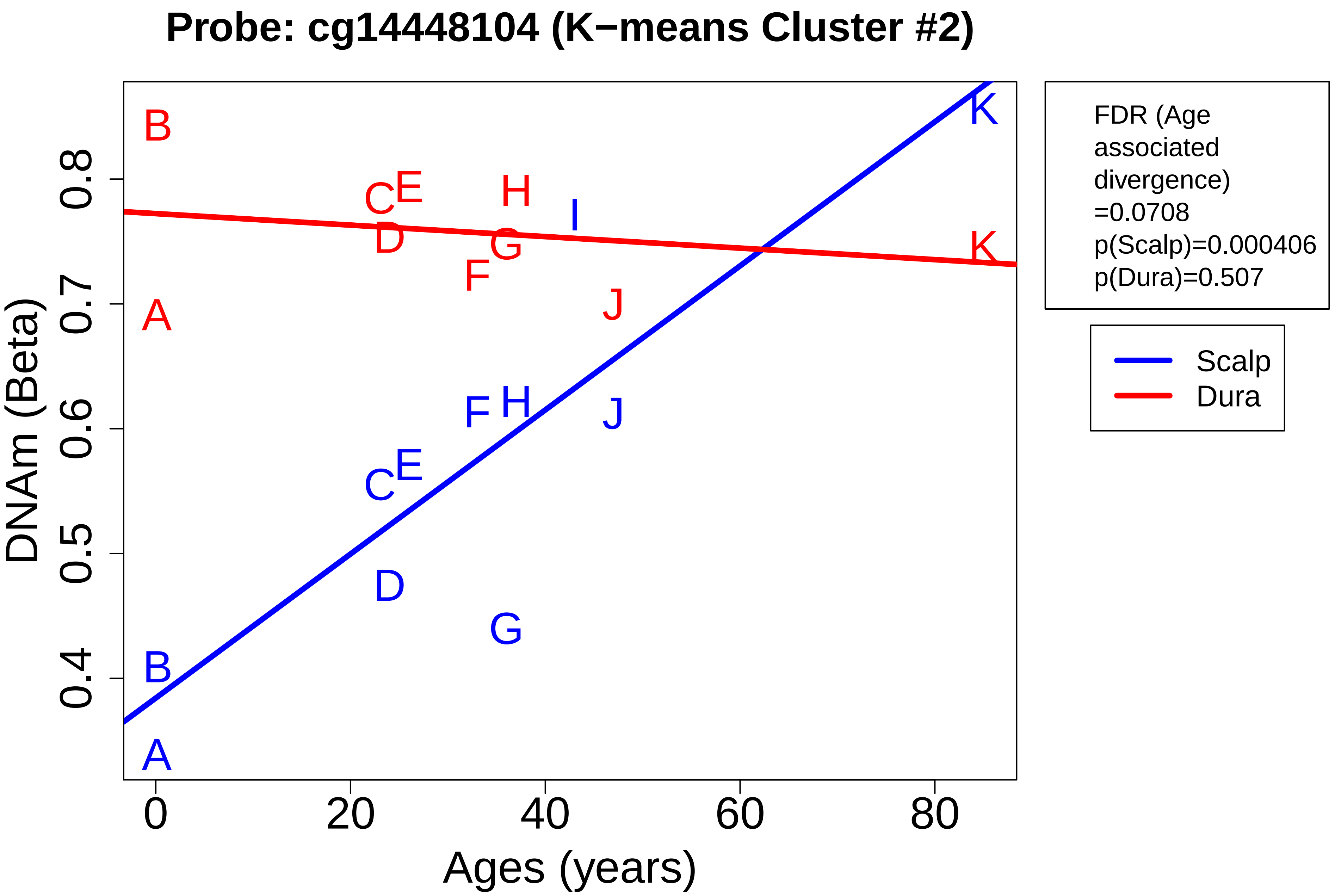

Probe: cg14448104 (K−means Cluster #2)
DNAm (Beta)
Ages (years)
0.4
0.5
0.6
0.7
0.8
0
20
40
60
80
FDR (Age associated divergence) =0.0708
p(Scalp)=0.000406
p(Dura)=0.507
Scalp
Dura
A
B
C
D
E
F
G
H
I
J
K

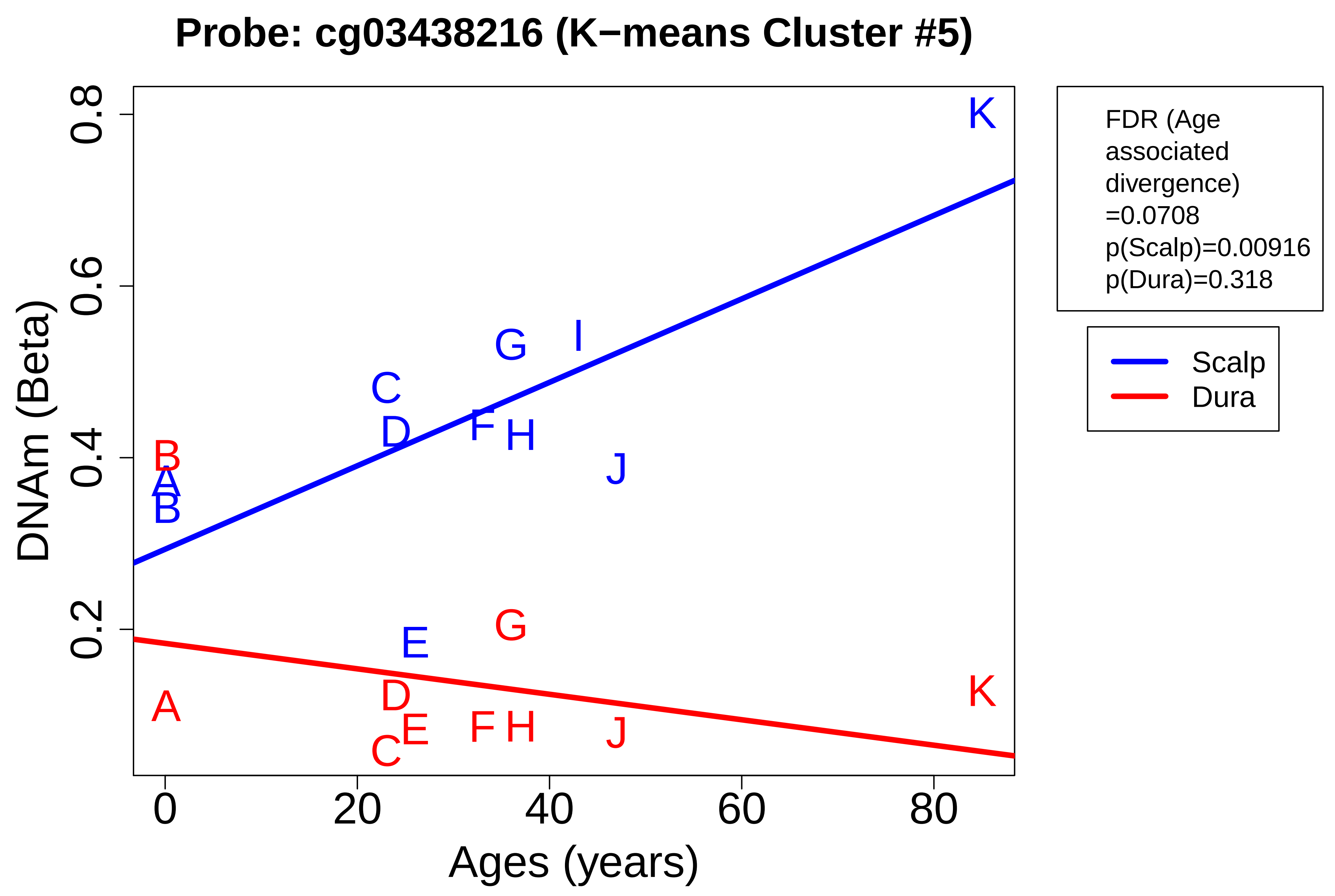

Probe: cg03438216 (K−means Cluster #5)
DNAm (Beta)
0.2
0.4
0.6
0.8
0
20
40
60
80
Ages (years)
FDR (Age associated divergence) =0.0708
p(Scalp)=0.00916
p(Dura)=0.318
Scalp
Dura
A
B
C
D
E
F
G
H
I
J
K

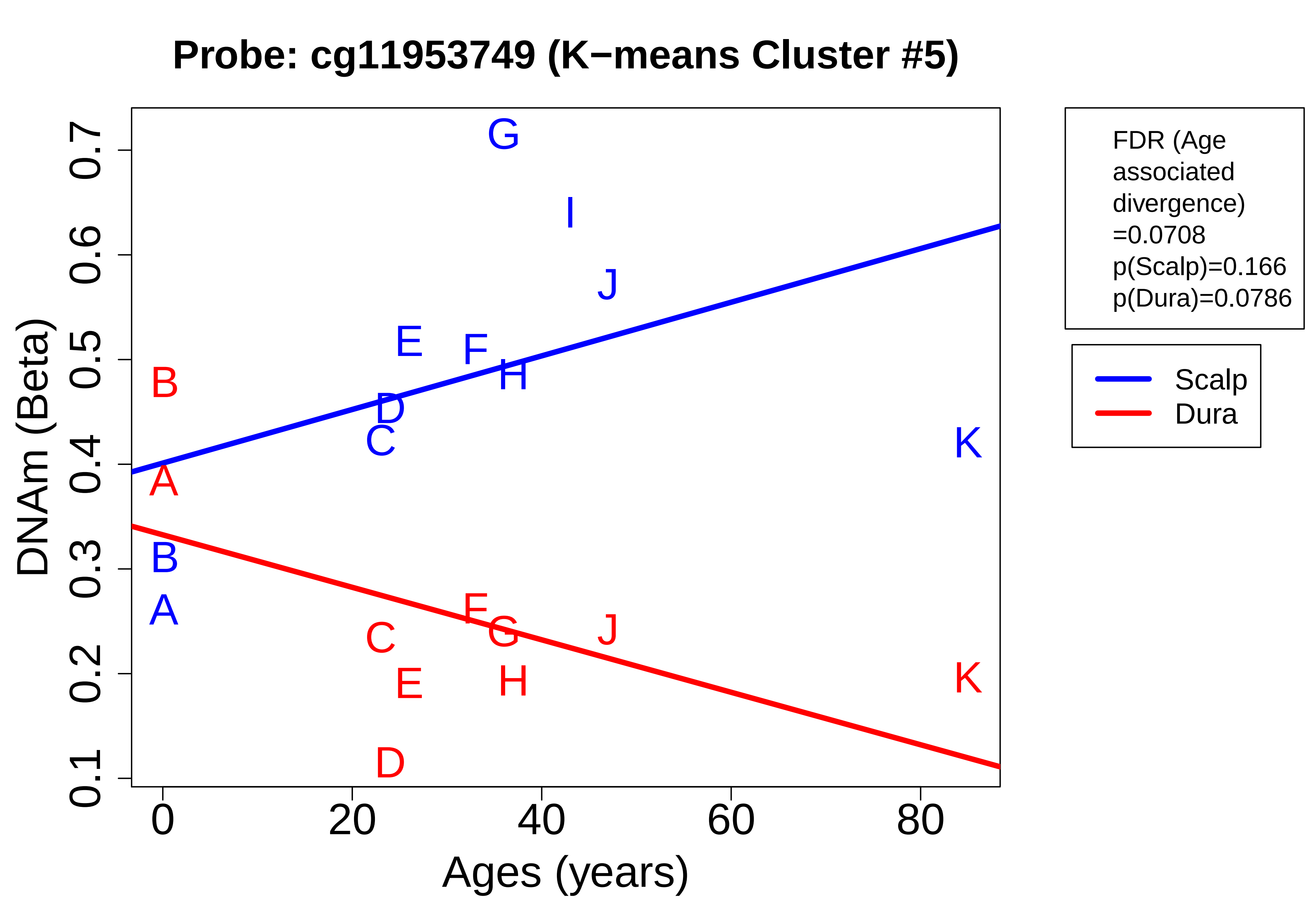

Probe: cg11953749 (K−means Cluster #5)
DNAm (Beta)
Ages (years)
0.1
0.2
0.3
0.4
0.5
0.6
0.7
0
20
40
60
80
FDR (Age associated divergence) =0.0708
p(Scalp)=0.166
p(Dura)=0.0786
Scalp
Dura
A
B
C
D
E
F
G
H
I
J
K

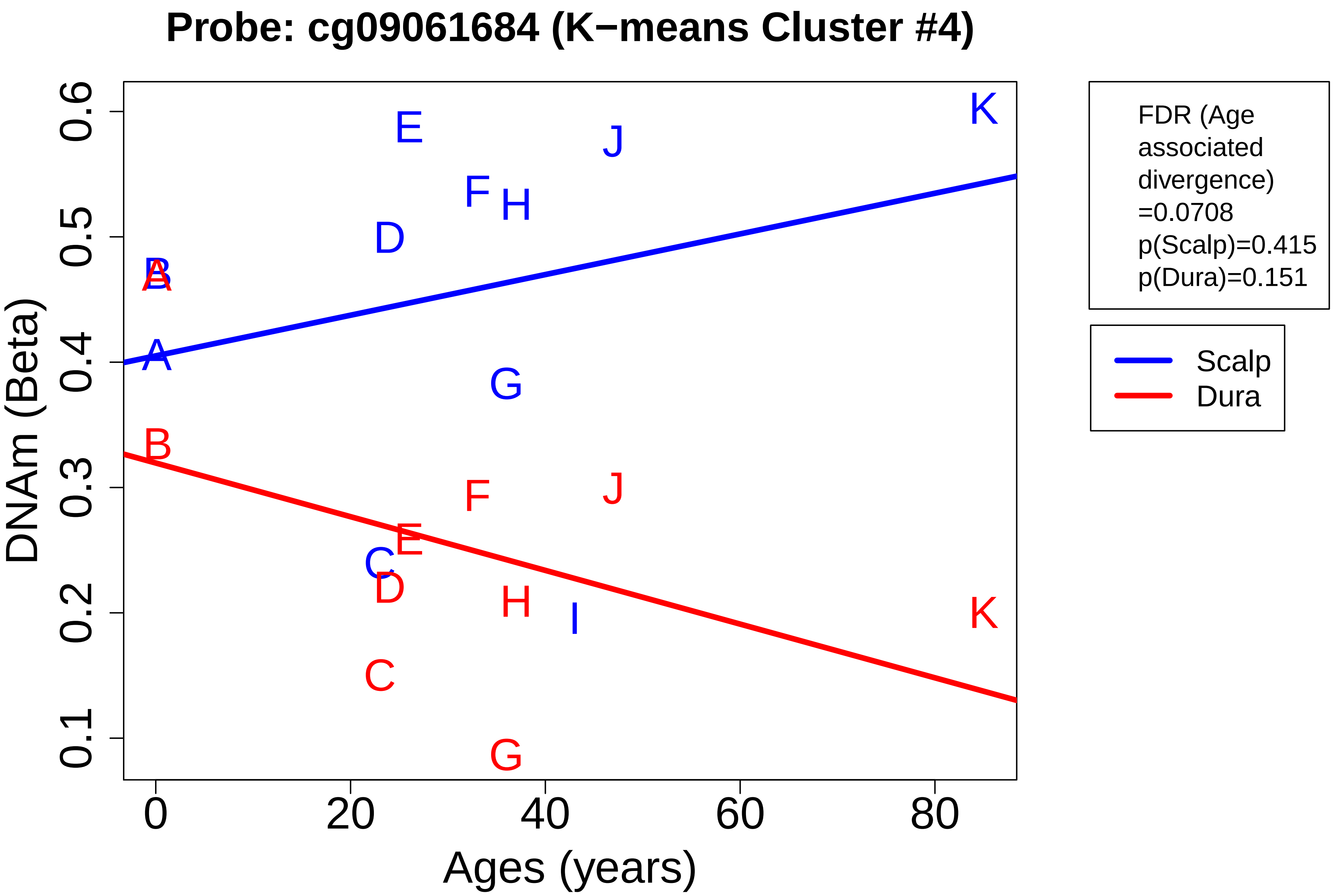
Probe: cg09061684 (K−means Cluster #4)
DNAm (Beta)
Ages (years)
0.1
0.2
0.3
0.4
0.5
0.6
0
20
40
60
80
FDR (Age associated divergence) =0.0708
p(Scalp)=0.415
p(Dura)=0.151
Scalp
Dura
A
B
C
D
E
F
G
H
I
J
K

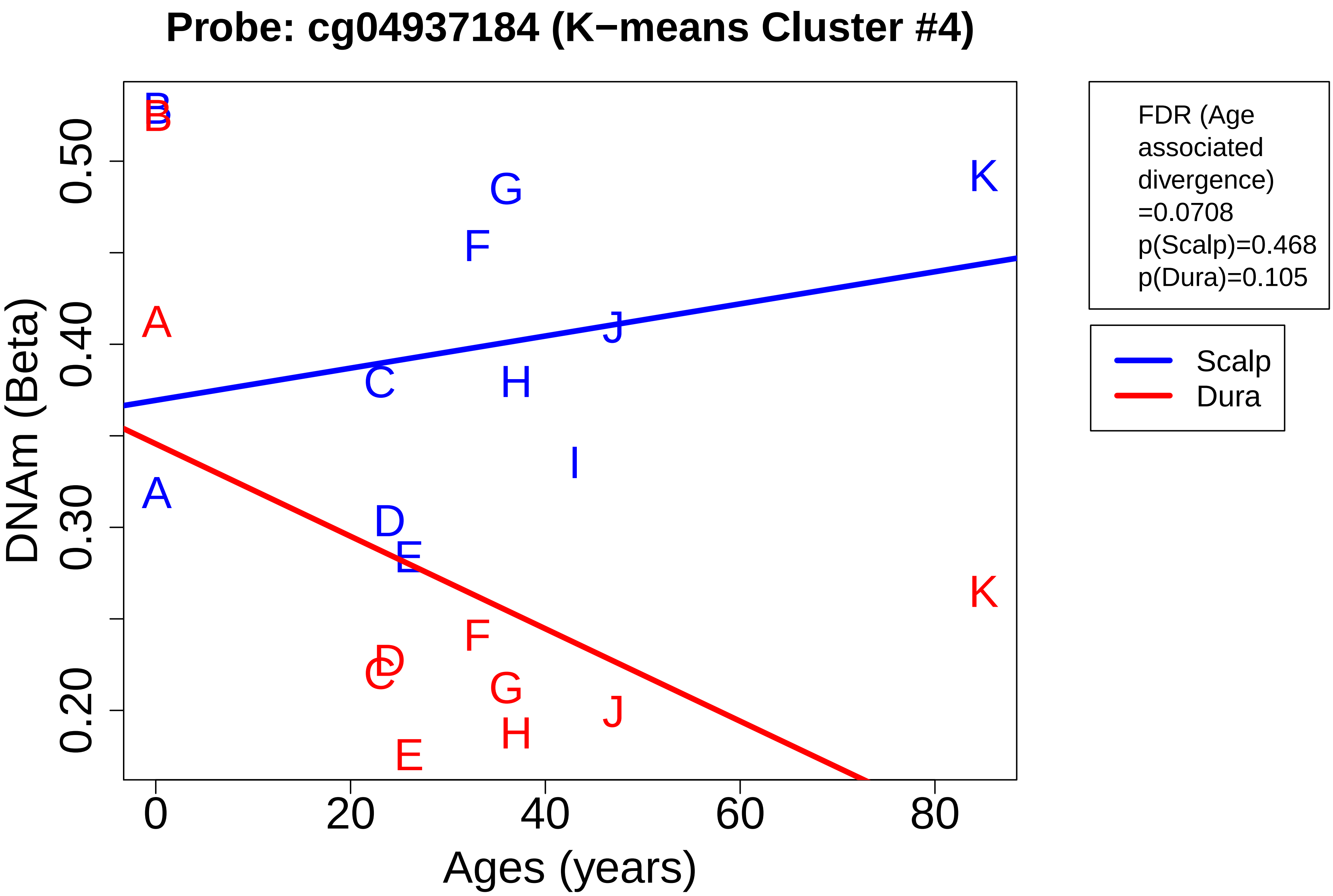

Probe: cg04937184 (K−means Cluster #4)
DNAm (Beta)
0.20
0.30
0.40
0.50
0
20
40
60
80
Ages (years)
FDR (Age associated divergence) =0.0708
p(Scalp)=0.468
p(Dura)=0.105
Scalp
Dura
A
B
C
D
E
F
G
H
I
J
K

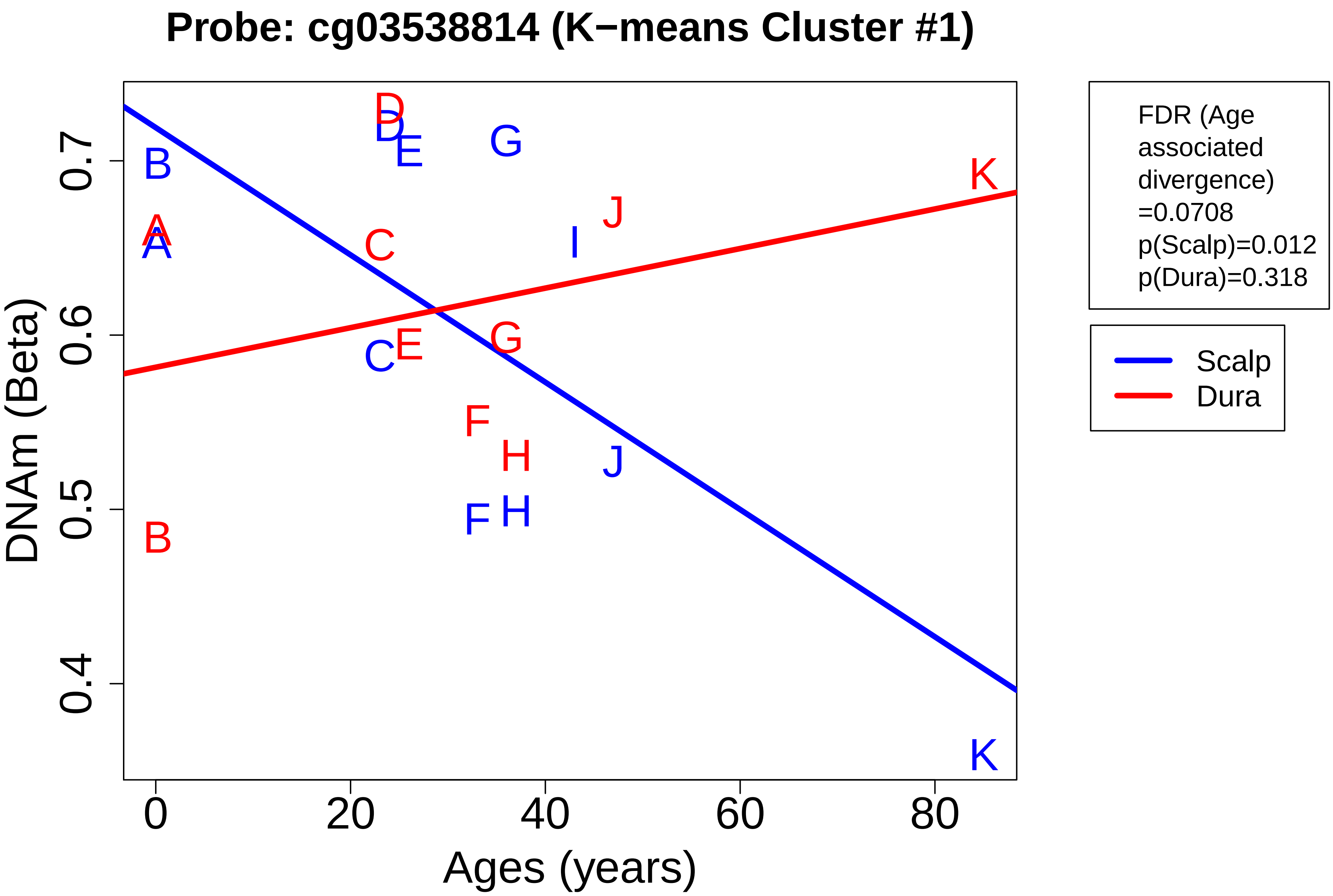
Probe: cg03538814 (K−means Cluster #1)
DNAm (Beta)
0.4
0.5
0.6
0.7
0
20
40
60
80
Ages (years)
A
B
C
D
E
F
G
H
I
J
K
FDR (Age associated divergence) =0.0708
p(Scalp)=0.012
p(Dura)=0.318
Scalp
Dura

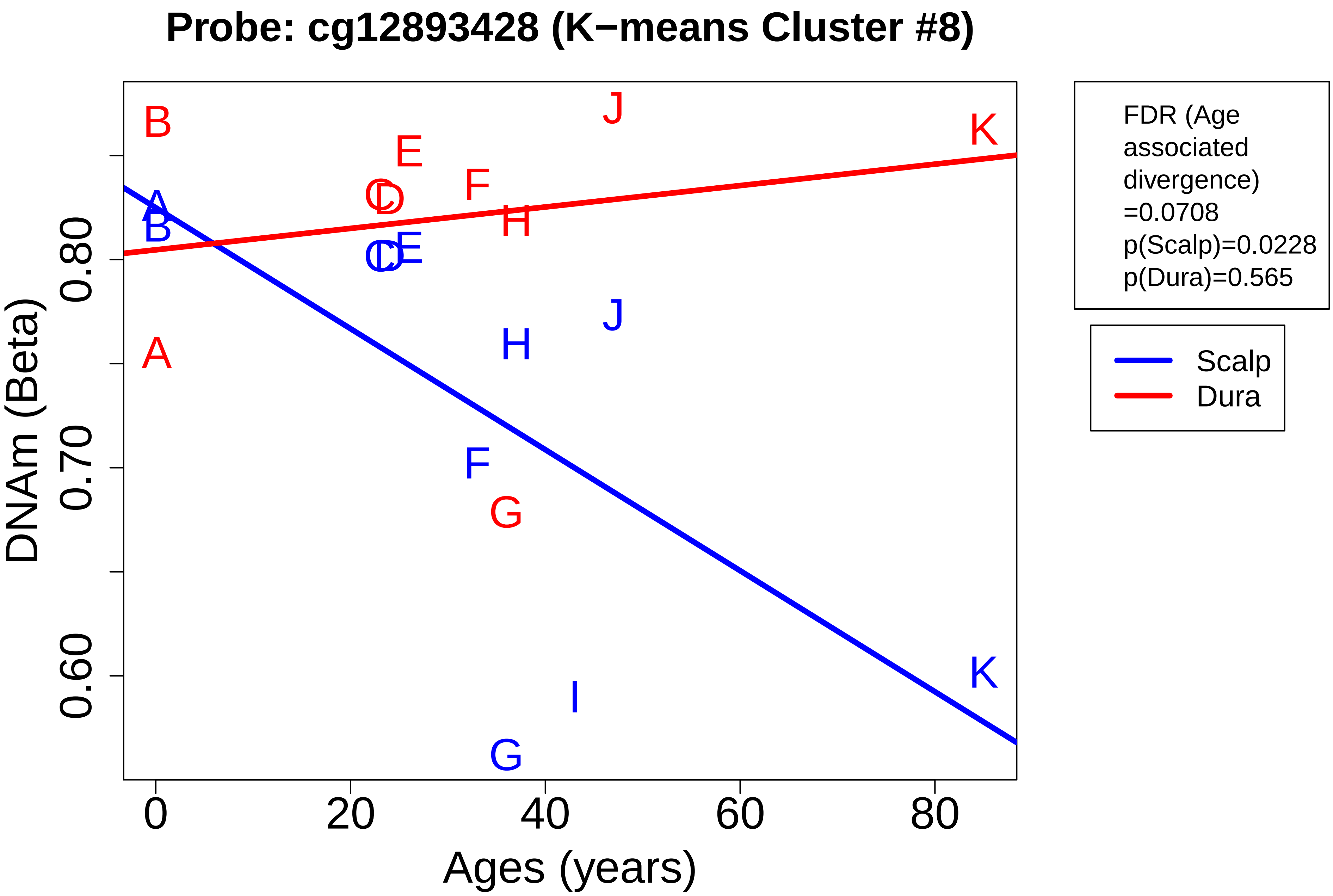

Probe: cg12893428 (K−means Cluster #8)
DNAm (Beta)
0.60
0.70
0.80
0
20
40
60
80
Ages (years)
FDR (Age associated divergence) =0.0708
p(Scalp)=0.0228
p(Dura)=0.565
Scalp
Dura

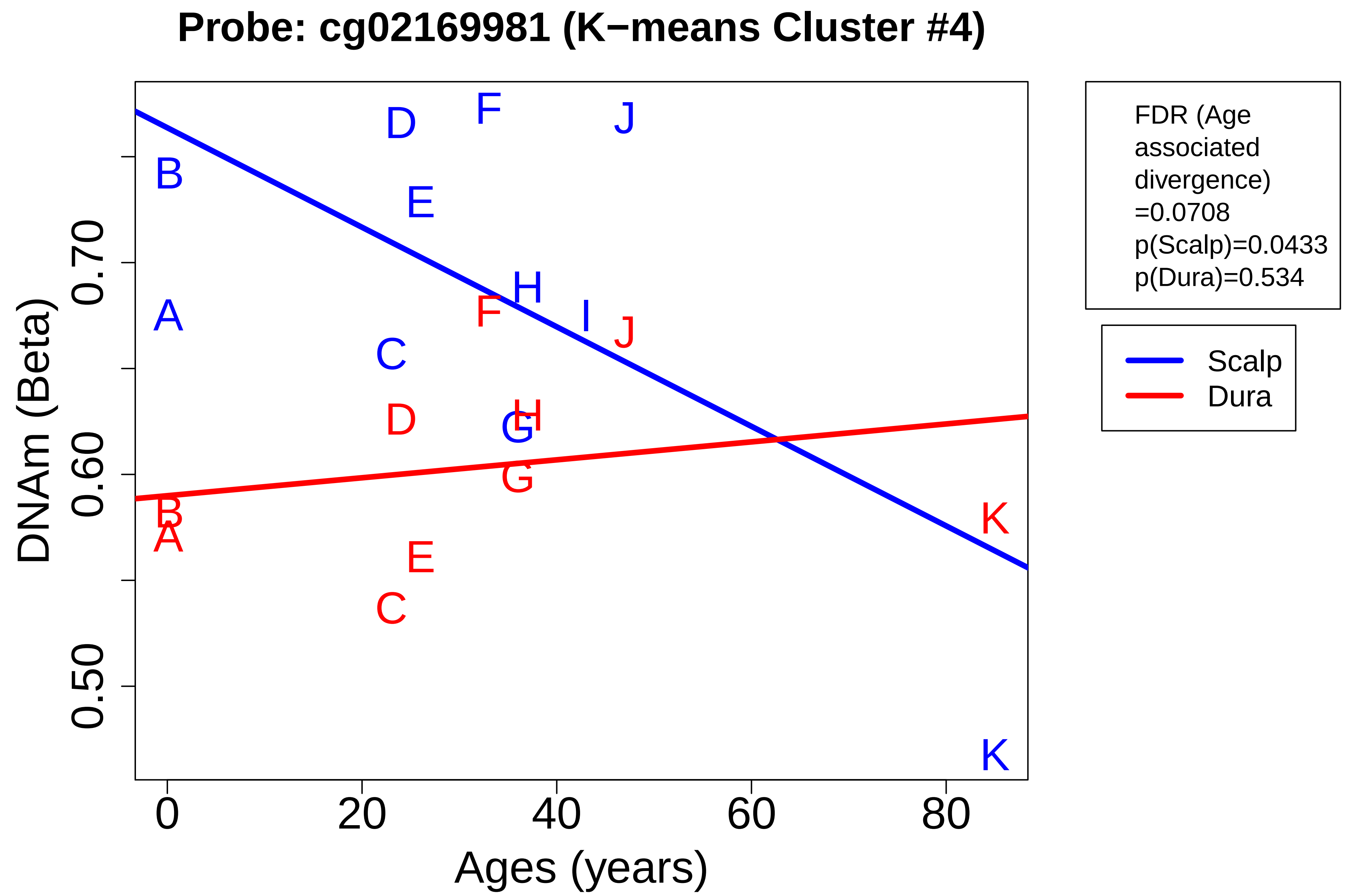

Probe: cg02169981 (K−means Cluster #4)
DNAm (Beta)
0.50
0.60
0.70
Ages (years)
0
20
40
60
80
FDR (Age associated divergence) =0.0708
p(Scalp)=0.0433
p(Dura)=0.534
Scalp
Dura

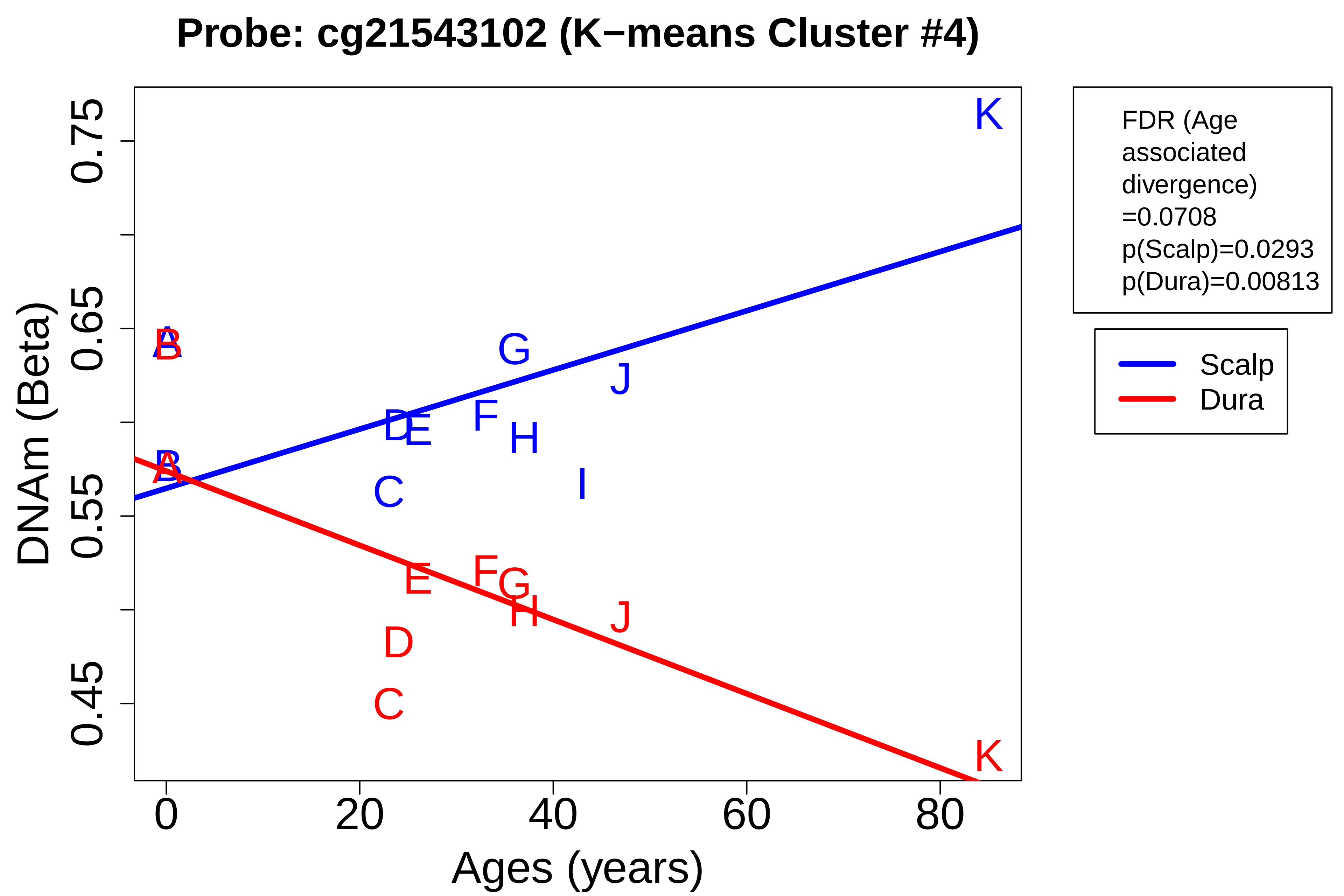

Probe: cg21543102 (K−means Cluster #4)
FDR (Age associated divergence) =0.0708
p(Scalp)=0.0293
p(Dura)=0.00813
Scalp
Dura
DNAm (Beta)
0.45
0.55
0.65
0.75
0
20
40
60
80
Ages (years)
A
B
C
D
E
F
G
H
I
J
K

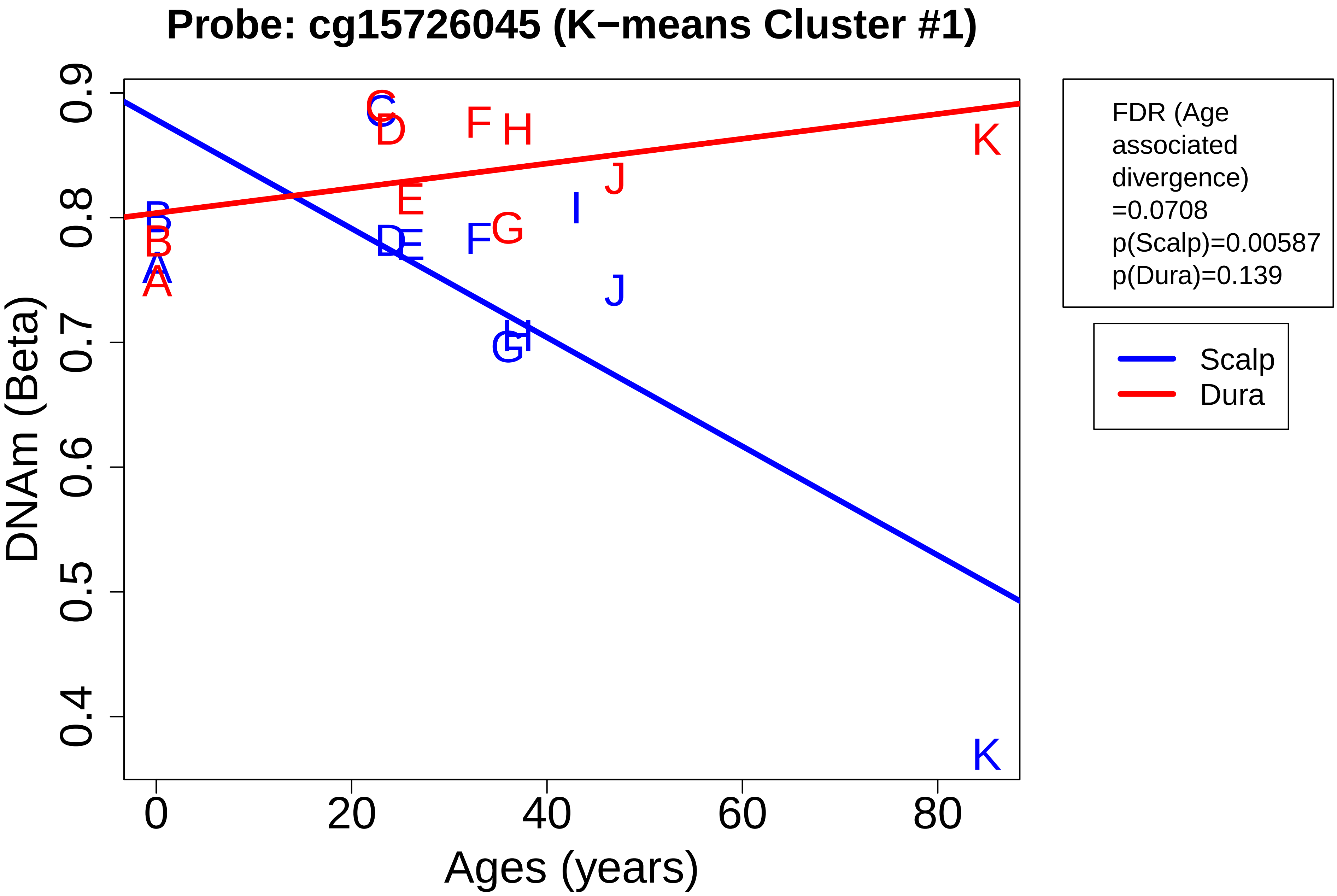

Probe: cg15726045 (K−means Cluster #1)
DNAm (Beta)
0.4
0.5
0.6
0.7
0.8
0.9
0
20
40
60
80
Ages (years)
FDR (Age associated divergence) =0.0708
p(Scalp)=0.00587
p(Dura)=0.139
Scalp
Dura

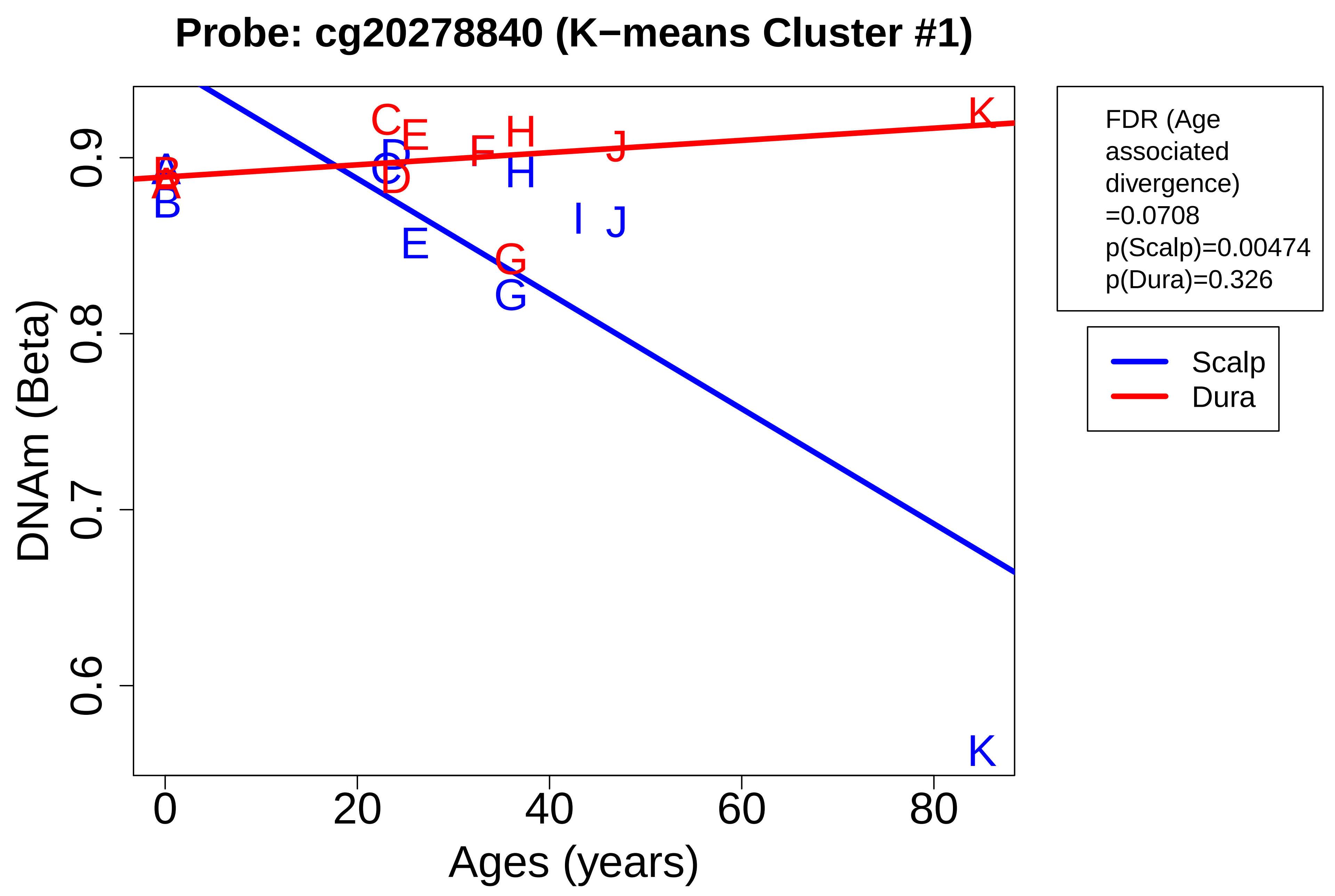
Probe: cg20278840 (K−means Cluster #1)
DNAm (Beta)
0.6
0.7
0.8
0.9
0
20
40
60
80
Ages (years)
A
B
C
D
E
F
G
H
I
J
K
FDR (Age associated divergence) =0.0708
p(Scalp)=0.00474
p(Dura)=0.326
Scalp
Dura

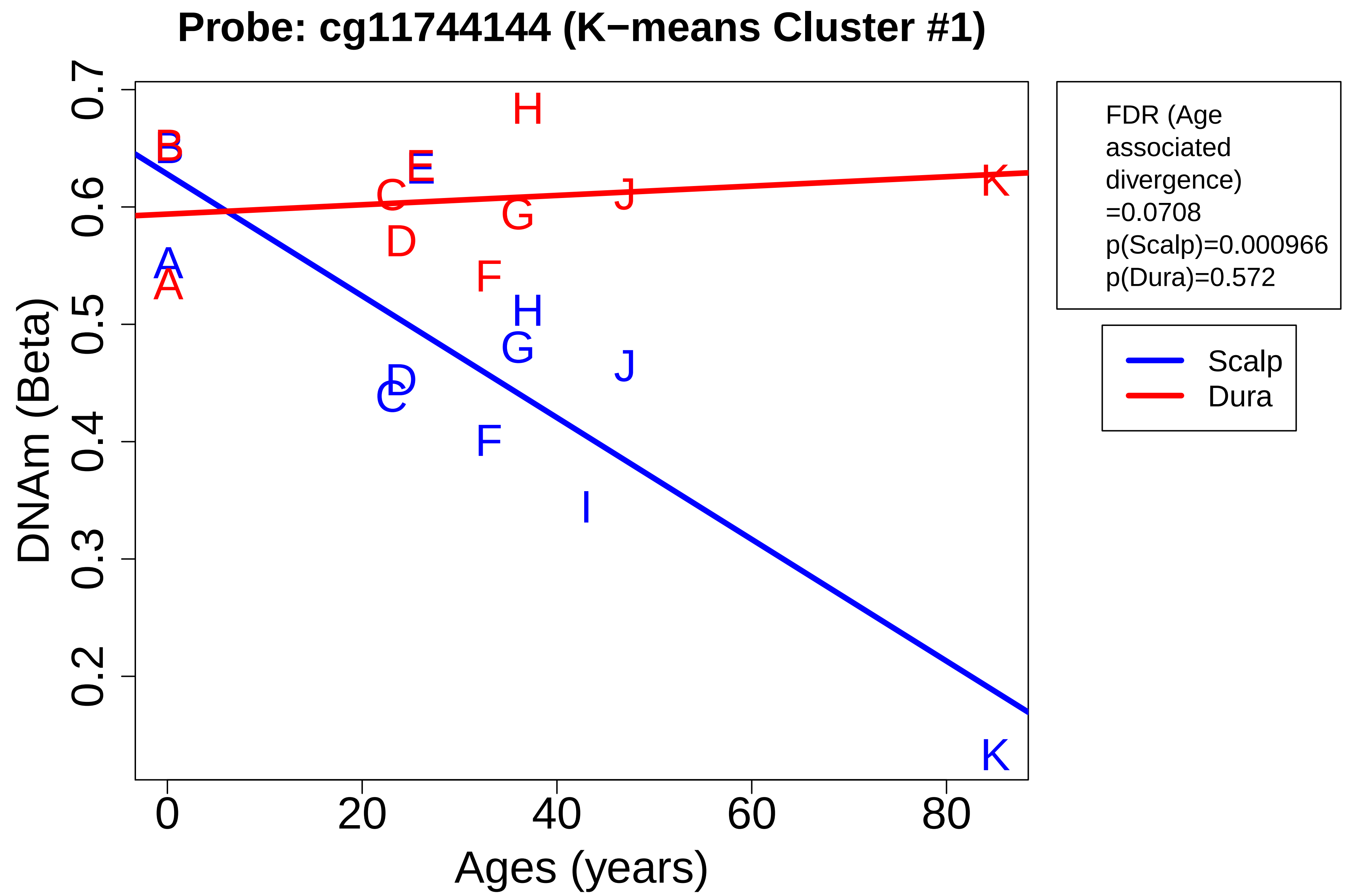

Probe: cg11744144 (K−means Cluster #1)
DNAm (Beta)
0.2
0.3
0.4
0.5
0.6
0.7
Ages (years)
0
20
40
60
80
A
B
C
D
E
F
G
H
I
J
K
FDR (Age associated divergence) =0.0708
p(Scalp)=0.000966
p(Dura)=0.572
Scalp
Dura

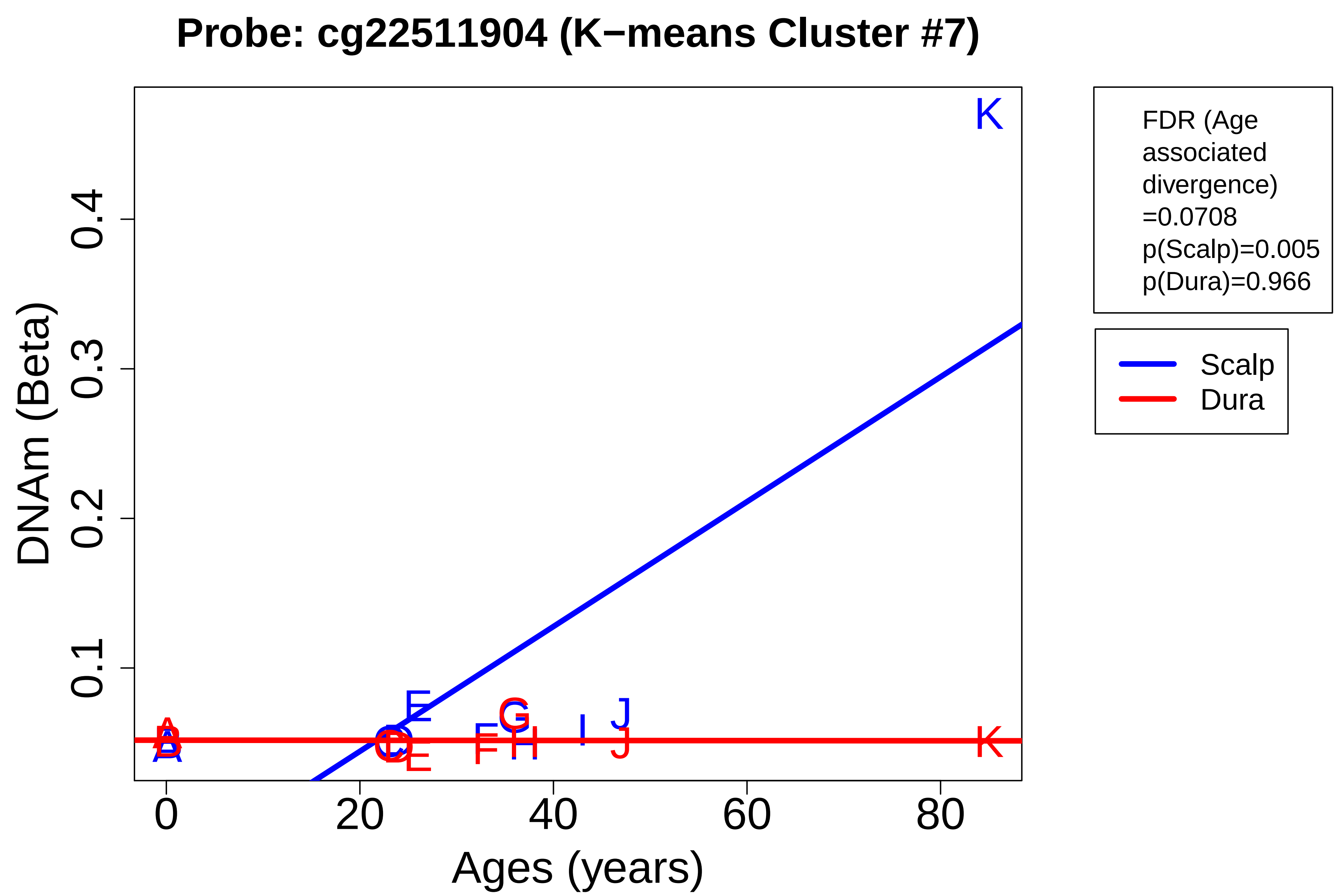
Probe: cg22511904 (K−means Cluster #7)
DNAm (Beta)
0.1
0.2
0.3
0.4
Ages (years)
0
20
40
60
80
FDR (Age associated divergence) =0.0708
p(Scalp)=0.005
p(Dura)=0.966
Scalp
Dura
A
B
C
D
E
F
G
H
I
J
K

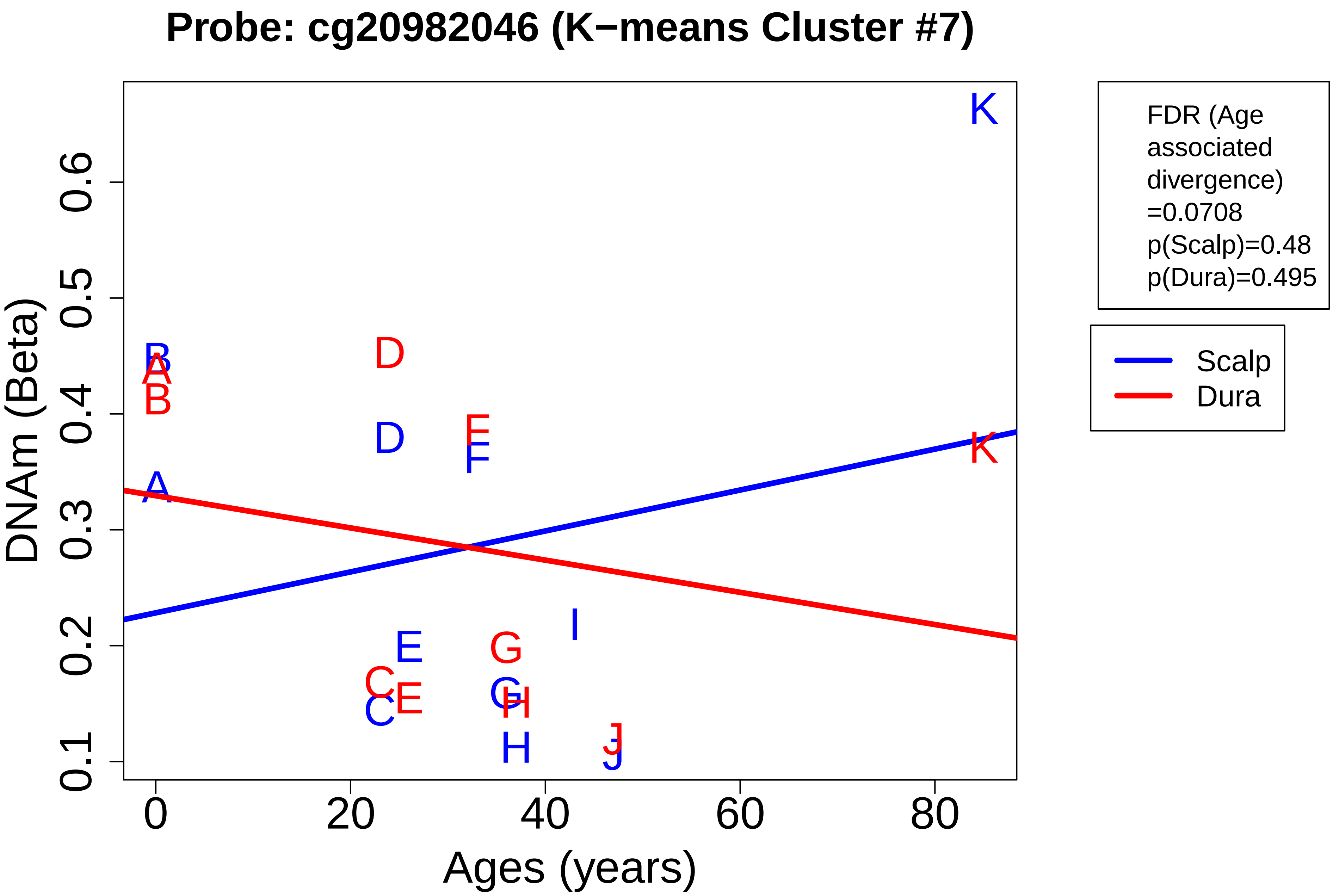

Probe: cg20982046 (K−means Cluster #7)
DNAm (Beta)
0.1
0.2
0.3
0.4
0.5
0.6
0
20
40
60
80
Ages (years)
FDR (Age associated divergence) =0.0708
p(Scalp)=0.48
p(Dura)=0.495
Scalp
Dura

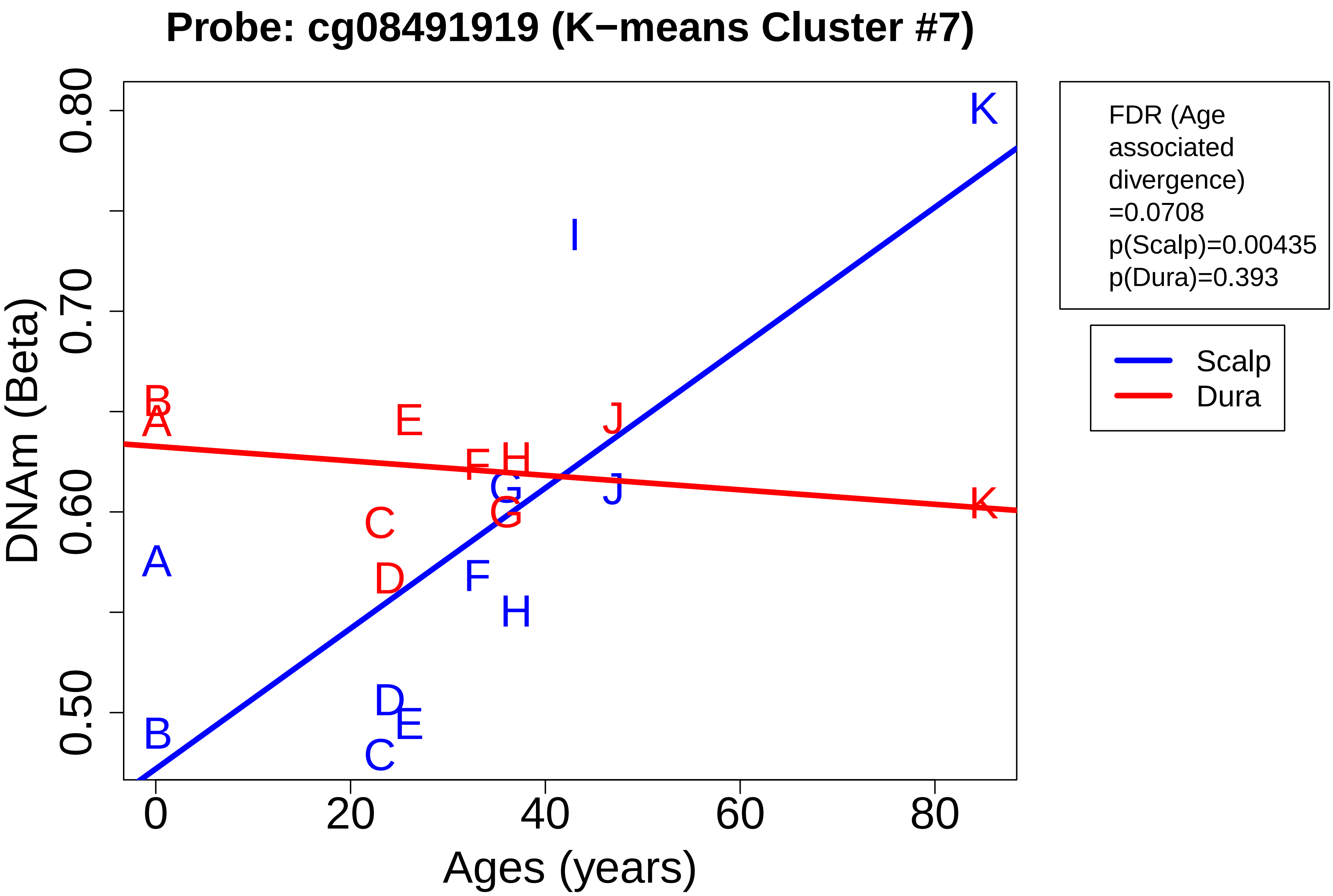

Probe: cg08491919 (K−means Cluster #7)
DNAm (Beta)
0.50
0.60
0.70
0.80
Ages (years)
0
20
40
60
80
FDR (Age associated divergence) =0.0708
p(Scalp)=0.00435
p(Dura)=0.393
Scalp
Dura
A
B
C
D
E
F
G
H
I
J
K

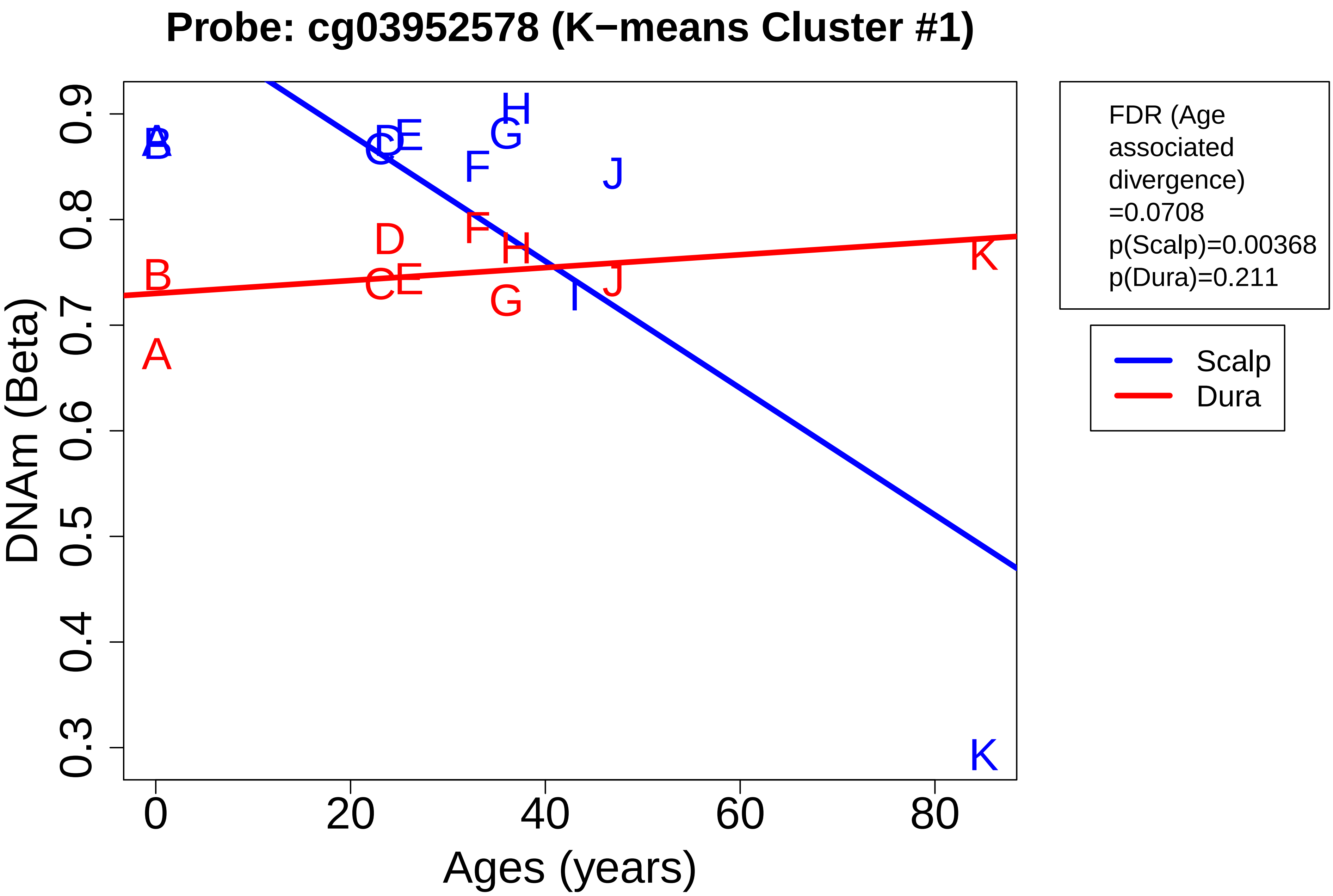

Probe: cg03952578 (K−means Cluster #1)
DNAm (Beta)
0.3
0.4
0.5
0.6
0.7
0.8
0.9
0
20
40
60
80
Ages (years)
FDR (Age associated divergence) =0.0708
p(Scalp)=0.00368
p(Dura)=0.211
Scalp
Dura

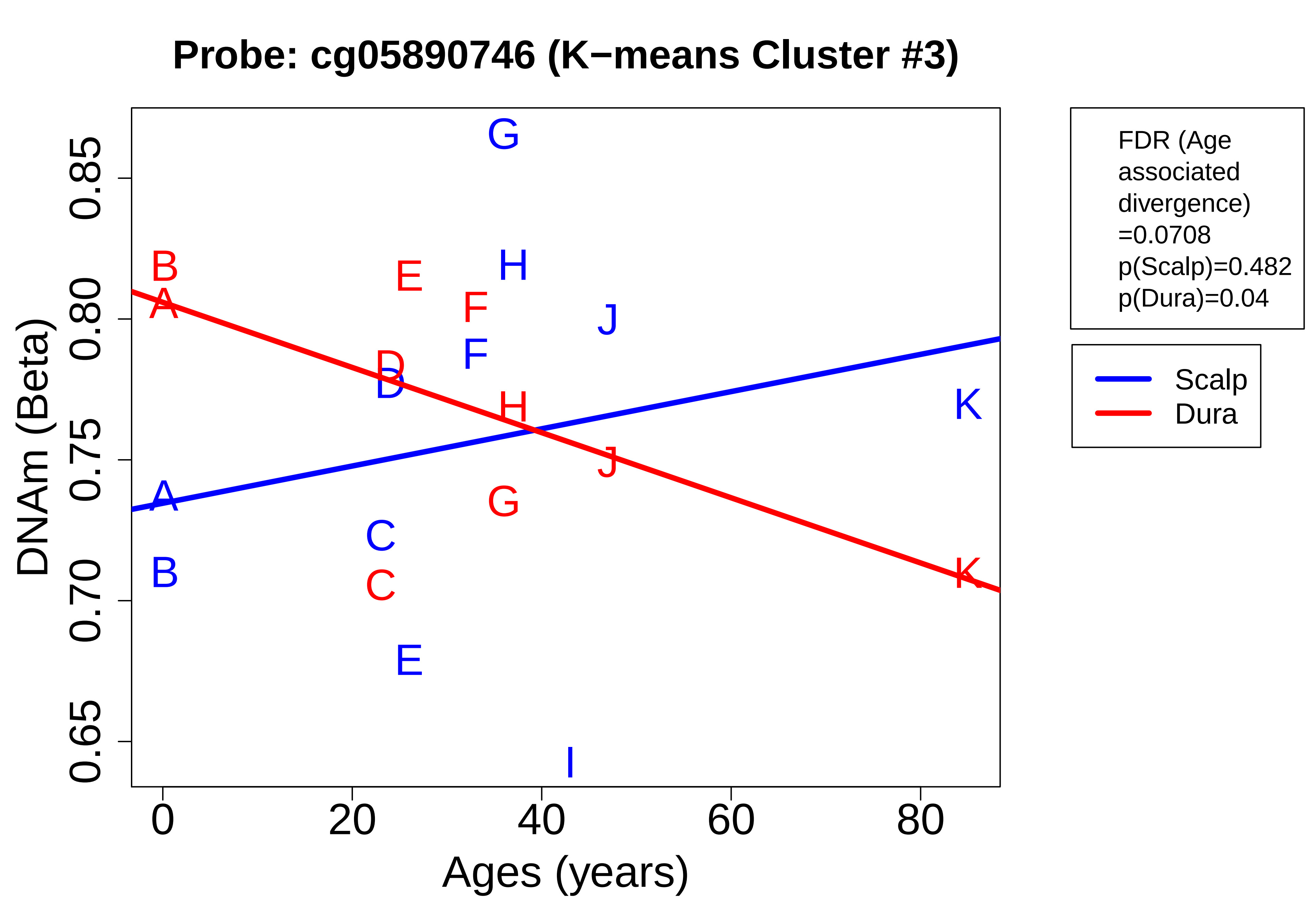

Probe: cg05890746 (K−means Cluster #3)
DNAm (Beta)
Ages (years)
0.65
0.70
0.75
0.80
0.85
0
20
40
60
80
FDR (Age associated divergence) =0.0708
p(Scalp)=0.482
p(Dura)=0.04
Scalp
Dura

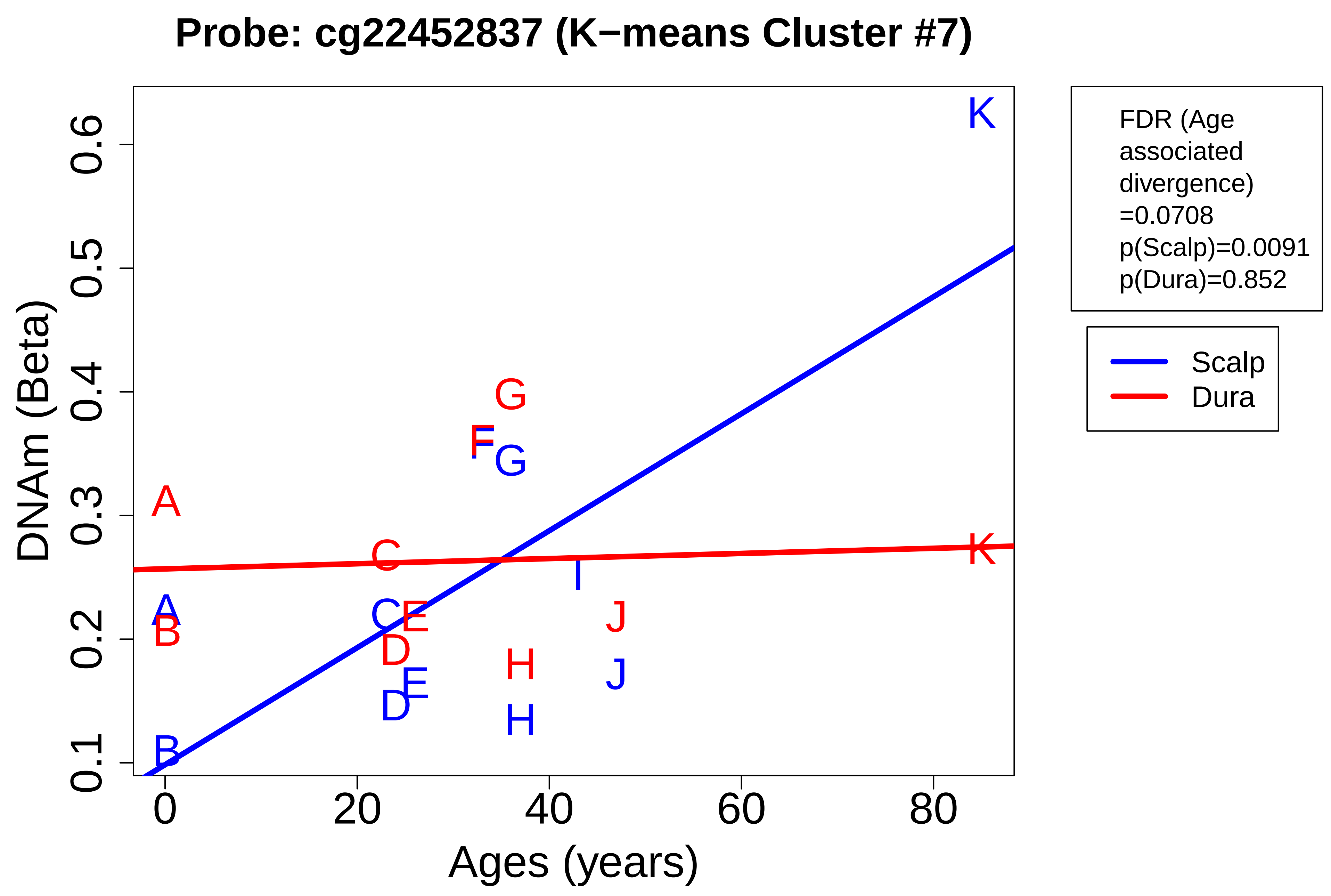

Probe: cg22452837 (K−means Cluster #7)
DNAm (Beta)
0.1
0.2
0.3
0.4
0.5
0.6
0
20
40
60
80
Ages (years)
A
B
C
D
E
F
G
H
I
J
K
FDR (Age associated divergence) =0.0708
p(Scalp)=0.0091
p(Dura)=0.852
Scalp
Dura

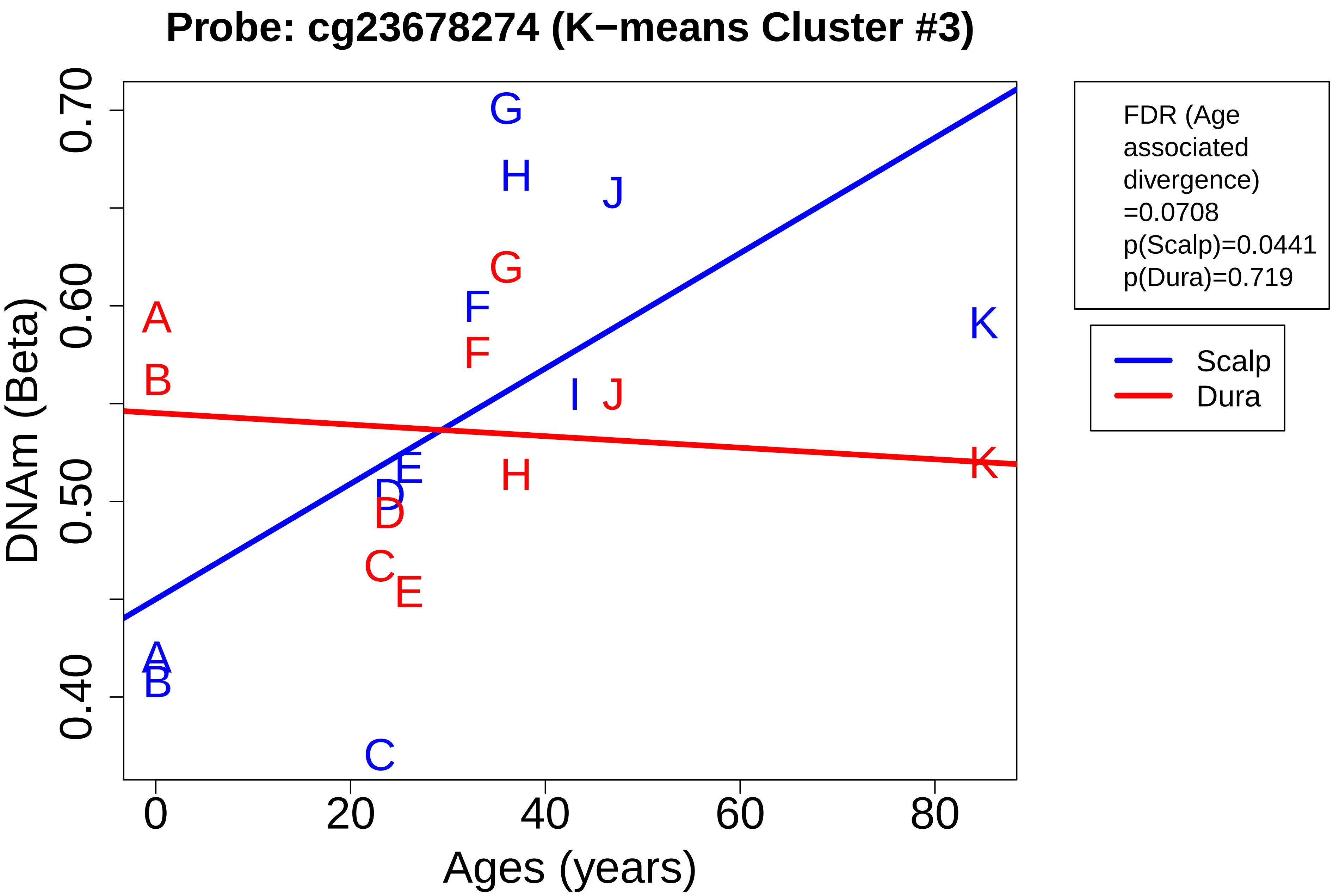

Probe: cg23678274 (K−means Cluster #3)
DNAm (Beta)
0.40
0.50
0.60
0.70
Ages (years)
0
20
40
60
80
FDR (Age associated divergence) =0.0708
p(Scalp)=0.0441
p(Dura)=0.719
Scalp
Dura

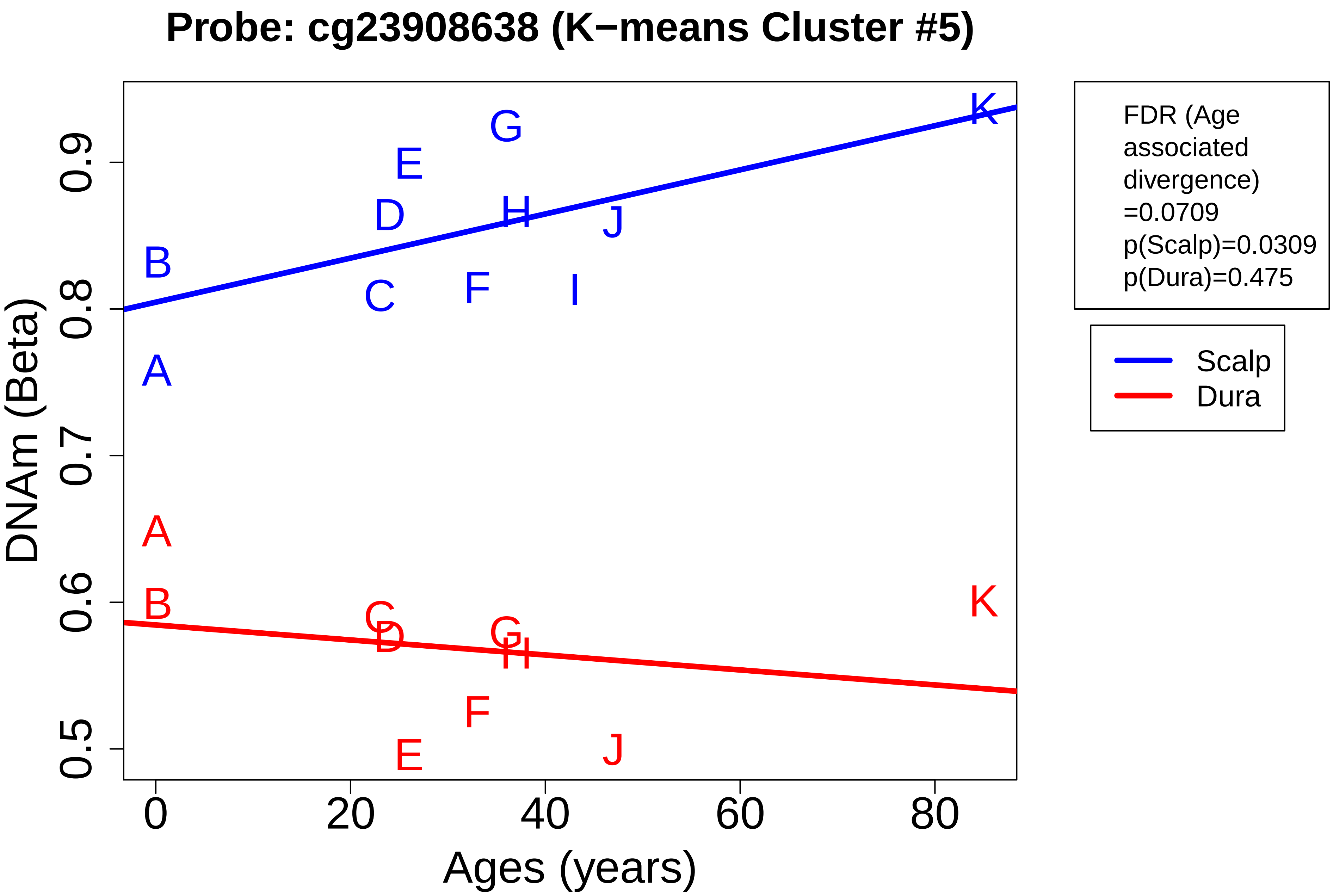

Probe: cg23908638 (K−means Cluster #5)
DNAm (Beta)
0.5
0.6
0.7
0.8
0.9
Ages (years)
0
20
40
60
80
A
B
C
D
E
F
G
H
I
J
K
FDR (Age associated divergence) =0.0709
p(Scalp)=0.0309
p(Dura)=0.475
Scalp
Dura

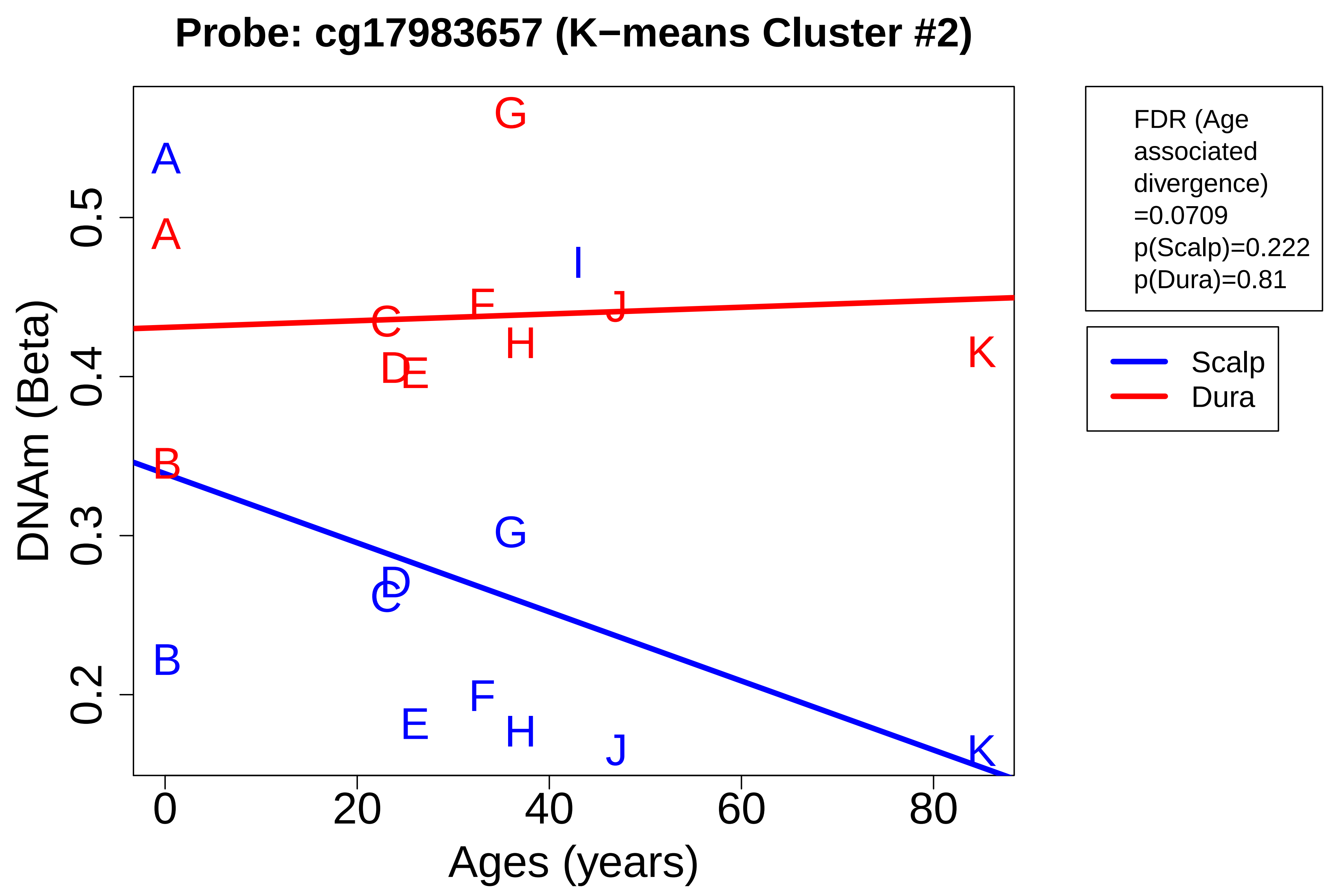

Probe: cg17983657 (K−means Cluster #2)
FDR (Age associated divergence) =0.0709
p(Scalp)=0.222
p(Dura)=0.81
Scalp
Dura
DNAm (Beta)
0.2
0.3
0.4
0.5
0
20
40
60
80
Ages (years)
A
A
B
B
C
C
D
D
E
E
F
F
G
G
H
H
I
J
J
K
K

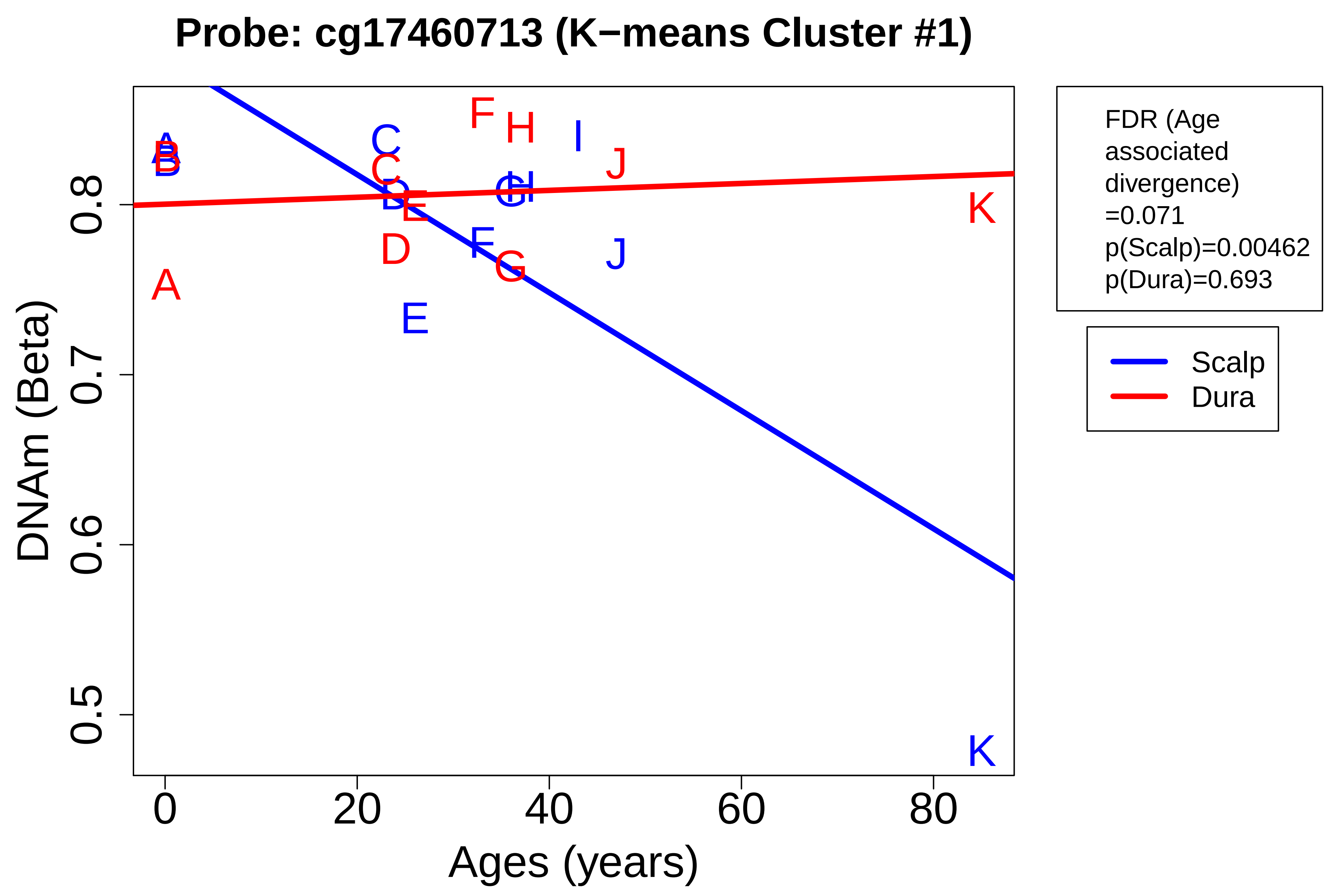

Probe: cg17460713 (K−means Cluster #1)
DNAm (Beta)
0.5
0.6
0.7
0.8
0
20
40
60
80
Ages (years)
A
B
C
D
E
F
G
H
I
J
K
FDR (Age associated divergence) =0.071
p(Scalp)=0.00462
p(Dura)=0.693
Scalp
Dura

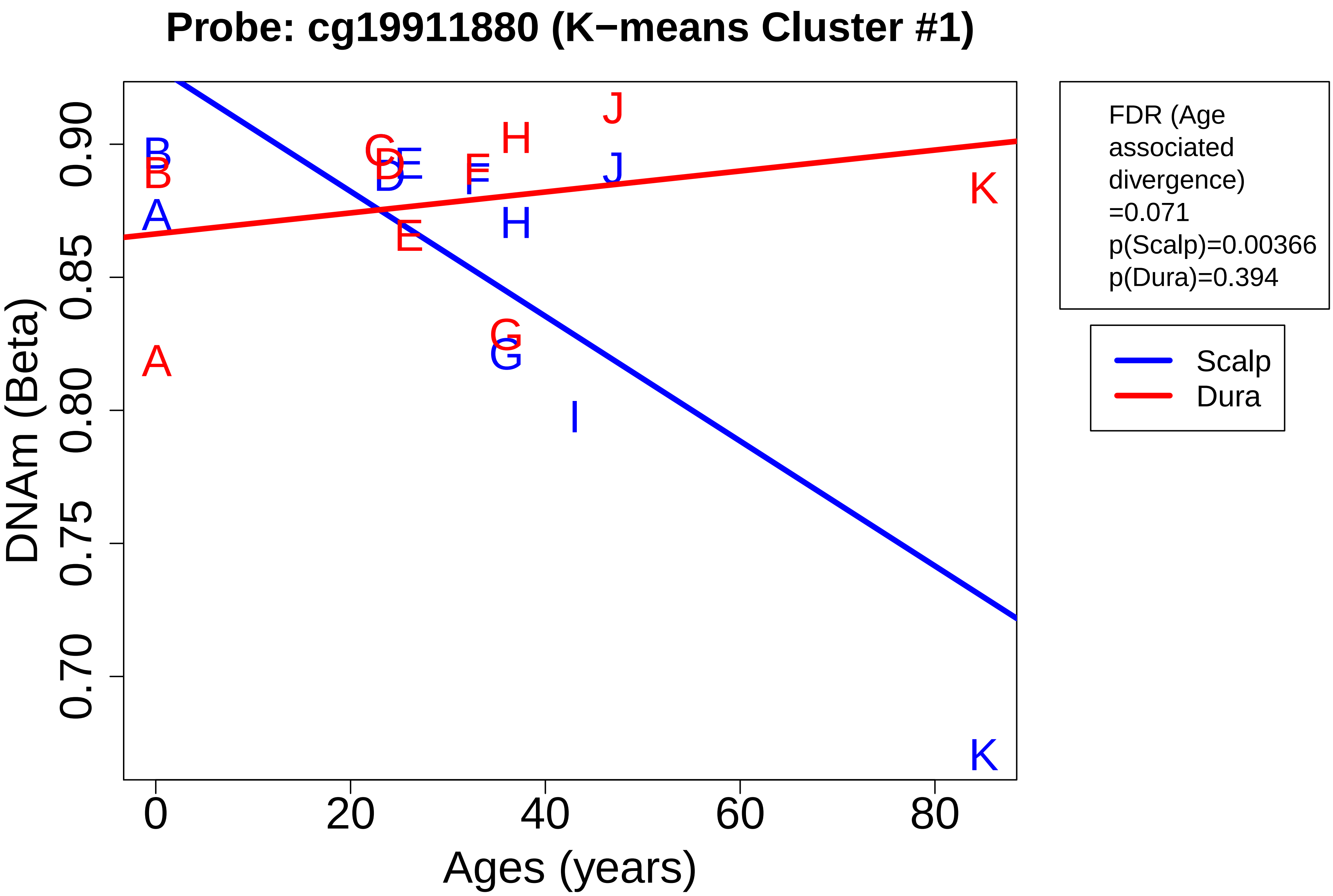

Probe: cg19911880 (K−means Cluster #1)
DNAm (Beta)
0.70
0.75
0.80
0.85
0.90
0
20
40
60
80
Ages (years)
A
B
C
D
E
F
G
H
I
J
K
FDR (Age associated divergence) =0.071
p(Scalp)=0.00366
p(Dura)=0.394
Scalp
Dura

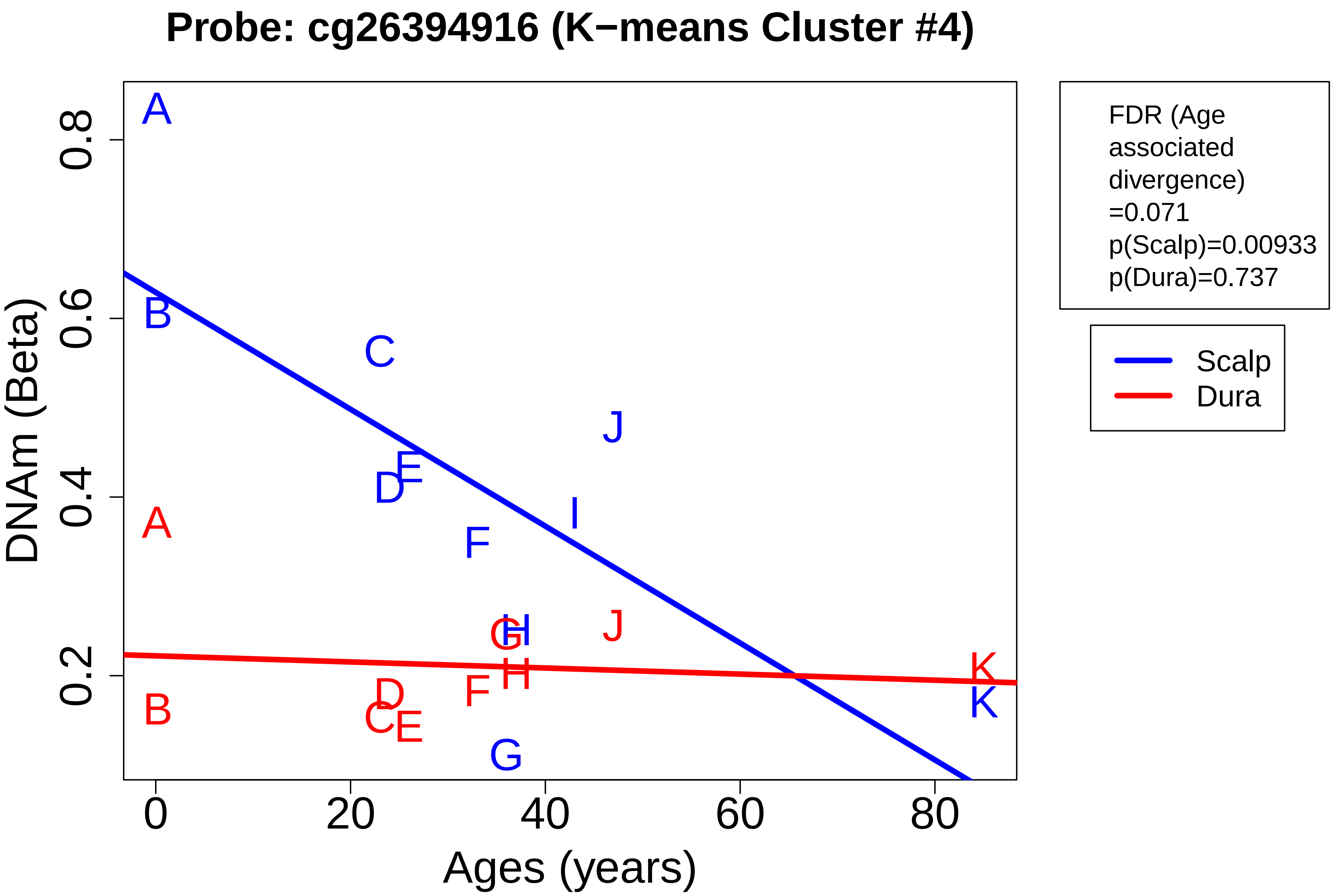

Probe: cg26394916 (K−means Cluster #4)
DNAm (Beta)
Ages (years)
0.2
0.4
0.6
0.8
0
20
40
60
80
FDR (Age associated divergence) =0.071
p(Scalp)=0.00933
p(Dura)=0.737
Scalp
Dura
A
B
C
D
E
F
G
H
I
J
K

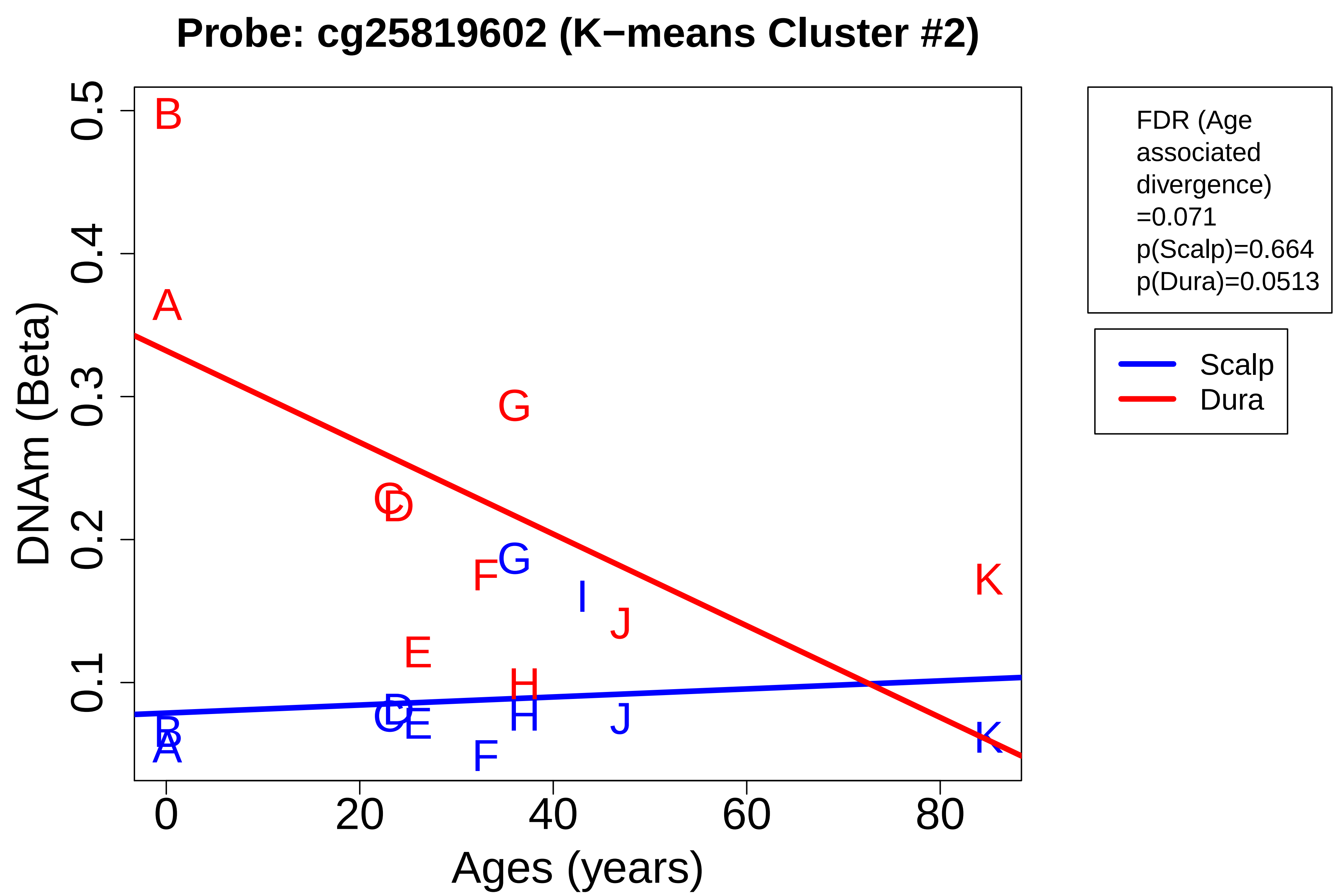

Probe: cg25819602 (K−means Cluster #2)
DNAm (Beta)
0.1
0.2
0.3
0.4
0.5
0
20
40
60
80
Ages (years)
FDR (Age associated divergence) =0.071
p(Scalp)=0.664
p(Dura)=0.0513
Scalp
Dura

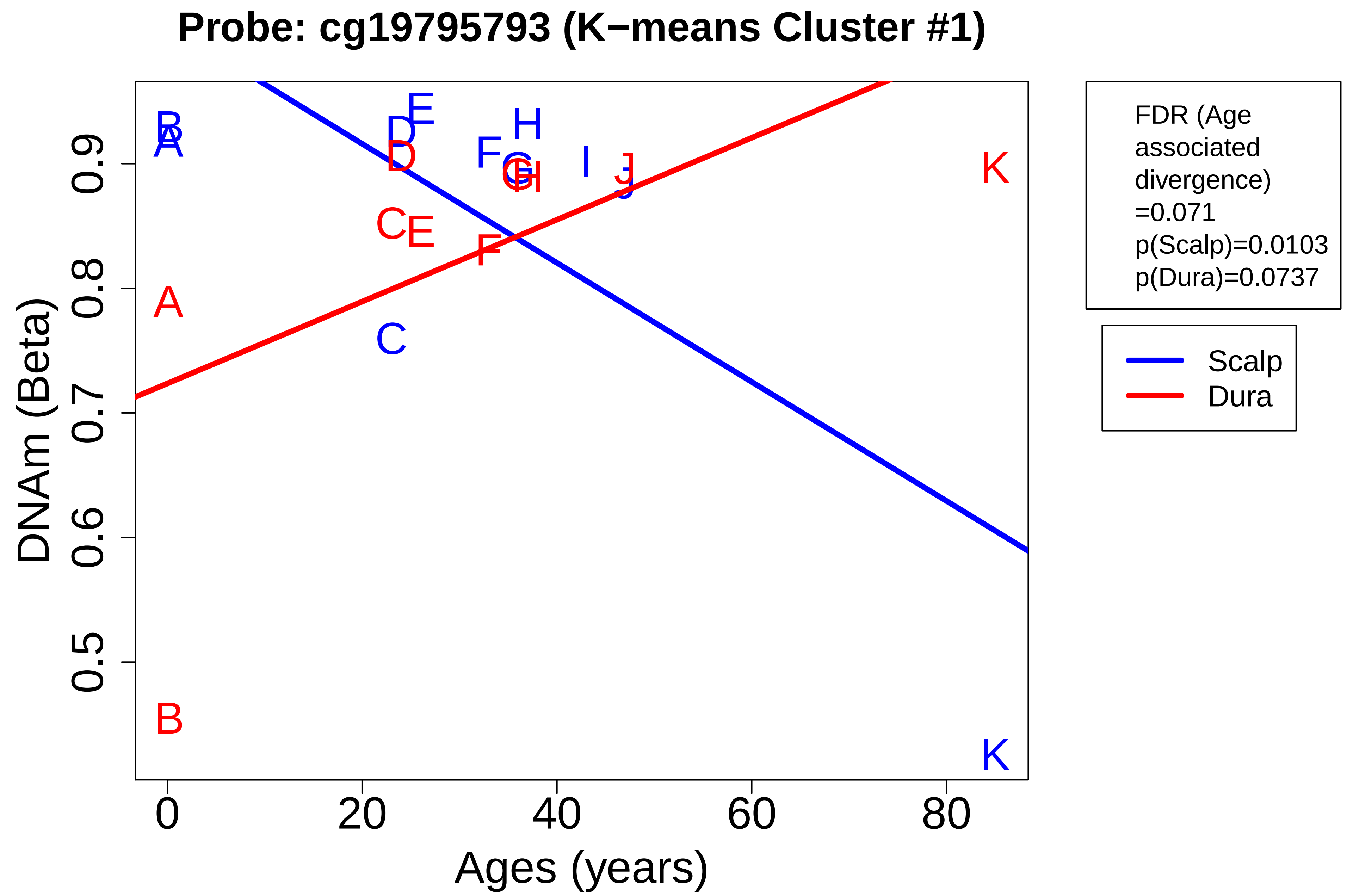
Probe: cg19795793 (K−means Cluster #1)
DNAm (Beta)
Ages (years)
FDR (Age associated divergence) =0.071
p(Scalp)=0.0103
p(Dura)=0.0737
Scalp
Dura

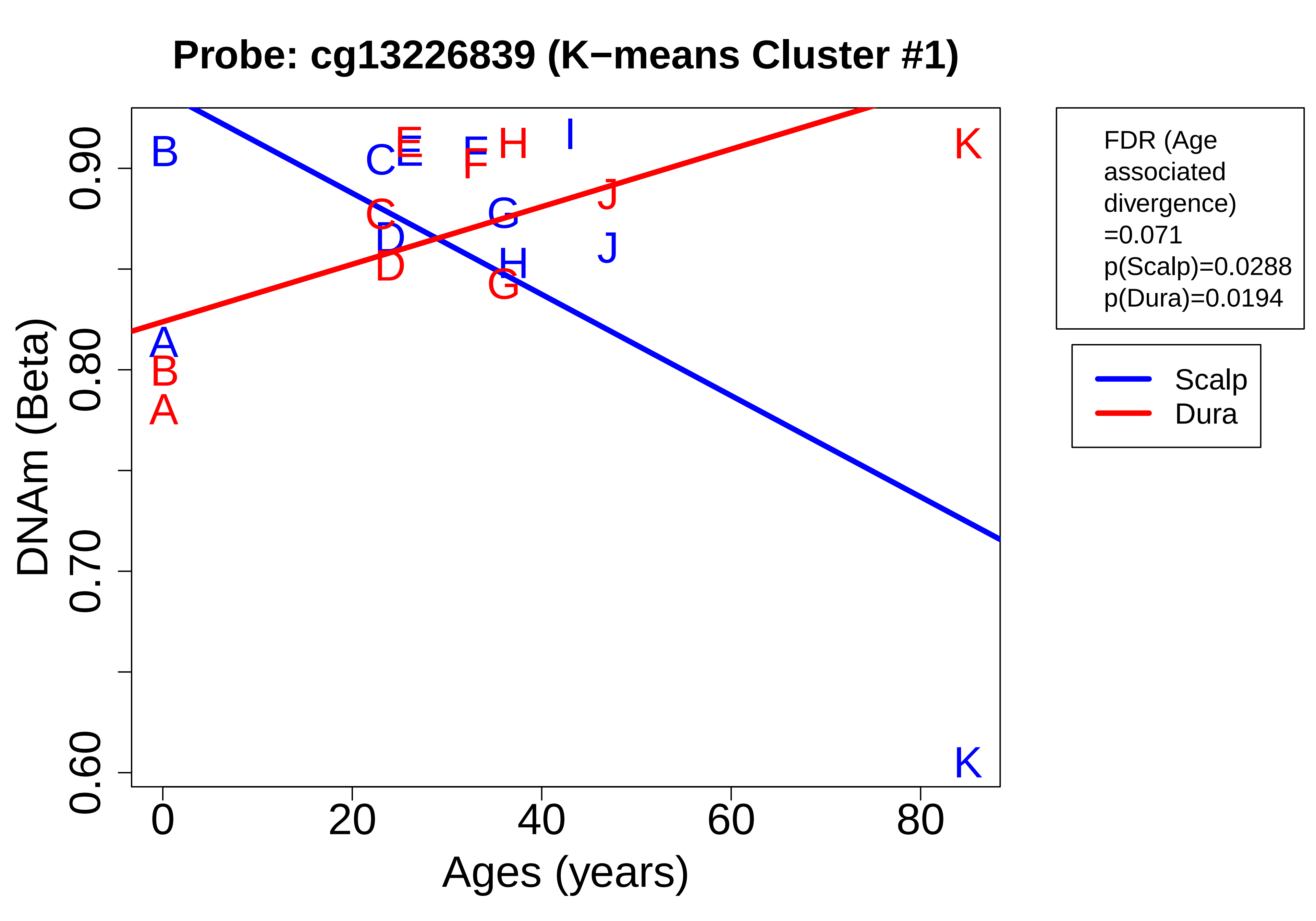
Probe: cg13226839 (K−means Cluster #1)
DNAm (Beta)
0.60
0.70
0.80
0.90
Ages (years)
0
20
40
60
80
A
B
C
D
E
F
G
H
I
J
K
FDR (Age associated divergence) =0.071
p(Scalp)=0.0288
p(Dura)=0.0194
Scalp
Dura

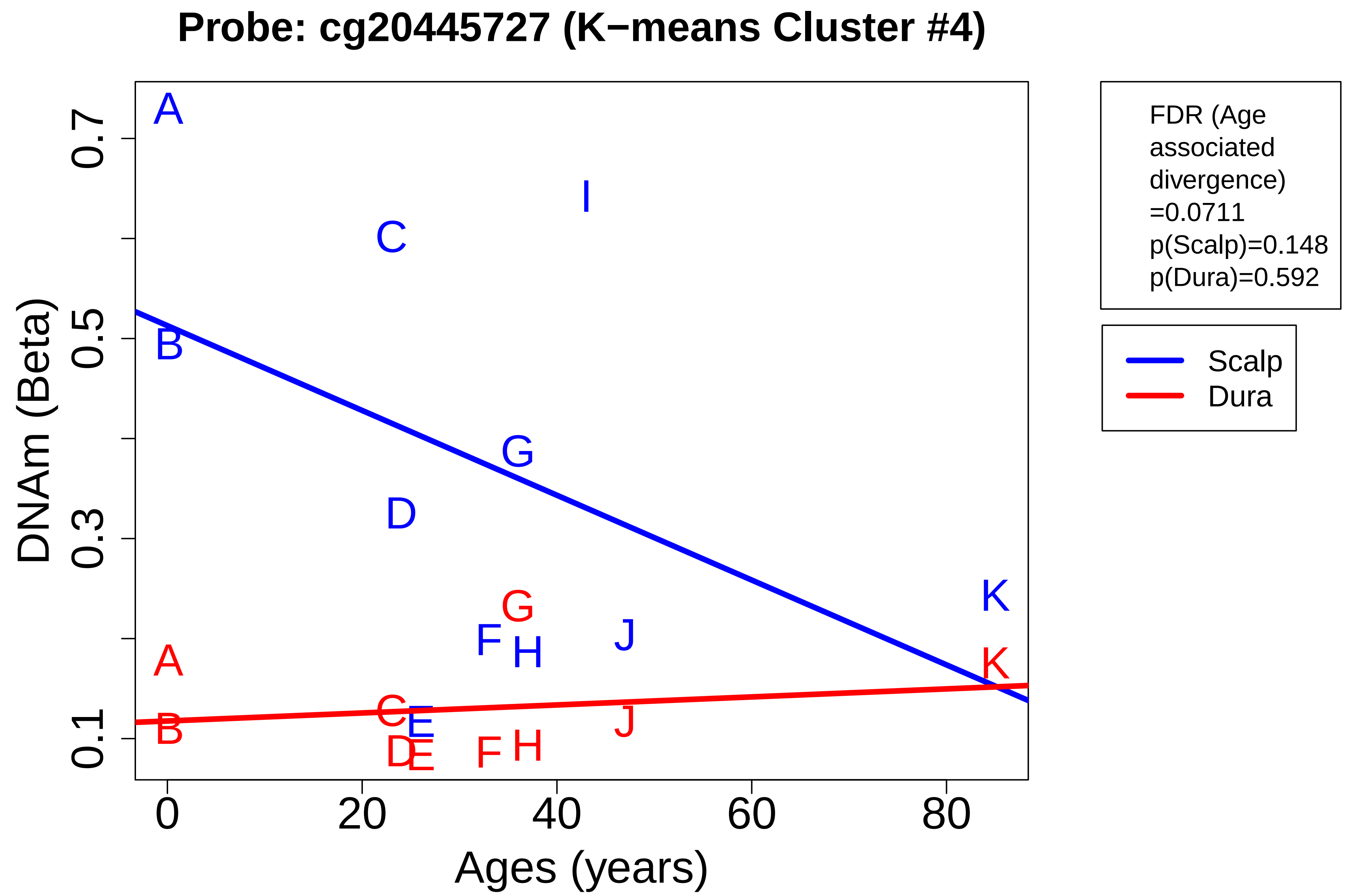

Probe: cg20445727 (K−means Cluster #4)
DNAm (Beta)
0.1
0.3
0.5
0.7
0
20
40
60
80
Ages (years)
A
B
C
D
E
F
G
H
I
J
K
FDR (Age associated divergence) =0.0711
p(Scalp)=0.148
p(Dura)=0.592
Scalp
Dura

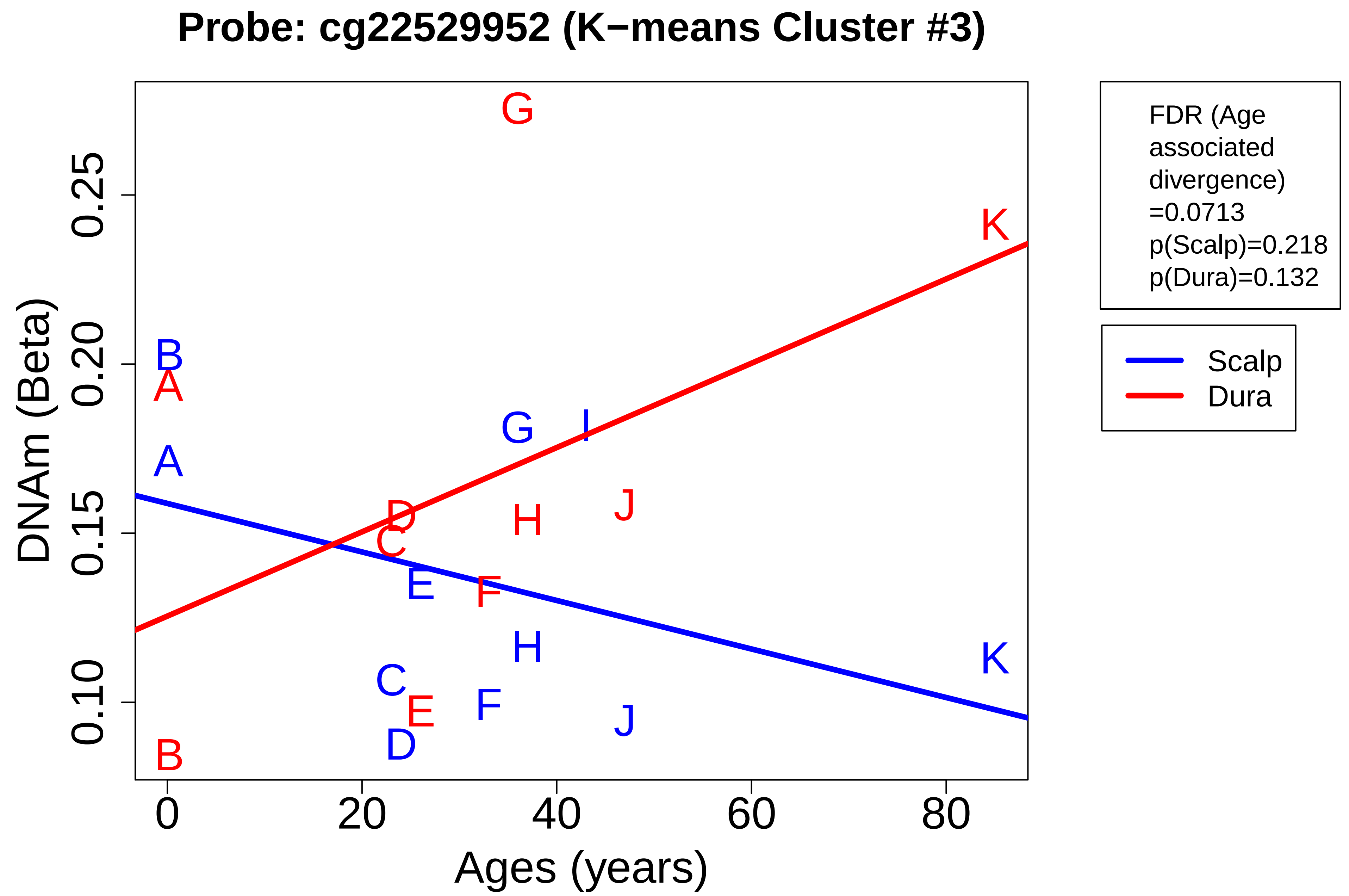
Probe: cg22529952 (K−means Cluster #3)
FDR (Age associated divergence) =0.0713
p(Scalp)=0.218
p(Dura)=0.132
Scalp
Dura
DNAm (Beta)
0.10
0.15
0.20
0.25
Ages (years)
0
20
40
60
80
A
B
C
D
E
F
G
H
I
J
K

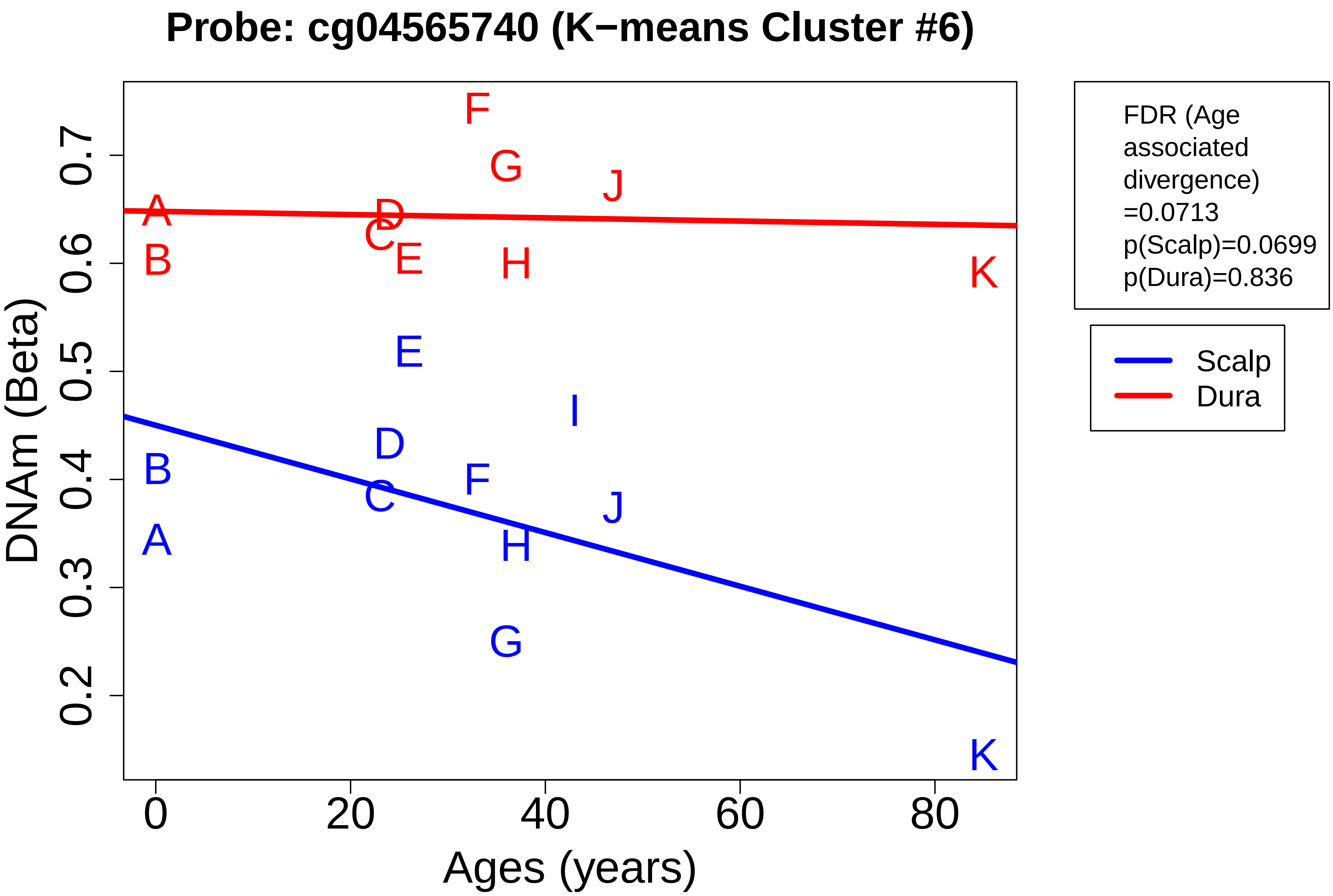

Probe: cg04565740 (K−means Cluster #6)
DNAm (Beta)
Ages (years)
0.2
0.3
0.4
0.5
0.6
0.7
0
20
40
60
80
FDR (Age associated divergence) =0.0713
p(Scalp)=0.0699
p(Dura)=0.836
Scalp
Dura
A
B
C
D
E
F
G
H
I
J
K

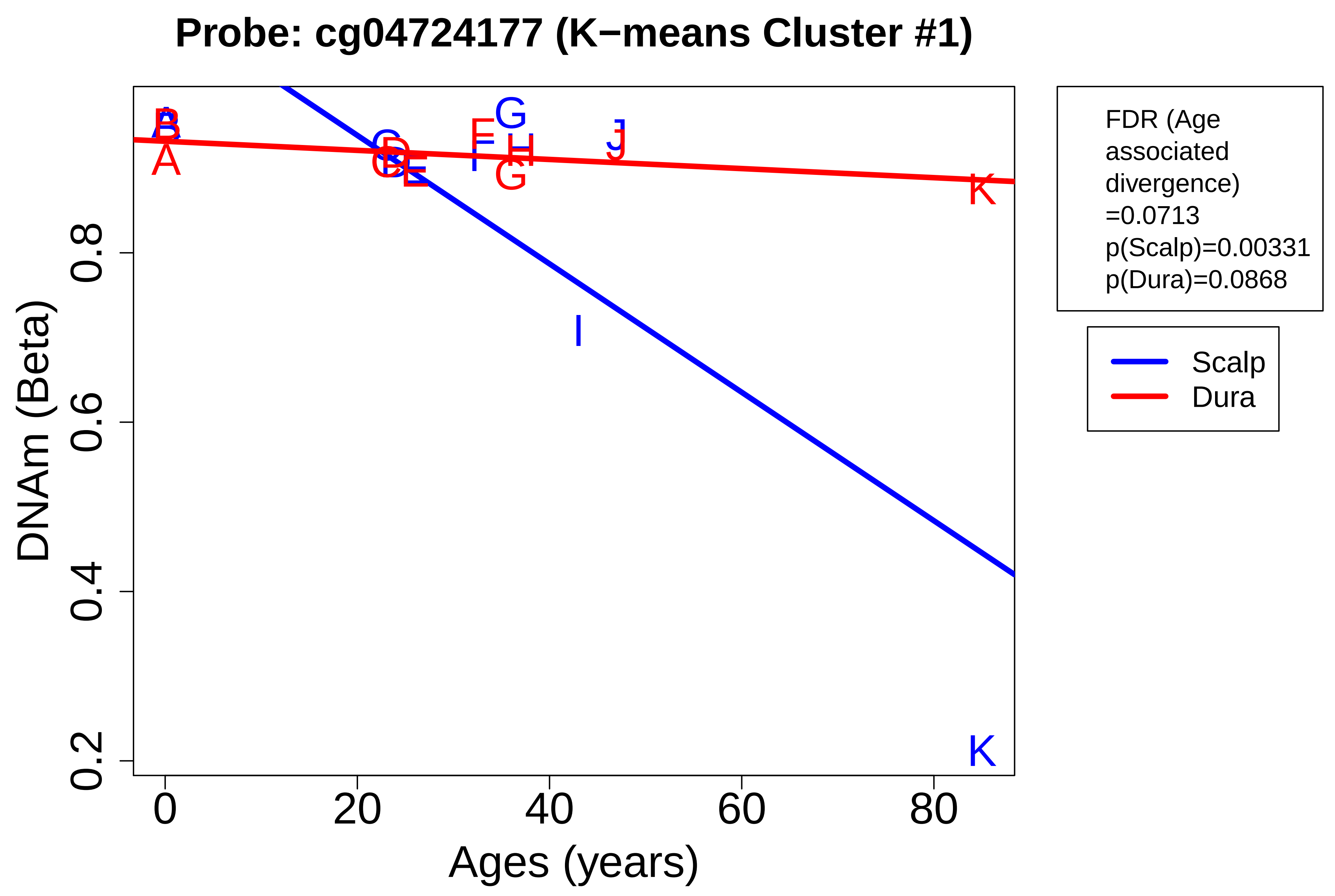

Probe: cg04724177 (K−means Cluster #1)
DNAm (Beta)
0.2
0.4
0.6
0.8
0
20
40
60
80
Ages (years)
FDR (Age associated divergence) =0.0713
p(Scalp)=0.00331
p(Dura)=0.0868
Scalp
Dura

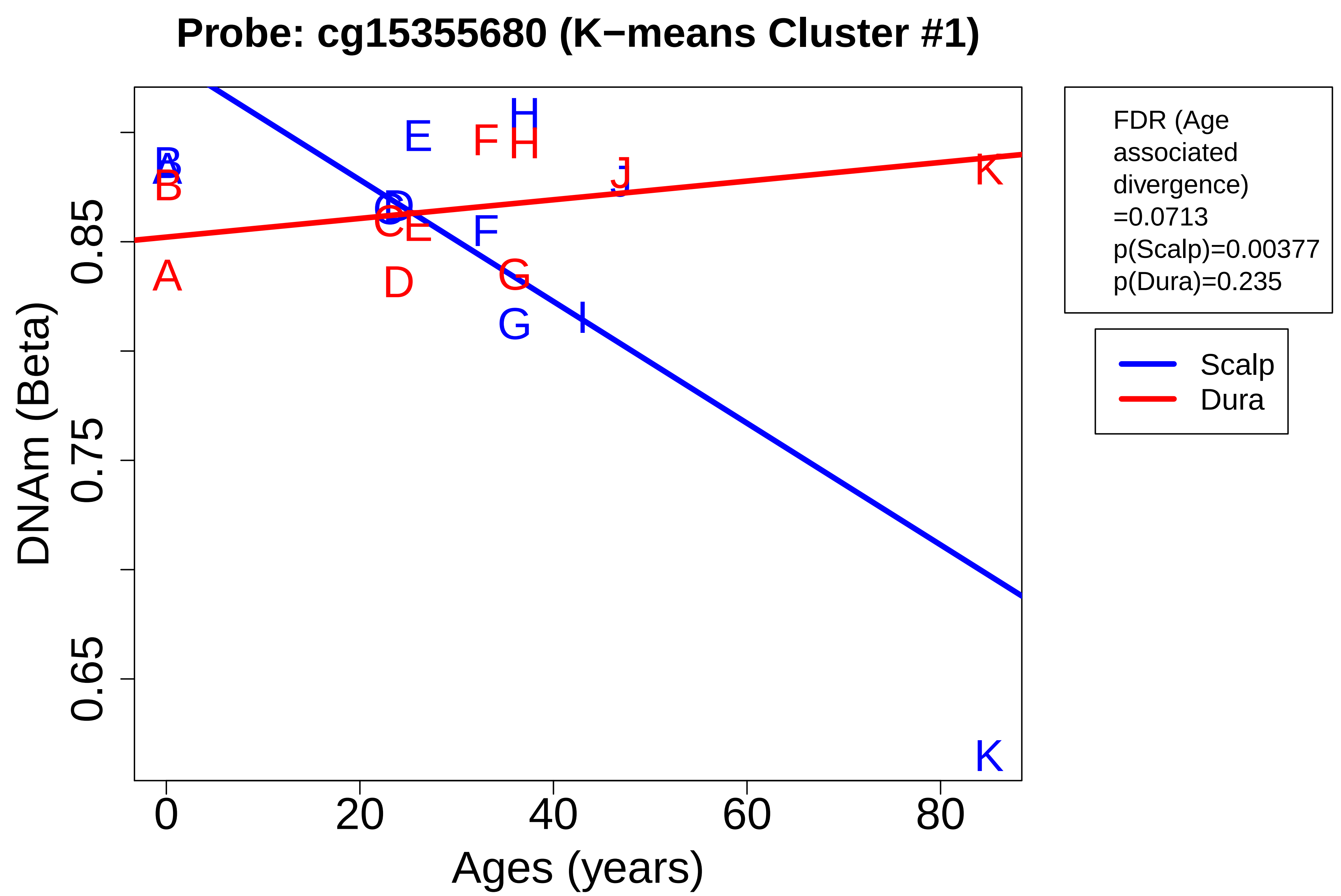

Probe: cg15355680 (K−means Cluster #1)
FDR (Age associated divergence) =0.0713
p(Scalp)=0.00377
p(Dura)=0.235
Scalp
Dura
DNAm (Beta)
0.65
0.75
0.85
Ages (years)
0
20
40
60
80
A B C D E F G H I J K

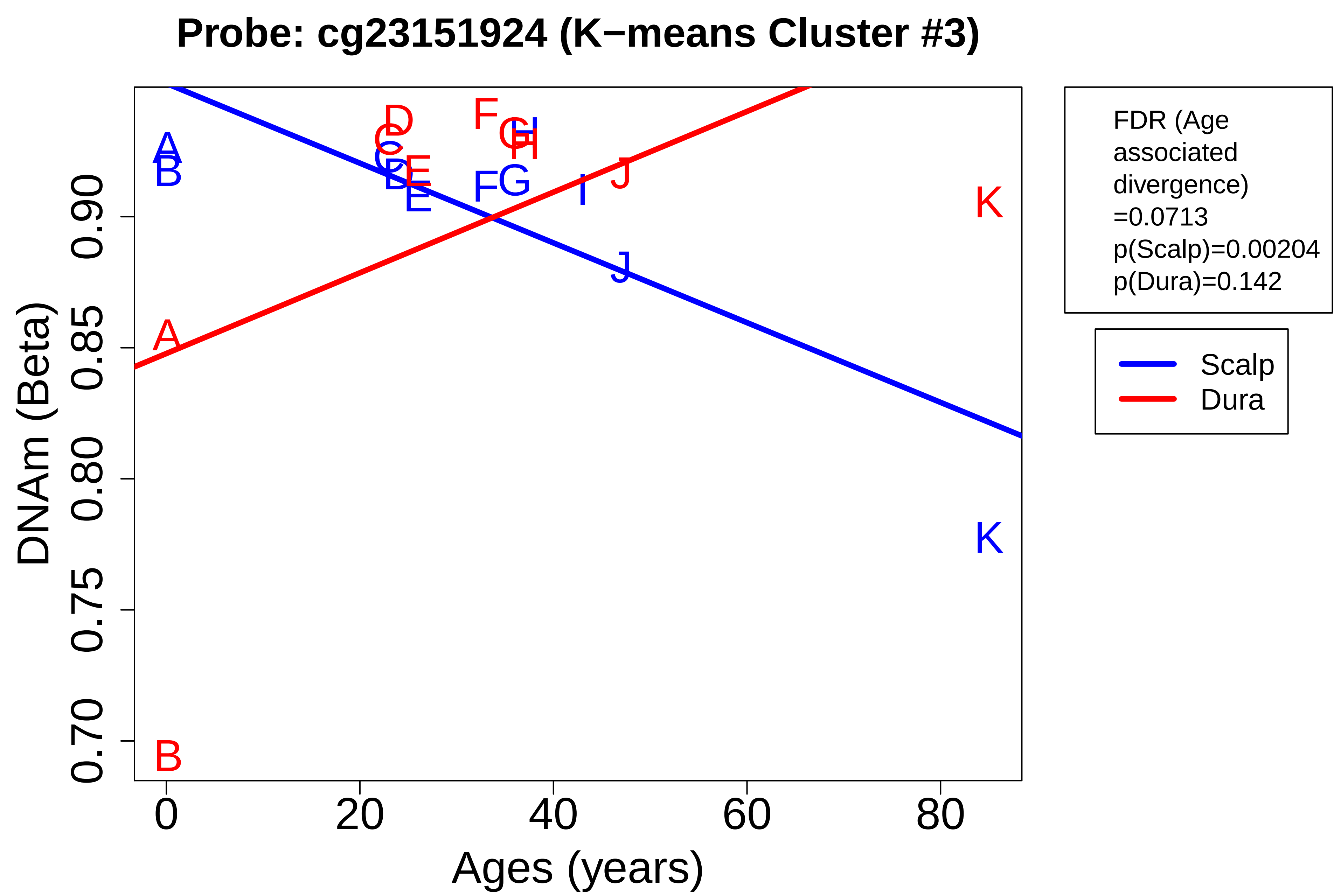

Probe: cg23151924 (K−means Cluster #3)
DNAm (Beta)
0.70
0.75
0.80
0.85
0.90
0
20
40
60
80
Ages (years)
FDR (Age associated divergence) =0.0713
p(Scalp)=0.00204
p(Dura)=0.142
Scalp
Dura
A
B
C
D
E
F
G
H
I
J
K

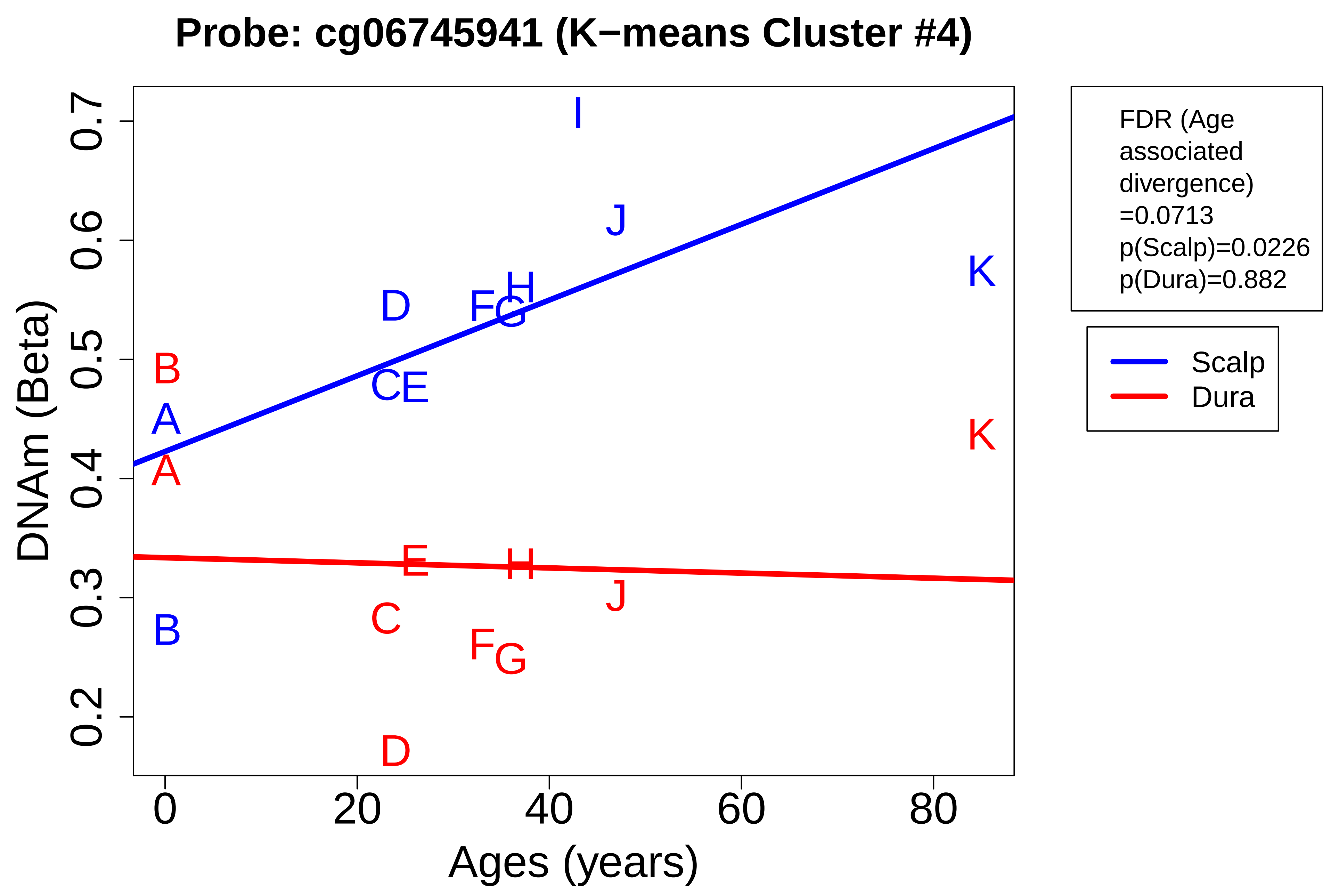

Probe: cg06745941 (K−means Cluster #4)
DNAm (Beta)
0.2
0.3
0.4
0.5
0.6
0.7
0
20
40
60
80
Ages (years)
A
B
C
D
E
F
G
H
I
J
K
FDR (Age associated divergence) =0.0713
p(Scalp)=0.0226
p(Dura)=0.882
Scalp
Dura

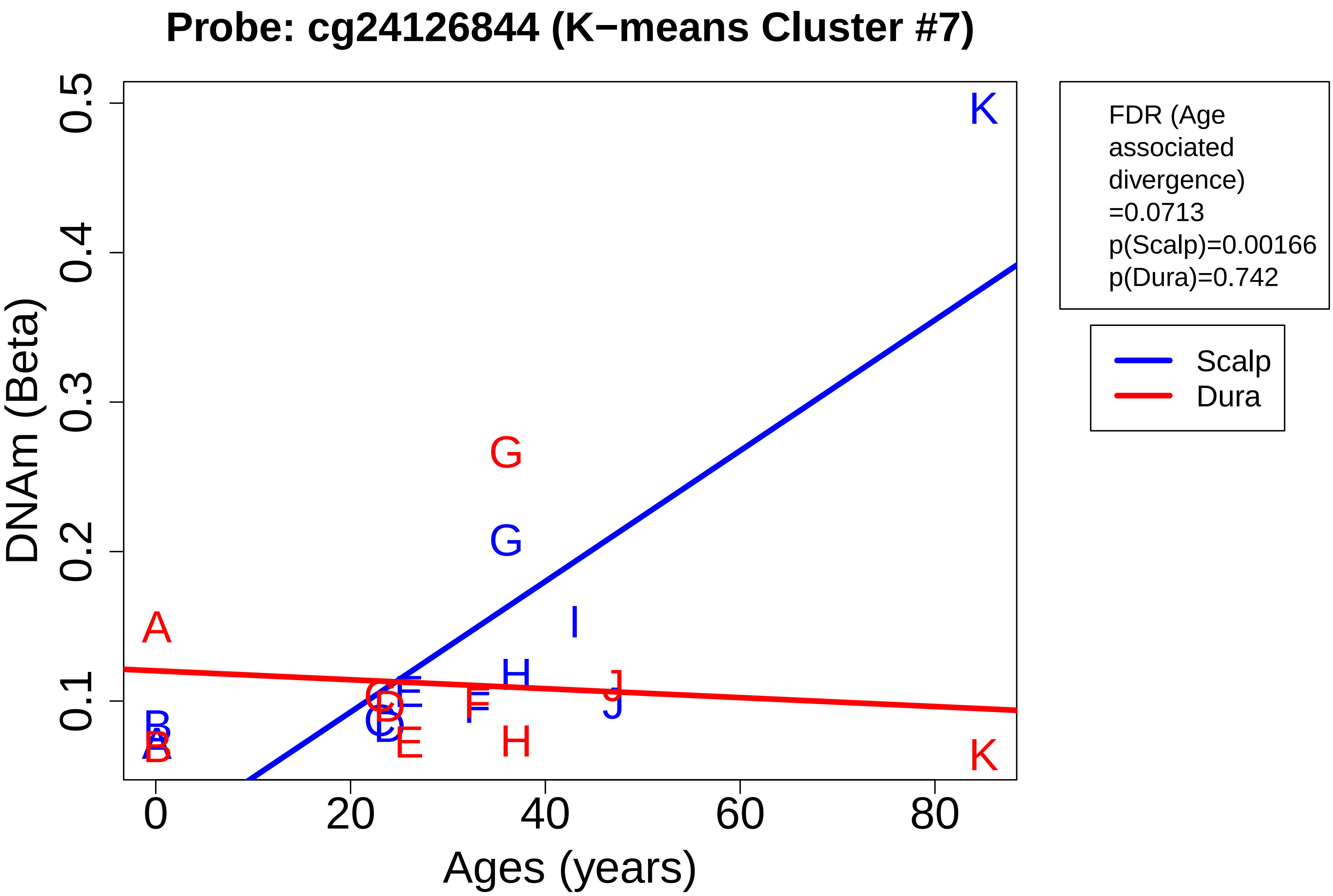

Probe: cg24126844 (K−means Cluster #7)
DNAm (Beta)
0.1
0.2
0.3
0.4
0.5
Ages (years)
0
20
40
60
80
FDR (Age associated divergence) =0.0713
p(Scalp)=0.00166
p(Dura)=0.742
Scalp
Dura

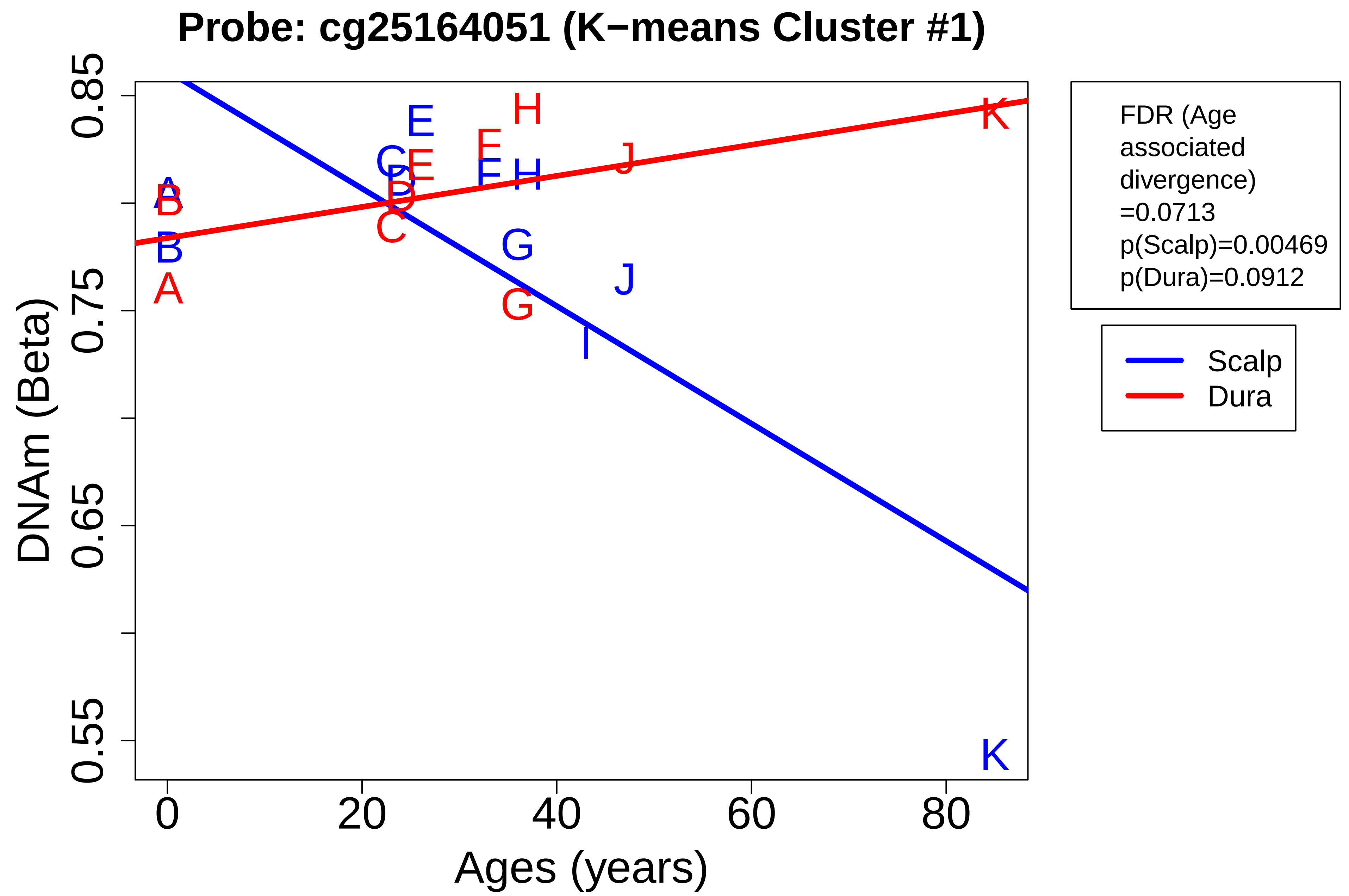

Probe: cg25164051 (K−means Cluster #1)
DNAm (Beta)
0.55
0.65
0.75
0.85
Ages (years)
0
20
40
60
80
FDR (Age associated divergence) =0.0713
p(Scalp)=0.00469
p(Dura)=0.0912
Scalp
Dura

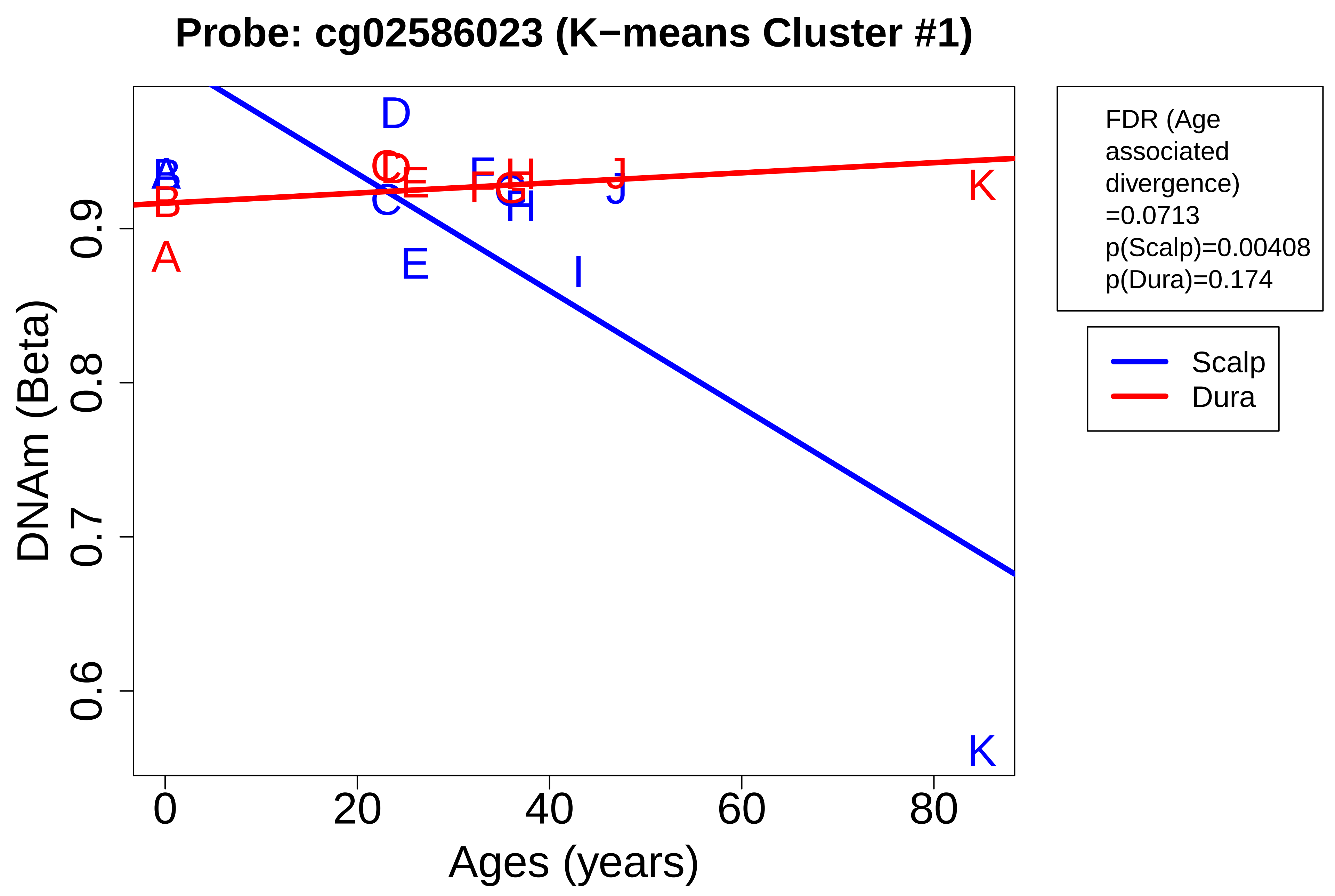
Probe: cg02586023 (K−means Cluster #1)
DNAm (Beta)
0.6
0.7
0.8
0.9
0
20
40
60
80
Ages (years)
FDR (Age associated divergence) =0.0713
p(Scalp)=0.00408
p(Dura)=0.174
Scalp
Dura
A
B
C
D
E
F
G
H
I
J
K

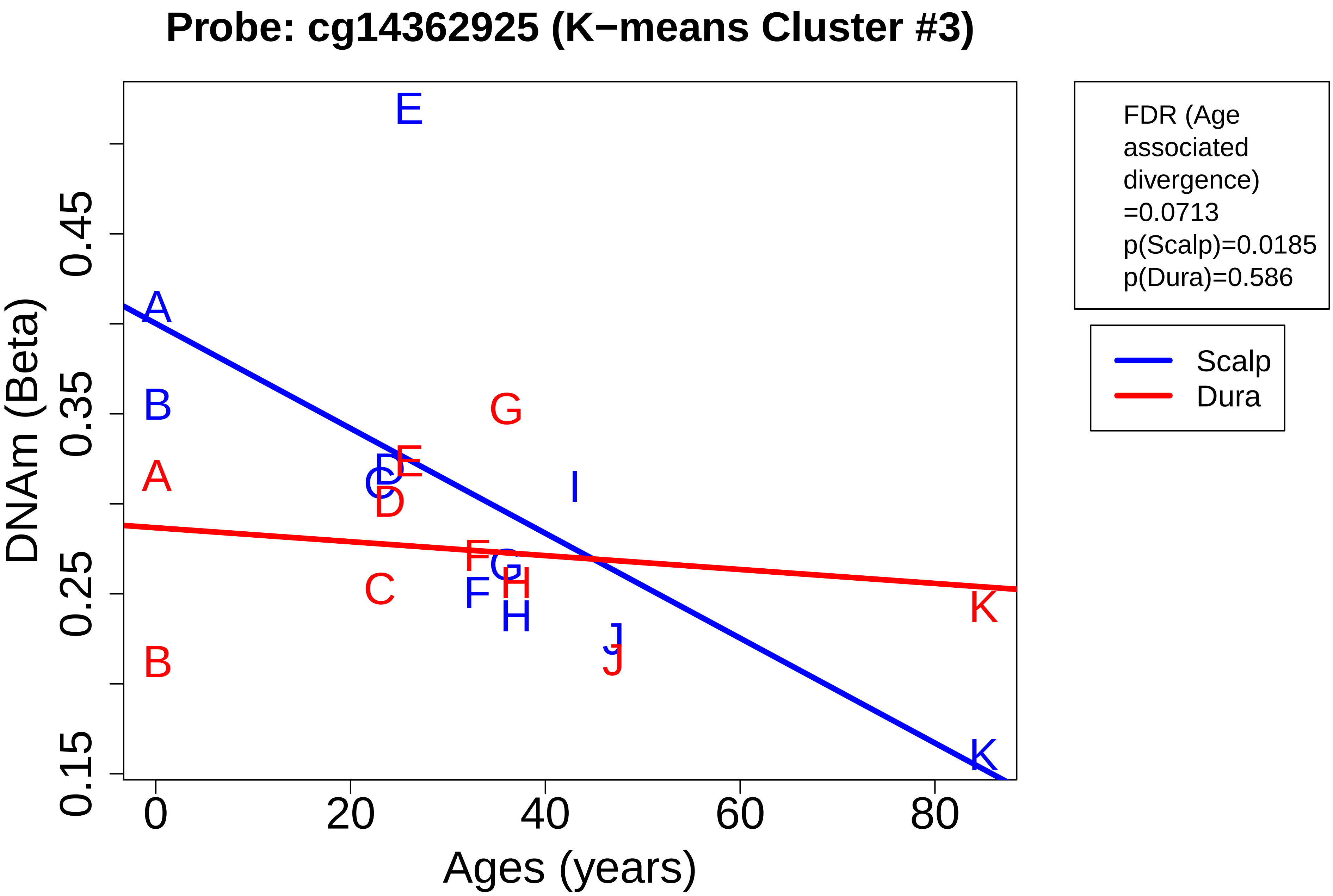

Probe: cg14362925 (K−means Cluster #3)
DNAm (Beta)
Ages (years)
0.15
0.25
0.35
0.45
0
20
40
60
80
FDR (Age associated divergence) =0.0713
p(Scalp)=0.0185
p(Dura)=0.586
Scalp
Dura

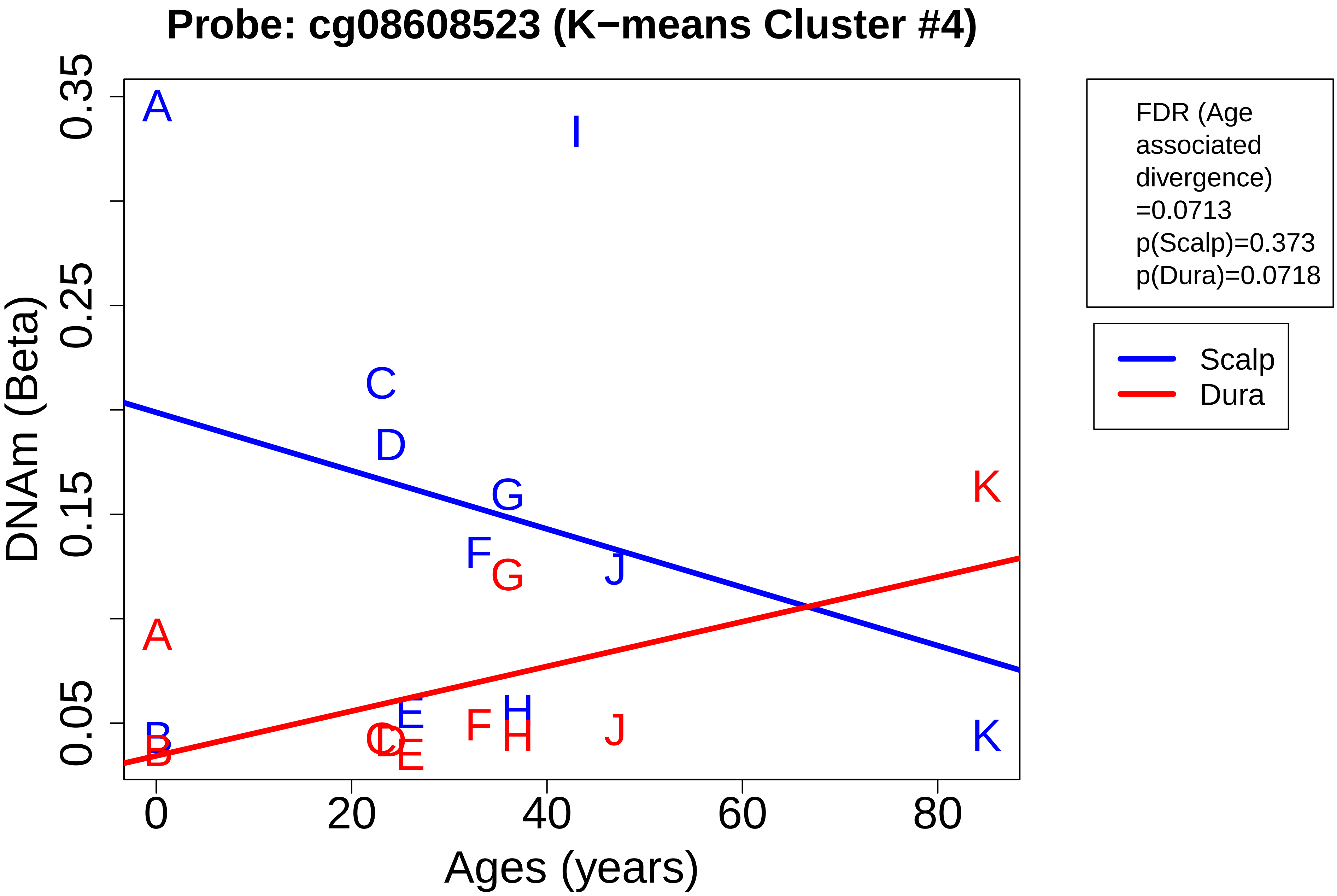

Probe: cg08608523 (K−means Cluster #4)
DNAm (Beta)
0.05
0.15
0.25
0.35
Ages (years)
0
20
40
60
80
A
I
C
D
G
K
F
G
J
A
E
H
B
B
C
D
F
H
J
K
E
FDR (Age associated divergence) =0.0713
p(Scalp)=0.373
p(Dura)=0.0718
Scalp
Dura

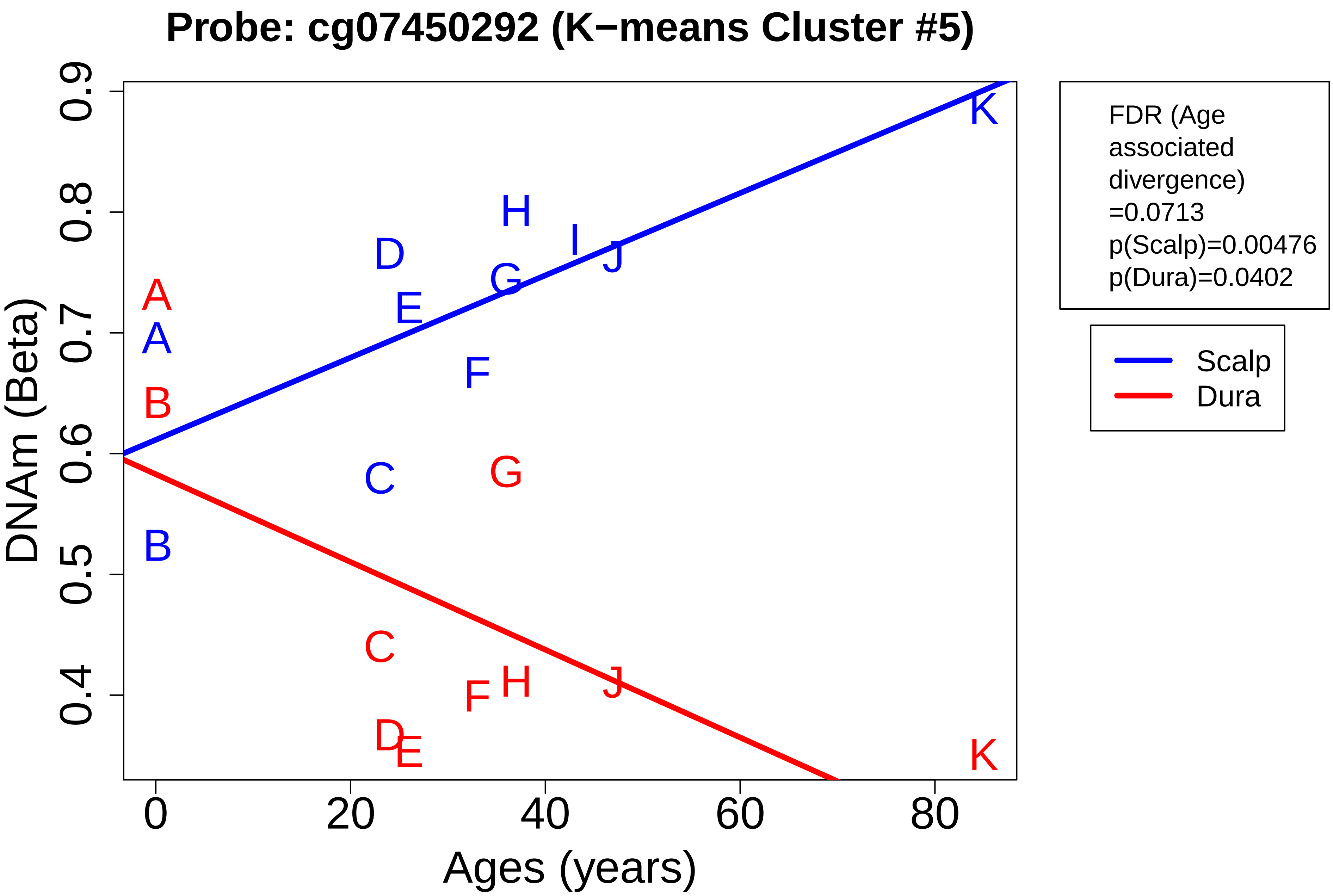

Probe: cg07450292 (K−means Cluster #5)
DNAm (Beta)
0.4
0.5
0.6
0.7
0.8
0.9
Ages (years)
0
20
40
60
80
FDR (Age associated divergence) =0.0713
p(Scalp)=0.00476
p(Dura)=0.0402
Scalp
Dura
A
B
C
D
E
F
G
H
I
J
K

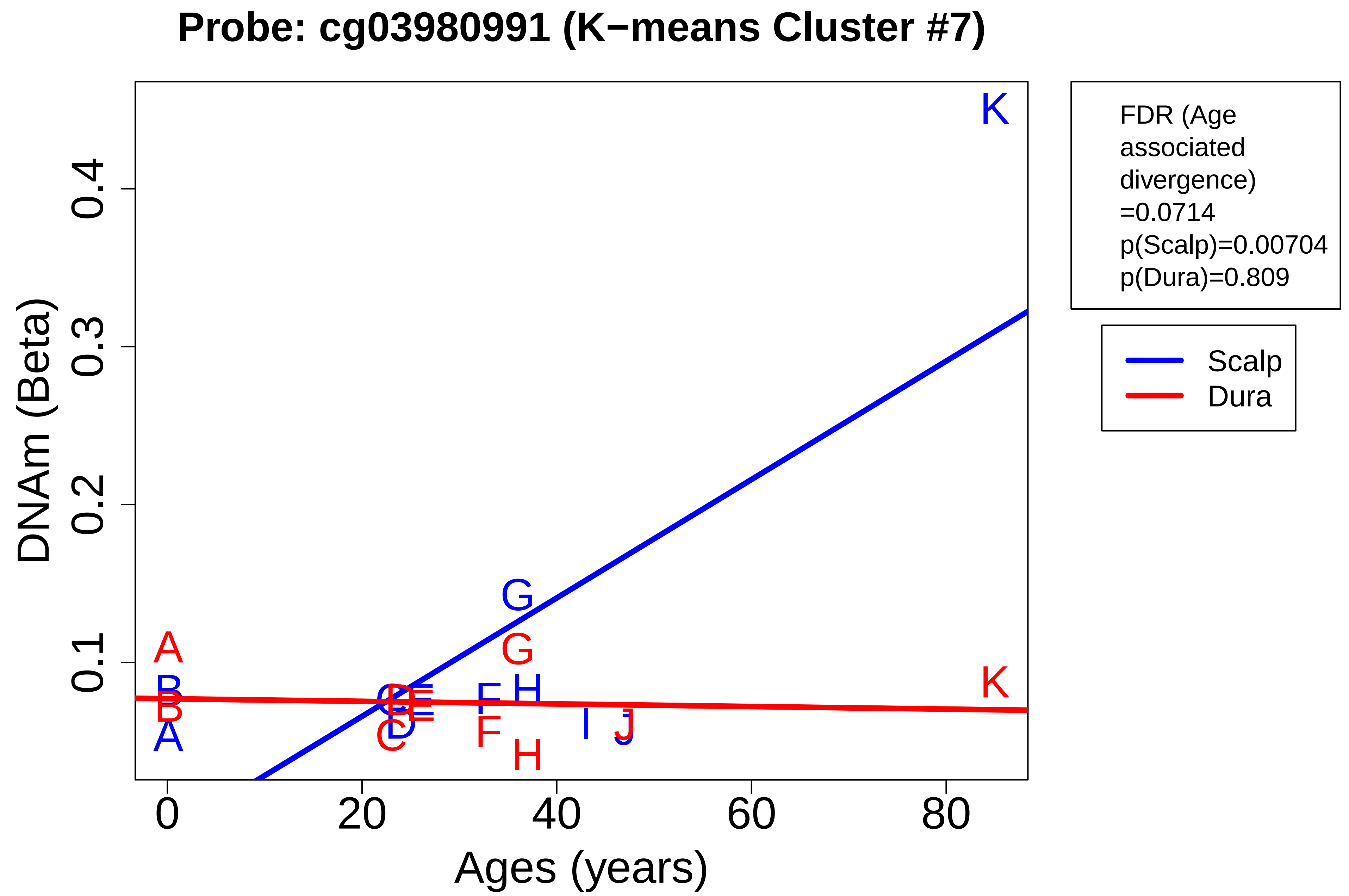

Probe: cg03980991 (K−means Cluster #7)
DNAm (Beta)
0.1
0.2
0.3
0.4
Ages (years)
0
20
40
60
80
FDR (Age associated divergence) =0.0714
p(Scalp)=0.00704
p(Dura)=0.809
Scalp
Dura

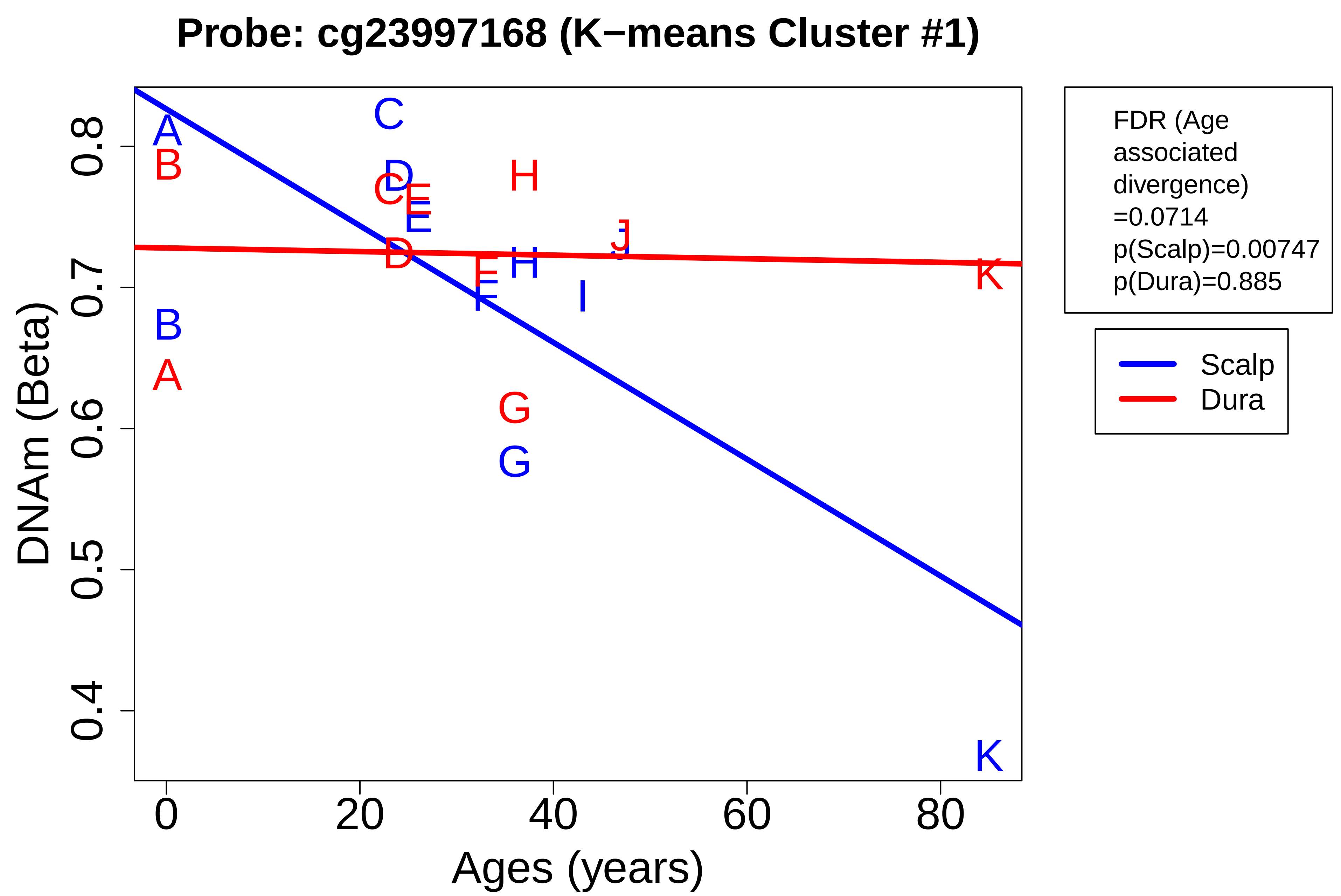

Probe: cg23997168 (K−means Cluster #1)
DNAm (Beta)
0.4
0.5
0.6
0.7
0.8
0
20
40
60
80
Ages (years)
A
B
C
D
E
F
G
H
I
J
K
FDR (Age associated divergence) =0.0714
p(Scalp)=0.00747
p(Dura)=0.885
Scalp
Dura

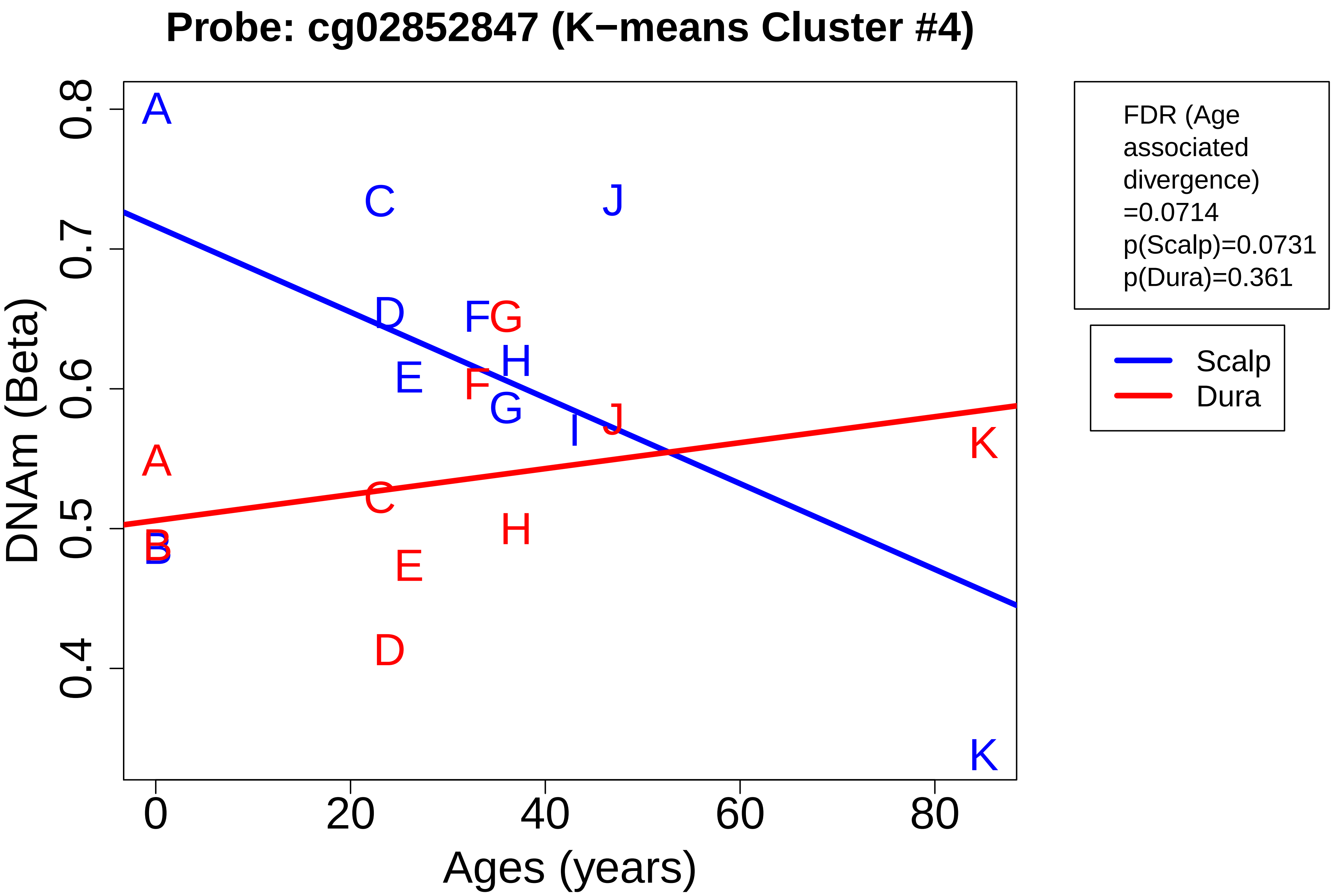

Probe: cg02852847 (K−means Cluster #4)
DNAm (Beta)
0.4
0.5
0.6
0.7
0.8
Ages (years)
0
20
40
60
80
FDR (Age associated divergence) =0.0714
p(Scalp)=0.0731
p(Dura)=0.361
Scalp
Dura

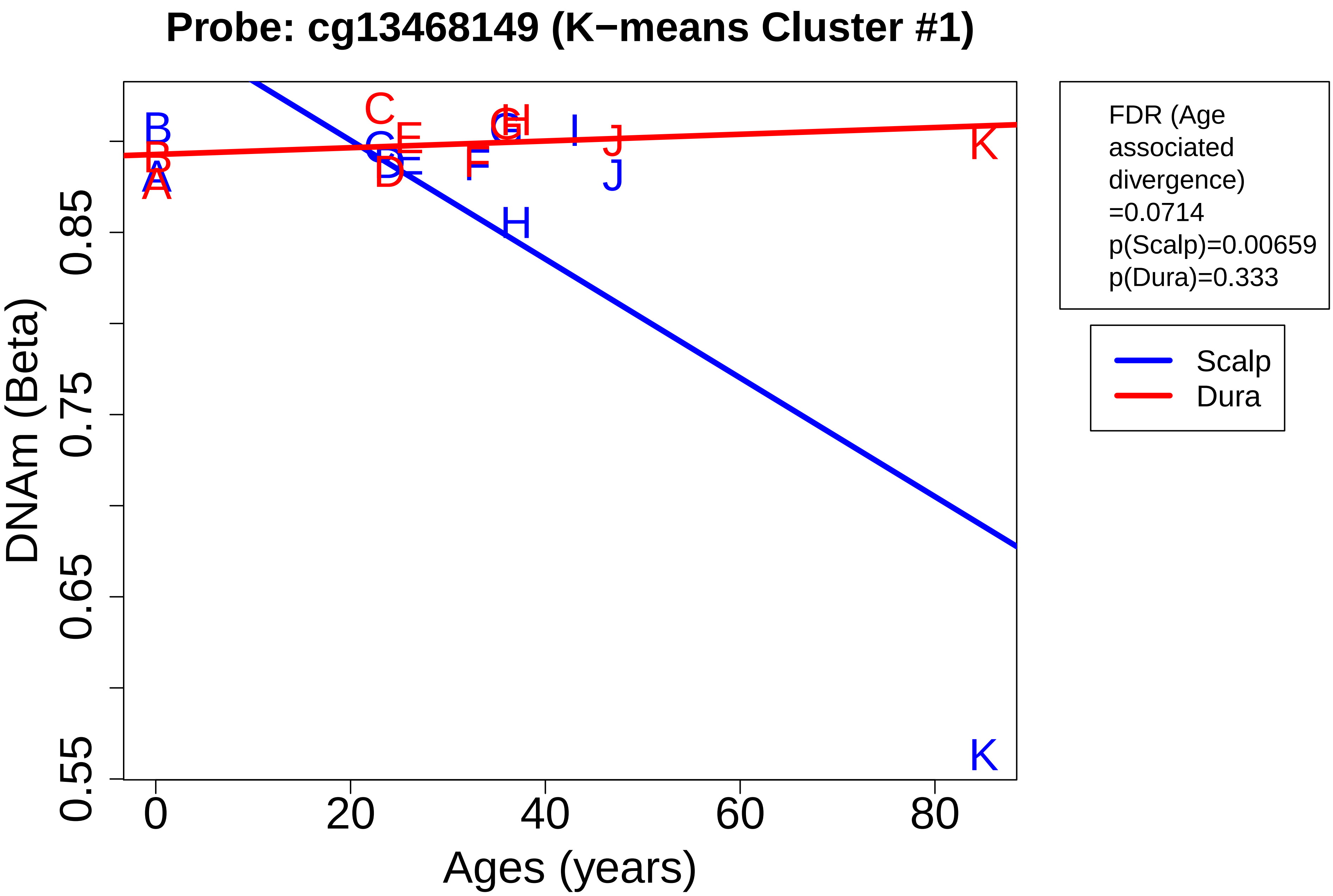

Probe: cg13468149 (K−means Cluster #1)
DNAm (Beta)
0.55
0.65
0.75
0.85
Ages (years)
0
20
40
60
80
FDR (Age associated divergence) =0.0714
p(Scalp)=0.00659
p(Dura)=0.333
Scalp
Dura

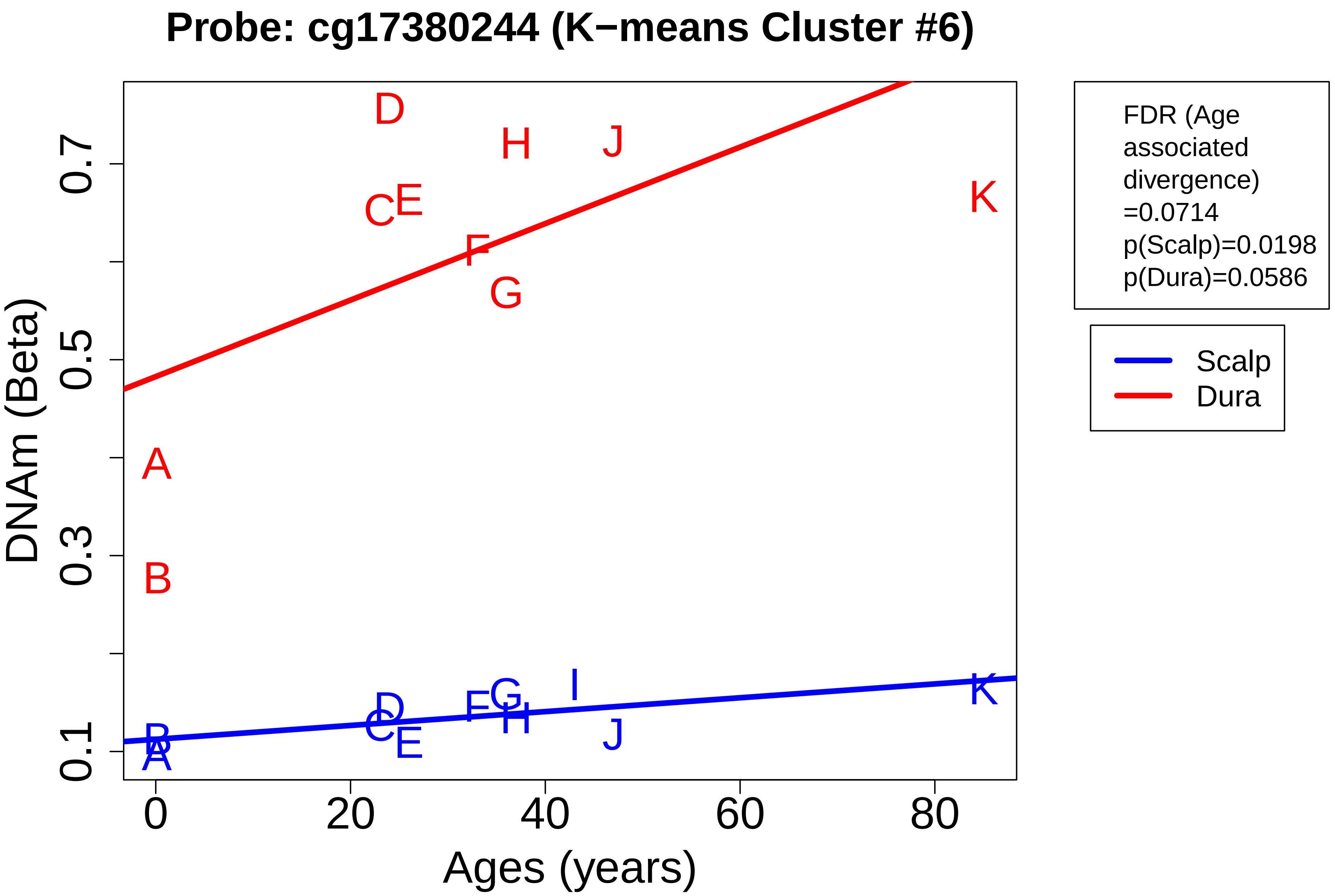

Probe: cg17380244 (K−means Cluster #6)
DNAm (Beta)
0.1
0.3
0.5
0.7
Ages (years)
0
20
40
60
80
FDR (Age associated divergence) =0.0714
p(Scalp)=0.0198
p(Dura)=0.0586
Scalp
Dura
A
B
C
D
E
F
G
H
I
J
K

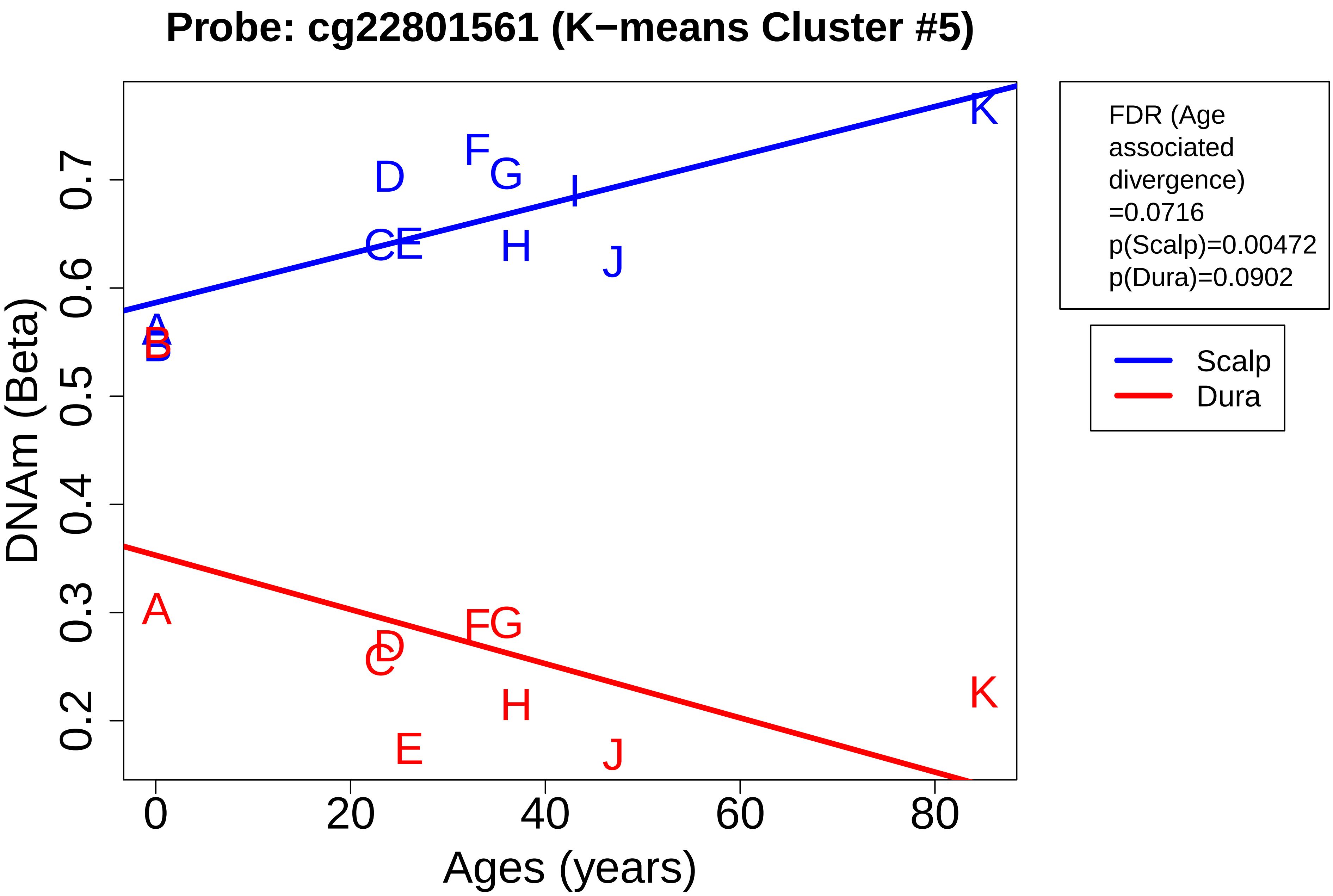

Probe: cg22801561 (K−means Cluster #5)
DNAm (Beta)
0.2
0.3
0.4
0.5
0.6
0.7
Ages (years)
0
20
40
60
80
A
B
C
D
E
F
G
H
I
J
K
FDR (Age associated divergence) =0.0716
p(Scalp)=0.00472
p(Dura)=0.0902
Scalp
Dura

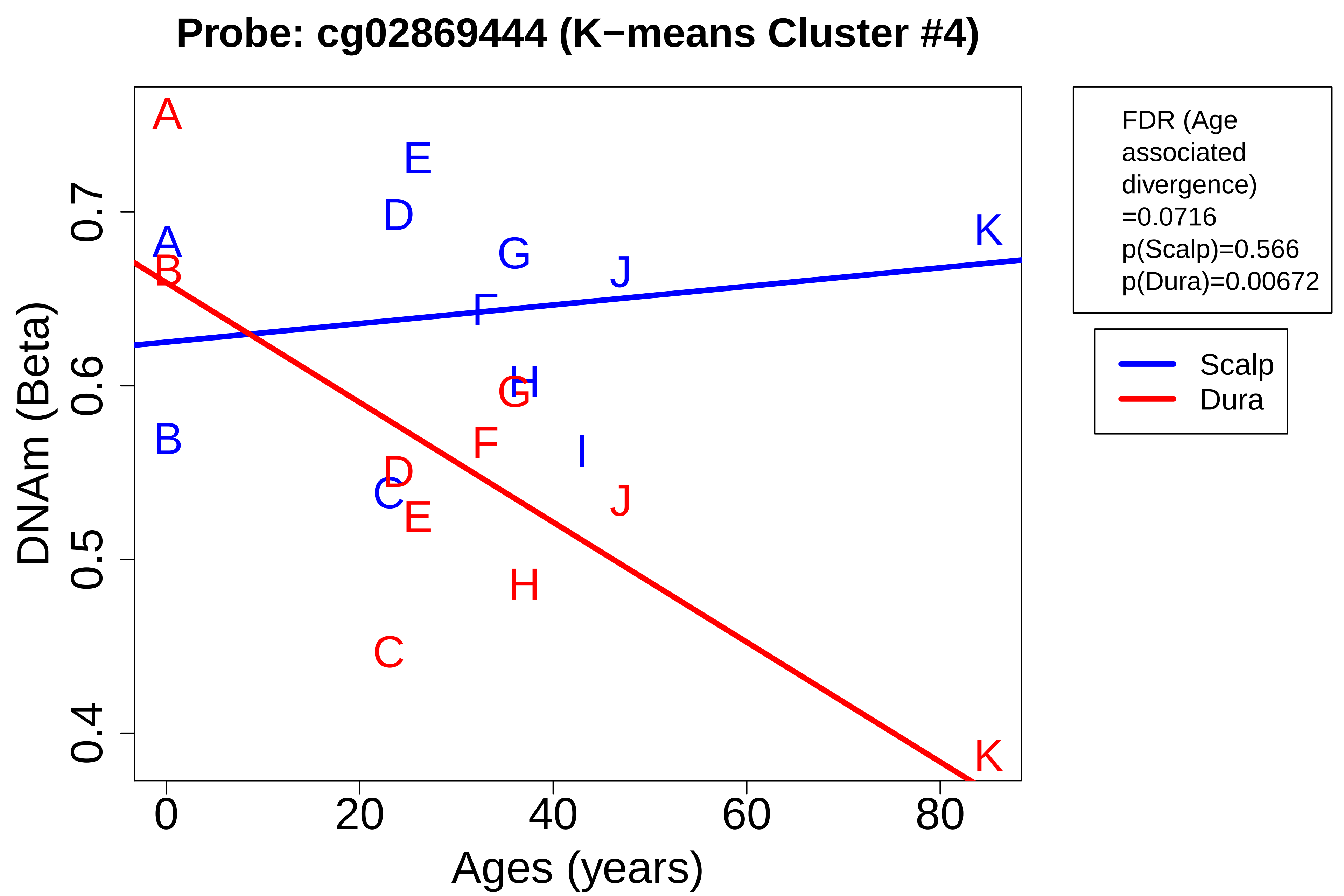

Probe: cg02869444 (K−means Cluster #4)
DNAm (Beta)
0.4
0.5
0.6
0.7
0
20
40
60
80
Ages (years)
A
B
C
D
E
F
G
H
I
J
K
FDR (Age associated divergence) =0.0716
p(Scalp)=0.566
p(Dura)=0.00672
Scalp
Dura

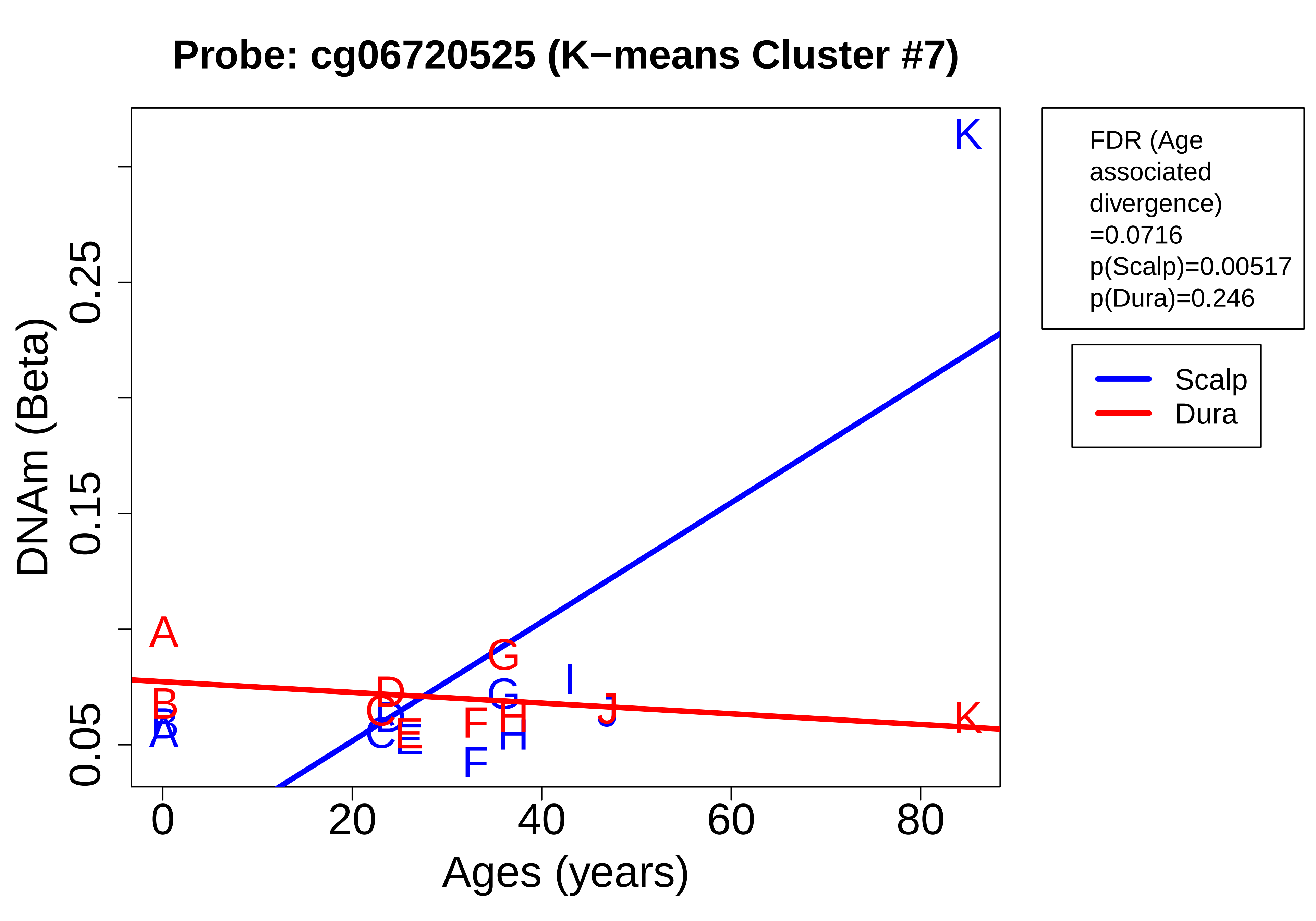

Probe: cg06720525 (K−means Cluster #7)
DNAm (Beta)
0.05
0.15
0.25
Ages (years)
0
20
40
60
80
FDR (Age associated divergence) =0.0716
p(Scalp)=0.00517
p(Dura)=0.246
Scalp
Dura

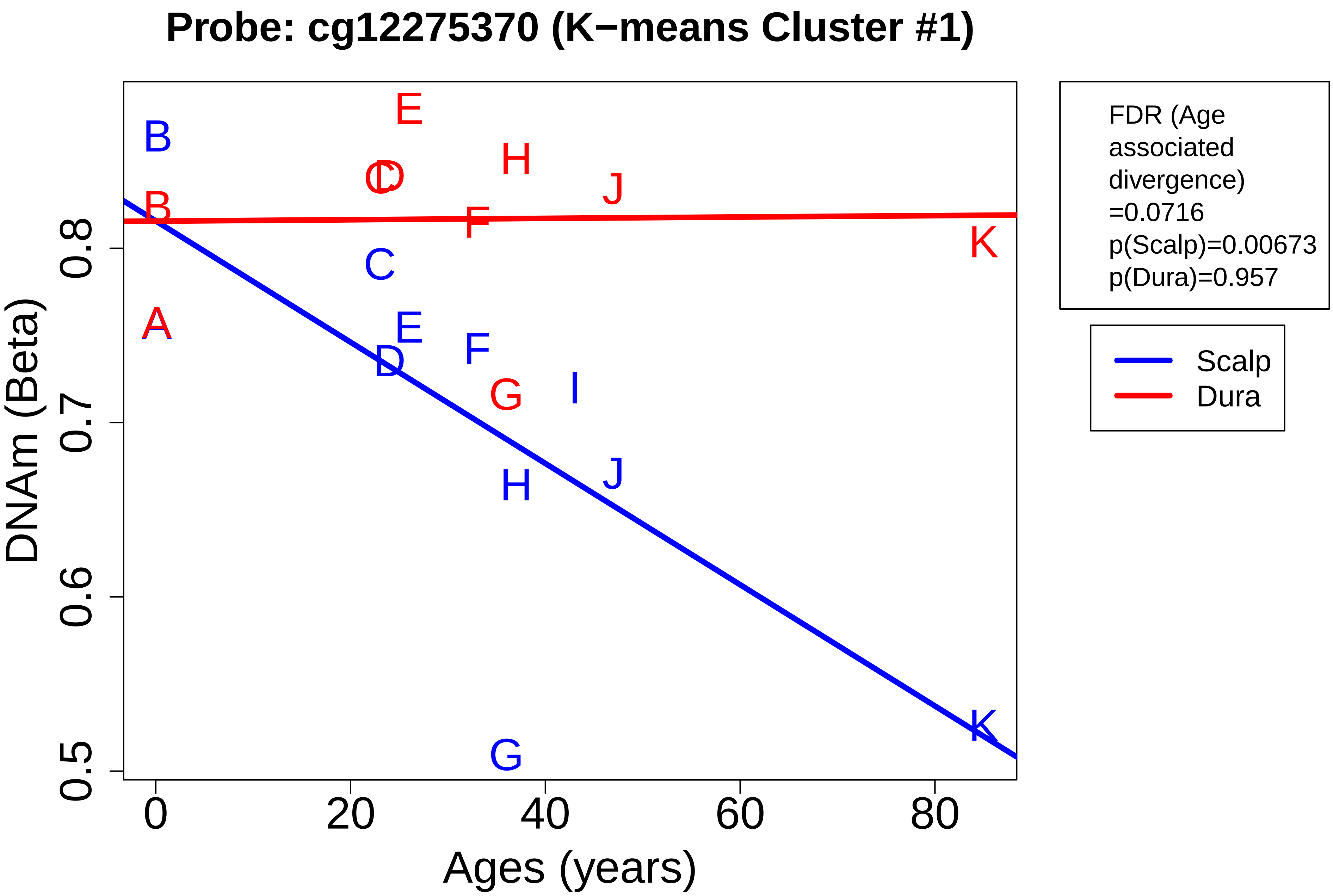

Probe: cg12275370 (K−means Cluster #1)
FDR (Age associated divergence) =0.0716
p(Scalp)=0.00673
p(Dura)=0.957
Scalp
Dura
DNAm (Beta)
Ages (years)
0.5
0.6
0.7
0.8
0
20
40
60
80

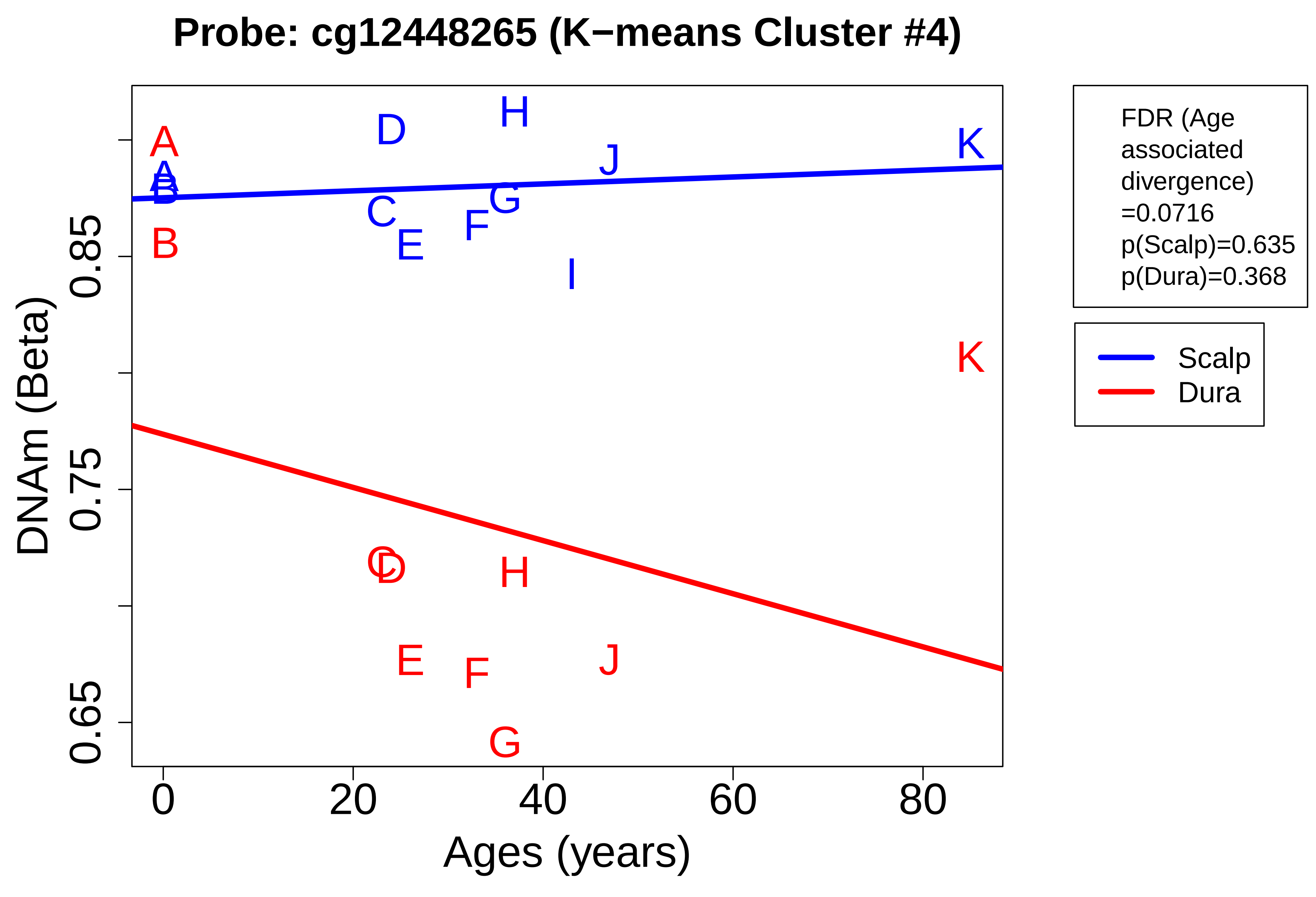

Probe: cg12448265 (K−means Cluster #4)
DNAm (Beta)
0.65
0.75
0.85
0
20
40
60
80
Ages (years)
A
B
C
D
E
F
G
H
I
J
K
FDR (Age associated divergence) =0.0716
p(Scalp)=0.635
p(Dura)=0.368
Scalp
Dura

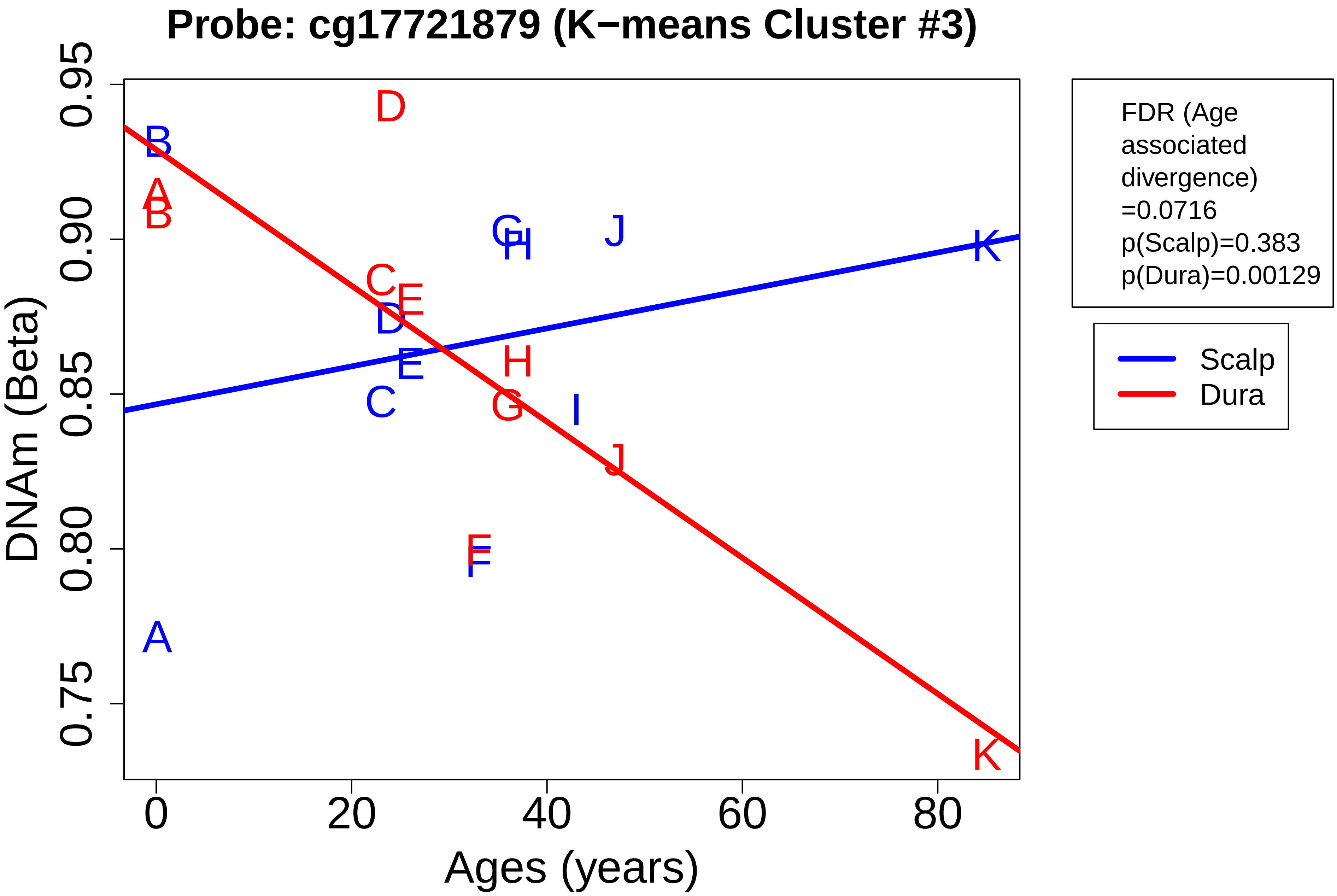

Probe: cg17721879 (K−means Cluster #3)
DNAm (Beta)
0.75
0.80
0.85
0.90
0.95
Ages (years)
0
20
40
60
80
A
B
C
D
E
F
G
H
I
J
K
FDR (Age associated divergence) =0.0716
p(Scalp)=0.383
p(Dura)=0.00129
Scalp
Dura

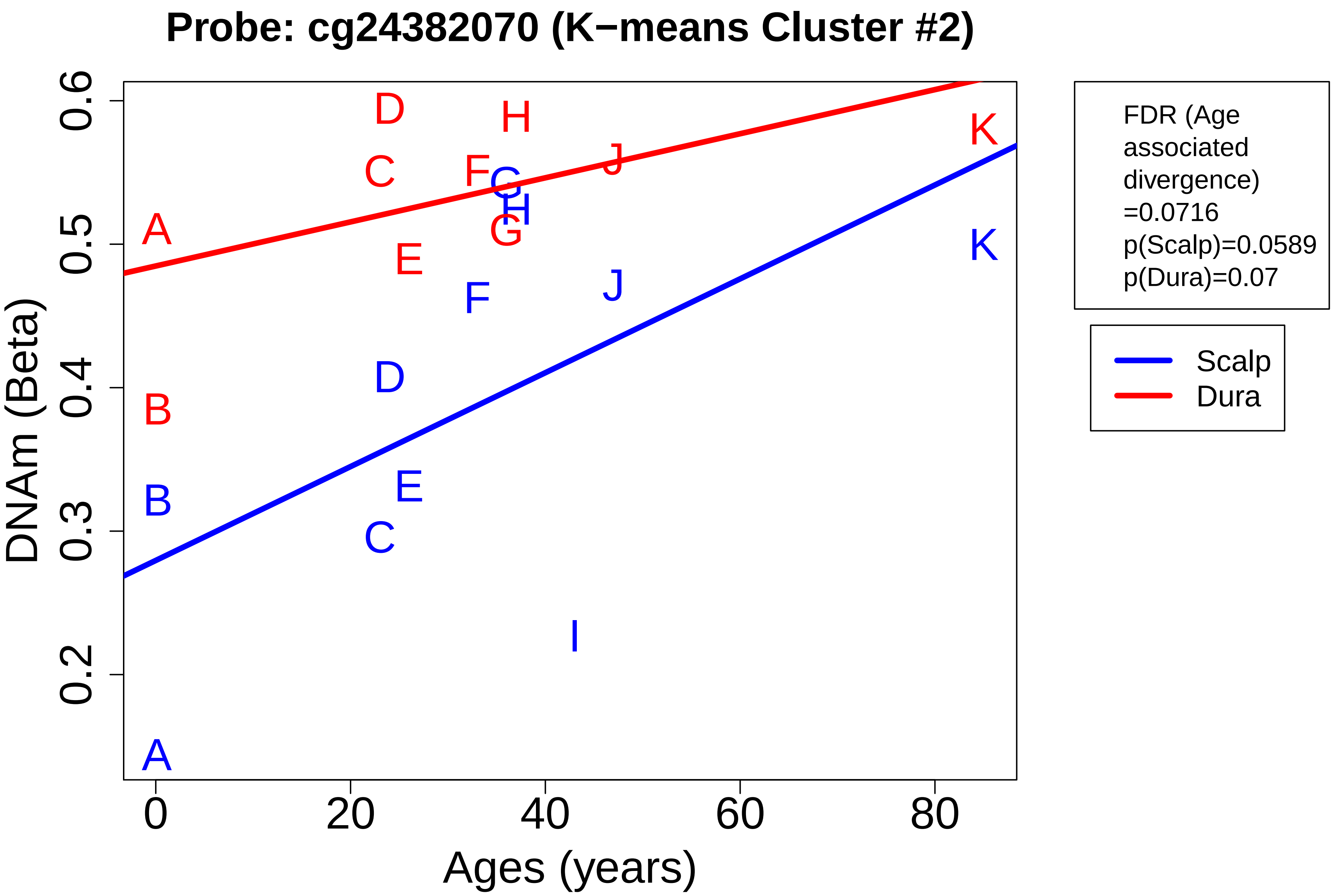

Probe: cg24382070 (K−means Cluster #2)
DNAm (Beta)
0.2
0.3
0.4
0.5
0.6
Ages (years)
0
20
40
60
80
A
B
C
D
E
F
G
H
I
J
K
FDR (Age associated divergence) =0.0716
p(Scalp)=0.0589
p(Dura)=0.07
Scalp
Dura

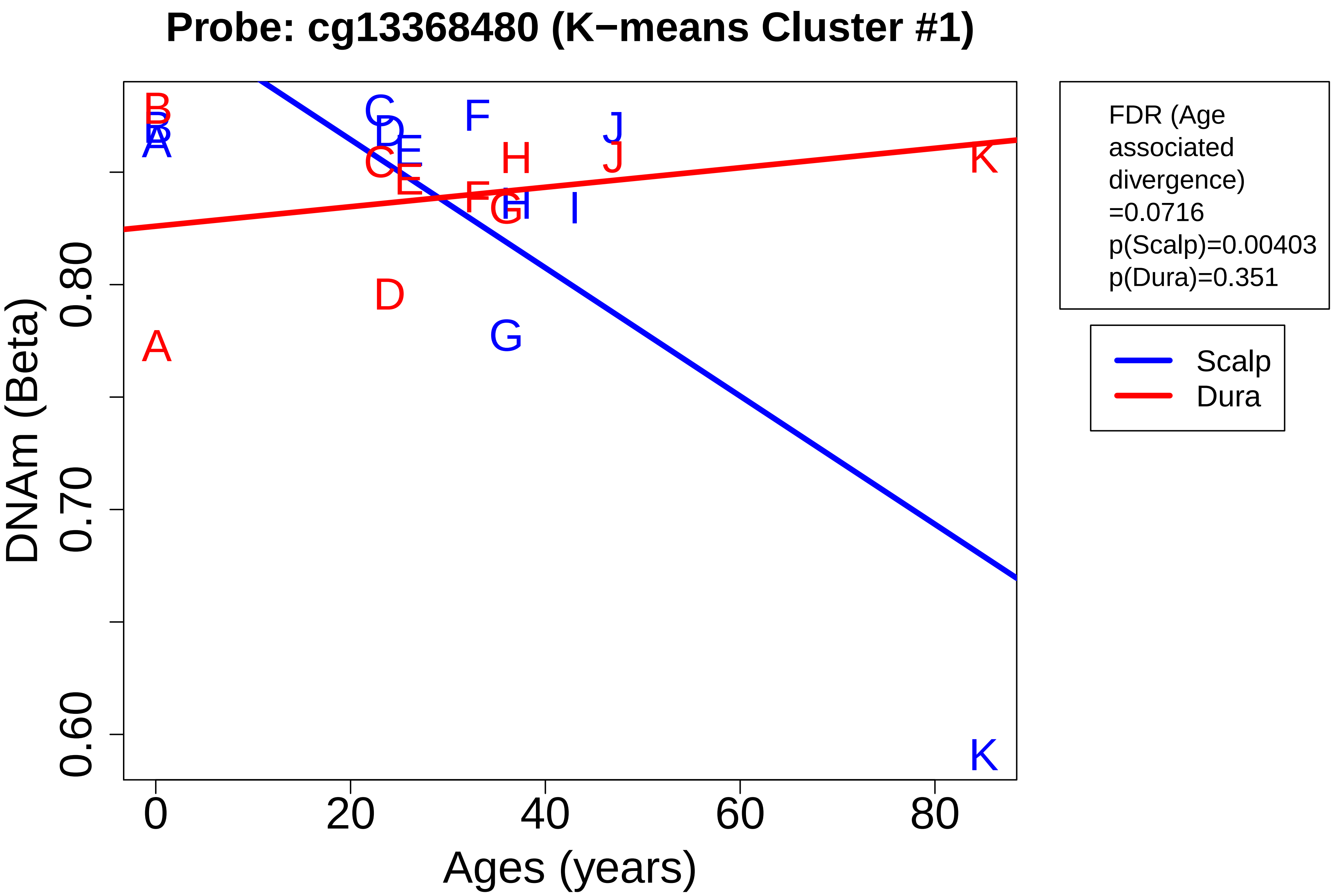

Probe: cg13368480 (K−means Cluster #1)
DNAm (Beta)
0.60
0.70
0.80
0
20
40
60
80
Ages (years)
A
B
C
D
E
F
G
H
I
J
K
FDR (Age associated divergence) =0.0716
p(Scalp)=0.00403
p(Dura)=0.351
Scalp
Dura

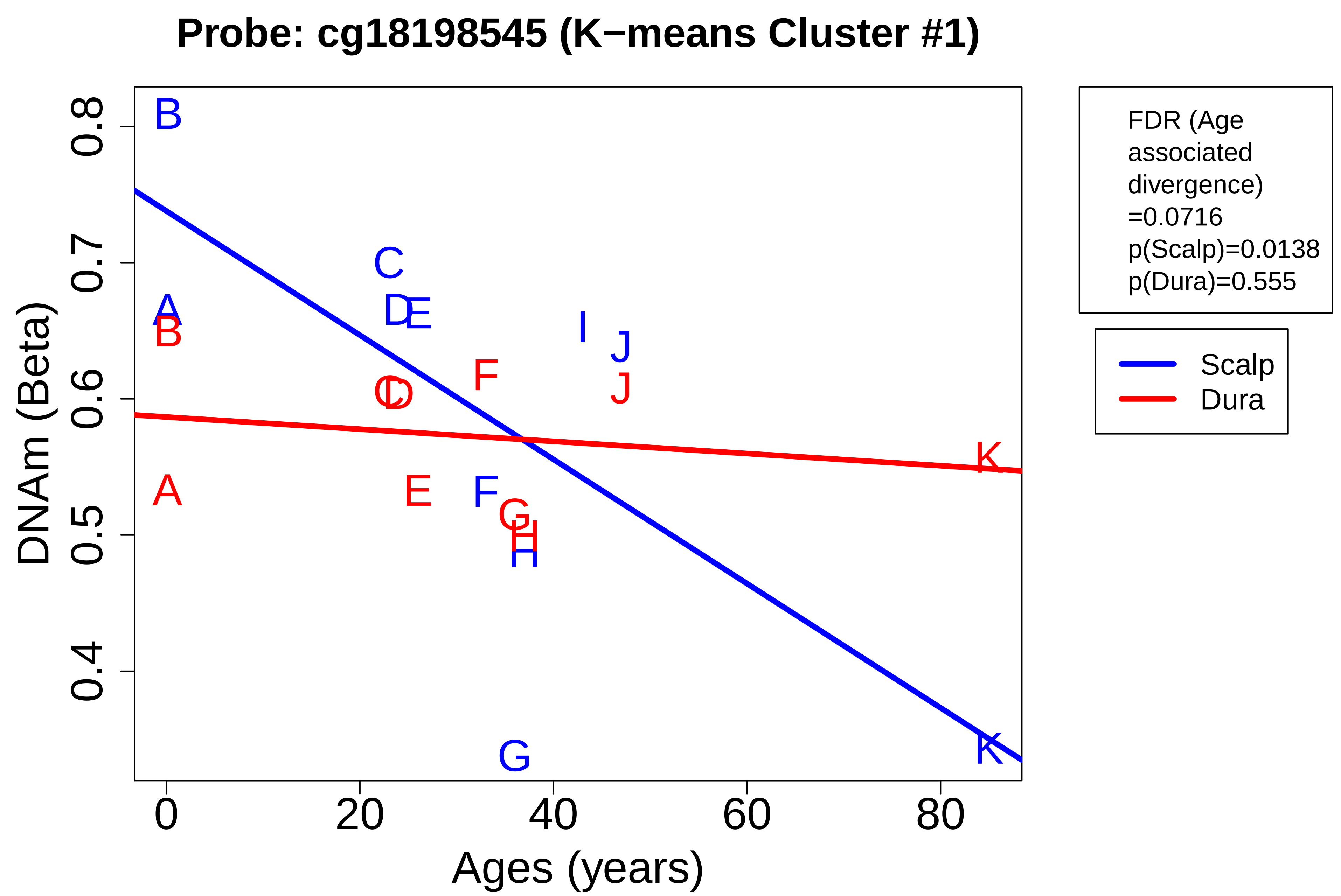

Probe: cg18198545 (K−means Cluster #1)
DNAm (Beta)
0.4
0.5
0.6
0.7
0.8
0
20
40
60
80
Ages (years)
B
C
A
D
E
B
I
J
F
C
D
J
K
A
E
F
G
H
H
G
K
FDR (Age associated divergence) =0.0716
p(Scalp)=0.0138
p(Dura)=0.555
Scalp
Dura

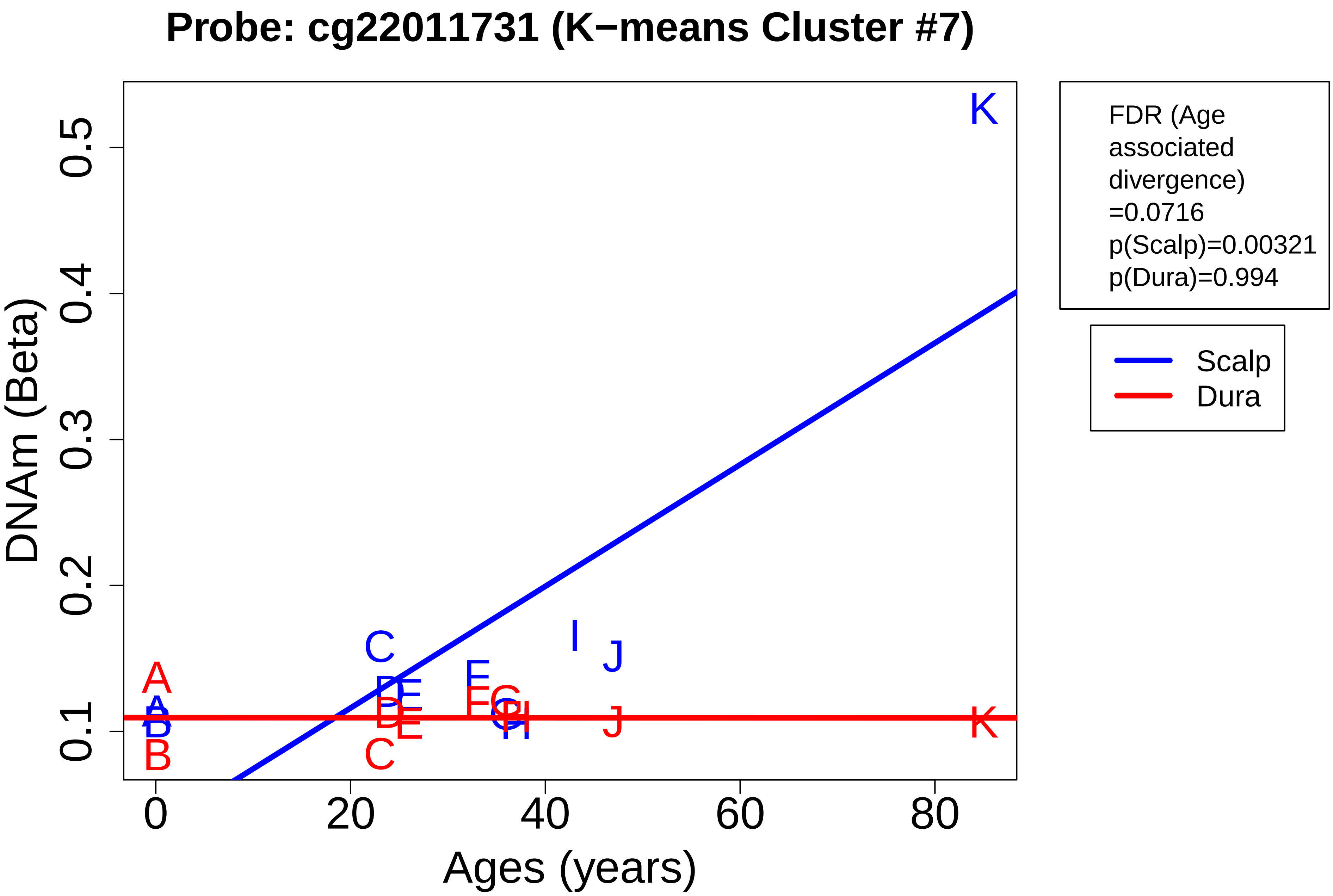

Probe: cg22011731 (K−means Cluster #7)
DNAm (Beta)
0.1
0.2
0.3
0.4
0.5
0
20
40
60
80
Ages (years)
FDR (Age associated divergence) =0.0716
p(Scalp)=0.00321
p(Dura)=0.994
Scalp
Dura

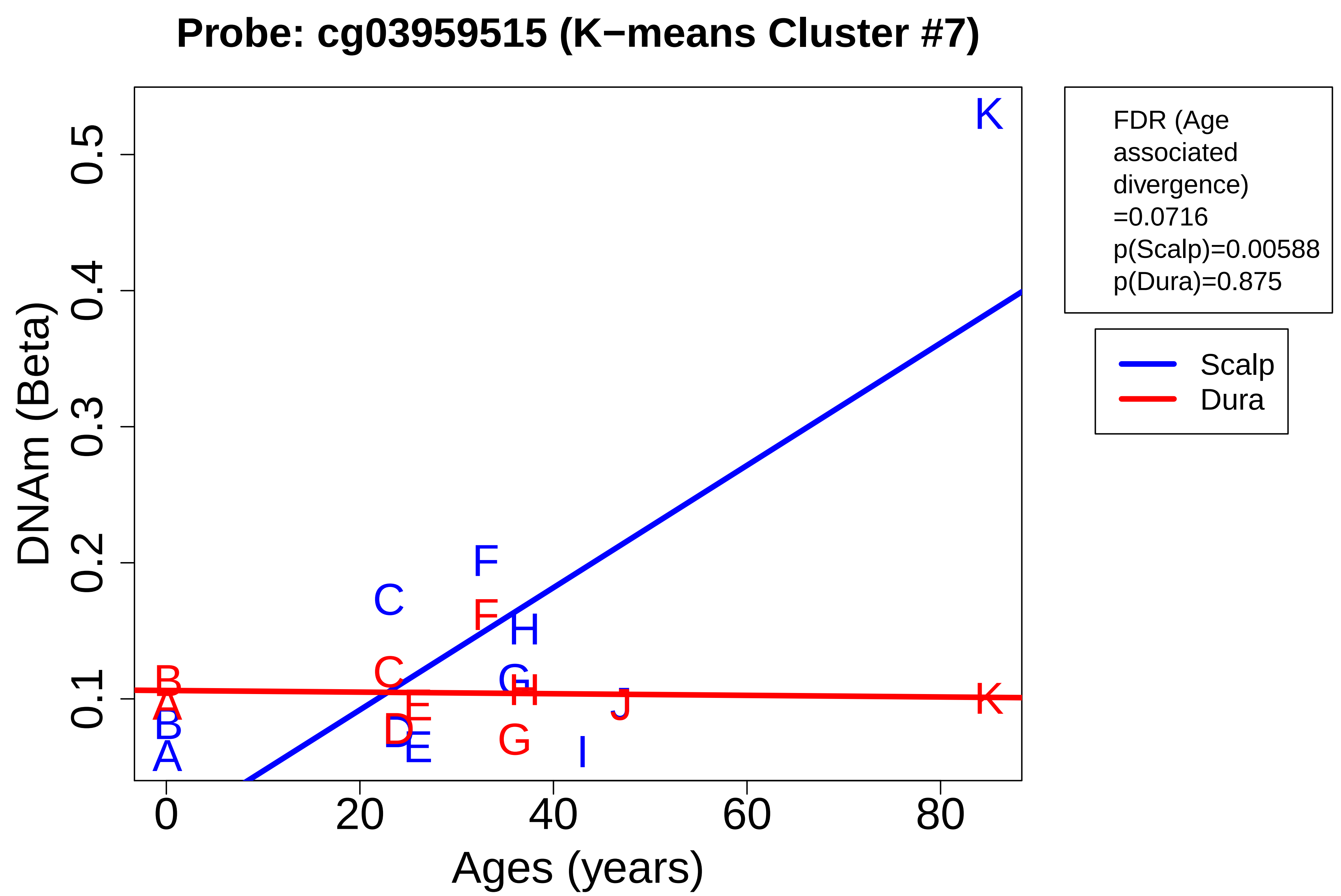

Probe: cg03959515 (K−means Cluster #7)
FDR (Age associated divergence) =0.0716
p(Scalp)=0.00588
p(Dura)=0.875
Scalp
Dura
DNAm (Beta)
0.1
0.2
0.3
0.4
0.5
0
20
40
60
80
Ages (years)
A
B
C
D
E
F
G
H
I
J
K

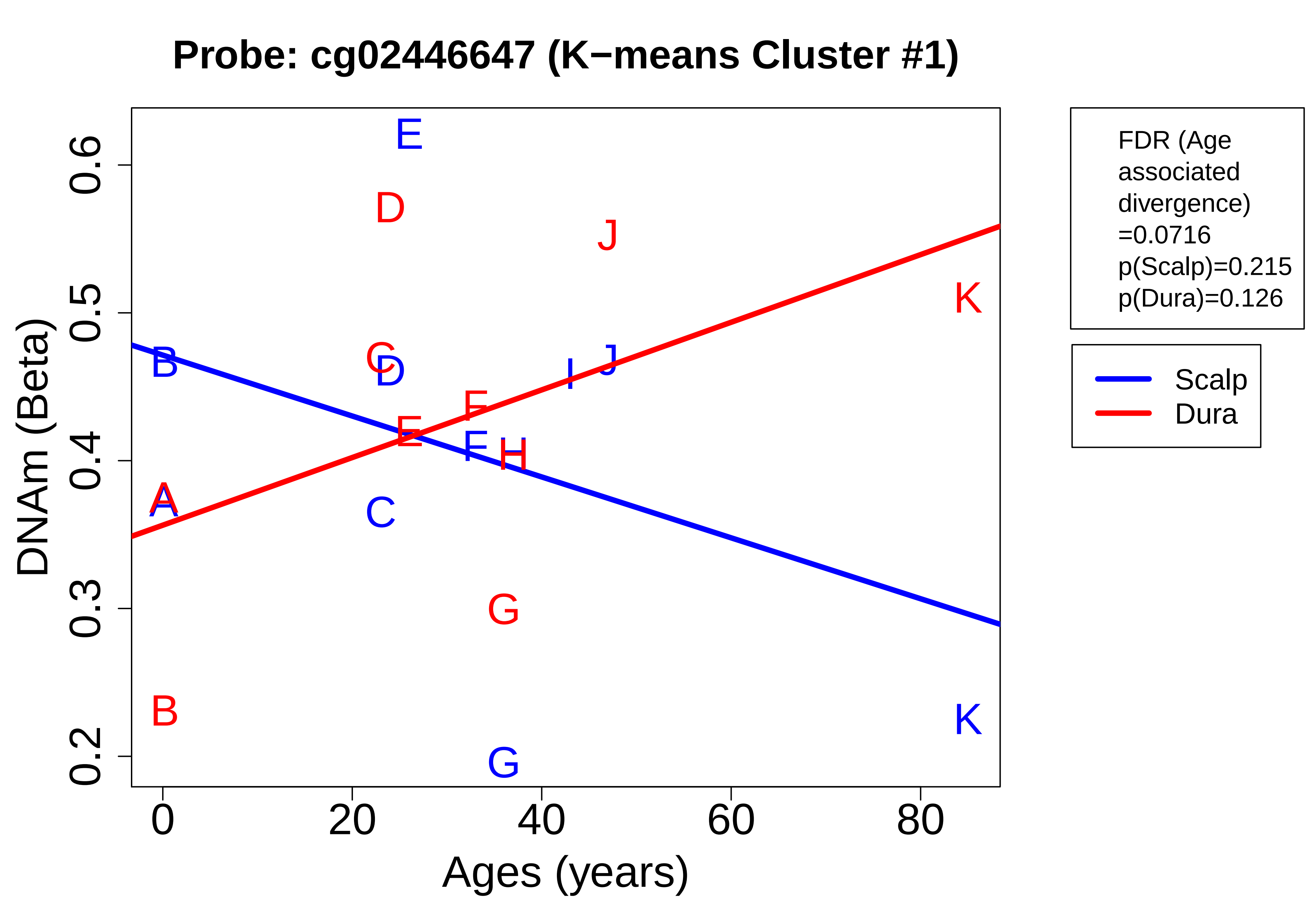
Probe: cg02446647 (K−means Cluster #1)
DNAm (Beta)
Ages (years)
0.2
0.3
0.4
0.5
0.6
0
20
40
60
80
FDR (Age associated divergence) =0.0716
p(Scalp)=0.215
p(Dura)=0.126
Scalp
Dura

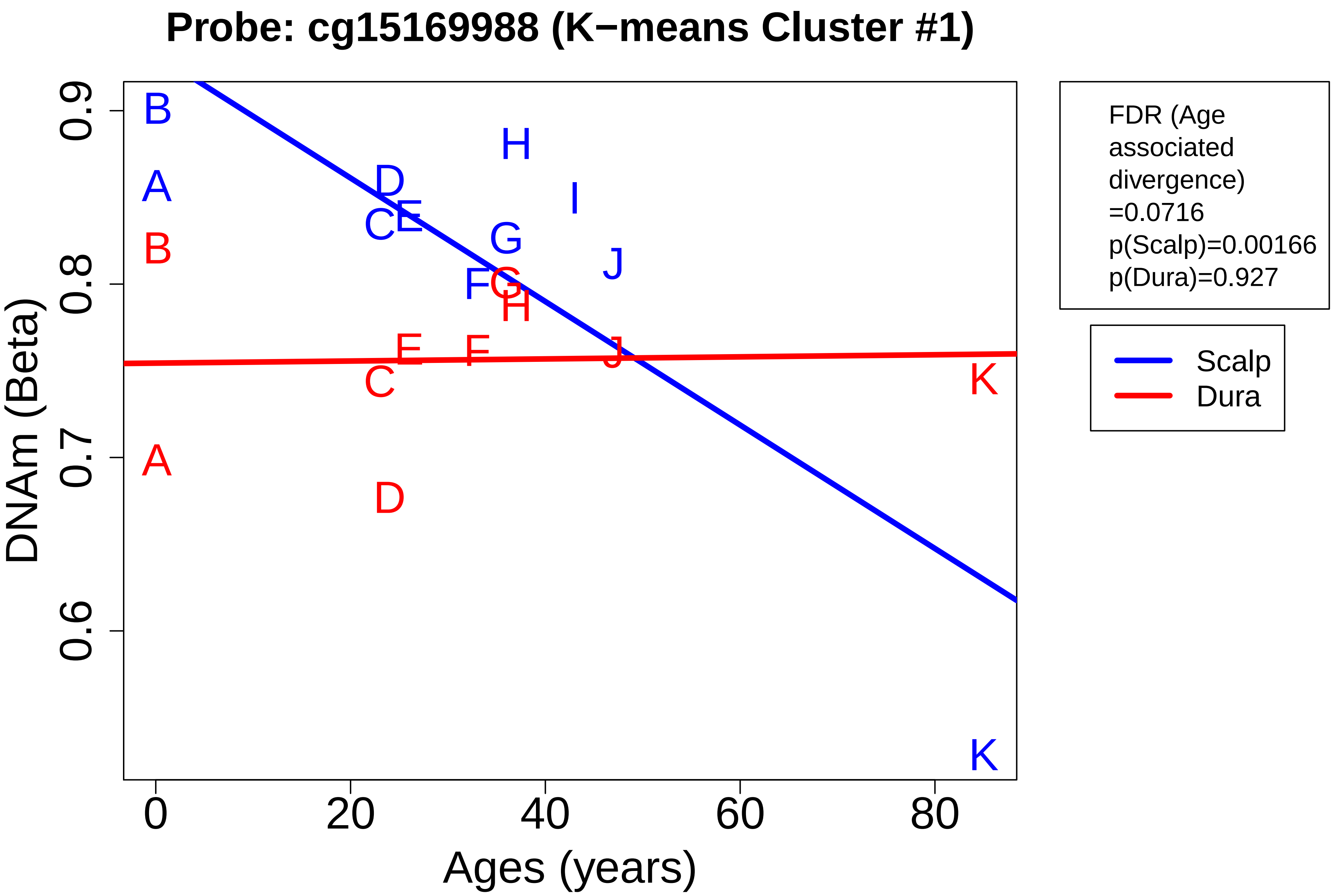

Probe: cg15169988 (K−means Cluster #1)
FDR (Age associated divergence) =0.0716
p(Scalp)=0.00166
p(Dura)=0.927
Scalp
Dura
DNAm (Beta)
0.6
0.7
0.8
0.9
Ages (years)
0
20
40
60
80

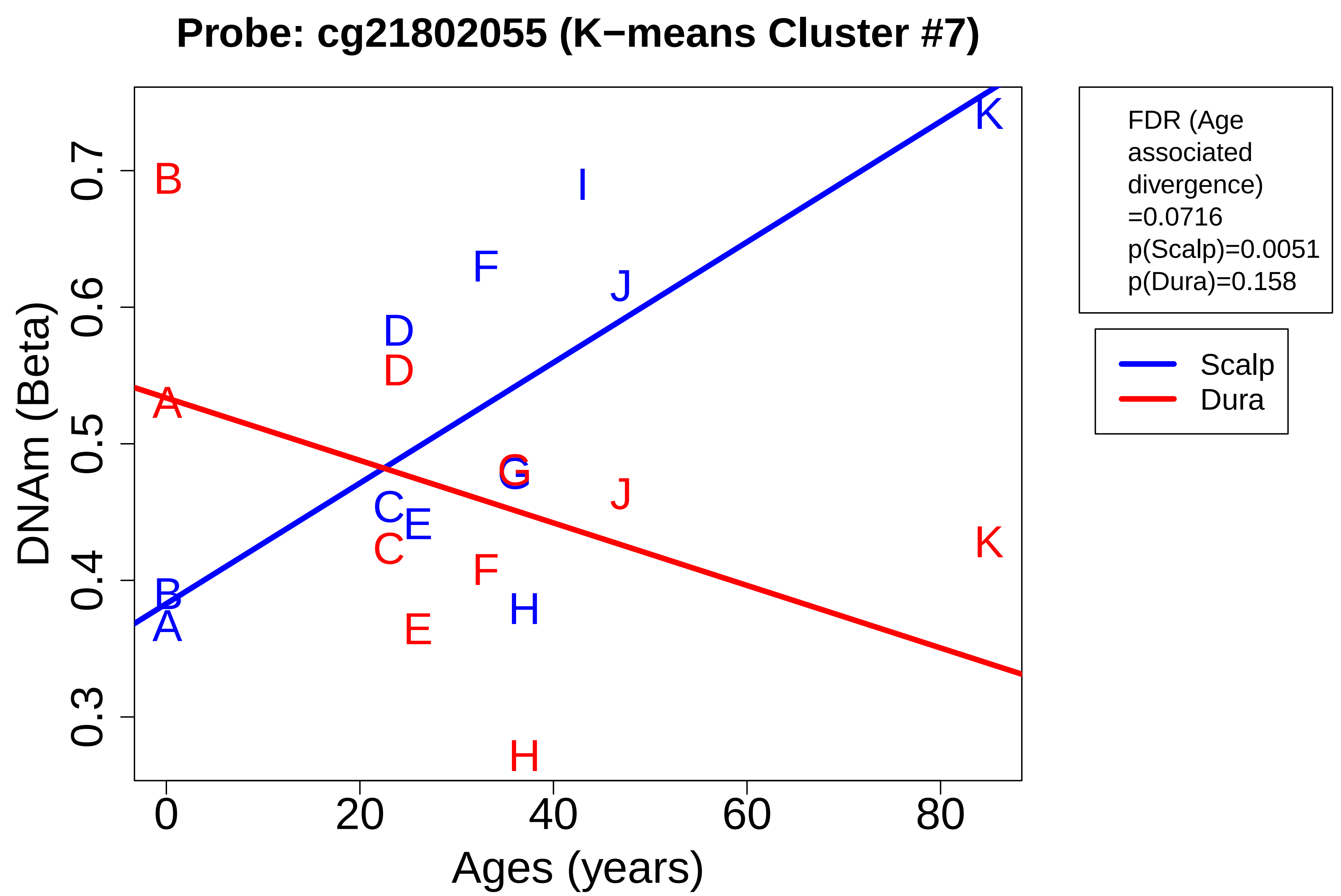

Probe: cg21802055 (K−means Cluster #7)
DNAm (Beta)
0.3
0.4
0.5
0.6
0.7
Ages (years)
0
20
40
60
80
FDR (Age associated divergence) =0.0716
p(Scalp)=0.0051
p(Dura)=0.158
Scalp
Dura

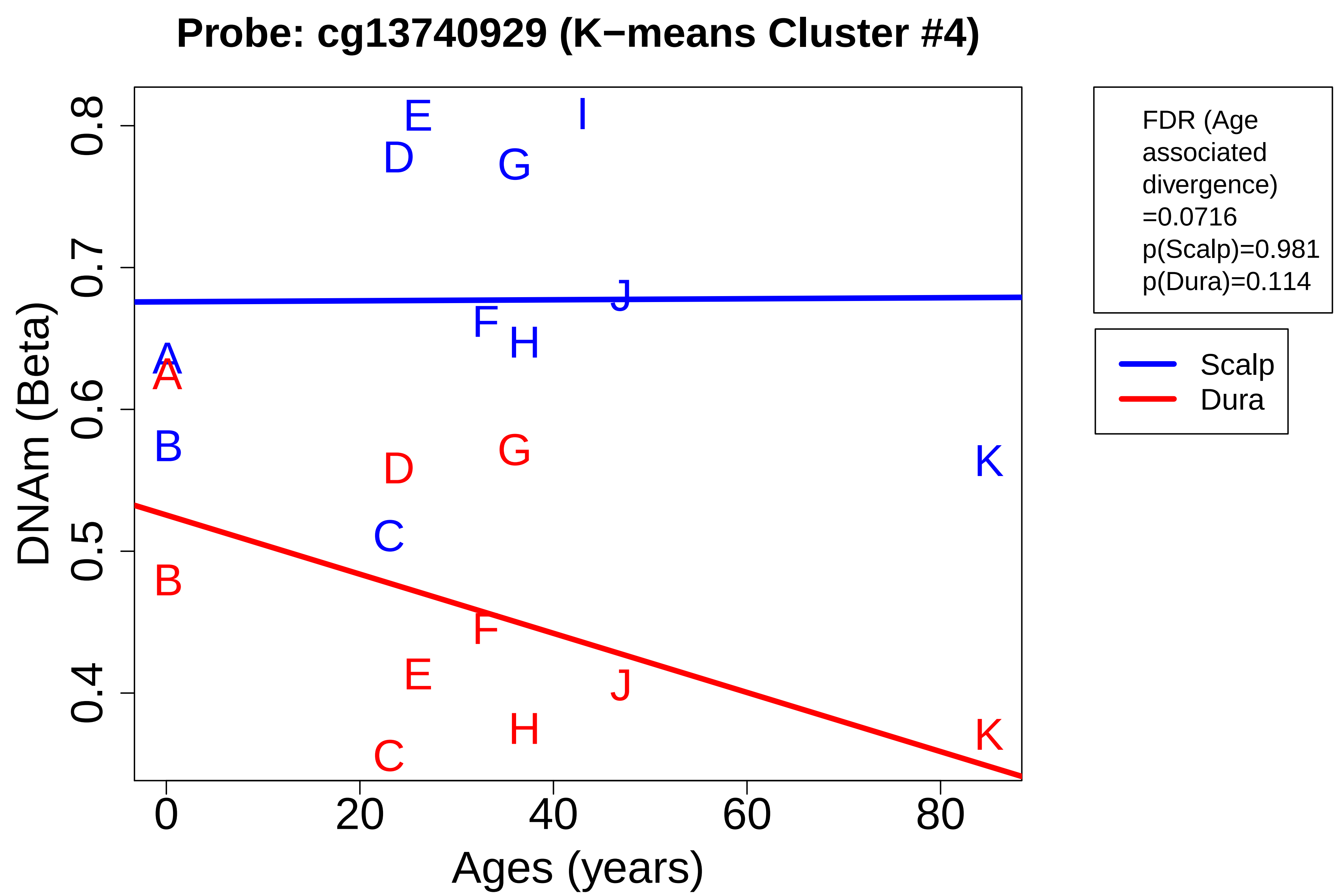

Probe: cg13740929 (K−means Cluster #4)
DNAm (Beta)
0.4
0.5
0.6
0.7
0.8
0
20
40
60
80
Ages (years)
A
B
C
D
E
F
G
H
I
J
K
A
B
C
D
E
F
G
H
J
K
FDR (Age associated divergence) =0.0716
p(Scalp)=0.981
p(Dura)=0.114
Scalp
Dura

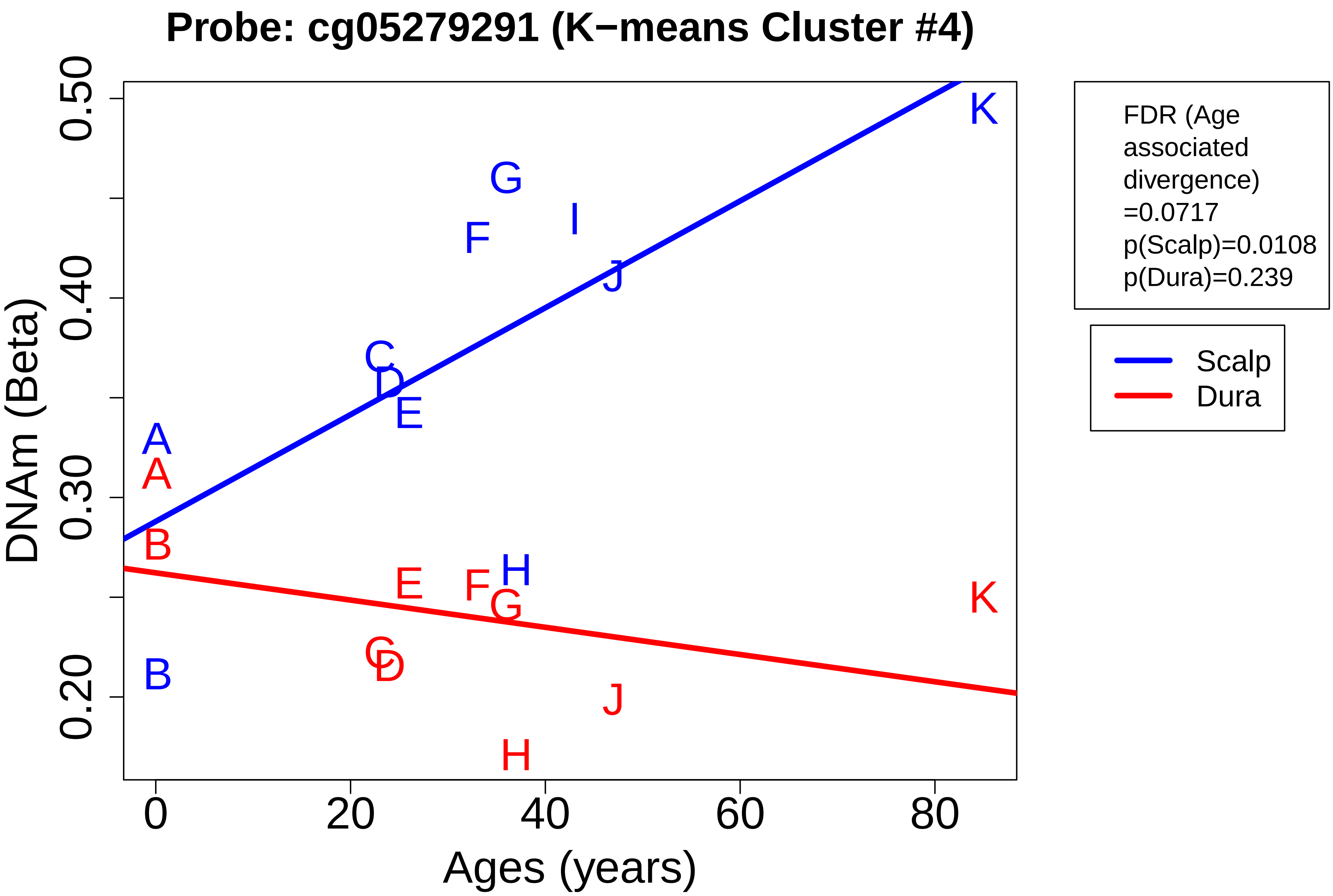

Probe: cg05279291 (K−means Cluster #4)
DNAm (Beta)
0.20
0.30
0.40
0.50
Ages (years)
0
20
40
60
80
A
B
C
D
E
F
G
H
I
J
K
FDR (Age associated divergence) =0.0717
p(Scalp)=0.0108
p(Dura)=0.239
Scalp
Dura

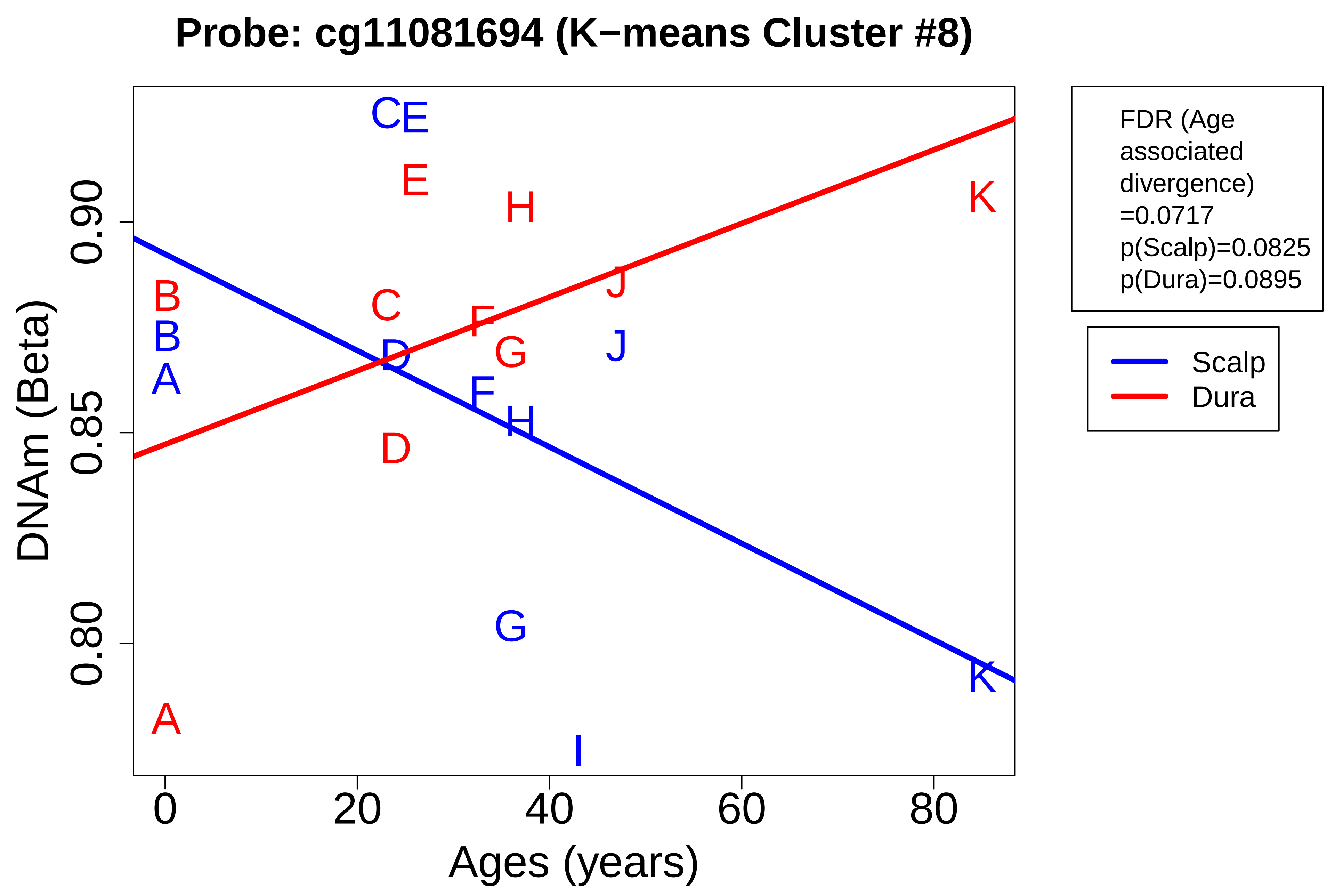

Probe: cg11081694 (K−means Cluster #8)
FDR (Age associated divergence) =0.0717
p(Scalp)=0.0825
p(Dura)=0.0895
Scalp
Dura
DNAm (Beta)
0.80
0.85
0.90
0
20
40
60
80
Ages (years)

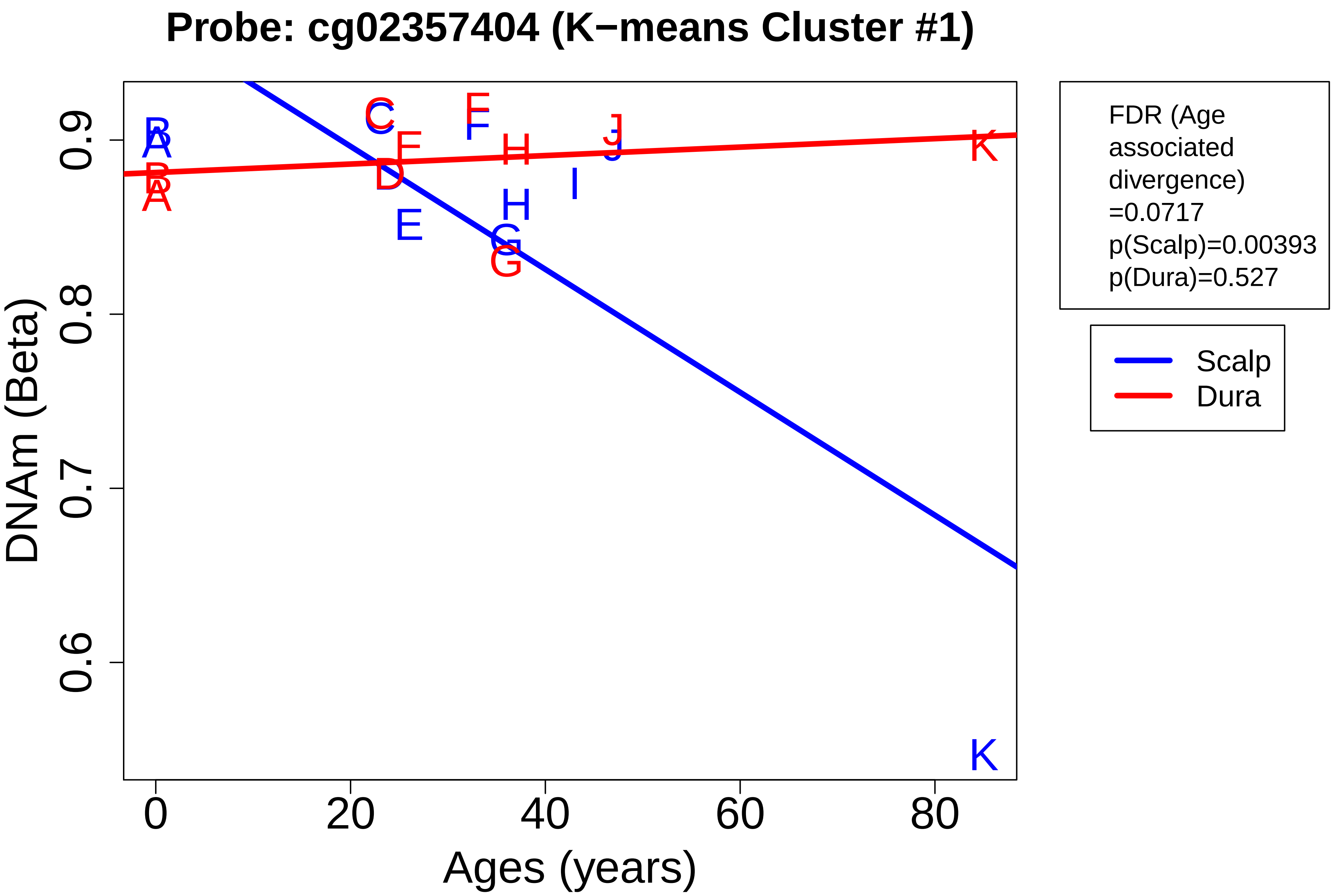

Probe: cg02357404 (K−means Cluster #1)
DNAm (Beta)
0.6
0.7
0.8
0.9
Ages (years)
0
20
40
60
80
FDR (Age associated divergence) =0.0717
p(Scalp)=0.00393
p(Dura)=0.527
Scalp
Dura

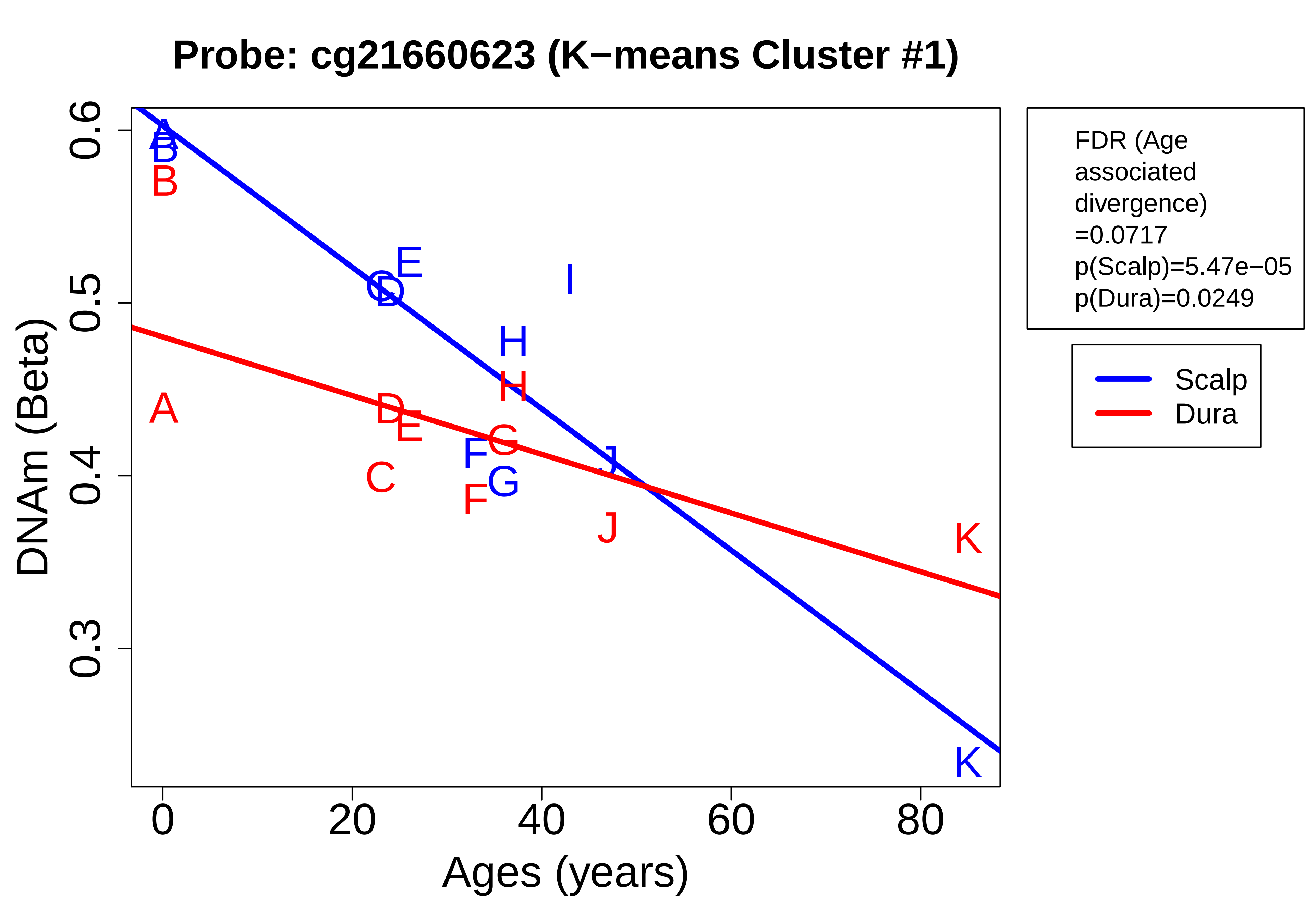

Probe: cg21660623 (K−means Cluster #1)
DNAm (Beta)
Ages (years)
0.3
0.4
0.5
0.6
0
20
40
60
80
FDR (Age associated divergence) =0.0717
p(Scalp)=5.47e−05
p(Dura)=0.0249
Scalp
Dura

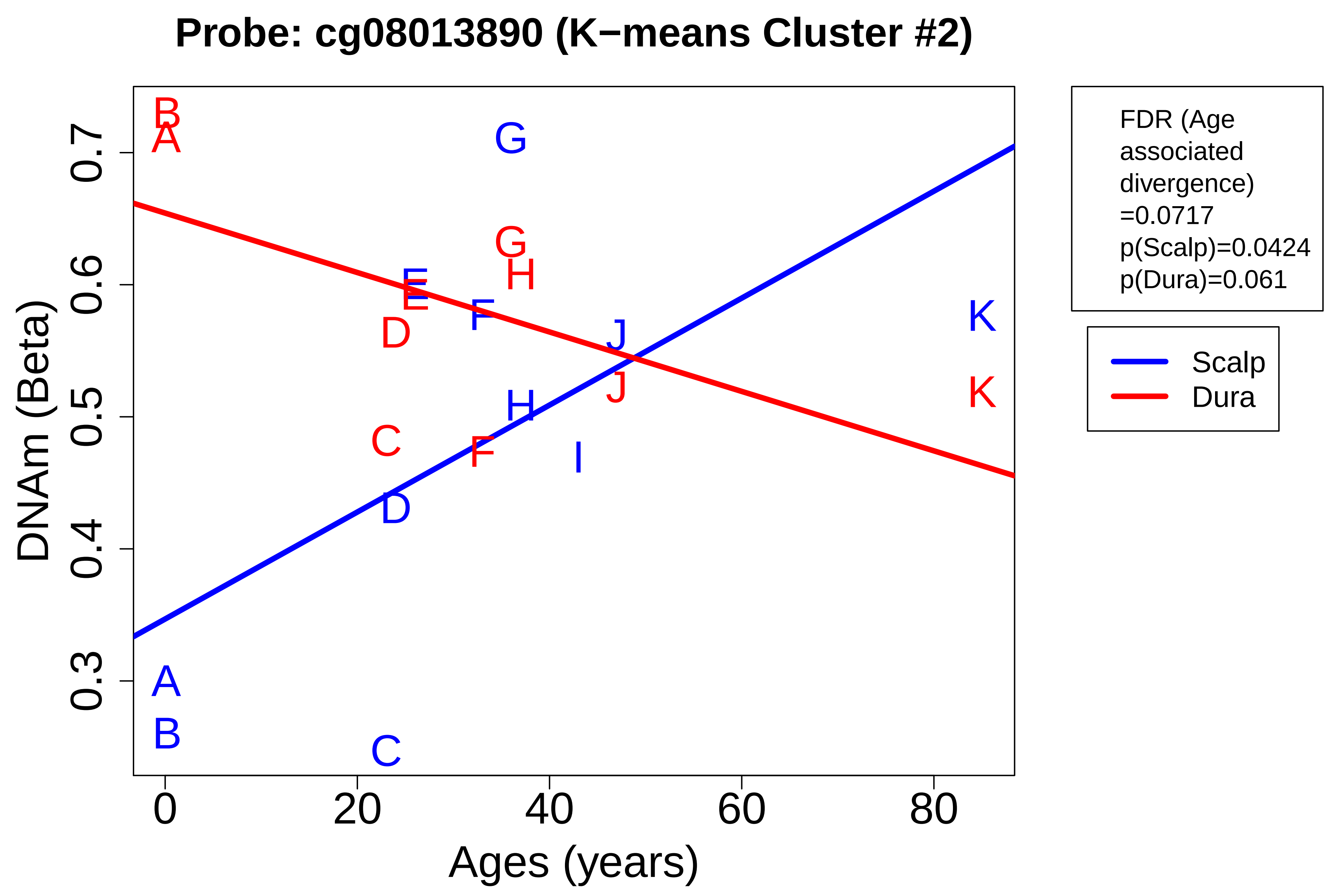

Probe: cg08013890 (K−means Cluster #2)
DNAm (Beta)
0.3
0.4
0.5
0.6
0.7
Ages (years)
0
20
40
60
80
FDR (Age associated divergence) =0.0717
p(Scalp)=0.0424
p(Dura)=0.061
Scalp
Dura
A
B
C
D
E
F
G
H
I
J
K

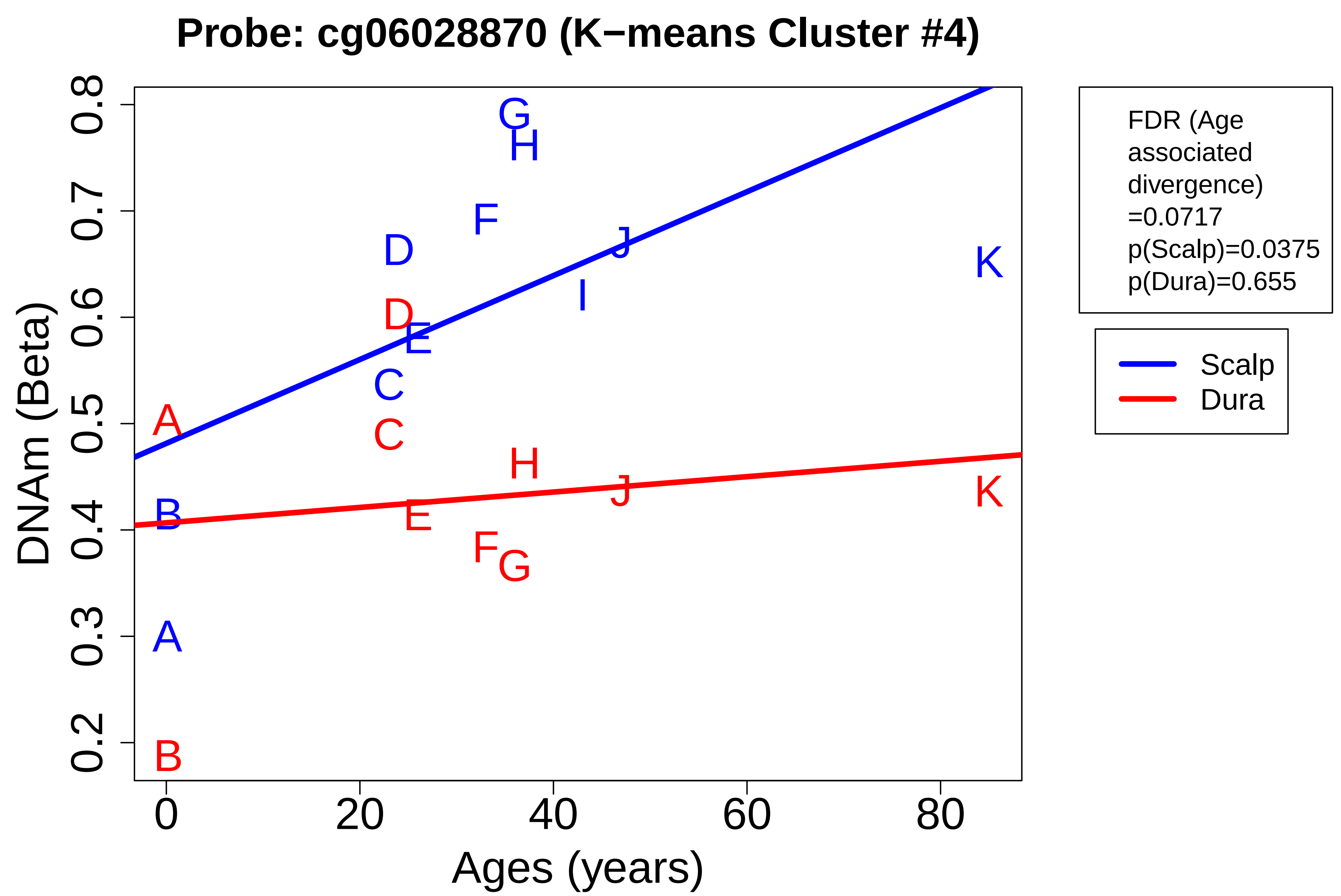

Probe: cg06028870 (K−means Cluster #4)
DNAm (Beta)
0.2
0.3
0.4
0.5
0.6
0.7
0.8
Ages (years)
0
20
40
60
80
A
B
C
D
E
F
G
H
I
J
K
FDR (Age associated divergence) =0.0717
p(Scalp)=0.0375
p(Dura)=0.655
Scalp
Dura

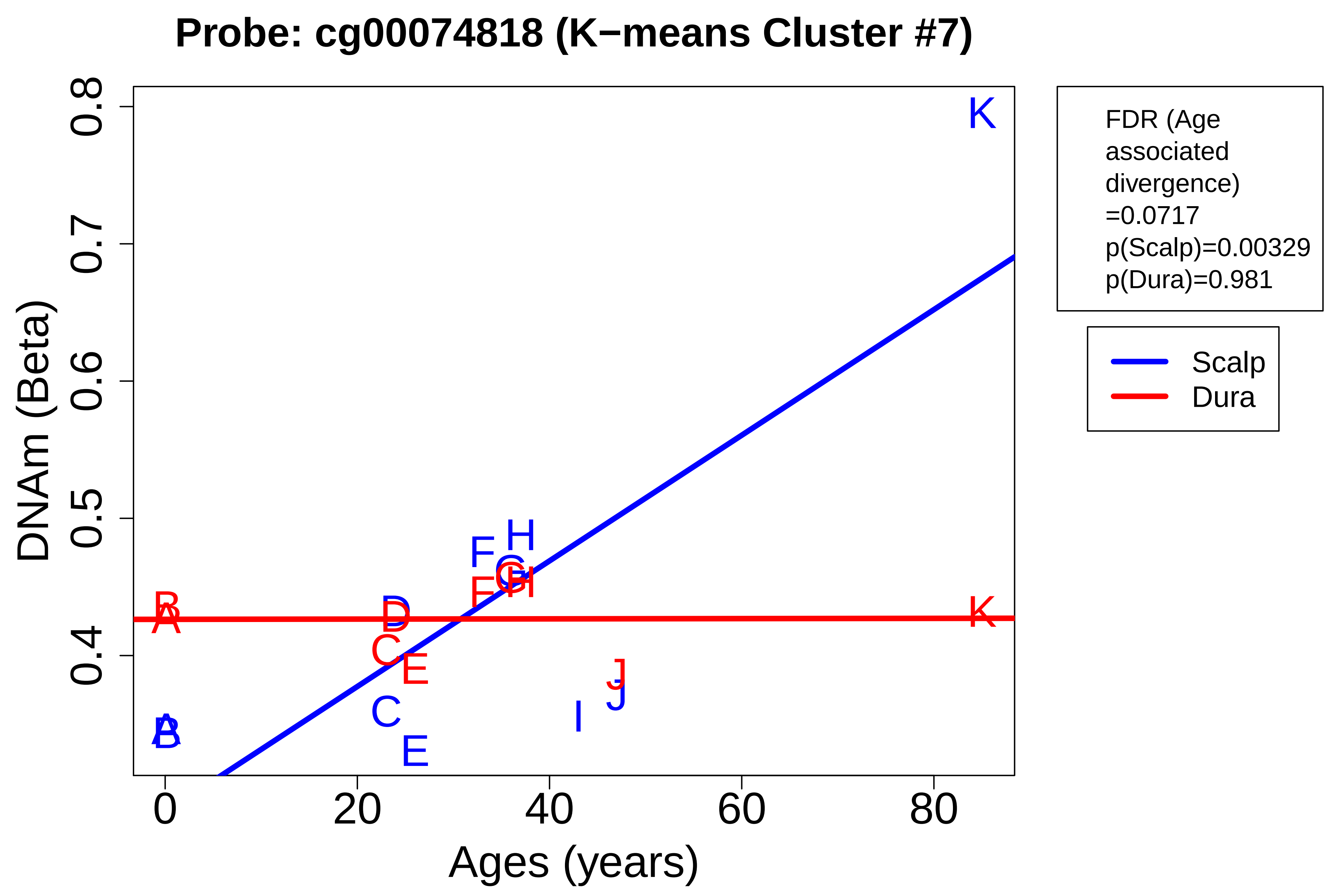

Probe: cg00074818 (K−means Cluster #7)
DNAm (Beta)
Ages (years)
0.4
0.5
0.6
0.7
0.8
0
20
40
60
80
FDR (Age associated divergence) =0.0717
p(Scalp)=0.00329
p(Dura)=0.981
Scalp
Dura

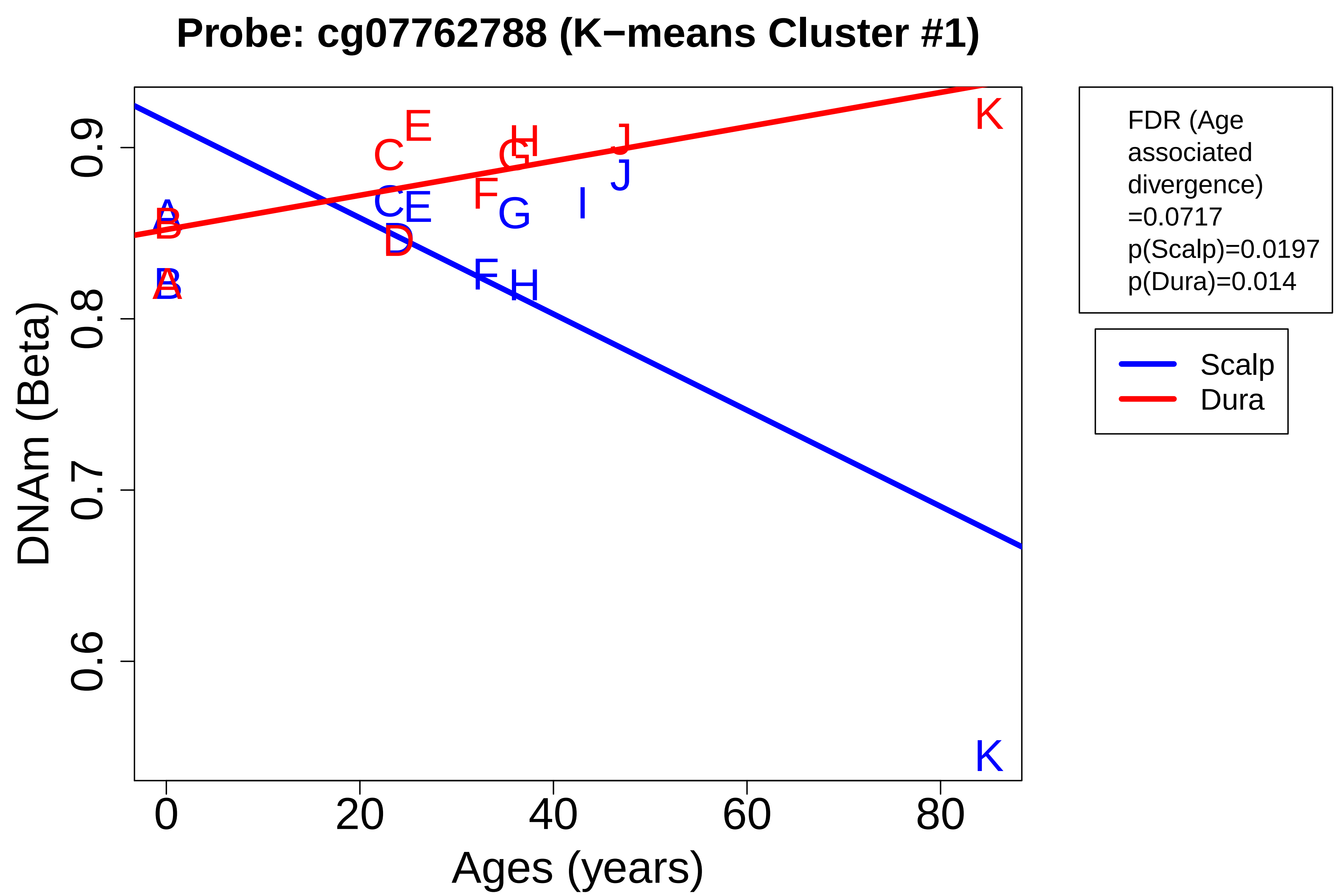
Probe: cg07762788 (K−means Cluster #1)
DNAm (Beta)
0.6
0.7
0.8
0.9
0
20
40
60
80
Ages (years)
A
B
C
D
E
F
G
H
I
J
K
FDR (Age associated divergence) =0.0717
p(Scalp)=0.0197
p(Dura)=0.014
Scalp
Dura

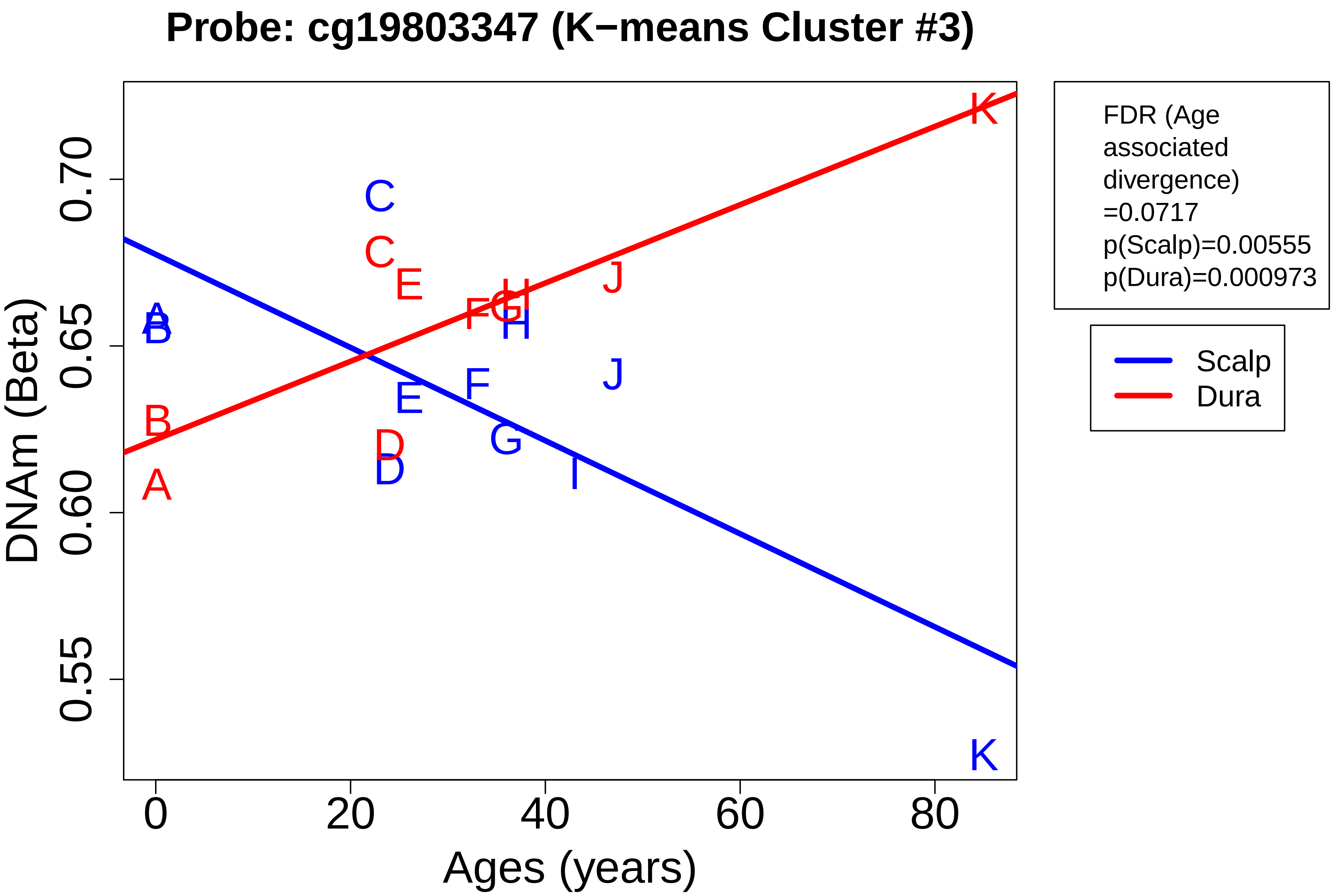
Probe: cg19803347 (K−means Cluster #3)
FDR (Age associated divergence) =0.0717
p(Scalp)=0.00555
p(Dura)=0.000973
Scalp
Dura
DNAm (Beta)
0.55
0.60
0.65
0.70
Ages (years)
0
20
40
60
80
A
B
C
D
E
F
G
H
I
J
K

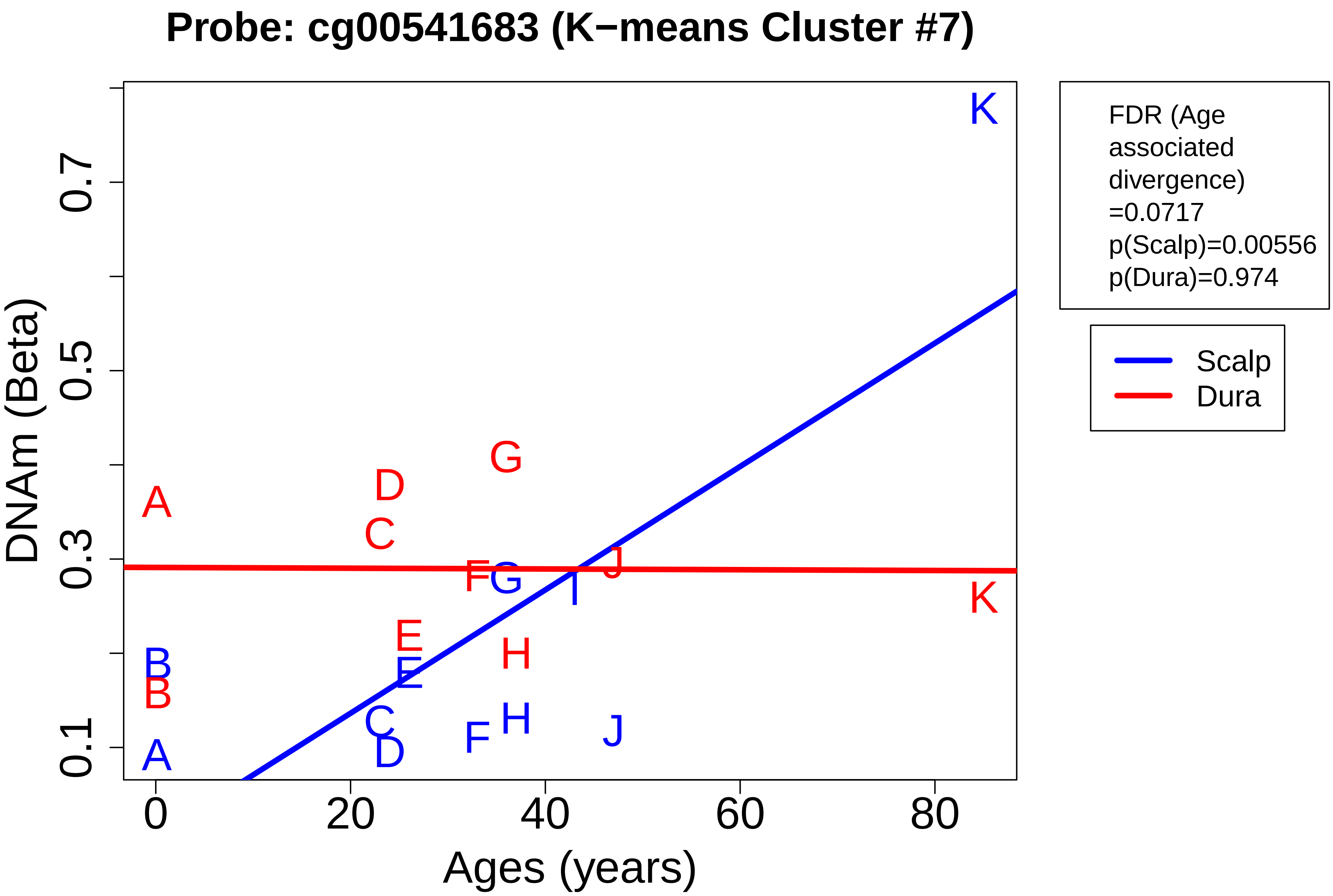
Probe: cg00541683 (K−means Cluster #7)
DNAm (Beta)
0.1
0.3
0.5
0.7
0
20
40
60
80
Ages (years)
FDR (Age associated divergence) =0.0717
p(Scalp)=0.00556
p(Dura)=0.974
Scalp
Dura

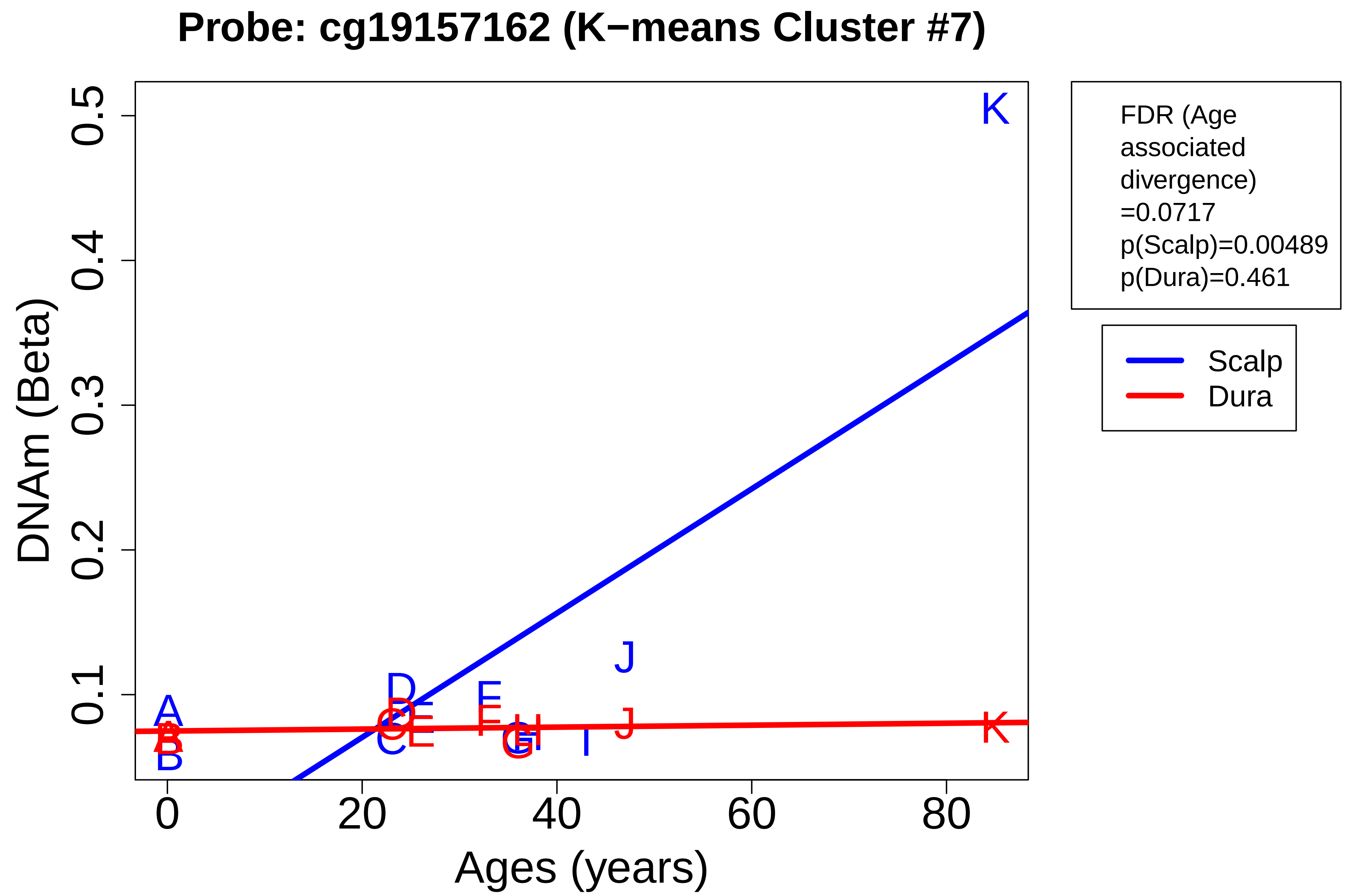

Probe: cg19157162 (K−means Cluster #7)
DNAm (Beta)
0.1
0.2
0.3
0.4
0.5
Ages (years)
0
20
40
60
80
A
B
C
D
E
F
G
H
I
J
K
FDR (Age associated divergence) =0.0717
p(Scalp)=0.00489
p(Dura)=0.461
Scalp
Dura

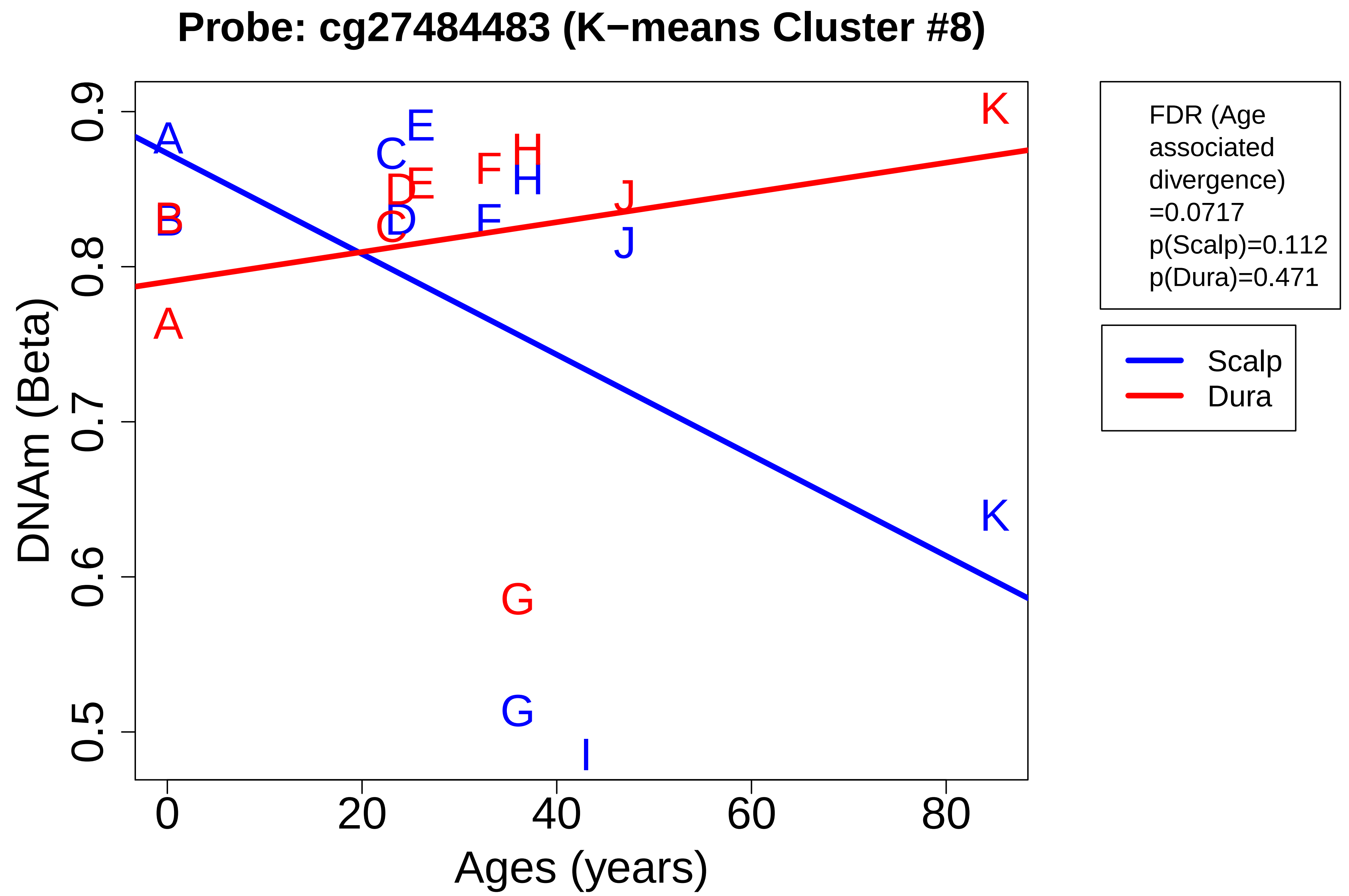

Probe: cg27484483 (K−means Cluster #8)
DNAm (Beta)
0.5
0.6
0.7
0.8
0.9
0
20
40
60
80
Ages (years)
A B C D E F G H I J K
A B C D E F G H J K
FDR (Age associated divergence) =0.0717
p(Scalp)=0.112
p(Dura)=0.471
Scalp
Dura

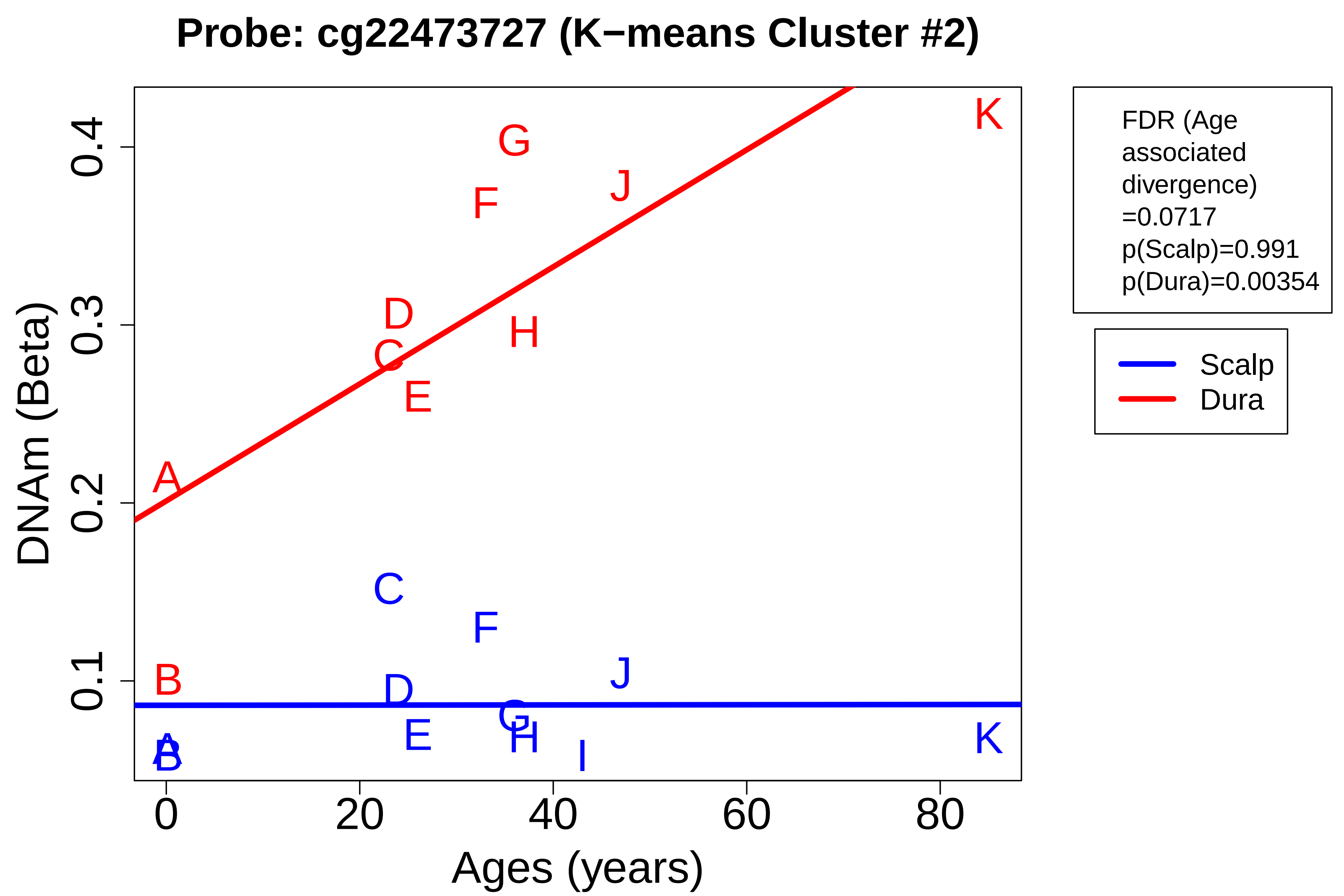

Probe: cg22473727 (K−means Cluster #2)
DNAm (Beta)
0.1
0.2
0.3
0.4
0
20
40
60
80
Ages (years)
FDR (Age associated divergence) =0.0717
p(Scalp)=0.991
p(Dura)=0.00354
Scalp
Dura
A
B
C
D
E
F
G
H
I
J
K

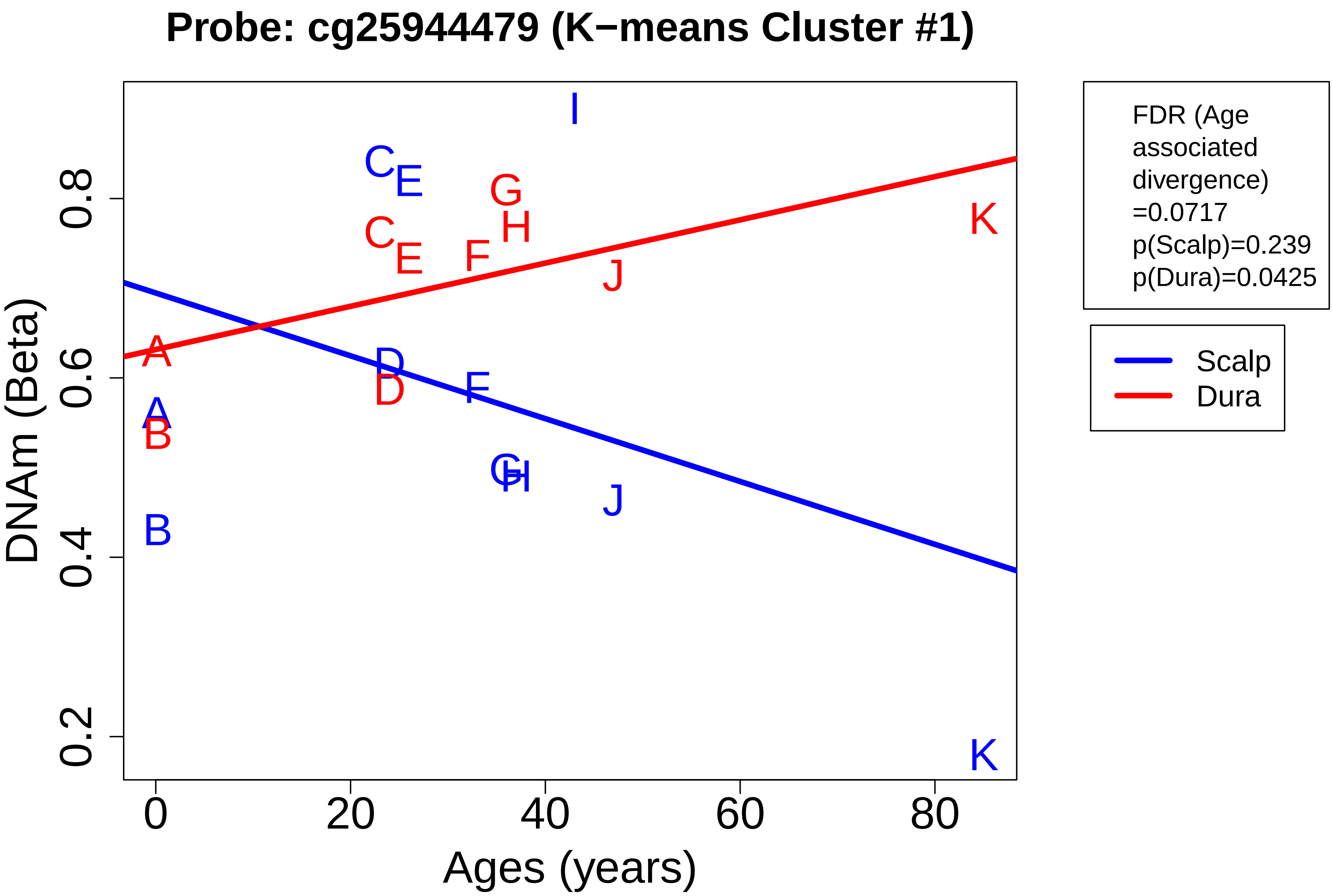

Probe: cg25944479 (K−means Cluster #1)
FDR (Age associated divergence) =0.0717
p(Scalp)=0.239
p(Dura)=0.0425
Scalp
Dura
DNAm (Beta)
0.2
0.4
0.6
0.8
0
20
40
60
80
Ages (years)
A
B
C
D
E
F
G
H
I
J
K

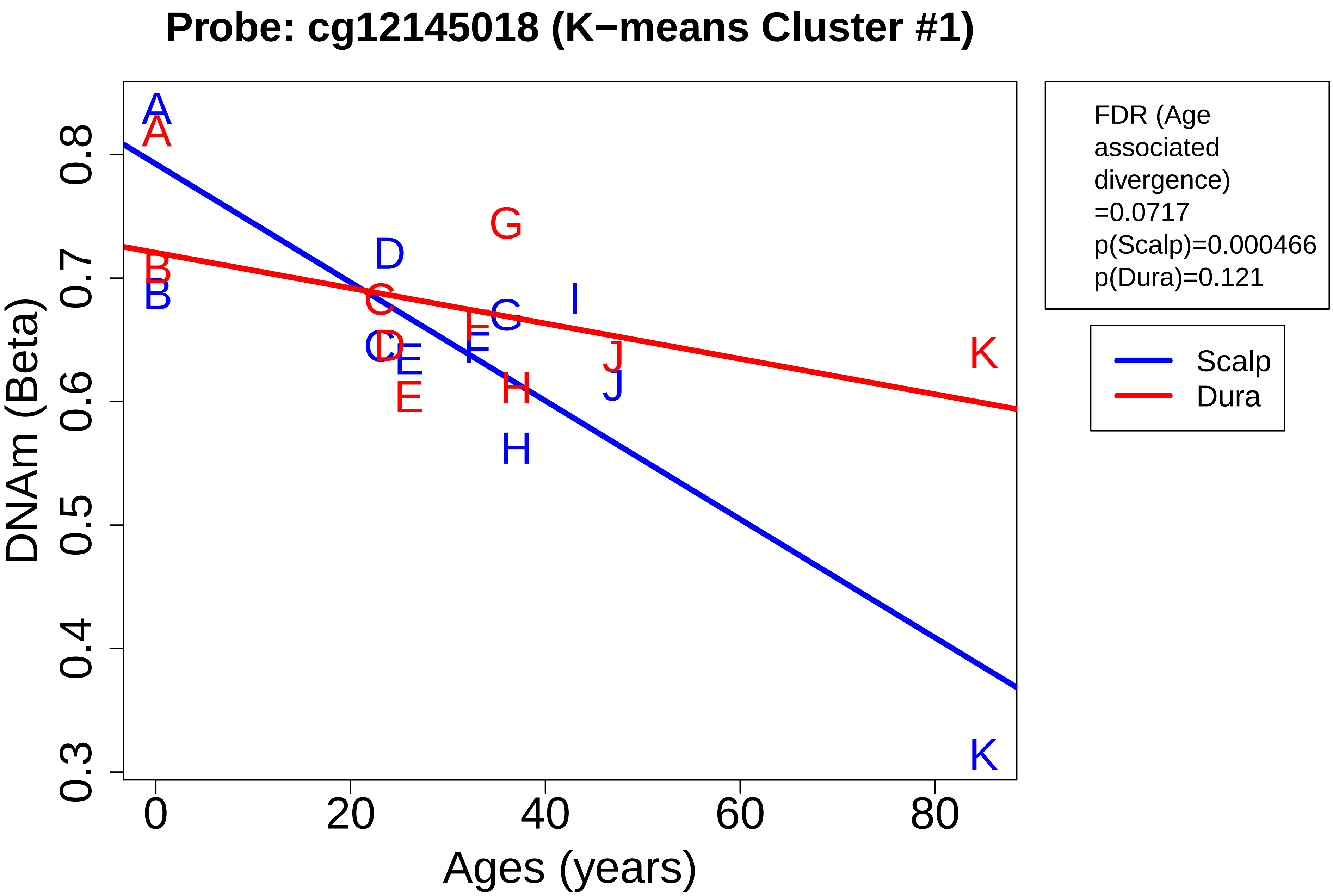

Probe: cg12145018 (K−means Cluster #1)
DNAm (Beta)
0.3
0.4
0.5
0.6
0.7
0.8
Ages (years)
0
20
40
60
80
A
B
C
D
E
F
G
H
I
J
K
FDR (Age associated divergence) =0.0717
p(Scalp)=0.000466
p(Dura)=0.121
Scalp
Dura

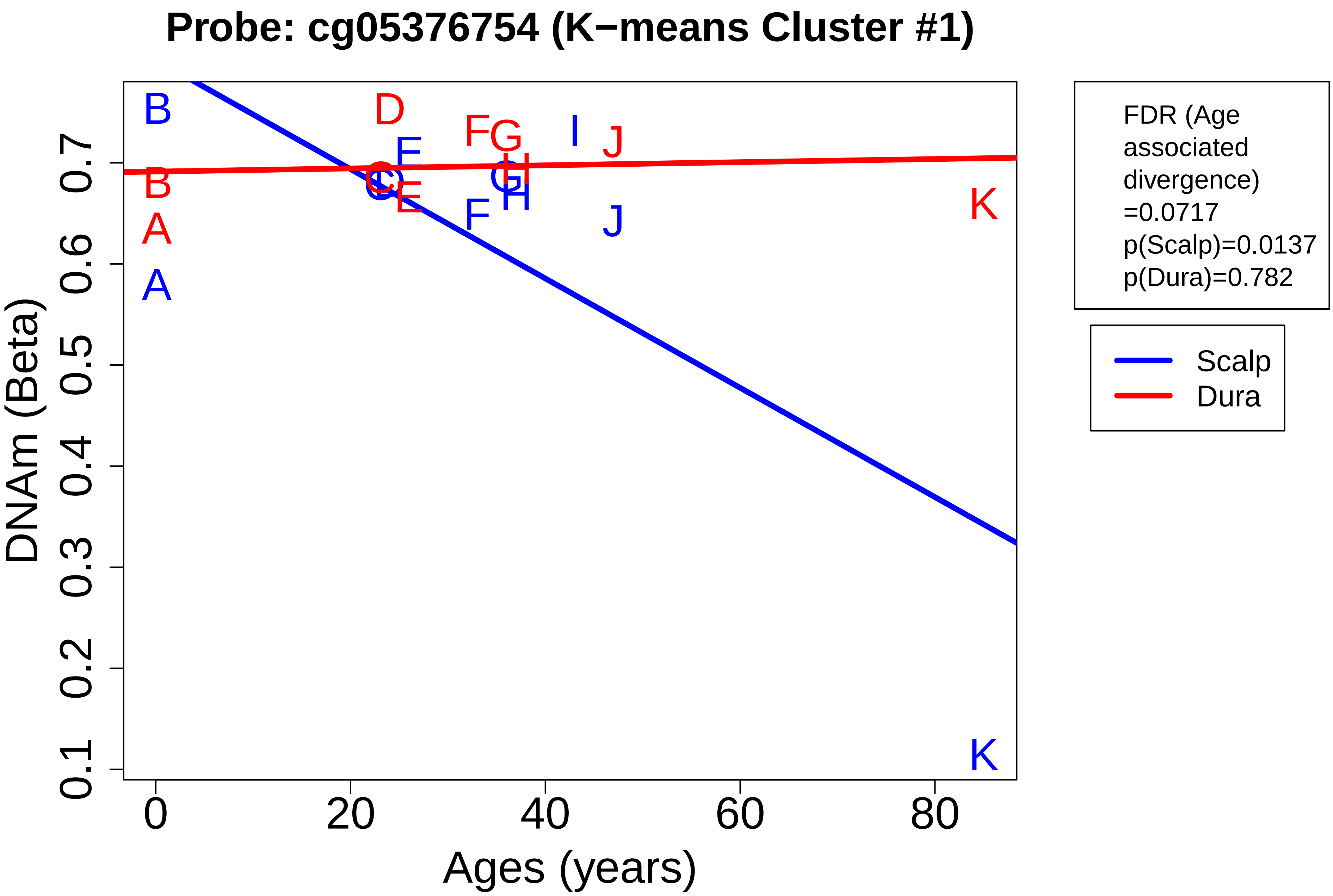

Probe: cg05376754 (K−means Cluster #1)
DNAm (Beta)
0.1
0.2
0.3
0.4
0.5
0.6
0.7
Ages (years)
0
20
40
60
80
B
D
F
G
I
J
E
H
B
C
C
G
E
H
A
F
J
K
A
K
FDR (Age associated divergence) =0.0717
p(Scalp)=0.0137
p(Dura)=0.782
Scalp
Dura

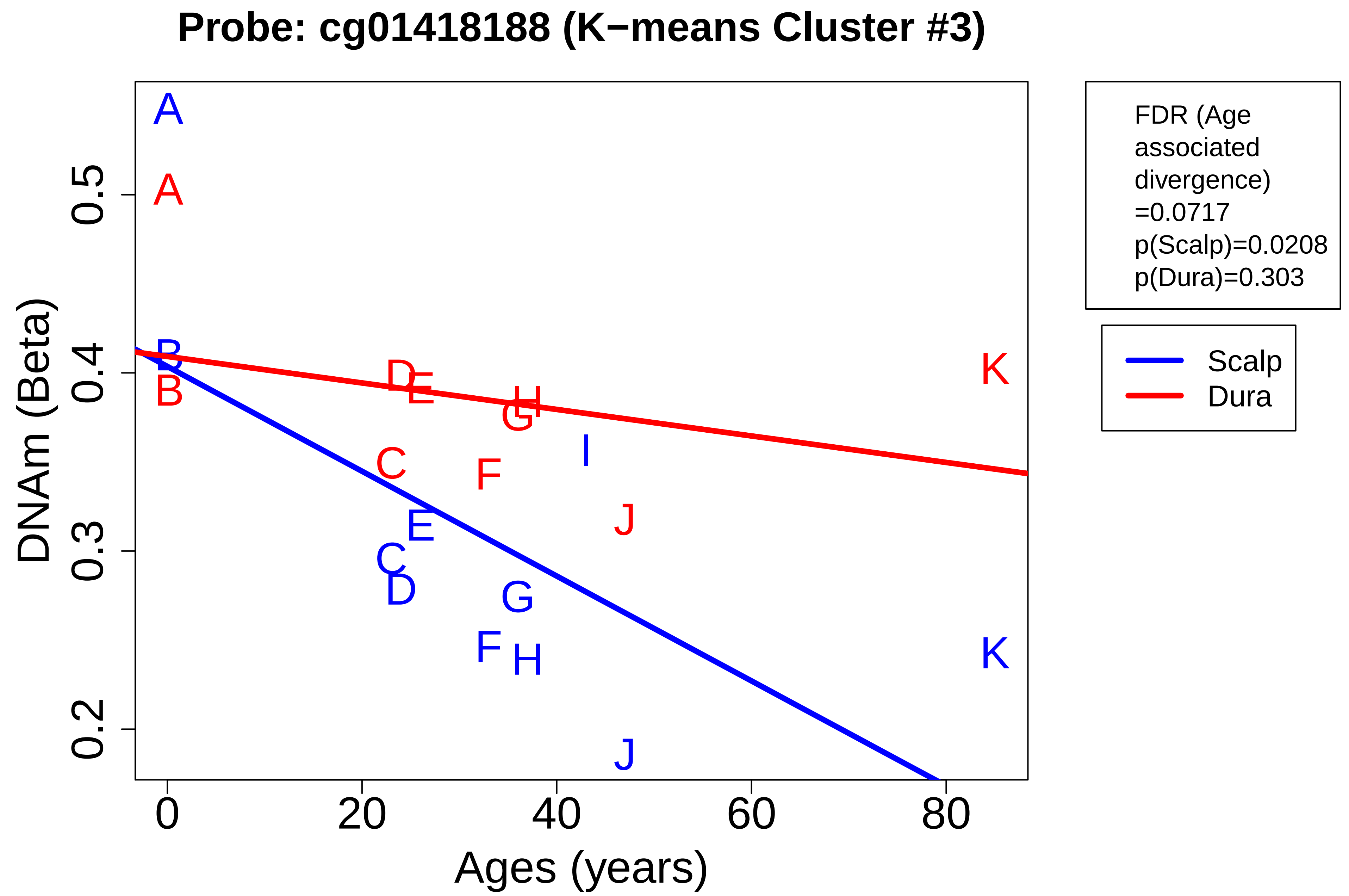

Probe: cg01418188 (K−means Cluster #3)
DNAm (Beta)
0.2
0.3
0.4
0.5
0
20
40
60
80
Ages (years)
A
A
B
B
C
C
D
D
E
E
F
F
G
G
H
H
I
I
J
J
K
K
FDR (Age associated divergence) =0.0717
p(Scalp)=0.0208
p(Dura)=0.303
Scalp
Dura

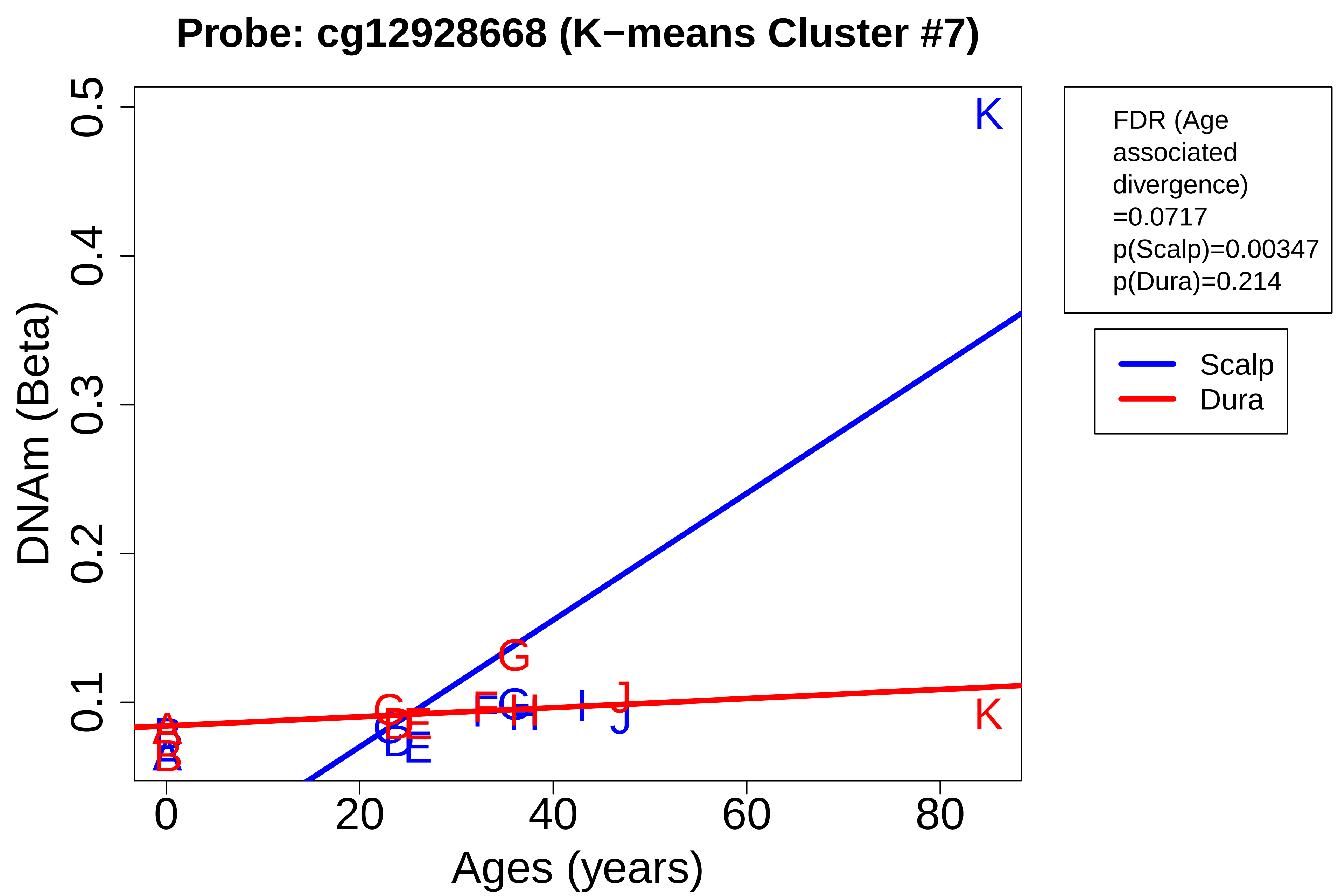
Probe: cg12928668 (K−means Cluster #7)
DNAm (Beta)
0.1
0.2
0.3
0.4
0.5
Ages (years)
0
20
40
60
80
FDR (Age associated divergence) =0.0717
p(Scalp)=0.00347
p(Dura)=0.214
Scalp
Dura

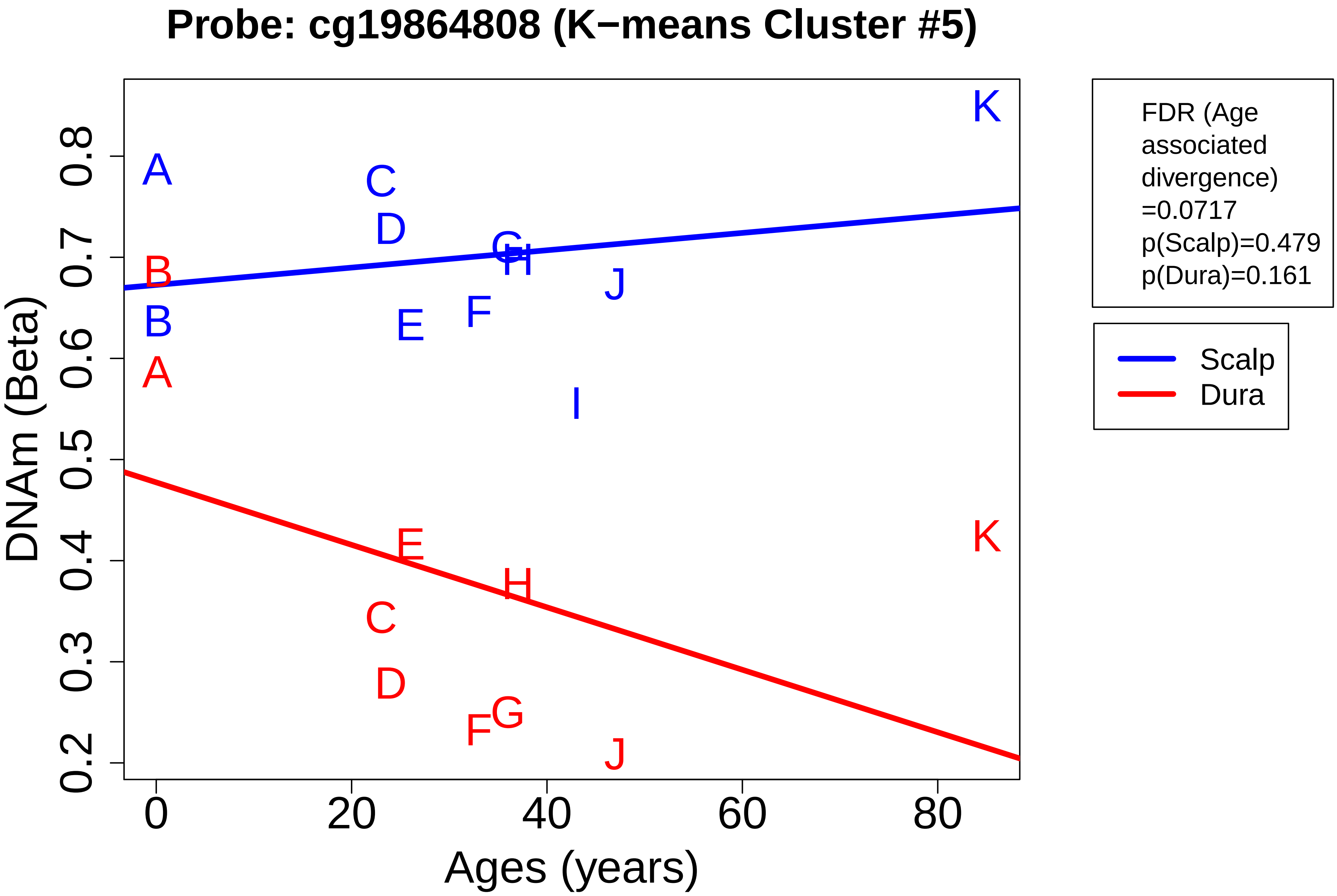
Probe: cg19864808 (K−means Cluster #5)
DNAm (Beta)
0.2
0.3
0.4
0.5
0.6
0.7
0.8
0
20
40
60
80
Ages (years)
A
B
C
D
E
F
G
H
I
J
K
FDR (Age associated divergence) =0.0717
p(Scalp)=0.479
p(Dura)=0.161
Scalp
Dura

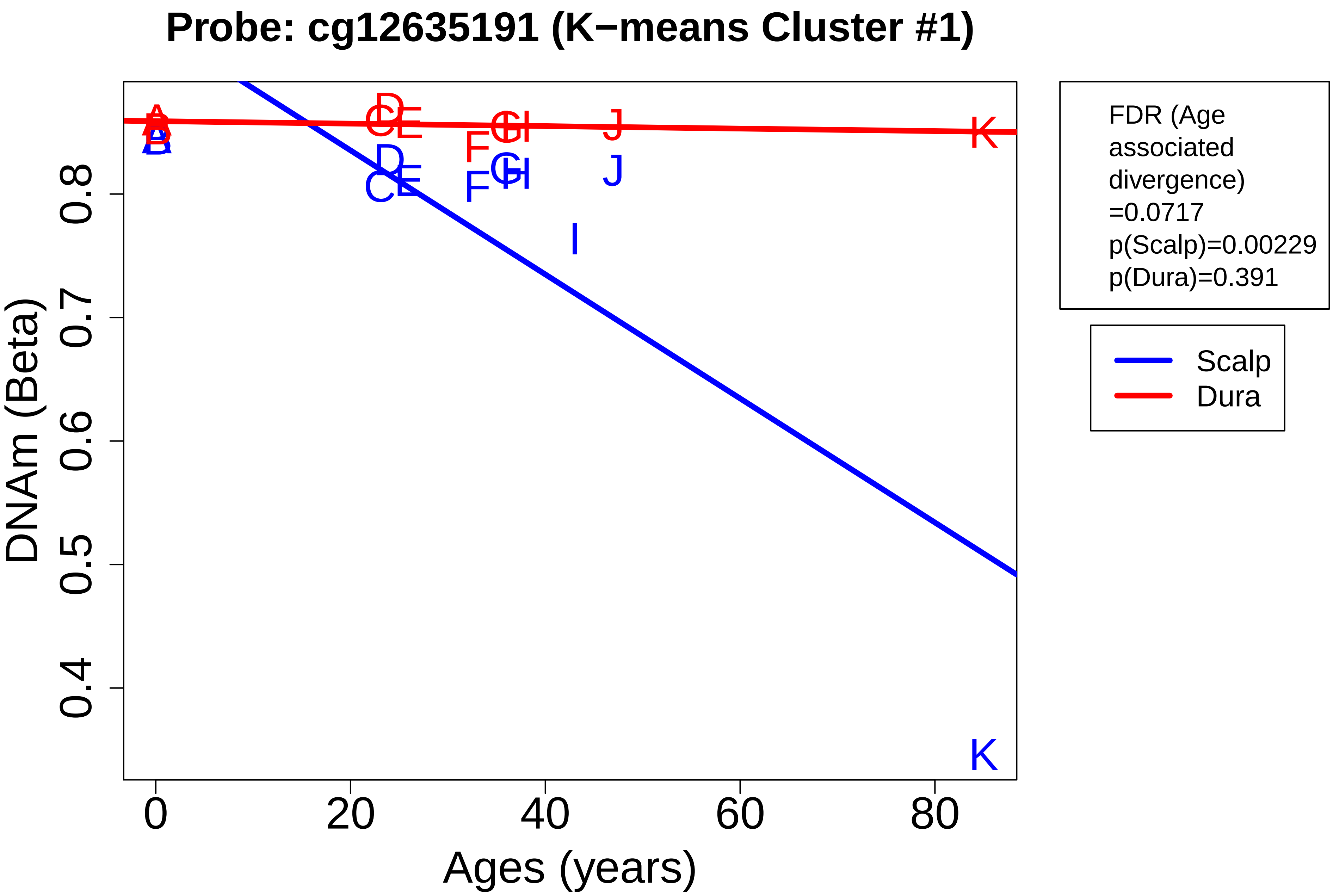

Probe: cg12635191 (K−means Cluster #1)
DNAm (Beta)
Ages (years)
0.4
0.5
0.6
0.7
0.8
0
20
40
60
80
FDR (Age associated divergence) =0.0717
p(Scalp)=0.00229
p(Dura)=0.391
Scalp
Dura

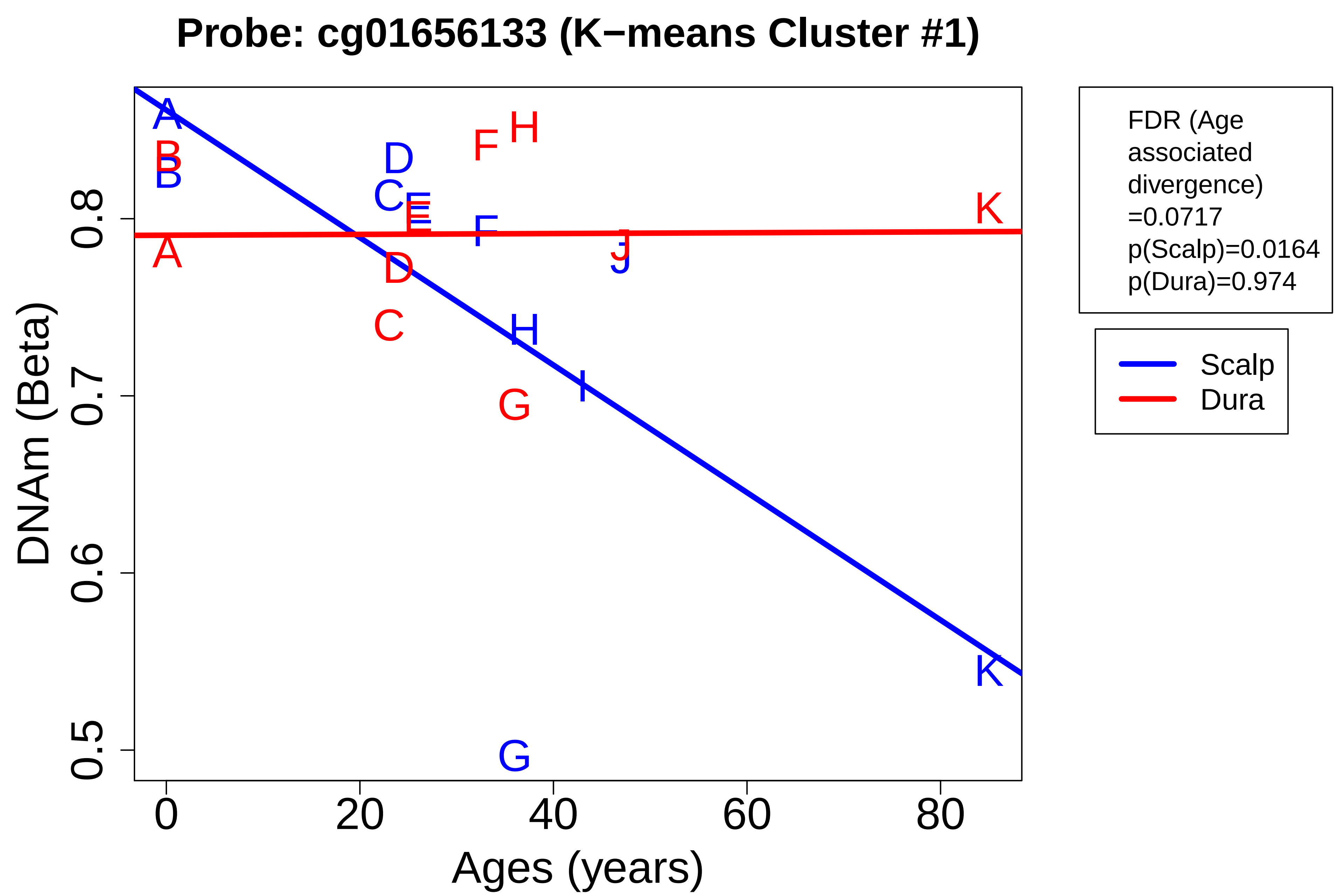

Probe: cg01656133 (K−means Cluster #1)
DNAm (Beta)
0.5
0.6
0.7
0.8
0
20
40
60
80
Ages (years)
A
B
C
D
E
F
G
H
I
J
K
FDR (Age associated divergence) =0.0717
p(Scalp)=0.0164
p(Dura)=0.974
Scalp
Dura

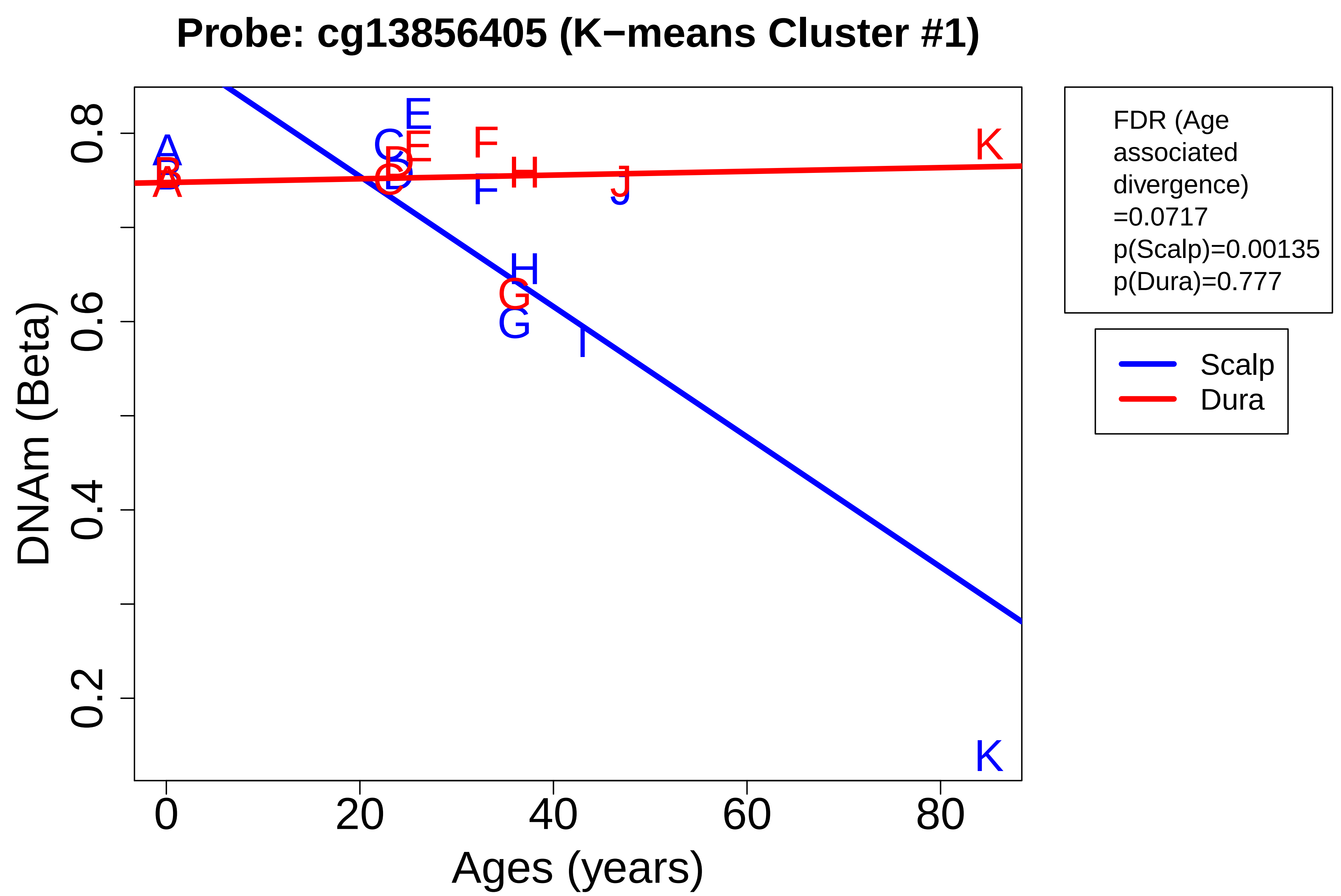

Probe: cg13856405 (K−means Cluster #1)
DNAm (Beta)
0.2
0.4
0.6
0.8
0
20
40
60
80
Ages (years)
FDR (Age associated divergence) =0.0717
p(Scalp)=0.00135
p(Dura)=0.777
Scalp
Dura

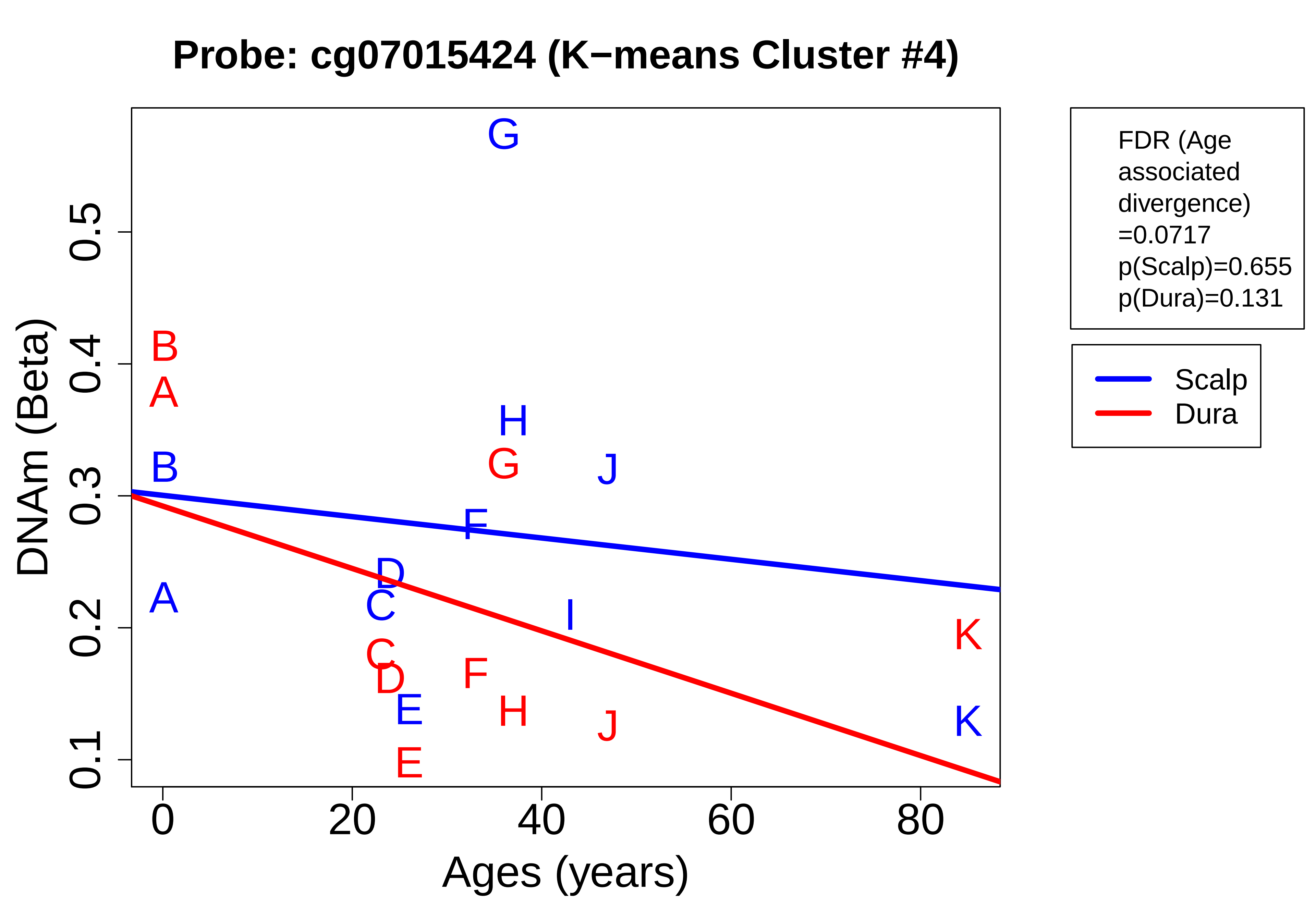

Probe: cg07015424 (K−means Cluster #4)
DNAm (Beta)
0.1
0.2
0.3
0.4
0.5
Ages (years)
0
20
40
60
80
FDR (Age associated divergence) =0.0717
p(Scalp)=0.655
p(Dura)=0.131
Scalp
Dura
A
B
C
D
E
F
G
H
I
J
K

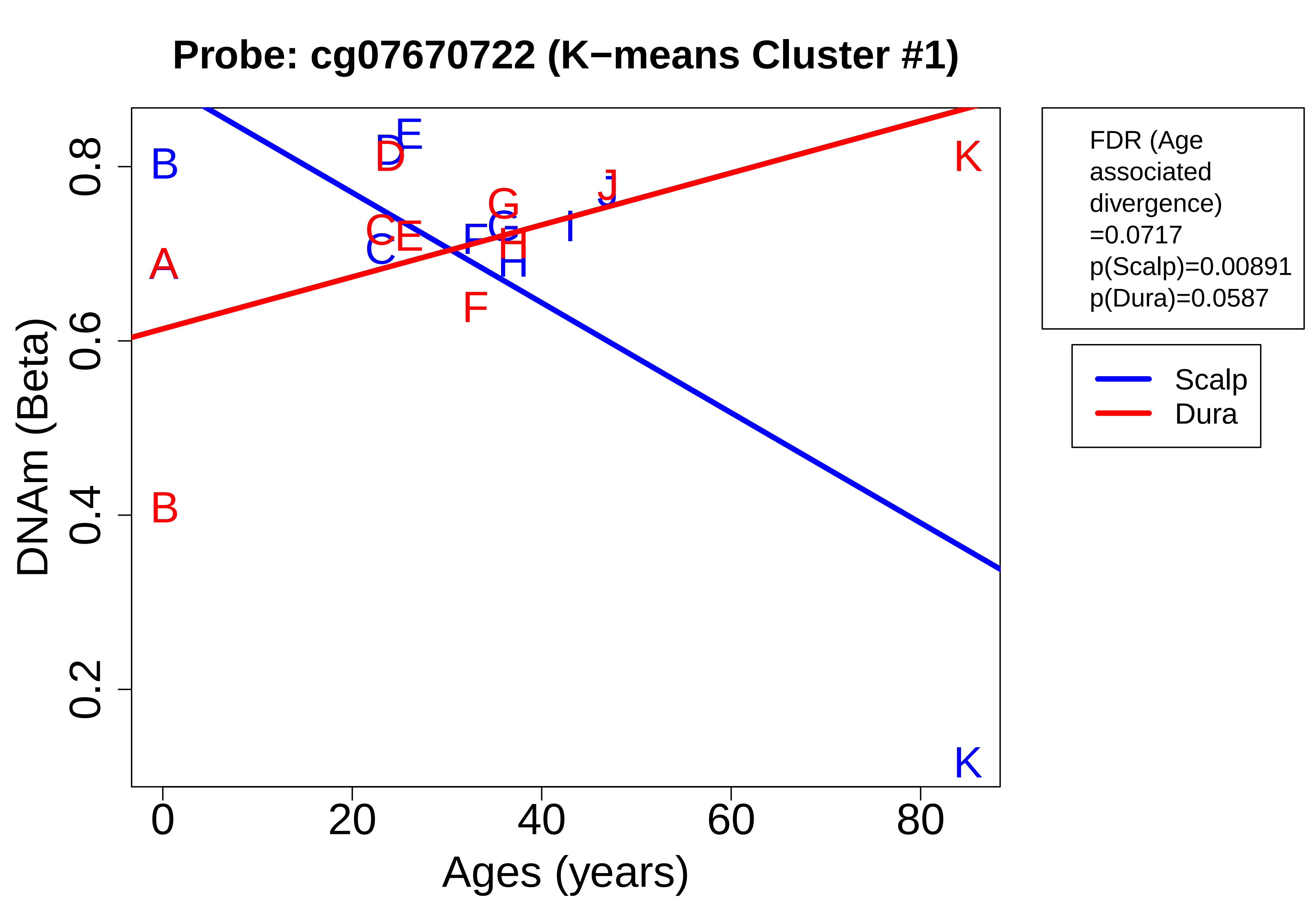

Probe: cg07670722 (K−means Cluster #1)
DNAm (Beta)
0.2
0.4
0.6
0.8
0
20
40
60
80
Ages (years)
A
B
C
D
E
F
G
H
I
J
K
FDR (Age associated divergence) =0.0717
p(Scalp)=0.00891
p(Dura)=0.0587
Scalp
Dura

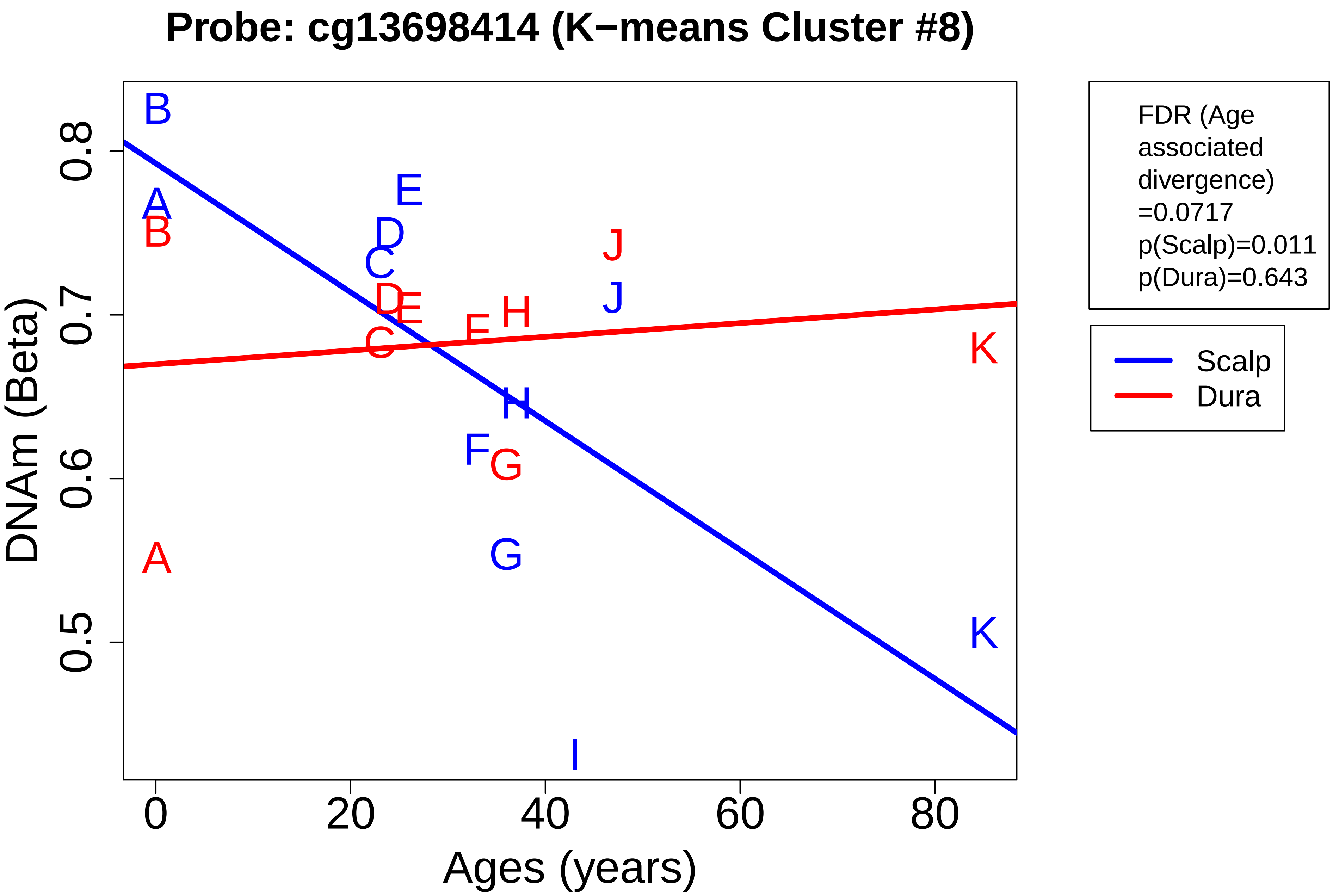

Probe: cg13698414 (K−means Cluster #8)
DNAm (Beta)
0.5
0.6
0.7
0.8
Ages (years)
0
20
40
60
80
FDR (Age associated divergence) =0.0717
p(Scalp)=0.011
p(Dura)=0.643
Scalp
Dura
A
B
C
D
E
F
G
H
I
J
K

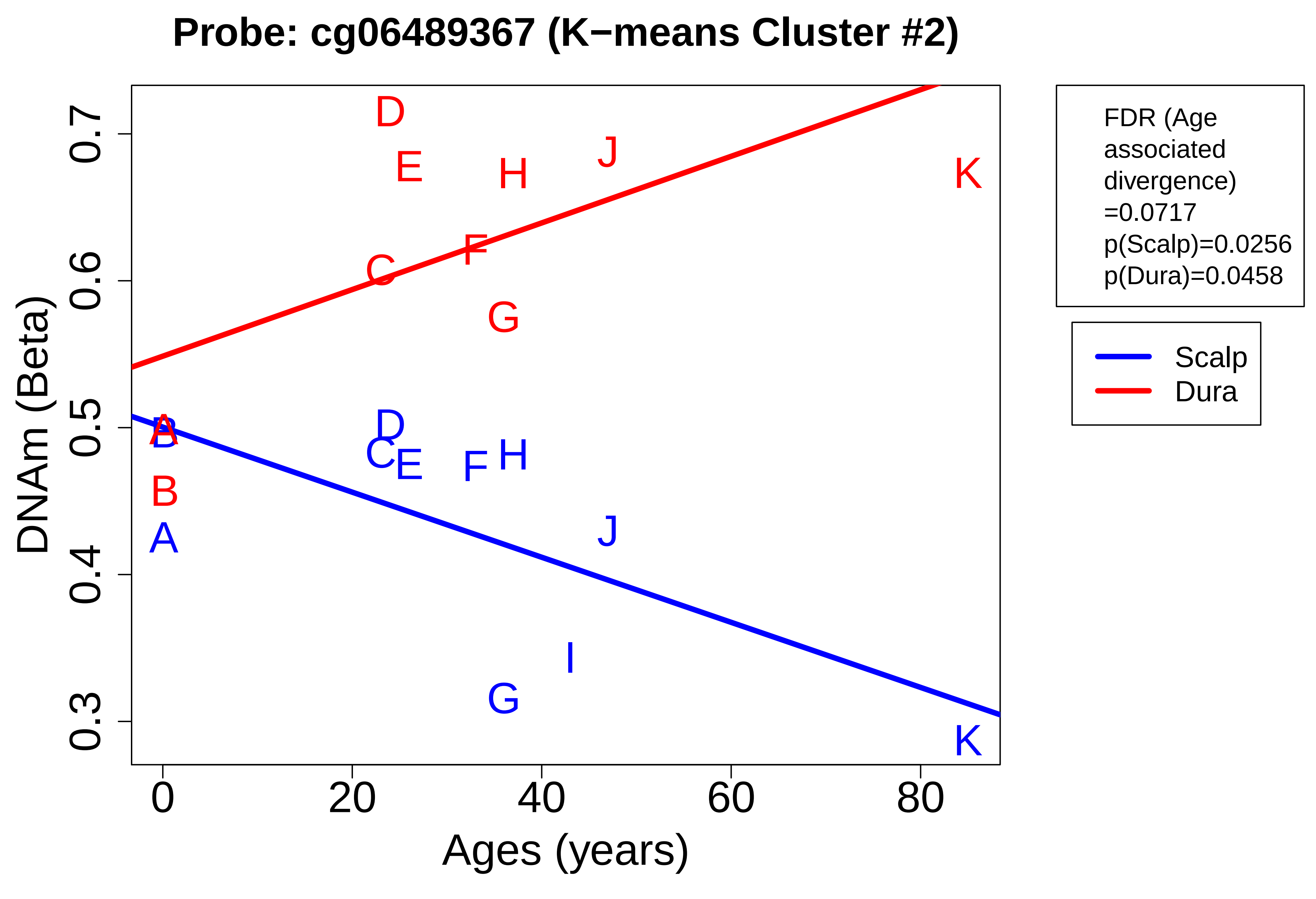
Probe: cg06489367 (K−means Cluster #2)
DNAm (Beta)
Ages (years)
0.3
0.4
0.5
0.6
0.7
0
20
40
60
80
FDR (Age associated divergence) =0.0717
p(Scalp)=0.0256
p(Dura)=0.0458
Scalp
Dura
A
B
C
D
E
F
G
H
I
J
K

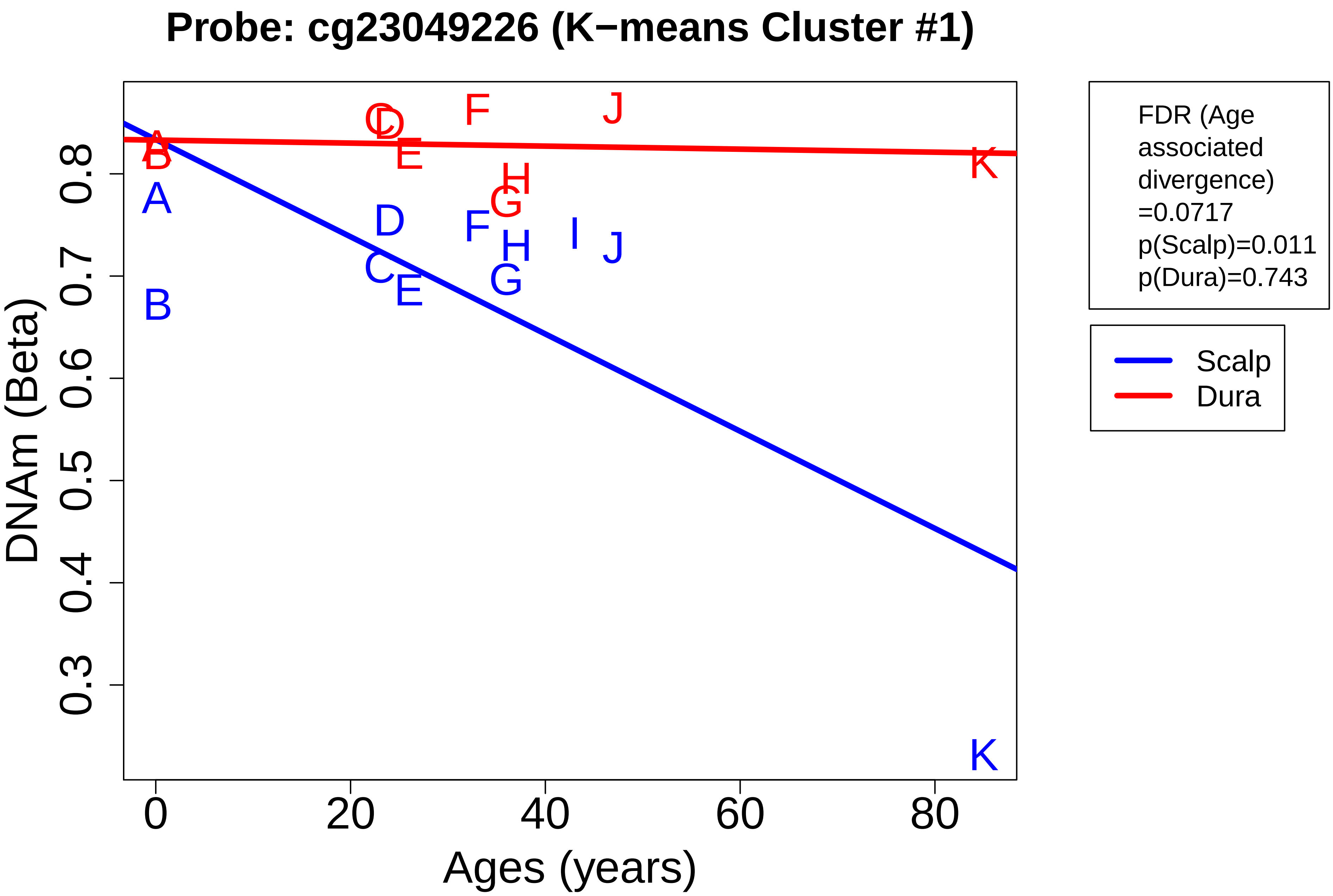

Probe: cg23049226 (K−means Cluster #1)
DNAm (Beta)
0.3
0.4
0.5
0.6
0.7
0.8
0
20
40
60
80
Ages (years)
A
B
C
D
E
F
G
H
I
J
K
FDR (Age associated divergence) =0.0717
p(Scalp)=0.011
p(Dura)=0.743
Scalp
Dura

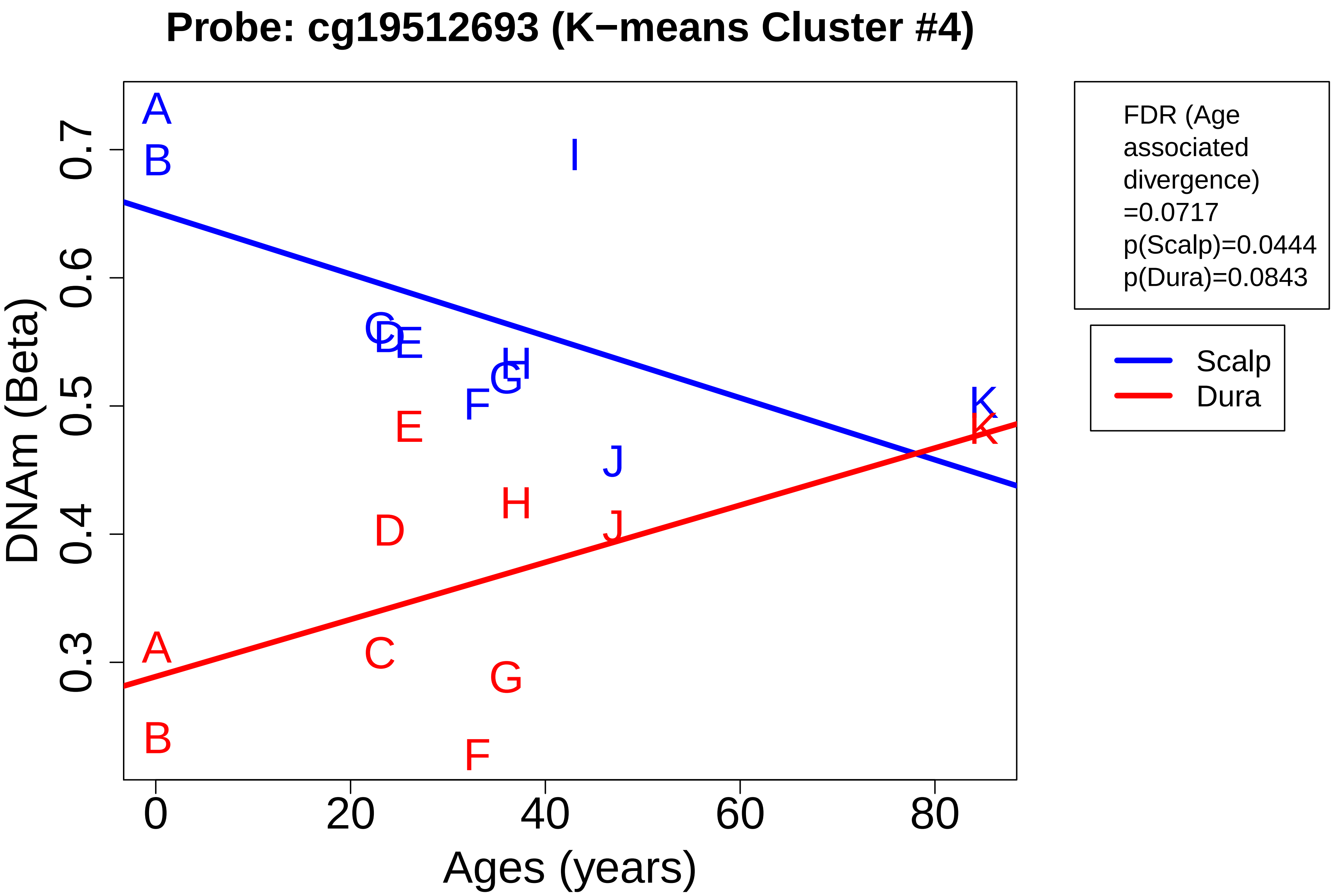

Probe: cg19512693 (K−means Cluster #4)
DNAm (Beta)
Ages (years)
0.3
0.4
0.5
0.6
0.7
0
20
40
60
80
FDR (Age associated divergence) =0.0717
p(Scalp)=0.0444
p(Dura)=0.0843
Scalp
Dura

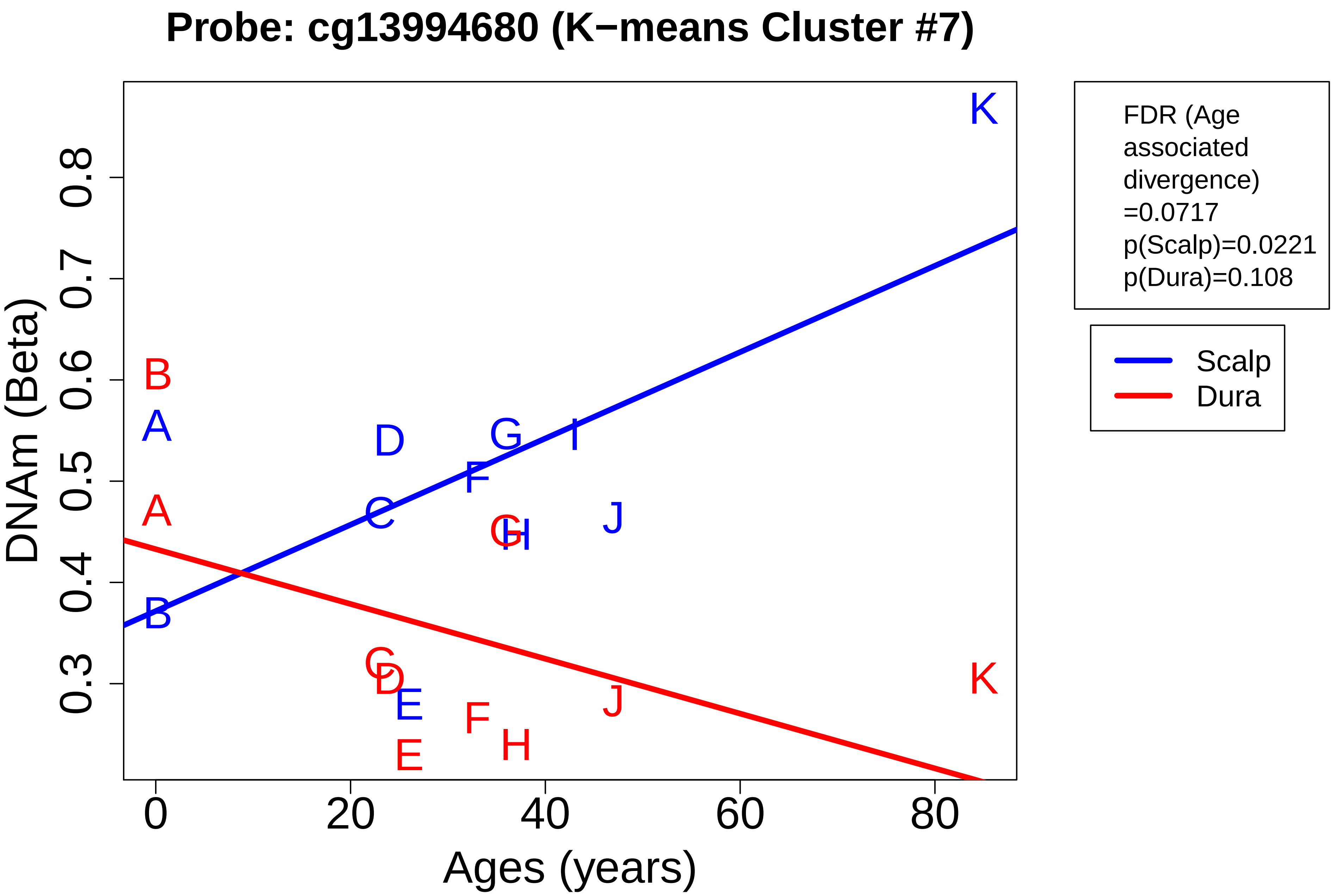

Probe: cg13994680 (K−means Cluster #7)
DNAm (Beta)
0.3
0.4
0.5
0.6
0.7
0.8
0
20
40
60
80
Ages (years)
FDR (Age associated divergence) =0.0717
p(Scalp)=0.0221
p(Dura)=0.108
Scalp
Dura

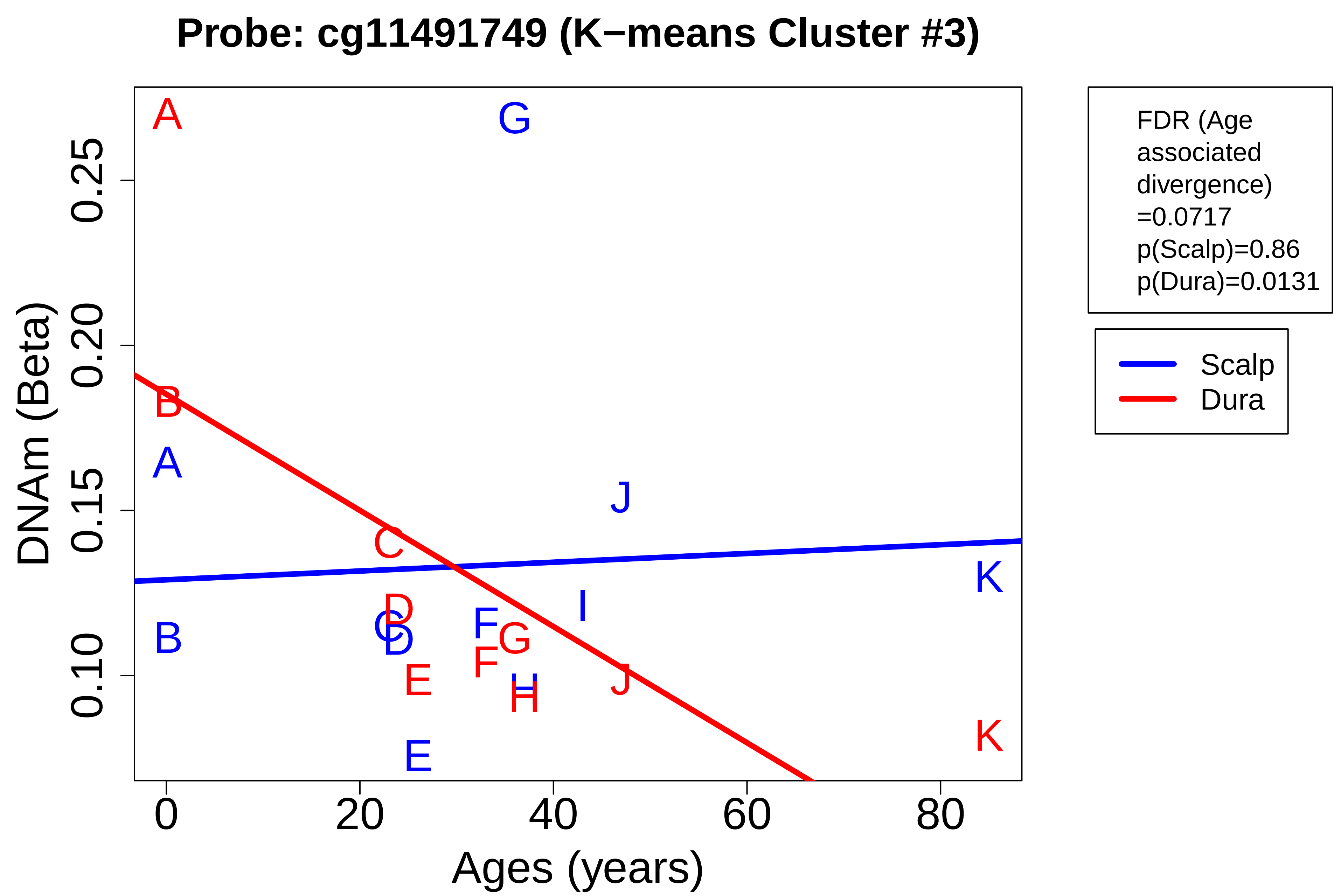

Probe: cg11491749 (K−means Cluster #3)
DNAm (Beta)
0.10
0.15
0.20
0.25
Ages (years)
0
20
40
60
80
A
B
C
D
E
F
G
H
I
J
K
FDR (Age associated divergence) =0.0717
p(Scalp)=0.86
p(Dura)=0.0131
Scalp
Dura

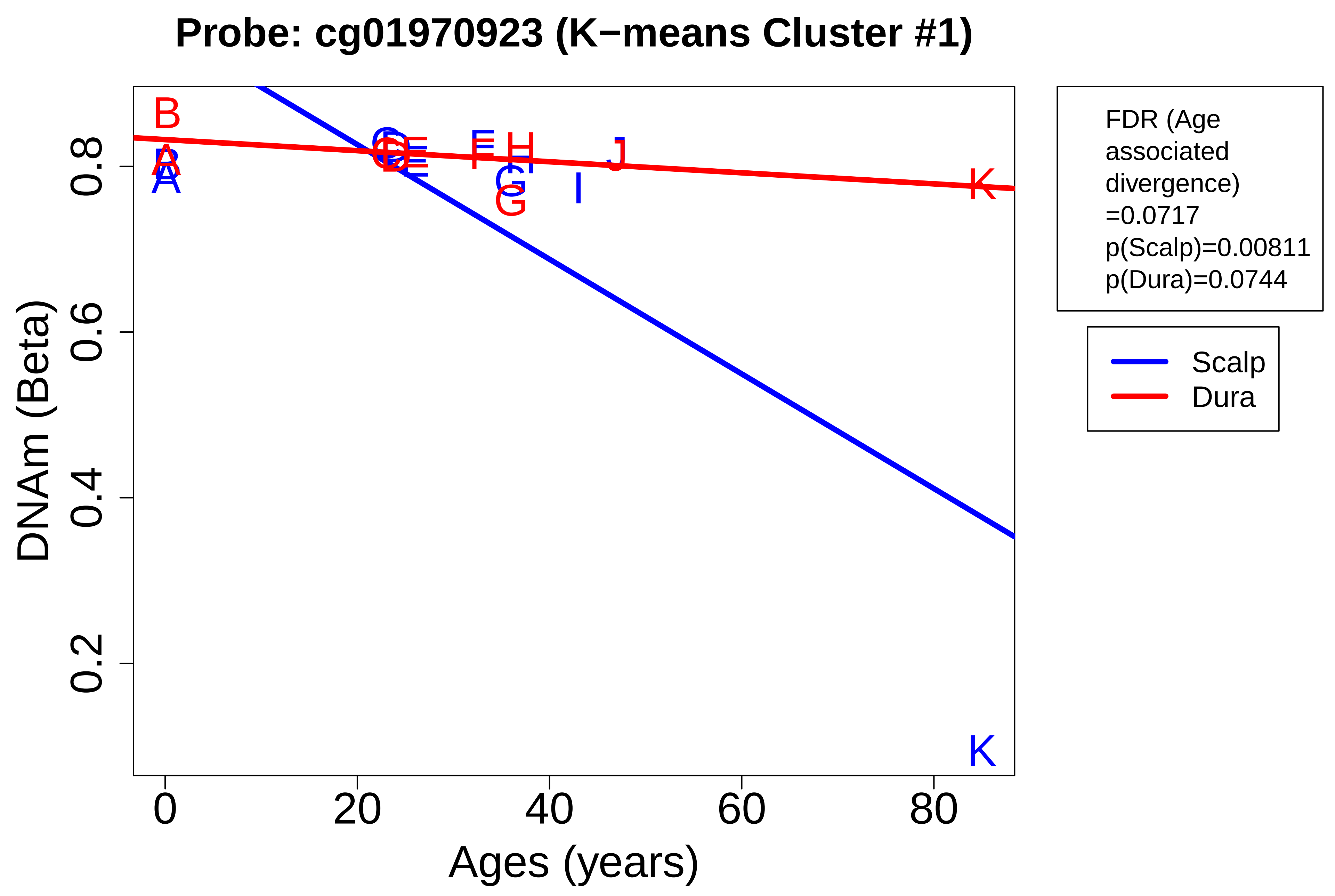

Probe: cg01970923 (K−means Cluster #1)
DNAm (Beta)
0.2
0.4
0.6
0.8
Ages (years)
0
20
40
60
80
FDR (Age associated divergence) =0.0717
p(Scalp)=0.00811
p(Dura)=0.0744
Scalp
Dura

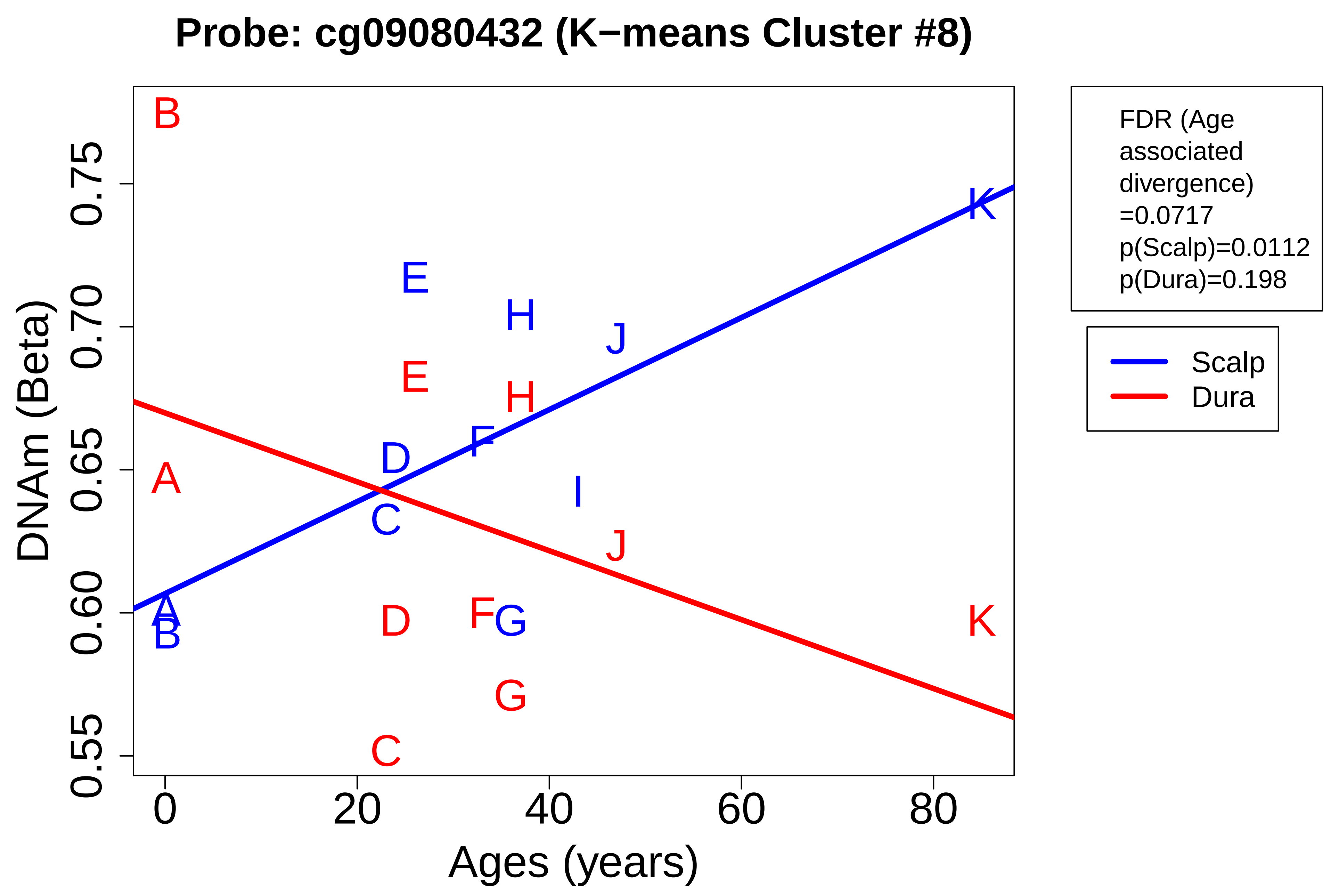
Probe: cg09080432 (K−means Cluster #8)
DNAm (Beta)
0.55
0.60
0.65
0.70
0.75
0
20
40
60
80
Ages (years)
FDR (Age associated divergence) =0.0717
p(Scalp)=0.0112
p(Dura)=0.198
Scalp
Dura

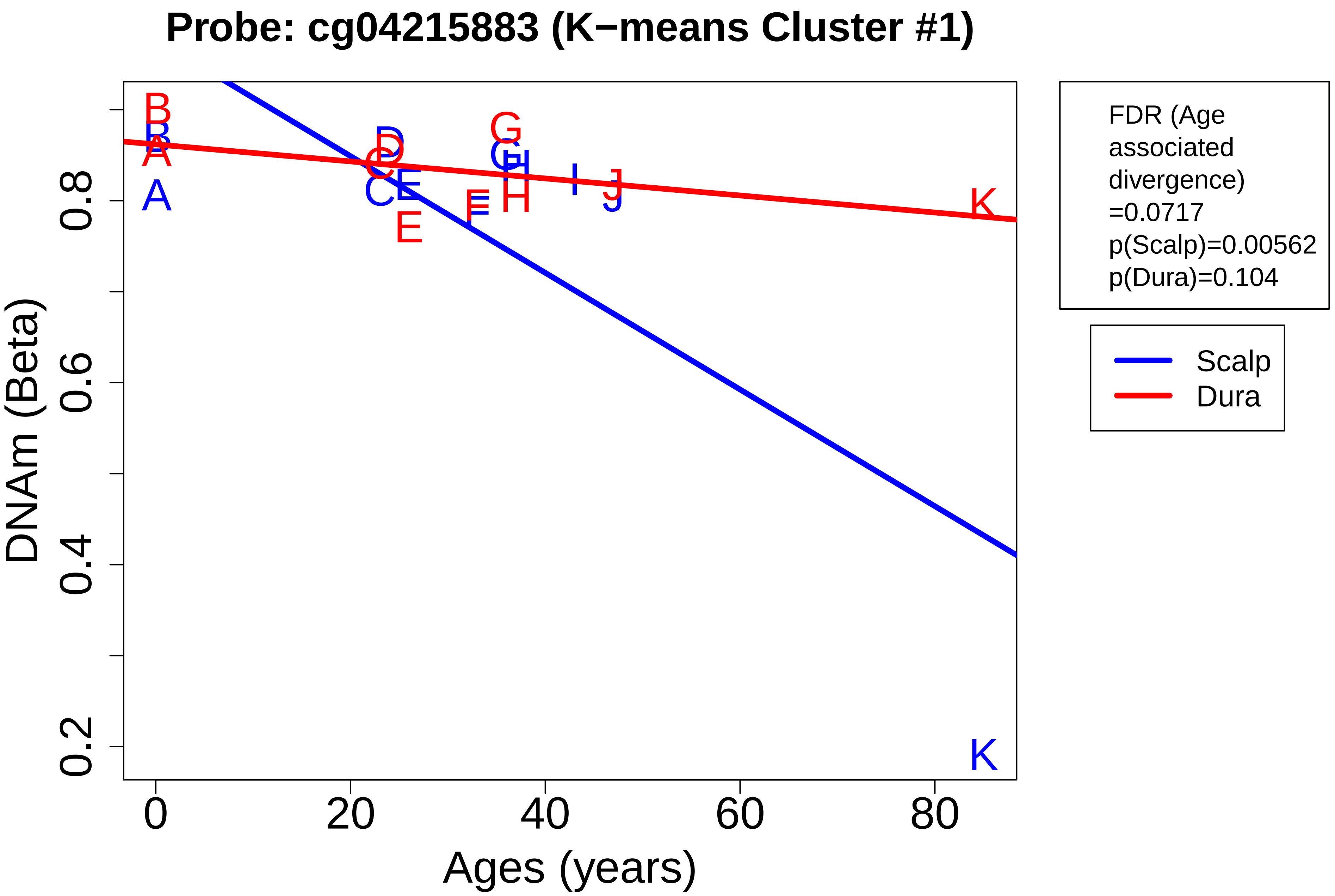

Probe: cg04215883 (K−means Cluster #1)
DNAm (Beta)
0.2
0.4
0.6
0.8
0
20
40
60
80
Ages (years)
FDR (Age associated divergence) =0.0717
p(Scalp)=0.00562
p(Dura)=0.104
Scalp
Dura

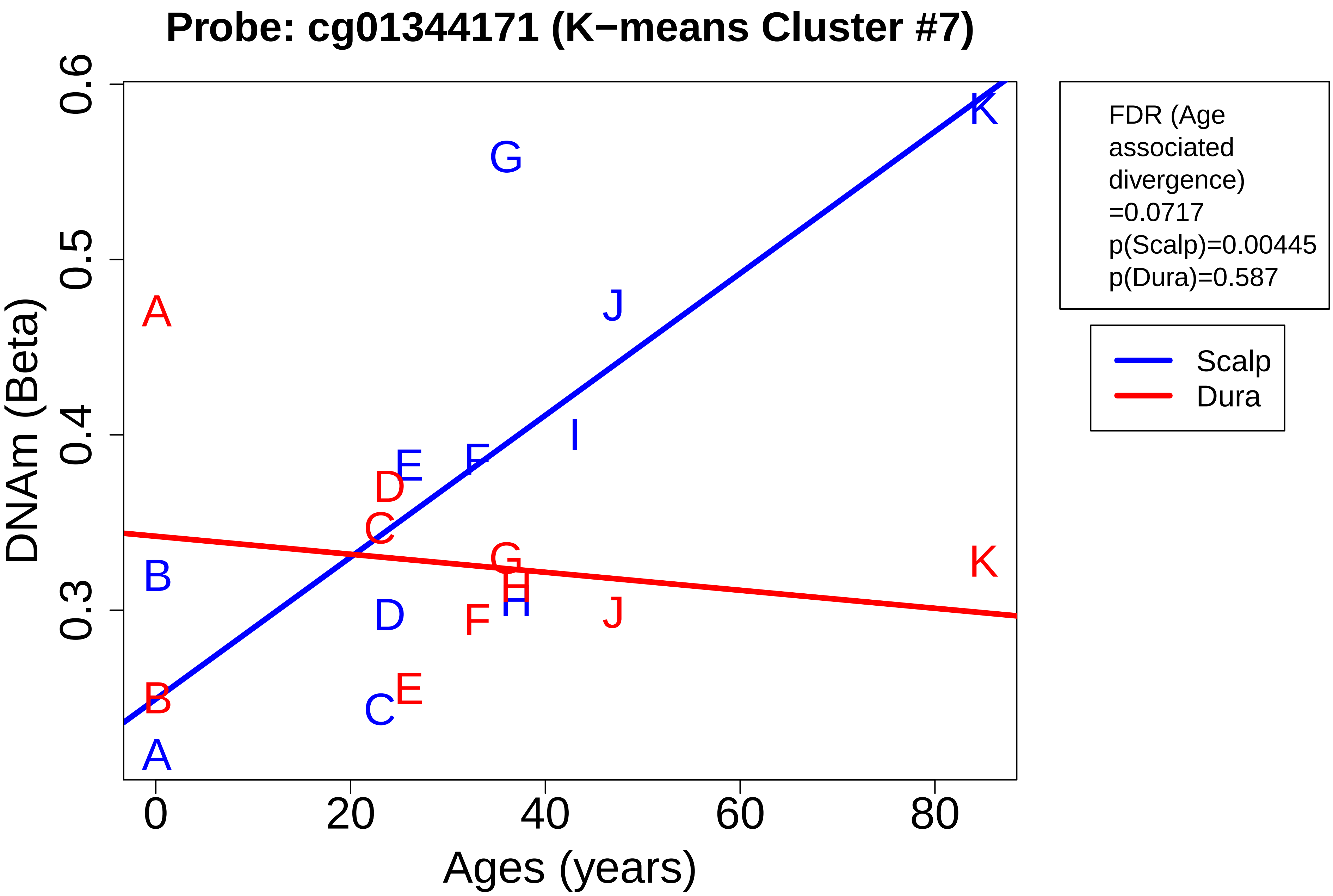

Probe: cg01344171 (K−means Cluster #7)
DNAm (Beta)
0.3
0.4
0.5
0.6
0
20
40
60
80
Ages (years)
FDR (Age associated divergence) =0.0717
p(Scalp)=0.00445
p(Dura)=0.587
Scalp
Dura
A
B
C
D
E
F
G
H
I
J
K

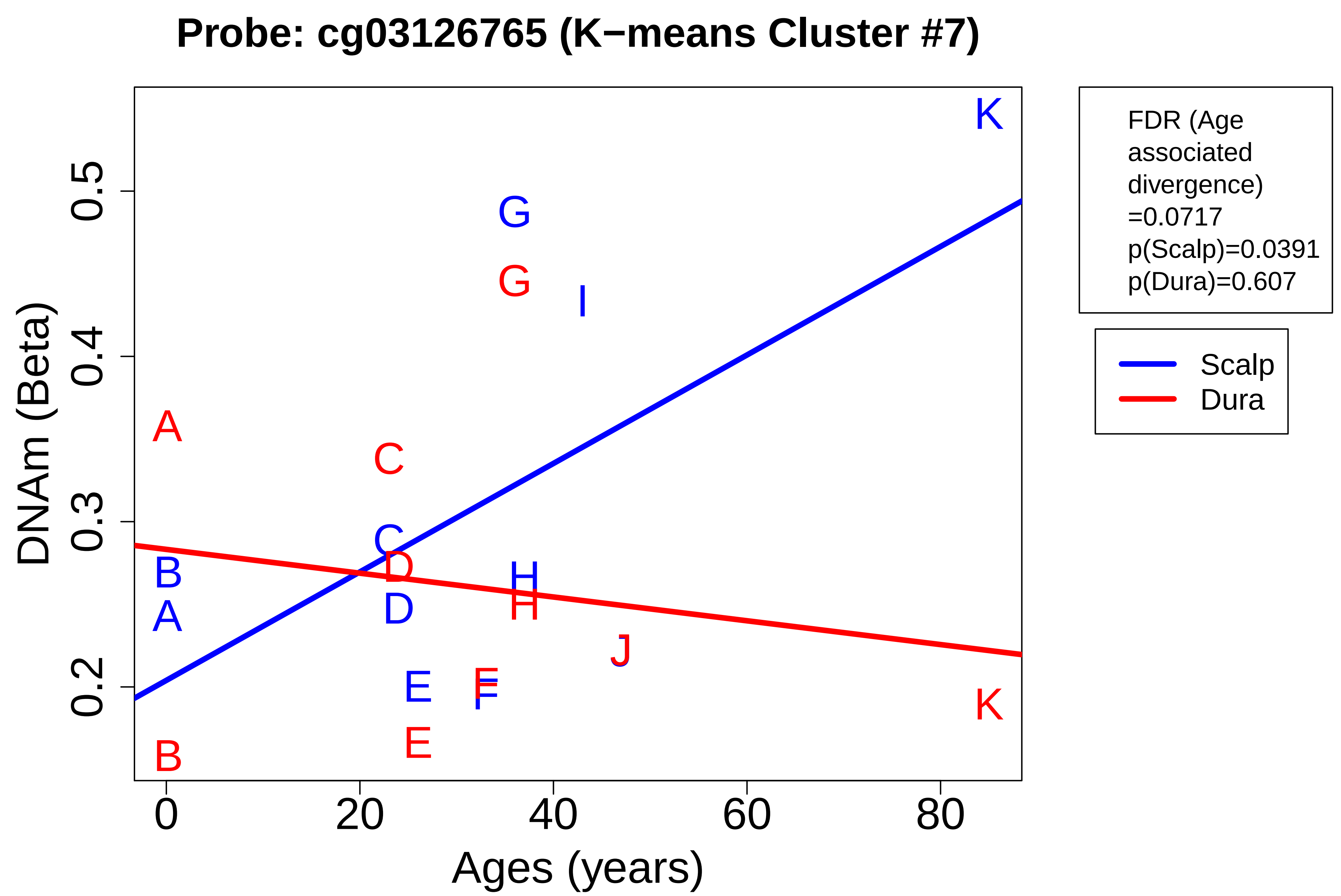

Probe: cg03126765 (K−means Cluster #7)
DNAm (Beta)
0.2
0.3
0.4
0.5
Ages (years)
0
20
40
60
80
A
B
C
D
E
F
G
H
I
J
K
FDR (Age associated divergence) =0.0717
p(Scalp)=0.0391
p(Dura)=0.607
Scalp
Dura

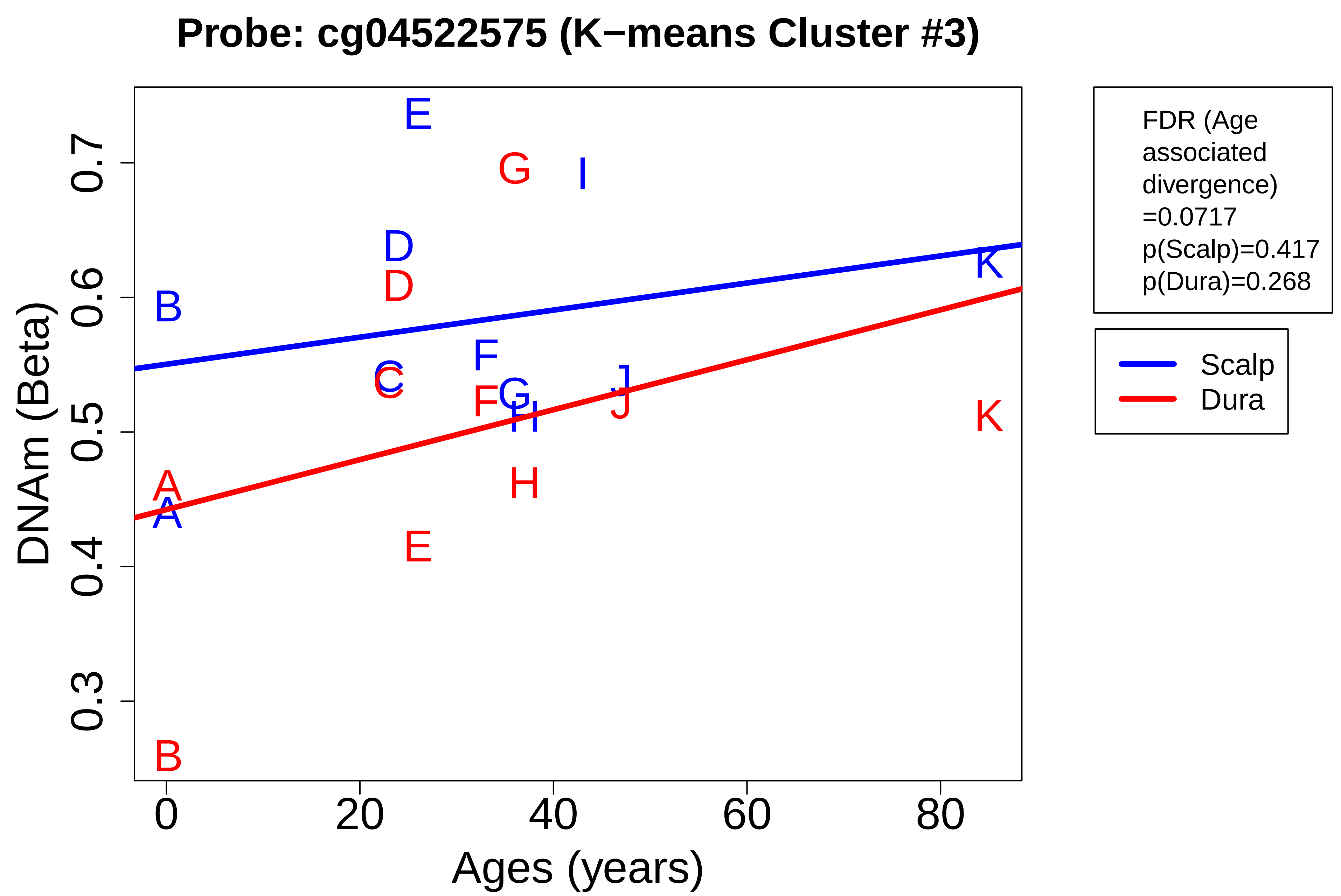

Probe: cg04522575 (K−means Cluster #3)
DNAm (Beta)
0.3
0.4
0.5
0.6
0.7
0
20
40
60
80
Ages (years)
A
B
C
D
E
F
G
H
I
J
K
FDR (Age associated divergence) =0.0717
p(Scalp)=0.417
p(Dura)=0.268
Scalp
Dura

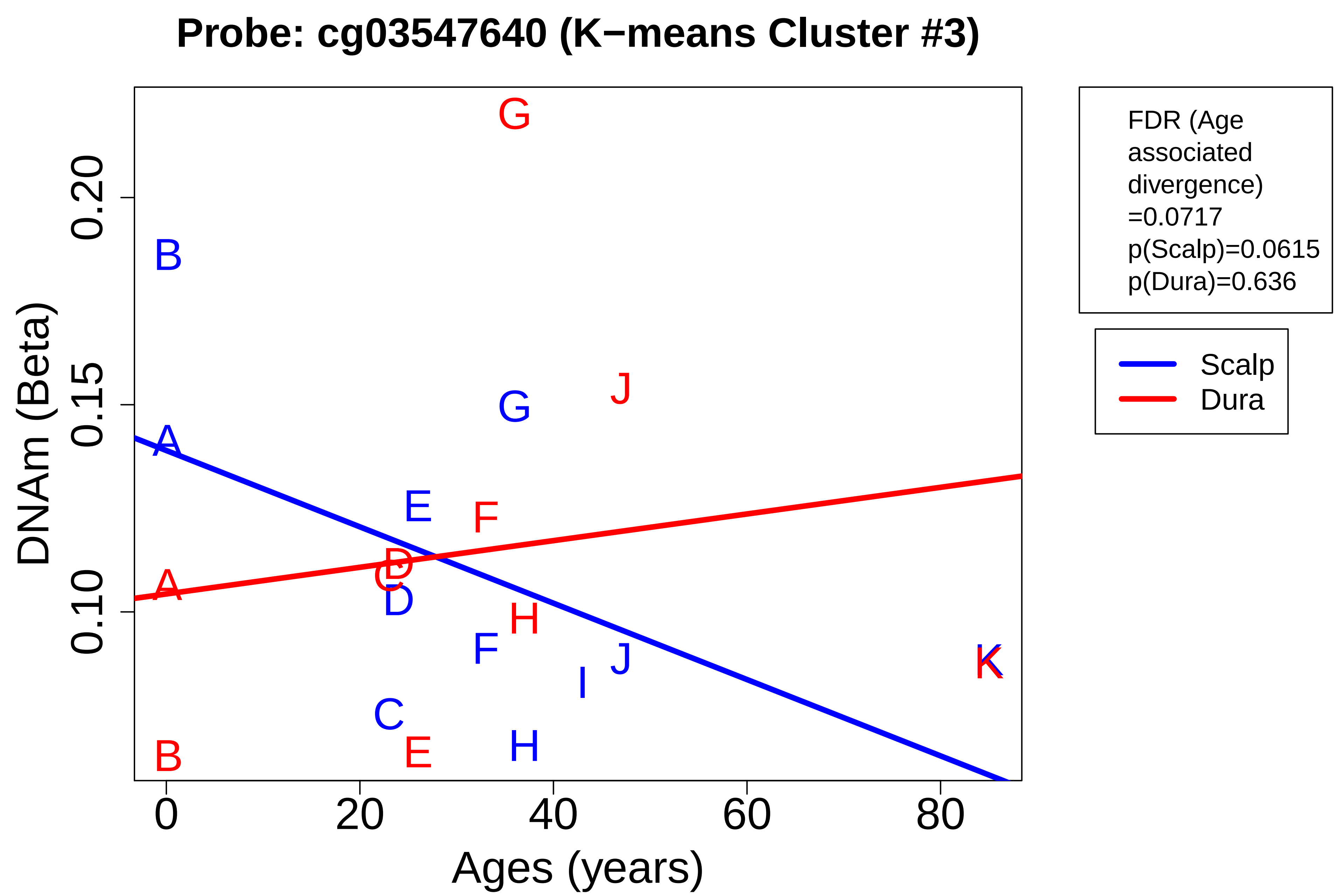

Probe: cg03547640 (K−means Cluster #3)
DNAm (Beta)
0.10
0.15
0.20
Ages (years)
0
20
40
60
80
FDR (Age associated divergence) =0.0717
p(Scalp)=0.0615
p(Dura)=0.636
Scalp
Dura

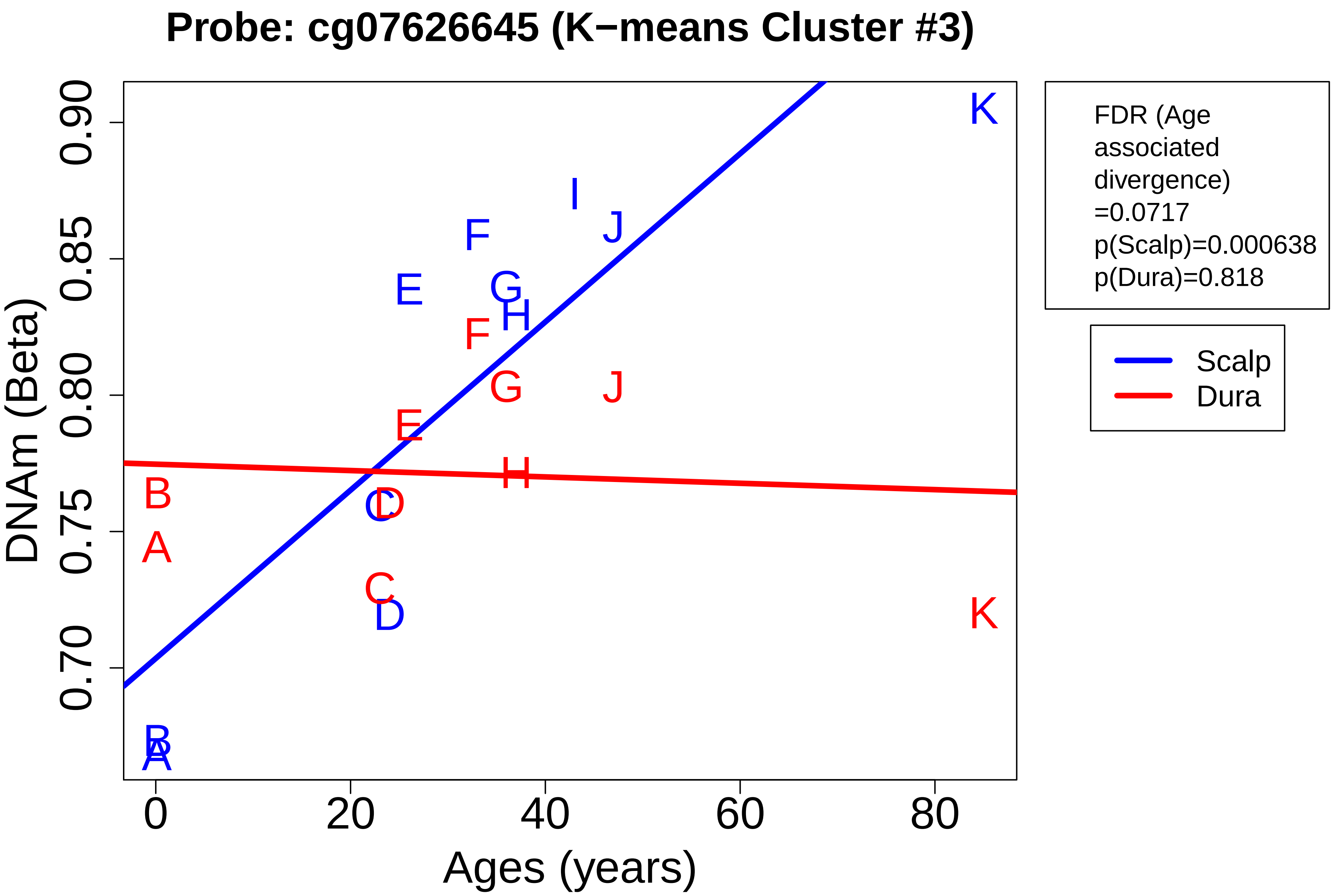

Probe: cg07626645 (K−means Cluster #3)
DNAm (Beta)
0.70
0.75
0.80
0.85
0.90
0
20
40
60
80
Ages (years)
FDR (Age associated divergence) =0.0717
p(Scalp)=0.000638
p(Dura)=0.818
Scalp
Dura

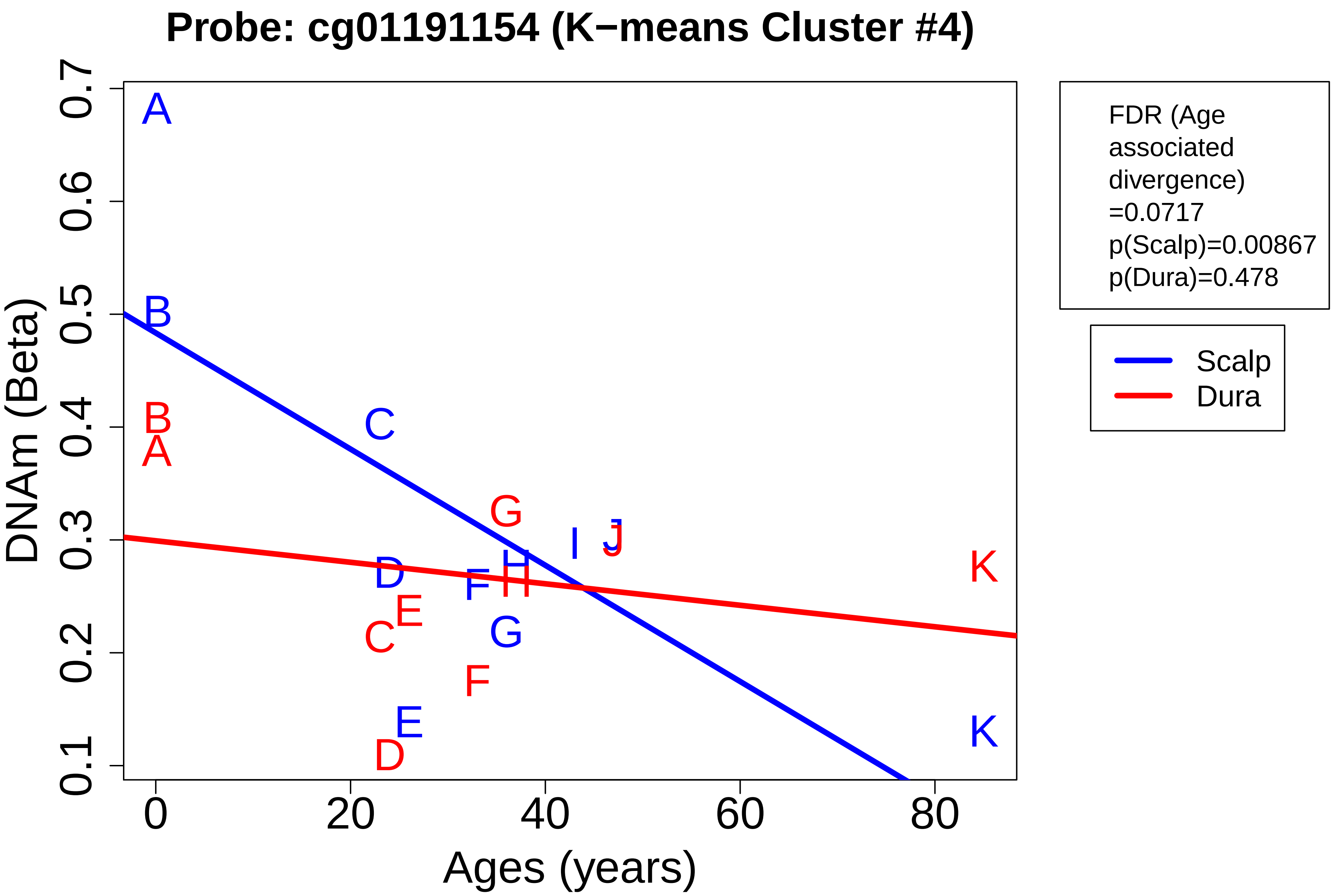

Probe: cg01191154 (K−means Cluster #4)
DNAm (Beta)
0.1
0.2
0.3
0.4
0.5
0.6
0.7
Ages (years)
0
20
40
60
80
A
B
C
D
E
F
G
H
I
J
K
FDR (Age associated divergence) =0.0717
p(Scalp)=0.00867
p(Dura)=0.478
Scalp
Dura

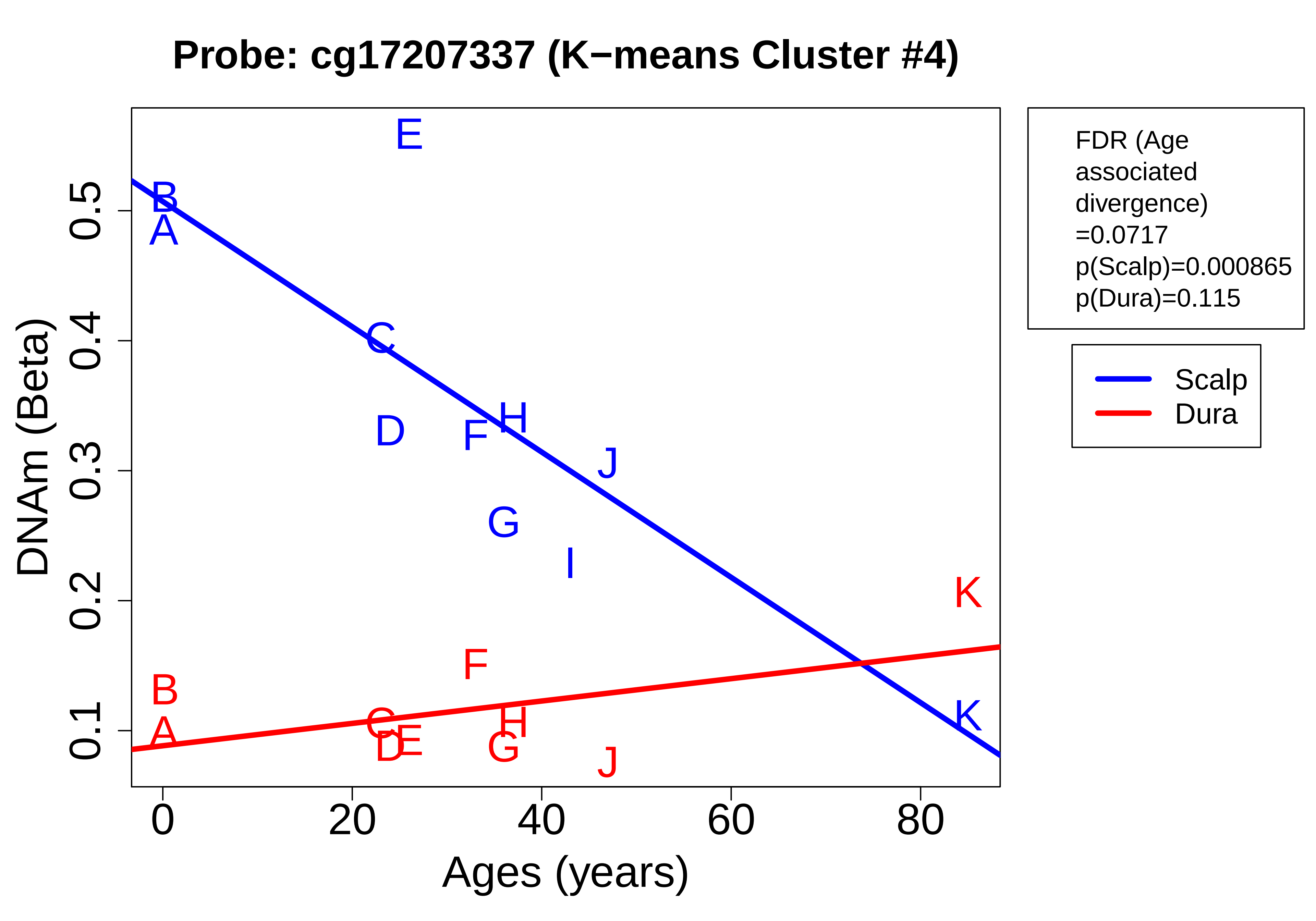

Probe: cg17207337 (K−means Cluster #4)
FDR (Age associated divergence) =0.0717
p(Scalp)=0.000865
p(Dura)=0.115
Scalp
Dura
DNAm (Beta)
0.1
0.2
0.3
0.4
0.5
0
20
40
60
80
Ages (years)
A
B
C
D
E
F
G
H
I
J
K

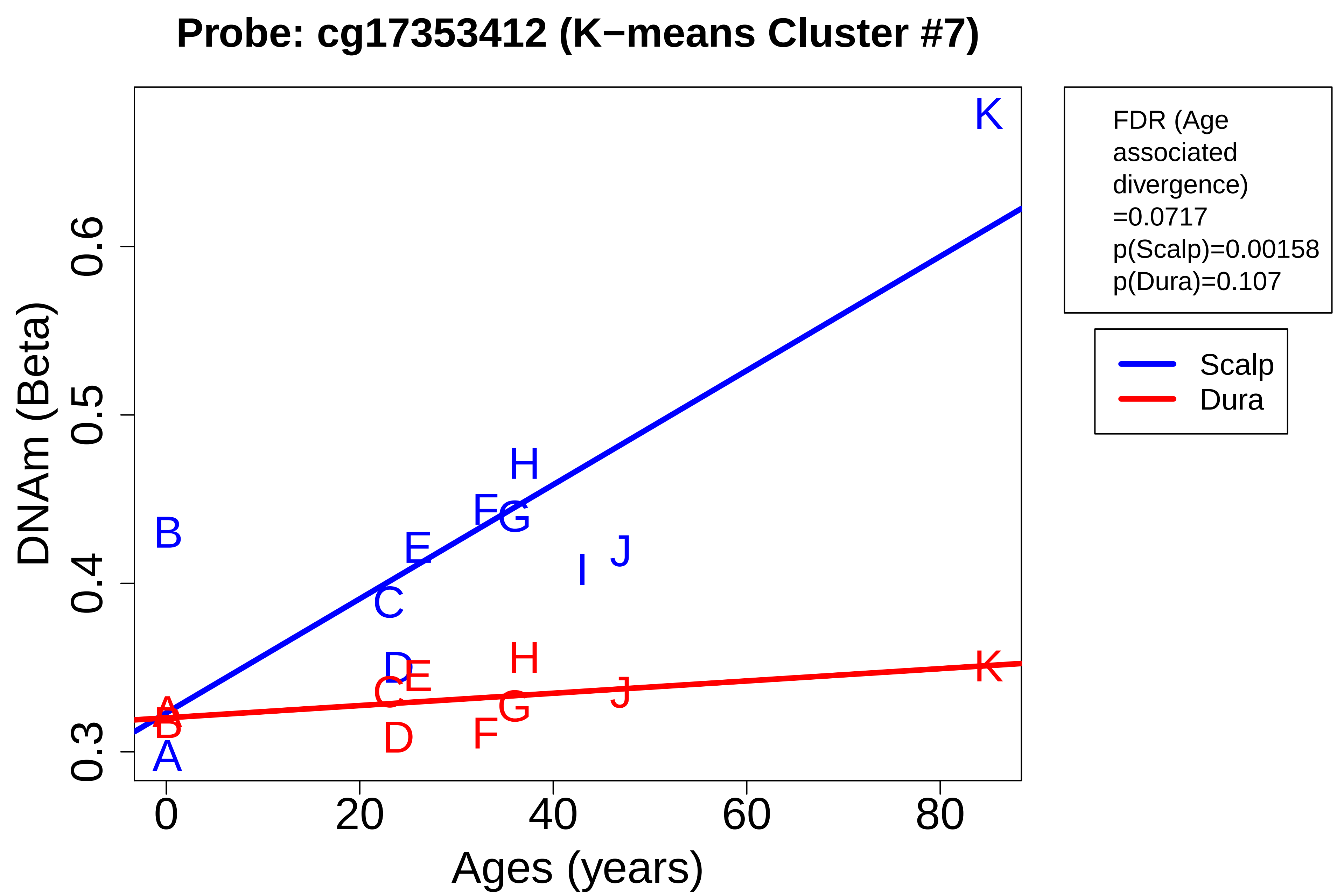

Probe: cg17353412 (K−means Cluster #7)
DNAm (Beta)
0.3
0.4
0.5
0.6
0
20
40
60
80
Ages (years)
FDR (Age associated divergence) =0.0717
p(Scalp)=0.00158
p(Dura)=0.107
Scalp
Dura
A
B
C
D
E
F
G
H
I
J
K

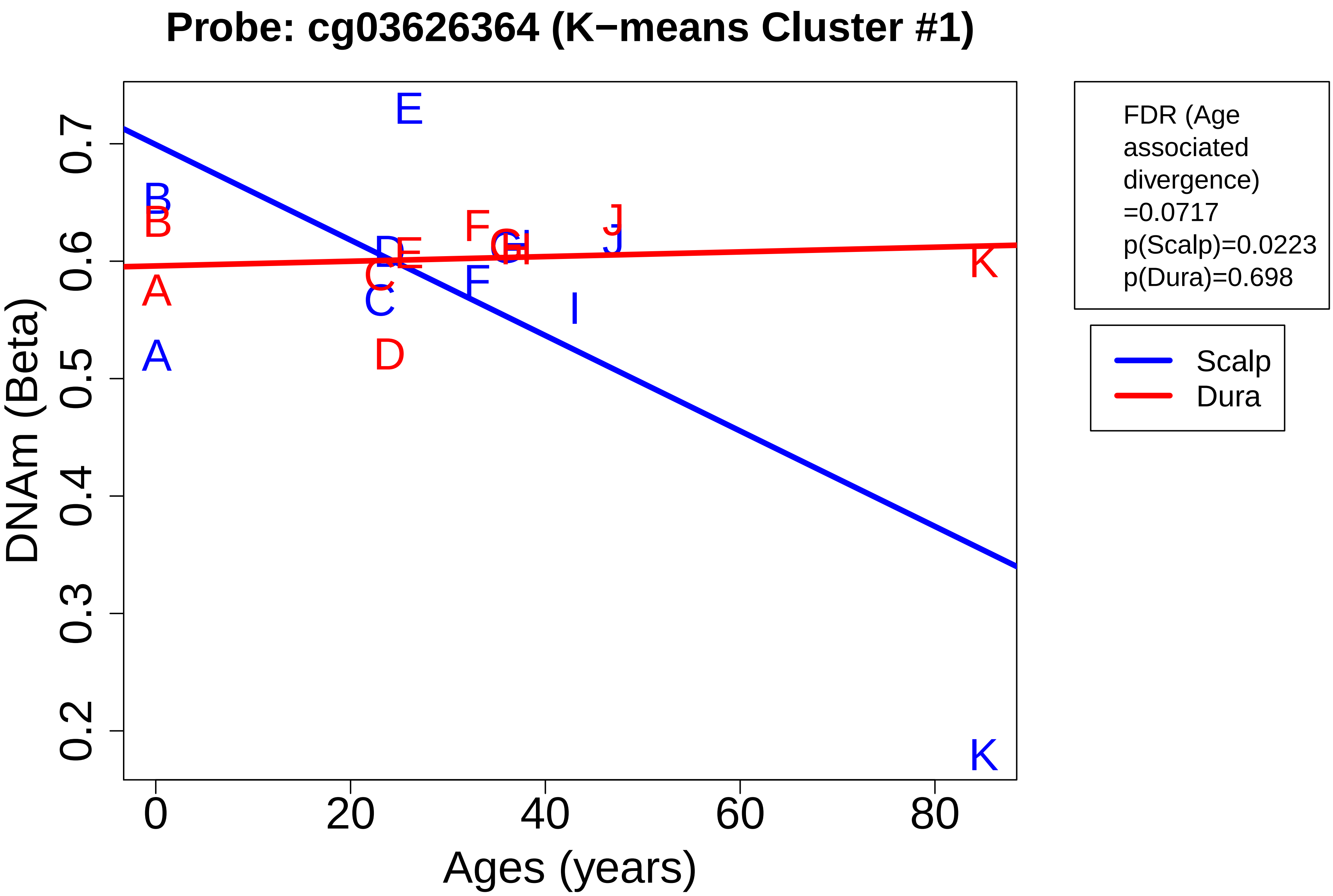

Probe: cg03626364 (K−means Cluster #1)
DNAm (Beta)
0.2
0.3
0.4
0.5
0.6
0.7
0
20
40
60
80
Ages (years)
FDR (Age associated divergence) =0.0717
p(Scalp)=0.0223
p(Dura)=0.698
Scalp
Dura

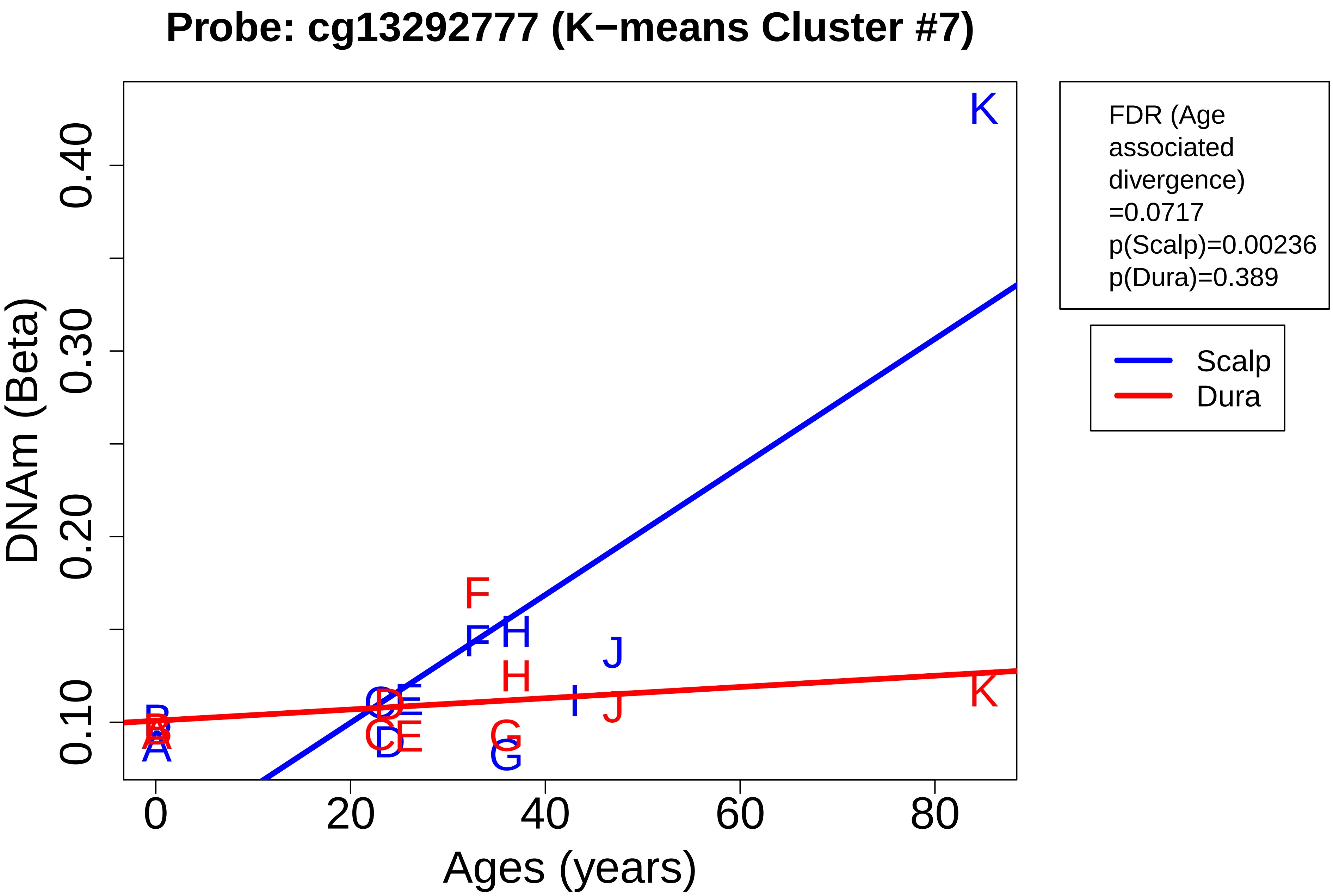
Probe: cg13292777 (K−means Cluster #7)
DNAm (Beta)
0.10
0.20
0.30
0.40
Ages (years)
0
20
40
60
80
FDR (Age associated divergence) =0.0717
p(Scalp)=0.00236
p(Dura)=0.389
Scalp
Dura

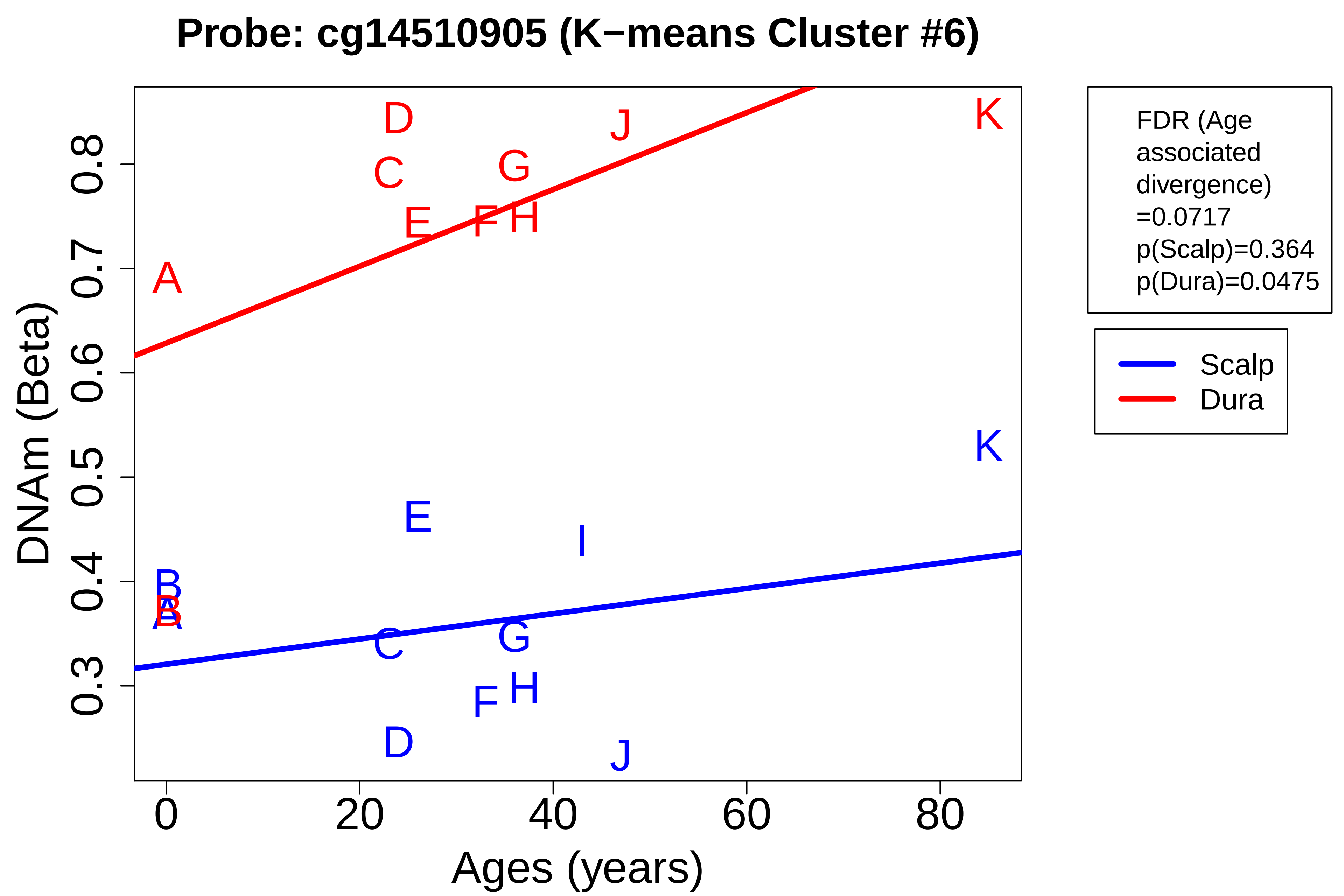

Probe: cg14510905 (K−means Cluster #6)
DNAm (Beta)
0.3
0.4
0.5
0.6
0.7
0.8
0
20
40
60
80
Ages (years)
FDR (Age associated divergence) =0.0717
p(Scalp)=0.364
p(Dura)=0.0475
Scalp
Dura

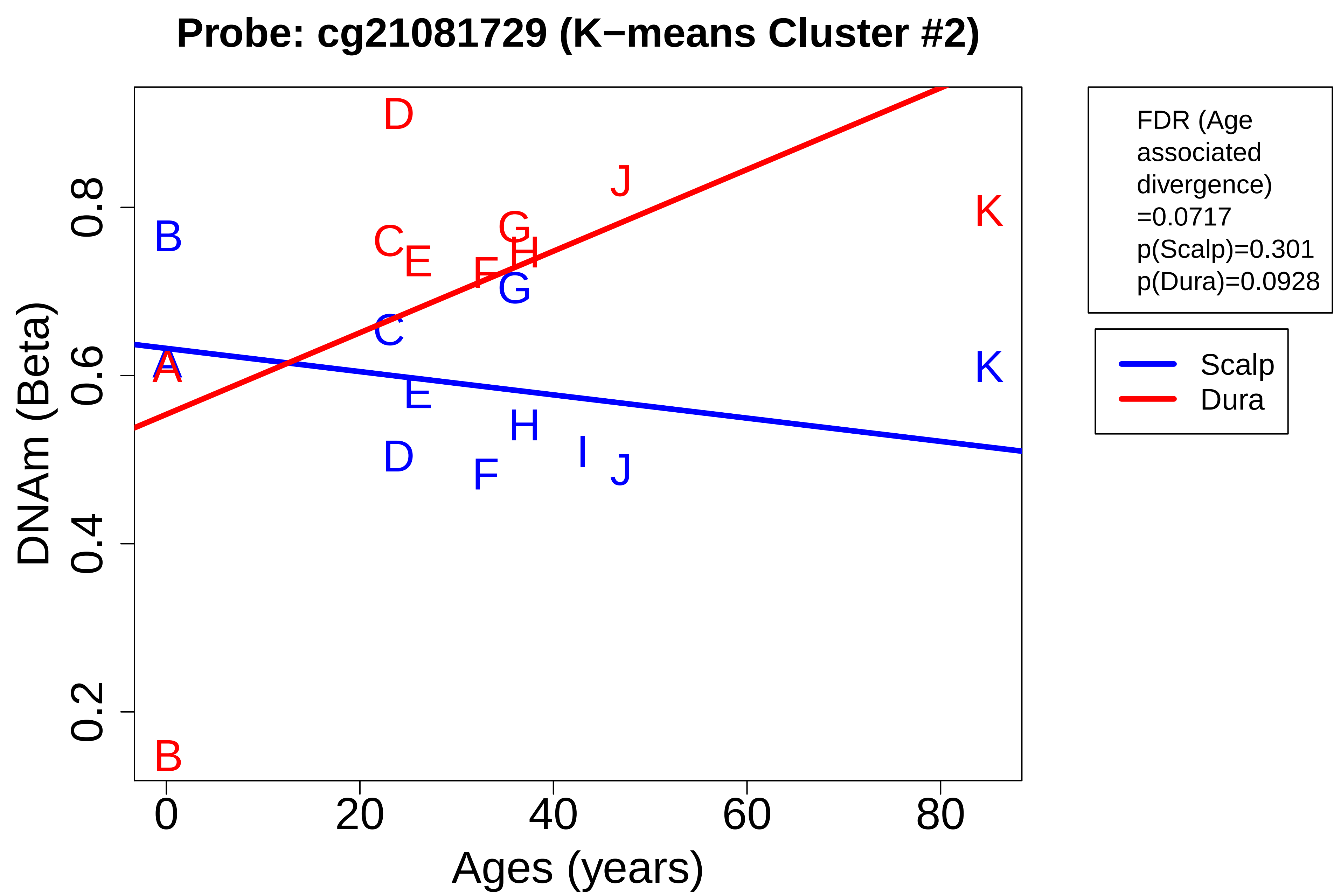

Probe: cg21081729 (K−means Cluster #2)
FDR (Age associated divergence) =0.0717
p(Scalp)=0.301
p(Dura)=0.0928
Scalp
Dura
DNAm (Beta)
0.2
0.4
0.6
0.8
0
20
40
60
80
Ages (years)
A
B
C
D
E
F
G
H
I
J
K

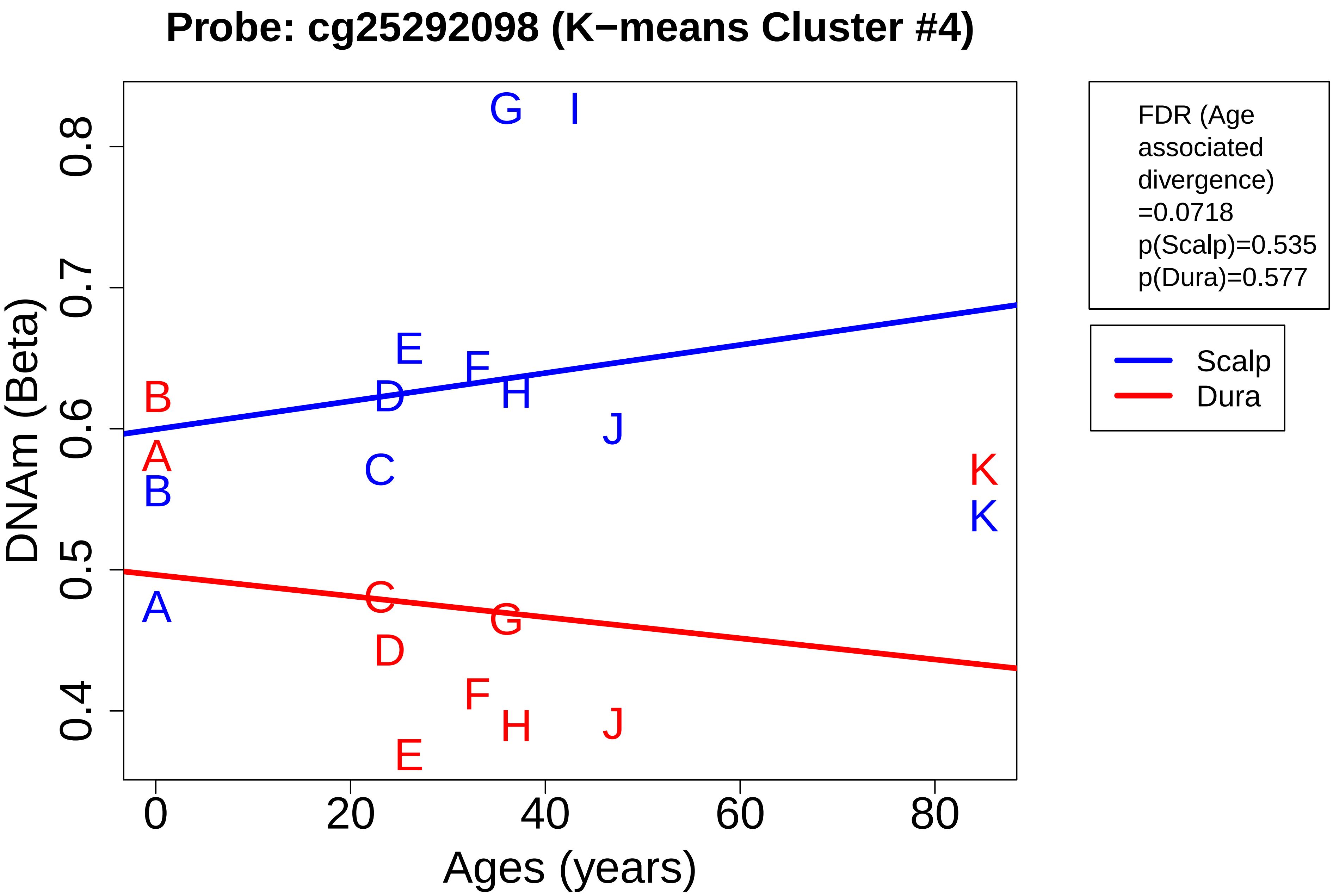

Probe: cg25292098 (K−means Cluster #4)
DNAm (Beta)
0.4
0.5
0.6
0.7
0.8
Ages (years)
0
20
40
60
80
FDR (Age associated divergence) =0.0718
p(Scalp)=0.535
p(Dura)=0.577
Scalp
Dura
A
B
C
D
E
F
G
H
I
J
K

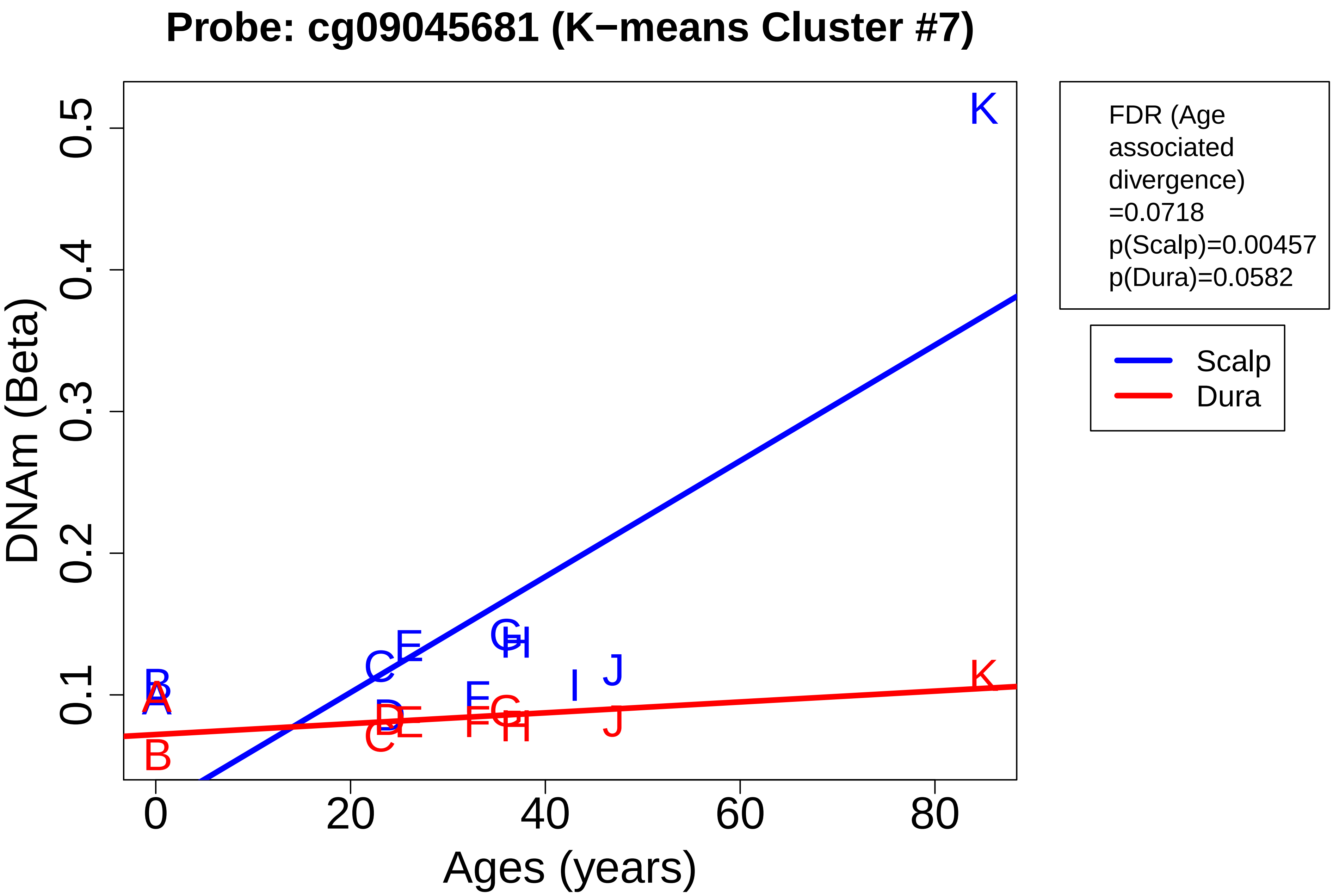

Probe: cg09045681 (K−means Cluster #7)
DNAm (Beta)
0.1
0.2
0.3
0.4
0.5
0
20
40
60
80
Ages (years)
A
B
C
D
E
F
G
H
I
J
K
FDR (Age associated divergence) =0.0718
p(Scalp)=0.00457
p(Dura)=0.0582
Scalp
Dura

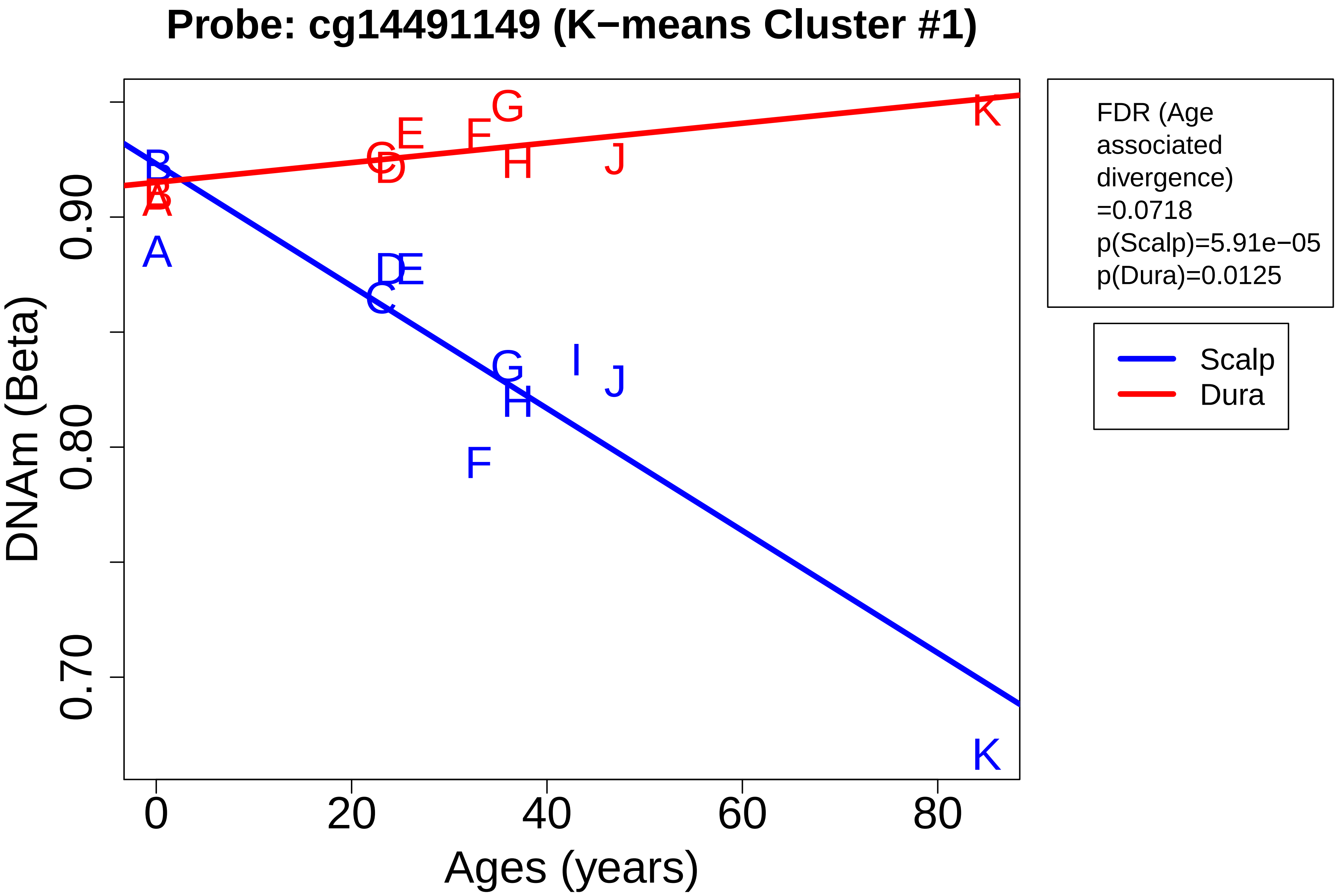

Probe: cg14491149 (K−means Cluster #1)
FDR (Age associated divergence) =0.0718
p(Scalp)=5.91e−05
p(Dura)=0.0125
Scalp
Dura
DNAm (Beta)
0.70
0.80
0.90
Ages (years)
0
20
40
60
80

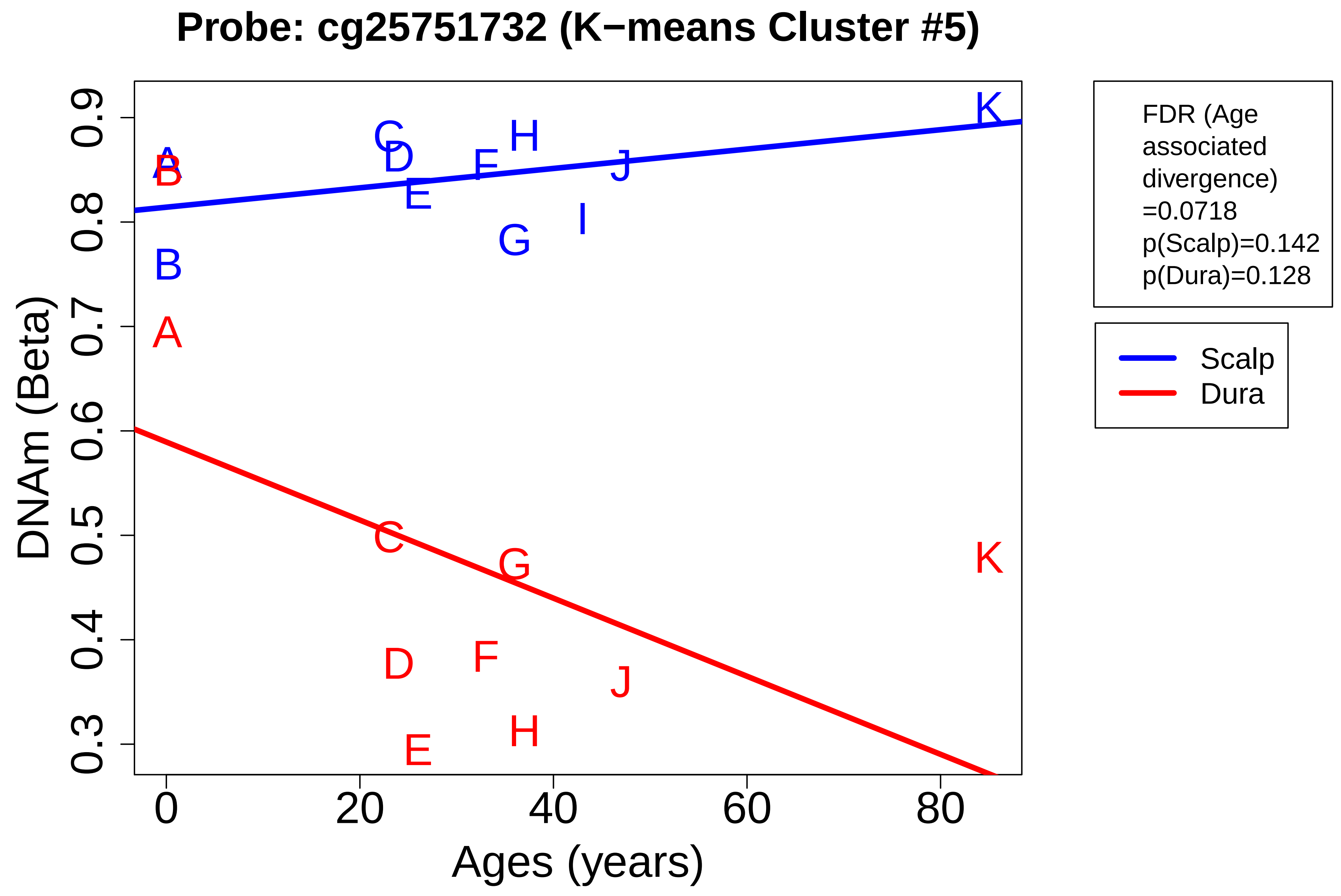
Probe: cg25751732 (K−means Cluster #5)
DNAm (Beta)
0.3
0.4
0.5
0.6
0.7
0.8
0.9
0
20
40
60
80
Ages (years)
FDR (Age associated divergence) =0.0718
p(Scalp)=0.142
p(Dura)=0.128
Scalp
Dura
A
B
C
D
E
F
G
H
I
J
K

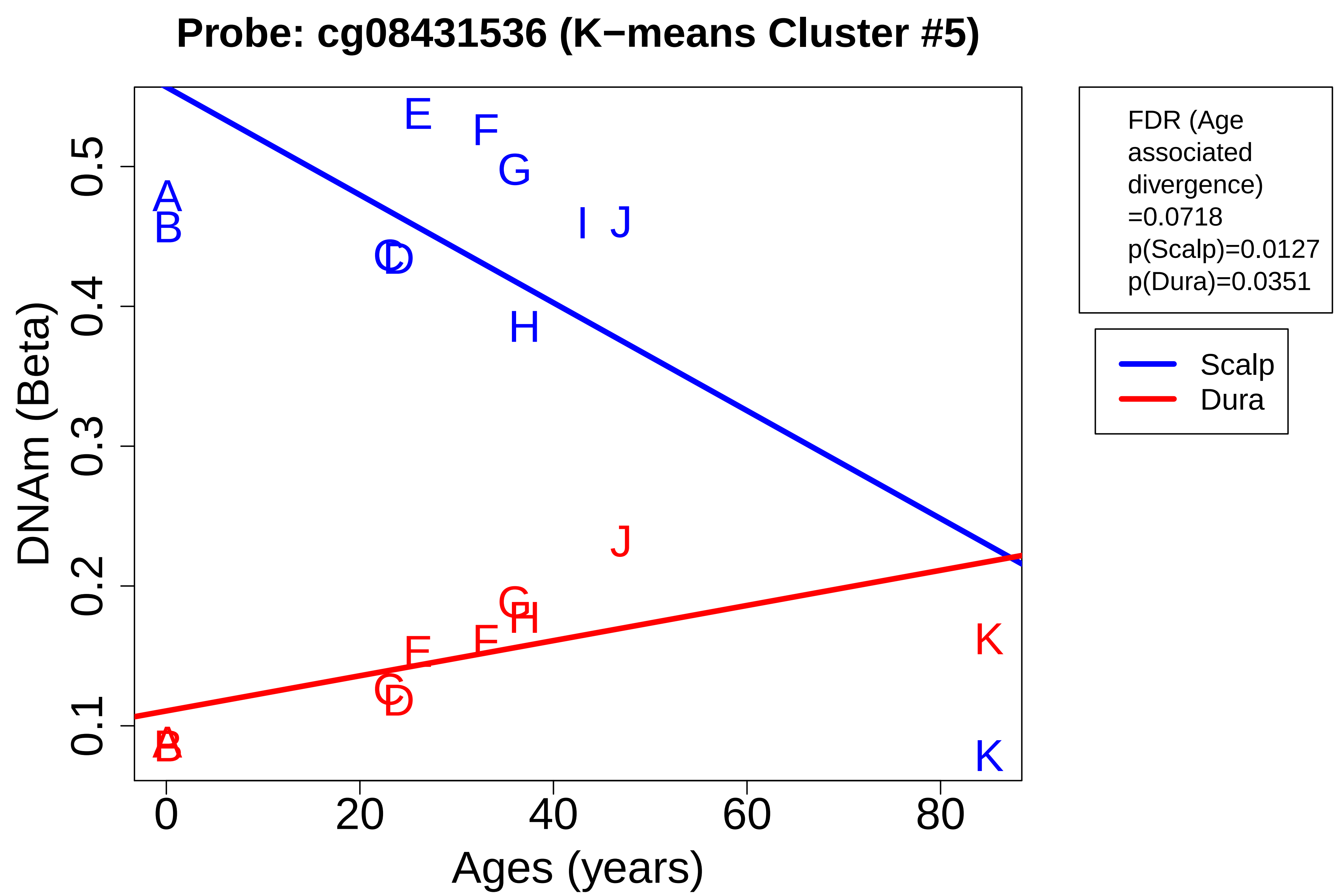

Probe: cg08431536 (K−means Cluster #5)
DNAm (Beta)
0.1
0.2
0.3
0.4
0.5
Ages (years)
0
20
40
60
80
A
B
C
D
E
F
G
H
I
J
K
FDR (Age associated divergence) =0.0718
p(Scalp)=0.0127
p(Dura)=0.0351
Scalp
Dura

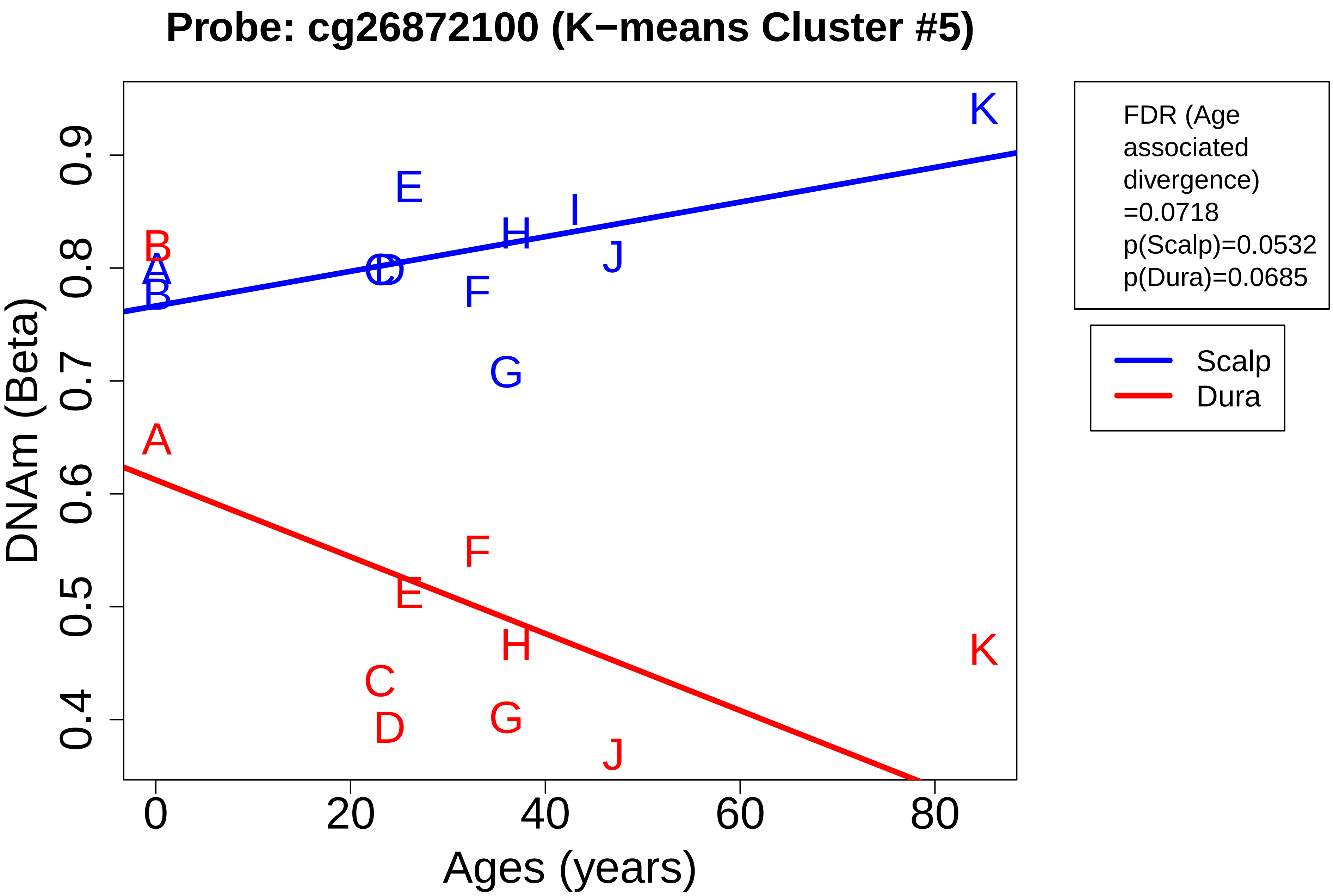

Probe: cg26872100 (K−means Cluster #5)
DNAm (Beta)
0.4
0.5
0.6
0.7
0.8
0.9
0
20
40
60
80
Ages (years)
A
B
C
D
E
F
G
H
I
J
K
FDR (Age associated divergence) =0.0718
p(Scalp)=0.0532
p(Dura)=0.0685
Scalp
Dura

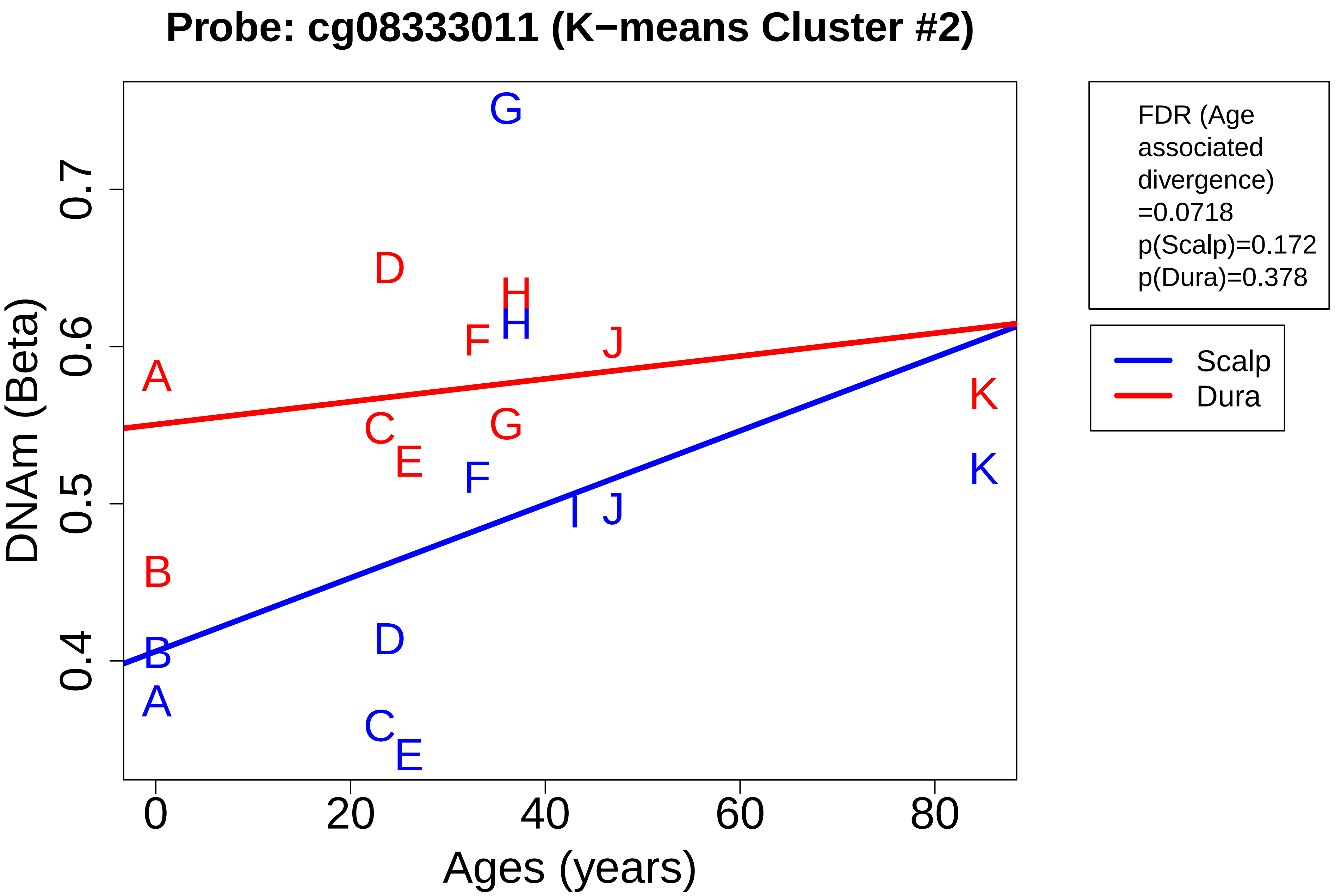

Probe: cg08333011 (K−means Cluster #2)
DNAm (Beta)
0.4
0.5
0.6
0.7
Ages (years)
0
20
40
60
80
FDR (Age associated divergence) =0.0718
p(Scalp)=0.172
p(Dura)=0.378
Scalp
Dura
A
B
C
D
E
F
G
H
I
J
K

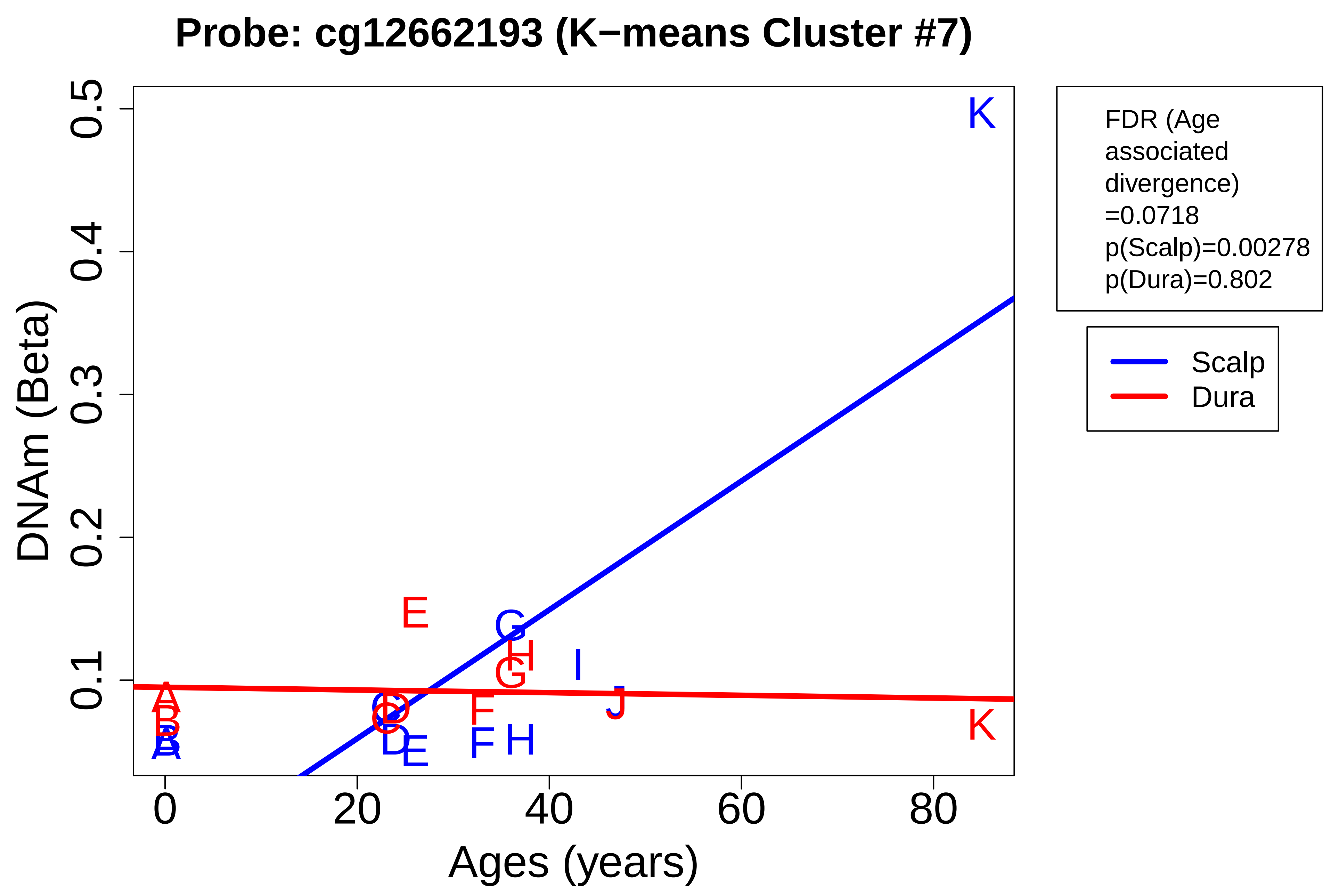

Probe: cg12662193 (K−means Cluster #7)
DNAm (Beta)
Ages (years)
0.1
0.2
0.3
0.4
0.5
0
20
40
60
80
FDR (Age associated divergence) =0.0718
p(Scalp)=0.00278
p(Dura)=0.802
Scalp
Dura

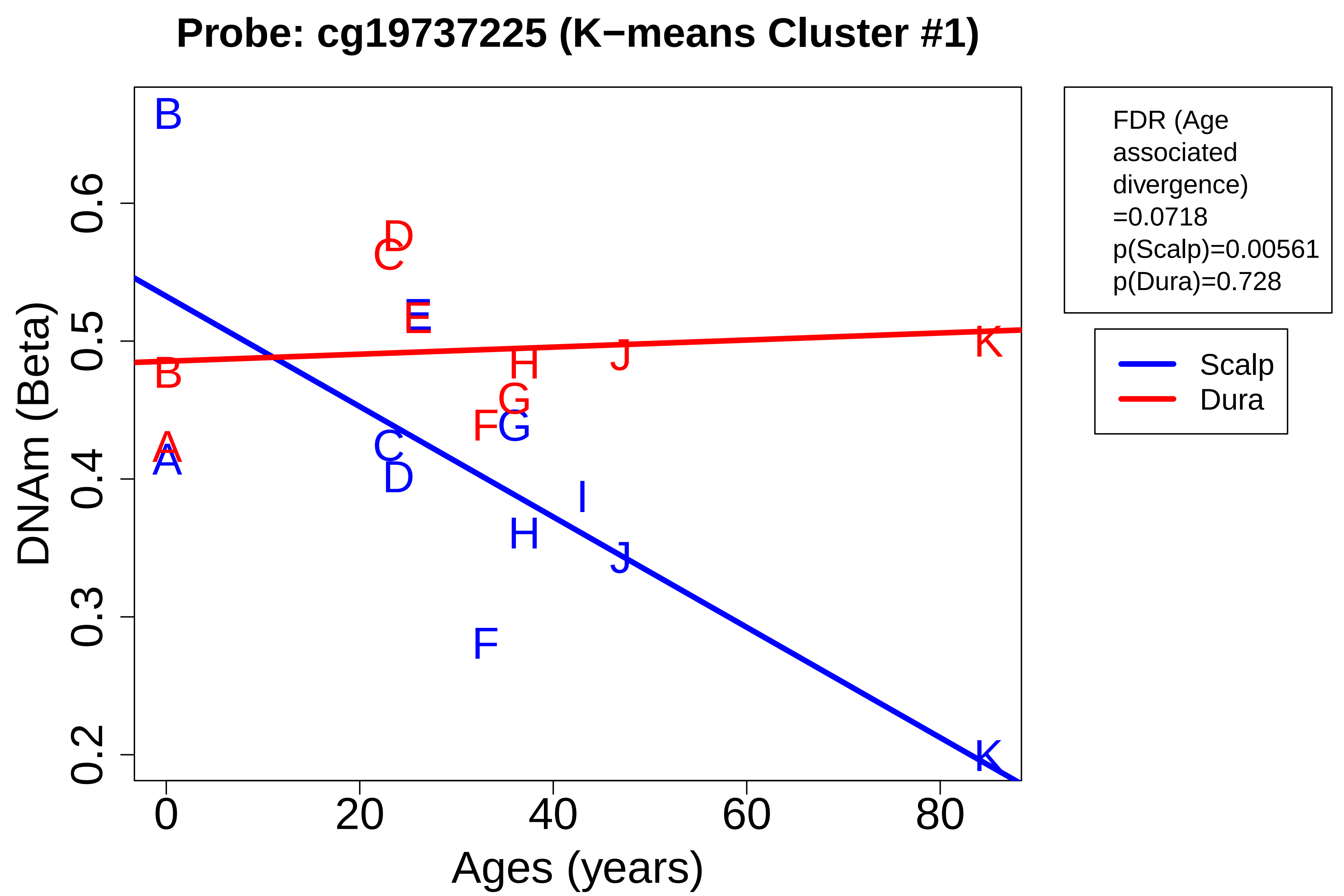
Probe: cg19737225 (K−means Cluster #1)
DNAm (Beta)
0.2
0.3
0.4
0.5
0.6
Ages (years)
0
20
40
60
80
A
B
C
D
E
F
G
H
I
J
K
FDR (Age associated divergence) =0.0718
p(Scalp)=0.00561
p(Dura)=0.728
Scalp
Dura

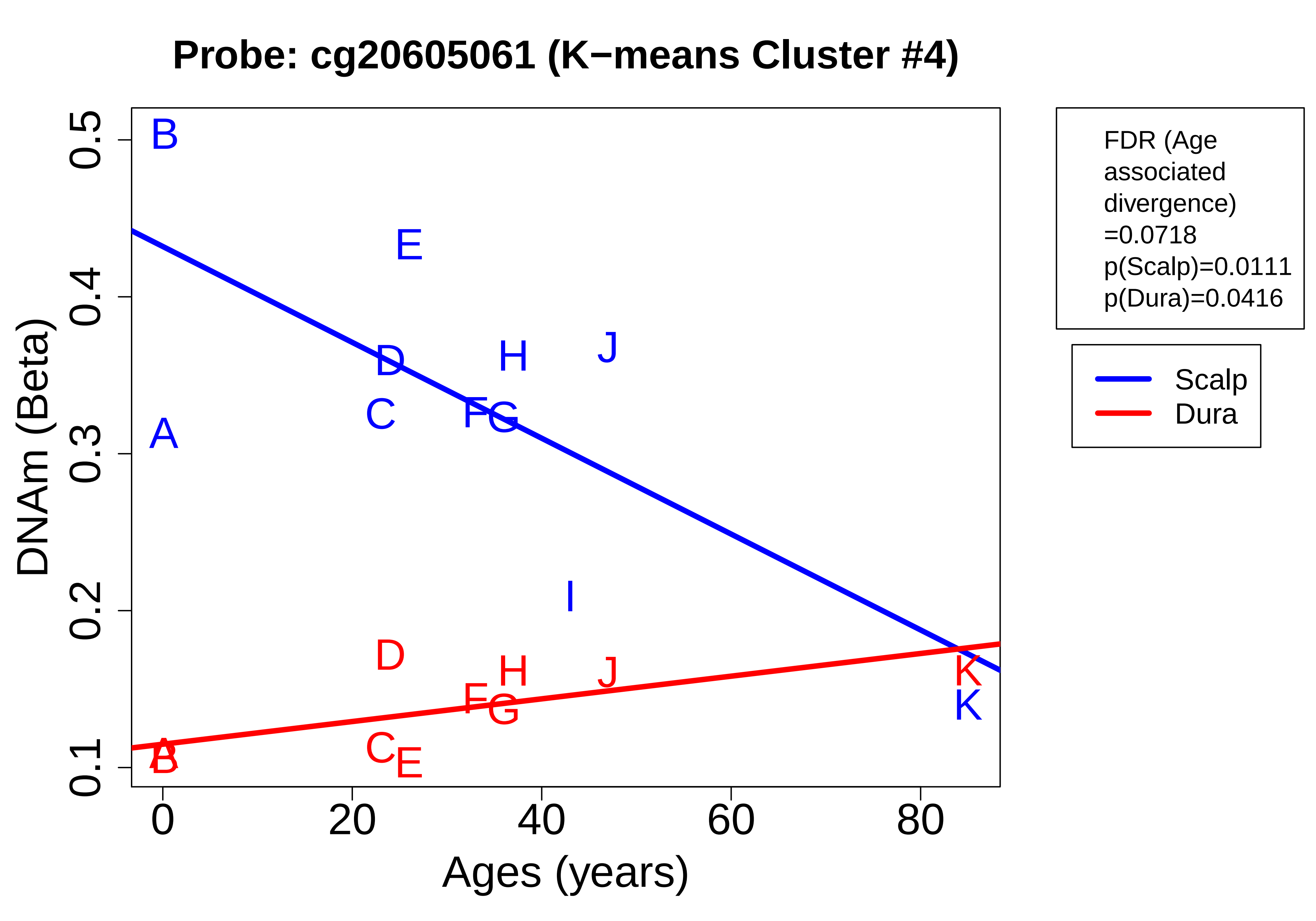

Probe: cg20605061 (K−means Cluster #4)
DNAm (Beta)
0.1
0.2
0.3
0.4
0.5
Ages (years)
0
20
40
60
80
FDR (Age associated divergence) =0.0718
p(Scalp)=0.0111
p(Dura)=0.0416
Scalp
Dura
A
B
C
D
E
F
G
H
I
J
K

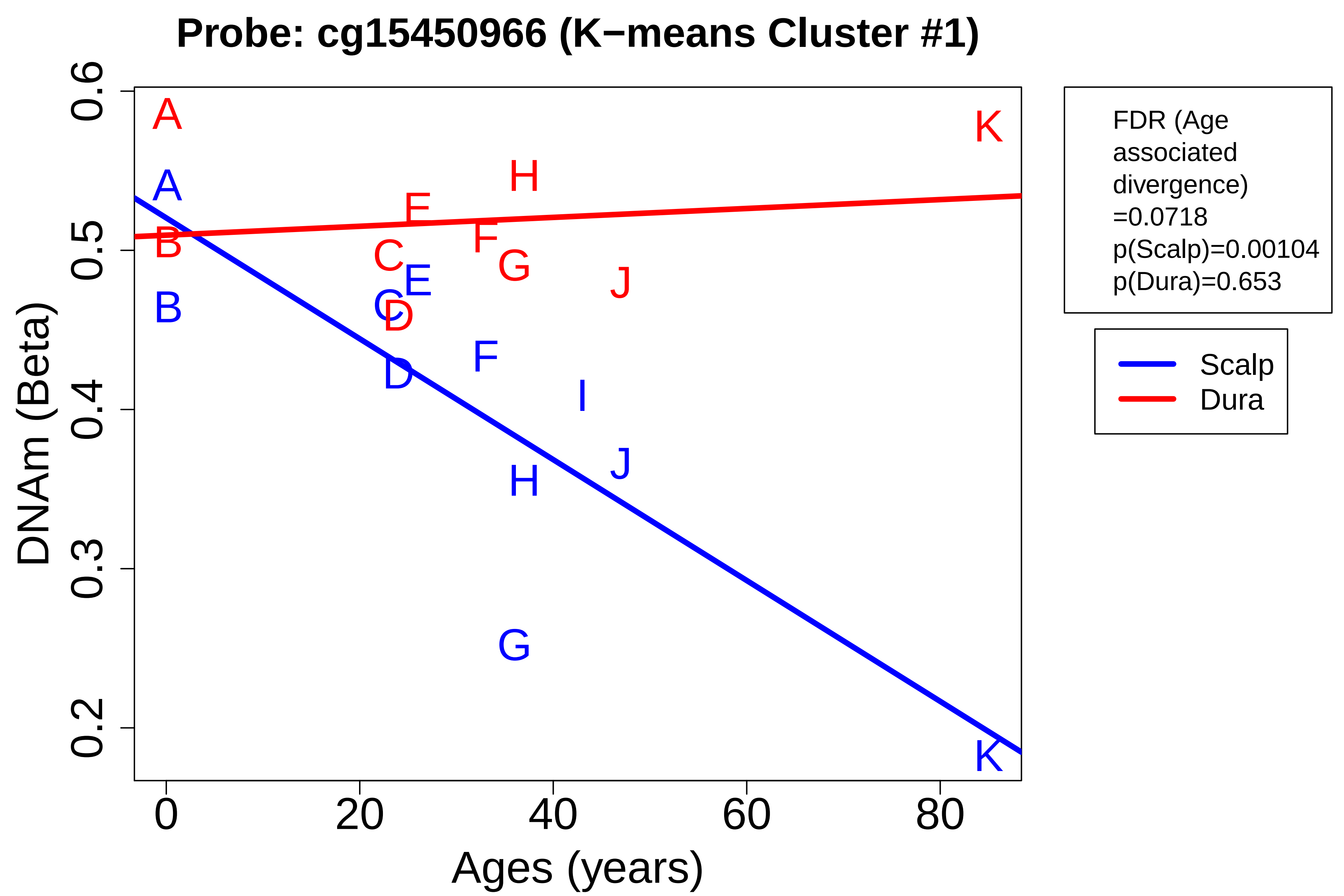

Probe: cg15450966 (K−means Cluster #1)
DNAm (Beta)
0.2
0.3
0.4
0.5
0.6
Ages (years)
0
20
40
60
80
A
B
C
D
E
F
G
H
I
J
K
FDR (Age associated divergence) =0.0718
p(Scalp)=0.00104
p(Dura)=0.653
Scalp
Dura

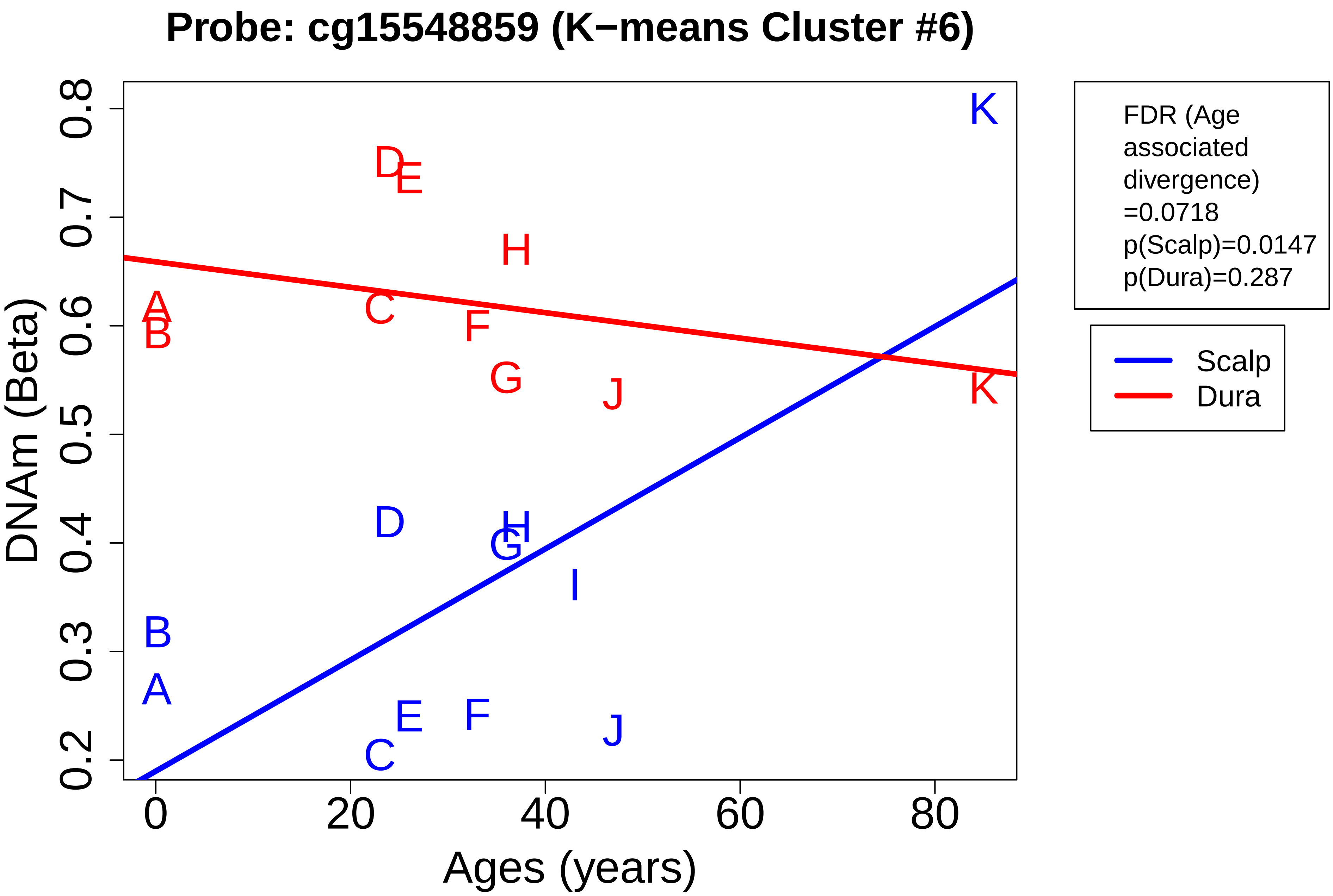

Probe: cg15548859 (K−means Cluster #6)
DNAm (Beta)
0.2
0.3
0.4
0.5
0.6
0.7
0.8
Ages (years)
0
20
40
60
80
A
B
C
D
E
F
G
H
I
J
K
FDR (Age associated divergence) =0.0718
p(Scalp)=0.0147
p(Dura)=0.287
Scalp
Dura

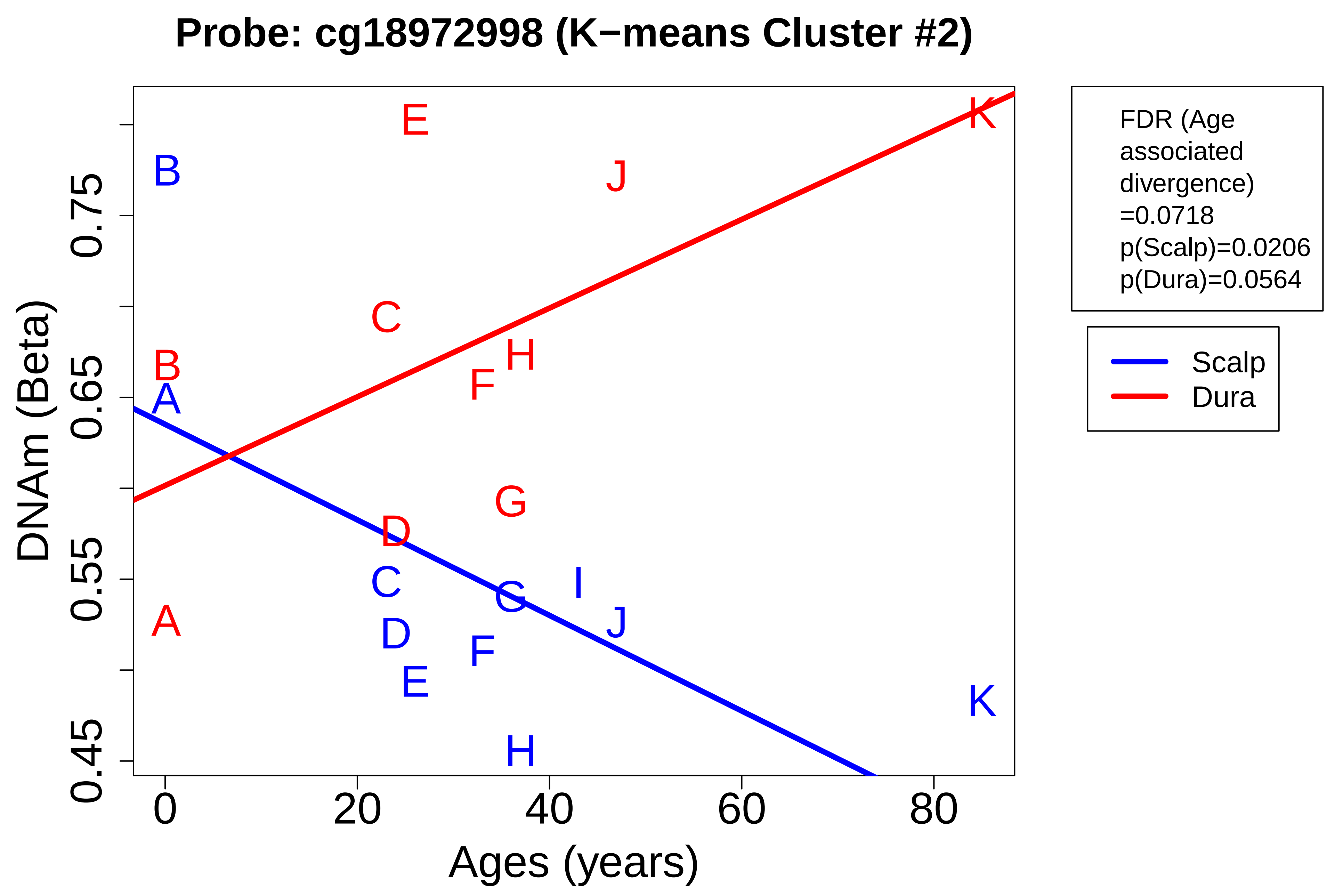

Probe: cg18972998 (K−means Cluster #2)
FDR (Age associated divergence) =0.0718
p(Scalp)=0.0206
p(Dura)=0.0564
Scalp
Dura
DNAm (Beta)
0.45
0.55
0.65
0.75
0
20
40
60
80
Ages (years)
A B C D E F G H I J K

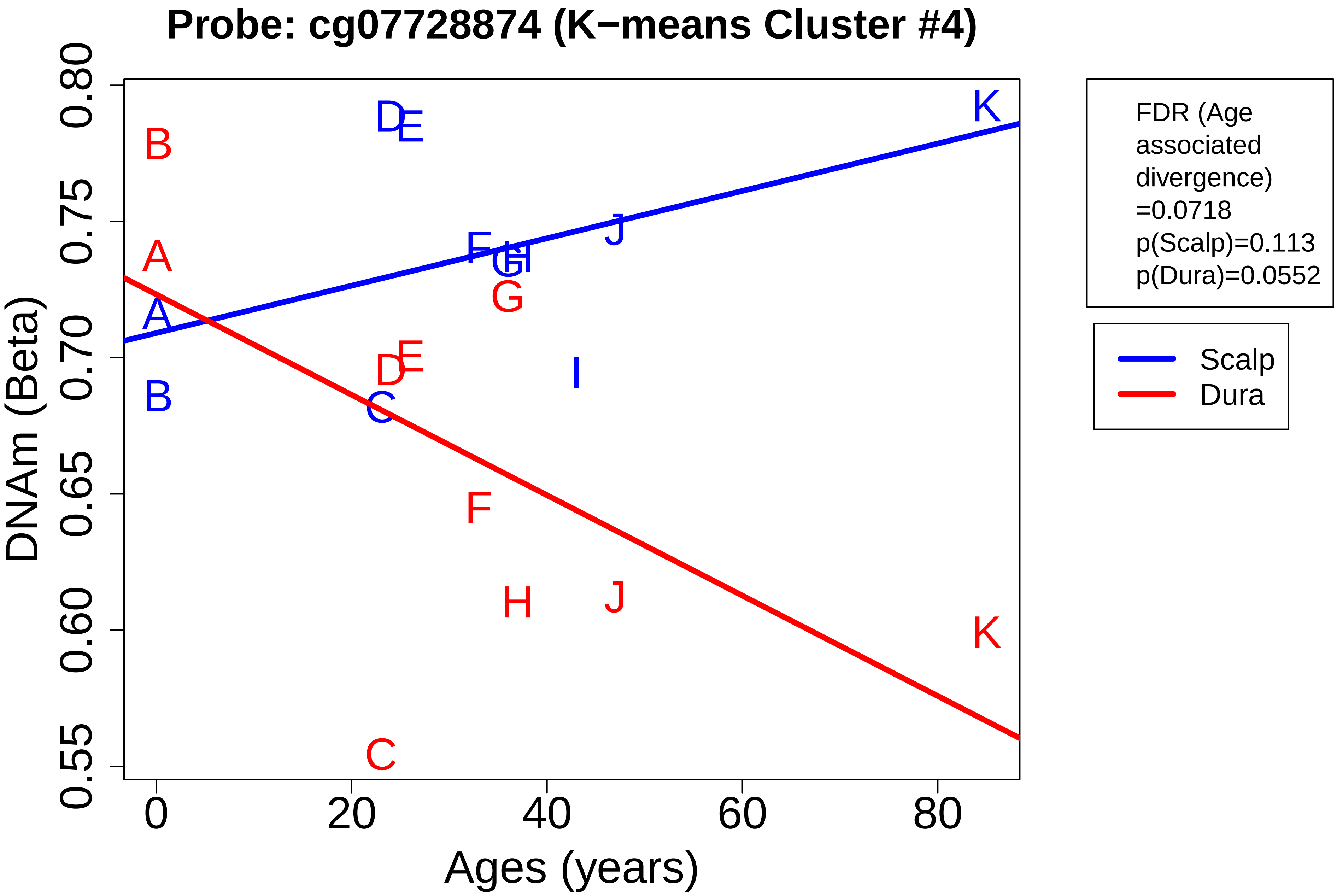

Probe: cg07728874 (K−means Cluster #4)
DNAm (Beta)
0.55
0.60
0.65
0.70
0.75
0.80
0
20
40
60
80
Ages (years)
FDR (Age associated divergence) =0.0718
p(Scalp)=0.113
p(Dura)=0.0552
Scalp
Dura

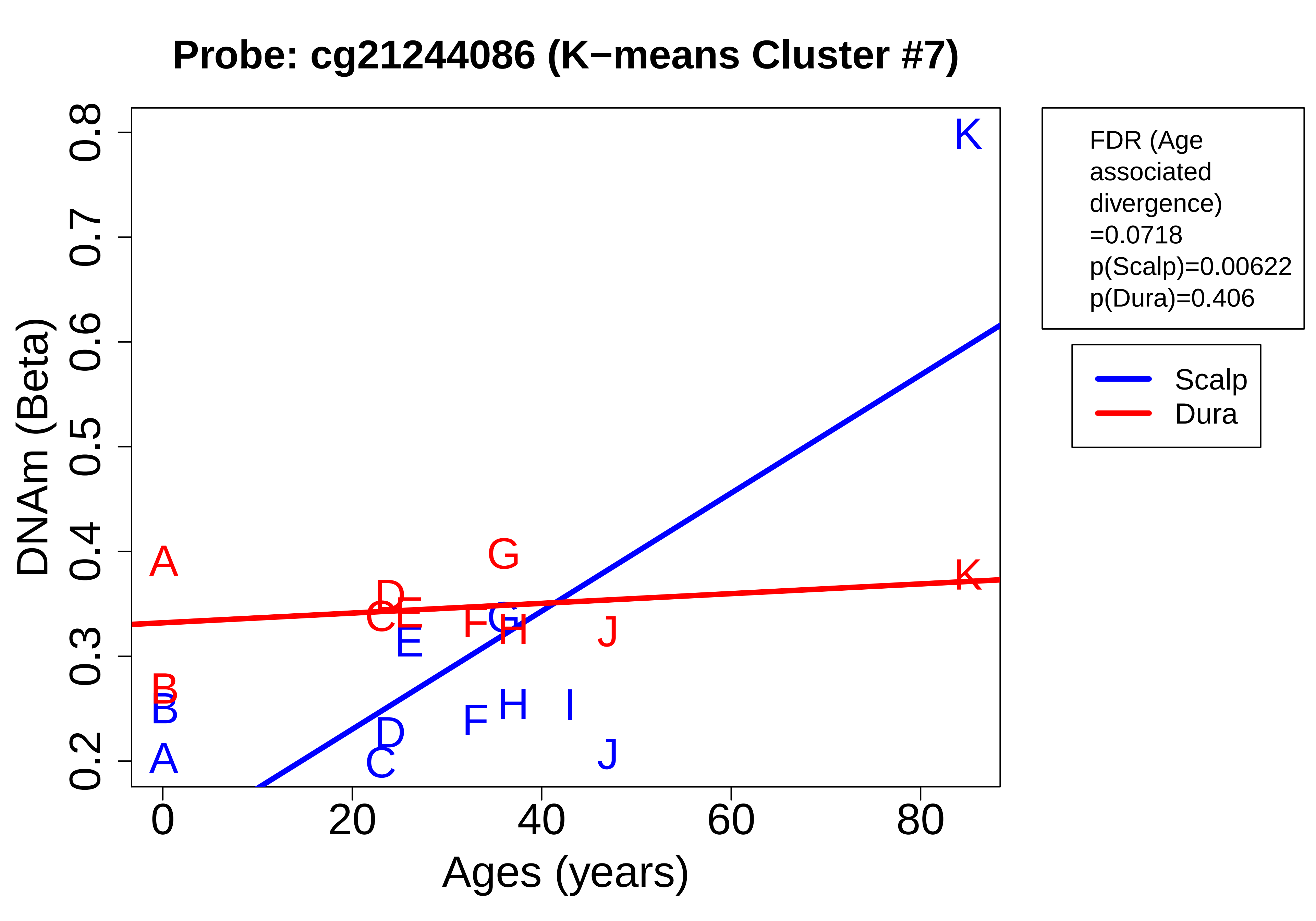

Probe: cg21244086 (K−means Cluster #7)
DNAm (Beta)
0.2
0.3
0.4
0.5
0.6
0.7
0.8
Ages (years)
0
20
40
60
80
FDR (Age associated divergence) =0.0718
p(Scalp)=0.00622
p(Dura)=0.406
Scalp
Dura

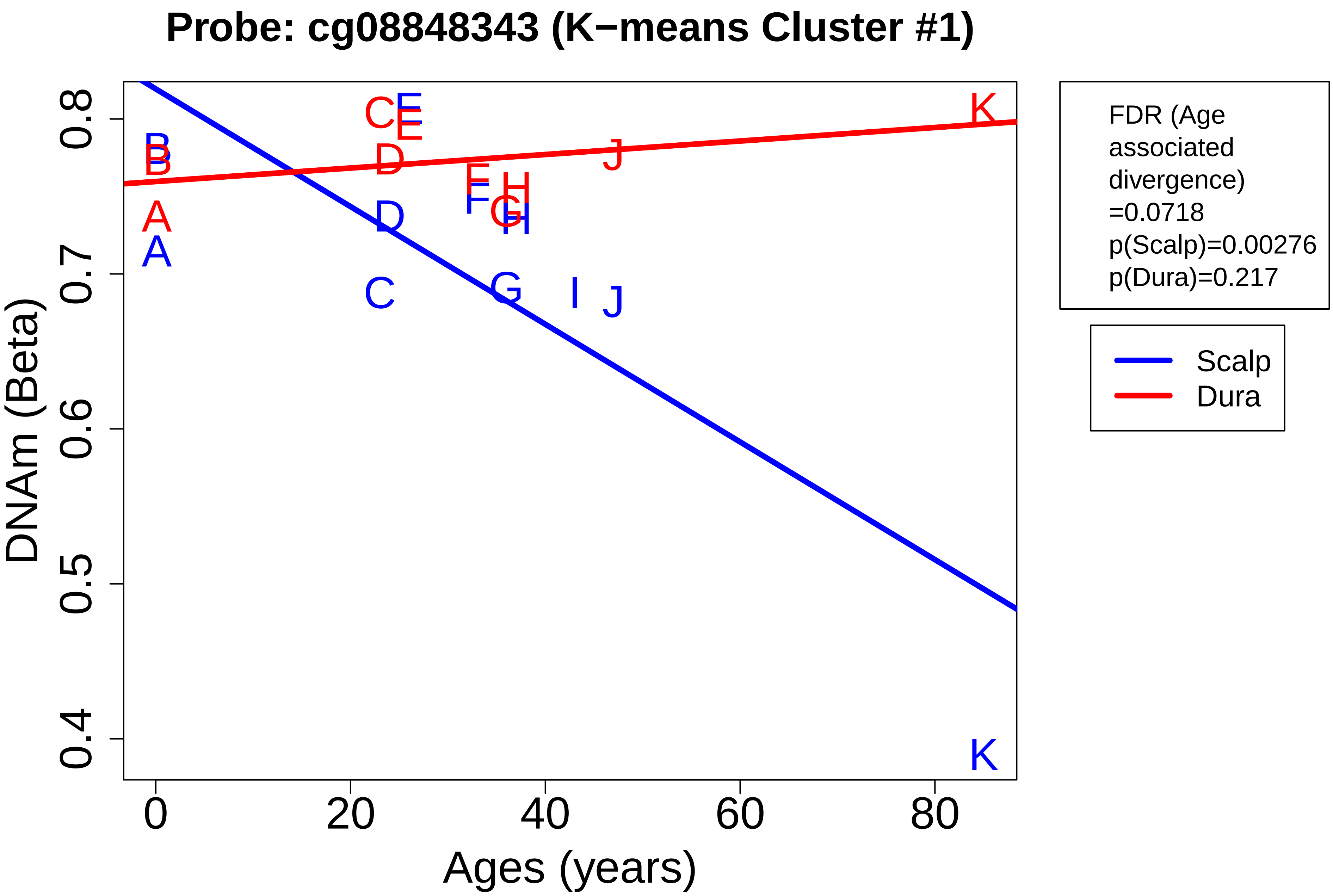

Probe: cg08848343 (K−means Cluster #1)
DNAm (Beta)
0.4
0.5
0.6
0.7
0.8
0
20
40
60
80
Ages (years)
A
B
C
D
E
F
G
H
I
J
K
FDR (Age associated divergence) =0.0718
p(Scalp)=0.00276
p(Dura)=0.217
Scalp
Dura

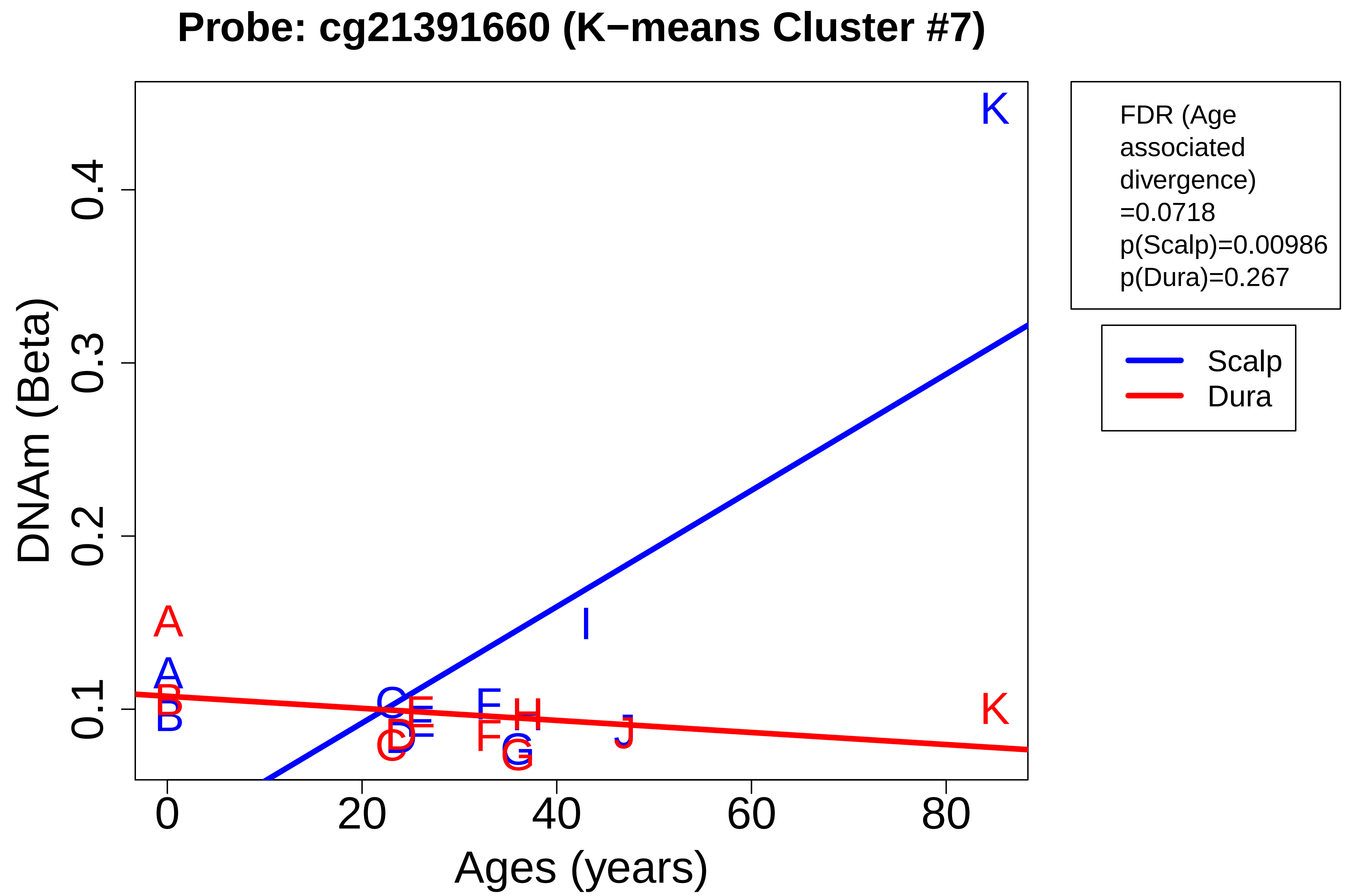

Probe: cg21391660 (K−means Cluster #7)
DNAm (Beta)
0.1
0.2
0.3
0.4
0
20
40
60
80
Ages (years)
A
B
C
D
E
F
G
H
I
J
K
FDR (Age associated divergence) =0.0718
p(Scalp)=0.00986
p(Dura)=0.267
Scalp
Dura

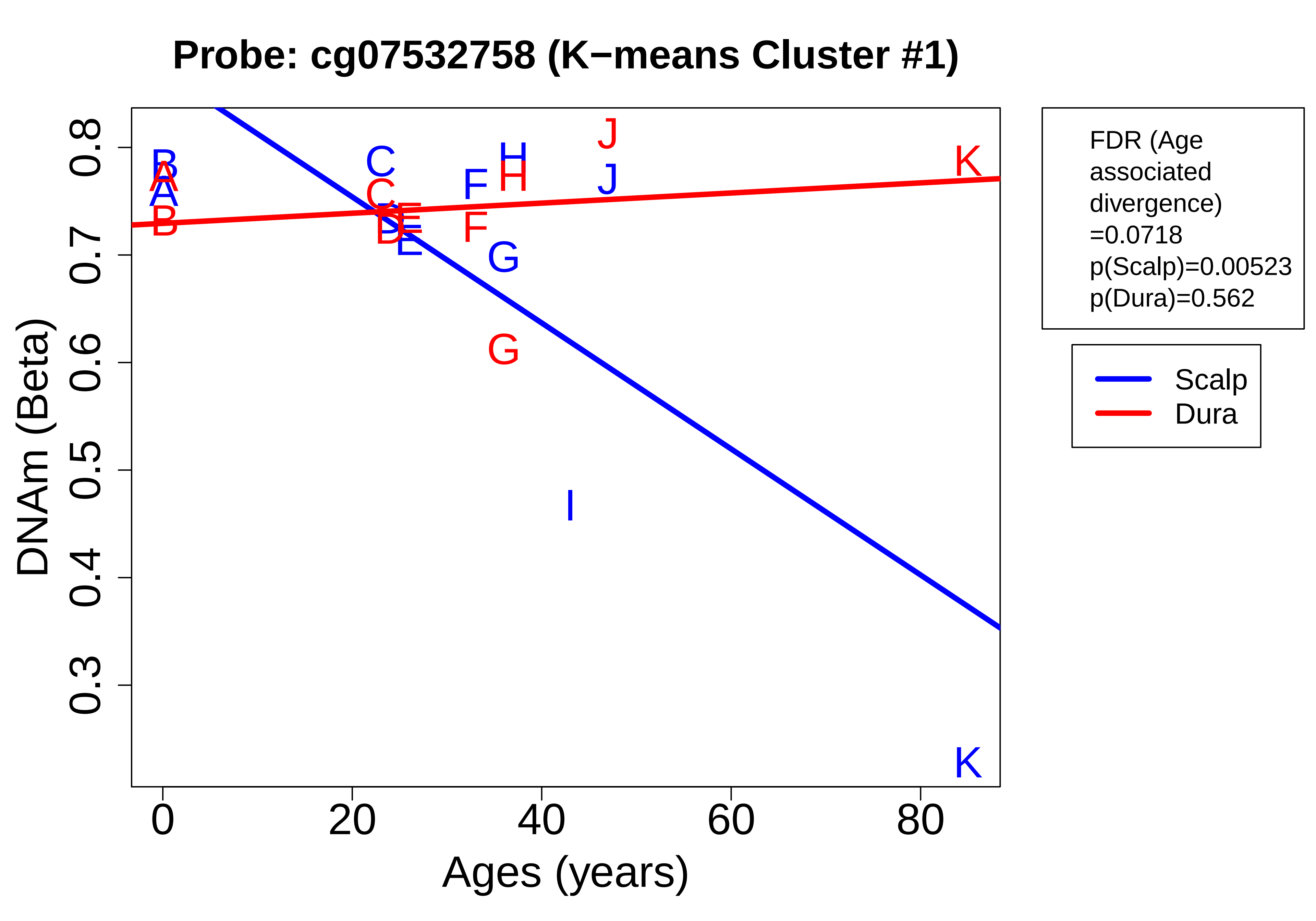

Probe: cg07532758 (K−means Cluster #1)
DNAm (Beta)
0.3
0.4
0.5
0.6
0.7
0.8
0
20
40
60
80
Ages (years)
A
B
C
D
E
F
G
H
I
J
K
FDR (Age associated divergence) =0.0718
p(Scalp)=0.00523
p(Dura)=0.562
Scalp
Dura

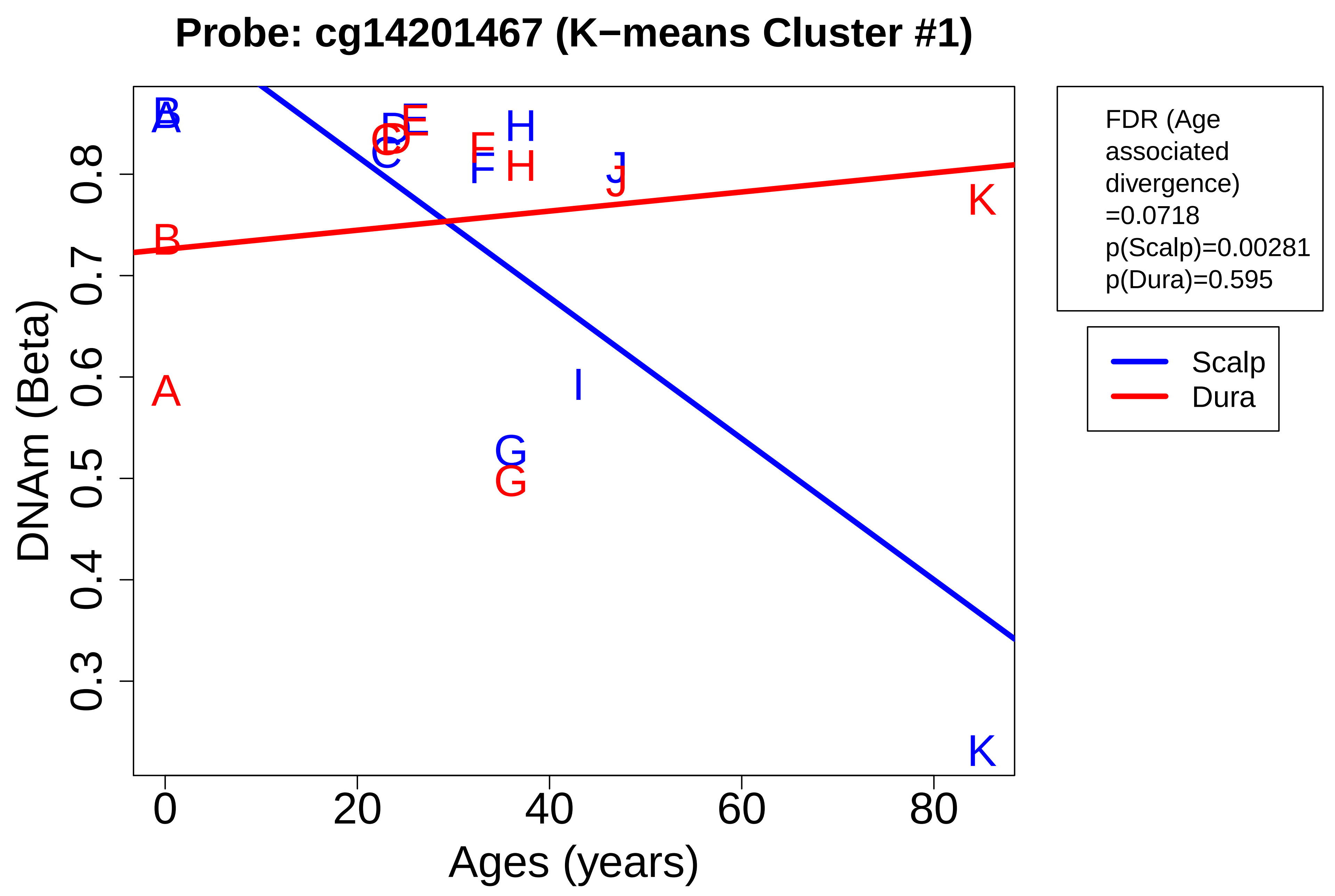

Probe: cg14201467 (K−means Cluster #1)
DNAm (Beta)
0.3
0.4
0.5
0.6
0.7
0.8
0
20
40
60
80
Ages (years)
A
B
C
D
E
F
G
H
I
J
K
FDR (Age associated divergence) =0.0718
p(Scalp)=0.00281
p(Dura)=0.595
Scalp
Dura

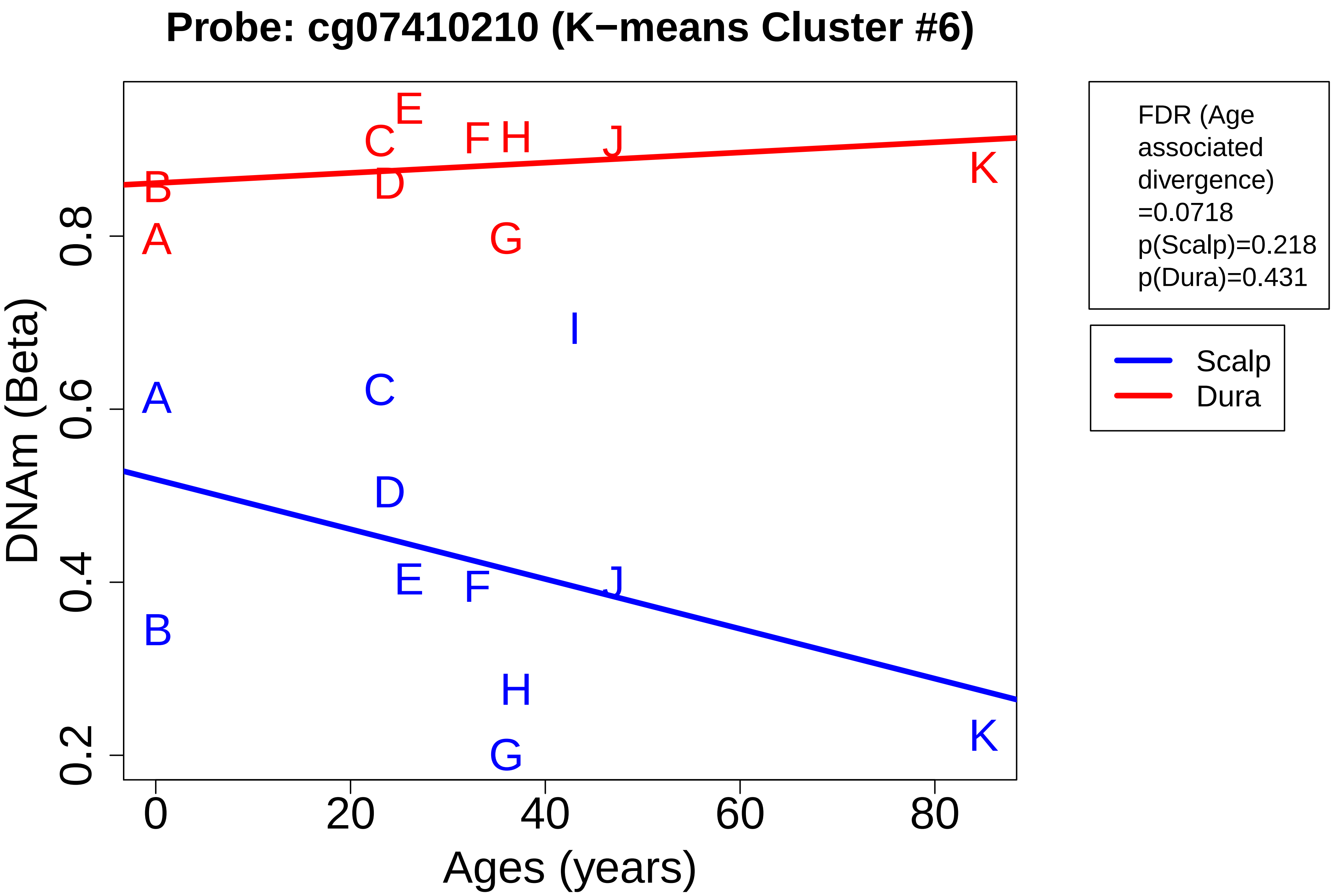

Probe: cg07410210 (K−means Cluster #6)
DNAm (Beta)
0.2
0.4
0.6
0.8
0
20
40
60
80
Ages (years)
FDR (Age associated divergence) =0.0718
p(Scalp)=0.218
p(Dura)=0.431
Scalp
Dura

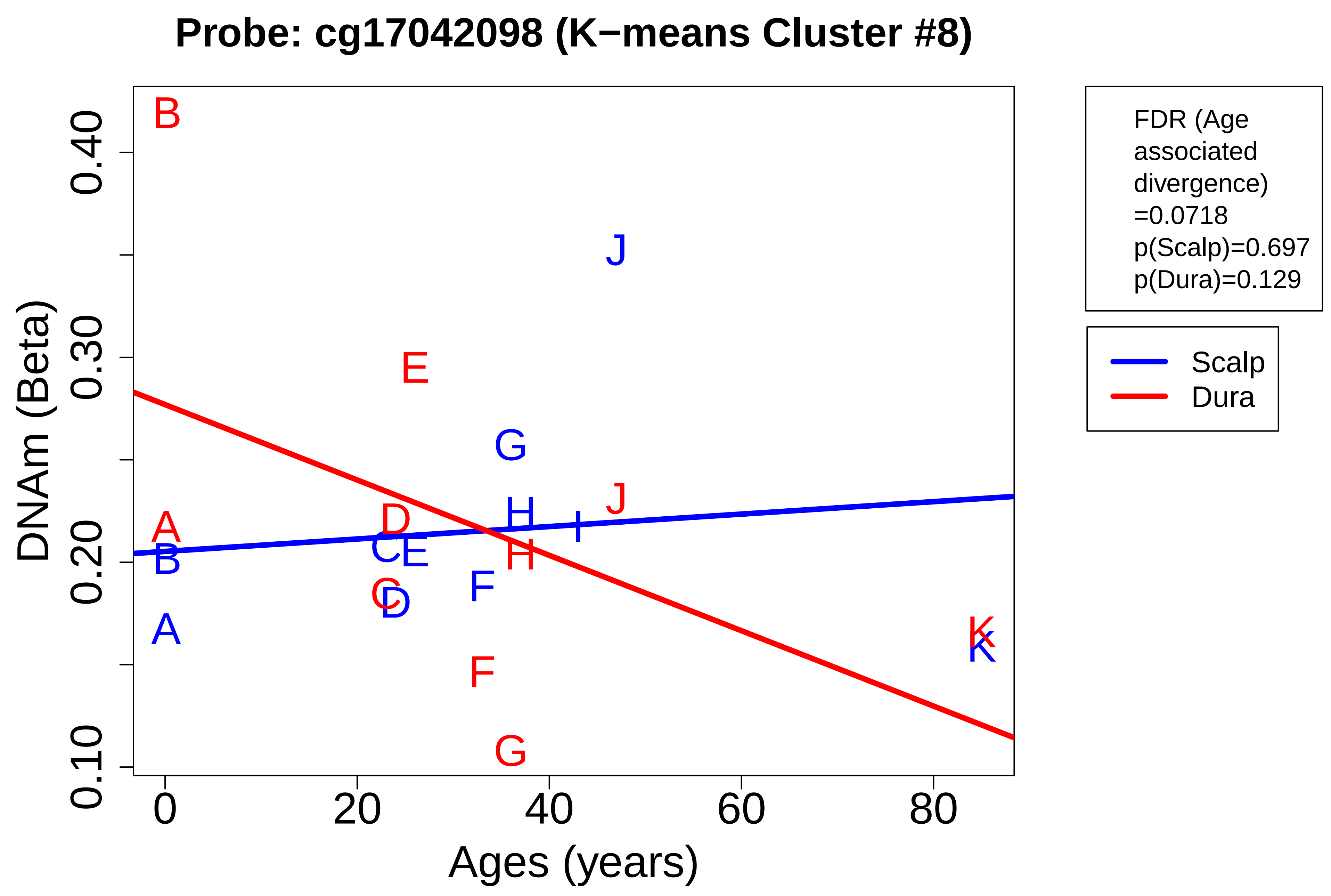
Probe: cg17042098 (K−means Cluster #8)
DNAm (Beta)
0.10
0.20
0.30
0.40
Ages (years)
0
20
40
60
80
FDR (Age associated divergence) =0.0718
p(Scalp)=0.697
p(Dura)=0.129
Scalp
Dura
A
B
C
D
E
F
G
H
I
J
K

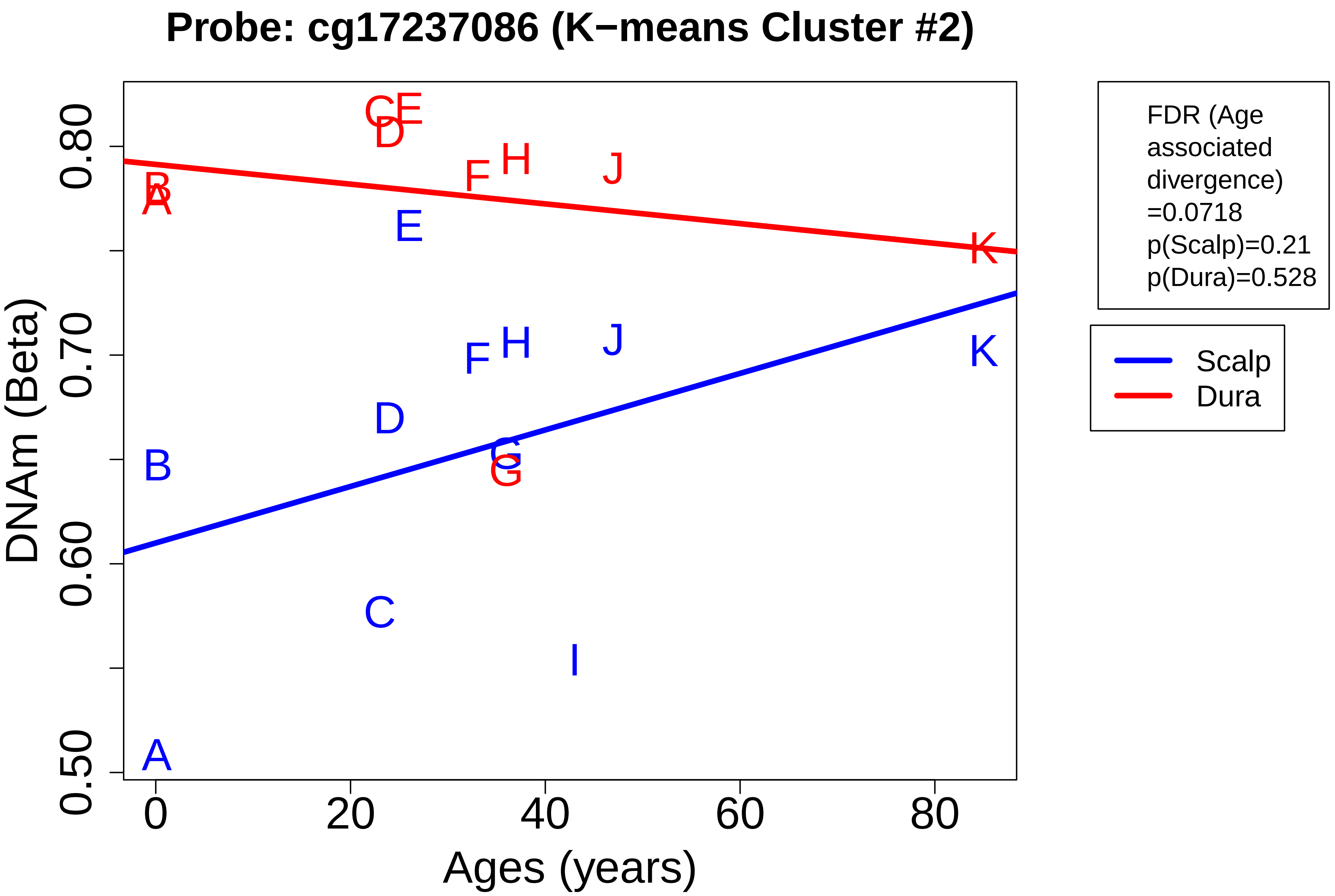
Probe: cg17237086 (K−means Cluster #2)
DNAm (Beta)
0.50
0.60
0.70
0.80
Ages (years)
0
20
40
60
80
FDR (Age associated divergence) =0.0718
p(Scalp)=0.21
p(Dura)=0.528
Scalp
Dura
A
B
C
D
E
F
G
H
I
J
K

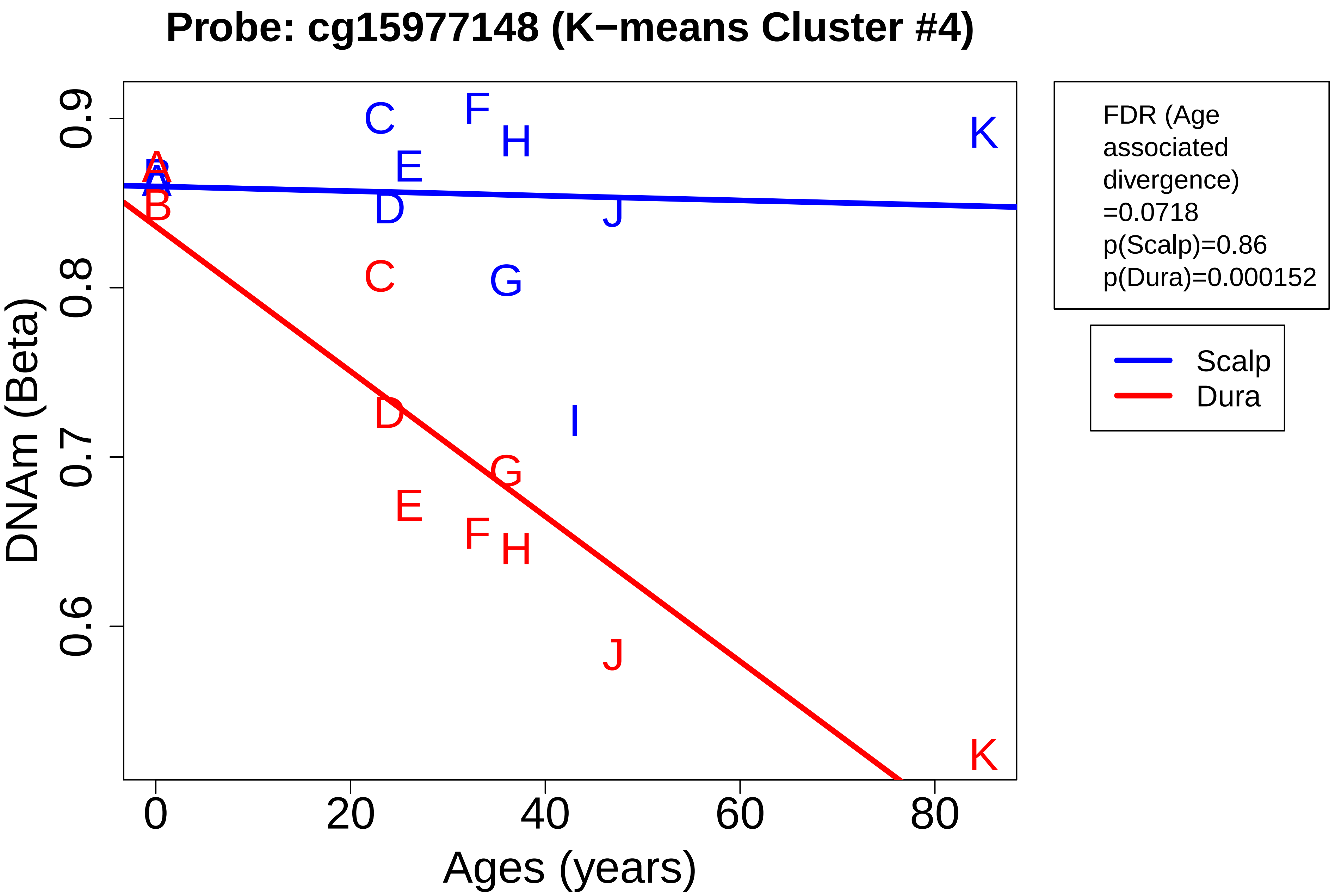

Probe: cg15977148 (K−means Cluster #4)
DNAm (Beta)
0.6
0.7
0.8
0.9
Ages (years)
0
20
40
60
80
A
B
C
D
E
F
G
H
I
J
K
FDR (Age associated divergence) =0.0718
p(Scalp)=0.86
p(Dura)=0.000152
Scalp
Dura

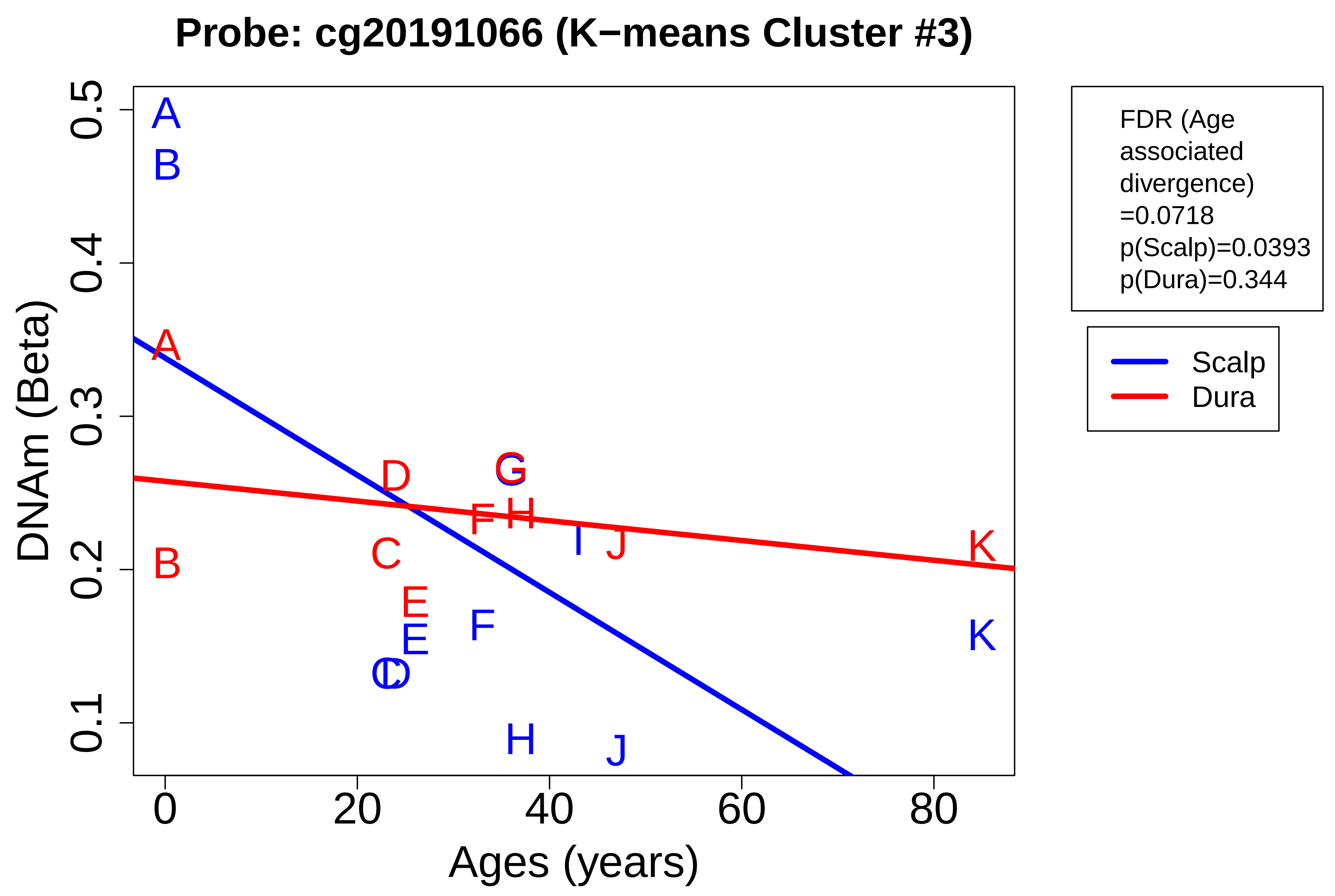
Probe: cg20191066 (K−means Cluster #3)
DNAm (Beta)
Ages (years)
0.1
0.2
0.3
0.4
0.5
0
20
40
60
80
FDR (Age associated divergence) =0.0718
p(Scalp)=0.0393
p(Dura)=0.344
Scalp
Dura
A
B
C
D
E
F
G
H
I
J
K

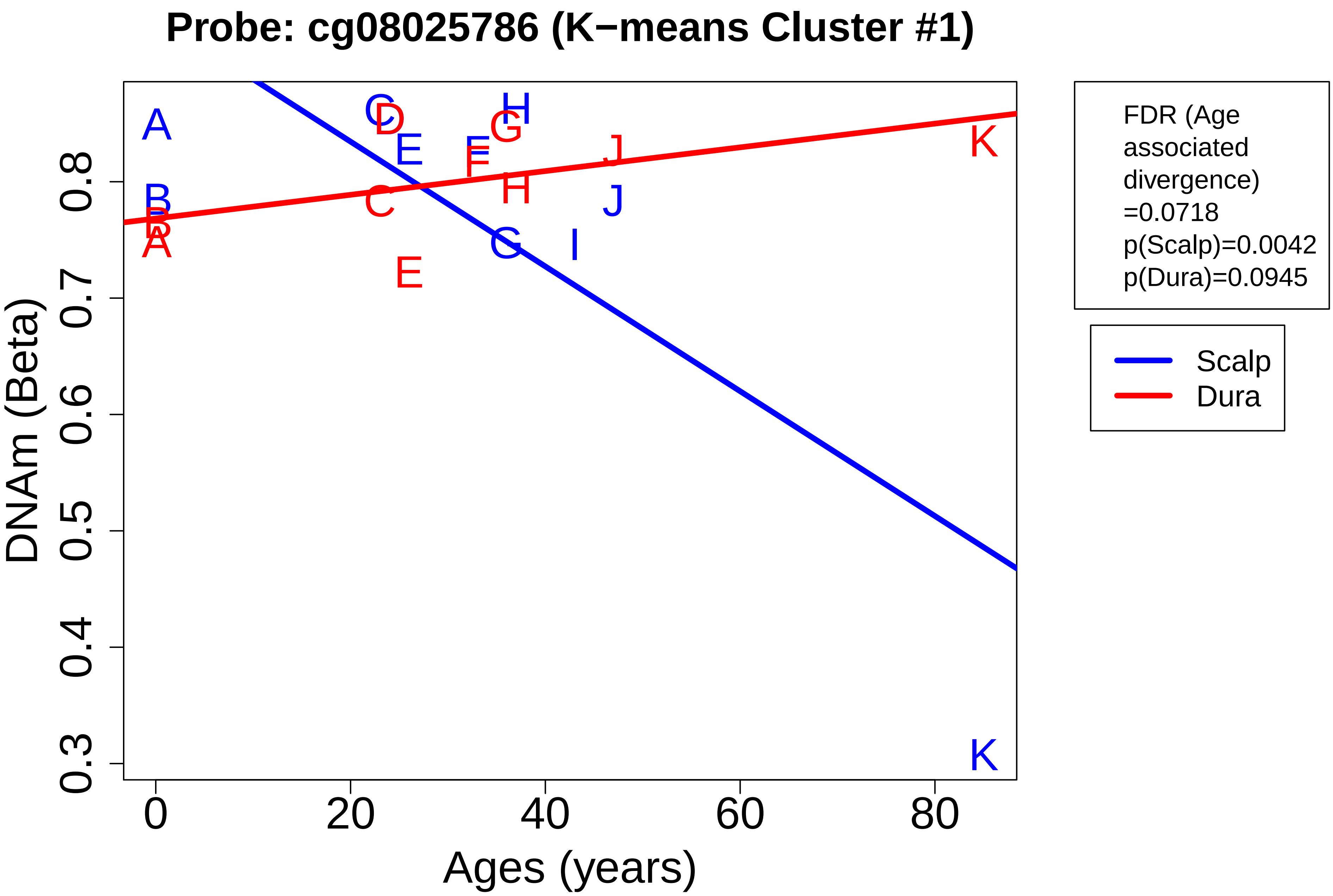

Probe: cg08025786 (K−means Cluster #1)
DNAm (Beta)
0.3
0.4
0.5
0.6
0.7
0.8
0
20
40
60
80
Ages (years)
FDR (Age associated divergence) =0.0718
p(Scalp)=0.0042
p(Dura)=0.0945
Scalp
Dura
A
B
C
D
E
F
G
H
I
J
K

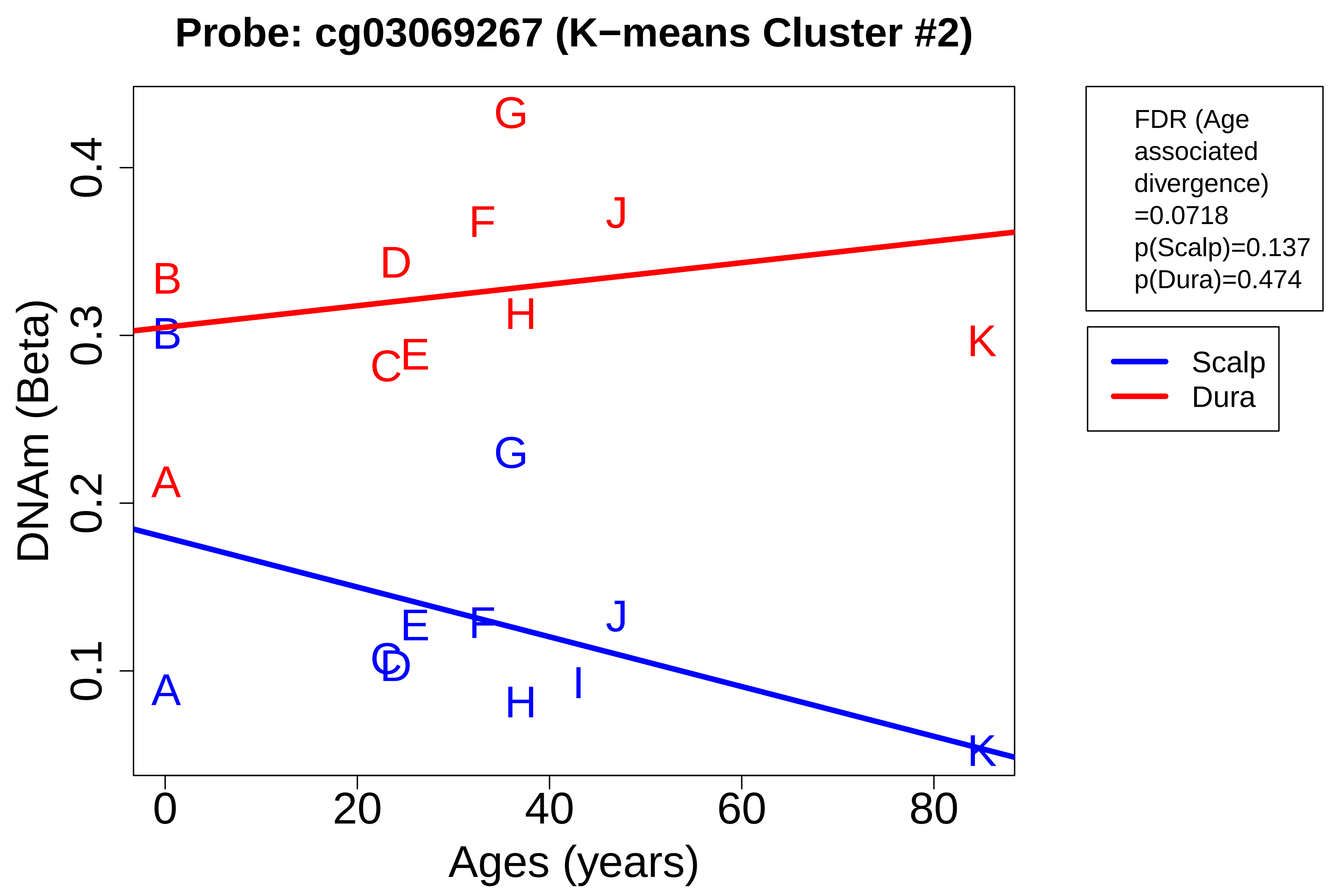

Probe: cg03069267 (K−means Cluster #2)
DNAm (Beta)
0.1
0.2
0.3
0.4
0
20
40
60
80
Ages (years)
G
F
J
D
B
B
H
K
C
E
G
A
E
F
J
C
D
I
A
H
K
FDR (Age associated divergence) =0.0718
p(Scalp)=0.137
p(Dura)=0.474
Scalp
Dura

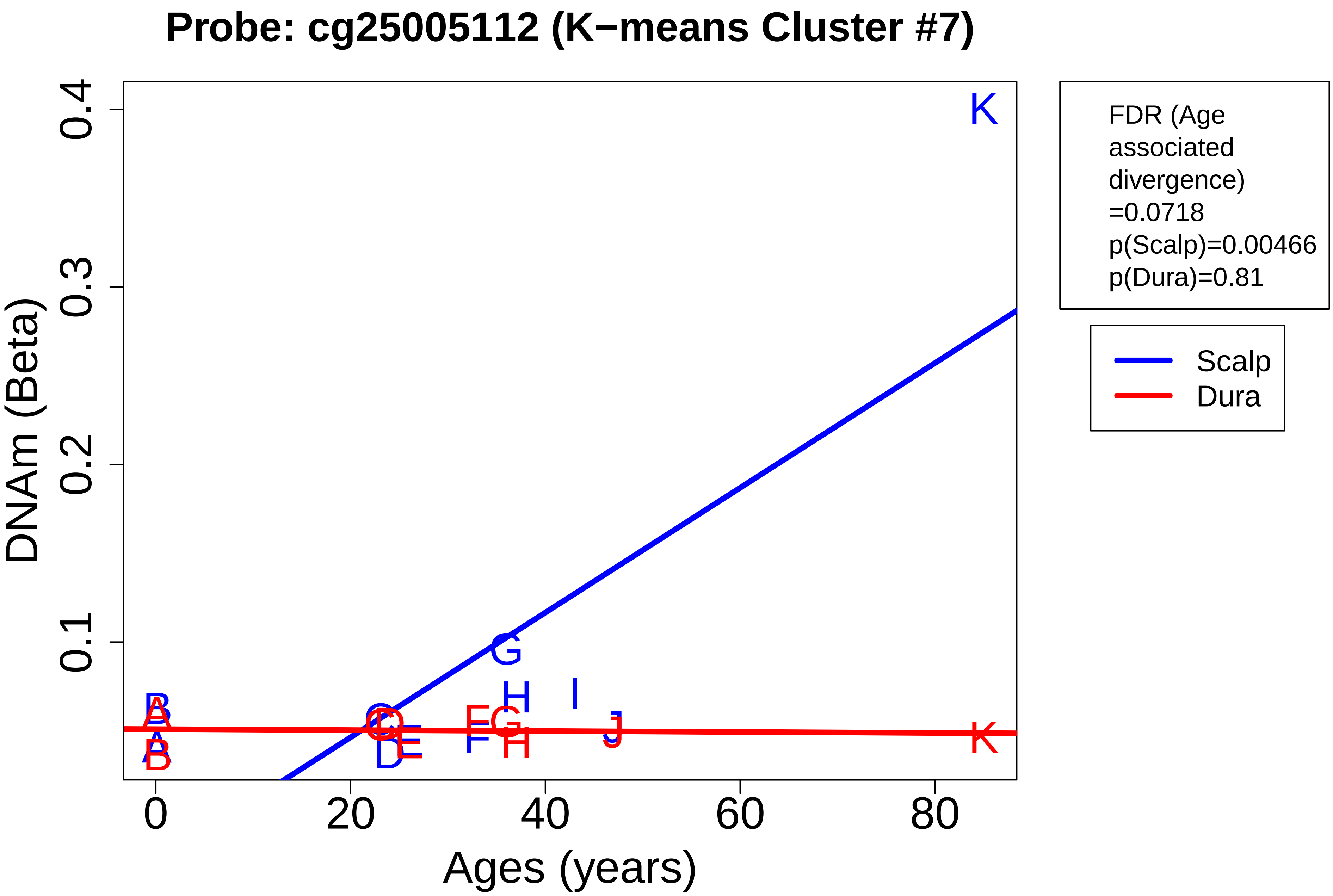

Probe: cg25005112 (K−means Cluster #7)
DNAm (Beta)
0.1
0.2
0.3
0.4
0
20
40
60
80
Ages (years)
FDR (Age associated divergence) =0.0718
p(Scalp)=0.00466
p(Dura)=0.81
Scalp
Dura

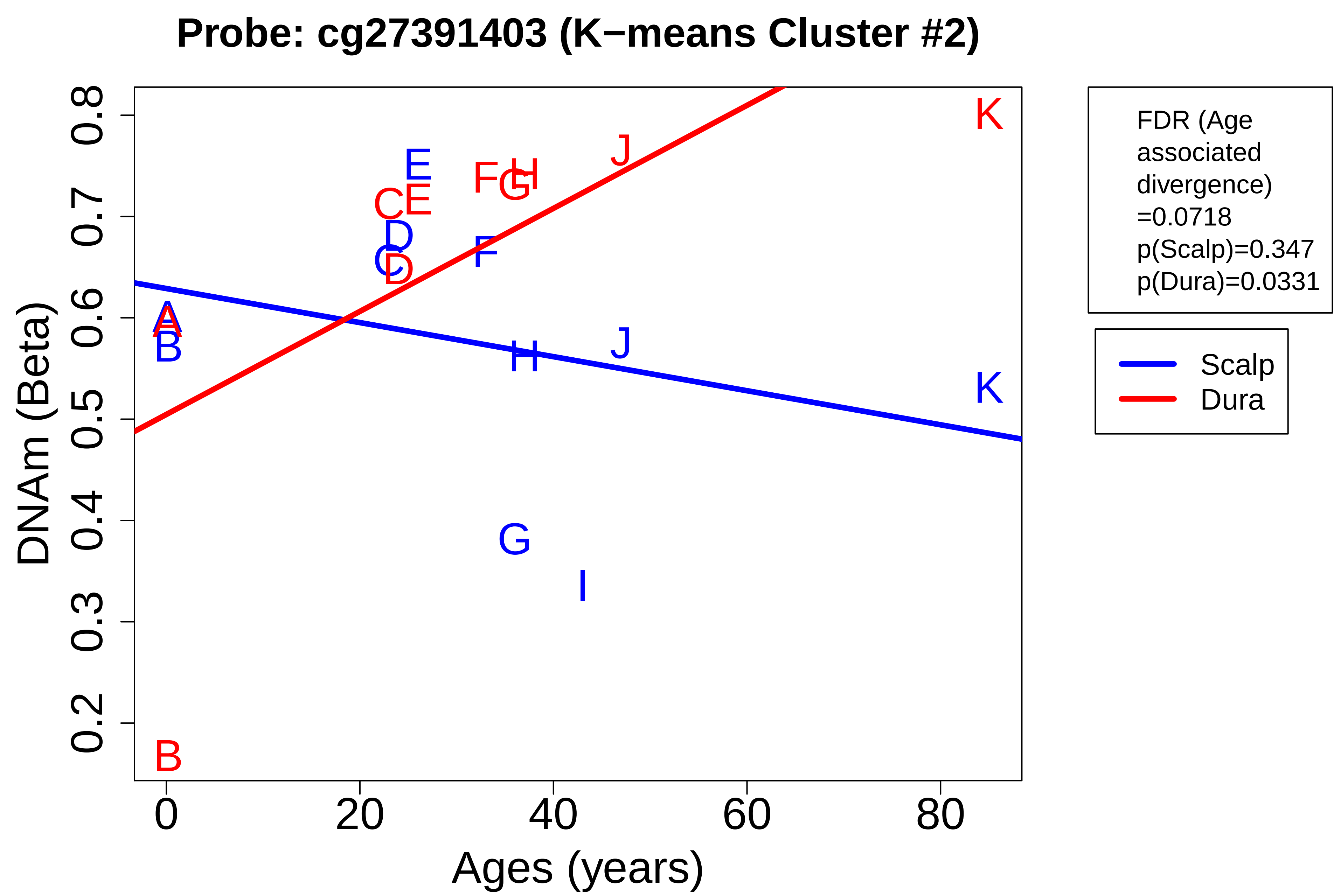

Probe: cg27391403 (K−means Cluster #2)
DNAm (Beta)
Ages (years)
0.2
0.3
0.4
0.5
0.6
0.7
0.8
0
20
40
60
80
FDR (Age associated divergence) =0.0718
p(Scalp)=0.347
p(Dura)=0.0331
Scalp
Dura

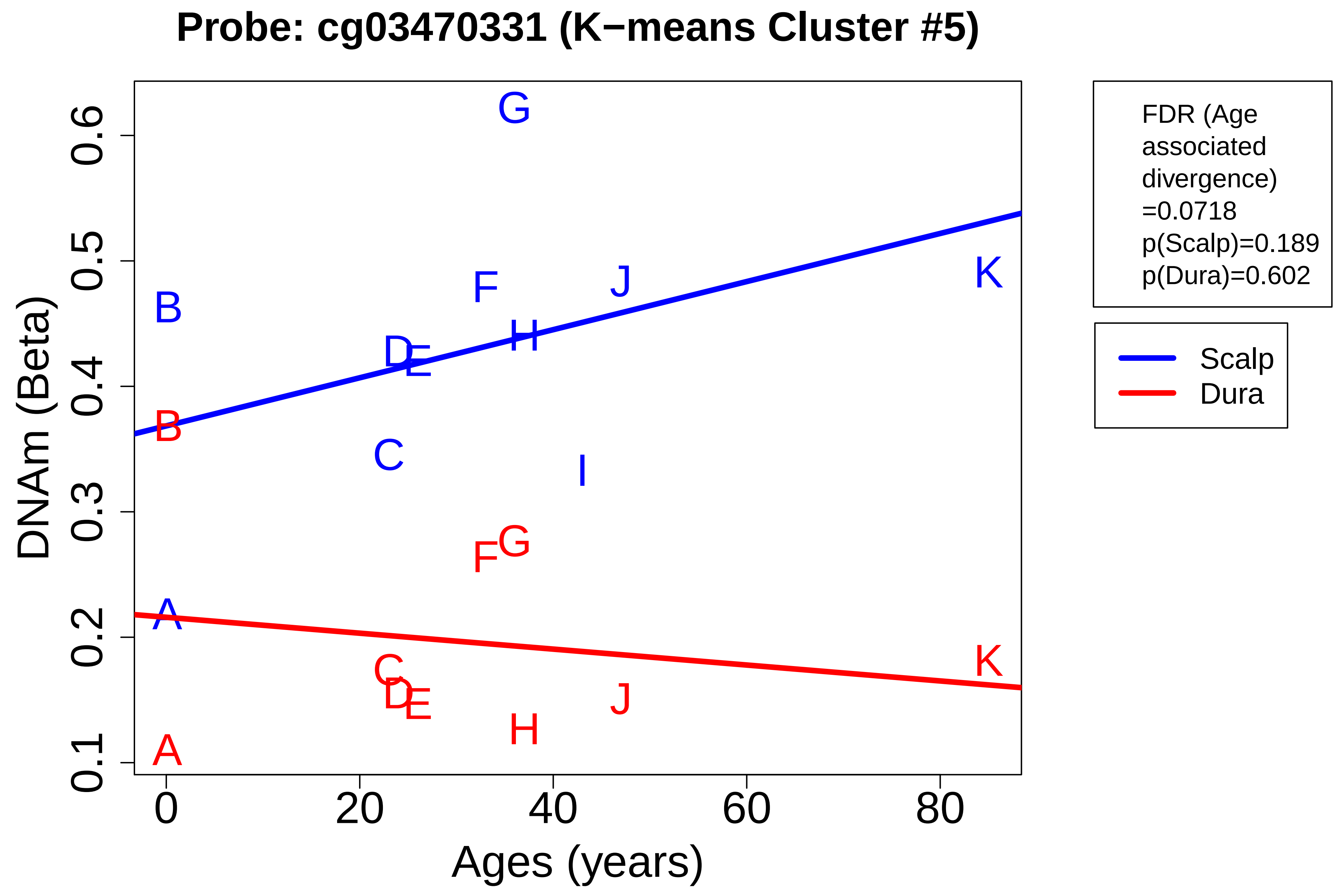

Probe: cg03470331 (K−means Cluster #5)
FDR (Age associated divergence) =0.0718
p(Scalp)=0.189
p(Dura)=0.602
Scalp
Dura
DNAm (Beta)
0.1
0.2
0.3
0.4
0.5
0.6
0
20
40
60
80
Ages (years)
A B C D E F G H I J K

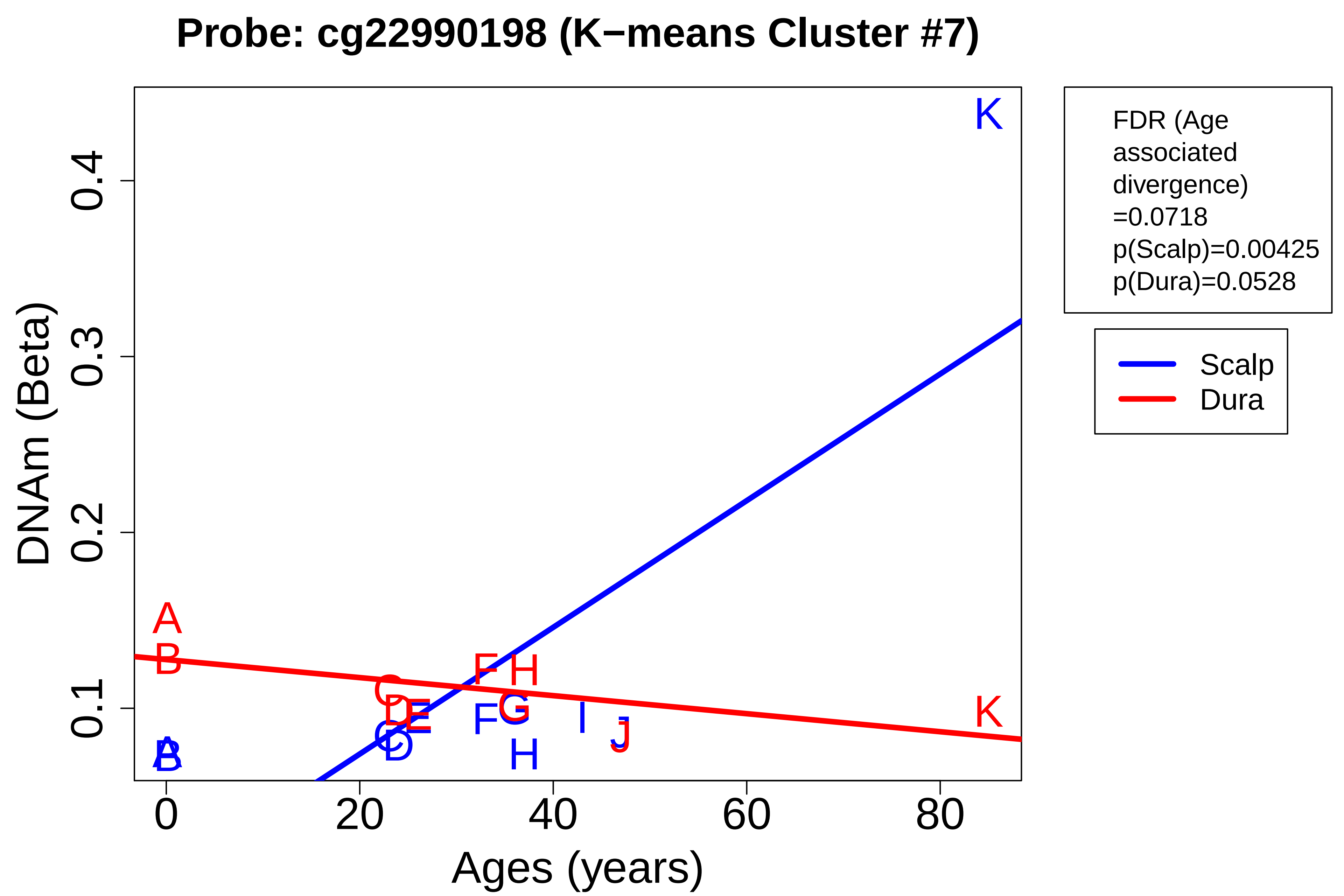
Probe: cg22990198 (K−means Cluster #7)
DNAm (Beta)
0.1
0.2
0.3
0.4
Ages (years)
0
20
40
60
80
FDR (Age associated divergence) =0.0718
p(Scalp)=0.00425
p(Dura)=0.0528
Scalp
Dura
A
B
C
D
E
F
G
H
I
J
K

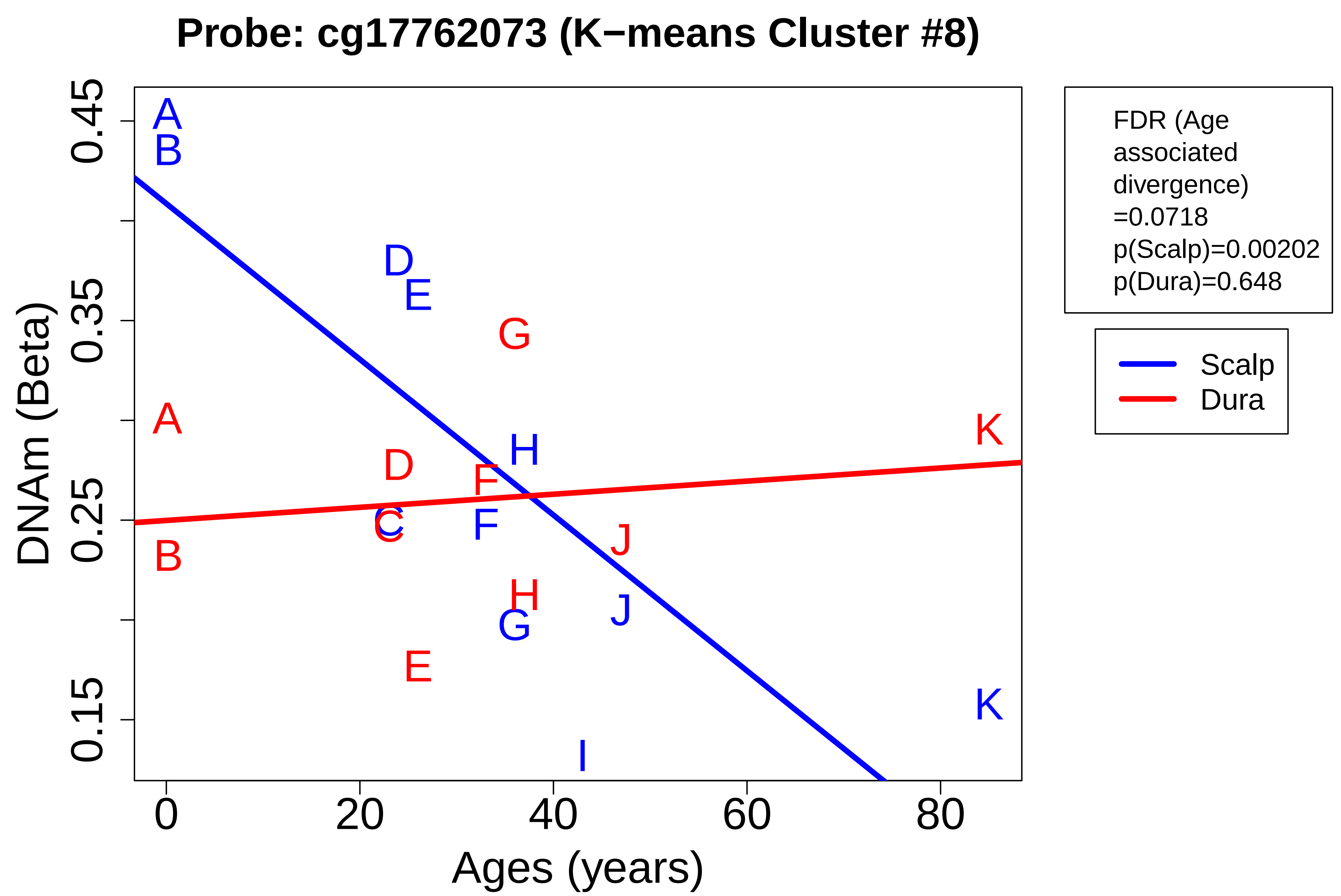

Probe: cg17762073 (K−means Cluster #8)
DNAm (Beta)
0.15
0.25
0.35
0.45
Ages (years)
0
20
40
60
80
A
B
C
D
E
F
G
H
I
J
K
FDR (Age associated divergence) =0.0718
p(Scalp)=0.00202
p(Dura)=0.648
Scalp
Dura

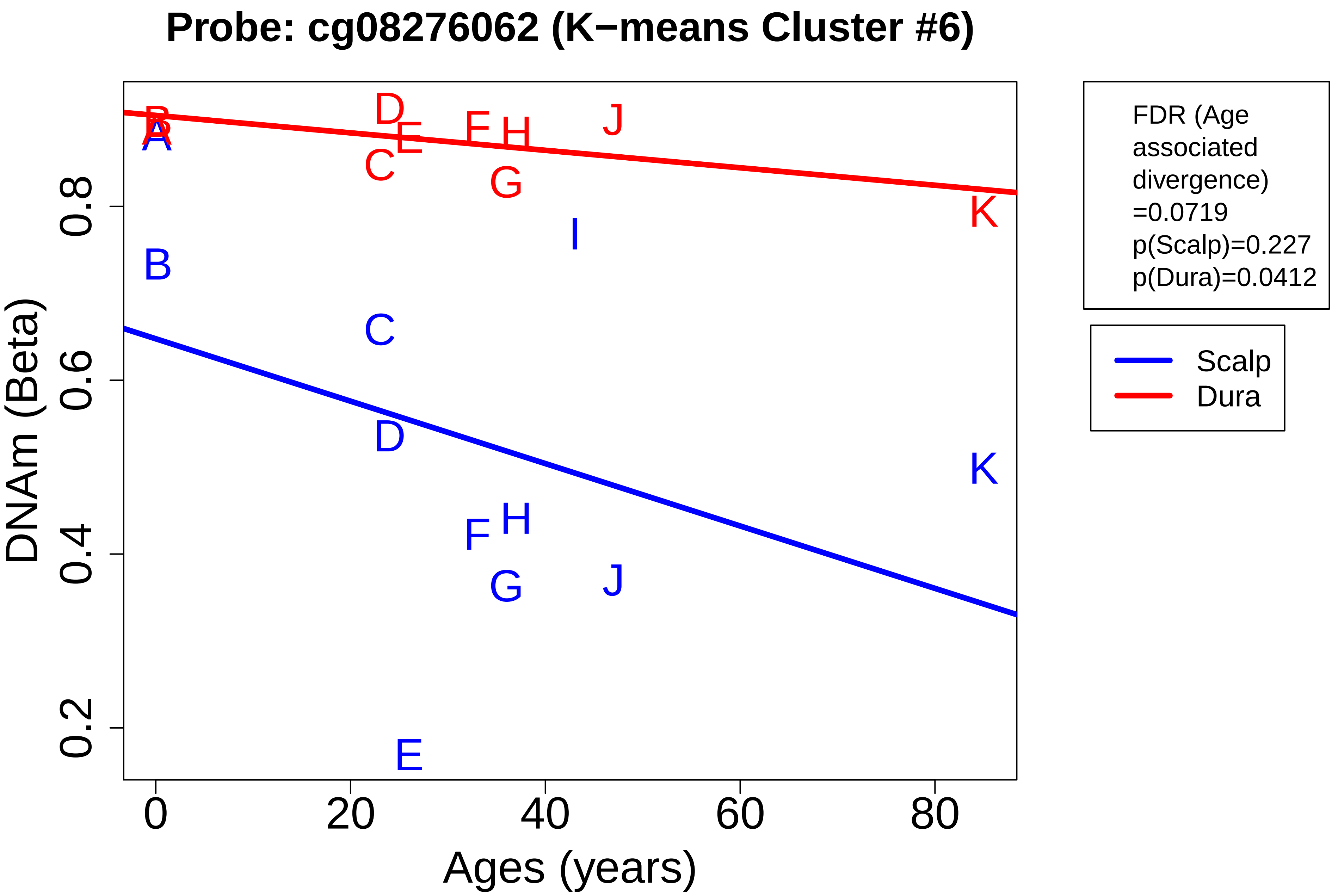

Probe: cg08276062 (K−means Cluster #6)
DNAm (Beta)
0.2
0.4
0.6
0.8
0
20
40
60
80
Ages (years)
FDR (Age associated divergence) =0.0719
p(Scalp)=0.227
p(Dura)=0.0412
Scalp
Dura

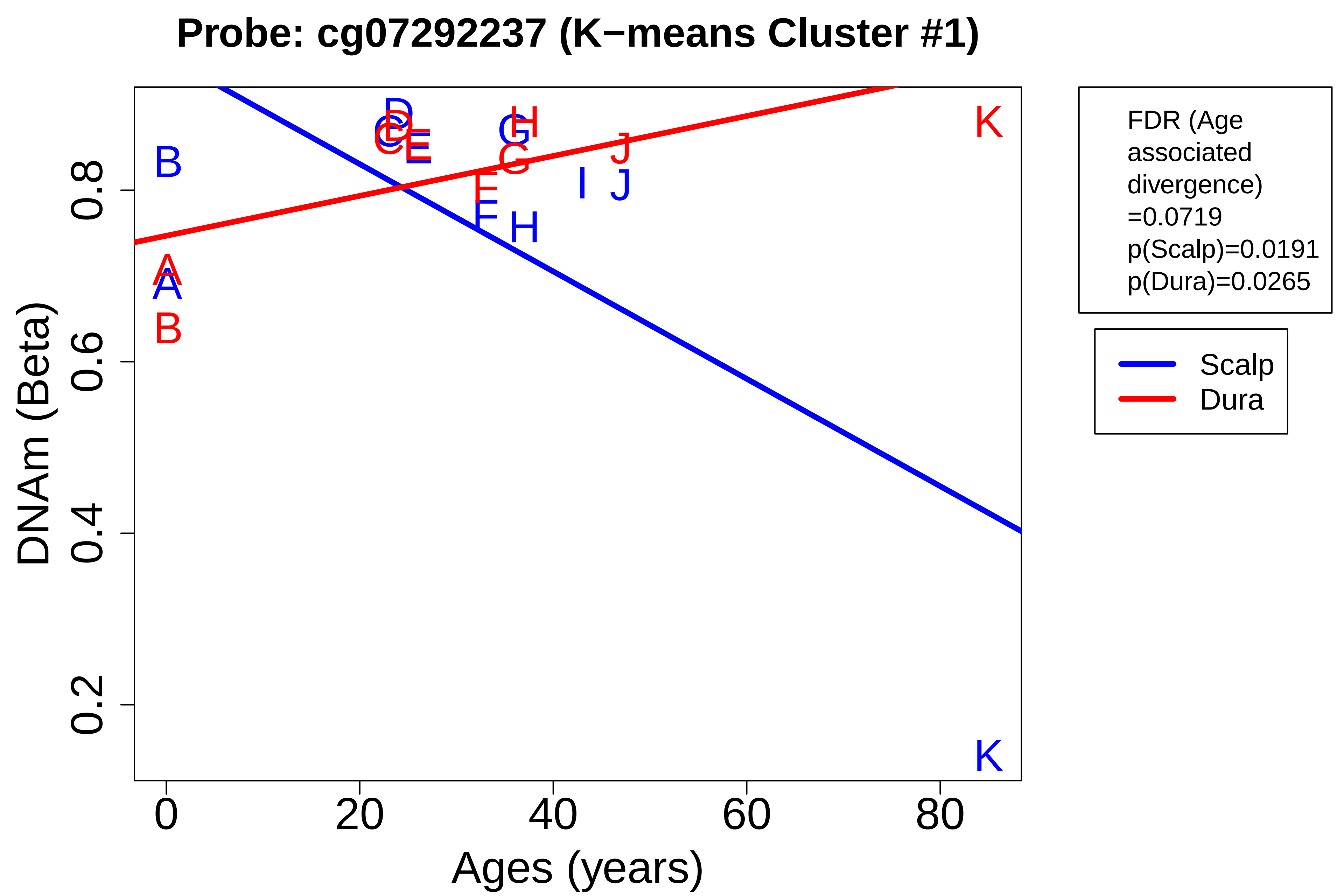

Probe: cg07292237 (K−means Cluster #1)
DNAm (Beta)
0.2
0.4
0.6
0.8
0
20
40
60
80
Ages (years)
A
B
C
D
E
F
G
H
I
J
K
FDR (Age associated divergence) =0.0719
p(Scalp)=0.0191
p(Dura)=0.0265
Scalp
Dura

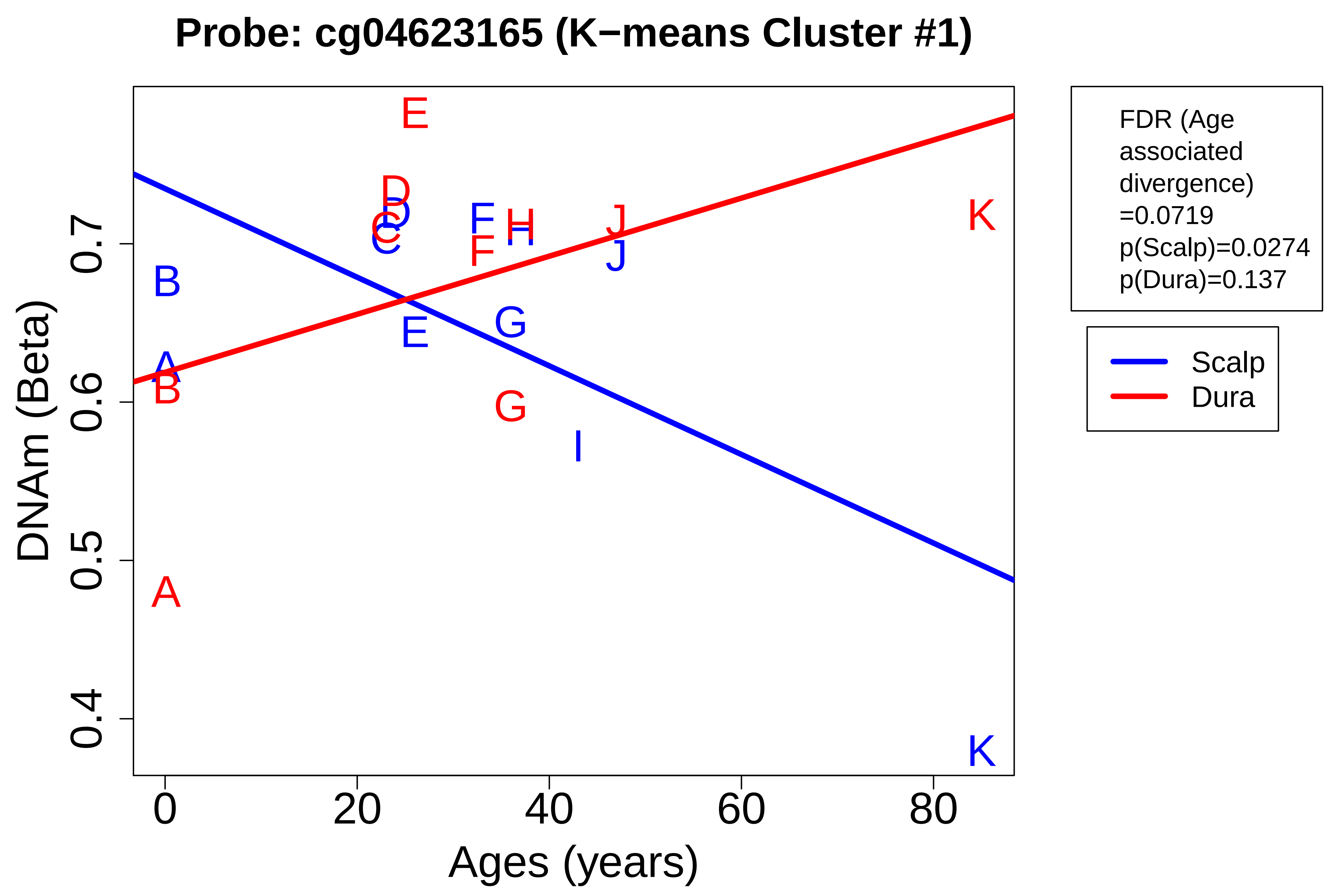

Probe: cg04623165 (K−means Cluster #1)
DNAm (Beta)
0.4
0.5
0.6
0.7
0
20
40
60
80
Ages (years)
FDR (Age associated divergence) =0.0719
p(Scalp)=0.0274
p(Dura)=0.137
Scalp
Dura

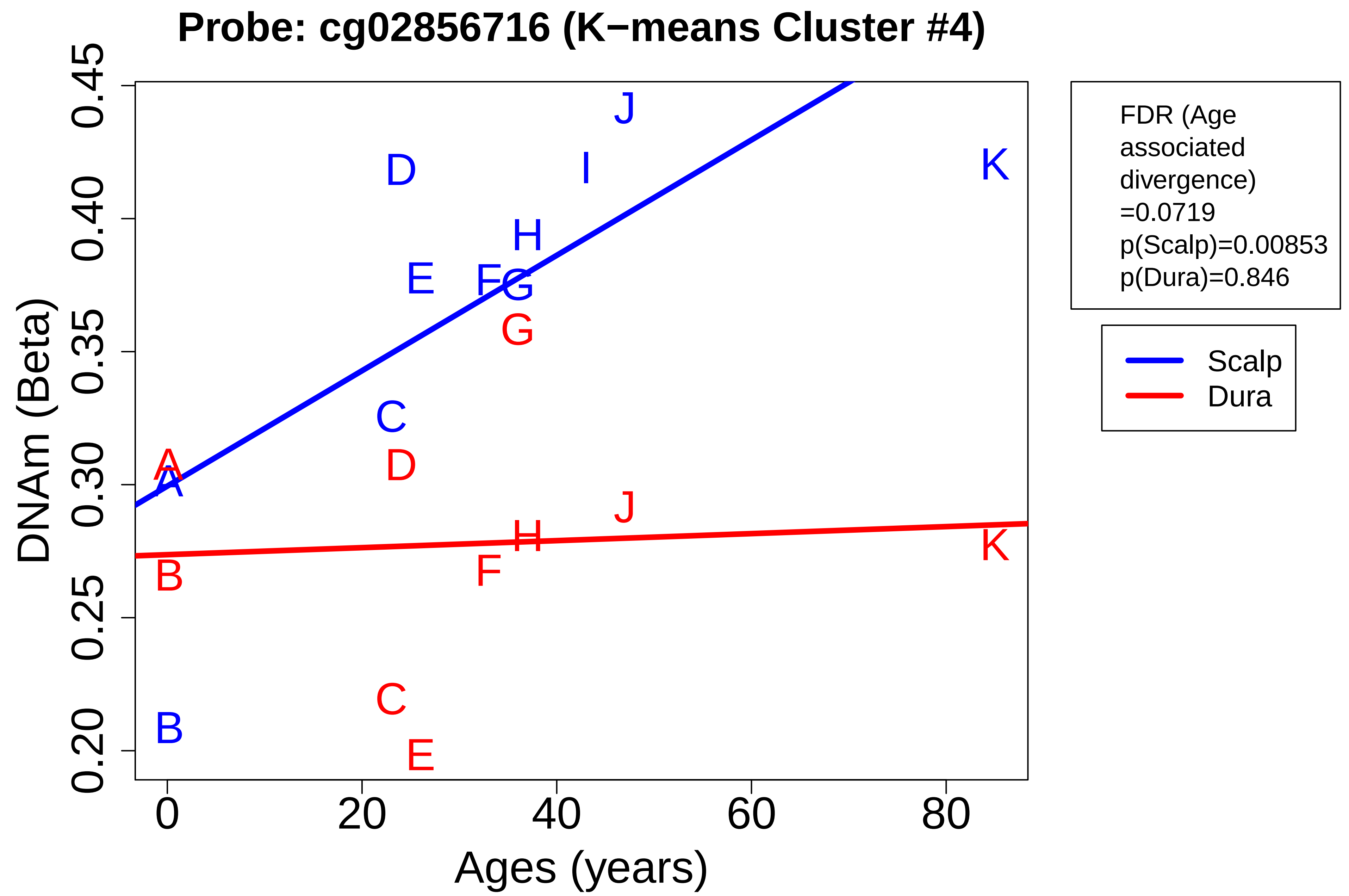

Probe: cg02856716 (K−means Cluster #4)
DNAm (Beta)
0.20
0.25
0.30
0.35
0.40
0.45
0
20
40
60
80
Ages (years)
FDR (Age associated divergence) =0.0719
p(Scalp)=0.00853
p(Dura)=0.846
Scalp
Dura

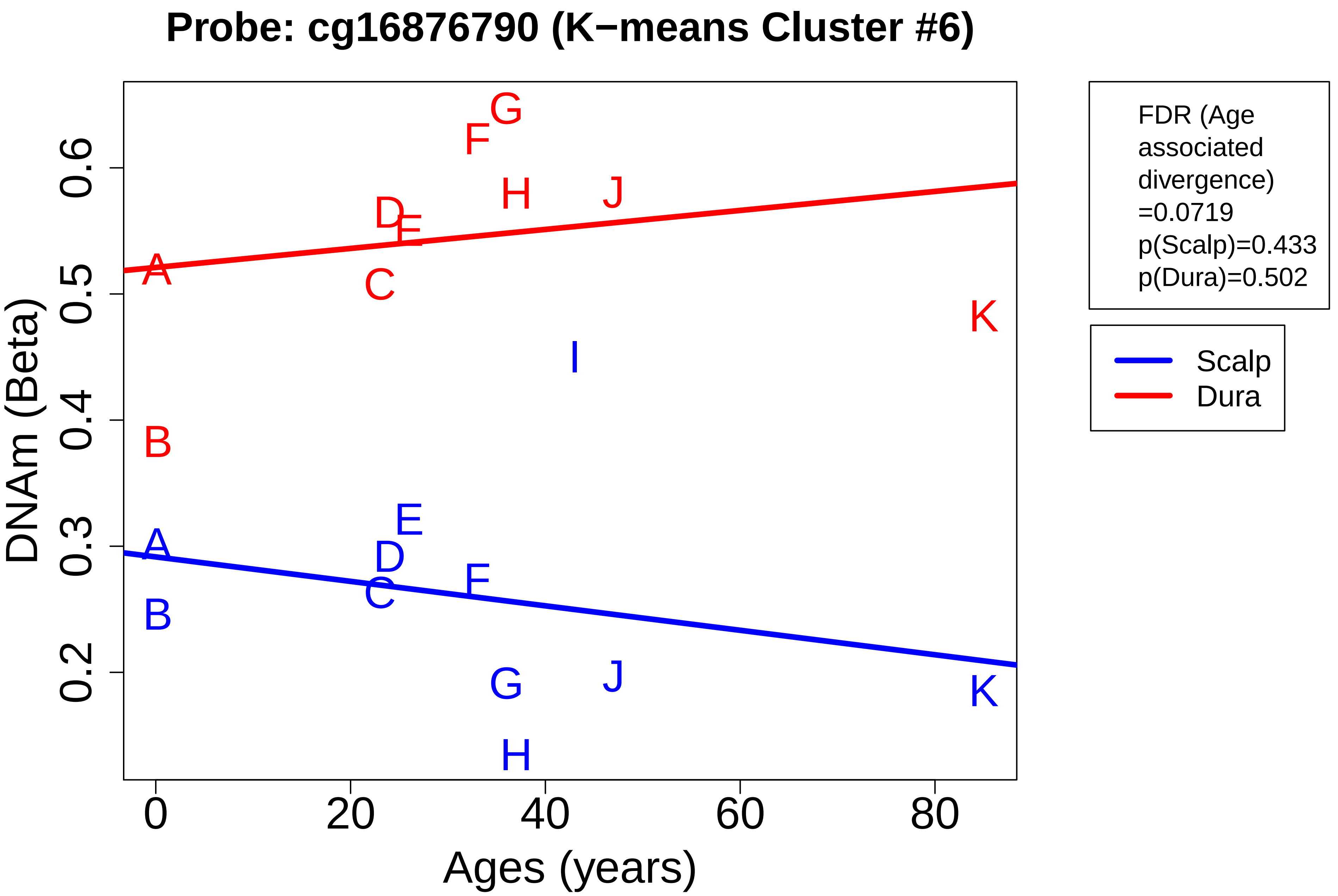

Probe: cg16876790 (K−means Cluster #6)
DNAm (Beta)
0.2
0.3
0.4
0.5
0.6
Ages (years)
0
20
40
60
80
FDR (Age associated divergence) =0.0719
p(Scalp)=0.433
p(Dura)=0.502
Scalp
Dura

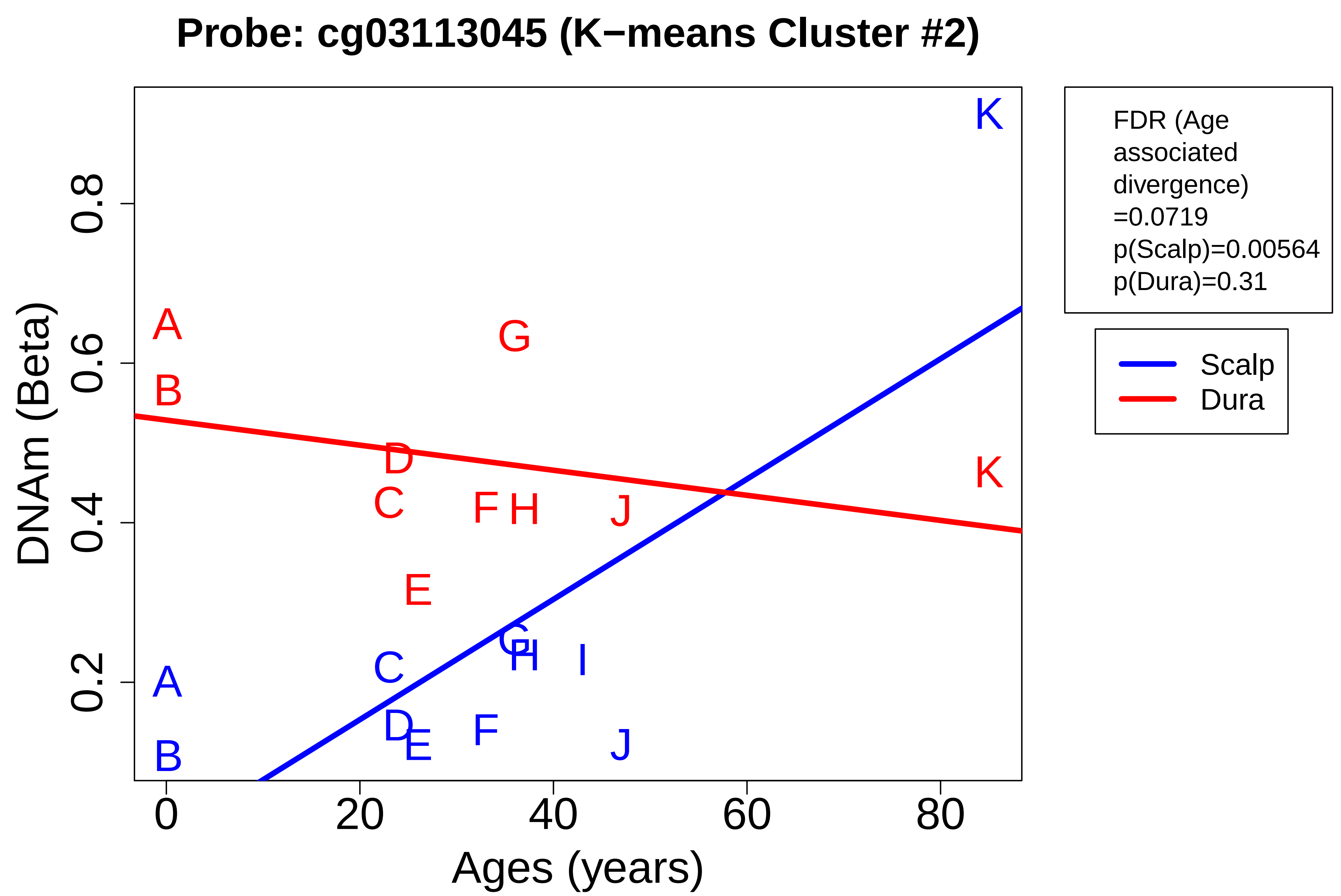
Probe: cg03113045 (K−means Cluster #2)
FDR (Age associated divergence) =0.0719
p(Scalp)=0.00564
p(Dura)=0.31
Scalp
Dura
DNAm (Beta)
0.2
0.4
0.6
0.8
0
20
40
60
80
Ages (years)
A
B
C
D
E
F
G
H
I
J
K

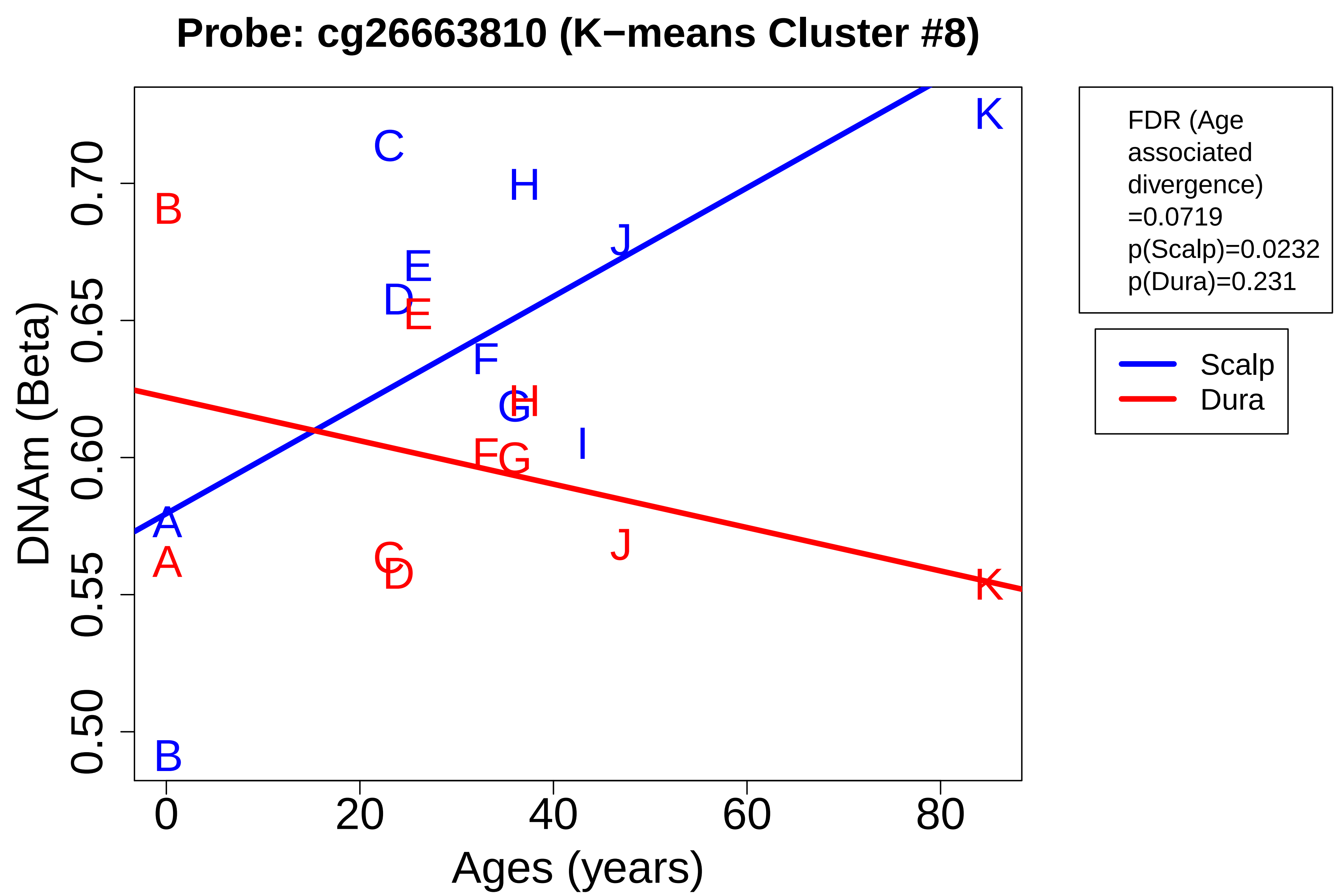

Probe: cg26663810 (K−means Cluster #8)
DNAm (Beta)
0.50
0.55
0.60
0.65
0.70
Ages (years)
0
20
40
60
80
FDR (Age associated divergence) =0.0719
p(Scalp)=0.0232
p(Dura)=0.231
Scalp
Dura

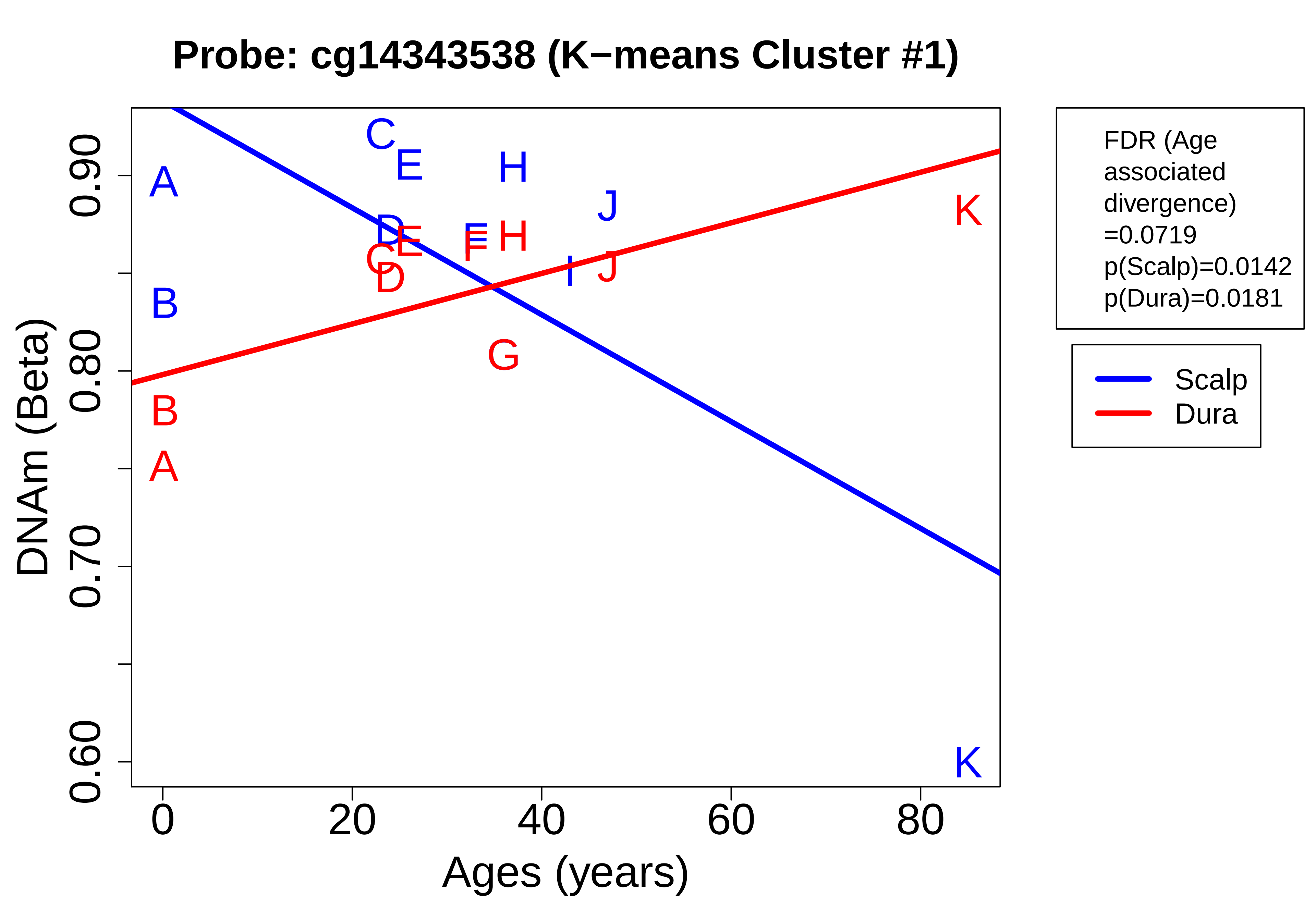

Probe: cg14343538 (K−means Cluster #1)
DNAm (Beta)
0.60
0.70
0.80
0.90
Ages (years)
0
20
40
60
80
A
B
C
D
E
F
G
H
I
J
K
FDR (Age associated divergence) =0.0719
p(Scalp)=0.0142
p(Dura)=0.0181
Scalp
Dura

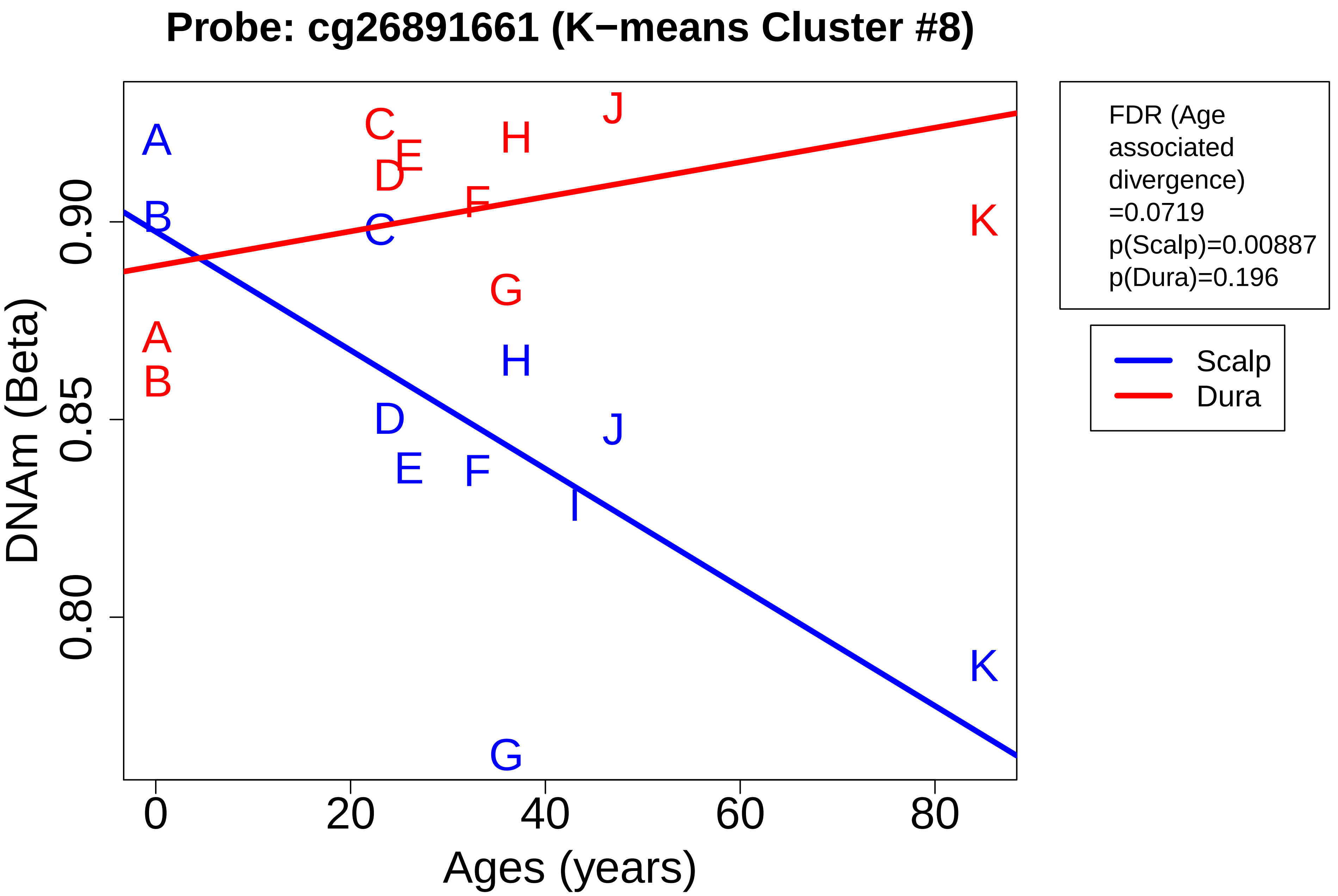

Probe: cg26891661 (K−means Cluster #8)
DNAm (Beta)
0.80
0.85
0.90
Ages (years)
0
20
40
60
80
FDR (Age associated divergence) =0.0719
p(Scalp)=0.00887
p(Dura)=0.196
Scalp
Dura

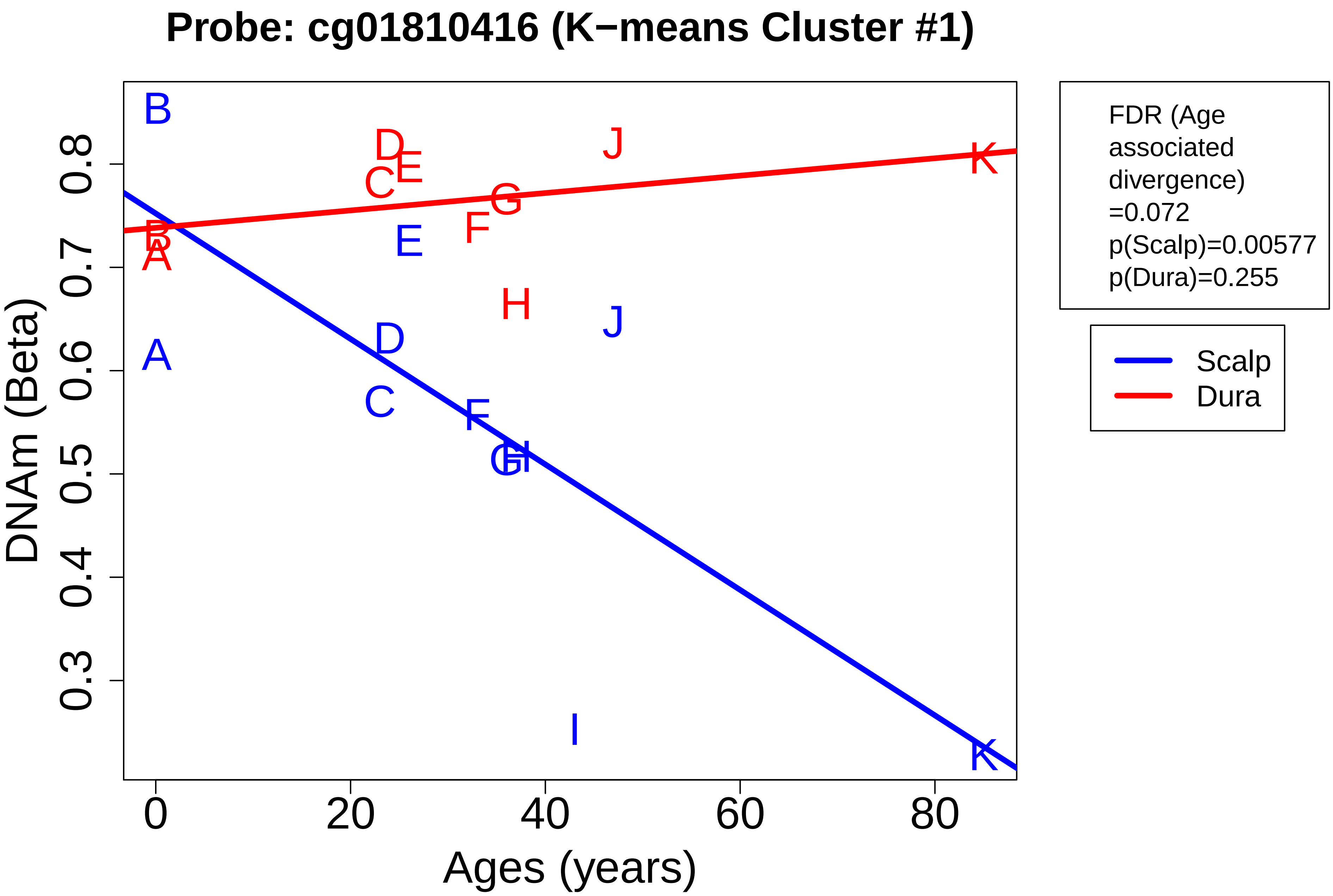

Probe: cg01810416 (K−means Cluster #1)
DNAm (Beta)
0.3
0.4
0.5
0.6
0.7
0.8
0
20
40
60
80
Ages (years)
FDR (Age associated divergence) =0.072
p(Scalp)=0.00577
p(Dura)=0.255
Scalp
Dura

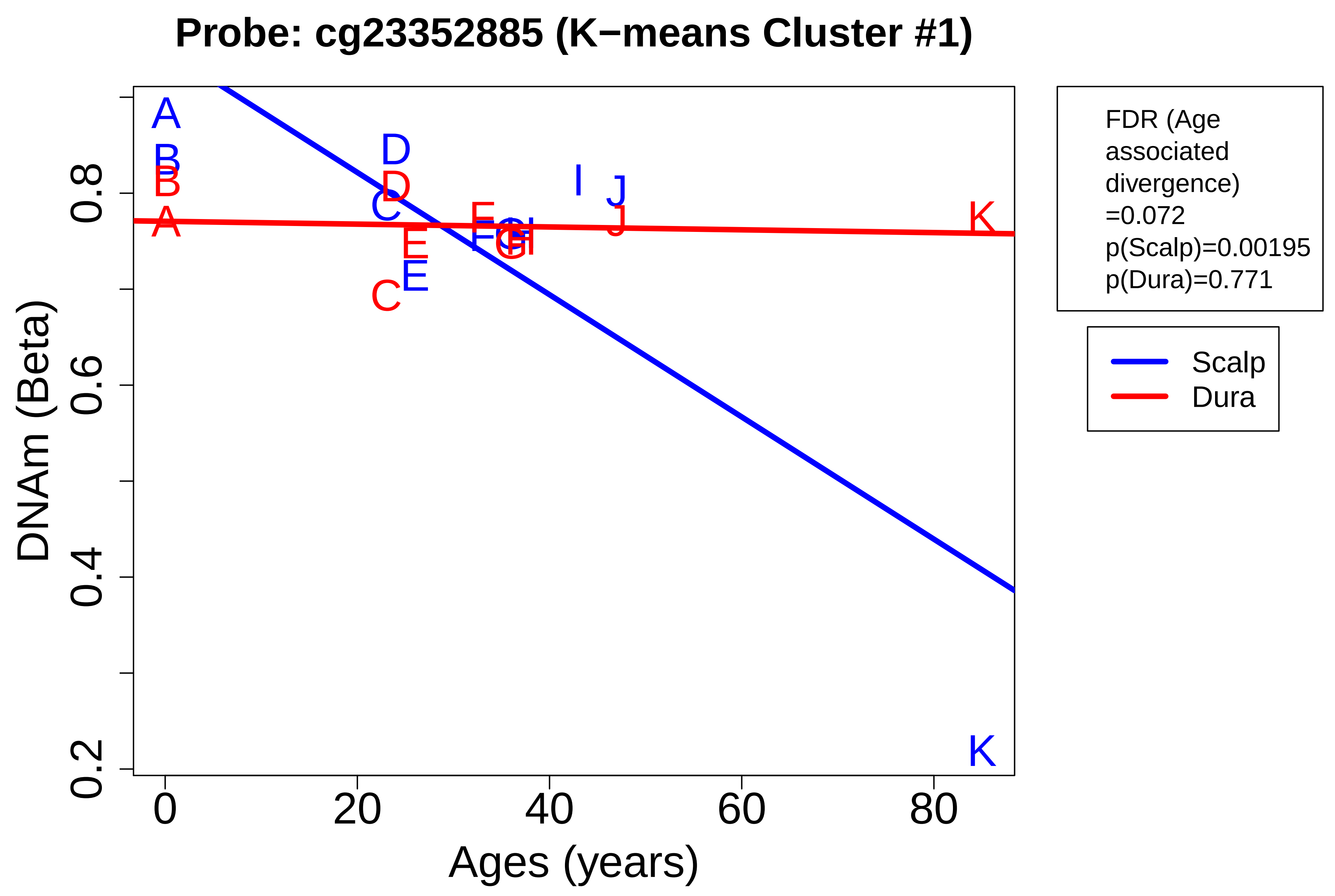

Probe: cg23352885 (K−means Cluster #1)
DNAm (Beta)
0.2
0.4
0.6
0.8
Ages (years)
0
20
40
60
80
FDR (Age associated divergence) =0.072
p(Scalp)=0.00195
p(Dura)=0.771
Scalp
Dura
A
B
C
D
E
F
G
H
I
J
K

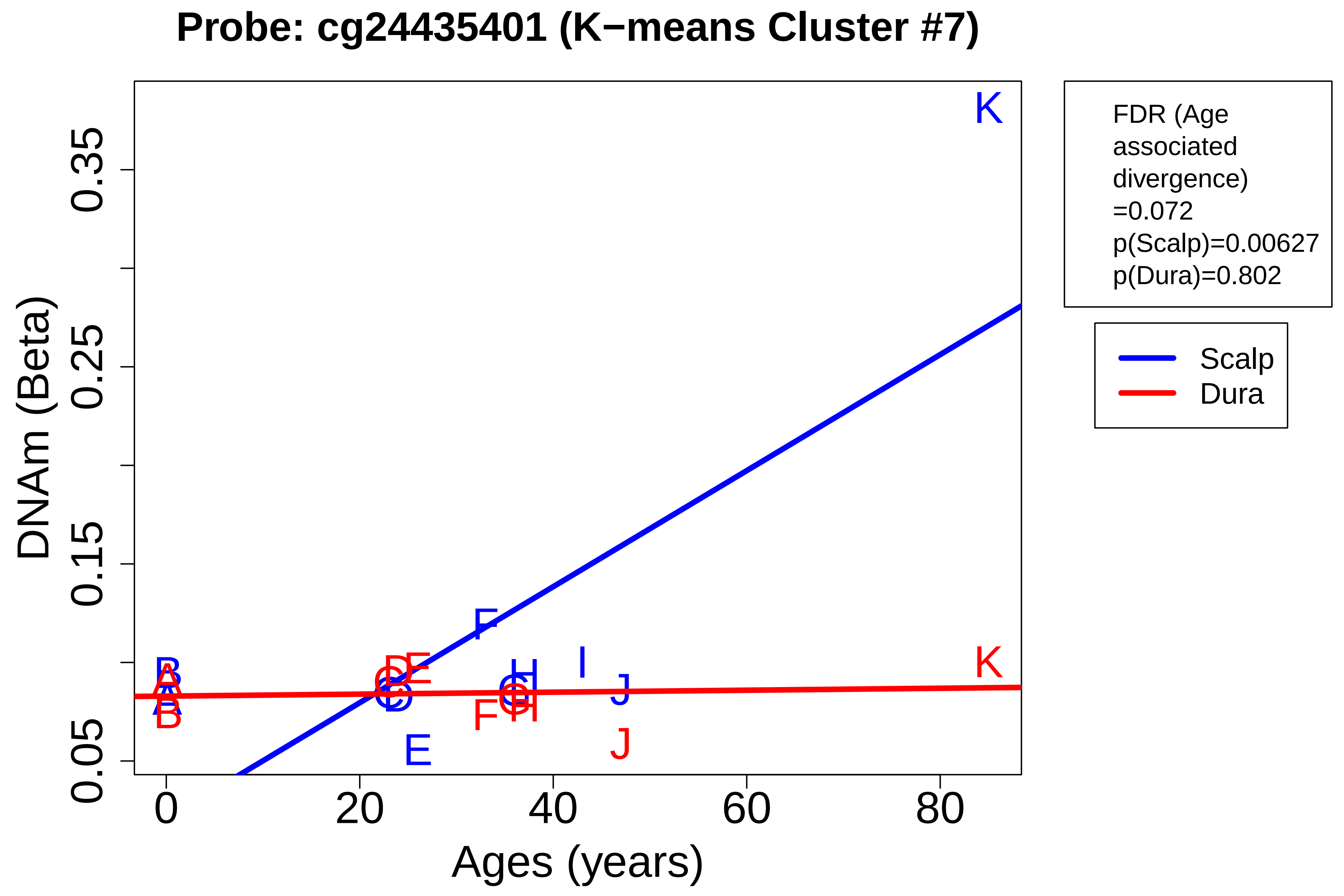

Probe: cg24435401 (K−means Cluster #7)
FDR (Age associated divergence) =0.072
p(Scalp)=0.00627
p(Dura)=0.802
Scalp
Dura
DNAm (Beta)
Ages (years)
0.05
0.15
0.25
0.35
0
20
40
60
80

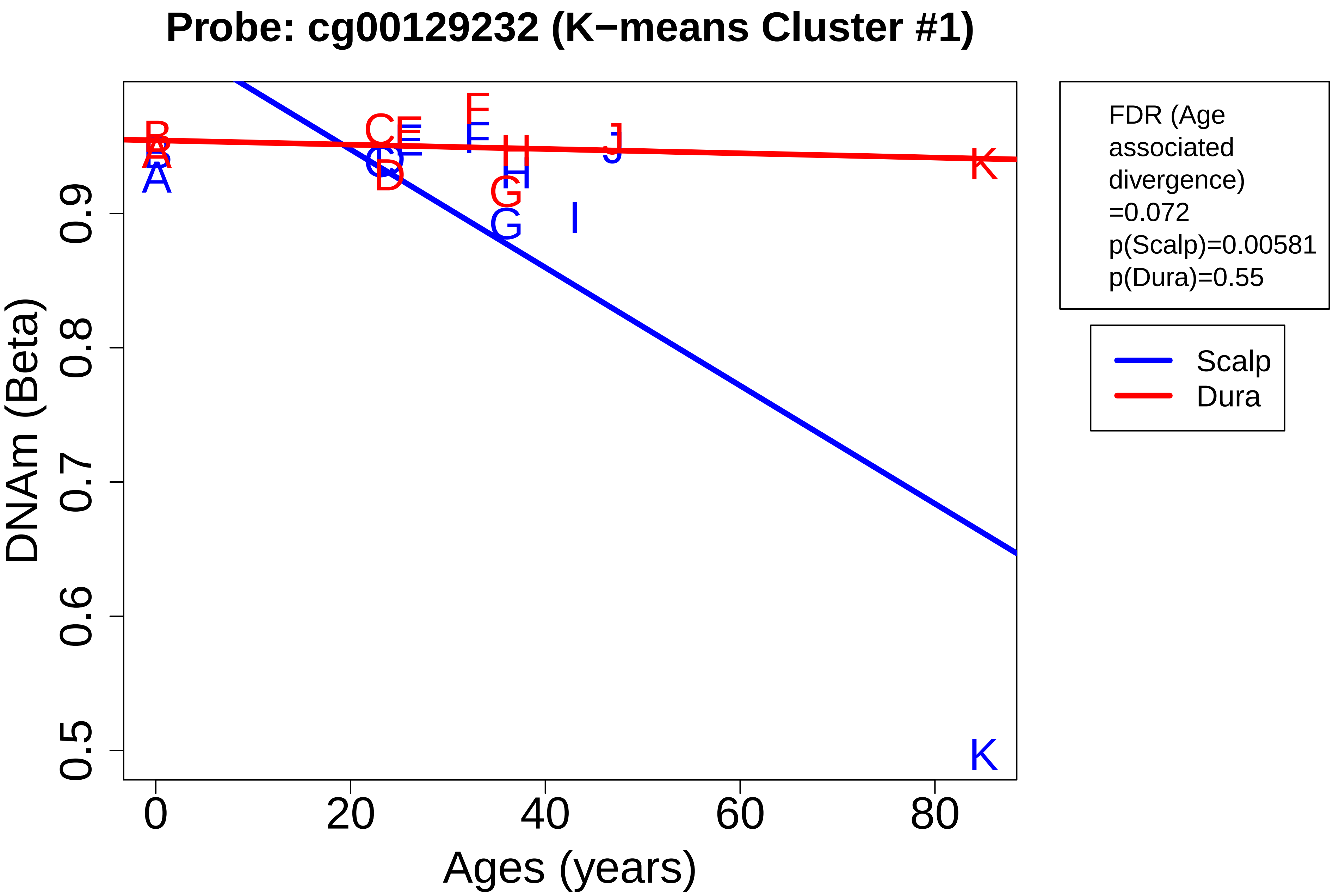

Probe: cg00129232 (K−means Cluster #1)
DNAm (Beta)
0.5
0.6
0.7
0.8
0.9
0
20
40
60
80
Ages (years)
FDR (Age associated divergence) =0.072
p(Scalp)=0.00581
p(Dura)=0.55
Scalp
Dura
A
B
C
D
E
F
G
H
I
J
K

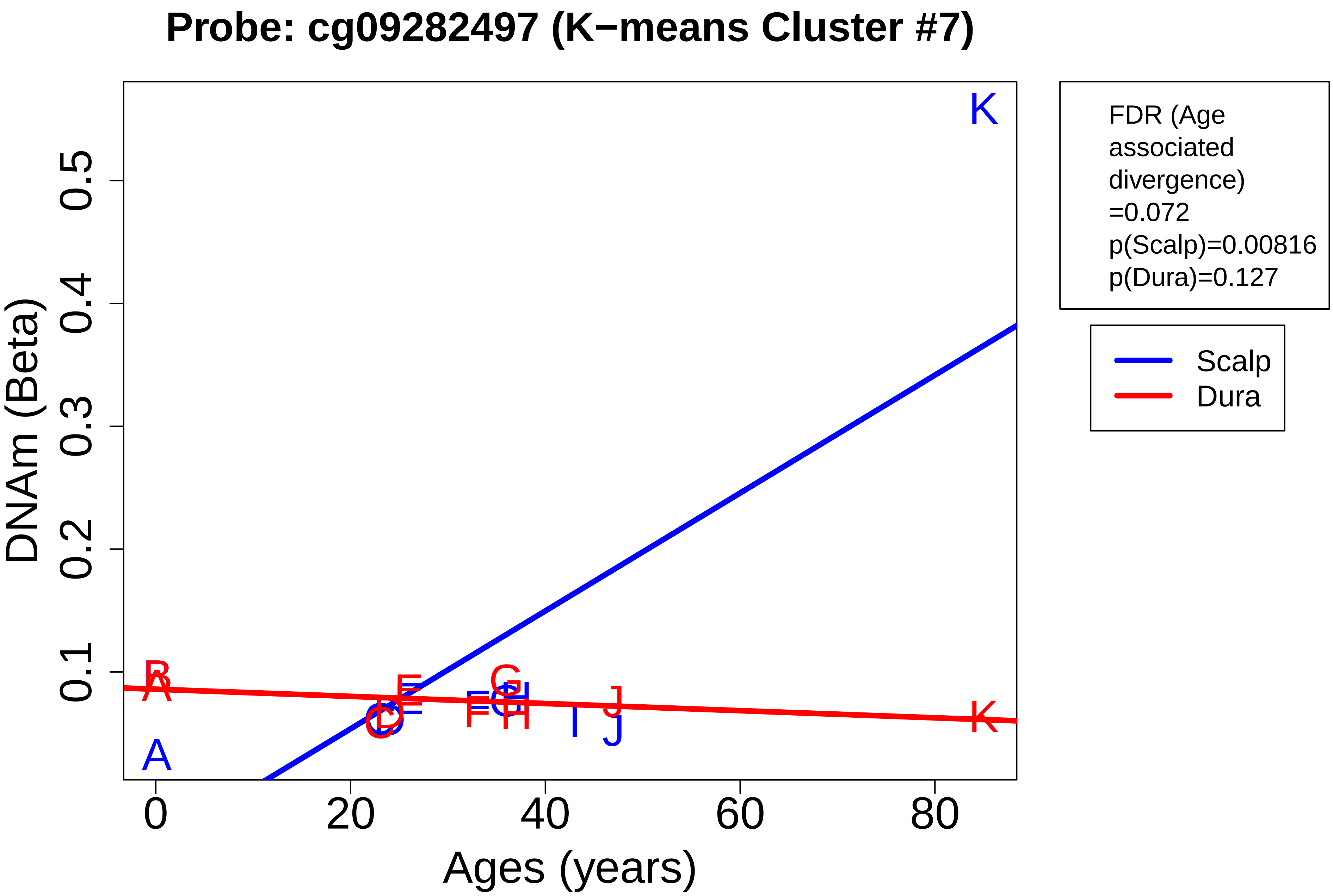

Probe: cg09282497 (K−means Cluster #7)
DNAm (Beta)
0.1
0.2
0.3
0.4
0.5
0
20
40
60
80
Ages (years)
FDR (Age associated divergence) =0.072
p(Scalp)=0.00816
p(Dura)=0.127
Scalp
Dura

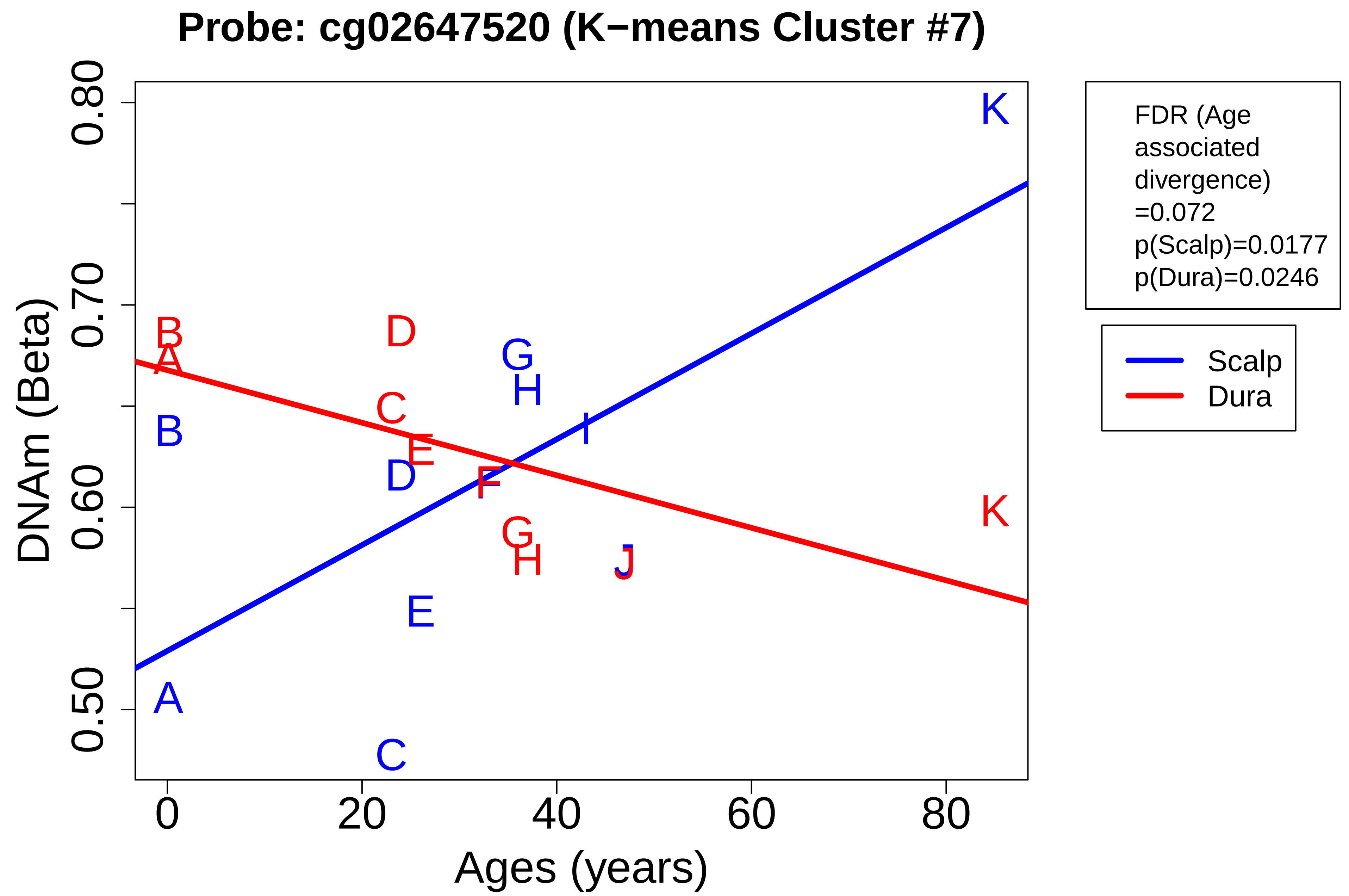

Probe: cg02647520 (K−means Cluster #7)
DNAm (Beta)
0.50
0.60
0.70
0.80
Ages (years)
0
20
40
60
80
A
B
C
D
E
F
G
H
I
J
K
FDR (Age associated divergence) =0.072
p(Scalp)=0.0177
p(Dura)=0.0246
Scalp
Dura

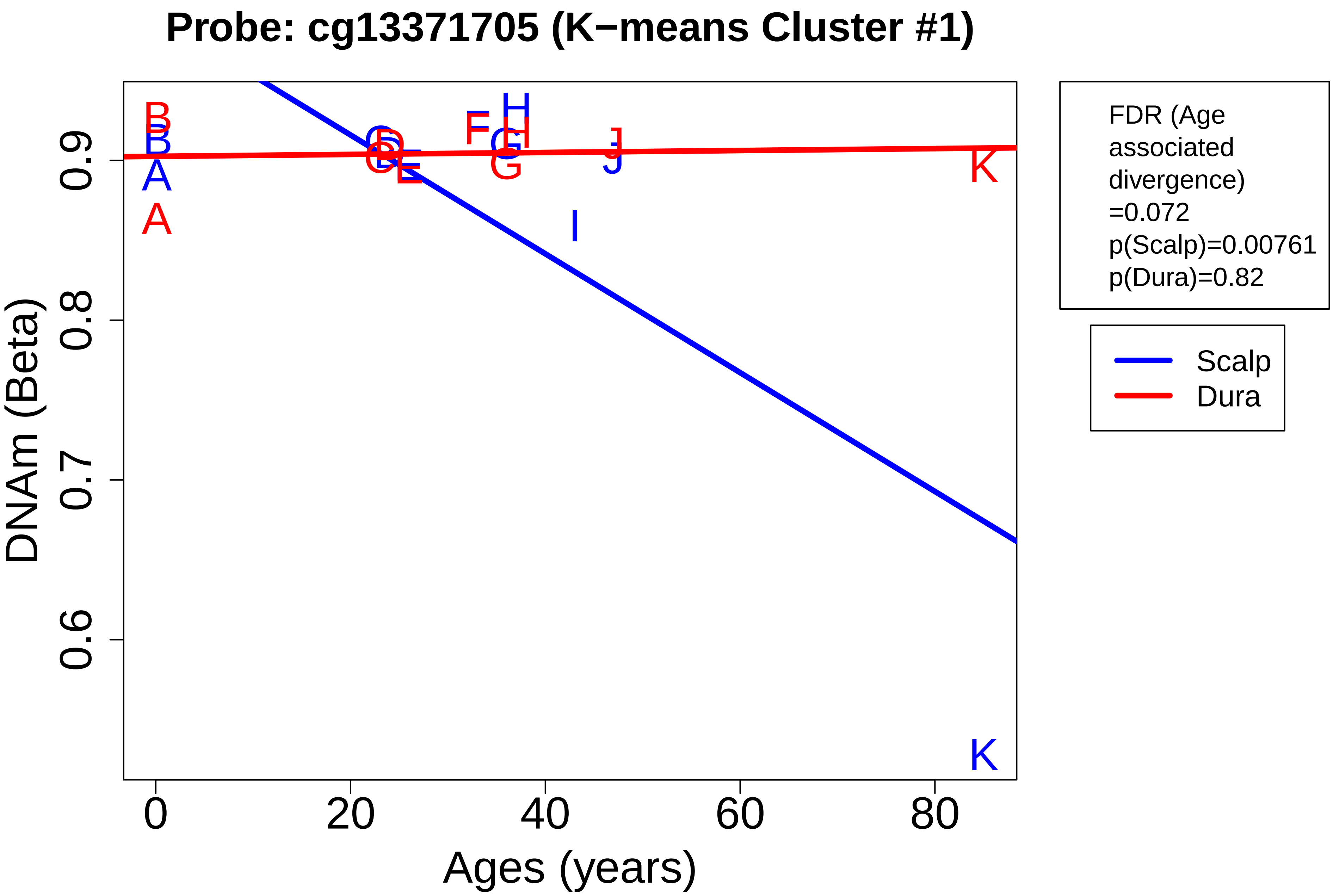

Probe: cg13371705 (K−means Cluster #1)
DNAm (Beta)
0.6
0.7
0.8
0.9
0
20
40
60
80
Ages (years)
FDR (Age associated divergence) =0.072
p(Scalp)=0.00761
p(Dura)=0.82
Scalp
Dura

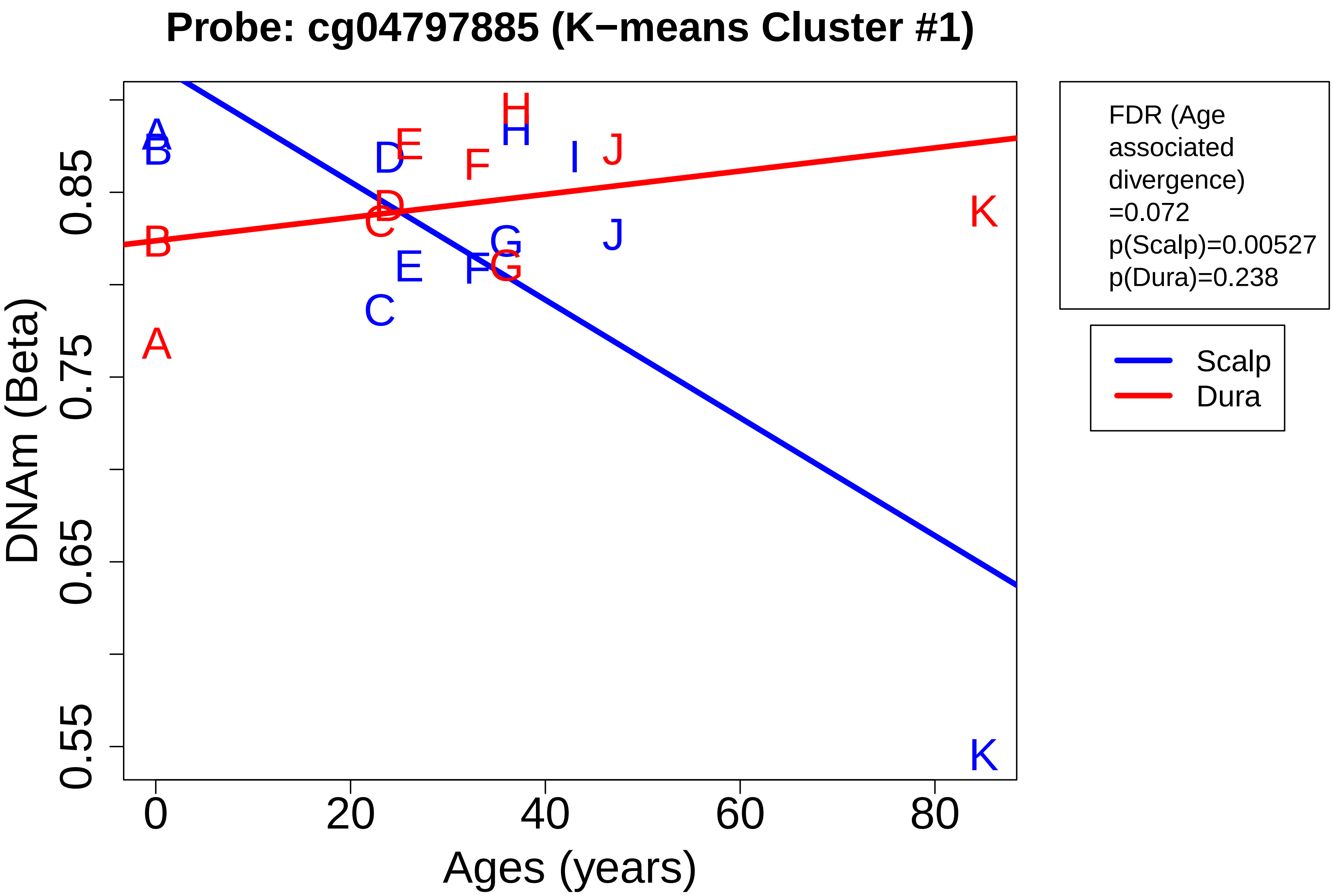

Probe: cg04797885 (K−means Cluster #1)
DNAm (Beta)
0.55
0.65
0.75
0.85
Ages (years)
0
20
40
60
80
A
B
C
D
E
F
G
H
I
J
K
FDR (Age associated divergence) =0.072
p(Scalp)=0.00527
p(Dura)=0.238
Scalp
Dura

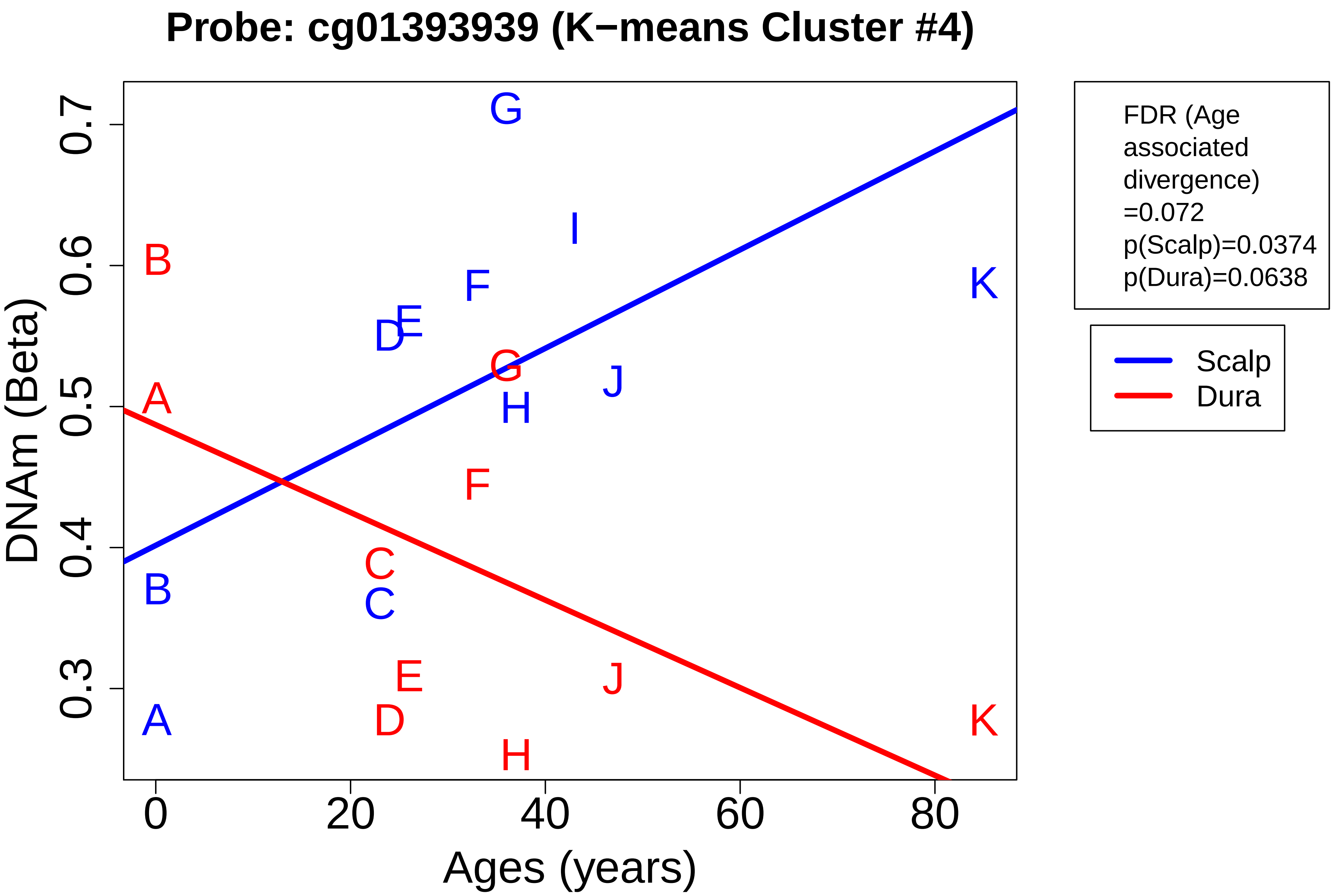
Probe: cg01393939 (K−means Cluster #4)
DNAm (Beta)
Ages (years)
0.3
0.4
0.5
0.6
0.7
0
20
40
60
80
FDR (Age associated divergence) =0.072
p(Scalp)=0.0374
p(Dura)=0.0638
Scalp
Dura
A B C D E F G H I J K

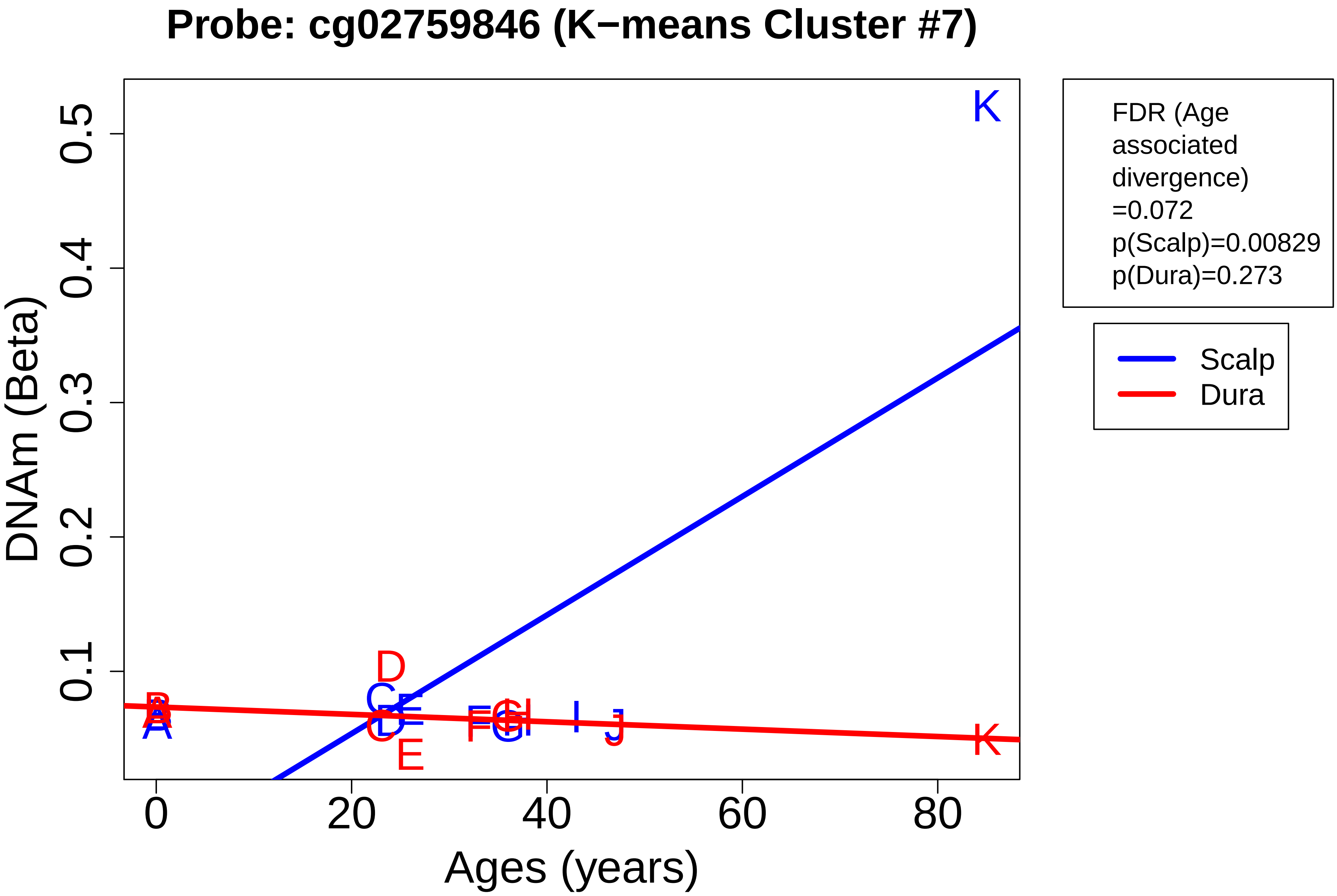

Probe: cg02759846 (K−means Cluster #7)
DNAm (Beta)
0.1
0.2
0.3
0.4
0.5
Ages (years)
0
20
40
60
80
FDR (Age associated divergence) =0.072
p(Scalp)=0.00829
p(Dura)=0.273
Scalp
Dura

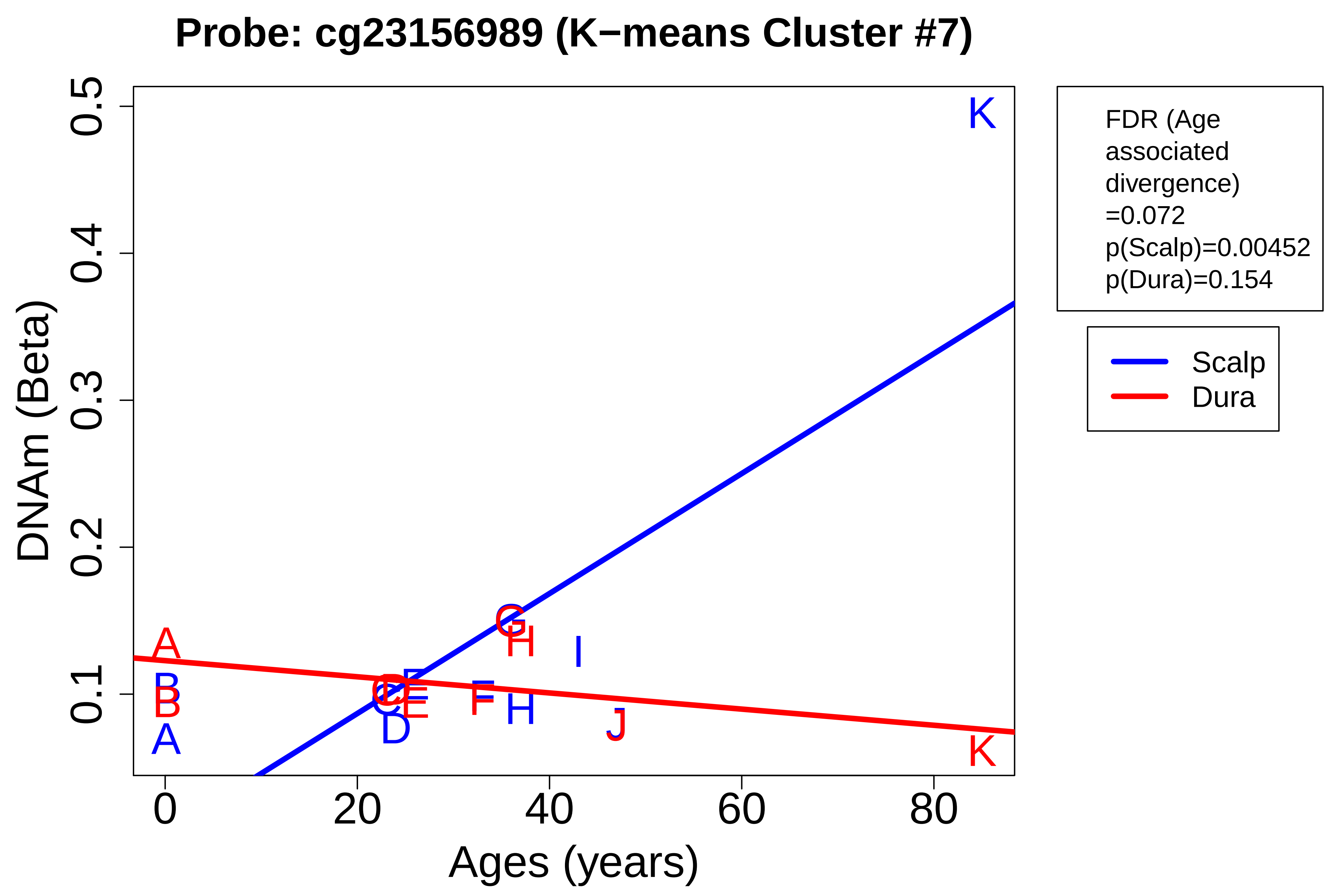
Probe: cg23156989 (K−means Cluster #7)
DNAm (Beta)
0.1
0.2
0.3
0.4
0.5
Ages (years)
0
20
40
60
80
FDR (Age associated divergence) =0.072
p(Scalp)=0.00452
p(Dura)=0.154
Scalp
Dura
A
B
C
D
E
F
G
H
I
J
K

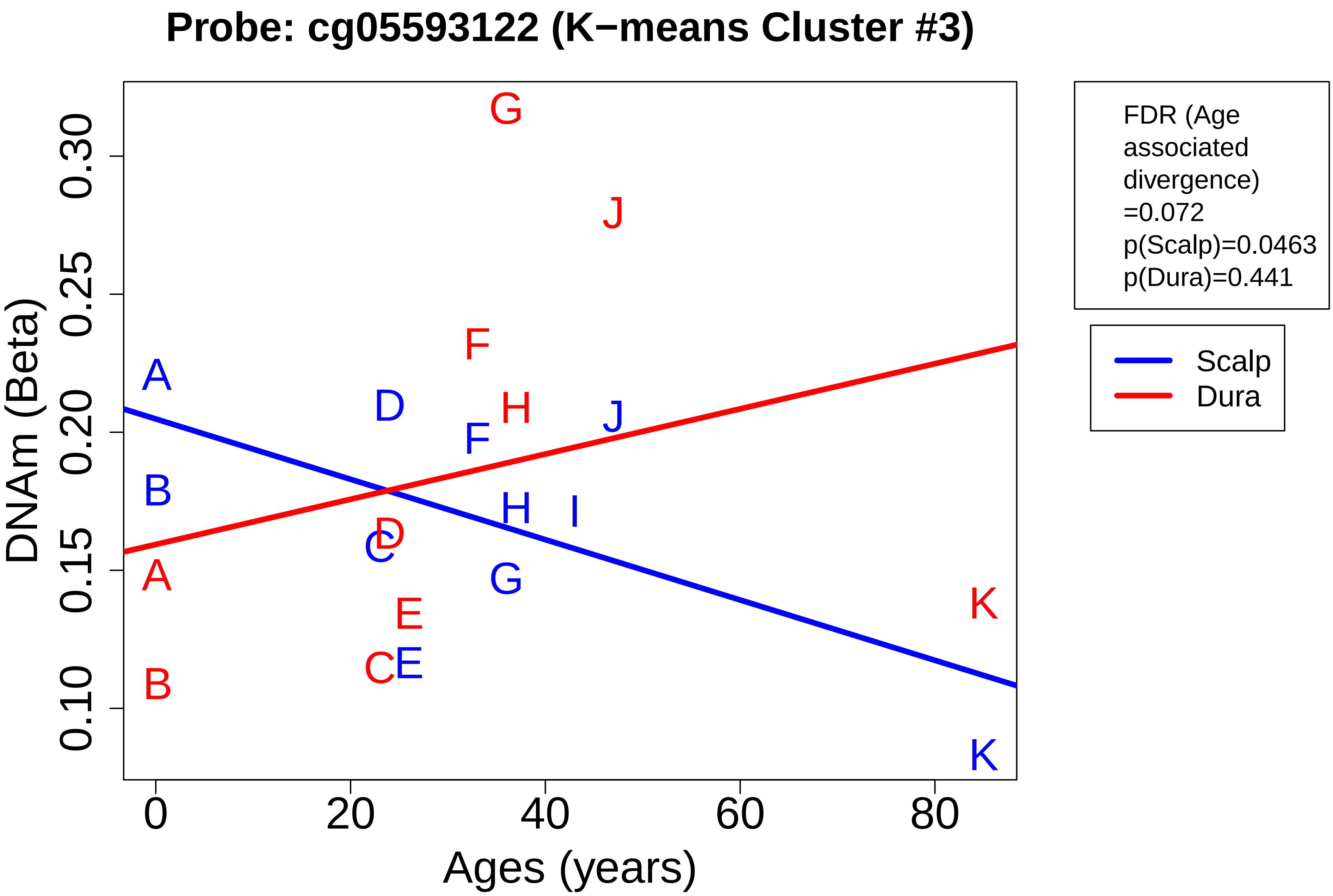

Probe: cg05593122 (K−means Cluster #3)
DNAm (Beta)
0.10
0.15
0.20
0.25
0.30
Ages (years)
0
20
40
60
80
A
B
C
D
E
F
G
H
I
J
K
FDR (Age associated divergence) =0.072
p(Scalp)=0.0463
p(Dura)=0.441
Scalp
Dura

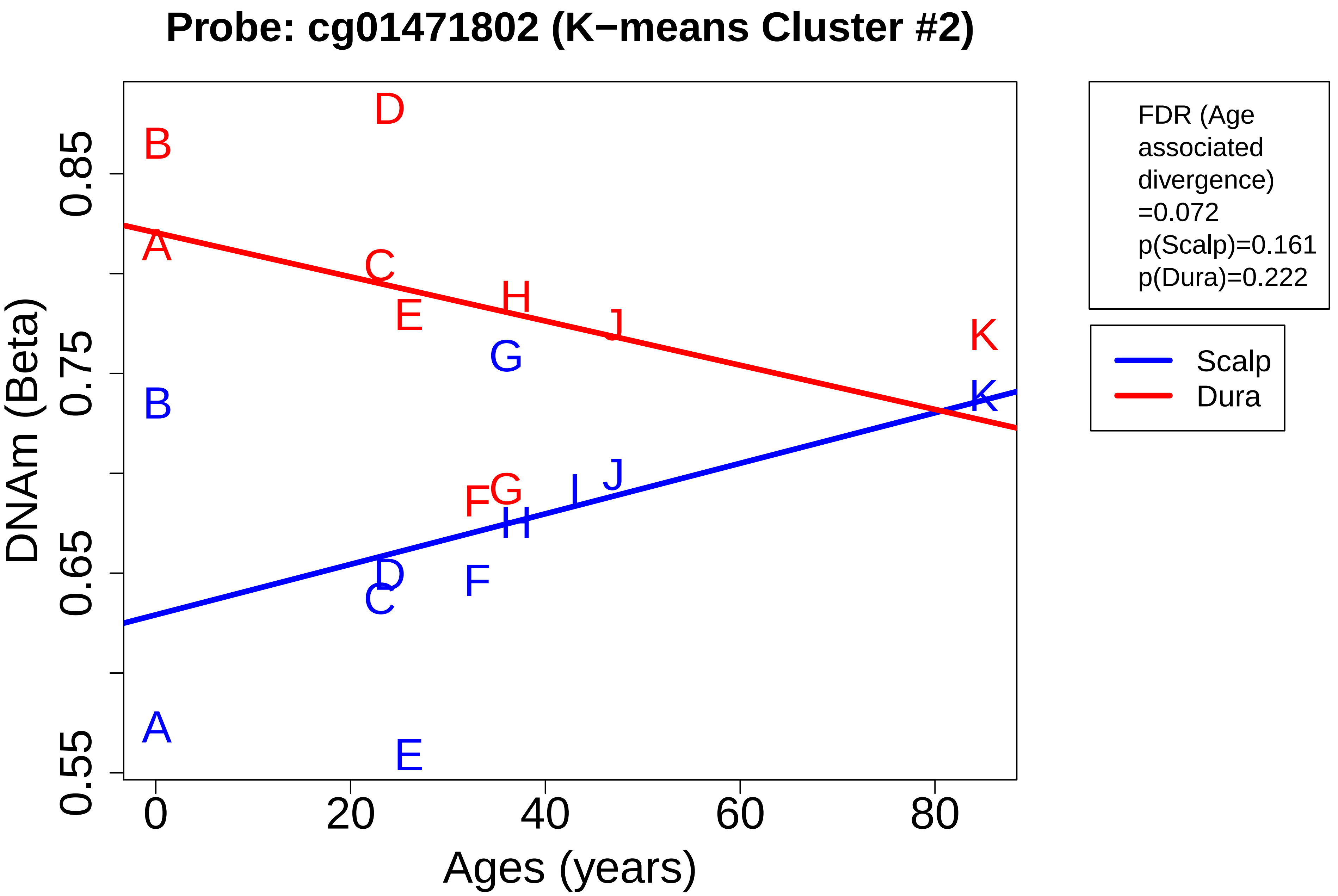

Probe: cg01471802 (K−means Cluster #2)
DNAm (Beta)
0.55
0.65
0.75
0.85
0
20
40
60
80
Ages (years)
FDR (Age associated divergence) =0.072
p(Scalp)=0.161
p(Dura)=0.222
Scalp
Dura
A
B
C
D
E
F
G
H
I
J
K

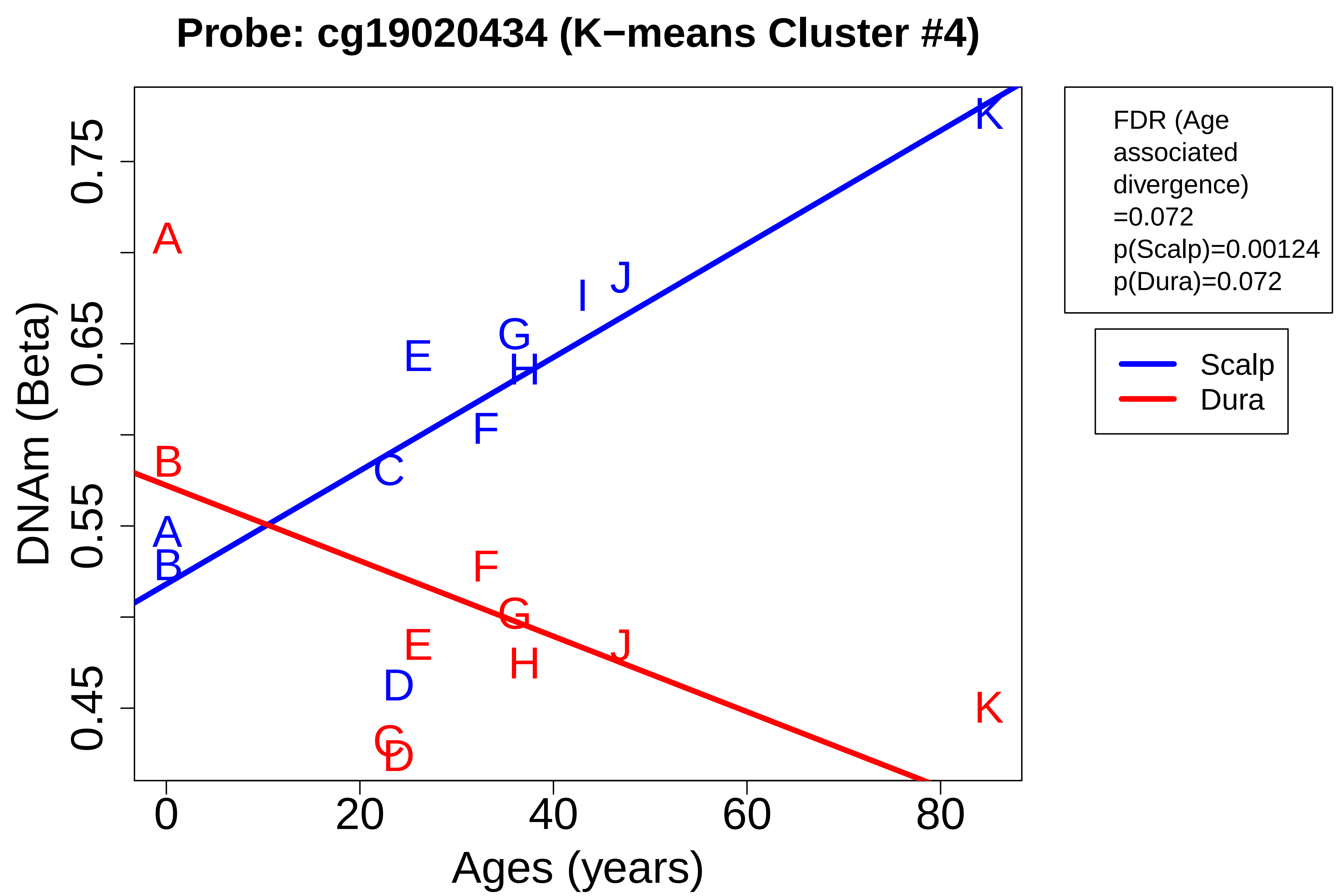

Probe: cg19020434 (K−means Cluster #4)
DNAm (Beta)
0.45
0.55
0.65
0.75
Ages (years)
0
20
40
60
80
FDR (Age associated divergence) =0.072
p(Scalp)=0.00124
p(Dura)=0.072
Scalp
Dura
A
B
C
D
E
F
G
H
I
J
K

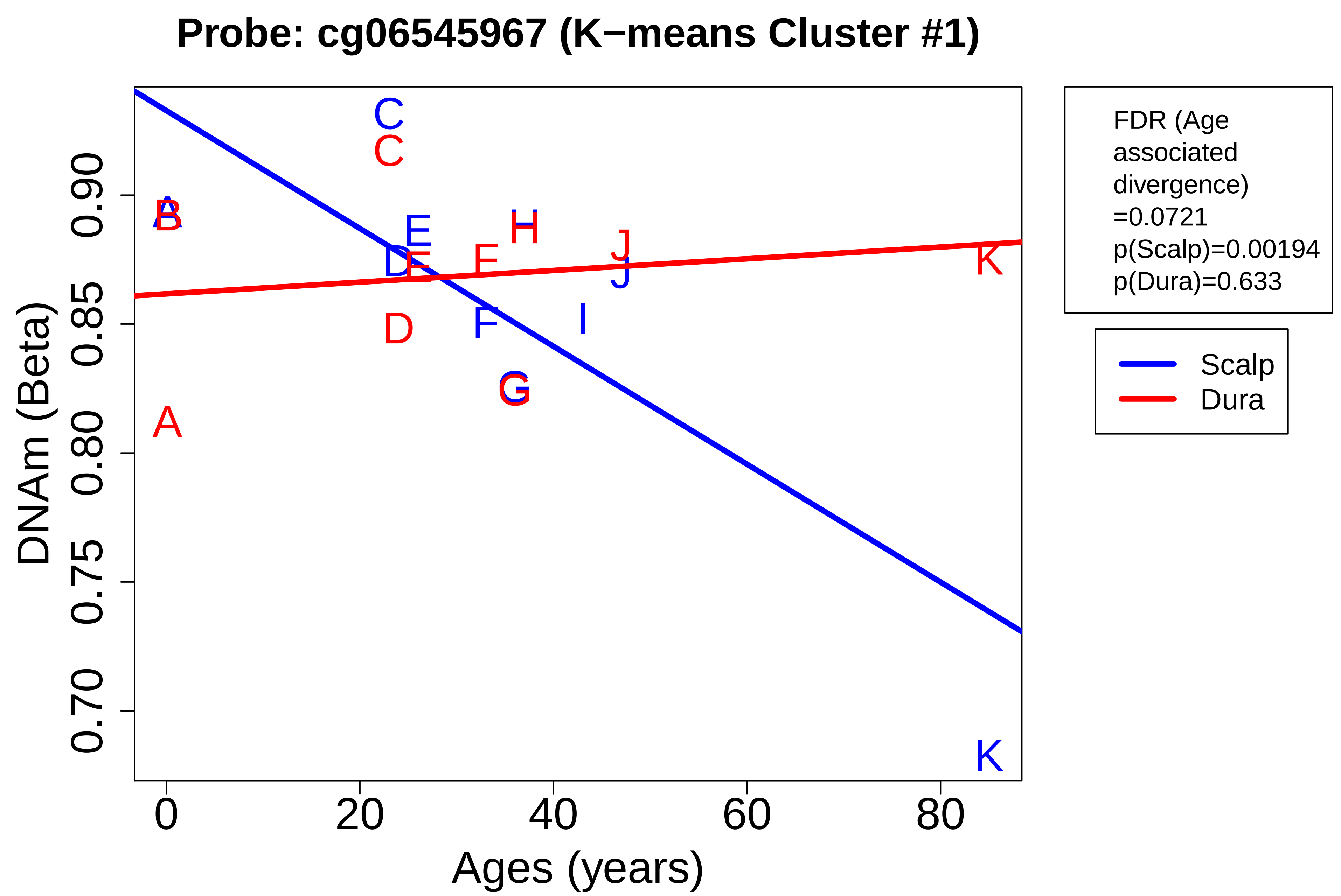

Probe: cg06545967 (K−means Cluster #1)
DNAm (Beta)
0.70
0.75
0.80
0.85
0.90
Ages (years)
0
20
40
60
80
FDR (Age associated divergence) =0.0721
p(Scalp)=0.00194
p(Dura)=0.633
Scalp
Dura

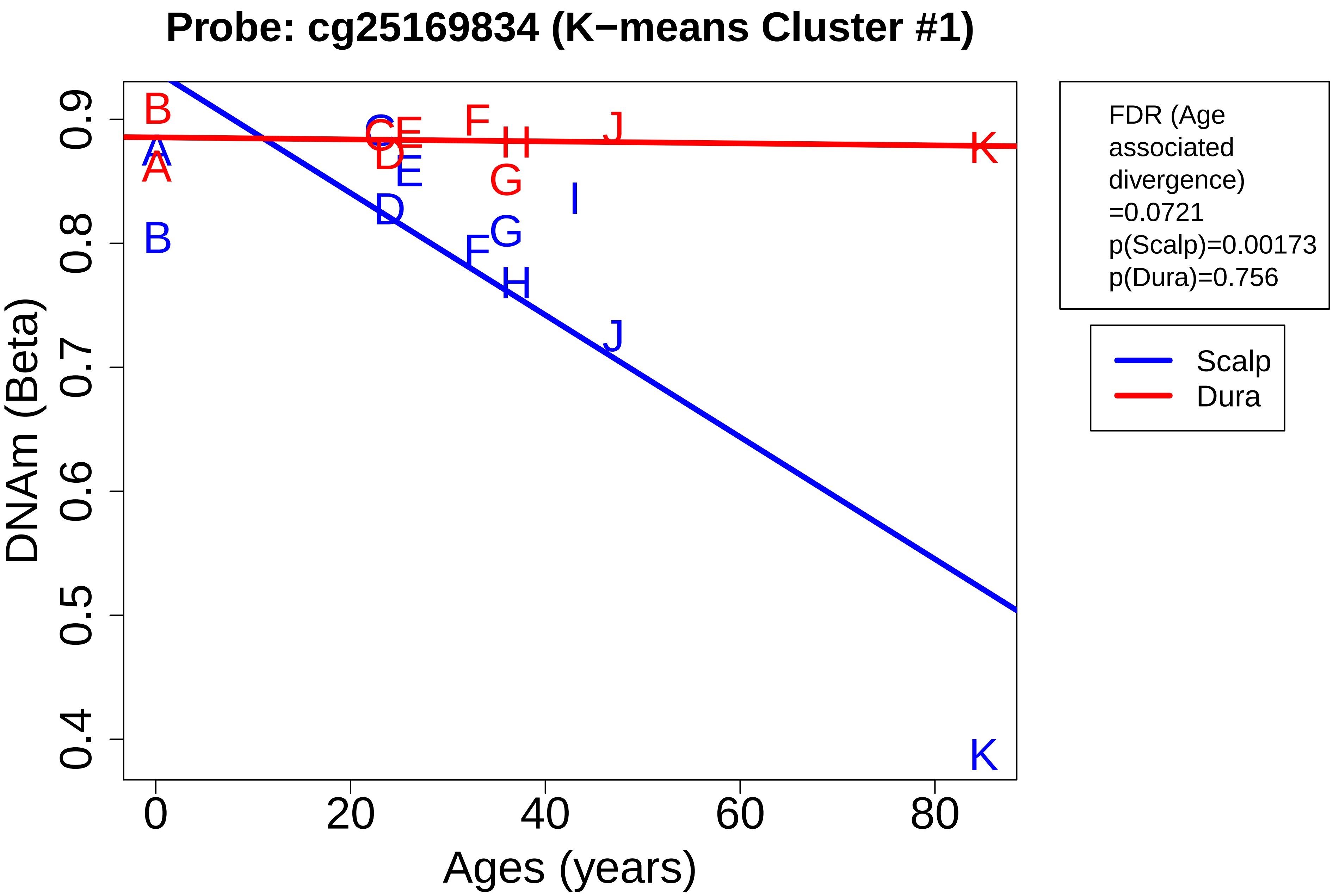

Probe: cg25169834 (K−means Cluster #1)
DNAm (Beta)
0.4
0.5
0.6
0.7
0.8
0.9
0
20
40
60
80
Ages (years)
FDR (Age associated divergence) =0.0721
p(Scalp)=0.00173
p(Dura)=0.756
Scalp
Dura

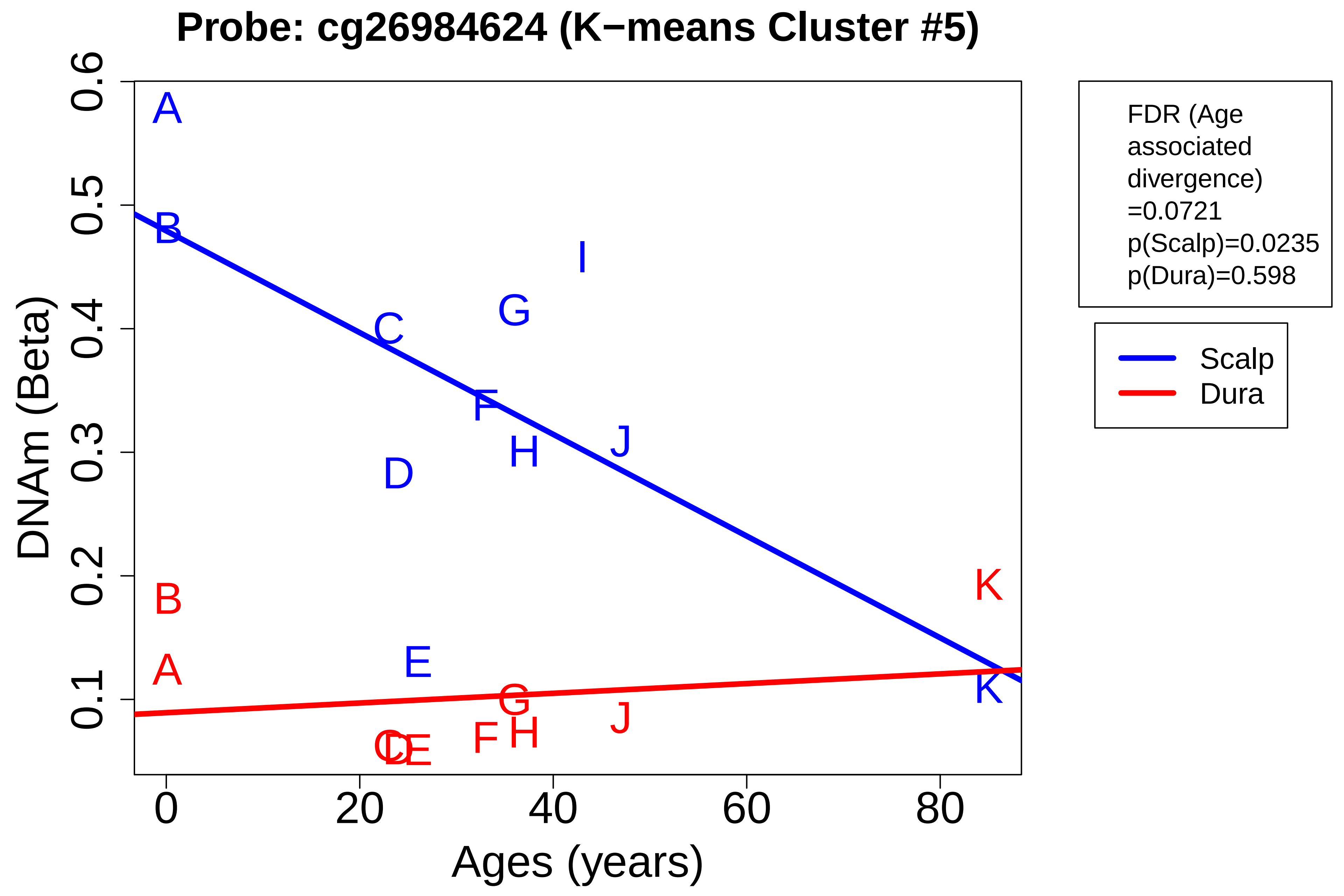

Probe: cg26984624 (K−means Cluster #5)
DNAm (Beta)
0.1
0.2
0.3
0.4
0.5
0.6
Ages (years)
0
20
40
60
80
A
B
C
D
E
F
G
H
I
J
K
FDR (Age associated divergence) =0.0721
p(Scalp)=0.0235
p(Dura)=0.598
Scalp
Dura

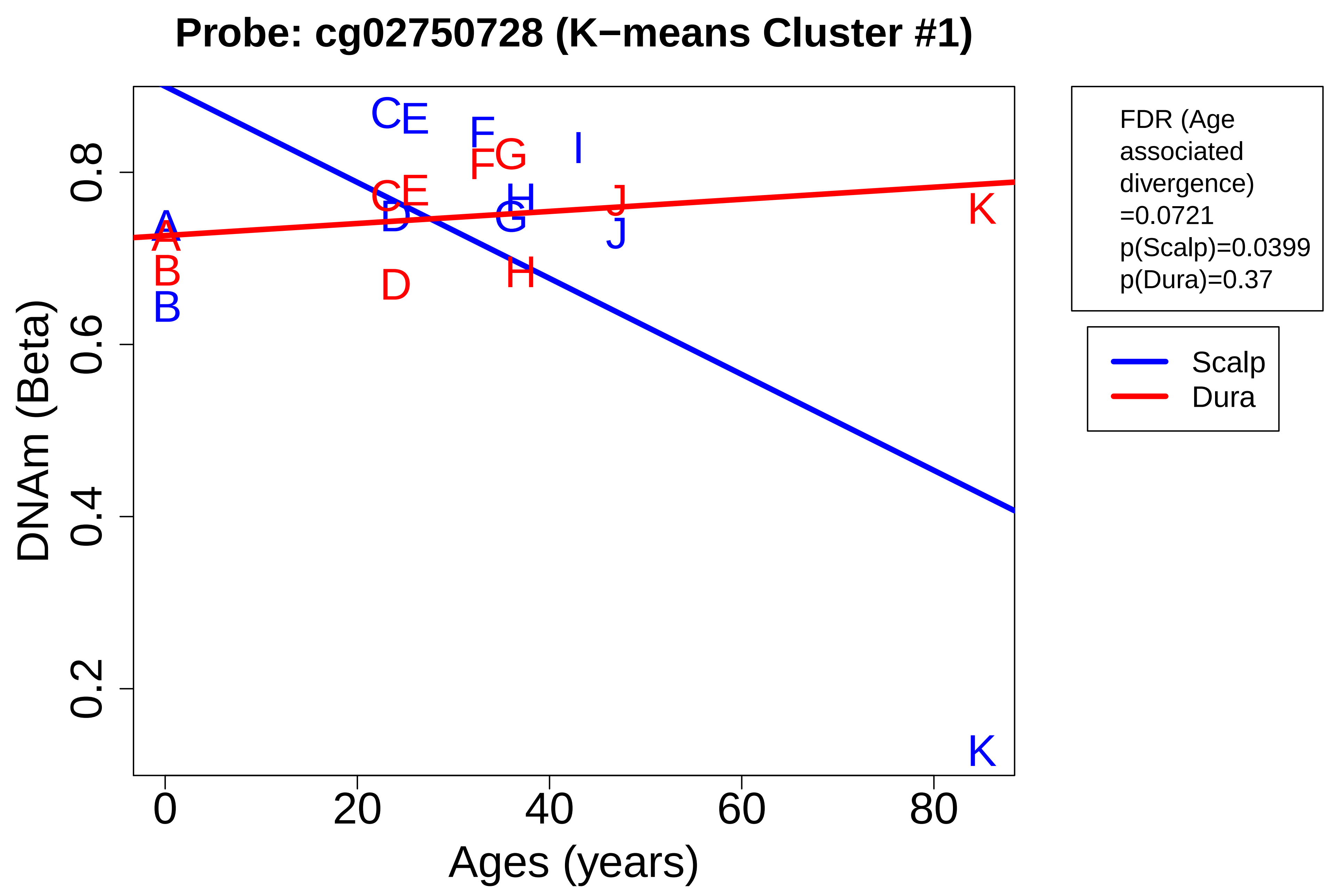

Probe: cg02750728 (K−means Cluster #1)
DNAm (Beta)
0.2
0.4
0.6
0.8
0
20
40
60
80
Ages (years)
A
B
C
D
E
F
G
H
I
J
K
FDR (Age associated divergence) =0.0721
p(Scalp)=0.0399
p(Dura)=0.37
Scalp
Dura

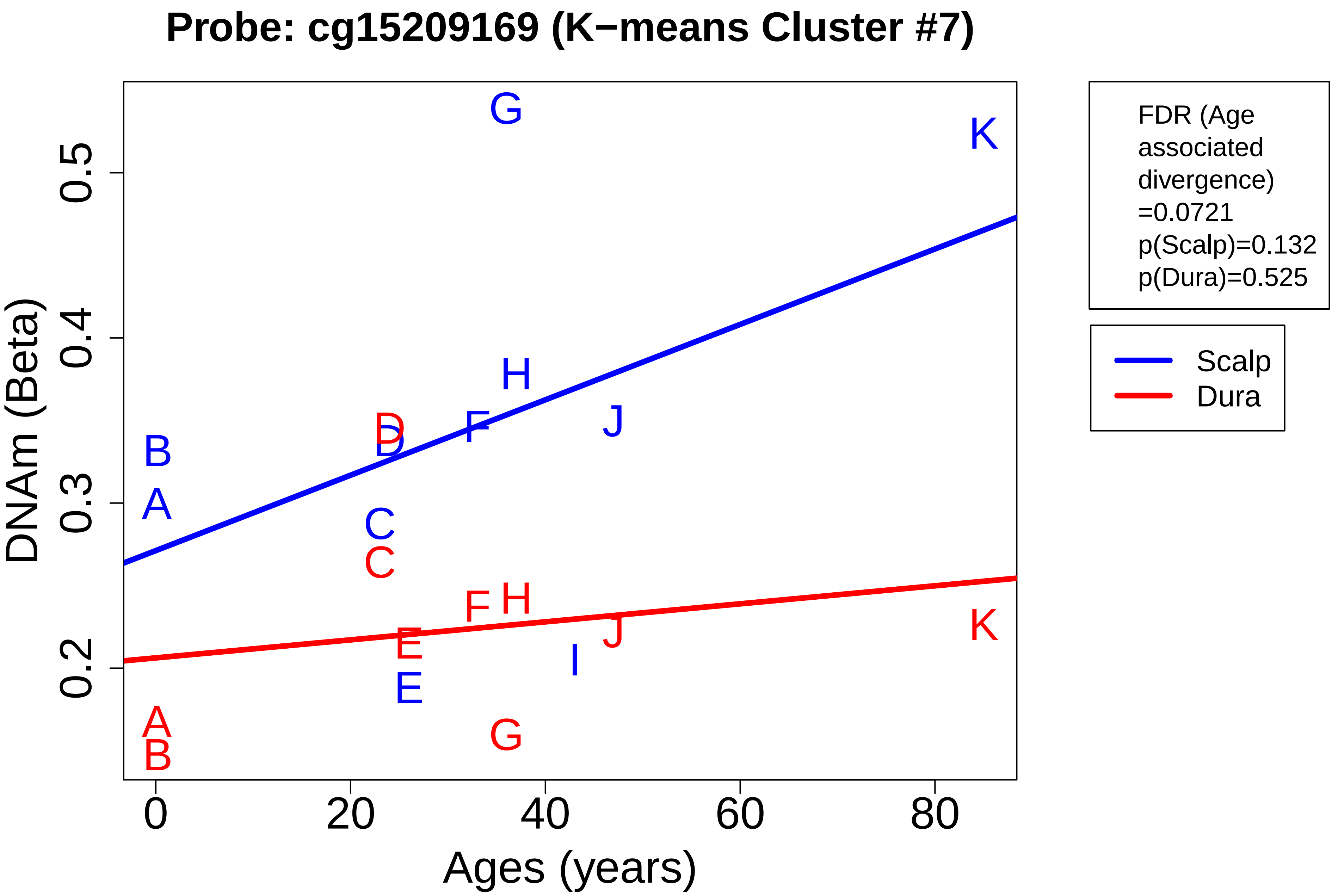
Probe: cg15209169 (K−means Cluster #7)
FDR (Age associated divergence) =0.0721
p(Scalp)=0.132
p(Dura)=0.525
Scalp
Dura
DNAm (Beta)
0.2
0.3
0.4
0.5
0
20
40
60
80
Ages (years)
A
B
C
D
E
F
G
H
I
J
K

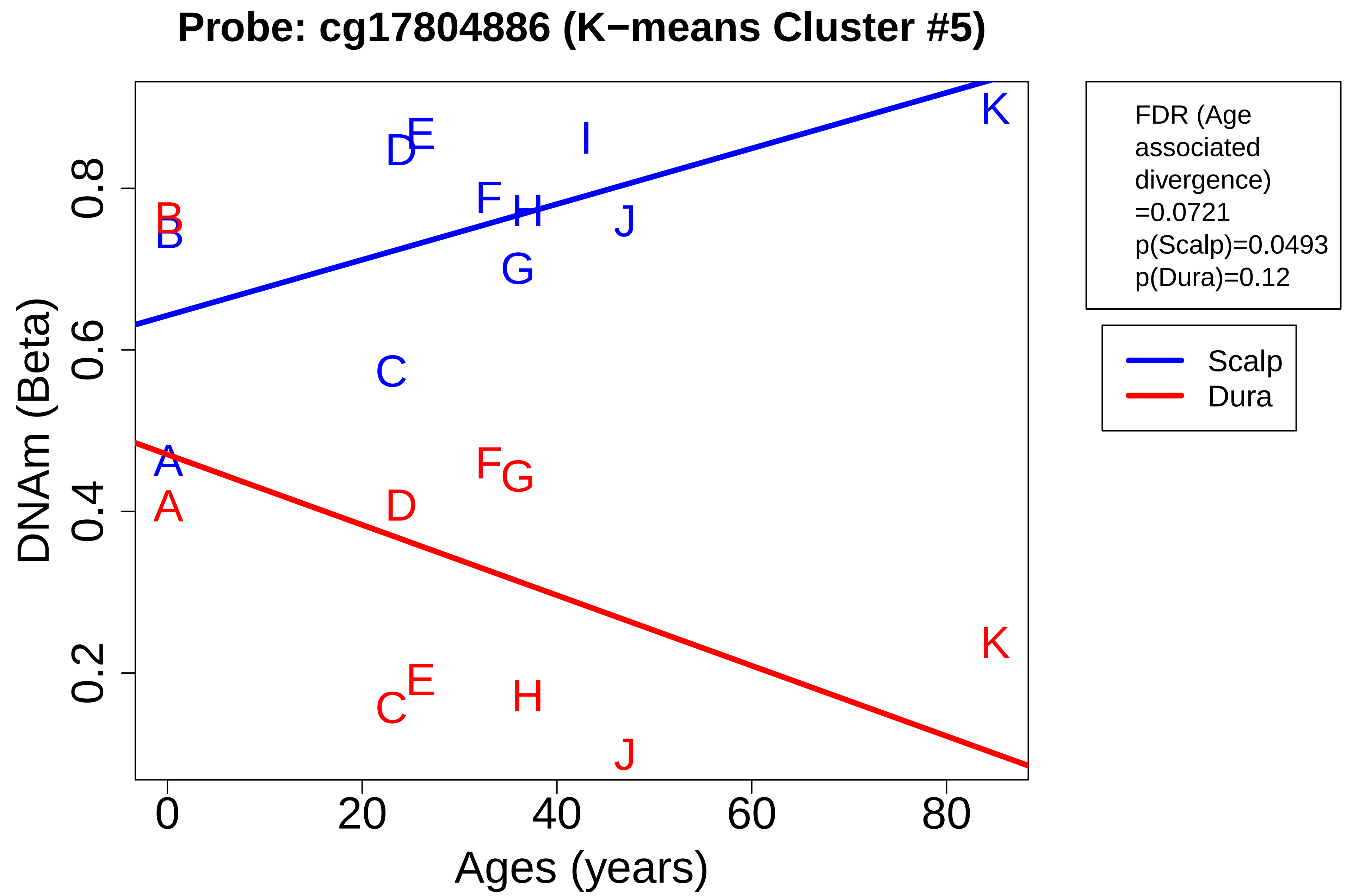

Probe: cg17804886 (K−means Cluster #5)
DNAm (Beta)
0.2
0.4
0.6
0.8
Ages (years)
0
20
40
60
80
FDR (Age associated divergence) =0.0721
p(Scalp)=0.0493
p(Dura)=0.12
Scalp
Dura
A B C D E F G H I J K

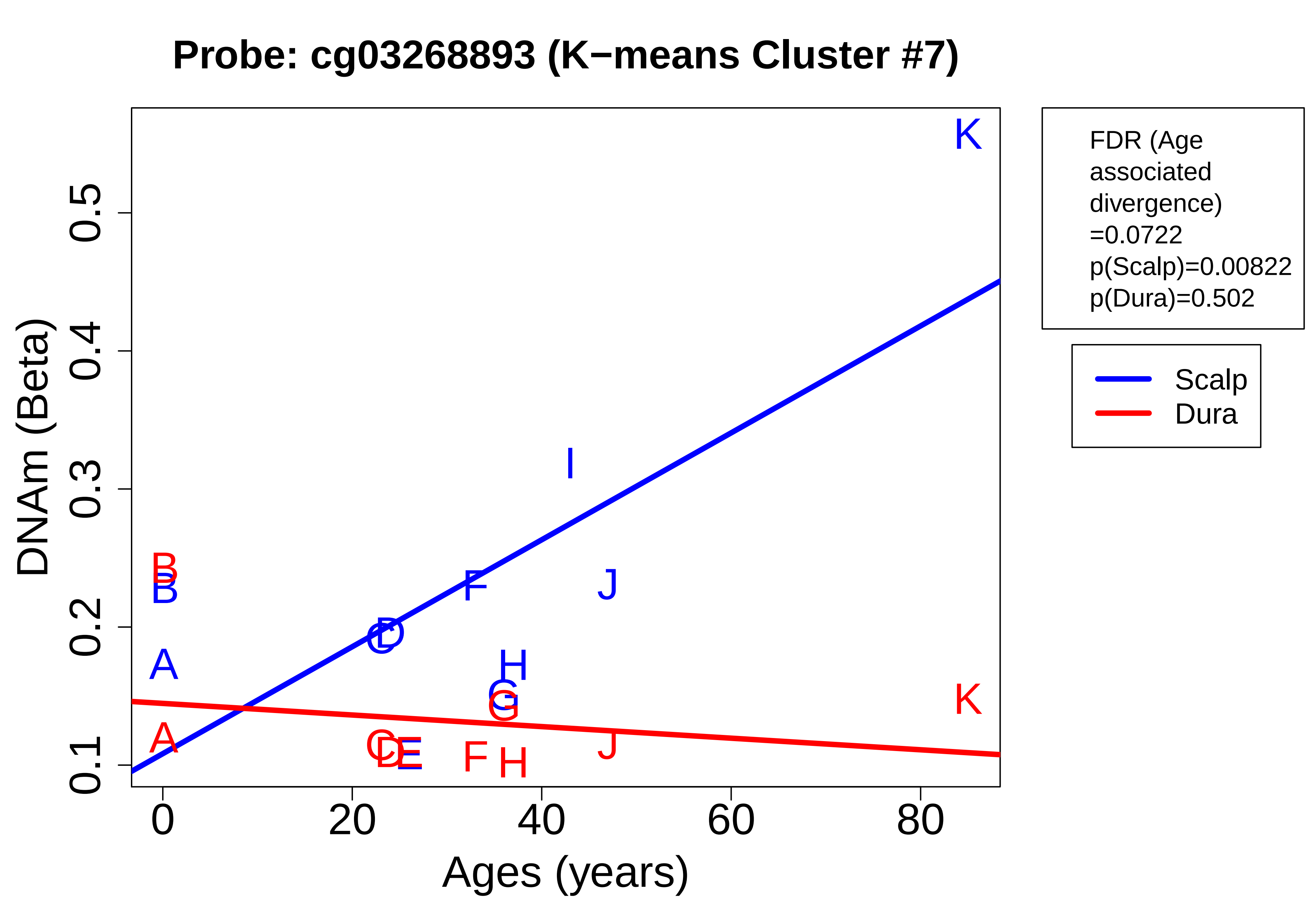

Probe: cg03268893 (K−means Cluster #7)
FDR (Age associated divergence) =0.0722
p(Scalp)=0.00822
p(Dura)=0.502
Scalp
Dura
DNAm (Beta)
0.1
0.2
0.3
0.4
0.5
0
20
40
60
80
Ages (years)
A
B
C
D
E
F
G
H
I
J
K

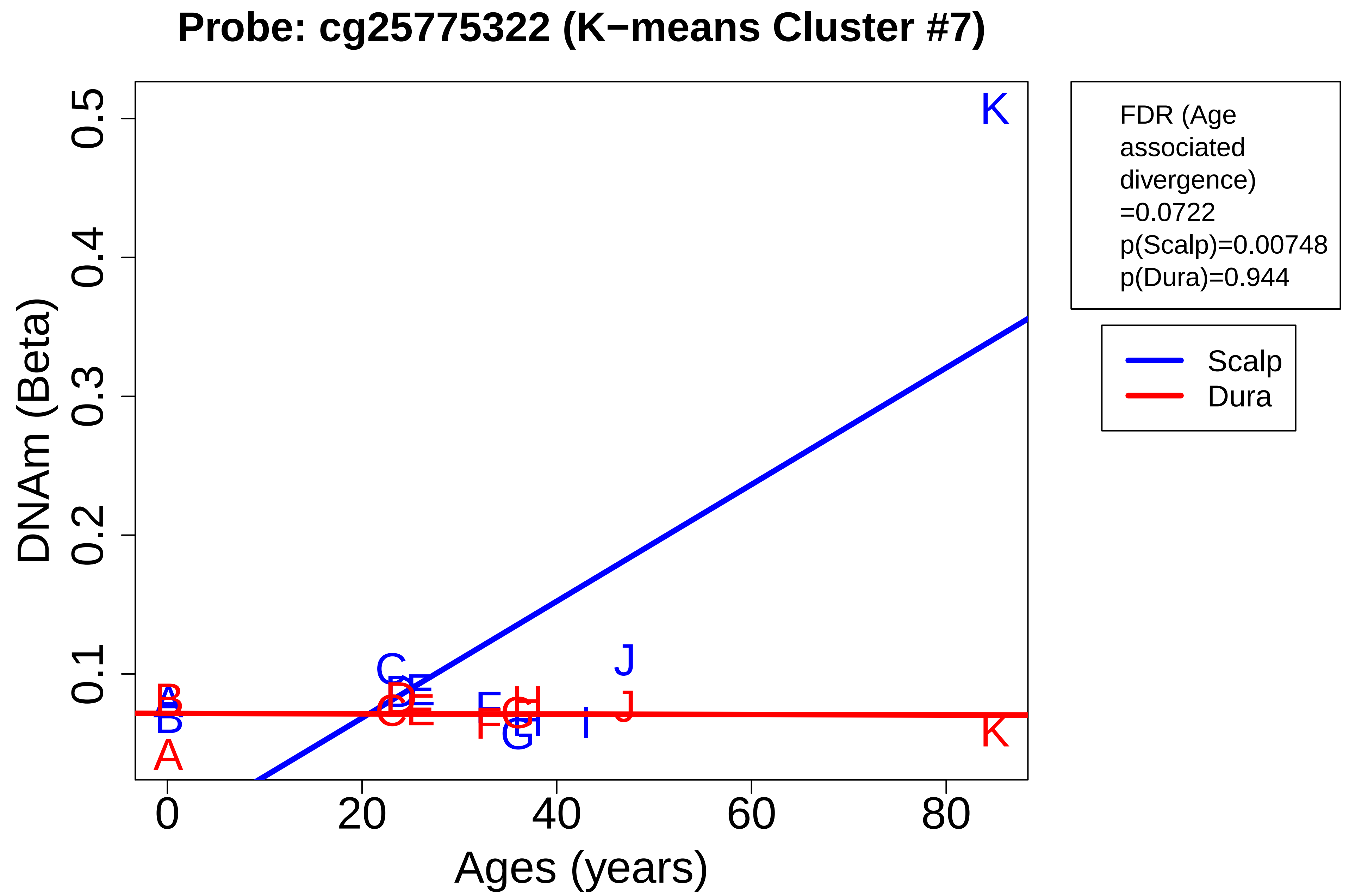

Probe: cg25775322 (K−means Cluster #7)
DNAm (Beta)
0.1
0.2
0.3
0.4
0.5
Ages (years)
0
20
40
60
80
FDR (Age associated divergence) =0.0722
p(Scalp)=0.00748
p(Dura)=0.944
Scalp
Dura

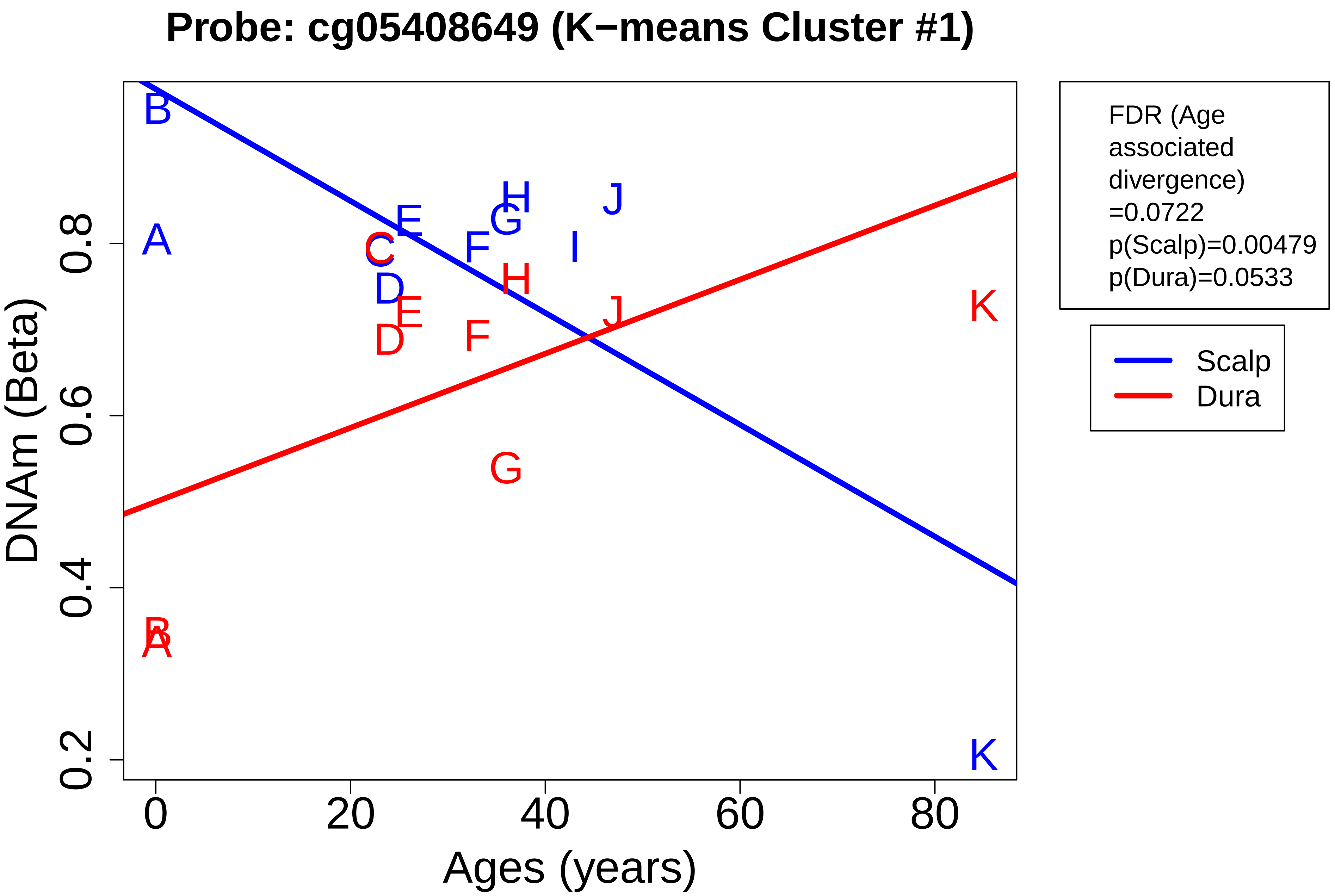

Probe: cg05408649 (K−means Cluster #1)
DNAm (Beta)
0.2
0.4
0.6
0.8
0
20
40
60
80
Ages (years)
FDR (Age associated divergence) =0.0722
p(Scalp)=0.00479
p(Dura)=0.0533
Scalp
Dura

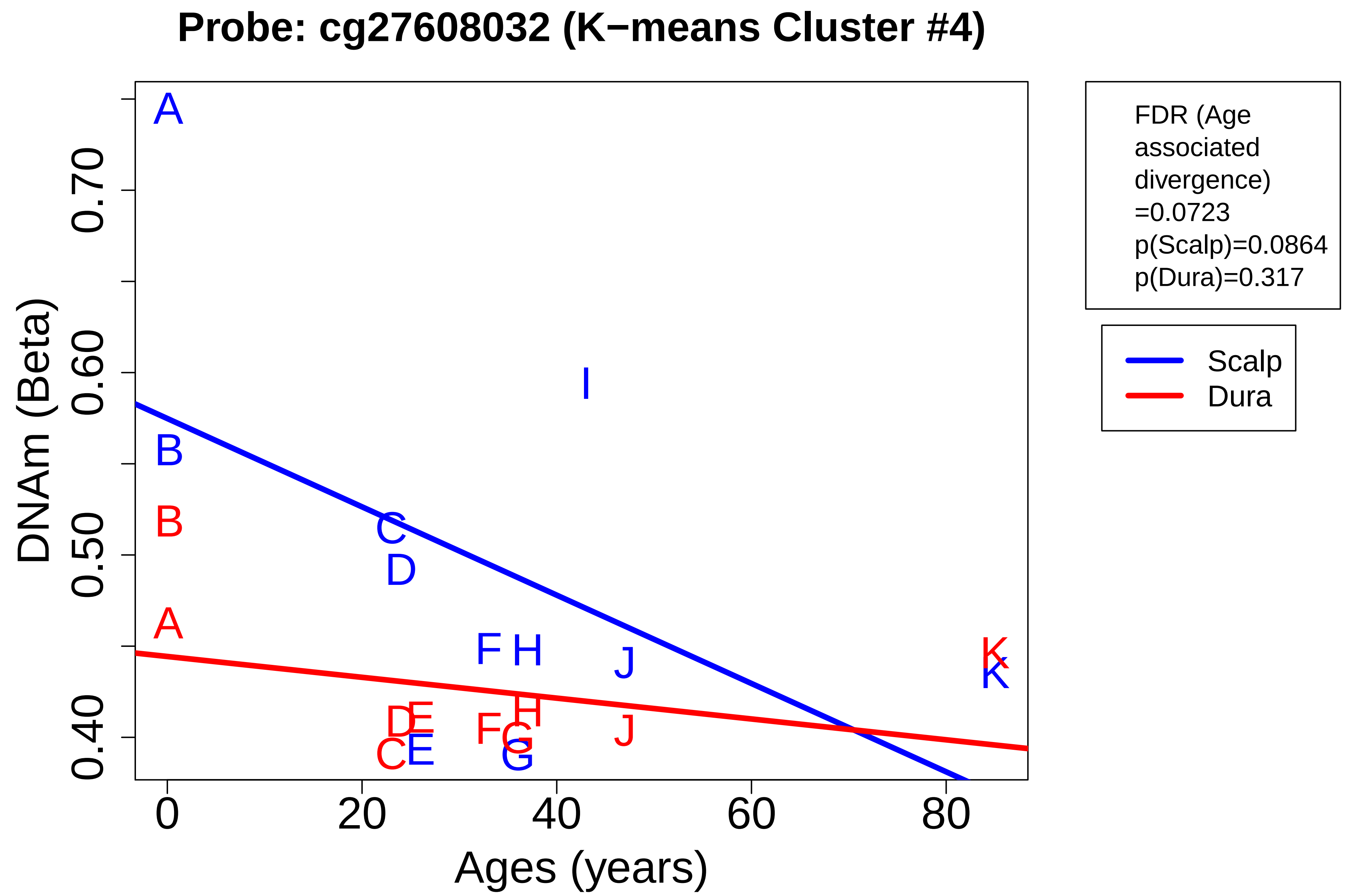

Probe: cg27608032 (K−means Cluster #4)
FDR (Age associated divergence) =0.0723
p(Scalp)=0.0864
p(Dura)=0.317
Scalp
Dura
DNAm (Beta)
0.40
0.50
0.60
0.70
0
20
40
60
80
Ages (years)
A
B
C
D
E
F
G
H
I
J
K

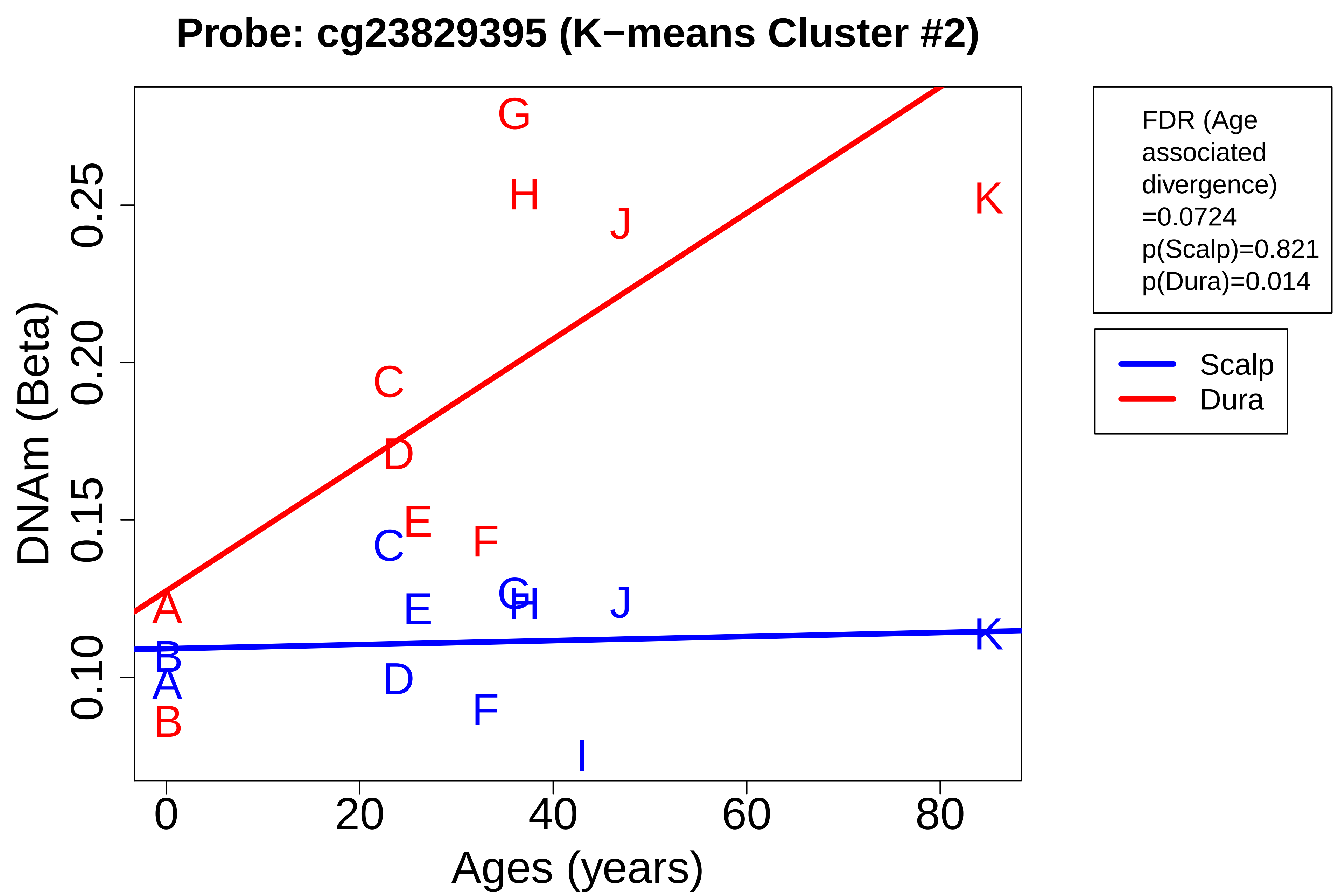
Probe: cg23829395 (K−means Cluster #2)
DNAm (Beta)
0.10
0.15
0.20
0.25
0
20
40
60
80
Ages (years)
A
B
C
D
E
F
G
H
I
J
K
FDR (Age associated divergence) =0.0724
p(Scalp)=0.821
p(Dura)=0.014
Scalp
Dura

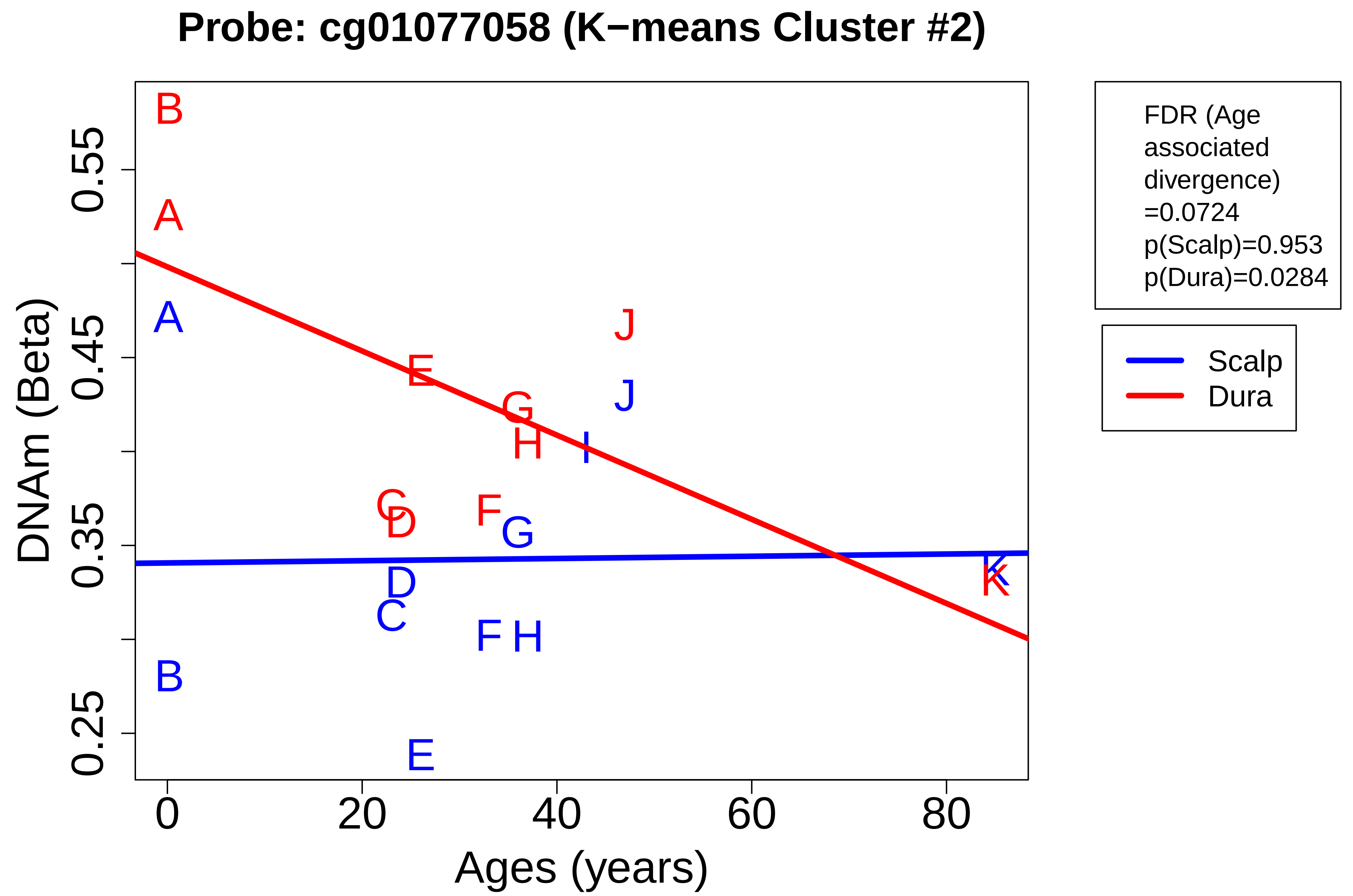

Probe: cg01077058 (K−means Cluster #2)
DNAm (Beta)
0.25
0.35
0.45
0.55
Ages (years)
0
20
40
60
80
FDR (Age associated divergence) =0.0724
p(Scalp)=0.953
p(Dura)=0.0284
Scalp
Dura
A
B
C
D
E
F
G
H
I
J
K

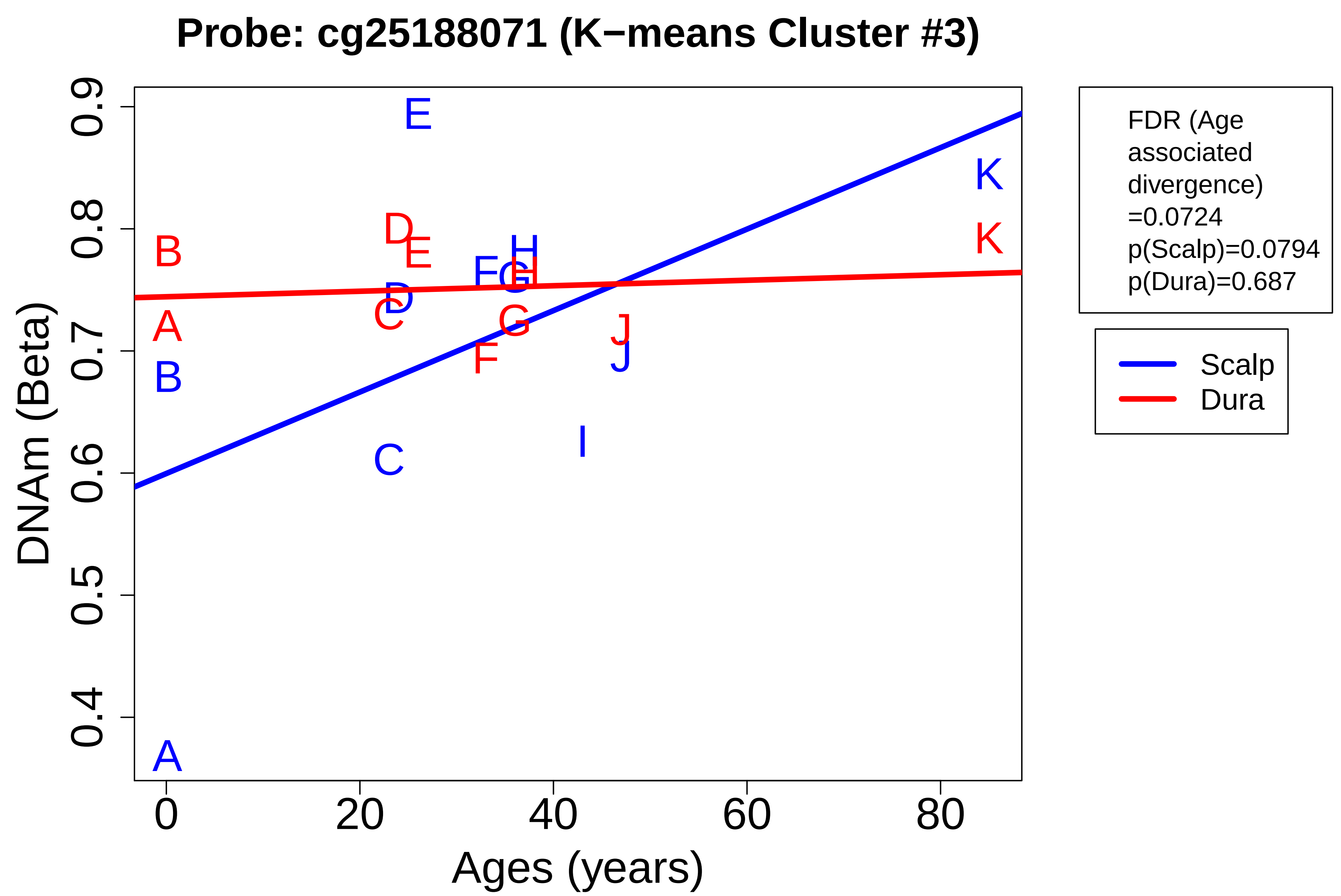

Probe: cg25188071 (K−means Cluster #3)
DNAm (Beta)
Ages (years)
0.4
0.5
0.6
0.7
0.8
0.9
0
20
40
60
80
FDR (Age associated divergence) =0.0724
p(Scalp)=0.0794
p(Dura)=0.687
Scalp
Dura

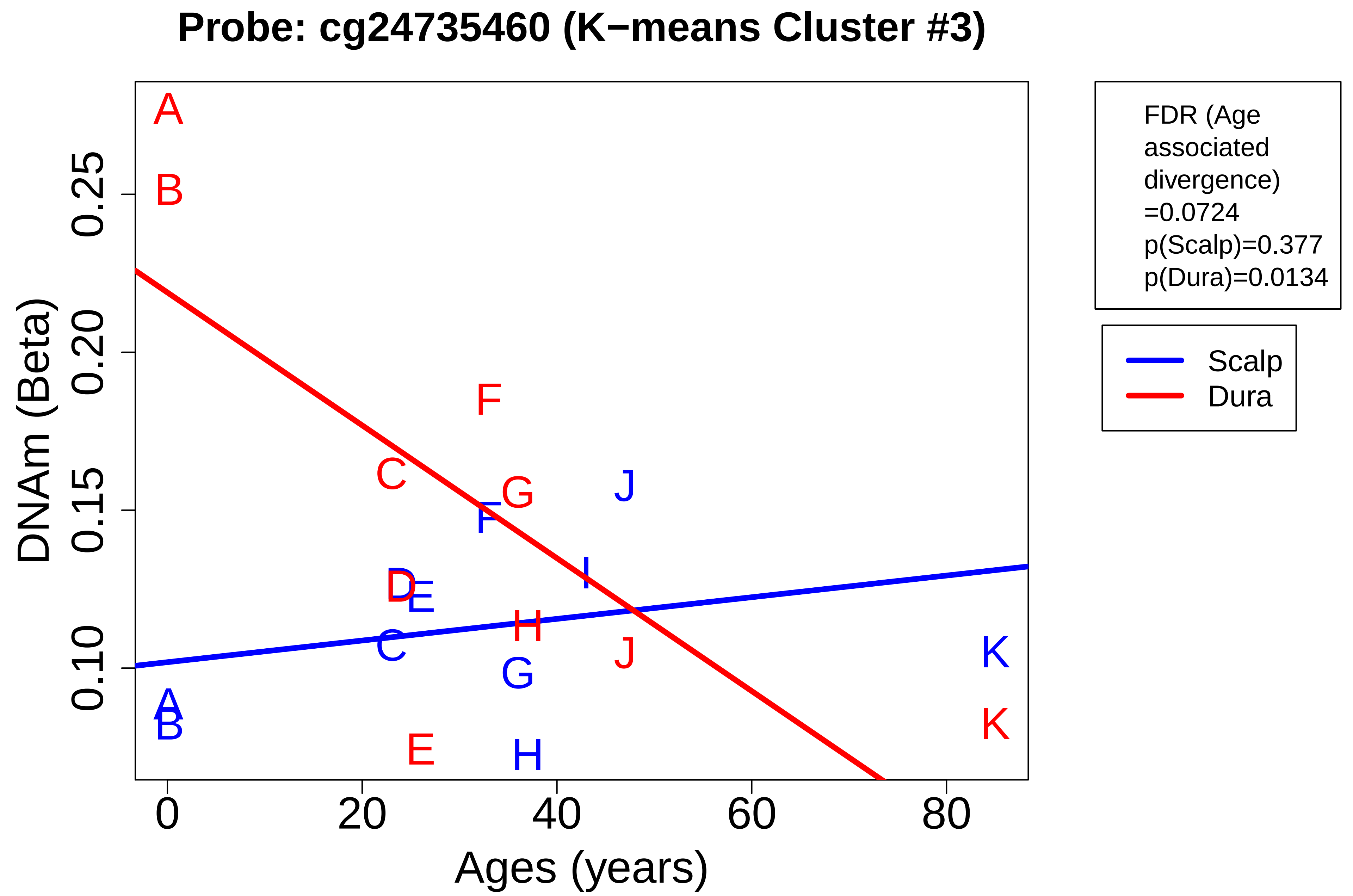

Probe: cg24735460 (K−means Cluster #3)
DNAm (Beta)
0.10
0.15
0.20
0.25
Ages (years)
0
20
40
60
80
A
B
C
D
E
F
G
H
I
J
K
FDR (Age associated divergence) =0.0724
p(Scalp)=0.377
p(Dura)=0.0134
Scalp
Dura

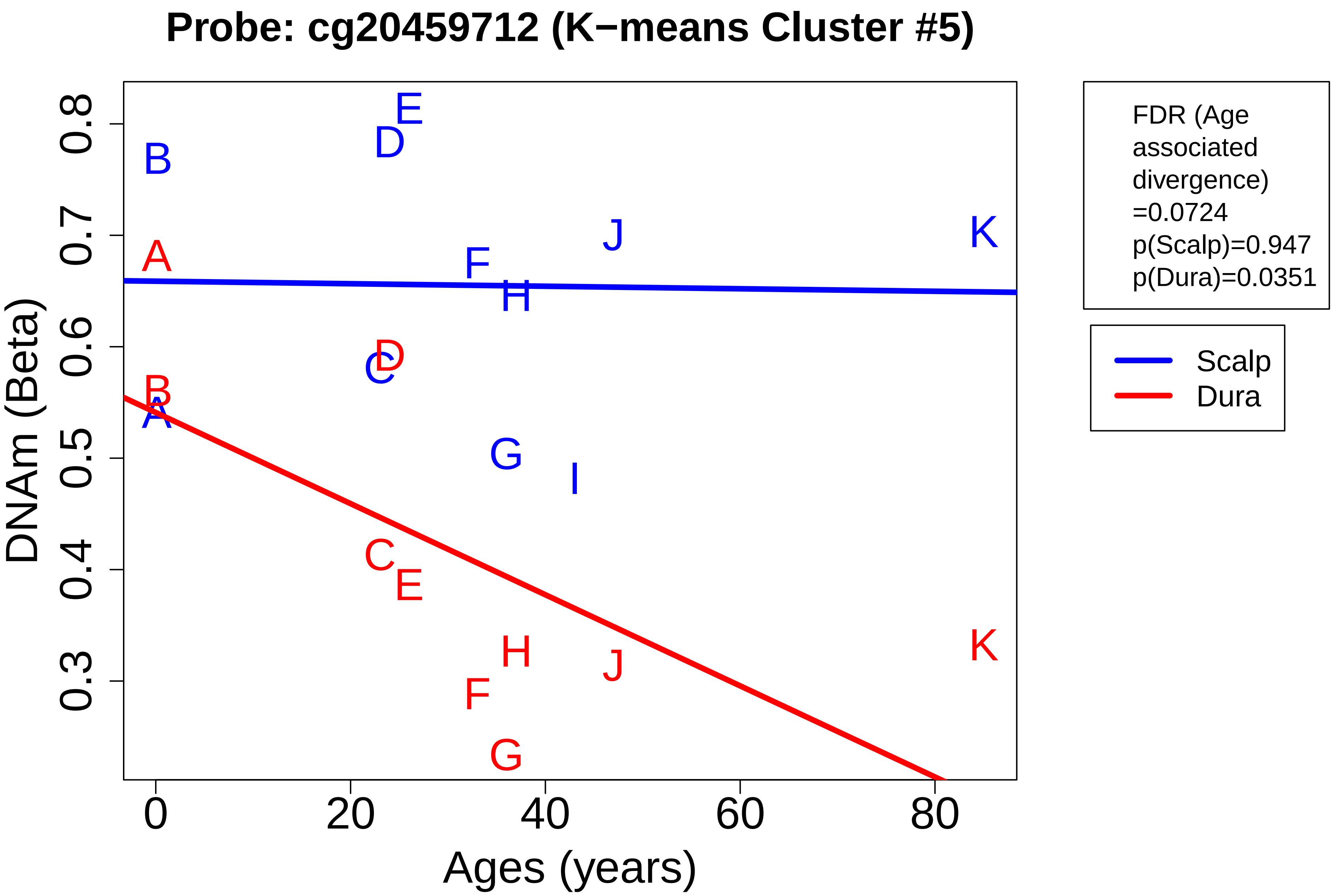
Probe: cg20459712 (K−means Cluster #5)
DNAm (Beta)
0.3
0.4
0.5
0.6
0.7
0.8
0
20
40
60
80
Ages (years)
A
B
C
D
E
F
G
H
I
J
K
A
B
C
D
E
F
G
H
J
K
FDR (Age
associated
divergence)
=0.0724
p(Scalp)=0.947
p(Dura)=0.0351
Scalp
Dura

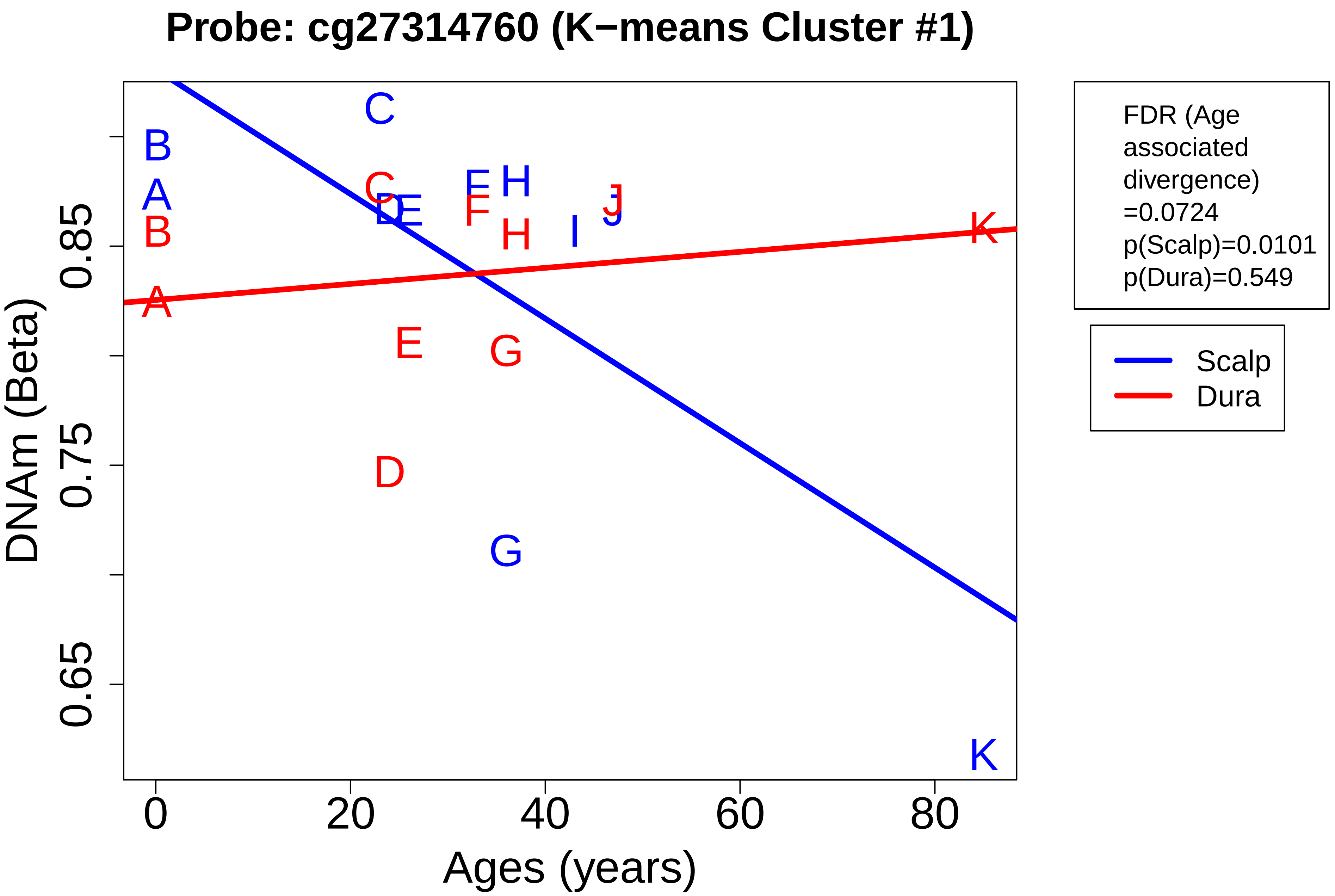

Probe: cg27314760 (K−means Cluster #1)
DNAm (Beta)
0.65
0.75
0.85
0
20
40
60
80
Ages (years)
A
B
C
D
E
F
G
H
I
J
K
FDR (Age associated divergence) =0.0724
p(Scalp)=0.0101
p(Dura)=0.549
Scalp
Dura

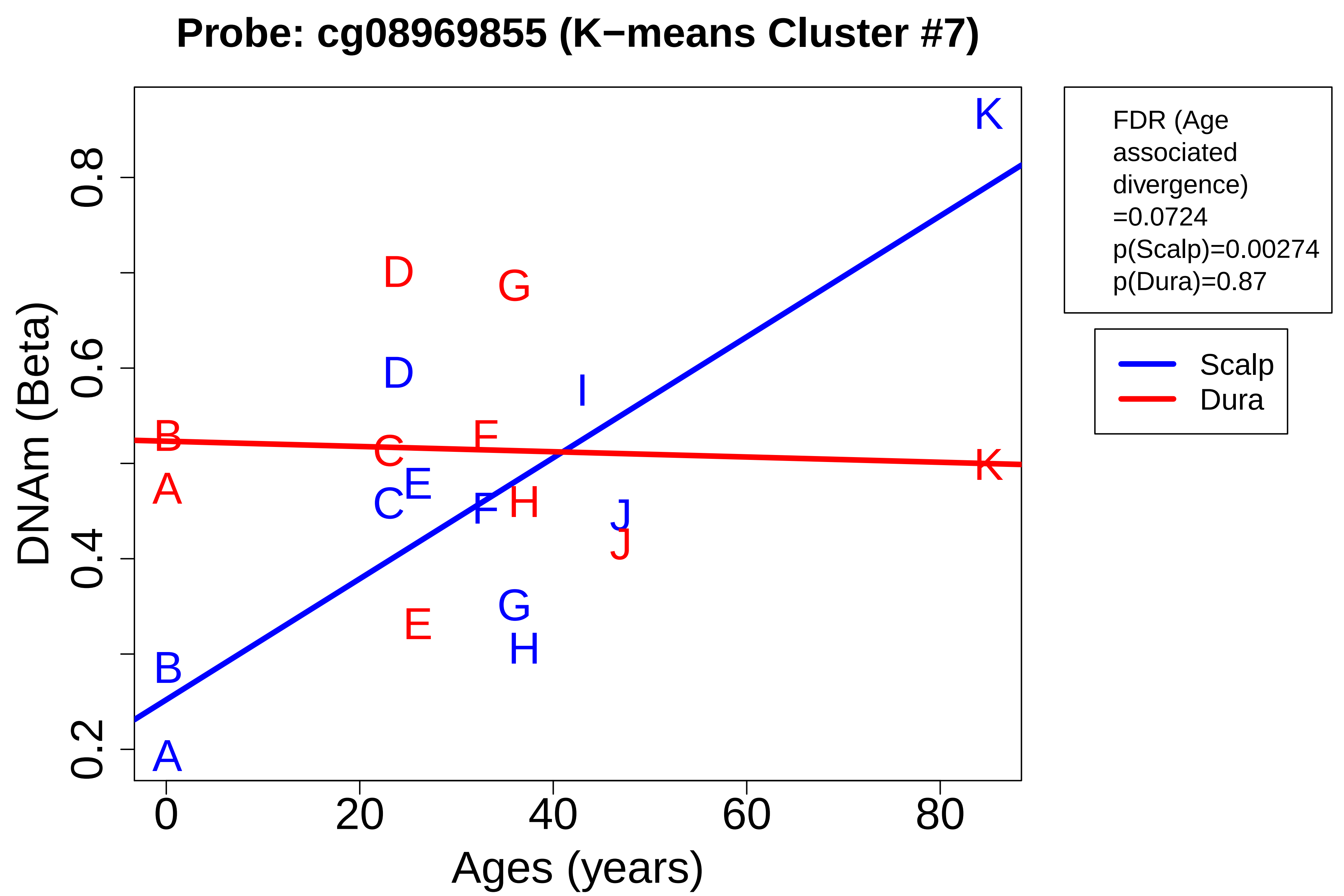

Probe: cg08969855 (K−means Cluster #7)
DNAm (Beta)
Ages (years)
0.2
0.4
0.6
0.8
0
20
40
60
80
FDR (Age associated divergence) =0.0724
p(Scalp)=0.00274
p(Dura)=0.87
Scalp
Dura

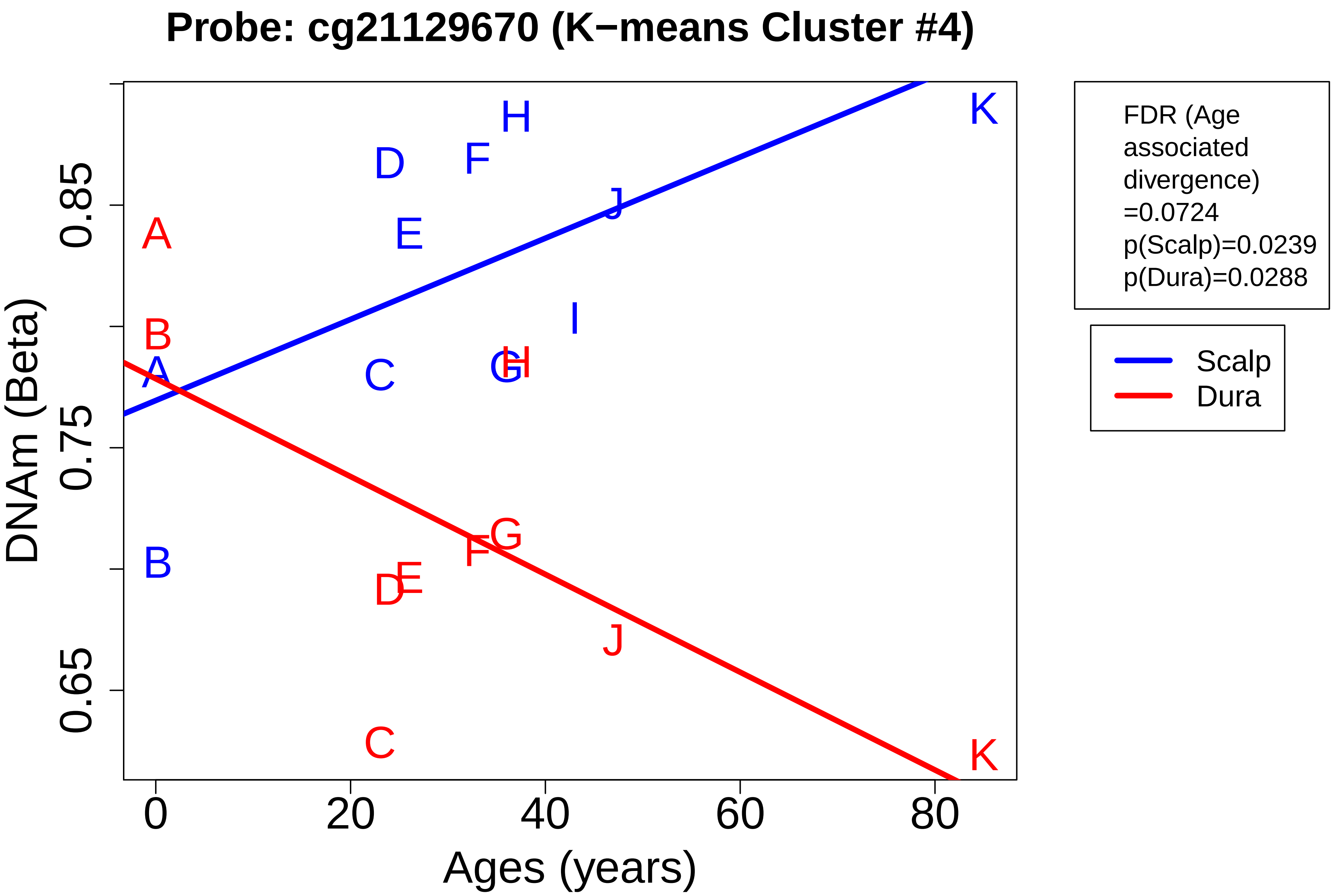

Probe: cg21129670 (K−means Cluster #4)
DNAm (Beta)
0.65
0.75
0.85
Ages (years)
0
20
40
60
80
A
B
C
D
E
F
G
H
I
J
K
FDR (Age associated divergence) =0.0724
p(Scalp)=0.0239
p(Dura)=0.0288
Scalp
Dura

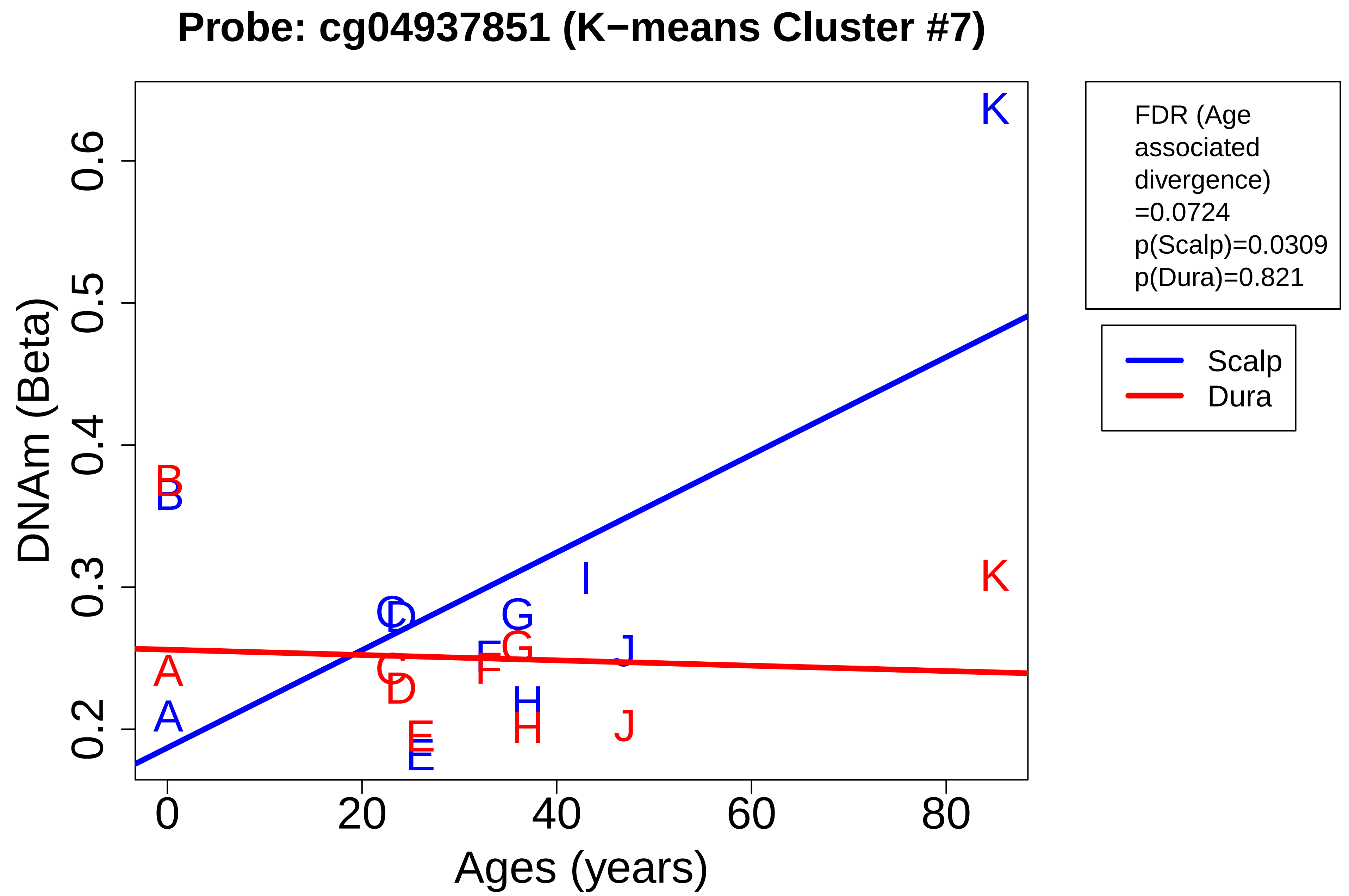

Probe: cg04937851 (K−means Cluster #7)
DNAm (Beta)
0.2
0.3
0.4
0.5
0.6
0
20
40
60
80
Ages (years)
FDR (Age associated divergence) =0.0724
p(Scalp)=0.0309
p(Dura)=0.821
Scalp
Dura

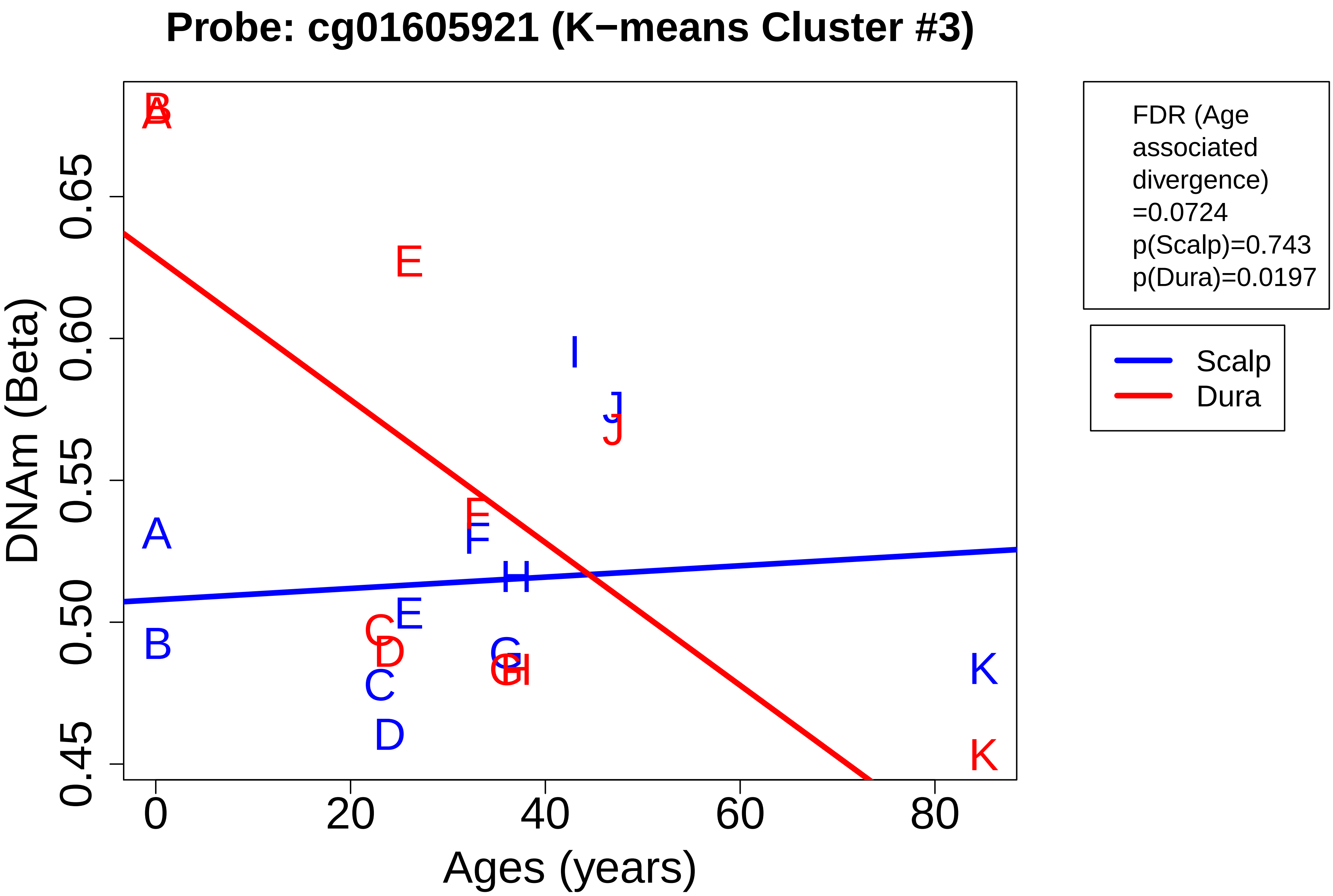

Probe: cg01605921 (K−means Cluster #3)
DNAm (Beta)
Ages (years)
0.45
0.50
0.55
0.60
0.65
0
20
40
60
80
FDR (Age associated divergence) =0.0724
p(Scalp)=0.743
p(Dura)=0.0197
Scalp
Dura

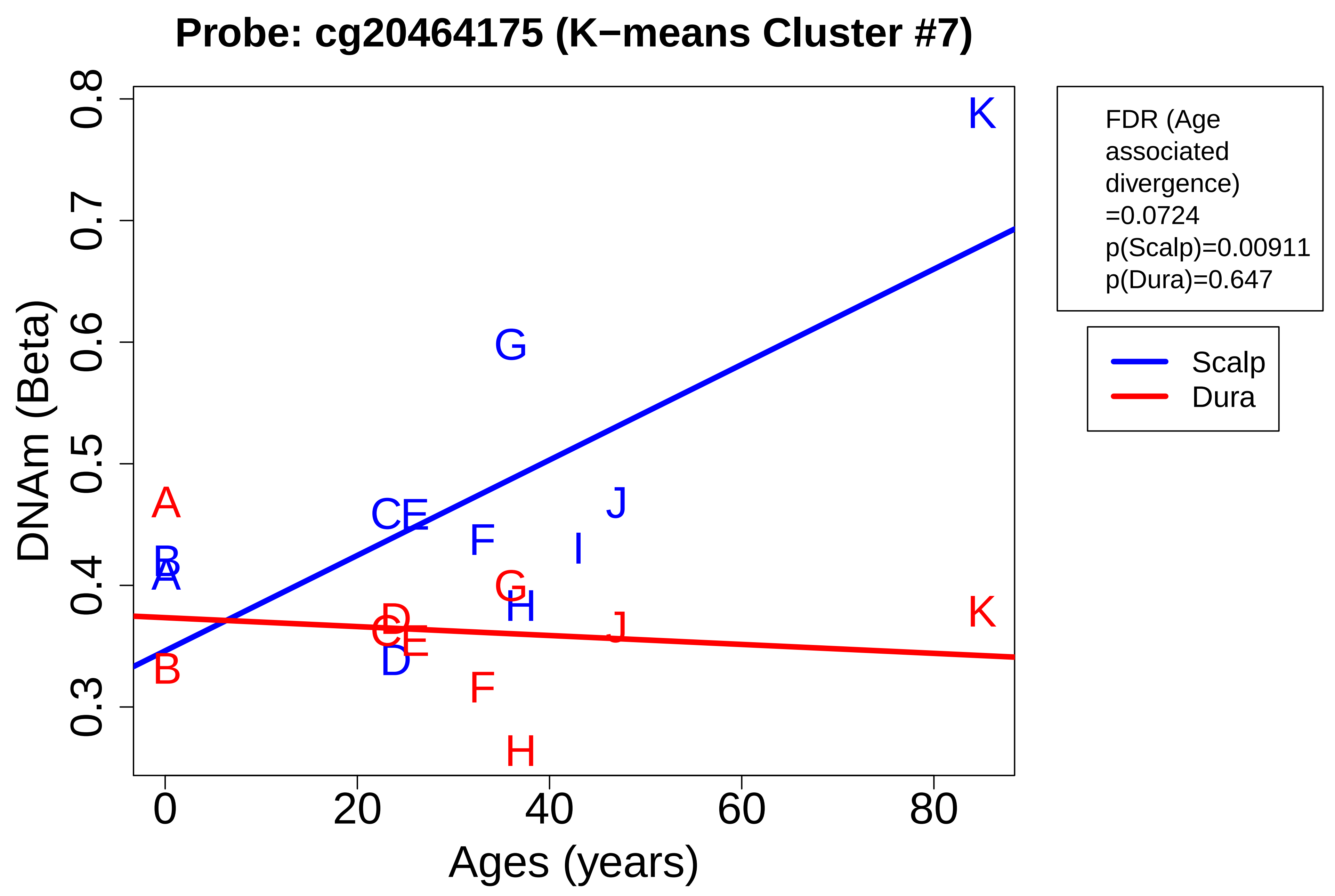

Probe: cg20464175 (K−means Cluster #7)
DNAm (Beta)
0.3
0.4
0.5
0.6
0.7
0.8
Ages (years)
0
20
40
60
80
FDR (Age associated divergence) =0.0724
p(Scalp)=0.00911
p(Dura)=0.647
Scalp
Dura

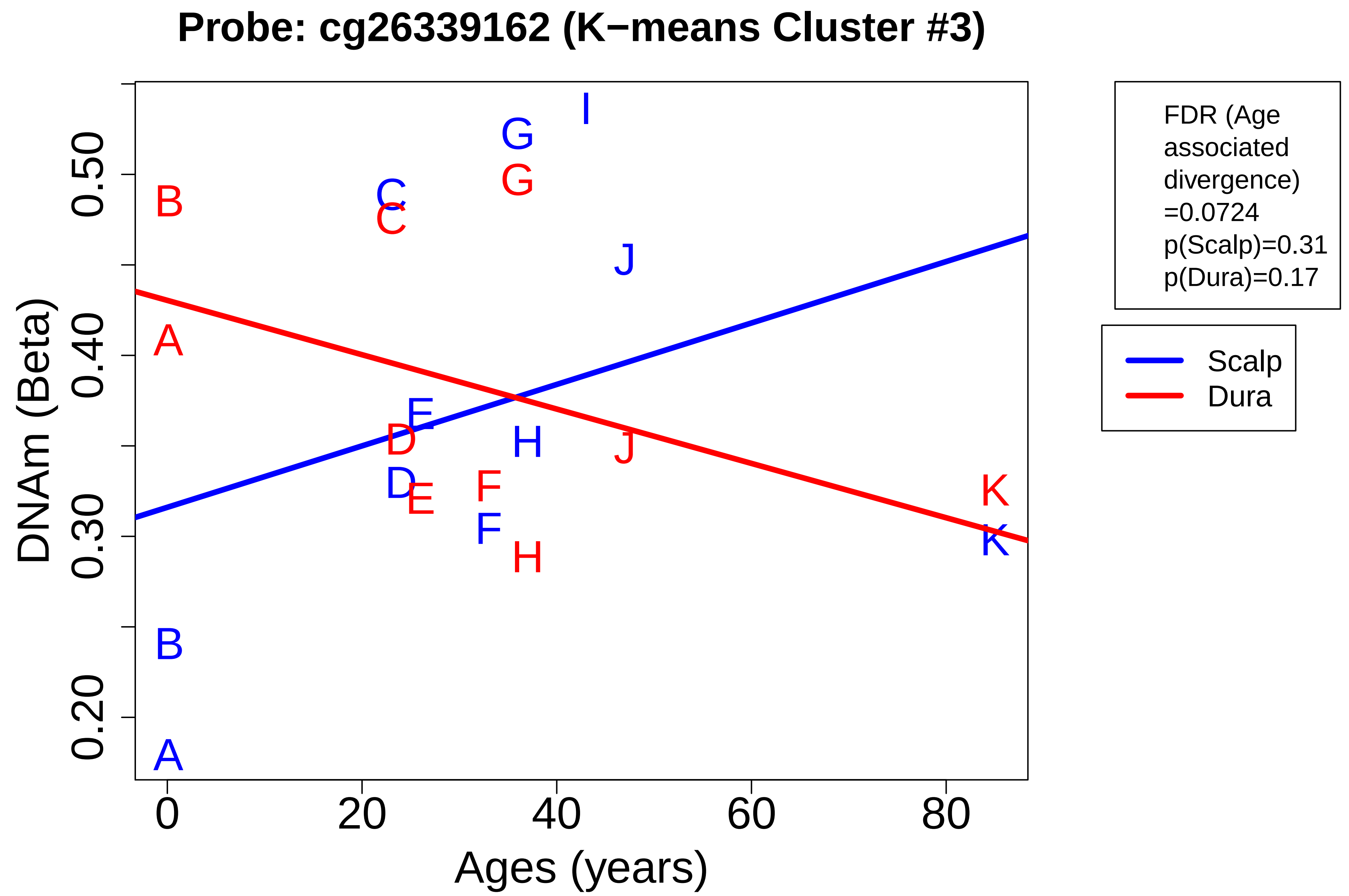

Probe: cg26339162 (K−means Cluster #3)
DNAm (Beta)
0.20
0.30
0.40
0.50
0
20
40
60
80
Ages (years)
A
B
C
D
E
F
G
H
I
J
K
FDR (Age associated divergence) =0.0724
p(Scalp)=0.31
p(Dura)=0.17
Scalp
Dura

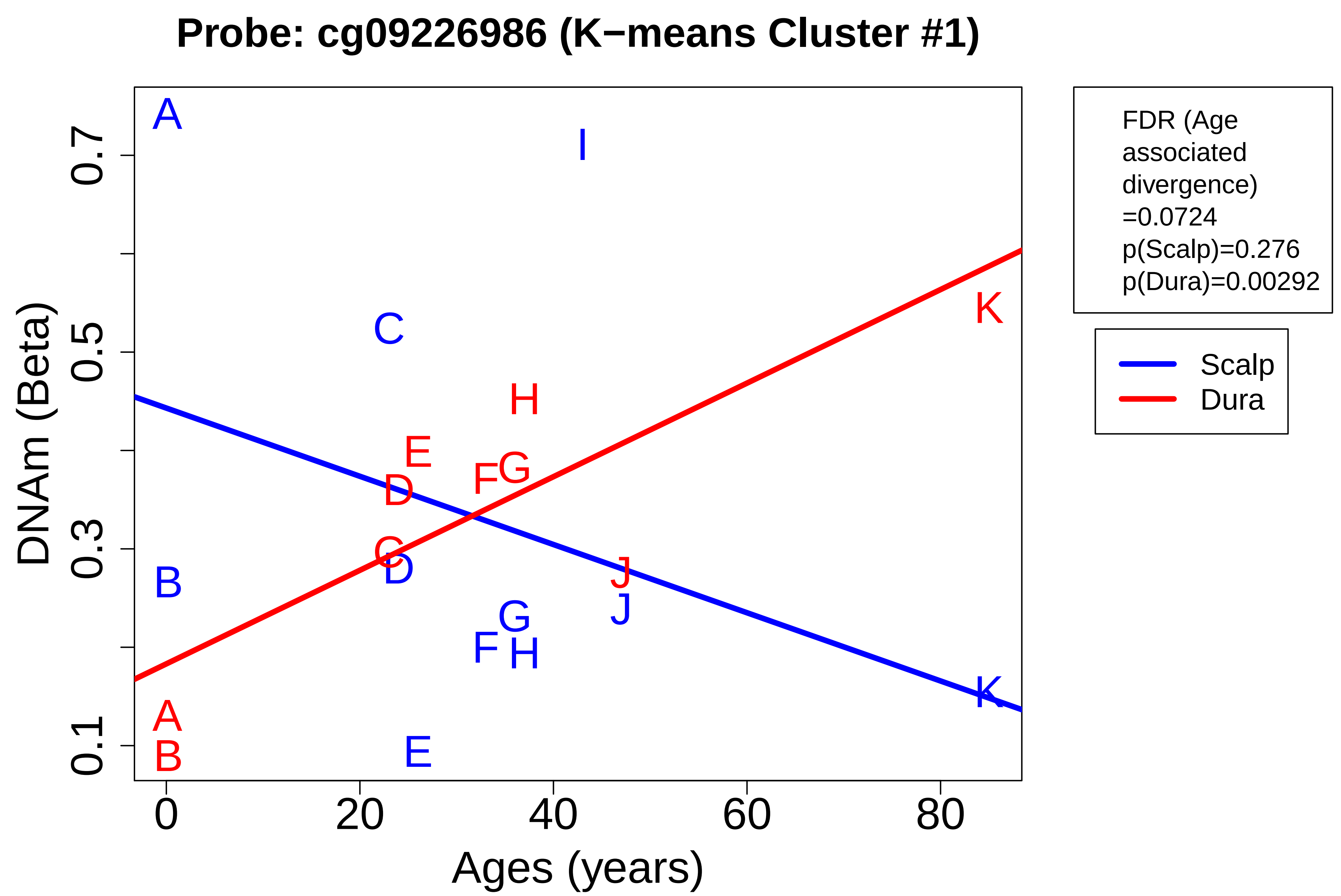

Probe: cg09226986 (K−means Cluster #1)
DNAm (Beta)
Ages (years)
0.1
0.3
0.5
0.7
0
20
40
60
80
FDR (Age associated divergence) =0.0724
p(Scalp)=0.276
p(Dura)=0.00292
Scalp
Dura
A
B
C
D
E
F
G
H
I
J
K

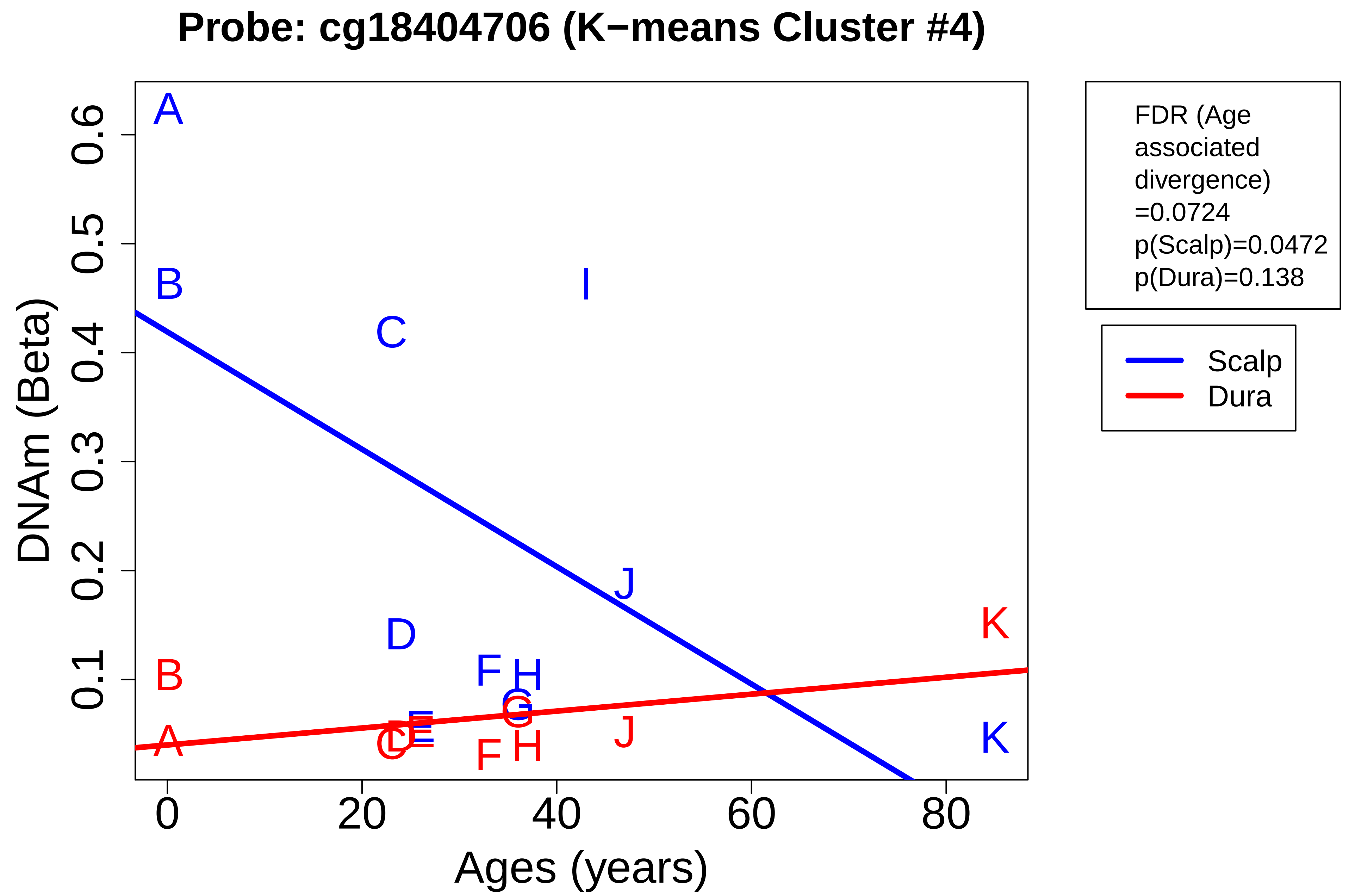

Probe: cg18404706 (K−means Cluster #4)
DNAm (Beta)
0.1
0.2
0.3
0.4
0.5
0.6
0
20
40
60
80
Ages (years)
A
B
C
D
E
F
G
H
I
J
K
FDR (Age associated divergence) =0.0724
p(Scalp)=0.0472
p(Dura)=0.138
Scalp
Dura

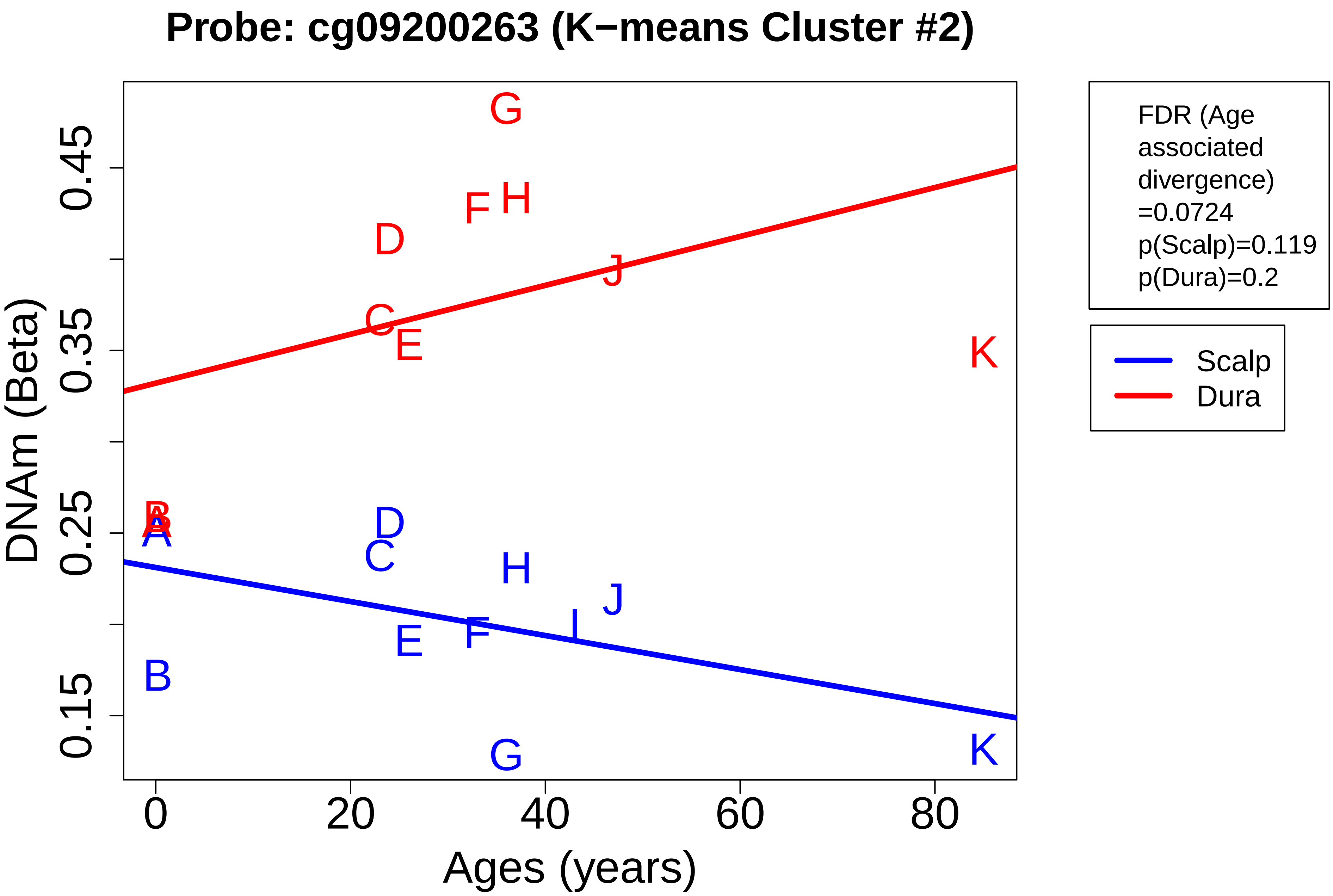

Probe: cg09200263 (K−means Cluster #2)
DNAm (Beta)
0.15
0.25
0.35
0.45
0
20
40
60
80
Ages (years)
FDR (Age associated divergence) =0.0724
p(Scalp)=0.119
p(Dura)=0.2
Scalp
Dura

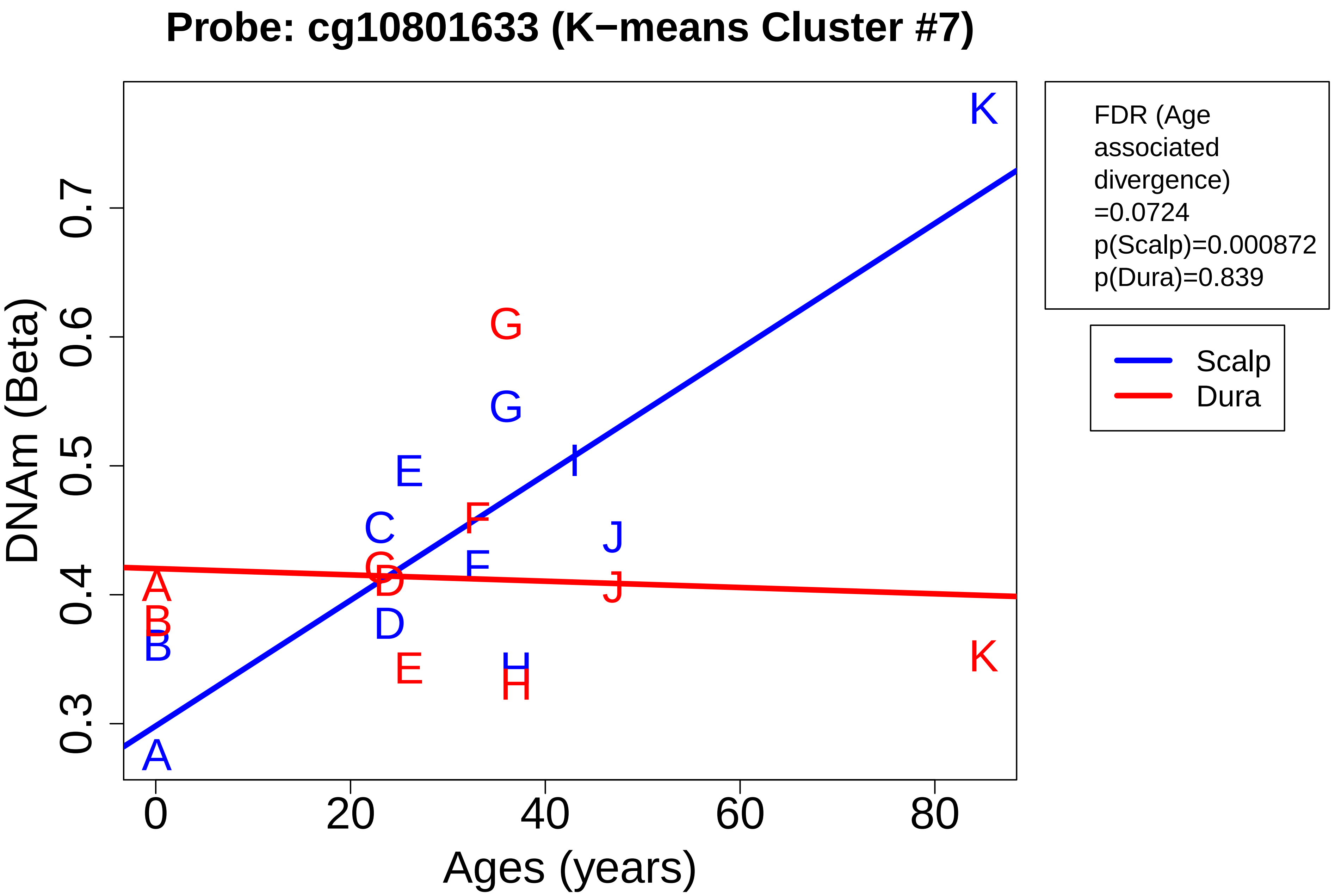

Probe: cg10801633 (K−means Cluster #7)
DNAm (Beta)
0.3
0.4
0.5
0.6
0.7
0
20
40
60
80
Ages (years)
FDR (Age associated divergence) =0.0724
p(Scalp)=0.000872
p(Dura)=0.839
Scalp
Dura
A
B
C
D
E
F
G
H
I
J
K

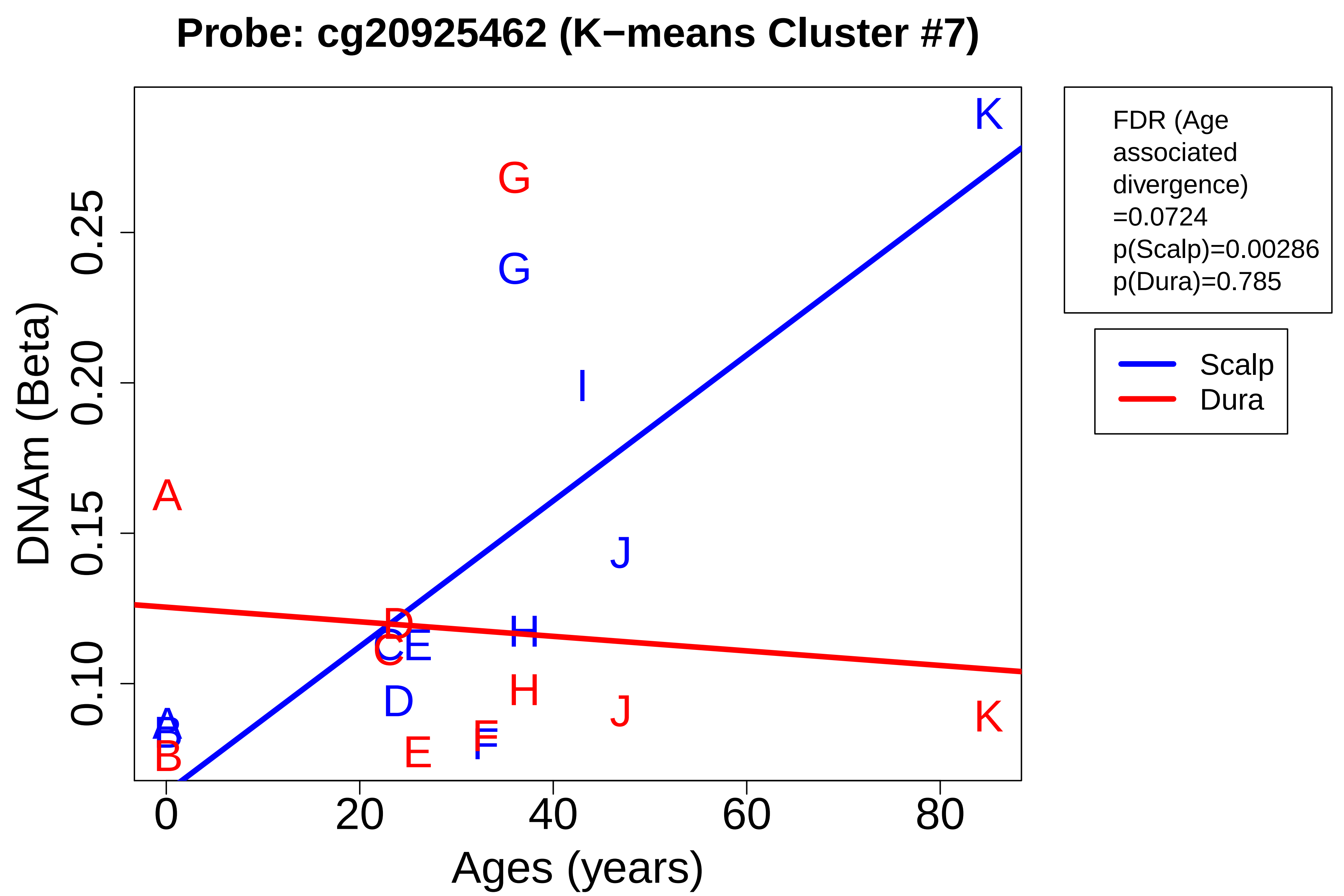

Probe: cg20925462 (K−means Cluster #7)
DNAm (Beta)
0.10
0.15
0.20
0.25
0
20
40
60
80
Ages (years)
A
B
C
D
E
F
G
H
I
J
K
FDR (Age associated divergence) =0.0724
p(Scalp)=0.00286
p(Dura)=0.785
Scalp
Dura

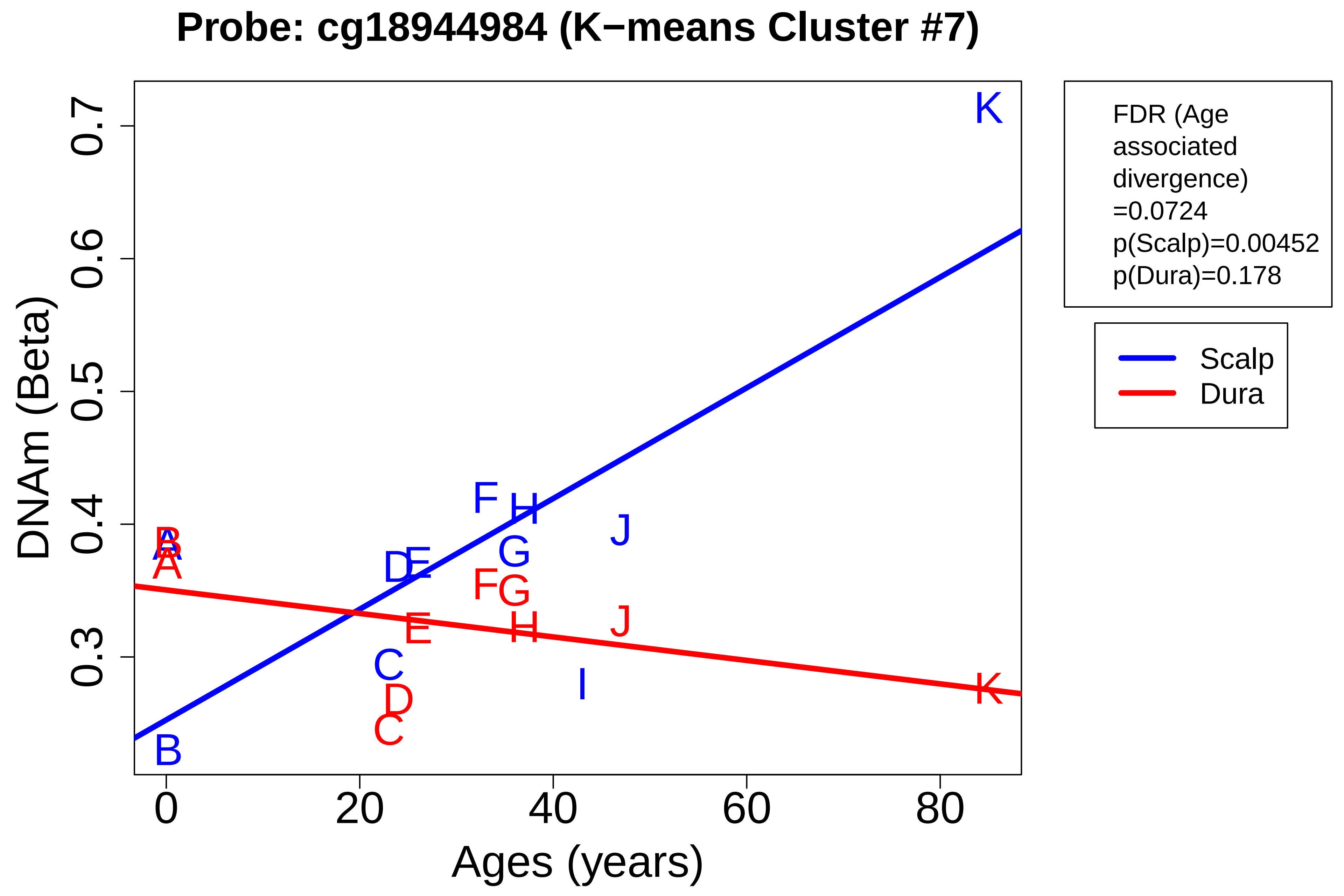

Probe: cg18944984 (K−means Cluster #7)
DNAm (Beta)
0.3
0.4
0.5
0.6
0.7
0
20
40
60
80
Ages (years)
A
B
C
D
E
F
G
H
I
J
K
FDR (Age associated divergence) =0.0724
p(Scalp)=0.00452
p(Dura)=0.178
Scalp
Dura

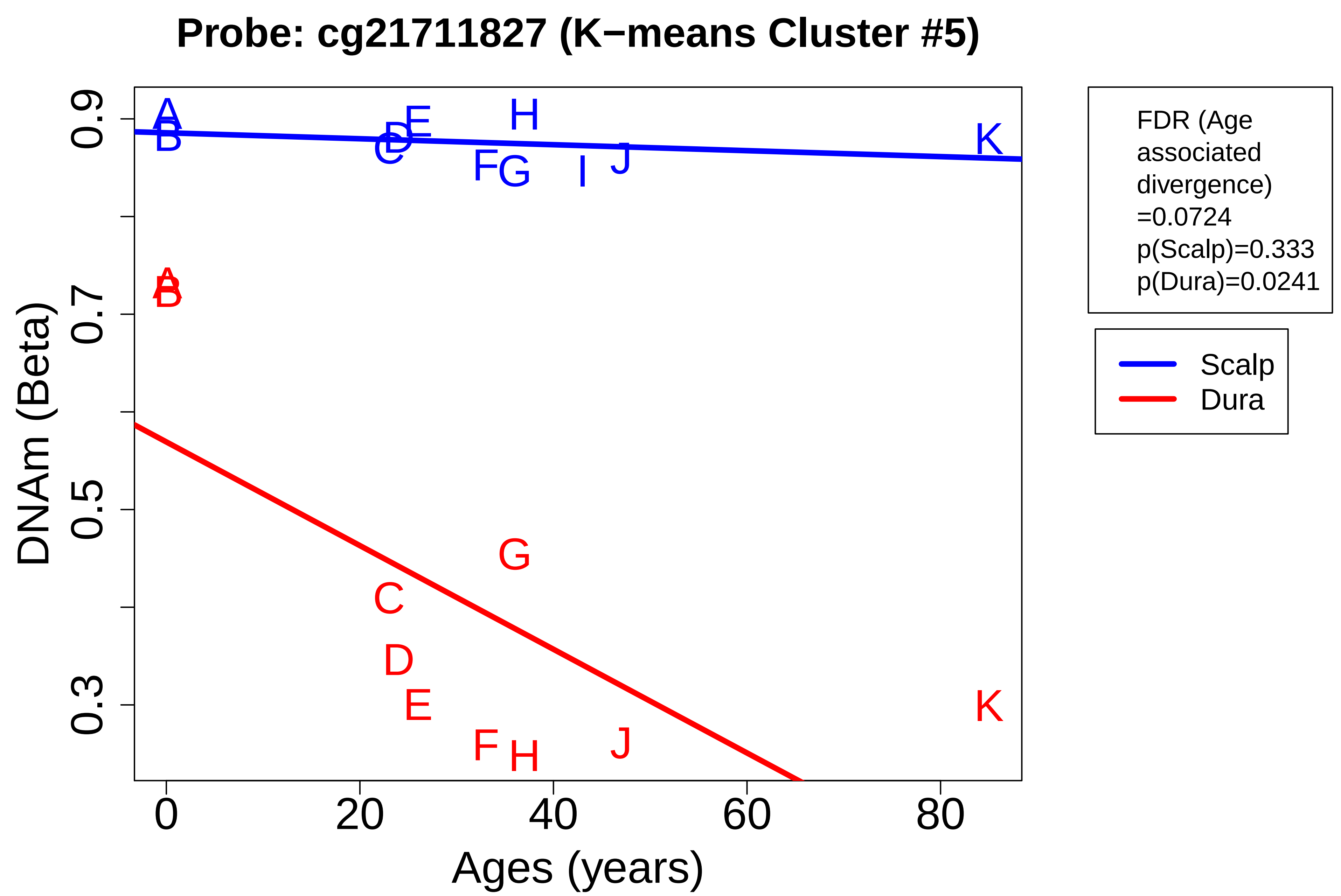

Probe: cg21711827 (K−means Cluster #5)
DNAm (Beta)
0.3
0.5
0.7
0.9
Ages (years)
0
20
40
60
80
A
B
C
D
E
F
G
H
I
J
K
FDR (Age associated divergence) =0.0724
p(Scalp)=0.333
p(Dura)=0.0241
Scalp
Dura

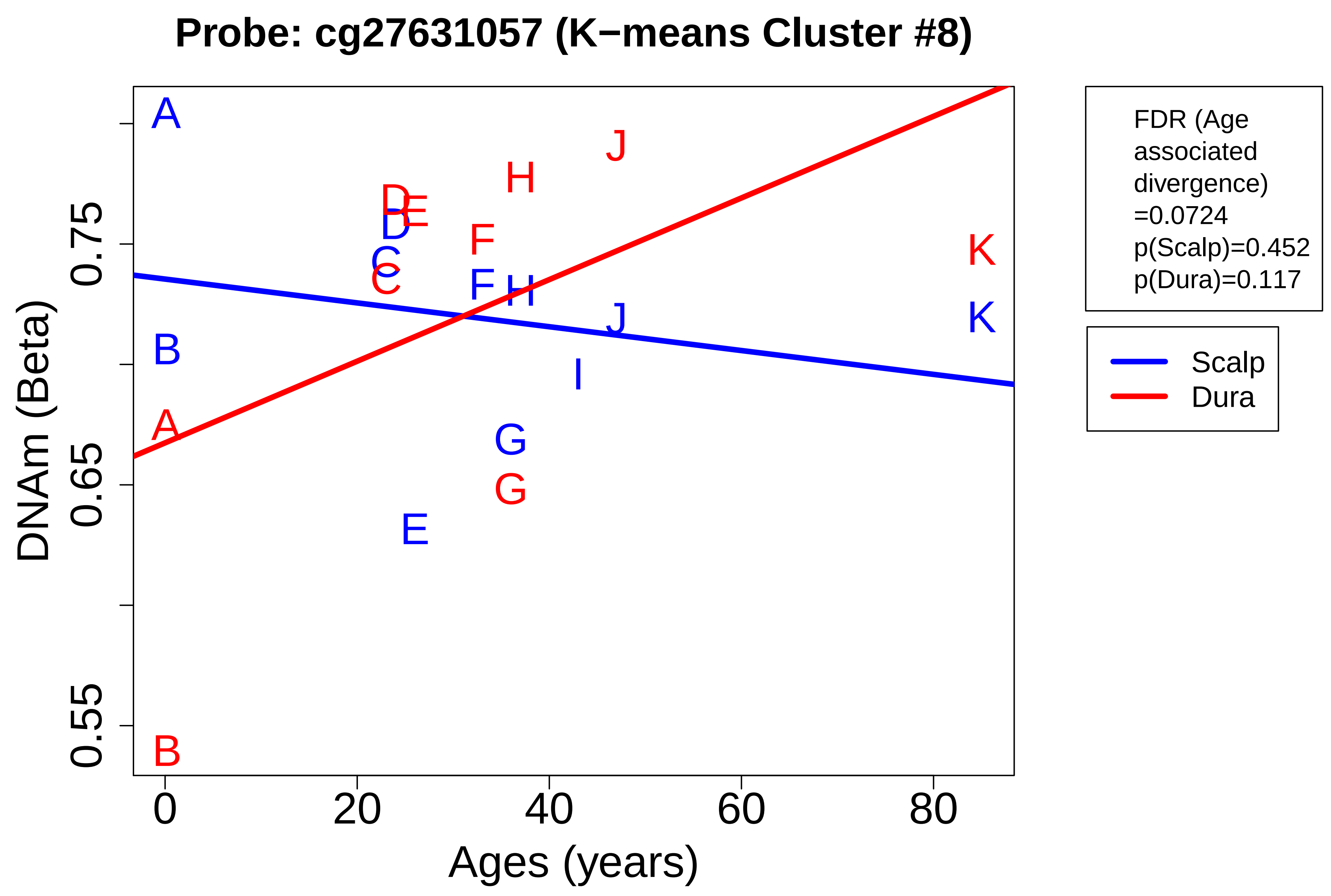

Probe: cg27631057 (K−means Cluster #8)
DNAm (Beta)
0.55
0.65
0.75
Ages (years)
0
20
40
60
80
FDR (Age associated divergence) =0.0724
p(Scalp)=0.452
p(Dura)=0.117
Scalp
Dura

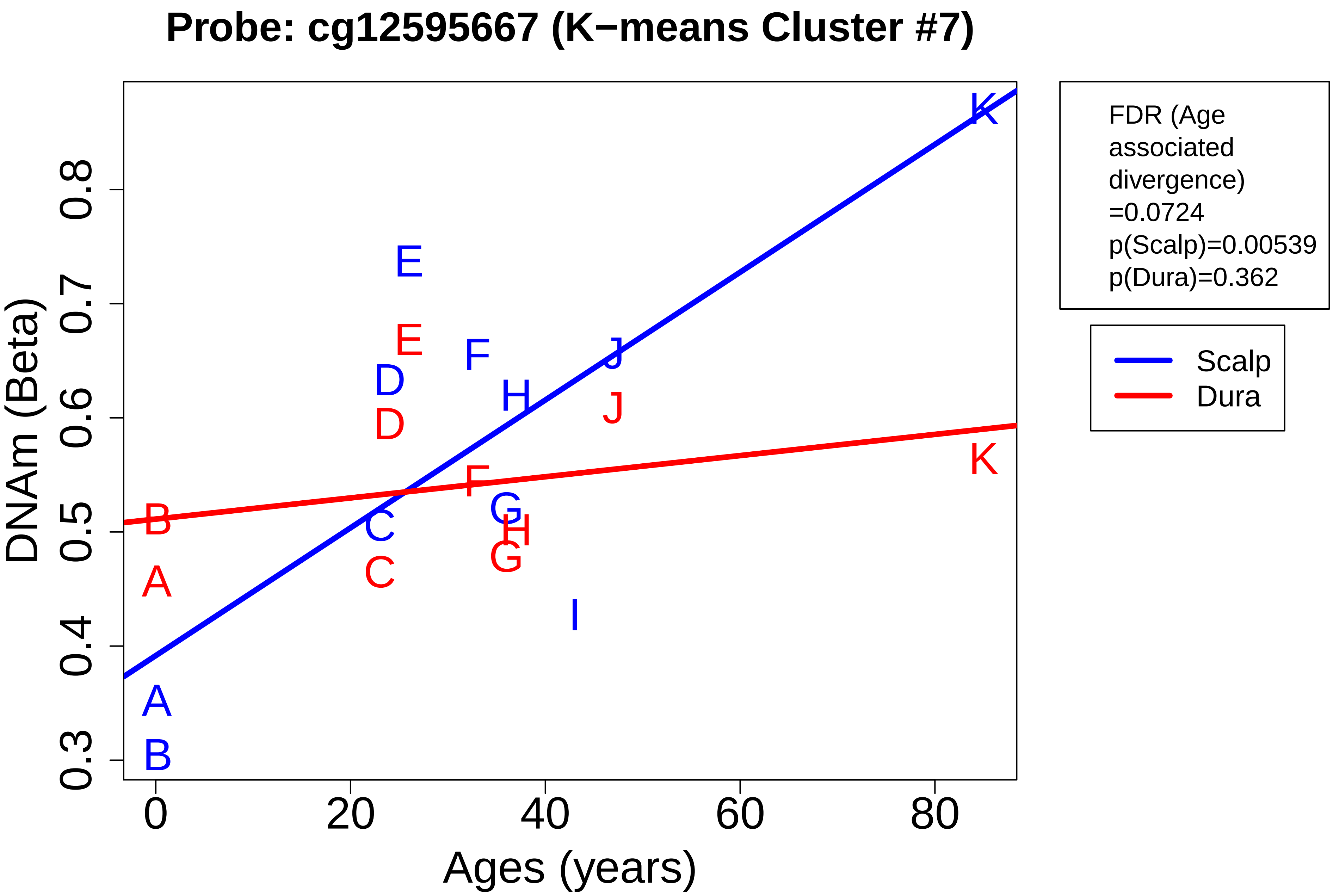

Probe: cg12595667 (K−means Cluster #7)
DNAm (Beta)
0.3
0.4
0.5
0.6
0.7
0.8
0
20
40
60
80
Ages (years)
A
B
C
D
E
F
G
H
I
J
K
FDR (Age associated divergence) =0.0724
p(Scalp)=0.00539
p(Dura)=0.362
Scalp
Dura

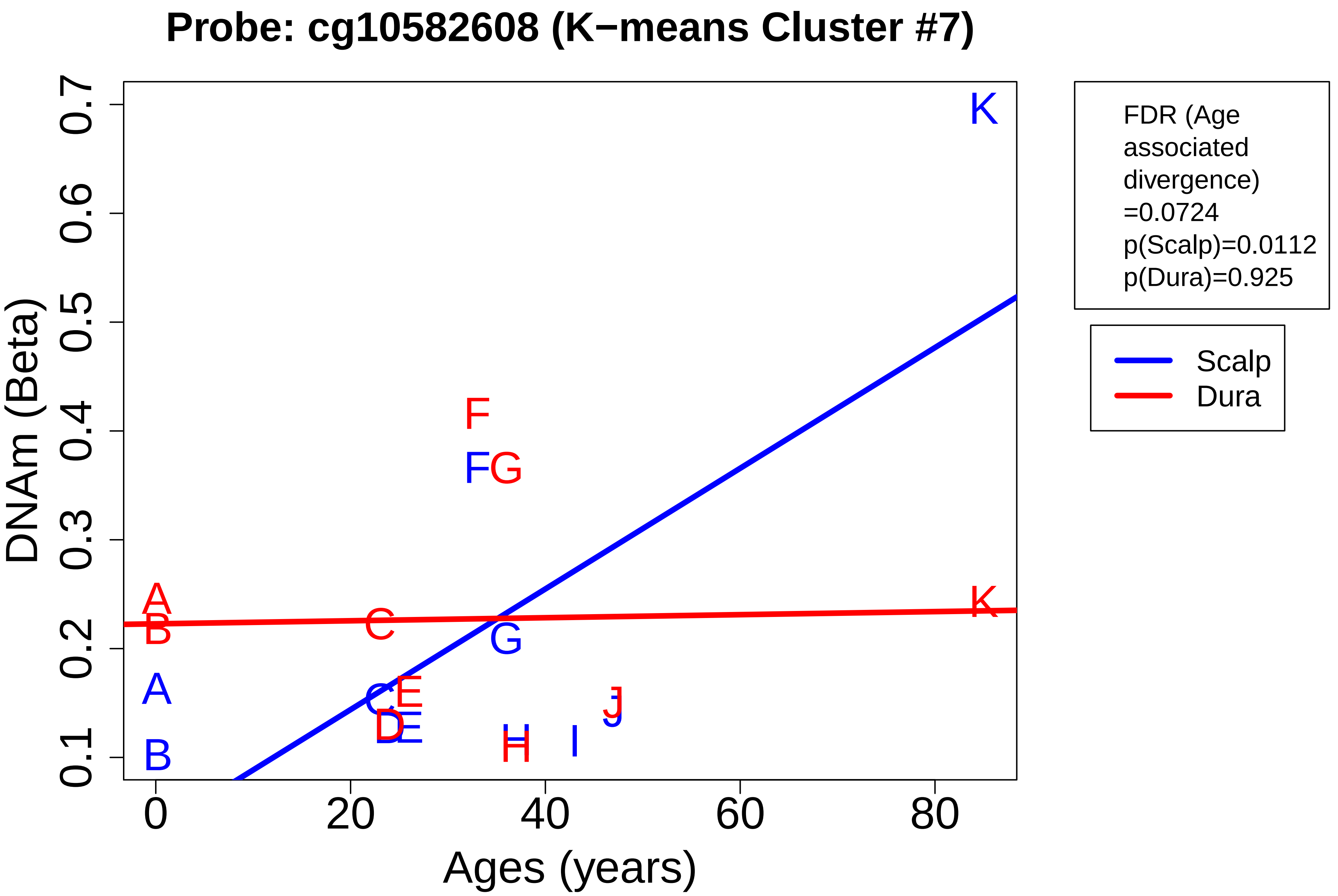
Probe: cg10582608 (K−means Cluster #7)
DNAm (Beta)
0.1
0.2
0.3
0.4
0.5
0.6
0.7
Ages (years)
0
20
40
60
80
FDR (Age associated divergence) =0.0724
p(Scalp)=0.0112
p(Dura)=0.925
Scalp
Dura

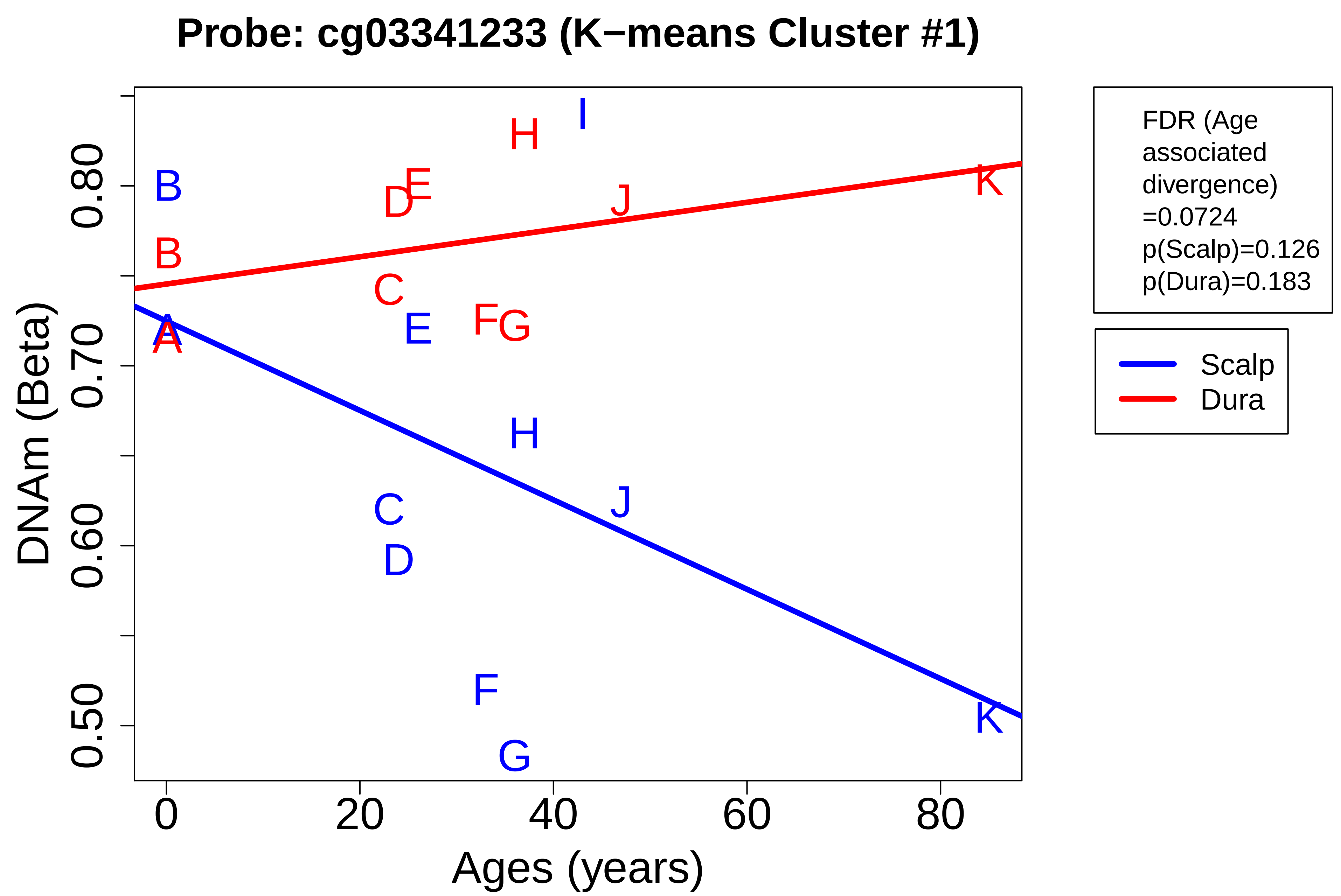

Probe: cg03341233 (K−means Cluster #1)
DNAm (Beta)
0.50
0.60
0.70
0.80
0
20
40
60
80
Ages (years)
FDR (Age associated divergence) =0.0724
p(Scalp)=0.126
p(Dura)=0.183
Scalp
Dura

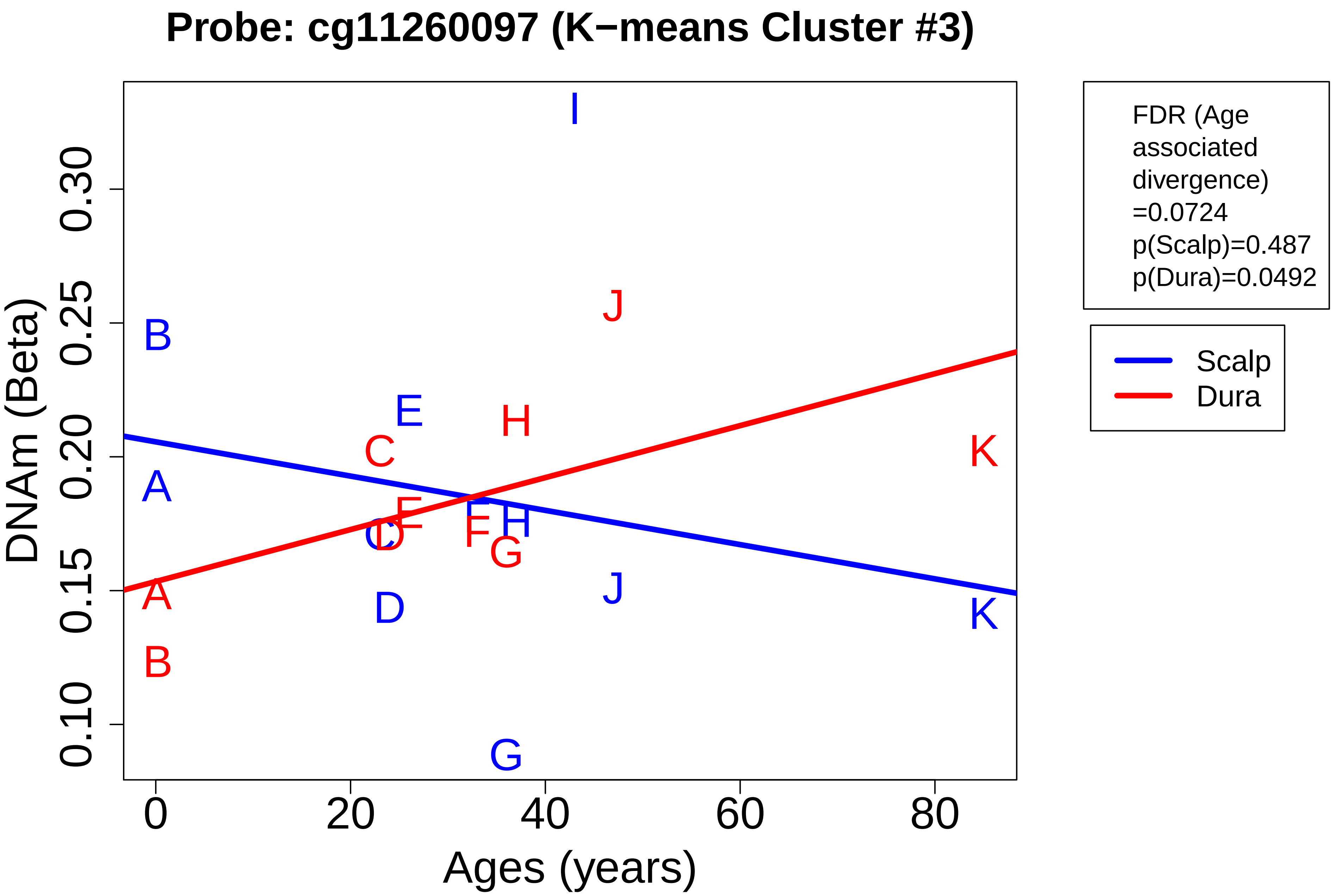

Probe: cg11260097 (K−means Cluster #3)
DNAm (Beta)
0.10
0.15
0.20
0.25
0.30
Ages (years)
0
20
40
60
80
FDR (Age associated divergence) =0.0724
p(Scalp)=0.487
p(Dura)=0.0492
Scalp
Dura

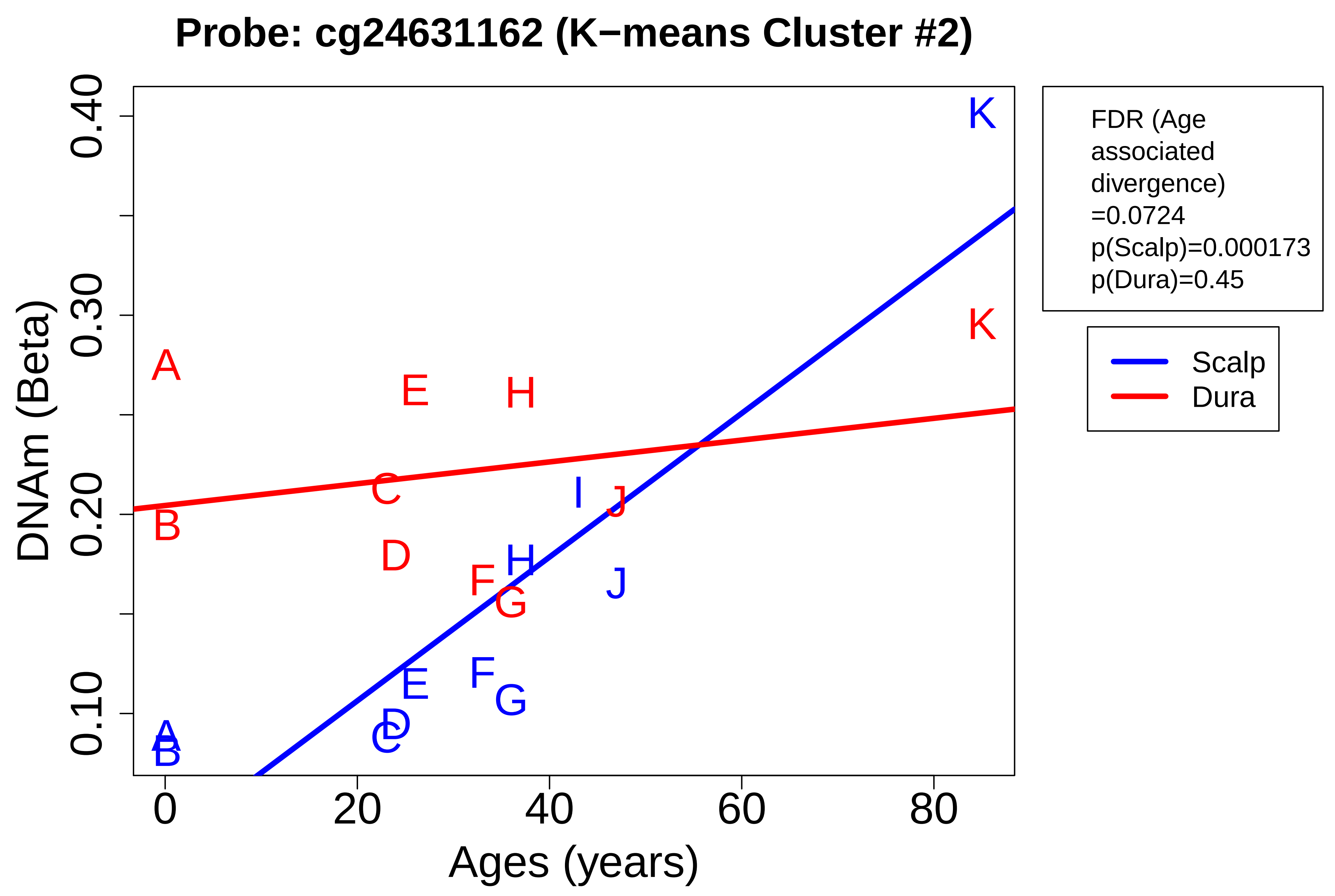

Probe: cg24631162 (K−means Cluster #2)
DNAm (Beta)
0.10
0.20
0.30
0.40
Ages (years)
0
20
40
60
80
A
B
C
D
E
F
G
H
I
J
K
FDR (Age associated divergence) =0.0724
p(Scalp)=0.000173
p(Dura)=0.45
Scalp
Dura

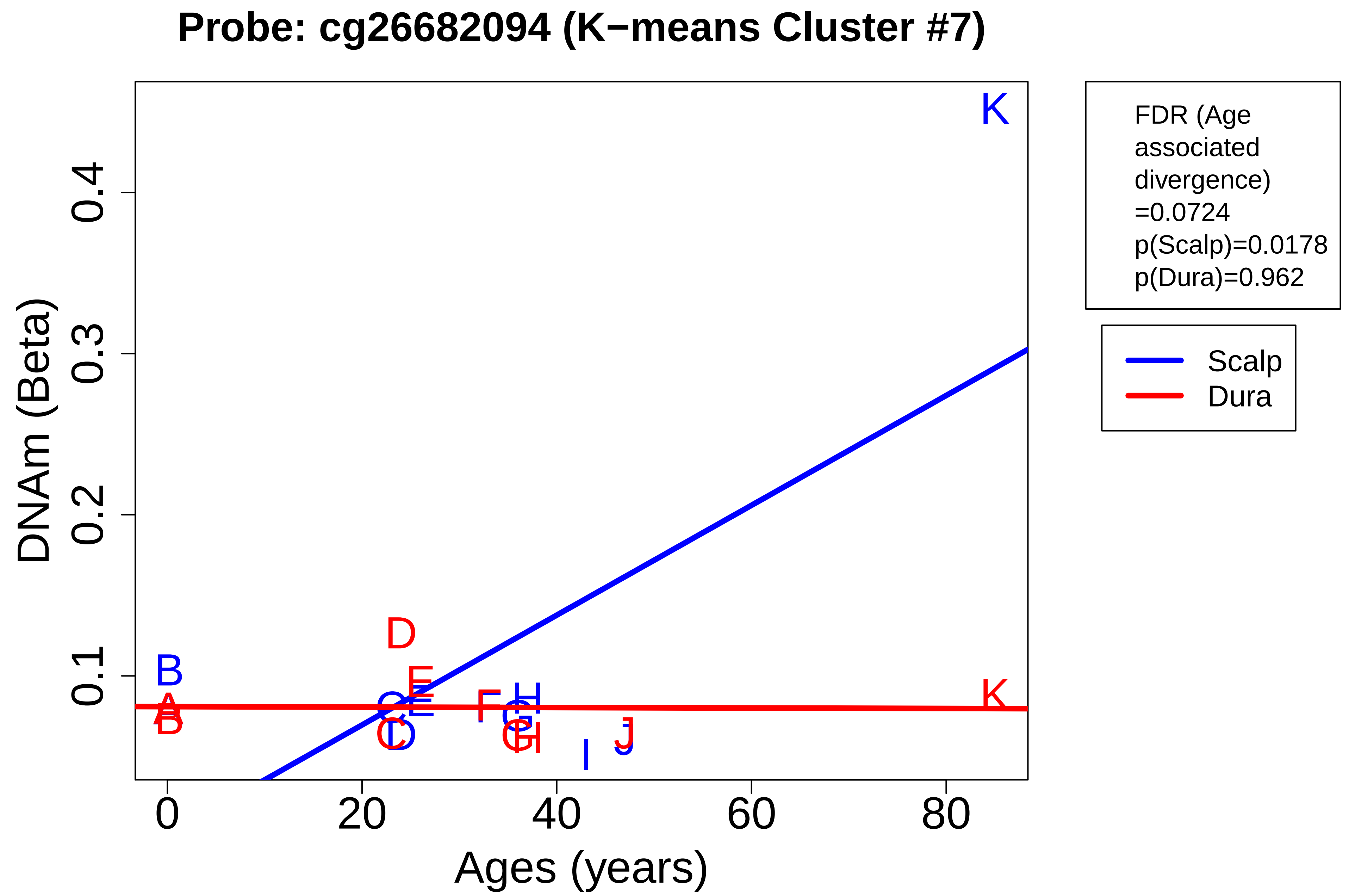

Probe: cg26682094 (K−means Cluster #7)
DNAm (Beta)
0.1
0.2
0.3
0.4
0
20
40
60
80
Ages (years)
A
B
C
D
E
F
G
H
I
J
K
FDR (Age associated divergence) =0.0724
p(Scalp)=0.0178
p(Dura)=0.962
Scalp
Dura

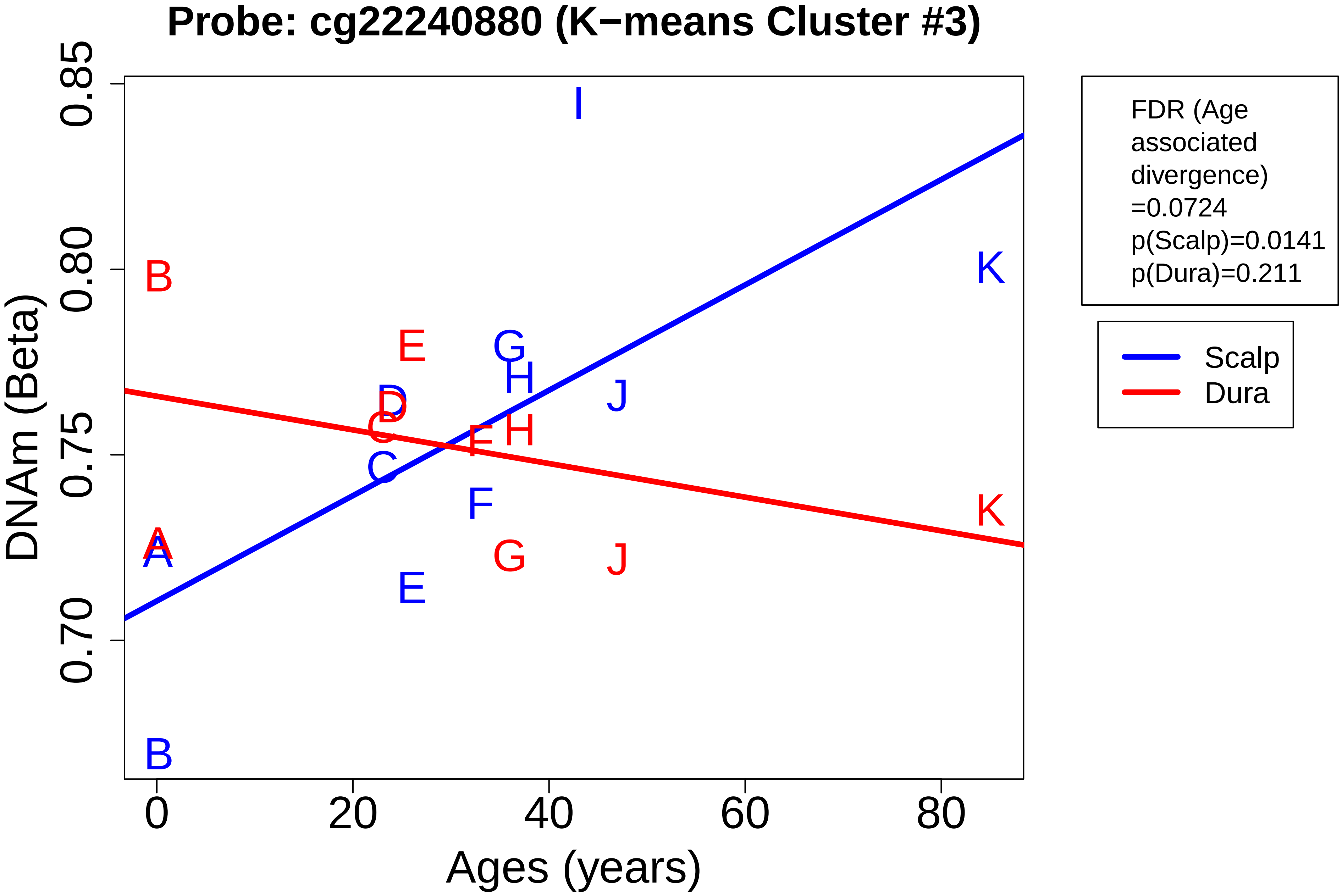

Probe: cg22240880 (K−means Cluster #3)
DNAm (Beta)
0.70
0.75
0.80
0.85
Ages (years)
0
20
40
60
80
FDR (Age associated divergence) =0.0724
p(Scalp)=0.0141
p(Dura)=0.211
Scalp
Dura
A
B
C
D
E
F
G
H
I
J
K

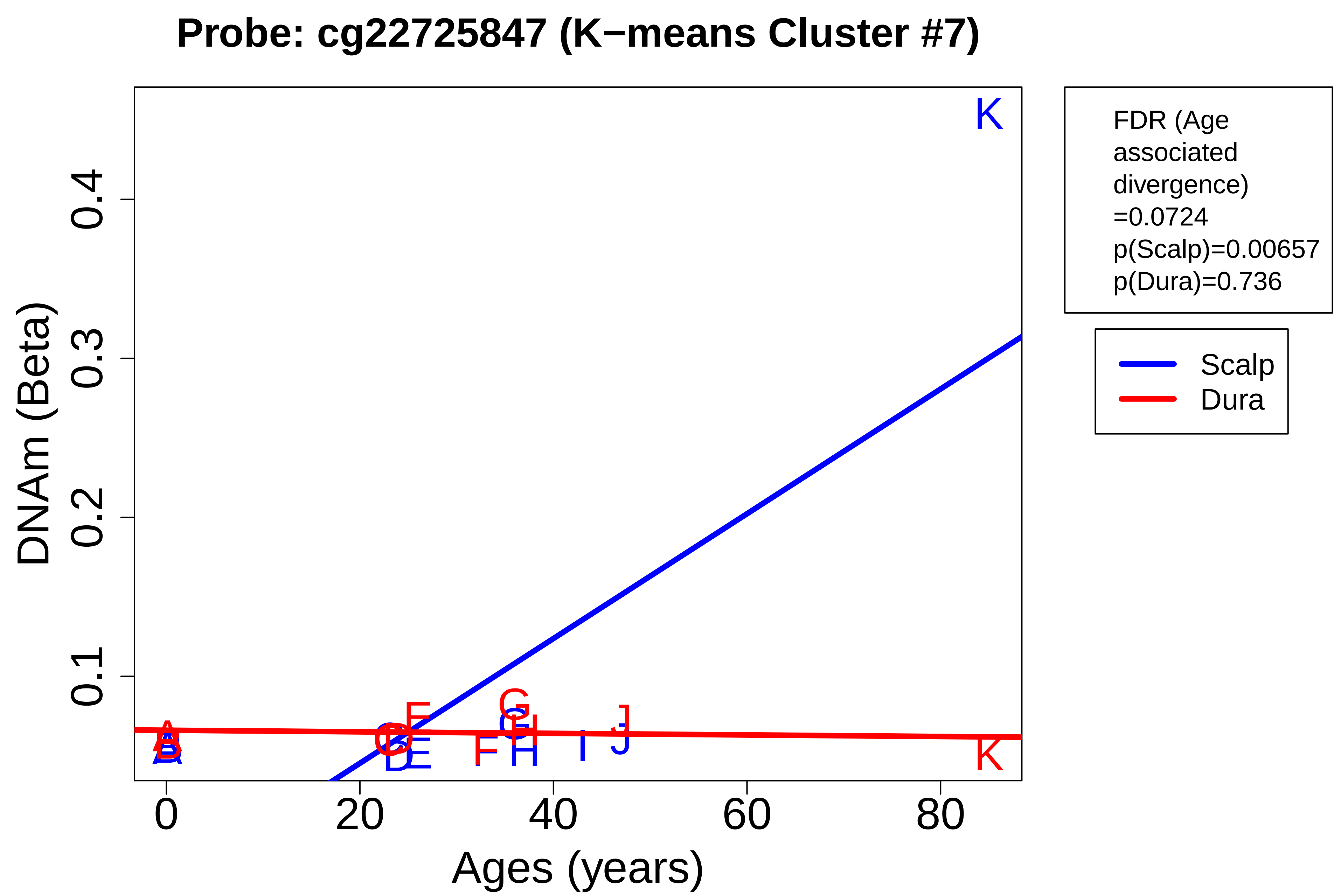

Probe: cg22725847 (K−means Cluster #7)
DNAm (Beta)
0.1
0.2
0.3
0.4
0
20
40
60
80
Ages (years)
A
B
C
D
E
F
G
H
I
J
K
FDR (Age associated divergence) =0.0724
p(Scalp)=0.00657
p(Dura)=0.736
Scalp
Dura

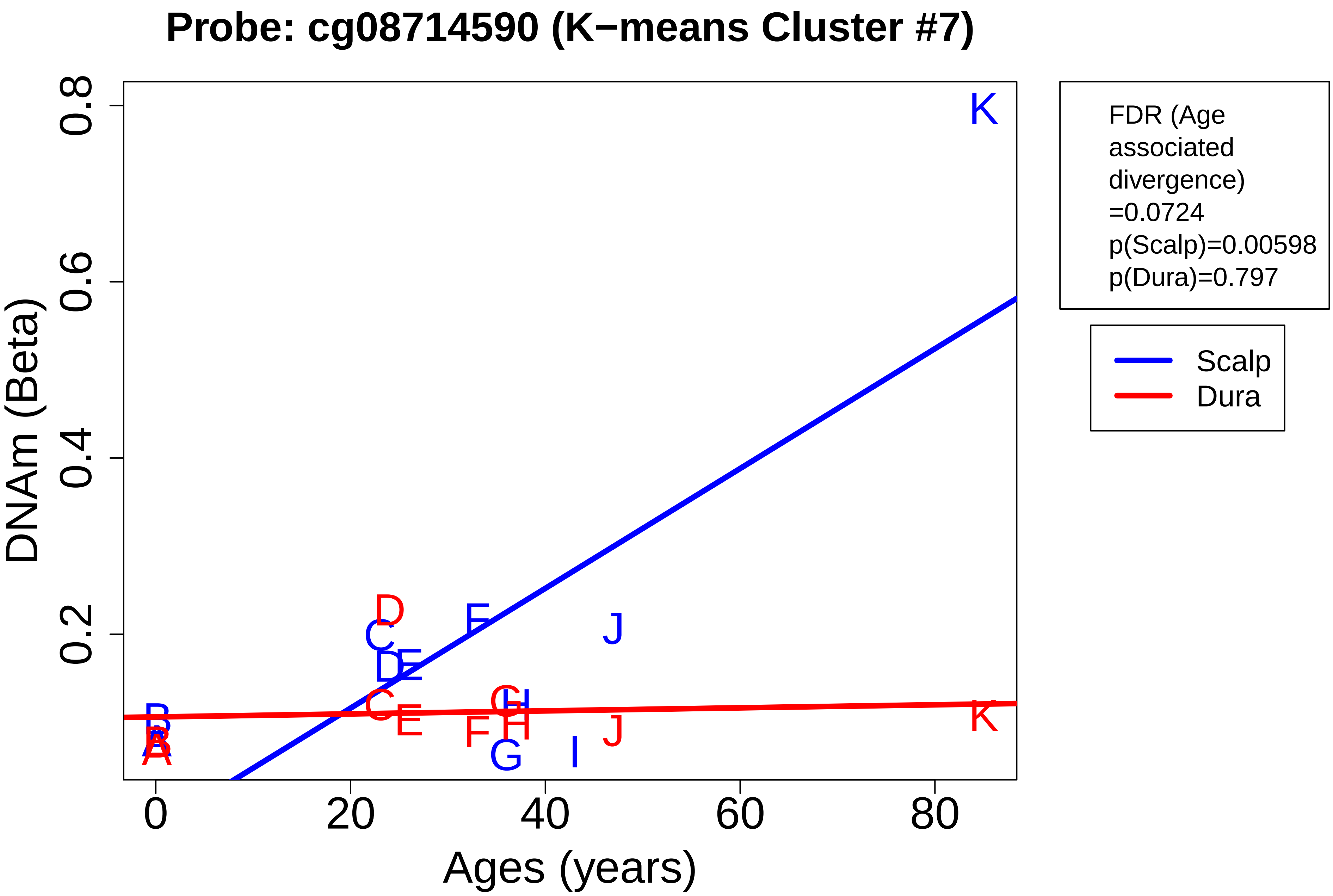

Probe: cg08714590 (K−means Cluster #7)
DNAm (Beta)
0.2
0.4
0.6
0.8
0
20
40
60
80
Ages (years)
FDR (Age associated divergence) =0.0724
p(Scalp)=0.00598
p(Dura)=0.797
Scalp
Dura

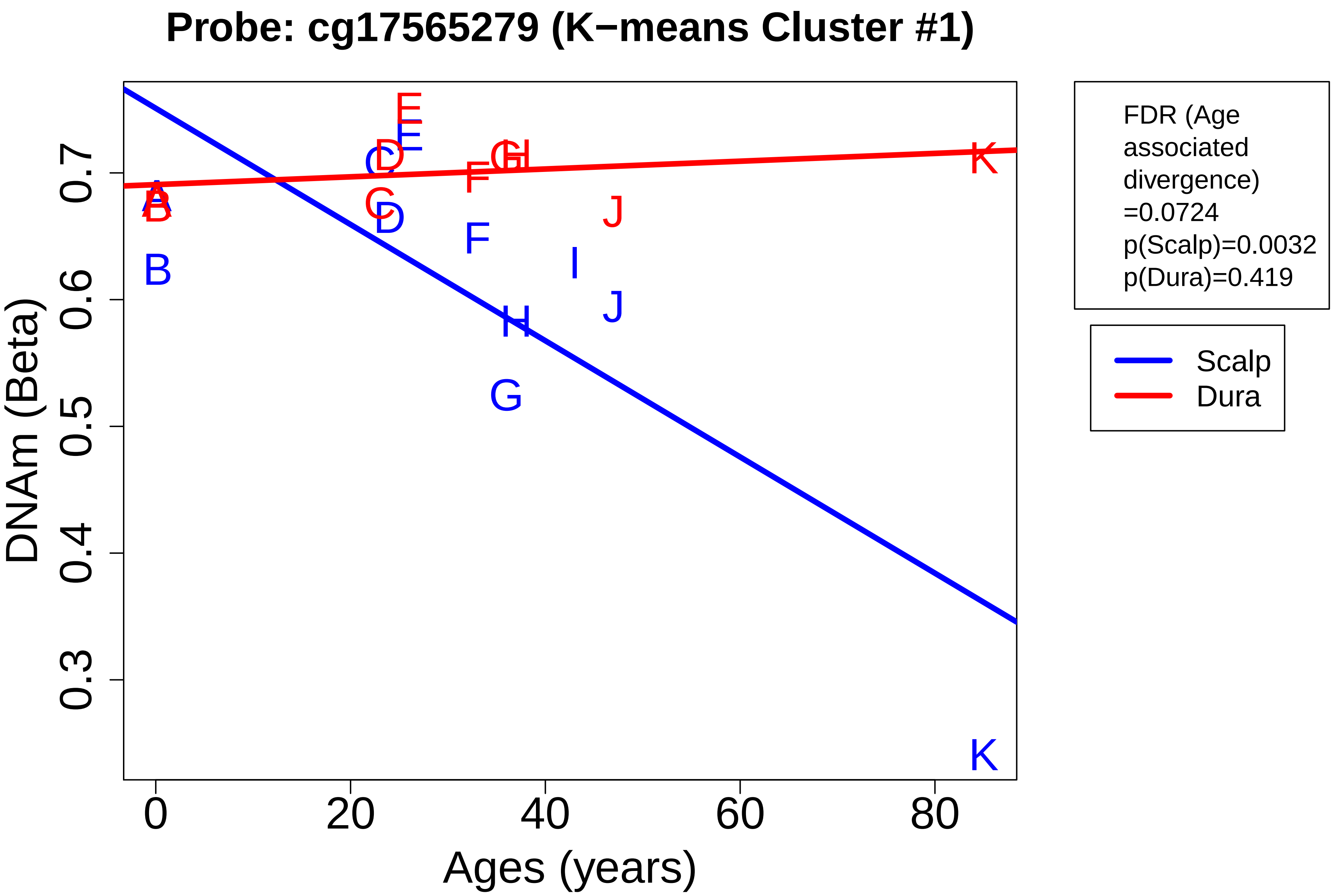

Probe: cg17565279 (K−means Cluster #1)
DNAm (Beta)
Ages (years)
FDR (Age associated divergence) =0.0724
p(Scalp)=0.0032
p(Dura)=0.419
Scalp
Dura

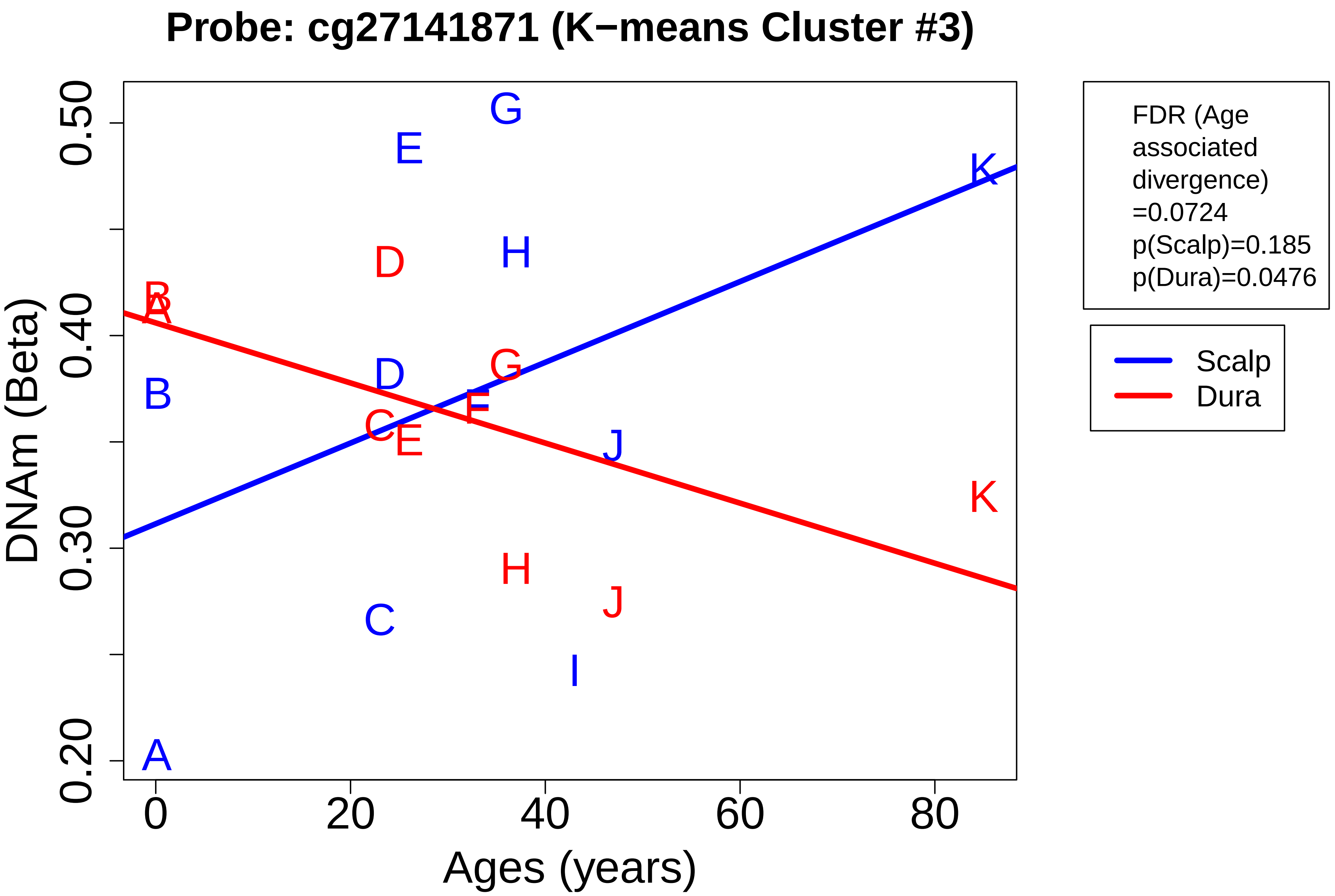

Probe: cg27141871 (K−means Cluster #3)
DNAm (Beta)
0.20
0.30
0.40
0.50
Ages (years)
0
20
40
60
80
FDR (Age associated divergence) =0.0724
p(Scalp)=0.185
p(Dura)=0.0476
Scalp
Dura

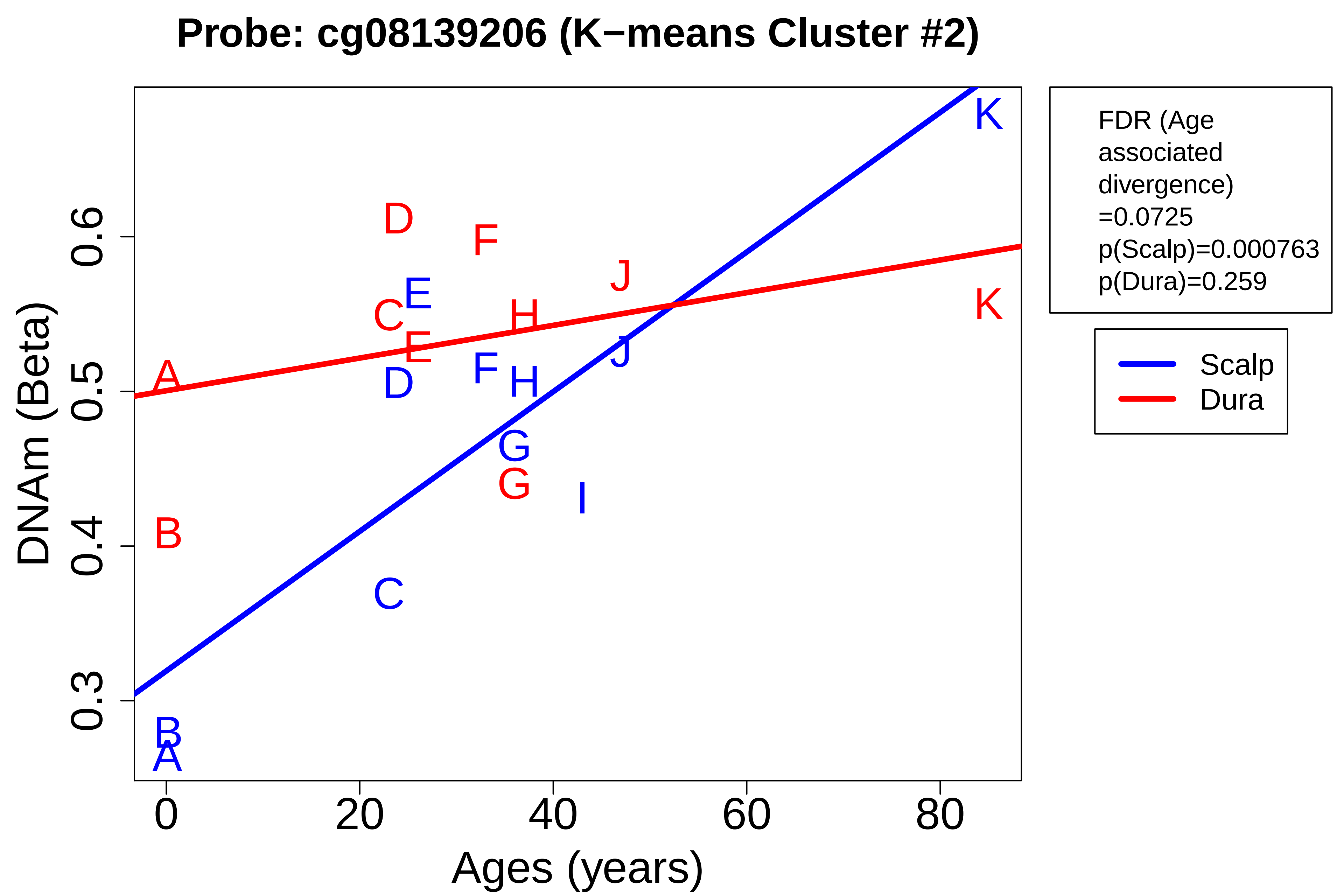

Probe: cg08139206 (K−means Cluster #2)
FDR (Age associated divergence) =0.0725
p(Scalp)=0.000763
p(Dura)=0.259
Scalp
Dura
DNAm (Beta)
0.3
0.4
0.5
0.6
0
20
40
60
80
Ages (years)

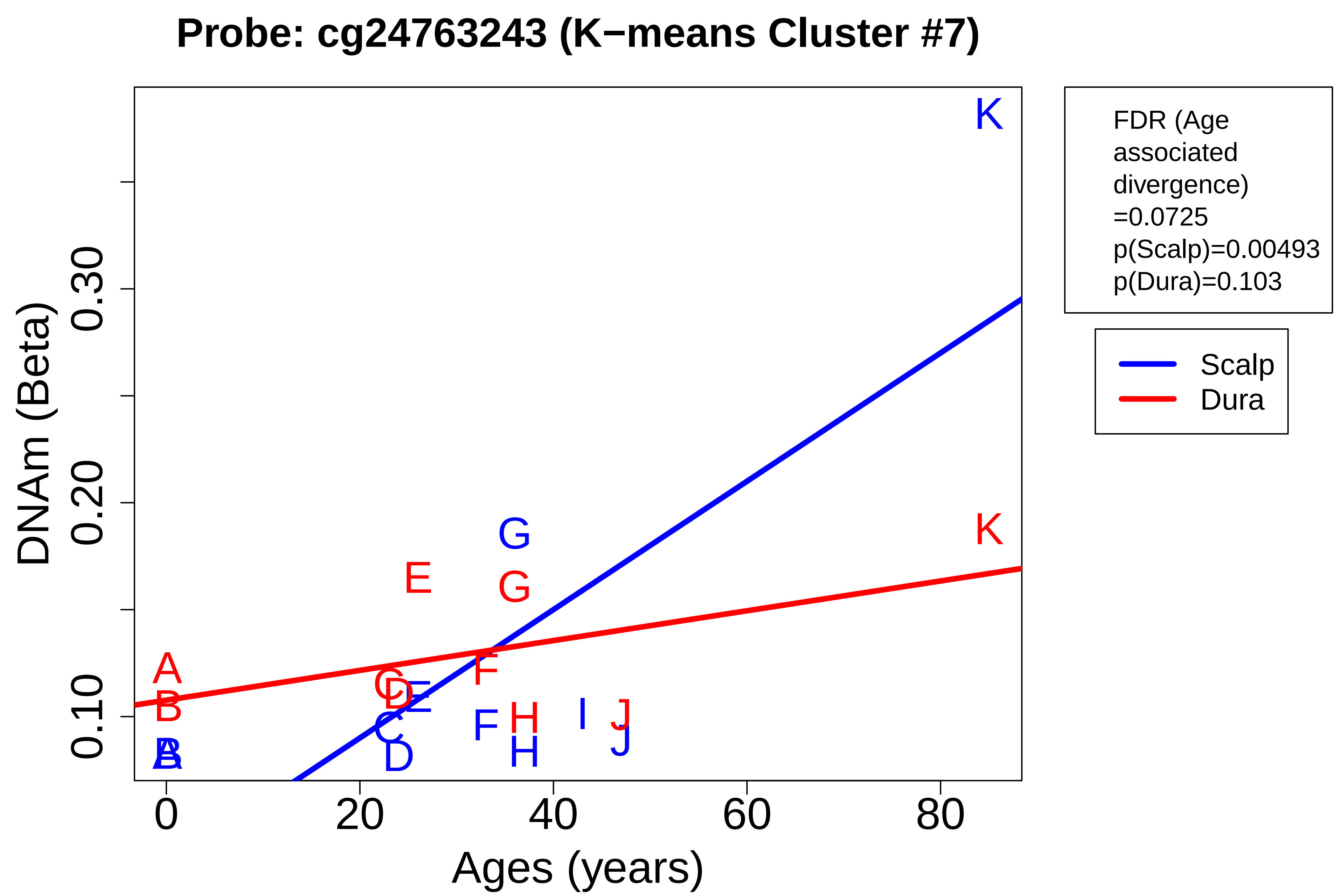

Probe: cg24763243 (K−means Cluster #7)
DNAm (Beta)
0.10
0.20
0.30
Ages (years)
0
20
40
60
80
FDR (Age associated divergence) =0.0725
p(Scalp)=0.00493
p(Dura)=0.103
Scalp
Dura
A
B
C
D
E
F
G
H
I
J
K

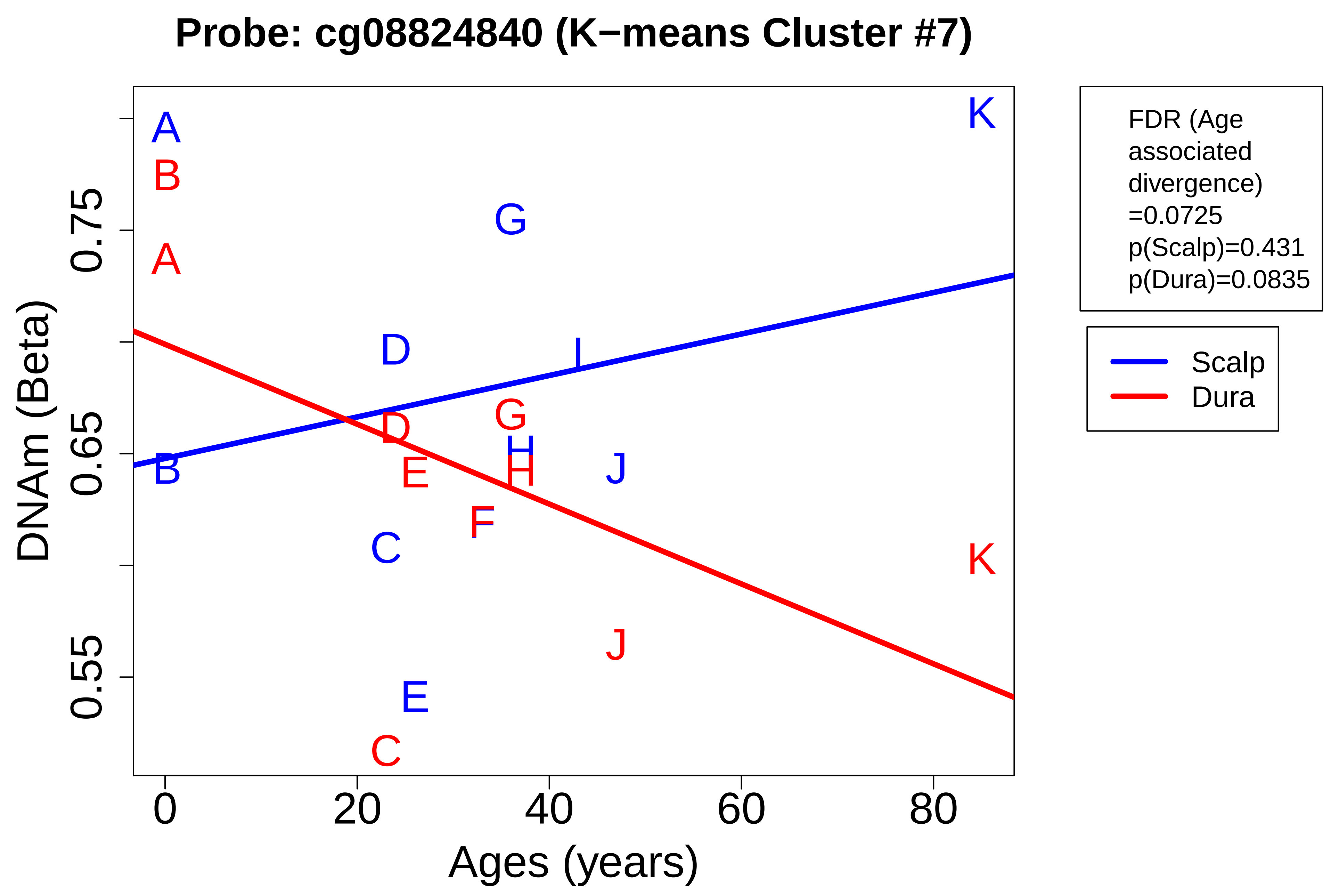

Probe: cg08824840 (K−means Cluster #7)
DNAm (Beta)
0.55
0.65
0.75
Ages (years)
0
20
40
60
80
A
B
A
B
C
C
D
D
E
E
F
G
G
H
H
I
J
J
K
K
FDR (Age associated divergence) =0.0725
p(Scalp)=0.431
p(Dura)=0.0835
Scalp
Dura

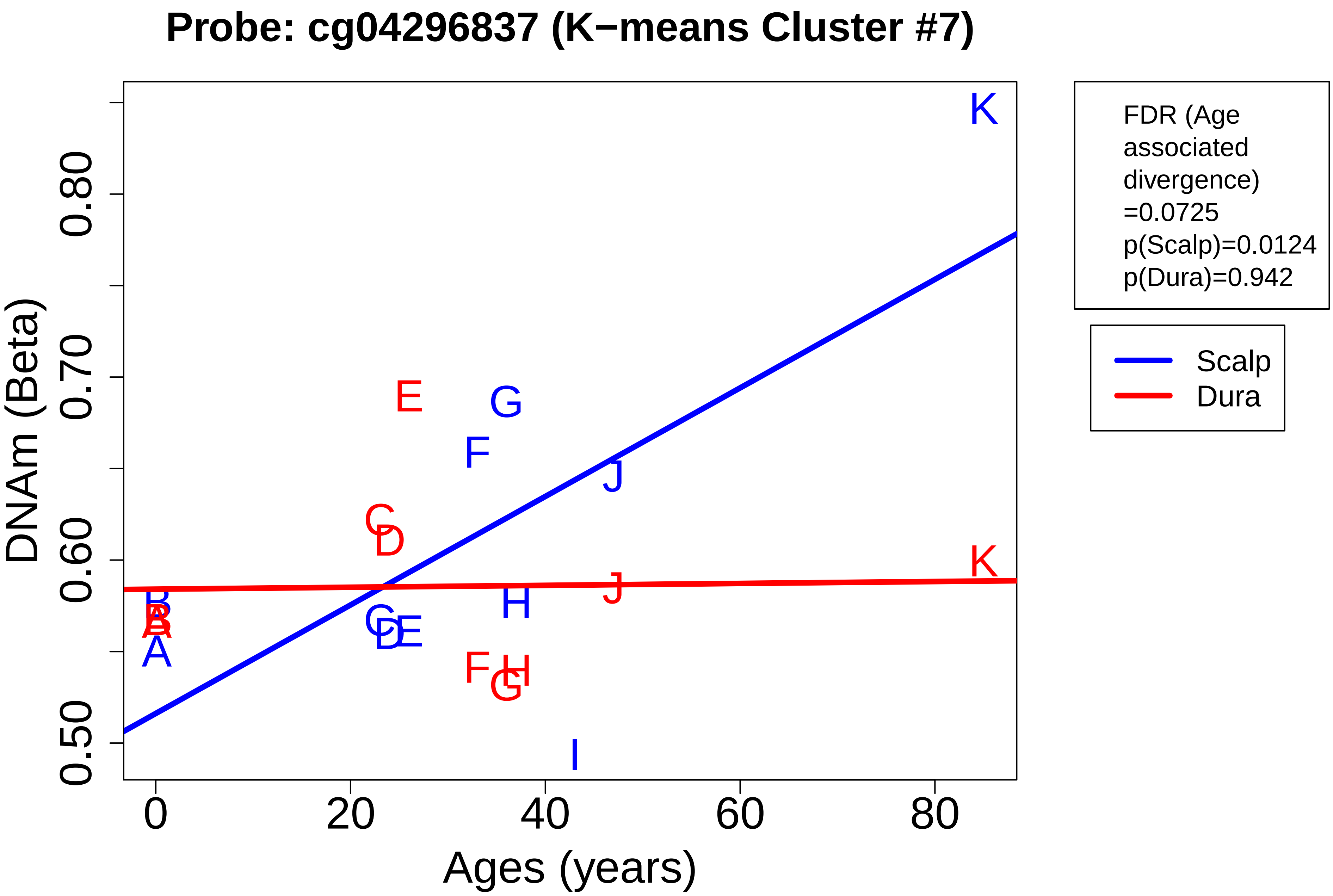

Probe: cg04296837 (K−means Cluster #7)
FDR (Age associated divergence) =0.0725
p(Scalp)=0.0124
p(Dura)=0.942
Scalp
Dura
DNAm (Beta)
0.50
0.60
0.70
0.80
0
20
40
60
80
Ages (years)
A
B
C
D
E
F
G
H
I
J
K

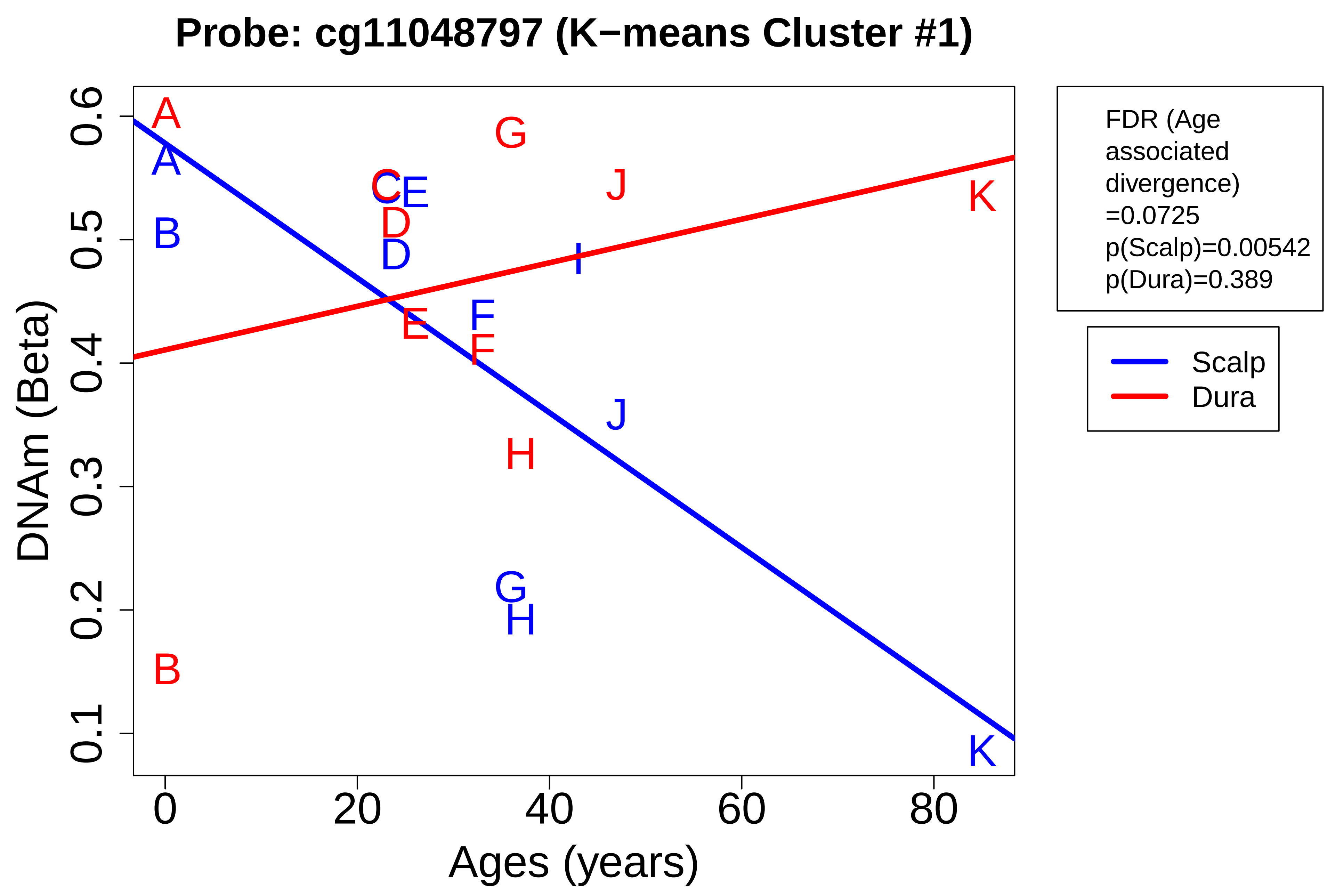

Probe: cg11048797 (K−means Cluster #1)
DNAm (Beta)
0.1
0.2
0.3
0.4
0.5
0.6
0
20
40
60
80
Ages (years)
A
B
C
D
E
F
G
H
I
J
K
A
B
C
D
E
F
G
H
J
K
FDR (Age associated divergence) =0.0725
p(Scalp)=0.00542
p(Dura)=0.389
Scalp
Dura

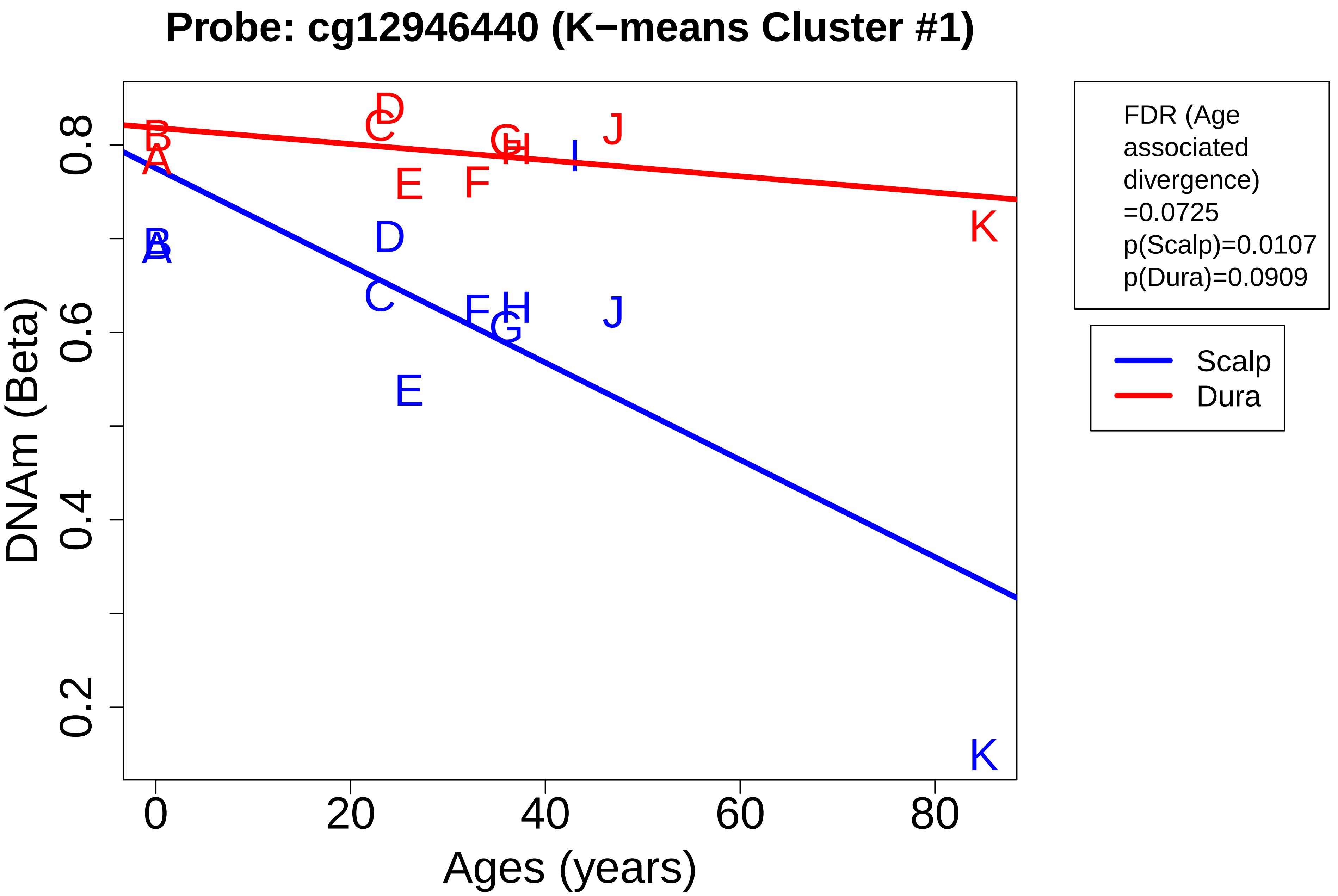
Probe: cg12946440 (K−means Cluster #1)
DNAm (Beta)
Ages (years)
0.2
0.4
0.6
0.8
0
20
40
60
80
FDR (Age associated divergence) =0.0725
p(Scalp)=0.0107
p(Dura)=0.0909
Scalp
Dura
A B C D E F G H I J K

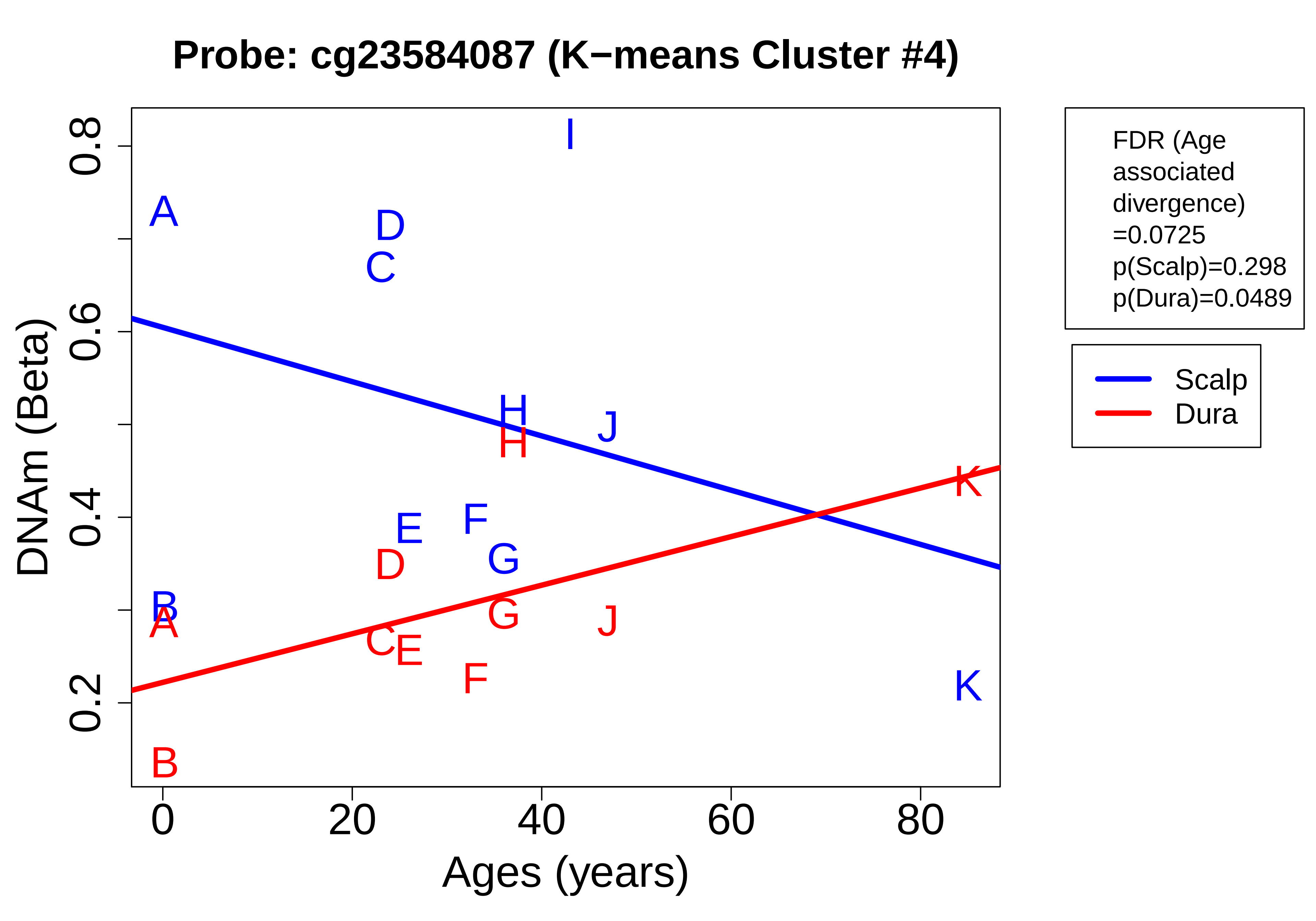
Probe: cg23584087 (K−means Cluster #4)
DNAm (Beta)
0.2
0.4
0.6
0.8
Ages (years)
0
20
40
60
80
FDR (Age associated divergence) =0.0725
p(Scalp)=0.298
p(Dura)=0.0489
Scalp
Dura
A
B
C
D
E
F
G
H
I
J
K

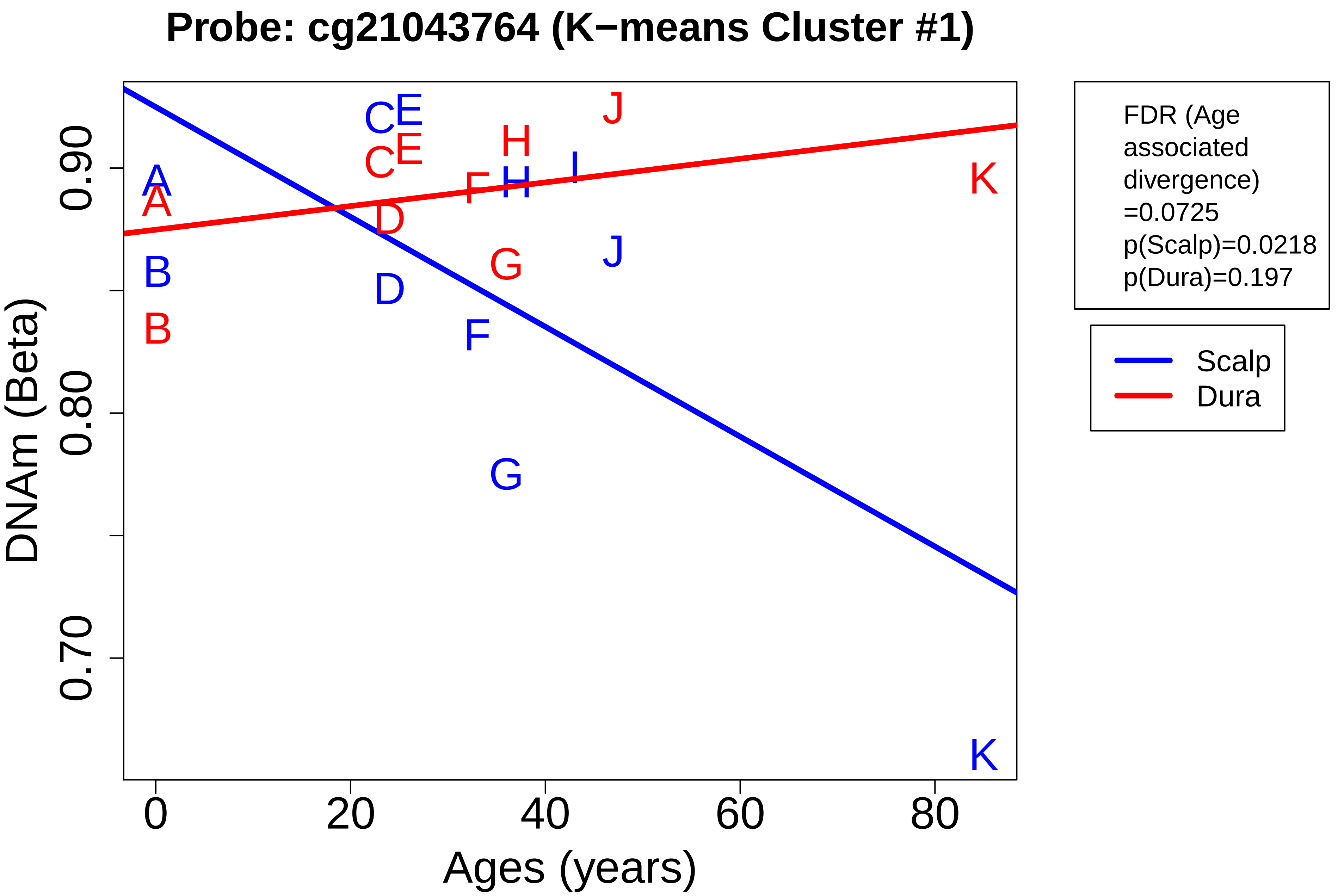

Probe: cg21043764 (K−means Cluster #1)
DNAm (Beta)
0.70
0.80
0.90
Ages (years)
0
20
40
60
80
FDR (Age associated divergence) =0.0725
p(Scalp)=0.0218
p(Dura)=0.197
Scalp
Dura
A
B
C
D
E
F
G
H
I
J
K

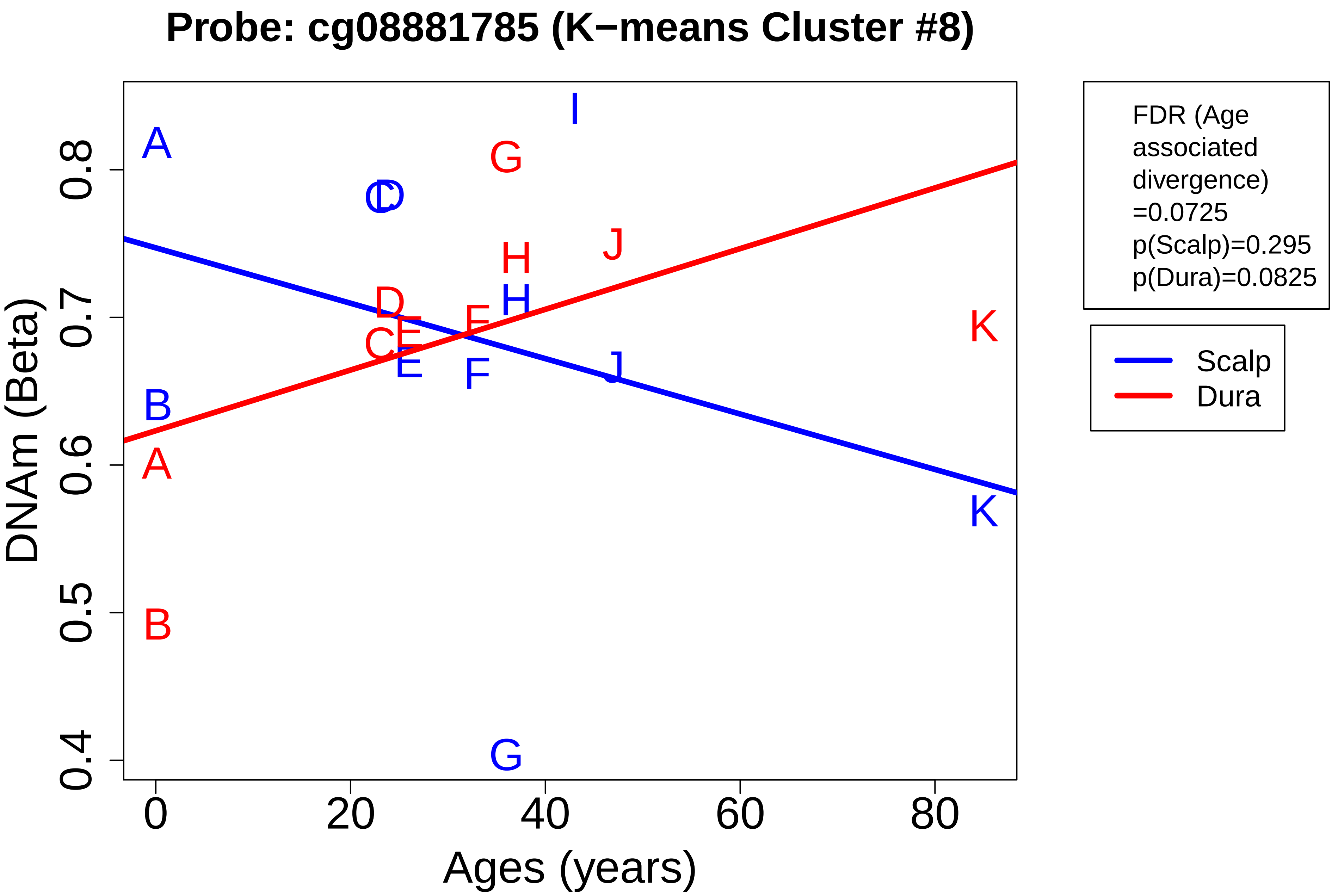

Probe: cg08881785 (K−means Cluster #8)
DNAm (Beta)
0.4
0.5
0.6
0.7
0.8
Ages (years)
0
20
40
60
80
FDR (Age associated divergence) =0.0725
p(Scalp)=0.295
p(Dura)=0.0825
Scalp
Dura

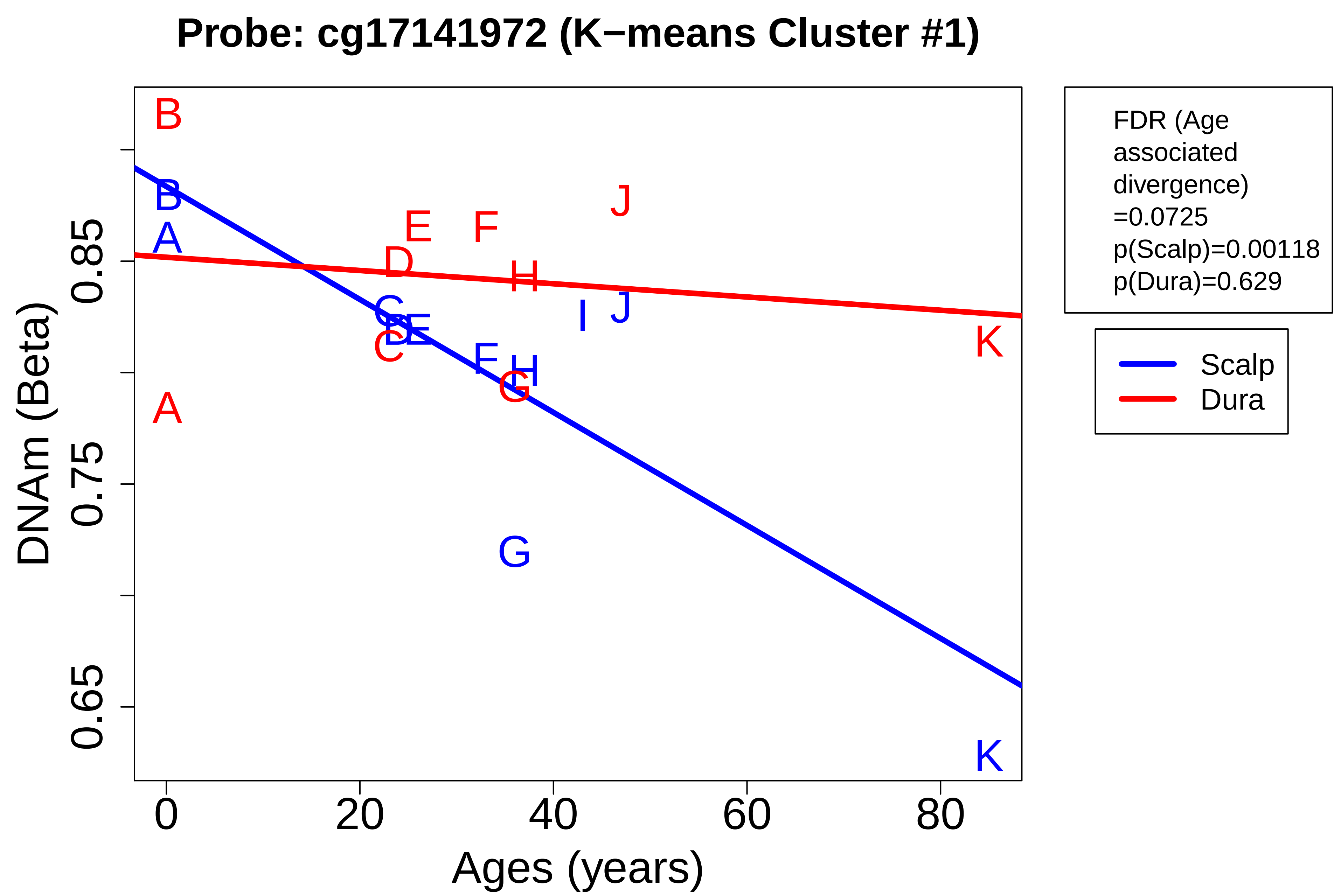

Probe: cg17141972 (K−means Cluster #1)
DNAm (Beta)
0.65
0.75
0.85
Ages (years)
0
20
40
60
80
FDR (Age associated divergence) =0.0725
p(Scalp)=0.00118
p(Dura)=0.629
Scalp
Dura
A B C D E F G H I J K

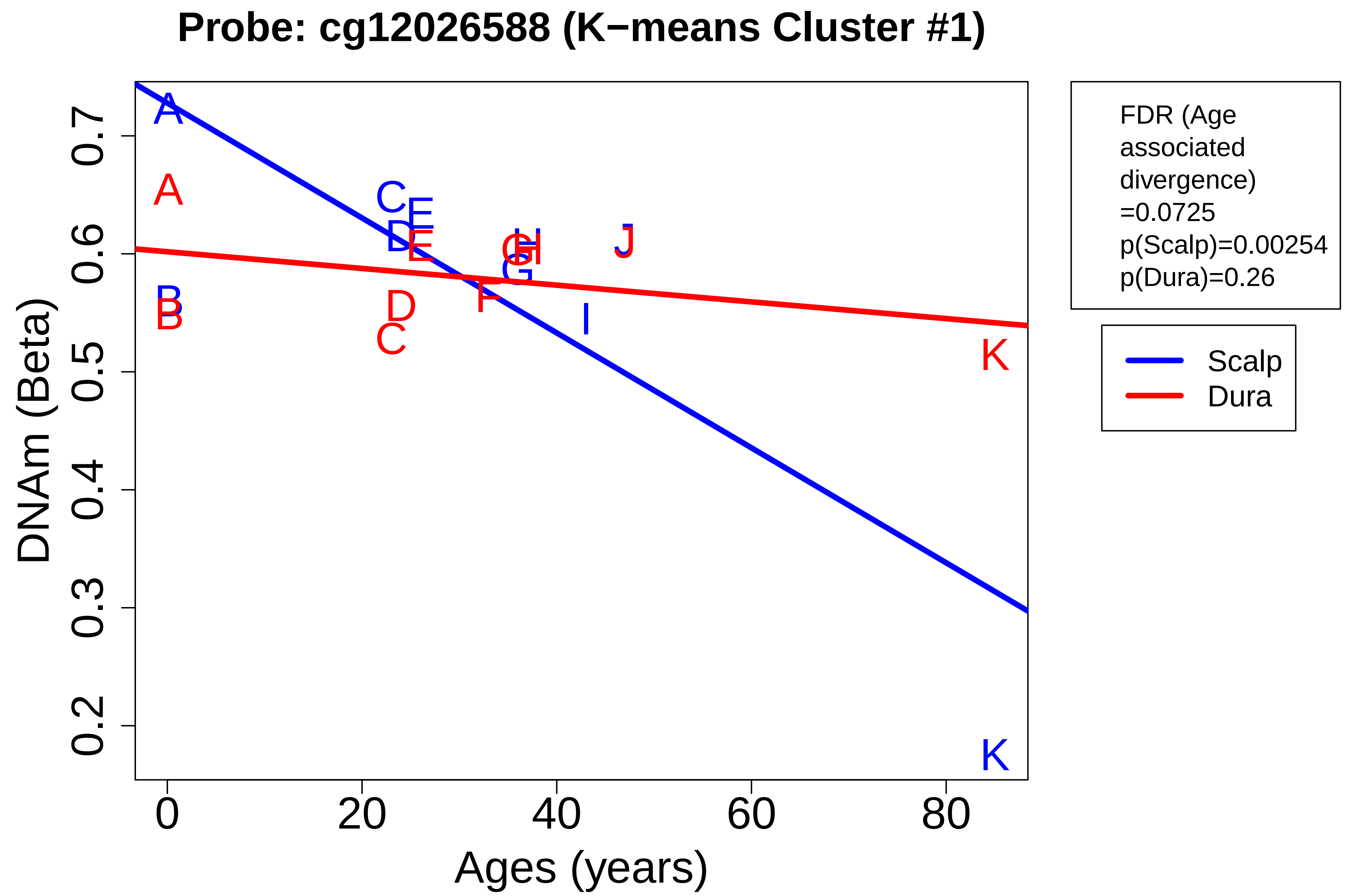
Probe: cg12026588 (K−means Cluster #1)
DNAm (Beta)
Ages (years)
0.2
0.3
0.4
0.5
0.6
0.7
0
20
40
60
80
A
B
C
D
E
F
G
H
I
J
K
FDR (Age associated divergence) =0.0725
p(Scalp)=0.00254
p(Dura)=0.26
Scalp
Dura

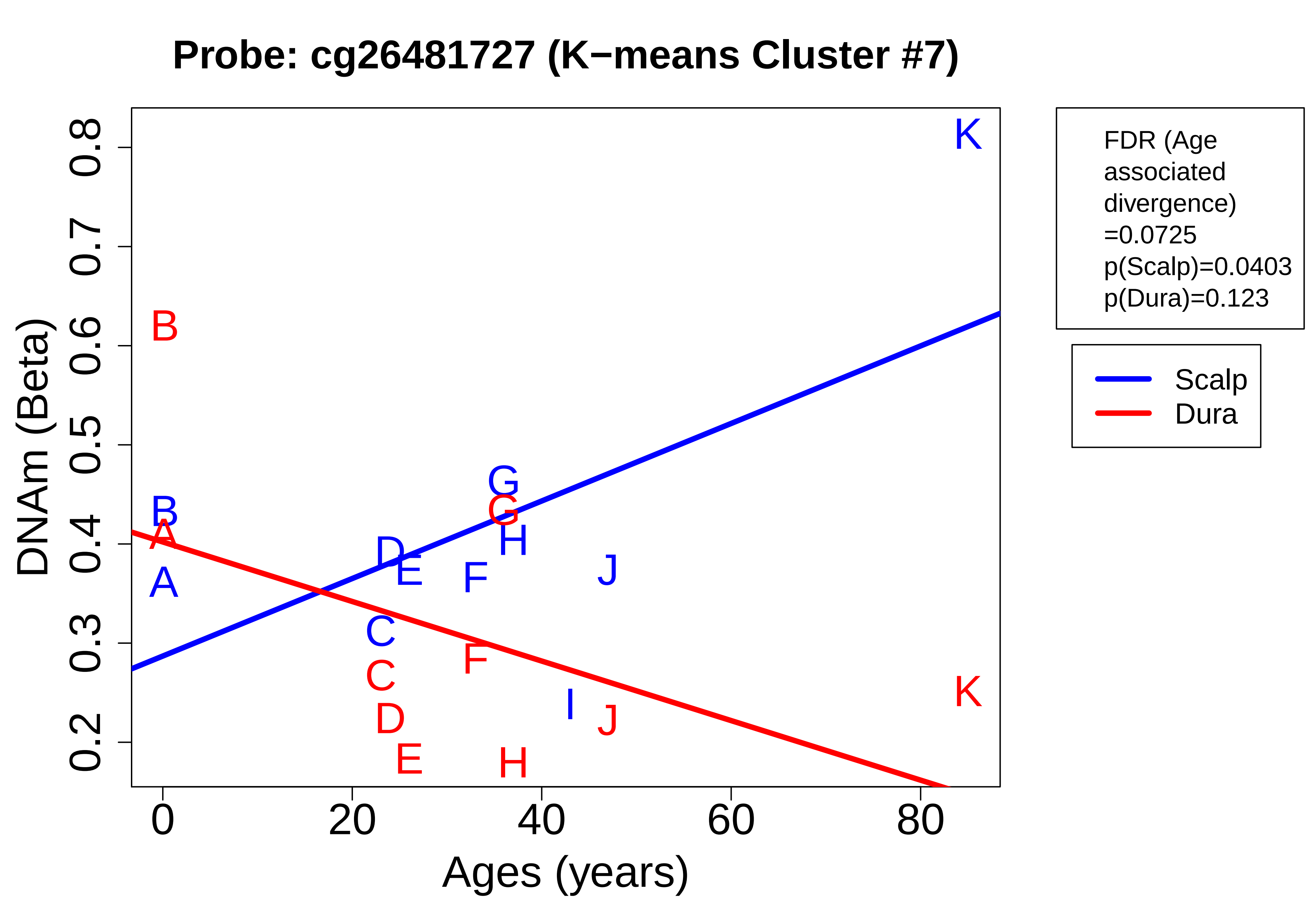

Probe: cg26481727 (K−means Cluster #7)
DNAm (Beta)
0.2
0.3
0.4
0.5
0.6
0.7
0.8
0
20
40
60
80
Ages (years)
FDR (Age associated divergence) =0.0725
p(Scalp)=0.0403
p(Dura)=0.123
Scalp
Dura
A
B
C
D
E
F
G
H
I
J
K

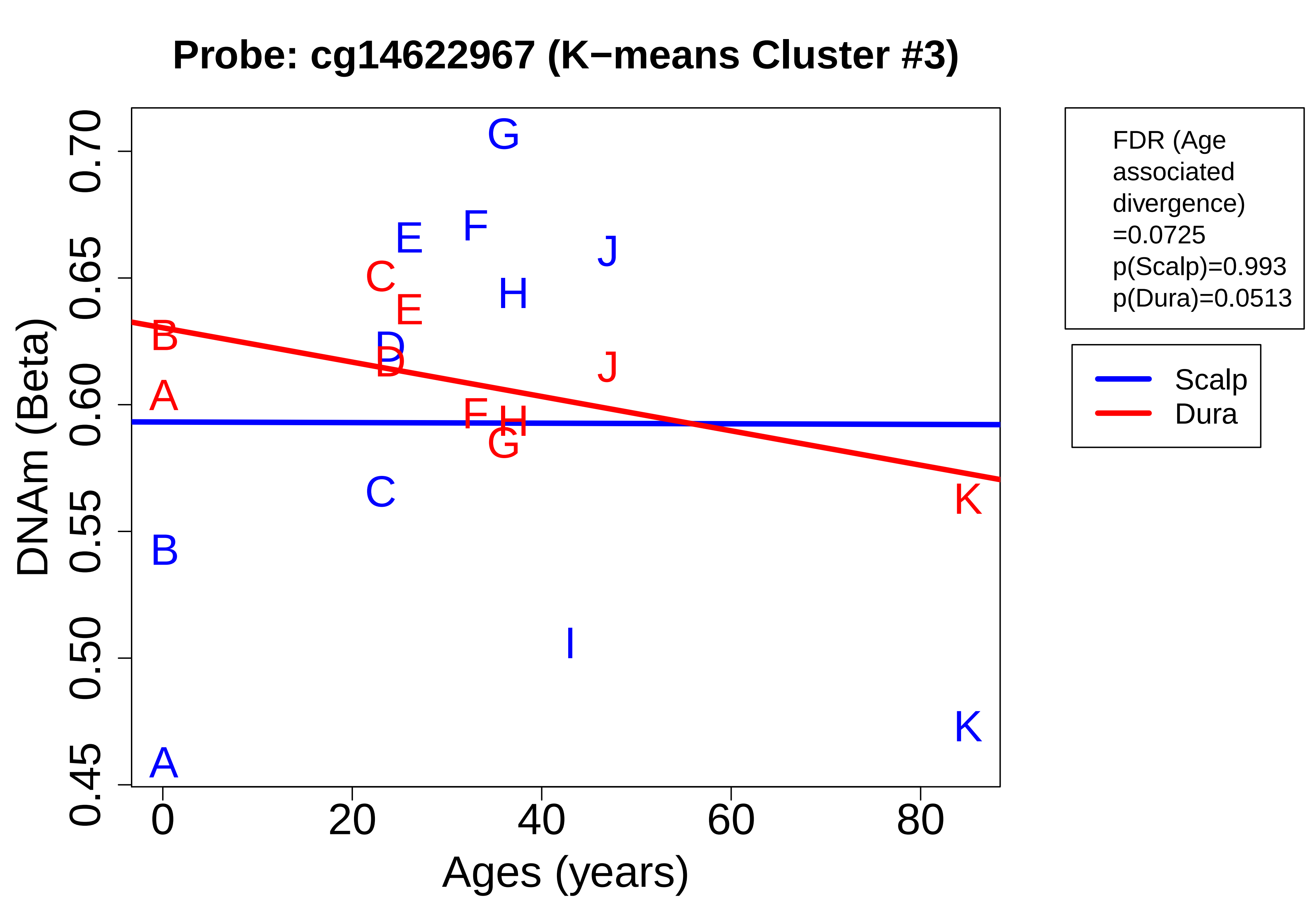

Probe: cg14622967 (K−means Cluster #3)
DNAm (Beta)
0.45
0.50
0.55
0.60
0.65
0.70
0
20
40
60
80
Ages (years)
FDR (Age associated divergence) =0.0725
p(Scalp)=0.993
p(Dura)=0.0513
Scalp
Dura

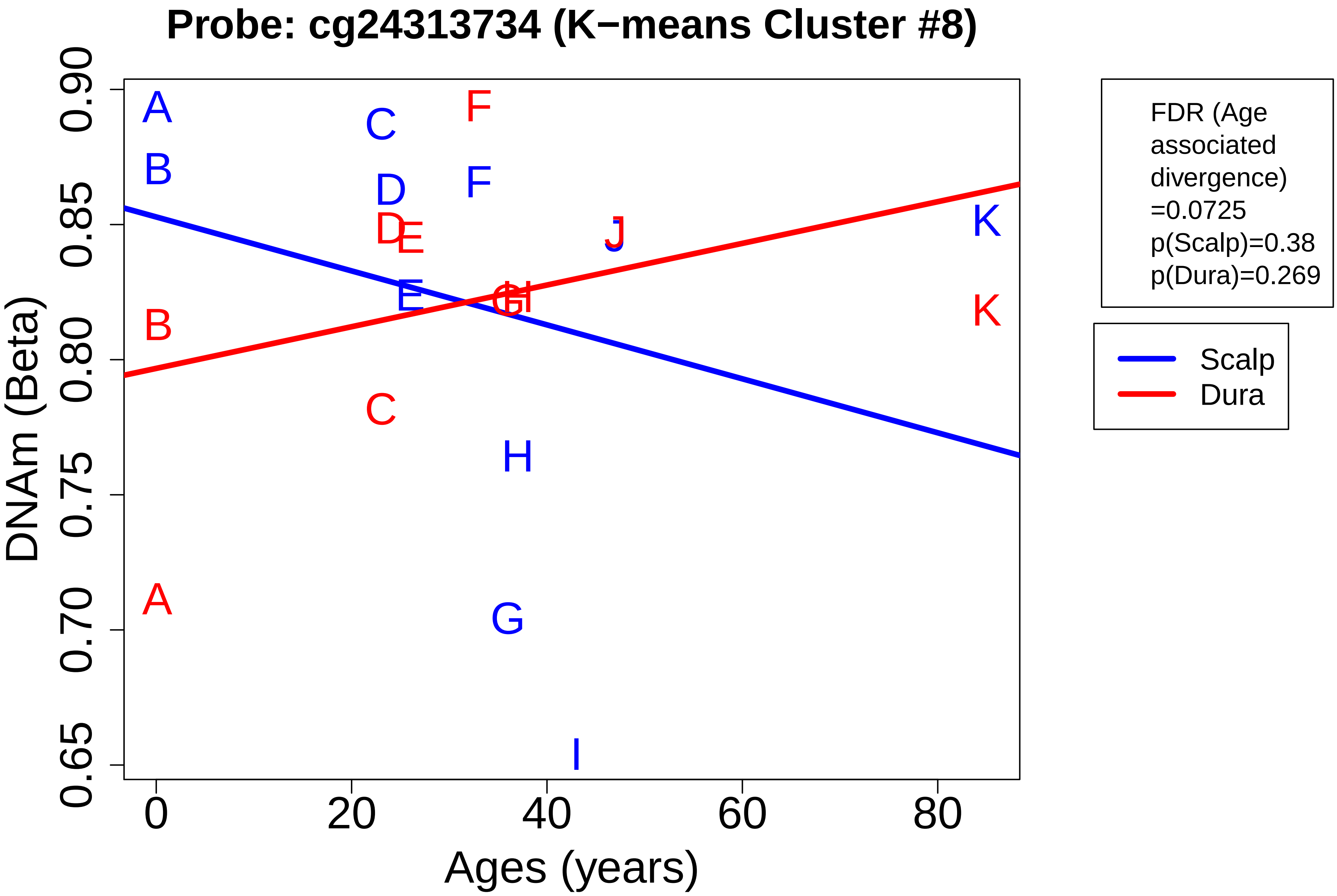

Probe: cg24313734 (K−means Cluster #8)
DNAm (Beta)
0.65
0.70
0.75
0.80
0.85
0.90
0
20
40
60
80
Ages (years)
FDR (Age associated divergence) =0.0725
p(Scalp)=0.38
p(Dura)=0.269
Scalp
Dura

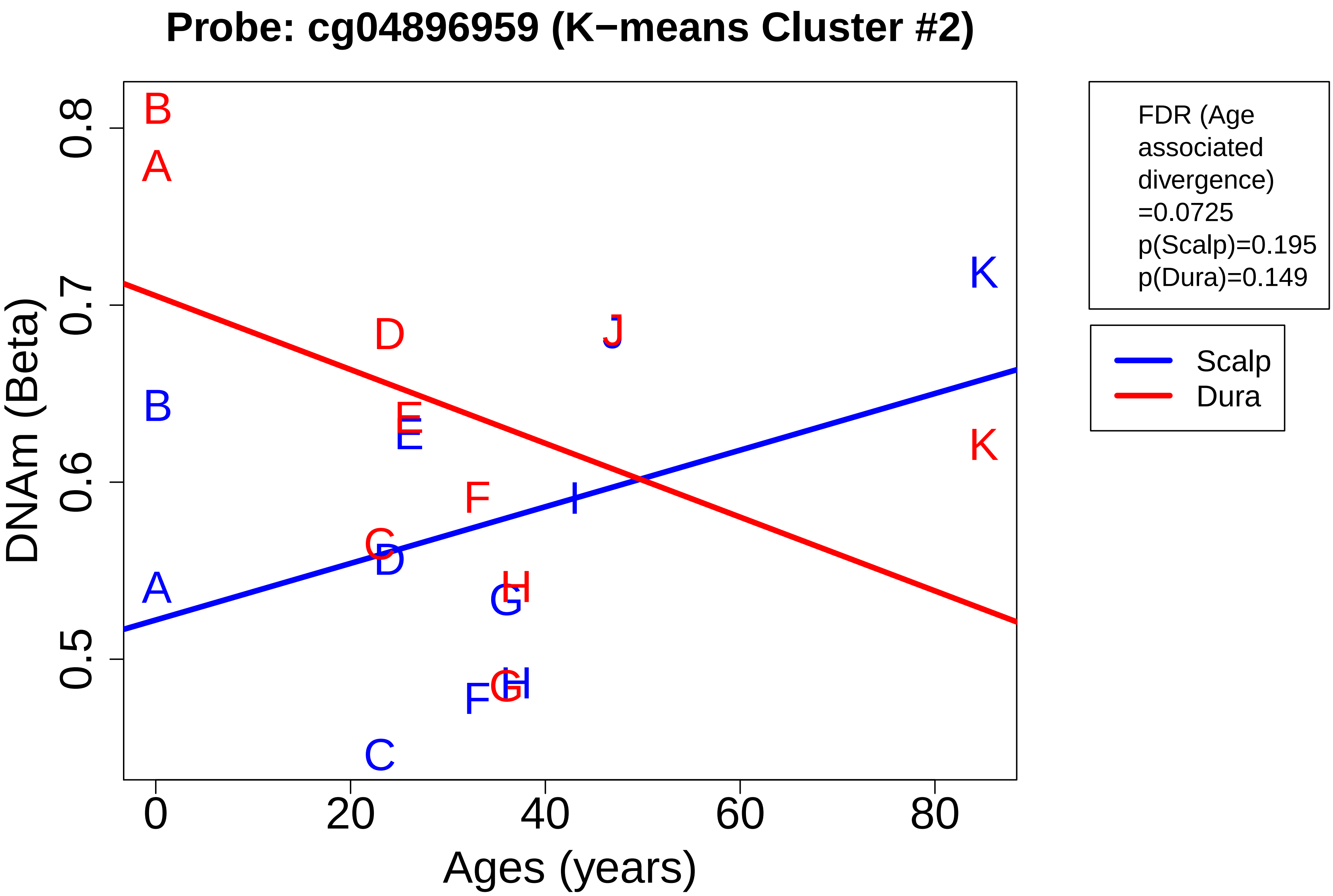

Probe: cg04896959 (K−means Cluster #2)
DNAm (Beta)
0.5
0.6
0.7
0.8
Ages (years)
0
20
40
60
80
B
A
K
D
J
B
E
E
K
F
I
C
D
A
H
G
F
G
H
C
FDR (Age associated divergence) =0.0725
p(Scalp)=0.195
p(Dura)=0.149
Scalp
Dura

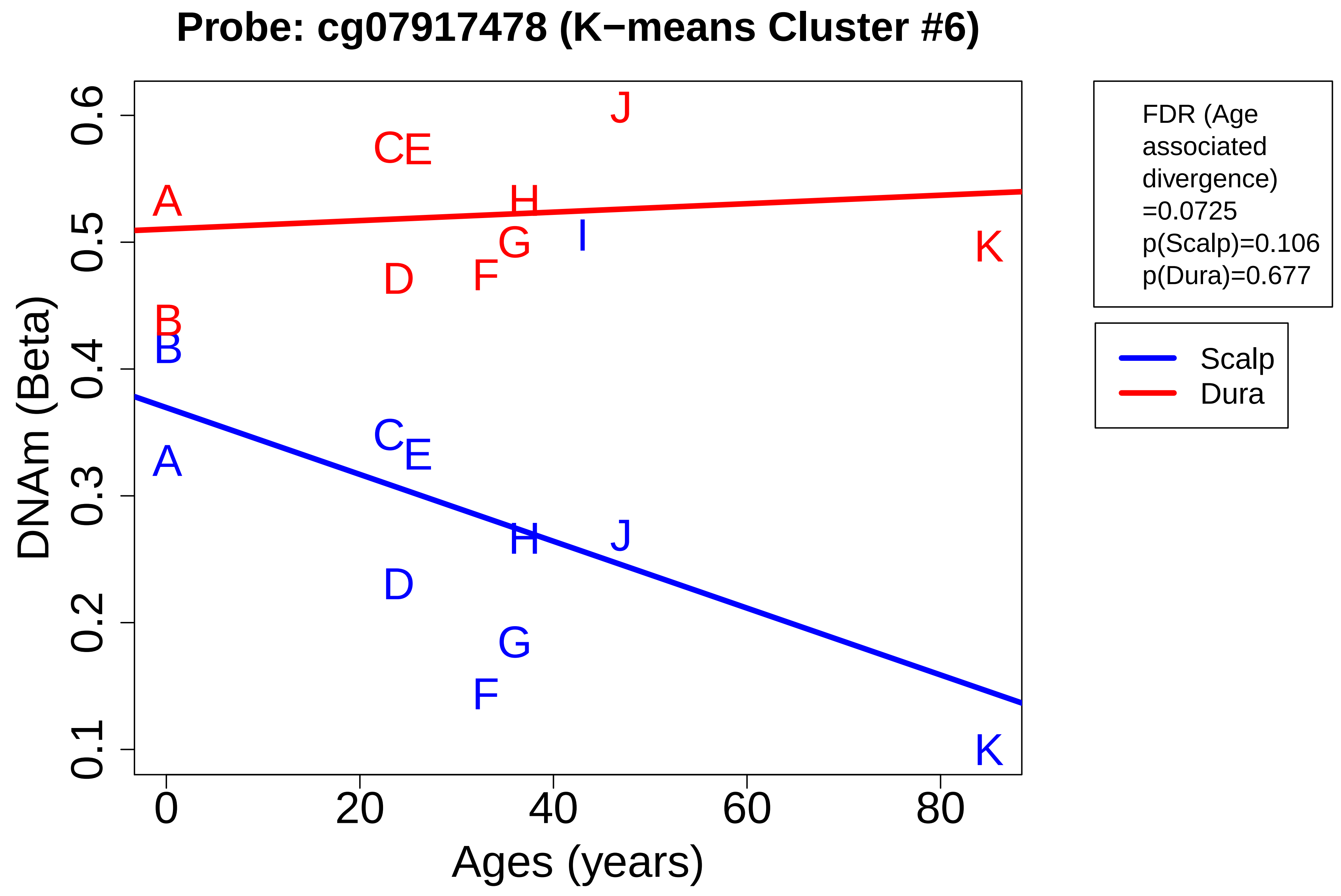

Probe: cg07917478 (K−means Cluster #6)
DNAm (Beta)
0.1
0.2
0.3
0.4
0.5
0.6
0
20
40
60
80
Ages (years)
A
B
C
D
E
F
G
H
I
J
K
FDR (Age associated divergence) =0.0725
p(Scalp)=0.106
p(Dura)=0.677
Scalp
Dura

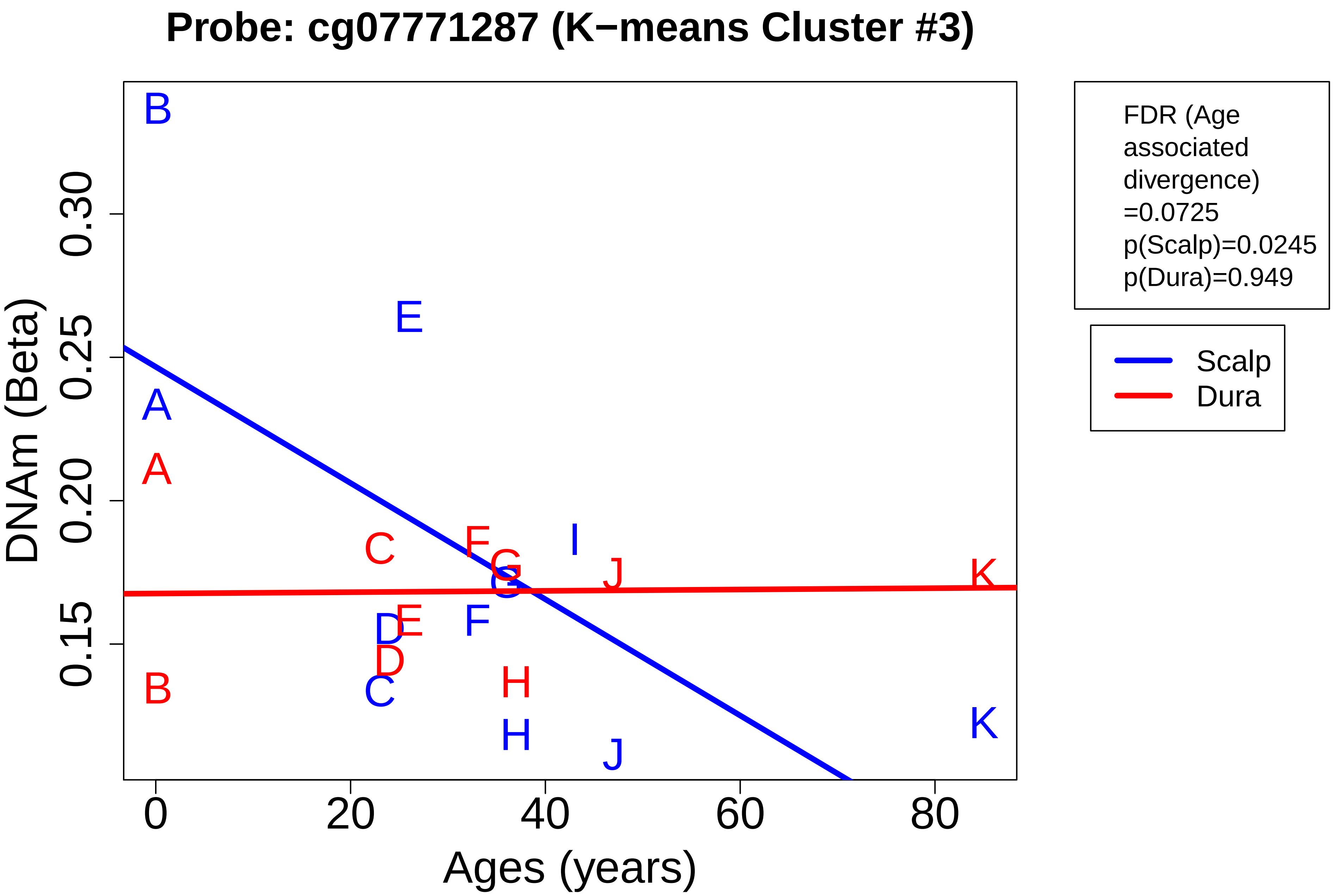

Probe: cg07771287 (K−means Cluster #3)
DNAm (Beta)
0.15
0.20
0.25
0.30
Ages (years)
0
20
40
60
80
FDR (Age associated divergence) =0.0725
p(Scalp)=0.0245
p(Dura)=0.949
Scalp
Dura

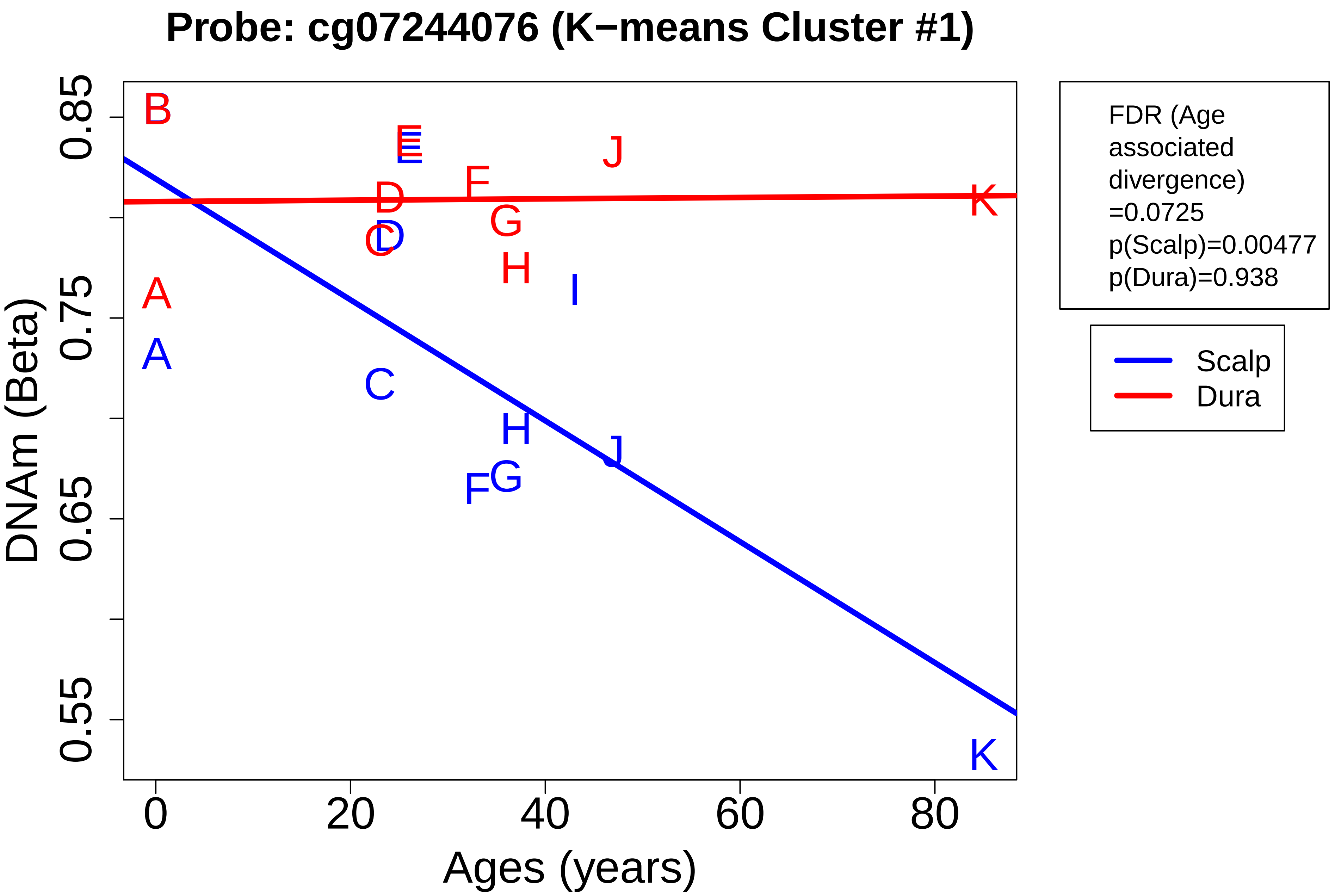

Probe: cg07244076 (K−means Cluster #1)
DNAm (Beta)
0.55
0.65
0.75
0.85
Ages (years)
0
20
40
60
80
FDR (Age associated divergence) =0.0725
p(Scalp)=0.00477
p(Dura)=0.938
Scalp
Dura
A
B
C
D
E
F
G
H
I
J
K

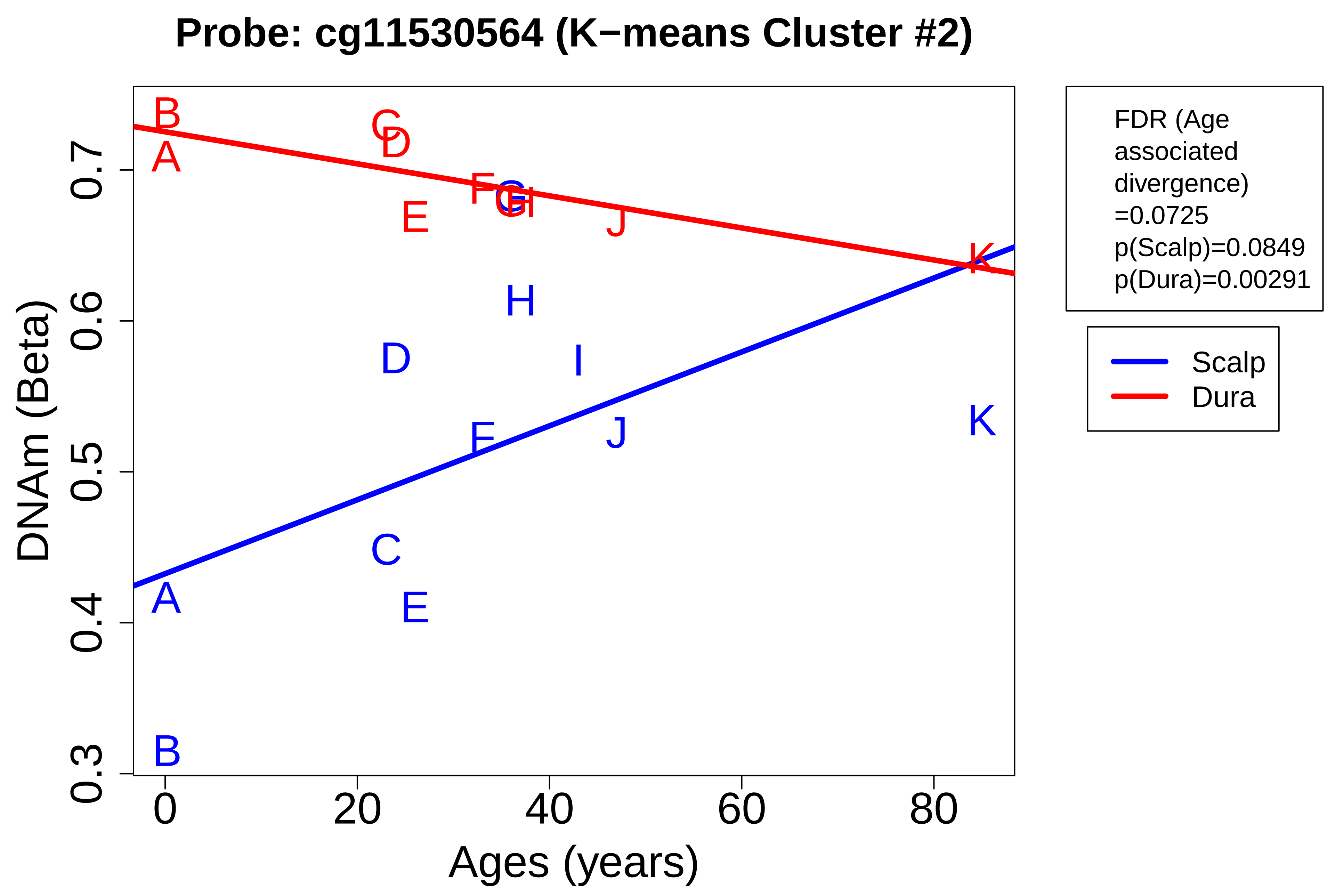

Probe: cg11530564 (K−means Cluster #2)
DNAm (Beta)
0.3
0.4
0.5
0.6
0.7
0
20
40
60
80
Ages (years)
FDR (Age associated divergence) =0.0725
p(Scalp)=0.0849
p(Dura)=0.00291
Scalp
Dura

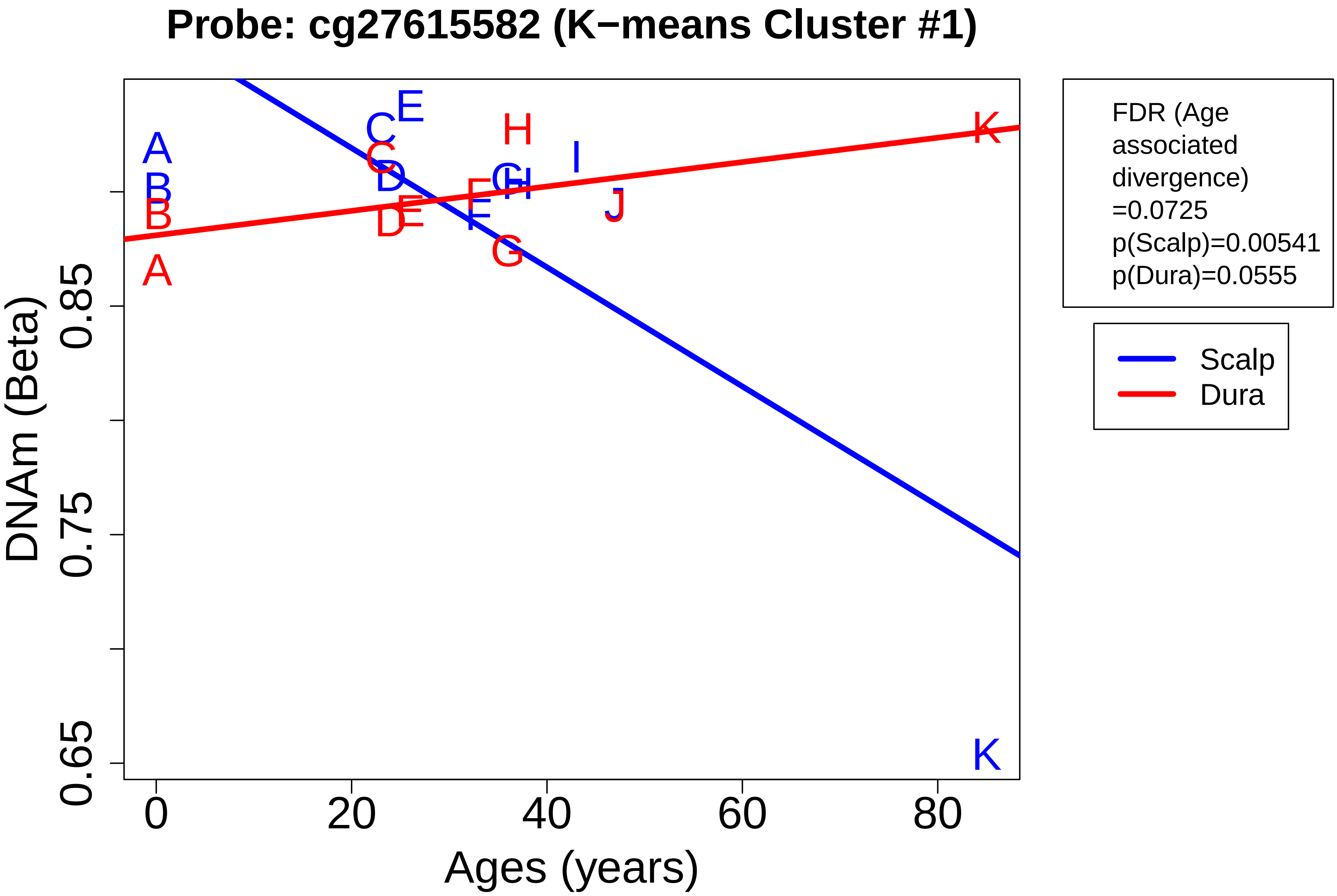

Probe: cg27615582 (K−means Cluster #1)
DNAm (Beta)
0.65
0.75
0.85
Ages (years)
0
20
40
60
80
FDR (Age associated divergence) =0.0725
p(Scalp)=0.00541
p(Dura)=0.0555
Scalp
Dura

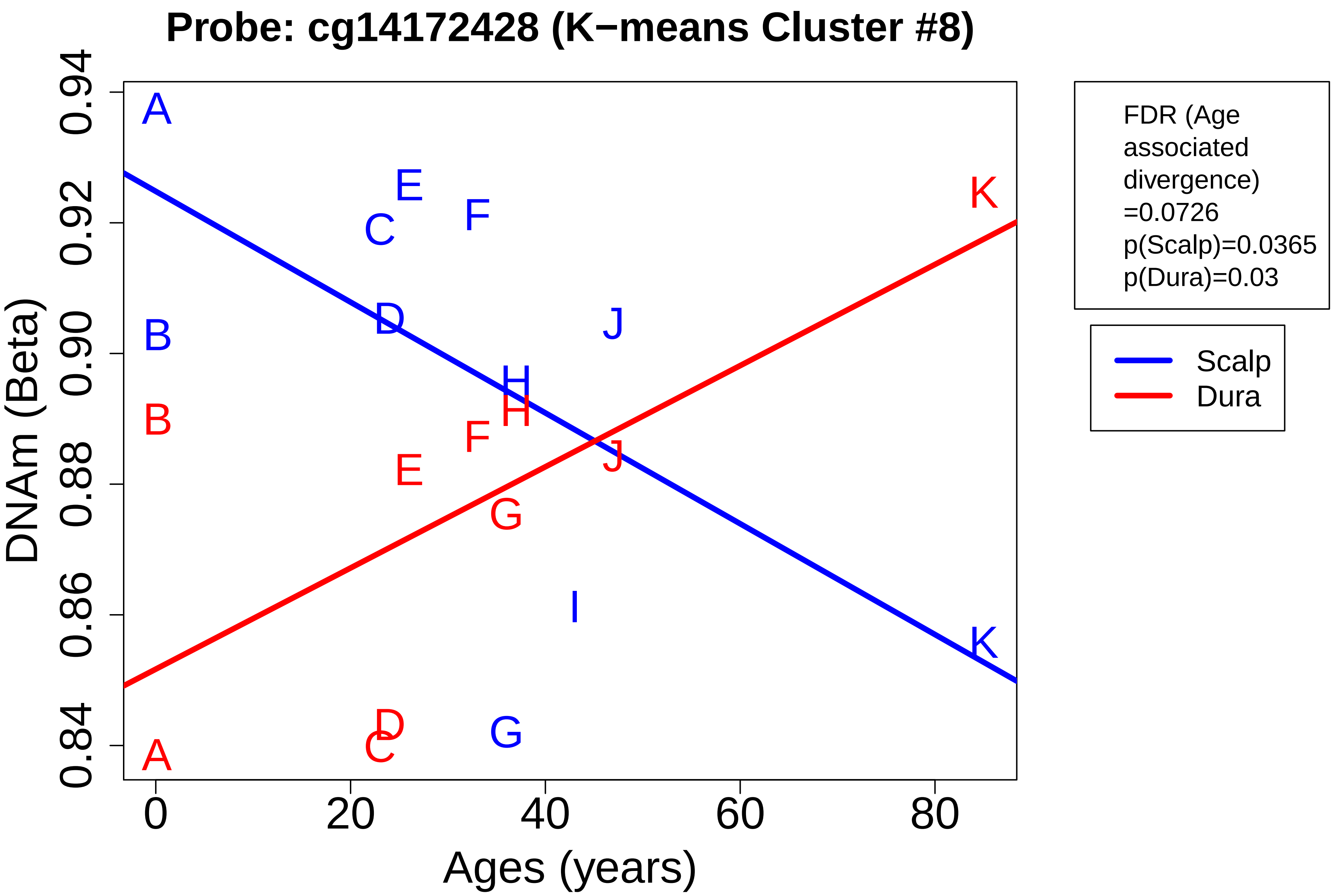

Probe: cg14172428 (K−means Cluster #8)
DNAm (Beta)
0.84
0.86
0.88
0.90
0.92
0.94
0
20
40
60
80
Ages (years)
A
B
C
D
E
F
G
H
I
J
K
FDR (Age associated divergence) =0.0726
p(Scalp)=0.0365
p(Dura)=0.03
Scalp
Dura

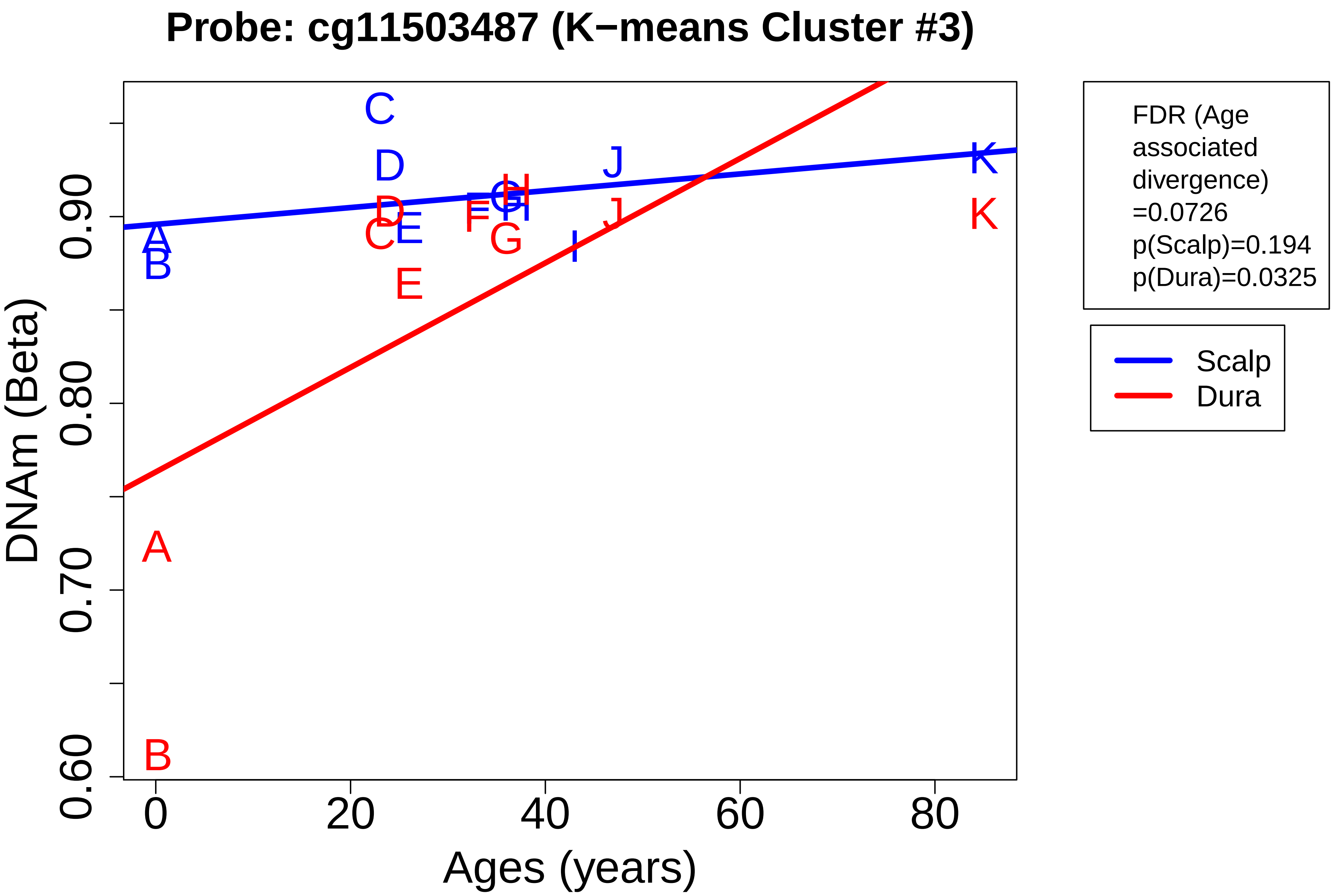

Probe: cg11503487 (K−means Cluster #3)
DNAm (Beta)
0.60
0.70
0.80
0.90
Ages (years)
0
20
40
60
80
FDR (Age associated divergence) =0.0726
p(Scalp)=0.194
p(Dura)=0.0325
Scalp
Dura
A
B
C
D
E
F
G
H
I
J
K

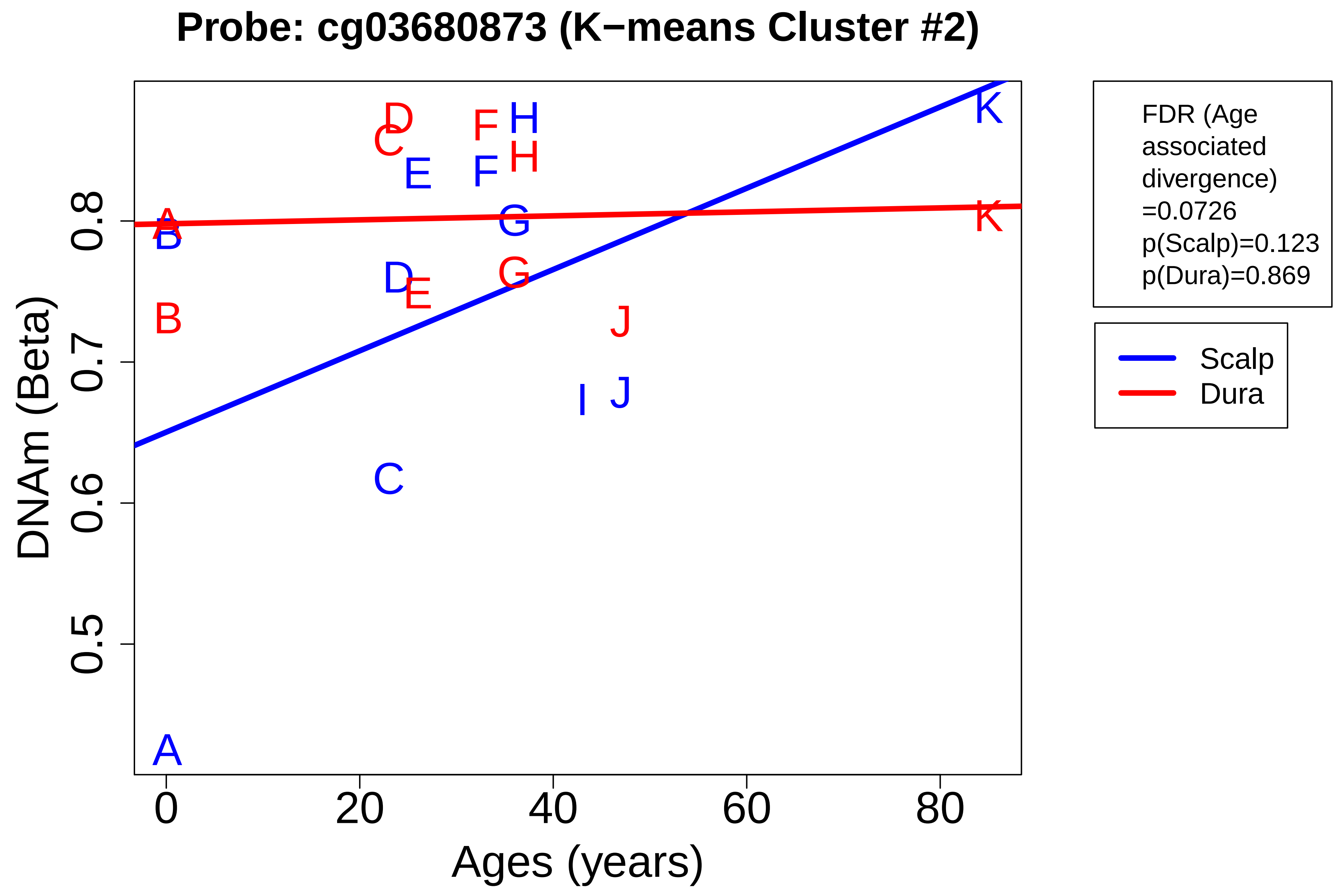
Probe: cg03680873 (K−means Cluster #2)
DNAm (Beta)
0.5
0.6
0.7
0.8
Ages (years)
0
20
40
60
80
A
B
C
D
E
F
G
H
I
J
K
FDR (Age associated divergence) =0.0726
p(Scalp)=0.123
p(Dura)=0.869
Scalp
Dura

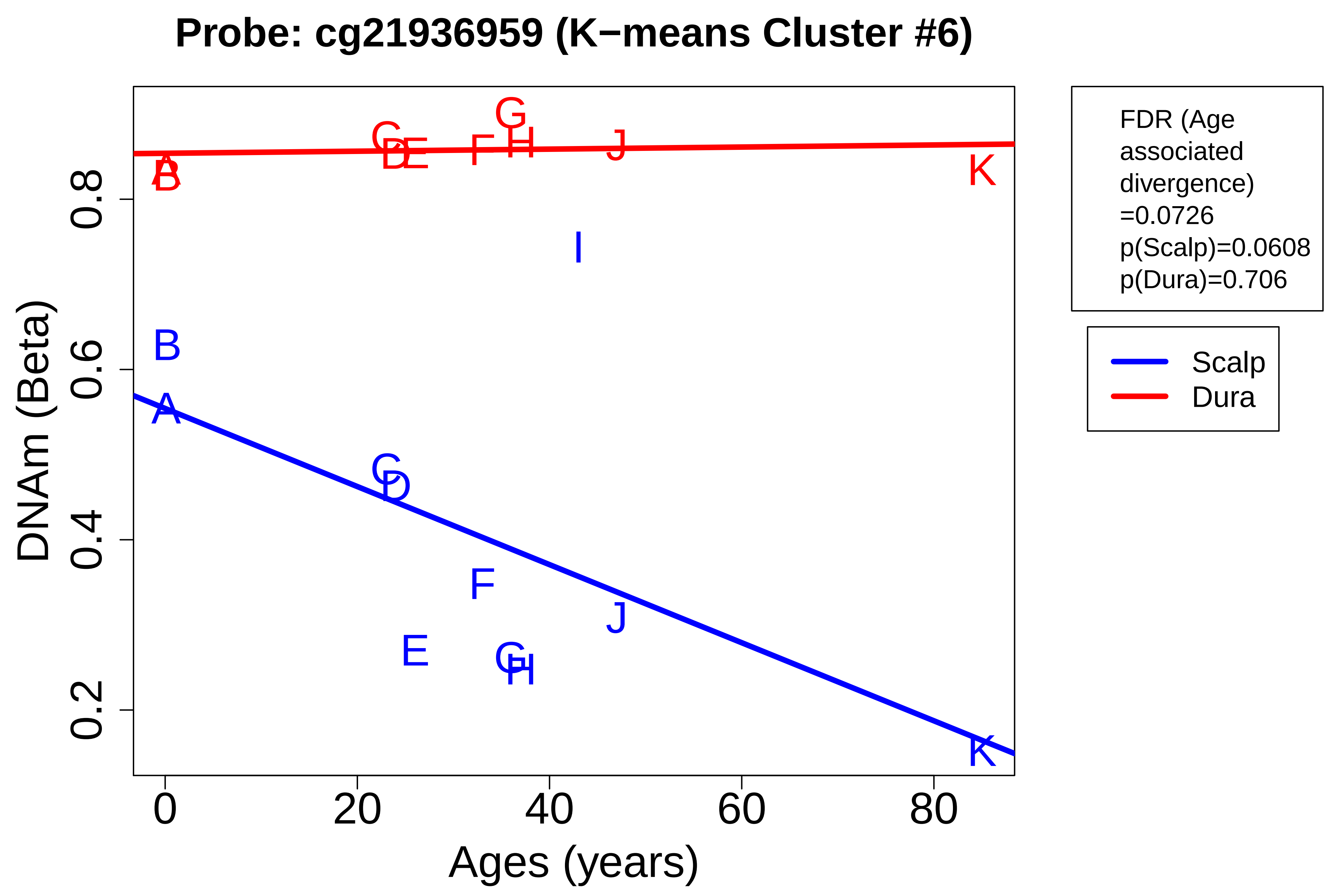

Probe: cg21936959 (K−means Cluster #6)
FDR (Age associated divergence) =0.0726
p(Scalp)=0.0608
p(Dura)=0.706
Scalp
Dura
DNAm (Beta)
0.2
0.4
0.6
0.8
0
20
40
60
80
Ages (years)

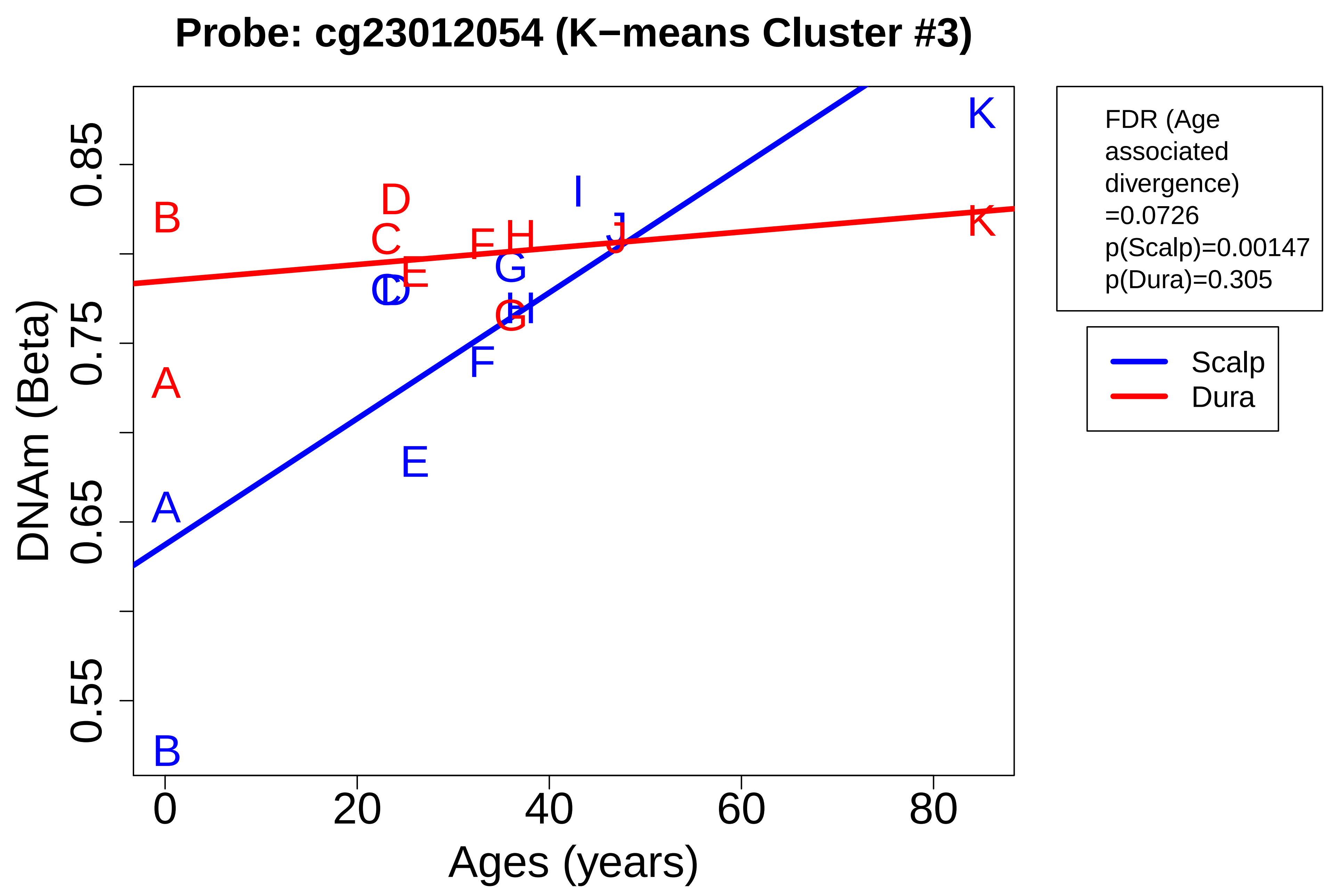

Probe: cg23012054 (K−means Cluster #3)
DNAm (Beta)
0.55
0.65
0.75
0.85
0
20
40
60
80
Ages (years)
FDR (Age associated divergence) =0.0726
p(Scalp)=0.00147
p(Dura)=0.305
Scalp
Dura

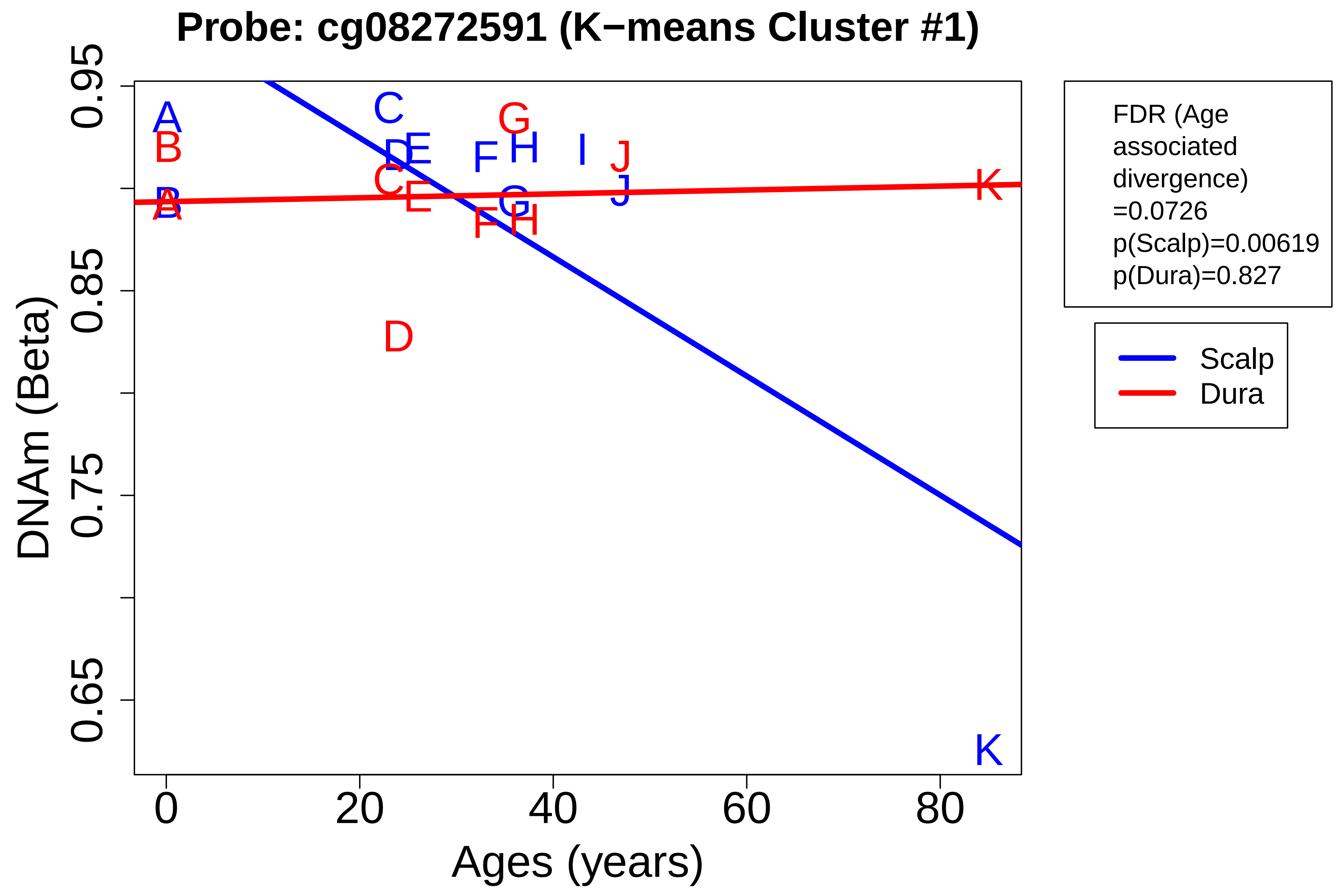

Probe: cg08272591 (K−means Cluster #1)
DNAm (Beta)
0.95
0.85
0.75
0.65
Ages (years)
0
20
40
60
80
A
B
C
D
E
F
G
H
I
J
K
FDR (Age associated divergence) =0.0726
p(Scalp)=0.00619
p(Dura)=0.827
Scalp
Dura

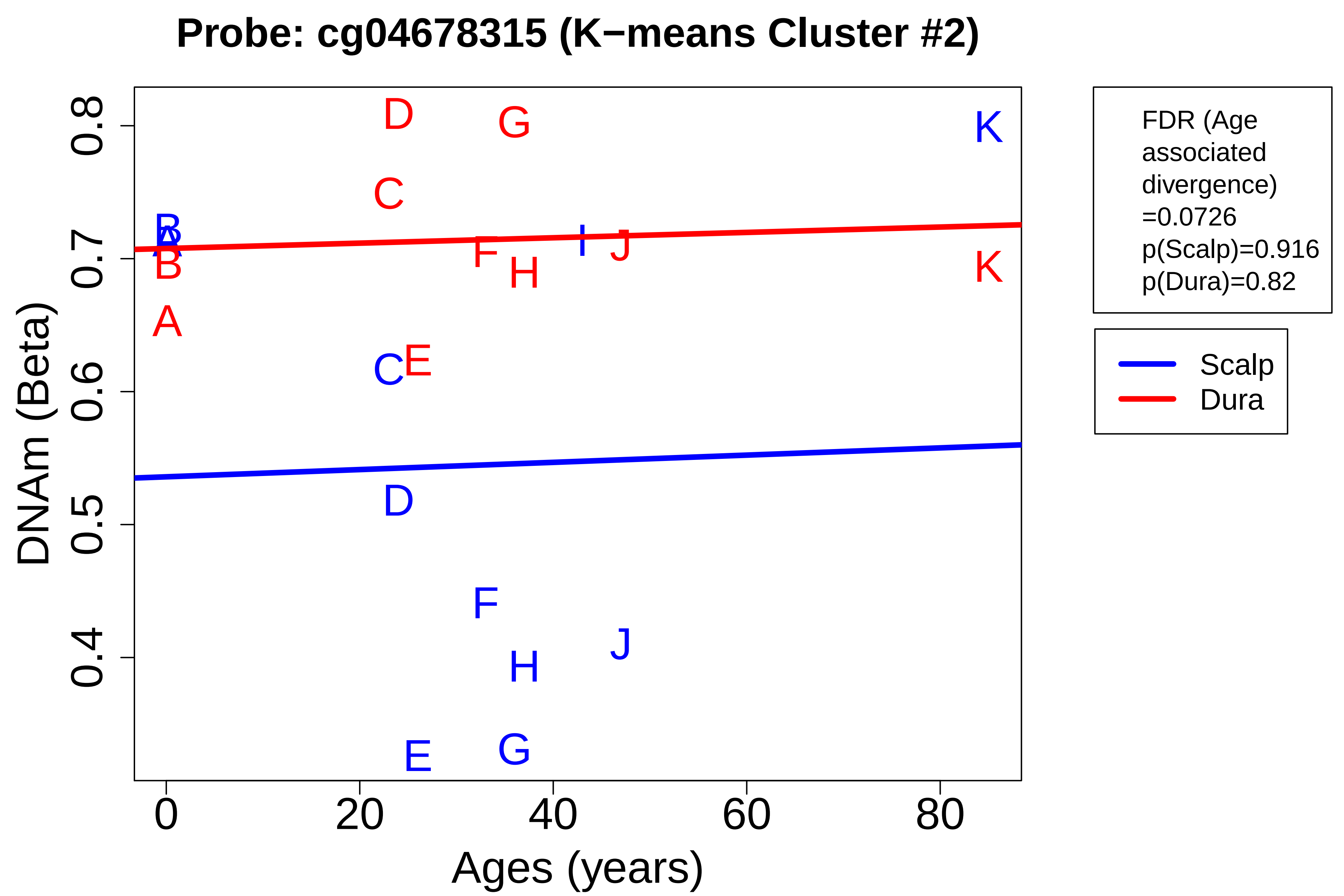

Probe: cg04678315 (K−means Cluster #2)
DNAm (Beta)
0.4
0.5
0.6
0.7
0.8
Ages (years)
0
20
40
60
80
FDR (Age associated divergence) =0.0726
p(Scalp)=0.916
p(Dura)=0.82
Scalp
Dura
A
B
C
D
E
F
G
H
I
J
K

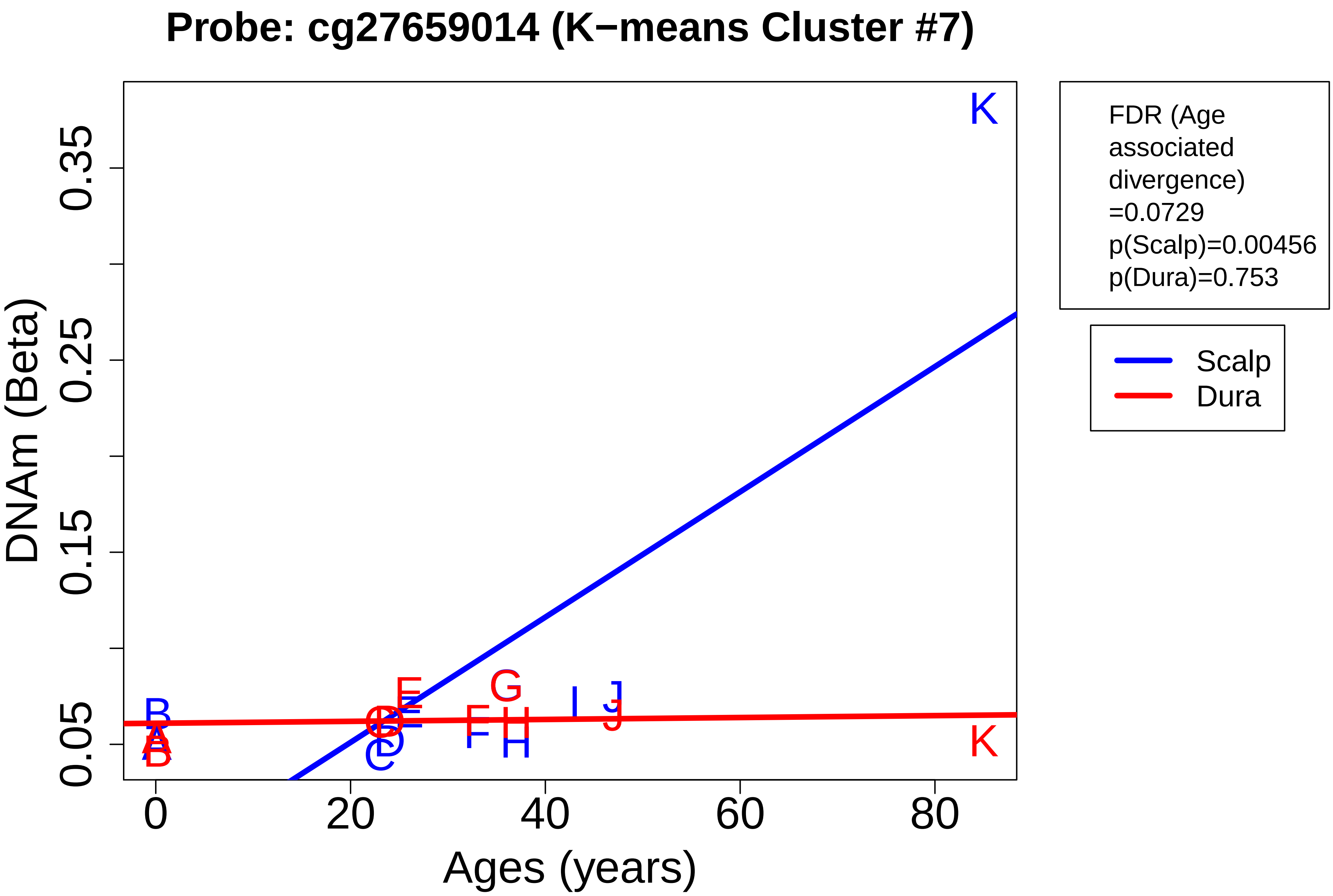

Probe: cg27659014 (K−means Cluster #7)
DNAm (Beta)
0.05
0.15
0.25
0.35
0
20
40
60
80
Ages (years)
A
B
C
D
E
F
G
H
I
J
K
FDR (Age associated divergence) =0.0729
p(Scalp)=0.00456
p(Dura)=0.753
Scalp
Dura

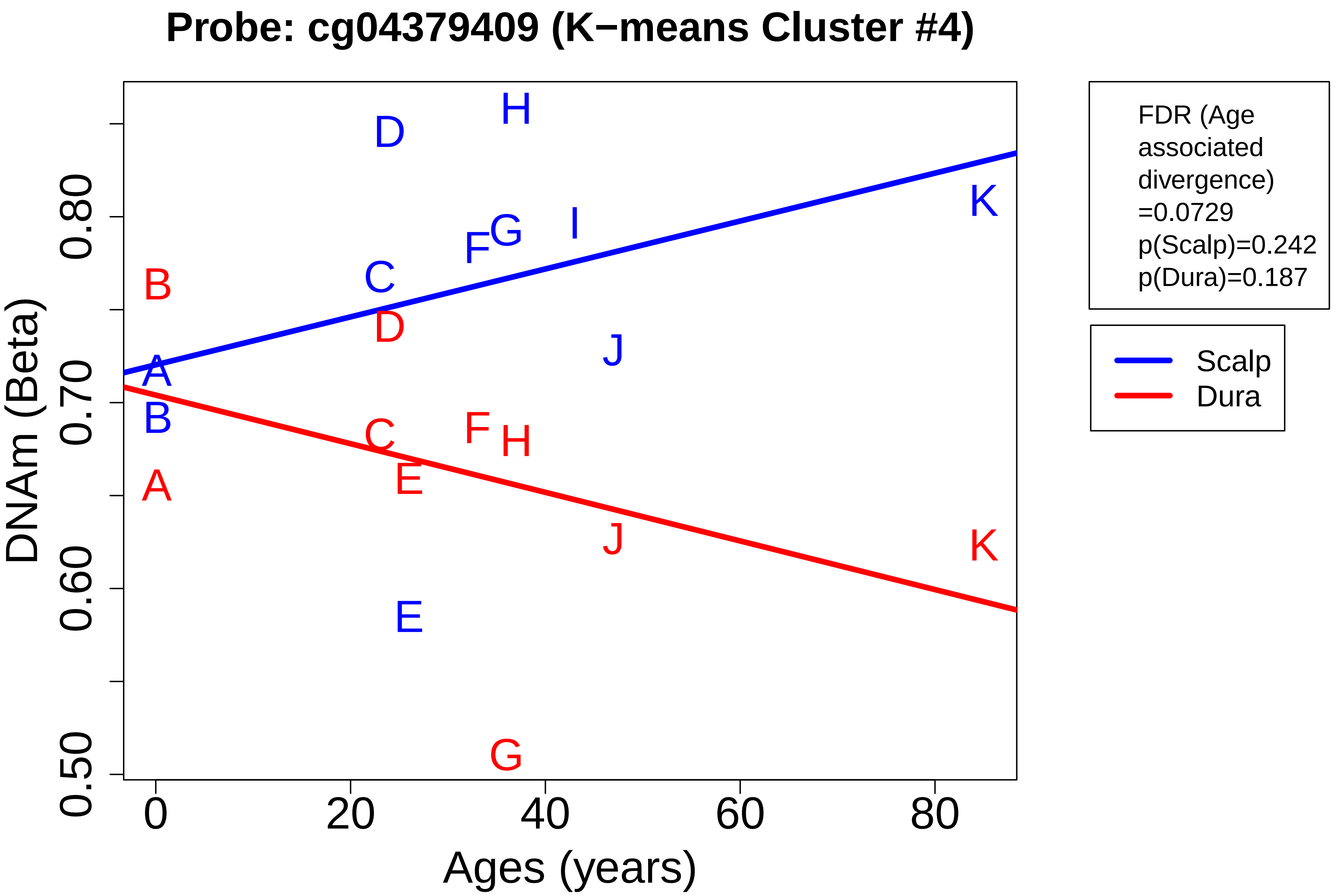

Probe: cg04379409 (K−means Cluster #4)
DNAm (Beta)
0.50
0.60
0.70
0.80
0
20
40
60
80
Ages (years)
FDR (Age associated divergence) =0.0729
p(Scalp)=0.242
p(Dura)=0.187
Scalp
Dura

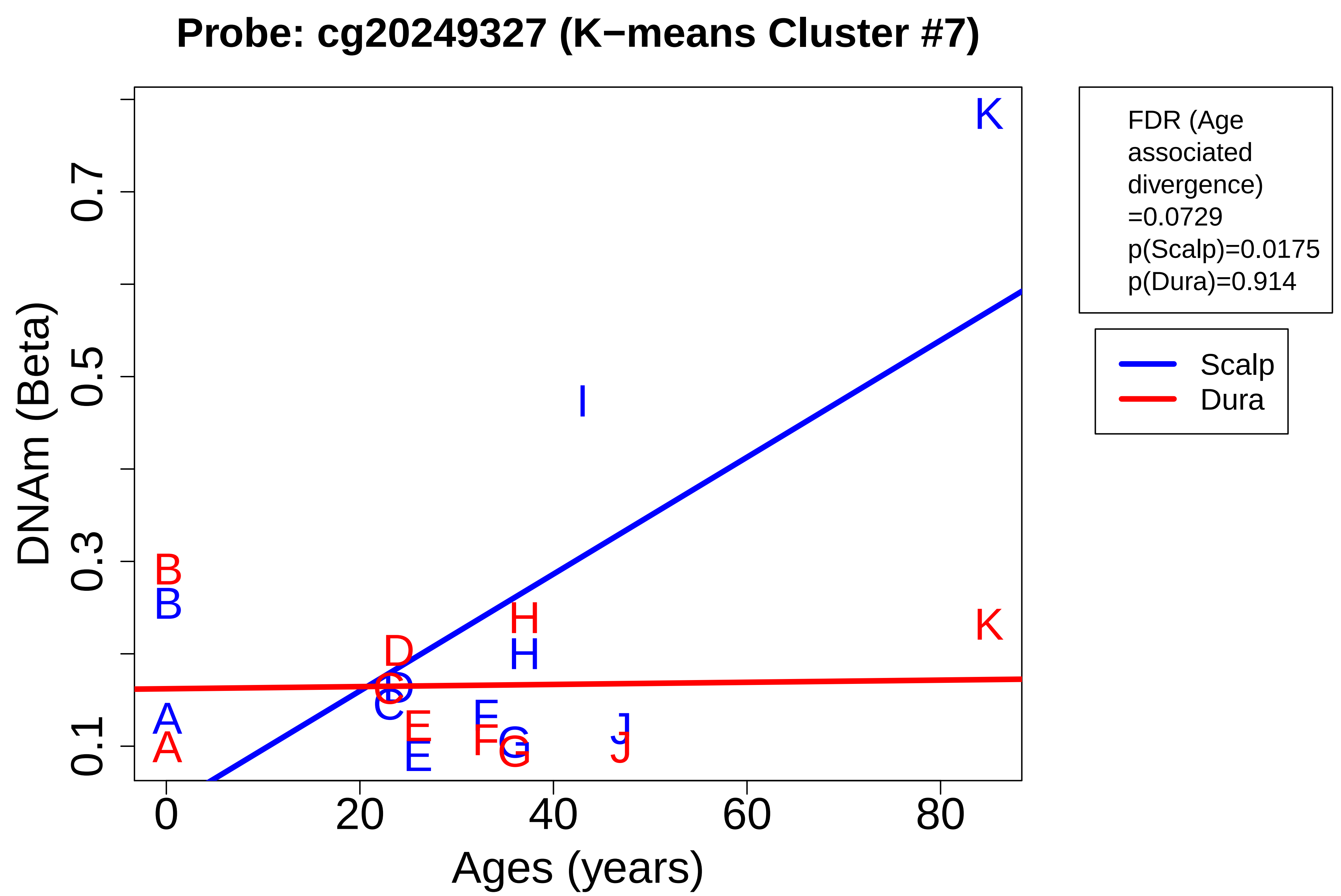
Probe: cg20249327 (K−means Cluster #7)
DNAm (Beta)
0.1
0.3
0.5
0.7
Ages (years)
0
20
40
60
80
A
B
C
D
E
F
G
H
I
J
K
FDR (Age associated divergence) =0.0729
p(Scalp)=0.0175
p(Dura)=0.914
Scalp
Dura

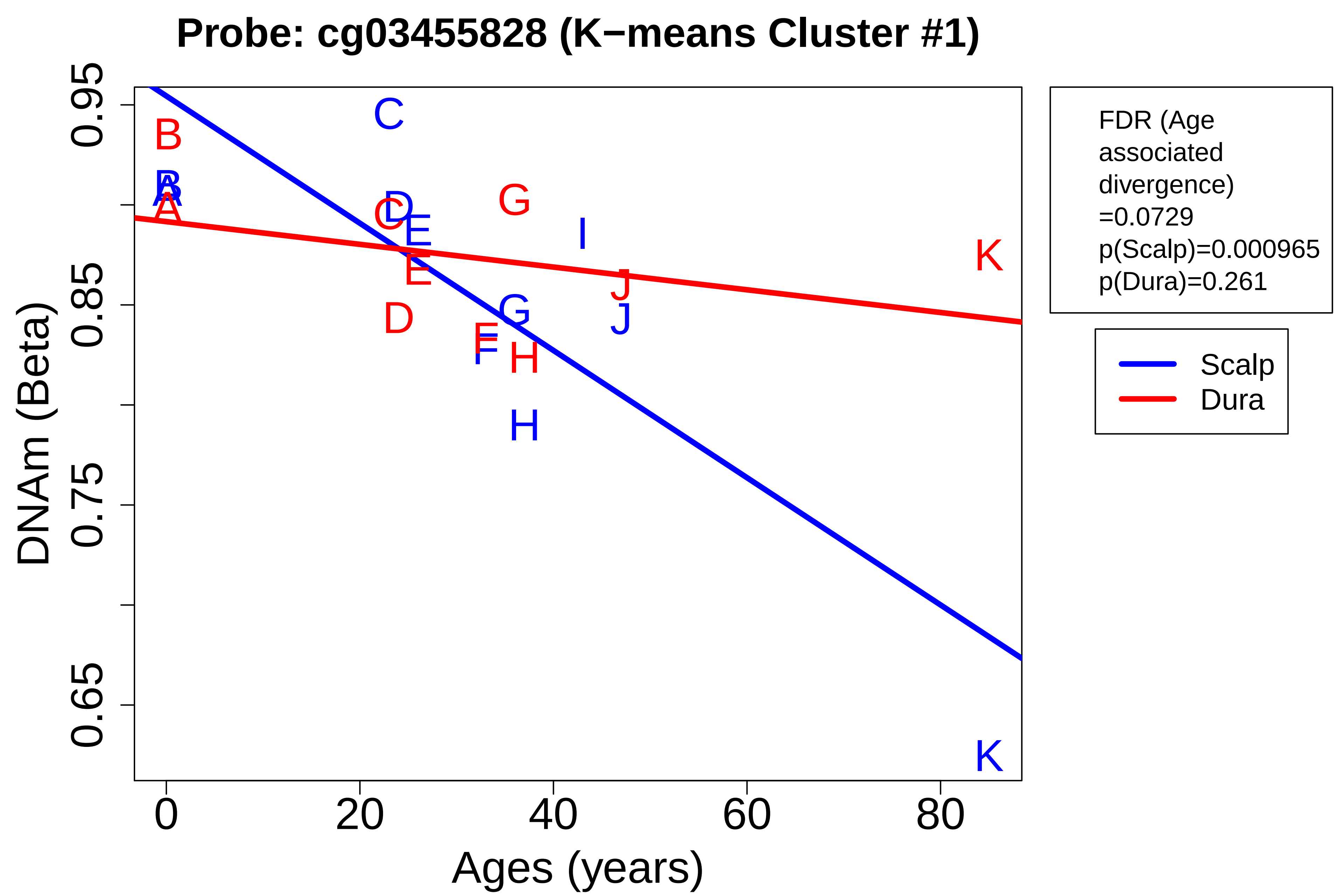

Probe: cg03455828 (K−means Cluster #1)
DNAm (Beta)
0.65
0.75
0.85
0.95
Ages (years)
0
20
40
60
80
FDR (Age associated divergence) =0.0729
p(Scalp)=0.000965
p(Dura)=0.261
Scalp
Dura
A
B
C
D
E
F
G
H
I
J
K

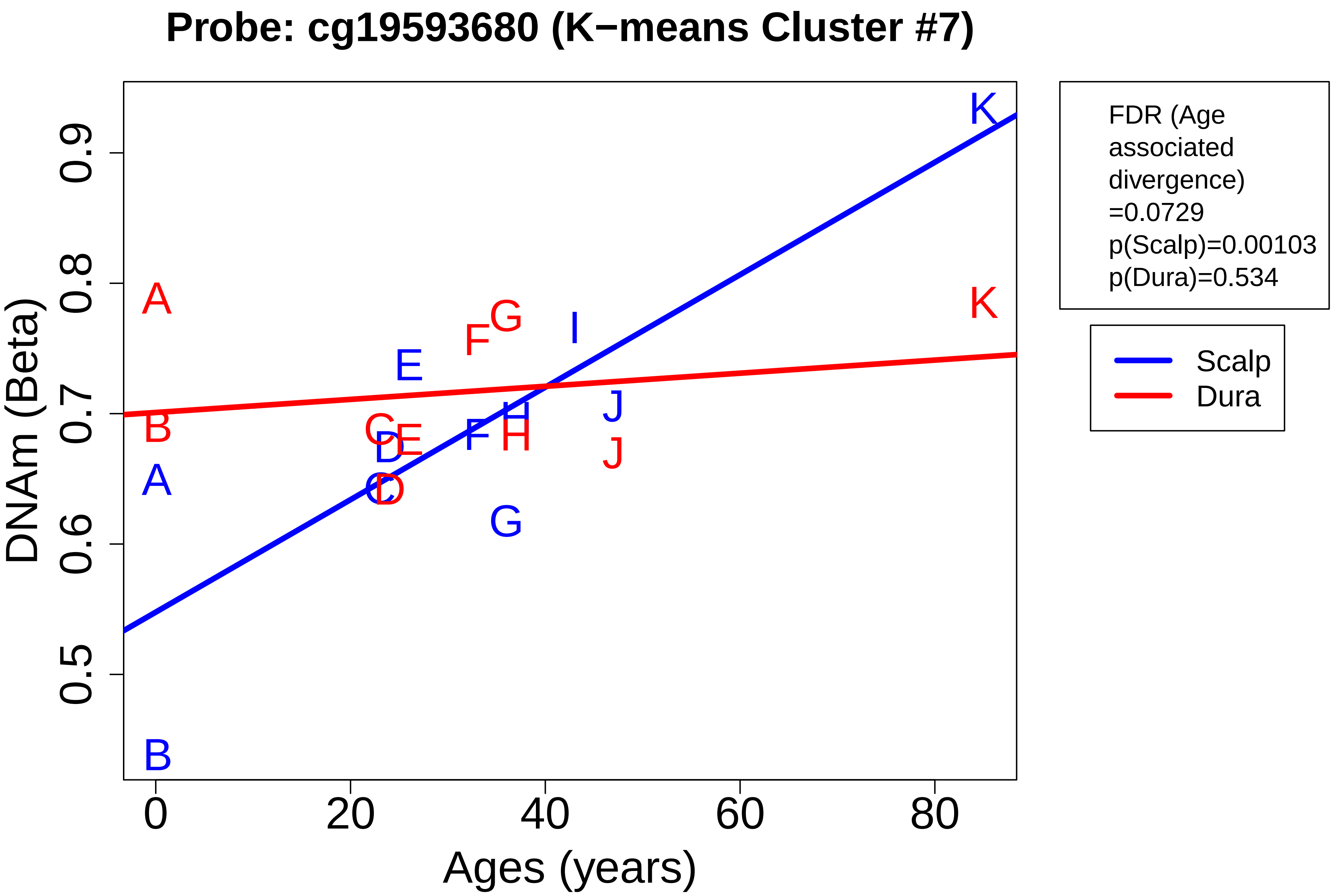

Probe: cg19593680 (K−means Cluster #7)
DNAm (Beta)
0.5
0.6
0.7
0.8
0.9
Ages (years)
0
20
40
60
80
A
B
C
D
E
F
G
H
I
J
K
FDR (Age associated divergence) =0.0729
p(Scalp)=0.00103
p(Dura)=0.534
Scalp
Dura

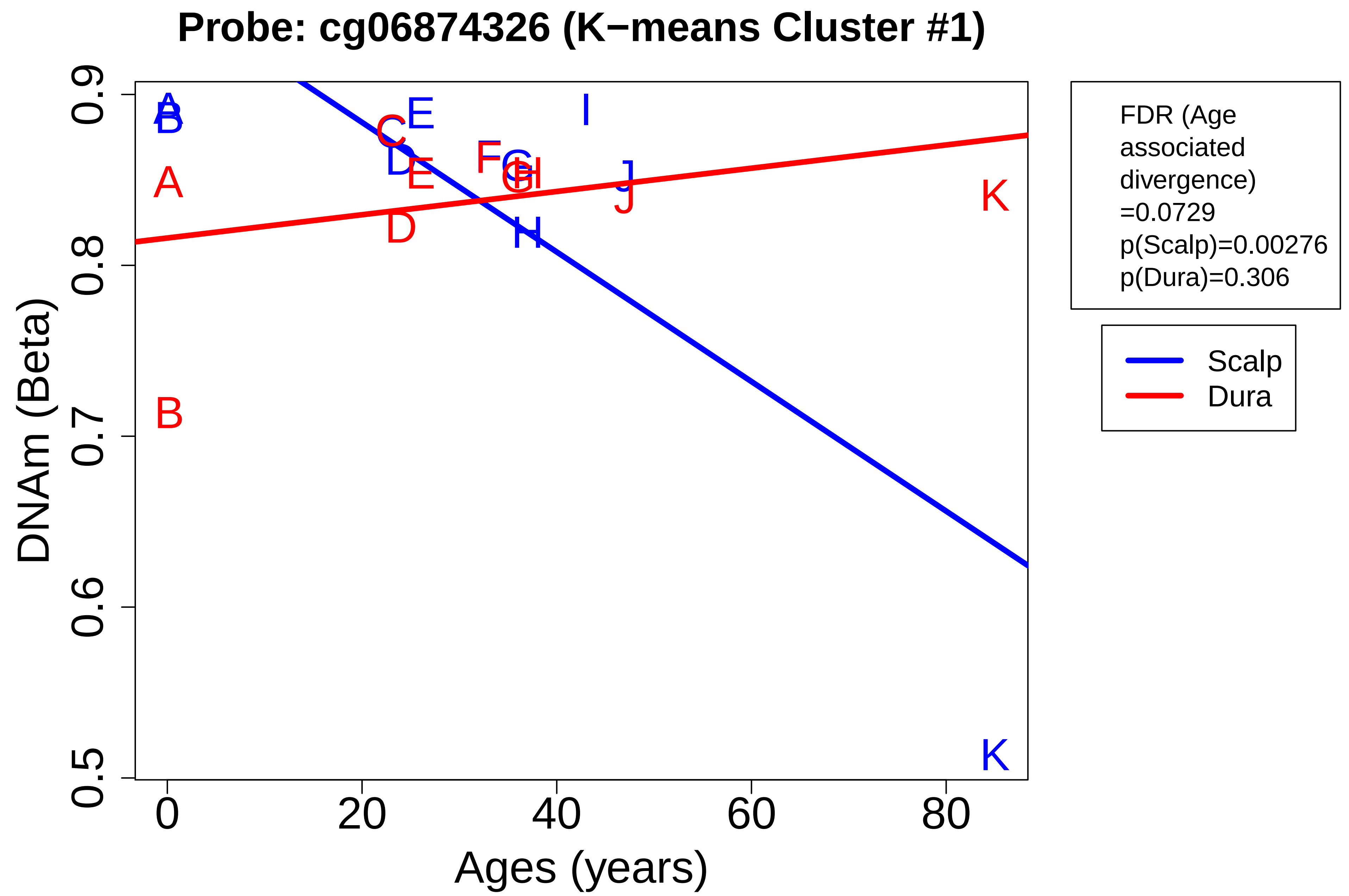

Probe: cg06874326 (K−means Cluster #1)
DNAm (Beta)
0.5
0.6
0.7
0.8
0.9
0
20
40
60
80
Ages (years)
FDR (Age associated divergence) =0.0729
p(Scalp)=0.00276
p(Dura)=0.306
Scalp
Dura

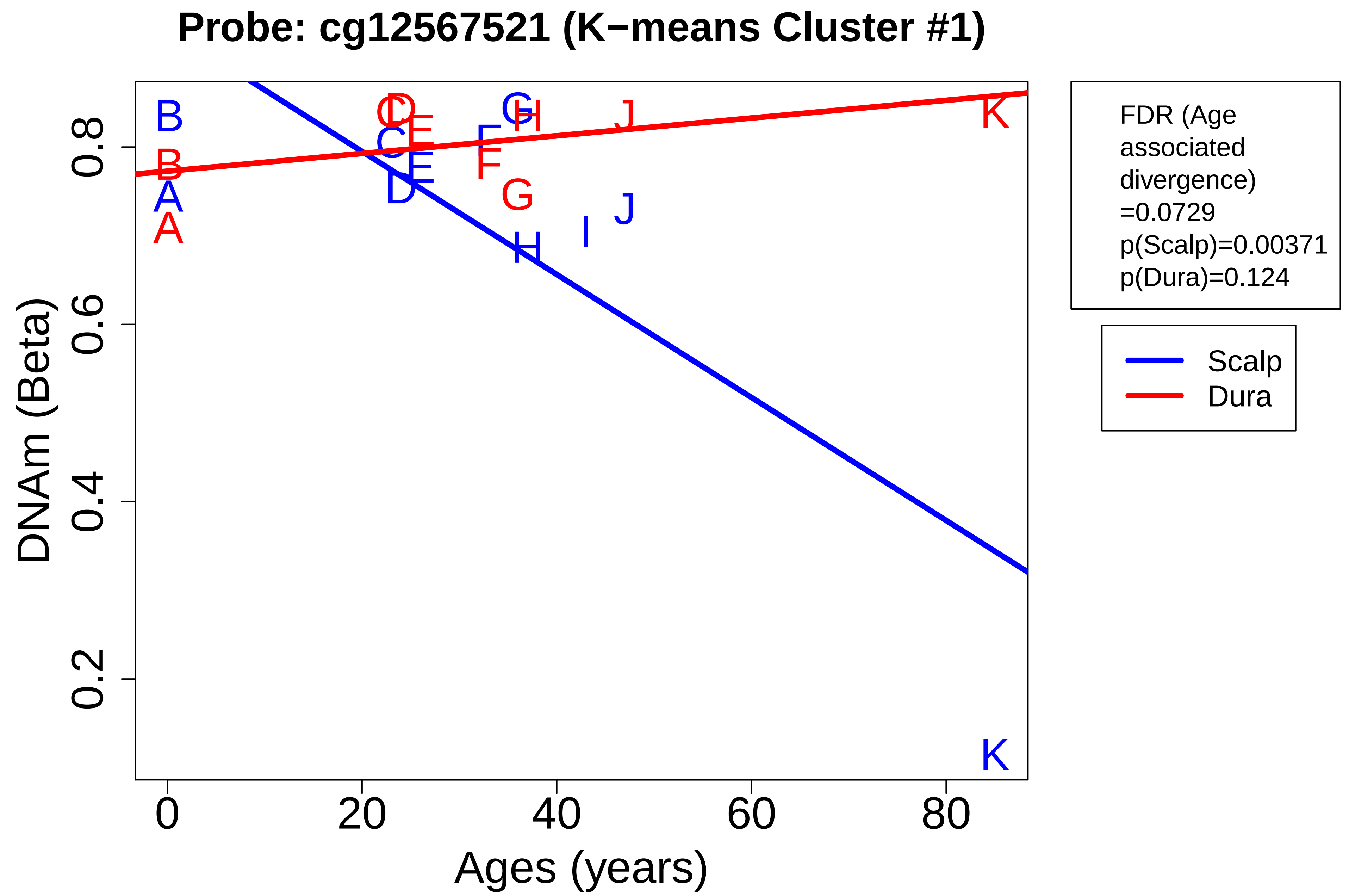

Probe: cg12567521 (K−means Cluster #1)
DNAm (Beta)
0.2
0.4
0.6
0.8
Ages (years)
0
20
40
60
80
A
B
C
D
E
F
G
H
I
J
K
FDR (Age associated divergence) =0.0729
p(Scalp)=0.00371
p(Dura)=0.124
Scalp
Dura

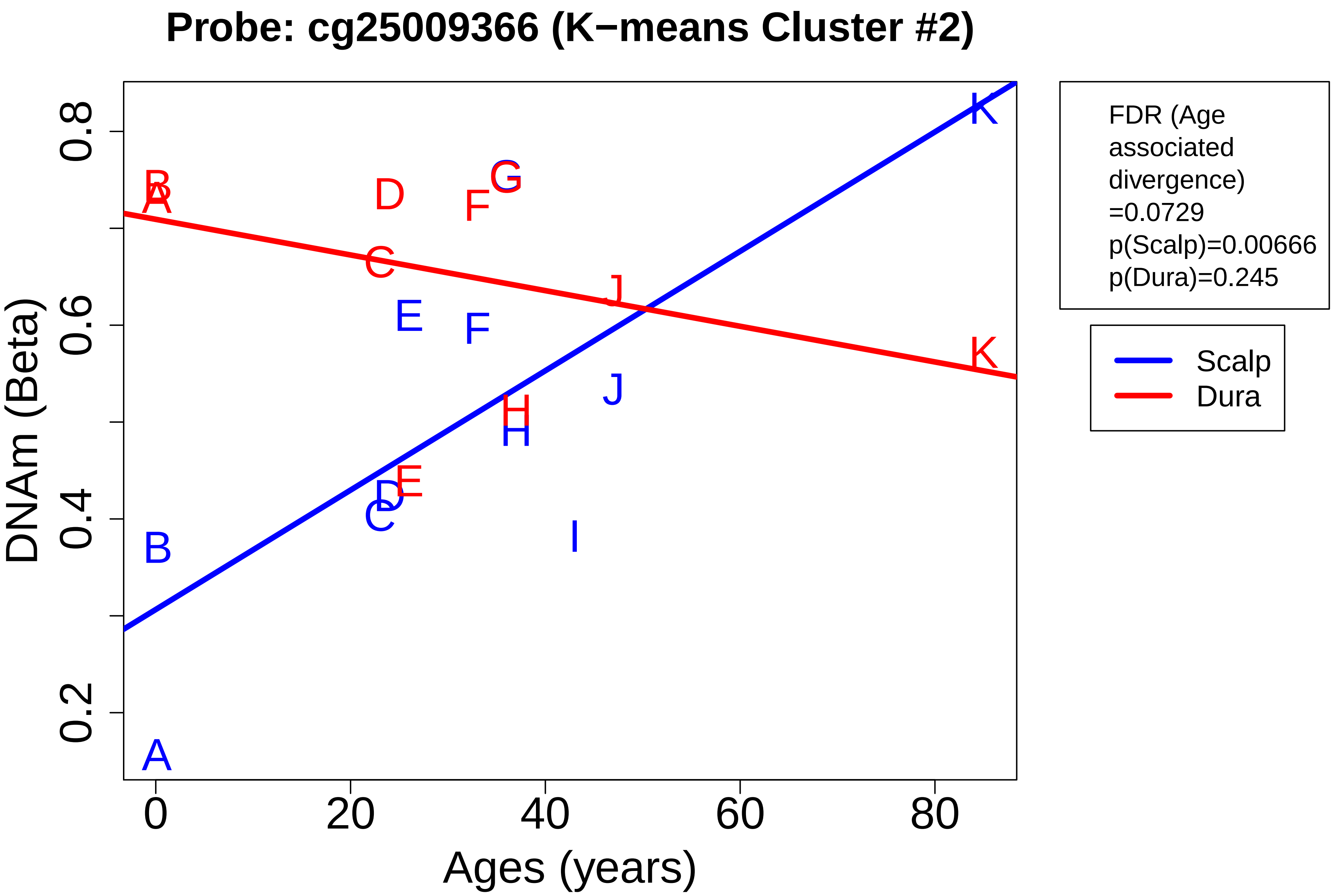

Probe: cg25009366 (K−means Cluster #2)
DNAm (Beta)
0.2
0.4
0.6
0.8
0
20
40
60
80
Ages (years)
FDR (Age associated divergence) =0.0729
p(Scalp)=0.00666
p(Dura)=0.245
Scalp
Dura

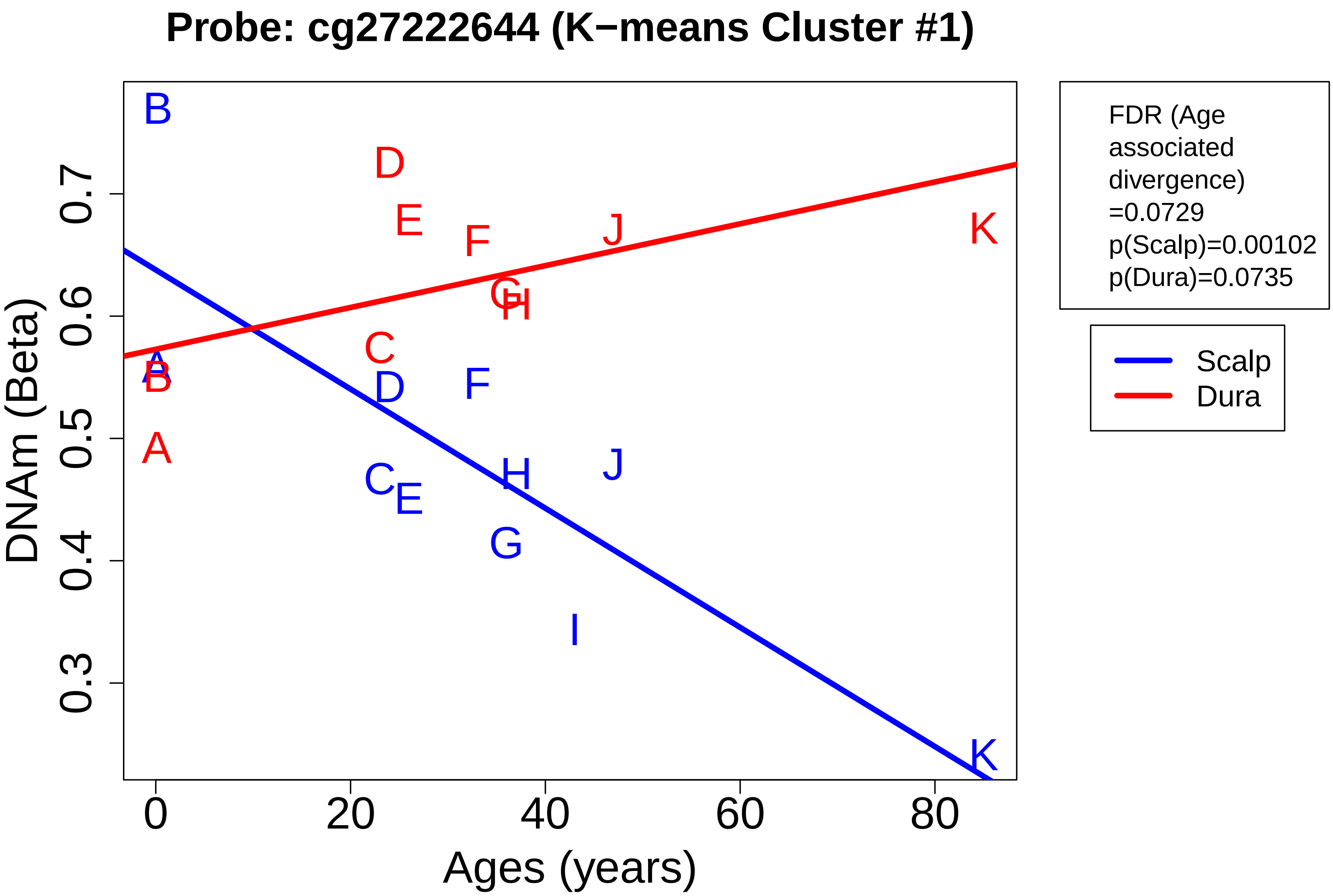

Probe: cg27222644 (K−means Cluster #1)
FDR (Age associated divergence) =0.0729
p(Scalp)=0.00102
p(Dura)=0.0735
Scalp
Dura
DNAm (Beta)
Ages (years)
0.3
0.4
0.5
0.6
0.7
0
20
40
60
80

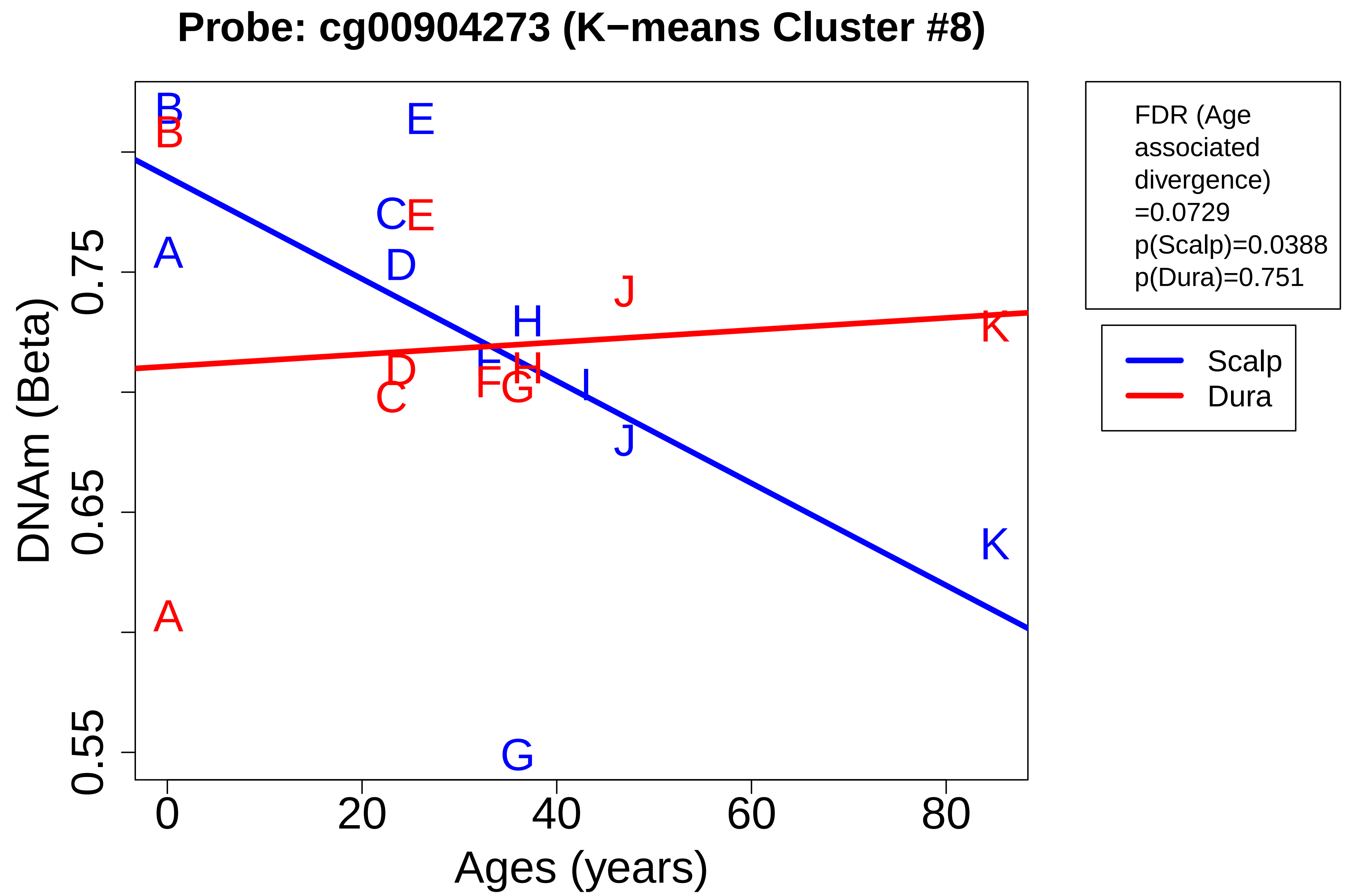

Probe: cg00904273 (K−means Cluster #8)
DNAm (Beta)
0.55
0.65
0.75
0
20
40
60
80
Ages (years)
FDR (Age associated divergence) =0.0729
p(Scalp)=0.0388
p(Dura)=0.751
Scalp
Dura

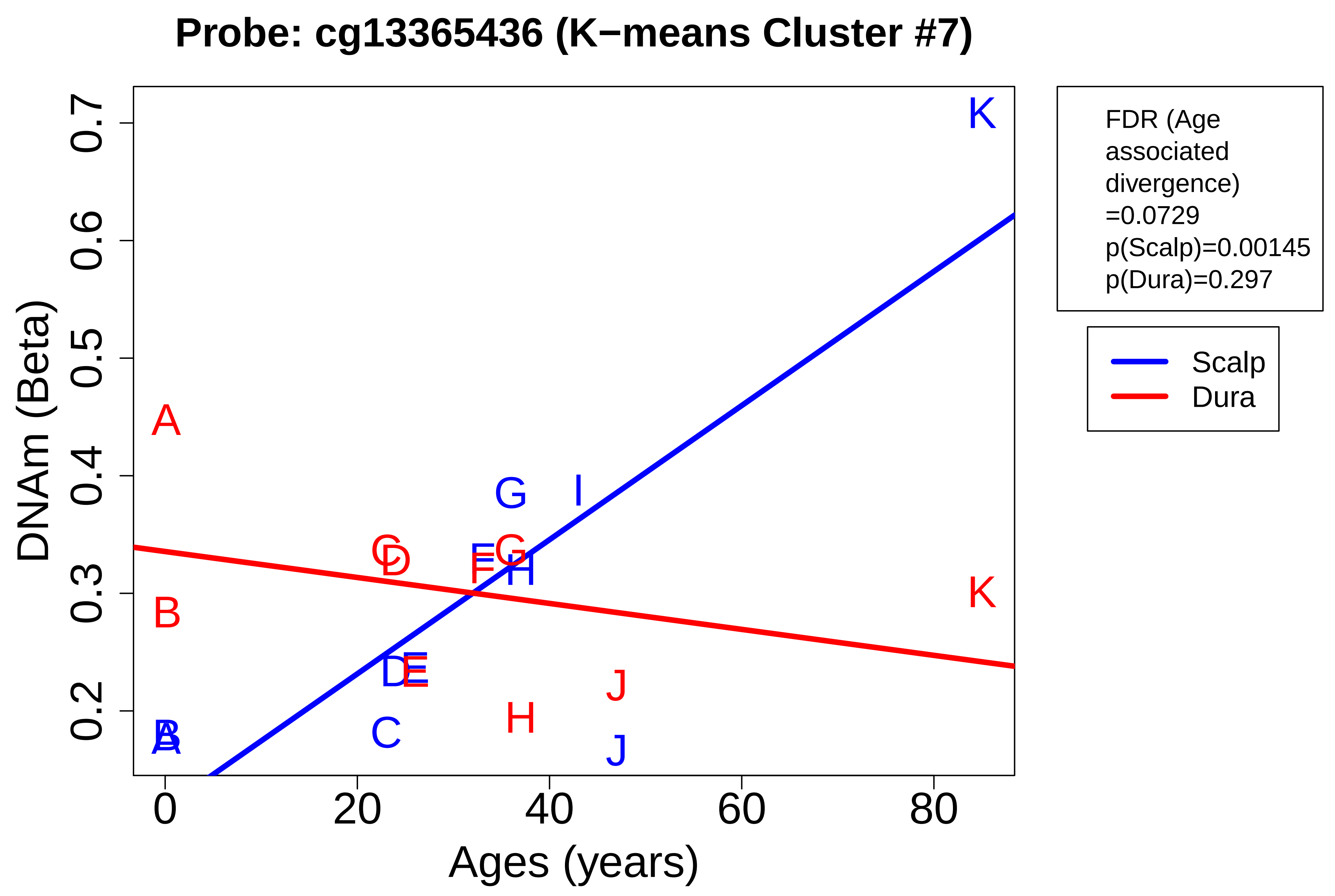

Probe: cg13365436 (K−means Cluster #7)
DNAm (Beta)
0.2
0.3
0.4
0.5
0.6
0.7
Ages (years)
0
20
40
60
80
FDR (Age associated divergence) =0.0729
p(Scalp)=0.00145
p(Dura)=0.297
Scalp
Dura

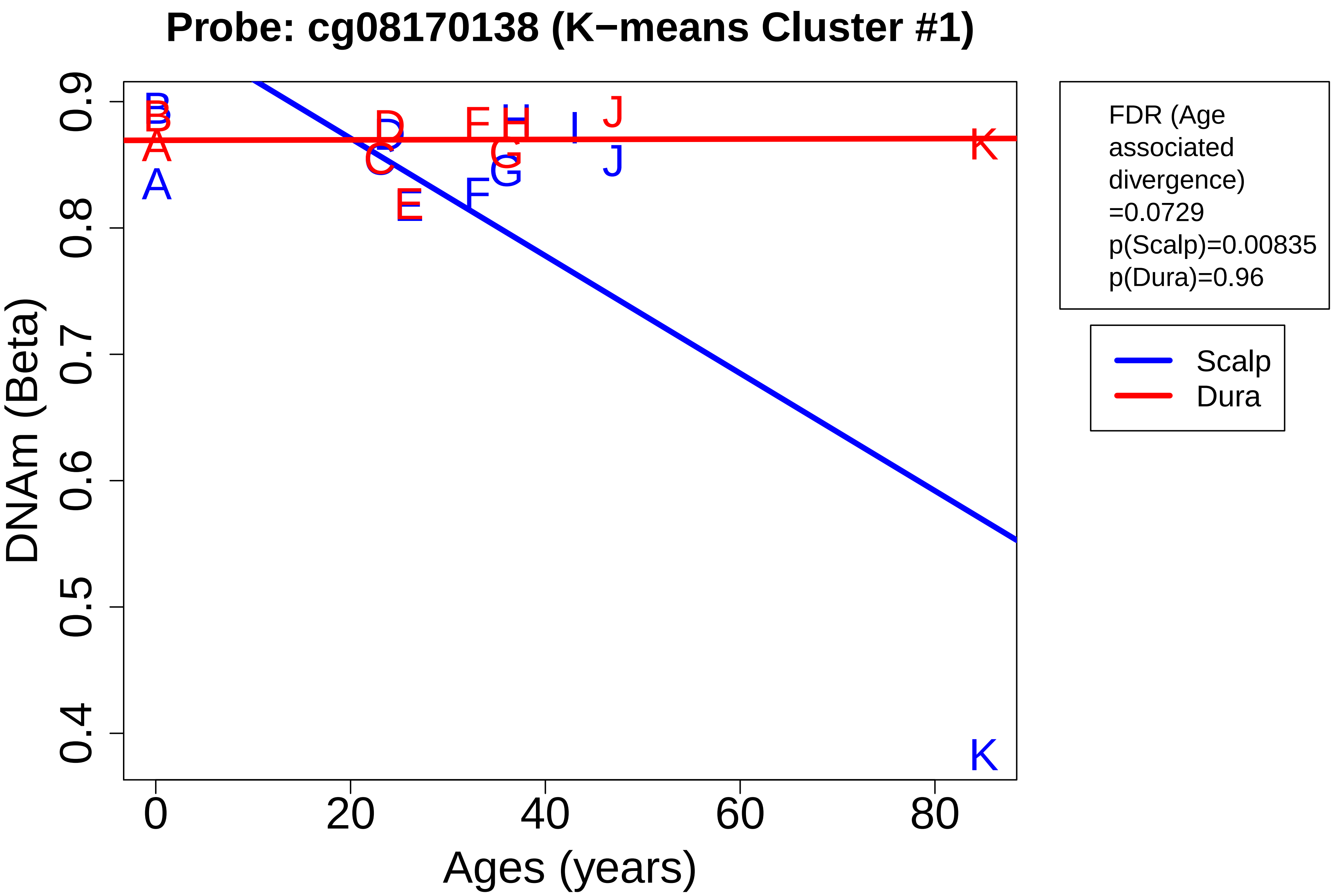
Probe: cg08170138 (K−means Cluster #1)
DNAm (Beta)
Ages (years)
0.4
0.5
0.6
0.7
0.8
0.9
0
20
40
60
80
FDR (Age associated divergence) =0.0729
p(Scalp)=0.00835
p(Dura)=0.96
Scalp
Dura
A
B
C
D
E
F
G
H
I
J
K

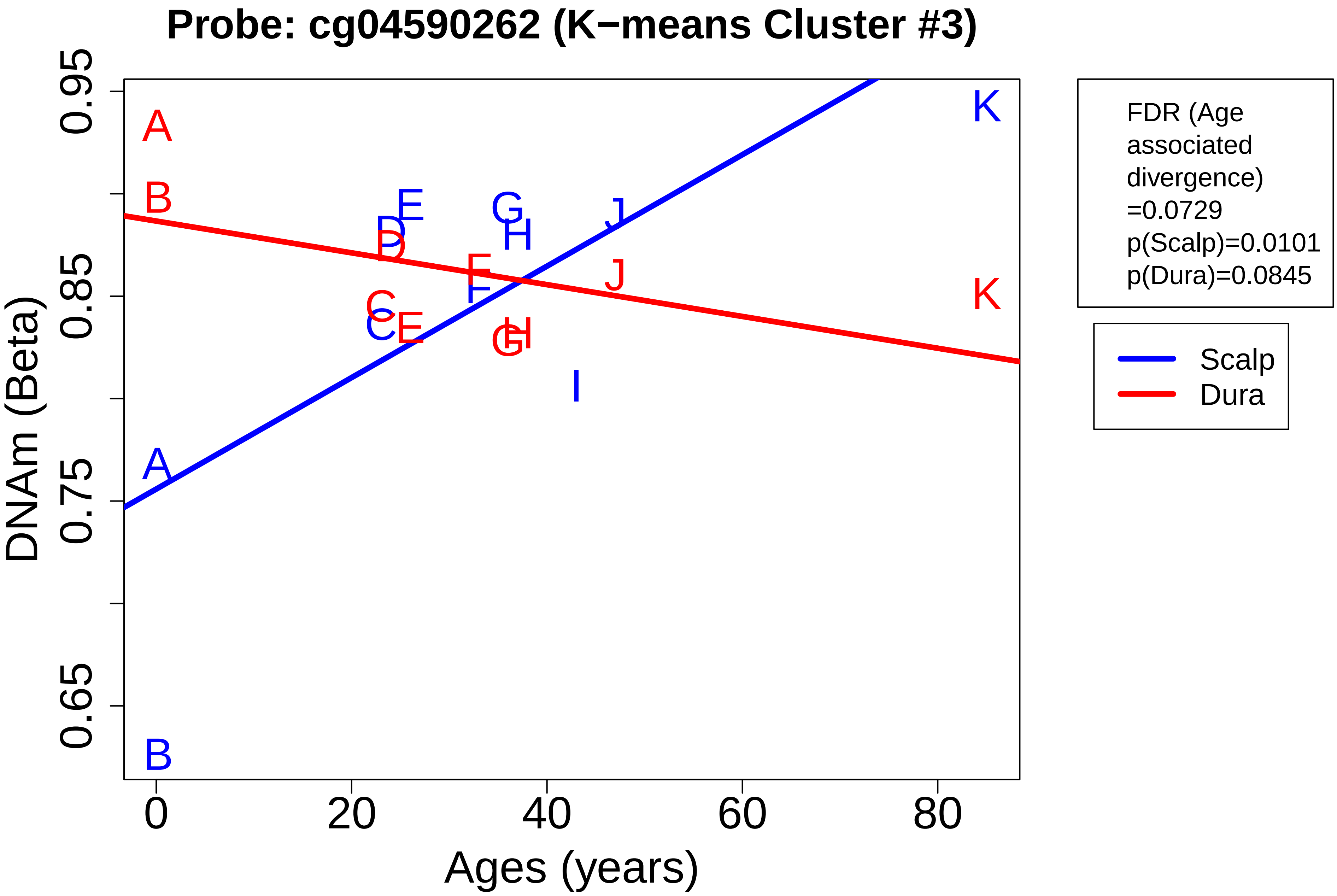

Probe: cg04590262 (K−means Cluster #3)
DNAm (Beta)
0.65
0.75
0.85
0.95
Ages (years)
0
20
40
60
80
A
B
C
D
E
F
G
H
I
J
K
FDR (Age associated divergence) =0.0729
p(Scalp)=0.0101
p(Dura)=0.0845
Scalp
Dura

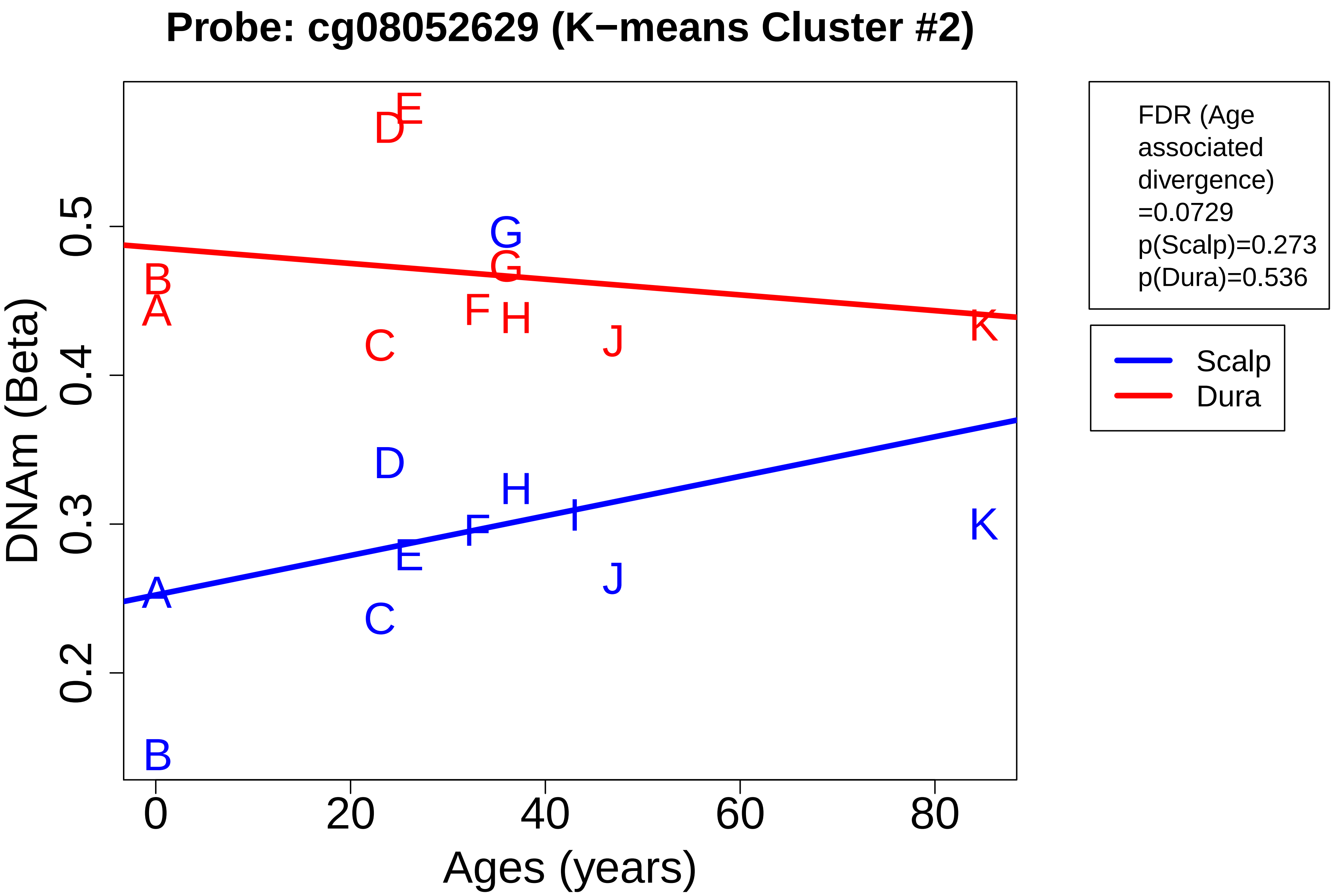
Probe: cg08052629 (K−means Cluster #2)
DNAm (Beta)
0.2
0.3
0.4
0.5
Ages (years)
0
20
40
60
80
A
B
C
D
E
F
G
H
I
J
K
FDR (Age associated divergence) =0.0729
p(Scalp)=0.273
p(Dura)=0.536
Scalp
Dura

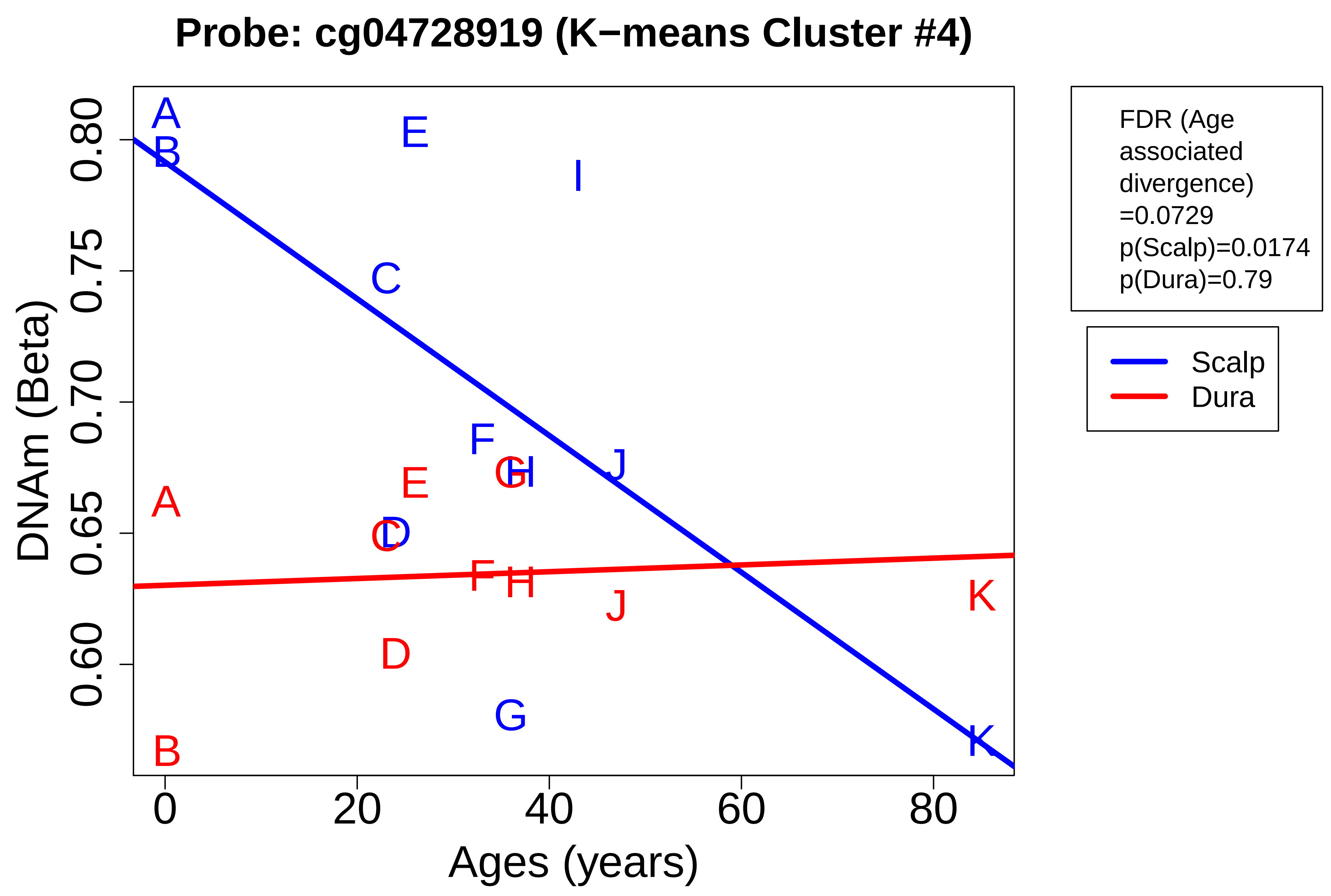

Probe: cg04728919 (K−means Cluster #4)
DNAm (Beta)
0.60
0.65
0.70
0.75
0.80
0
20
40
60
80
Ages (years)
A
B
C
D
E
F
G
H
I
J
K
FDR (Age associated divergence) =0.0729
p(Scalp)=0.0174
p(Dura)=0.79
Scalp
Dura

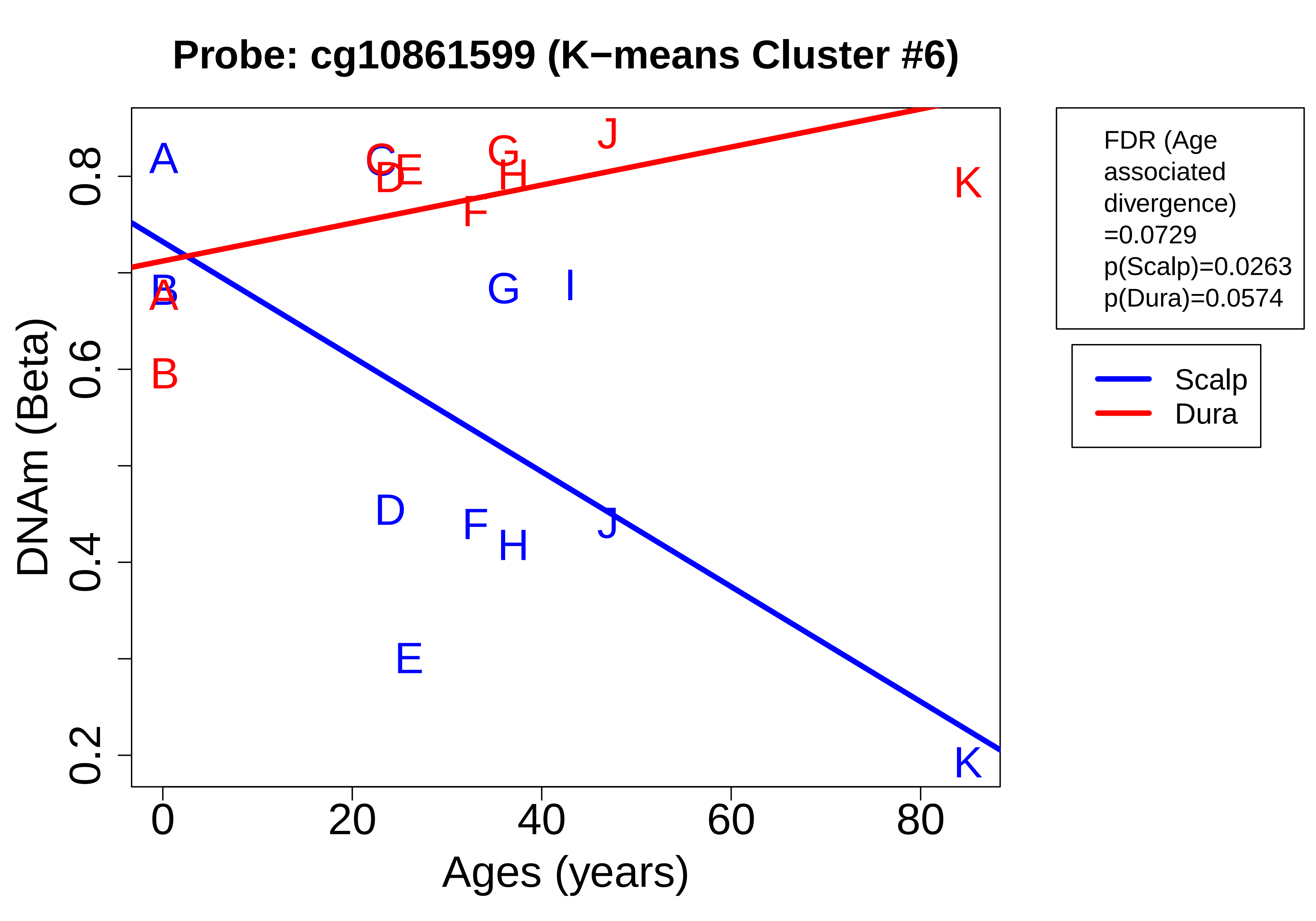

Probe: cg10861599 (K−means Cluster #6)
DNAm (Beta)
0.2
0.4
0.6
0.8
0
20
40
60
80
Ages (years)
A
B
C
D
E
F
G
H
I
J
K
A
B
C
D
E
F
G
H
J
K
FDR (Age associated divergence) =0.0729
p(Scalp)=0.0263
p(Dura)=0.0574
Scalp
Dura

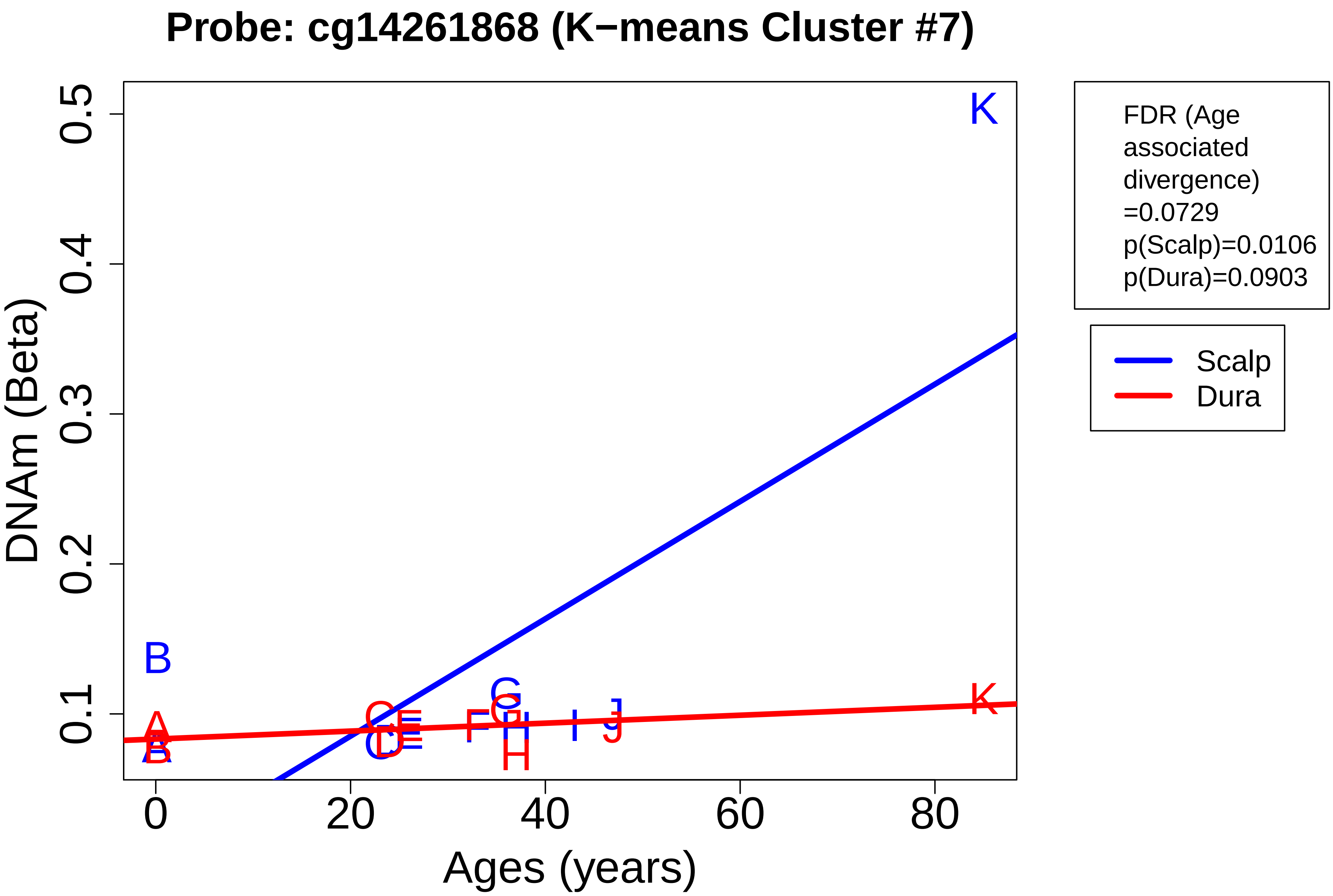

Probe: cg14261868 (K−means Cluster #7)
DNAm (Beta)
0.1
0.2
0.3
0.4
0.5
0
20
40
60
80
Ages (years)
FDR (Age associated divergence) =0.0729
p(Scalp)=0.0106
p(Dura)=0.0903
Scalp
Dura

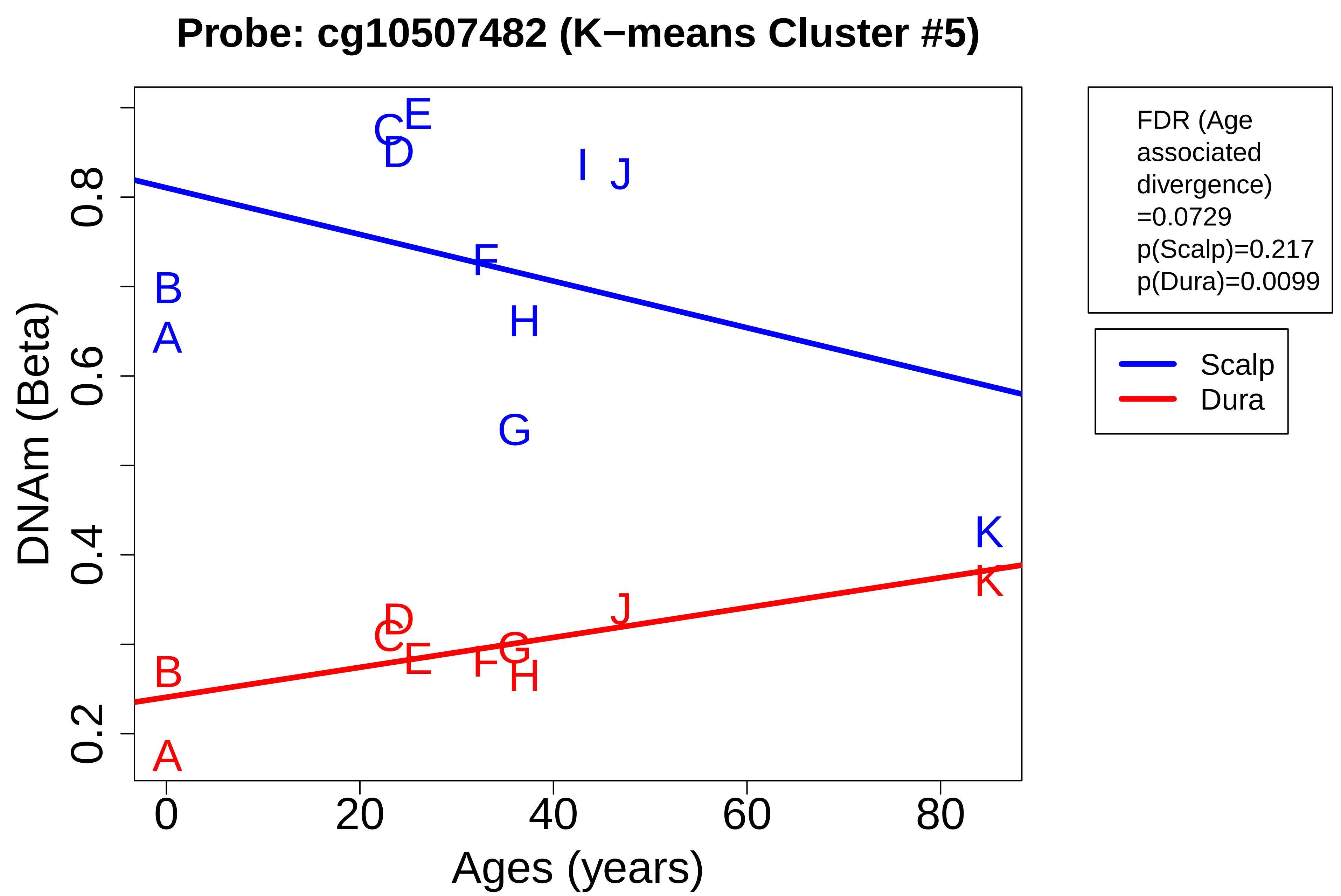

Probe: cg10507482 (K−means Cluster #5)
DNAm (Beta)
0.2
0.4
0.6
0.8
0
20
40
60
80
Ages (years)
A
B
C
D
E
F
G
H
I
J
K
FDR (Age associated divergence) =0.0729
p(Scalp)=0.217
p(Dura)=0.0099
Scalp
Dura

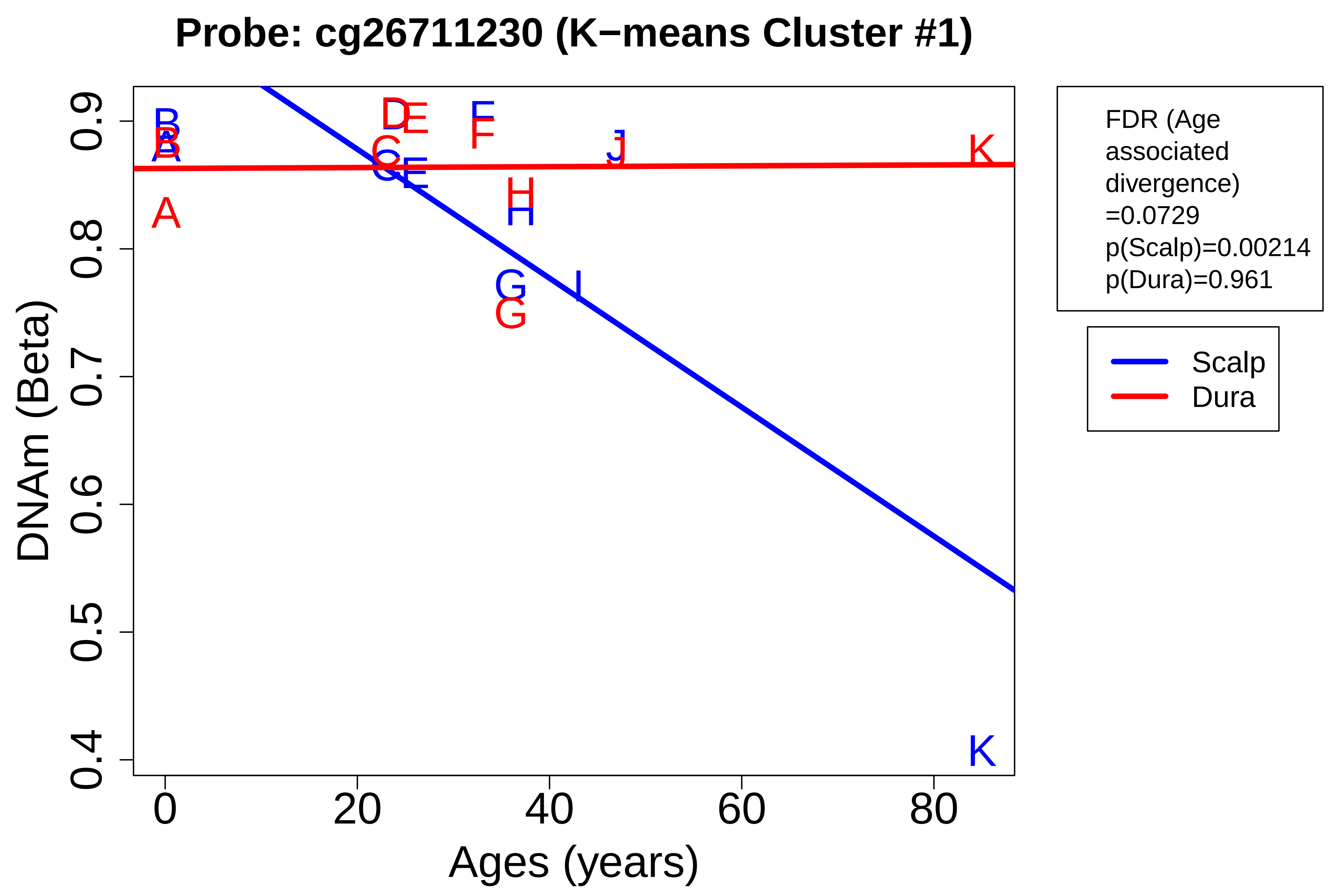

Probe: cg26711230 (K−means Cluster #1)
DNAm (Beta)
0.4
0.5
0.6
0.7
0.8
0.9
0
20
40
60
80
Ages (years)
A
B
C
D
E
F
G
H
I
J
K
FDR (Age associated divergence) =0.0729
p(Scalp)=0.00214
p(Dura)=0.961
Scalp
Dura

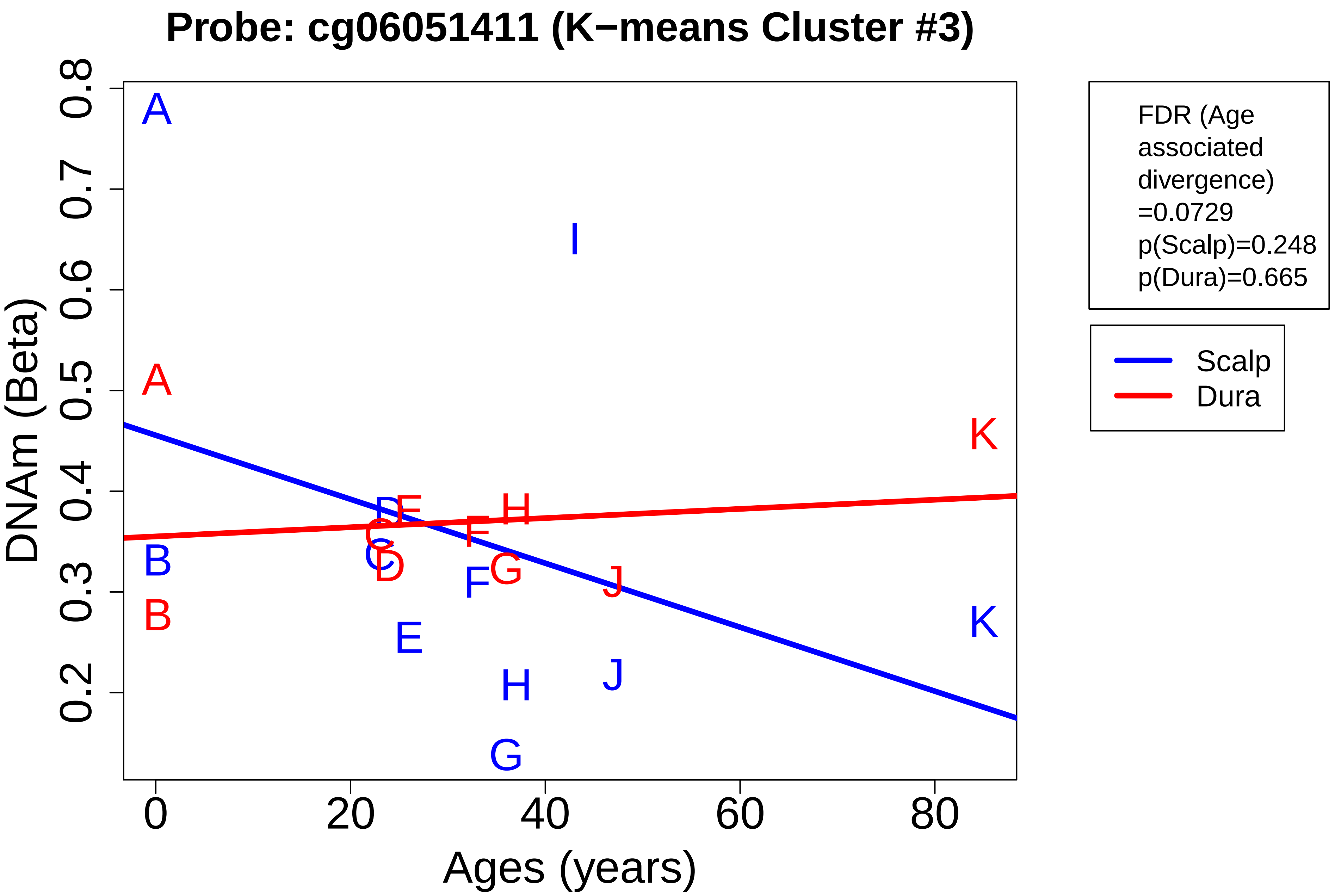

Probe: cg06051411 (K−means Cluster #3)
DNAm (Beta)
0.2
0.3
0.4
0.5
0.6
0.7
0.8
0
20
40
60
80
Ages (years)
FDR (Age associated divergence) =0.0729
p(Scalp)=0.248
p(Dura)=0.665
Scalp
Dura

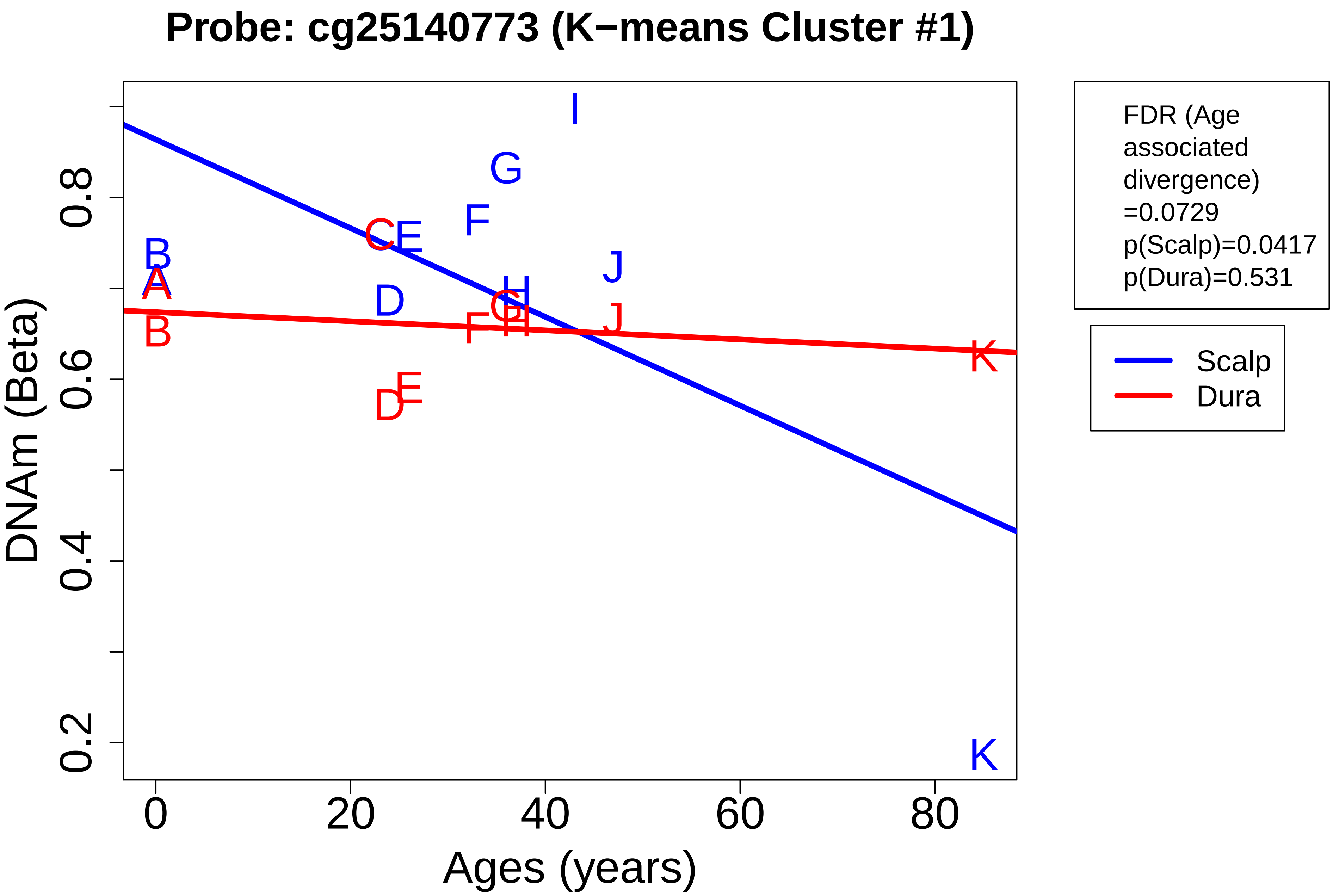

Probe: cg25140773 (K−means Cluster #1)
FDR (Age associated divergence) =0.0729
p(Scalp)=0.0417
p(Dura)=0.531
Scalp
Dura
DNAm (Beta)
0.2
0.4
0.6
0.8
0
20
40
60
80
Ages (years)

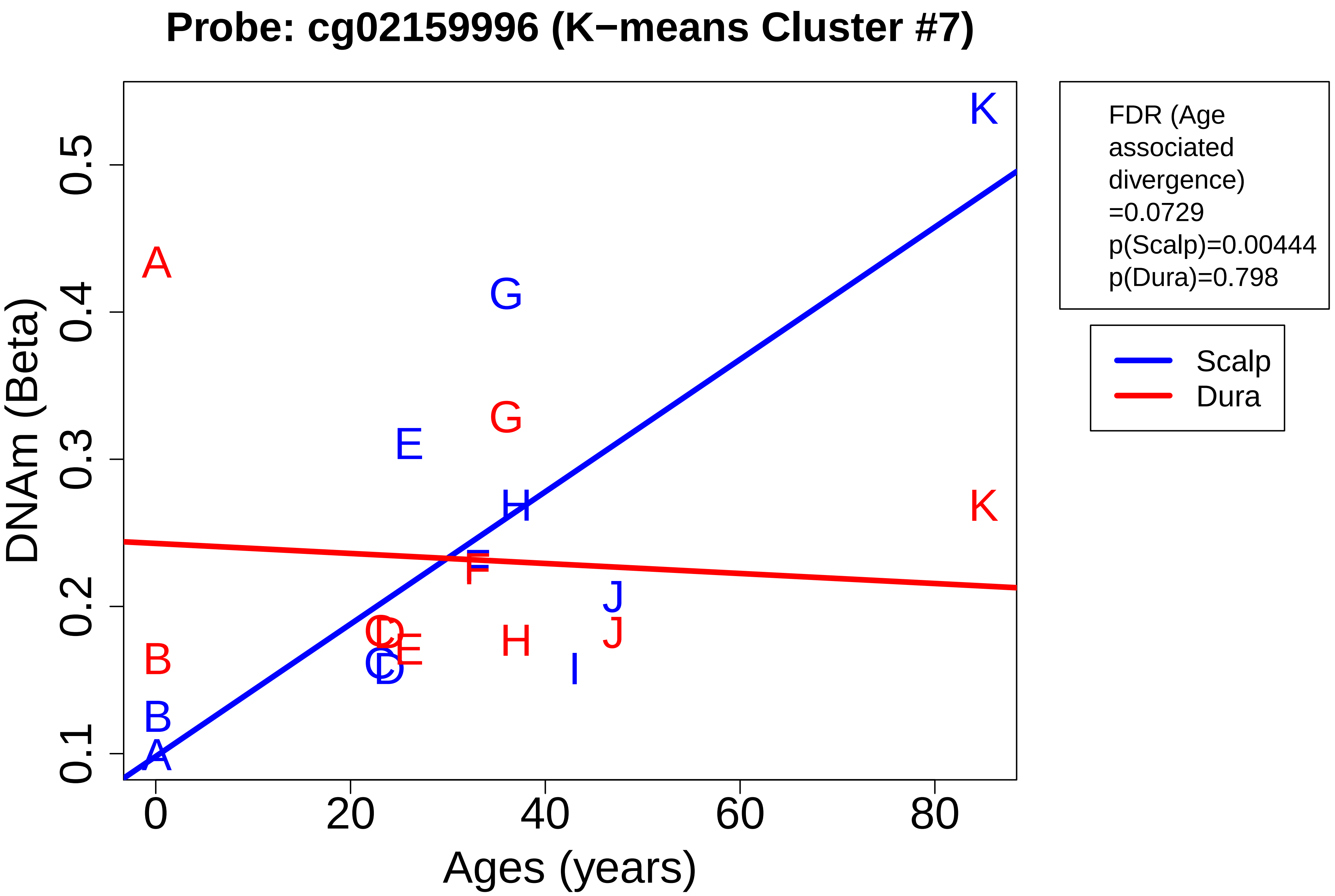

Probe: cg02159996 (K−means Cluster #7)
DNAm (Beta)
Ages (years)
0.1
0.2
0.3
0.4
0.5
0
20
40
60
80
FDR (Age associated divergence) =0.0729
p(Scalp)=0.00444
p(Dura)=0.798
Scalp
Dura

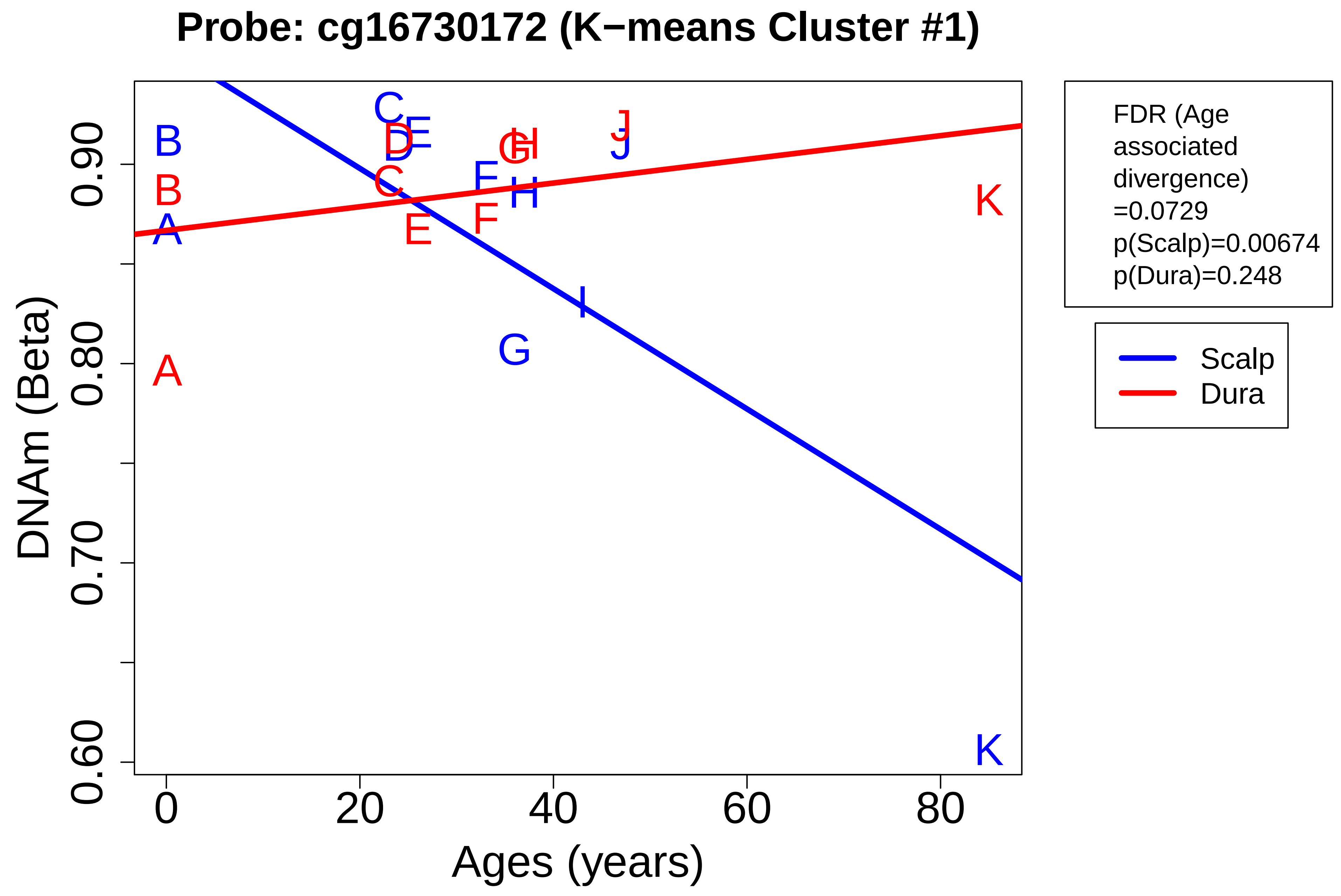

Probe: cg16730172 (K−means Cluster #1)
DNAm (Beta)
0.60
0.70
0.80
0.90
Ages (years)
0
20
40
60
80
FDR (Age associated divergence) =0.0729
p(Scalp)=0.00674
p(Dura)=0.248
Scalp
Dura

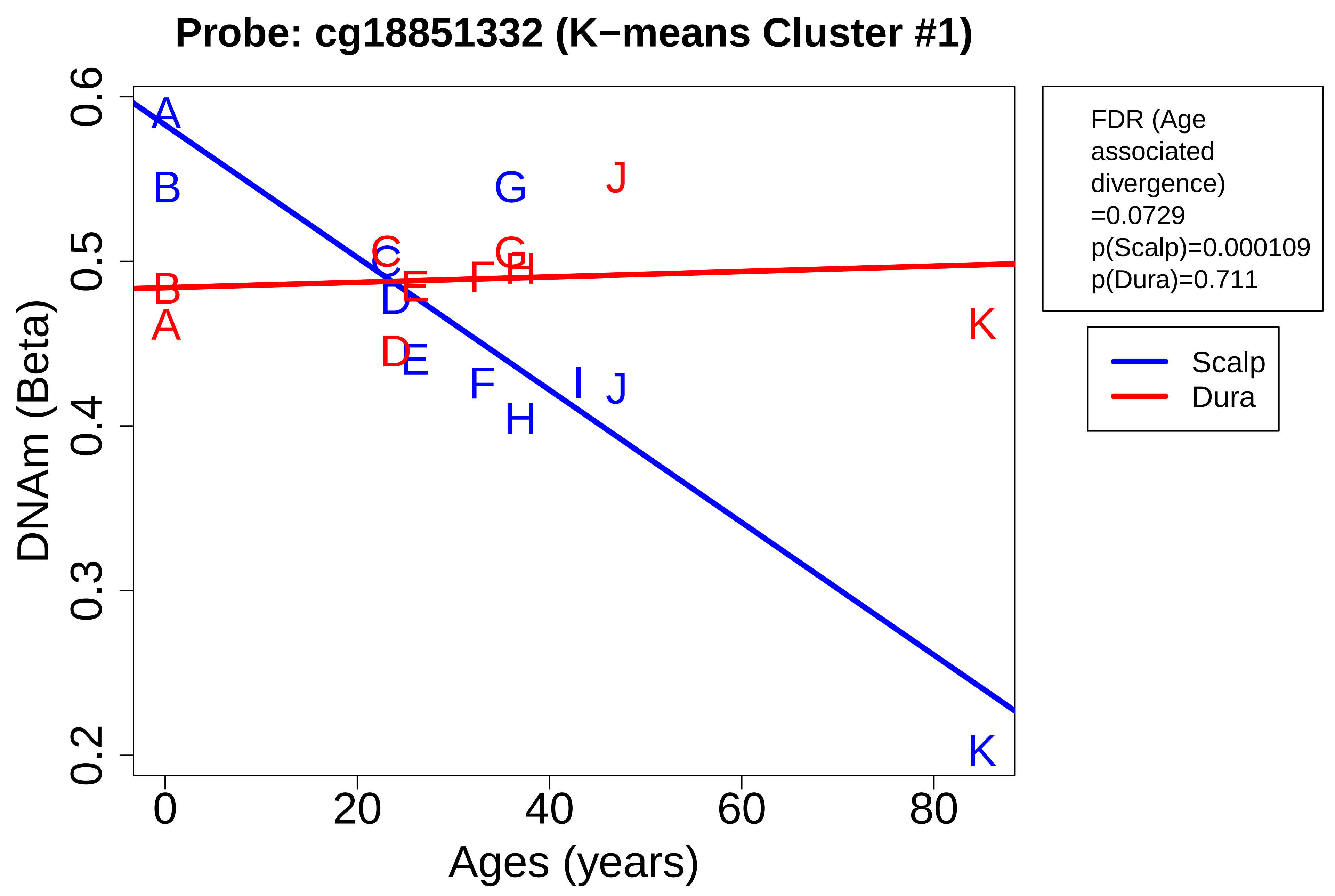

Probe: cg18851332 (K−means Cluster #1)
FDR (Age associated divergence) =0.0729
p(Scalp)=0.000109
p(Dura)=0.711
Scalp
Dura
DNAm (Beta)
Ages (years)
0.2
0.3
0.4
0.5
0.6
0
20
40
60
80

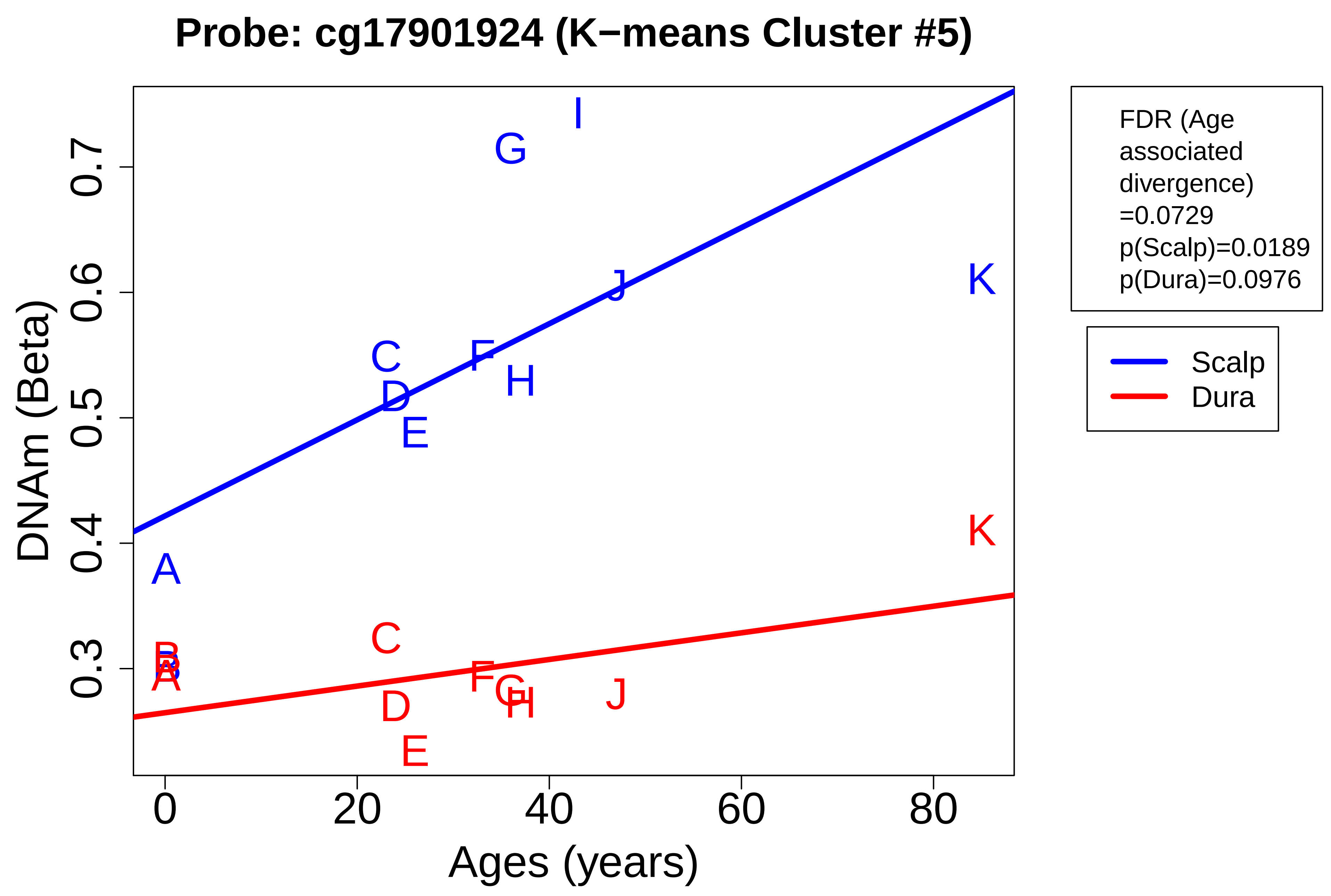
Probe: cg17901924 (K−means Cluster #5)
DNAm (Beta)
0.3
0.4
0.5
0.6
0.7
0
20
40
60
80
Ages (years)
FDR (Age associated divergence) =0.0729
p(Scalp)=0.0189
p(Dura)=0.0976
Scalp
Dura
A
B
C
D
E
F
G
H
I
J
K

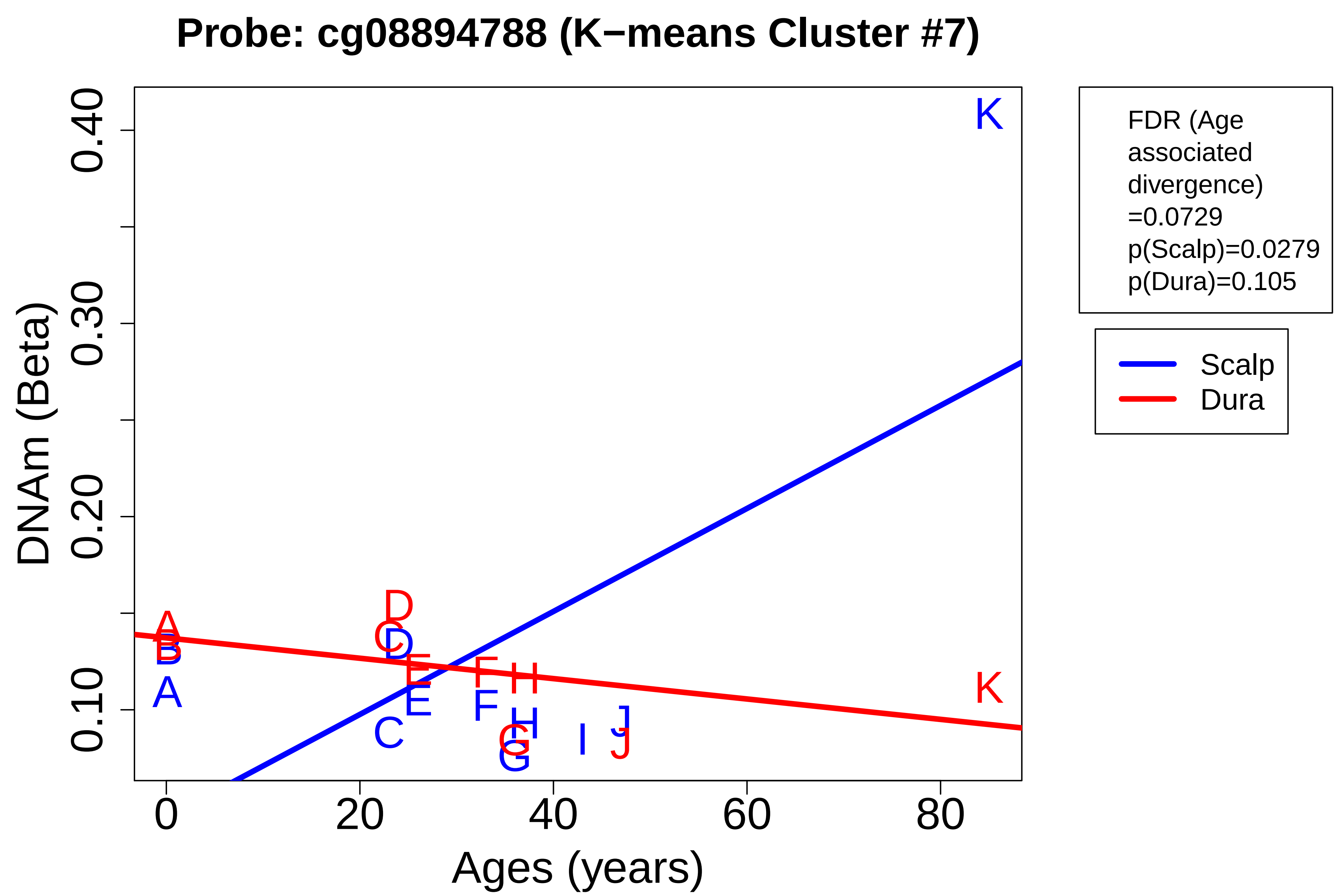

Probe: cg08894788 (K−means Cluster #7)
DNAm (Beta)
0.10
0.20
0.30
0.40
Ages (years)
0
20
40
60
80
A
B
C
D
E
F
G
H
I
J
K
FDR (Age associated divergence) =0.0729
p(Scalp)=0.0279
p(Dura)=0.105
Scalp
Dura

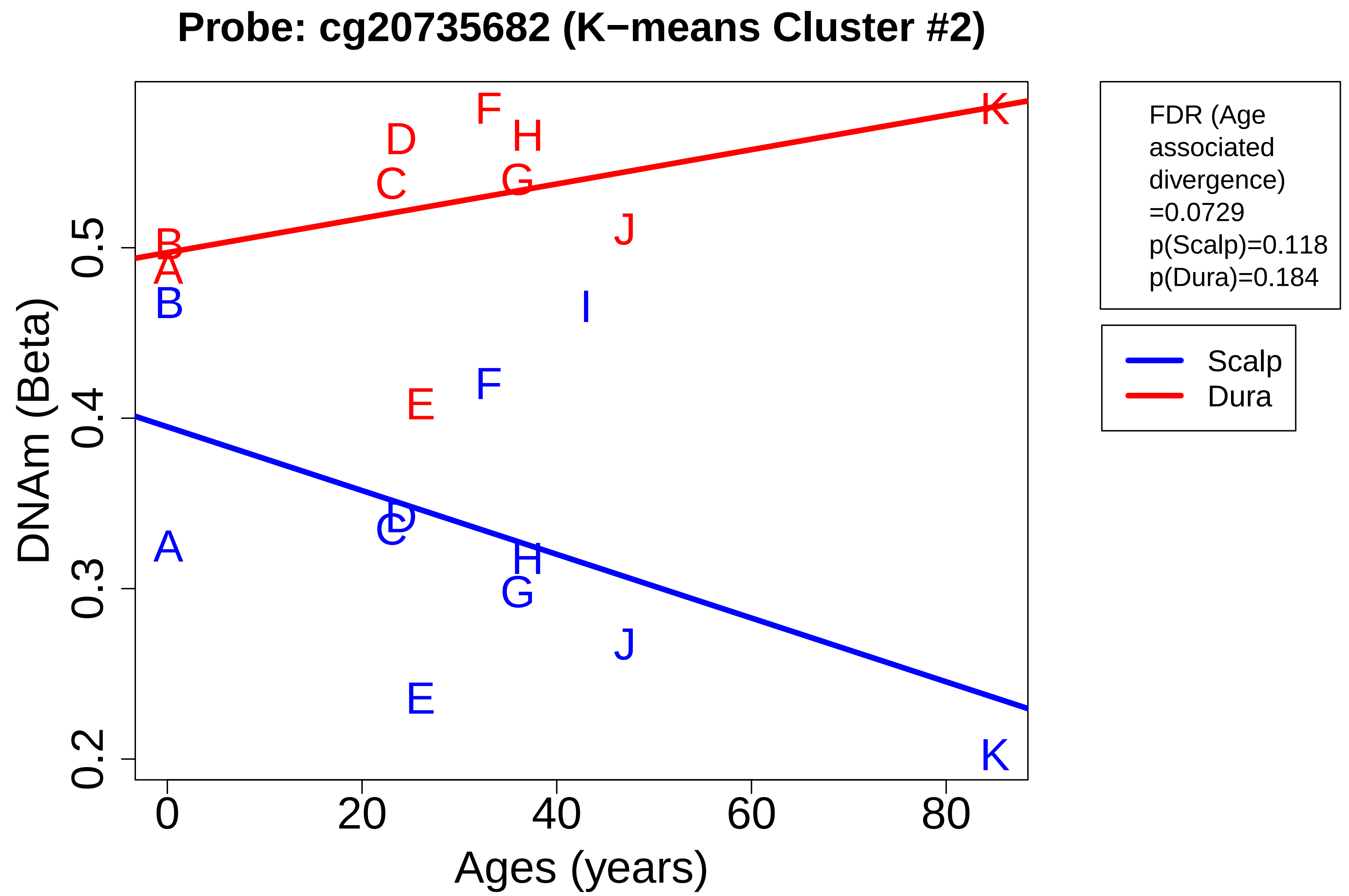

Probe: cg20735682 (K−means Cluster #2)
DNAm (Beta)
0.2
0.3
0.4
0.5
0
20
40
60
80
Ages (years)
FDR (Age associated divergence) =0.0729
p(Scalp)=0.118
p(Dura)=0.184
Scalp
Dura

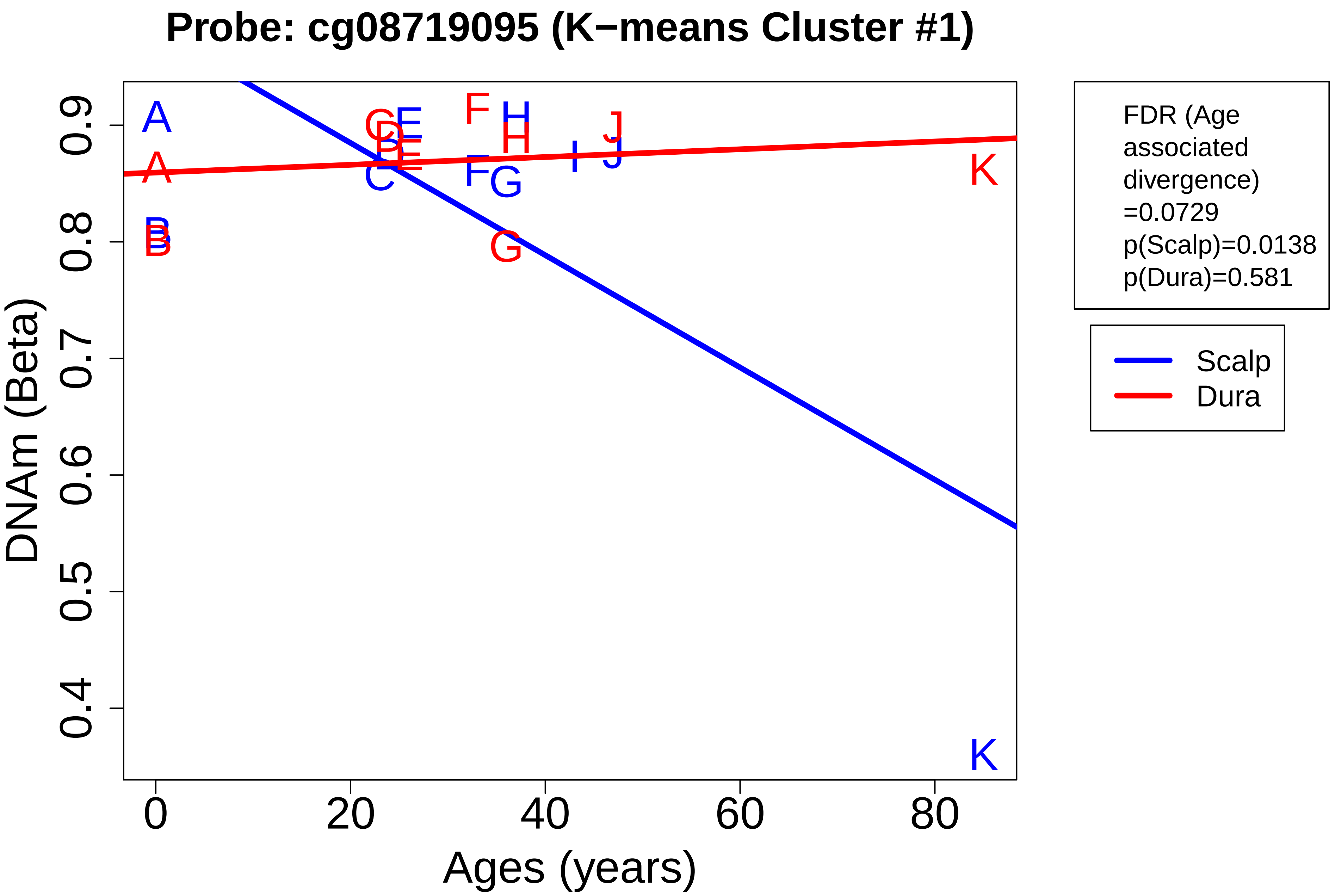

Probe: cg08719095 (K−means Cluster #1)
DNAm (Beta)
0.4
0.5
0.6
0.7
0.8
0.9
0
20
40
60
80
Ages (years)
A
B
C
D
E
F
G
H
I
J
K
FDR (Age
associated
divergence)
=0.0729
p(Scalp)=0.0138
p(Dura)=0.581
Scalp
Dura

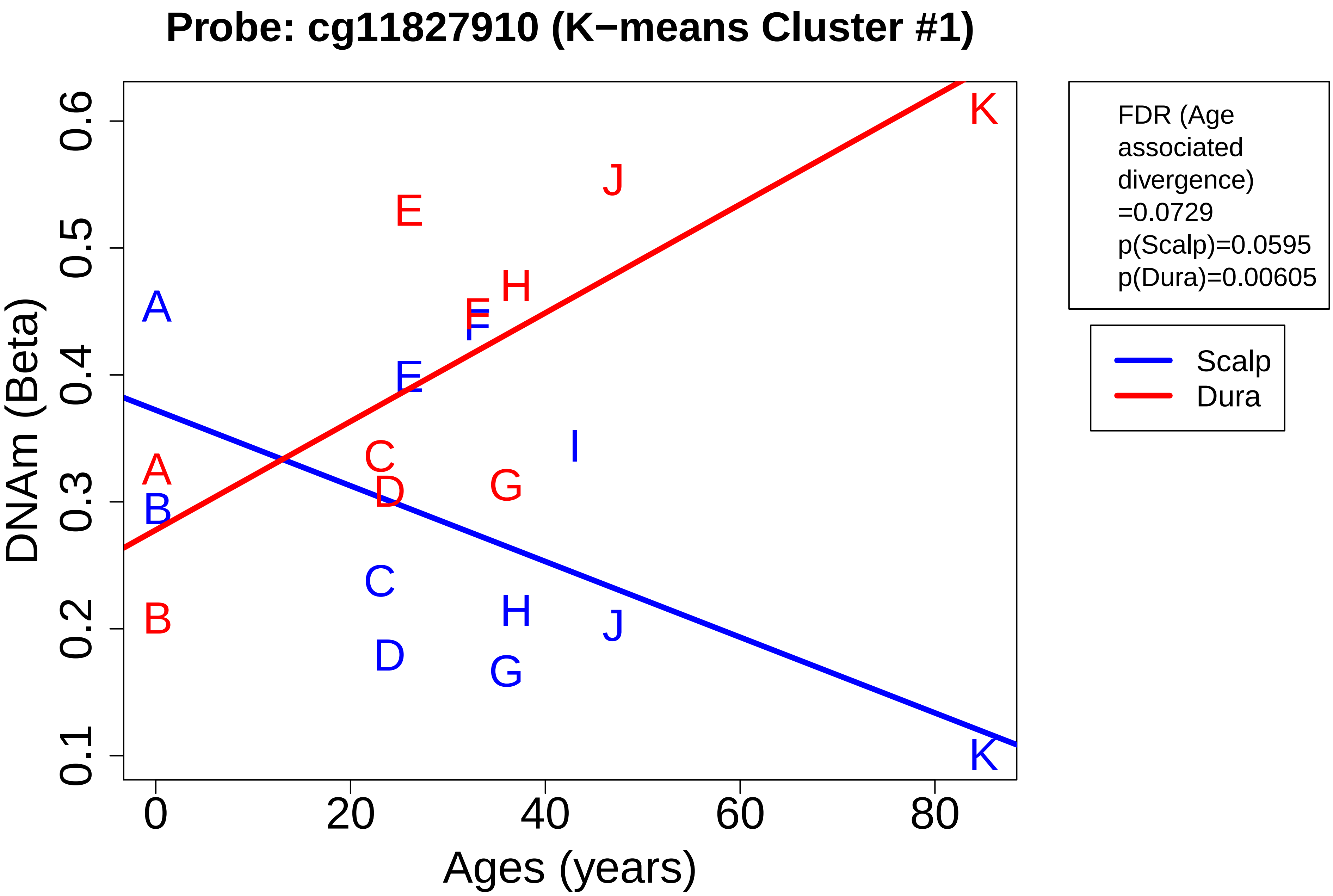

Probe: cg11827910 (K−means Cluster #1)
DNAm (Beta)
0.1
0.2
0.3
0.4
0.5
0.6
0
20
40
60
80
Ages (years)
FDR (Age associated divergence) =0.0729
p(Scalp)=0.0595
p(Dura)=0.00605
Scalp
Dura
A
B
C
D
E
F
G
H
I
J
K

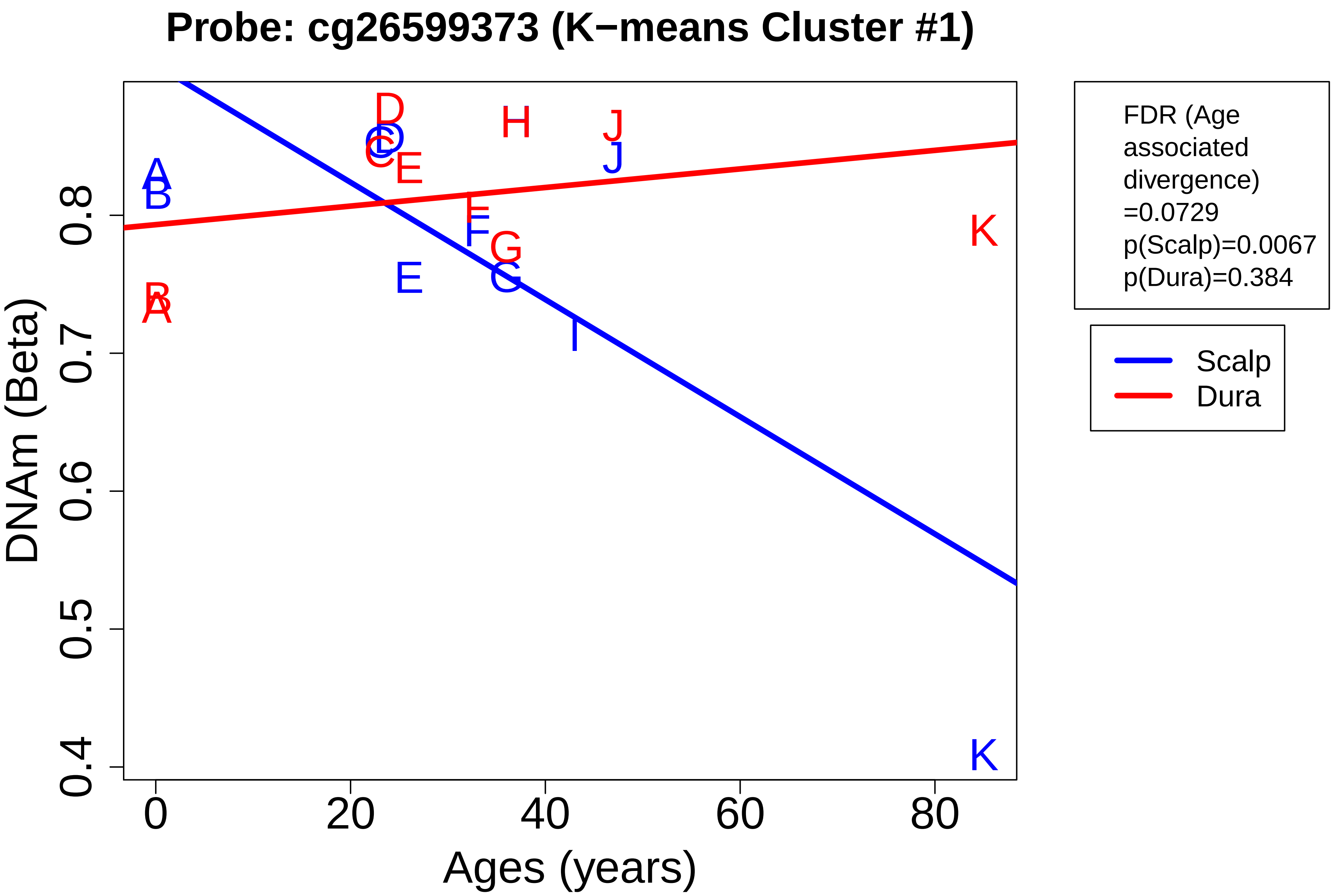
Probe: cg26599373 (K−means Cluster #1)
DNAm (Beta)
0.4
0.5
0.6
0.7
0.8
0
20
40
60
80
Ages (years)
FDR (Age associated divergence) =0.0729
p(Scalp)=0.0067
p(Dura)=0.384
Scalp
Dura

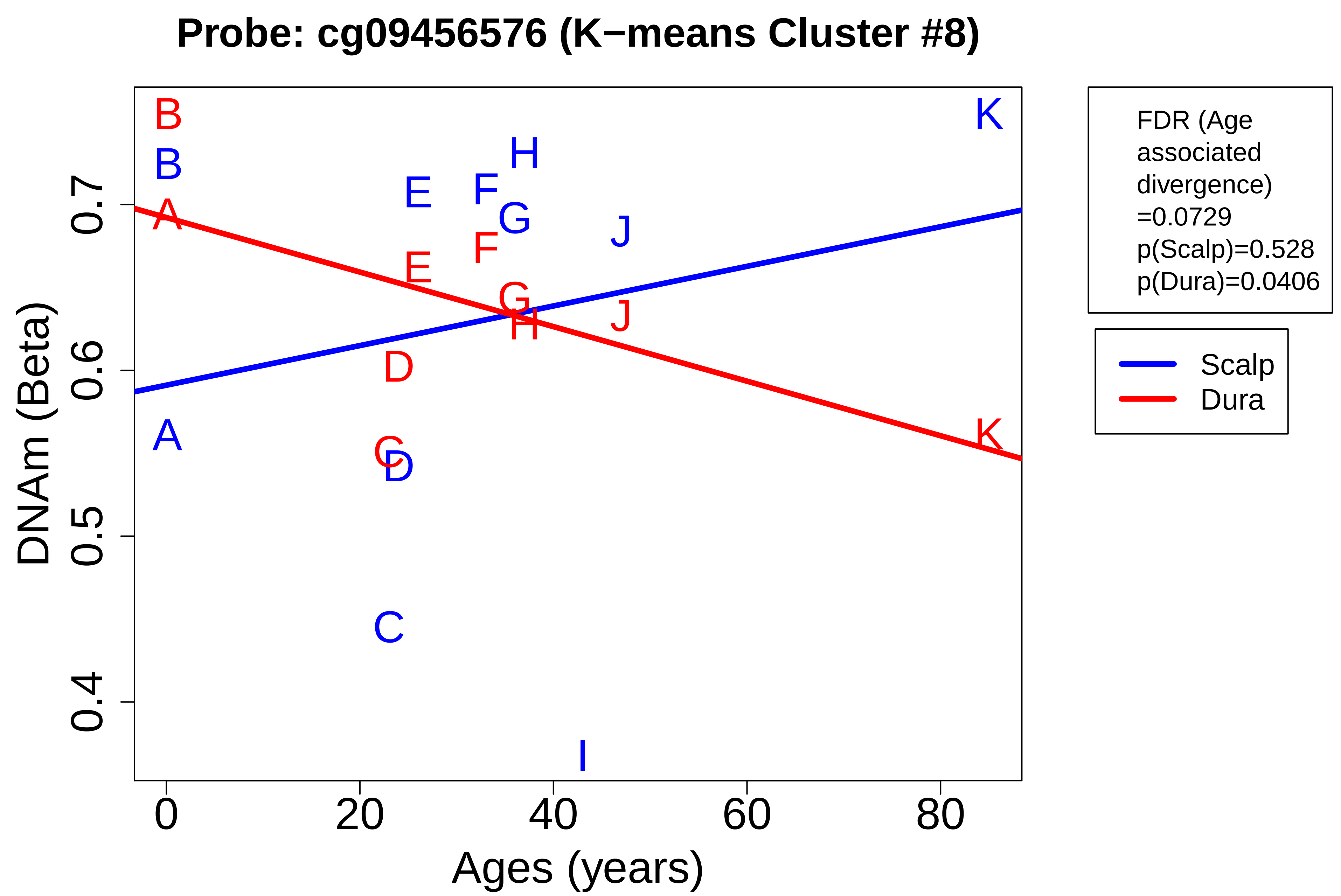
Probe: cg09456576 (K−means Cluster #8)
DNAm (Beta)
0.4
0.5
0.6
0.7
Ages (years)
0
20
40
60
80
FDR (Age associated divergence) =0.0729
p(Scalp)=0.528
p(Dura)=0.0406
Scalp
Dura

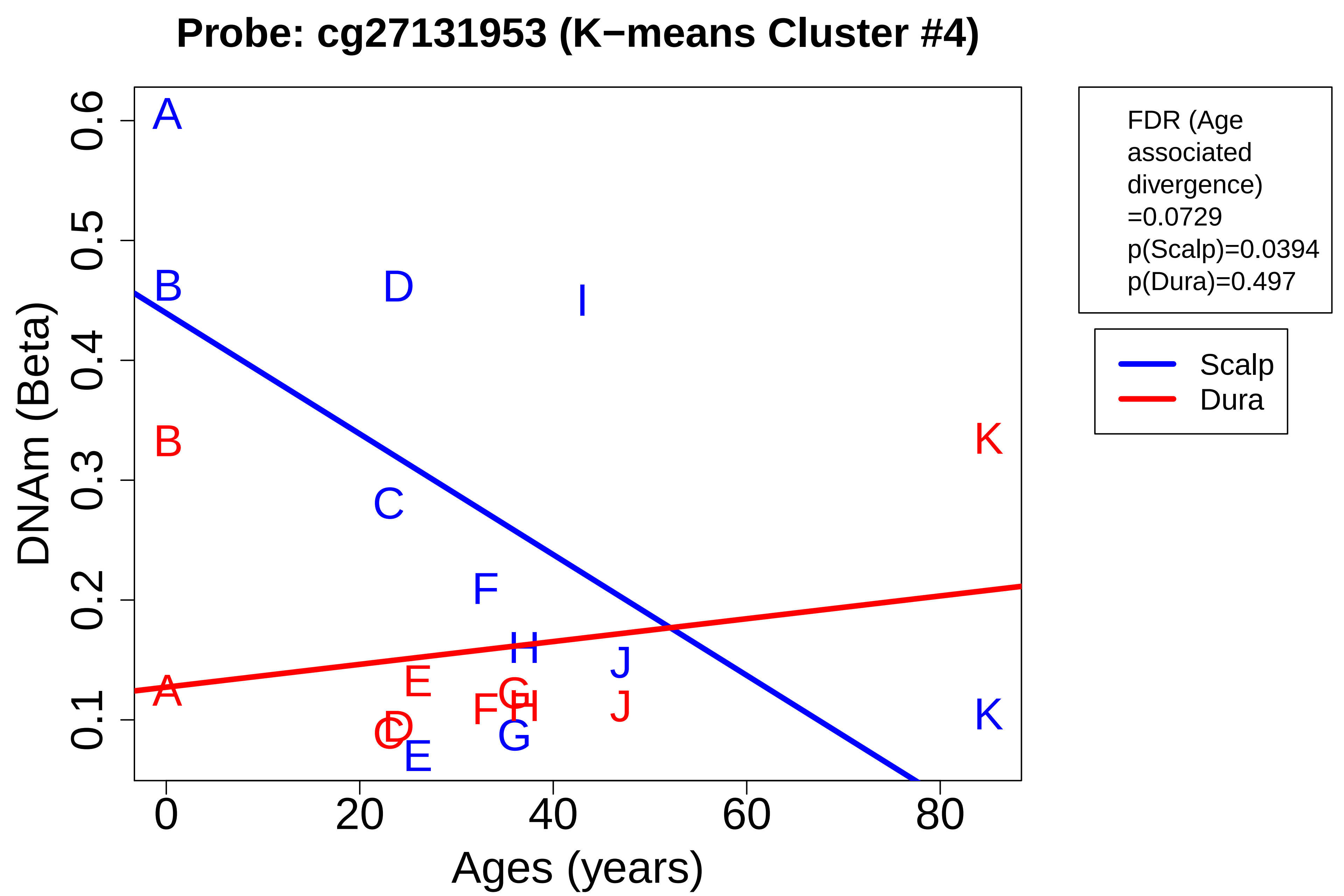

Probe: cg27131953 (K−means Cluster #4)
DNAm (Beta)
Ages (years)
0.1
0.2
0.3
0.4
0.5
0.6
0
20
40
60
80
FDR (Age associated divergence) =0.0729
p(Scalp)=0.0394
p(Dura)=0.497
Scalp
Dura

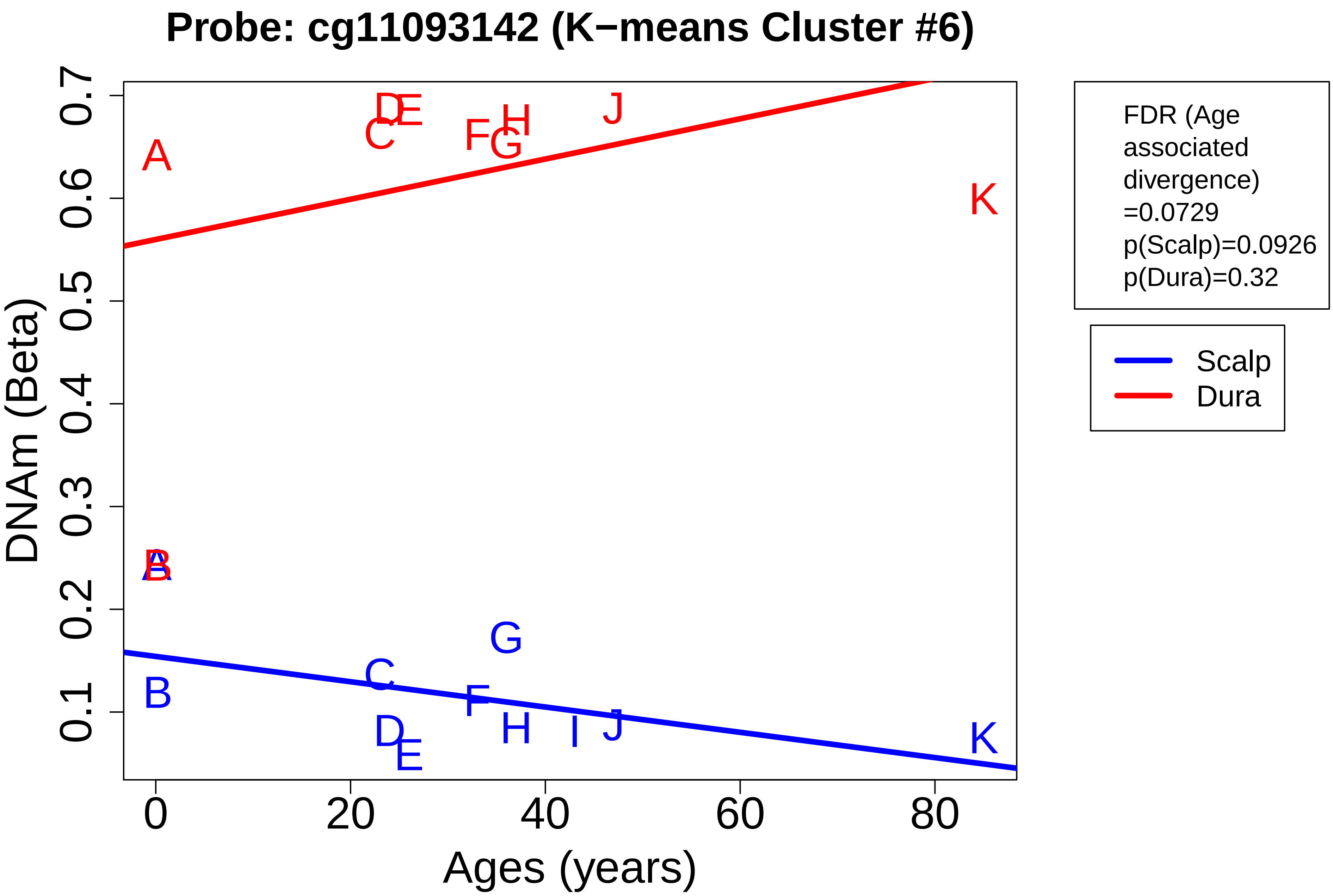

Probe: cg11093142 (K−means Cluster #6)
DNAm (Beta)
0.1
0.2
0.3
0.4
0.5
0.6
0.7
0
20
40
60
80
Ages (years)
FDR (Age associated divergence) =0.0729
p(Scalp)=0.0926
p(Dura)=0.32
Scalp
Dura
A
B
C
D
E
F
G
H
I
J
K

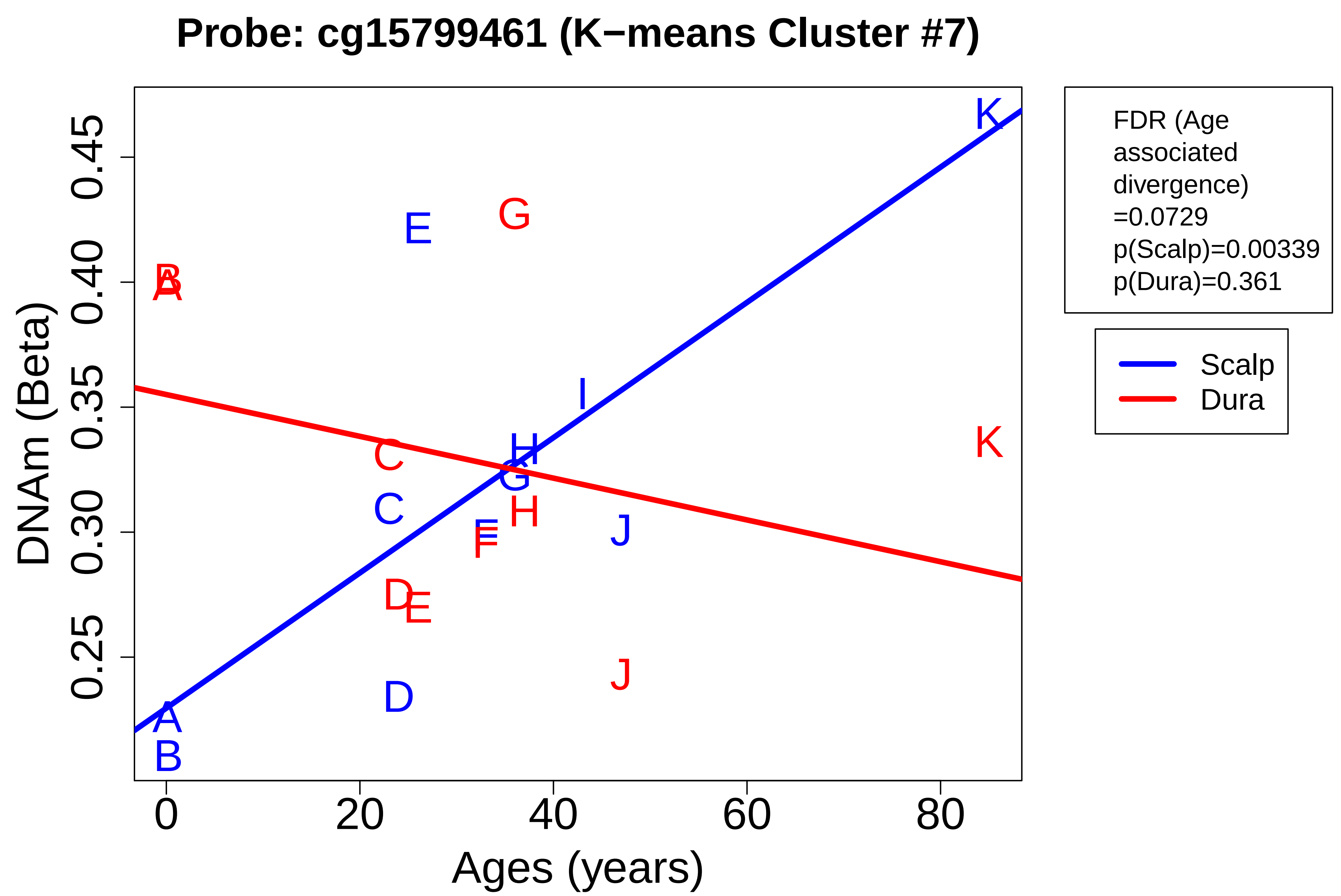

Probe: cg15799461 (K−means Cluster #7)
DNAm (Beta)
0.25
0.30
0.35
0.40
0.45
0
20
40
60
80
Ages (years)
A
B
C
D
E
F
G
H
I
J
K
FDR (Age associated divergence) =0.0729
p(Scalp)=0.00339
p(Dura)=0.361
Scalp
Dura

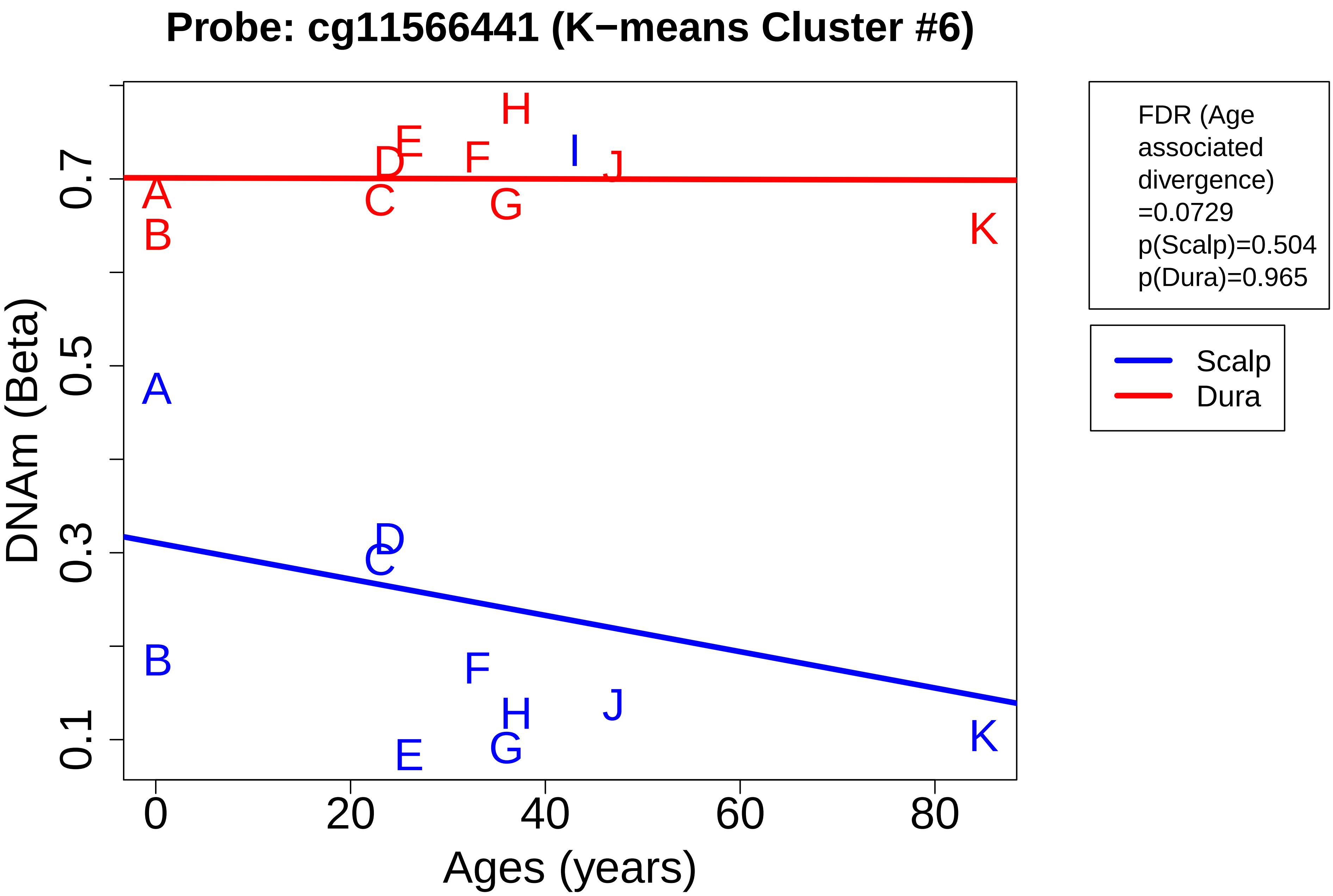

Probe: cg11566441 (K−means Cluster #6)
DNAm (Beta)
0.1
0.3
0.5
0.7
Ages (years)
0
20
40
60
80
FDR (Age associated divergence) =0.0729
p(Scalp)=0.504
p(Dura)=0.965
Scalp
Dura
A
B
C
D
E
F
G
H
I
J
K

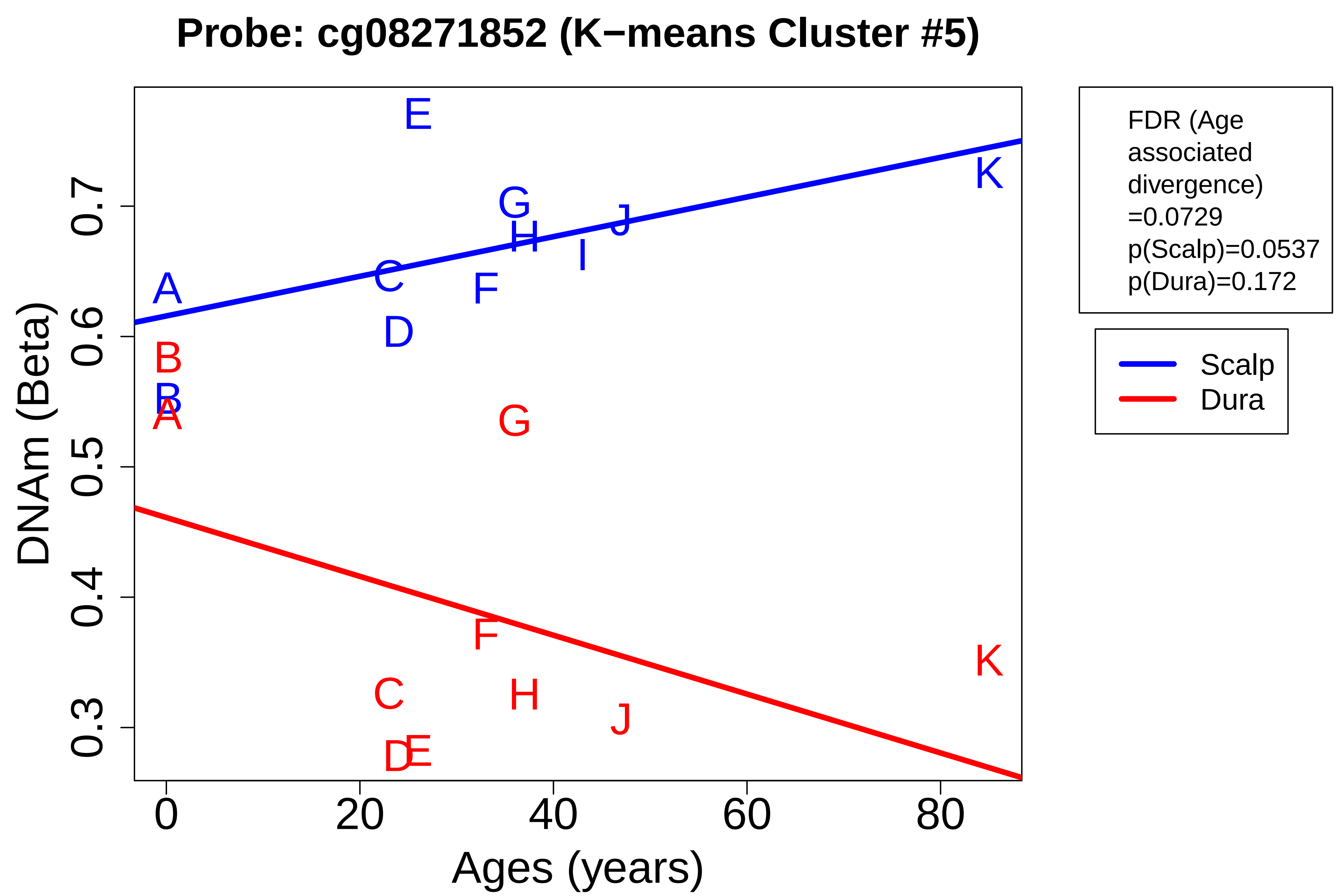

Probe: cg08271852 (K−means Cluster #5)
DNAm (Beta)
0.3
0.4
0.5
0.6
0.7
Ages (years)
0
20
40
60
80
FDR (Age associated divergence) =0.0729
p(Scalp)=0.0537
p(Dura)=0.172
Scalp
Dura

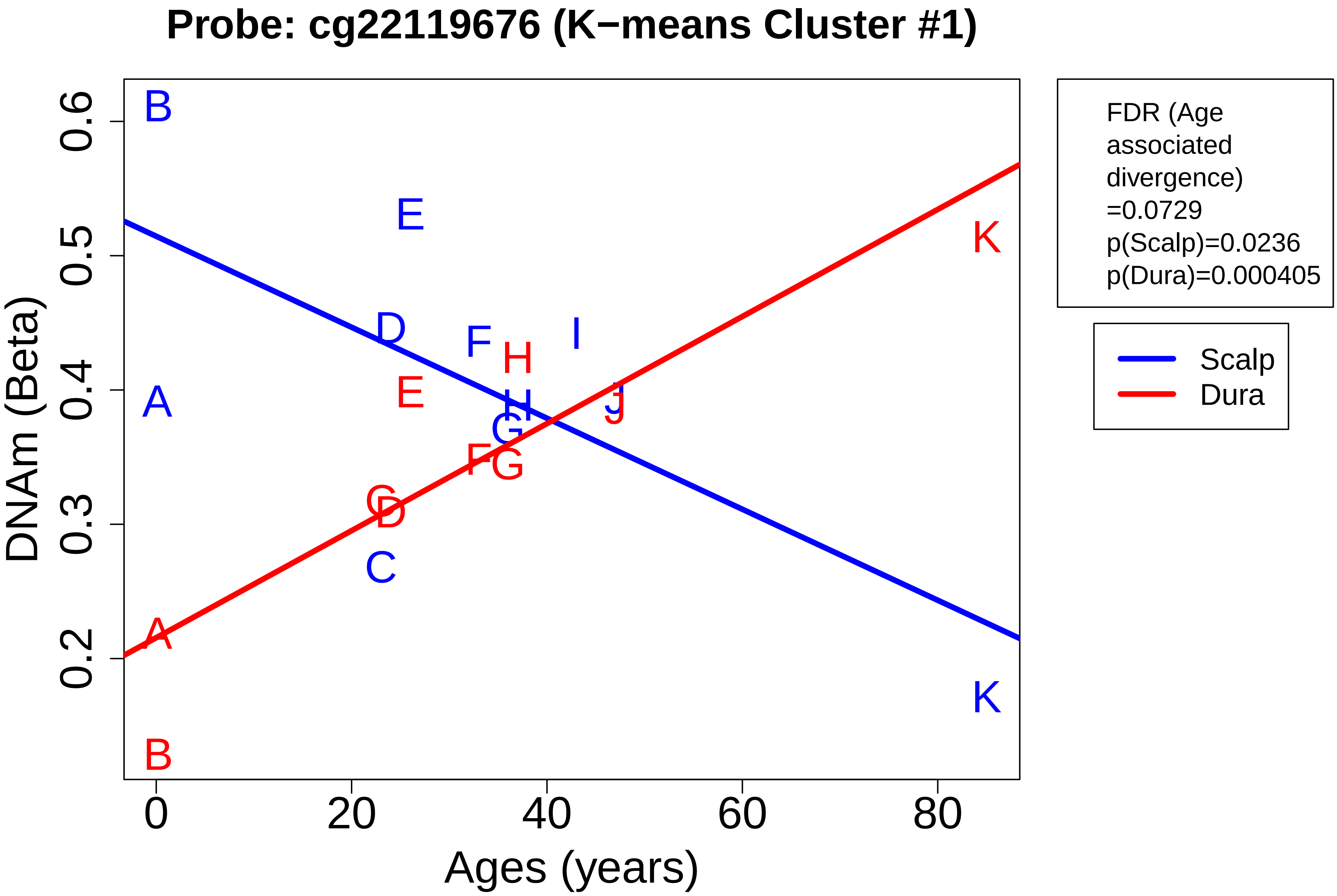

Probe: cg22119676 (K−means Cluster #1)
DNAm (Beta)
0.2
0.3
0.4
0.5
0.6
0
20
40
60
80
Ages (years)
FDR (Age associated divergence) =0.0729
p(Scalp)=0.0236
p(Dura)=0.000405
Scalp
Dura

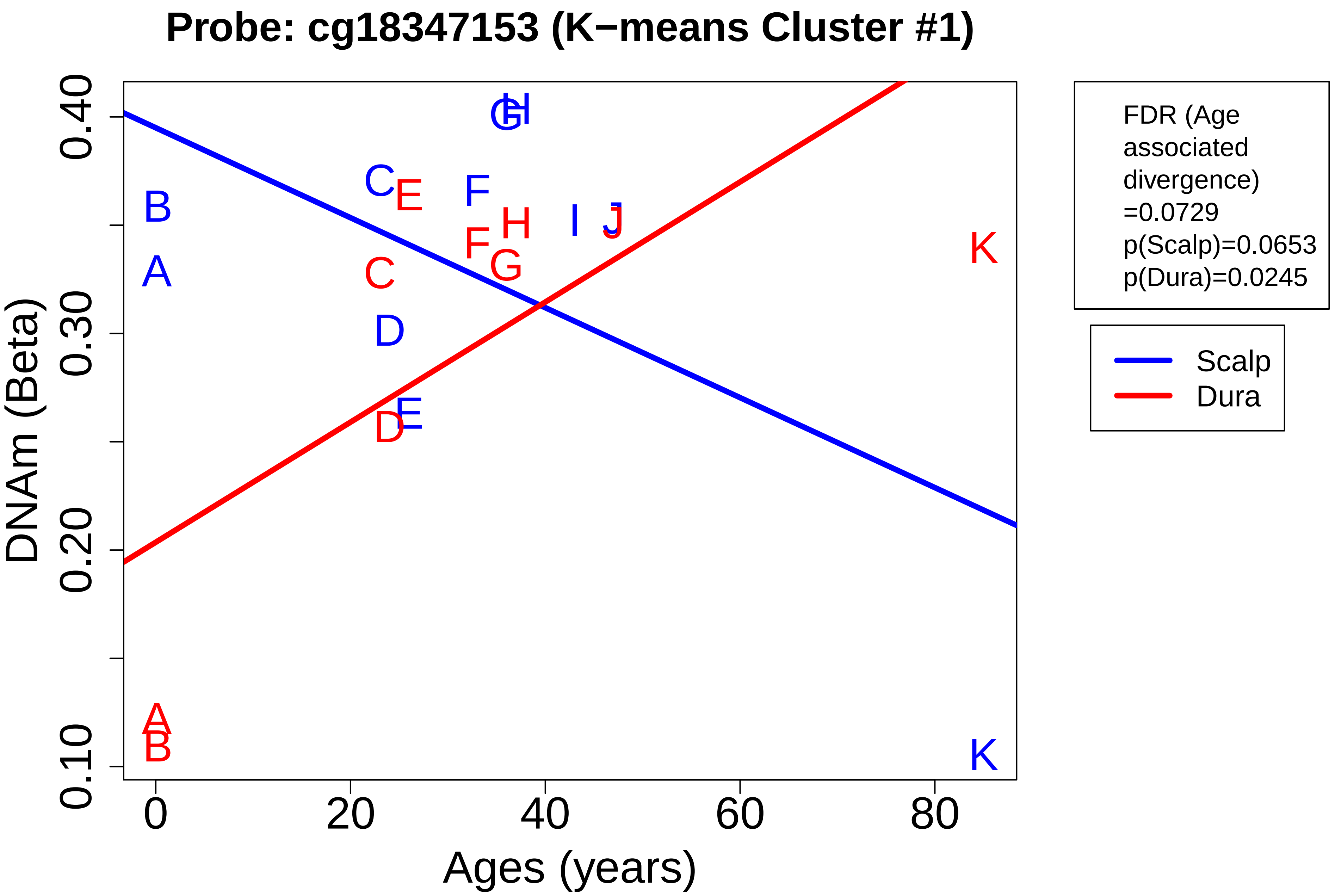

Probe: cg18347153 (K−means Cluster #1)
DNAm (Beta)
0.10
0.20
0.30
0.40
Ages (years)
0
20
40
60
80
FDR (Age associated divergence) =0.0729
p(Scalp)=0.0653
p(Dura)=0.0245
Scalp
Dura

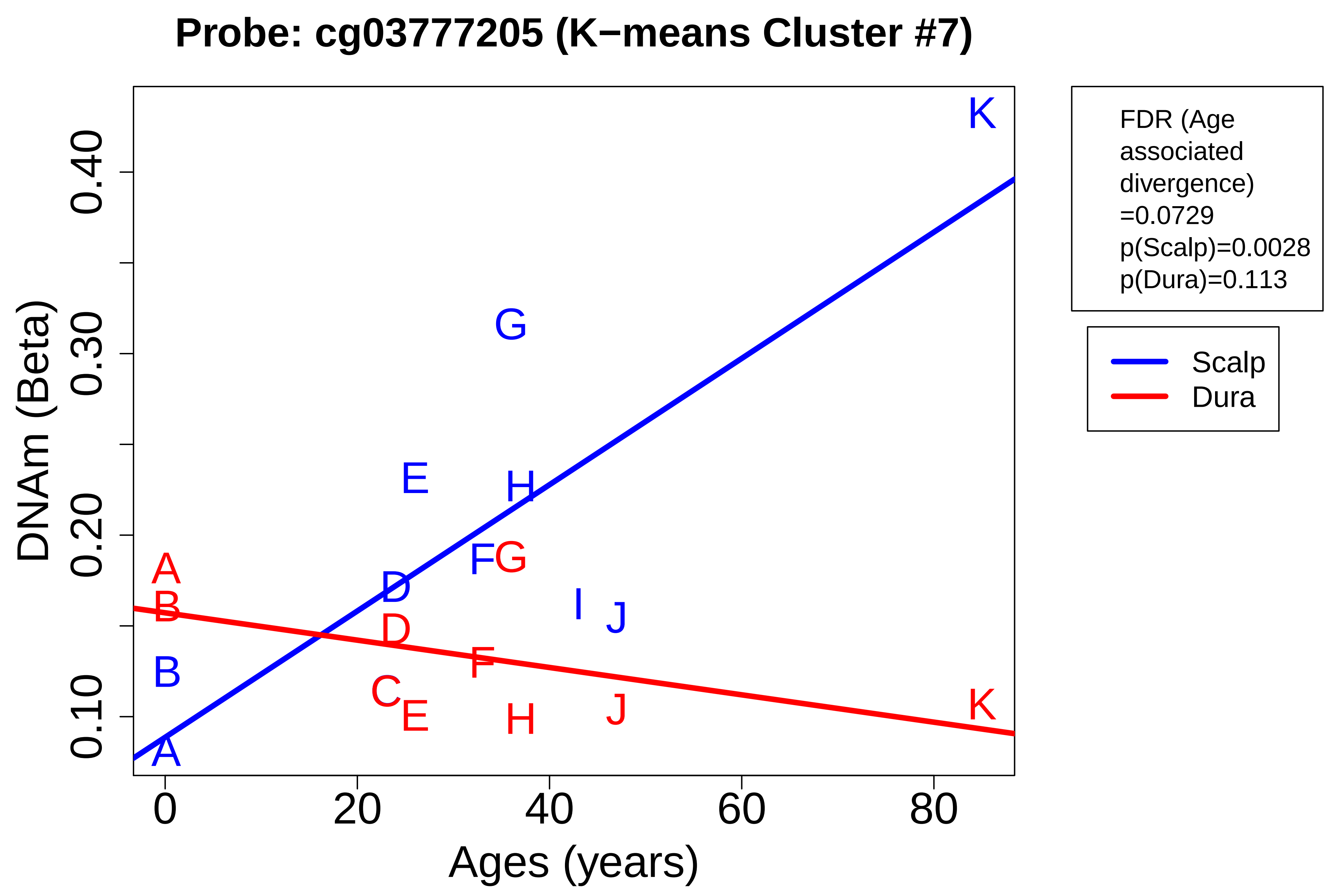

Probe: cg03777205 (K−means Cluster #7)
DNAm (Beta)
0.10
0.20
0.30
0.40
Ages (years)
0
20
40
60
80
FDR (Age associated divergence) =0.0729
p(Scalp)=0.0028
p(Dura)=0.113
Scalp
Dura
A
B
C
D
E
F
G
H
I
J
K

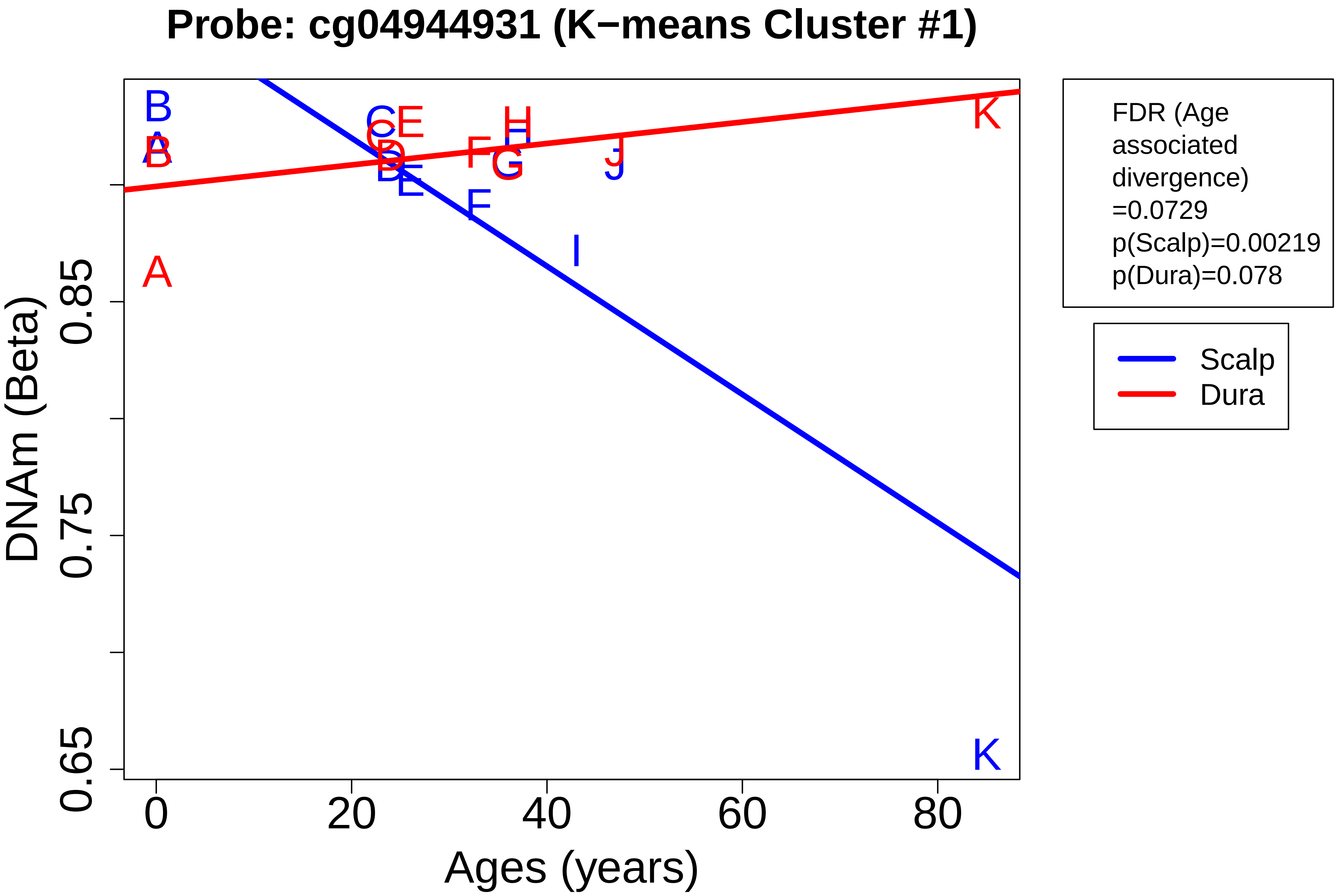

Probe: cg04944931 (K−means Cluster #1)
DNAm (Beta)
0.65
0.75
0.85
Ages (years)
0
20
40
60
80
FDR (Age associated divergence) =0.0729
p(Scalp)=0.00219
p(Dura)=0.078
Scalp
Dura

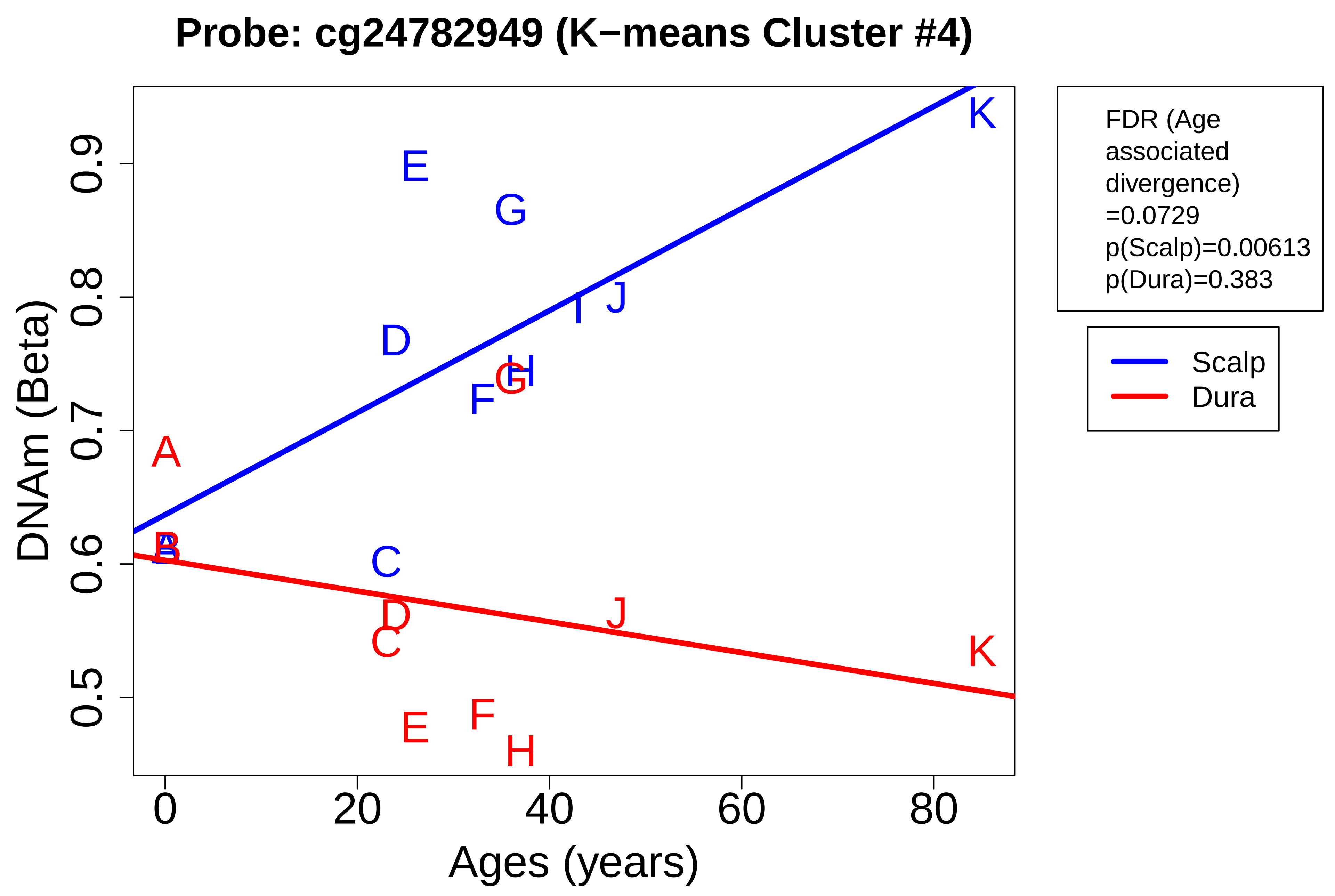

Probe: cg24782949 (K−means Cluster #4)
DNAm (Beta)
0.5
0.6
0.7
0.8
0.9
0
20
40
60
80
Ages (years)
FDR (Age associated divergence) =0.0729
p(Scalp)=0.00613
p(Dura)=0.383
Scalp
Dura

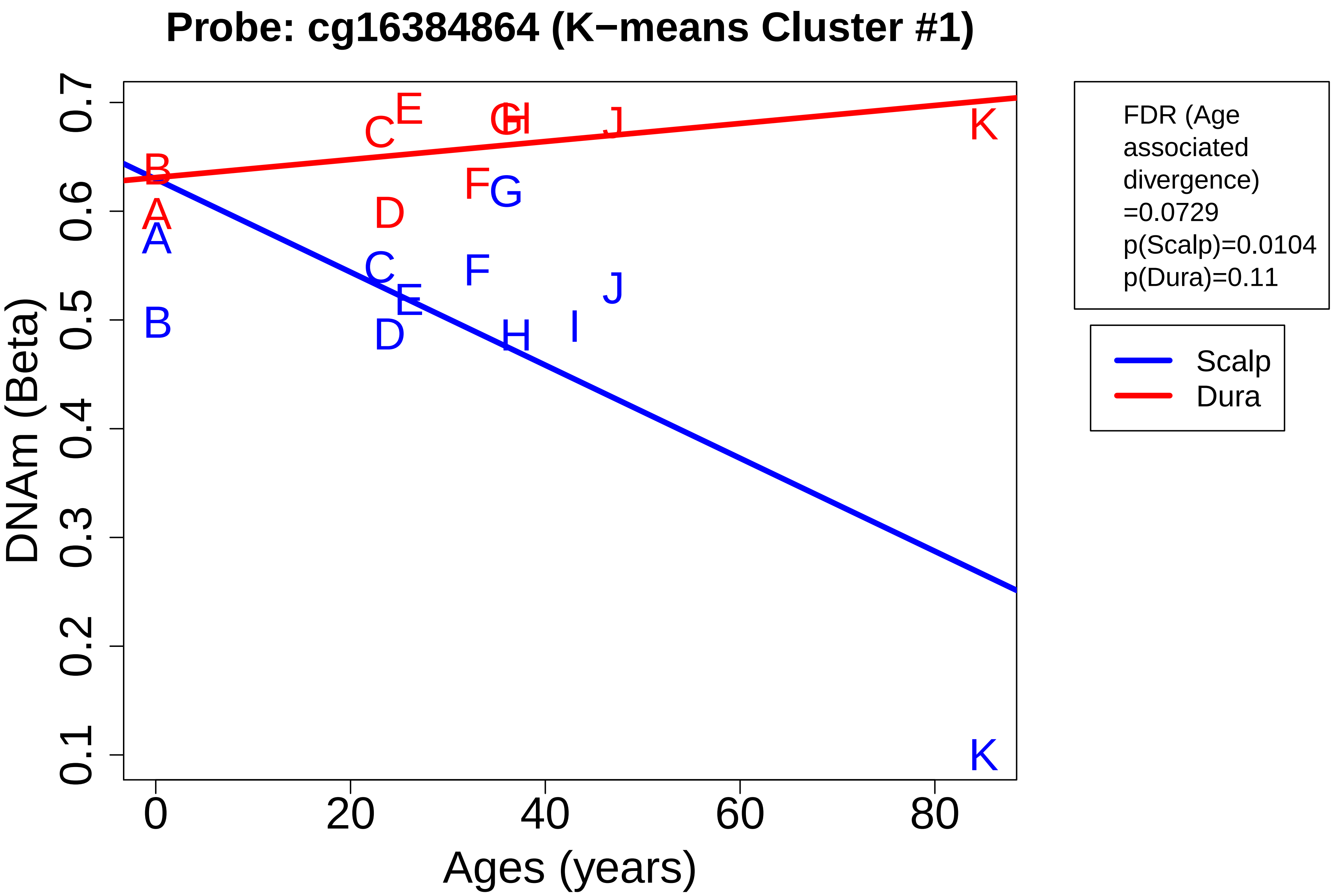
Probe: cg16384864 (K−means Cluster #1)
DNAm (Beta)
0.1
0.2
0.3
0.4
0.5
0.6
0.7
Ages (years)
0
20
40
60
80
A
B
C
D
E
F
G
H
I
J
K
FDR (Age associated divergence) =0.0729
p(Scalp)=0.0104
p(Dura)=0.11
Scalp
Dura

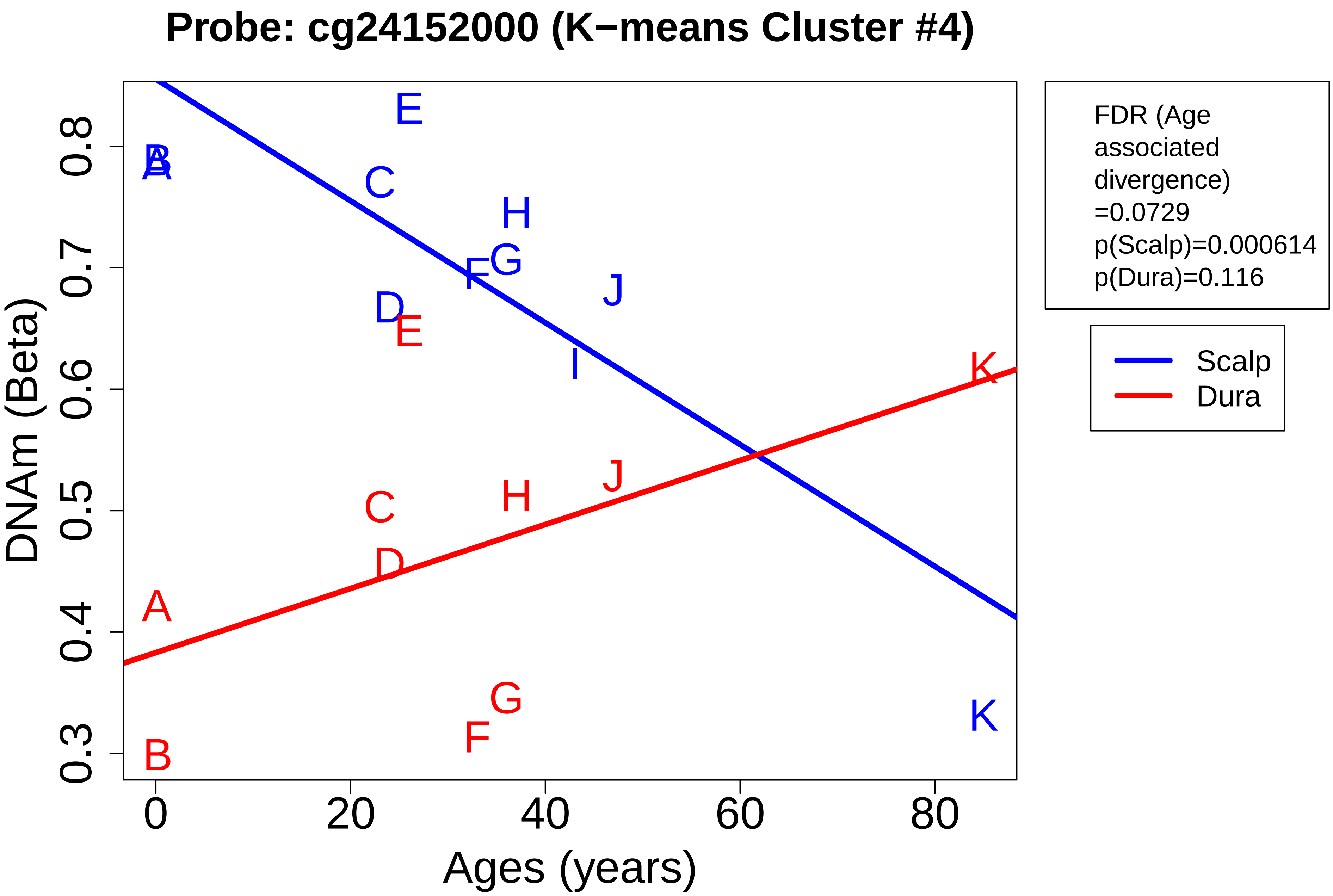
Probe: cg24152000 (K−means Cluster #4)
DNAm (Beta)
0.3
0.4
0.5
0.6
0.7
0.8
0
20
40
60
80
Ages (years)
E
B
A
C
H
F
G
J
D
E
I
K
J
C
H
D
A
G
F
B
K
FDR (Age associated divergence) =0.0729
p(Scalp)=0.000614
p(Dura)=0.116
Scalp
Dura

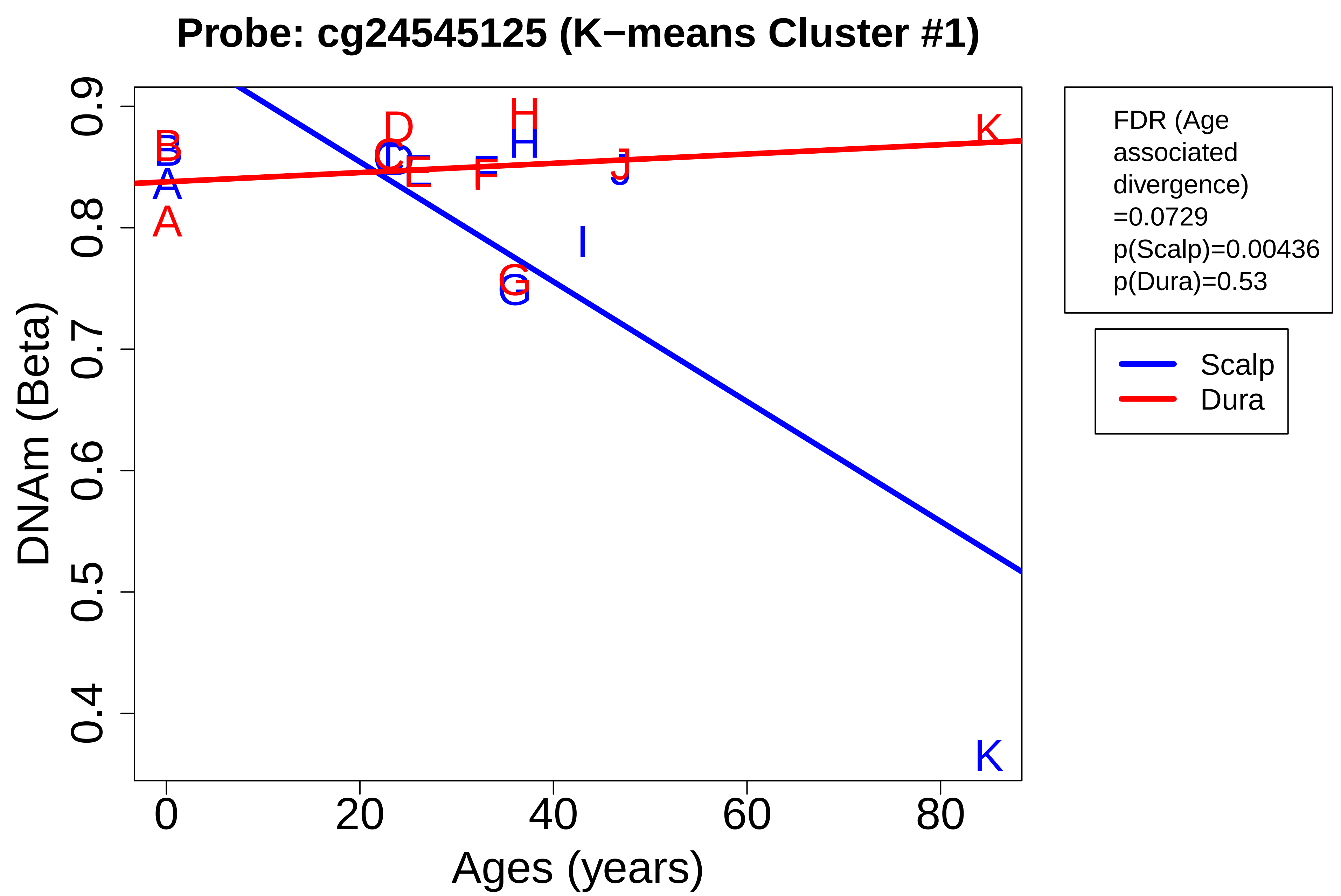

Probe: cg24545125 (K−means Cluster #1)
DNAm (Beta)
0.4
0.5
0.6
0.7
0.8
0.9
Ages (years)
0
20
40
60
80
A
B
C
D
E
F
G
H
I
J
K
FDR (Age associated divergence) =0.0729
p(Scalp)=0.00436
p(Dura)=0.53
Scalp
Dura

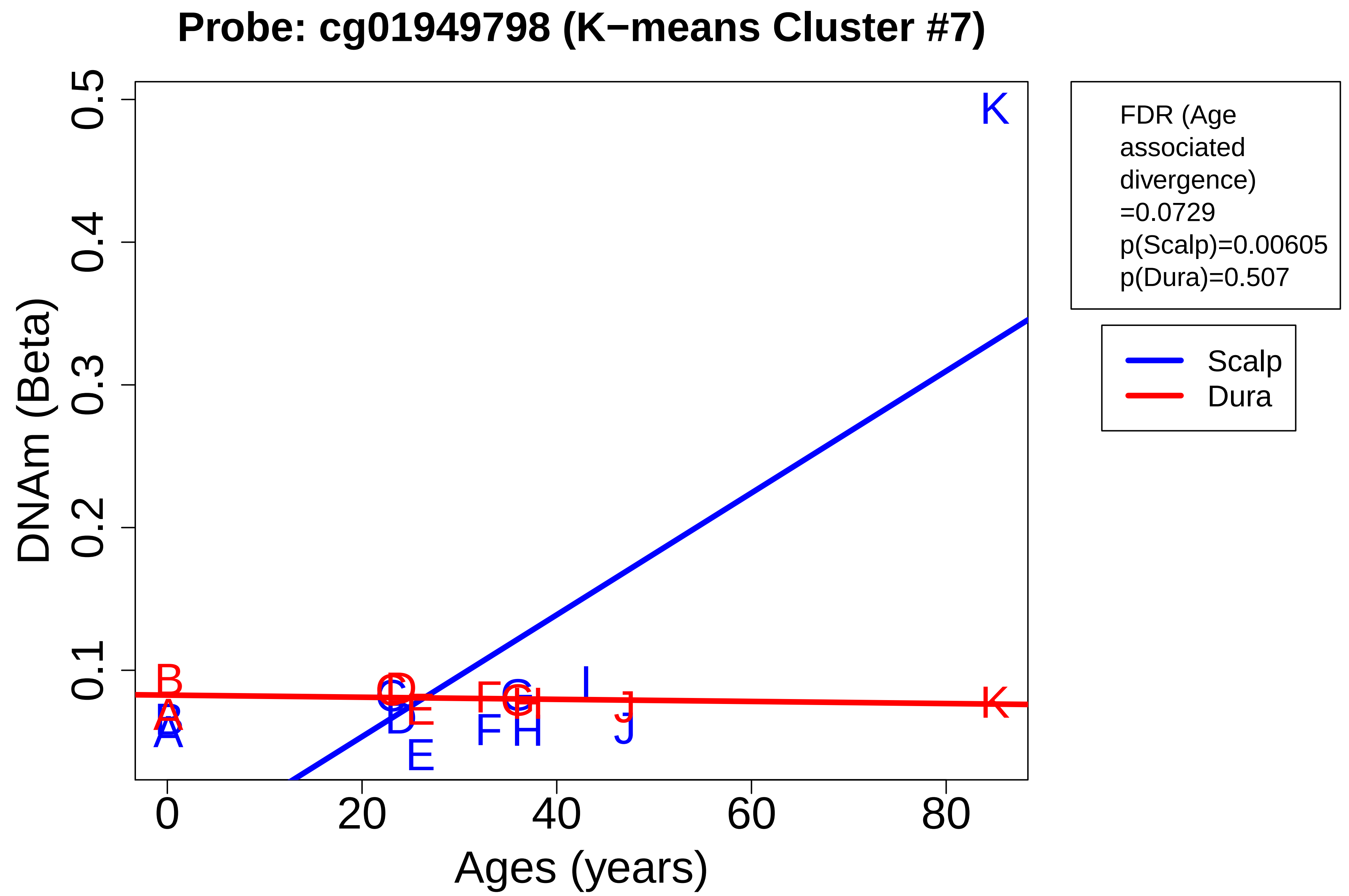

Probe: cg01949798 (K−means Cluster #7)
DNAm (Beta)
0.1
0.2
0.3
0.4
0.5
0
20
40
60
80
Ages (years)
FDR (Age associated divergence) =0.0729
p(Scalp)=0.00605
p(Dura)=0.507
Scalp
Dura

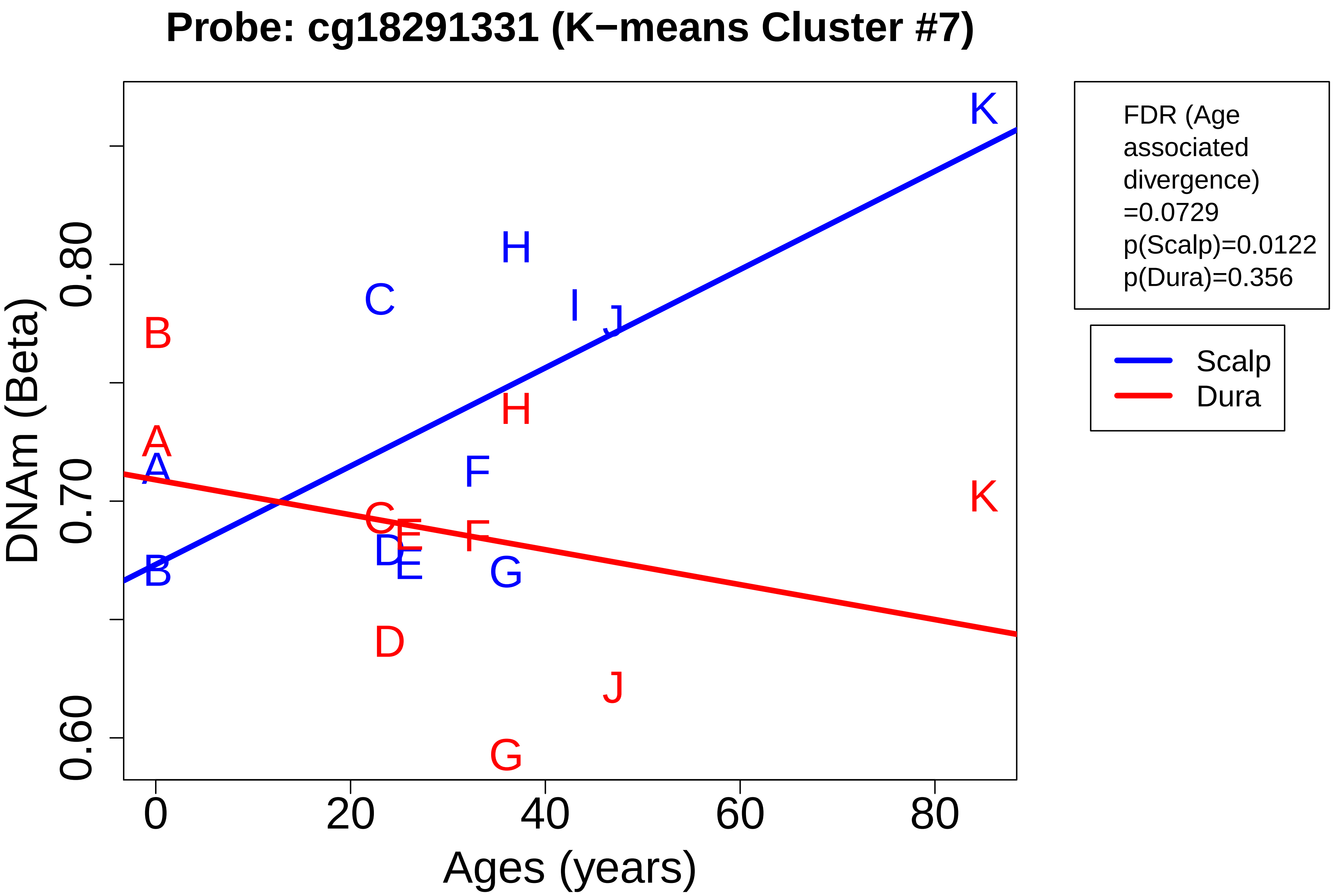

Probe: cg18291331 (K−means Cluster #7)
DNAm (Beta)
0.60
0.70
0.80
Ages (years)
0
20
40
60
80
FDR (Age associated divergence) =0.0729
p(Scalp)=0.0122
p(Dura)=0.356
Scalp
Dura

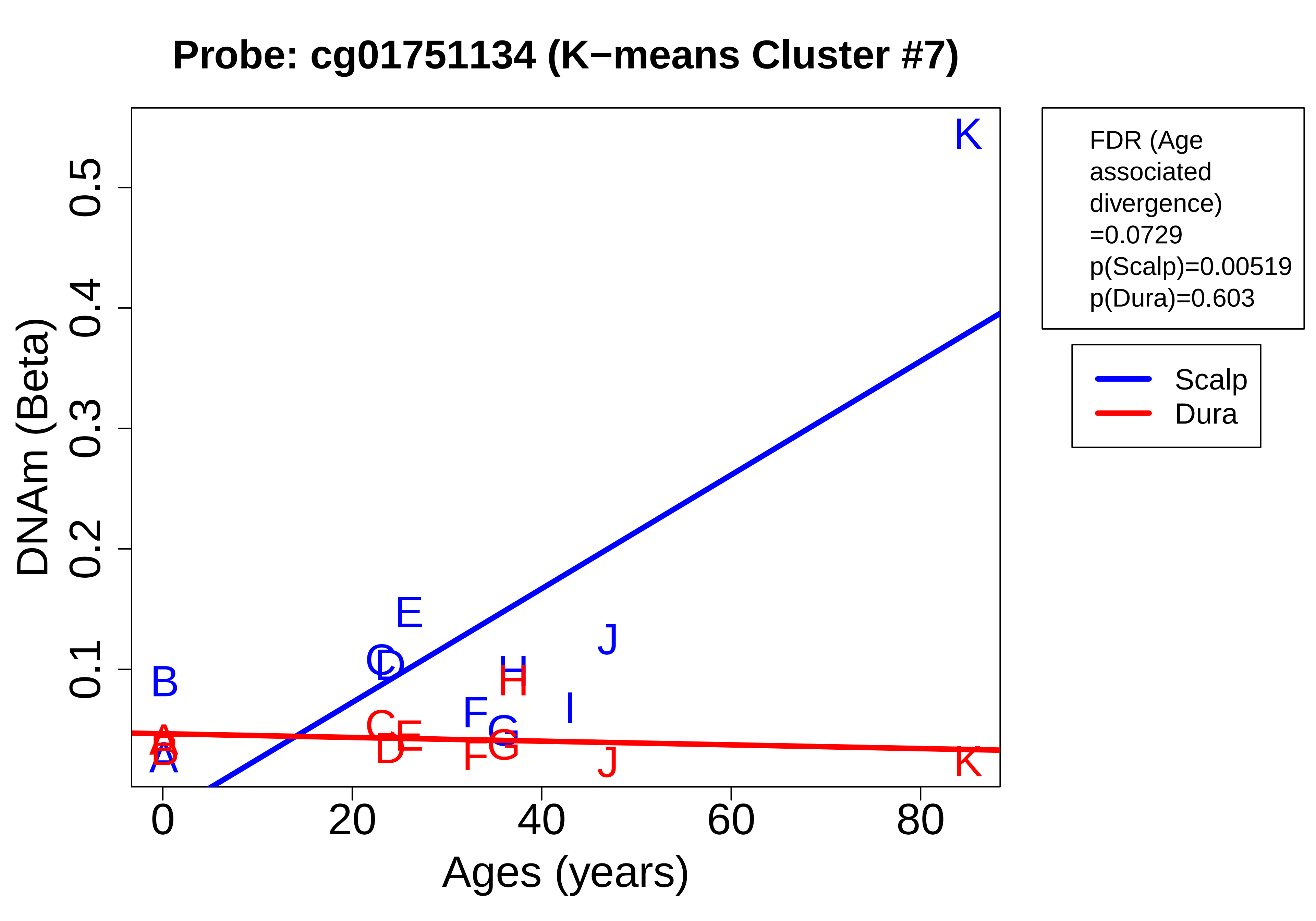

Probe: cg01751134 (K−means Cluster #7)
DNAm (Beta)
0.1
0.2
0.3
0.4
0.5
0
20
40
60
80
Ages (years)
FDR (Age associated divergence) =0.0729
p(Scalp)=0.00519
p(Dura)=0.603
Scalp
Dura

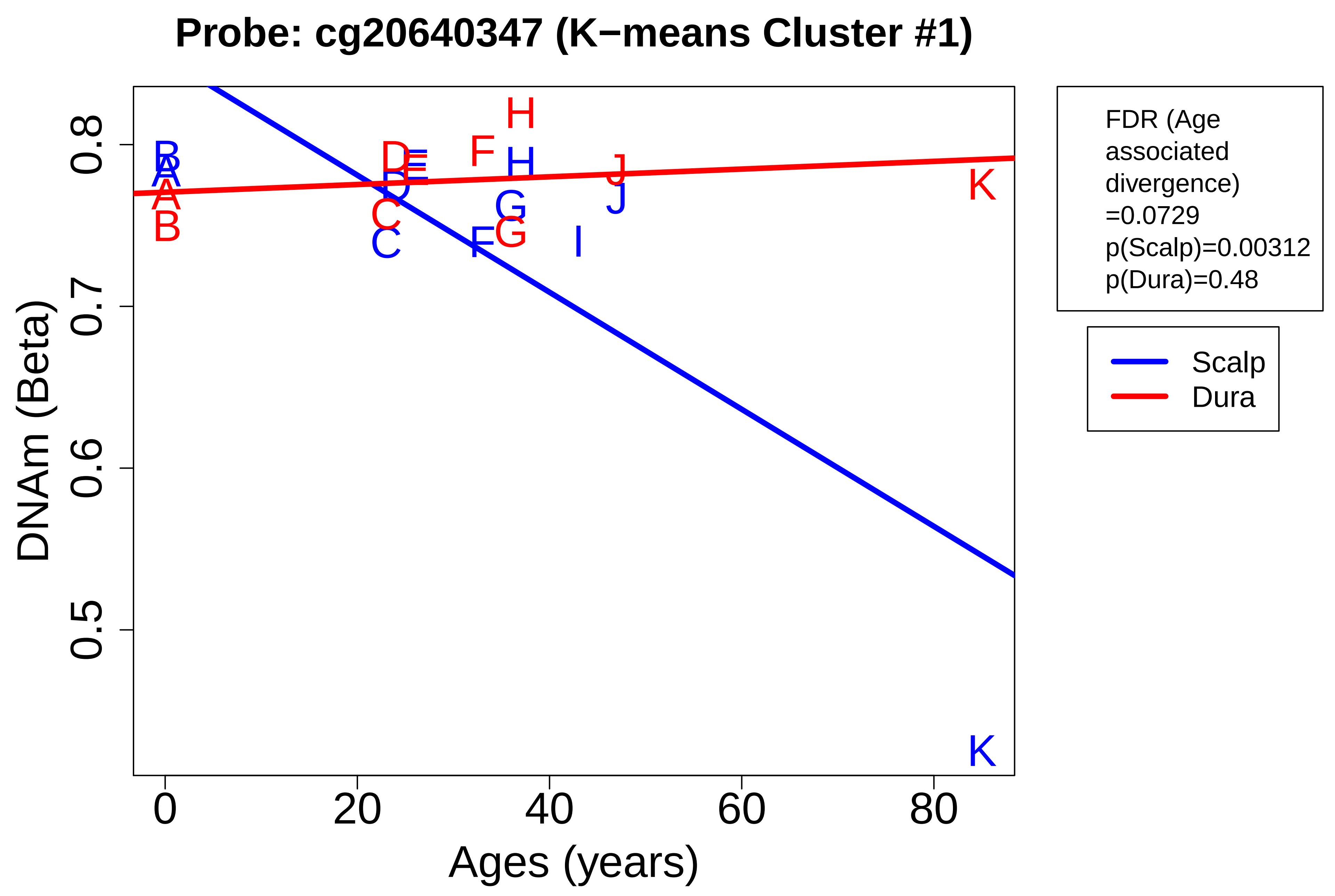

Probe: cg20640347 (K−means Cluster #1)
DNAm (Beta)
0.5
0.6
0.7
0.8
Ages (years)
0
20
40
60
80
FDR (Age associated divergence) =0.0729
p(Scalp)=0.00312
p(Dura)=0.48
Scalp
Dura
A
B
C
D
E
F
G
H
I
J
K

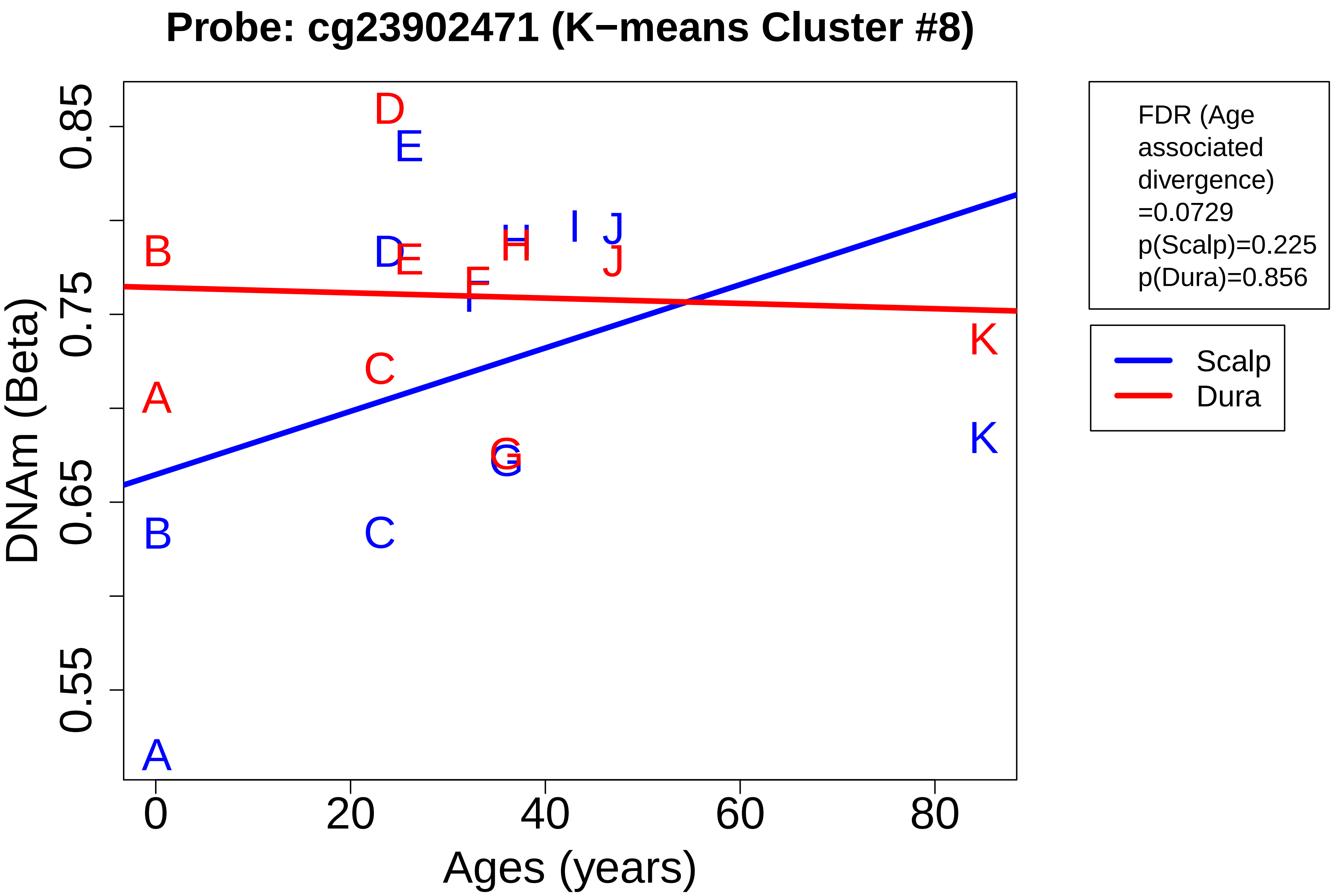

Probe: cg23902471 (K−means Cluster #8)
DNAm (Beta)
0.55
0.65
0.75
0.85
Ages (years)
0
20
40
60
80
FDR (Age associated divergence) =0.0729
p(Scalp)=0.225
p(Dura)=0.856
Scalp
Dura

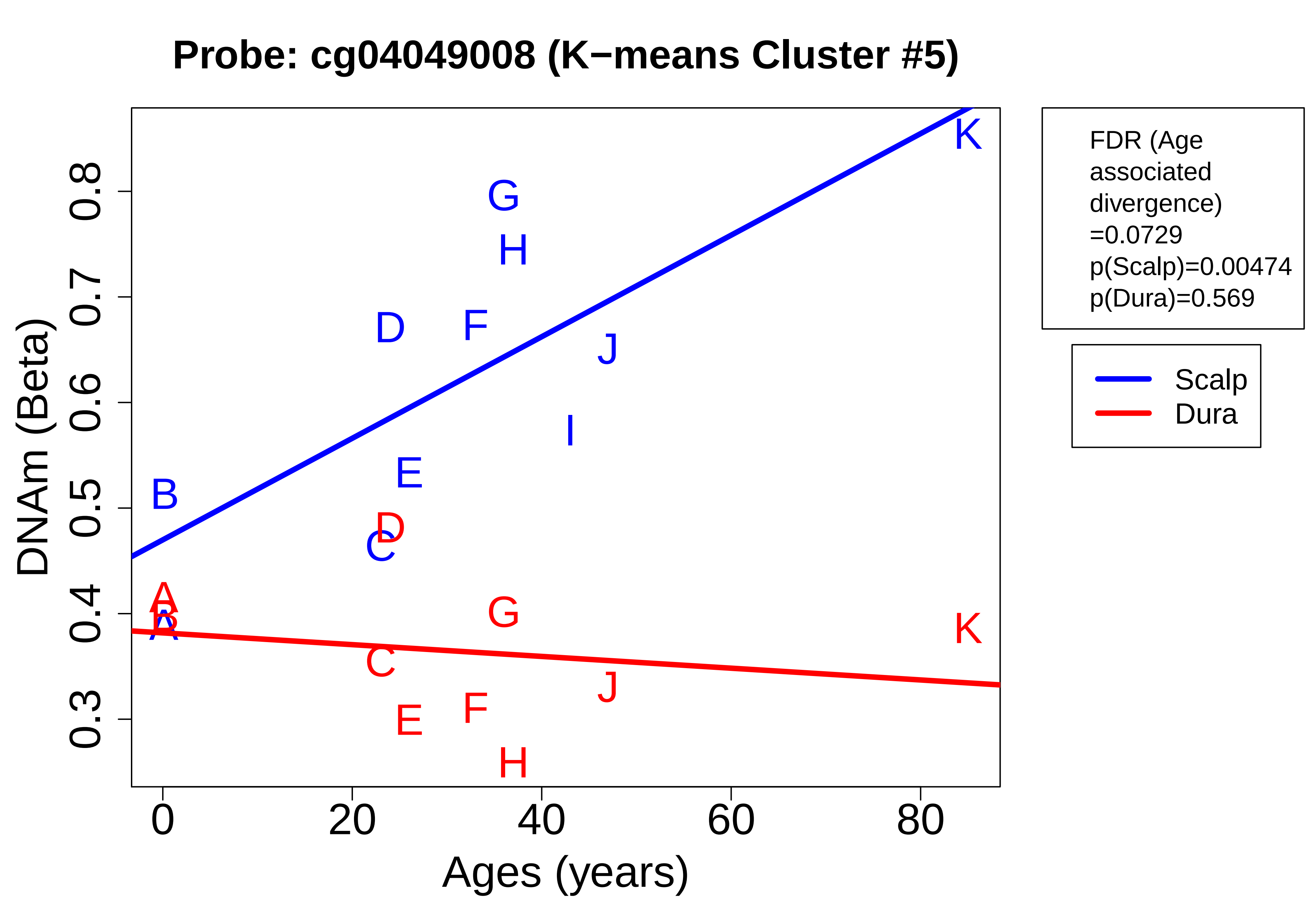

Probe: cg04049008 (K−means Cluster #5)
DNAm (Beta)
0.3
0.4
0.5
0.6
0.7
0.8
0
20
40
60
80
Ages (years)
A
B
C
D
E
F
G
H
I
J
K
FDR (Age associated divergence) =0.0729
p(Scalp)=0.00474
p(Dura)=0.569
Scalp
Dura

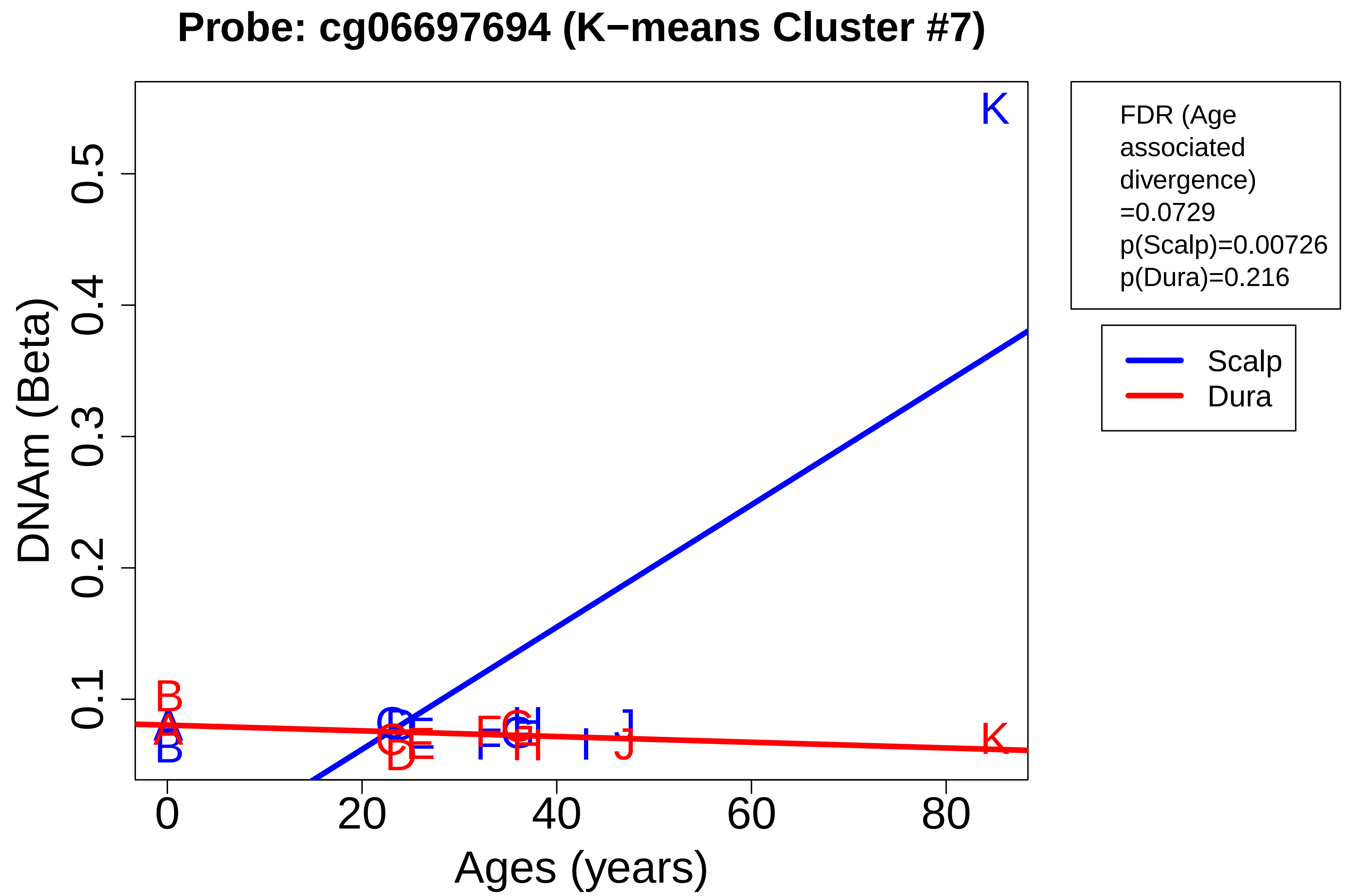

Probe: cg06697694 (K−means Cluster #7)
DNAm (Beta)
0.1
0.2
0.3
0.4
0.5
0
20
40
60
80
Ages (years)
FDR (Age associated divergence) =0.0729
p(Scalp)=0.00726
p(Dura)=0.216
Scalp
Dura

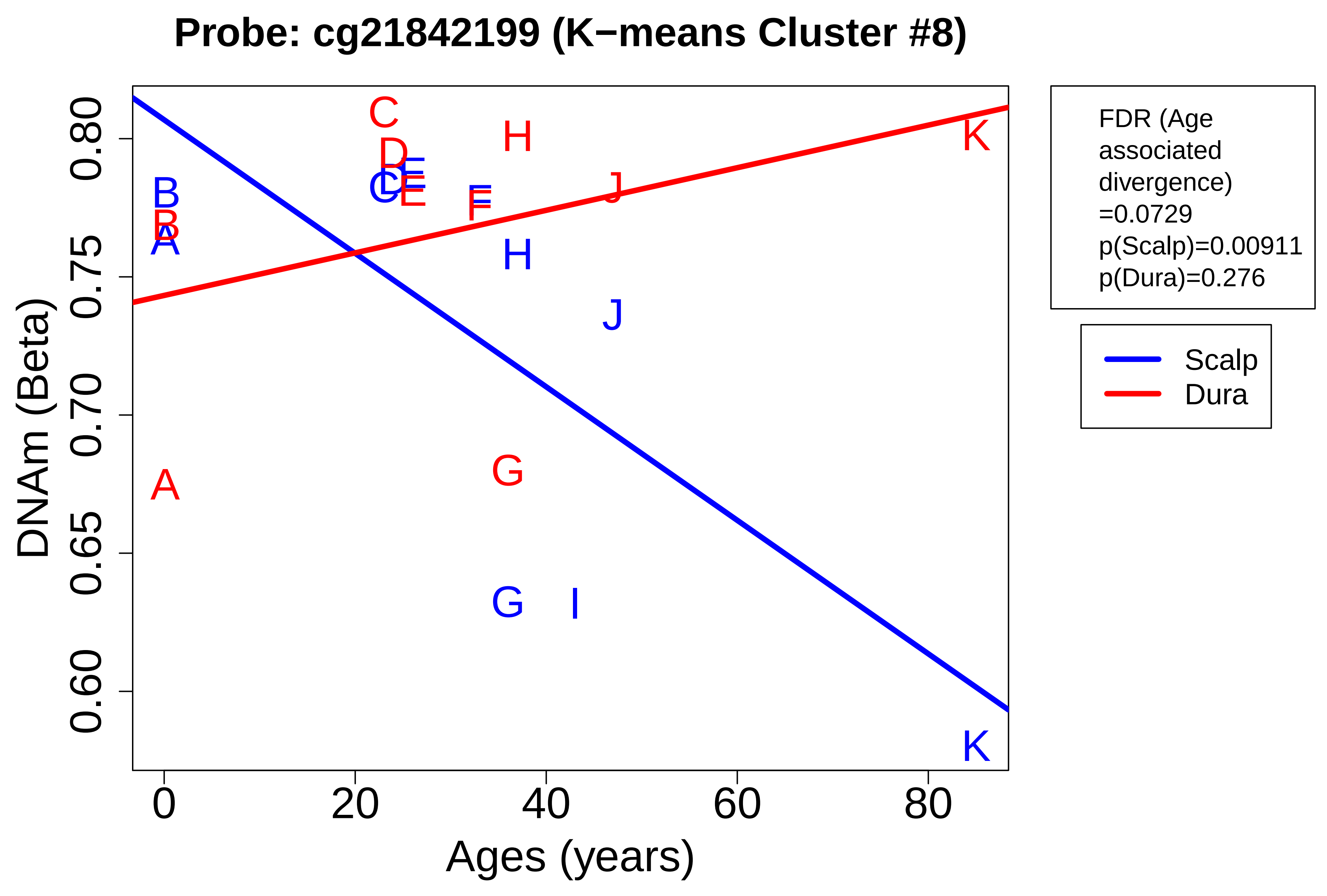

Probe: cg21842199 (K−means Cluster #8)
DNAm (Beta)
0.60
0.65
0.70
0.75
0.80
0
20
40
60
80
Ages (years)
FDR (Age associated divergence) =0.0729
p(Scalp)=0.00911
p(Dura)=0.276
Scalp
Dura

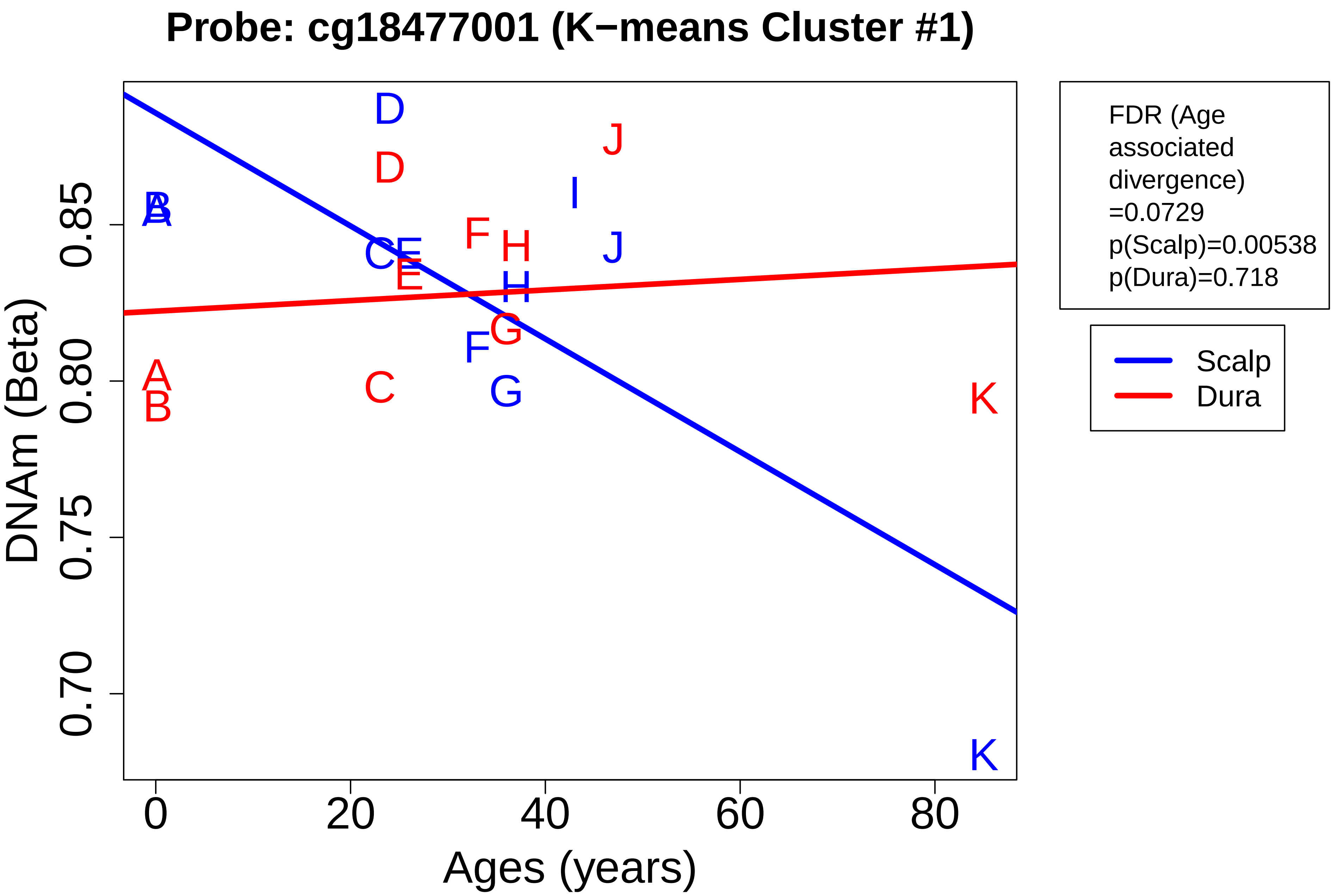

Probe: cg18477001 (K−means Cluster #1)
DNAm (Beta)
0.70
0.75
0.80
0.85
0
20
40
60
80
Ages (years)
A
B
C
D
E
F
G
H
I
J
K
FDR (Age associated divergence) =0.0729
p(Scalp)=0.00538
p(Dura)=0.718
Scalp
Dura

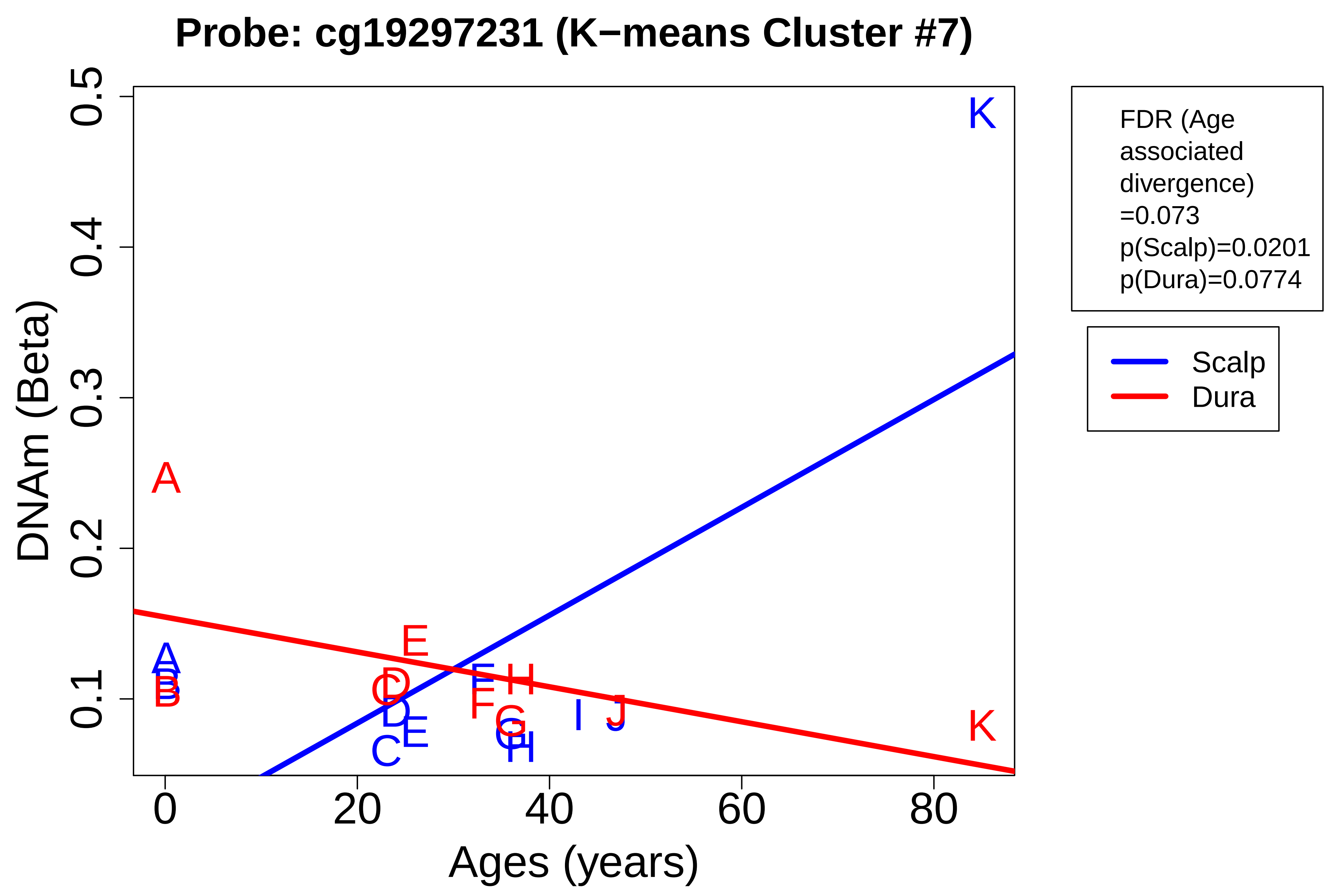

Probe: cg19297231 (K−means Cluster #7)
DNAm (Beta)
0.1
0.2
0.3
0.4
0.5
0
20
40
60
80
Ages (years)
FDR (Age associated divergence) =0.073
p(Scalp)=0.0201
p(Dura)=0.0774
Scalp
Dura

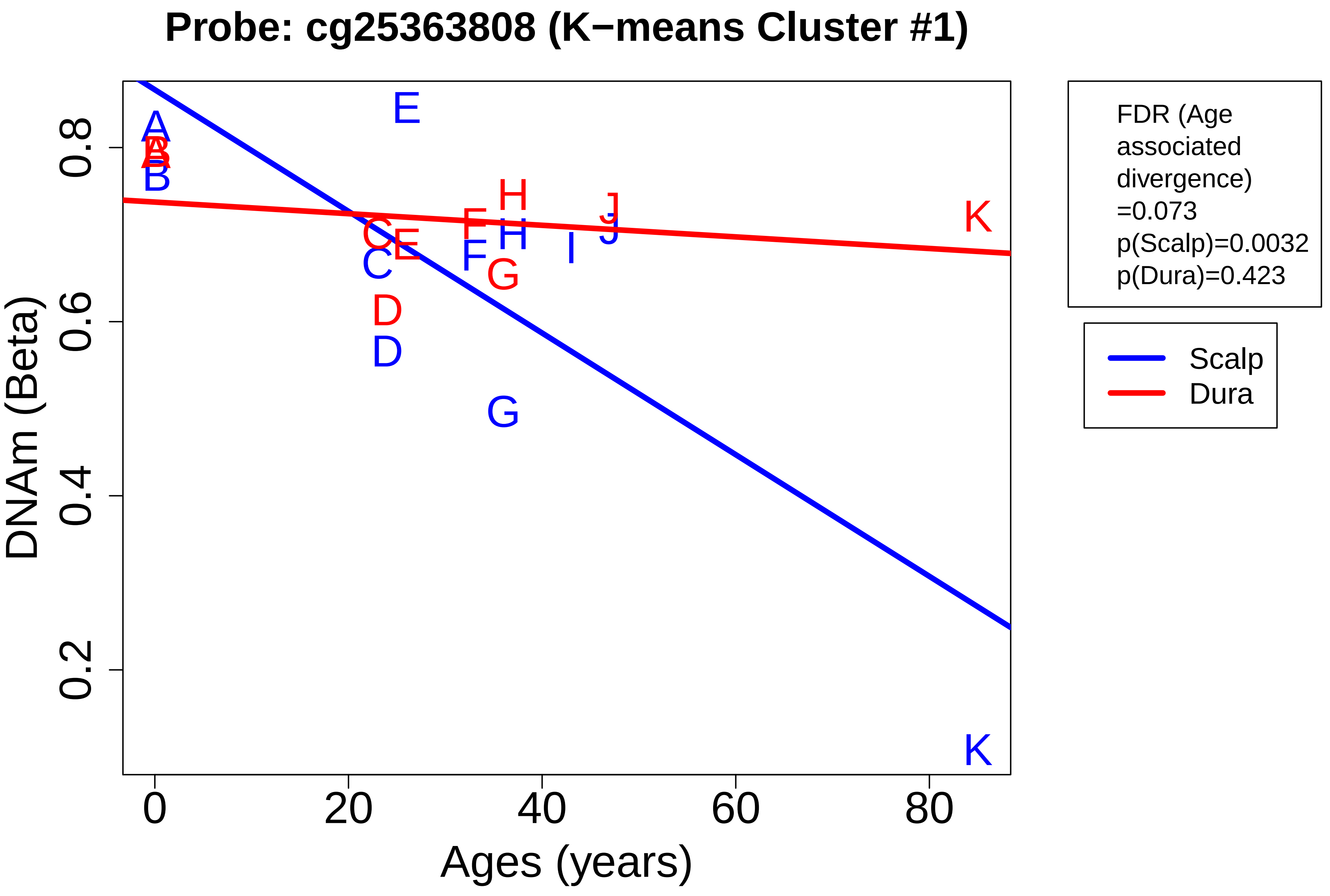

Probe: cg25363808 (K−means Cluster #1)
DNAm (Beta)
0.2
0.4
0.6
0.8
Ages (years)
0
20
40
60
80
FDR (Age associated divergence) =0.073
p(Scalp)=0.0032
p(Dura)=0.423
Scalp
Dura

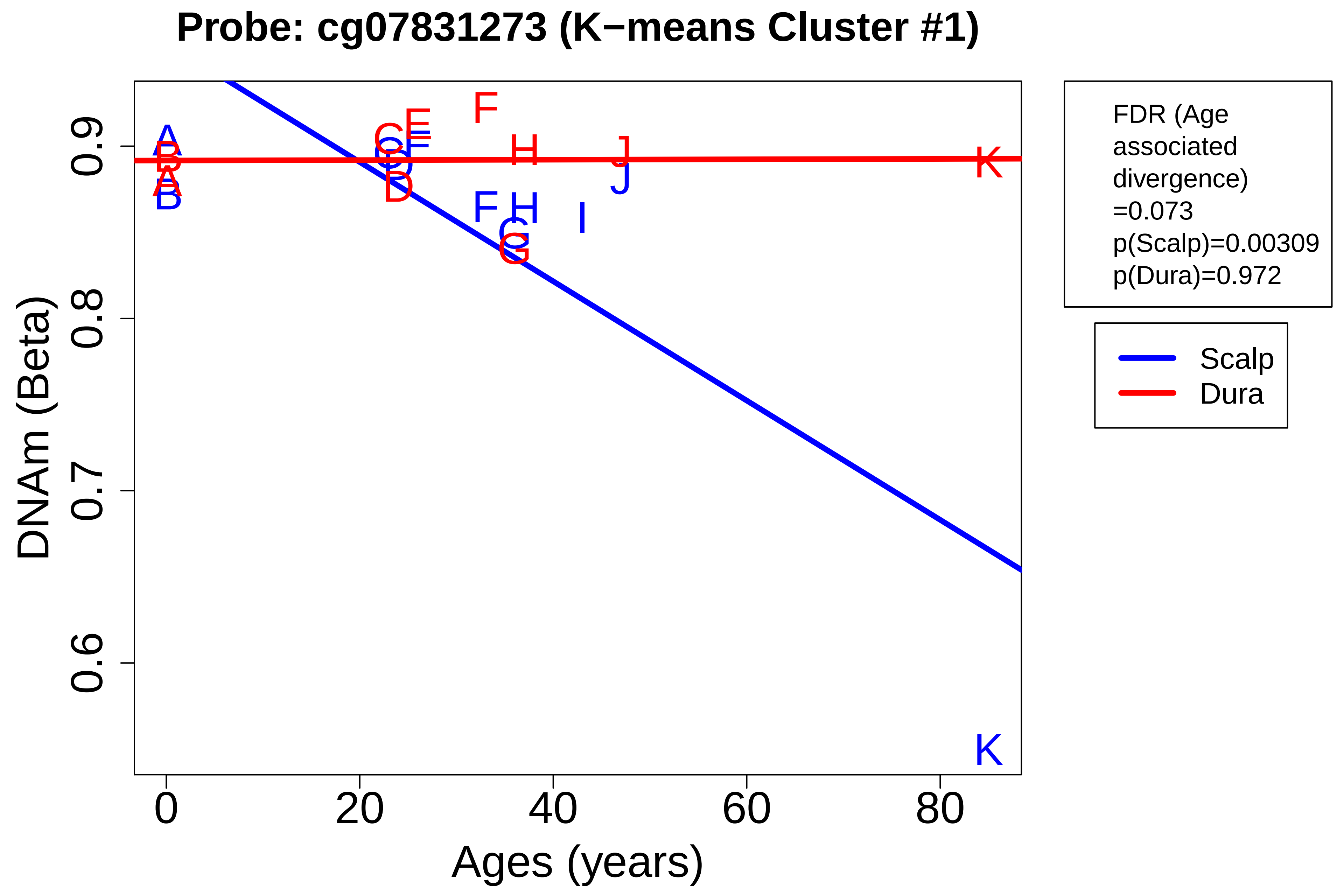
Probe: cg07831273 (K−means Cluster #1)
FDR (Age associated divergence) =0.073
p(Scalp)=0.00309
p(Dura)=0.972
Scalp
Dura
DNAm (Beta)
Ages (years)
0.6
0.7
0.8
0.9
0
20
40
60
80

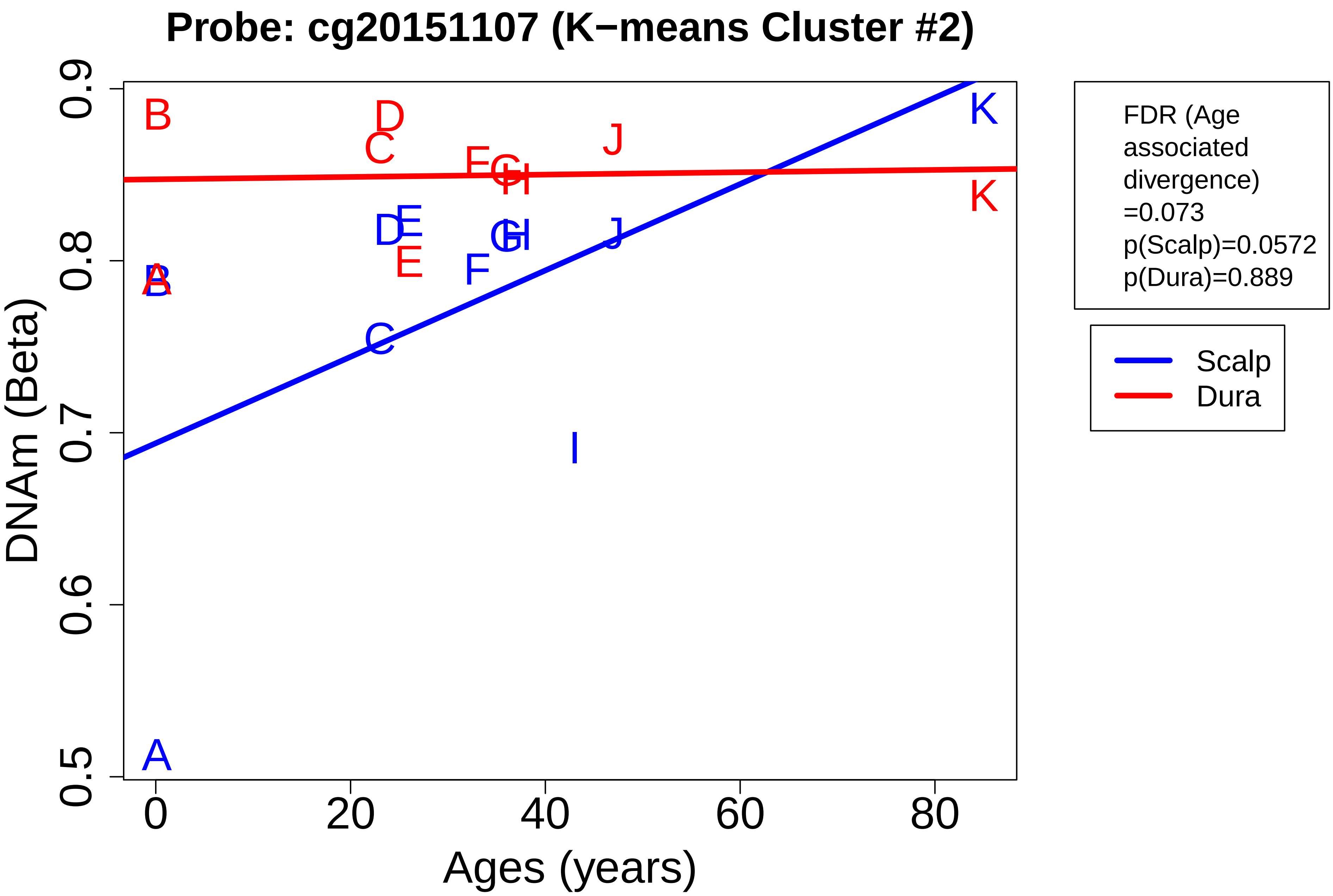

Probe: cg20151107 (K−means Cluster #2)
DNAm (Beta)
0.5
0.6
0.7
0.8
0.9
0
20
40
60
80
Ages (years)
FDR (Age associated divergence) =0.073
p(Scalp)=0.0572
p(Dura)=0.889
Scalp
Dura

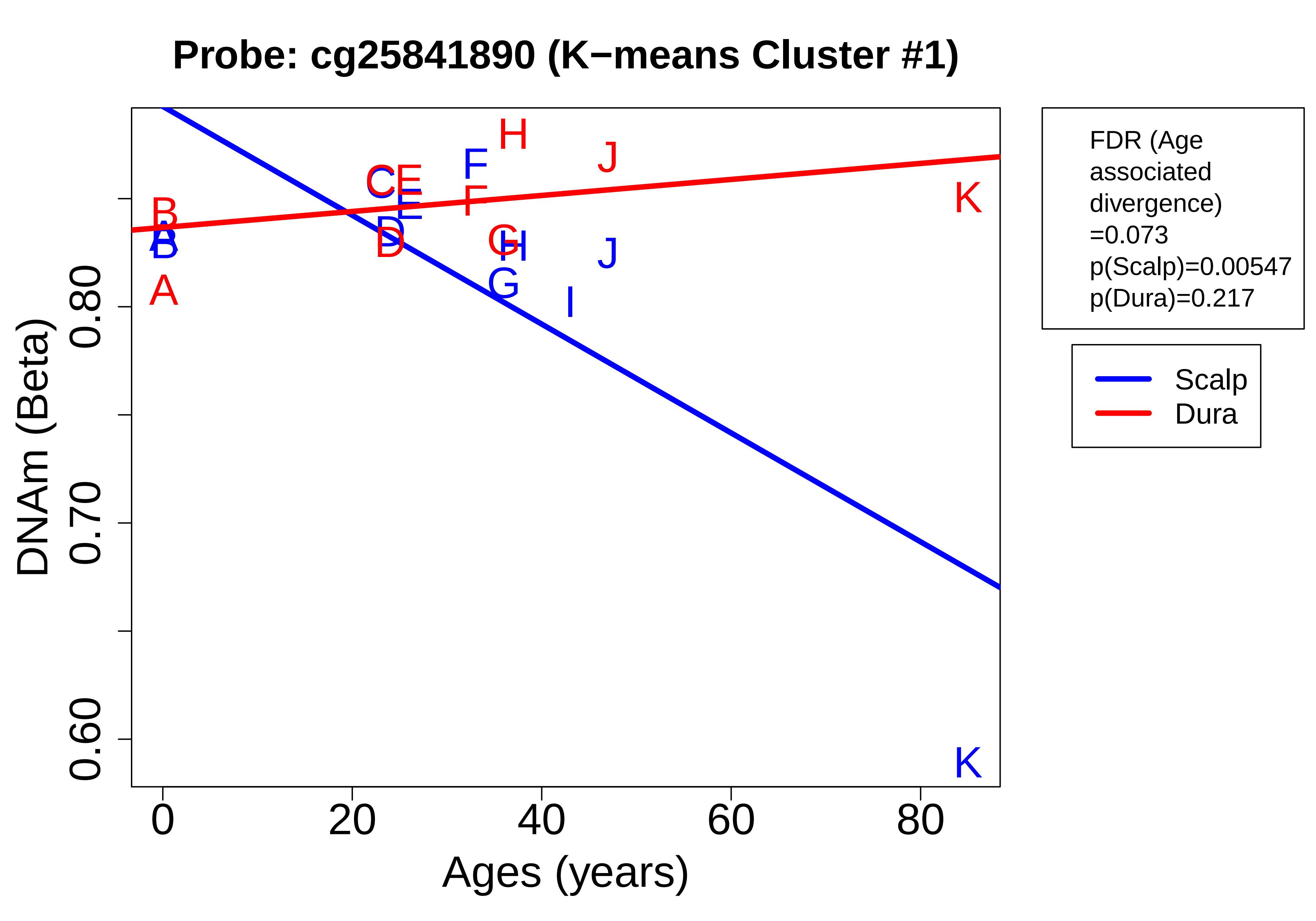

Probe: cg25841890 (K−means Cluster #1)
DNAm (Beta)
0.60
0.70
0.80
0
20
40
60
80
Ages (years)
FDR (Age associated divergence) =0.073
p(Scalp)=0.00547
p(Dura)=0.217
Scalp
Dura

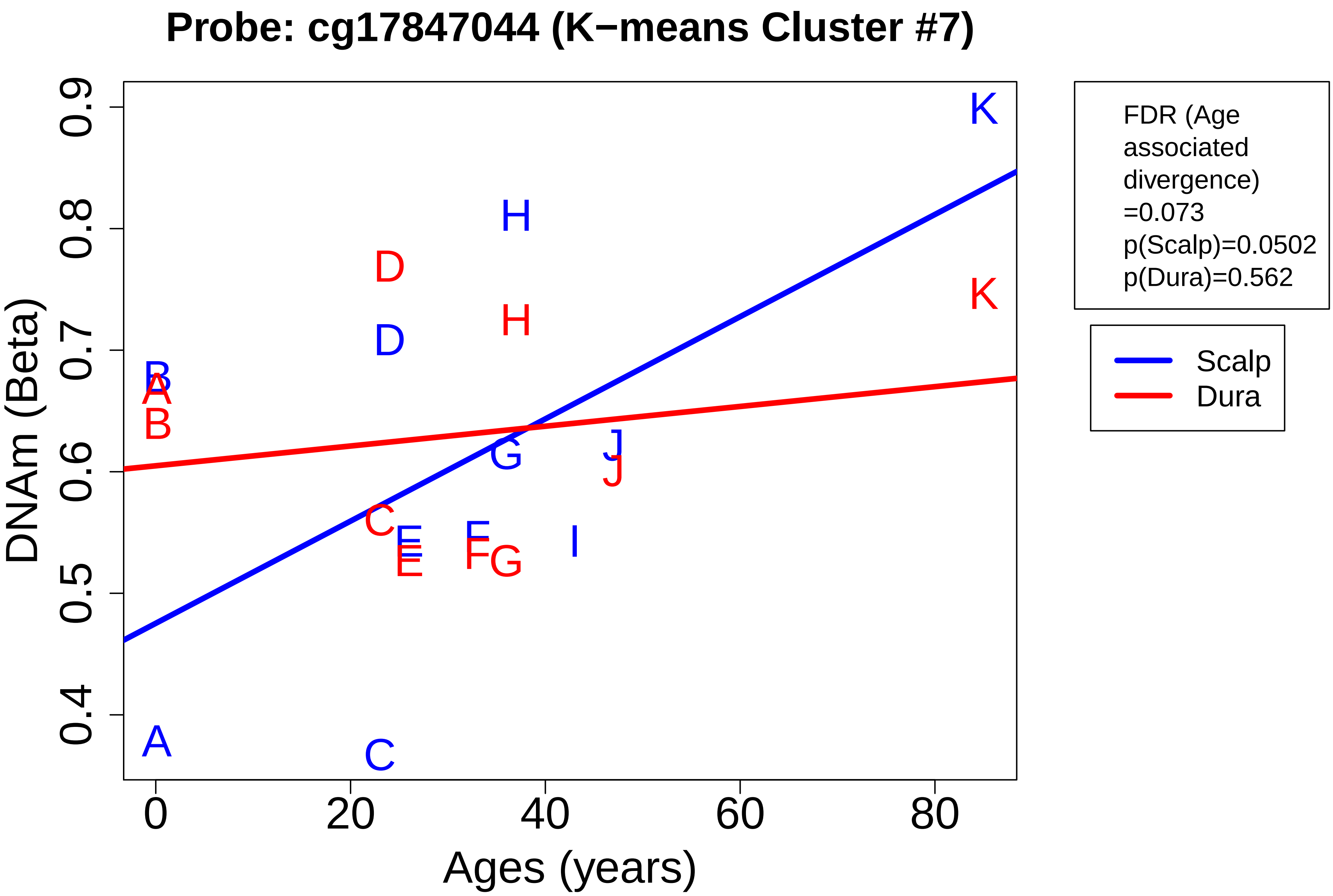

Probe: cg17847044 (K−means Cluster #7)
DNAm (Beta)
0.4
0.5
0.6
0.7
0.8
0.9
Ages (years)
0
20
40
60
80
A
B
C
D
E
F
G
H
I
J
K
FDR (Age associated divergence) =0.073
p(Scalp)=0.0502
p(Dura)=0.562
Scalp
Dura

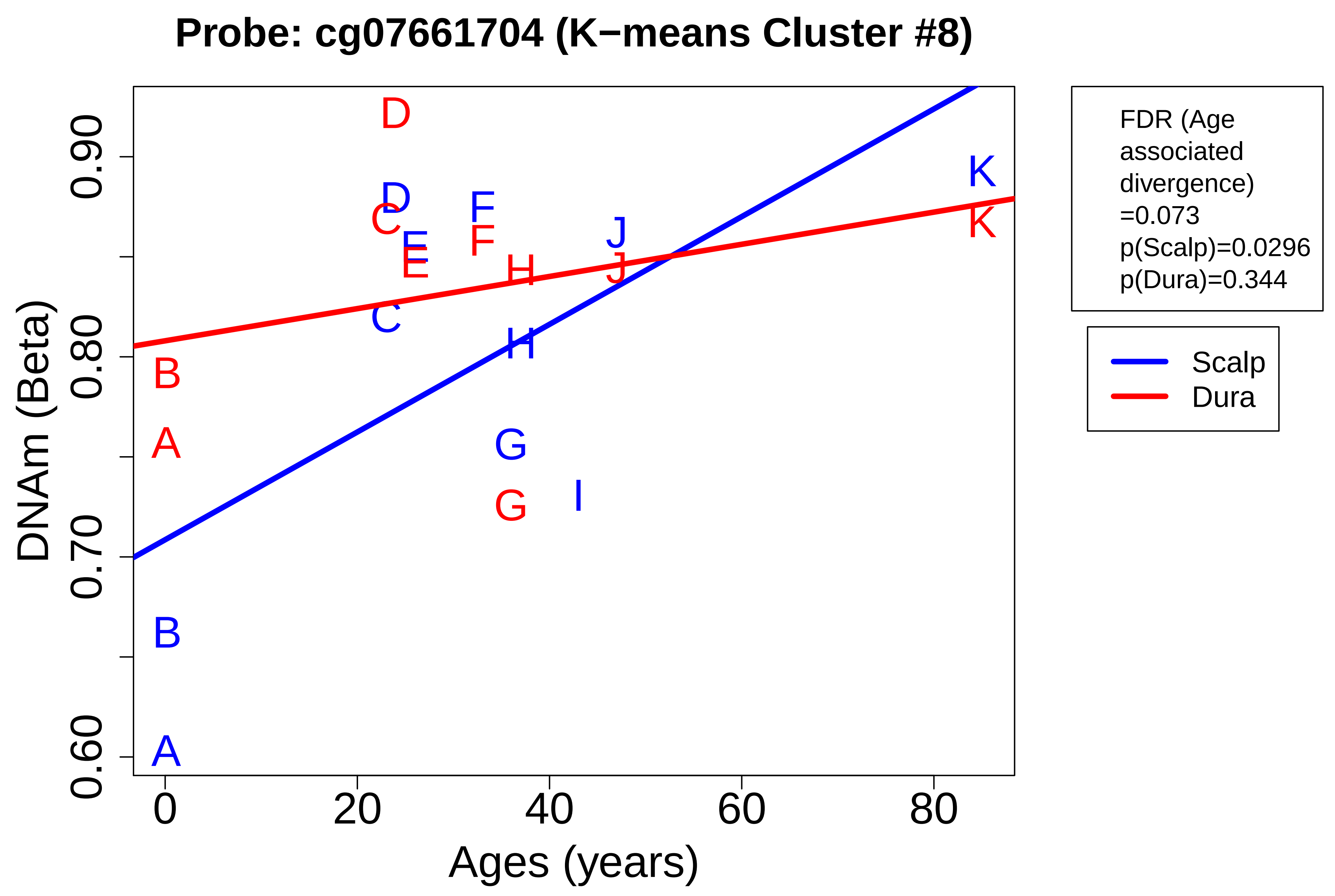

Probe: cg07661704 (K−means Cluster #8)
DNAm (Beta)
0.60
0.70
0.80
0.90
0
20
40
60
80
Ages (years)
FDR (Age associated divergence) =0.073
p(Scalp)=0.0296
p(Dura)=0.344
Scalp
Dura
A B C D E F G H I J K
A B C D E F G H J K

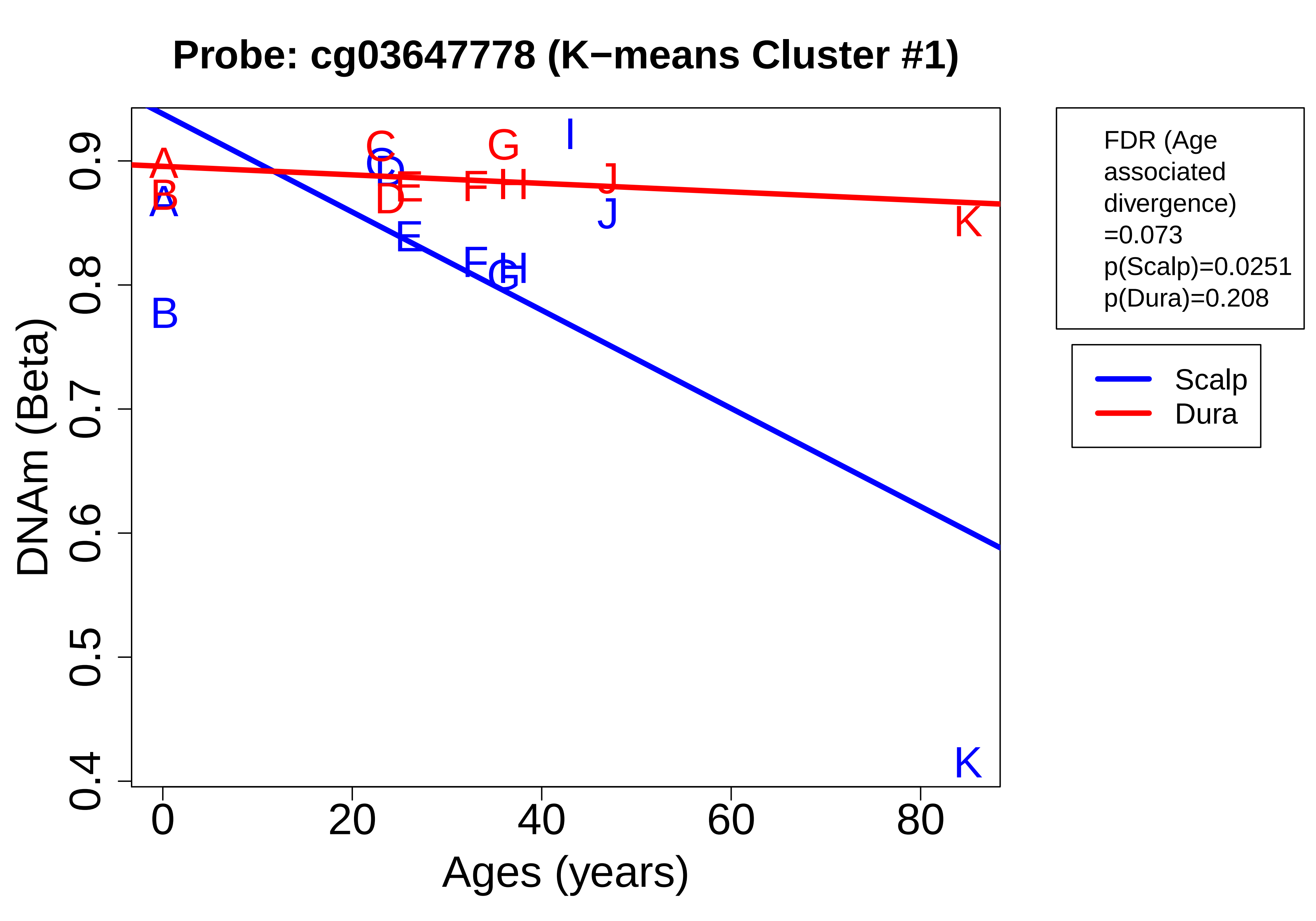

Probe: cg03647778 (K−means Cluster #1)
DNAm (Beta)
Ages (years)
0.4
0.5
0.6
0.7
0.8
0.9
0
20
40
60
80
FDR (Age associated divergence) =0.073
p(Scalp)=0.0251
p(Dura)=0.208
Scalp
Dura

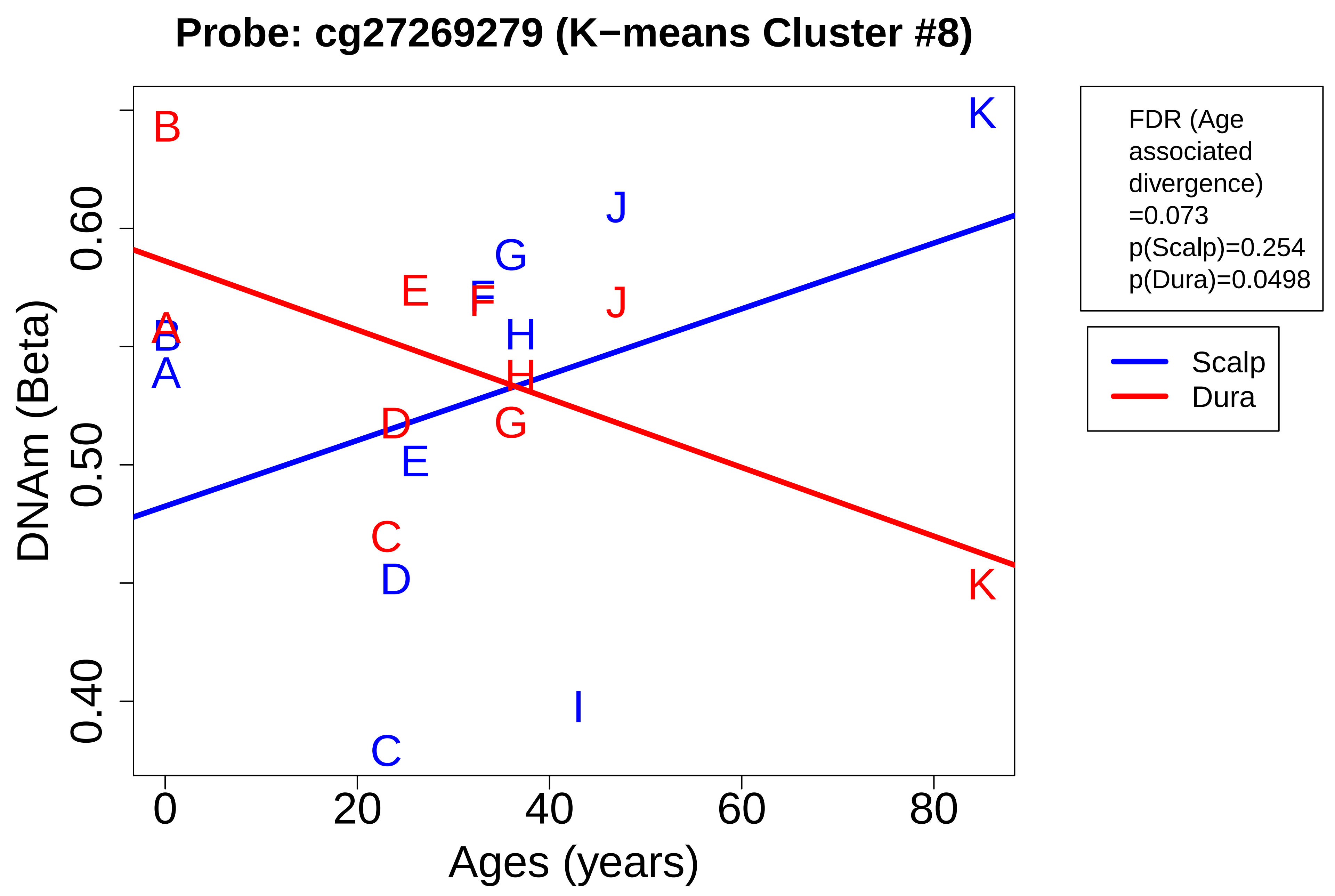

Probe: cg27269279 (K−means Cluster #8)
DNAm (Beta)
0.40
0.50
0.60
0
20
40
60
80
Ages (years)
FDR (Age associated divergence) =0.073
p(Scalp)=0.254
p(Dura)=0.0498
Scalp
Dura
A
B
C
D
E
F
G
H
I
J
K

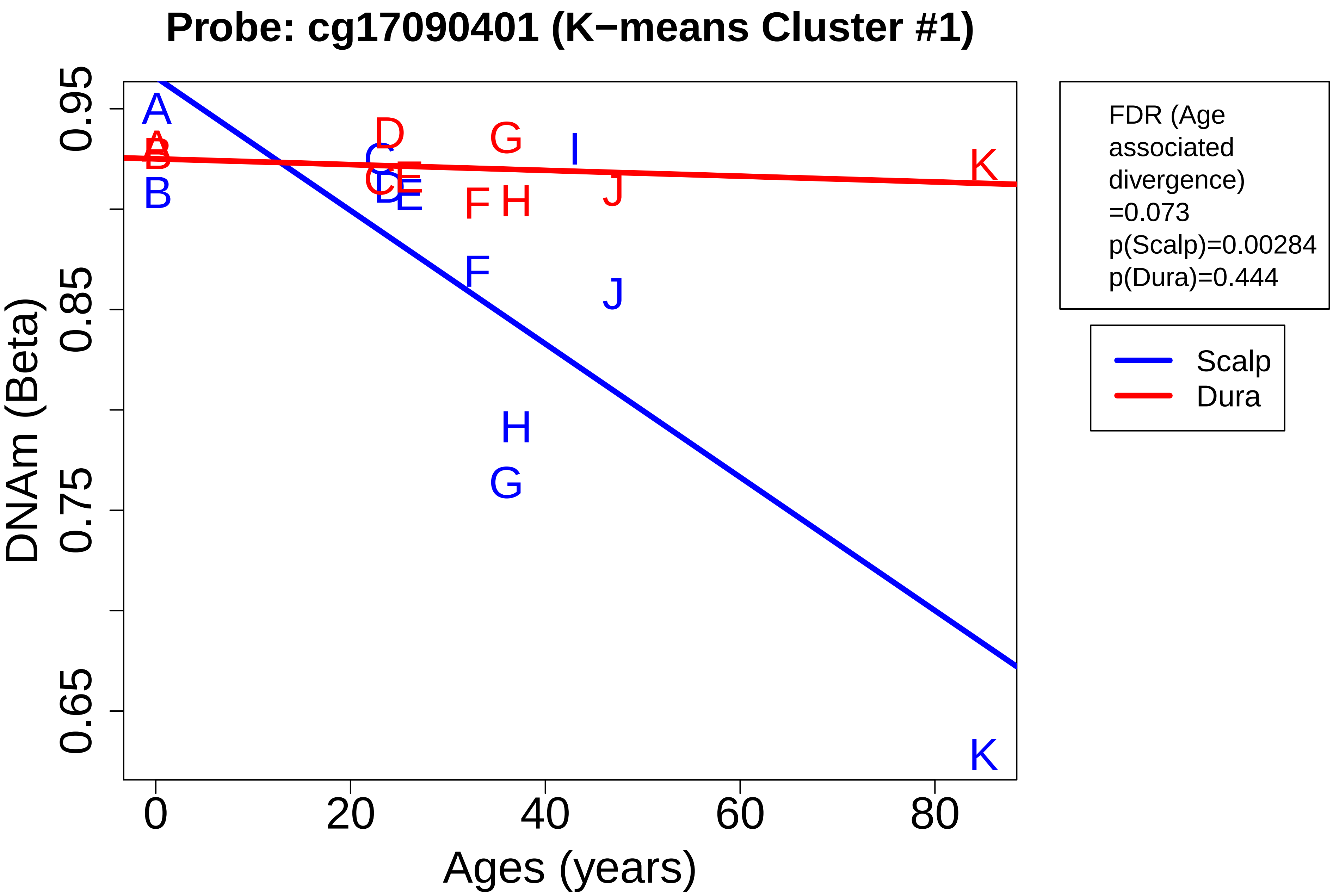

Probe: cg17090401 (K−means Cluster #1)
DNAm (Beta)
0.65
0.75
0.85
0.95
Ages (years)
0
20
40
60
80
FDR (Age associated divergence) =0.073
p(Scalp)=0.00284
p(Dura)=0.444
Scalp
Dura
A B C D E F G H I J K

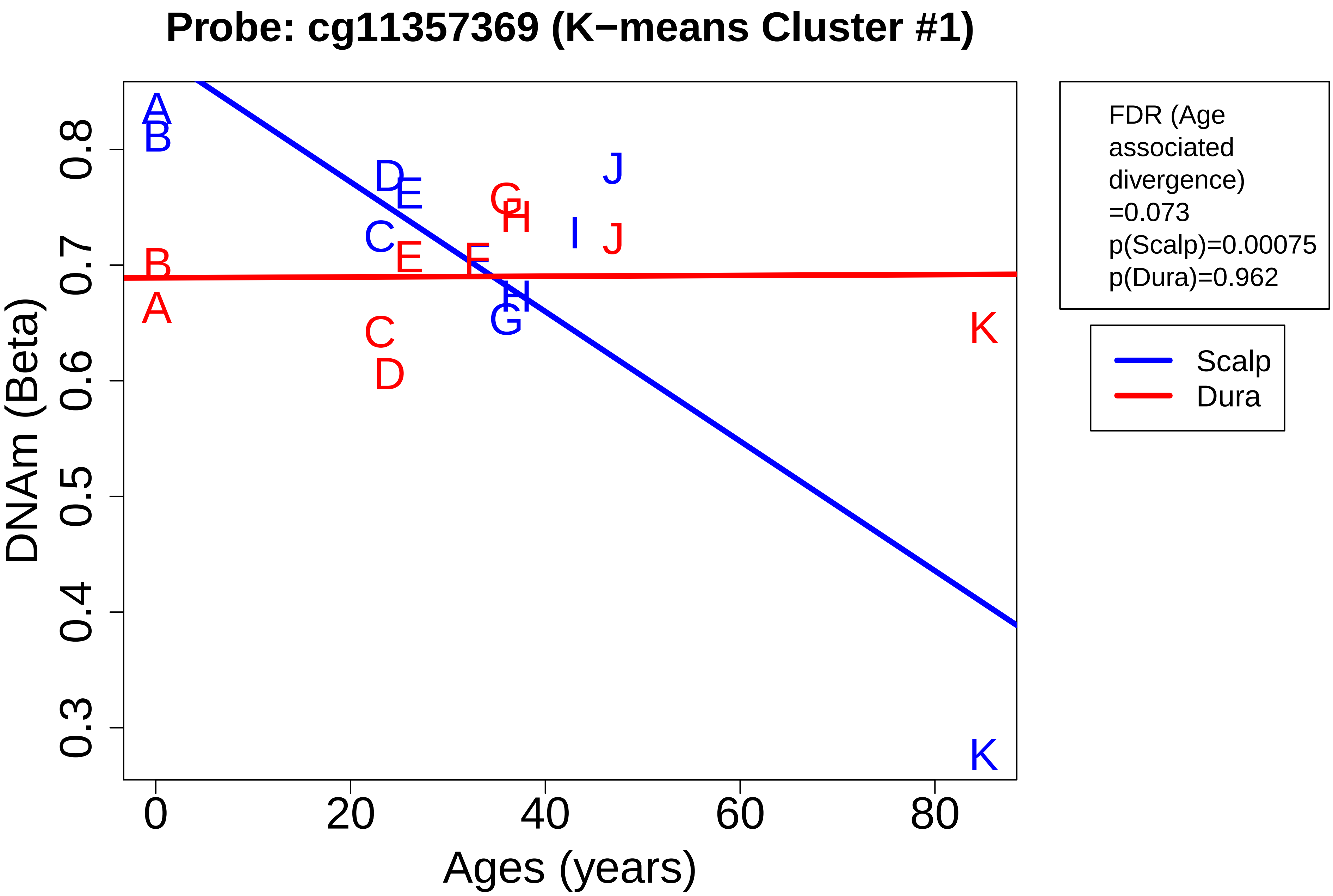

Probe: cg11357369 (K−means Cluster #1)
DNAm (Beta)
0.3
0.4
0.5
0.6
0.7
0.8
0
20
40
60
80
Ages (years)
FDR (Age associated divergence) =0.073
p(Scalp)=0.00075
p(Dura)=0.962
Scalp
Dura

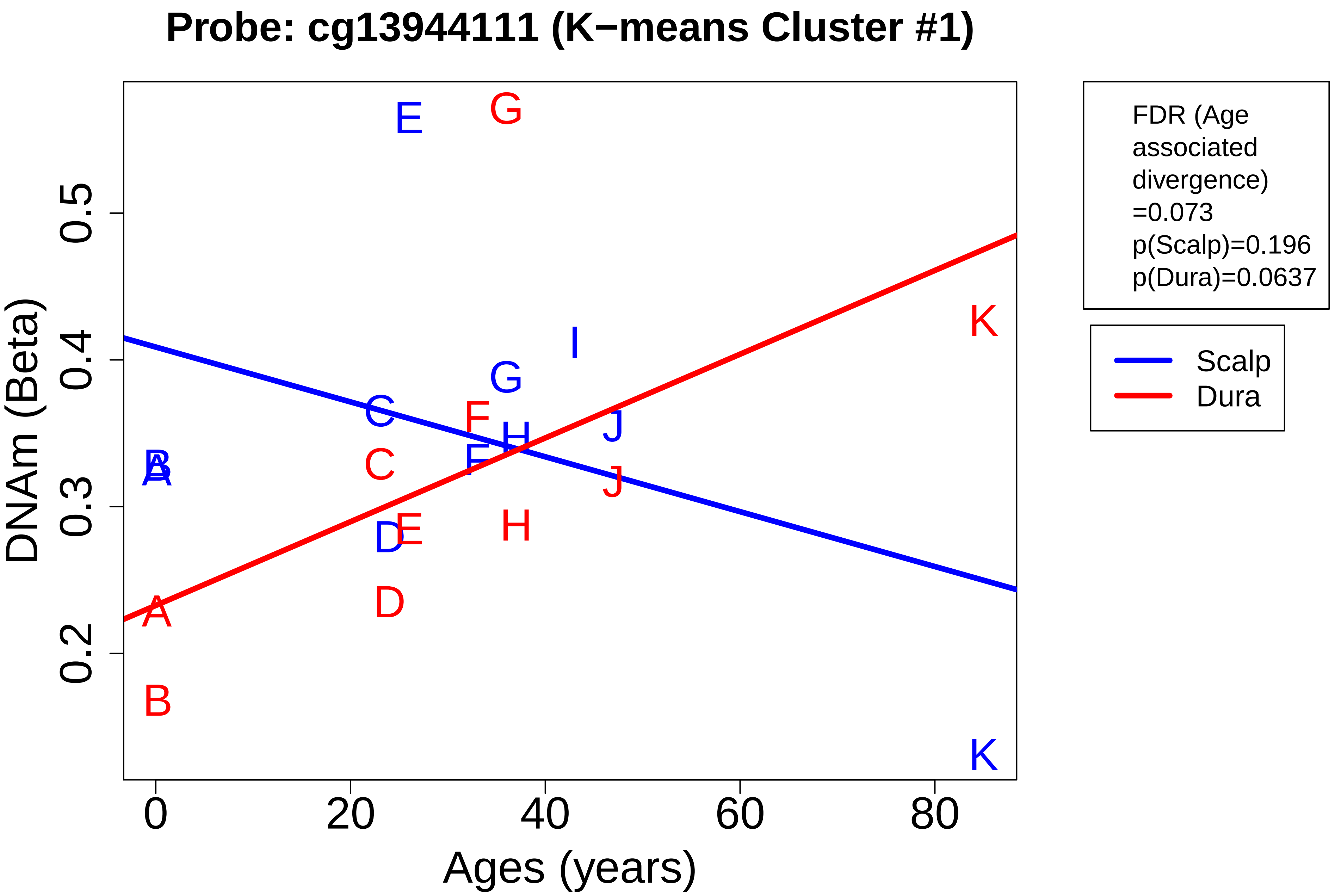

Probe: cg13944111 (K−means Cluster #1)
DNAm (Beta)
0.2
0.3
0.4
0.5
0
20
40
60
80
Ages (years)
FDR (Age associated divergence) =0.073
p(Scalp)=0.196
p(Dura)=0.0637
Scalp
Dura

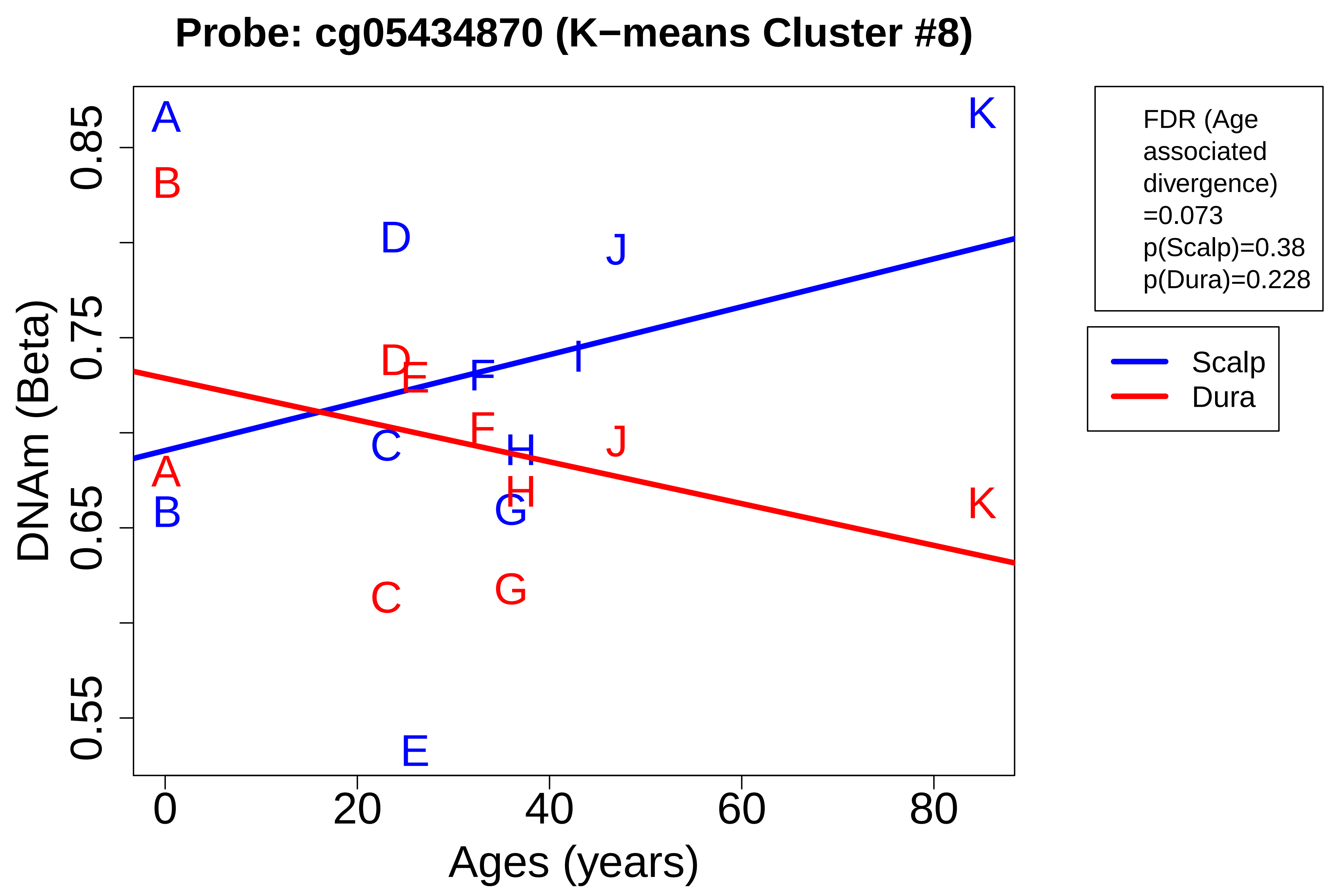

Probe: cg05434870 (K−means Cluster #8)
DNAm (Beta)
0.55
0.65
0.75
0.85
0
20
40
60
80
Ages (years)
A
B
C
D
E
F
G
H
I
J
K
A
B
C
D
E
F
G
H
J
K
FDR (Age associated divergence) =0.073
p(Scalp)=0.38
p(Dura)=0.228
Scalp
Dura

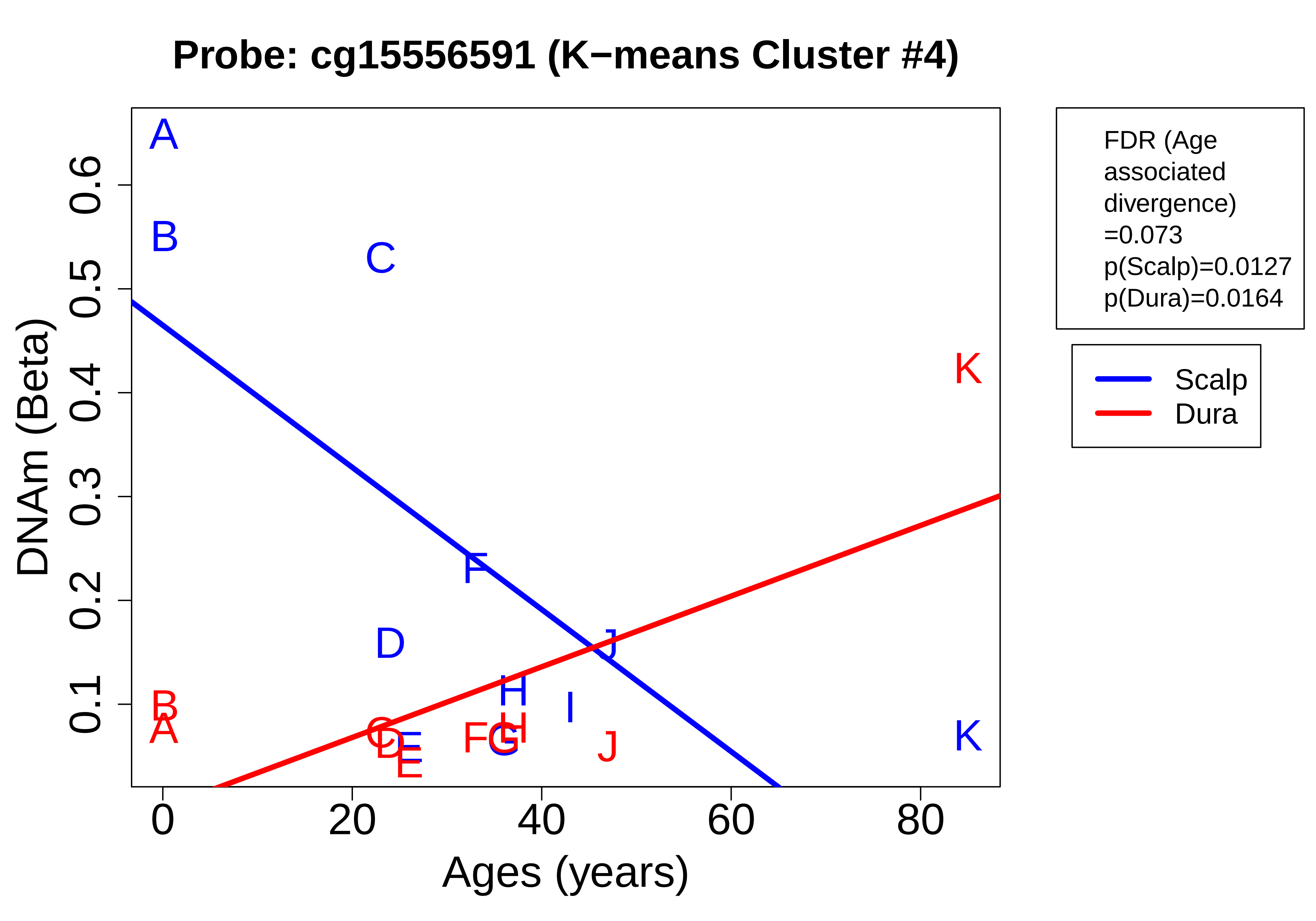

Probe: cg15556591 (K−means Cluster #4)
DNAm (Beta)
0.1
0.2
0.3
0.4
0.5
0.6
0
20
40
60
80
Ages (years)
A
B
C
D
E
F
G
H
I
J
K
FDR (Age associated divergence) =0.073
p(Scalp)=0.0127
p(Dura)=0.0164
Scalp
Dura

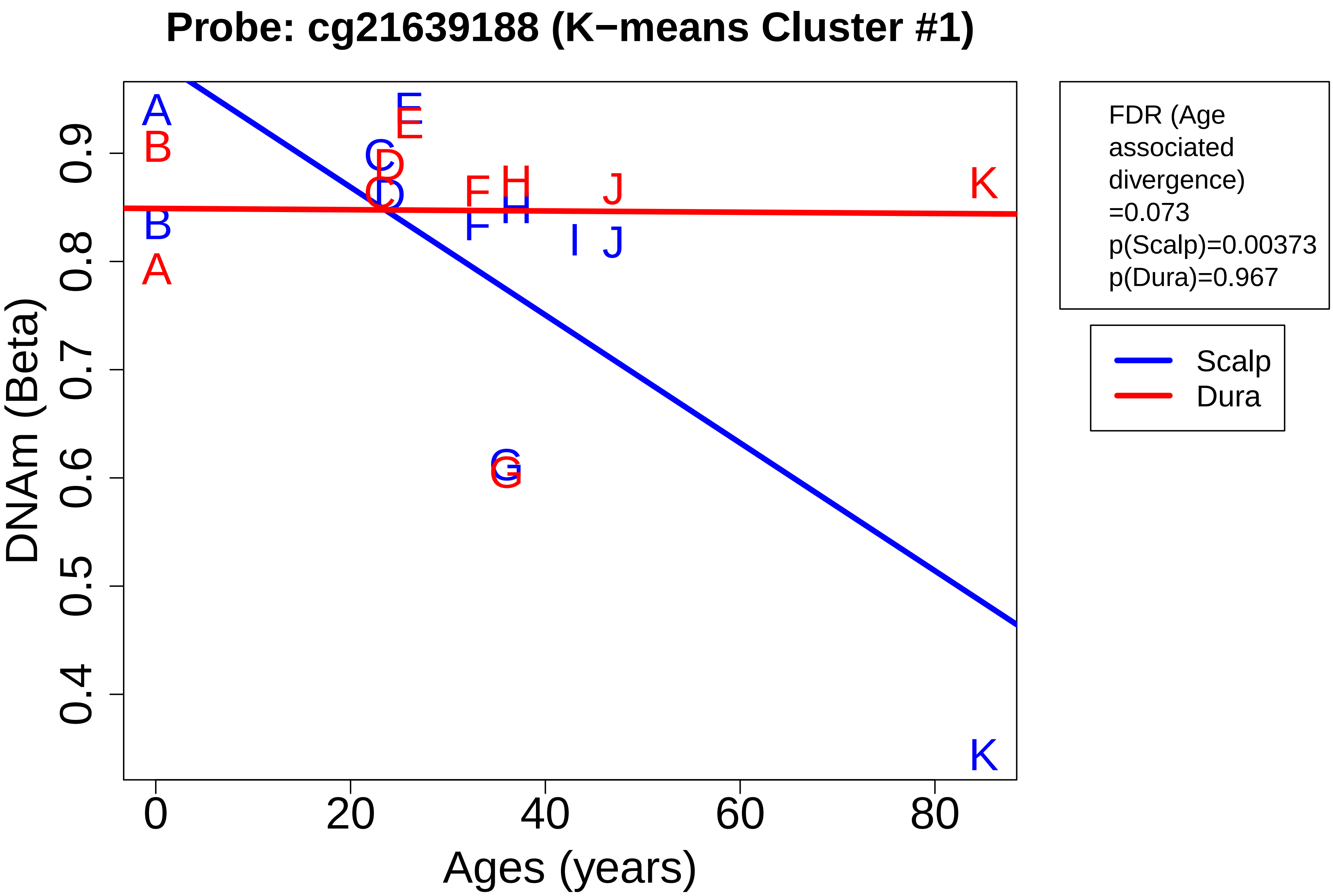

Probe: cg21639188 (K−means Cluster #1)
DNAm (Beta)
Ages (years)
0.4
0.5
0.6
0.7
0.8
0.9
0
20
40
60
80
FDR (Age associated divergence) =0.073
p(Scalp)=0.00373
p(Dura)=0.967
Scalp
Dura

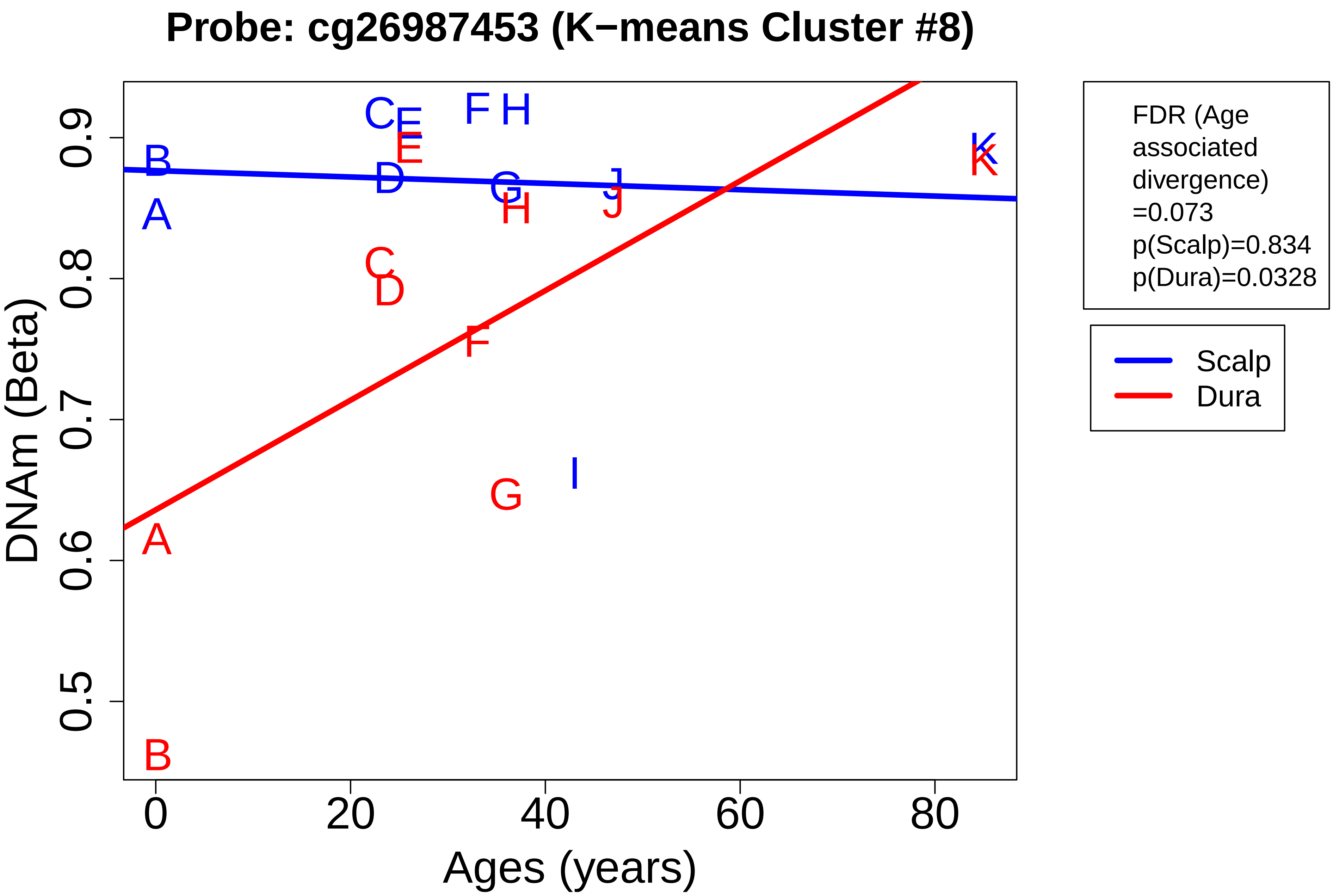

Probe: cg26987453 (K−means Cluster #8)
DNAm (Beta)
0.5
0.6
0.7
0.8
0.9
Ages (years)
0
20
40
60
80
FDR (Age associated divergence) =0.073
p(Scalp)=0.834
p(Dura)=0.0328
Scalp
Dura

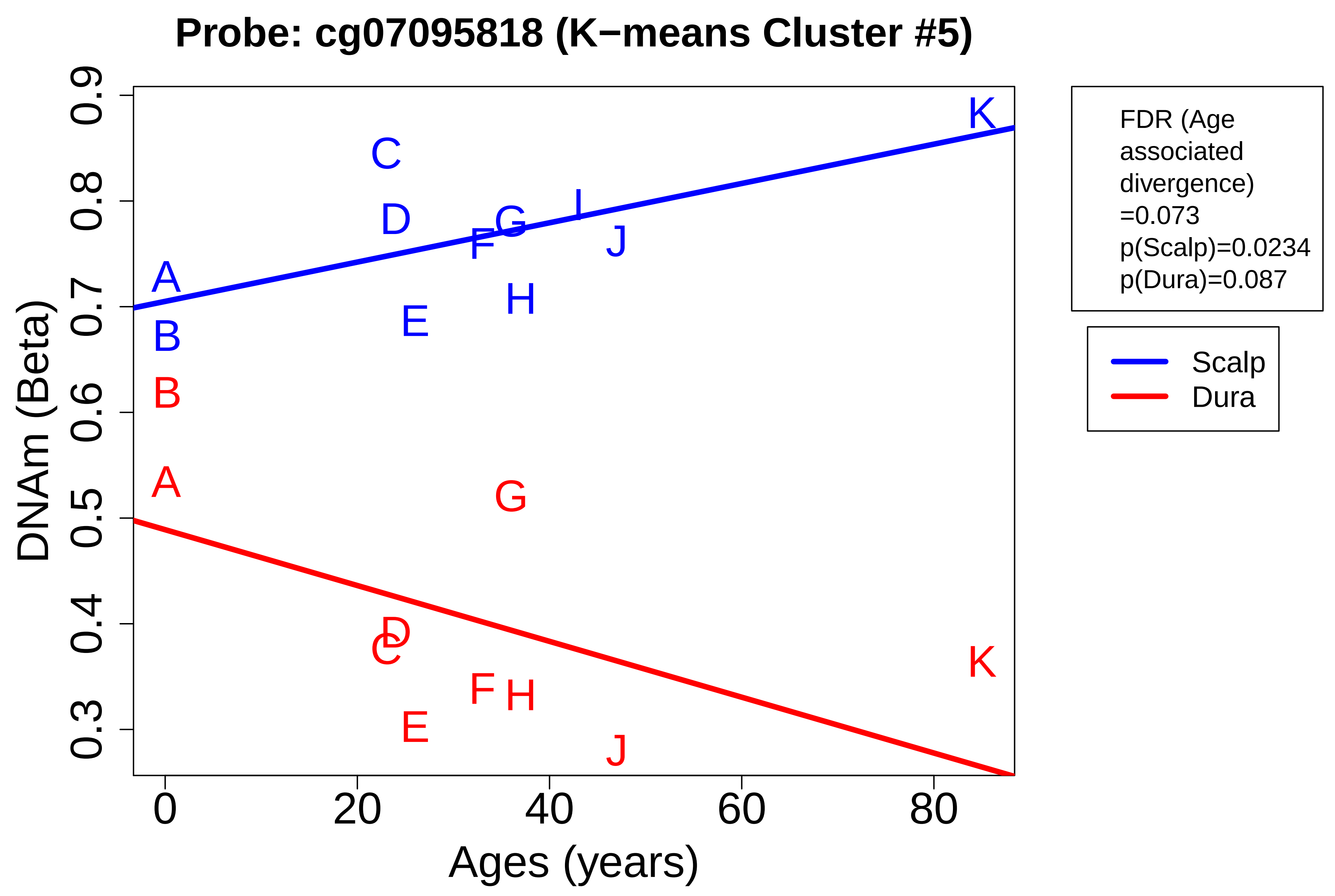

Probe: cg07095818 (K−means Cluster #5)
DNAm (Beta)
0.3
0.4
0.5
0.6
0.7
0.8
0.9
0
20
40
60
80
Ages (years)
A
B
C
D
E
F
G
H
I
J
K
A
B
C
D
E
F
G
H
J
K
FDR (Age associated divergence) =0.073
p(Scalp)=0.0234
p(Dura)=0.087
Scalp
Dura

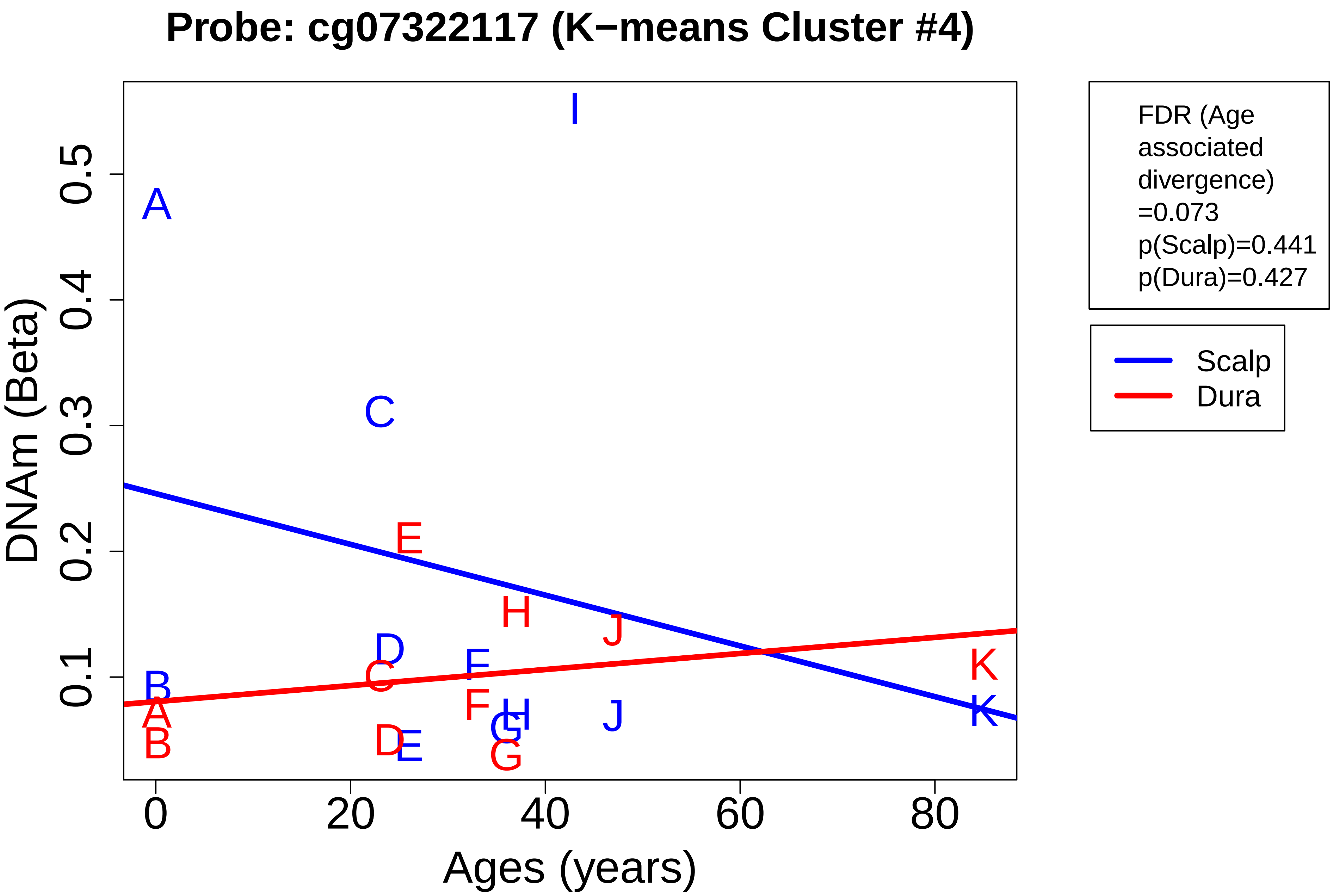

Probe: cg07322117 (K−means Cluster #4)
FDR (Age associated divergence) =0.073
p(Scalp)=0.441
p(Dura)=0.427
Scalp
Dura
DNAm (Beta)
0.1
0.2
0.3
0.4
0.5
0
20
40
60
80
Ages (years)

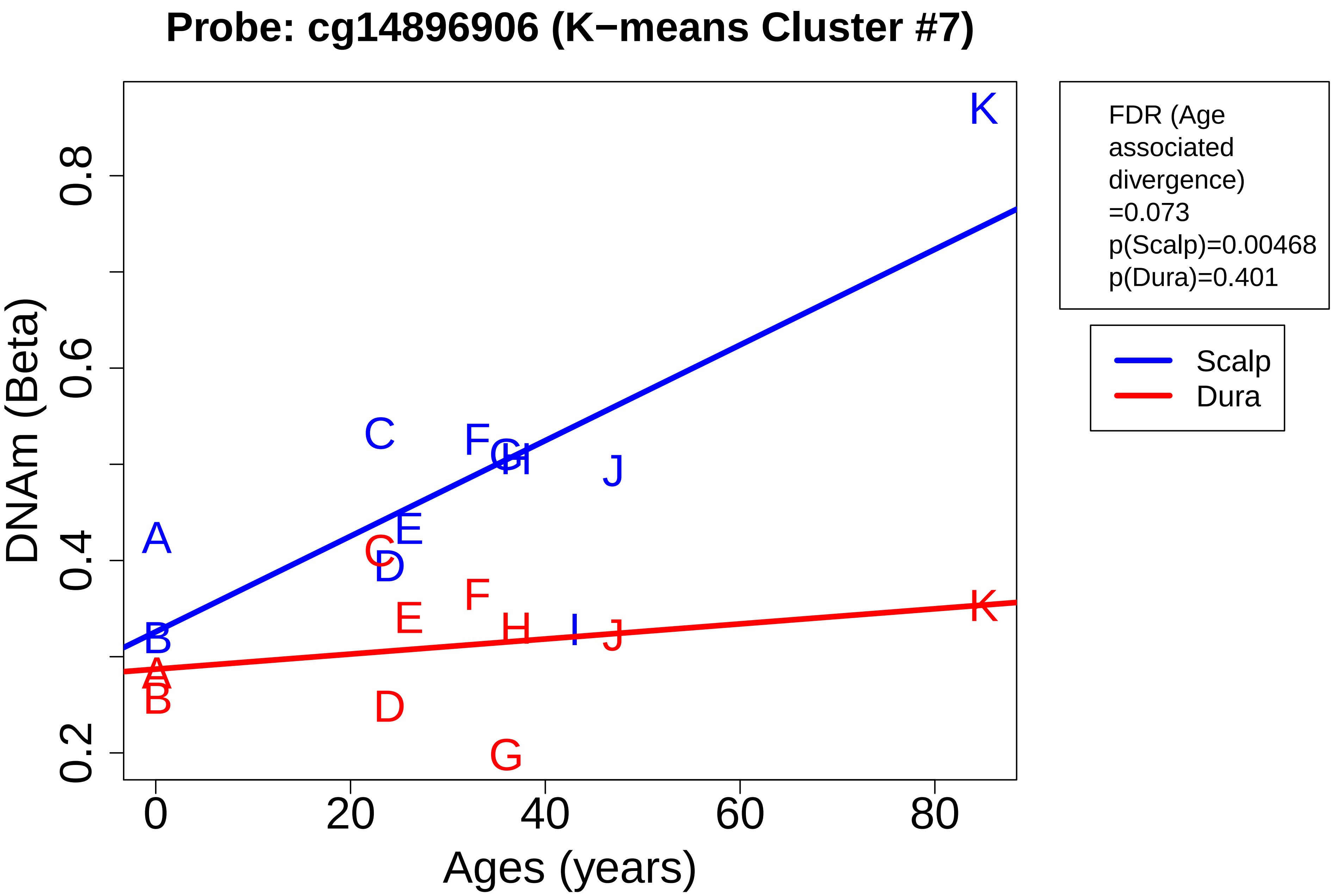

Probe: cg14896906 (K−means Cluster #7)
DNAm (Beta)
0.2
0.4
0.6
0.8
Ages (years)
0
20
40
60
80
FDR (Age associated divergence) =0.073
p(Scalp)=0.00468
p(Dura)=0.401
Scalp
Dura

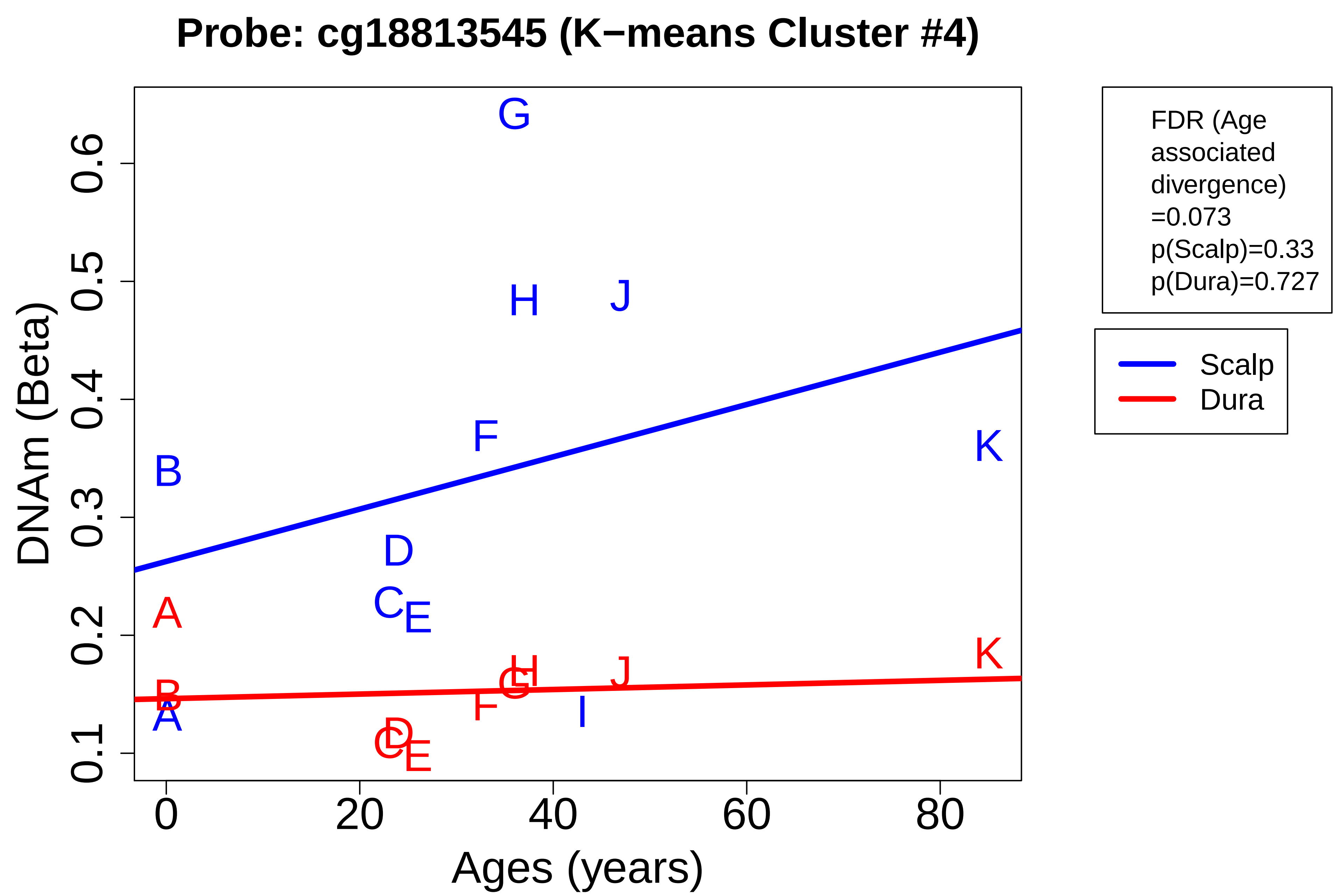
Probe: cg18813545 (K−means Cluster #4)
FDR (Age associated divergence) =0.073
p(Scalp)=0.33
p(Dura)=0.727
Scalp
Dura
DNAm (Beta)
Ages (years)
0.1
0.2
0.3
0.4
0.5
0.6
0
20
40
60
80

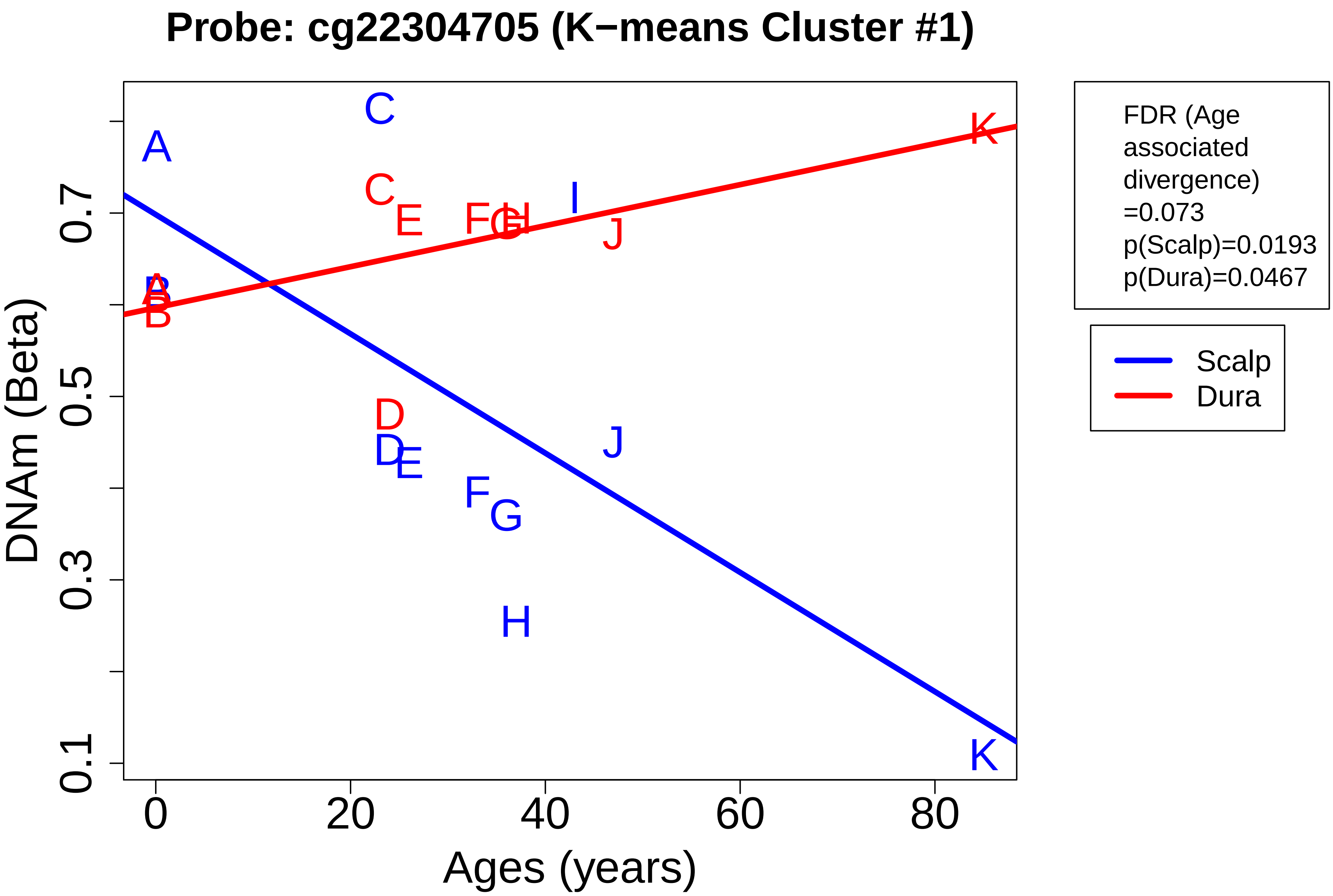

Probe: cg22304705 (K−means Cluster #1)
DNAm (Beta)
0.1
0.3
0.5
0.7
0
20
40
60
80
Ages (years)
A
B
C
D
E
F
G
H
I
J
K
FDR (Age associated divergence) =0.073
p(Scalp)=0.0193
p(Dura)=0.0467
Scalp
Dura

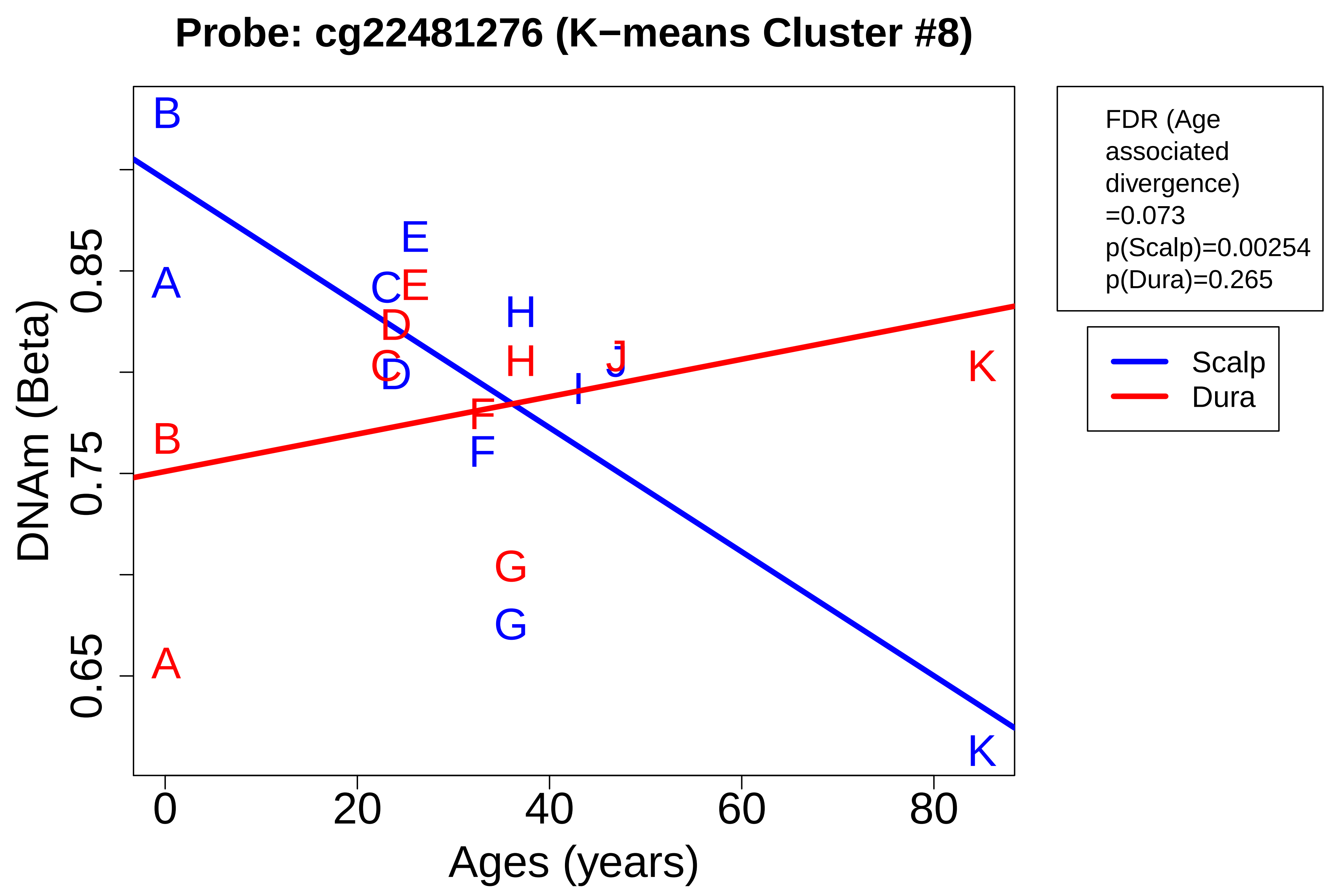

Probe: cg22481276 (K−means Cluster #8)
FDR (Age associated divergence) =0.073
p(Scalp)=0.00254
p(Dura)=0.265
Scalp
Dura
DNAm (Beta)
0.65
0.75
0.85
0
20
40
60
80
Ages (years)
A
B
C
D
E
F
G
H
I
J
K

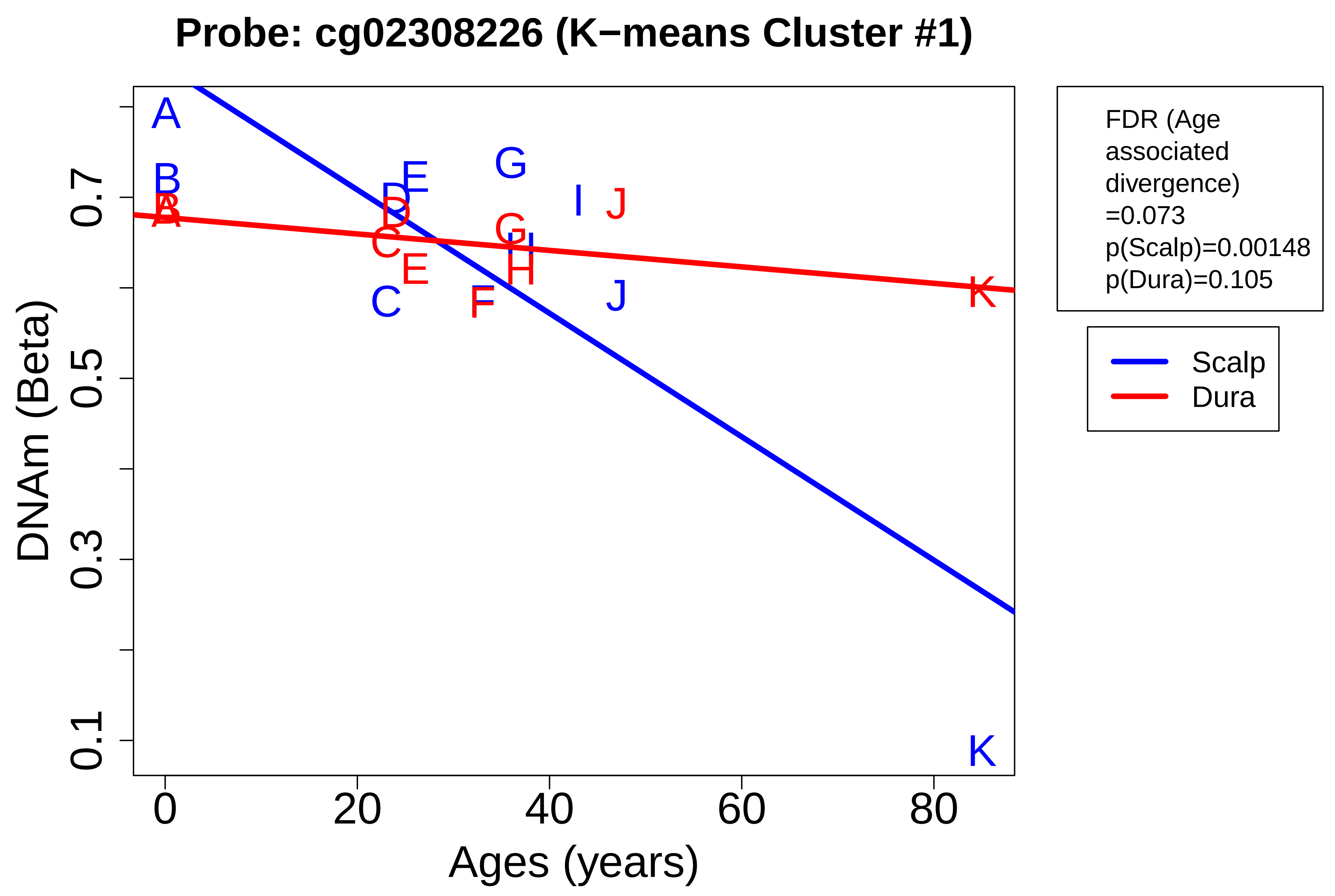

Probe: cg02308226 (K−means Cluster #1)
DNAm (Beta)
0.1
0.3
0.5
0.7
Ages (years)
0
20
40
60
80
A
B
C
D
E
F
G
H
I
J
K
FDR (Age associated divergence) =0.073
p(Scalp)=0.00148
p(Dura)=0.105
Scalp
Dura

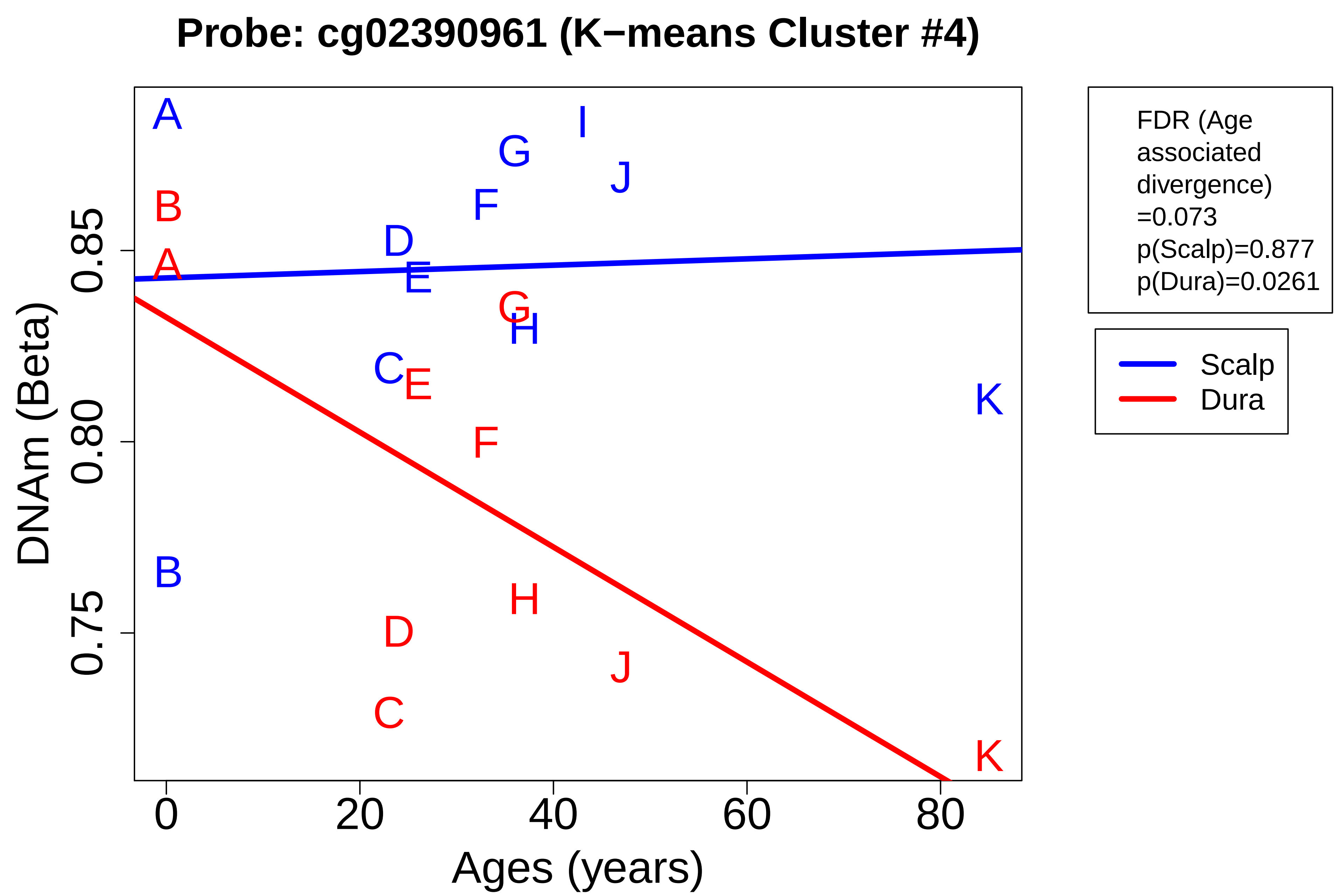

Probe: cg02390961 (K−means Cluster #4)
DNAm (Beta)
0.75
0.80
0.85
Ages (years)
0
20
40
60
80
A
B
C
D
E
F
G
H
I
J
K
FDR (Age associated divergence) =0.073
p(Scalp)=0.877
p(Dura)=0.0261
Scalp
Dura

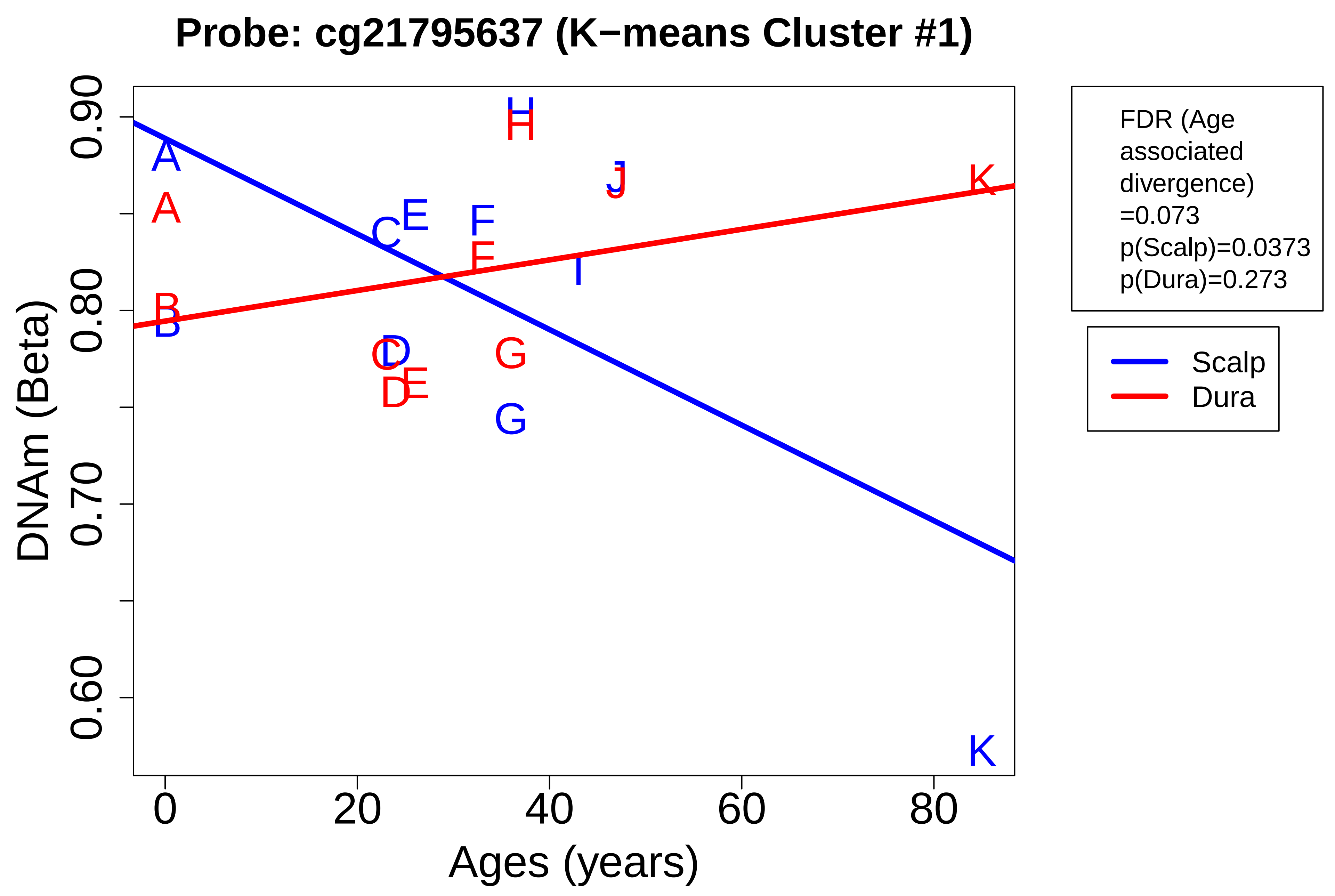

Probe: cg21795637 (K−means Cluster #1)
DNAm (Beta)
0.60
0.70
0.80
0.90
Ages (years)
0
20
40
60
80
FDR (Age associated divergence) =0.073
p(Scalp)=0.0373
p(Dura)=0.273
Scalp
Dura

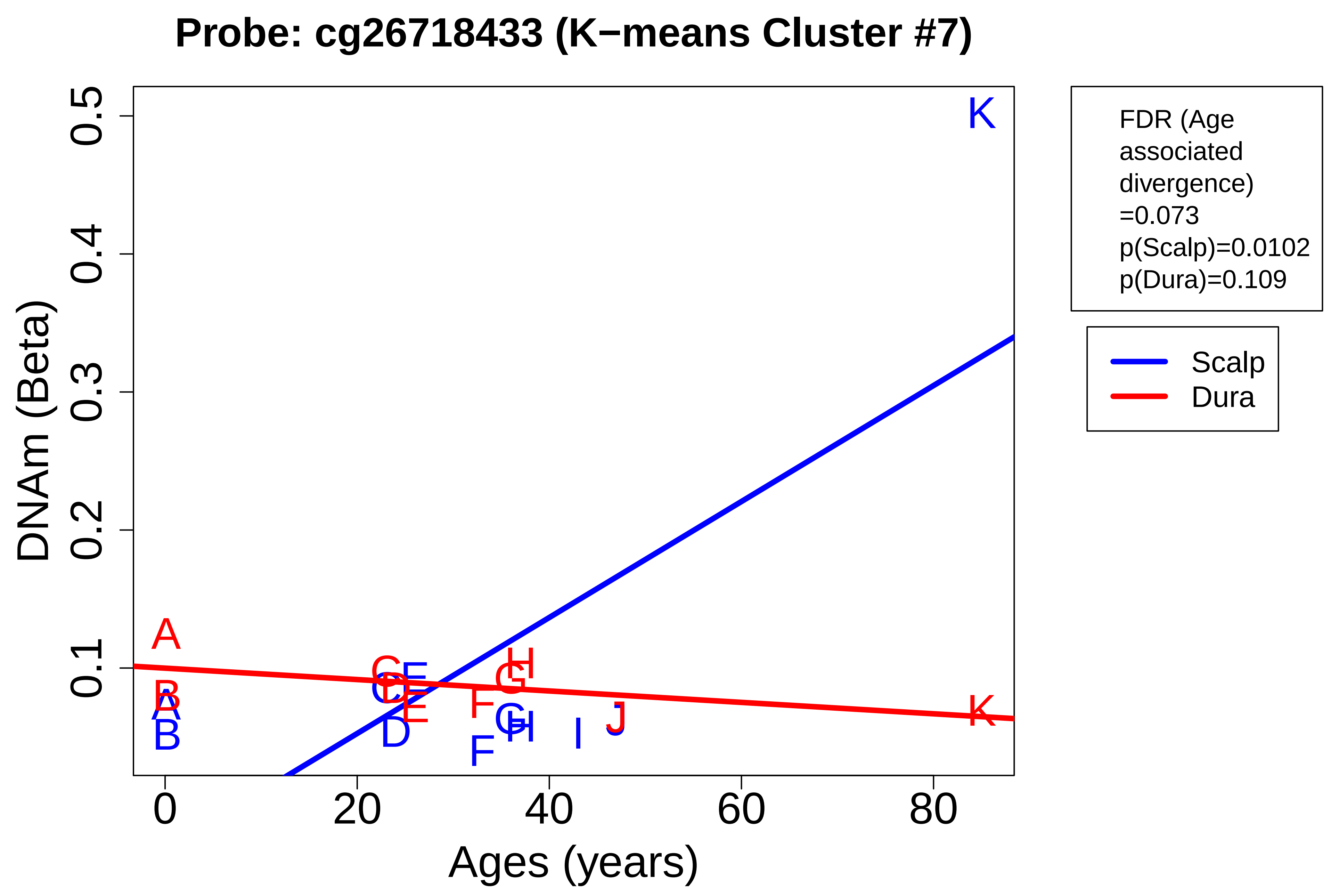

Probe: cg26718433 (K−means Cluster #7)
DNAm (Beta)
0.1
0.2
0.3
0.4
0.5
0
20
40
60
80
Ages (years)
FDR (Age associated divergence) =0.073
p(Scalp)=0.0102
p(Dura)=0.109
Scalp
Dura
A
B
C
D
E
F
G
H
I
J
K

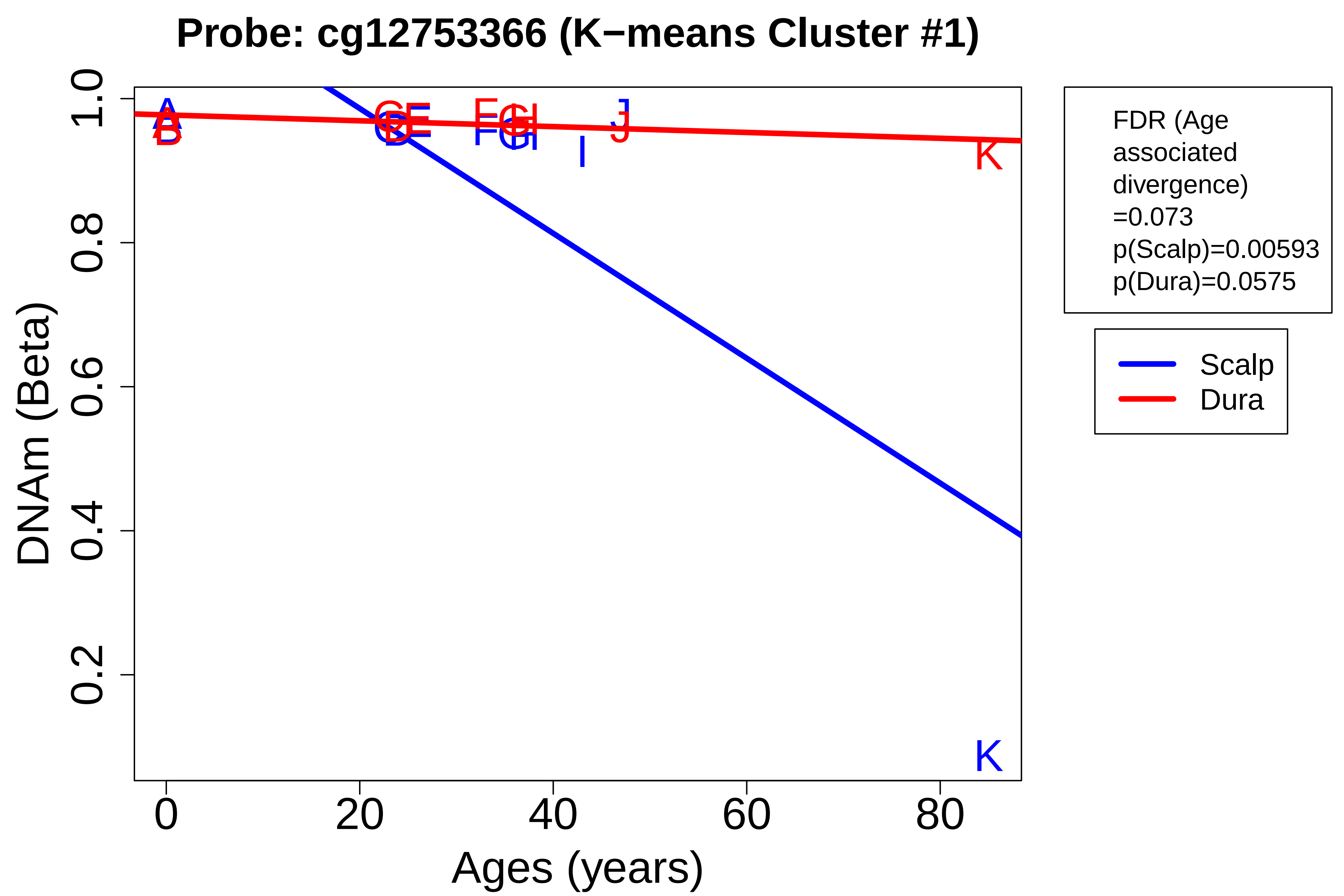

Probe: cg12753366 (K−means Cluster #1)
DNAm (Beta)
1.0
0.8
0.6
0.4
0.2
0
20
40
60
80
Ages (years)
FDR (Age associated divergence) =0.073
p(Scalp)=0.00593
p(Dura)=0.0575
Scalp
Dura

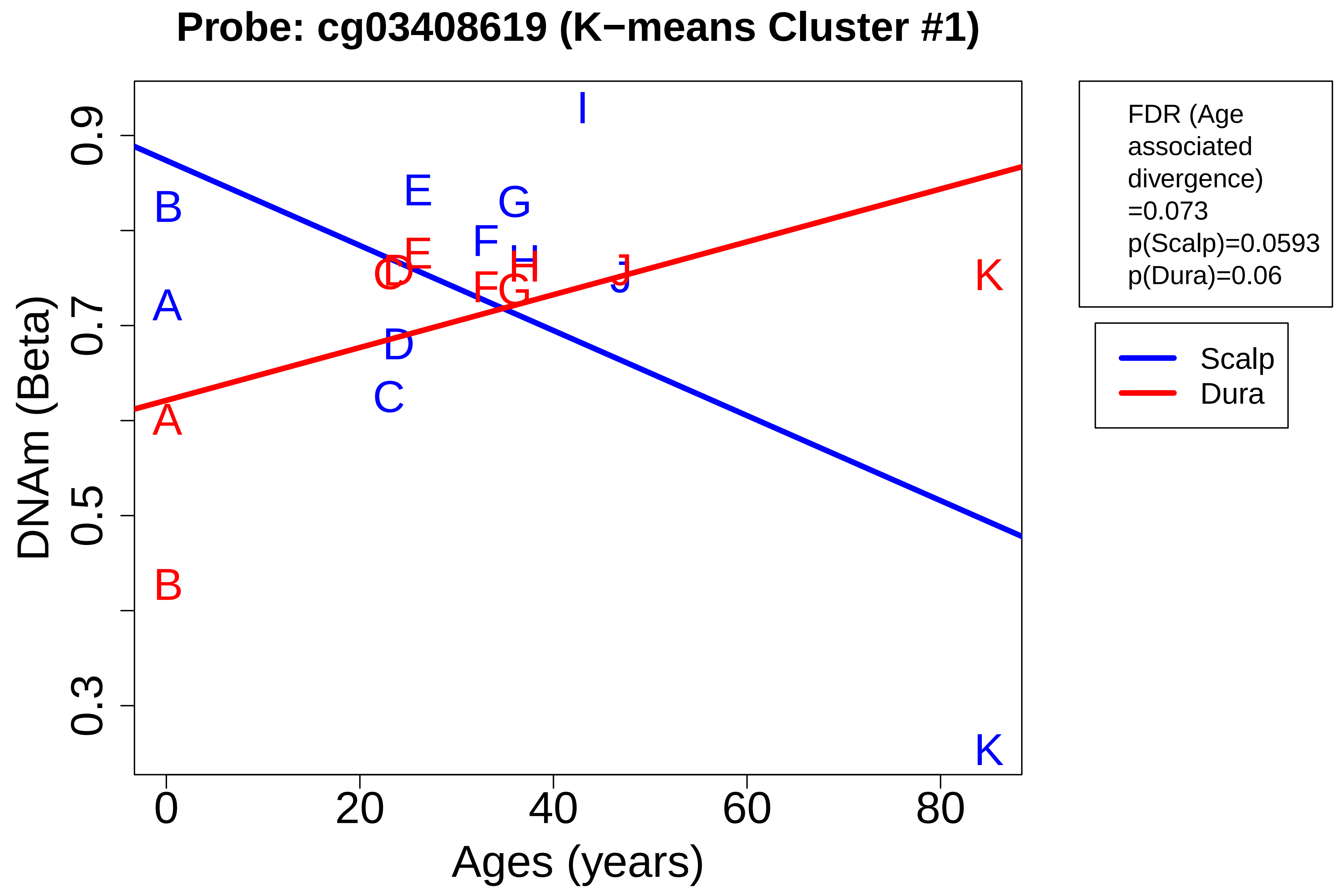

Probe: cg03408619 (K−means Cluster #1)
DNAm (Beta)
0.3
0.5
0.7
0.9
Ages (years)
0
20
40
60
80
A
B
C
D
E
F
G
H
I
J
K
A
B
CD
E
FG
H
J
K
FDR (Age associated divergence) =0.073
p(Scalp)=0.0593
p(Dura)=0.06
Scalp
Dura

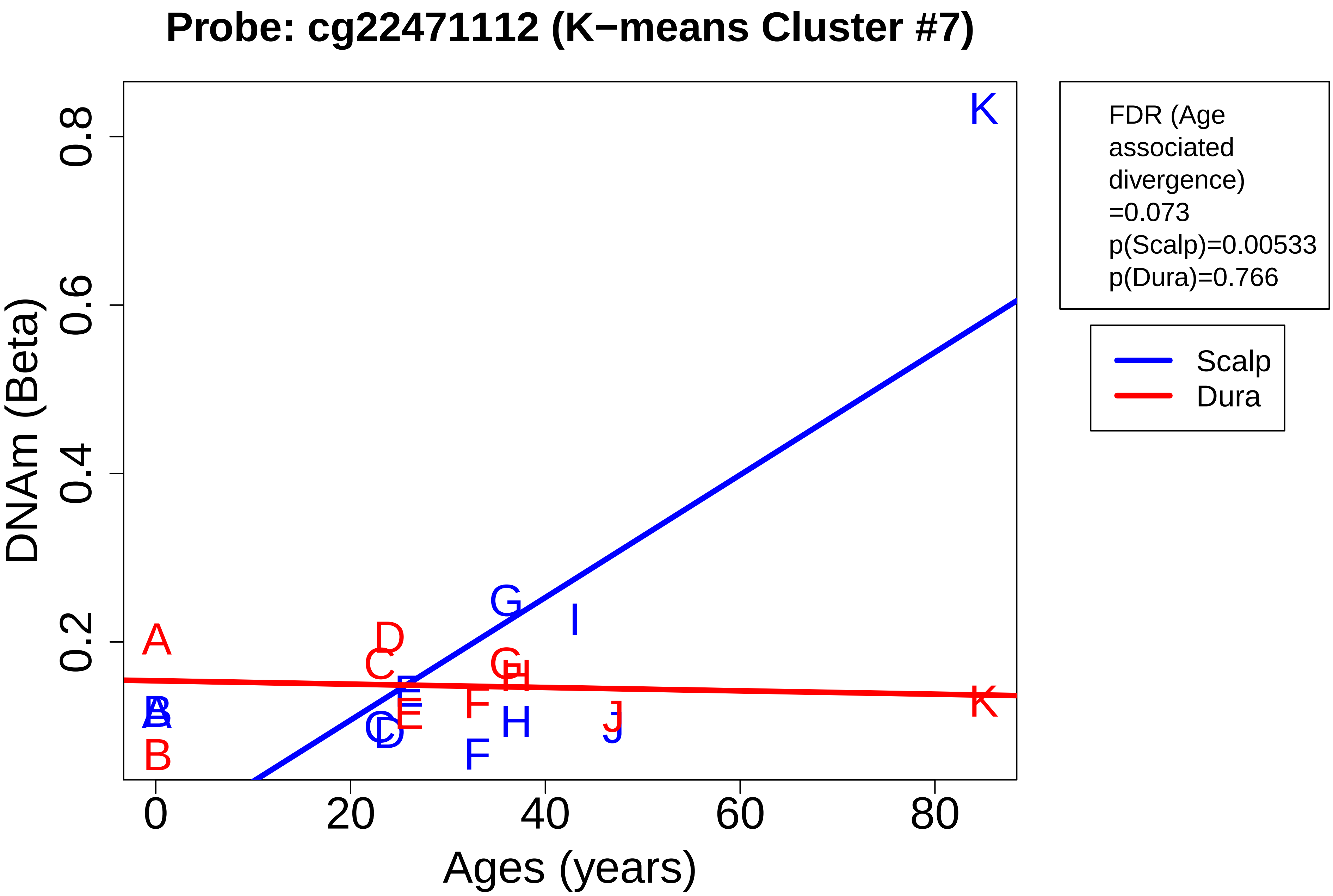

Probe: cg22471112 (K−means Cluster #7)
DNAm (Beta)
0.2
0.4
0.6
0.8
Ages (years)
0
20
40
60
80
FDR (Age associated divergence) =0.073
p(Scalp)=0.00533
p(Dura)=0.766
Scalp
Dura
A
B
C
D
E
F
G
H
I
J
K

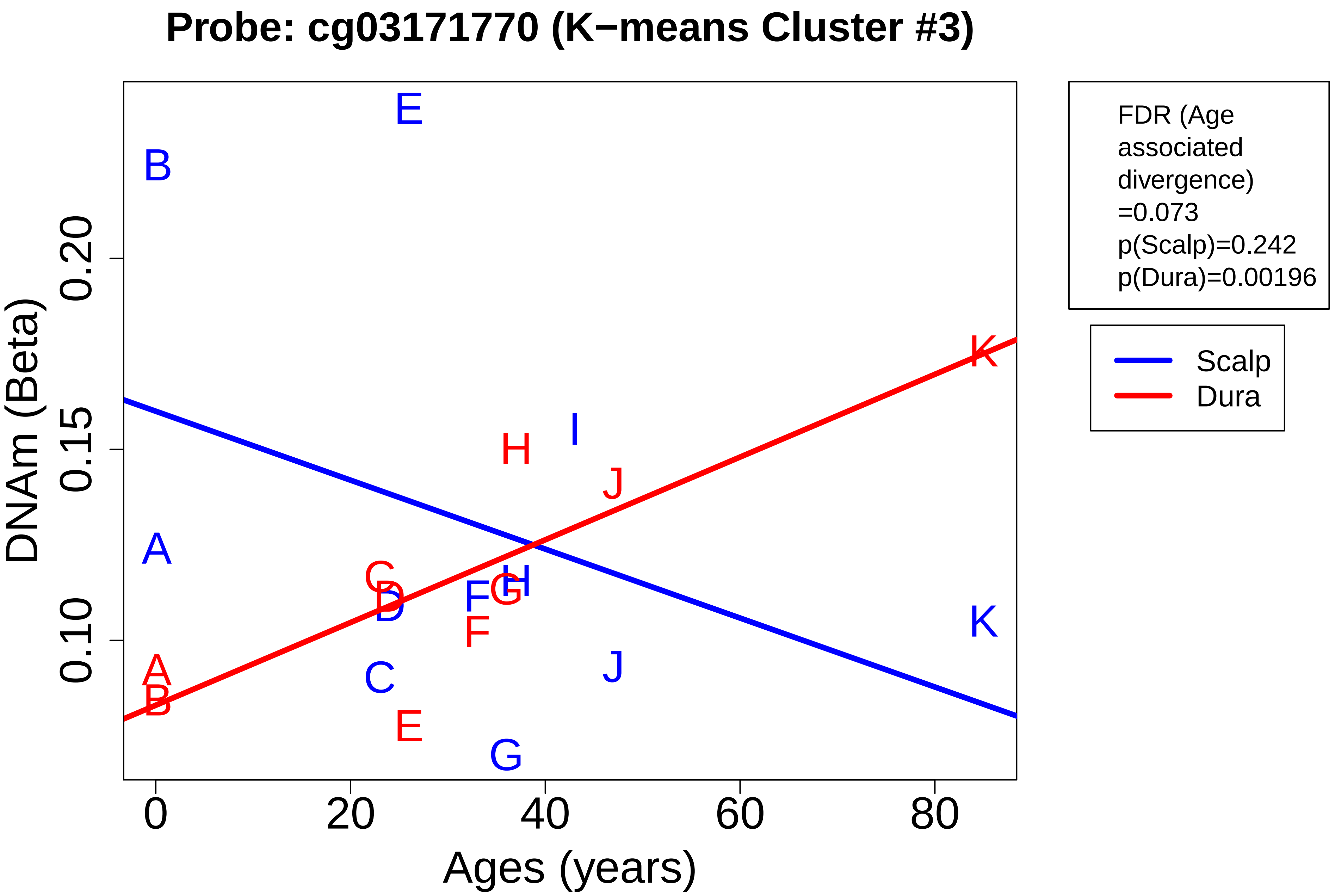
Probe: cg03171770 (K−means Cluster #3)
FDR (Age associated divergence) =0.073
p(Scalp)=0.242
p(Dura)=0.00196
Scalp
Dura
DNAm (Beta)
0.10
0.15
0.20
Ages (years)
0
20
40
60
80
A
B
C
D
E
F
G
H
I
J
K

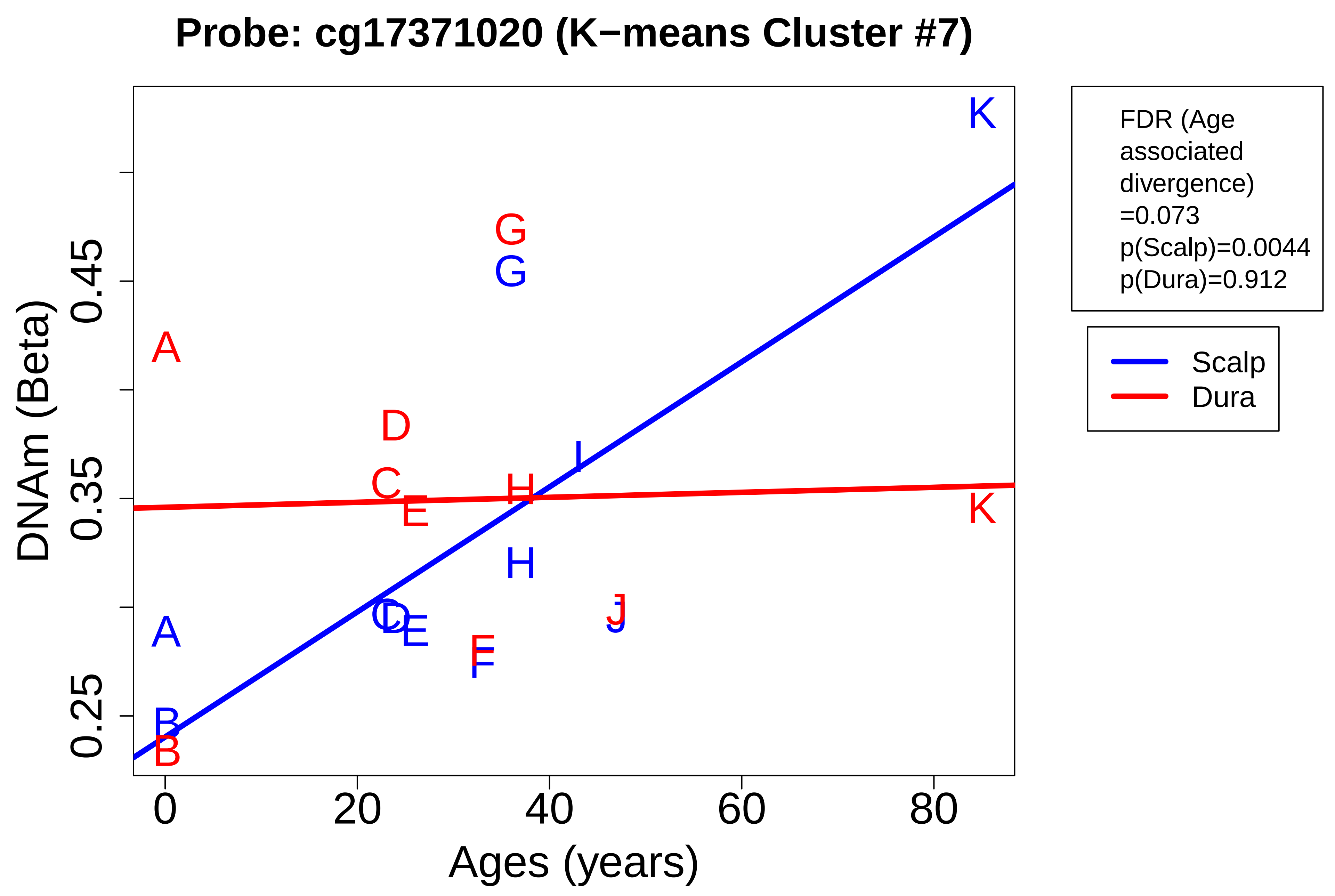

Probe: cg17371020 (K−means Cluster #7)
DNAm (Beta)
0.25
0.35
0.45
Ages (years)
0
20
40
60
80
A
B
C
D
E
F
G
H
I
J
K
FDR (Age associated divergence) =0.073
p(Scalp)=0.0044
p(Dura)=0.912
Scalp
Dura

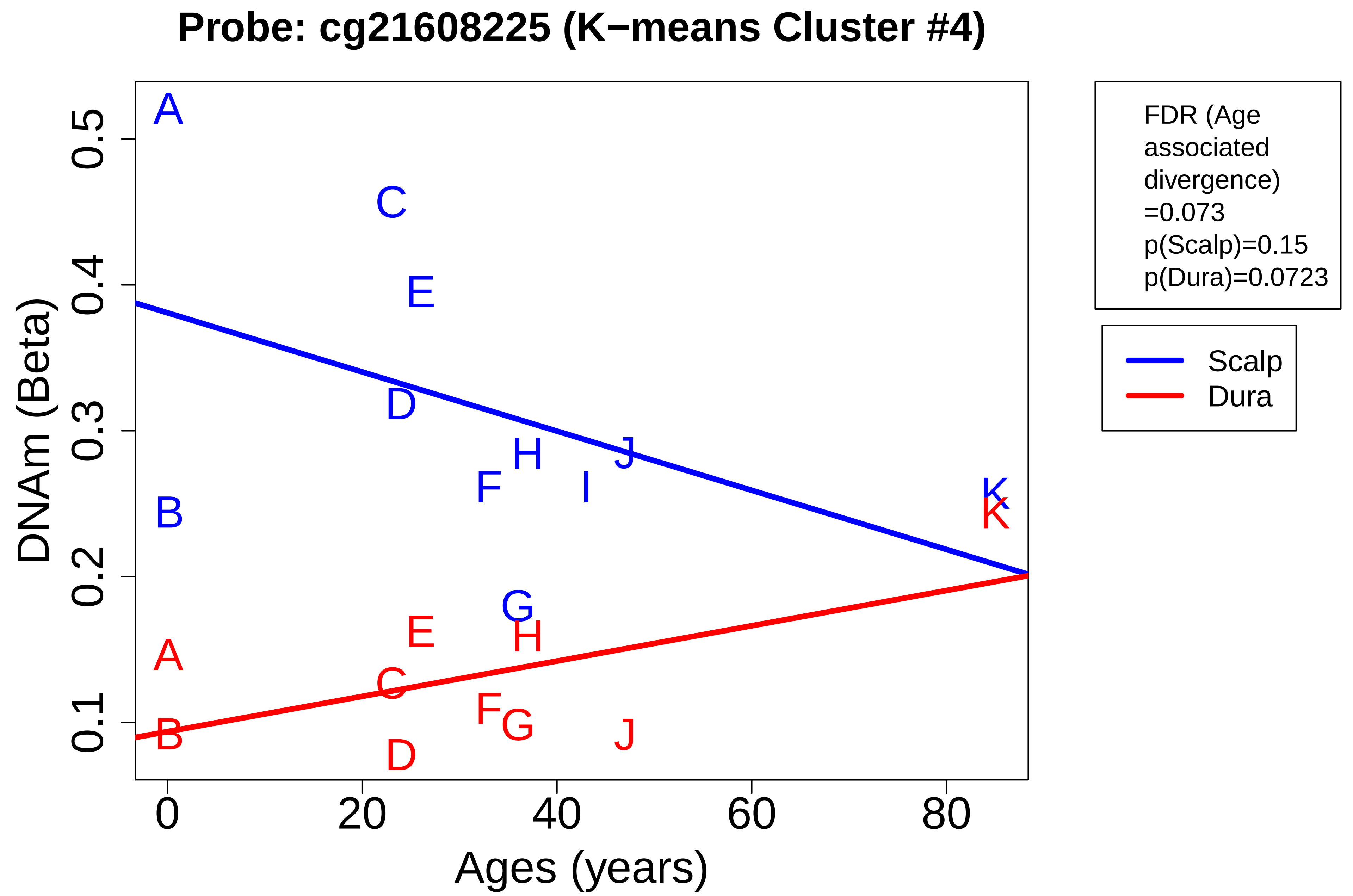

Probe: cg21608225 (K−means Cluster #4)
DNAm (Beta)
Ages (years)
0.1
0.2
0.3
0.4
0.5
0
20
40
60
80
FDR (Age associated divergence) =0.073
p(Scalp)=0.15
p(Dura)=0.0723
Scalp
Dura
A
B
C
D
E
F
G
H
I
J
K

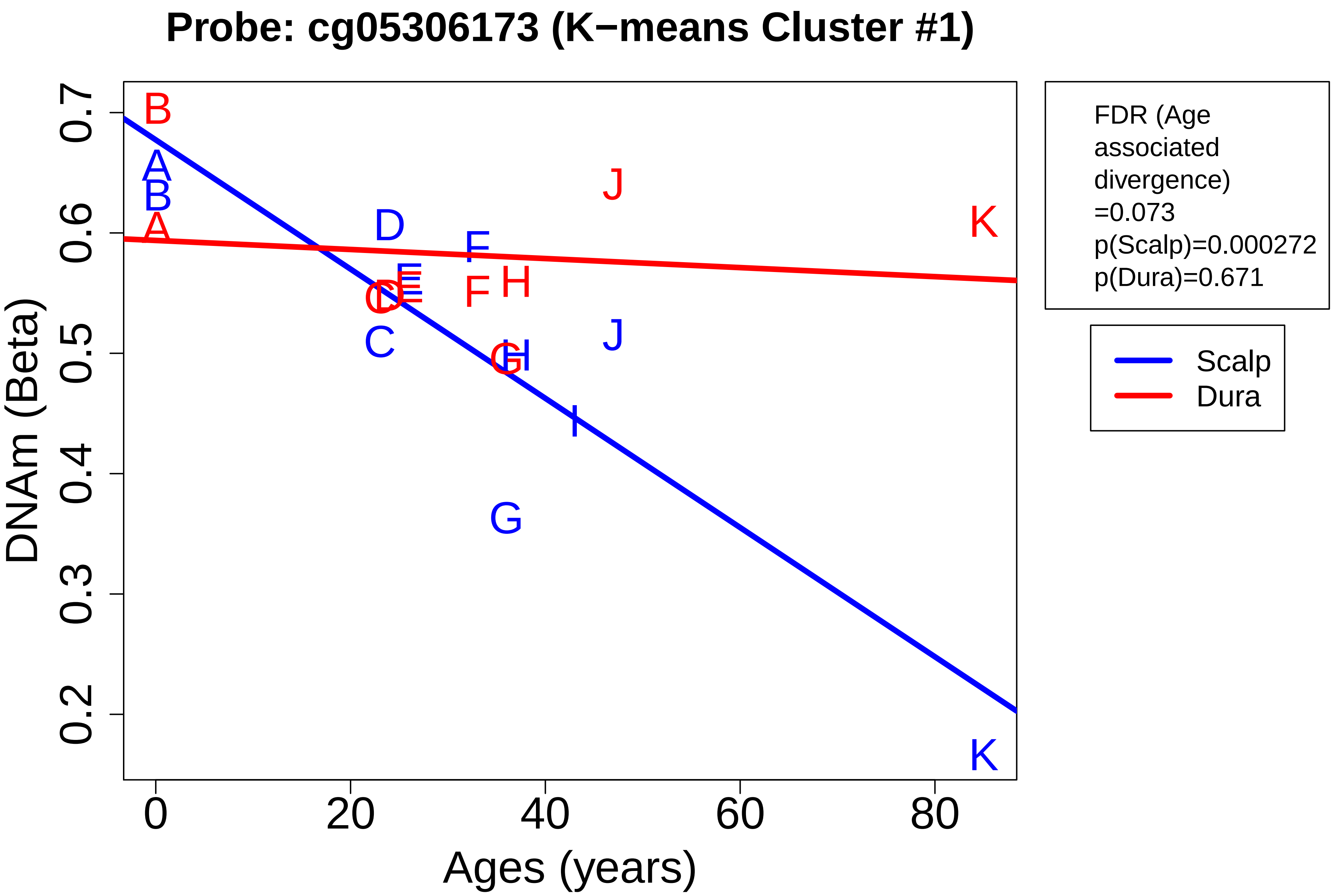

Probe: cg05306173 (K−means Cluster #1)
DNAm (Beta)
0.2
0.3
0.4
0.5
0.6
0.7
Ages (years)
0
20
40
60
80
FDR (Age associated divergence) =0.073
p(Scalp)=0.000272
p(Dura)=0.671
Scalp
Dura

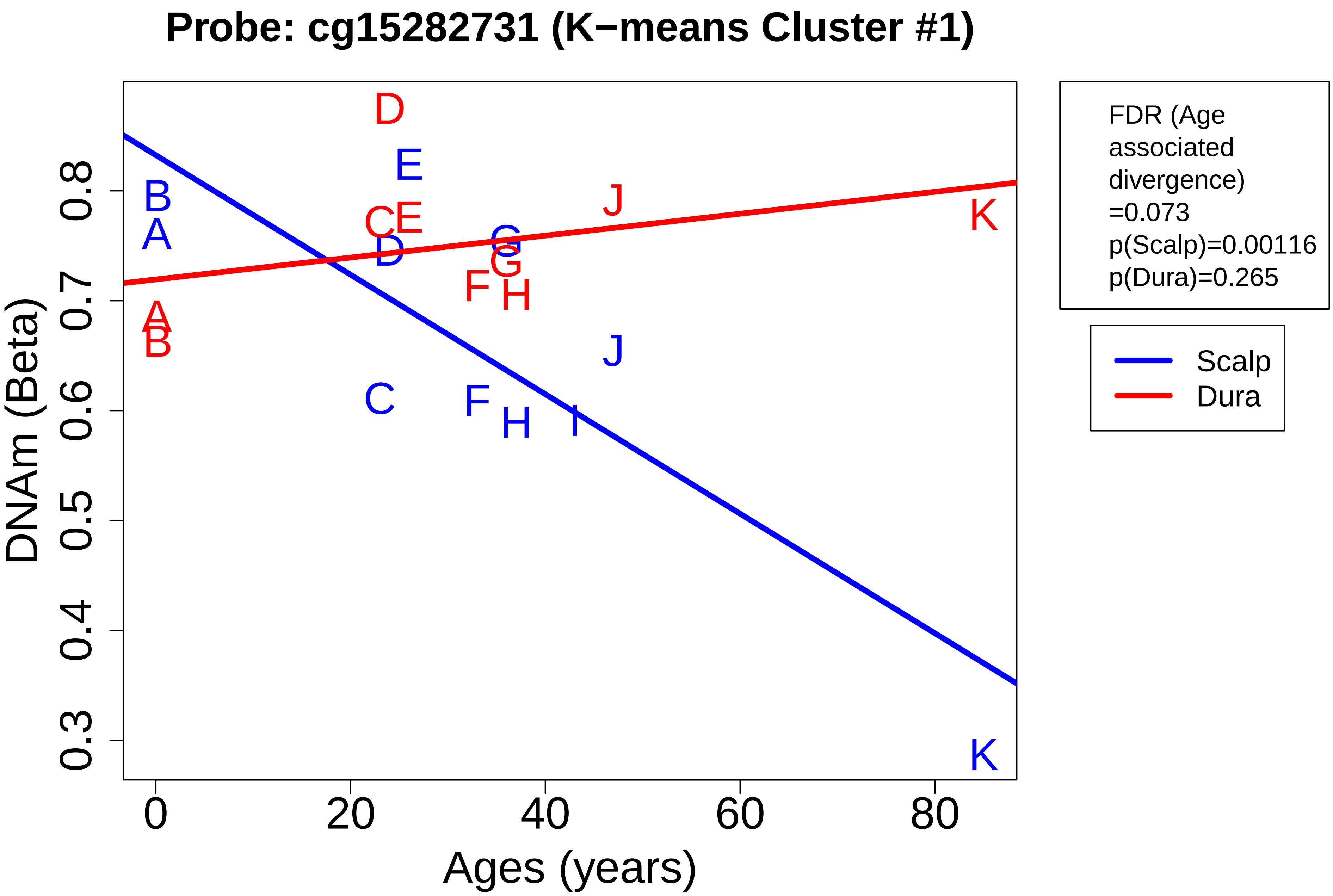
Probe: cg15282731 (K−means Cluster #1)
DNAm (Beta)
0.3
0.4
0.5
0.6
0.7
0.8
0
20
40
60
80
Ages (years)
A B C D E F G H I J K
A B C D E F G H J K
FDR (Age associated divergence) =0.073
p(Scalp)=0.00116
p(Dura)=0.265
Scalp
Dura

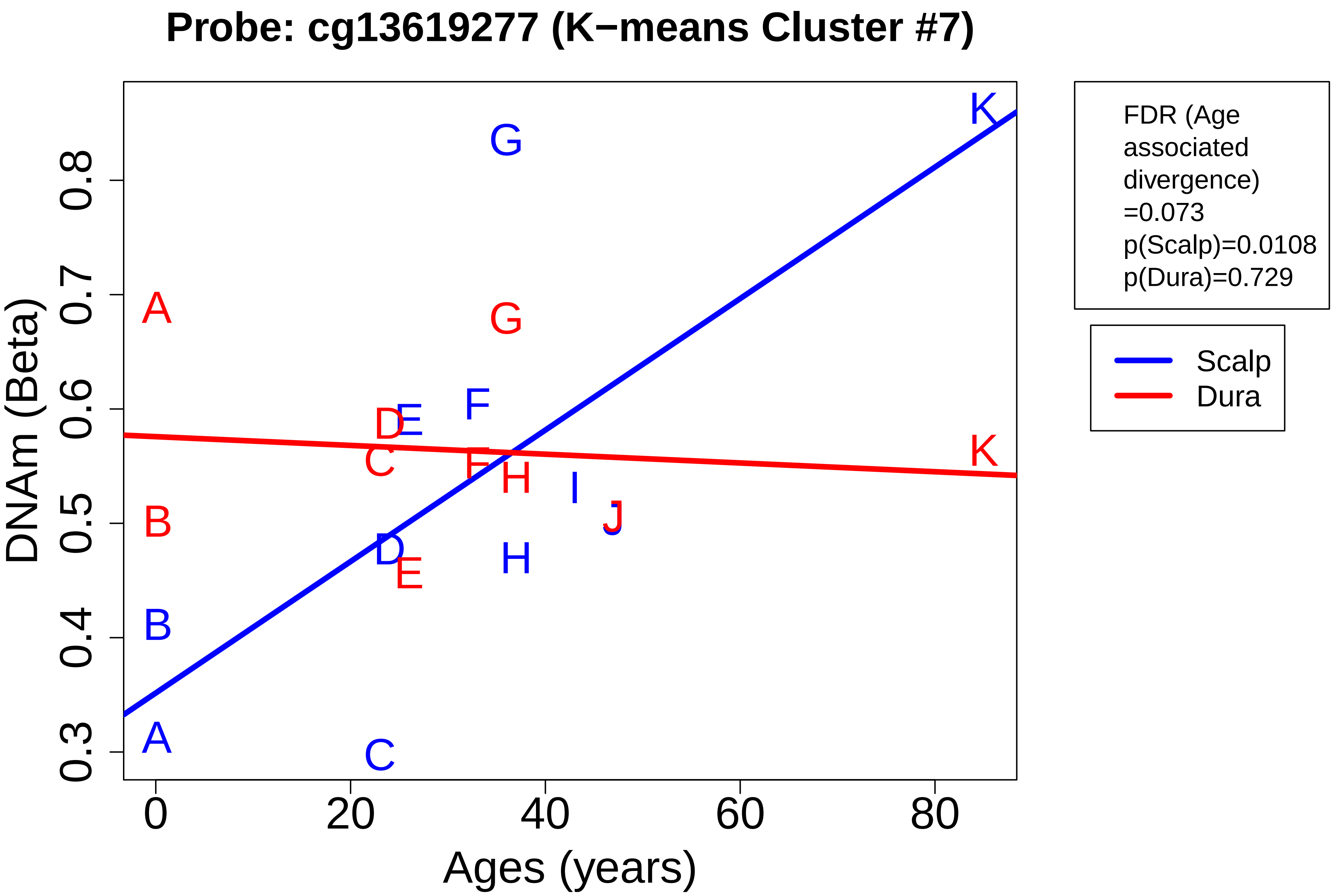

Probe: cg13619277 (K−means Cluster #7)
DNAm (Beta)
0.3
0.4
0.5
0.6
0.7
0.8
0
20
40
60
80
Ages (years)
A
B
C
D
E
F
G
H
I
J
K
FDR (Age associated divergence) =0.073
p(Scalp)=0.0108
p(Dura)=0.729
Scalp
Dura

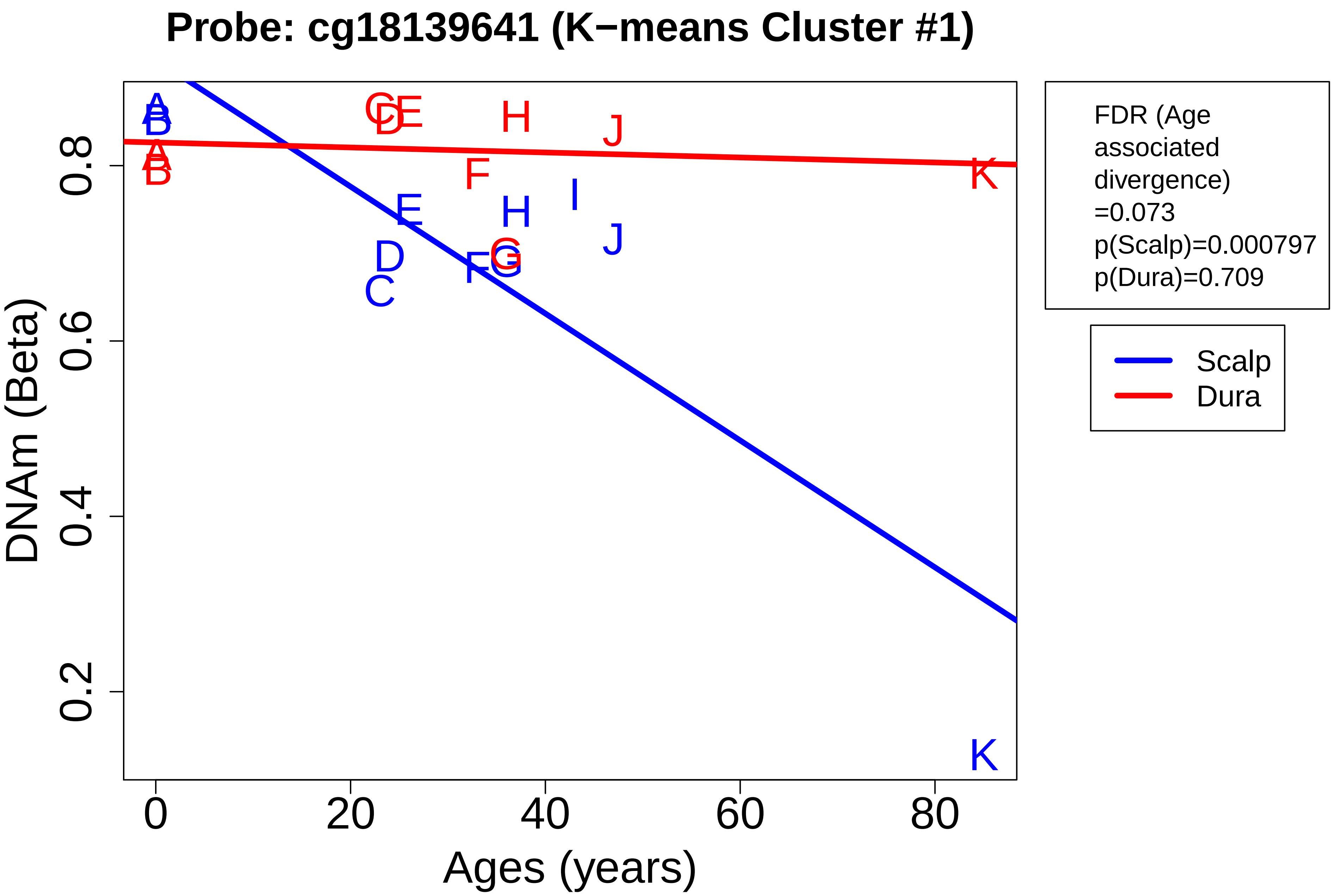

Probe: cg18139641 (K−means Cluster #1)
DNAm (Beta)
0.2
0.4
0.6
0.8
Ages (years)
0
20
40
60
80
FDR (Age associated divergence) =0.073
p(Scalp)=0.000797
p(Dura)=0.709
Scalp
Dura
A
B
C
D
E
F
G
H
I
J
K

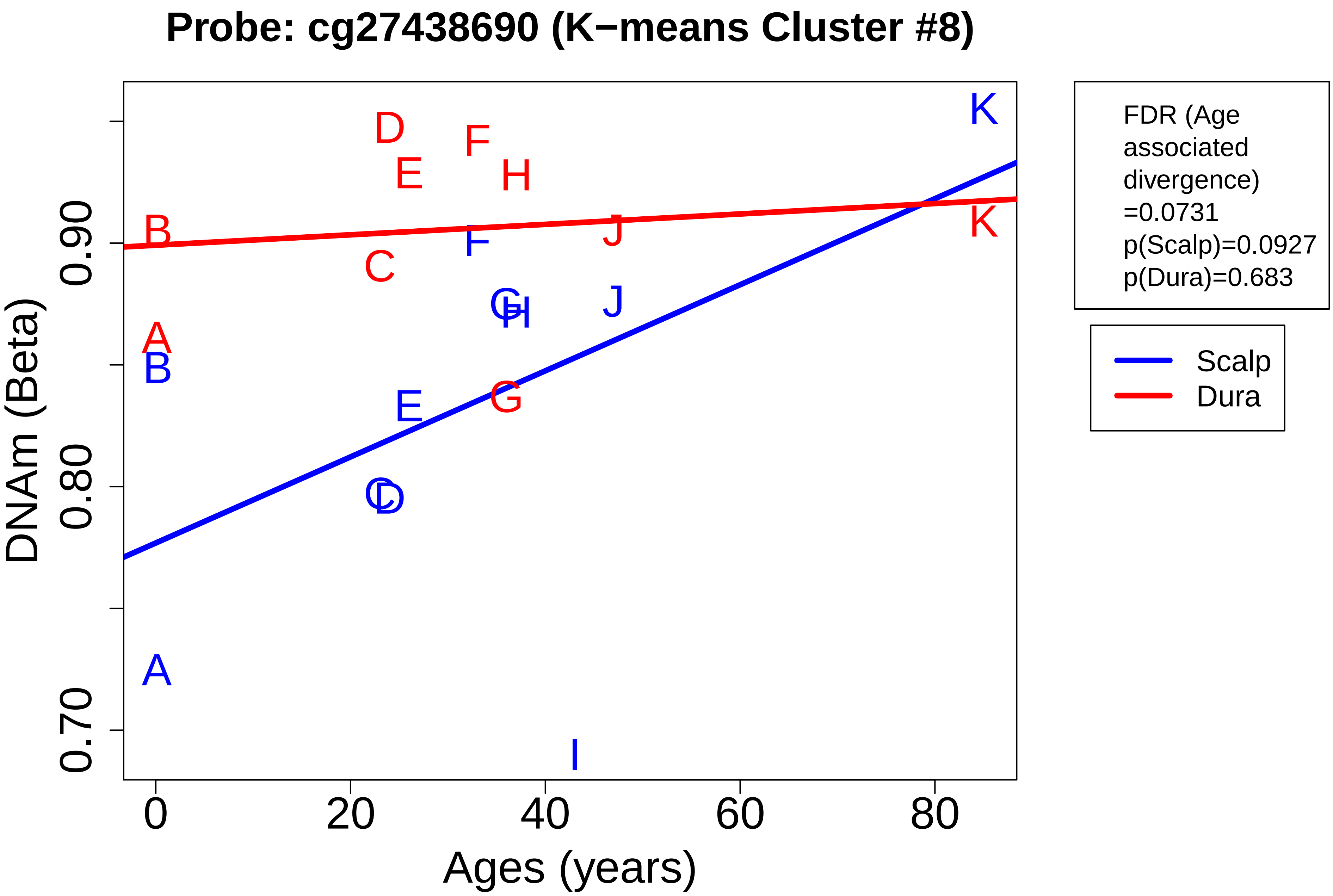

Probe: cg27438690 (K−means Cluster #8)
DNAm (Beta)
0.70
0.80
0.90
Ages (years)
0
20
40
60
80
FDR (Age associated divergence) =0.0731
p(Scalp)=0.0927
p(Dura)=0.683
Scalp
Dura

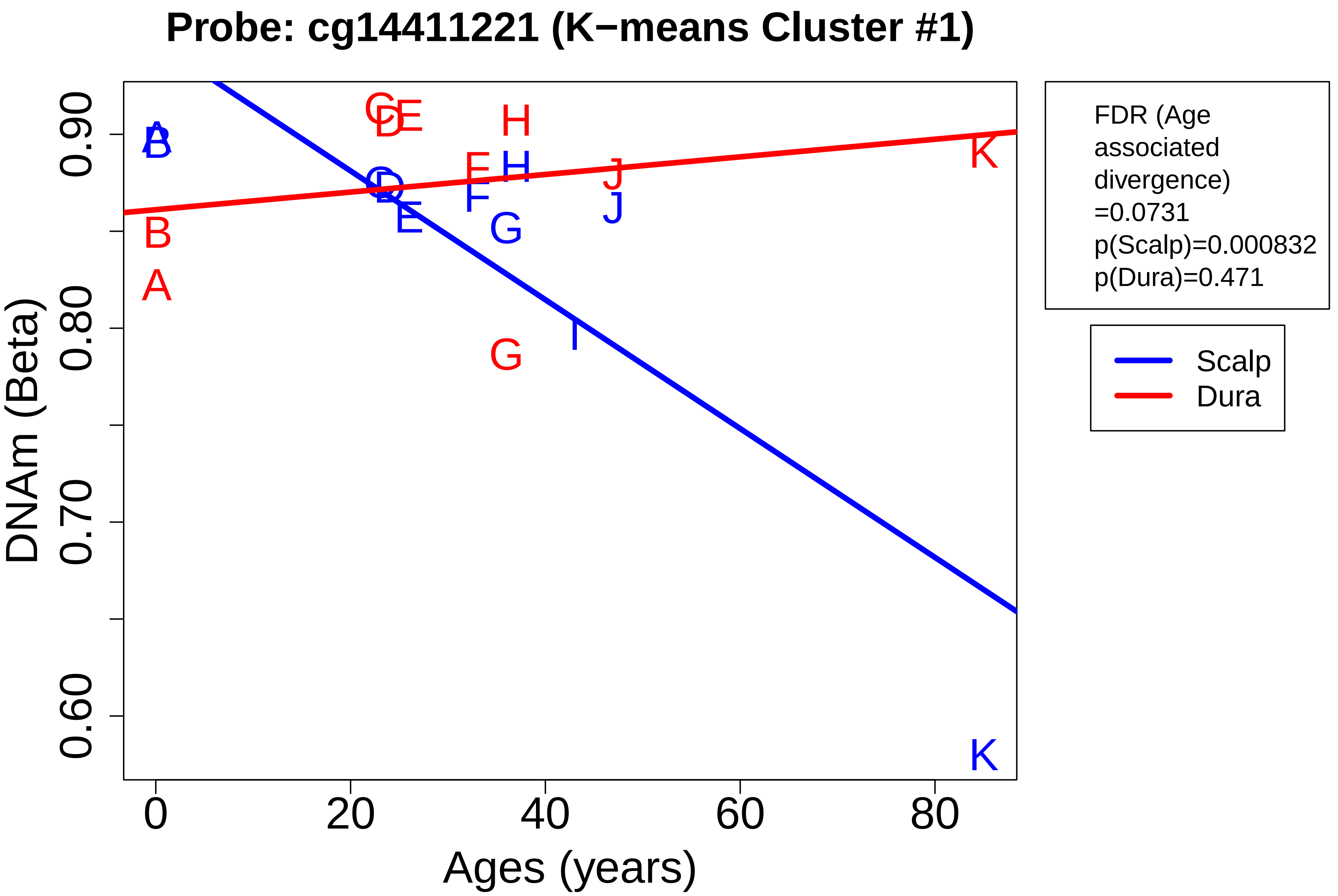

Probe: cg14411221 (K−means Cluster #1)
DNAm (Beta)
0.60
0.70
0.80
0.90
Ages (years)
0
20
40
60
80
FDR (Age associated divergence) =0.0731
p(Scalp)=0.000832
p(Dura)=0.471
Scalp
Dura

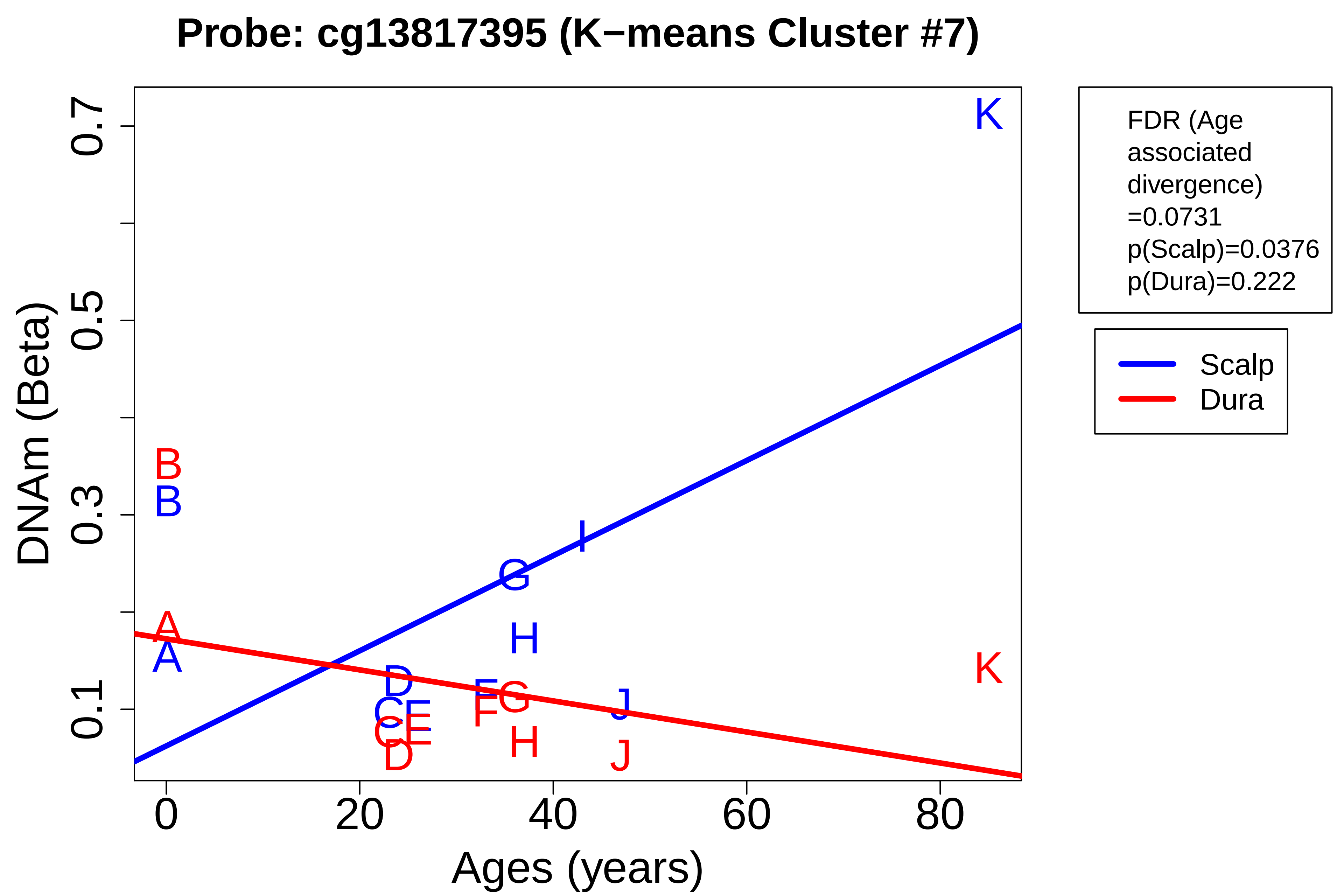

Probe: cg13817395 (K−means Cluster #7)
DNAm (Beta)
0.1
0.3
0.5
0.7
Ages (years)
0
20
40
60
80
FDR (Age associated divergence) =0.0731
p(Scalp)=0.0376
p(Dura)=0.222
Scalp
Dura

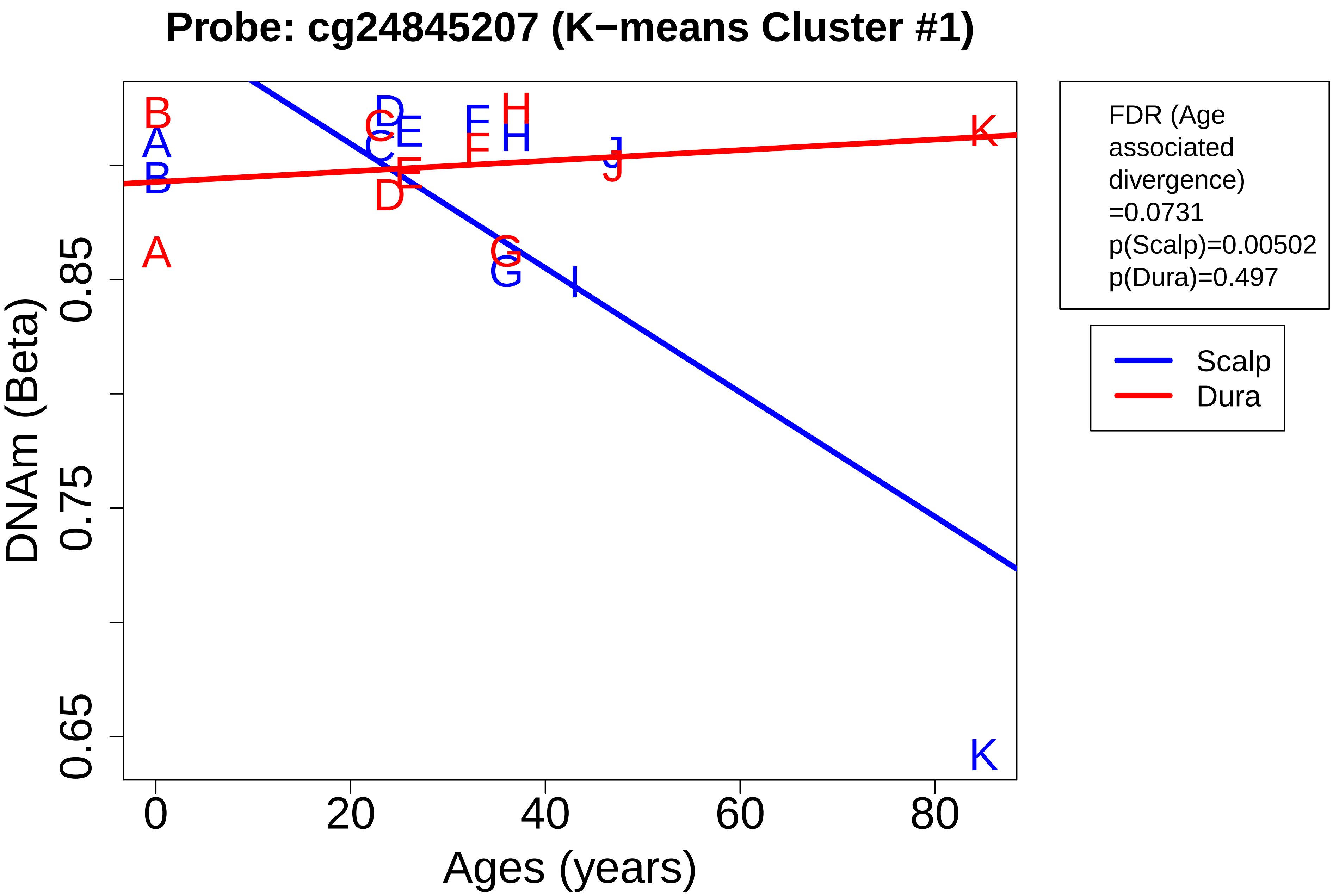

Probe: cg24845207 (K−means Cluster #1)
DNAm (Beta)
0.65
0.75
0.85
Ages (years)
0
20
40
60
80
FDR (Age associated divergence) =0.0731
p(Scalp)=0.00502
p(Dura)=0.497
Scalp
Dura

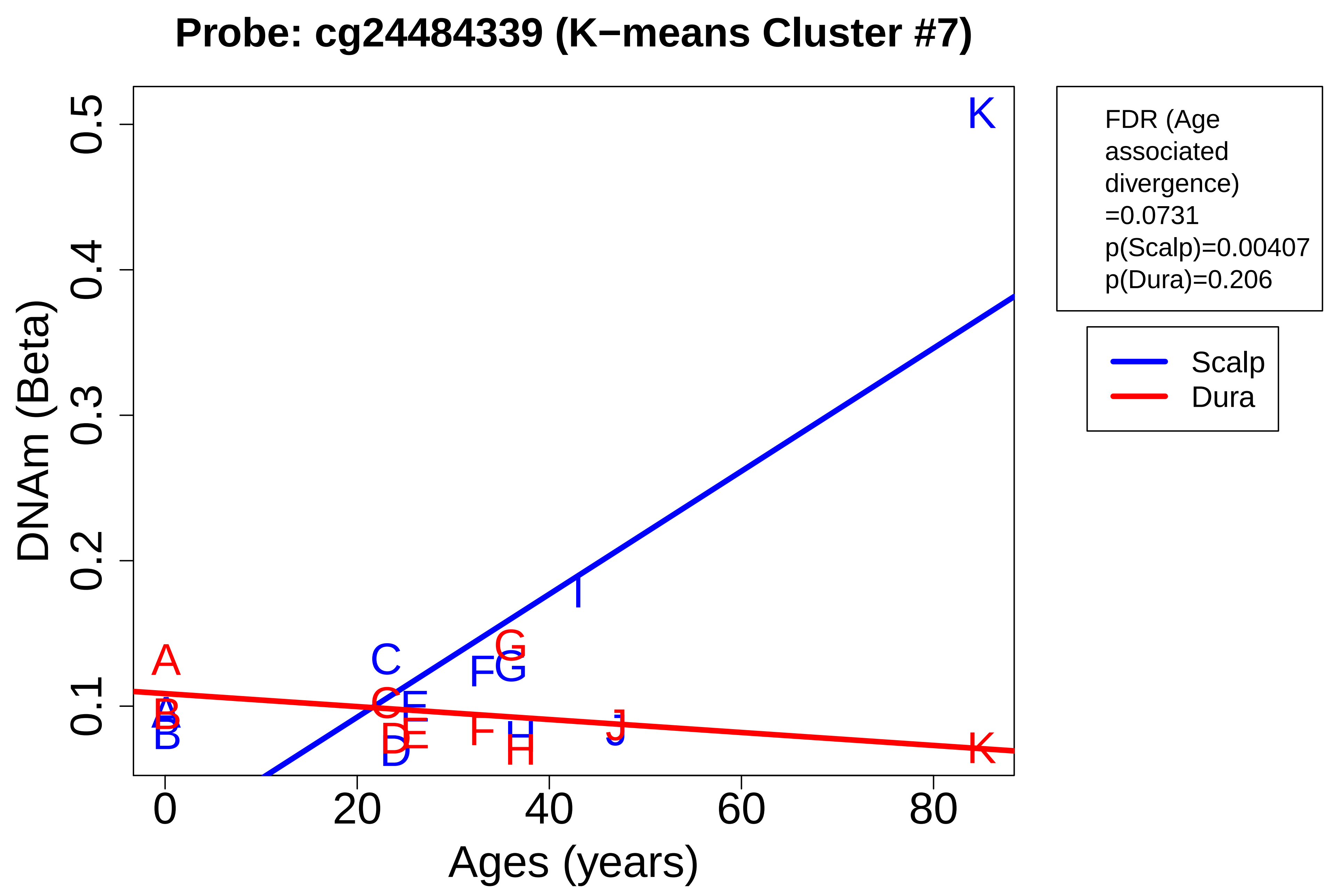

Probe: cg24484339 (K−means Cluster #7)
DNAm (Beta)
0.1
0.2
0.3
0.4
0.5
0
20
40
60
80
Ages (years)
FDR (Age associated divergence) =0.0731
p(Scalp)=0.00407
p(Dura)=0.206
Scalp
Dura

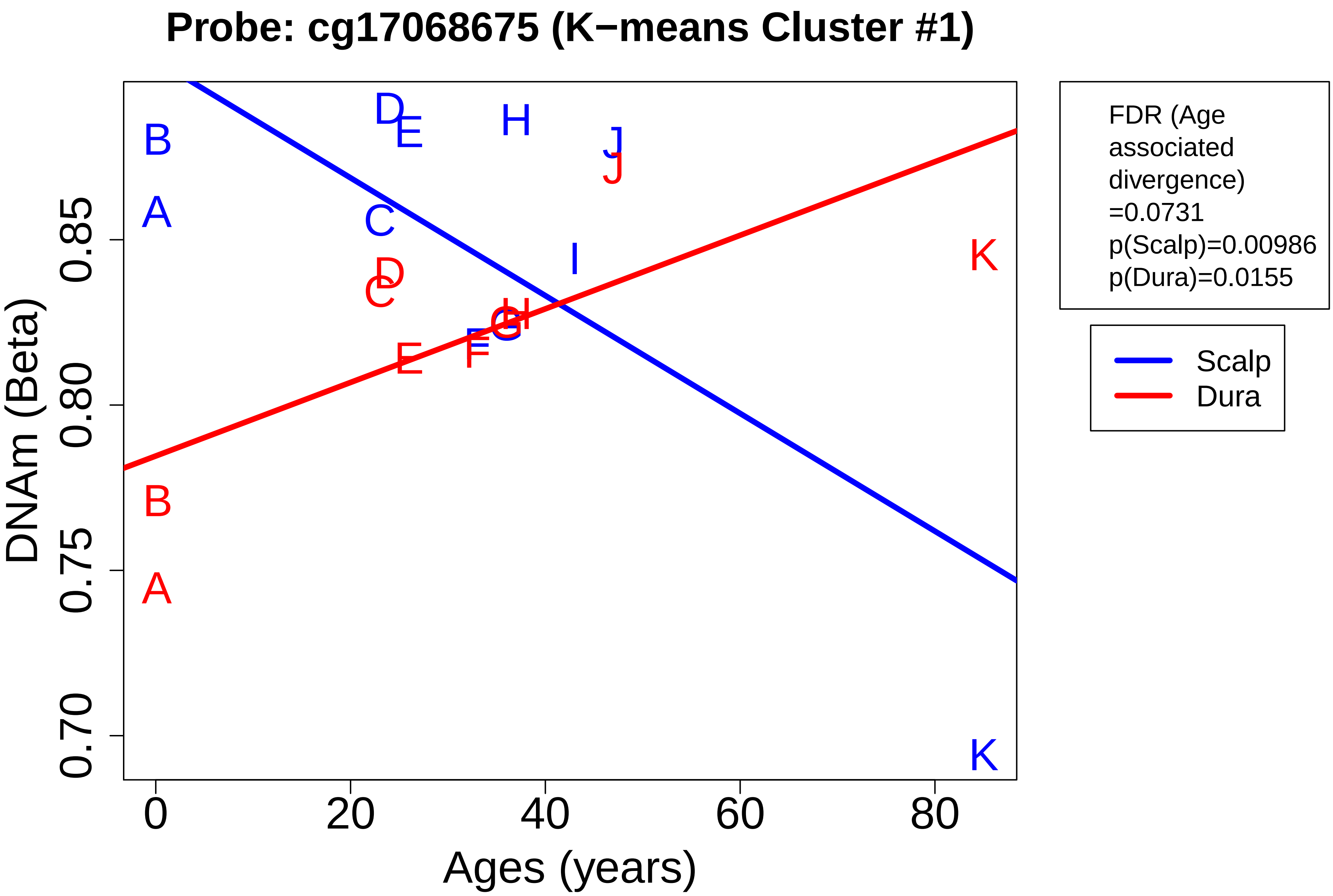

Probe: cg17068675 (K−means Cluster #1)
DNAm (Beta)
0.70
0.75
0.80
0.85
Ages (years)
0
20
40
60
80
FDR (Age associated divergence) =0.0731
p(Scalp)=0.00986
p(Dura)=0.0155
Scalp
Dura
A
B
C
D
E
F
G
H
I
J
K

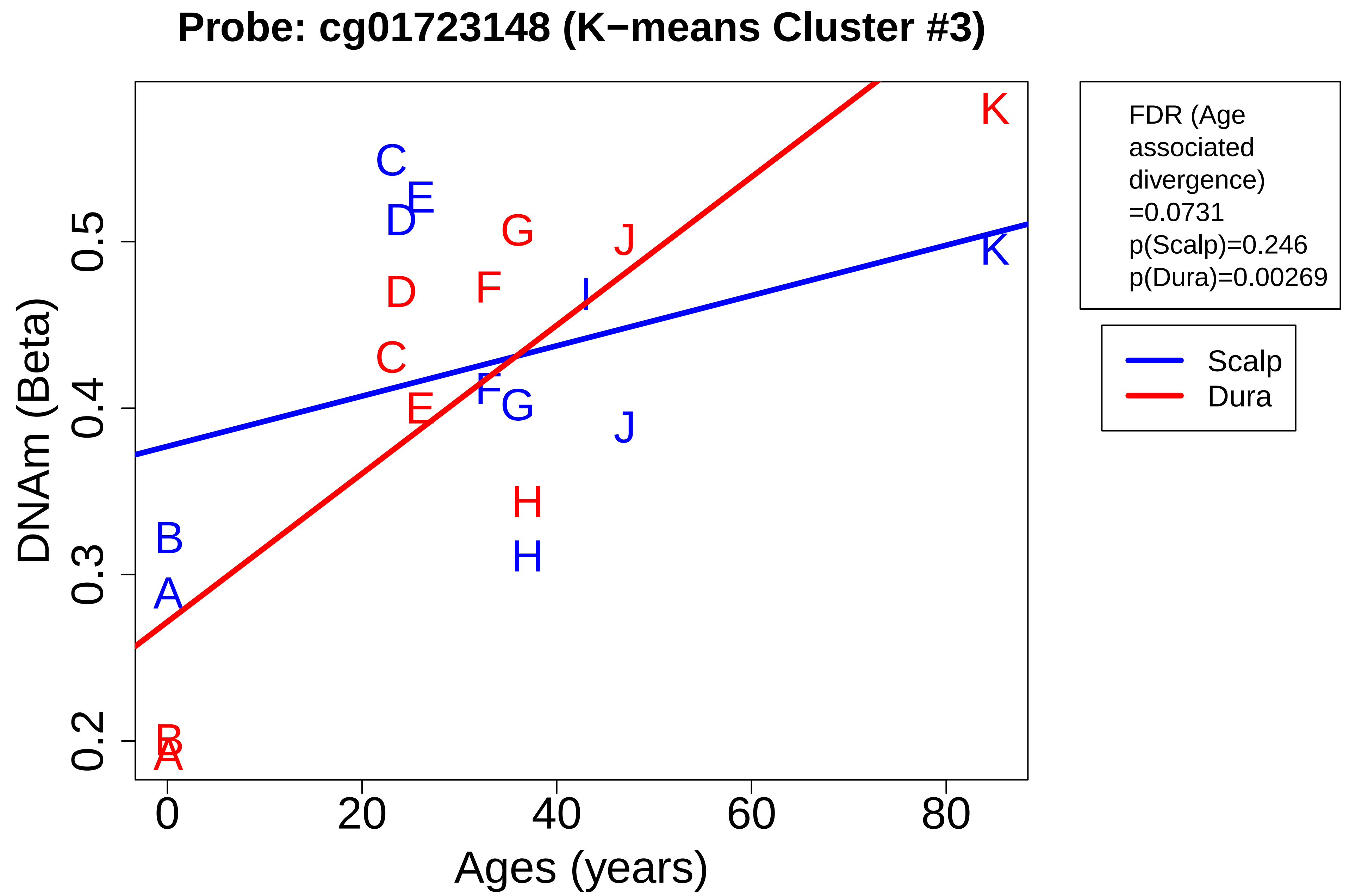

Probe: cg01723148 (K−means Cluster #3)
DNAm (Beta)
Ages (years)
0.2
0.3
0.4
0.5
0
20
40
60
80
FDR (Age associated divergence) =0.0731
p(Scalp)=0.246
p(Dura)=0.00269
Scalp
Dura

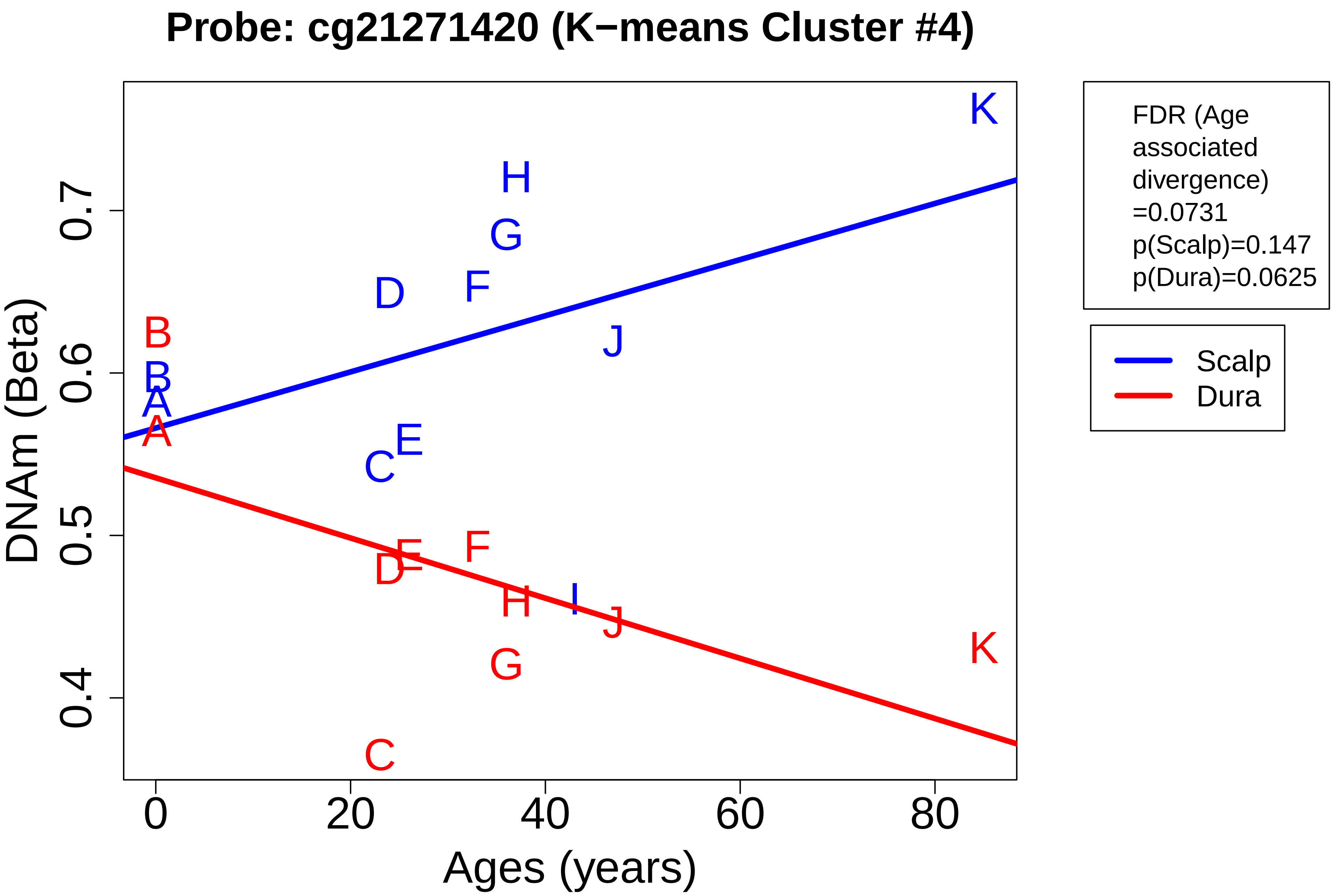

Probe: cg21271420 (K−means Cluster #4)
DNAm (Beta)
0.4
0.5
0.6
0.7
Ages (years)
0
20
40
60
80
FDR (Age associated divergence) =0.0731
p(Scalp)=0.147
p(Dura)=0.0625
Scalp
Dura
A
B
C
D
E
F
G
H
I
J
K

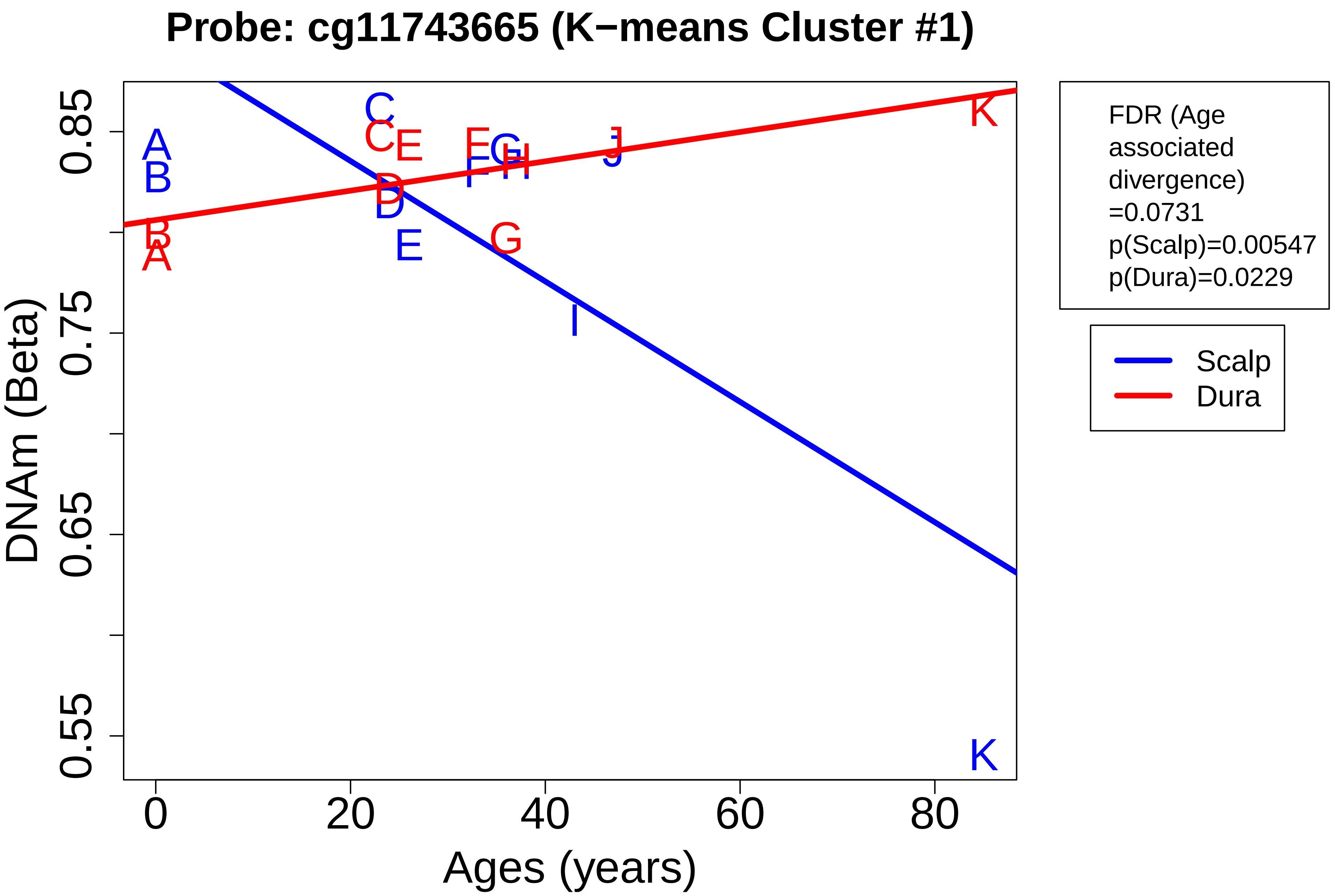

Probe: cg11743665 (K−means Cluster #1)
DNAm (Beta)
0.55
0.65
0.75
0.85
Ages (years)
0
20
40
60
80
FDR (Age associated divergence) =0.0731
p(Scalp)=0.00547
p(Dura)=0.0229
Scalp
Dura

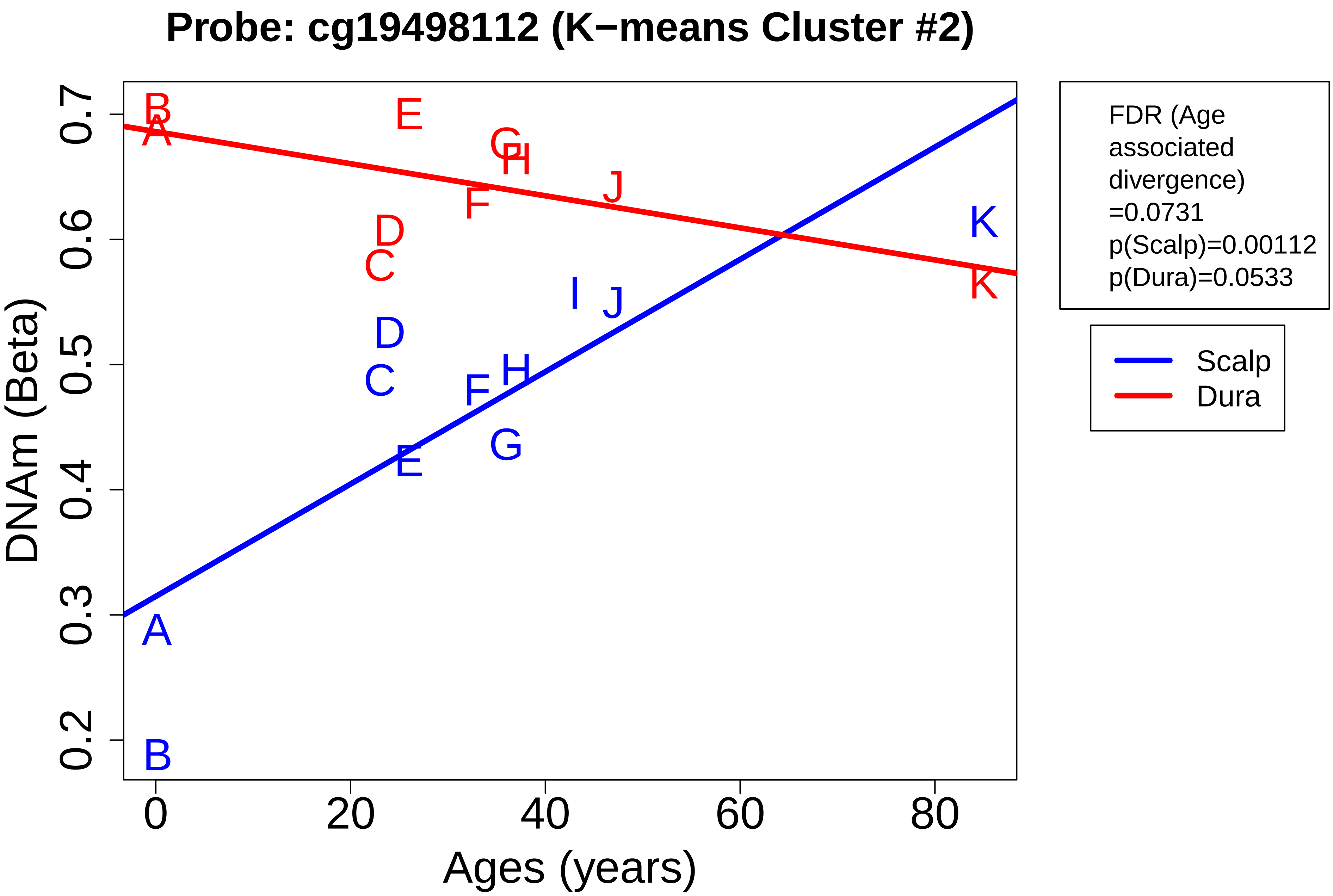
Probe: cg19498112 (K−means Cluster #2)
DNAm (Beta)
Ages (years)
0.2
0.3
0.4
0.5
0.6
0.7
0
20
40
60
80
FDR (Age associated divergence) =0.0731
p(Scalp)=0.00112
p(Dura)=0.0533
Scalp
Dura

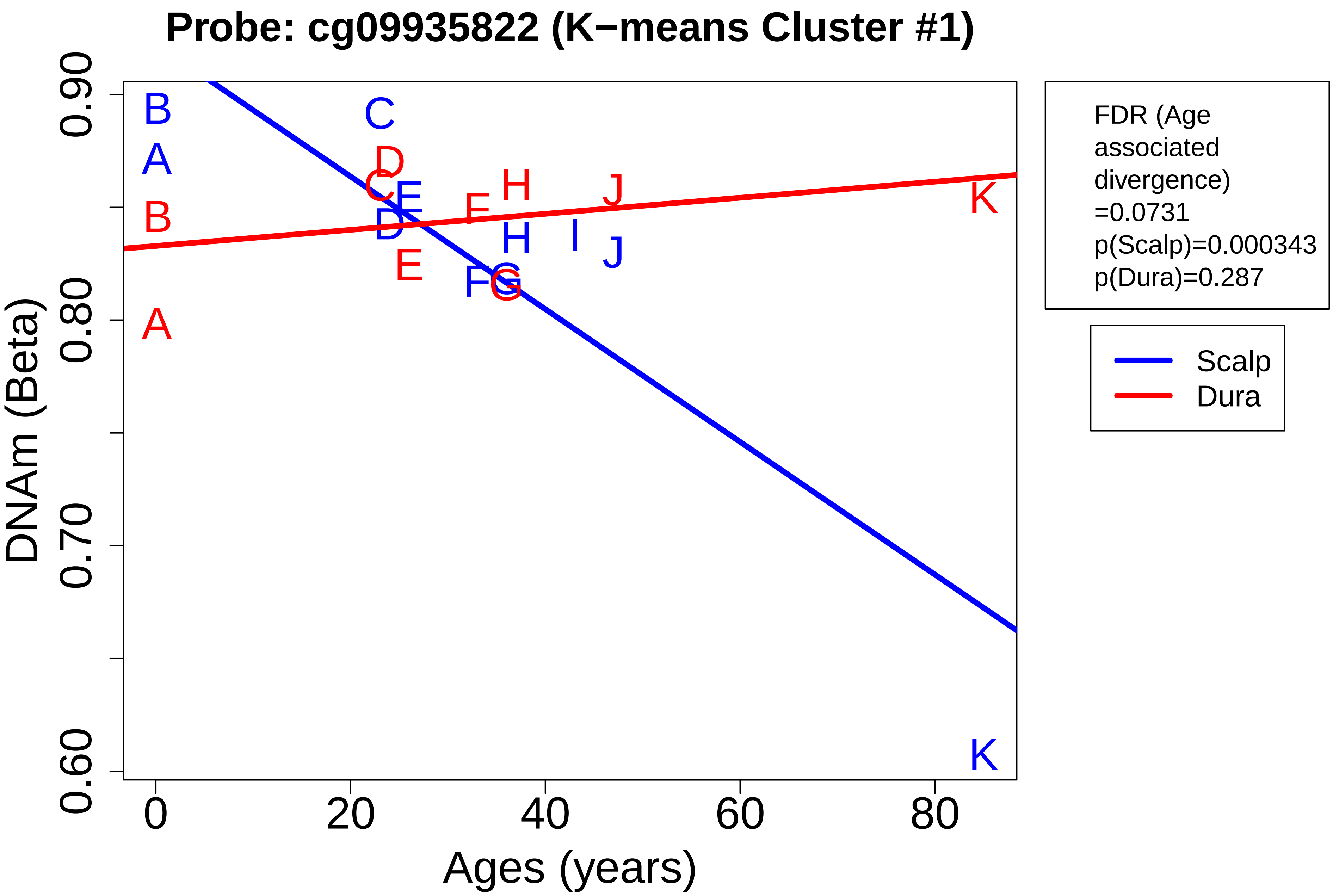

Probe: cg09935822 (K−means Cluster #1)
DNAm (Beta)
0.60
0.70
0.80
0.90
Ages (years)
0
20
40
60
80
FDR (Age associated divergence) =0.0731
p(Scalp)=0.000343
p(Dura)=0.287
Scalp
Dura

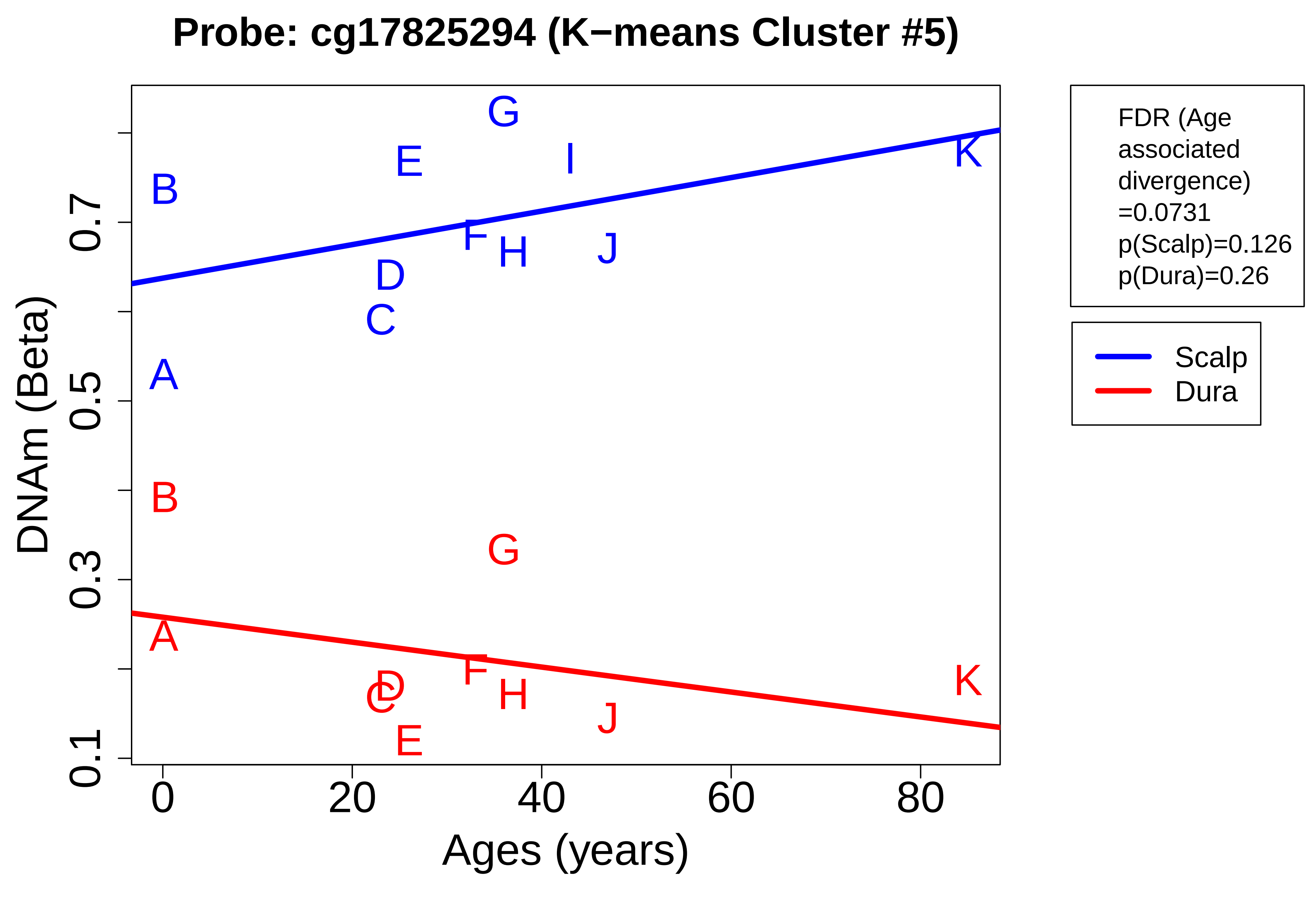

Probe: cg17825294 (K−means Cluster #5)
DNAm (Beta)
0.1
0.3
0.5
0.7
0
20
40
60
80
Ages (years)
FDR (Age associated divergence) =0.0731
p(Scalp)=0.126
p(Dura)=0.26
Scalp
Dura

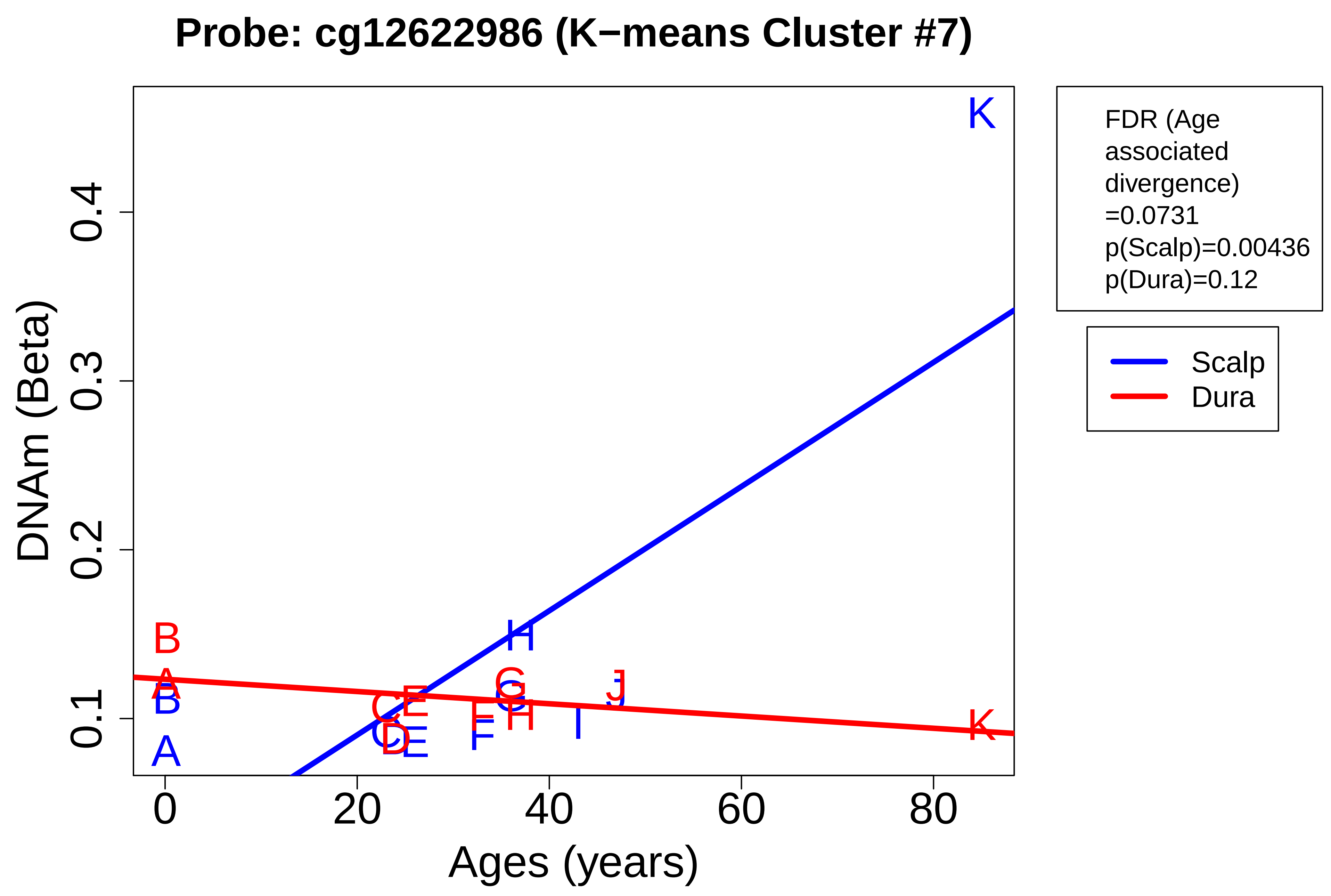

Probe: cg12622986 (K−means Cluster #7)
DNAm (Beta)
0.1
0.2
0.3
0.4
0
20
40
60
80
Ages (years)
FDR (Age associated divergence) =0.0731
p(Scalp)=0.00436
p(Dura)=0.12
Scalp
Dura

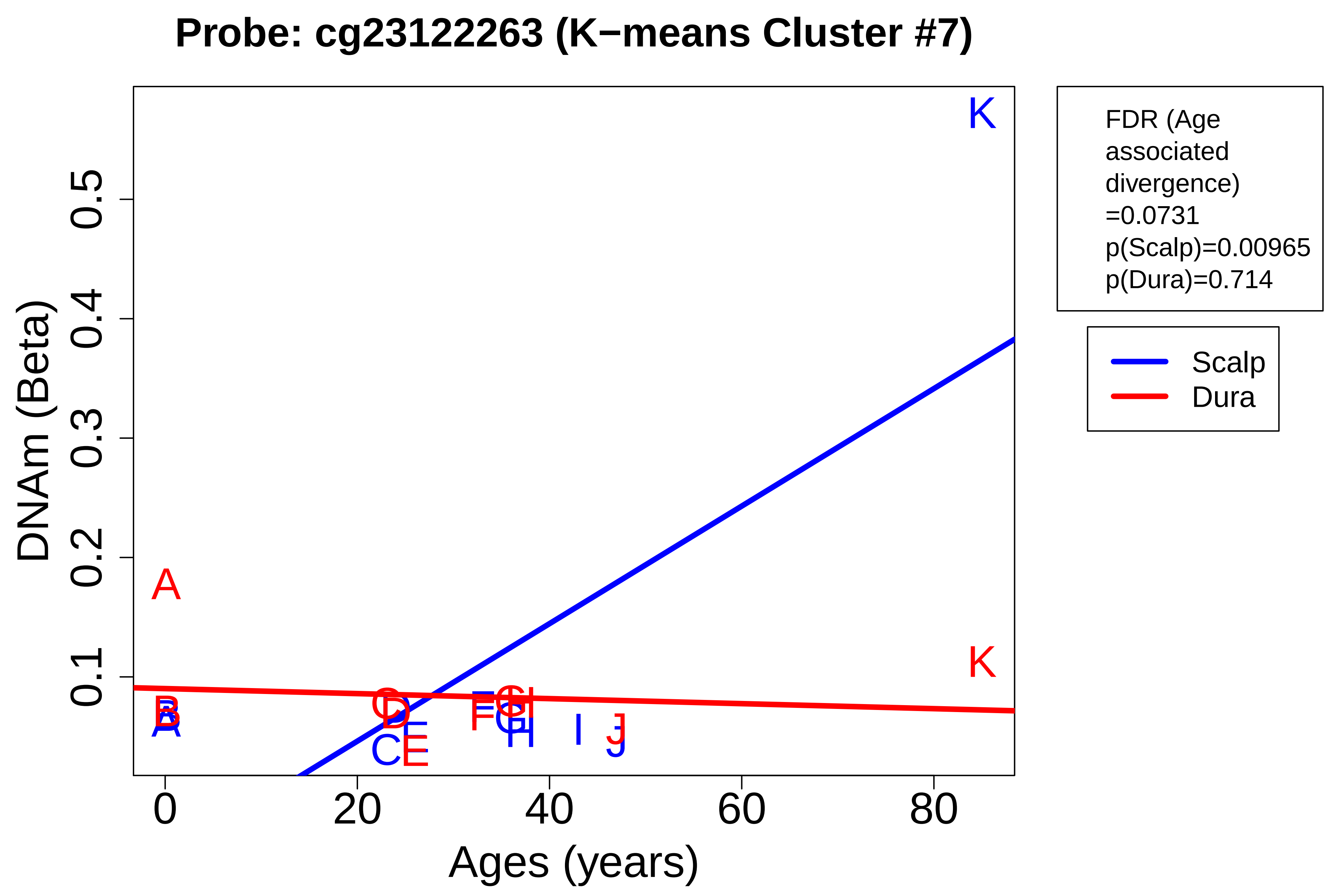

Probe: cg23122263 (K−means Cluster #7)
DNAm (Beta)
0.1
0.2
0.3
0.4
0.5
Ages (years)
0
20
40
60
80
FDR (Age associated divergence) =0.0731
p(Scalp)=0.00965
p(Dura)=0.714
Scalp
Dura

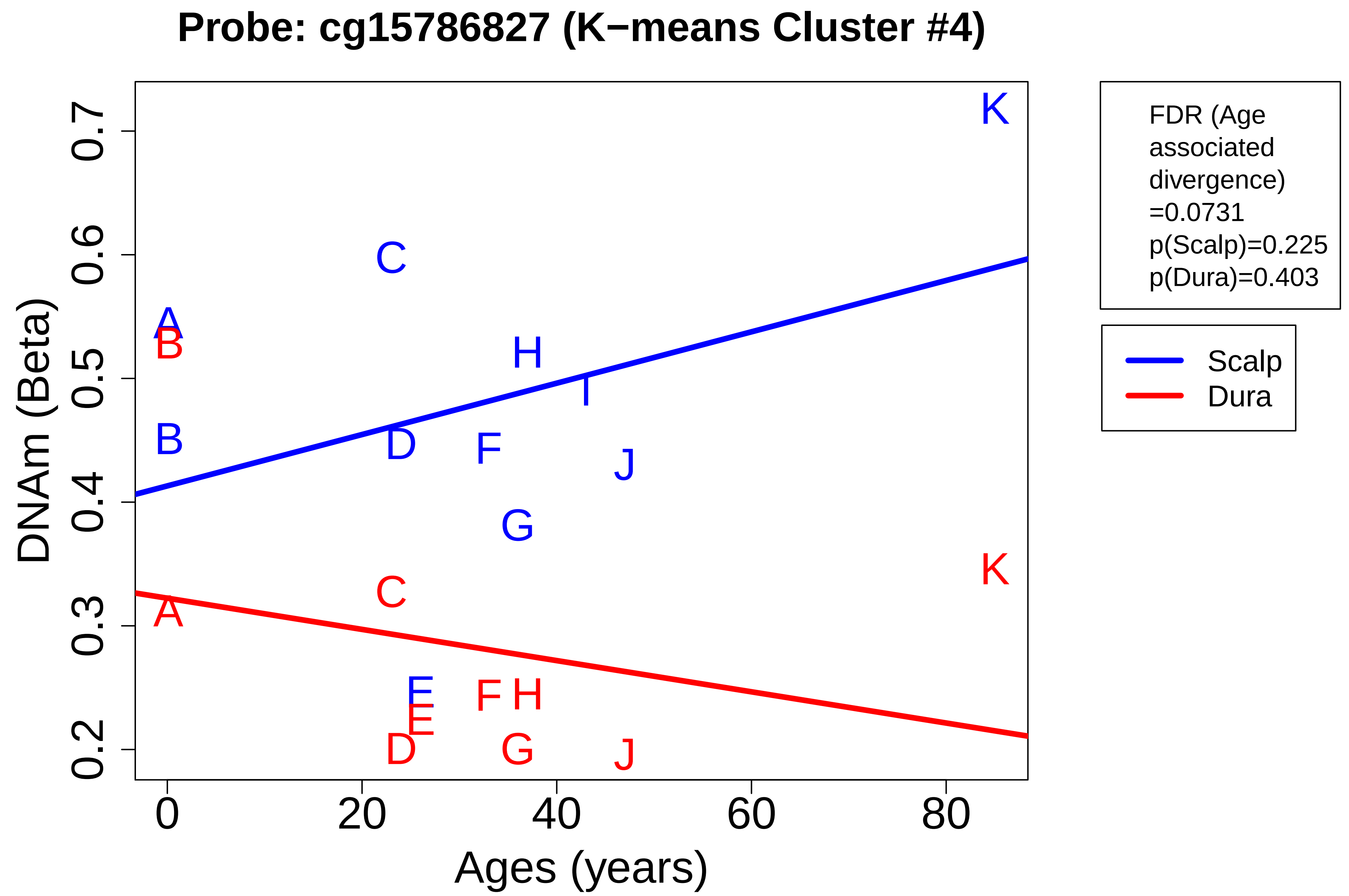

Probe: cg15786827 (K−means Cluster #4)
DNAm (Beta)
0.2
0.3
0.4
0.5
0.6
0.7
0
20
40
60
80
Ages (years)
A
B
C
D
E
F
G
H
I
J
K
FDR (Age associated divergence) =0.0731
p(Scalp)=0.225
p(Dura)=0.403
Scalp
Dura

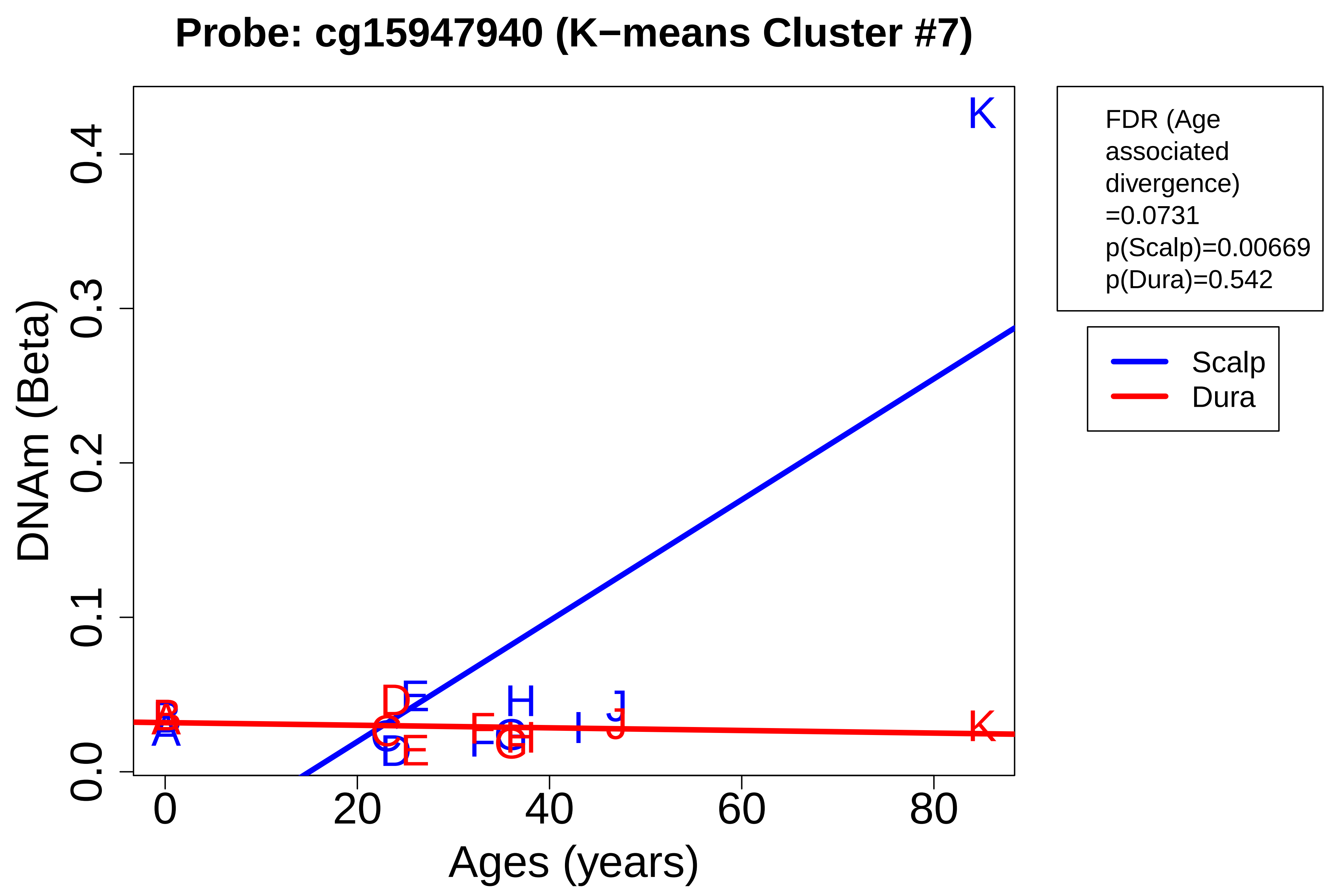

Probe: cg15947940 (K−means Cluster #7)
DNAm (Beta)
0.0
0.1
0.2
0.3
0.4
Ages (years)
0
20
40
60
80
FDR (Age associated divergence) =0.0731
p(Scalp)=0.00669
p(Dura)=0.542
Scalp
Dura

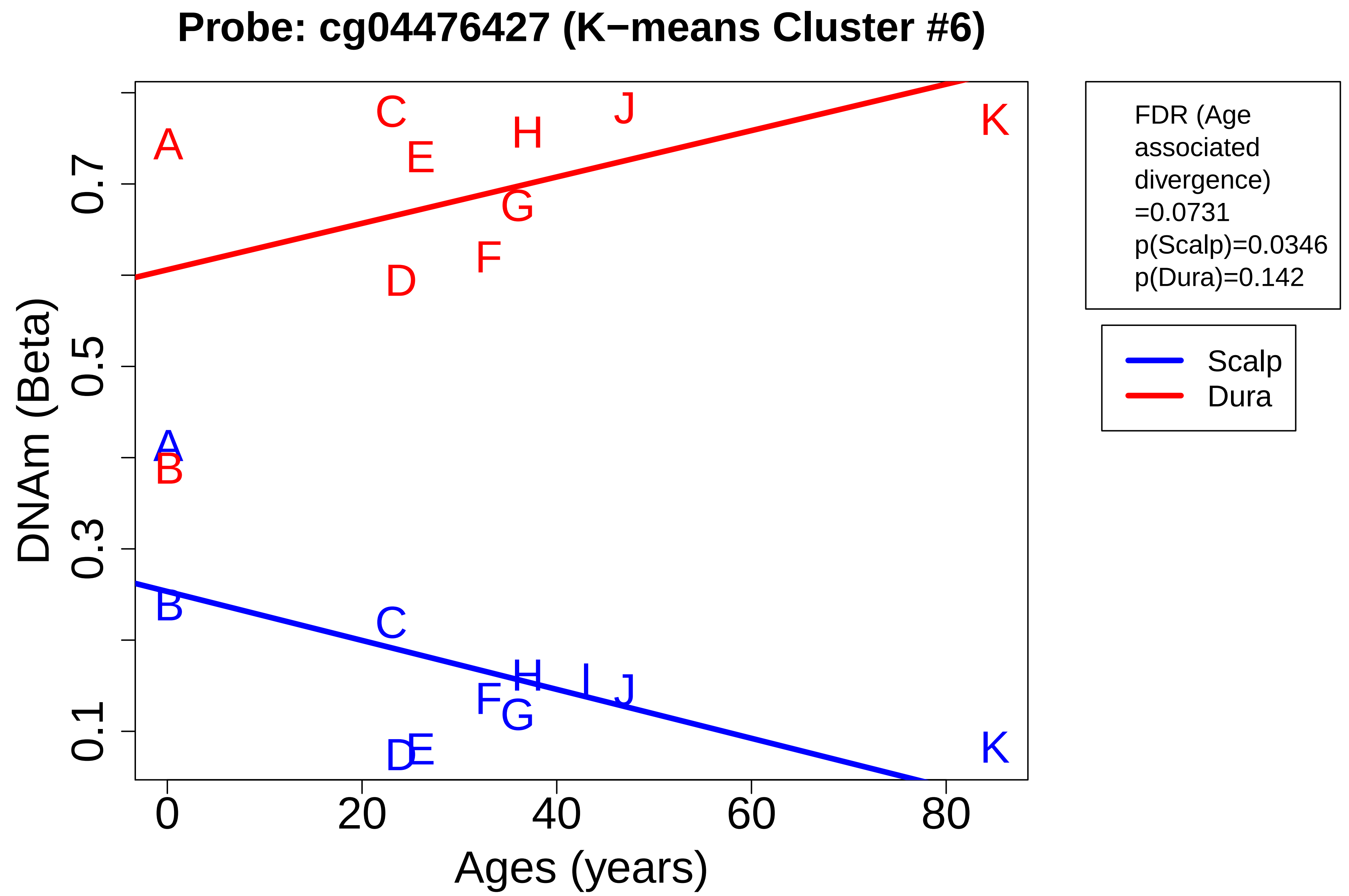

Probe: cg04476427 (K−means Cluster #6)
DNAm (Beta)
0.1
0.3
0.5
0.7
0
20
40
60
80
Ages (years)
FDR (Age associated divergence) =0.0731
p(Scalp)=0.0346
p(Dura)=0.142
Scalp
Dura

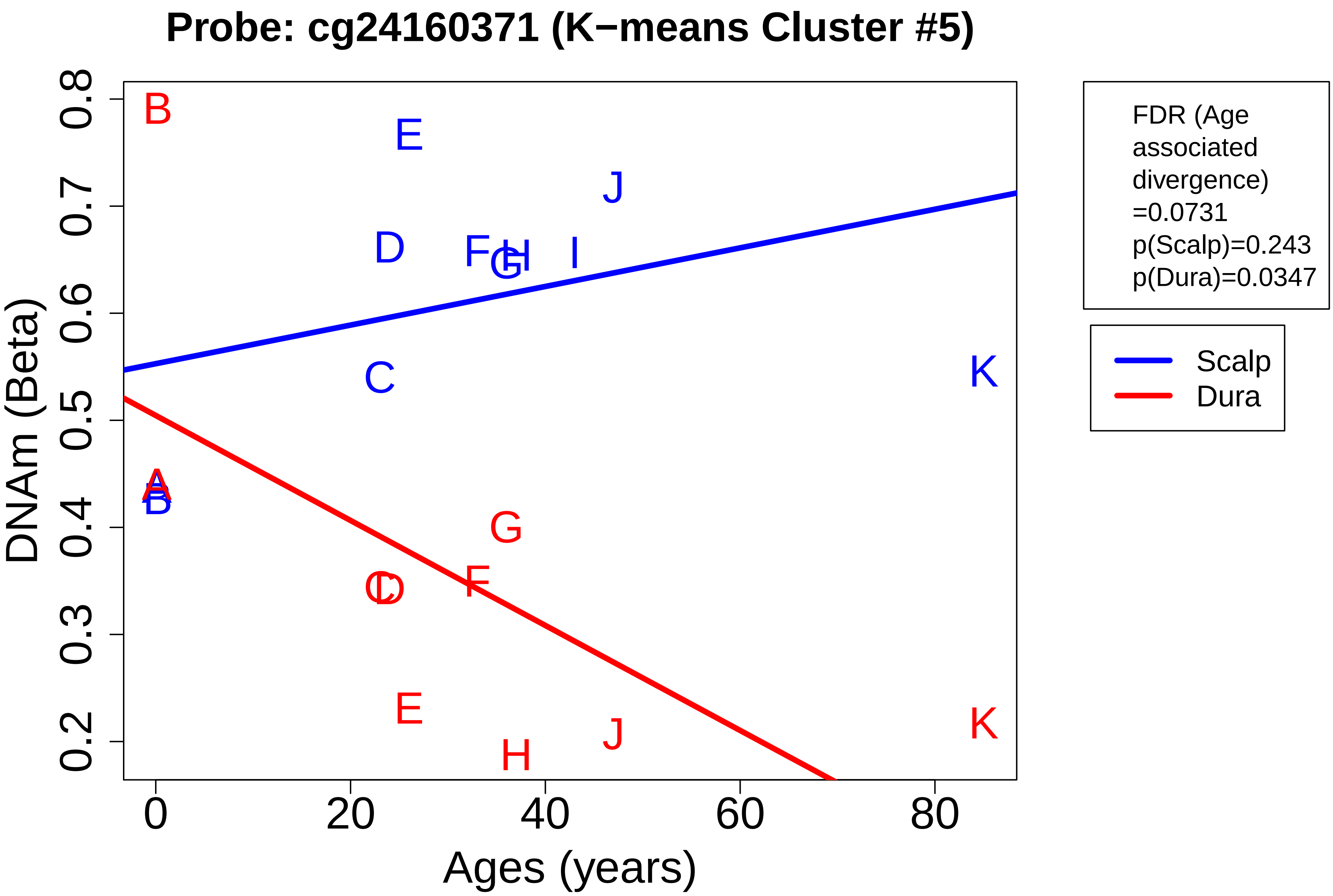
Probe: cg24160371 (K−means Cluster #5)
DNAm (Beta)
Ages (years)
0.2
0.3
0.4
0.5
0.6
0.7
0.8
0
20
40
60
80
FDR (Age associated divergence) =0.0731
p(Scalp)=0.243
p(Dura)=0.0347
Scalp
Dura

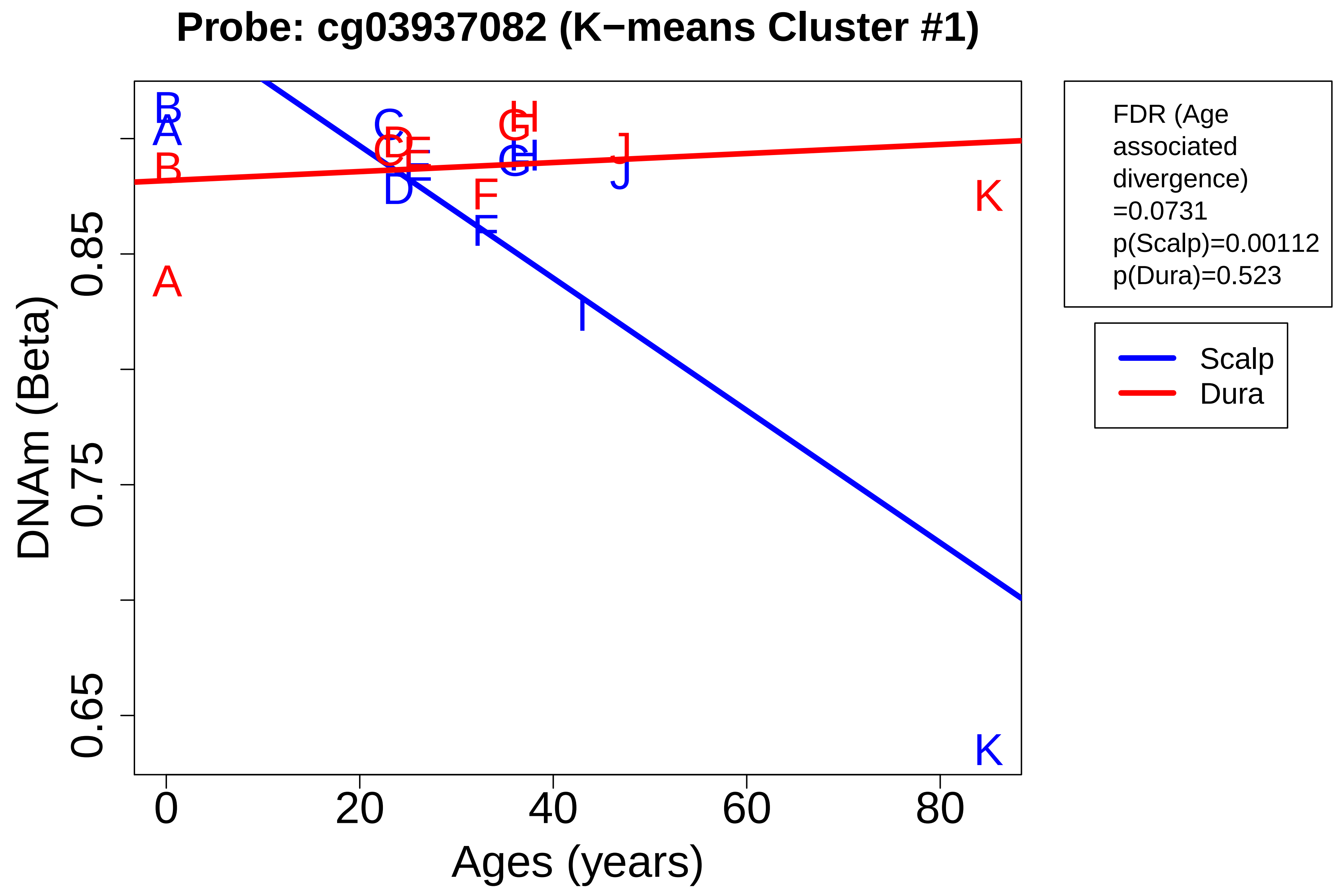

Probe: cg03937082 (K−means Cluster #1)
FDR (Age associated divergence) =0.0731
p(Scalp)=0.00112
p(Dura)=0.523
Scalp
Dura
DNAm (Beta)
0.65
0.75
0.85
Ages (years)
0
20
40
60
80

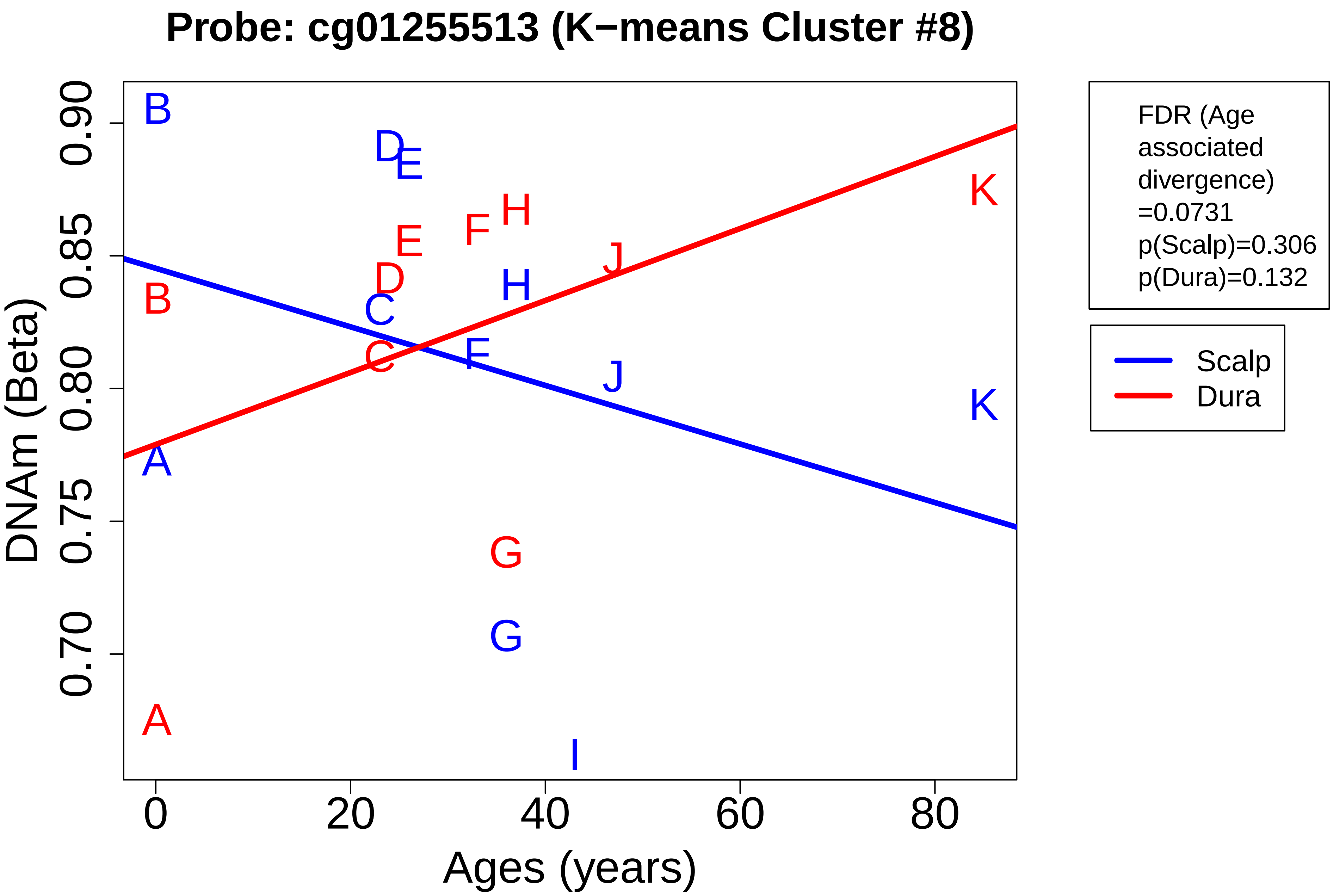
Probe: cg01255513 (K−means Cluster #8)
DNAm (Beta)
0.70
0.75
0.80
0.85
0.90
0
20
40
60
80
Ages (years)
A
B
C
D
E
F
G
H
I
J
K
A
B
C
D
E
F
G
H
J
K
FDR (Age associated divergence) =0.0731
p(Scalp)=0.306
p(Dura)=0.132
Scalp
Dura

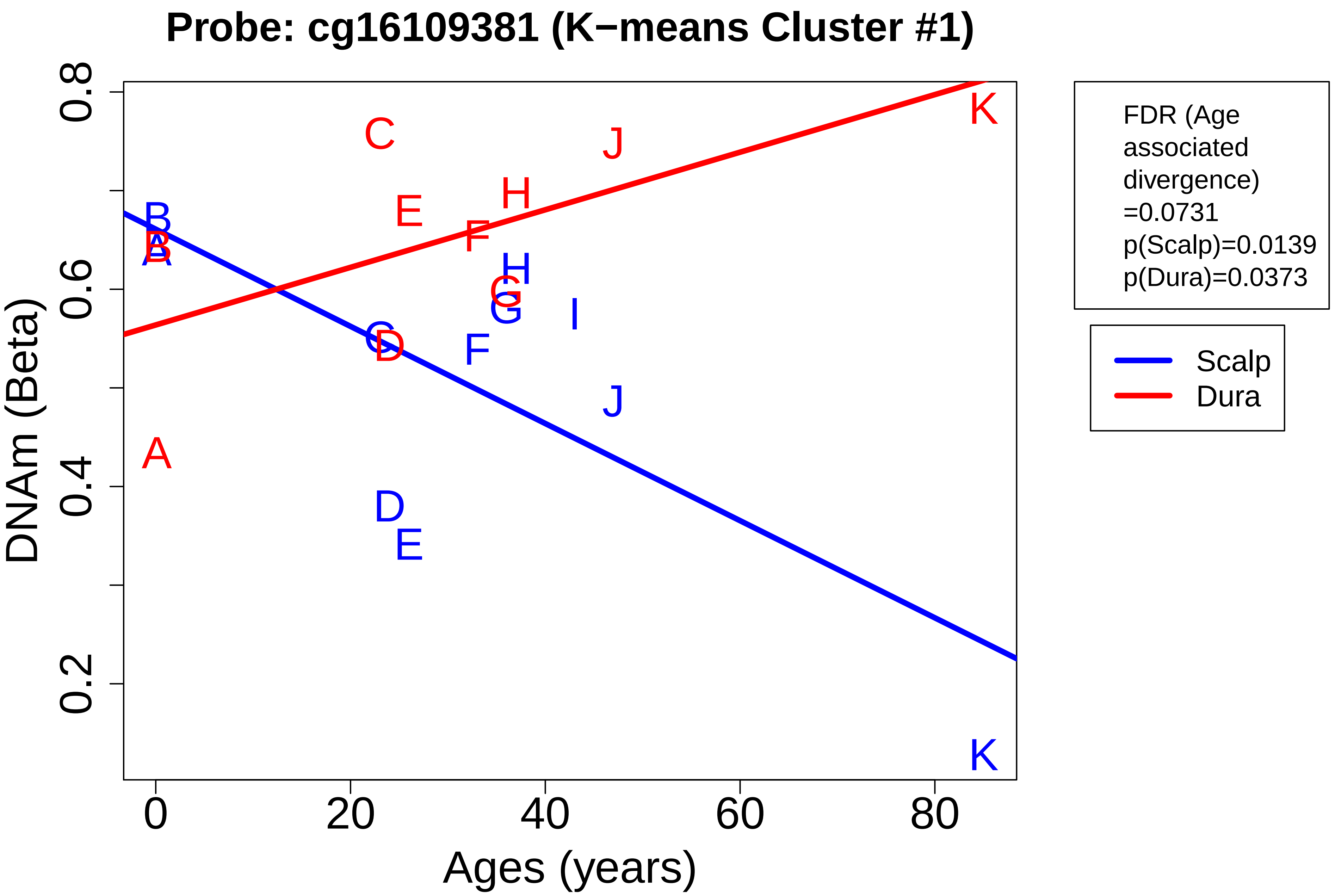
Probe: cg16109381 (K−means Cluster #1)
DNAm (Beta)
0.2
0.4
0.6
0.8
0
20
40
60
80
Ages (years)
A
B
C
D
E
F
G
H
I
J
K
FDR (Age associated divergence) =0.0731
p(Scalp)=0.0139
p(Dura)=0.0373
Scalp
Dura

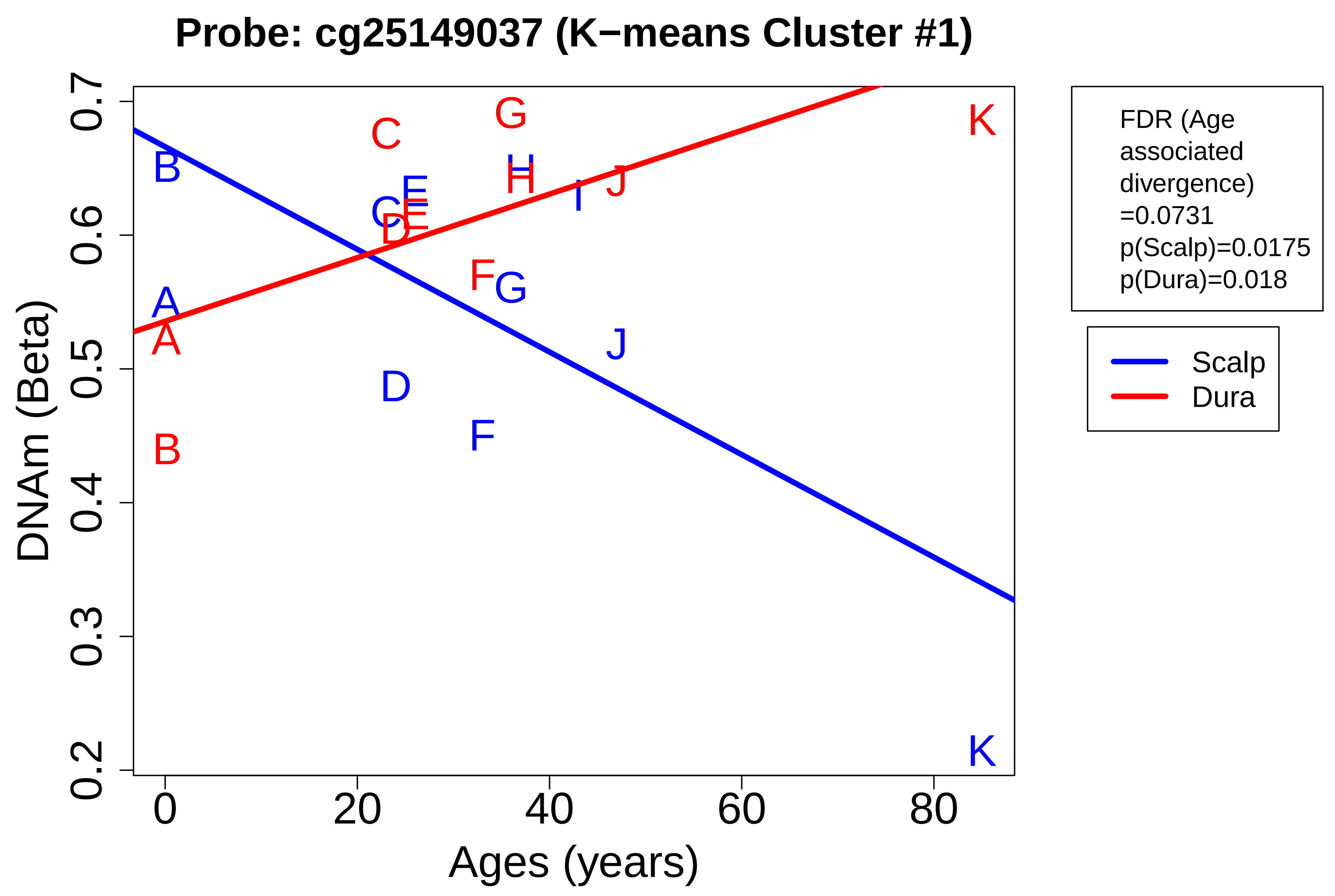
Probe: cg25149037 (K−means Cluster #1)
DNAm (Beta)
0.2
0.3
0.4
0.5
0.6
0.7
Ages (years)
0
20
40
60
80
FDR (Age associated divergence) =0.0731
p(Scalp)=0.0175
p(Dura)=0.018
Scalp
Dura
A
B
C
D
E
F
G
H
I
J
K

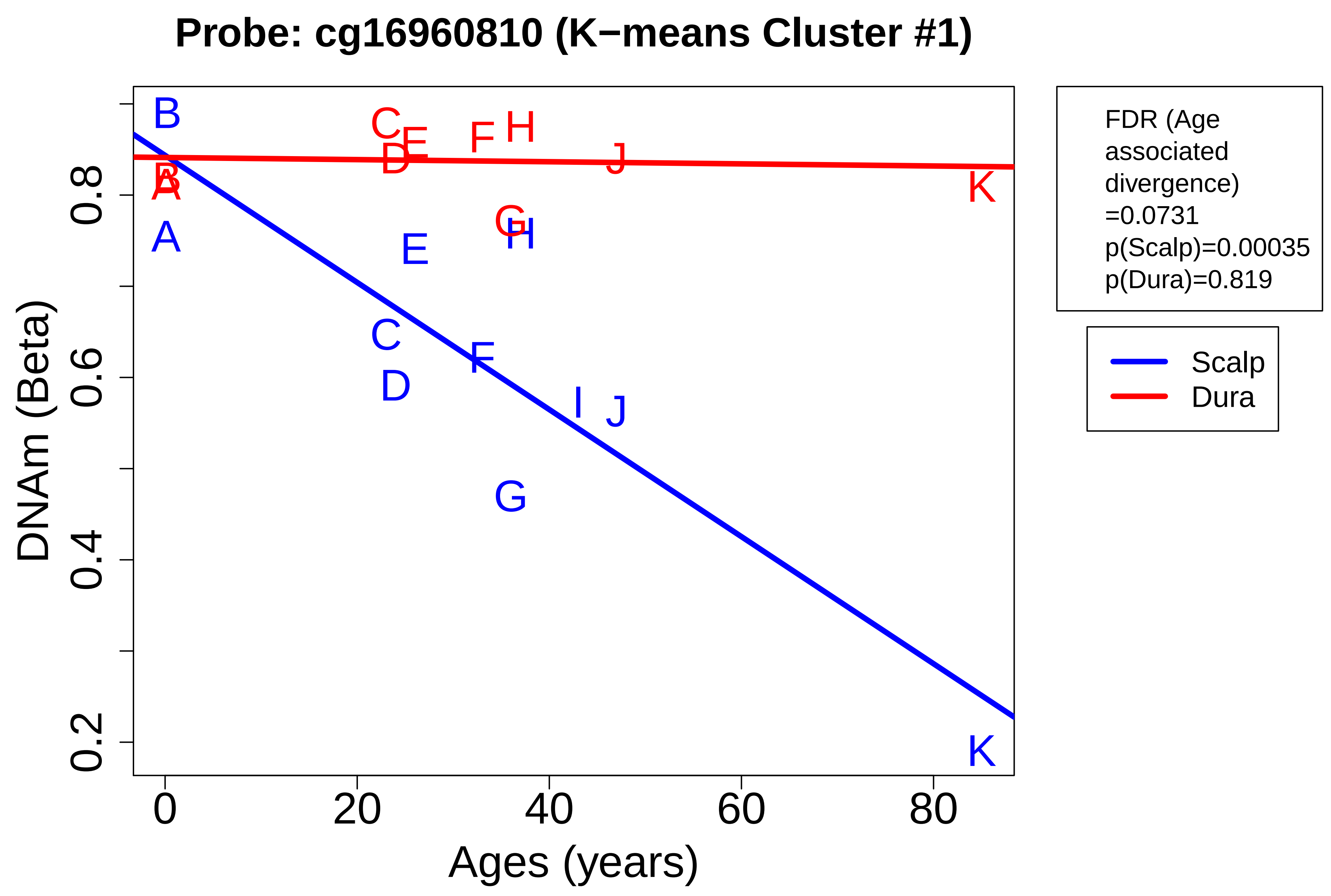

Probe: cg16960810 (K−means Cluster #1)
DNAm (Beta)
0.2
0.4
0.6
0.8
0
20
40
60
80
Ages (years)
FDR (Age associated divergence) =0.0731
p(Scalp)=0.00035
p(Dura)=0.819
Scalp
Dura

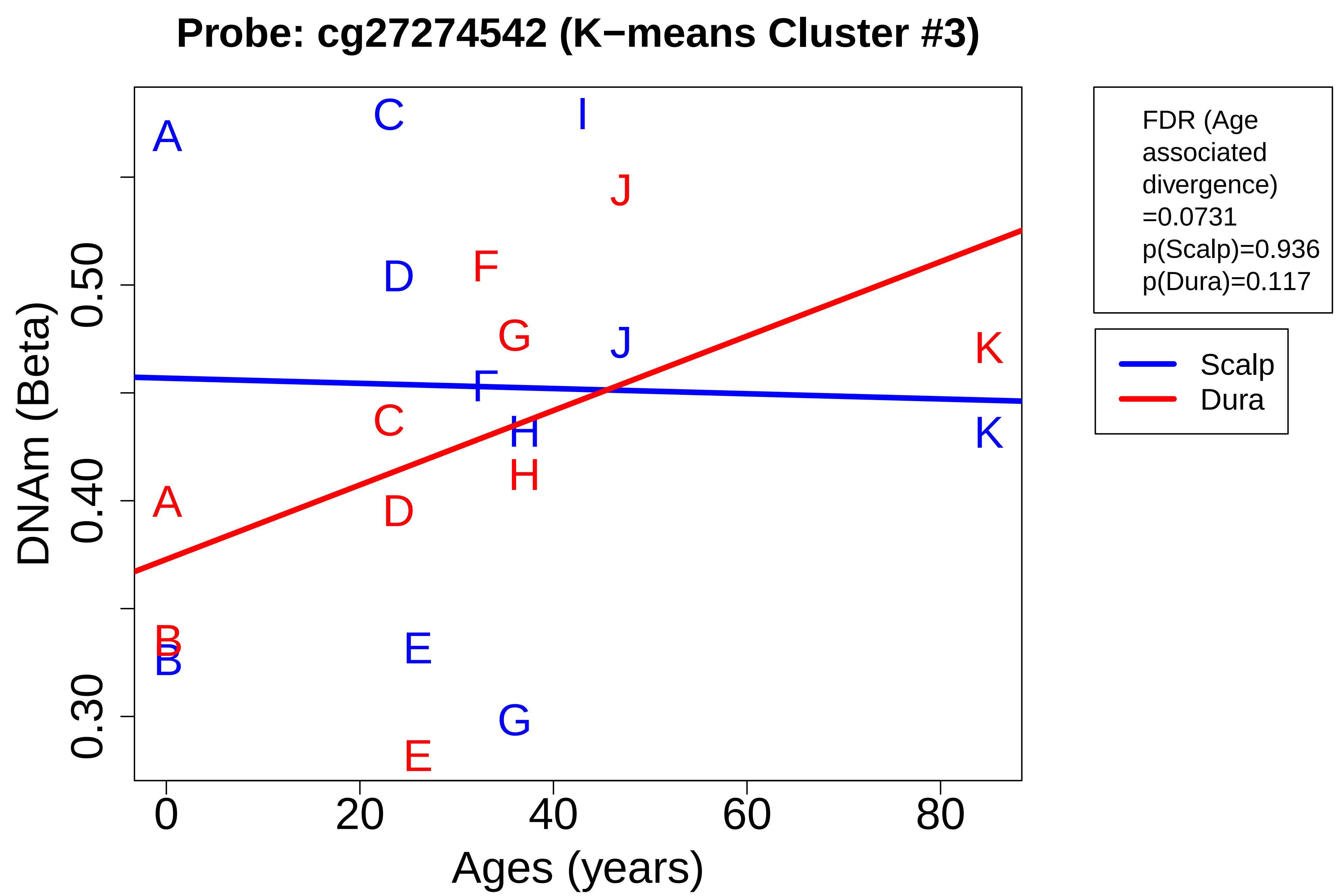

Probe: cg27274542 (K−means Cluster #3)
FDR (Age associated divergence) =0.0731
p(Scalp)=0.936
p(Dura)=0.117
Scalp
Dura
DNAm (Beta)
0.30
0.40
0.50
0
20
40
60
80
Ages (years)
A
B
C
D
E
F
G
H
I
J
K

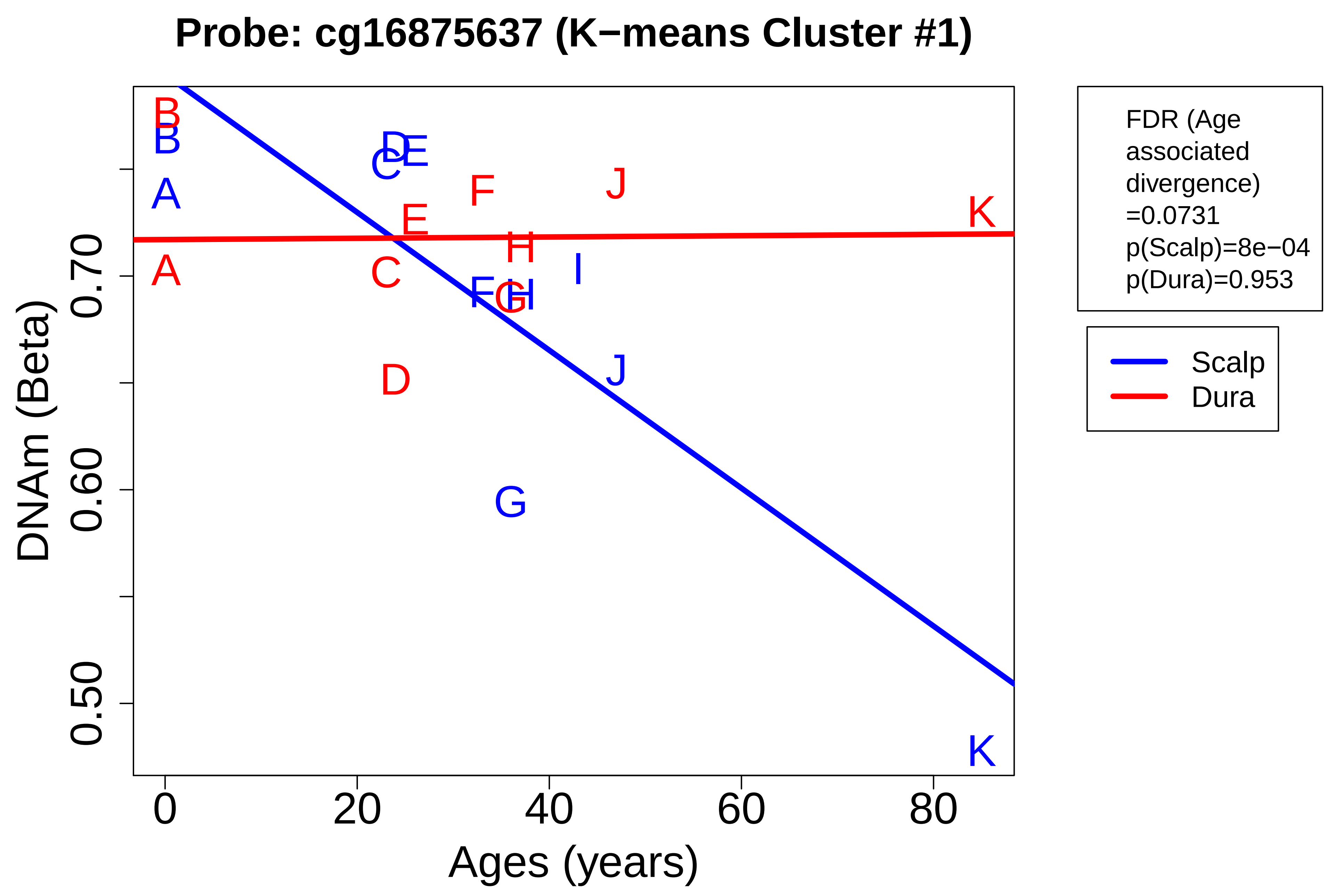

Probe: cg16875637 (K−means Cluster #1)
DNAm (Beta)
0.50
0.60
0.70
Ages (years)
0
20
40
60
80
A
B
C
D
E
F
G
H
I
J
K
FDR (Age associated divergence) =0.0731
p(Scalp)=8e−04
p(Dura)=0.953
Scalp
Dura

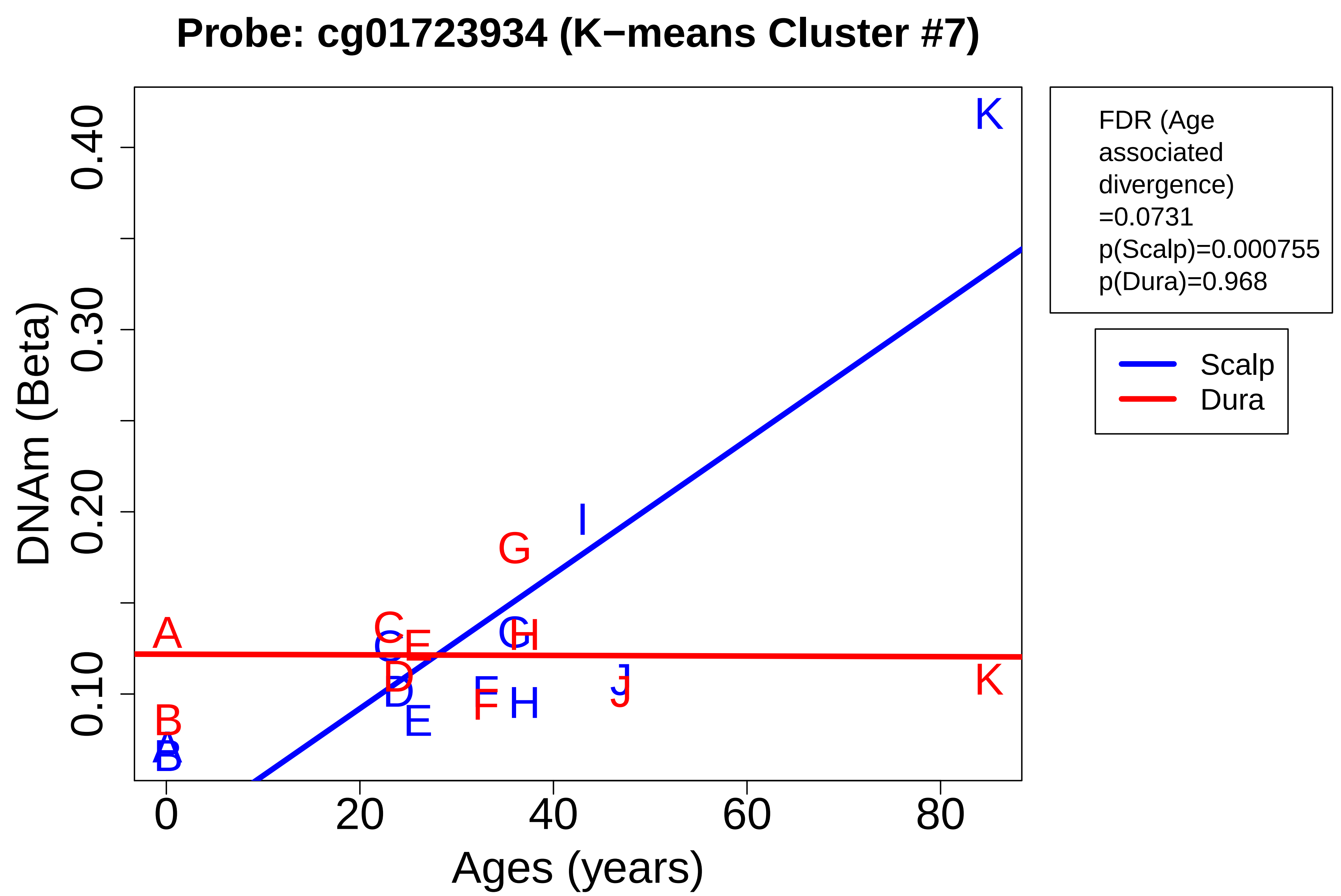

Probe: cg01723934 (K−means Cluster #7)
DNAm (Beta)
0.10
0.20
0.30
0.40
Ages (years)
0
20
40
60
80
FDR (Age associated divergence) =0.0731
p(Scalp)=0.000755
p(Dura)=0.968
Scalp
Dura
A
B
C
D
E
F
G
H
I
J
K

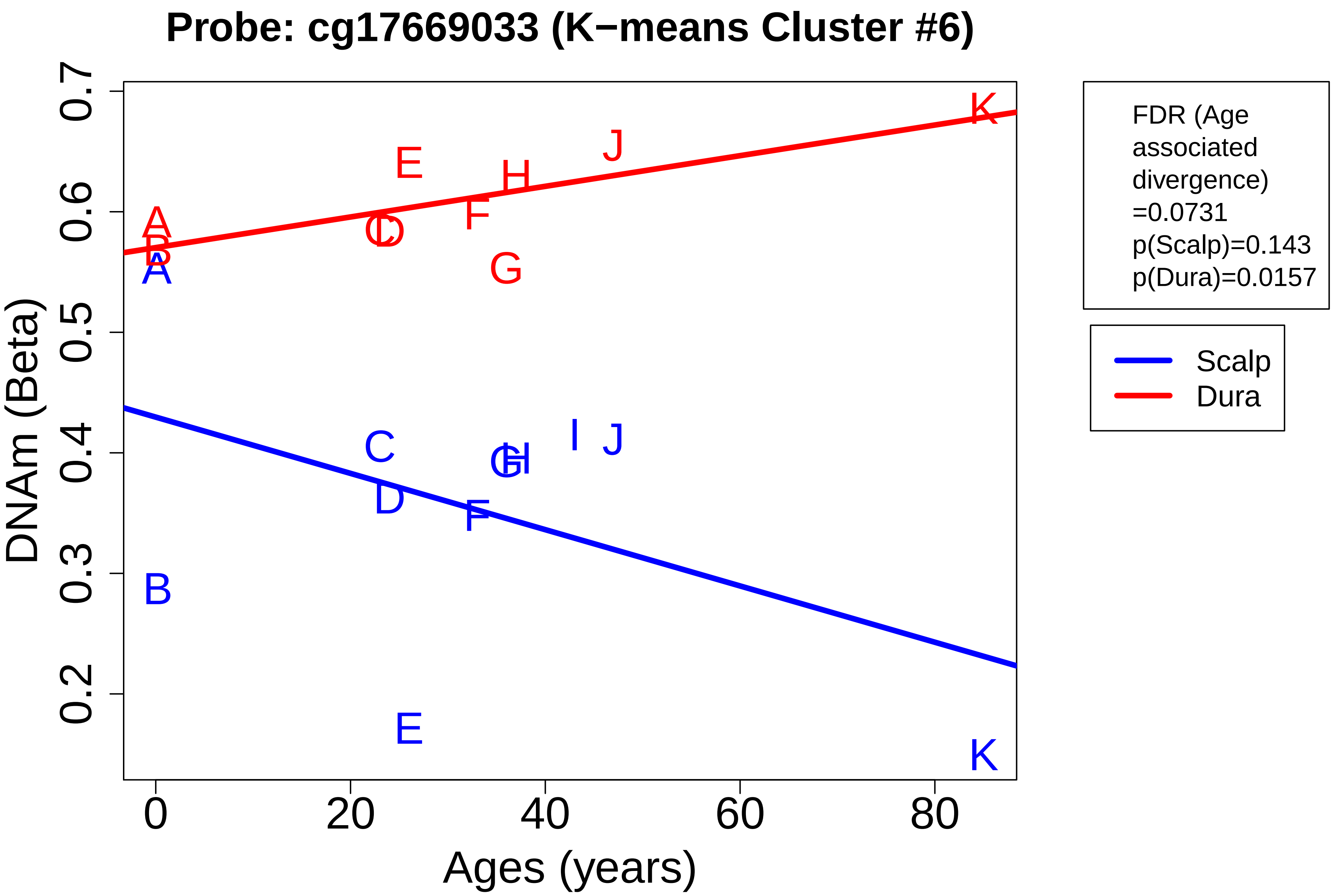

Probe: cg17669033 (K−means Cluster #6)
DNAm (Beta)
0.2
0.3
0.4
0.5
0.6
0.7
Ages (years)
0
20
40
60
80
FDR (Age associated divergence) =0.0731
p(Scalp)=0.143
p(Dura)=0.0157
Scalp
Dura
A B C D E F G H I J K

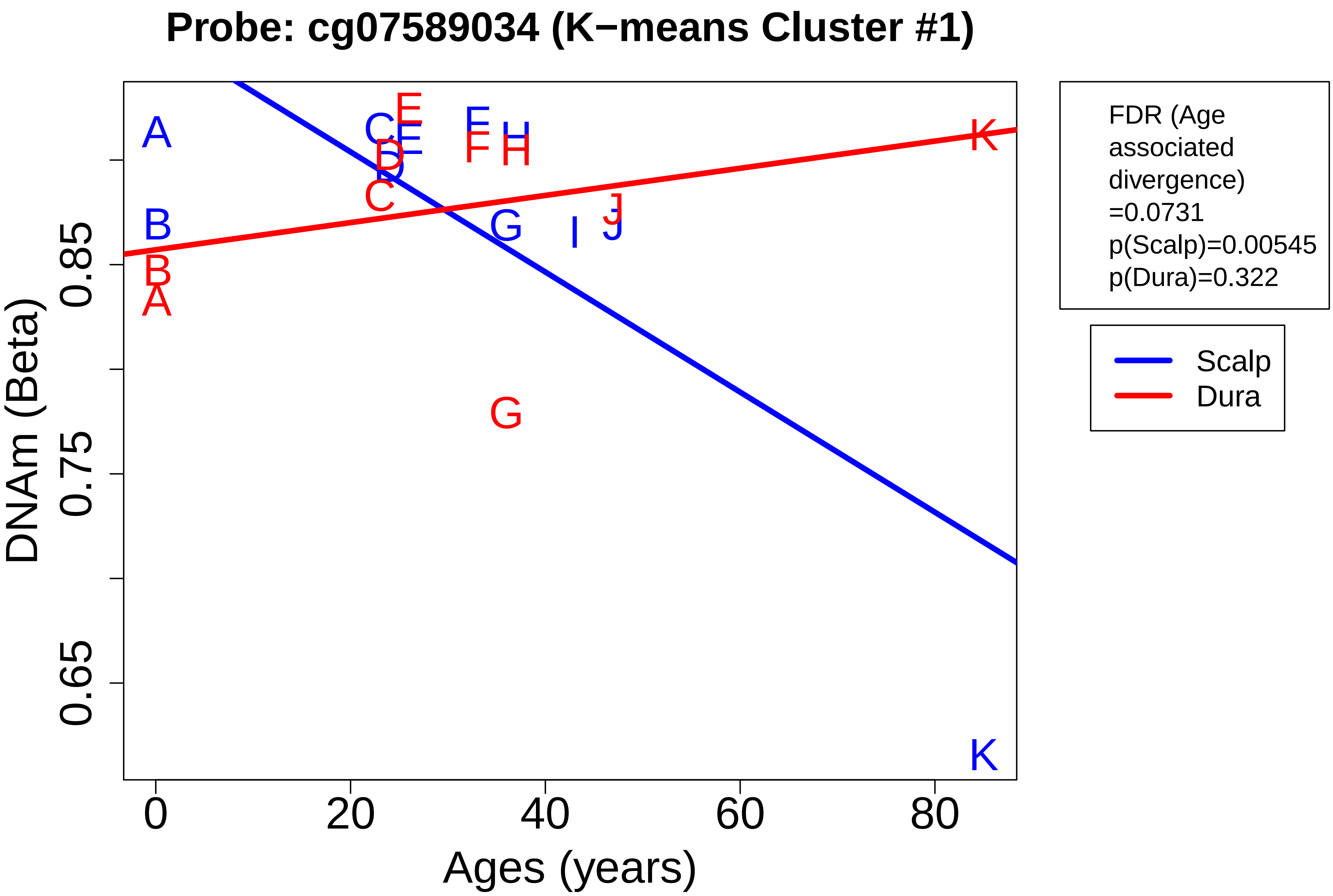

Probe: cg07589034 (K−means Cluster #1)
FDR (Age associated divergence) =0.0731
p(Scalp)=0.00545
p(Dura)=0.322
Scalp
Dura
DNAm (Beta)
0.65
0.75
0.85
Ages (years)
0
20
40
60
80

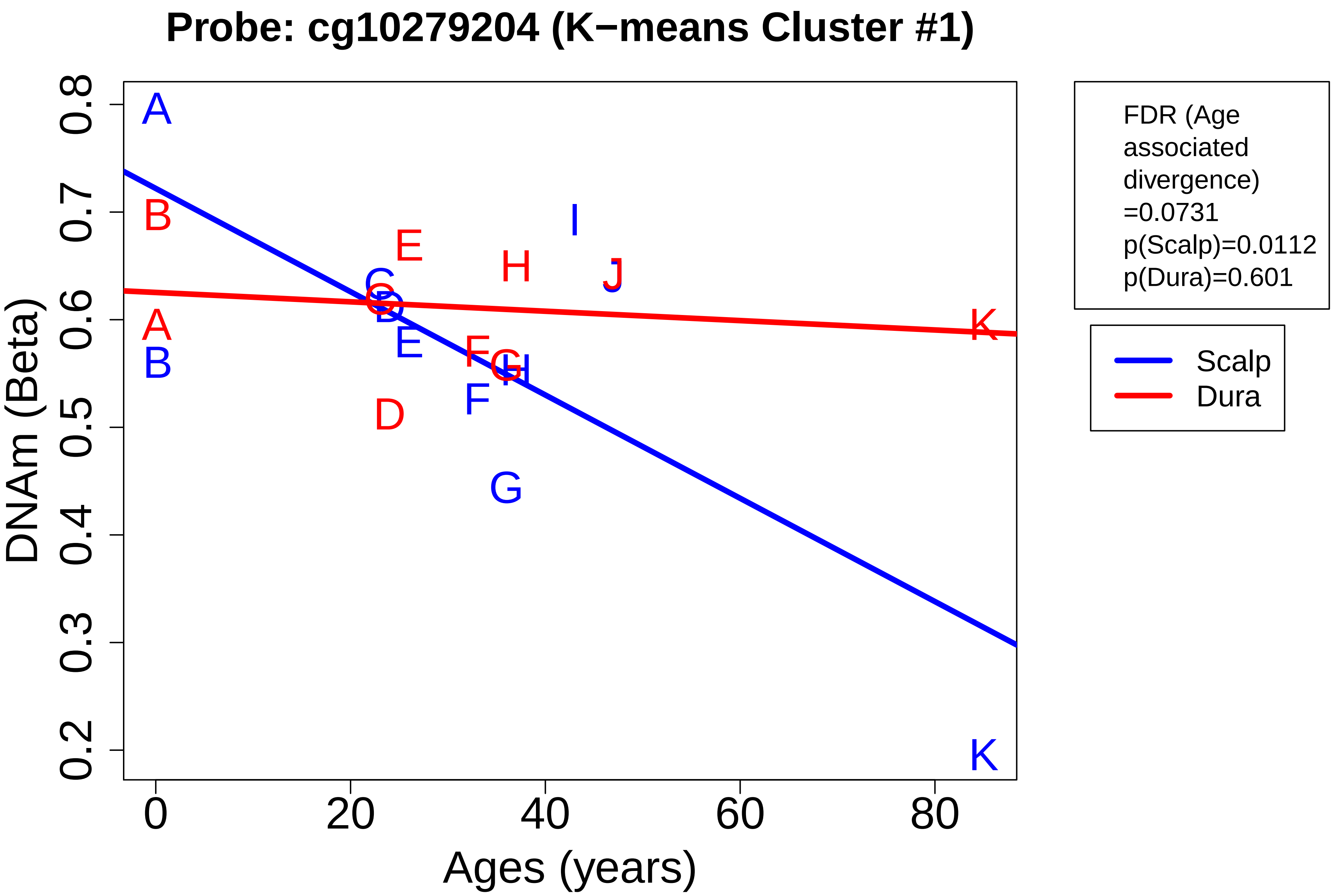
Probe: cg10279204 (K−means Cluster #1)
DNAm (Beta)
0.2
0.3
0.4
0.5
0.6
0.7
0.8
Ages (years)
0
20
40
60
80
FDR (Age associated divergence) =0.0731
p(Scalp)=0.0112
p(Dura)=0.601
Scalp
Dura

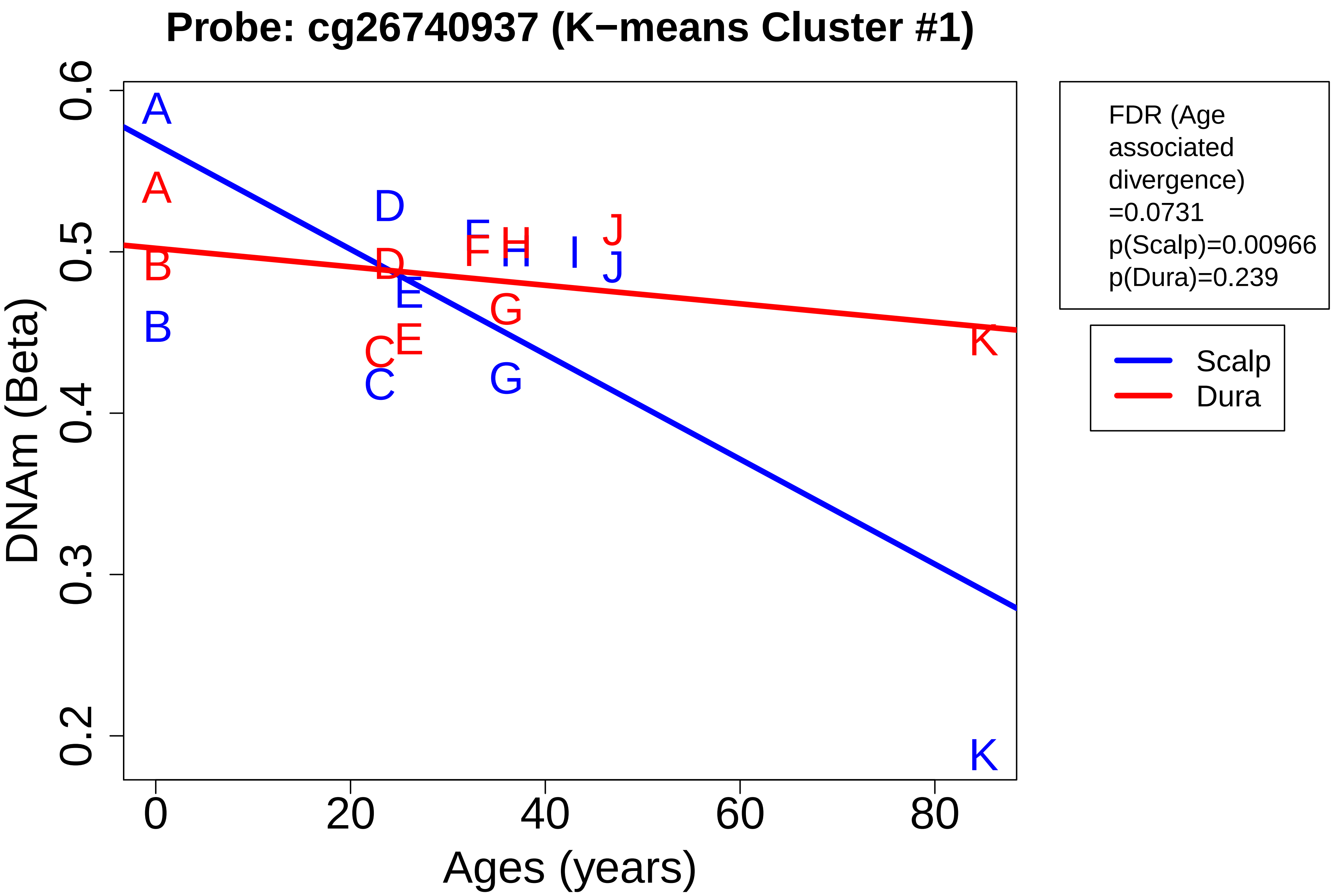
Probe: cg26740937 (K−means Cluster #1)
DNAm (Beta)
0.2
0.3
0.4
0.5
0.6
Ages (years)
0
20
40
60
80
A
B
C
D
E
F
G
H
I
J
K
FDR (Age associated divergence) =0.0731
p(Scalp)=0.00966
p(Dura)=0.239
Scalp
Dura

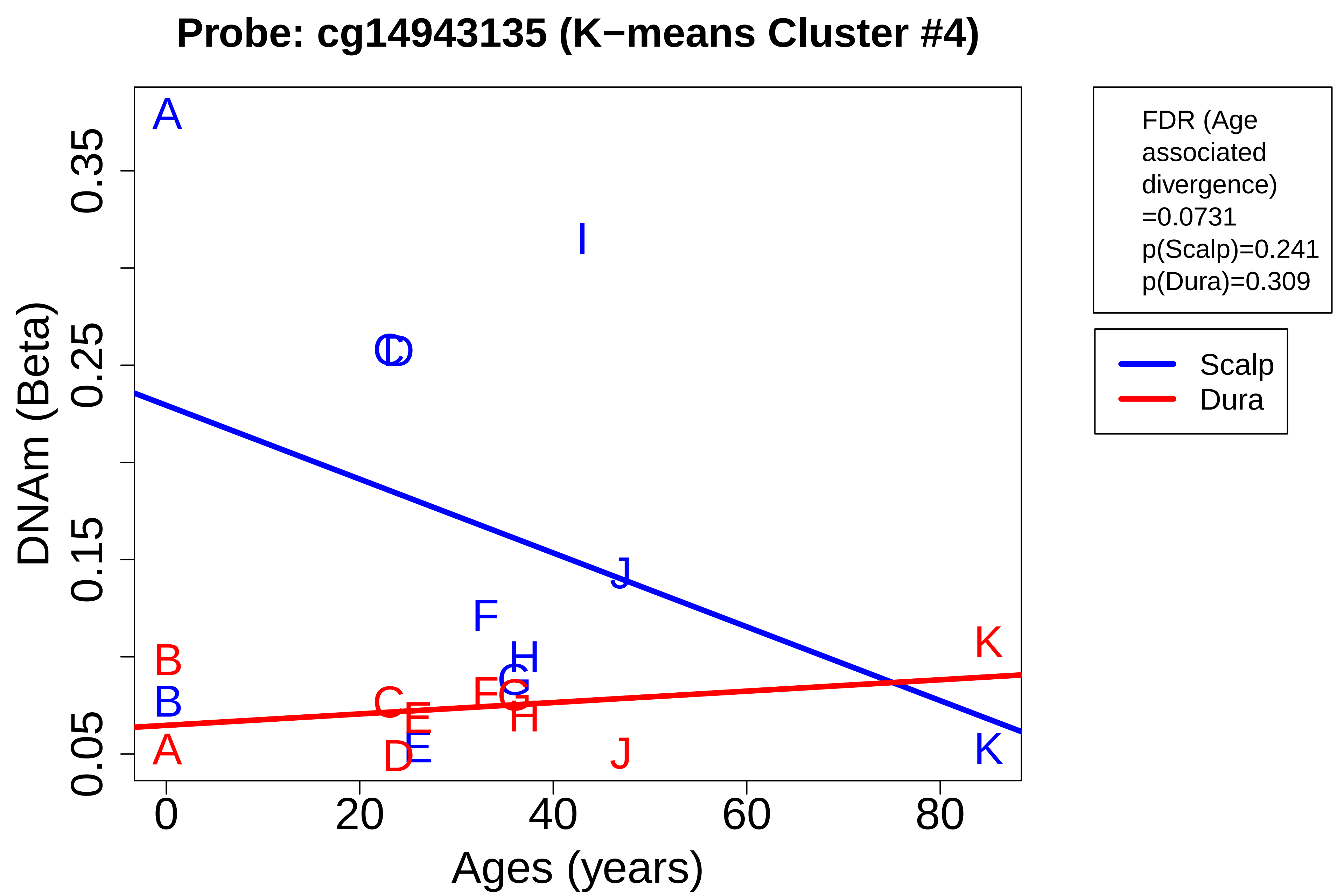
Probe: cg14943135 (K−means Cluster #4)
DNAm (Beta)
0.05
0.15
0.25
0.35
Ages (years)
0
20
40
60
80
A
B
C
D
E
F
G
H
I
J
K
FDR (Age associated divergence) =0.0731
p(Scalp)=0.241
p(Dura)=0.309
Scalp
Dura

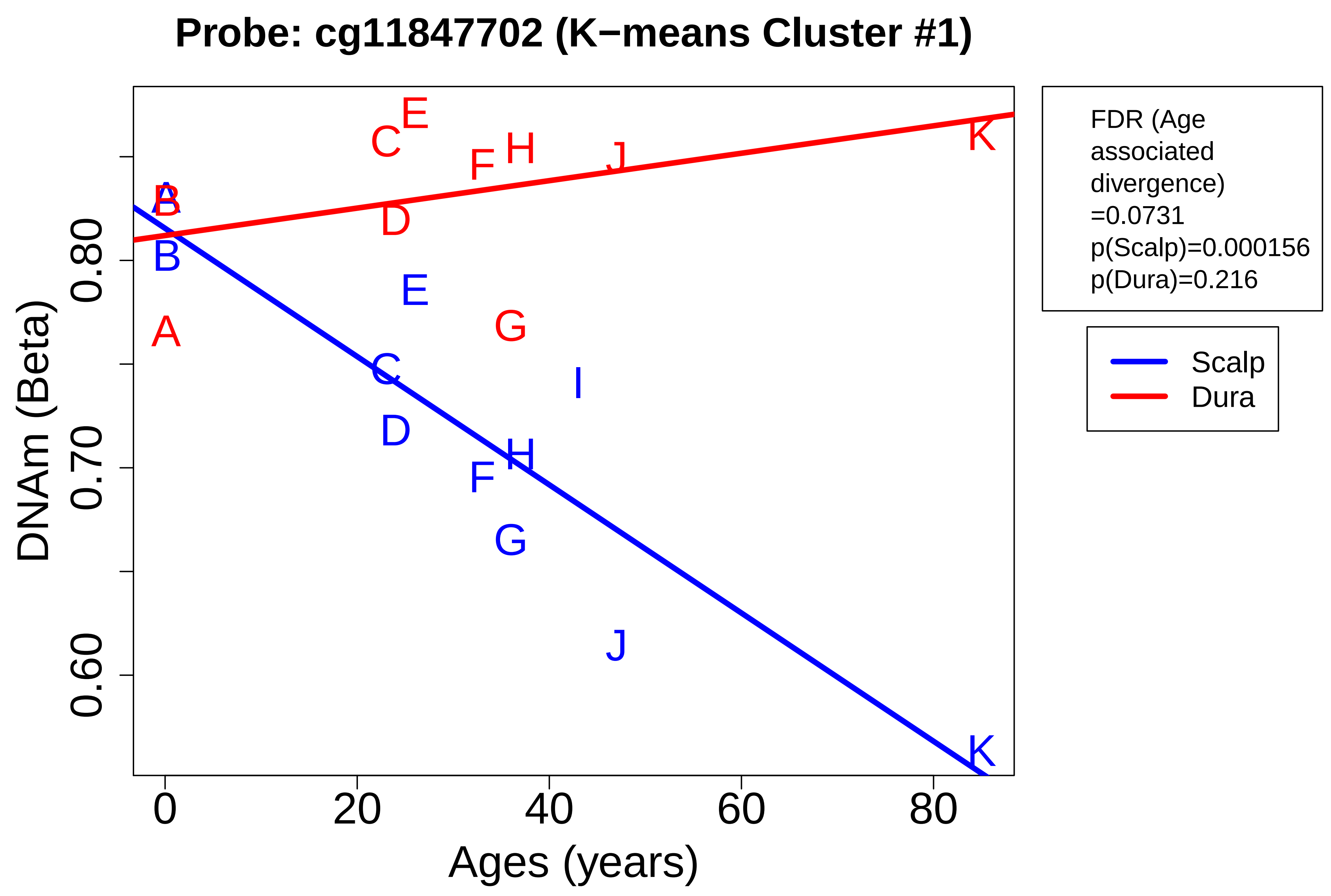

Probe: cg11847702 (K−means Cluster #1)
DNAm (Beta)
0.60
0.70
0.80
0
20
40
60
80
Ages (years)
A
B
C
D
E
F
G
H
I
J
K
FDR (Age associated divergence) =0.0731
p(Scalp)=0.000156
p(Dura)=0.216
Scalp
Dura

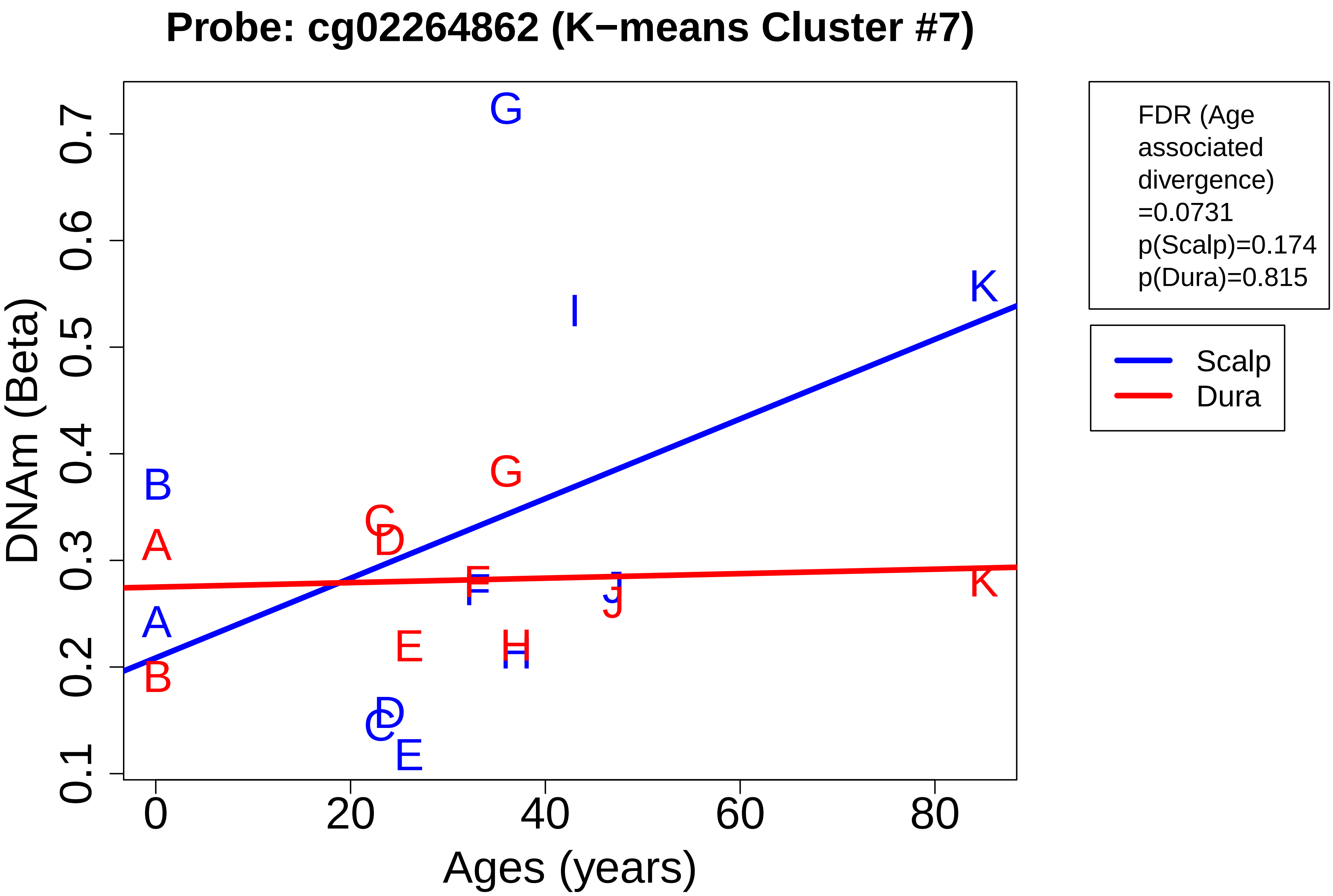

Probe: cg02264862 (K−means Cluster #7)
DNAm (Beta)
0.1
0.2
0.3
0.4
0.5
0.6
0.7
0
20
40
60
80
Ages (years)
FDR (Age associated divergence) =0.0731
p(Scalp)=0.174
p(Dura)=0.815
Scalp
Dura
A
B
C
D
E
F
G
H
I
J
K

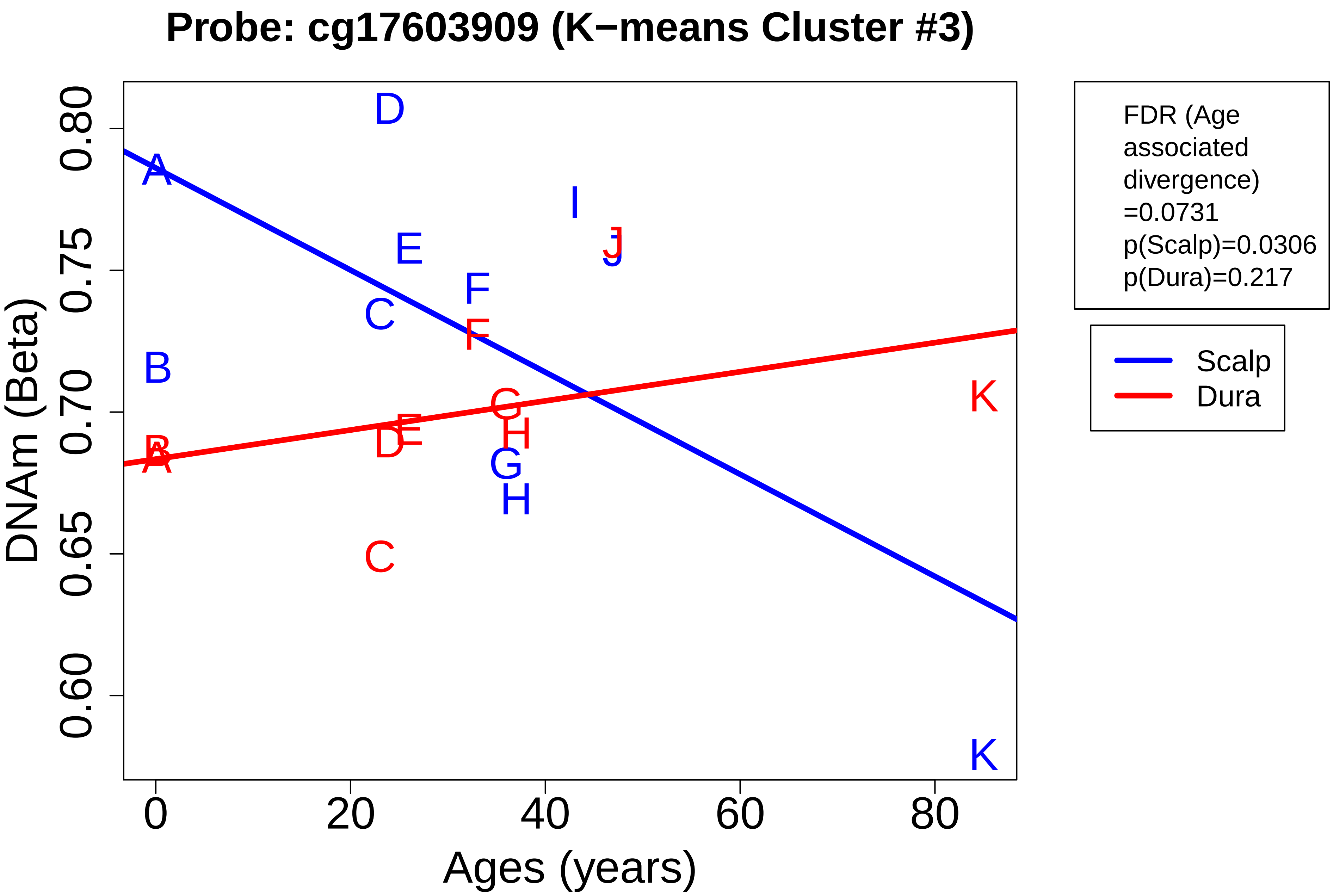
Probe: cg17603909 (K−means Cluster #3)
DNAm (Beta)
Ages (years)
0.60
0.65
0.70
0.75
0.80
0
20
40
60
80
FDR (Age associated divergence) =0.0731
p(Scalp)=0.0306
p(Dura)=0.217
Scalp
Dura

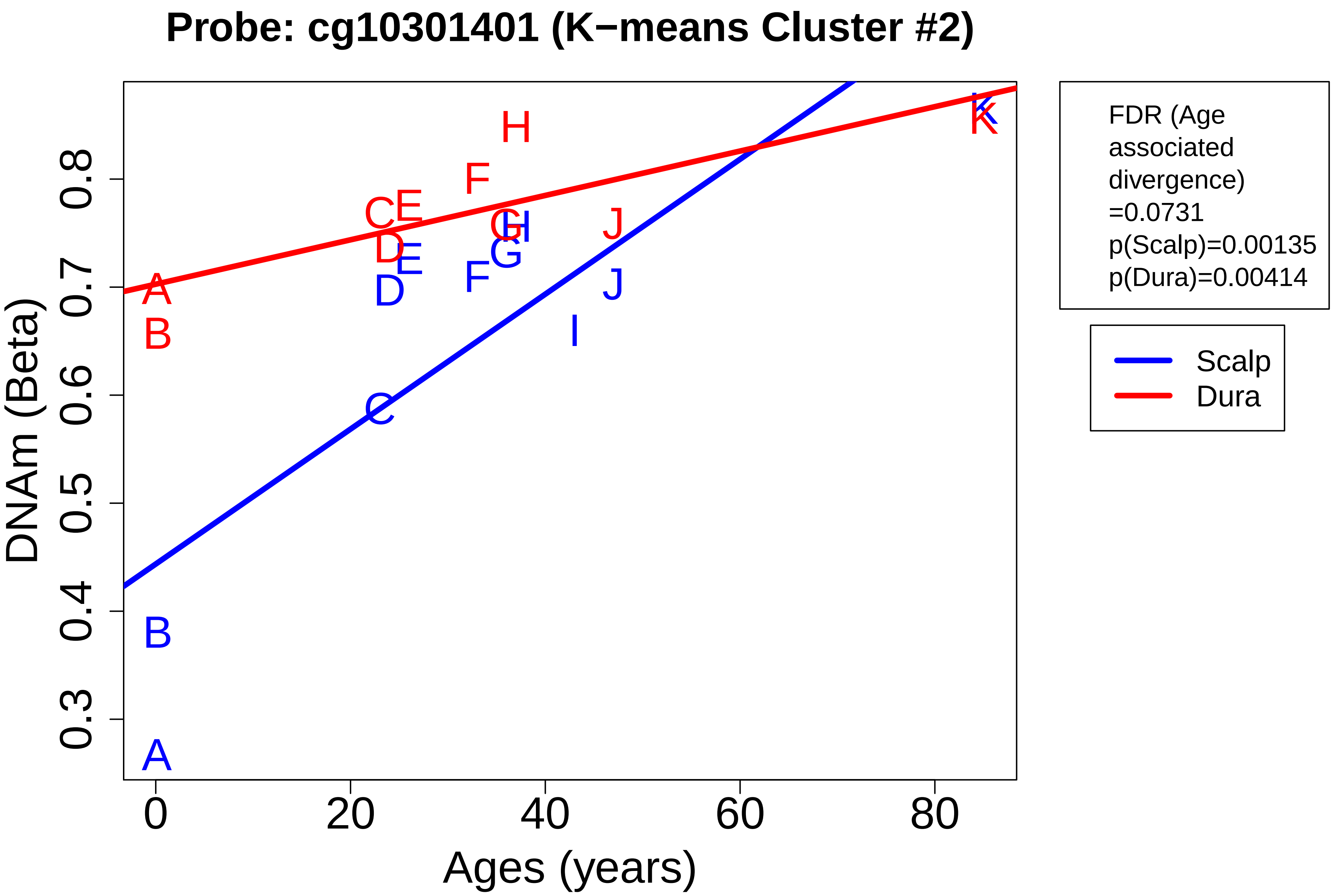

Probe: cg10301401 (K−means Cluster #2)
DNAm (Beta)
Ages (years)
0.3
0.4
0.5
0.6
0.7
0.8
0
20
40
60
80
FDR (Age associated divergence) =0.0731
p(Scalp)=0.00135
p(Dura)=0.00414
Scalp
Dura
A
B
C
D
E
F
G
H
I
J
K

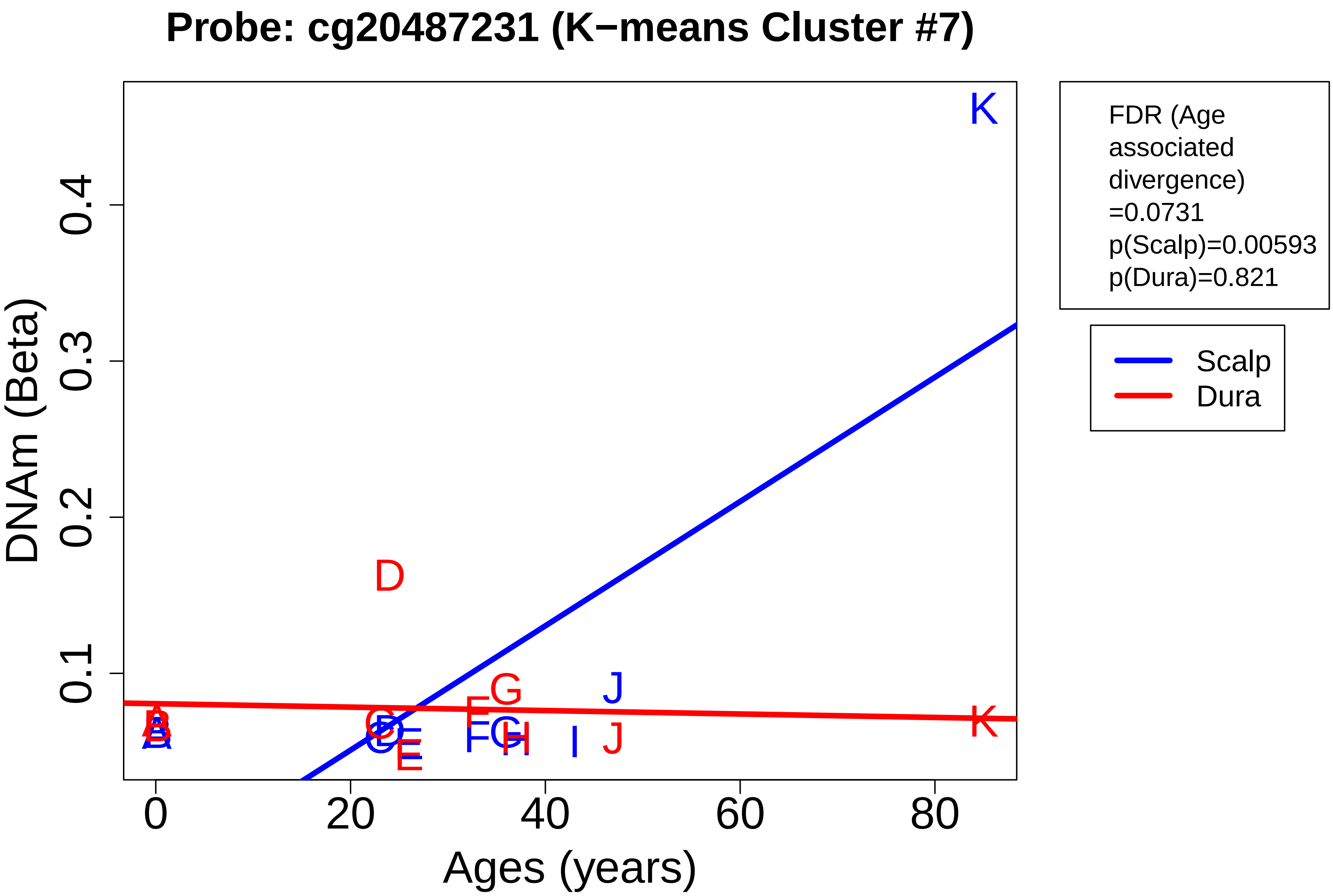

Probe: cg20487231 (K−means Cluster #7)
DNAm (Beta)
0.1
0.2
0.3
0.4
0
20
40
60
80
Ages (years)
FDR (Age associated divergence) =0.0731
p(Scalp)=0.00593
p(Dura)=0.821
Scalp
Dura

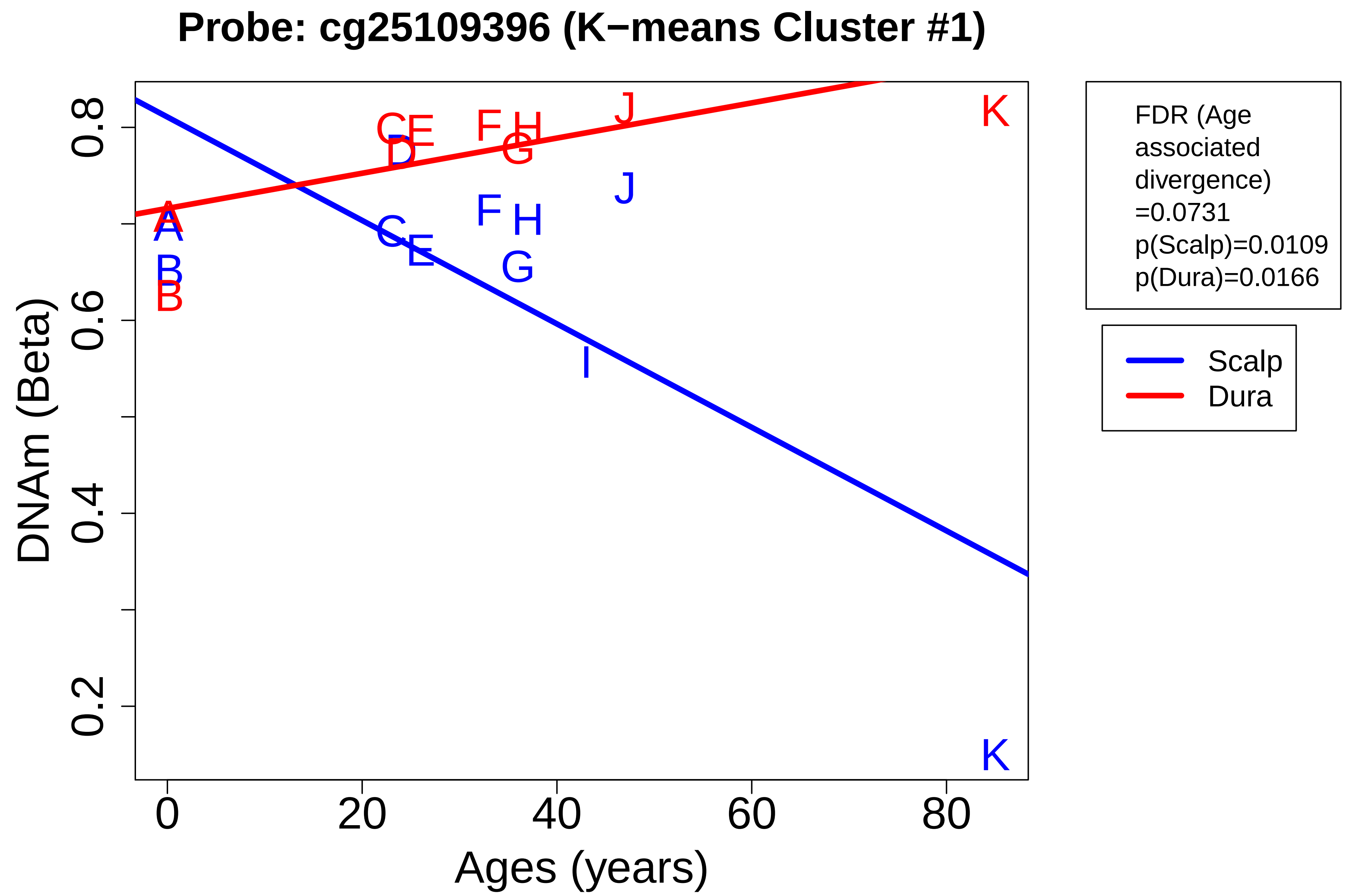

Probe: cg25109396 (K−means Cluster #1)
DNAm (Beta)
0.2
0.4
0.6
0.8
0
20
40
60
80
Ages (years)
FDR (Age associated divergence) =0.0731
p(Scalp)=0.0109
p(Dura)=0.0166
Scalp
Dura

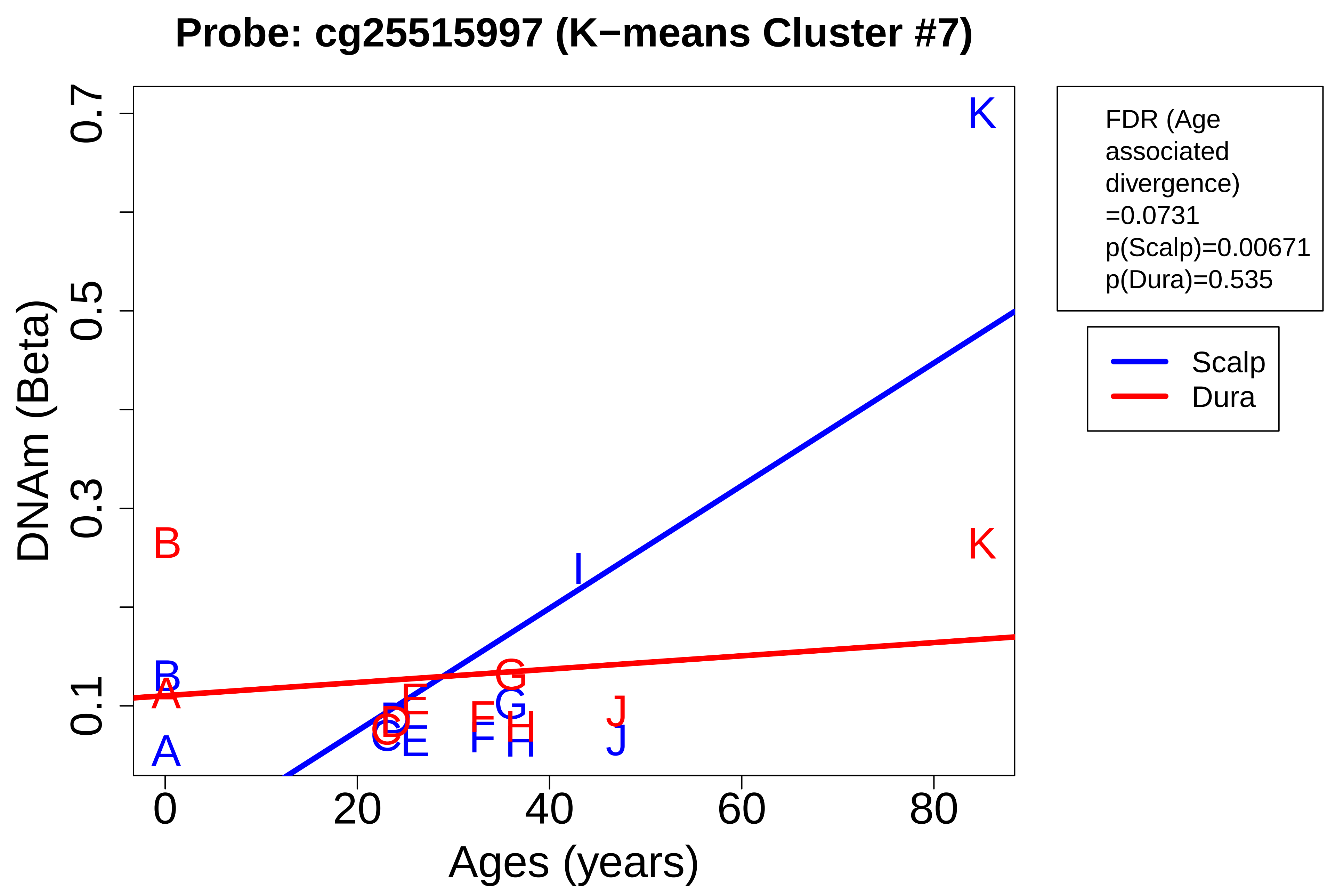

Probe: cg25515997 (K−means Cluster #7)
DNAm (Beta)
0.1
0.3
0.5
0.7
Ages (years)
0
20
40
60
80
A
B
C
D
E
F
G
H
I
J
K
FDR (Age associated divergence) =0.0731
p(Scalp)=0.00671
p(Dura)=0.535
Scalp
Dura

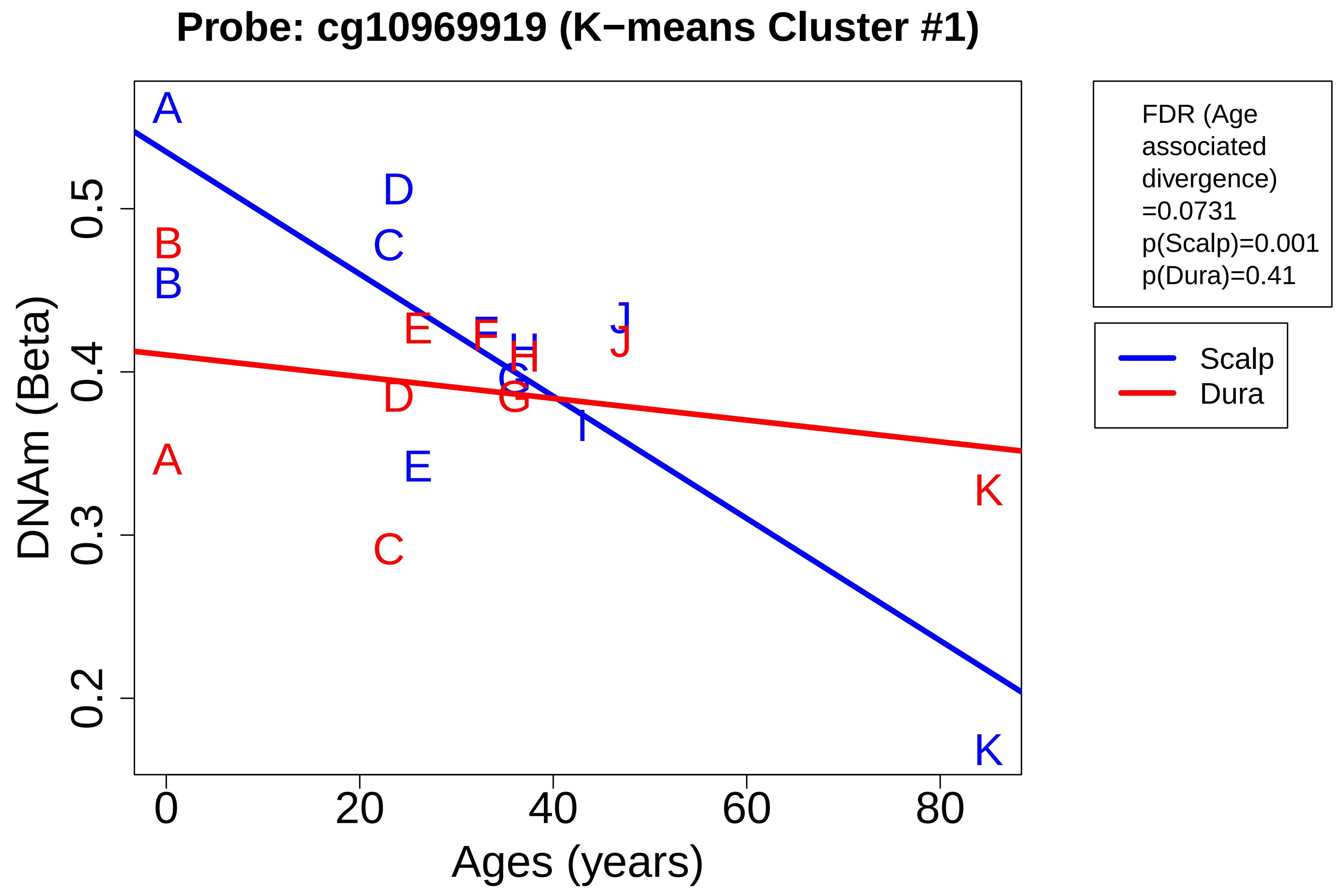
Probe: cg10969919 (K−means Cluster #1)
DNAm (Beta)
Ages (years)
0.2
0.3
0.4
0.5
0
20
40
60
80
FDR (Age associated divergence) =0.0731
p(Scalp)=0.001
p(Dura)=0.41
Scalp
Dura
A
B
C
D
E
F
G
H
I
J
K

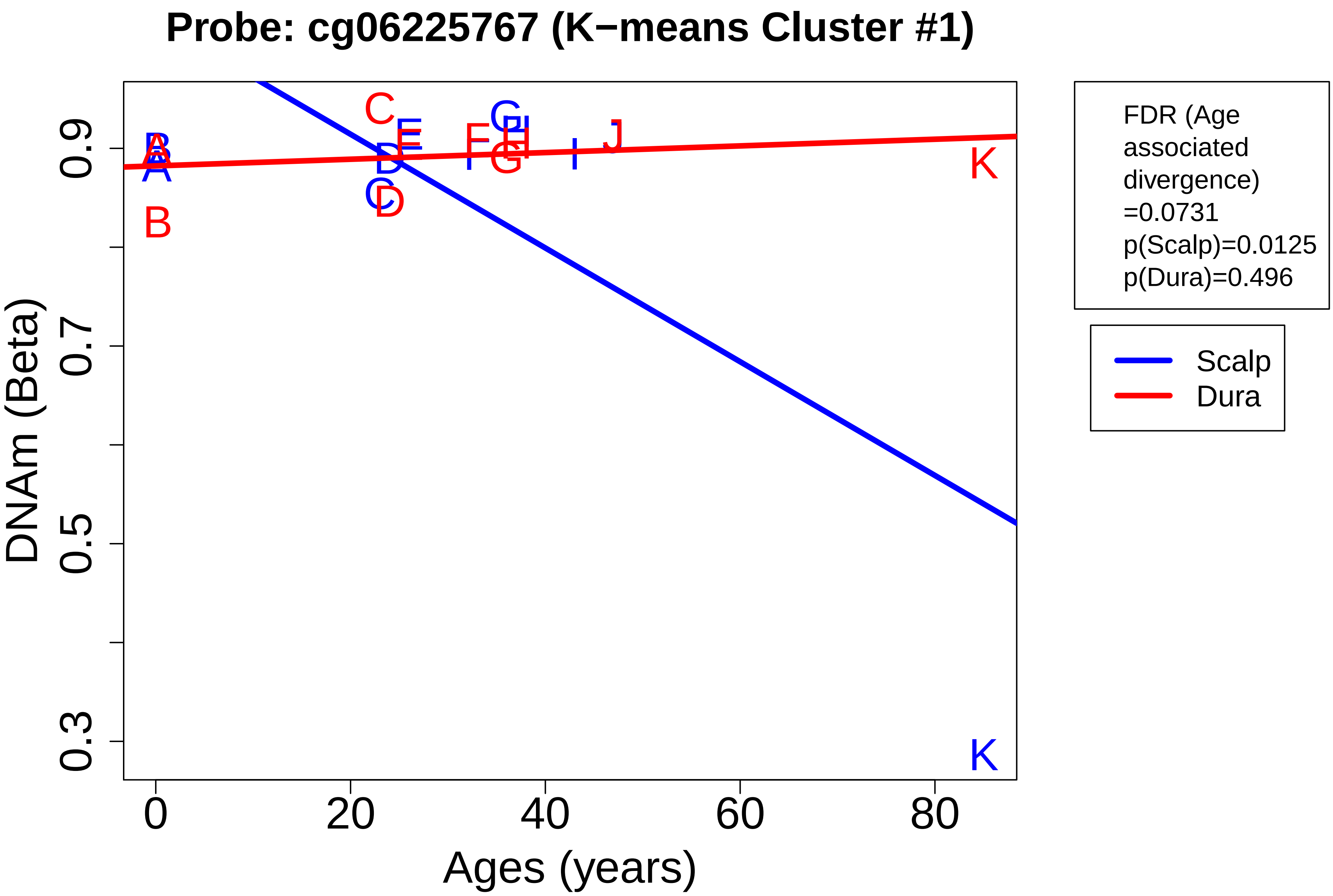

Probe: cg06225767 (K−means Cluster #1)
DNAm (Beta)
0.3
0.5
0.7
0.9
Ages (years)
0
20
40
60
80
FDR (Age associated divergence) =0.0731
p(Scalp)=0.0125
p(Dura)=0.496
Scalp
Dura

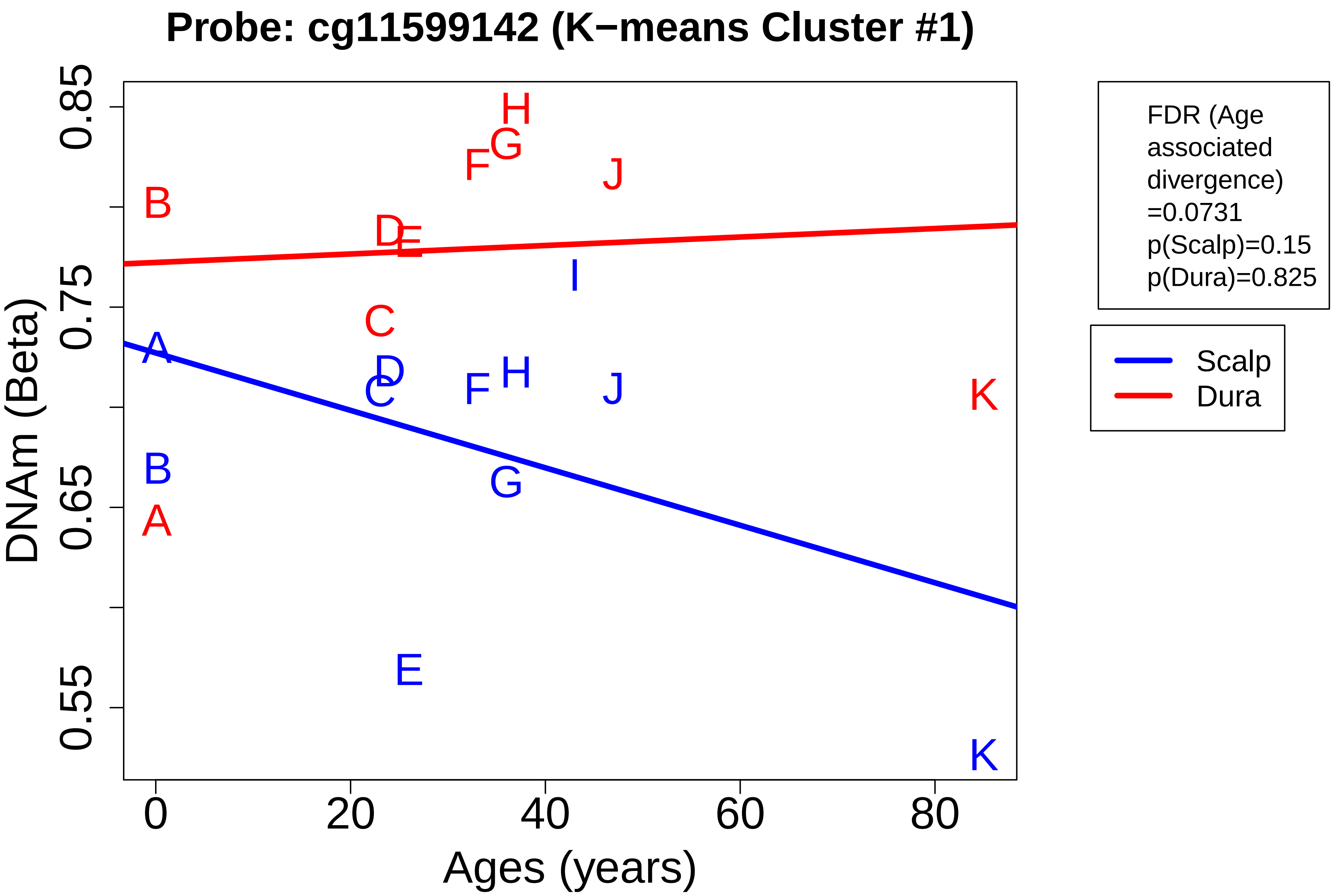
Probe: cg11599142 (K−means Cluster #1)
DNAm (Beta)
0.55
0.65
0.75
0.85
0
20
40
60
80
Ages (years)
A
B
C
D
E
F
G
H
I
J
K
FDR (Age associated divergence) =0.0731
p(Scalp)=0.15
p(Dura)=0.825
Scalp
Dura

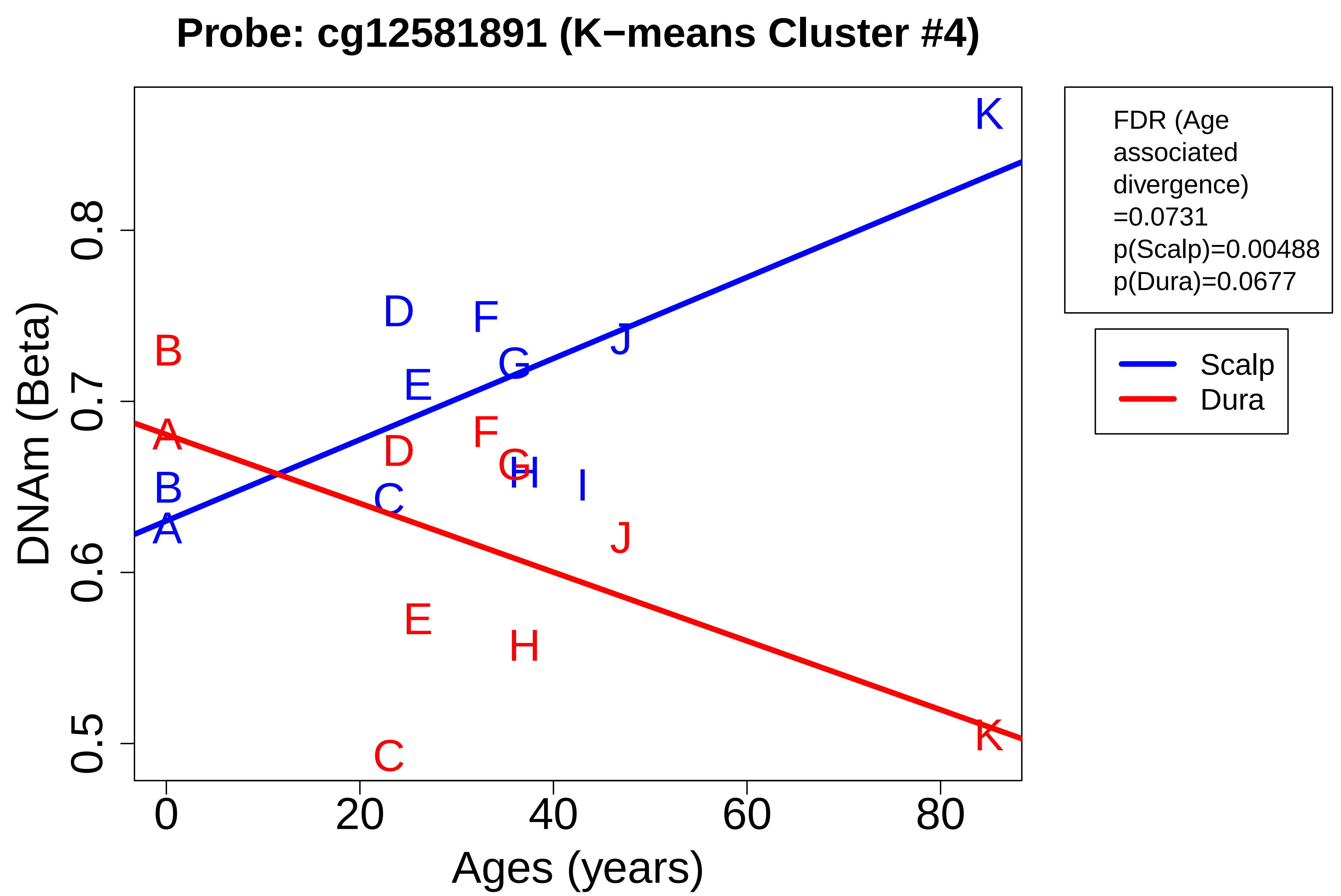

Probe: cg12581891 (K−means Cluster #4)
DNAm (Beta)
0.5
0.6
0.7
0.8
0
20
40
60
80
Ages (years)
FDR (Age associated divergence) =0.0731
p(Scalp)=0.00488
p(Dura)=0.0677
Scalp
Dura

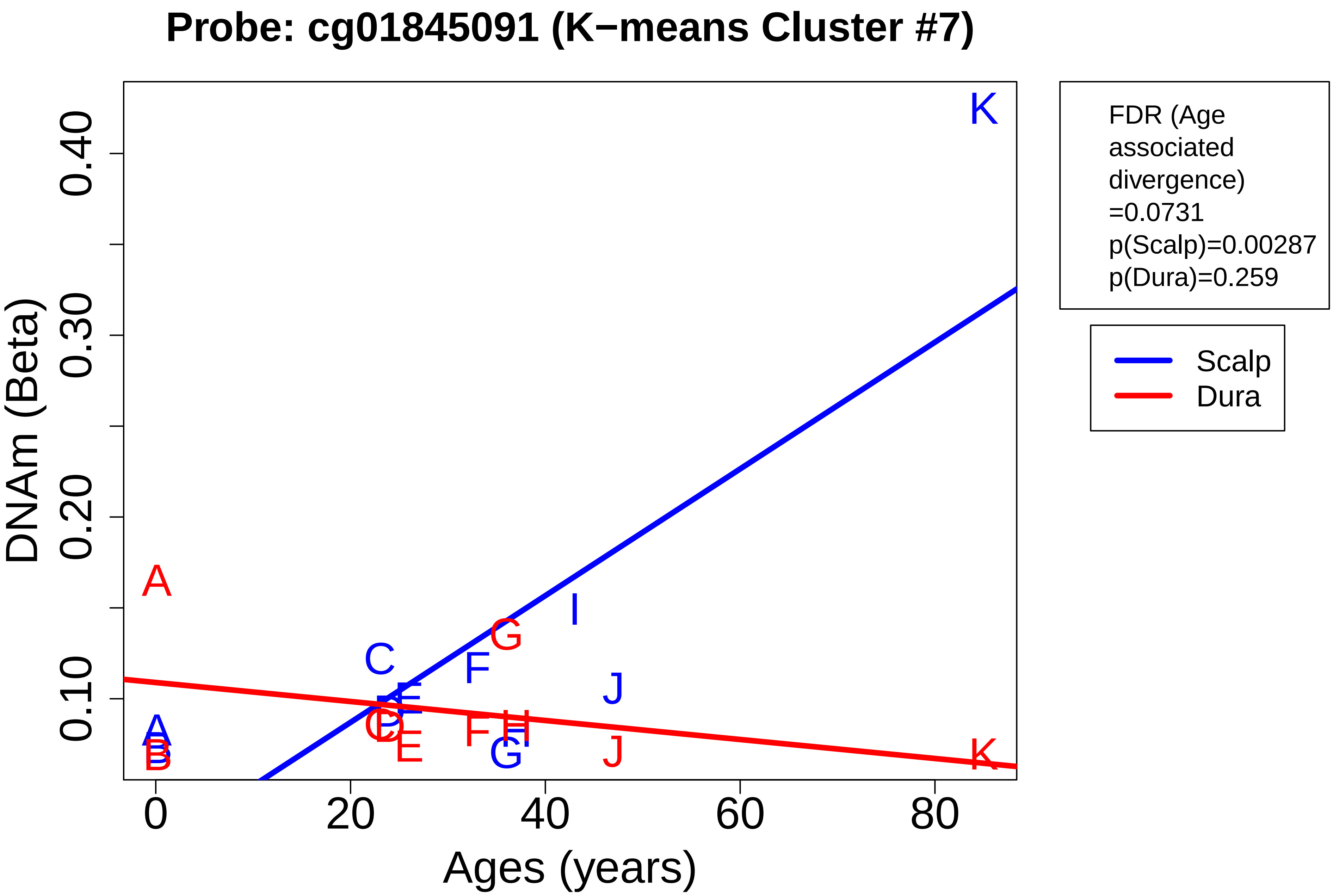

Probe: cg01845091 (K−means Cluster #7)
DNAm (Beta)
0.10
0.20
0.30
0.40
Ages (years)
0
20
40
60
80
FDR (Age associated divergence) =0.0731
p(Scalp)=0.00287
p(Dura)=0.259
Scalp
Dura
A
B
C
D
E
F
G
H
I
J
K

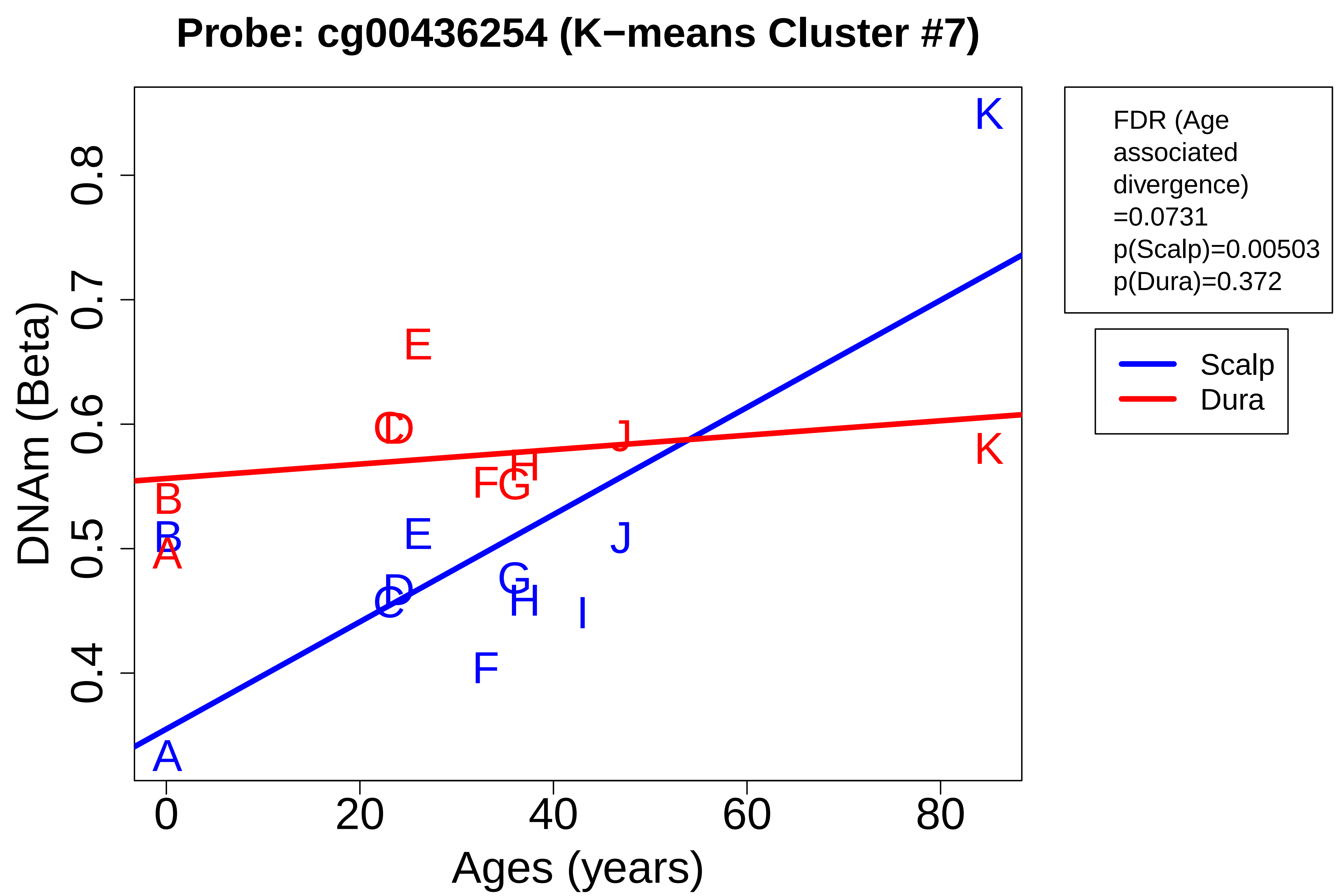
Probe: cg00436254 (K−means Cluster #7)
FDR (Age associated divergence) =0.0731
p(Scalp)=0.00503
p(Dura)=0.372
Scalp
Dura
DNAm (Beta)
Ages (years)
0.4
0.5
0.6
0.7
0.8
0
20
40
60
80
A
B
C
D
E
F
G
H
I
J
K

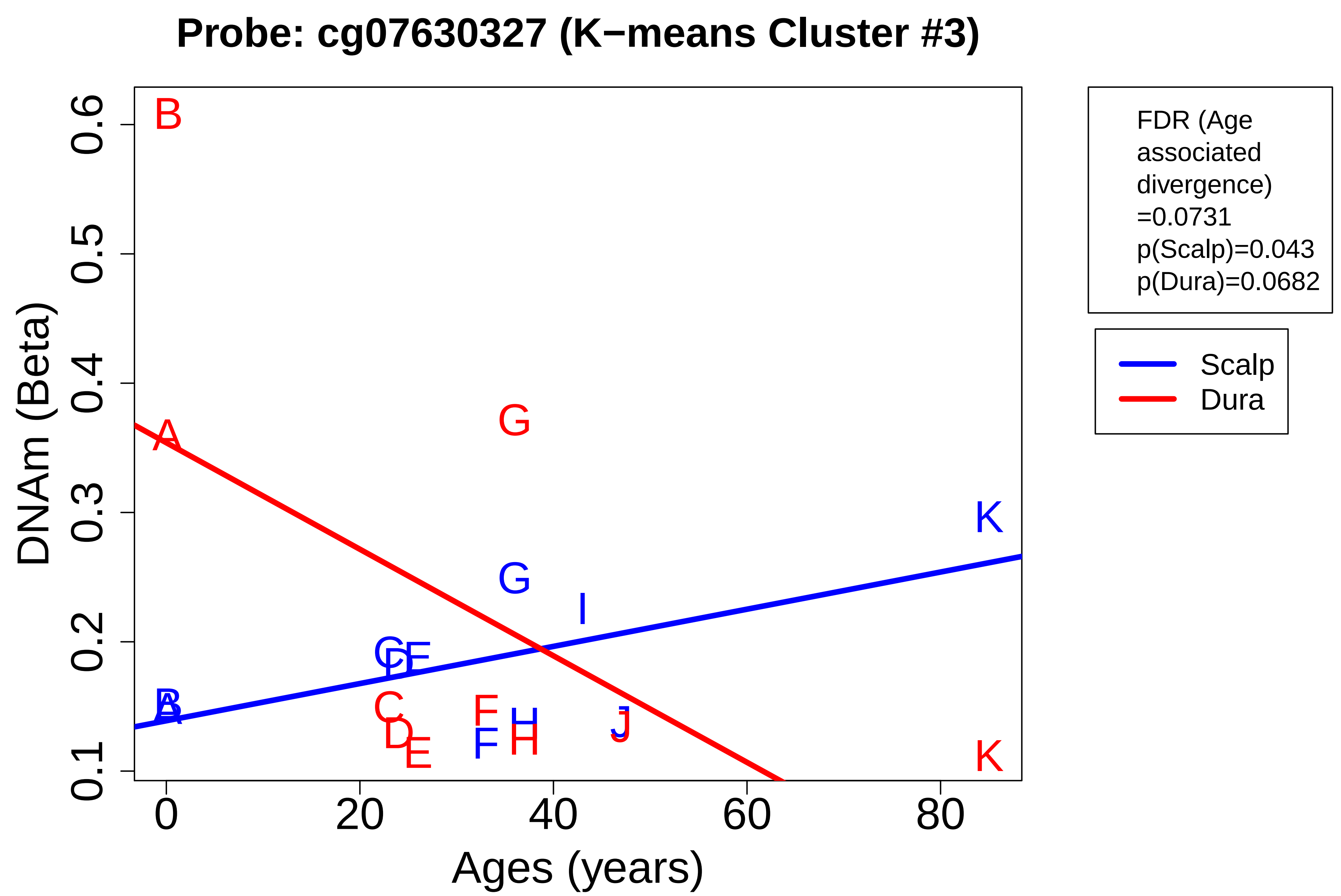
Probe: cg07630327 (K−means Cluster #3)
DNAm (Beta)
Ages (years)
0.1
0.2
0.3
0.4
0.5
0.6
0
20
40
60
80
FDR (Age associated divergence) =0.0731
p(Scalp)=0.043
p(Dura)=0.0682
Scalp
Dura

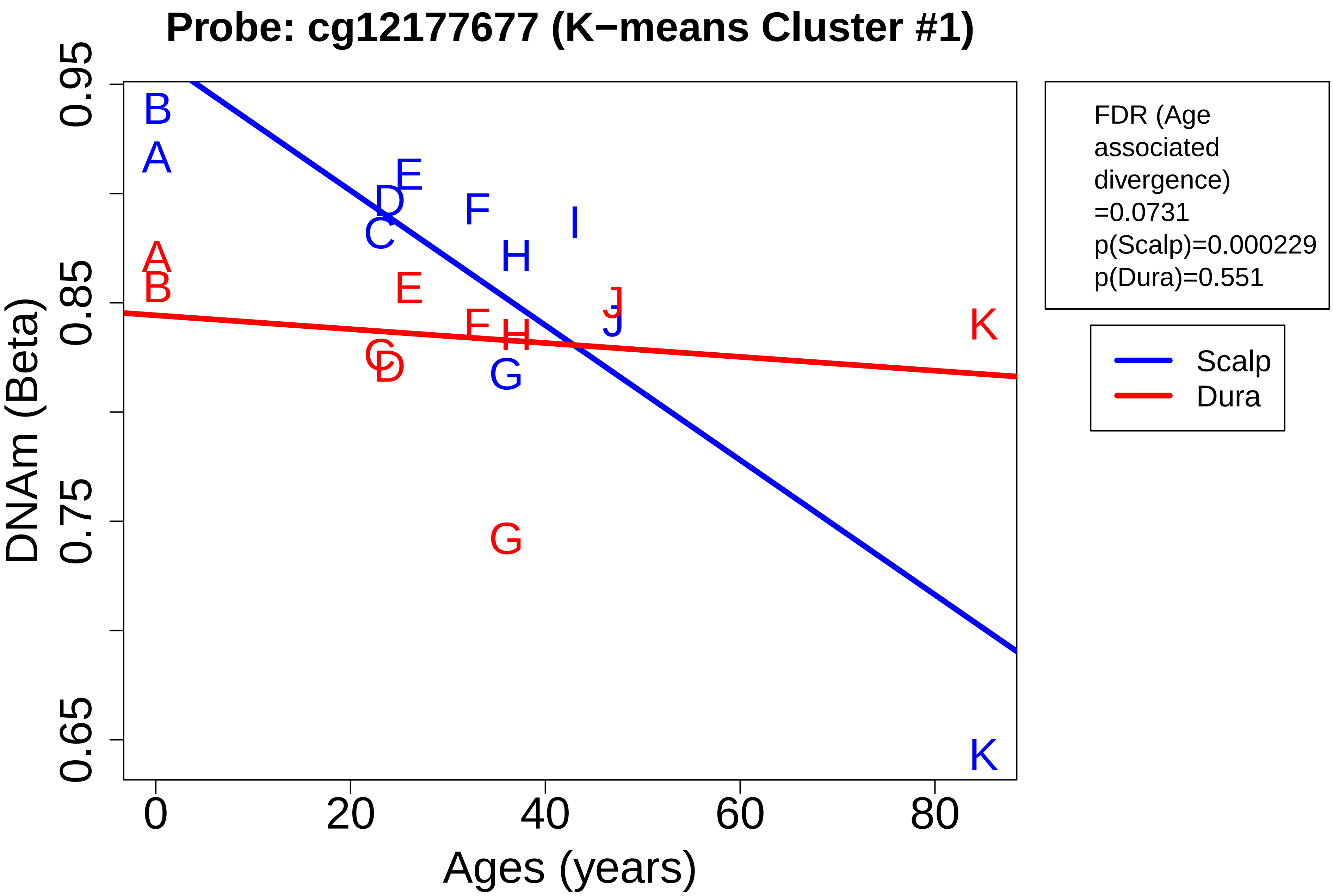

Probe: cg12177677 (K−means Cluster #1)
DNAm (Beta)
0.95
0.85
0.75
0.65
Ages (years)
0
20
40
60
80
FDR (Age associated divergence) =0.0731
p(Scalp)=0.000229
p(Dura)=0.551
Scalp
Dura

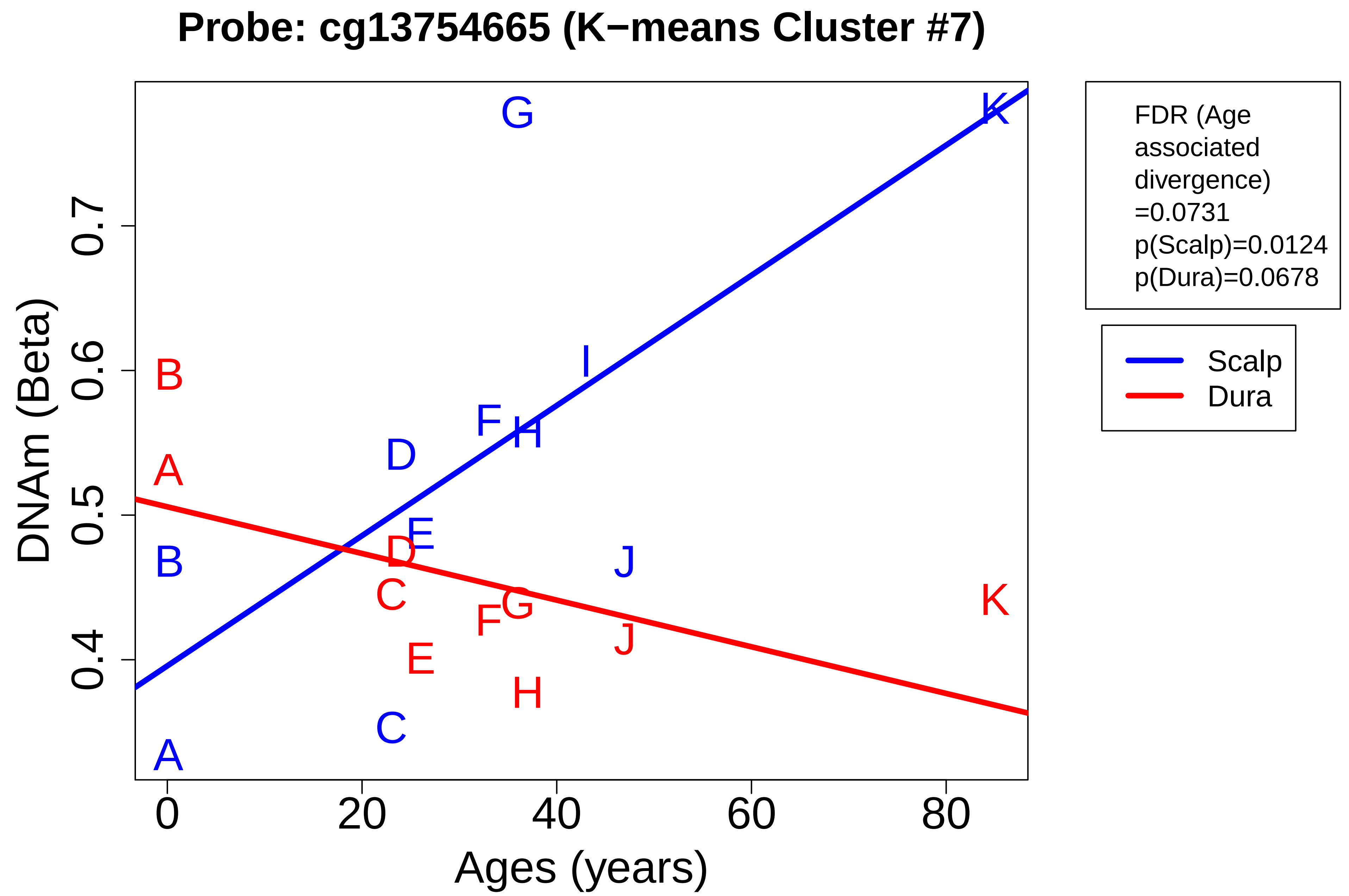
Probe: cg13754665 (K−means Cluster #7)
DNAm (Beta)
0.4
0.5
0.6
0.7
Ages (years)
0
20
40
60
80
FDR (Age associated divergence) =0.0731
p(Scalp)=0.0124
p(Dura)=0.0678
Scalp
Dura
A
B
C
D
E
F
G
H
I
J
K

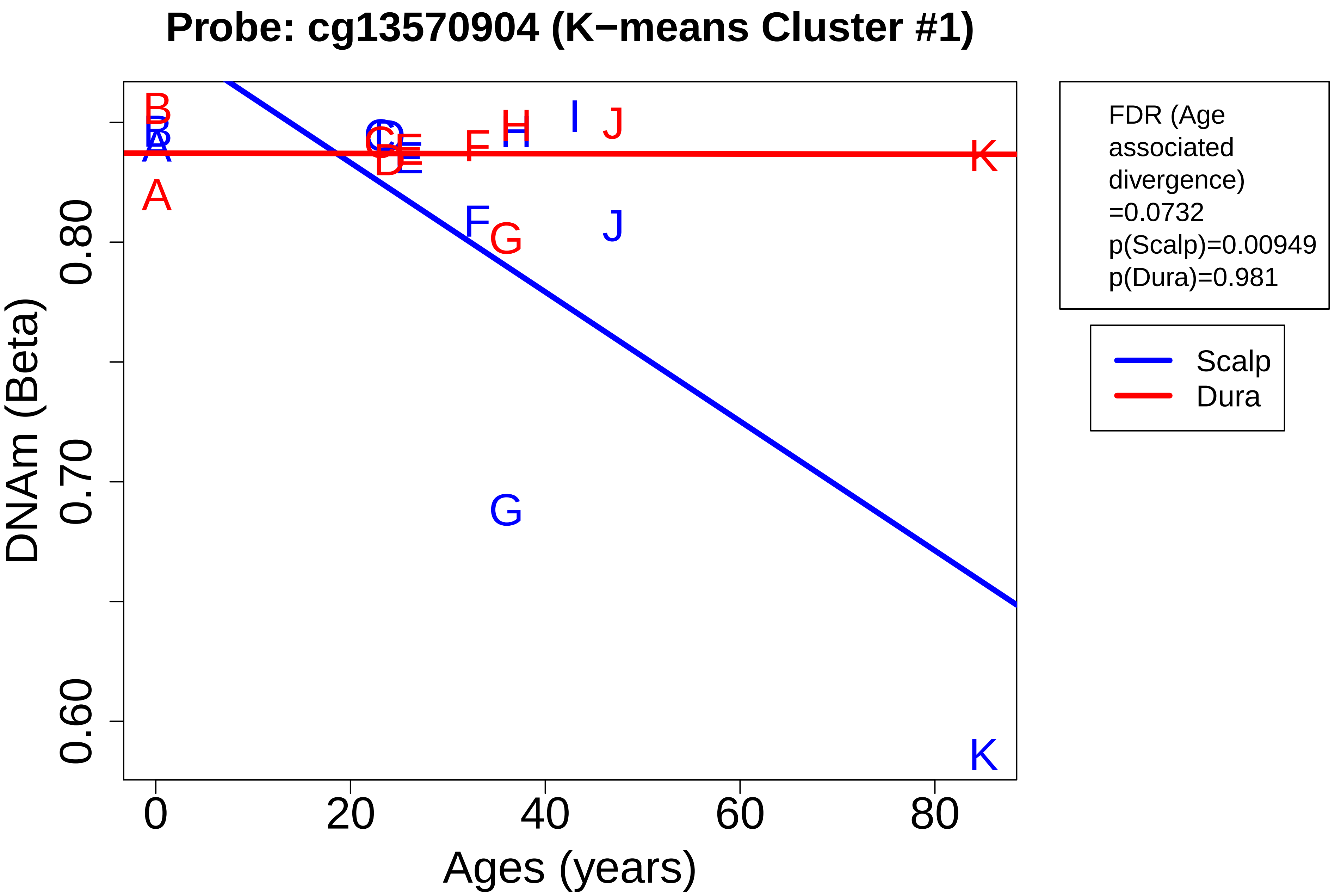

Probe: cg13570904 (K−means Cluster #1)
DNAm (Beta)
0.60
0.70
0.80
0
20
40
60
80
Ages (years)
FDR (Age associated divergence) =0.0732
p(Scalp)=0.00949
p(Dura)=0.981
Scalp
Dura

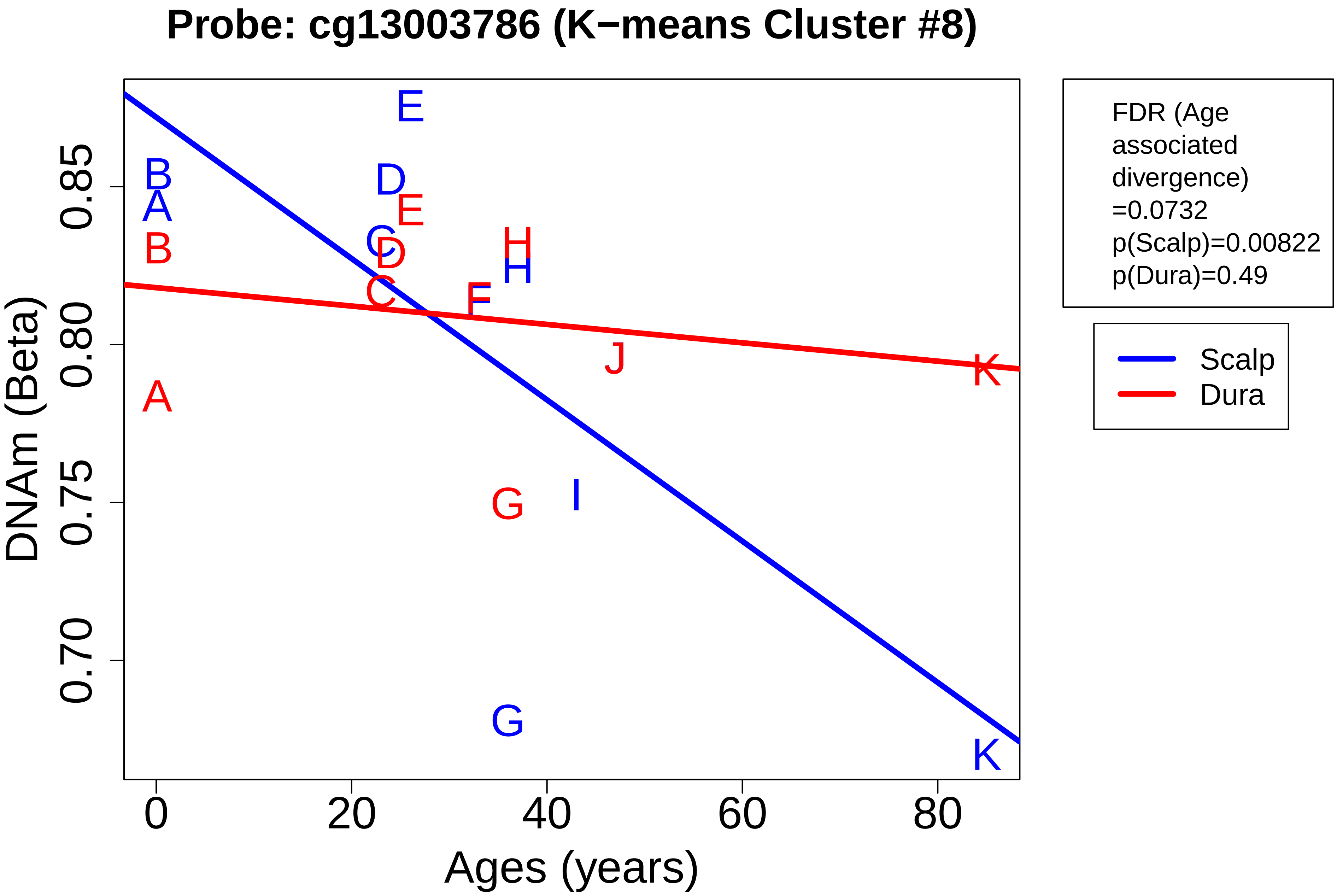

Probe: cg13003786 (K−means Cluster #8)
DNAm (Beta)
0.70
0.75
0.80
0.85
0
20
40
60
80
Ages (years)
A
B
C
D
E
F
G
H
I
J
K
FDR (Age associated divergence) =0.0732
p(Scalp)=0.00822
p(Dura)=0.49
Scalp
Dura

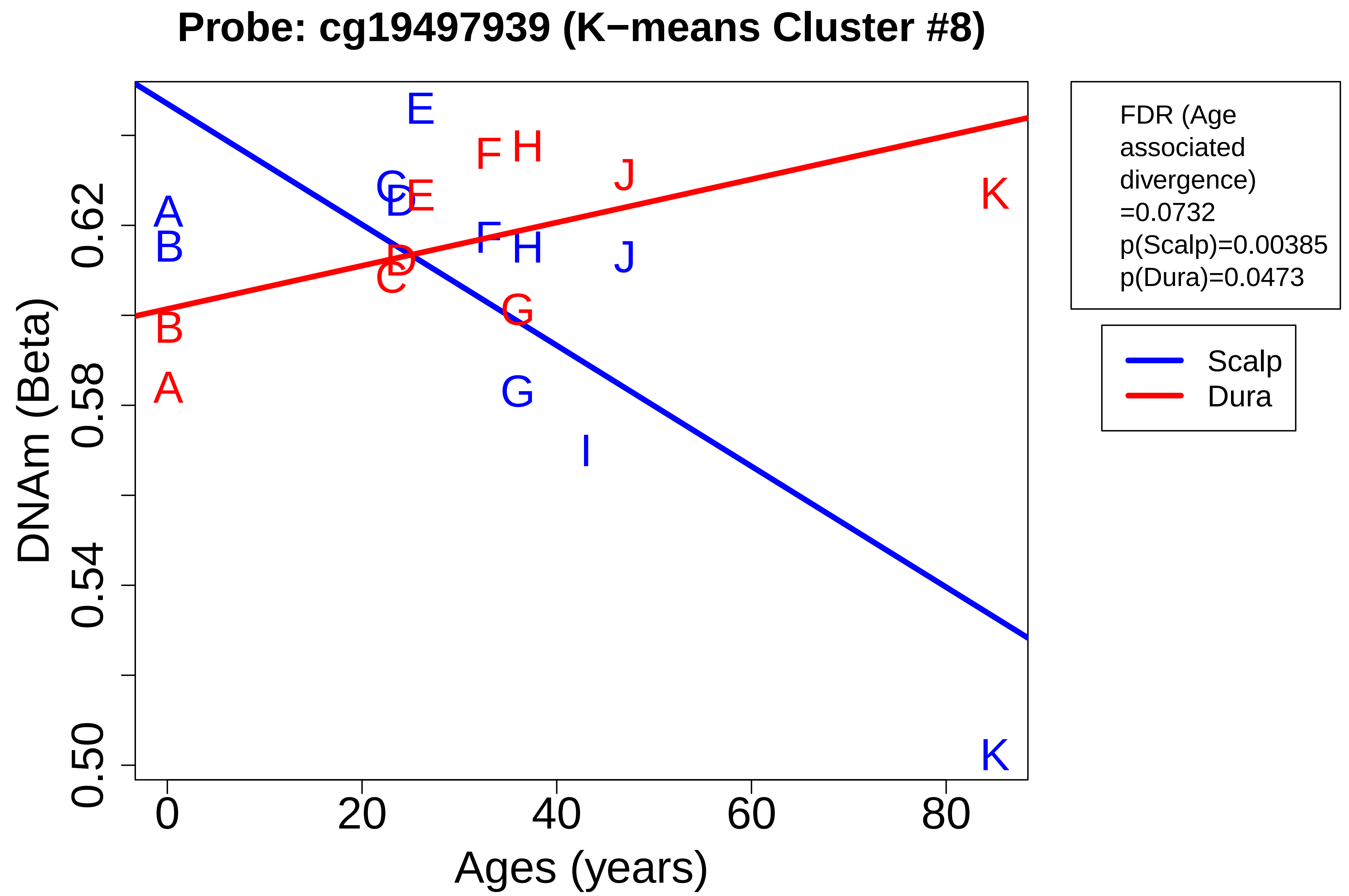

Probe: cg19497939 (K−means Cluster #8)
DNAm (Beta)
0.50
0.54
0.58
0.62
Ages (years)
0
20
40
60
80
FDR (Age associated divergence) =0.0732
p(Scalp)=0.00385
p(Dura)=0.0473
Scalp
Dura

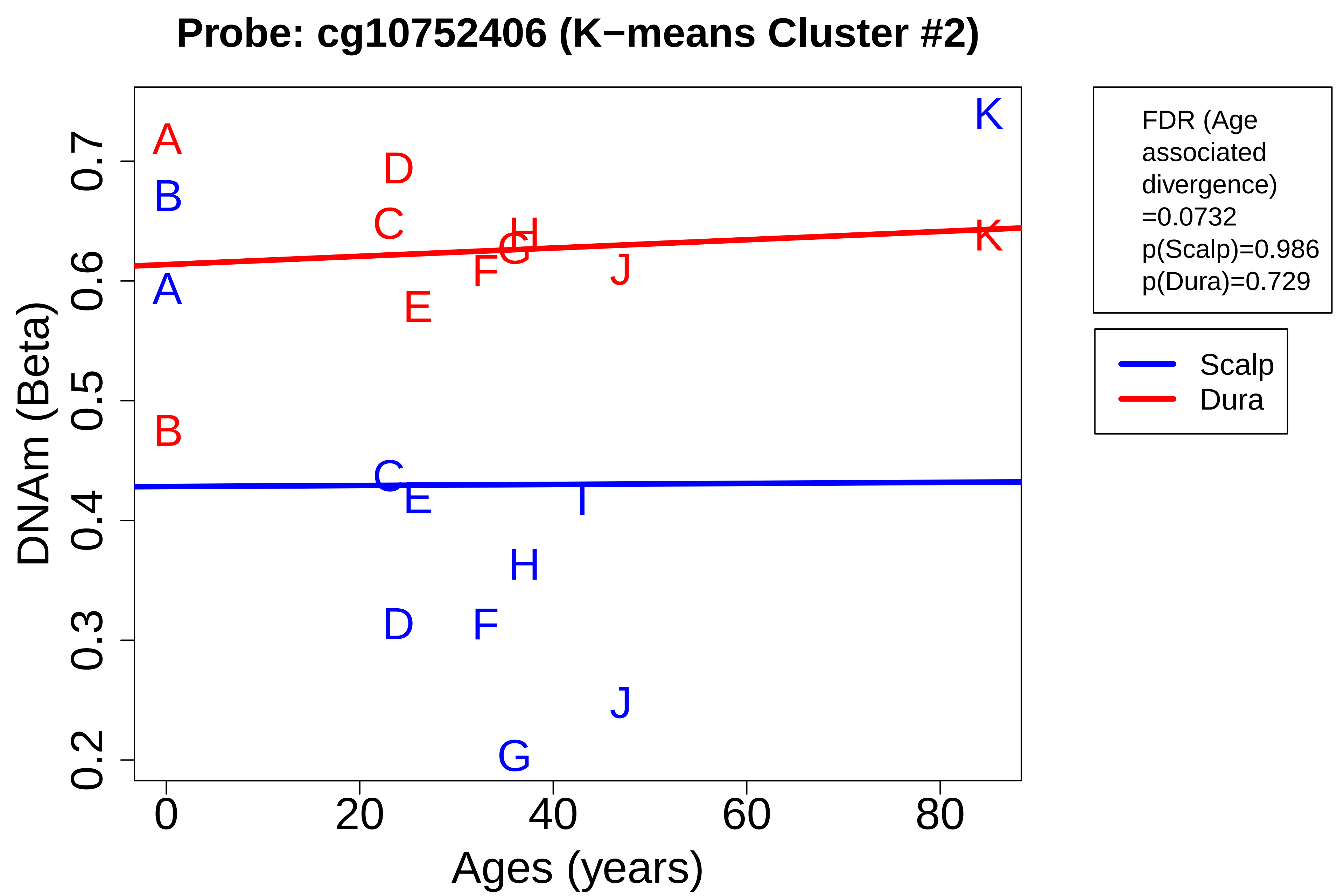

Probe: cg10752406 (K−means Cluster #2)
DNAm (Beta)
0.2
0.3
0.4
0.5
0.6
0.7
0
20
40
60
80
Ages (years)
A
B
C
D
E
F
G
H
I
J
K
FDR (Age associated divergence) =0.0732
p(Scalp)=0.986
p(Dura)=0.729
Scalp
Dura

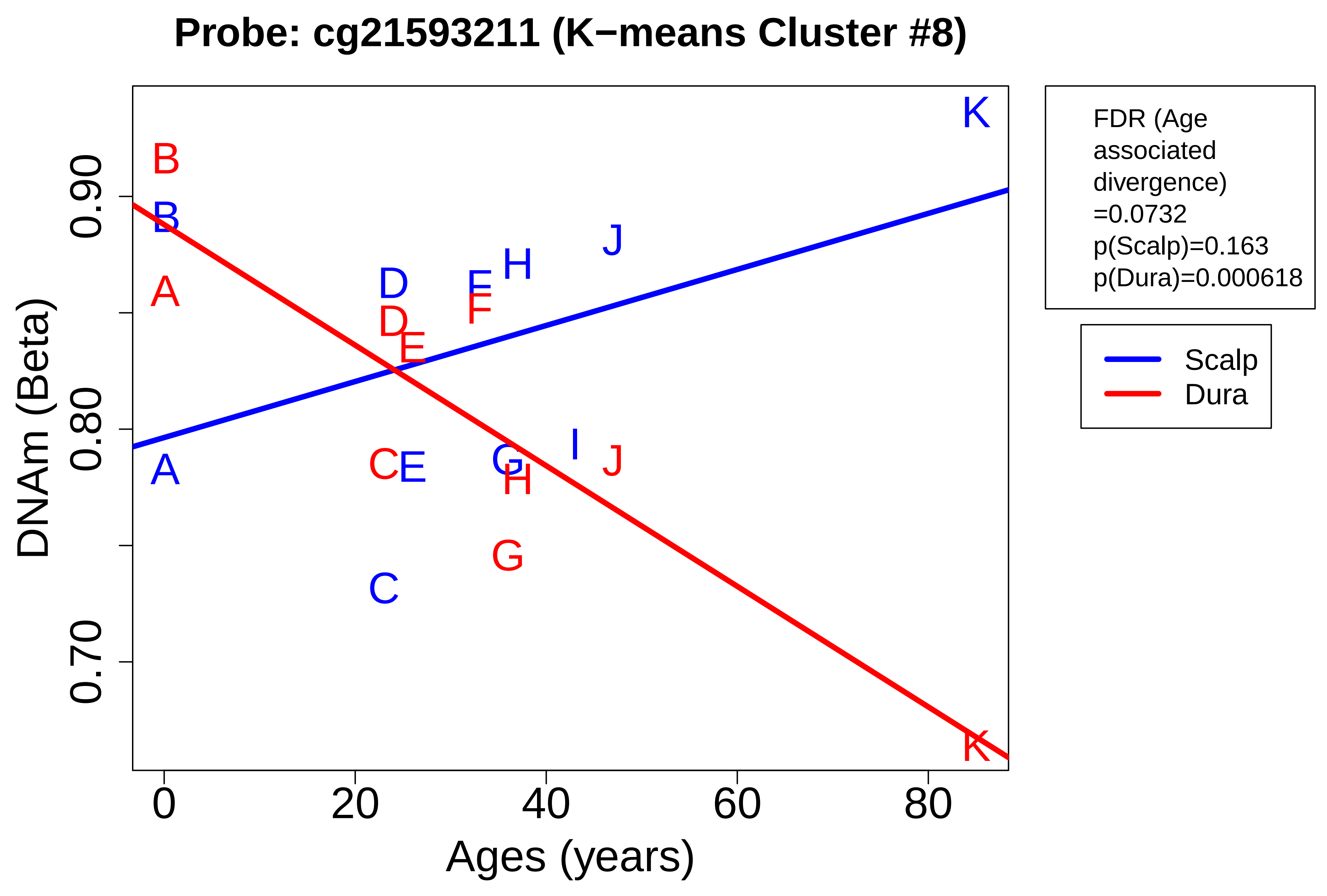

Probe: cg21593211 (K−means Cluster #8)
DNAm (Beta)
0.70
0.80
0.90
Ages (years)
0
20
40
60
80
A
B
C
D
E
F
G
H
I
J
K
FDR (Age associated divergence) =0.0732
p(Scalp)=0.163
p(Dura)=0.000618
Scalp
Dura

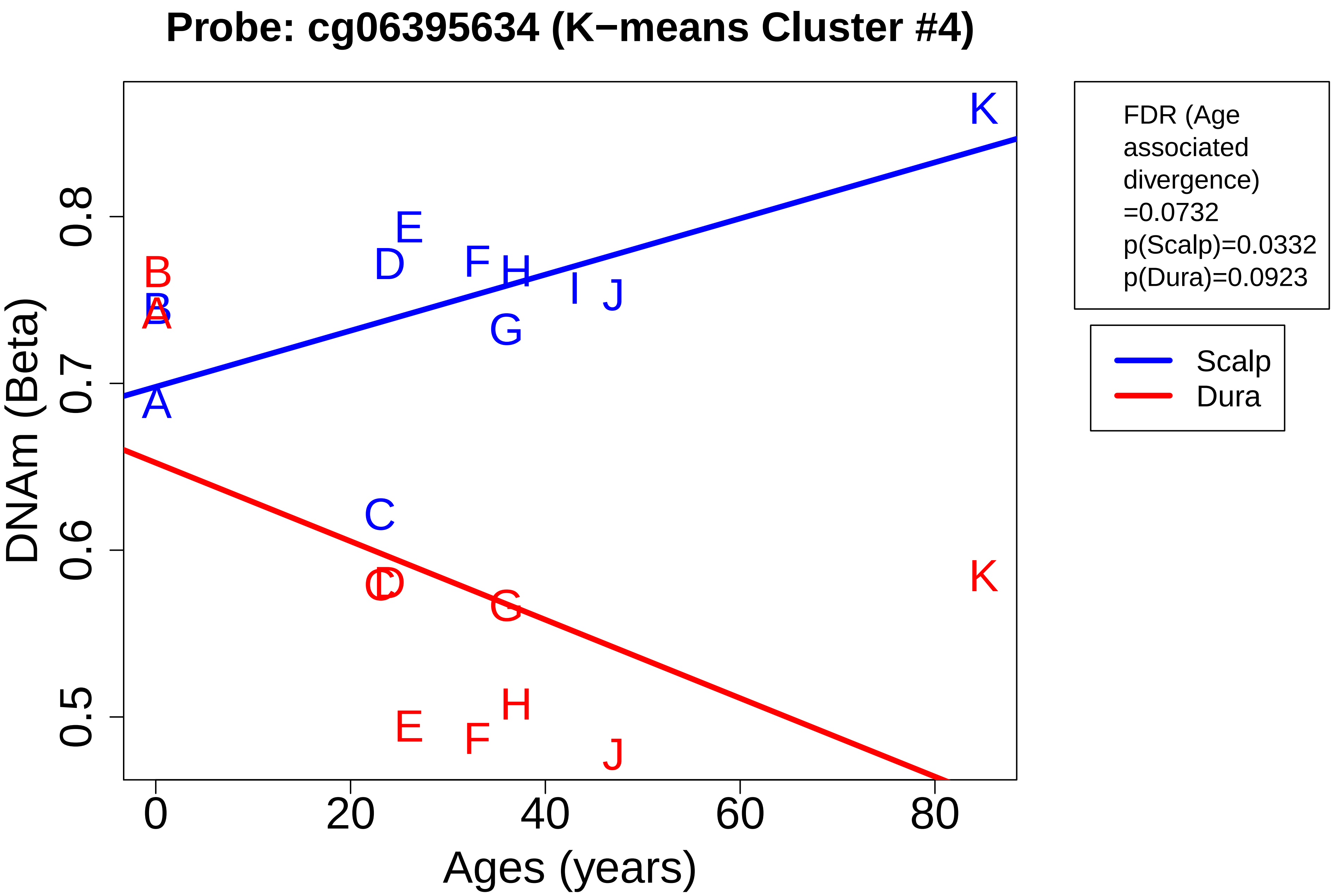

Probe: cg06395634 (K−means Cluster #4)
DNAm (Beta)
0.5
0.6
0.7
0.8
Ages (years)
0
20
40
60
80
FDR (Age associated divergence) =0.0732
p(Scalp)=0.0332
p(Dura)=0.0923
Scalp
Dura

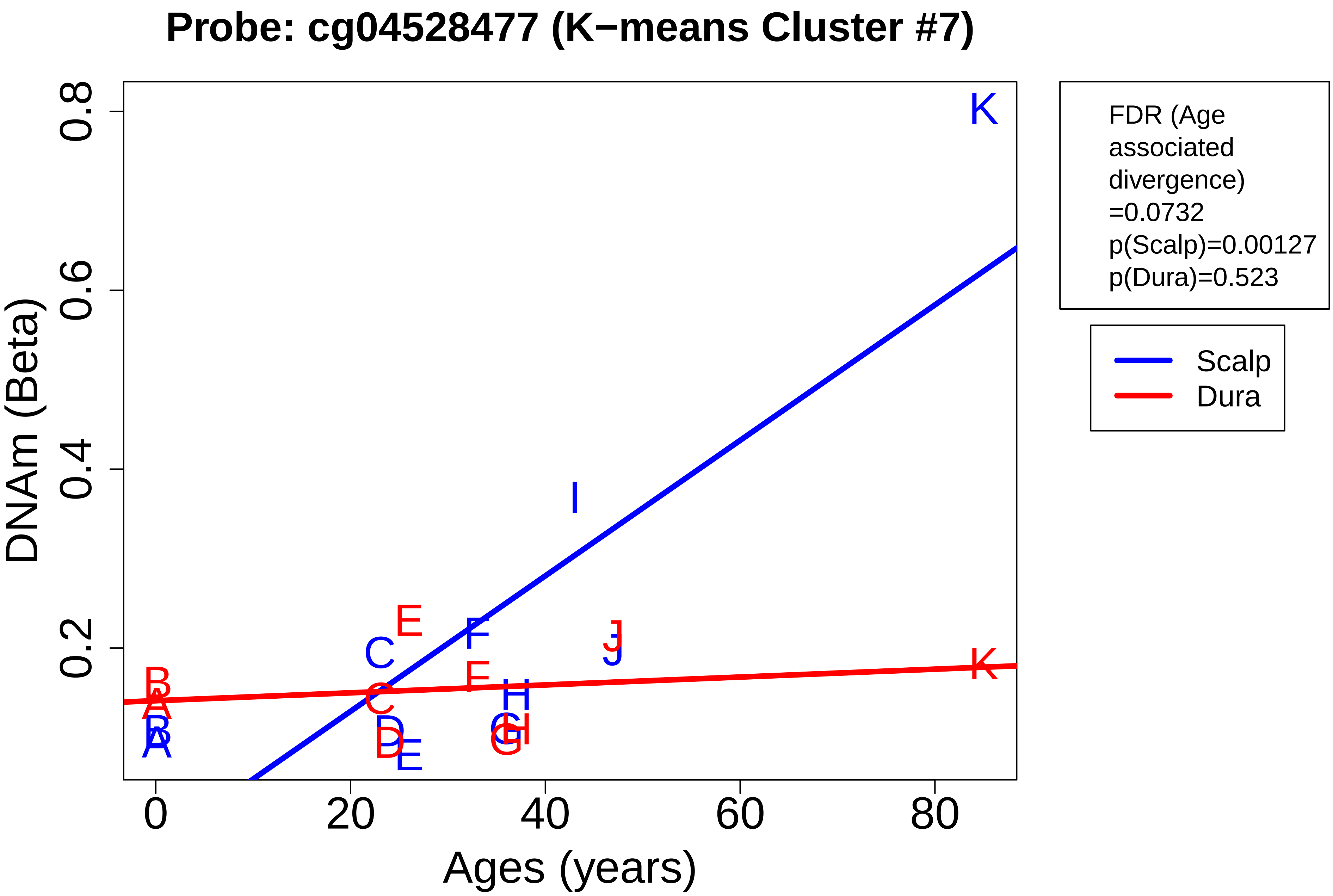

Probe: cg04528477 (K−means Cluster #7)
DNAm (Beta)
Ages (years)
0.2
0.4
0.6
0.8
0
20
40
60
80
FDR (Age associated divergence) =0.0732
p(Scalp)=0.00127
p(Dura)=0.523
Scalp
Dura
A
B
C
D
E
F
G
H
I
J
K

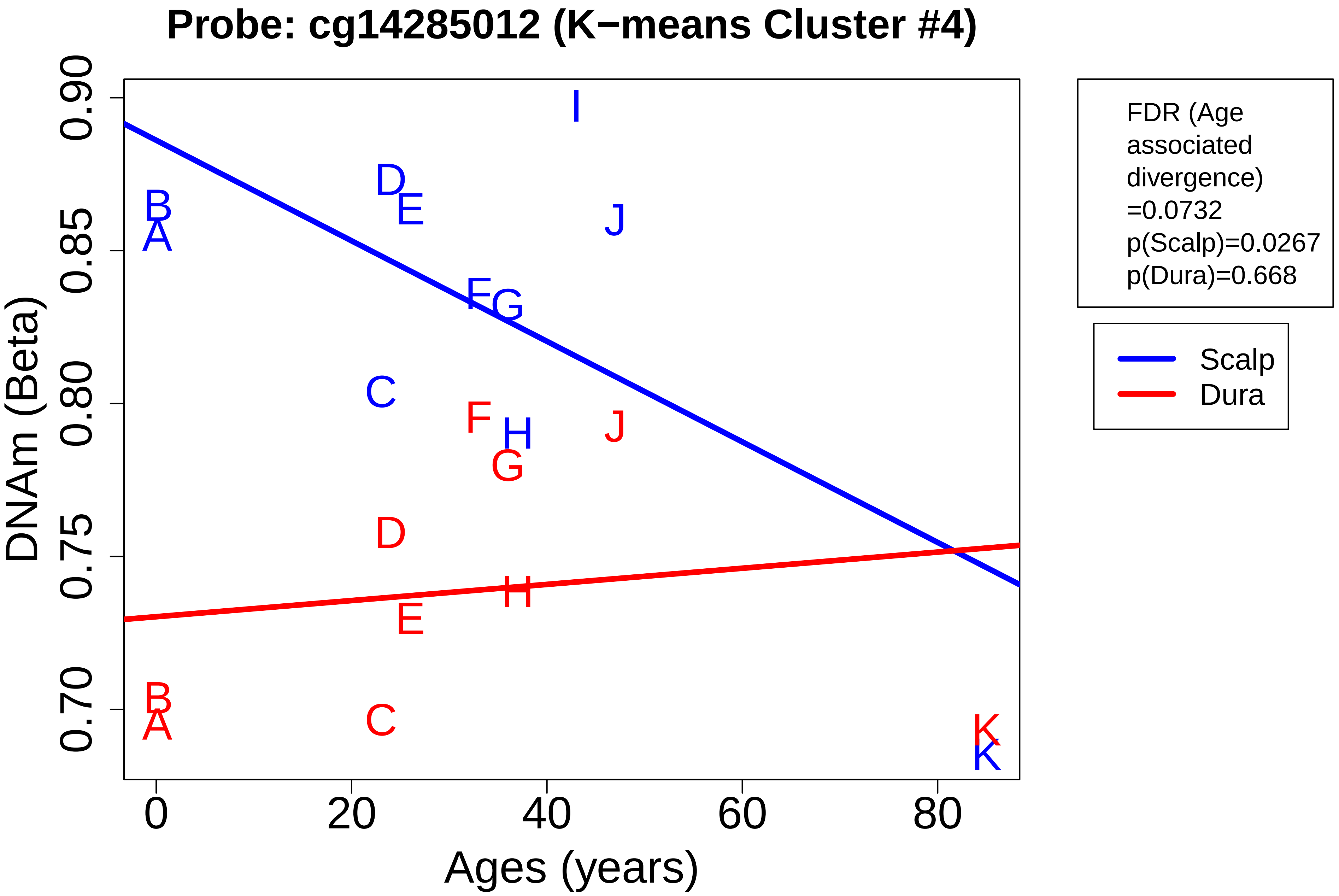

Probe: cg14285012 (K−means Cluster #4)
DNAm (Beta)
0.70
0.75
0.80
0.85
0.90
Ages (years)
0
20
40
60
80
FDR (Age associated divergence) =0.0732
p(Scalp)=0.0267
p(Dura)=0.668
Scalp
Dura

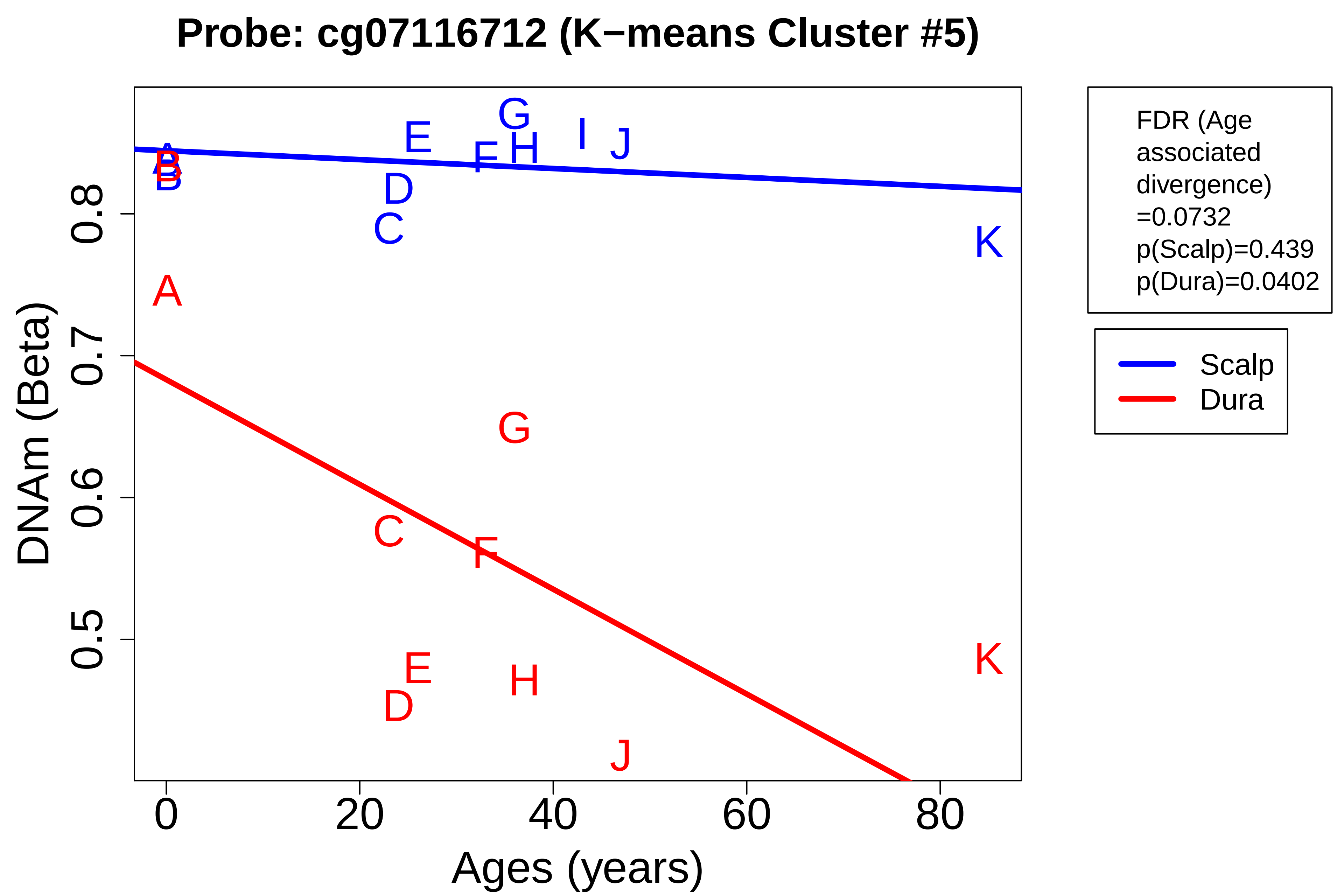

Probe: cg07116712 (K−means Cluster #5)
DNAm (Beta)
Ages (years)
0.5
0.6
0.7
0.8
0
20
40
60
80
FDR (Age associated divergence) =0.0732
p(Scalp)=0.439
p(Dura)=0.0402
Scalp
Dura

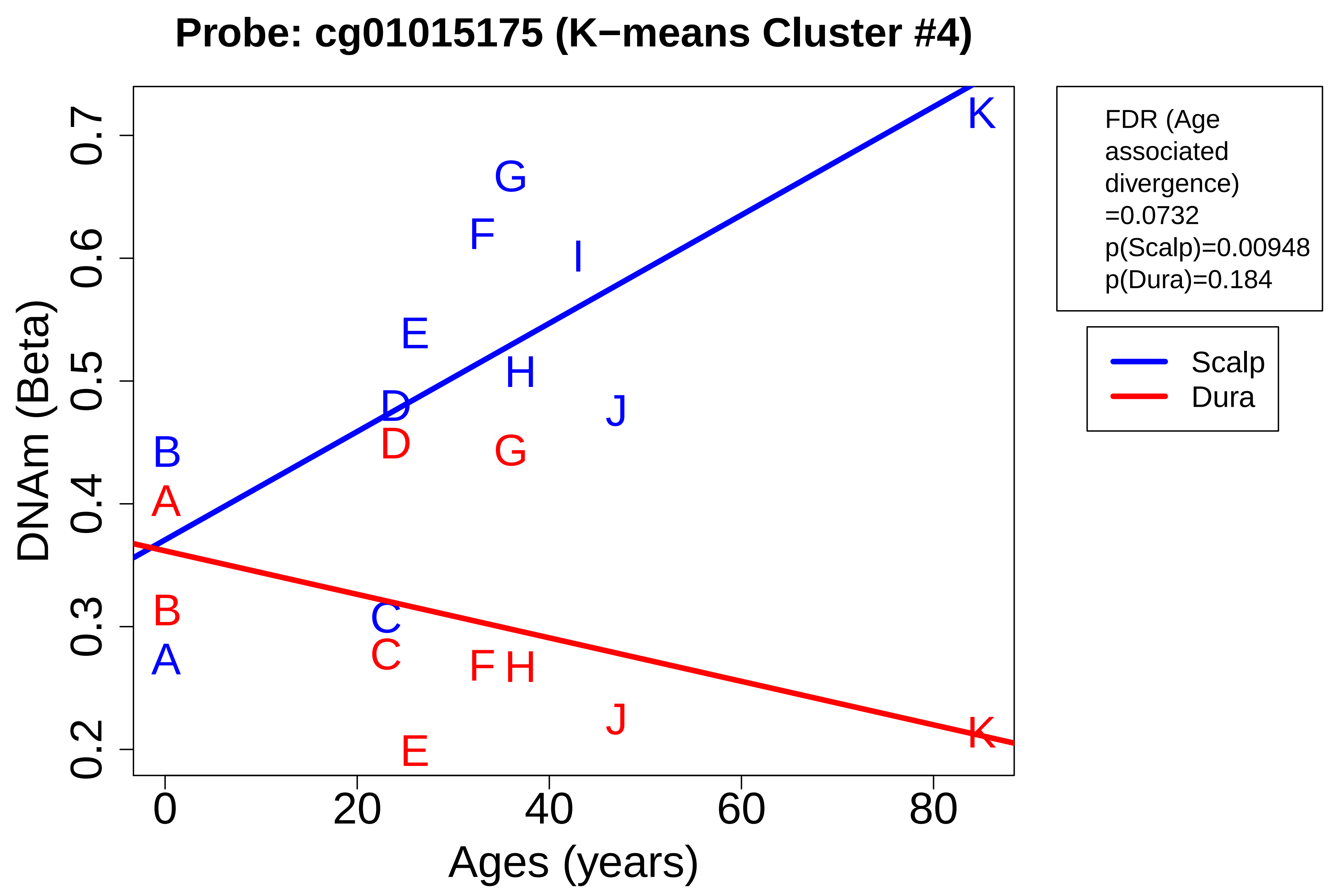

Probe: cg01015175 (K−means Cluster #4)
FDR (Age associated divergence) =0.0732
p(Scalp)=0.00948
p(Dura)=0.184
Scalp
Dura
DNAm (Beta)
0.2
0.3
0.4
0.5
0.6
0.7
Ages (years)
0
20
40
60
80
A
B
C
D
E
F
G
H
I
J
K

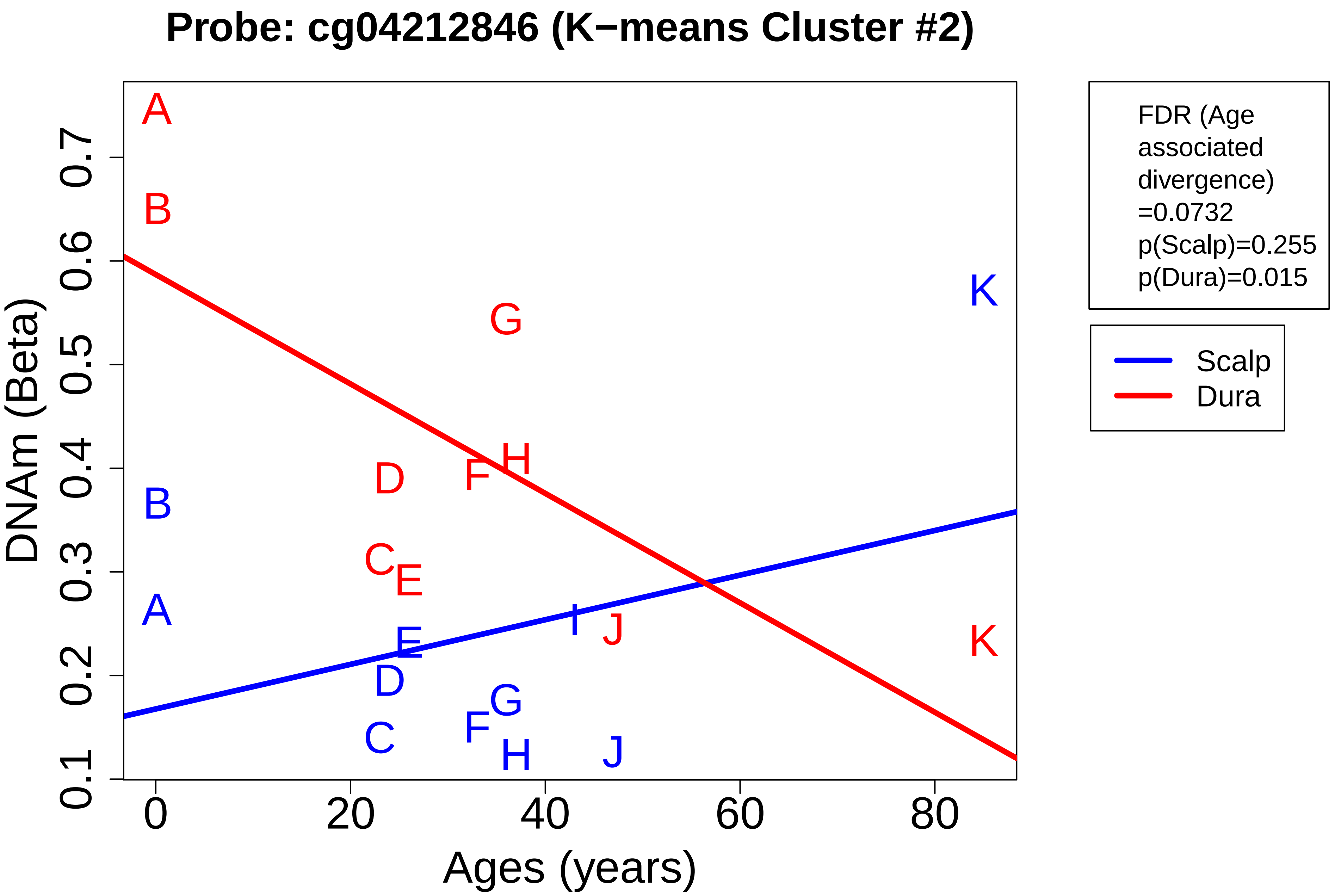
Probe: cg04212846 (K−means Cluster #2)
DNAm (Beta)
Ages (years)
0.1
0.2
0.3
0.4
0.5
0.6
0.7
0
20
40
60
80
FDR (Age associated divergence) =0.0732
p(Scalp)=0.255
p(Dura)=0.015
Scalp
Dura
A
B
C
D
E
F
G
H
I
J
K

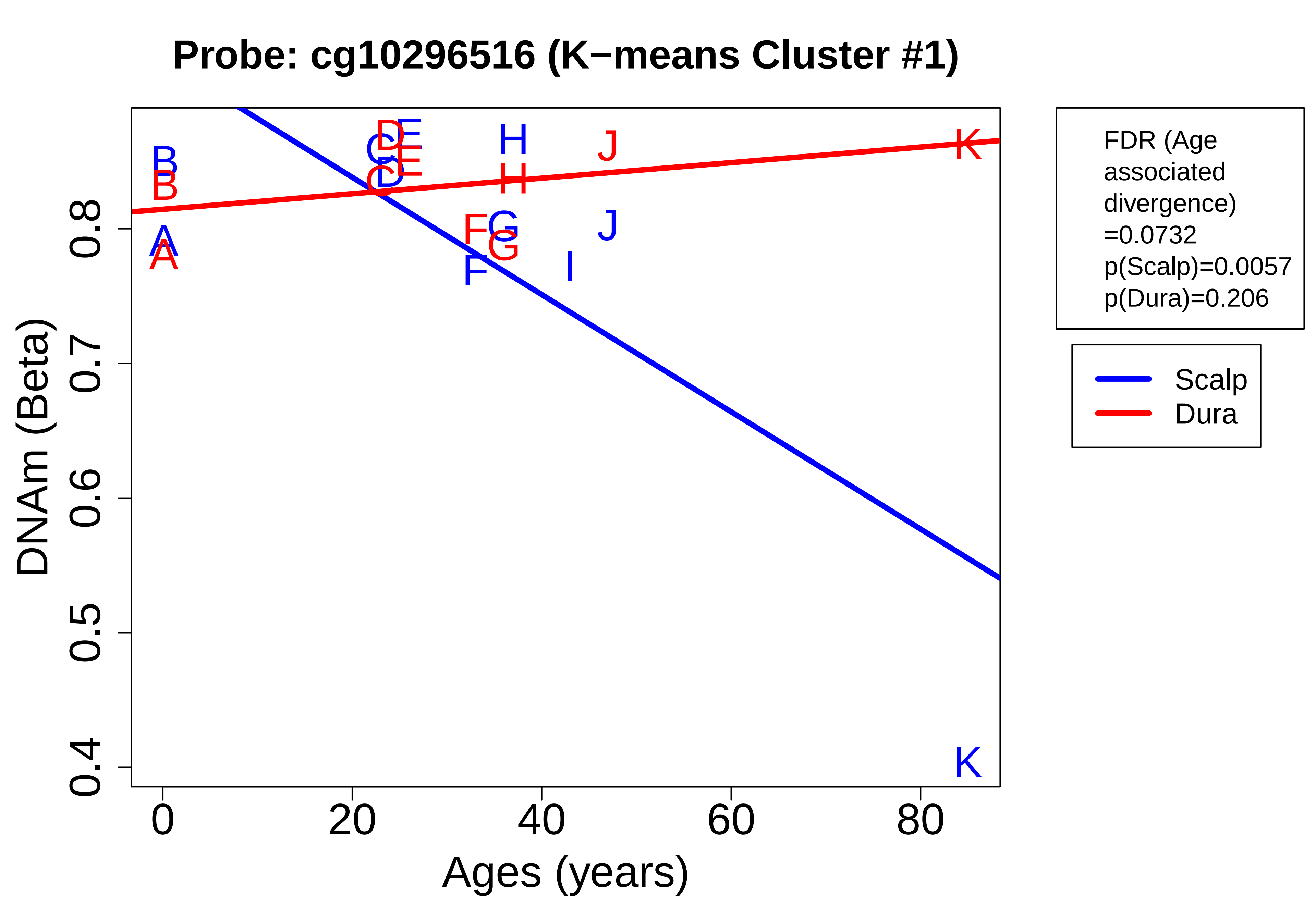

Probe: cg10296516 (K−means Cluster #1)
DNAm (Beta)
0.4
0.5
0.6
0.7
0.8
0
20
40
60
80
Ages (years)
A
B
C
D
E
F
G
H
I
J
K
FDR (Age associated divergence) =0.0732
p(Scalp)=0.0057
p(Dura)=0.206
Scalp
Dura

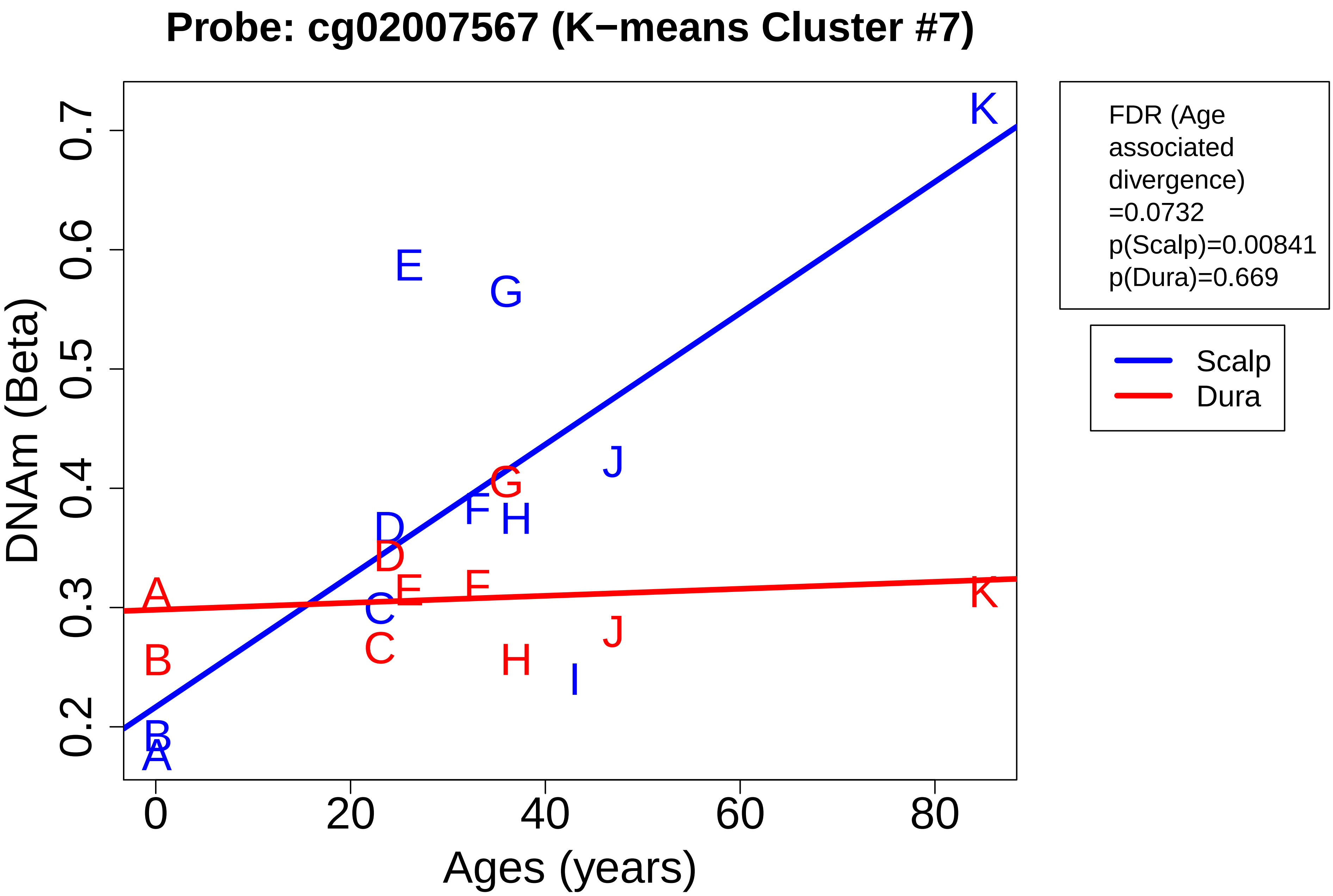
Probe: cg02007567 (K−means Cluster #7)
DNAm (Beta)
0.2
0.3
0.4
0.5
0.6
0.7
Ages (years)
0
20
40
60
80
FDR (Age associated divergence) =0.0732
p(Scalp)=0.00841
p(Dura)=0.669
Scalp
Dura

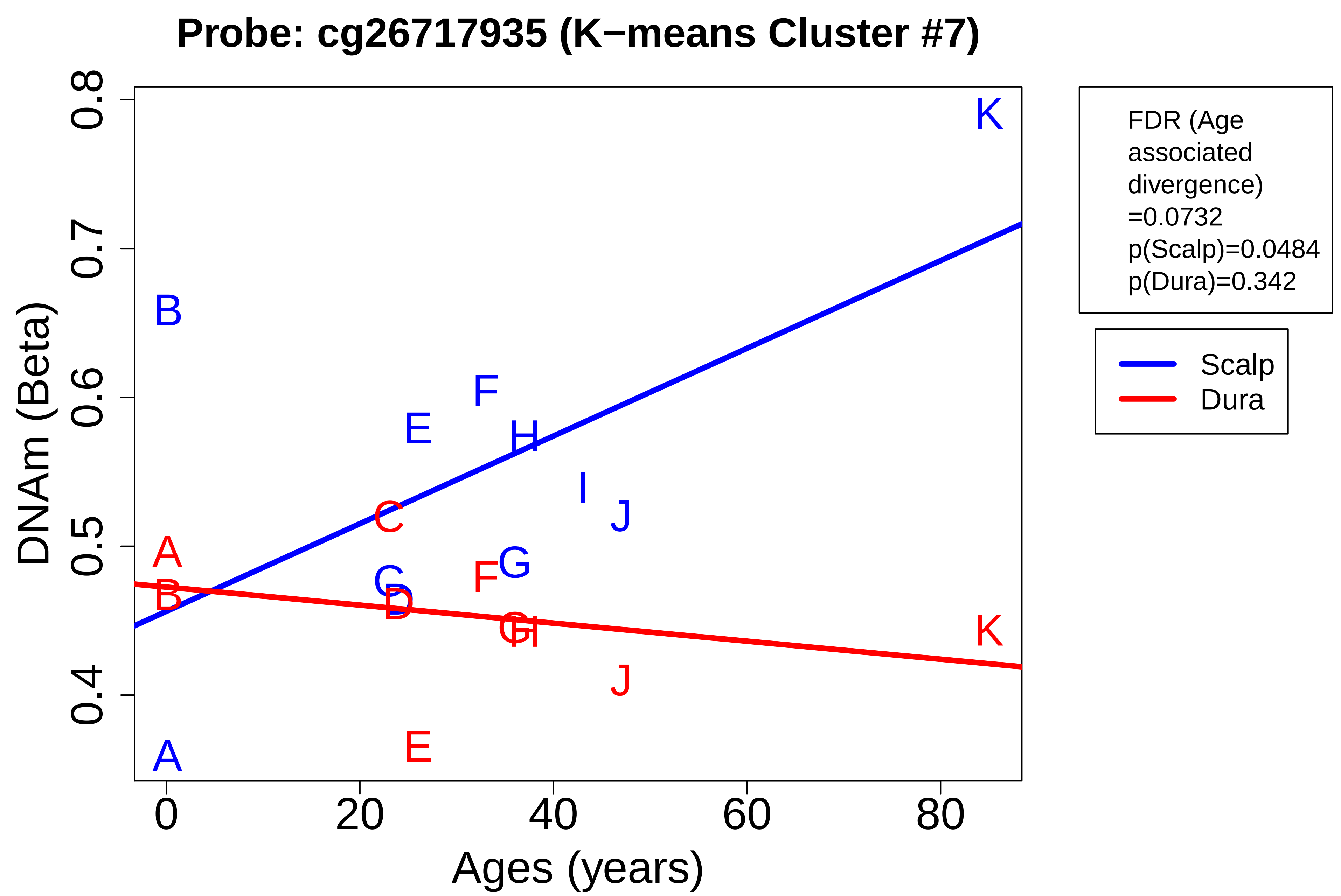

Probe: cg26717935 (K−means Cluster #7)
DNAm (Beta)
0.4
0.5
0.6
0.7
0.8
Ages (years)
0
20
40
60
80
FDR (Age associated divergence) =0.0732
p(Scalp)=0.0484
p(Dura)=0.342
Scalp
Dura

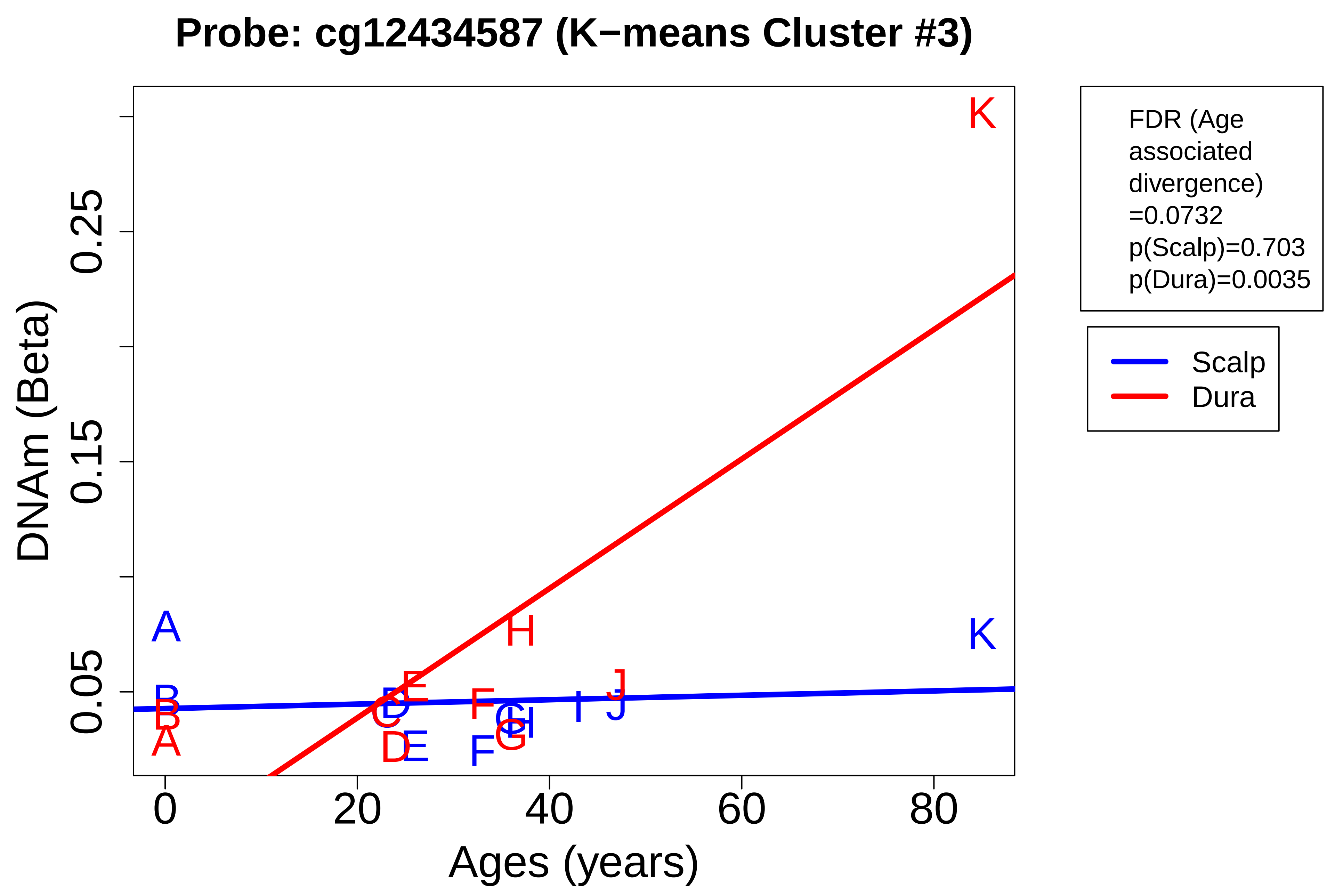
Probe: cg12434587 (K−means Cluster #3)
FDR (Age associated divergence) =0.0732
p(Scalp)=0.703
p(Dura)=0.0035
Scalp
Dura
DNAm (Beta)
0.05
0.15
0.25
0
20
40
60
80
Ages (years)
A
B
C
D
E
F
G
H
I
J
K

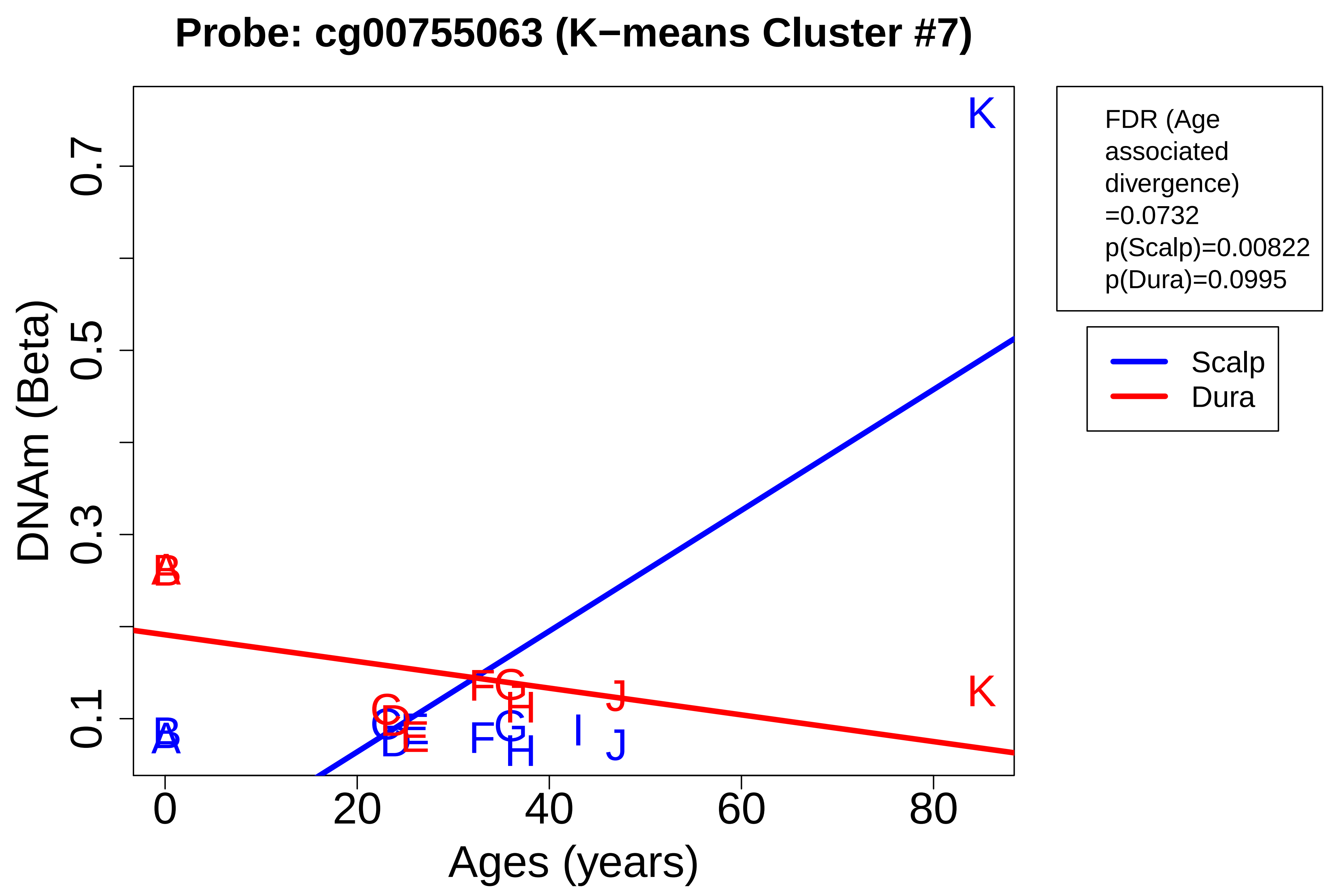

Probe: cg00755063 (K−means Cluster #7)
DNAm (Beta)
0.1
0.3
0.5
0.7
0
20
40
60
80
Ages (years)
FDR (Age associated divergence) =0.0732
p(Scalp)=0.00822
p(Dura)=0.0995
Scalp
Dura

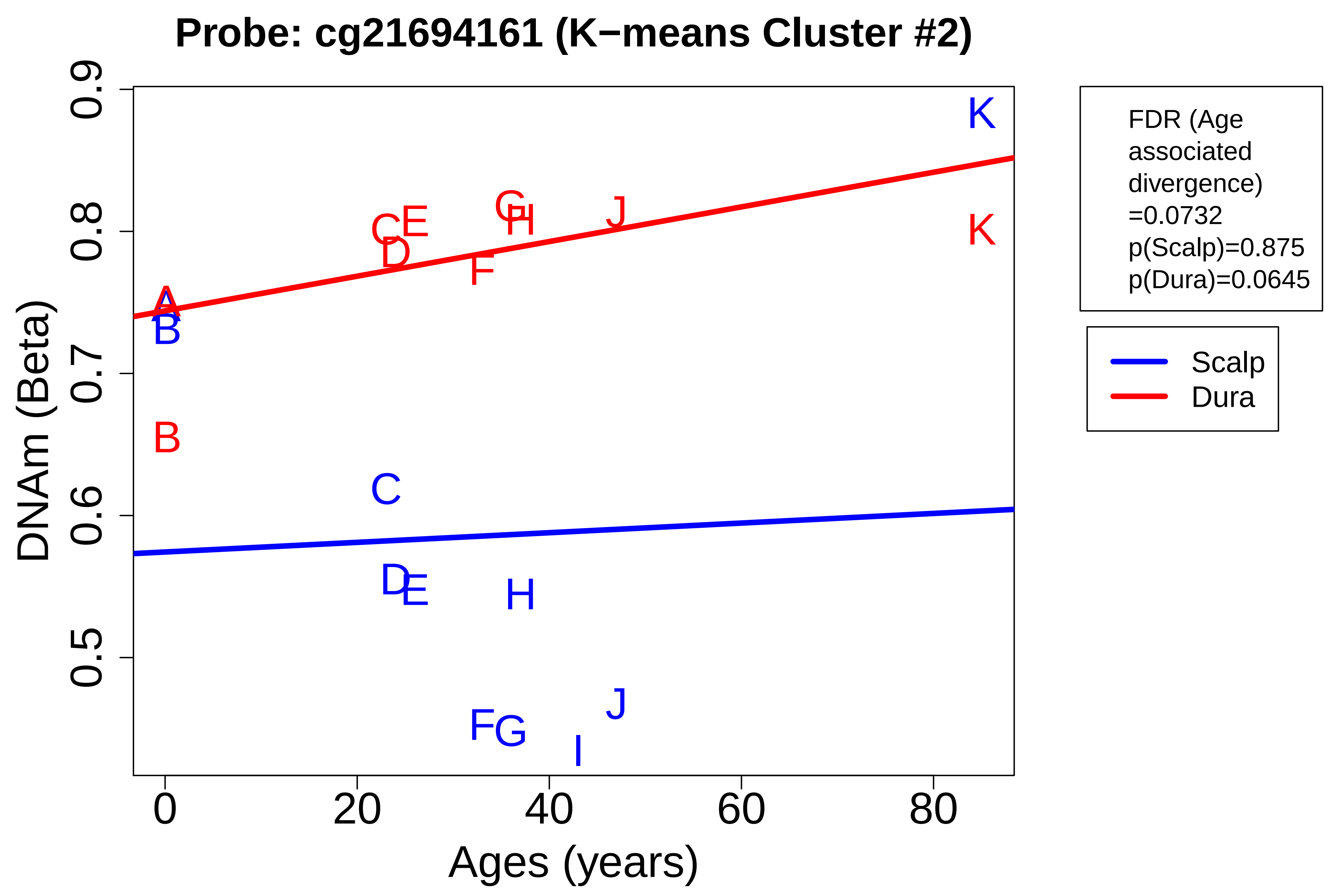

Probe: cg21694161 (K−means Cluster #2)
DNAm (Beta)
0.5
0.6
0.7
0.8
0.9
0
20
40
60
80
Ages (years)
A
B
C
D
E
F
G
H
I
J
K
FDR (Age associated divergence) =0.0732
p(Scalp)=0.875
p(Dura)=0.0645
Scalp
Dura

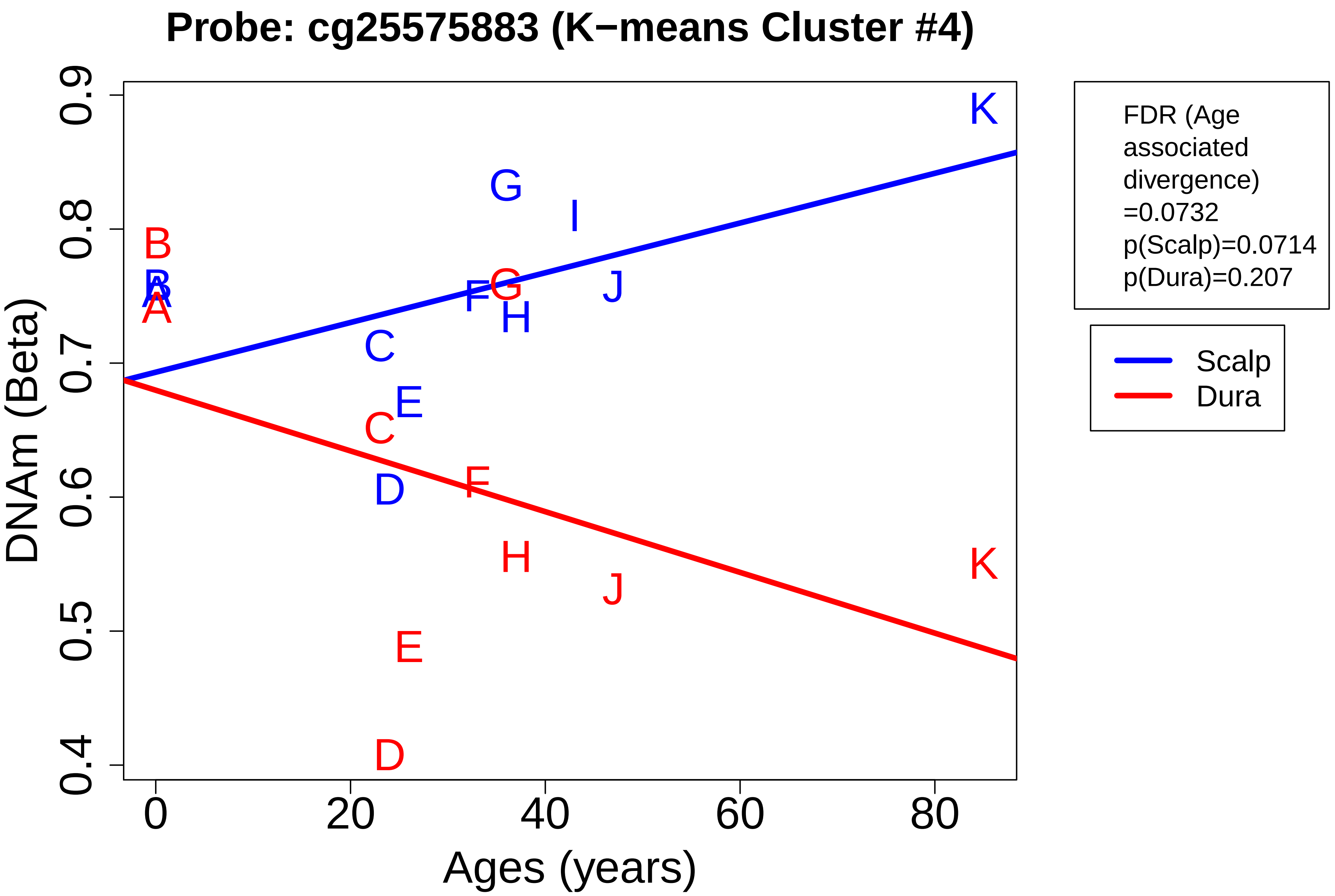

Probe: cg25575883 (K−means Cluster #4)
DNAm (Beta)
0.4
0.5
0.6
0.7
0.8
0.9
0
20
40
60
80
Ages (years)
FDR (Age associated divergence) =0.0732
p(Scalp)=0.0714
p(Dura)=0.207
Scalp
Dura
A
B
C
D
E
F
G
H
I
J
K

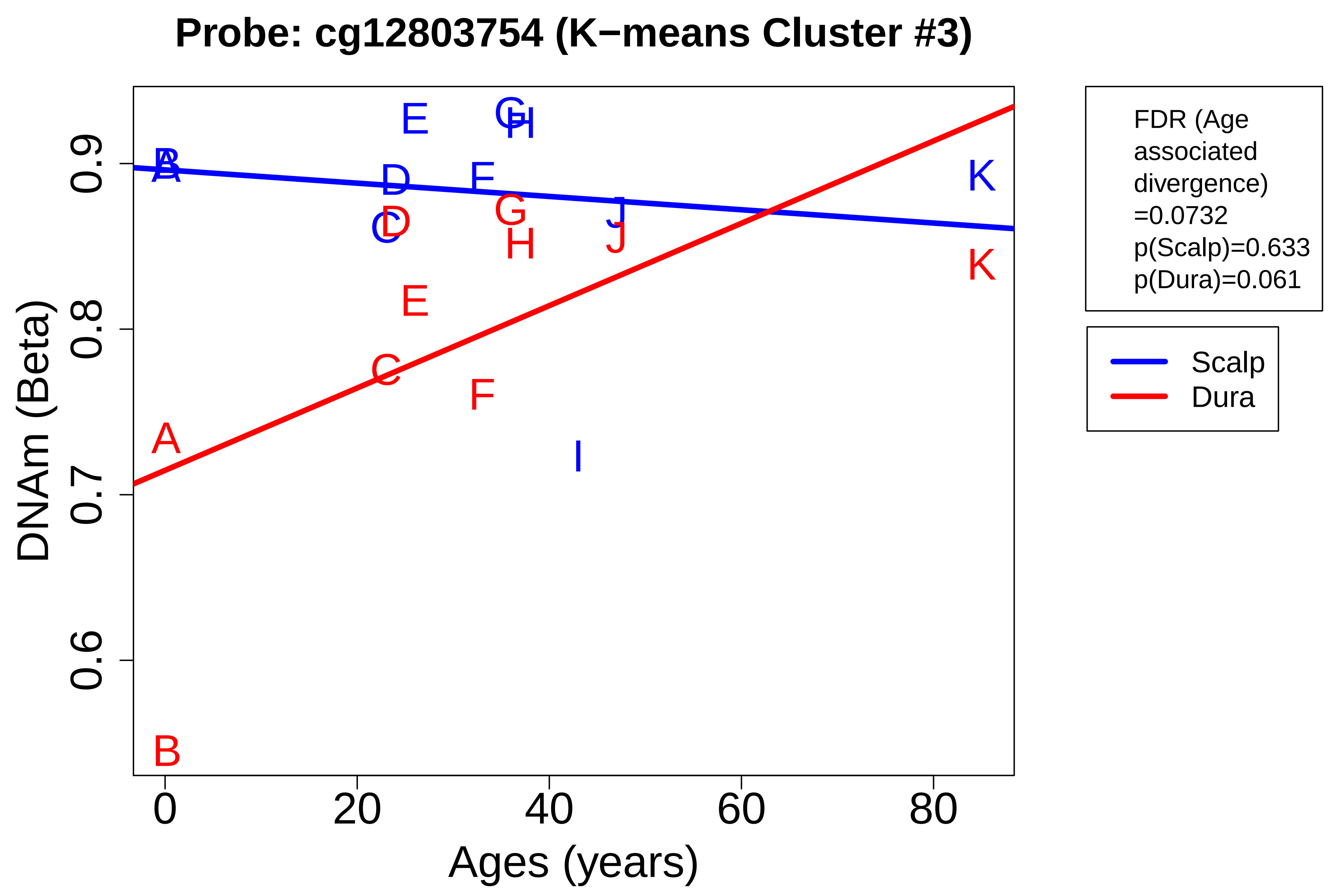

Probe: cg12803754 (K−means Cluster #3)
DNAm (Beta)
0.6
0.7
0.8
0.9
0
20
40
60
80
Ages (years)
FDR (Age associated divergence) =0.0732
p(Scalp)=0.633
p(Dura)=0.061
Scalp
Dura

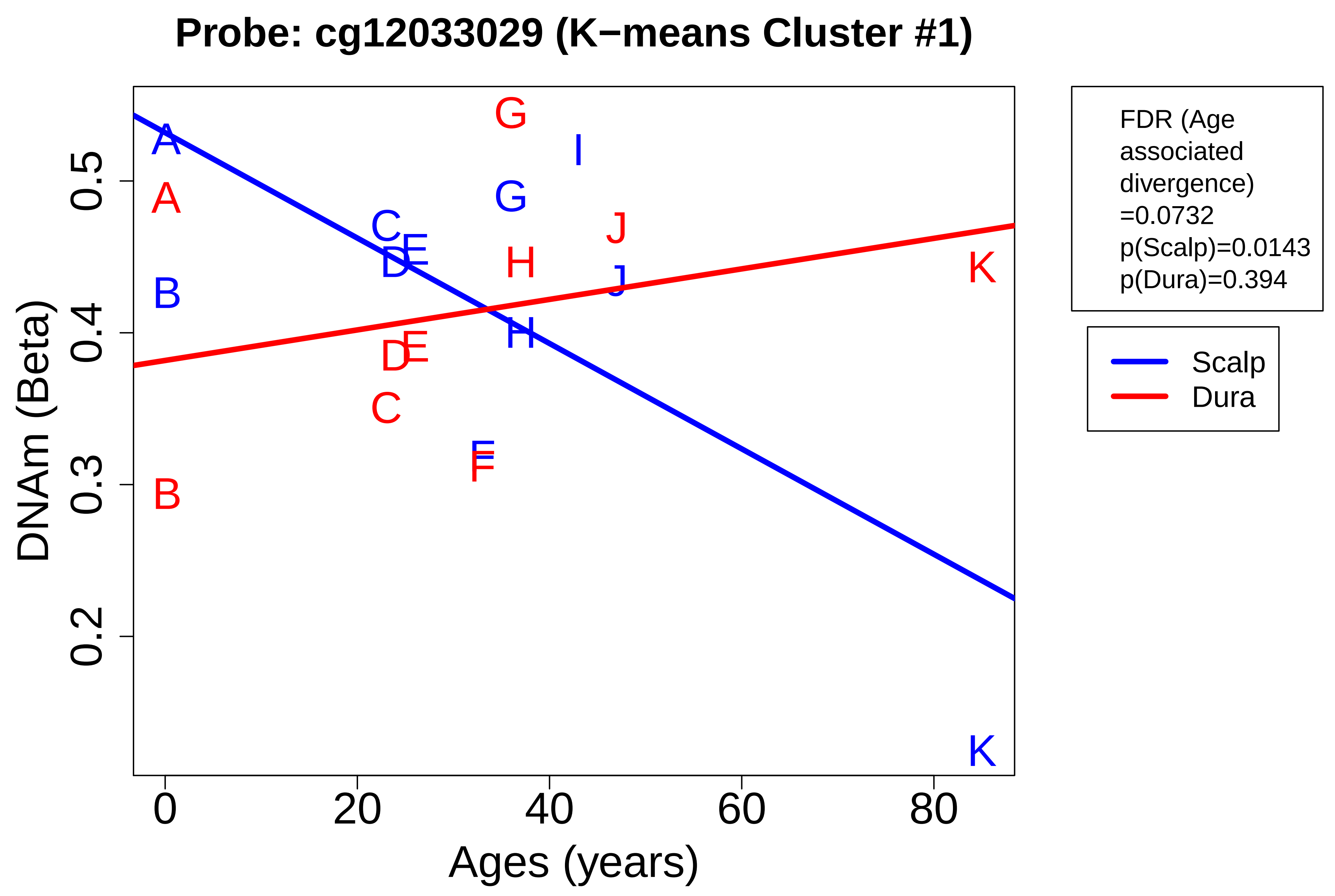

Probe: cg12033029 (K−means Cluster #1)
DNAm (Beta)
0.2
0.3
0.4
0.5
0
20
40
60
80
Ages (years)
A
B
C
D
E
F
G
H
I
J
K
FDR (Age associated divergence) =0.0732
p(Scalp)=0.0143
p(Dura)=0.394
Scalp
Dura

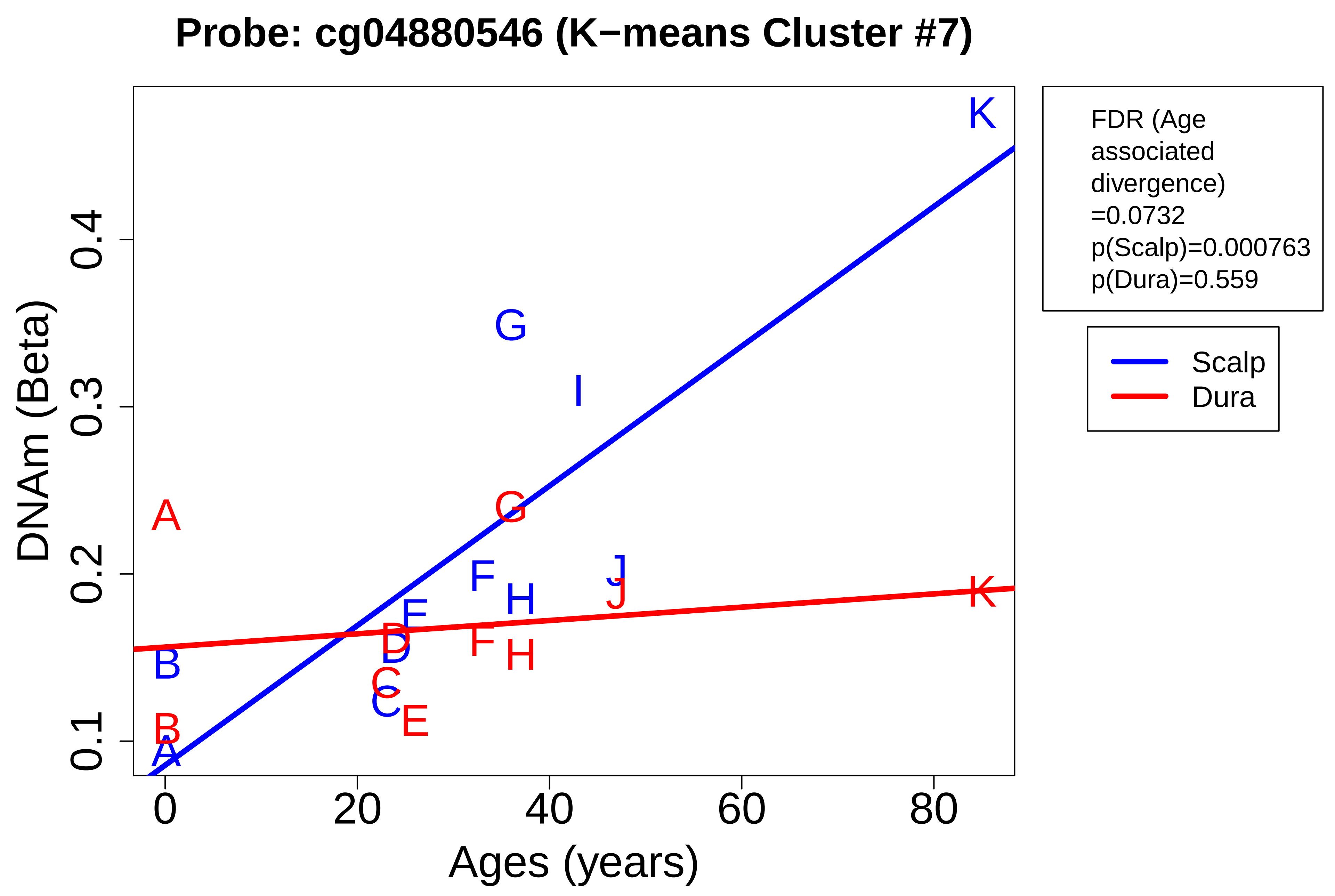

Probe: cg04880546 (K−means Cluster #7)
DNAm (Beta)
0.1
0.2
0.3
0.4
0
20
40
60
80
Ages (years)
FDR (Age associated divergence) =0.0732
p(Scalp)=0.000763
p(Dura)=0.559
Scalp
Dura
A
B
C
D
E
F
G
H
I
J
K

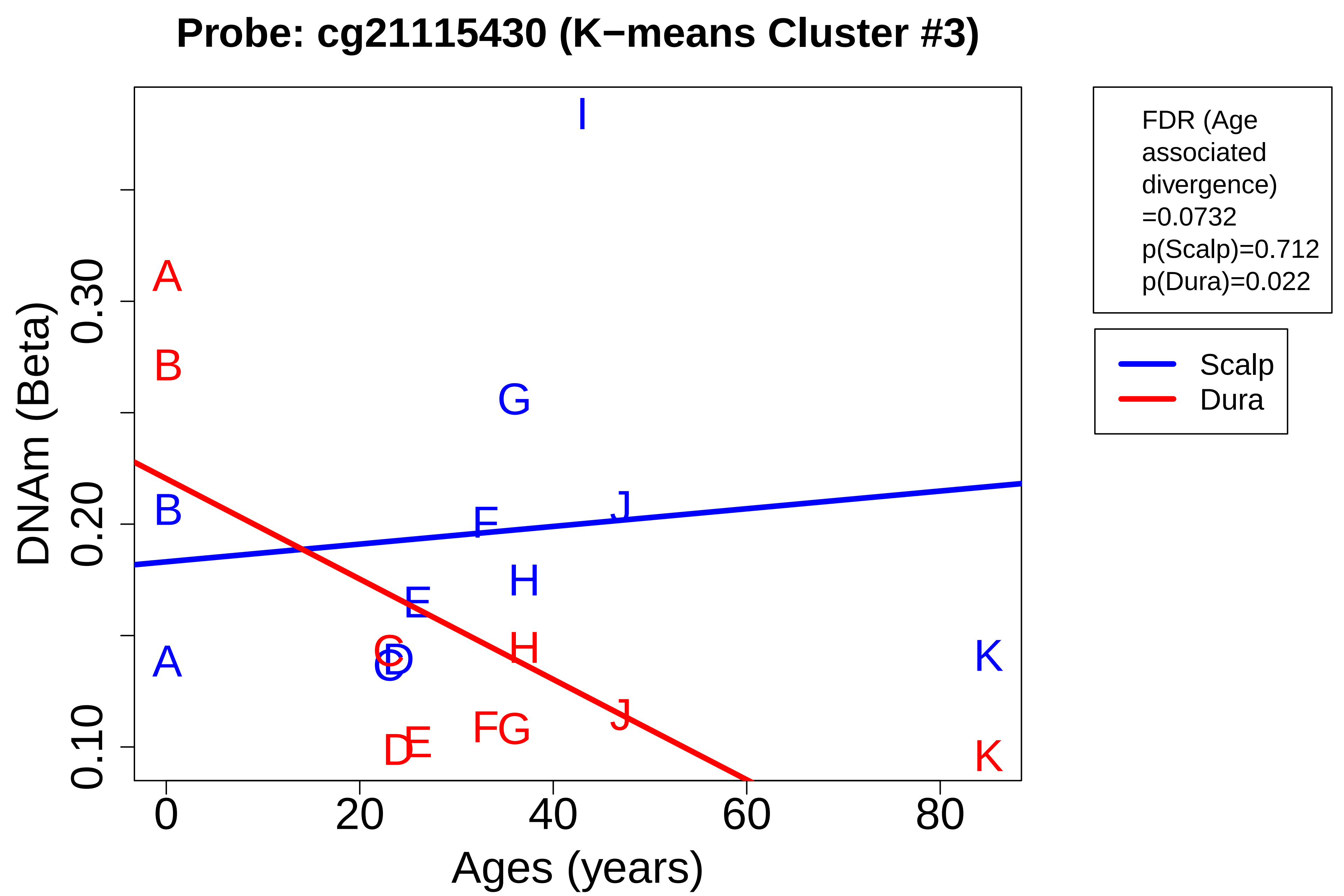

Probe: cg21115430 (K−means Cluster #3)
DNAm (Beta)
0.10
0.20
0.30
0
20
40
60
80
Ages (years)
FDR (Age associated divergence) =0.0732
p(Scalp)=0.712
p(Dura)=0.022
Scalp
Dura

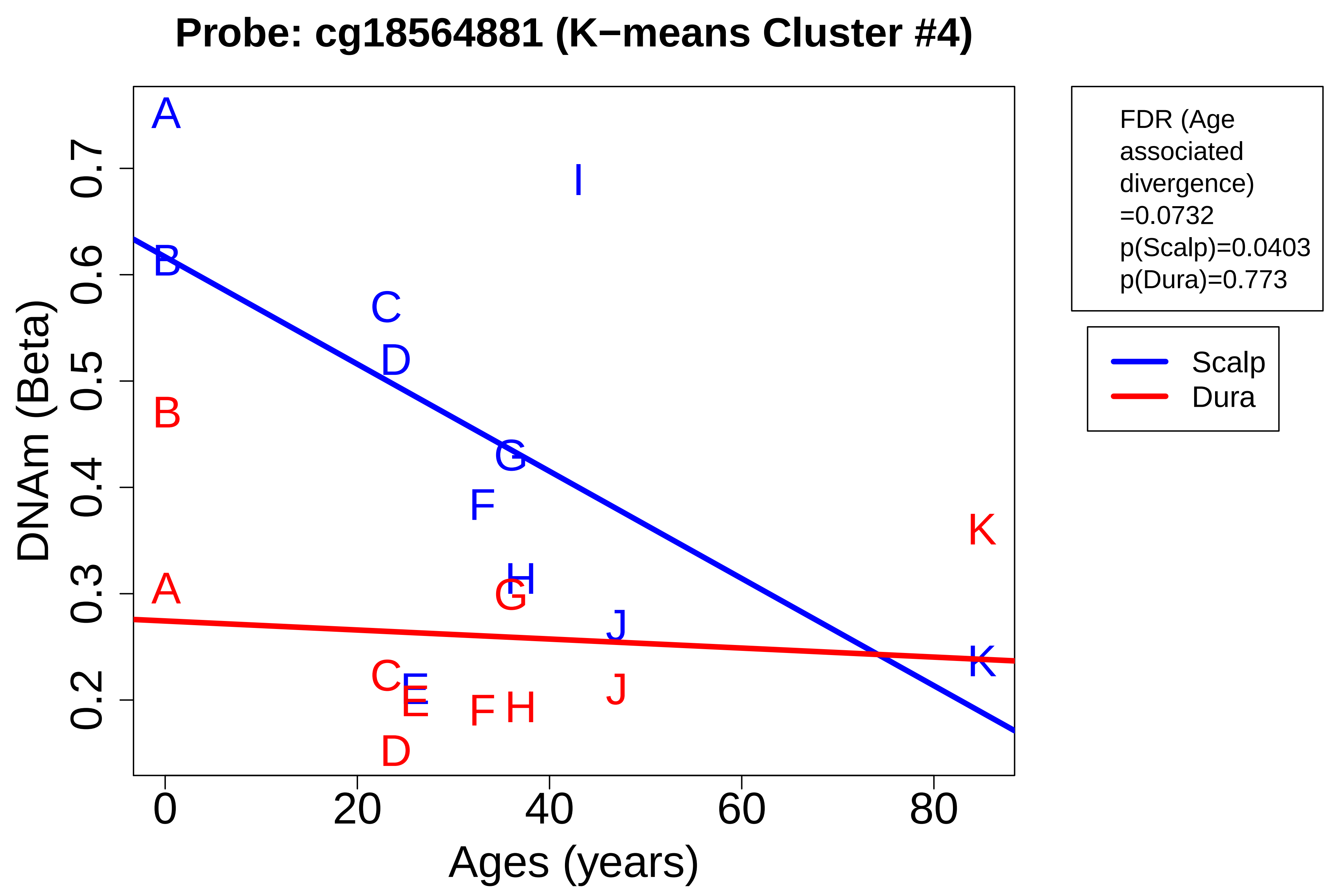

Probe: cg18564881 (K−means Cluster #4)
DNAm (Beta)
0.2
0.3
0.4
0.5
0.6
0.7
0
20
40
60
80
Ages (years)
A
B
C
D
E
F
G
H
I
J
K
A
B
C
D
E
F
G
H
J
K
FDR (Age associated divergence) =0.0732
p(Scalp)=0.0403
p(Dura)=0.773
Scalp
Dura

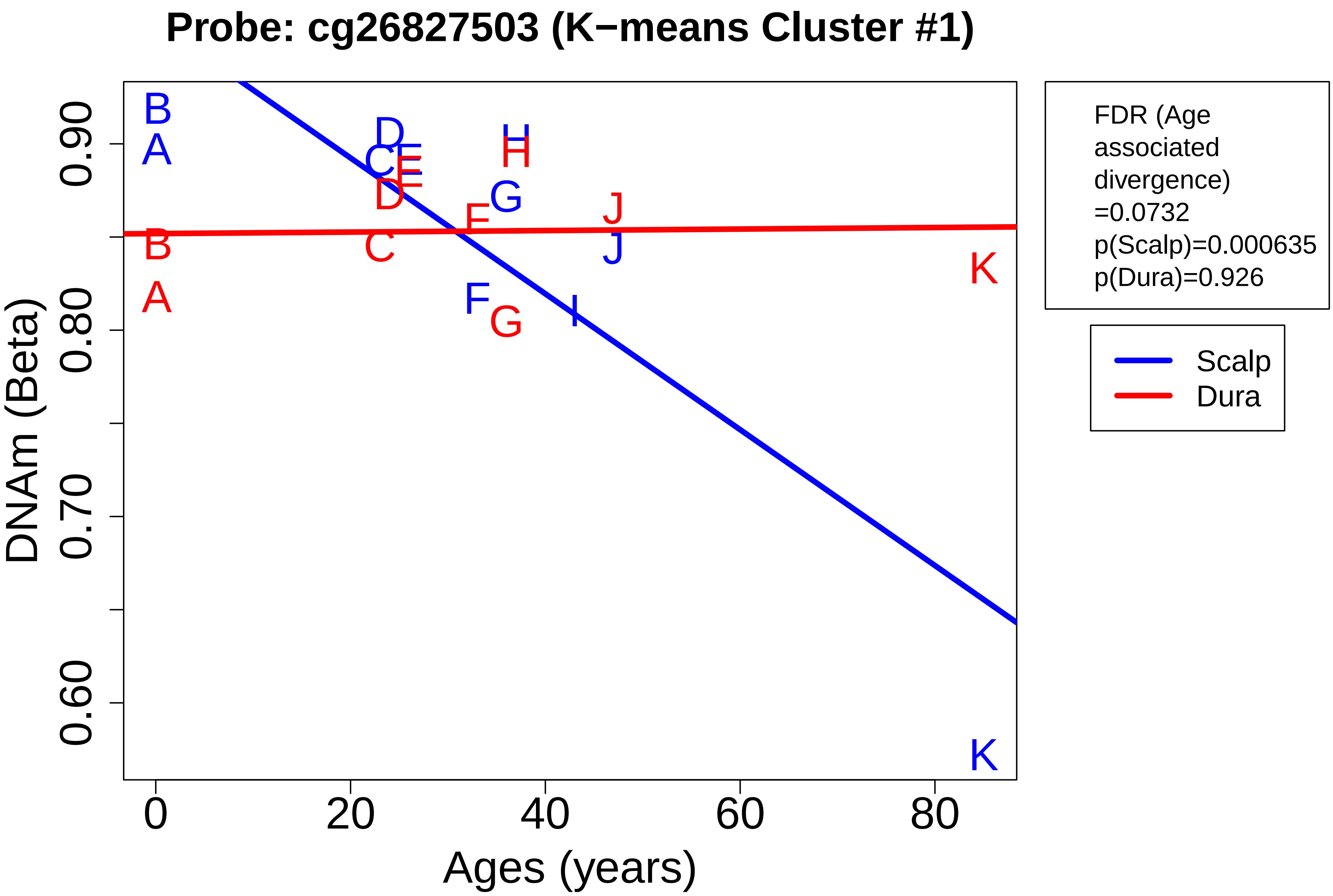

Probe: cg26827503 (K−means Cluster #1)
DNAm (Beta)
0.60
0.70
0.80
0.90
0
20
40
60
80
Ages (years)
FDR (Age associated divergence) =0.0732
p(Scalp)=0.000635
p(Dura)=0.926
Scalp
Dura

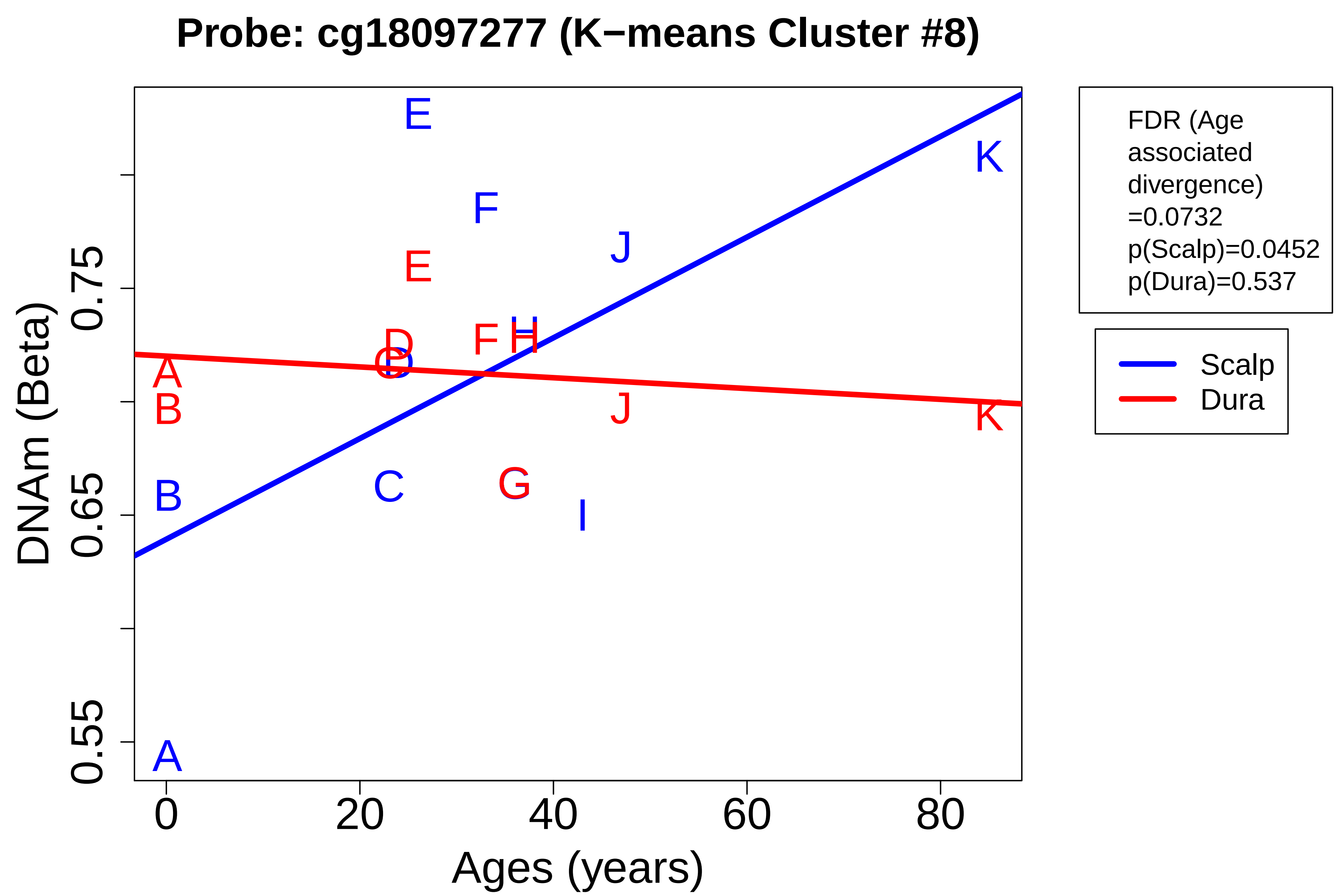

Probe: cg18097277 (K−means Cluster #8)
DNAm (Beta)
0.55
0.65
0.75
0
20
40
60
80
Ages (years)
A
B
C
D
E
F
G
H
I
J
K
FDR (Age associated divergence) =0.0732
p(Scalp)=0.0452
p(Dura)=0.537
Scalp
Dura

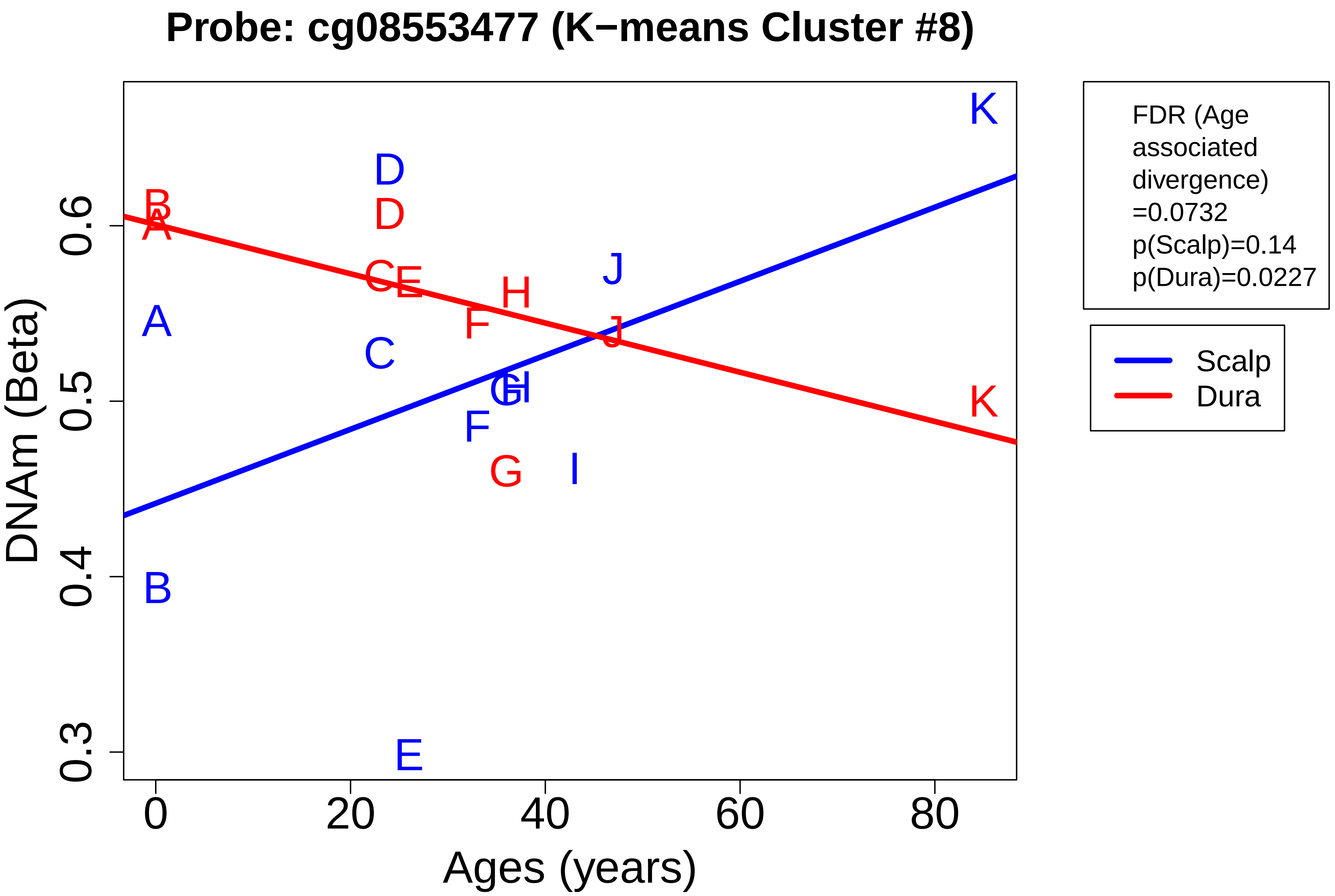

Probe: cg08553477 (K−means Cluster #8)
FDR (Age associated divergence) =0.0732
p(Scalp)=0.14
p(Dura)=0.0227
Scalp
Dura
DNAm (Beta)
0.3
0.4
0.5
0.6
Ages (years)
0
20
40
60
80

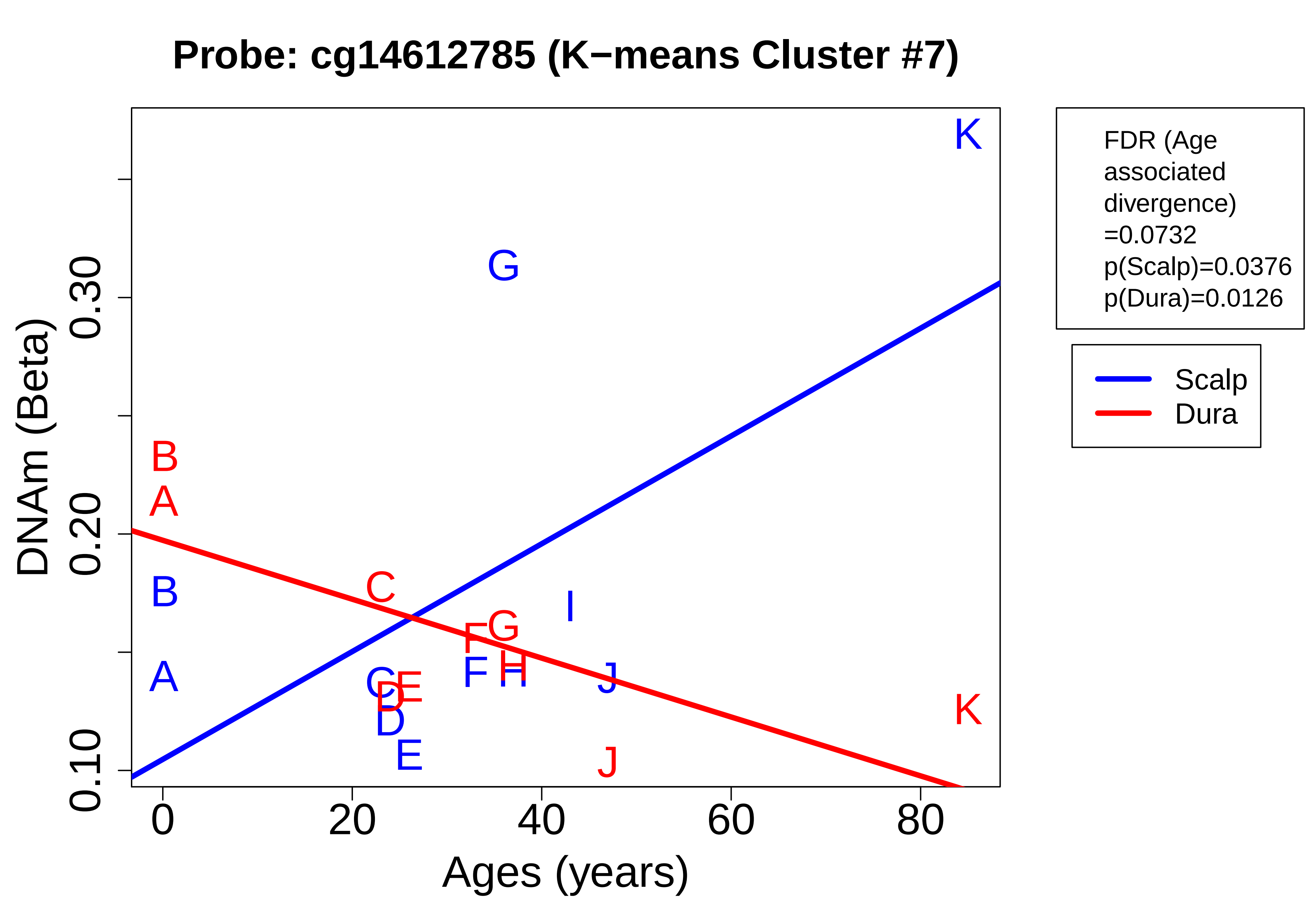

Probe: cg14612785 (K−means Cluster #7)
DNAm (Beta)
0.10
0.20
0.30
Ages (years)
0
20
40
60
80
FDR (Age associated divergence) =0.0732
p(Scalp)=0.0376
p(Dura)=0.0126
Scalp
Dura
A
B
C
D
E
F
G
H
I
J
K

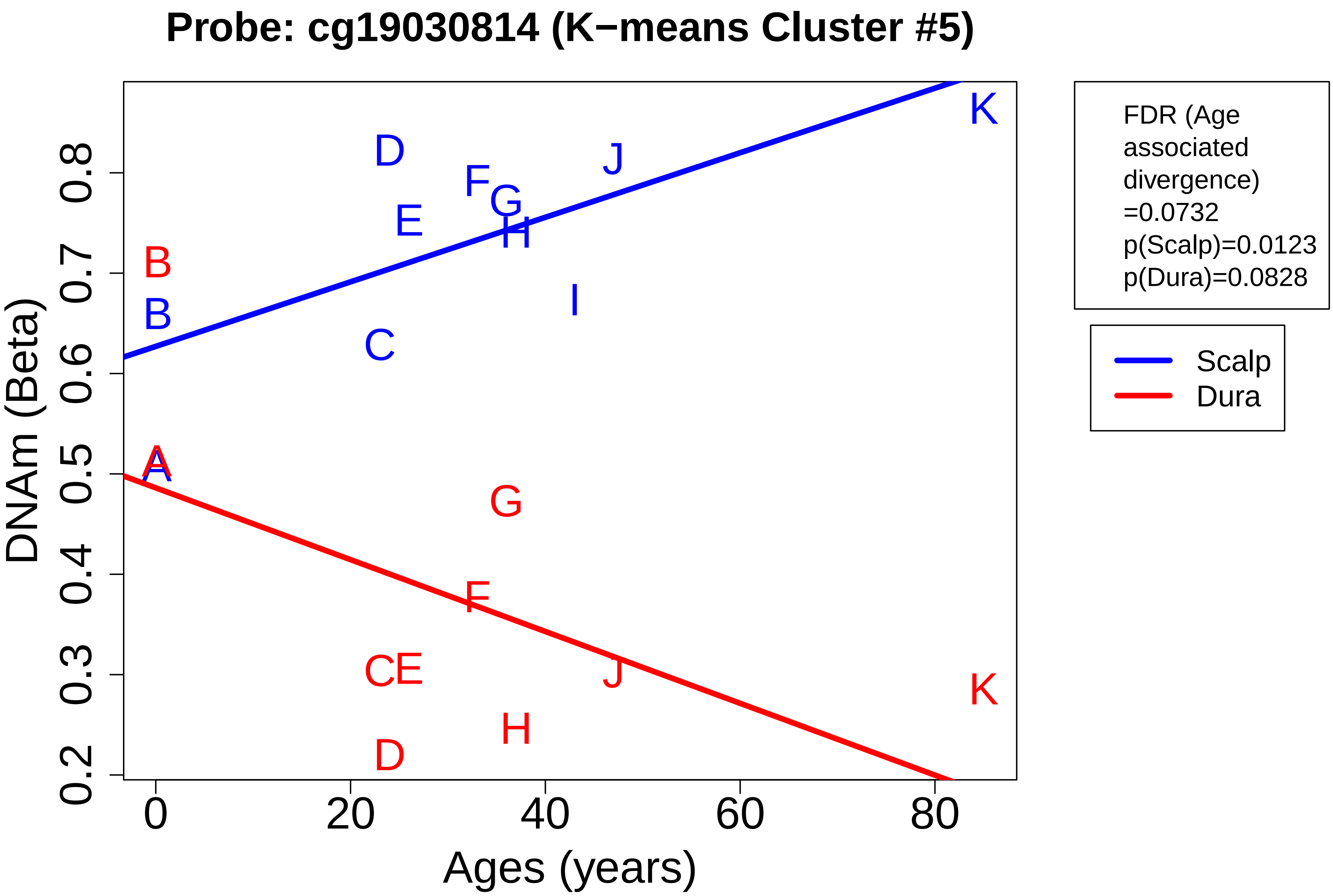

Probe: cg19030814 (K−means Cluster #5)
DNAm (Beta)
Ages (years)
0.2
0.3
0.4
0.5
0.6
0.7
0.8
0
20
40
60
80
A
B
C
D
E
F
G
H
I
J
K
FDR (Age associated divergence) =0.0732
p(Scalp)=0.0123
p(Dura)=0.0828
Scalp
Dura

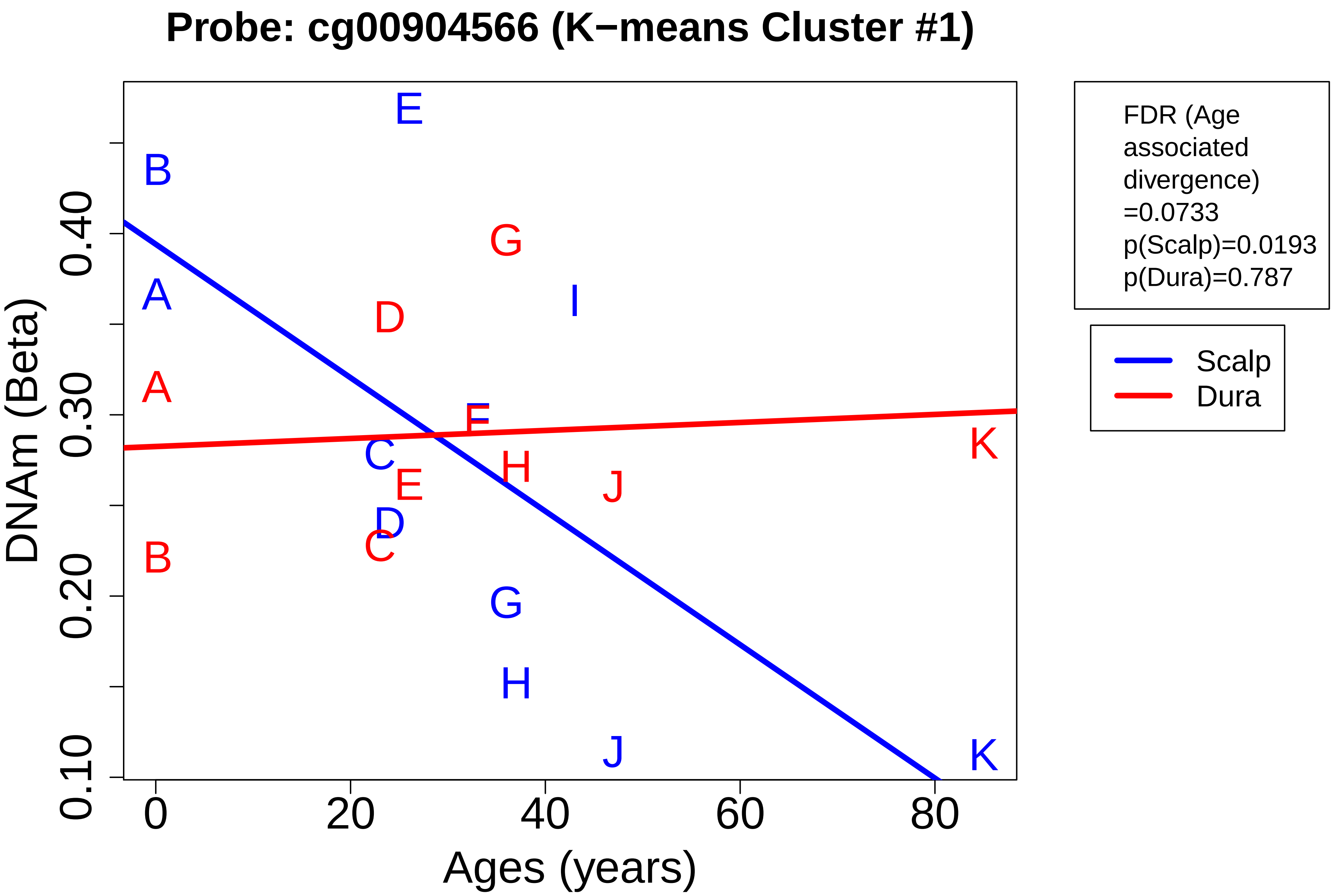

Probe: cg00904566 (K−means Cluster #1)
DNAm (Beta)
Ages (years)
0.10
0.20
0.30
0.40
0
20
40
60
80
FDR (Age associated divergence) =0.0733
p(Scalp)=0.0193
p(Dura)=0.787
Scalp
Dura
A
B
C
D
E
F
G
H
I
J
K

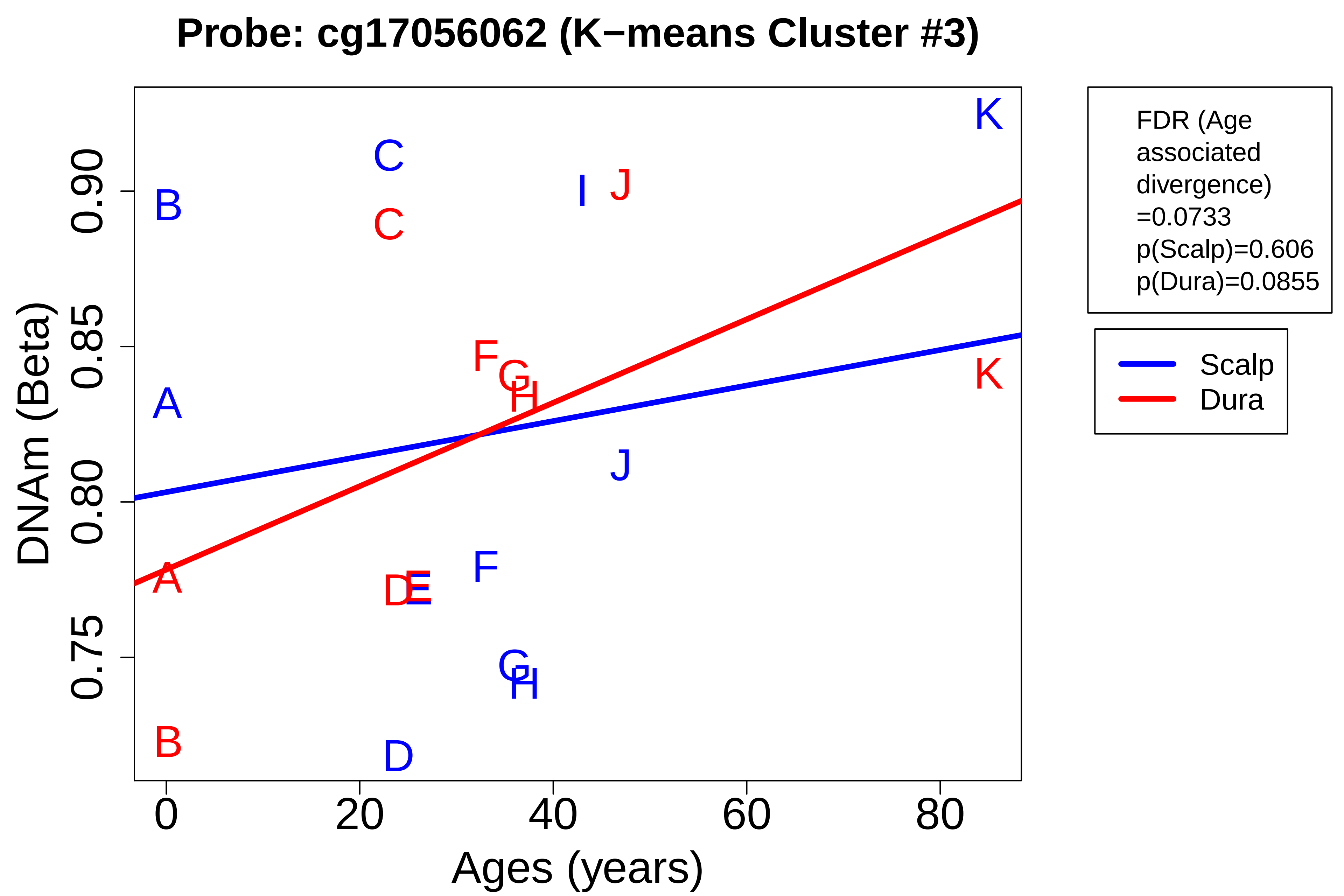
Probe: cg17056062 (K−means Cluster #3)
DNAm (Beta)
0.75
0.80
0.85
0.90
Ages (years)
0
20
40
60
80
FDR (Age associated divergence) =0.0733
p(Scalp)=0.606
p(Dura)=0.0855
Scalp
Dura
A
B
C
D
E
F
G
H
I
J
K

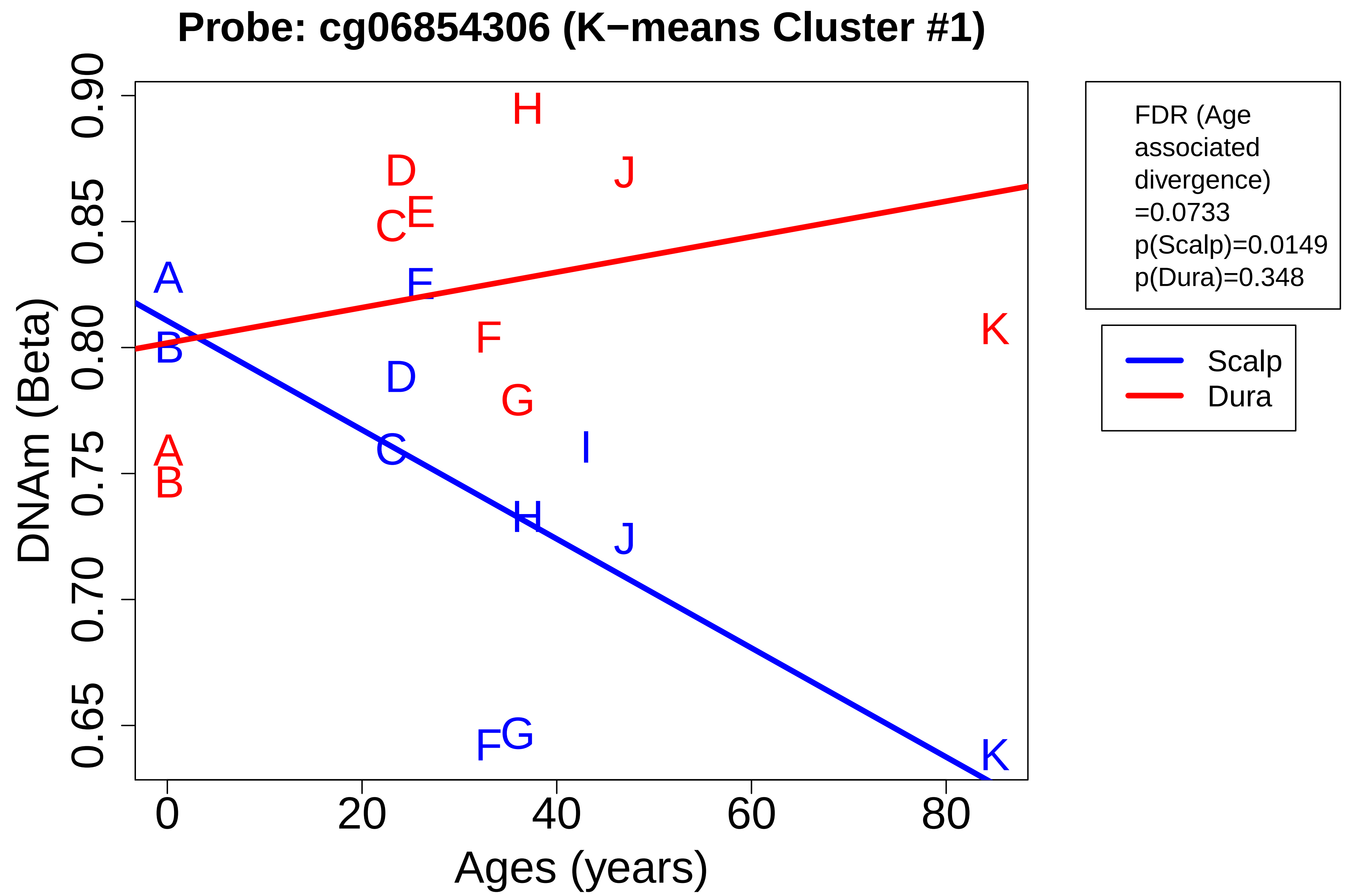

Probe: cg06854306 (K−means Cluster #1)
DNAm (Beta)
0.65
0.70
0.75
0.80
0.85
0.90
0
20
40
60
80
Ages (years)
FDR (Age associated divergence) =0.0733
p(Scalp)=0.0149
p(Dura)=0.348
Scalp
Dura

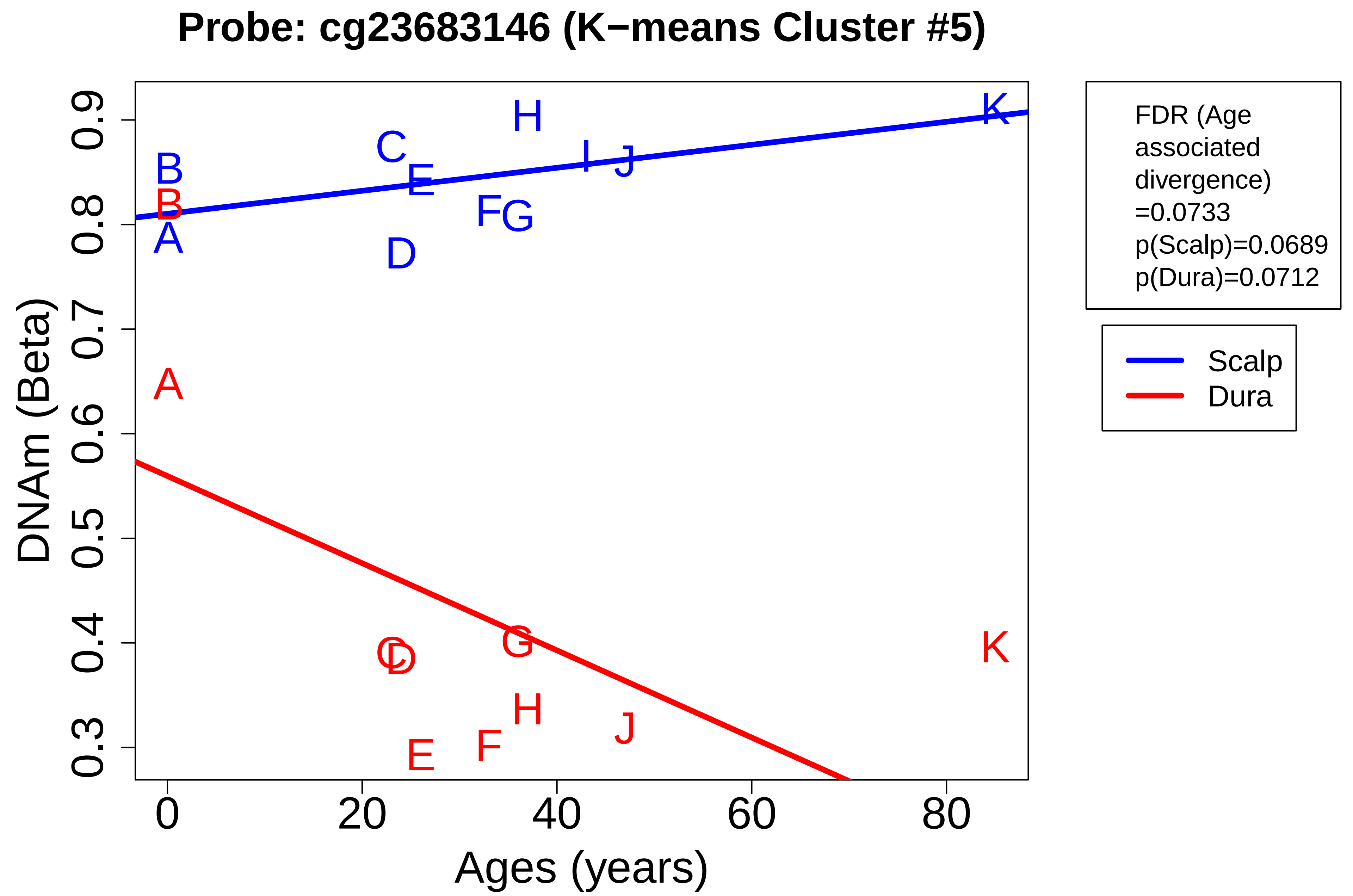

Probe: cg23683146 (K−means Cluster #5)
DNAm (Beta)
0.3
0.4
0.5
0.6
0.7
0.8
0.9
0
20
40
60
80
Ages (years)
FDR (Age associated divergence) =0.0733
p(Scalp)=0.0689
p(Dura)=0.0712
Scalp
Dura
A
B
C
D
E
F
G
H
I
J
K

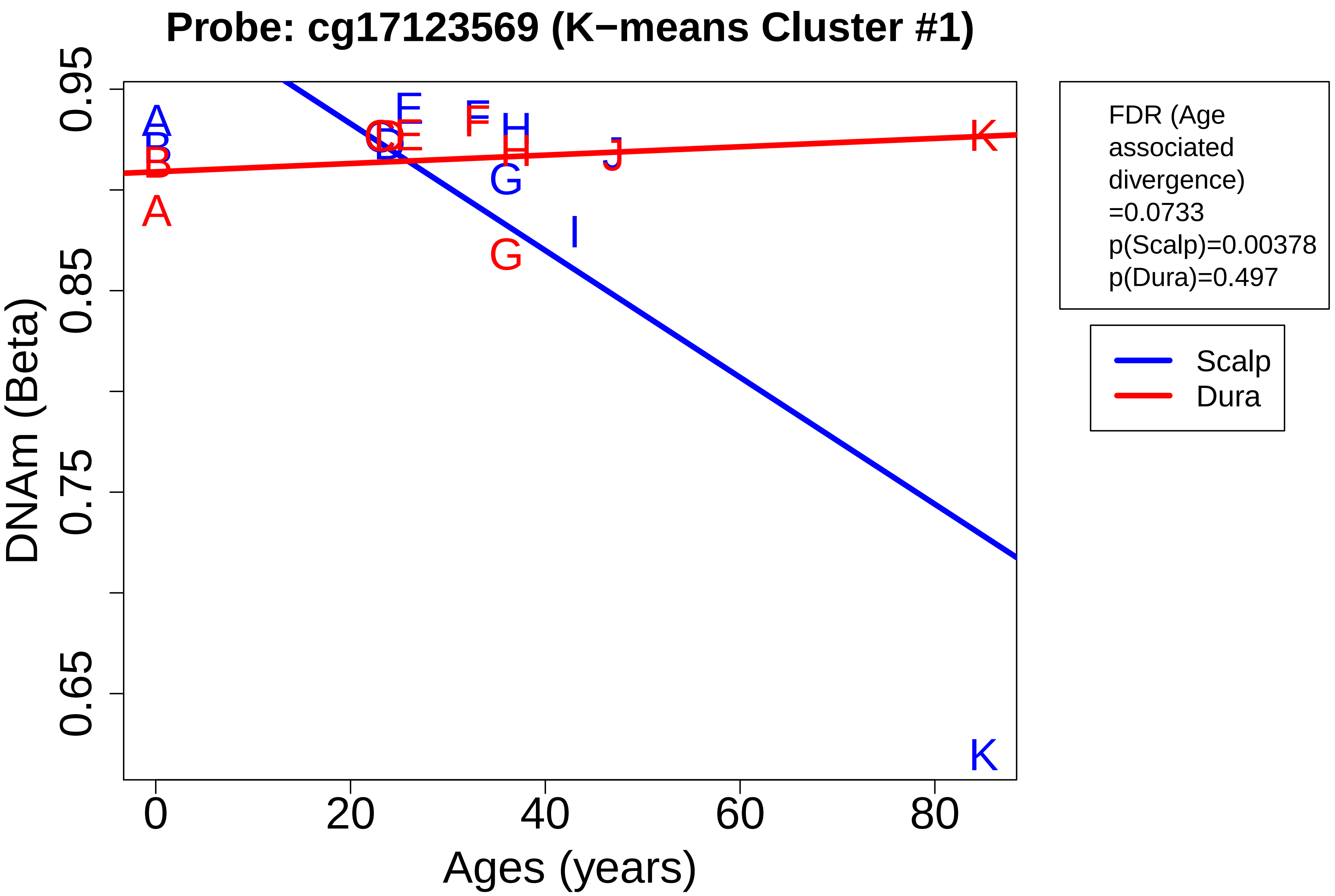

Probe: cg17123569 (K−means Cluster #1)
DNAm (Beta)
0.65
0.75
0.85
0.95
0
20
40
60
80
Ages (years)
FDR (Age associated divergence) =0.0733
p(Scalp)=0.00378
p(Dura)=0.497
Scalp
Dura

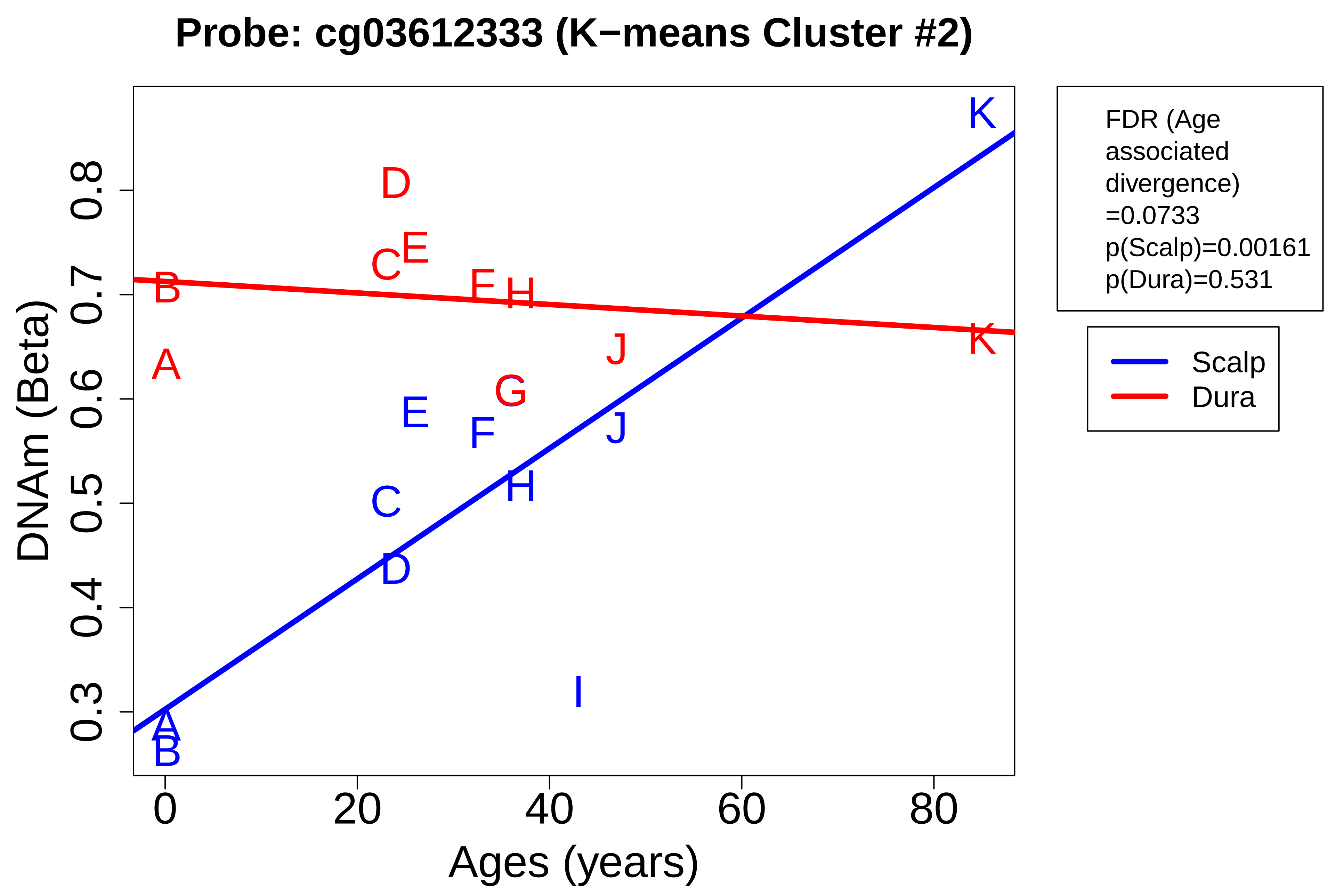

Probe: cg03612333 (K−means Cluster #2)
DNAm (Beta)
0.3
0.4
0.5
0.6
0.7
0.8
Ages (years)
0
20
40
60
80
A
B
C
D
E
F
G
H
I
J
K
FDR (Age associated divergence) =0.0733
p(Scalp)=0.00161
p(Dura)=0.531
Scalp
Dura

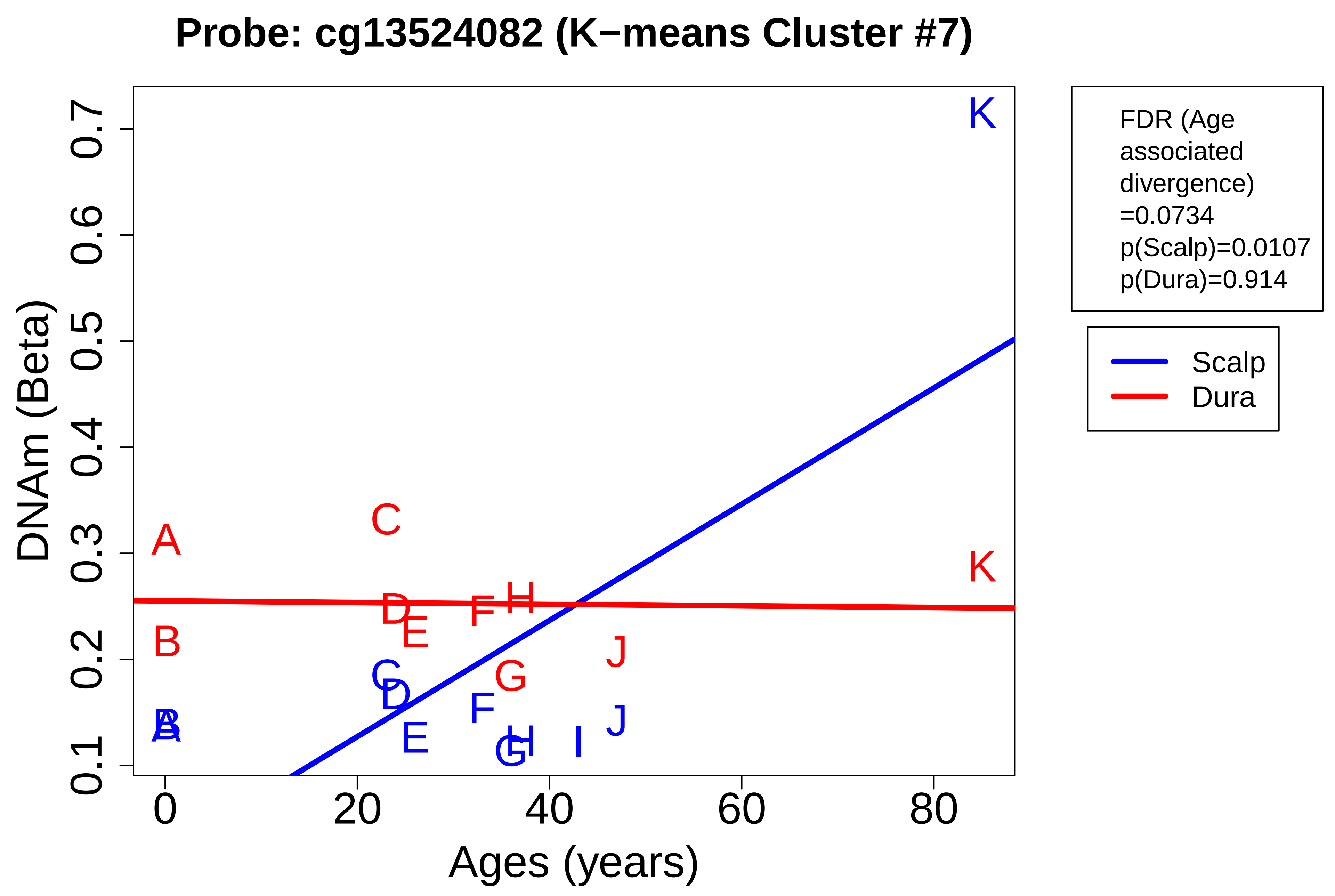
Probe: cg13524082 (K−means Cluster #7)
DNAm (Beta)
0.1
0.2
0.3
0.4
0.5
0.6
0.7
Ages (years)
0
20
40
60
80
FDR (Age associated divergence) =0.0734
p(Scalp)=0.0107
p(Dura)=0.914
Scalp
Dura

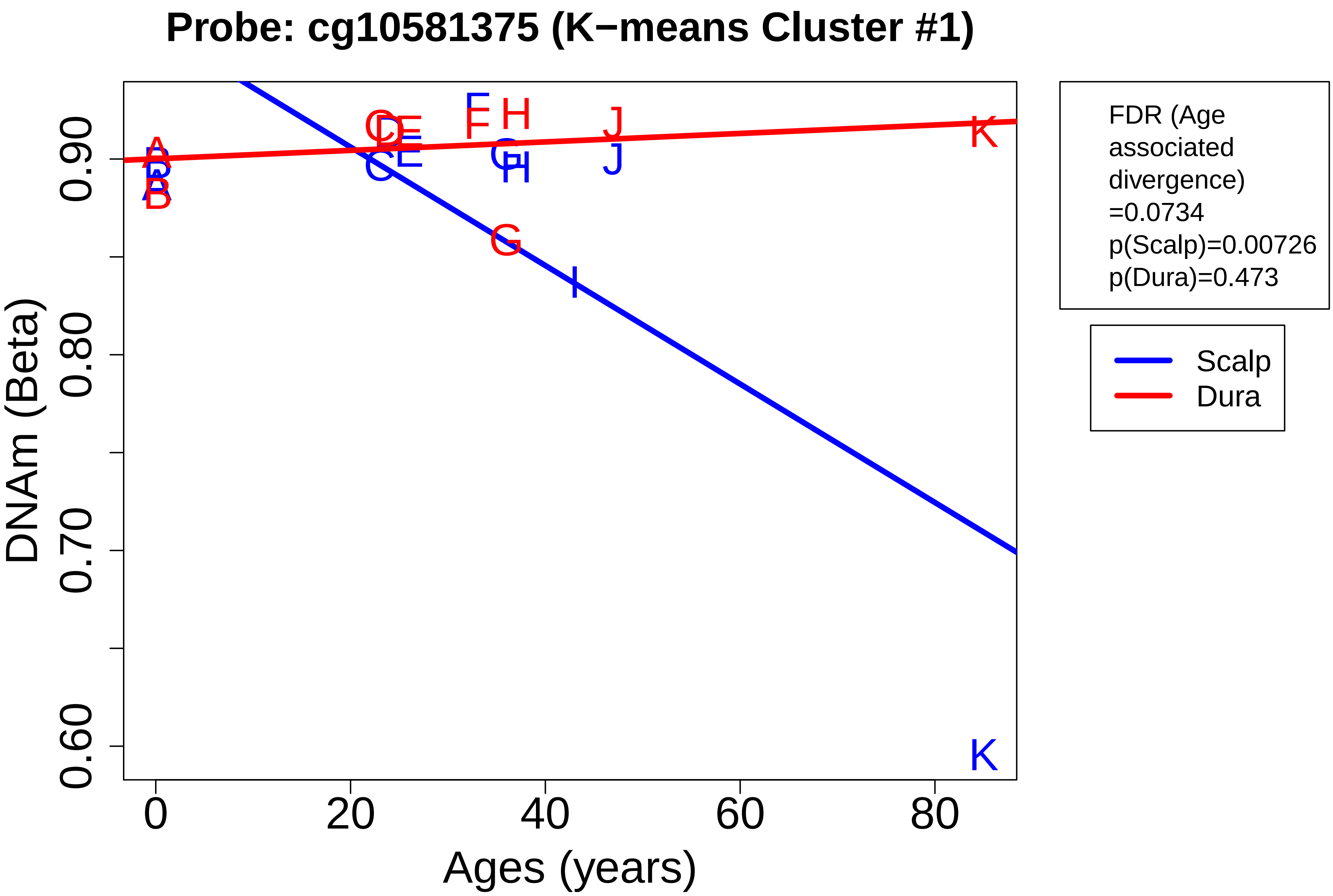

Probe: cg10581375 (K−means Cluster #1)
DNAm (Beta)
0.60
0.70
0.80
0.90
0
20
40
60
80
Ages (years)
FDR (Age associated divergence) =0.0734
p(Scalp)=0.00726
p(Dura)=0.473
Scalp
Dura
A
B
C
D
E
F
G
H
I
J
K

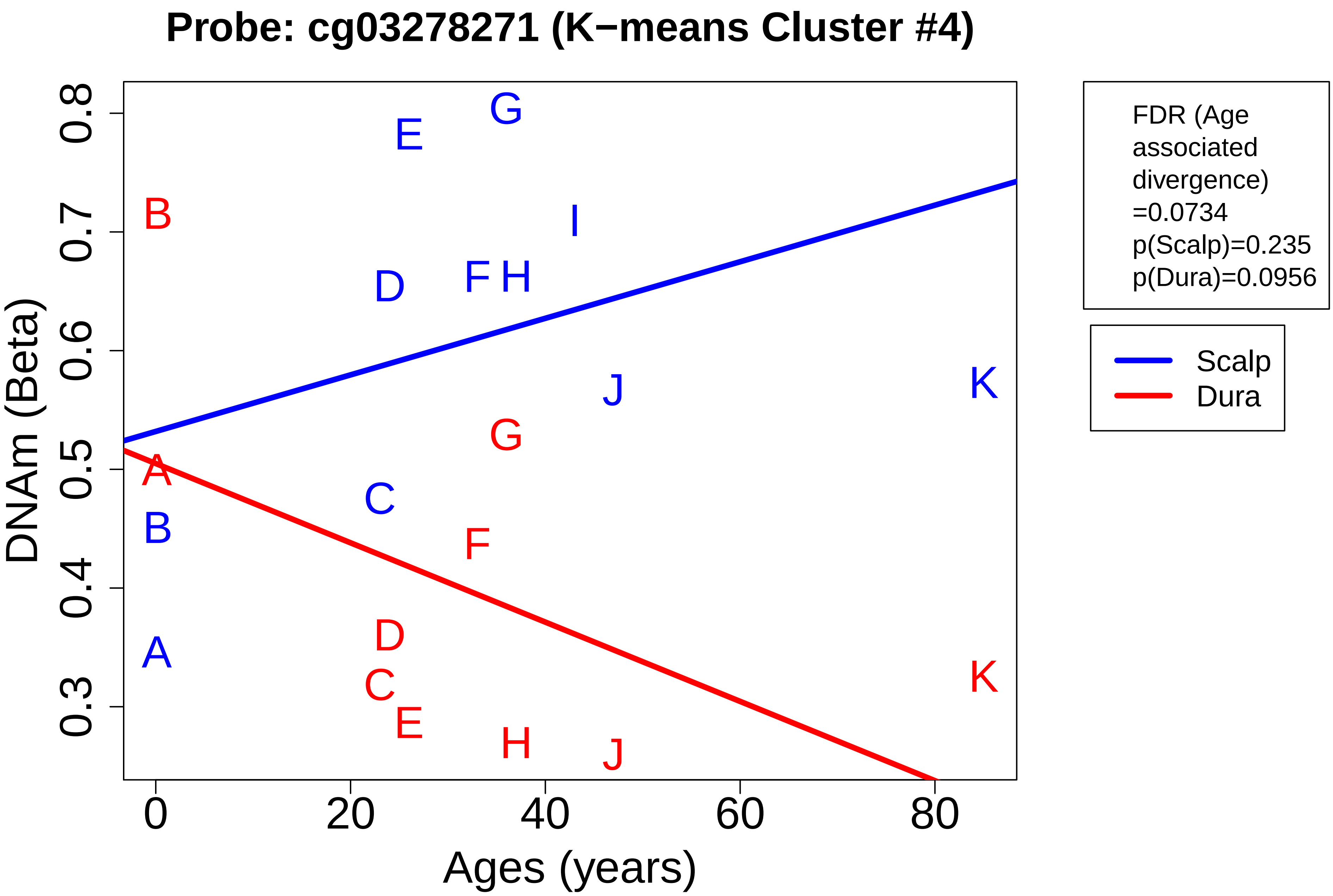
Probe: cg03278271 (K−means Cluster #4)
FDR (Age associated divergence) =0.0734
p(Scalp)=0.235
p(Dura)=0.0956
Scalp
Dura
DNAm (Beta)
0.3
0.4
0.5
0.6
0.7
0.8
0
20
40
60
80
Ages (years)
A B C D E F G H I J K
A B C D E F G H J K

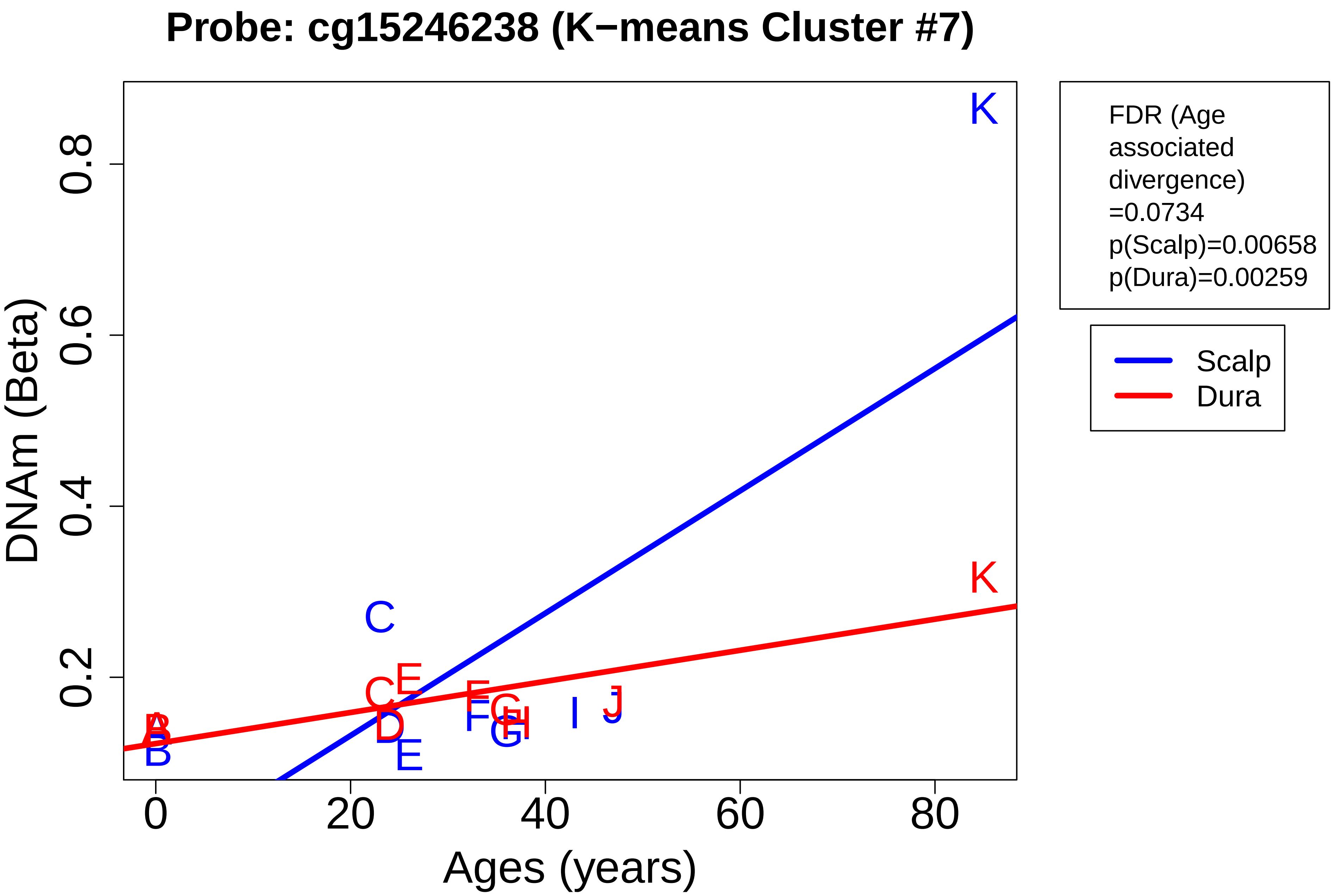

Probe: cg15246238 (K−means Cluster #7)
FDR (Age associated divergence) =0.0734
p(Scalp)=0.00658
p(Dura)=0.00259
Scalp
Dura
DNAm (Beta)
0.2
0.4
0.6
0.8
0
20
40
60
80
Ages (years)
A
B
C
D
E
F
G
H
I
J
K

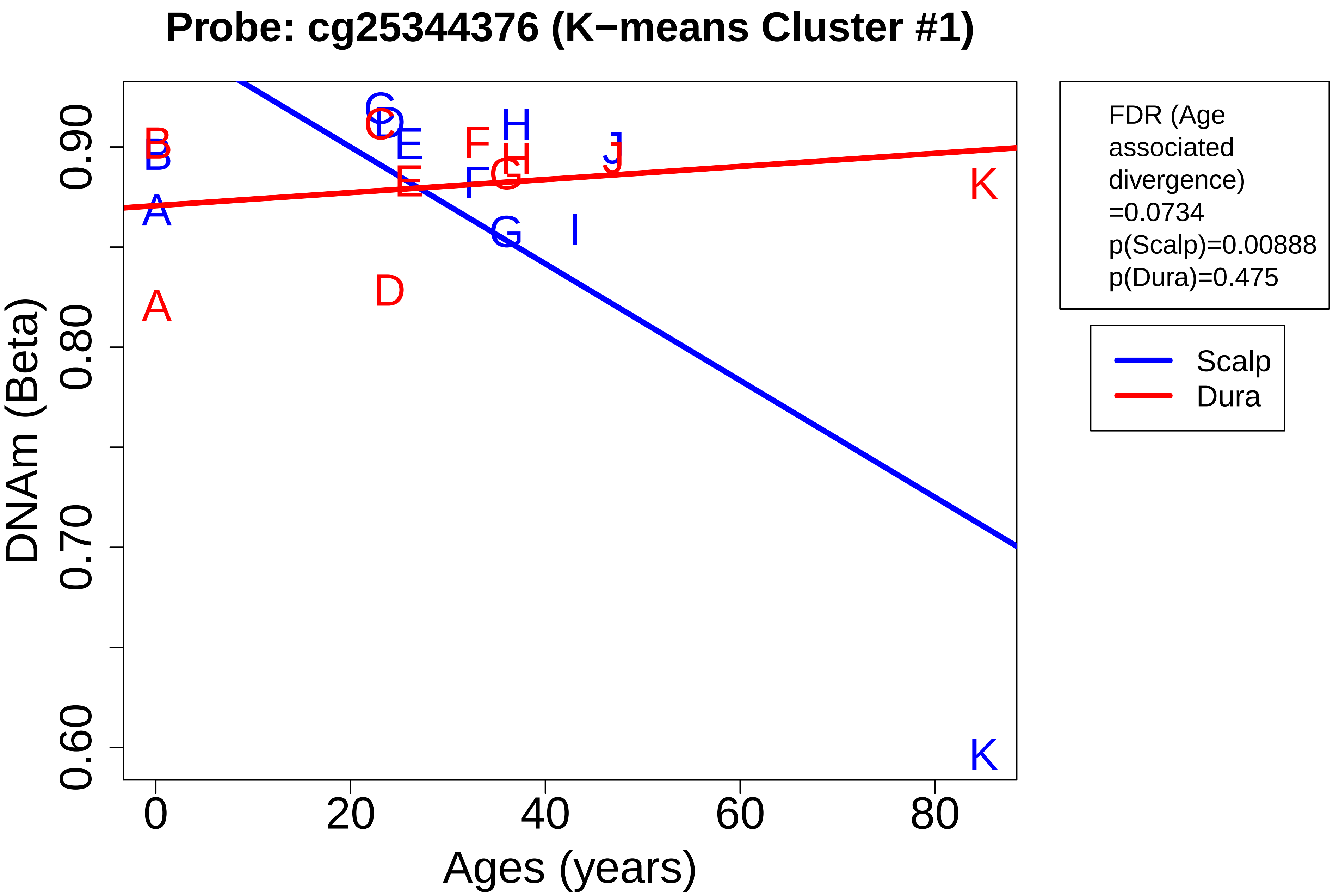

Probe: cg25344376 (K−means Cluster #1)
FDR (Age associated divergence) =0.0734
p(Scalp)=0.00888
p(Dura)=0.475
Scalp
Dura
DNAm (Beta)
0.60
0.70
0.80
0.90
Ages (years)
0
20
40
60
80
A
B
C
D
E
F
G
H
I
J
K

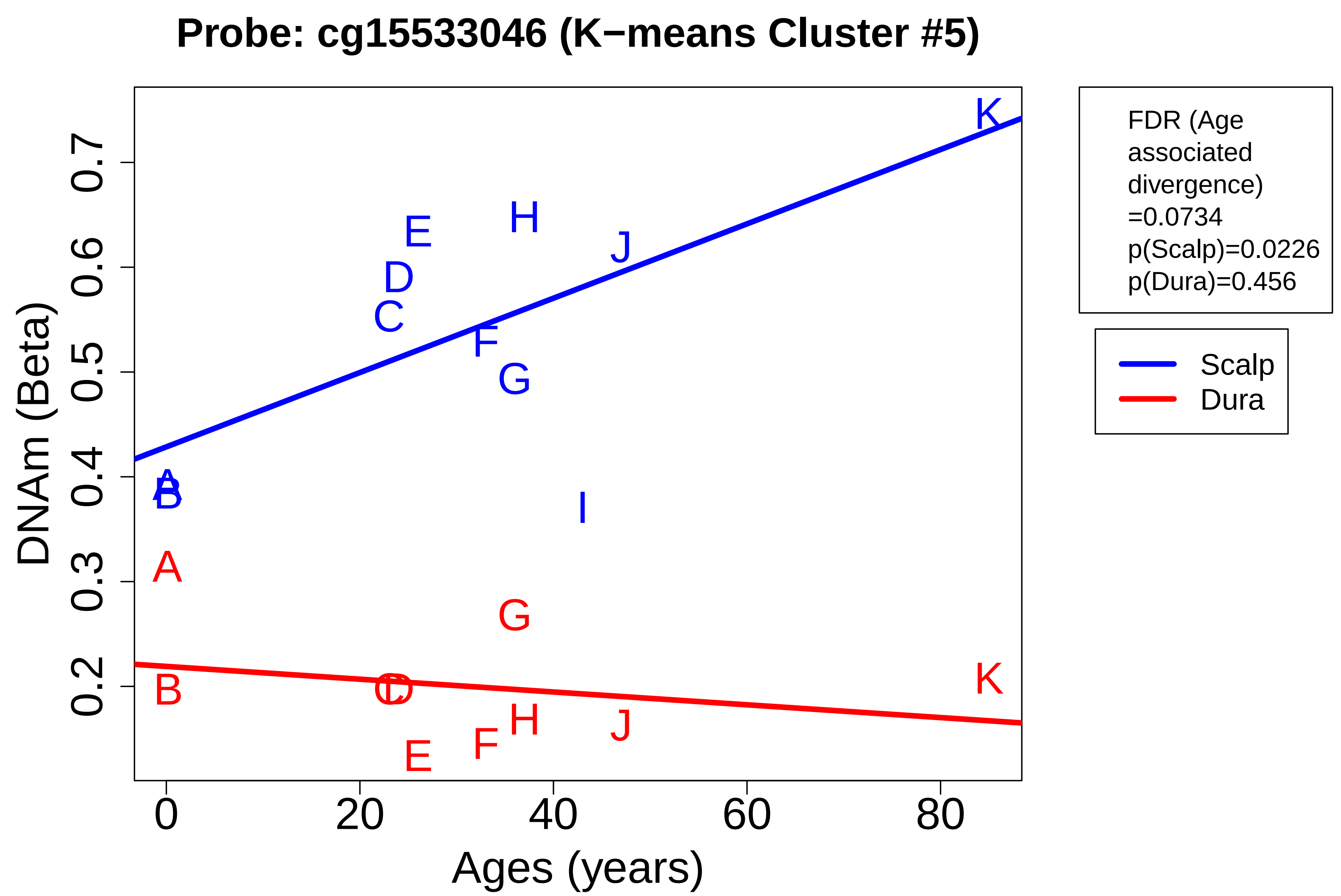

Probe: cg15533046 (K−means Cluster #5)
DNAm (Beta)
0.2
0.3
0.4
0.5
0.6
0.7
0
20
40
60
80
Ages (years)
FDR (Age associated divergence) =0.0734
p(Scalp)=0.0226
p(Dura)=0.456
Scalp
Dura

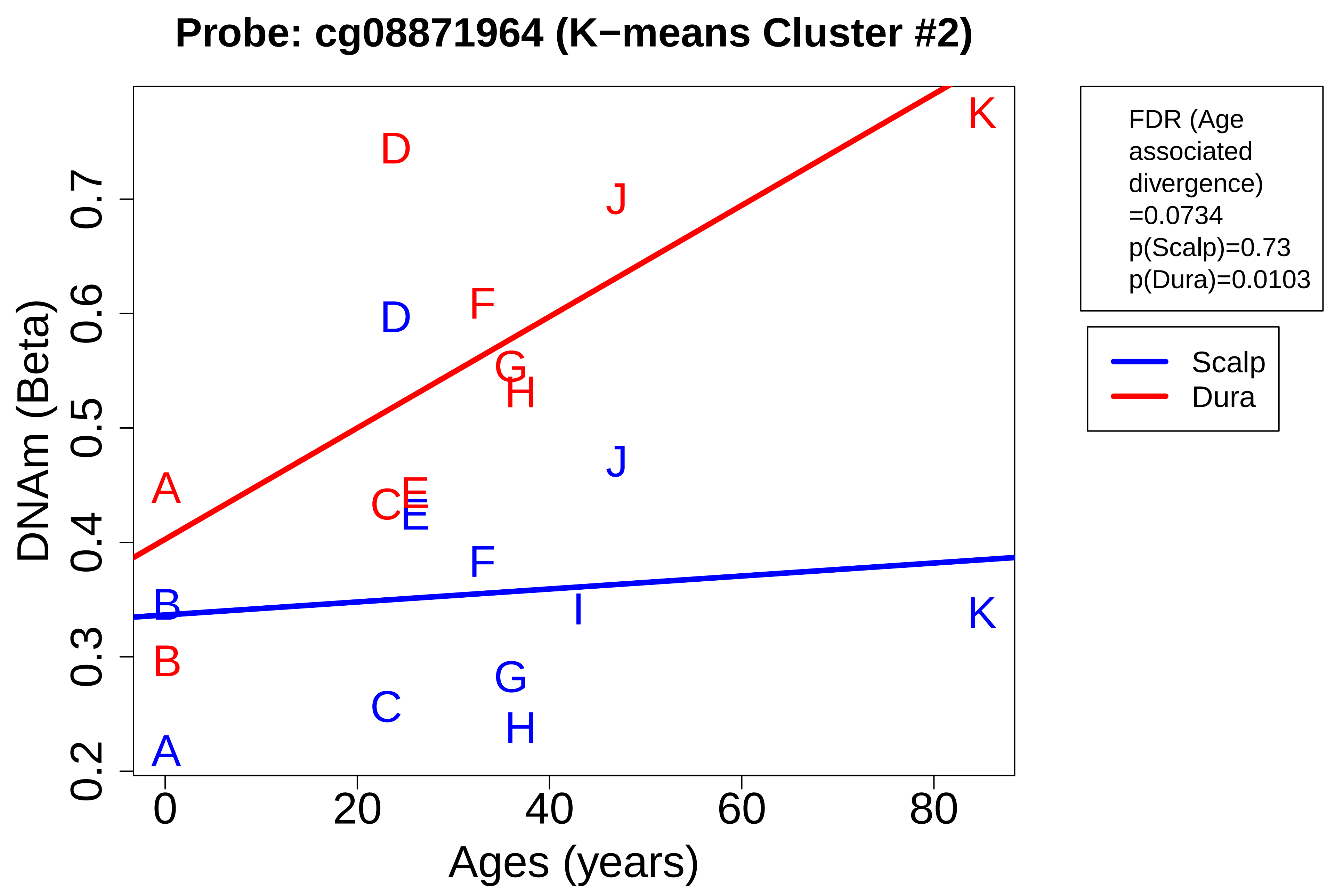

Probe: cg08871964 (K−means Cluster #2)
DNAm (Beta)
Ages (years)
0.2
0.3
0.4
0.5
0.6
0.7
0
20
40
60
80
A
B
C
D
E
F
G
H
I
J
K
FDR (Age associated divergence) =0.0734
p(Scalp)=0.73
p(Dura)=0.0103
Scalp
Dura

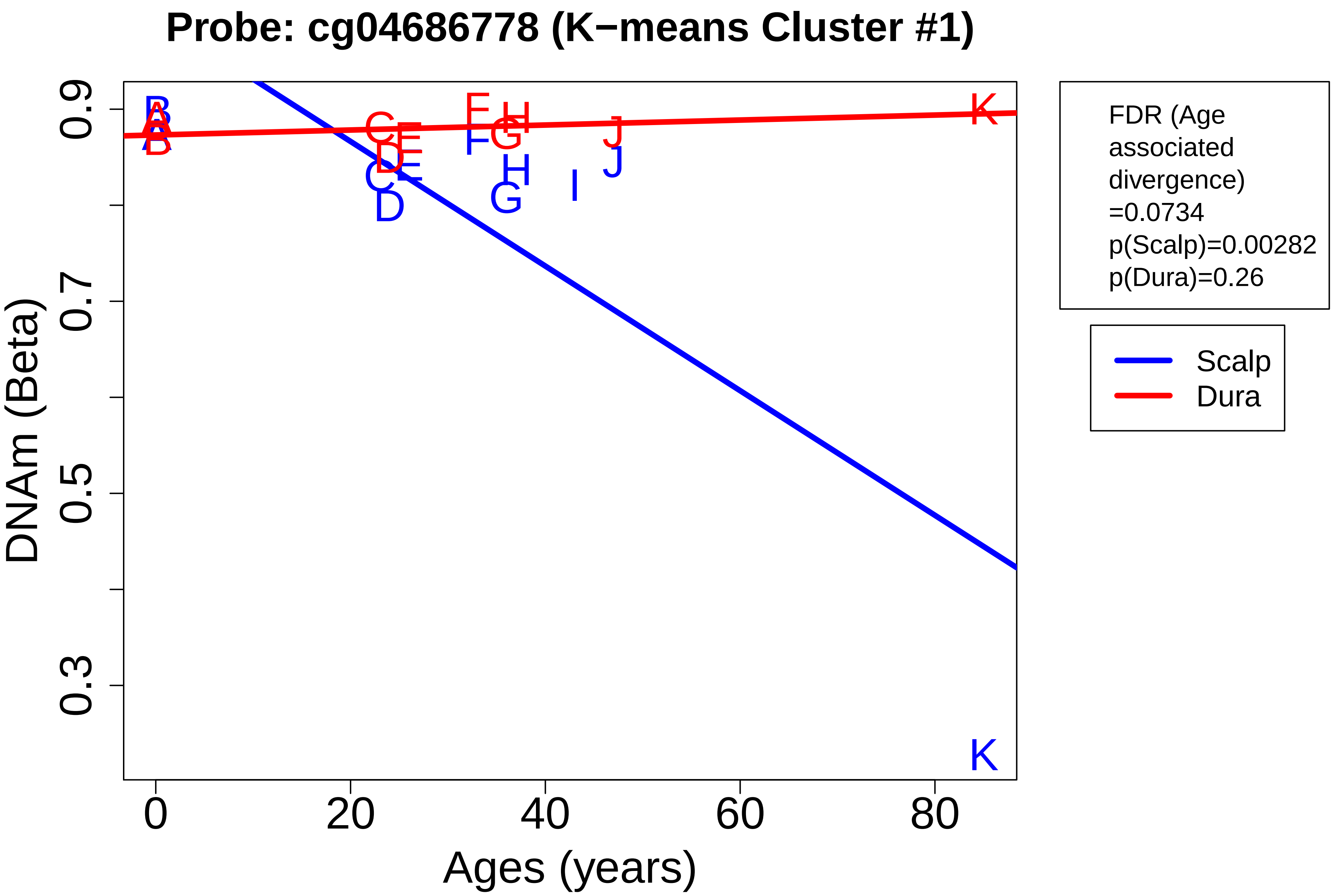

Probe: cg04686778 (K−means Cluster #1)
DNAm (Beta)
0.3
0.5
0.7
0.9
Ages (years)
0
20
40
60
80
FDR (Age associated divergence) =0.0734
p(Scalp)=0.00282
p(Dura)=0.26
Scalp
Dura

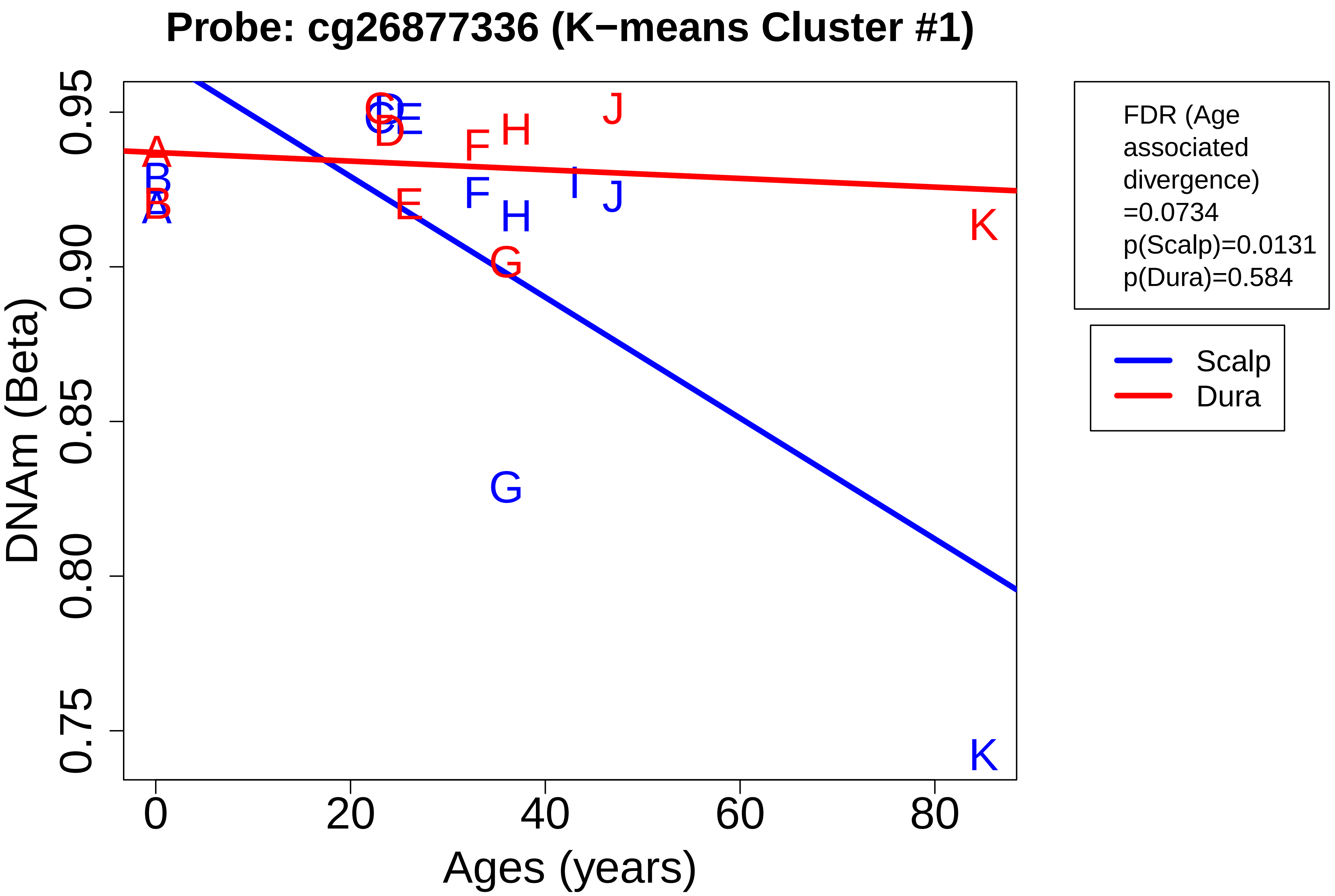

Probe: cg26877336 (K−means Cluster #1)
DNAm (Beta)
0.75
0.80
0.85
0.90
0.95
Ages (years)
0
20
40
60
80
FDR (Age associated divergence) =0.0734
p(Scalp)=0.0131
p(Dura)=0.584
Scalp
Dura

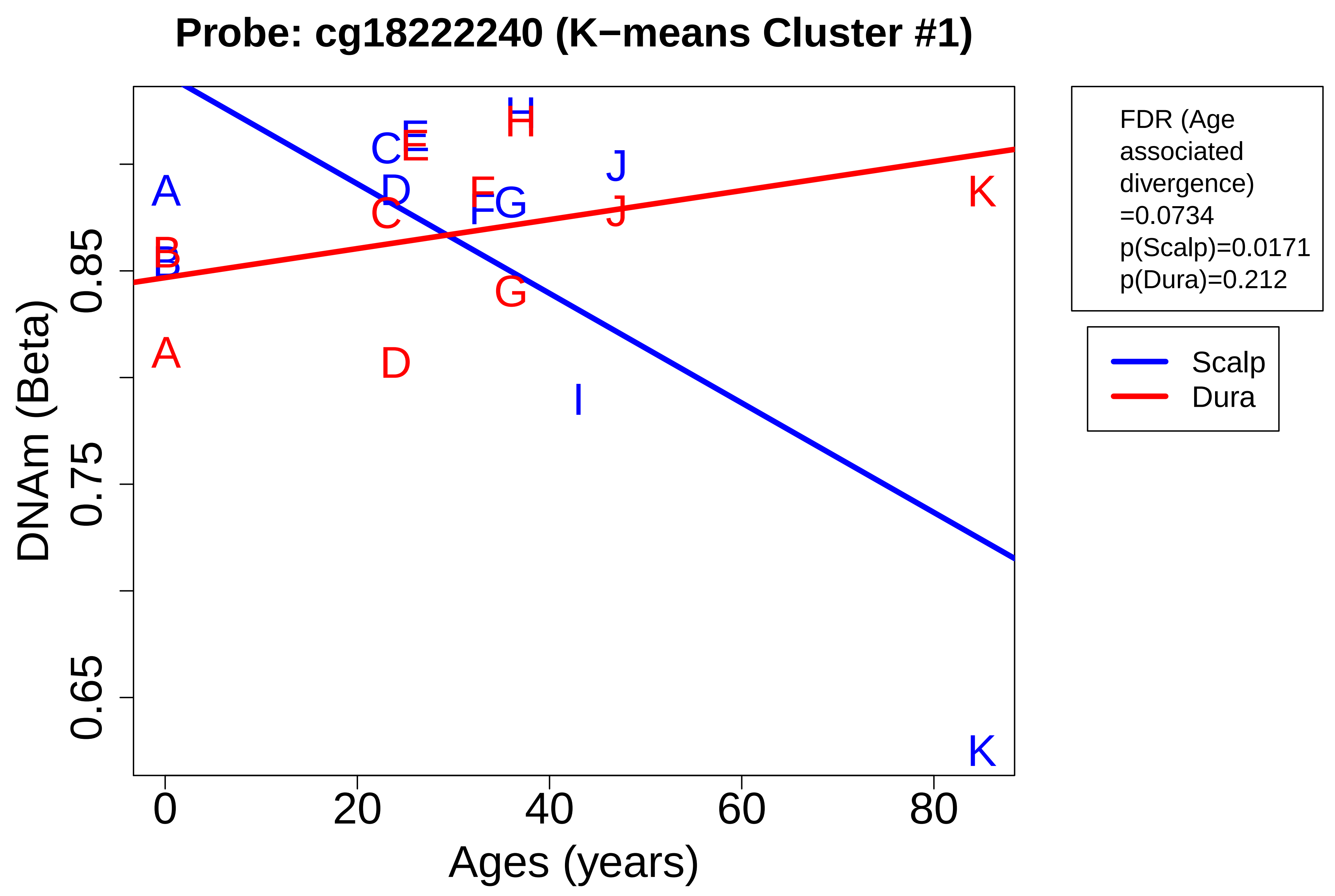

Probe: cg18222240 (K−means Cluster #1)
DNAm (Beta)
0.65
0.75
0.85
Ages (years)
0
20
40
60
80
A
B
C
D
E
F
G
H
I
J
K
FDR (Age associated divergence) =0.0734
p(Scalp)=0.0171
p(Dura)=0.212
Scalp
Dura

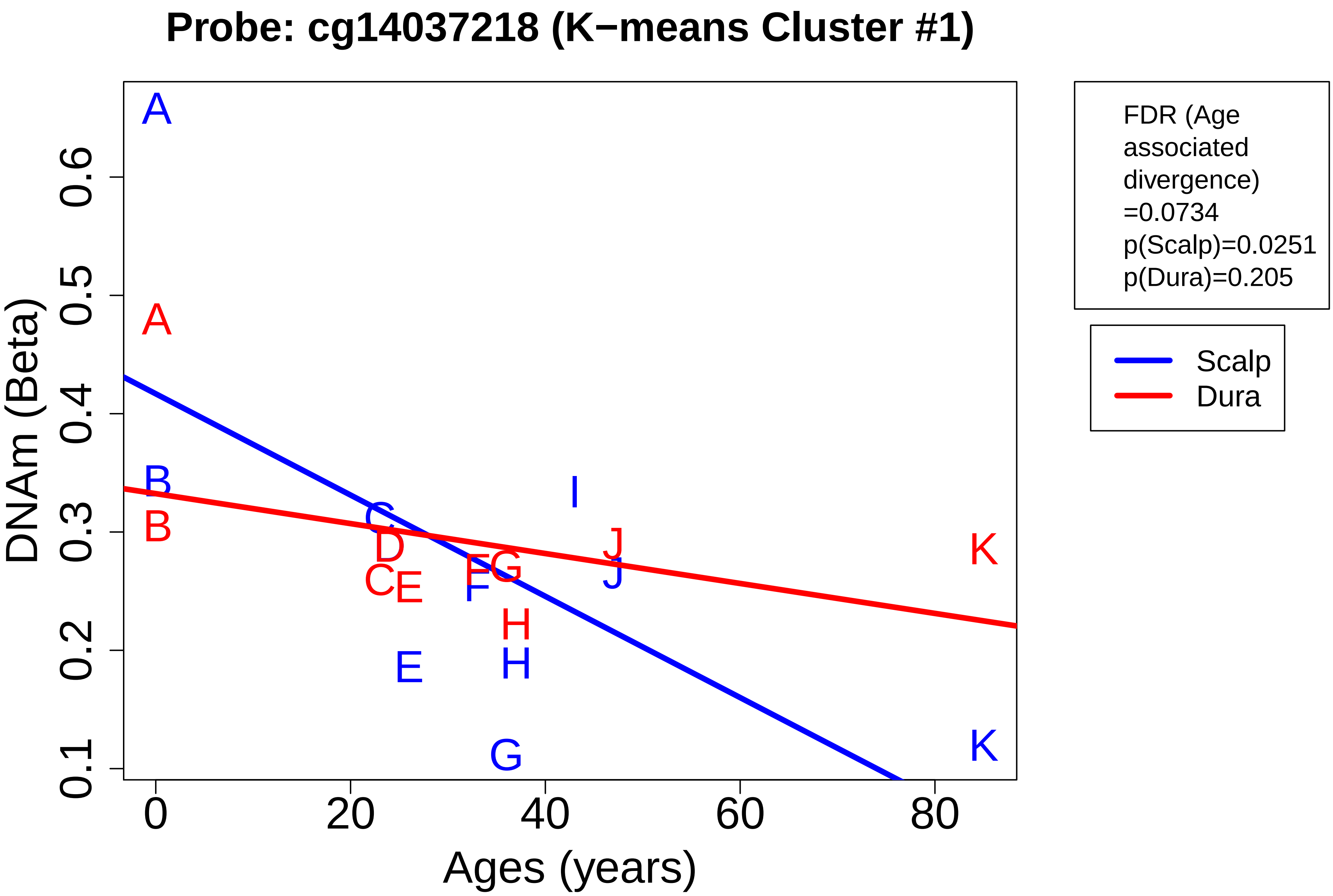

Probe: cg14037218 (K−means Cluster #1)
DNAm (Beta)
0.1
0.2
0.3
0.4
0.5
0.6
0
20
40
60
80
Ages (years)
A
A
B
B
C
C
D
E
E
F
F
G
G
H
H
I
J
J
K
K
FDR (Age associated divergence) =0.0734
p(Scalp)=0.0251
p(Dura)=0.205
Scalp
Dura

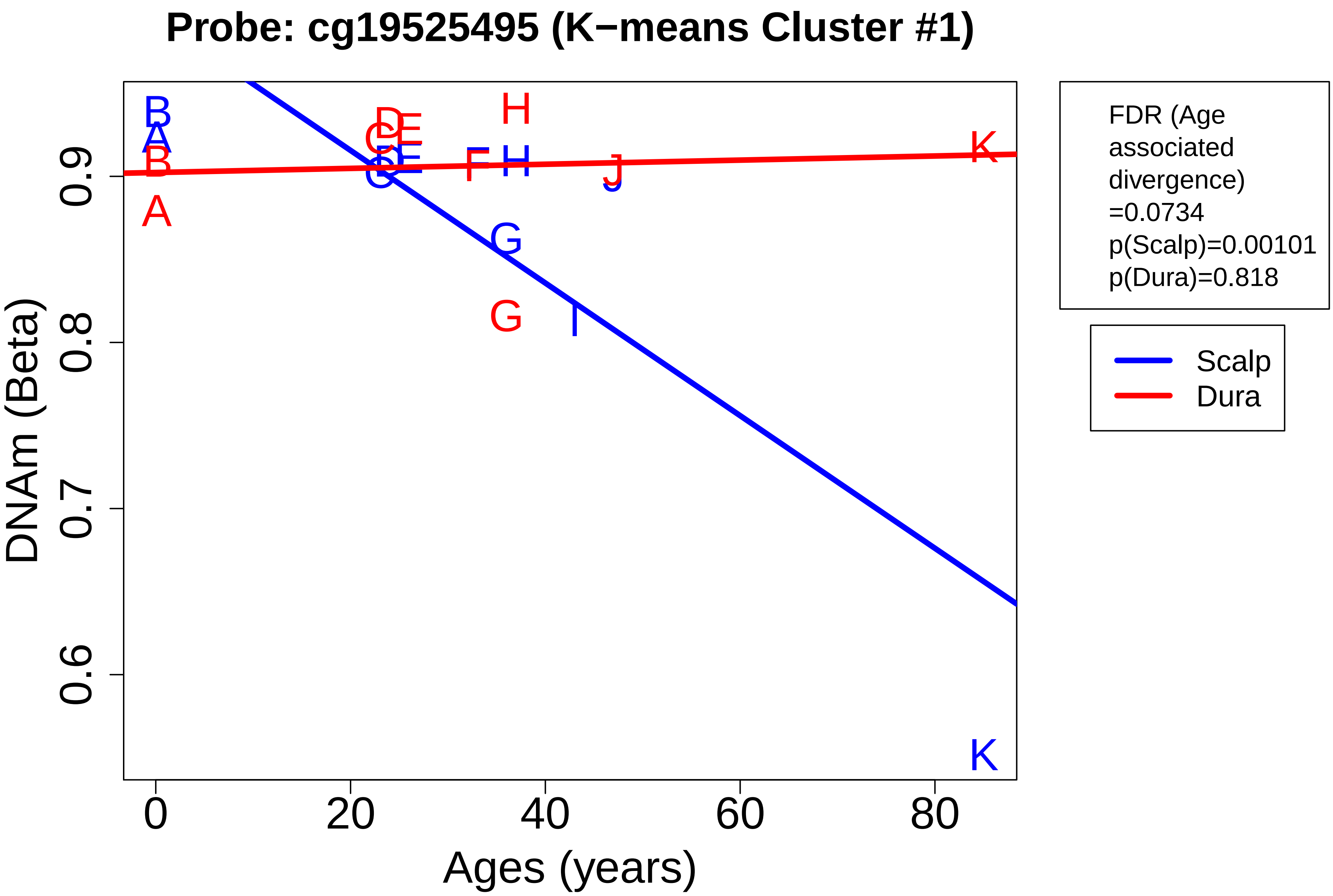

Probe: cg19525495 (K−means Cluster #1)
DNAm (Beta)
0.6
0.7
0.8
0.9
0
20
40
60
80
Ages (years)
FDR (Age associated divergence) =0.0734
p(Scalp)=0.00101
p(Dura)=0.818
Scalp
Dura

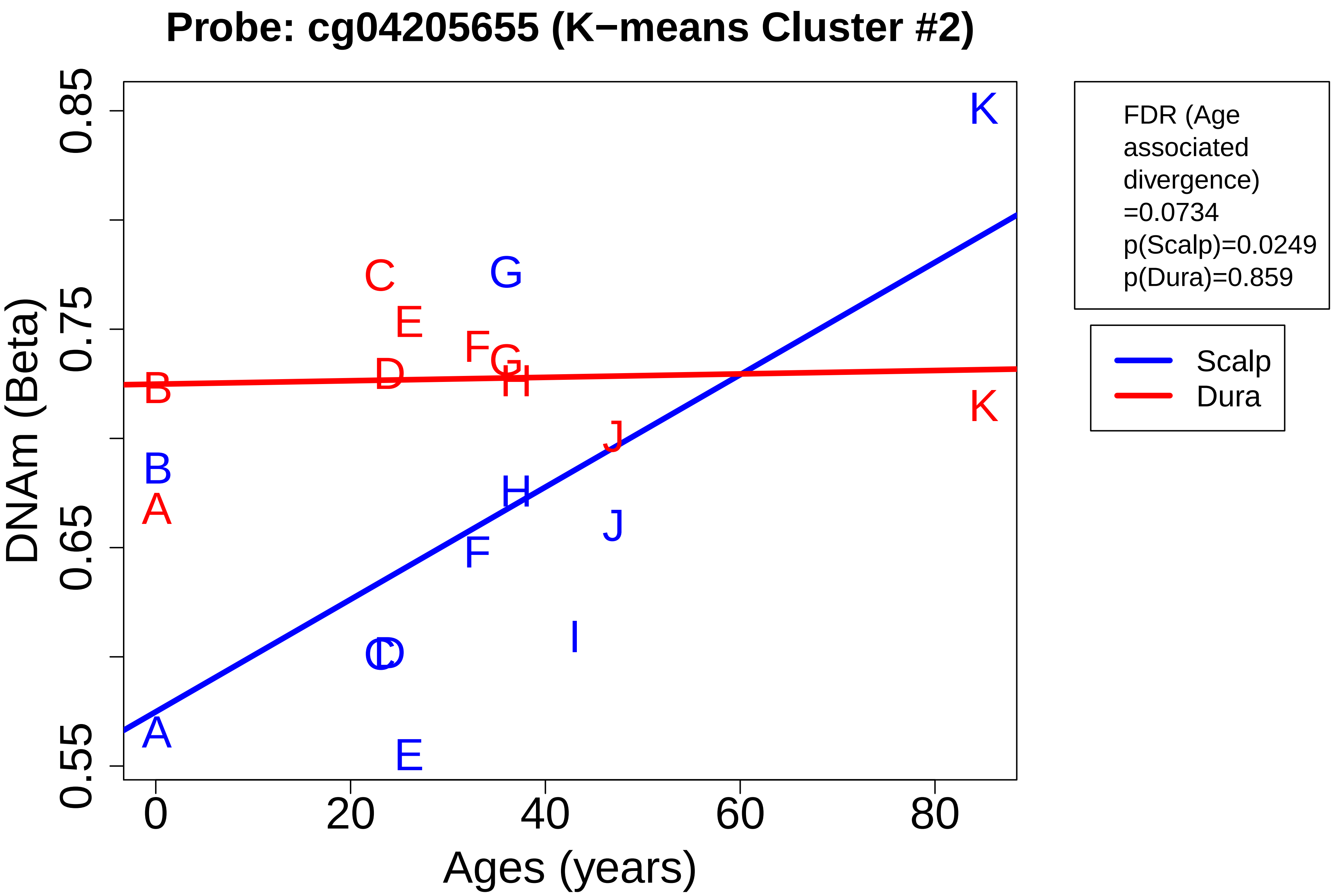
Probe: cg04205655 (K−means Cluster #2)
DNAm (Beta)
0.55
0.65
0.75
0.85
Ages (years)
0
20
40
60
80
FDR (Age associated divergence) =0.0734
p(Scalp)=0.0249
p(Dura)=0.859
Scalp
Dura
A
B
C
D
E
F
G
H
I
J
K

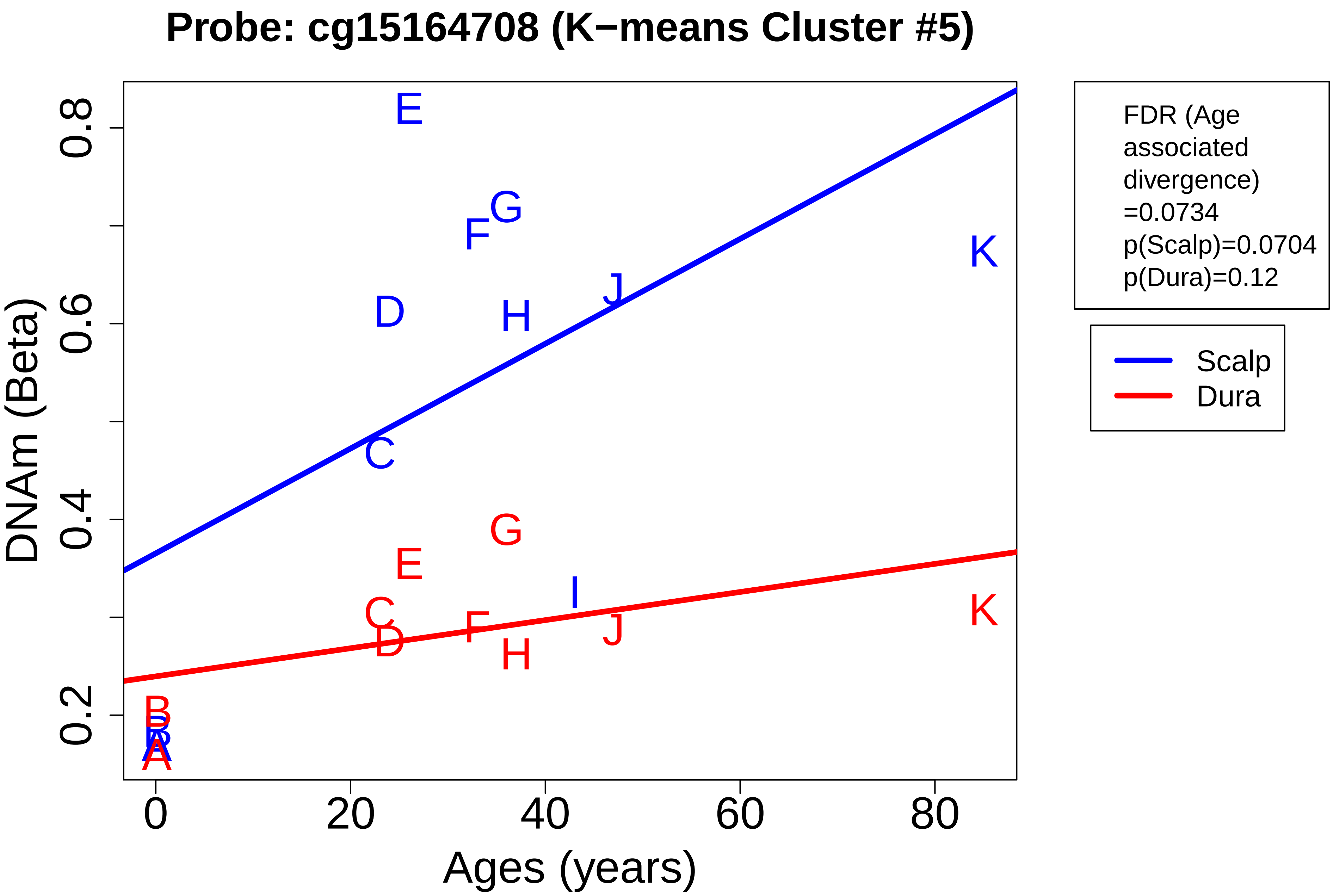

Probe: cg15164708 (K−means Cluster #5)
DNAm (Beta)
0.2
0.4
0.6
0.8
0
20
40
60
80
Ages (years)
FDR (Age associated divergence) =0.0734
p(Scalp)=0.0704
p(Dura)=0.12
Scalp
Dura

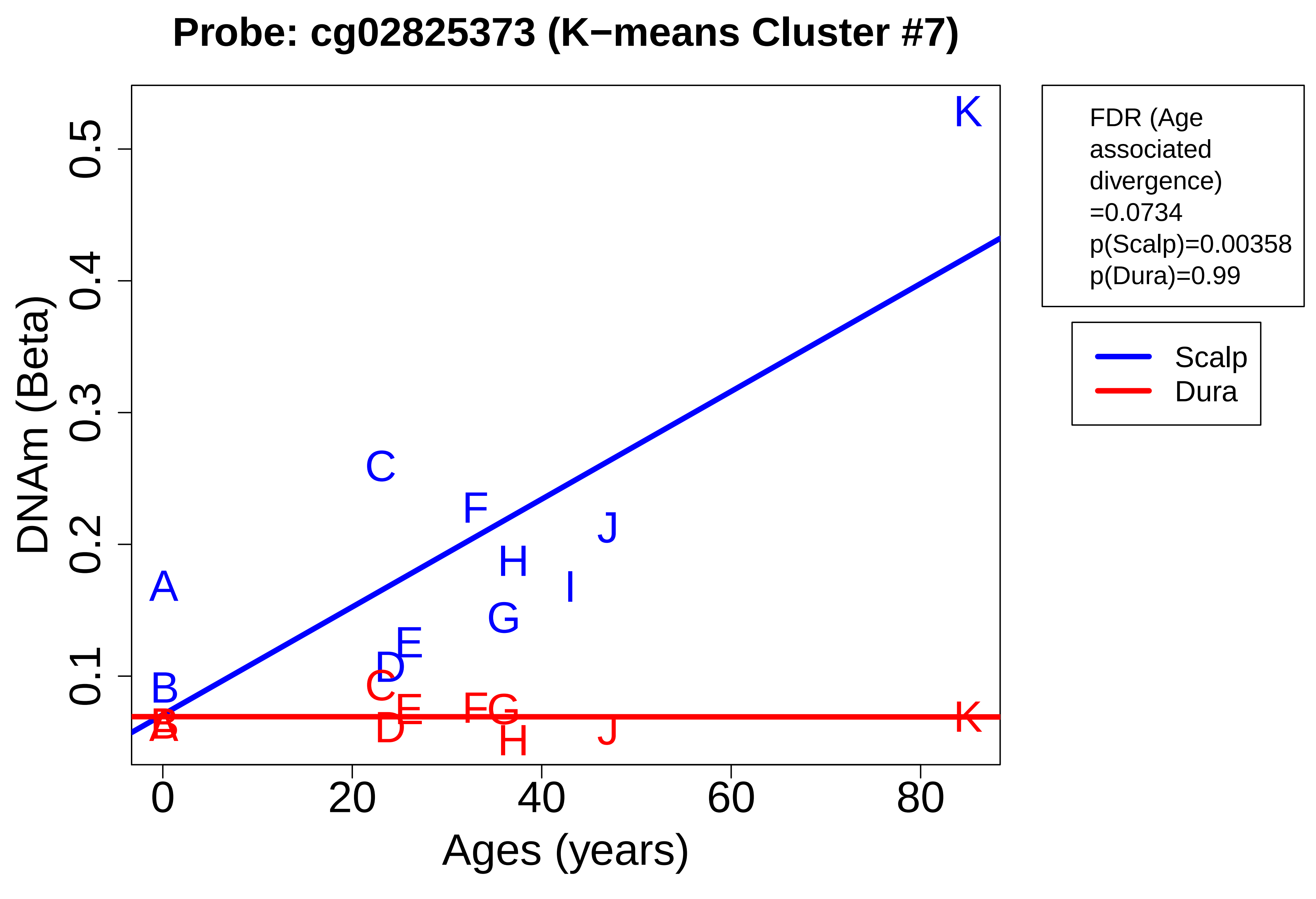

Probe: cg02825373 (K−means Cluster #7)
DNAm (Beta)
0.1
0.2
0.3
0.4
0.5
0
20
40
60
80
Ages (years)
FDR (Age associated divergence) =0.0734
p(Scalp)=0.00358
p(Dura)=0.99
Scalp
Dura

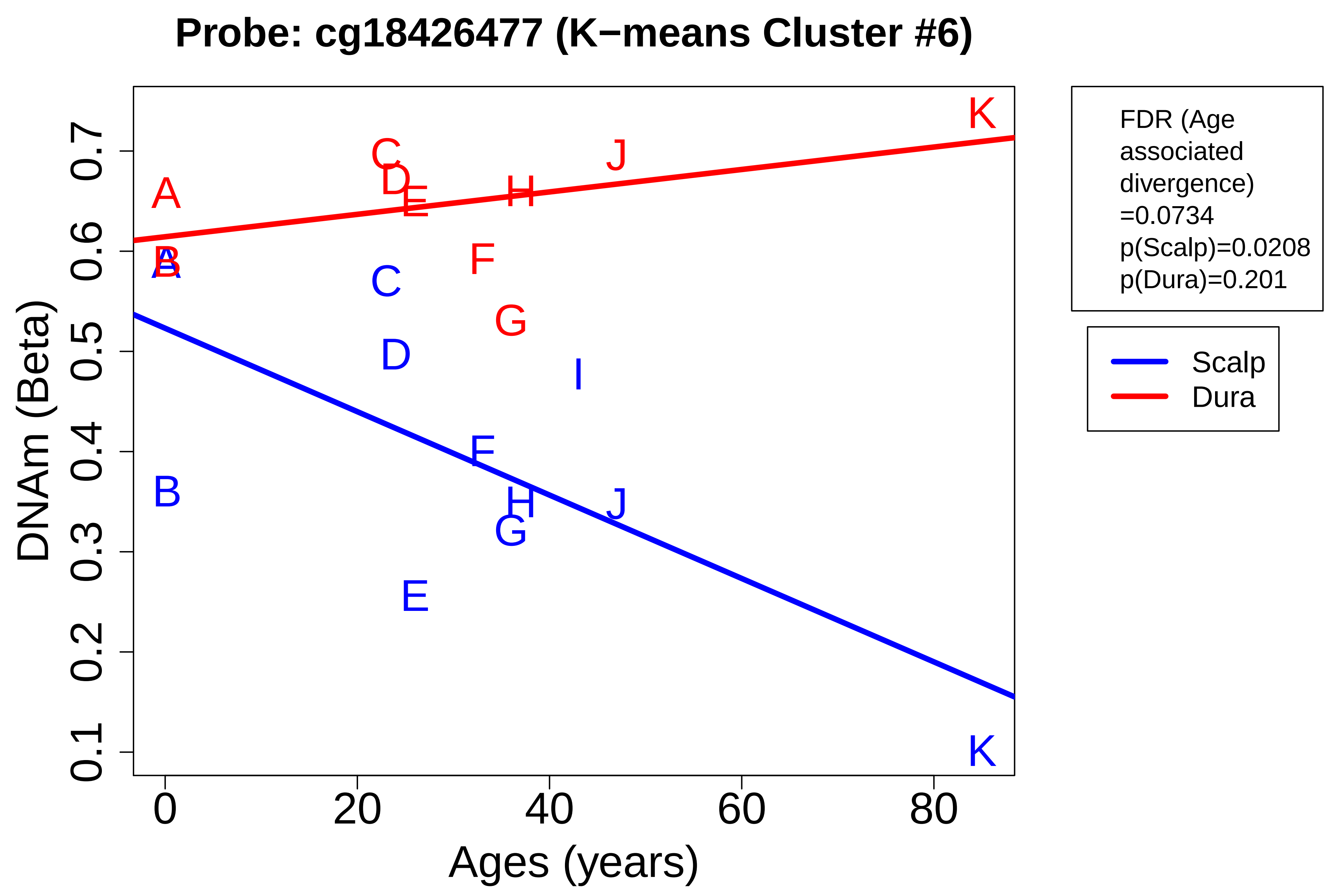
Probe: cg18426477 (K−means Cluster #6)
DNAm (Beta)
0.1
0.2
0.3
0.4
0.5
0.6
0.7
0
20
40
60
80
Ages (years)
A
B
C
D
E
F
G
H
I
J
K
FDR (Age associated divergence) =0.0734
p(Scalp)=0.0208
p(Dura)=0.201
Scalp
Dura

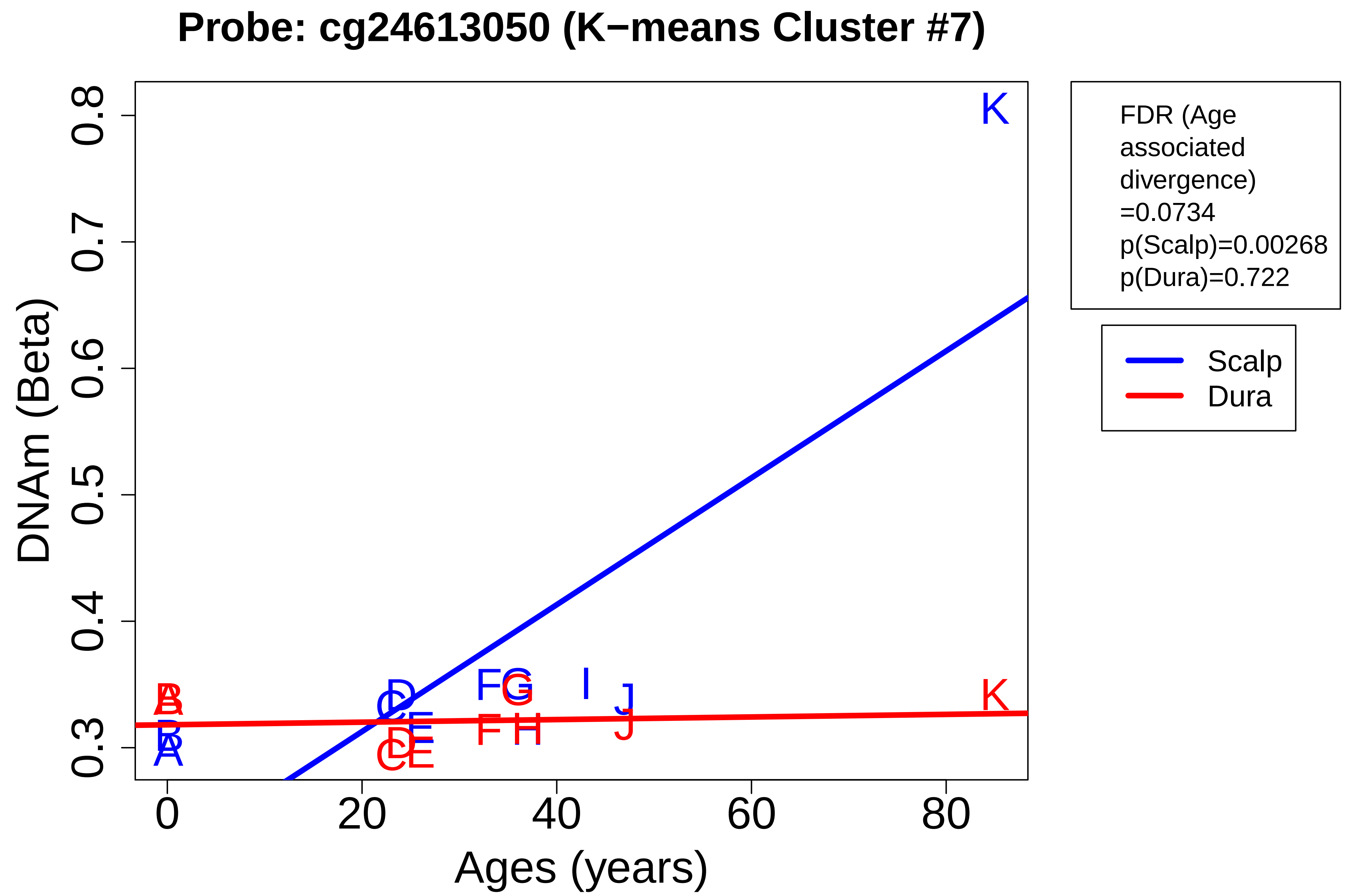

Probe: cg24613050 (K−means Cluster #7)
DNAm (Beta)
0.3
0.4
0.5
0.6
0.7
0.8
0
20
40
60
80
Ages (years)
FDR (Age associated divergence) =0.0734
p(Scalp)=0.00268
p(Dura)=0.722
Scalp
Dura

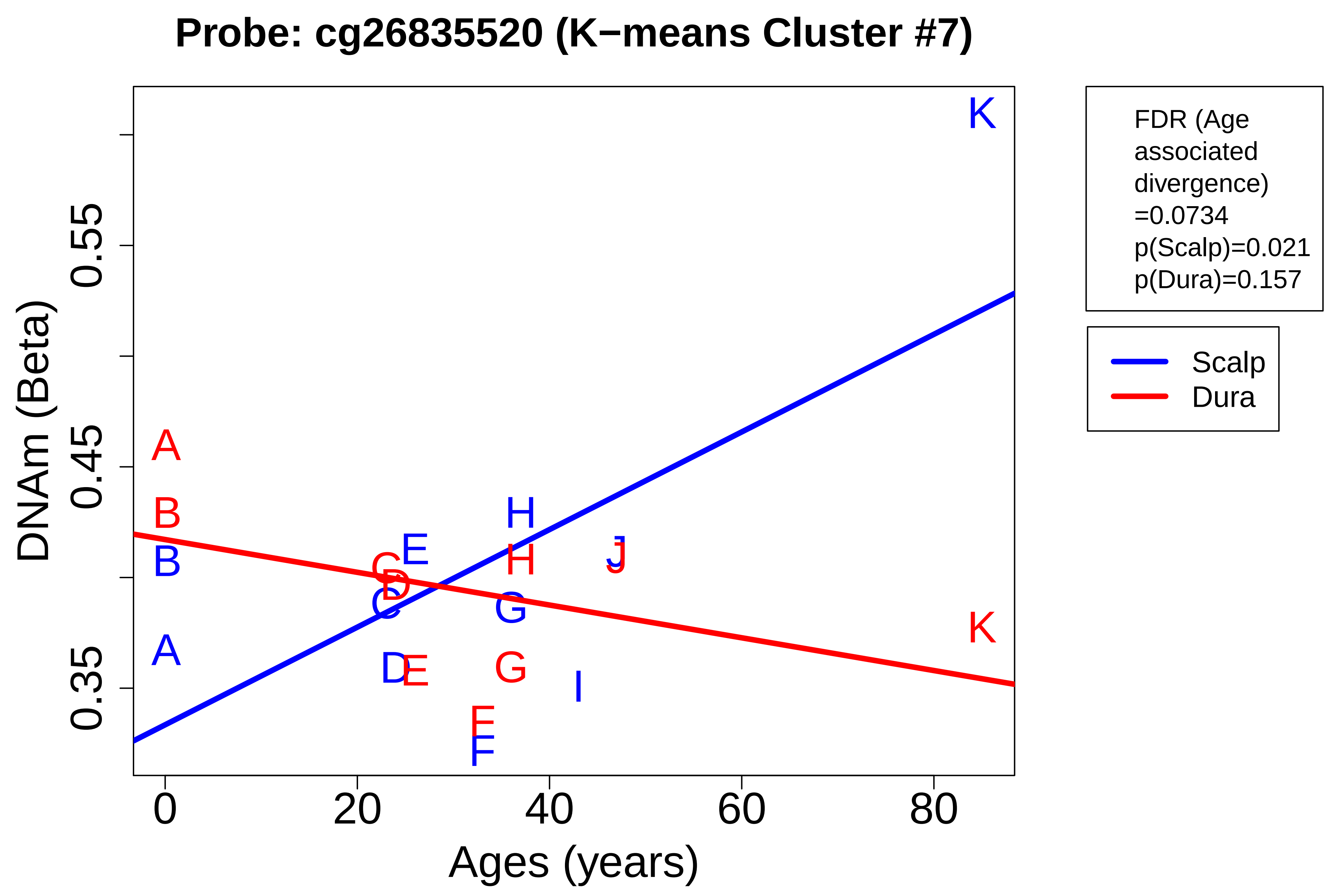

Probe: cg26835520 (K−means Cluster #7)
DNAm (Beta)
0.35
0.45
0.55
Ages (years)
0
20
40
60
80
FDR (Age associated divergence) =0.0734
p(Scalp)=0.021
p(Dura)=0.157
Scalp
Dura
A
B
C
D
E
F
G
H
I
J
K

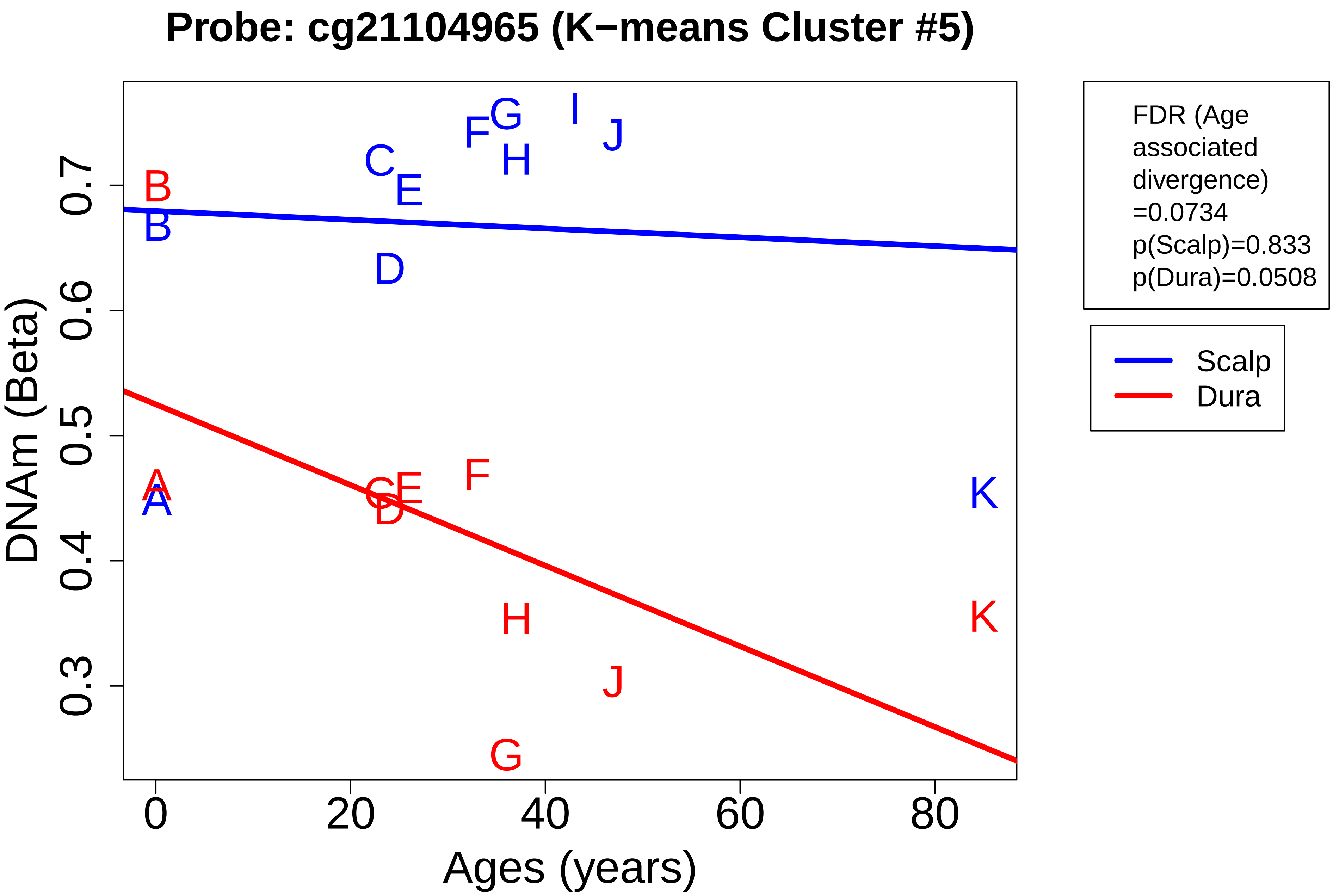

Probe: cg21104965 (K−means Cluster #5)
DNAm (Beta)
0.3
0.4
0.5
0.6
0.7
0
20
40
60
80
Ages (years)
A B C D E F G H I J K
FDR (Age associated divergence) =0.0734
p(Scalp)=0.833
p(Dura)=0.0508
Scalp
Dura

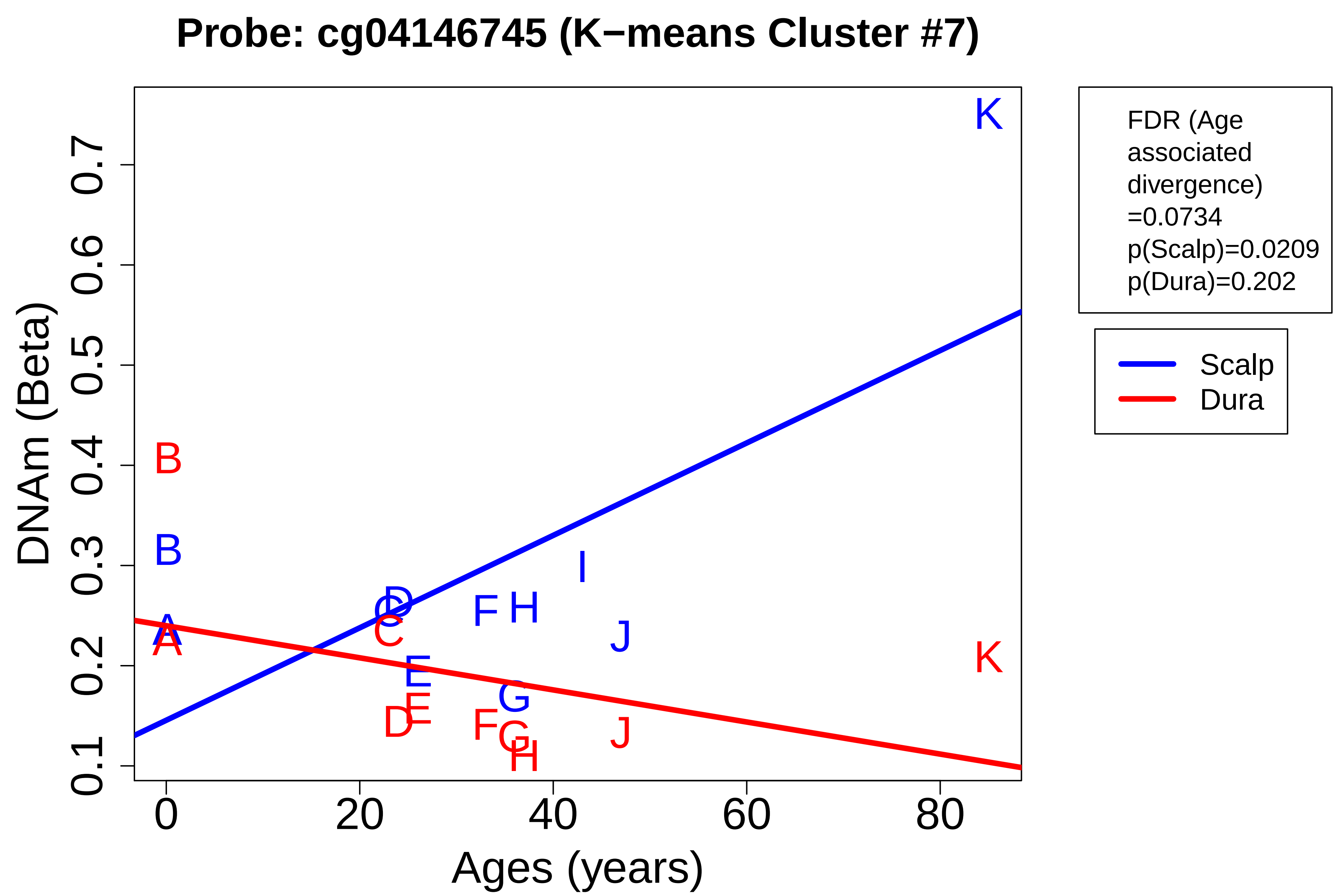

Probe: cg04146745 (K−means Cluster #7)
DNAm (Beta)
Ages (years)
0.1
0.2
0.3
0.4
0.5
0.6
0.7
0
20
40
60
80
FDR (Age associated divergence) =0.0734
p(Scalp)=0.0209
p(Dura)=0.202
Scalp
Dura
A
B
C
D
E
F
G
H
I
J
K

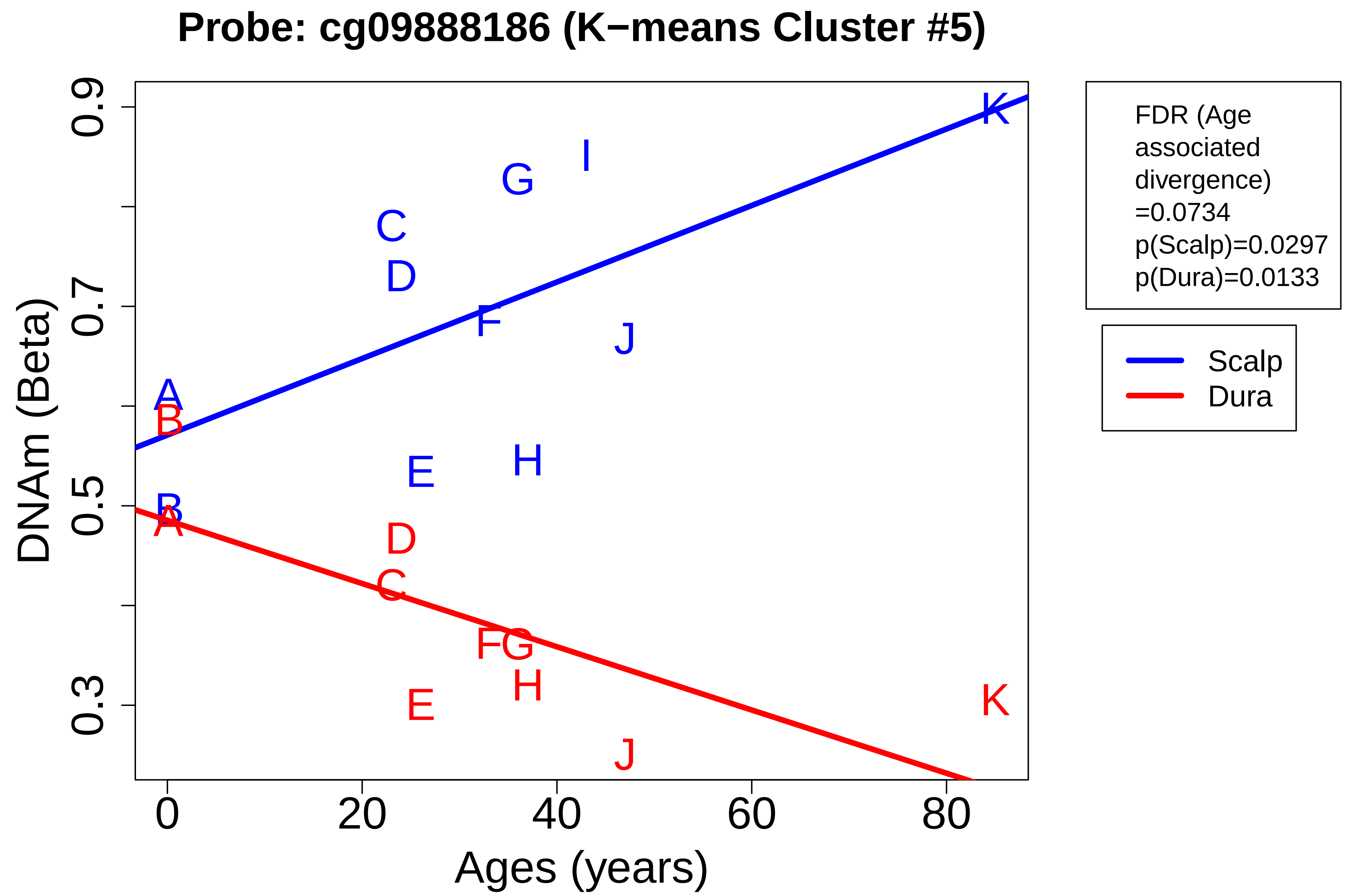

Probe: cg09888186 (K−means Cluster #5)
DNAm (Beta)
0.3
0.5
0.7
0.9
Ages (years)
0
20
40
60
80
A
B
C
D
E
F
G
H
I
J
K
FDR (Age associated divergence) =0.0734
p(Scalp)=0.0297
p(Dura)=0.0133
Scalp
Dura

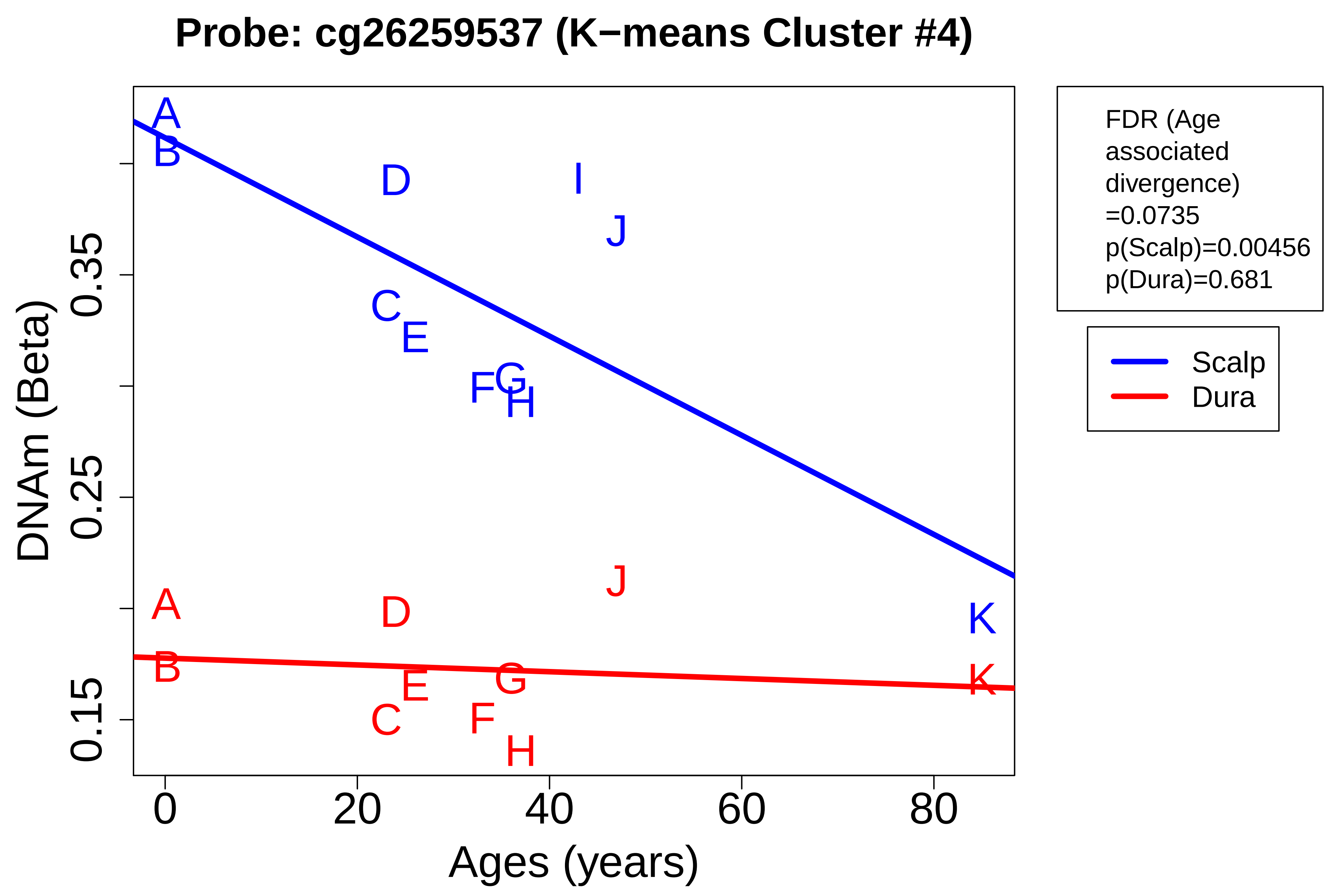

Probe: cg26259537 (K−means Cluster #4)
FDR (Age associated divergence) =0.0735
p(Scalp)=0.00456
p(Dura)=0.681
Scalp
Dura
DNAm (Beta)
0.15
0.25
0.35
Ages (years)
0
20
40
60
80
A
B
C
D
E
F
G
H
I
J
K

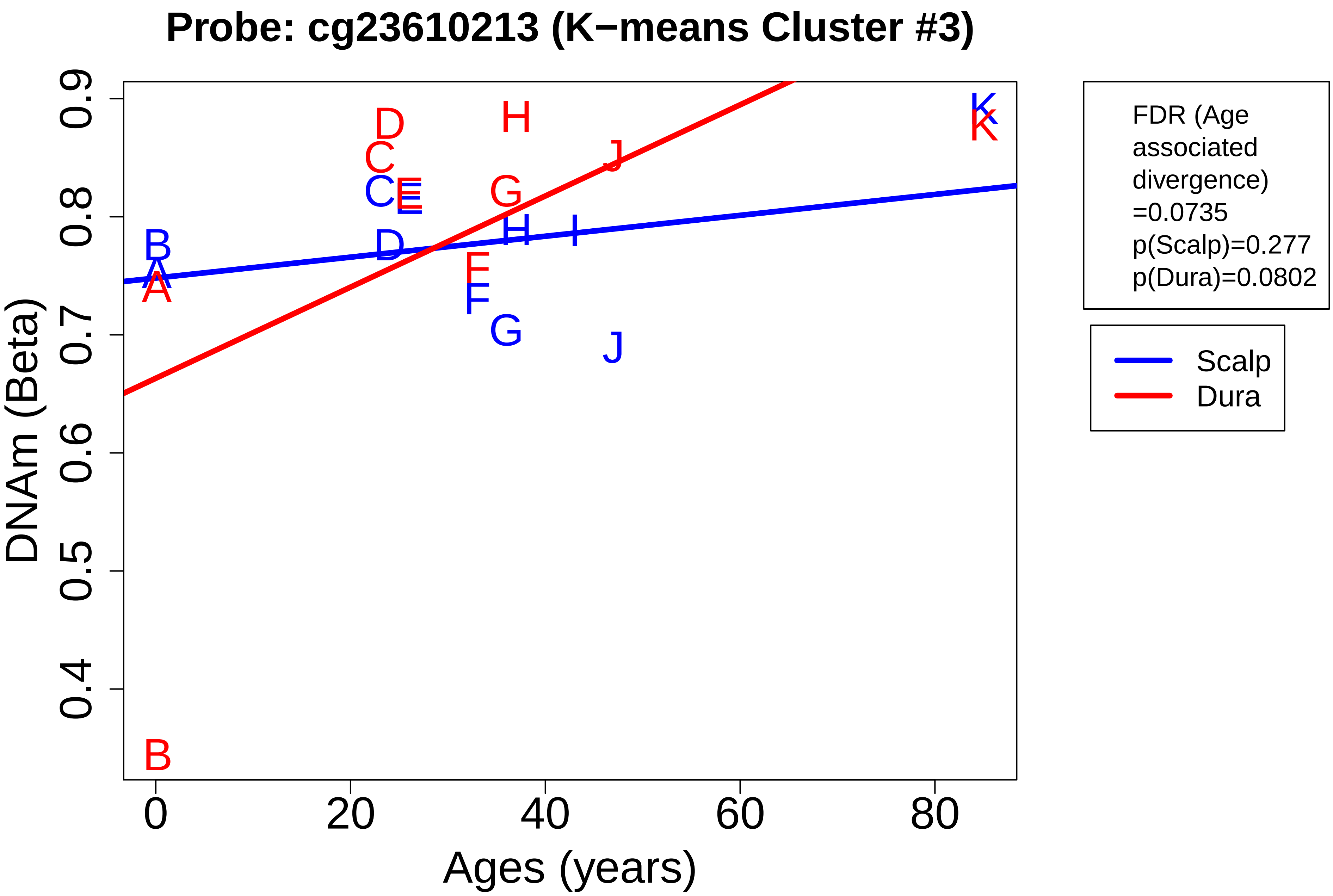

Probe: cg23610213 (K−means Cluster #3)
DNAm (Beta)
Ages (years)
0.4
0.5
0.6
0.7
0.8
0.9
0
20
40
60
80
FDR (Age associated divergence) =0.0735
p(Scalp)=0.277
p(Dura)=0.0802
Scalp
Dura

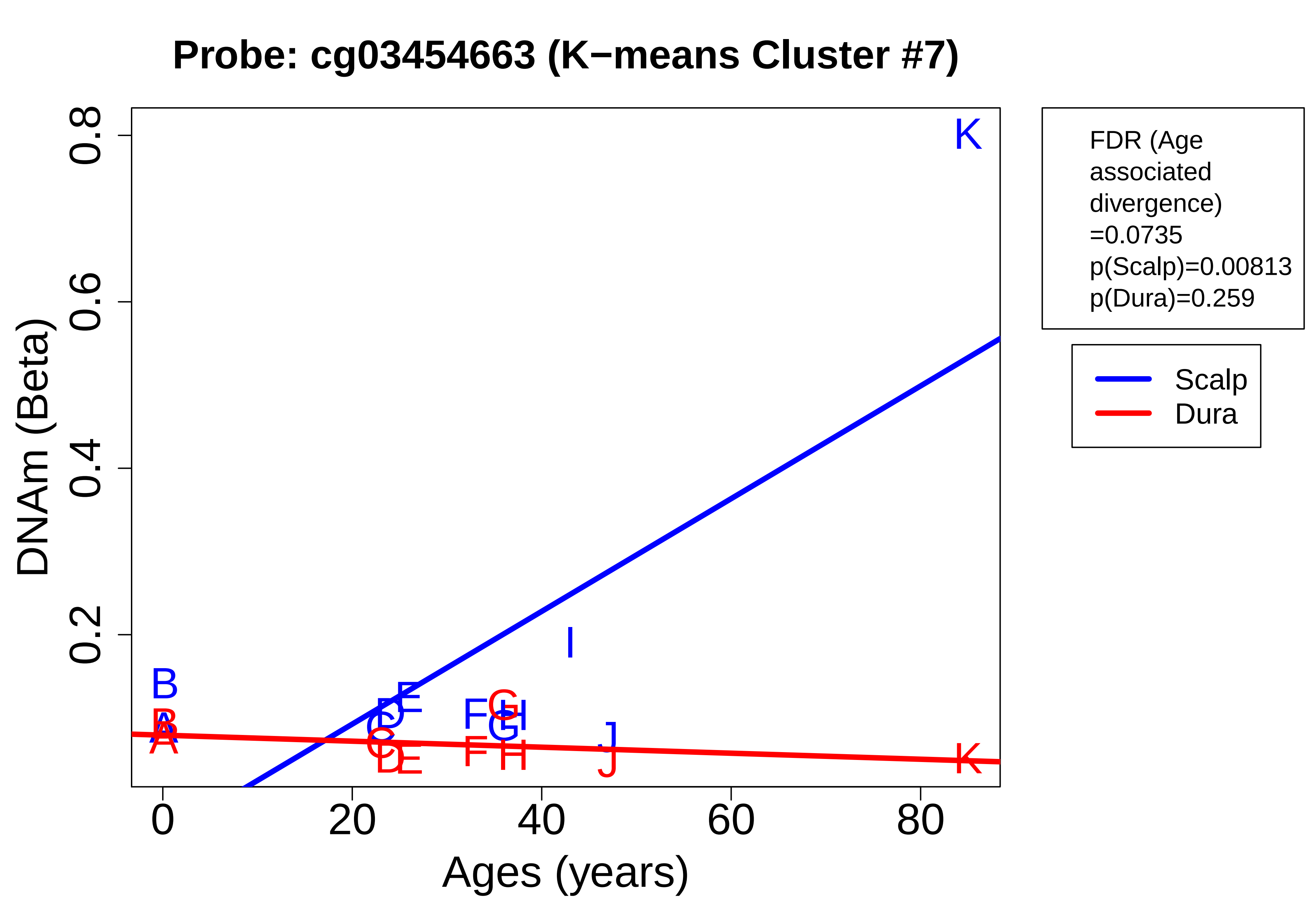
Probe: cg03454663 (K−means Cluster #7)
DNAm (Beta)
0.2
0.4
0.6
0.8
Ages (years)
0
20
40
60
80
FDR (Age associated divergence) =0.0735
p(Scalp)=0.00813
p(Dura)=0.259
Scalp
Dura

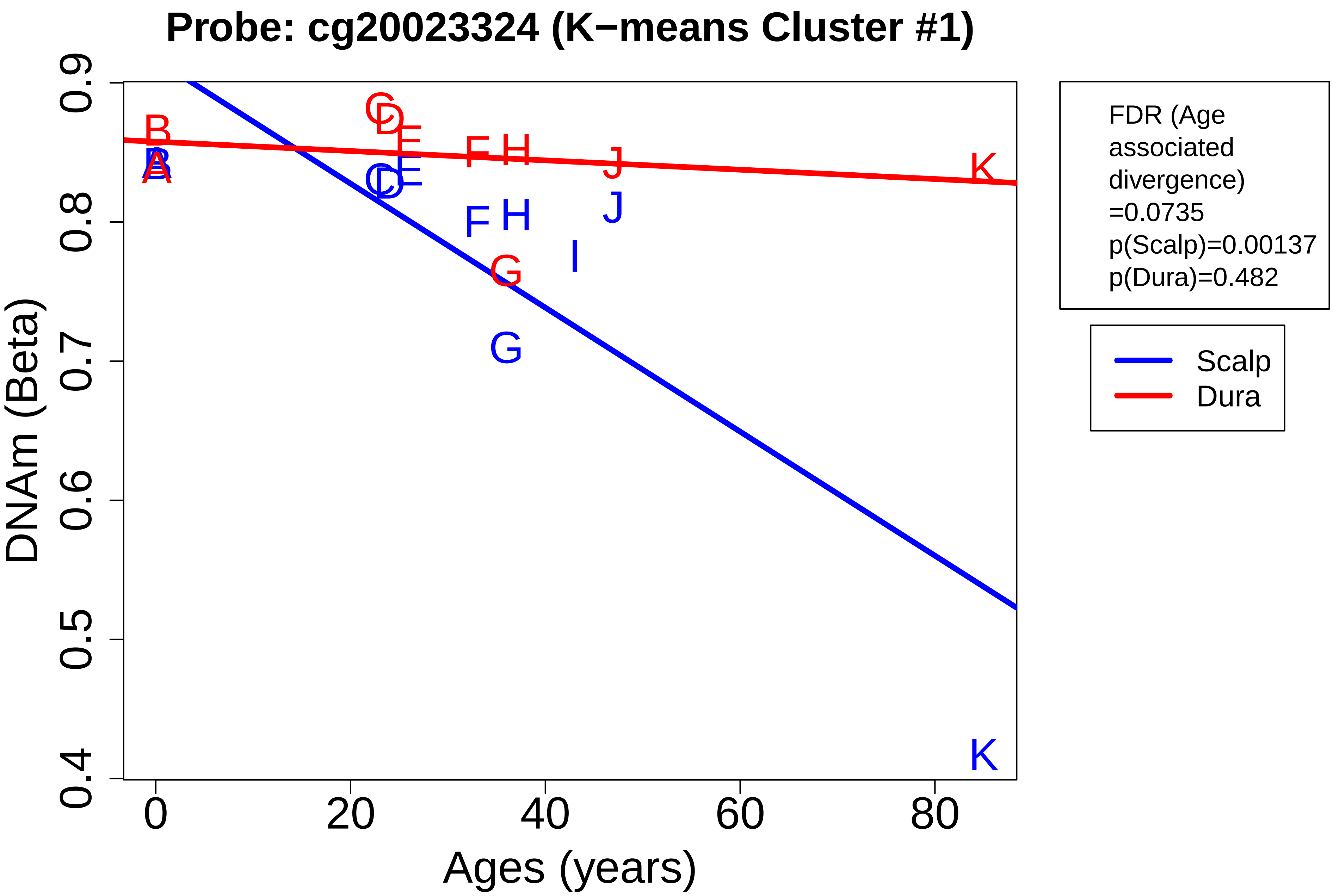

Probe: cg20023324 (K−means Cluster #1)
DNAm (Beta)
0.4
0.5
0.6
0.7
0.8
0.9
0
20
40
60
80
Ages (years)
FDR (Age associated divergence) =0.0735
p(Scalp)=0.00137
p(Dura)=0.482
Scalp
Dura

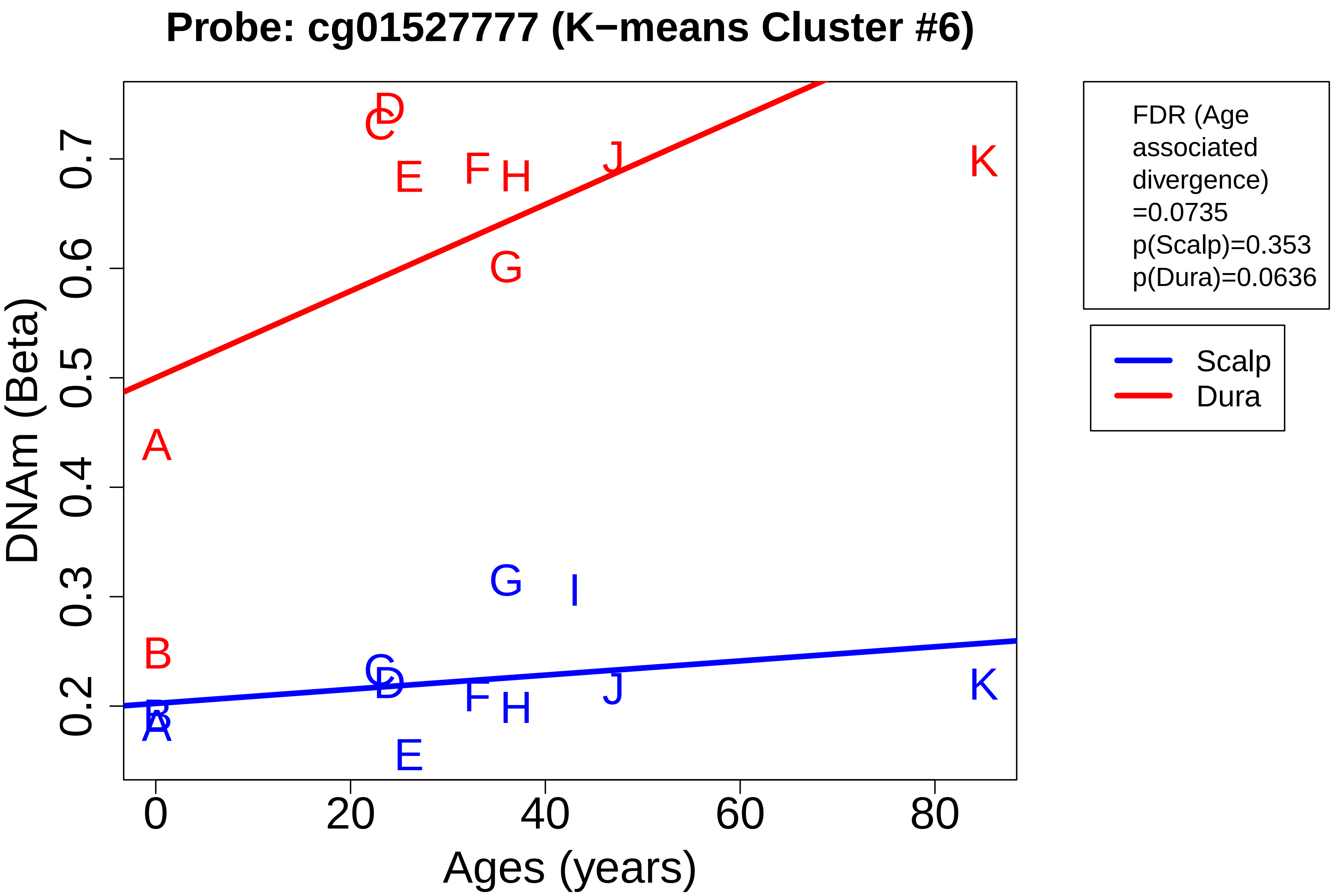

Probe: cg01527777 (K−means Cluster #6)
DNAm (Beta)
Ages (years)
0.2
0.3
0.4
0.5
0.6
0.7
0
20
40
60
80
FDR (Age associated divergence) =0.0735
p(Scalp)=0.353
p(Dura)=0.0636
Scalp
Dura

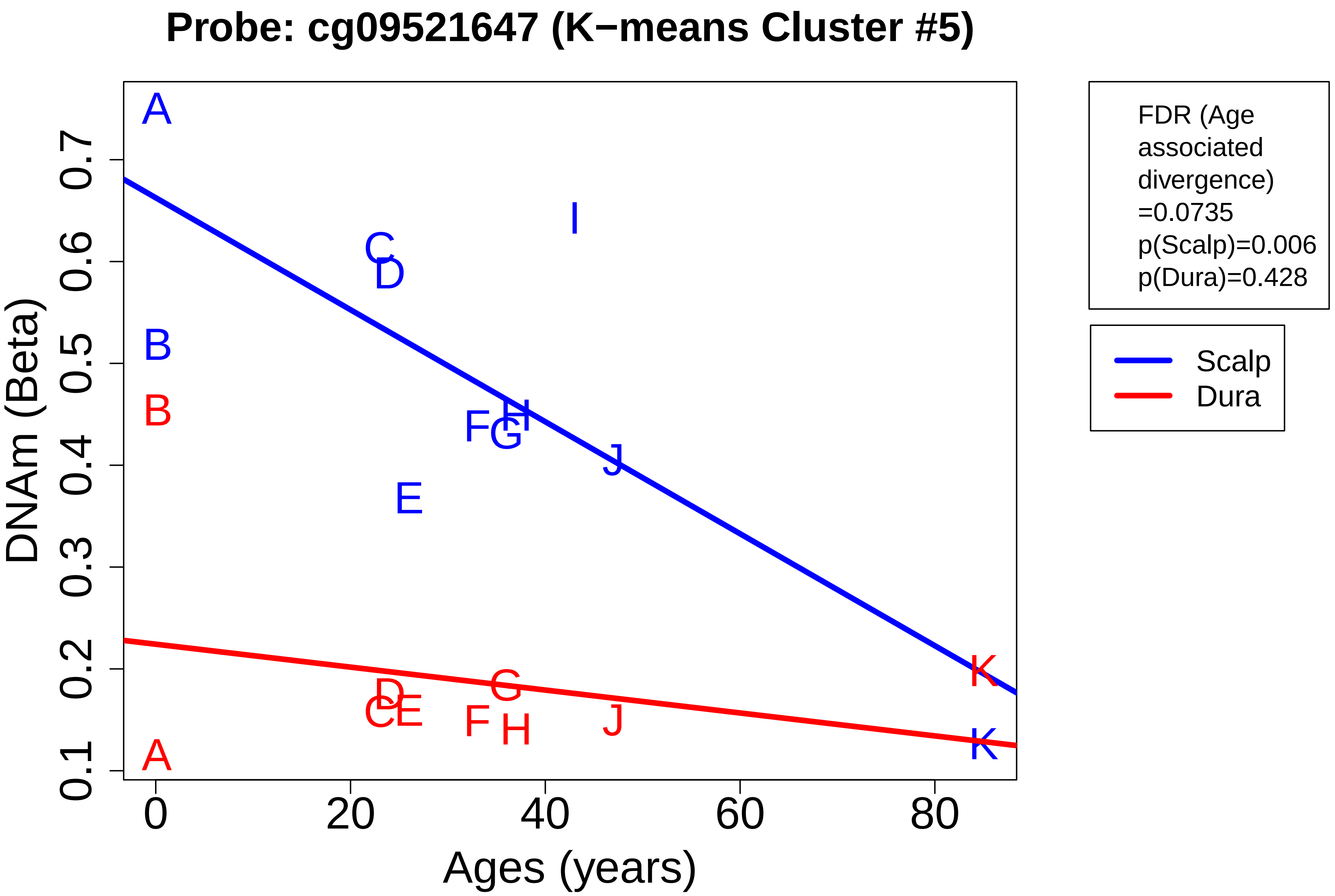

Probe: cg09521647 (K−means Cluster #5)
DNAm (Beta)
0.1
0.2
0.3
0.4
0.5
0.6
0.7
Ages (years)
0
20
40
60
80
A
B
C
D
E
F
G
H
I
J
K
FDR (Age associated divergence) =0.0735
p(Scalp)=0.006
p(Dura)=0.428
Scalp
Dura

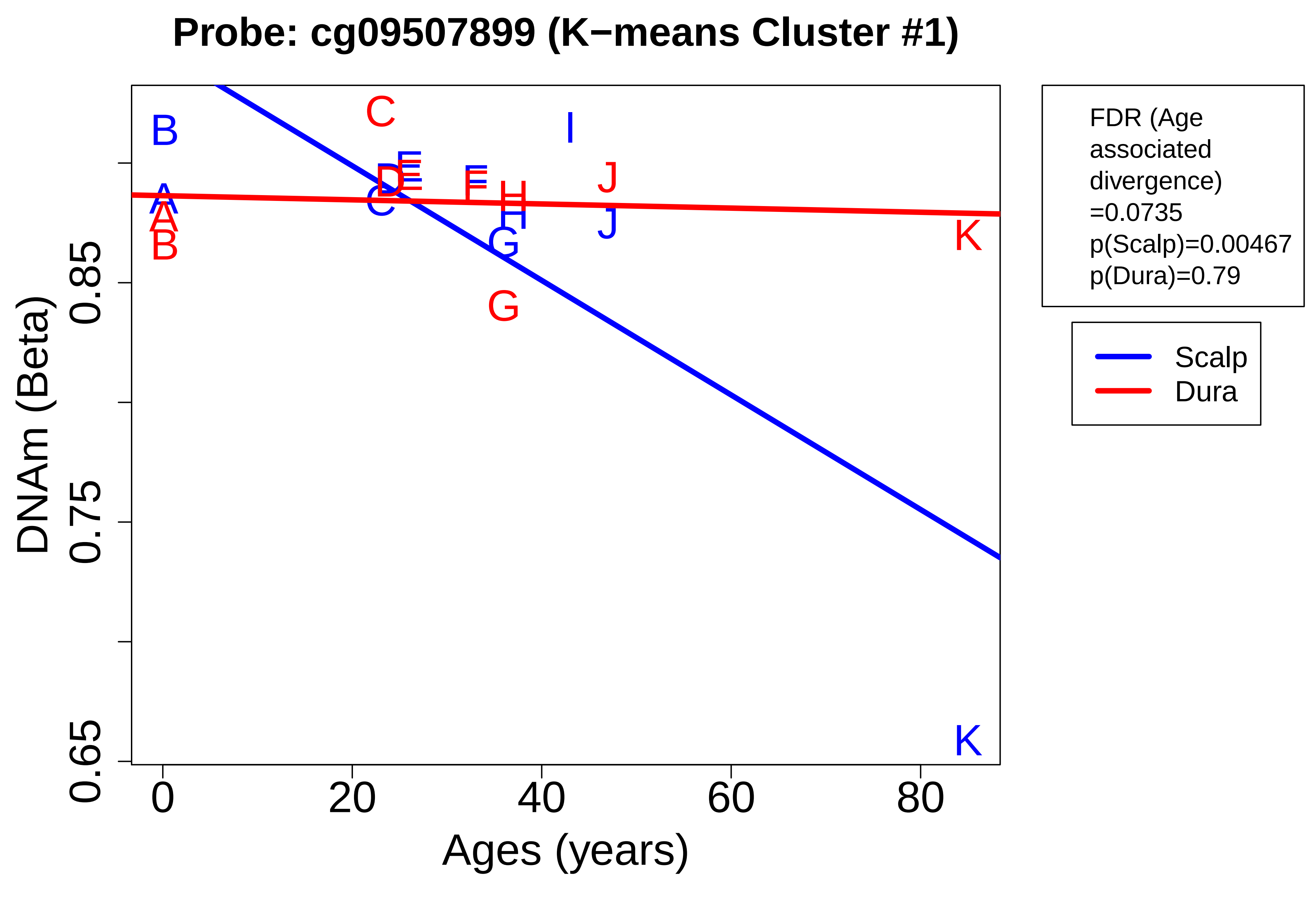

Probe: cg09507899 (K−means Cluster #1)
DNAm (Beta)
0.65
0.75
0.85
0
20
40
60
80
Ages (years)
A
B
C
D
E
F
G
H
I
J
K
FDR (Age associated divergence) =0.0735
p(Scalp)=0.00467
p(Dura)=0.79
Scalp
Dura

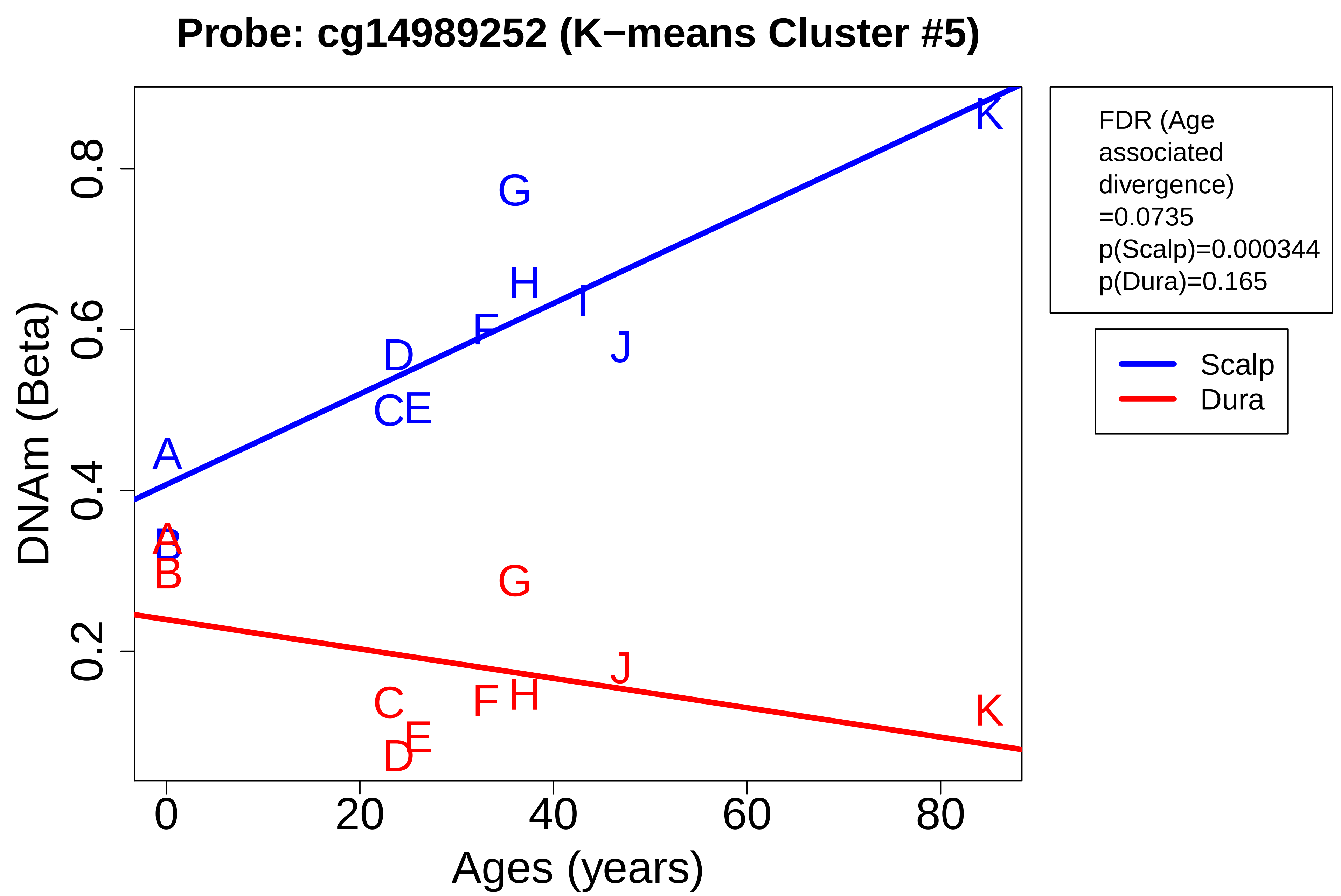
Probe: cg14989252 (K−means Cluster #5)
FDR (Age associated divergence) =0.0735
p(Scalp)=0.000344
p(Dura)=0.165
Scalp
Dura
DNAm (Beta)
0.2
0.4
0.6
0.8
0
20
40
60
80
Ages (years)
A
B
C
D
E
F
G
H
I
J
K

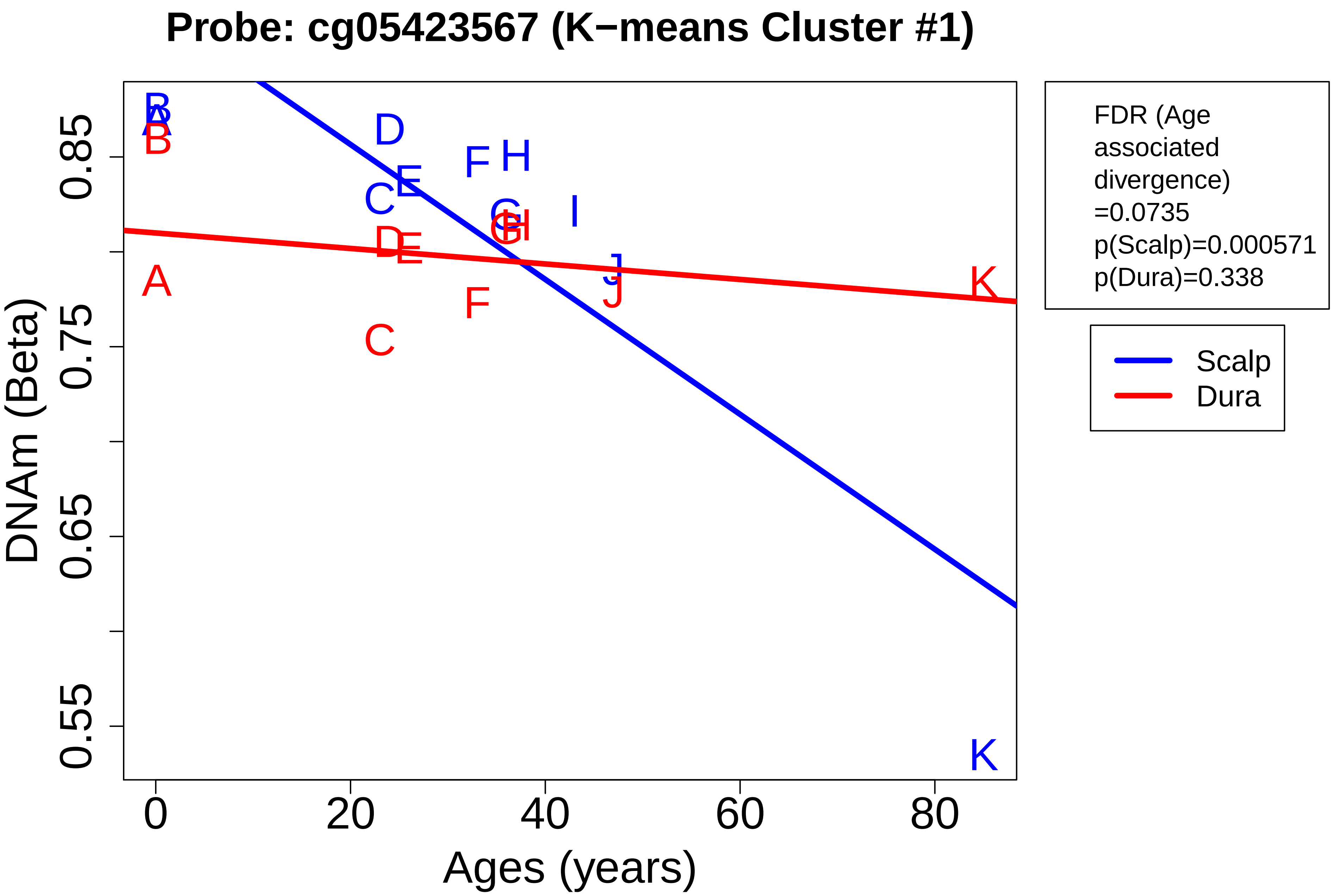

Probe: cg05423567 (K−means Cluster #1)
DNAm (Beta)
0.55
0.65
0.75
0.85
Ages (years)
0
20
40
60
80
FDR (Age associated divergence) =0.0735
p(Scalp)=0.000571
p(Dura)=0.338
Scalp
Dura

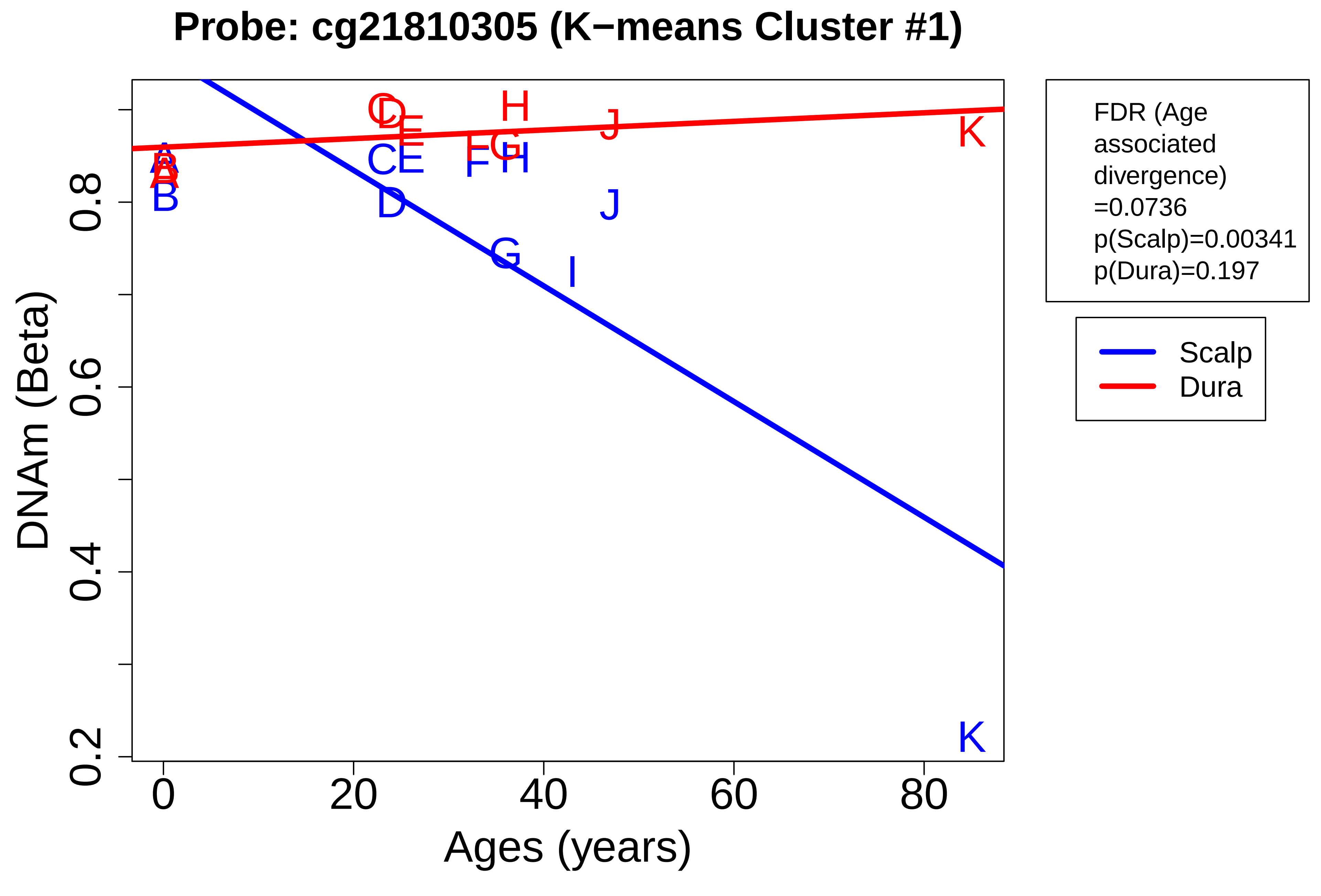

Probe: cg21810305 (K−means Cluster #1)
DNAm (Beta)
0.2
0.4
0.6
0.8
0
20
40
60
80
Ages (years)
A
B
C
D
E
F
G
H
I
J
K
FDR (Age associated divergence) =0.0736
p(Scalp)=0.00341
p(Dura)=0.197
Scalp
Dura

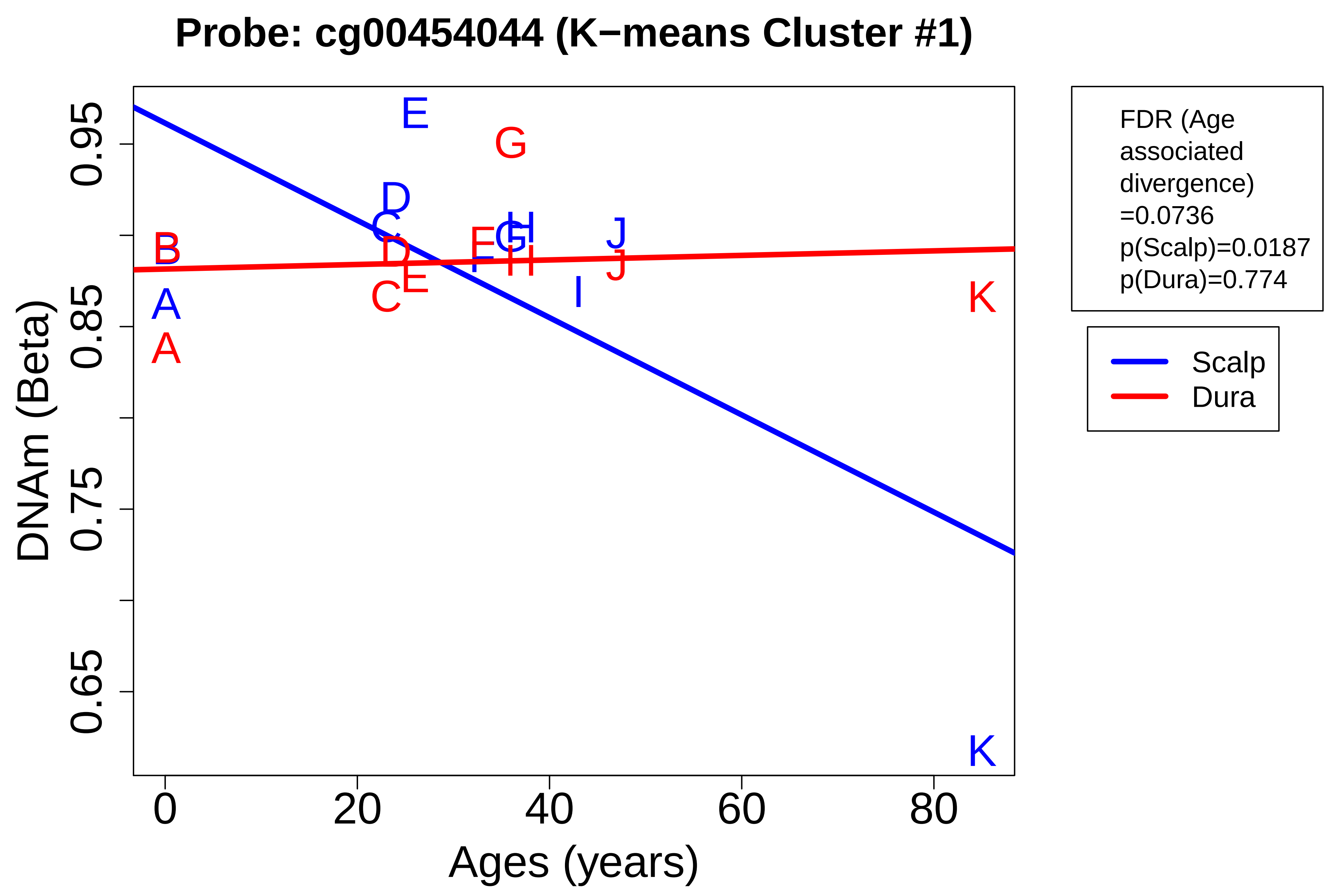

Probe: cg00454044 (K−means Cluster #1)
DNAm (Beta)
0.65
0.75
0.85
0.95
Ages (years)
0
20
40
60
80
A
B
C
D
E
F
G
H
I
J
K
FDR (Age associated divergence) =0.0736
p(Scalp)=0.0187
p(Dura)=0.774
Scalp
Dura

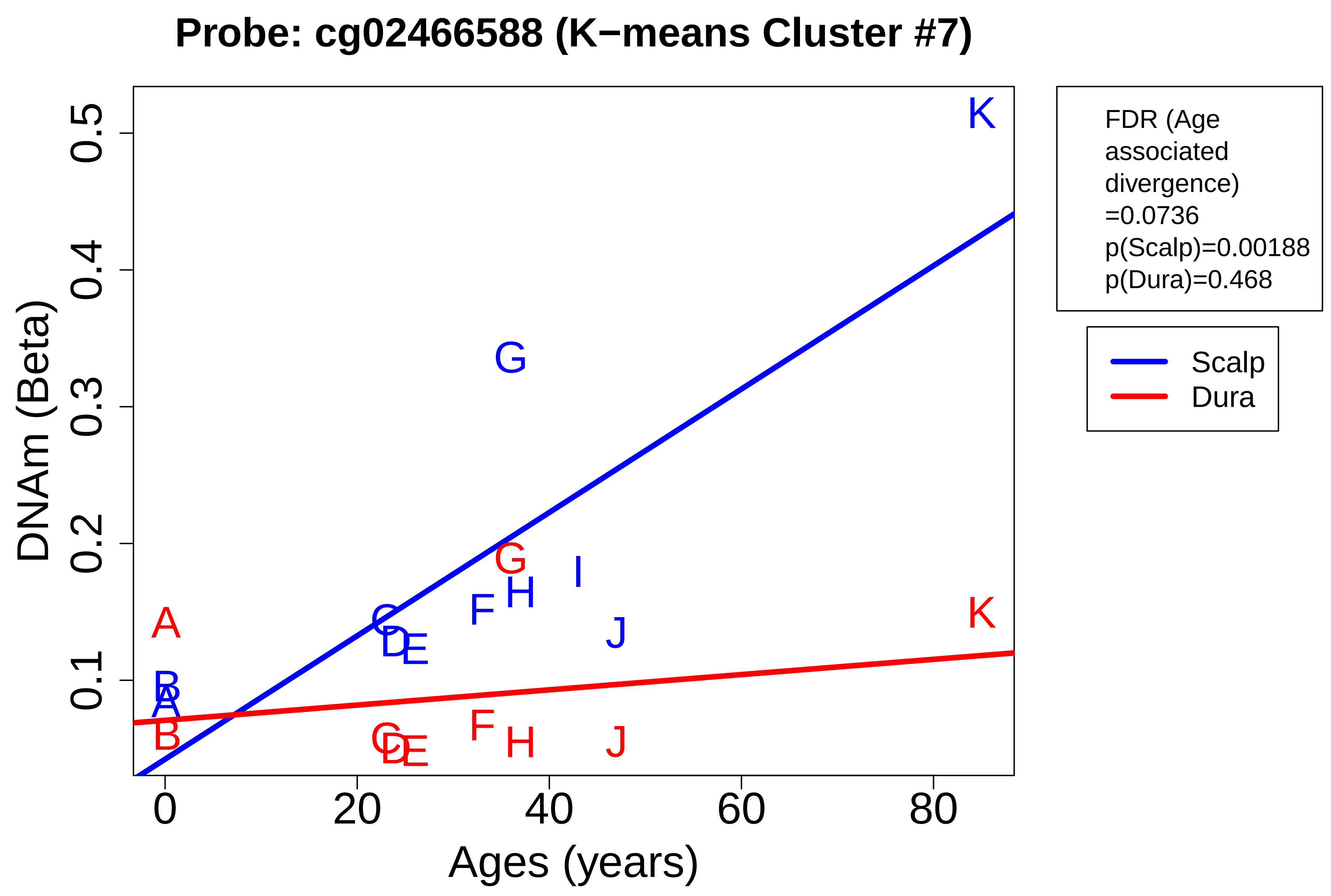

Probe: cg02466588 (K−means Cluster #7)
DNAm (Beta)
0.1
0.2
0.3
0.4
0.5
0
20
40
60
80
Ages (years)
K
G
I
H
F
C
D
E
J
B
A
A
B
C
D
E
F
H
J
K
FDR (Age associated divergence) =0.0736
p(Scalp)=0.00188
p(Dura)=0.468
Scalp
Dura

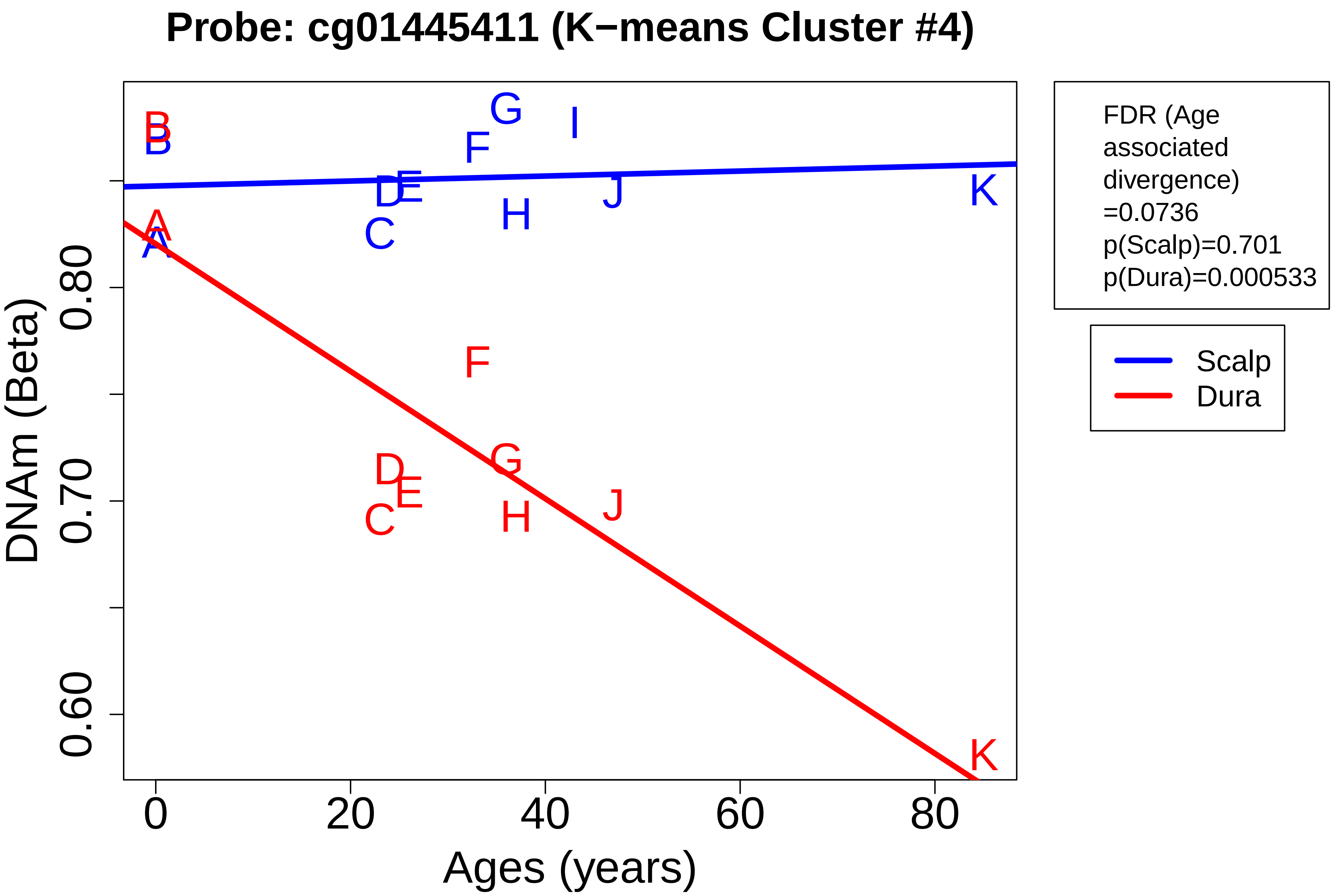

Probe: cg01445411 (K−means Cluster #4)
FDR (Age associated divergence) =0.0736
p(Scalp)=0.701
p(Dura)=0.000533
Scalp
Dura
DNAm (Beta)
0.60
0.70
0.80
Ages (years)
0
20
40
60
80
A
B
C
D
E
F
G
H
I
J
K

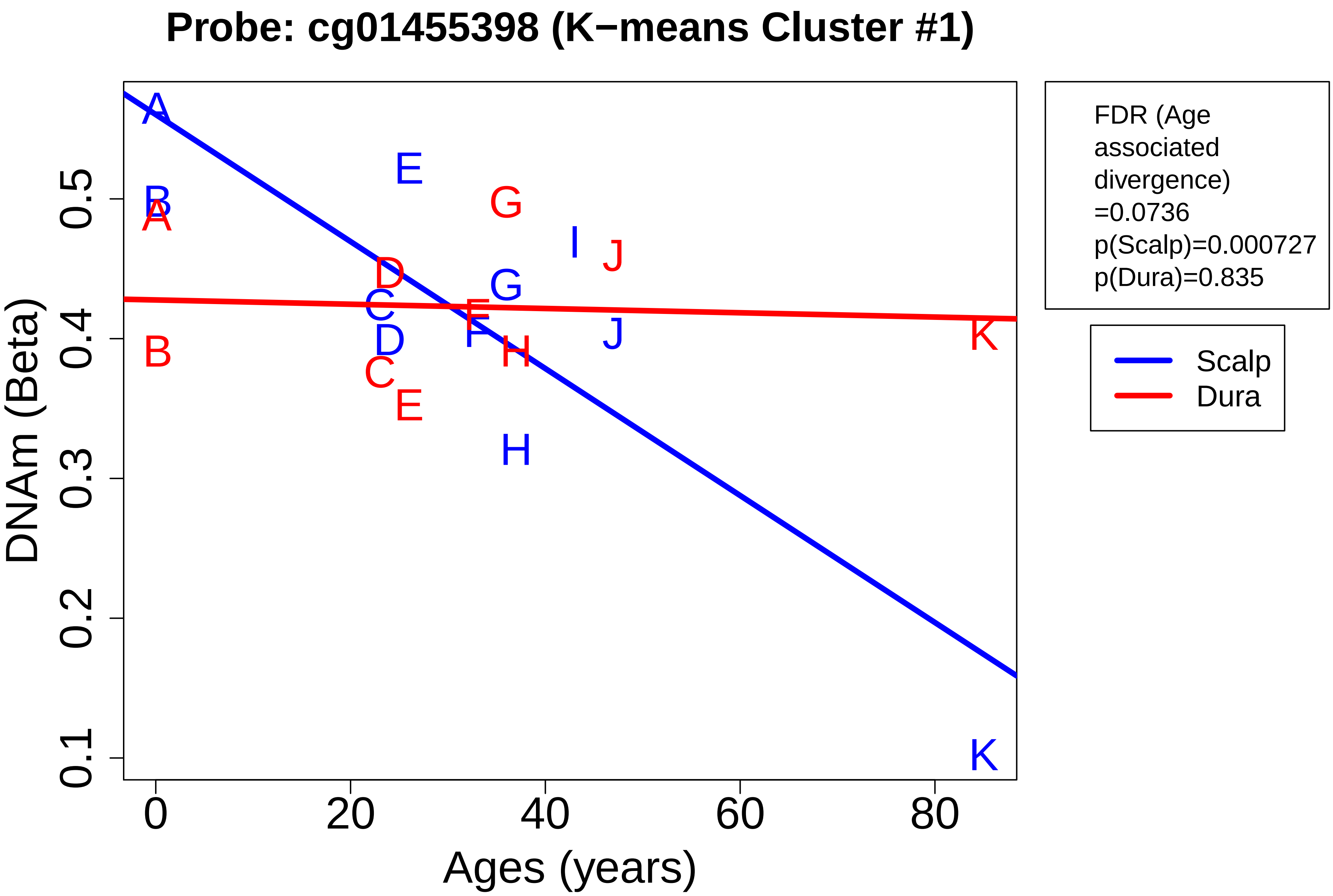

Probe: cg01455398 (K−means Cluster #1)
DNAm (Beta)
0.1
0.2
0.3
0.4
0.5
0
20
40
60
80
Ages (years)
FDR (Age associated divergence) =0.0736
p(Scalp)=0.000727
p(Dura)=0.835
Scalp
Dura
A
B
C
D
E
F
G
H
I
J
K

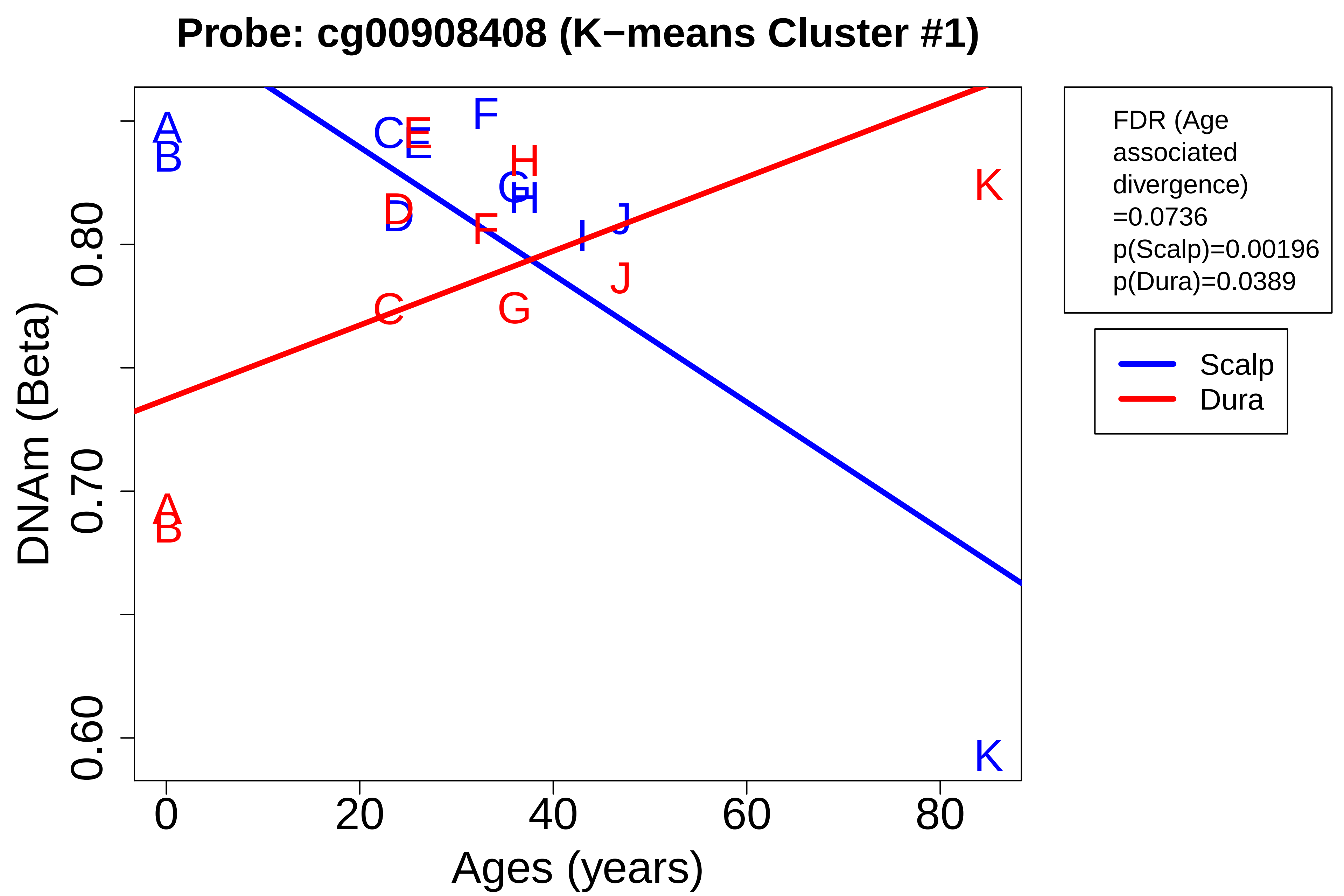

Probe: cg00908408 (K−means Cluster #1)
DNAm (Beta)
0.60
0.70
0.80
Ages (years)
0
20
40
60
80
FDR (Age associated divergence) =0.0736
p(Scalp)=0.00196
p(Dura)=0.0389
Scalp
Dura

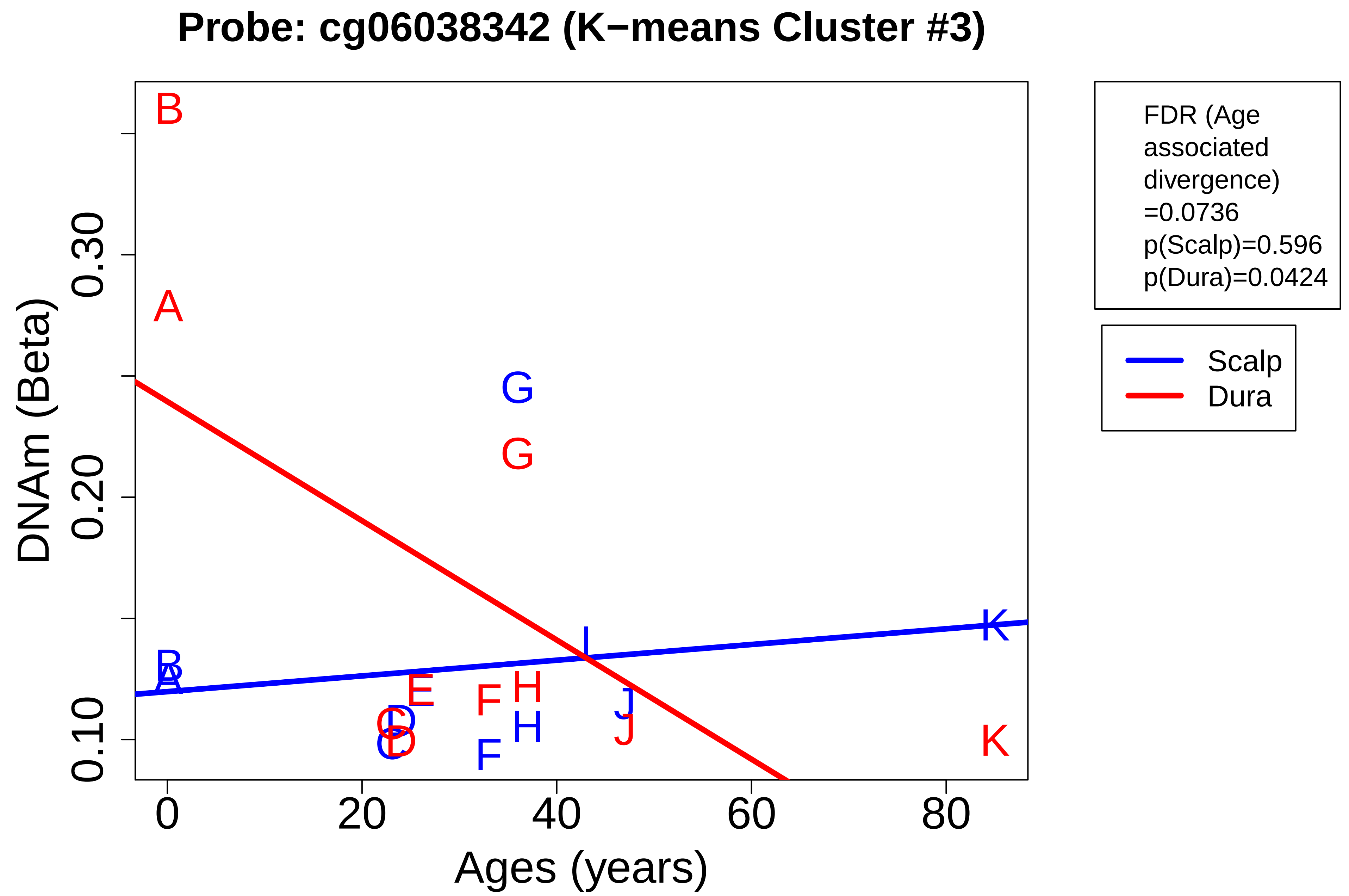
Probe: cg06038342 (K−means Cluster #3)
DNAm (Beta)
0.10
0.20
0.30
0
20
40
60
80
Ages (years)
FDR (Age associated divergence) =0.0736
p(Scalp)=0.596
p(Dura)=0.0424
Scalp
Dura

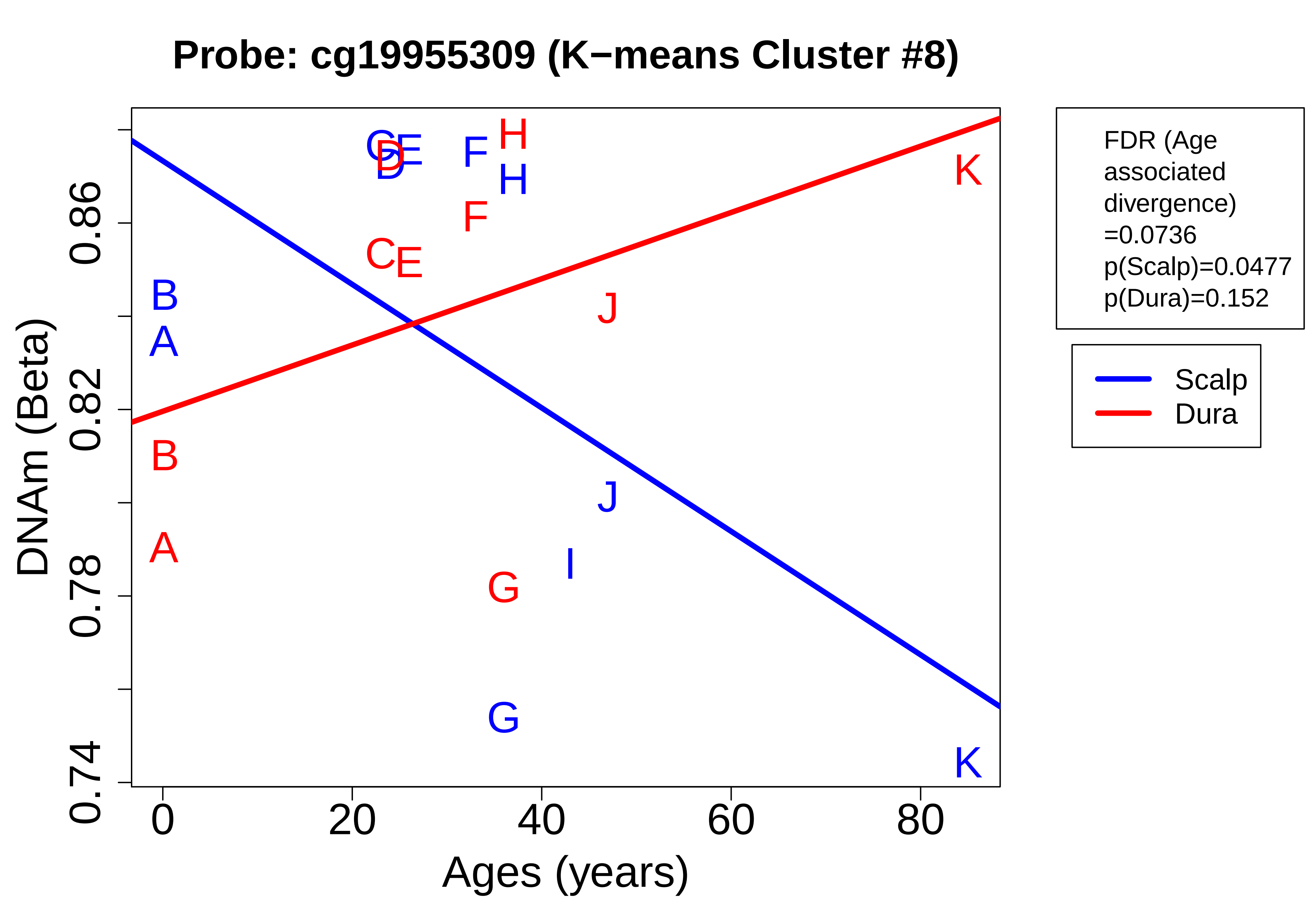

Probe: cg19955309 (K−means Cluster #8)
DNAm (Beta)
0.74
0.78
0.82
0.86
Ages (years)
0
20
40
60
80
FDR (Age associated divergence) =0.0736
p(Scalp)=0.0477
p(Dura)=0.152
Scalp
Dura

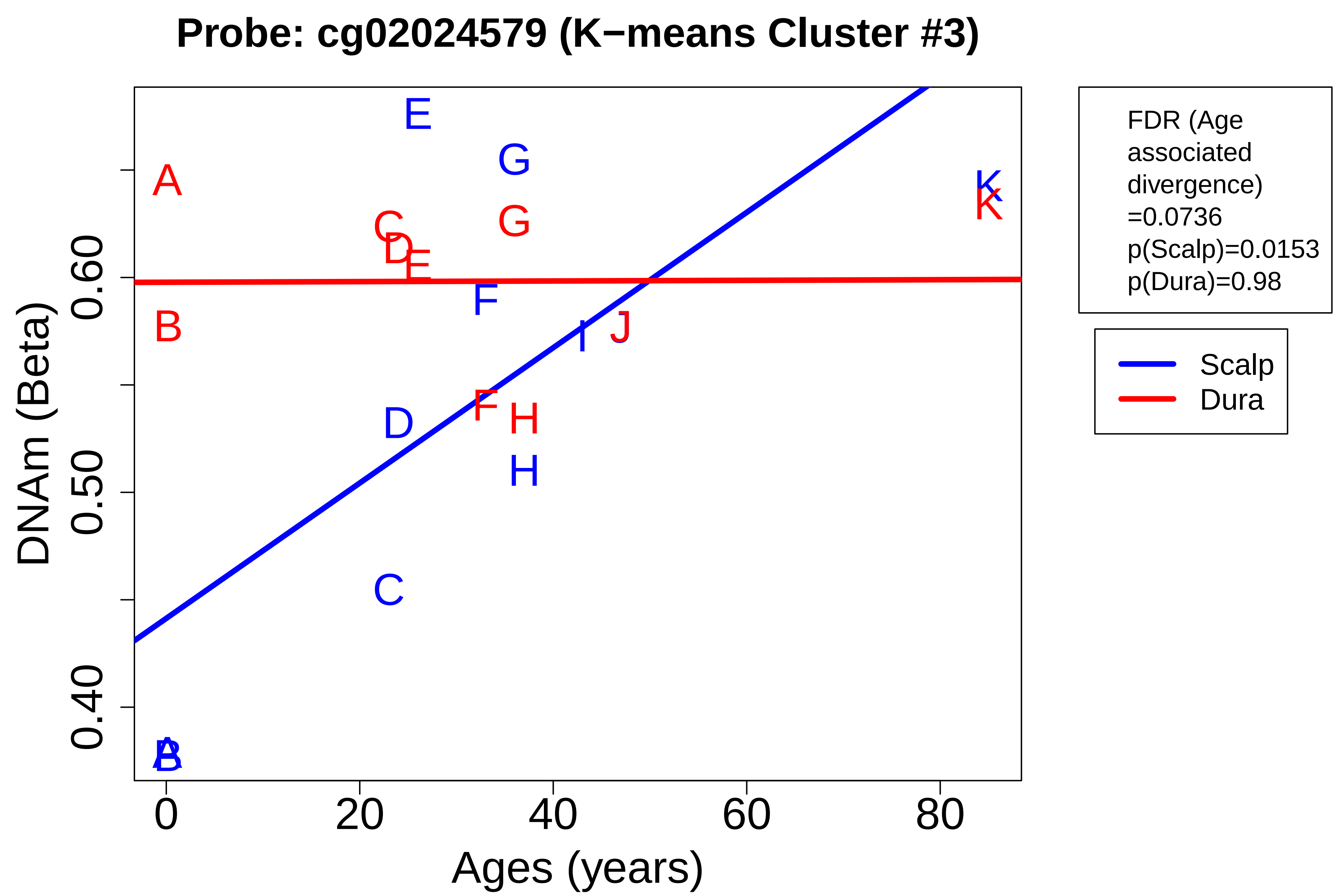

Probe: cg02024579 (K−means Cluster #3)
DNAm (Beta)
0.40
0.50
0.60
0
20
40
60
80
Ages (years)
FDR (Age associated divergence) =0.0736
p(Scalp)=0.0153
p(Dura)=0.98
Scalp
Dura

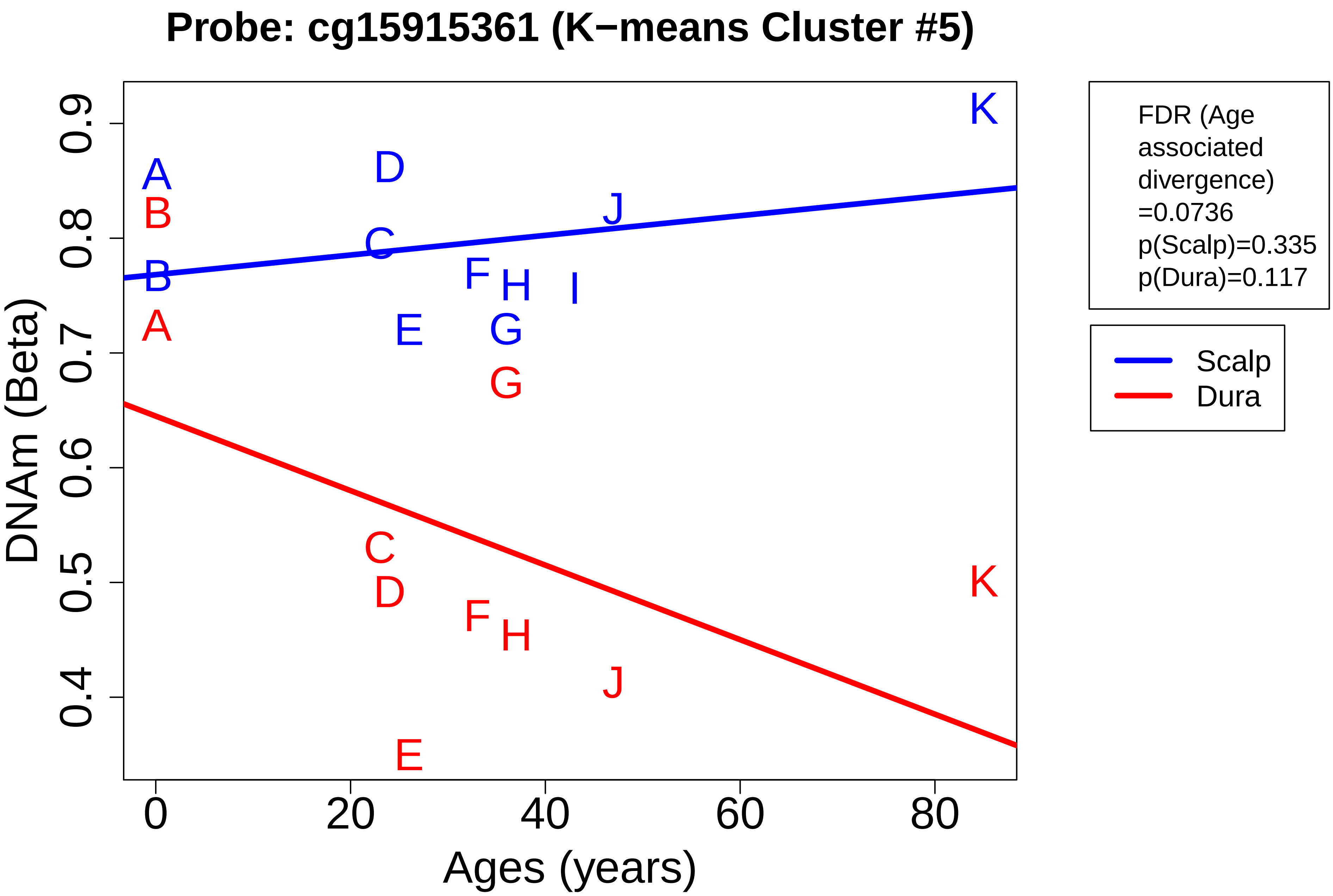

Probe: cg15915361 (K−means Cluster #5)
DNAm (Beta)
0.4
0.5
0.6
0.7
0.8
0.9
0
20
40
60
80
Ages (years)
A
B
C
D
E
F
G
H
I
J
K
FDR (Age associated divergence) =0.0736
p(Scalp)=0.335
p(Dura)=0.117
Scalp
Dura

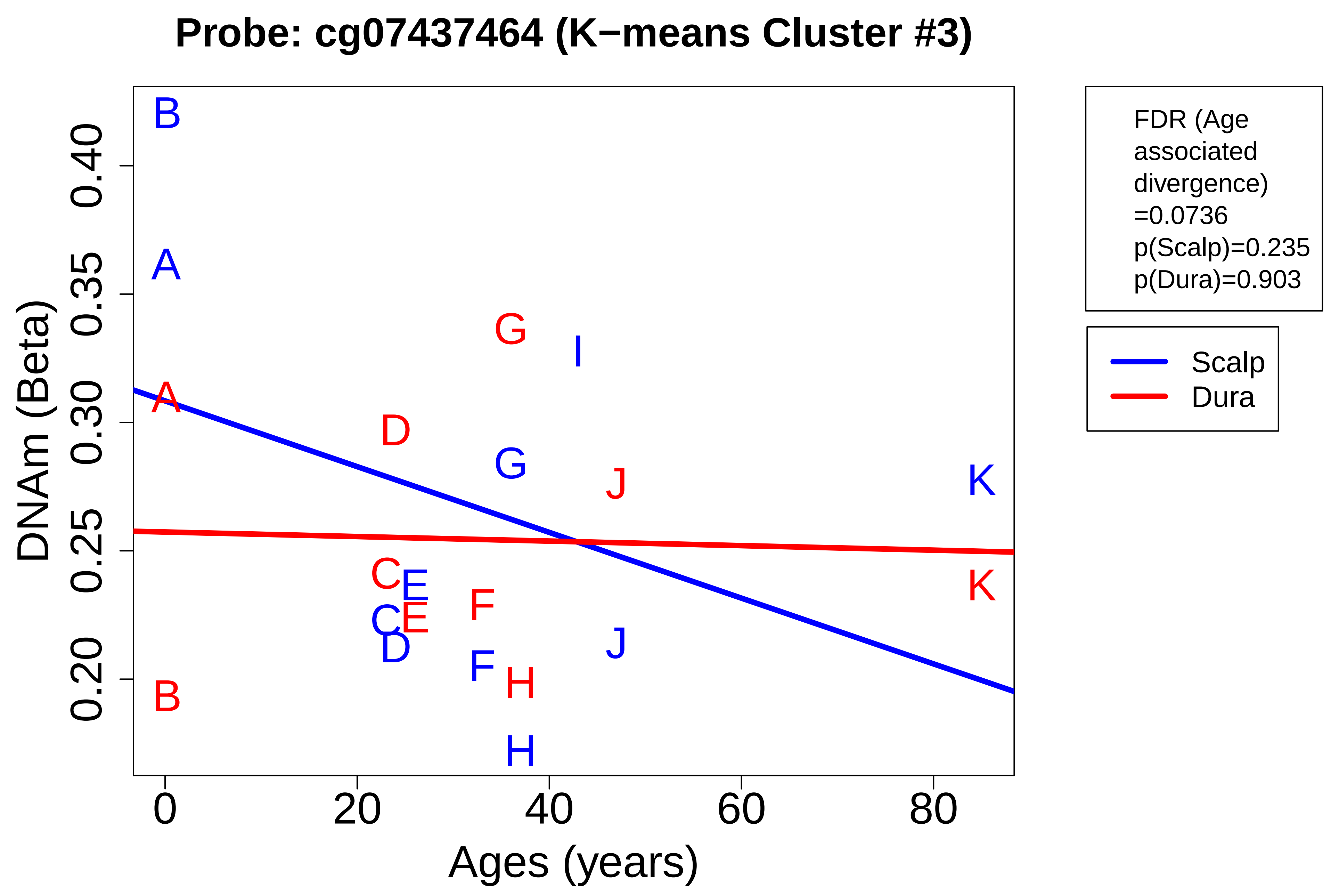

Probe: cg07437464 (K−means Cluster #3)
DNAm (Beta)
0.20
0.25
0.30
0.35
0.40
Ages (years)
0
20
40
60
80
B
A
G
I
A
D
G
J
K
C
E
C
E
F
D
J
F
H
K
B
H
FDR (Age associated divergence) =0.0736
p(Scalp)=0.235
p(Dura)=0.903
Scalp
Dura

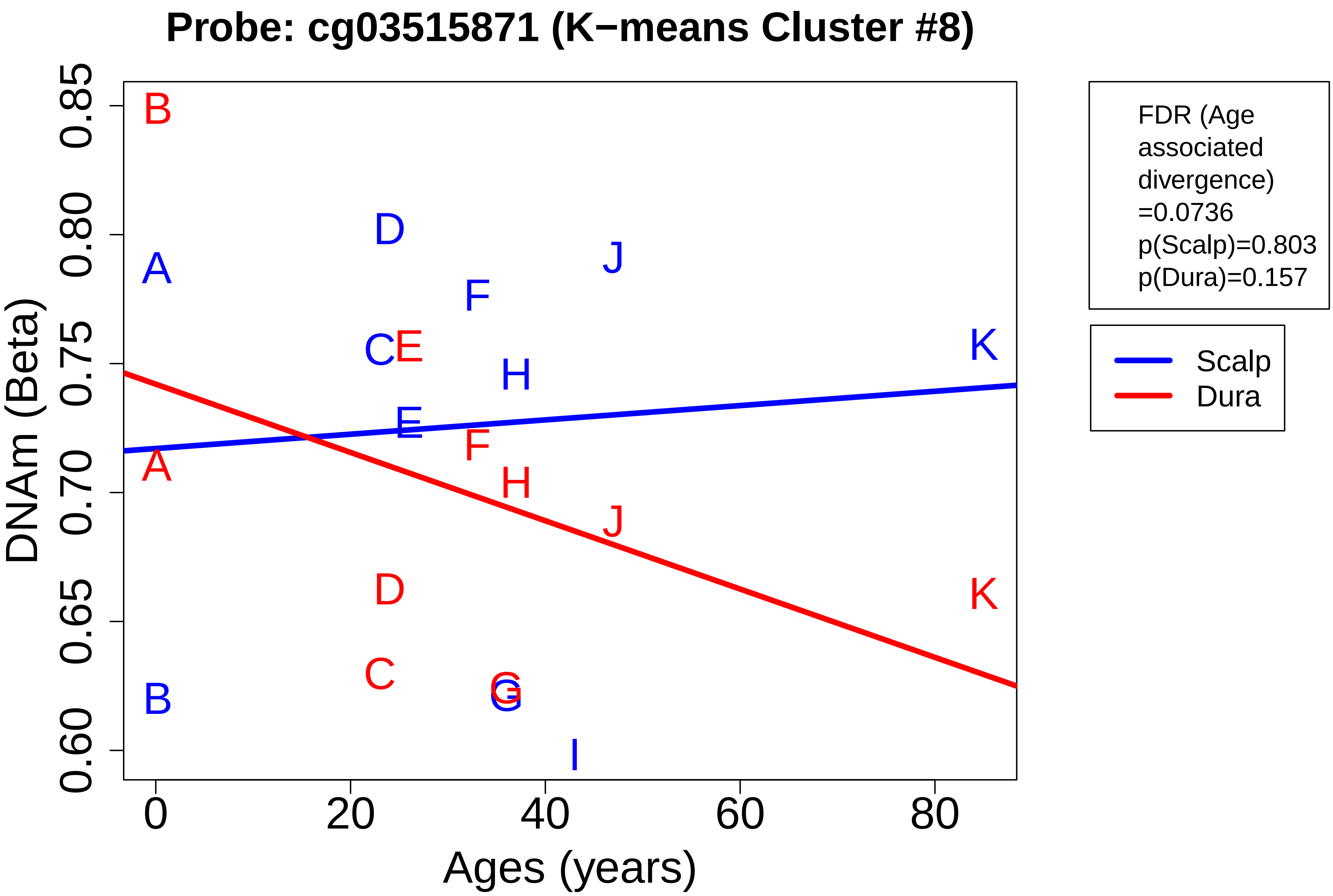

Probe: cg03515871 (K−means Cluster #8)
DNAm (Beta)
0.60
0.65
0.70
0.75
0.80
0.85
Ages (years)
0
20
40
60
80
FDR (Age associated divergence) =0.0736
p(Scalp)=0.803
p(Dura)=0.157
Scalp
Dura

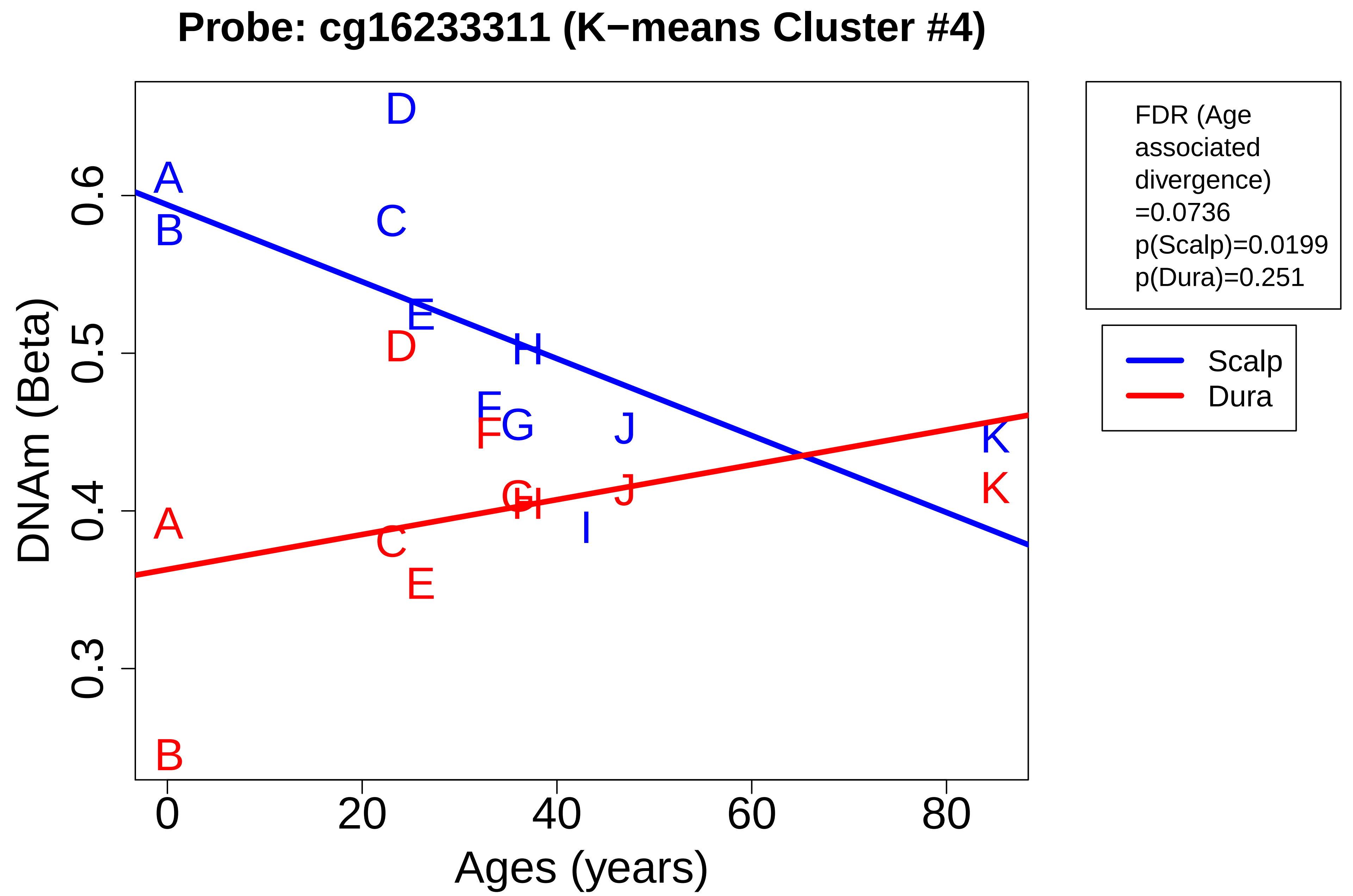

Probe: cg16233311 (K−means Cluster #4)
DNAm (Beta)
0.3
0.4
0.5
0.6
0
20
40
60
80
Ages (years)
FDR (Age associated divergence) =0.0736
p(Scalp)=0.0199
p(Dura)=0.251
Scalp
Dura

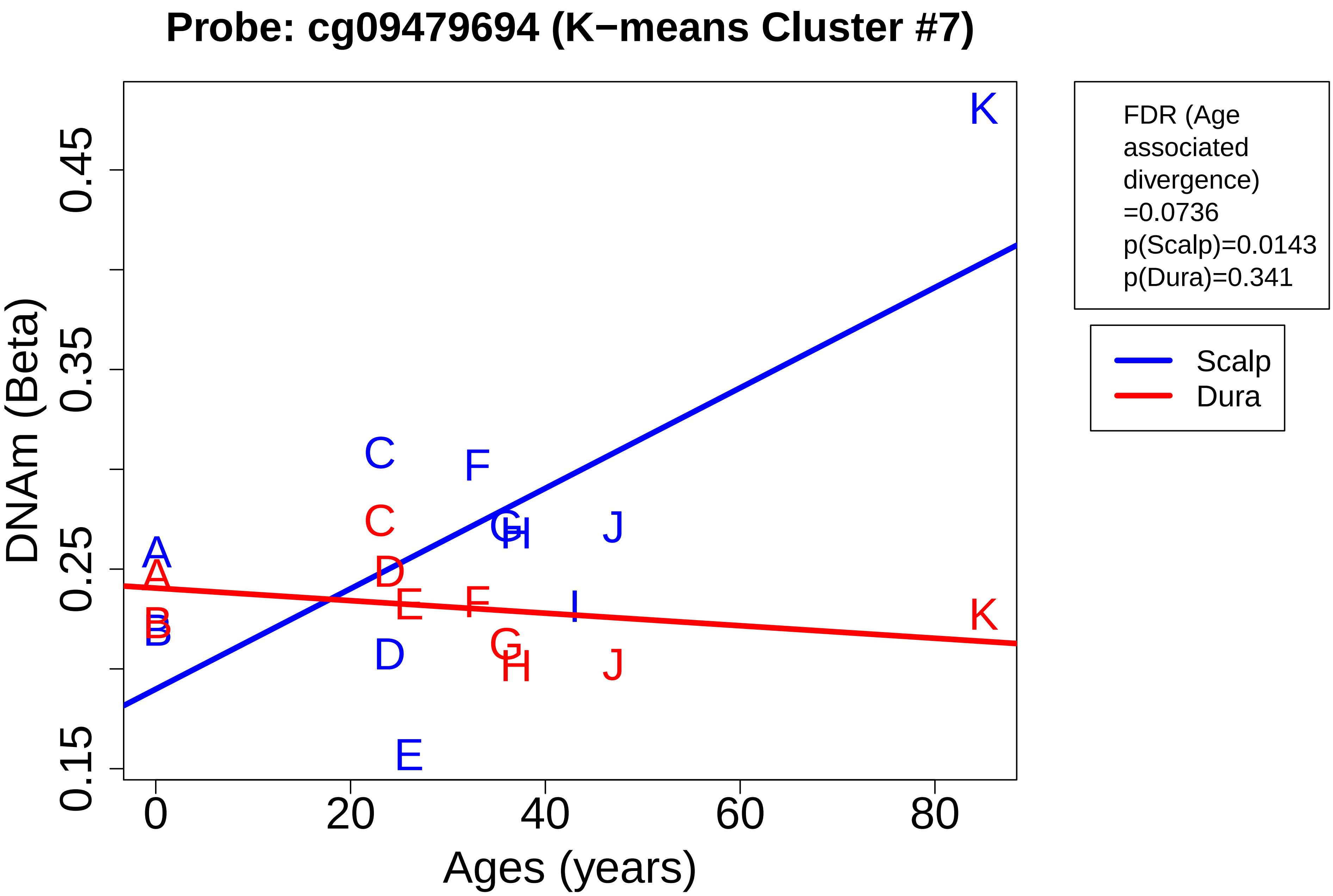
Probe: cg09479694 (K−means Cluster #7)
DNAm (Beta)
0.15
0.25
0.35
0.45
0
20
40
60
80
Ages (years)
A
B
C
D
E
F
G
H
I
J
K
FDR (Age associated divergence) =0.0736
p(Scalp)=0.0143
p(Dura)=0.341
Scalp
Dura

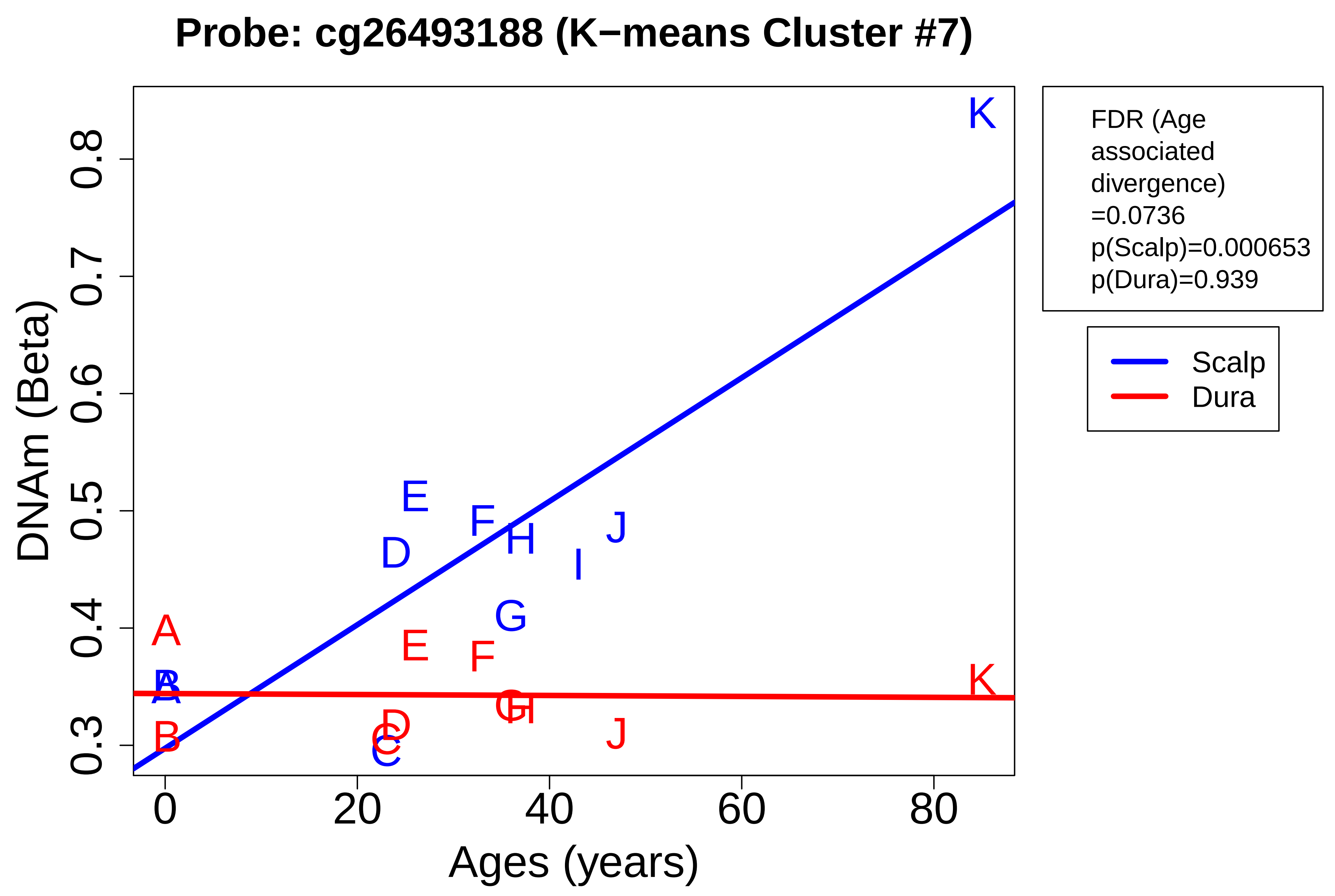
Probe: cg26493188 (K−means Cluster #7)
FDR (Age associated divergence) =0.0736
p(Scalp)=0.000653
p(Dura)=0.939
Scalp
Dura
DNAm (Beta)
Ages (years)
0.3
0.4
0.5
0.6
0.7
0.8
0
20
40
60
80
A
B
C
D
E
F
G
H
I
J
K

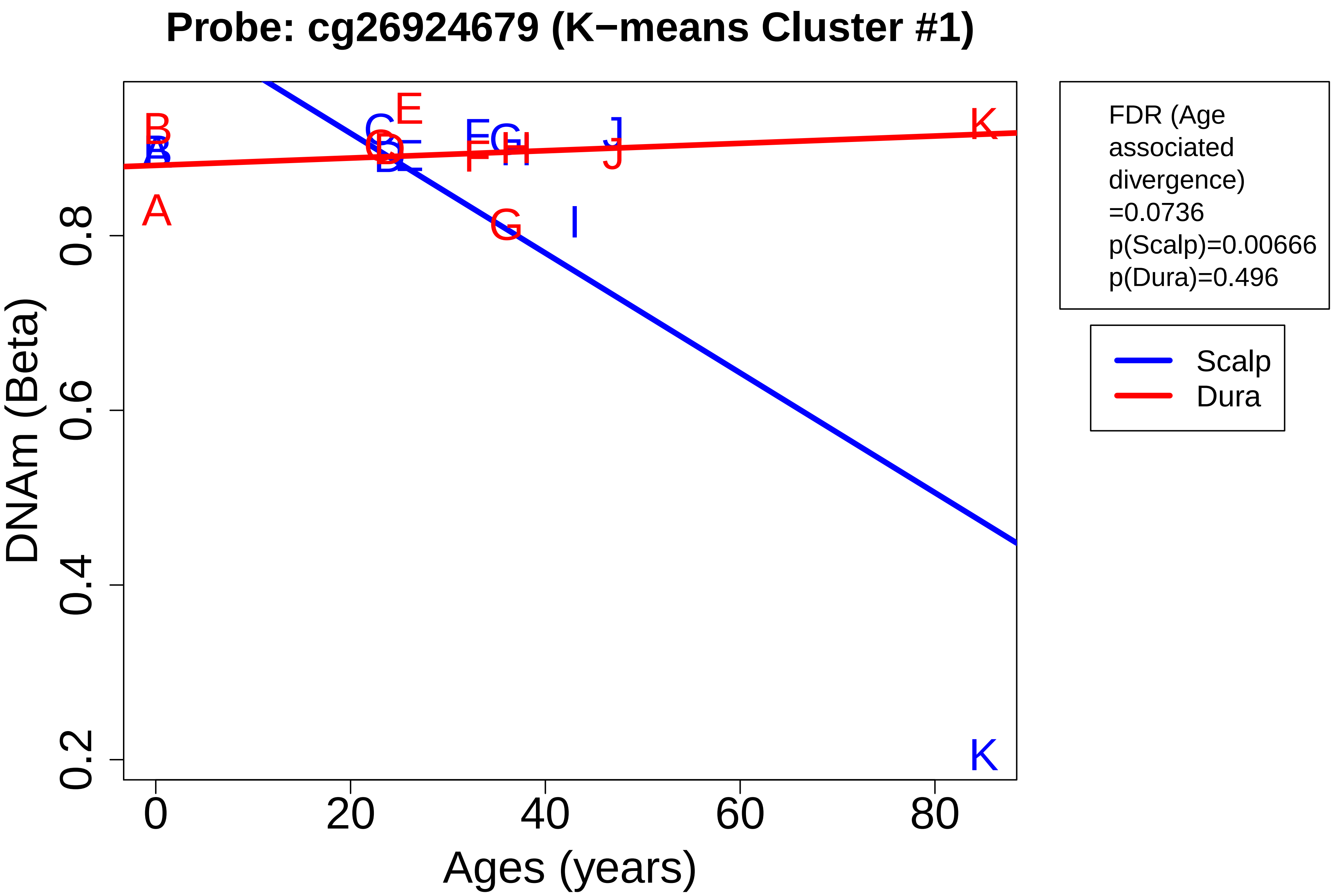
Probe: cg26924679 (K−means Cluster #1)
DNAm (Beta)
0.2
0.4
0.6
0.8
0
20
40
60
80
Ages (years)
FDR (Age associated divergence) =0.0736
p(Scalp)=0.00666
p(Dura)=0.496
Scalp
Dura

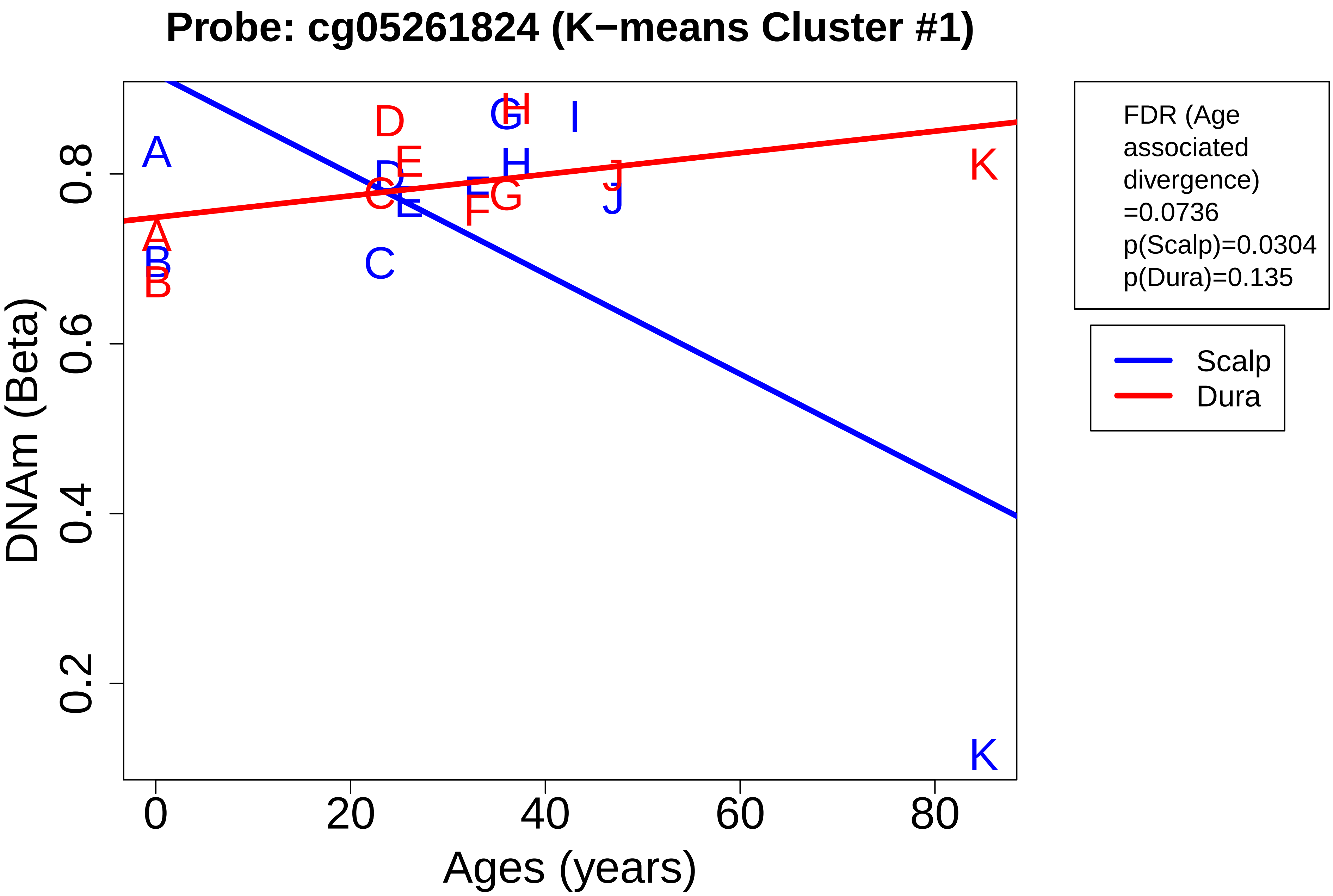

Probe: cg05261824 (K−means Cluster #1)
DNAm (Beta)
0.2
0.4
0.6
0.8
0
20
40
60
80
Ages (years)
FDR (Age associated divergence) =0.0736
p(Scalp)=0.0304
p(Dura)=0.135
Scalp
Dura

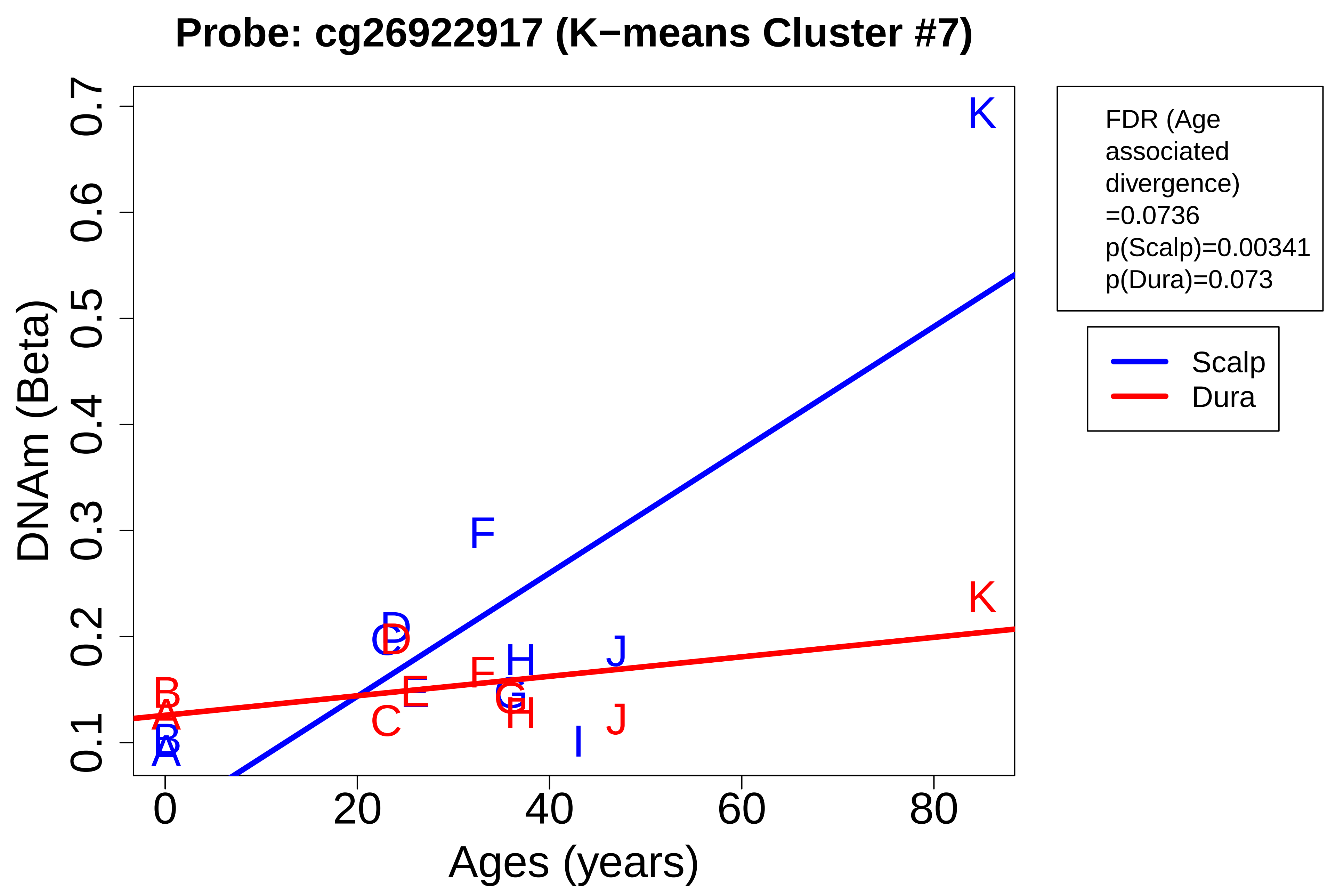

Probe: cg26922917 (K−means Cluster #7)
DNAm (Beta)
0.1
0.2
0.3
0.4
0.5
0.6
0.7
0
20
40
60
80
Ages (years)
FDR (Age associated divergence) =0.0736
p(Scalp)=0.00341
p(Dura)=0.073
Scalp
Dura

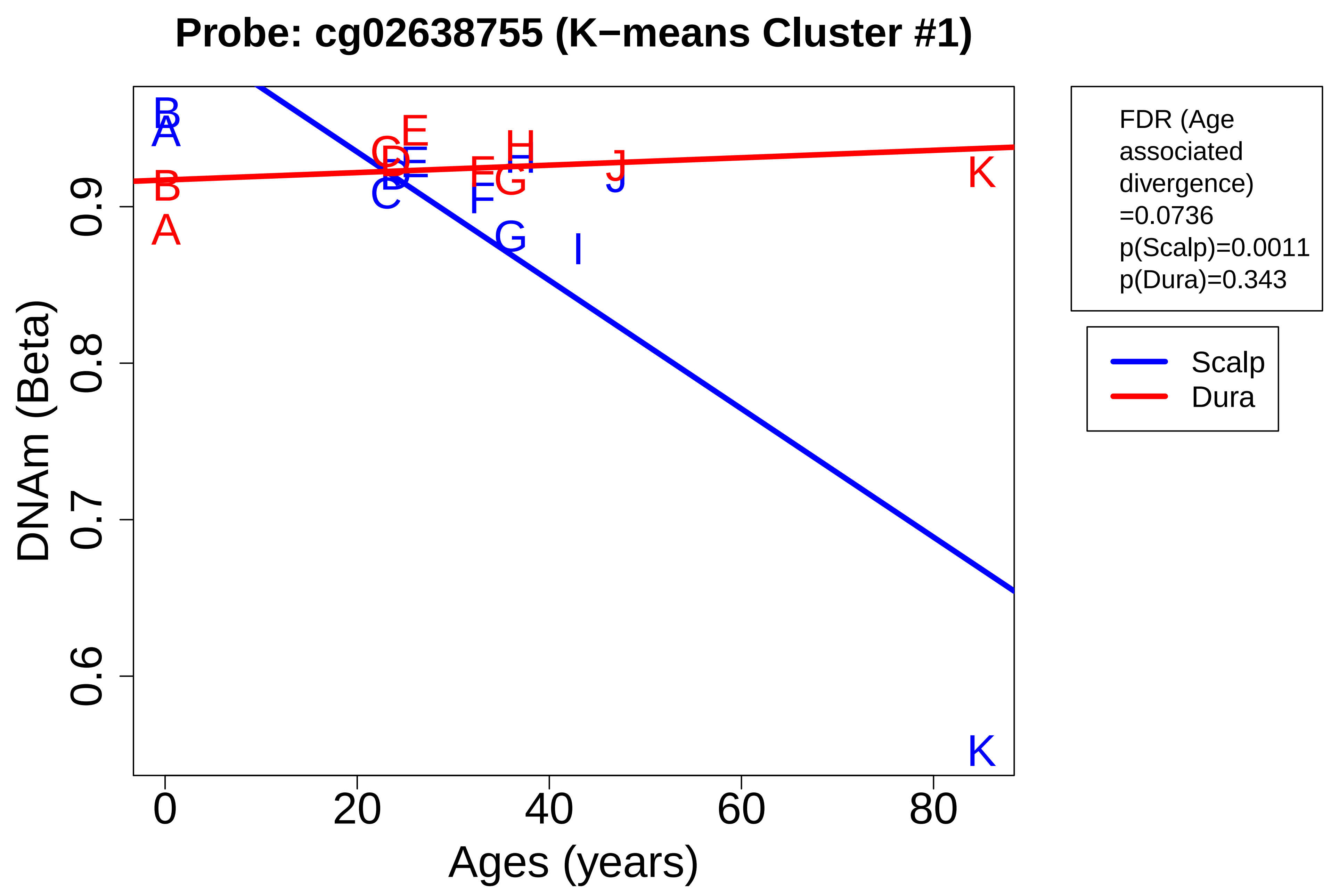

Probe: cg02638755 (K−means Cluster #1)
DNAm (Beta)
0.6
0.7
0.8
0.9
0
20
40
60
80
Ages (years)
FDR (Age associated divergence) =0.0736
p(Scalp)=0.0011
p(Dura)=0.343
Scalp
Dura

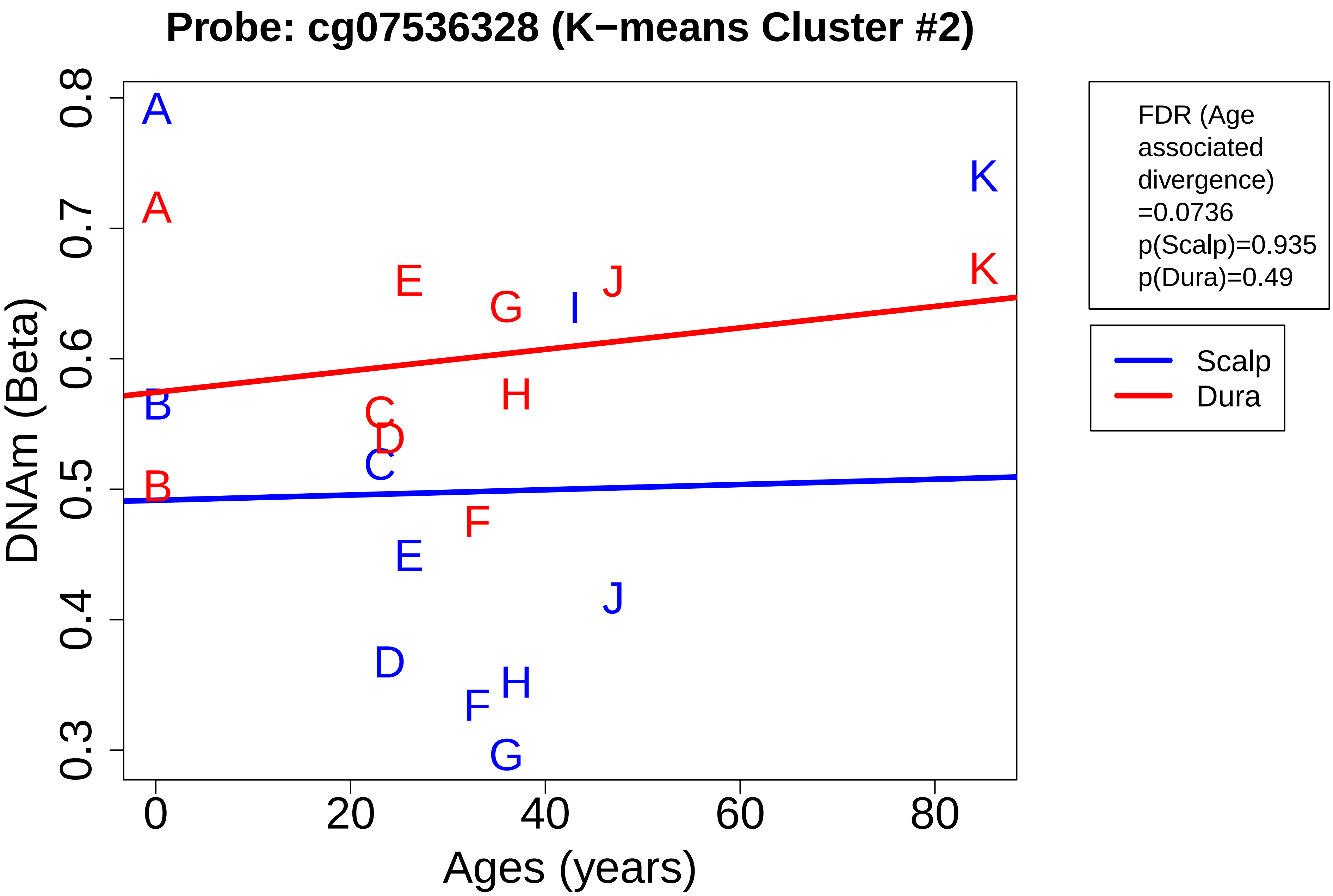

Probe: cg07536328 (K−means Cluster #2)
DNAm (Beta)
0.3
0.4
0.5
0.6
0.7
0.8
Ages (years)
0
20
40
60
80
A
B
C
D
E
F
G
H
I
J
K
FDR (Age associated divergence) =0.0736
p(Scalp)=0.935
p(Dura)=0.49
Scalp
Dura

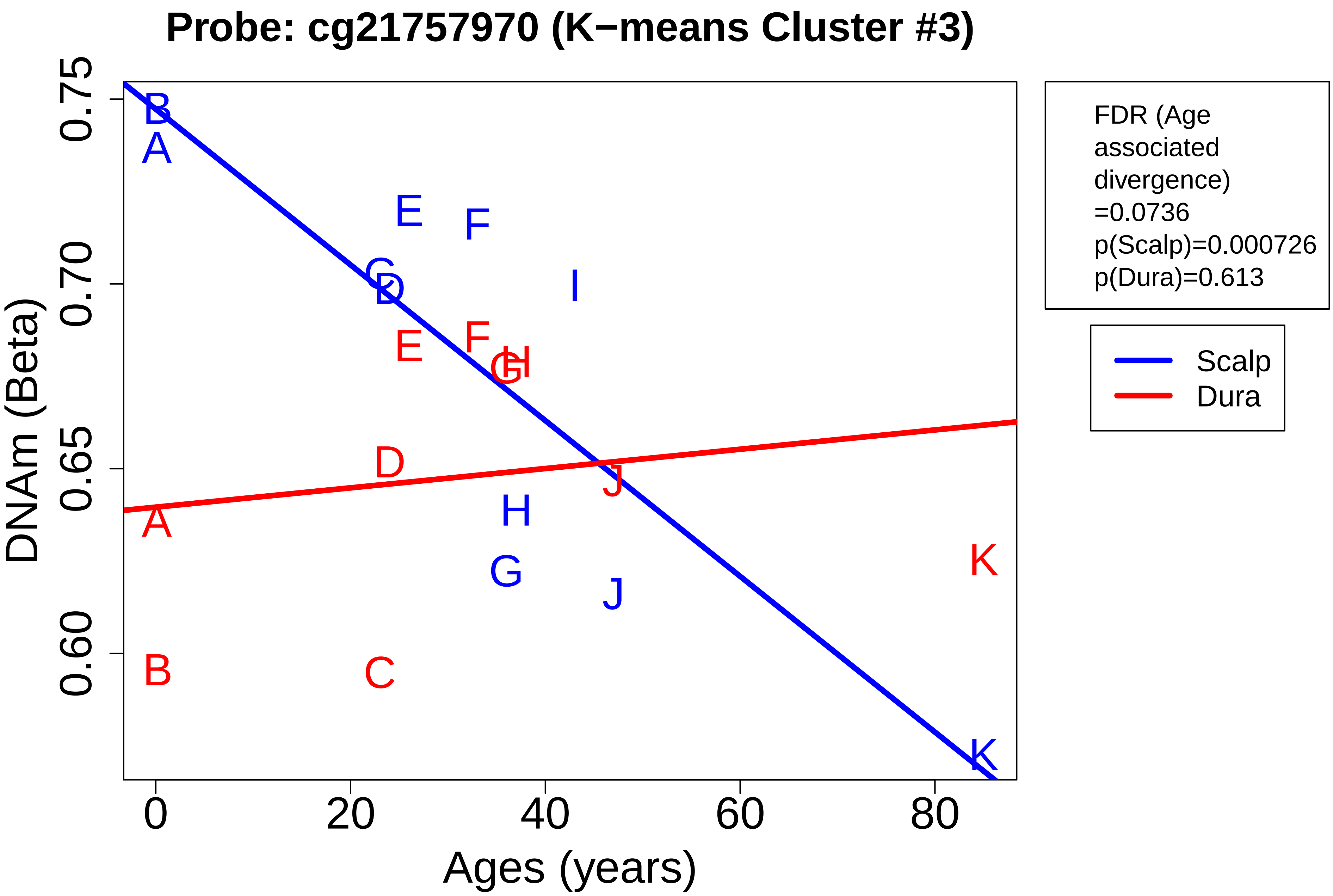

Probe: cg21757970 (K−means Cluster #3)
DNAm (Beta)
0.60
0.65
0.70
0.75
0
20
40
60
80
Ages (years)
FDR (Age associated divergence) =0.0736
p(Scalp)=0.000726
p(Dura)=0.613
Scalp
Dura

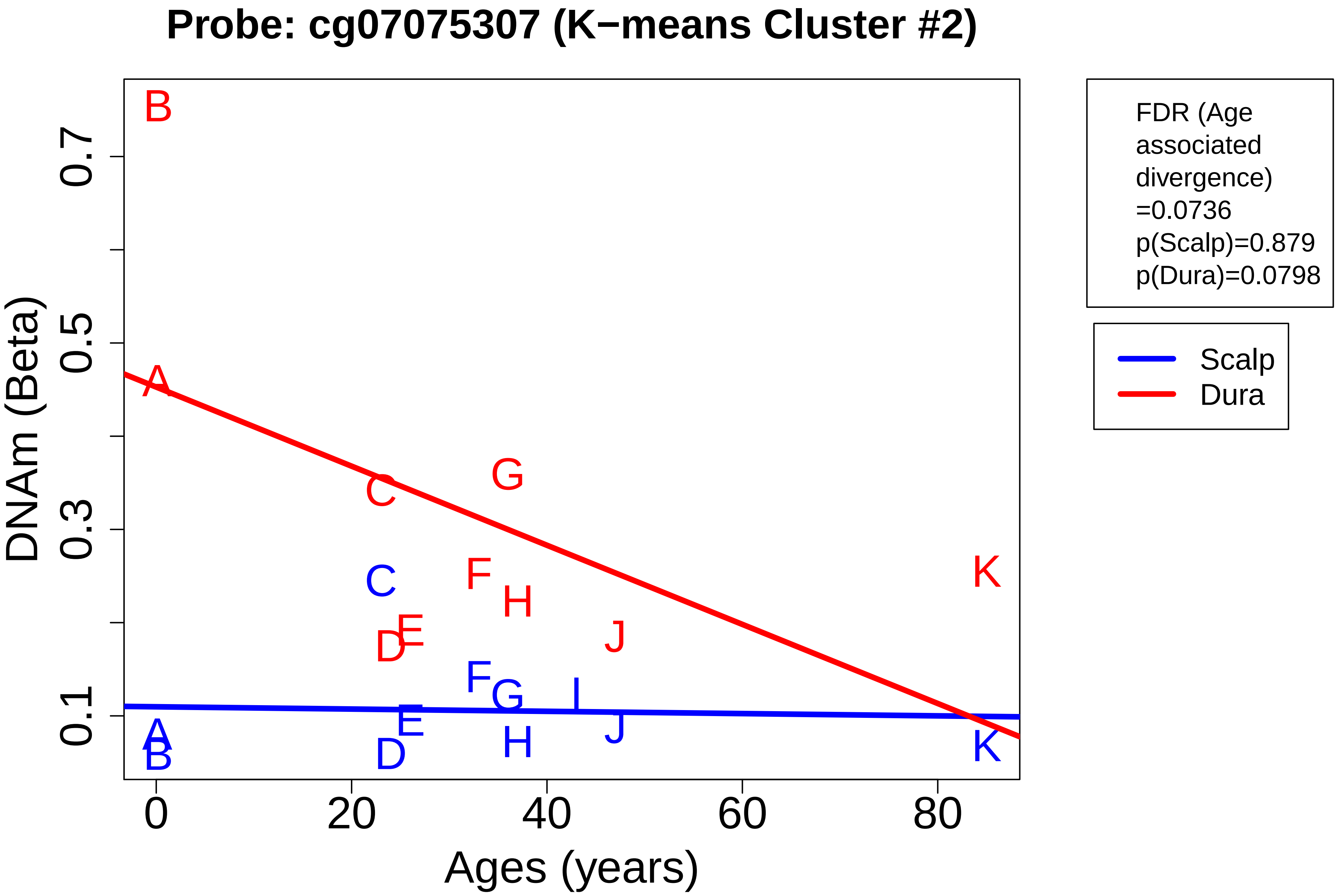

Probe: cg07075307 (K−means Cluster #2)
DNAm (Beta)
0.1
0.3
0.5
0.7
Ages (years)
0
20
40
60
80
A
B
C
D
E
F
G
H
I
J
K
FDR (Age associated divergence) =0.0736
p(Scalp)=0.879
p(Dura)=0.0798
Scalp
Dura

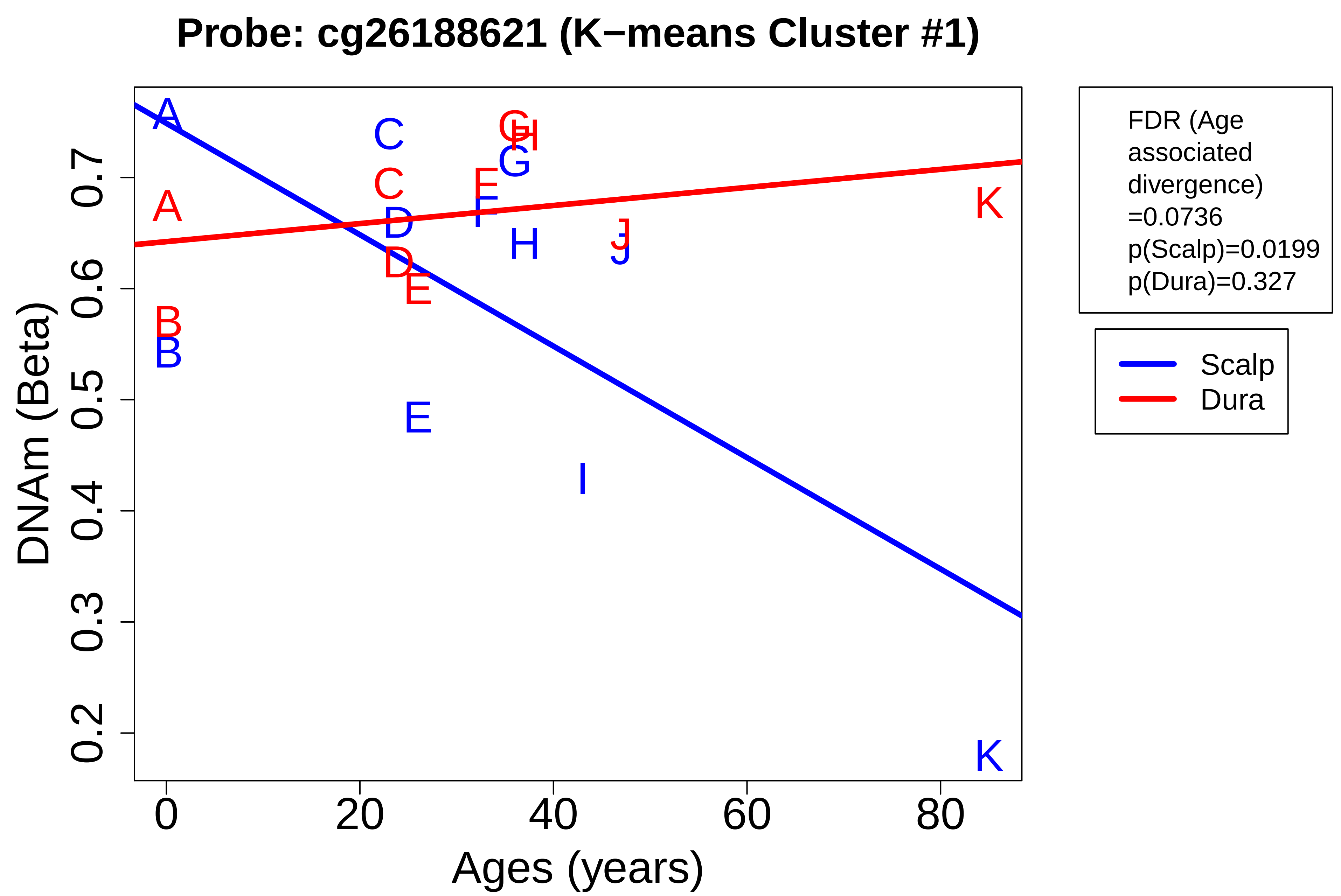

Probe: cg26188621 (K−means Cluster #1)
DNAm (Beta)
0.2
0.3
0.4
0.5
0.6
0.7
0
20
40
60
80
Ages (years)
A
B
C
D
E
F
G
H
I
J
K
FDR (Age associated divergence) =0.0736
p(Scalp)=0.0199
p(Dura)=0.327
Scalp
Dura

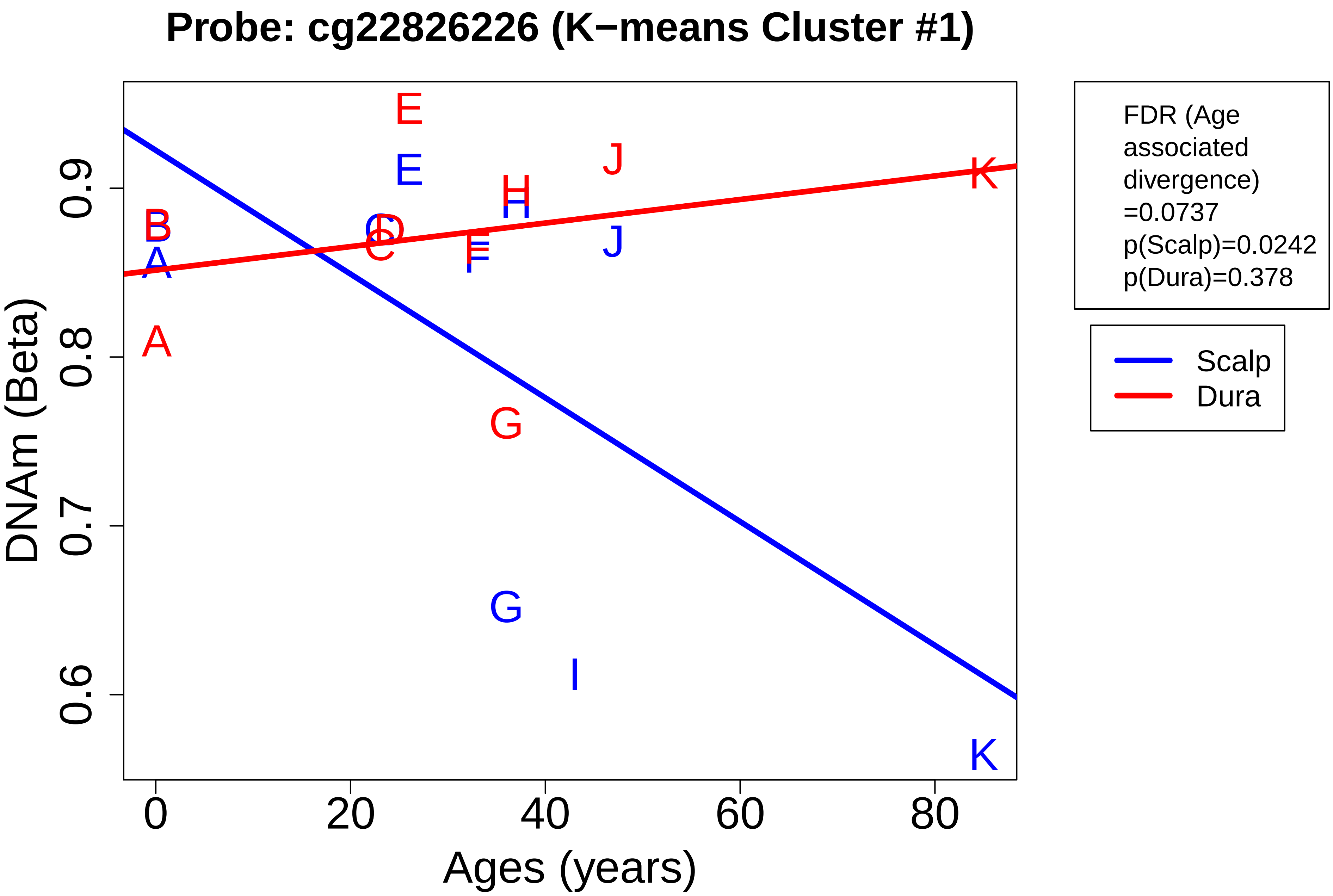

Probe: cg22826226 (K−means Cluster #1)
DNAm (Beta)
0.6
0.7
0.8
0.9
0
20
40
60
80
Ages (years)
FDR (Age associated divergence) =0.0737
p(Scalp)=0.0242
p(Dura)=0.378
Scalp
Dura
A
B
C
D
E
F
G
H
I
J
K

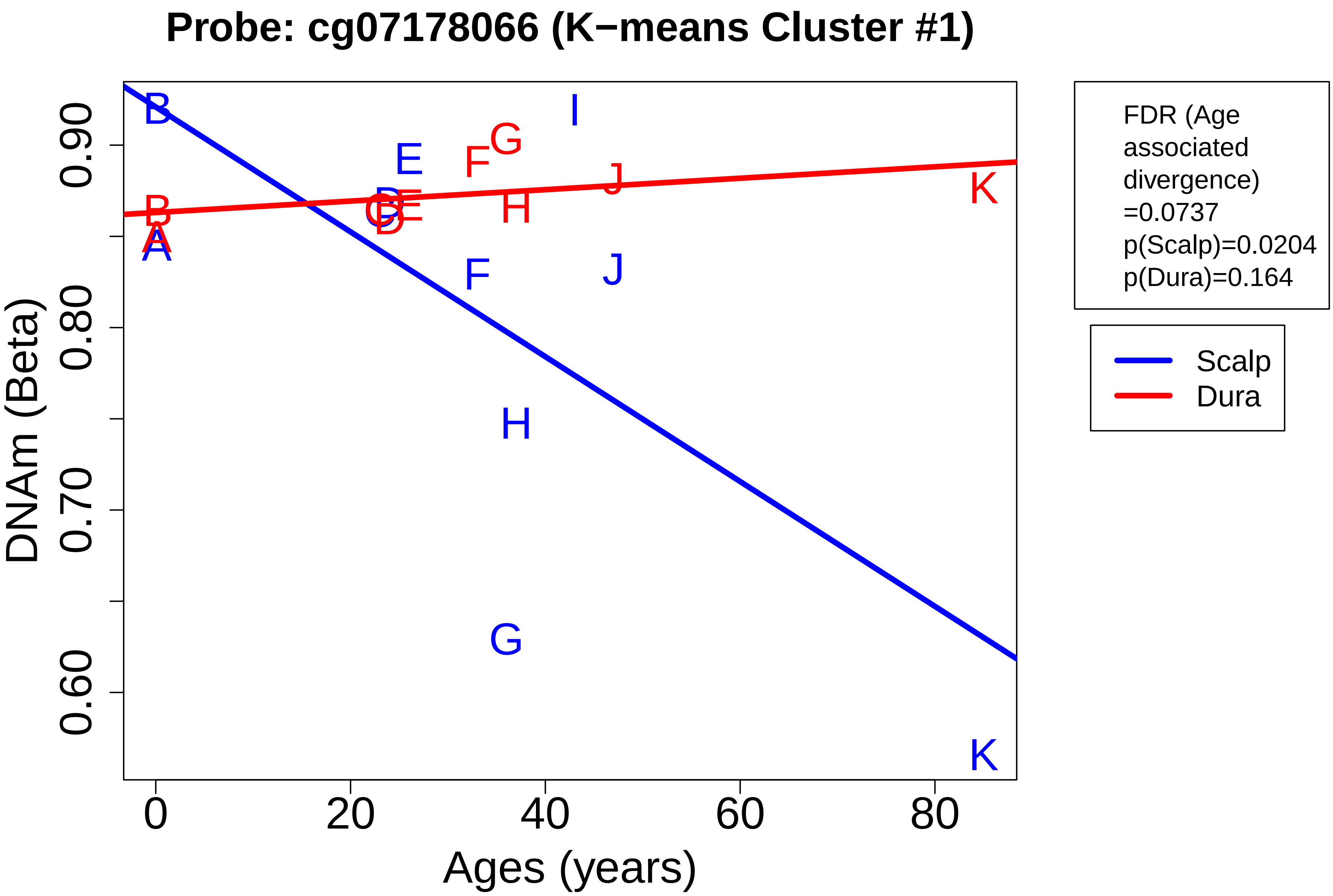

Probe: cg07178066 (K−means Cluster #1)
DNAm (Beta)
0.60
0.70
0.80
0.90
0
20
40
60
80
Ages (years)
FDR (Age associated divergence) =0.0737
p(Scalp)=0.0204
p(Dura)=0.164
Scalp
Dura

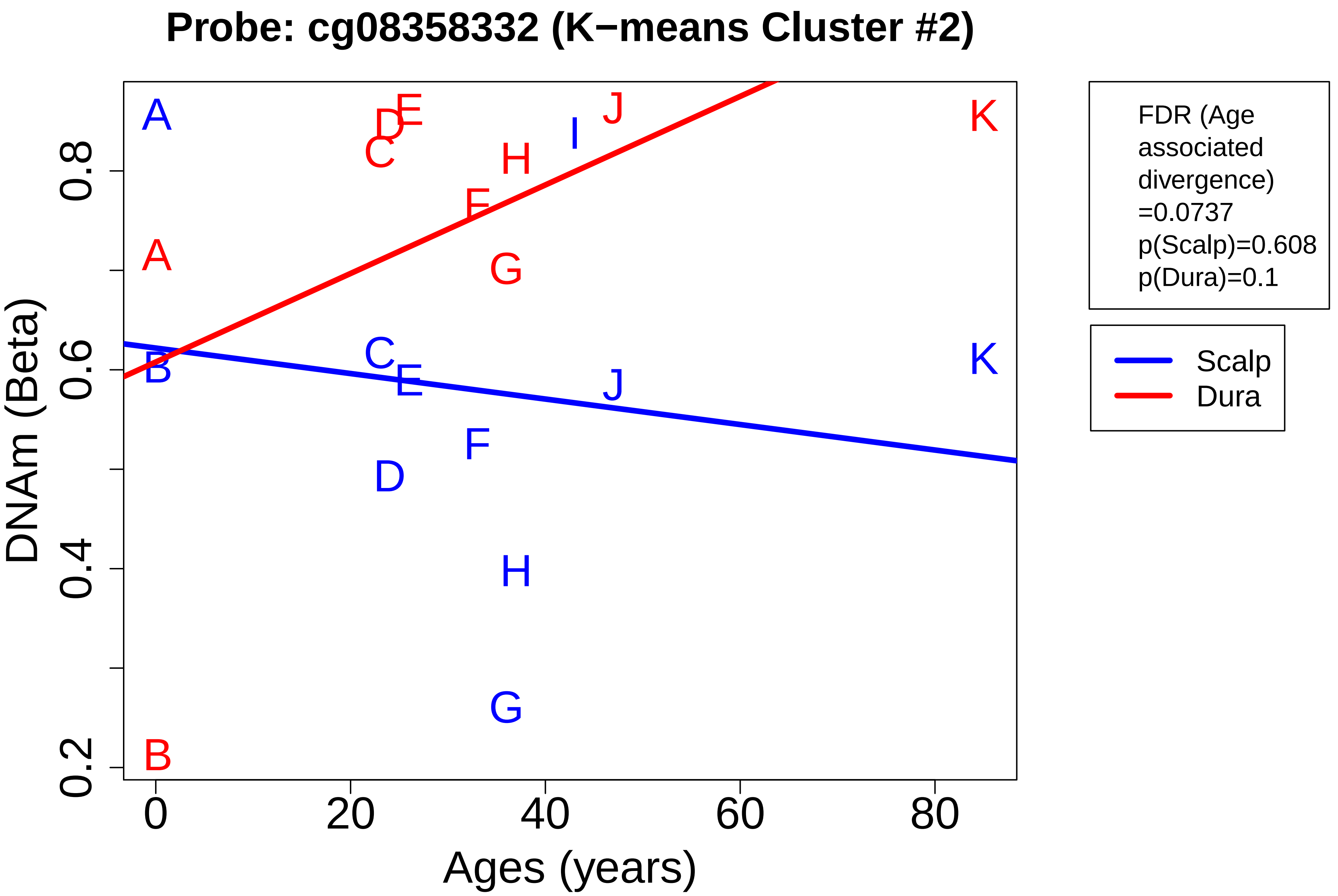

Probe: cg08358332 (K−means Cluster #2)
DNAm (Beta)
0.2
0.4
0.6
0.8
Ages (years)
0
20
40
60
80
FDR (Age associated divergence) =0.0737
p(Scalp)=0.608
p(Dura)=0.1
Scalp
Dura
A B C D E F G H I J K

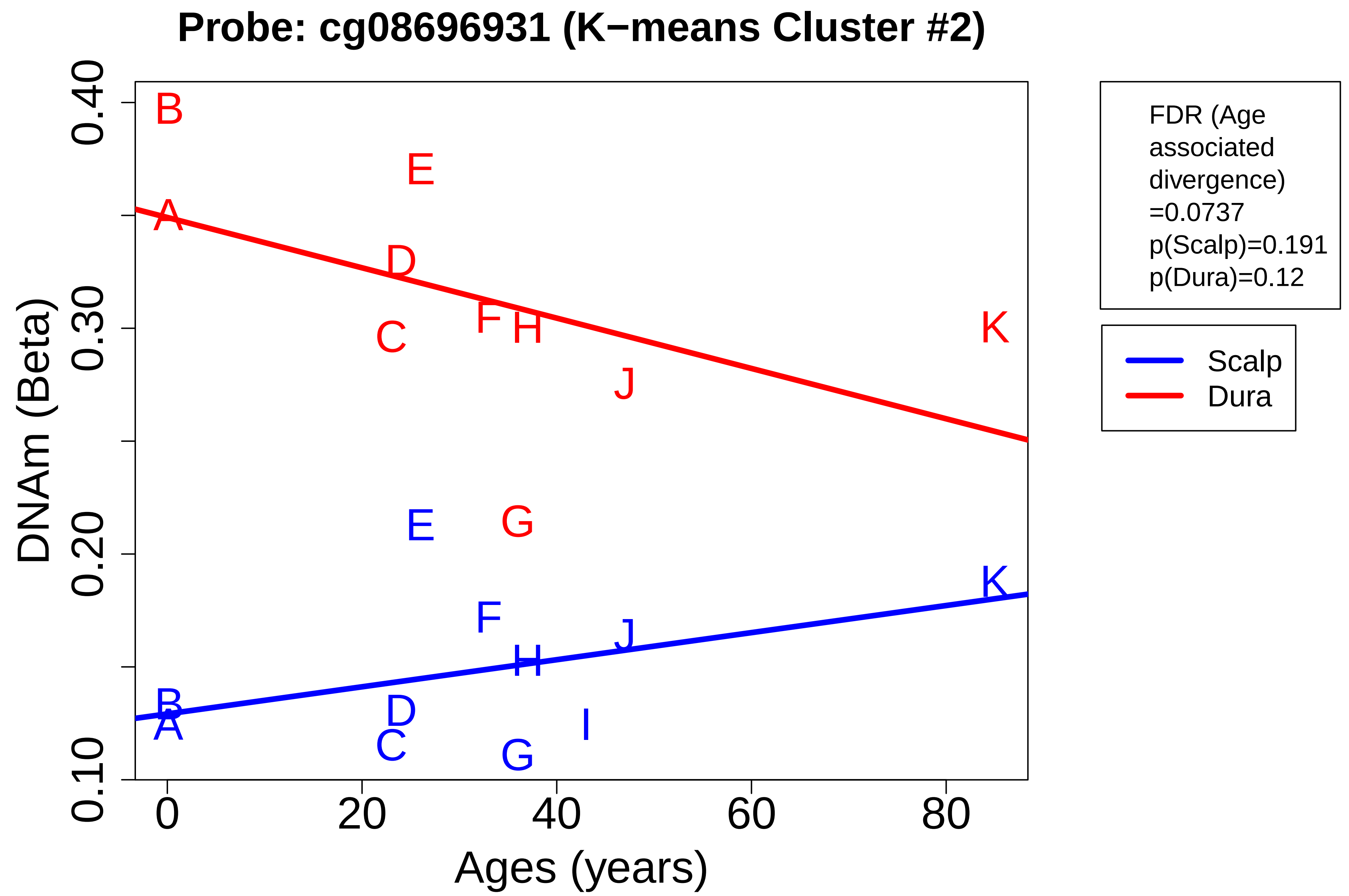

Probe: cg08696931 (K−means Cluster #2)
DNAm (Beta)
0.10
0.20
0.30
0.40
0
20
40
60
80
Ages (years)
FDR (Age associated divergence) =0.0737
p(Scalp)=0.191
p(Dura)=0.12
Scalp
Dura

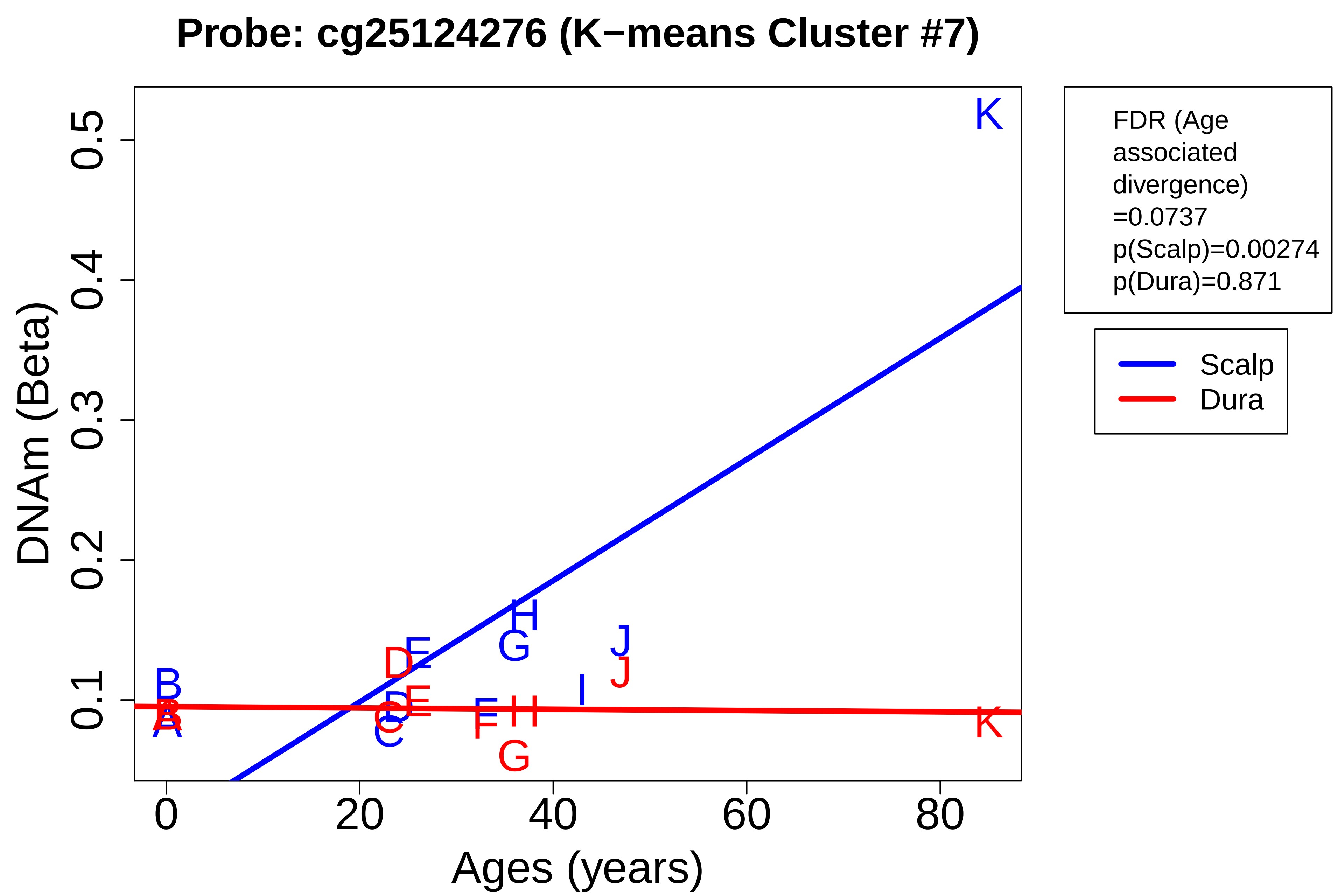

Probe: cg25124276 (K−means Cluster #7)
DNAm (Beta)
0.1
0.2
0.3
0.4
0.5
0
20
40
60
80
Ages (years)
FDR (Age associated divergence) =0.0737
p(Scalp)=0.00274
p(Dura)=0.871
Scalp
Dura

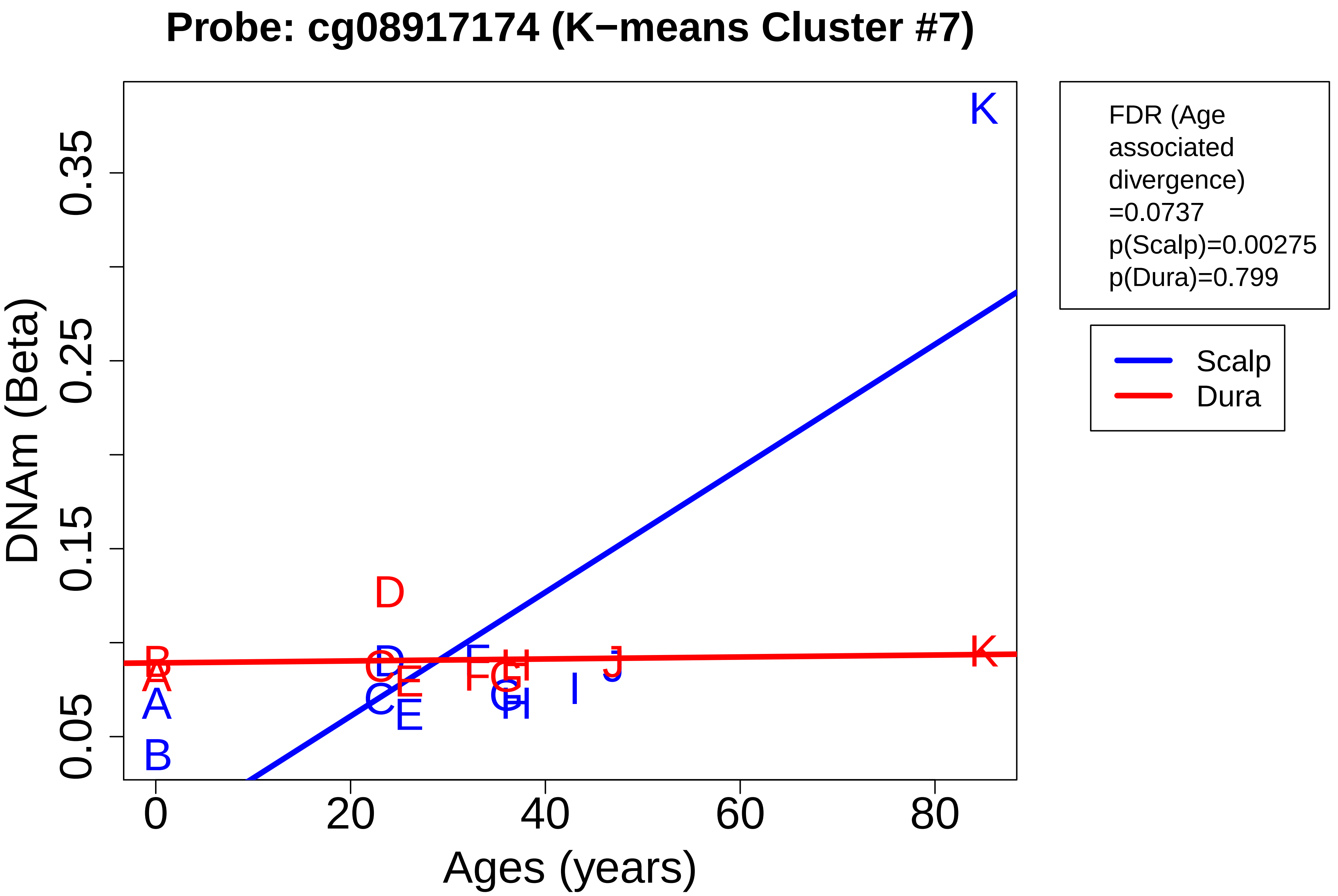
Probe: cg08917174 (K−means Cluster #7)
DNAm (Beta)
0.05
0.15
0.25
0.35
0
20
40
60
80
Ages (years)
FDR (Age associated divergence) =0.0737
p(Scalp)=0.00275
p(Dura)=0.799
Scalp
Dura

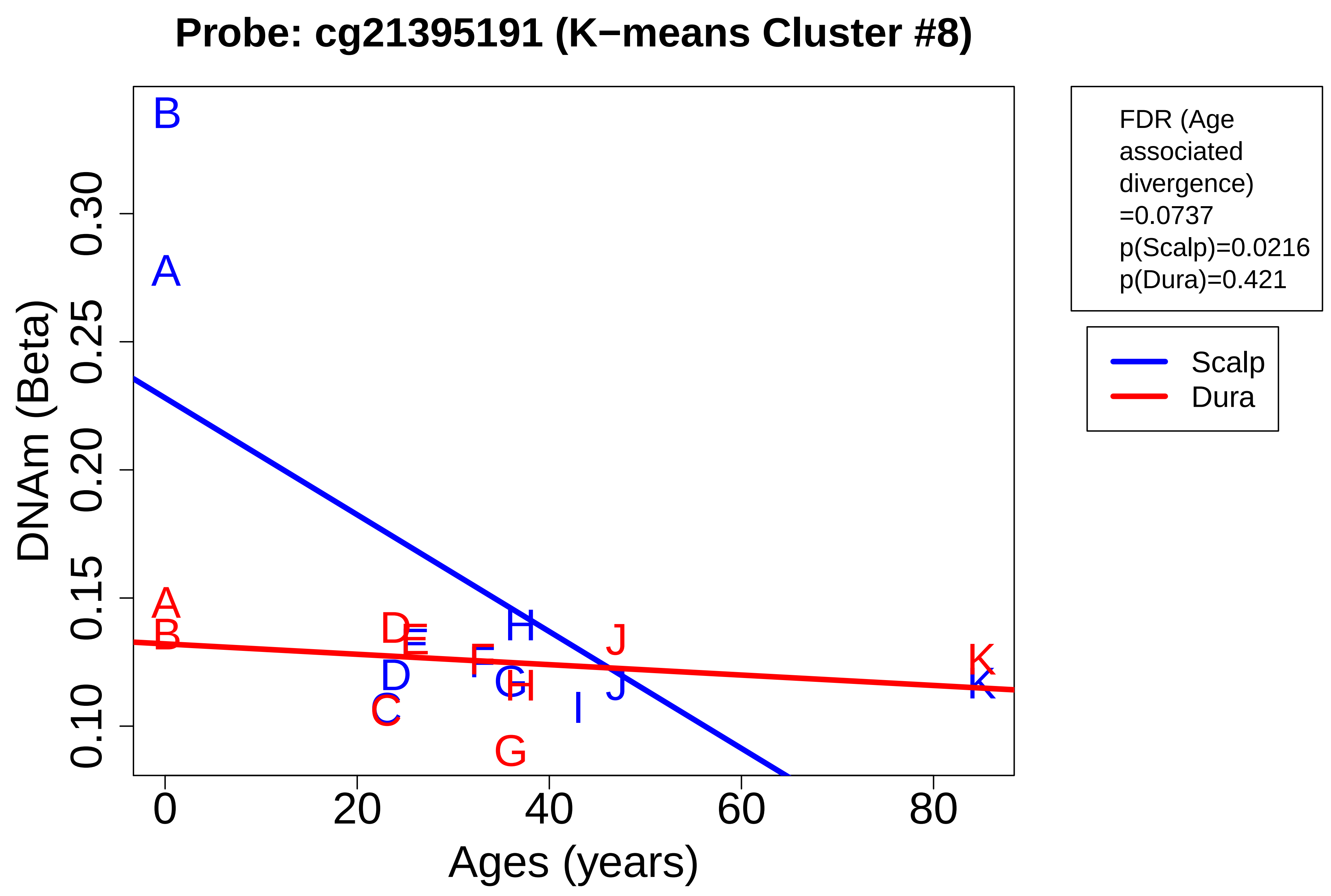
Probe: cg21395191 (K−means Cluster #8)
DNAm (Beta)
0.10
0.15
0.20
0.25
0.30
Ages (years)
0
20
40
60
80
FDR (Age associated divergence) =0.0737
p(Scalp)=0.0216
p(Dura)=0.421
Scalp
Dura

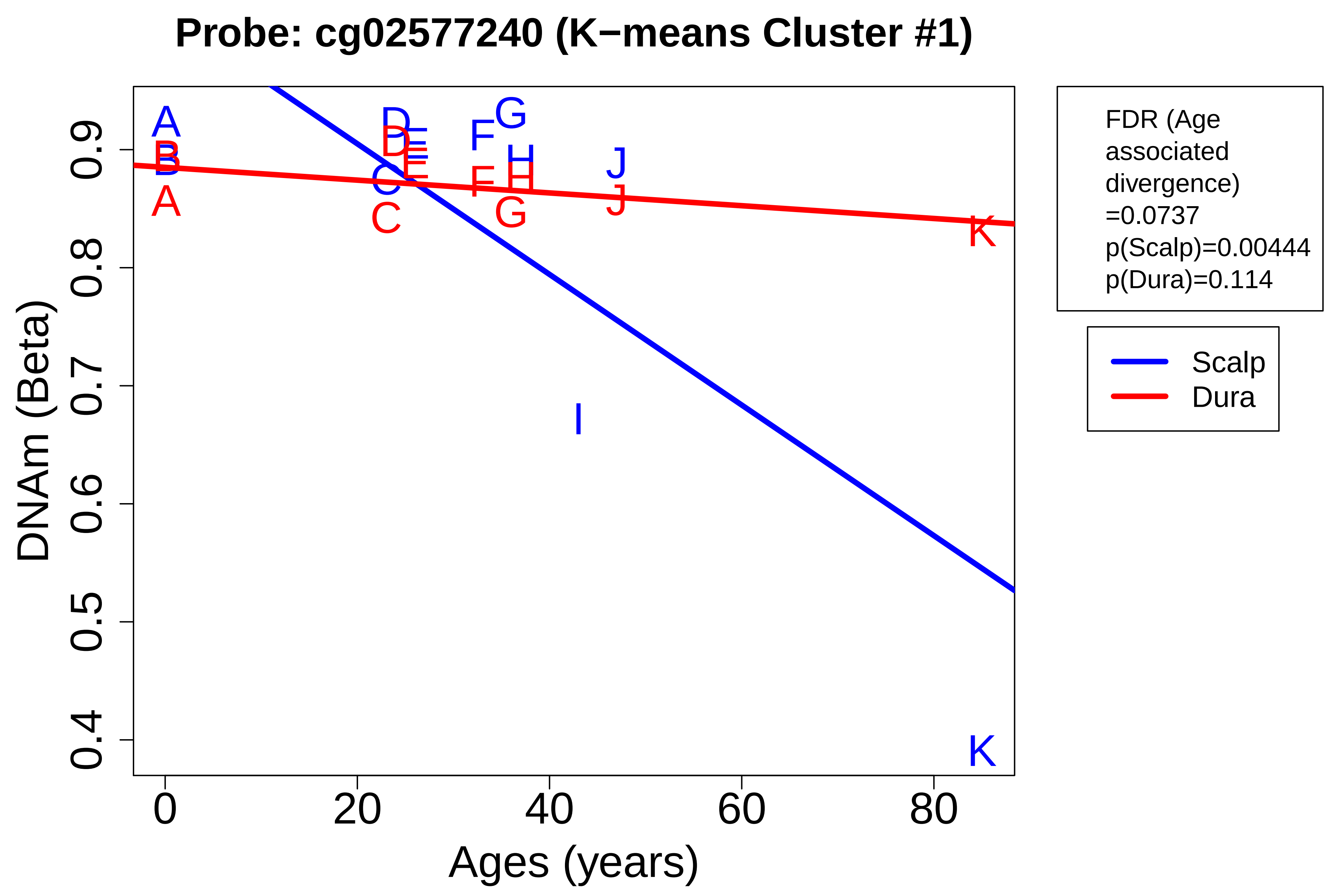

Probe: cg02577240 (K−means Cluster #1)
DNAm (Beta)
0.4
0.5
0.6
0.7
0.8
0.9
Ages (years)
0
20
40
60
80
A
B
C
D
E
F
G
H
I
J
K
FDR (Age
associated
divergence)
=0.0737
p(Scalp)=0.00444
p(Dura)=0.114
Scalp
Dura

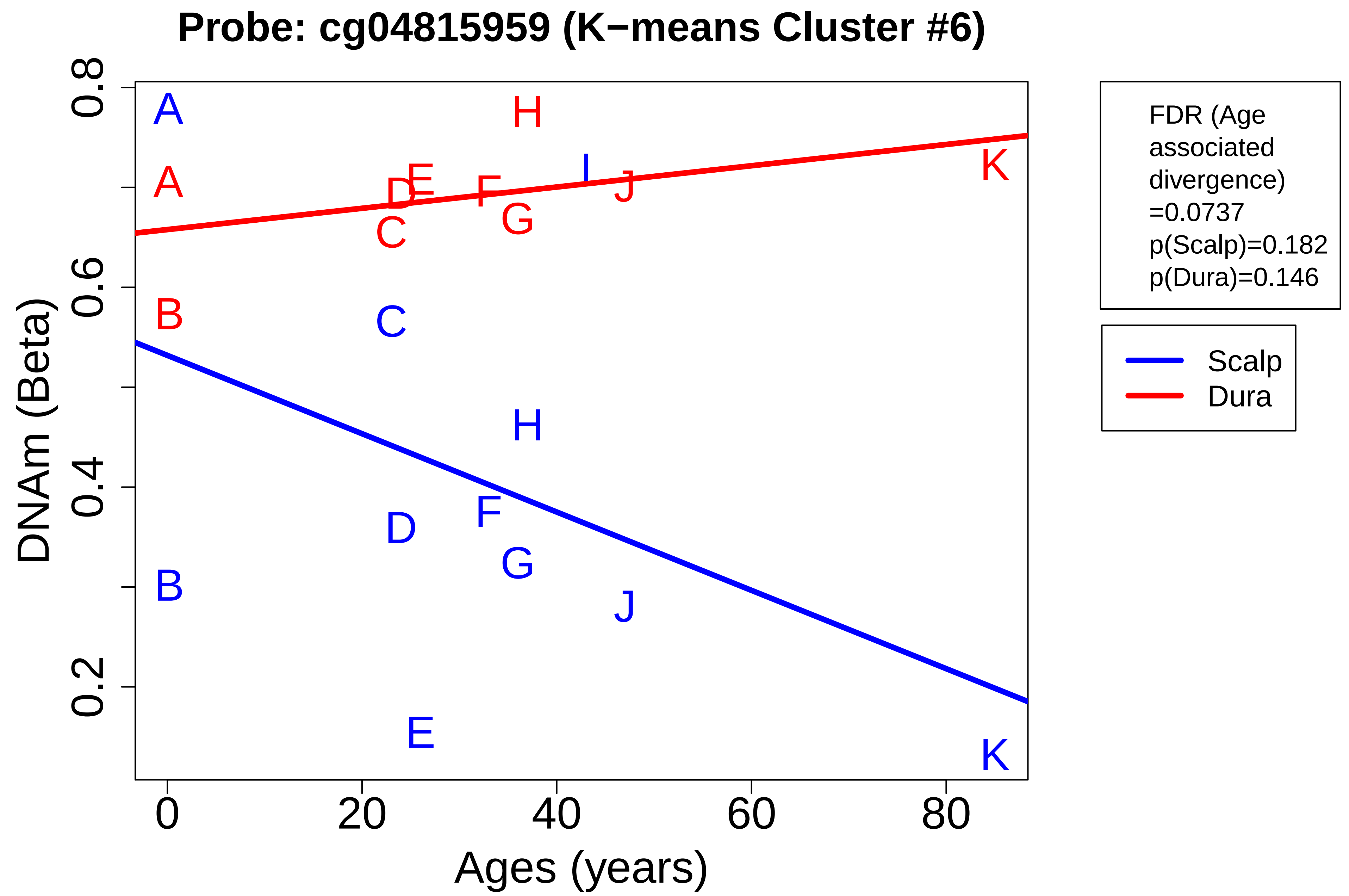

Probe: cg04815959 (K−means Cluster #6)
DNAm (Beta)
0.2
0.4
0.6
0.8
Ages (years)
0
20
40
60
80
A
B
C
D
E
F
G
H
I
J
K
FDR (Age associated divergence) =0.0737
p(Scalp)=0.182
p(Dura)=0.146
Scalp
Dura

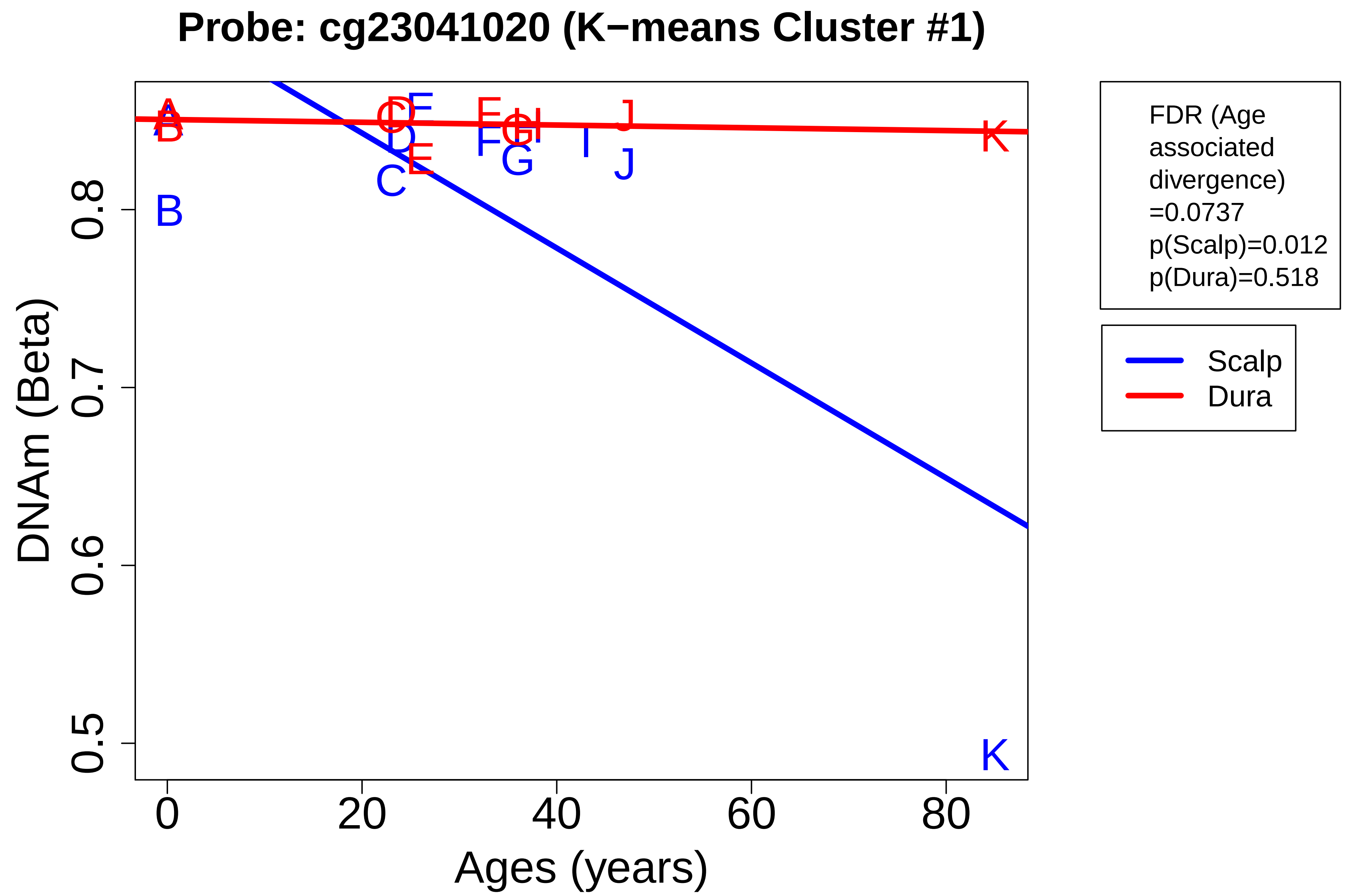

Probe: cg23041020 (K−means Cluster #1)
DNAm (Beta)
0.5
0.6
0.7
0.8
0
20
40
60
80
Ages (years)
A
B
C
D
E
F
G
H
I
J
K
FDR (Age associated divergence) =0.0737
p(Scalp)=0.012
p(Dura)=0.518
Scalp
Dura

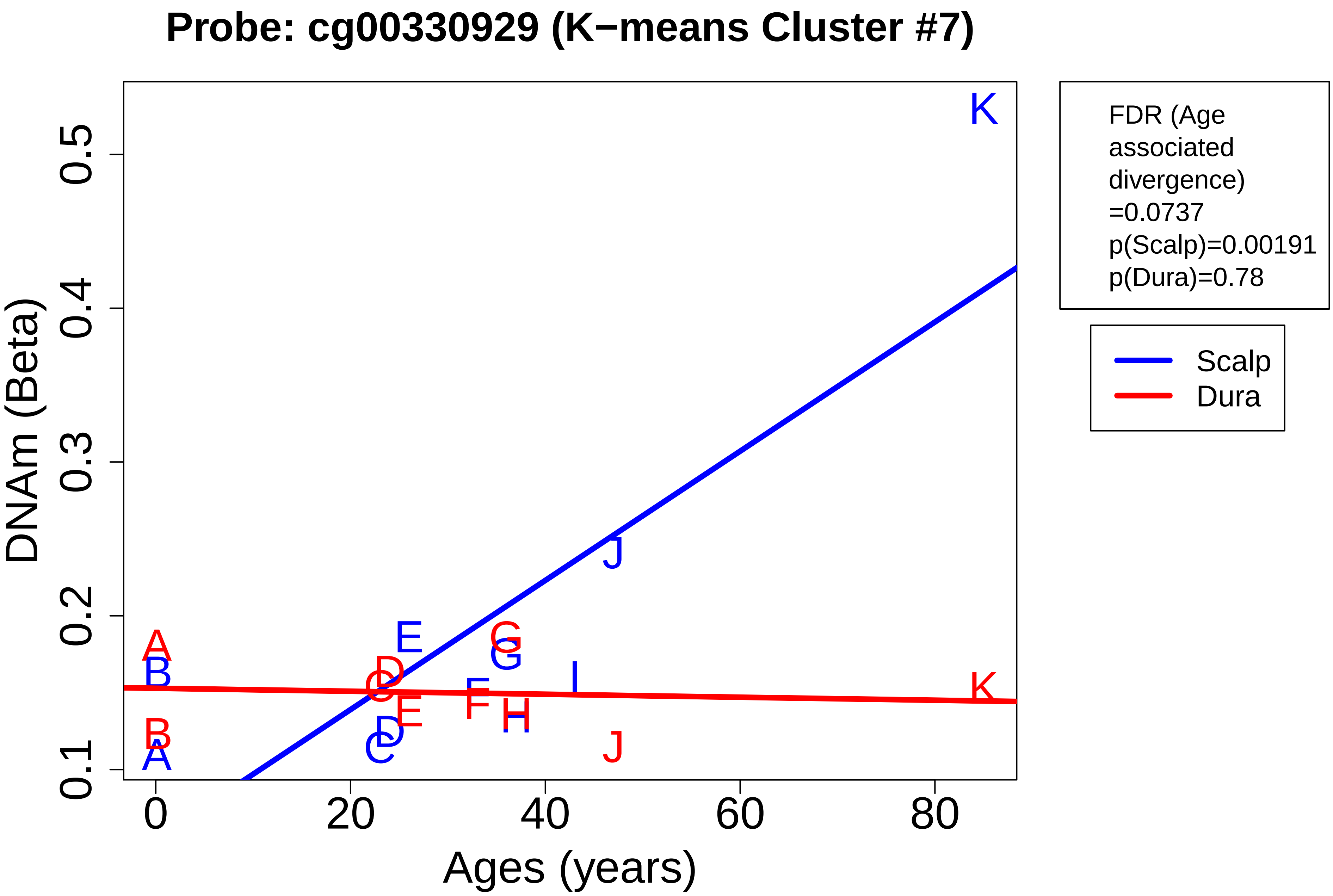

Probe: cg00330929 (K−means Cluster #7)
DNAm (Beta)
0.1
0.2
0.3
0.4
0.5
0
20
40
60
80
Ages (years)
FDR (Age associated divergence) =0.0737
p(Scalp)=0.00191
p(Dura)=0.78
Scalp
Dura

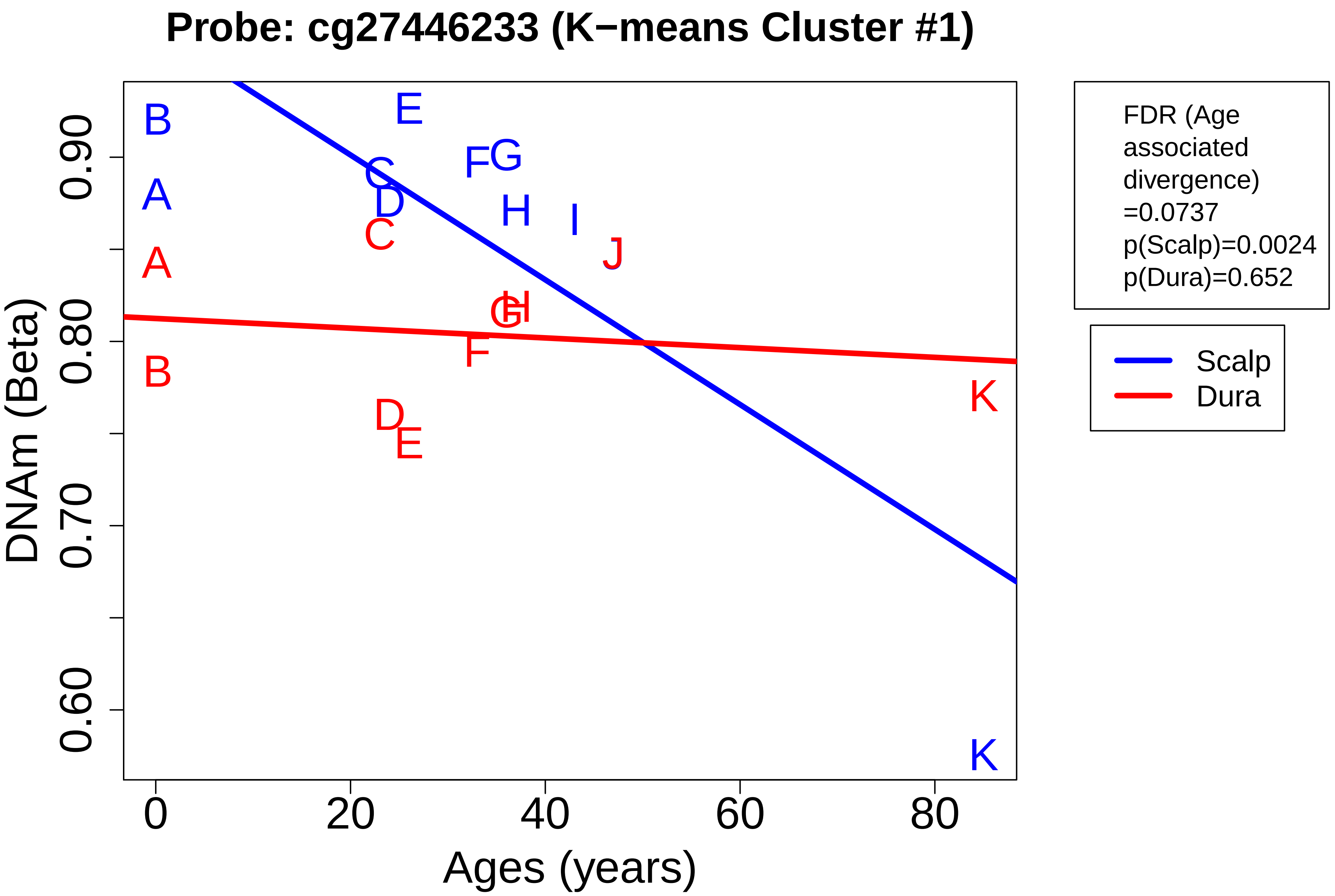

Probe: cg27446233 (K−means Cluster #1)
DNAm (Beta)
0.60
0.70
0.80
0.90
Ages (years)
0
20
40
60
80
FDR (Age associated divergence) =0.0737
p(Scalp)=0.0024
p(Dura)=0.652
Scalp
Dura

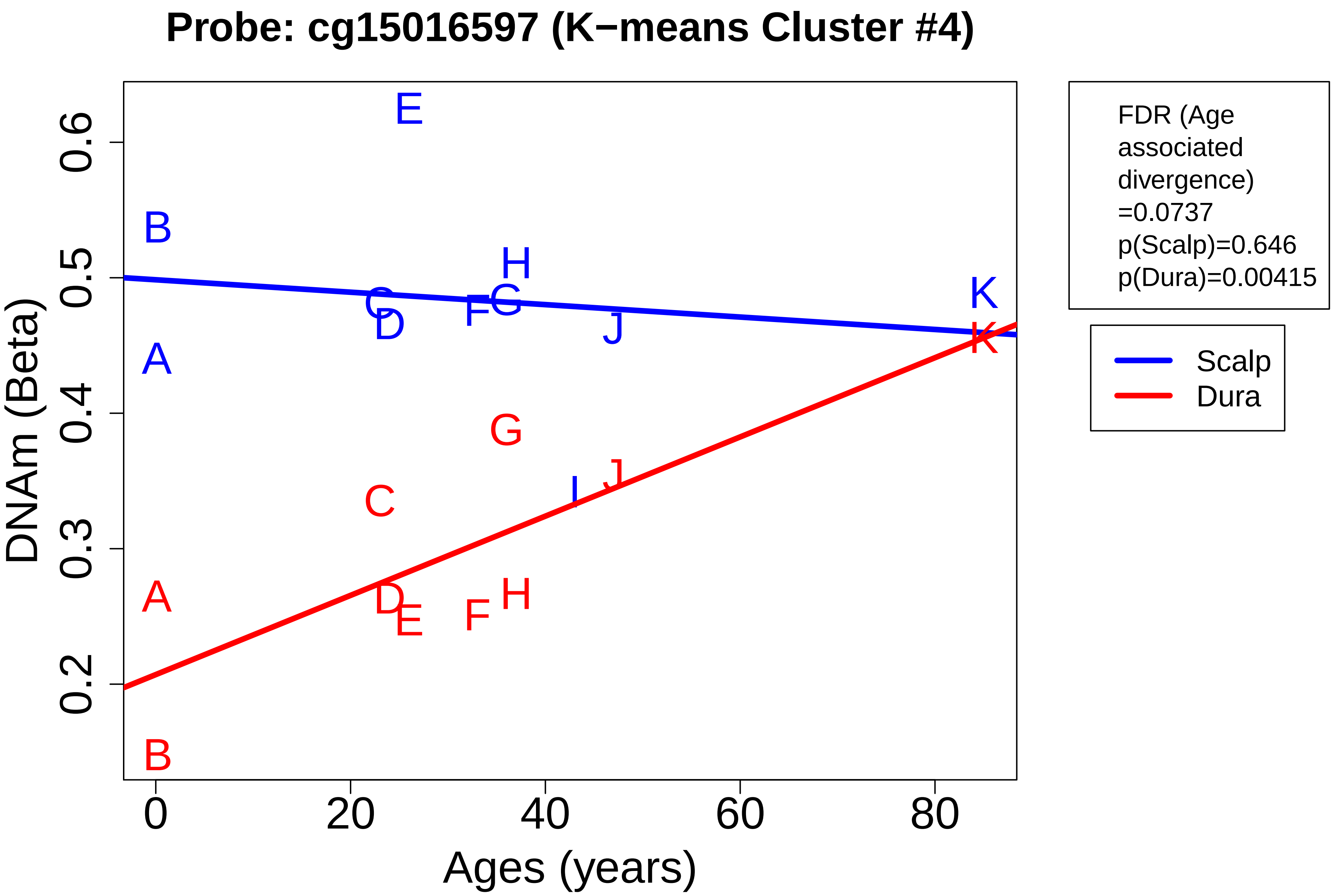

Probe: cg15016597 (K−means Cluster #4)
DNAm (Beta)
0.2
0.3
0.4
0.5
0.6
Ages (years)
0
20
40
60
80
FDR (Age associated divergence) =0.0737
p(Scalp)=0.646
p(Dura)=0.00415
Scalp
Dura
A
B
C
D
E
F
G
H
I
J
K

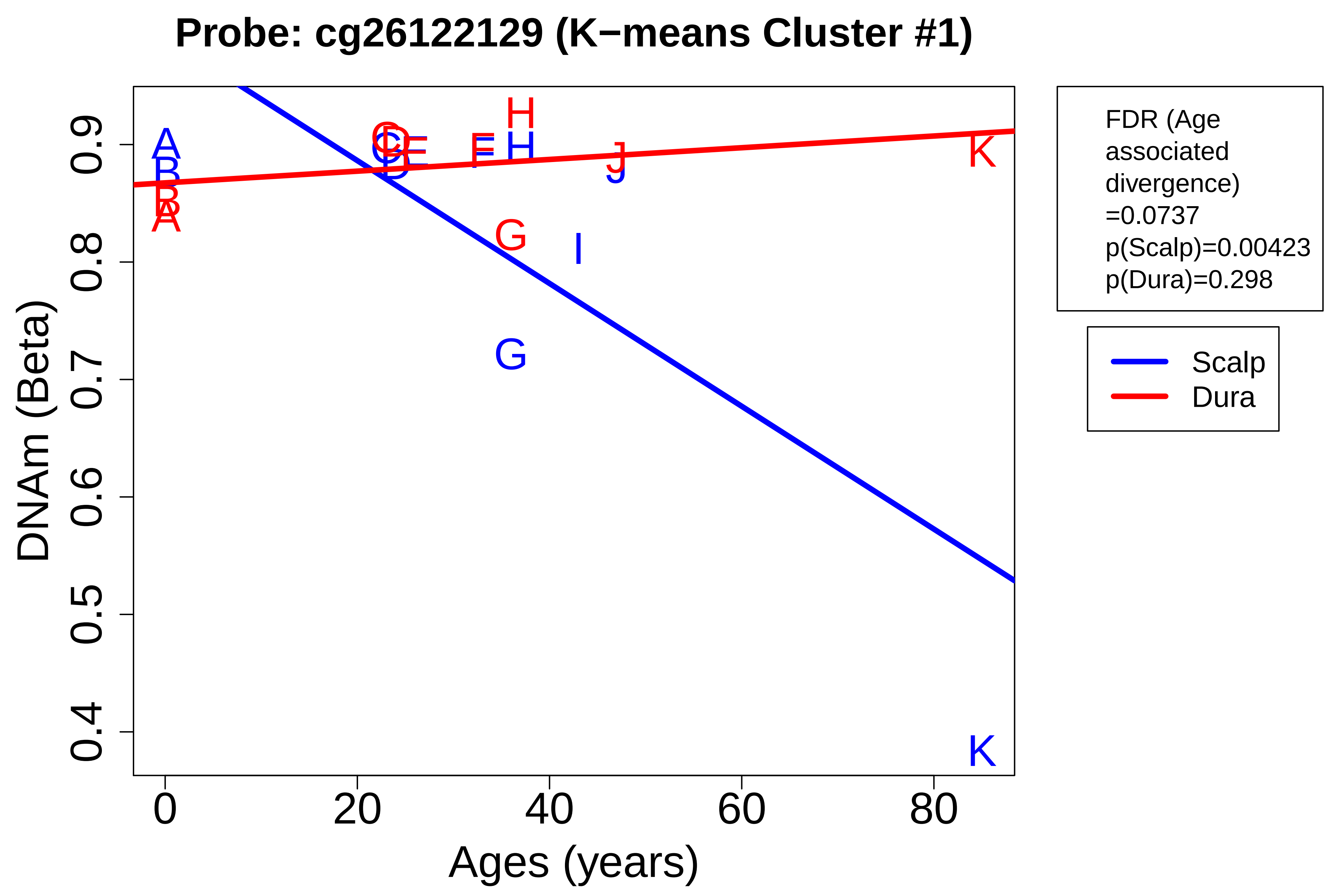

Probe: cg26122129 (K−means Cluster #1)
FDR (Age associated divergence) =0.0737
p(Scalp)=0.00423
p(Dura)=0.298
Scalp
Dura
DNAm (Beta)
0.4
0.5
0.6
0.7
0.8
0.9
0
20
40
60
80
Ages (years)
A
B
C
D
E
F
G
H
I
J
K

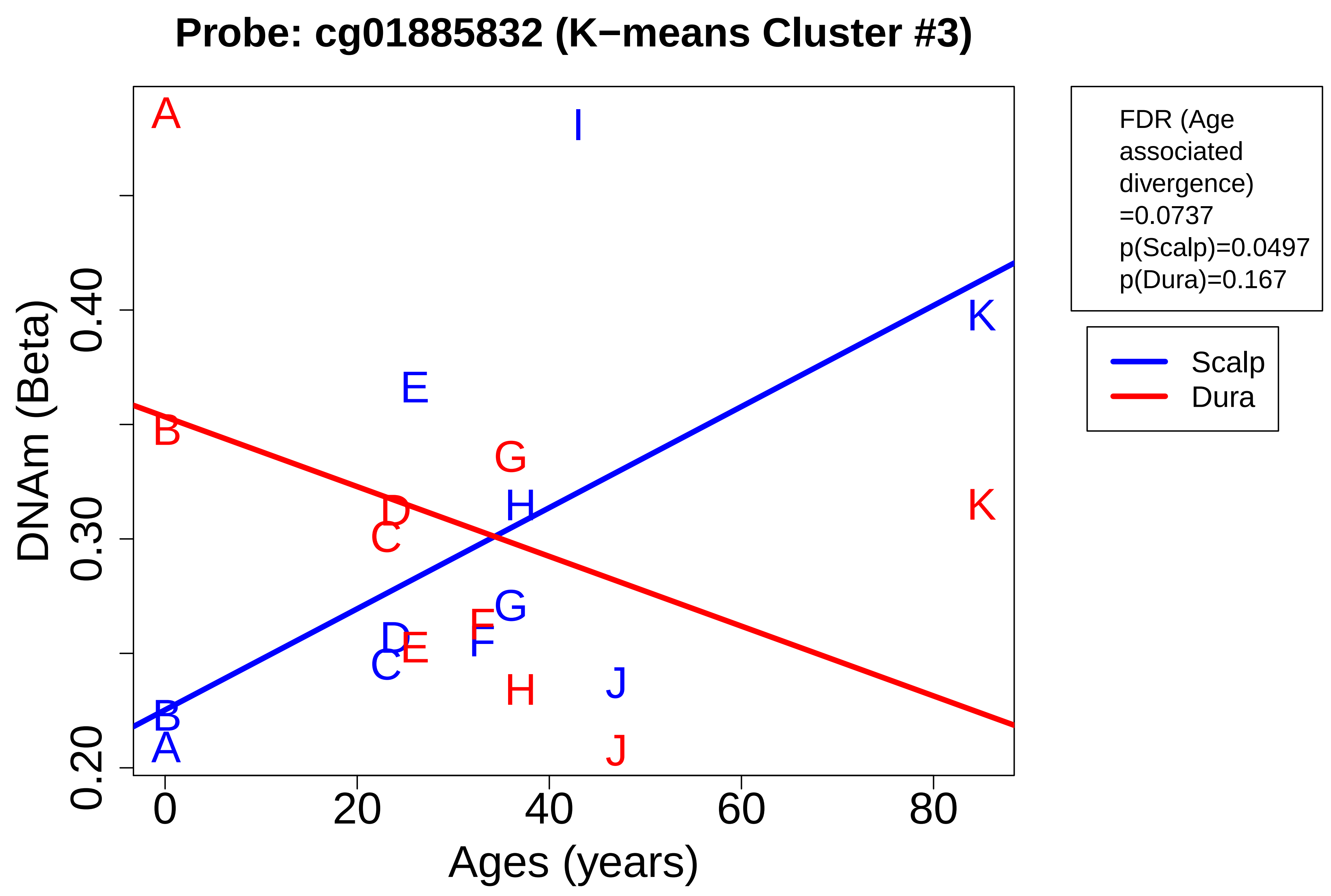

Probe: cg01885832 (K−means Cluster #3)
DNAm (Beta)
0.20
0.30
0.40
Ages (years)
0
20
40
60
80
A
B
C
D
E
F
G
H
I
J
K
FDR (Age associated divergence) =0.0737
p(Scalp)=0.0497
p(Dura)=0.167
Scalp
Dura

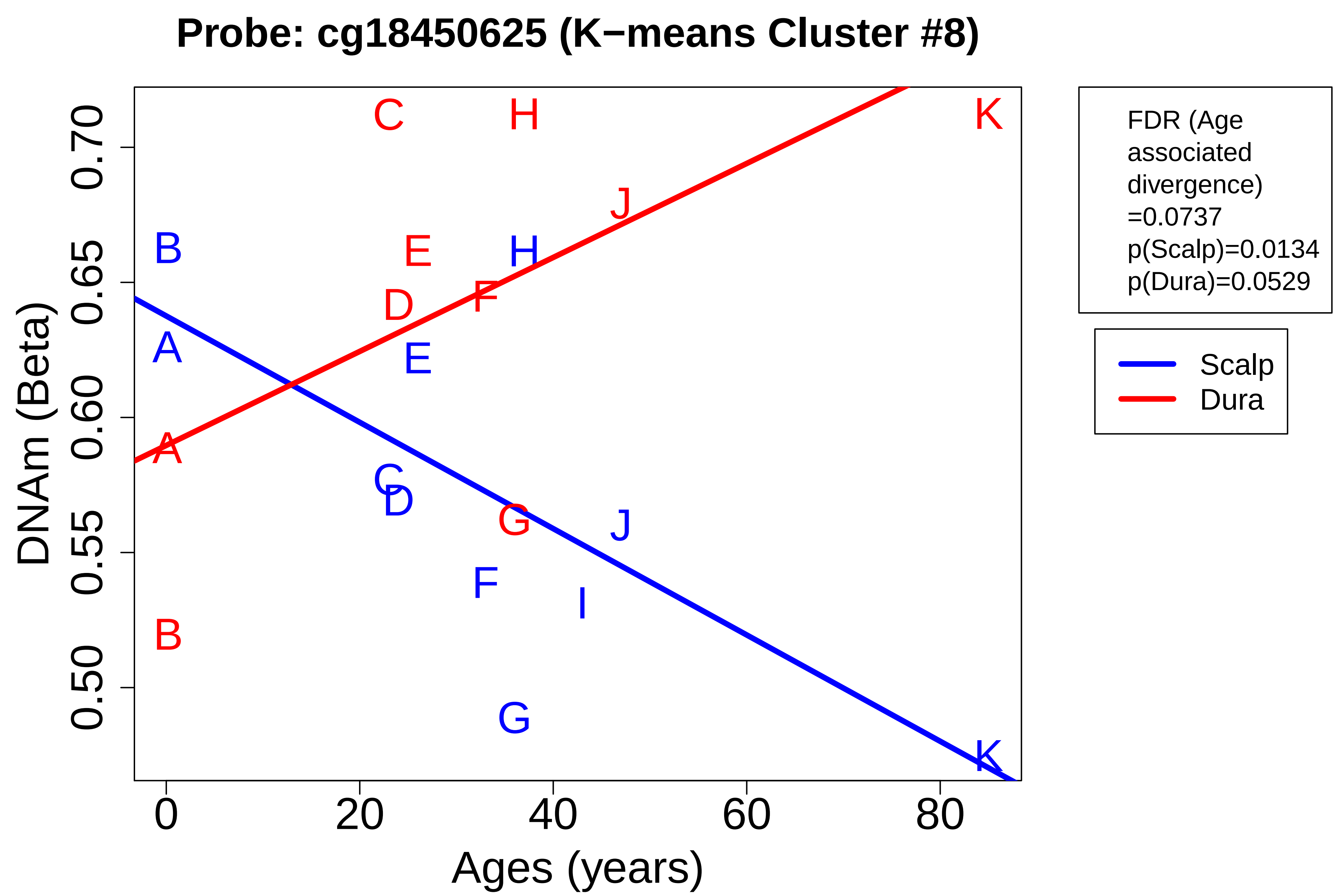

Probe: cg18450625 (K−means Cluster #8)
DNAm (Beta)
0.50
0.55
0.60
0.65
0.70
Ages (years)
0
20
40
60
80
FDR (Age associated divergence) =0.0737
p(Scalp)=0.0134
p(Dura)=0.0529
Scalp
Dura
A
B
C
D
E
F
G
H
I
J
K

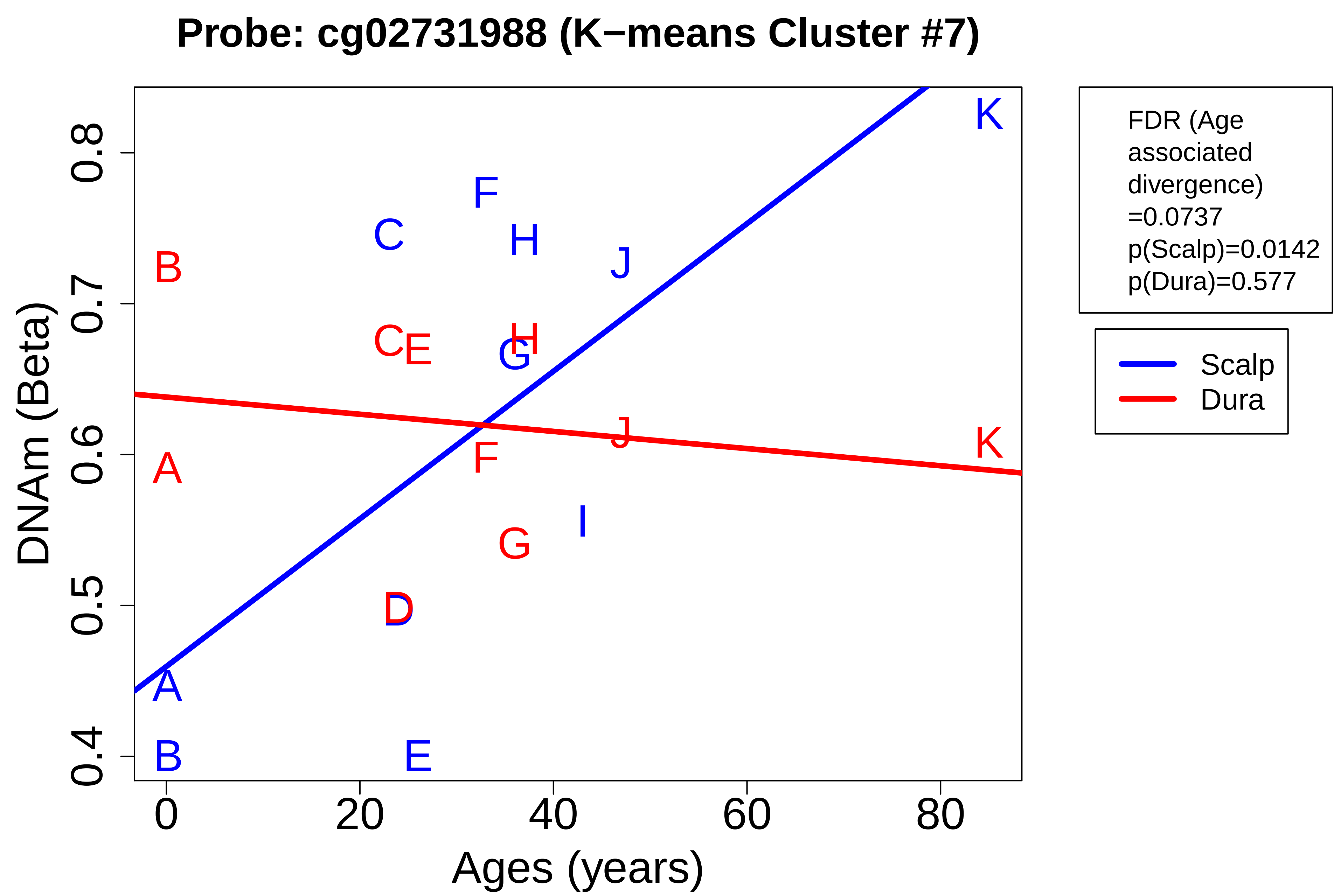

Probe: cg02731988 (K−means Cluster #7)
DNAm (Beta)
0.4
0.5
0.6
0.7
0.8
0
20
40
60
80
Ages (years)
FDR (Age associated divergence) =0.0737
p(Scalp)=0.0142
p(Dura)=0.577
Scalp
Dura
A
B
C
D
E
F
G
H
I
J
K

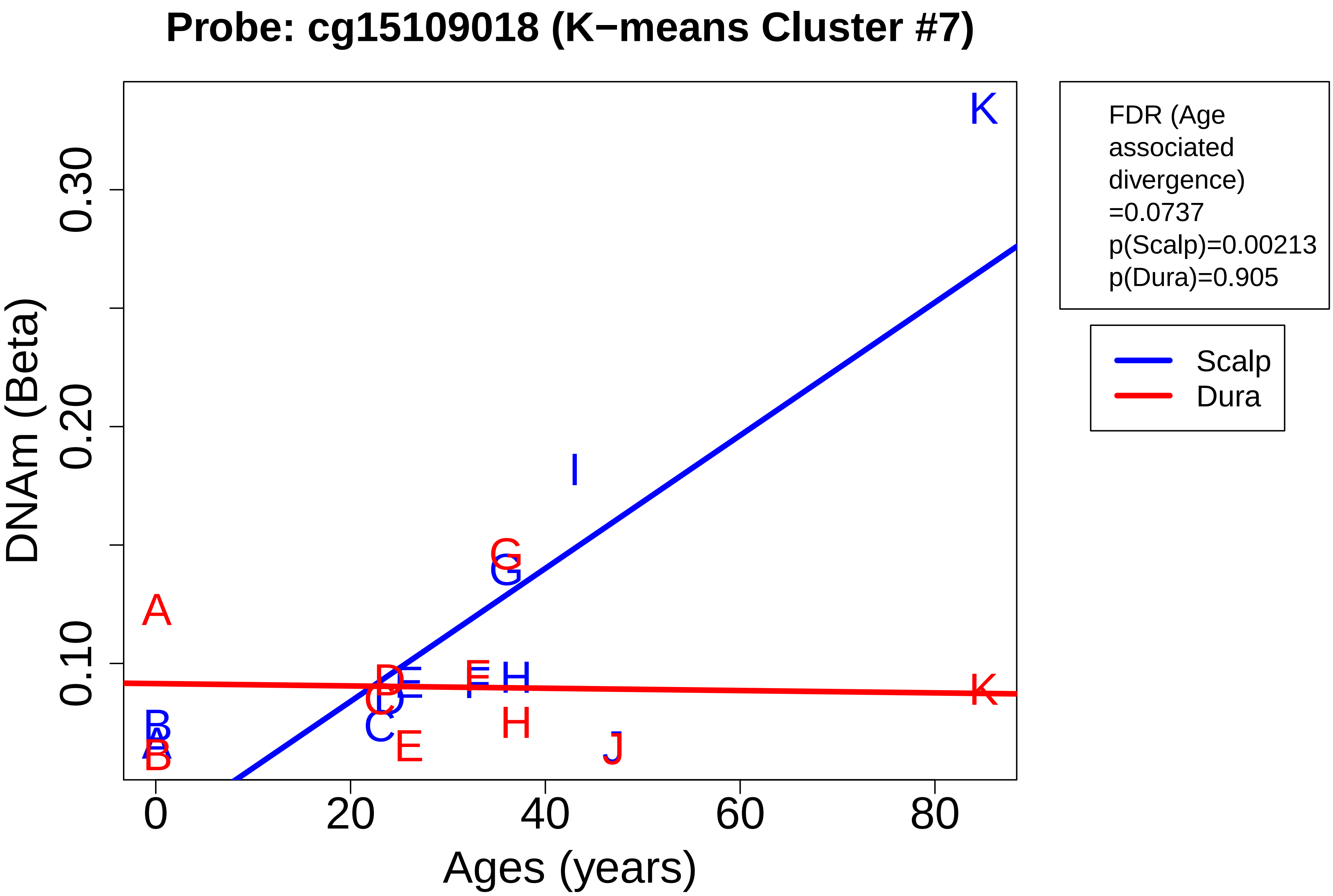
Probe: cg15109018 (K−means Cluster #7)
DNAm (Beta)
0.10
0.20
0.30
Ages (years)
0
20
40
60
80
FDR (Age associated divergence) =0.0737
p(Scalp)=0.00213
p(Dura)=0.905
Scalp
Dura
A
B
C
D
E
F
G
H
I
J
K

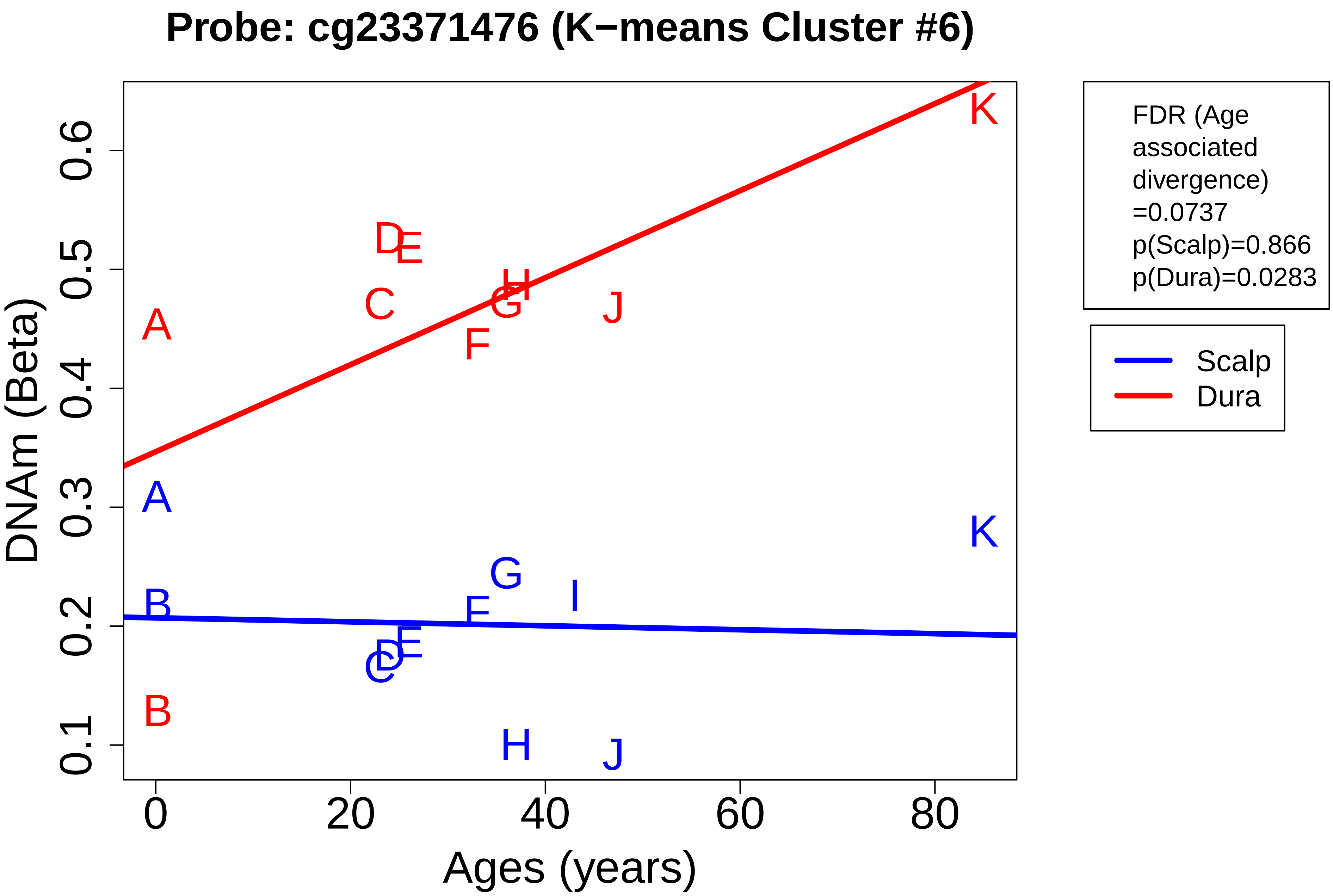

Probe: cg23371476 (K−means Cluster #6)
DNAm (Beta)
0.1
0.2
0.3
0.4
0.5
0.6
Ages (years)
0
20
40
60
80
FDR (Age associated divergence) =0.0737
p(Scalp)=0.866
p(Dura)=0.0283
Scalp
Dura

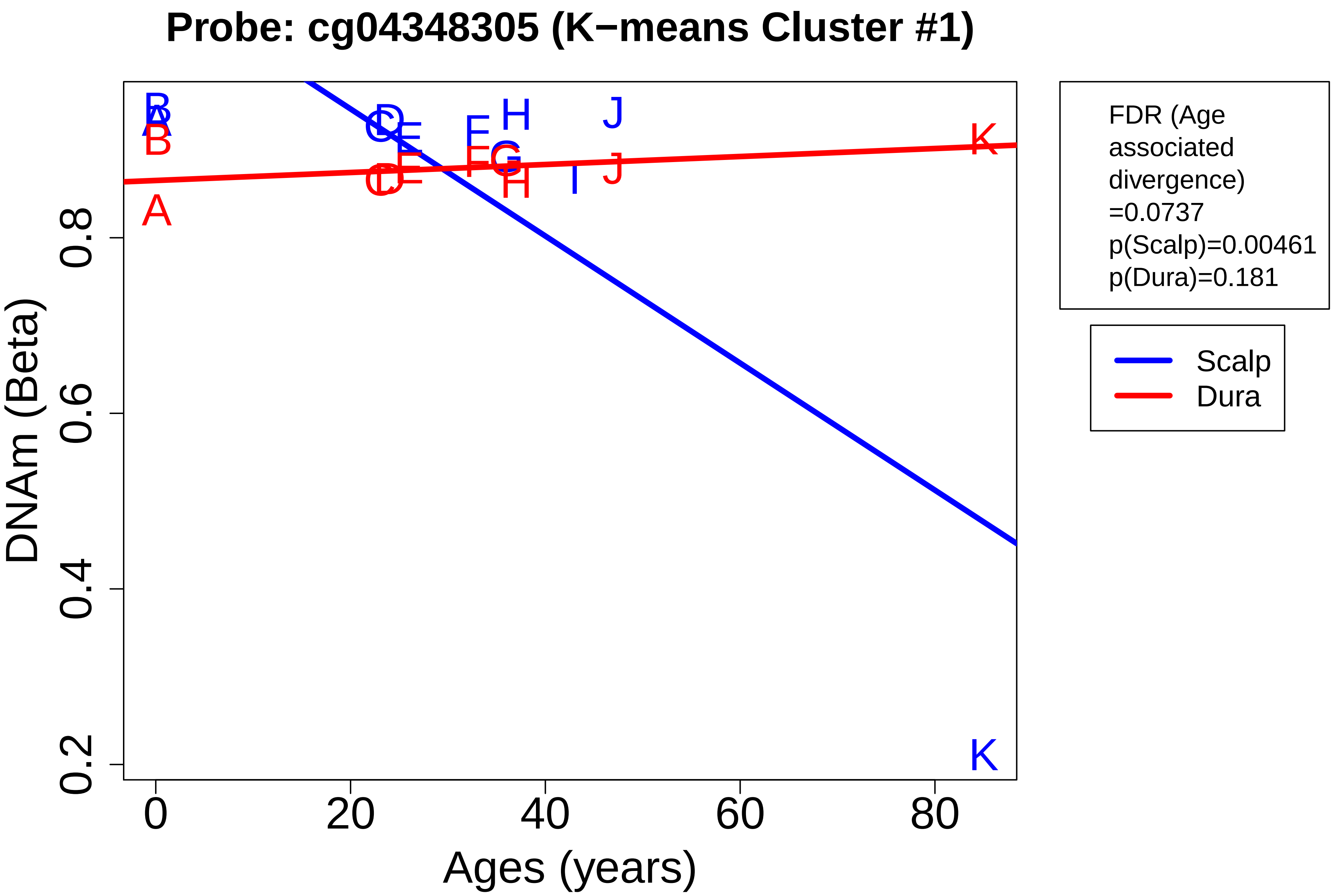

Probe: cg04348305 (K−means Cluster #1)
DNAm (Beta)
0.2
0.4
0.6
0.8
Ages (years)
0
20
40
60
80
FDR (Age associated divergence) =0.0737
p(Scalp)=0.00461
p(Dura)=0.181
Scalp
Dura

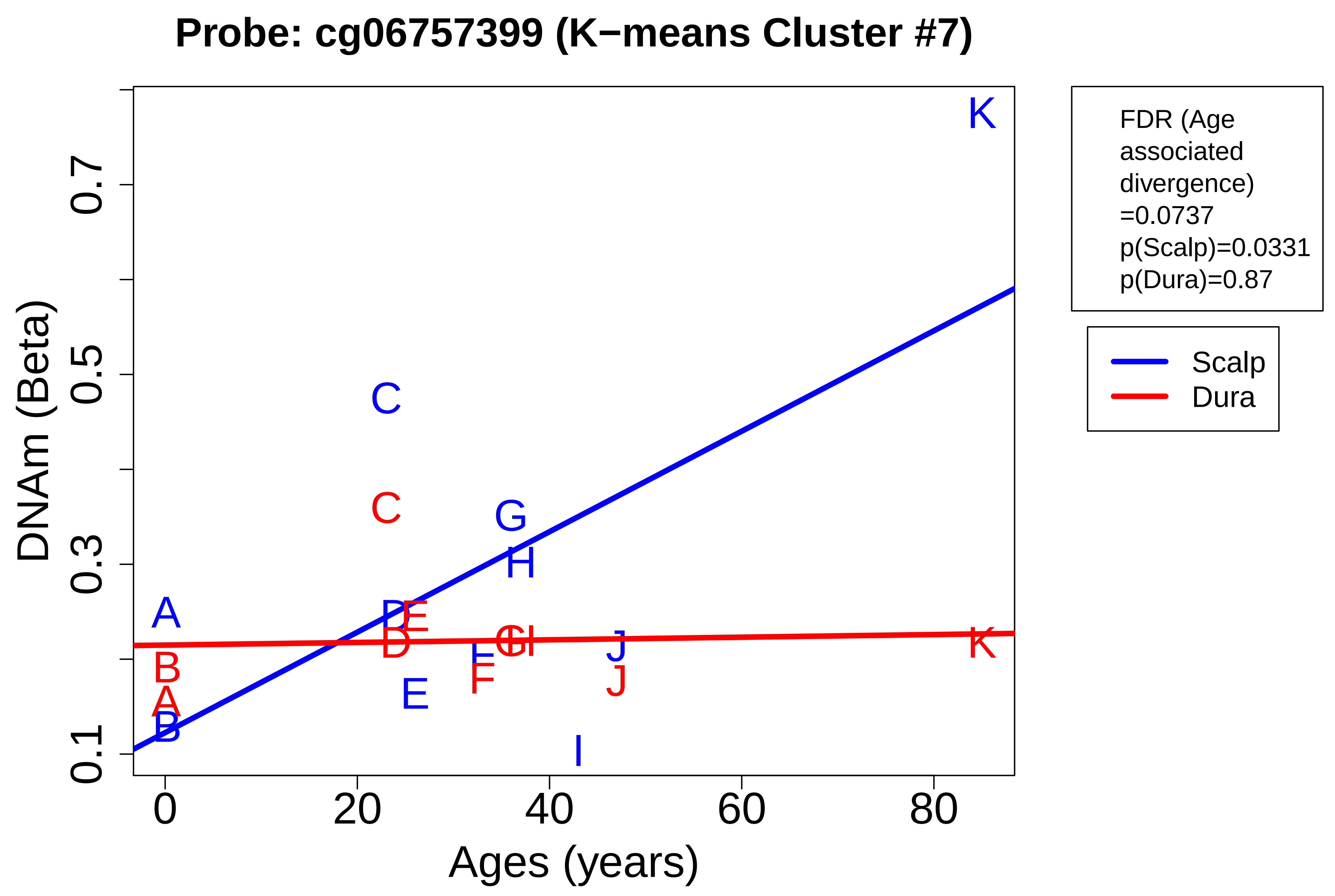

Probe: cg06757399 (K−means Cluster #7)
DNAm (Beta)
0.1
0.3
0.5
0.7
Ages (years)
0
20
40
60
80
FDR (Age associated divergence) =0.0737
p(Scalp)=0.0331
p(Dura)=0.87
Scalp
Dura

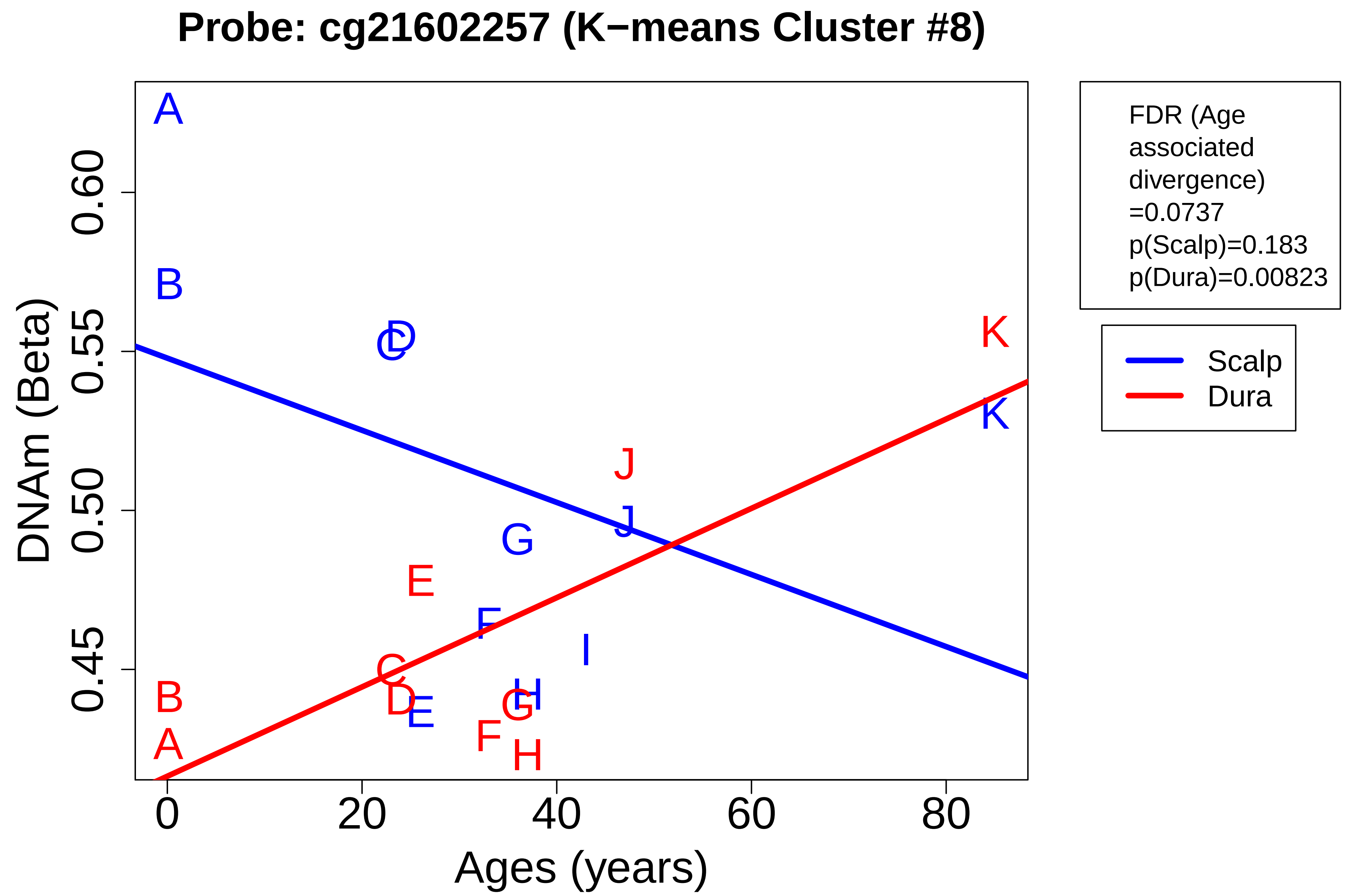

Probe: cg21602257 (K−means Cluster #8)
DNAm (Beta)
0.45
0.50
0.55
0.60
Ages (years)
0
20
40
60
80
FDR (Age associated divergence) =0.0737
p(Scalp)=0.183
p(Dura)=0.00823
Scalp
Dura
A
B
C
D
E
F
G
H
I
J
K

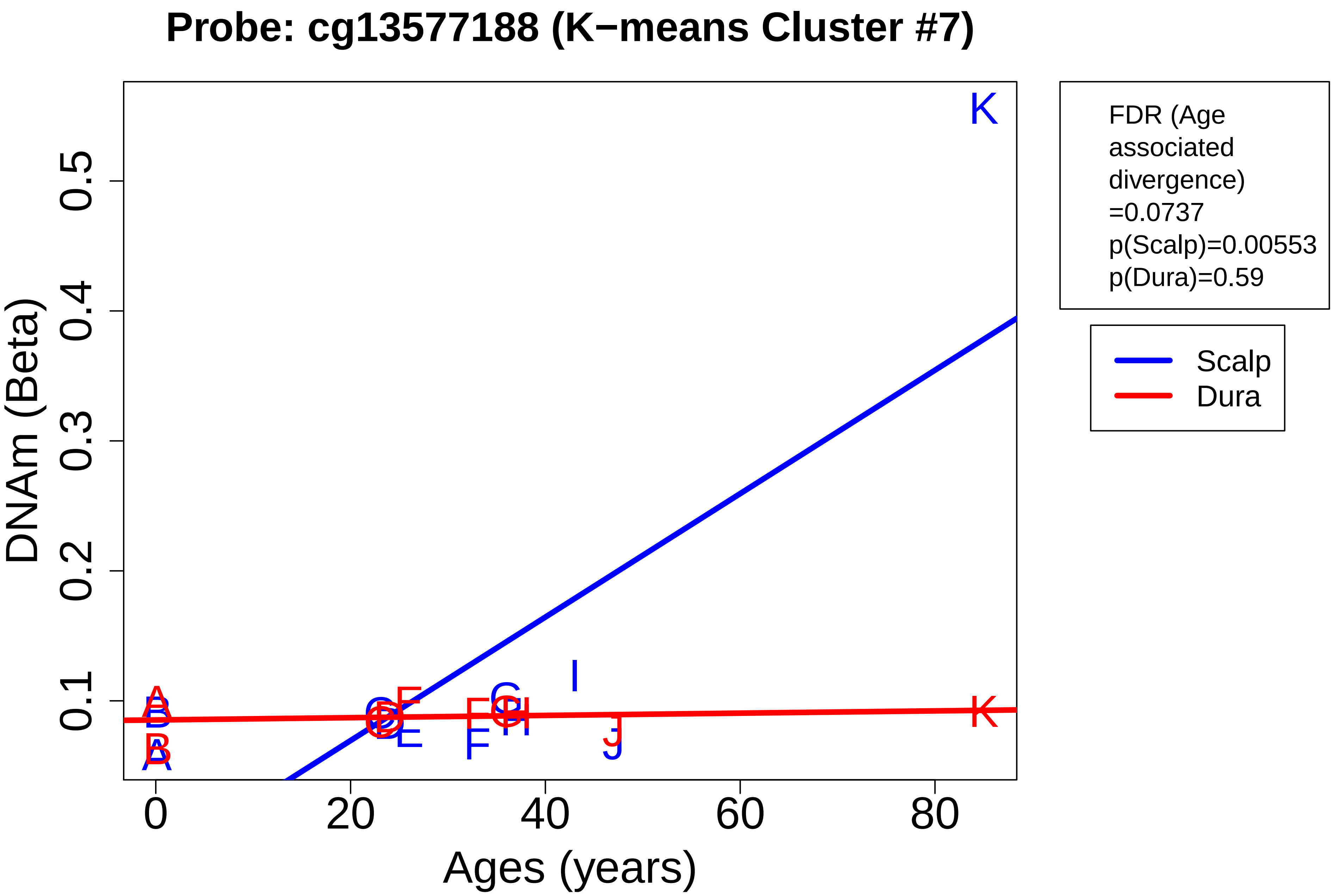

Probe: cg13577188 (K−means Cluster #7)
FDR (Age associated divergence) =0.0737
p(Scalp)=0.00553
p(Dura)=0.59
Scalp
Dura
DNAm (Beta)
0.1
0.2
0.3
0.4
0.5
Ages (years)
0
20
40
60
80
A
B
C
D
E
F
G
H
I
J
K

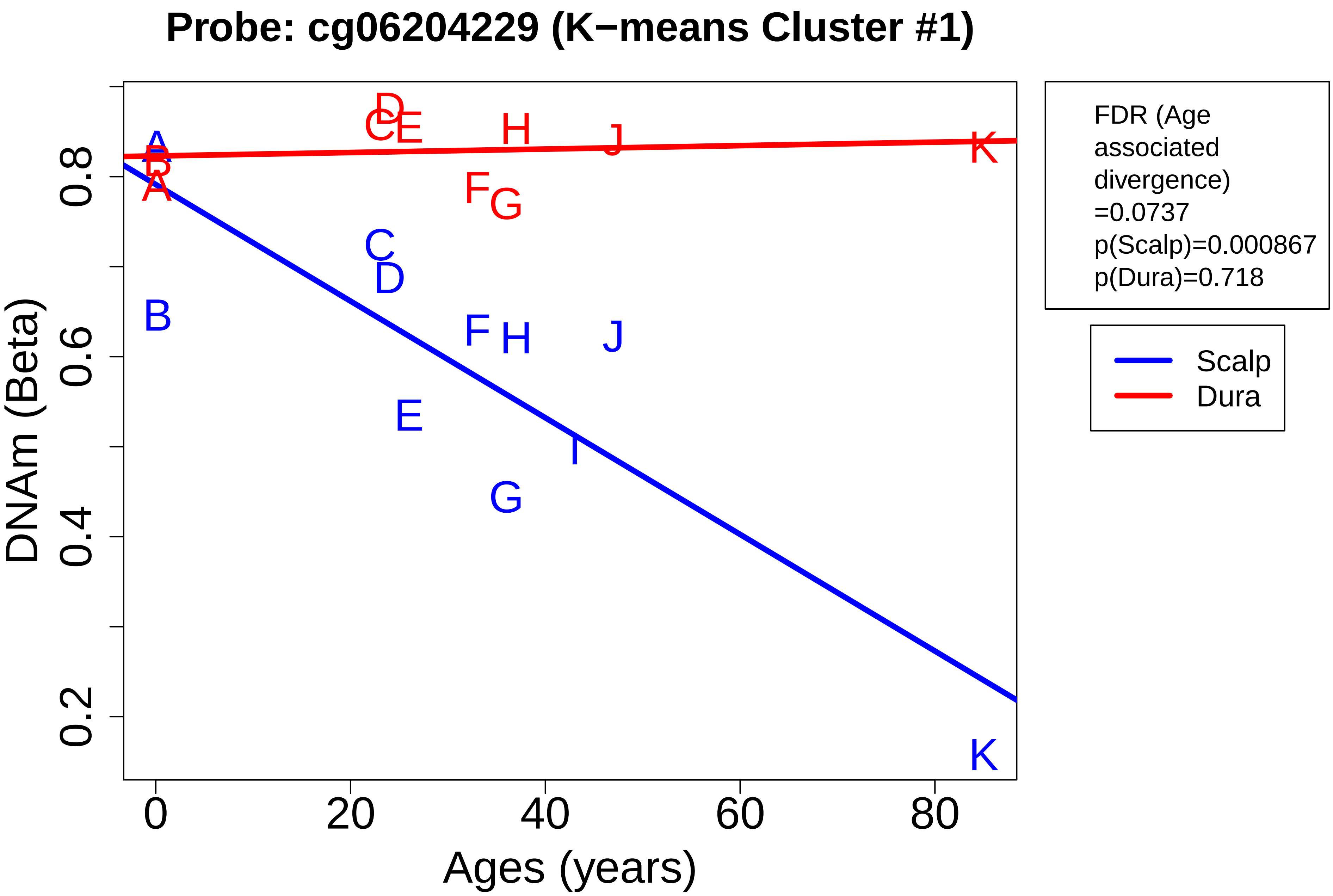

Probe: cg06204229 (K−means Cluster #1)
FDR (Age associated divergence) =0.0737
p(Scalp)=0.000867
p(Dura)=0.718
Scalp
Dura
DNAm (Beta)
0.2
0.4
0.6
0.8
Ages (years)
0
20
40
60
80

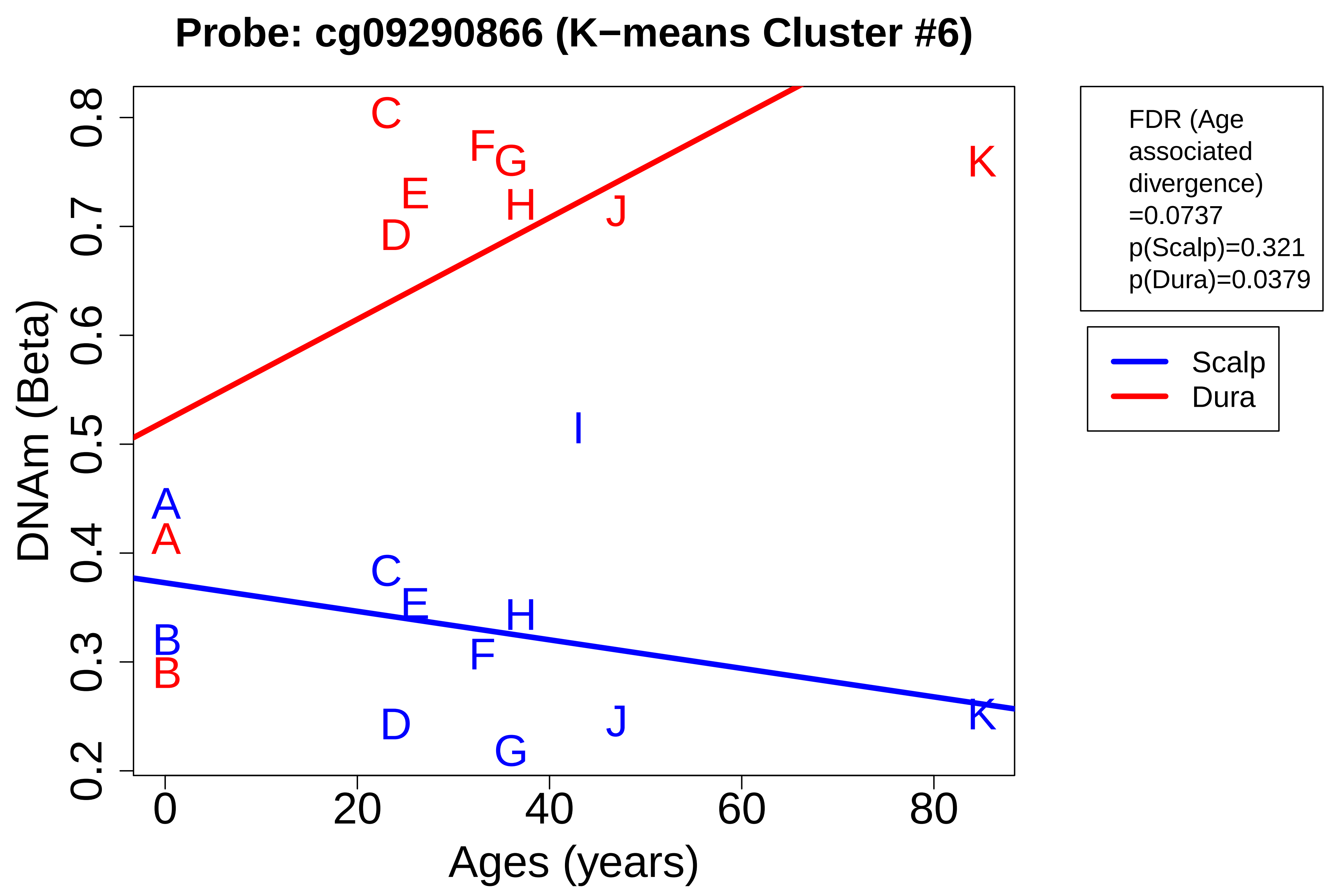
Probe: cg09290866 (K−means Cluster #6)
DNAm (Beta)
0.2
0.3
0.4
0.5
0.6
0.7
0.8
Ages (years)
0
20
40
60
80
FDR (Age associated divergence) =0.0737
p(Scalp)=0.321
p(Dura)=0.0379
Scalp
Dura
A
B
C
D
E
F
G
H
I
J
K

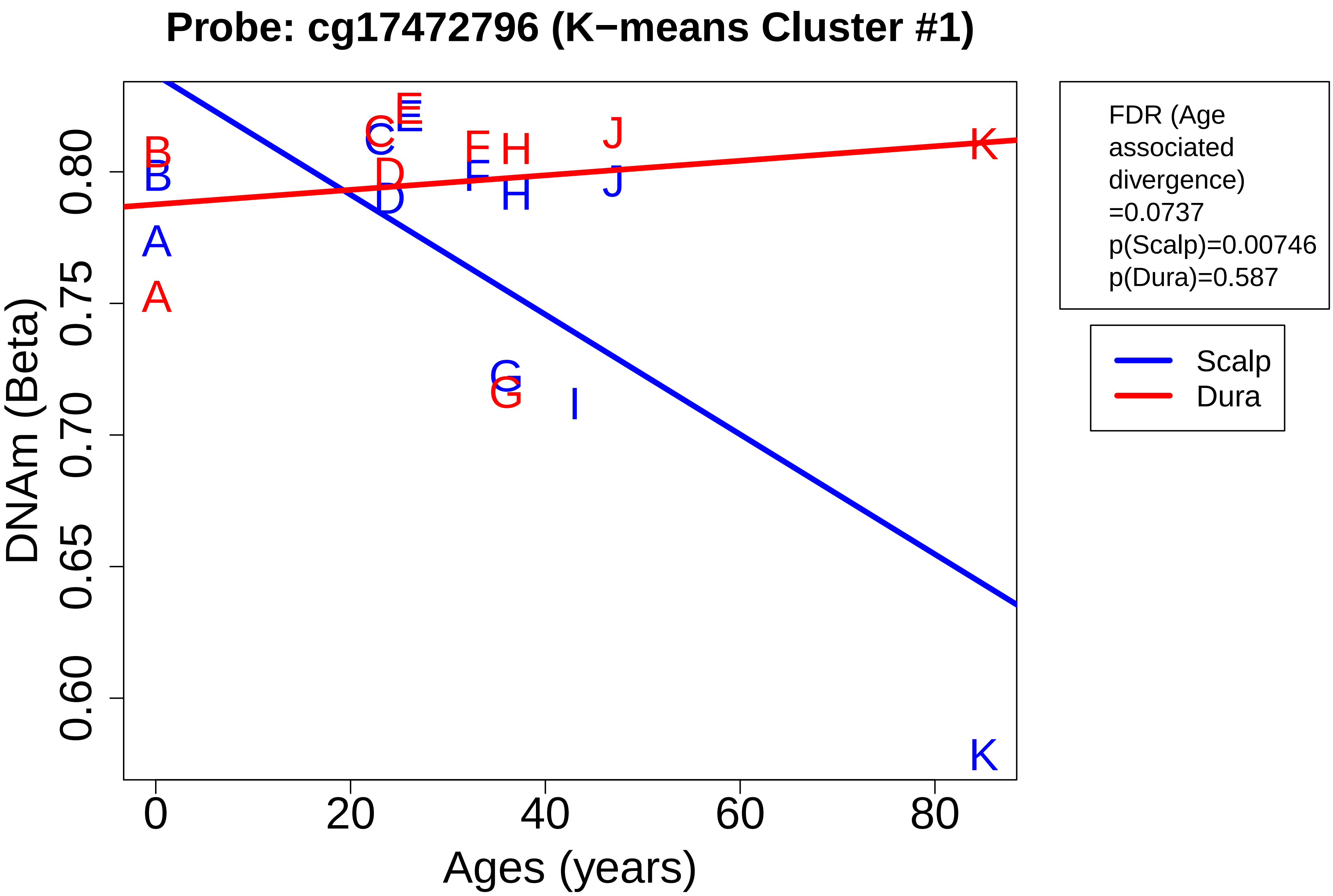

Probe: cg17472796 (K−means Cluster #1)
DNAm (Beta)
0.60
0.65
0.70
0.75
0.80
0
20
40
60
80
Ages (years)
A
B
C
D
E
F
G
H
I
J
K
FDR (Age associated divergence) =0.0737
p(Scalp)=0.00746
p(Dura)=0.587
Scalp
Dura

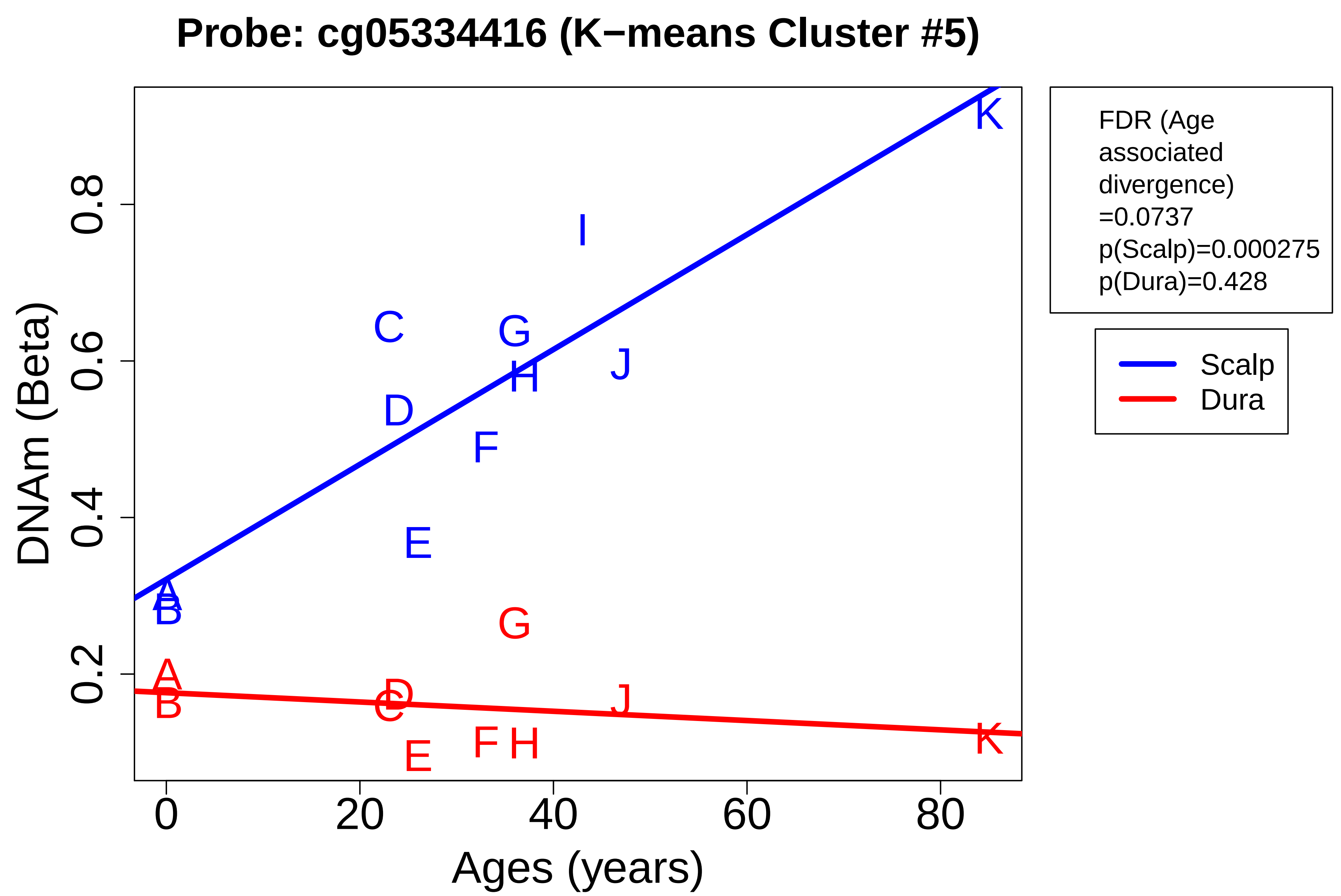

Probe: cg05334416 (K−means Cluster #5)
DNAm (Beta)
0.2
0.4
0.6
0.8
0
20
40
60
80
Ages (years)
FDR (Age associated divergence) =0.0737
p(Scalp)=0.000275
p(Dura)=0.428
Scalp
Dura
A
B
C
D
E
F
G
H
I
J
K

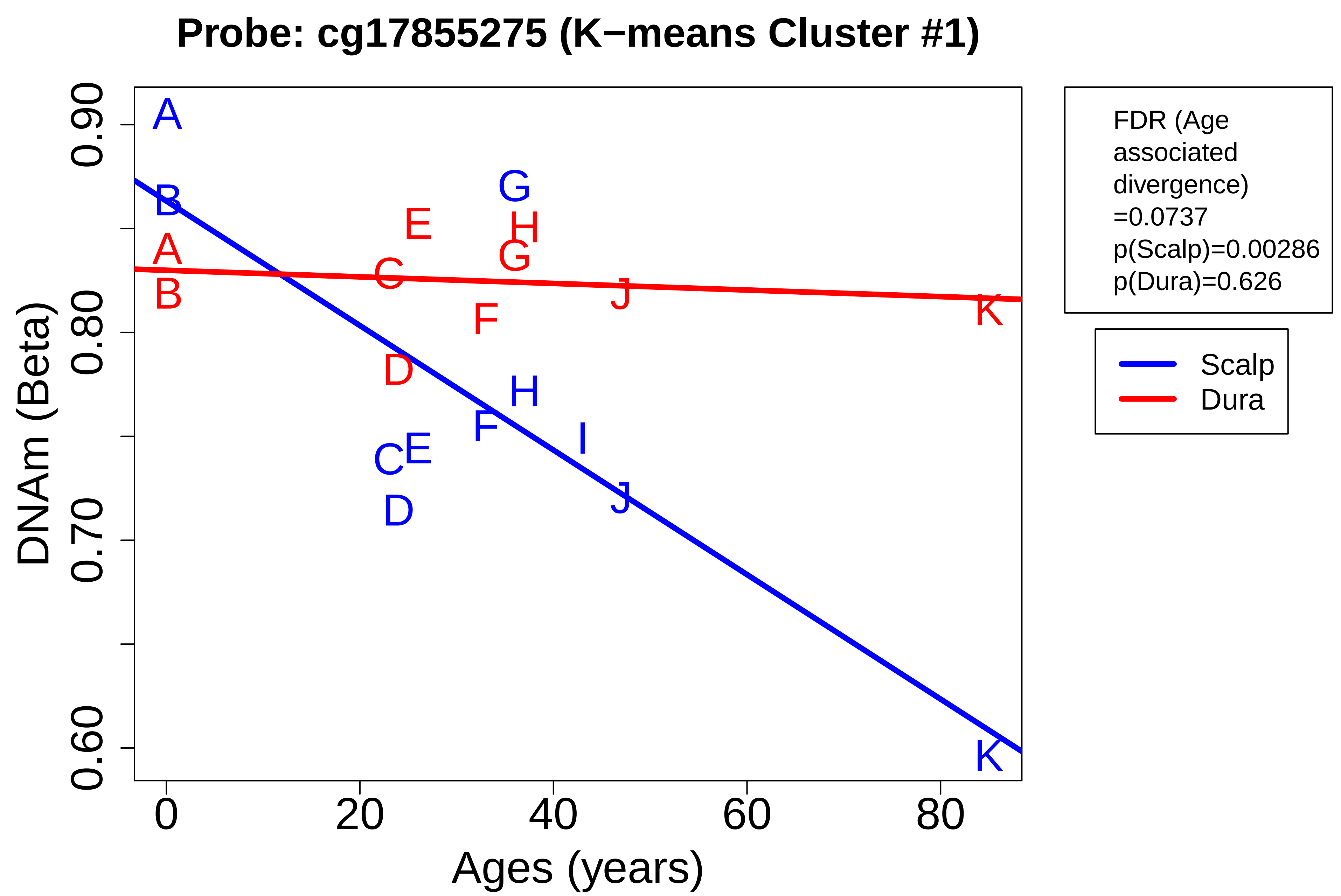

Probe: cg17855275 (K−means Cluster #1)
DNAm (Beta)
0.60
0.70
0.80
0.90
0
20
40
60
80
Ages (years)
FDR (Age associated divergence) =0.0737
p(Scalp)=0.00286
p(Dura)=0.626
Scalp
Dura

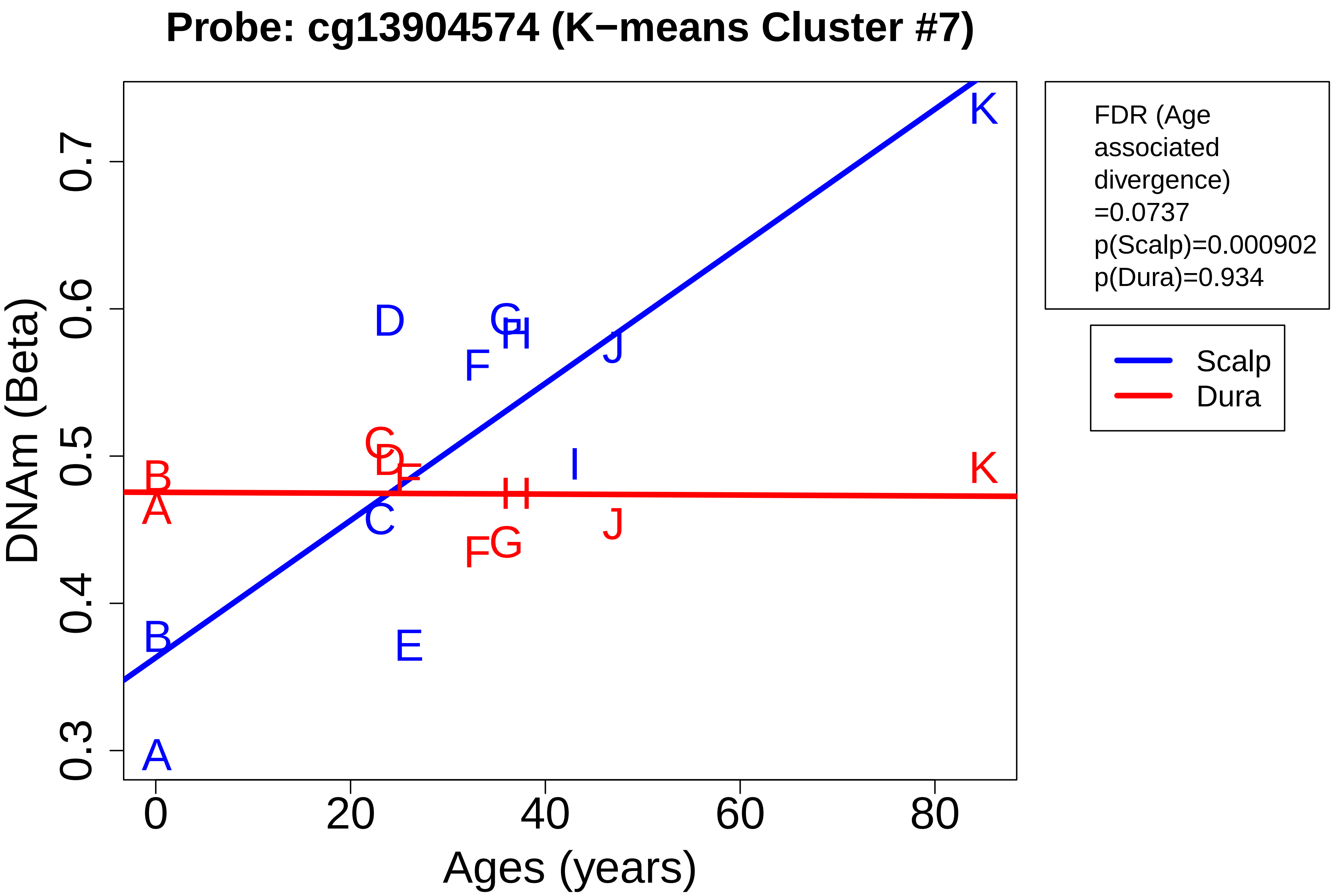

Probe: cg13904574 (K−means Cluster #7)
DNAm (Beta)
0.3
0.4
0.5
0.6
0.7
0
20
40
60
80
Ages (years)
FDR (Age associated divergence) =0.0737
p(Scalp)=0.000902
p(Dura)=0.934
Scalp
Dura

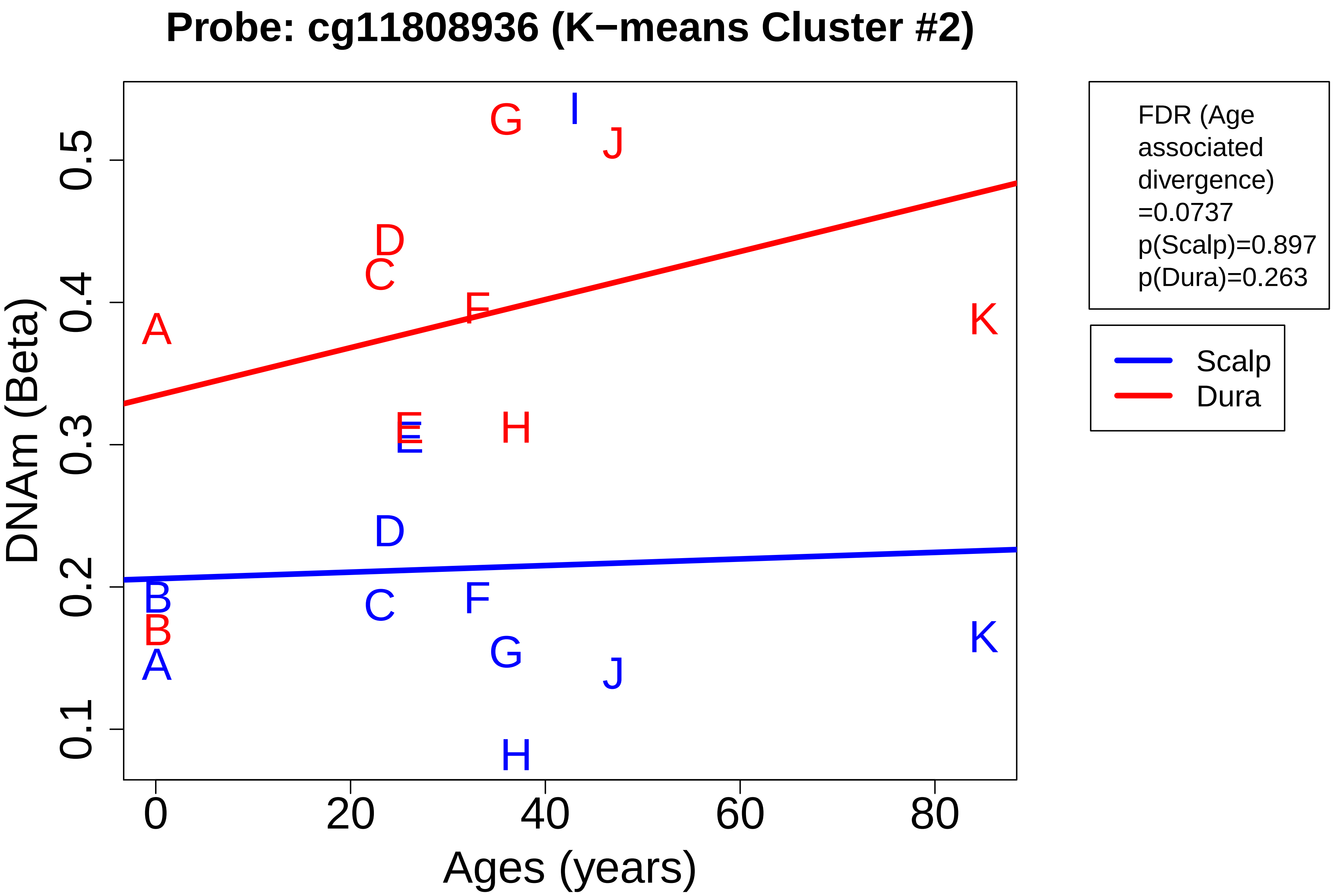

Probe: cg11808936 (K−means Cluster #2)
DNAm (Beta)
0.1
0.2
0.3
0.4
0.5
0
20
40
60
80
Ages (years)
FDR (Age associated divergence) =0.0737
p(Scalp)=0.897
p(Dura)=0.263
Scalp
Dura

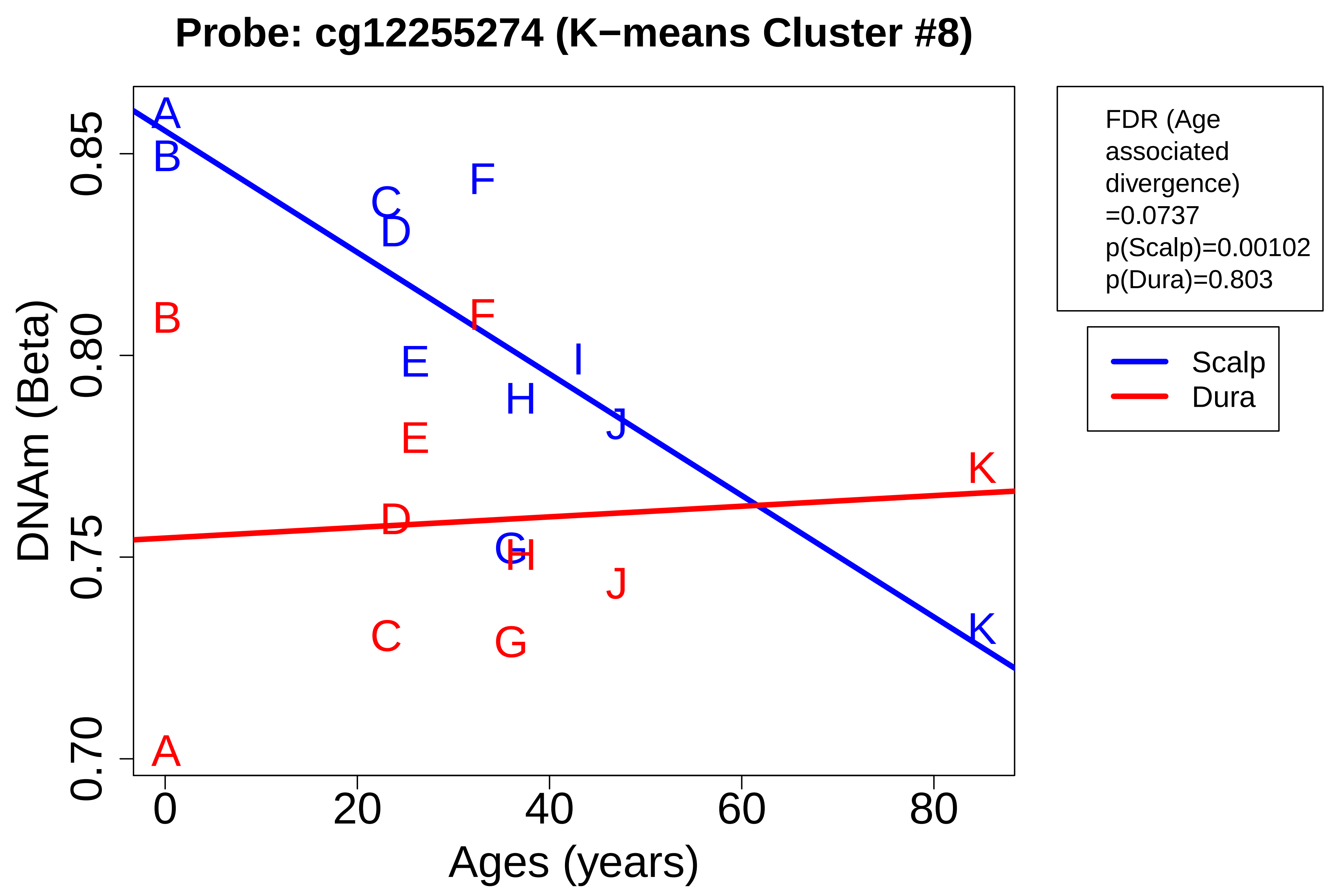

Probe: cg12255274 (K−means Cluster #8)
FDR (Age associated divergence) =0.0737
p(Scalp)=0.00102
p(Dura)=0.803
Scalp
Dura
DNAm (Beta)
0.70
0.75
0.80
0.85
0
20
40
60
80
Ages (years)
A
B
C
D
E
F
G
H
I
J
K

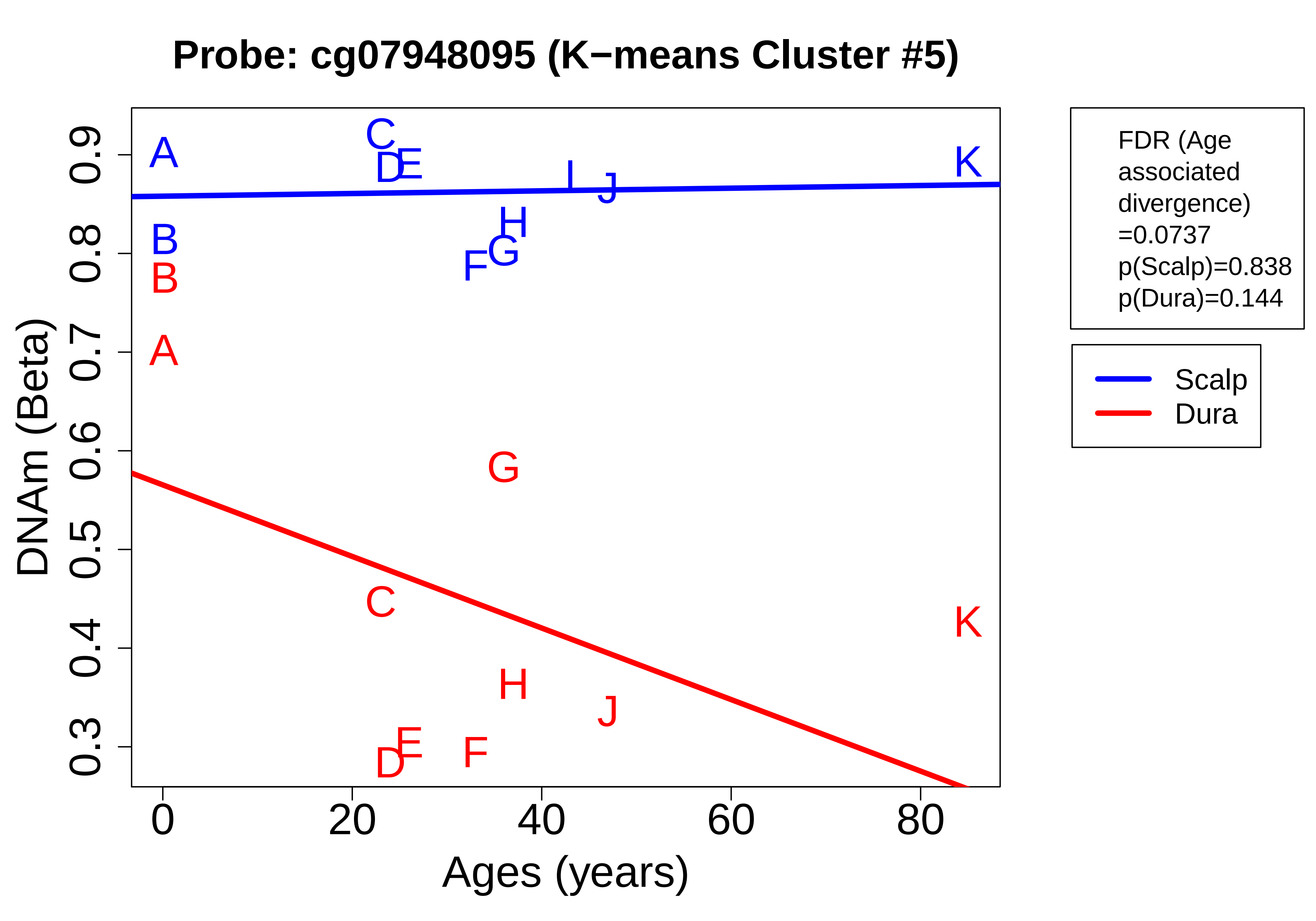

Probe: cg07948095 (K−means Cluster #5)
DNAm (Beta)
0.3
0.4
0.5
0.6
0.7
0.8
0.9
Ages (years)
0
20
40
60
80
A
B
C
D
E
F
G
H
I
J
K
FDR (Age associated divergence) =0.0737
p(Scalp)=0.838
p(Dura)=0.144
Scalp
Dura

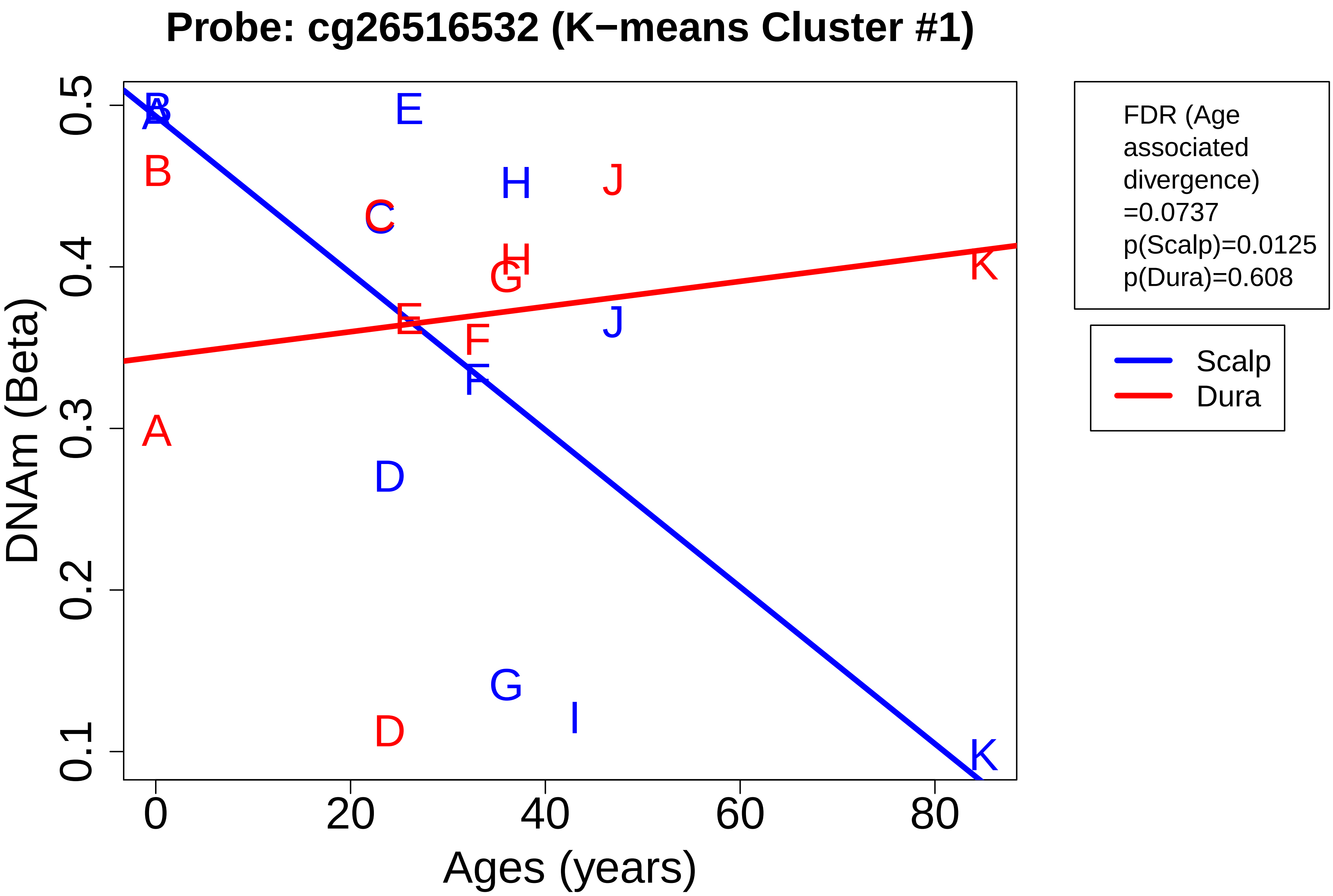
Probe: cg26516532 (K−means Cluster #1)
DNAm (Beta)
Ages (years)
0.1
0.2
0.3
0.4
0.5
0
20
40
60
80
FDR (Age associated divergence) =0.0737
p(Scalp)=0.0125
p(Dura)=0.608
Scalp
Dura

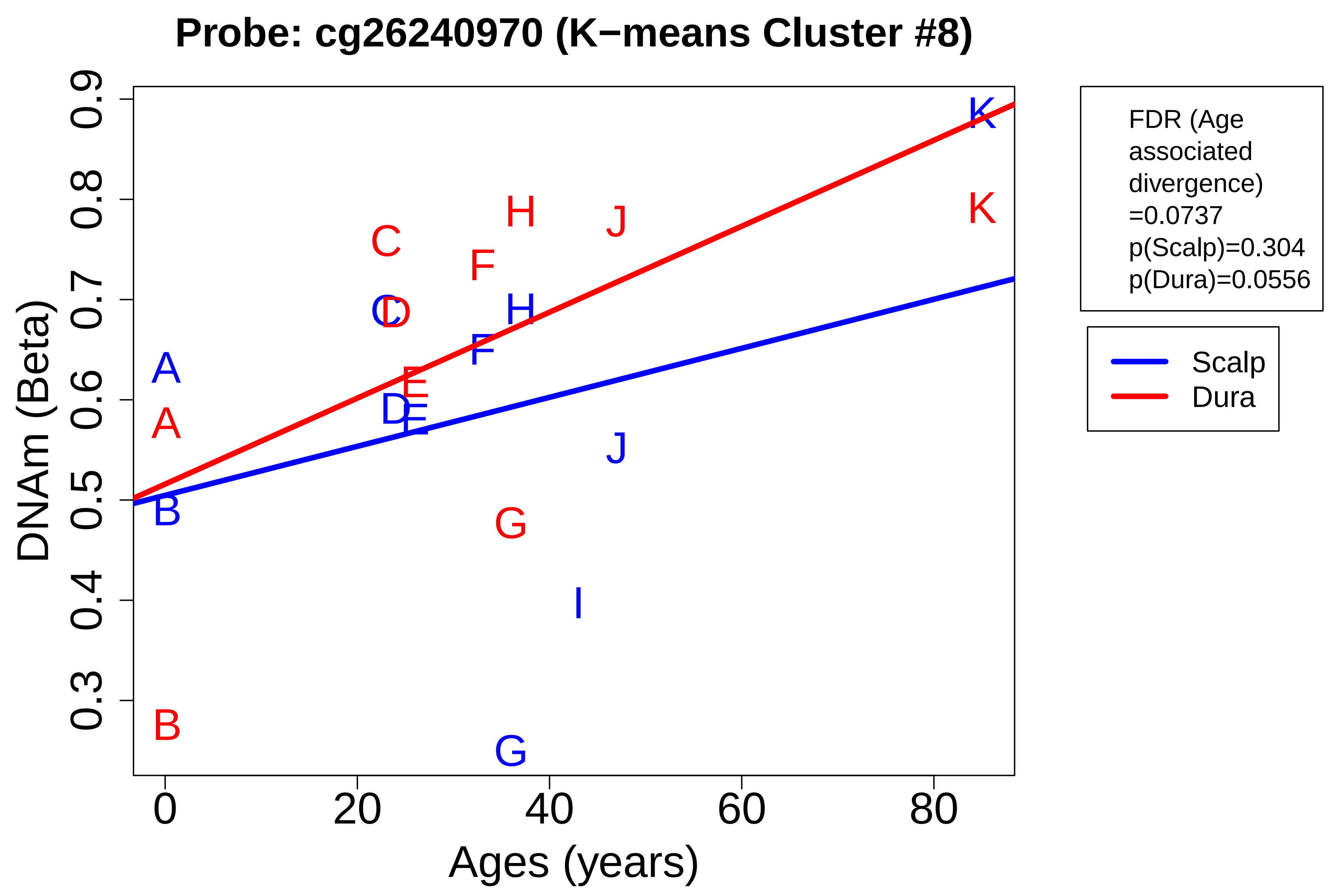

Probe: cg26240970 (K−means Cluster #8)
DNAm (Beta)
0.3
0.4
0.5
0.6
0.7
0.8
0.9
Ages (years)
0
20
40
60
80
FDR (Age associated divergence) =0.0737
p(Scalp)=0.304
p(Dura)=0.0556
Scalp
Dura

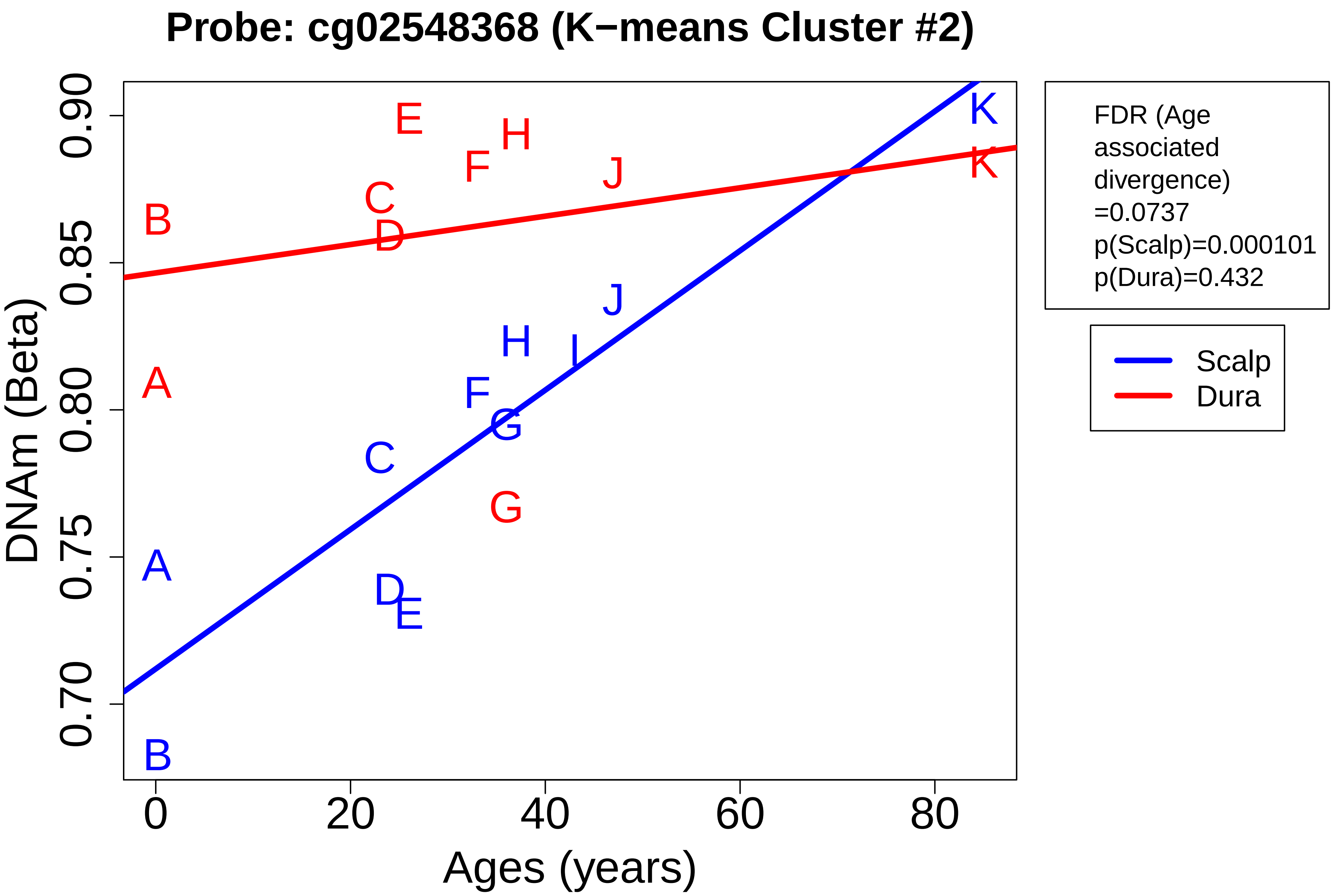
Probe: cg02548368 (K−means Cluster #2)
DNAm (Beta)
0.70
0.75
0.80
0.85
0.90
0
20
40
60
80
Ages (years)
A
B
C
D
E
F
G
H
I
J
K
FDR (Age associated divergence) =0.0737
p(Scalp)=0.000101
p(Dura)=0.432
Scalp
Dura

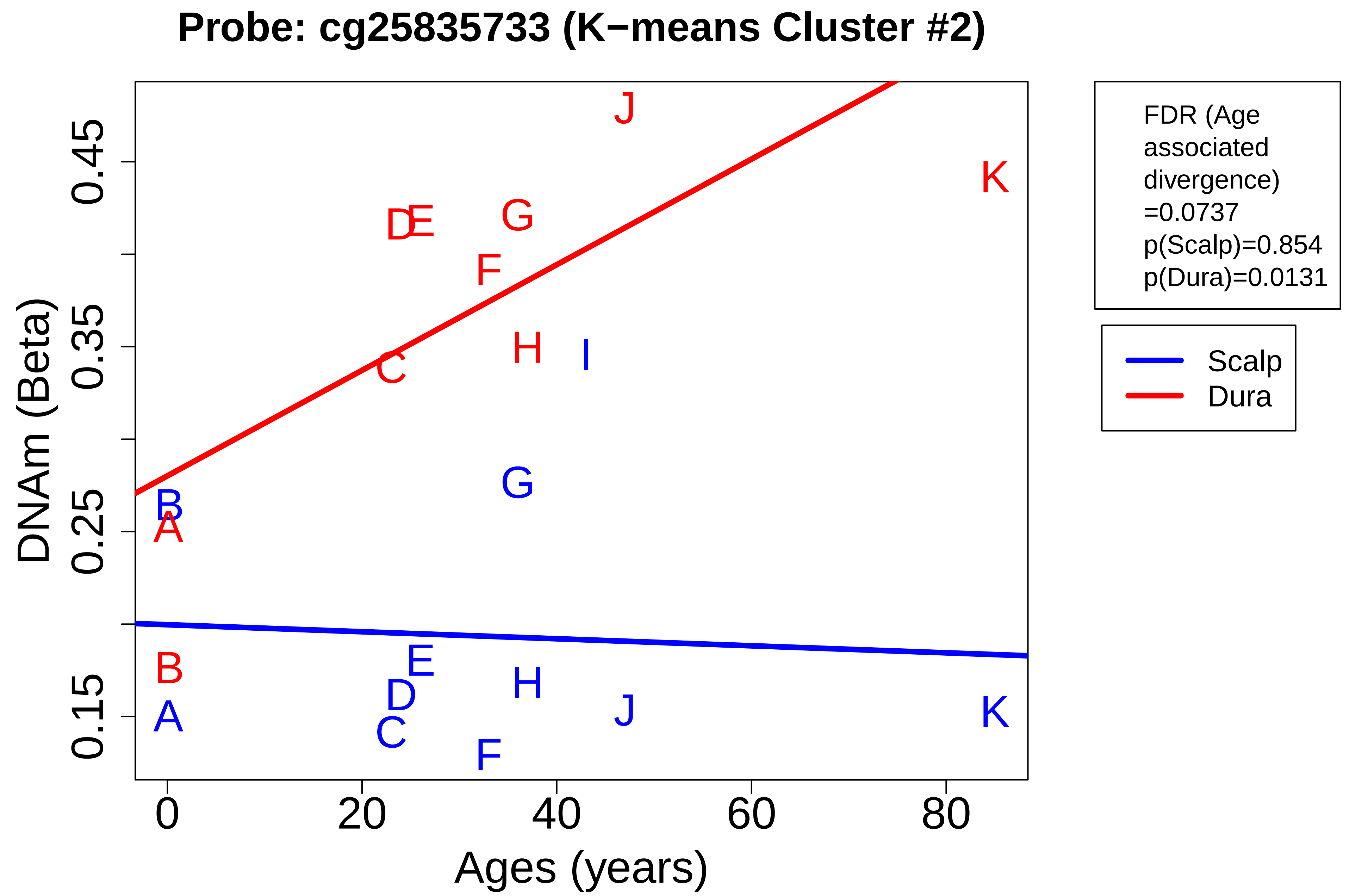

Probe: cg25835733 (K−means Cluster #2)
DNAm (Beta)
0.15
0.25
0.35
0.45
0
20
40
60
80
Ages (years)
A
B
C
D
E
F
G
H
I
J
K
FDR (Age associated divergence) =0.0737
p(Scalp)=0.854
p(Dura)=0.0131
Scalp
Dura

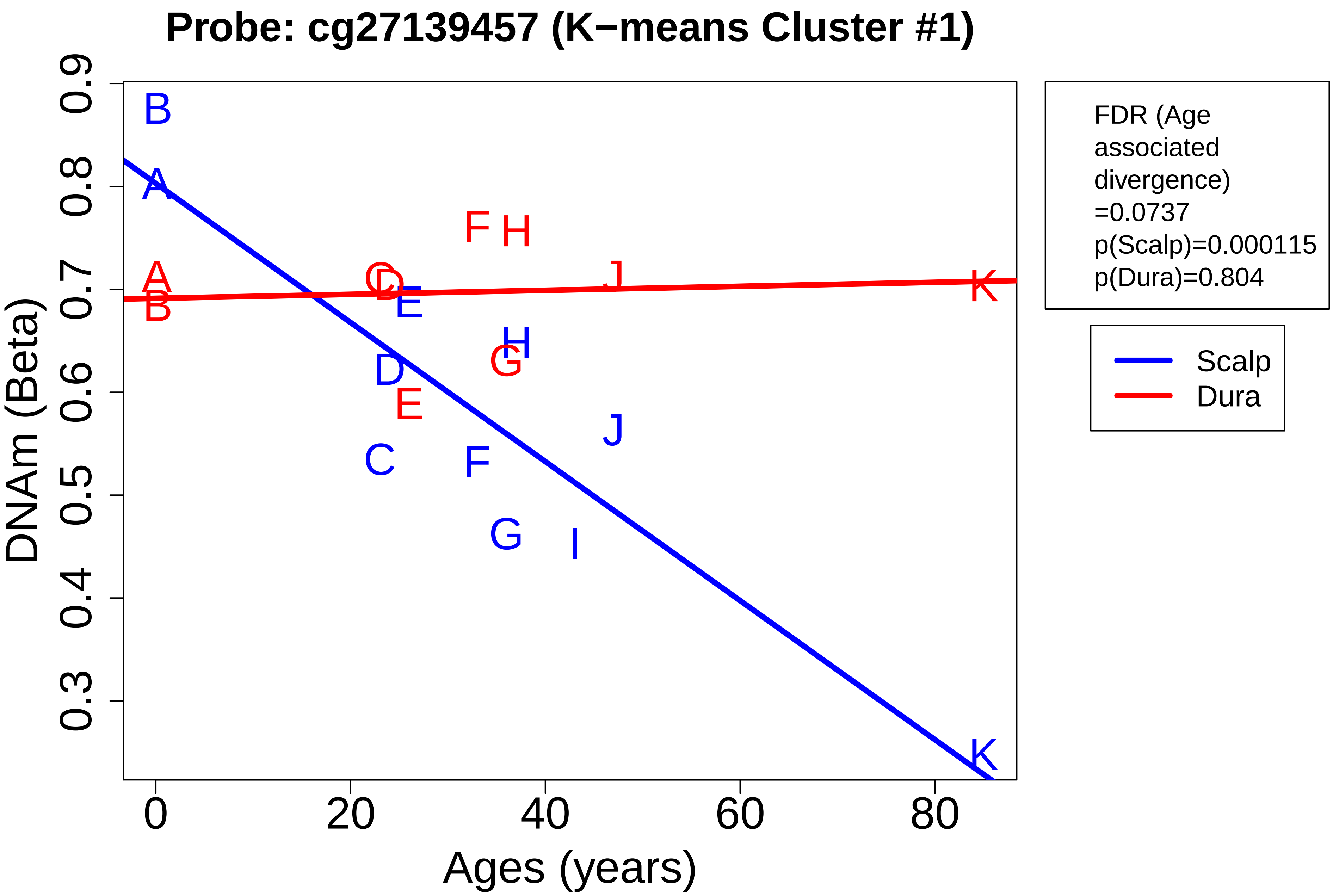

Probe: cg27139457 (K−means Cluster #1)
DNAm (Beta)
0.3
0.4
0.5
0.6
0.7
0.8
0.9
0
20
40
60
80
Ages (years)
FDR (Age associated divergence) =0.0737
p(Scalp)=0.000115
p(Dura)=0.804
Scalp
Dura

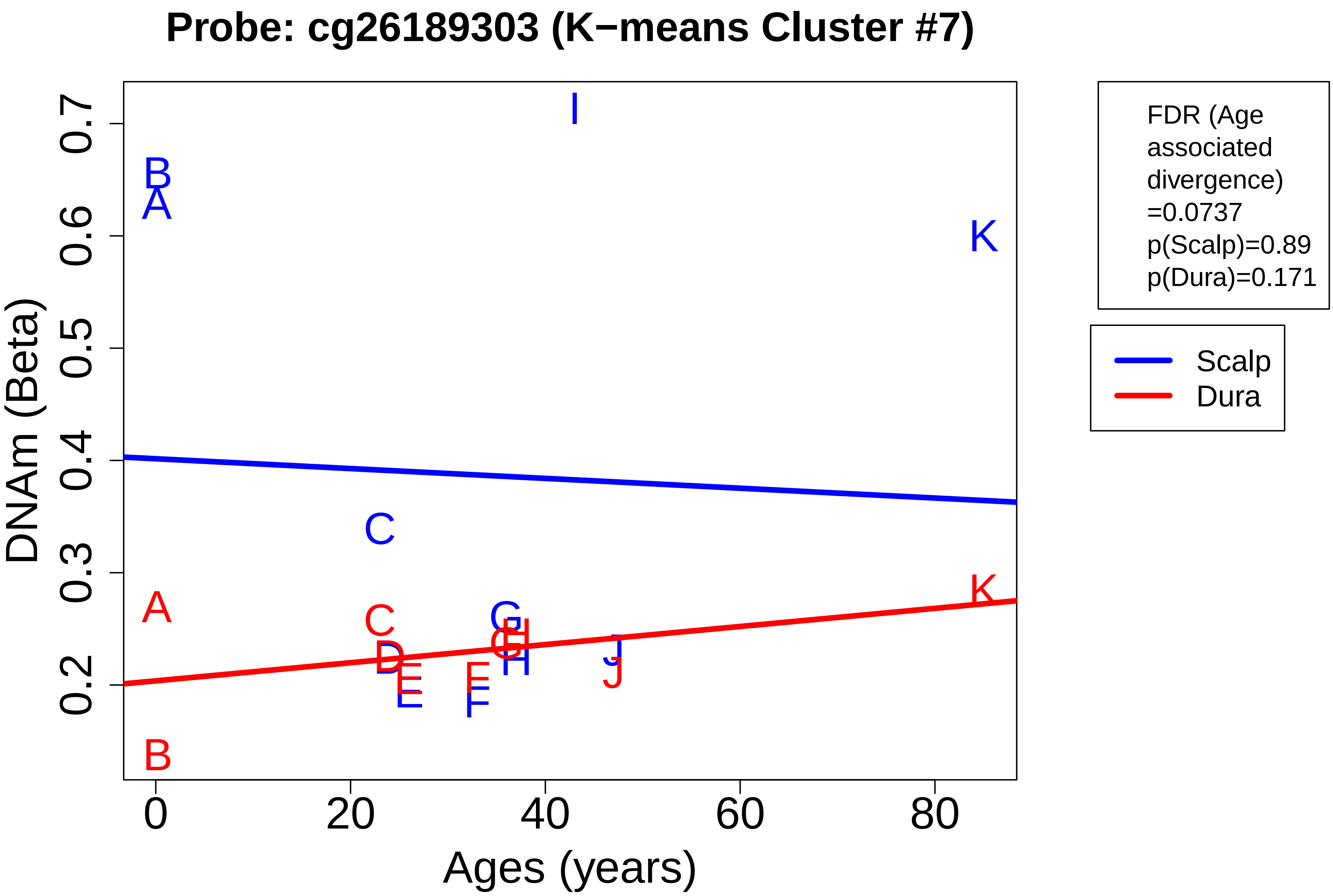

Probe: cg26189303 (K−means Cluster #7)
DNAm (Beta)
Ages (years)
FDR (Age associated divergence) =0.0737
p(Scalp)=0.89
p(Dura)=0.171
Scalp
Dura

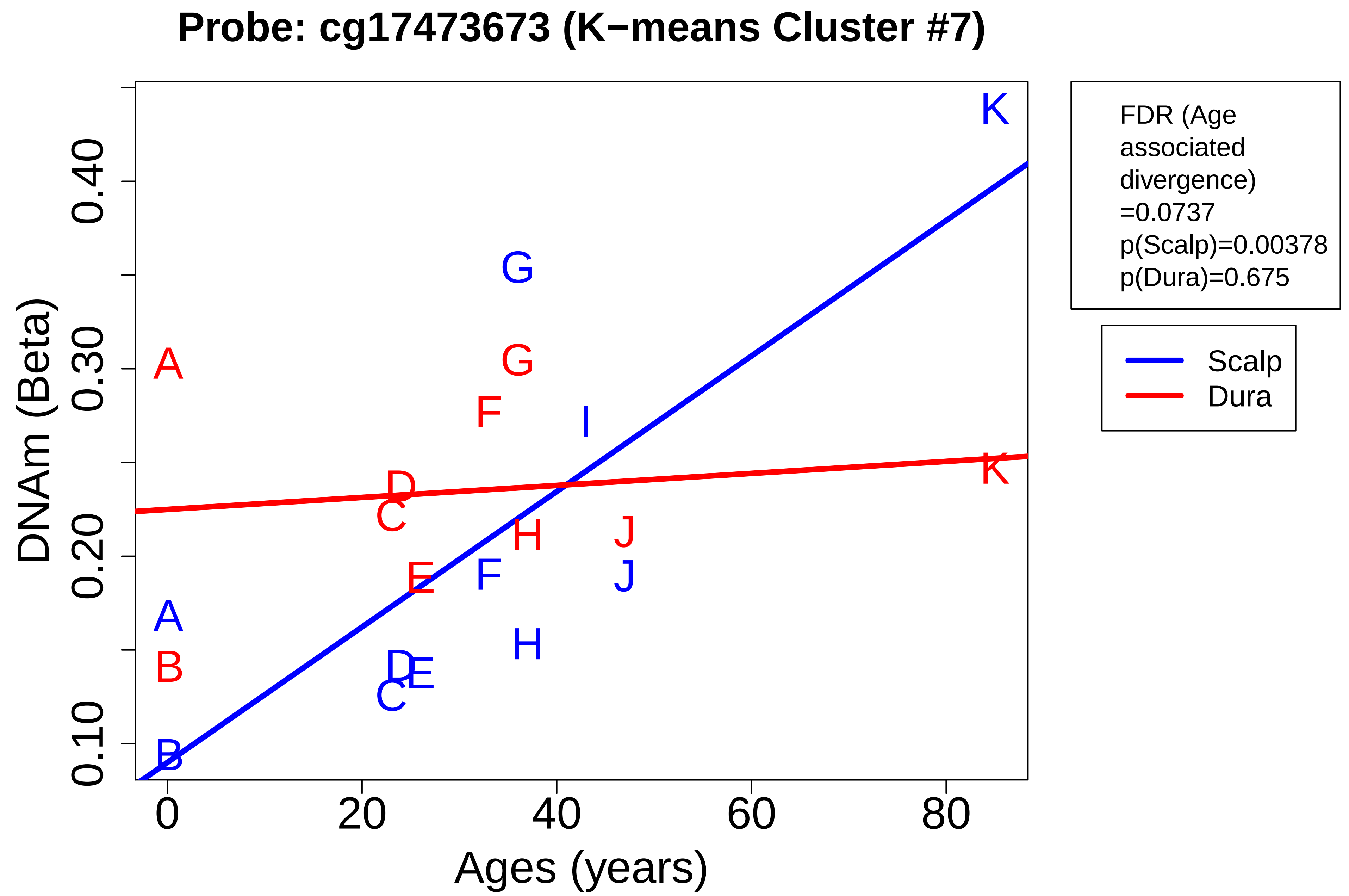
Probe: cg17473673 (K−means Cluster #7)
DNAm (Beta)
0.10
0.20
0.30
0.40
0
20
40
60
80
Ages (years)
FDR (Age associated divergence) =0.0737
p(Scalp)=0.00378
p(Dura)=0.675
Scalp
Dura
A
B
C
D
E
F
G
H
I
J
K

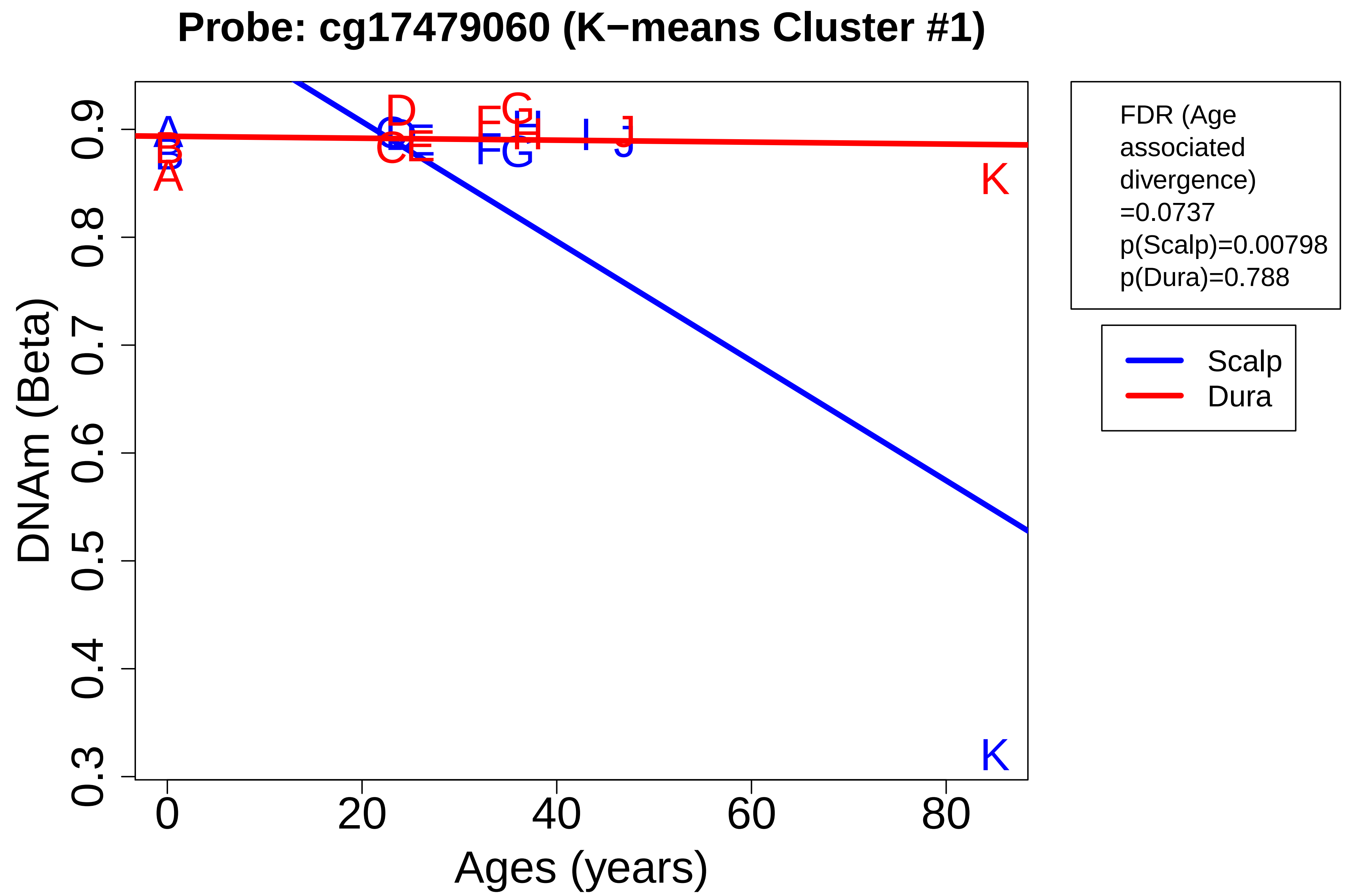

Probe: cg17479060 (K−means Cluster #1)
DNAm (Beta)
0.3
0.4
0.5
0.6
0.7
0.8
0.9
0
20
40
60
80
Ages (years)
A
B
C
D
E
F
G
H
I
J
K
FDR (Age associated divergence) =0.0737
p(Scalp)=0.00798
p(Dura)=0.788
Scalp
Dura

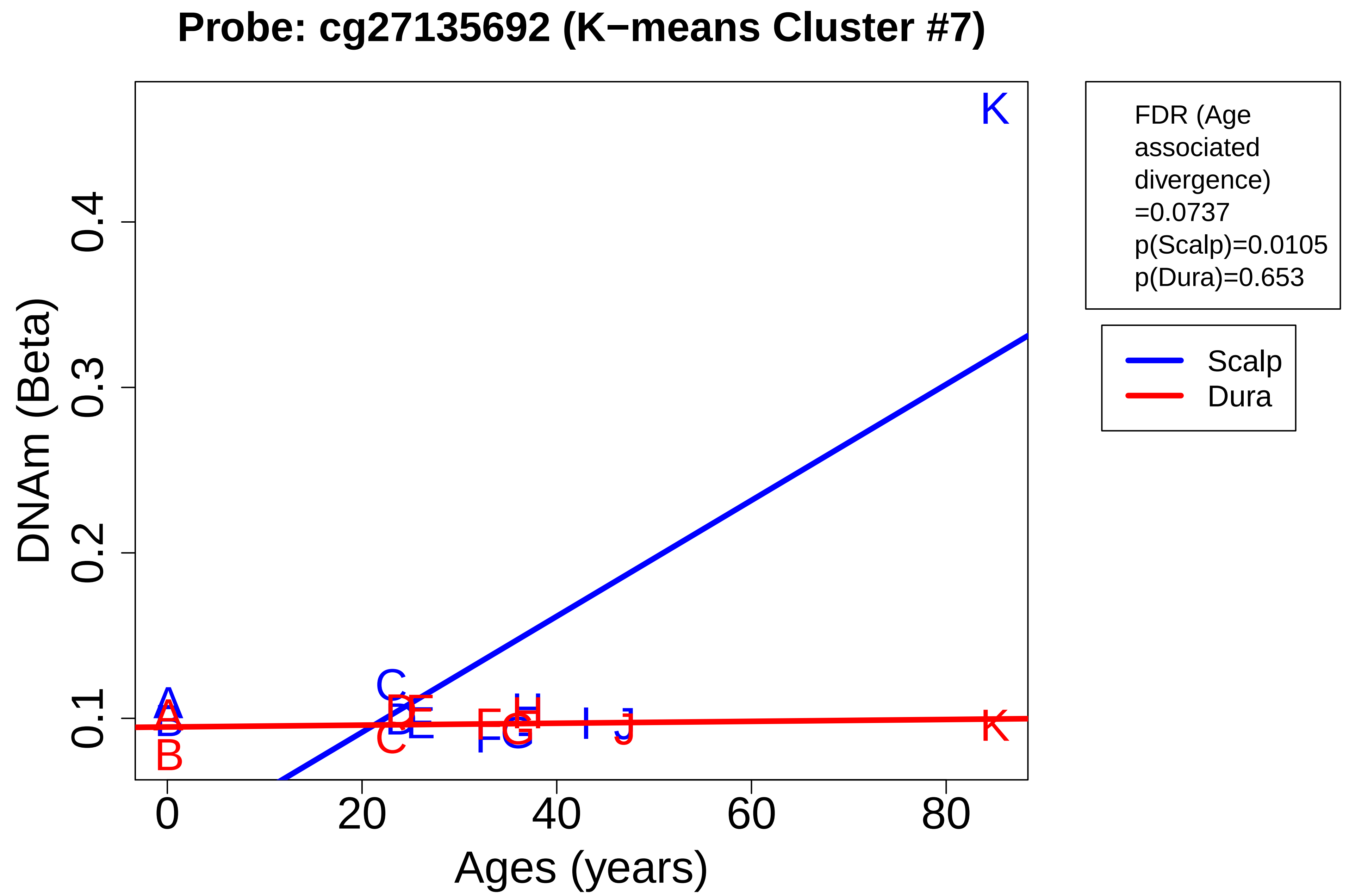

Probe: cg27135692 (K−means Cluster #7)
DNAm (Beta)
0.1
0.2
0.3
0.4
0
20
40
60
80
Ages (years)
FDR (Age associated divergence) =0.0737
p(Scalp)=0.0105
p(Dura)=0.653
Scalp
Dura

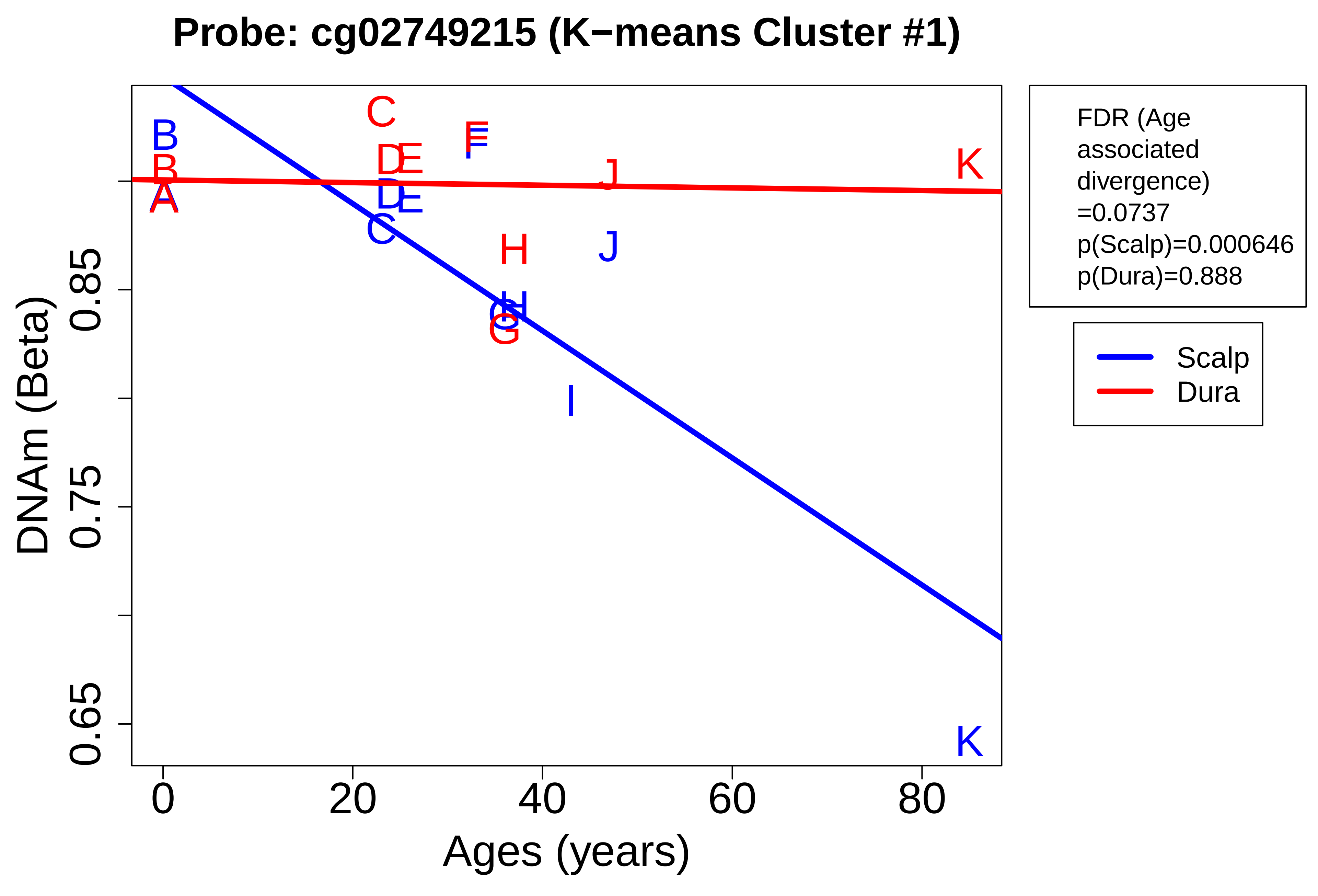

Probe: cg02749215 (K−means Cluster #1)
DNAm (Beta)
0.65
0.75
0.85
Ages (years)
0
20
40
60
80
FDR (Age associated divergence) =0.0737
p(Scalp)=0.000646
p(Dura)=0.888
Scalp
Dura

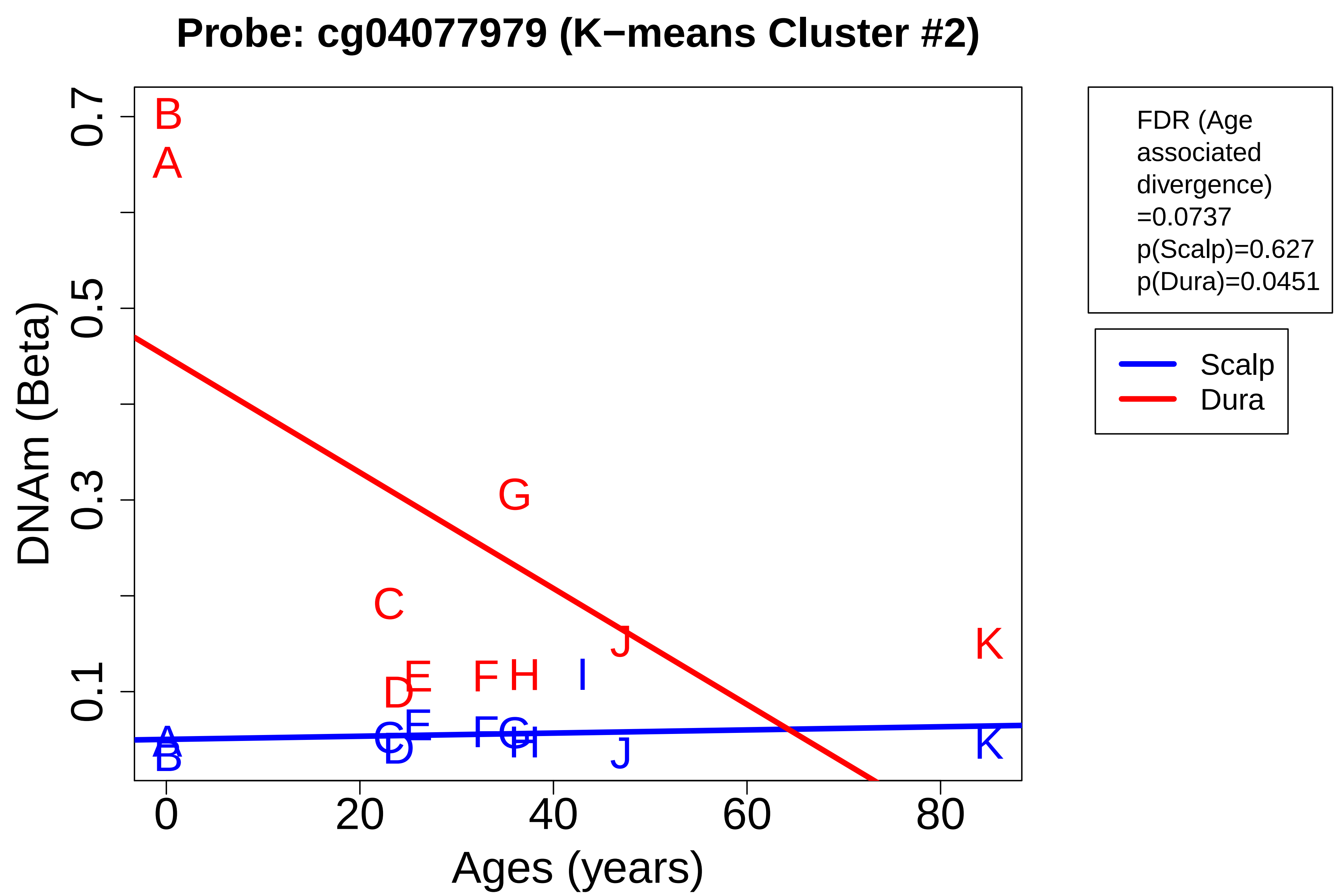

Probe: cg04077979 (K−means Cluster #2)
FDR (Age associated divergence) =0.0737
p(Scalp)=0.627
p(Dura)=0.0451
Scalp
Dura
DNAm (Beta)
0.1
0.3
0.5
0.7
Ages (years)
0
20
40
60
80
A
B
C
D
E
F
G
H
I
J
K

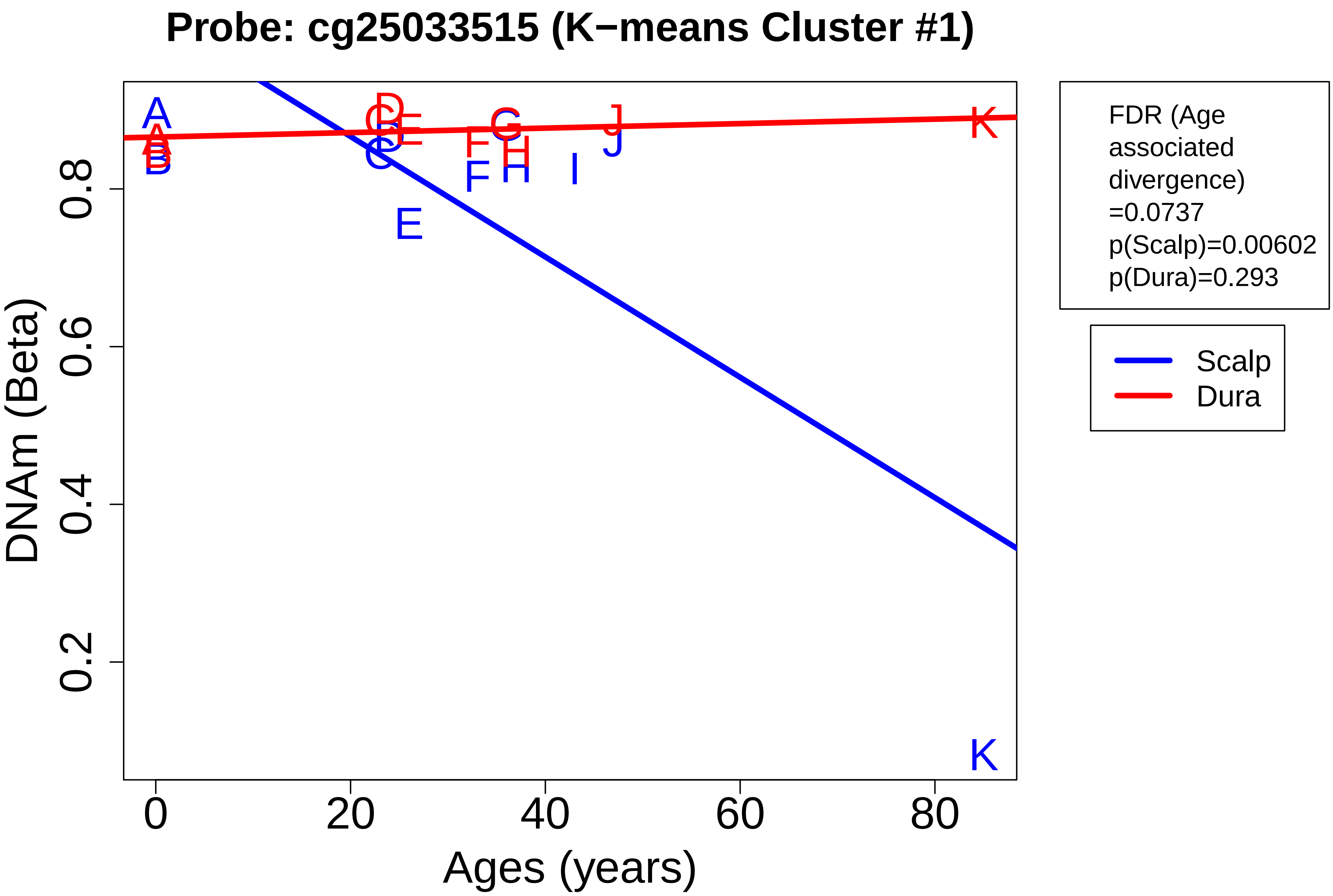

Probe: cg25033515 (K−means Cluster #1)
FDR (Age associated divergence) =0.0737
p(Scalp)=0.00602
p(Dura)=0.293
Scalp
Dura
DNAm (Beta)
0.2
0.4
0.6
0.8
0
20
40
60
80
Ages (years)

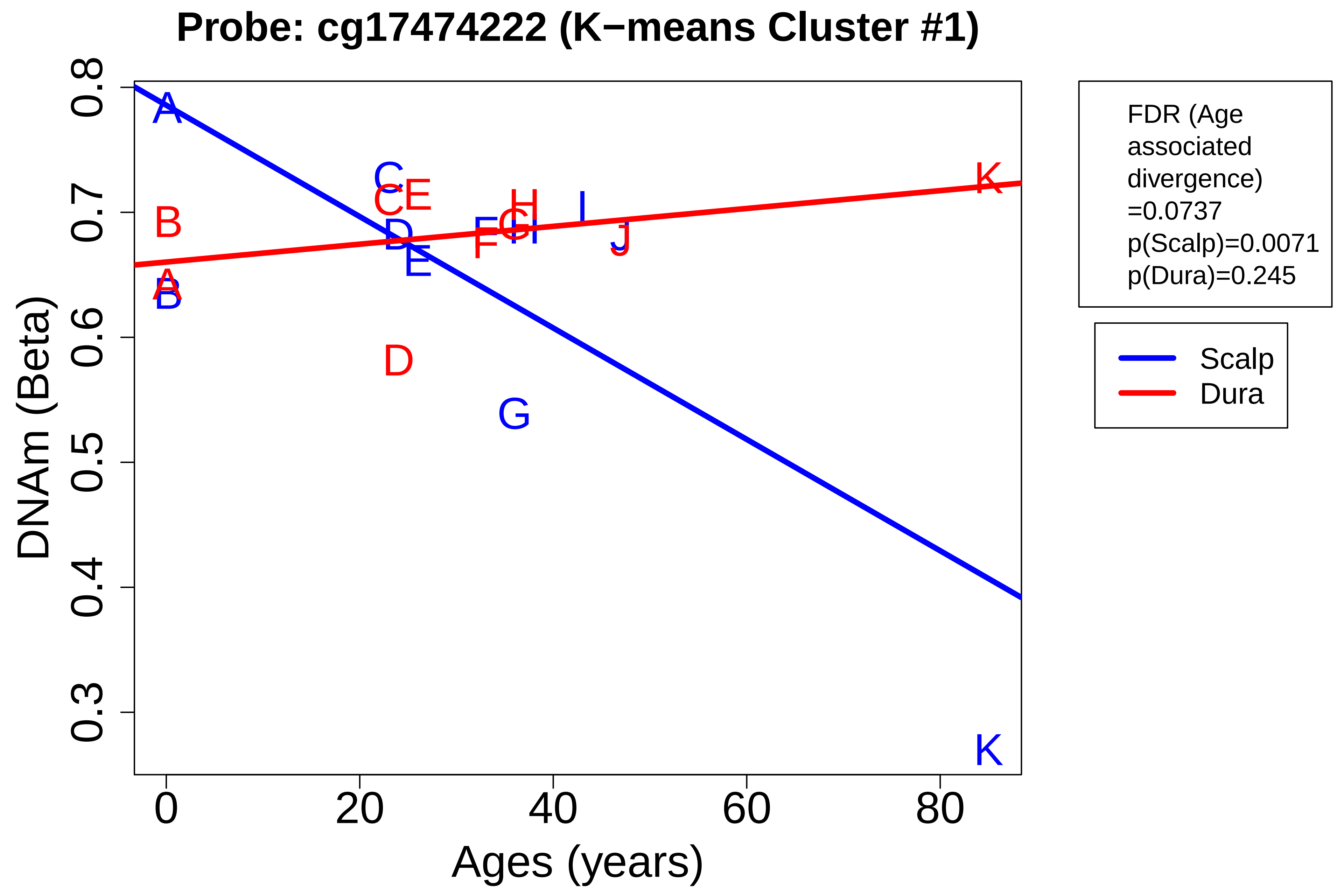

Probe: cg17474222 (K−means Cluster #1)
DNAm (Beta)
0.3
0.4
0.5
0.6
0.7
0.8
0
20
40
60
80
Ages (years)
A
B
C
D
E
F
G
H
I
J
K
FDR (Age associated divergence) =0.0737
p(Scalp)=0.0071
p(Dura)=0.245
Scalp
Dura

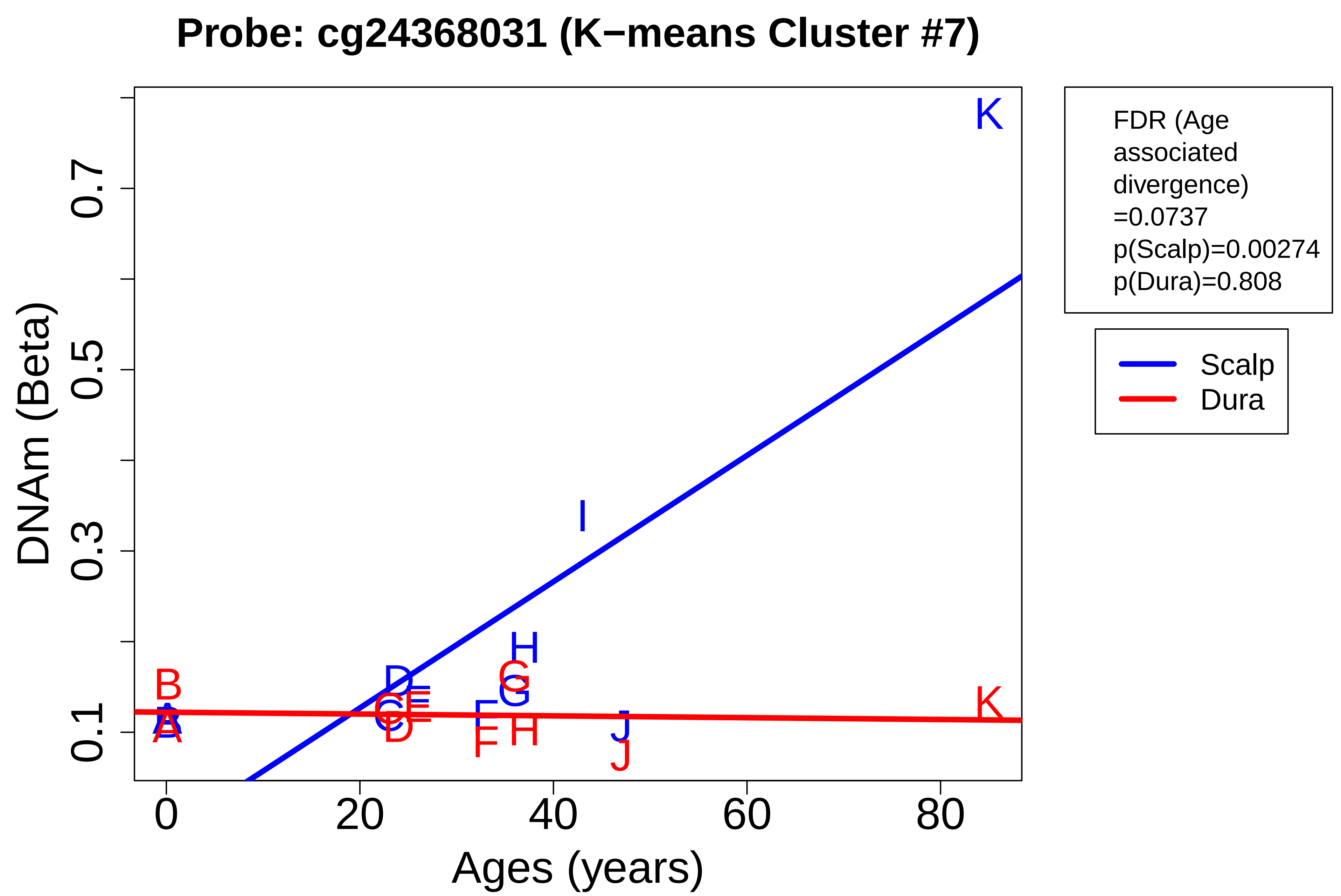

Probe: cg24368031 (K−means Cluster #7)
DNAm (Beta)
0.1
0.3
0.5
0.7
0
20
40
60
80
Ages (years)
FDR (Age associated divergence) =0.0737
p(Scalp)=0.00274
p(Dura)=0.808
Scalp
Dura

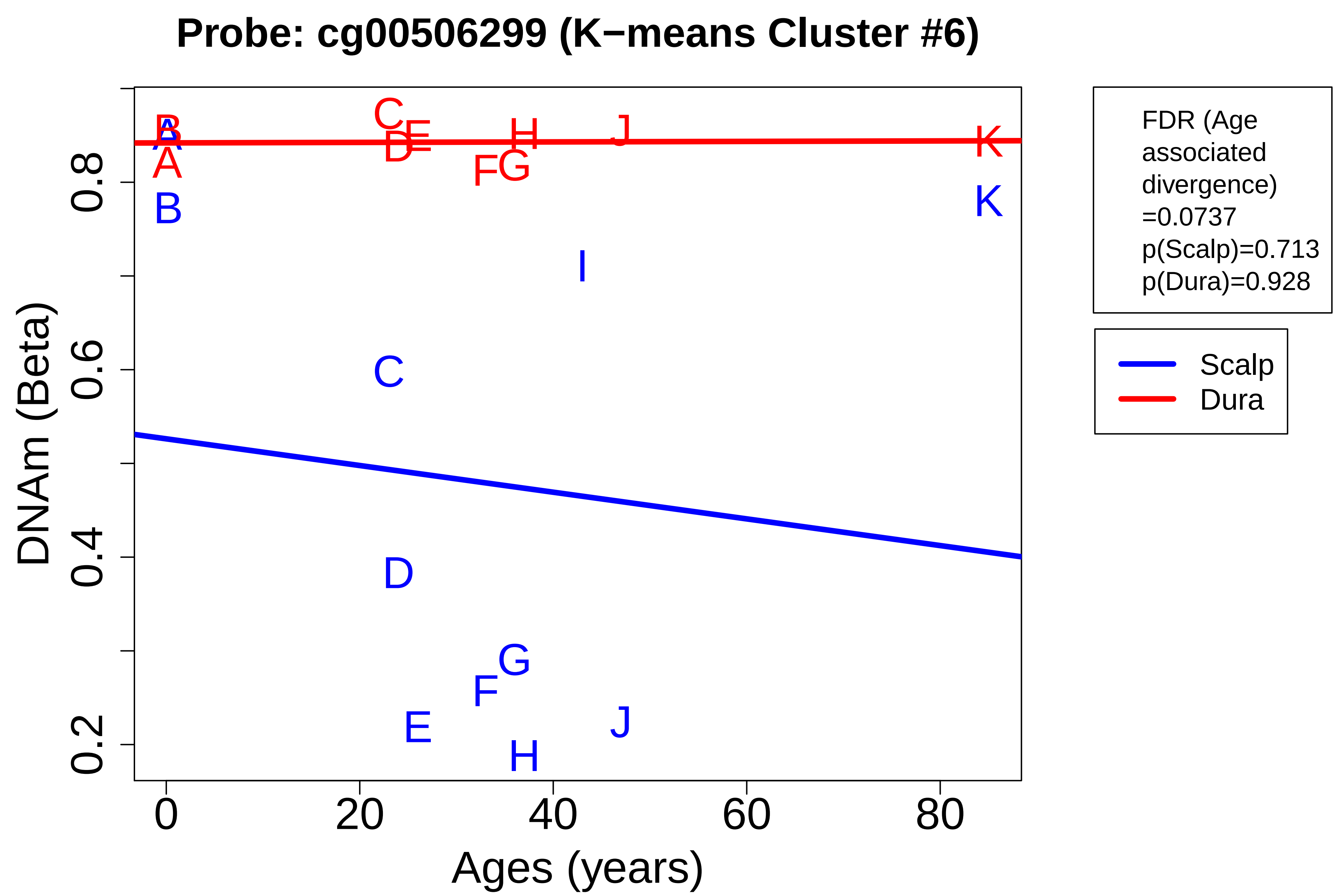

Probe: cg00506299 (K−means Cluster #6)
DNAm (Beta)
0.2
0.4
0.6
0.8
0
20
40
60
80
Ages (years)
FDR (Age associated divergence) =0.0737
p(Scalp)=0.713
p(Dura)=0.928
Scalp
Dura
A B C D E F G H I J K

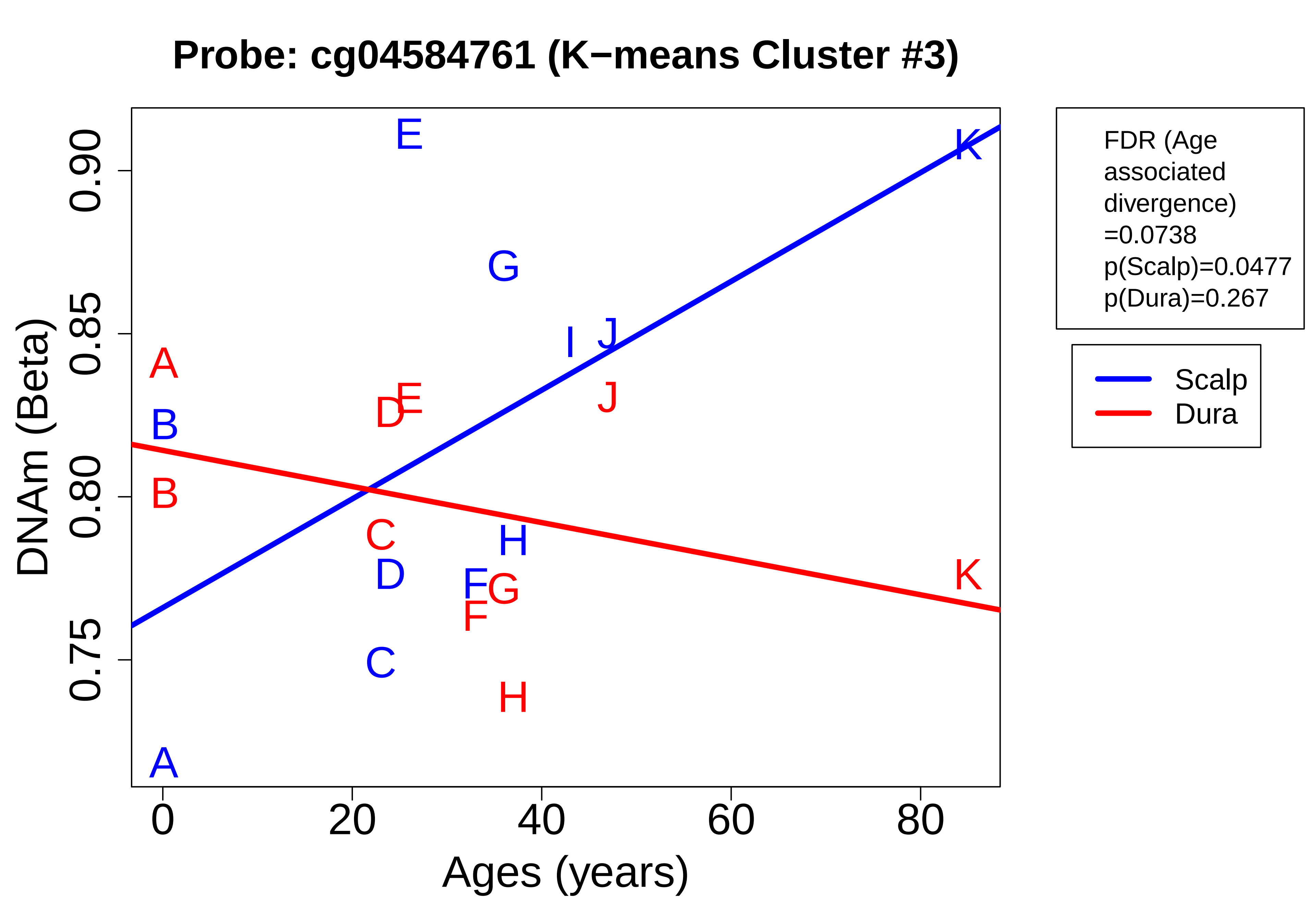
Probe: cg04584761 (K−means Cluster #3)
DNAm (Beta)
0.75
0.80
0.85
0.90
Ages (years)
0
20
40
60
80
FDR (Age associated divergence) =0.0738
p(Scalp)=0.0477
p(Dura)=0.267
Scalp
Dura
A
B
C
D
E
F
G
H
I
J
K

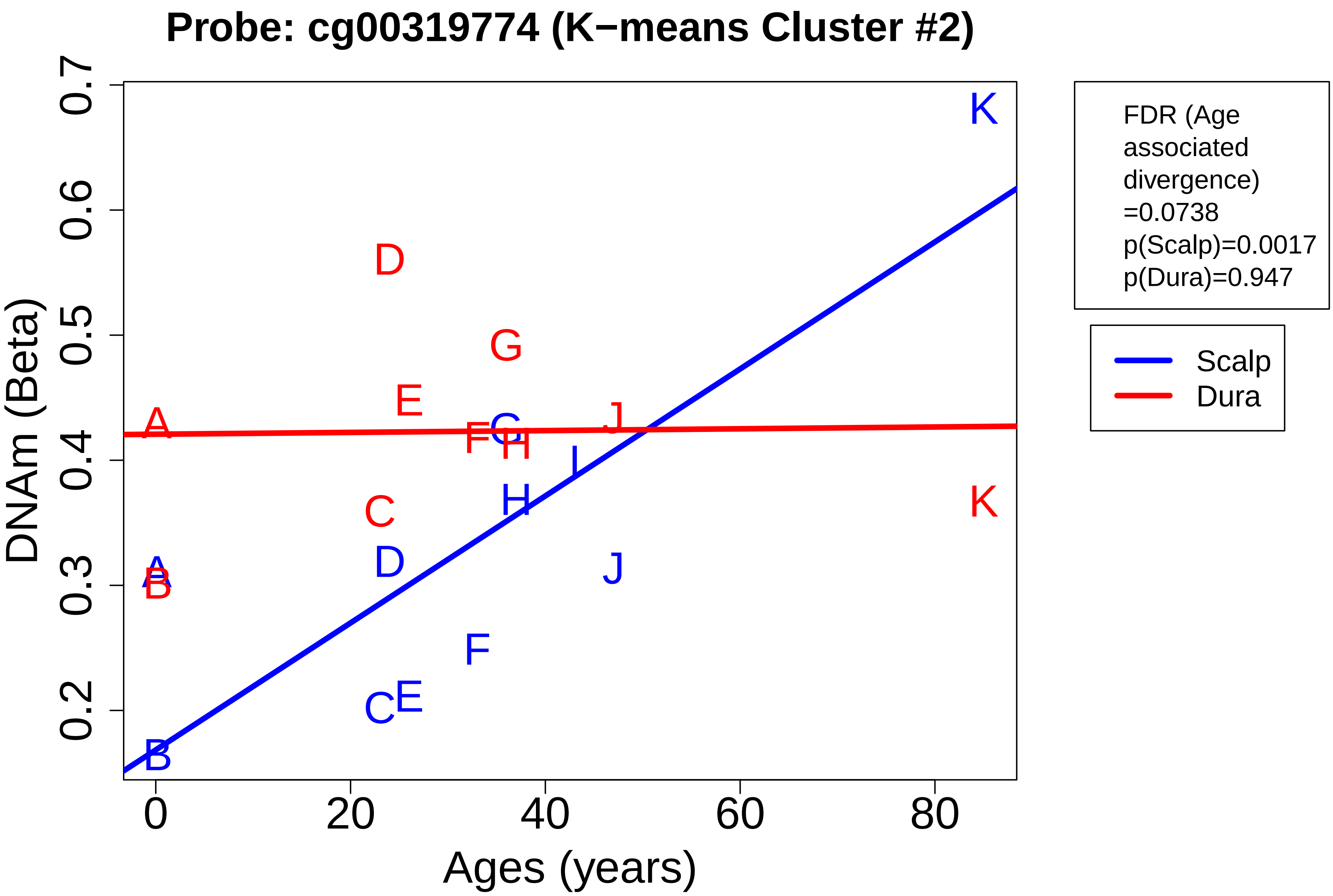

Probe: cg00319774 (K−means Cluster #2)
DNAm (Beta)
0.2
0.3
0.4
0.5
0.6
0.7
Ages (years)
0
20
40
60
80
FDR (Age associated divergence) =0.0738
p(Scalp)=0.0017
p(Dura)=0.947
Scalp
Dura
A
B
C
D
E
F
G
H
I
J
K

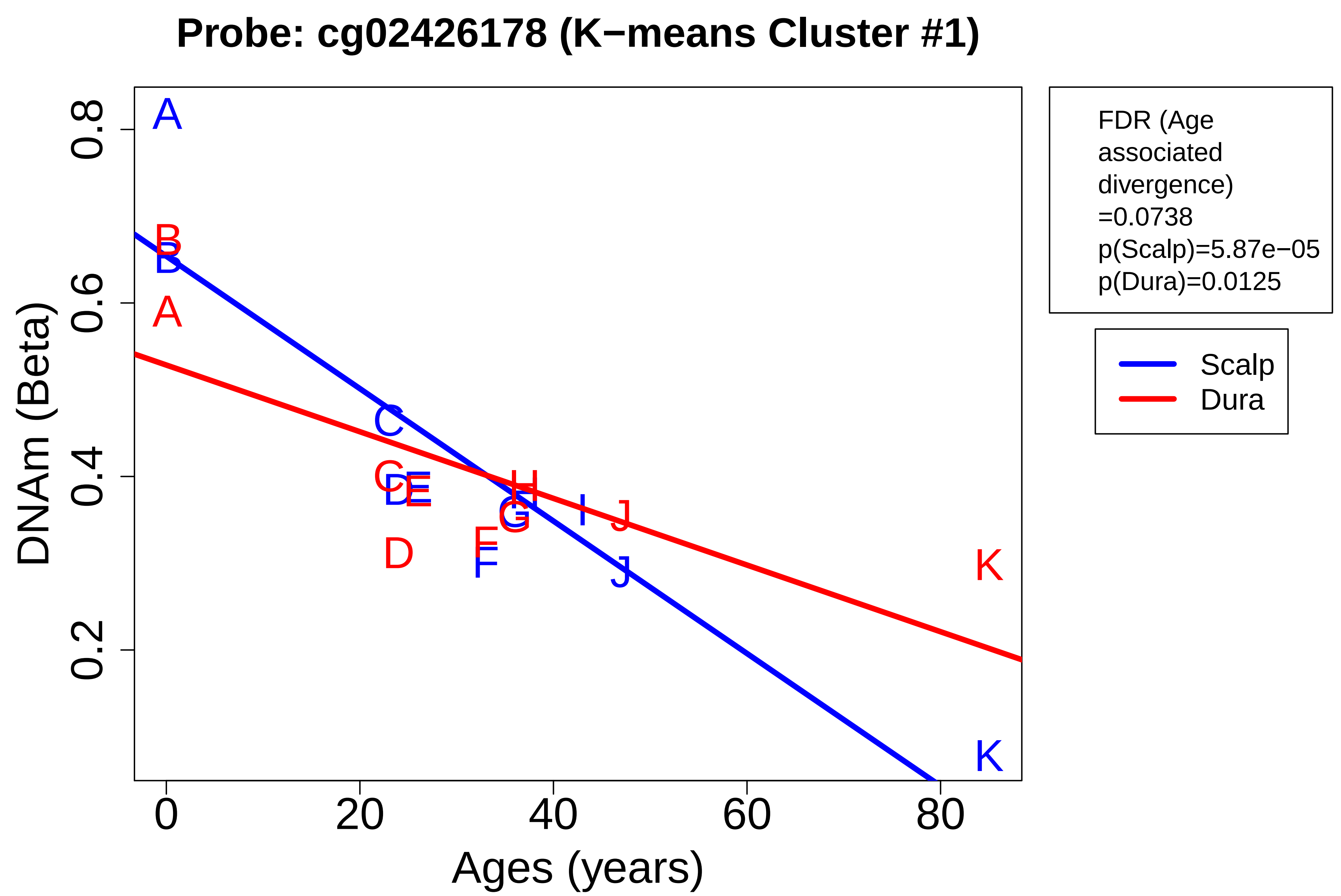

Probe: cg02426178 (K−means Cluster #1)
DNAm (Beta)
0.2
0.4
0.6
0.8
0
20
40
60
80
Ages (years)
A
B
C
D
E
F
G
H
I
J
K
FDR (Age associated divergence) =0.0738
p(Scalp)=5.87e−05
p(Dura)=0.0125
Scalp
Dura

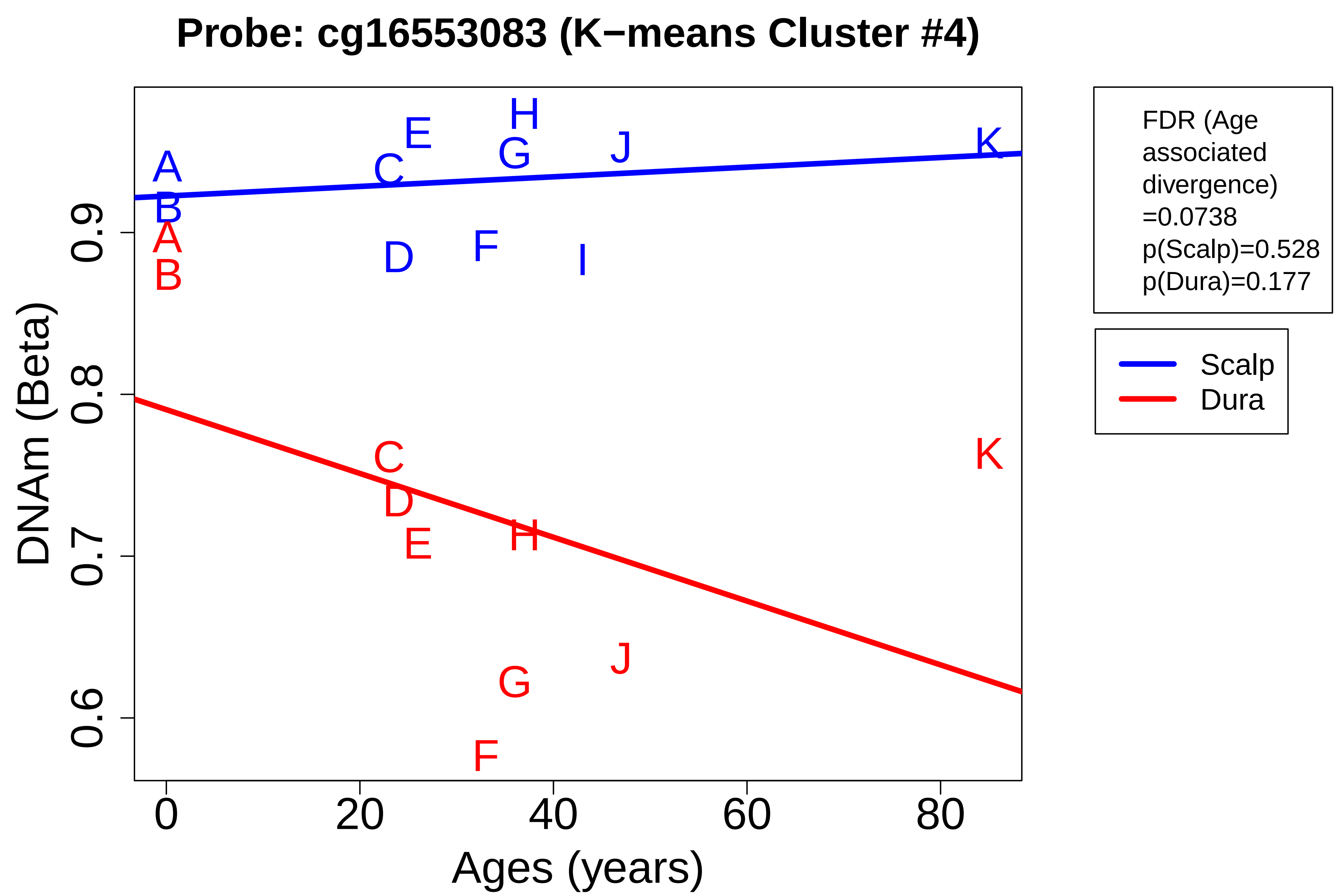

Probe: cg16553083 (K−means Cluster #4)
DNAm (Beta)
0.6
0.7
0.8
0.9
0
20
40
60
80
Ages (years)
A
B
C
D
E
F
G
H
I
J
K
FDR (Age associated divergence) =0.0738
p(Scalp)=0.528
p(Dura)=0.177
Scalp
Dura

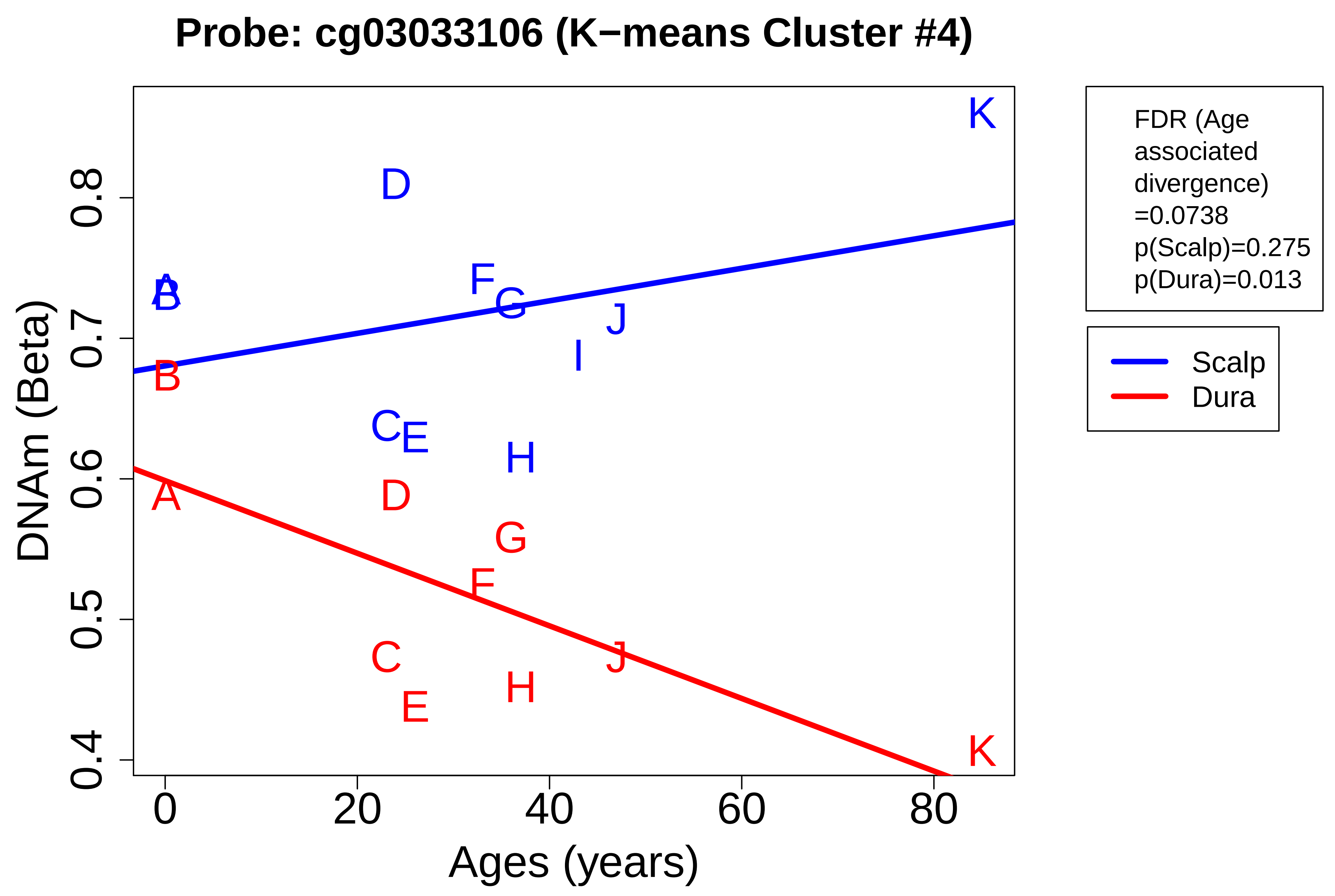

Probe: cg03033106 (K−means Cluster #4)
FDR (Age associated divergence) =0.0738
p(Scalp)=0.275
p(Dura)=0.013
Scalp
Dura
DNAm (Beta)
Ages (years)
0.4
0.5
0.6
0.7
0.8
0
20
40
60
80

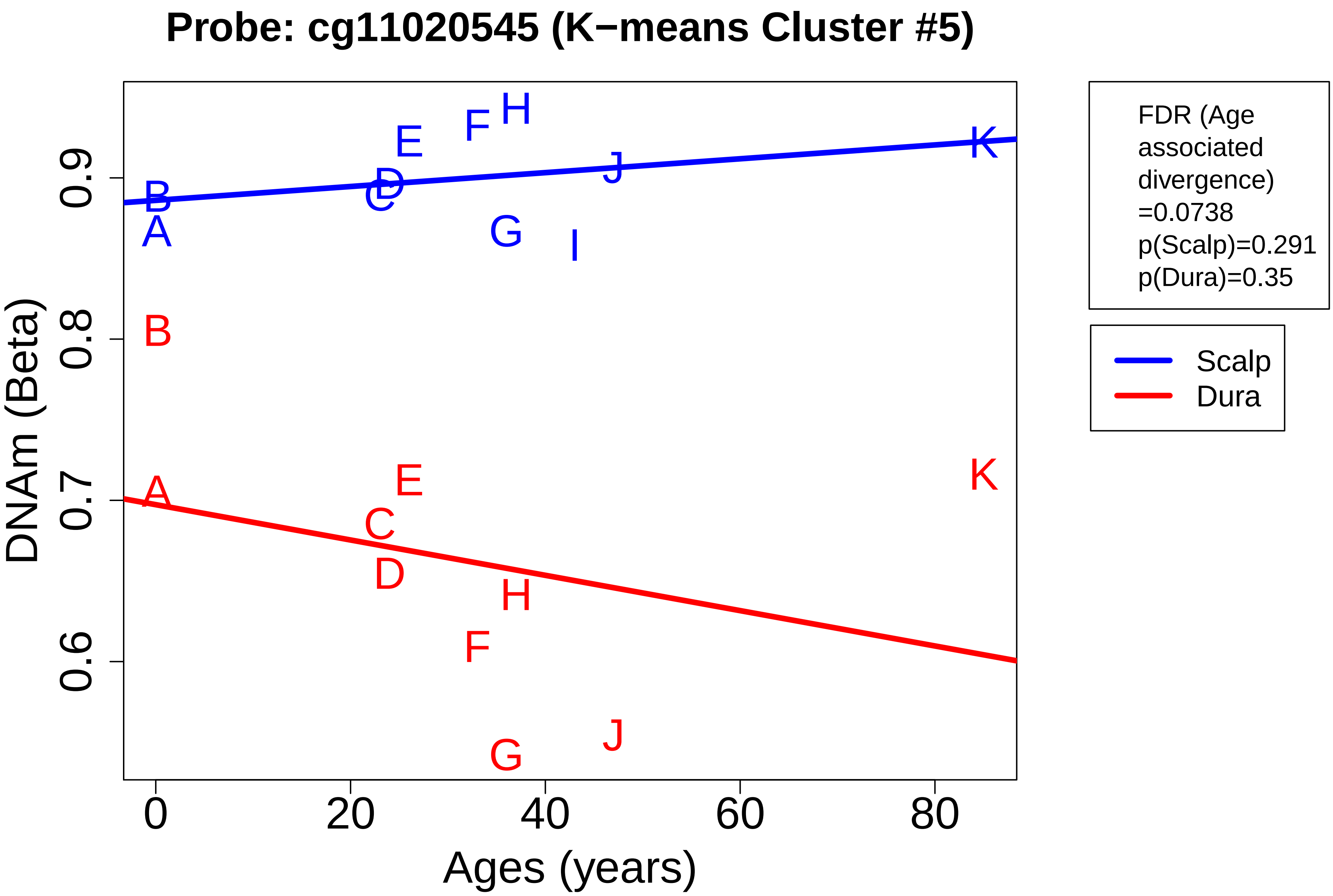

Probe: cg11020545 (K−means Cluster #5)
DNAm (Beta)
0.6
0.7
0.8
0.9
0
20
40
60
80
Ages (years)
FDR (Age associated divergence) =0.0738
p(Scalp)=0.291
p(Dura)=0.35
Scalp
Dura

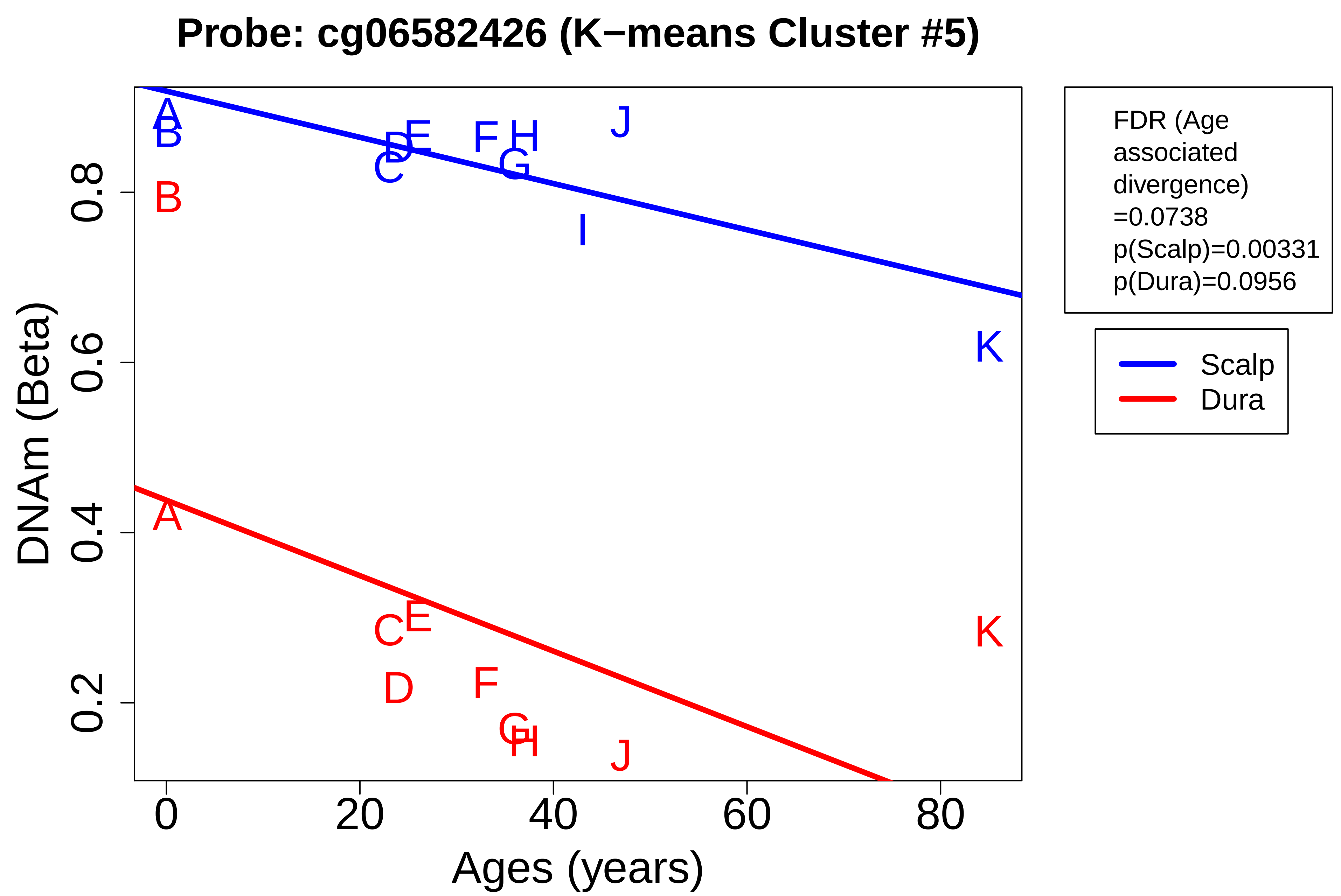

Probe: cg06582426 (K−means Cluster #5)
DNAm (Beta)
0.2
0.4
0.6
0.8
Ages (years)
0
20
40
60
80
A
B
C
D
E
F
G
H
I
J
K
FDR (Age associated divergence) =0.0738
p(Scalp)=0.00331
p(Dura)=0.0956
Scalp
Dura

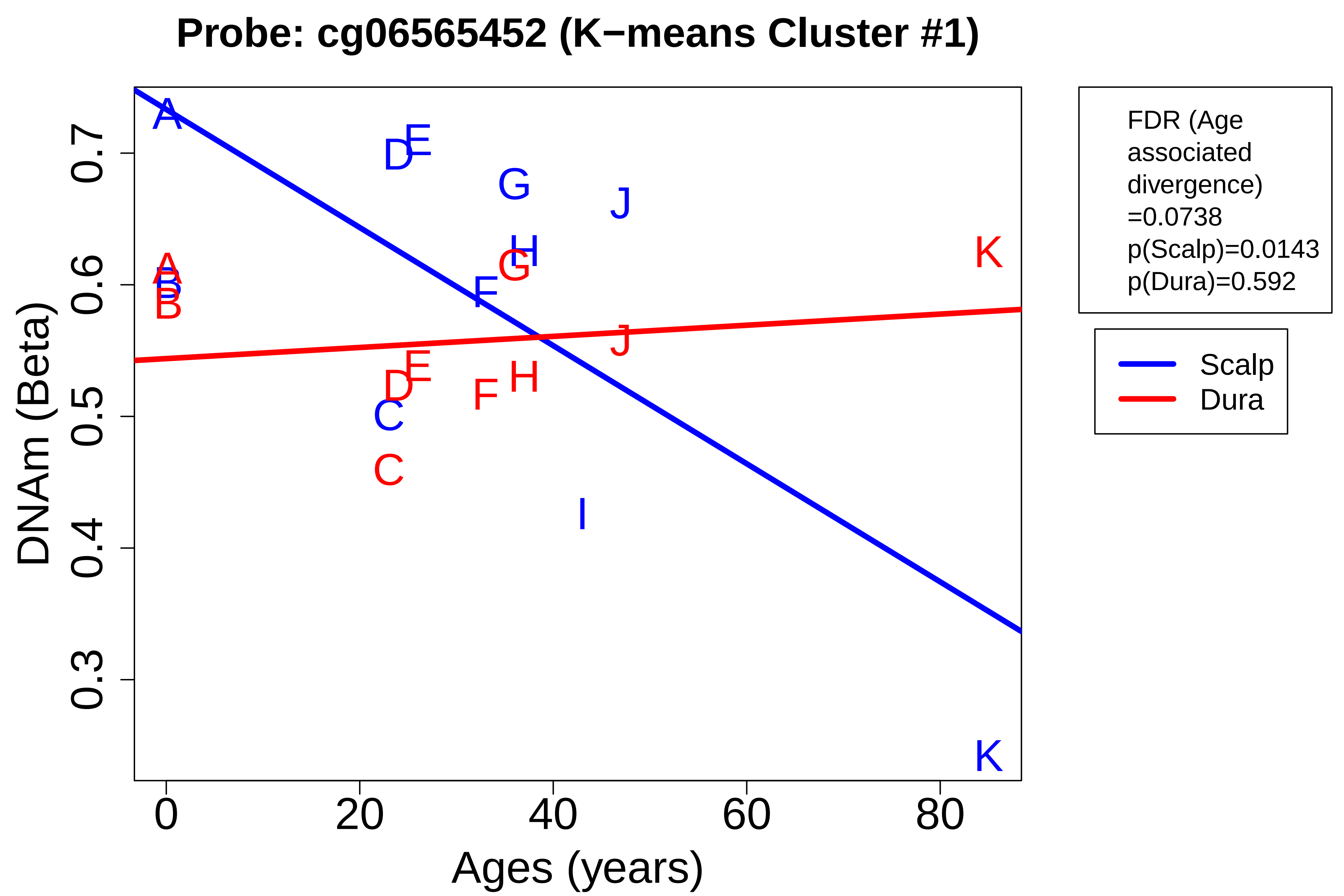

Probe: cg06565452 (K−means Cluster #1)
DNAm (Beta)
0.3
0.4
0.5
0.6
0.7
Ages (years)
0
20
40
60
80
A
B
C
D
E
F
G
H
I
J
K
FDR (Age associated divergence) =0.0738
p(Scalp)=0.0143
p(Dura)=0.592
Scalp
Dura

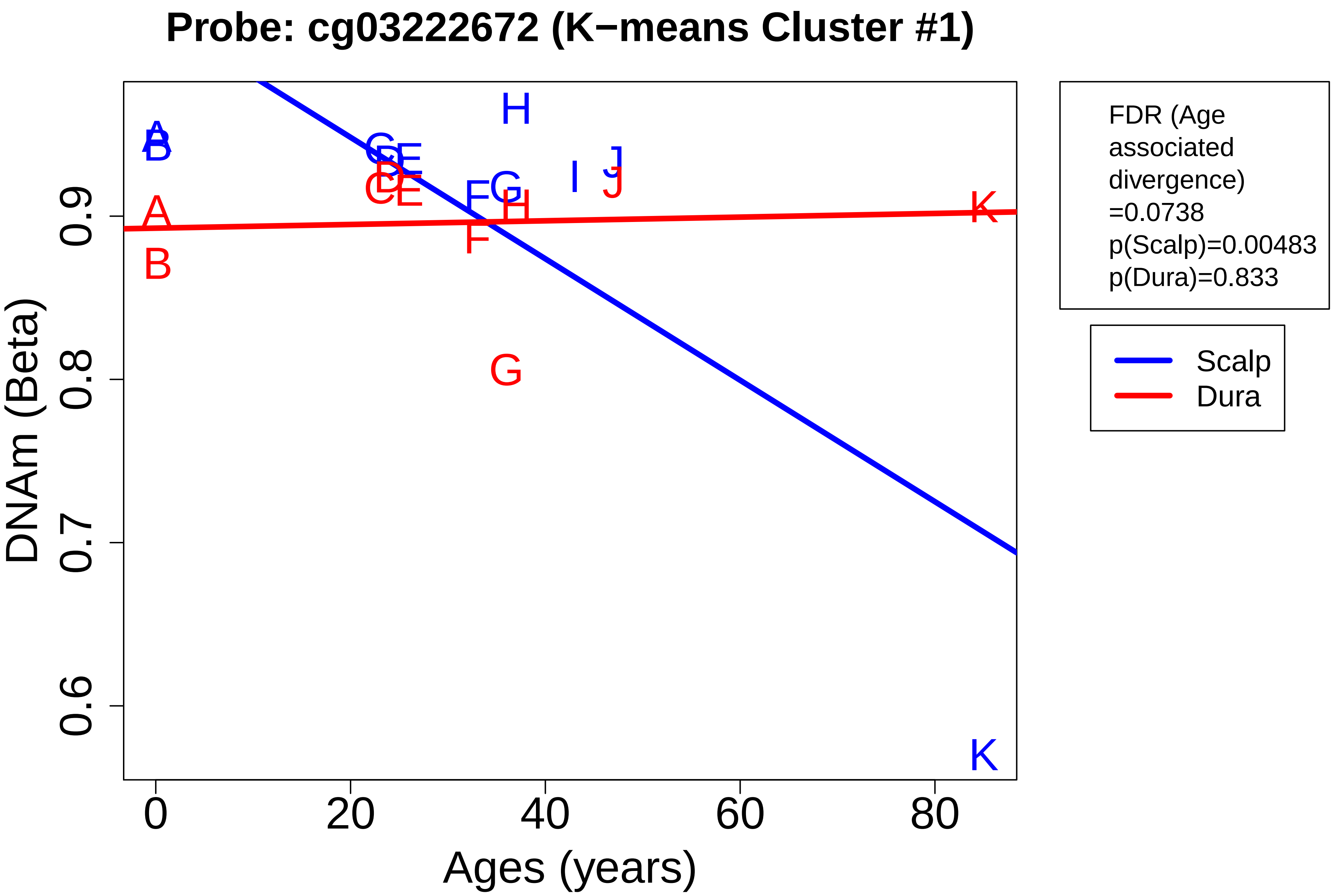

Probe: cg03222672 (K−means Cluster #1)
DNAm (Beta)
0.6
0.7
0.8
0.9
Ages (years)
0
20
40
60
80
A
B
C
D
E
F
G
H
I
J
K
FDR (Age associated divergence) =0.0738
p(Scalp)=0.00483
p(Dura)=0.833
Scalp
Dura

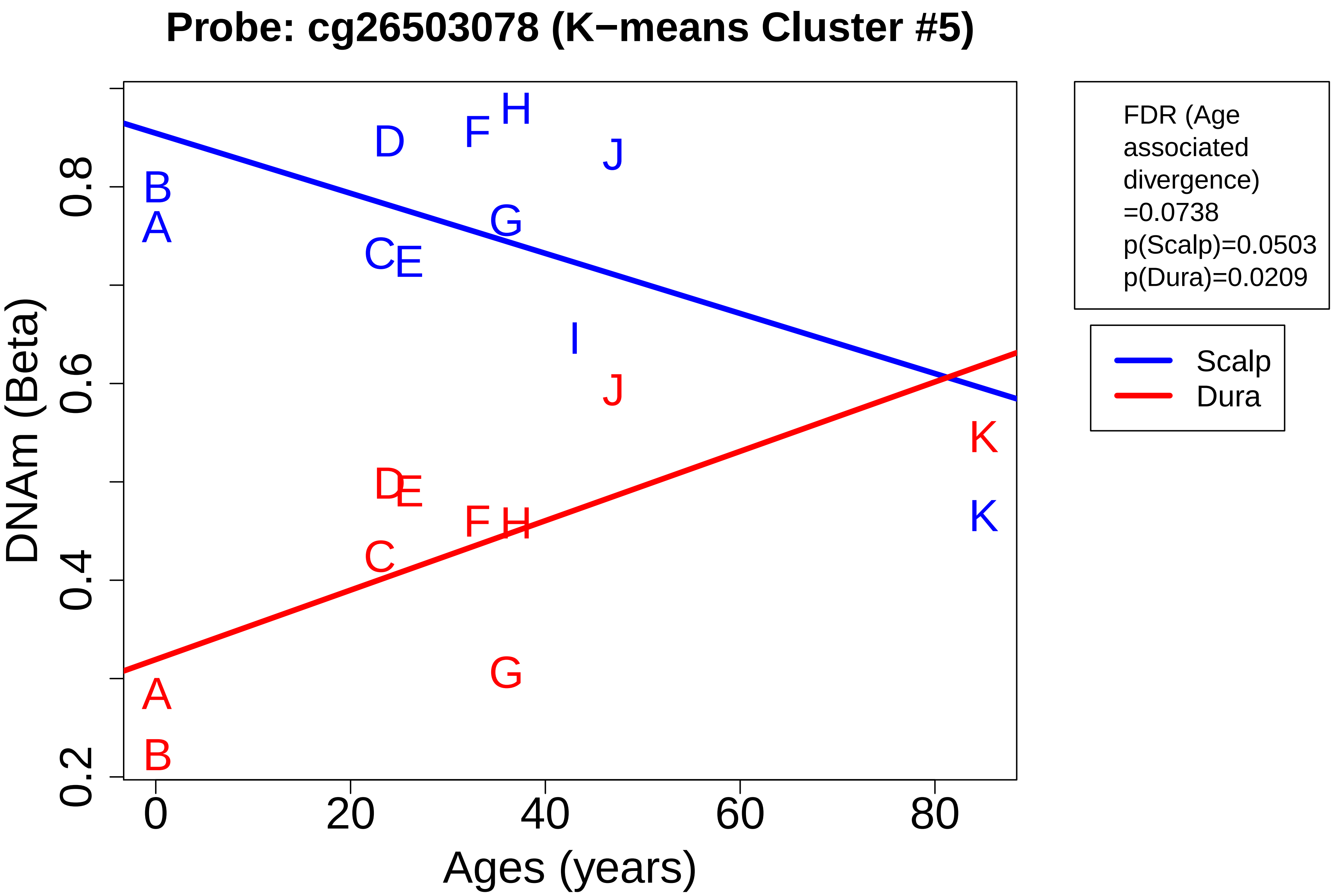

Probe: cg26503078 (K−means Cluster #5)
DNAm (Beta)
0.2
0.4
0.6
0.8
0
20
40
60
80
Ages (years)
FDR (Age associated divergence) =0.0738
p(Scalp)=0.0503
p(Dura)=0.0209
Scalp
Dura
A
B
C
D
E
F
G
H
I
J
K

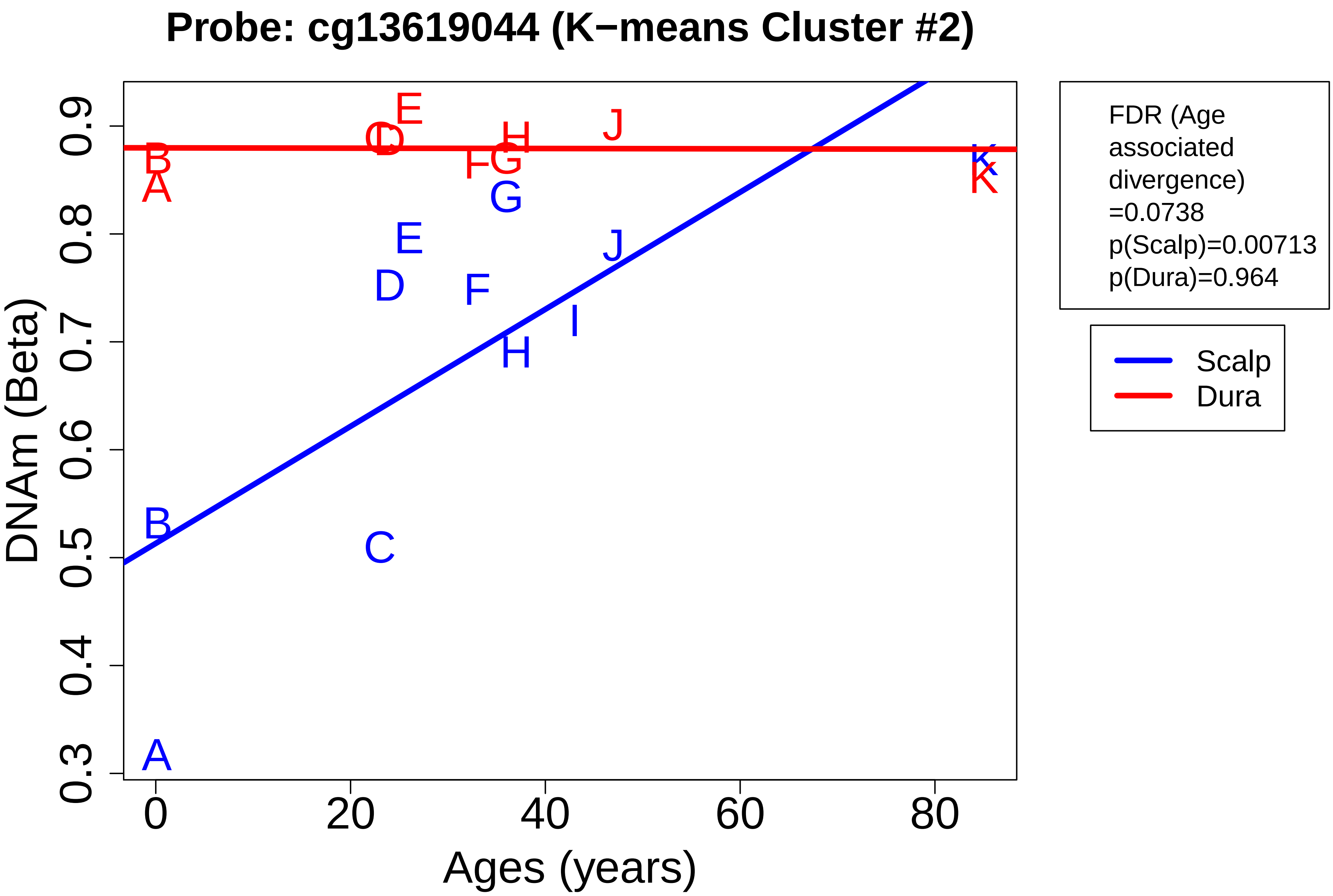

Probe: cg13619044 (K−means Cluster #2)
DNAm (Beta)
Ages (years)
0.3
0.4
0.5
0.6
0.7
0.8
0.9
0
20
40
60
80
FDR (Age associated divergence) =0.0738
p(Scalp)=0.00713
p(Dura)=0.964
Scalp
Dura

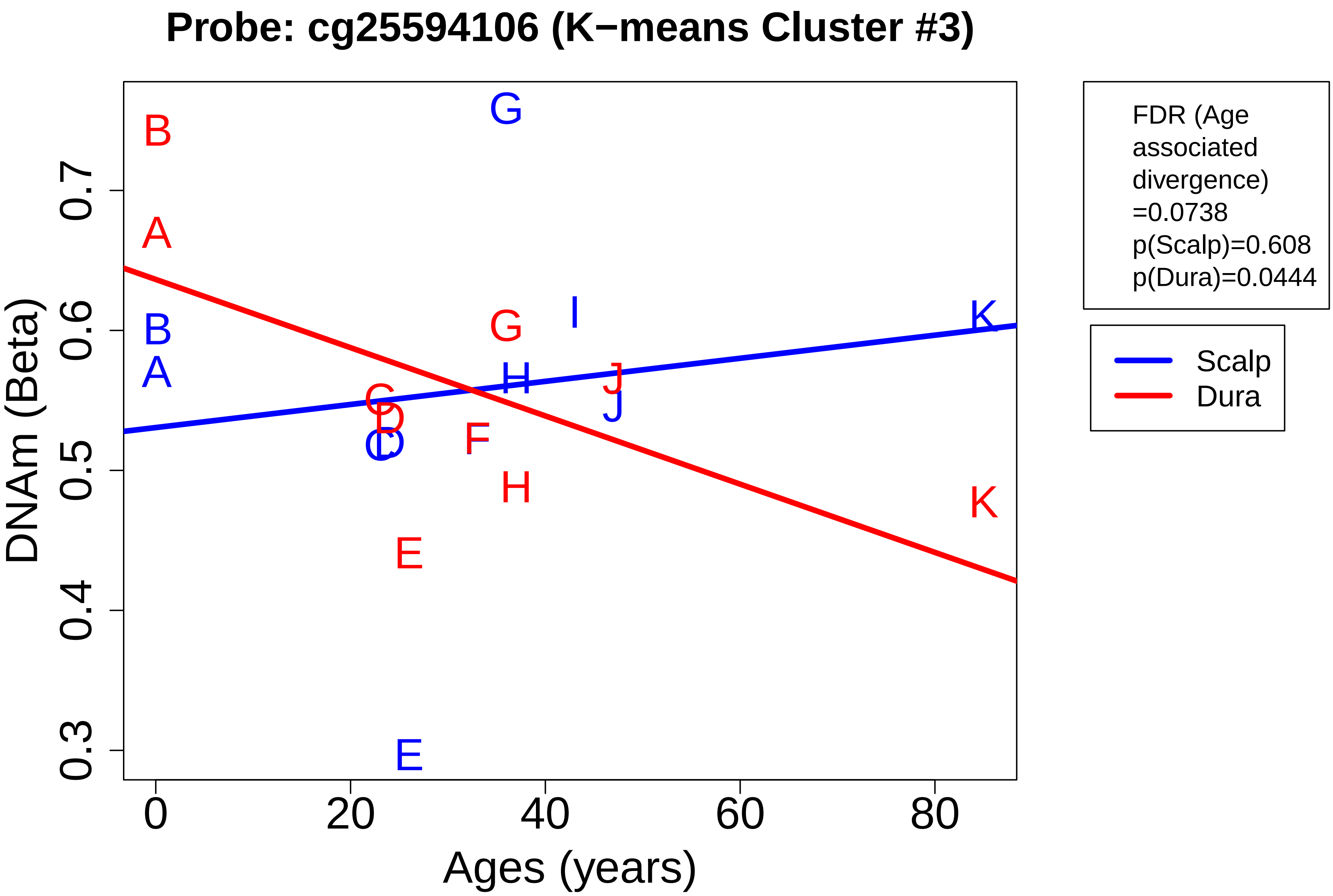

Probe: cg25594106 (K−means Cluster #3)
FDR (Age associated divergence) =0.0738
p(Scalp)=0.608
p(Dura)=0.0444
Scalp
Dura
DNAm (Beta)
0.3
0.4
0.5
0.6
0.7
0
20
40
60
80
Ages (years)

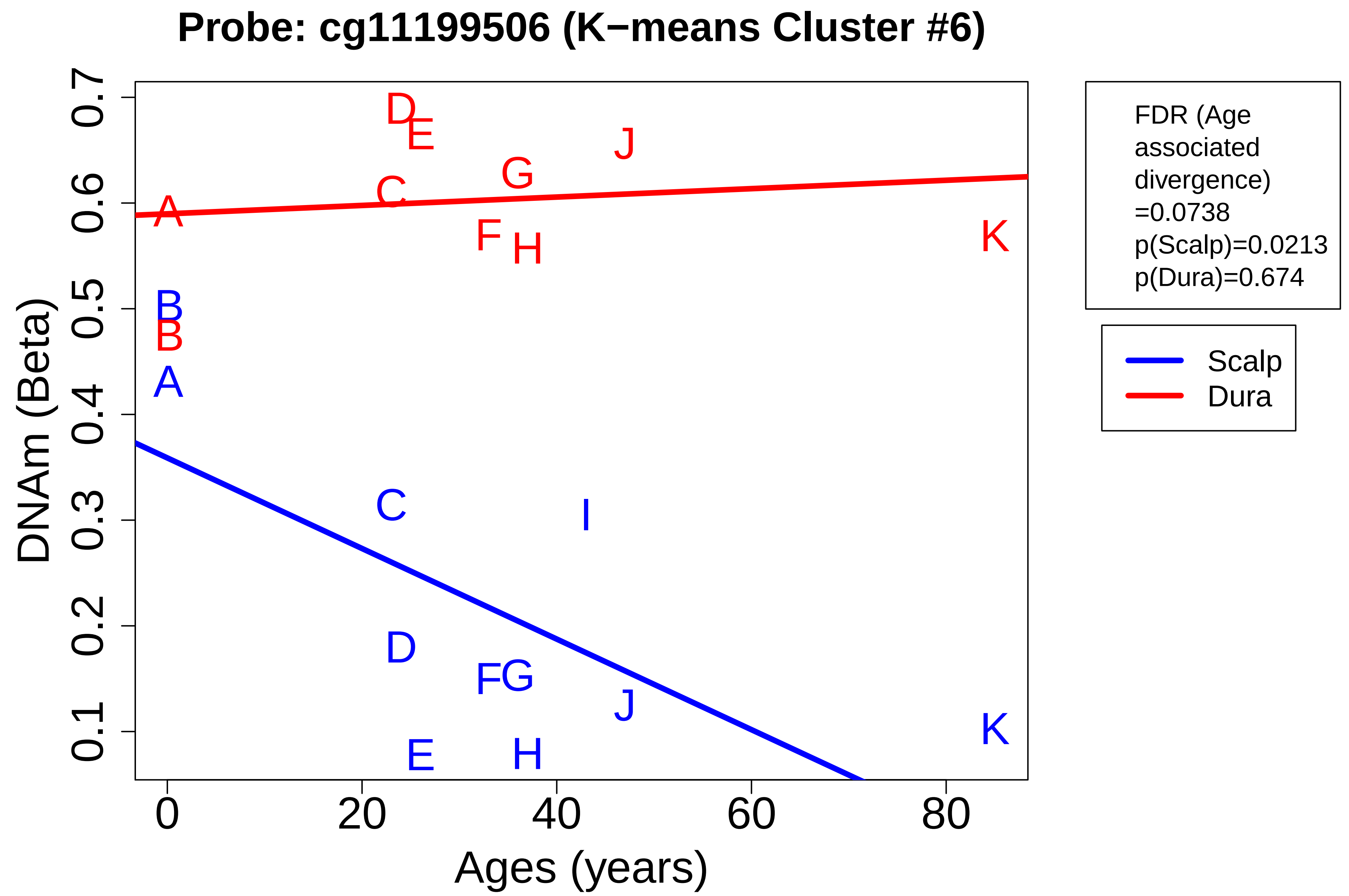

Probe: cg11199506 (K−means Cluster #6)
DNAm (Beta)
0.1
0.2
0.3
0.4
0.5
0.6
0.7
Ages (years)
0
20
40
60
80
A
B
C
D
E
F
G
H
I
J
K
FDR (Age associated divergence) =0.0738
p(Scalp)=0.0213
p(Dura)=0.674
Scalp
Dura

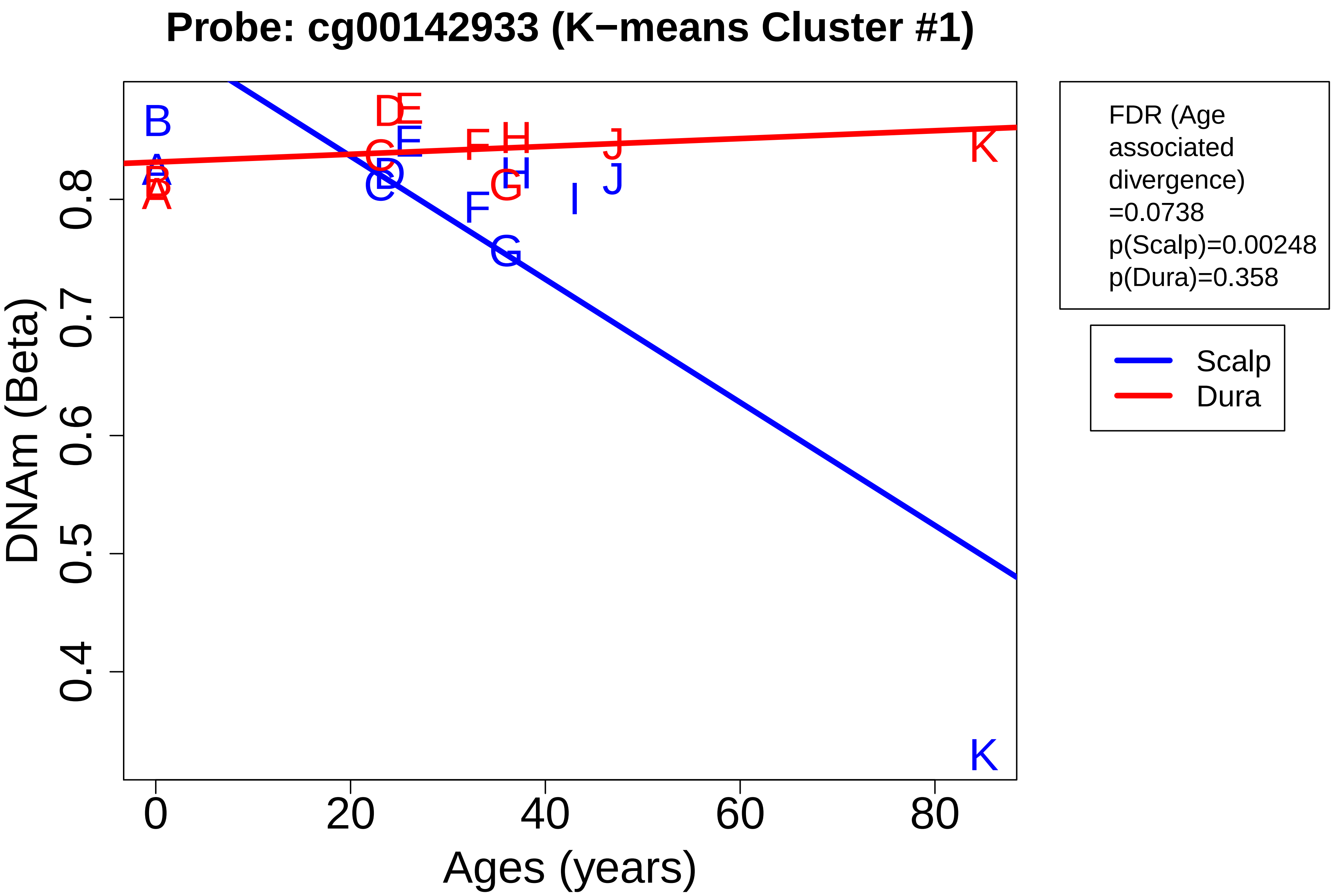

Probe: cg00142933 (K−means Cluster #1)
DNAm (Beta)
0.4
0.5
0.6
0.7
0.8
Ages (years)
0
20
40
60
80
FDR (Age associated divergence) =0.0738
p(Scalp)=0.00248
p(Dura)=0.358
Scalp
Dura

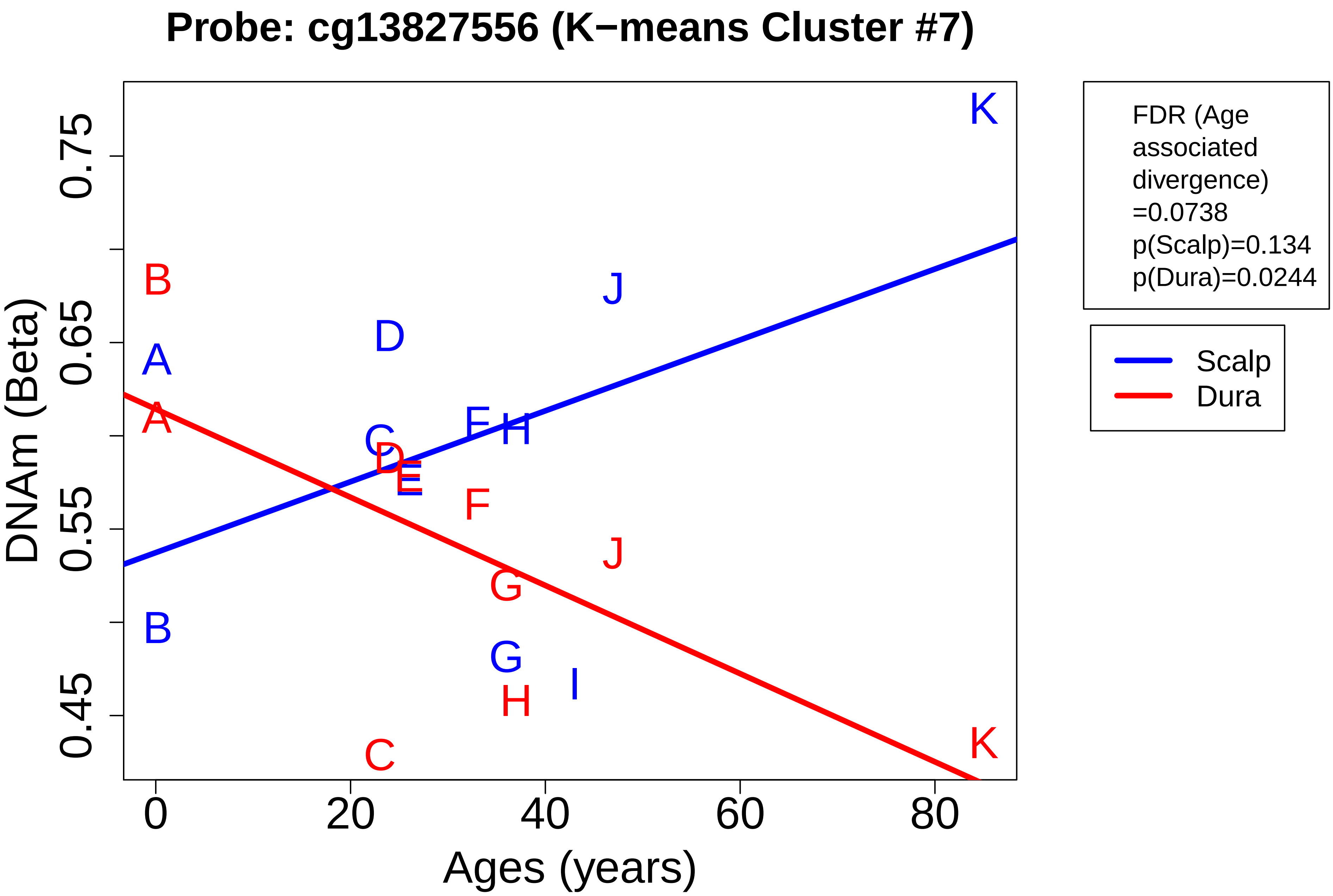

Probe: cg13827556 (K−means Cluster #7)
DNAm (Beta)
0.45
0.55
0.65
0.75
Ages (years)
0
20
40
60
80
FDR (Age associated divergence) =0.0738
p(Scalp)=0.134
p(Dura)=0.0244
Scalp
Dura

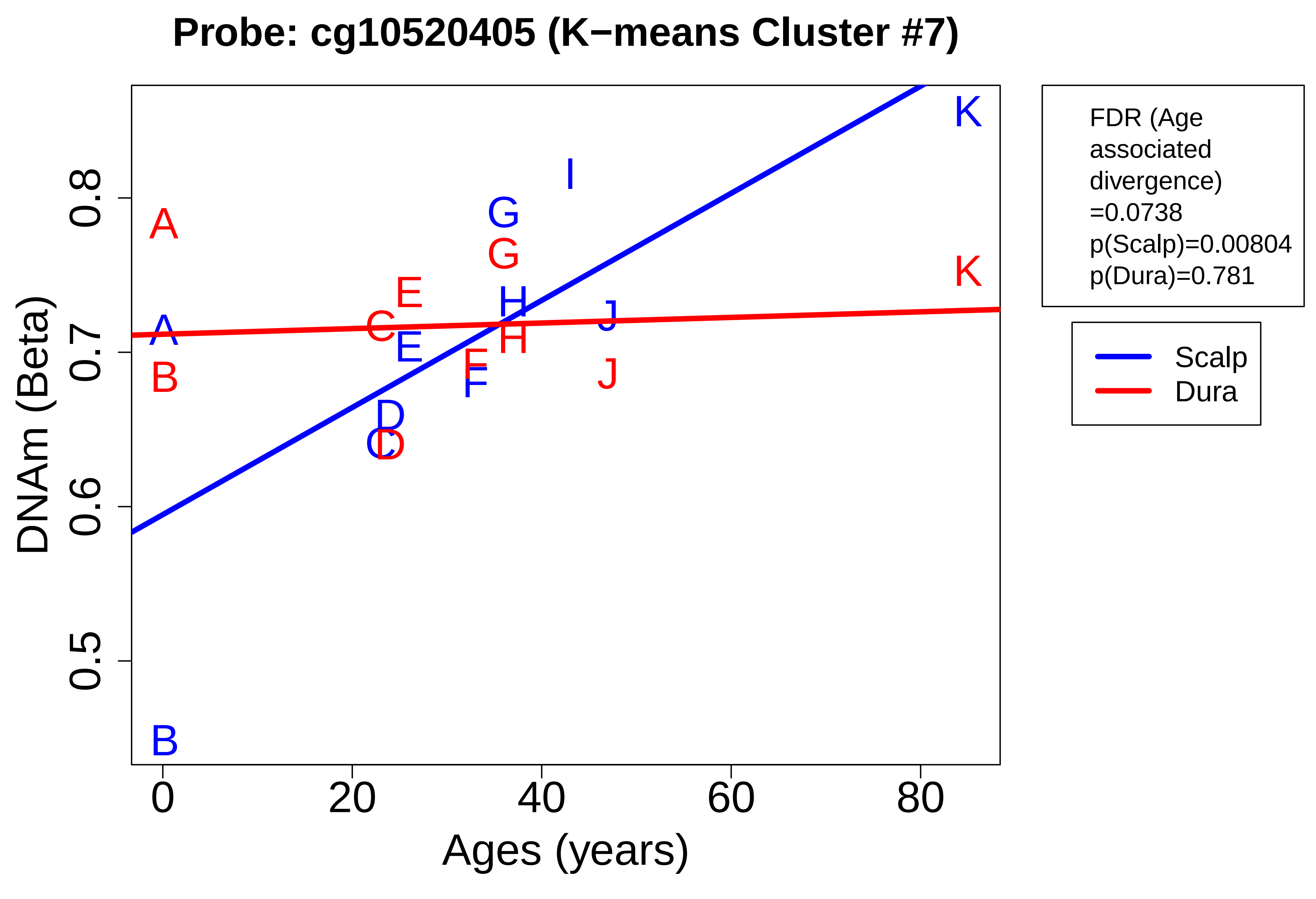

Probe: cg10520405 (K−means Cluster #7)
DNAm (Beta)
0.5
0.6
0.7
0.8
0
20
40
60
80
Ages (years)
A
B
C
D
E
F
G
H
I
J
K
FDR (Age associated divergence) =0.0738
p(Scalp)=0.00804
p(Dura)=0.781
Scalp
Dura

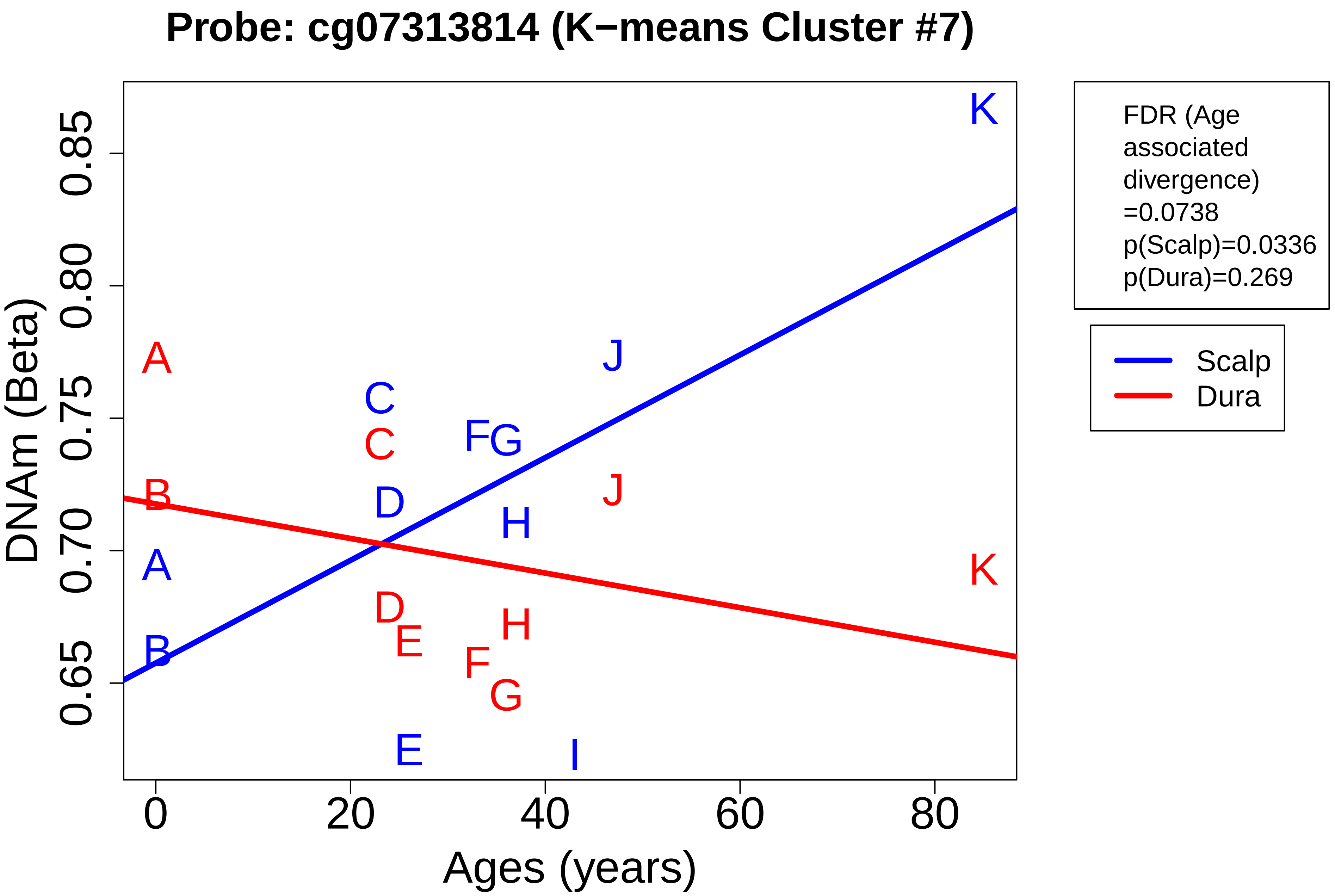

Probe: cg07313814 (K−means Cluster #7)
DNAm (Beta)
0.65
0.70
0.75
0.80
0.85
Ages (years)
0
20
40
60
80
FDR (Age associated divergence) =0.0738
p(Scalp)=0.0336
p(Dura)=0.269
Scalp
Dura
A
B
C
D
E
F
G
H
I
J
K

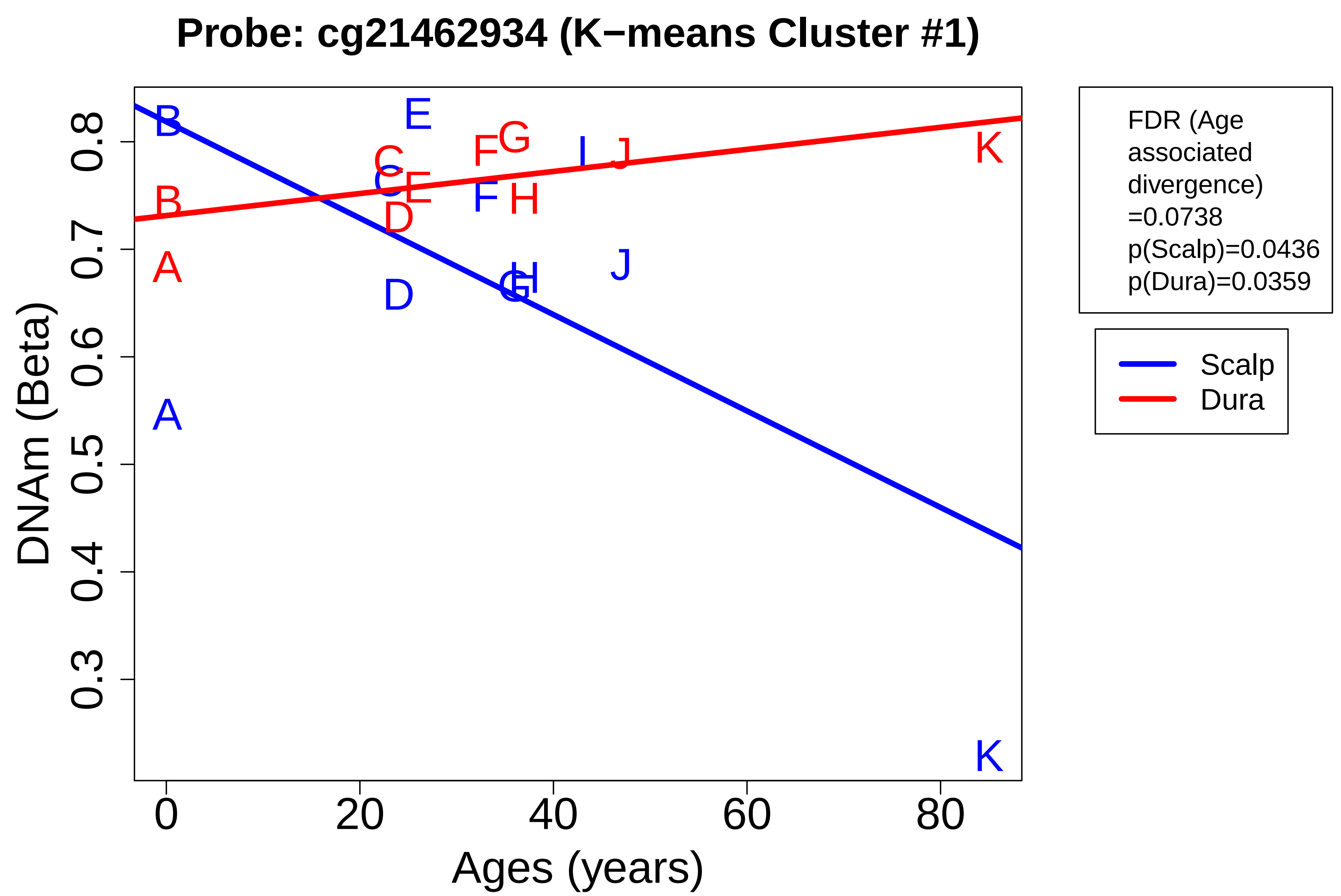

Probe: cg21462934 (K−means Cluster #1)
DNAm (Beta)
0.3
0.4
0.5
0.6
0.7
0.8
0
20
40
60
80
Ages (years)
A
B
C
D
E
F
G
H
I
J
K
FDR (Age associated divergence) =0.0738
p(Scalp)=0.0436
p(Dura)=0.0359
Scalp
Dura

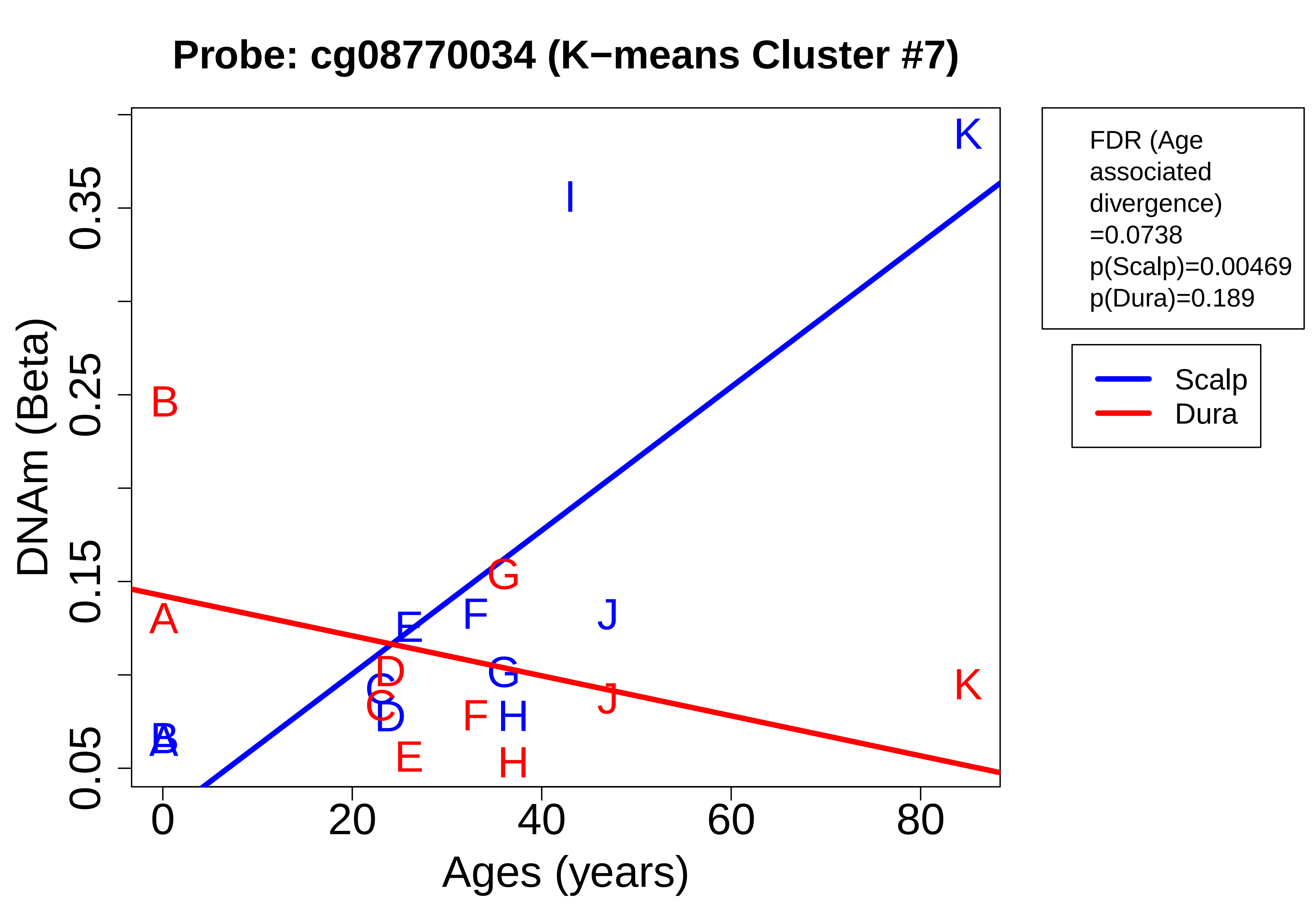

Probe: cg08770034 (K−means Cluster #7)
FDR (Age associated divergence) =0.0738
p(Scalp)=0.00469
p(Dura)=0.189
Scalp
Dura
DNAm (Beta)
0.05
0.15
0.25
0.35
Ages (years)
0
20
40
60
80
A
B
C
D
E
F
G
H
I
J
K

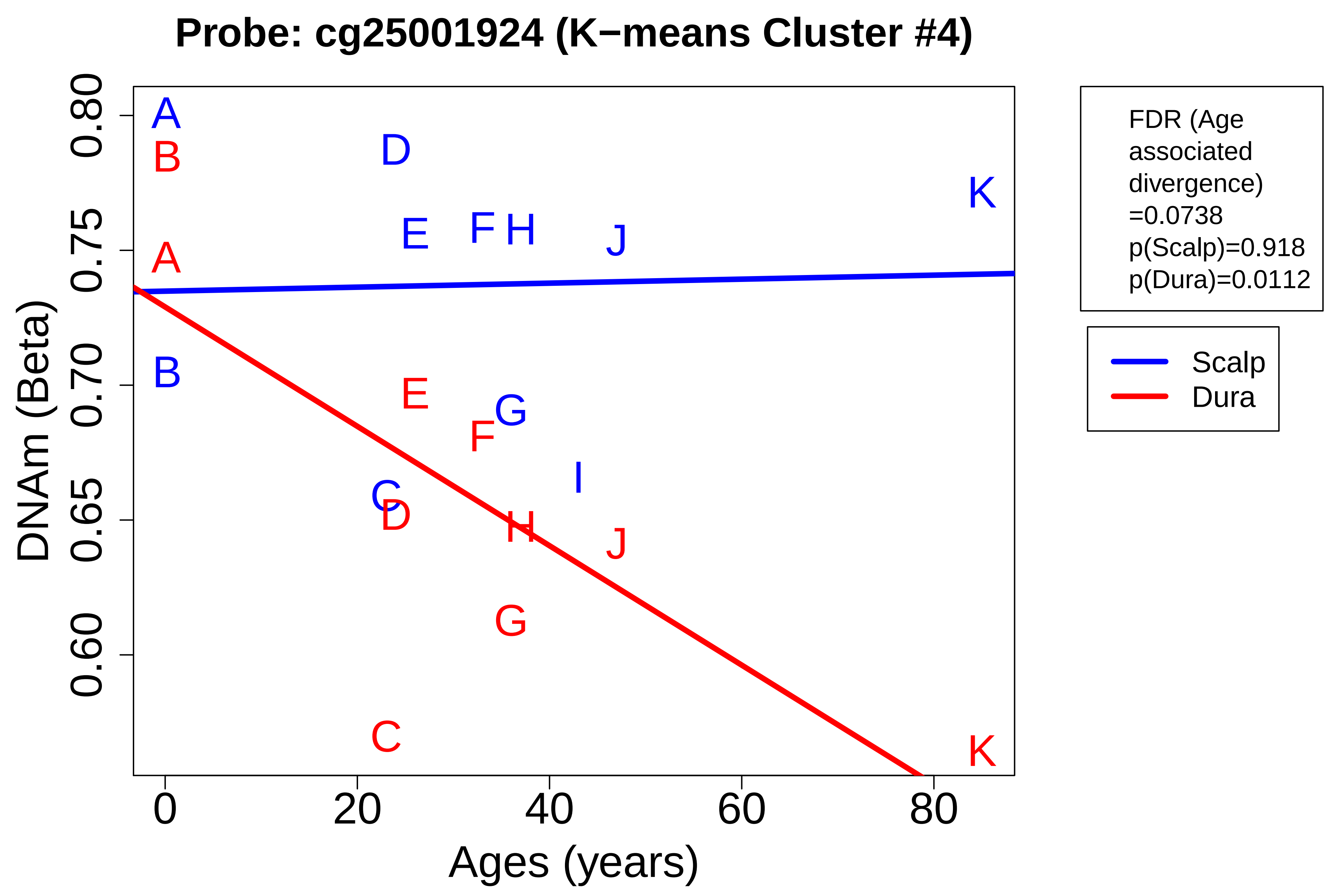

Probe: cg25001924 (K−means Cluster #4)
DNAm (Beta)
0.60
0.65
0.70
0.75
0.80
Ages (years)
0
20
40
60
80
FDR (Age associated divergence) =0.0738
p(Scalp)=0.918
p(Dura)=0.0112
Scalp
Dura

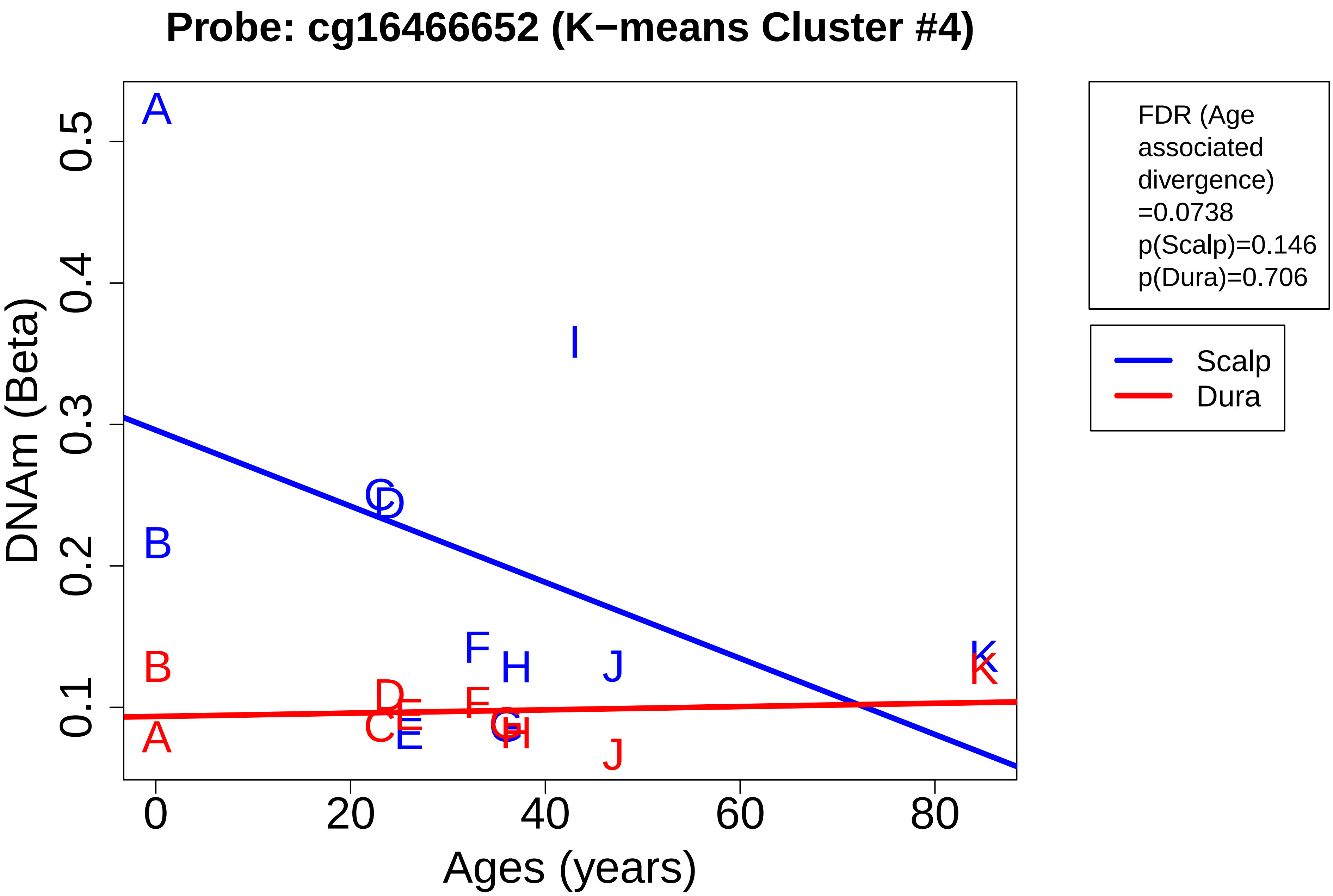

Probe: cg16466652 (K−means Cluster #4)
DNAm (Beta)
Ages (years)
0.1
0.2
0.3
0.4
0.5
0
20
40
60
80
FDR (Age associated divergence) =0.0738
p(Scalp)=0.146
p(Dura)=0.706
Scalp
Dura

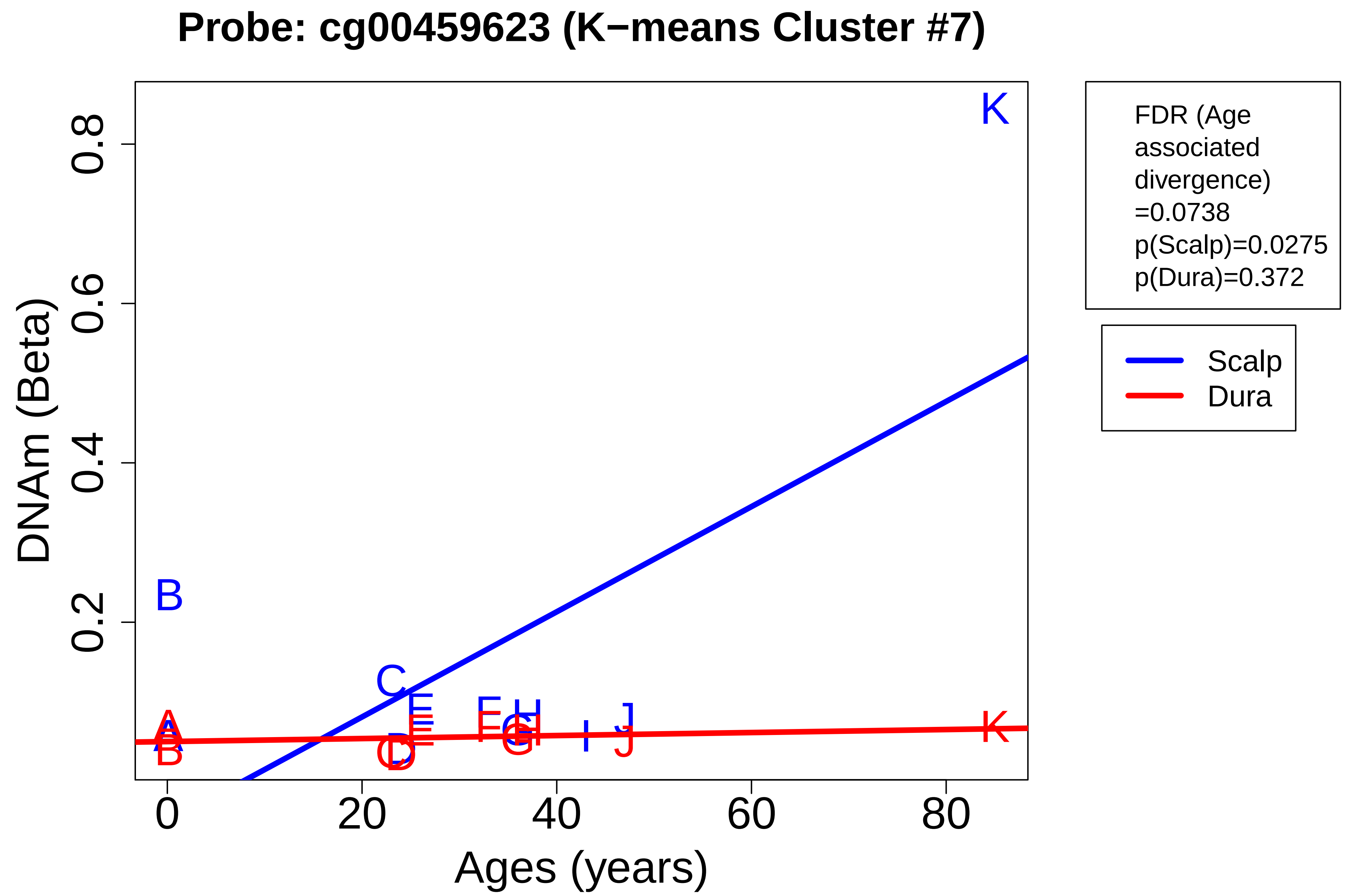

Probe: cg00459623 (K−means Cluster #7)
DNAm (Beta)
0.2
0.4
0.6
0.8
0
20
40
60
80
Ages (years)
FDR (Age associated divergence) =0.0738
p(Scalp)=0.0275
p(Dura)=0.372
Scalp
Dura

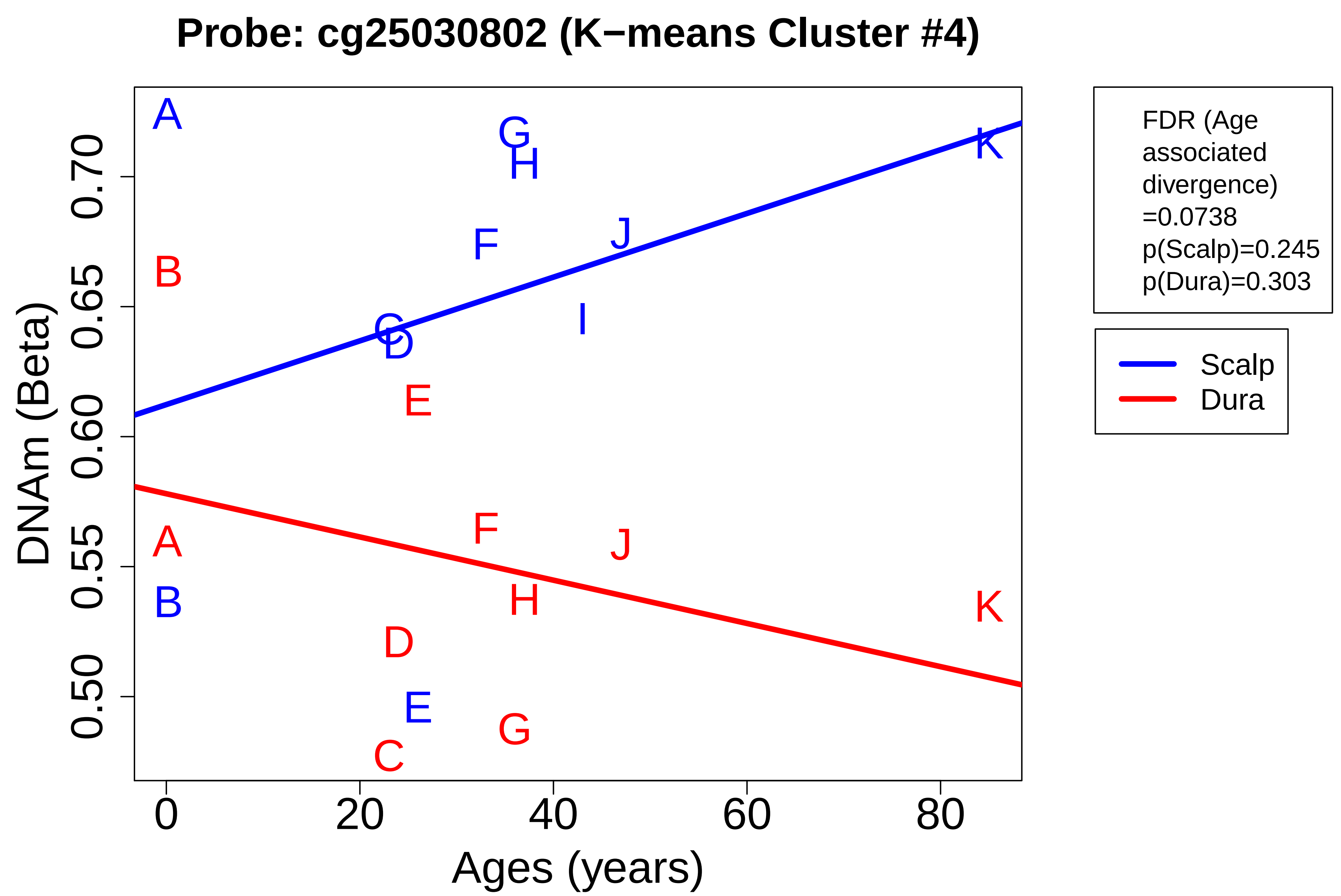

Probe: cg25030802 (K−means Cluster #4)
DNAm (Beta)
0.50
0.55
0.60
0.65
0.70
0
20
40
60
80
Ages (years)
A
B
C
D
E
F
G
H
I
J
K
FDR (Age associated divergence) =0.0738
p(Scalp)=0.245
p(Dura)=0.303
Scalp
Dura

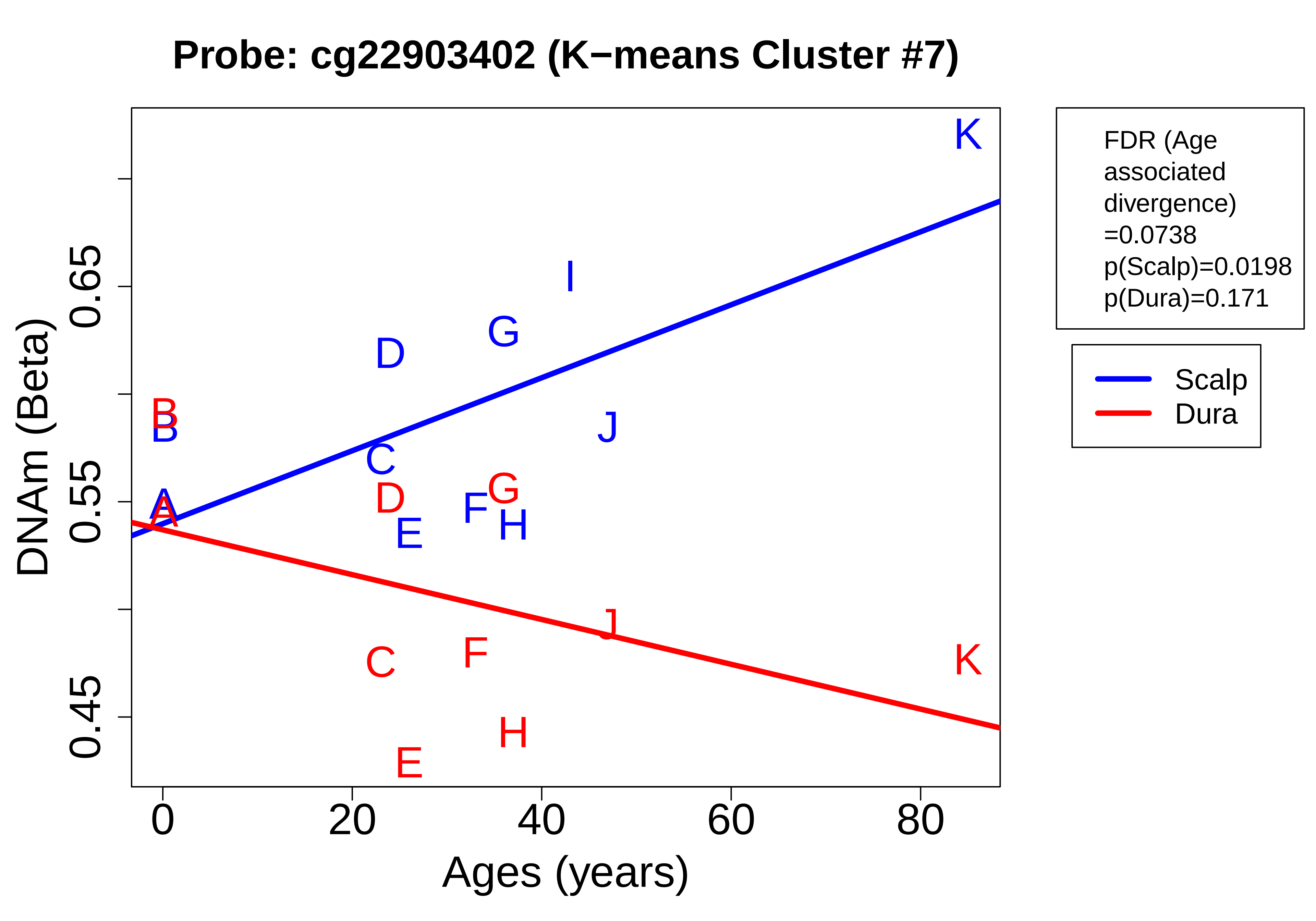
Probe: cg22903402 (K−means Cluster #7)
DNAm (Beta)
0.45
0.55
0.65
Ages (years)
0
20
40
60
80
FDR (Age associated divergence) =0.0738
p(Scalp)=0.0198
p(Dura)=0.171
Scalp
Dura
A
B
C
D
E
F
G
H
I
J
K

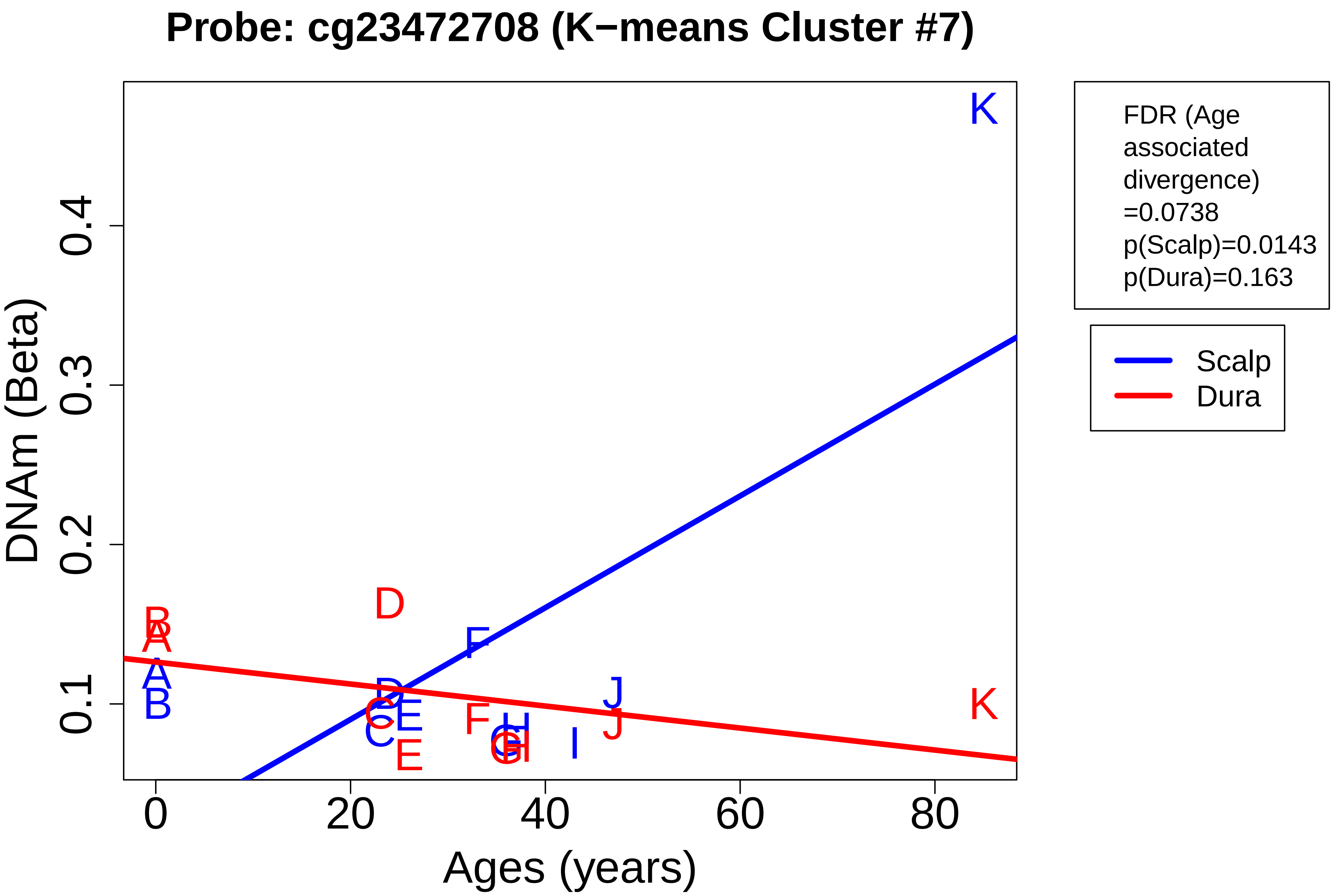

Probe: cg23472708 (K−means Cluster #7)
DNAm (Beta)
0.1
0.2
0.3
0.4
0
20
40
60
80
Ages (years)
FDR (Age associated divergence) =0.0738
p(Scalp)=0.0143
p(Dura)=0.163
Scalp
Dura
A
B
C
D
E
F
G
H
I
J
K

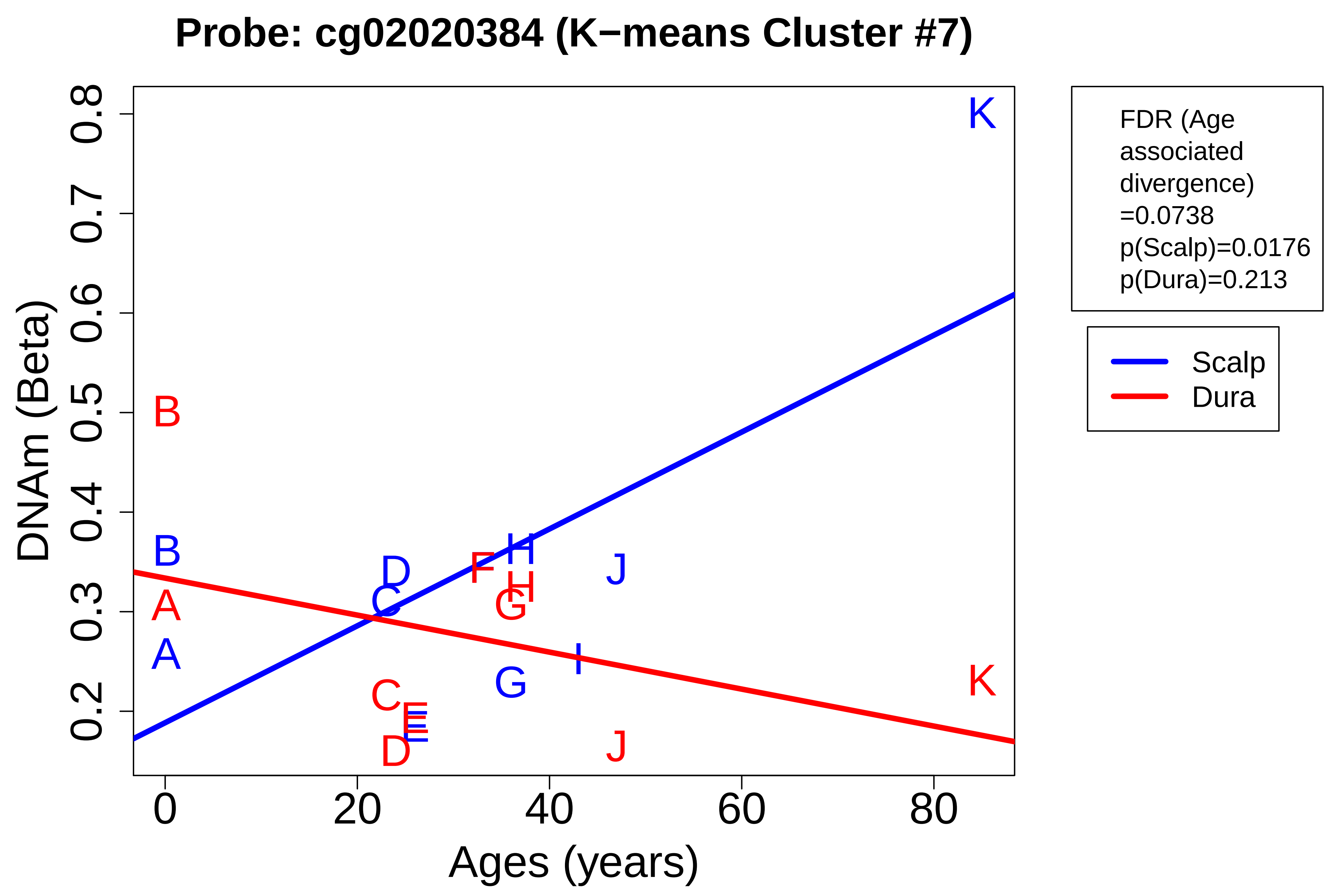
Probe: cg02020384 (K−means Cluster #7)
DNAm (Beta)
0.2
0.3
0.4
0.5
0.6
0.7
0.8
0
20
40
60
80
Ages (years)
K
B
B
A
A
D
C
F
H
H
G
J
I
G
K
C
E
E
D
J
FDR (Age associated divergence) =0.0738
p(Scalp)=0.0176
p(Dura)=0.213
Scalp
Dura

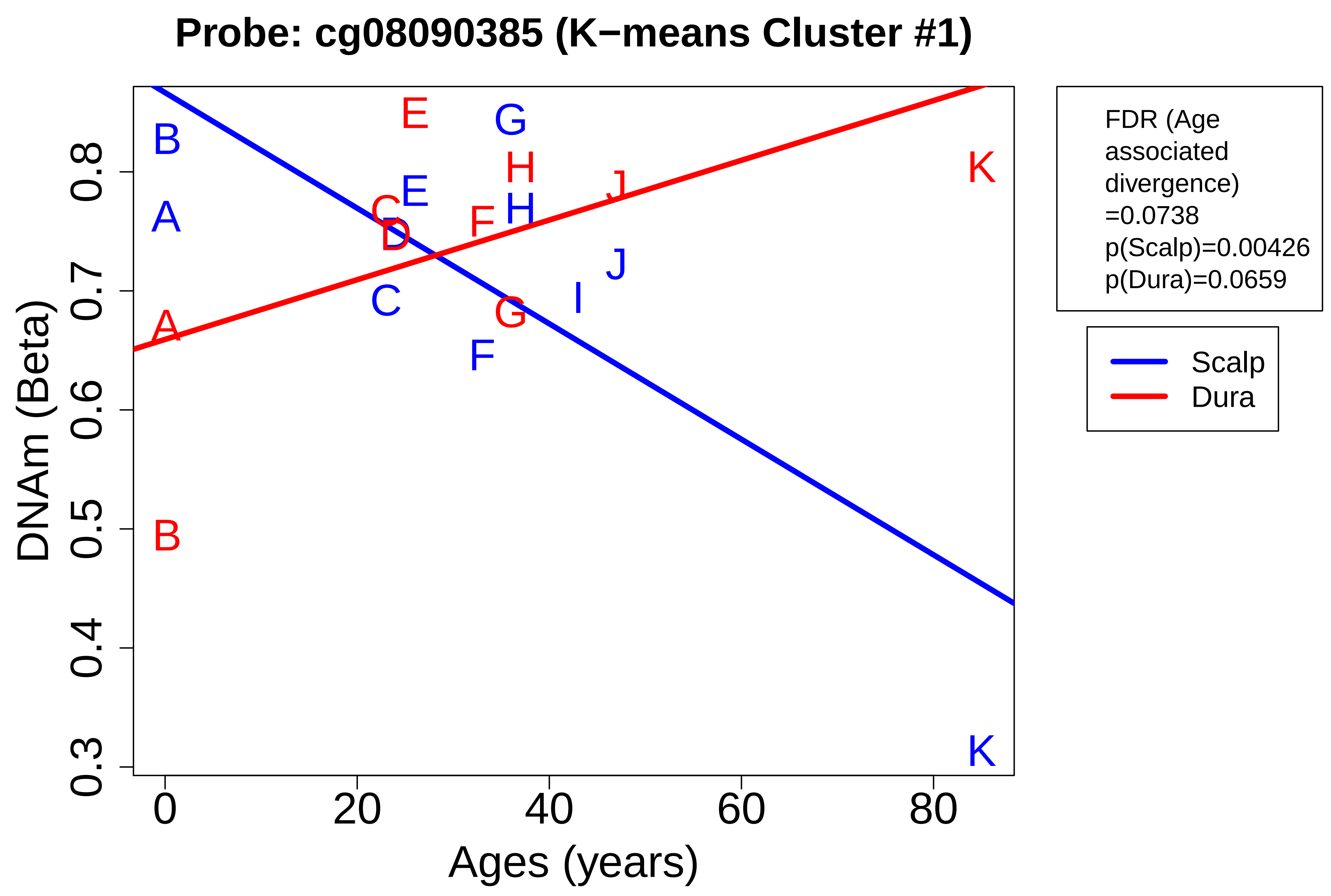

Probe: cg08090385 (K−means Cluster #1)
DNAm (Beta)
0.3
0.4
0.5
0.6
0.7
0.8
0
20
40
60
80
Ages (years)
FDR (Age associated divergence) =0.0738
p(Scalp)=0.00426
p(Dura)=0.0659
Scalp
Dura

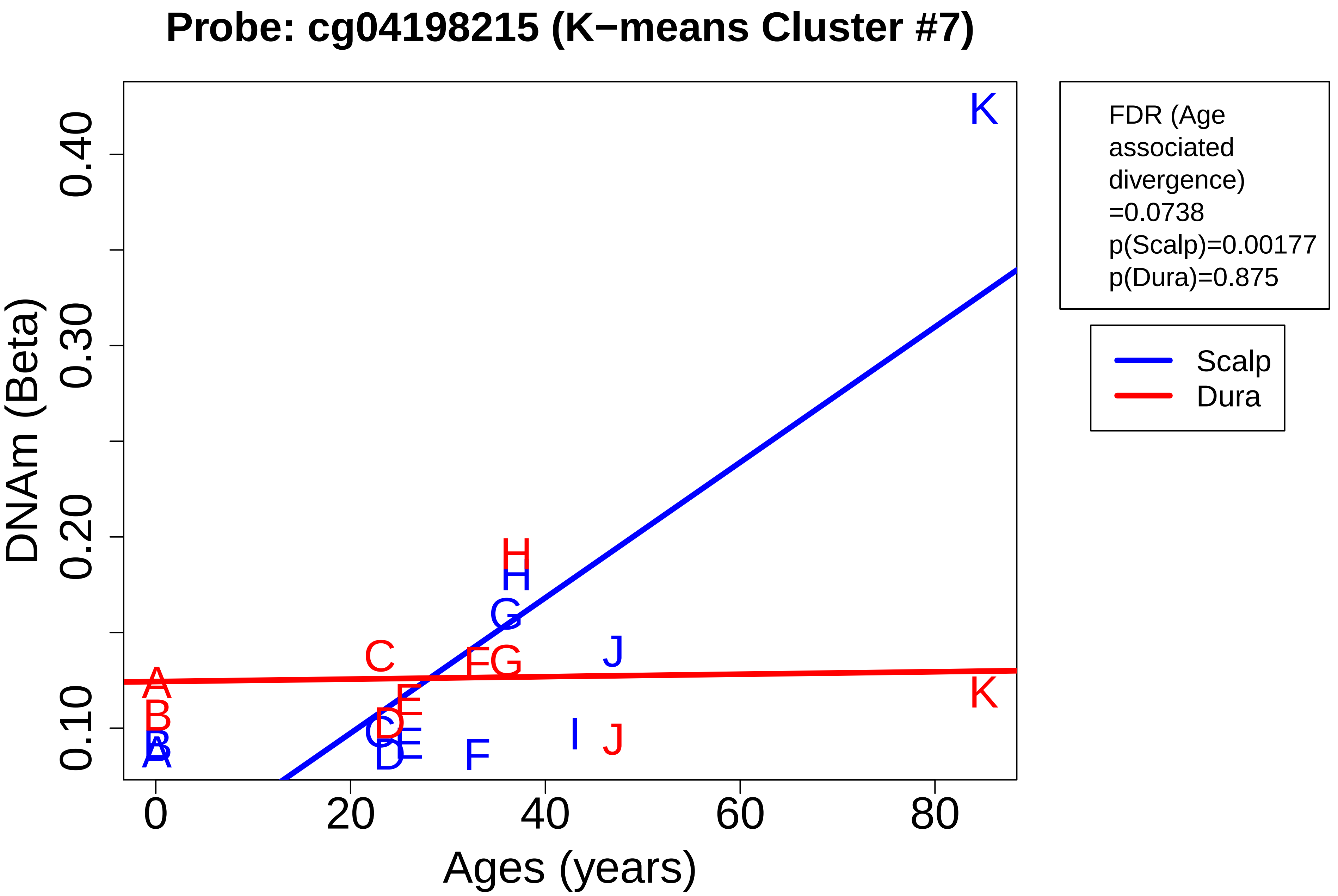

Probe: cg04198215 (K−means Cluster #7)
DNAm (Beta)
0.10
0.20
0.30
0.40
Ages (years)
0
20
40
60
80
FDR (Age associated divergence) =0.0738
p(Scalp)=0.00177
p(Dura)=0.875
Scalp
Dura

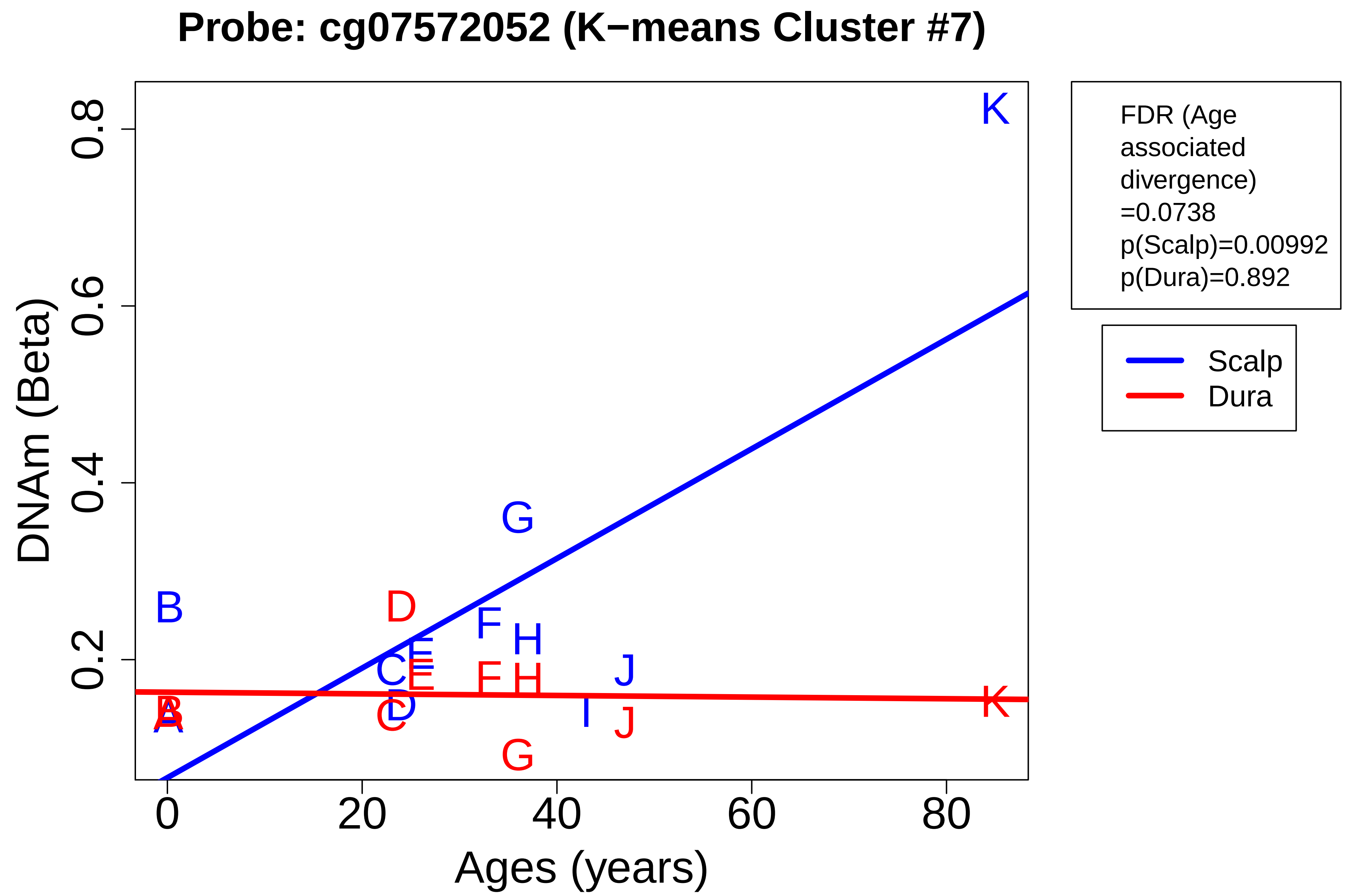

Probe: cg07572052 (K−means Cluster #7)
DNAm (Beta)
0.2
0.4
0.6
0.8
0
20
40
60
80
Ages (years)
FDR (Age associated divergence) =0.0738
p(Scalp)=0.00992
p(Dura)=0.892
Scalp
Dura

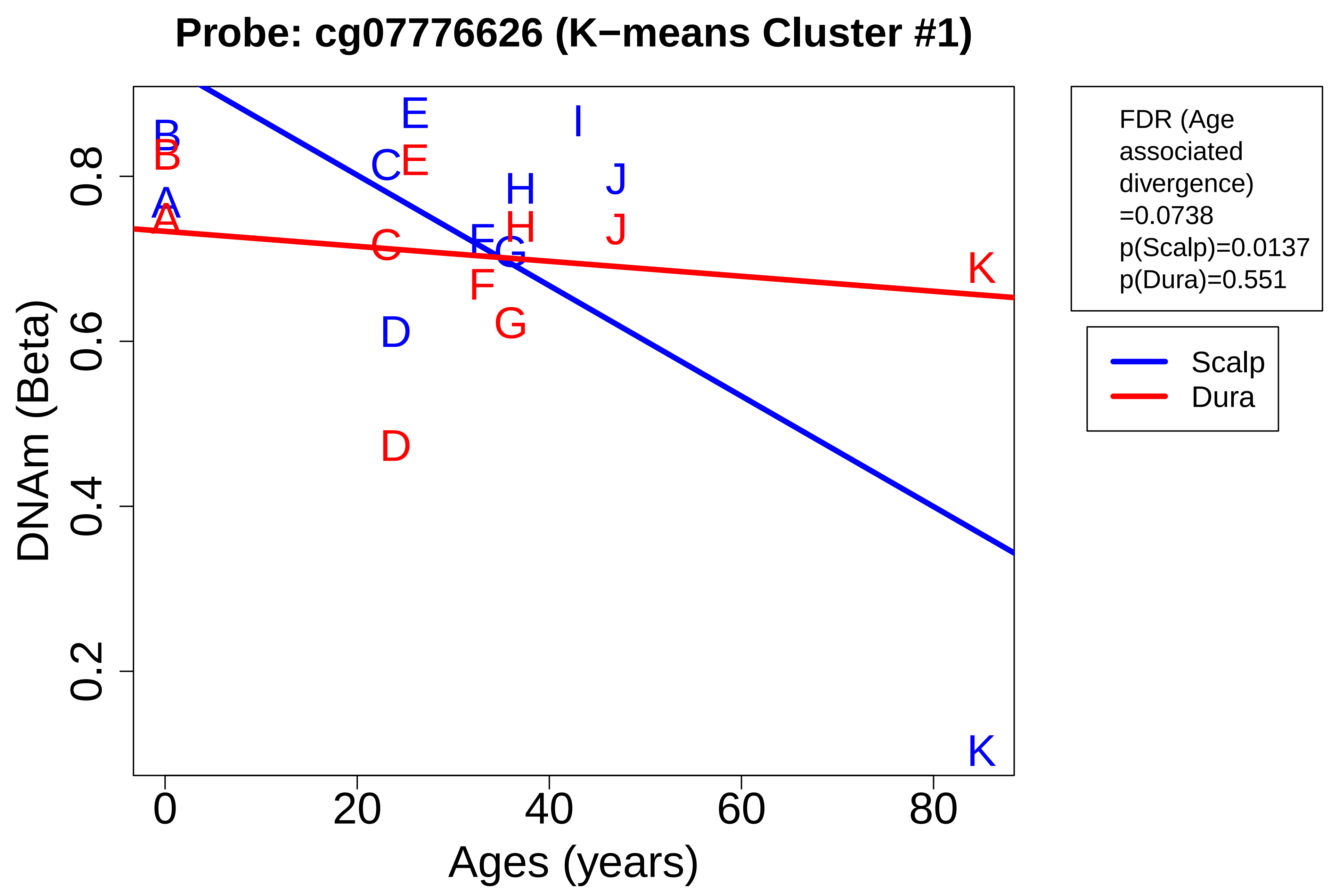

Probe: cg07776626 (K−means Cluster #1)
DNAm (Beta)
0.2
0.4
0.6
0.8
0
20
40
60
80
Ages (years)
A
B
C
D
E
F
G
H
I
J
K
FDR (Age associated divergence) =0.0738
p(Scalp)=0.0137
p(Dura)=0.551
Scalp
Dura

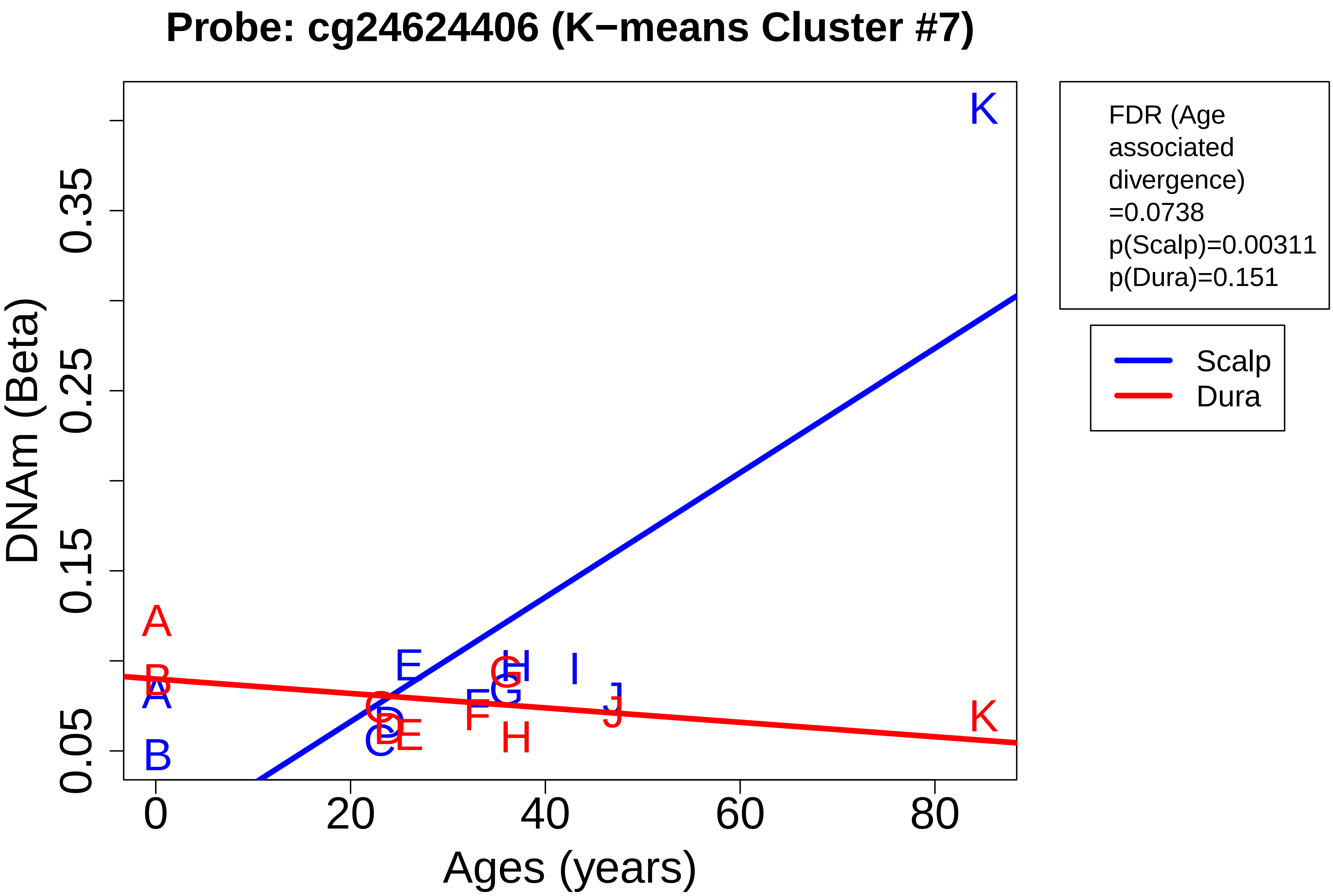
Probe: cg24624406 (K−means Cluster #7)
DNAm (Beta)
Ages (years)
0.05
0.15
0.25
0.35
0
20
40
60
80
FDR (Age associated divergence) =0.0738
p(Scalp)=0.00311
p(Dura)=0.151
Scalp
Dura

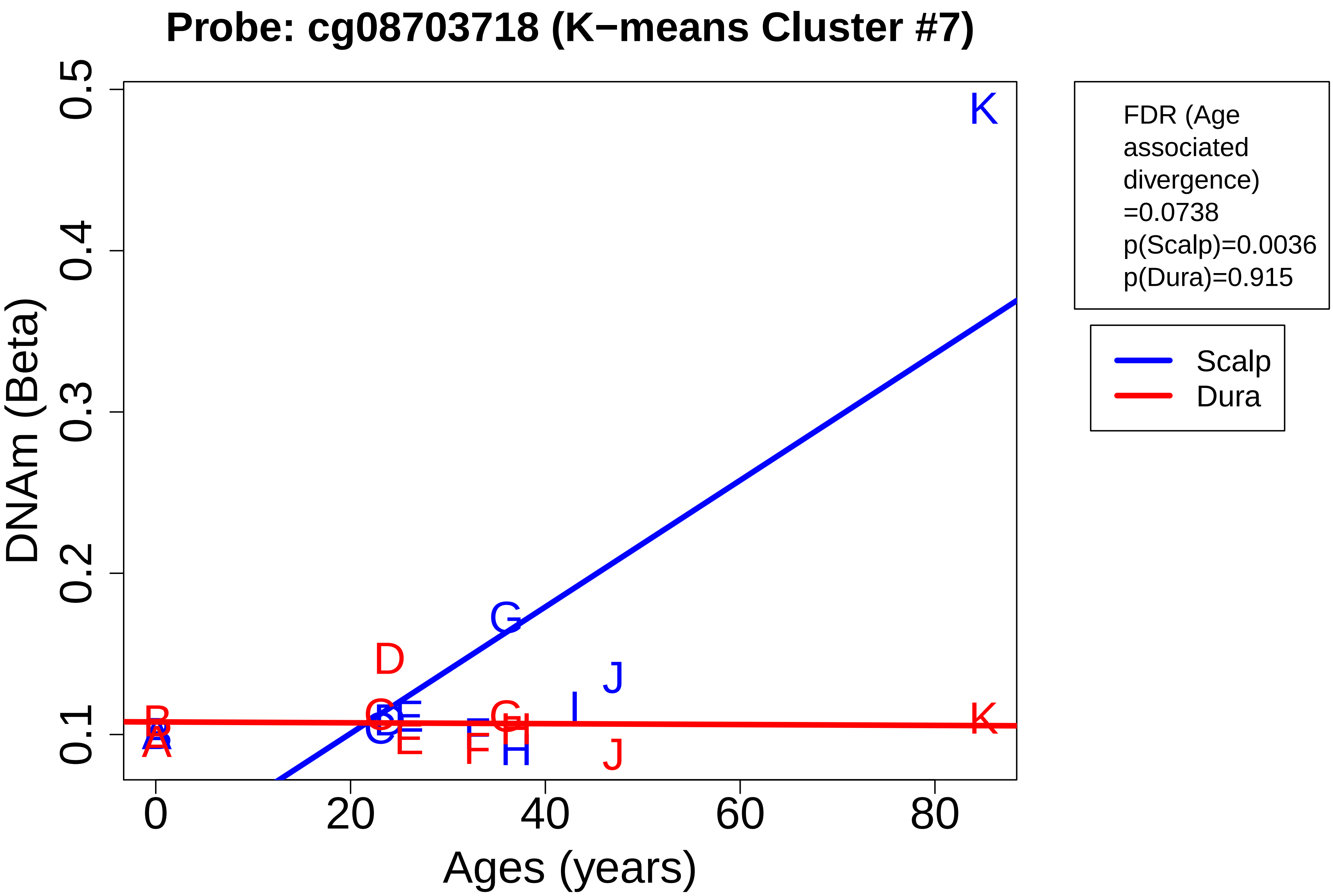

Probe: cg08703718 (K−means Cluster #7)
DNAm (Beta)
0.1
0.2
0.3
0.4
0.5
0
20
40
60
80
Ages (years)
FDR (Age associated divergence) =0.0738
p(Scalp)=0.0036
p(Dura)=0.915
Scalp
Dura

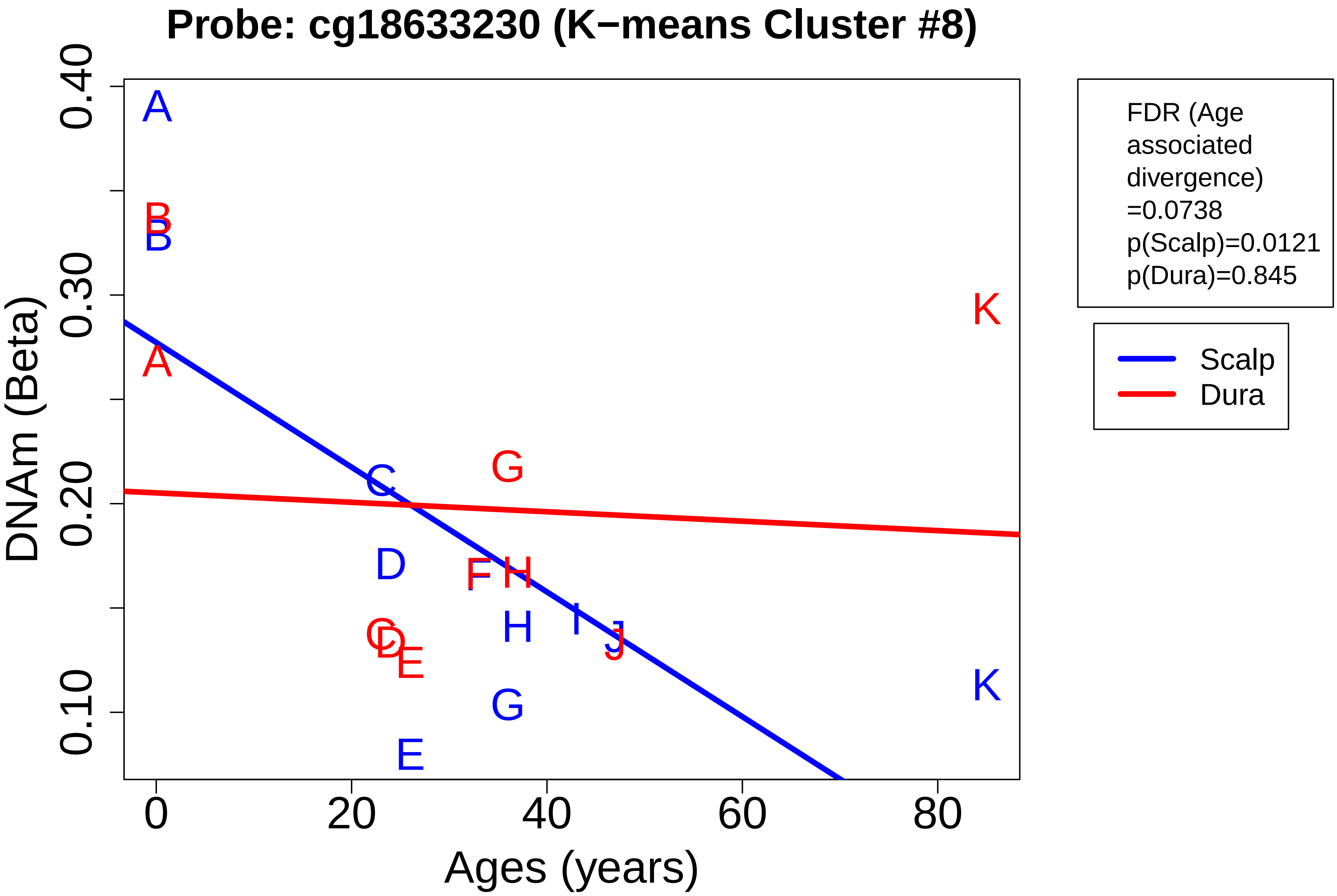

Probe: cg18633230 (K−means Cluster #8)
DNAm (Beta)
0.10
0.20
0.30
0.40
Ages (years)
0
20
40
60
80
A
B
C
D
E
F
G
H
I
J
K
FDR (Age associated divergence) =0.0738
p(Scalp)=0.0121
p(Dura)=0.845
Scalp
Dura

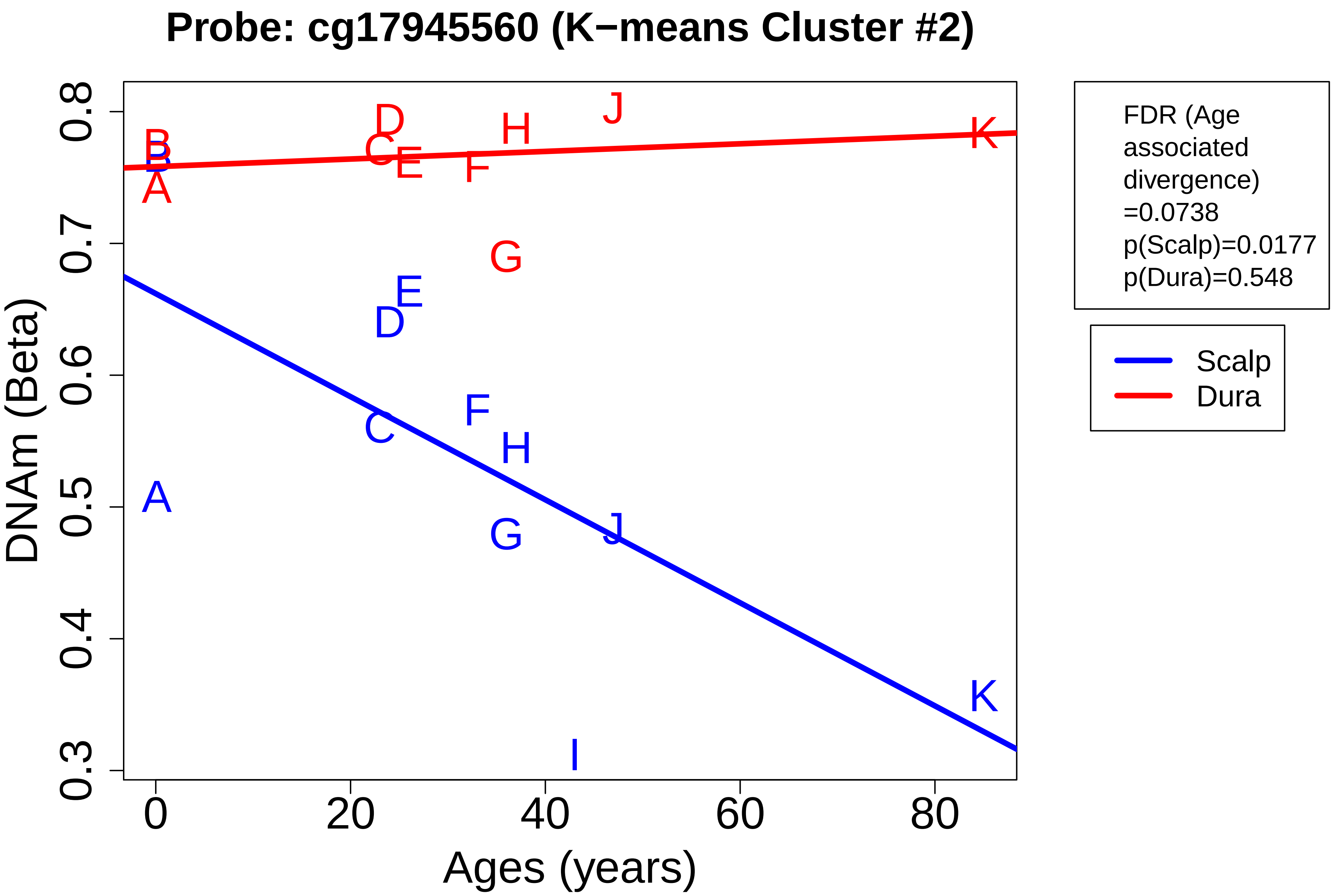

Probe: cg17945560 (K−means Cluster #2)
DNAm (Beta)
Ages (years)
0.3
0.4
0.5
0.6
0.7
0.8
0
20
40
60
80
FDR (Age associated divergence) =0.0738
p(Scalp)=0.0177
p(Dura)=0.548
Scalp
Dura
A
B
C
D
E
F
G
H
I
J
K

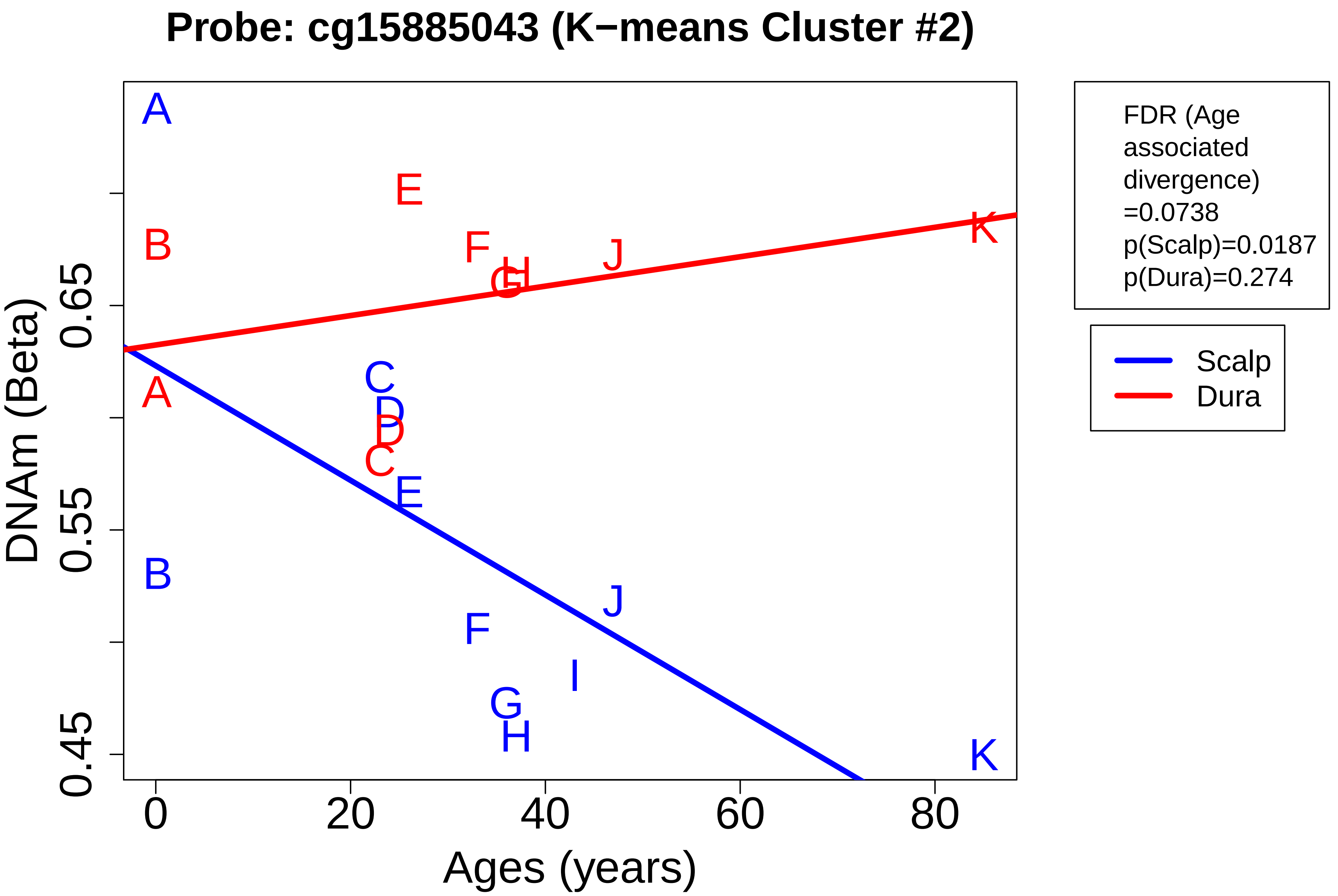

Probe: cg15885043 (K−means Cluster #2)
DNAm (Beta)
0.45
0.55
0.65
0
20
40
60
80
Ages (years)
FDR (Age associated divergence) =0.0738
p(Scalp)=0.0187
p(Dura)=0.274
Scalp
Dura

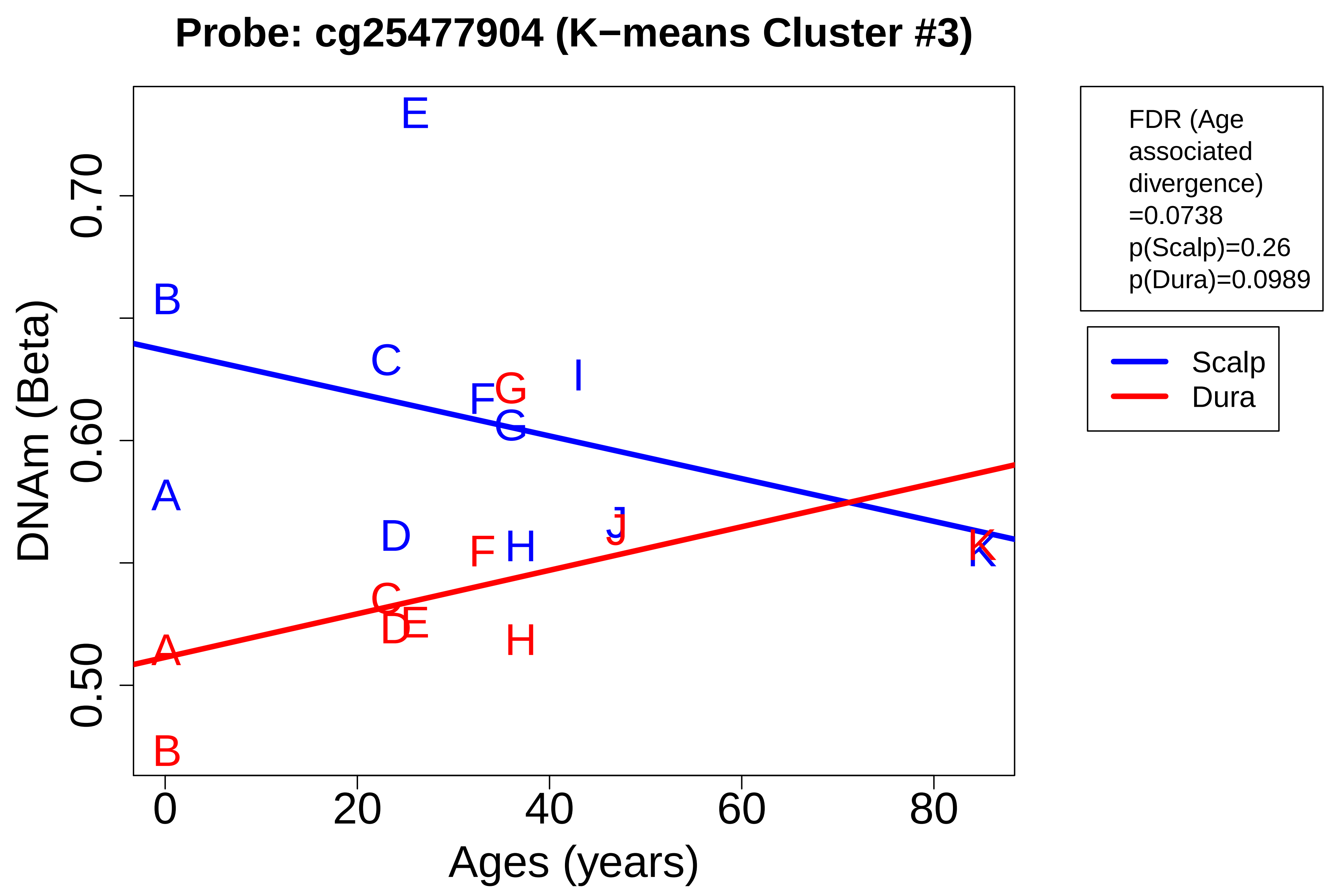

Probe: cg25477904 (K−means Cluster #3)
DNAm (Beta)
0.50
0.60
0.70
Ages (years)
0
20
40
60
80
FDR (Age associated divergence) =0.0738
p(Scalp)=0.26
p(Dura)=0.0989
Scalp
Dura

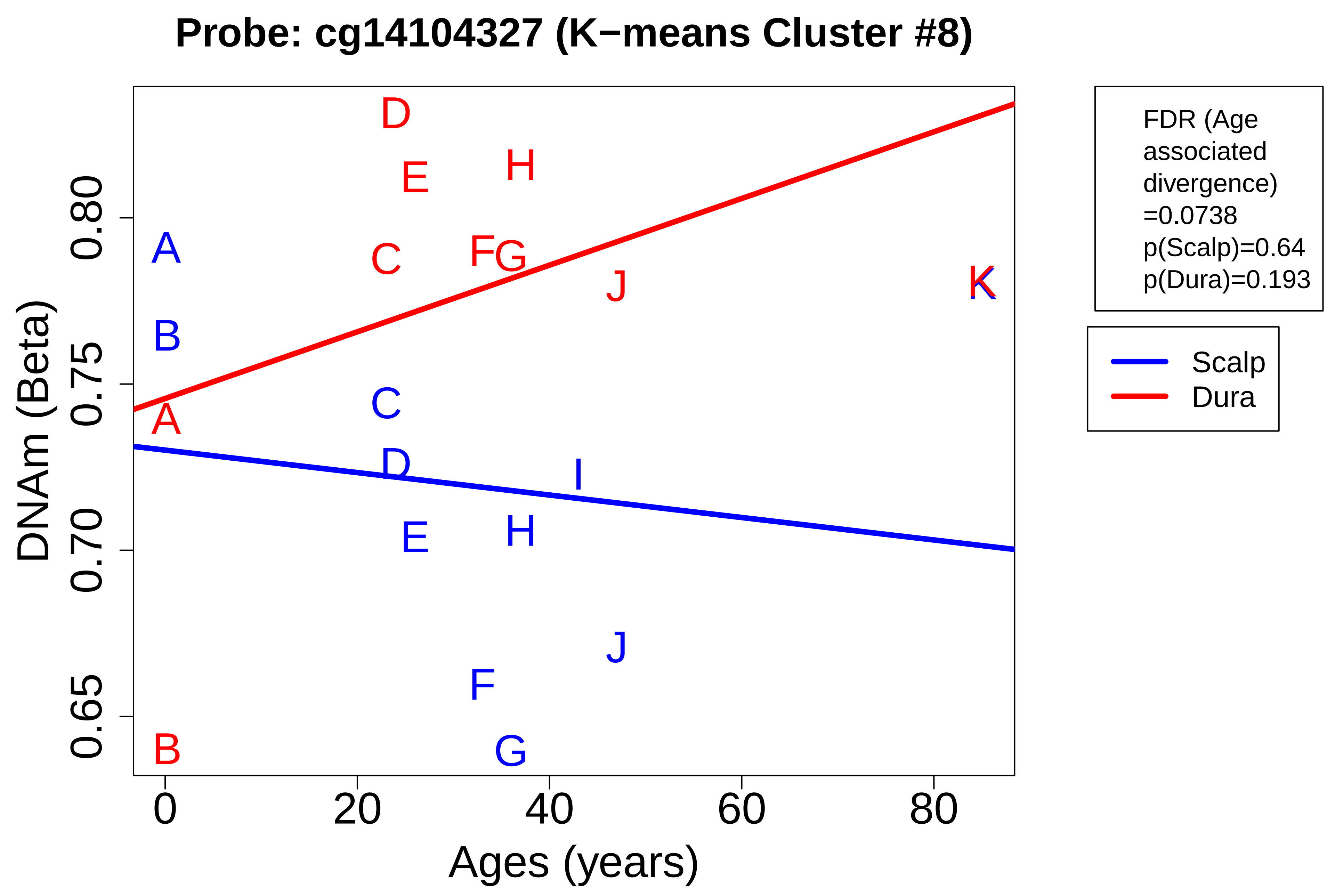

Probe: cg14104327 (K−means Cluster #8)
DNAm (Beta)
0.65
0.70
0.75
0.80
Ages (years)
0
20
40
60
80
FDR (Age associated divergence) =0.0738
p(Scalp)=0.64
p(Dura)=0.193
Scalp
Dura

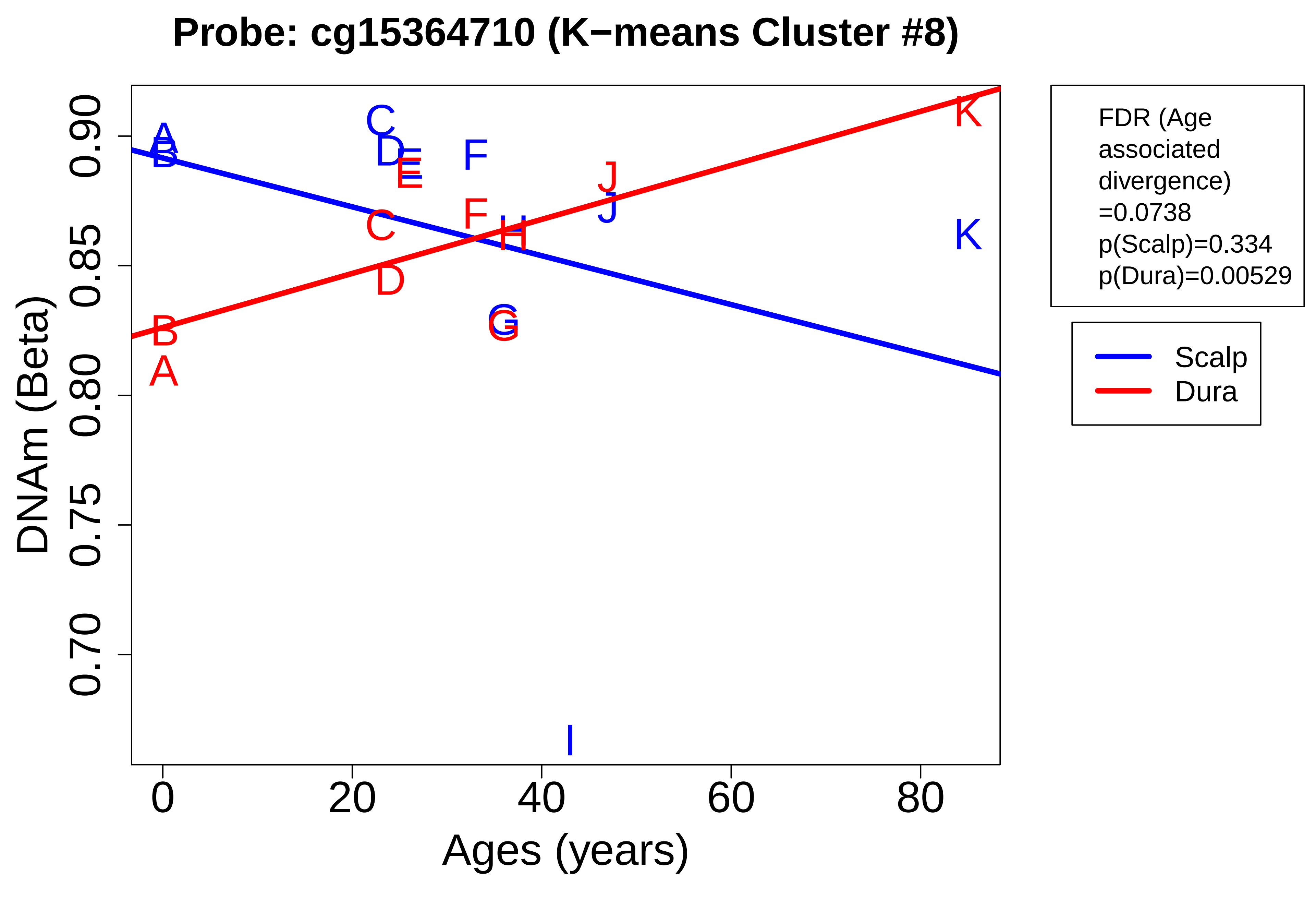

Probe: cg15364710 (K−means Cluster #8)
DNAm (Beta)
0.70
0.75
0.80
0.85
0.90
Ages (years)
0
20
40
60
80
FDR (Age associated divergence) =0.0738
p(Scalp)=0.334
p(Dura)=0.00529
Scalp
Dura

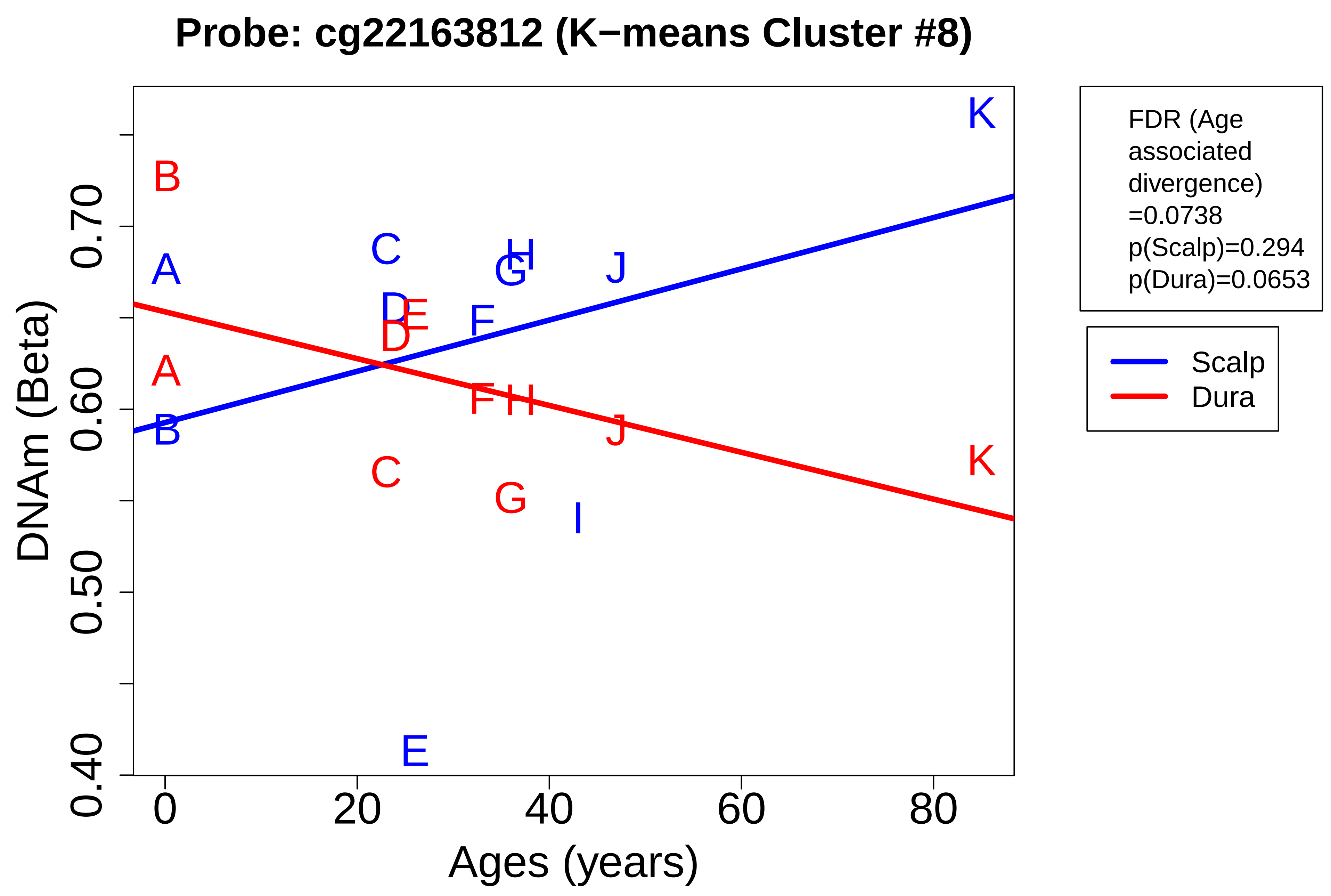

Probe: cg22163812 (K−means Cluster #8)
DNAm (Beta)
0.40
0.50
0.60
0.70
Ages (years)
0
20
40
60
80
A
B
C
D
E
F
G
H
I
J
K
FDR (Age associated divergence) =0.0738
p(Scalp)=0.294
p(Dura)=0.0653
Scalp
Dura

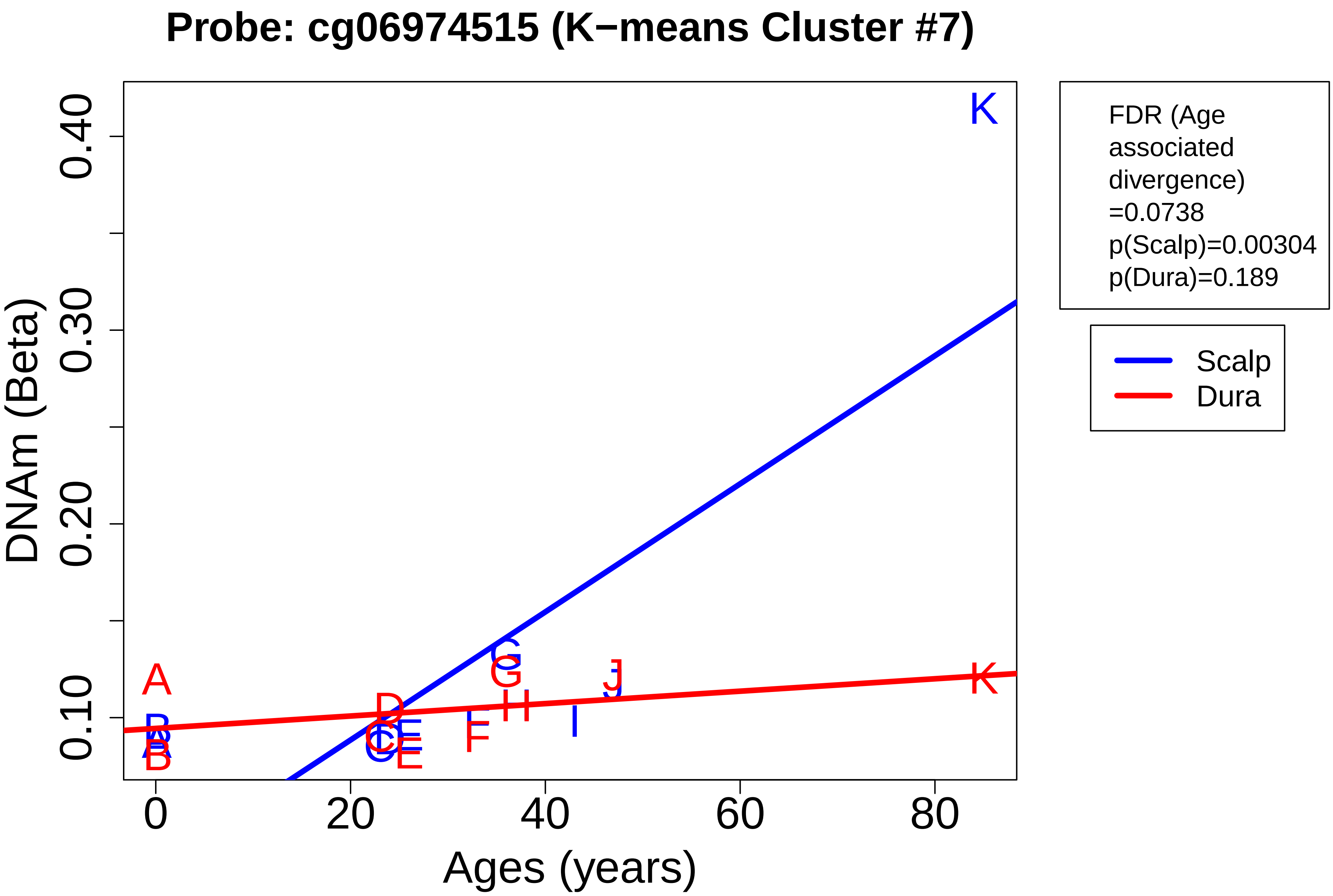

Probe: cg06974515 (K−means Cluster #7)
DNAm (Beta)
0.10
0.20
0.30
0.40
0
20
40
60
80
Ages (years)
FDR (Age associated divergence) =0.0738
p(Scalp)=0.00304
p(Dura)=0.189
Scalp
Dura
A
B
C
D
E
F
G
H
I
J
K

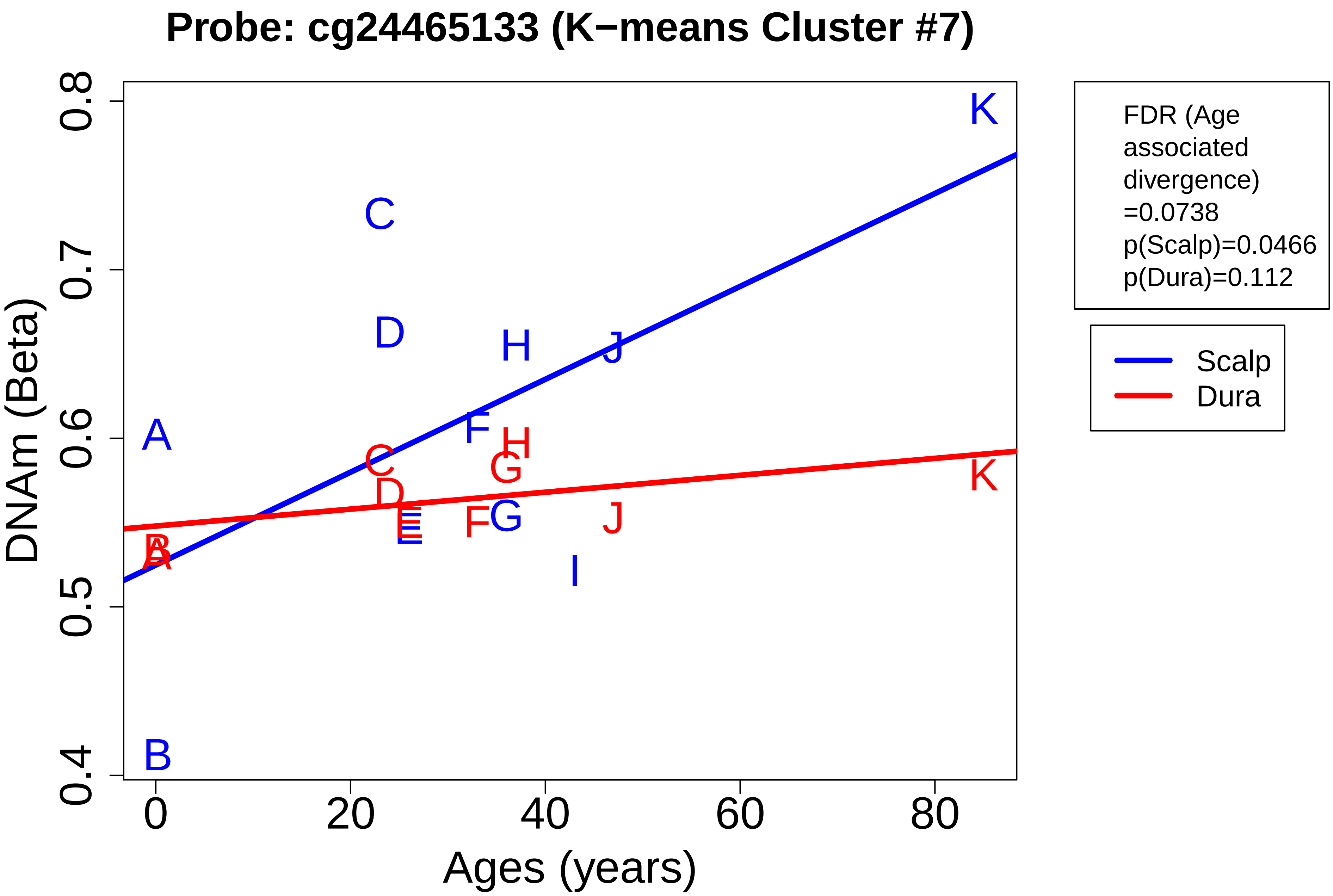

Probe: cg24465133 (K−means Cluster #7)
DNAm (Beta)
Ages (years)
0.4
0.5
0.6
0.7
0.8
0
20
40
60
80
FDR (Age associated divergence) =0.0738
p(Scalp)=0.0466
p(Dura)=0.112
Scalp
Dura

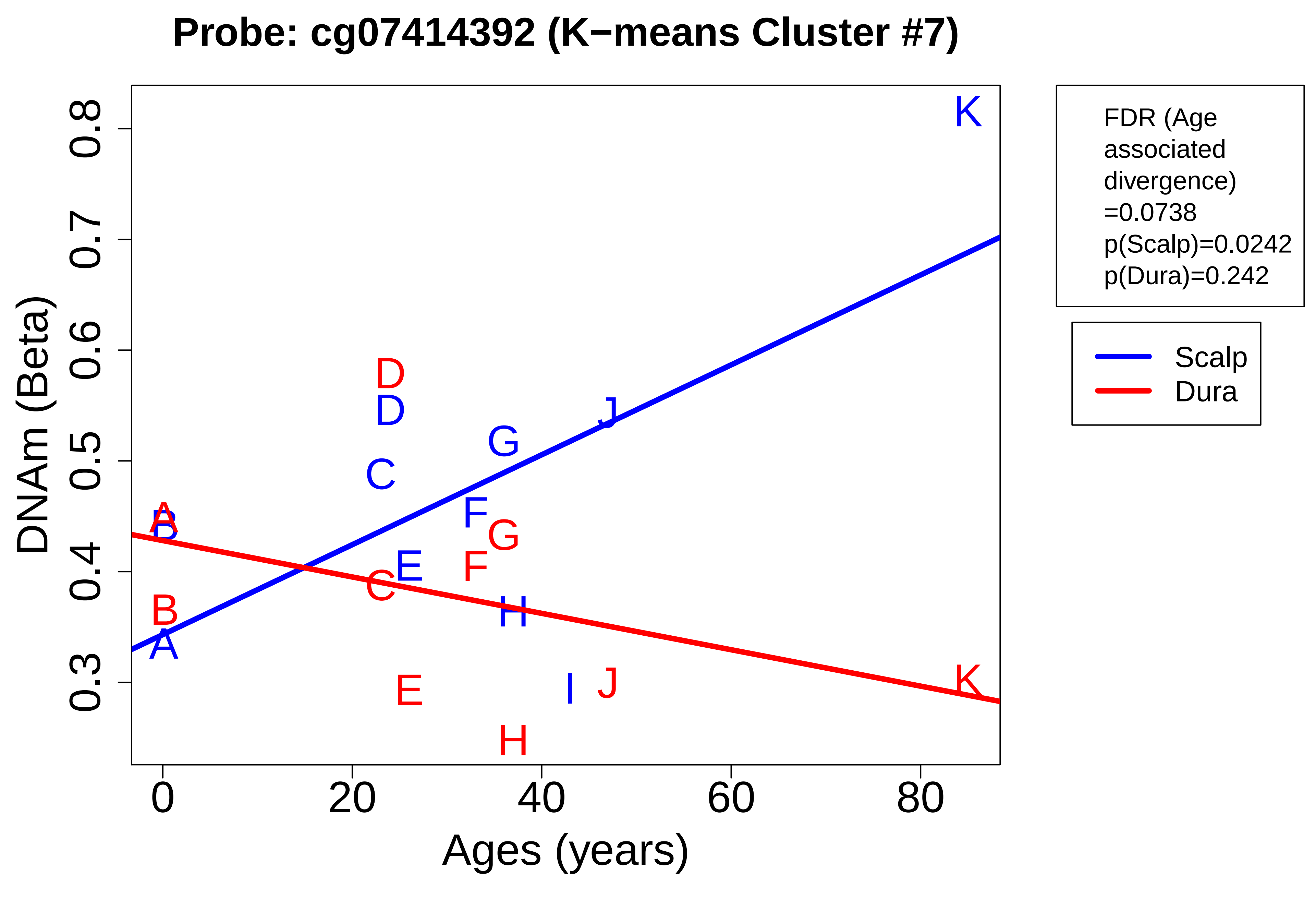

Probe: cg07414392 (K−means Cluster #7)
DNAm (Beta)
0.3
0.4
0.5
0.6
0.7
0.8
0
20
40
60
80
Ages (years)
FDR (Age associated divergence) =0.0738
p(Scalp)=0.0242
p(Dura)=0.242
Scalp
Dura

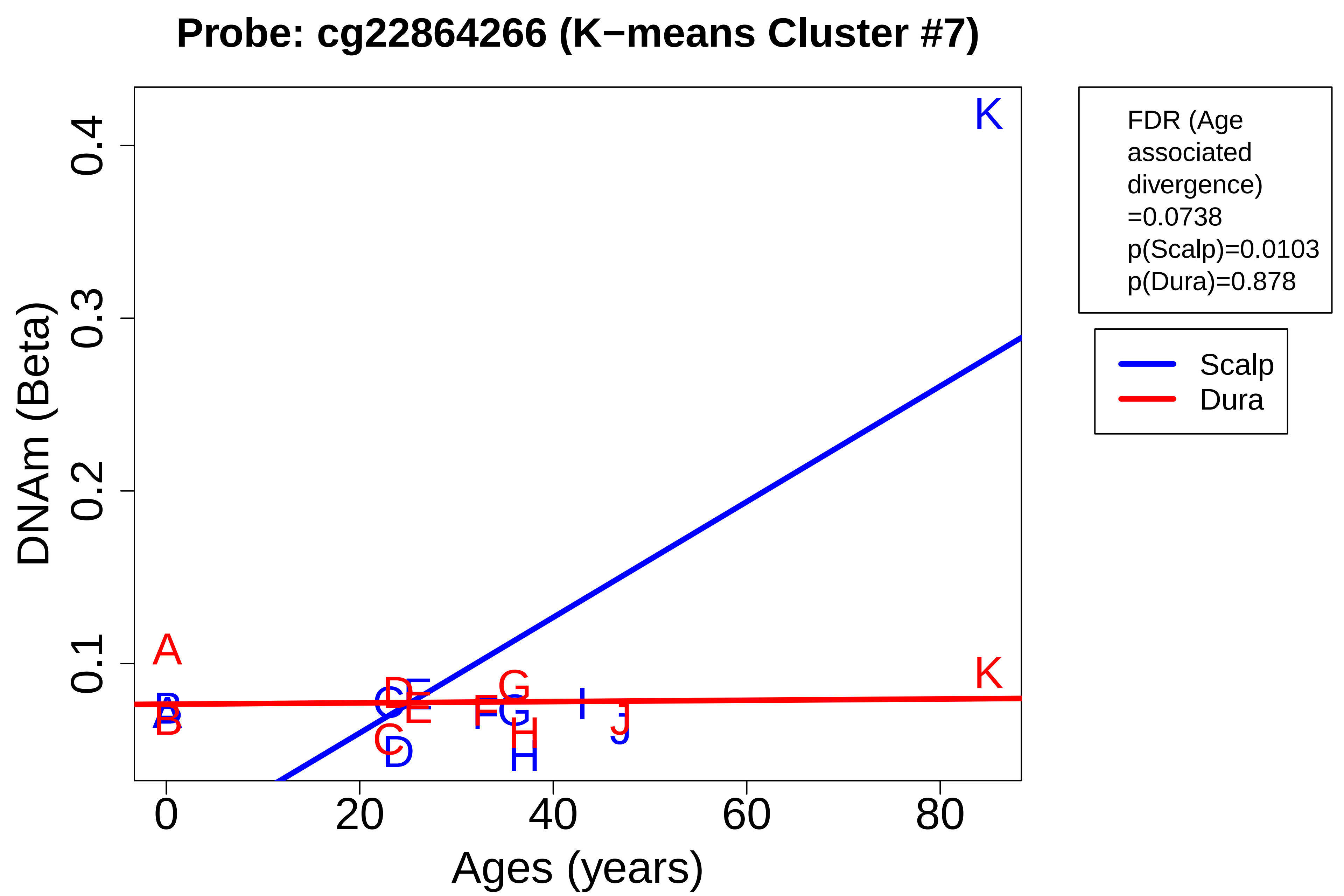

Probe: cg22864266 (K−means Cluster #7)
DNAm (Beta)
0.1
0.2
0.3
0.4
0
20
40
60
80
Ages (years)
FDR (Age associated divergence) =0.0738
p(Scalp)=0.0103
p(Dura)=0.878
Scalp
Dura
A
B
C
D
E
F
G
H
I
J
K

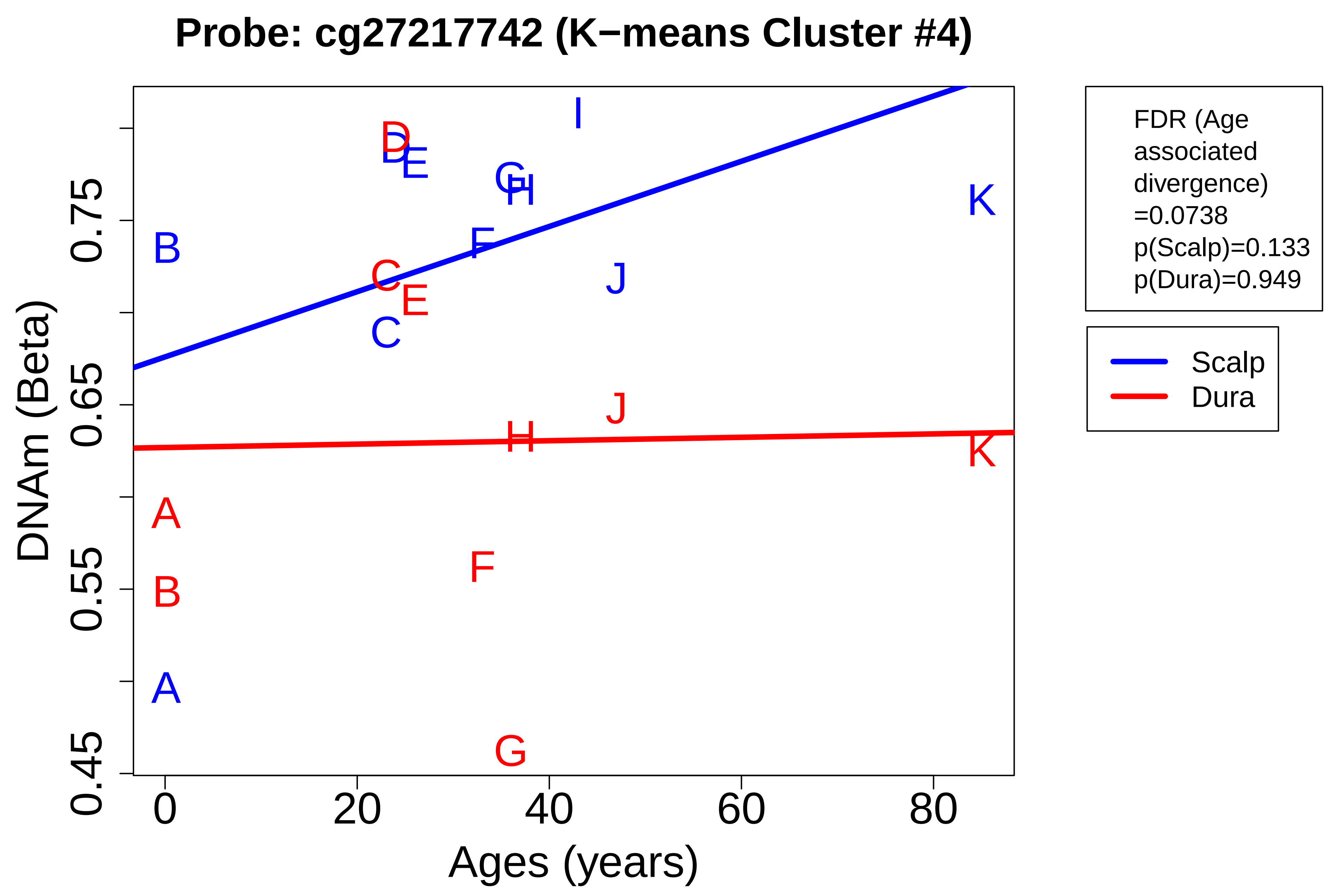

Probe: cg27217742 (K−means Cluster #4)
DNAm (Beta)
0.45
0.55
0.65
0.75
Ages (years)
0
20
40
60
80
A
B
C
D
E
F
G
H
I
J
K
FDR (Age associated divergence) =0.0738
p(Scalp)=0.133
p(Dura)=0.949
Scalp
Dura

Probe: cg02459016 (K−means Cluster #1)

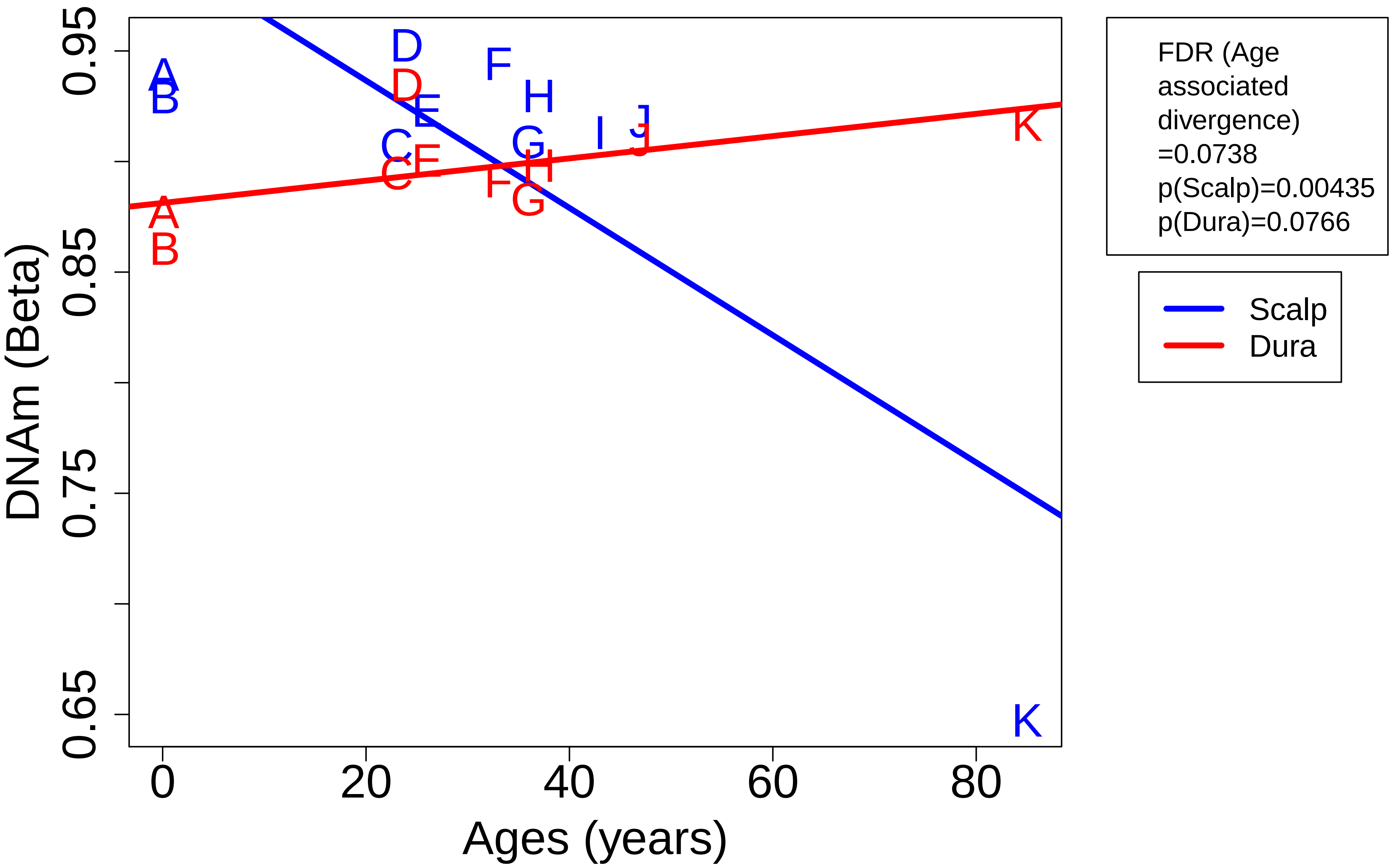

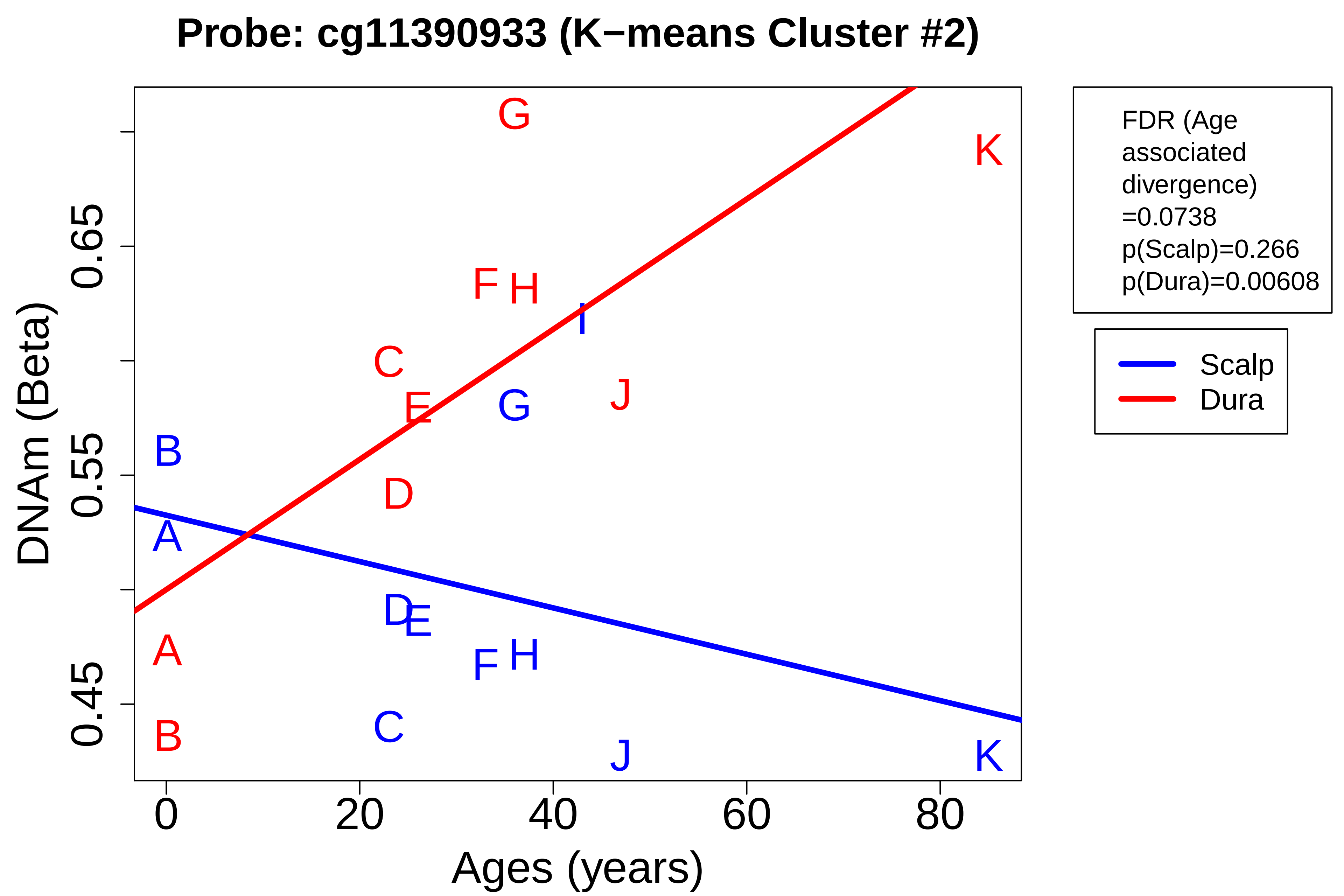

Probe: cg11390933 (K−means Cluster #2)
DNAm (Beta)
0.45
0.55
0.65
Ages (years)
0
20
40
60
80
FDR (Age associated divergence) =0.0738
p(Scalp)=0.266
p(Dura)=0.00608
Scalp
Dura

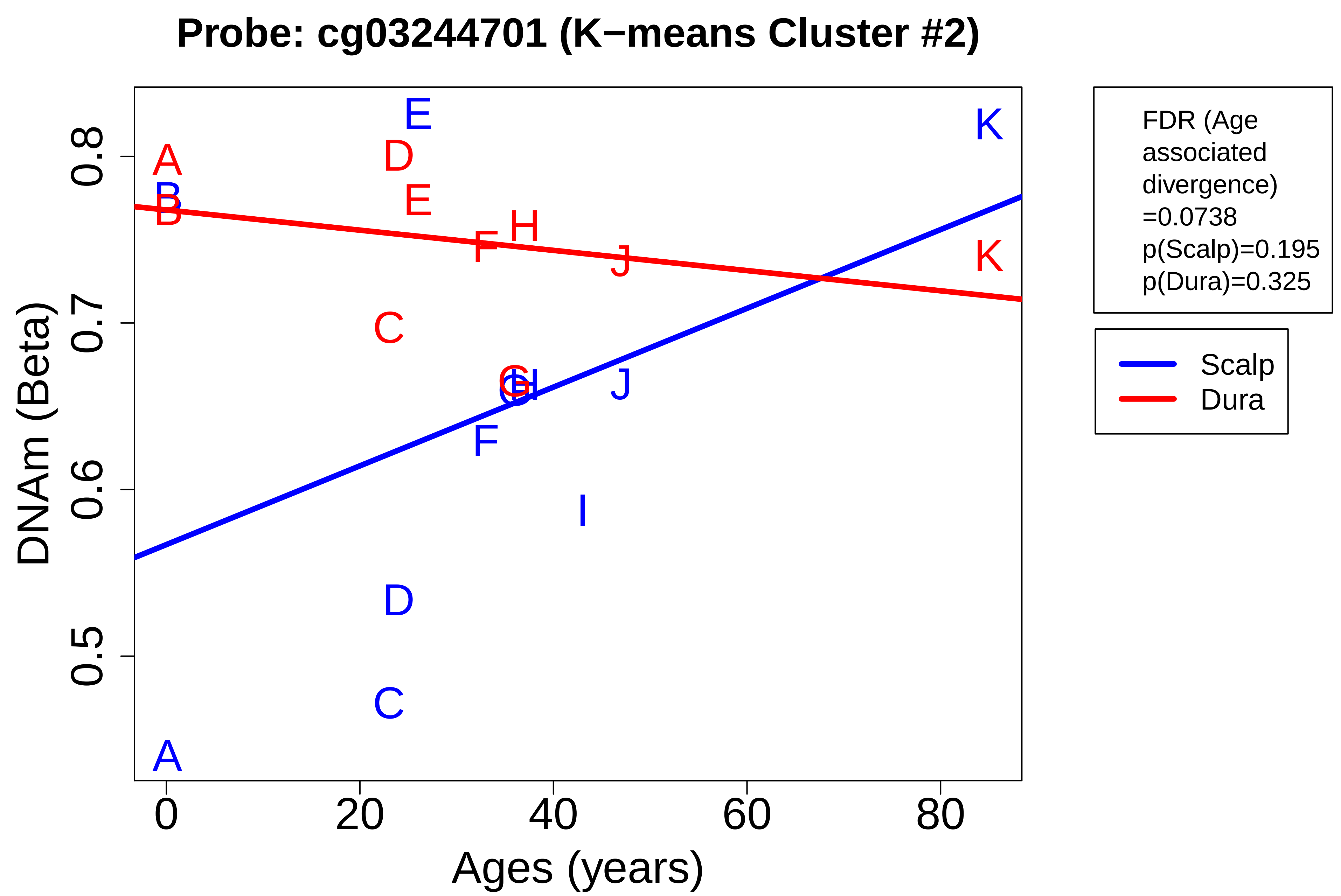

Probe: cg03244701 (K−means Cluster #2)
DNAm (Beta)
0.5
0.6
0.7
0.8
0
20
40
60
80
Ages (years)
A
B
C
D
E
F
G
H
I
J
K
FDR (Age associated divergence) =0.0738
p(Scalp)=0.195
p(Dura)=0.325
Scalp
Dura

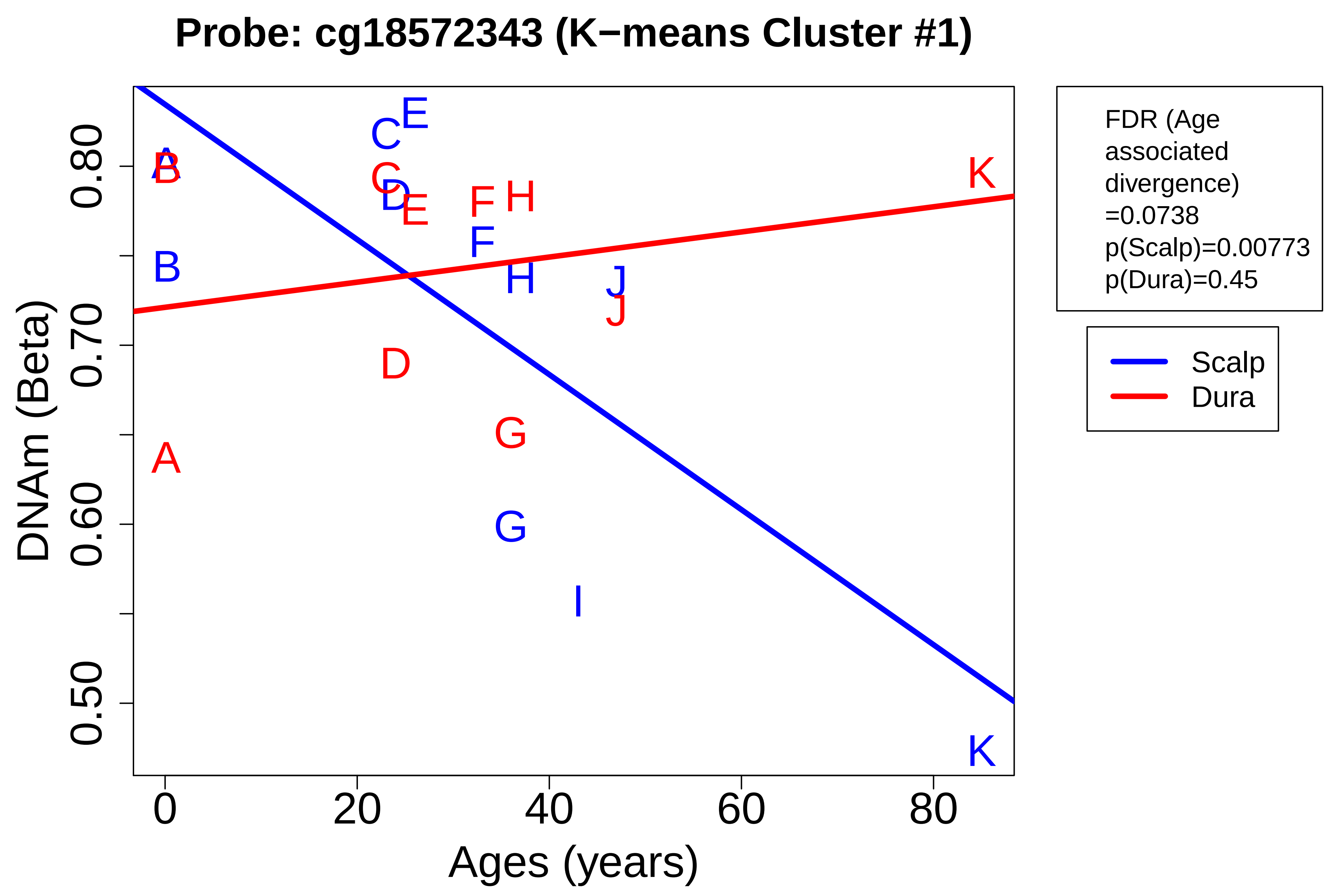

Probe: cg18572343 (K−means Cluster #1)
DNAm (Beta)
0.50
0.60
0.70
0.80
0
20
40
60
80
Ages (years)
FDR (Age associated divergence) =0.0738
p(Scalp)=0.00773
p(Dura)=0.45
Scalp
Dura
A
B
C
D
E
F
G
H
I
J
K

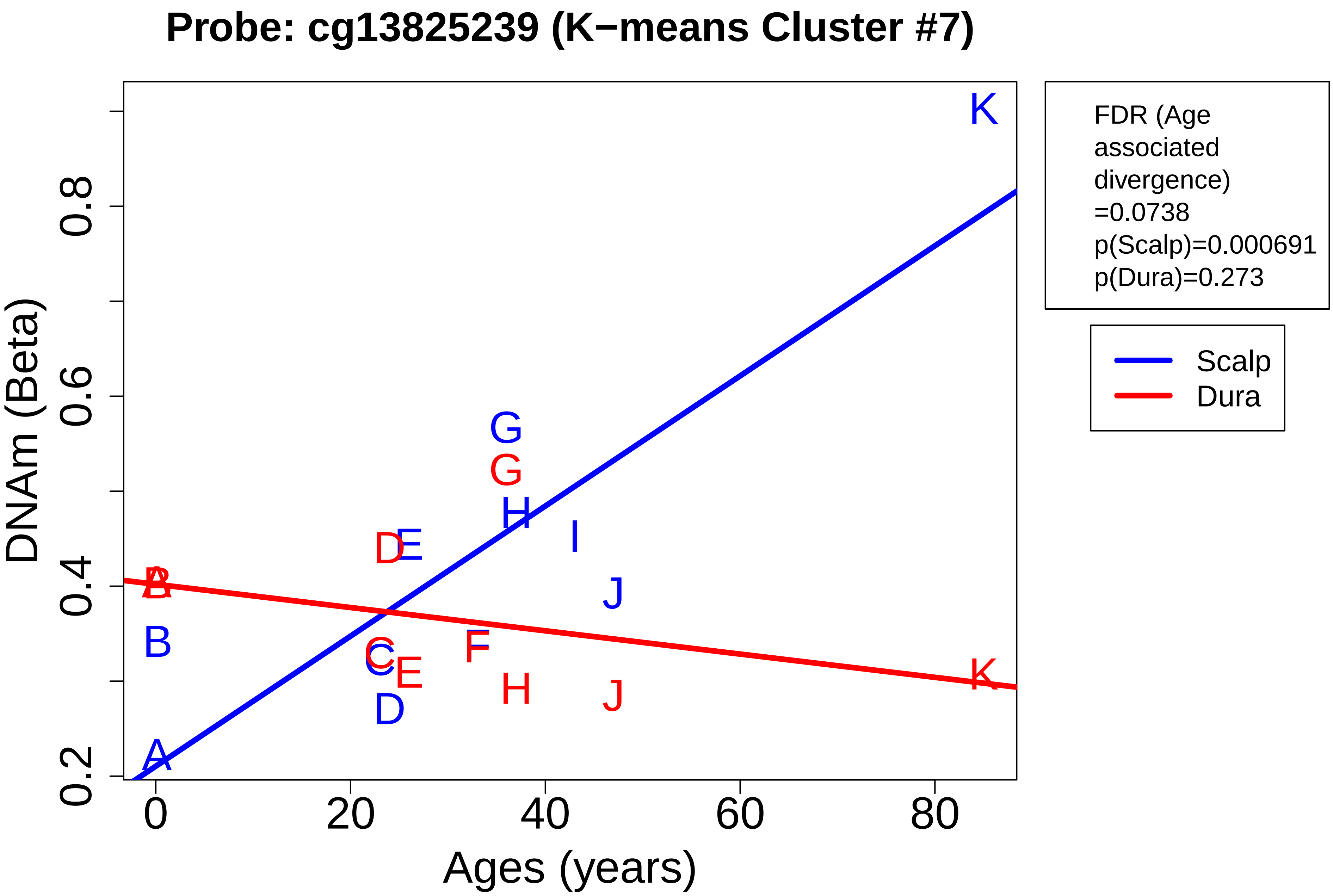

Probe: cg13825239 (K−means Cluster #7)
DNAm (Beta)
0.2
0.4
0.6
0.8
0
20
40
60
80
Ages (years)
A
B
C
D
E
F
G
H
I
J
K
FDR (Age
associated
divergence)
=0.0738
p(Scalp)=0.000691
p(Dura)=0.273
Scalp
Dura

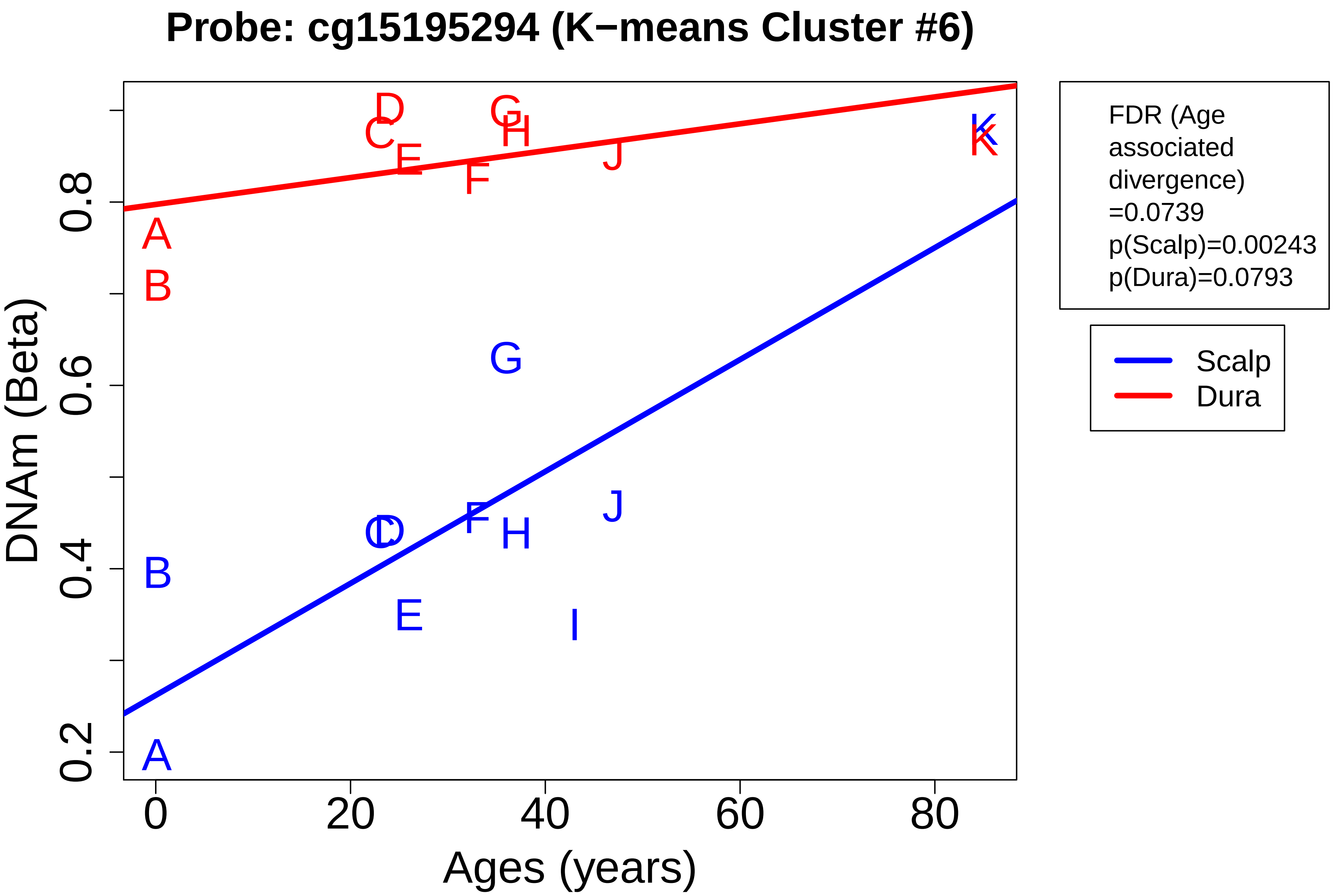

Probe: cg15195294 (K−means Cluster #6)
DNAm (Beta)
0.2
0.4
0.6
0.8
0
20
40
60
80
Ages (years)
FDR (Age associated divergence) =0.0739
p(Scalp)=0.00243
p(Dura)=0.0793
Scalp
Dura

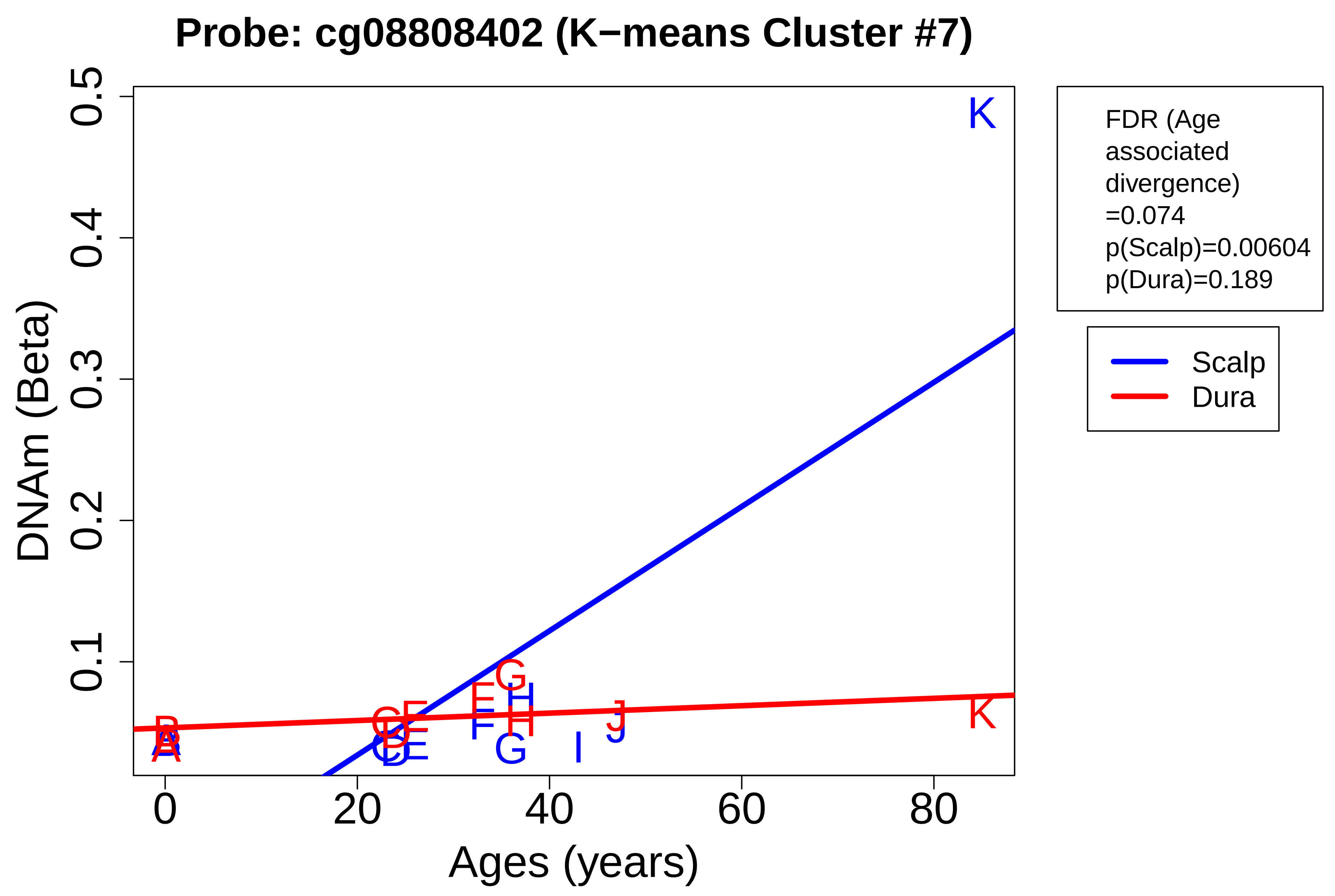
Probe: cg08808402 (K−means Cluster #7)
DNAm (Beta)
0.1
0.2
0.3
0.4
0.5
Ages (years)
0
20
40
60
80
FDR (Age associated divergence) =0.074
p(Scalp)=0.00604
p(Dura)=0.189
Scalp
Dura
A
B
C
D
E
F
G
H
I
J
K

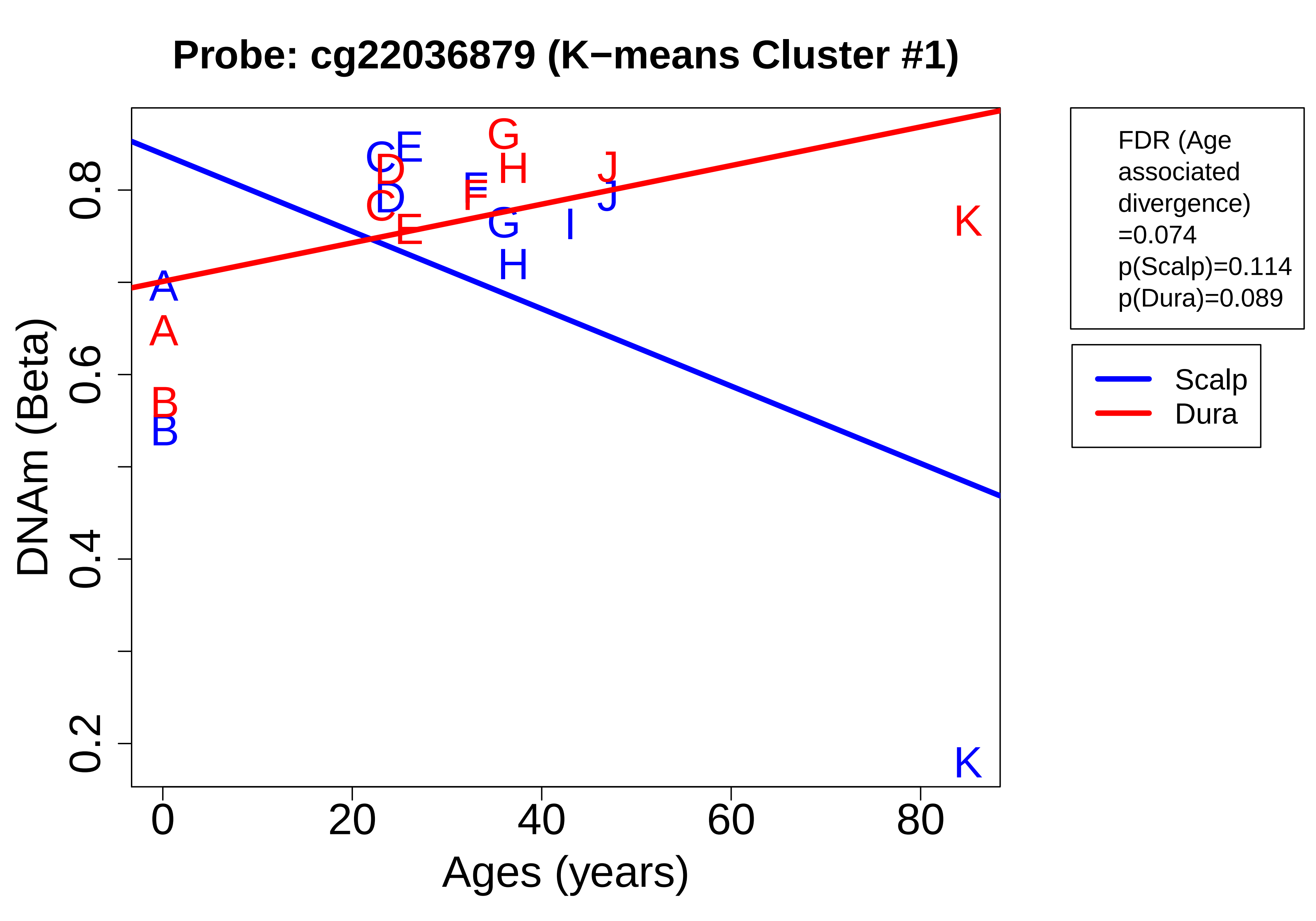
Probe: cg22036879 (K−means Cluster #1)
DNAm (Beta)
0.2
0.4
0.6
0.8
Ages (years)
0
20
40
60
80
FDR (Age associated divergence) =0.074
p(Scalp)=0.114
p(Dura)=0.089
Scalp
Dura

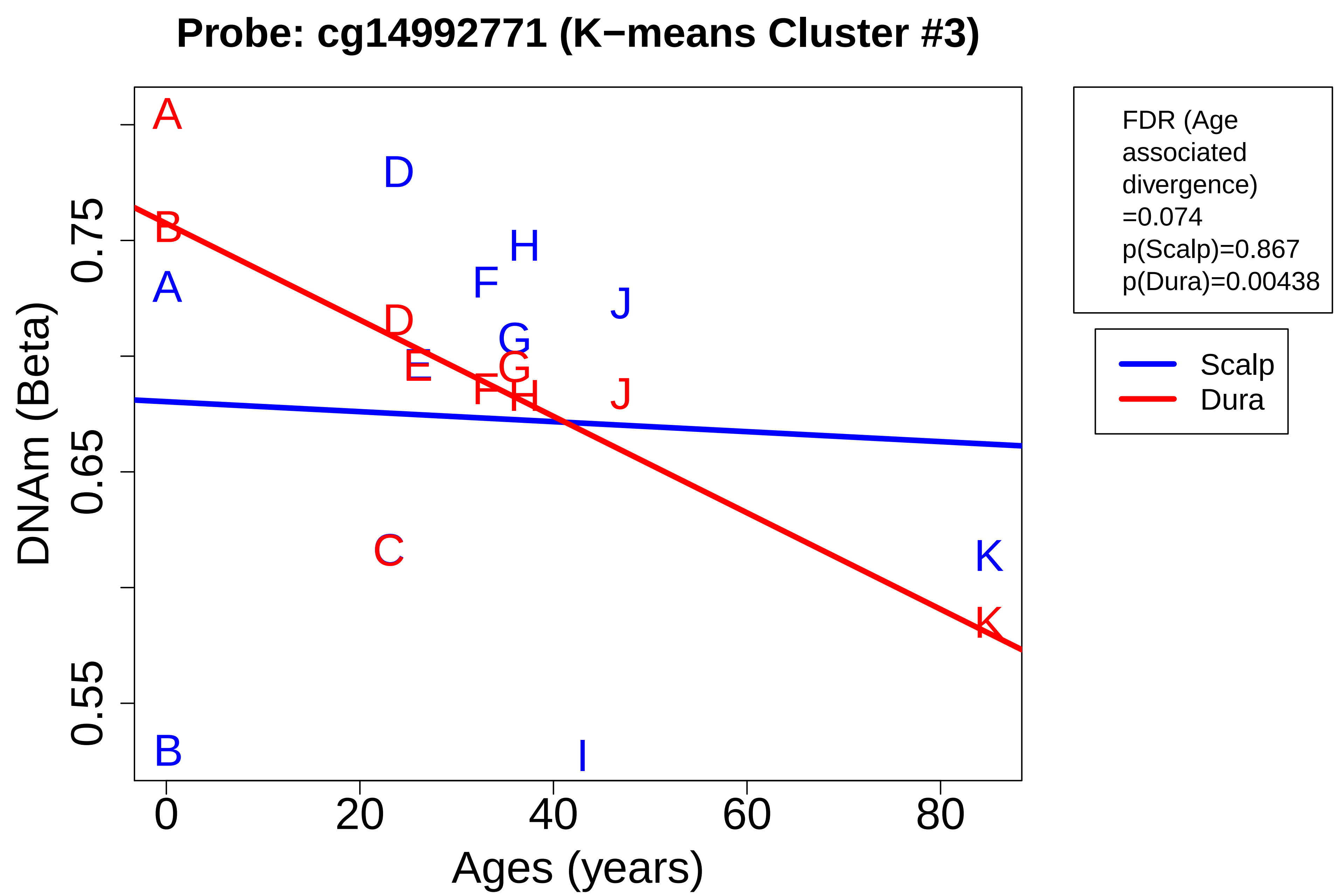

Probe: cg14992771 (K−means Cluster #3)
DNAm (Beta)
0.55
0.65
0.75
Ages (years)
0
20
40
60
80
A
B
C
D
E
F
G
H
I
J
K
FDR (Age associated divergence) =0.074
p(Scalp)=0.867
p(Dura)=0.00438
Scalp
Dura

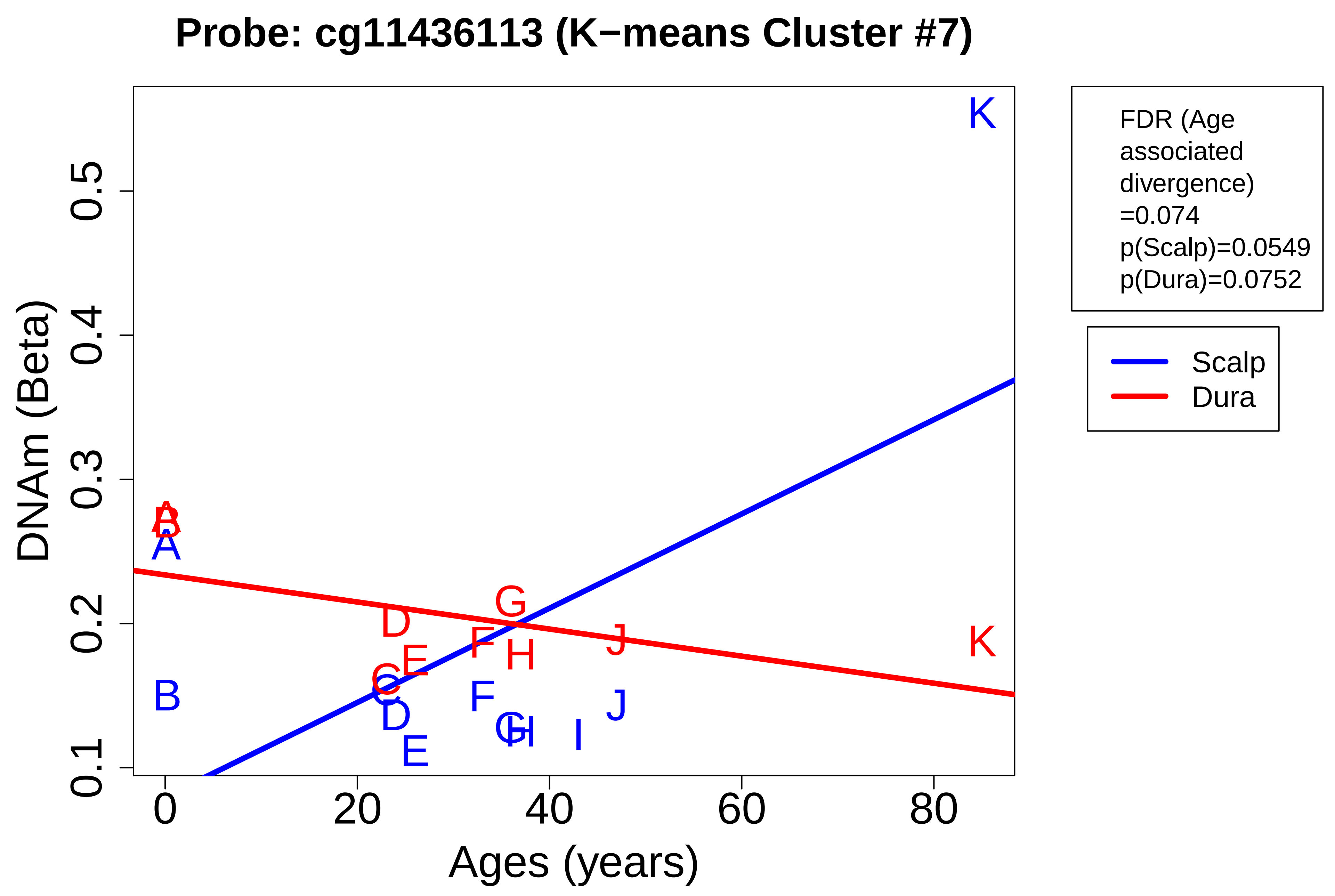

Probe: cg11436113 (K−means Cluster #7)
DNAm (Beta)
0.1
0.2
0.3
0.4
0.5
0
20
40
60
80
Ages (years)
FDR (Age associated divergence) =0.074
p(Scalp)=0.0549
p(Dura)=0.0752
Scalp
Dura

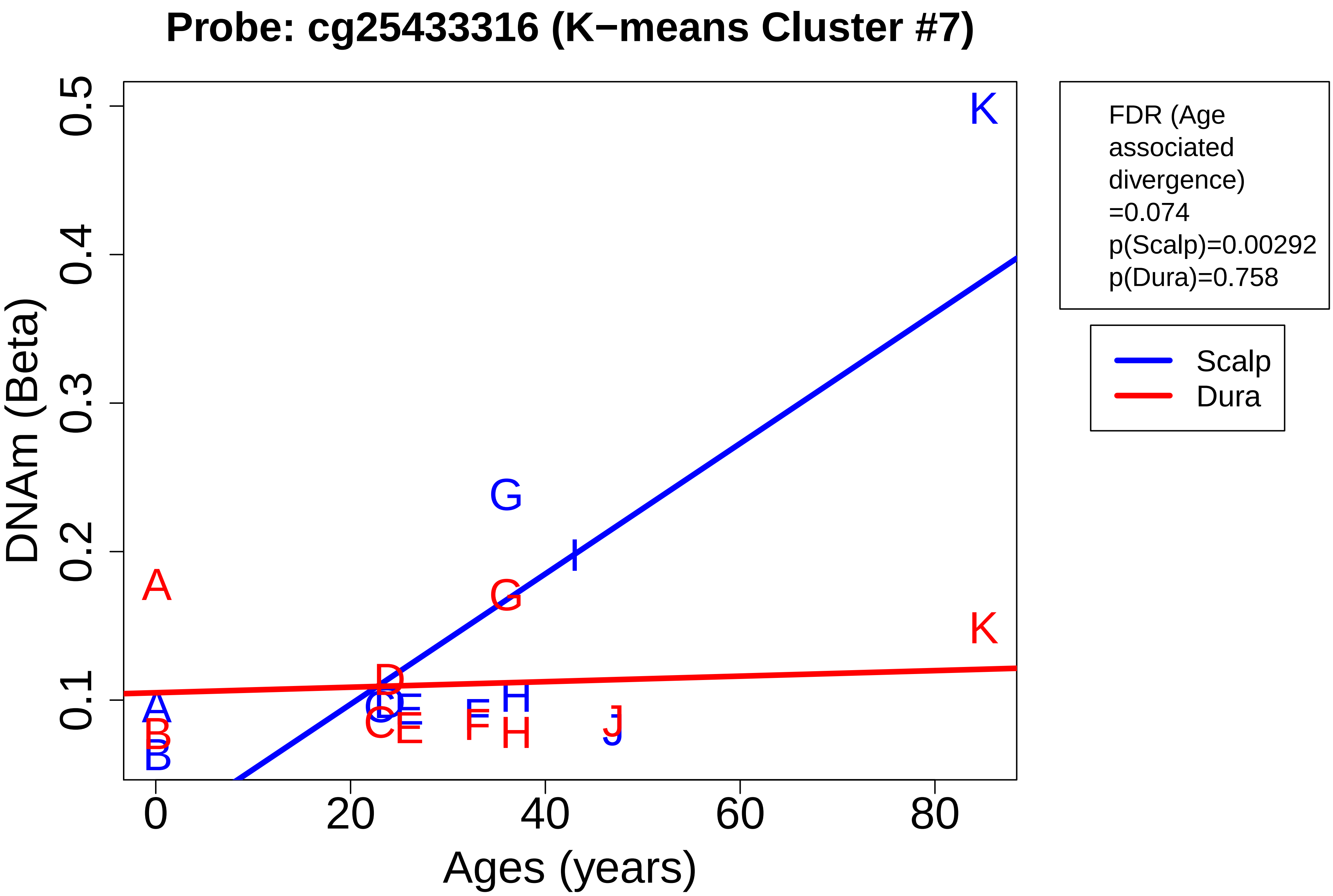

Probe: cg25433316 (K−means Cluster #7)
DNAm (Beta)
0.1
0.2
0.3
0.4
0.5
Ages (years)
0
20
40
60
80
A
B
C
D
E
F
G
H
I
J
K
FDR (Age associated divergence) =0.074
p(Scalp)=0.00292
p(Dura)=0.758
Scalp
Dura

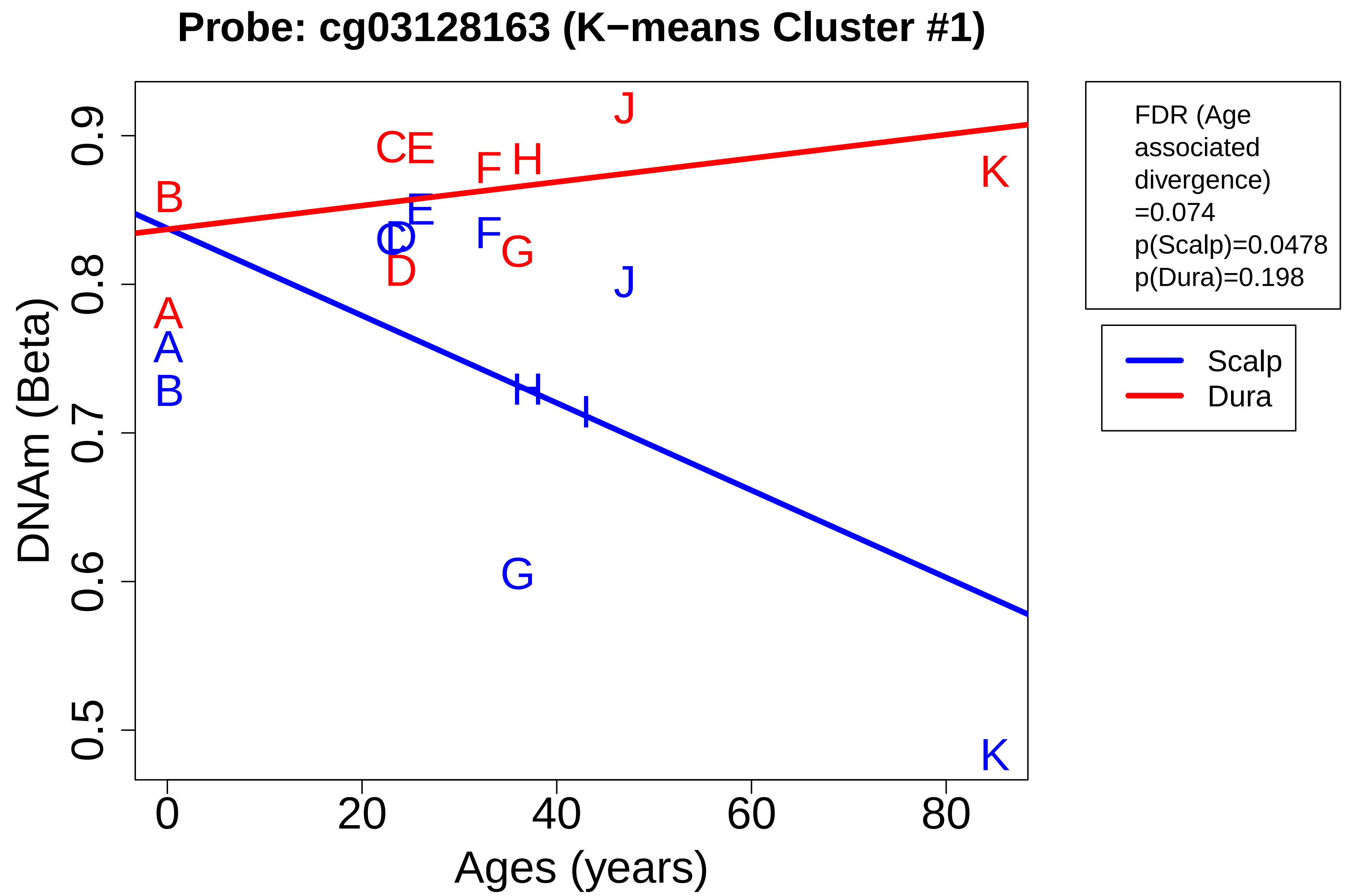

Probe: cg03128163 (K−means Cluster #1)
DNAm (Beta)
0.5
0.6
0.7
0.8
0.9
Ages (years)
0
20
40
60
80
FDR (Age associated divergence) =0.074
p(Scalp)=0.0478
p(Dura)=0.198
Scalp
Dura
A
B
C
D
E
F
G
H
I
J
K

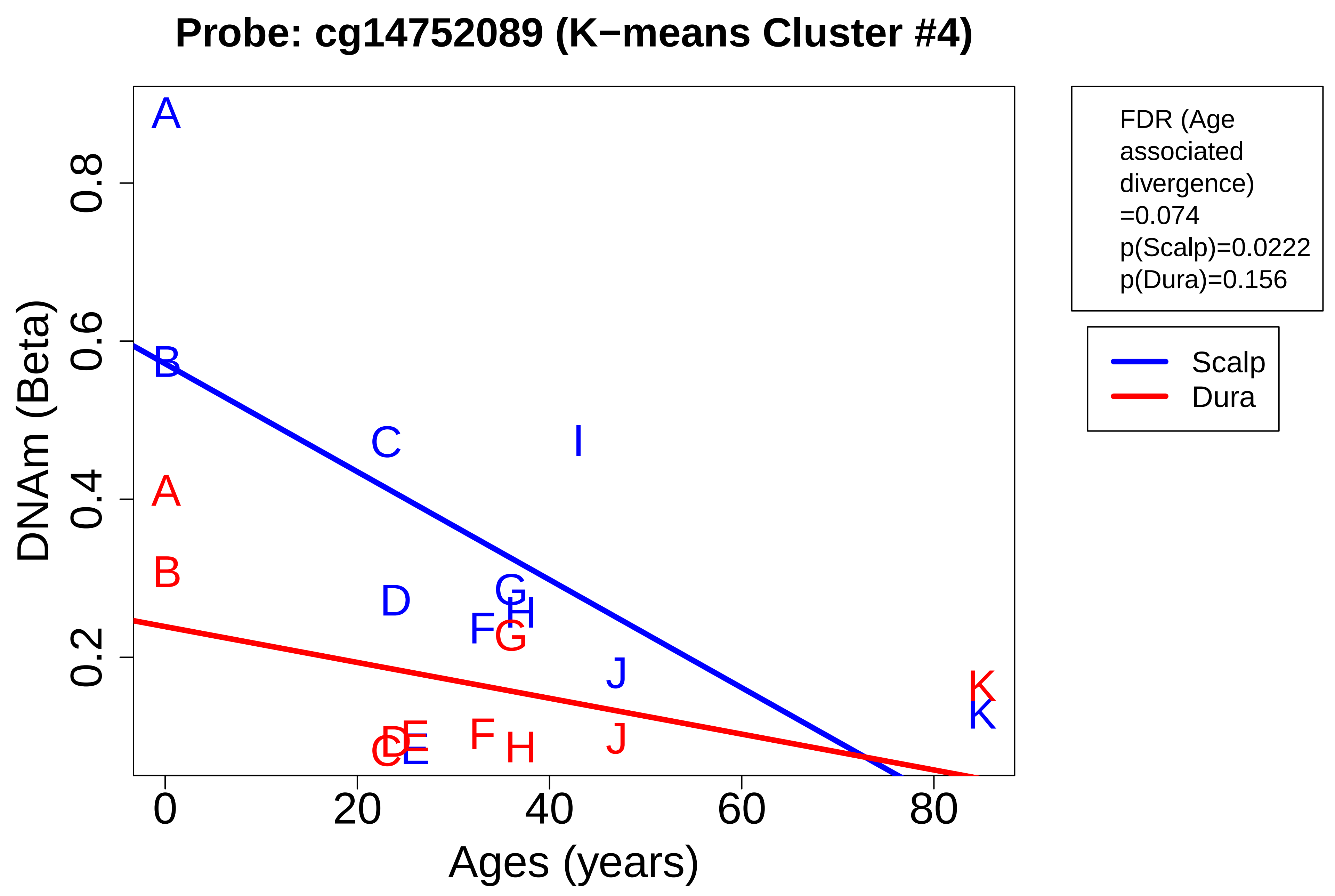

Probe: cg14752089 (K−means Cluster #4)
DNAm (Beta)
0.2
0.4
0.6
0.8
Ages (years)
0
20
40
60
80
A
B
C
D
E
F
G
H
I
J
K
FDR (Age associated divergence) =0.074
p(Scalp)=0.0222
p(Dura)=0.156
Scalp
Dura

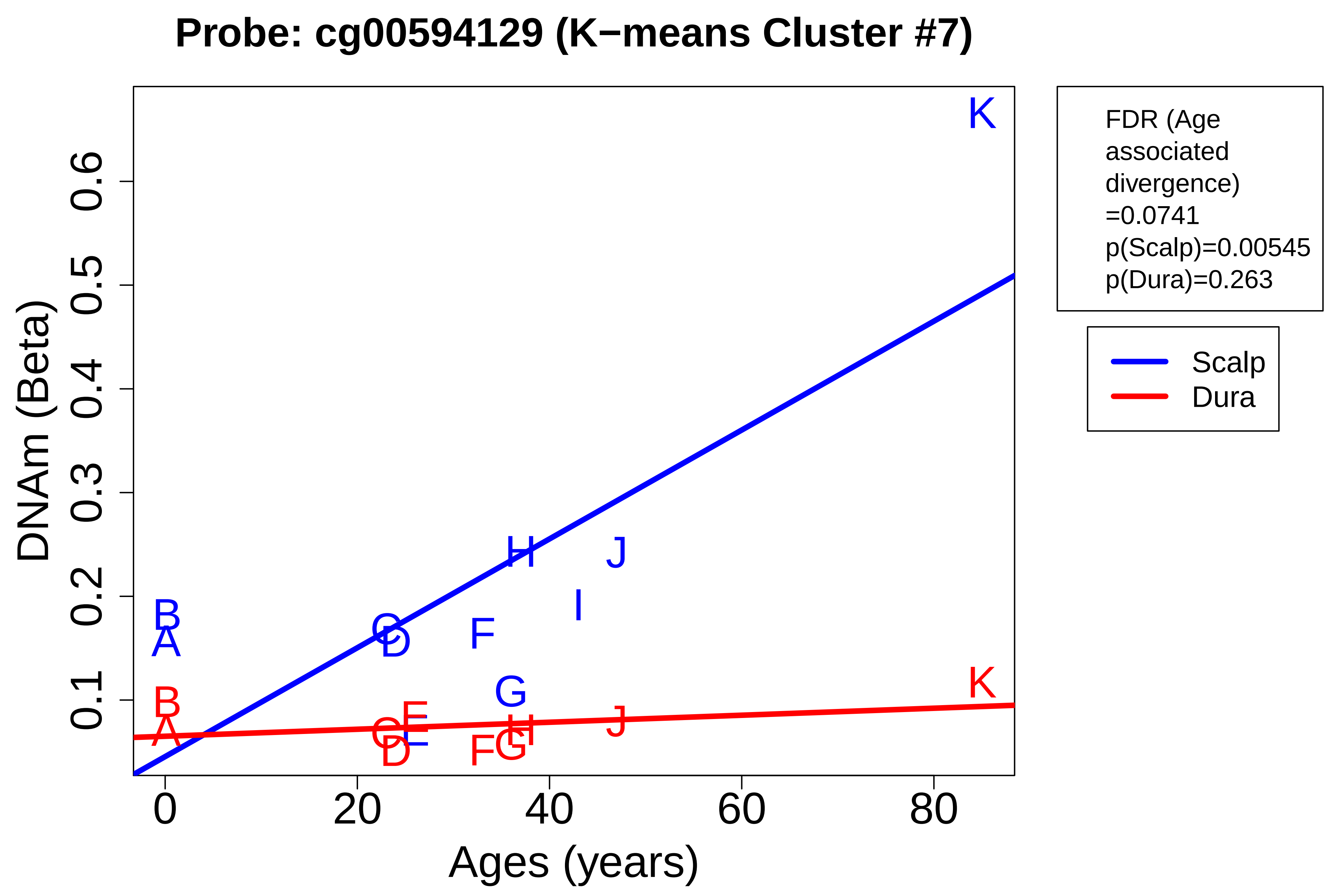

Probe: cg00594129 (K−means Cluster #7)
DNAm (Beta)
0.1
0.2
0.3
0.4
0.5
0.6
Ages (years)
0
20
40
60
80
FDR (Age associated divergence) =0.0741
p(Scalp)=0.00545
p(Dura)=0.263
Scalp
Dura
A
B
C
D
E
F
G
H
I
J
K

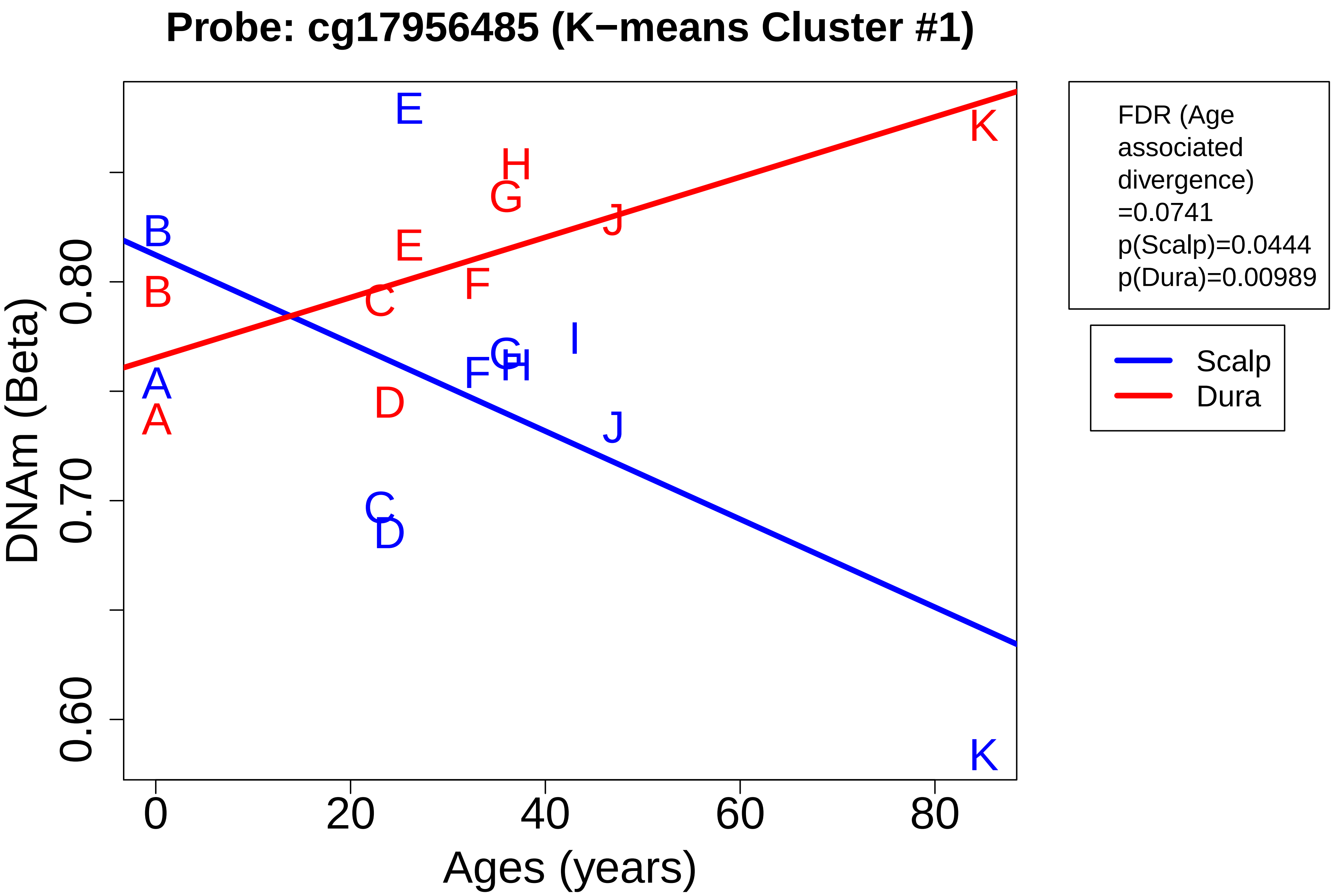
Probe: cg17956485 (K−means Cluster #1)
DNAm (Beta)
0.60
0.70
0.80
Ages (years)
0
20
40
60
80
A
B
C
D
E
F
G
H
I
J
K
FDR (Age associated divergence) =0.0741
p(Scalp)=0.0444
p(Dura)=0.00989
Scalp
Dura

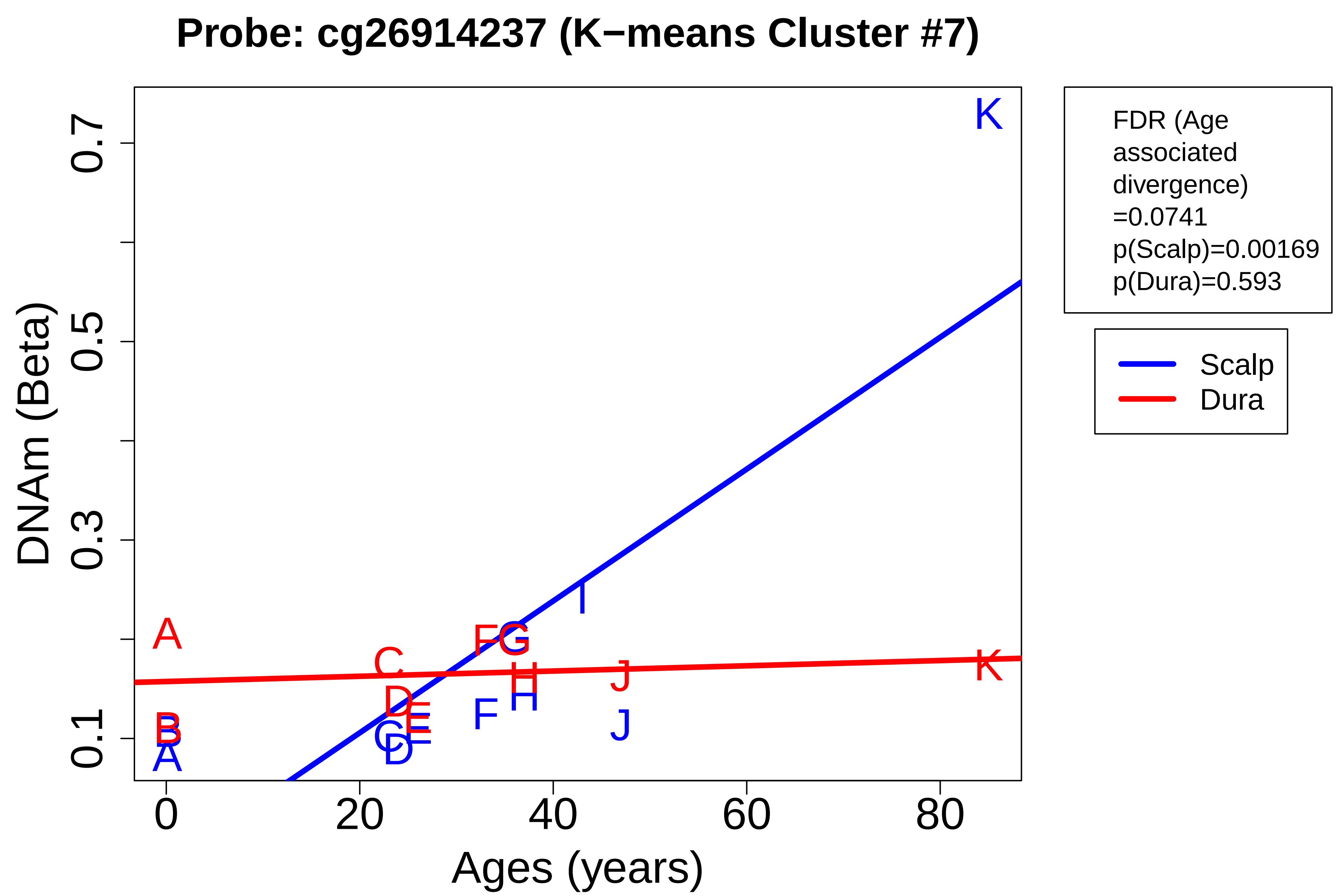

Probe: cg26914237 (K−means Cluster #7)
DNAm (Beta)
0.1
0.3
0.5
0.7
Ages (years)
0
20
40
60
80
FDR (Age associated divergence) =0.0741
p(Scalp)=0.00169
p(Dura)=0.593
Scalp
Dura

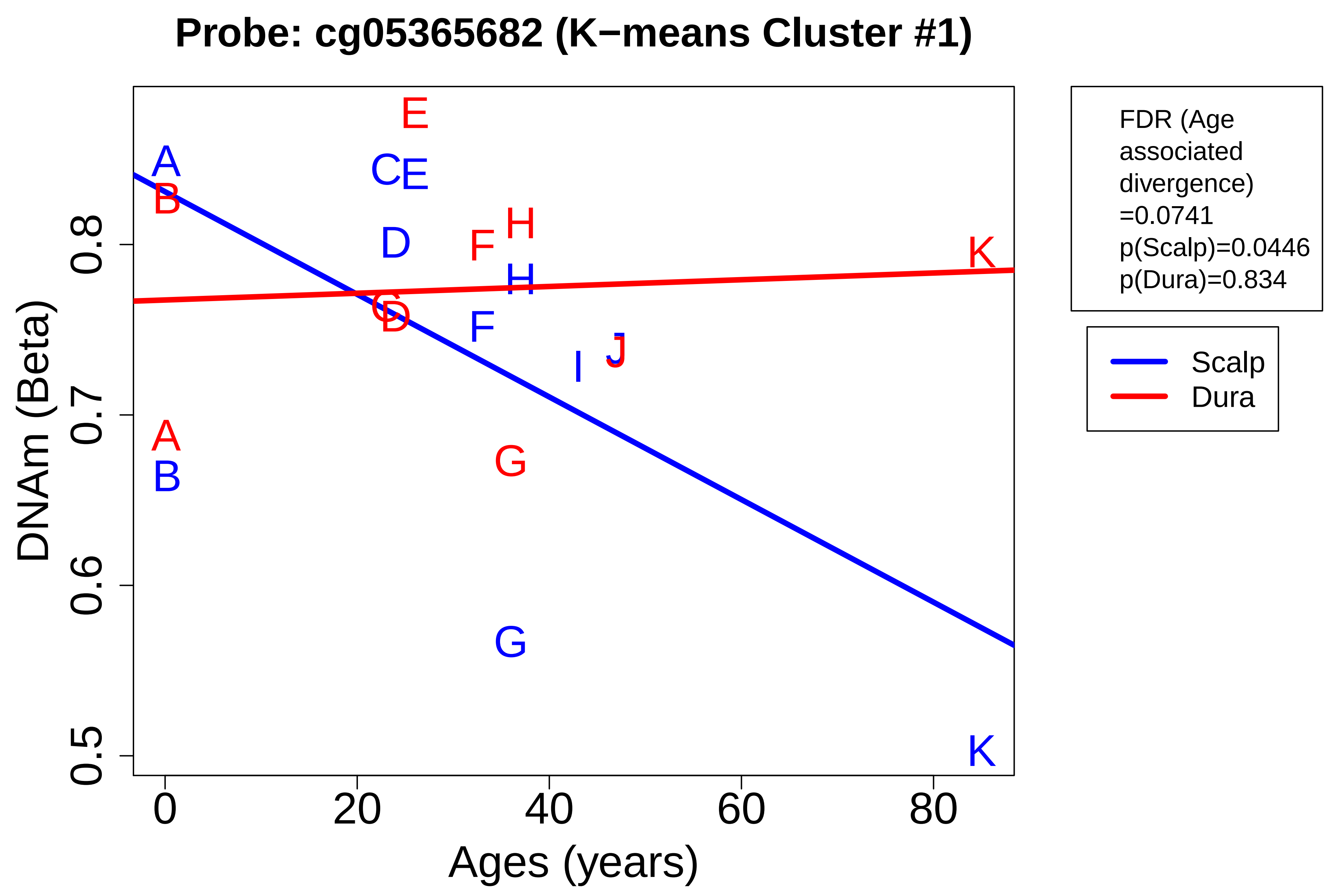

Probe: cg05365682 (K−means Cluster #1)
DNAm (Beta)
0.5
0.6
0.7
0.8
0
20
40
60
80
Ages (years)
FDR (Age associated divergence) =0.0741
p(Scalp)=0.0446
p(Dura)=0.834
Scalp
Dura

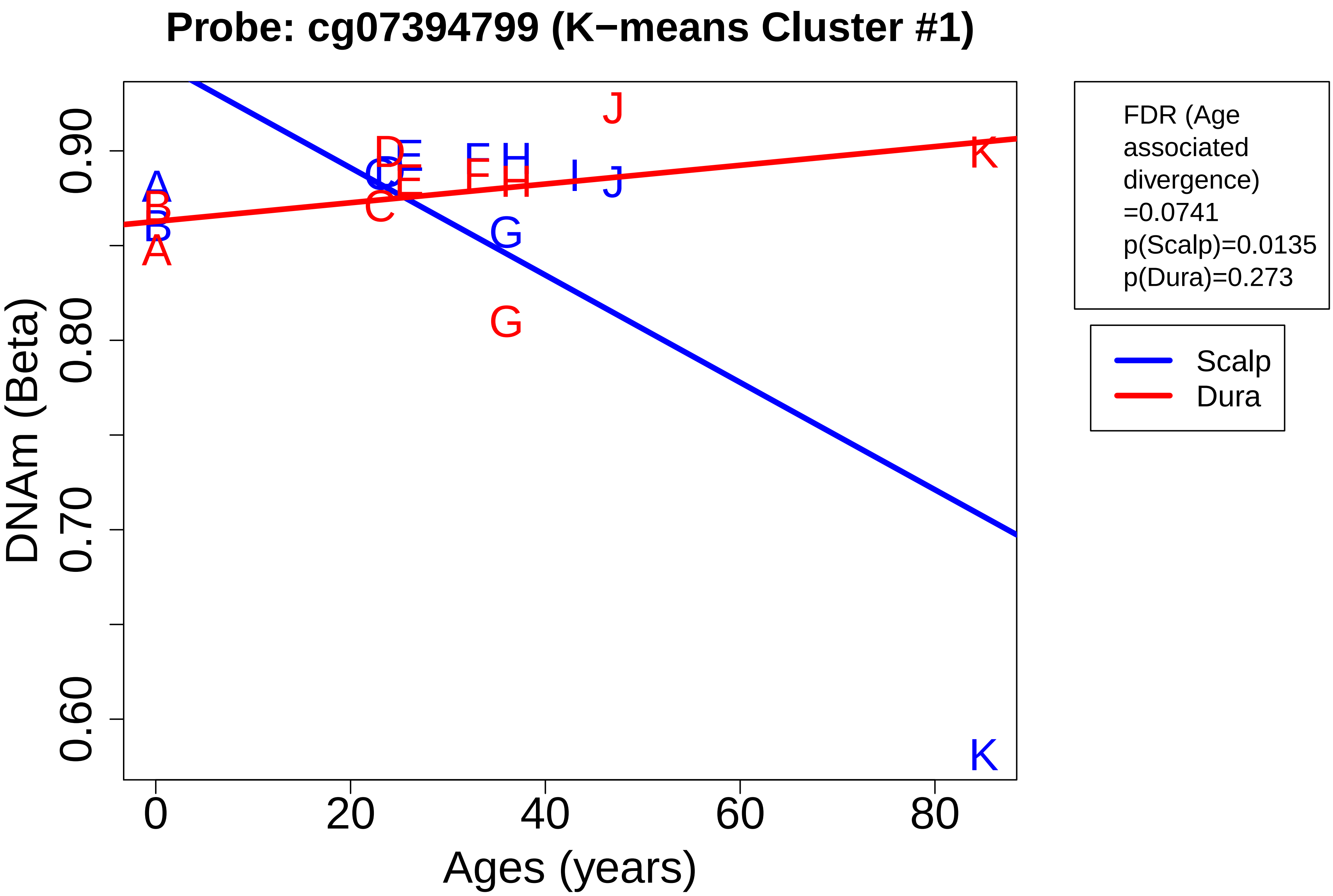
Probe: cg07394799 (K−means Cluster #1)
DNAm (Beta)
0.60
0.70
0.80
0.90
0
20
40
60
80
Ages (years)
A
B
C
D
E
F
G
H
I
J
K
FDR (Age associated divergence) =0.0741
p(Scalp)=0.0135
p(Dura)=0.273
Scalp
Dura

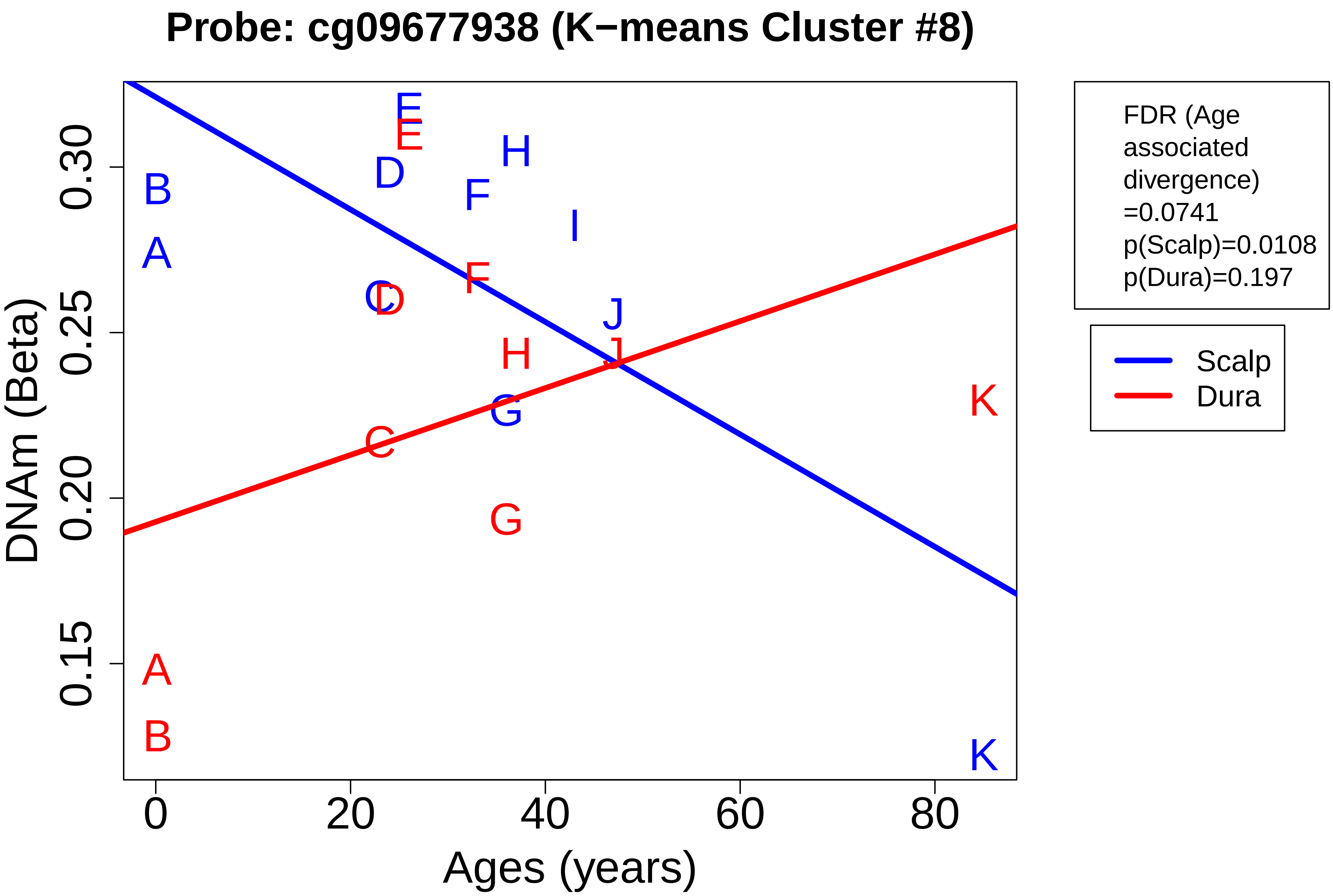

Probe: cg09677938 (K−means Cluster #8)
DNAm (Beta)
0.15
0.20
0.25
0.30
0
20
40
60
80
Ages (years)
FDR (Age associated divergence) =0.0741
p(Scalp)=0.0108
p(Dura)=0.197
Scalp
Dura

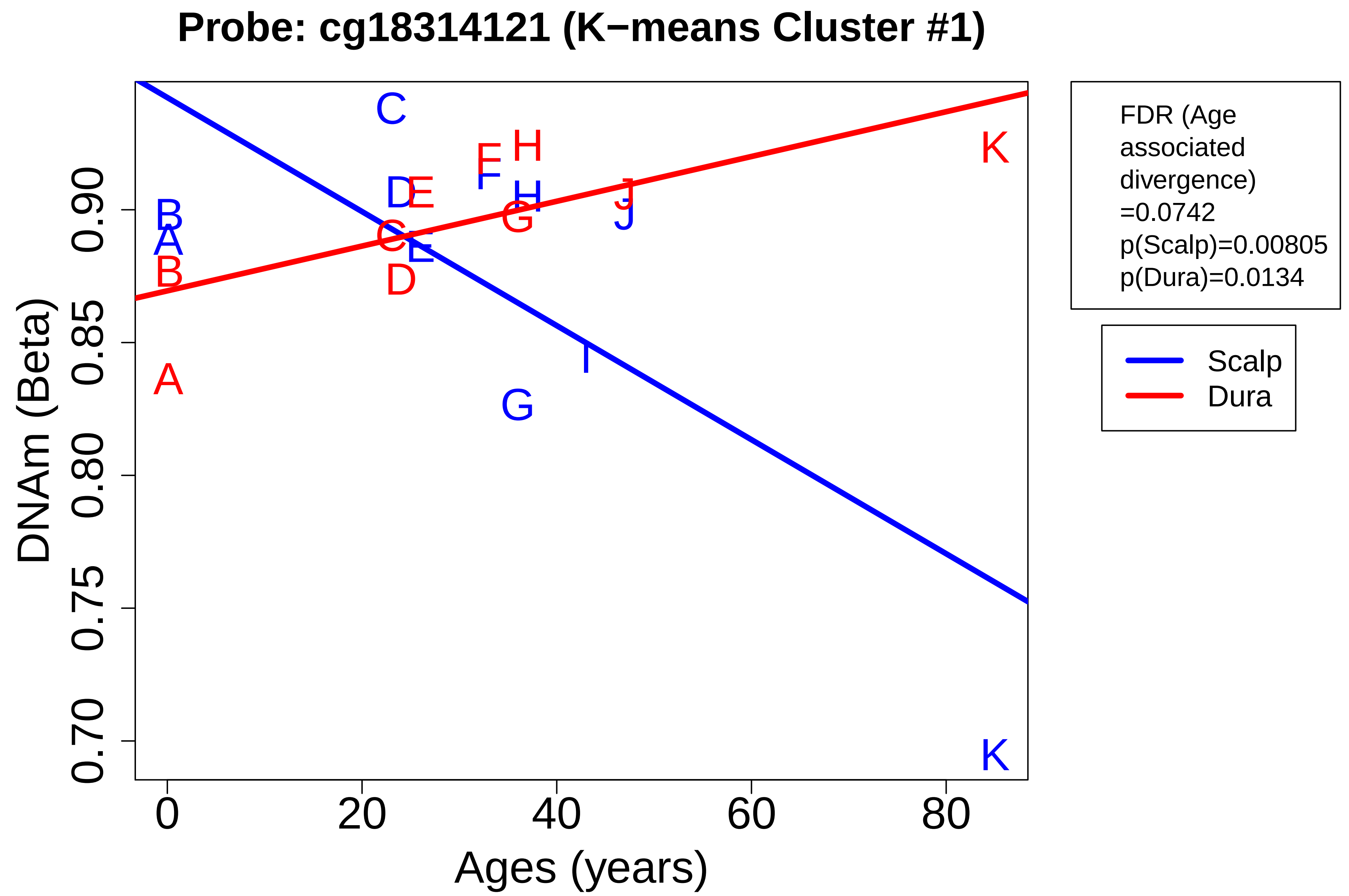

Probe: cg18314121 (K−means Cluster #1)
DNAm (Beta)
0.70
0.75
0.80
0.85
0.90
0
20
40
60
80
Ages (years)
FDR (Age associated divergence) =0.0742
p(Scalp)=0.00805
p(Dura)=0.0134
Scalp
Dura

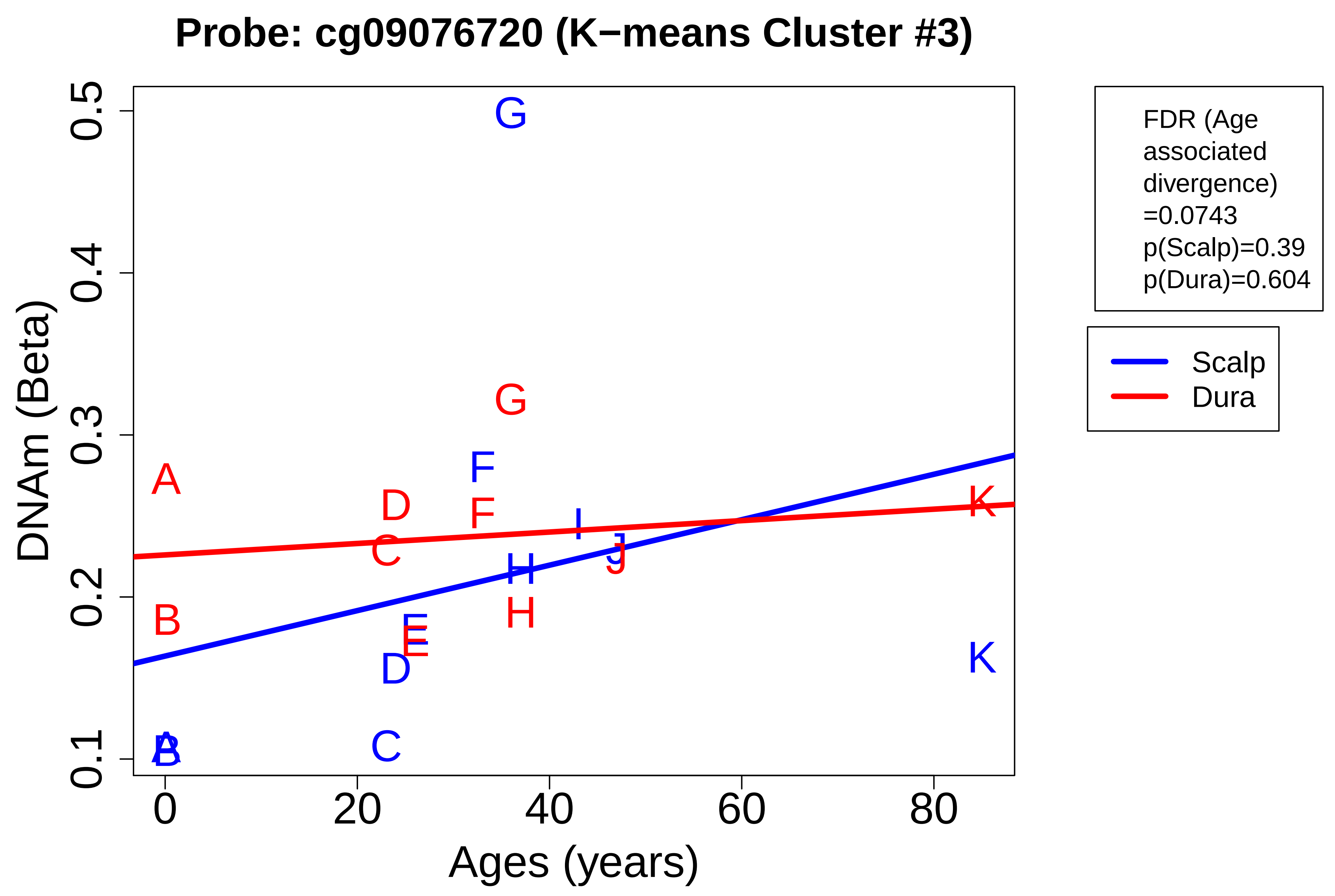

Probe: cg09076720 (K−means Cluster #3)
DNAm (Beta)
Ages (years)
0.1
0.2
0.3
0.4
0.5
0
20
40
60
80
FDR (Age associated divergence) =0.0743
p(Scalp)=0.39
p(Dura)=0.604
Scalp
Dura

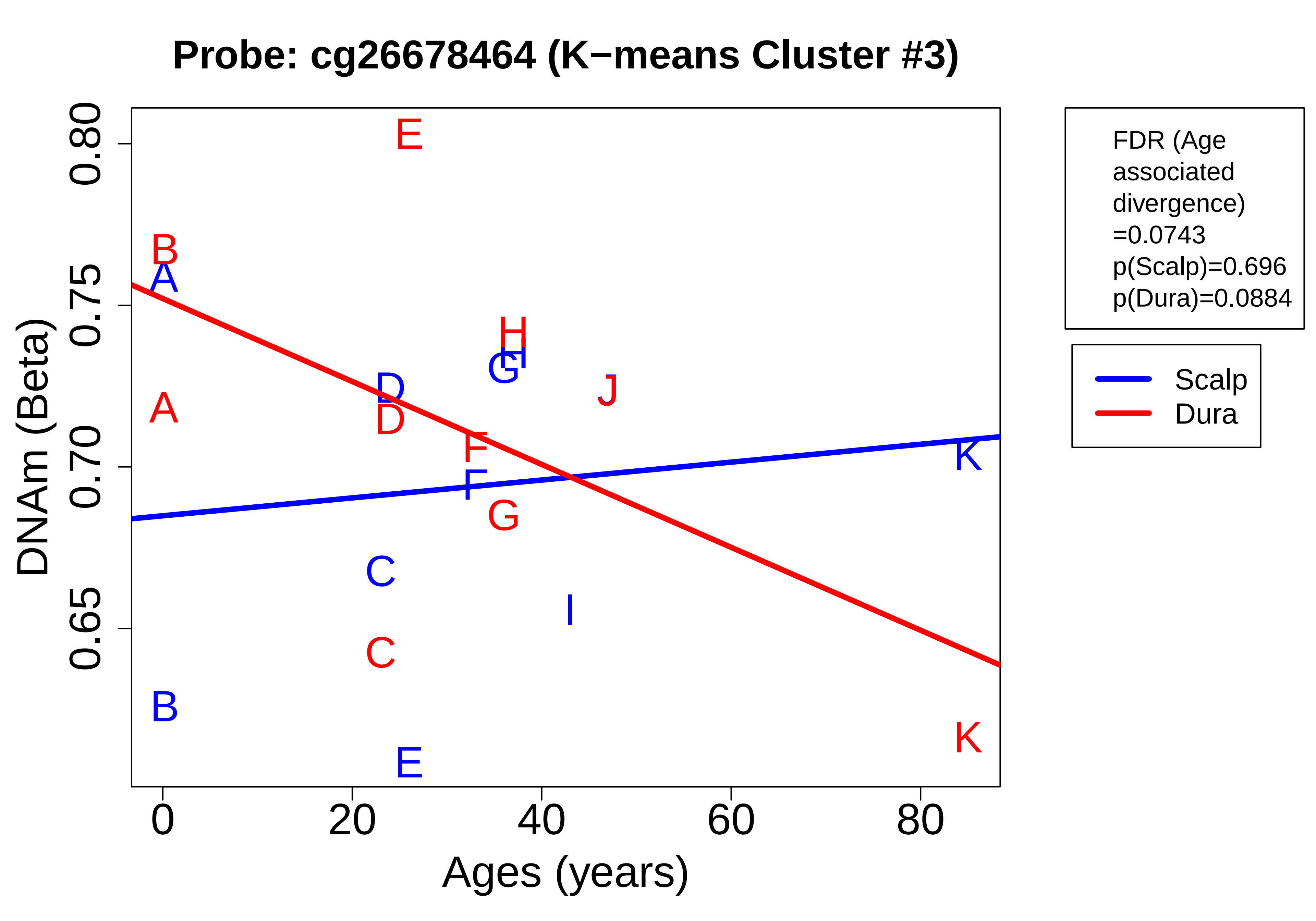

Probe: cg26678464 (K−means Cluster #3)
DNAm (Beta)
0.65
0.70
0.75
0.80
Ages (years)
0
20
40
60
80
A
B
C
D
E
F
G
H
I
J
K
FDR (Age associated divergence) =0.0743
p(Scalp)=0.696
p(Dura)=0.0884
Scalp
Dura

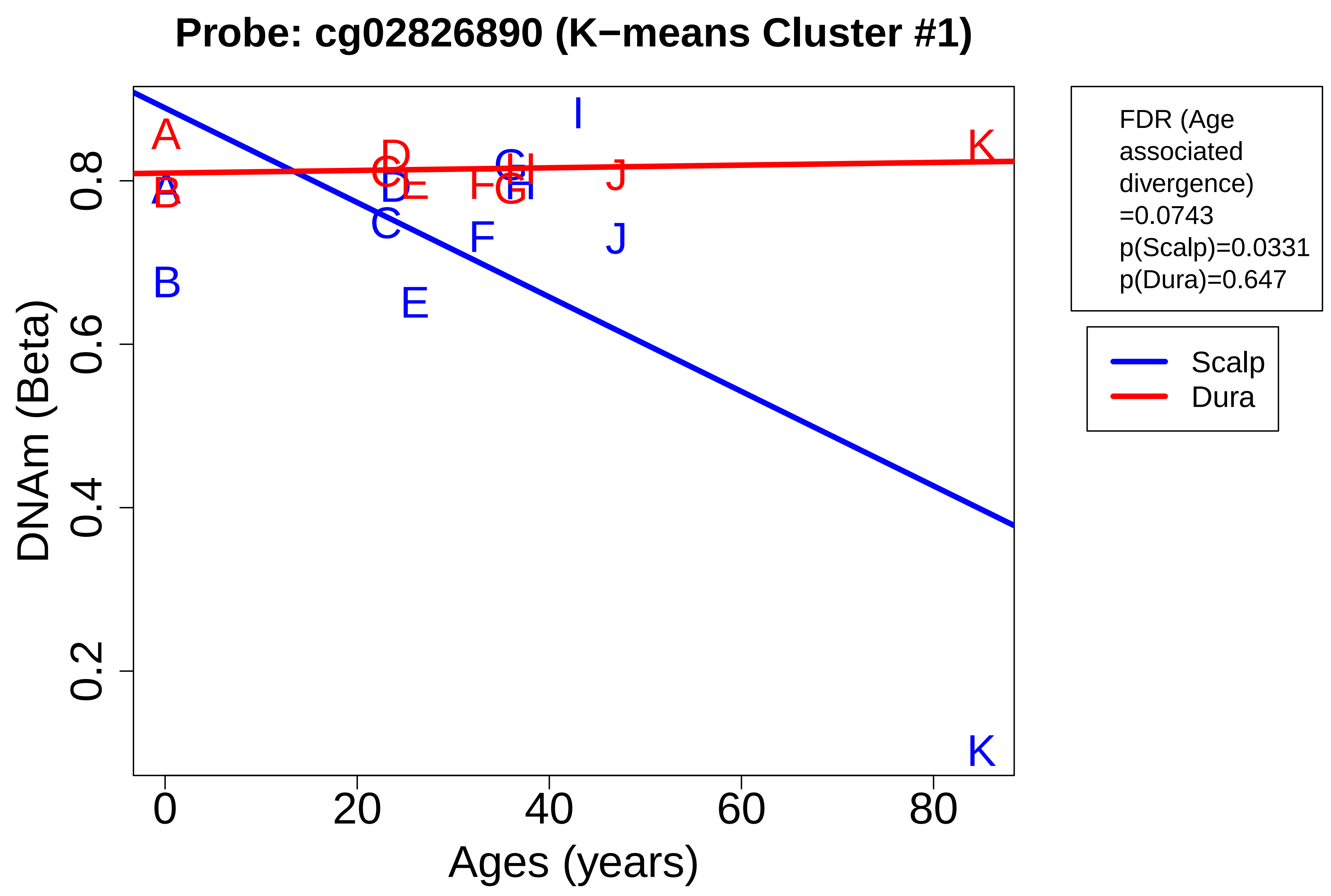

Probe: cg02826890 (K−means Cluster #1)
DNAm (Beta)
0.2
0.4
0.6
0.8
Ages (years)
0
20
40
60
80
A
B
C
D
E
F
G
H
I
J
K
FDR (Age associated divergence) =0.0743
p(Scalp)=0.0331
p(Dura)=0.647
Scalp
Dura

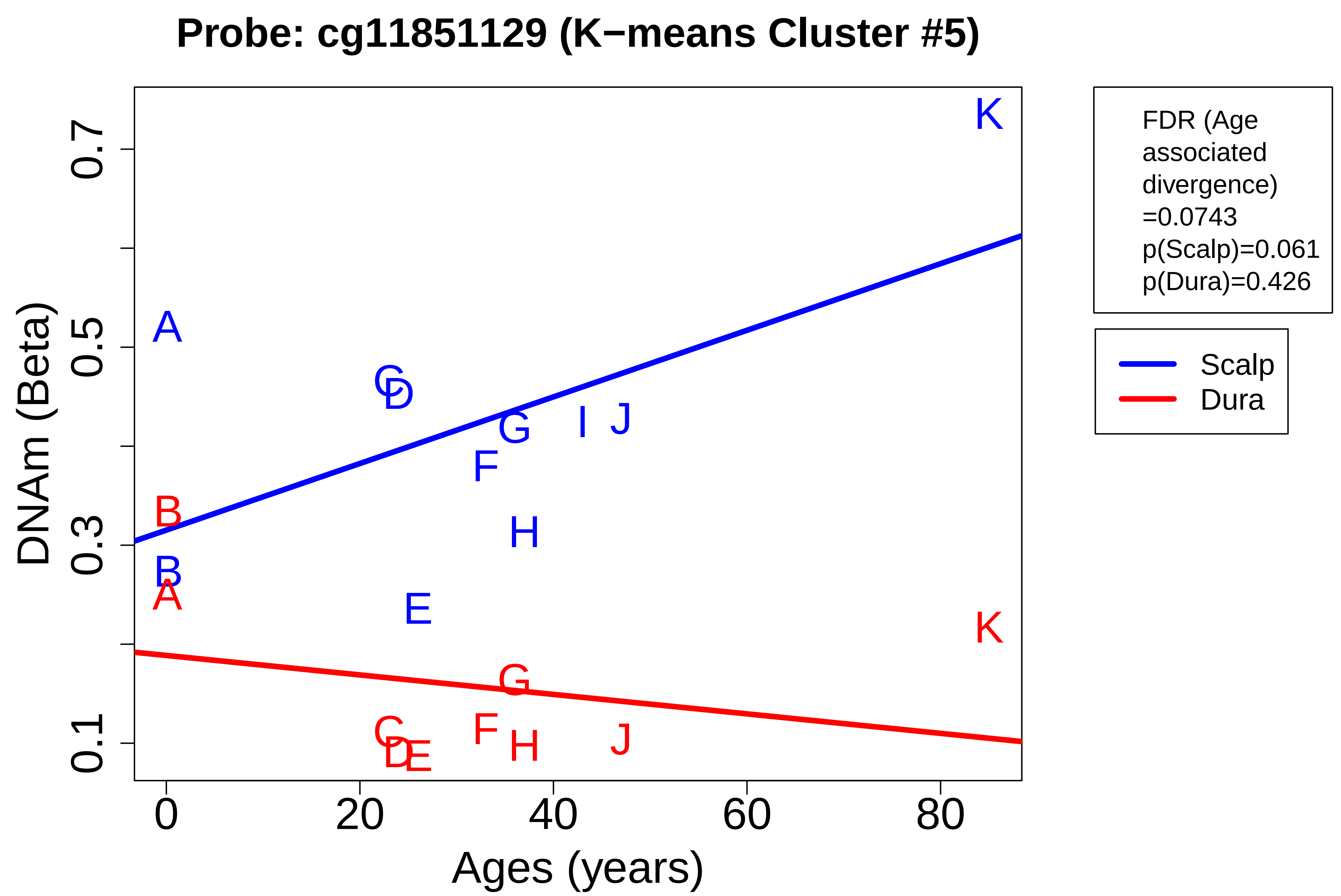
Probe: cg11851129 (K−means Cluster #5)
DNAm (Beta)
0.1
0.3
0.5
0.7
Ages (years)
0
20
40
60
80
FDR (Age associated divergence) =0.0743
p(Scalp)=0.061
p(Dura)=0.426
Scalp
Dura
A
B
C
D
E
F
G
H
I
J
K

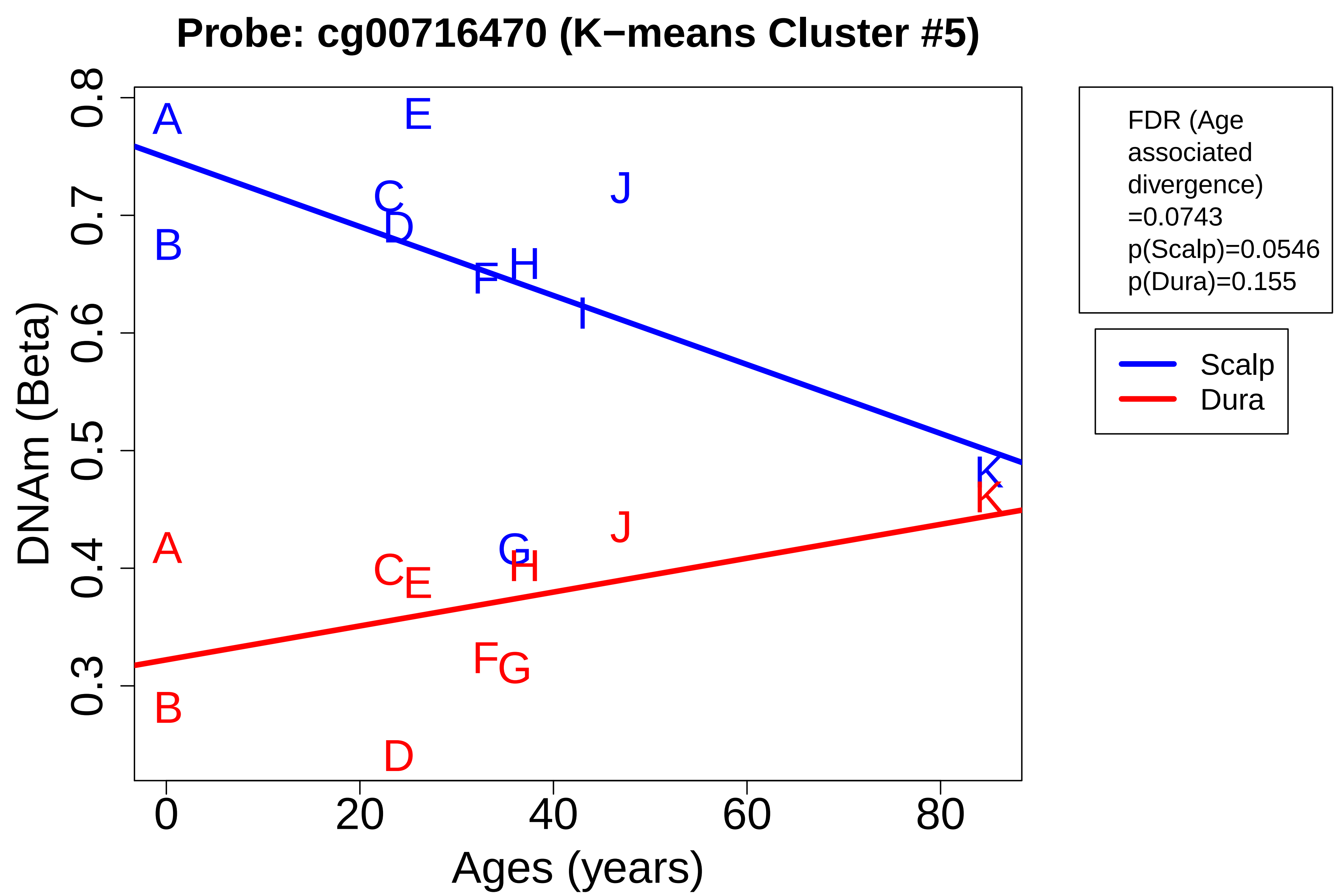

Probe: cg00716470 (K−means Cluster #5)
DNAm (Beta)
0.3
0.4
0.5
0.6
0.7
0.8
Ages (years)
0
20
40
60
80
A
B
C
D
E
F
G
H
I
J
K
FDR (Age associated divergence) =0.0743
p(Scalp)=0.0546
p(Dura)=0.155
Scalp
Dura

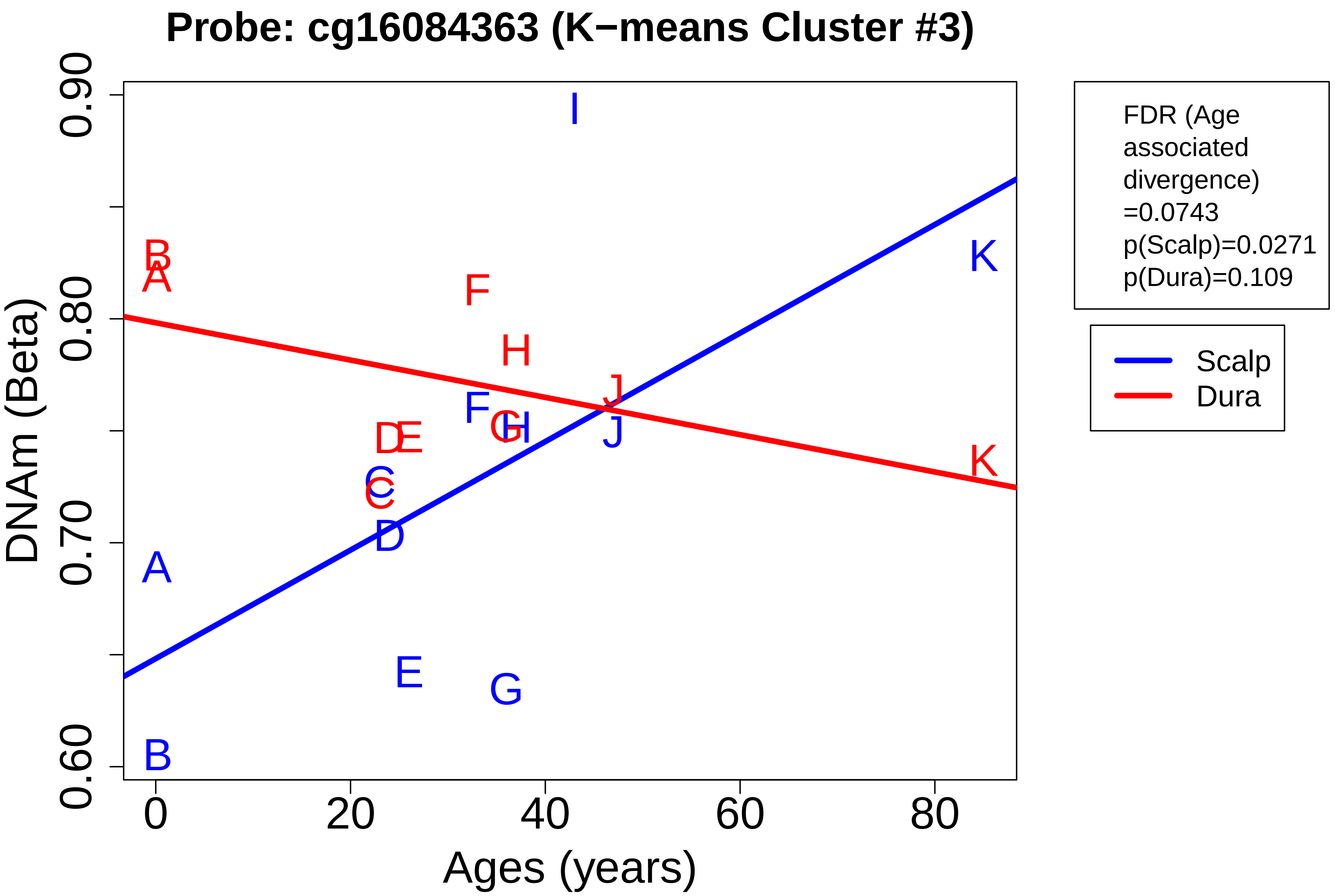

Probe: cg16084363 (K−means Cluster #3)
DNAm (Beta)
0.60
0.70
0.80
0.90
Ages (years)
0
20
40
60
80
FDR (Age associated divergence) =0.0743
p(Scalp)=0.0271
p(Dura)=0.109
Scalp
Dura
A
B
C
D
E
F
G
H
I
J
K

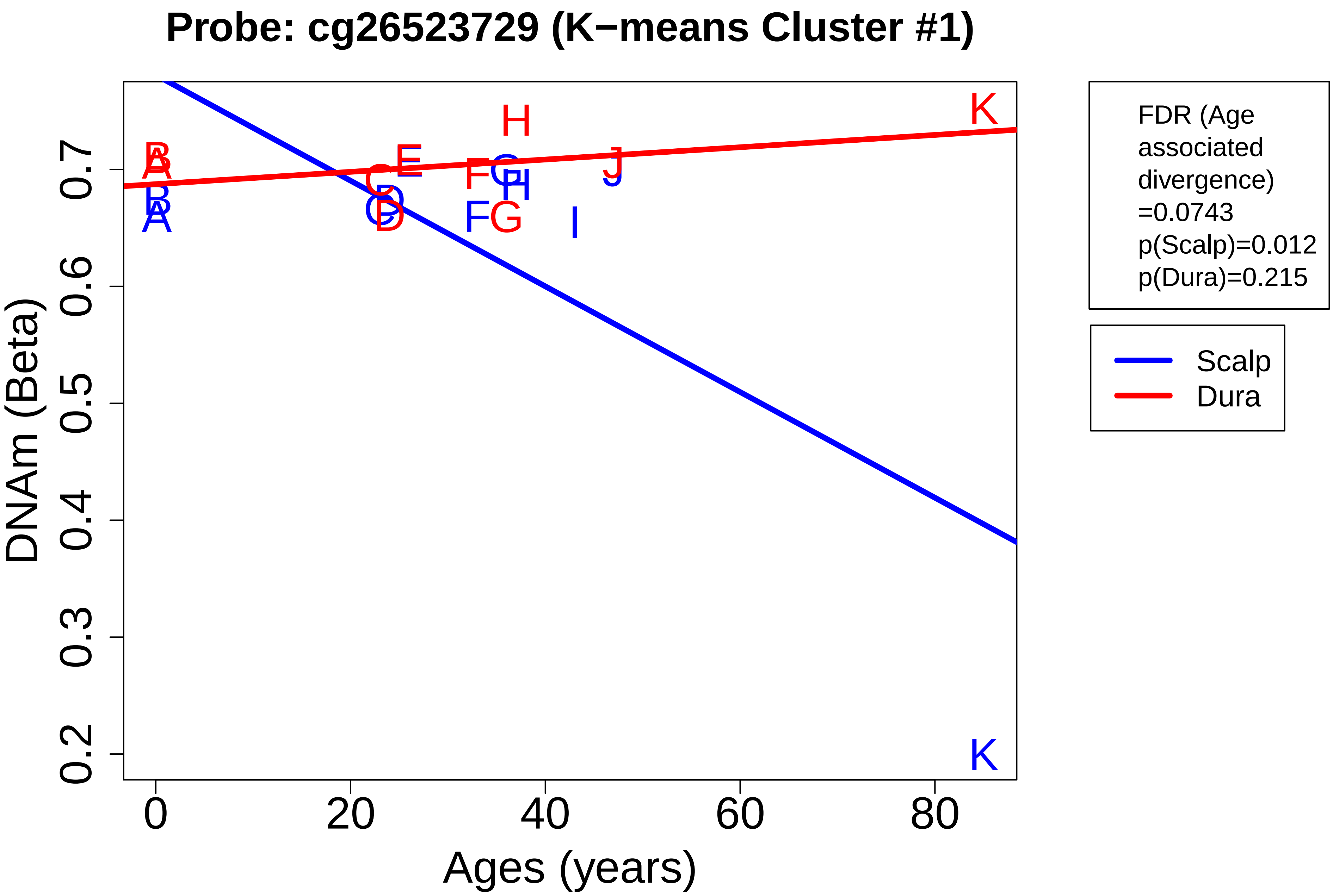

Probe: cg26523729 (K−means Cluster #1)
DNAm (Beta)
0.2
0.3
0.4
0.5
0.6
0.7
0
20
40
60
80
Ages (years)
FDR (Age associated divergence) =0.0743
p(Scalp)=0.012
p(Dura)=0.215
Scalp
Dura

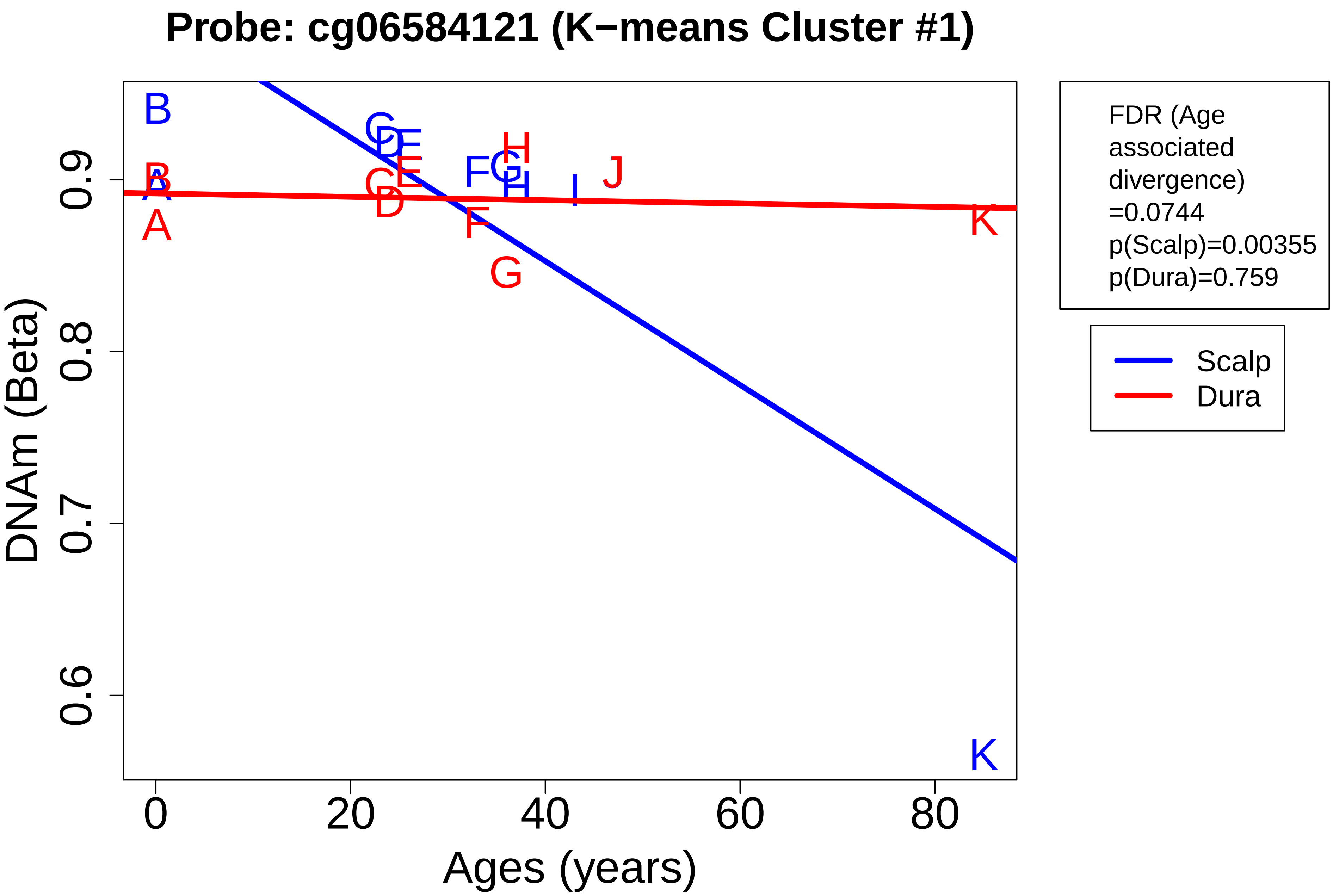

Probe: cg06584121 (K−means Cluster #1)
DNAm (Beta)
0.6
0.7
0.8
0.9
Ages (years)
0
20
40
60
80
FDR (Age associated divergence) =0.0744
p(Scalp)=0.00355
p(Dura)=0.759
Scalp
Dura

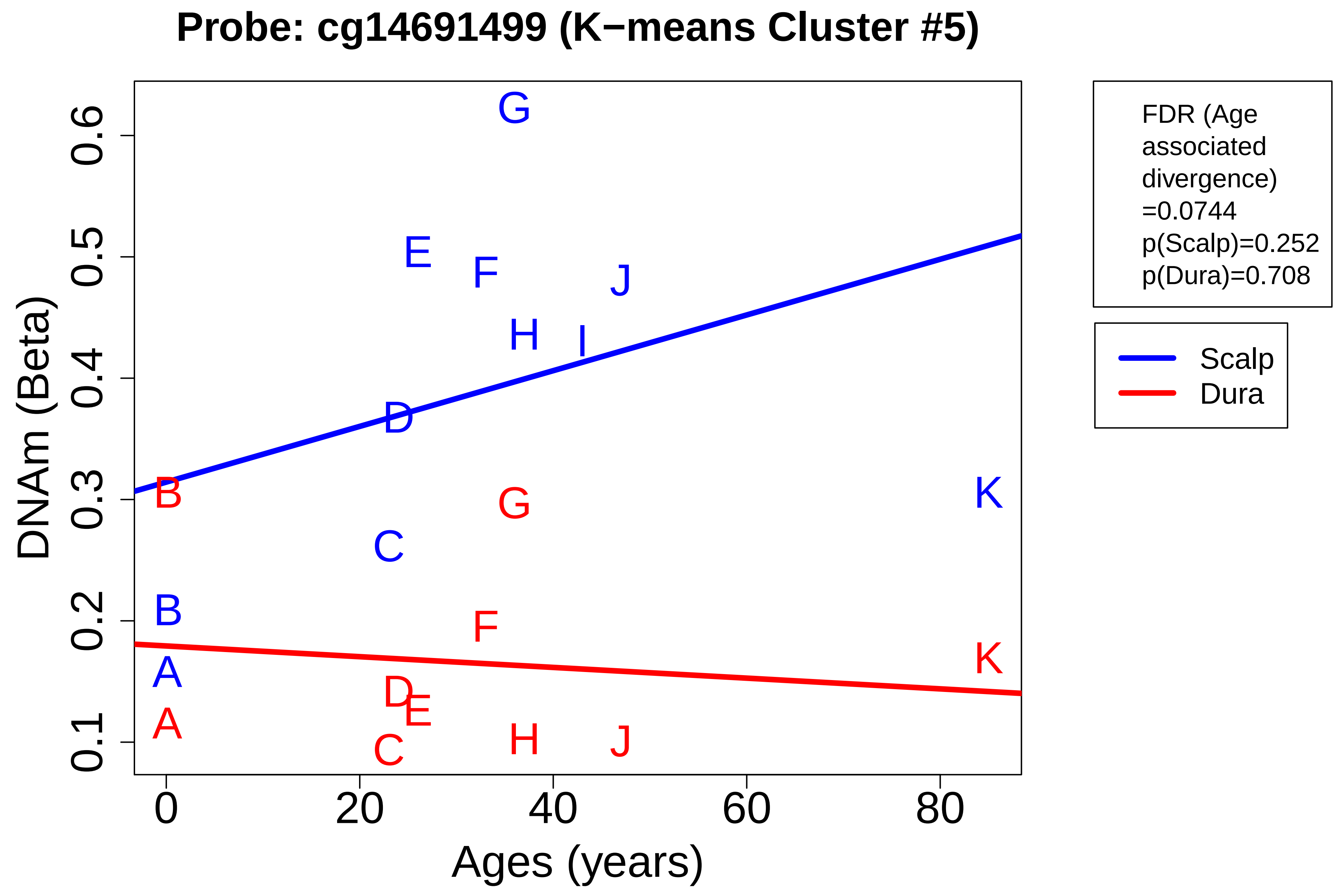

Probe: cg14691499 (K−means Cluster #5)
DNAm (Beta)
0.1
0.2
0.3
0.4
0.5
0.6
0
20
40
60
80
Ages (years)
FDR (Age associated divergence) =0.0744
p(Scalp)=0.252
p(Dura)=0.708
Scalp
Dura
A
B
C
D
E
F
G
H
I
J
K

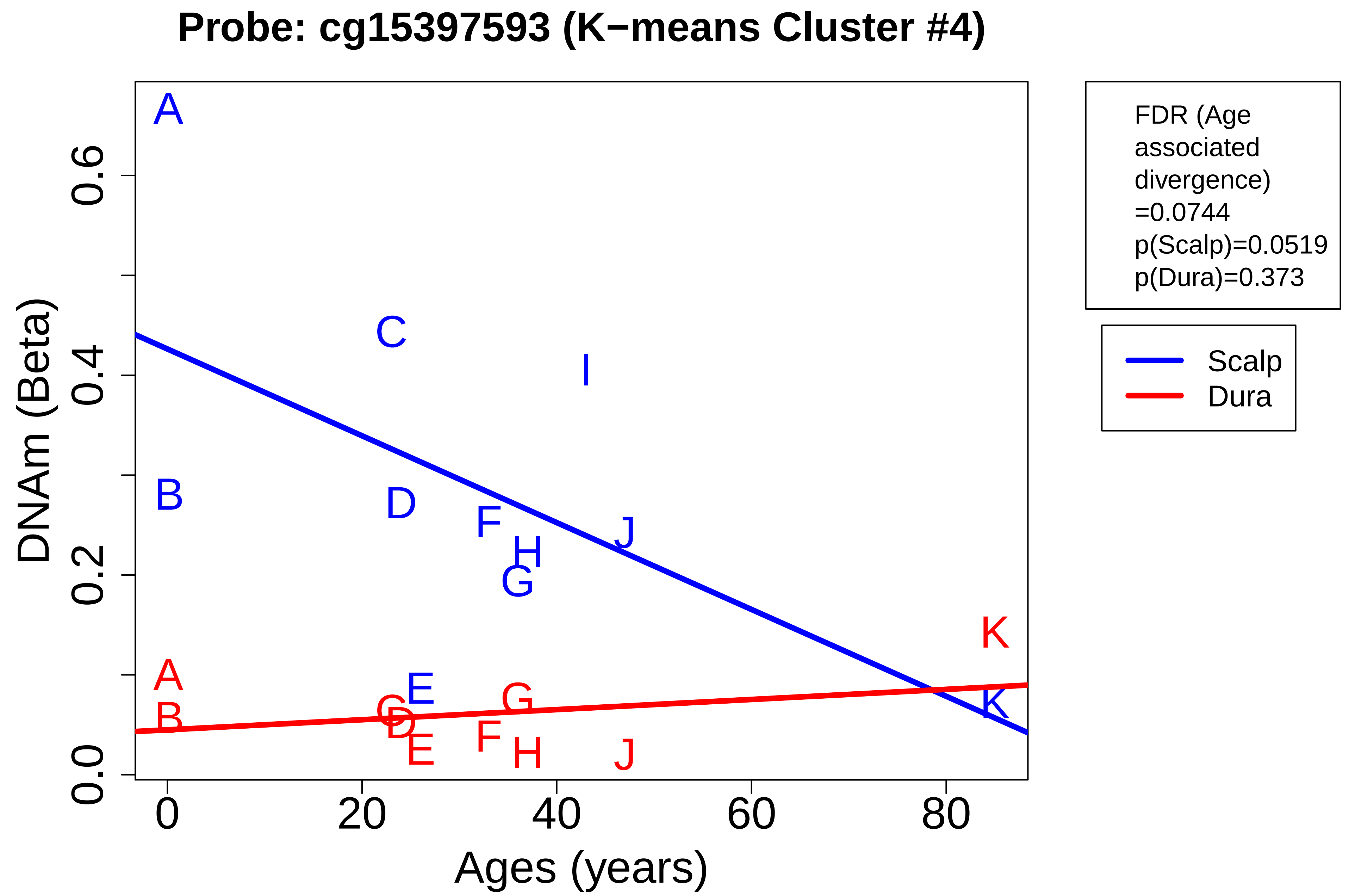

Probe: cg15397593 (K−means Cluster #4)
DNAm (Beta)
0.0
0.2
0.4
0.6
0
20
40
60
80
Ages (years)
FDR (Age associated divergence) =0.0744
p(Scalp)=0.0519
p(Dura)=0.373
Scalp
Dura

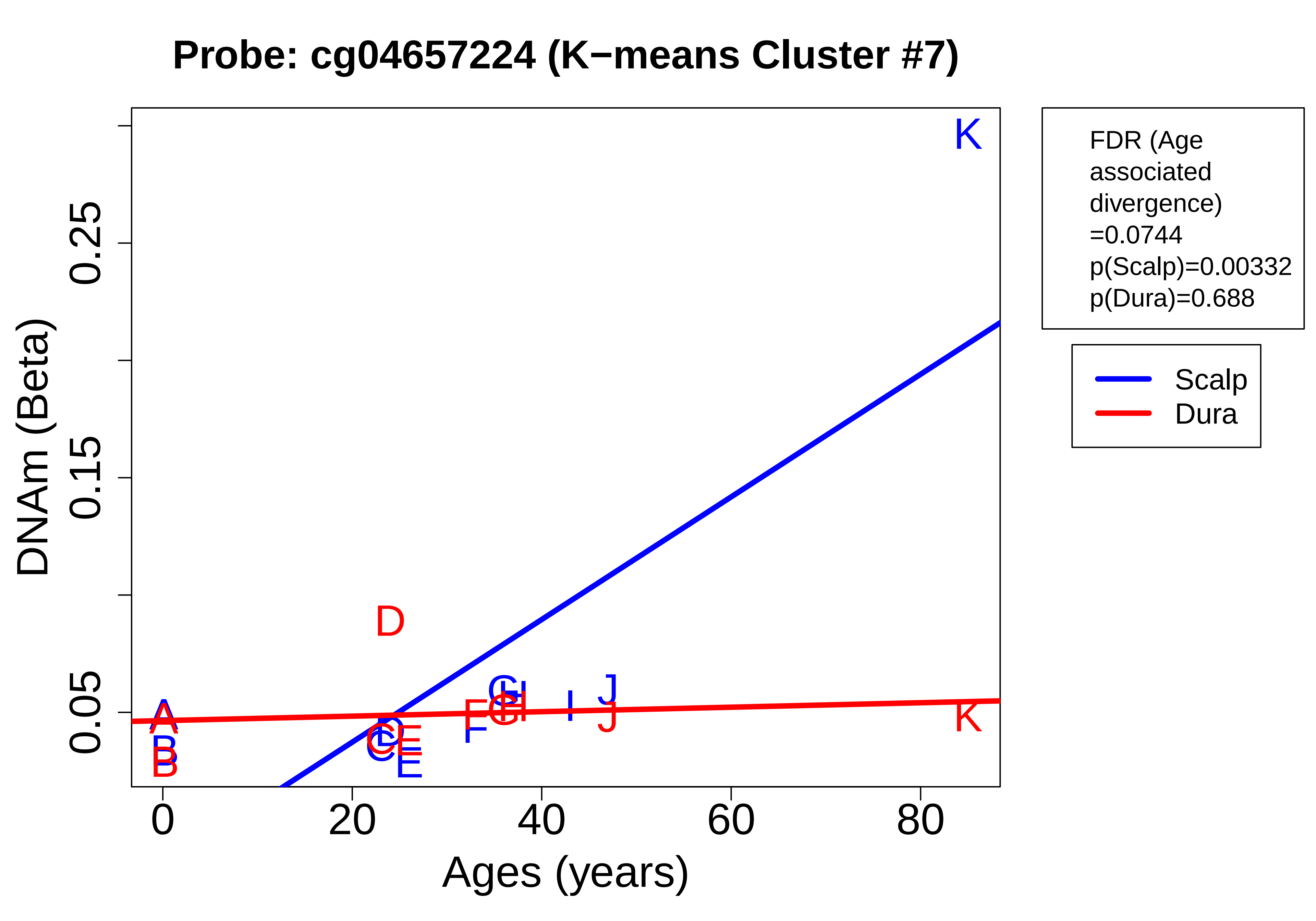

Probe: cg04657224 (K−means Cluster #7)
DNAm (Beta)
0.05
0.15
0.25
Ages (years)
0
20
40
60
80
FDR (Age associated divergence) =0.0744
p(Scalp)=0.00332
p(Dura)=0.688
Scalp
Dura
A
B
C
D
E
F
G
H
I
J
K

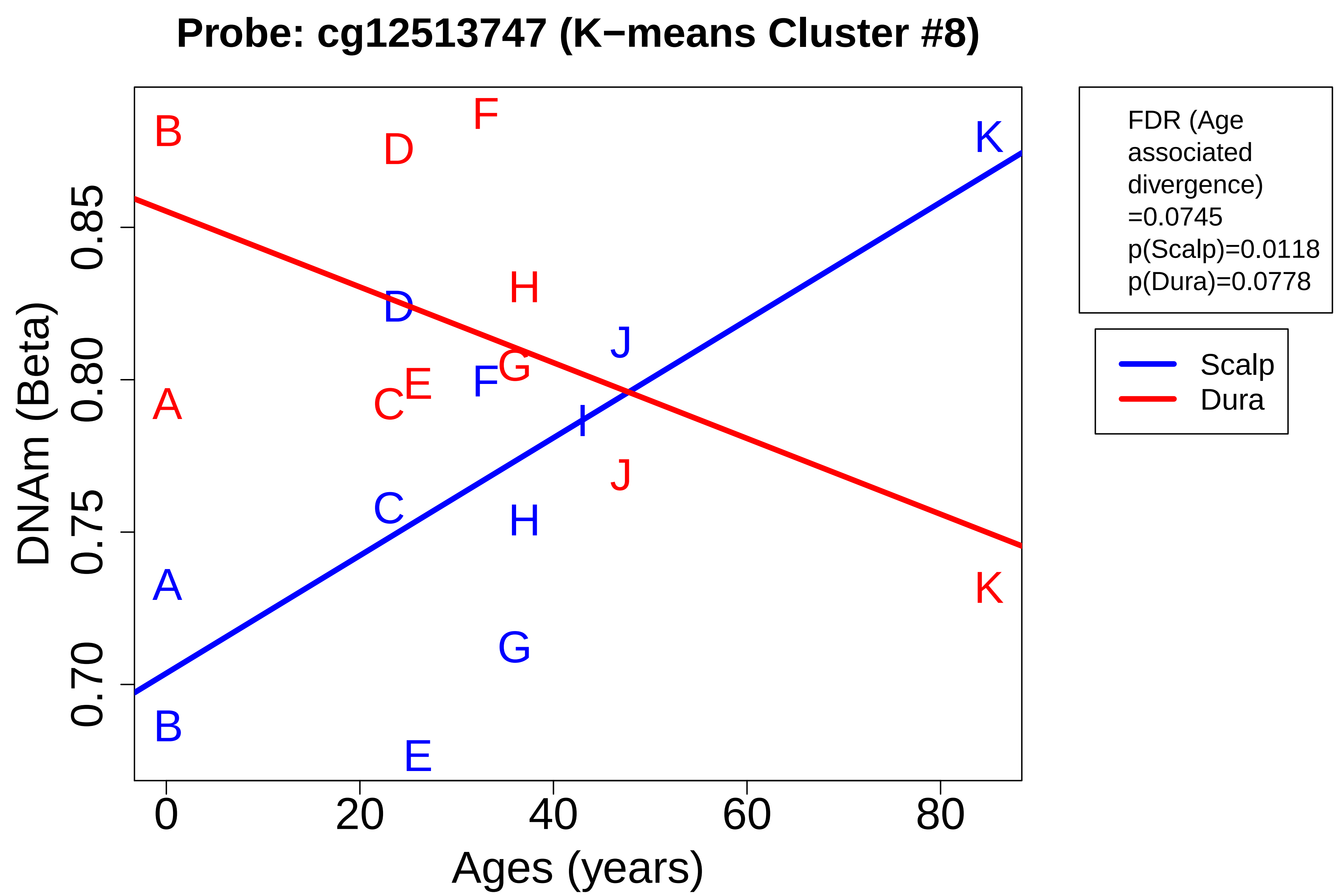
Probe: cg12513747 (K−means Cluster #8)
DNAm (Beta)
0.70
0.75
0.80
0.85
Ages (years)
0
20
40
60
80
FDR (Age associated divergence) =0.0745
p(Scalp)=0.0118
p(Dura)=0.0778
Scalp
Dura
A B C D E F G H I J K

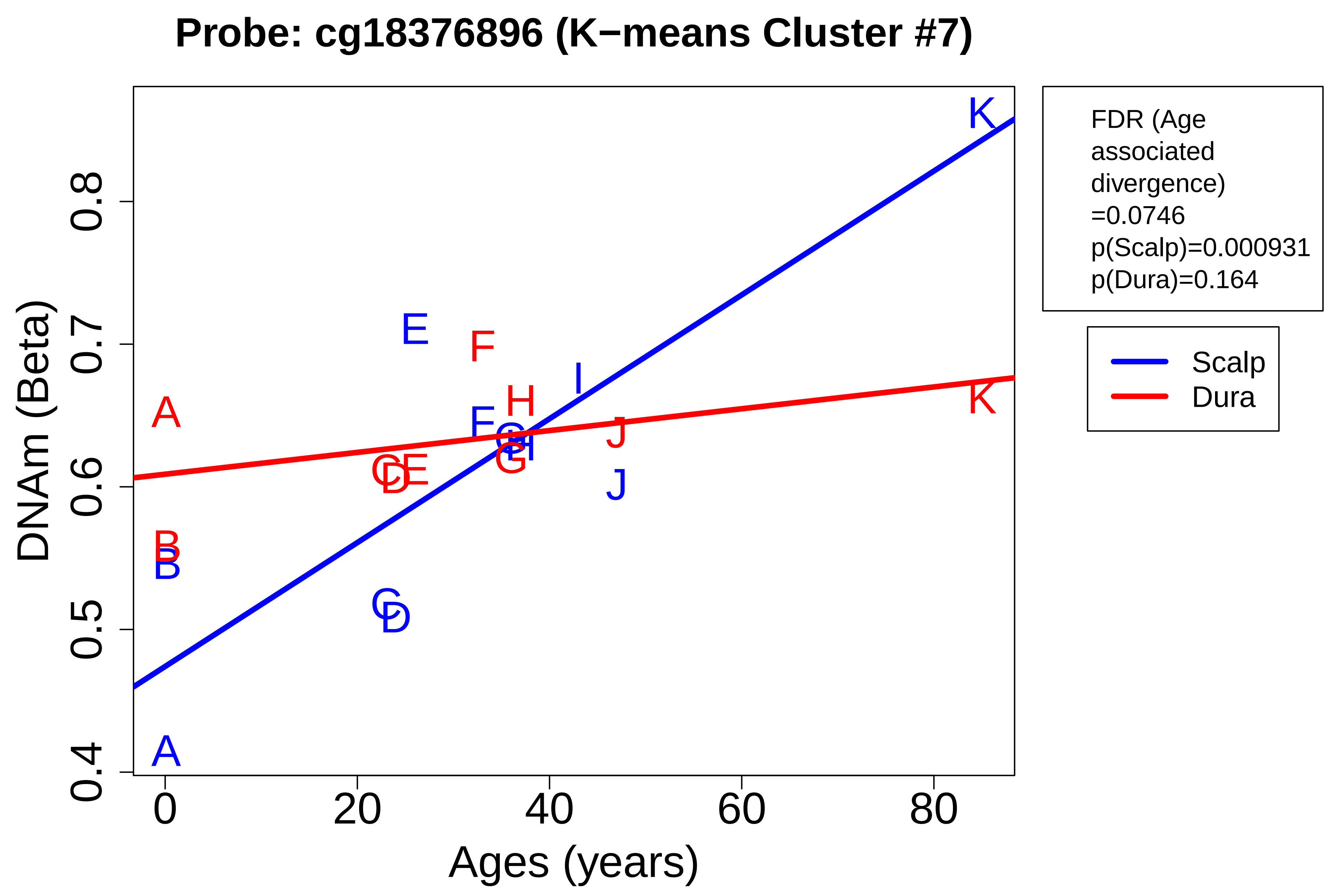

Probe: cg18376896 (K−means Cluster #7)
DNAm (Beta)
Ages (years)
0.4
0.5
0.6
0.7
0.8
0
20
40
60
80
FDR (Age associated divergence) =0.0746
p(Scalp)=0.000931
p(Dura)=0.164
Scalp
Dura

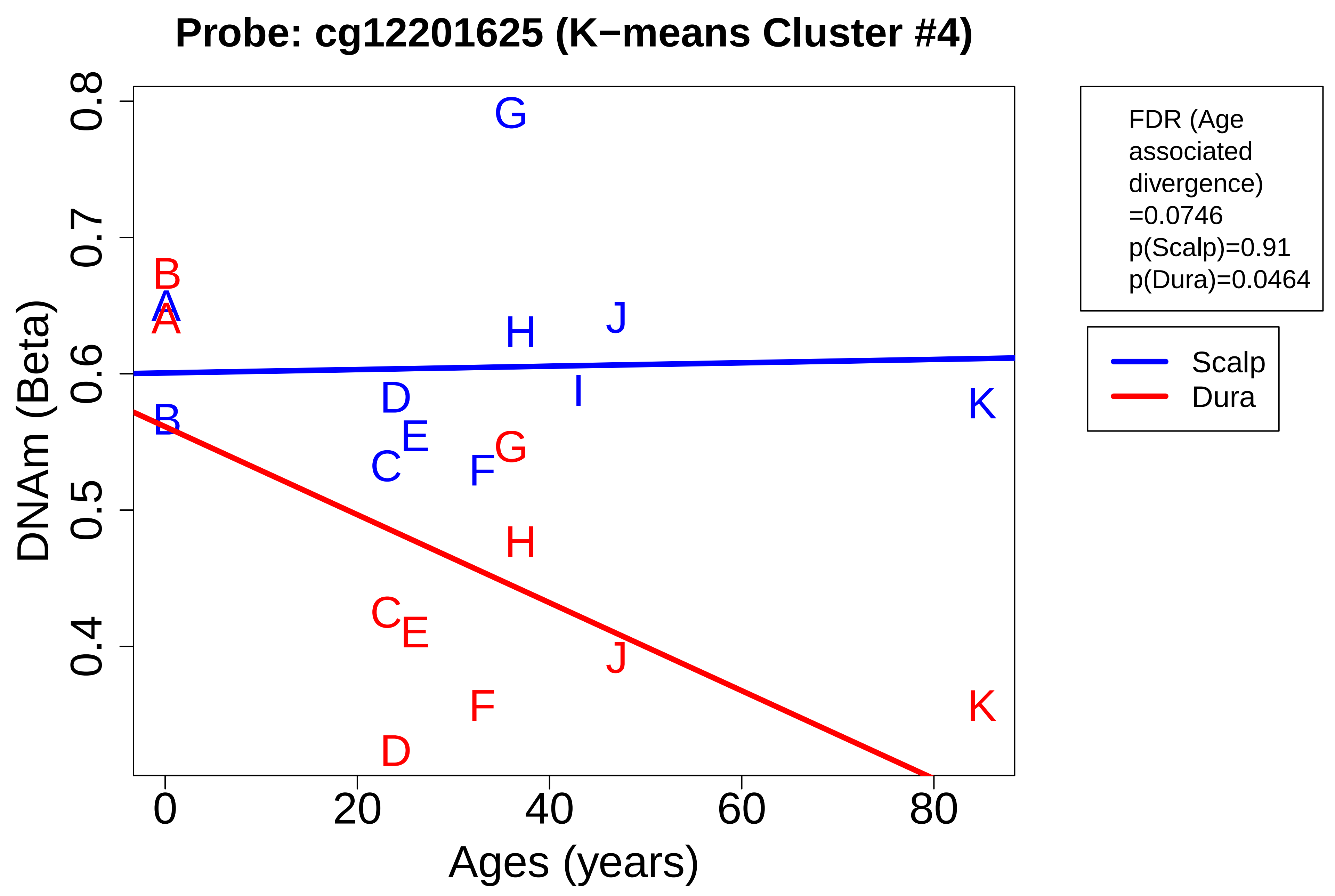

Probe: cg12201625 (K−means Cluster #4)
DNAm (Beta)
0.4
0.5
0.6
0.7
0.8
0
20
40
60
80
Ages (years)
A
B
C
D
E
F
G
H
I
J
K
A
B
C
D
E
F
G
H
J
K
FDR (Age
associated
divergence)
=0.0746
p(Scalp)=0.91
p(Dura)=0.0464
Scalp
Dura

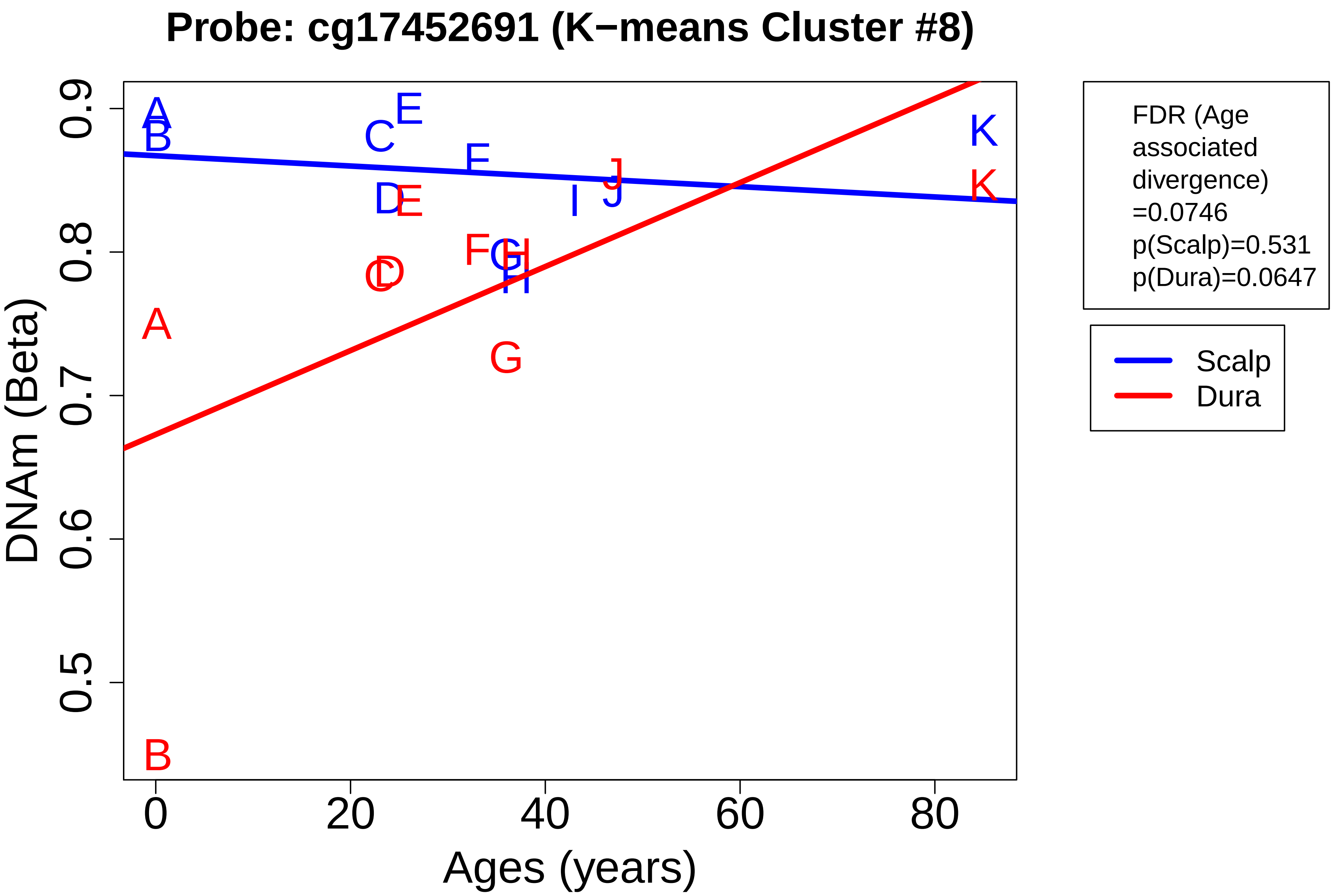

Probe: cg17452691 (K−means Cluster #8)
DNAm (Beta)
0.5
0.6
0.7
0.8
0.9
0
20
40
60
80
Ages (years)
FDR (Age associated divergence) =0.0746
p(Scalp)=0.531
p(Dura)=0.0647
Scalp
Dura

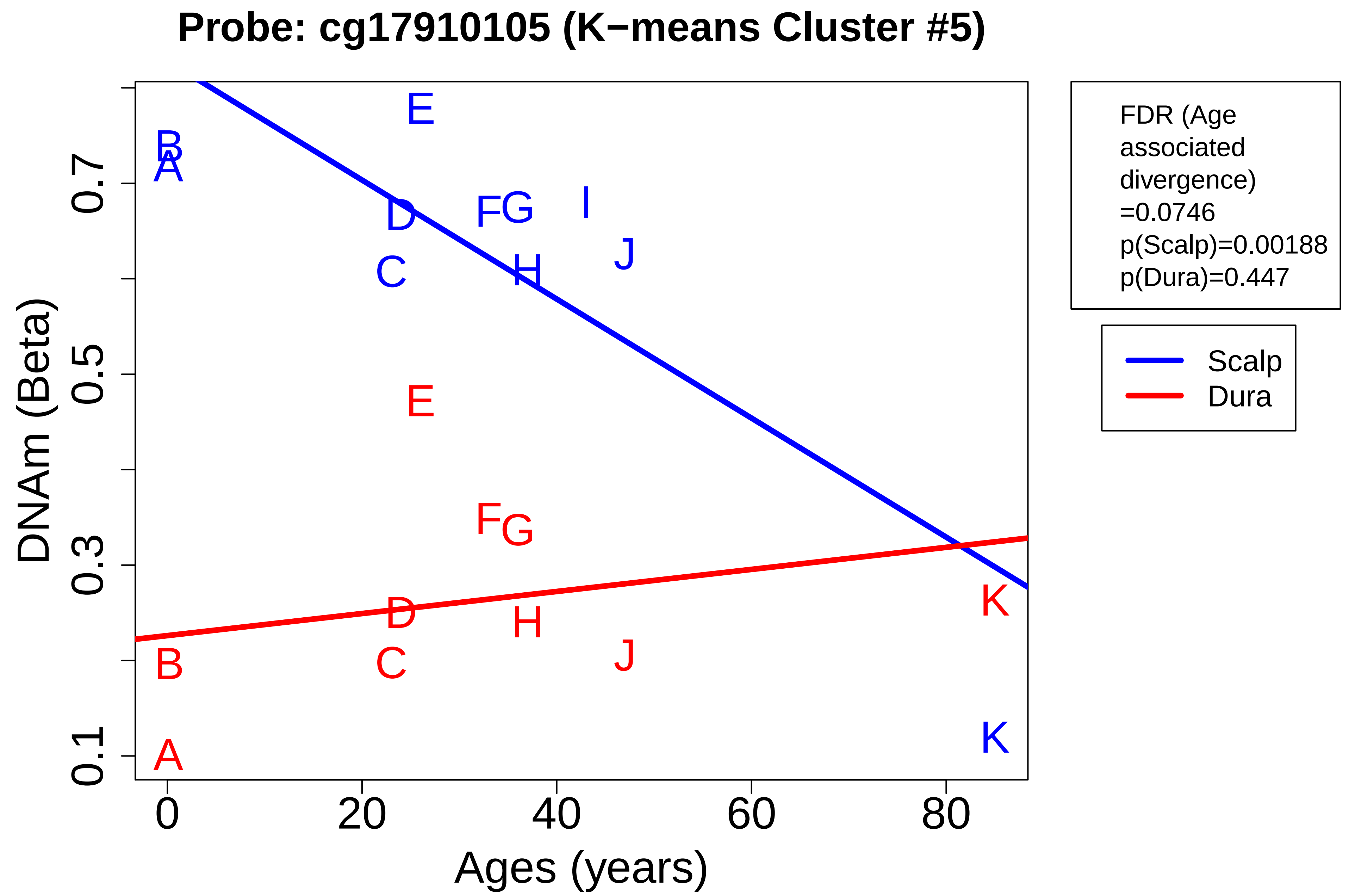

Probe: cg17910105 (K−means Cluster #5)
DNAm (Beta)
0.1
0.3
0.5
0.7
Ages (years)
0
20
40
60
80
A B C D E F G H I J K
FDR (Age associated divergence) =0.0746
p(Scalp)=0.00188
p(Dura)=0.447
Scalp
Dura

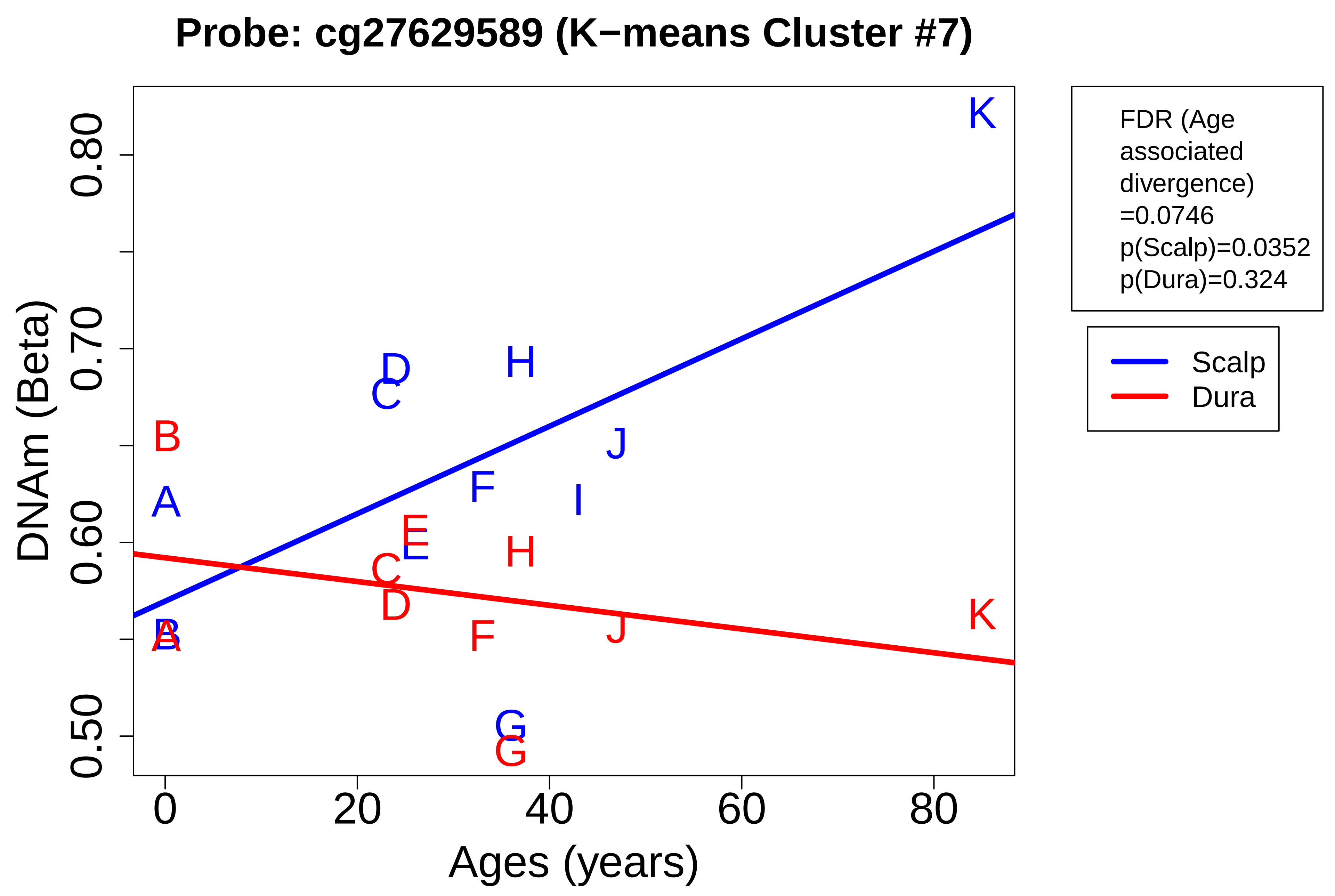

Probe: cg27629589 (K−means Cluster #7)
DNAm (Beta)
0.50
0.60
0.70
0.80
Ages (years)
0
20
40
60
80
FDR (Age associated divergence) =0.0746
p(Scalp)=0.0352
p(Dura)=0.324
Scalp
Dura

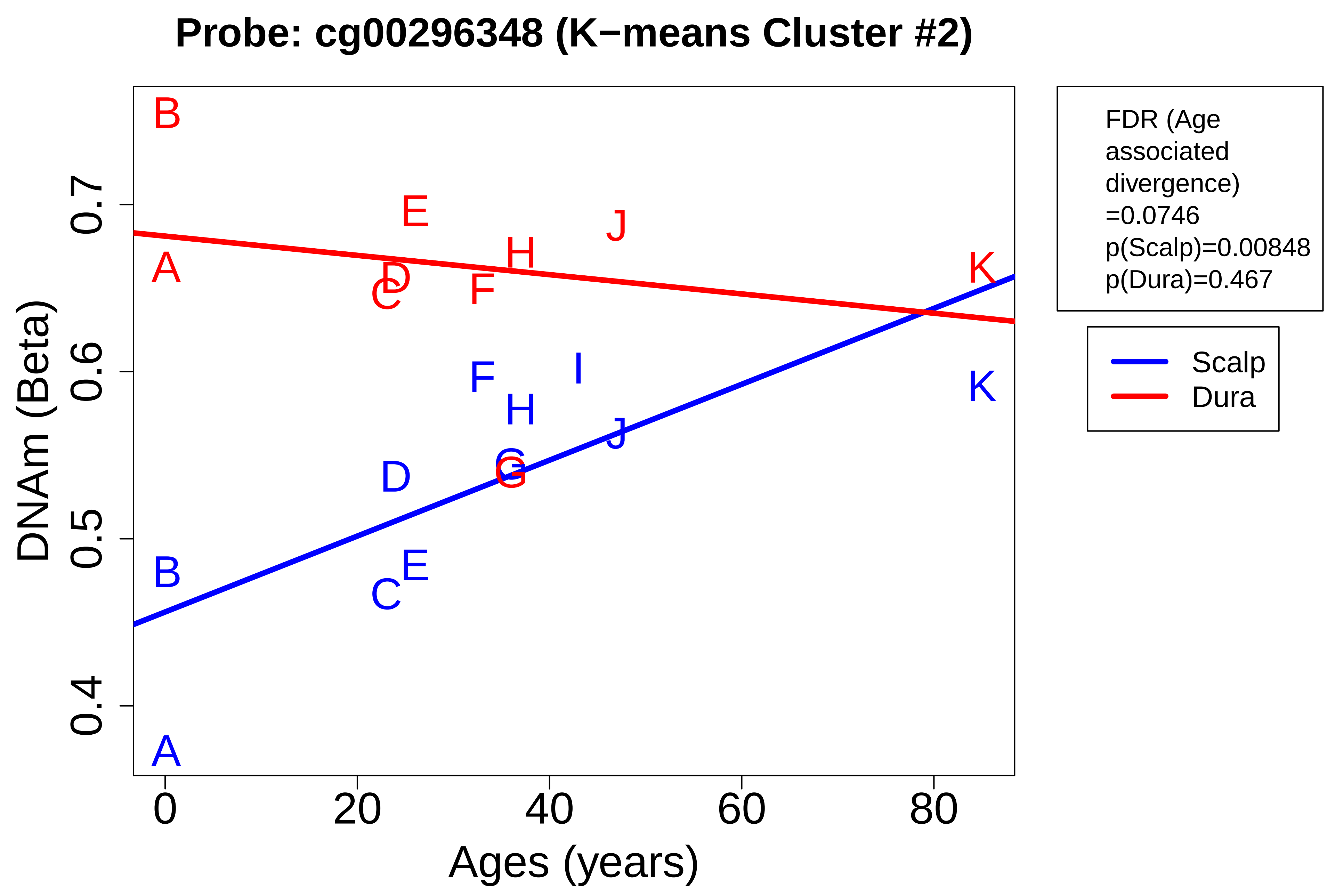

Probe: cg00296348 (K−means Cluster #2)
DNAm (Beta)
0.4
0.5
0.6
0.7
0
20
40
60
80
Ages (years)
A
B
C
D
E
F
G
H
I
J
K
FDR (Age associated divergence) =0.0746
p(Scalp)=0.00848
p(Dura)=0.467
Scalp
Dura

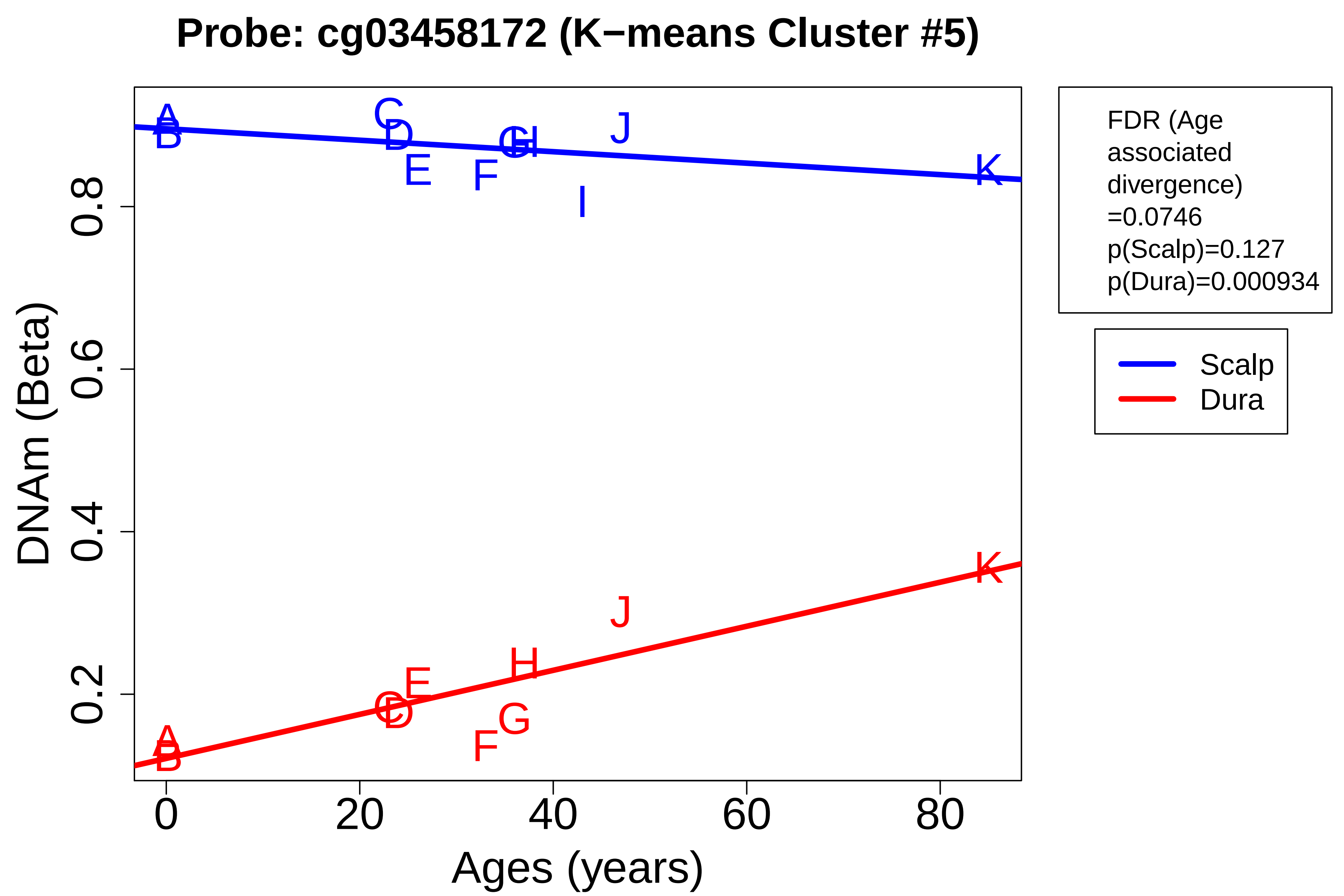

Probe: cg03458172 (K−means Cluster #5)
DNAm (Beta)
0.2
0.4
0.6
0.8
0
20
40
60
80
Ages (years)
A
B
C
D
E
F
G
H
I
J
K
FDR (Age associated divergence) =0.0746
p(Scalp)=0.127
p(Dura)=0.000934
Scalp
Dura

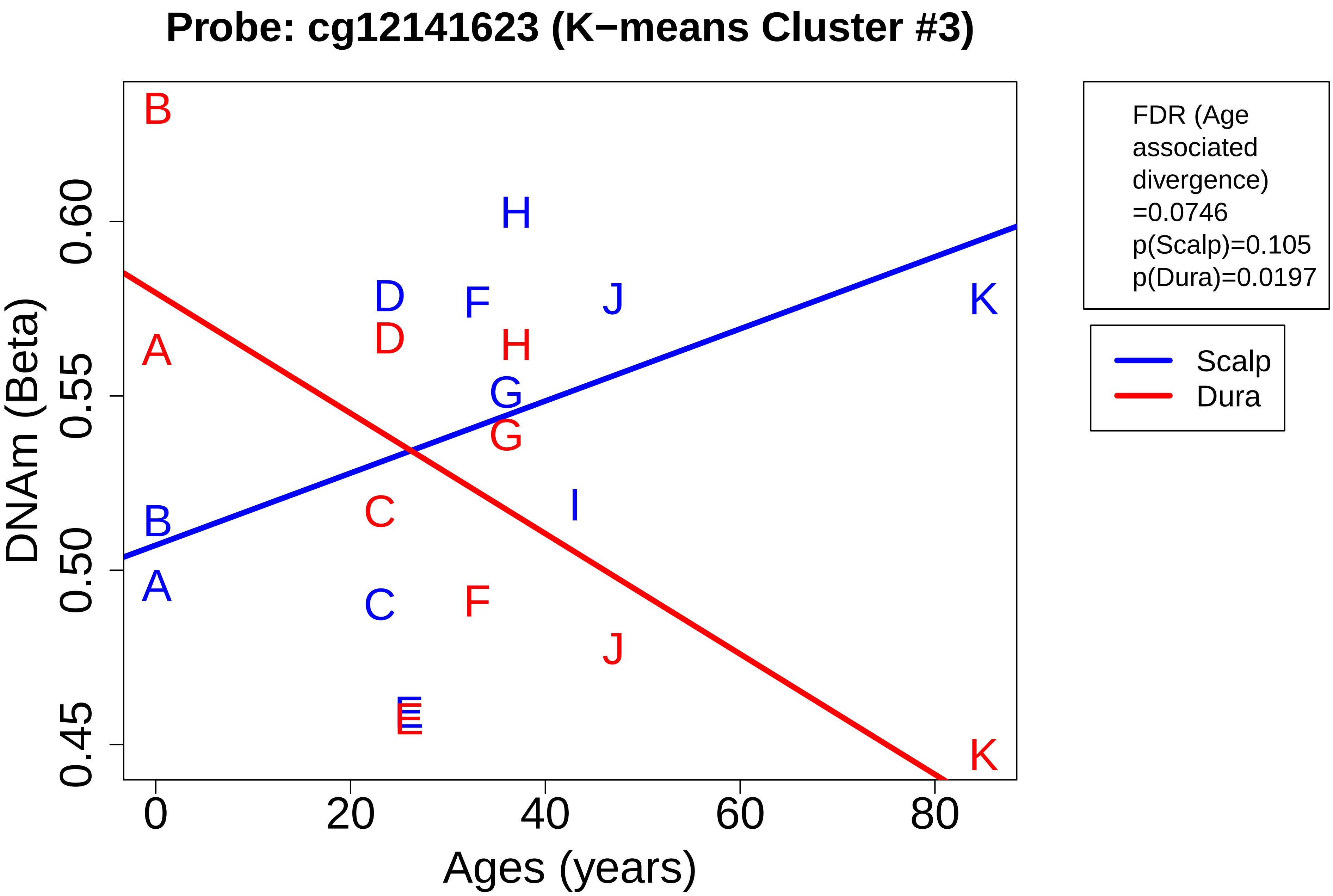
Probe: cg12141623 (K−means Cluster #3)
DNAm (Beta)
Ages (years)
0.45
0.50
0.55
0.60
0
20
40
60
80
FDR (Age associated divergence) =0.0746
p(Scalp)=0.105
p(Dura)=0.0197
Scalp
Dura

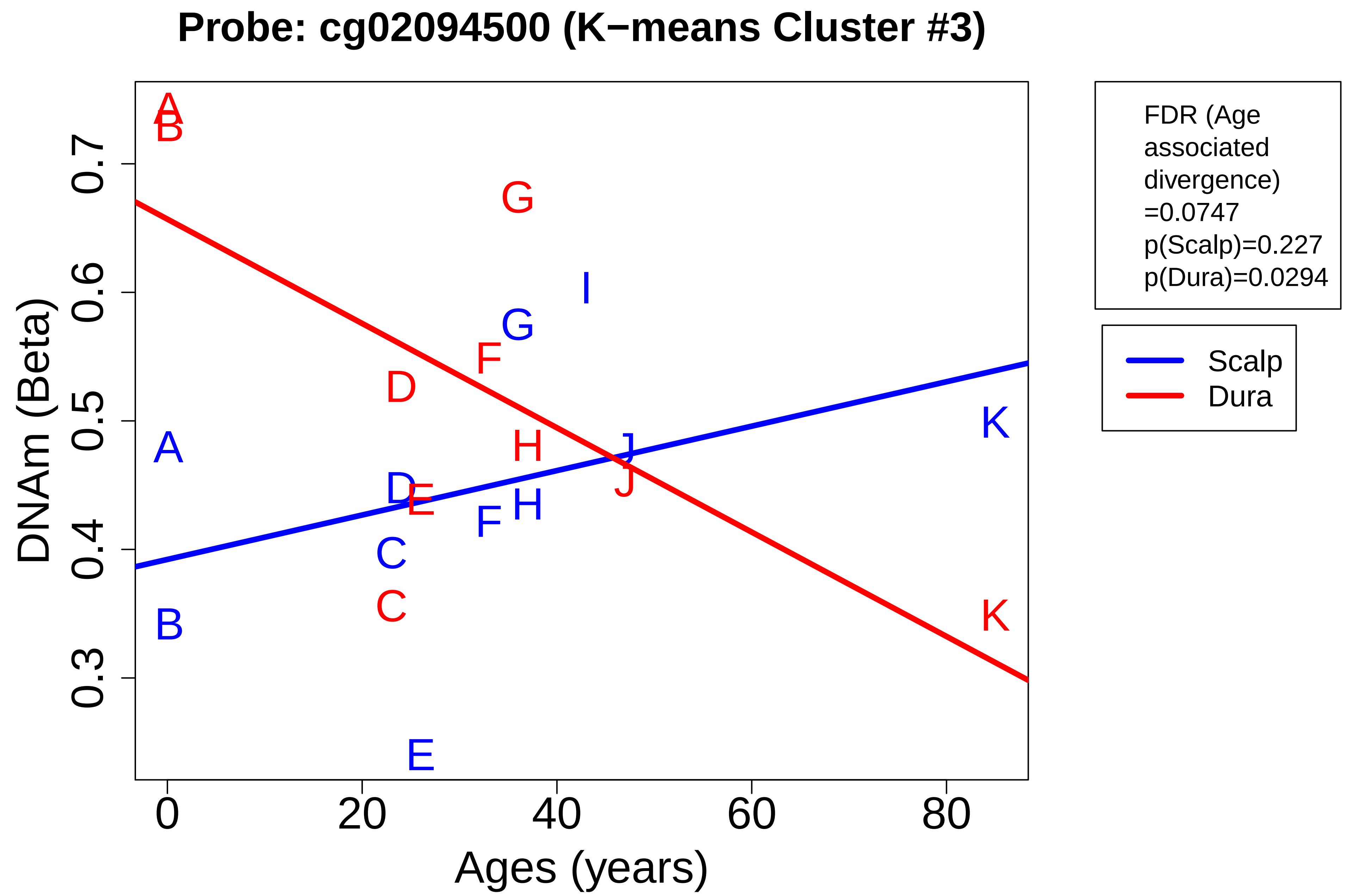
Probe: cg02094500 (K−means Cluster #3)
DNAm (Beta)
0.3
0.4
0.5
0.6
0.7
Ages (years)
0
20
40
60
80
A
B
C
D
E
F
G
H
I
J
K
FDR (Age associated divergence) =0.0747
p(Scalp)=0.227
p(Dura)=0.0294
Scalp
Dura

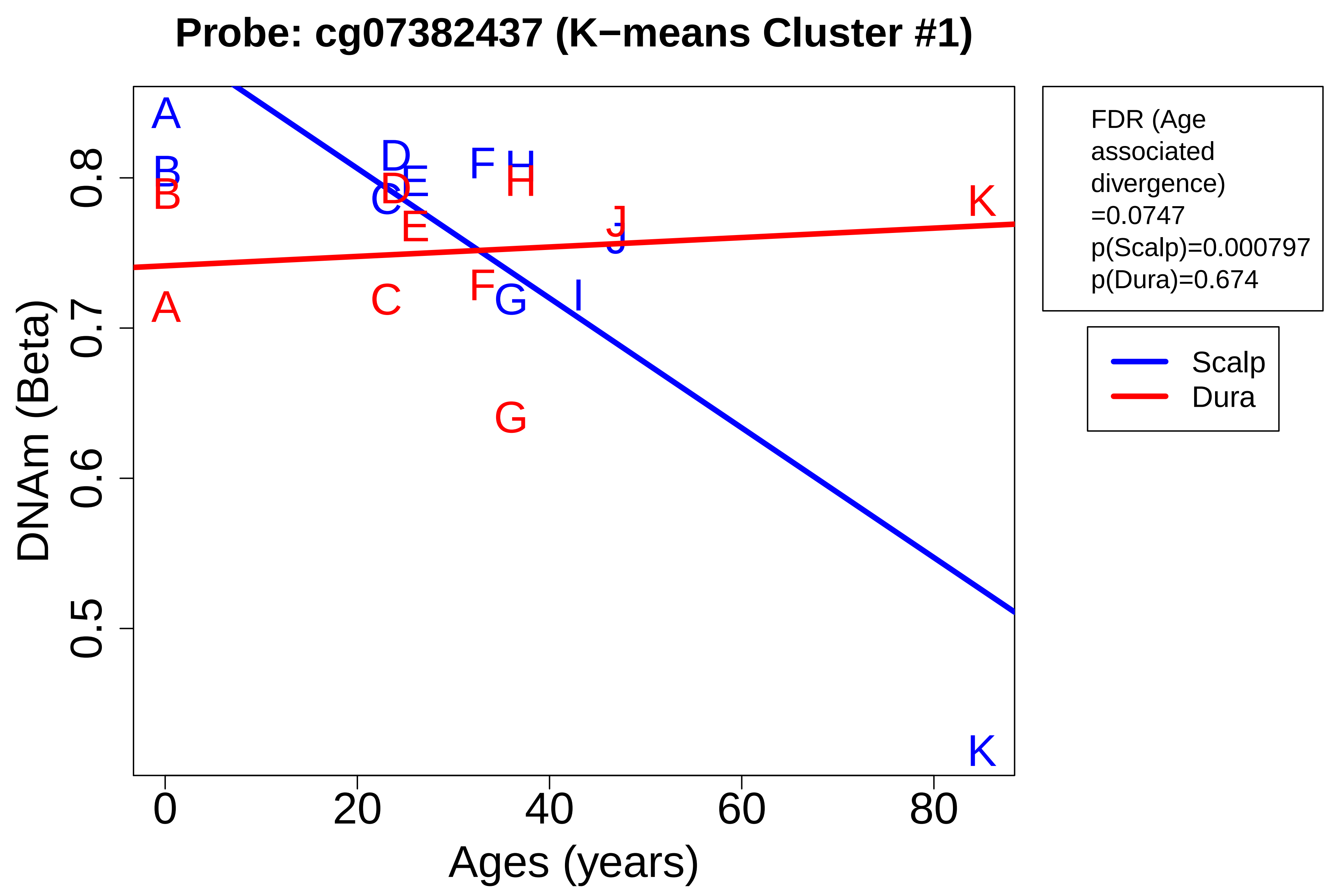

Probe: cg07382437 (K−means Cluster #1)
DNAm (Beta)
0.5
0.6
0.7
0.8
0
20
40
60
80
Ages (years)
A
B
C
D
E
F
G
H
I
J
K
FDR (Age associated divergence) =0.0747
p(Scalp)=0.000797
p(Dura)=0.674
Scalp
Dura

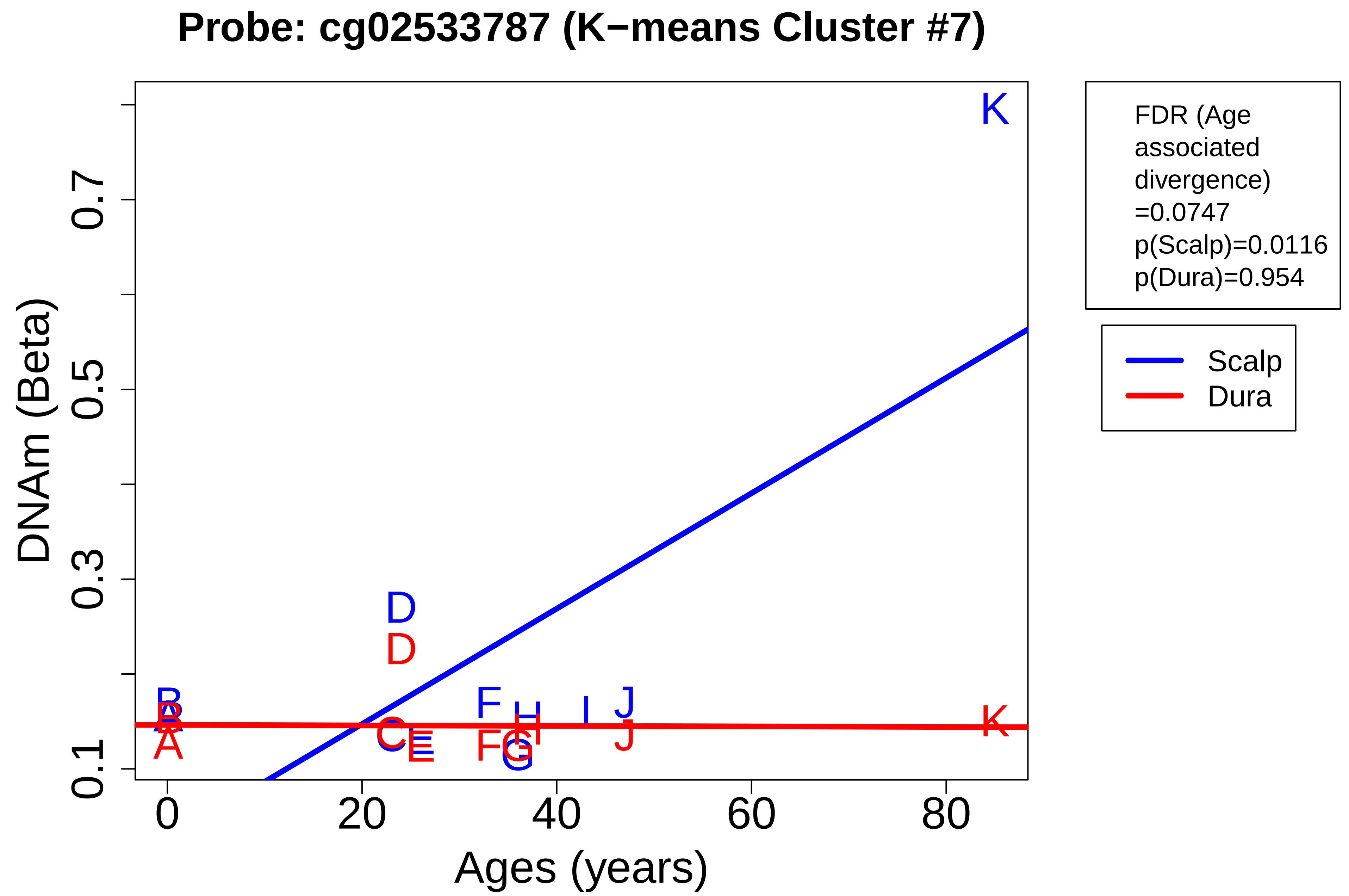

Probe: cg02533787 (K−means Cluster #7)
DNAm (Beta)
0.1
0.3
0.5
0.7
0
20
40
60
80
Ages (years)
A
B
C
D
E
F
G
H
I
J
K
FDR (Age associated divergence) =0.0747
p(Scalp)=0.0116
p(Dura)=0.954
Scalp
Dura

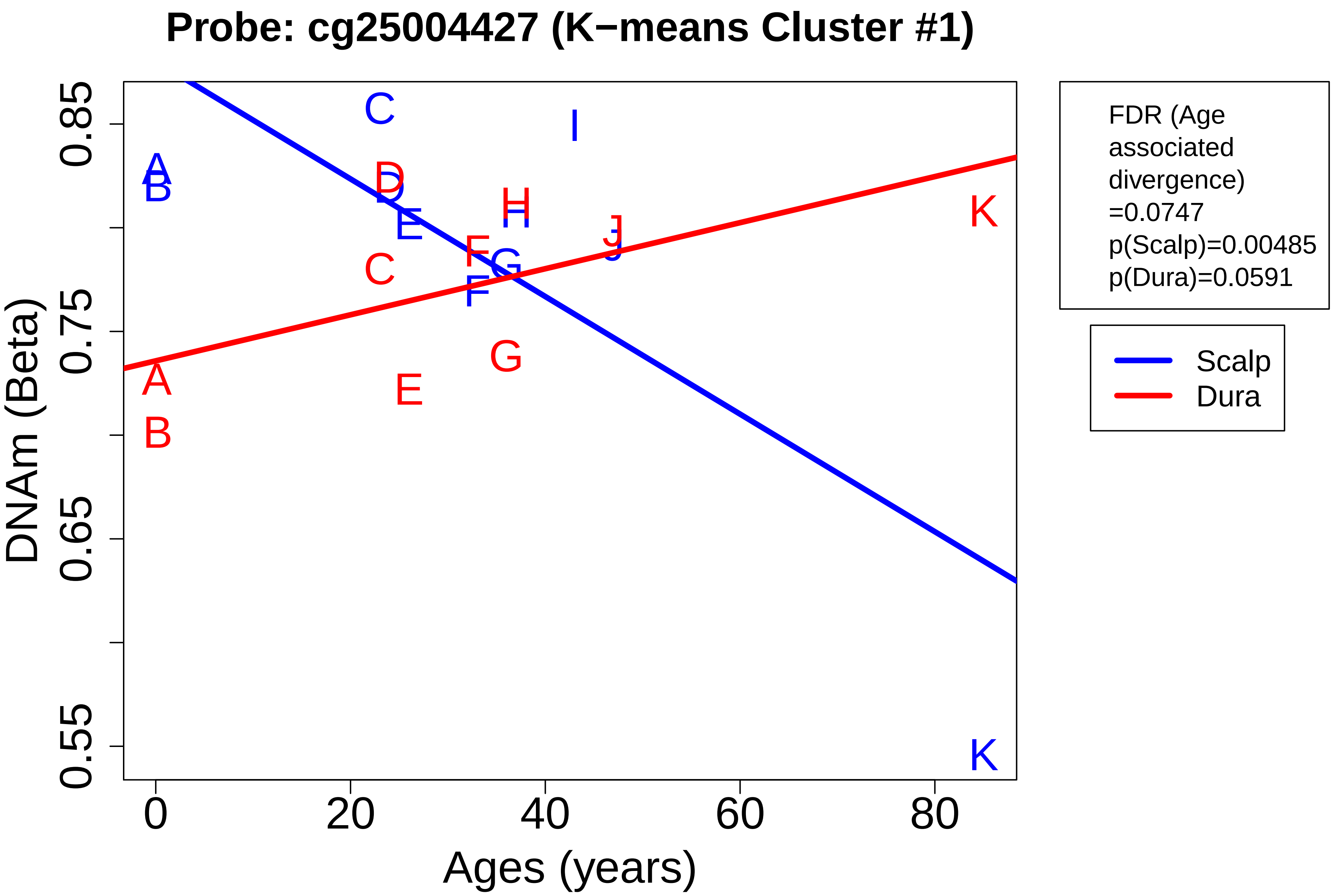

Probe: cg25004427 (K−means Cluster #1)
DNAm (Beta)
0.55
0.65
0.75
0.85
0
20
40
60
80
Ages (years)
A
B
C
D
E
F
G
H
I
J
K
FDR (Age associated divergence) =0.0747
p(Scalp)=0.00485
p(Dura)=0.0591
Scalp
Dura

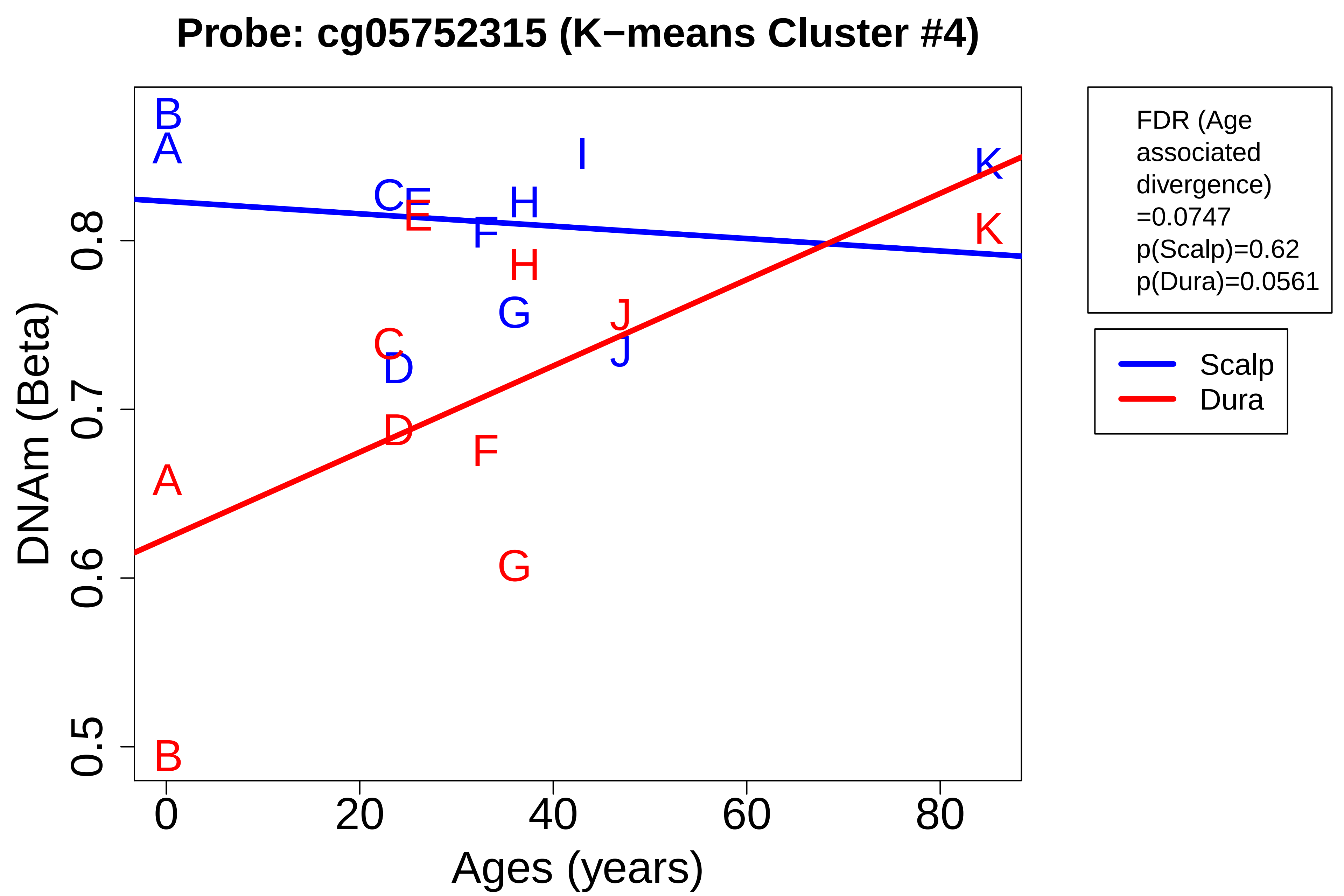

Probe: cg05752315 (K−means Cluster #4)
DNAm (Beta)
0.5
0.6
0.7
0.8
0
20
40
60
80
Ages (years)
B
A
I
K
C
E
H
F
K
H
G
J
C
J
D
D
F
A
G
B
FDR (Age associated divergence) =0.0747
p(Scalp)=0.62
p(Dura)=0.0561
Scalp
Dura

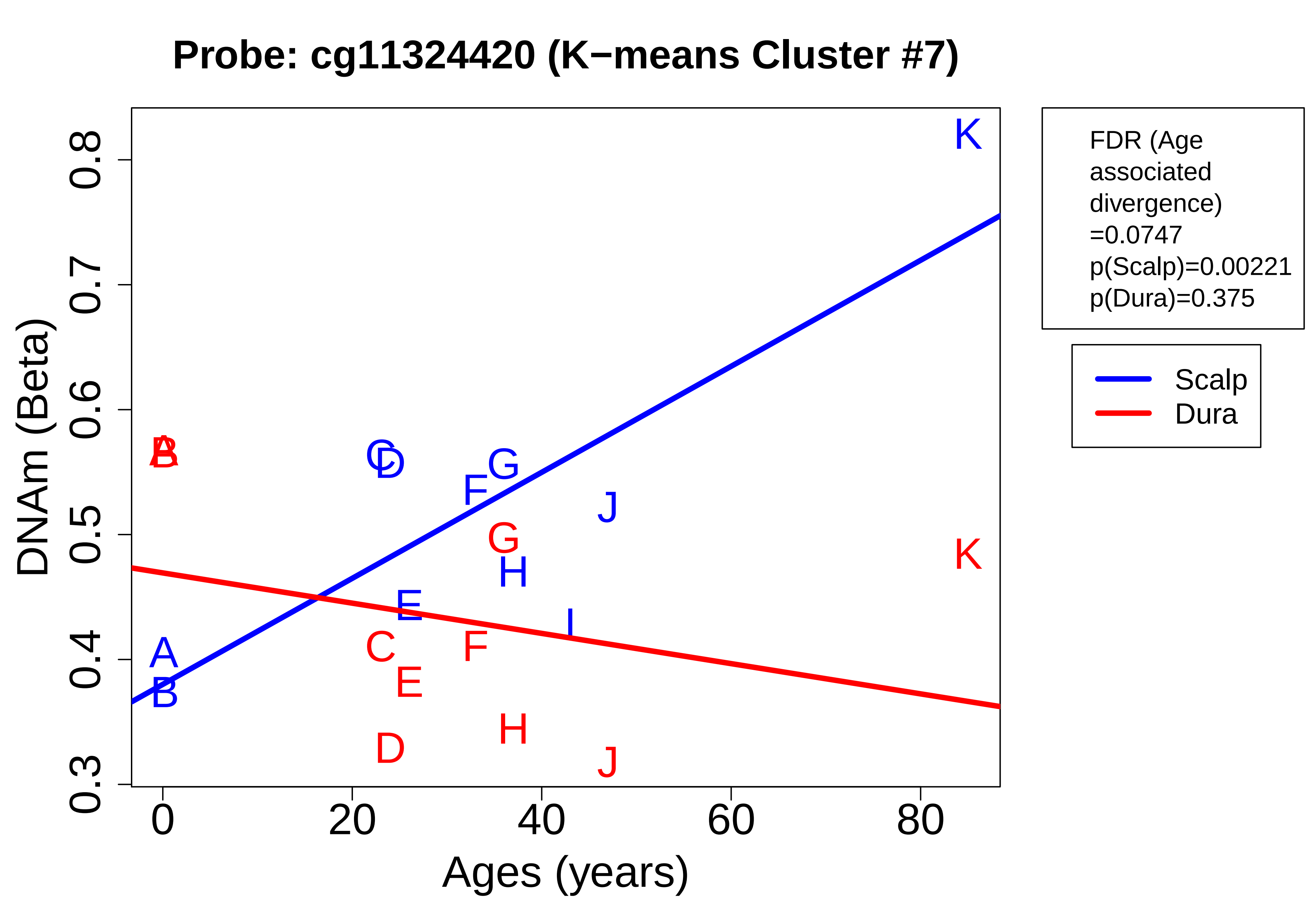
Probe: cg11324420 (K−means Cluster #7)
DNAm (Beta)
0.3
0.4
0.5
0.6
0.7
0.8
Ages (years)
0
20
40
60
80
FDR (Age associated divergence) =0.0747
p(Scalp)=0.00221
p(Dura)=0.375
Scalp
Dura
A
B
C
D
E
F
G
H
I
J
K

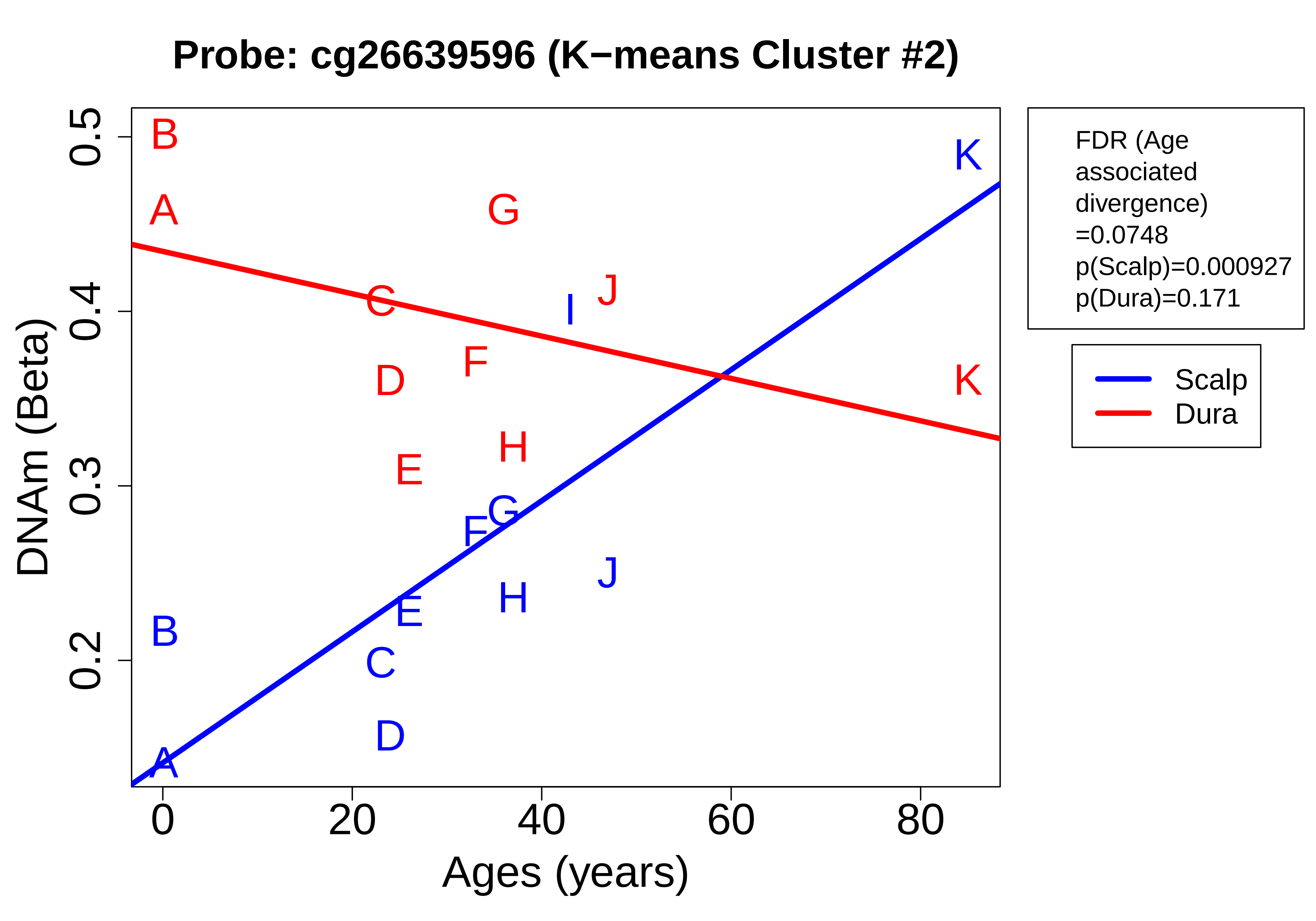

Probe: cg26639596 (K−means Cluster #2)
DNAm (Beta)
0.2
0.3
0.4
0.5
Ages (years)
0
20
40
60
80
FDR (Age associated divergence) =0.0748
p(Scalp)=0.000927
p(Dura)=0.171
Scalp
Dura
A
B
C
D
E
F
G
H
I
J
K

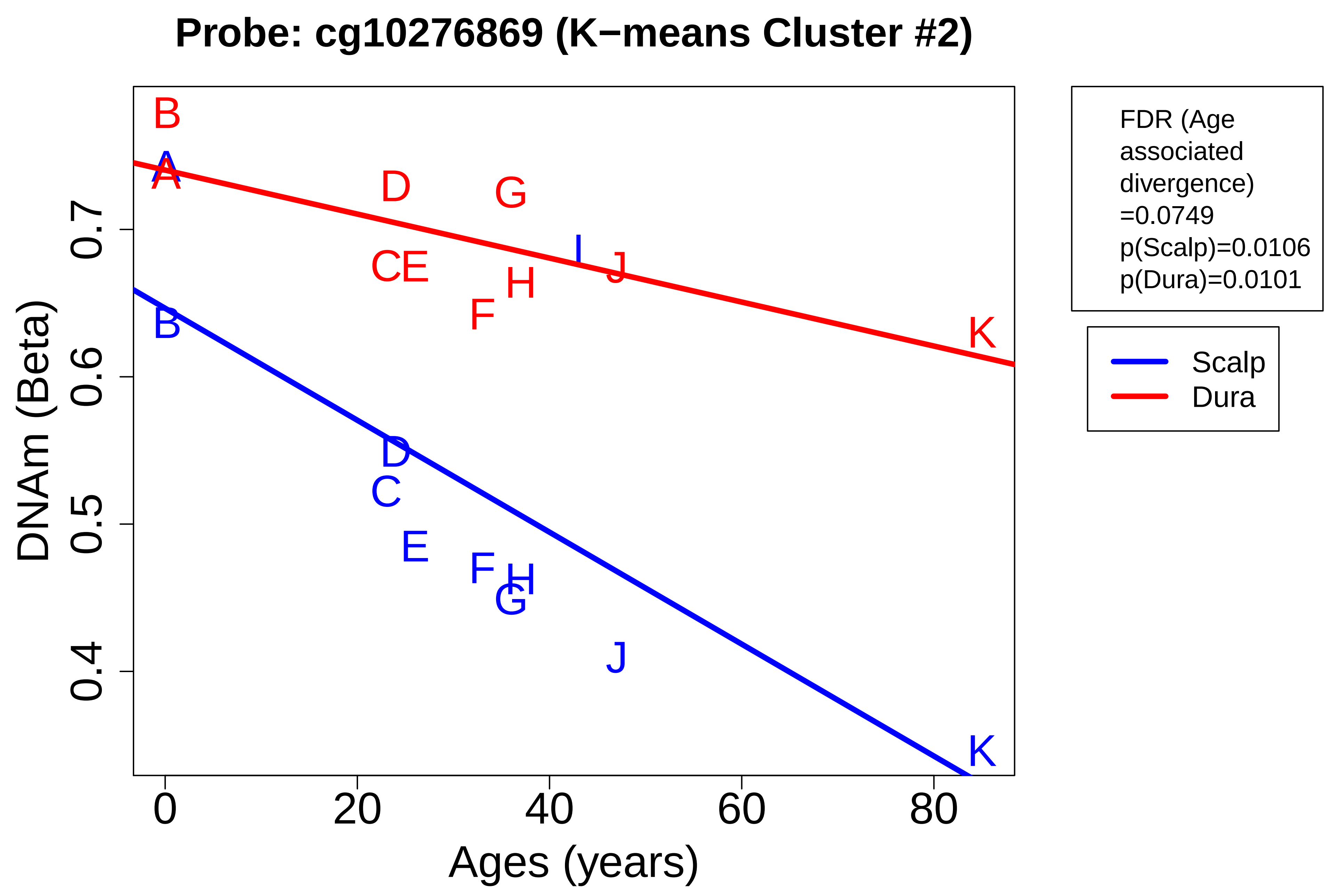
Probe: cg10276869 (K−means Cluster #2)
DNAm (Beta)
0.4
0.5
0.6
0.7
0
20
40
60
80
Ages (years)
FDR (Age associated divergence) =0.0749
p(Scalp)=0.0106
p(Dura)=0.0101
Scalp
Dura
A
B
C
D
E
F
G
H
I
J
K
A
B
CE
D
F
G
H
J
K

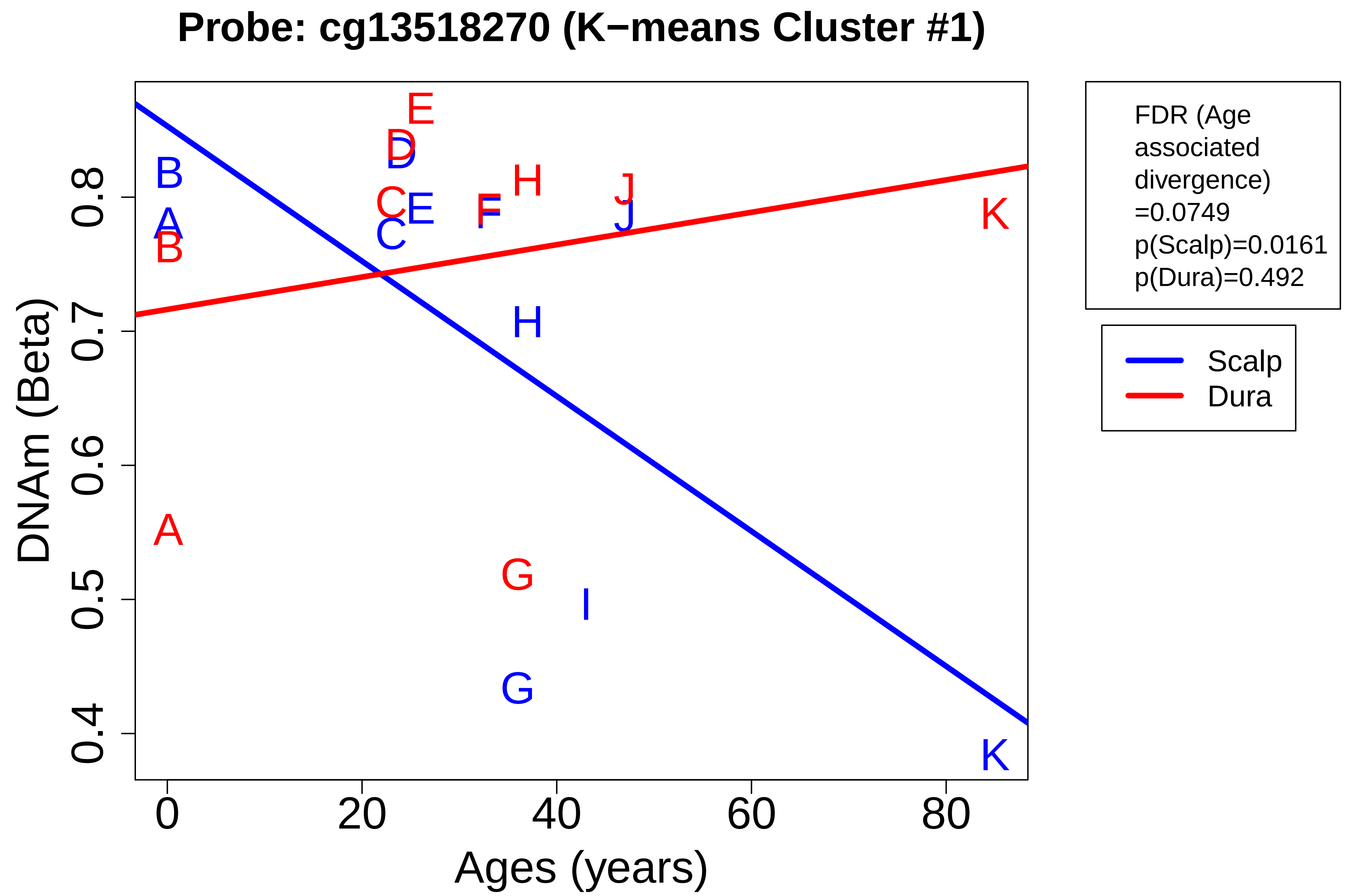

Probe: cg13518270 (K−means Cluster #1)
DNAm (Beta)
0.4
0.5
0.6
0.7
0.8
Ages (years)
0
20
40
60
80
FDR (Age associated divergence) =0.0749
p(Scalp)=0.0161
p(Dura)=0.492
Scalp
Dura

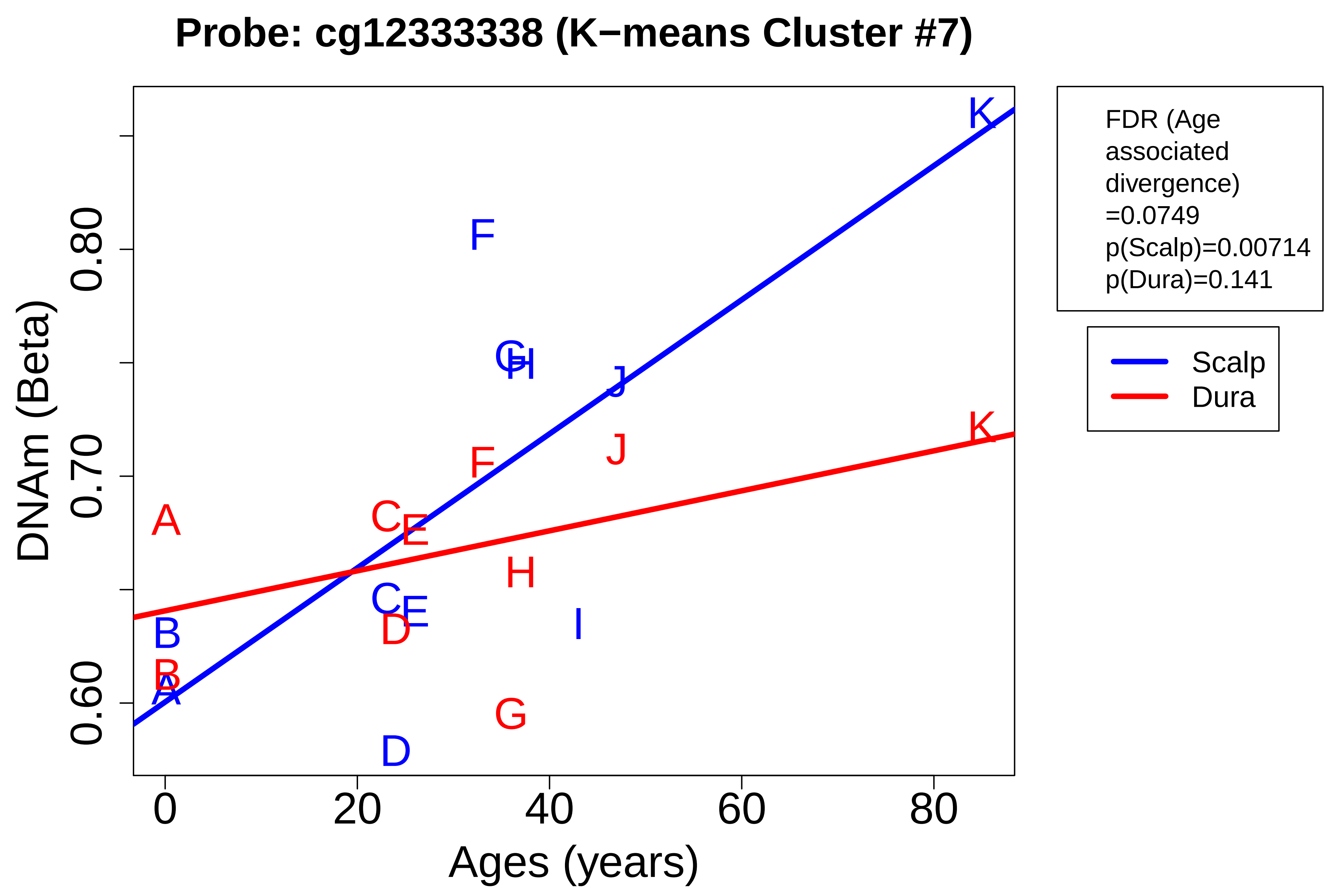
Probe: cg12333338 (K−means Cluster #7)
DNAm (Beta)
0.60
0.70
0.80
Ages (years)
0
20
40
60
80
FDR (Age associated divergence) =0.0749
p(Scalp)=0.00714
p(Dura)=0.141
Scalp
Dura

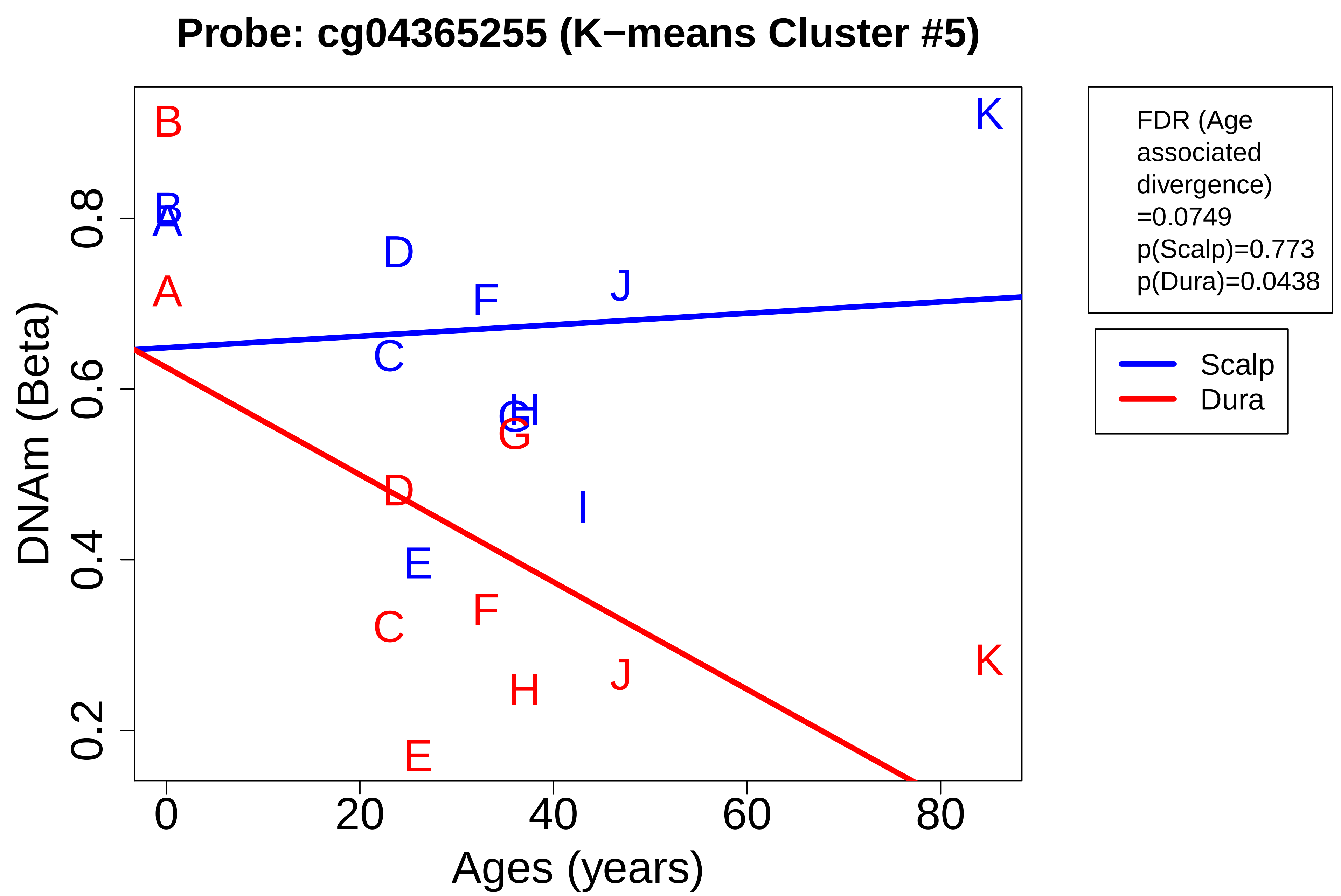

Probe: cg04365255 (K−means Cluster #5)
DNAm (Beta)
0.2
0.4
0.6
0.8
0
20
40
60
80
Ages (years)
B
K
B
A
D
A
J
F
C
H
G
G
D
I
E
F
C
K
J
H
E
FDR (Age associated divergence) =0.0749
p(Scalp)=0.773
p(Dura)=0.0438
Scalp
Dura

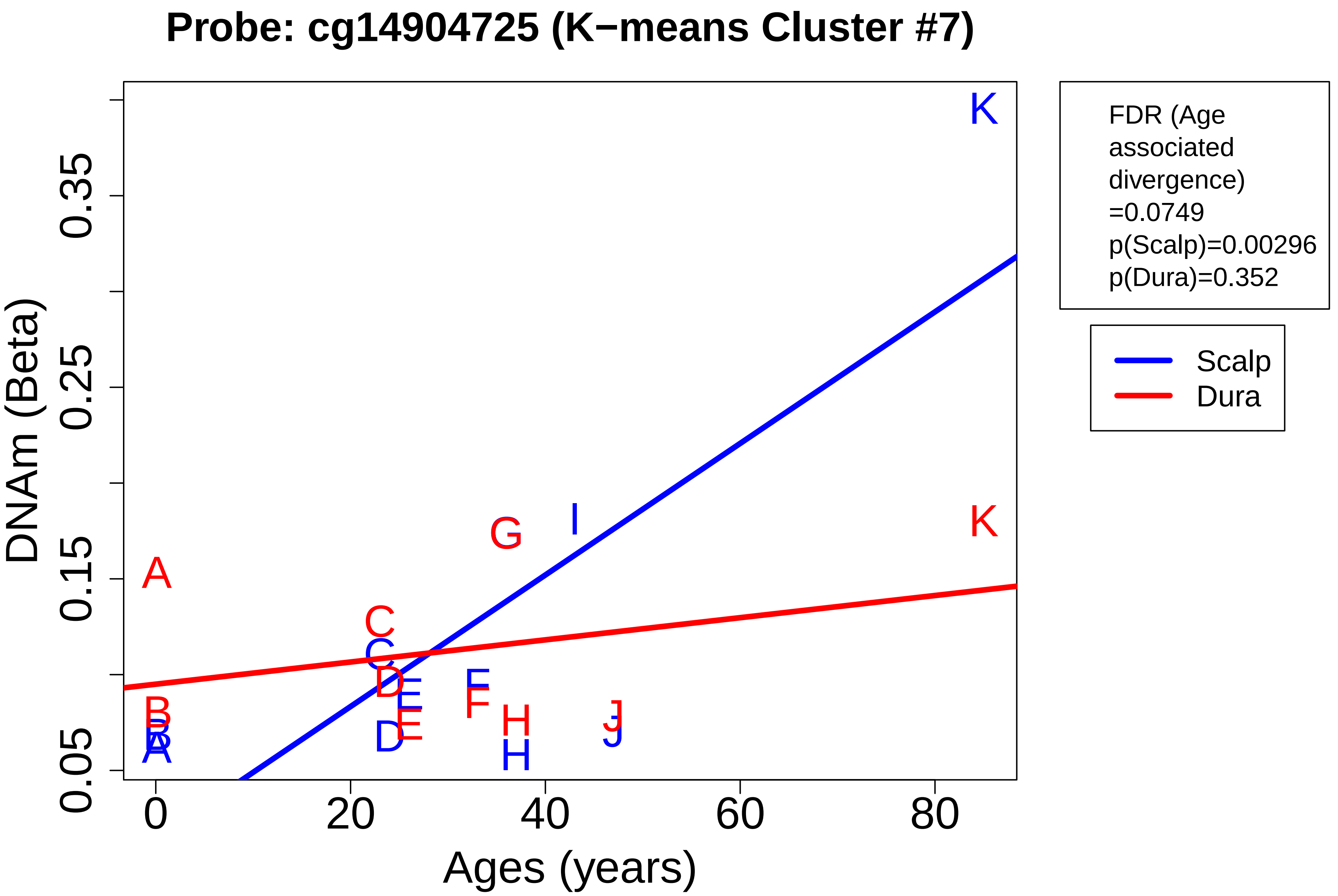

Probe: cg14904725 (K−means Cluster #7)
DNAm (Beta)
0.05
0.15
0.25
0.35
0
20
40
60
80
Ages (years)
FDR (Age associated divergence) =0.0749
p(Scalp)=0.00296
p(Dura)=0.352
Scalp
Dura

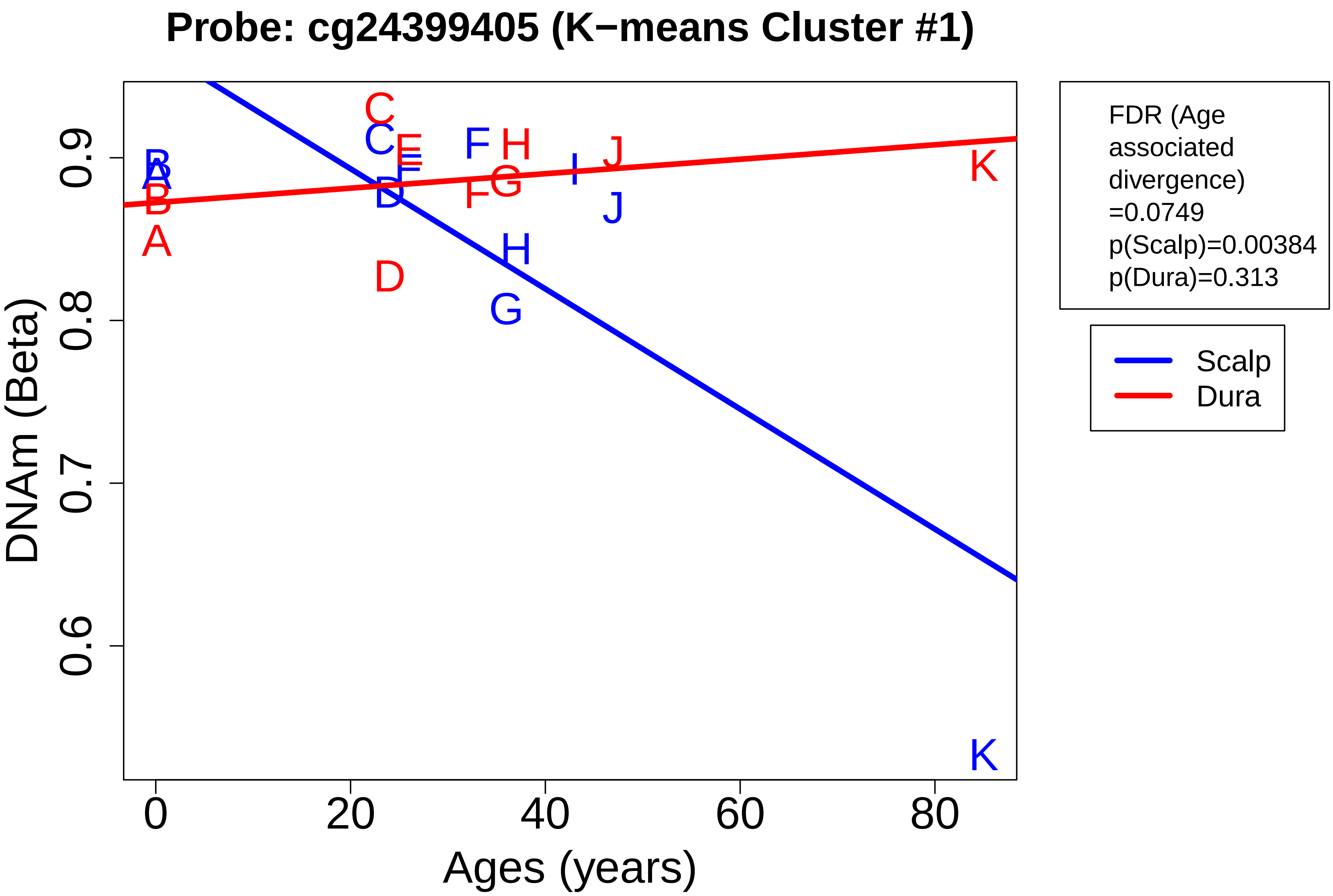
Probe: cg24399405 (K−means Cluster #1)
DNAm (Beta)
0.6
0.7
0.8
0.9
0
20
40
60
80
Ages (years)
FDR (Age associated divergence) =0.0749
p(Scalp)=0.00384
p(Dura)=0.313
Scalp
Dura

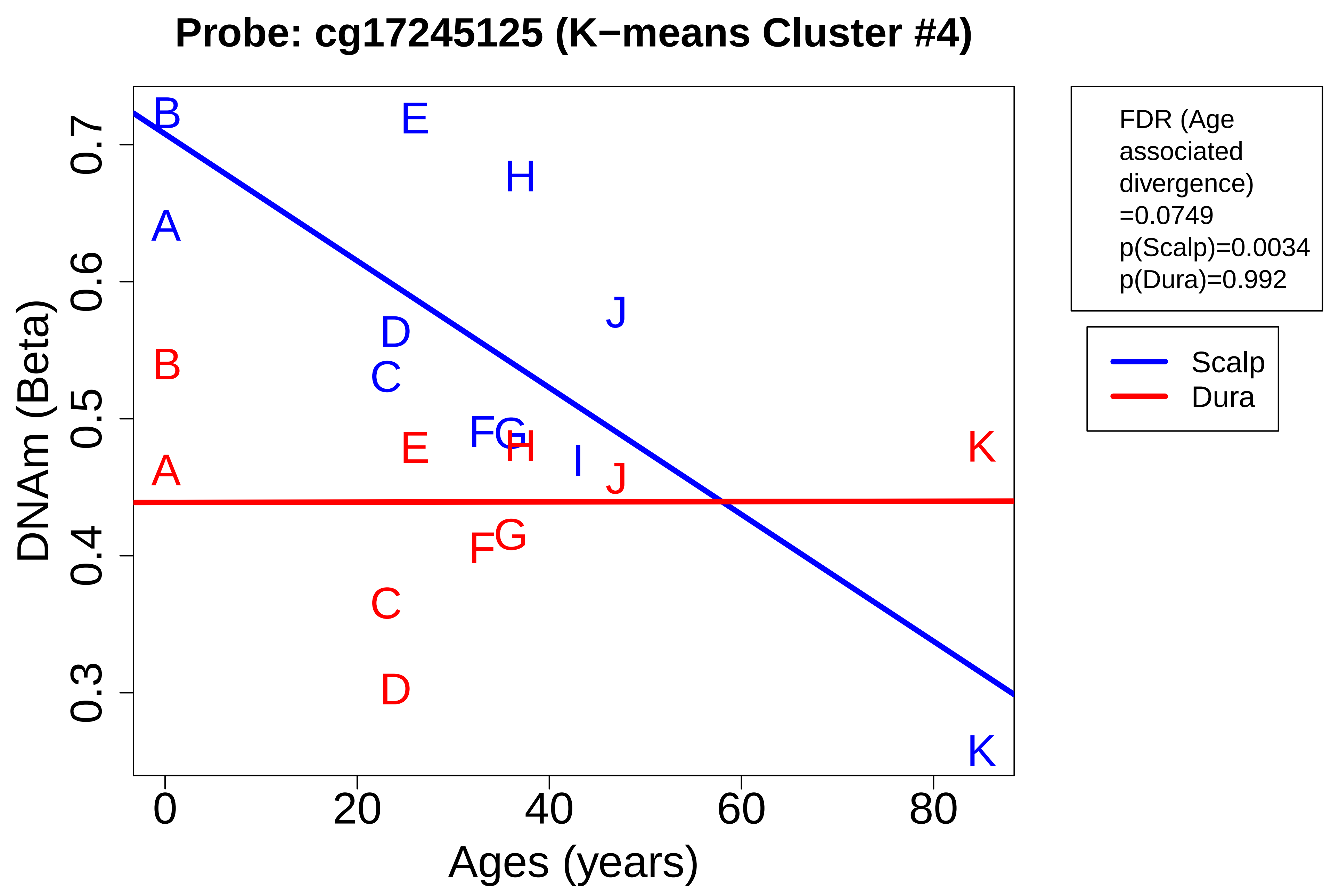

Probe: cg17245125 (K−means Cluster #4)
FDR (Age associated divergence) =0.0749
p(Scalp)=0.0034
p(Dura)=0.992
Scalp
Dura
DNAm (Beta)
0.3
0.4
0.5
0.6
0.7
0
20
40
60
80
Ages (years)
A B C D E F G H I J K

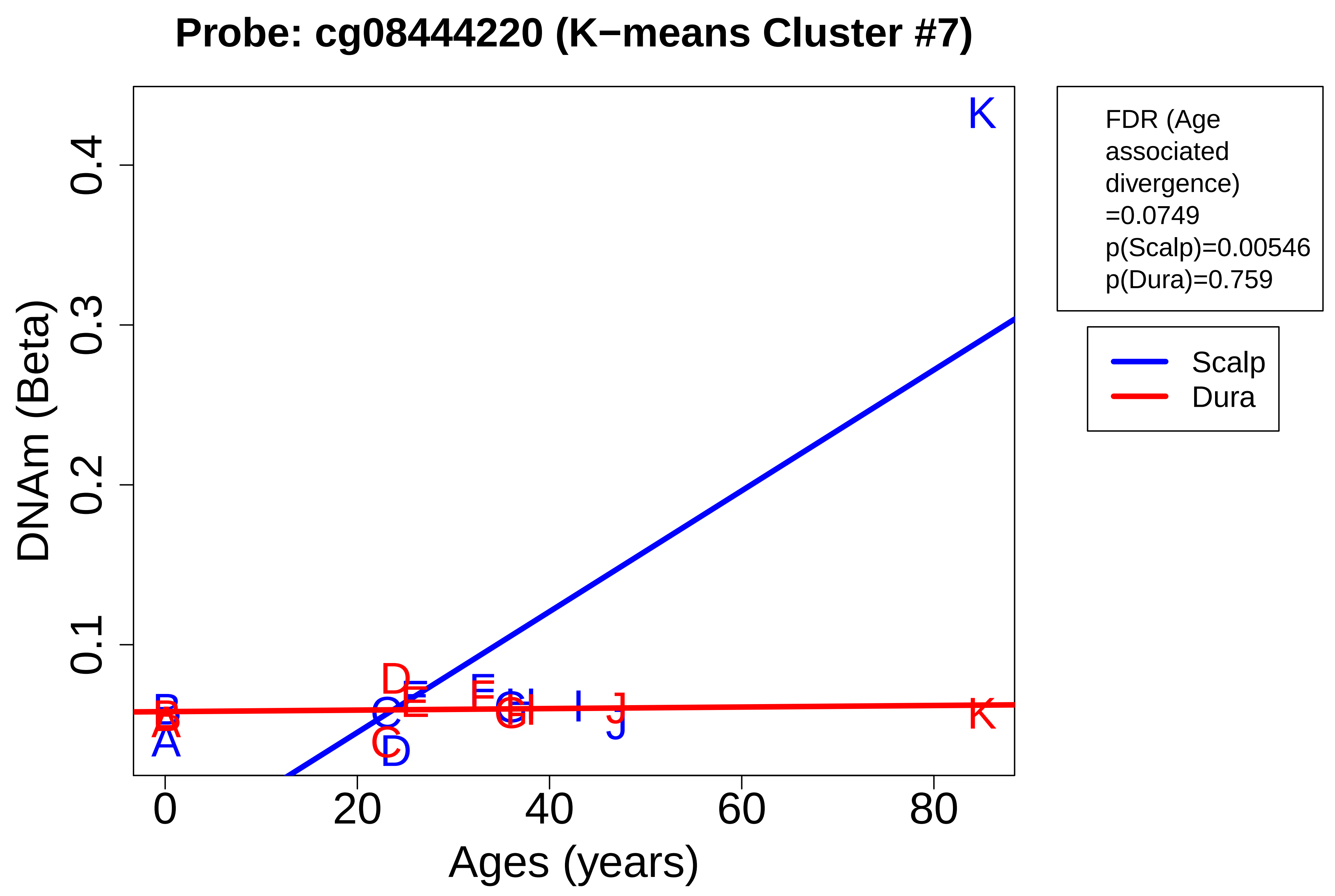

Probe: cg08444220 (K−means Cluster #7)
DNAm (Beta)
0.1
0.2
0.3
0.4
0
20
40
60
80
Ages (years)
A
B
C
D
E
F
G
H
I
J
K
FDR (Age associated divergence) =0.0749
p(Scalp)=0.00546
p(Dura)=0.759
Scalp
Dura

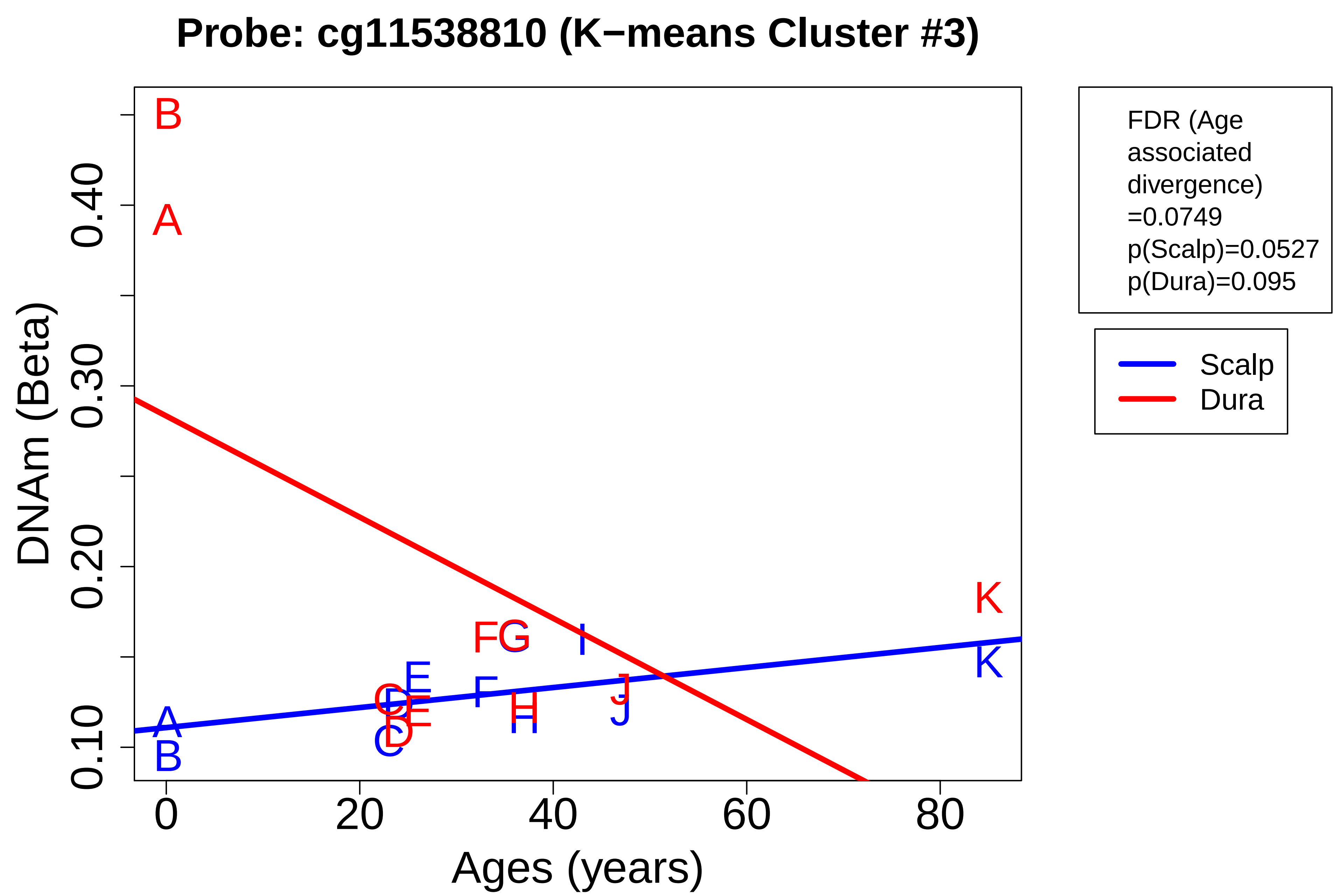

Probe: cg11538810 (K−means Cluster #3)
DNAm (Beta)
0.10
0.20
0.30
0.40
Ages (years)
0
20
40
60
80
FDR (Age associated divergence) =0.0749
p(Scalp)=0.0527
p(Dura)=0.095
Scalp
Dura

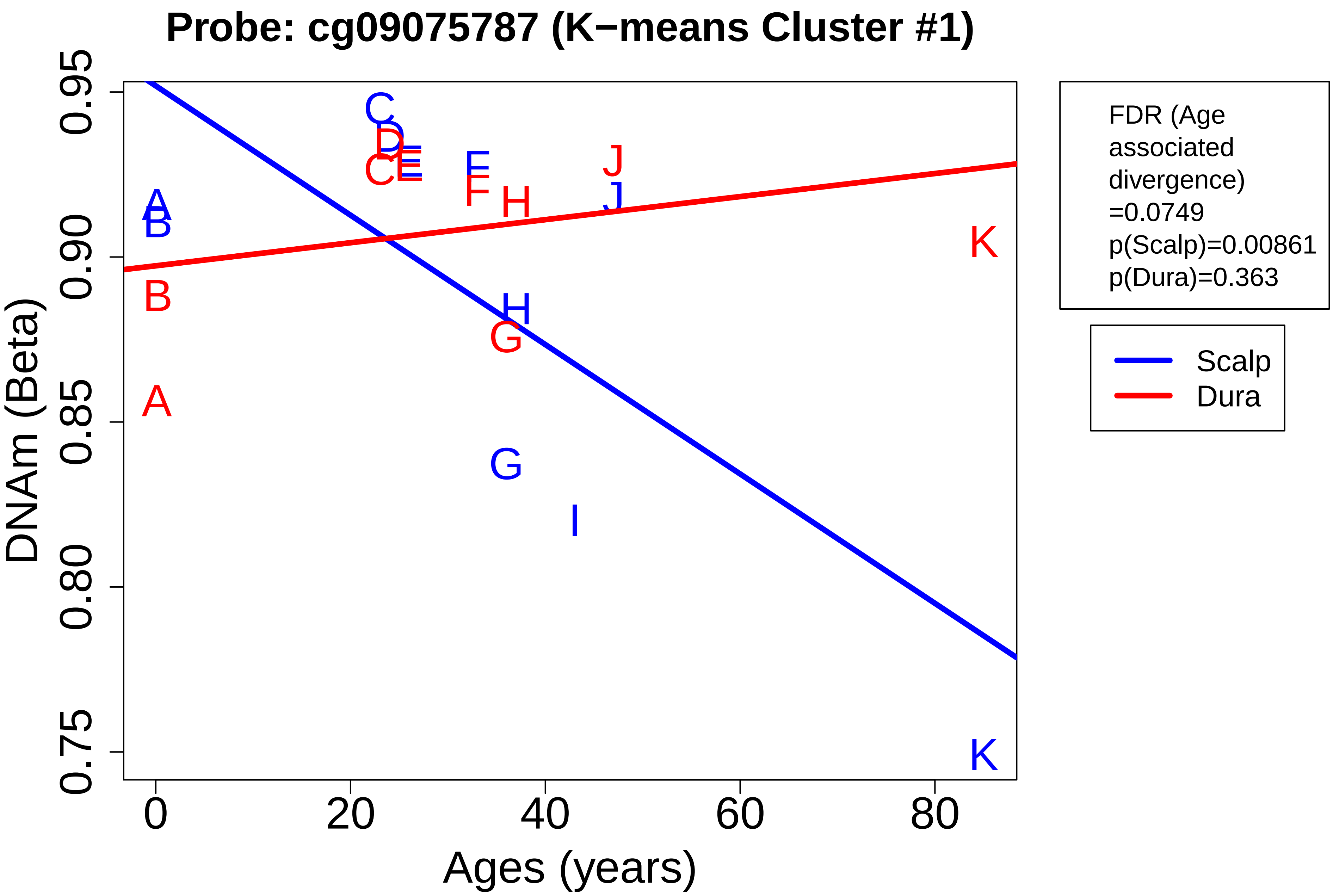

Probe: cg09075787 (K−means Cluster #1)
DNAm (Beta)
0.75
0.80
0.85
0.90
0.95
0
20
40
60
80
Ages (years)
FDR (Age associated divergence) =0.0749
p(Scalp)=0.00861
p(Dura)=0.363
Scalp
Dura

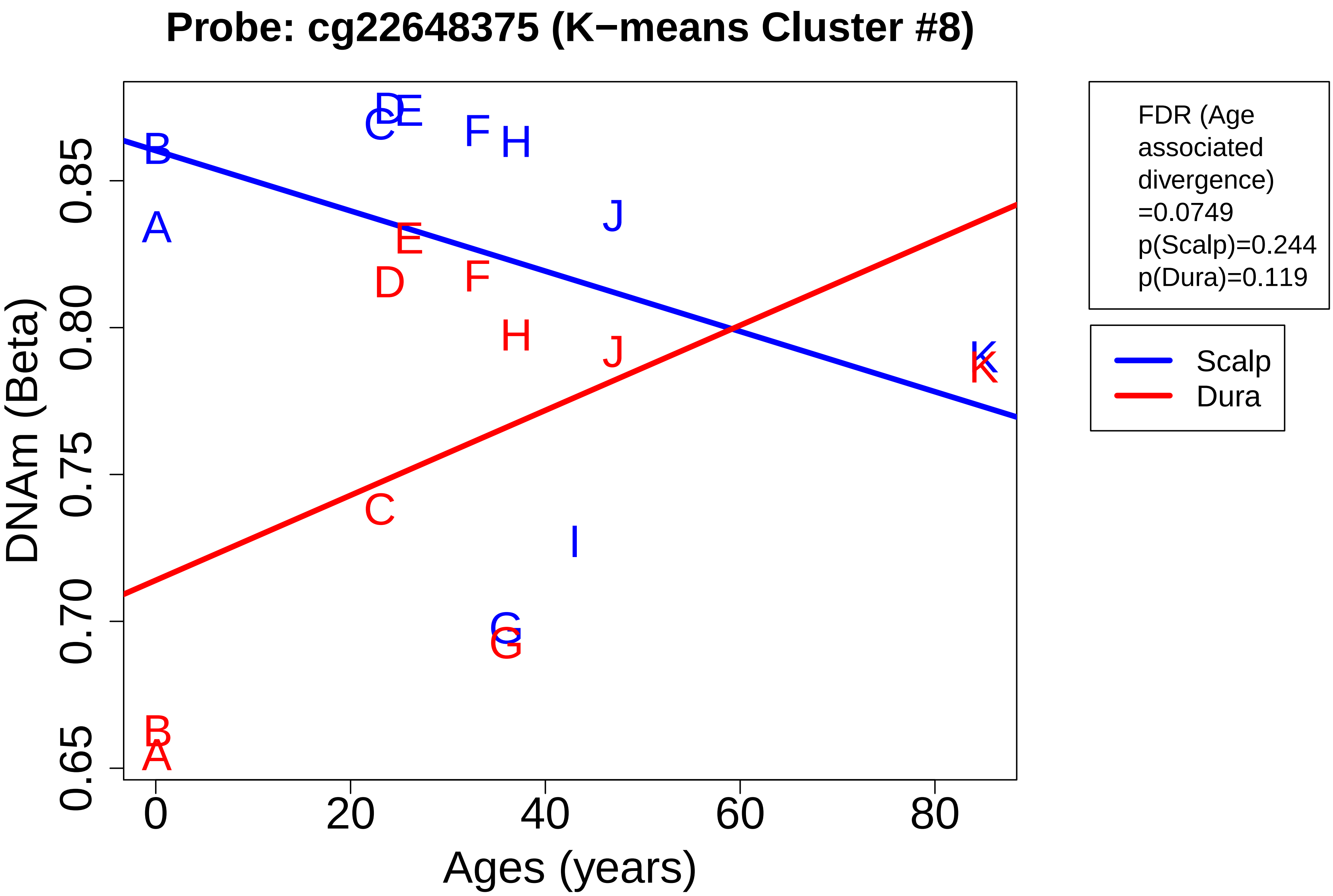

Probe: cg22648375 (K−means Cluster #8)
DNAm (Beta)
0.65
0.70
0.75
0.80
0.85
0
20
40
60
80
Ages (years)
FDR (Age associated divergence) =0.0749
p(Scalp)=0.244
p(Dura)=0.119
Scalp
Dura

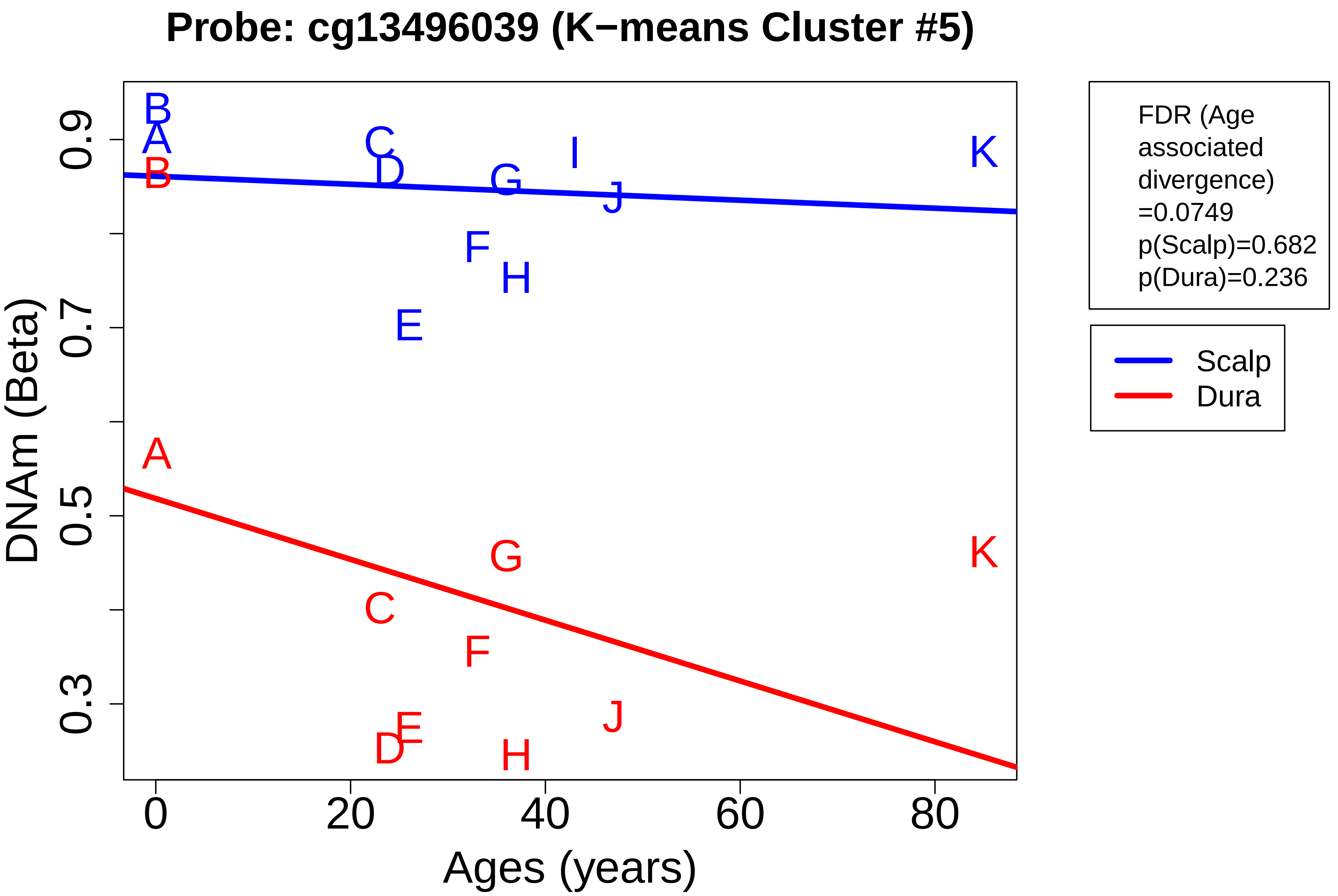

Probe: cg13496039 (K−means Cluster #5)
DNAm (Beta)
0.3
0.5
0.7
0.9
Ages (years)
0
20
40
60
80
FDR (Age associated divergence) =0.0749
p(Scalp)=0.682
p(Dura)=0.236
Scalp
Dura

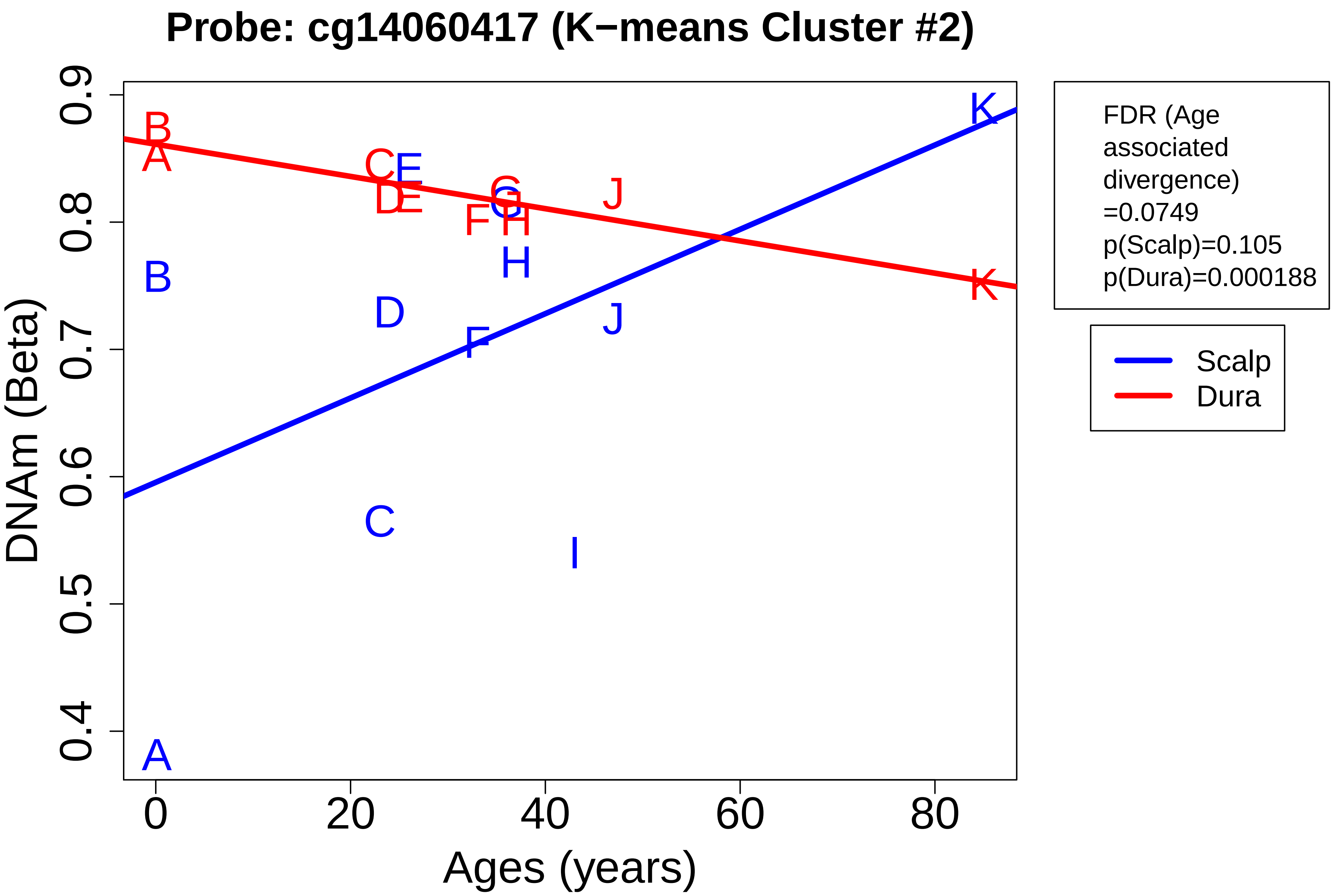

Probe: cg14060417 (K−means Cluster #2)
DNAm (Beta)
0.4
0.5
0.6
0.7
0.8
0.9
0
20
40
60
80
Ages (years)
FDR (Age associated divergence) =0.0749
p(Scalp)=0.105
p(Dura)=0.000188
Scalp
Dura

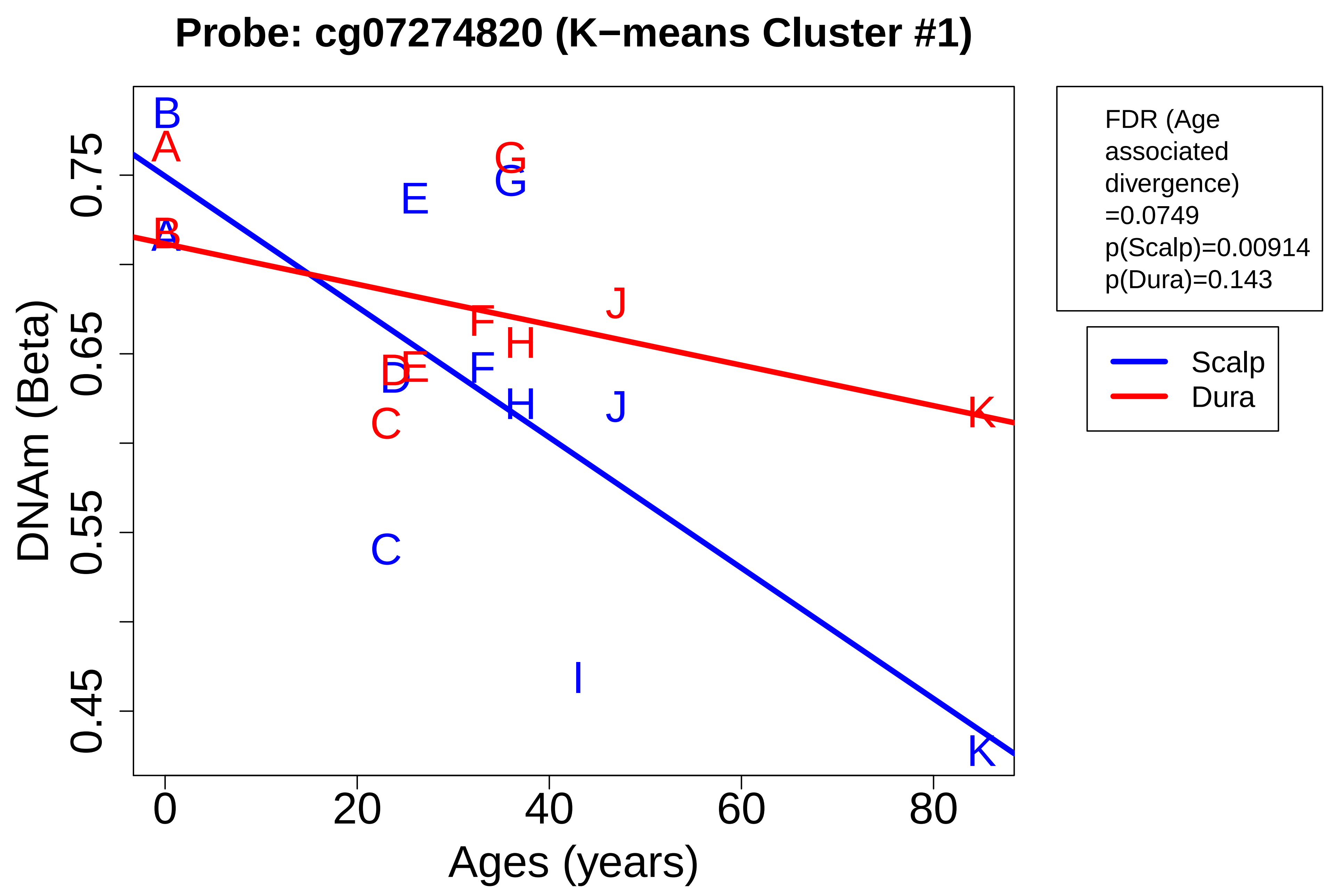
Probe: cg07274820 (K−means Cluster #1)
FDR (Age associated divergence) =0.0749
p(Scalp)=0.00914
p(Dura)=0.143
Scalp
Dura
DNAm (Beta)
0.45
0.55
0.65
0.75
Ages (years)
0
20
40
60
80

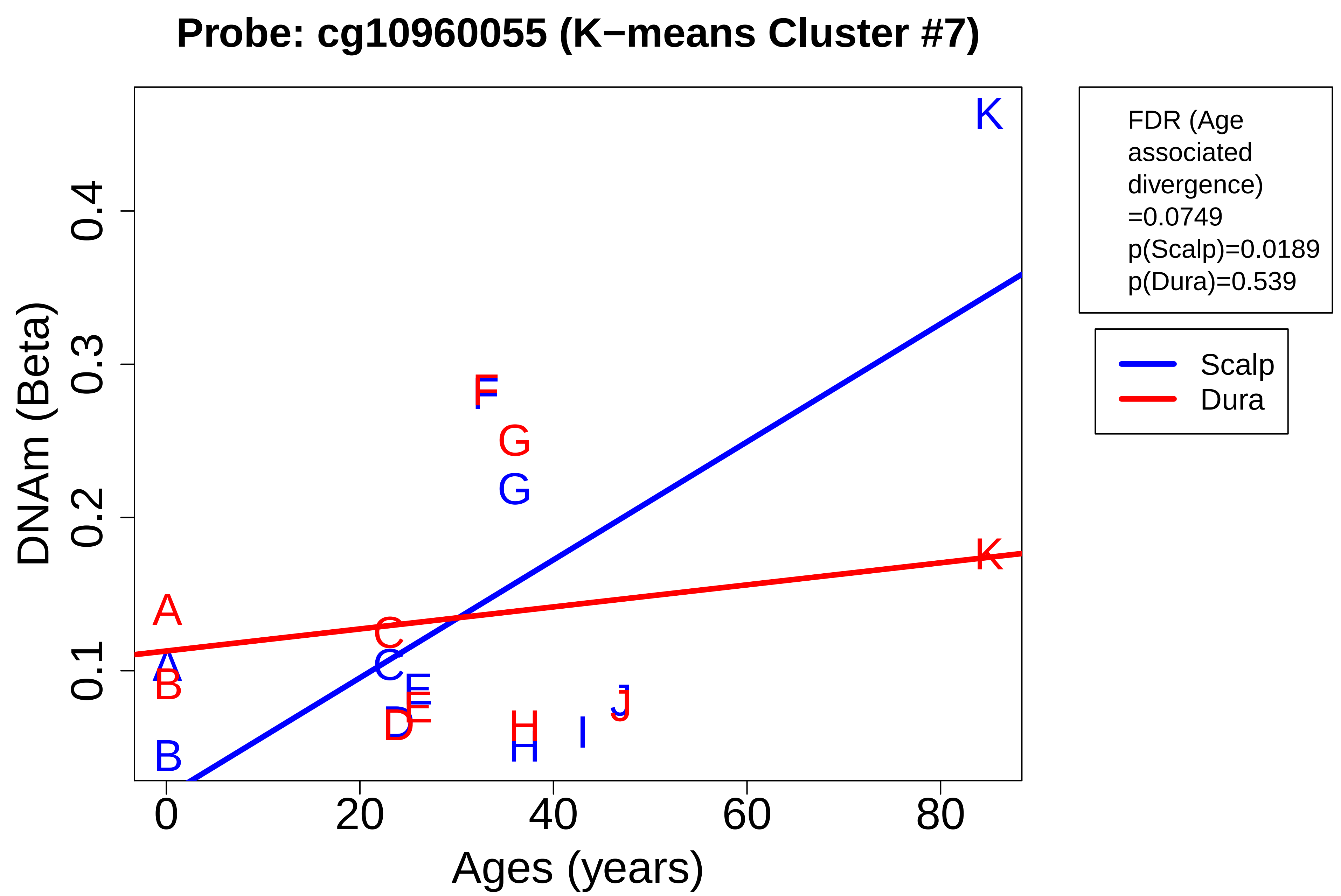

Probe: cg10960055 (K−means Cluster #7)
DNAm (Beta)
0.1
0.2
0.3
0.4
Ages (years)
0
20
40
60
80
A
B
C
D
E
F
G
H
I
J
K
FDR (Age associated divergence) =0.0749
p(Scalp)=0.0189
p(Dura)=0.539
Scalp
Dura

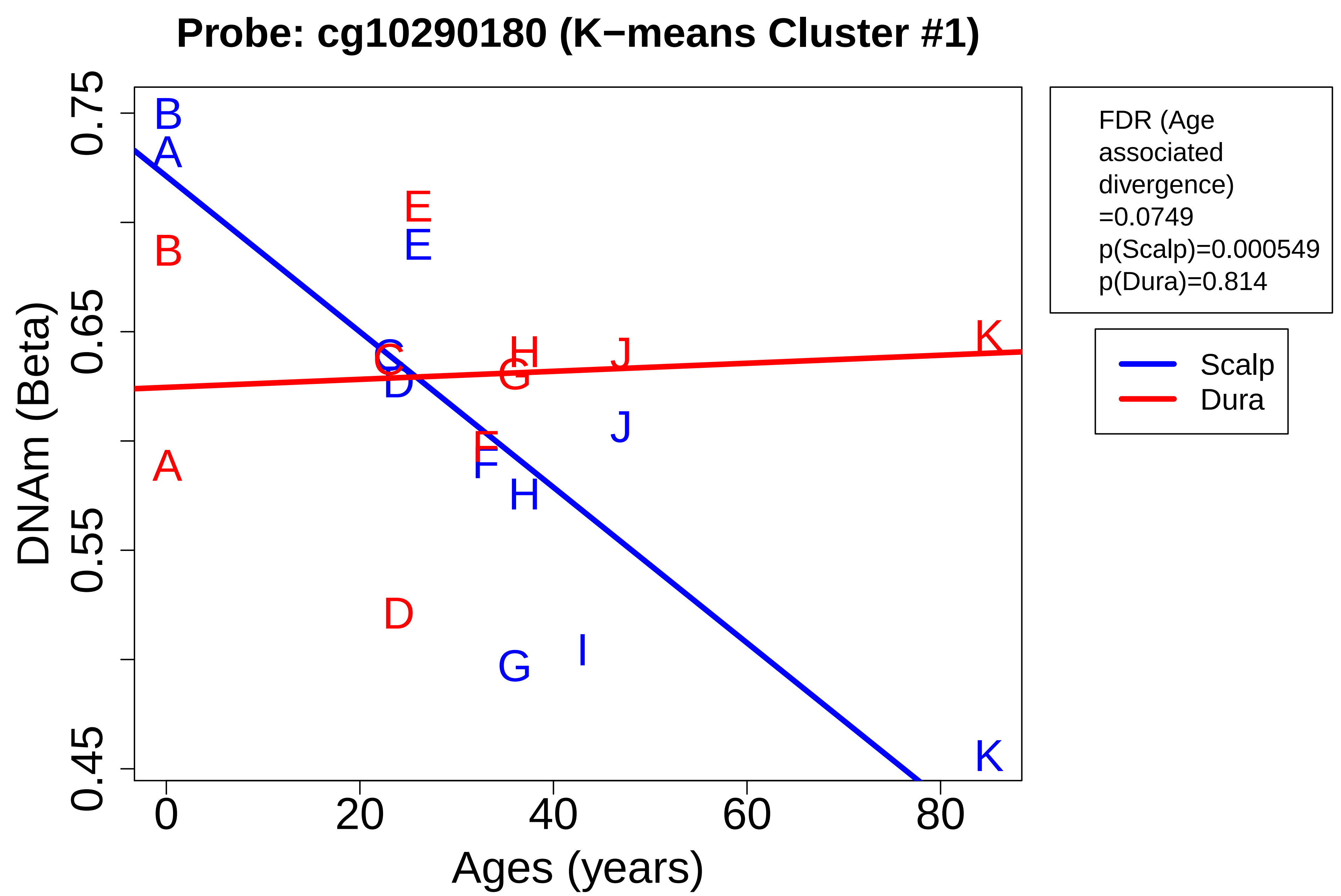

Probe: cg10290180 (K−means Cluster #1)
DNAm (Beta)
0.45
0.55
0.65
0.75
0
20
40
60
80
Ages (years)
B
A
E
E
B
C
C
H
J
K
G
D
J
F
F
A
H
D
I
G
K
FDR (Age associated divergence) =0.0749
p(Scalp)=0.000549
p(Dura)=0.814
Scalp
Dura

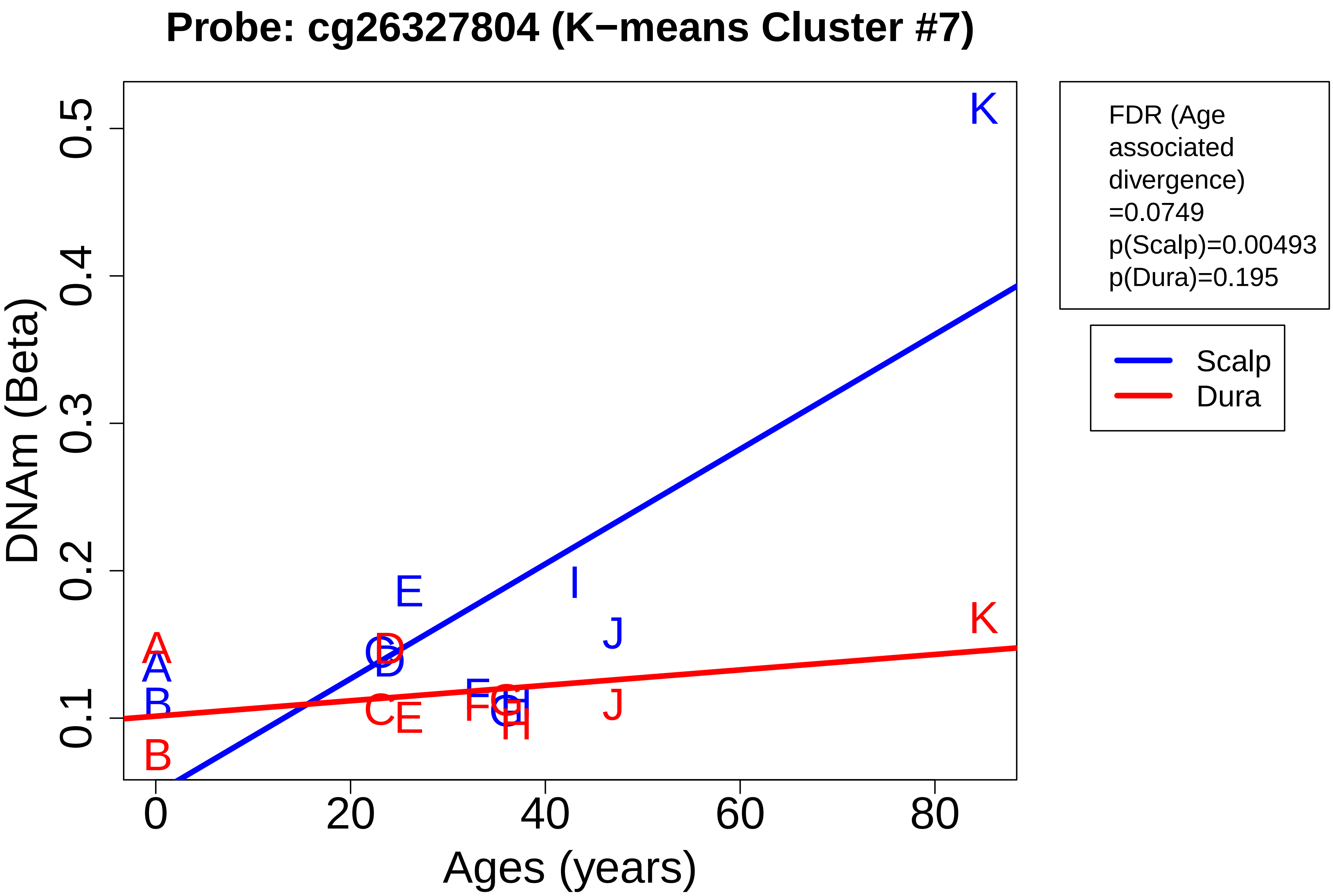

Probe: cg26327804 (K−means Cluster #7)
DNAm (Beta)
Ages (years)
0.1
0.2
0.3
0.4
0.5
0
20
40
60
80
FDR (Age associated divergence) =0.0749
p(Scalp)=0.00493
p(Dura)=0.195
Scalp
Dura

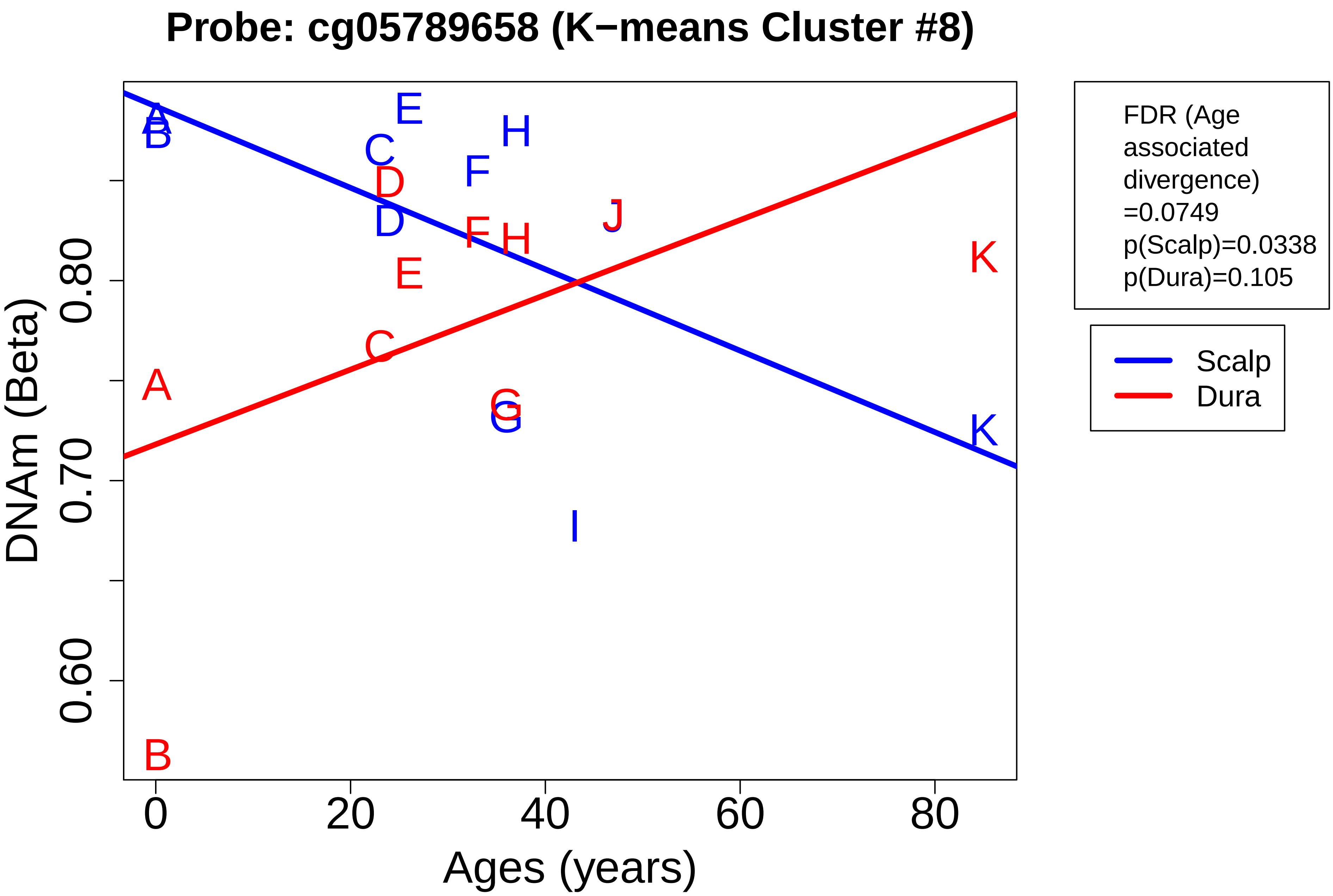

Probe: cg05789658 (K−means Cluster #8)
FDR (Age associated divergence) =0.0749
p(Scalp)=0.0338
p(Dura)=0.105
Scalp
Dura
DNAm (Beta)
0.60
0.70
0.80
0
20
40
60
80
Ages (years)
A
B
C
D
E
F
G
H
I
J
K

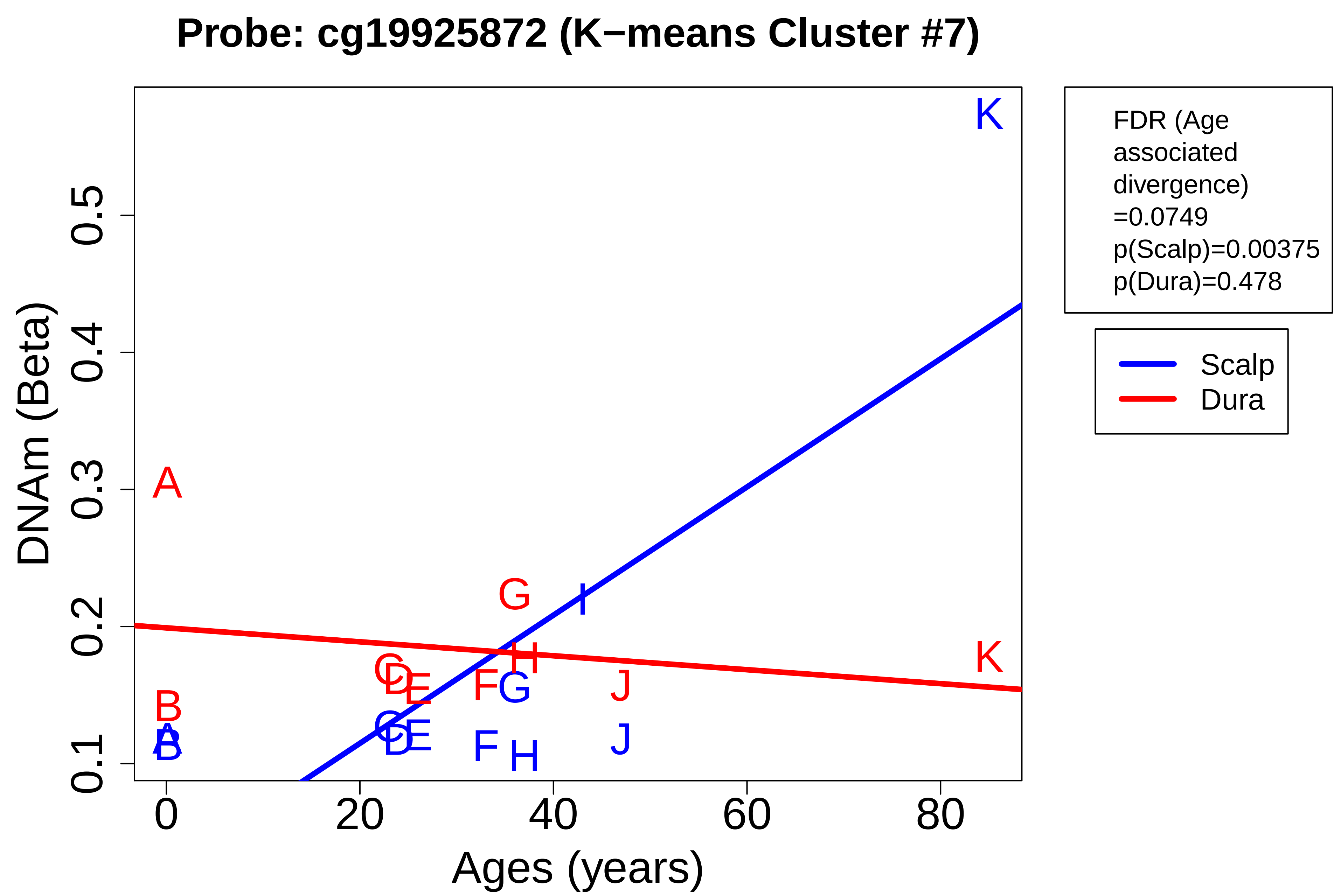
Probe: cg19925872 (K−means Cluster #7)
DNAm (Beta)
0.1
0.2
0.3
0.4
0.5
0
20
40
60
80
Ages (years)
A
B
C
D
E
F
G
H
I
J
K
FDR (Age associated divergence) =0.0749
p(Scalp)=0.00375
p(Dura)=0.478
Scalp
Dura

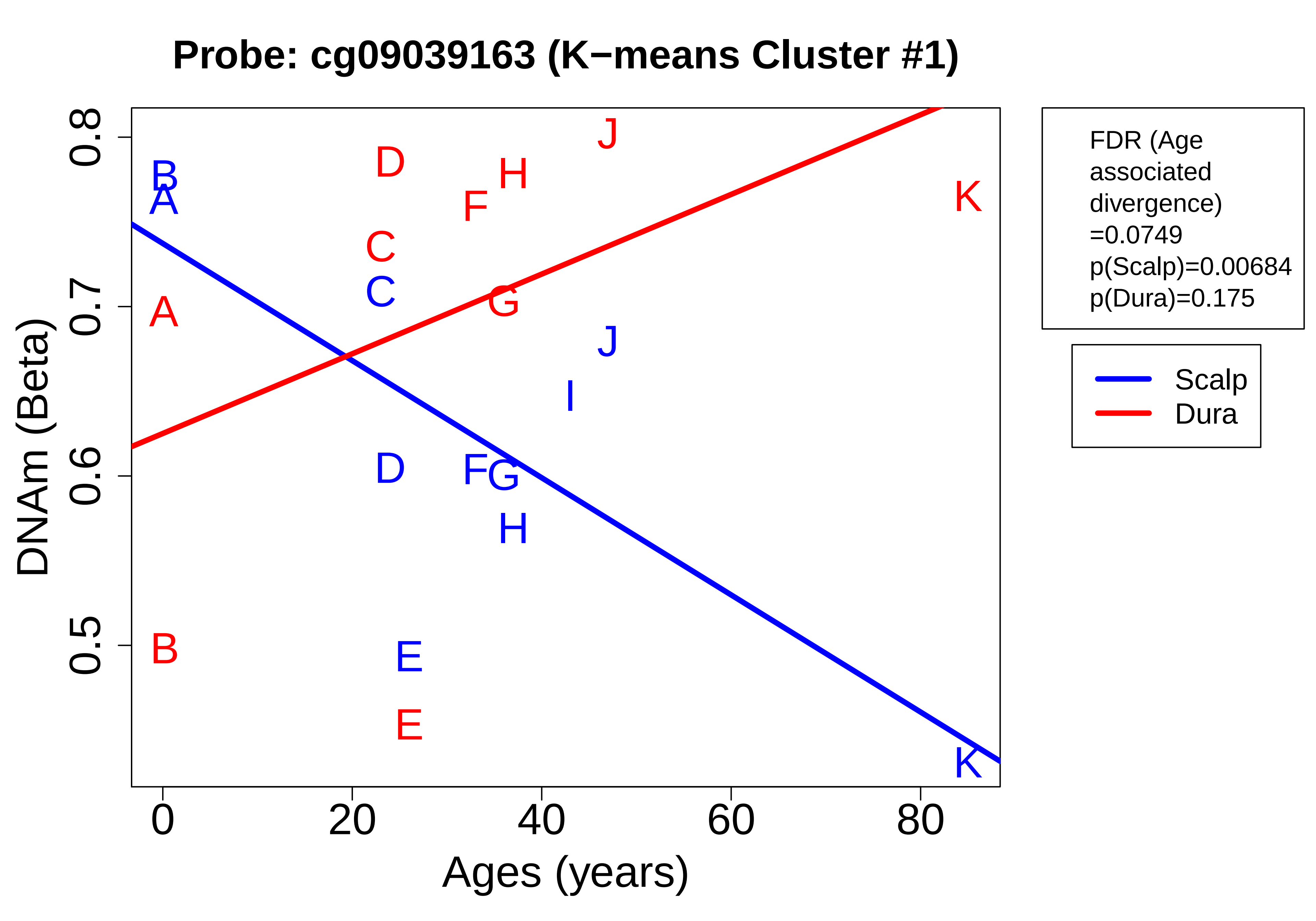

Probe: cg09039163 (K−means Cluster #1)
DNAm (Beta)
0.5
0.6
0.7
0.8
0
20
40
60
80
Ages (years)
A
B
C
D
E
F
G
H
I
J
K
FDR (Age associated divergence) =0.0749
p(Scalp)=0.00684
p(Dura)=0.175
Scalp
Dura

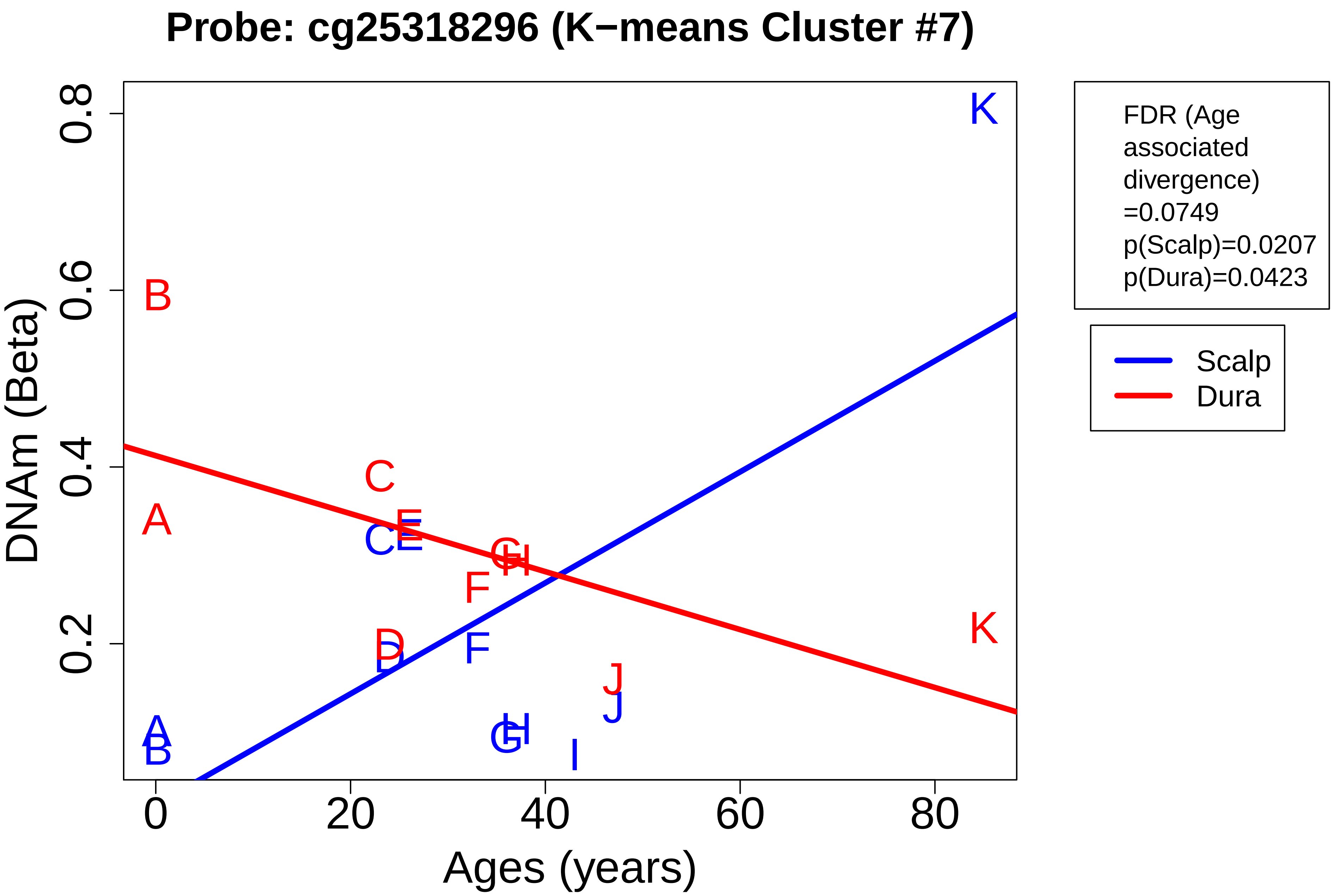

Probe: cg25318296 (K−means Cluster #7)
DNAm (Beta)
0.2
0.4
0.6
0.8
0
20
40
60
80
Ages (years)
FDR (Age associated divergence) =0.0749
p(Scalp)=0.0207
p(Dura)=0.0423
Scalp
Dura
A
B
C
D
E
F
G
H
I
J
K

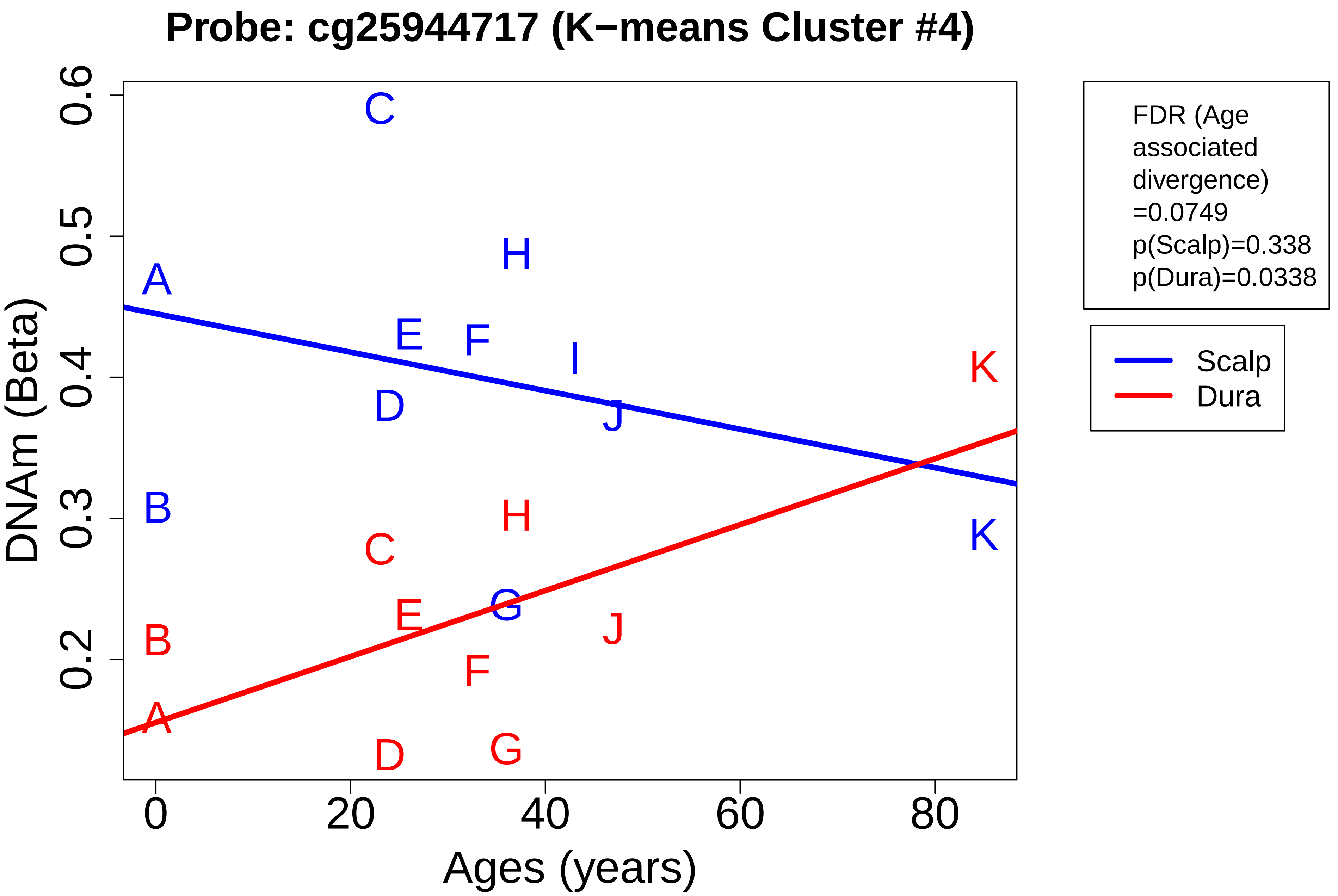
Probe: cg25944717 (K−means Cluster #4)
DNAm (Beta)
0.2
0.3
0.4
0.5
0.6
Ages (years)
0
20
40
60
80
A
B
C
D
E
F
G
H
I
J
K
FDR (Age associated divergence) =0.0749
p(Scalp)=0.338
p(Dura)=0.0338
Scalp
Dura

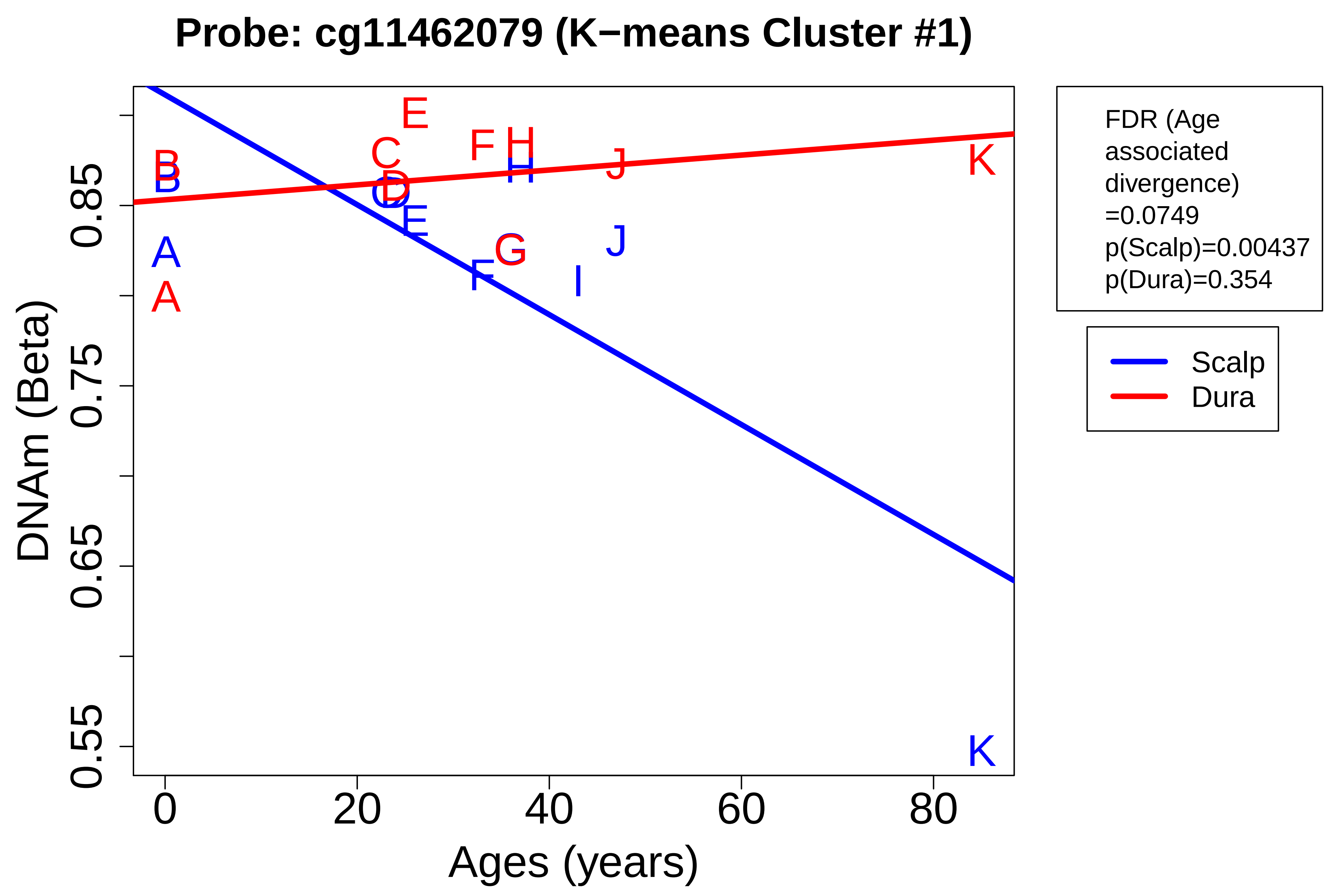

Probe: cg11462079 (K−means Cluster #1)
DNAm (Beta)
0.55
0.65
0.75
0.85
0
20
40
60
80
Ages (years)
A
B
C
D
E
F
G
H
I
J
K
FDR (Age associated divergence) =0.0749
p(Scalp)=0.00437
p(Dura)=0.354
Scalp
Dura

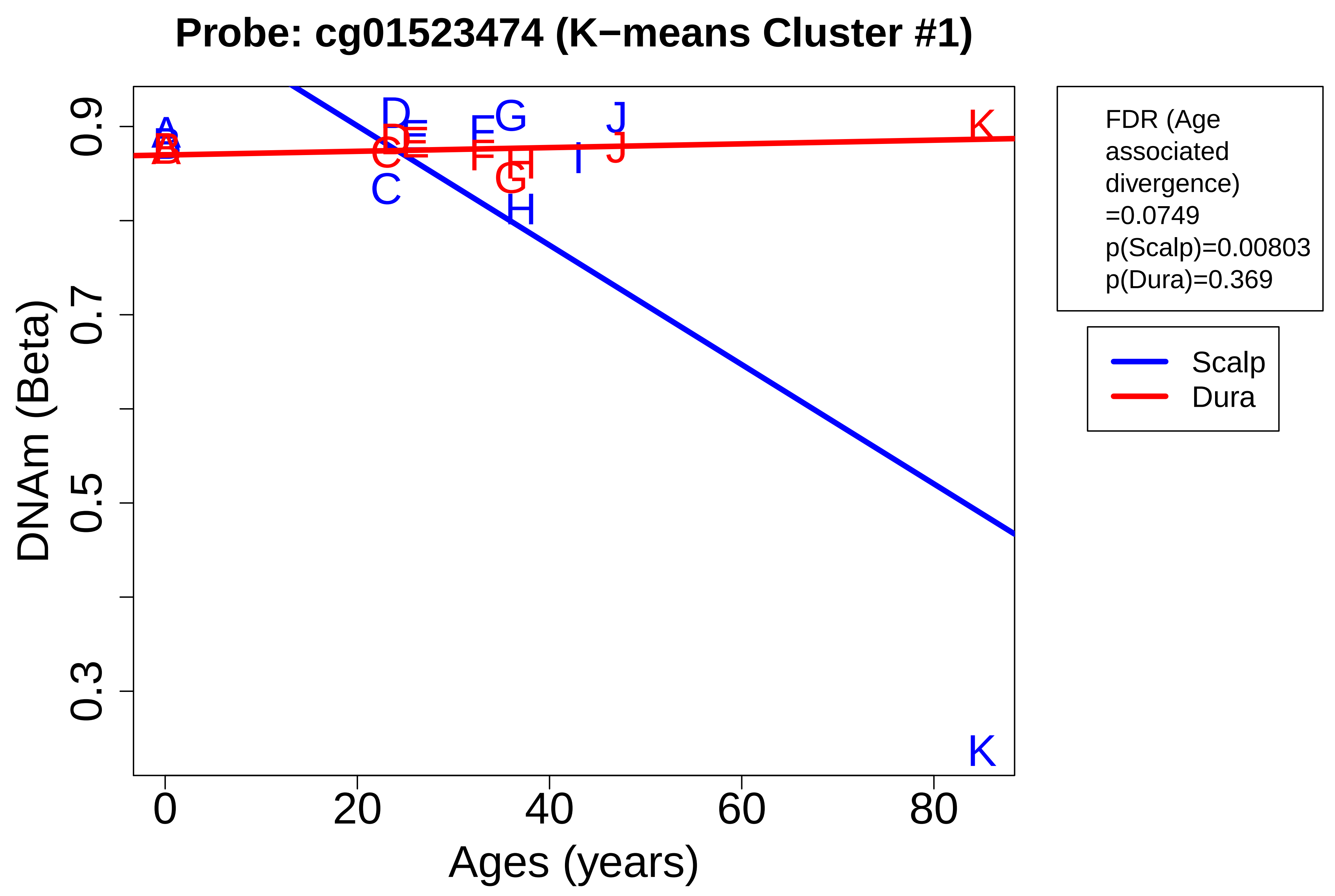

Probe: cg01523474 (K−means Cluster #1)
DNAm (Beta)
0.3
0.5
0.7
0.9
Ages (years)
0
20
40
60
80
FDR (Age associated divergence) =0.0749
p(Scalp)=0.00803
p(Dura)=0.369
Scalp
Dura

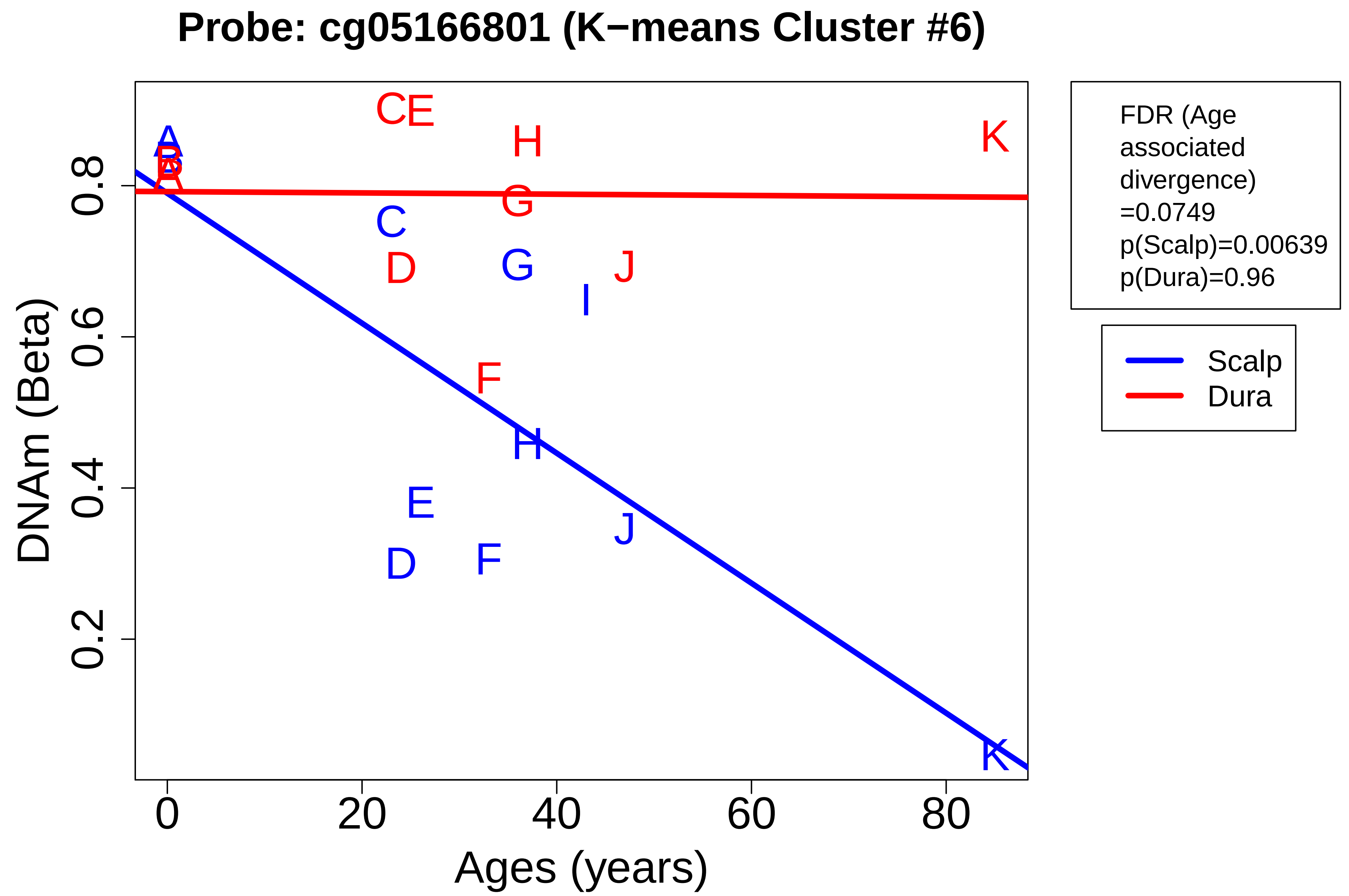
Probe: cg05166801 (K−means Cluster #6)
DNAm (Beta)
0.2
0.4
0.6
0.8
0
20
40
60
80
Ages (years)
FDR (Age associated divergence) =0.0749
p(Scalp)=0.00639
p(Dura)=0.96
Scalp
Dura

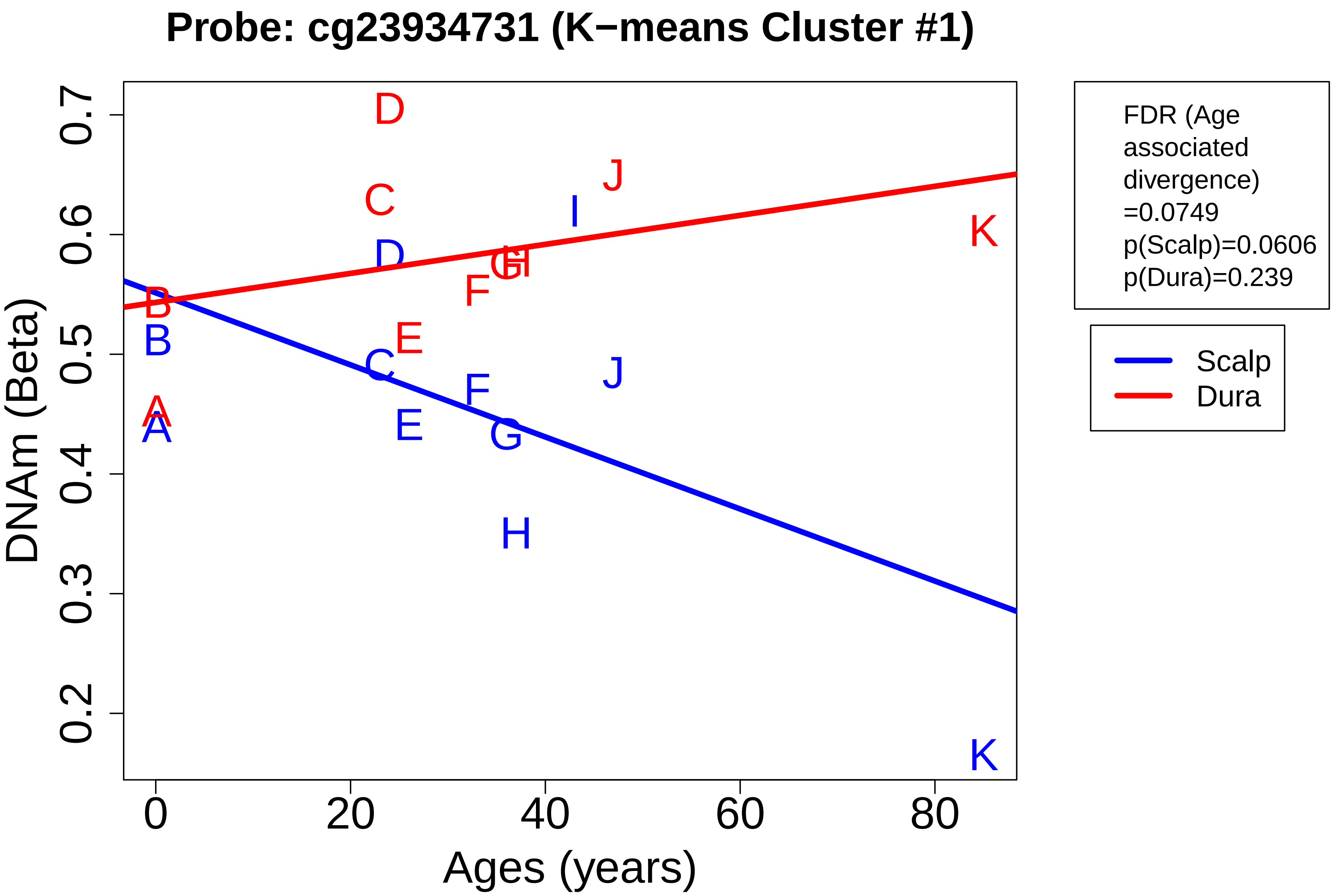

Probe: cg23934731 (K−means Cluster #1)
DNAm (Beta)
0.2
0.3
0.4
0.5
0.6
0.7
0
20
40
60
80
Ages (years)
FDR (Age associated divergence) =0.0749
p(Scalp)=0.0606
p(Dura)=0.239
Scalp
Dura

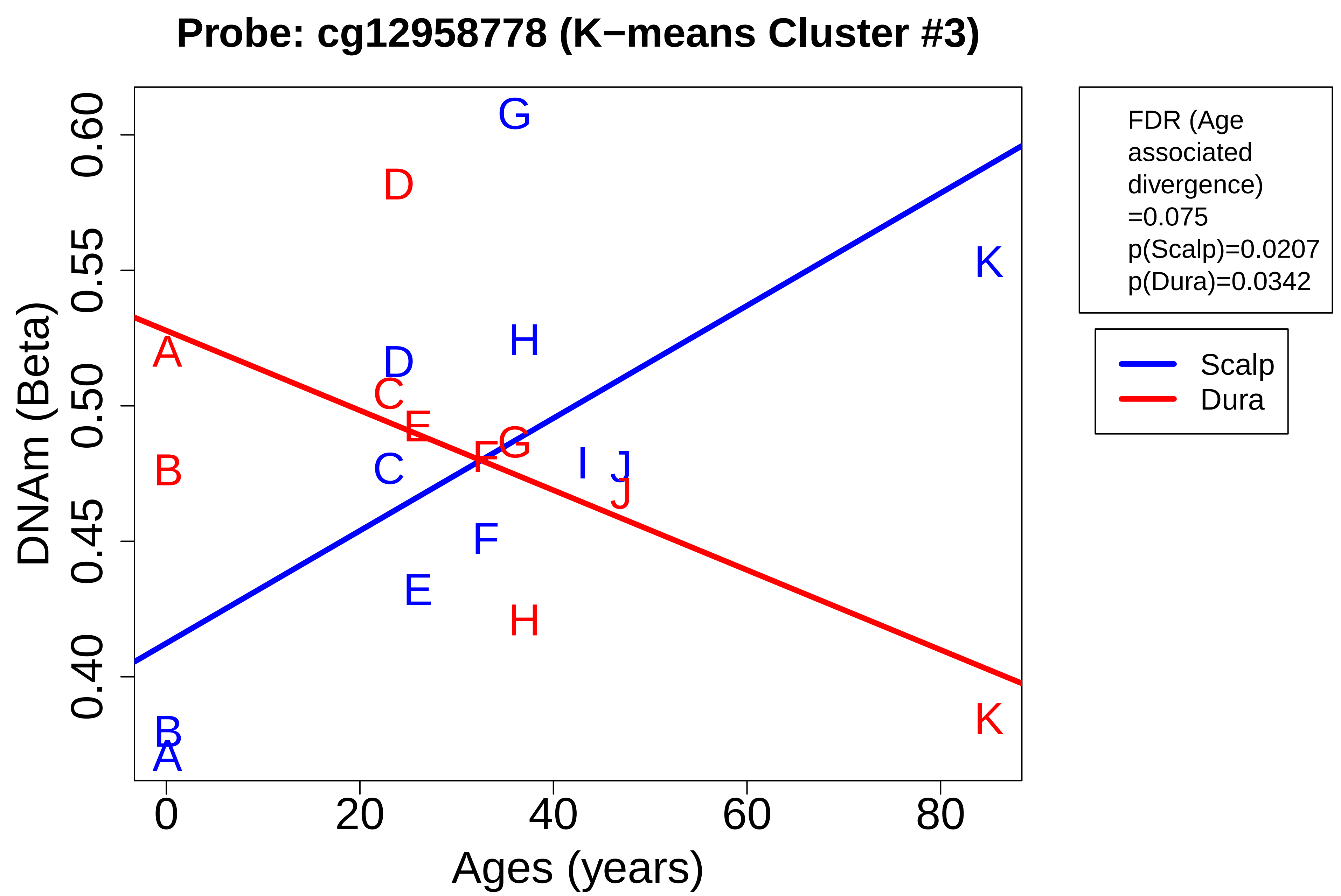

Probe: cg12958778 (K−means Cluster #3)
DNAm (Beta)
0.40
0.45
0.50
0.55
0.60
Ages (years)
0
20
40
60
80
FDR (Age associated divergence) =0.075
p(Scalp)=0.0207
p(Dura)=0.0342
Scalp
Dura

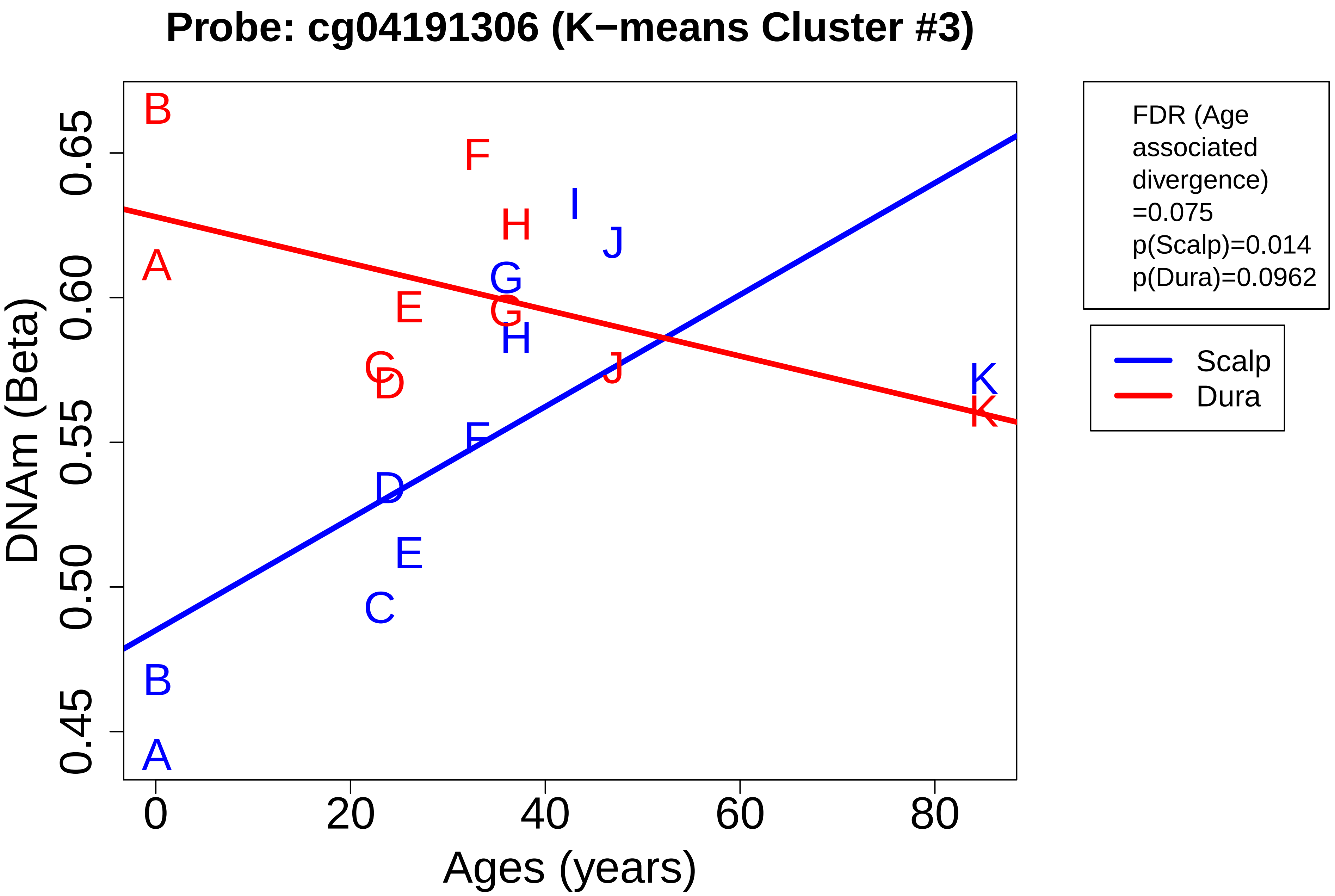
Probe: cg04191306 (K−means Cluster #3)
DNAm (Beta)
0.45
0.50
0.55
0.60
0.65
0
20
40
60
80
Ages (years)
FDR (Age associated divergence) =0.075
p(Scalp)=0.014
p(Dura)=0.0962
Scalp
Dura
A
B
C
D
E
F
G
H
I
J
K

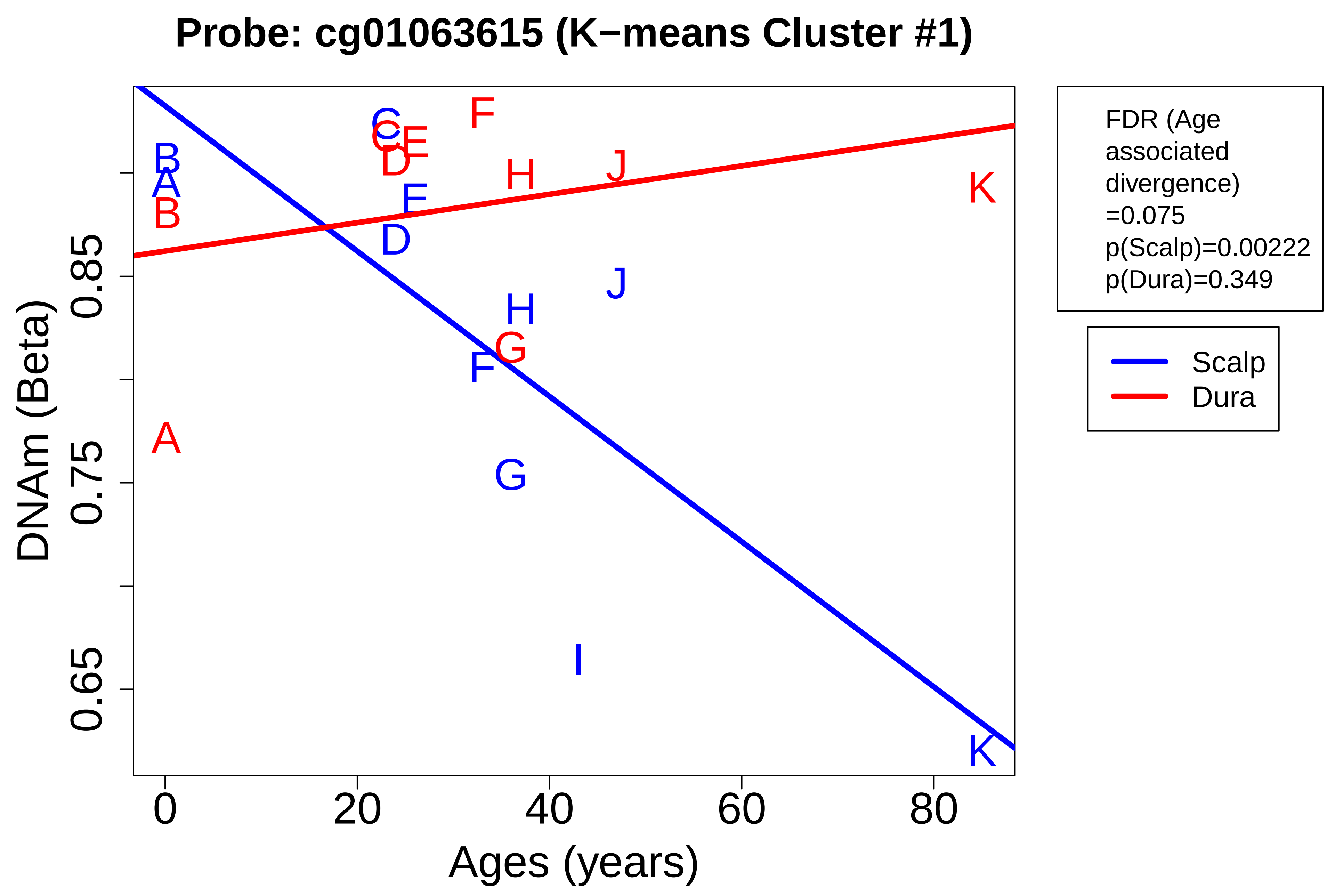

Probe: cg01063615 (K−means Cluster #1)
DNAm (Beta)
0.65
0.75
0.85
0
20
40
60
80
Ages (years)
FDR (Age associated divergence) =0.075
p(Scalp)=0.00222
p(Dura)=0.349
Scalp
Dura
A
B
C
D
E
F
G
H
I
J
K

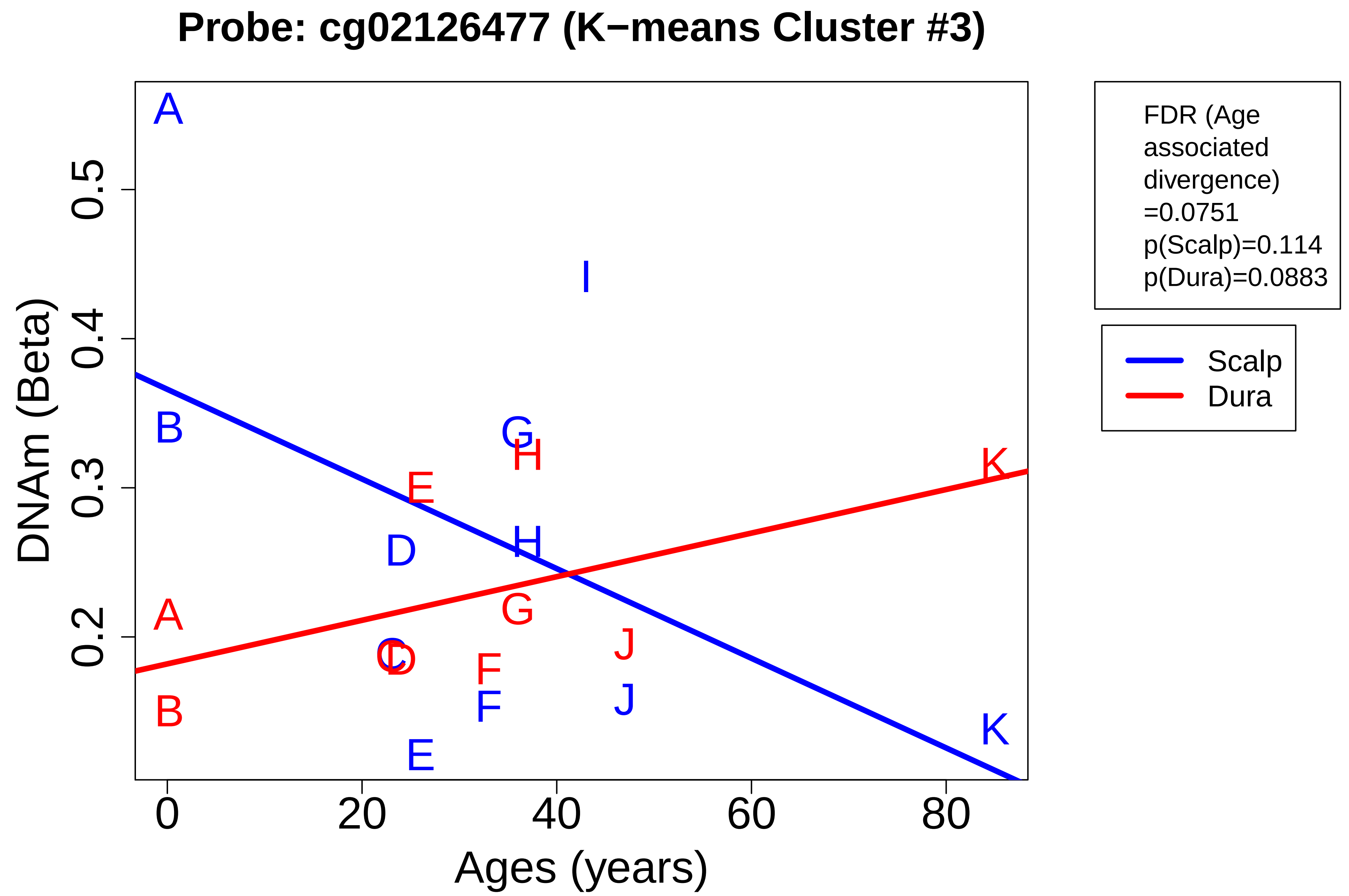

Probe: cg02126477 (K−means Cluster #3)
DNAm (Beta)
0.2
0.3
0.4
0.5
0
20
40
60
80
Ages (years)
A
B
C
D
E
F
G
H
I
J
K
A
B
C
D
E
F
G
H
J
K
FDR (Age associated divergence) =0.0751
p(Scalp)=0.114
p(Dura)=0.0883
Scalp
Dura

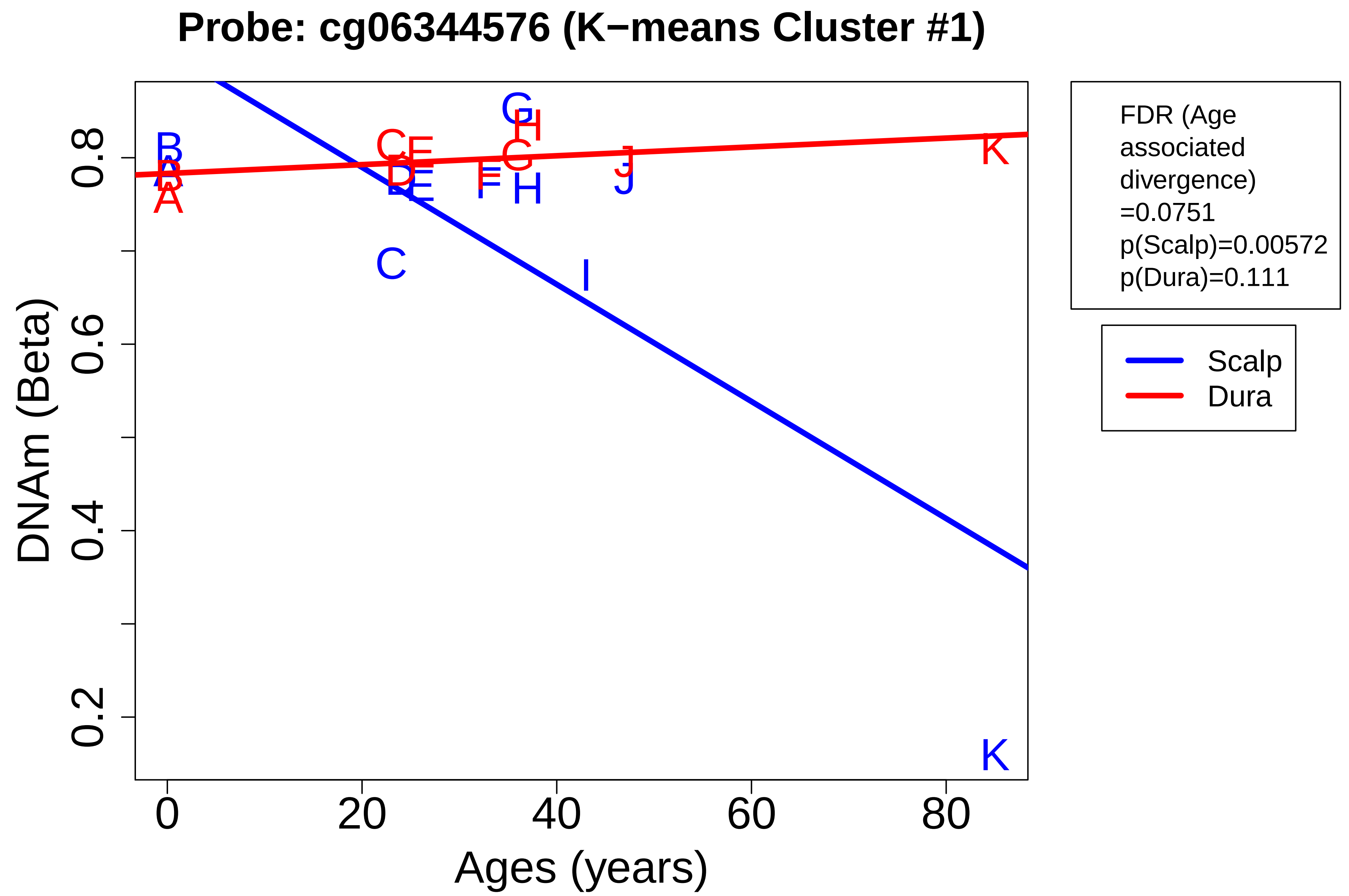

Probe: cg06344576 (K−means Cluster #1)
FDR (Age associated divergence) =0.0751
p(Scalp)=0.00572
p(Dura)=0.111
Scalp
Dura
DNAm (Beta)
0.2
0.4
0.6
0.8
Ages (years)
0
20
40
60
80

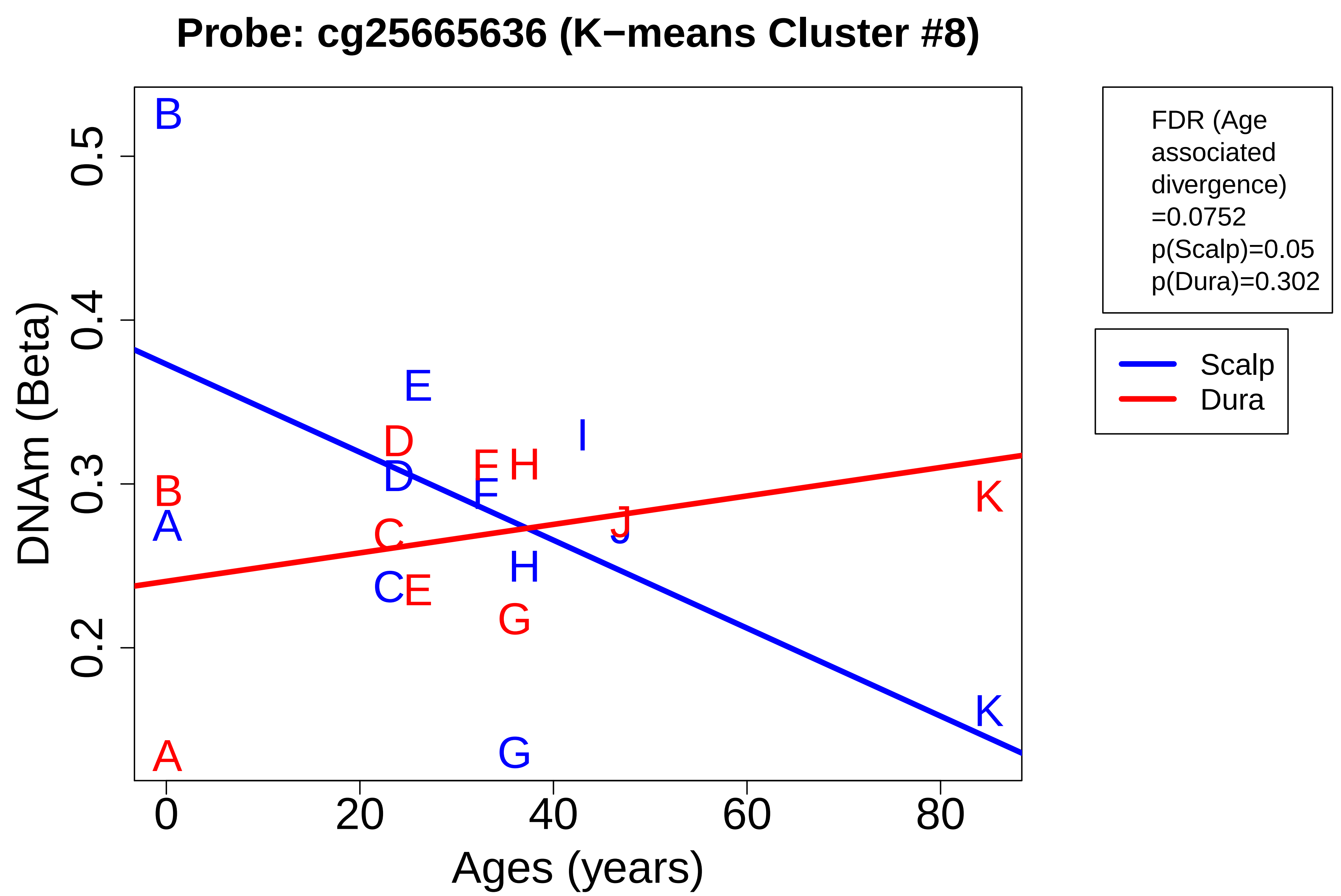

Probe: cg25665636 (K−means Cluster #8)
DNAm (Beta)
0.2
0.3
0.4
0.5
0
20
40
60
80
Ages (years)
FDR (Age associated divergence) =0.0752
p(Scalp)=0.05
p(Dura)=0.302
Scalp
Dura

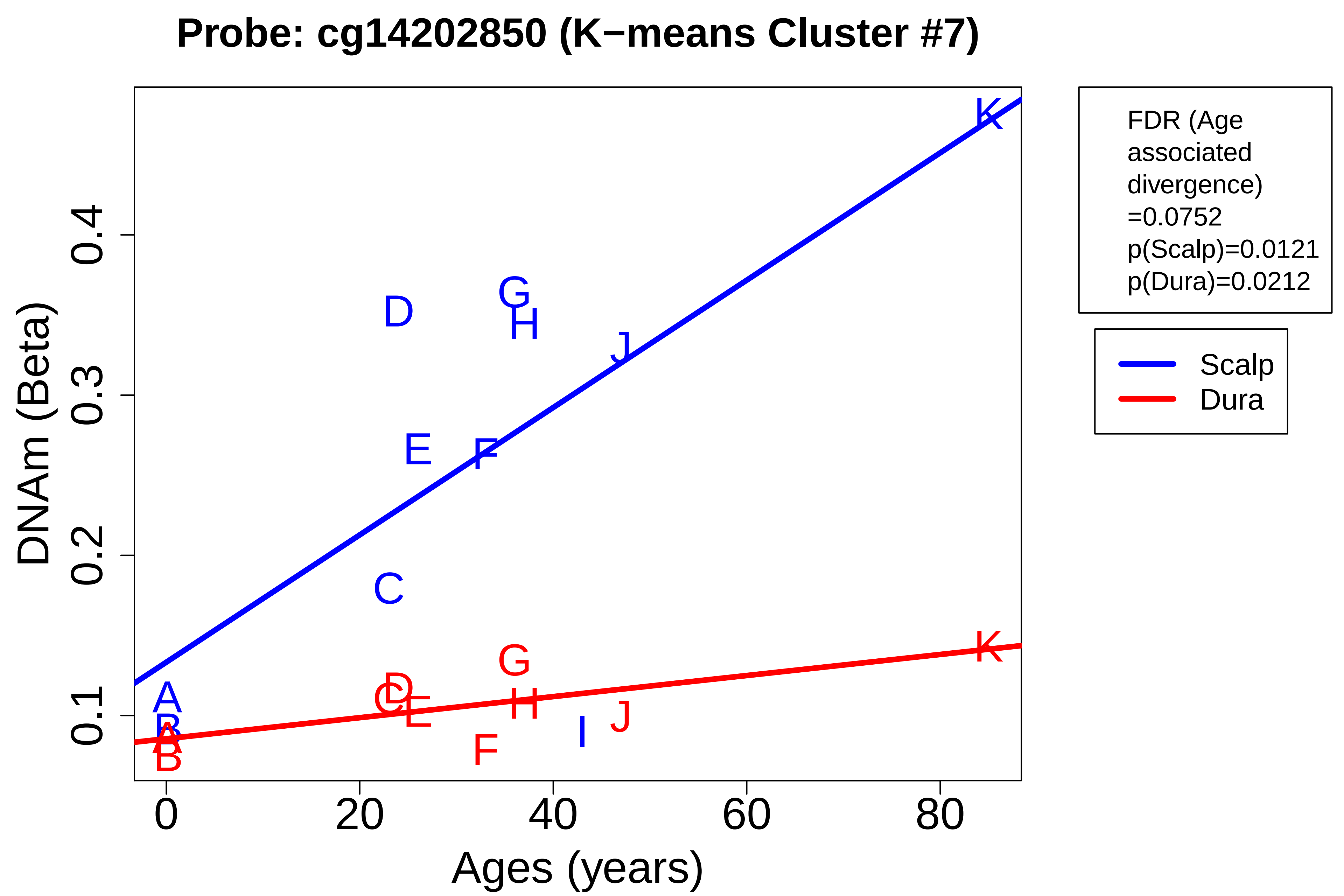
Probe: cg14202850 (K−means Cluster #7)
DNAm (Beta)
Ages (years)
0.1
0.2
0.3
0.4
0
20
40
60
80
A
B
C
D
E
F
G
H
I
J
K
FDR (Age associated divergence) =0.0752
p(Scalp)=0.0121
p(Dura)=0.0212
Scalp
Dura

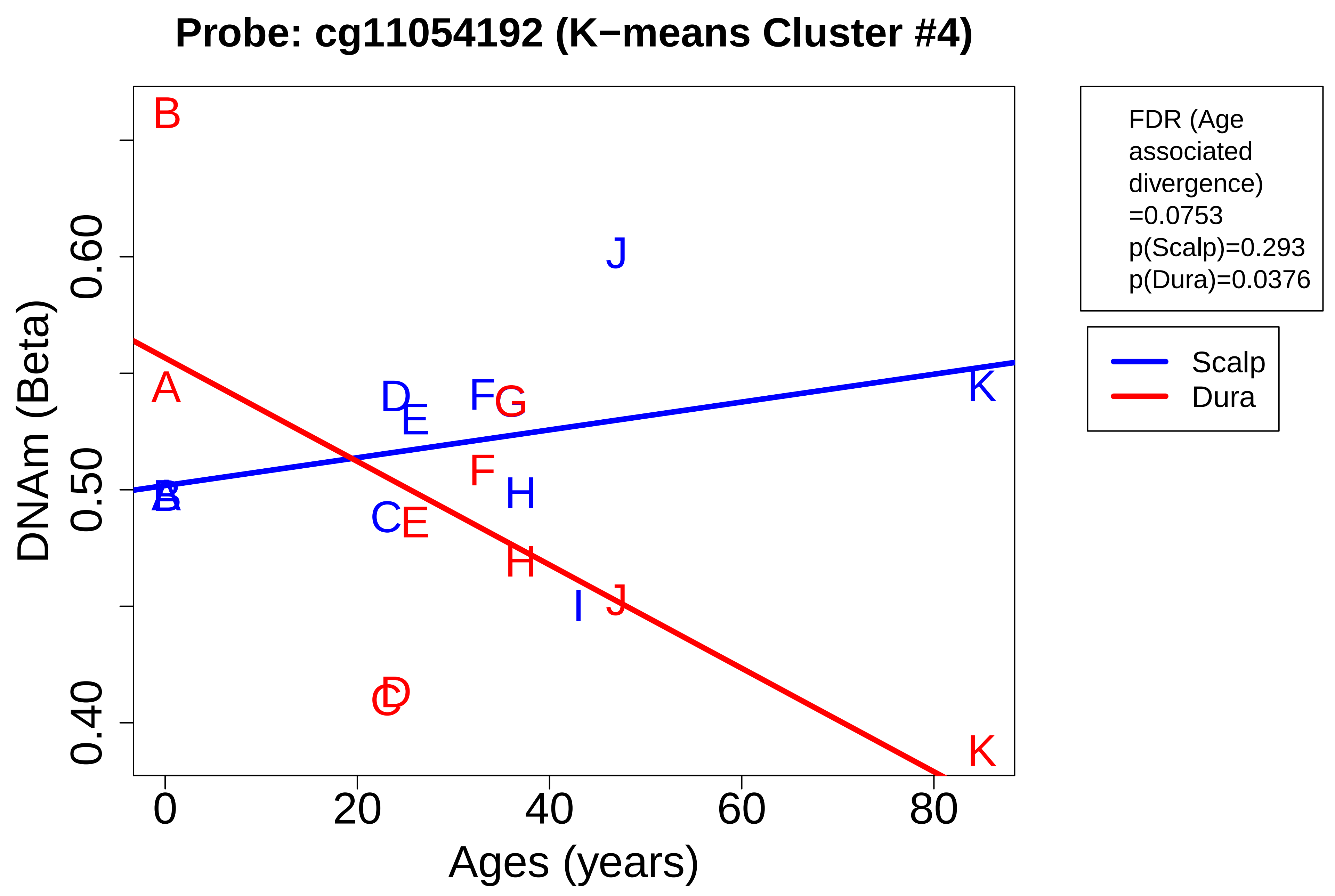

Probe: cg11054192 (K−means Cluster #4)
DNAm (Beta)
0.40
0.50
0.60
Ages (years)
0
20
40
60
80
FDR (Age associated divergence) =0.0753
p(Scalp)=0.293
p(Dura)=0.0376
Scalp
Dura

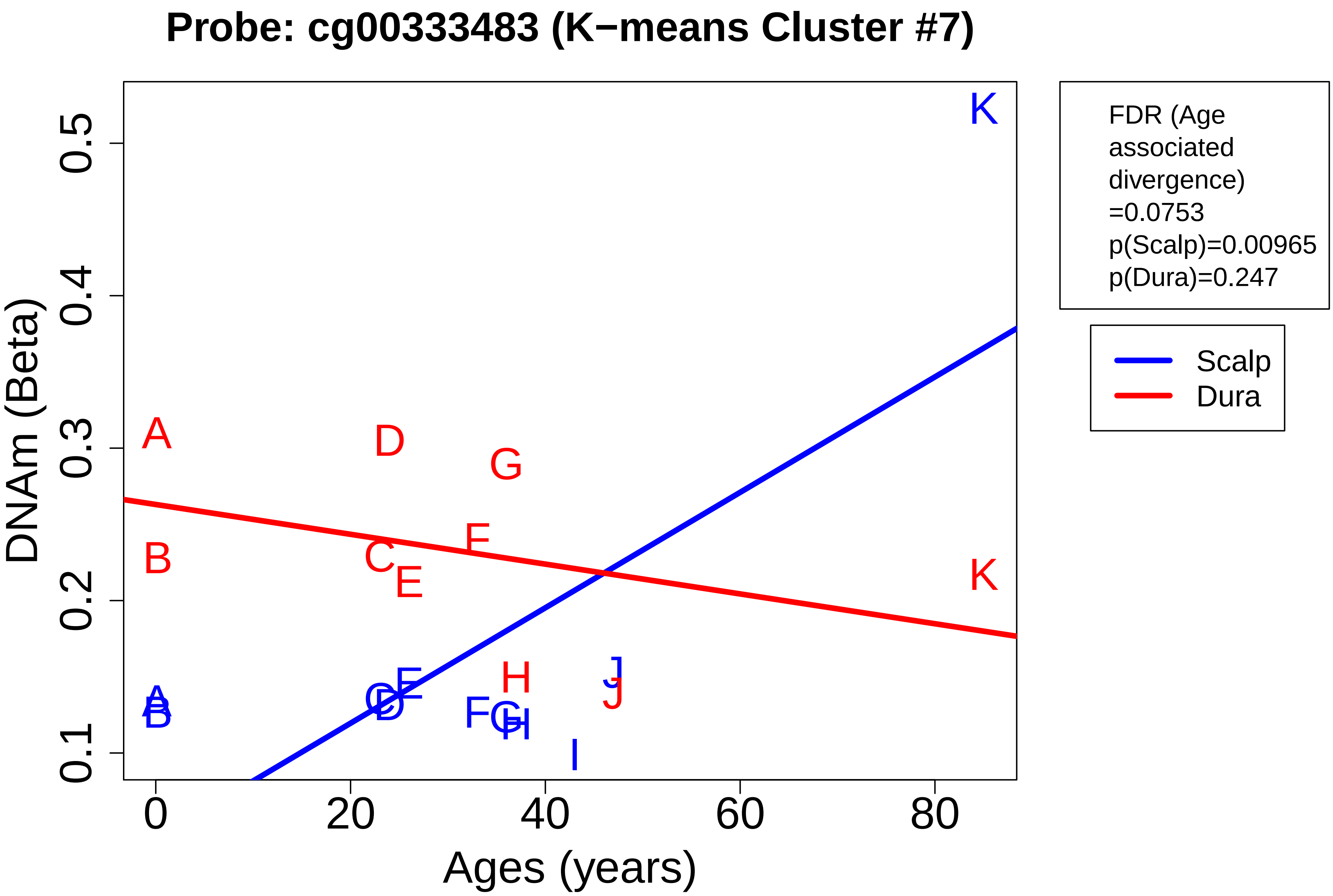

Probe: cg00333483 (K−means Cluster #7)
DNAm (Beta)
Ages (years)
0.1
0.2
0.3
0.4
0.5
0
20
40
60
80
FDR (Age associated divergence) =0.0753
p(Scalp)=0.00965
p(Dura)=0.247
Scalp
Dura

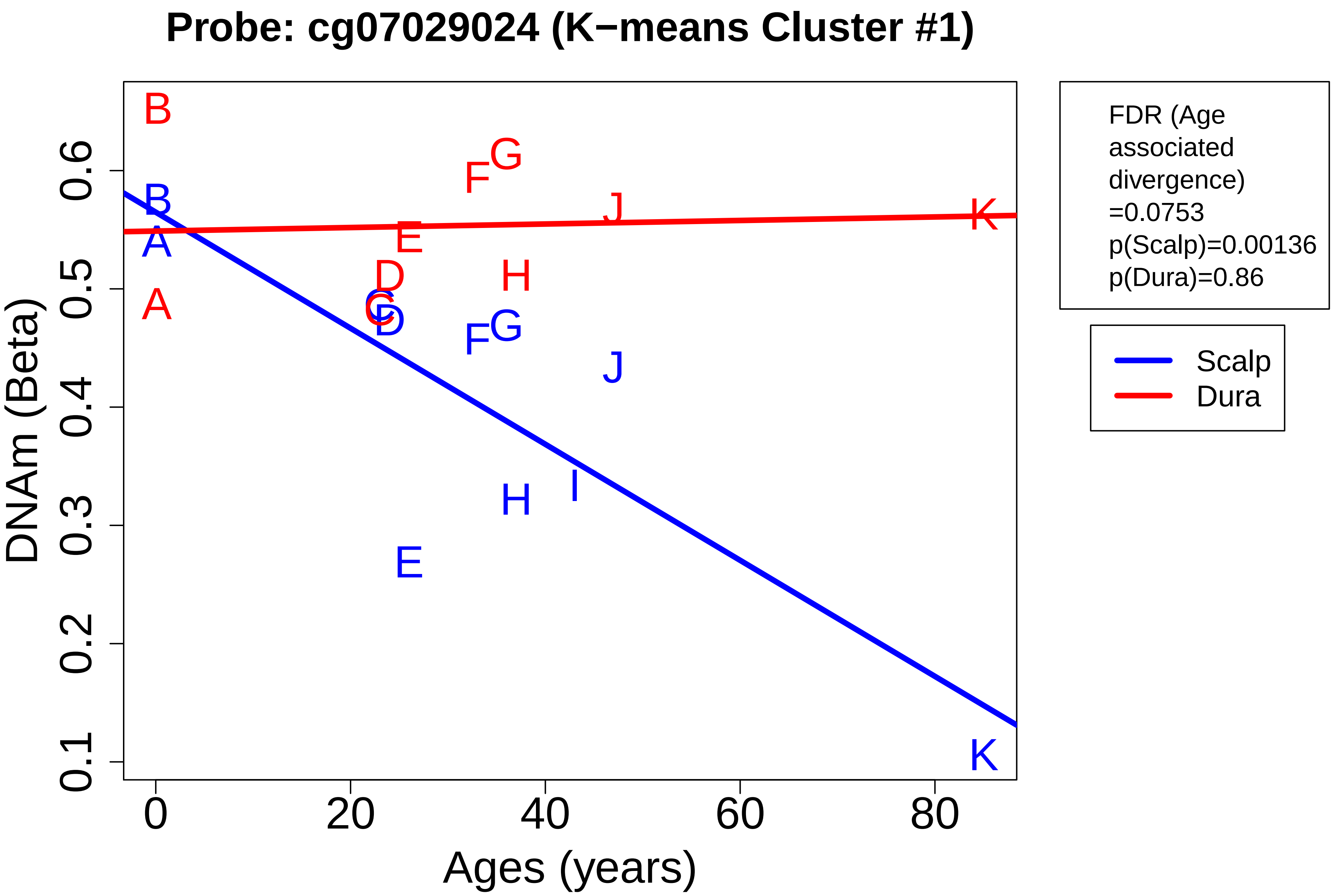

Probe: cg07029024 (K−means Cluster #1)
DNAm (Beta)
0.1
0.2
0.3
0.4
0.5
0.6
0
20
40
60
80
Ages (years)
FDR (Age associated divergence) =0.0753
p(Scalp)=0.00136
p(Dura)=0.86
Scalp
Dura

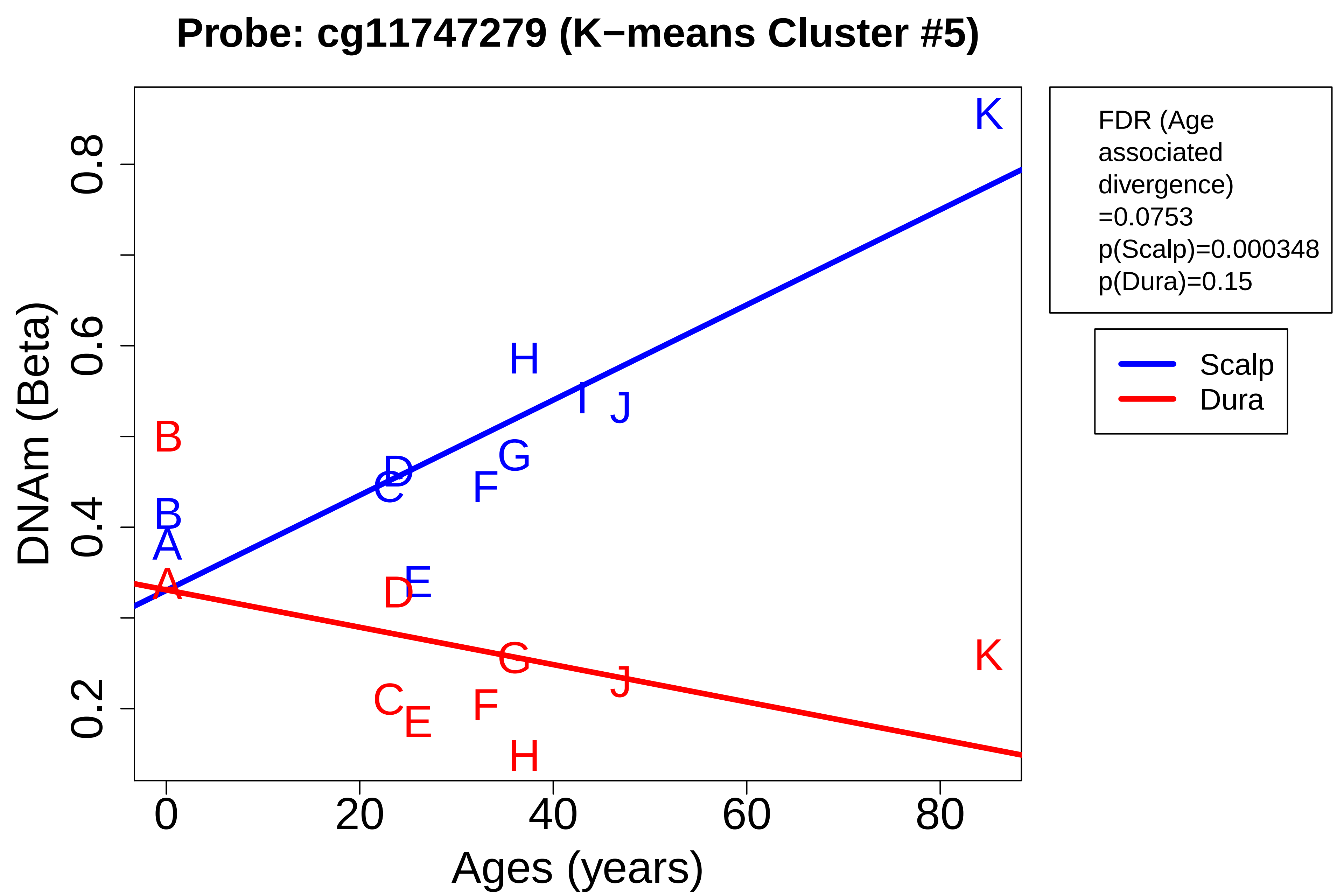
Probe: cg11747279 (K−means Cluster #5)
DNAm (Beta)
0.2
0.4
0.6
0.8
Ages (years)
0
20
40
60
80
FDR (Age associated divergence) =0.0753
p(Scalp)=0.000348
p(Dura)=0.15
Scalp
Dura
A
B
C
D
E
F
G
H
I
J
K

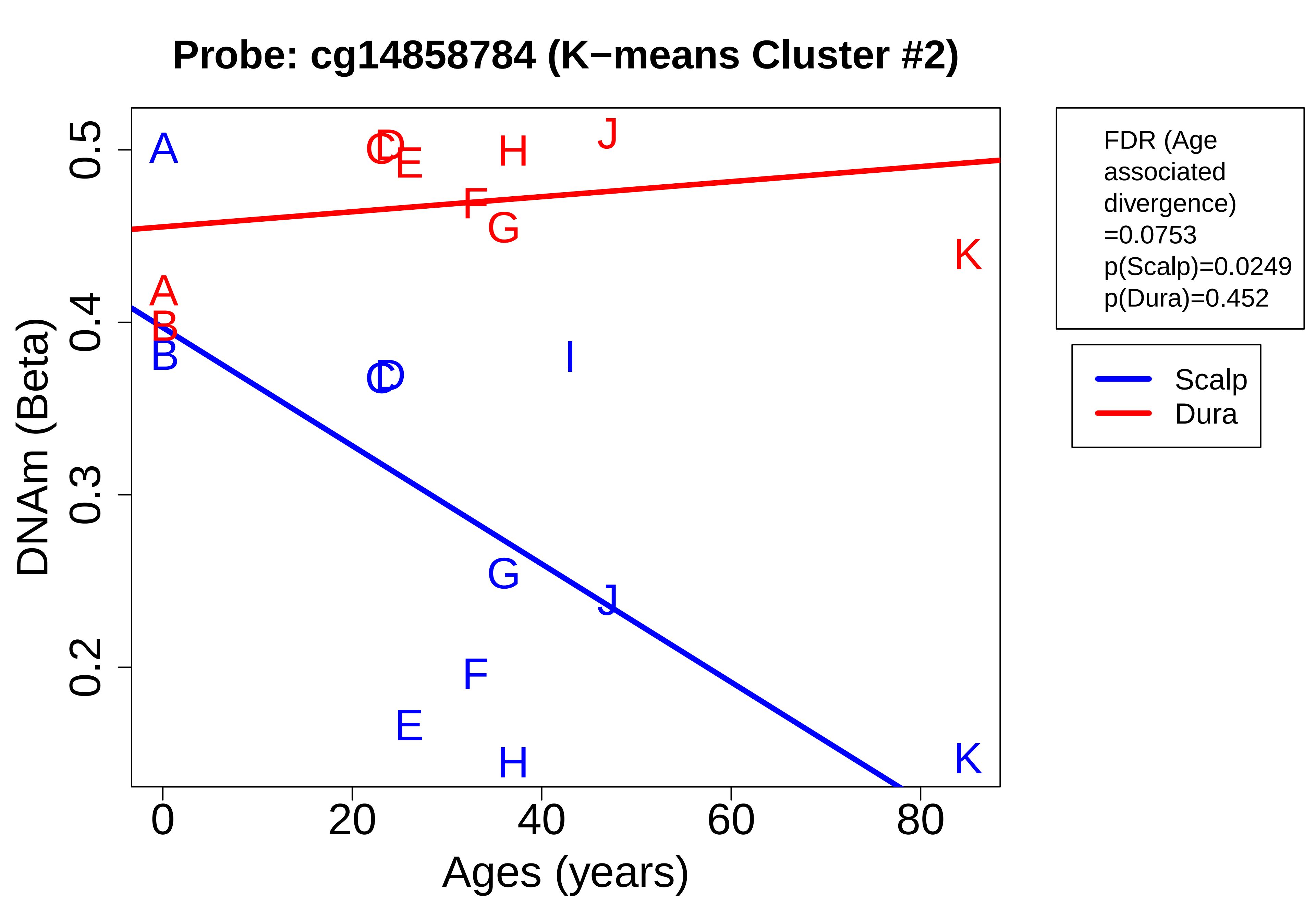

Probe: cg14858784 (K−means Cluster #2)
DNAm (Beta)
0.2
0.3
0.4
0.5
0
20
40
60
80
Ages (years)
A
B
C
D
E
F
G
H
I
J
K
FDR (Age associated divergence) =0.0753
p(Scalp)=0.0249
p(Dura)=0.452
Scalp
Dura

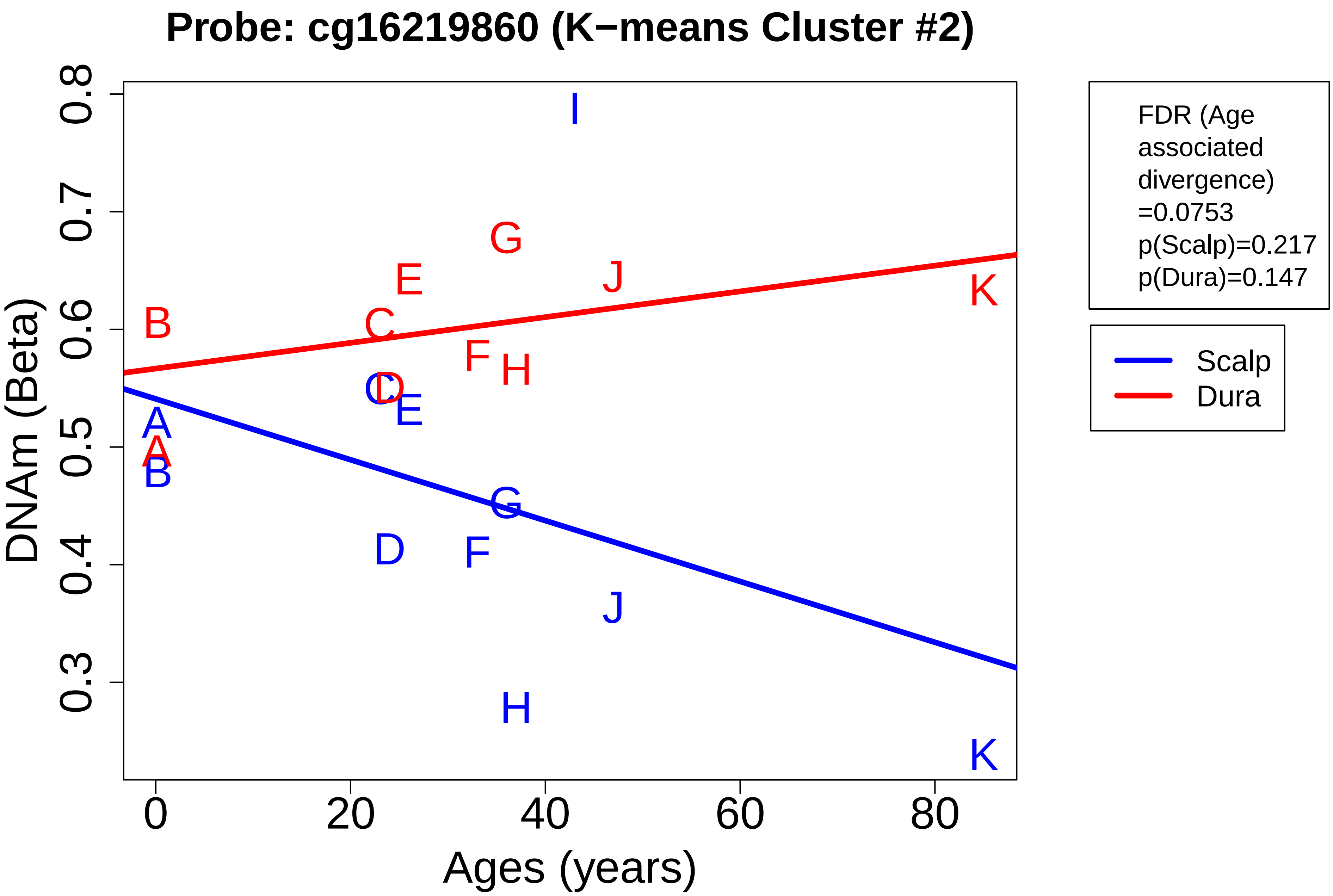

Probe: cg16219860 (K−means Cluster #2)
DNAm (Beta)
0.3
0.4
0.5
0.6
0.7
0.8
0
20
40
60
80
Ages (years)
FDR (Age associated divergence) =0.0753
p(Scalp)=0.217
p(Dura)=0.147
Scalp
Dura

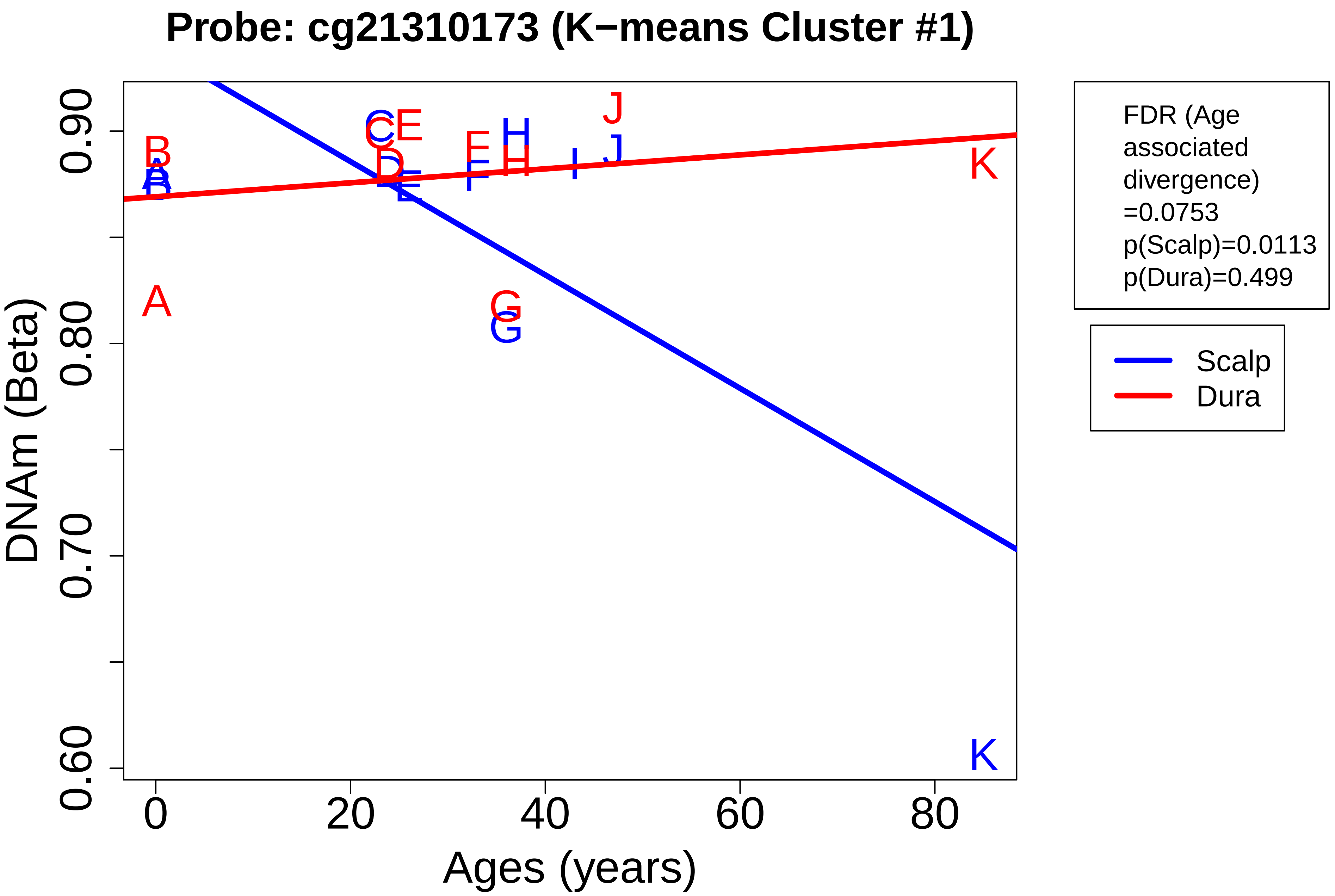

Probe: cg21310173 (K−means Cluster #1)
DNAm (Beta)
0.60
0.70
0.80
0.90
Ages (years)
0
20
40
60
80
FDR (Age associated divergence) =0.0753
p(Scalp)=0.0113
p(Dura)=0.499
Scalp
Dura

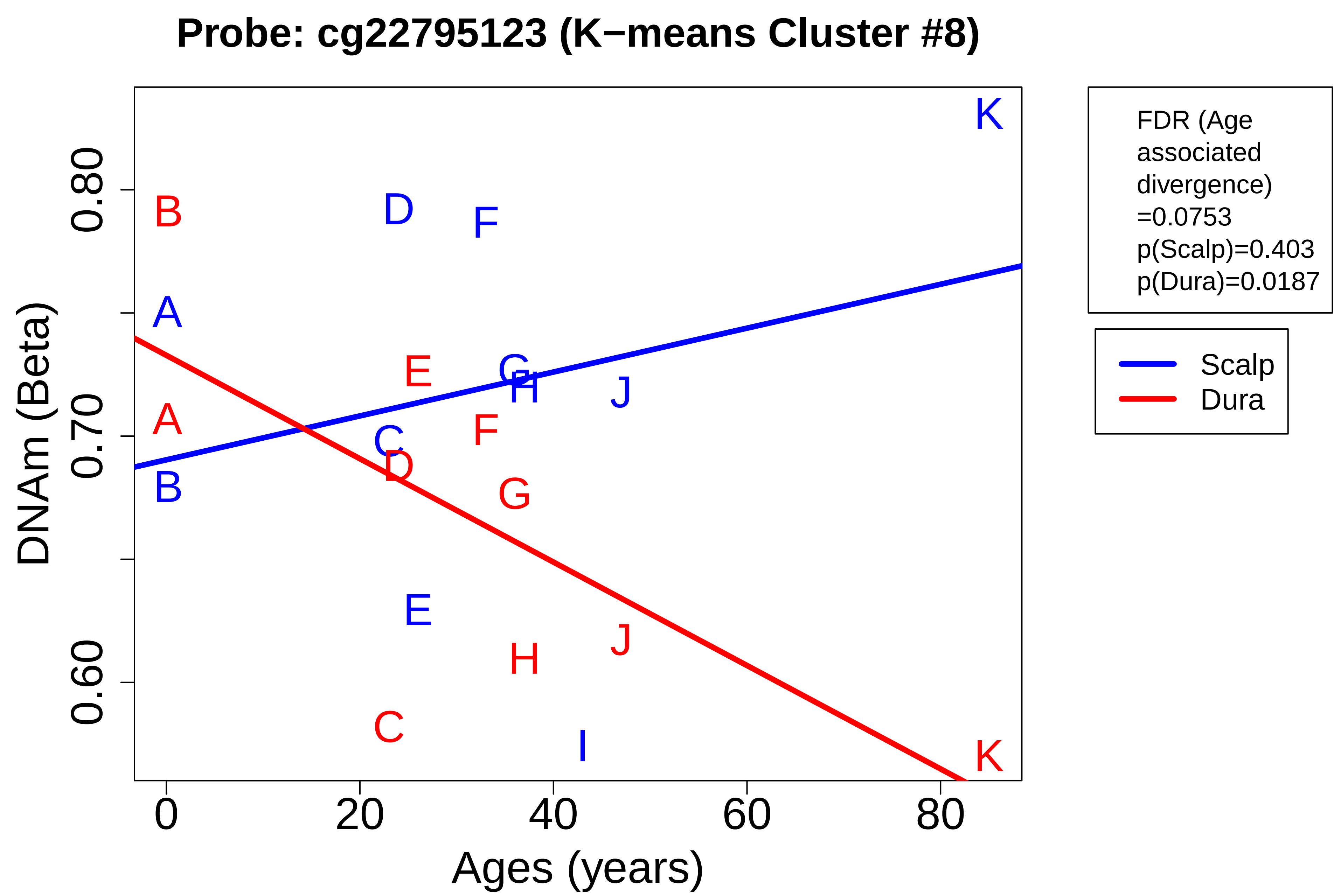

Probe: cg22795123 (K−means Cluster #8)
DNAm (Beta)
0.60
0.70
0.80
Ages (years)
0
20
40
60
80
A
B
C
D
E
F
G
H
I
J
K
A
B
C
D
E
F
G
H
J
K
FDR (Age associated divergence) =0.0753
p(Scalp)=0.403
p(Dura)=0.0187
Scalp
Dura

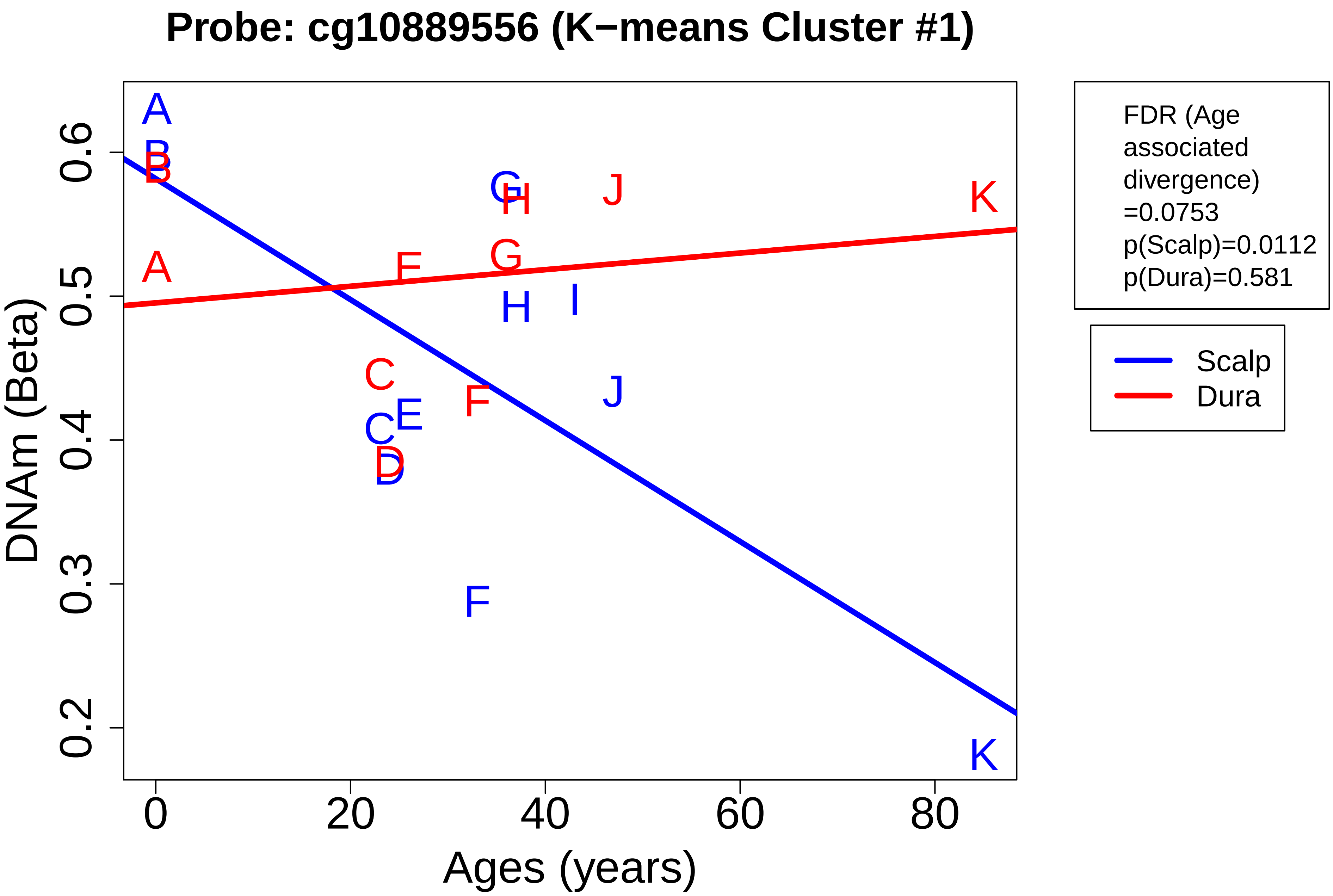

Probe: cg10889556 (K−means Cluster #1)
DNAm (Beta)
0.2
0.3
0.4
0.5
0.6
0
20
40
60
80
Ages (years)
FDR (Age associated divergence) =0.0753
p(Scalp)=0.0112
p(Dura)=0.581
Scalp
Dura
A
B
C
D
E
F
G
H
I
J
K

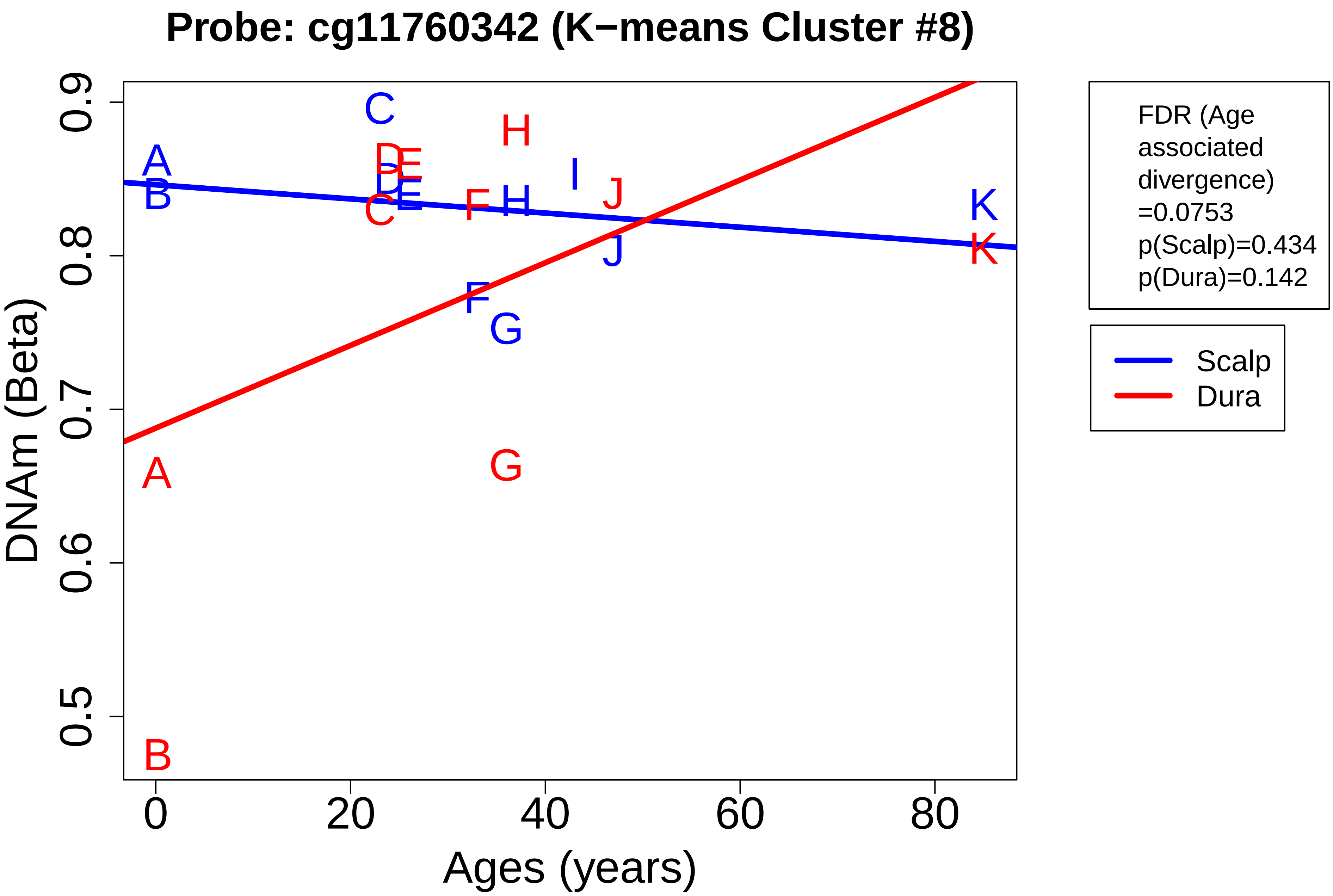
Probe: cg11760342 (K−means Cluster #8)
DNAm (Beta)
Ages (years)
0.5
0.6
0.7
0.8
0.9
0
20
40
60
80
FDR (Age associated divergence) =0.0753
p(Scalp)=0.434
p(Dura)=0.142
Scalp
Dura
A
B
C
D
E
F
G
H
I
J
K

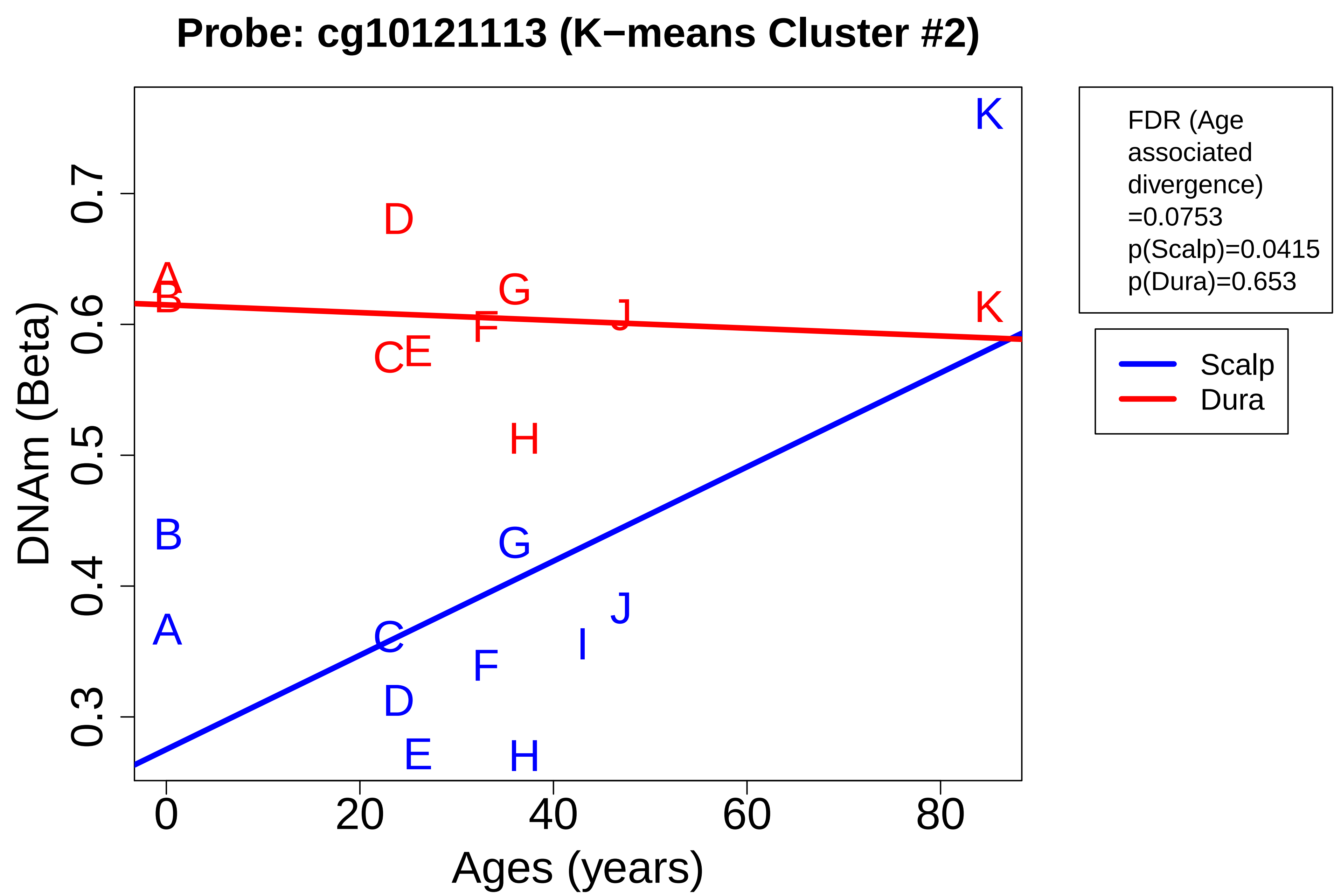

Probe: cg10121113 (K−means Cluster #2)
DNAm (Beta)
0.3
0.4
0.5
0.6
0.7
Ages (years)
0
20
40
60
80
FDR (Age associated divergence) =0.0753
p(Scalp)=0.0415
p(Dura)=0.653
Scalp
Dura
A
B
C
D
E
F
G
H
I
J
K

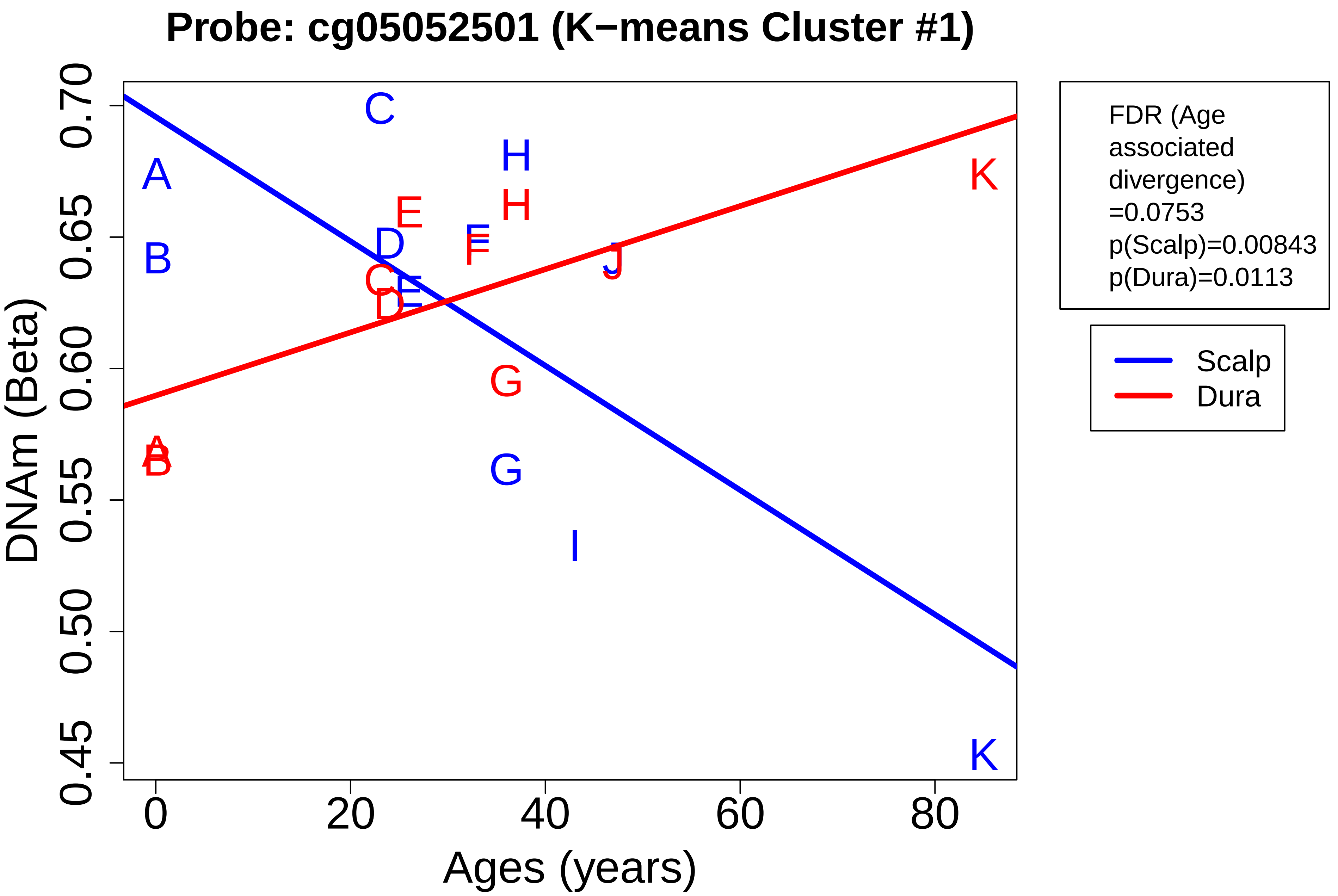

Probe: cg05052501 (K−means Cluster #1)
FDR (Age associated divergence) =0.0753
p(Scalp)=0.00843
p(Dura)=0.0113
Scalp
Dura
DNAm (Beta)
0.45
0.50
0.55
0.60
0.65
0.70
Ages (years)
0
20
40
60
80
A
B
C
D
E
F
G
H
I
J
K

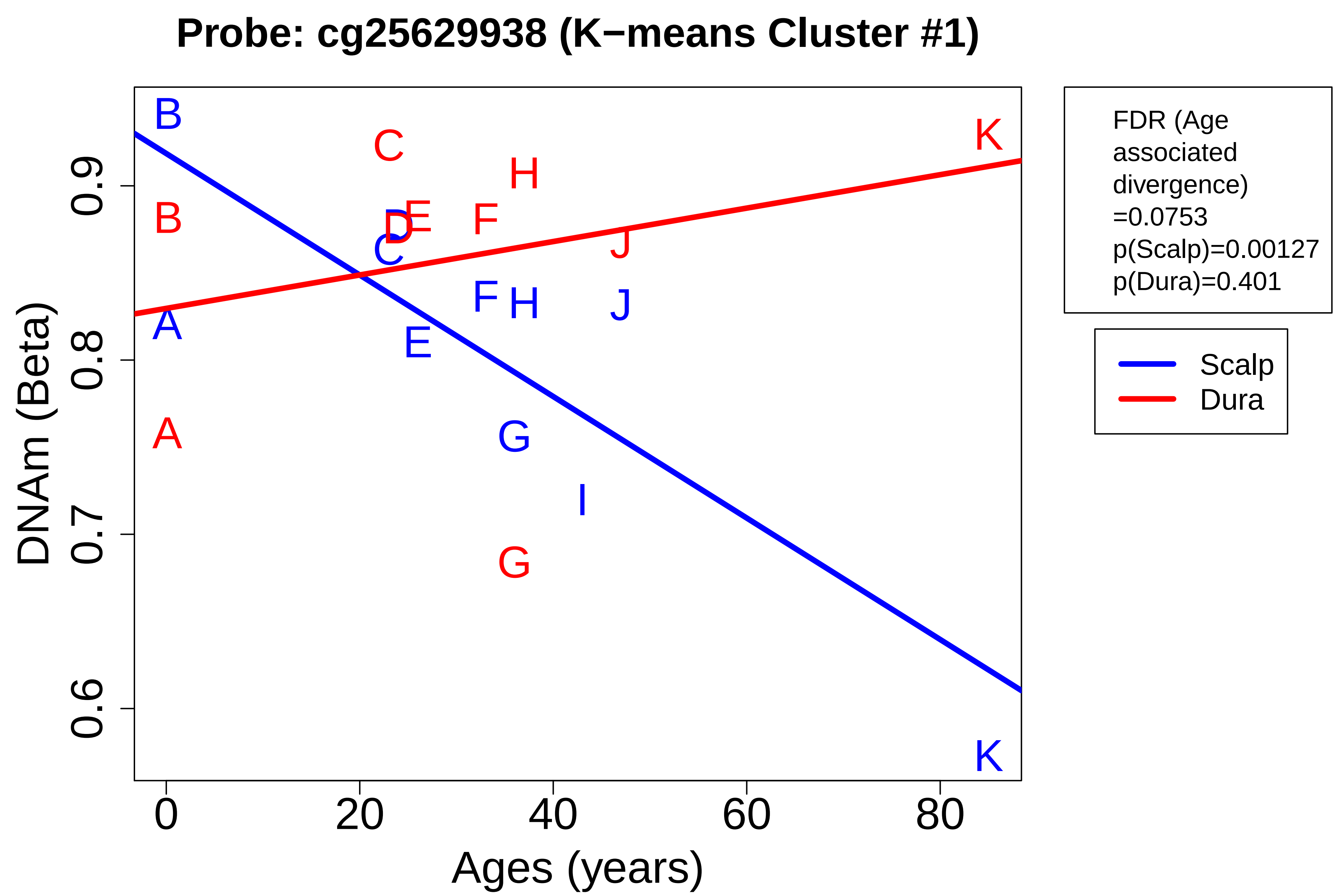
Probe: cg25629938 (K−means Cluster #1)
FDR (Age associated divergence) =0.0753
p(Scalp)=0.00127
p(Dura)=0.401
Scalp
Dura
DNAm (Beta)
0.6
0.7
0.8
0.9
0
20
40
60
80
Ages (years)
A
B
C
D
E
F
G
H
I
J
K

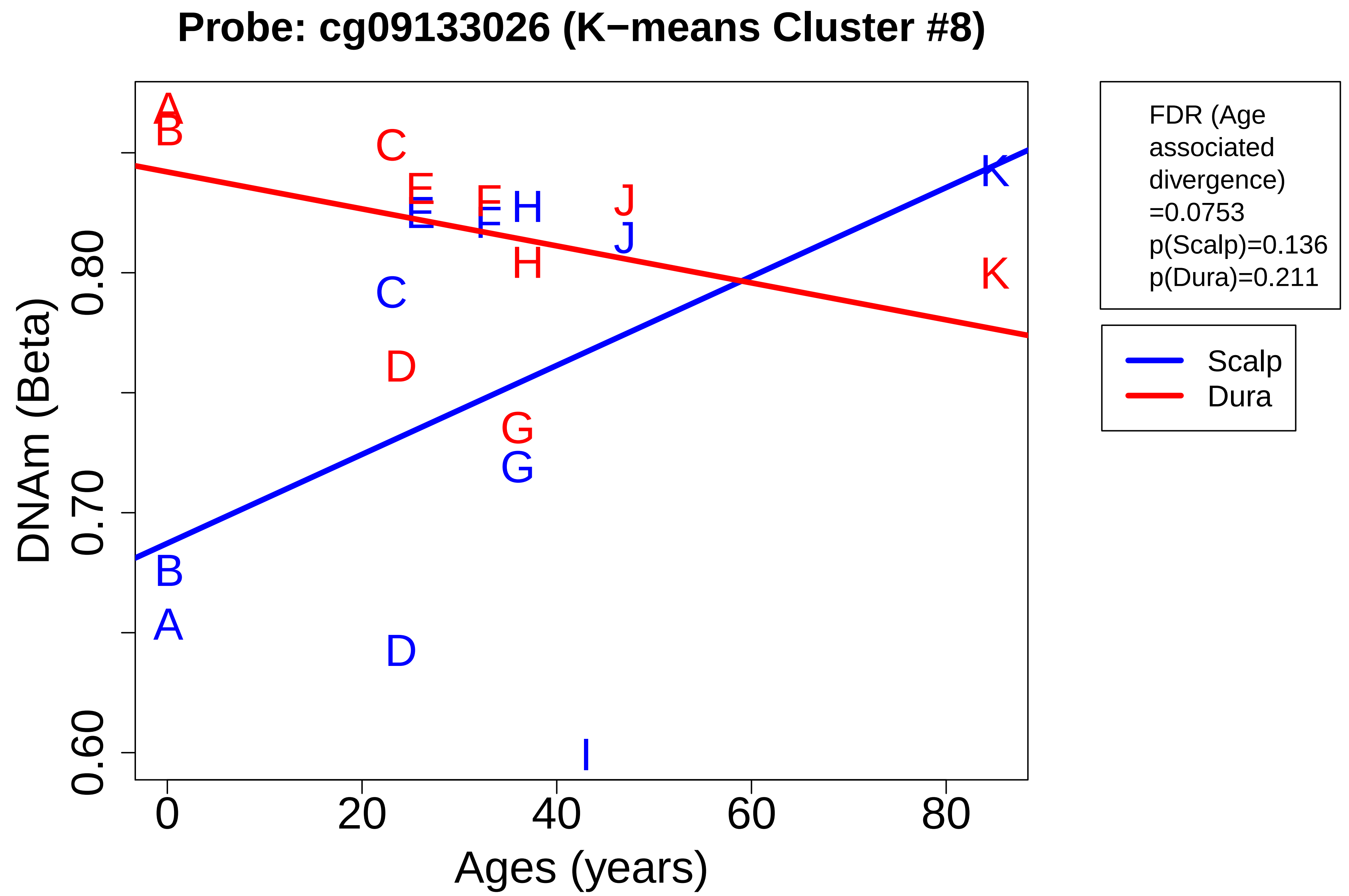

Probe: cg09133026 (K−means Cluster #8)
FDR (Age associated divergence) =0.0753
p(Scalp)=0.136
p(Dura)=0.211
Scalp
Dura
DNAm (Beta)
0.60
0.70
0.80
0
20
40
60
80
Ages (years)
A
B
C
D
E
F
G
H
I
J
K

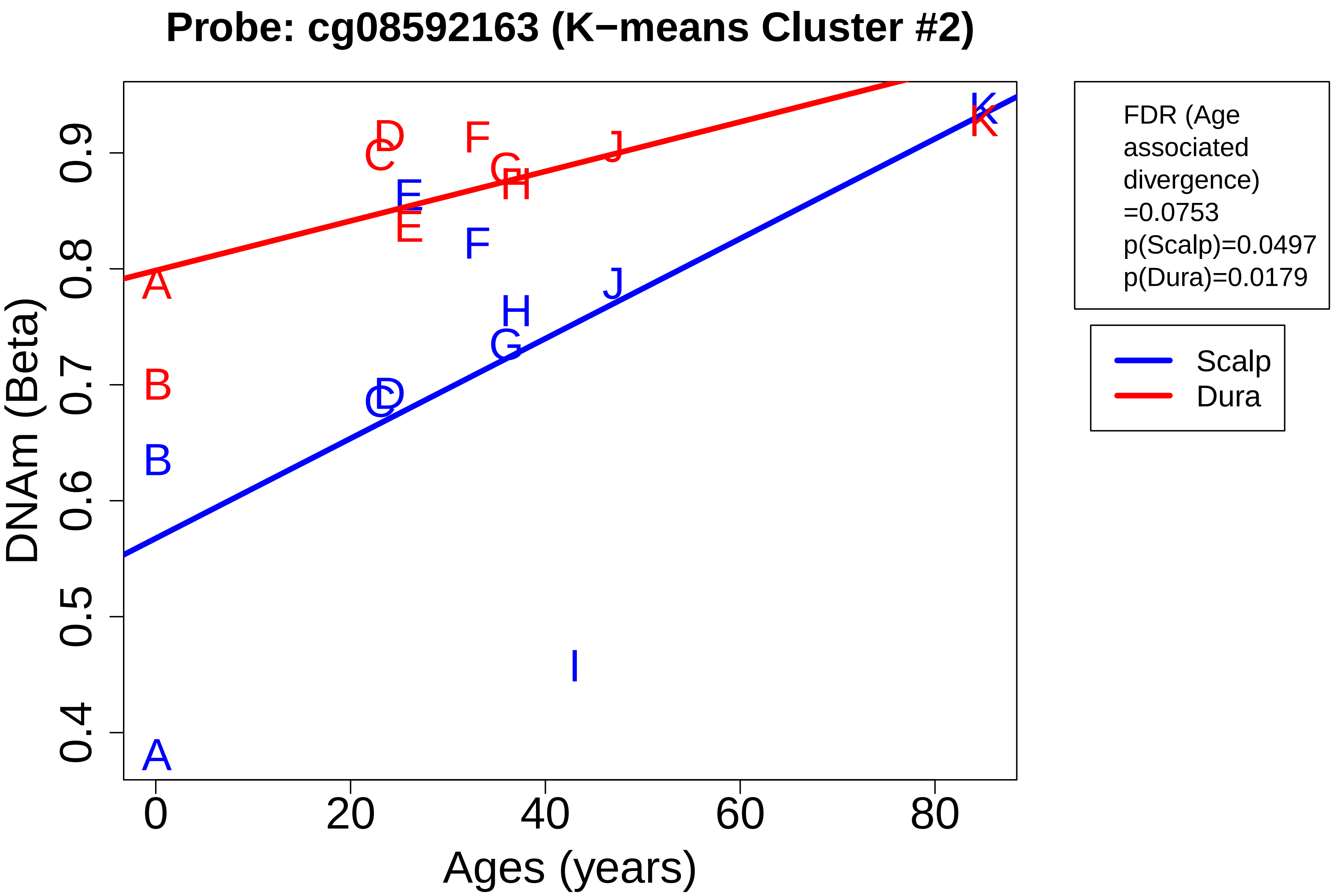

Probe: cg08592163 (K−means Cluster #2)
DNAm (Beta)
0.4
0.5
0.6
0.7
0.8
0.9
0
20
40
60
80
Ages (years)
FDR (Age associated divergence) =0.0753
p(Scalp)=0.0497
p(Dura)=0.0179
Scalp
Dura

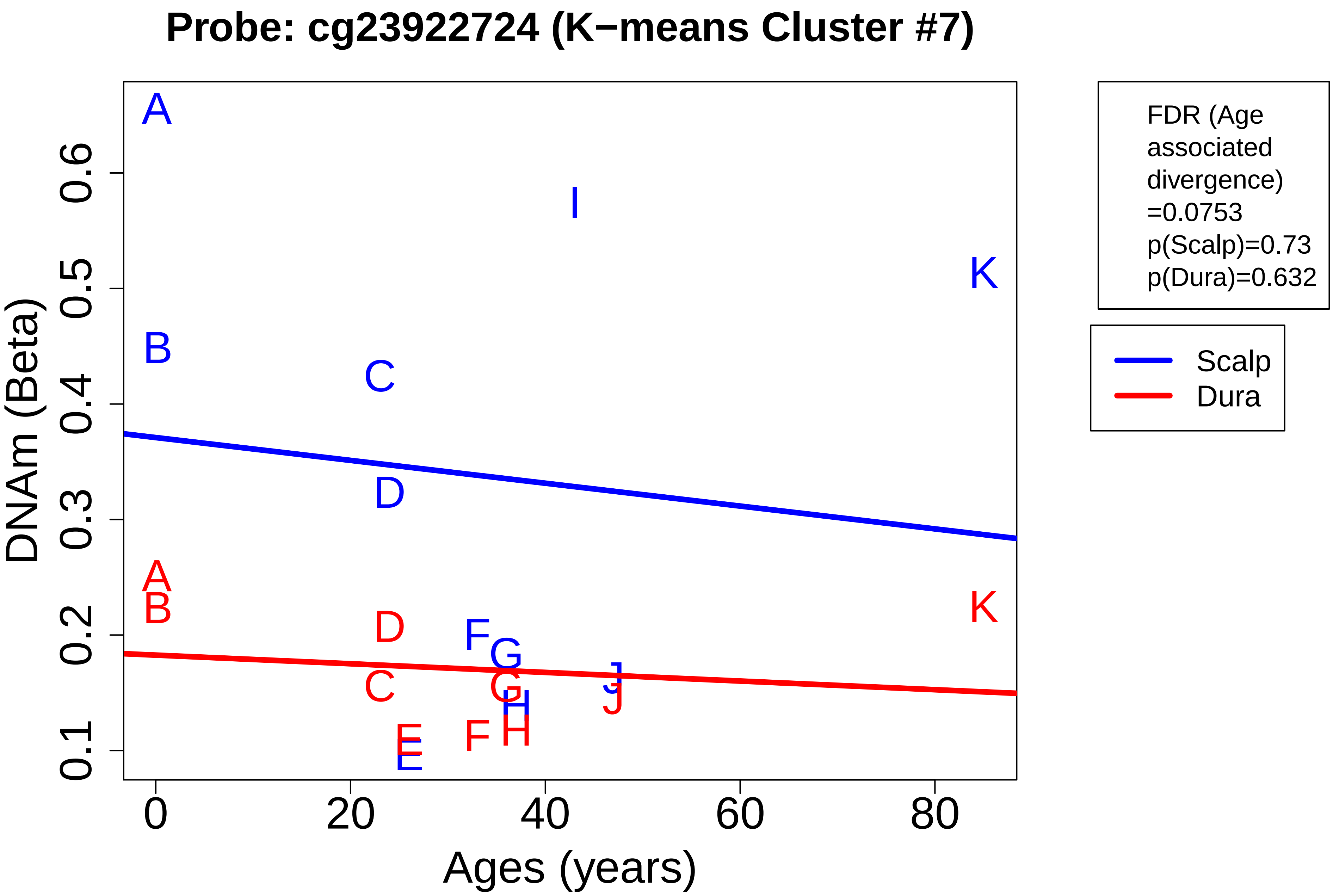

Probe: cg23922724 (K−means Cluster #7)
DNAm (Beta)
0.1
0.2
0.3
0.4
0.5
0.6
0
20
40
60
80
Ages (years)
A
B
C
D
E
F
G
H
I
J
K
FDR (Age associated divergence) =0.0753
p(Scalp)=0.73
p(Dura)=0.632
Scalp
Dura

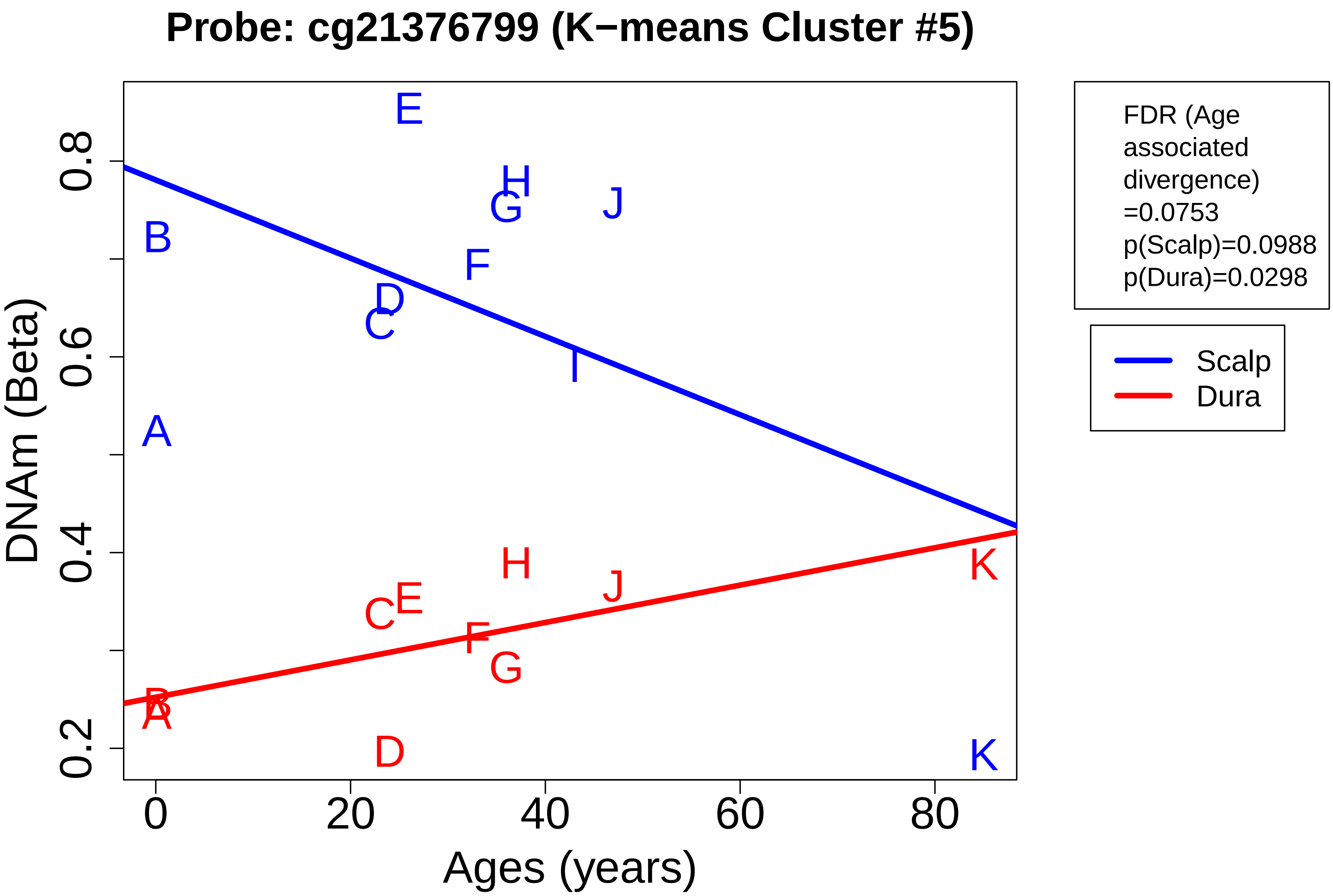
Probe: cg21376799 (K−means Cluster #5)
DNAm (Beta)
0.2
0.4
0.6
0.8
0
20
40
60
80
Ages (years)
FDR (Age associated divergence) =0.0753
p(Scalp)=0.0988
p(Dura)=0.0298
Scalp
Dura
A
B
C
D
E
F
G
H
I
J
K

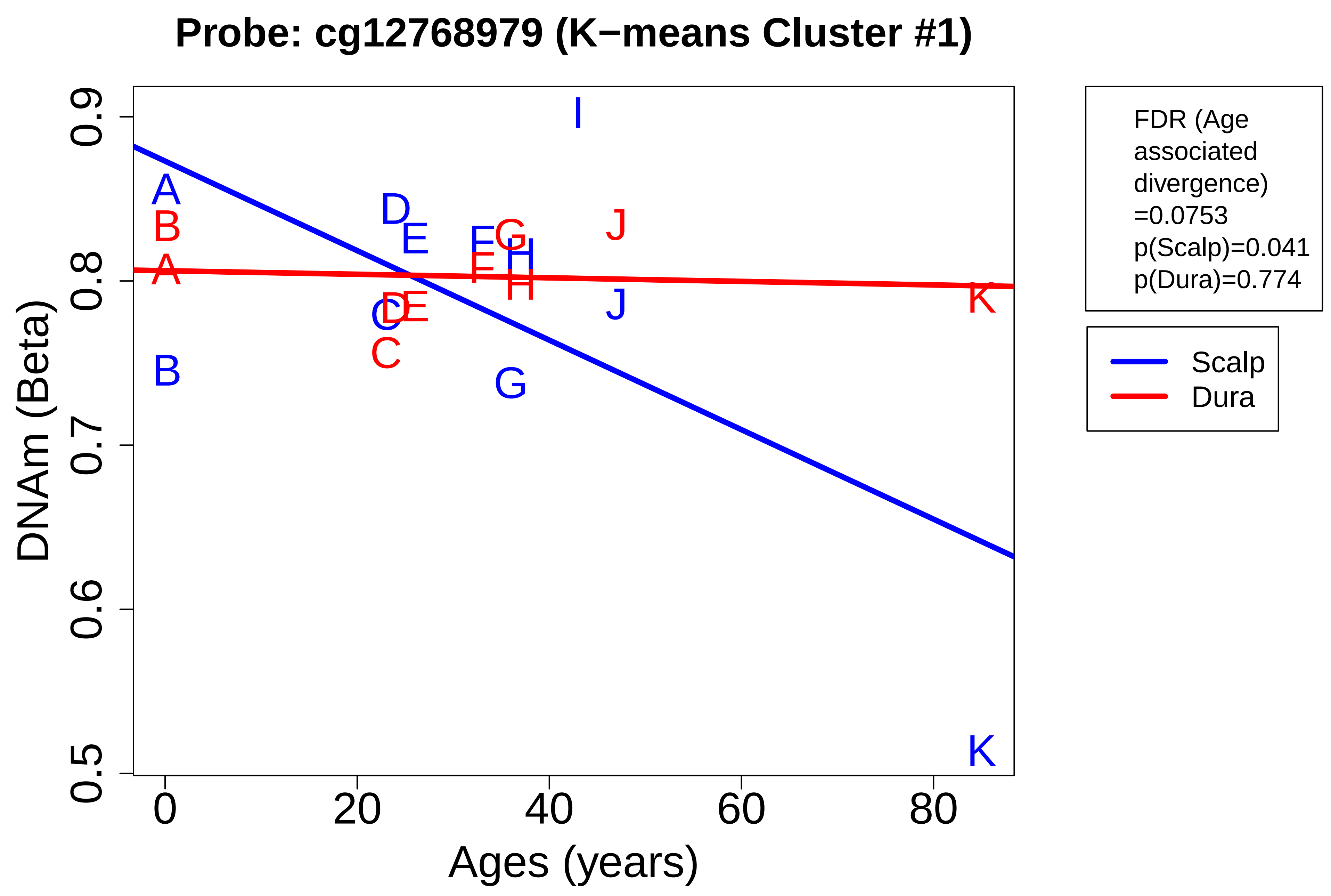

Probe: cg12768979 (K−means Cluster #1)
DNAm (Beta)
0.5
0.6
0.7
0.8
0.9
0
20
40
60
80
Ages (years)
FDR (Age associated divergence) =0.0753
p(Scalp)=0.041
p(Dura)=0.774
Scalp
Dura
A
B
C
D
E
F
G
H
I
J
K

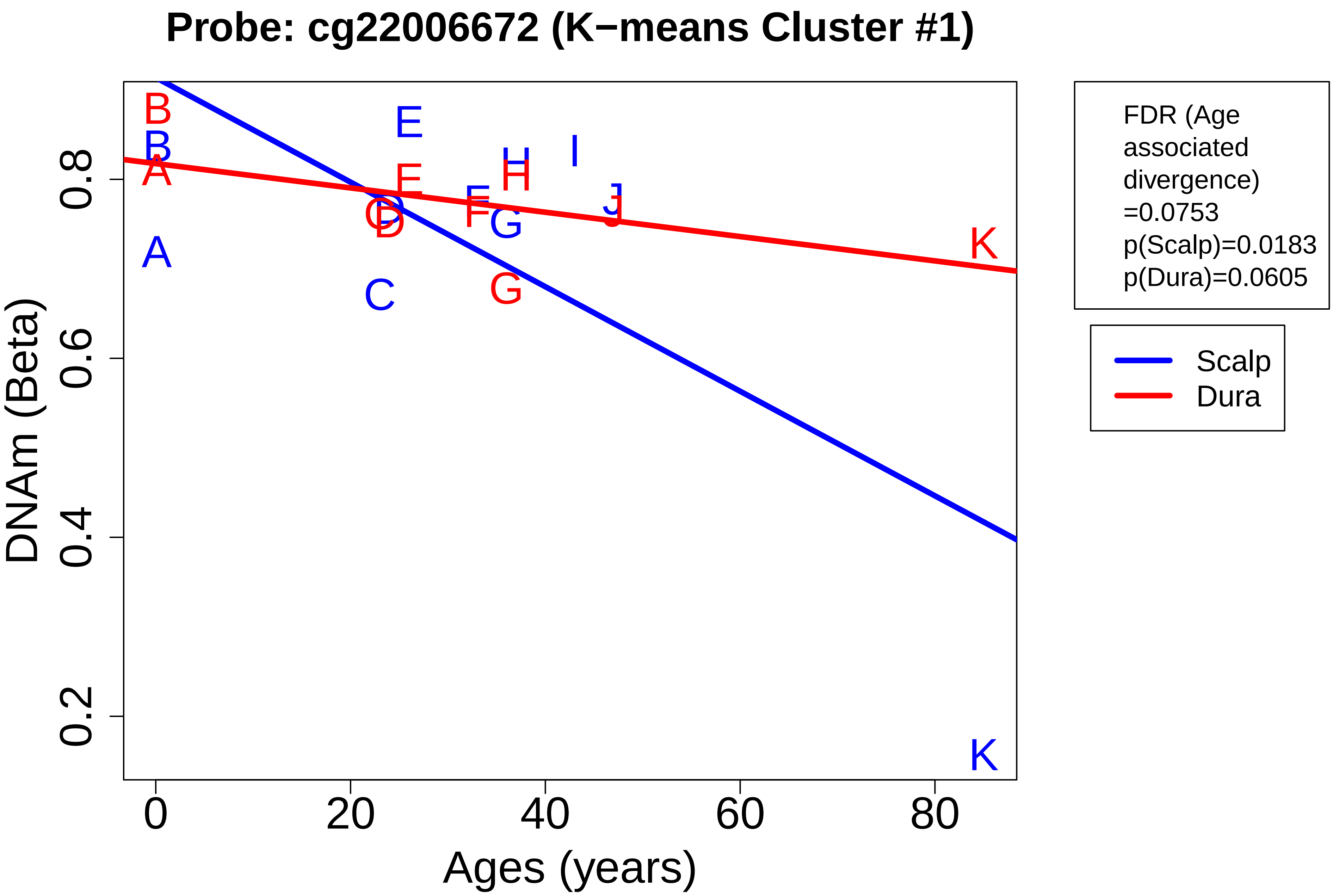
Probe: cg22006672 (K−means Cluster #1)
DNAm (Beta)
0.2
0.4
0.6
0.8
Ages (years)
0
20
40
60
80
FDR (Age associated divergence) =0.0753
p(Scalp)=0.0183
p(Dura)=0.0605
Scalp
Dura

Probe: cg07143158 (K−means Cluster #6)

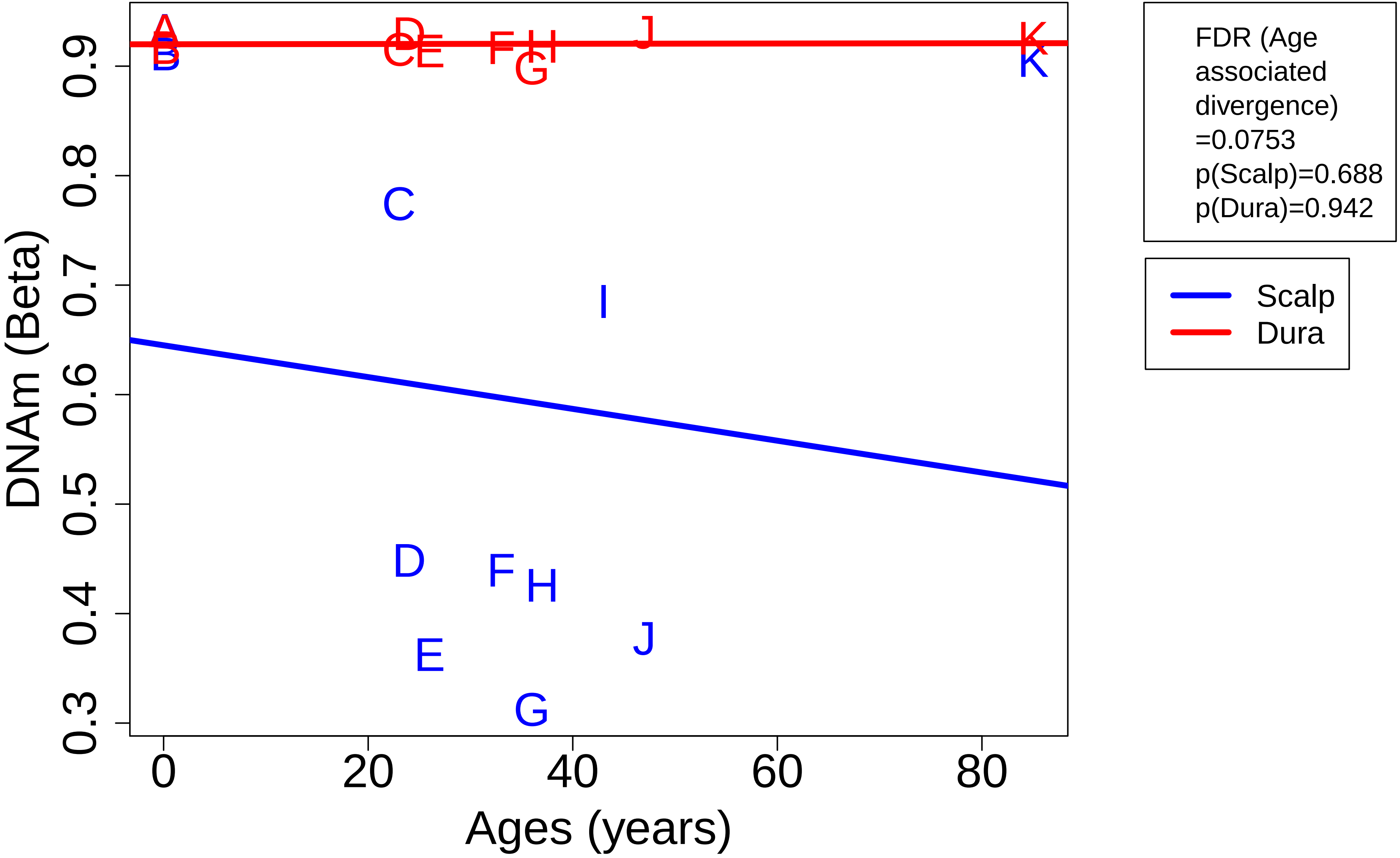

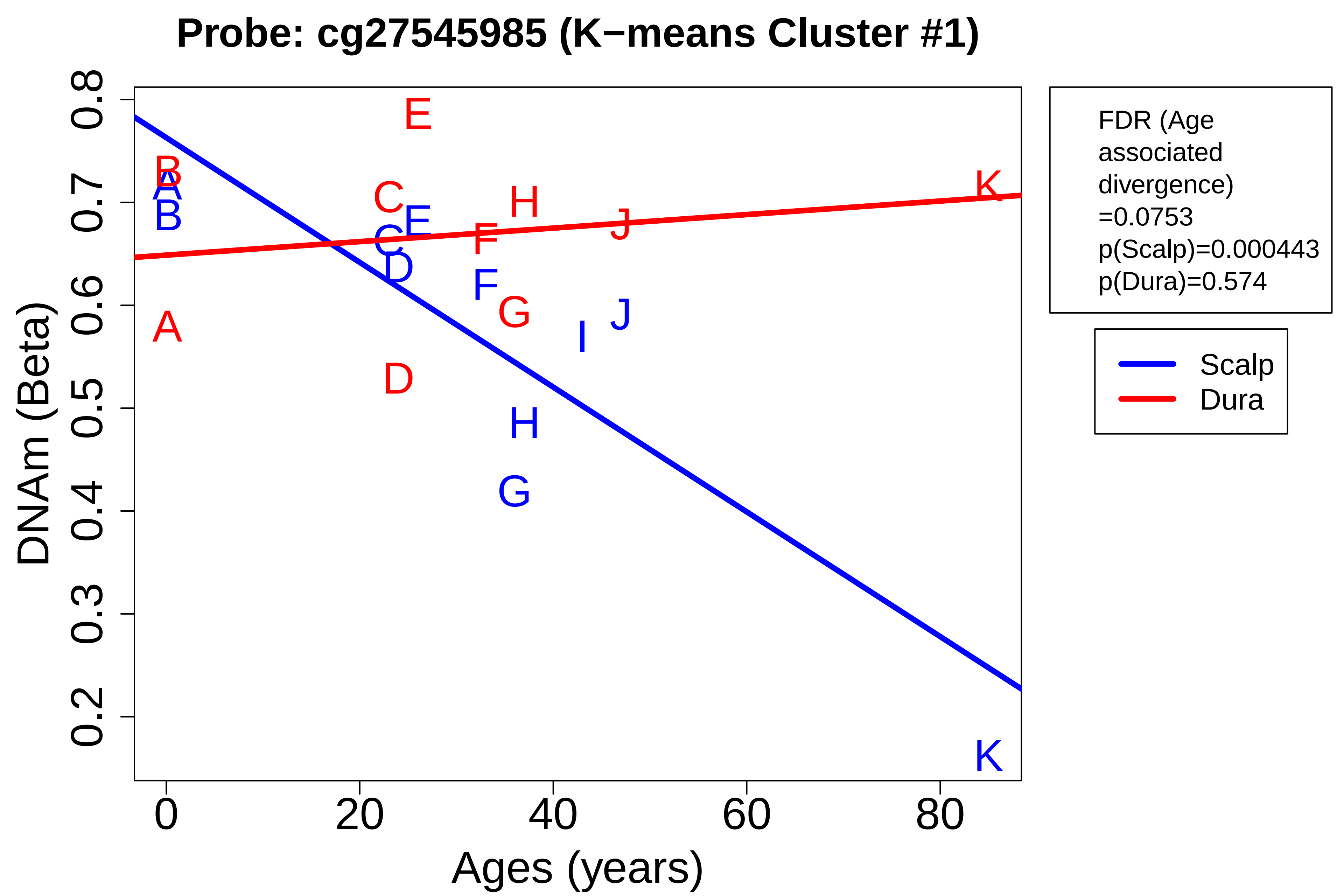

Probe: cg27545985 (K−means Cluster #1)
DNAm (Beta)
Ages (years)
0.2
0.3
0.4
0.5
0.6
0.7
0.8
0
20
40
60
80
FDR (Age associated divergence) =0.0753
p(Scalp)=0.000443
p(Dura)=0.574
Scalp
Dura

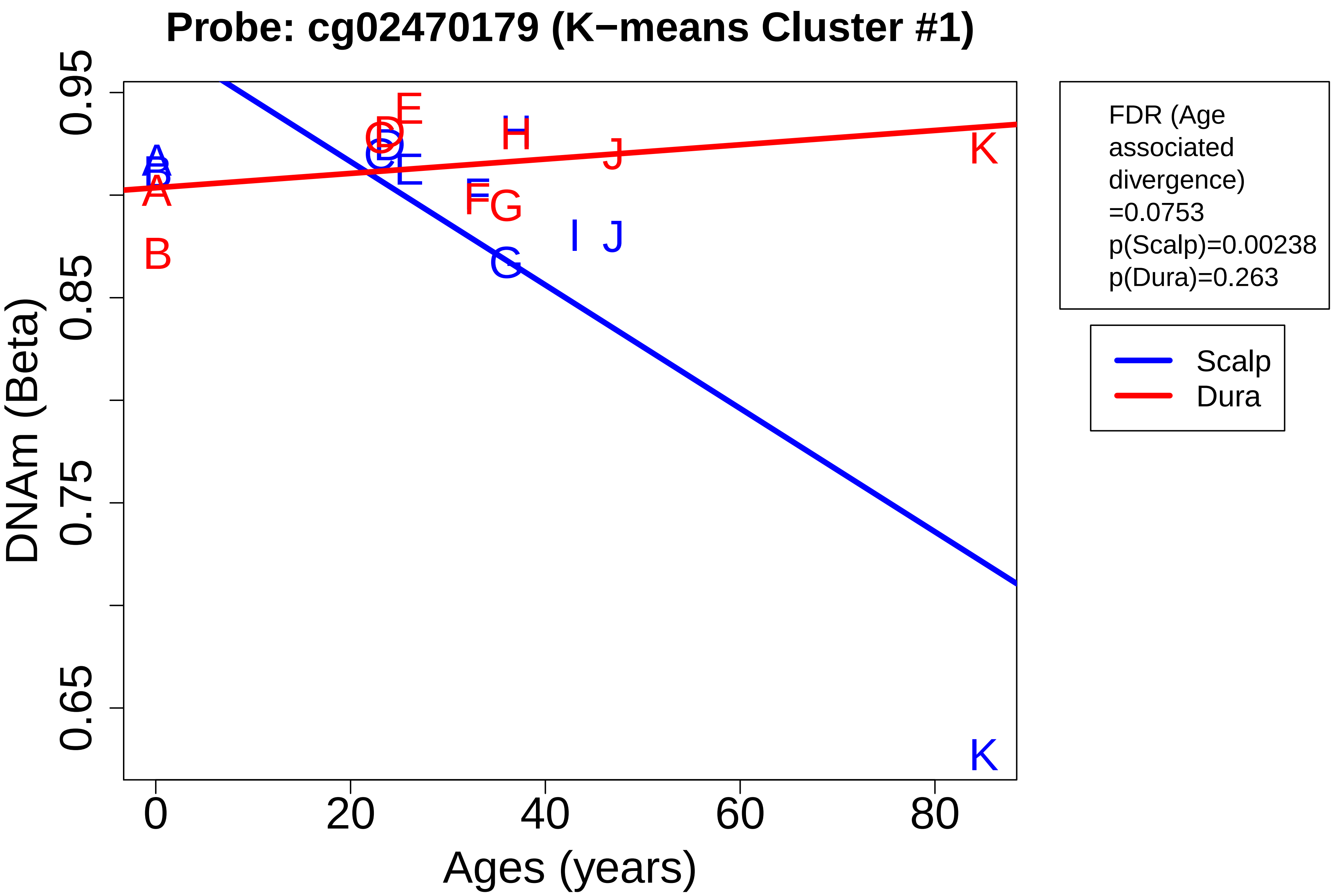

Probe: cg02470179 (K−means Cluster #1)
DNAm (Beta)
0.95
0.85
0.75
0.65
0
20
40
60
80
Ages (years)
FDR (Age associated divergence) =0.0753
p(Scalp)=0.00238
p(Dura)=0.263
Scalp
Dura

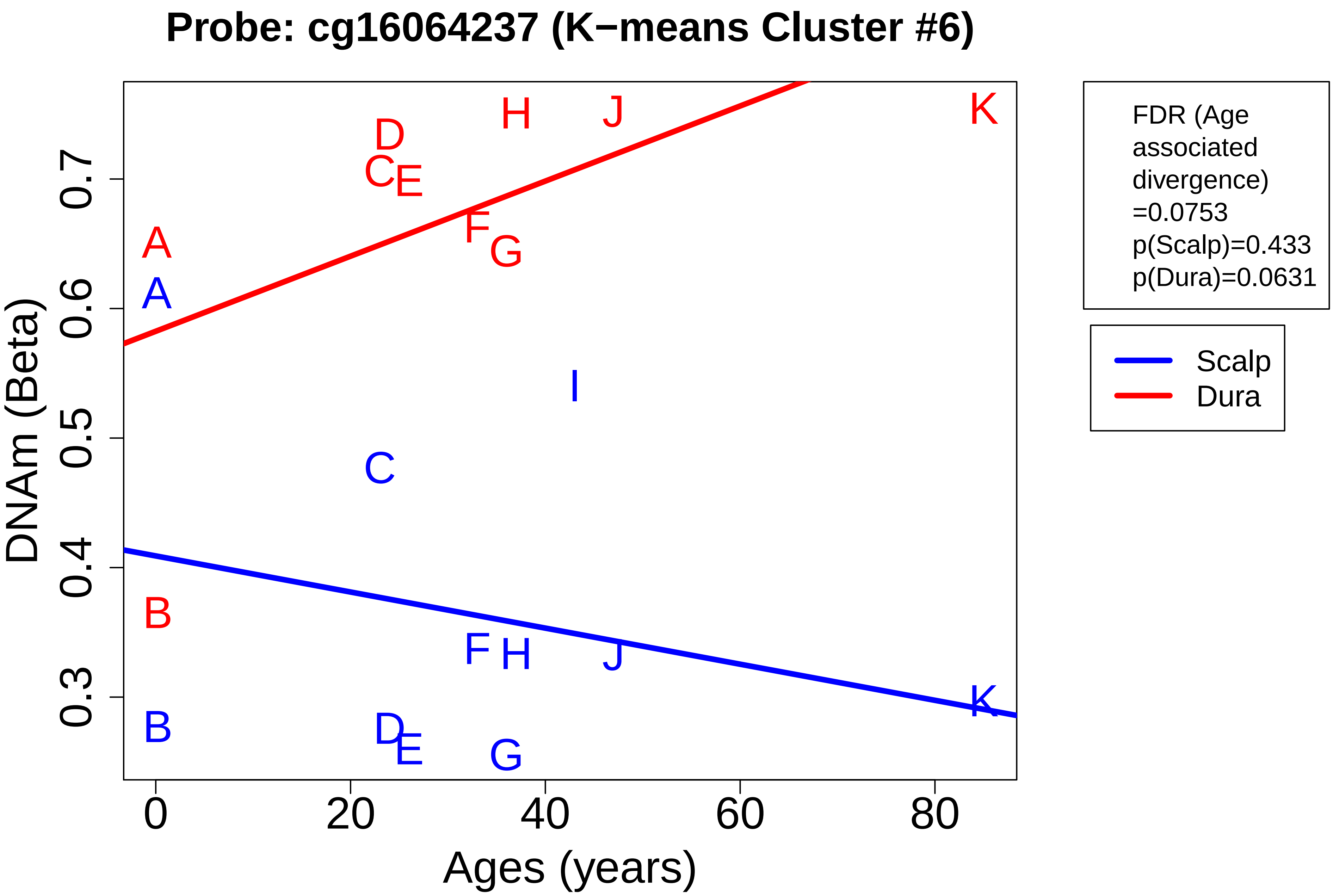
Probe: cg16064237 (K−means Cluster #6)
DNAm (Beta)
0.3
0.4
0.5
0.6
0.7
Ages (years)
0
20
40
60
80
FDR (Age associated divergence) =0.0753
p(Scalp)=0.433
p(Dura)=0.0631
Scalp
Dura

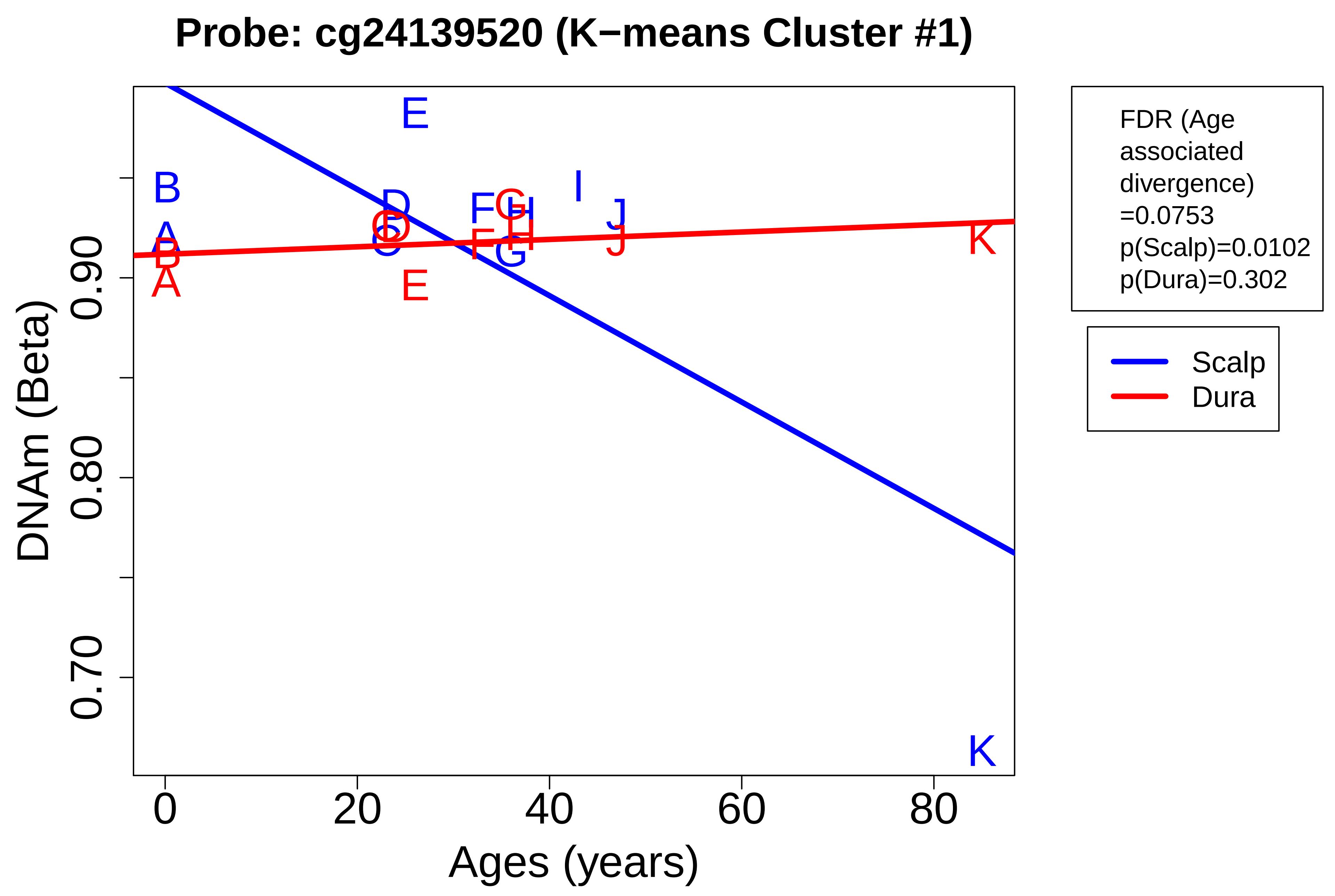

Probe: cg24139520 (K−means Cluster #1)
DNAm (Beta)
0.70
0.80
0.90
0
20
40
60
80
Ages (years)
FDR (Age associated divergence) =0.0753
p(Scalp)=0.0102
p(Dura)=0.302
Scalp
Dura

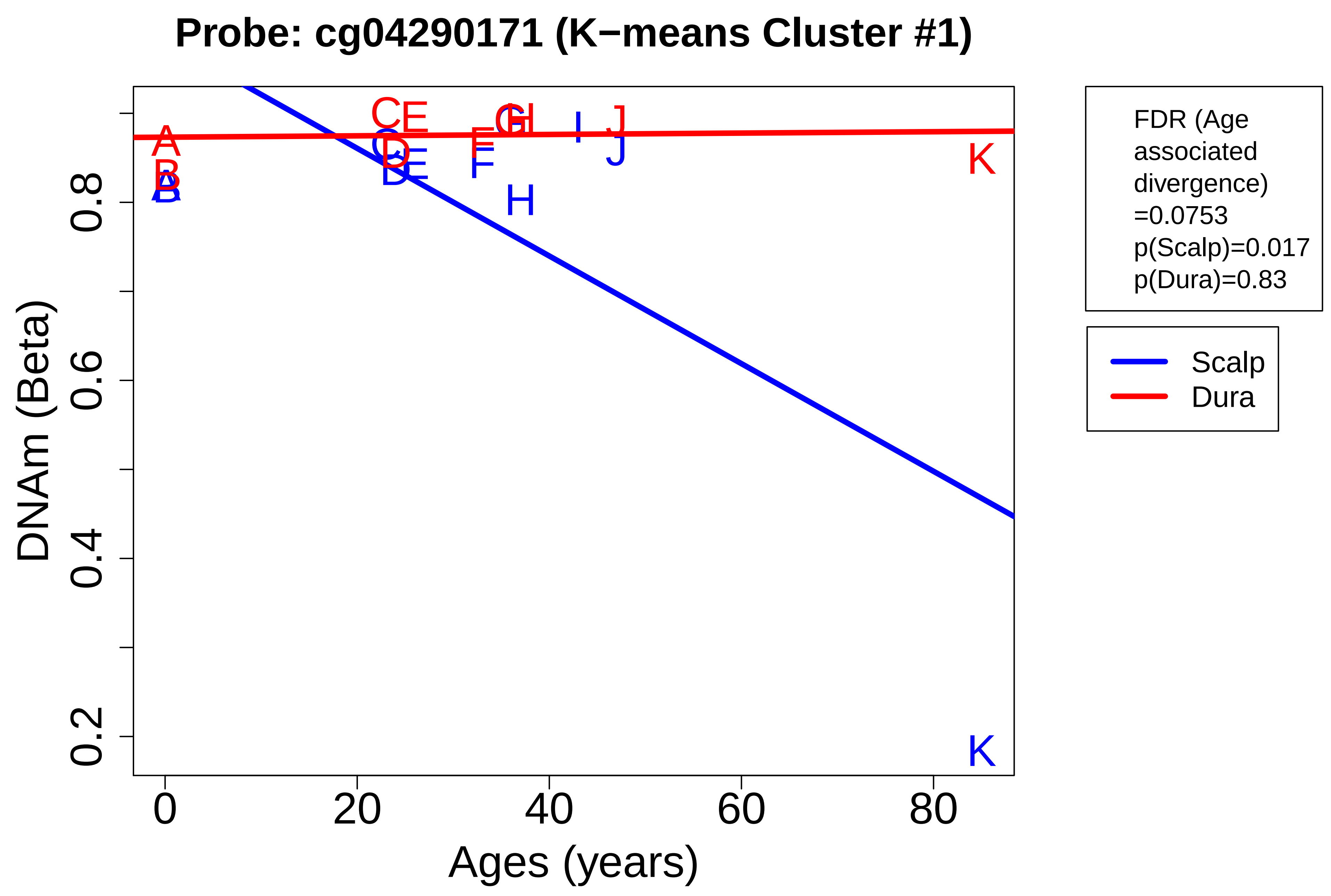

Probe: cg04290171 (K−means Cluster #1)
DNAm (Beta)
0.2
0.4
0.6
0.8
Ages (years)
0
20
40
60
80
FDR (Age associated divergence) =0.0753
p(Scalp)=0.017
p(Dura)=0.83
Scalp
Dura

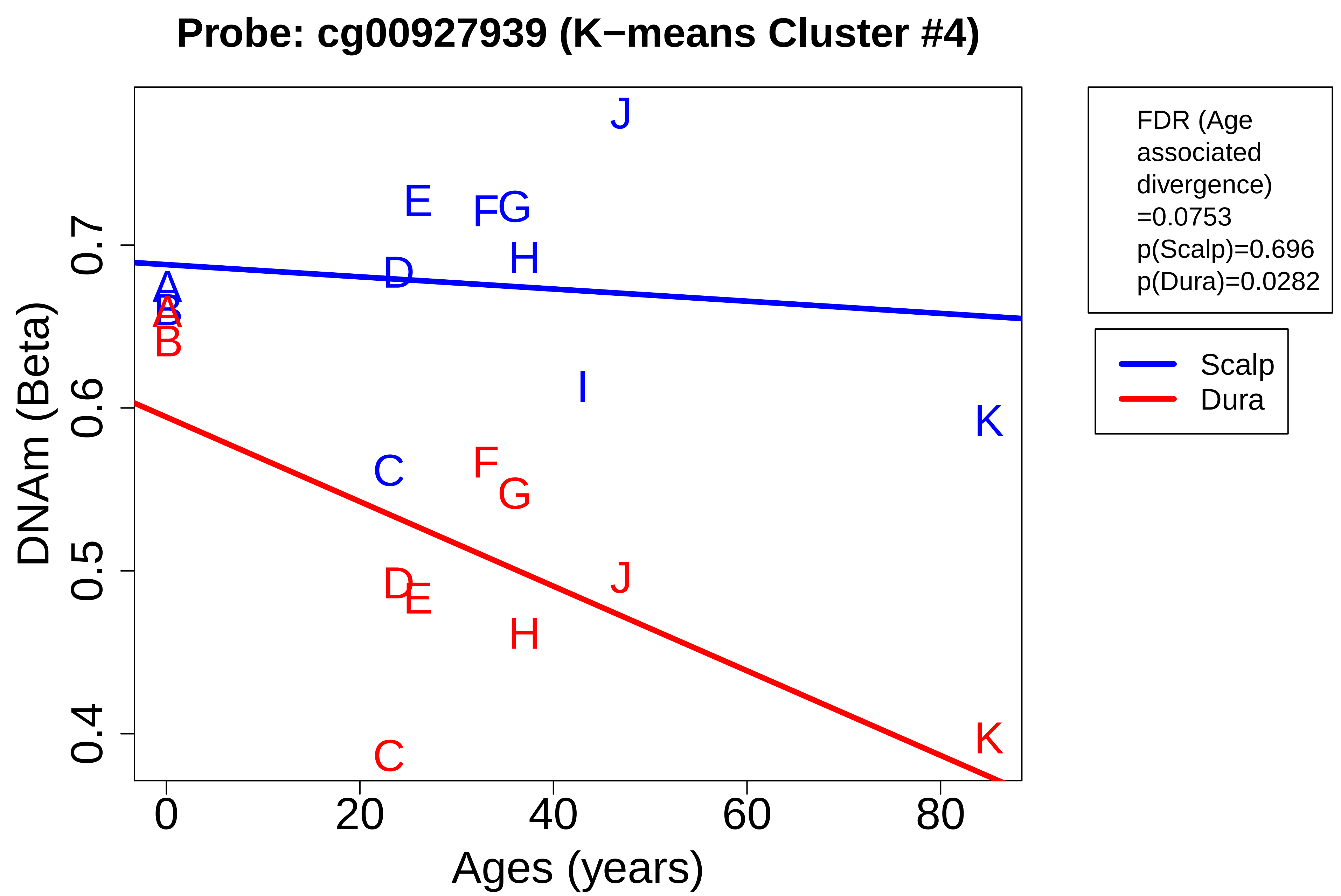
Probe: cg00927939 (K−means Cluster #4)
DNAm (Beta)
0.4
0.5
0.6
0.7
0
20
40
60
80
Ages (years)
A
B
C
D
E
F
G
H
I
J
K
FDR (Age associated divergence) =0.0753
p(Scalp)=0.696
p(Dura)=0.0282
Scalp
Dura

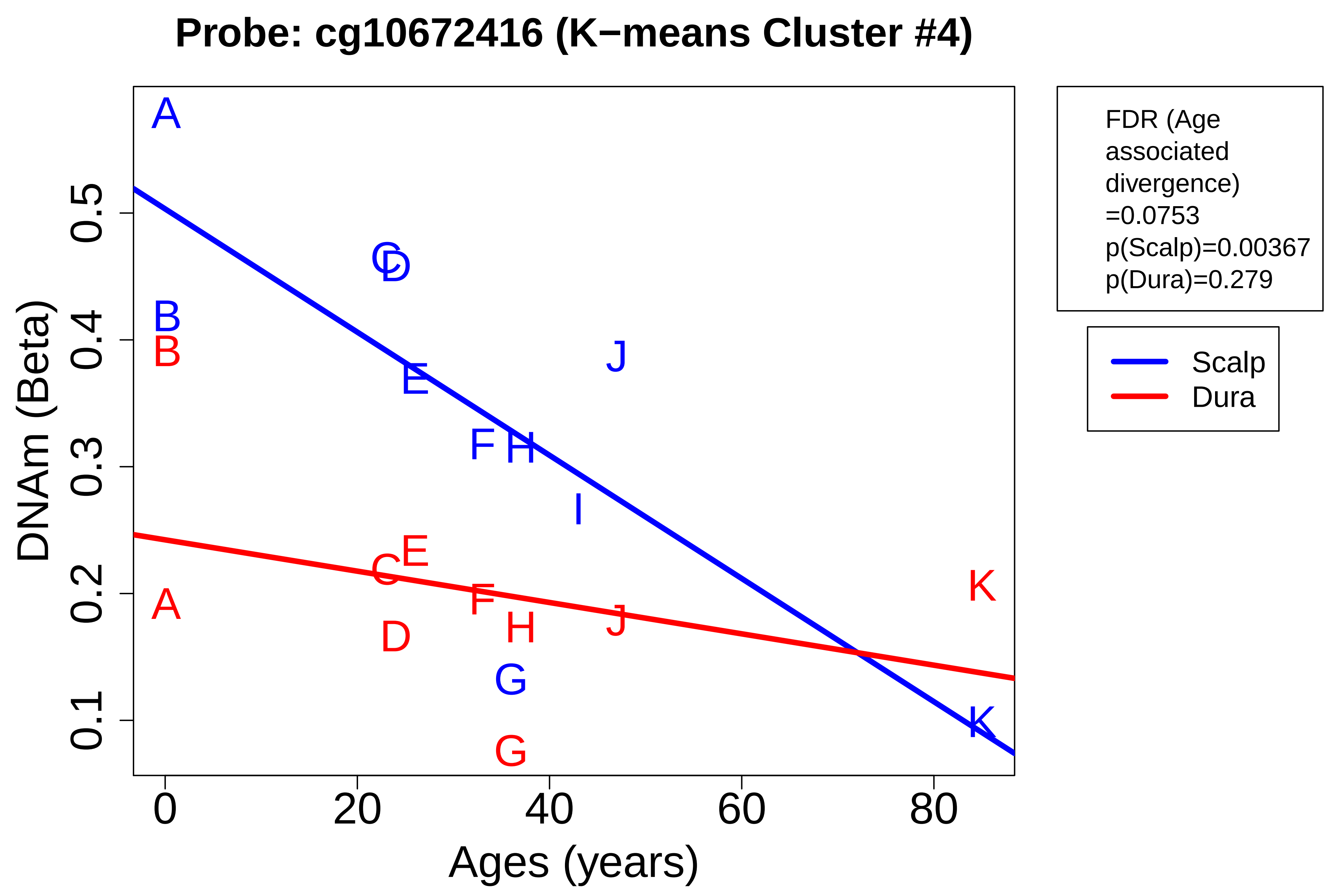

Probe: cg10672416 (K−means Cluster #4)
DNAm (Beta)
Ages (years)
0.1
0.2
0.3
0.4
0.5
0
20
40
60
80
FDR (Age associated divergence) =0.0753
p(Scalp)=0.00367
p(Dura)=0.279
Scalp
Dura
A
B
C
D
E
F
G
H
I
J
K

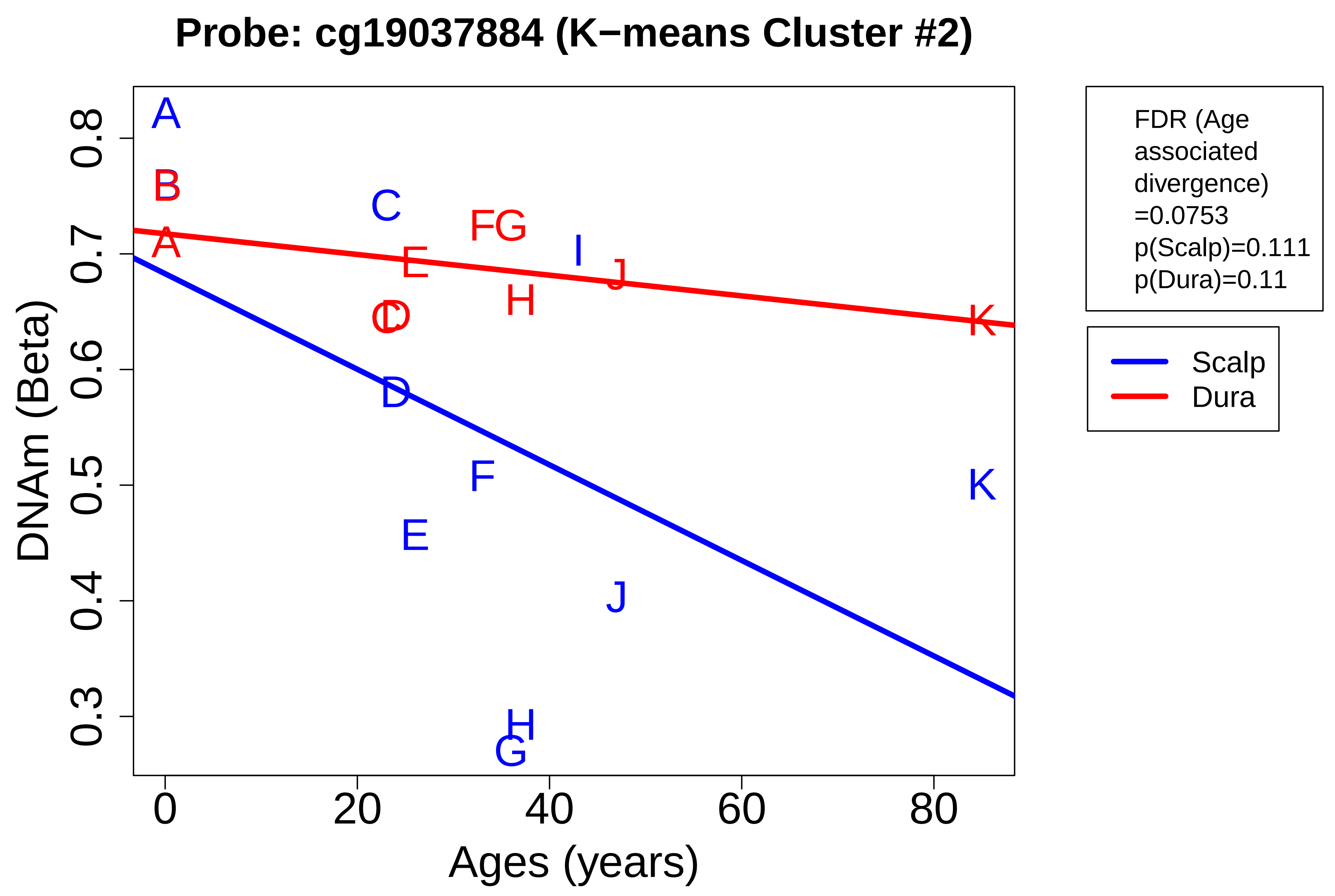

Probe: cg19037884 (K−means Cluster #2)
DNAm (Beta)
0.3
0.4
0.5
0.6
0.7
0.8
Ages (years)
0
20
40
60
80
A
B
C
D
E
F
G
H
I
J
K
FDR (Age associated divergence) =0.0753
p(Scalp)=0.111
p(Dura)=0.11
Scalp
Dura

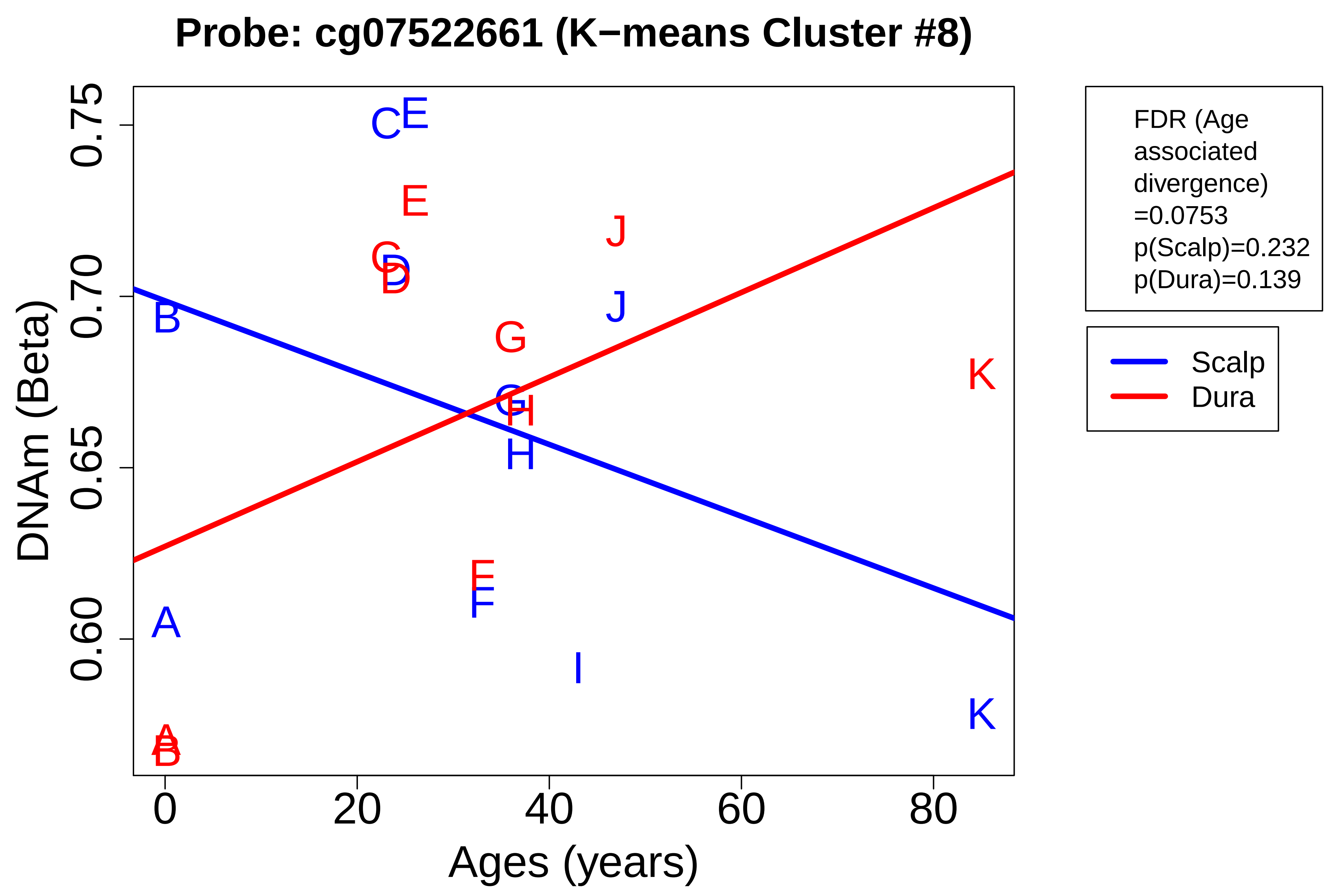

Probe: cg07522661 (K−means Cluster #8)
DNAm (Beta)
0.60
0.65
0.70
0.75
Ages (years)
0
20
40
60
80
FDR (Age associated divergence) =0.0753
p(Scalp)=0.232
p(Dura)=0.139
Scalp
Dura

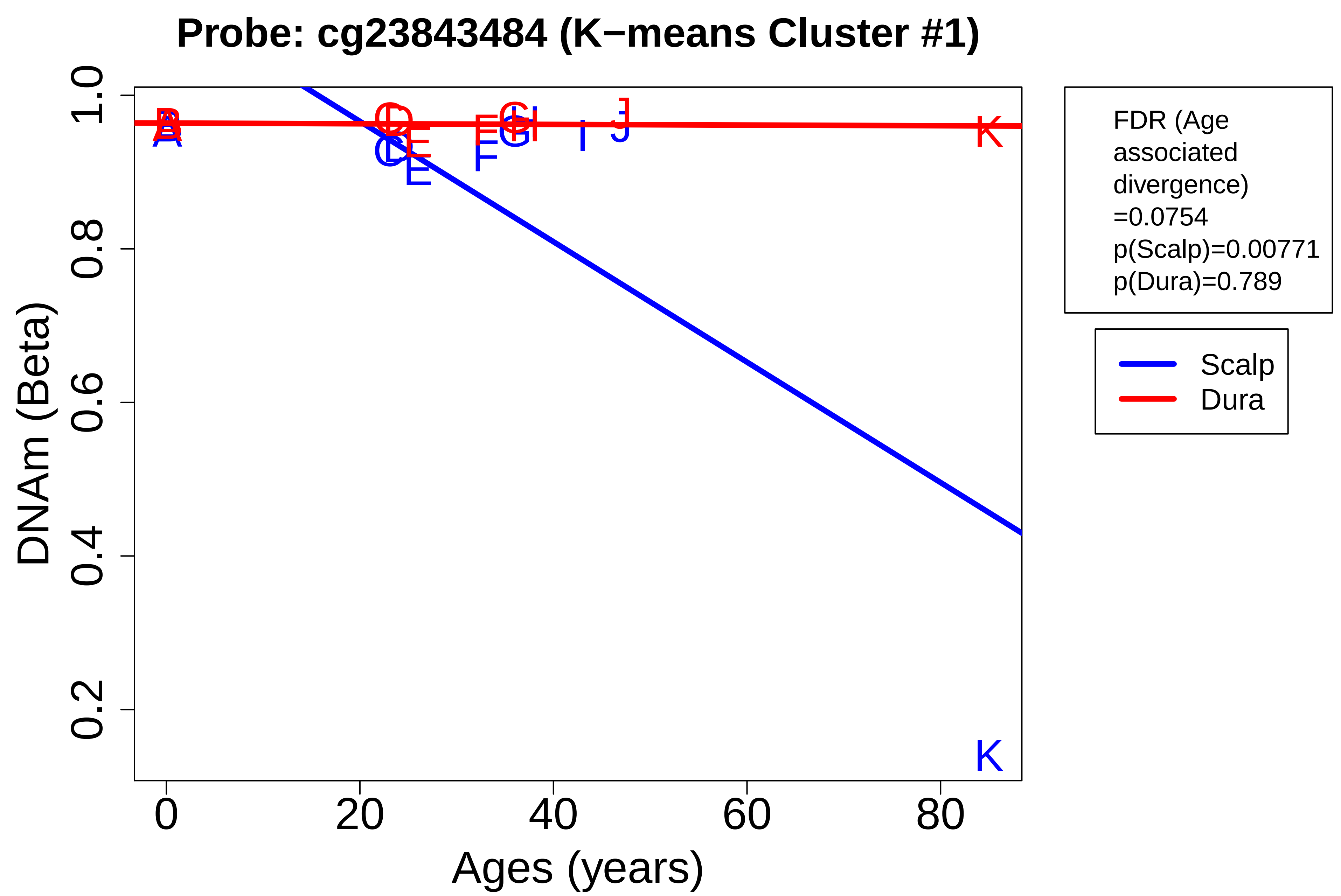

Probe: cg23843484 (K−means Cluster #1)
DNAm (Beta)
1.0
0.8
0.6
0.4
0.2
0
20
40
60
80
Ages (years)
A
B
C
D
E
F
G
H
I
J
K
FDR (Age associated divergence) =0.0754
p(Scalp)=0.00771
p(Dura)=0.789
Scalp
Dura

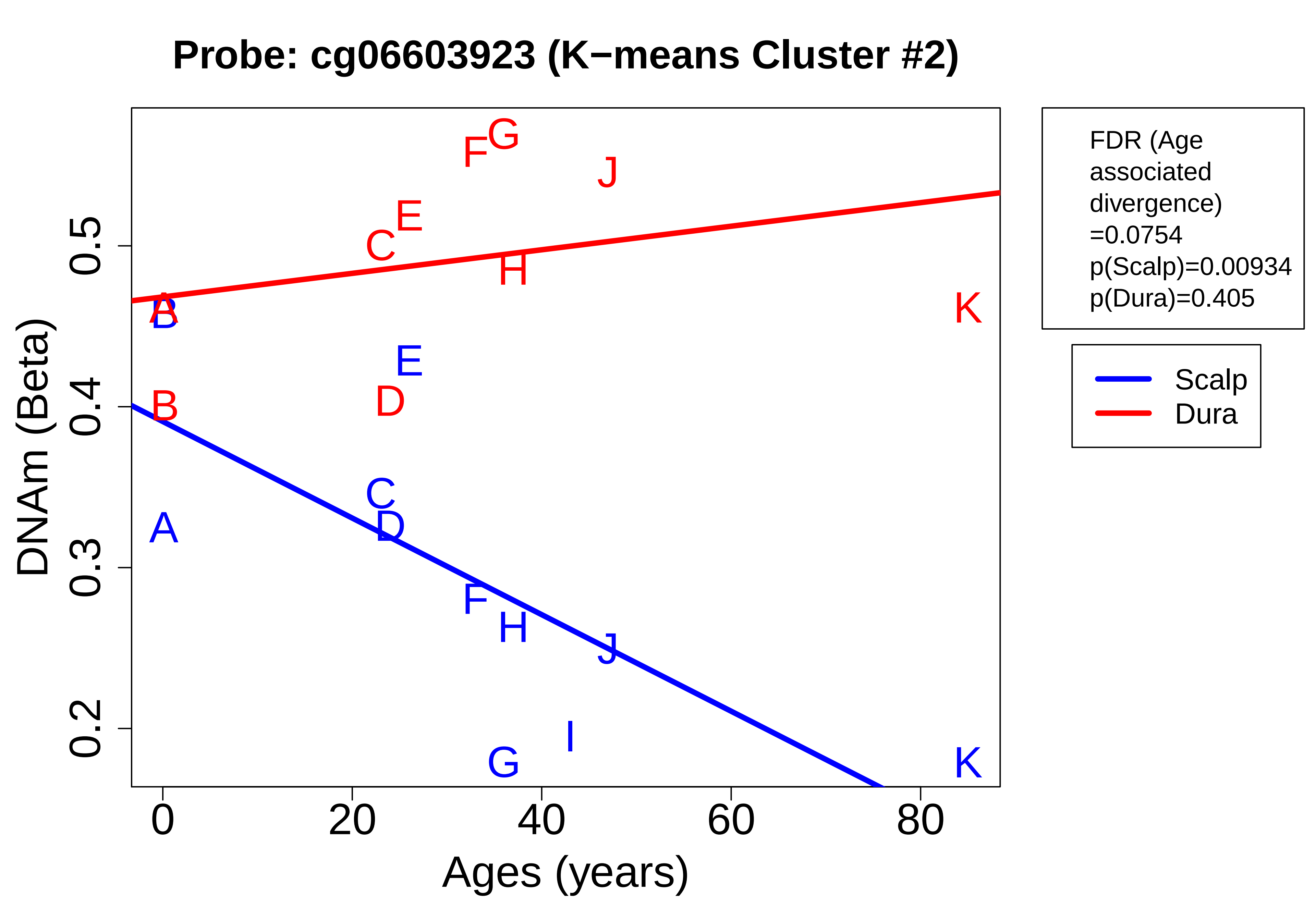
Probe: cg06603923 (K−means Cluster #2)
DNAm (Beta)
0.2
0.3
0.4
0.5
Ages (years)
0
20
40
60
80
A B C D E F G H I J K
FDR (Age associated divergence) =0.0754
p(Scalp)=0.00934
p(Dura)=0.405
Scalp
Dura

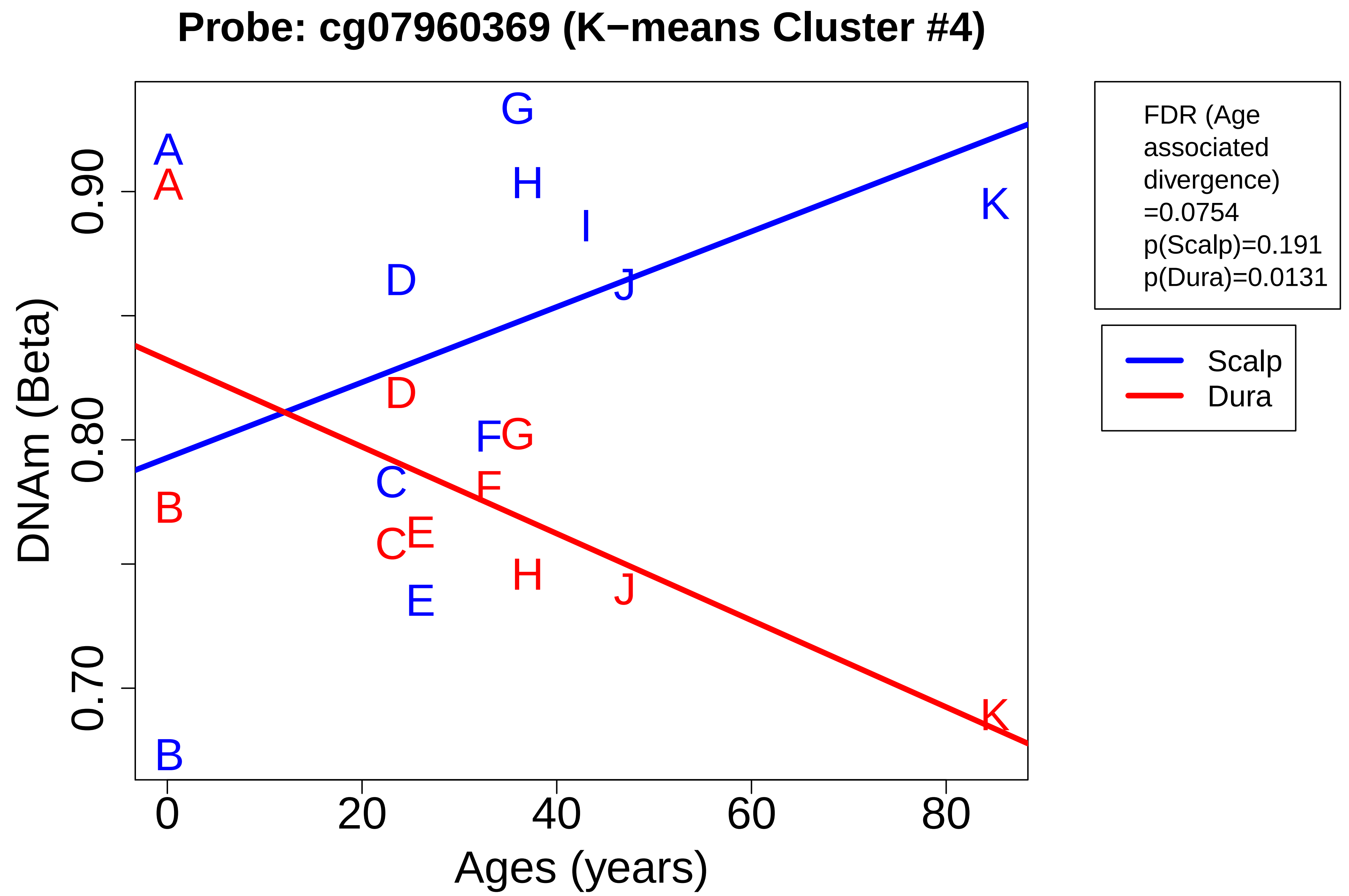

Probe: cg07960369 (K−means Cluster #4)
DNAm (Beta)
0.70
0.80
0.90
0
20
40
60
80
Ages (years)
A
A
B
B
C
C
D
D
E
E
F
F
G
G
H
H
I
J
J
K
K
FDR (Age associated divergence) =0.0754
p(Scalp)=0.191
p(Dura)=0.0131
Scalp
Dura

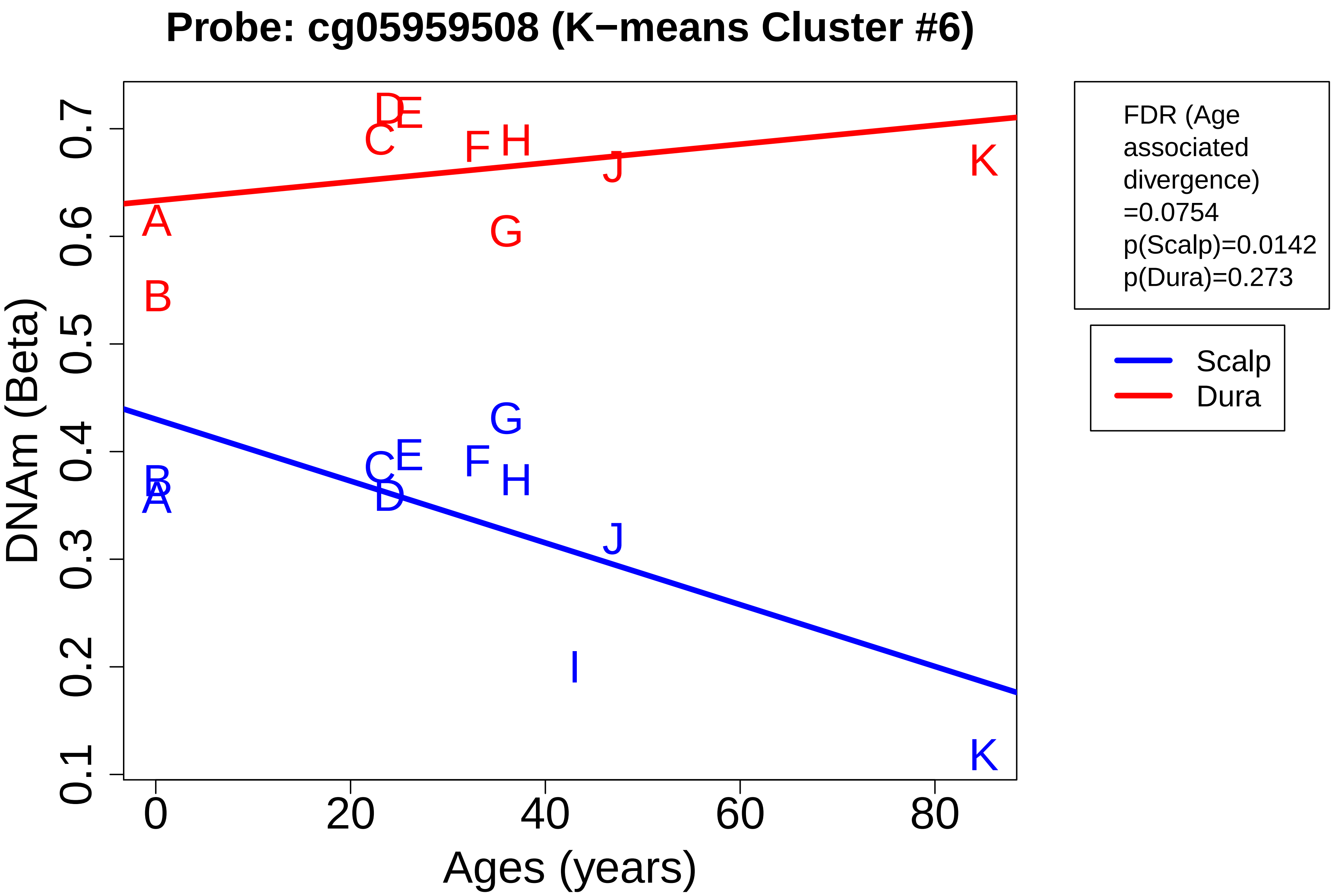

Probe: cg05959508 (K−means Cluster #6)
FDR (Age associated divergence) =0.0754
p(Scalp)=0.0142
p(Dura)=0.273
Scalp
Dura
DNAm (Beta)
Ages (years)
0.1
0.2
0.3
0.4
0.5
0.6
0.7
0
20
40
60
80

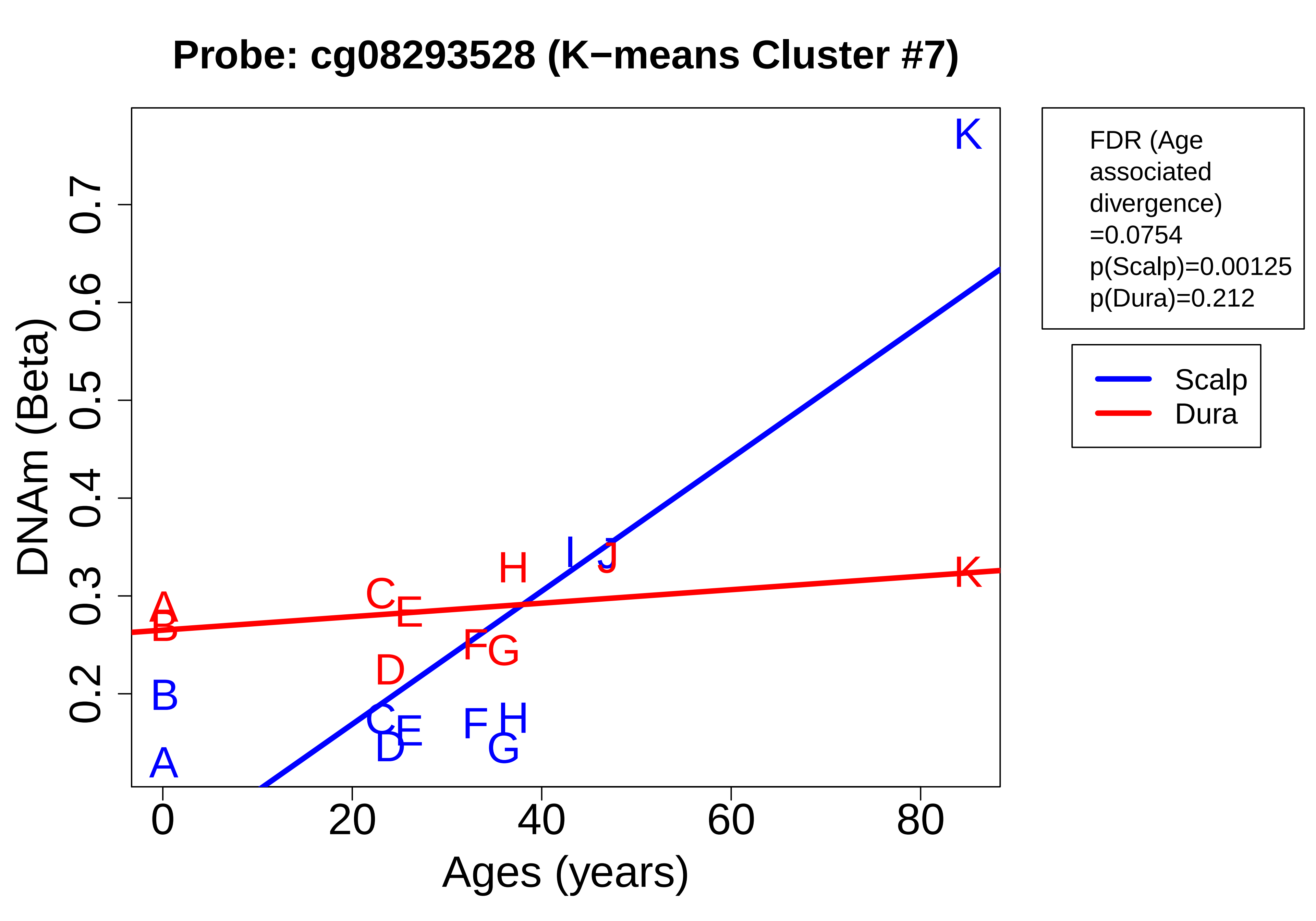

Probe: cg08293528 (K−means Cluster #7)
DNAm (Beta)
0.2
0.3
0.4
0.5
0.6
0.7
Ages (years)
0
20
40
60
80
FDR (Age associated divergence) =0.0754
p(Scalp)=0.00125
p(Dura)=0.212
Scalp
Dura
A
B
C
D
E
F
G
H
I
J
K

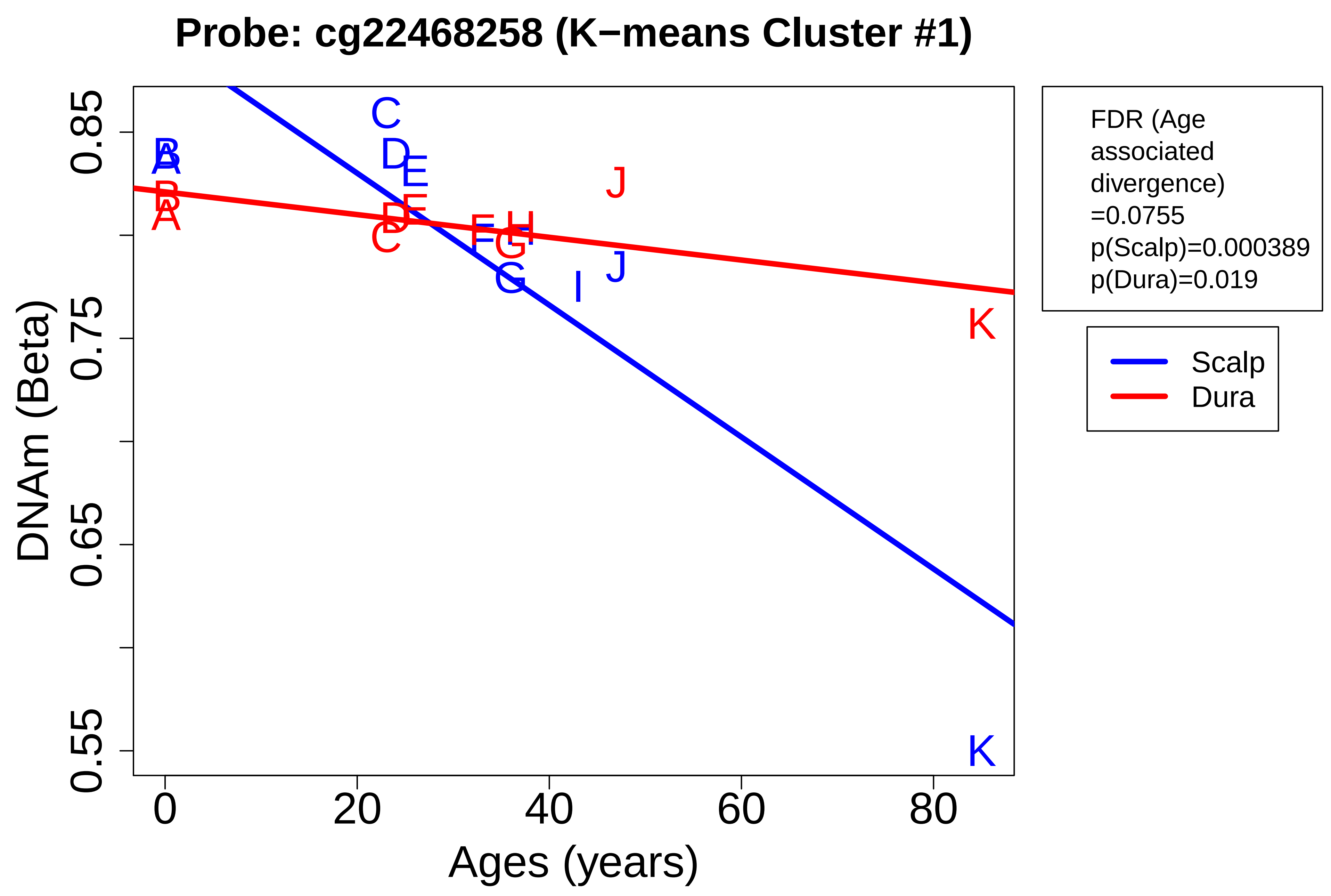

Probe: cg22468258 (K−means Cluster #1)
DNAm (Beta)
0.55
0.65
0.75
0.85
0
20
40
60
80
Ages (years)
FDR (Age associated divergence) =0.0755
p(Scalp)=0.000389
p(Dura)=0.019
Scalp
Dura

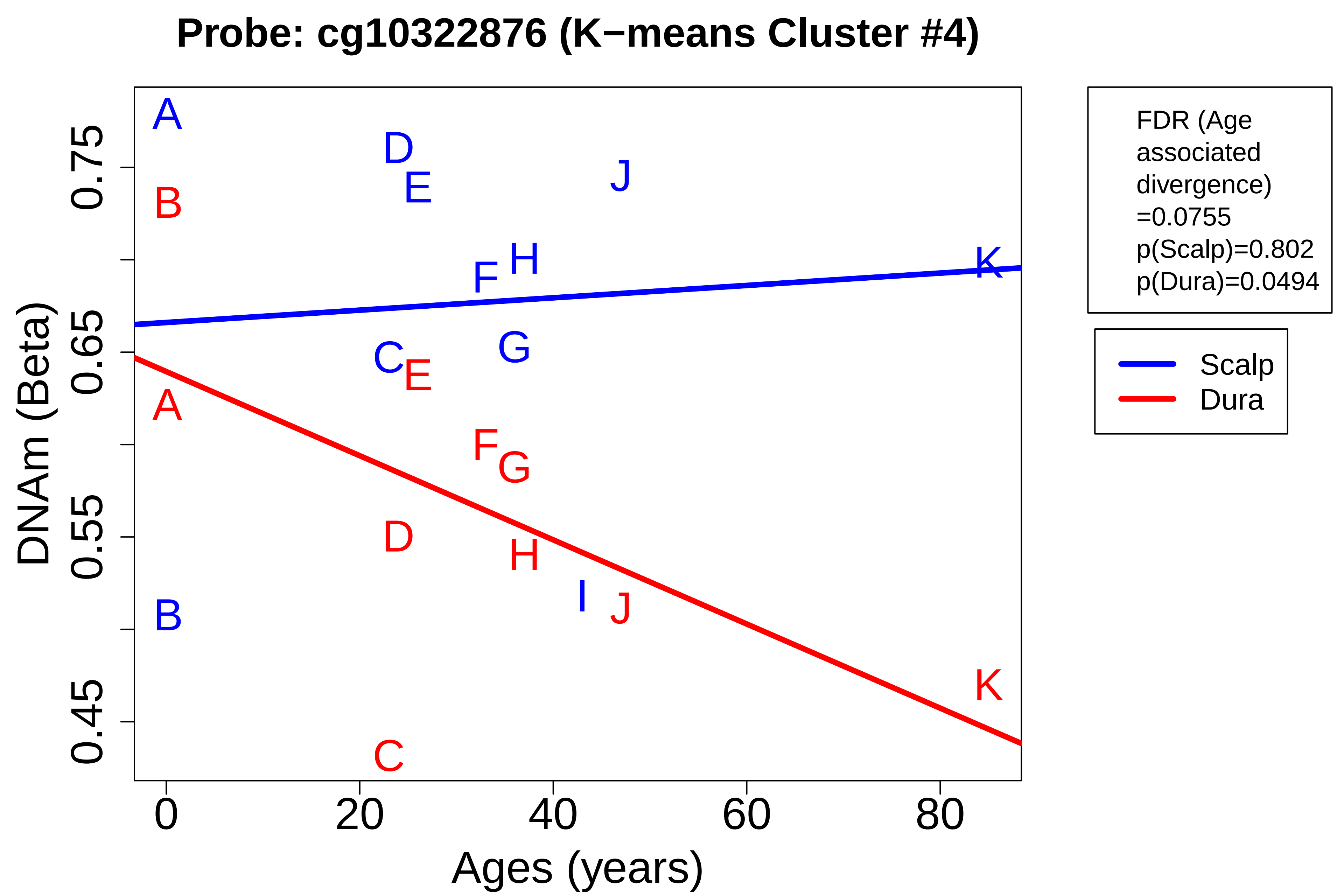

Probe: cg10322876 (K−means Cluster #4)
DNAm (Beta)
0.45
0.55
0.65
0.75
0
20
40
60
80
Ages (years)
FDR (Age associated divergence) =0.0755
p(Scalp)=0.802
p(Dura)=0.0494
Scalp
Dura
A
B
C
D
E
F
G
H
I
J
K

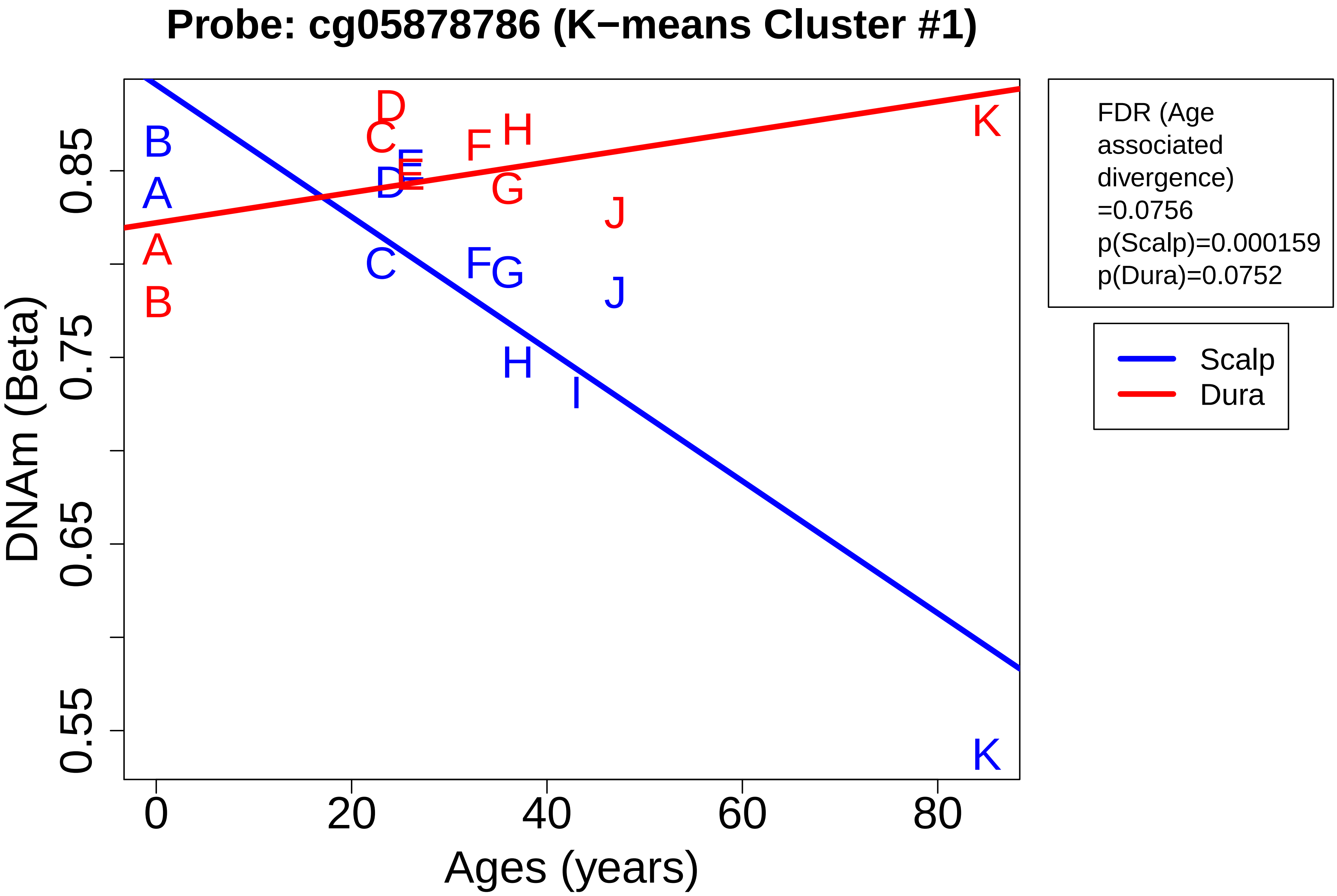
Probe: cg05878786 (K−means Cluster #1)
FDR (Age associated divergence) =0.0756
p(Scalp)=0.000159
p(Dura)=0.0752
Scalp
Dura
DNAm (Beta)
0.55
0.65
0.75
0.85
0
20
40
60
80
Ages (years)

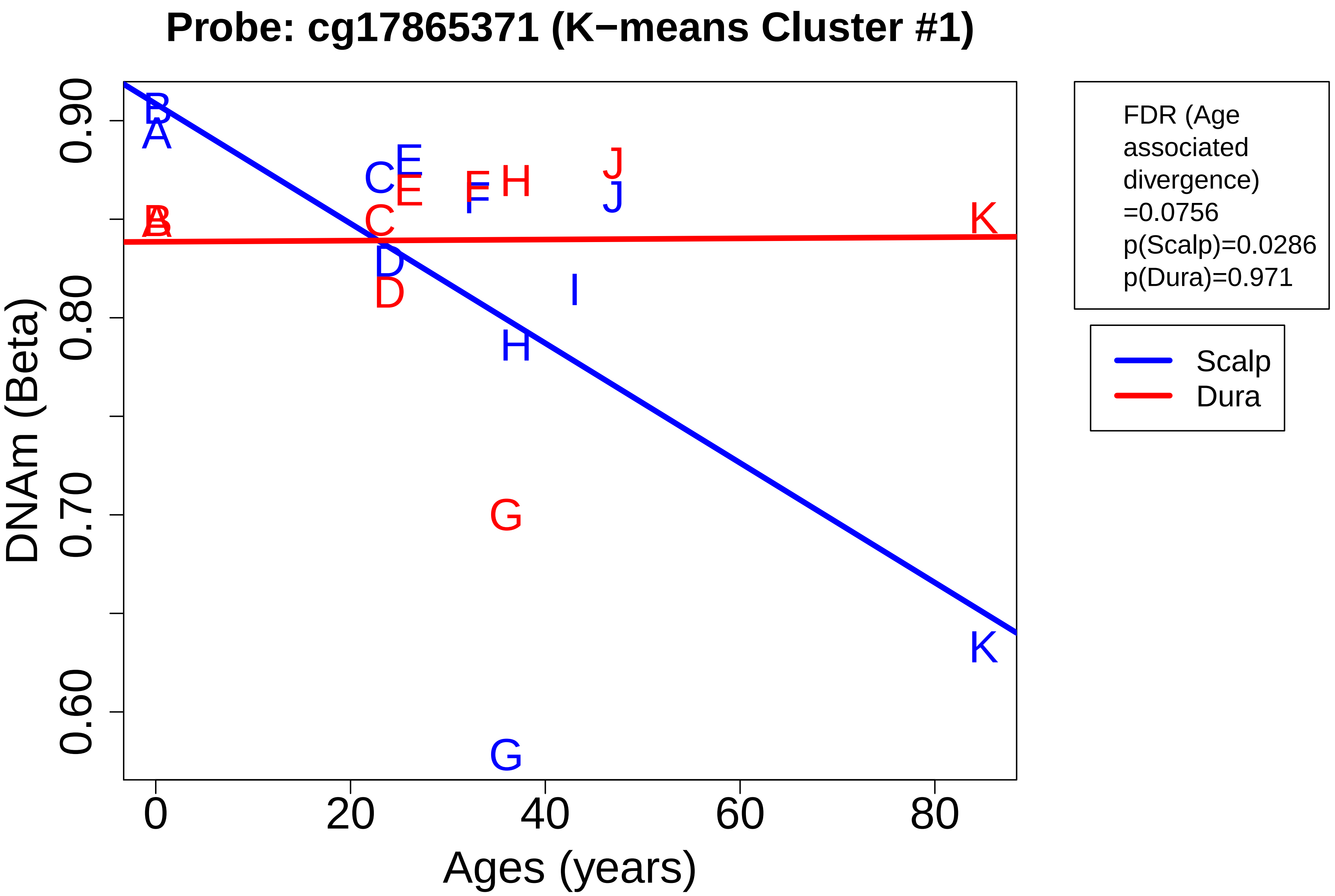

Probe: cg17865371 (K−means Cluster #1)
DNAm (Beta)
0.60
0.70
0.80
0.90
Ages (years)
0
20
40
60
80
B
A
C
E
F
H
J
I
D
G
K
FDR (Age associated divergence) =0.0756
p(Scalp)=0.0286
p(Dura)=0.971
Scalp
Dura

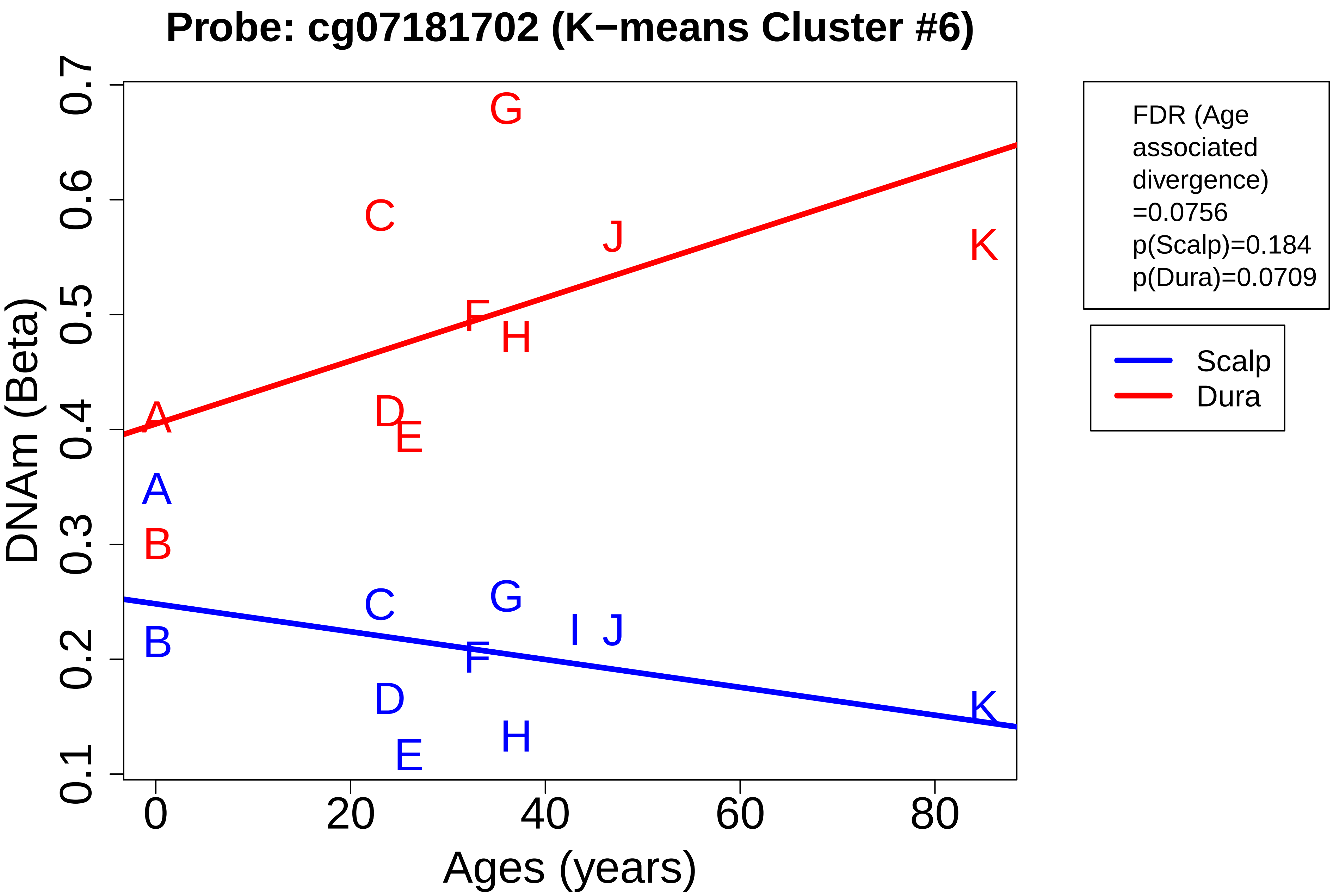

Probe: cg07181702 (K−means Cluster #6)
DNAm (Beta)
Ages (years)
0.1
0.2
0.3
0.4
0.5
0.6
0.7
0
20
40
60
80
FDR (Age associated divergence) =0.0756
p(Scalp)=0.184
p(Dura)=0.0709
Scalp
Dura

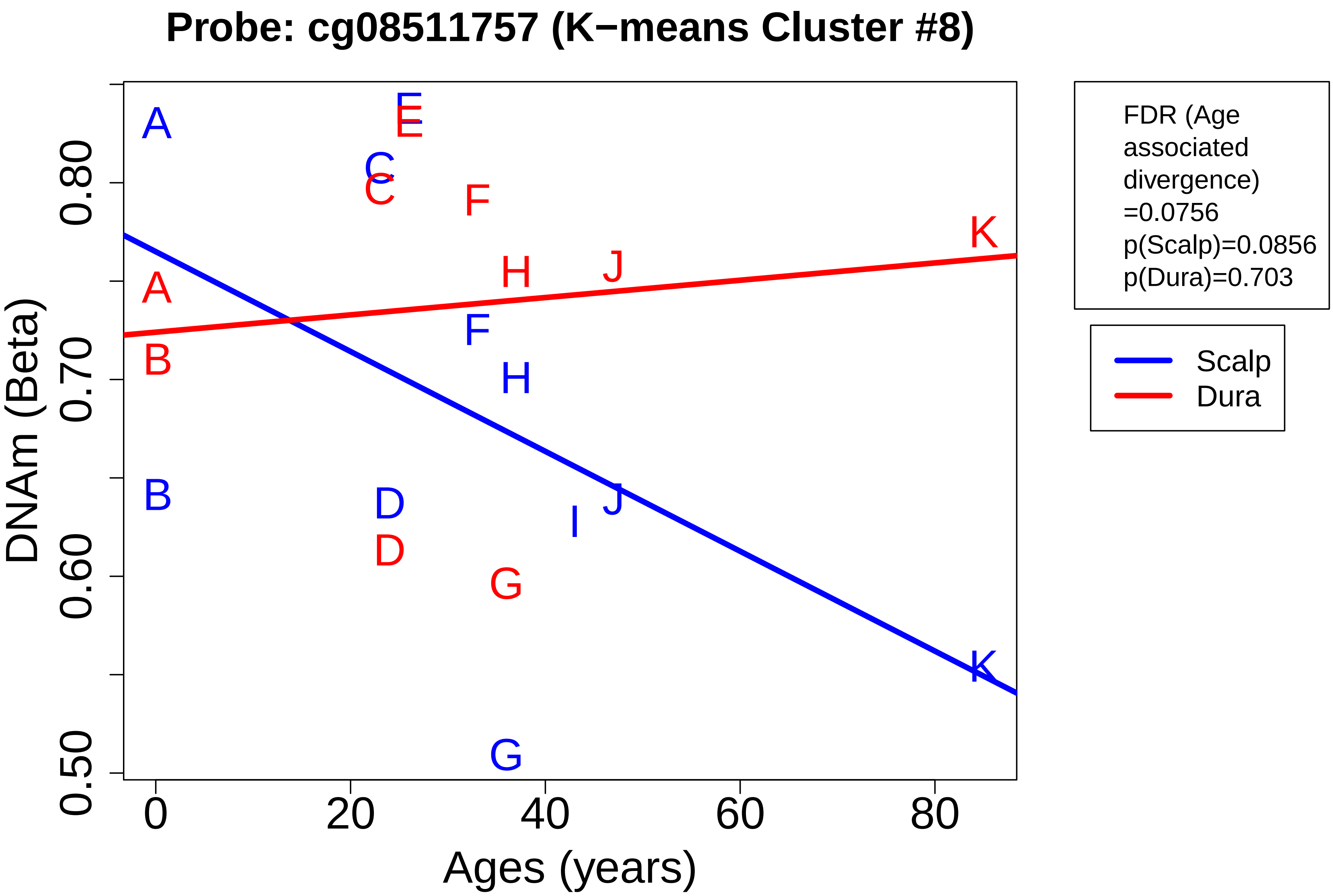

Probe: cg08511757 (K−means Cluster #8)
DNAm (Beta)
0.50
0.60
0.70
0.80
Ages (years)
0
20
40
60
80
FDR (Age associated divergence) =0.0756
p(Scalp)=0.0856
p(Dura)=0.703
Scalp
Dura
A
B
C
D
E
F
G
H
I
J
K

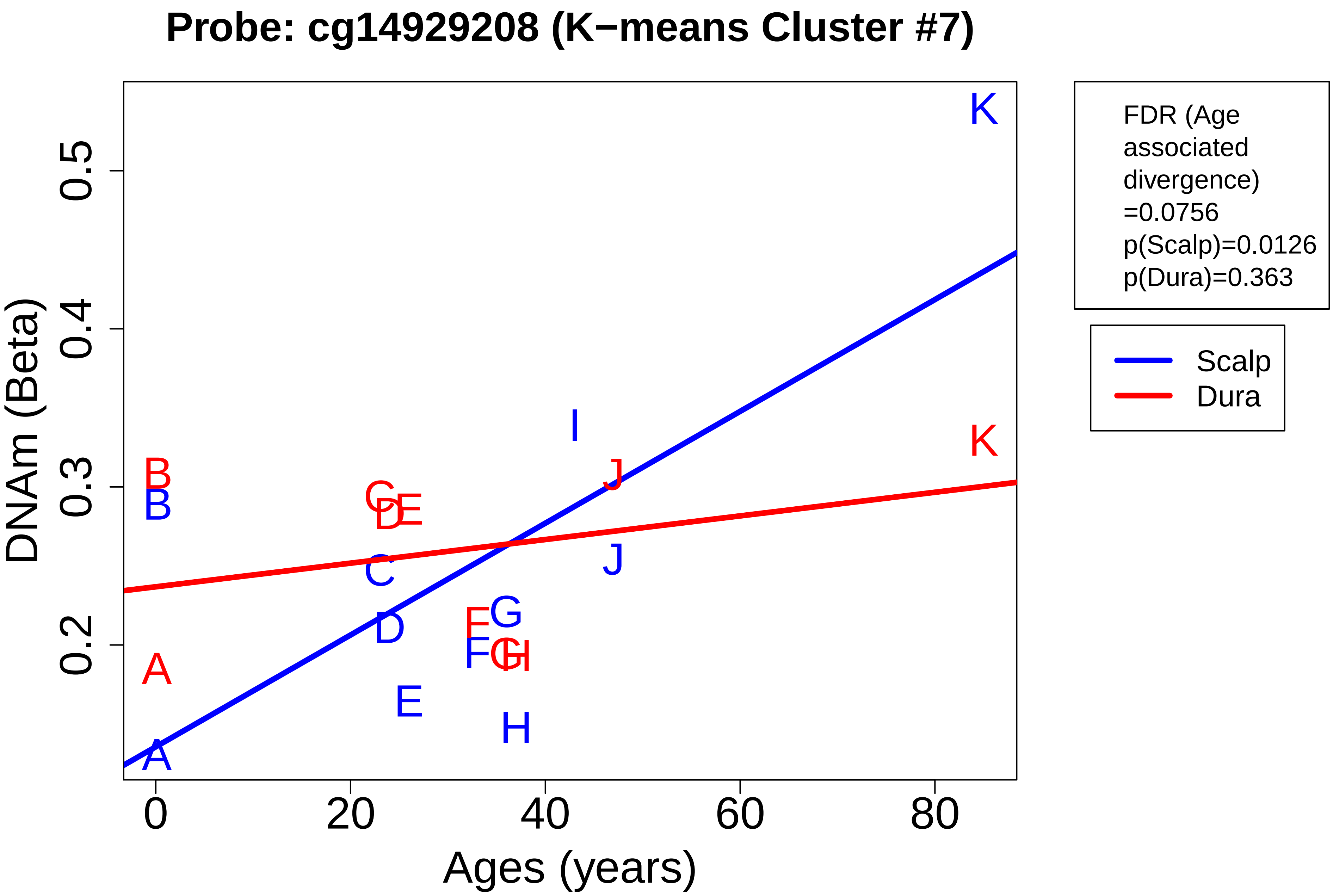

Probe: cg14929208 (K−means Cluster #7)
DNAm (Beta)
0.2
0.3
0.4
0.5
0
20
40
60
80
Ages (years)
FDR (Age associated divergence) =0.0756
p(Scalp)=0.0126
p(Dura)=0.363
Scalp
Dura

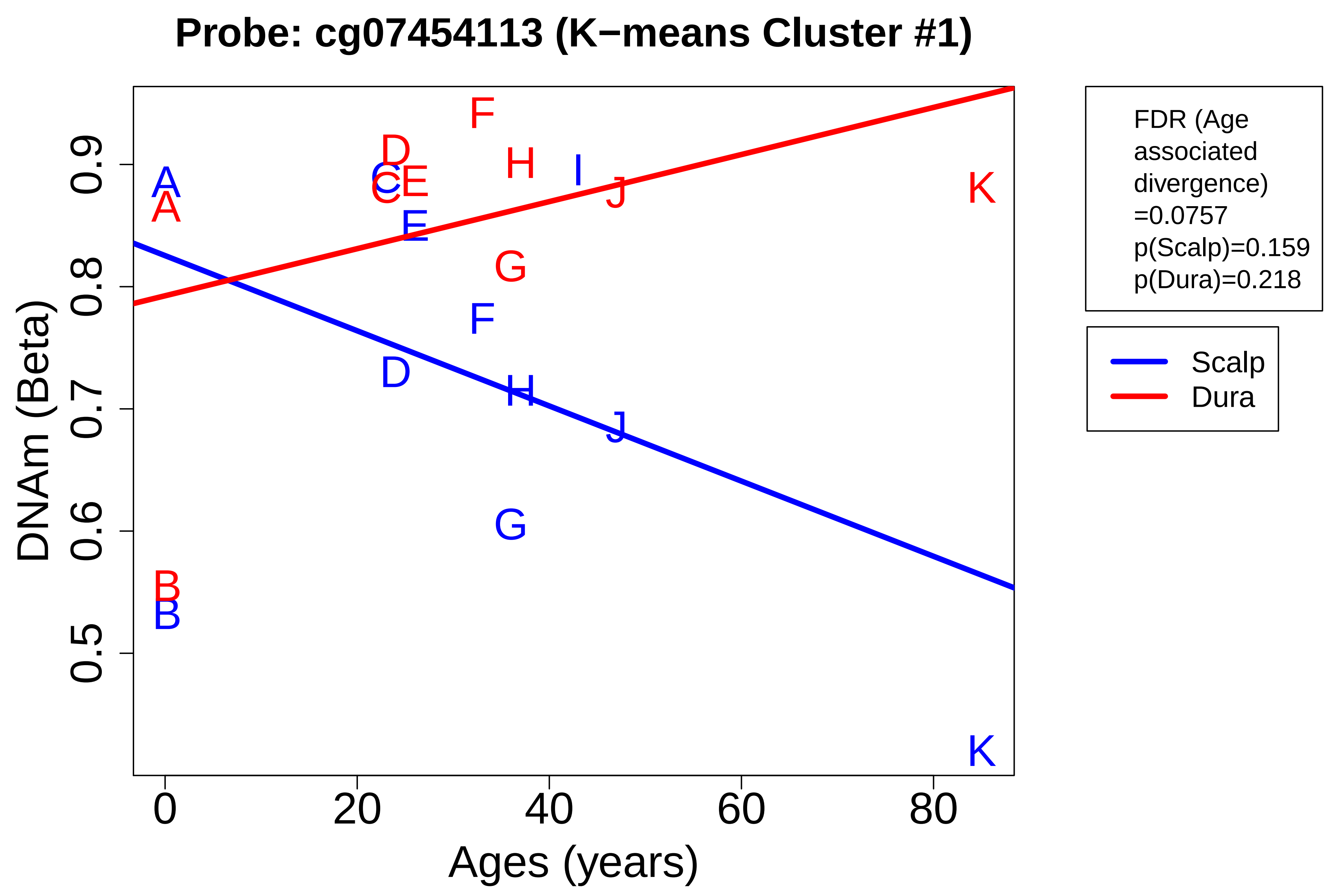

Probe: cg07454113 (K−means Cluster #1)
DNAm (Beta)
Ages (years)
0.5
0.6
0.7
0.8
0.9
0
20
40
60
80
FDR (Age associated divergence) =0.0757
p(Scalp)=0.159
p(Dura)=0.218
Scalp
Dura
A
B
C
D
E
F
G
H
I
J
K

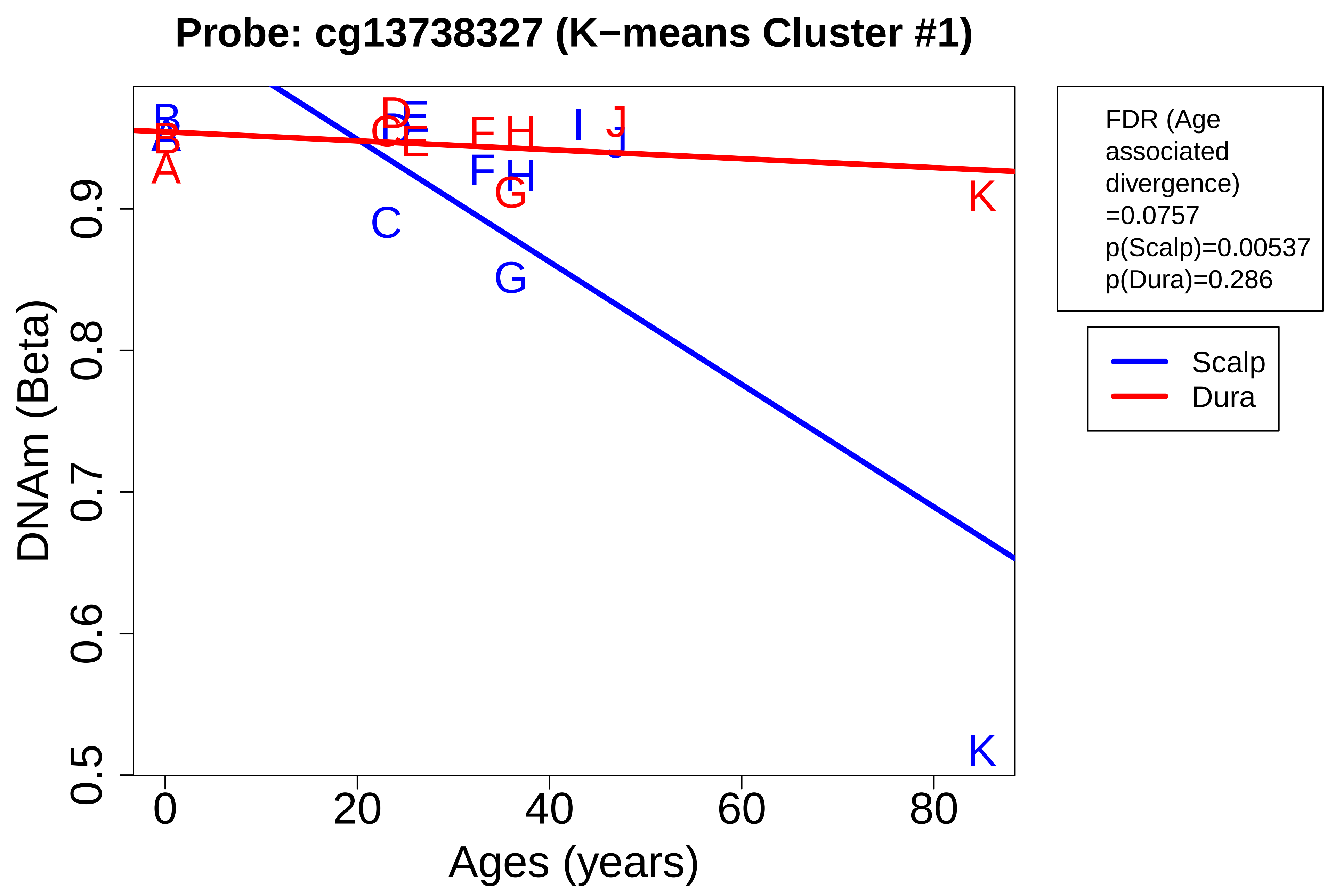

Probe: cg13738327 (K−means Cluster #1)
FDR (Age associated divergence) =0.0757
p(Scalp)=0.00537
p(Dura)=0.286
Scalp
Dura
DNAm (Beta)
0.5
0.6
0.7
0.8
0.9
Ages (years)
0
20
40
60
80

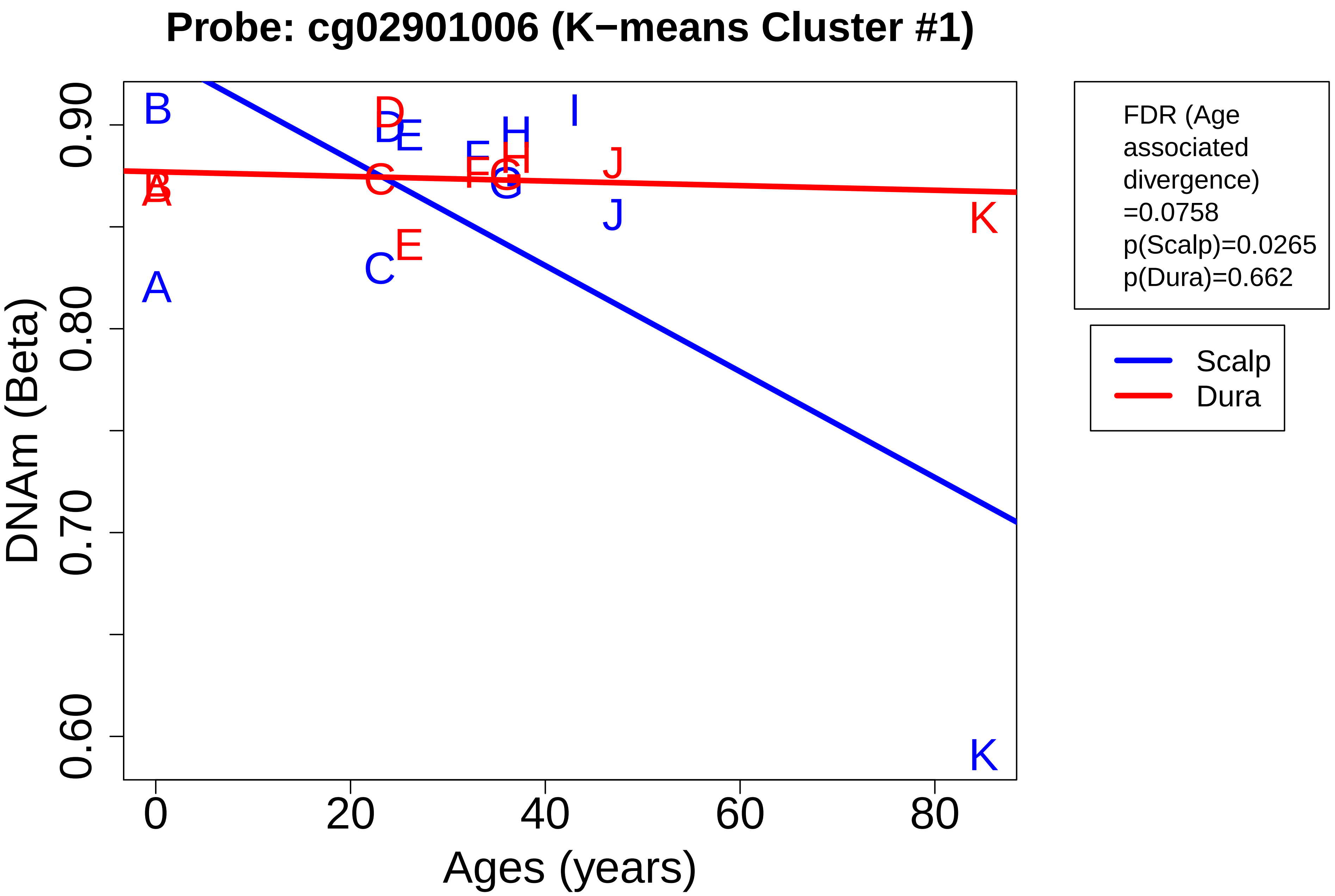

Probe: cg02901006 (K−means Cluster #1)
DNAm (Beta)
0.60
0.70
0.80
0.90
Ages (years)
0
20
40
60
80
A
B
C
D
E
F
G
H
I
J
K
FDR (Age associated divergence) =0.0758
p(Scalp)=0.0265
p(Dura)=0.662
Scalp
Dura

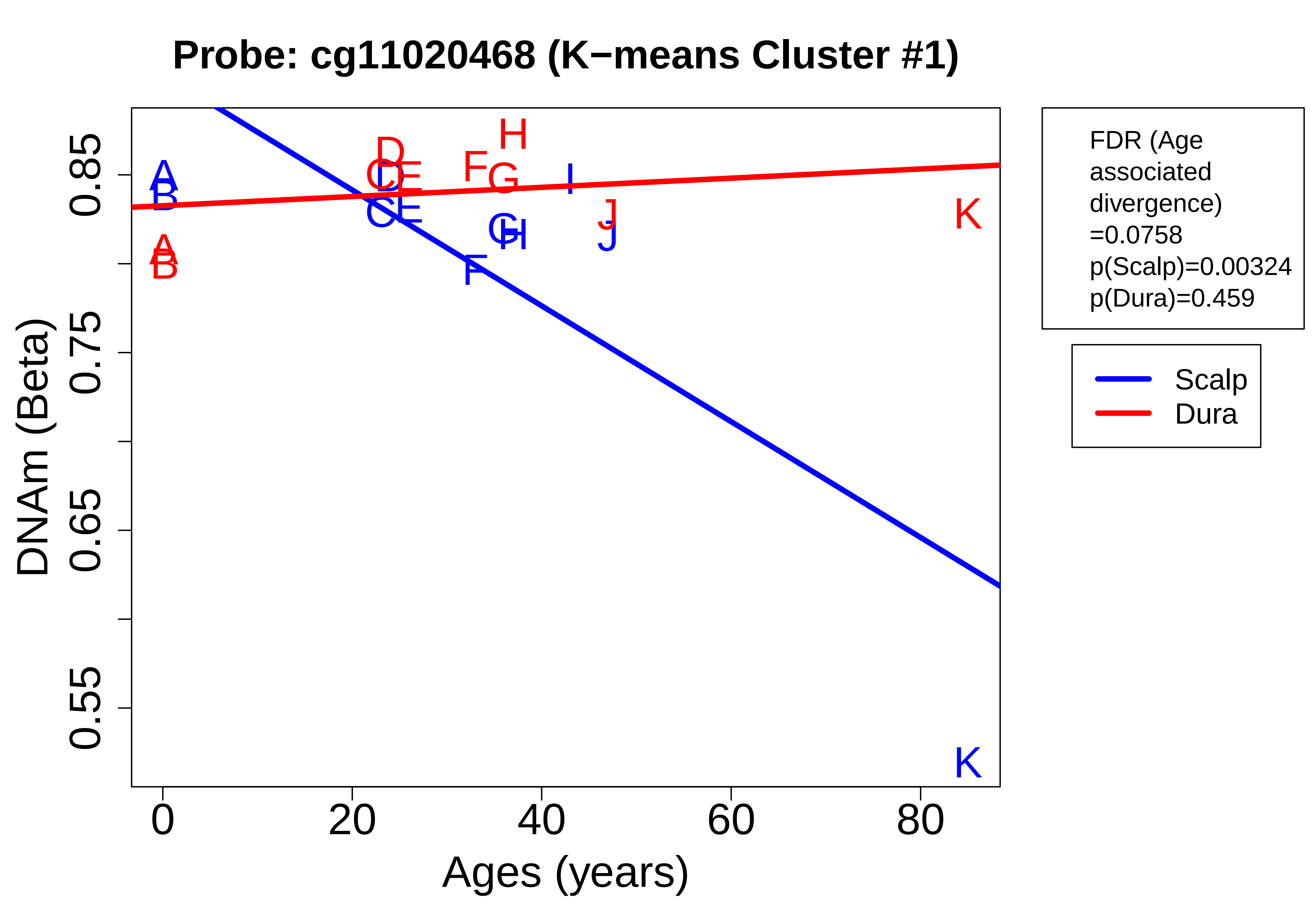

Probe: cg11020468 (K−means Cluster #1)
DNAm (Beta)
0.55
0.65
0.75
0.85
0
20
40
60
80
Ages (years)
FDR (Age associated divergence) =0.0758
p(Scalp)=0.00324
p(Dura)=0.459
Scalp
Dura

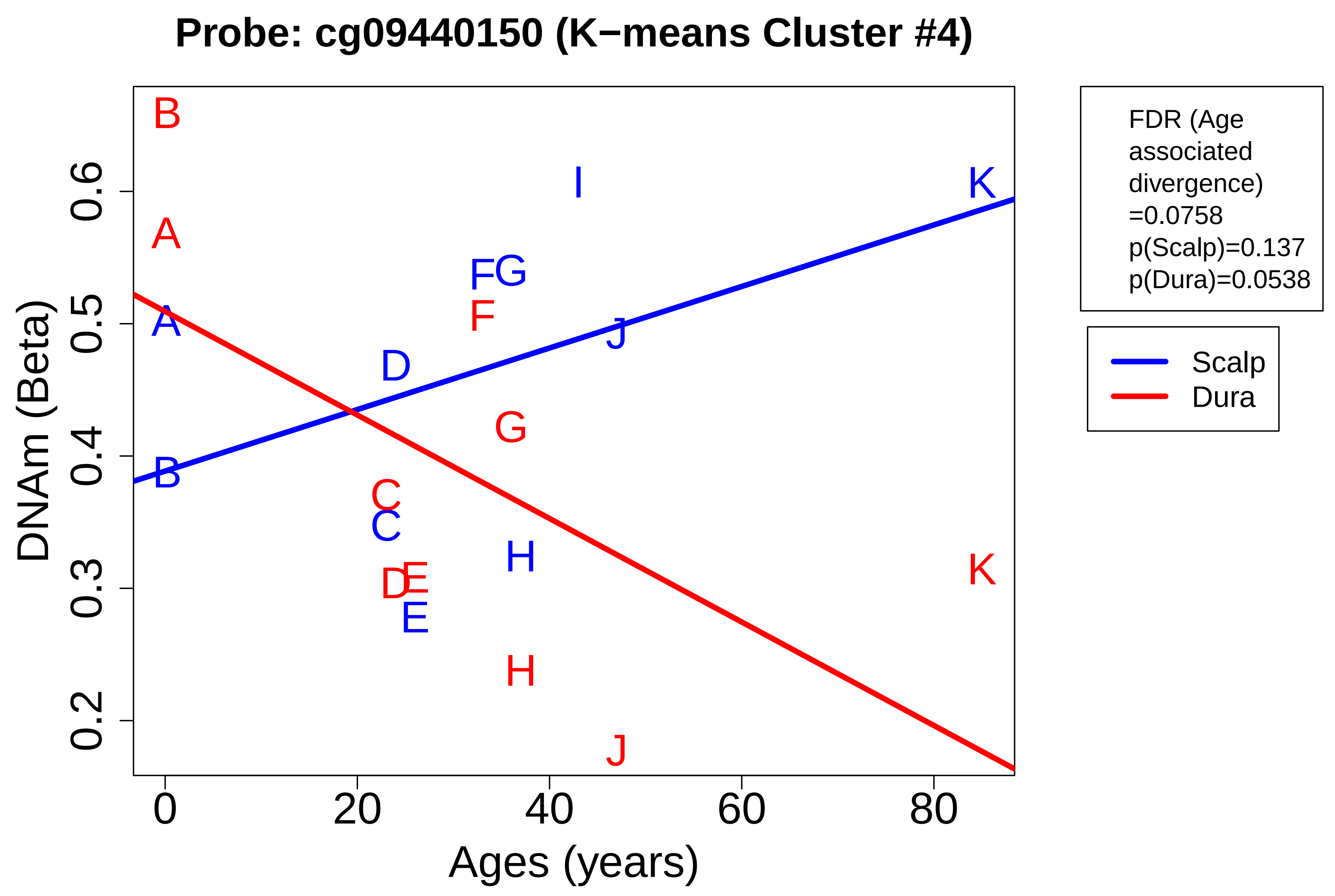
Probe: cg09440150 (K−means Cluster #4)
DNAm (Beta)
Ages (years)
0.2
0.3
0.4
0.5
0.6
0
20
40
60
80
FDR (Age associated divergence) =0.0758
p(Scalp)=0.137
p(Dura)=0.0538
Scalp
Dura

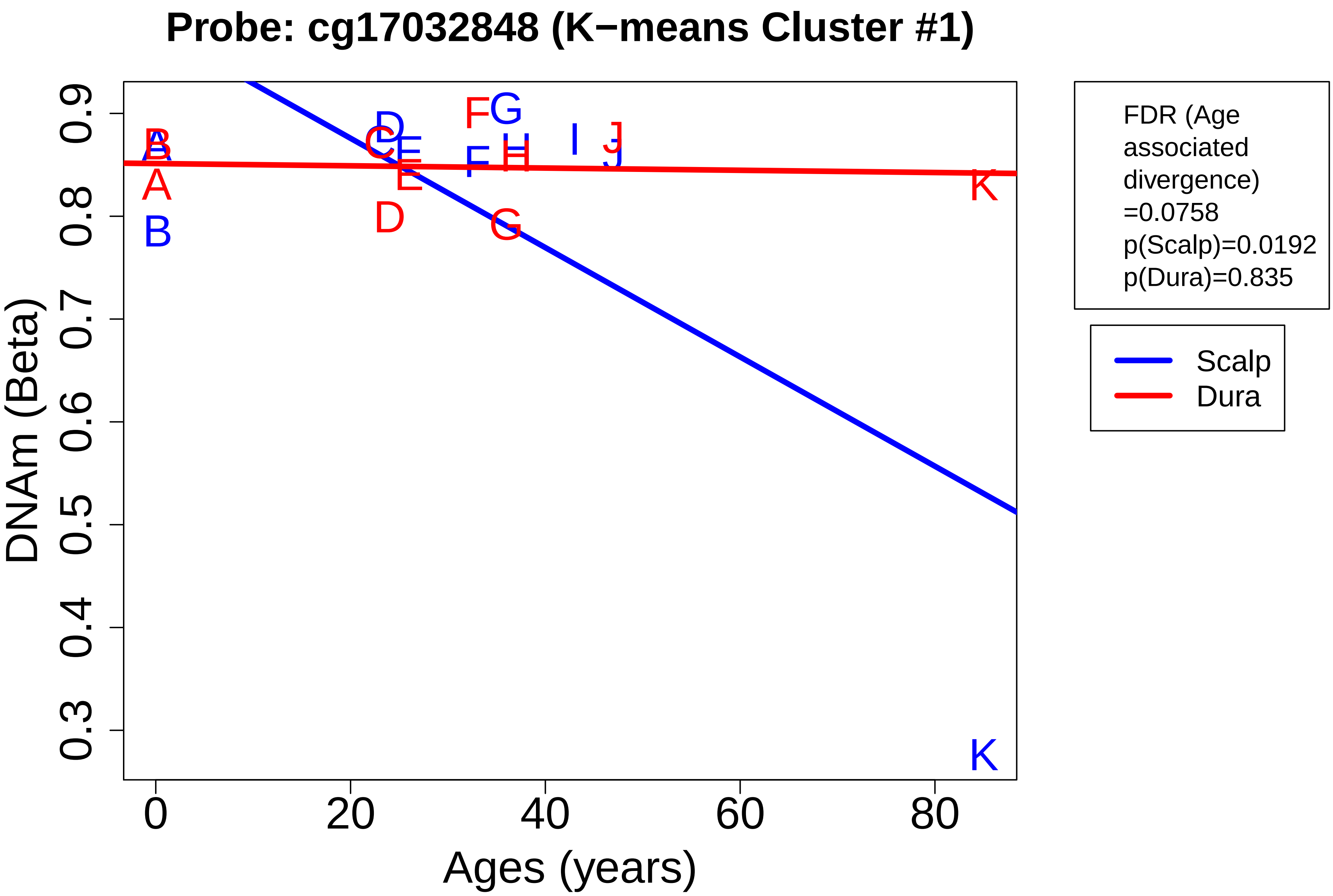
Probe: cg17032848 (K−means Cluster #1)
DNAm (Beta)
0.3
0.4
0.5
0.6
0.7
0.8
0.9
0
20
40
60
80
Ages (years)
A
B
C
D
E
F
G
H
I
J
K
FDR (Age associated divergence) =0.0758
p(Scalp)=0.0192
p(Dura)=0.835
Scalp
Dura

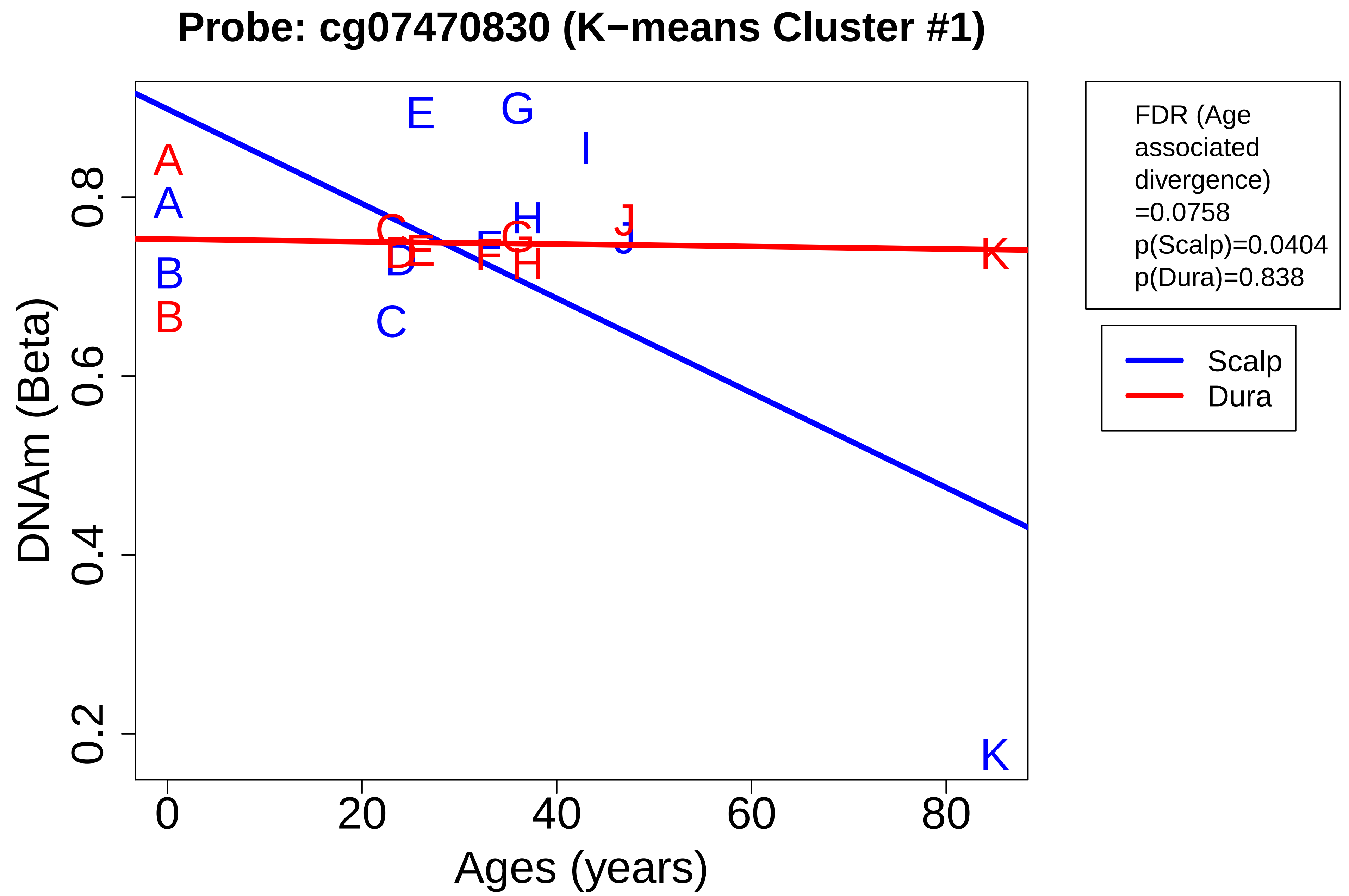

Probe: cg07470830 (K−means Cluster #1)
DNAm (Beta)
0.2
0.4
0.6
0.8
0
20
40
60
80
Ages (years)
FDR (Age associated divergence) =0.0758
p(Scalp)=0.0404
p(Dura)=0.838
Scalp
Dura

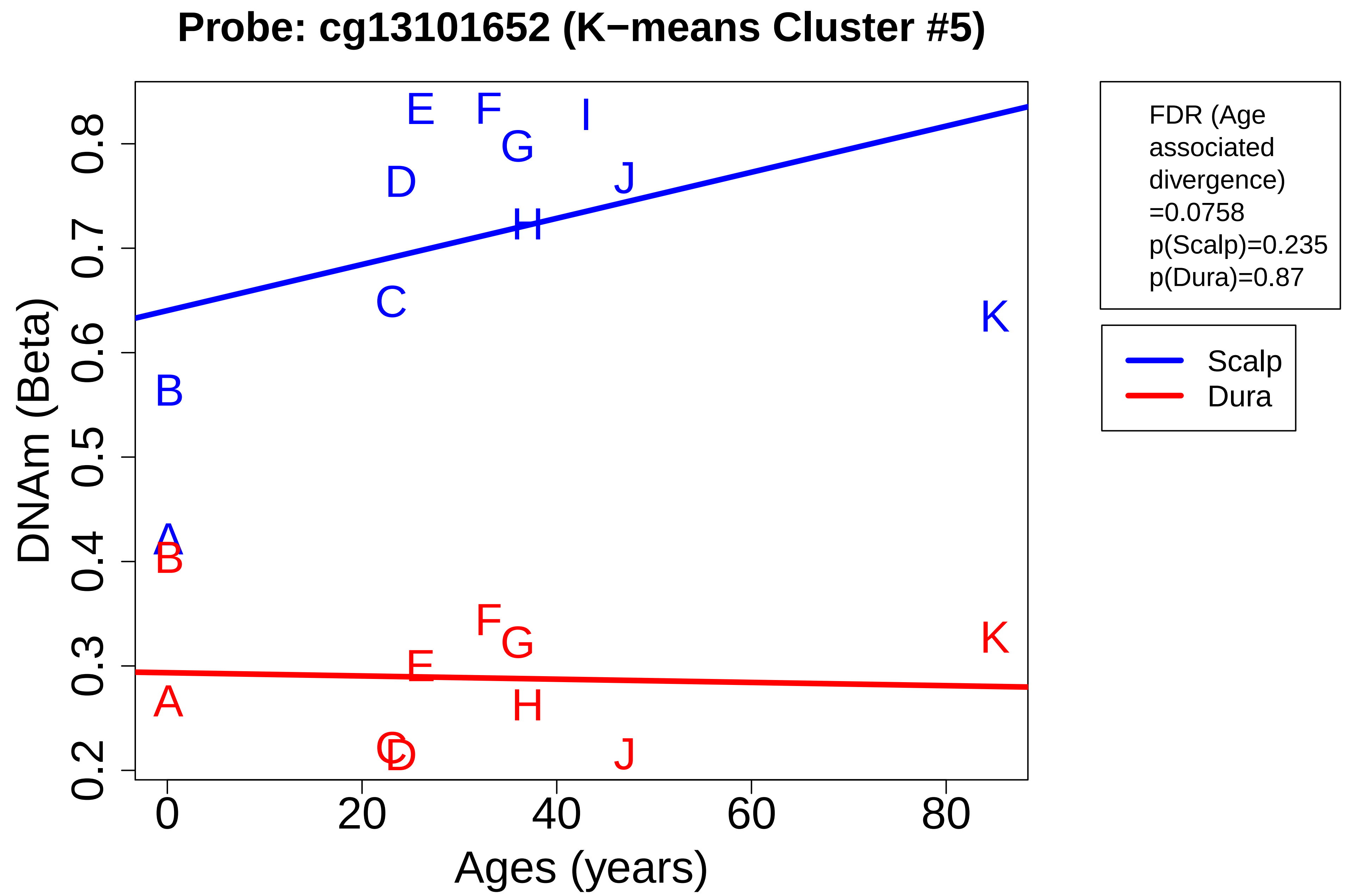

Probe: cg13101652 (K−means Cluster #5)
DNAm (Beta)
0.2
0.3
0.4
0.5
0.6
0.7
0.8
0
20
40
60
80
Ages (years)
FDR (Age associated divergence) =0.0758
p(Scalp)=0.235
p(Dura)=0.87
Scalp
Dura

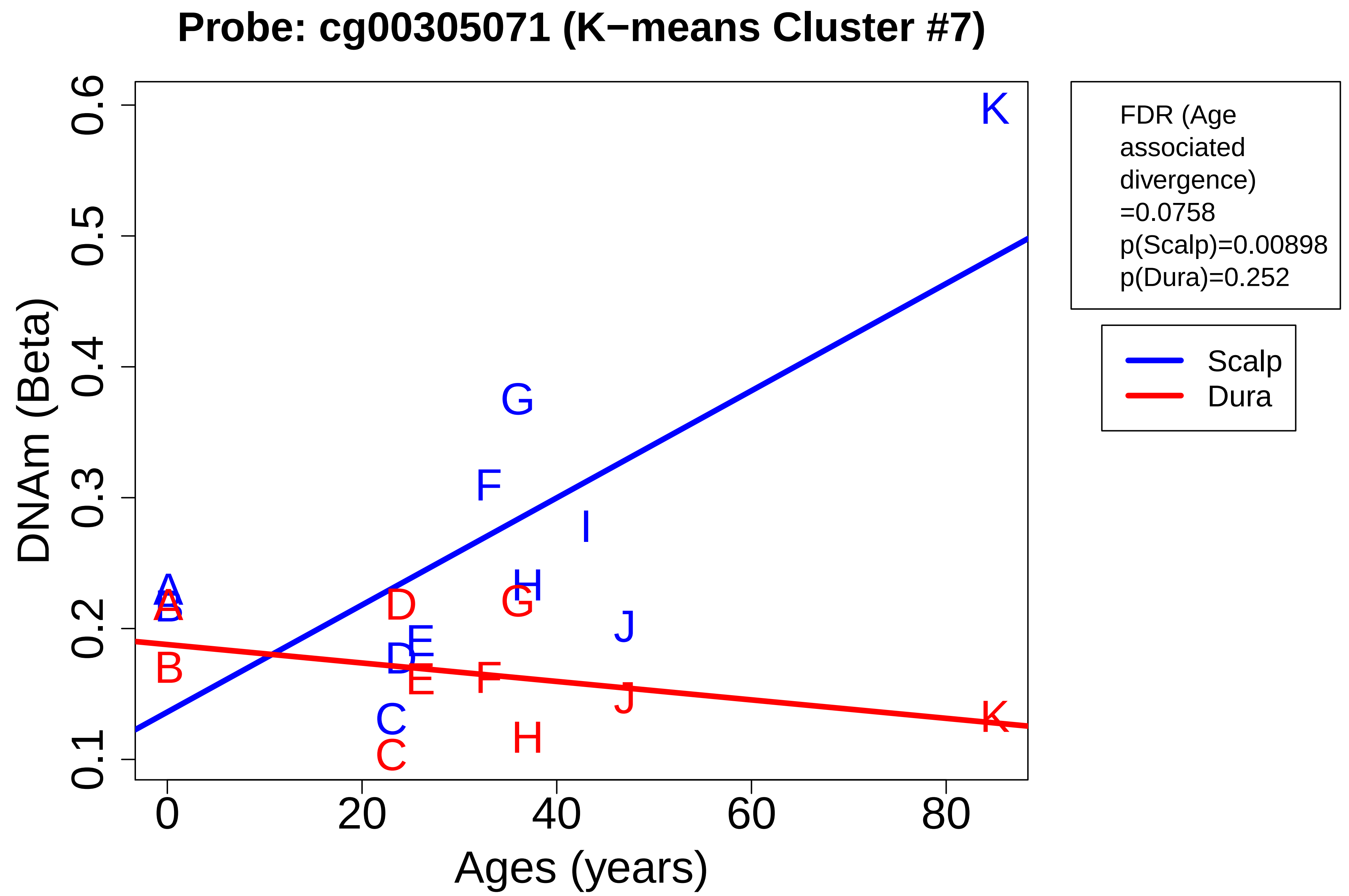

Probe: cg00305071 (K−means Cluster #7)
DNAm (Beta)
0.1
0.2
0.3
0.4
0.5
0.6
Ages (years)
0
20
40
60
80
FDR (Age associated divergence) =0.0758
p(Scalp)=0.00898
p(Dura)=0.252
Scalp
Dura

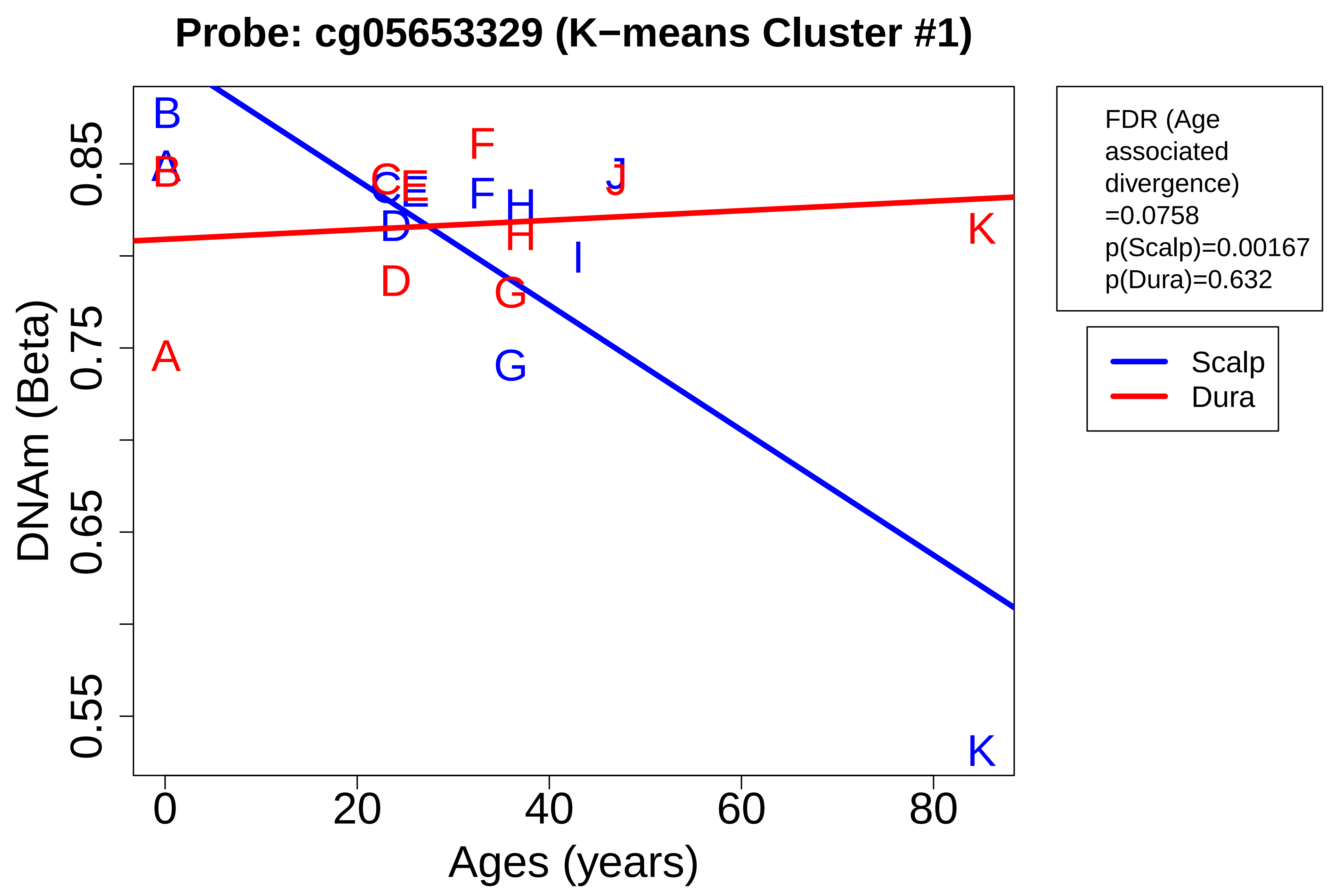

Probe: cg05653329 (K−means Cluster #1)
DNAm (Beta)
0.55
0.65
0.75
0.85
0
20
40
60
80
Ages (years)
A
B
C
D
E
F
G
H
I
J
K
FDR (Age associated divergence) =0.0758
p(Scalp)=0.00167
p(Dura)=0.632
Scalp
Dura

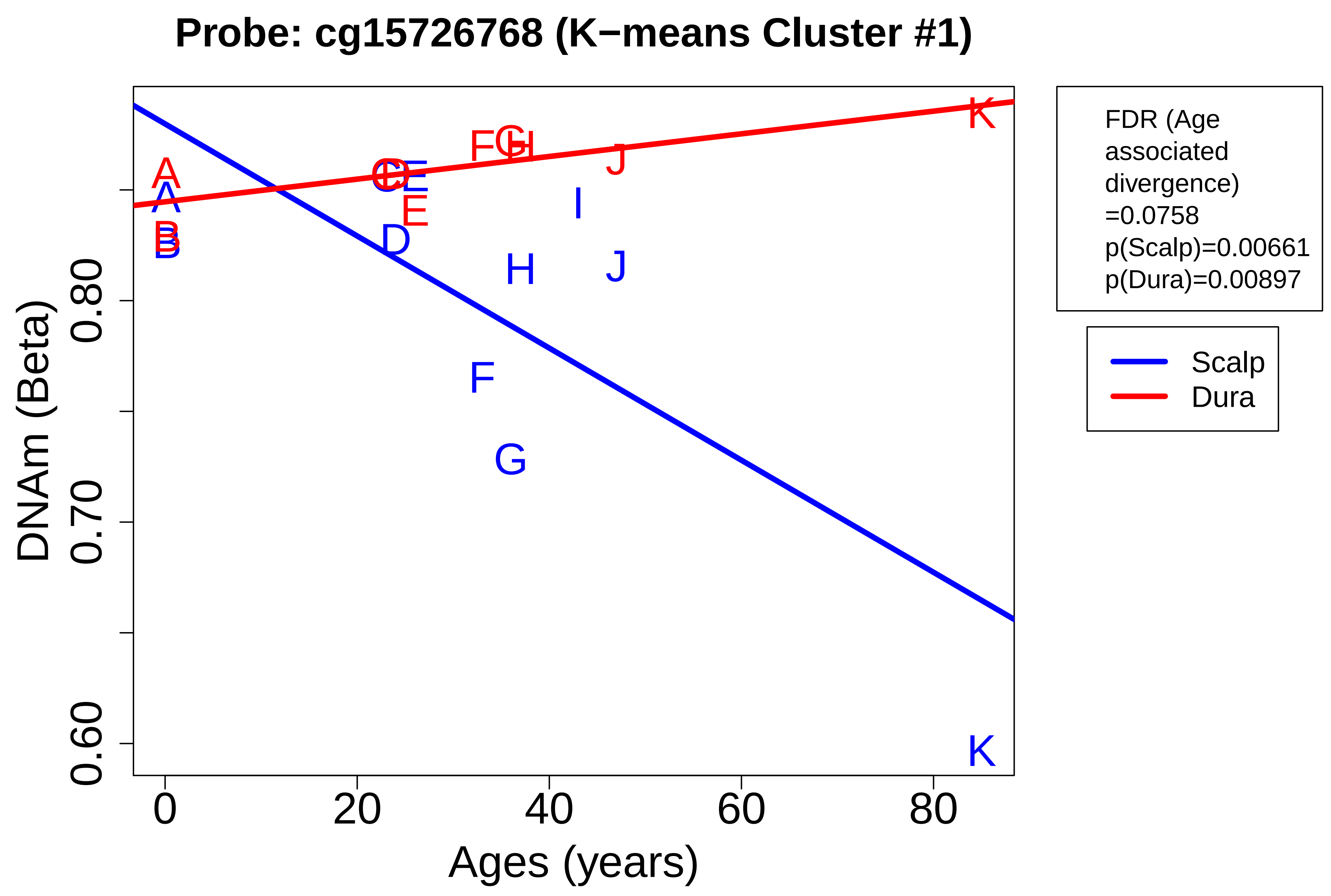

Probe: cg15726768 (K−means Cluster #1)
DNAm (Beta)
0.60
0.70
0.80
Ages (years)
0
20
40
60
80
FDR (Age associated divergence) =0.0758
p(Scalp)=0.00661
p(Dura)=0.00897
Scalp
Dura

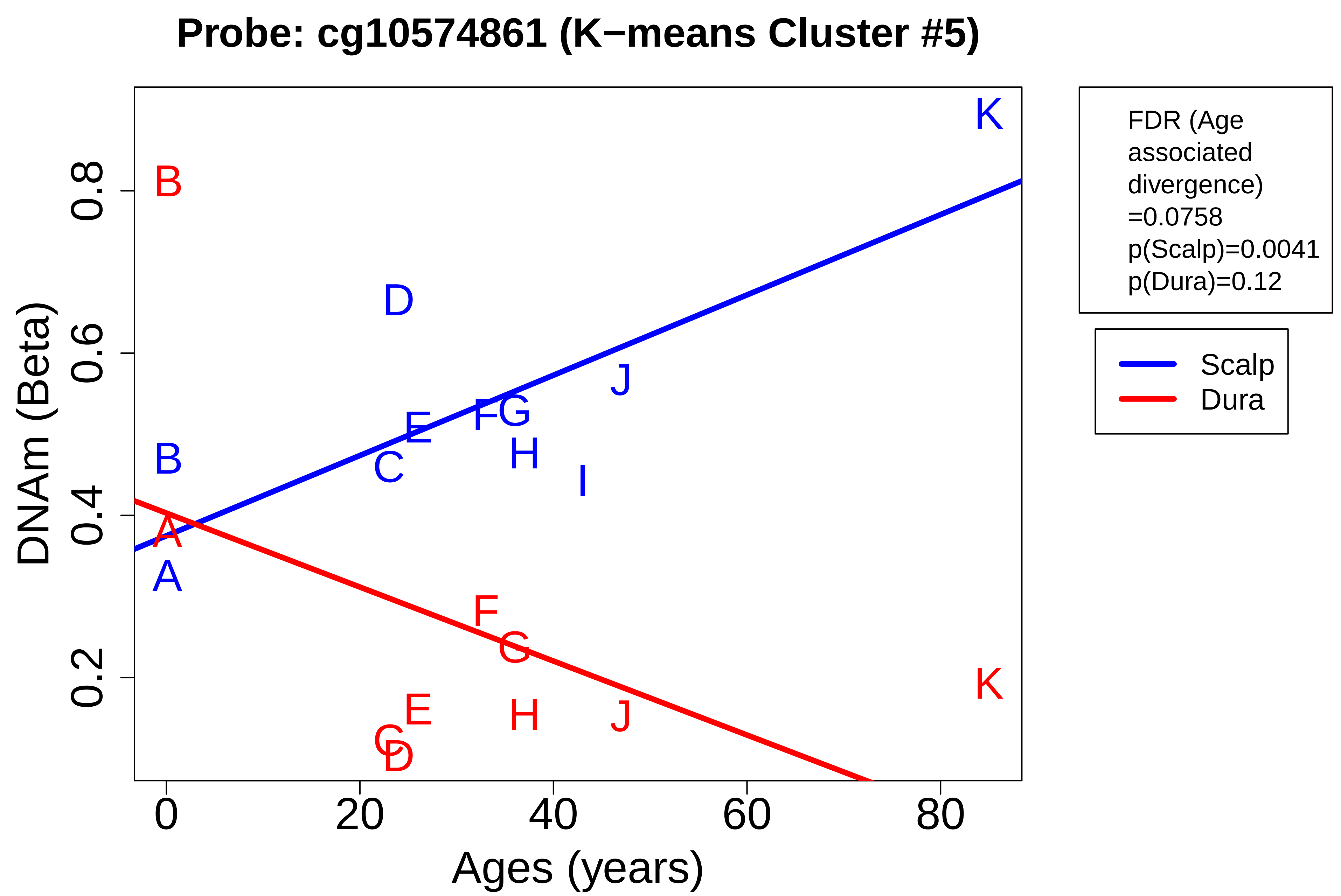

Probe: cg10574861 (K−means Cluster #5)
DNAm (Beta)
0.2
0.4
0.6
0.8
0
20
40
60
80
Ages (years)
FDR (Age associated divergence) =0.0758
p(Scalp)=0.0041
p(Dura)=0.12
Scalp
Dura
A
B
C
D
E
F
G
H
I
J
K

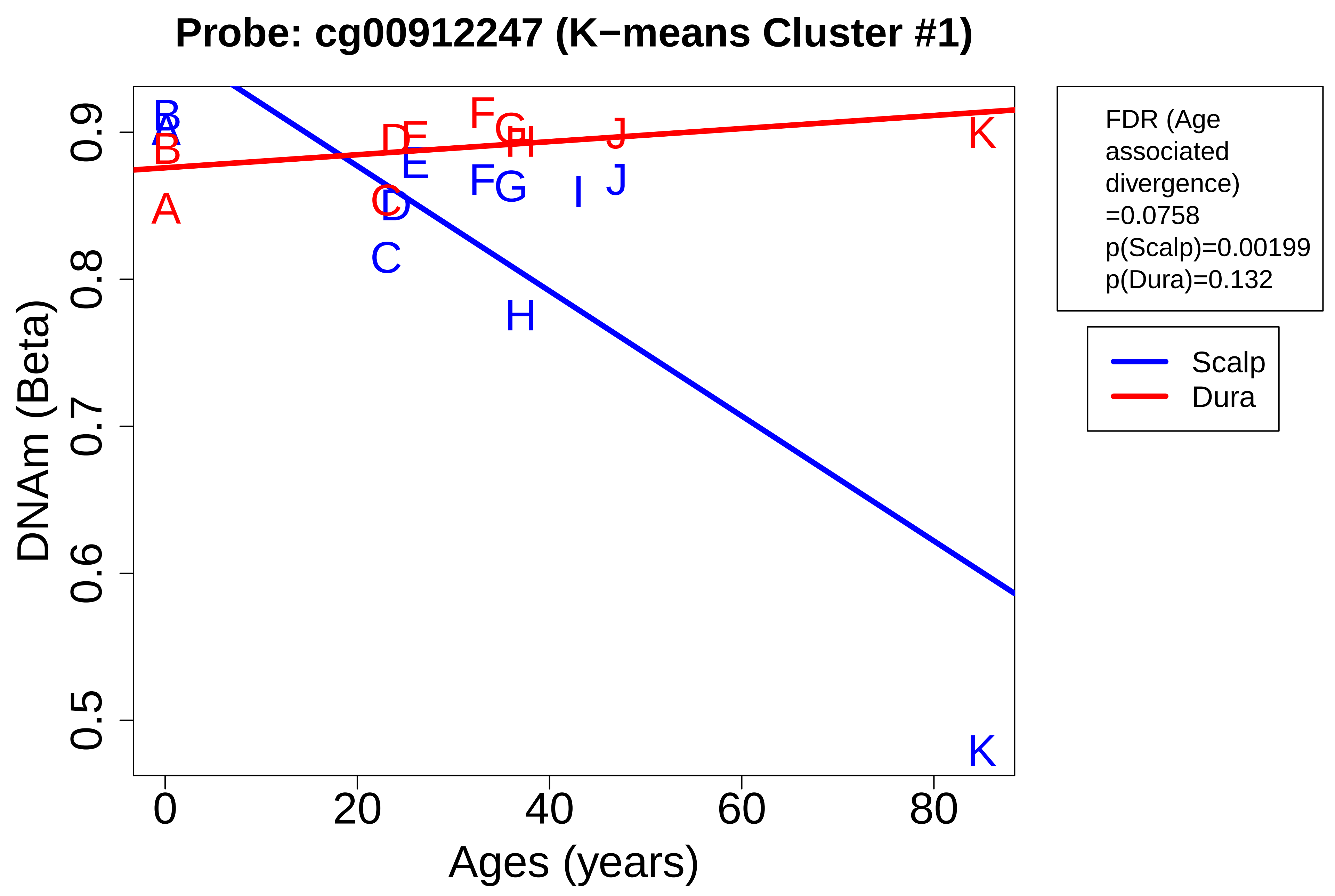

Probe: cg00912247 (K−means Cluster #1)
DNAm (Beta)
0.5
0.6
0.7
0.8
0.9
Ages (years)
0
20
40
60
80
A
B
C
D
E
F
G
H
I
J
K
FDR (Age associated divergence) =0.0758
p(Scalp)=0.00199
p(Dura)=0.132
Scalp
Dura

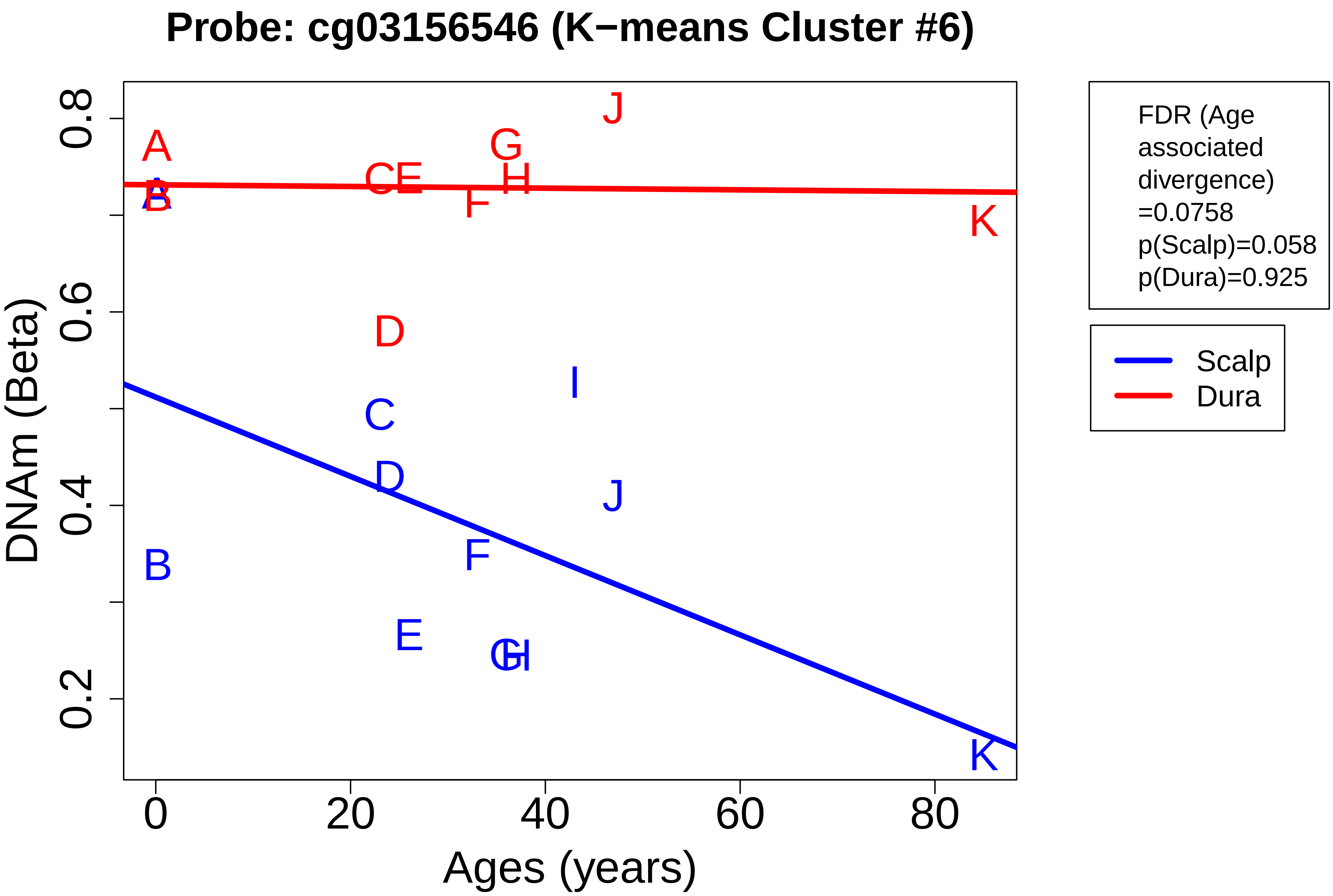

Probe: cg03156546 (K−means Cluster #6)
DNAm (Beta)
0.2
0.4
0.6
0.8
Ages (years)
0
20
40
60
80
A
B
C
D
E
F
G
H
I
J
K
FDR (Age associated divergence) =0.0758
p(Scalp)=0.058
p(Dura)=0.925
Scalp
Dura

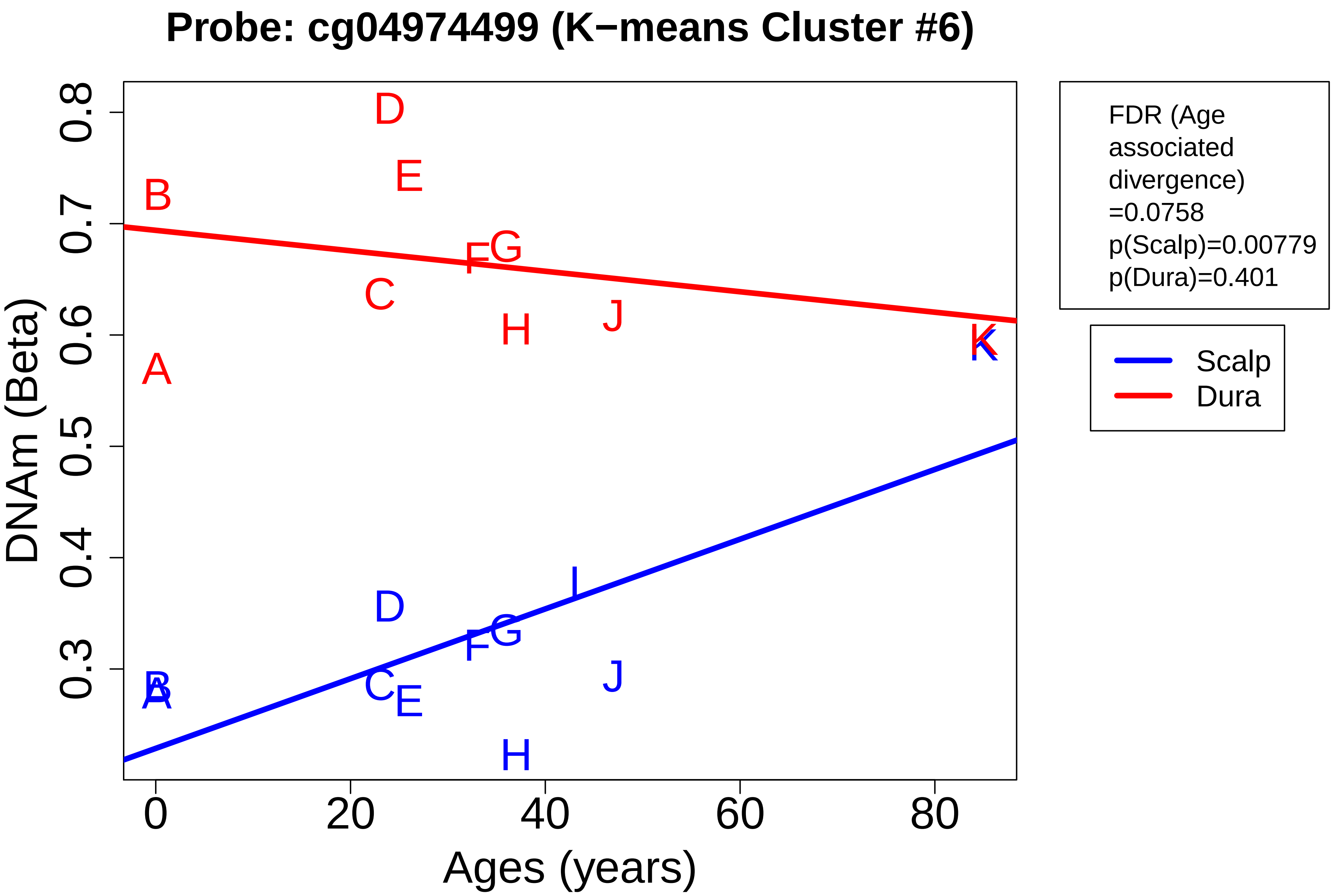

Probe: cg04974499 (K−means Cluster #6)
DNAm (Beta)
0.3
0.4
0.5
0.6
0.7
0.8
Ages (years)
0
20
40
60
80
A
B
C
D
E
F
G
H
I
J
K
FDR (Age associated divergence) =0.0758
p(Scalp)=0.00779
p(Dura)=0.401
Scalp
Dura

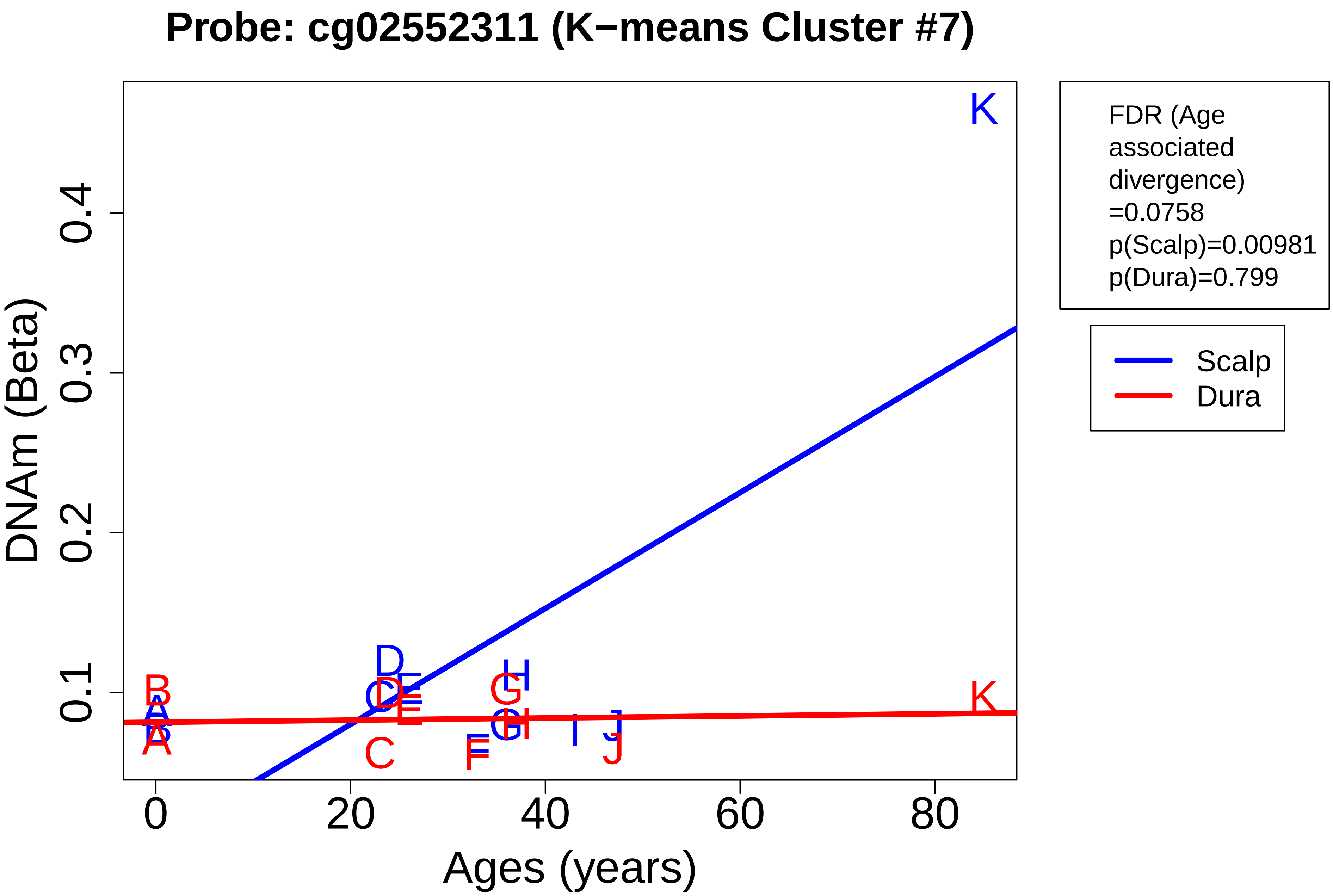

Probe: cg02552311 (K−means Cluster #7)
DNAm (Beta)
0.1
0.2
0.3
0.4
0
20
40
60
80
Ages (years)
FDR (Age associated divergence) =0.0758
p(Scalp)=0.00981
p(Dura)=0.799
Scalp
Dura

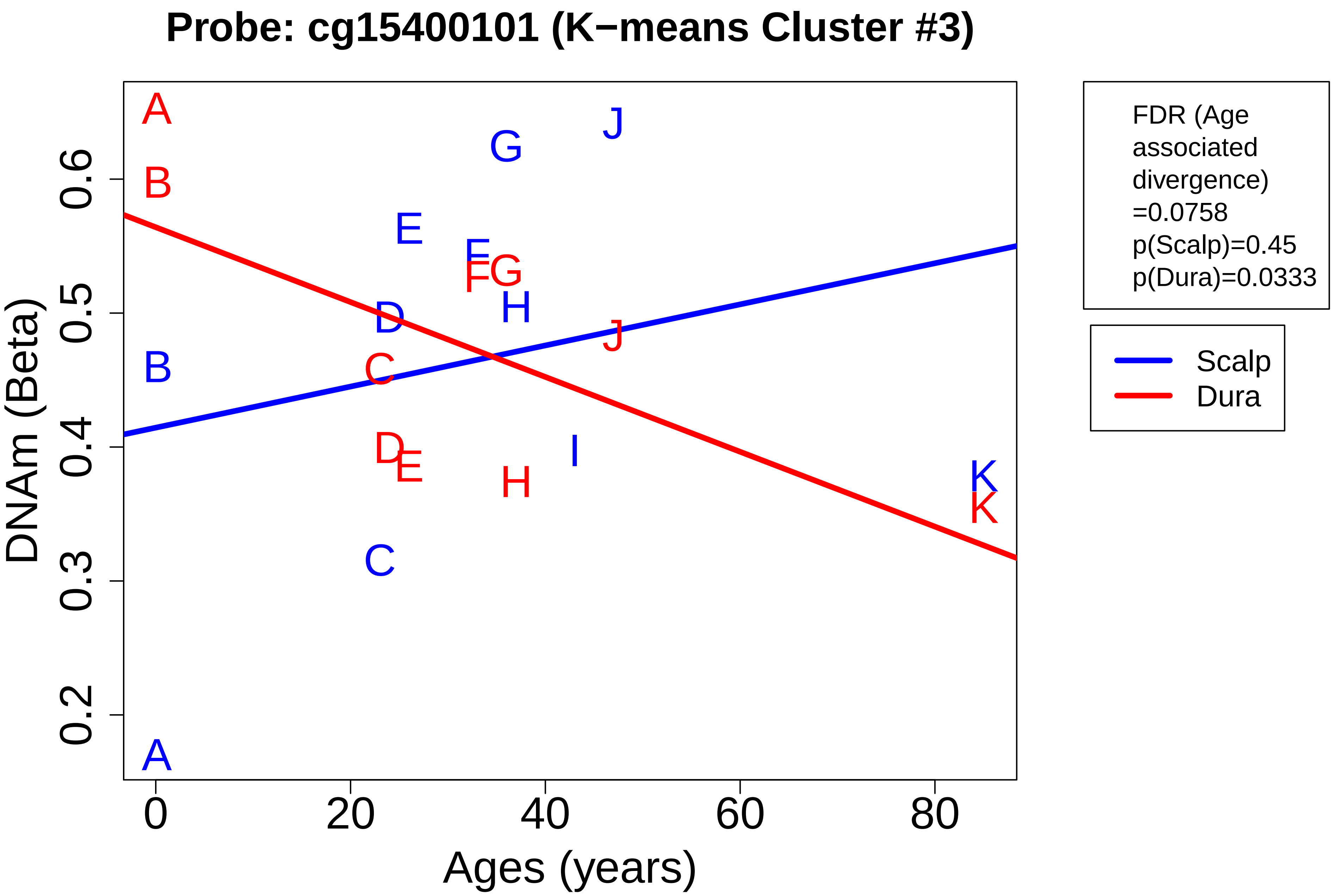

Probe: cg15400101 (K−means Cluster #3)
DNAm (Beta)
0.2
0.3
0.4
0.5
0.6
0
20
40
60
80
Ages (years)
FDR (Age associated divergence) =0.0758
p(Scalp)=0.45
p(Dura)=0.0333
Scalp
Dura

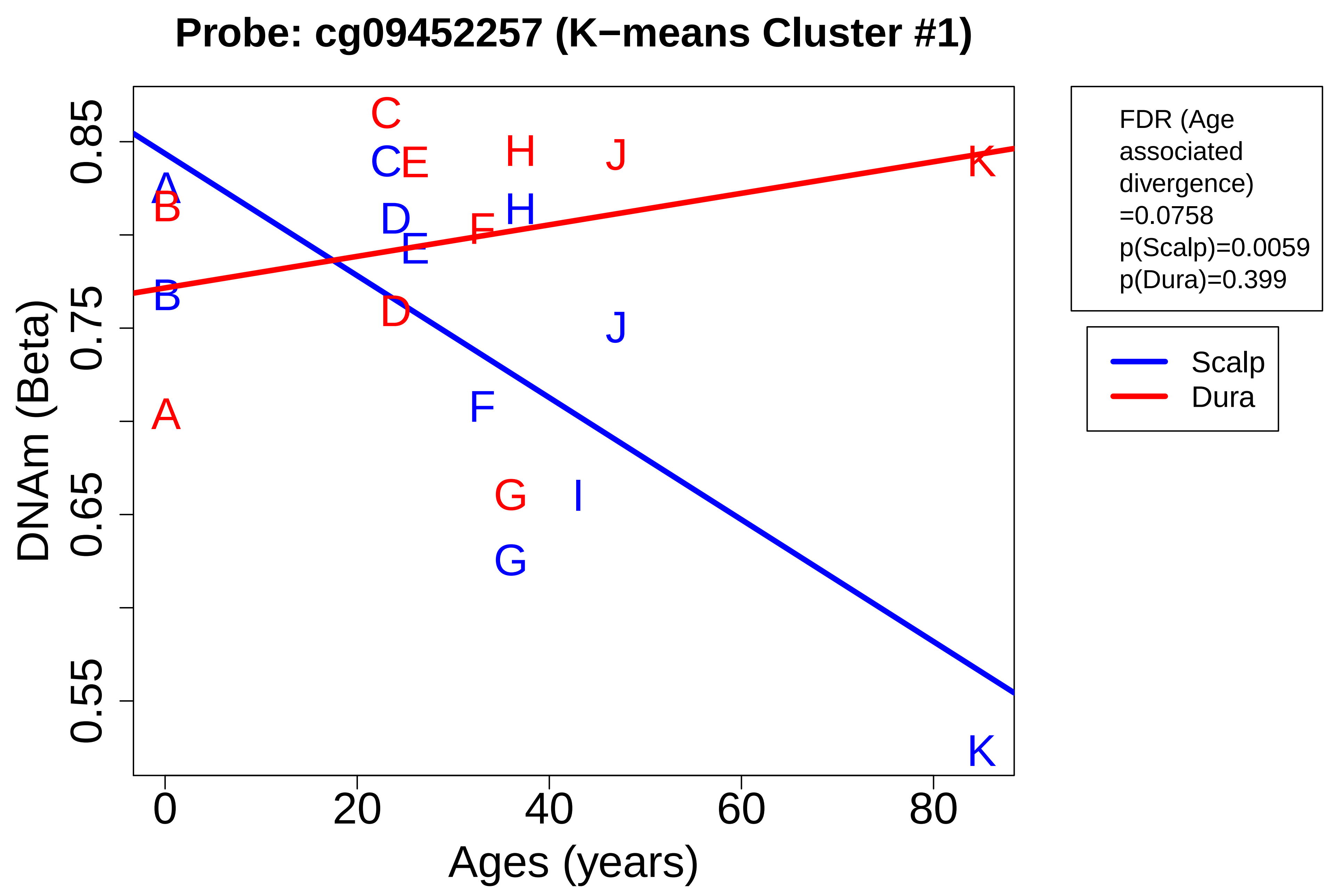

Probe: cg09452257 (K−means Cluster #1)
DNAm (Beta)
0.55
0.65
0.75
0.85
Ages (years)
0
20
40
60
80
FDR (Age associated divergence) =0.0758
p(Scalp)=0.0059
p(Dura)=0.399
Scalp
Dura
A B C D E F G H I J K

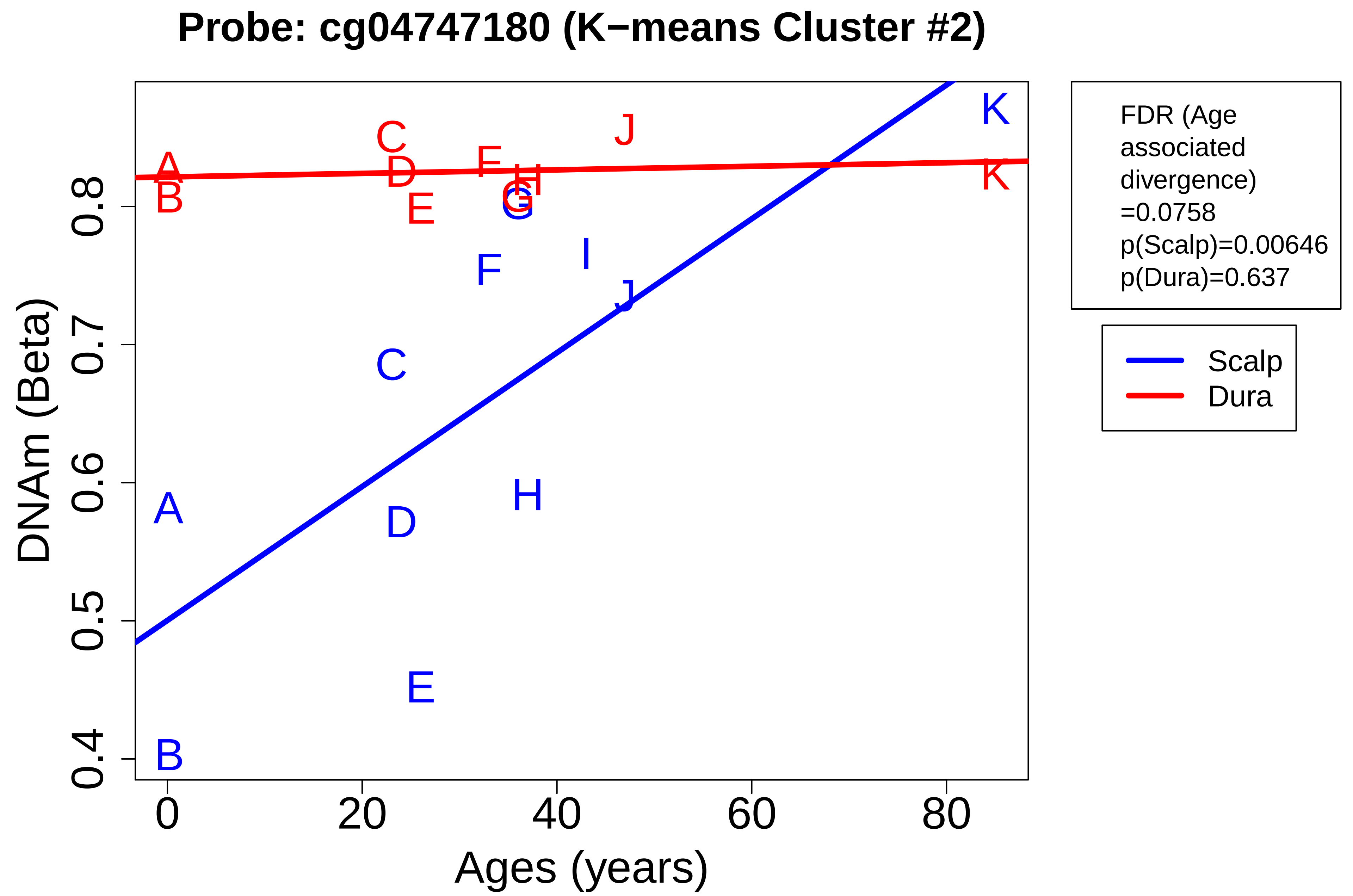

Probe: cg04747180 (K−means Cluster #2)
DNAm (Beta)
Ages (years)
0.4
0.5
0.6
0.7
0.8
0
20
40
60
80
FDR (Age associated divergence) =0.0758
p(Scalp)=0.00646
p(Dura)=0.637
Scalp
Dura

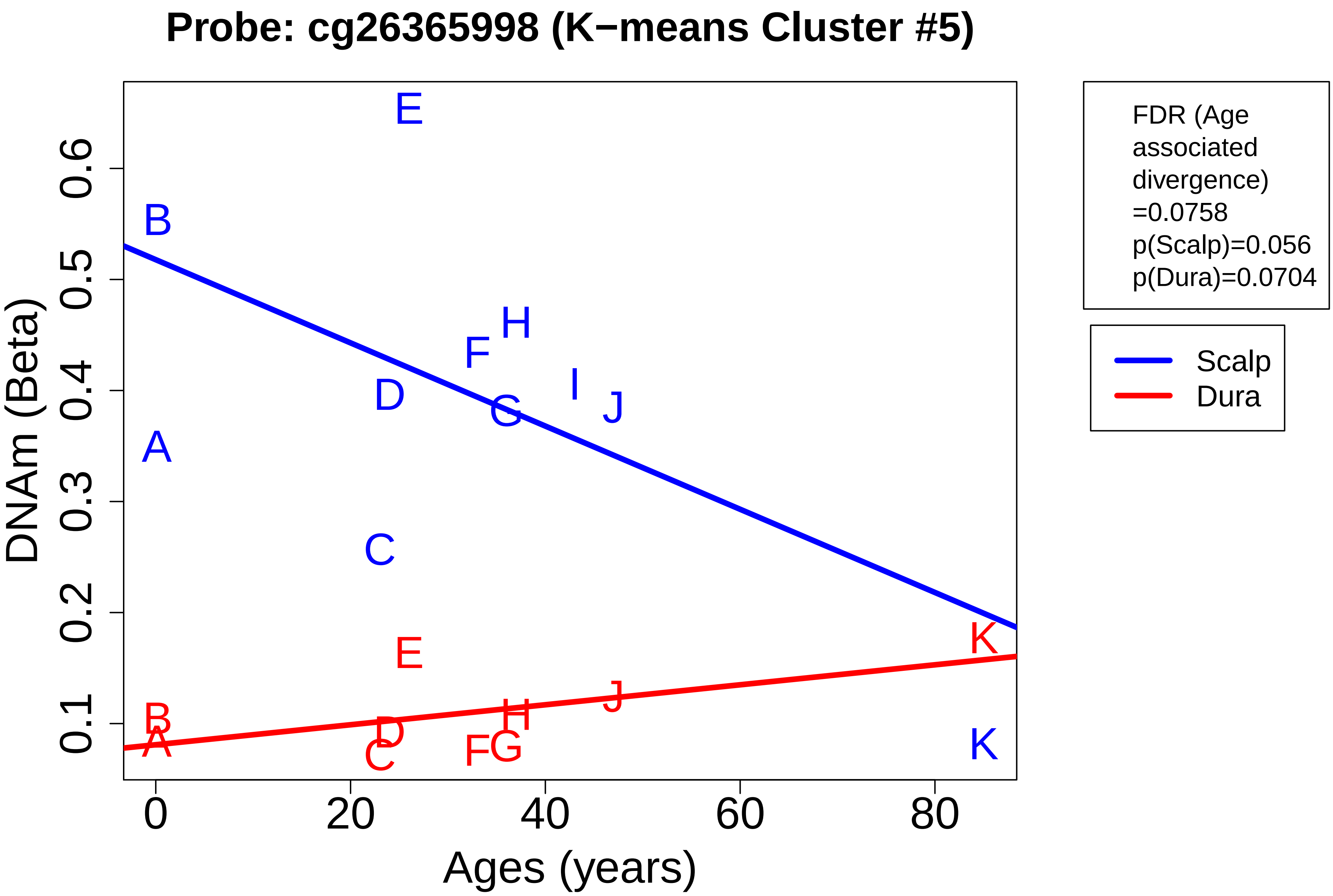
Probe: cg26365998 (K−means Cluster #5)
DNAm (Beta)
0.1
0.2
0.3
0.4
0.5
0.6
0
20
40
60
80
Ages (years)
A
B
C
D
E
F
G
H
I
J
K
FDR (Age associated divergence) =0.0758
p(Scalp)=0.056
p(Dura)=0.0704
Scalp
Dura

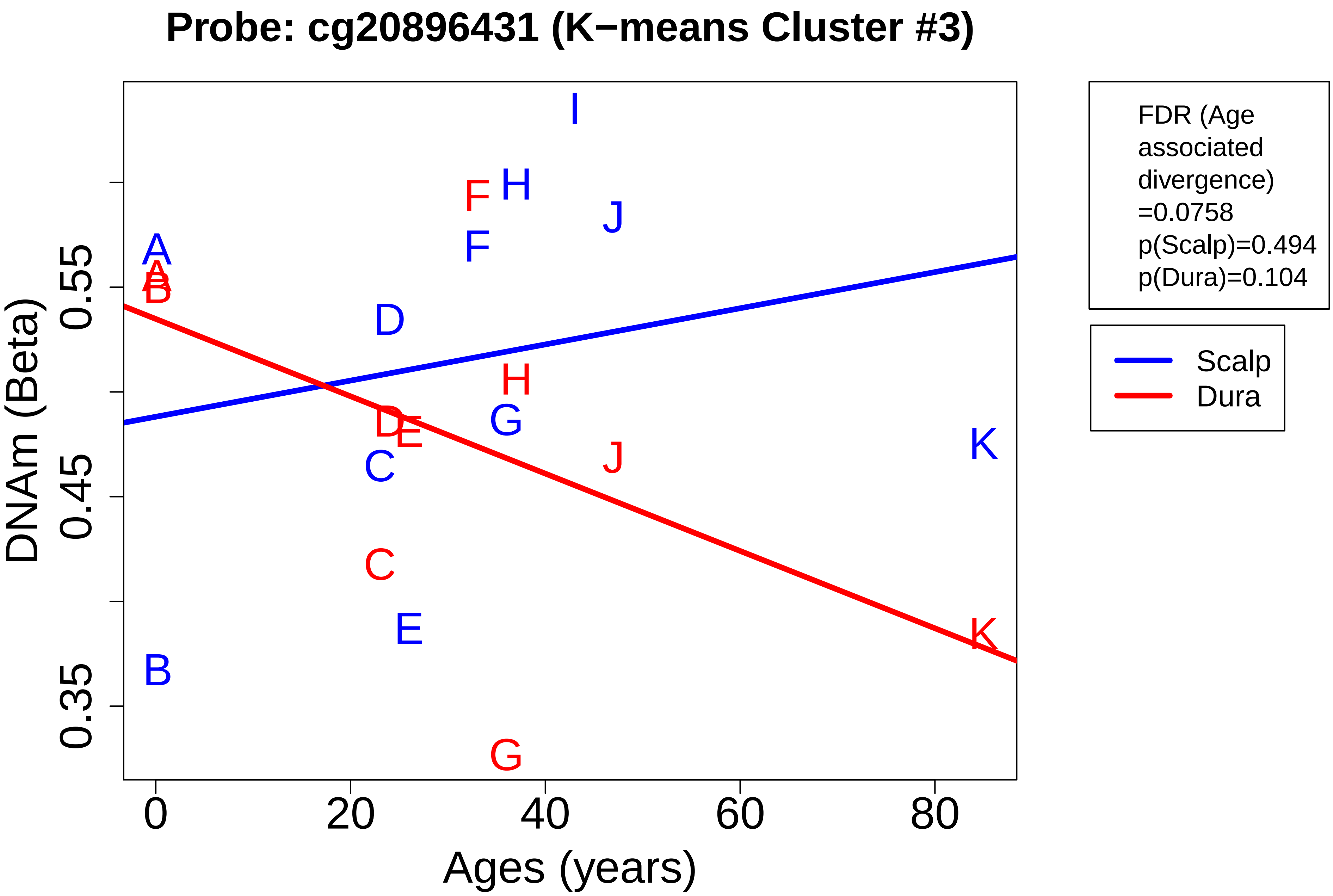

Probe: cg20896431 (K−means Cluster #3)
DNAm (Beta)
0.35
0.45
0.55
0
20
40
60
80
Ages (years)
A B C D E F G H I J K
A B C D E F G H J K
FDR (Age associated divergence) =0.0758
p(Scalp)=0.494
p(Dura)=0.104
Scalp
Dura

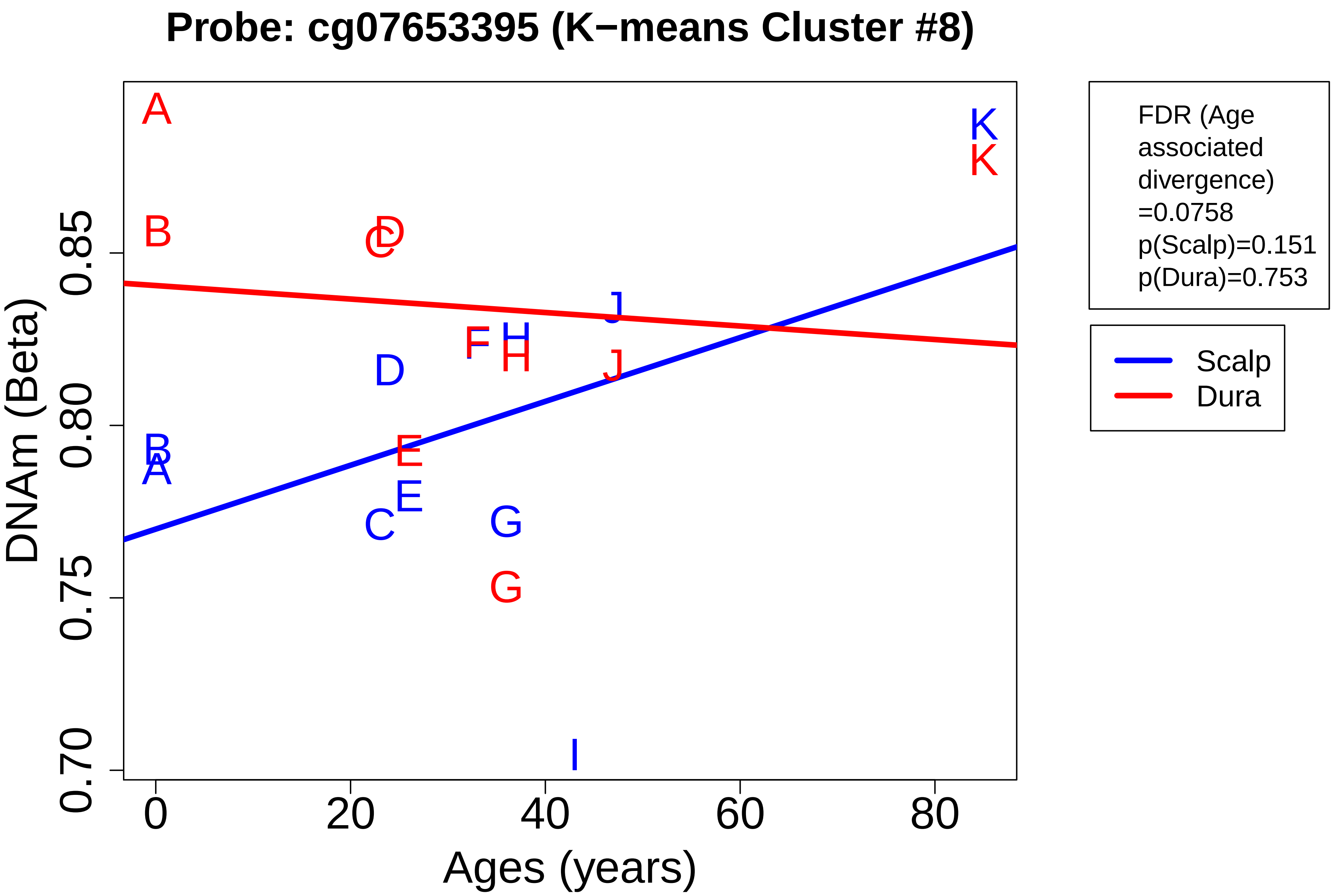

Probe: cg07653395 (K−means Cluster #8)
FDR (Age associated divergence) =0.0758
p(Scalp)=0.151
p(Dura)=0.753
Scalp
Dura
DNAm (Beta)
0.70
0.75
0.80
0.85
0
20
40
60
80
Ages (years)

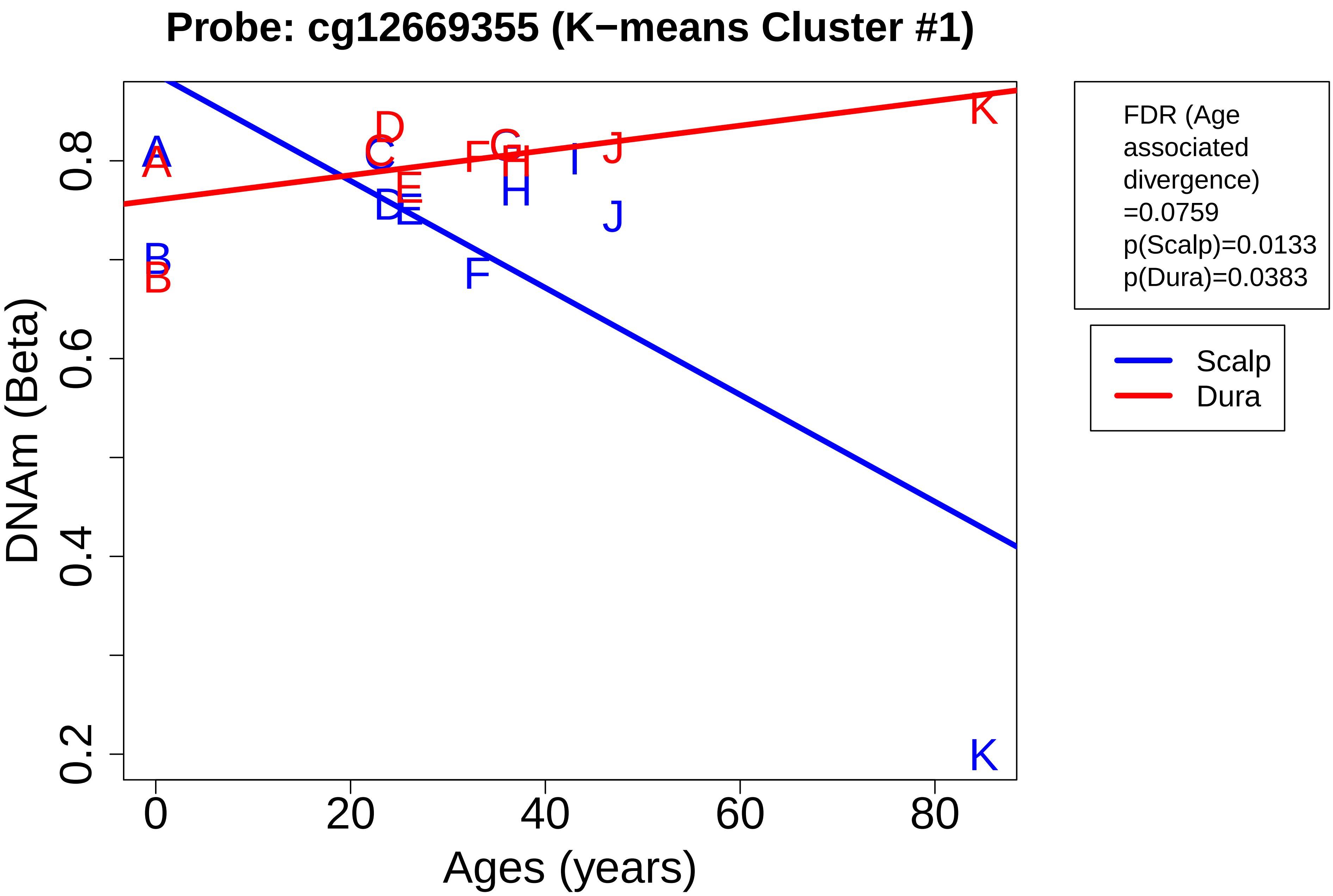

Probe: cg12669355 (K−means Cluster #1)
DNAm (Beta)
0.2
0.4
0.6
0.8
Ages (years)
0
20
40
60
80
A
B
C
D
E
F
G
H
I
J
K
FDR (Age associated divergence) =0.0759
p(Scalp)=0.0133
p(Dura)=0.0383
Scalp
Dura

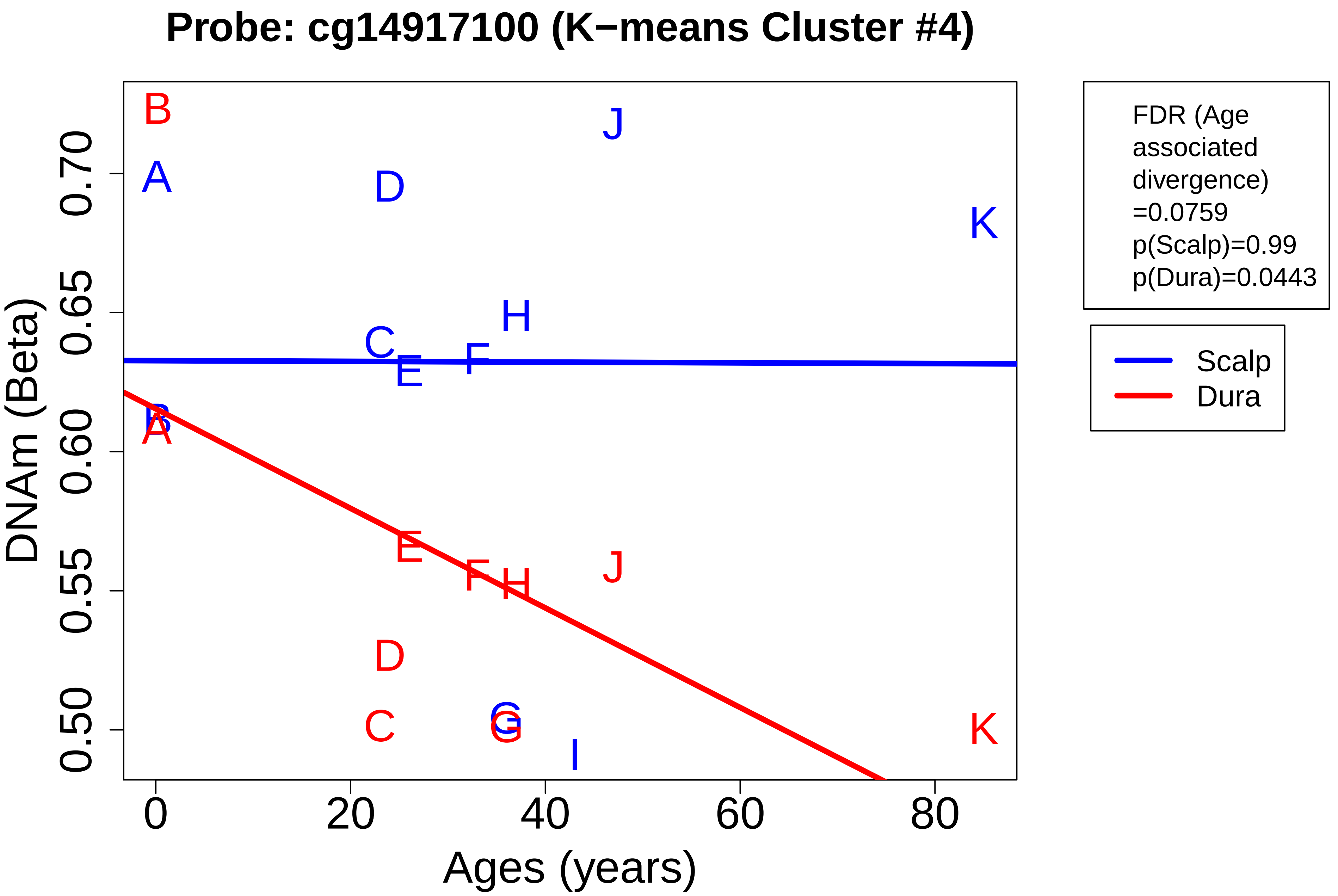
Probe: cg14917100 (K−means Cluster #4)
DNAm (Beta)
Ages (years)
0.50
0.55
0.60
0.65
0.70
0
20
40
60
80
FDR (Age associated divergence) =0.0759
p(Scalp)=0.99
p(Dura)=0.0443
Scalp
Dura
A
B
C
D
E
F
G
H
I
J
K

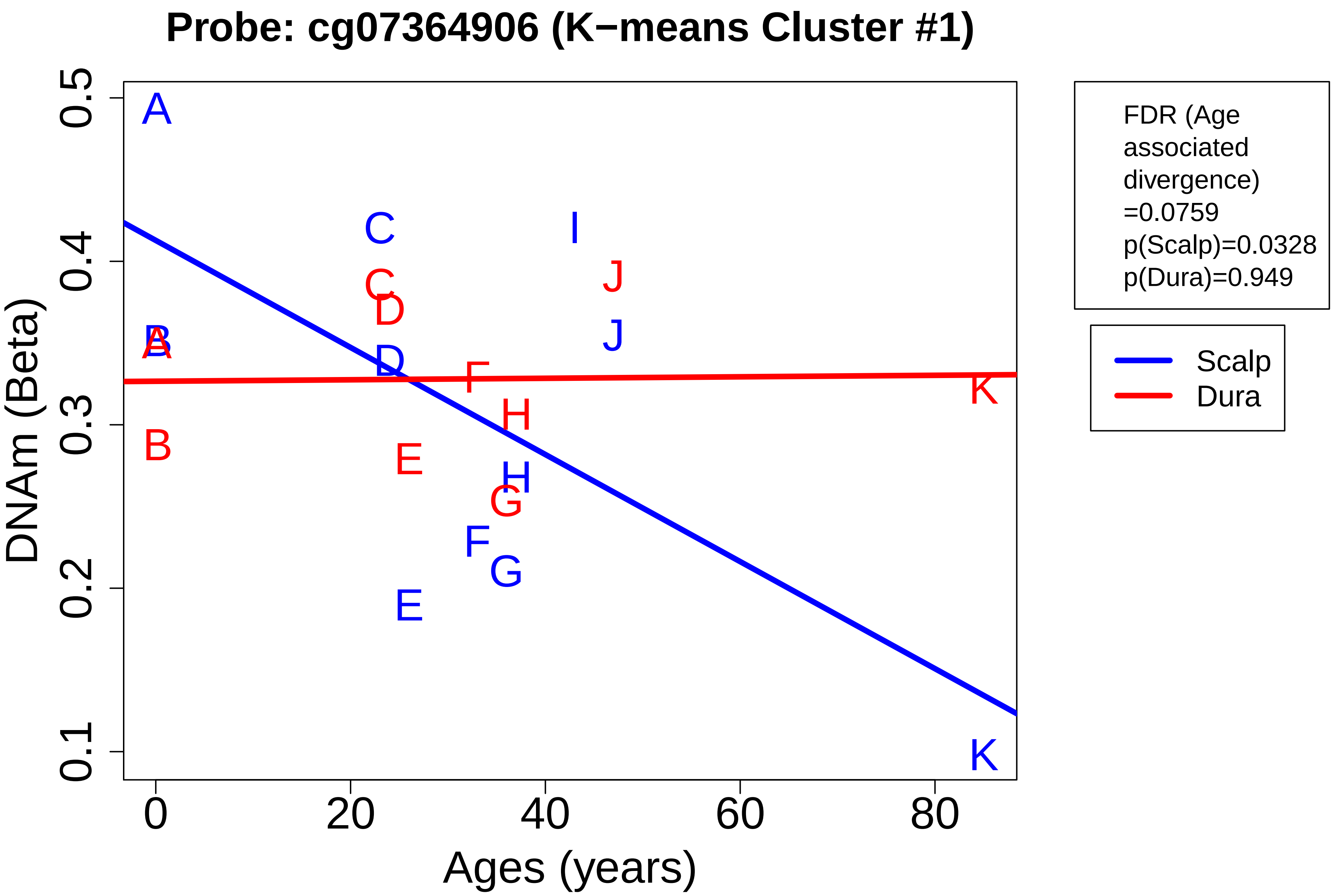

Probe: cg07364906 (K−means Cluster #1)
DNAm (Beta)
0.1
0.2
0.3
0.4
0.5
Ages (years)
0
20
40
60
80
A
B
C
D
E
F
G
H
I
J
K
FDR (Age associated divergence) =0.0759
p(Scalp)=0.0328
p(Dura)=0.949
Scalp
Dura

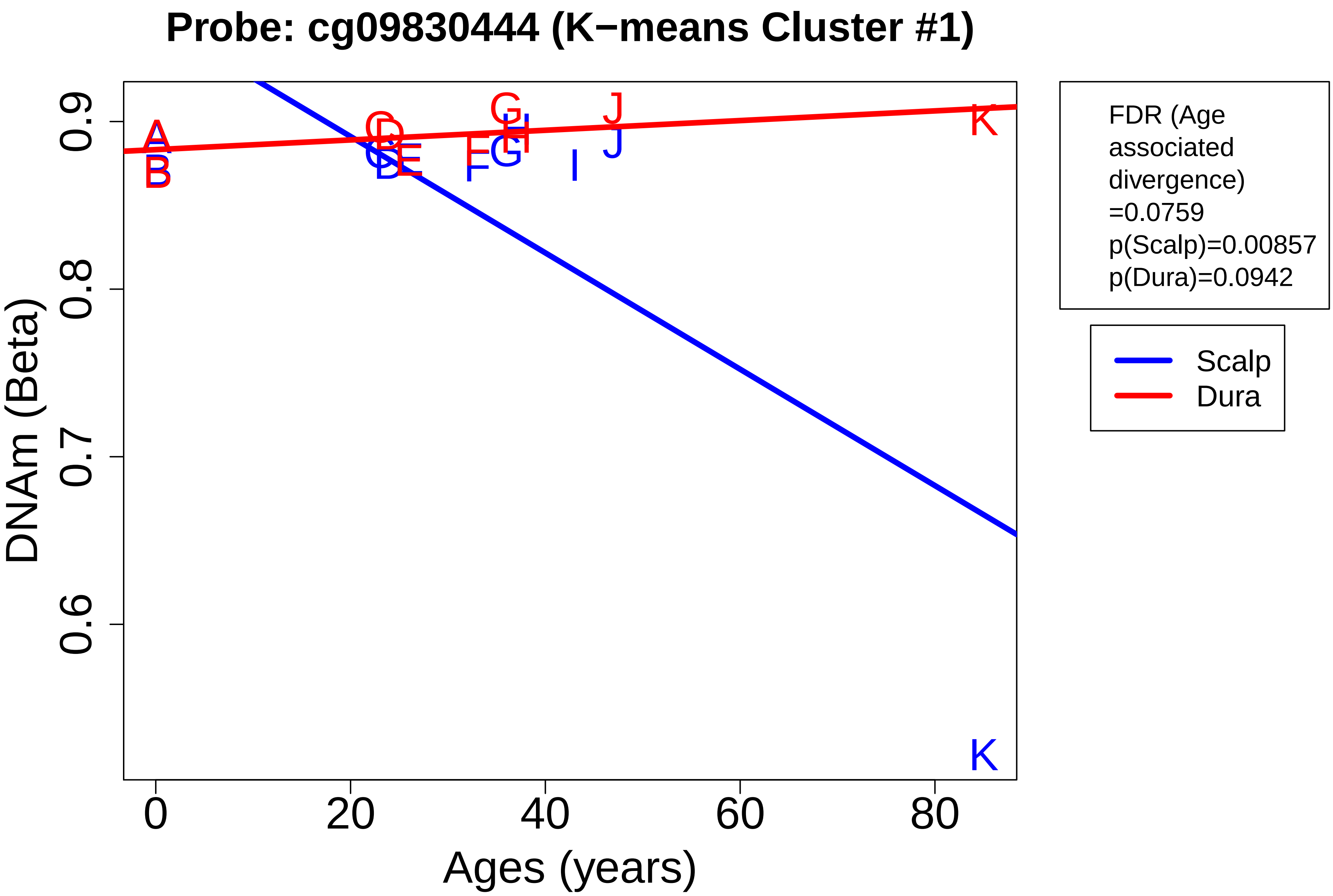
Probe: cg09830444 (K−means Cluster #1)
DNAm (Beta)
0.6
0.7
0.8
0.9
Ages (years)
0
20
40
60
80
A
B
C
D
E
F
G
H
I
J
K
FDR (Age associated divergence) =0.0759
p(Scalp)=0.00857
p(Dura)=0.0942
Scalp
Dura

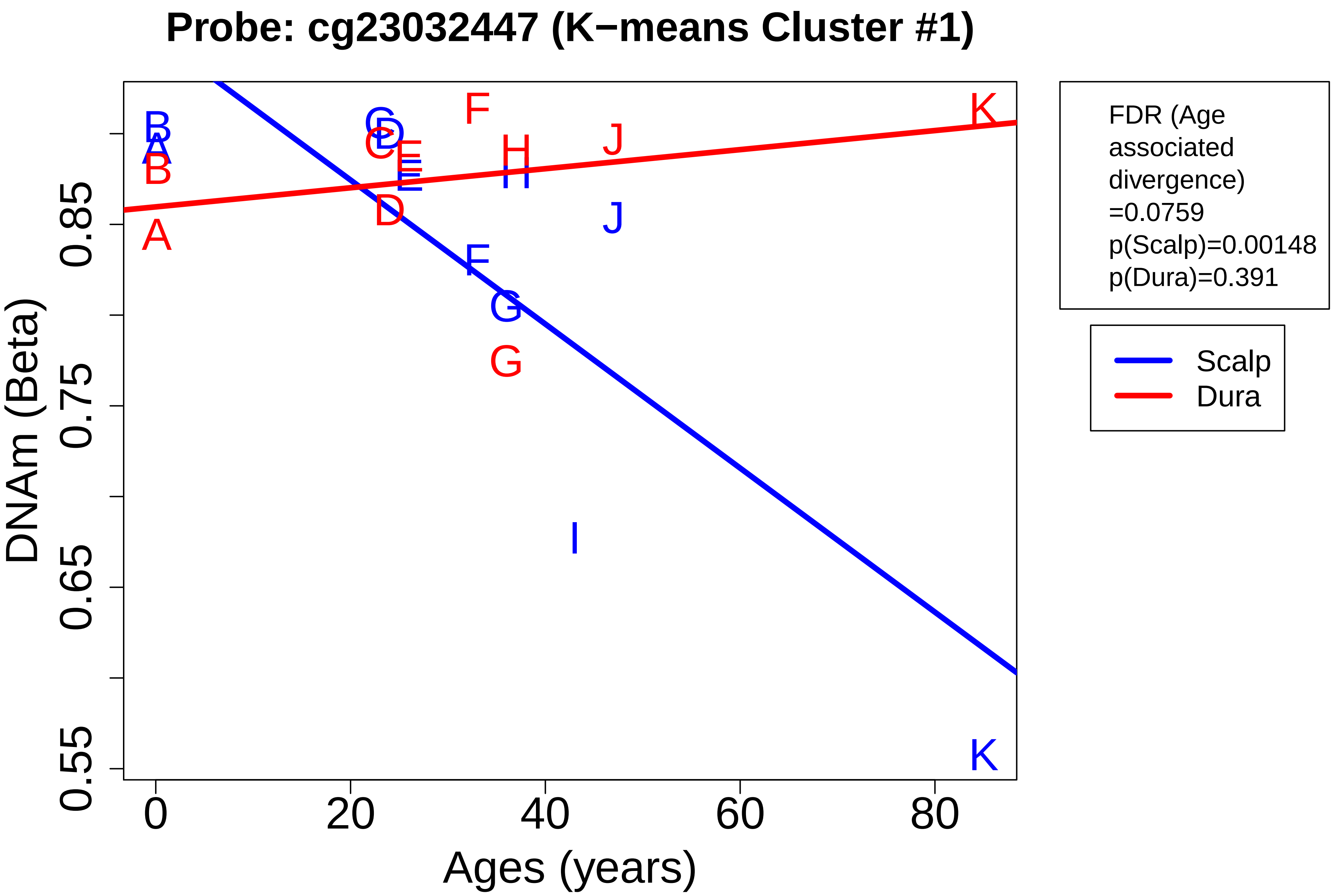

Probe: cg23032447 (K−means Cluster #1)
FDR (Age associated divergence) =0.0759
p(Scalp)=0.00148
p(Dura)=0.391
Scalp
Dura
DNAm (Beta)
0.55
0.65
0.75
0.85
Ages (years)
0
20
40
60
80

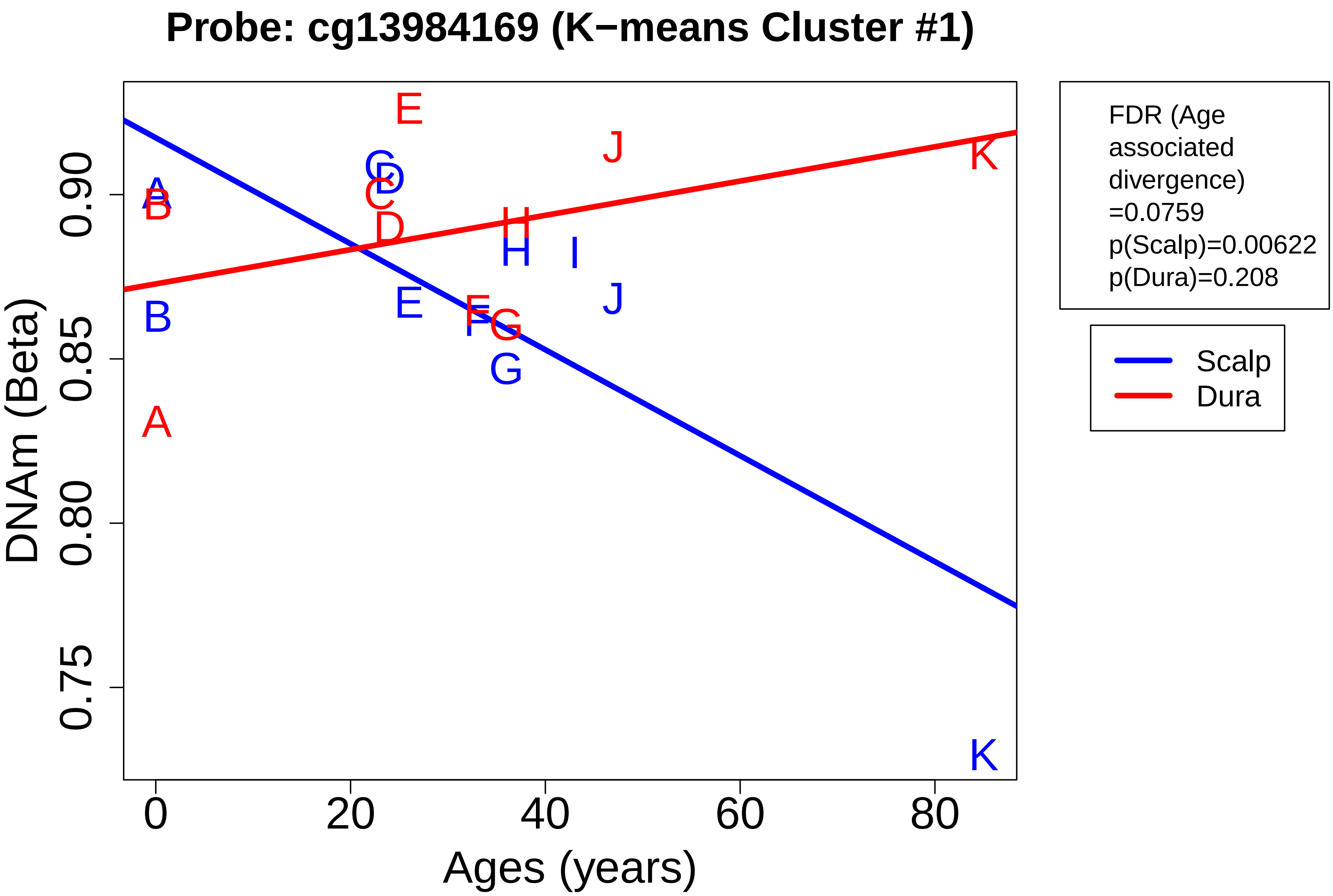

Probe: cg13984169 (K−means Cluster #1)
DNAm (Beta)
0.75
0.80
0.85
0.90
0
20
40
60
80
Ages (years)
FDR (Age associated divergence) =0.0759
p(Scalp)=0.00622
p(Dura)=0.208
Scalp
Dura

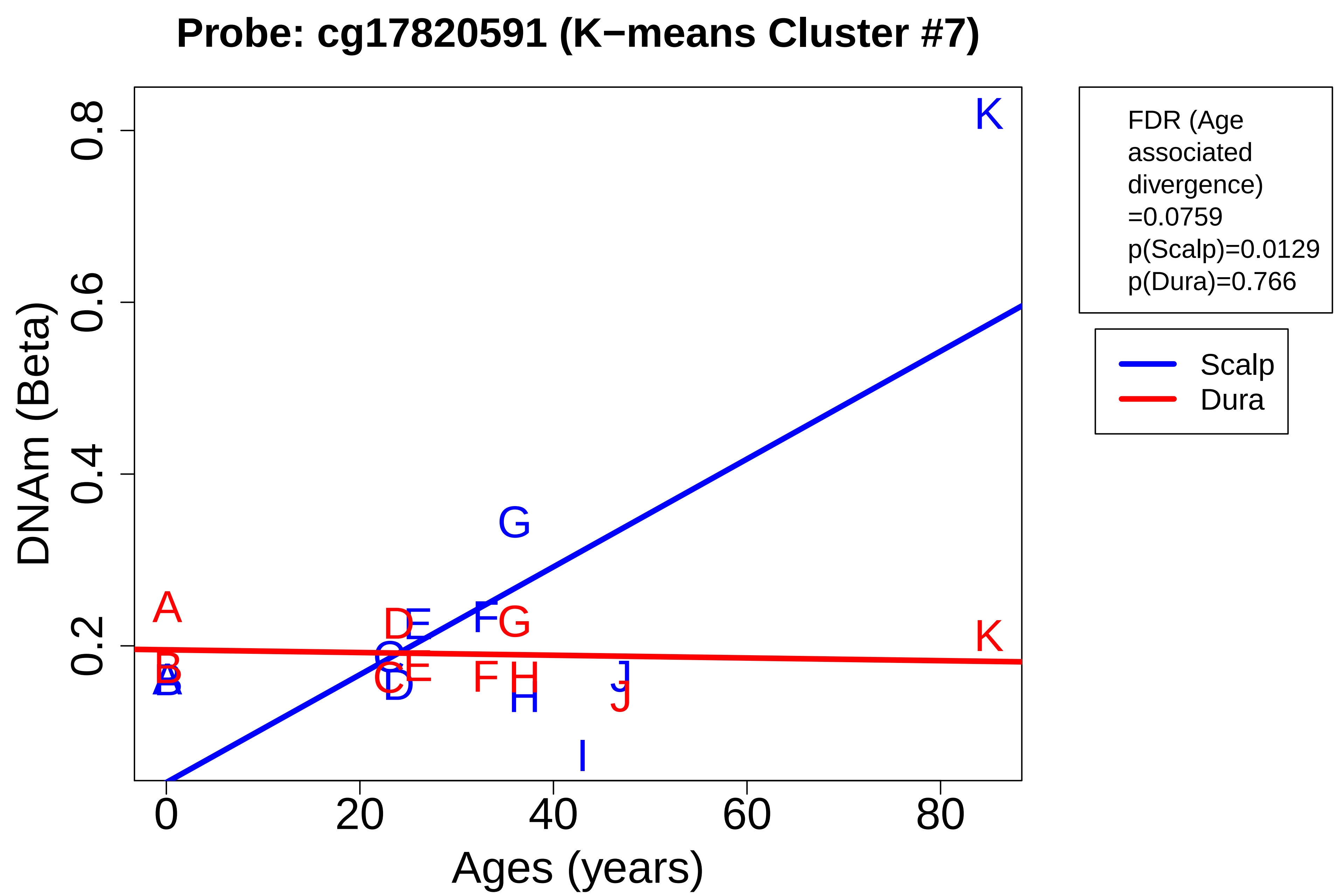
Probe: cg17820591 (K−means Cluster #7)
DNAm (Beta)
0.2
0.4
0.6
0.8
Ages (years)
0
20
40
60
80
A
B
C
D
E
F
G
H
I
J
K
FDR (Age associated divergence) =0.0759
p(Scalp)=0.0129
p(Dura)=0.766
Scalp
Dura

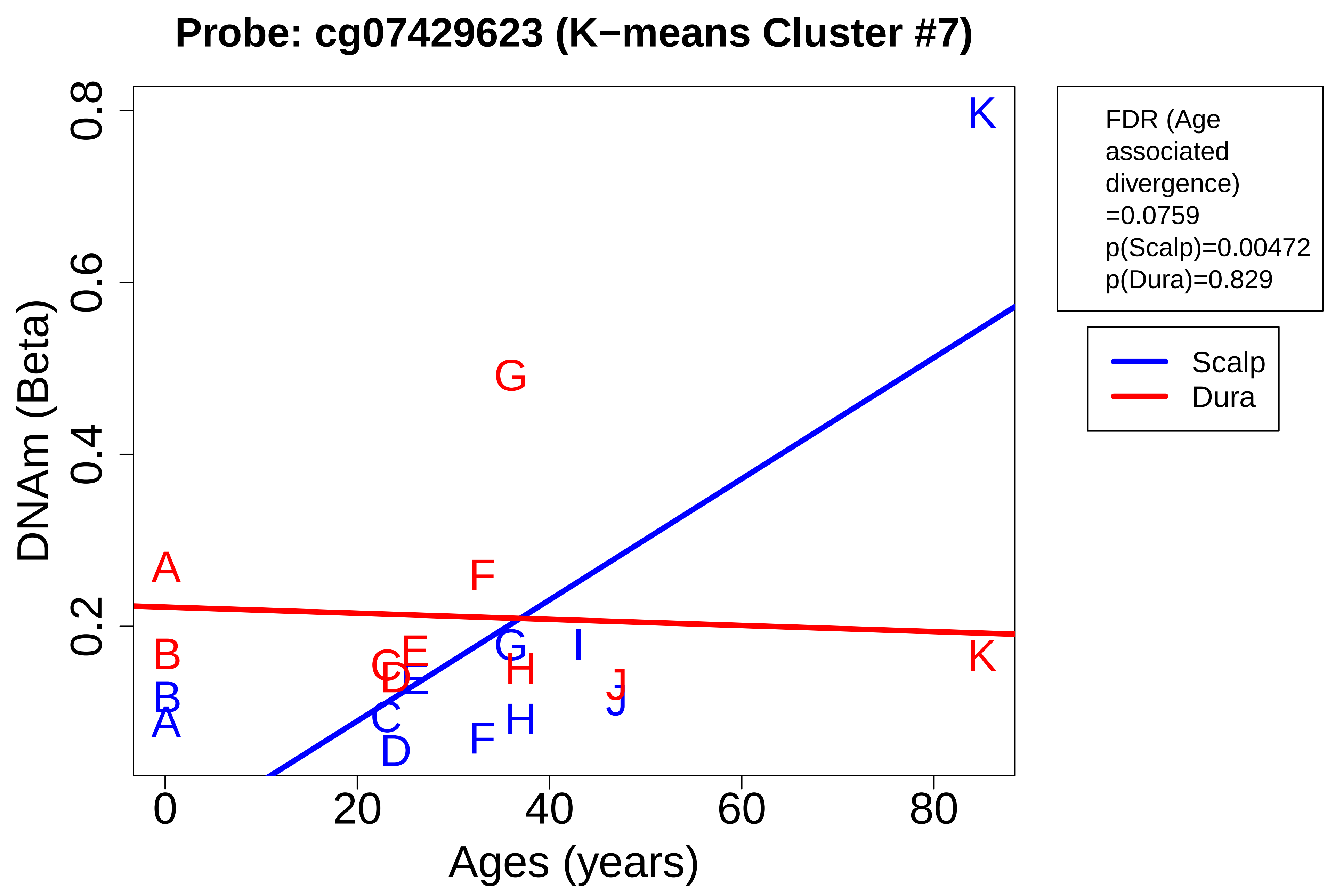

Probe: cg07429623 (K−means Cluster #7)
DNAm (Beta)
0.2
0.4
0.6
0.8
0
20
40
60
80
Ages (years)
FDR (Age associated divergence) =0.0759
p(Scalp)=0.00472
p(Dura)=0.829
Scalp
Dura
A
B
C
D
E
F
G
H
I
J
K

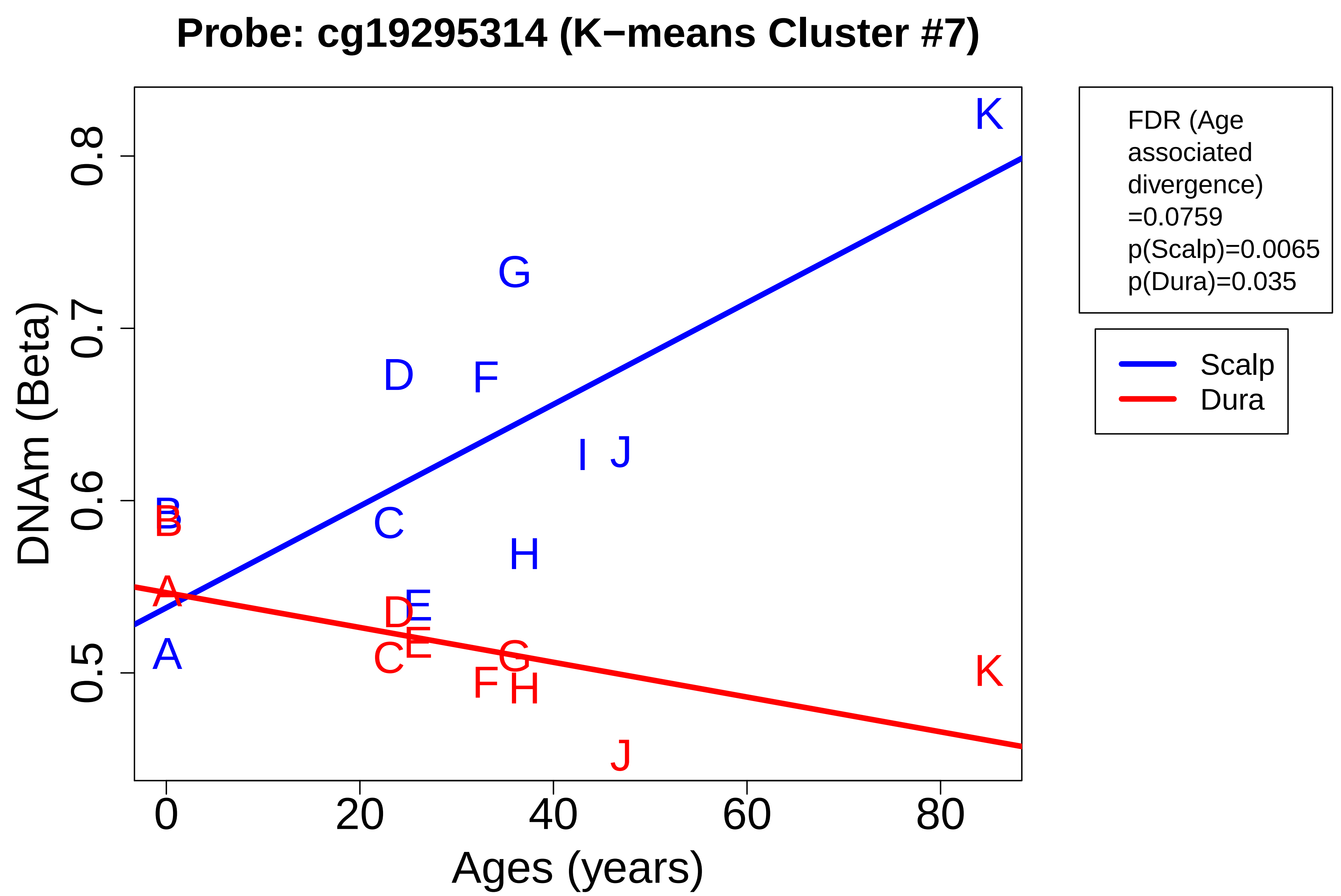
Probe: cg19295314 (K−means Cluster #7)
DNAm (Beta)
0.5
0.6
0.7
0.8
0
20
40
60
80
Ages (years)
FDR (Age associated divergence) =0.0759
p(Scalp)=0.0065
p(Dura)=0.035
Scalp
Dura
A
B
C
D
E
F
G
H
I
J
K

Probe: cg25511335 (K−means Cluster #1)

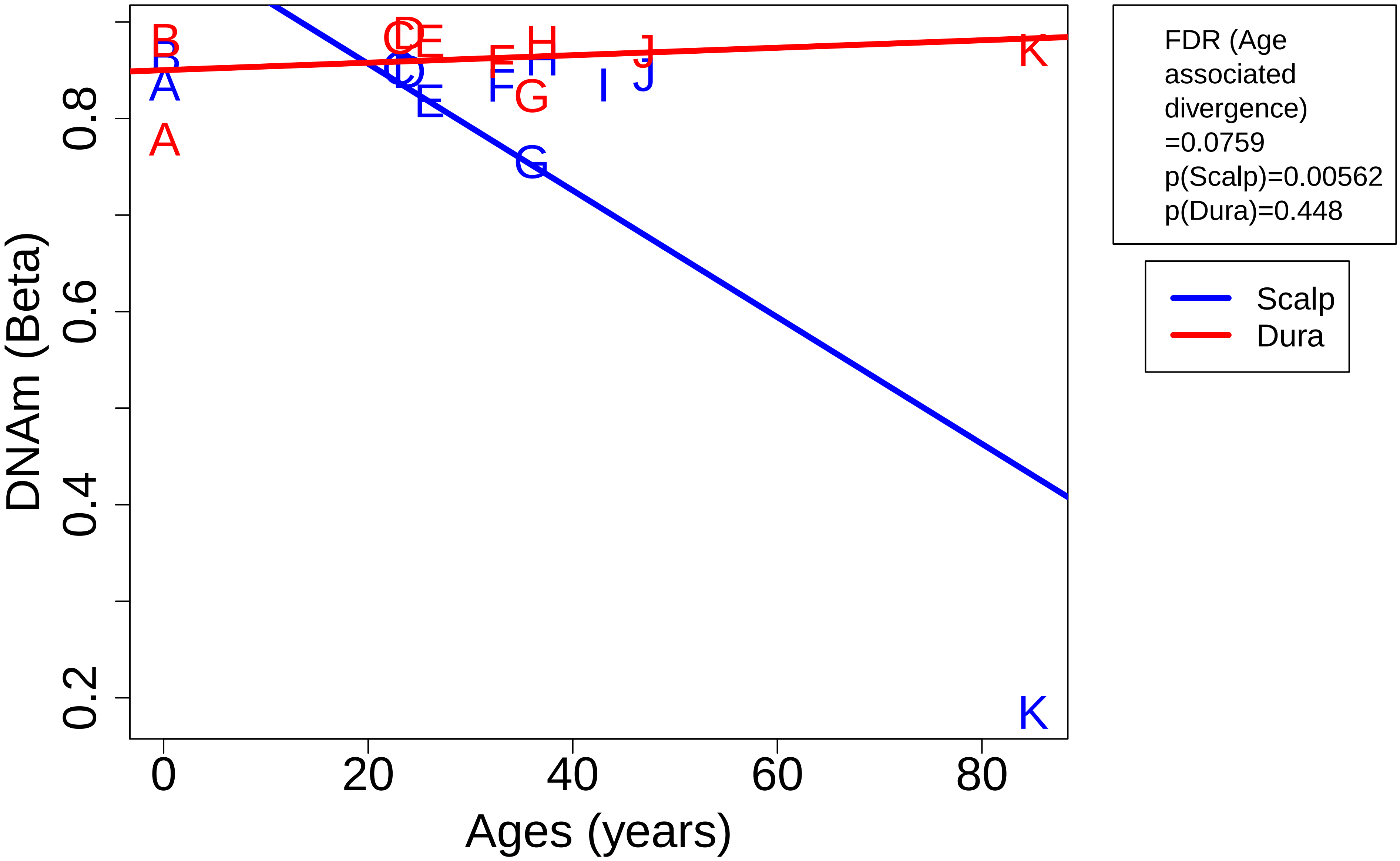

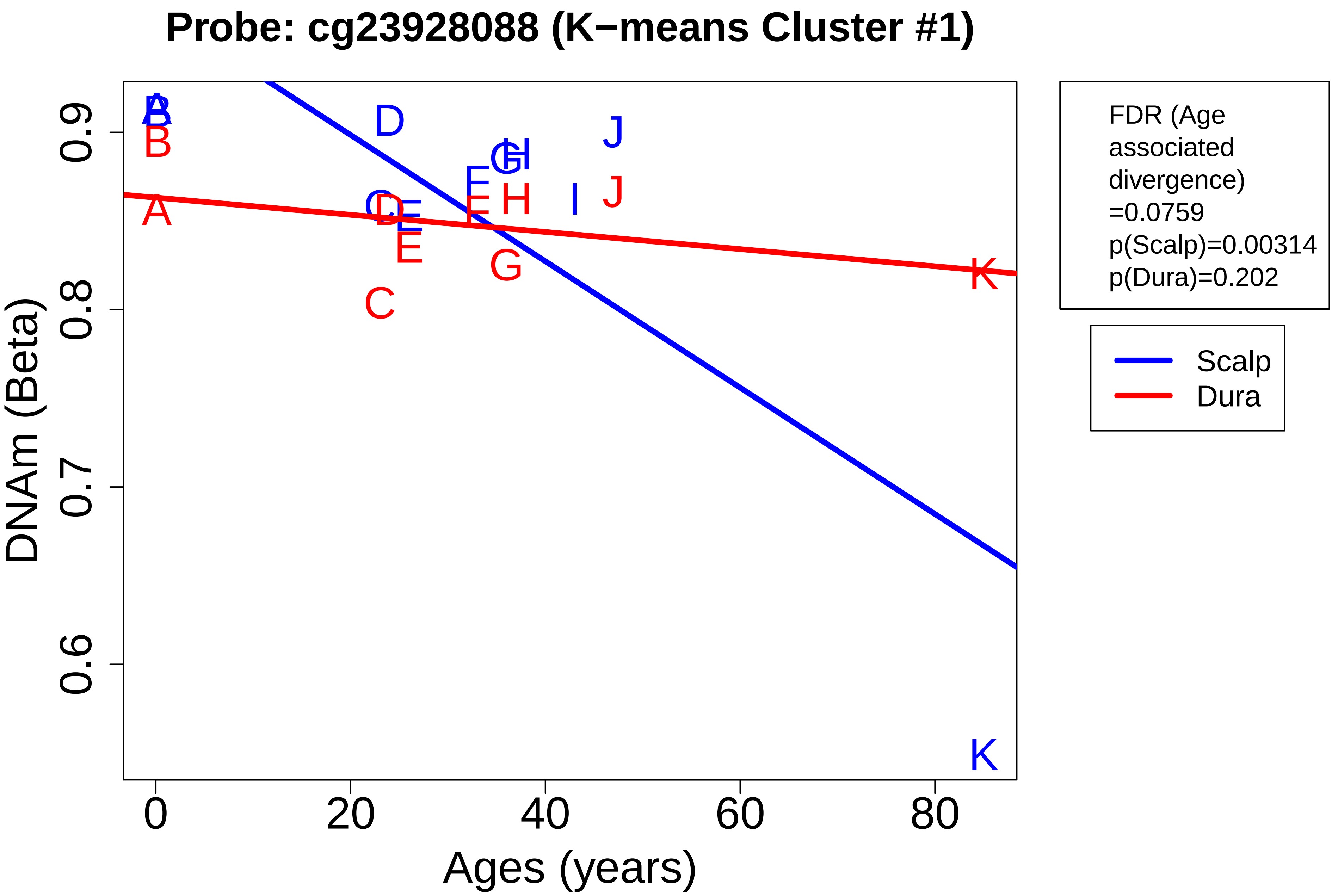

Probe: cg23928088 (K−means Cluster #1)
DNAm (Beta)
0.6
0.7
0.8
0.9
Ages (years)
0
20
40
60
80
FDR (Age associated divergence) =0.0759
p(Scalp)=0.00314
p(Dura)=0.202
Scalp
Dura

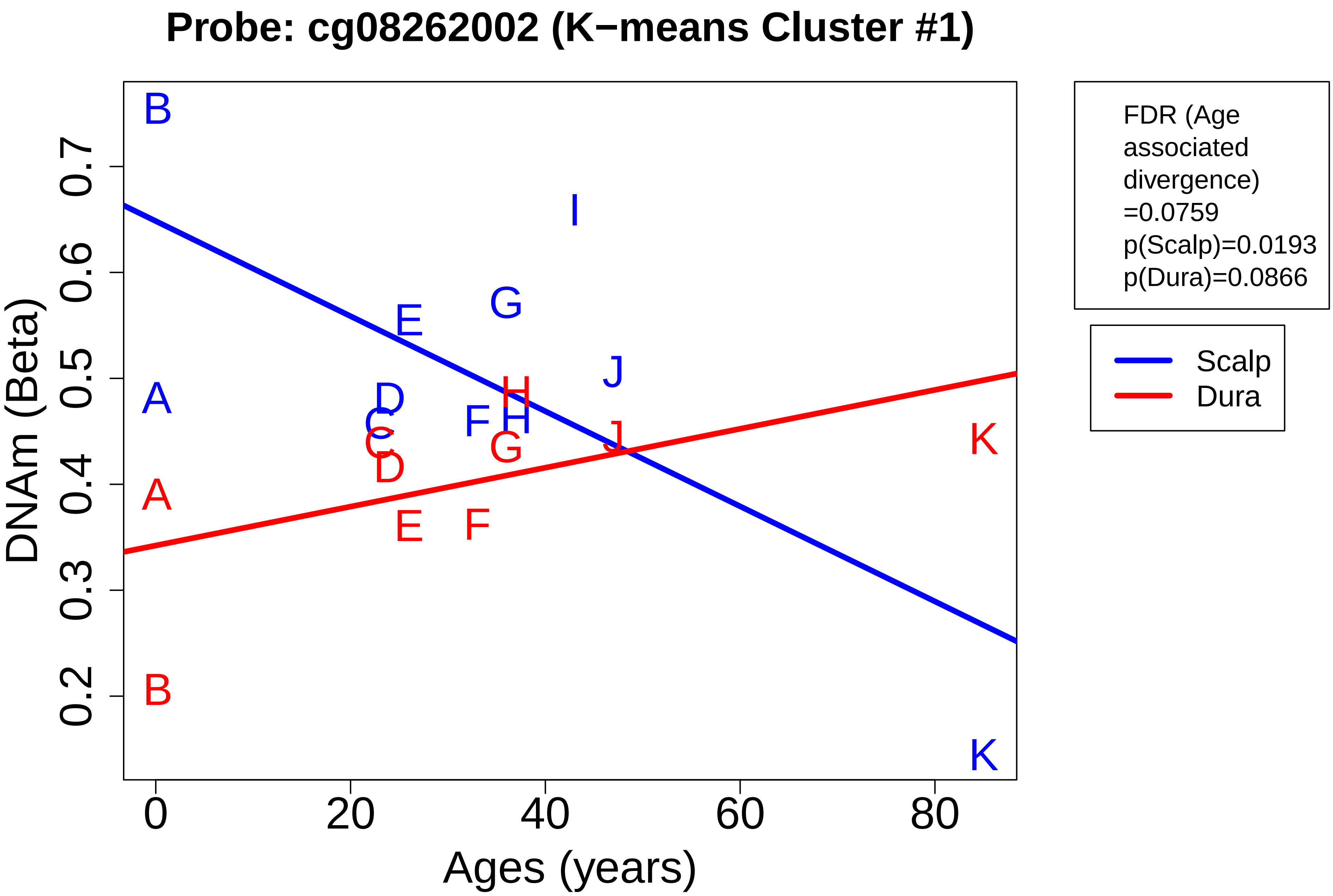

Probe: cg08262002 (K−means Cluster #1)
DNAm (Beta)
Ages (years)
0.2
0.3
0.4
0.5
0.6
0.7
0
20
40
60
80
FDR (Age associated divergence) =0.0759
p(Scalp)=0.0193
p(Dura)=0.0866
Scalp
Dura

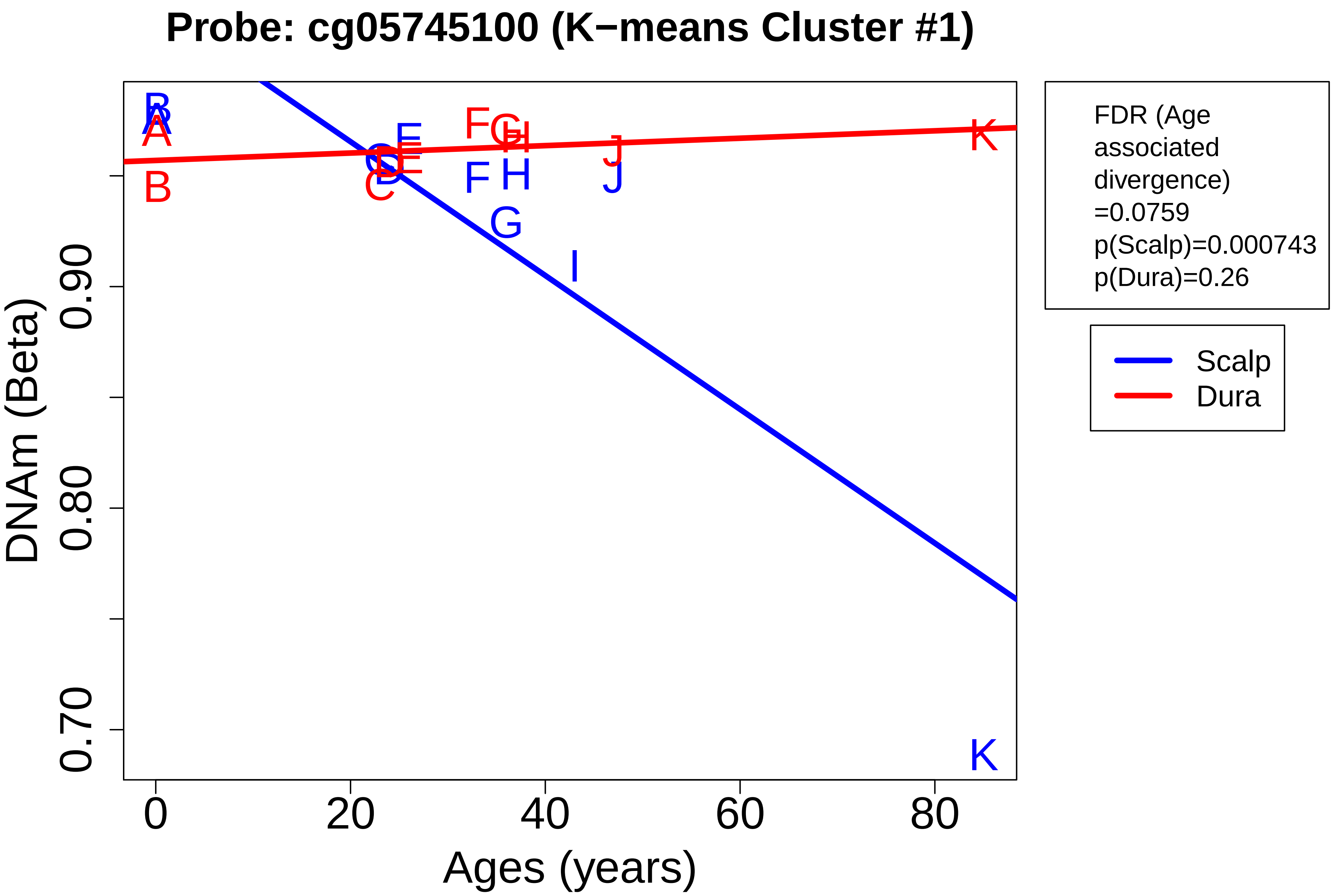

Probe: cg05745100 (K−means Cluster #1)
DNAm (Beta)
0.70
0.80
0.90
Ages (years)
0
20
40
60
80
FDR (Age associated divergence) =0.0759
p(Scalp)=0.000743
p(Dura)=0.26
Scalp
Dura

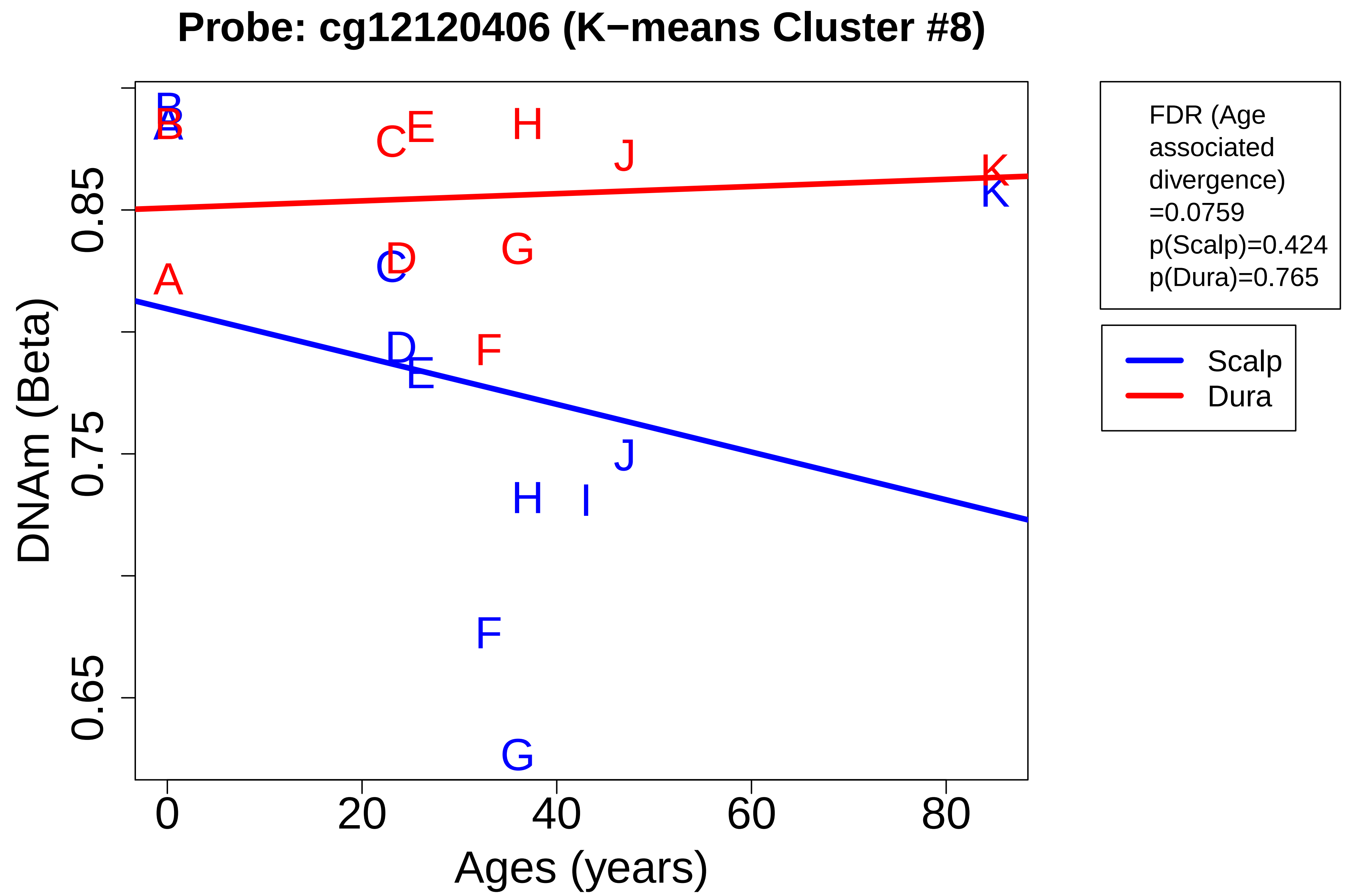

Probe: cg12120406 (K−means Cluster #8)
DNAm (Beta)
0.65
0.75
0.85
Ages (years)
0
20
40
60
80
FDR (Age associated divergence) =0.0759
p(Scalp)=0.424
p(Dura)=0.765
Scalp
Dura

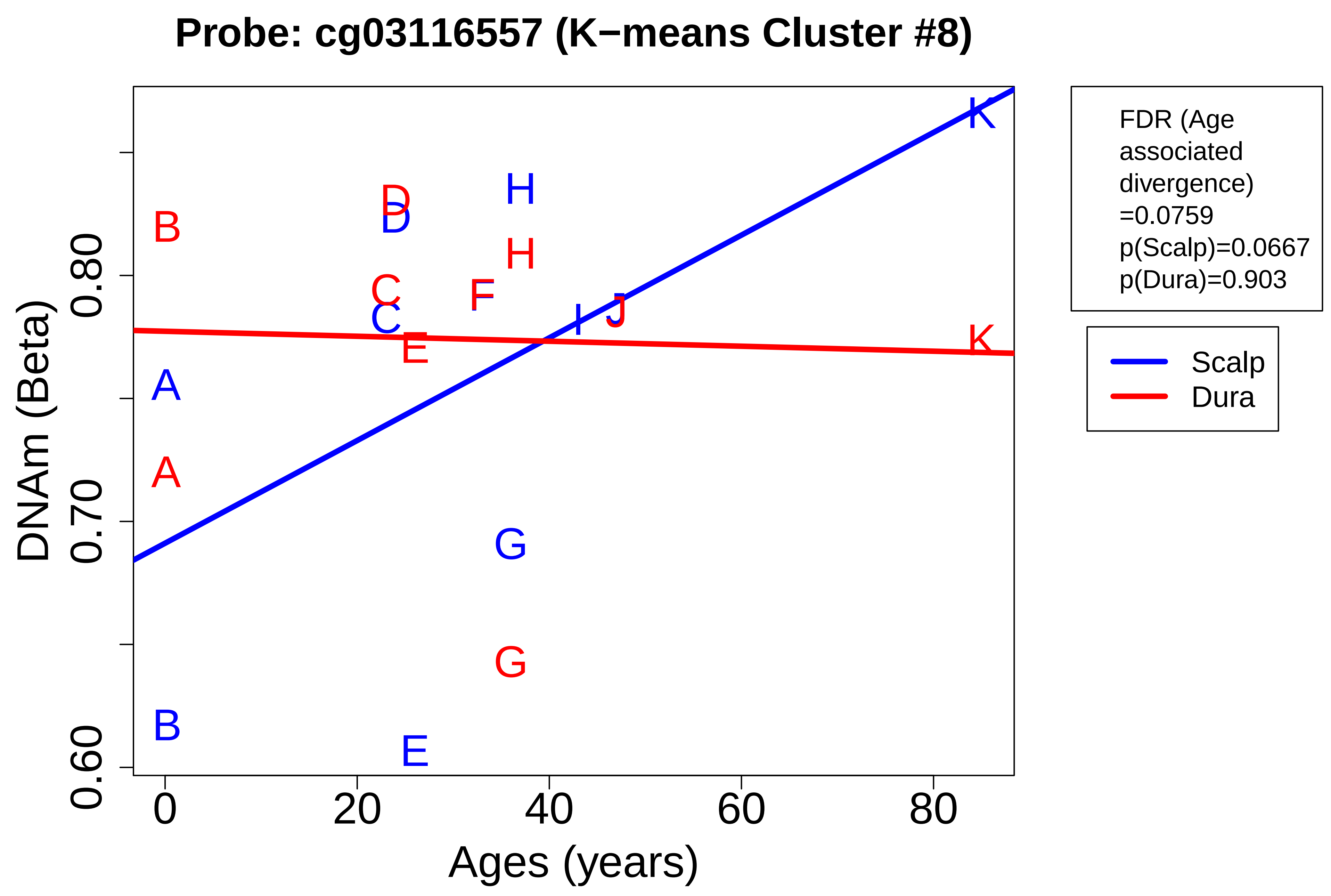

Probe: cg03116557 (K−means Cluster #8)
DNAm (Beta)
0.60
0.70
0.80
0
20
40
60
80
Ages (years)
A
B
C
D
E
F
G
H
I
J
K
FDR (Age associated divergence) =0.0759
p(Scalp)=0.0667
p(Dura)=0.903
Scalp
Dura

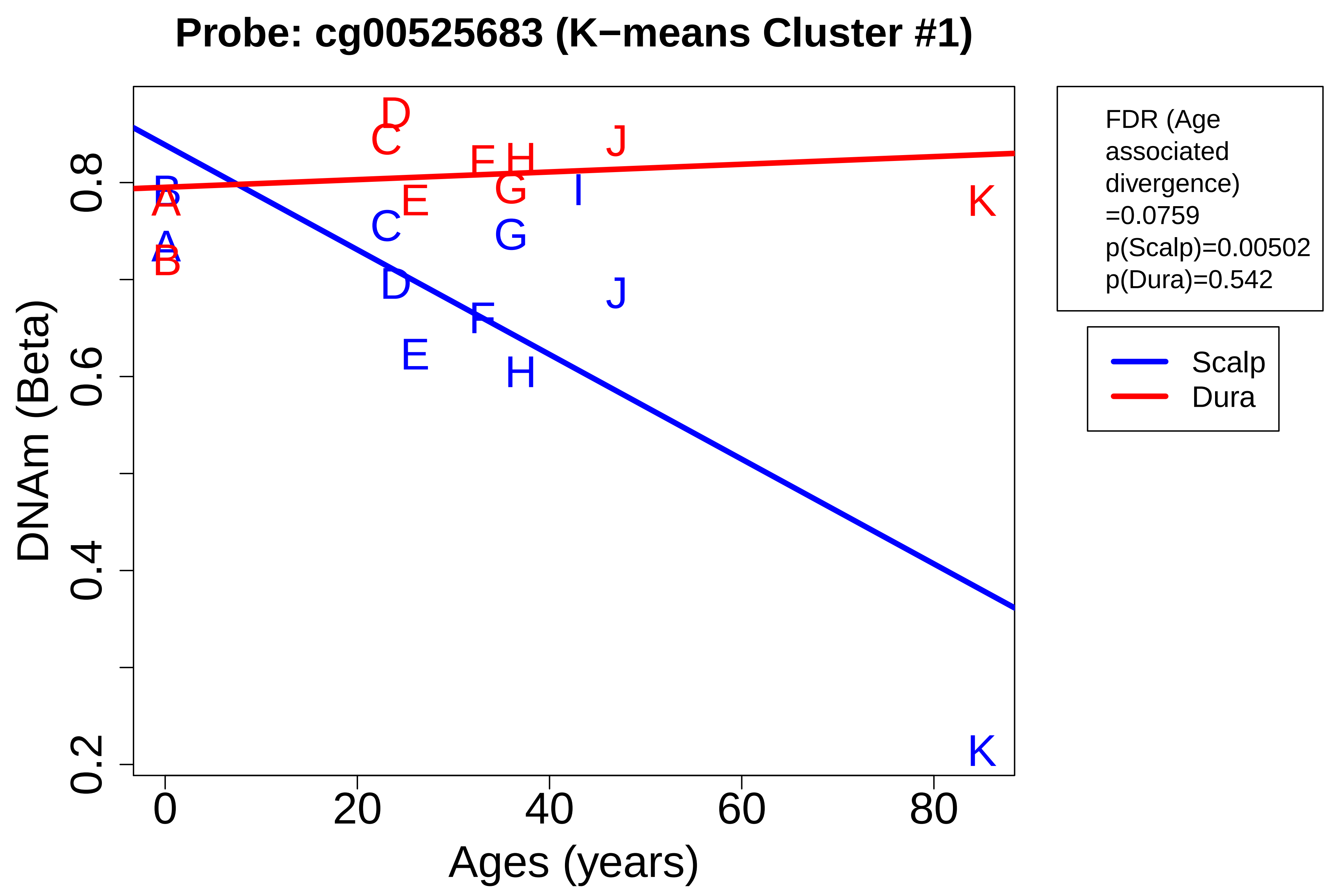

Probe: cg00525683 (K−means Cluster #1)
DNAm (Beta)
0.2
0.4
0.6
0.8
0
20
40
60
80
Ages (years)
FDR (Age associated divergence) =0.0759
p(Scalp)=0.00502
p(Dura)=0.542
Scalp
Dura

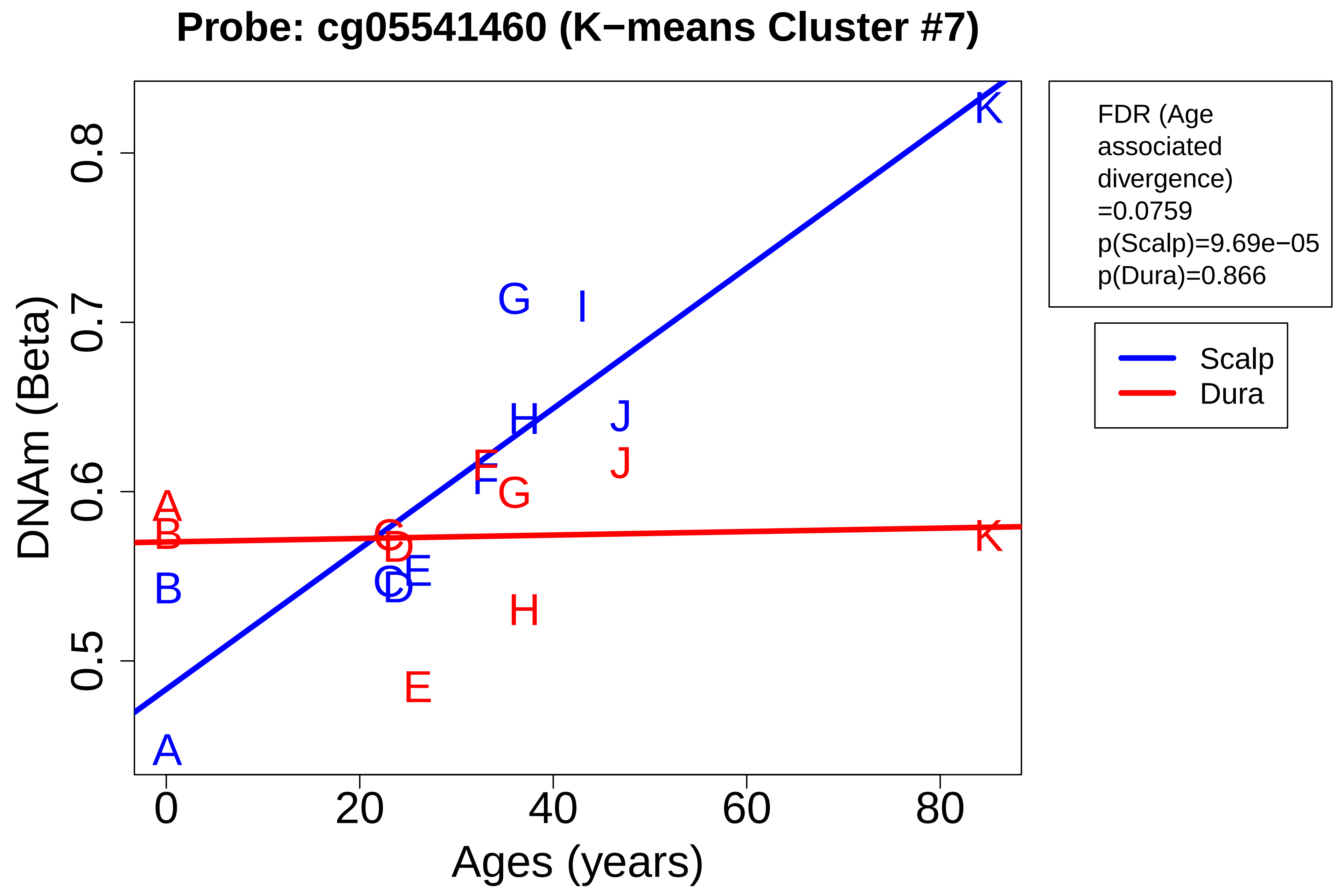

Probe: cg05541460 (K−means Cluster #7)
DNAm (Beta)
0.5
0.6
0.7
0.8
0
20
40
60
80
Ages (years)
FDR (Age associated divergence) =0.0759
p(Scalp)=9.69e−05
p(Dura)=0.866
Scalp
Dura

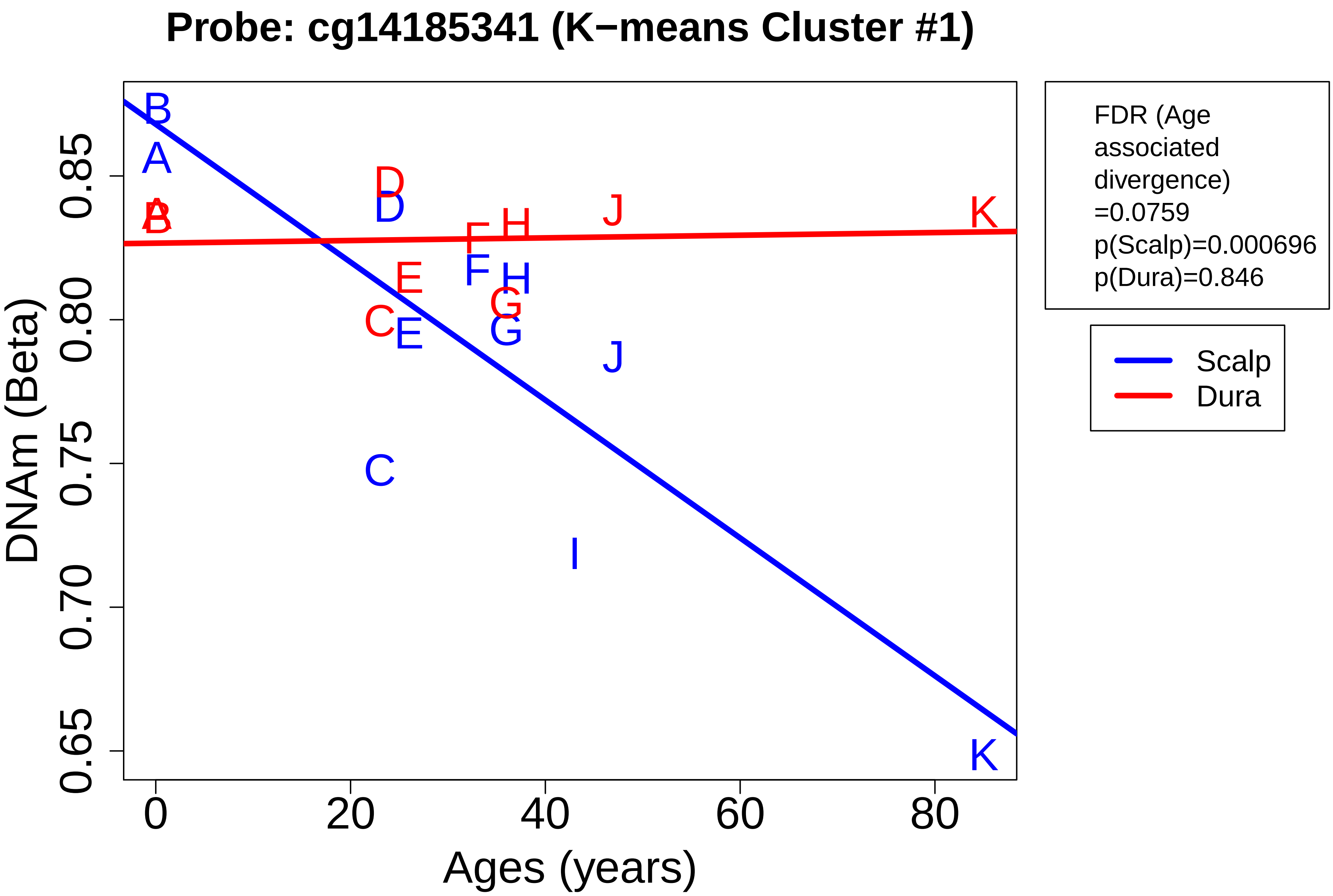

Probe: cg14185341 (K−means Cluster #1)
DNAm (Beta)
0.65
0.70
0.75
0.80
0.85
0
20
40
60
80
Ages (years)
FDR (Age associated divergence) =0.0759
p(Scalp)=0.000696
p(Dura)=0.846
Scalp
Dura

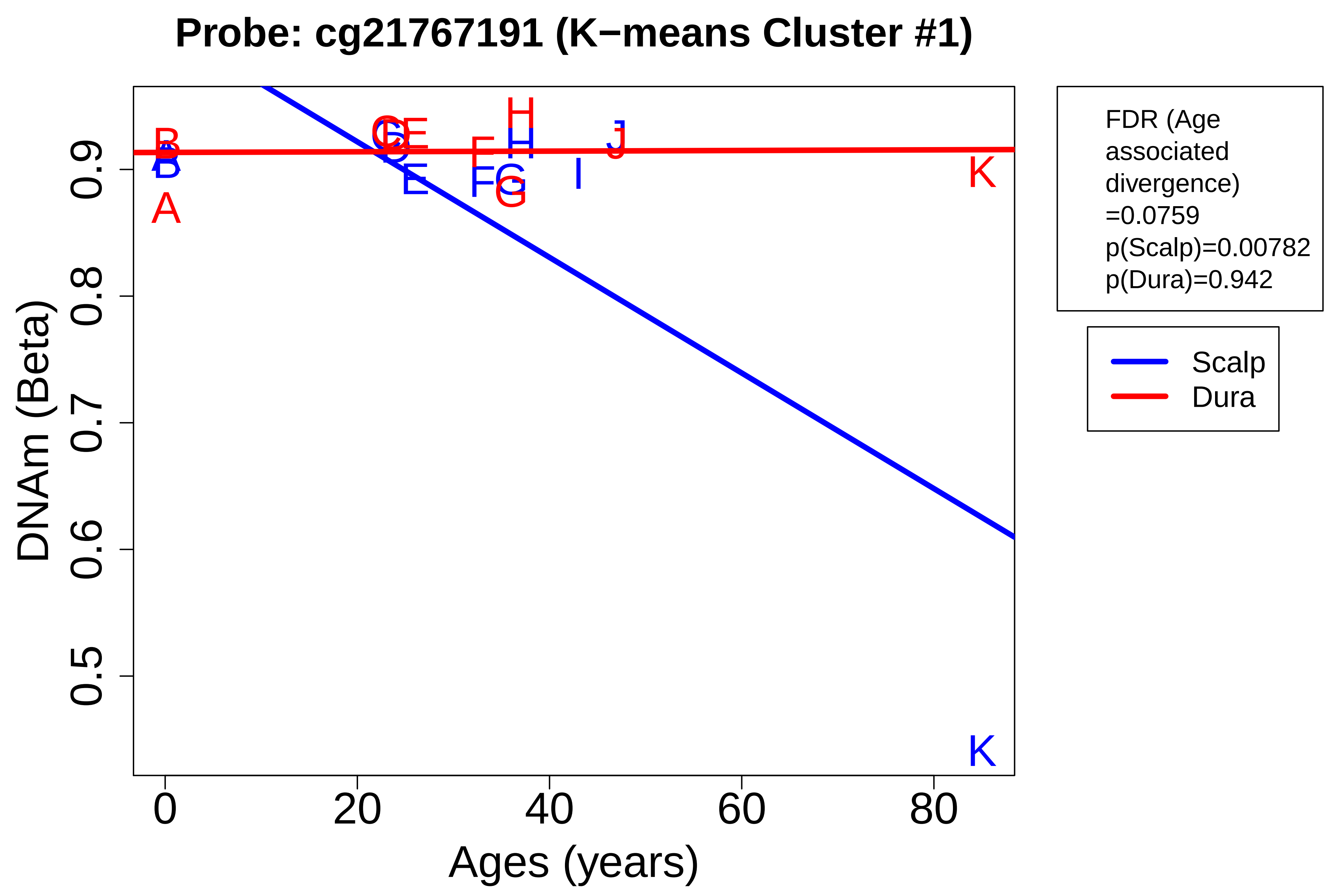

Probe: cg21767191 (K−means Cluster #1)
DNAm (Beta)
0.5
0.6
0.7
0.8
0.9
0
20
40
60
80
Ages (years)
FDR (Age associated divergence) =0.0759
p(Scalp)=0.00782
p(Dura)=0.942
Scalp
Dura

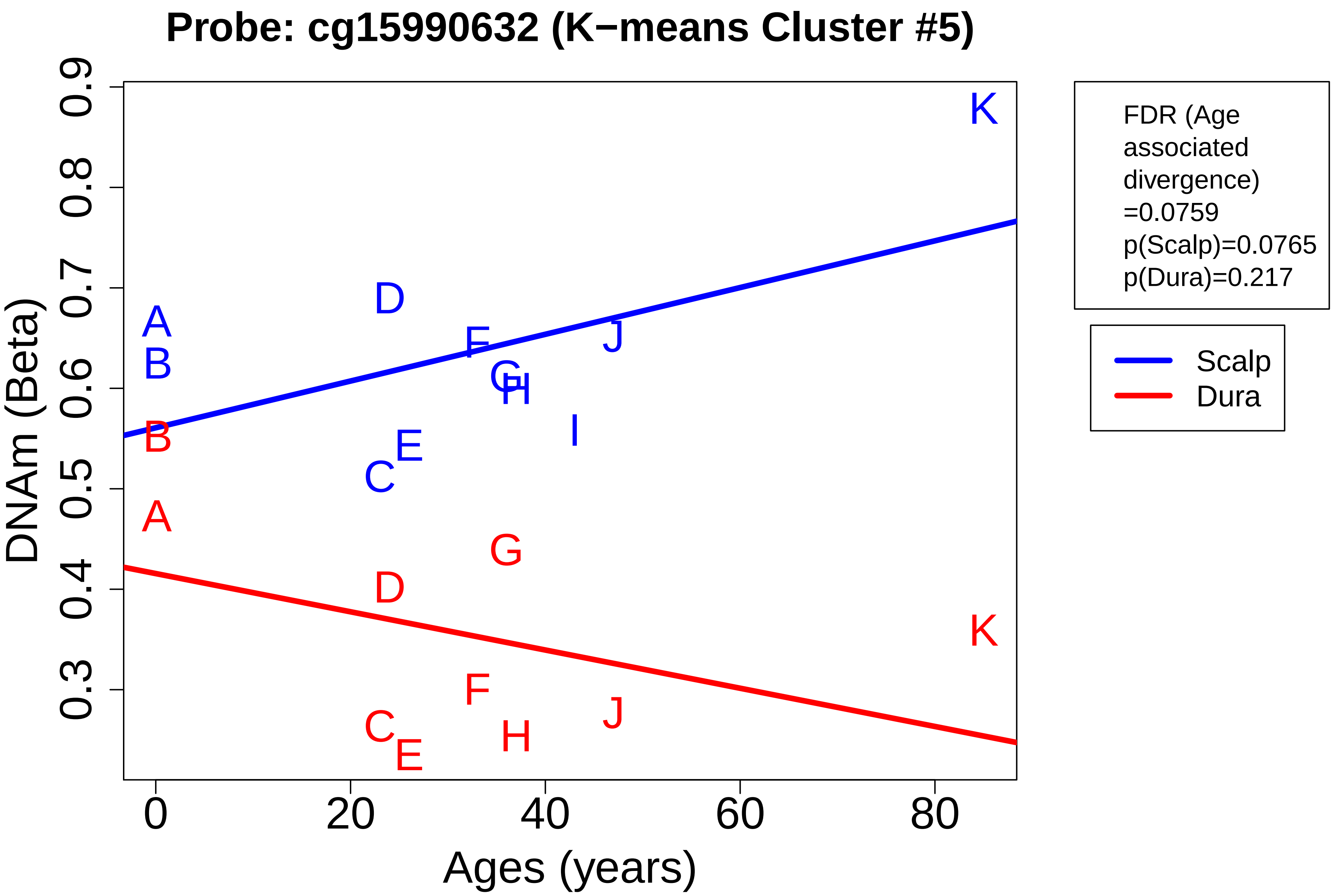

Probe: cg15990632 (K−means Cluster #5)
DNAm (Beta)
0.3
0.4
0.5
0.6
0.7
0.8
0.9
Ages (years)
0
20
40
60
80
A
B
C
D
E
F
G
H
I
J
K
FDR (Age associated divergence) =0.0759
p(Scalp)=0.0765
p(Dura)=0.217
Scalp
Dura

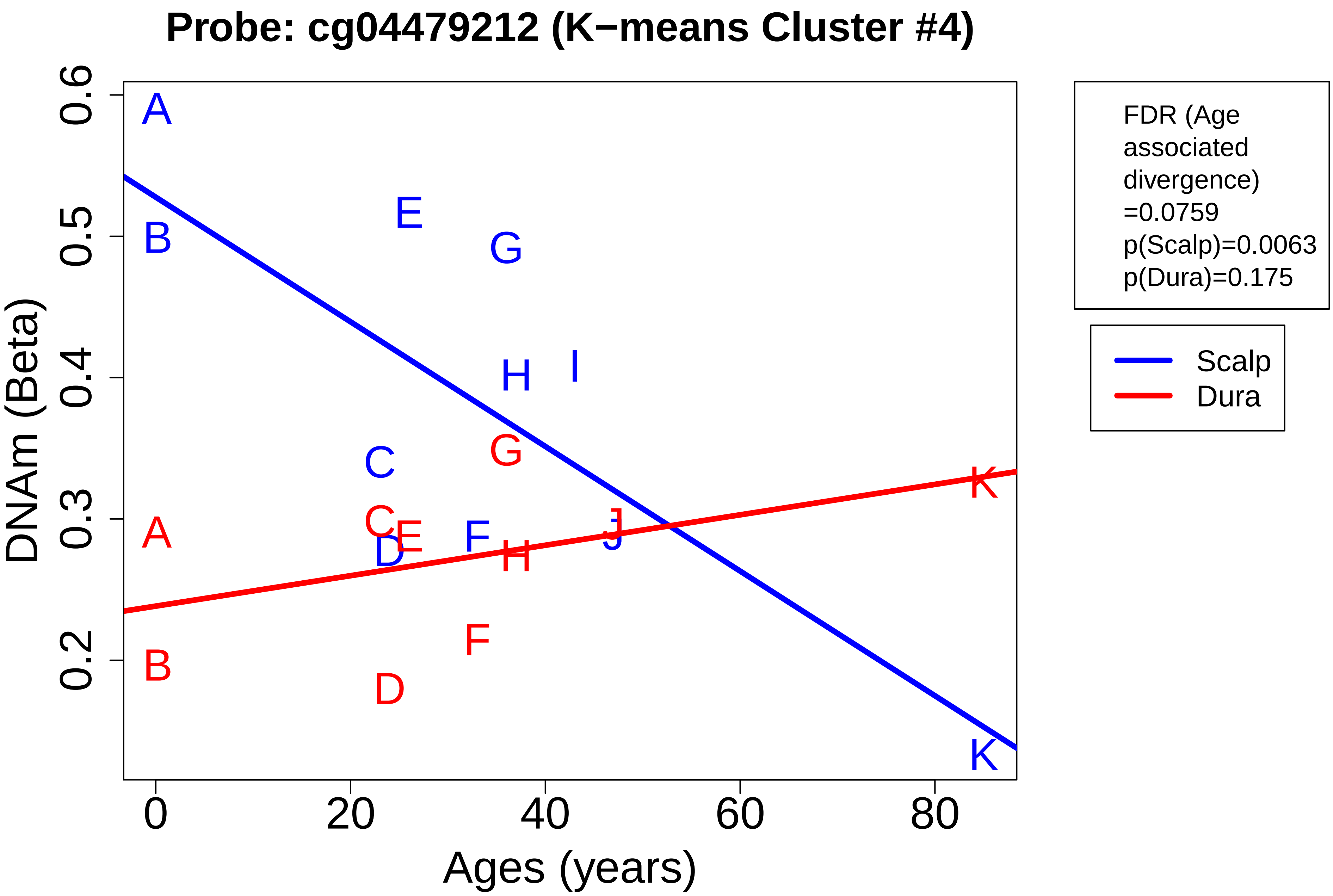
Probe: cg04479212 (K−means Cluster #4)
DNAm (Beta)
0.2
0.3
0.4
0.5
0.6
Ages (years)
0
20
40
60
80
FDR (Age associated divergence) =0.0759
p(Scalp)=0.0063
p(Dura)=0.175
Scalp
Dura

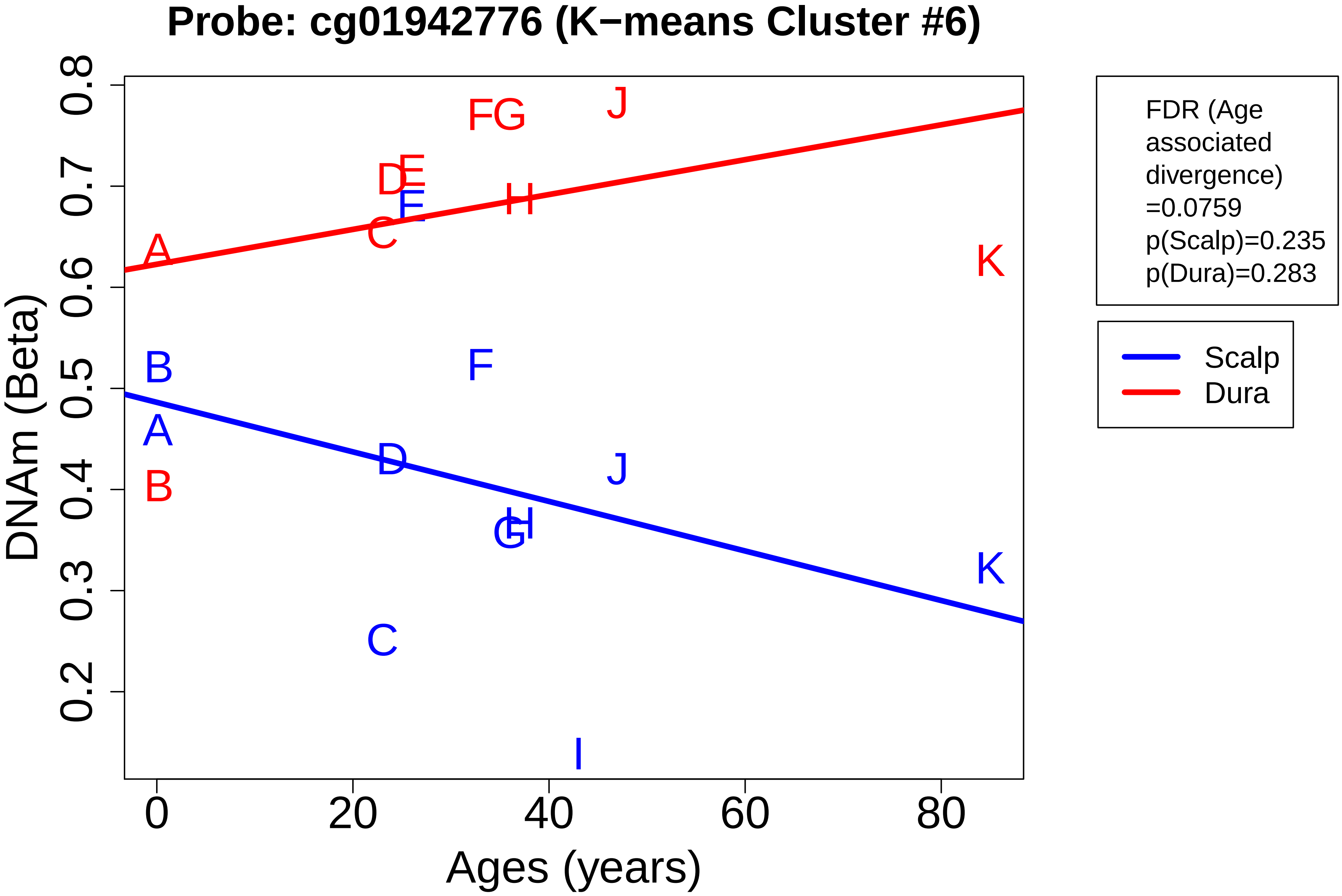

Probe: cg01942776 (K−means Cluster #6)
DNAm (Beta)
0.2
0.3
0.4
0.5
0.6
0.7
0.8
0
20
40
60
80
Ages (years)
FDR (Age associated divergence) =0.0759
p(Scalp)=0.235
p(Dura)=0.283
Scalp
Dura
A
B
C
D
E
F
G
H
I
J
K

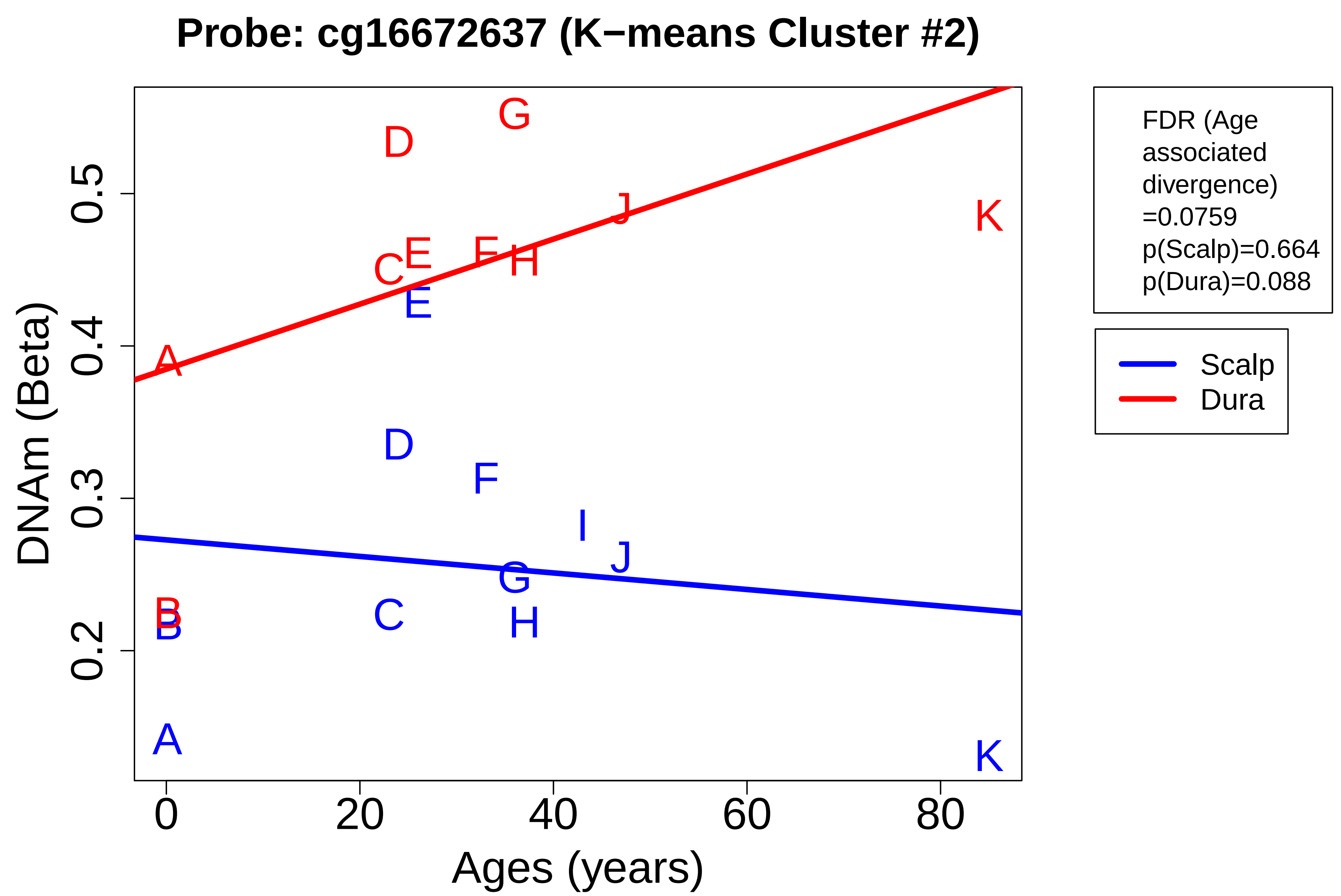

Probe: cg16672637 (K−means Cluster #2)
DNAm (Beta)
0.2
0.3
0.4
0.5
Ages (years)
0
20
40
60
80
FDR (Age associated divergence) =0.0759
p(Scalp)=0.664
p(Dura)=0.088
Scalp
Dura
A B C D E F G H I J K

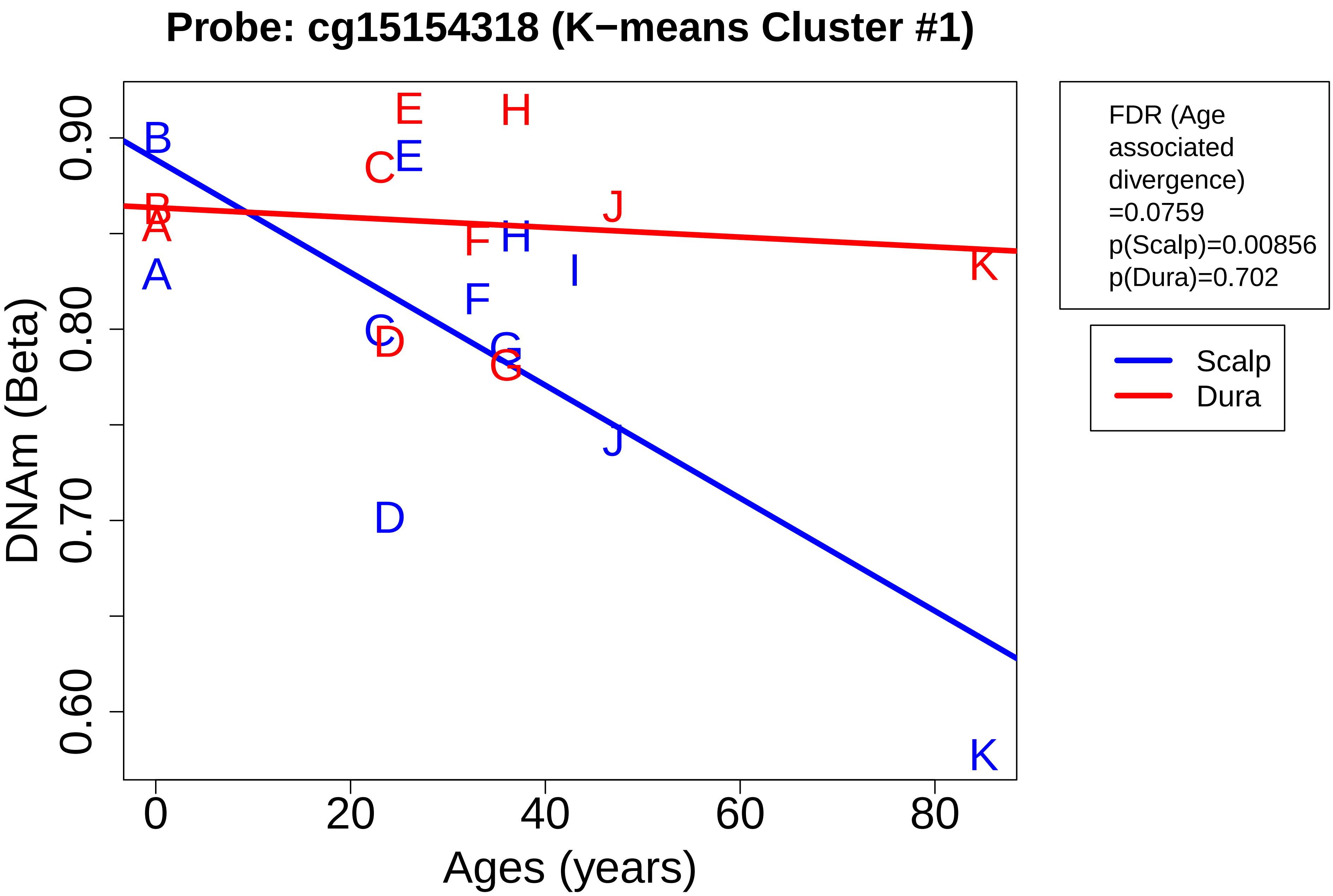
Probe: cg15154318 (K−means Cluster #1)
DNAm (Beta)
0.60
0.70
0.80
0.90
0
20
40
60
80
Ages (years)
FDR (Age associated divergence) =0.0759
p(Scalp)=0.00856
p(Dura)=0.702
Scalp
Dura

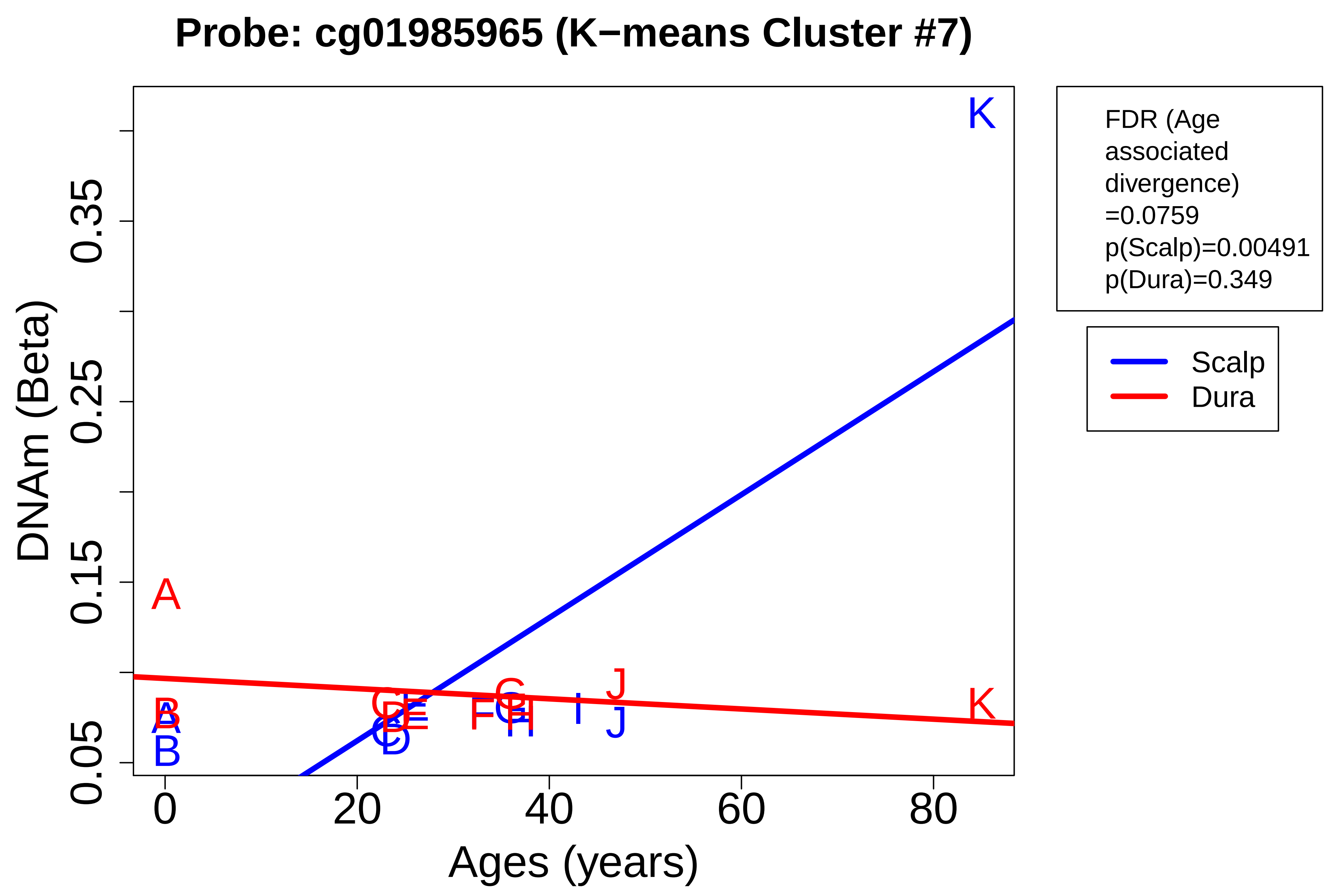

Probe: cg01985965 (K−means Cluster #7)
DNAm (Beta)
Ages (years)
0.05
0.15
0.25
0.35
0
20
40
60
80
FDR (Age associated divergence) =0.0759
p(Scalp)=0.00491
p(Dura)=0.349
Scalp
Dura

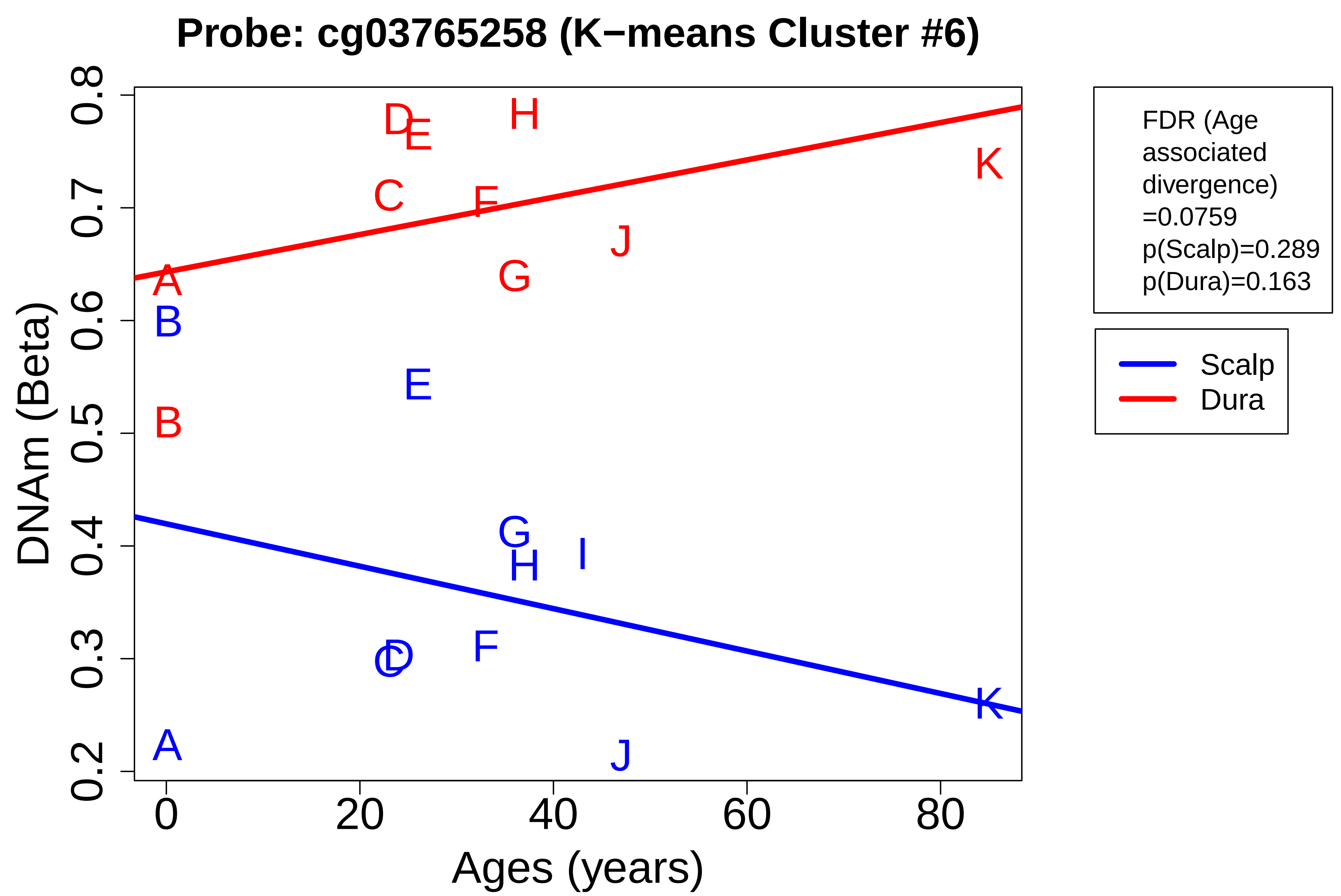

Probe: cg03765258 (K−means Cluster #6)
DNAm (Beta)
Ages (years)
0.2
0.3
0.4
0.5
0.6
0.7
0.8
0
20
40
60
80
A
B
C
D
E
F
G
H
I
J
K
FDR (Age associated divergence) =0.0759
p(Scalp)=0.289
p(Dura)=0.163
Scalp
Dura

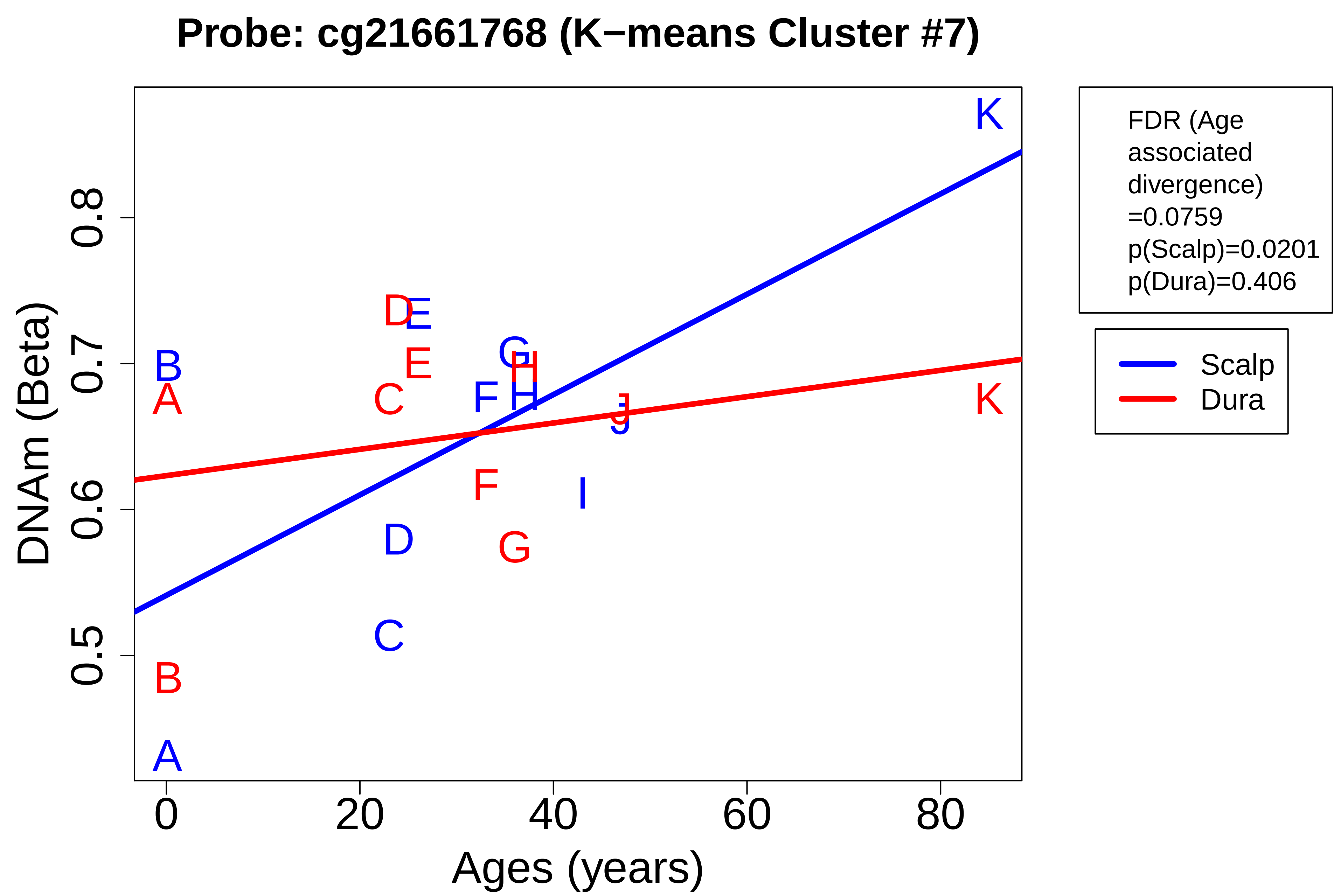

Probe: cg21661768 (K−means Cluster #7)
DNAm (Beta)
0.5
0.6
0.7
0.8
Ages (years)
0
20
40
60
80
A
B
C
D
E
F
G
H
I
J
K
FDR (Age associated divergence) =0.0759
p(Scalp)=0.0201
p(Dura)=0.406
Scalp
Dura

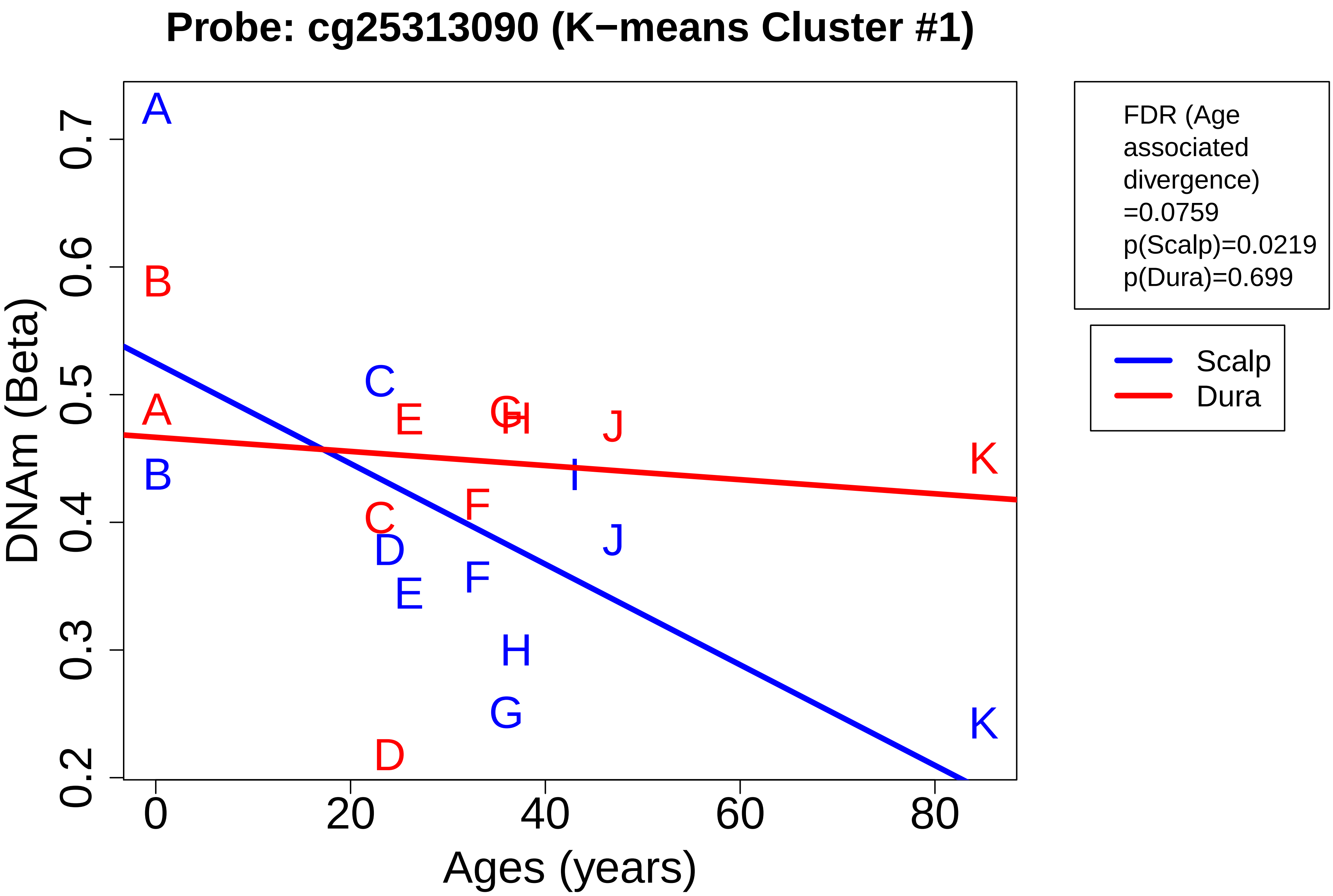

Probe: cg25313090 (K−means Cluster #1)
FDR (Age associated divergence) =0.0759
p(Scalp)=0.0219
p(Dura)=0.699
Scalp
Dura
DNAm (Beta)
Ages (years)
0.2
0.3
0.4
0.5
0.6
0.7
0
20
40
60
80
A
B
C
D
E
F
G
H
I
J
K

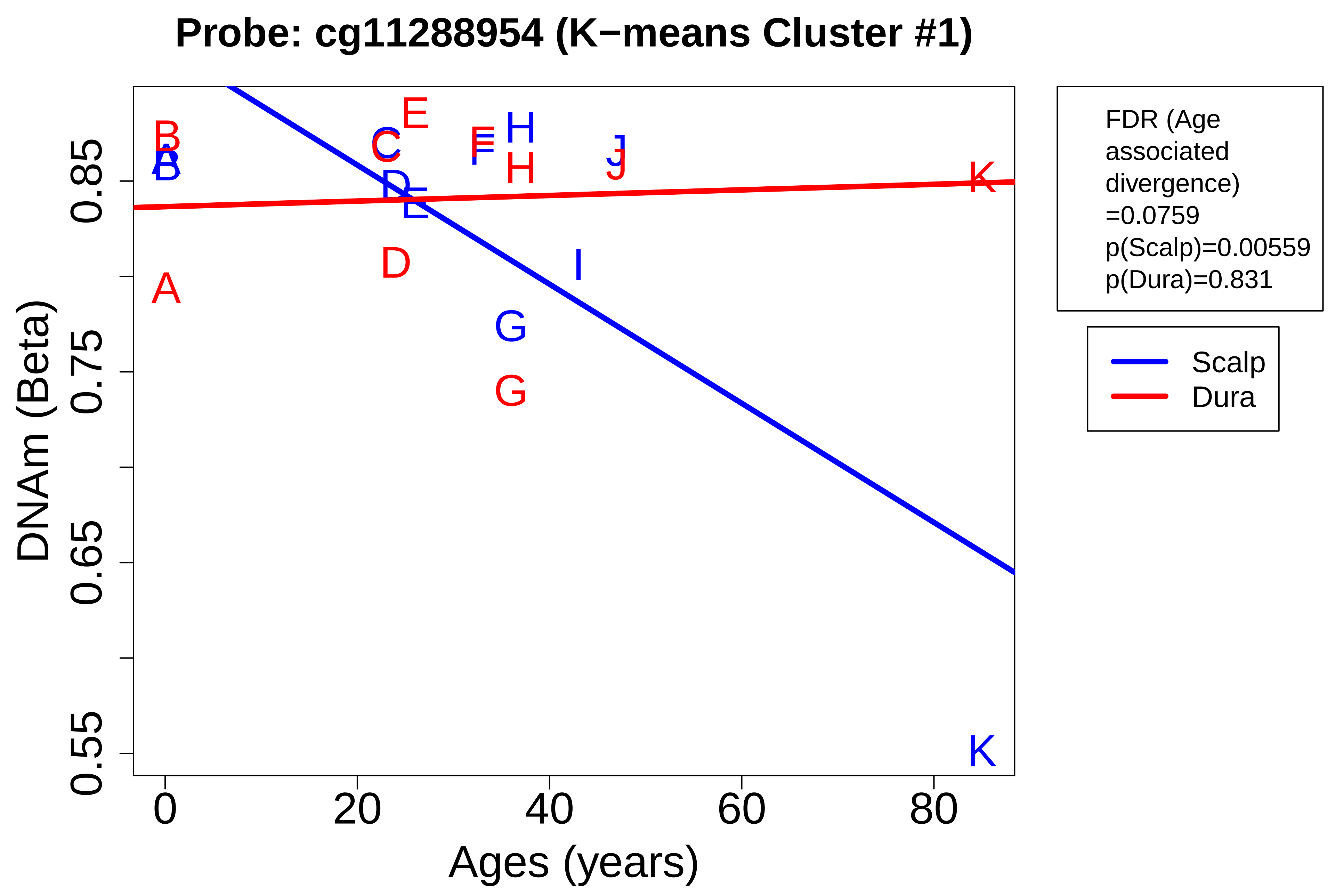

Probe: cg11288954 (K−means Cluster #1)
DNAm (Beta)
0.55
0.65
0.75
0.85
Ages (years)
0
20
40
60
80
A
B
C
D
E
F
G
H
I
J
K
FDR (Age associated divergence) =0.0759
p(Scalp)=0.00559
p(Dura)=0.831
Scalp
Dura

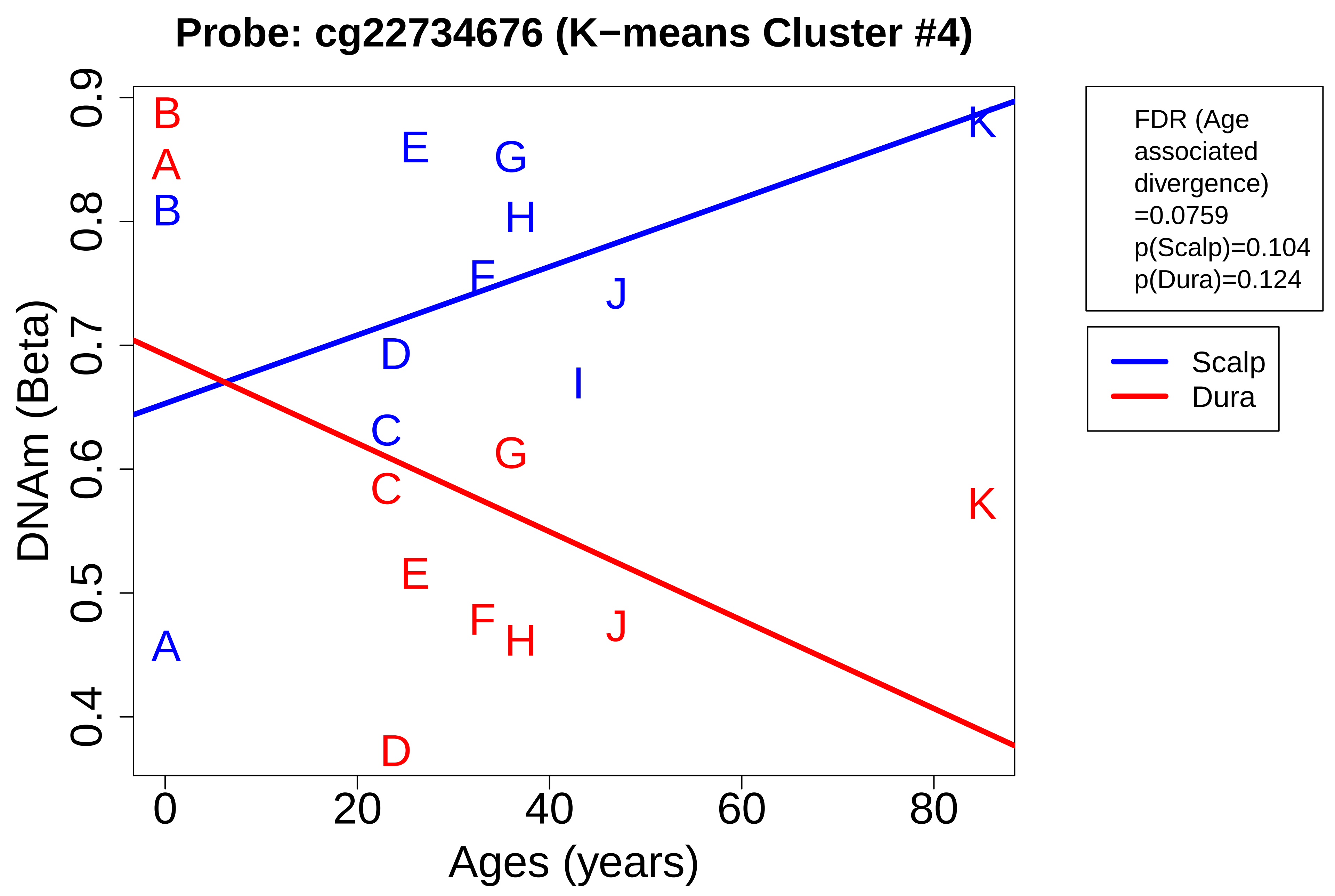

Probe: cg22734676 (K−means Cluster #4)
DNAm (Beta)
0.4
0.5
0.6
0.7
0.8
0.9
Ages (years)
0
20
40
60
80
A
B
C
D
E
F
G
H
I
J
K
FDR (Age associated divergence) =0.0759
p(Scalp)=0.104
p(Dura)=0.124
Scalp
Dura

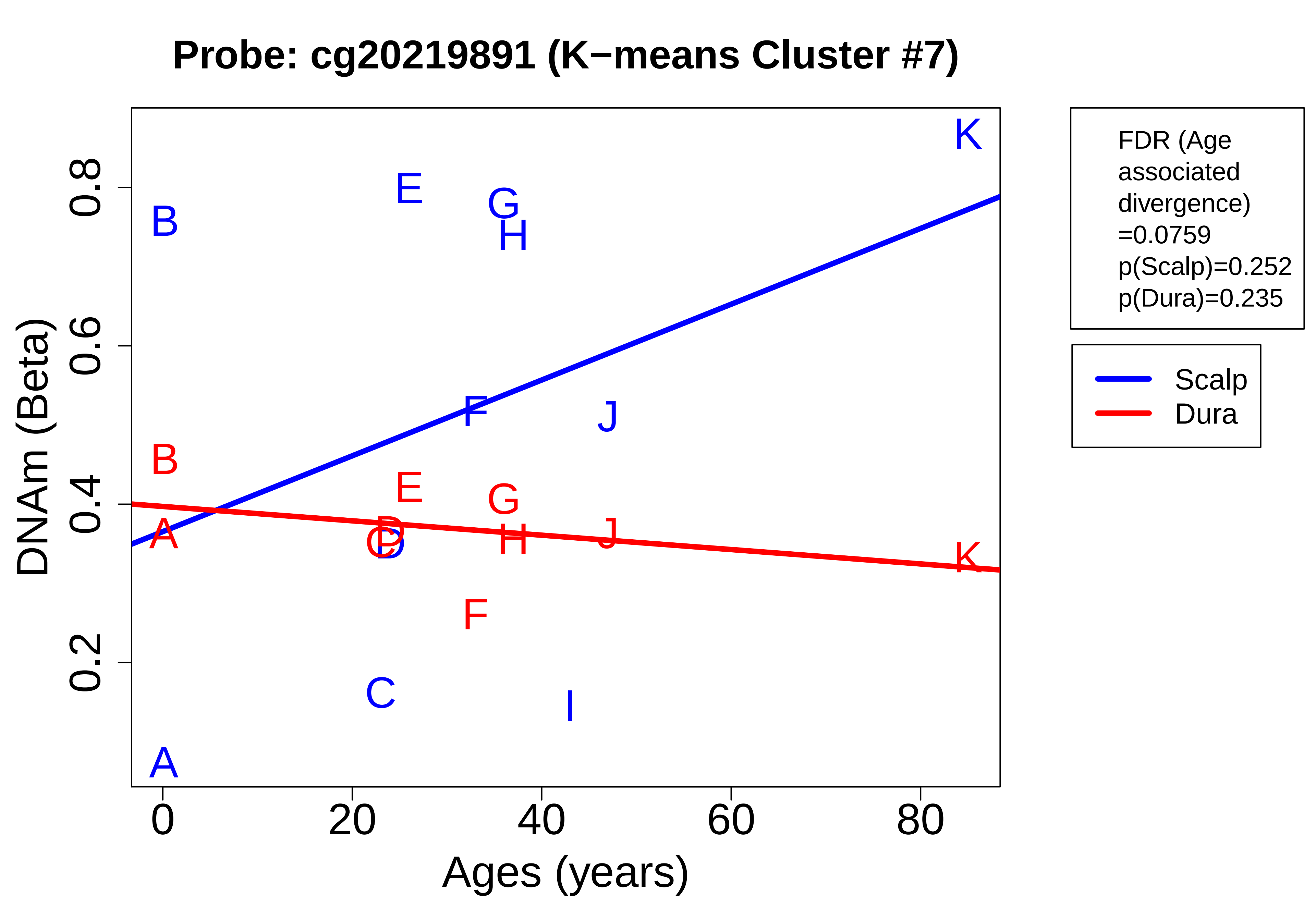

Probe: cg20219891 (K−means Cluster #7)
DNAm (Beta)
0.2
0.4
0.6
0.8
Ages (years)
0
20
40
60
80
A
B
C
D
E
F
G
H
I
J
K
FDR (Age associated divergence) =0.0759
p(Scalp)=0.252
p(Dura)=0.235
Scalp
Dura

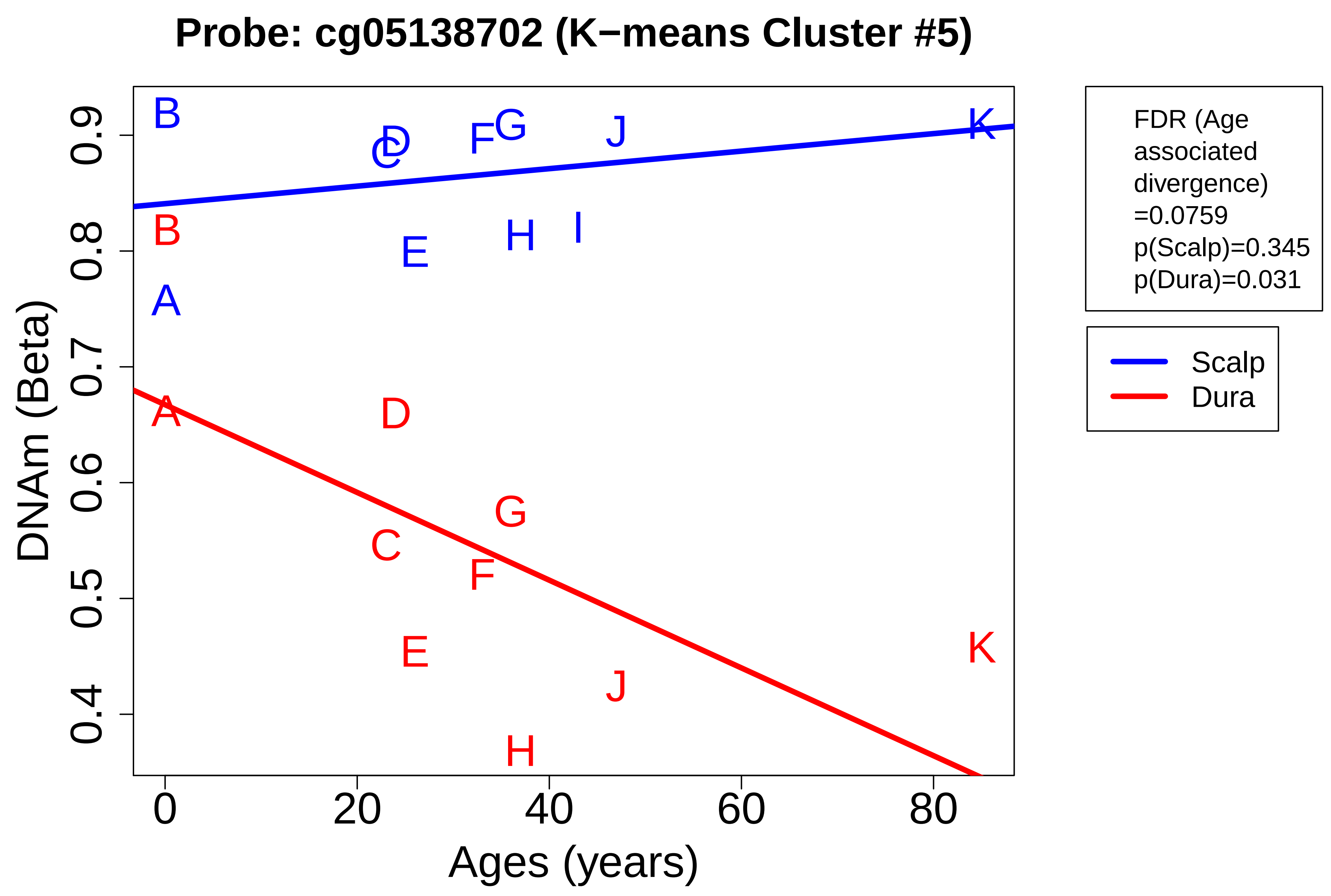

Probe: cg05138702 (K−means Cluster #5)
DNAm (Beta)
Ages (years)
0.4
0.5
0.6
0.7
0.8
0.9
0
20
40
60
80
FDR (Age associated divergence) =0.0759
p(Scalp)=0.345
p(Dura)=0.031
Scalp
Dura

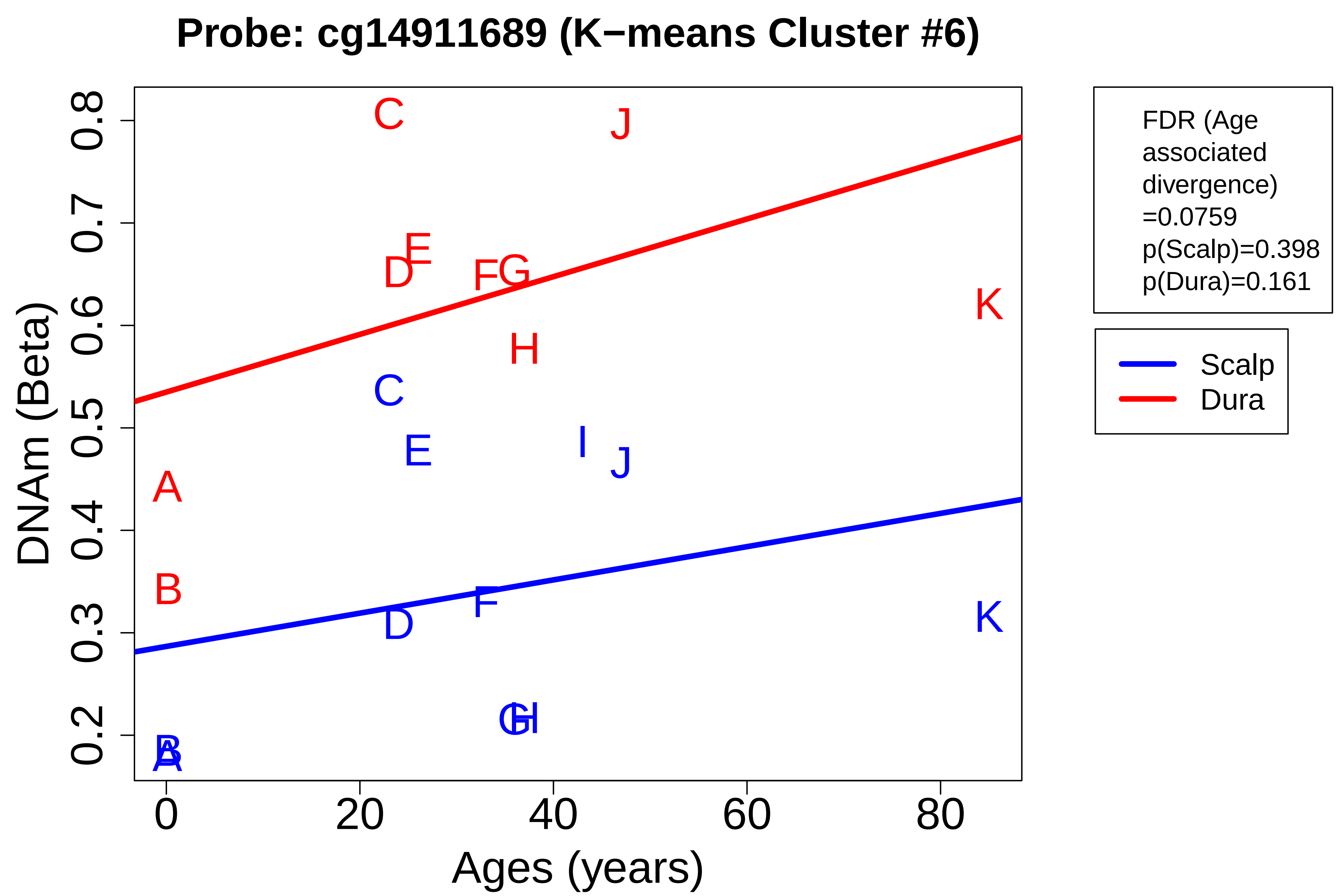

Probe: cg14911689 (K−means Cluster #6)
DNAm (Beta)
0.2
0.3
0.4
0.5
0.6
0.7
0.8
Ages (years)
0
20
40
60
80
FDR (Age associated divergence) =0.0759
p(Scalp)=0.398
p(Dura)=0.161
Scalp
Dura

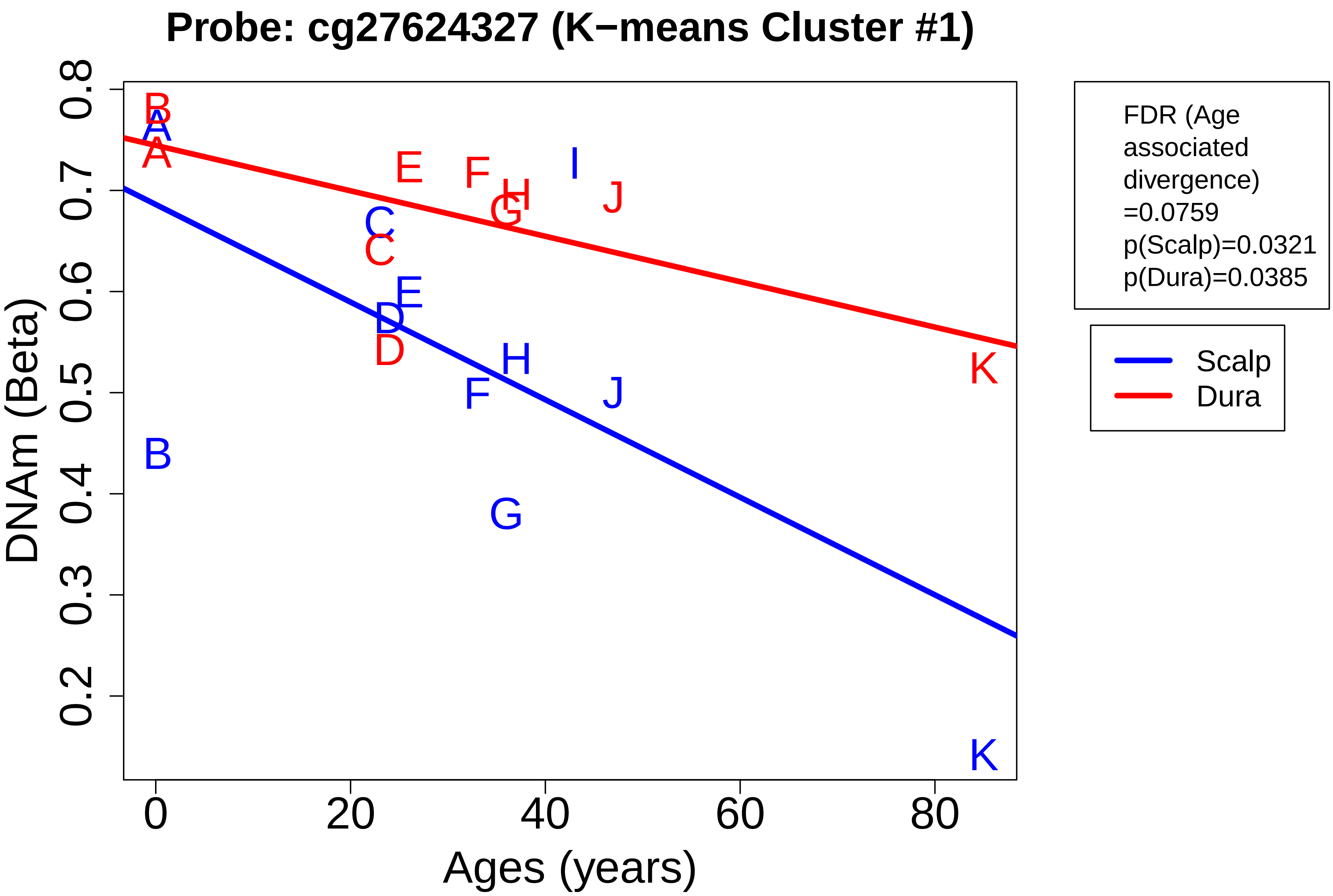

Probe: cg27624327 (K−means Cluster #1)
DNAm (Beta)
0.2
0.3
0.4
0.5
0.6
0.7
0.8
Ages (years)
0
20
40
60
80
A
B
C
D
E
F
G
H
I
J
K
FDR (Age associated divergence) =0.0759
p(Scalp)=0.0321
p(Dura)=0.0385
Scalp
Dura

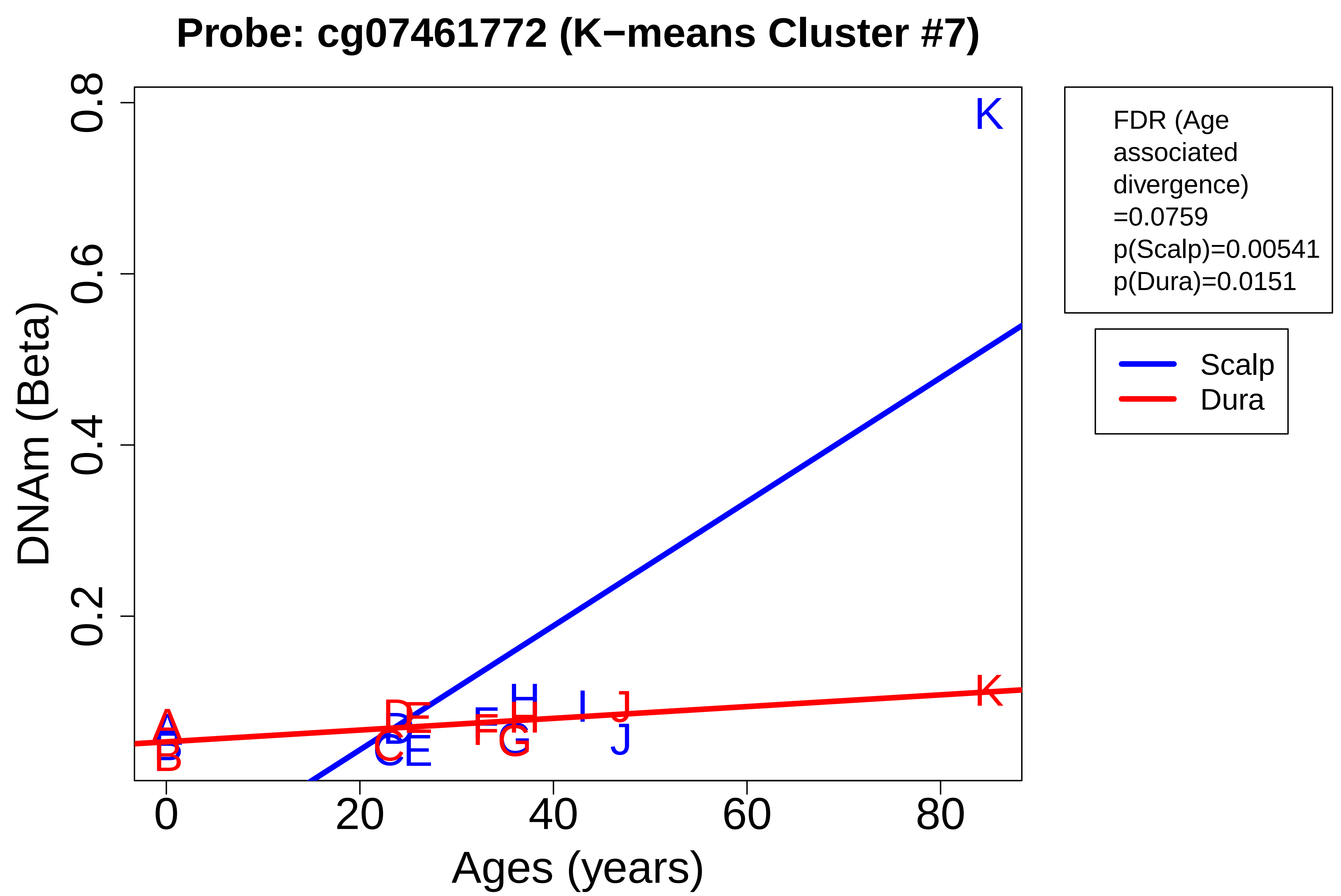

Probe: cg07461772 (K−means Cluster #7)
DNAm (Beta)
Ages (years)
0.2
0.4
0.6
0.8
0
20
40
60
80
FDR (Age associated divergence) =0.0759
p(Scalp)=0.00541
p(Dura)=0.0151
Scalp
Dura
A
B
C
D
E
F
G
H
I
J
K

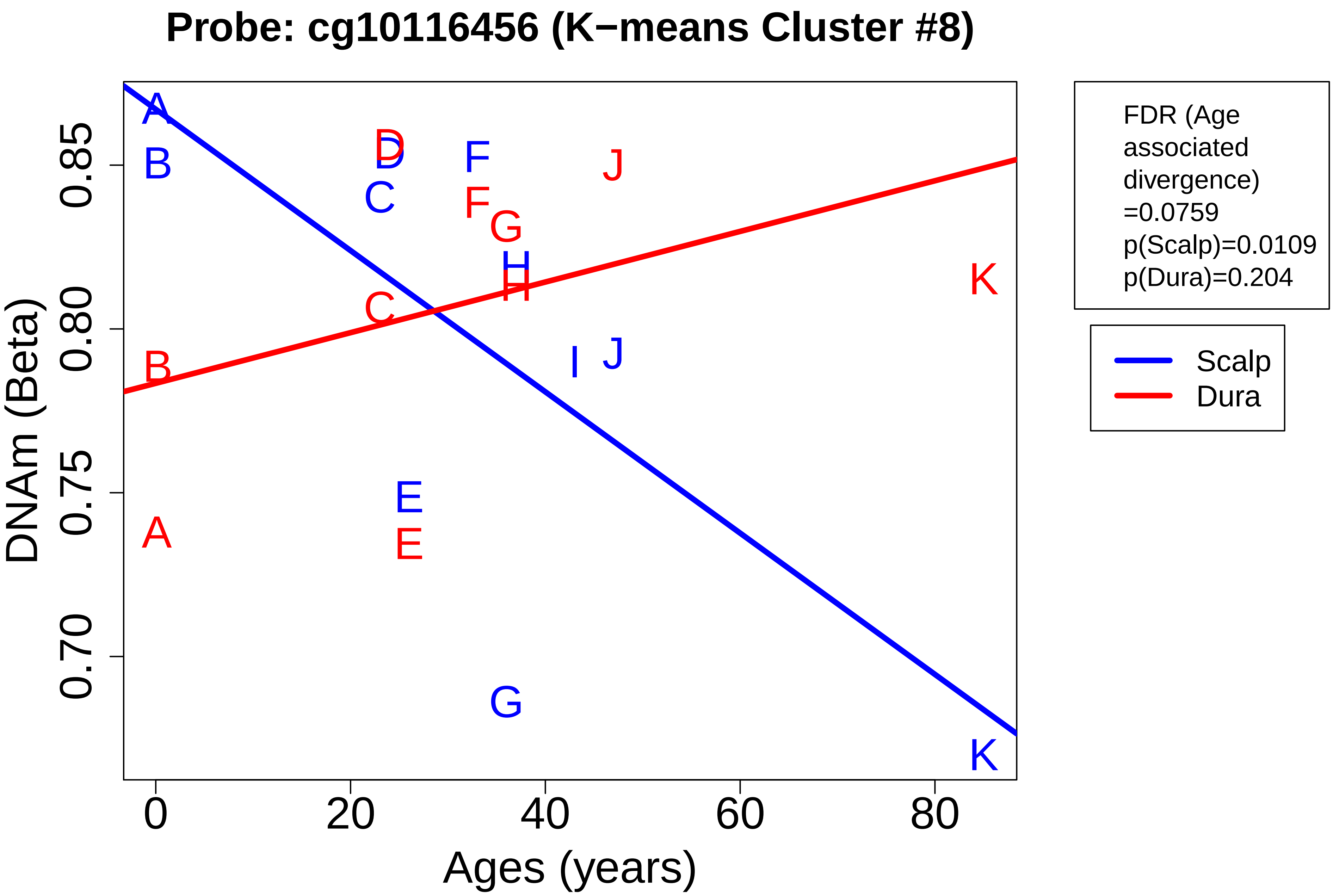
Probe: cg10116456 (K−means Cluster #8)
DNAm (Beta)
0.70
0.75
0.80
0.85
Ages (years)
0
20
40
60
80
A
B
C
D
E
F
G
H
I
J
K
FDR (Age associated divergence) =0.0759
p(Scalp)=0.0109
p(Dura)=0.204
Scalp
Dura

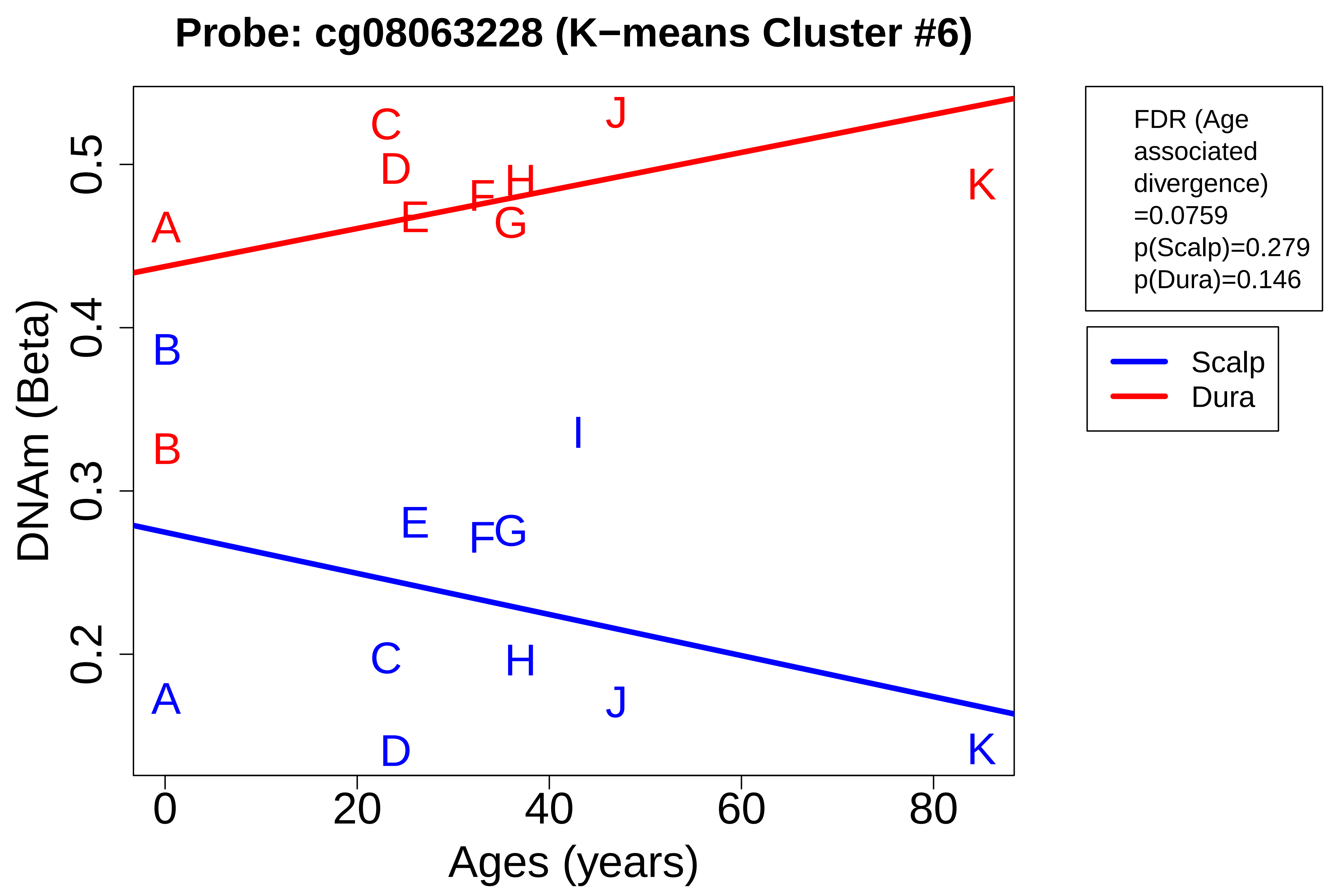

Probe: cg08063228 (K−means Cluster #6)
DNAm (Beta)
0.2
0.3
0.4
0.5
0
20
40
60
80
Ages (years)
A
B
C
D
E
F
G
H
I
J
K
FDR (Age associated divergence) =0.0759
p(Scalp)=0.279
p(Dura)=0.146
Scalp
Dura

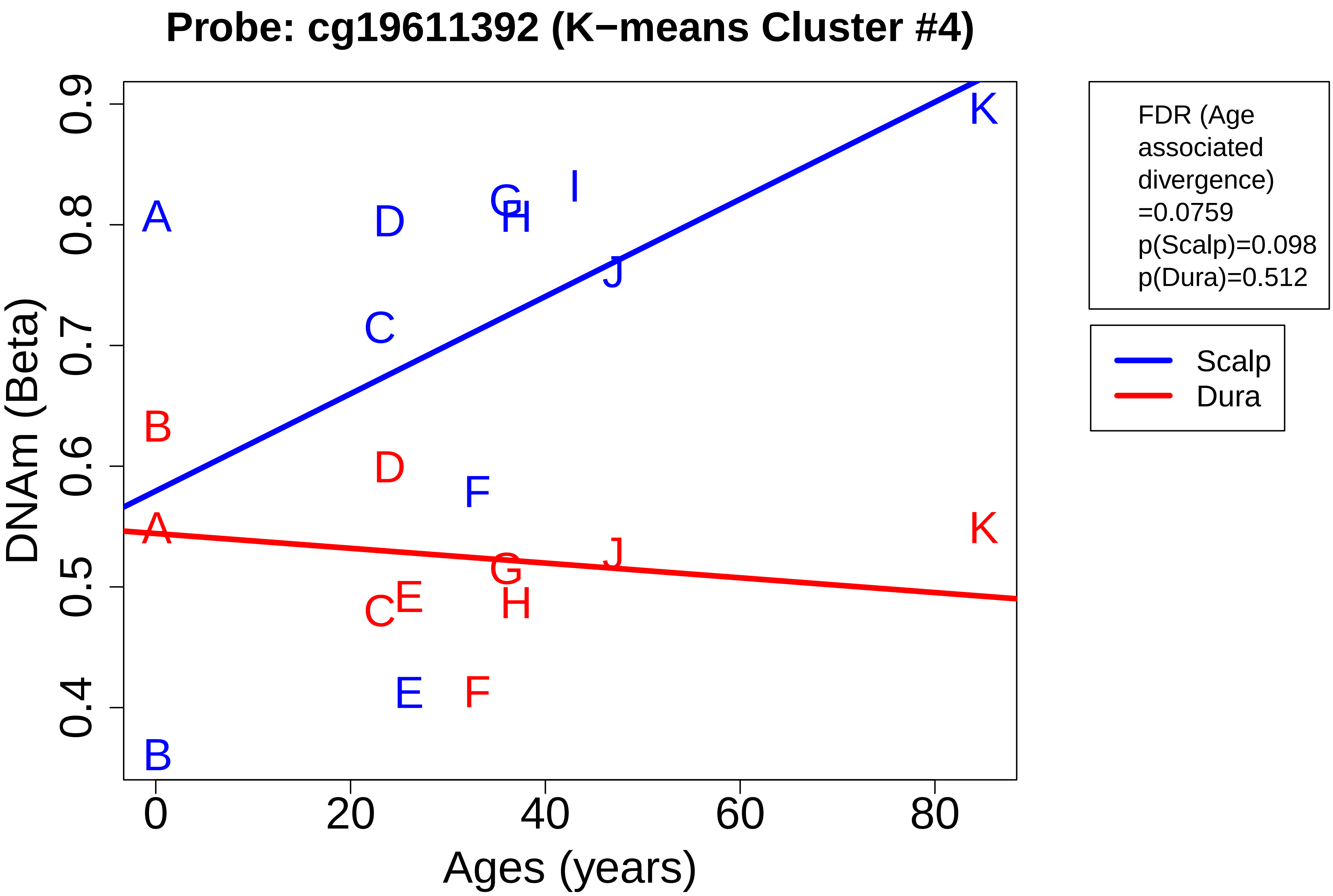

Probe: cg19611392 (K−means Cluster #4)
DNAm (Beta)
0.4
0.5
0.6
0.7
0.8
0.9
0
20
40
60
80
Ages (years)
A
B
C
D
E
F
G
H
I
J
K
FDR (Age associated divergence) =0.0759
p(Scalp)=0.098
p(Dura)=0.512
Scalp
Dura

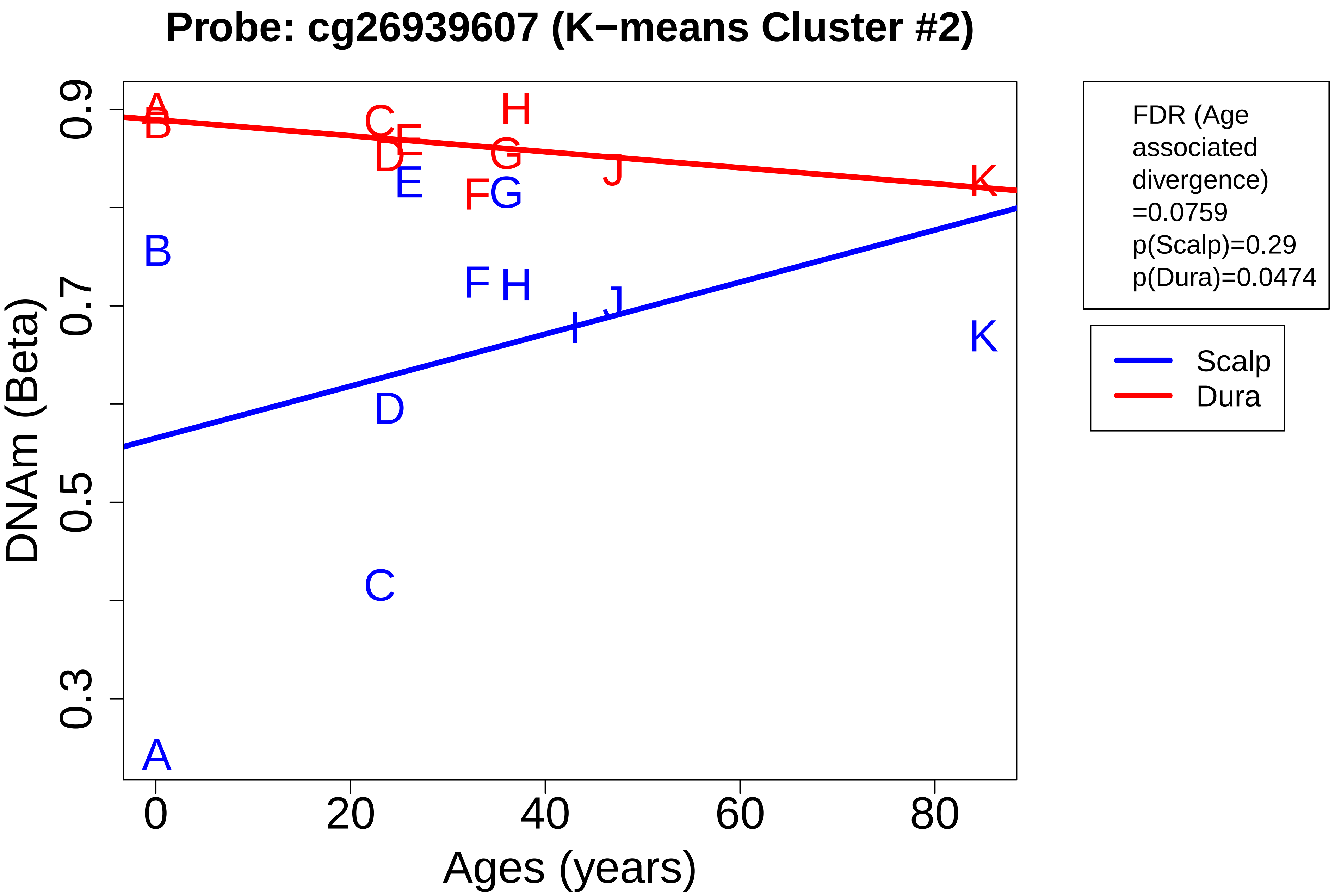

Probe: cg26939607 (K−means Cluster #2)
DNAm (Beta)
0.3
0.5
0.7
0.9
Ages (years)
0
20
40
60
80
A
B
C
D
E
F
G
H
I
J
K
FDR (Age associated divergence) =0.0759
p(Scalp)=0.29
p(Dura)=0.0474
Scalp
Dura

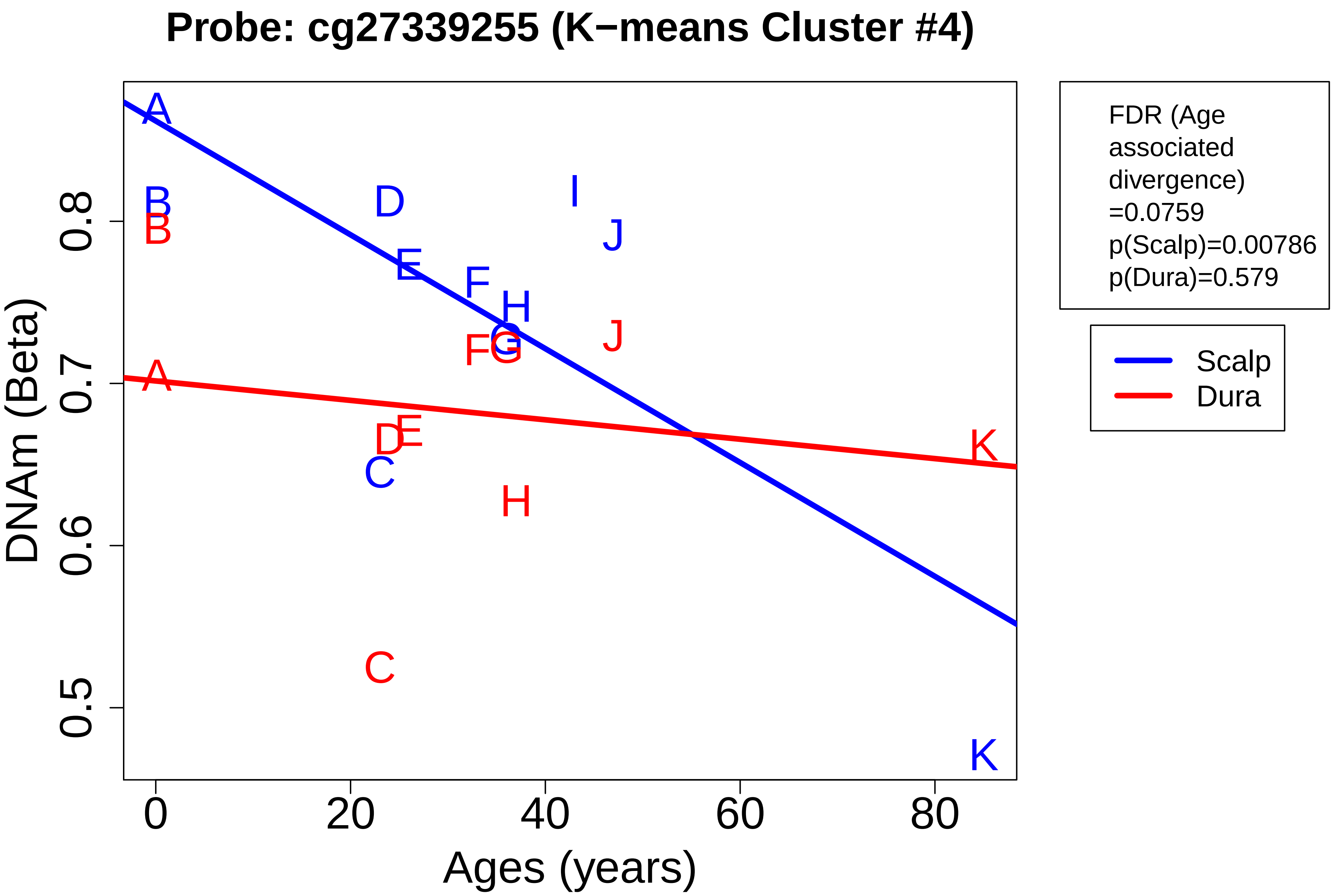

Probe: cg27339255 (K−means Cluster #4)
FDR (Age associated divergence) =0.0759
p(Scalp)=0.00786
p(Dura)=0.579
Scalp
Dura
DNAm (Beta)
0.5
0.6
0.7
0.8
0
20
40
60
80
Ages (years)

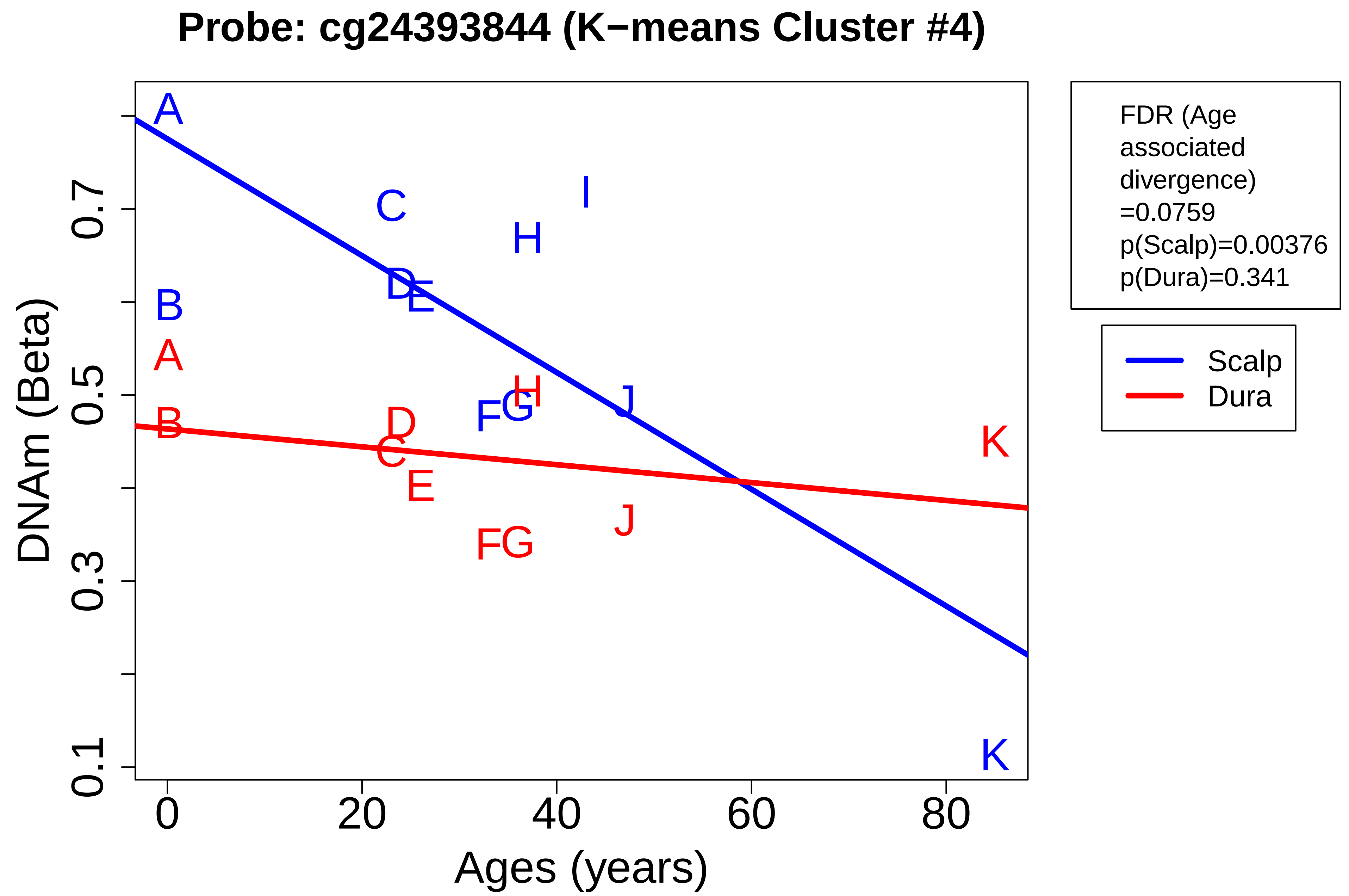

Probe: cg24393844 (K−means Cluster #4)
DNAm (Beta)
Ages (years)
0.1
0.3
0.5
0.7
0
20
40
60
80
FDR (Age associated divergence) =0.0759
p(Scalp)=0.00376
p(Dura)=0.341
Scalp
Dura

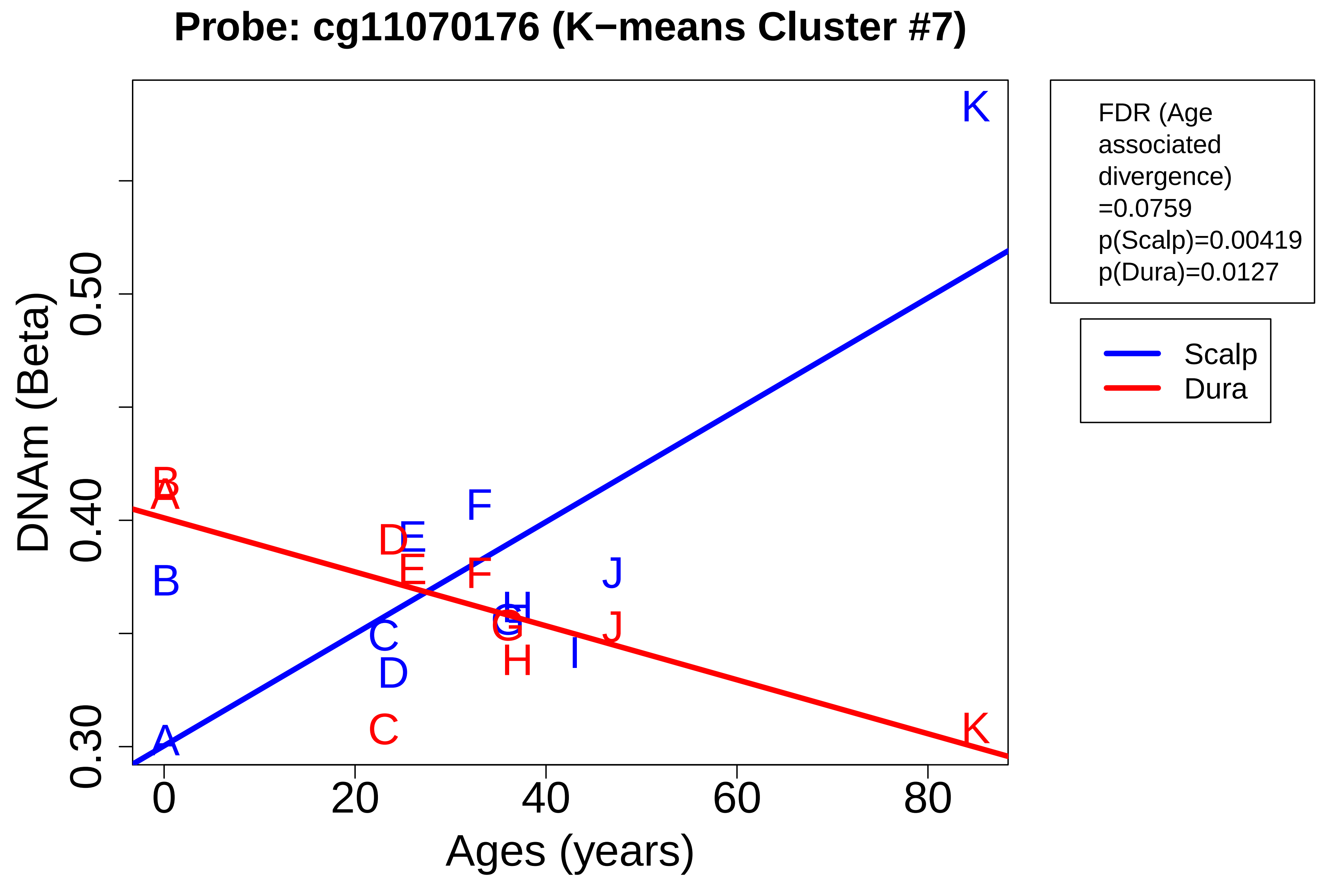

Probe: cg11070176 (K−means Cluster #7)
DNAm (Beta)
0.30
0.40
0.50
0
20
40
60
80
Ages (years)
FDR (Age associated divergence) =0.0759
p(Scalp)=0.00419
p(Dura)=0.0127
Scalp
Dura

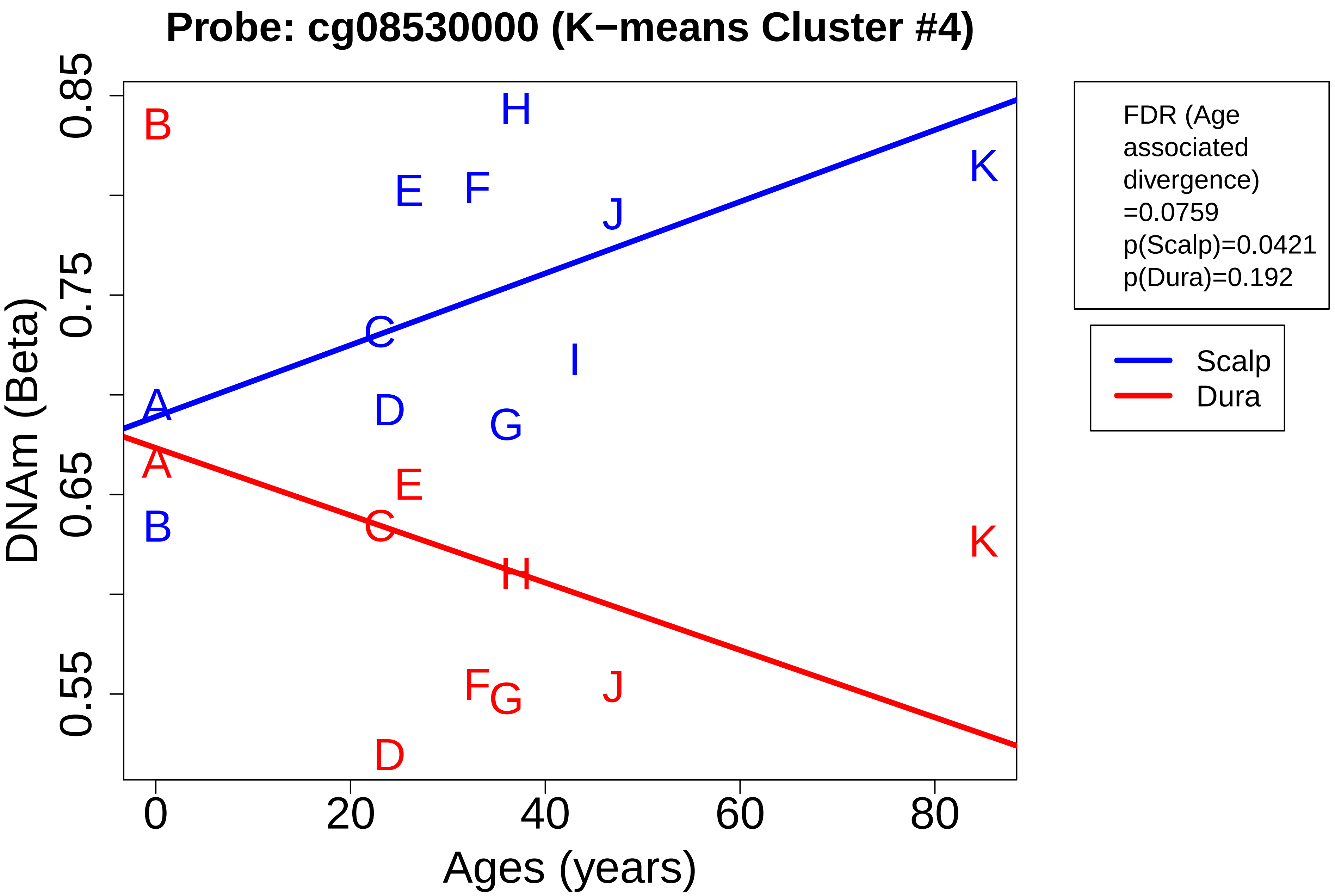

Probe: cg08530000 (K−means Cluster #4)
DNAm (Beta)
0.85
0.75
0.65
0.55
Ages (years)
0
20
40
60
80
FDR (Age associated divergence) =0.0759
p(Scalp)=0.0421
p(Dura)=0.192
Scalp
Dura
A
B
C
D
E
F
G
H
I
J
K

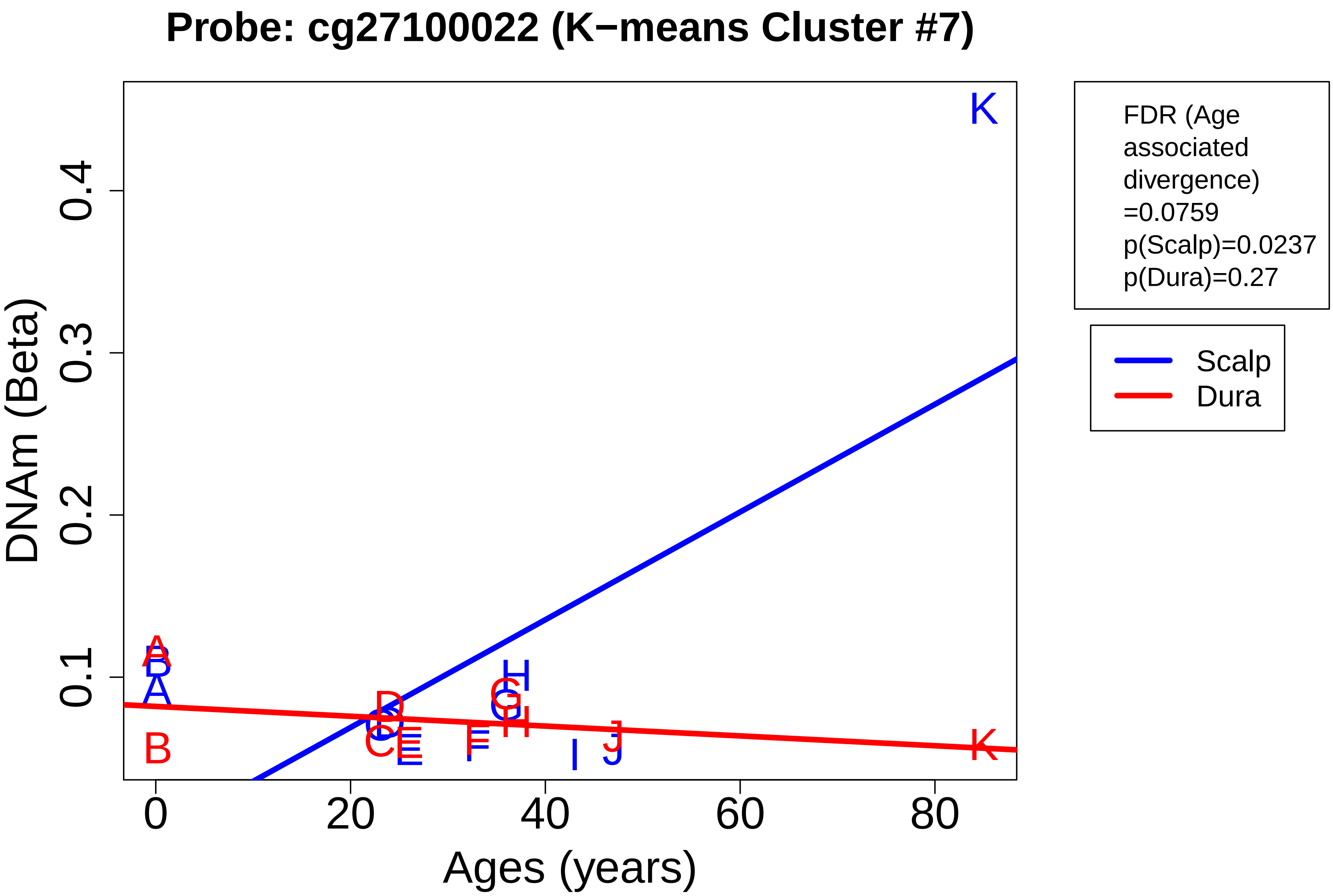

Probe: cg27100022 (K−means Cluster #7)
DNAm (Beta)
0.1
0.2
0.3
0.4
Ages (years)
0
20
40
60
80
FDR (Age associated divergence) =0.0759
p(Scalp)=0.0237
p(Dura)=0.27
Scalp
Dura

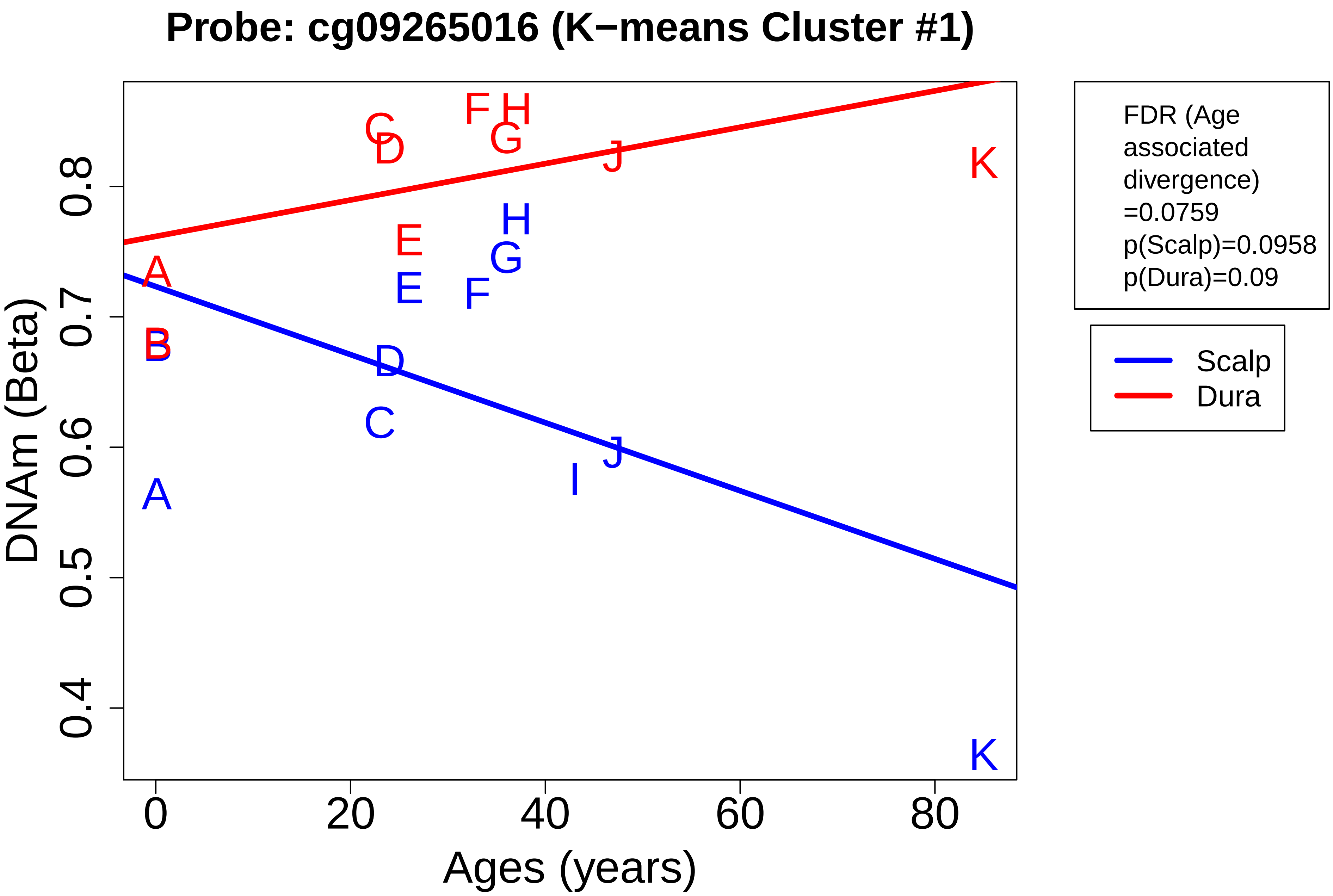

Probe: cg09265016 (K−means Cluster #1)
FDR (Age associated divergence) =0.0759
p(Scalp)=0.0958
p(Dura)=0.09
Scalp
Dura
DNAm (Beta)
0.4
0.5
0.6
0.7
0.8
0
20
40
60
80
Ages (years)
A
B
C
D
E
F
G
H
I
J
K

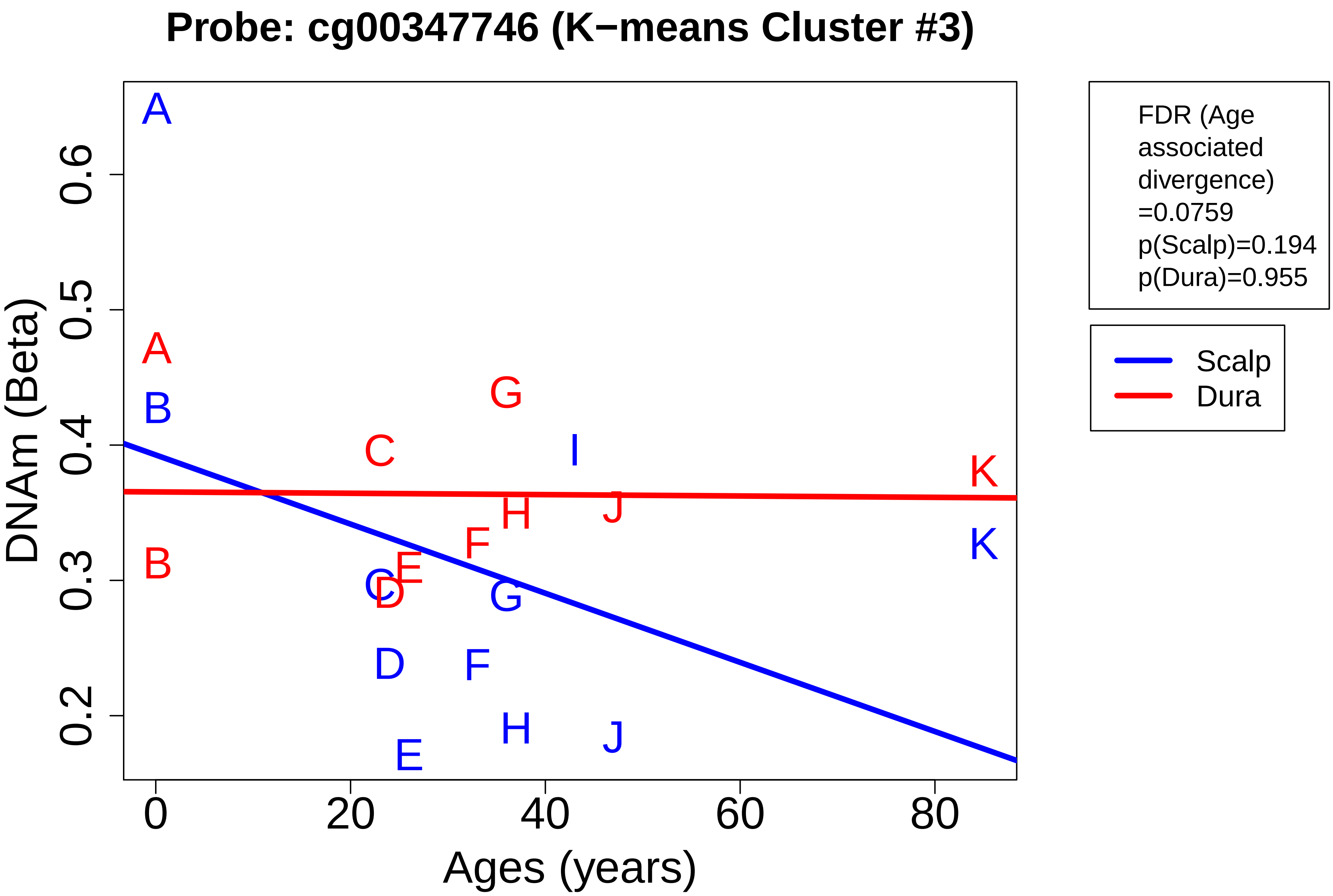

Probe: cg00347746 (K−means Cluster #3)
DNAm (Beta)
0.2
0.3
0.4
0.5
0.6
Ages (years)
0
20
40
60
80
FDR (Age associated divergence) =0.0759
p(Scalp)=0.194
p(Dura)=0.955
Scalp
Dura

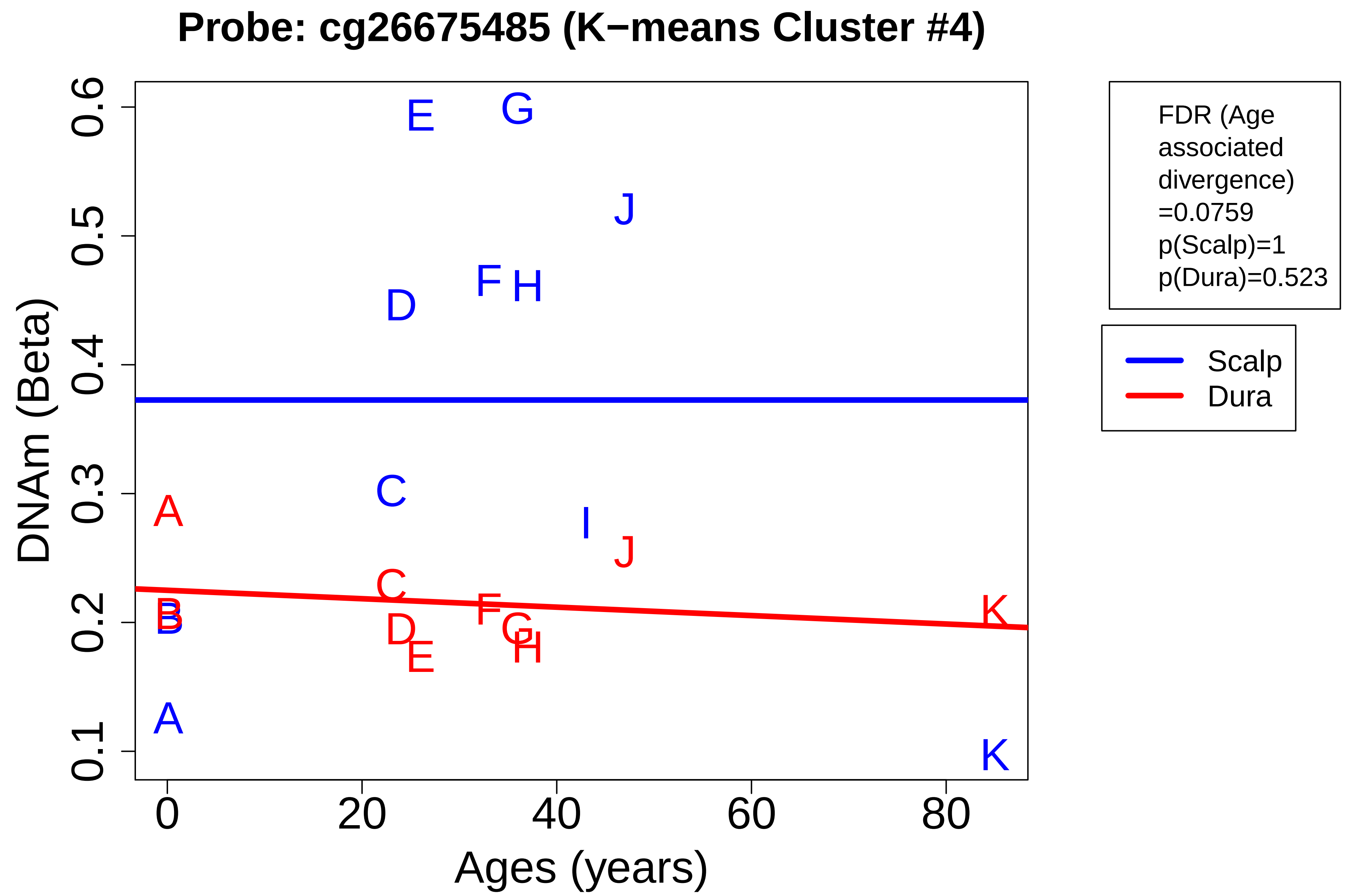

Probe: cg26675485 (K−means Cluster #4)
DNAm (Beta)
0.1
0.2
0.3
0.4
0.5
0.6
0
20
40
60
80
Ages (years)
E
G
J
F
H
D
C
A
I
J
C
F
B
B
D
G
E
H
K
A
K
FDR (Age associated divergence) =0.0759
p(Scalp)=1
p(Dura)=0.523
Scalp
Dura

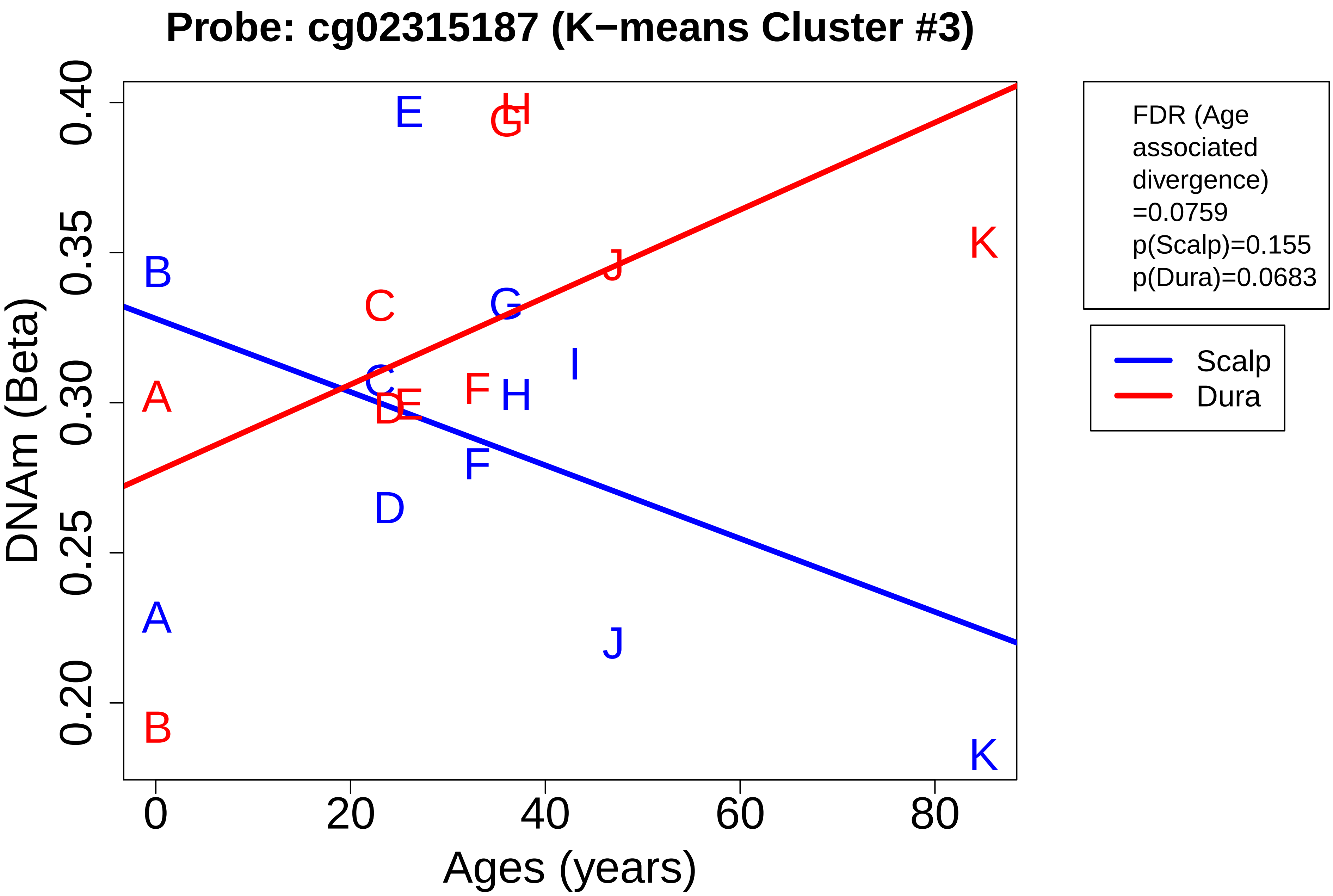
Probe: cg02315187 (K−means Cluster #3)
DNAm (Beta)
0.20
0.25
0.30
0.35
0.40
Ages (years)
0
20
40
60
80
FDR (Age associated divergence) =0.0759
p(Scalp)=0.155
p(Dura)=0.0683
Scalp
Dura

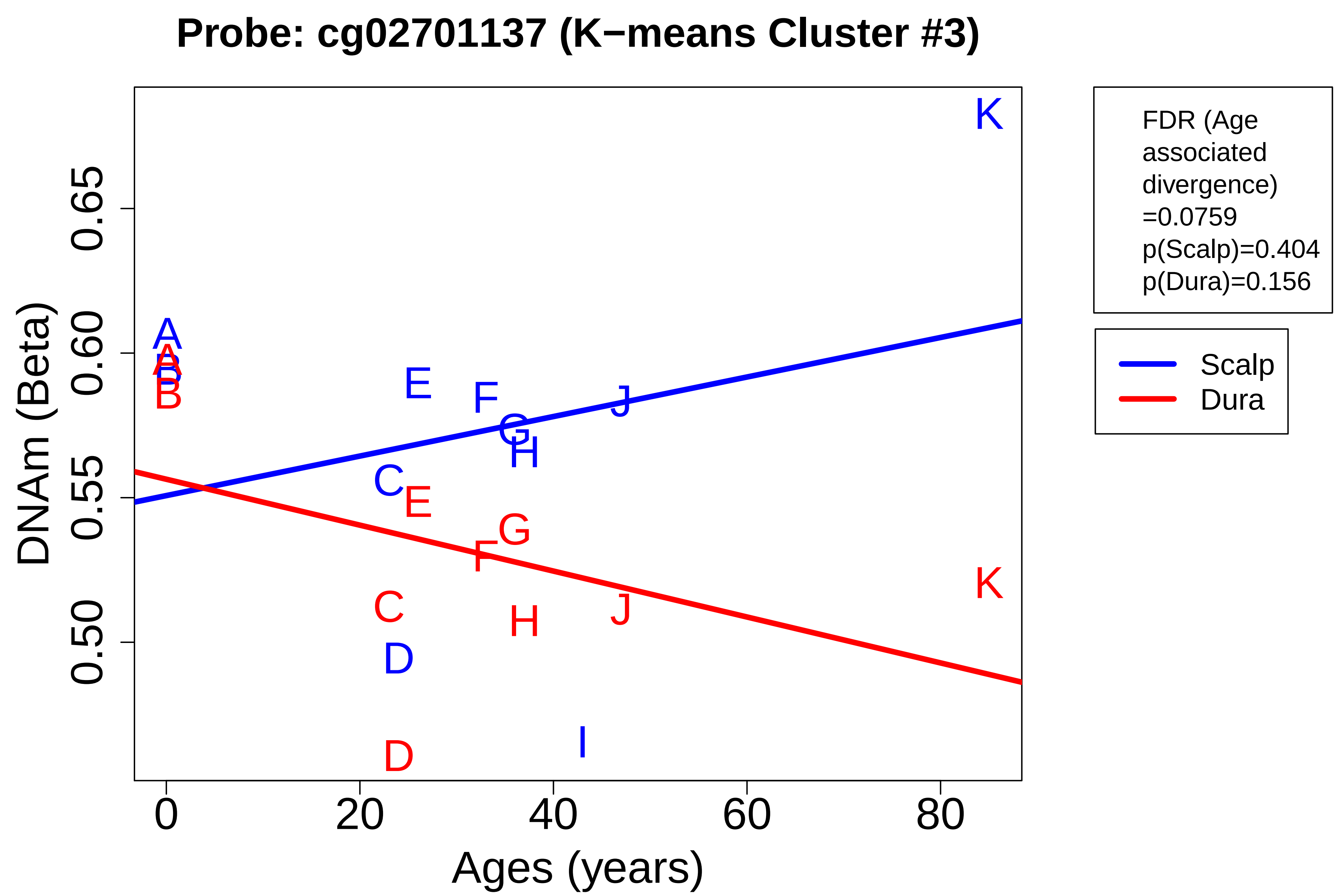

Probe: cg02701137 (K−means Cluster #3)
FDR (Age associated divergence) =0.0759
p(Scalp)=0.404
p(Dura)=0.156
Scalp
Dura
DNAm (Beta)
0.50
0.55
0.60
0.65
Ages (years)
0
20
40
60
80
A
B
C
D
E
F
G
H
I
J
K

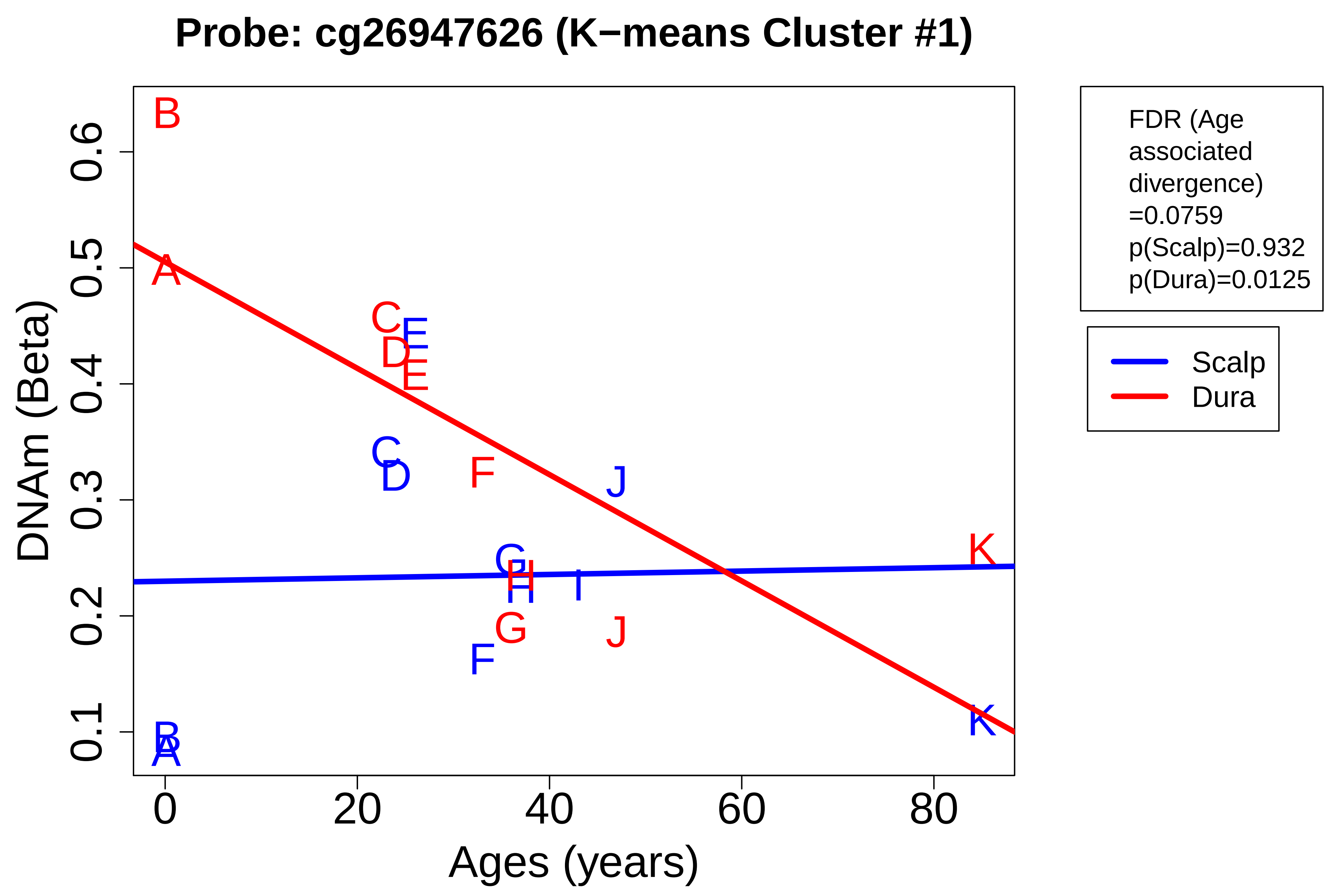

Probe: cg26947626 (K−means Cluster #1)
DNAm (Beta)
0.1
0.2
0.3
0.4
0.5
0.6
Ages (years)
0
20
40
60
80
B
A
C
D
E
F
G
H
I
J
K
FDR (Age associated divergence) =0.0759
p(Scalp)=0.932
p(Dura)=0.0125
Scalp
Dura

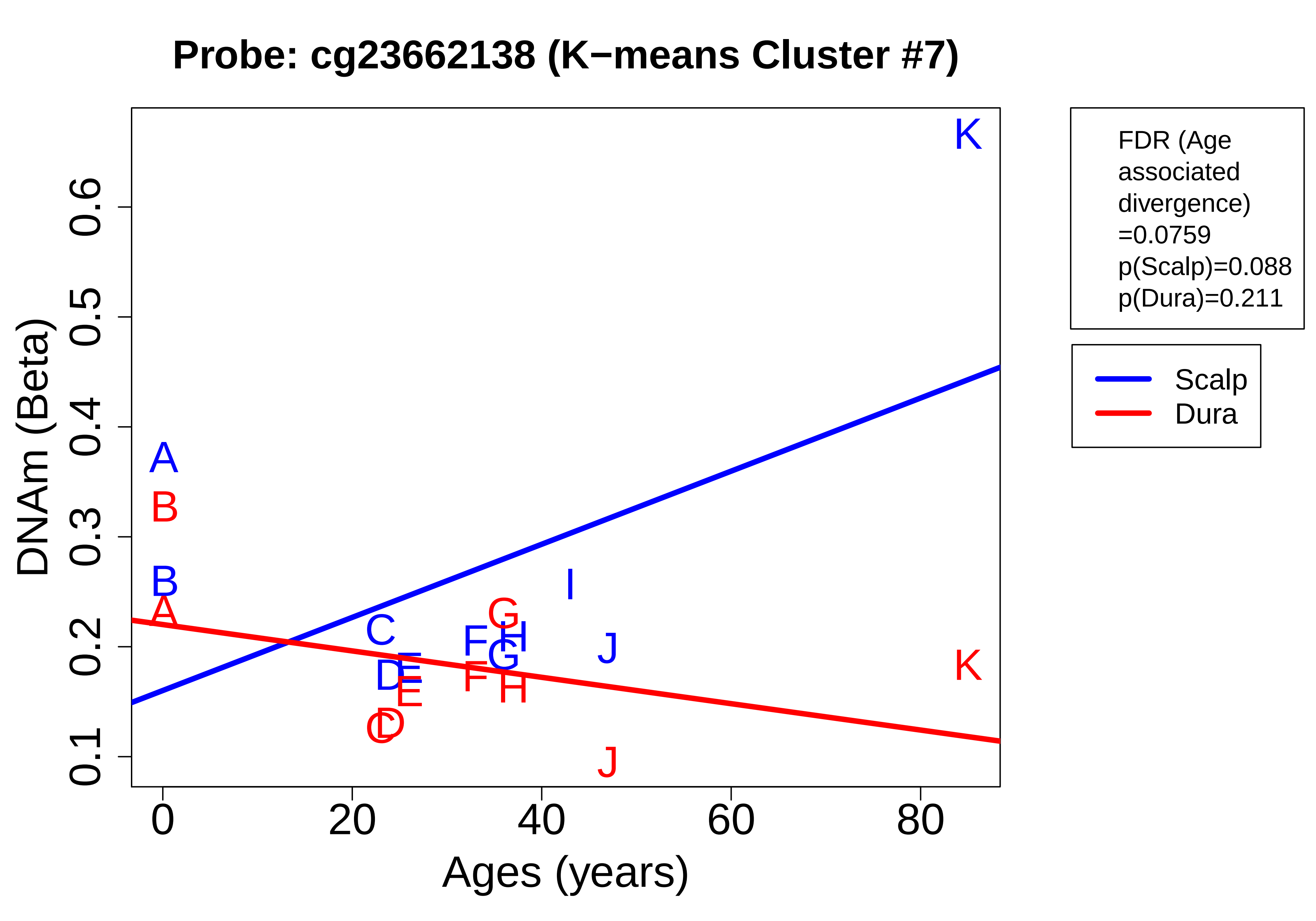

Probe: cg23662138 (K−means Cluster #7)
DNAm (Beta)
0.1
0.2
0.3
0.4
0.5
0.6
0
20
40
60
80
Ages (years)
FDR (Age associated divergence) =0.0759
p(Scalp)=0.088
p(Dura)=0.211
Scalp
Dura

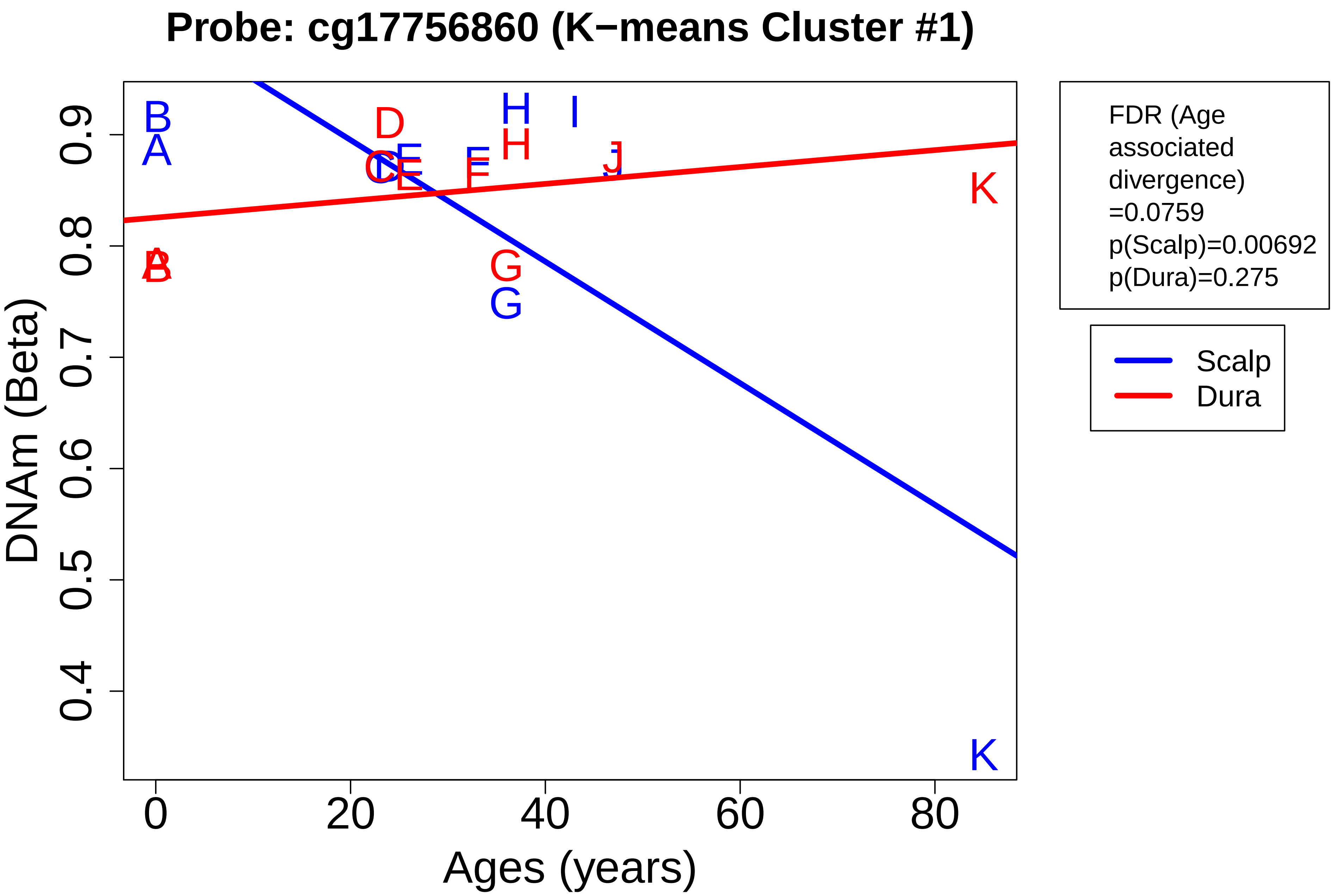

Probe: cg17756860 (K−means Cluster #1)
DNAm (Beta)
0.4
0.5
0.6
0.7
0.8
0.9
0
20
40
60
80
Ages (years)
FDR (Age associated divergence) =0.0759
p(Scalp)=0.00692
p(Dura)=0.275
Scalp
Dura

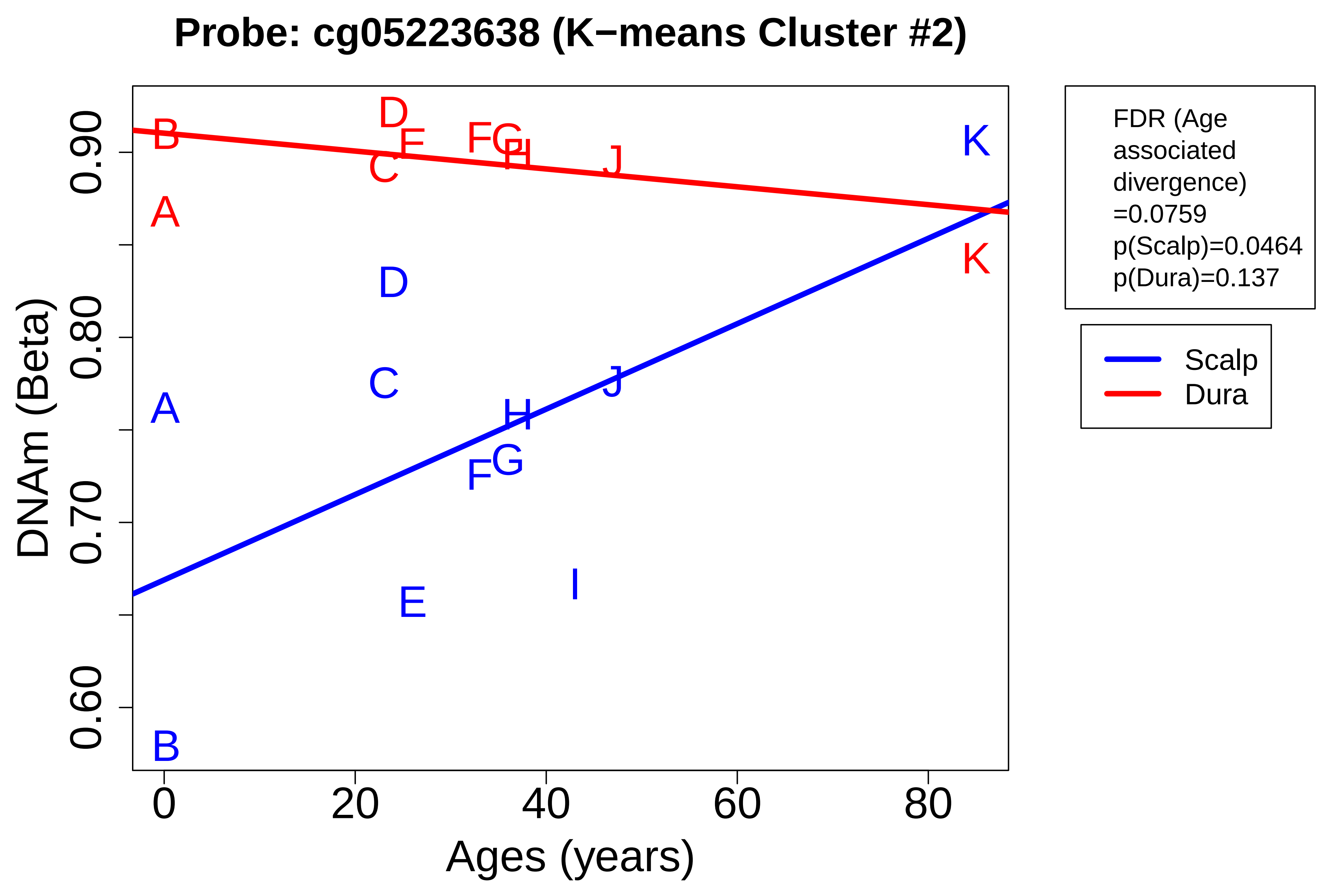

Probe: cg05223638 (K−means Cluster #2)
DNAm (Beta)
0.60
0.70
0.80
0.90
Ages (years)
0
20
40
60
80
FDR (Age associated divergence) =0.0759
p(Scalp)=0.0464
p(Dura)=0.137
Scalp
Dura
A
B
C
D
E
F
G
H
I
J
K

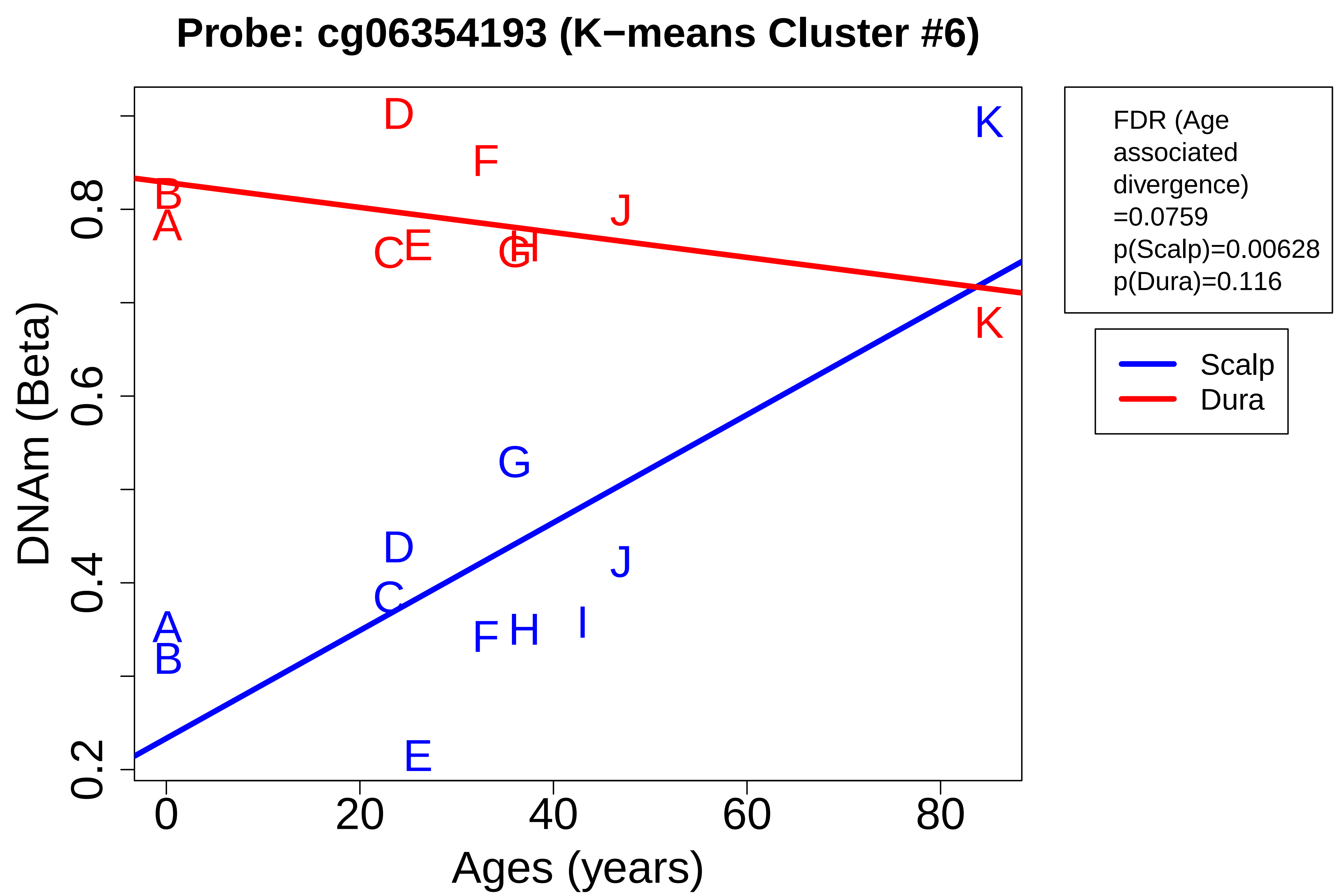

Probe: cg06354193 (K−means Cluster #6)
DNAm (Beta)
0.2
0.4
0.6
0.8
0
20
40
60
80
Ages (years)
FDR (Age associated divergence) =0.0759
p(Scalp)=0.00628
p(Dura)=0.116
Scalp
Dura

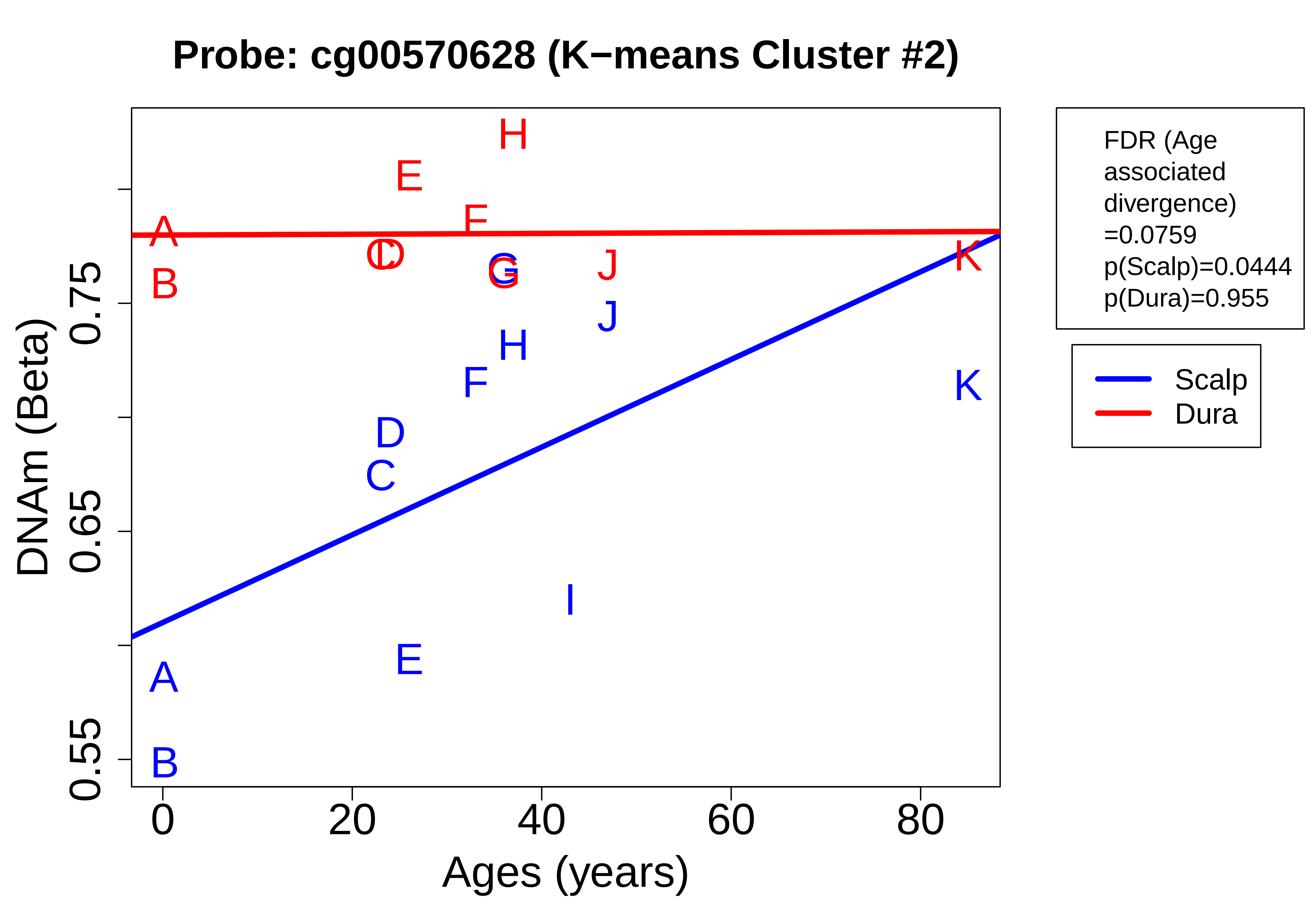
Probe: cg00570628 (K−means Cluster #2)
DNAm (Beta)
0.55
0.65
0.75
0
20
40
60
80
Ages (years)
FDR (Age associated divergence) =0.0759
p(Scalp)=0.0444
p(Dura)=0.955
Scalp
Dura
A
B
C
D
E
F
G
H
I
J
K

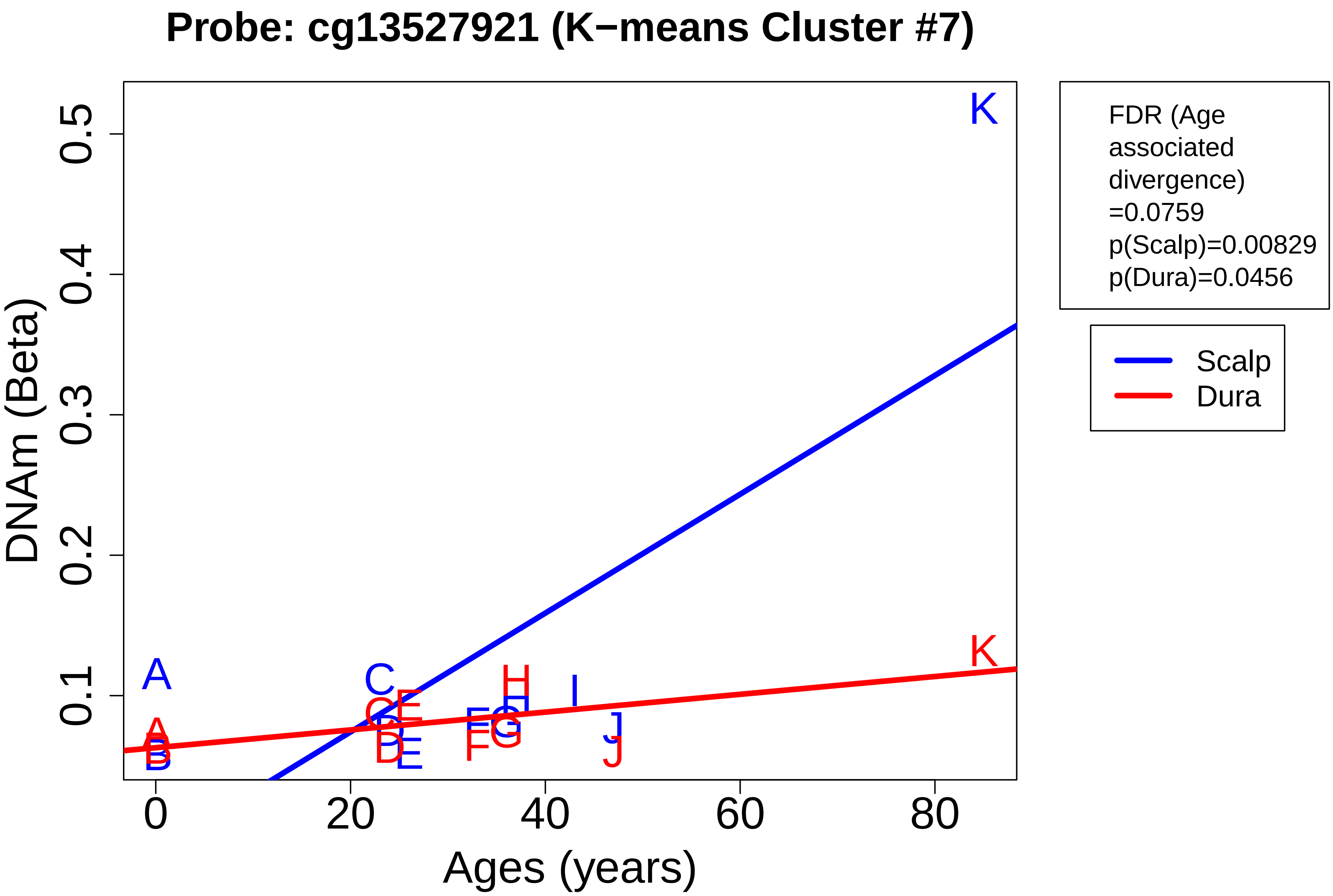
Probe: cg13527921 (K−means Cluster #7)
DNAm (Beta)
0.1
0.2
0.3
0.4
0.5
0
20
40
60
80
Ages (years)
FDR (Age associated divergence) =0.0759
p(Scalp)=0.00829
p(Dura)=0.0456
Scalp
Dura

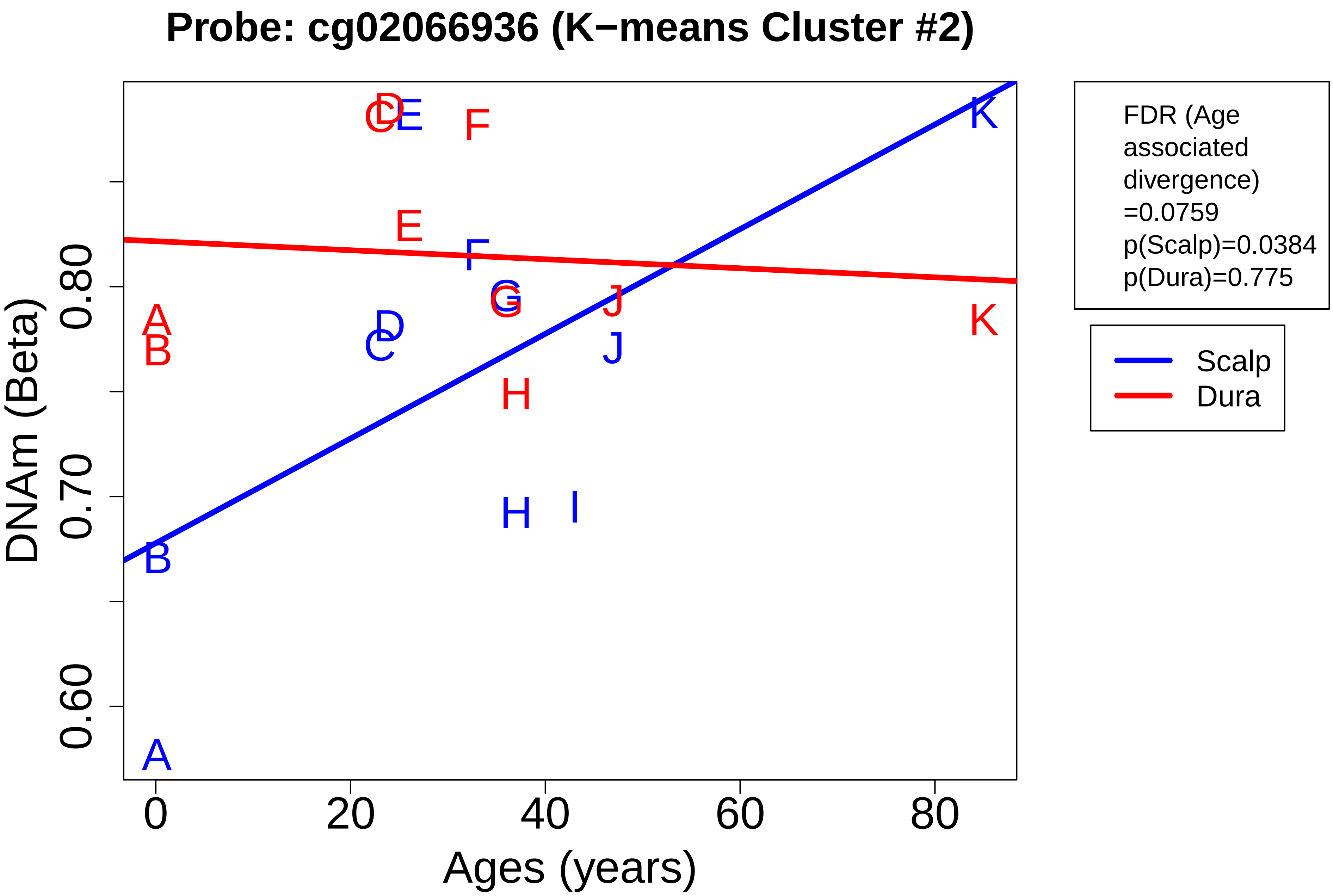

Probe: cg02066936 (K−means Cluster #2)
DNAm (Beta)
0.60
0.70
0.80
Ages (years)
0
20
40
60
80
FDR (Age associated divergence) =0.0759
p(Scalp)=0.0384
p(Dura)=0.775
Scalp
Dura

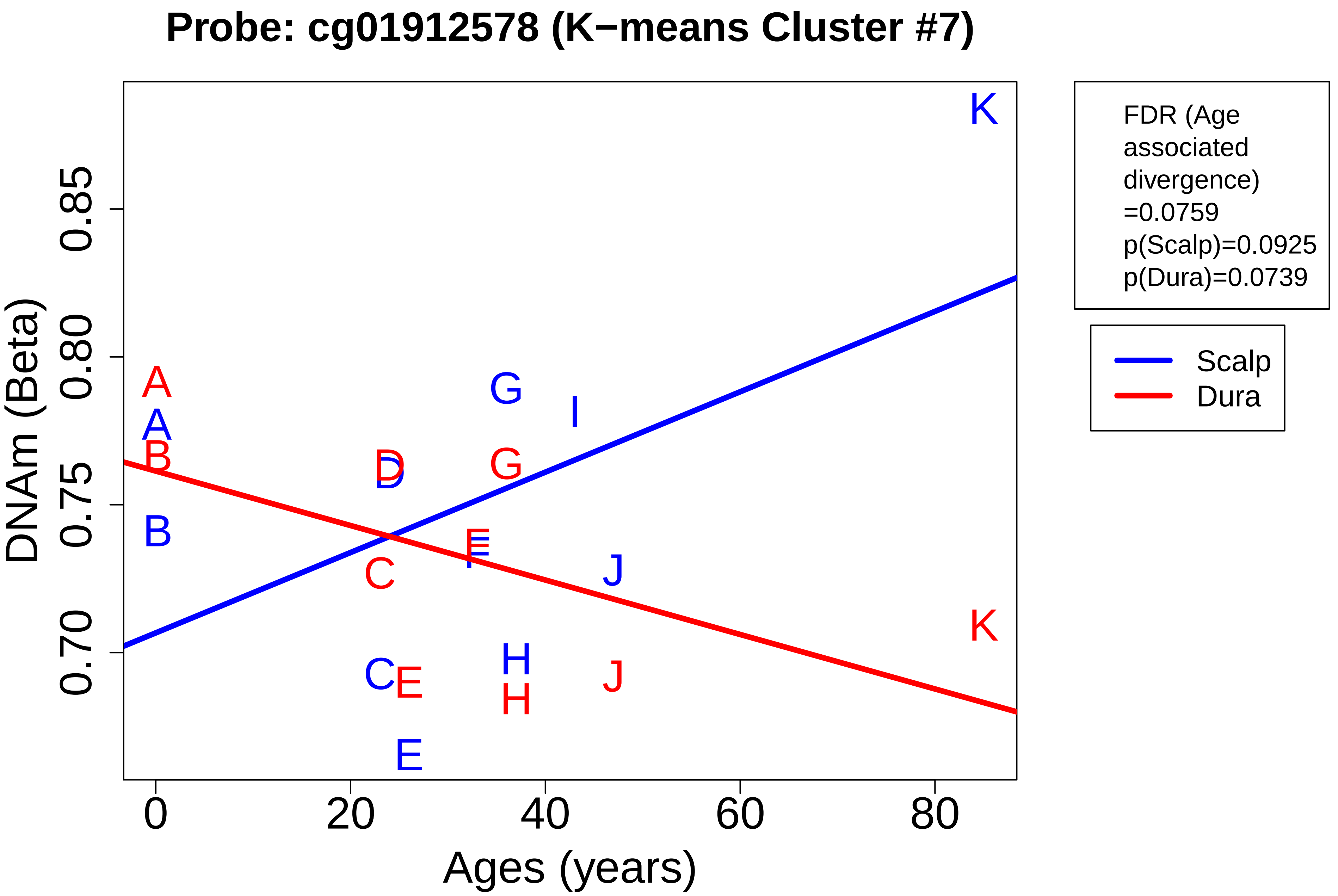

Probe: cg01912578 (K−means Cluster #7)
DNAm (Beta)
0.70
0.75
0.80
0.85
0
20
40
60
80
Ages (years)
FDR (Age associated divergence) =0.0759
p(Scalp)=0.0925
p(Dura)=0.0739
Scalp
Dura

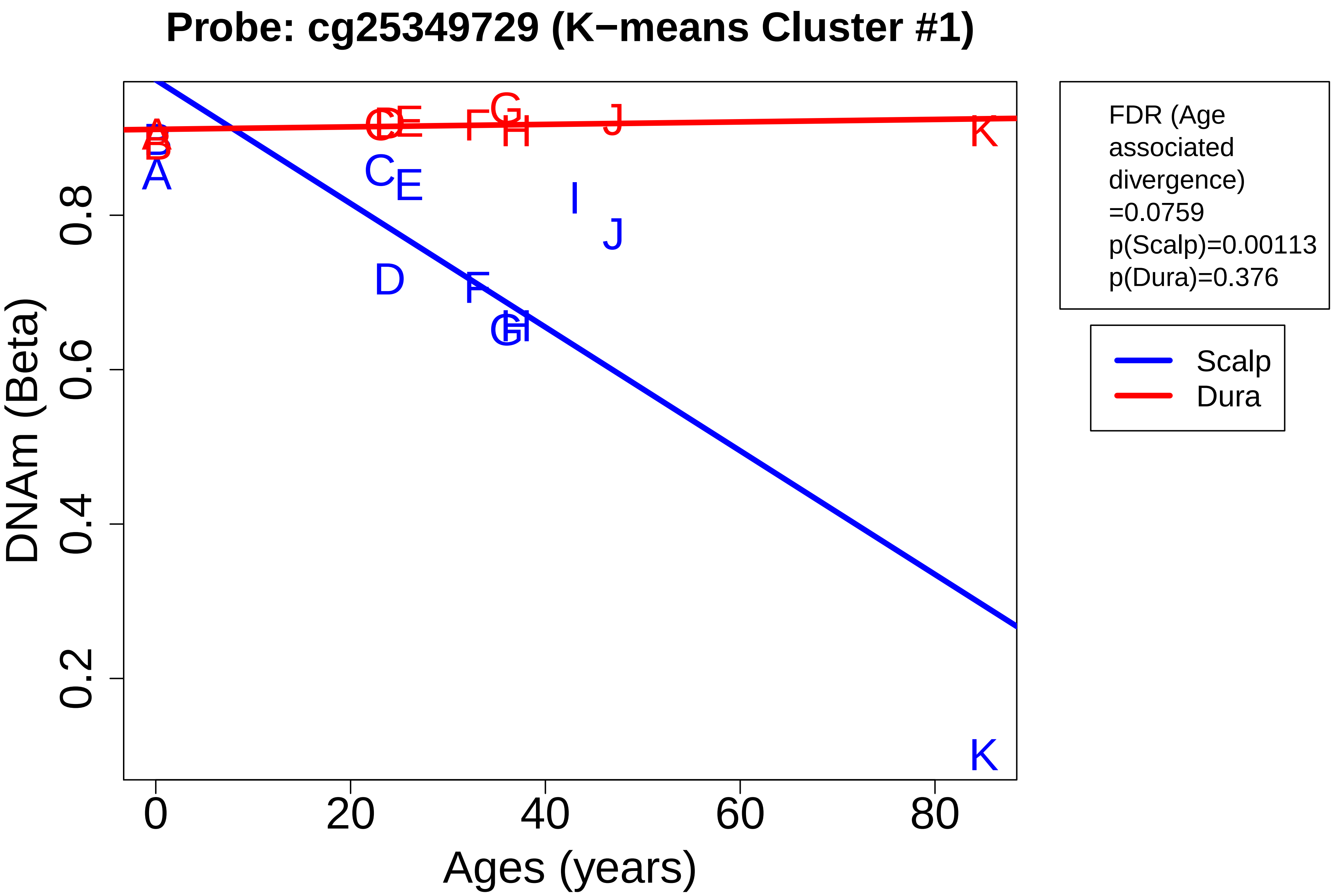

Probe: cg25349729 (K−means Cluster #1)
DNAm (Beta)
0.2
0.4
0.6
0.8
0
20
40
60
80
Ages (years)
A
B
C
D
E
F
G
H
I
J
K
FDR (Age associated divergence) =0.0759
p(Scalp)=0.00113
p(Dura)=0.376
Scalp
Dura

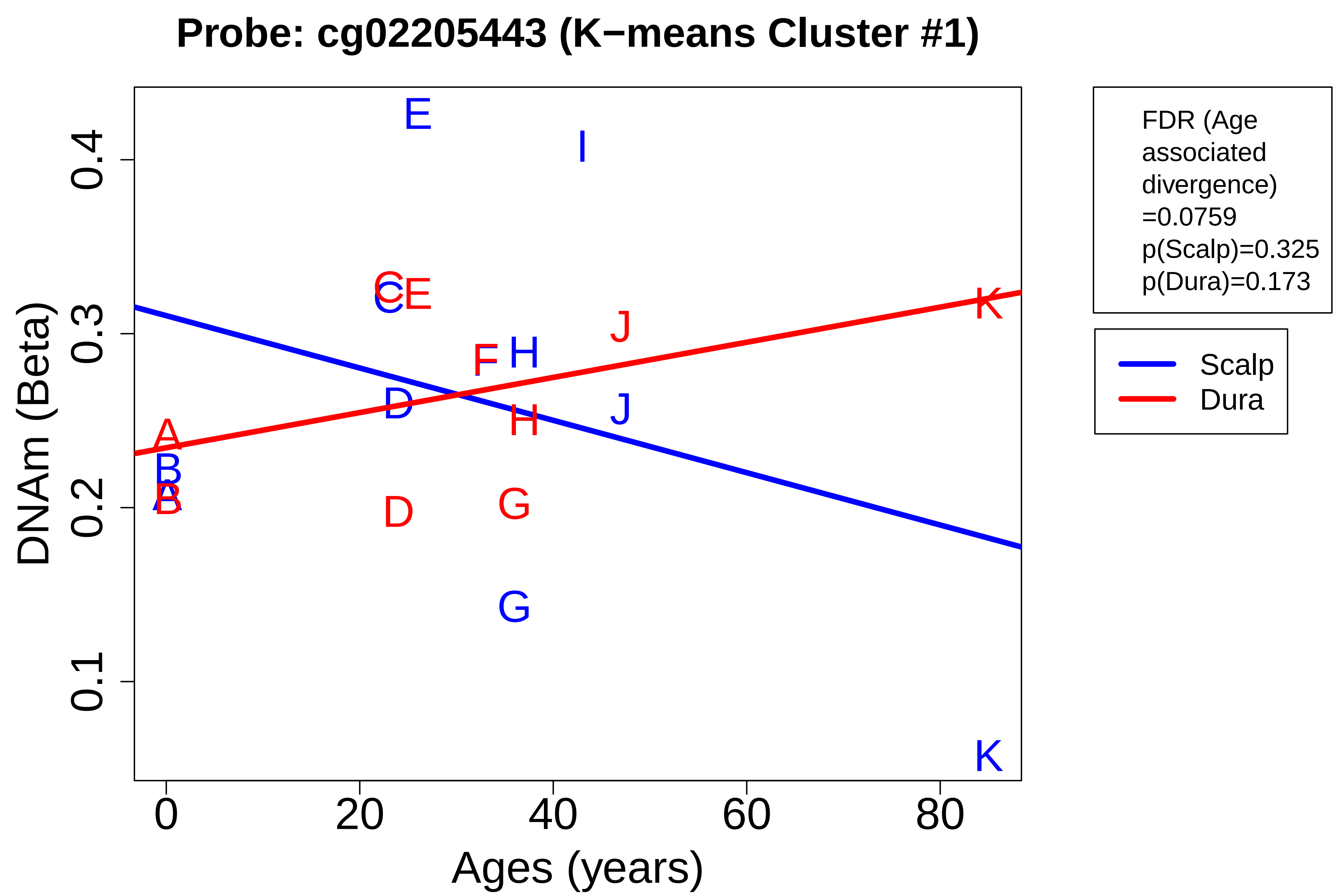

Probe: cg02205443 (K−means Cluster #1)
DNAm (Beta)
0.1
0.2
0.3
0.4
Ages (years)
0
20
40
60
80
FDR (Age associated divergence) =0.0759
p(Scalp)=0.325
p(Dura)=0.173
Scalp
Dura

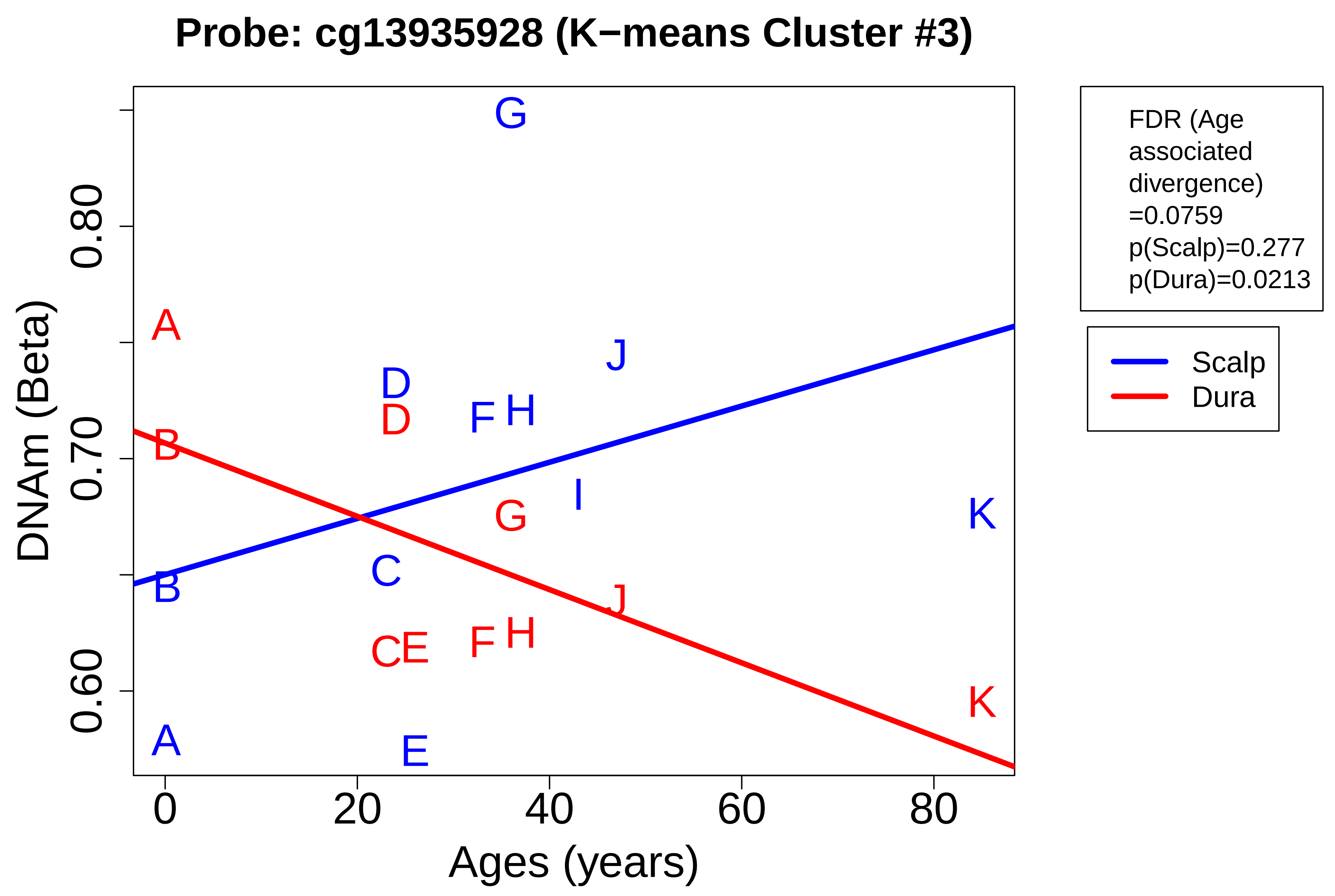
Probe: cg13935928 (K−means Cluster #3)
DNAm (Beta)
0.60
0.70
0.80
0
20
40
60
80
Ages (years)
FDR (Age associated divergence) =0.0759
p(Scalp)=0.277
p(Dura)=0.0213
Scalp
Dura
A
B
C
D
E
F
G
H
I
J
K

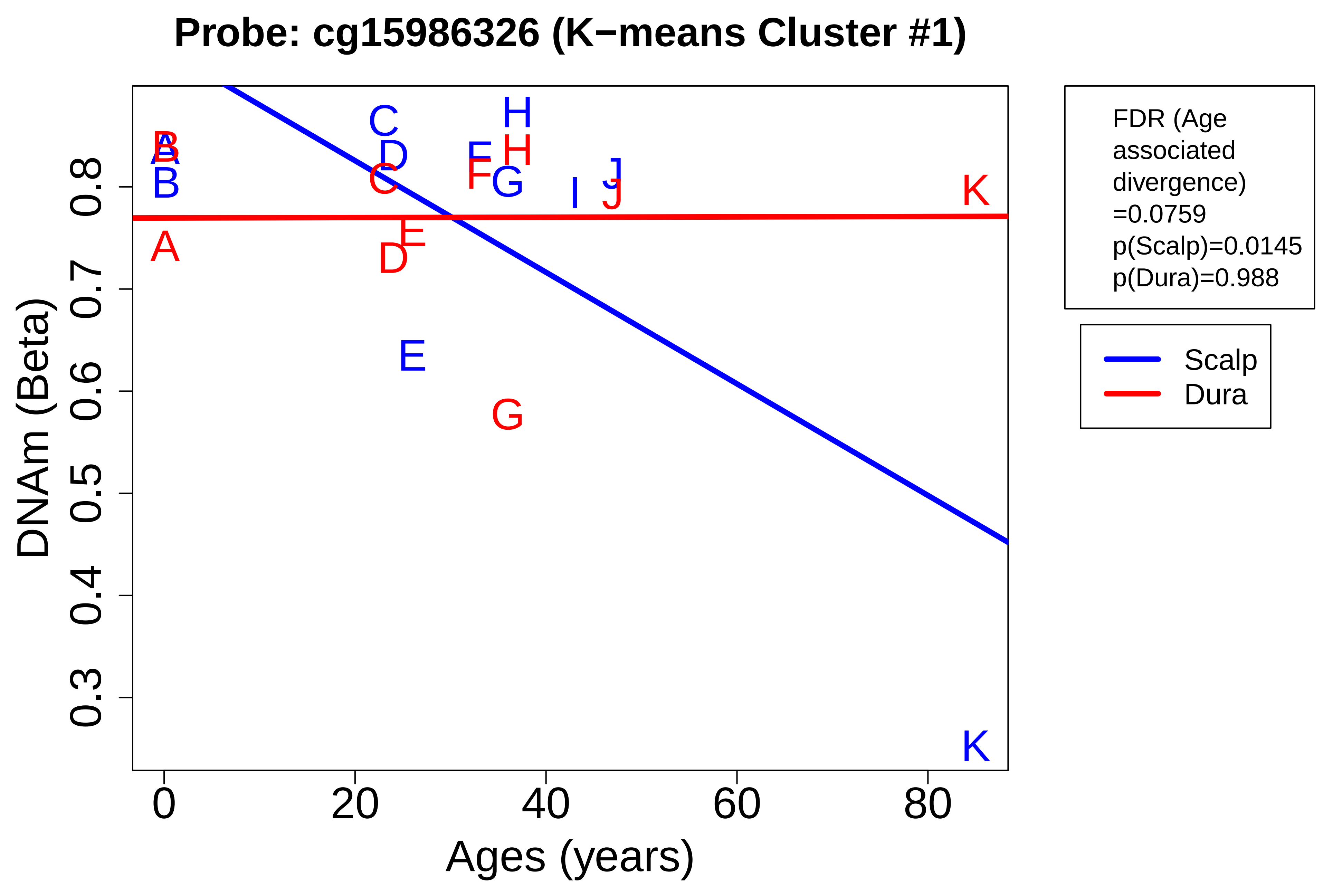

Probe: cg15986326 (K−means Cluster #1)
DNAm (Beta)
Ages (years)
0.3
0.4
0.5
0.6
0.7
0.8
0
20
40
60
80
FDR (Age associated divergence) =0.0759
p(Scalp)=0.0145
p(Dura)=0.988
Scalp
Dura
A
B
C
D
E
F
G
H
I
J
K

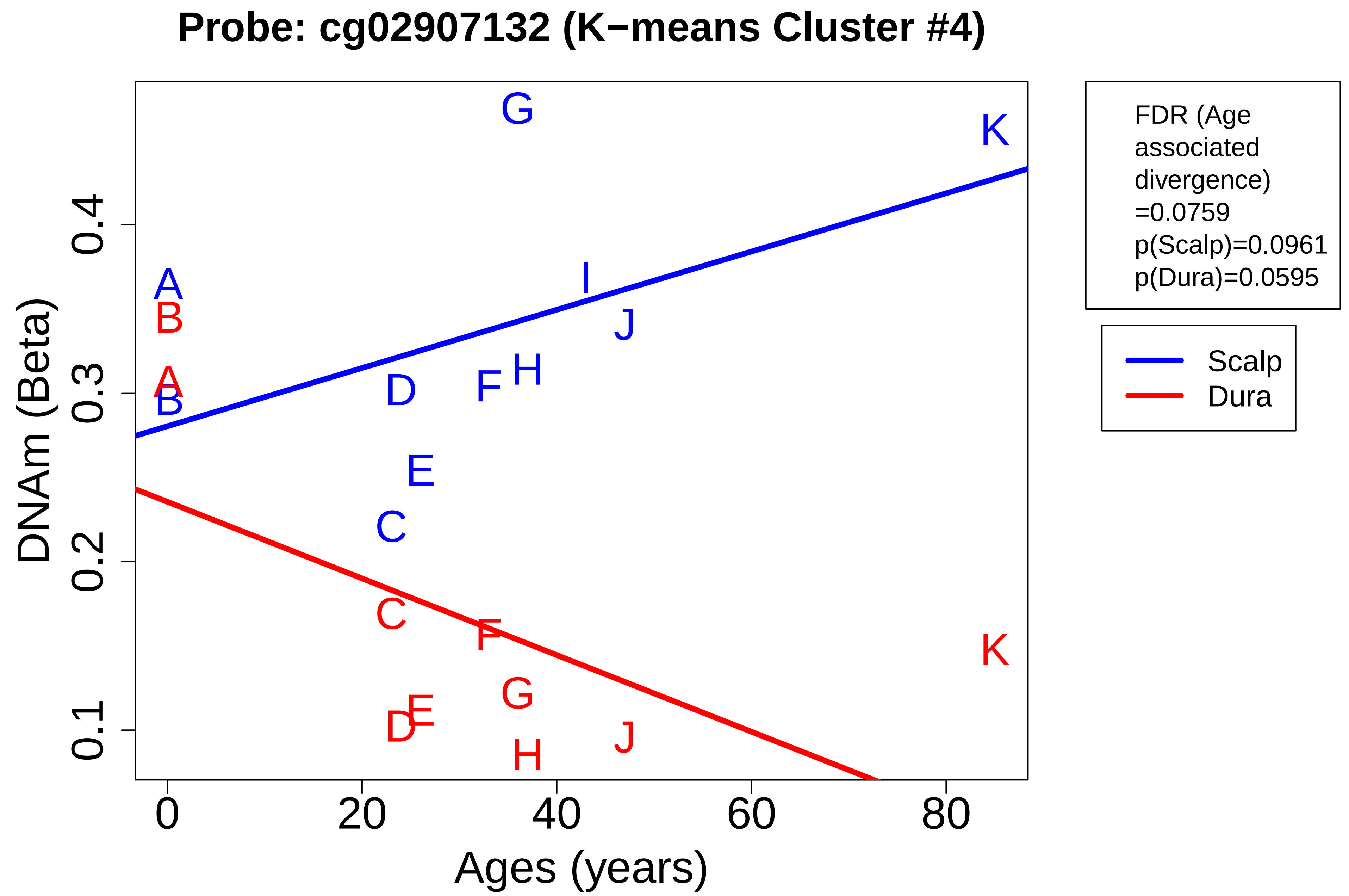

Probe: cg02907132 (K−means Cluster #4)
DNAm (Beta)
0.1
0.2
0.3
0.4
0
20
40
60
80
Ages (years)
A
B
C
D
E
F
G
H
I
J
K
FDR (Age associated divergence) =0.0759
p(Scalp)=0.0961
p(Dura)=0.0595
Scalp
Dura

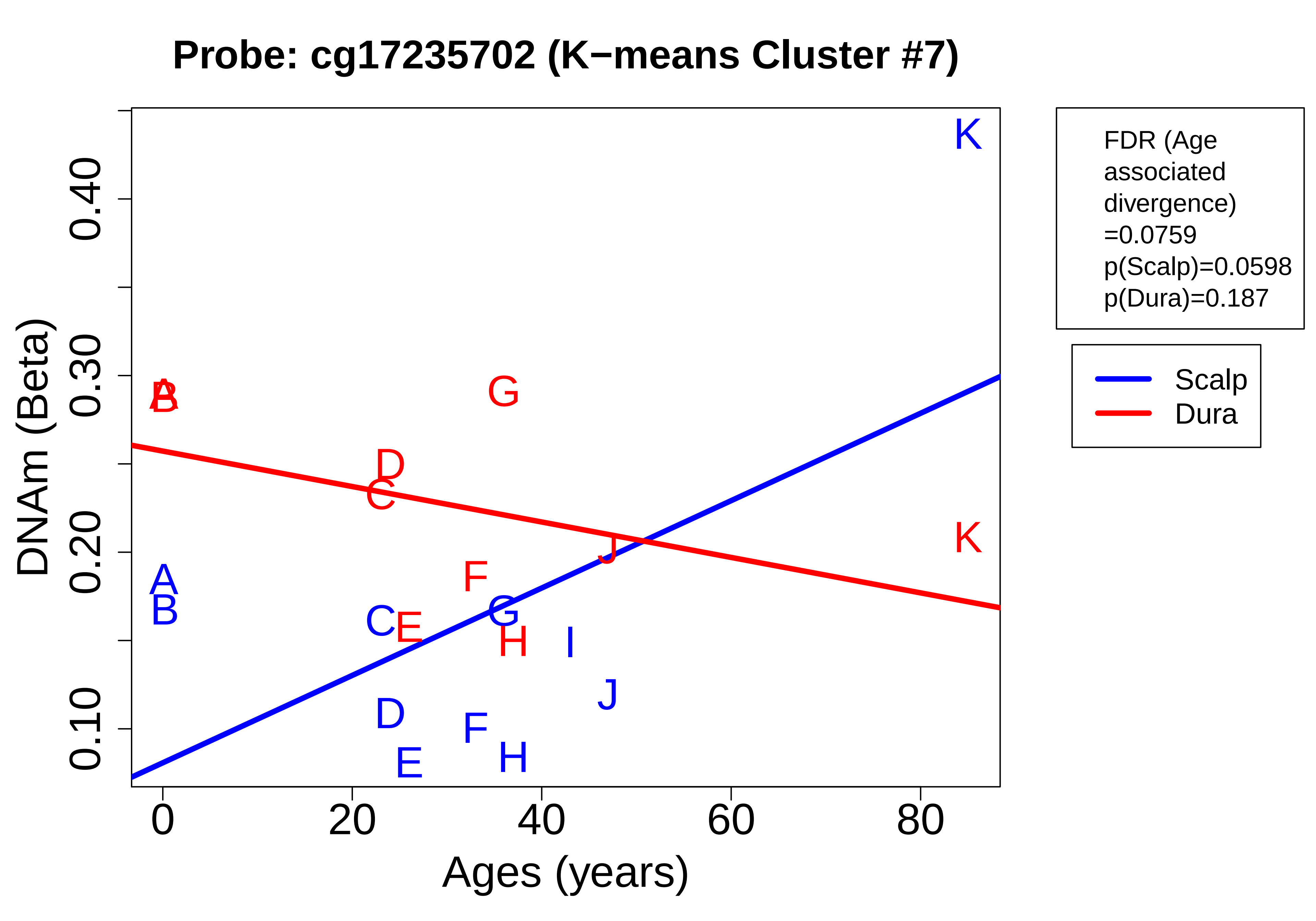

Probe: cg17235702 (K−means Cluster #7)
DNAm (Beta)
0.10
0.20
0.30
0.40
Ages (years)
0
20
40
60
80
FDR (Age associated divergence) =0.0759
p(Scalp)=0.0598
p(Dura)=0.187
Scalp
Dura

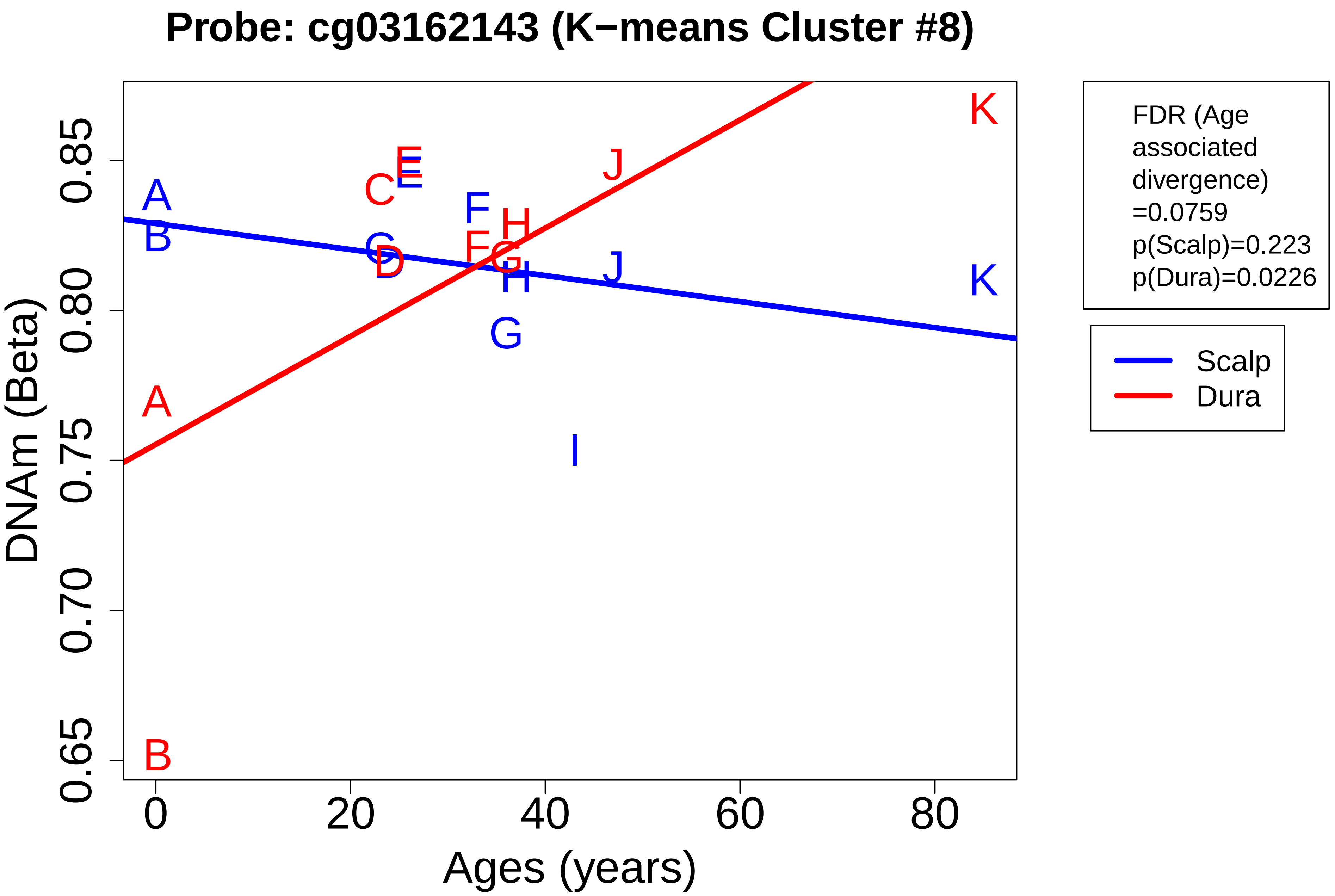

Probe: cg03162143 (K−means Cluster #8)
DNAm (Beta)
0.65
0.70
0.75
0.80
0.85
Ages (years)
0
20
40
60
80
A
B
C
D
E
F
G
H
I
J
K
FDR (Age associated divergence) =0.0759
p(Scalp)=0.223
p(Dura)=0.0226
Scalp
Dura

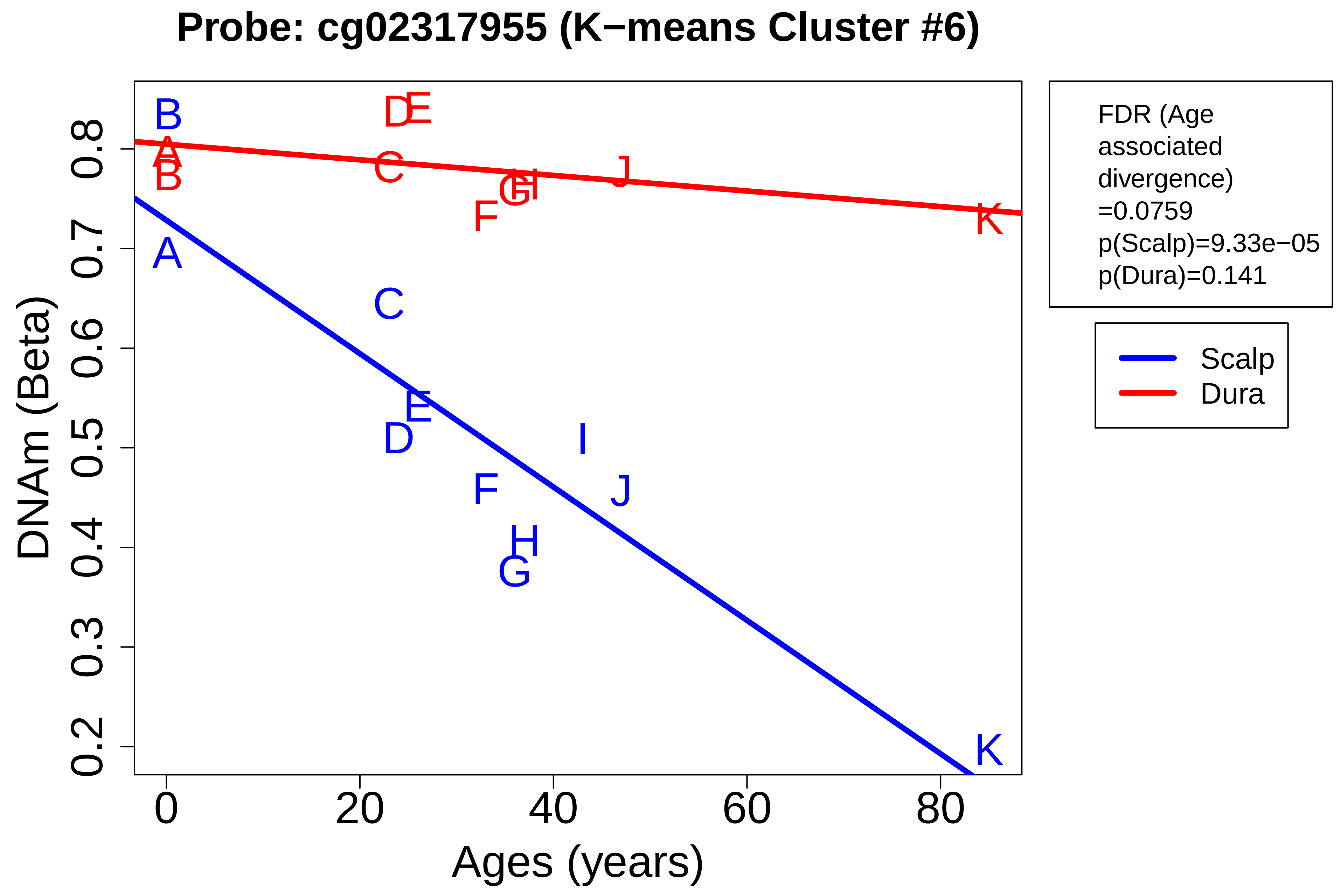

Probe: cg02317955 (K−means Cluster #6)
DNAm (Beta)
0.2
0.3
0.4
0.5
0.6
0.7
0.8
0
20
40
60
80
Ages (years)
FDR (Age associated divergence) =0.0759
p(Scalp)=9.33e−05
p(Dura)=0.141
Scalp
Dura

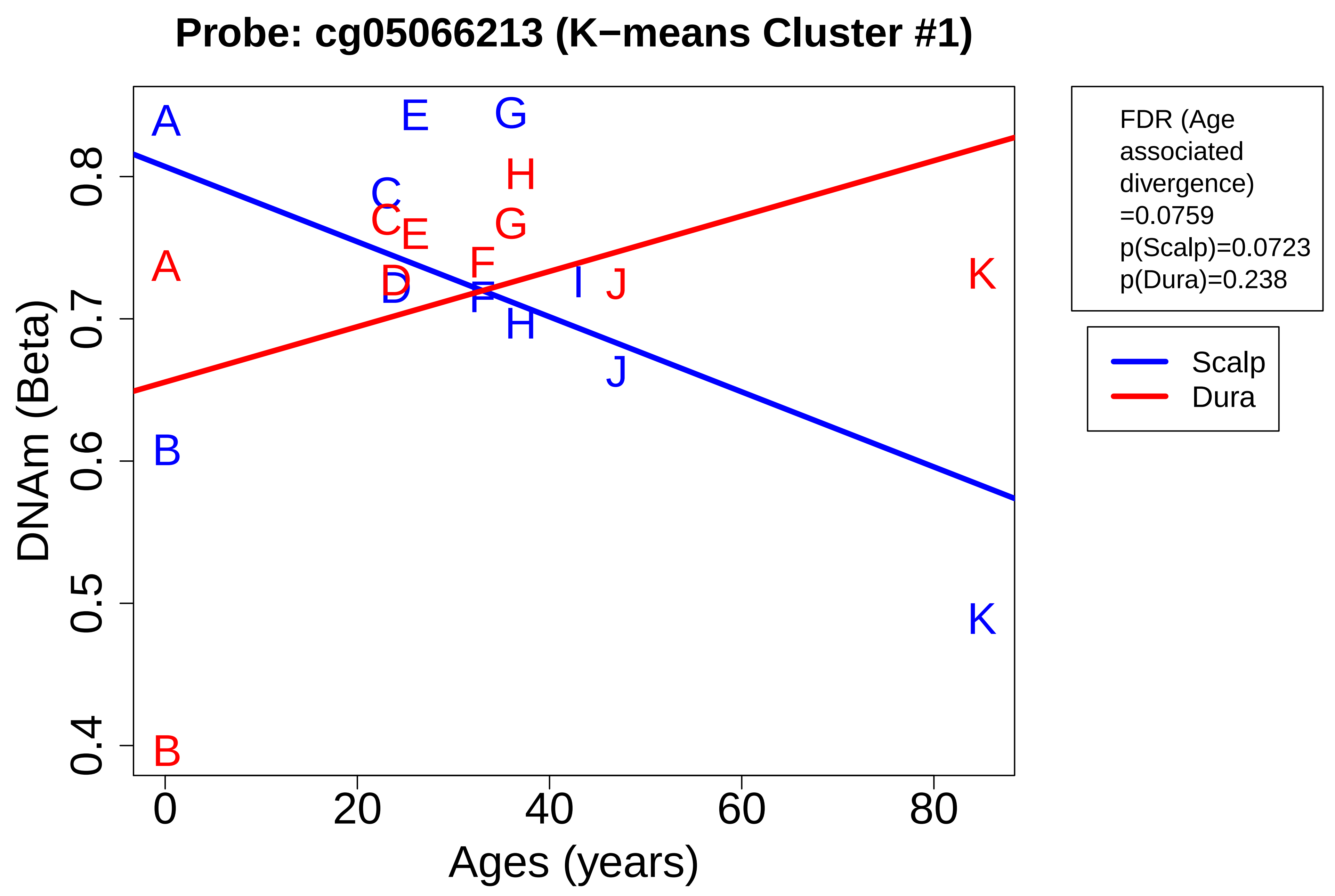

Probe: cg05066213 (K−means Cluster #1)
DNAm (Beta)
0.4
0.5
0.6
0.7
0.8
0
20
40
60
80
Ages (years)
A
B
C
D
E
F
G
H
I
J
K
A
B
C
D
E
F
G
H
J
K
FDR (Age associated divergence) =0.0759
p(Scalp)=0.0723
p(Dura)=0.238
Scalp
Dura

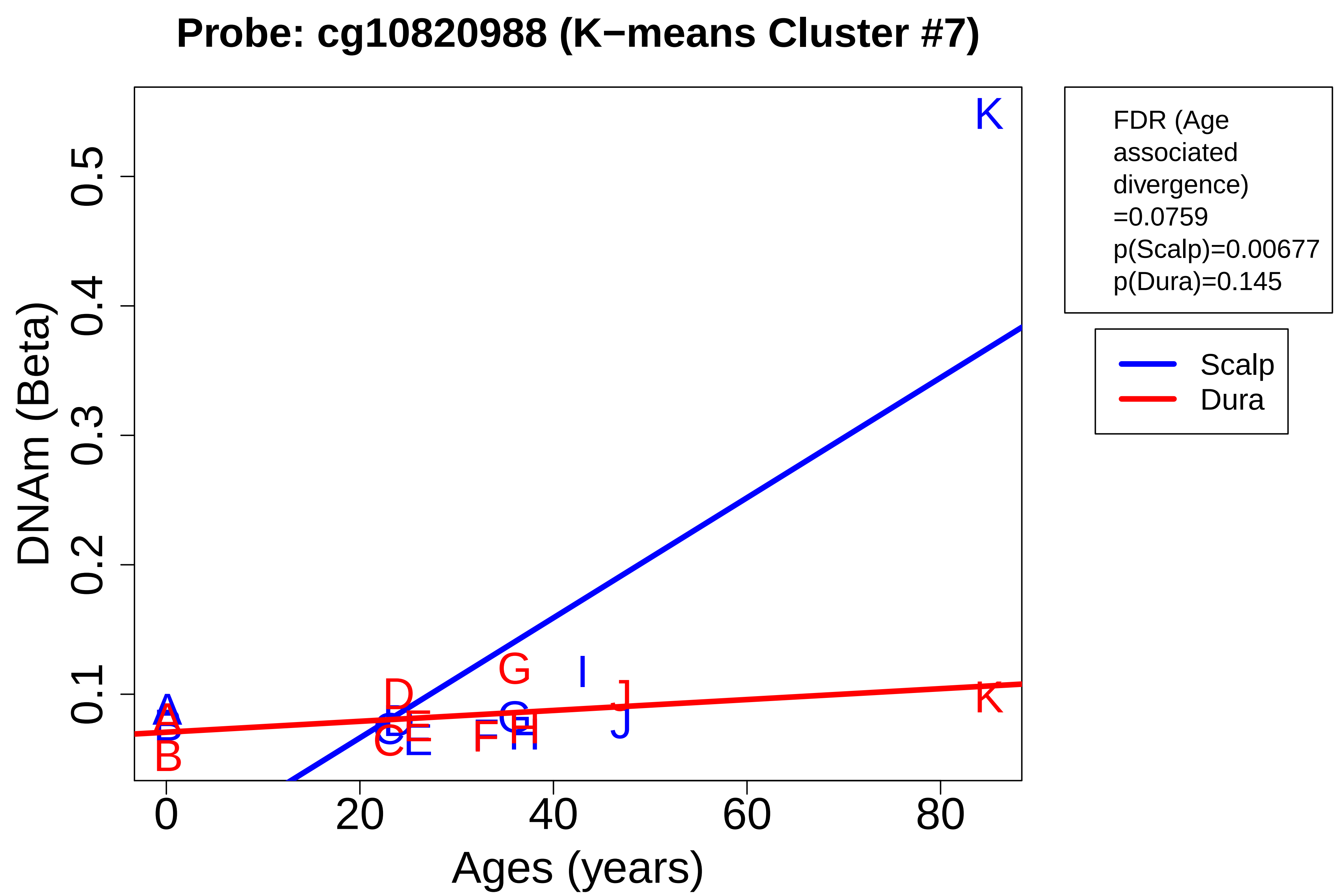

Probe: cg10820988 (K−means Cluster #7)
DNAm (Beta)
0.1
0.2
0.3
0.4
0.5
0
20
40
60
80
Ages (years)
FDR (Age associated divergence) =0.0759
p(Scalp)=0.00677
p(Dura)=0.145
Scalp
Dura

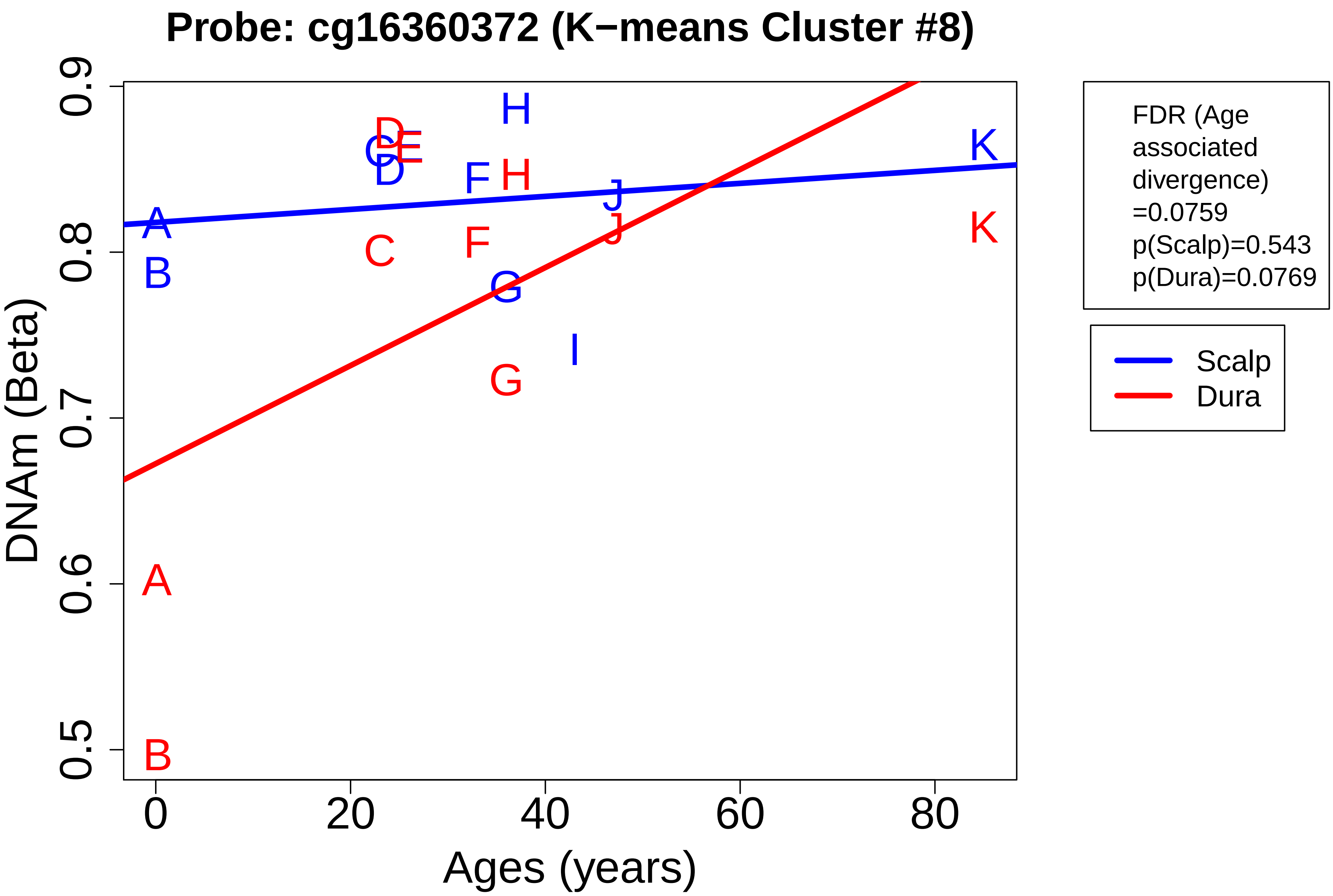

Probe: cg16360372 (K−means Cluster #8)
DNAm (Beta)
0.5
0.6
0.7
0.8
0.9
0
20
40
60
80
Ages (years)
FDR (Age associated divergence) =0.0759
p(Scalp)=0.543
p(Dura)=0.0769
Scalp
Dura

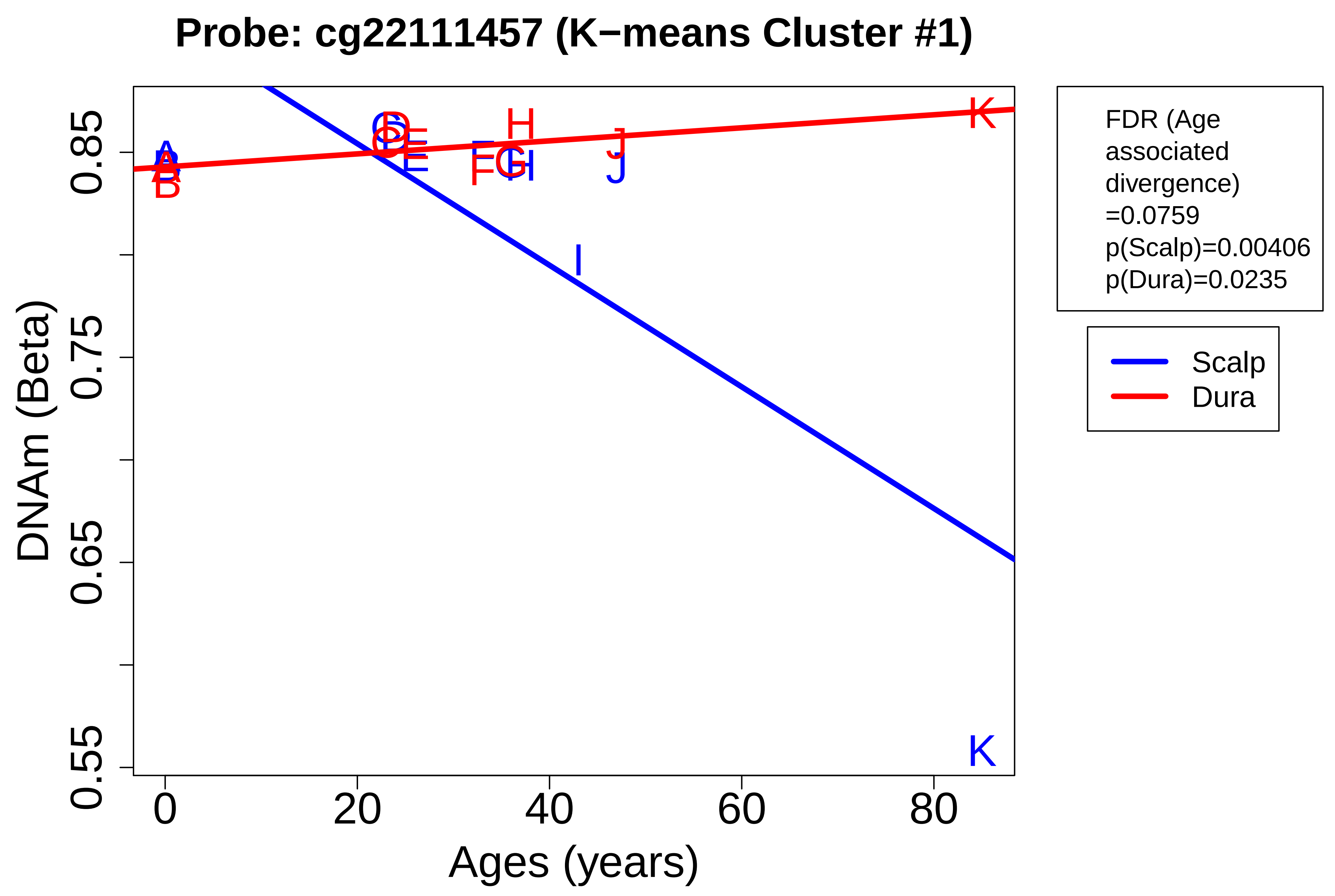

Probe: cg22111457 (K−means Cluster #1)
DNAm (Beta)
0.55
0.65
0.75
0.85
0
20
40
60
80
Ages (years)
FDR (Age associated divergence) =0.0759
p(Scalp)=0.00406
p(Dura)=0.0235
Scalp
Dura

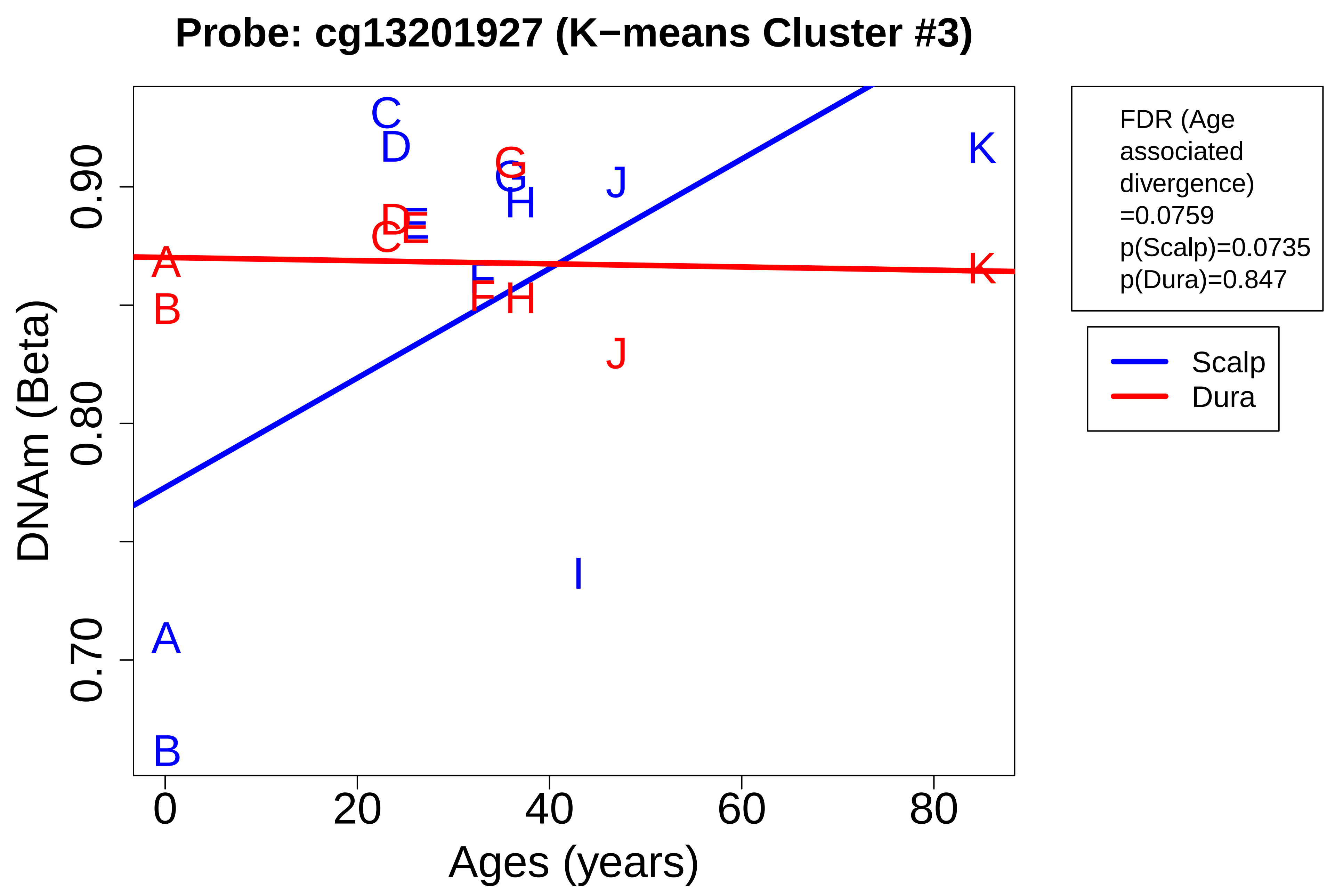
Probe: cg13201927 (K−means Cluster #3)
DNAm (Beta)
0.70
0.80
0.90
0
20
40
60
80
Ages (years)
FDR (Age associated divergence) =0.0759
p(Scalp)=0.0735
p(Dura)=0.847
Scalp
Dura
A
B
C
D
E
F
G
H
I
J
K

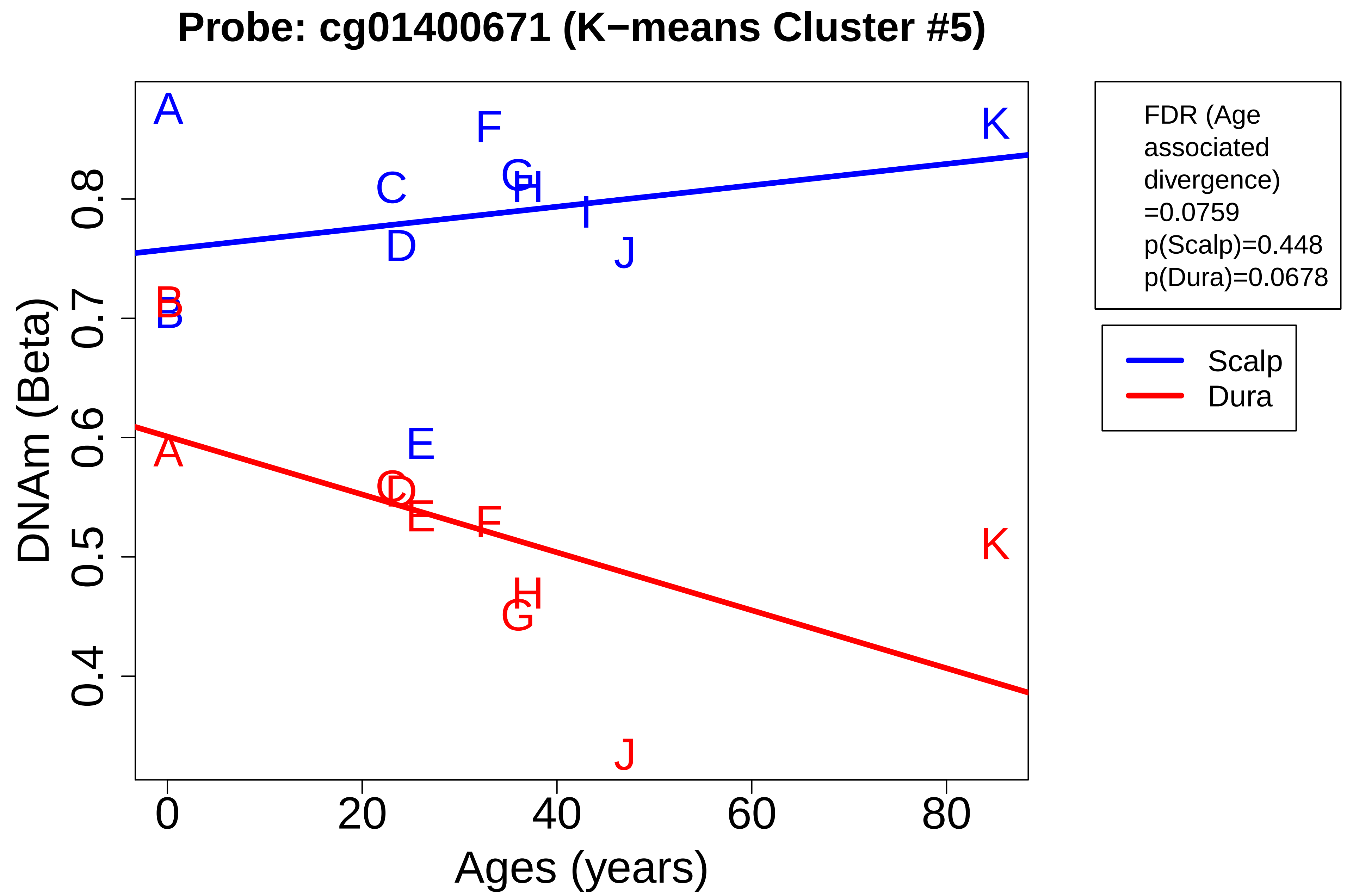

Probe: cg01400671 (K−means Cluster #5)
DNAm (Beta)
0.4
0.5
0.6
0.7
0.8
0
20
40
60
80
Ages (years)
A
B
C
D
E
F
G
H
I
J
K
A
B
C
D
E
F
G
H
J
K
FDR (Age associated divergence) =0.0759
p(Scalp)=0.448
p(Dura)=0.0678
Scalp
Dura

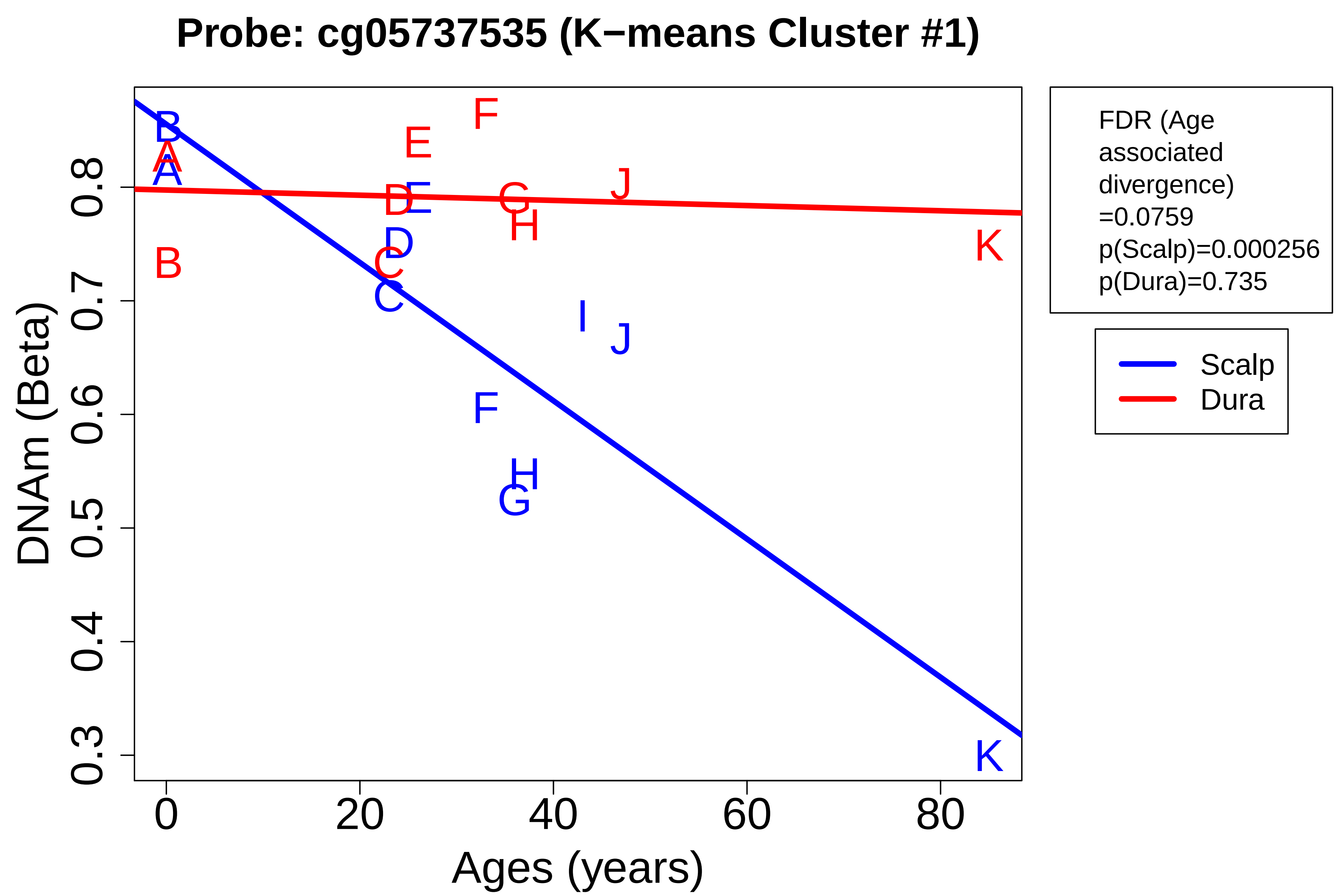

Probe: cg05737535 (K−means Cluster #1)
DNAm (Beta)
0.3
0.4
0.5
0.6
0.7
0.8
0
20
40
60
80
Ages (years)
FDR (Age associated divergence) =0.0759
p(Scalp)=0.000256
p(Dura)=0.735
Scalp
Dura

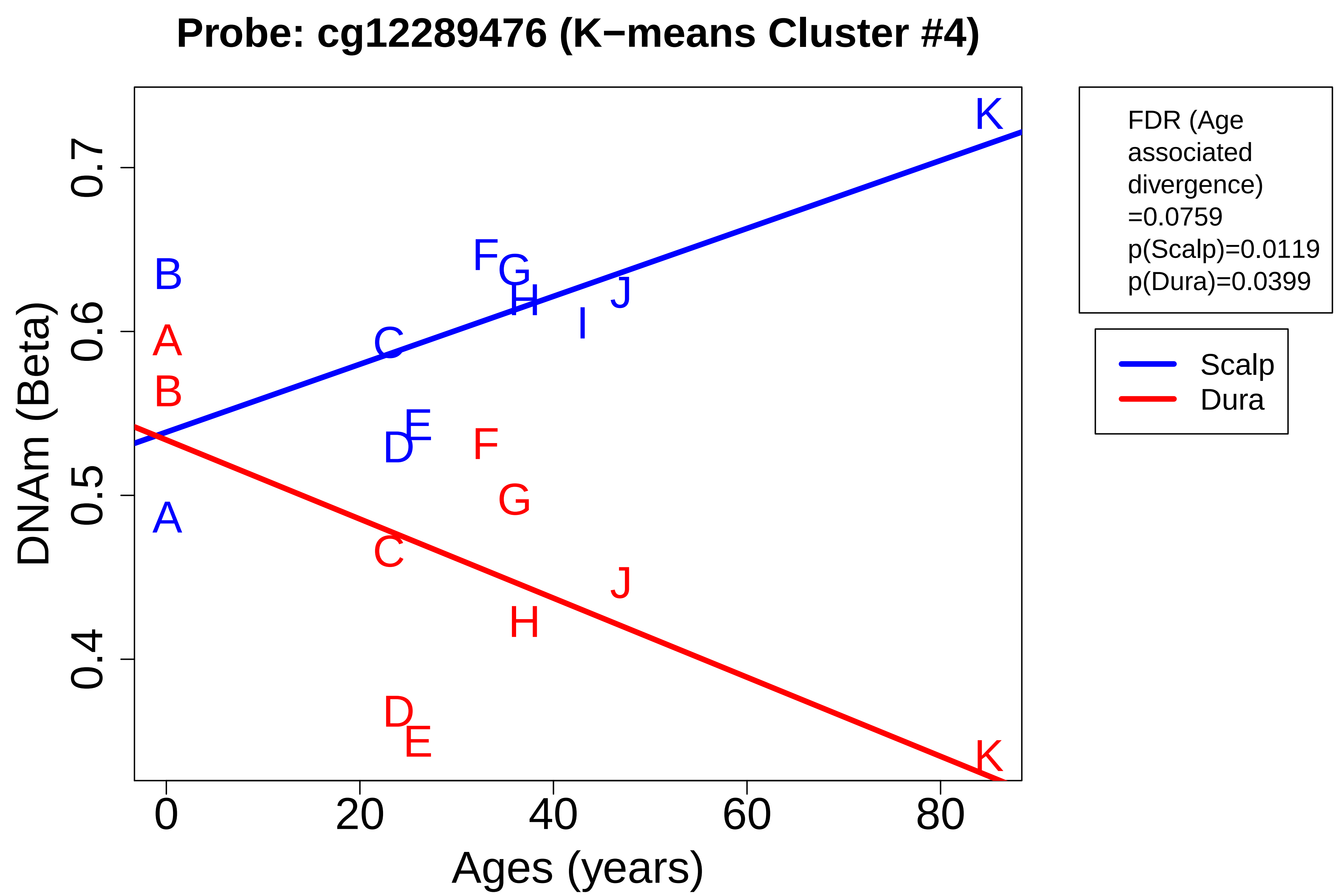

Probe: cg12289476 (K−means Cluster #4)
DNAm (Beta)
0.4
0.5
0.6
0.7
0
20
40
60
80
Ages (years)
FDR (Age associated divergence) =0.0759
p(Scalp)=0.0119
p(Dura)=0.0399
Scalp
Dura

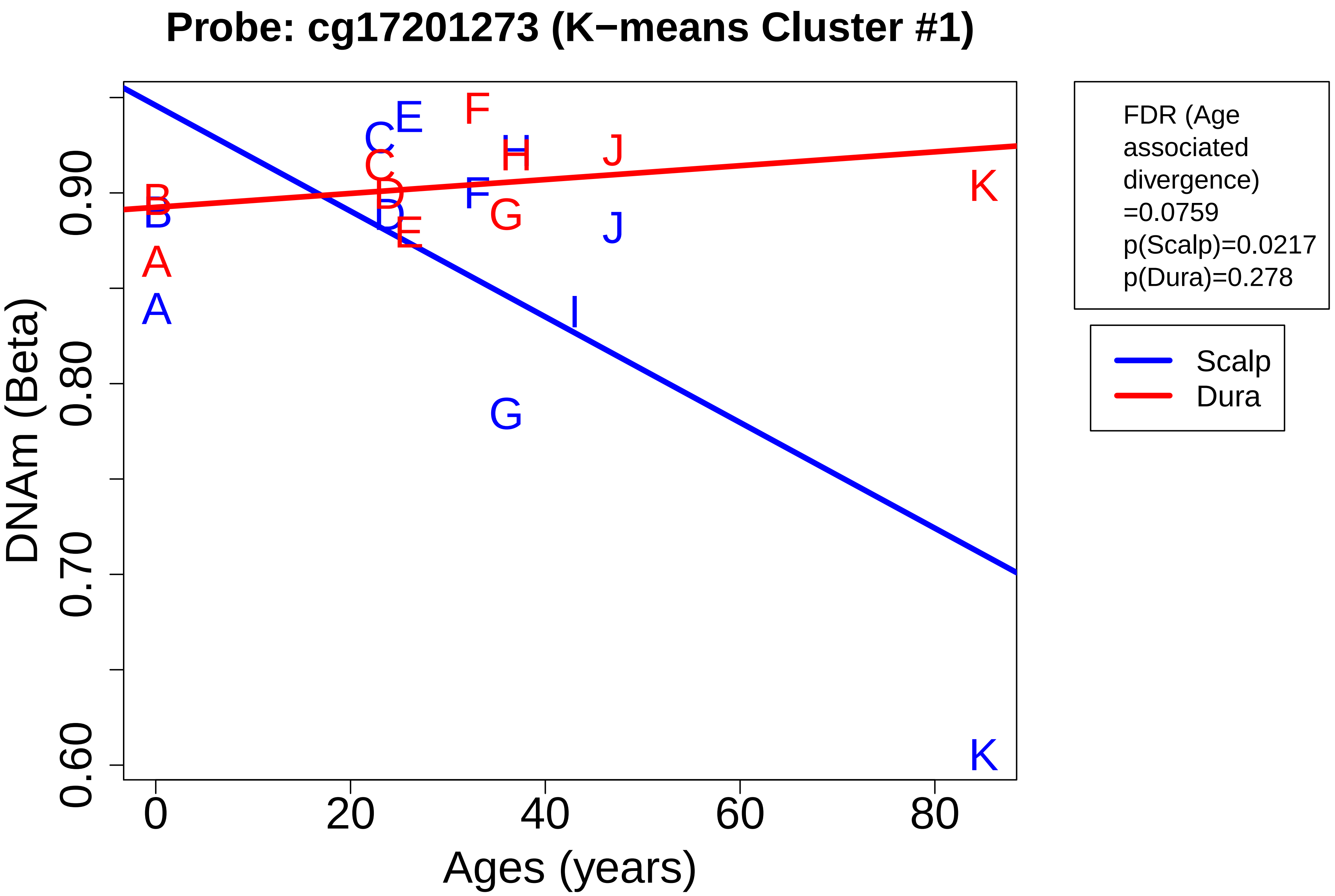
Probe: cg17201273 (K−means Cluster #1)
DNAm (Beta)
0.60
0.70
0.80
0.90
0
20
40
60
80
Ages (years)
A
B
C
D
E
F
G
H
I
J
K
FDR (Age associated divergence) =0.0759
p(Scalp)=0.0217
p(Dura)=0.278
Scalp
Dura

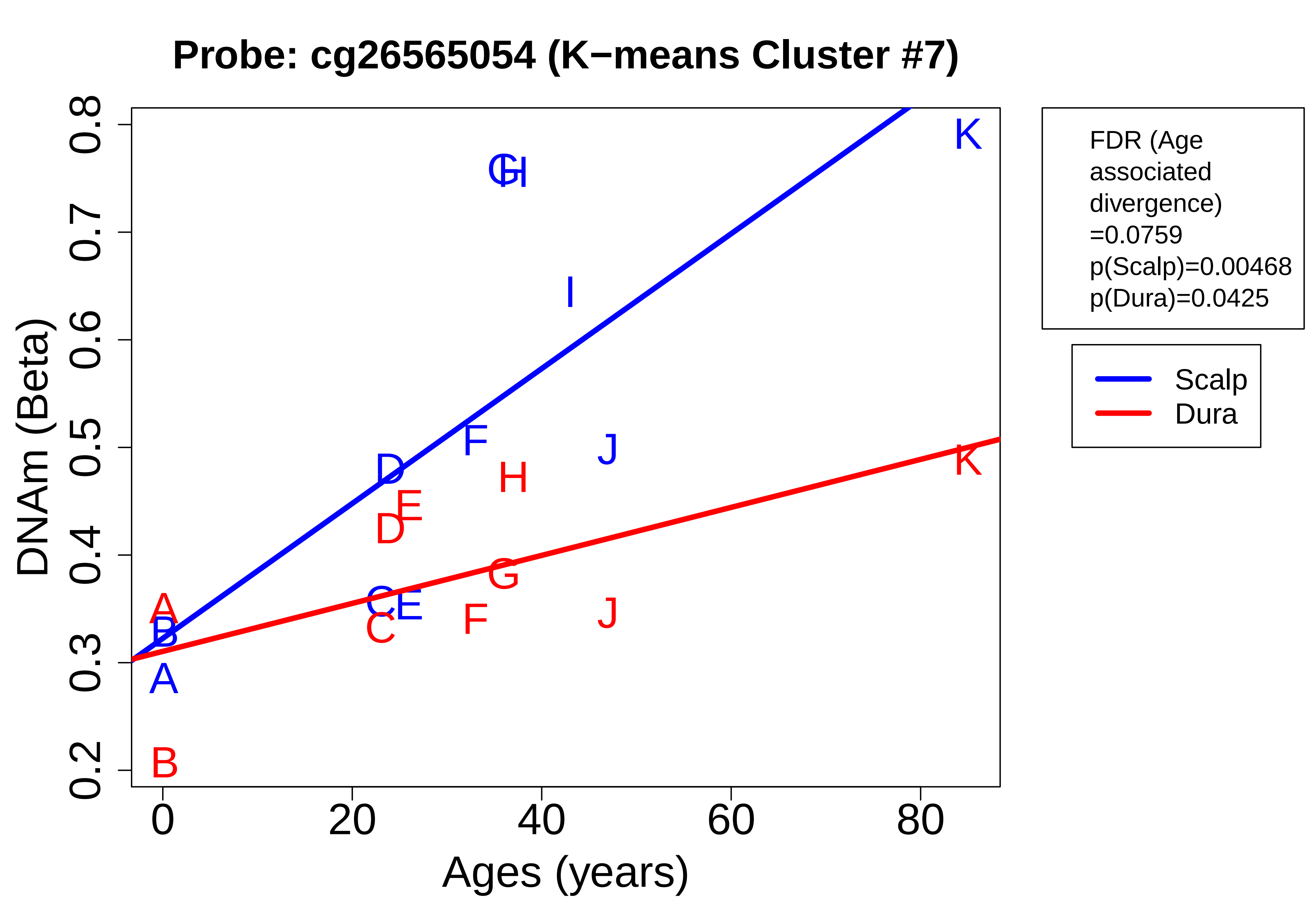

Probe: cg26565054 (K−means Cluster #7)
DNAm (Beta)
0.2
0.3
0.4
0.5
0.6
0.7
0.8
Ages (years)
0
20
40
60
80
FDR (Age associated divergence) =0.0759
p(Scalp)=0.00468
p(Dura)=0.0425
Scalp
Dura
A
B
C
D
E
F
G
H
I
J
K

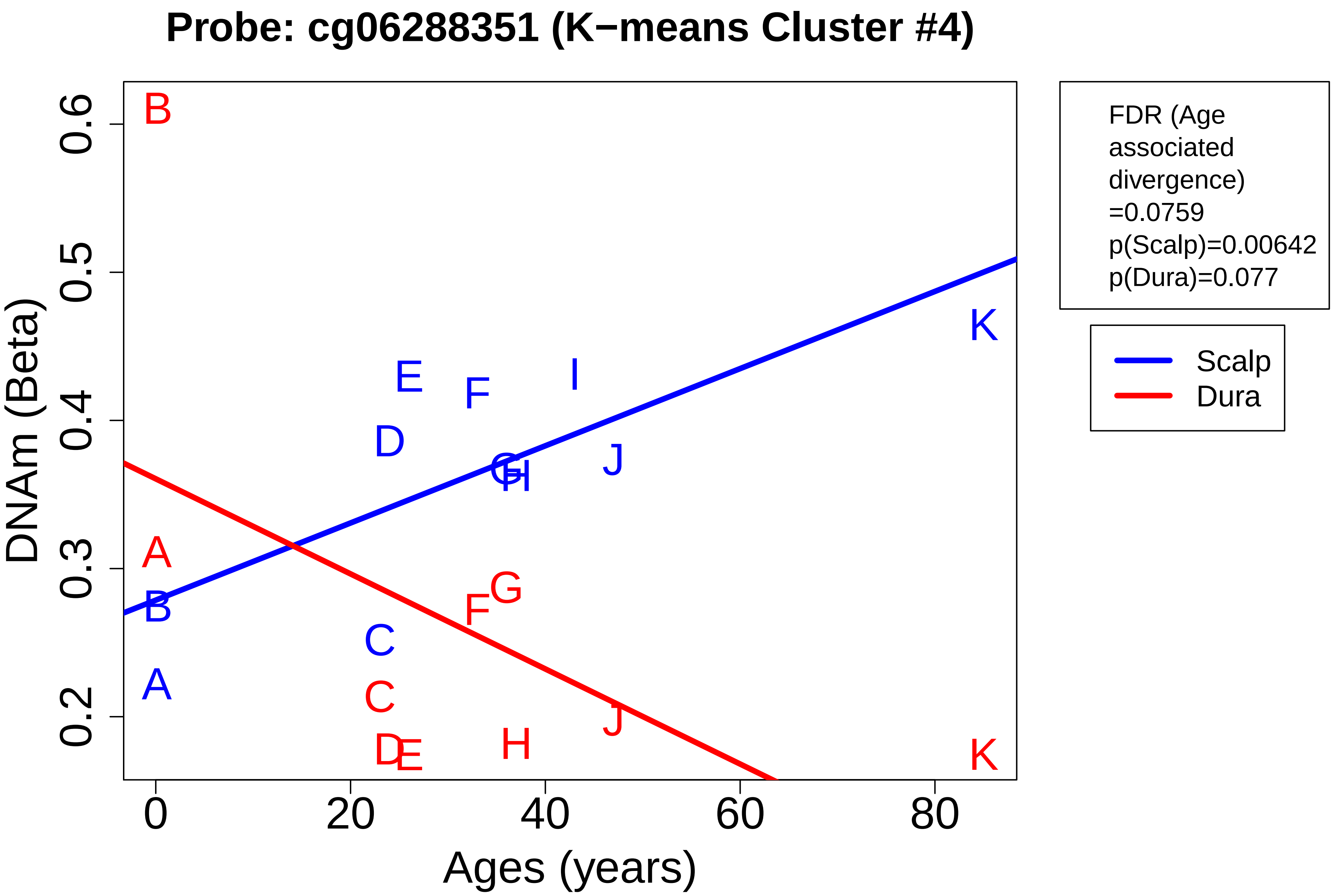

Probe: cg06288351 (K−means Cluster #4)
DNAm (Beta)
0.2
0.3
0.4
0.5
0.6
0
20
40
60
80
Ages (years)
FDR (Age associated divergence) =0.0759
p(Scalp)=0.00642
p(Dura)=0.077
Scalp
Dura

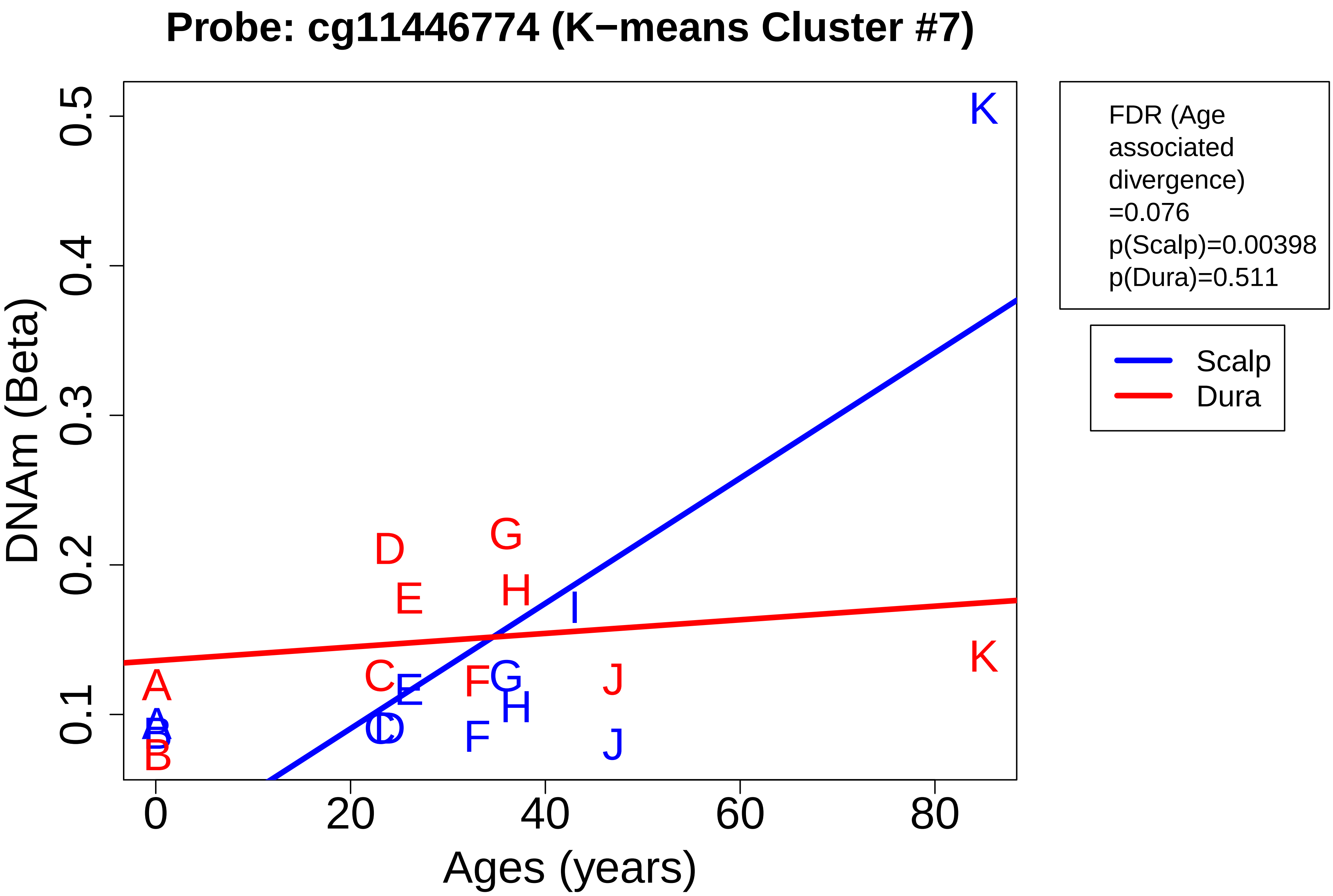

Probe: cg11446774 (K−means Cluster #7)
DNAm (Beta)
0.1
0.2
0.3
0.4
0.5
Ages (years)
0
20
40
60
80
FDR (Age associated divergence) =0.076
p(Scalp)=0.00398
p(Dura)=0.511
Scalp
Dura

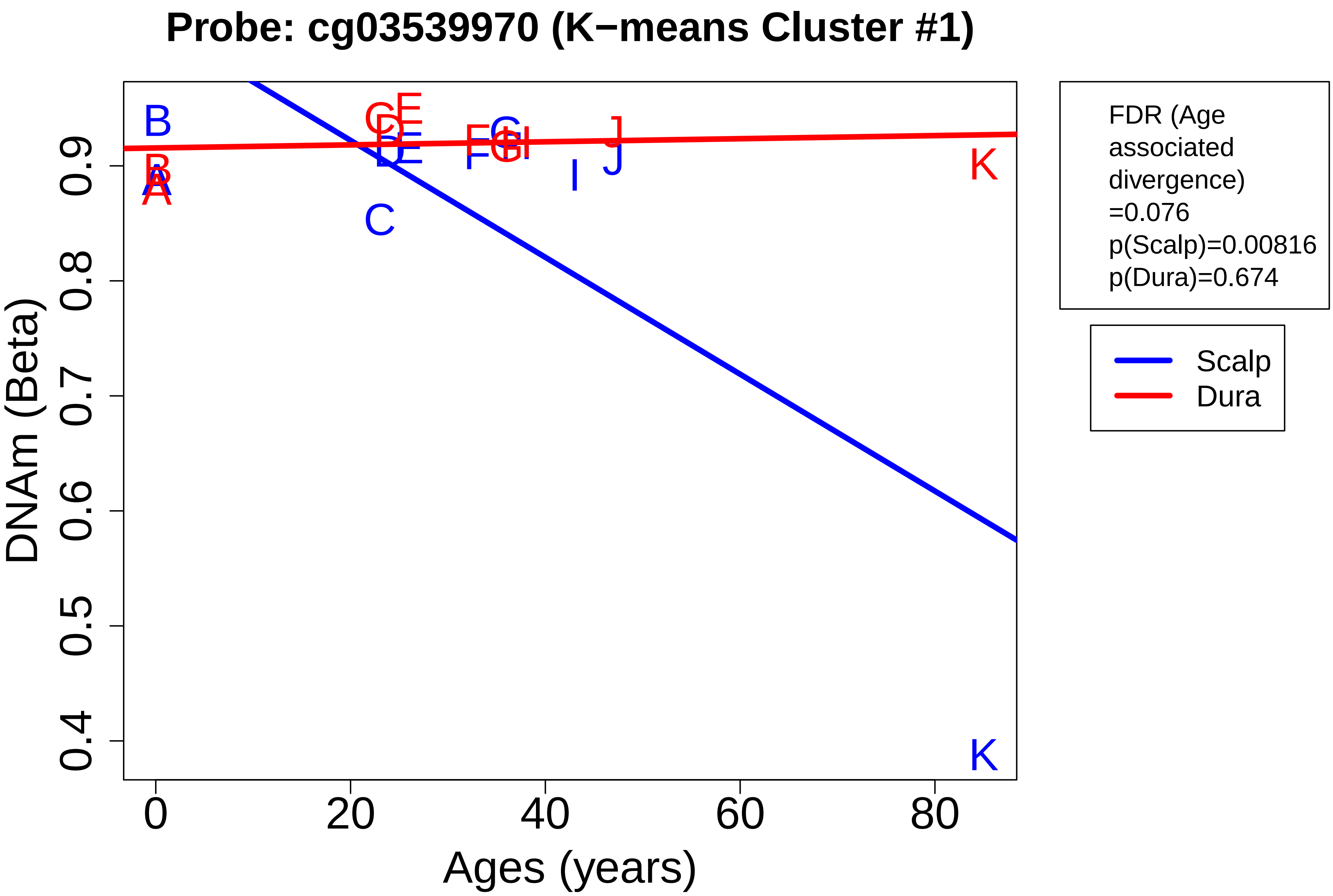

Probe: cg03539970 (K−means Cluster #1)
DNAm (Beta)
Ages (years)
0.4
0.5
0.6
0.7
0.8
0.9
0
20
40
60
80
FDR (Age associated divergence) =0.076
p(Scalp)=0.00816
p(Dura)=0.674
Scalp
Dura

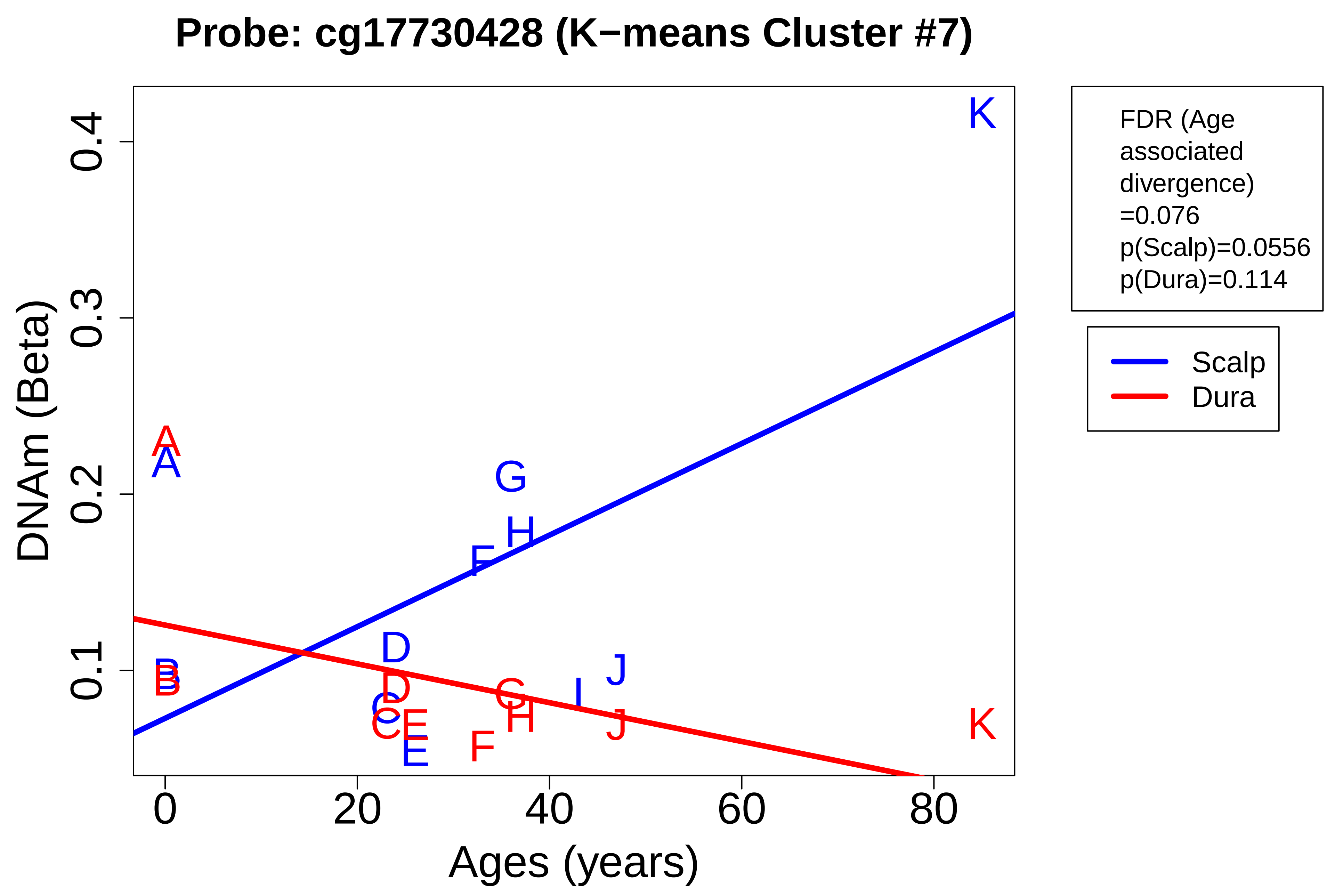

Probe: cg17730428 (K−means Cluster #7)
DNAm (Beta)
0.1
0.2
0.3
0.4
Ages (years)
0
20
40
60
80
FDR (Age associated divergence) =0.076
p(Scalp)=0.0556
p(Dura)=0.114
Scalp
Dura
A
B
C
D
E
F
G
H
I
J
K

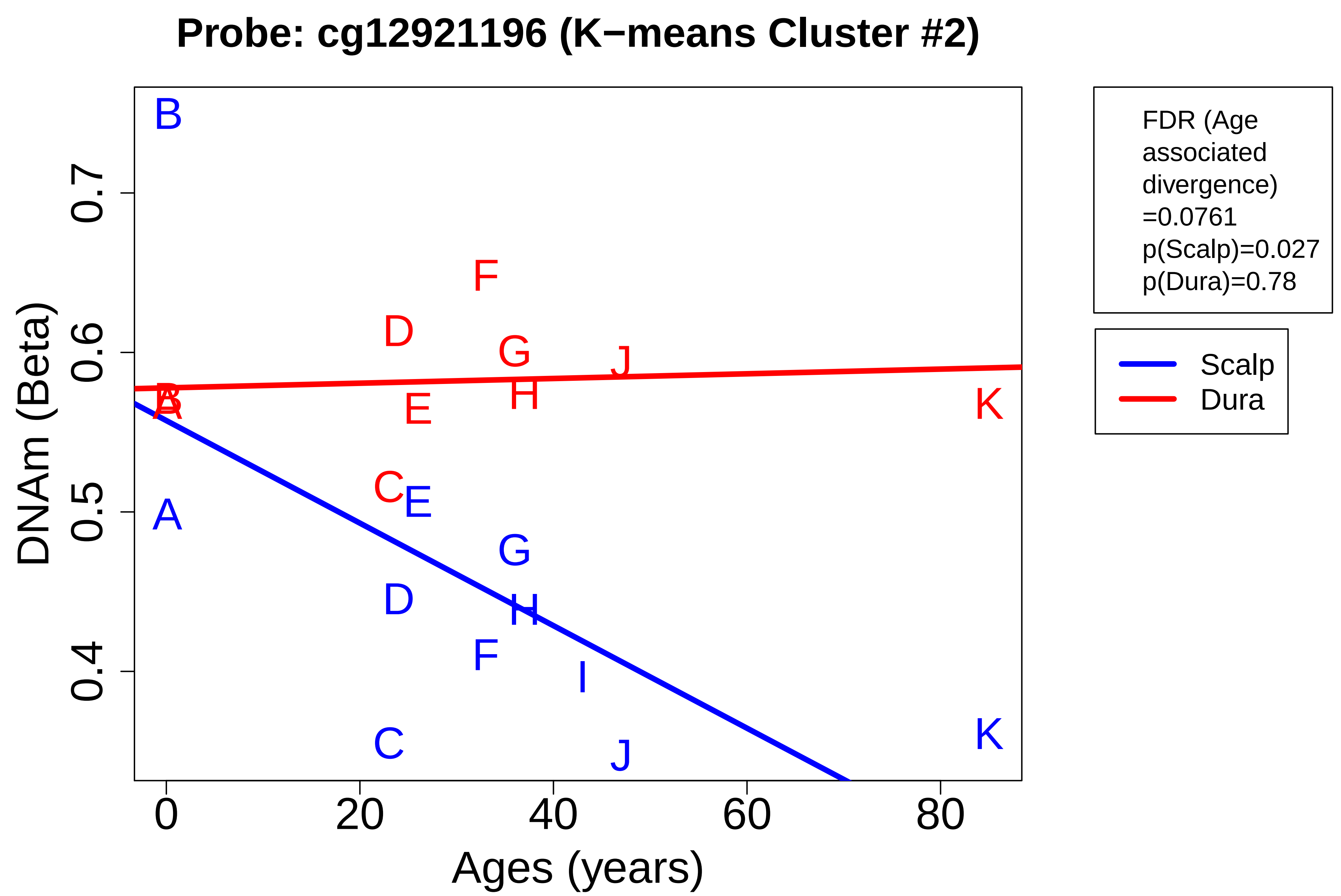

Probe: cg12921196 (K−means Cluster #2)
DNAm (Beta)
0.4
0.5
0.6
0.7
Ages (years)
0
20
40
60
80
FDR (Age associated divergence) =0.0761
p(Scalp)=0.027
p(Dura)=0.78
Scalp
Dura

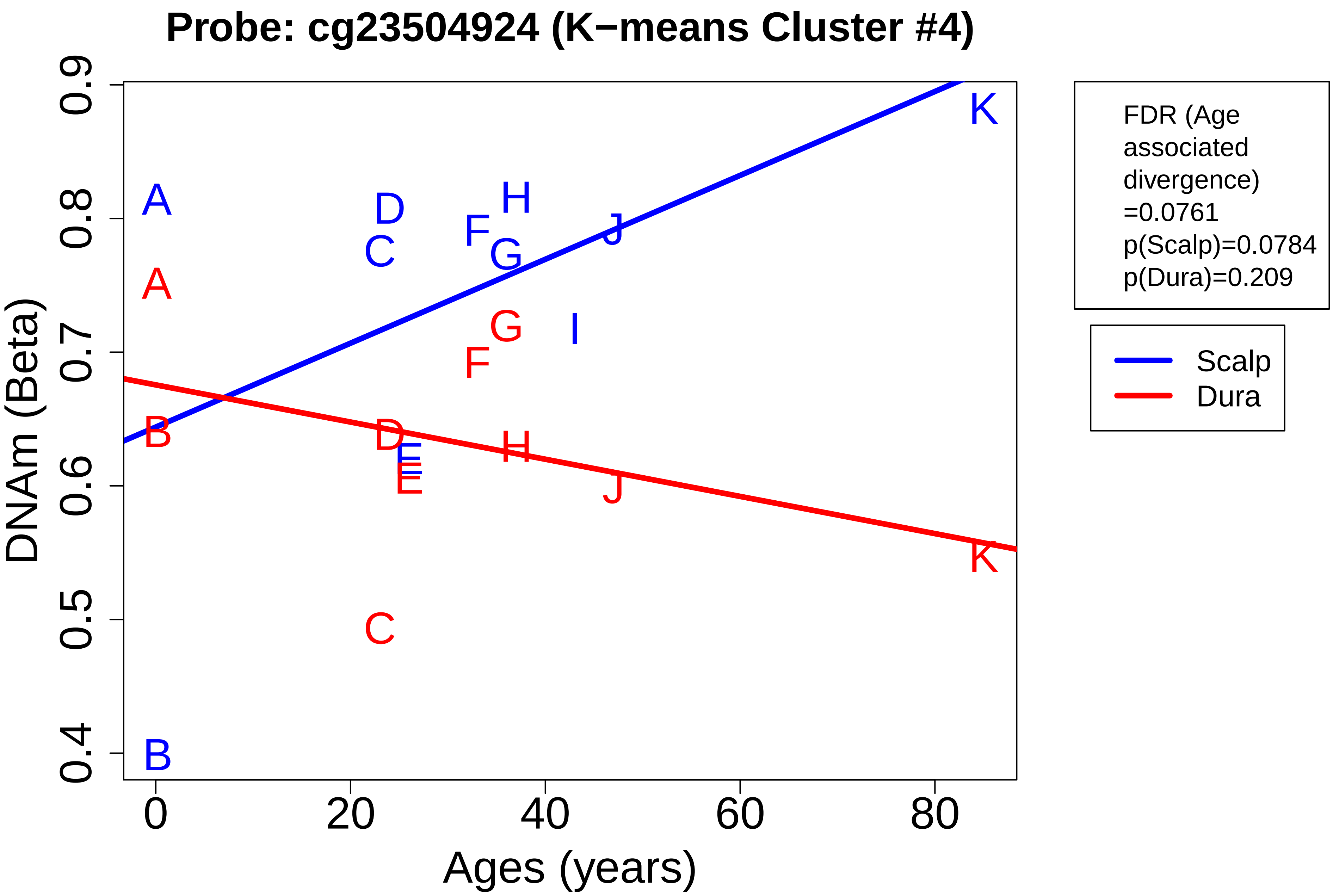

Probe: cg23504924 (K−means Cluster #4)
DNAm (Beta)
0.4
0.5
0.6
0.7
0.8
0.9
0
20
40
60
80
Ages (years)
A
B
C
D
E
F
G
H
I
J
K
A
B
C
D
E
F
G
H
J
K
FDR (Age associated divergence) =0.0761
p(Scalp)=0.0784
p(Dura)=0.209
Scalp
Dura

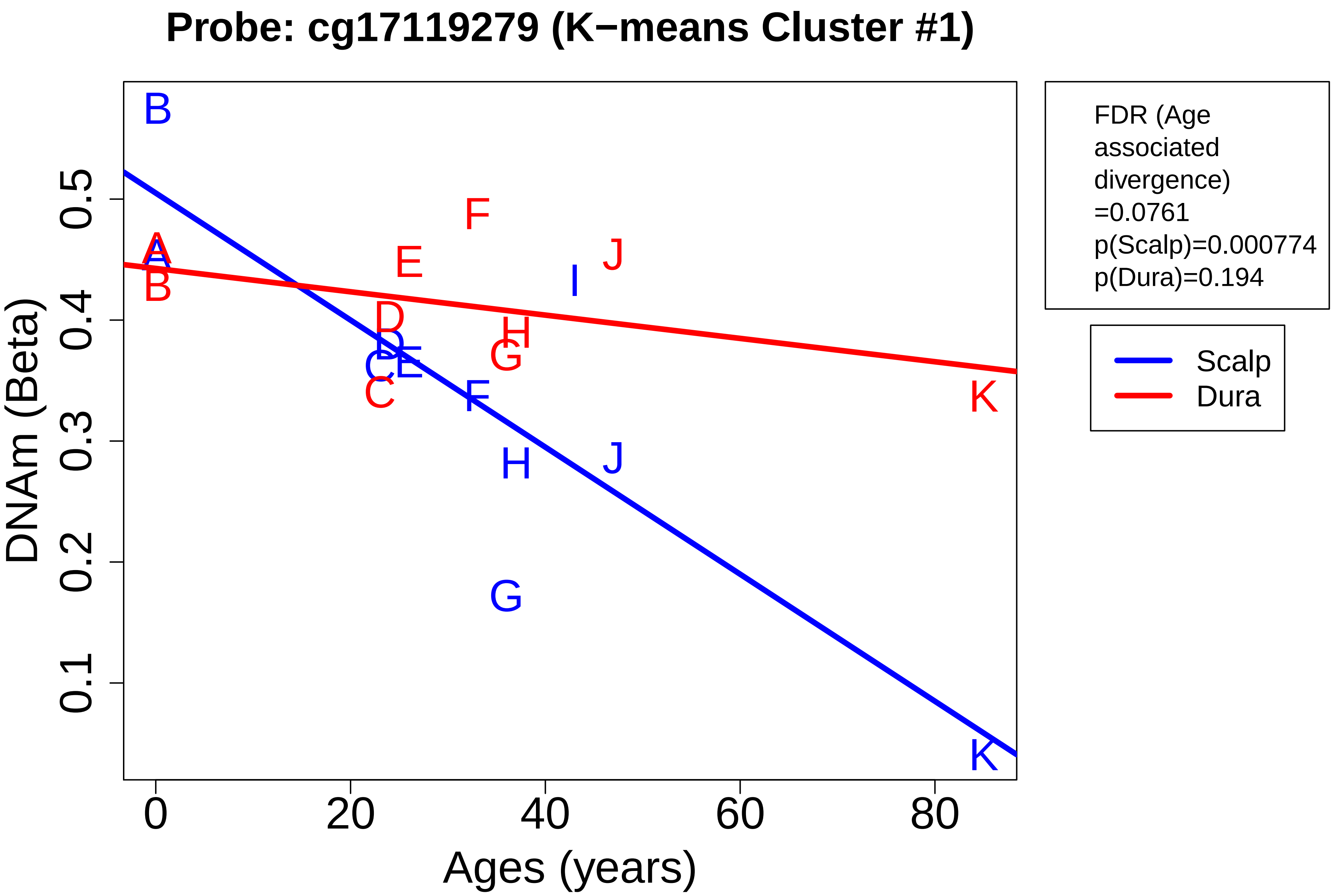

Probe: cg17119279 (K−means Cluster #1)
DNAm (Beta)
Ages (years)
0.1
0.2
0.3
0.4
0.5
0
20
40
60
80
FDR (Age associated divergence) =0.0761
p(Scalp)=0.000774
p(Dura)=0.194
Scalp
Dura

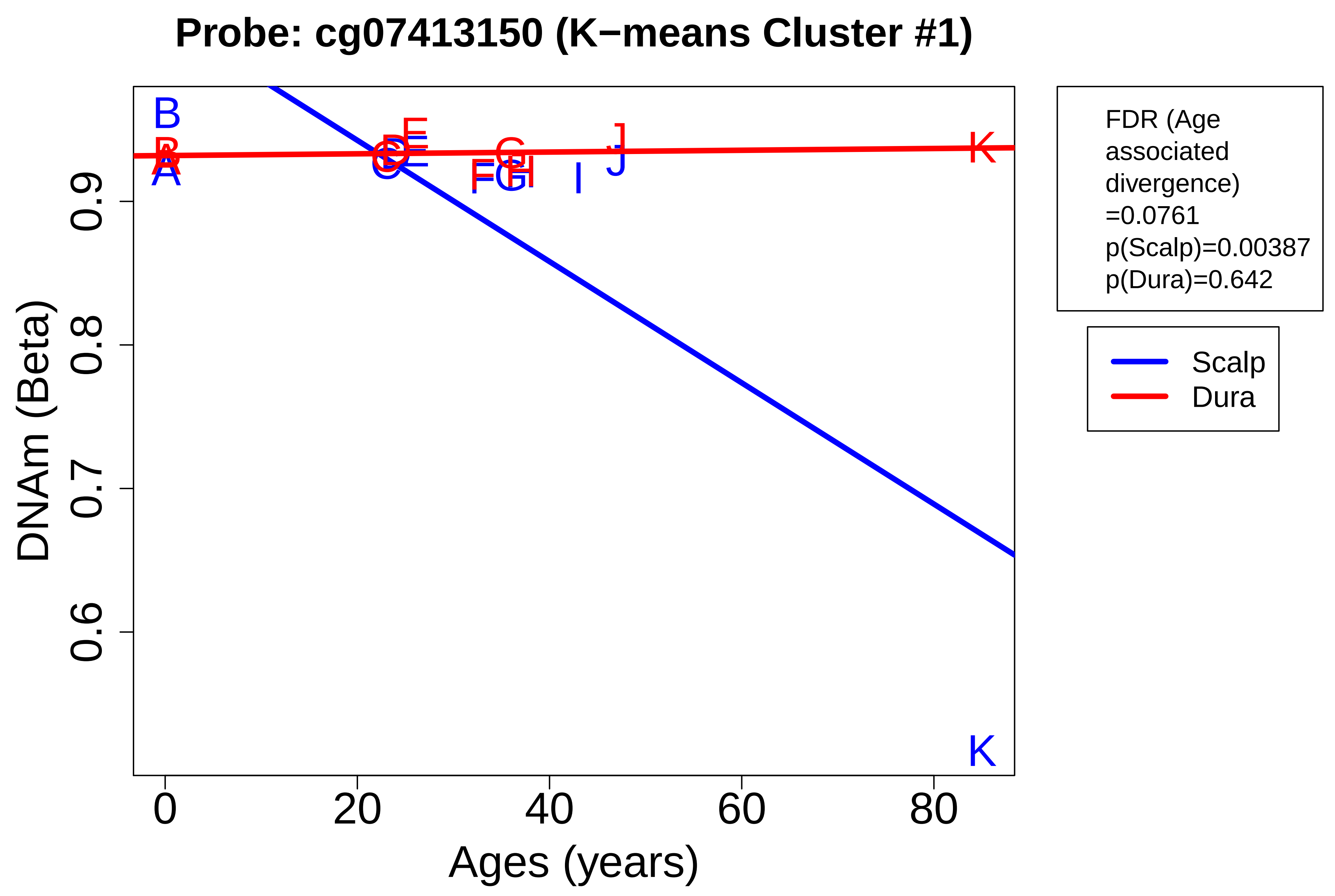

Probe: cg07413150 (K−means Cluster #1)
DNAm (Beta)
0.6
0.7
0.8
0.9
Ages (years)
0
20
40
60
80
FDR (Age associated divergence) =0.0761
p(Scalp)=0.00387
p(Dura)=0.642
Scalp
Dura

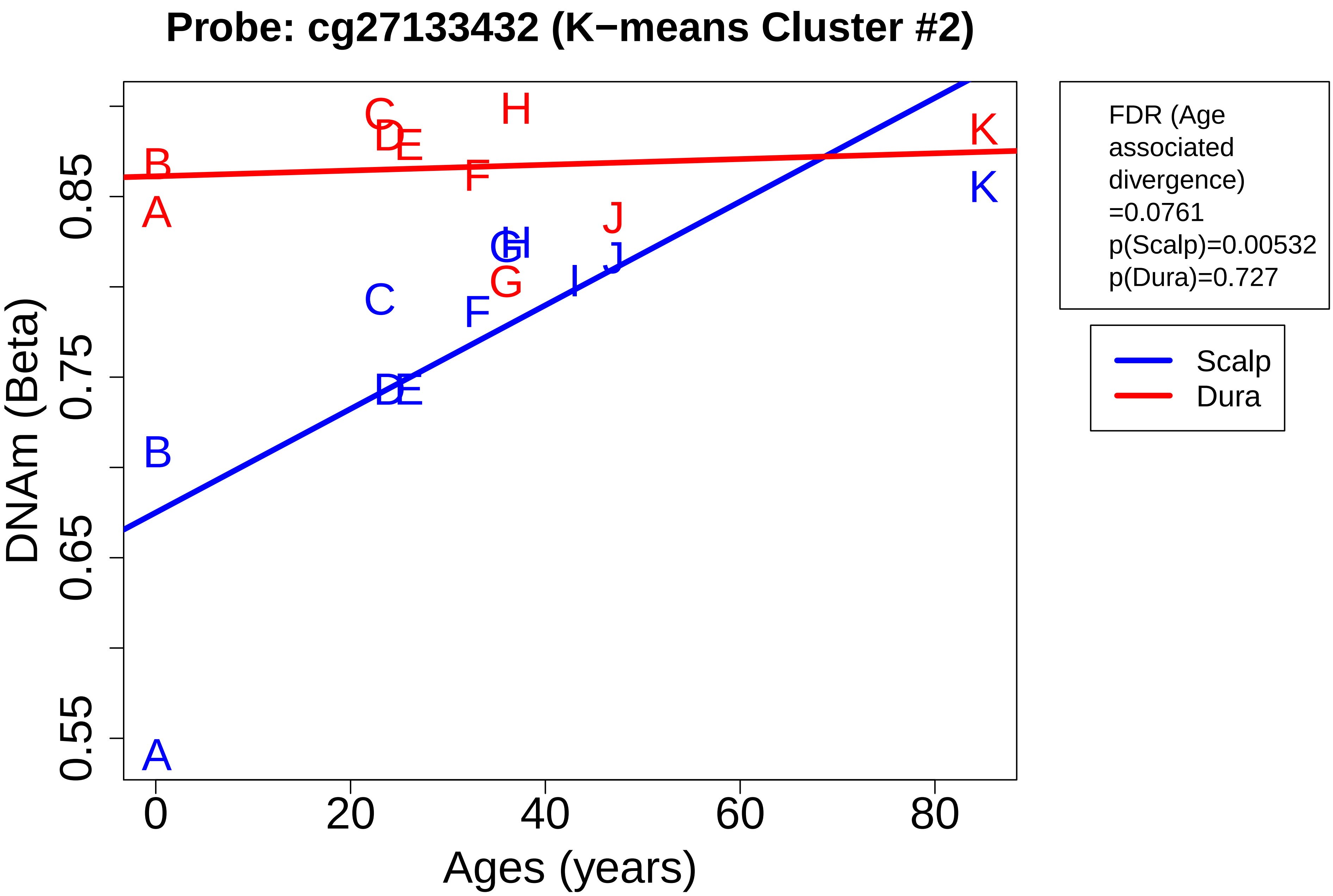

Probe: cg27133432 (K−means Cluster #2)
DNAm (Beta)
0.55
0.65
0.75
0.85
0
20
40
60
80
Ages (years)
A
B
C
D
E
F
G
H
I
J
K
FDR (Age associated divergence) =0.0761
p(Scalp)=0.00532
p(Dura)=0.727
Scalp
Dura

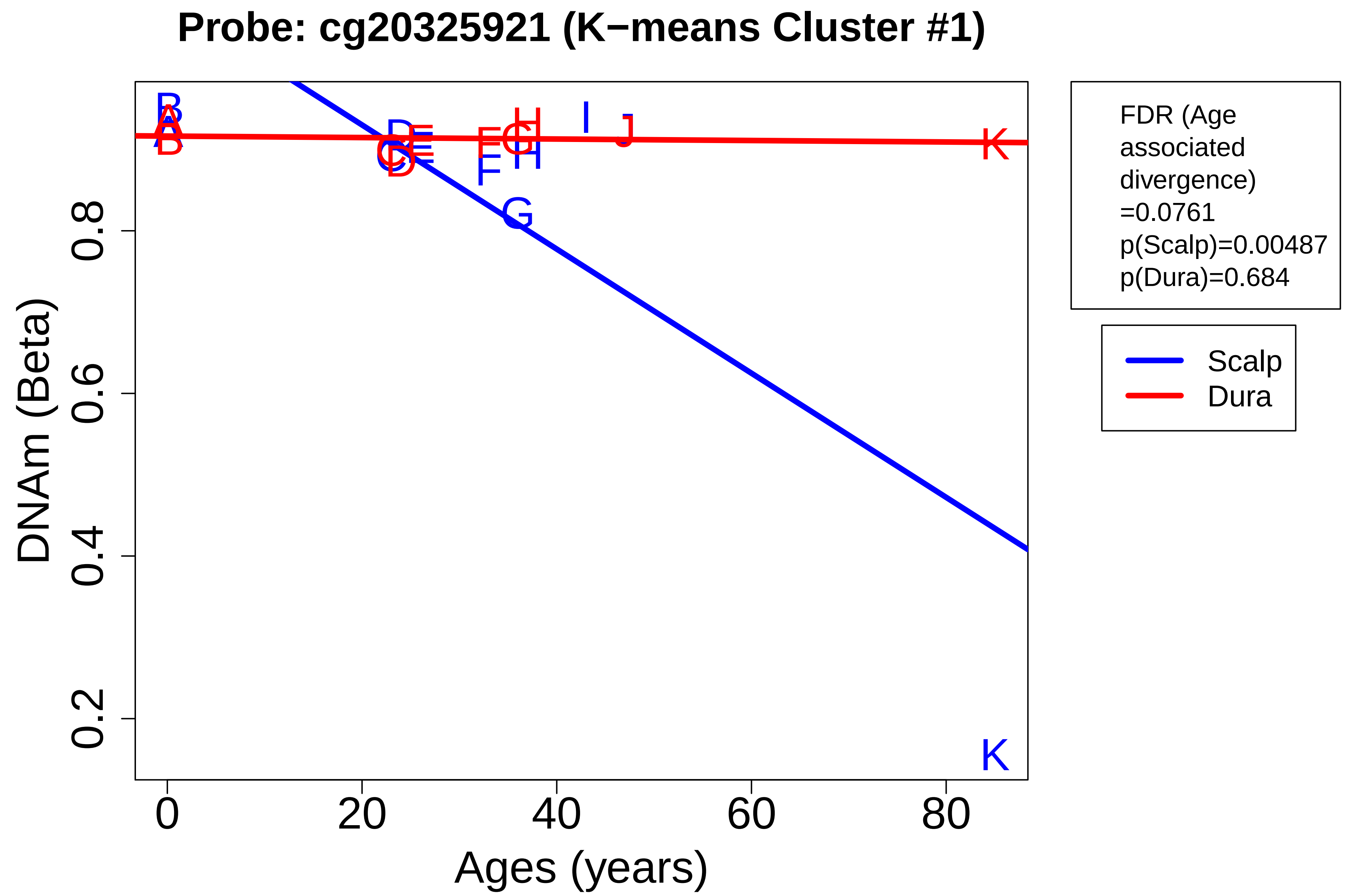

Probe: cg20325921 (K−means Cluster #1)
DNAm (Beta)
0.2
0.4
0.6
0.8
0
20
40
60
80
Ages (years)
FDR (Age associated divergence) =0.0761
p(Scalp)=0.00487
p(Dura)=0.684
Scalp
Dura

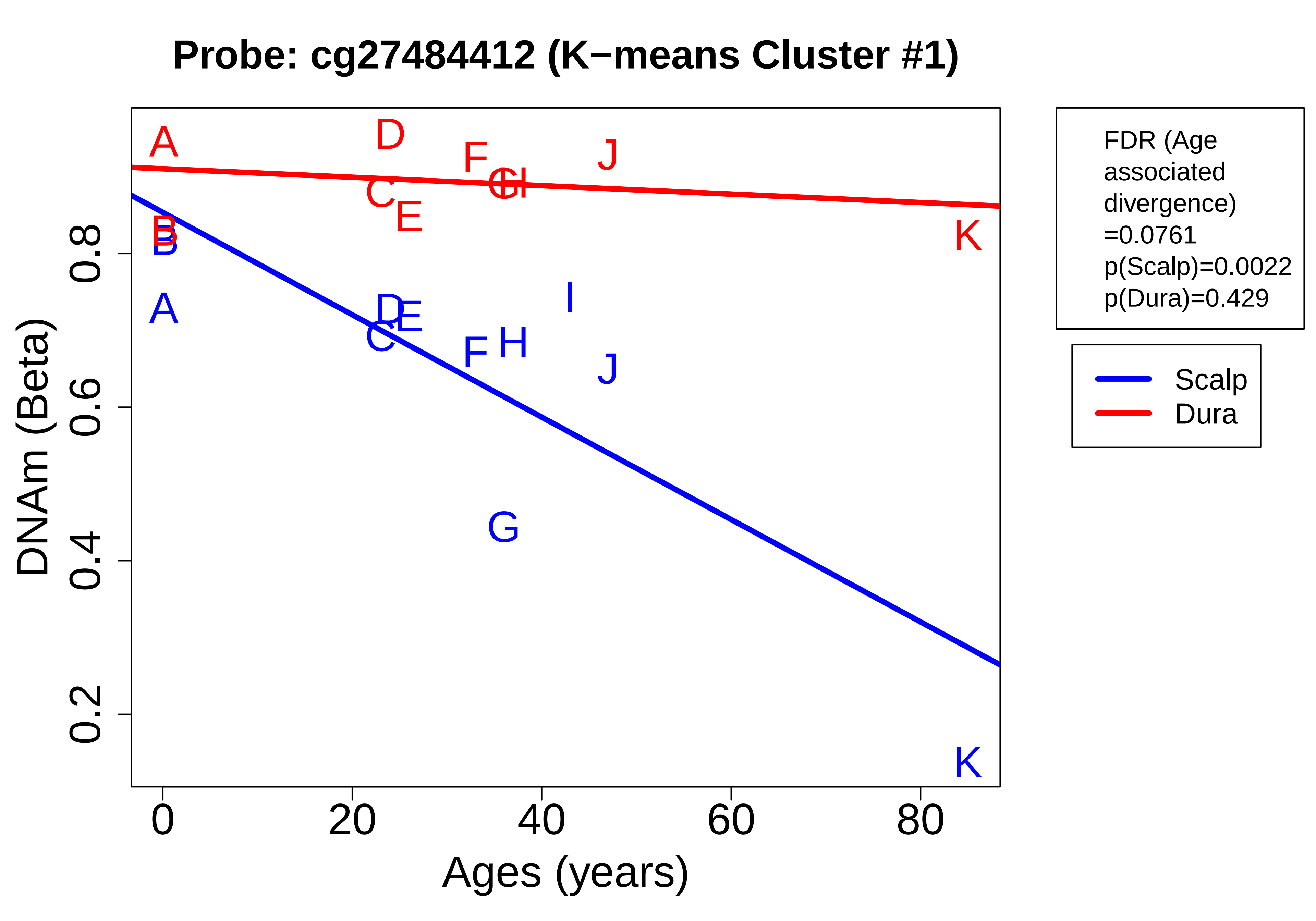

Probe: cg27484412 (K−means Cluster #1)
DNAm (Beta)
0.2
0.4
0.6
0.8
0
20
40
60
80
Ages (years)
A
B
C
D
E
F
G
H
I
J
K
FDR (Age associated divergence) =0.0761
p(Scalp)=0.0022
p(Dura)=0.429
Scalp
Dura

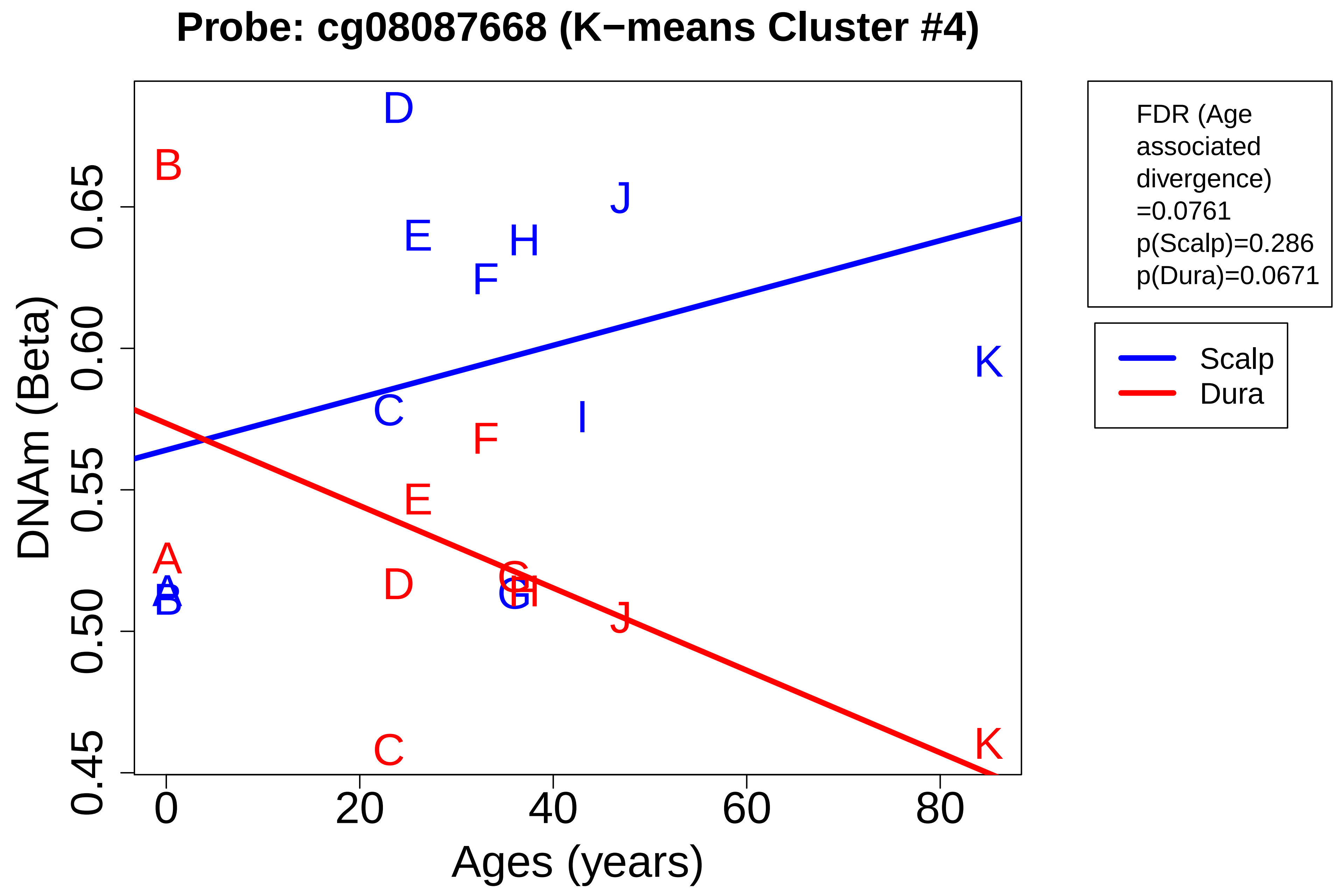

Probe: cg08087668 (K−means Cluster #4)
DNAm (Beta)
0.45
0.50
0.55
0.60
0.65
0
20
40
60
80
Ages (years)
FDR (Age associated divergence) =0.0761
p(Scalp)=0.286
p(Dura)=0.0671
Scalp
Dura

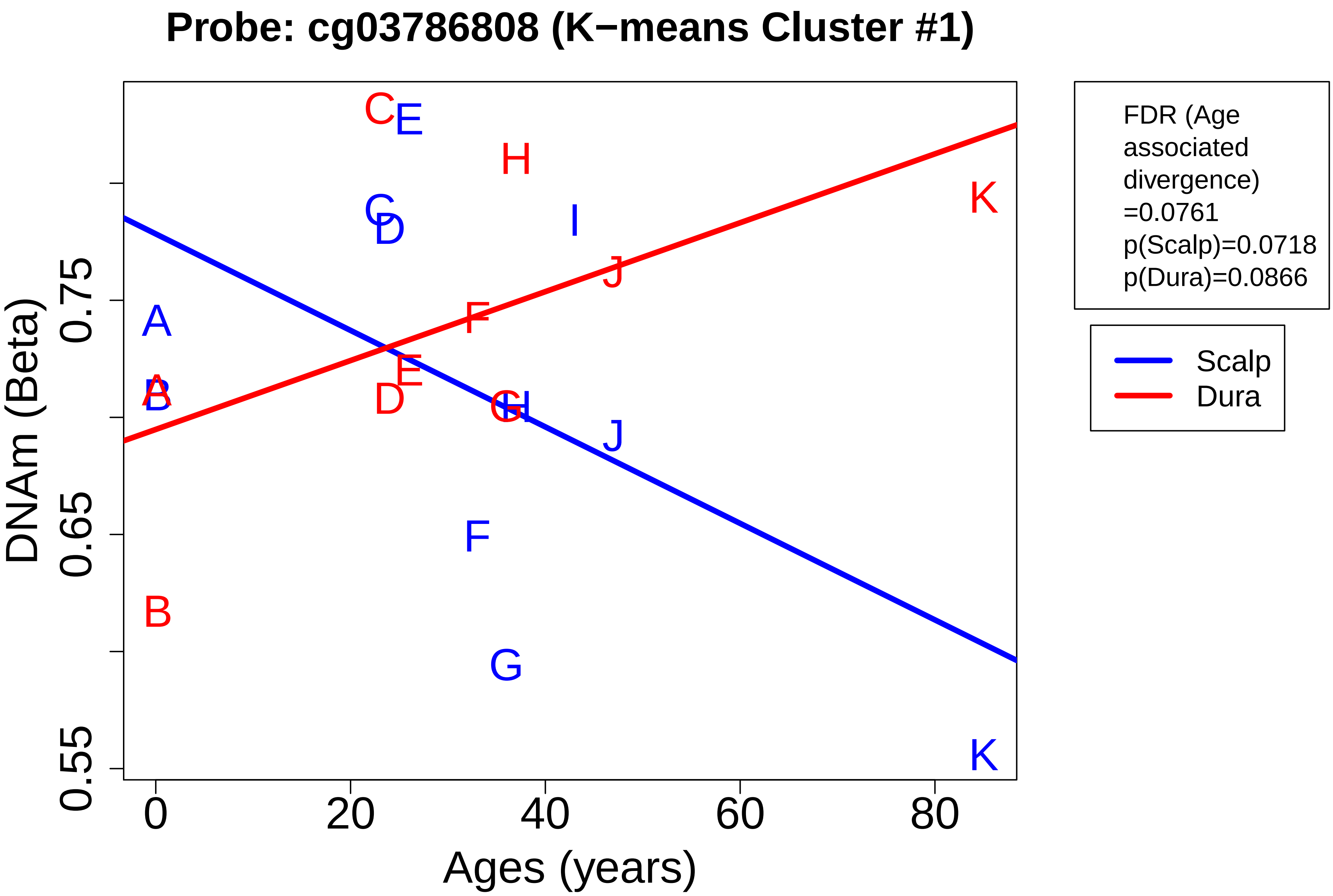

Probe: cg03786808 (K−means Cluster #1)
DNAm (Beta)
0.55
0.65
0.75
Ages (years)
0
20
40
60
80
FDR (Age associated divergence) =0.0761
p(Scalp)=0.0718
p(Dura)=0.0866
Scalp
Dura

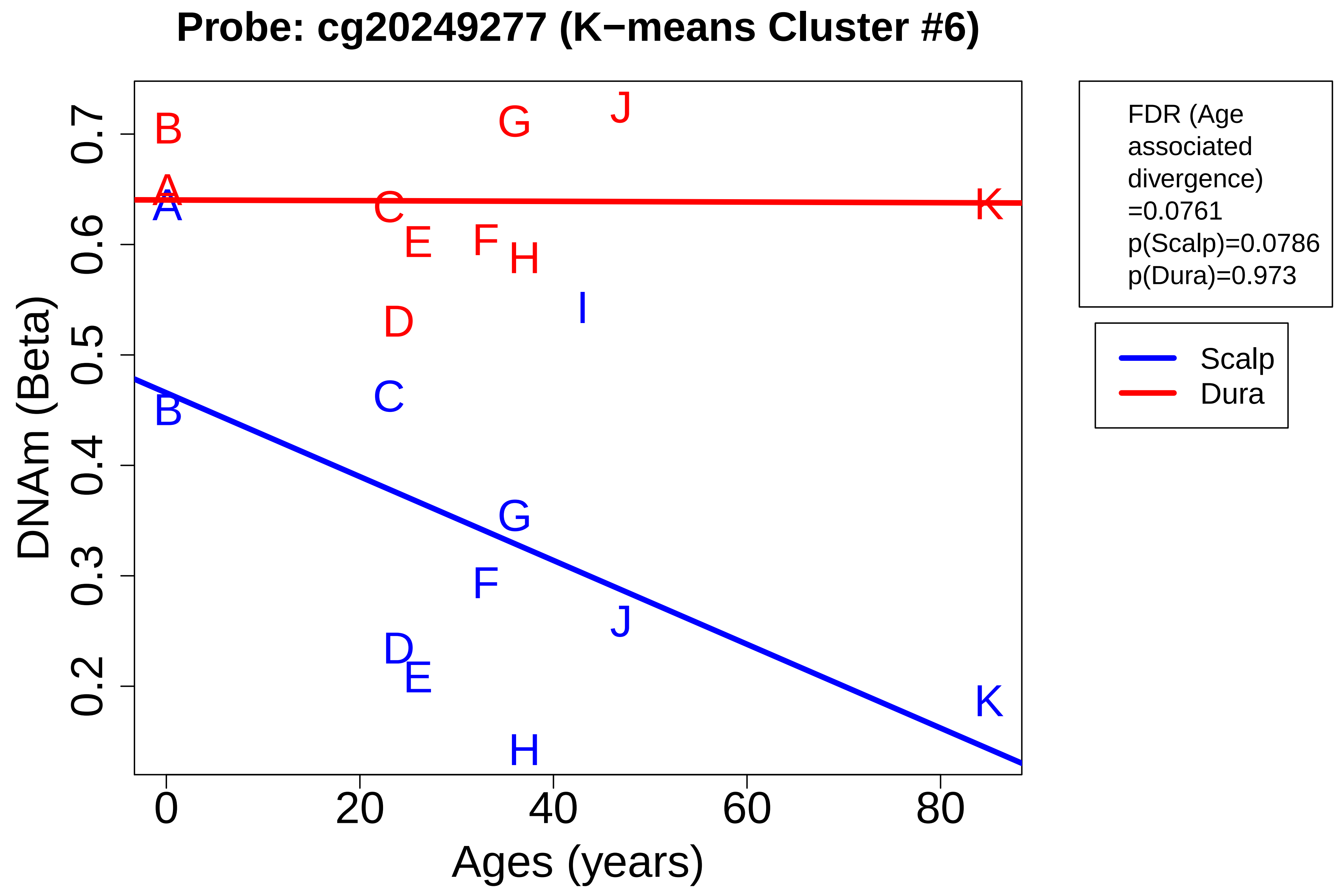

Probe: cg20249277 (K−means Cluster #6)
DNAm (Beta)
0.2
0.3
0.4
0.5
0.6
0.7
Ages (years)
0
20
40
60
80
A
B
C
D
E
F
G
H
I
J
K
FDR (Age associated divergence) =0.0761
p(Scalp)=0.0786
p(Dura)=0.973
Scalp
Dura

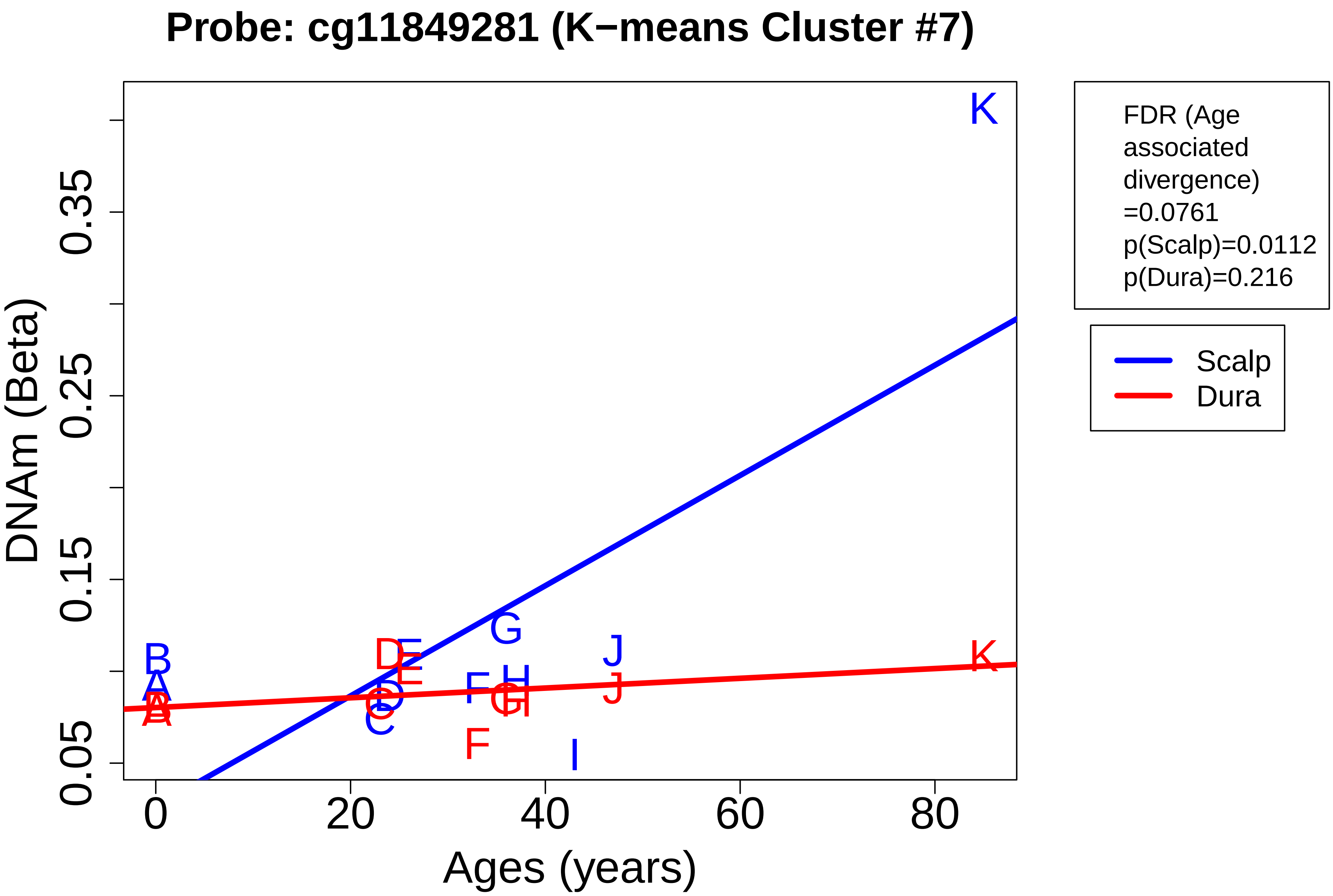

Probe: cg11849281 (K−means Cluster #7)
DNAm (Beta)
0.05
0.15
0.25
0.35
0
20
40
60
80
Ages (years)
FDR (Age associated divergence) =0.0761
p(Scalp)=0.0112
p(Dura)=0.216
Scalp
Dura

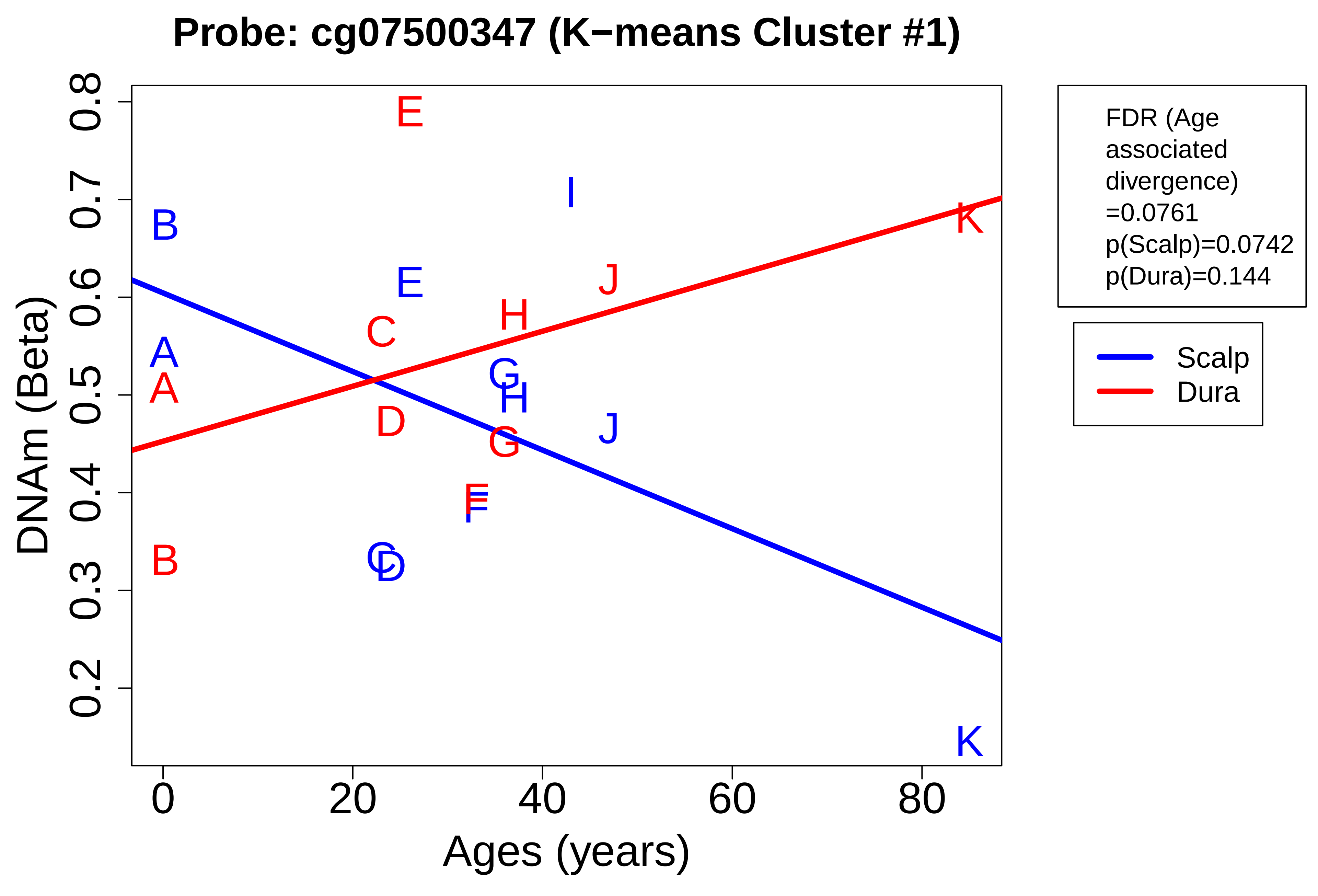

Probe: cg07500347 (K−means Cluster #1)
DNAm (Beta)
0.2
0.3
0.4
0.5
0.6
0.7
0.8
0
20
40
60
80
Ages (years)
A
B
C
D
E
F
G
H
I
J
K
FDR (Age associated divergence) =0.0761
p(Scalp)=0.0742
p(Dura)=0.144
Scalp
Dura

Probe: cg22593177 (K−means Cluster #4)

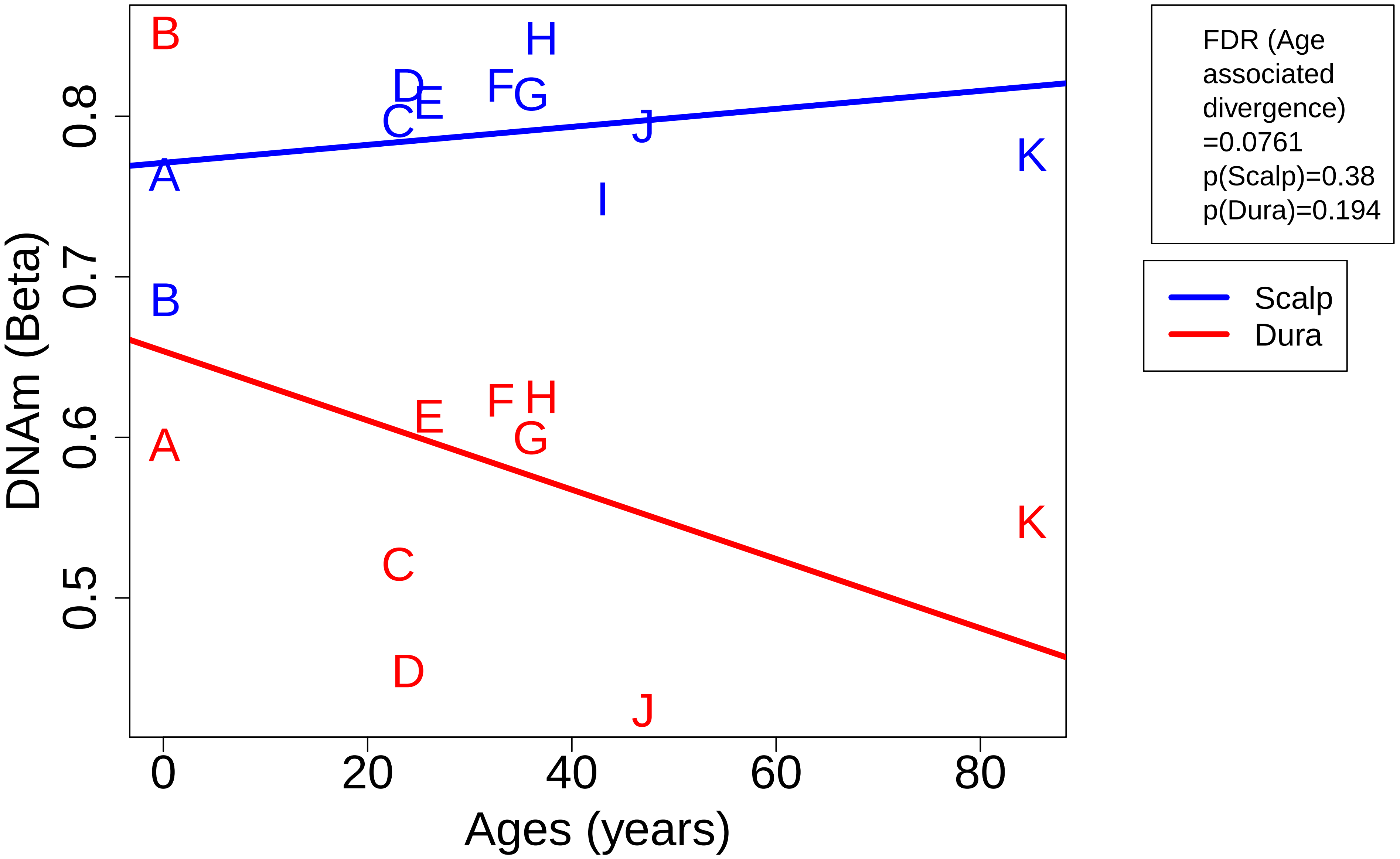

Probe: cg05760107 (K−means Cluster #7)

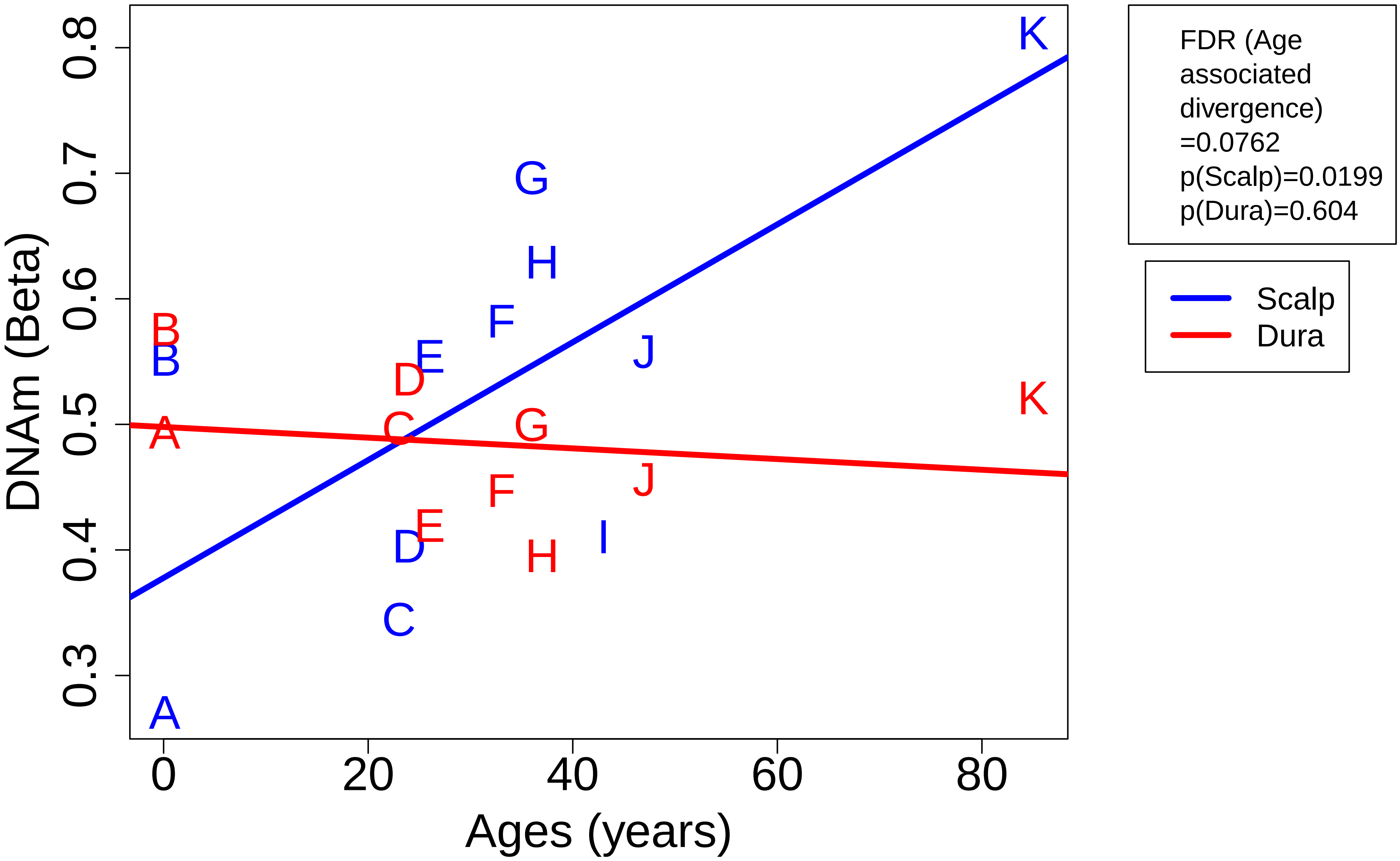

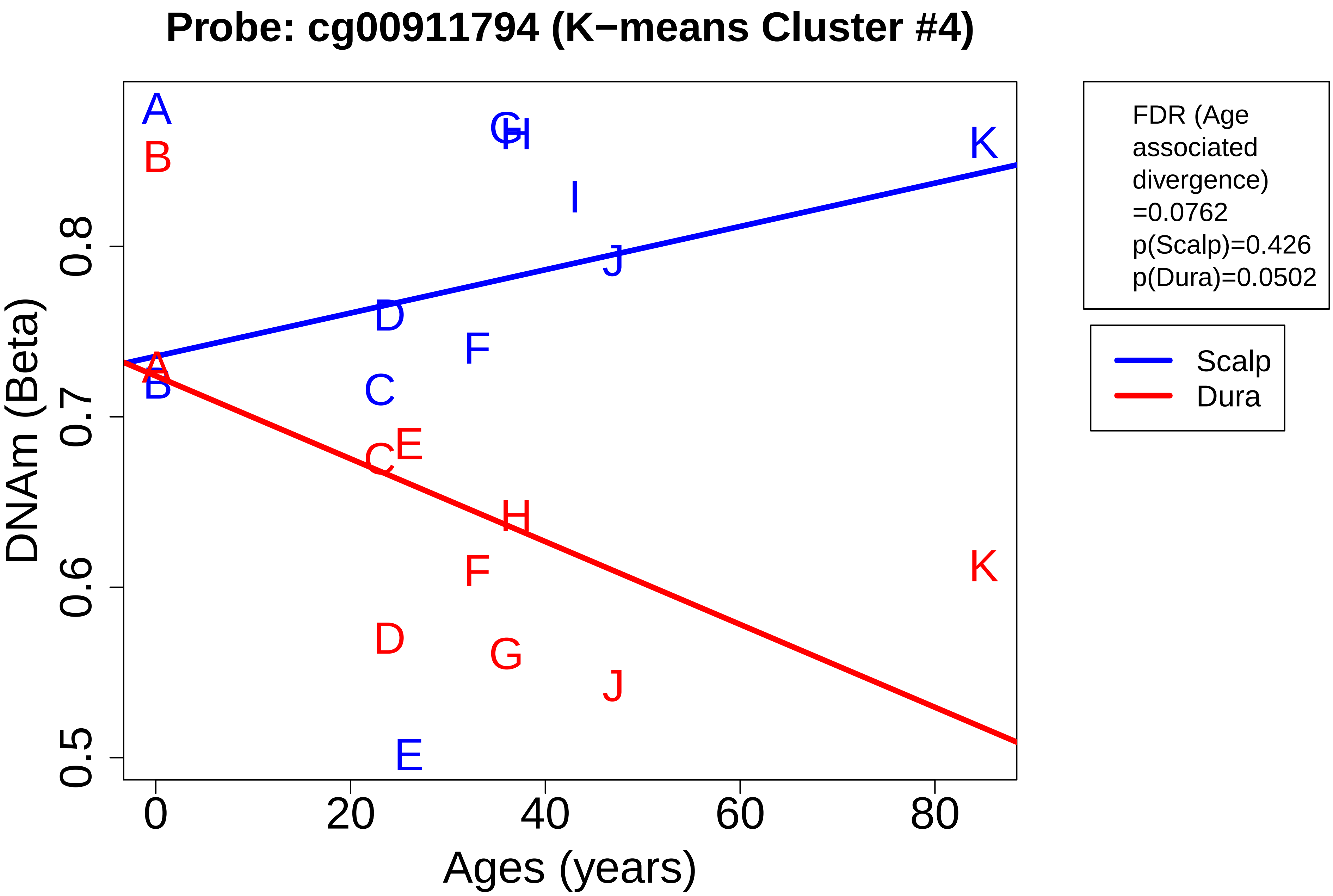

Probe: cg00911794 (K−means Cluster #4)
DNAm (Beta)
0.5
0.6
0.7
0.8
0
20
40
60
80
Ages (years)
A
B
C
D
E
F
G
H
I
J
K
FDR (Age associated divergence) =0.0762
p(Scalp)=0.426
p(Dura)=0.0502
Scalp
Dura

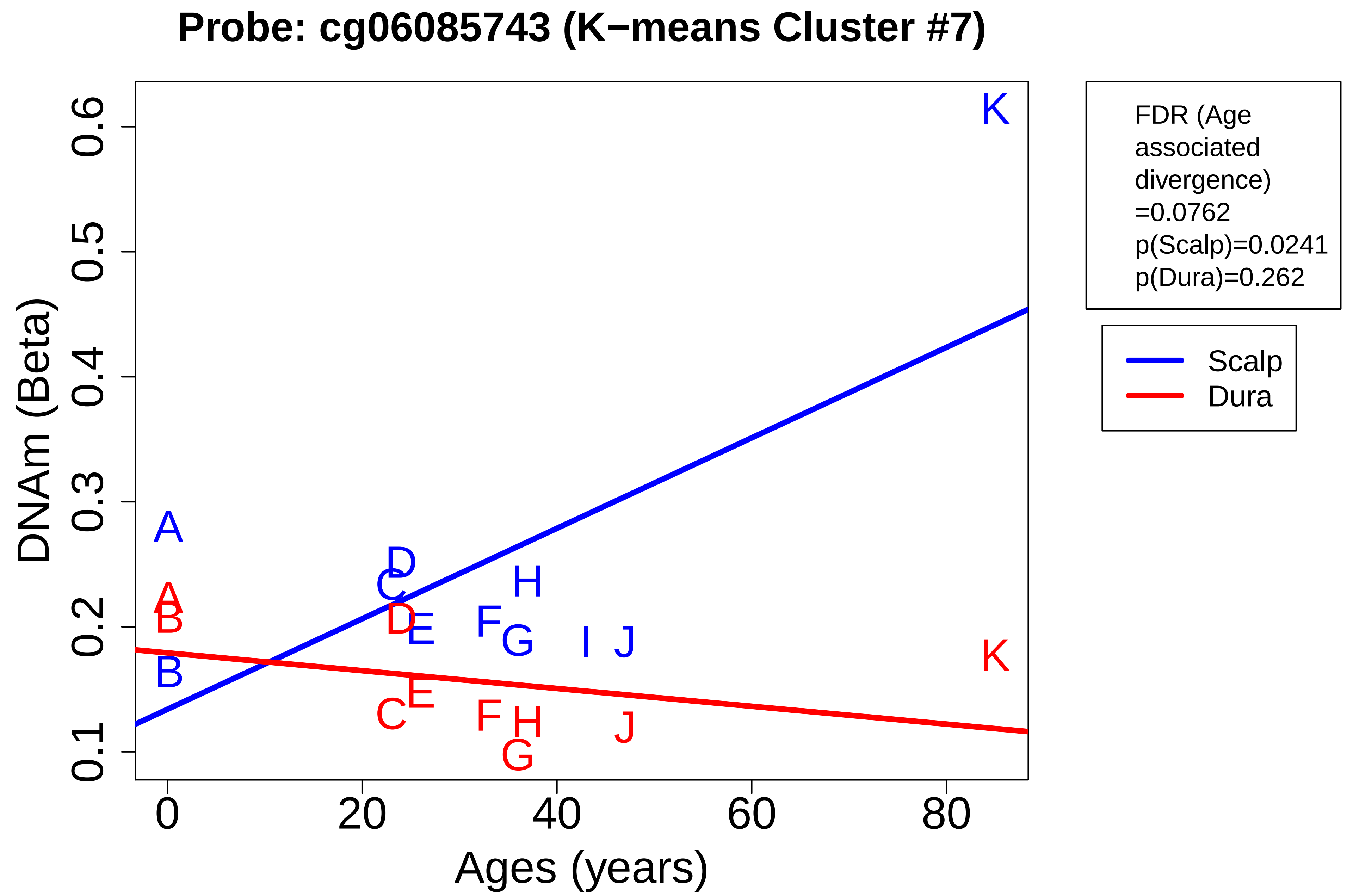

Probe: cg06085743 (K−means Cluster #7)
DNAm (Beta)
0.1
0.2
0.3
0.4
0.5
0.6
Ages (years)
0
20
40
60
80
FDR (Age associated divergence) =0.0762
p(Scalp)=0.0241
p(Dura)=0.262
Scalp
Dura
A B C D E F G H I J K
A B C D E F G H J K

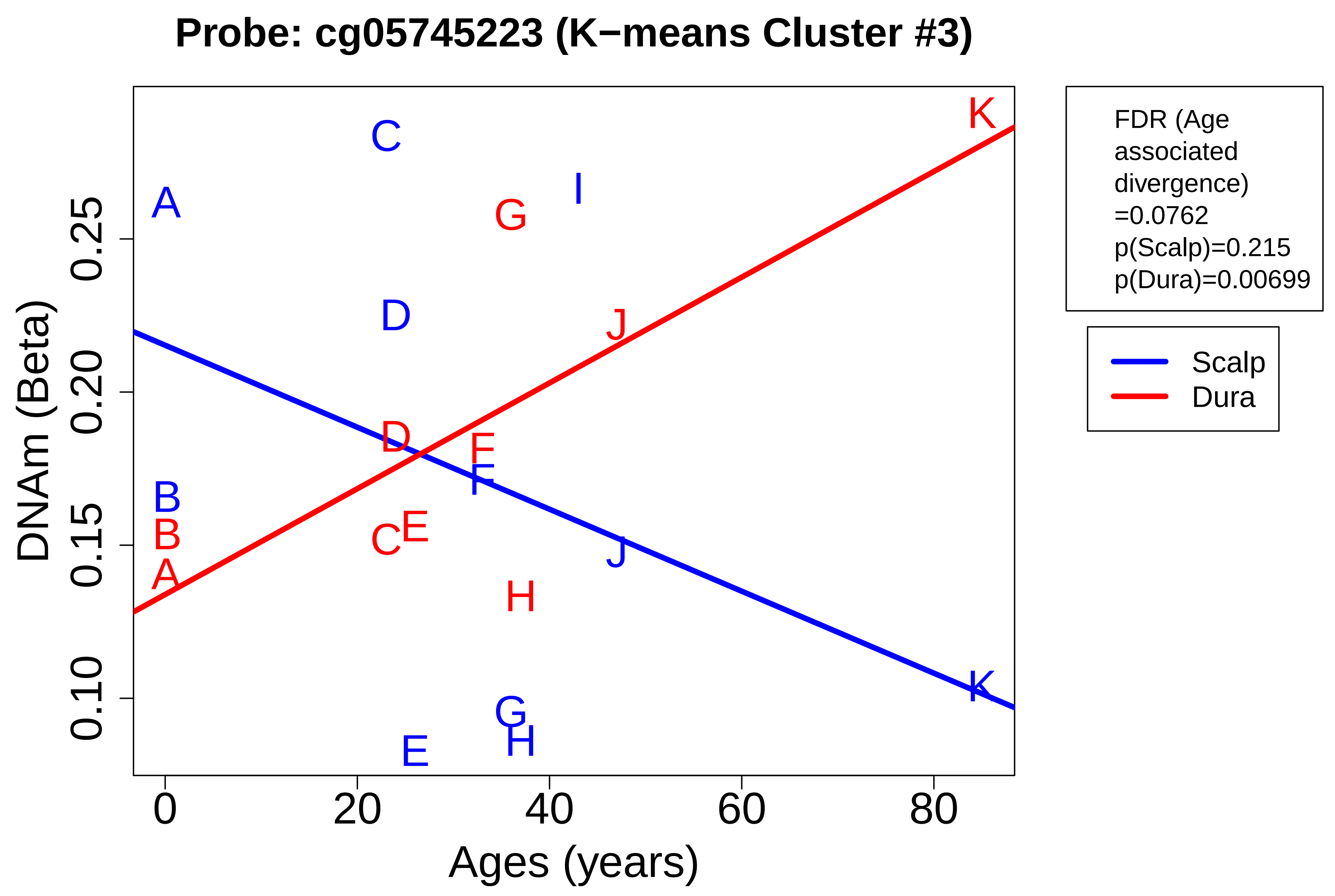

Probe: cg05745223 (K−means Cluster #3)
DNAm (Beta)
0.10
0.15
0.20
0.25
Ages (years)
0
20
40
60
80
FDR (Age associated divergence) =0.0762
p(Scalp)=0.215
p(Dura)=0.00699
Scalp
Dura

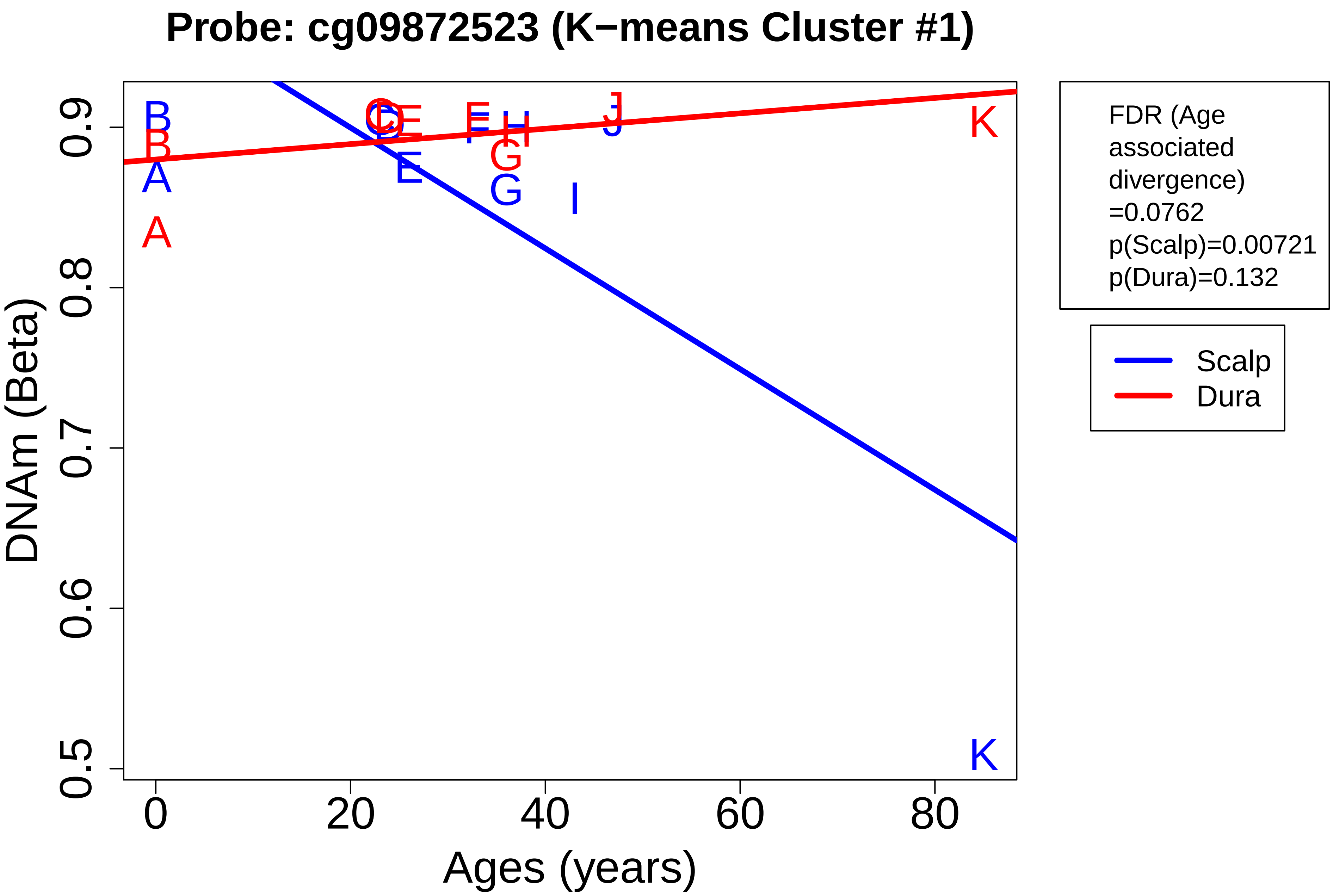

Probe: cg09872523 (K−means Cluster #1)
DNAm (Beta)
0.5
0.6
0.7
0.8
0.9
0
20
40
60
80
Ages (years)
FDR (Age associated divergence) =0.0762
p(Scalp)=0.00721
p(Dura)=0.132
Scalp
Dura

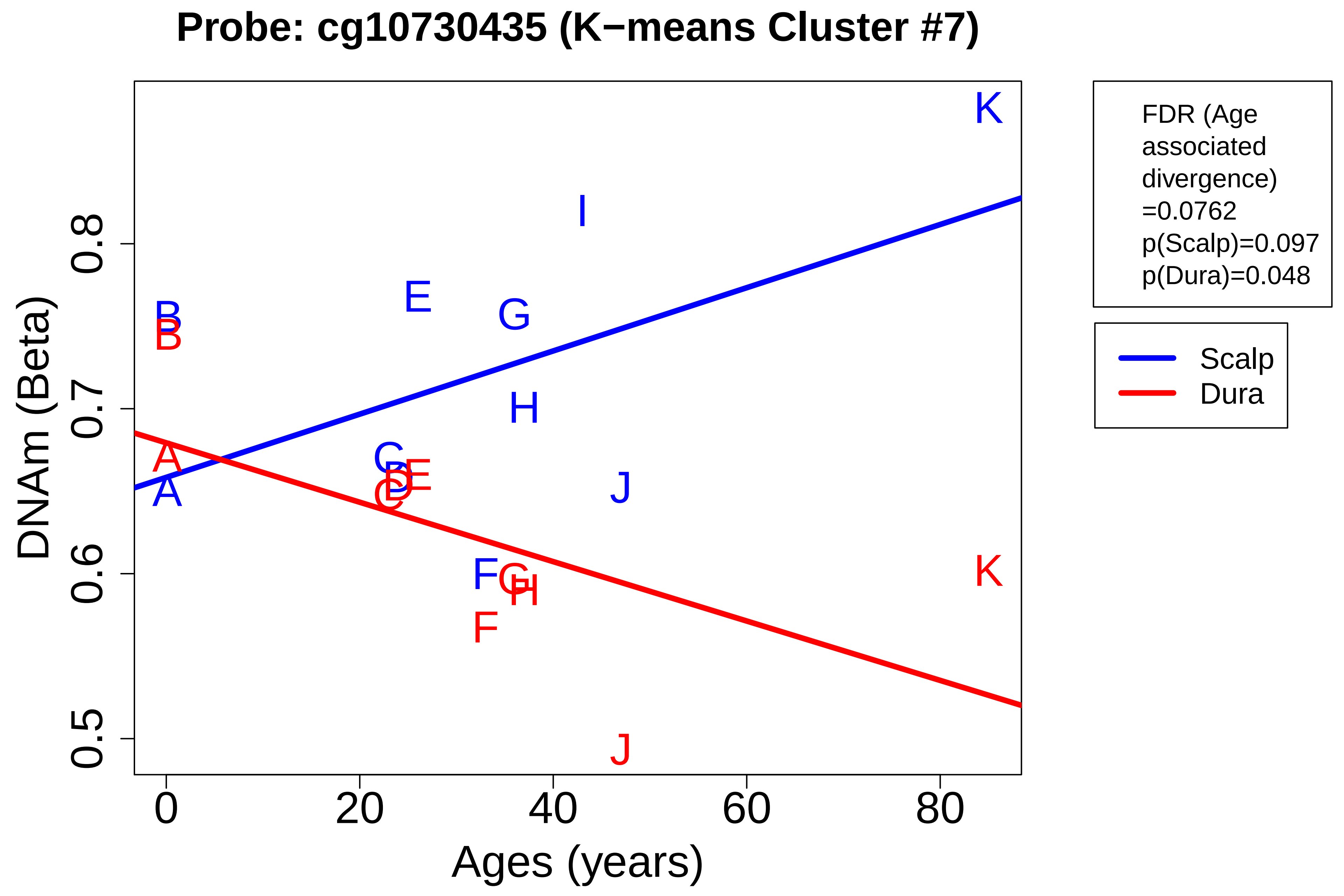

Probe: cg10730435 (K−means Cluster #7)
DNAm (Beta)
0.5
0.6
0.7
0.8
0
20
40
60
80
Ages (years)
FDR (Age associated divergence) =0.0762
p(Scalp)=0.097
p(Dura)=0.048
Scalp
Dura
A
B
C
D
E
F
G
H
I
J
K

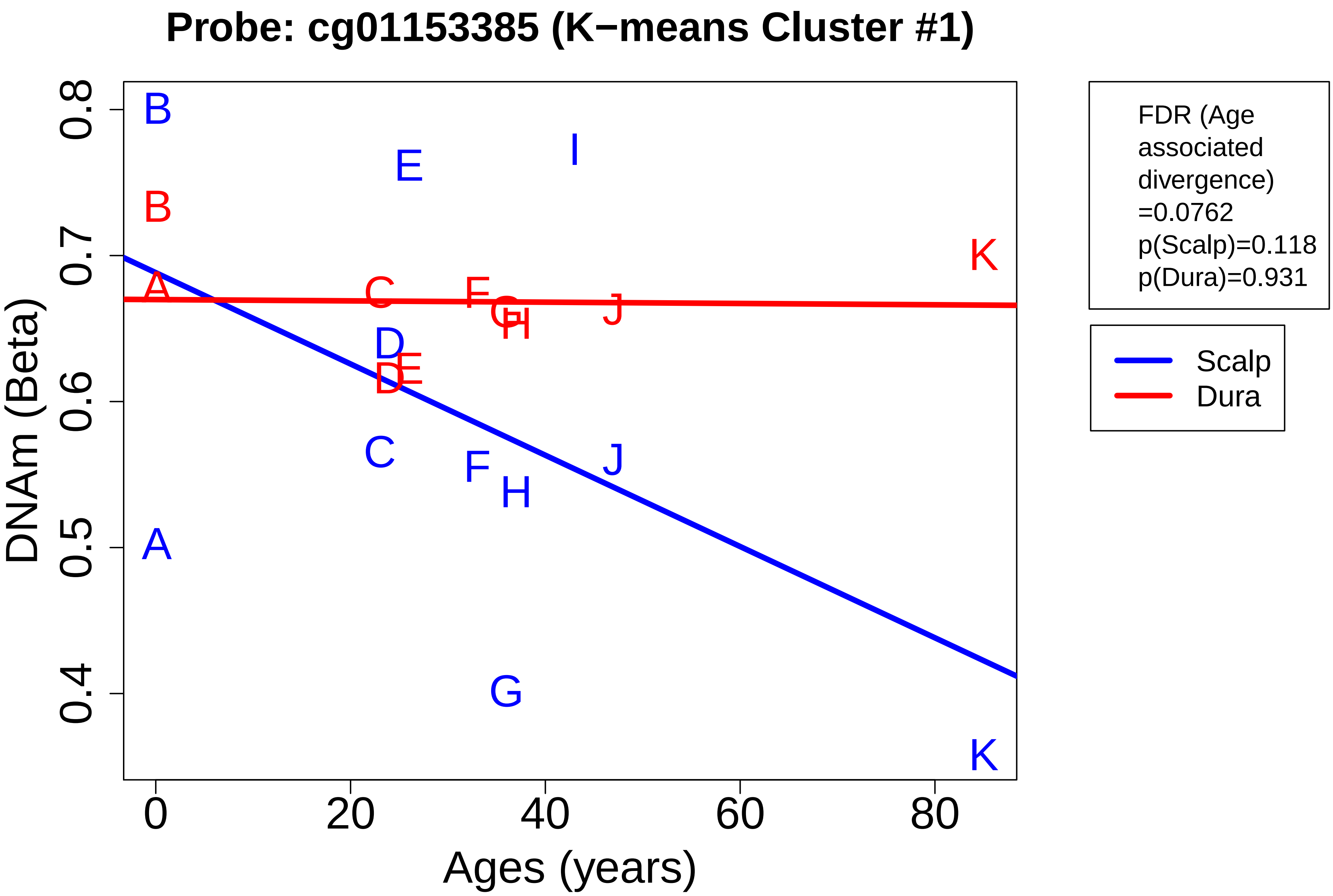

Probe: cg01153385 (K−means Cluster #1)
DNAm (Beta)
0.4
0.5
0.6
0.7
0.8
0
20
40
60
80
Ages (years)
FDR (Age associated divergence) =0.0762
p(Scalp)=0.118
p(Dura)=0.931
Scalp
Dura

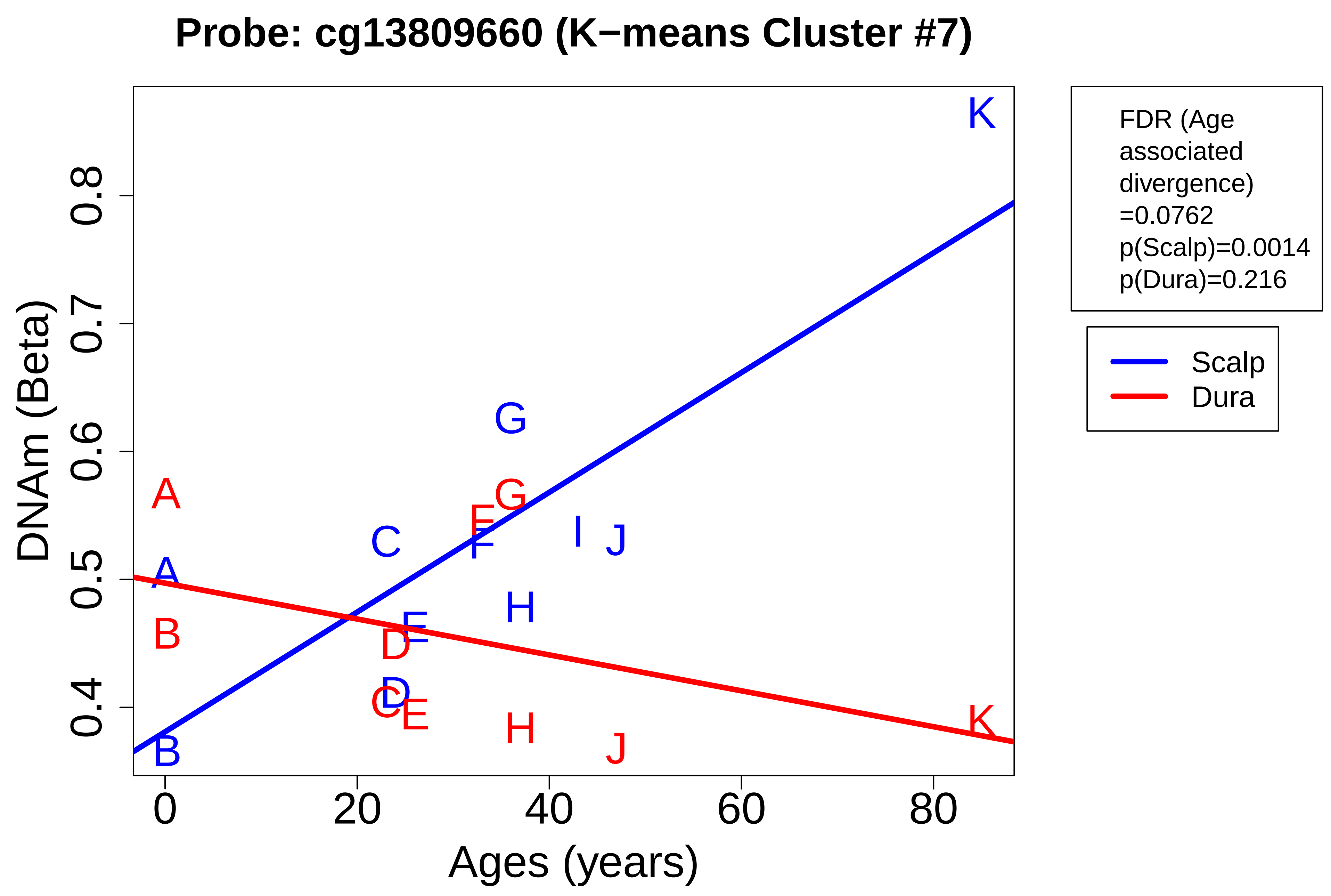

Probe: cg13809660 (K−means Cluster #7)
DNAm (Beta)
0.4
0.5
0.6
0.7
0.8
0
20
40
60
80
Ages (years)
FDR (Age associated divergence) =0.0762
p(Scalp)=0.0014
p(Dura)=0.216
Scalp
Dura

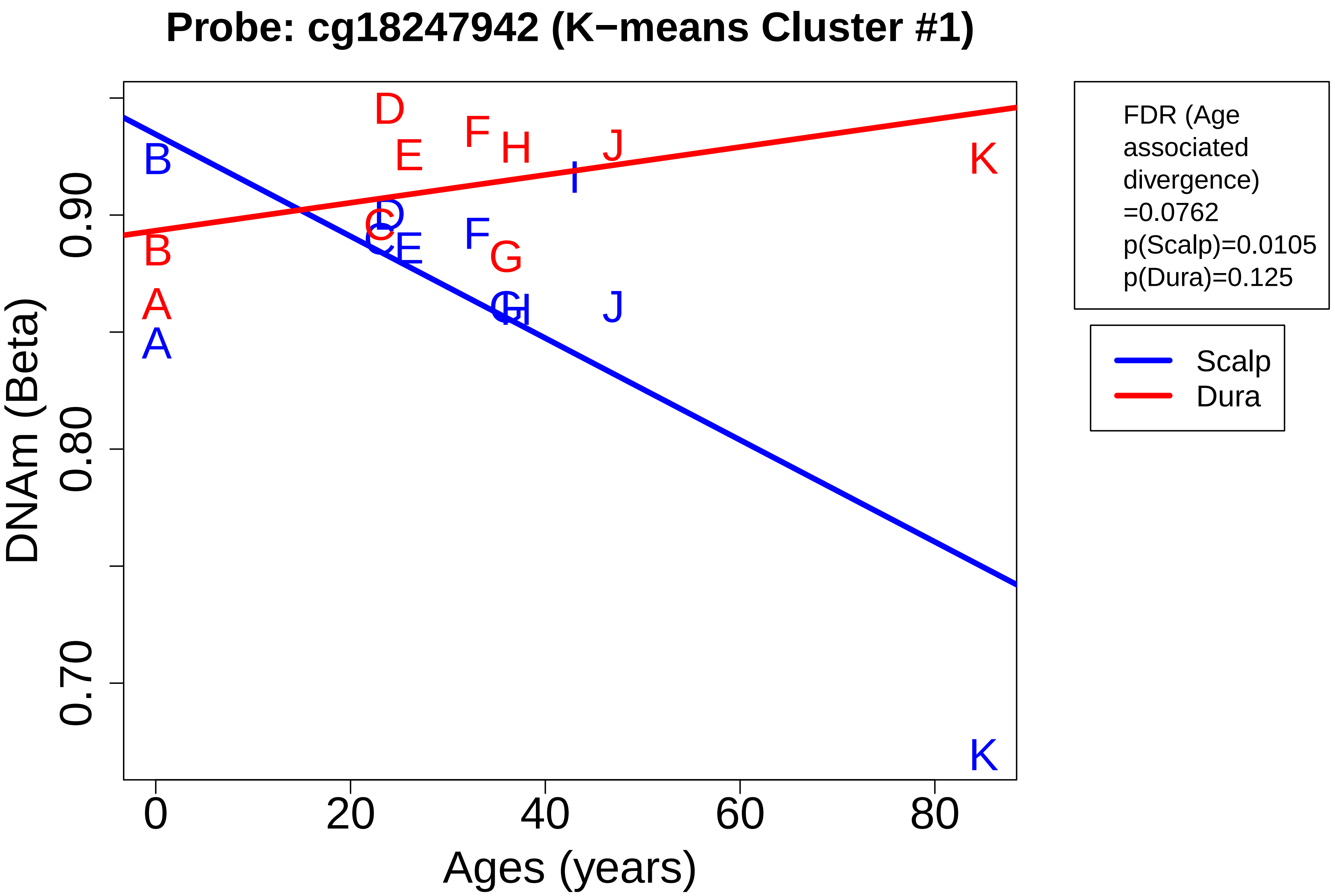
Probe: cg18247942 (K−means Cluster #1)
DNAm (Beta)
0.70
0.80
0.90
0
20
40
60
80
Ages (years)
FDR (Age associated divergence) =0.0762
p(Scalp)=0.0105
p(Dura)=0.125
Scalp
Dura
A
B
C
D
E
F
G
H
I
J
K

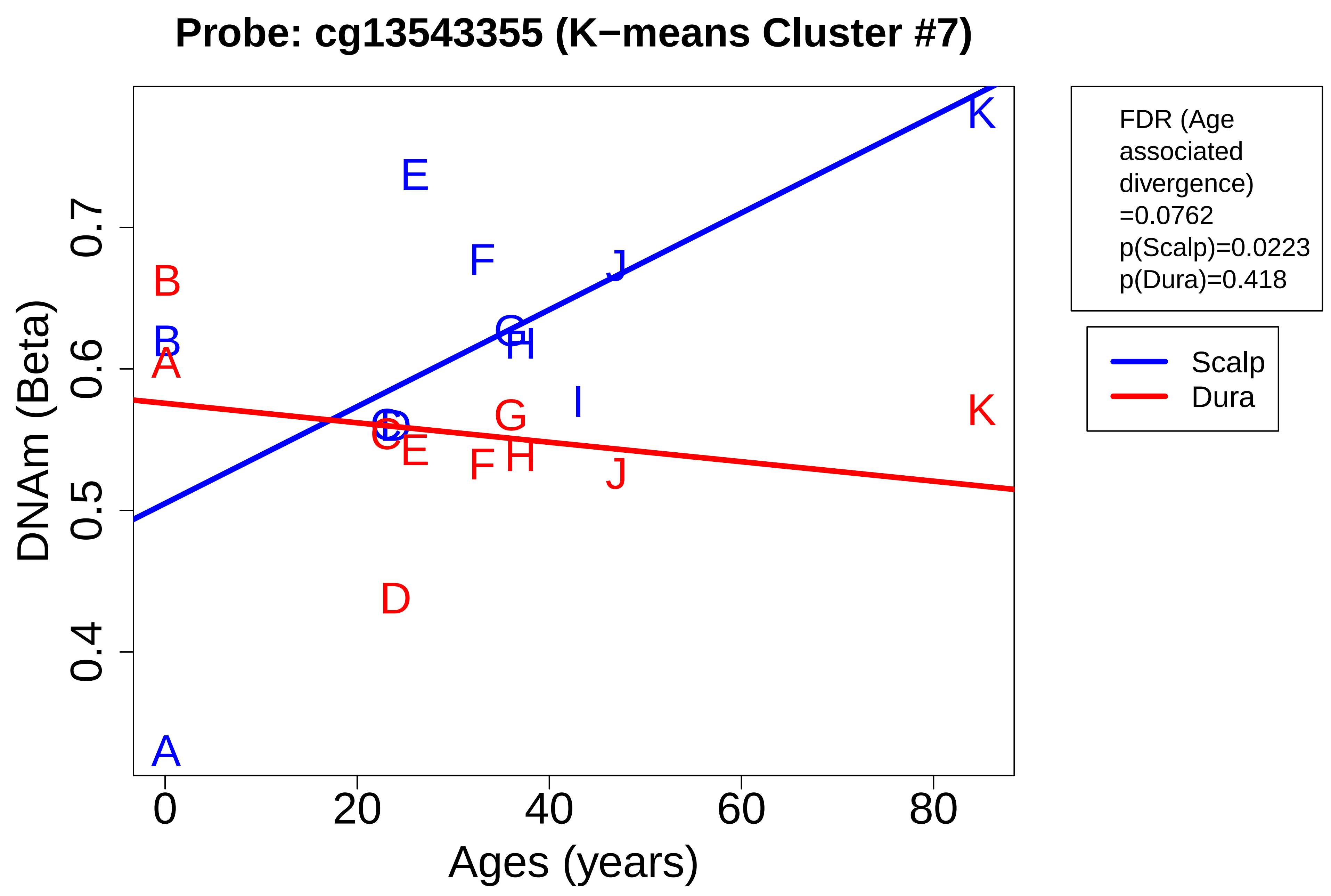

Probe: cg13543355 (K−means Cluster #7)
DNAm (Beta)
0.4
0.5
0.6
0.7
Ages (years)
0
20
40
60
80
A
B
C
D
E
F
G
H
I
J
K
FDR (Age associated divergence) =0.0762
p(Scalp)=0.0223
p(Dura)=0.418
Scalp
Dura

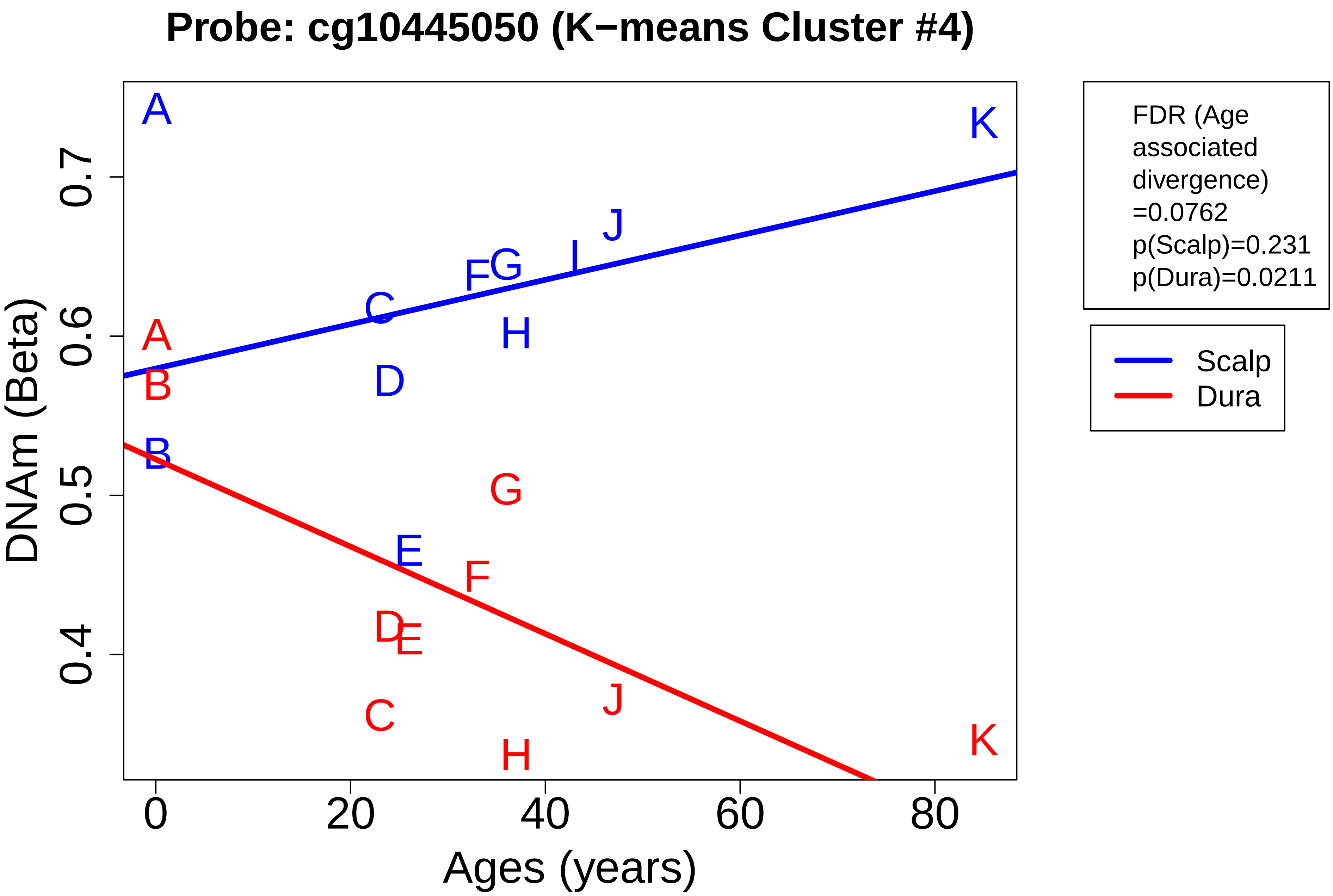

Probe: cg10445050 (K−means Cluster #4)
DNAm (Beta)
0.4
0.5
0.6
0.7
Ages (years)
0
20
40
60
80
FDR (Age associated divergence) =0.0762
p(Scalp)=0.231
p(Dura)=0.0211
Scalp
Dura
A
B
C
D
E
F
G
H
I
J
K

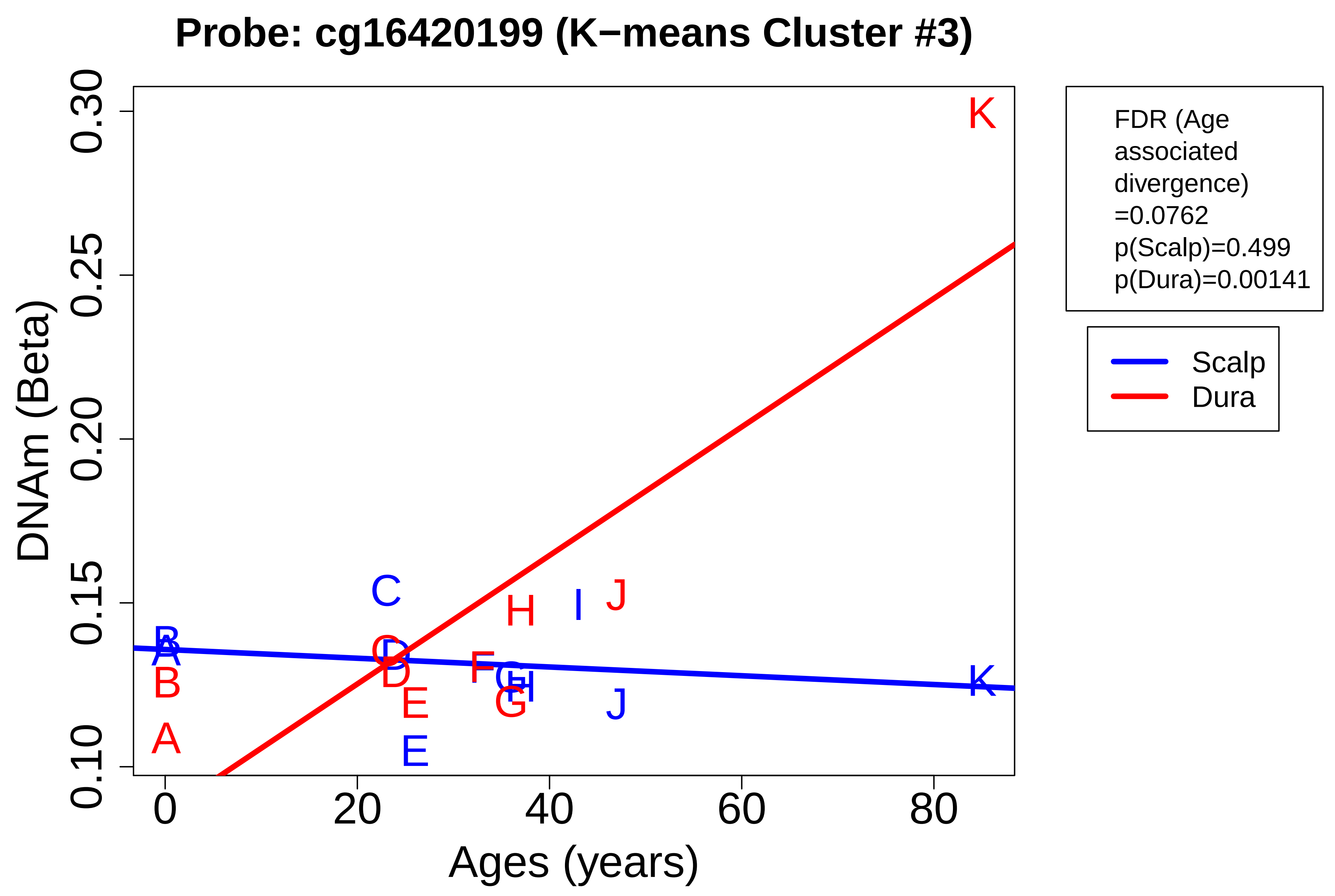

Probe: cg16420199 (K−means Cluster #3)
DNAm (Beta)
0.10
0.15
0.20
0.25
0.30
0
20
40
60
80
Ages (years)
FDR (Age associated divergence) =0.0762
p(Scalp)=0.499
p(Dura)=0.00141
Scalp
Dura
A
B
C
D
E
F
G
H
I
J
K

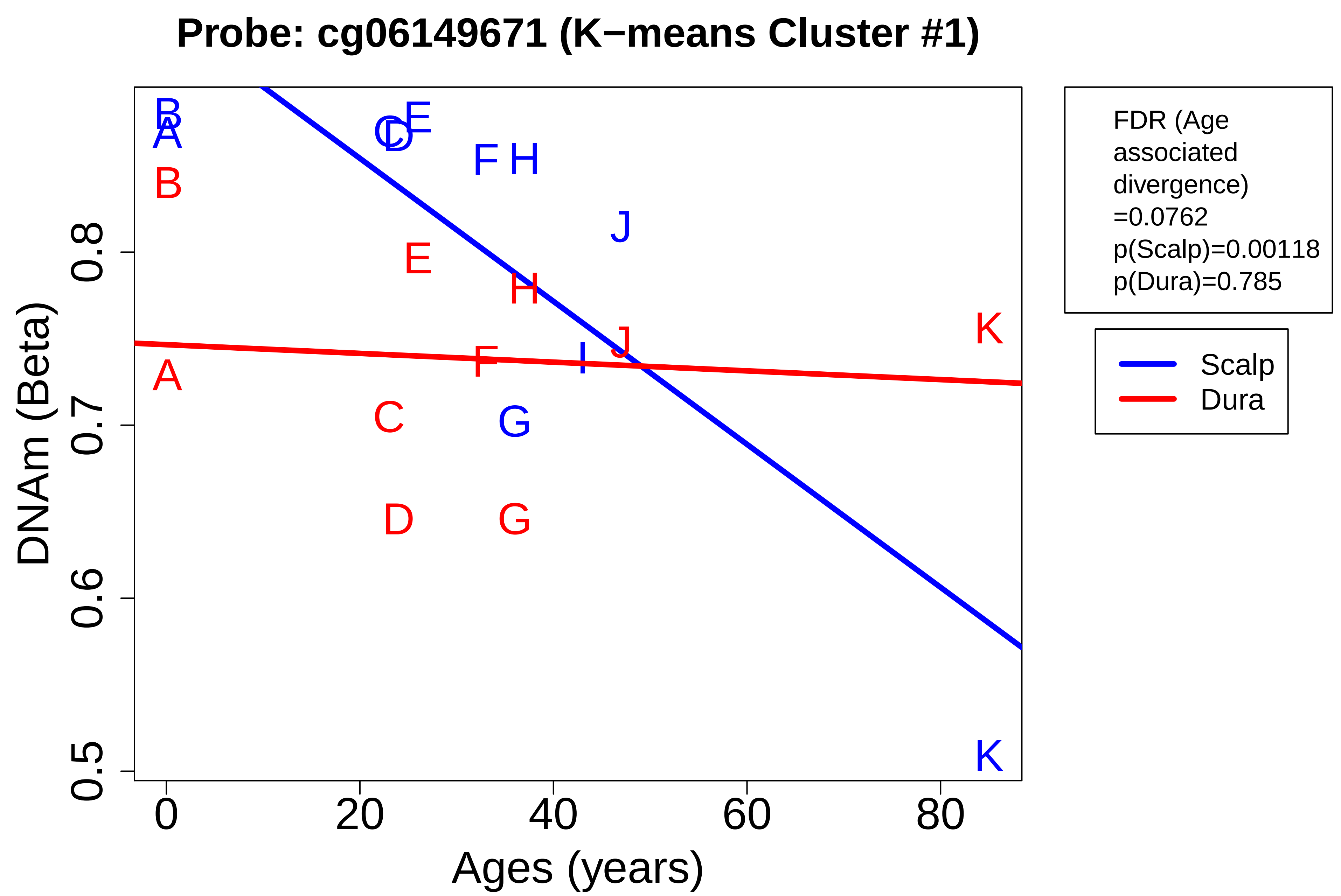

Probe: cg06149671 (K−means Cluster #1)
FDR (Age associated divergence) =0.0762
p(Scalp)=0.00118
p(Dura)=0.785
Scalp
Dura
DNAm (Beta)
0.5
0.6
0.7
0.8
0
20
40
60
80
Ages (years)
A
B
C
D
E
F
G
H
I
J
K

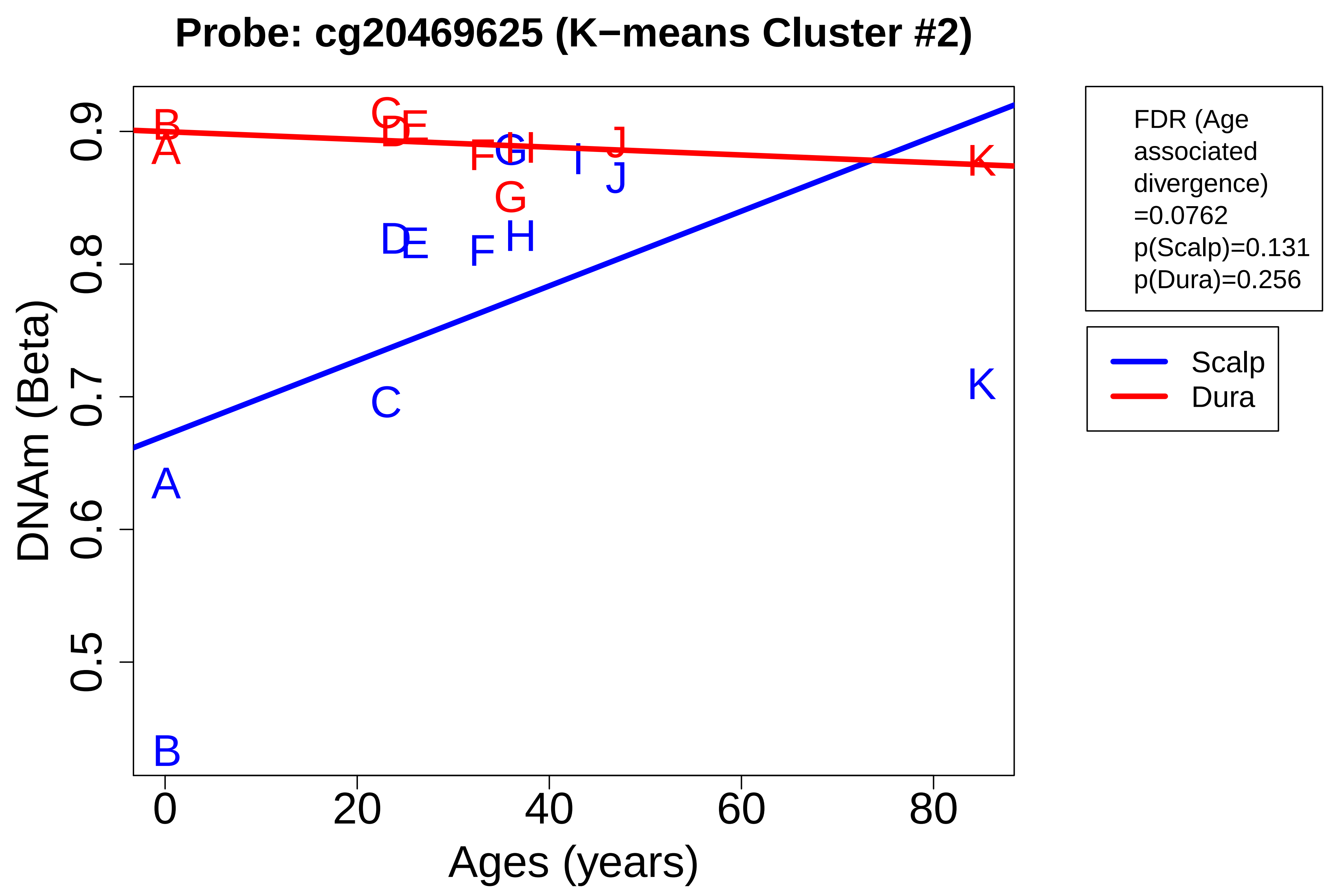

Probe: cg20469625 (K−means Cluster #2)
DNAm (Beta)
0.5
0.6
0.7
0.8
0.9
0
20
40
60
80
Ages (years)
A B C D E F G H I J K
FDR (Age associated divergence) =0.0762
p(Scalp)=0.131
p(Dura)=0.256
Scalp
Dura

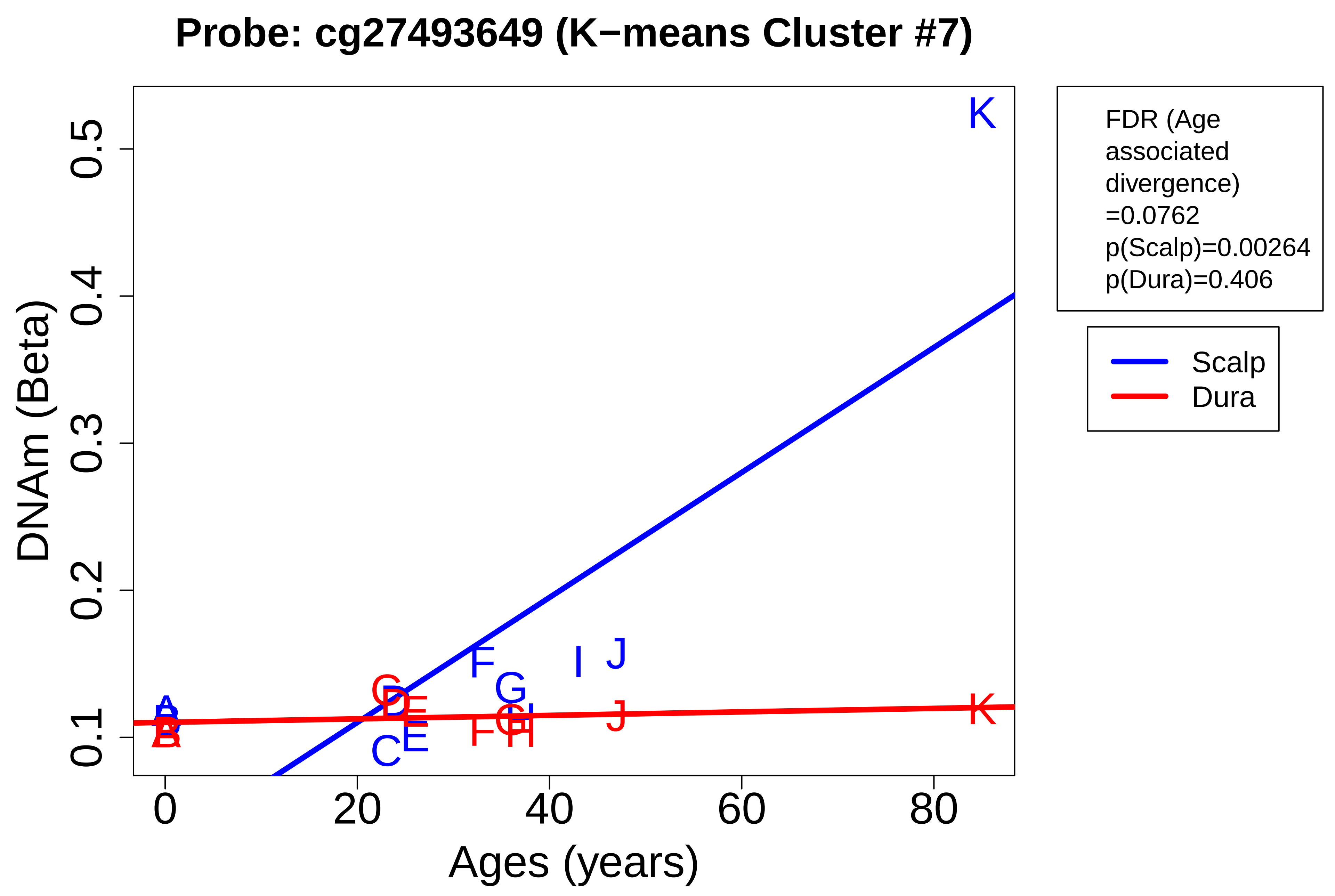

Probe: cg27493649 (K−means Cluster #7)
DNAm (Beta)
0.1
0.2
0.3
0.4
0.5
0
20
40
60
80
Ages (years)
FDR (Age associated divergence) =0.0762
p(Scalp)=0.00264
p(Dura)=0.406
Scalp
Dura

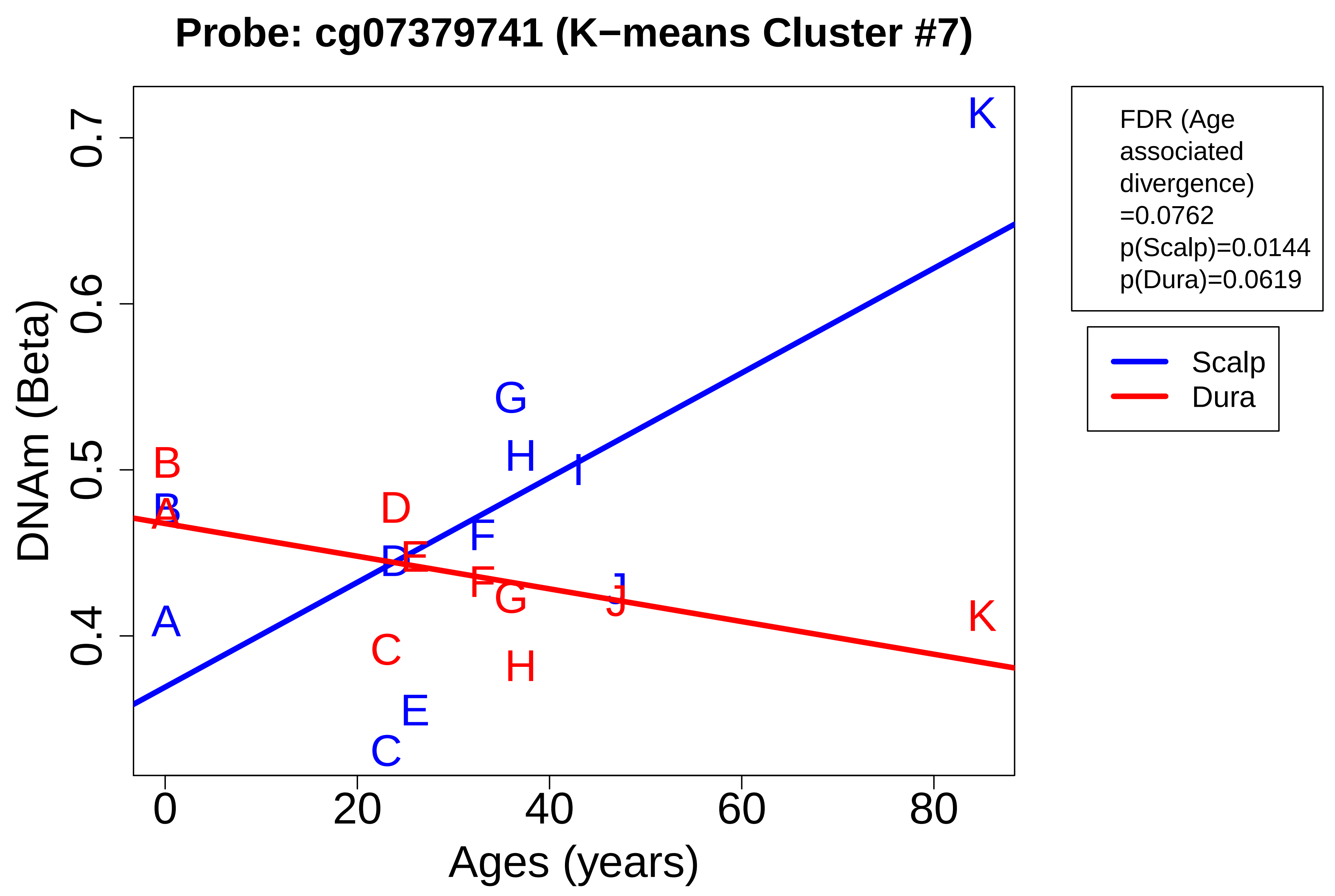

Probe: cg07379741 (K−means Cluster #7)
DNAm (Beta)
0.4
0.5
0.6
0.7
Ages (years)
0
20
40
60
80
FDR (Age associated divergence) =0.0762
p(Scalp)=0.0144
p(Dura)=0.0619
Scalp
Dura
A
B
C
D
E
F
G
H
I
J
K

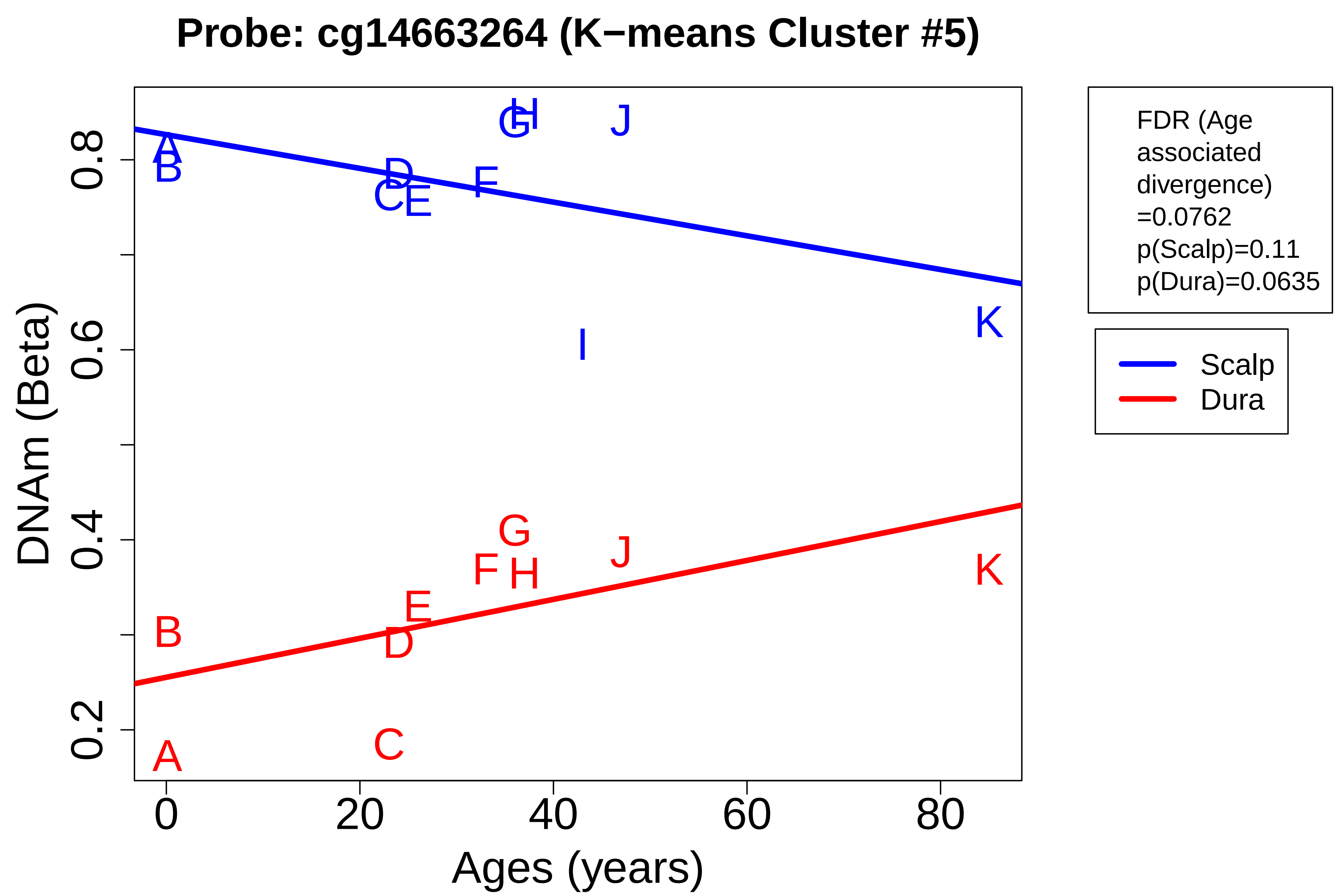

Probe: cg14663264 (K−means Cluster #5)
DNAm (Beta)
0.2
0.4
0.6
0.8
Ages (years)
0
20
40
60
80
A
B
C
D
E
F
G
H
I
J
K
FDR (Age associated divergence) =0.0762
p(Scalp)=0.11
p(Dura)=0.0635
Scalp
Dura

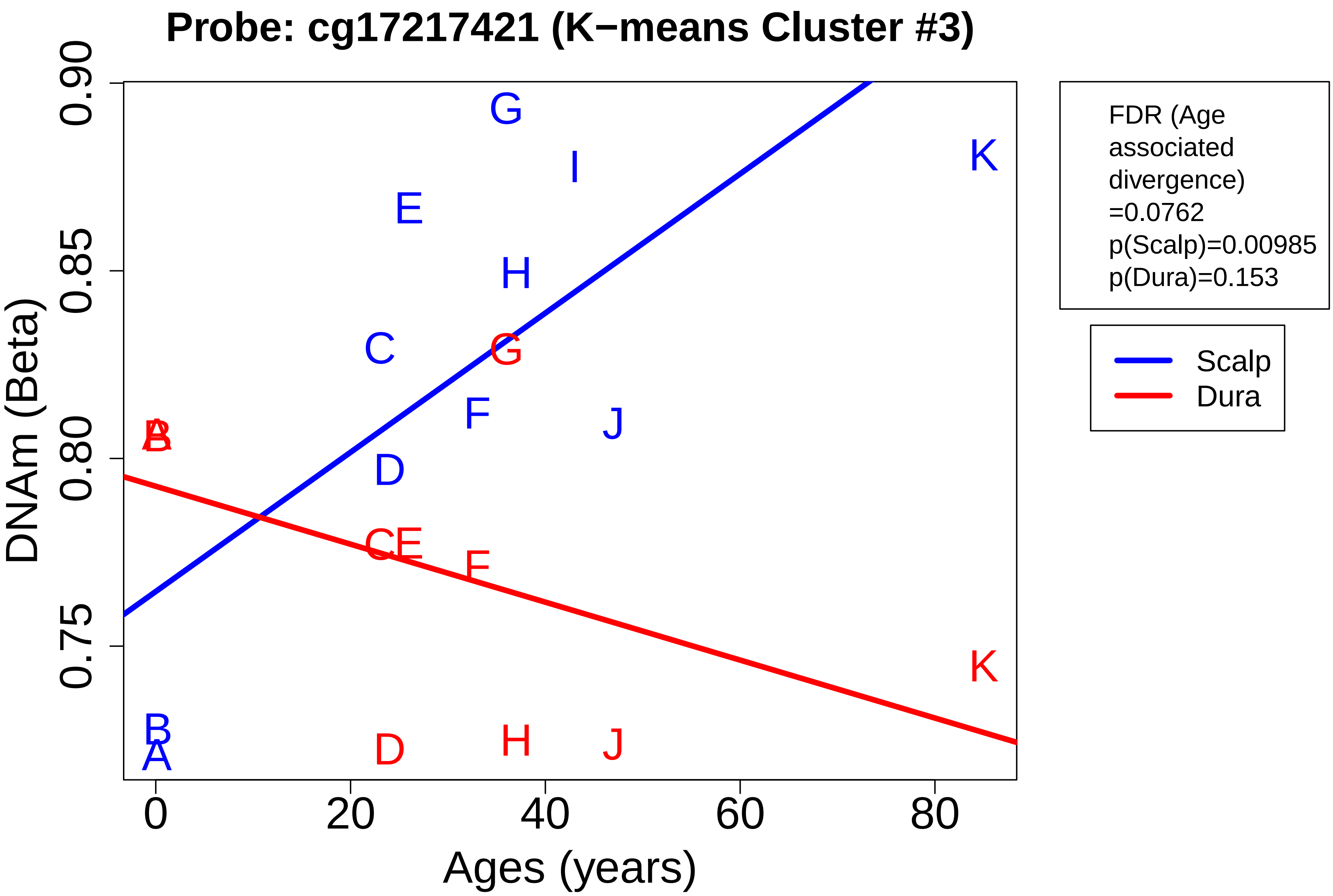

Probe: cg17217421 (K−means Cluster #3)
DNAm (Beta)
0.75
0.80
0.85
0.90
Ages (years)
0
20
40
60
80
FDR (Age associated divergence) =0.0762
p(Scalp)=0.00985
p(Dura)=0.153
Scalp
Dura
A
B
C
D
E
F
G
H
I
J
K

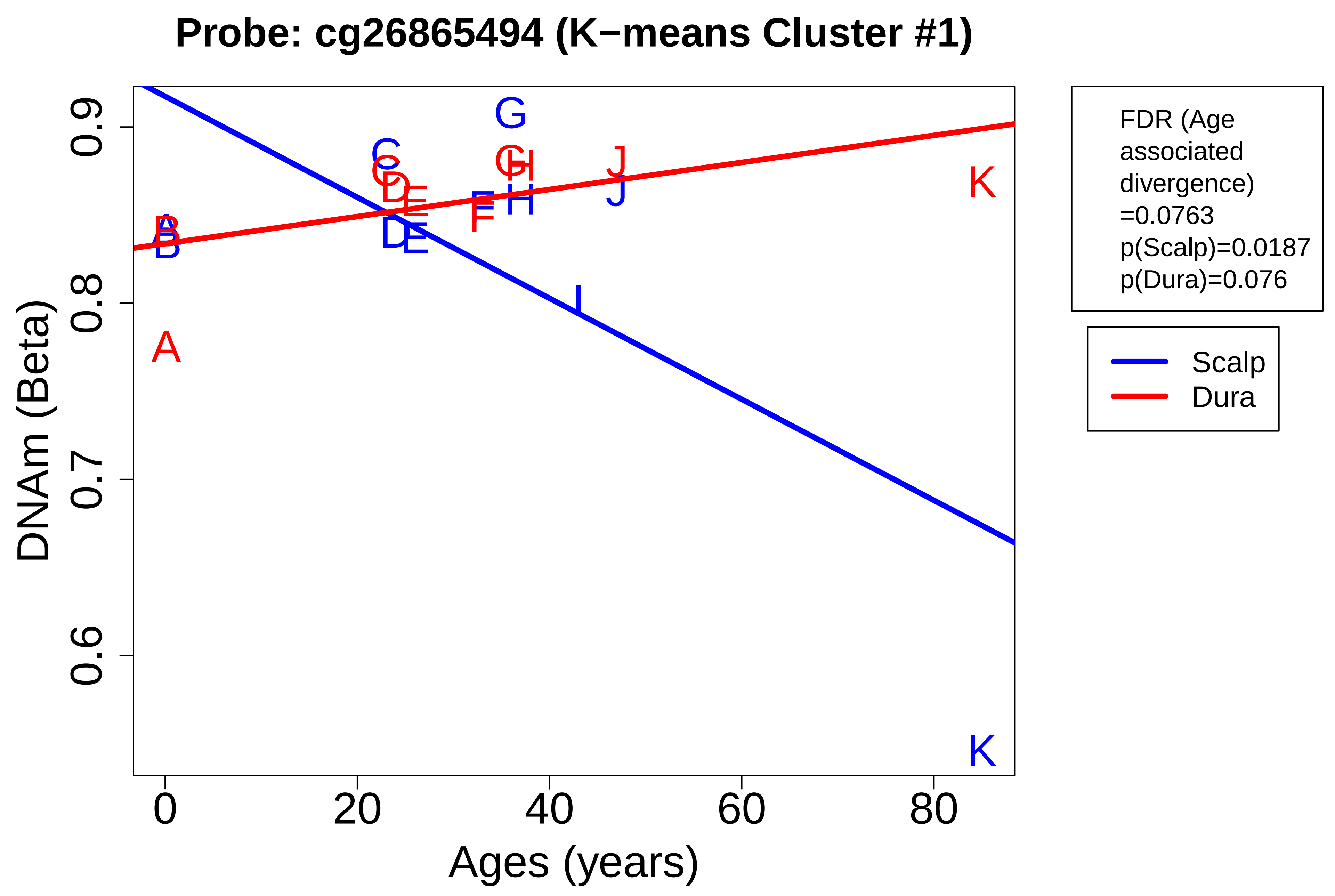

Probe: cg26865494 (K−means Cluster #1)
DNAm (Beta)
0.6
0.7
0.8
0.9
0
20
40
60
80
Ages (years)
FDR (Age associated divergence) =0.0763
p(Scalp)=0.0187
p(Dura)=0.076
Scalp
Dura

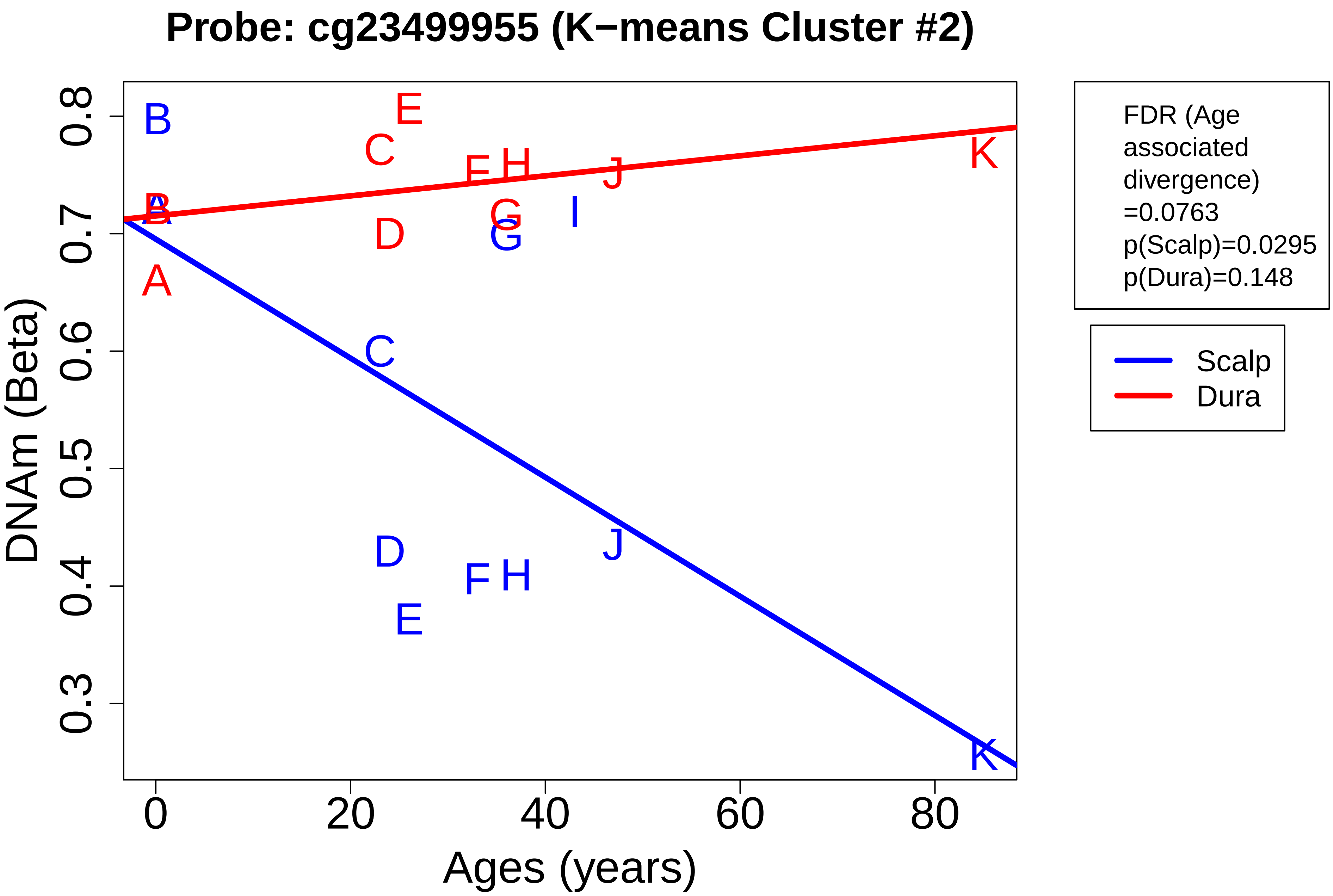

Probe: cg23499955 (K−means Cluster #2)
DNAm (Beta)
0.3
0.4
0.5
0.6
0.7
0.8
0
20
40
60
80
Ages (years)
FDR (Age associated divergence) =0.0763
p(Scalp)=0.0295
p(Dura)=0.148
Scalp
Dura

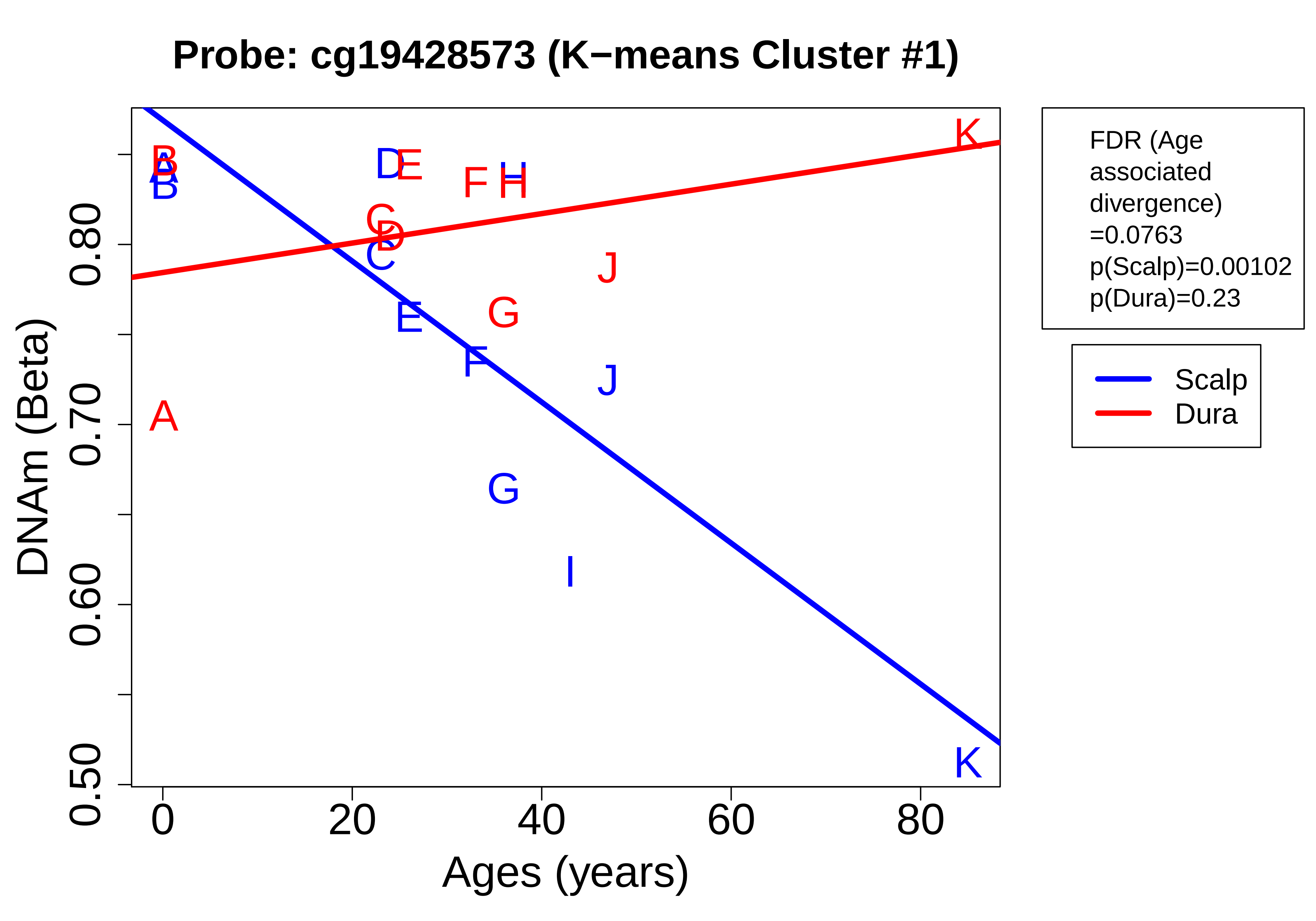

Probe: cg19428573 (K−means Cluster #1)
FDR (Age associated divergence) =0.0763
p(Scalp)=0.00102
p(Dura)=0.23
Scalp
Dura
DNAm (Beta)
0.50
0.60
0.70
0.80
0
20
40
60
80
Ages (years)
A
B
C
D
E
F
G
H
I
J
K

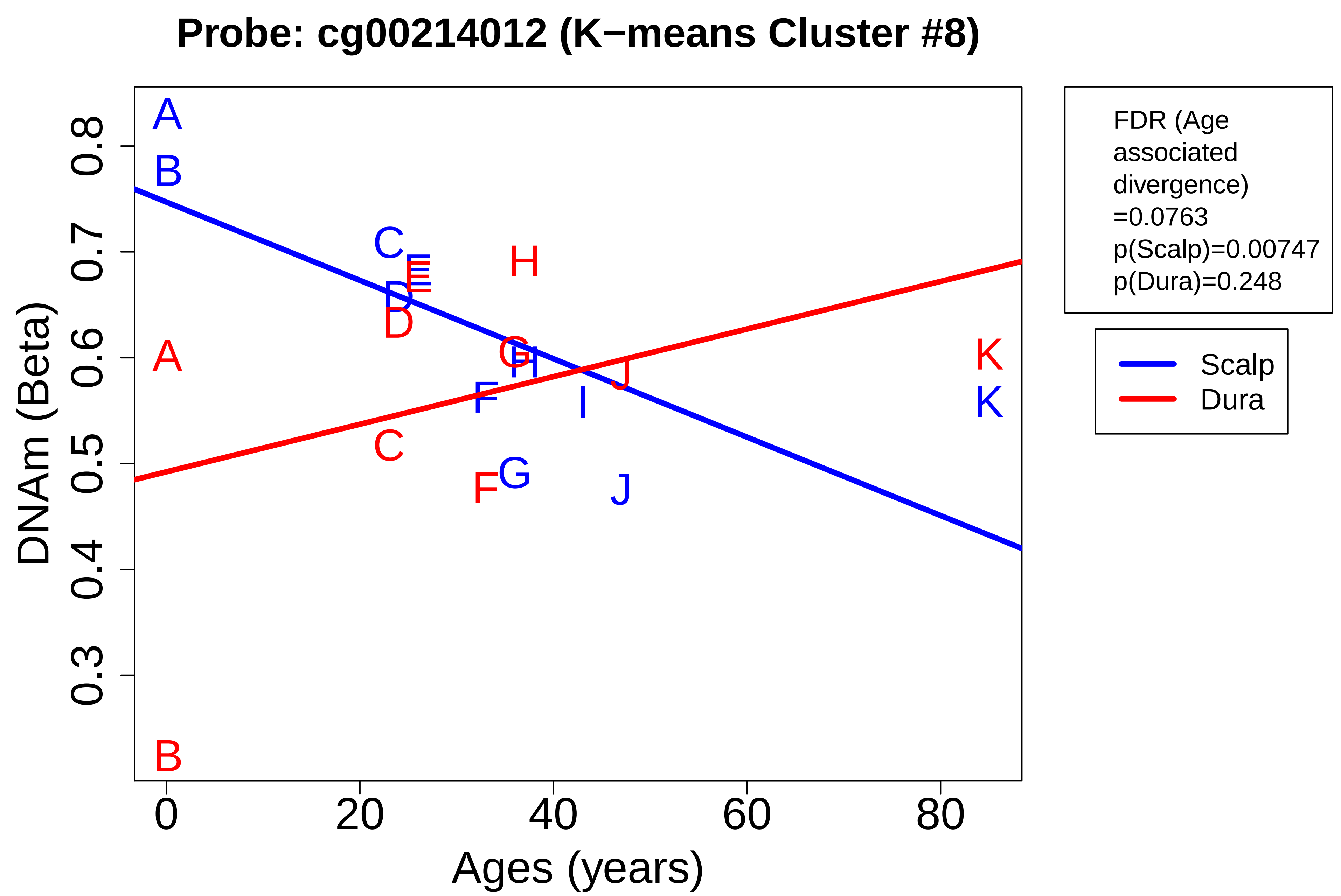
Probe: cg00214012 (K−means Cluster #8)
DNAm (Beta)
0.3
0.4
0.5
0.6
0.7
0.8
Ages (years)
0
20
40
60
80
A
B
C
D
E
F
G
H
I
J
K
FDR (Age associated divergence) =0.0763
p(Scalp)=0.00747
p(Dura)=0.248
Scalp
Dura

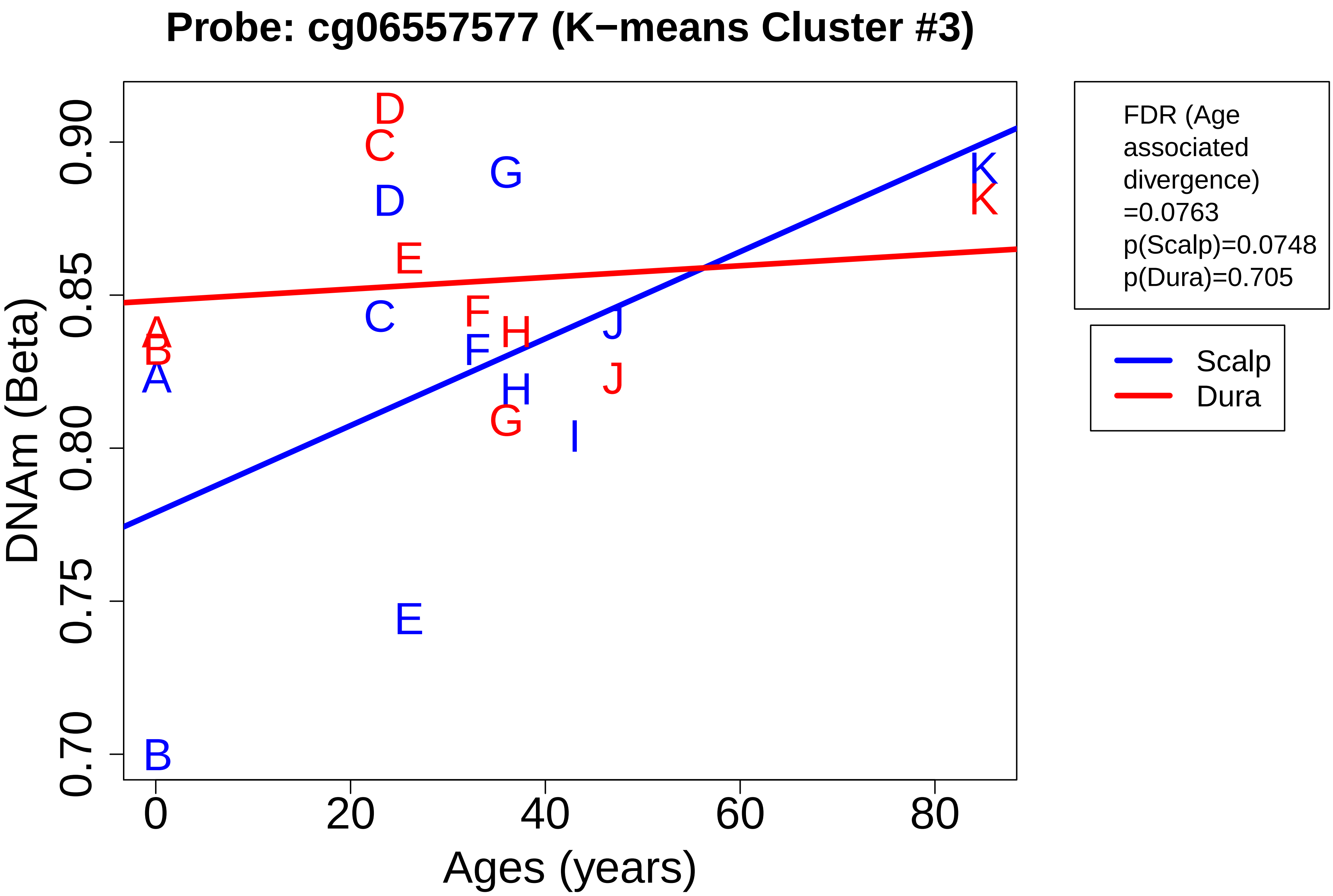

Probe: cg06557577 (K−means Cluster #3)
DNAm (Beta)
0.70
0.75
0.80
0.85
0.90
Ages (years)
0
20
40
60
80
FDR (Age associated divergence) =0.0763
p(Scalp)=0.0748
p(Dura)=0.705
Scalp
Dura

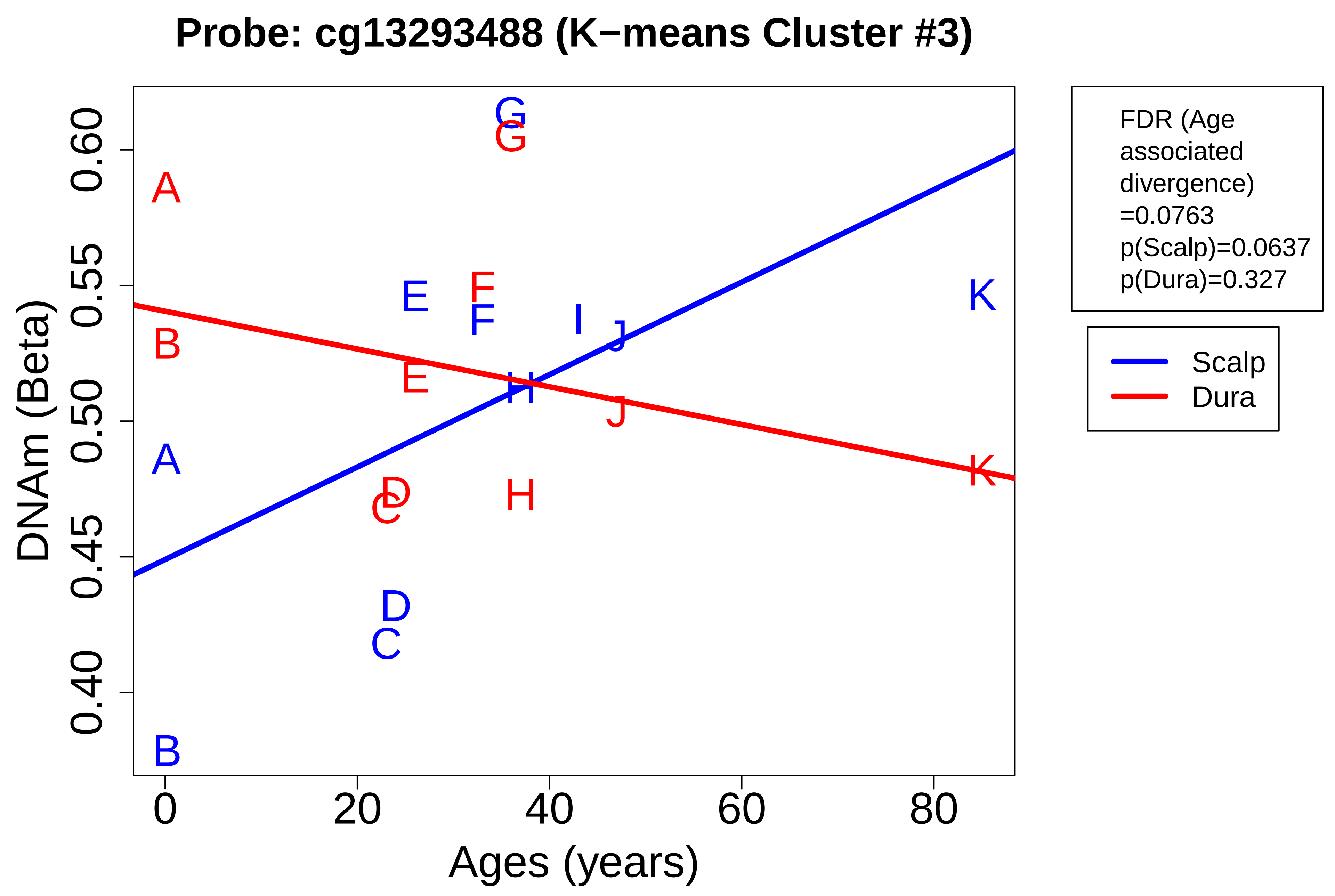
Probe: cg13293488 (K−means Cluster #3)
FDR (Age associated divergence) =0.0763
p(Scalp)=0.0637
p(Dura)=0.327
Scalp
Dura
DNAm (Beta)
0.40
0.45
0.50
0.55
0.60
0
20
40
60
80
Ages (years)
A
B
C
D
E
F
G
H
I
J
K

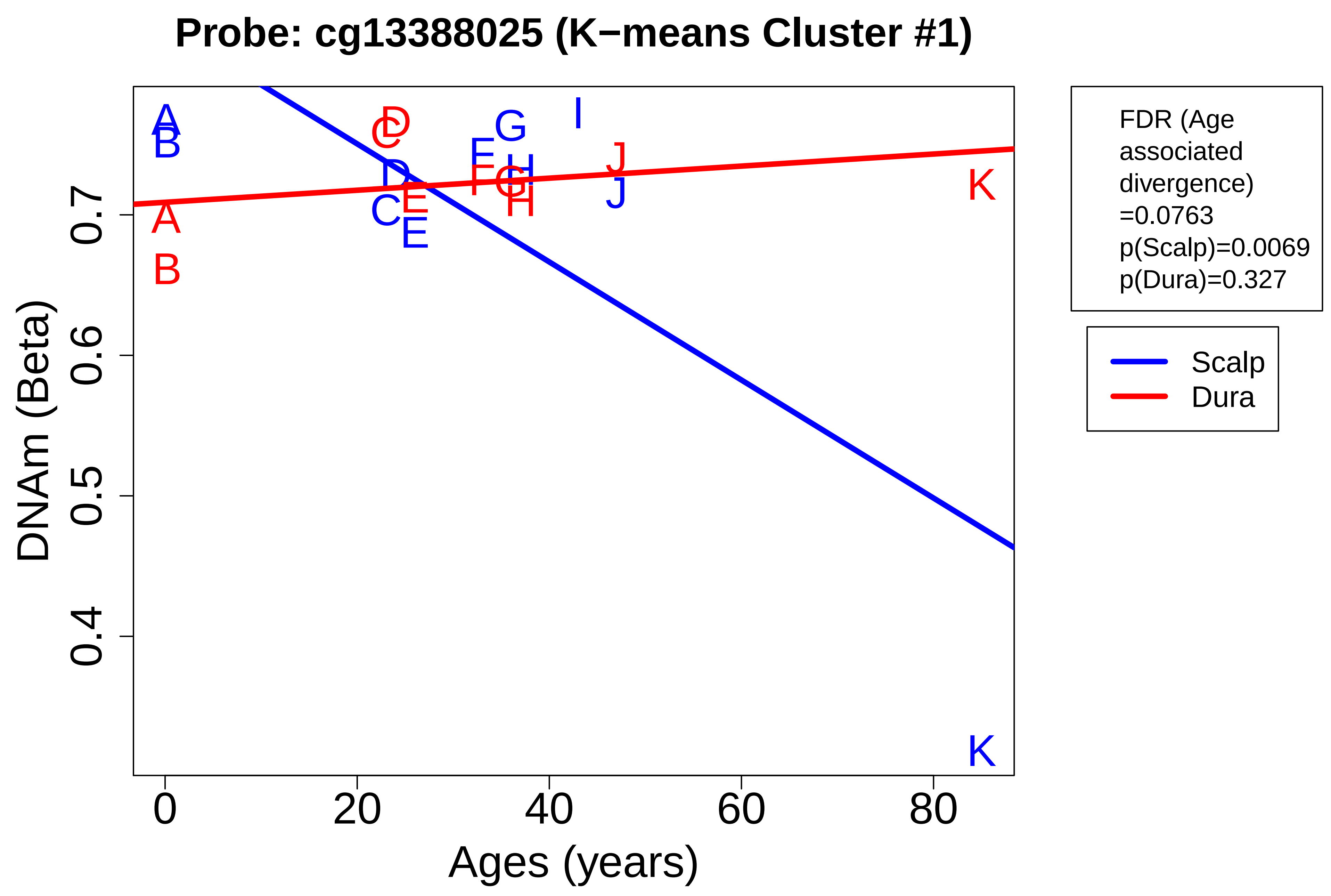

Probe: cg13388025 (K−means Cluster #1)
DNAm (Beta)
0.4
0.5
0.6
0.7
0
20
40
60
80
Ages (years)
A
B
C
D
E
F
G
H
I
J
K
FDR (Age associated divergence) =0.0763
p(Scalp)=0.0069
p(Dura)=0.327
Scalp
Dura

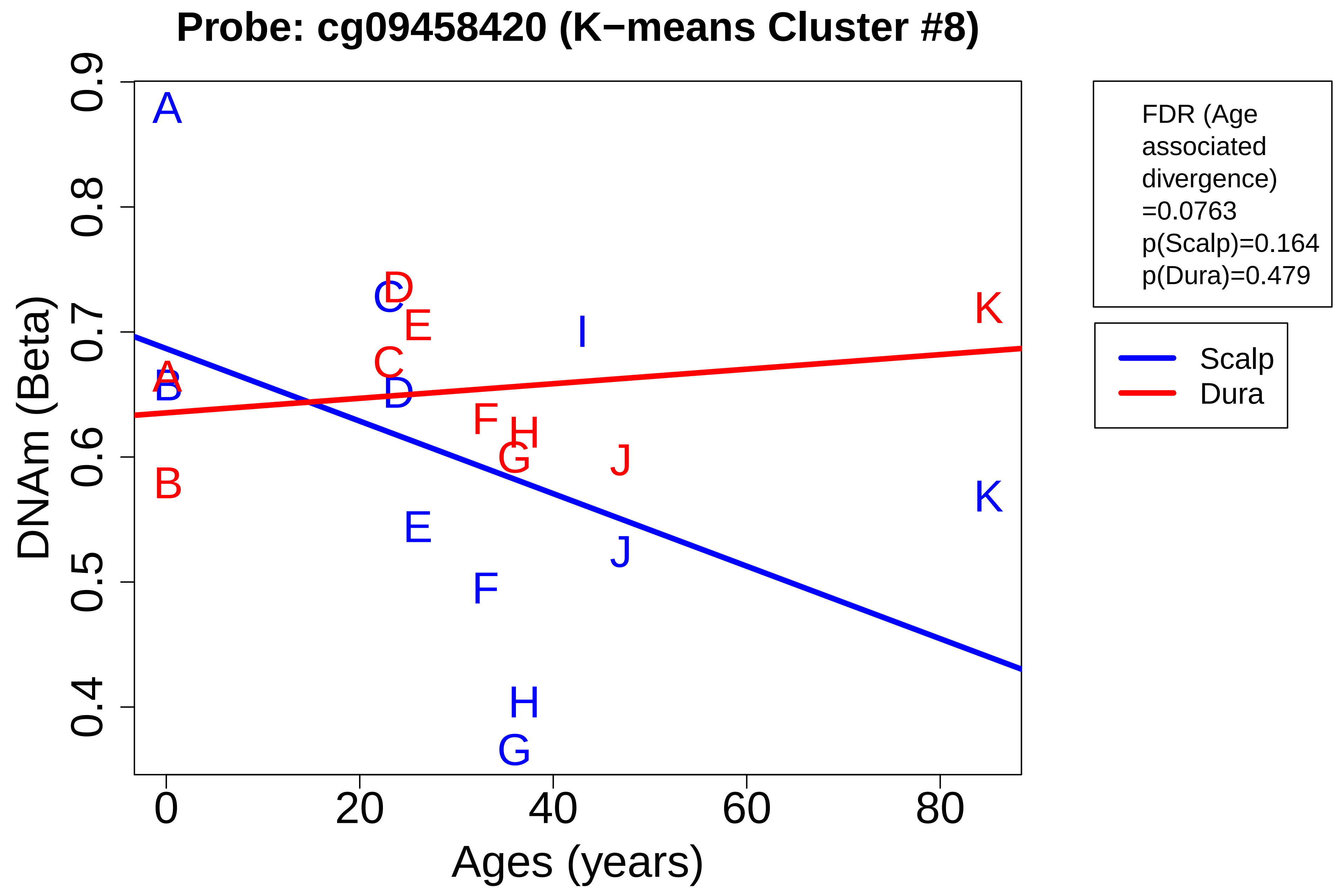

Probe: cg09458420 (K−means Cluster #8)
DNAm (Beta)
0.4
0.5
0.6
0.7
0.8
0.9
Ages (years)
0
20
40
60
80
A
B
C
D
E
F
G
H
I
J
K
FDR (Age associated divergence) =0.0763
p(Scalp)=0.164
p(Dura)=0.479
Scalp
Dura

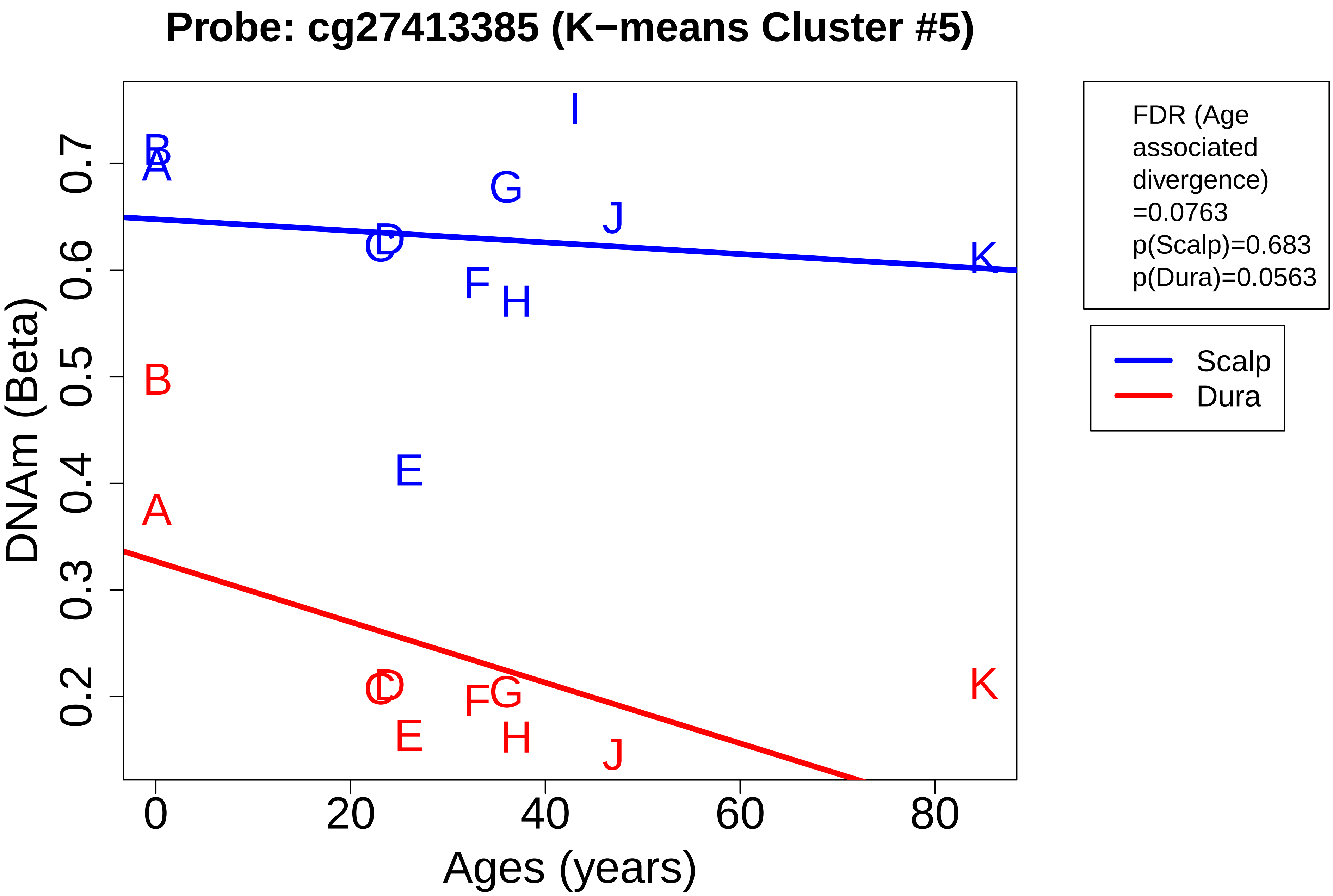

Probe: cg27413385 (K−means Cluster #5)
DNAm (Beta)
0.2
0.3
0.4
0.5
0.6
0.7
Ages (years)
0
20
40
60
80
FDR (Age associated divergence) =0.0763
p(Scalp)=0.683
p(Dura)=0.0563
Scalp
Dura

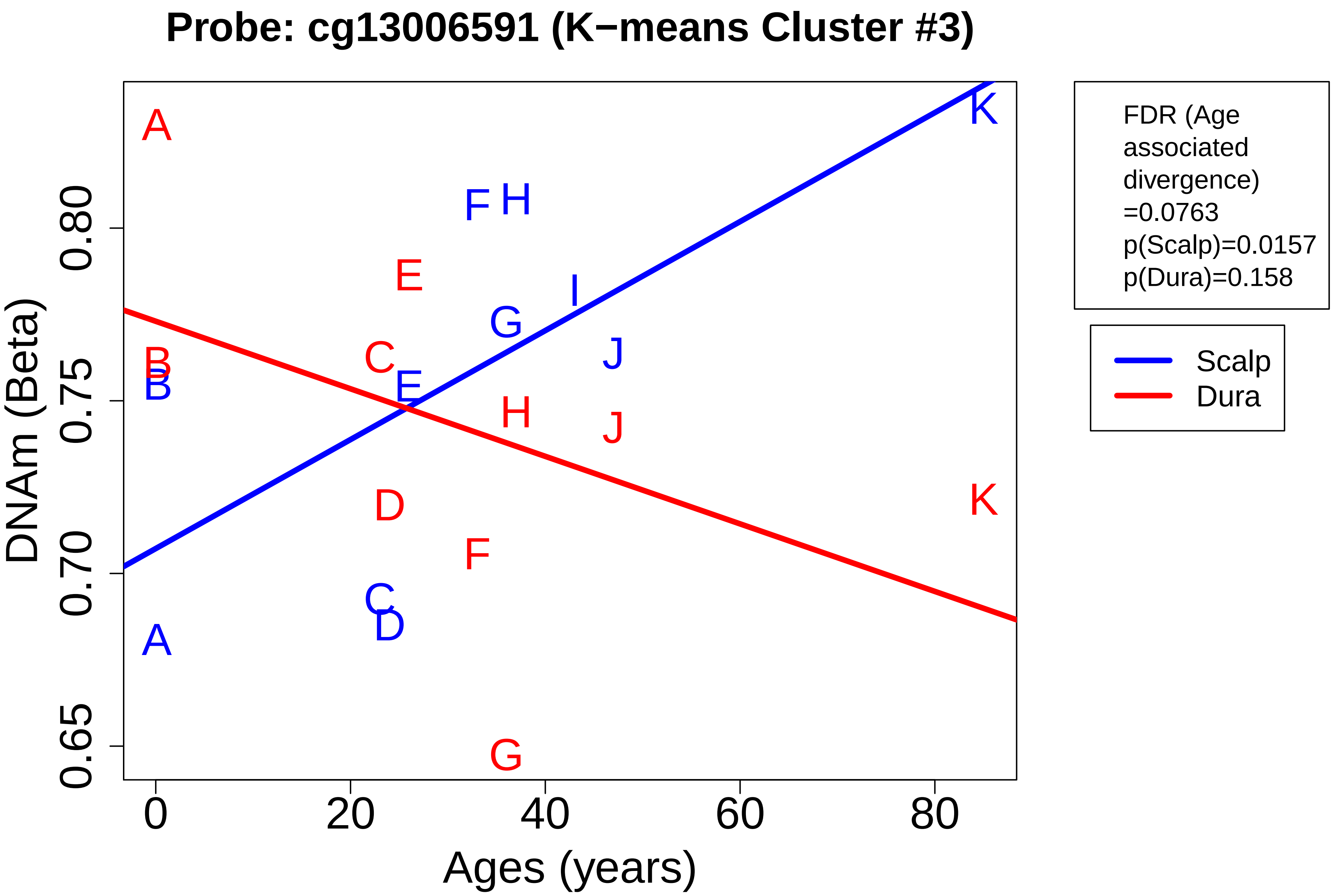

Probe: cg13006591 (K−means Cluster #3)
DNAm (Beta)
0.65
0.70
0.75
0.80
0
20
40
60
80
Ages (years)
FDR (Age associated divergence) =0.0763
p(Scalp)=0.0157
p(Dura)=0.158
Scalp
Dura
A
B
C
D
E
F
G
H
I
J
K

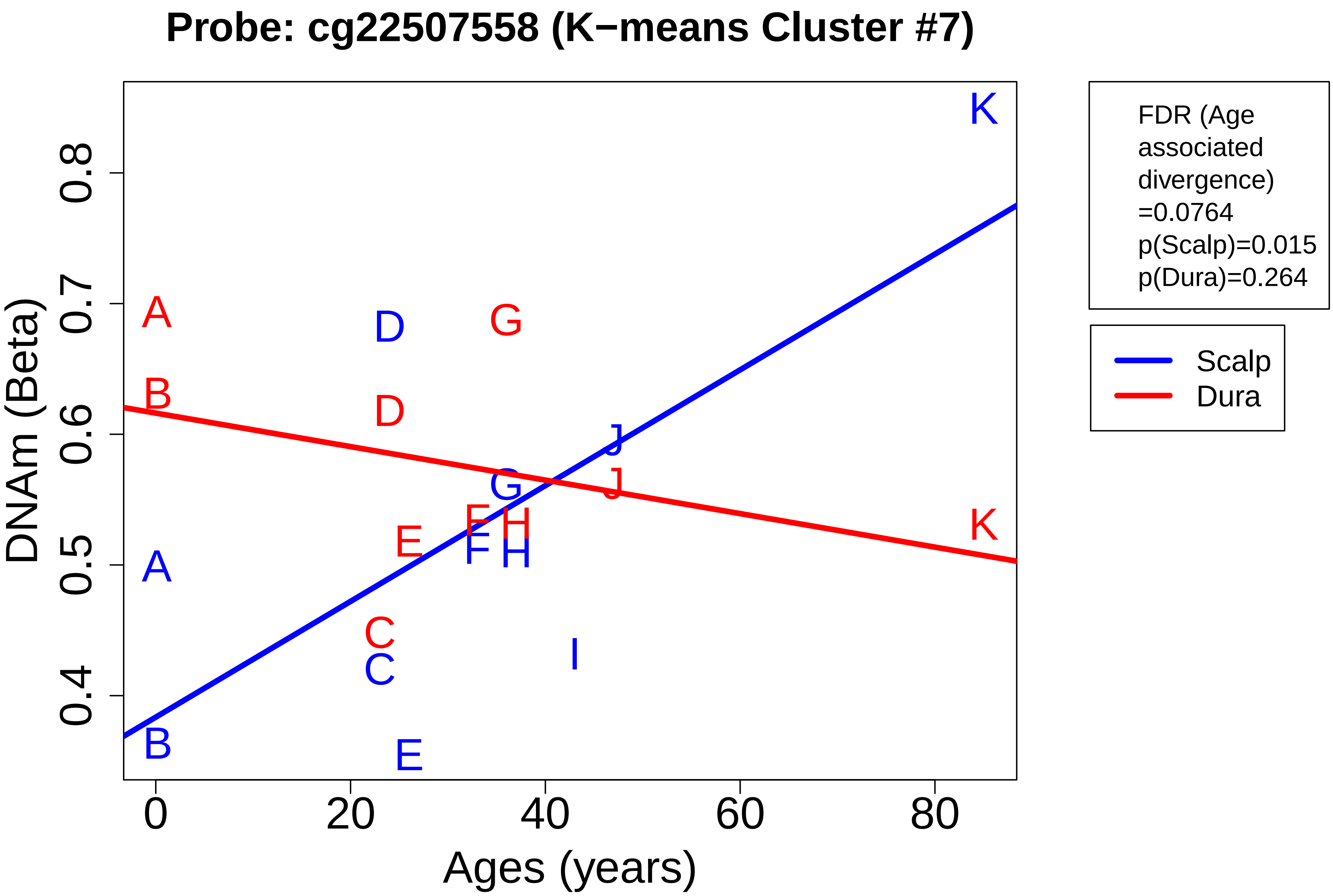

Probe: cg22507558 (K−means Cluster #7)
DNAm (Beta)
0.4
0.5
0.6
0.7
0.8
0
20
40
60
80
Ages (years)
FDR (Age associated divergence) =0.0764
p(Scalp)=0.015
p(Dura)=0.264
Scalp
Dura
A
B
C
D
E
F
G
H
I
J
K

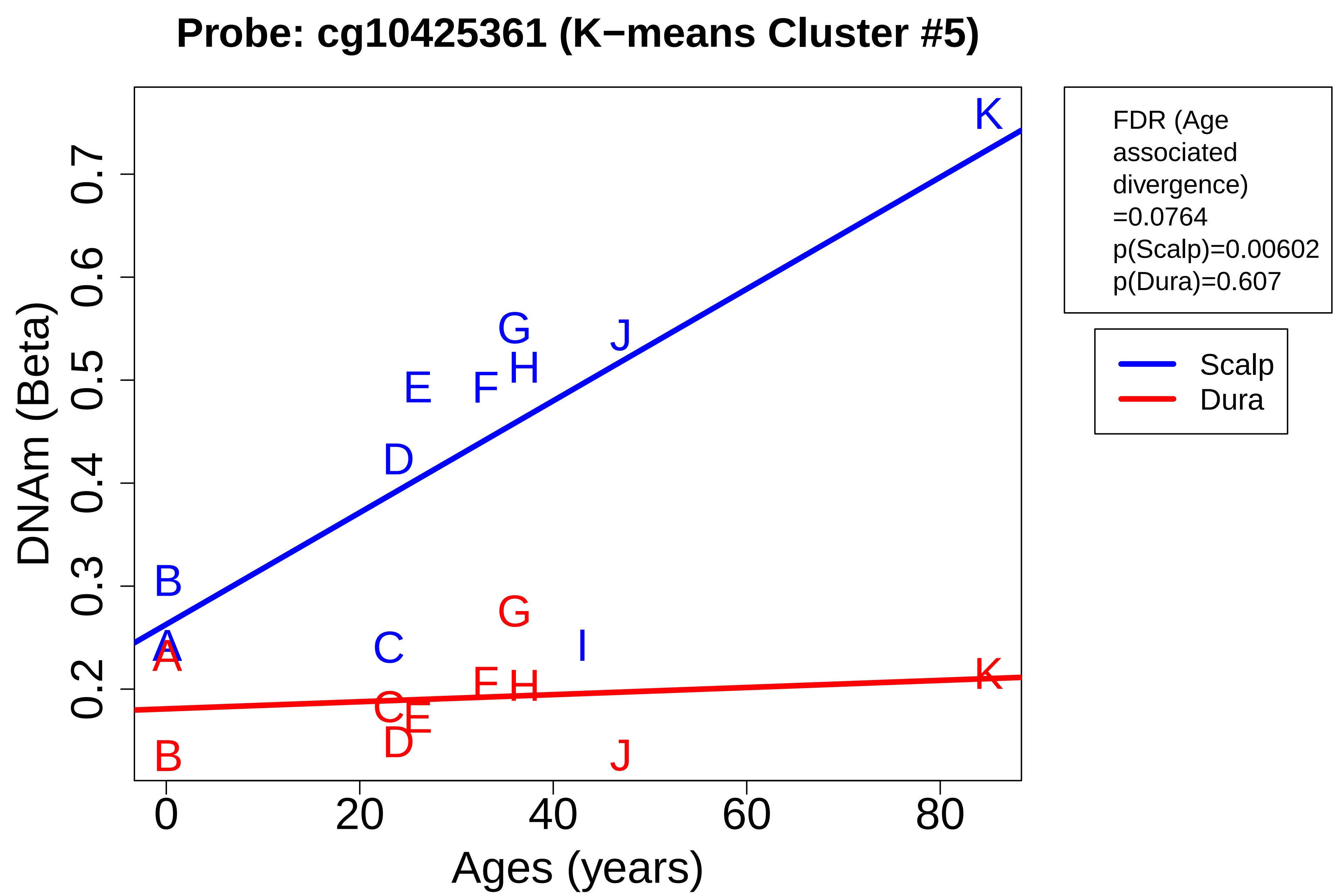

Probe: cg10425361 (K−means Cluster #5)
DNAm (Beta)
0.2
0.3
0.4
0.5
0.6
0.7
0
20
40
60
80
Ages (years)
A B C D E F G H I J K
A B C D E F G H J K
FDR (Age associated divergence) =0.0764
p(Scalp)=0.00602
p(Dura)=0.607
Scalp
Dura

Probe: cg01458504 (K−means Cluster #7)

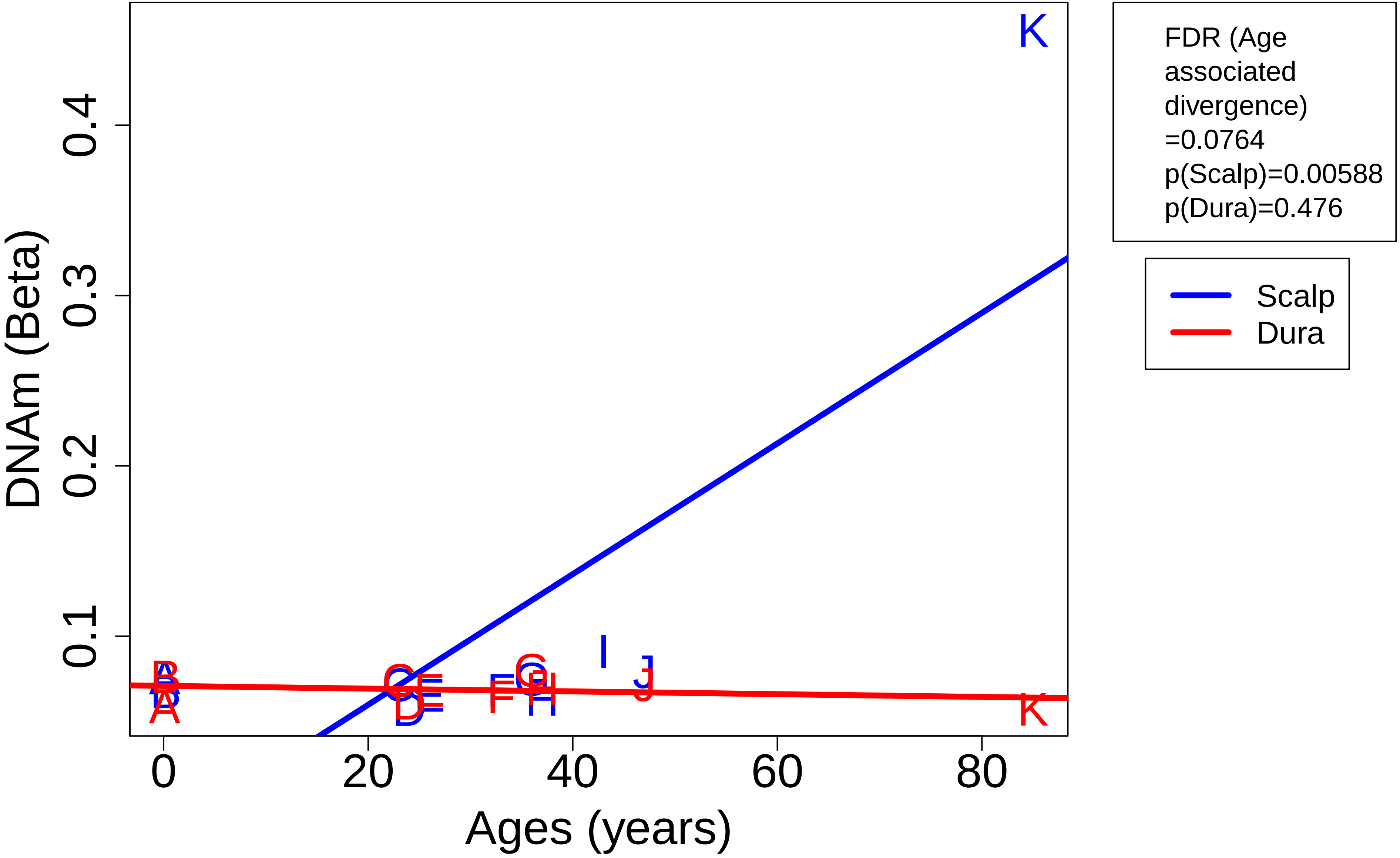

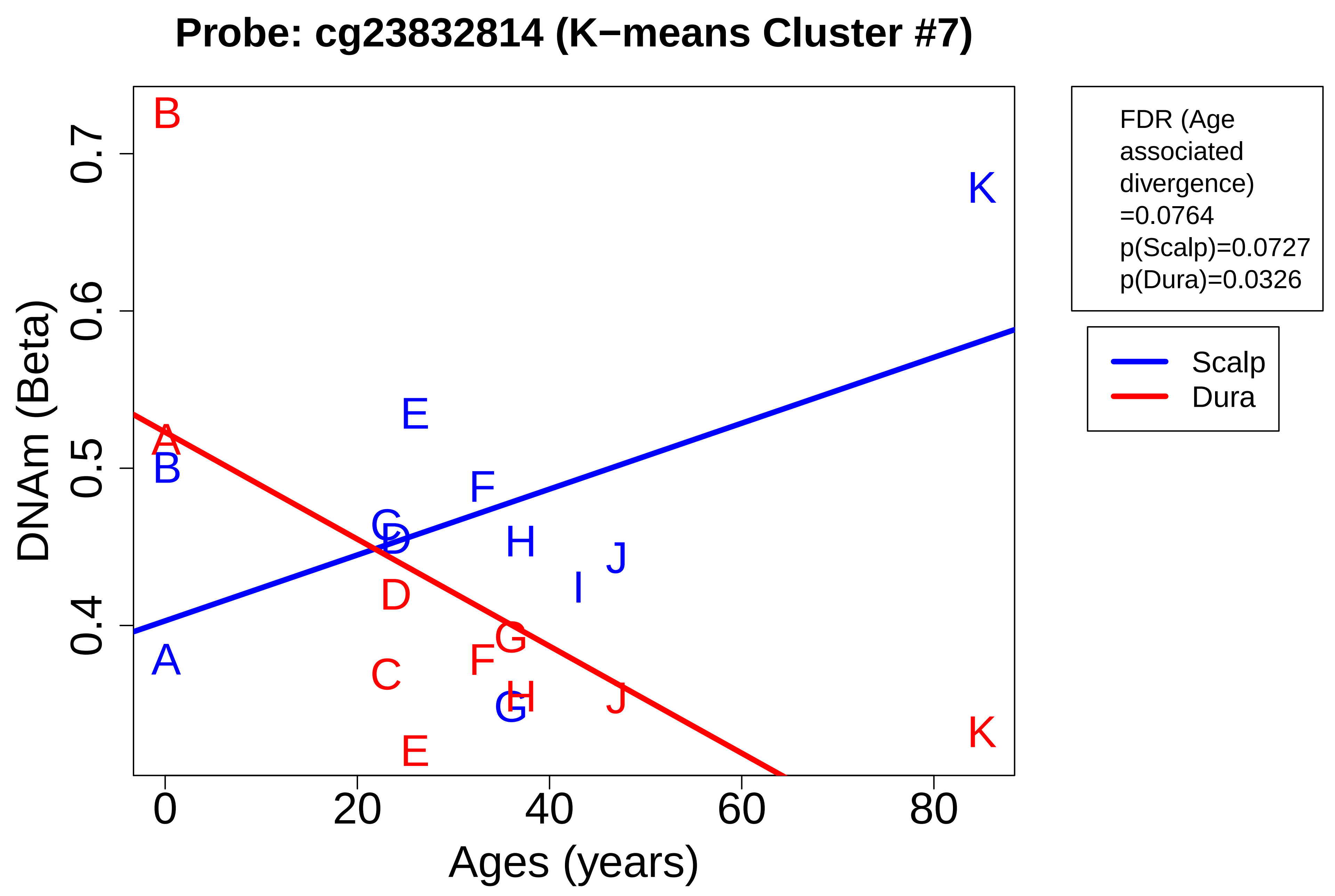

Probe: cg23832814 (K−means Cluster #7)
DNAm (Beta)
0.4
0.5
0.6
0.7
0
20
40
60
80
Ages (years)
FDR (Age associated divergence) =0.0764
p(Scalp)=0.0727
p(Dura)=0.0326
Scalp
Dura
A
B
C
D
E
F
G
H
I
J
K

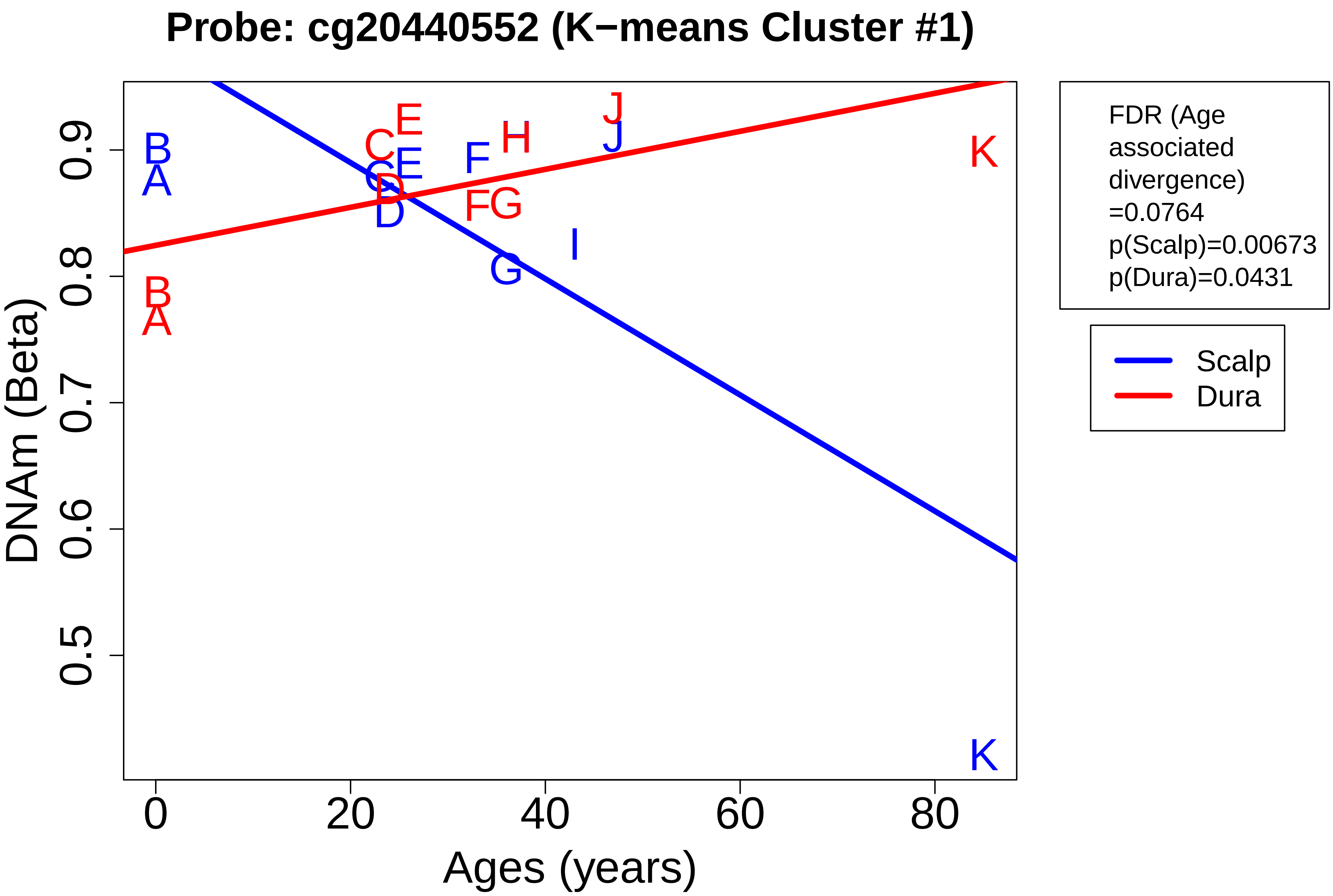

Probe: cg20440552 (K−means Cluster #1)
DNAm (Beta)
0.5
0.6
0.7
0.8
0.9
0
20
40
60
80
Ages (years)
FDR (Age associated divergence) =0.0764
p(Scalp)=0.00673
p(Dura)=0.0431
Scalp
Dura
A
B
C
D
E
F
G
H
I
J
K

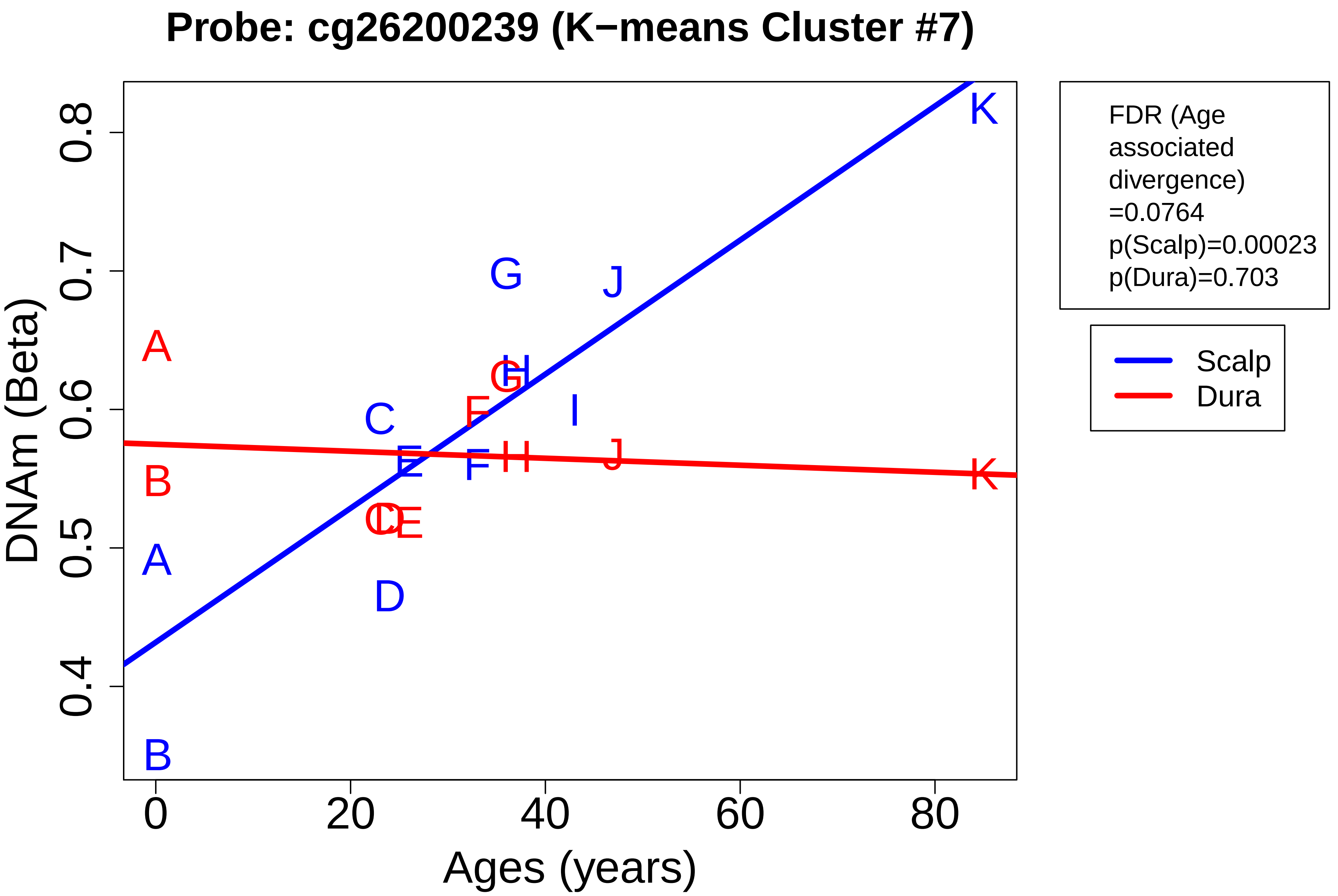
Probe: cg26200239 (K−means Cluster #7)
DNAm (Beta)
0.4
0.5
0.6
0.7
0.8
0
20
40
60
80
Ages (years)
A
B
C
D
E
F
G
H
I
J
K
FDR (Age associated divergence) =0.0764
p(Scalp)=0.00023
p(Dura)=0.703
Scalp
Dura

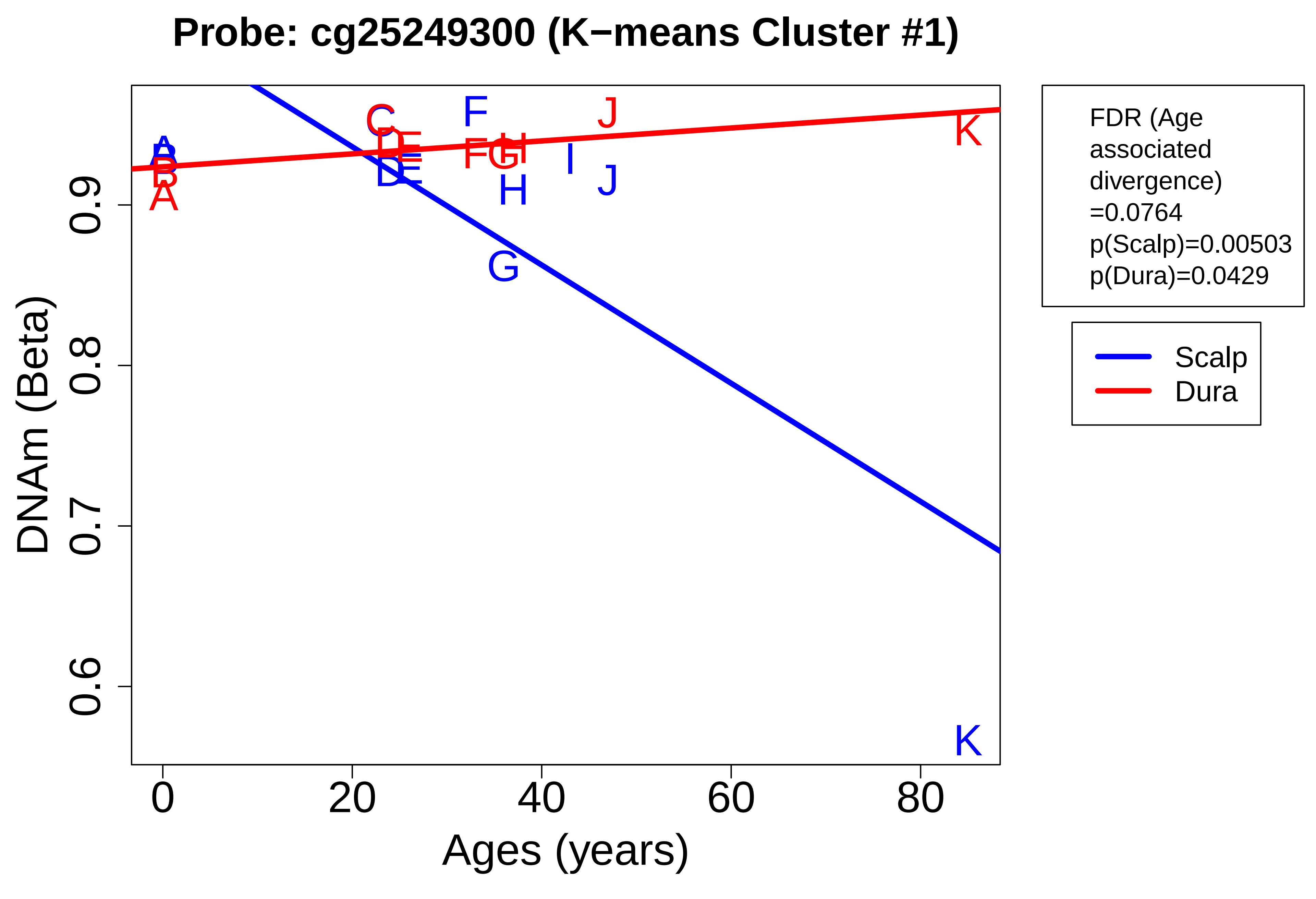

Probe: cg25249300 (K−means Cluster #1)
DNAm (Beta)
0.6
0.7
0.8
0.9
0
20
40
60
80
Ages (years)
A
B
C
D
E
F
G
H
I
J
K
FDR (Age associated divergence) =0.0764
p(Scalp)=0.00503
p(Dura)=0.0429
Scalp
Dura

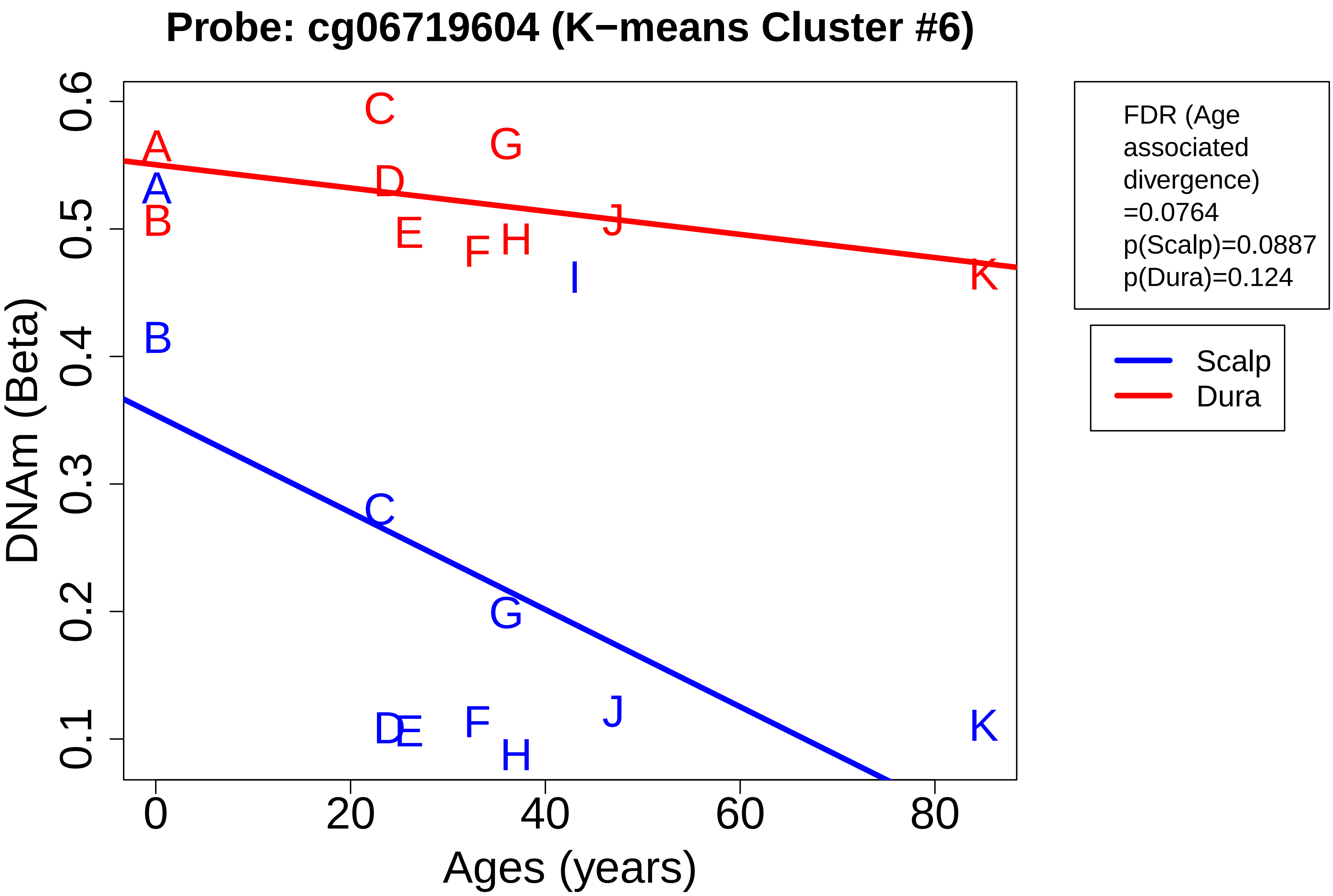

Probe: cg06719604 (K−means Cluster #6)
DNAm (Beta)
0.1
0.2
0.3
0.4
0.5
0.6
Ages (years)
0
20
40
60
80
A
B
C
D
E
F
G
H
I
J
K
FDR (Age associated divergence) =0.0764
p(Scalp)=0.0887
p(Dura)=0.124
Scalp
Dura

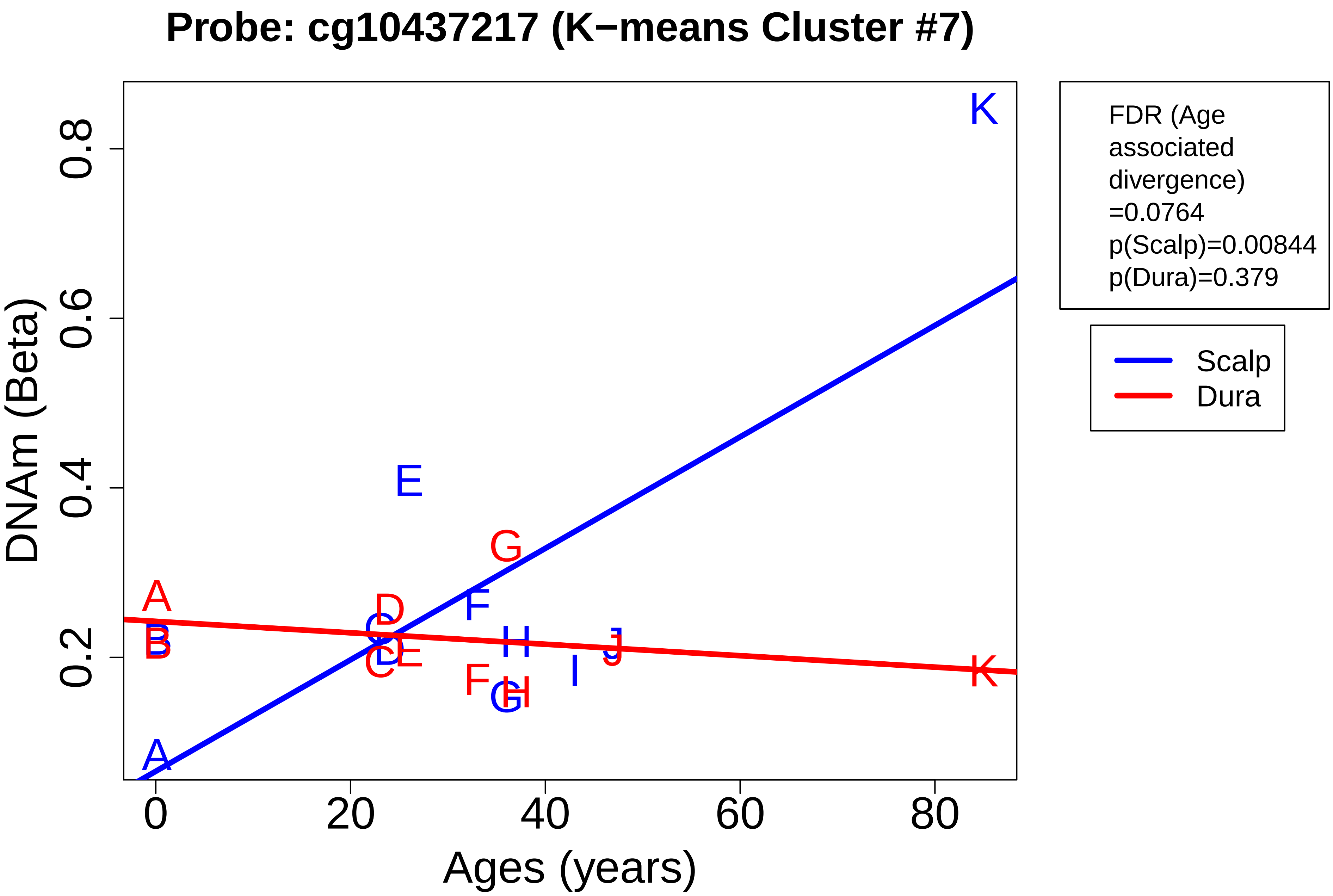

Probe: cg10437217 (K−means Cluster #7)
DNAm (Beta)
0.2
0.4
0.6
0.8
0
20
40
60
80
Ages (years)
FDR (Age associated divergence) =0.0764
p(Scalp)=0.00844
p(Dura)=0.379
Scalp
Dura

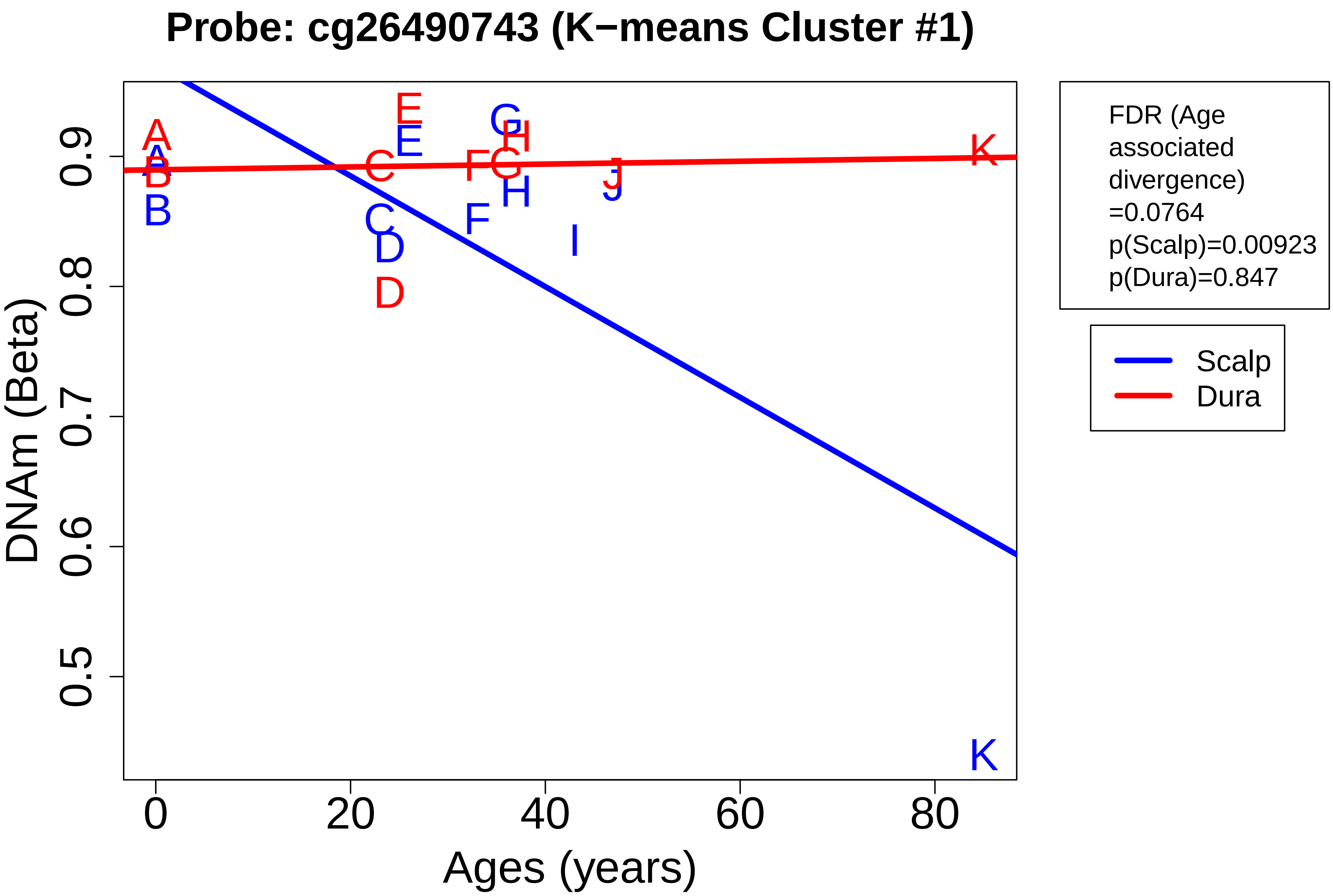

Probe: cg26490743 (K−means Cluster #1)
DNAm (Beta)
0.5
0.6
0.7
0.8
0.9
0
20
40
60
80
Ages (years)
FDR (Age associated divergence) =0.0764
p(Scalp)=0.00923
p(Dura)=0.847
Scalp
Dura

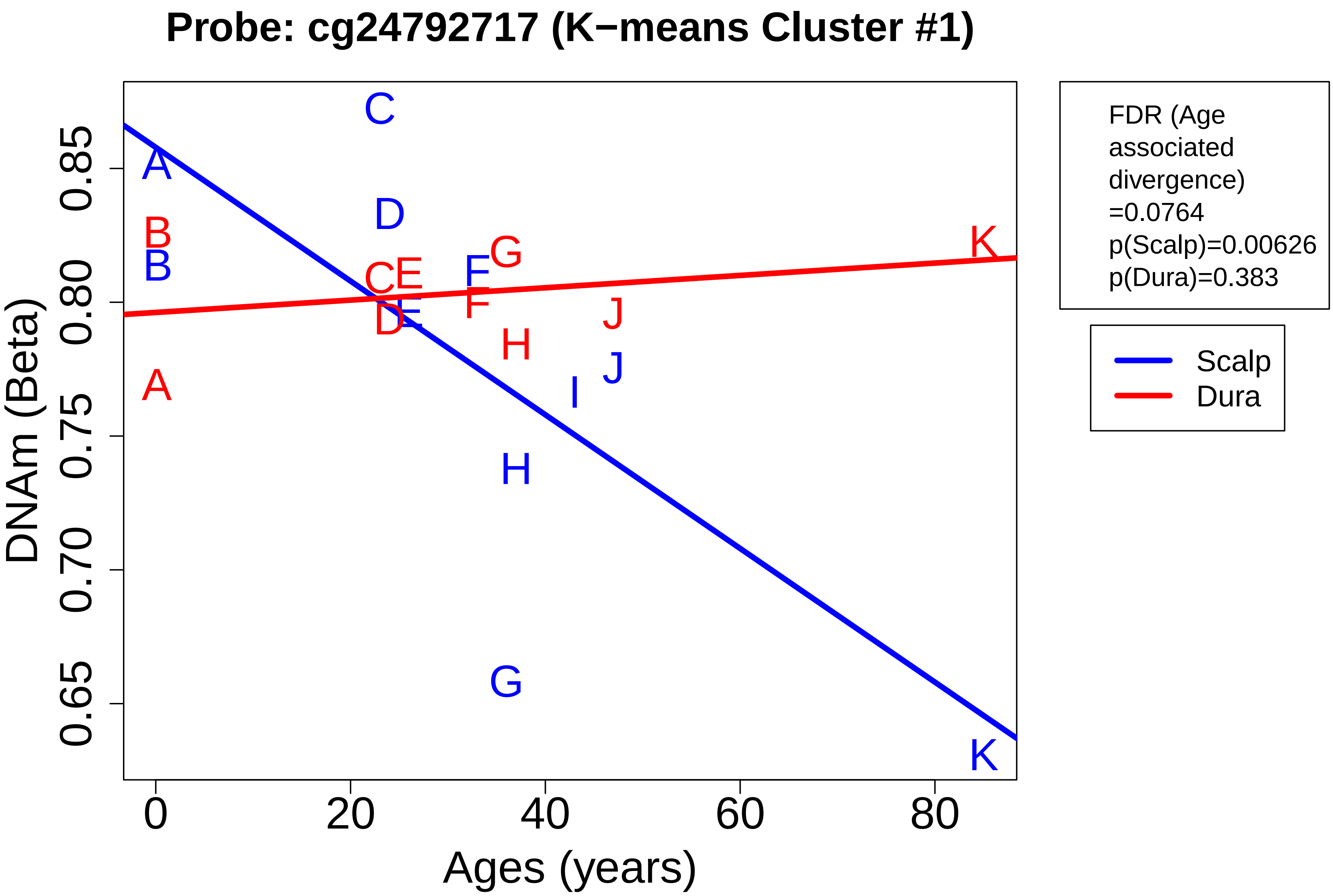

Probe: cg24792717 (K−means Cluster #1)
DNAm (Beta)
0.65
0.70
0.75
0.80
0.85
0
20
40
60
80
Ages (years)
A
B
C
D
E
F
G
H
I
J
K
FDR (Age associated divergence) =0.0764
p(Scalp)=0.00626
p(Dura)=0.383
Scalp
Dura

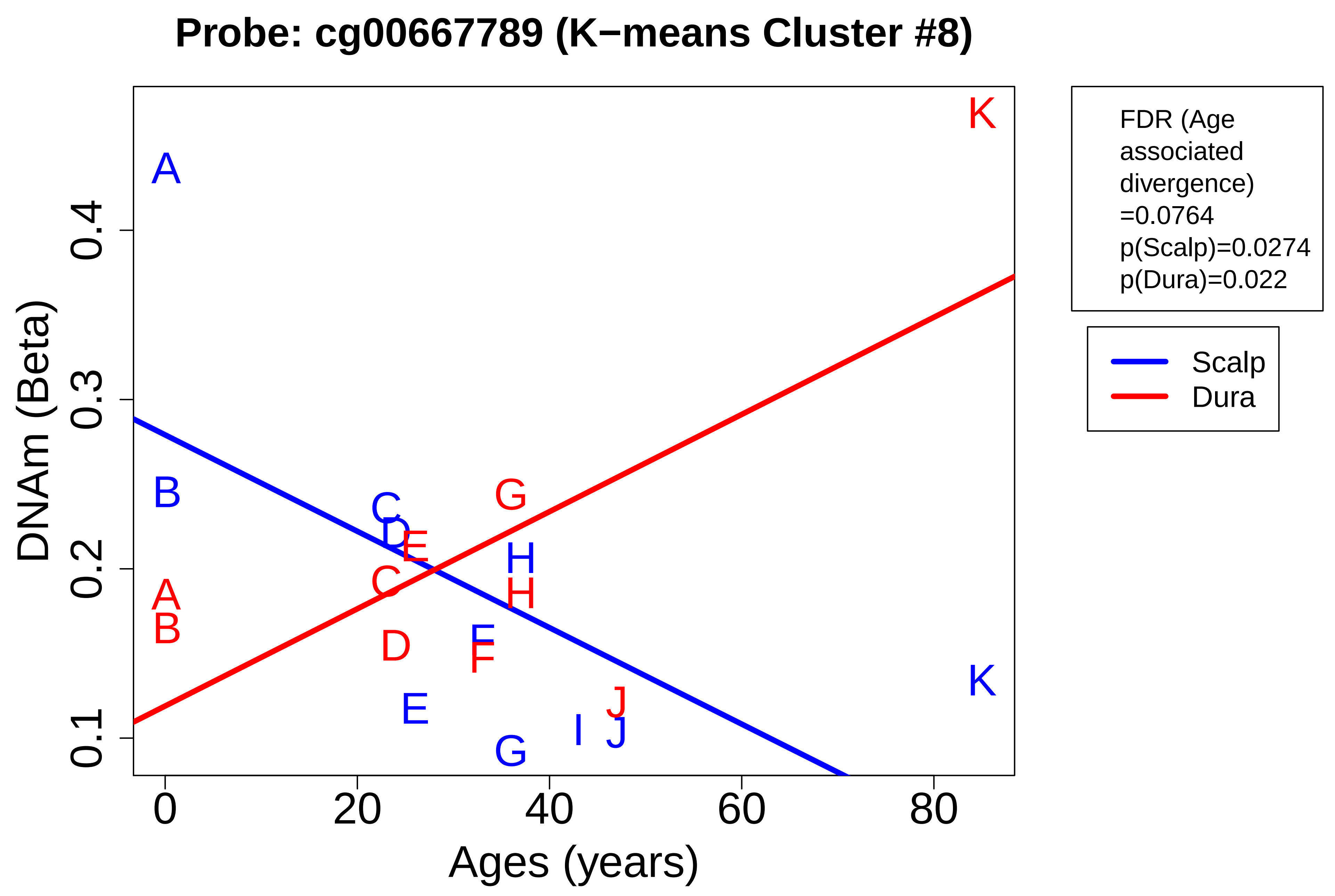

Probe: cg00667789 (K−means Cluster #8)
DNAm (Beta)
0.1
0.2
0.3
0.4
0
20
40
60
80
Ages (years)
A
B
C
D
E
F
G
H
I
J
K
FDR (Age associated divergence) =0.0764
p(Scalp)=0.0274
p(Dura)=0.022
Scalp
Dura

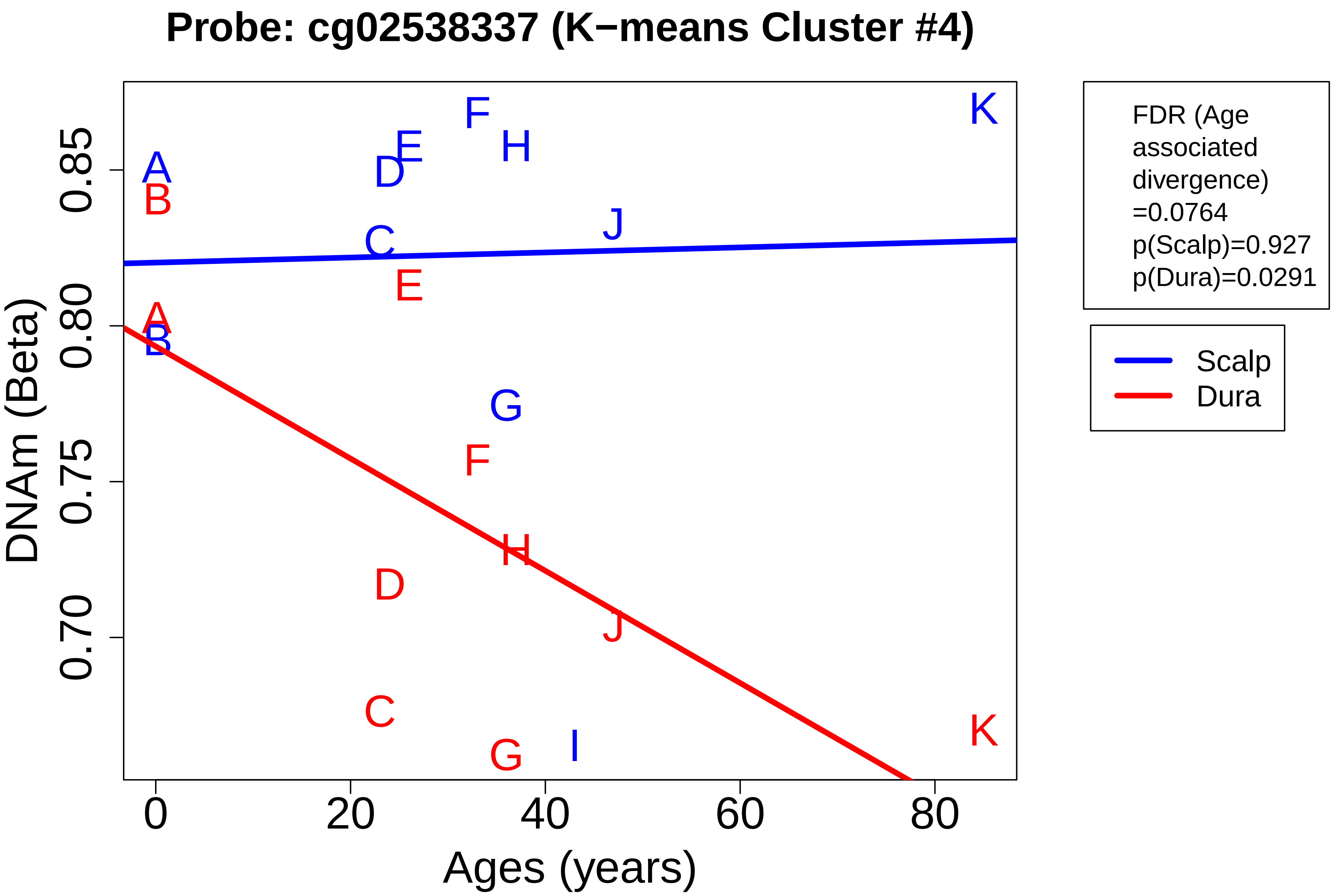

Probe: cg02538337 (K−means Cluster #4)
DNAm (Beta)
0.70
0.75
0.80
0.85
0
20
40
60
80
Ages (years)
A
B
C
D
E
F
G
H
I
J
K
FDR (Age associated divergence) =0.0764
p(Scalp)=0.927
p(Dura)=0.0291
Scalp
Dura

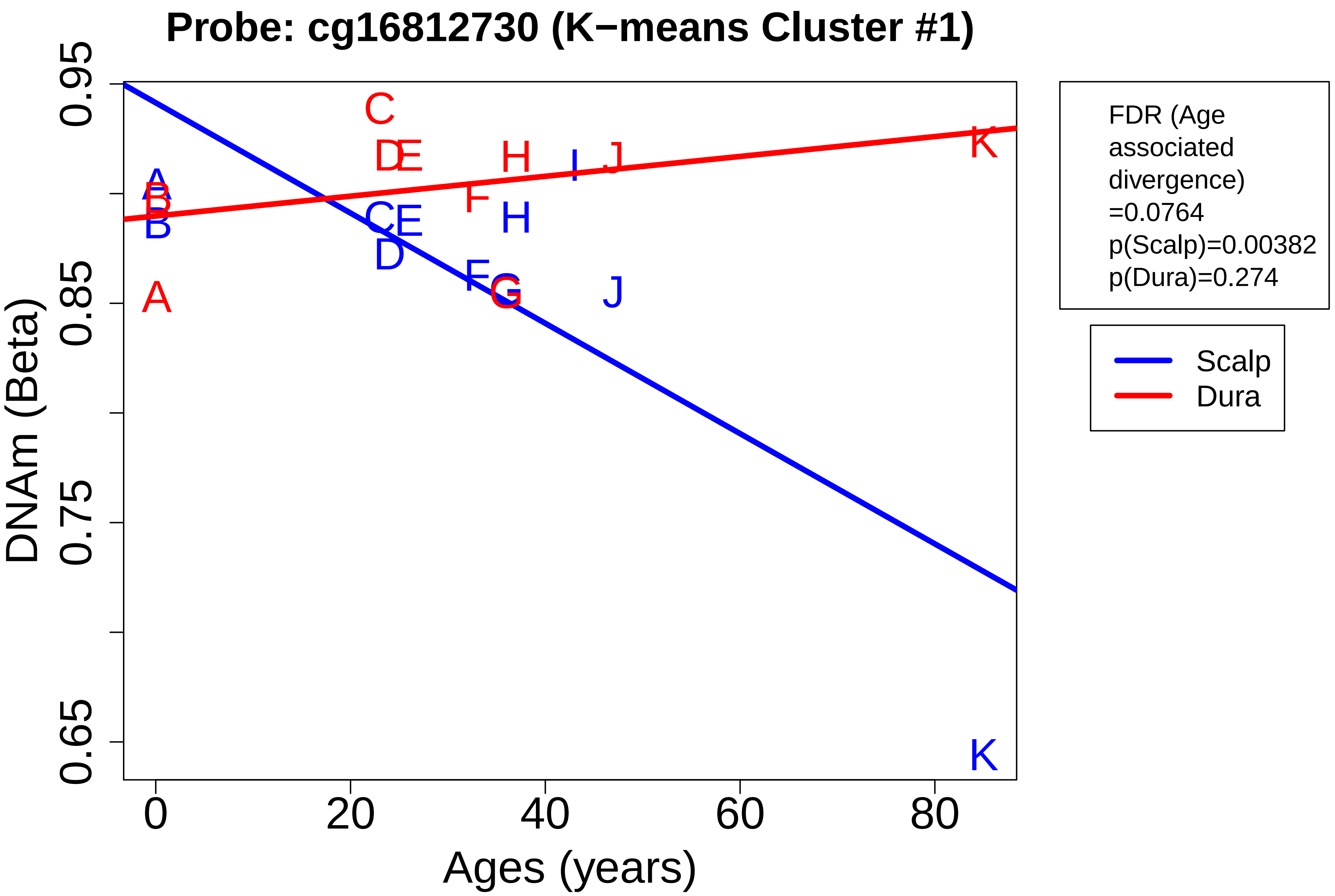

Probe: cg16812730 (K−means Cluster #1)
DNAm (Beta)
0.95
0.85
0.75
0.65
0
20
40
60
80
Ages (years)
FDR (Age associated divergence) =0.0764
p(Scalp)=0.00382
p(Dura)=0.274
Scalp
Dura

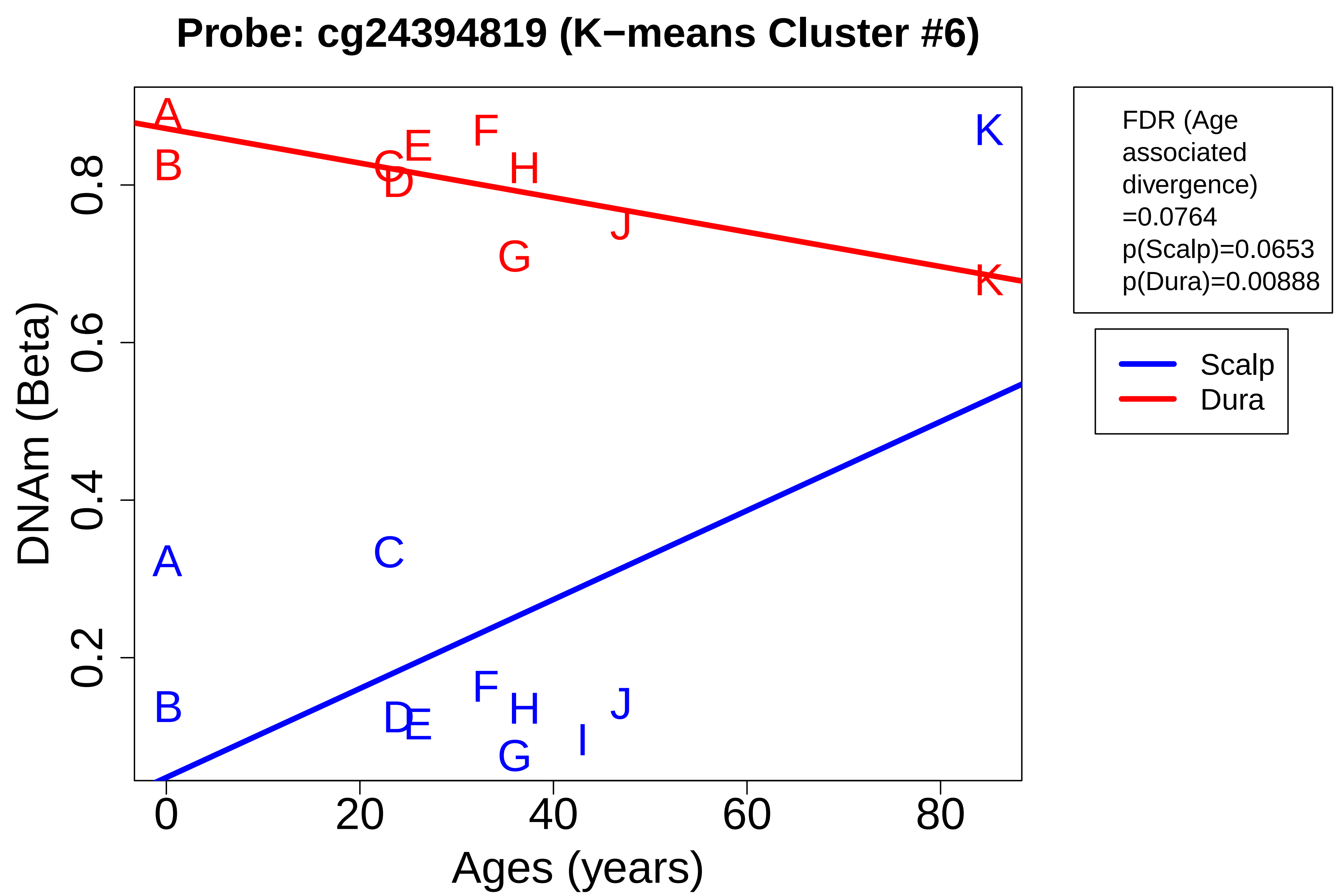

Probe: cg24394819 (K−means Cluster #6)
DNAm (Beta)
0.2
0.4
0.6
0.8
0
20
40
60
80
Ages (years)
A
B
C
D
E
F
G
H
I
J
K
FDR (Age associated divergence) =0.0764
p(Scalp)=0.0653
p(Dura)=0.00888
Scalp
Dura

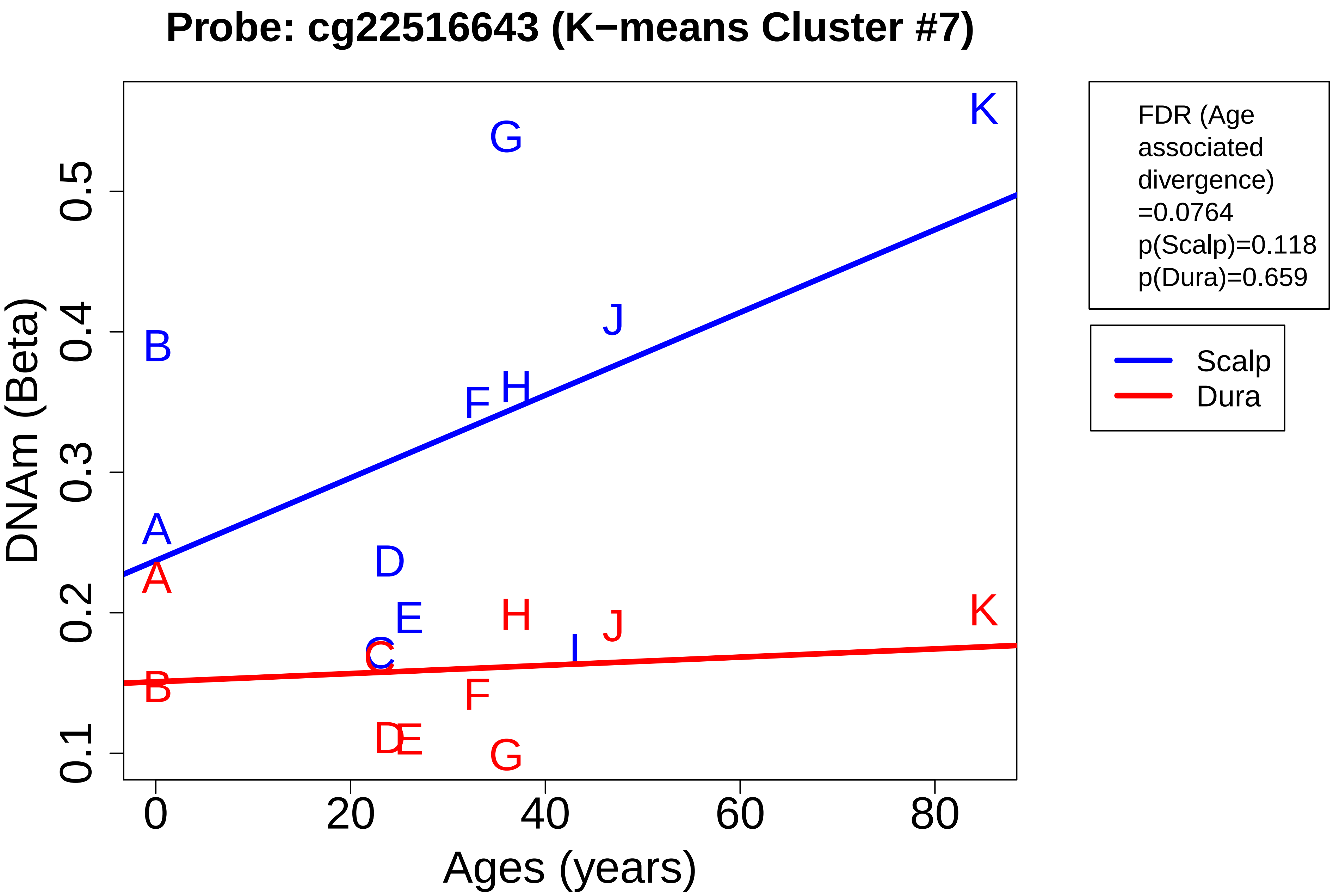

Probe: cg22516643 (K−means Cluster #7)
FDR (Age associated divergence) =0.0764
p(Scalp)=0.118
p(Dura)=0.659
Scalp
Dura
DNAm (Beta)
0.1
0.2
0.3
0.4
0.5
0
20
40
60
80
Ages (years)
A
B
C
D
E
F
G
H
I
J
K

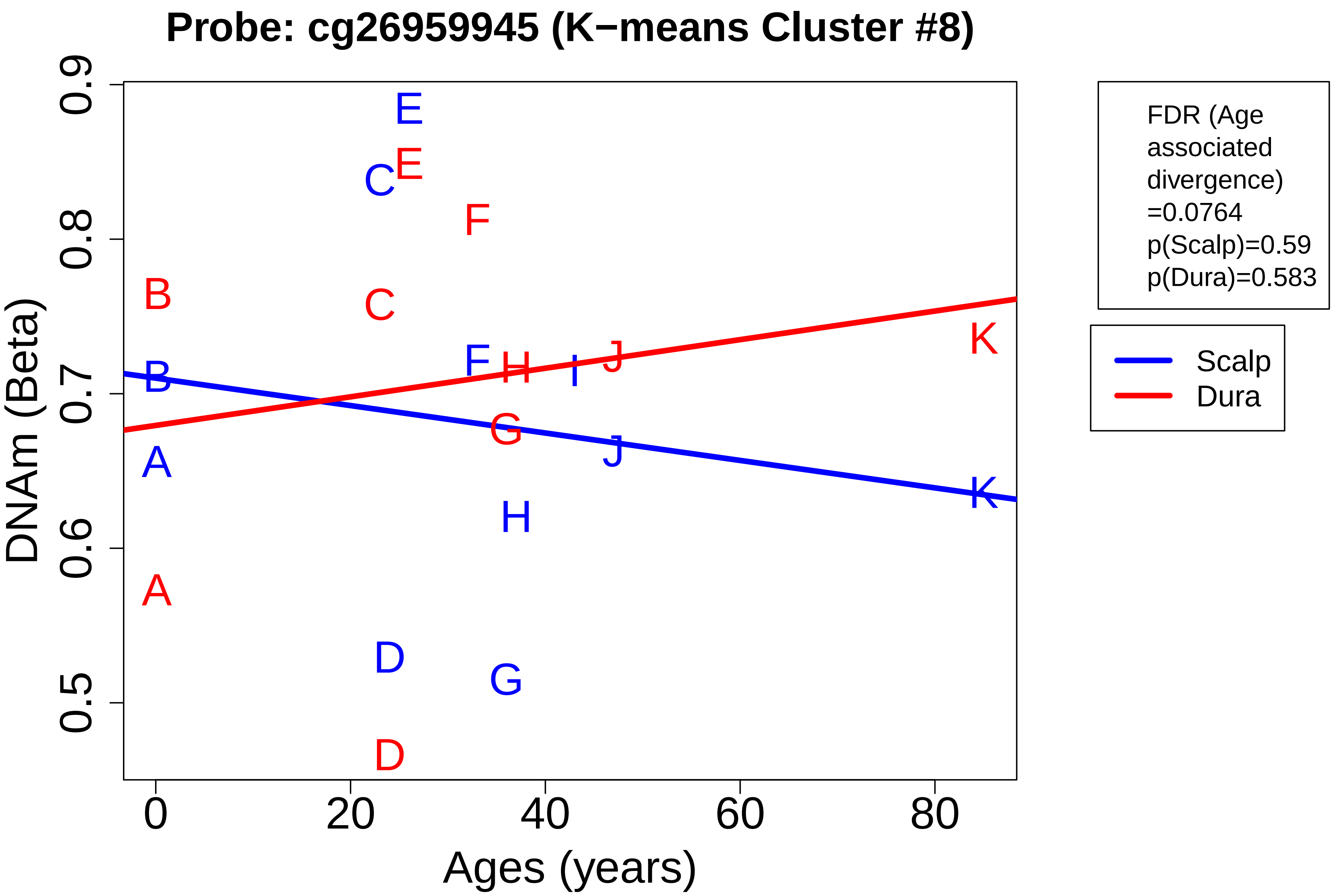
Probe: cg26959945 (K−means Cluster #8)
DNAm (Beta)
0.5
0.6
0.7
0.8
0.9
0
20
40
60
80
Ages (years)
A
B
C
D
E
F
G
H
I
J
K
A
B
C
D
E
F
G
H
J
K
FDR (Age
associated
divergence)
=0.0764
p(Scalp)=0.59
p(Dura)=0.583
Scalp
Dura

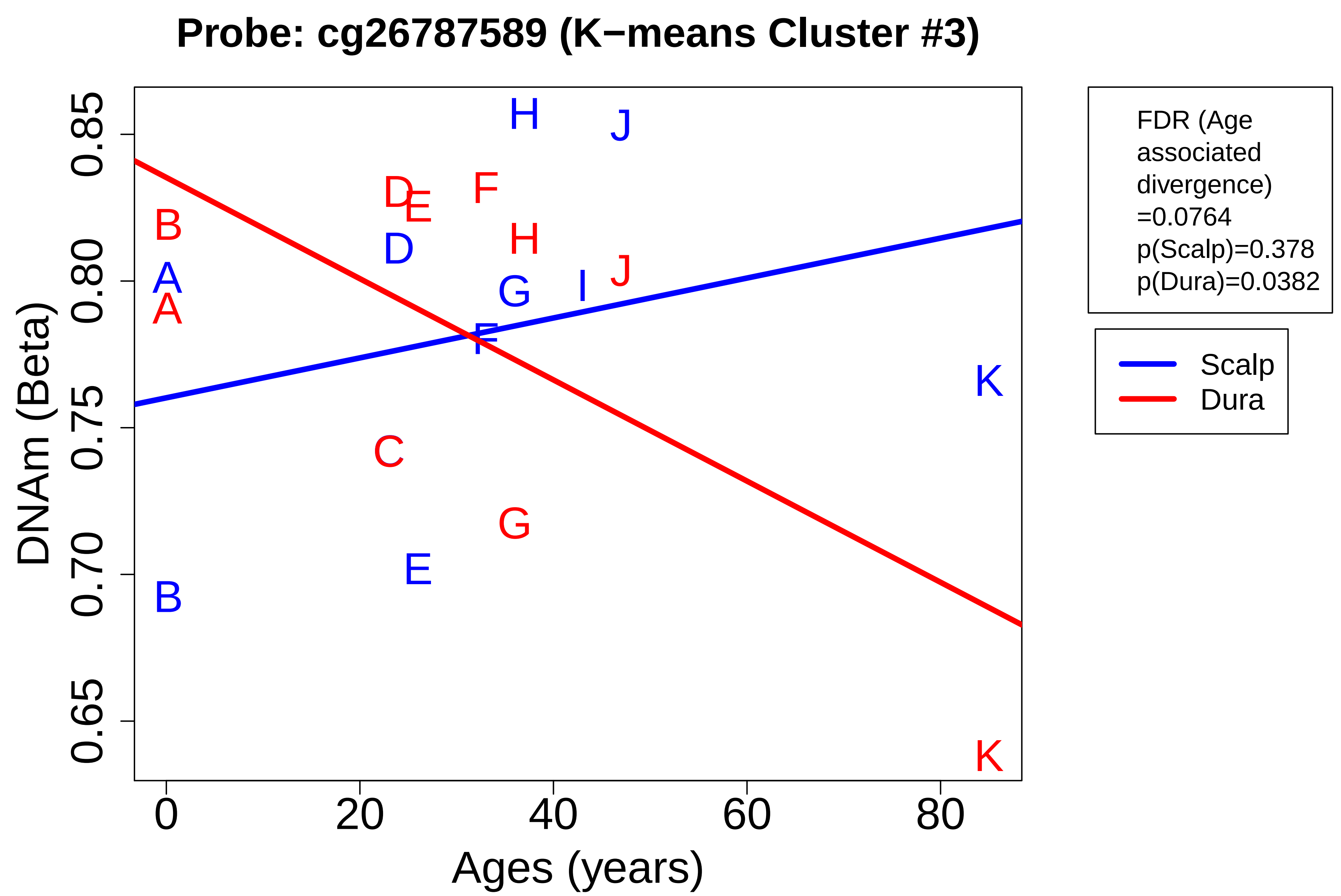

Probe: cg26787589 (K−means Cluster #3)
DNAm (Beta)
0.65
0.70
0.75
0.80
0.85
0
20
40
60
80
Ages (years)
A
B
C
D
E
F
G
H
I
J
K
FDR (Age associated divergence) =0.0764
p(Scalp)=0.378
p(Dura)=0.0382
Scalp
Dura

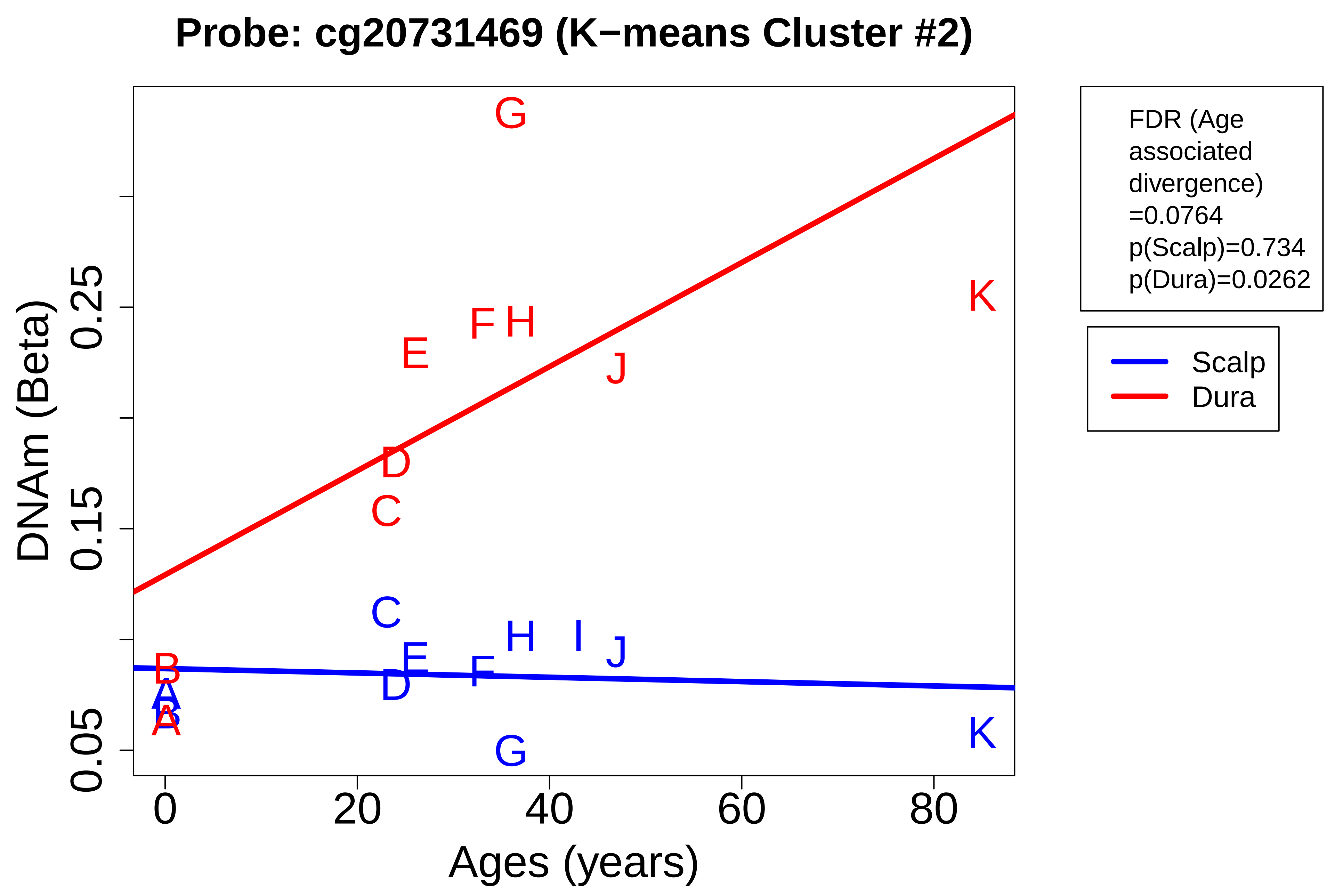

Probe: cg20731469 (K−means Cluster #2)
DNAm (Beta)
0.05
0.15
0.25
Ages (years)
0
20
40
60
80
FDR (Age associated divergence) =0.0764
p(Scalp)=0.734
p(Dura)=0.0262
Scalp
Dura
A
B
C
D
E
F
G
H
I
J
K

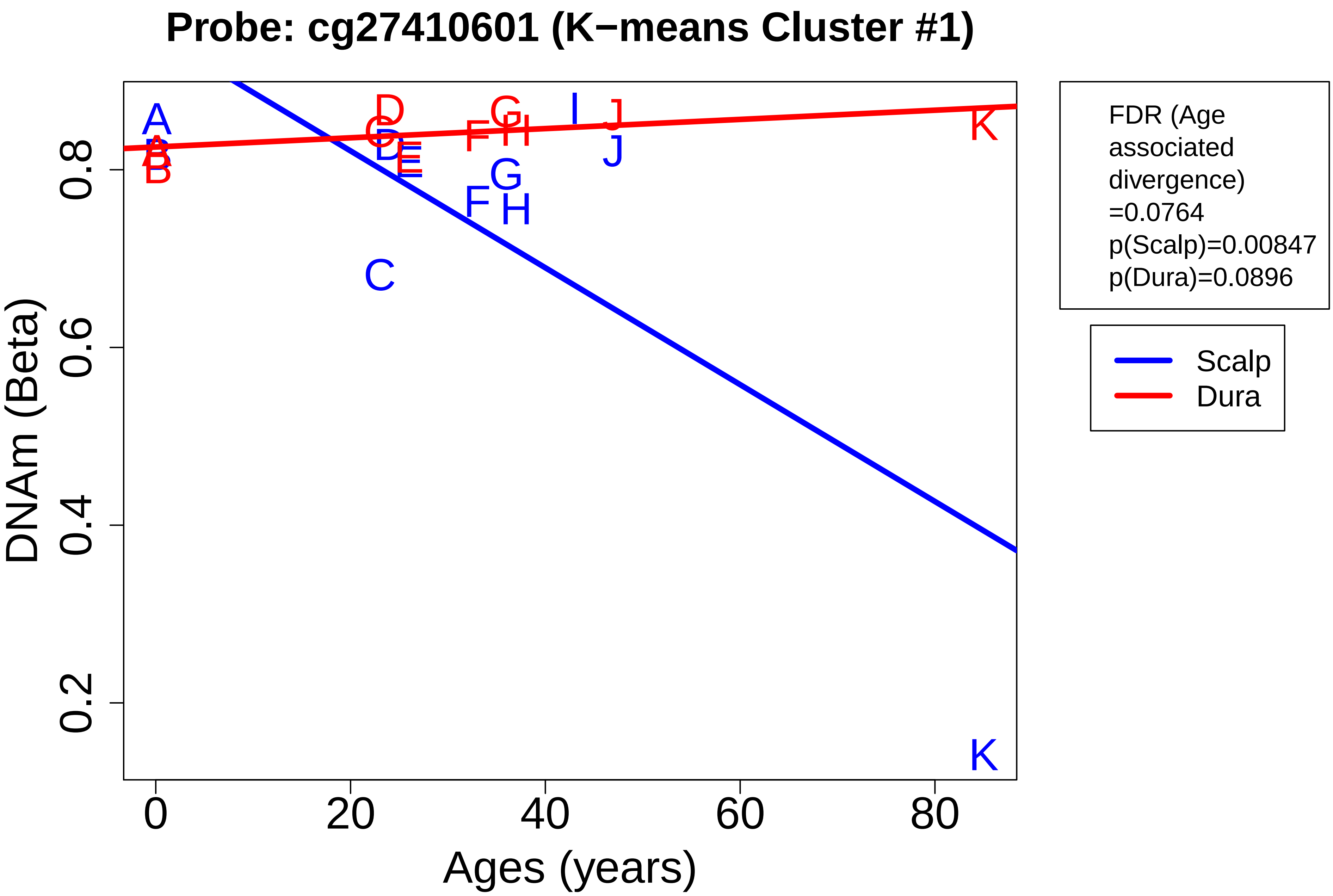
Probe: cg27410601 (K−means Cluster #1)
DNAm (Beta)
0.2
0.4
0.6
0.8
0
20
40
60
80
Ages (years)
A
B
C
D
E
F
G
H
I
J
K
FDR (Age associated divergence) =0.0764
p(Scalp)=0.00847
p(Dura)=0.0896
Scalp
Dura

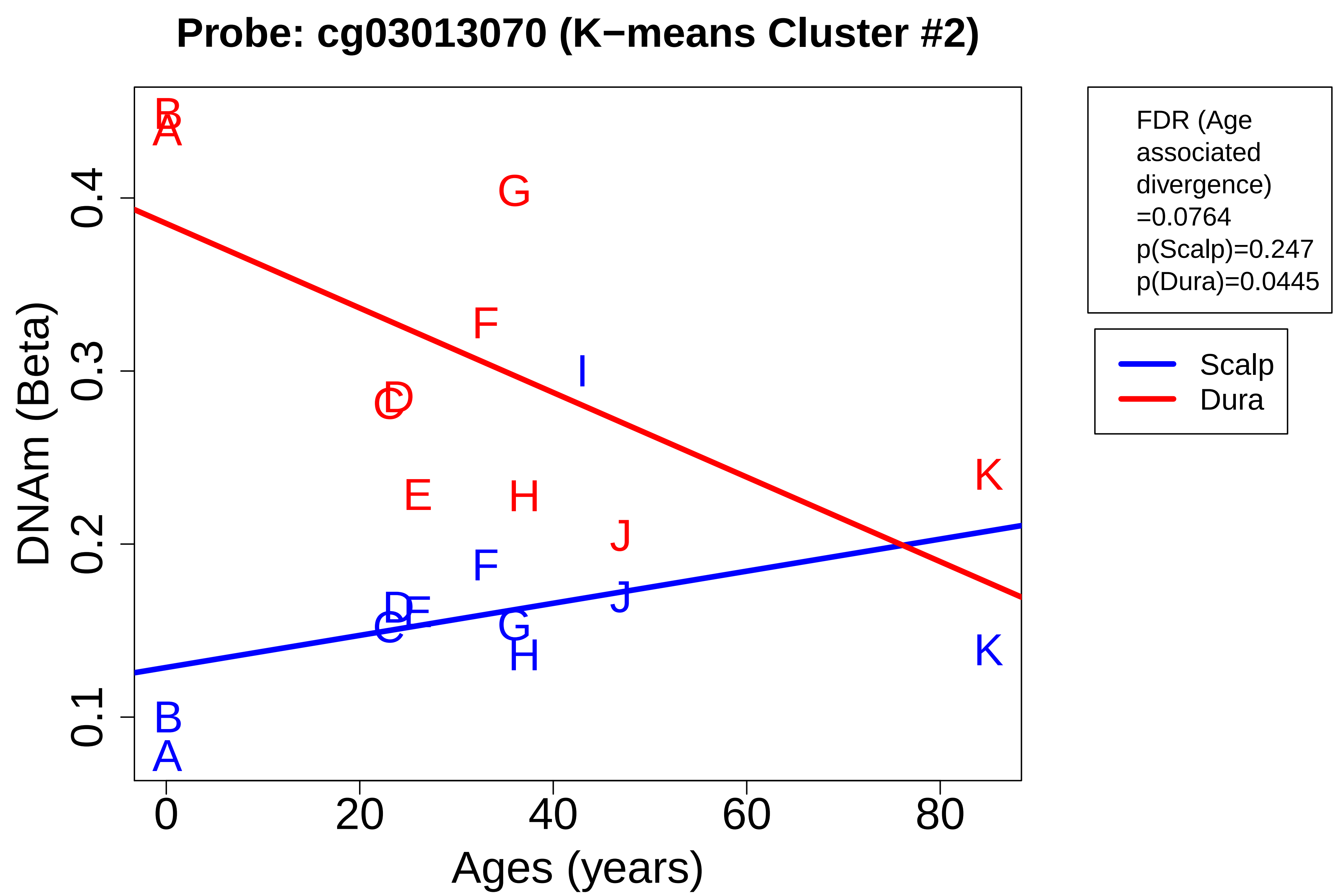

Probe: cg03013070 (K−means Cluster #2)
DNAm (Beta)
0.1
0.2
0.3
0.4
Ages (years)
0
20
40
60
80
FDR (Age associated divergence) =0.0764
p(Scalp)=0.247
p(Dura)=0.0445
Scalp
Dura

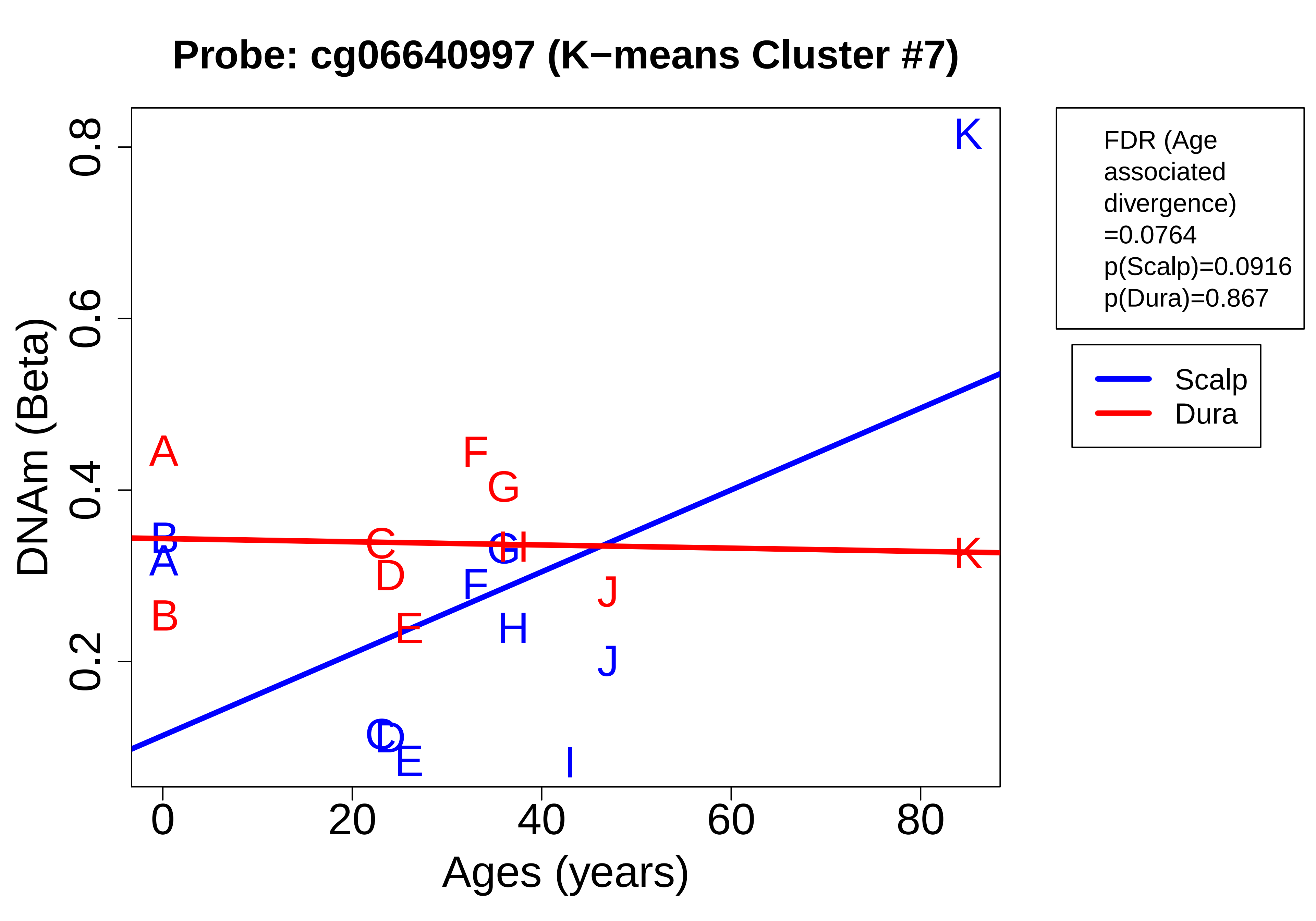
Probe: cg06640997 (K−means Cluster #7)
DNAm (Beta)
0.2
0.4
0.6
0.8
0
20
40
60
80
Ages (years)
FDR (Age associated divergence) =0.0764
p(Scalp)=0.0916
p(Dura)=0.867
Scalp
Dura
A
B
C
D
E
F
G
H
I
J
K

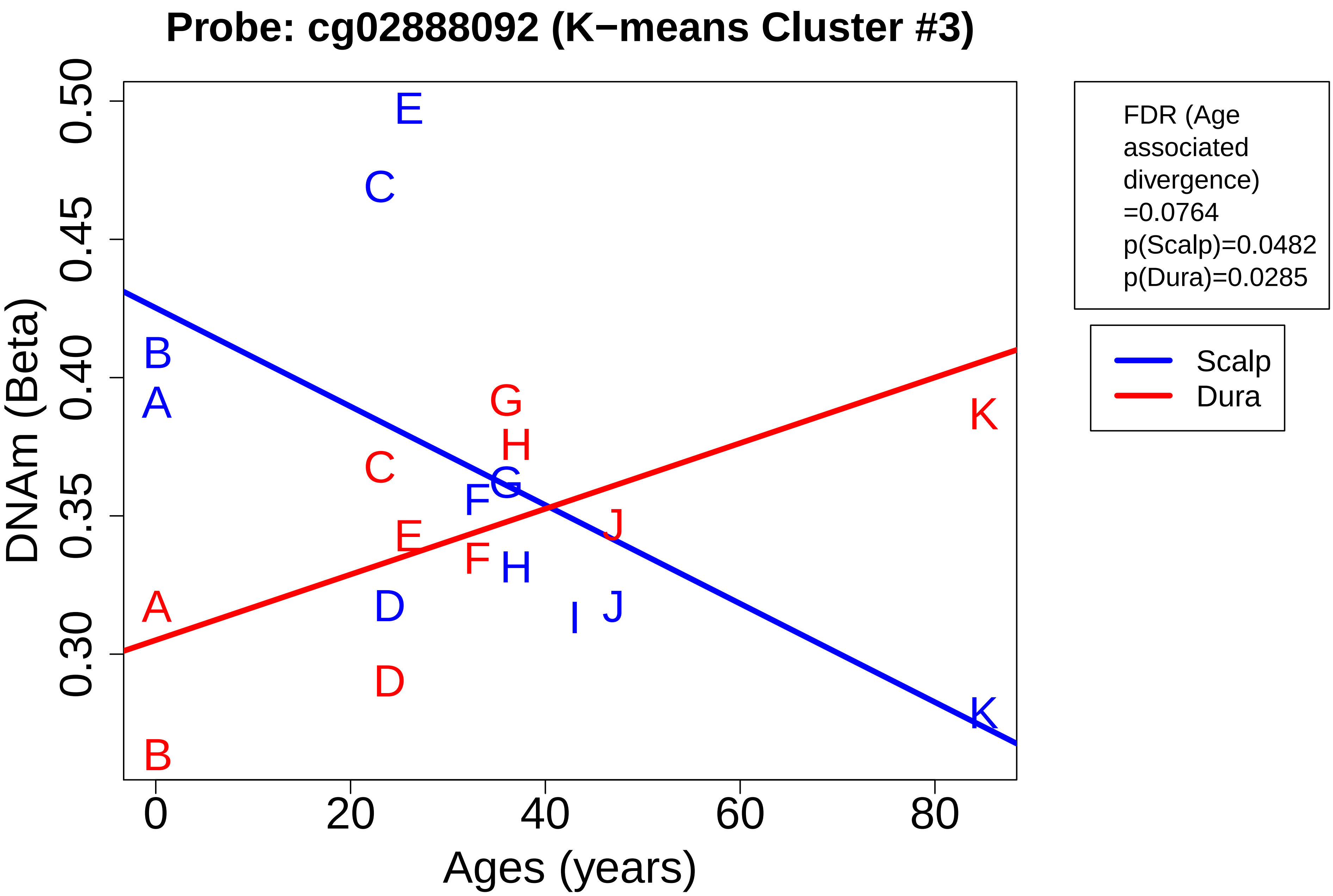

Probe: cg02888092 (K−means Cluster #3)
DNAm (Beta)
0.30
0.35
0.40
0.45
0.50
Ages (years)
0
20
40
60
80
FDR (Age associated divergence) =0.0764
p(Scalp)=0.0482
p(Dura)=0.0285
Scalp
Dura
A
B
C
D
E
F
G
H
I
J
K

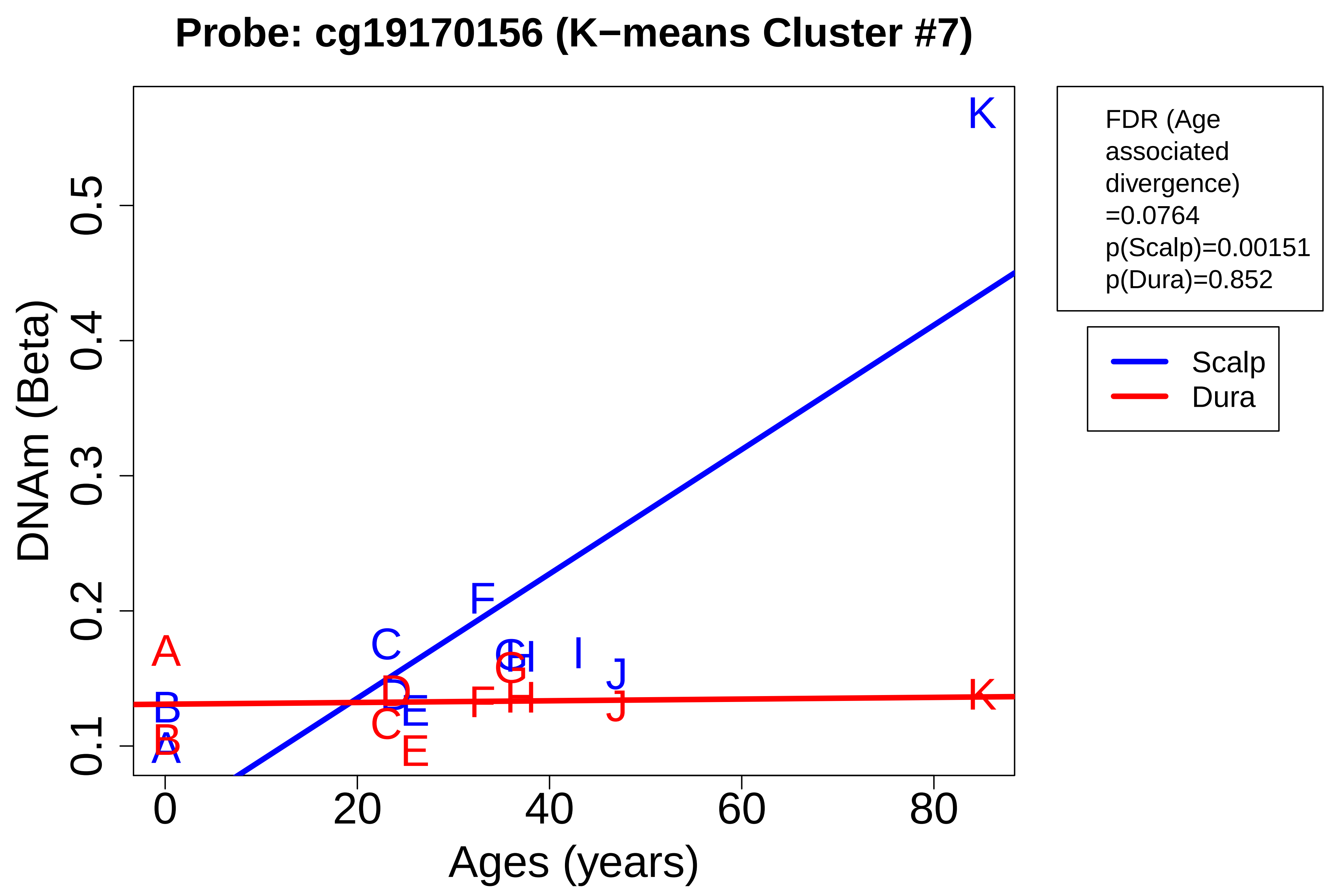

Probe: cg19170156 (K−means Cluster #7)
FDR (Age associated divergence) =0.0764
p(Scalp)=0.00151
p(Dura)=0.852
Scalp
Dura
DNAm (Beta)
Ages (years)
0.1
0.2
0.3
0.4
0.5
0
20
40
60
80

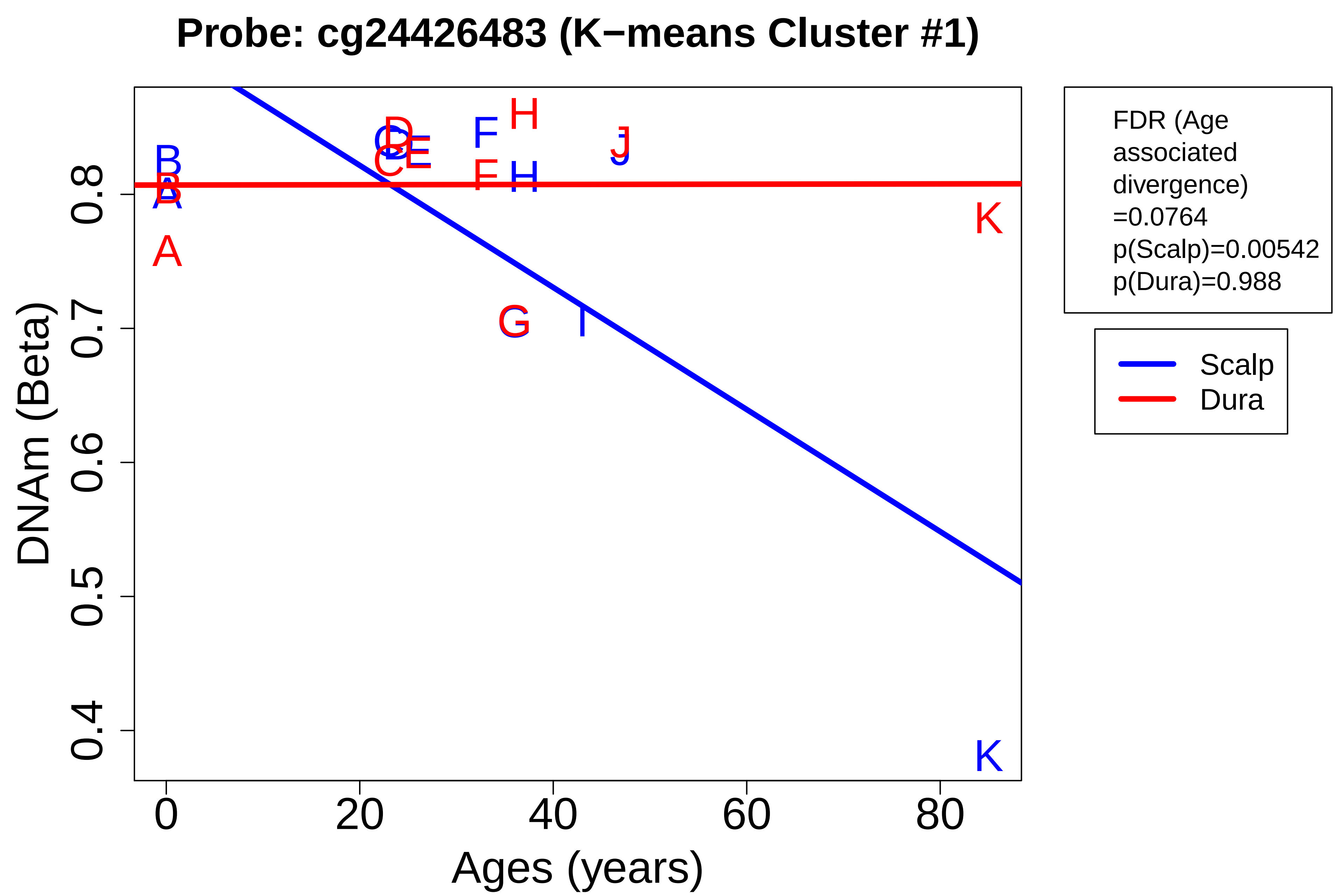

Probe: cg24426483 (K−means Cluster #1)
DNAm (Beta)
Ages (years)
0.4
0.5
0.6
0.7
0.8
0
20
40
60
80
A
B
C
D
E
F
G
H
I
J
K
FDR (Age associated divergence) =0.0764
p(Scalp)=0.00542
p(Dura)=0.988
Scalp
Dura

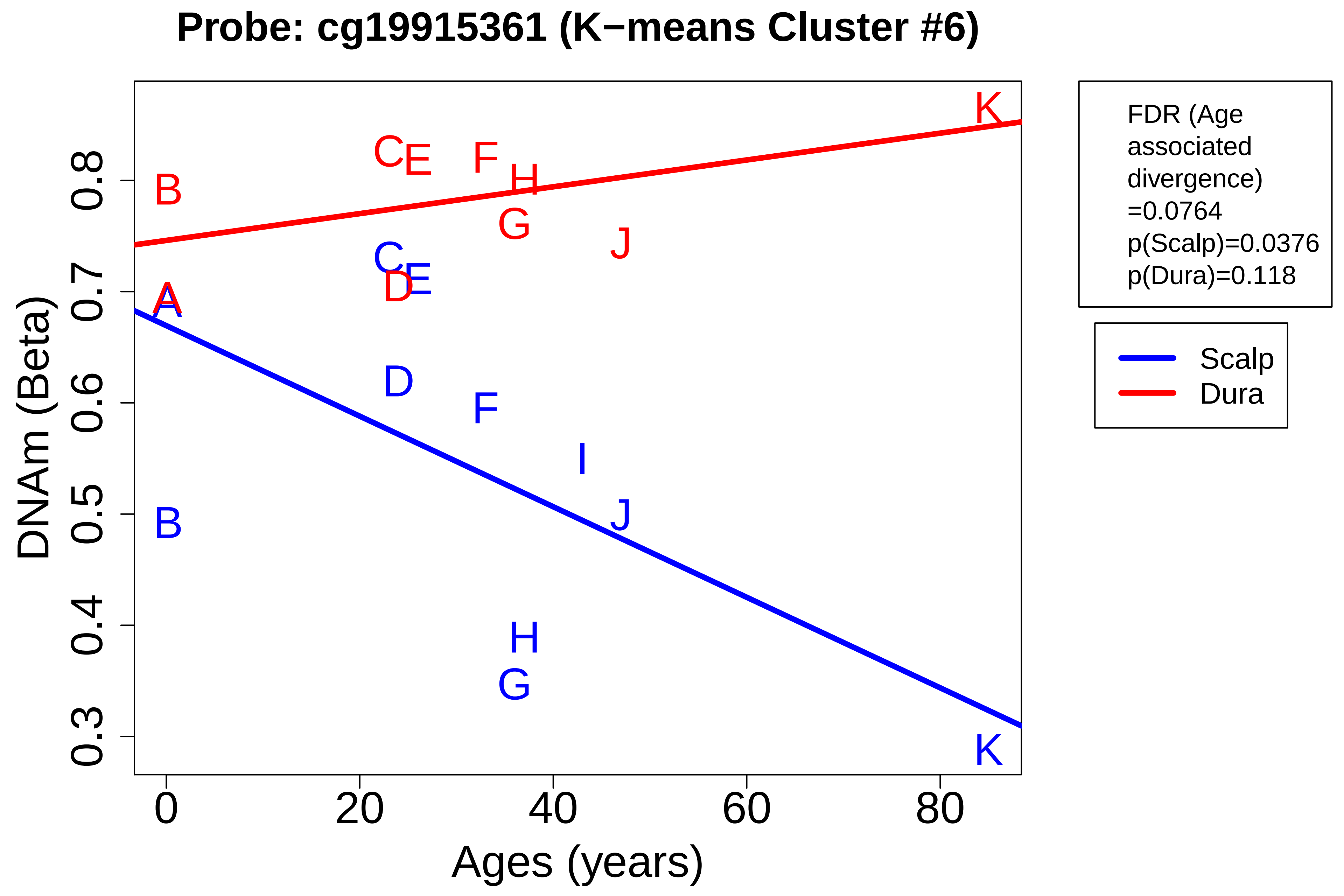

Probe: cg19915361 (K−means Cluster #6)
DNAm (Beta)
0.3
0.4
0.5
0.6
0.7
0.8
0
20
40
60
80
Ages (years)
FDR (Age associated divergence) =0.0764
p(Scalp)=0.0376
p(Dura)=0.118
Scalp
Dura

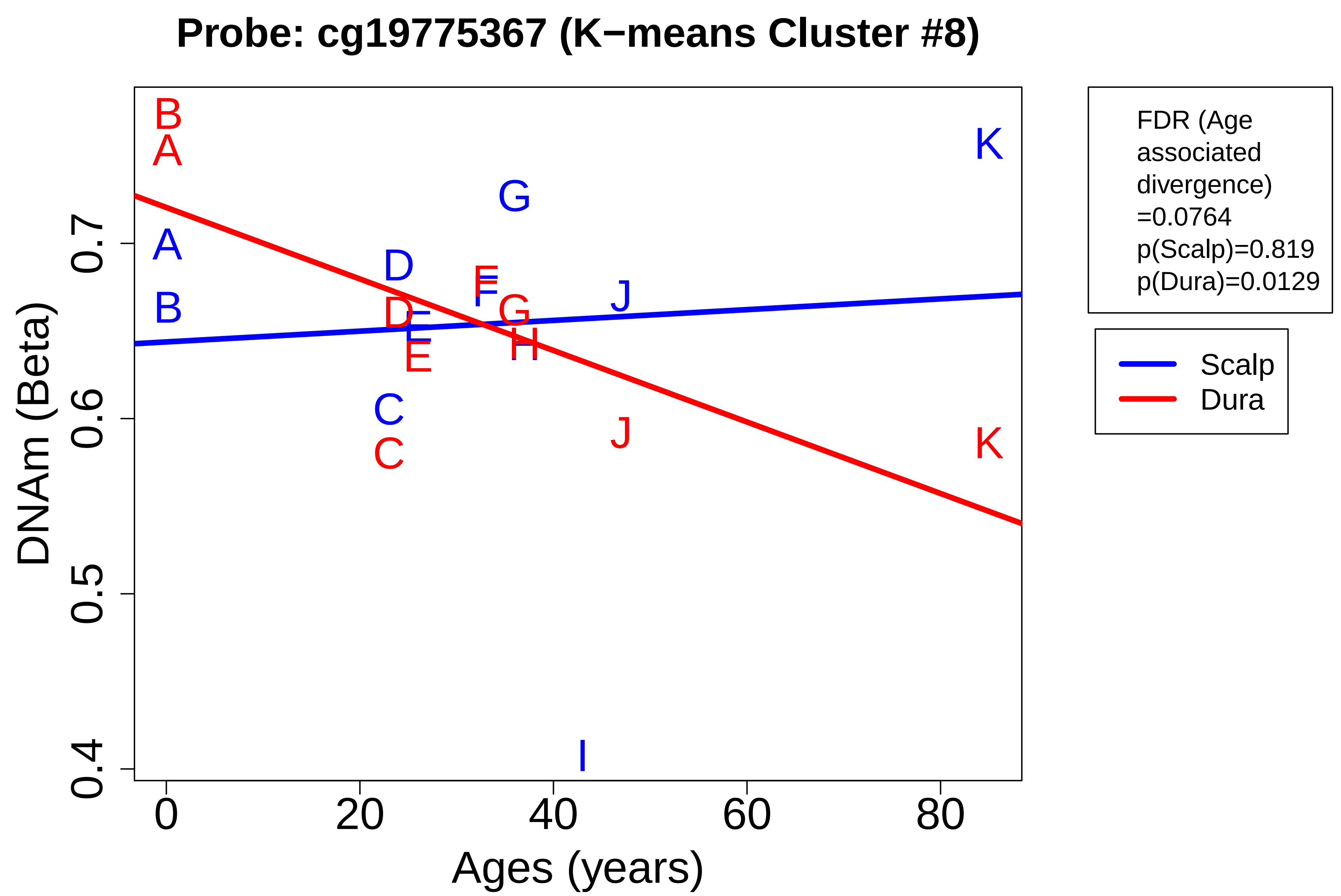

Probe: cg19775367 (K−means Cluster #8)
DNAm (Beta)
0.4
0.5
0.6
0.7
0
20
40
60
80
Ages (years)
FDR (Age associated divergence) =0.0764
p(Scalp)=0.819
p(Dura)=0.0129
Scalp
Dura

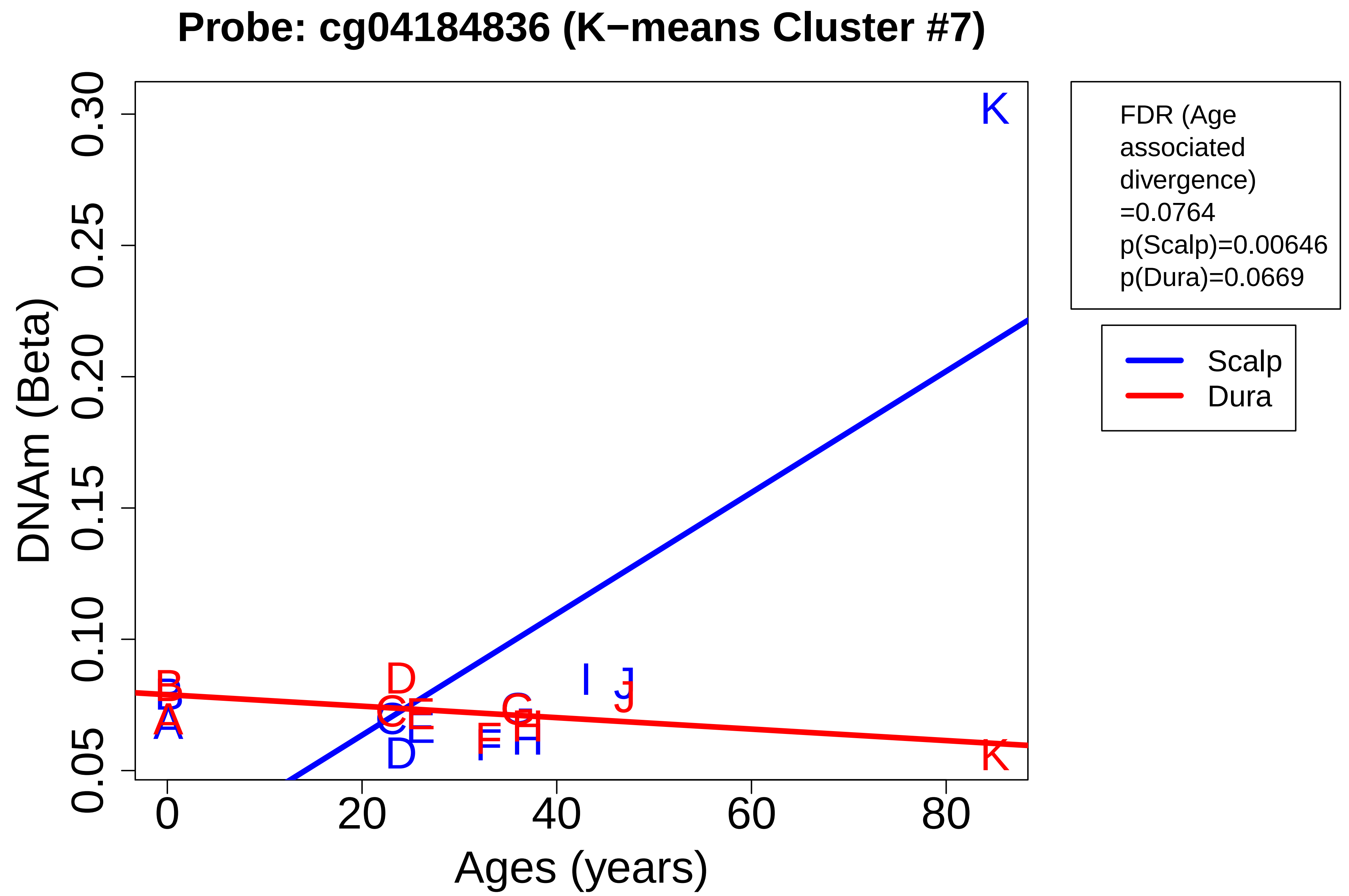

Probe: cg04184836 (K−means Cluster #7)
DNAm (Beta)
0.05
0.10
0.15
0.20
0.25
0.30
0
20
40
60
80
Ages (years)
FDR (Age associated divergence) =0.0764
p(Scalp)=0.00646
p(Dura)=0.0669
Scalp
Dura

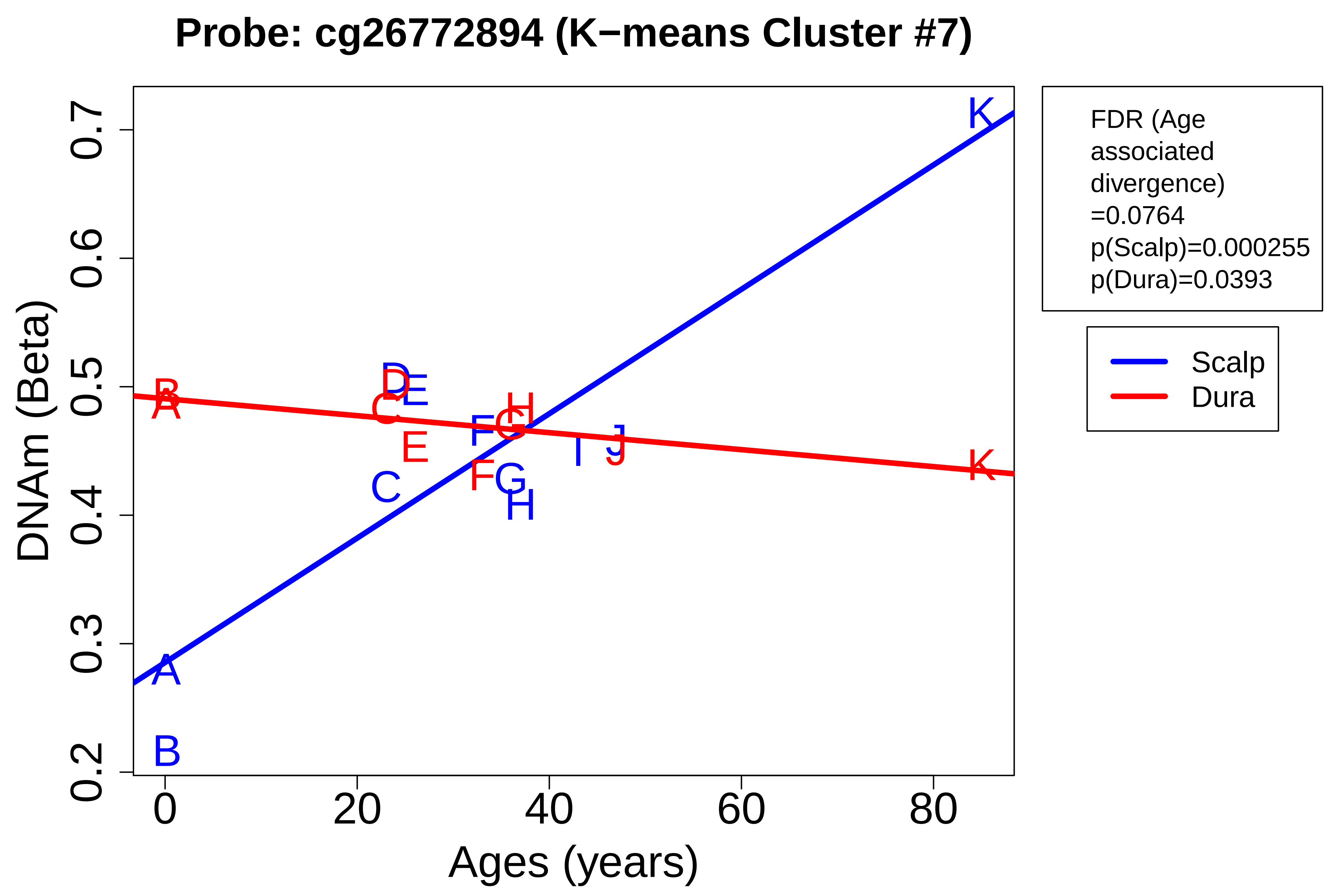

Probe: cg26772894 (K−means Cluster #7)
DNAm (Beta)
0.2
0.3
0.4
0.5
0.6
0.7
0
20
40
60
80
Ages (years)
FDR (Age associated divergence) =0.0764
p(Scalp)=0.000255
p(Dura)=0.0393
Scalp
Dura

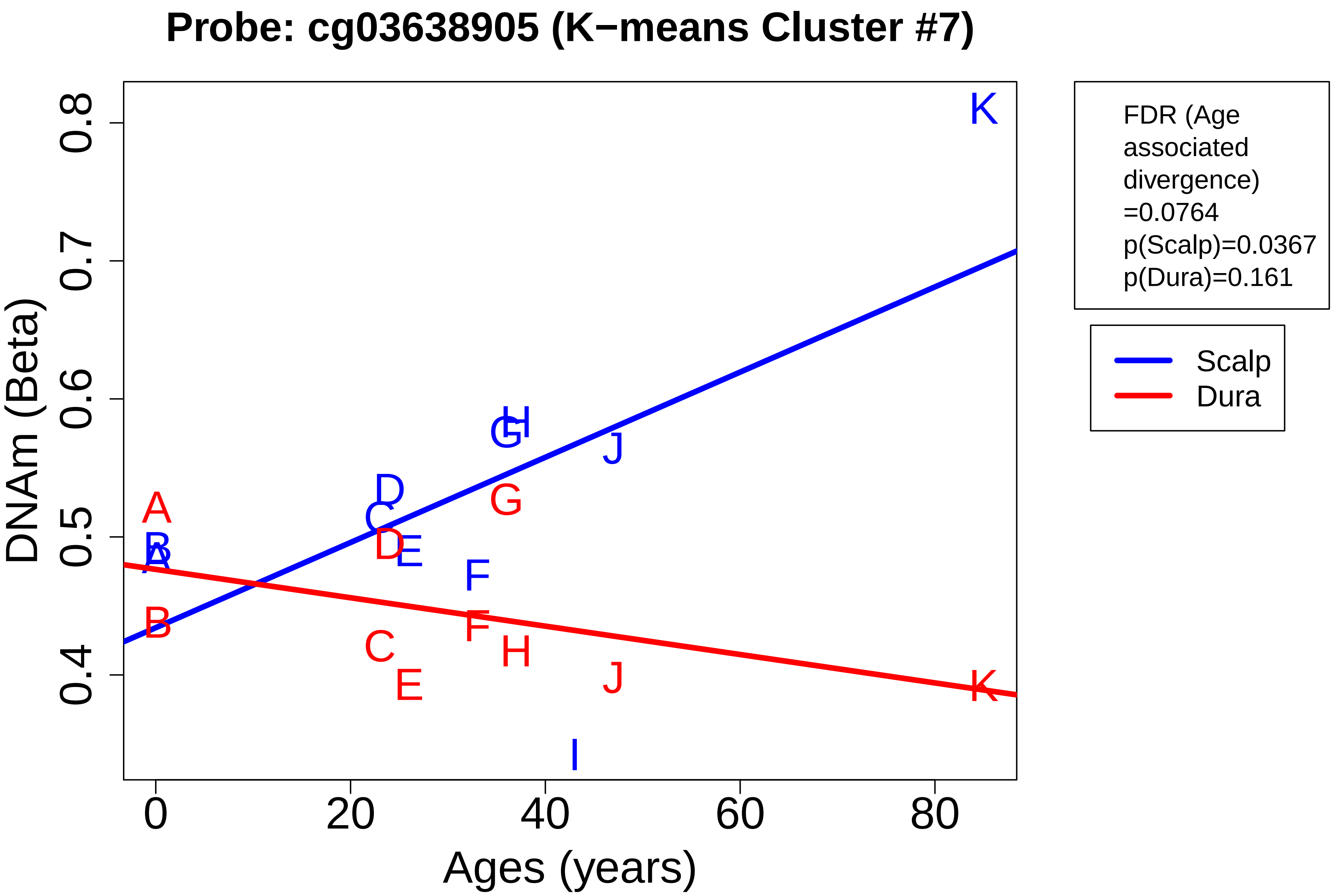
Probe: cg03638905 (K−means Cluster #7)
DNAm (Beta)
0.4
0.5
0.6
0.7
0.8
Ages (years)
0
20
40
60
80
FDR (Age associated divergence) =0.0764
p(Scalp)=0.0367
p(Dura)=0.161
Scalp
Dura

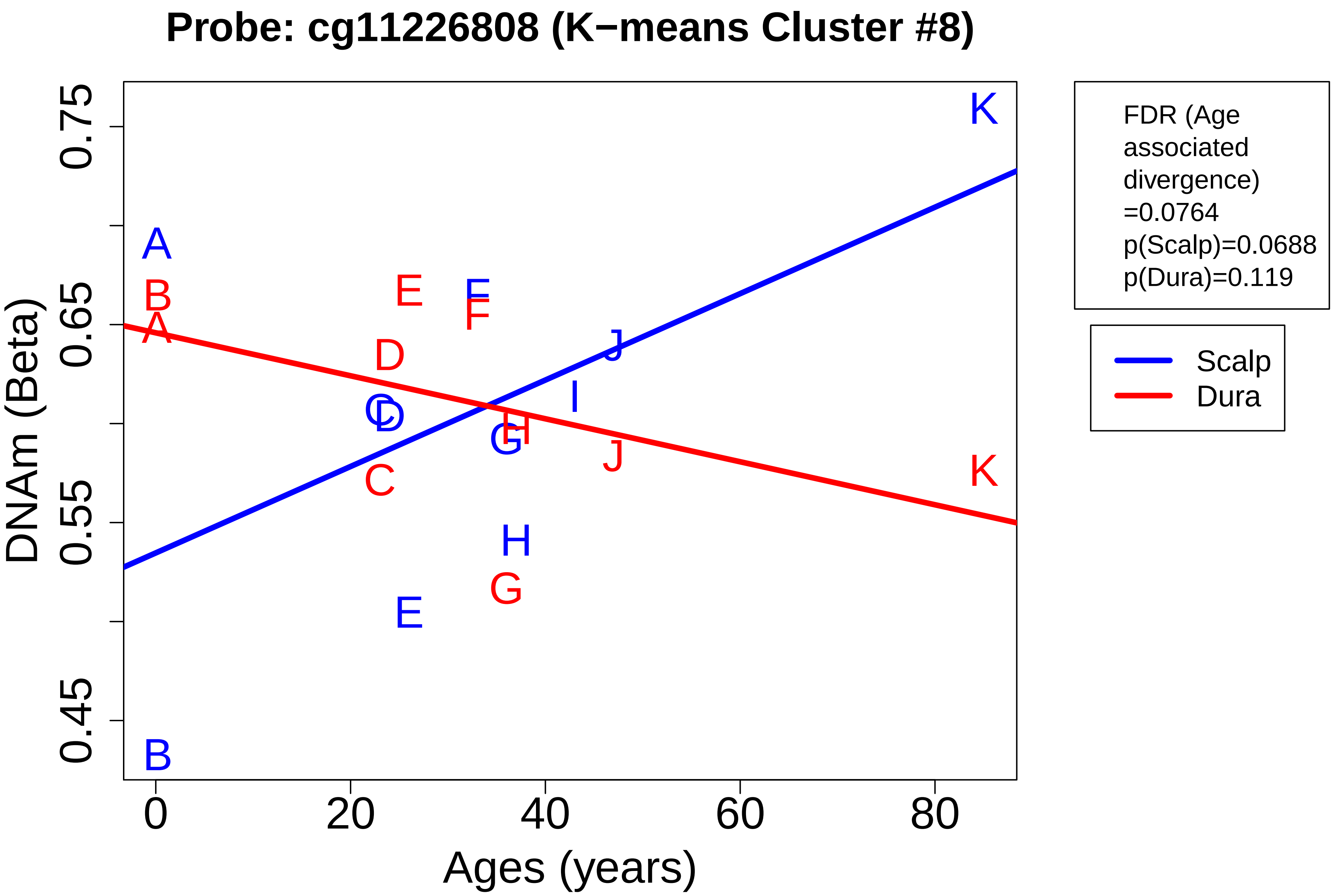
Probe: cg11226808 (K−means Cluster #8)
DNAm (Beta)
Ages (years)
0.45
0.55
0.65
0.75
0
20
40
60
80
FDR (Age associated divergence) =0.0764
p(Scalp)=0.0688
p(Dura)=0.119
Scalp
Dura

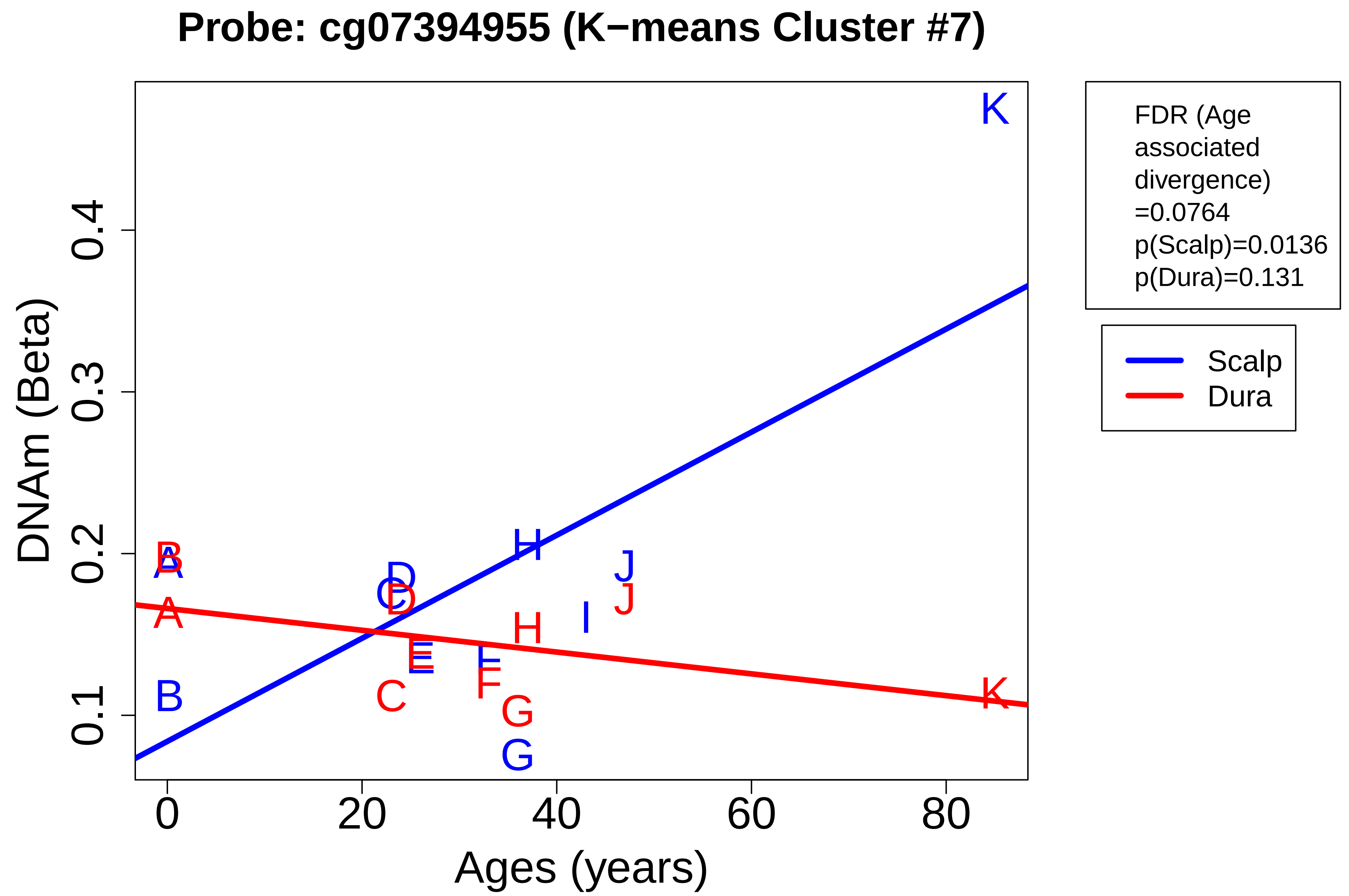

Probe: cg07394955 (K−means Cluster #7)
FDR (Age associated divergence) =0.0764
p(Scalp)=0.0136
p(Dura)=0.131
Scalp
Dura
DNAm (Beta)
Ages (years)
0.1
0.2
0.3
0.4
0
20
40
60
80

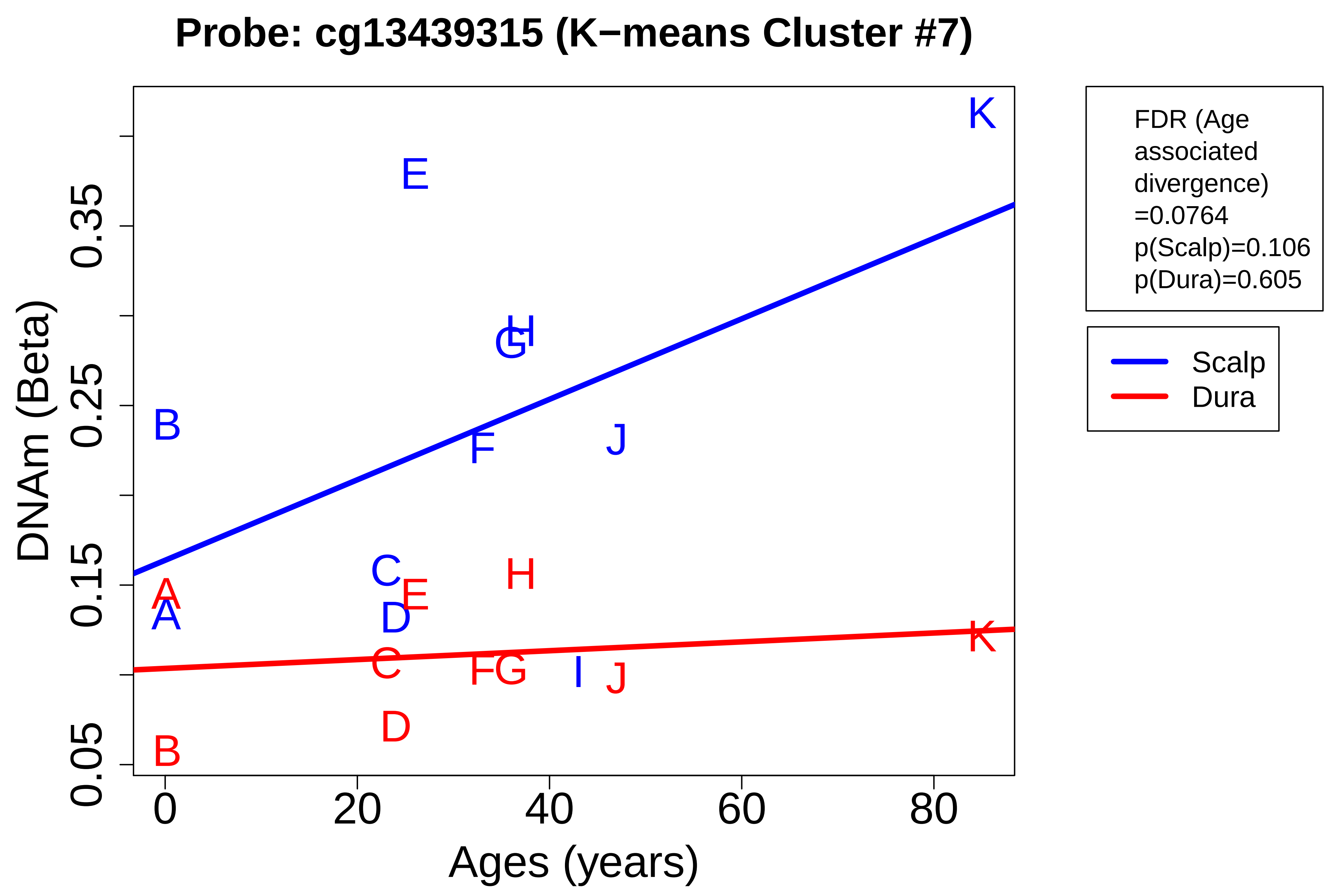
Probe: cg13439315 (K−means Cluster #7)
DNAm (Beta)
0.05
0.15
0.25
0.35
0
20
40
60
80
Ages (years)
FDR (Age associated divergence) =0.0764
p(Scalp)=0.106
p(Dura)=0.605
Scalp
Dura
A
B
C
D
E
F
G
H
I
J
K

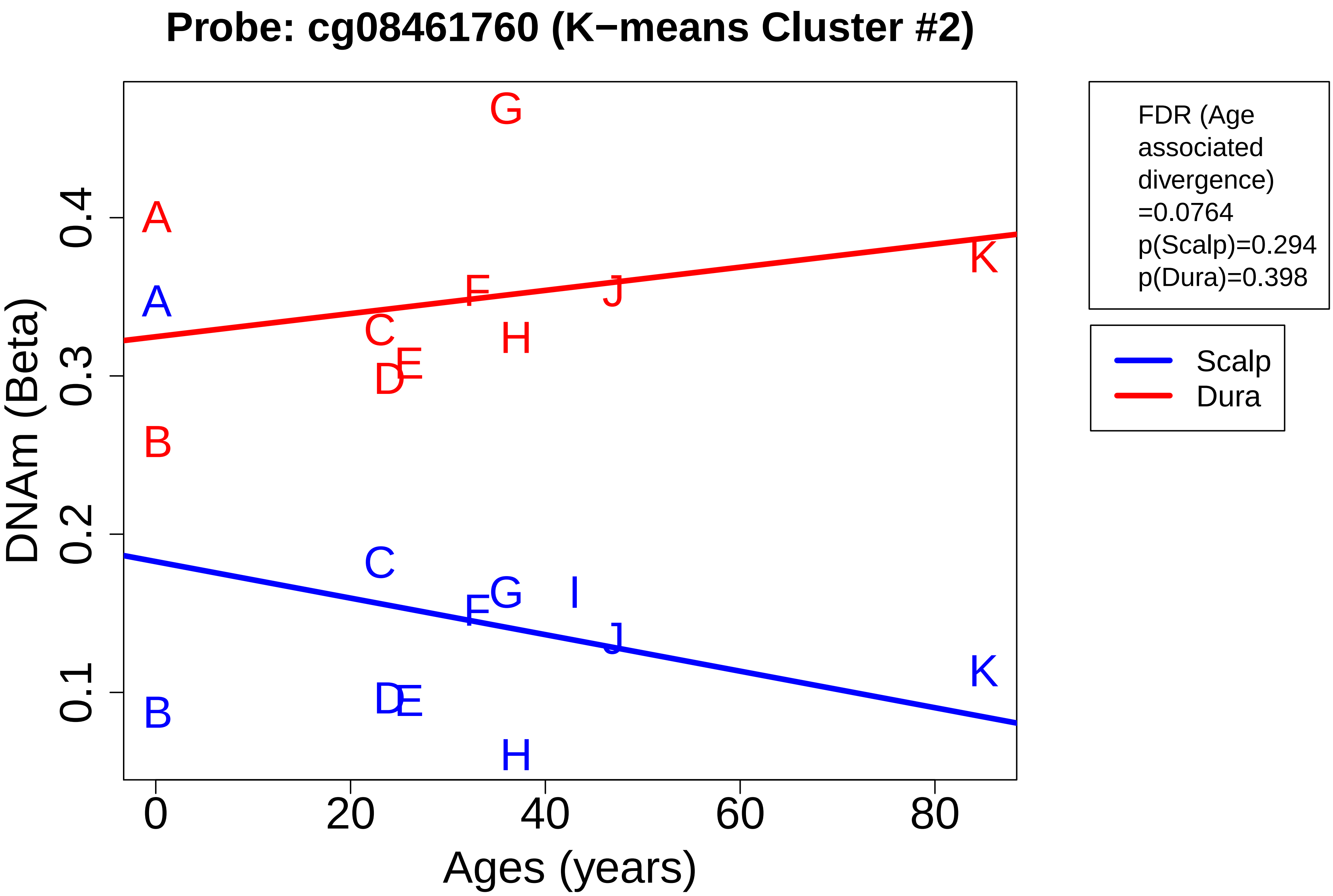

Probe: cg08461760 (K−means Cluster #2)
DNAm (Beta)
0.1
0.2
0.3
0.4
0
20
40
60
80
Ages (years)
A
B
C
D
E
F
G
H
J
K
A
B
C
D
E
F
G
H
I
J
K
FDR (Age associated divergence) =0.0764
p(Scalp)=0.294
p(Dura)=0.398
Scalp
Dura

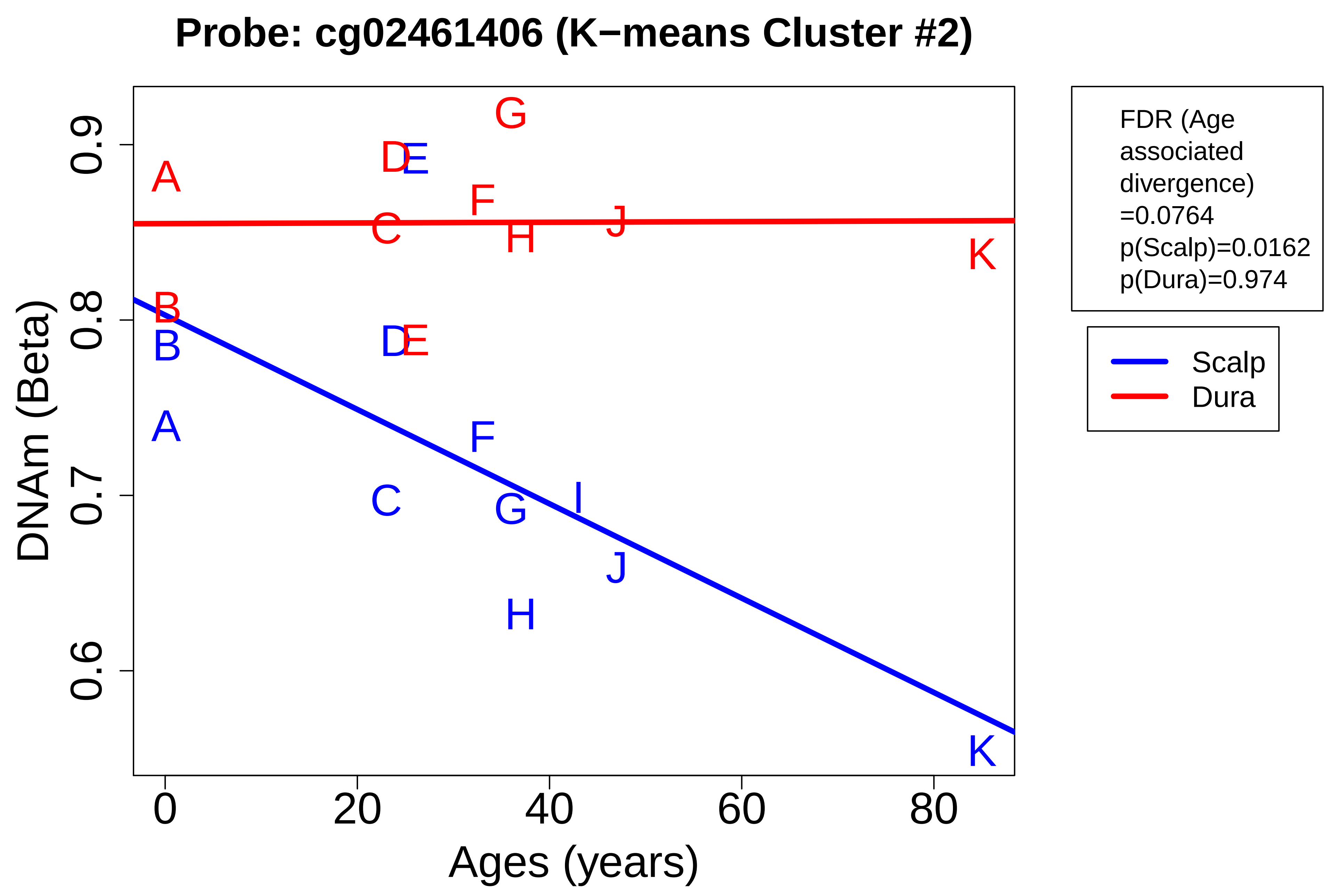
Probe: cg02461406 (K−means Cluster #2)
DNAm (Beta)
0.6
0.7
0.8
0.9
Ages (years)
0
20
40
60
80
A
B
C
D
E
F
G
H
I
J
K
FDR (Age associated divergence) =0.0764
p(Scalp)=0.0162
p(Dura)=0.974
Scalp
Dura

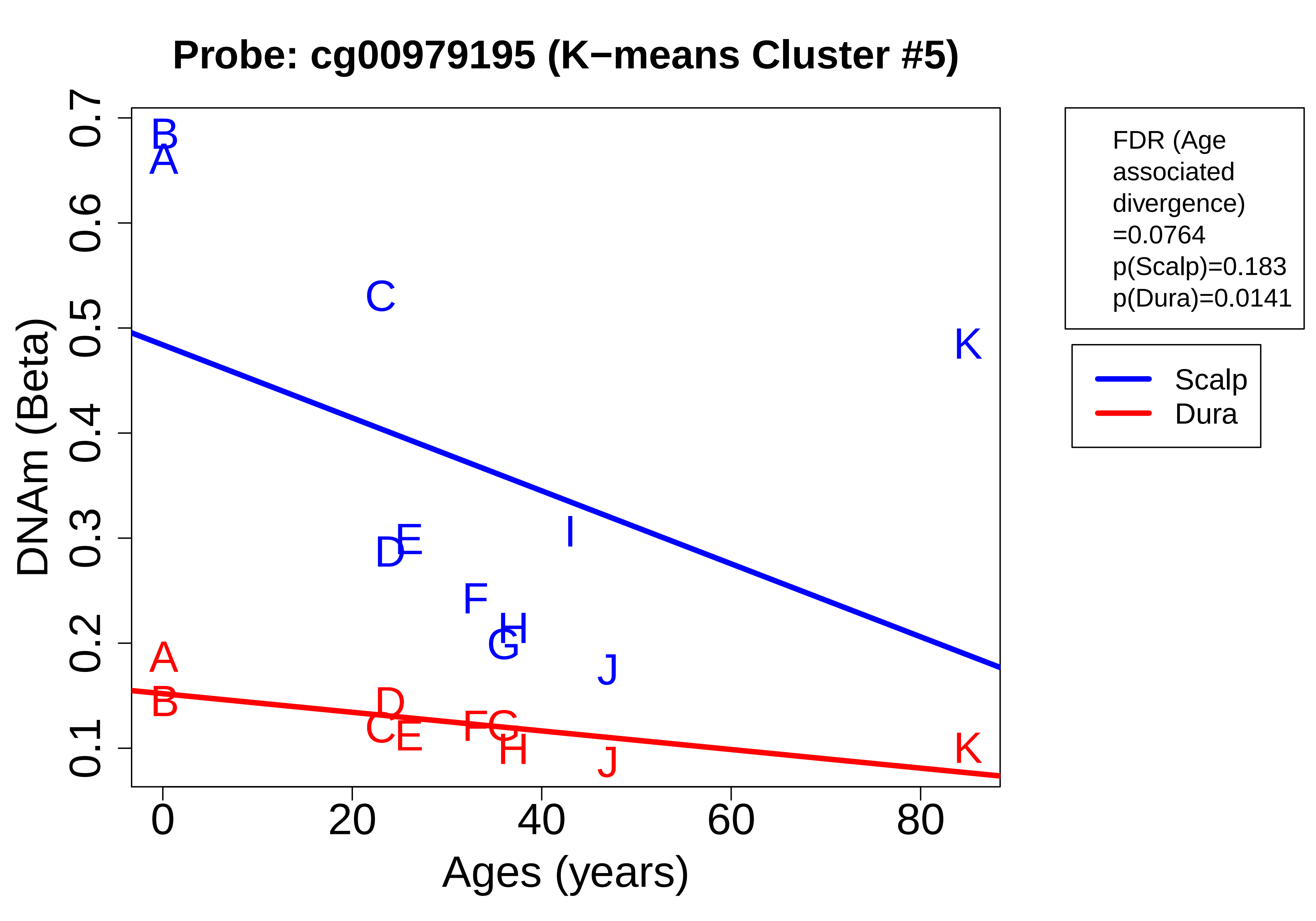

Probe: cg00979195 (K−means Cluster #5)
DNAm (Beta)
0.1
0.2
0.3
0.4
0.5
0.6
0.7
Ages (years)
0
20
40
60
80
FDR (Age associated divergence) =0.0764
p(Scalp)=0.183
p(Dura)=0.0141
Scalp
Dura

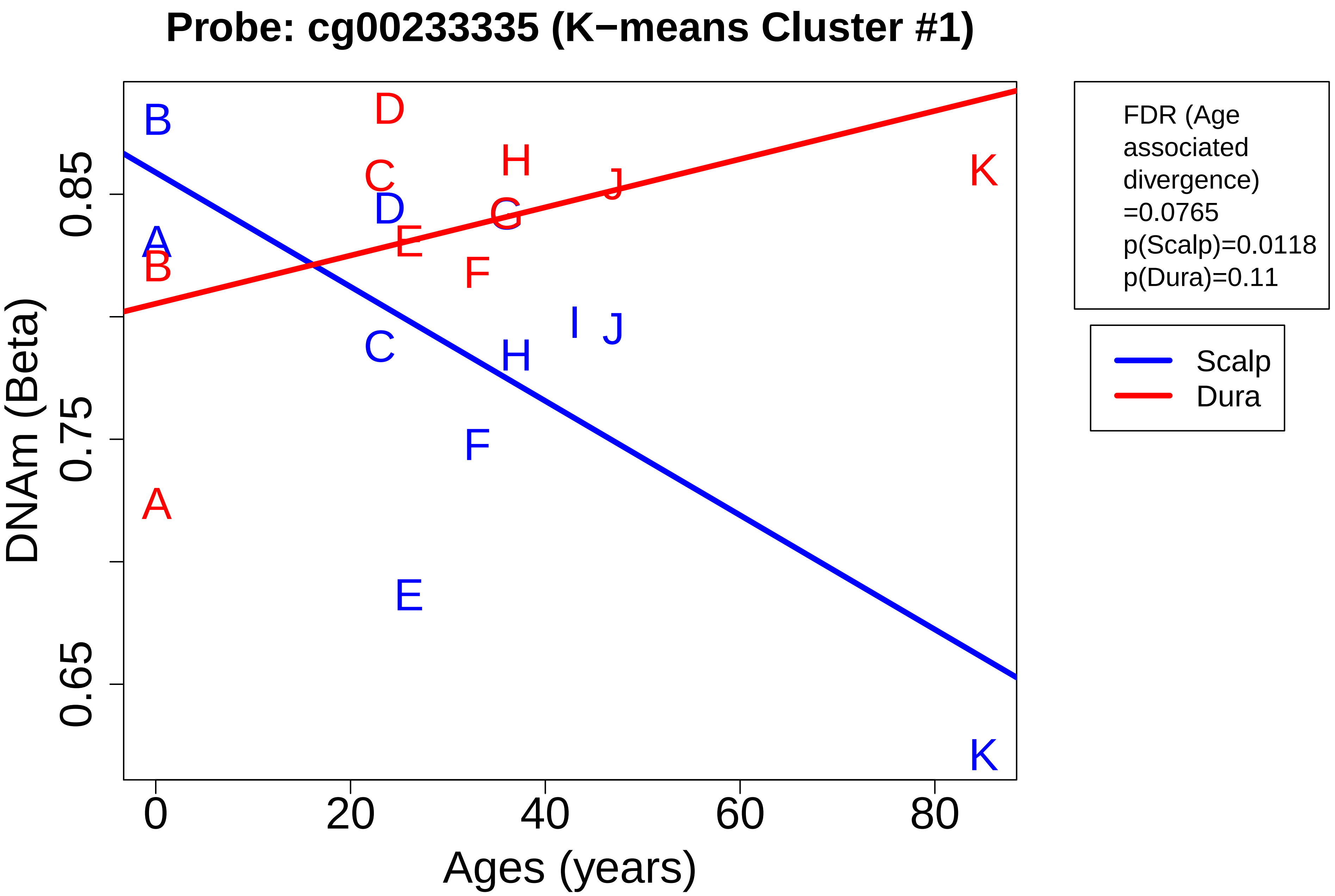

Probe: cg00233335 (K−means Cluster #1)
FDR (Age associated divergence) =0.0765
p(Scalp)=0.0118
p(Dura)=0.11
Scalp
Dura
DNAm (Beta)
0.65
0.75
0.85
0
20
40
60
80
Ages (years)
A
B
C
D
E
F
G
H
I
J
K

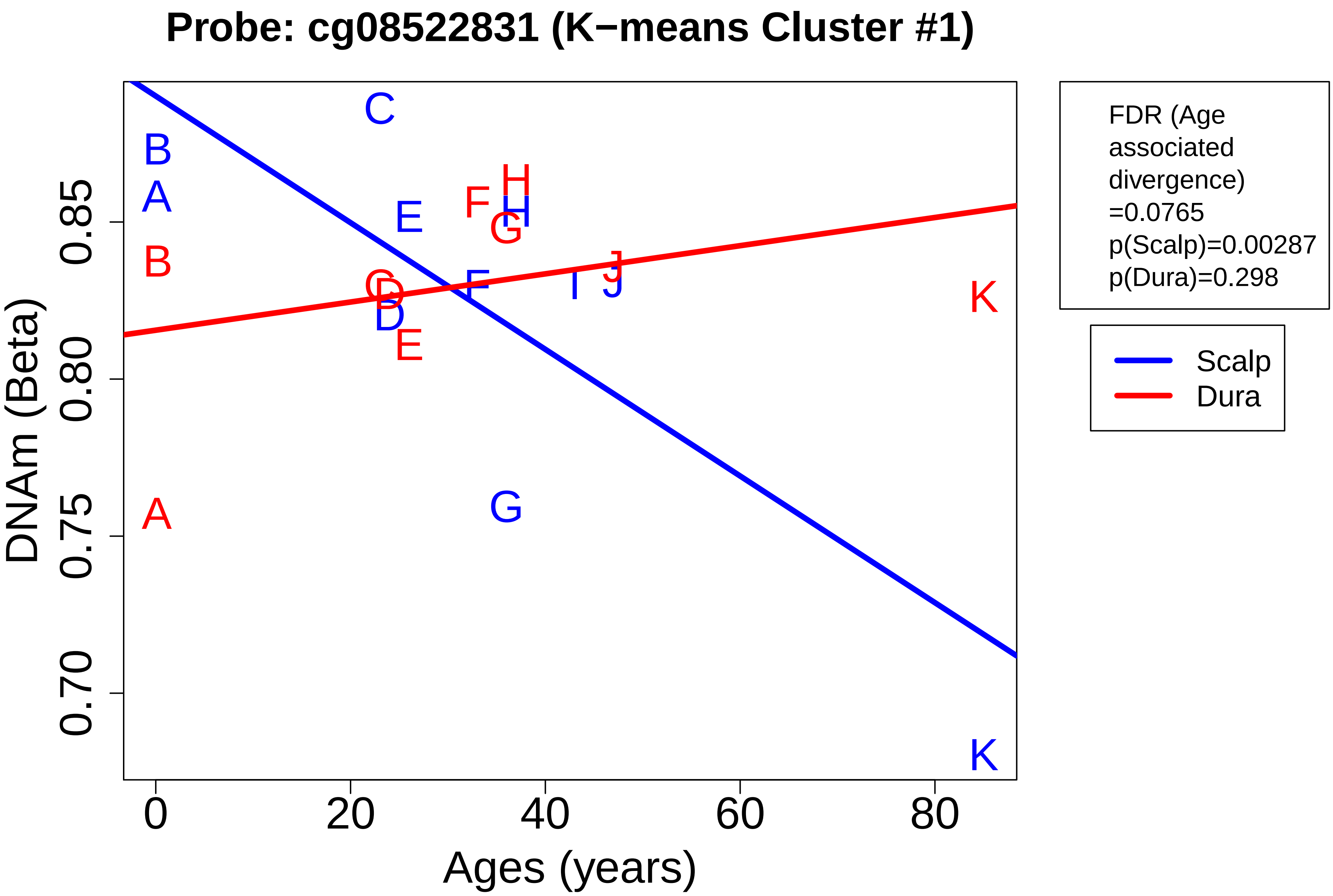

Probe: cg08522831 (K−means Cluster #1)
DNAm (Beta)
0.70
0.75
0.80
0.85
0
20
40
60
80
Ages (years)
A
B
C
D
E
F
G
H
I
J
K
FDR (Age associated divergence) =0.0765
p(Scalp)=0.00287
p(Dura)=0.298
Scalp
Dura

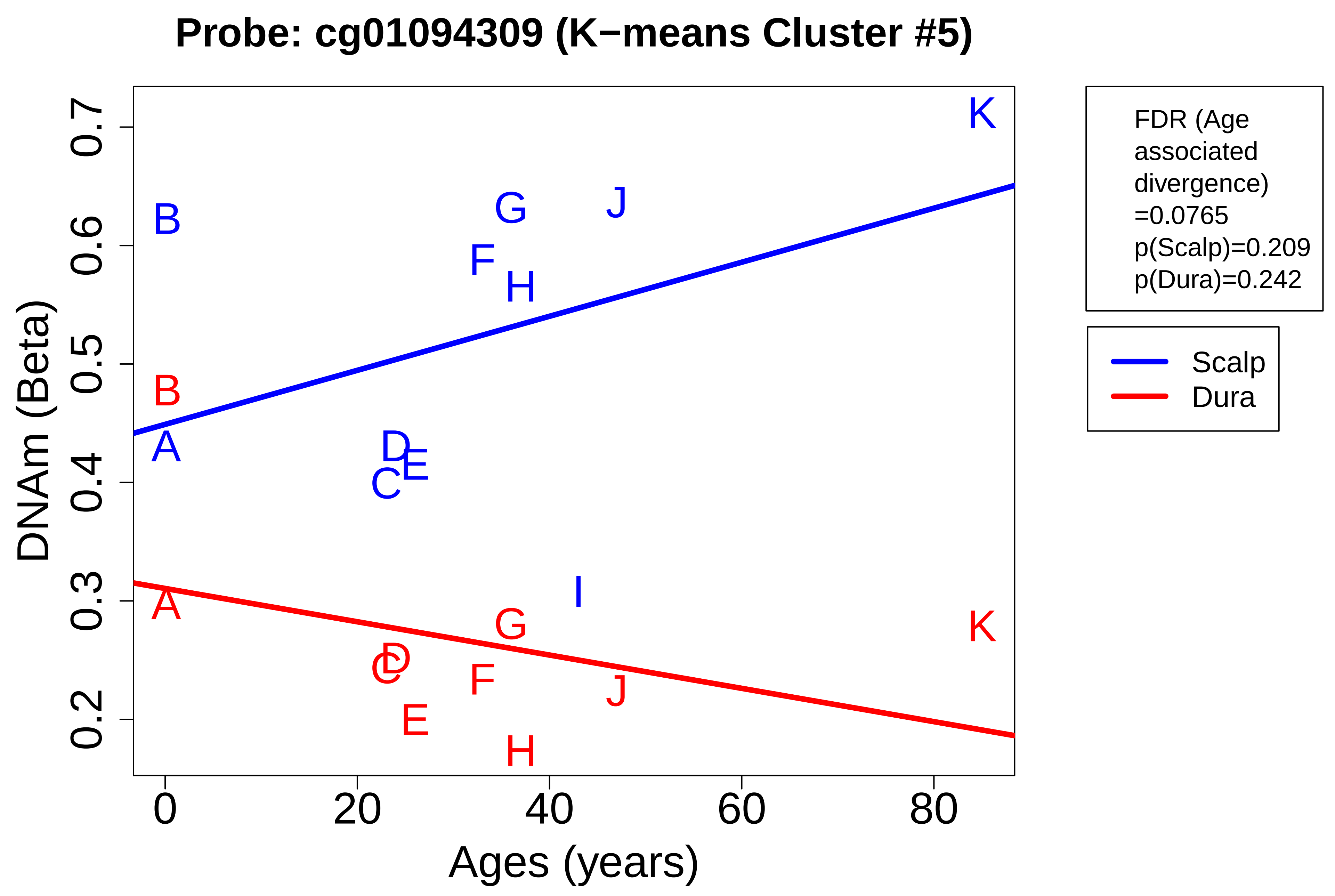

Probe: cg01094309 (K−means Cluster #5)
DNAm (Beta)
0.2
0.3
0.4
0.5
0.6
0.7
0
20
40
60
80
Ages (years)
FDR (Age associated divergence) =0.0765
p(Scalp)=0.209
p(Dura)=0.242
Scalp
Dura

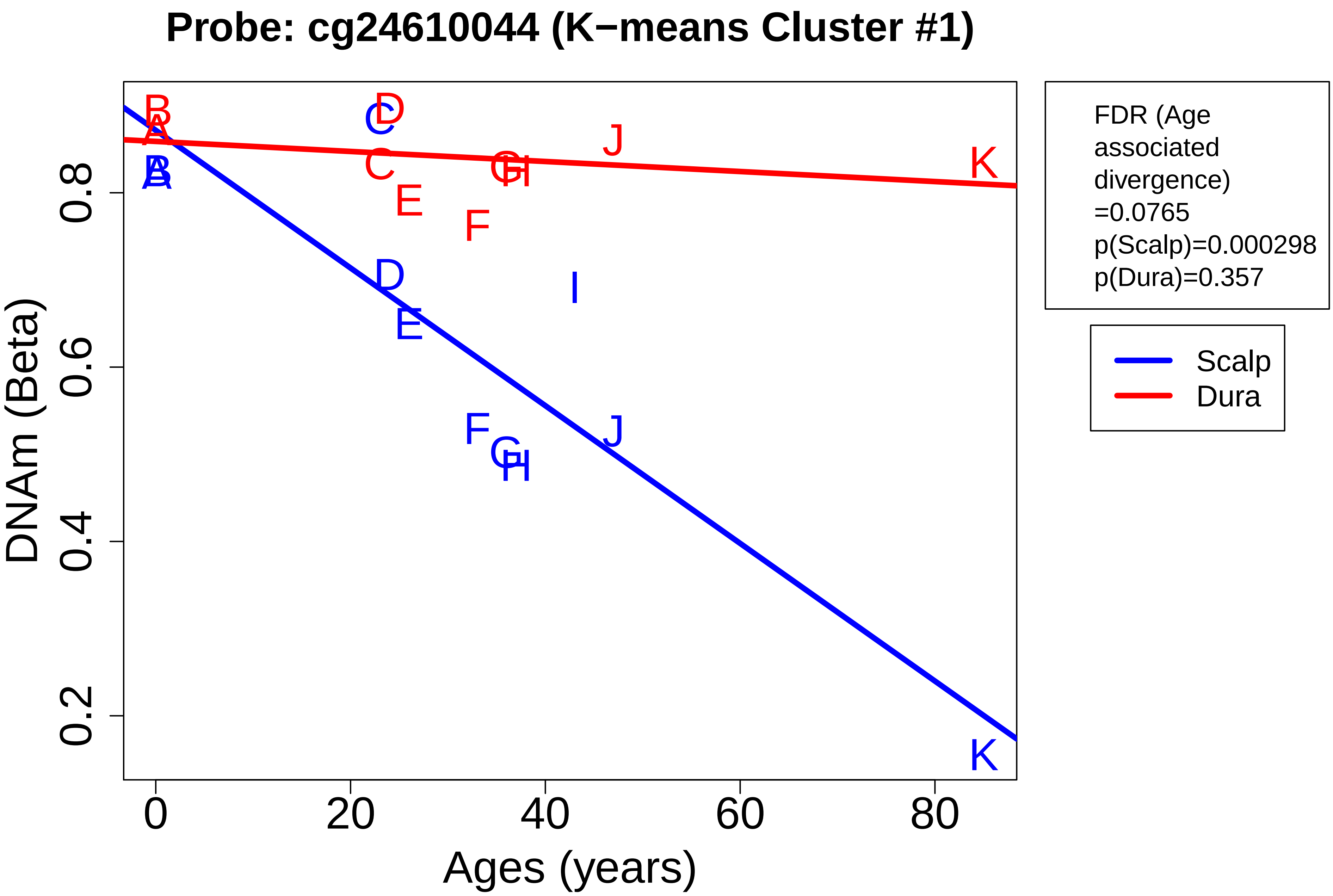

Probe: cg24610044 (K−means Cluster #1)
DNAm (Beta)
0.2
0.4
0.6
0.8
0
20
40
60
80
Ages (years)
FDR (Age associated divergence) =0.0765
p(Scalp)=0.000298
p(Dura)=0.357
Scalp
Dura

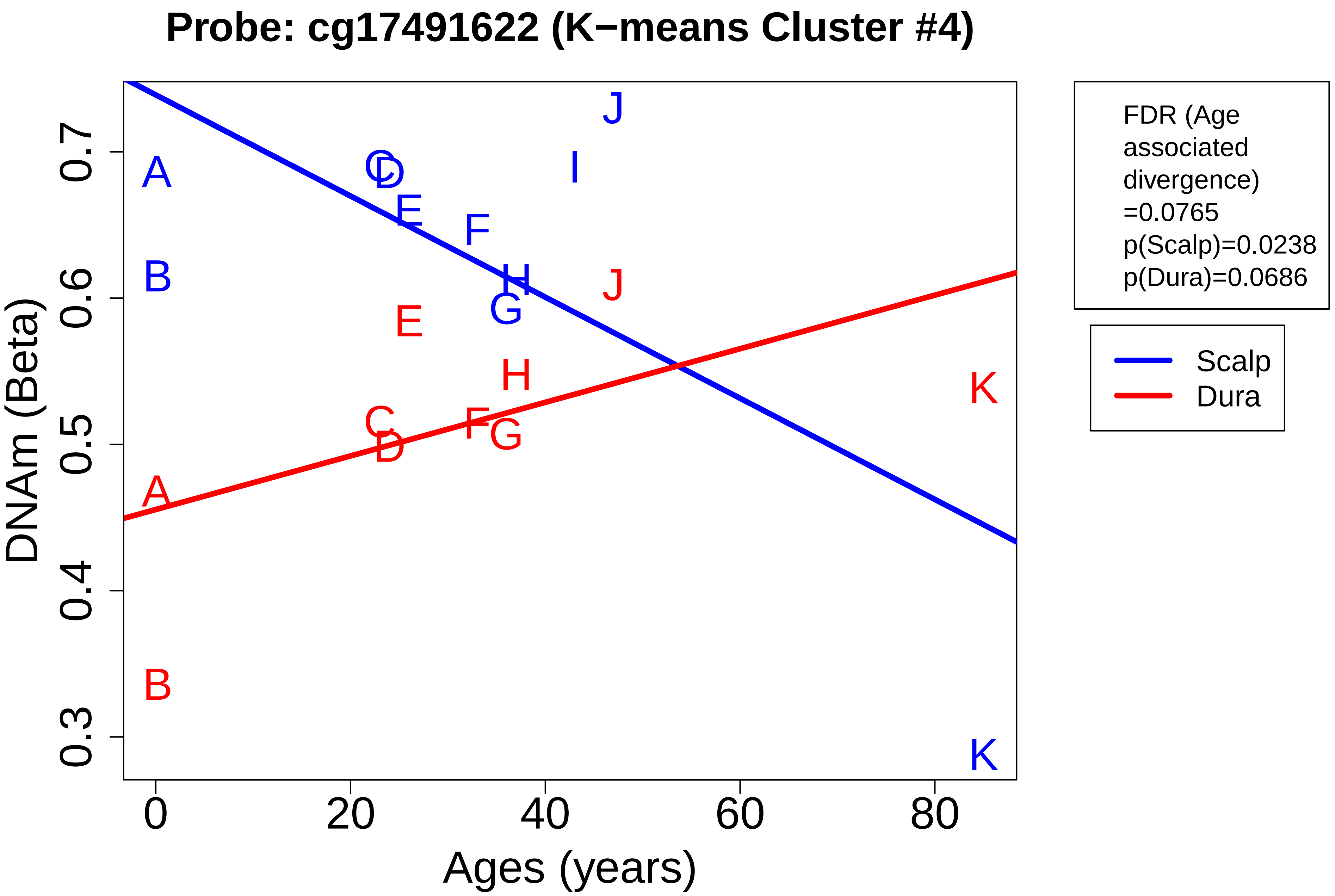

Probe: cg17491622 (K−means Cluster #4)
DNAm (Beta)
0.3
0.4
0.5
0.6
0.7
Ages (years)
0
20
40
60
80
A
B
C
D
E
F
G
H
I
J
K
FDR (Age associated divergence) =0.0765
p(Scalp)=0.0238
p(Dura)=0.0686
Scalp
Dura

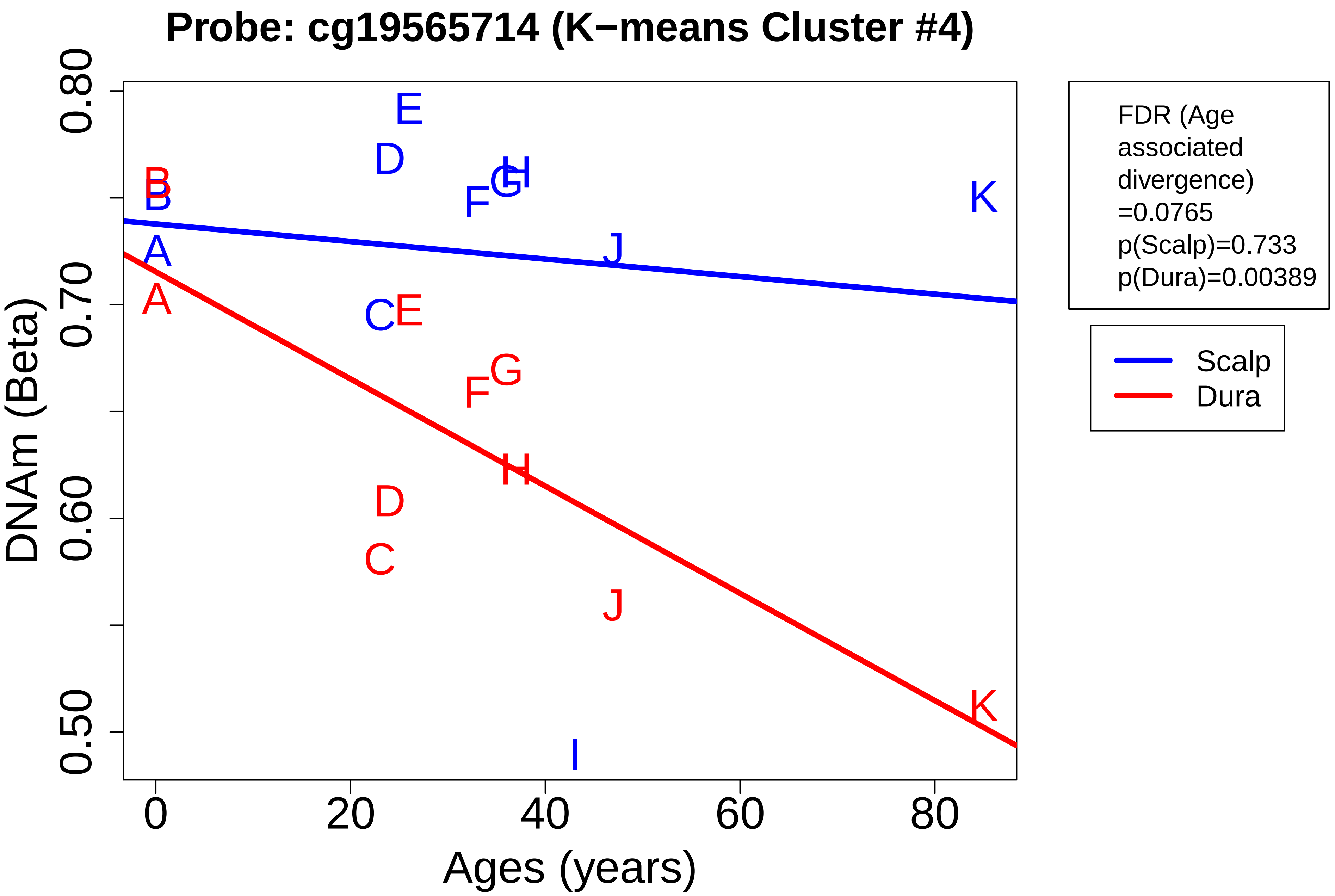

Probe: cg19565714 (K−means Cluster #4)
DNAm (Beta)
0.50
0.60
0.70
0.80
0
20
40
60
80
Ages (years)
FDR (Age associated divergence) =0.0765
p(Scalp)=0.733
p(Dura)=0.00389
Scalp
Dura

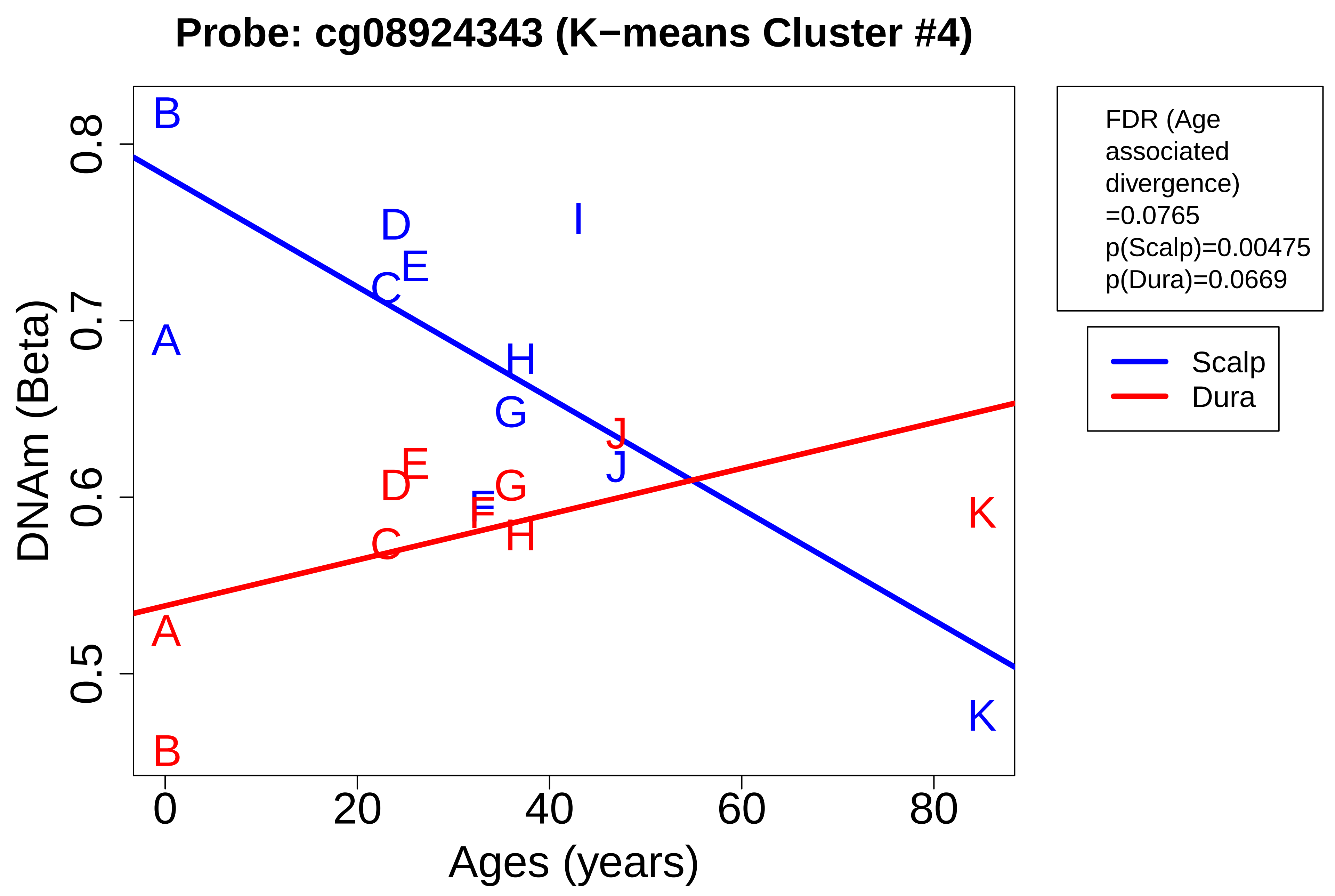

Probe: cg08924343 (K−means Cluster #4)
FDR (Age associated divergence) =0.0765
p(Scalp)=0.00475
p(Dura)=0.0669
Scalp
Dura
DNAm (Beta)
0.5
0.6
0.7
0.8
Ages (years)
0
20
40
60
80
A
B
C
D
E
F
G
H
I
J
K

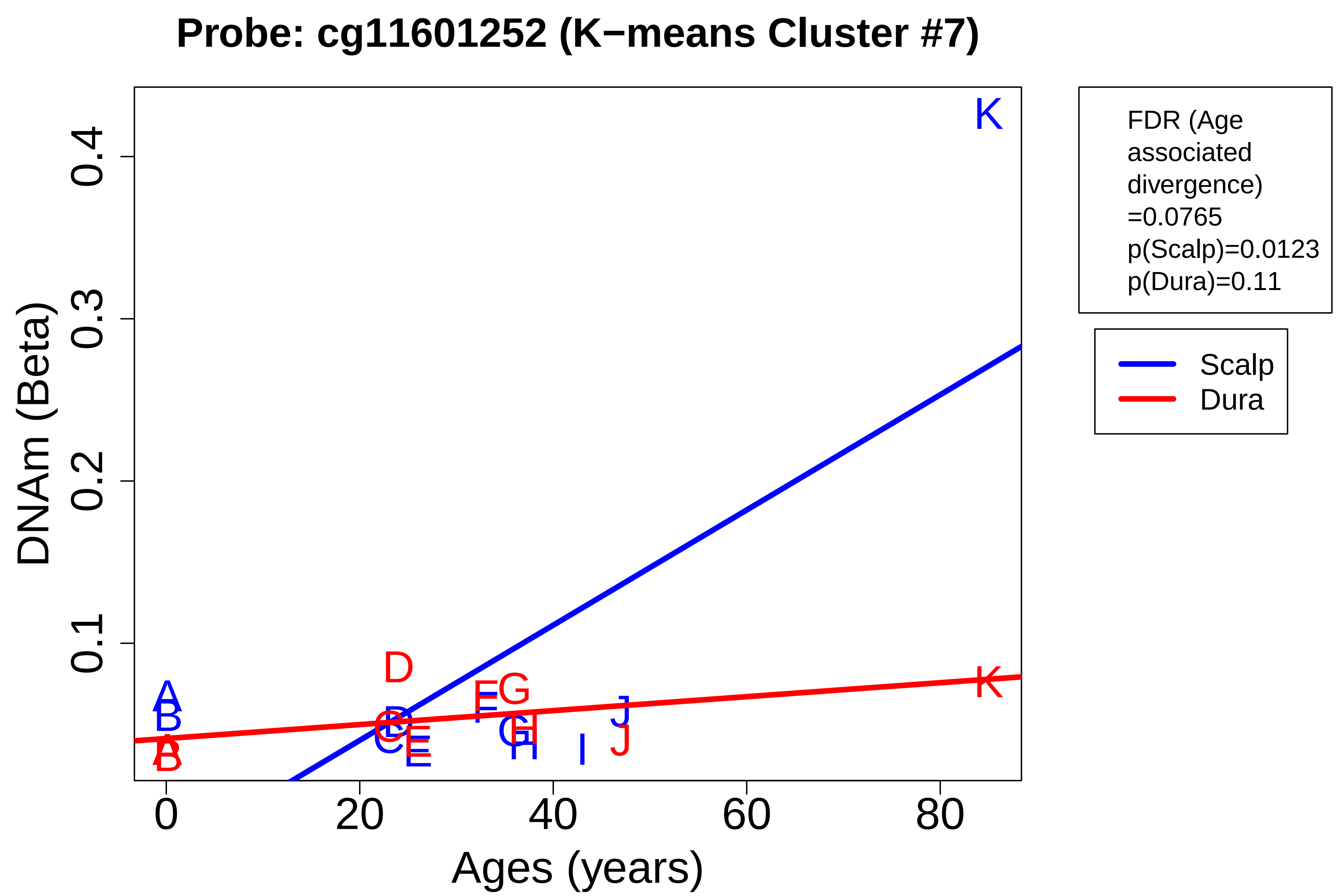

Probe: cg11601252 (K−means Cluster #7)
DNAm (Beta)
0.1
0.2
0.3
0.4
0
20
40
60
80
Ages (years)
A
B
C
D
E
F
G
H
I
J
K
FDR (Age associated divergence) =0.0765
p(Scalp)=0.0123
p(Dura)=0.11
Scalp
Dura

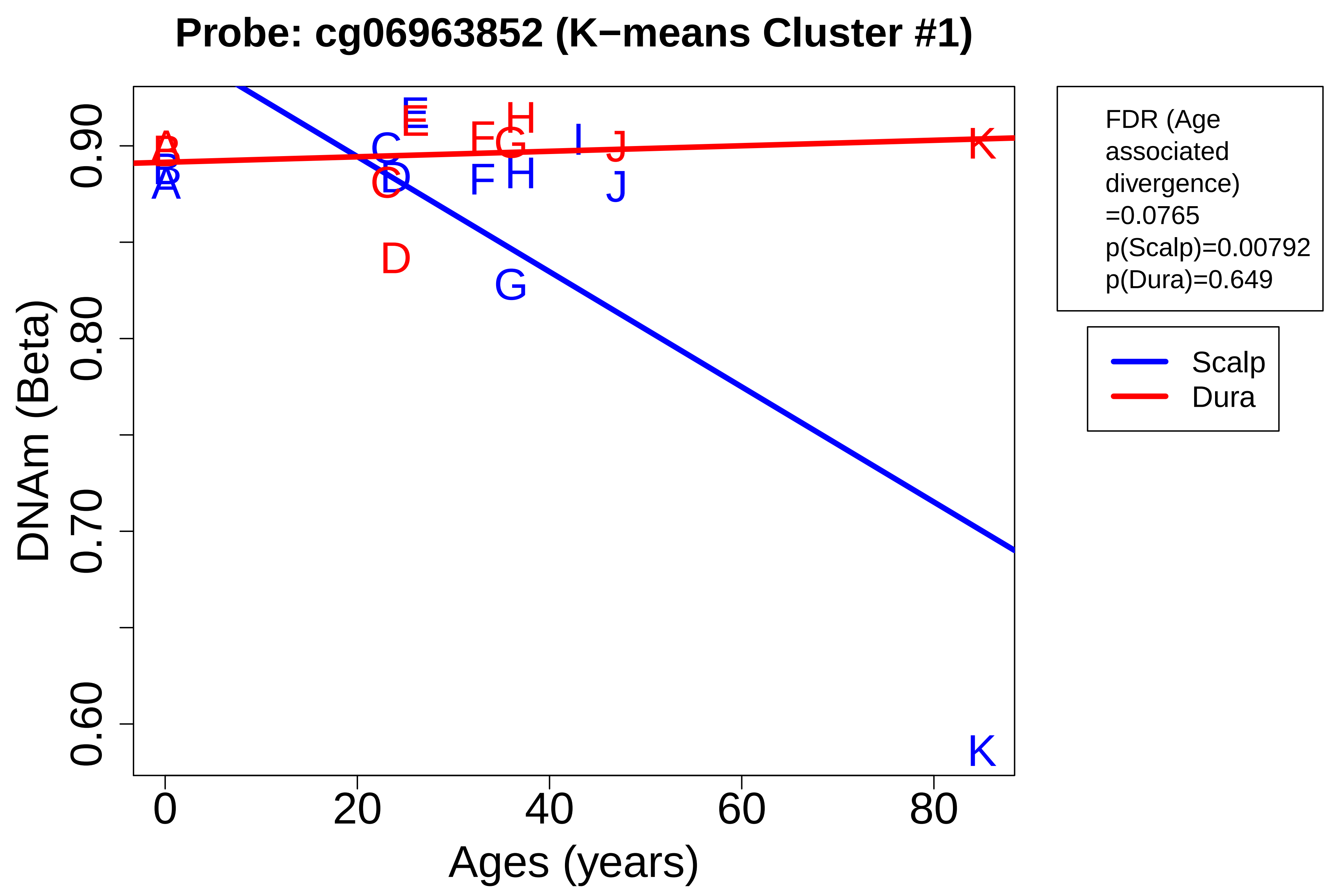

Probe: cg06963852 (K−means Cluster #1)
DNAm (Beta)
0.60
0.70
0.80
0.90
Ages (years)
0
20
40
60
80
FDR (Age associated divergence) =0.0765
p(Scalp)=0.00792
p(Dura)=0.649
Scalp
Dura

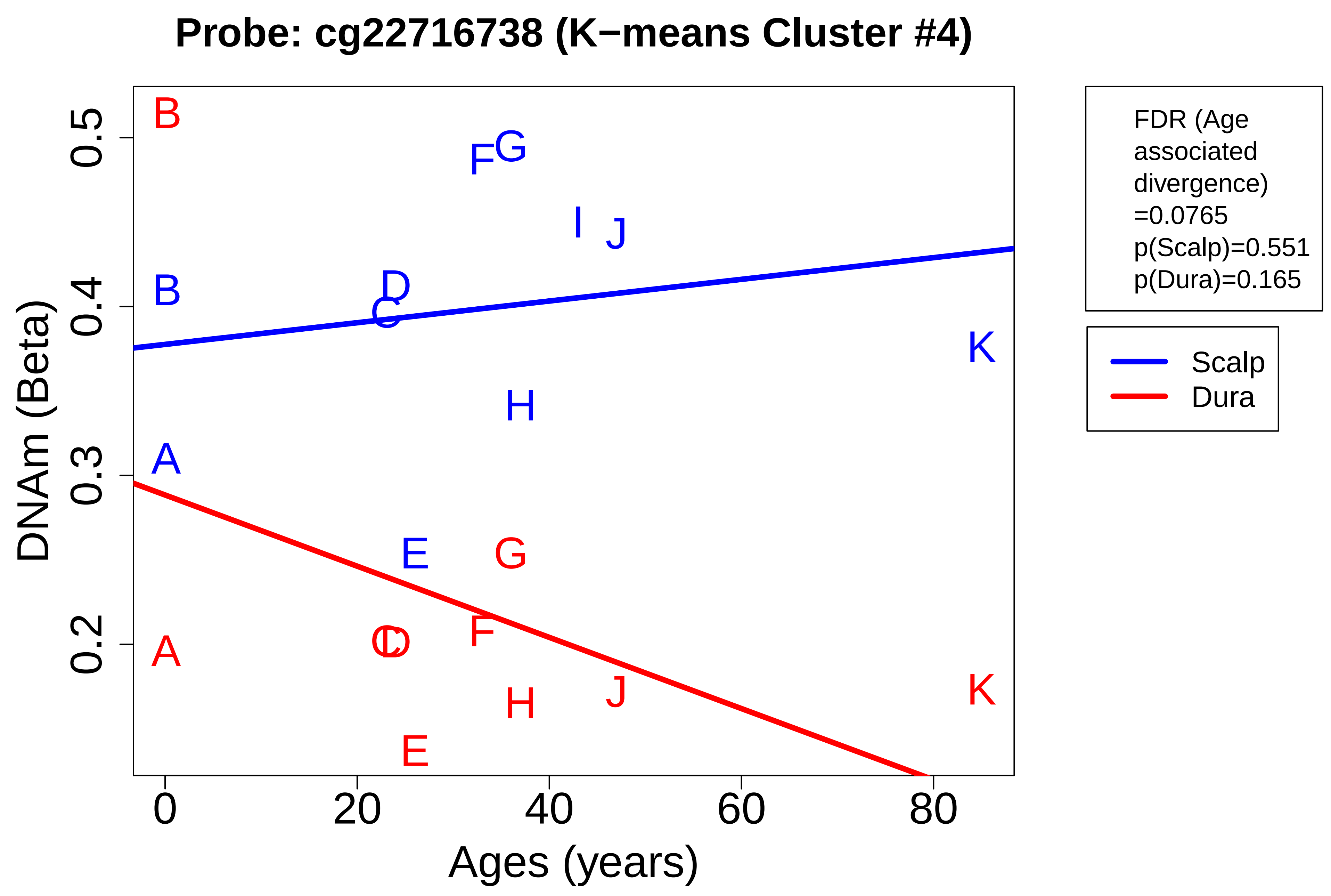

Probe: cg22716738 (K−means Cluster #4)
DNAm (Beta)
0.2
0.3
0.4
0.5
Ages (years)
0
20
40
60
80
FDR (Age associated divergence) =0.0765
p(Scalp)=0.551
p(Dura)=0.165
Scalp
Dura

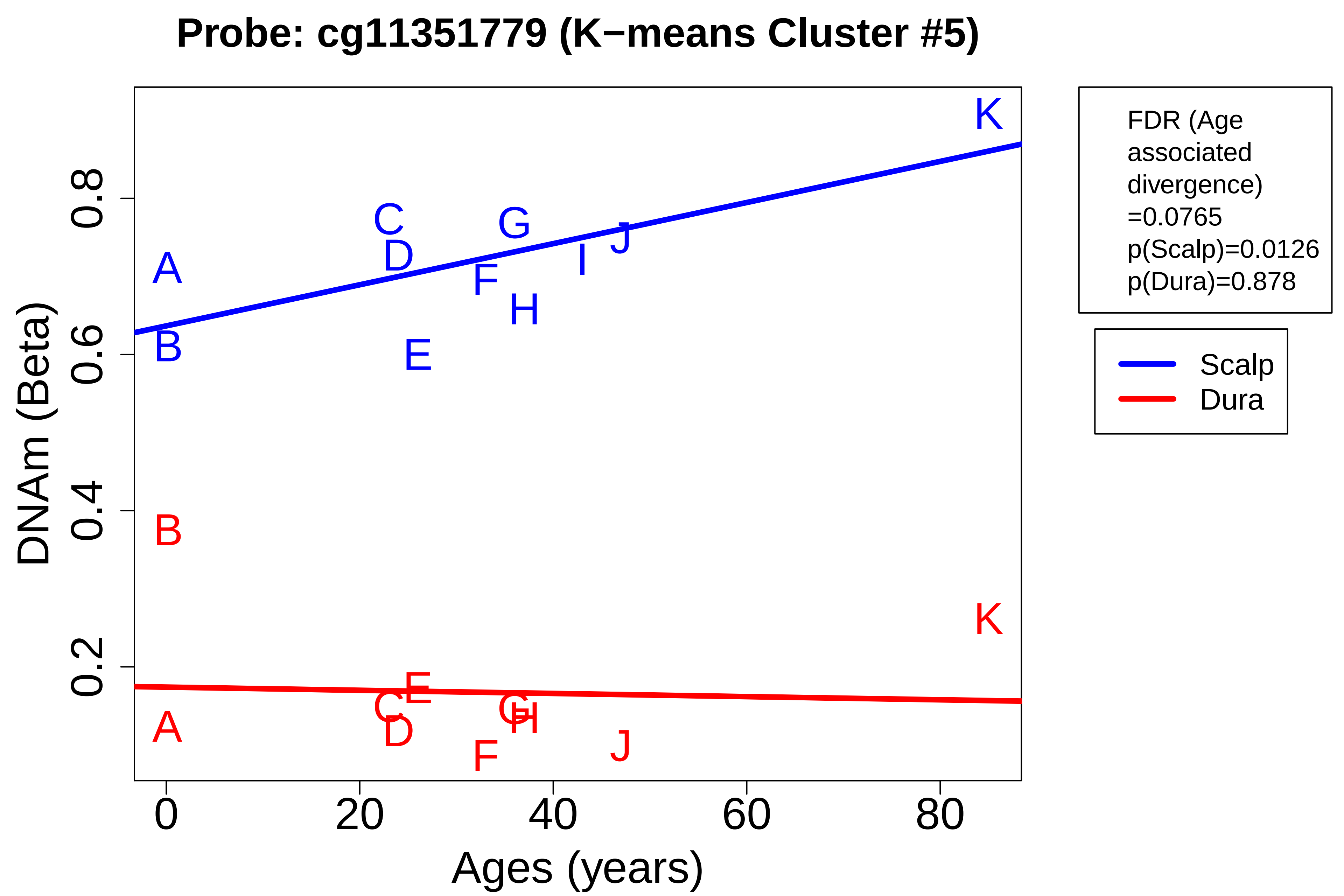

Probe: cg11351779 (K−means Cluster #5)
DNAm (Beta)
0.2
0.4
0.6
0.8
0
20
40
60
80
Ages (years)
FDR (Age associated divergence) =0.0765
p(Scalp)=0.0126
p(Dura)=0.878
Scalp
Dura

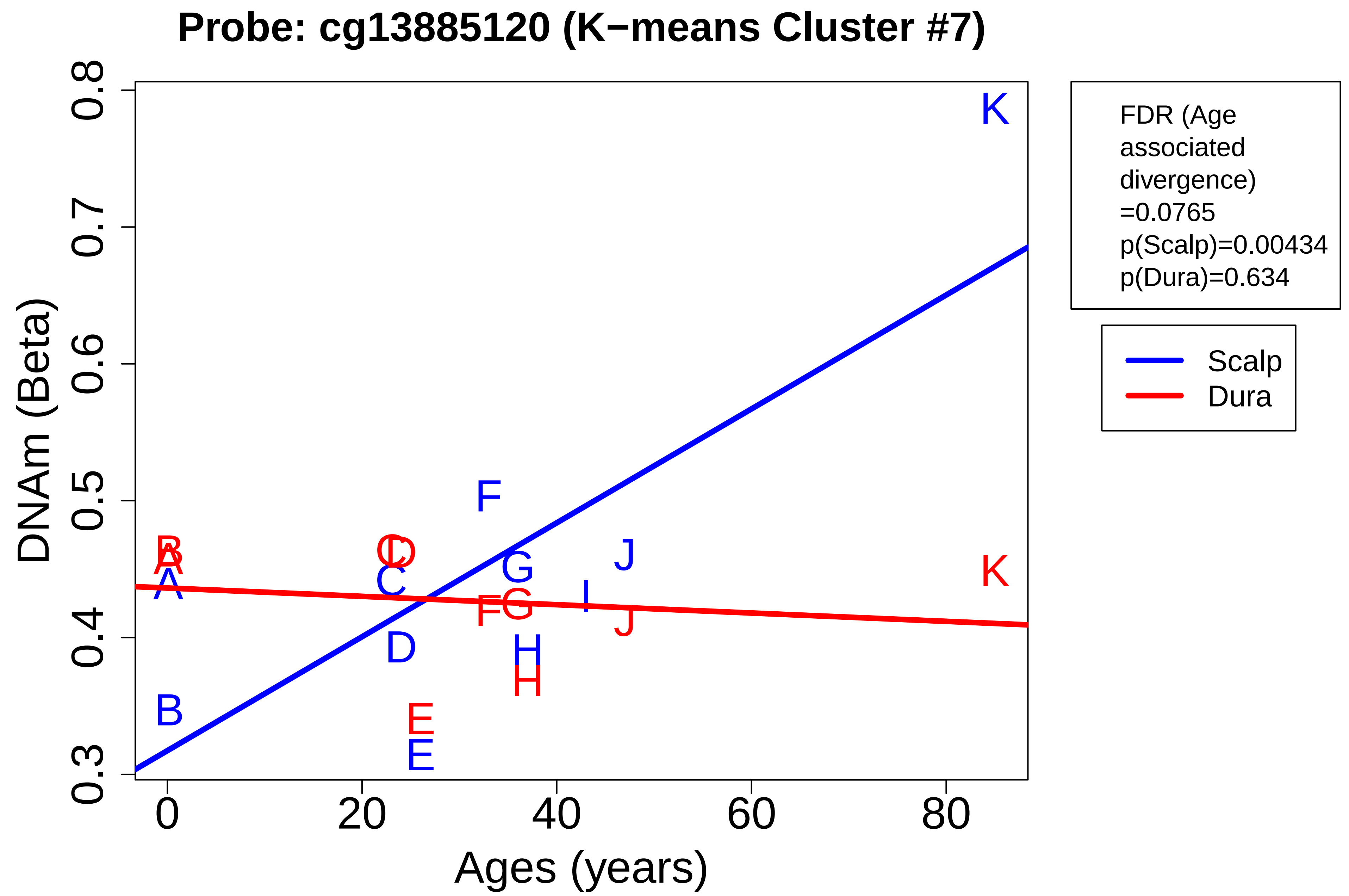

Probe: cg13885120 (K−means Cluster #7)
DNAm (Beta)
0.3
0.4
0.5
0.6
0.7
0.8
0
20
40
60
80
Ages (years)
FDR (Age associated divergence) =0.0765
p(Scalp)=0.00434
p(Dura)=0.634
Scalp
Dura

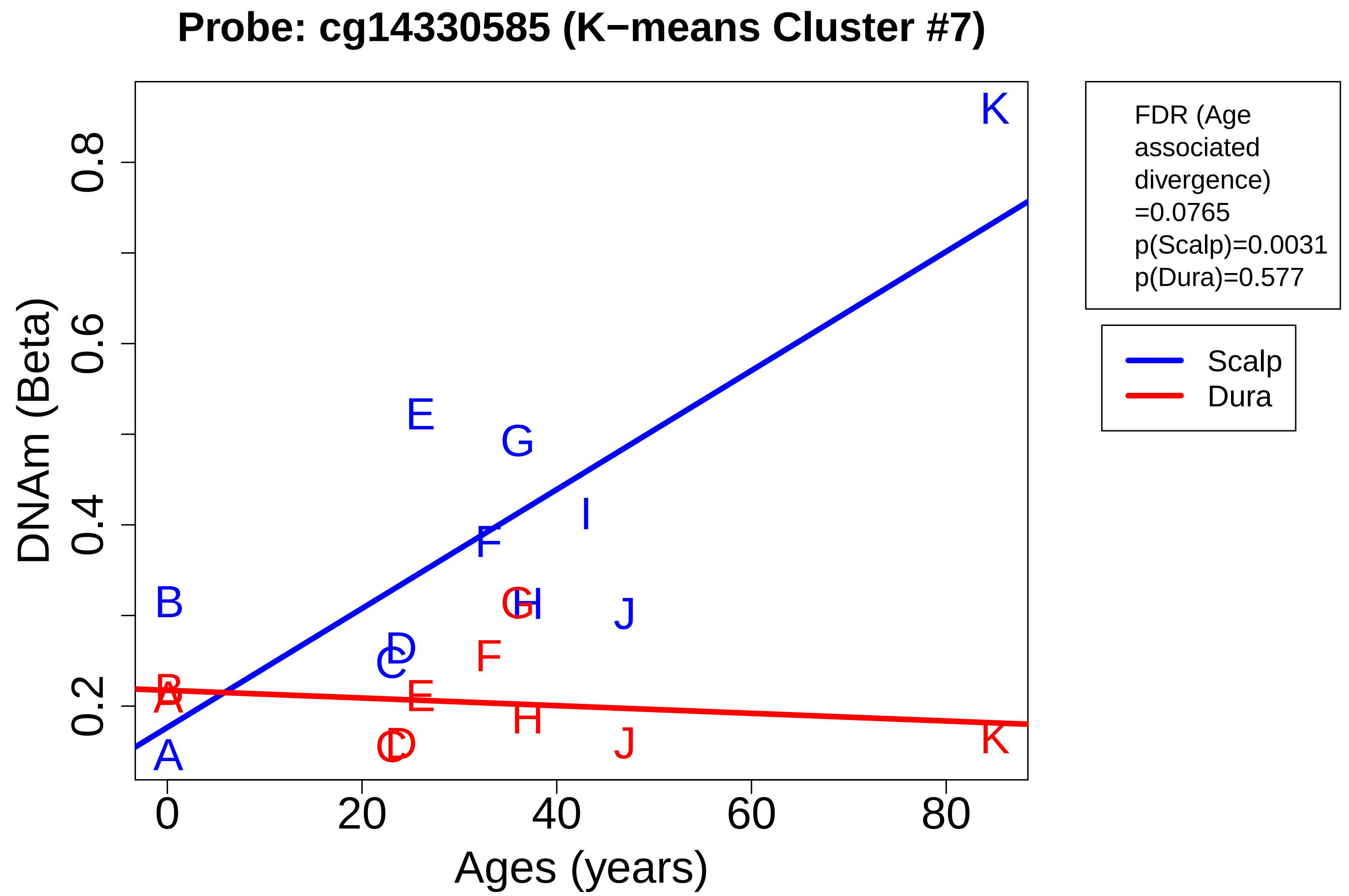

Probe: cg14330585 (K−means Cluster #7)
DNAm (Beta)
0.2
0.4
0.6
0.8
0
20
40
60
80
Ages (years)
FDR (Age associated divergence) =0.0765
p(Scalp)=0.0031
p(Dura)=0.577
Scalp
Dura

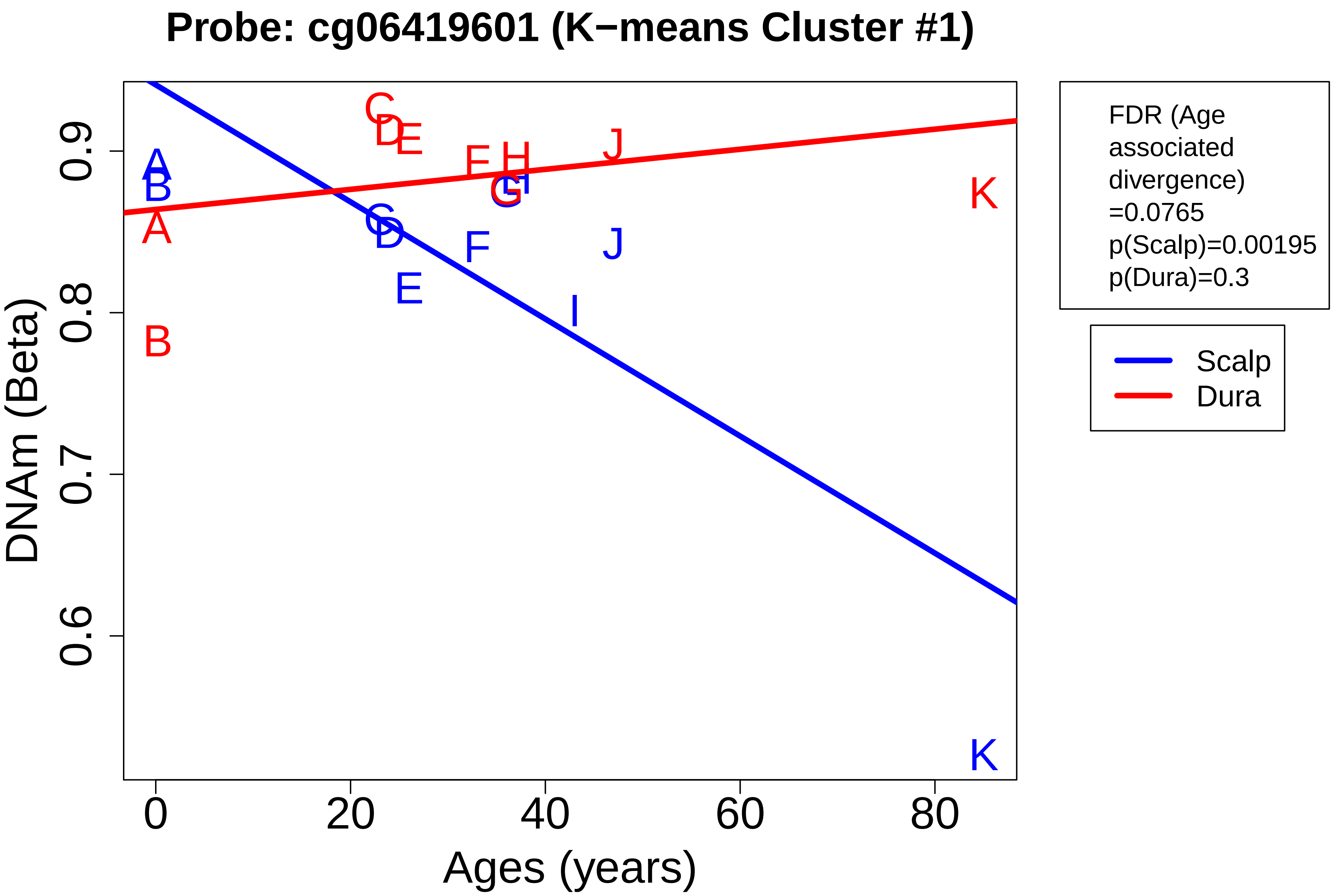

Probe: cg06419601 (K−means Cluster #1)
DNAm (Beta)
0.6
0.7
0.8
0.9
0
20
40
60
80
Ages (years)
FDR (Age associated divergence) =0.0765
p(Scalp)=0.00195
p(Dura)=0.3
Scalp
Dura

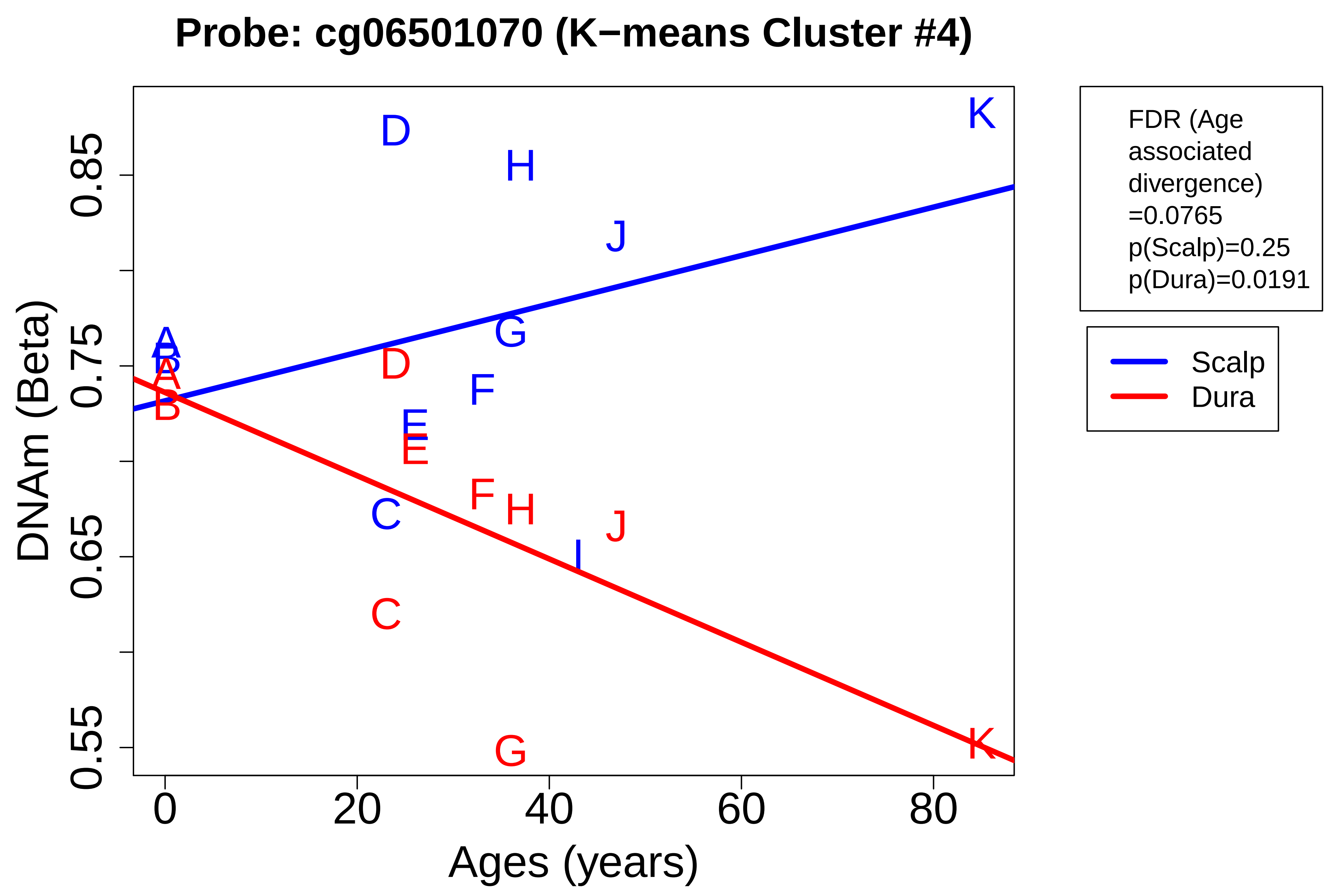

Probe: cg06501070 (K−means Cluster #4)
FDR (Age associated divergence) =0.0765
p(Scalp)=0.25
p(Dura)=0.0191
Scalp
Dura
DNAm (Beta)
0.55
0.65
0.75
0.85
0
20
40
60
80
Ages (years)
A
B
C
D
E
F
G
H
I
J
K

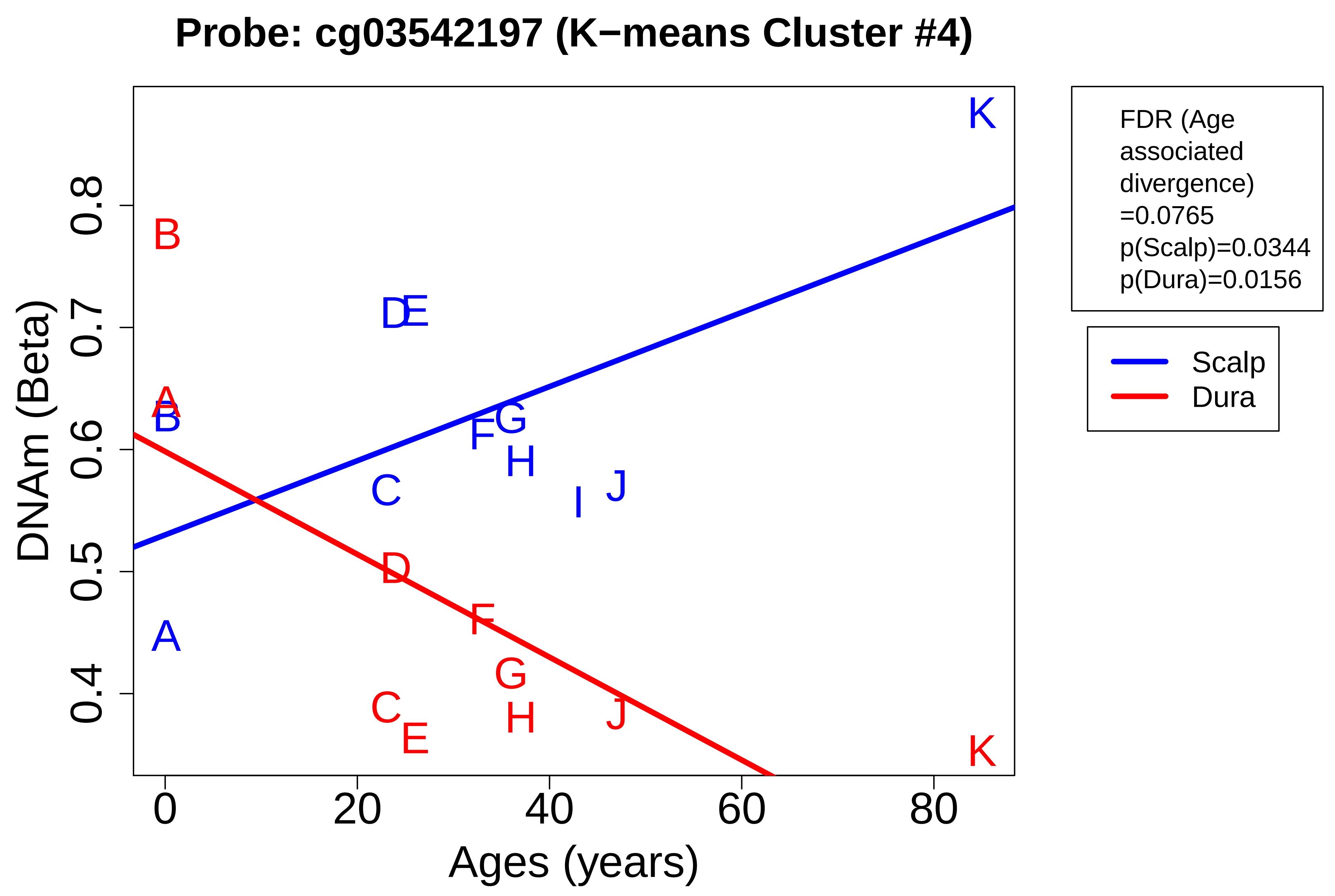

Probe: cg03542197 (K−means Cluster #4)
DNAm (Beta)
0.4
0.5
0.6
0.7
0.8
0
20
40
60
80
Ages (years)
FDR (Age associated divergence) =0.0765
p(Scalp)=0.0344
p(Dura)=0.0156
Scalp
Dura

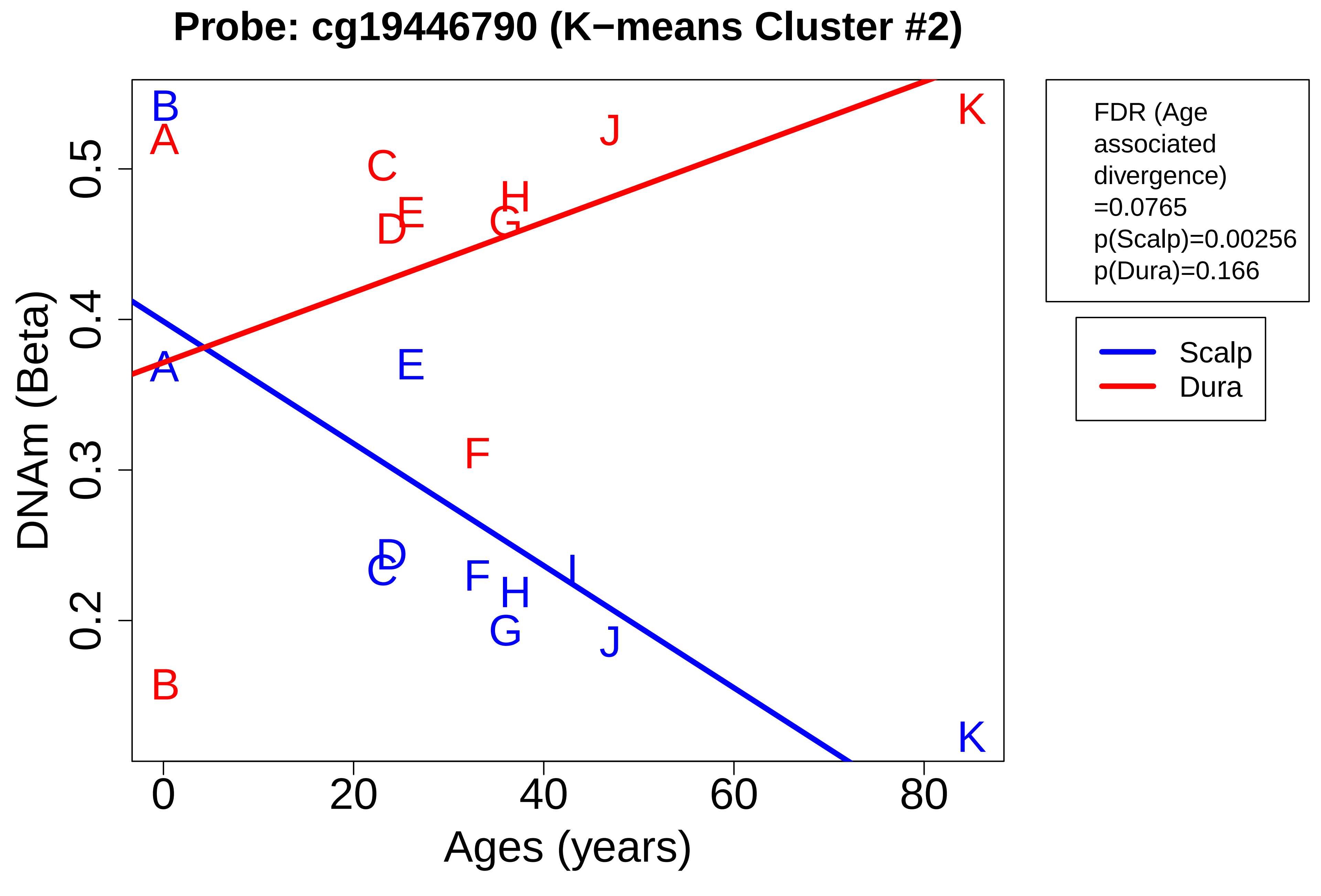

Probe: cg19446790 (K−means Cluster #2)
DNAm (Beta)
0.2
0.3
0.4
0.5
0
20
40
60
80
Ages (years)
FDR (Age associated divergence) =0.0765
p(Scalp)=0.00256
p(Dura)=0.166
Scalp
Dura

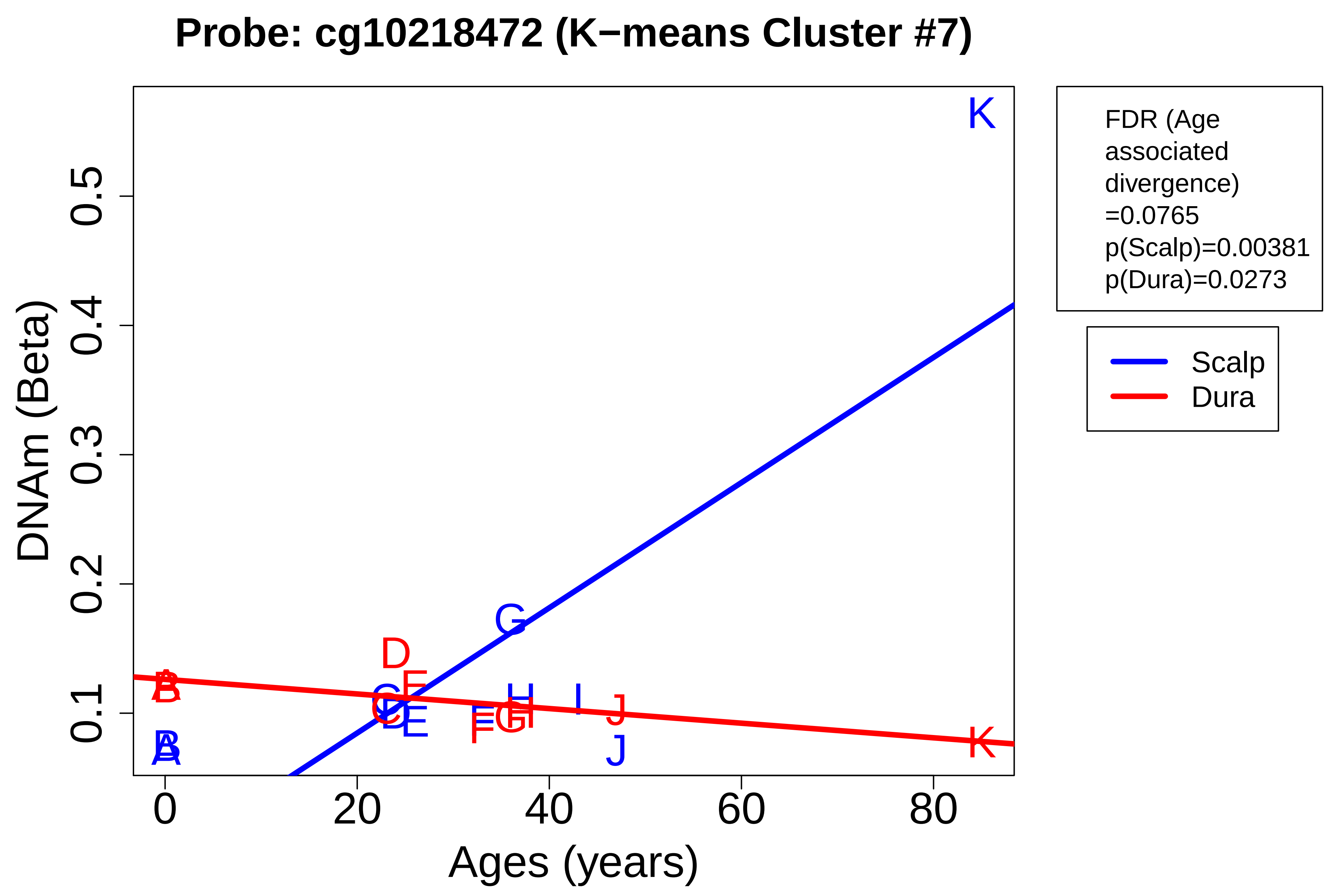

Probe: cg10218472 (K−means Cluster #7)
DNAm (Beta)
0.1
0.2
0.3
0.4
0.5
Ages (years)
0
20
40
60
80
FDR (Age associated divergence) =0.0765
p(Scalp)=0.00381
p(Dura)=0.0273
Scalp
Dura

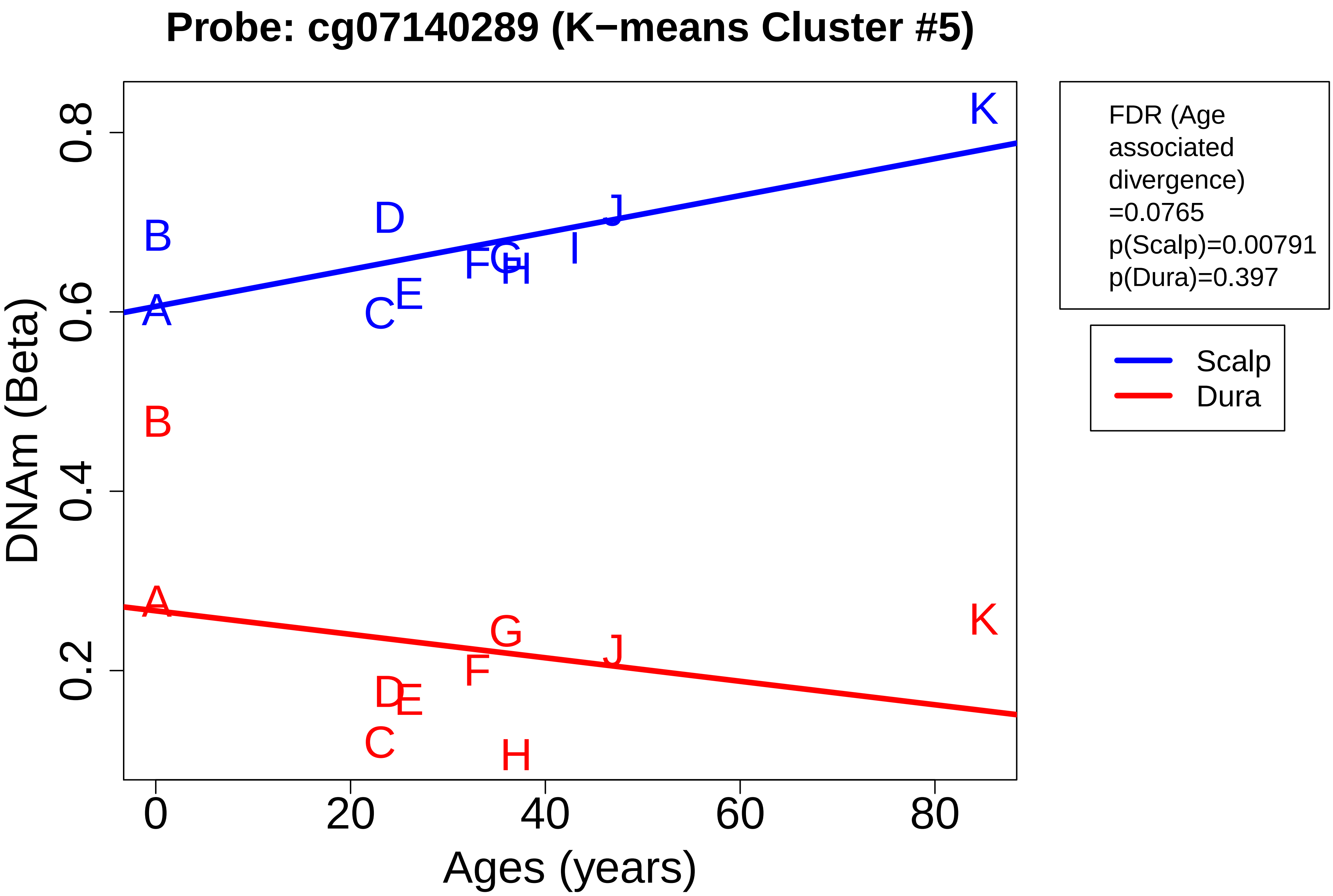

Probe: cg07140289 (K−means Cluster #5)
DNAm (Beta)
0.2
0.4
0.6
0.8
0
20
40
60
80
Ages (years)
A
B
C
D
E
F
G
H
I
J
K
FDR (Age associated divergence) =0.0765
p(Scalp)=0.00791
p(Dura)=0.397
Scalp
Dura

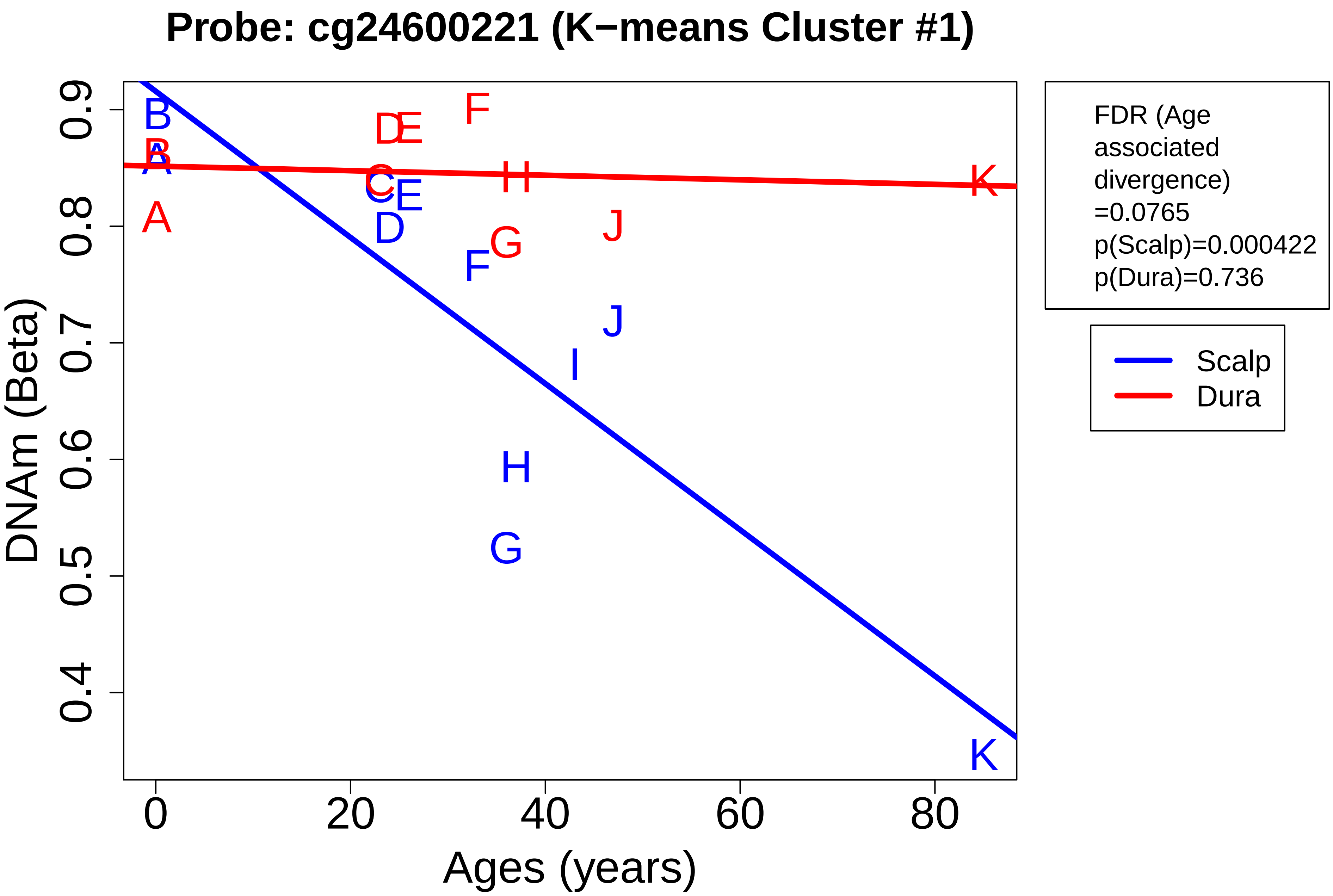

Probe: cg24600221 (K−means Cluster #1)
DNAm (Beta)
Ages (years)
0.4
0.5
0.6
0.7
0.8
0.9
0
20
40
60
80
FDR (Age associated divergence) =0.0765
p(Scalp)=0.000422
p(Dura)=0.736
Scalp
Dura

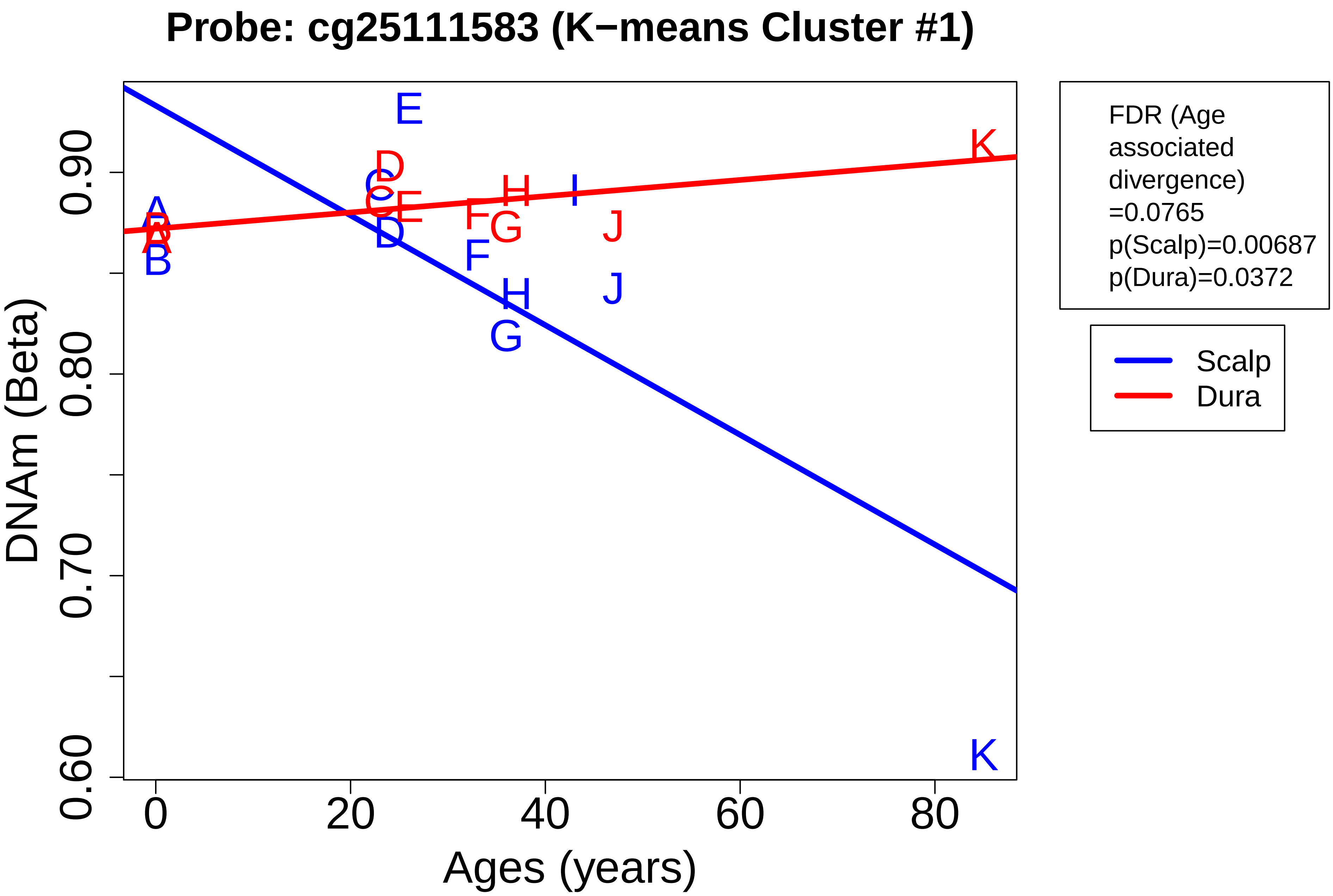

Probe: cg25111583 (K−means Cluster #1)
DNAm (Beta)
0.60
0.70
0.80
0.90
0
20
40
60
80
Ages (years)
FDR (Age associated divergence) =0.0765
p(Scalp)=0.00687
p(Dura)=0.0372
Scalp
Dura

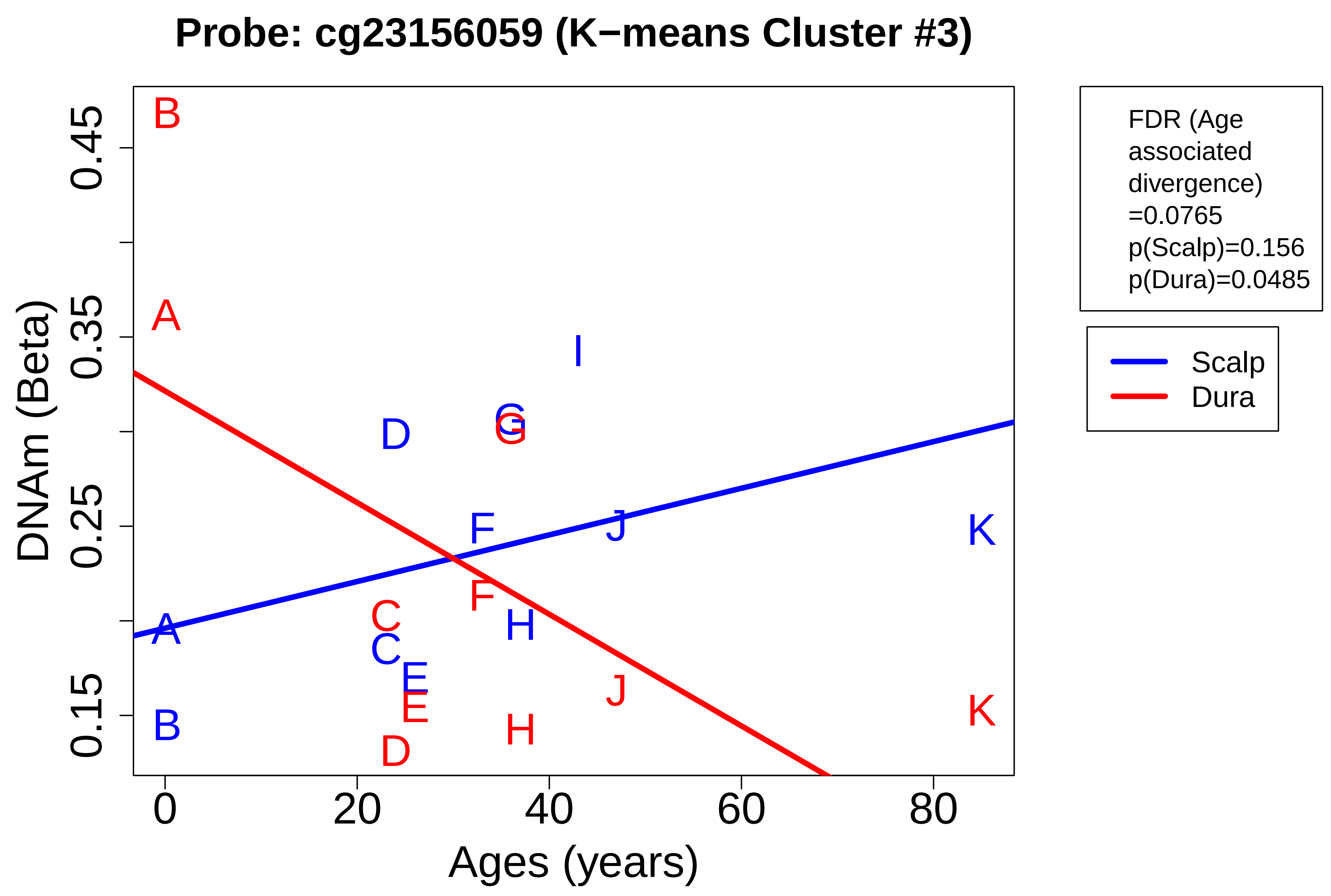

Probe: cg23156059 (K−means Cluster #3)
DNAm (Beta)
0.15
0.25
0.35
0.45
Ages (years)
0
20
40
60
80
FDR (Age associated divergence) =0.0765
p(Scalp)=0.156
p(Dura)=0.0485
Scalp
Dura

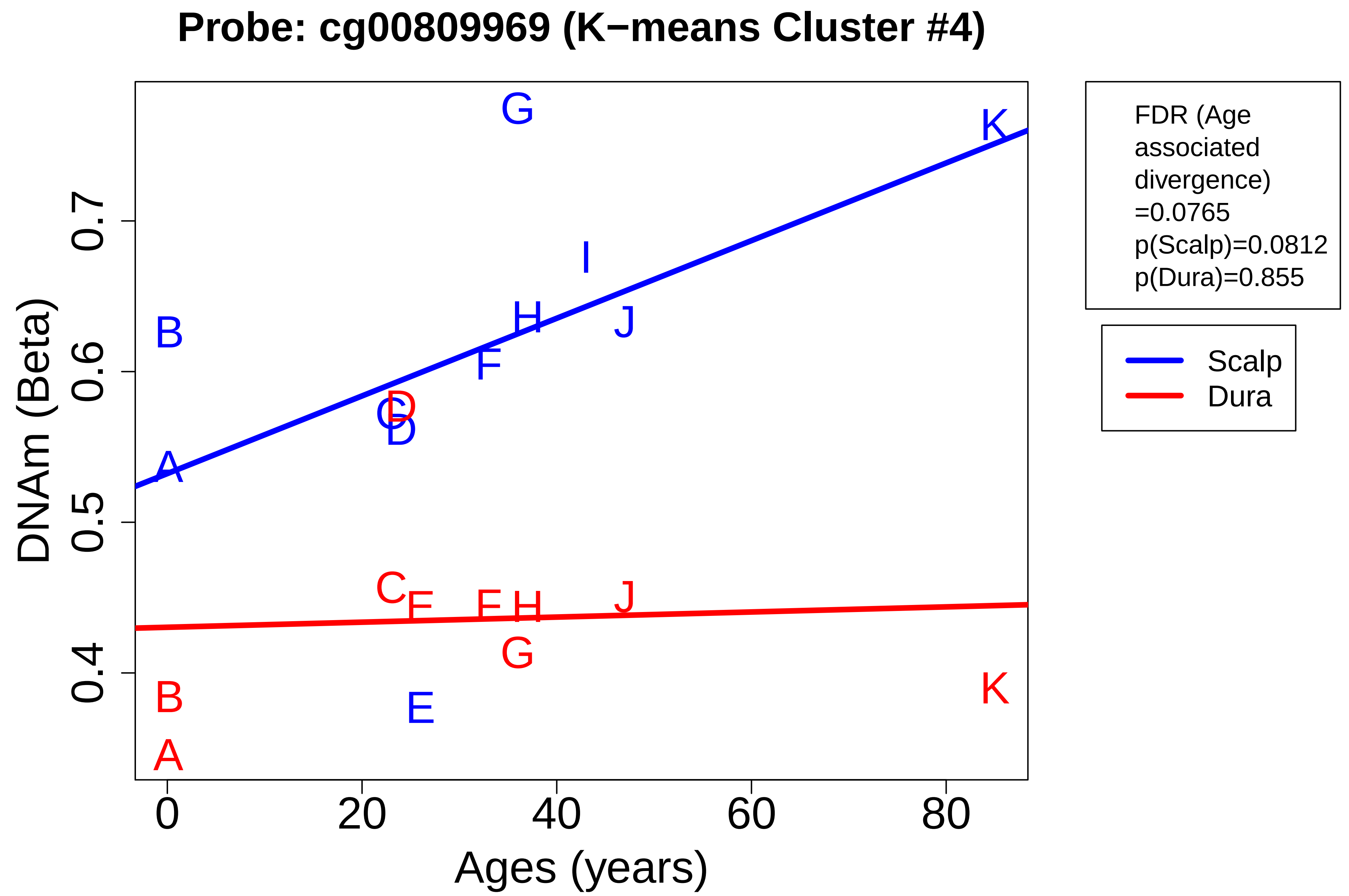

Probe: cg00809969 (K−means Cluster #4)
DNAm (Beta)
0.4
0.5
0.6
0.7
0
20
40
60
80
Ages (years)
FDR (Age associated divergence) =0.0765
p(Scalp)=0.0812
p(Dura)=0.855
Scalp
Dura

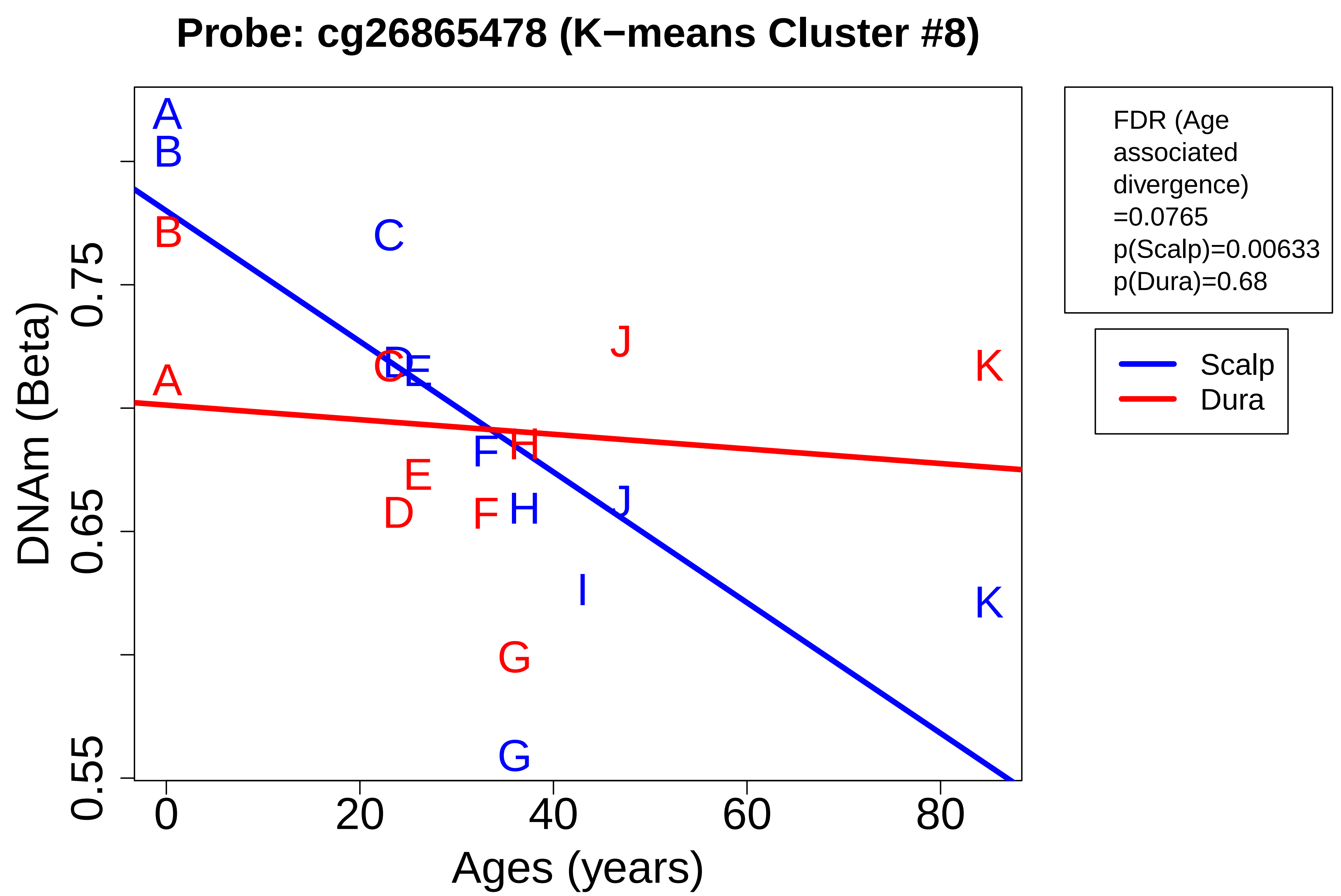

Probe: cg26865478 (K−means Cluster #8)
DNAm (Beta)
0.55
0.65
0.75
0
20
40
60
80
Ages (years)
A
B
B
C
D
C
E
A
F
H
E
D
F
H
J
J
I
K
K
G
G
FDR (Age associated divergence) =0.0765
p(Scalp)=0.00633
p(Dura)=0.68
Scalp
Dura

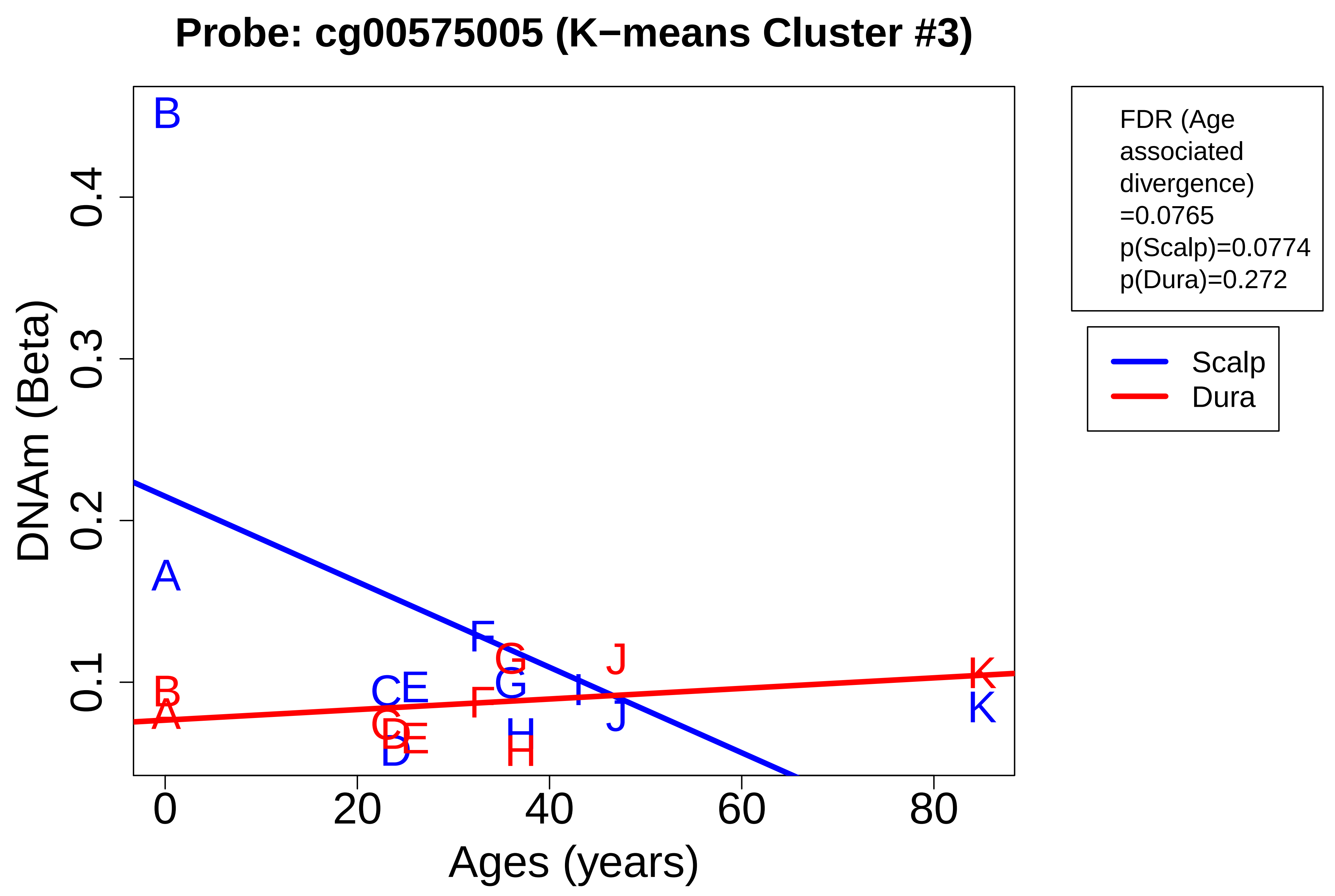

Probe: cg00575005 (K−means Cluster #3)
DNAm (Beta)
0.1
0.2
0.3
0.4
Ages (years)
0
20
40
60
80
FDR (Age associated divergence) =0.0765
p(Scalp)=0.0774
p(Dura)=0.272
Scalp
Dura

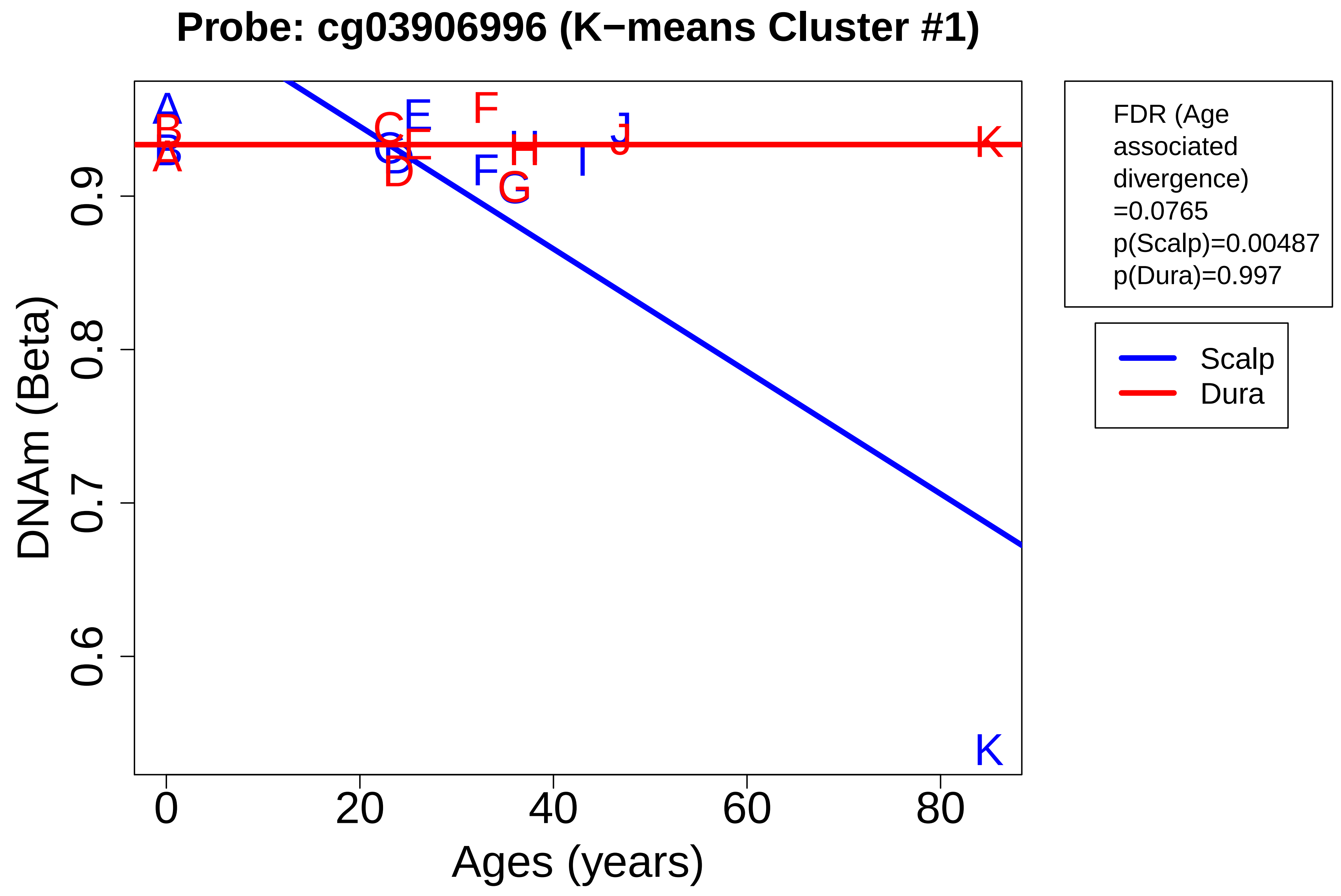

Probe: cg03906996 (K−means Cluster #1)
DNAm (Beta)
0.6
0.7
0.8
0.9
Ages (years)
0
20
40
60
80
FDR (Age associated divergence) =0.0765
p(Scalp)=0.00487
p(Dura)=0.997
Scalp
Dura

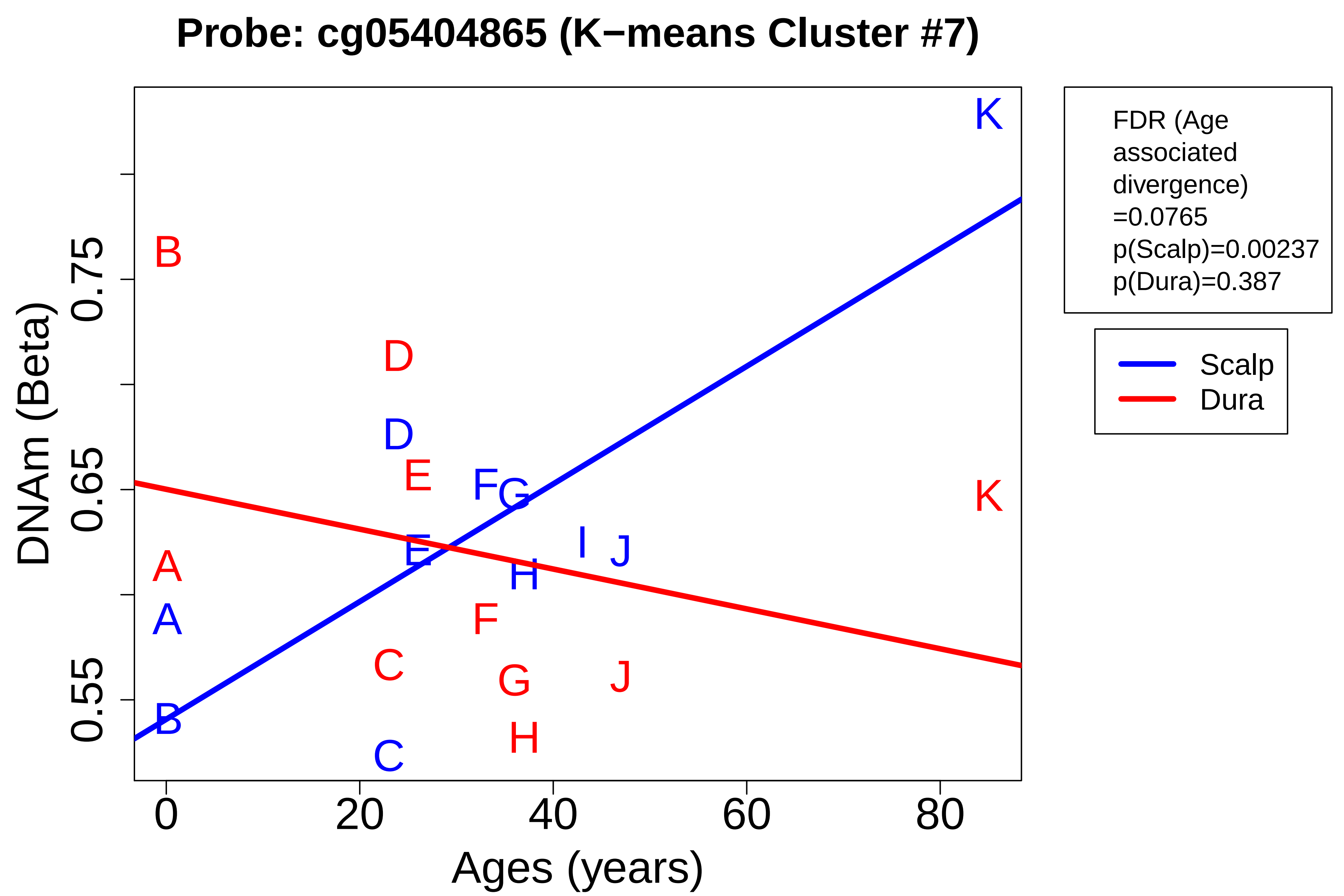

Probe: cg05404865 (K−means Cluster #7)
DNAm (Beta)
0.55
0.65
0.75
Ages (years)
0
20
40
60
80
FDR (Age associated divergence) =0.0765
p(Scalp)=0.00237
p(Dura)=0.387
Scalp
Dura
A
B
C
D
E
F
G
H
I
J
K

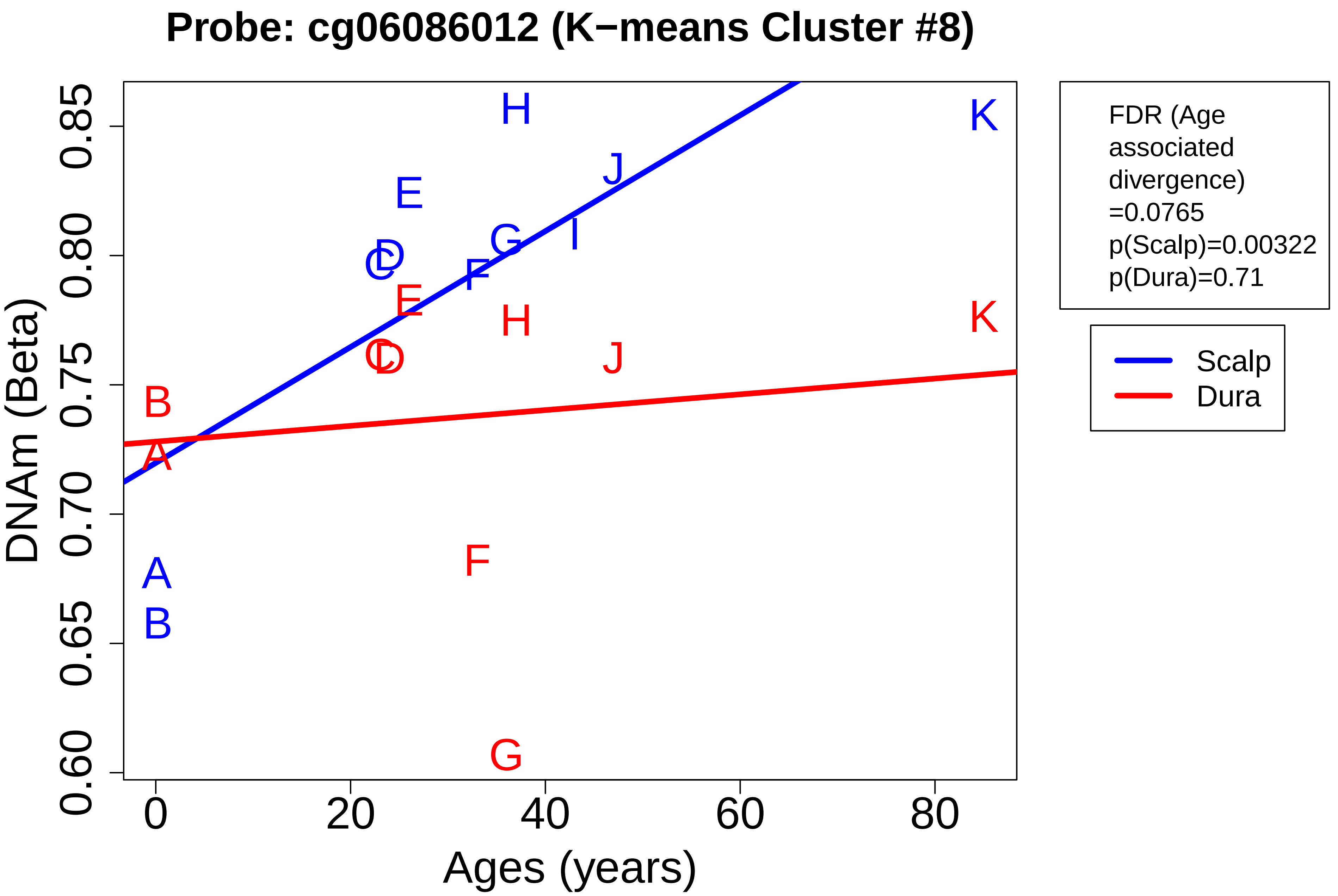

Probe: cg06086012 (K−means Cluster #8)
DNAm (Beta)
0.60
0.65
0.70
0.75
0.80
0.85
0
20
40
60
80
Ages (years)
FDR (Age associated divergence) =0.0765
p(Scalp)=0.00322
p(Dura)=0.71
Scalp
Dura

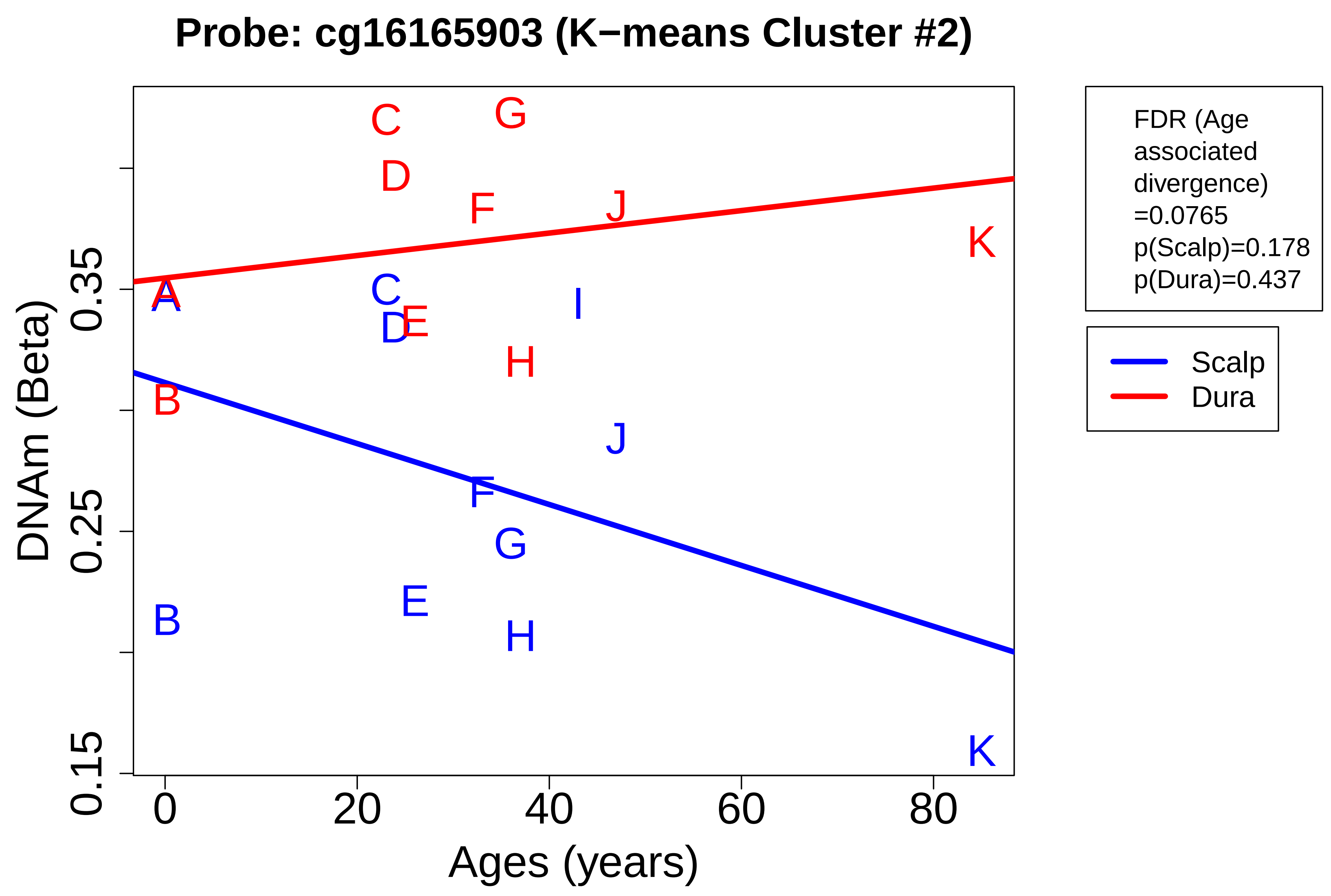

Probe: cg16165903 (K−means Cluster #2)
DNAm (Beta)
0.15
0.25
0.35
0
20
40
60
80
Ages (years)
FDR (Age associated divergence) =0.0765
p(Scalp)=0.178
p(Dura)=0.437
Scalp
Dura

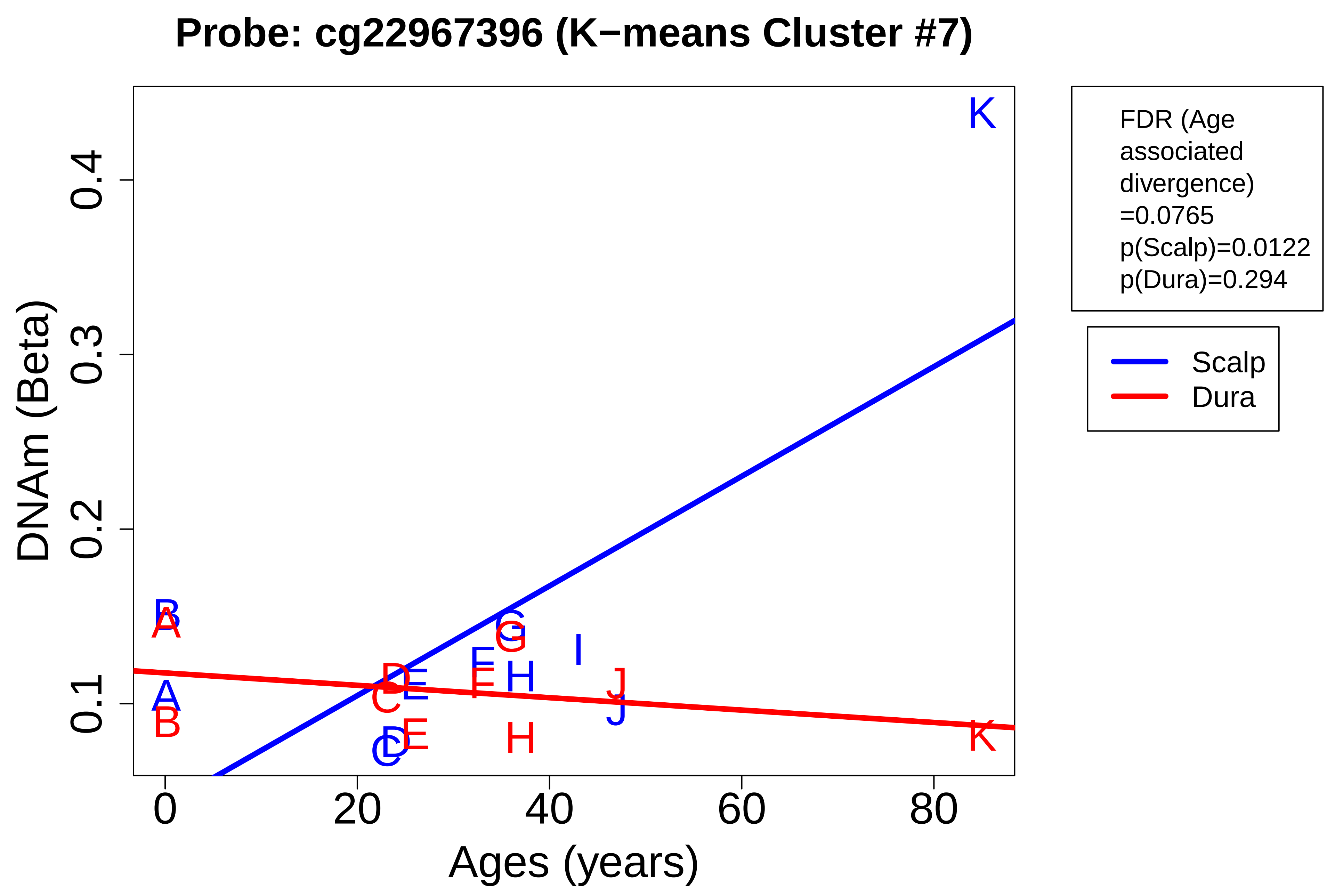

Probe: cg22967396 (K−means Cluster #7)
DNAm (Beta)
0.1
0.2
0.3
0.4
0
20
40
60
80
Ages (years)
FDR (Age associated divergence) =0.0765
p(Scalp)=0.0122
p(Dura)=0.294
Scalp
Dura
A
B
C
D
E
F
G
H
I
J
K

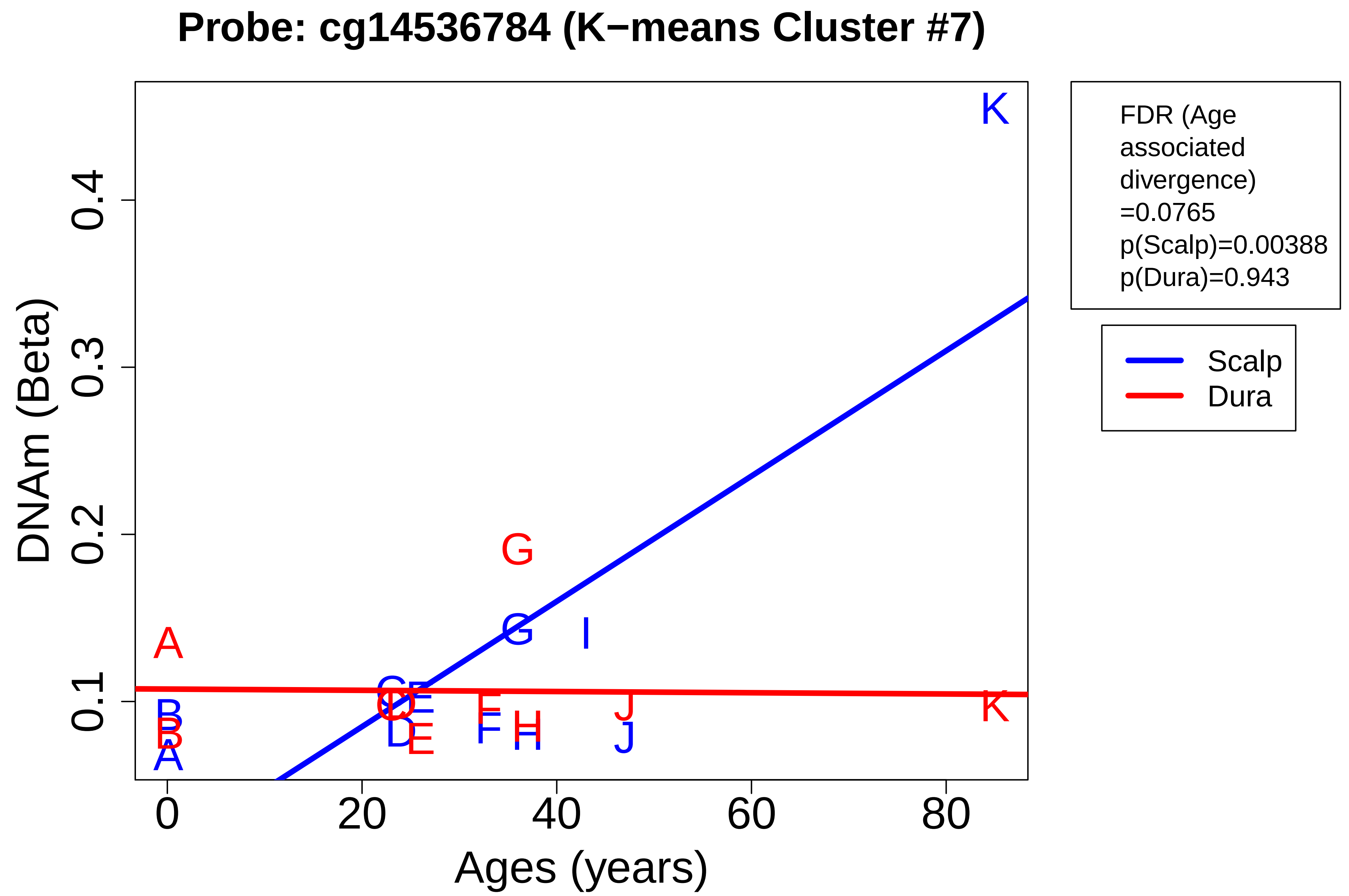

Probe: cg14536784 (K−means Cluster #7)
DNAm (Beta)
0.1
0.2
0.3
0.4
0
20
40
60
80
Ages (years)
A
B
C
D
E
F
G
H
I
J
K
FDR (Age associated divergence) =0.0765
p(Scalp)=0.00388
p(Dura)=0.943
Scalp
Dura

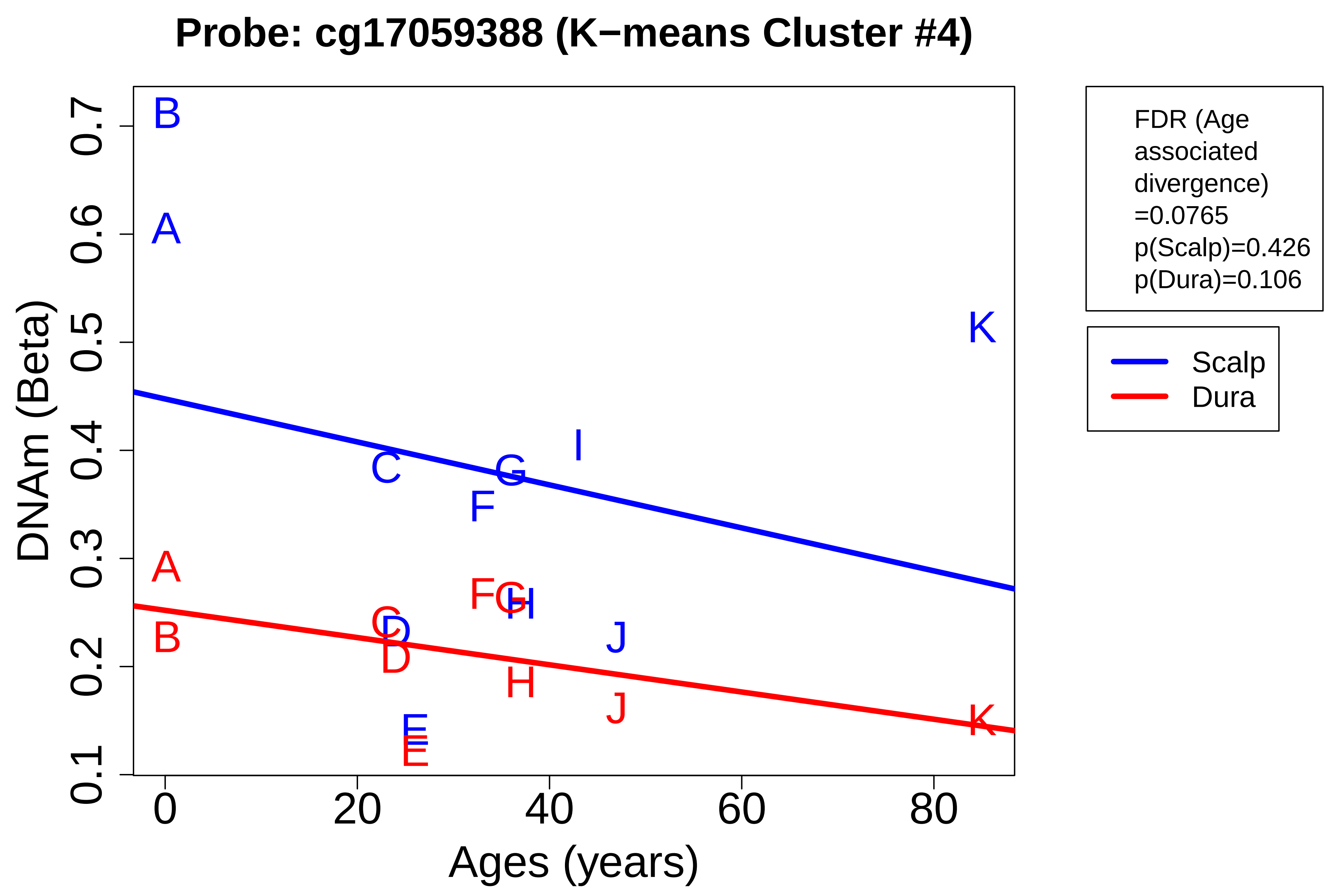

Probe: cg17059388 (K−means Cluster #4)
DNAm (Beta)
0.1
0.2
0.3
0.4
0.5
0.6
0.7
Ages (years)
0
20
40
60
80
B
A
K
I
C
G
F
A
FG
H
B
C
D
D
J
H
J
K
E
E
FDR (Age associated divergence) =0.0765
p(Scalp)=0.426
p(Dura)=0.106
Scalp
Dura

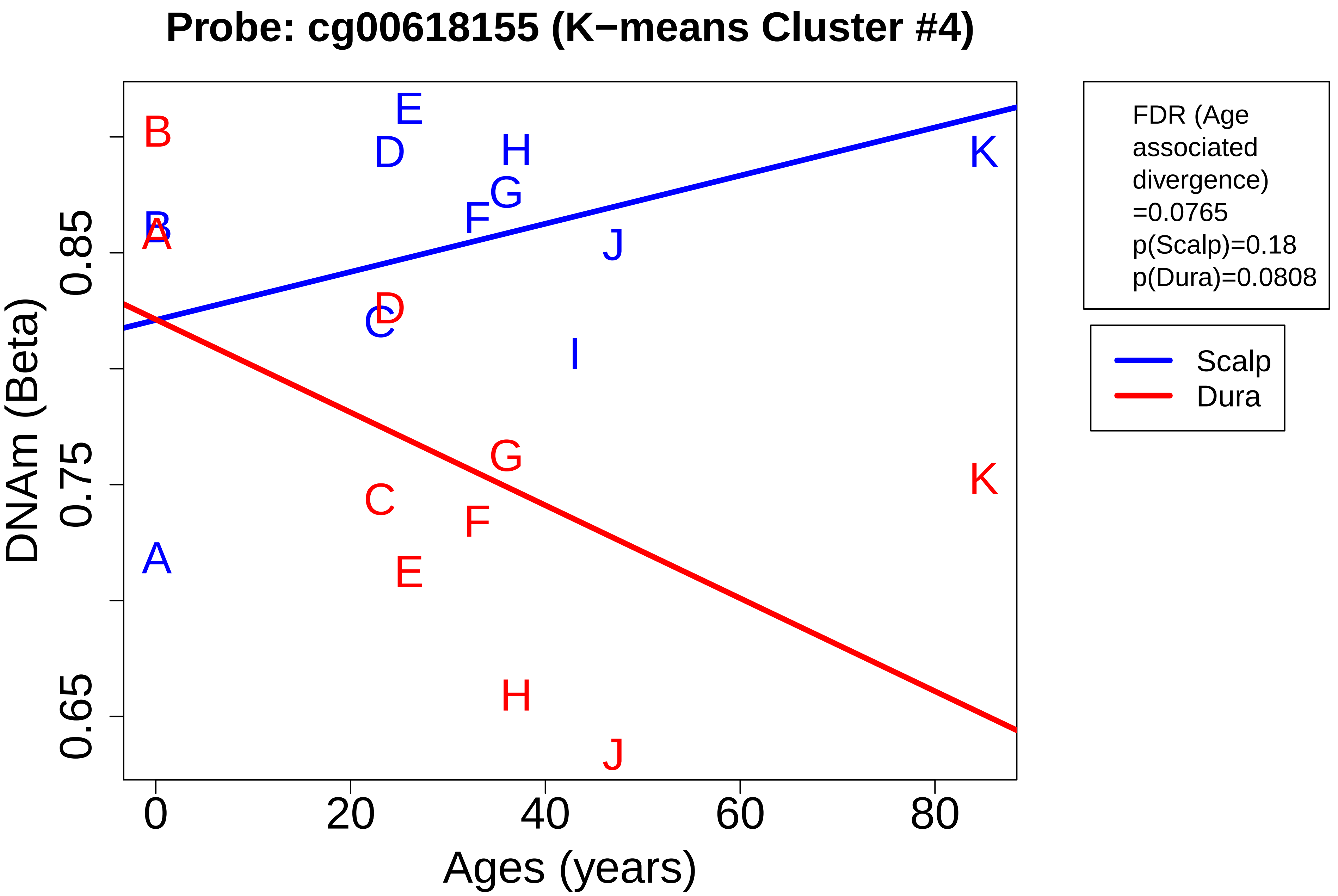

Probe: cg00618155 (K−means Cluster #4)
DNAm (Beta)
0.65
0.75
0.85
0
20
40
60
80
Ages (years)
FDR (Age associated divergence) =0.0765
p(Scalp)=0.18
p(Dura)=0.0808
Scalp
Dura
A
B
C
D
E
F
G
H
I
J
K

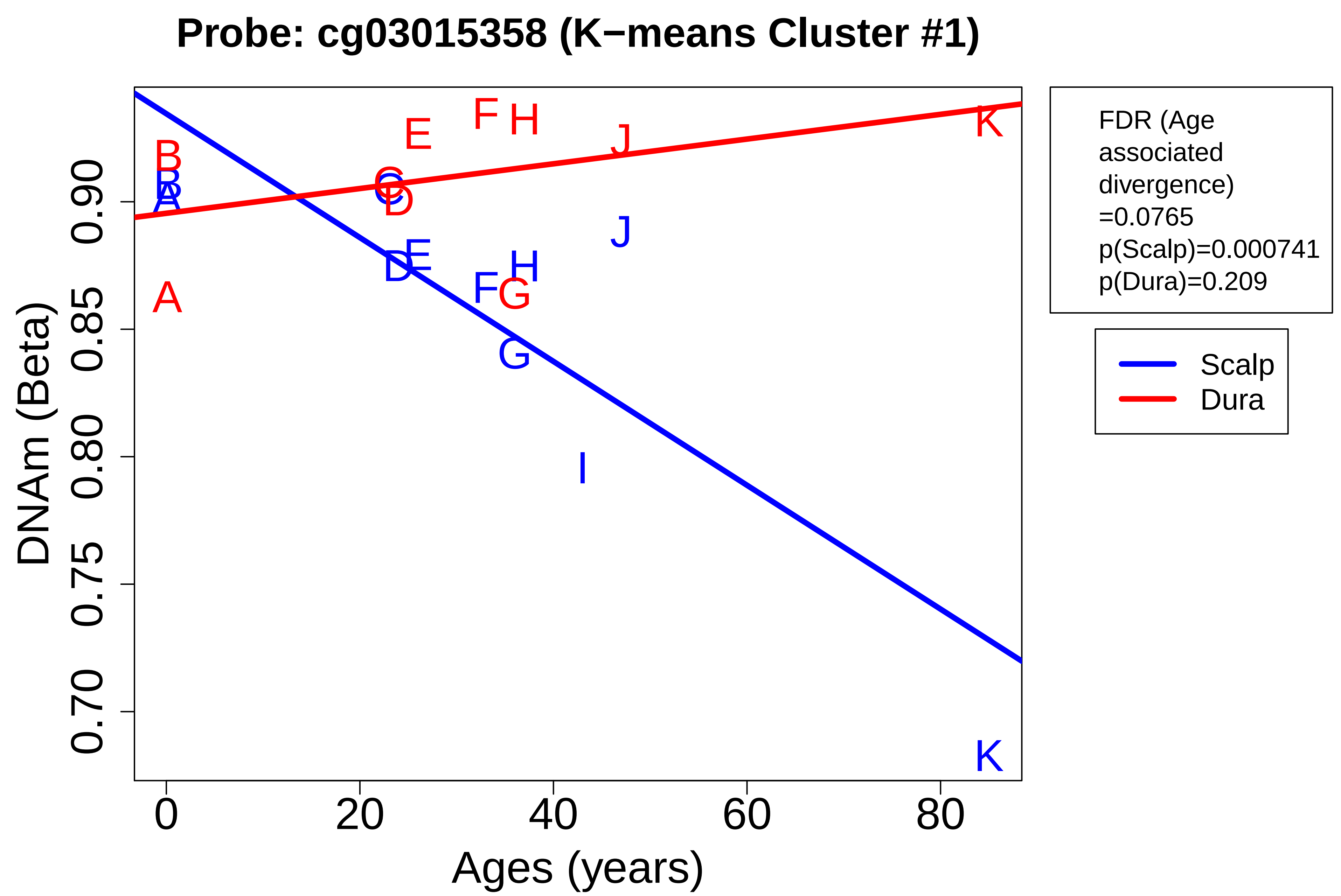

Probe: cg03015358 (K−means Cluster #1)
DNAm (Beta)
0.70
0.75
0.80
0.85
0.90
Ages (years)
0
20
40
60
80
FDR (Age associated divergence) =0.0765
p(Scalp)=0.000741
p(Dura)=0.209
Scalp
Dura

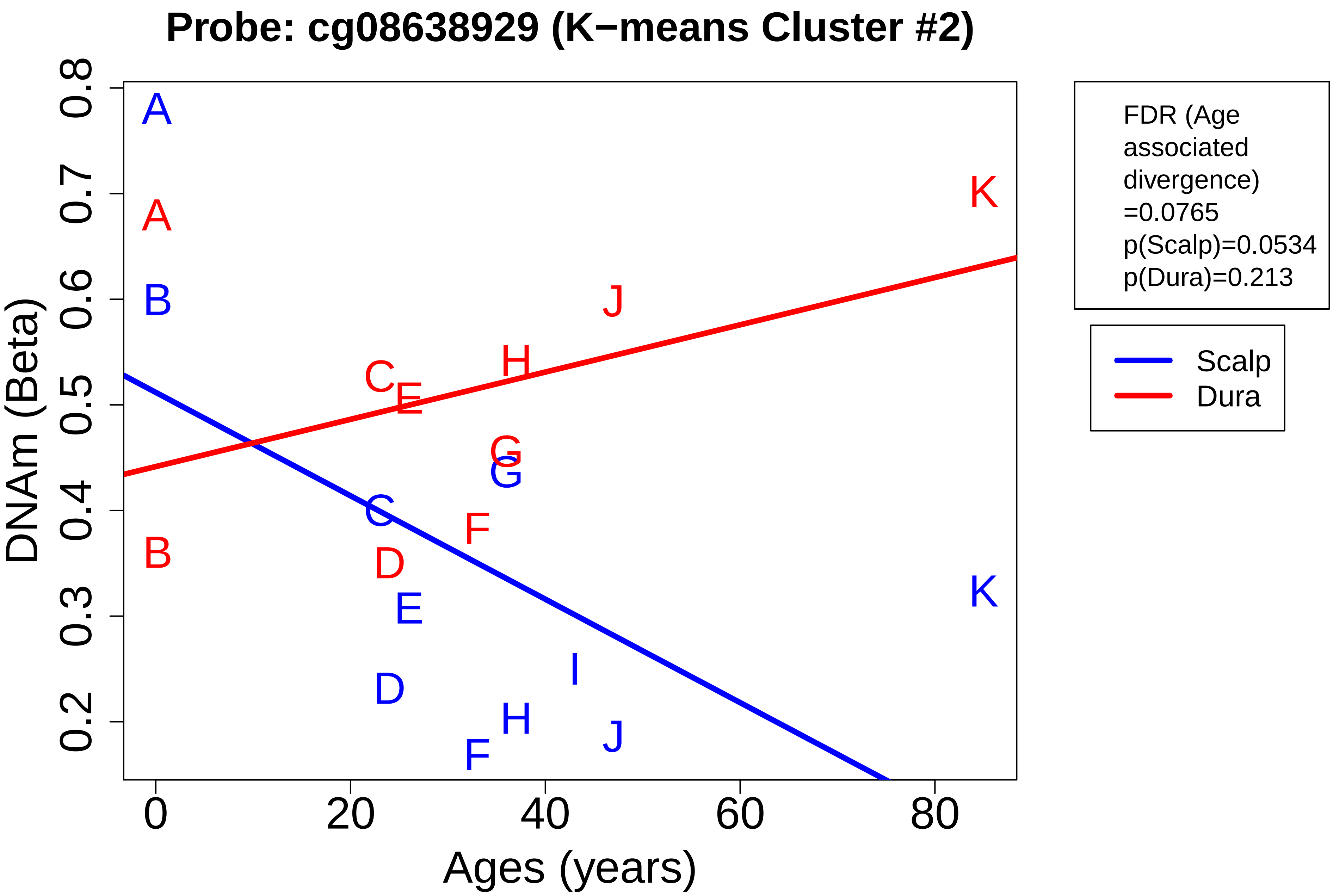

Probe: cg08638929 (K−means Cluster #2)
DNAm (Beta)
0.2
0.3
0.4
0.5
0.6
0.7
0.8
Ages (years)
0
20
40
60
80
A
A
B
B
C
C
D
D
E
E
F
F
G
G
H
H
I
J
J
K
K
FDR (Age associated divergence) =0.0765
p(Scalp)=0.0534
p(Dura)=0.213
Scalp
Dura

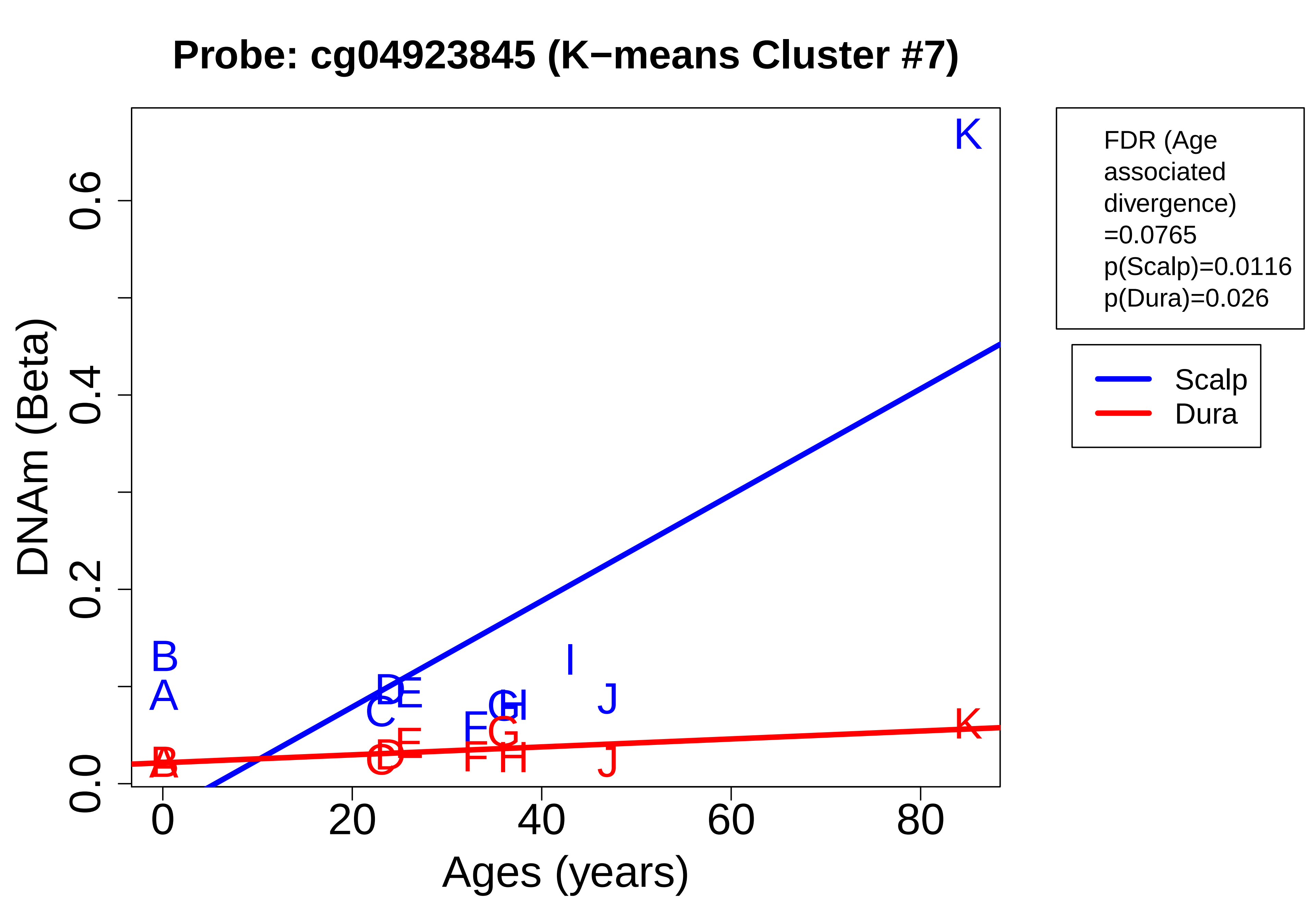

Probe: cg04923845 (K−means Cluster #7)
DNAm (Beta)
0.0
0.2
0.4
0.6
Ages (years)
0
20
40
60
80
FDR (Age associated divergence) =0.0765
p(Scalp)=0.0116
p(Dura)=0.026
Scalp
Dura
A
B
C
D
E
F
G
H
I
J
K

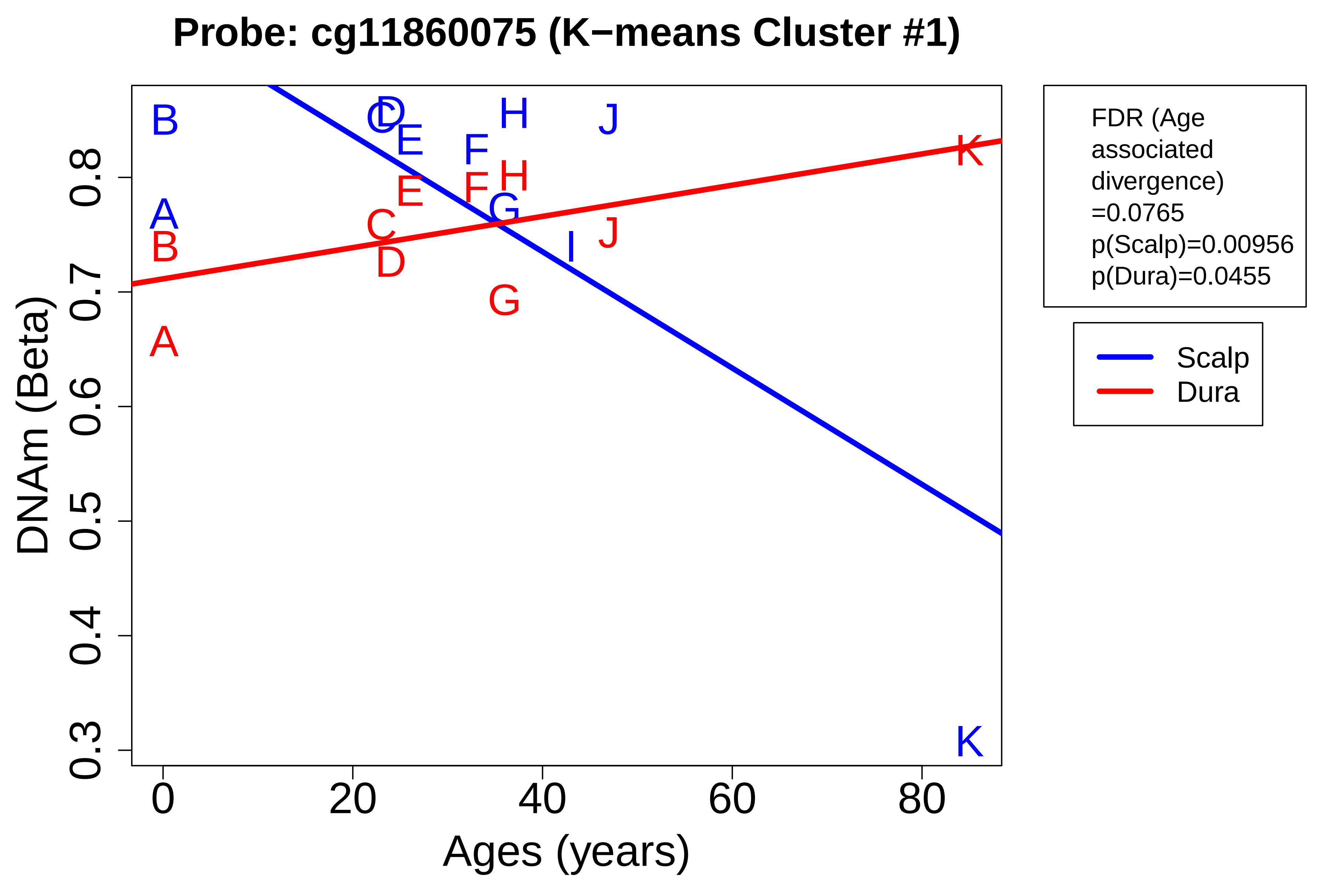

Probe: cg11860075 (K−means Cluster #1)
DNAm (Beta)
0.3
0.4
0.5
0.6
0.7
0.8
0
20
40
60
80
Ages (years)
A
B
C
D
E
F
G
H
I
J
K
FDR (Age associated divergence) =0.0765
p(Scalp)=0.00956
p(Dura)=0.0455
Scalp
Dura

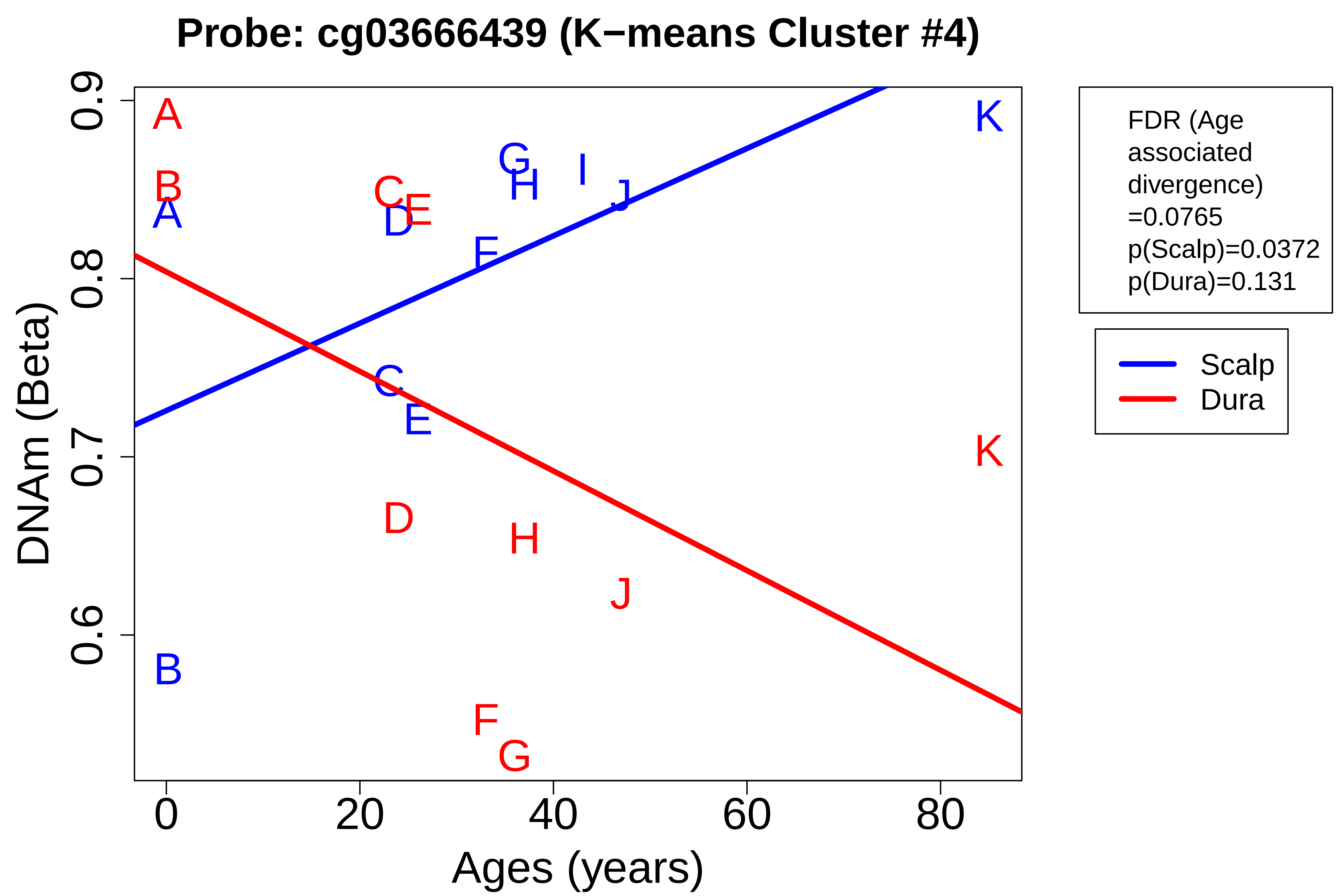

Probe: cg03666439 (K−means Cluster #4)
DNAm (Beta)
0.9
0.8
0.7
0.6
0
20
40
60
80
Ages (years)
A
B
A
C
E
D
G
H
I
J
F
K
C
E
K
D
H
J
B
F
G
FDR (Age associated divergence) =0.0765
p(Scalp)=0.0372
p(Dura)=0.131
Scalp
Dura

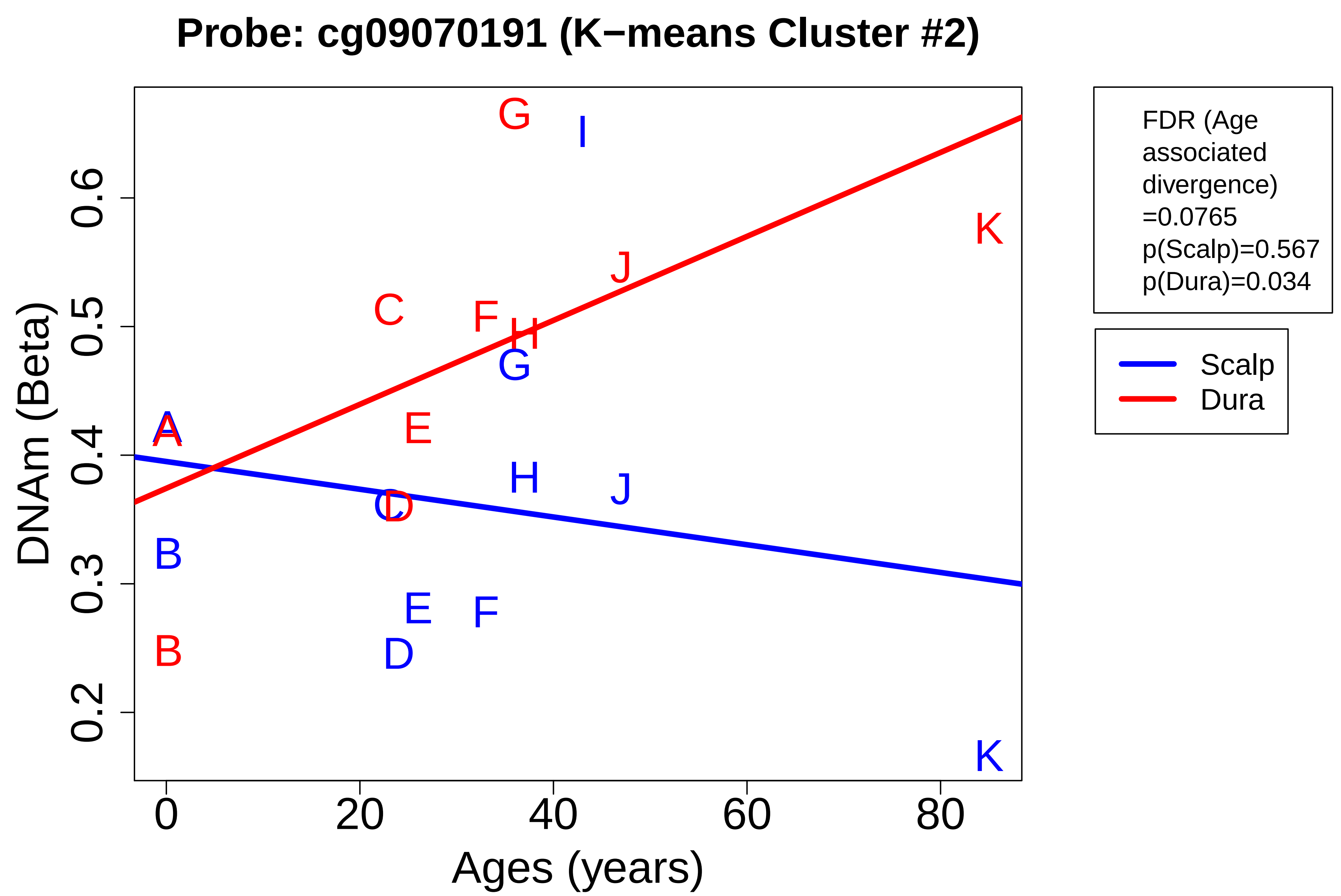

Probe: cg09070191 (K−means Cluster #2)
DNAm (Beta)
0.2
0.3
0.4
0.5
0.6
0
20
40
60
80
Ages (years)
A
B
C
D
E
F
G
H
I
J
K
FDR (Age associated divergence) =0.0765
p(Scalp)=0.567
p(Dura)=0.034
Scalp
Dura

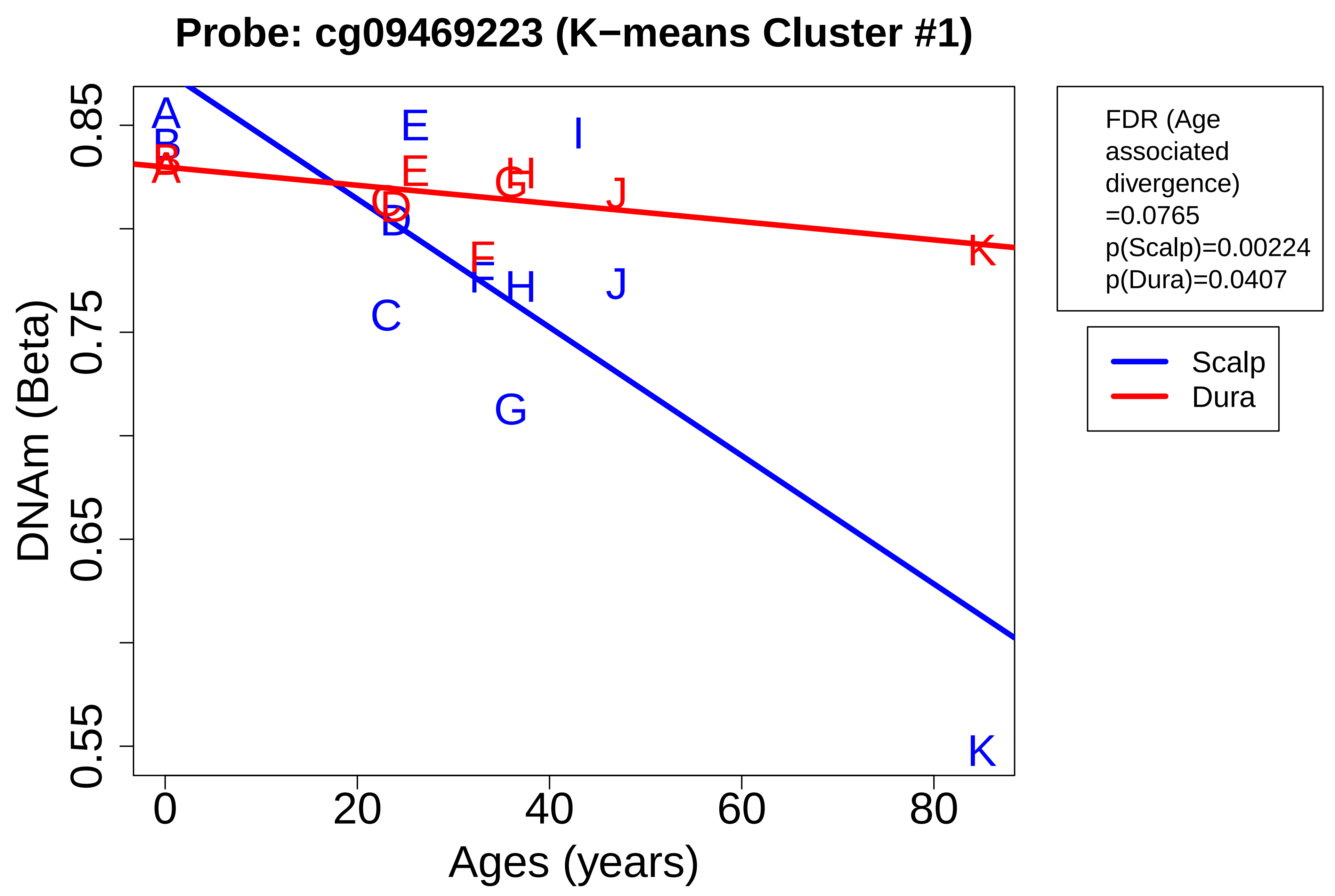
Probe: cg09469223 (K−means Cluster #1)
DNAm (Beta)
0.55
0.65
0.75
0.85
Ages (years)
0
20
40
60
80
A
B
C
D
E
F
G
H
I
J
K
FDR (Age associated divergence) =0.0765
p(Scalp)=0.00224
p(Dura)=0.0407
Scalp
Dura

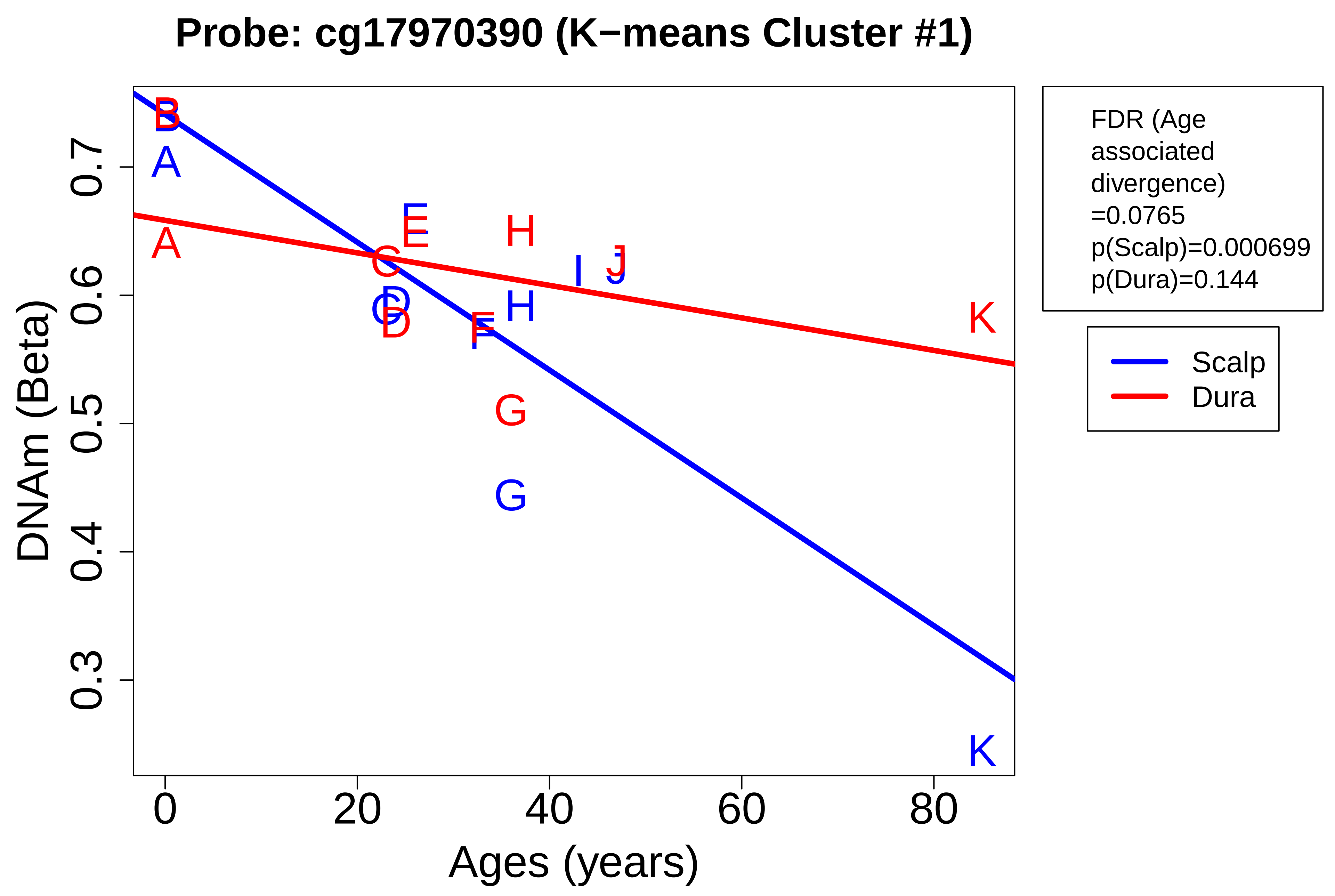

Probe: cg17970390 (K−means Cluster #1)
DNAm (Beta)
Ages (years)
0
20
40
60
80
0.3
0.4
0.5
0.6
0.7
FDR (Age associated divergence) =0.0765
p(Scalp)=0.000699
p(Dura)=0.144
Scalp
Dura

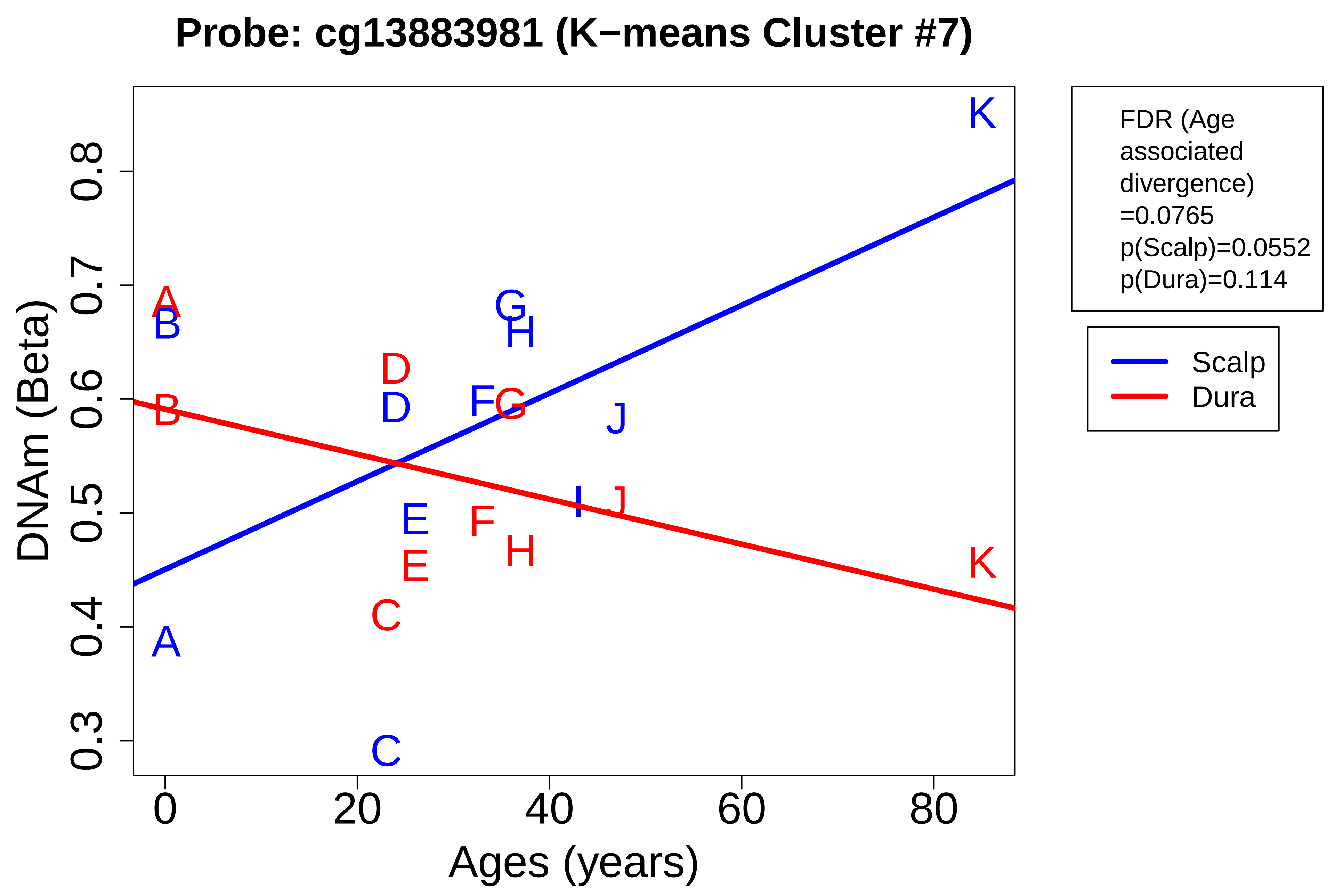
Probe: cg13883981 (K−means Cluster #7)
DNAm (Beta)
0.3
0.4
0.5
0.6
0.7
0.8
Ages (years)
0
20
40
60
80
A
B
C
D
E
F
G
H
I
J
K
FDR (Age associated divergence) =0.0765
p(Scalp)=0.0552
p(Dura)=0.114
Scalp
Dura

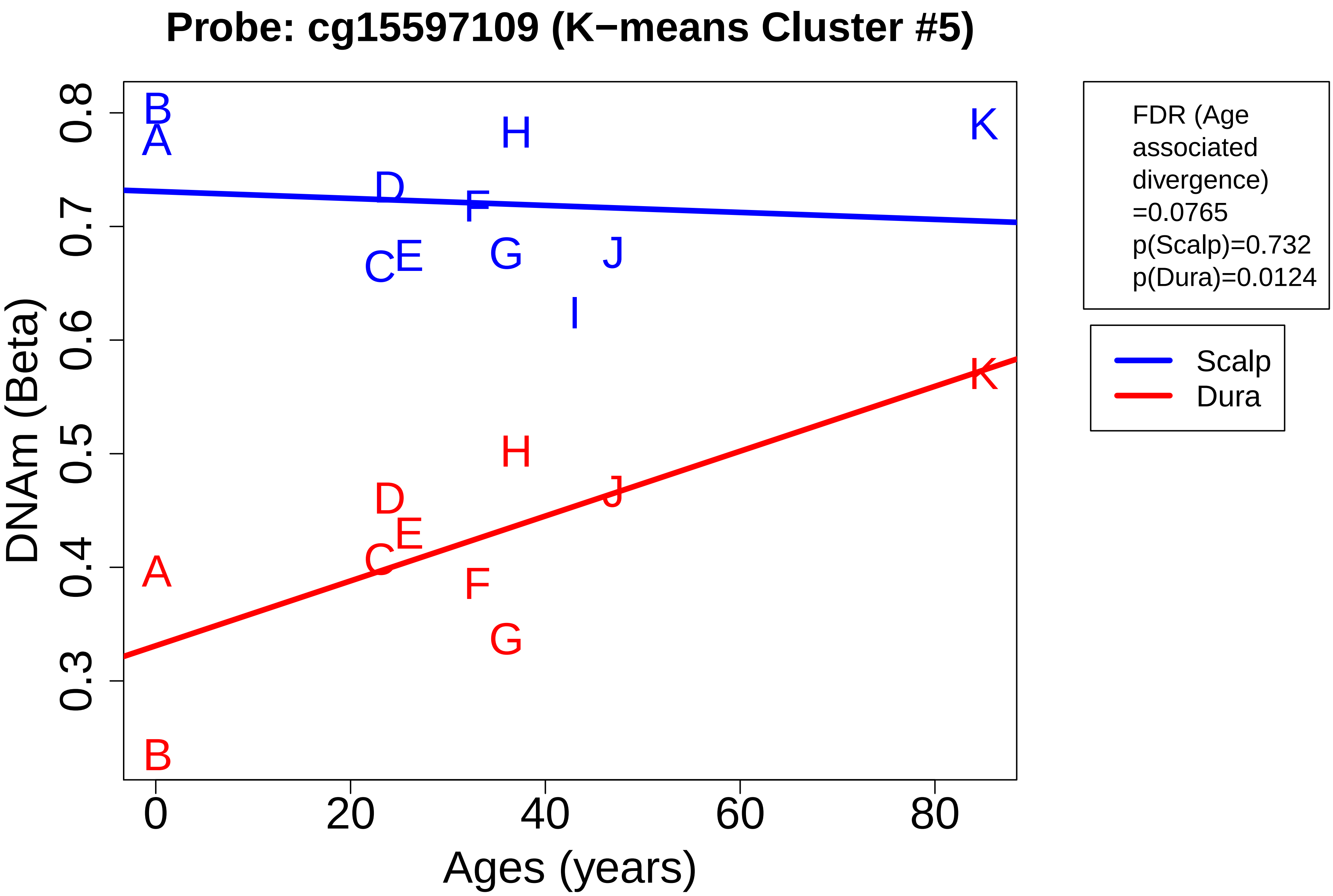
Probe: cg15597109 (K−means Cluster #5)
DNAm (Beta)
Ages (years)
0.3
0.4
0.5
0.6
0.7
0.8
0
20
40
60
80
FDR (Age associated divergence) =0.0765
p(Scalp)=0.732
p(Dura)=0.0124
Scalp
Dura

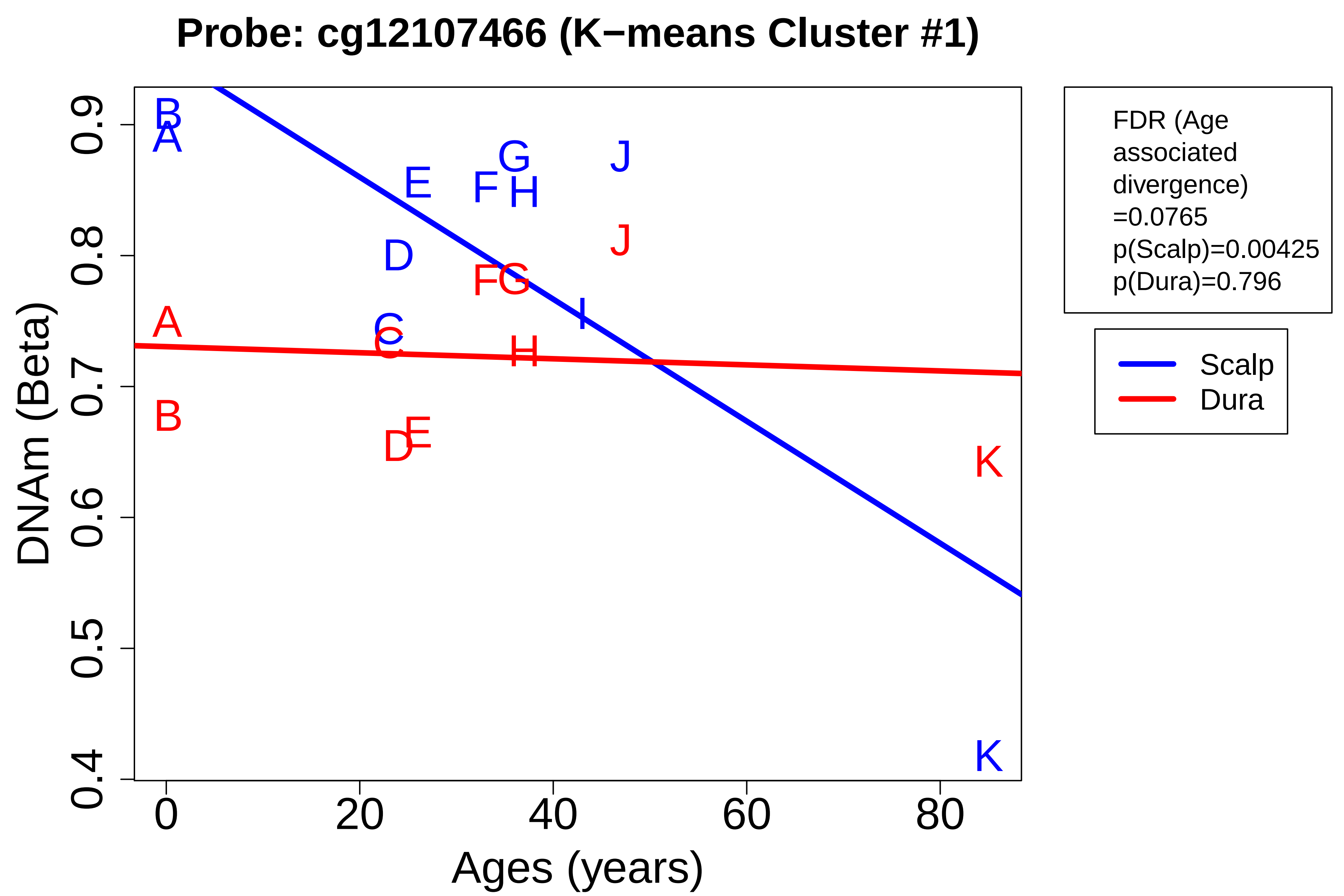

Probe: cg12107466 (K−means Cluster #1)
DNAm (Beta)
0.4
0.5
0.6
0.7
0.8
0.9
0
20
40
60
80
Ages (years)
FDR (Age associated divergence) =0.0765
p(Scalp)=0.00425
p(Dura)=0.796
Scalp
Dura
A
B
C
D
E
F
G
H
I
J
K

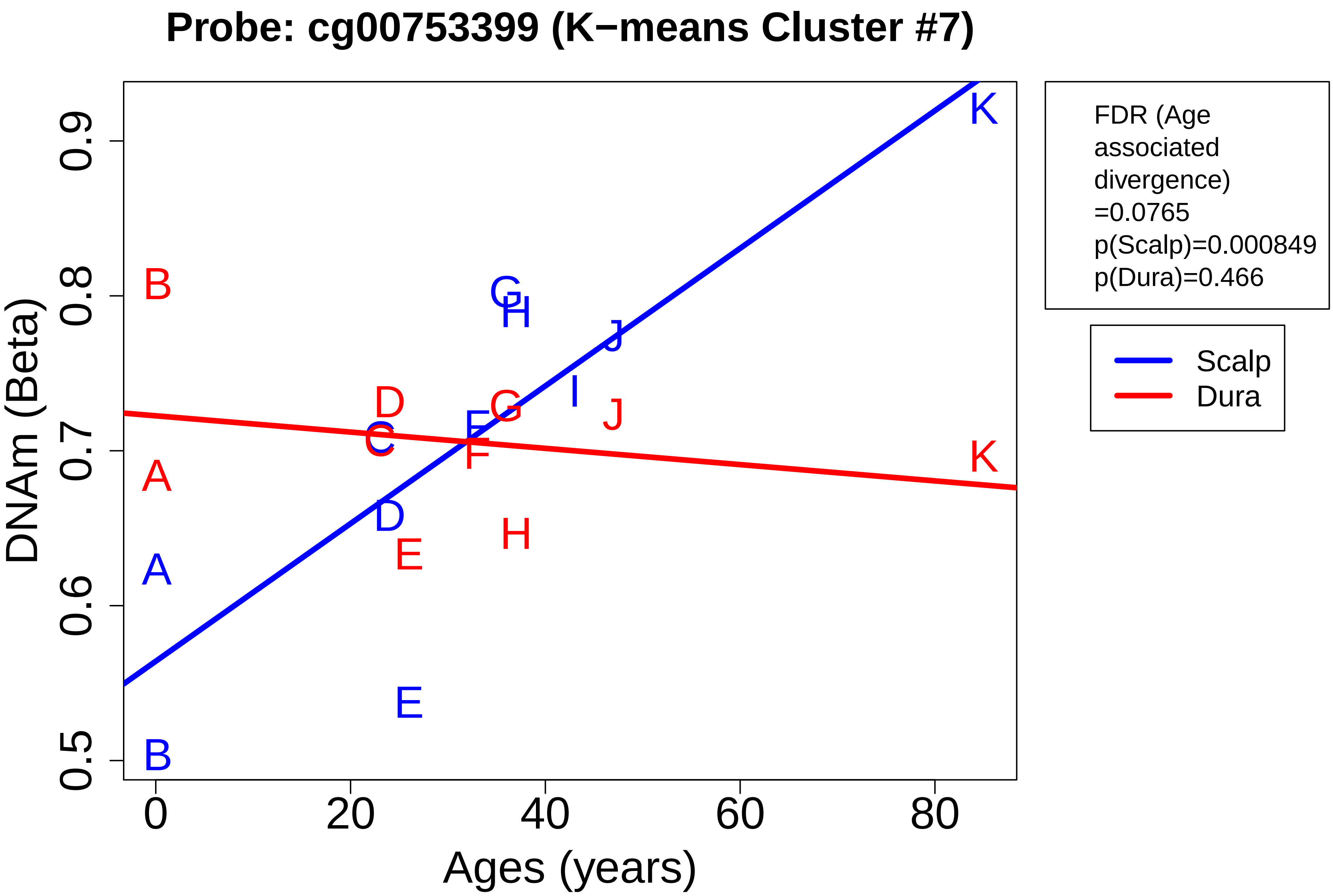

Probe: cg00753399 (K−means Cluster #7)
DNAm (Beta)
Ages (years)
0.5
0.6
0.7
0.8
0.9
0
20
40
60
80
FDR (Age associated divergence) =0.0765
p(Scalp)=0.000849
p(Dura)=0.466
Scalp
Dura

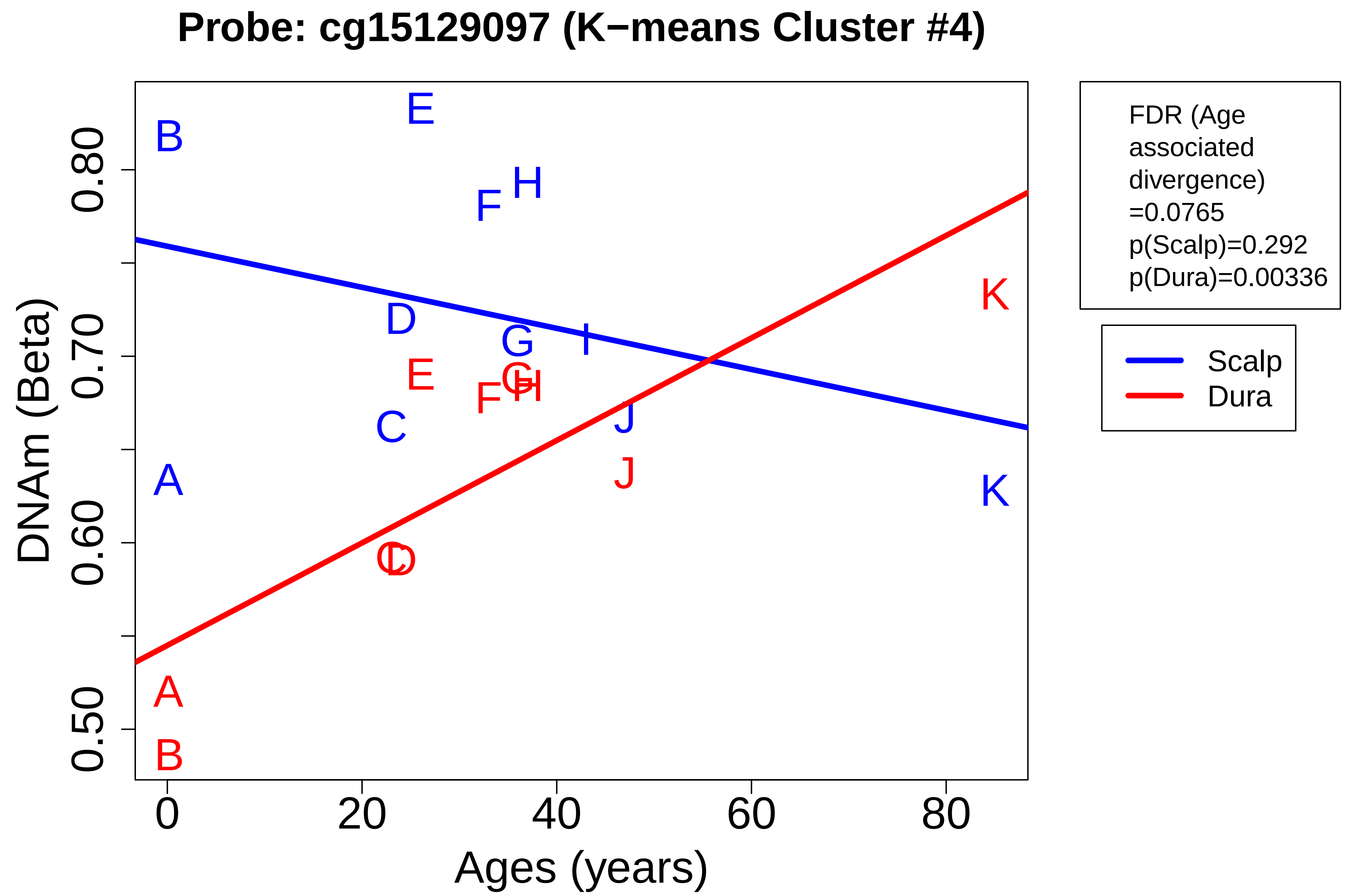

Probe: cg15129097 (K−means Cluster #4)
FDR (Age associated divergence) =0.0765
p(Scalp)=0.292
p(Dura)=0.00336
Scalp
Dura
DNAm (Beta)
0.50
0.60
0.70
0.80
0
20
40
60
80
Ages (years)
A
B
C
D
E
F
G
H
I
J
K

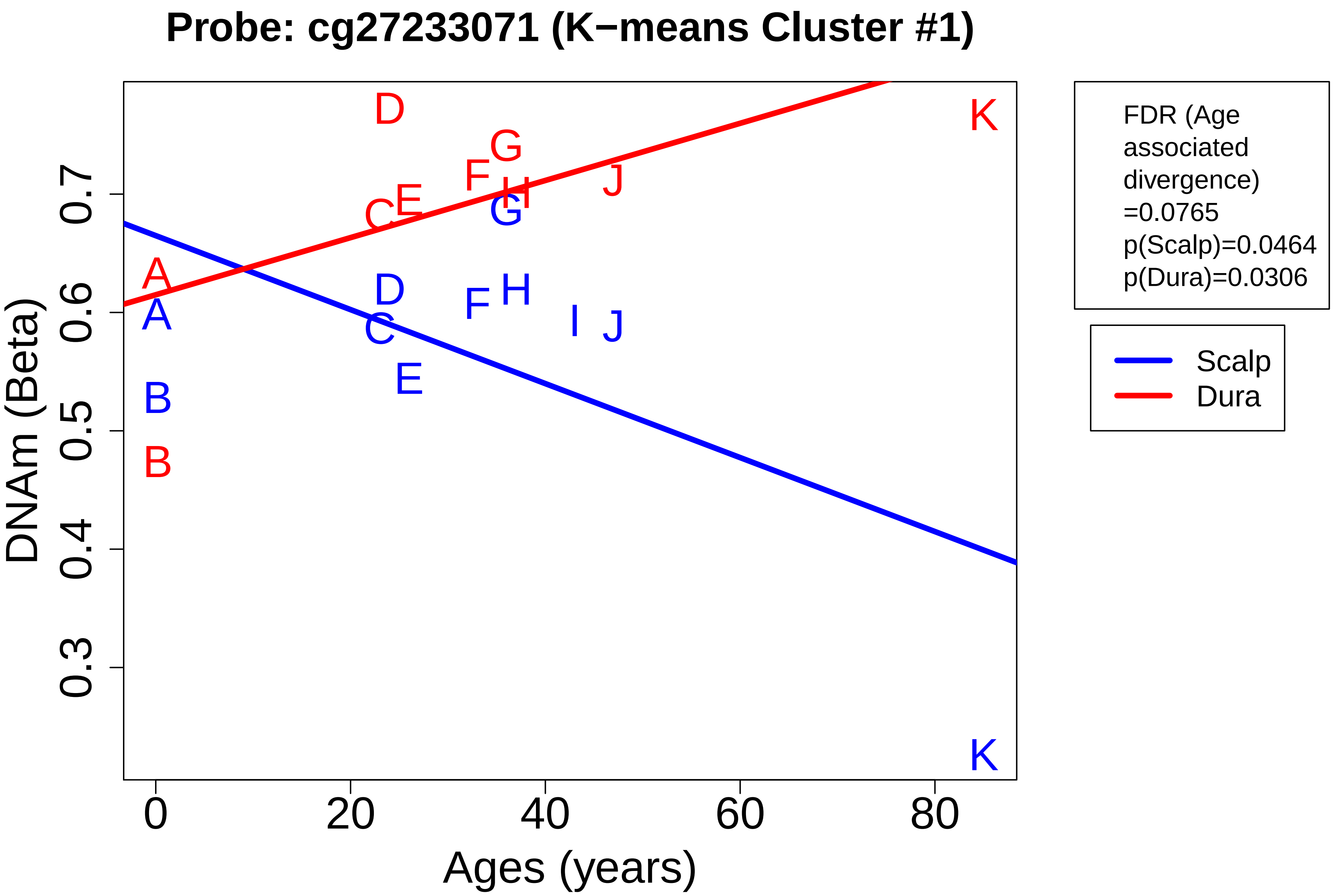

Probe: cg27233071 (K−means Cluster #1)
DNAm (Beta)
0.3
0.4
0.5
0.6
0.7
Ages (years)
0
20
40
60
80
FDR (Age associated divergence) =0.0765
p(Scalp)=0.0464
p(Dura)=0.0306
Scalp
Dura

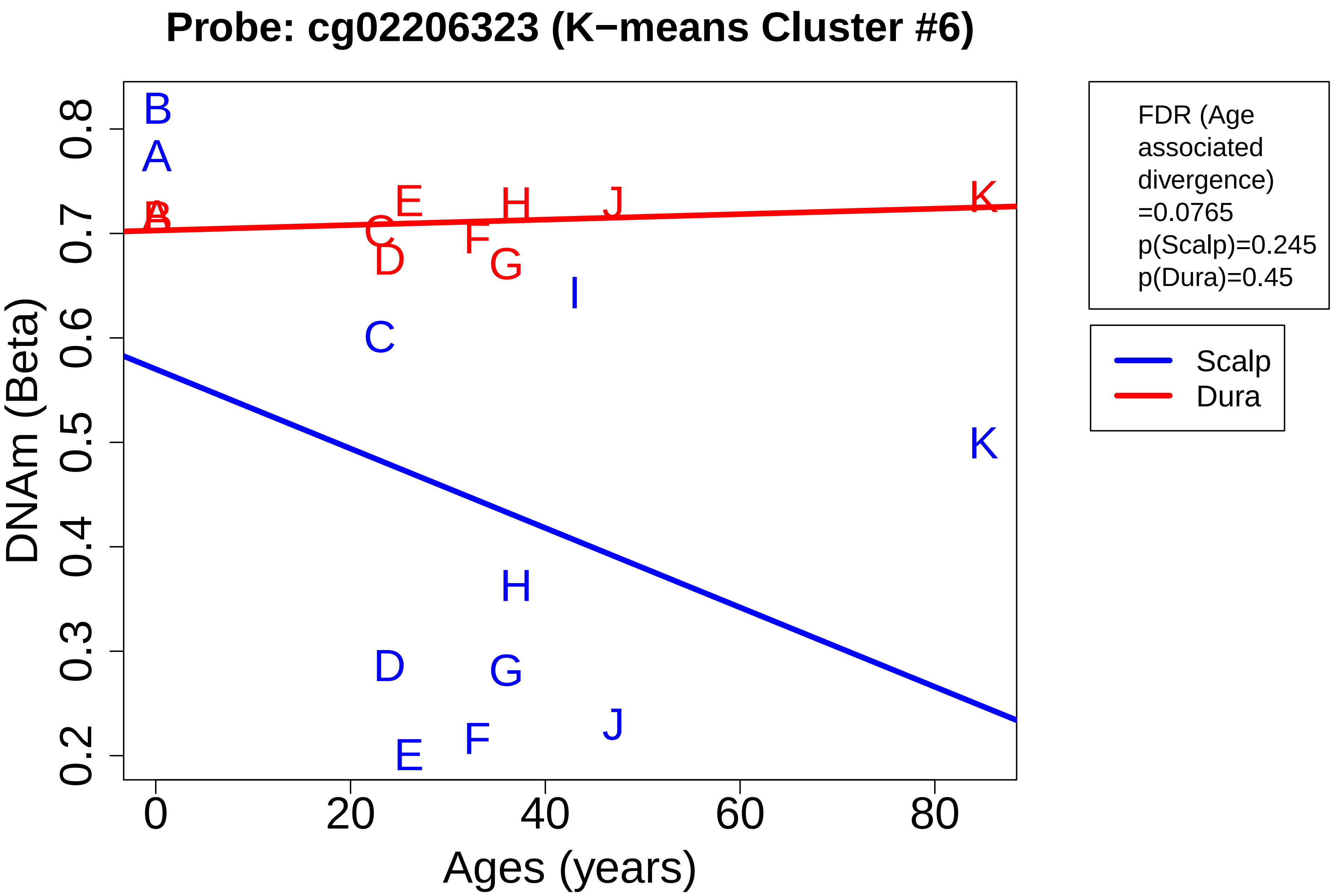

Probe: cg02206323 (K−means Cluster #6)
DNAm (Beta)
Ages (years)
0.2
0.3
0.4
0.5
0.6
0.7
0.8
0
20
40
60
80
FDR (Age associated divergence) =0.0765
p(Scalp)=0.245
p(Dura)=0.45
Scalp
Dura

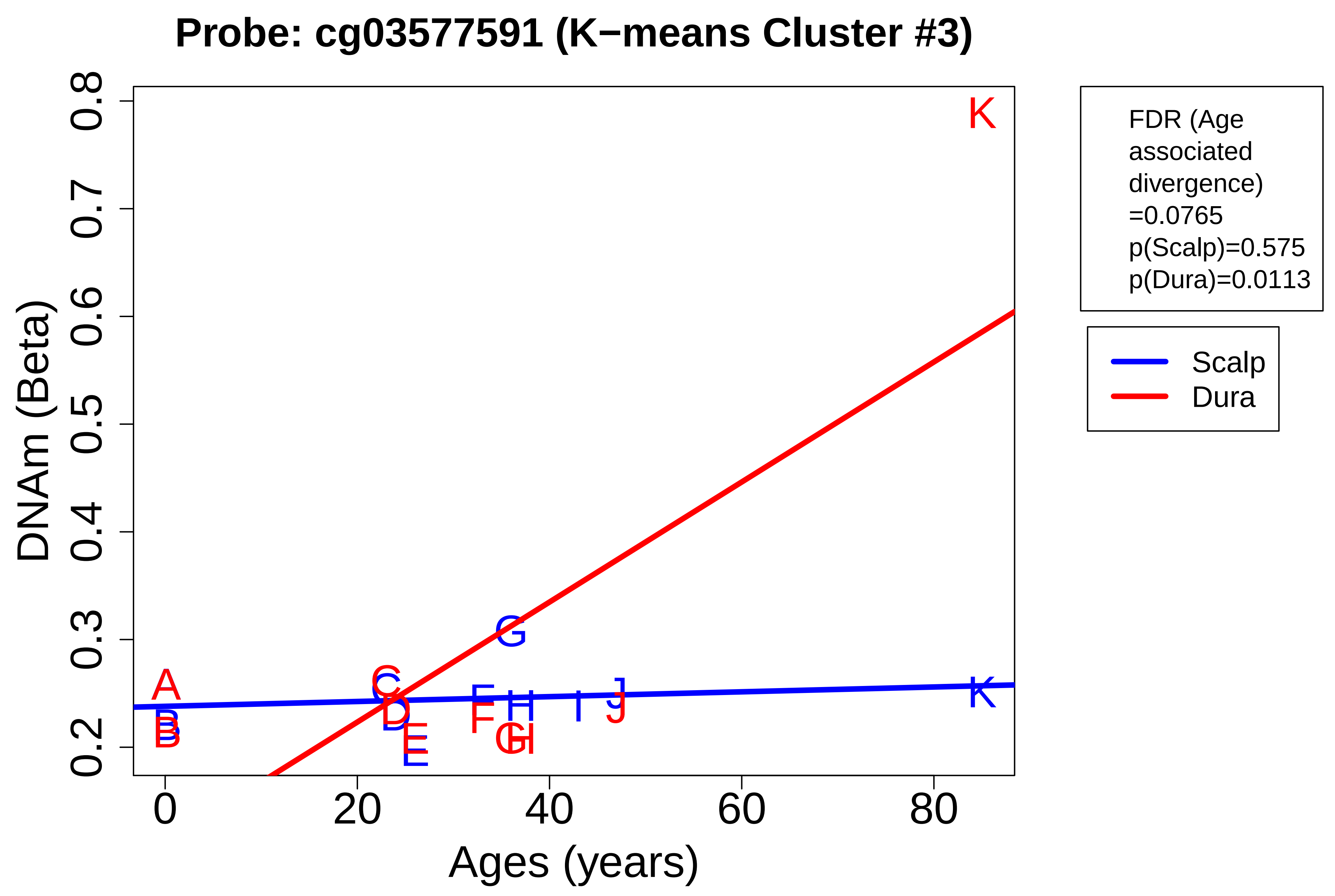

Probe: cg03577591 (K−means Cluster #3)
DNAm (Beta)
0.2
0.3
0.4
0.5
0.6
0.7
0.8
Ages (years)
0
20
40
60
80
FDR (Age associated divergence) =0.0765
p(Scalp)=0.575
p(Dura)=0.0113
Scalp
Dura

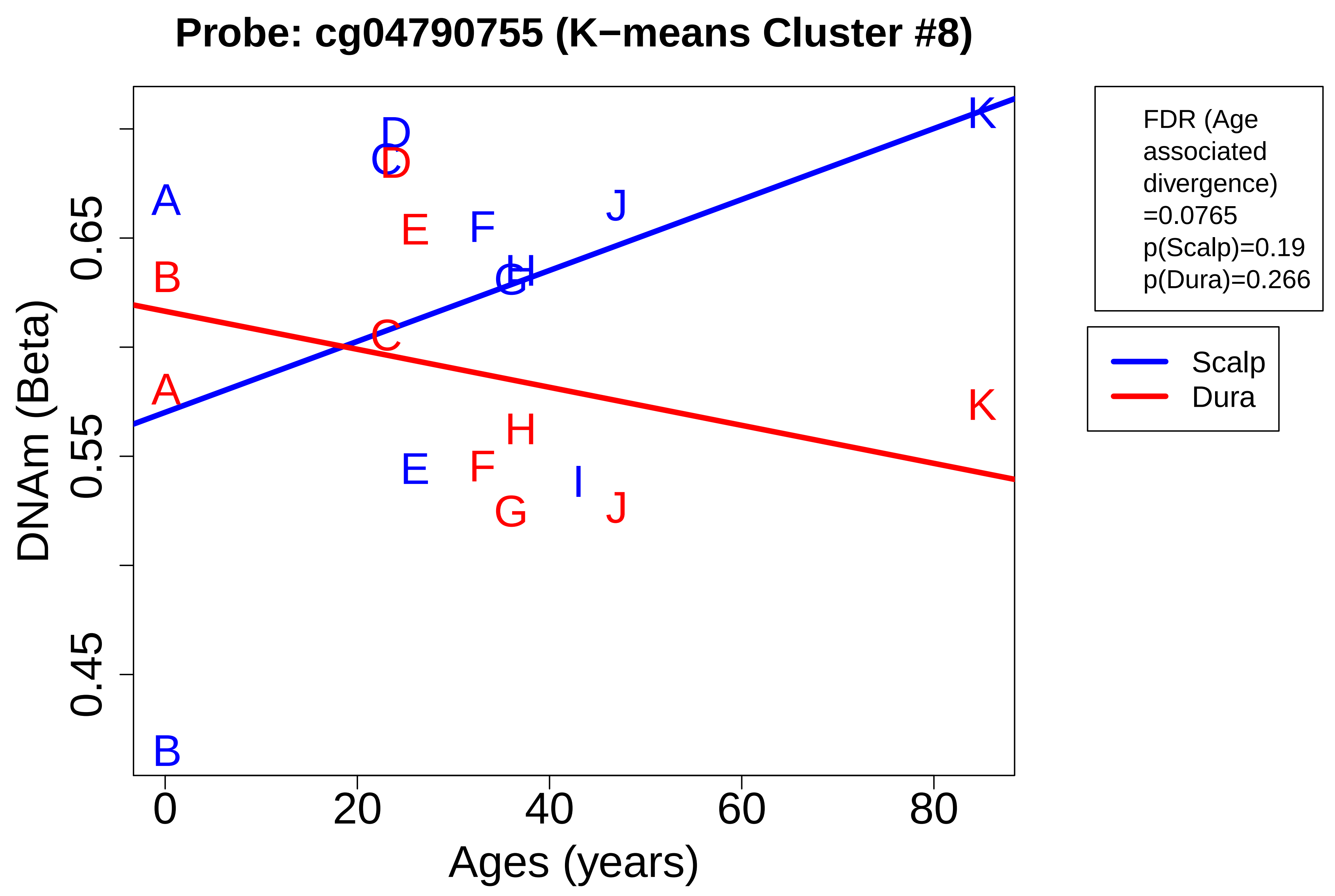

Probe: cg04790755 (K−means Cluster #8)
DNAm (Beta)
0.45
0.55
0.65
Ages (years)
0
20
40
60
80
A
B
C
D
E
F
G
H
I
J
K
FDR (Age associated divergence) =0.0765
p(Scalp)=0.19
p(Dura)=0.266
Scalp
Dura

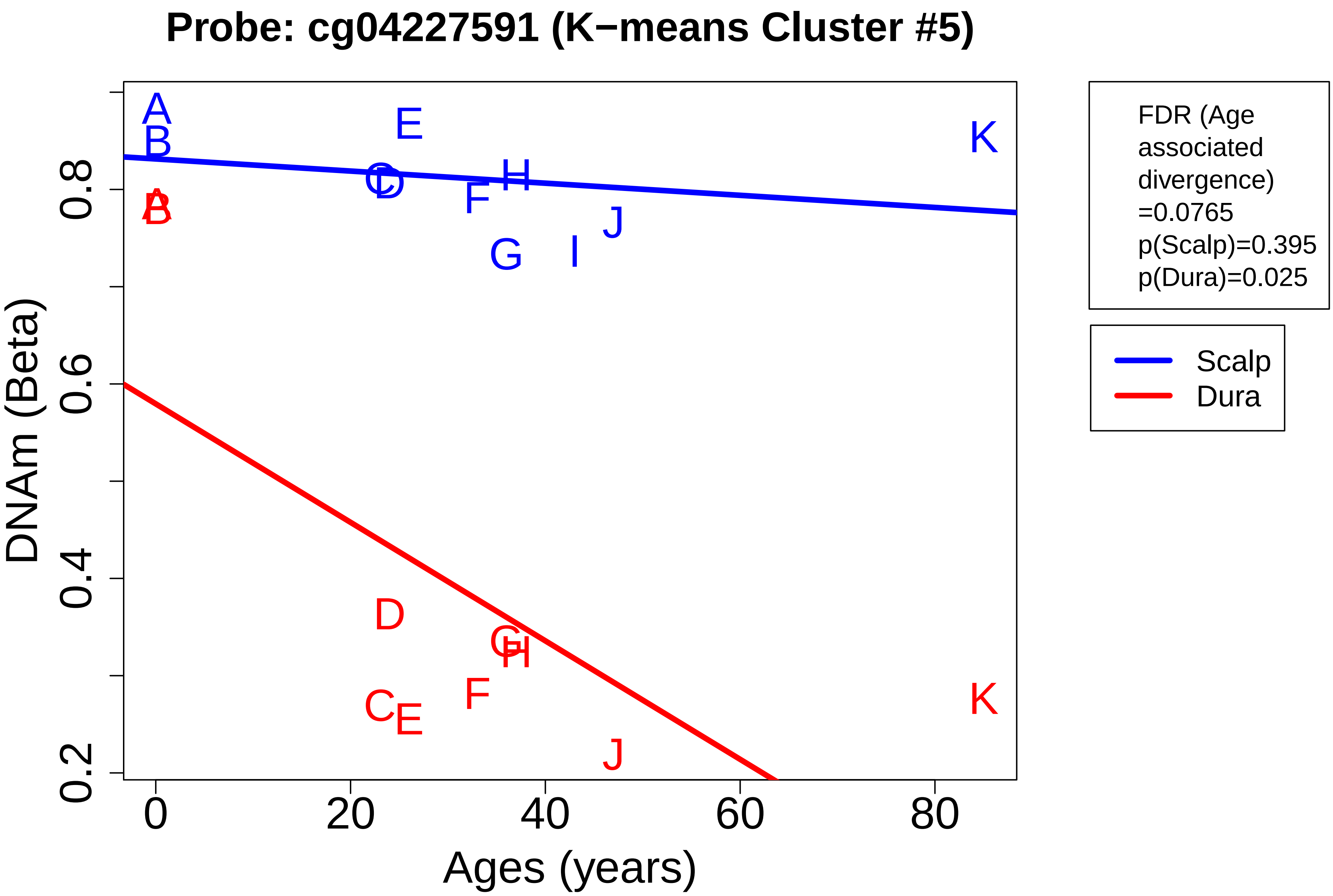

Probe: cg04227591 (K−means Cluster #5)
DNAm (Beta)
0.2
0.4
0.6
0.8
0
20
40
60
80
Ages (years)
FDR (Age associated divergence) =0.0765
p(Scalp)=0.395
p(Dura)=0.025
Scalp
Dura

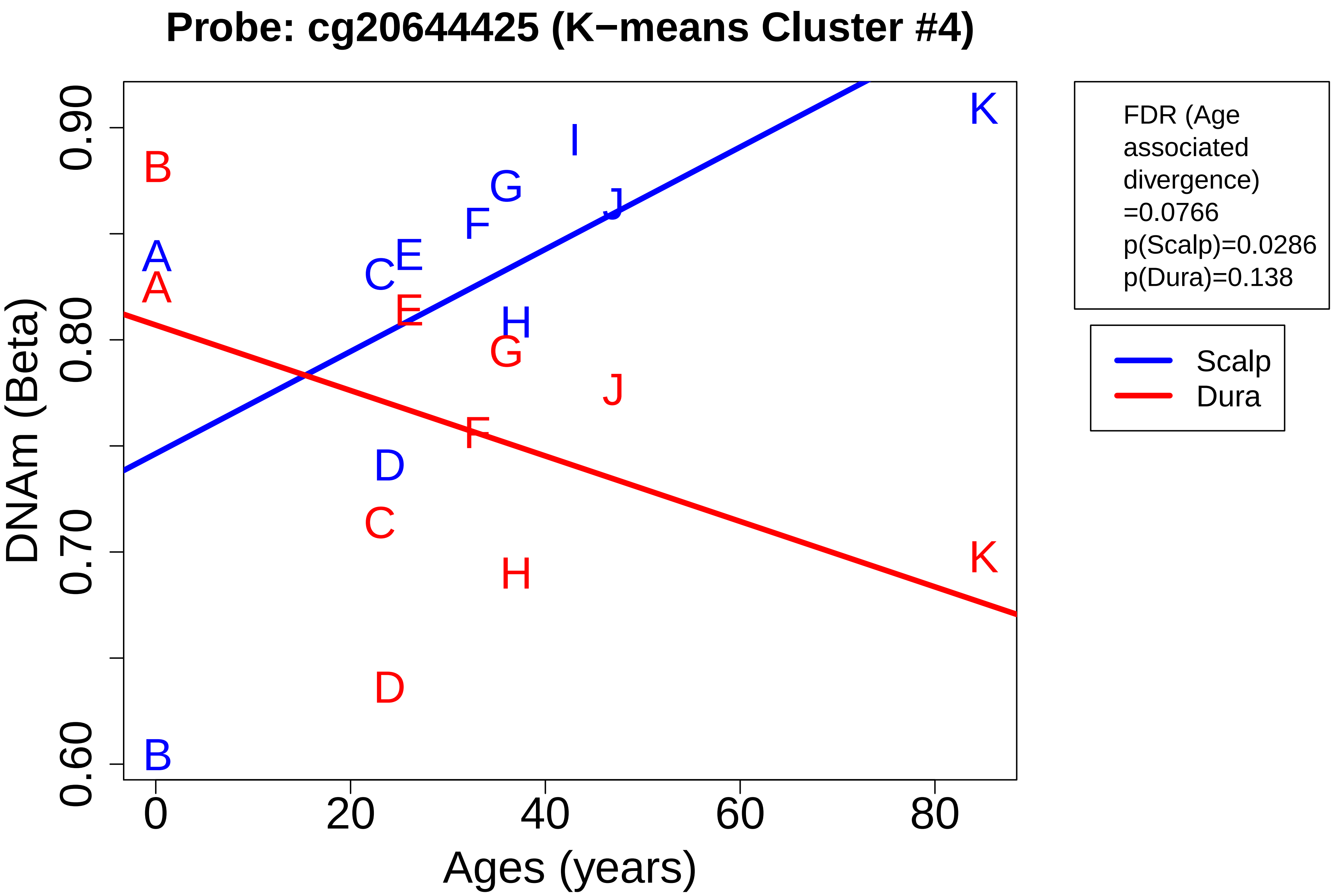
Probe: cg20644425 (K−means Cluster #4)
DNAm (Beta)
0.60
0.70
0.80
0.90
0
20
40
60
80
Ages (years)
FDR (Age associated divergence) =0.0766
p(Scalp)=0.0286
p(Dura)=0.138
Scalp
Dura
A
B
C
D
E
F
G
H
I
J
K

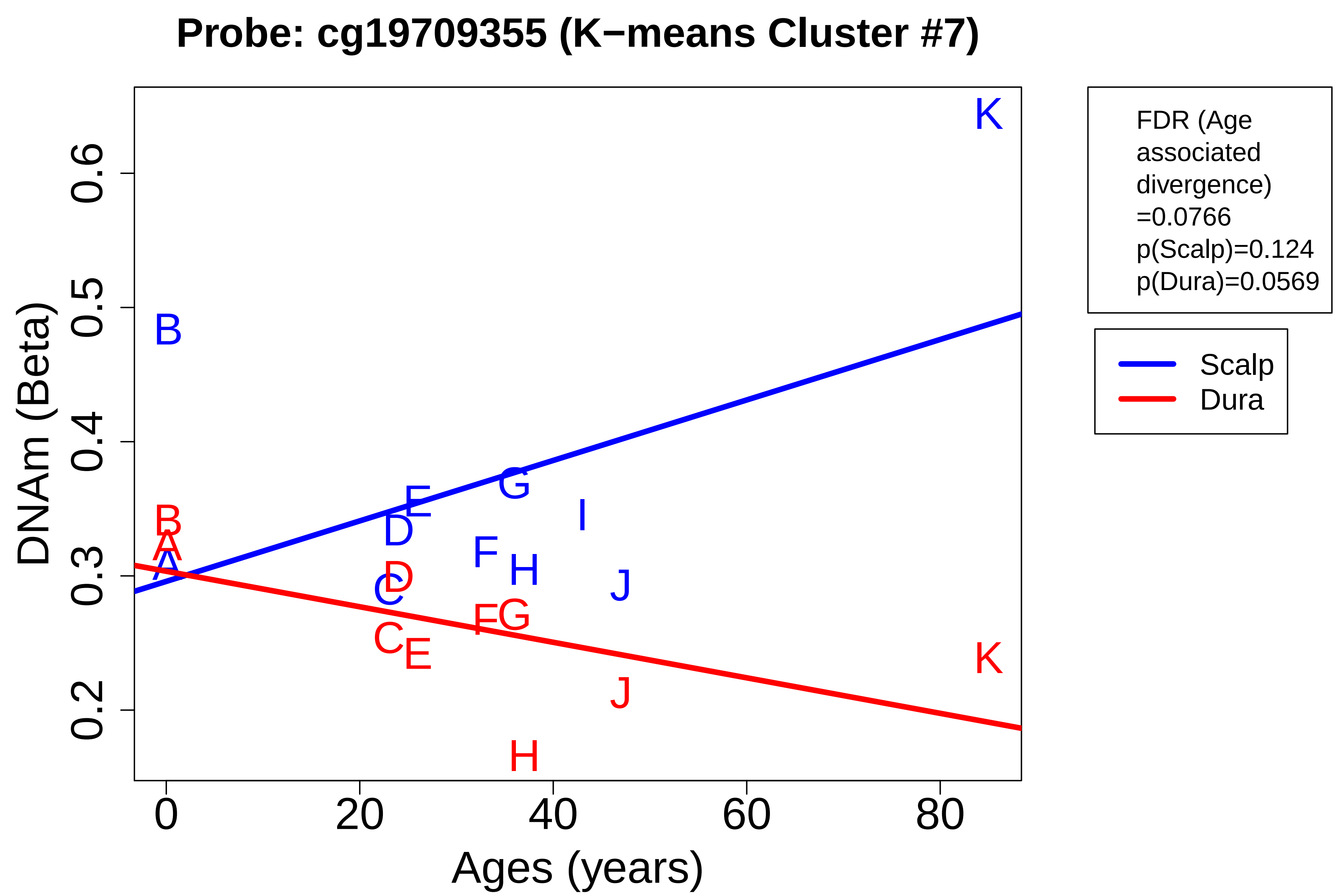

Probe: cg19709355 (K−means Cluster #7)
DNAm (Beta)
Ages (years)
0.2
0.3
0.4
0.5
0.6
0
20
40
60
80
FDR (Age associated divergence) =0.0766
p(Scalp)=0.124
p(Dura)=0.0569
Scalp
Dura
A
B
C
D
E
F
G
H
I
J
K

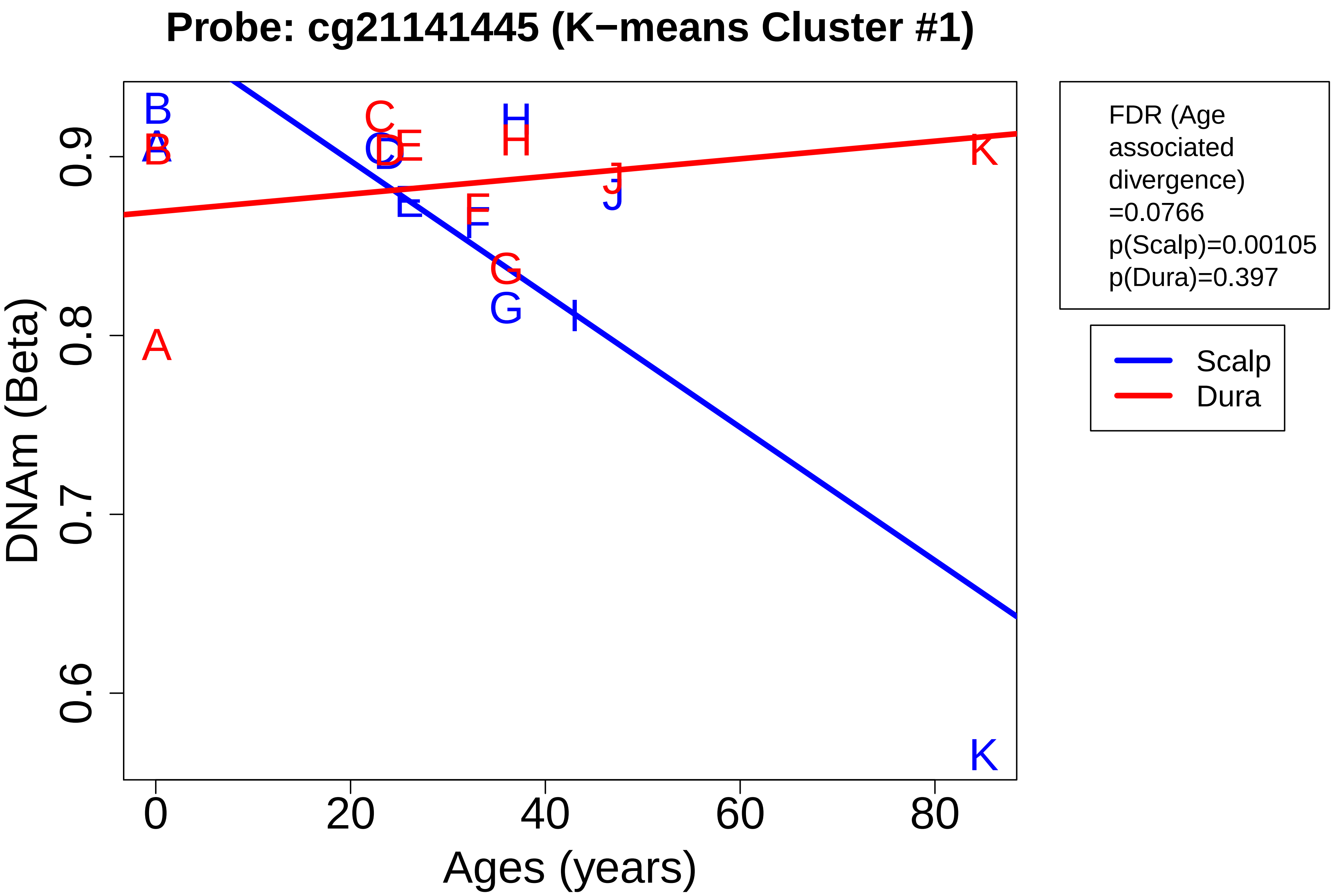

Probe: cg21141445 (K−means Cluster #1)
DNAm (Beta)
0.6
0.7
0.8
0.9
Ages (years)
0
20
40
60
80
FDR (Age associated divergence) =0.0766
p(Scalp)=0.00105
p(Dura)=0.397
Scalp
Dura

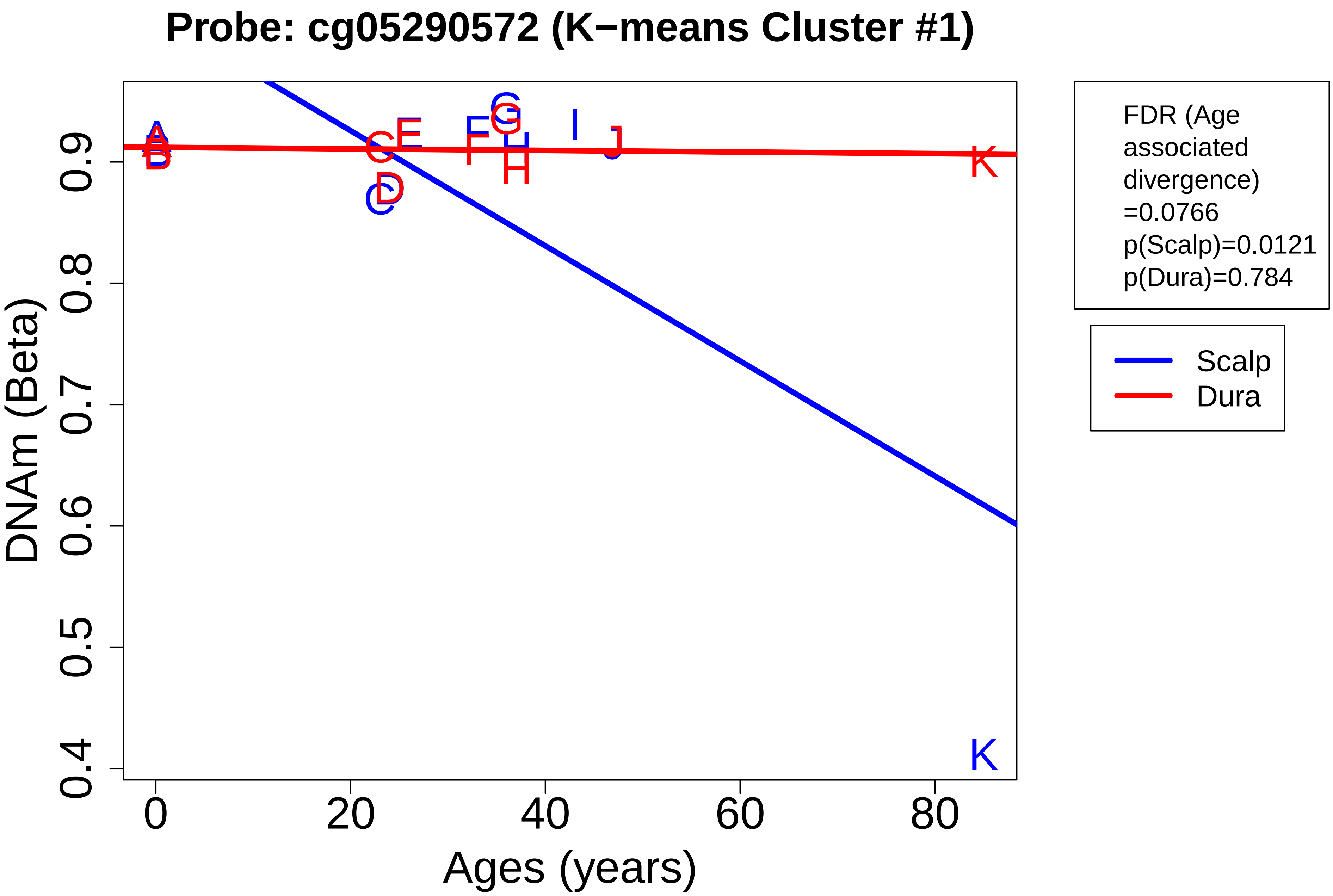

Probe: cg05290572 (K−means Cluster #1)
DNAm (Beta)
Ages (years)
0.4
0.5
0.6
0.7
0.8
0.9
0
20
40
60
80
A
B
C
D
E
F
G
H
I
J
K
FDR (Age associated divergence) =0.0766
p(Scalp)=0.0121
p(Dura)=0.784
Scalp
Dura

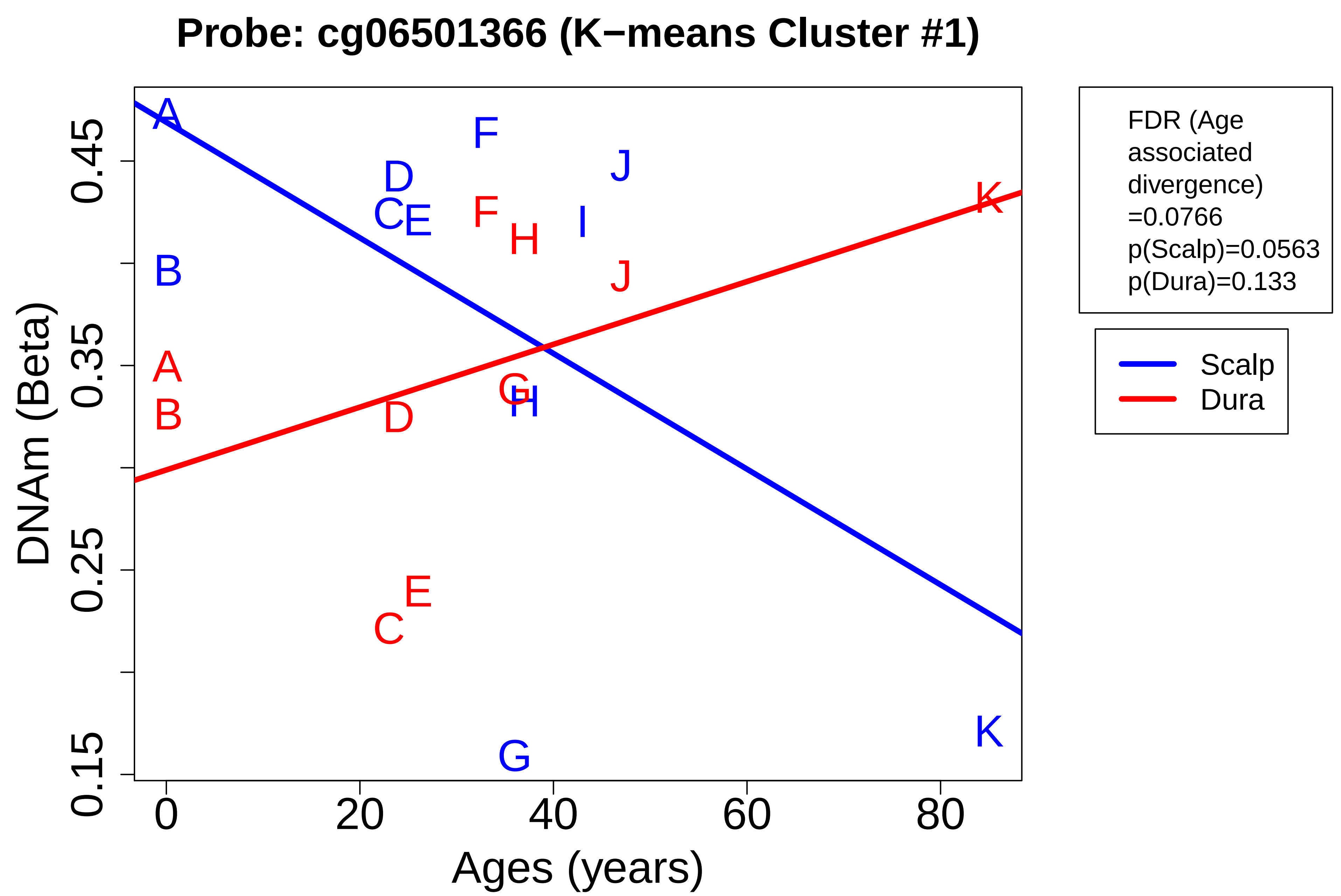

Probe: cg06501366 (K−means Cluster #1)
DNAm (Beta)
0.15
0.25
0.35
0.45
Ages (years)
0
20
40
60
80
A
B
C
D
E
F
G
H
I
J
K
FDR (Age associated divergence) =0.0766
p(Scalp)=0.0563
p(Dura)=0.133
Scalp
Dura

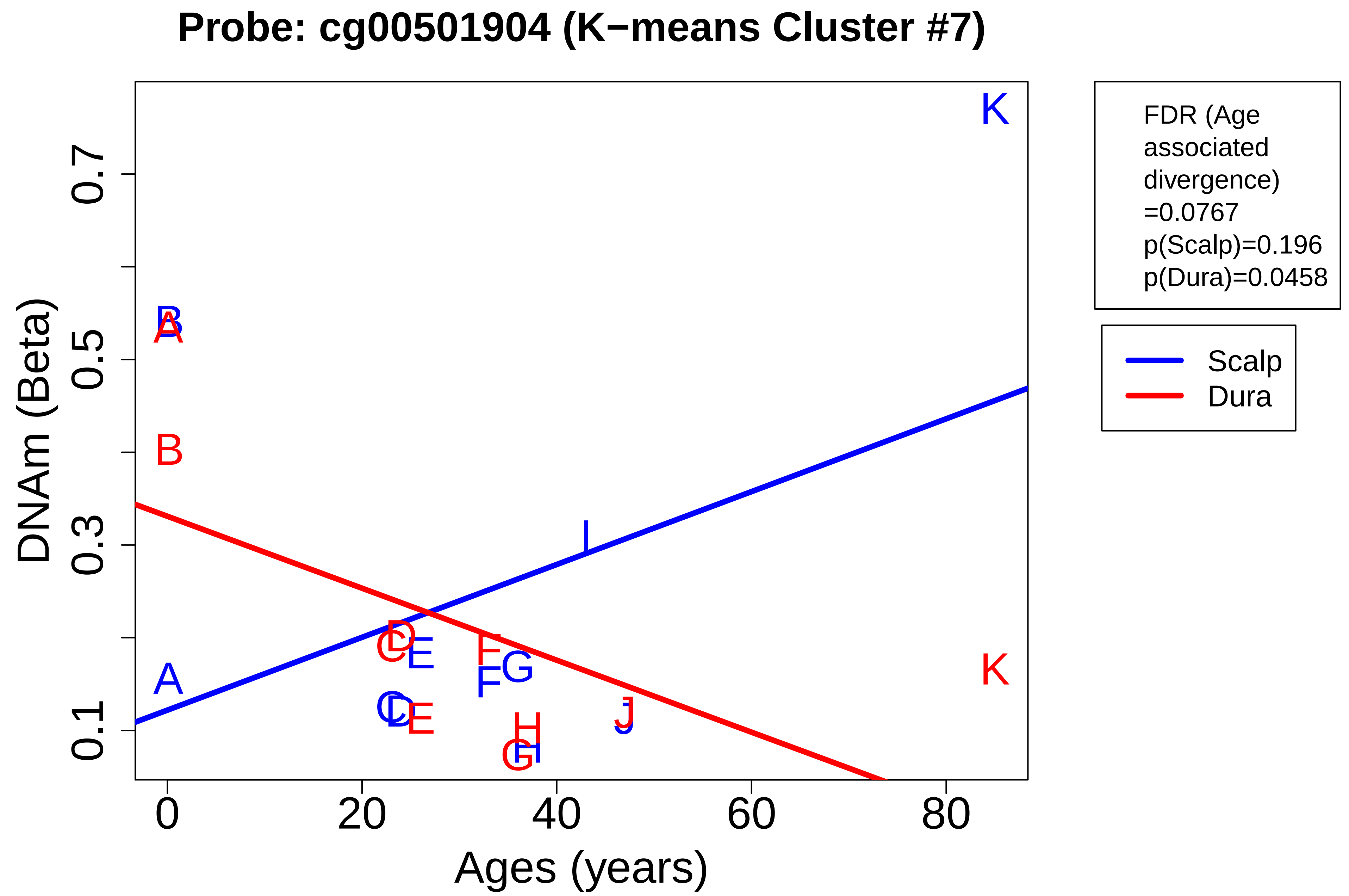

Probe: cg00501904 (K−means Cluster #7)
FDR (Age associated divergence) =0.0767
p(Scalp)=0.196
p(Dura)=0.0458
Scalp
Dura
DNAm (Beta)
Ages (years)
0.1
0.3
0.5
0.7
0
20
40
60
80

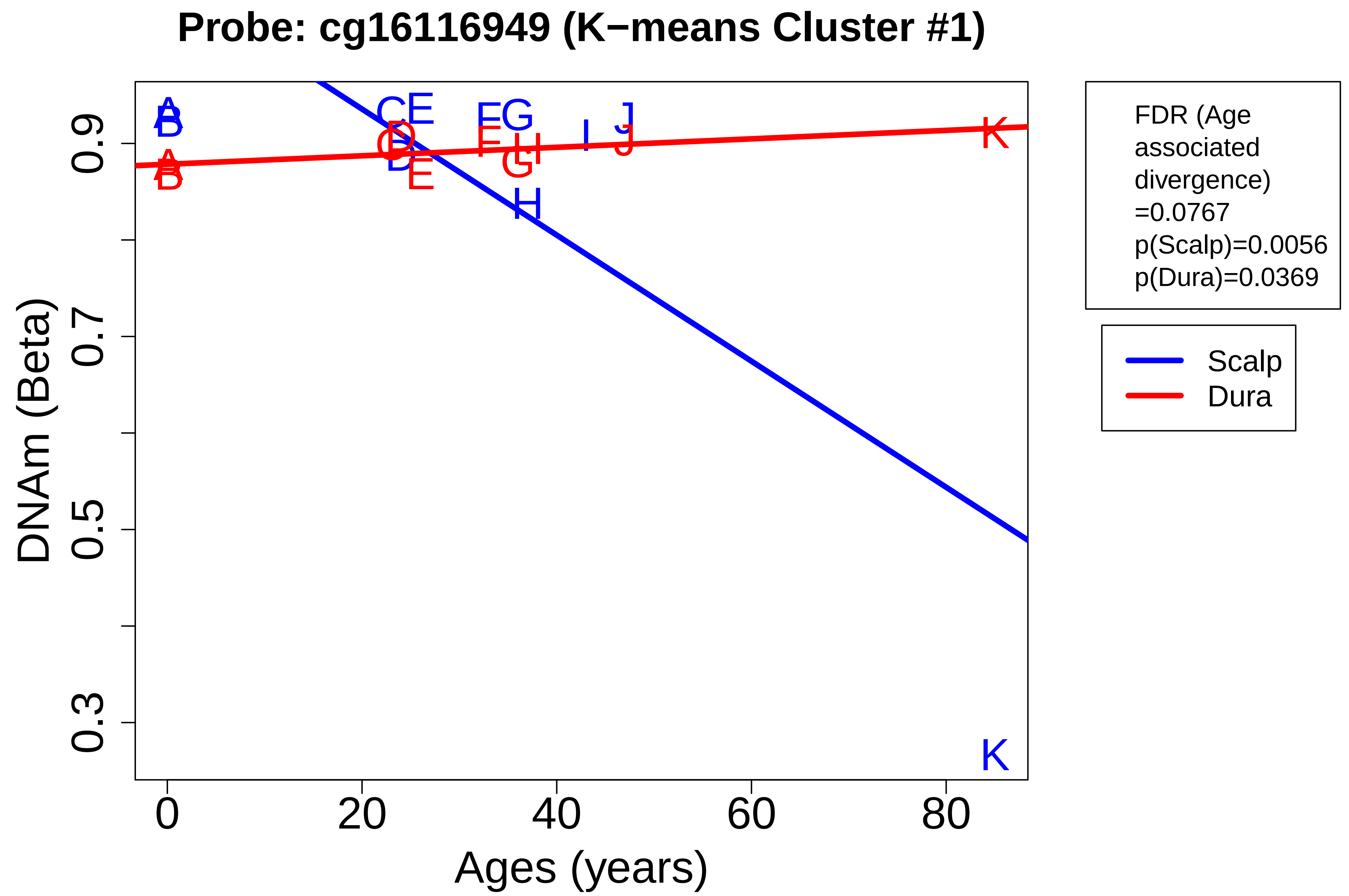

Probe: cg16116949 (K−means Cluster #1)
FDR (Age associated divergence) =0.0767
p(Scalp)=0.0056
p(Dura)=0.0369
Scalp
Dura
DNAm (Beta)
0.3
0.5
0.7
0.9
Ages (years)
0
20
40
60
80
A
B
C
D
E
F
G
H
I
J
K

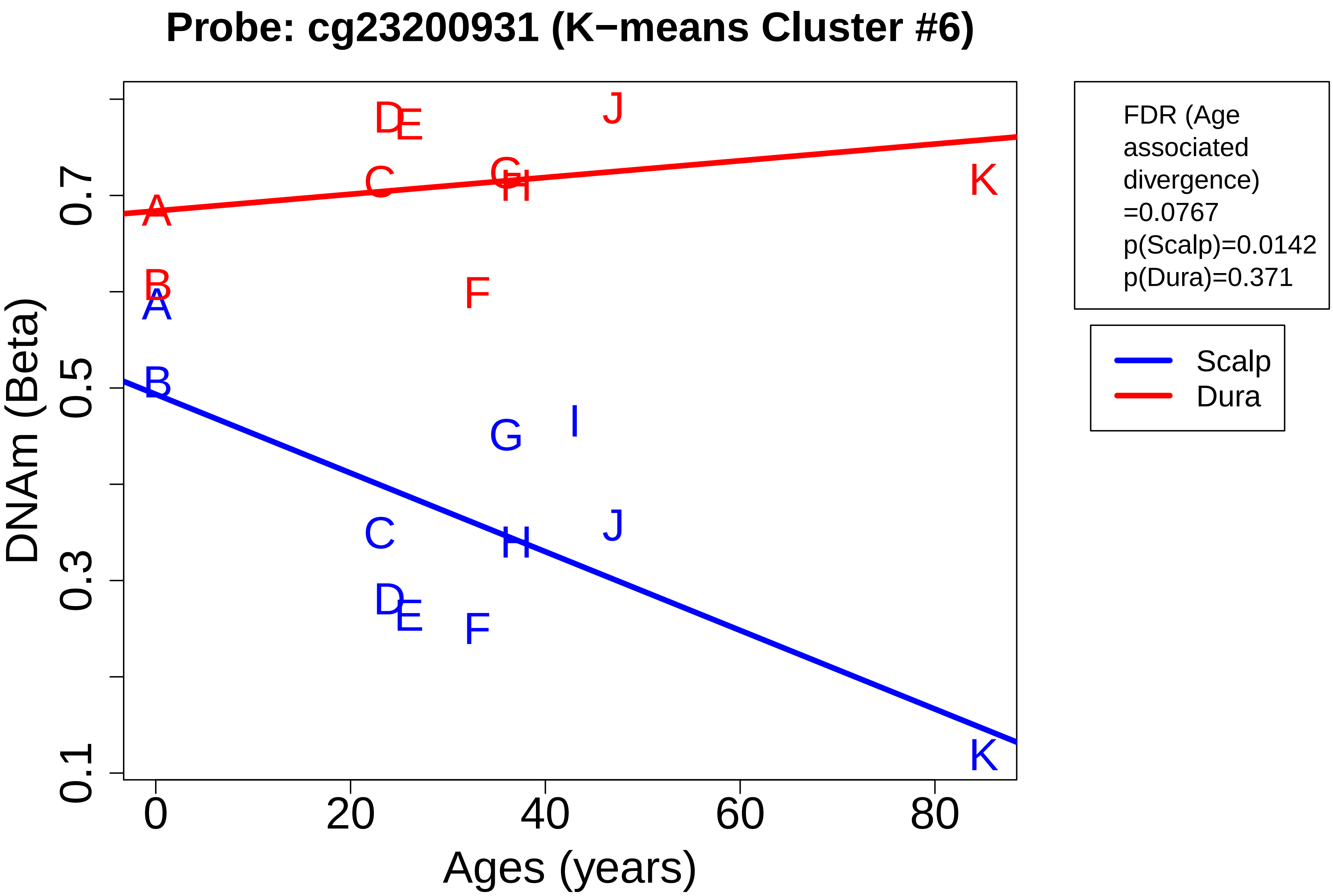
Probe: cg23200931 (K−means Cluster #6)
DNAm (Beta)
Ages (years)
0.1
0.3
0.5
0.7
0
20
40
60
80
FDR (Age associated divergence) =0.0767
p(Scalp)=0.0142
p(Dura)=0.371
Scalp
Dura
A
B
C
D
E
F
G
H
I
J
K

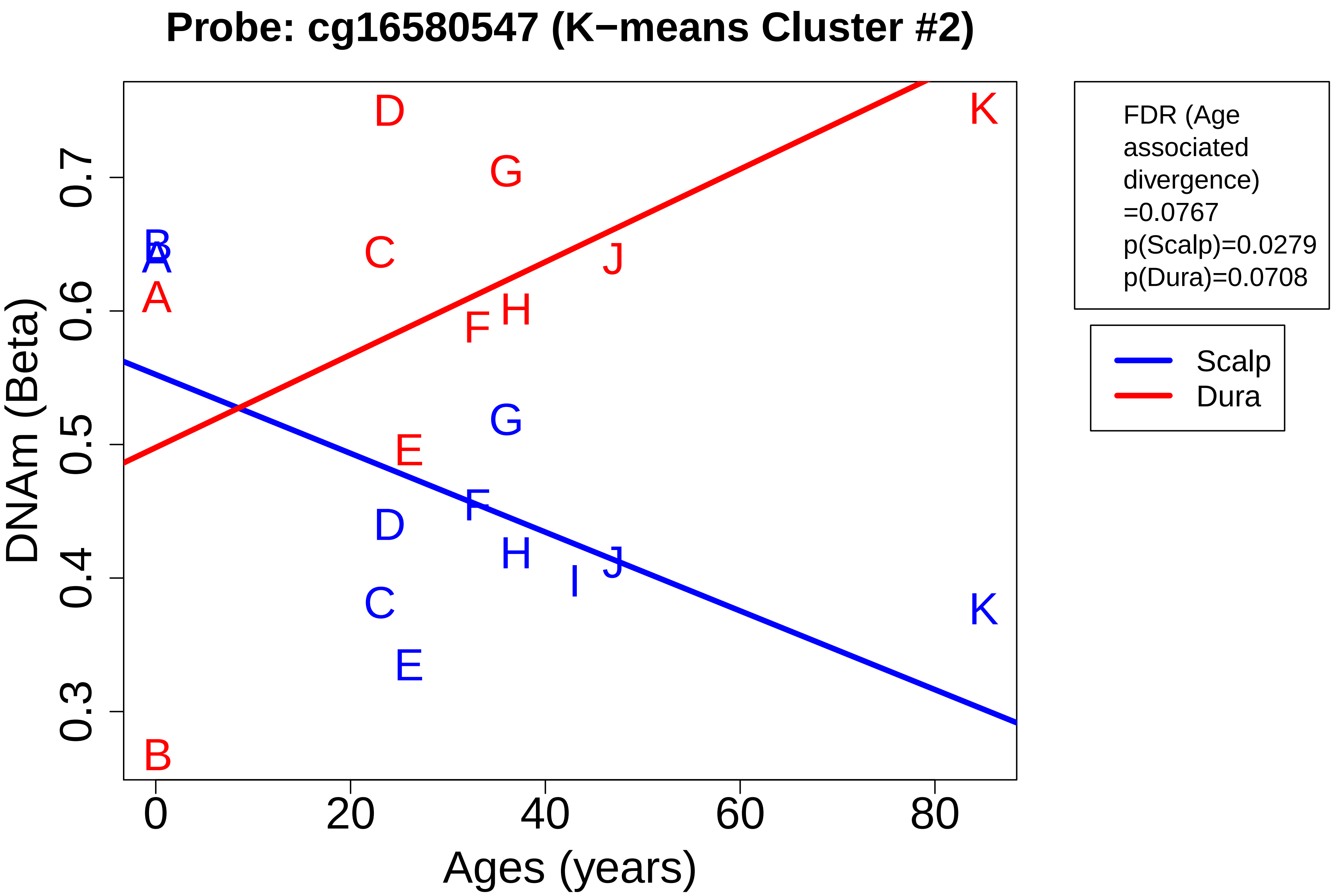

Probe: cg16580547 (K−means Cluster #2)
DNAm (Beta)
0.3
0.4
0.5
0.6
0.7
Ages (years)
0
20
40
60
80
FDR (Age associated divergence) =0.0767
p(Scalp)=0.0279
p(Dura)=0.0708
Scalp
Dura

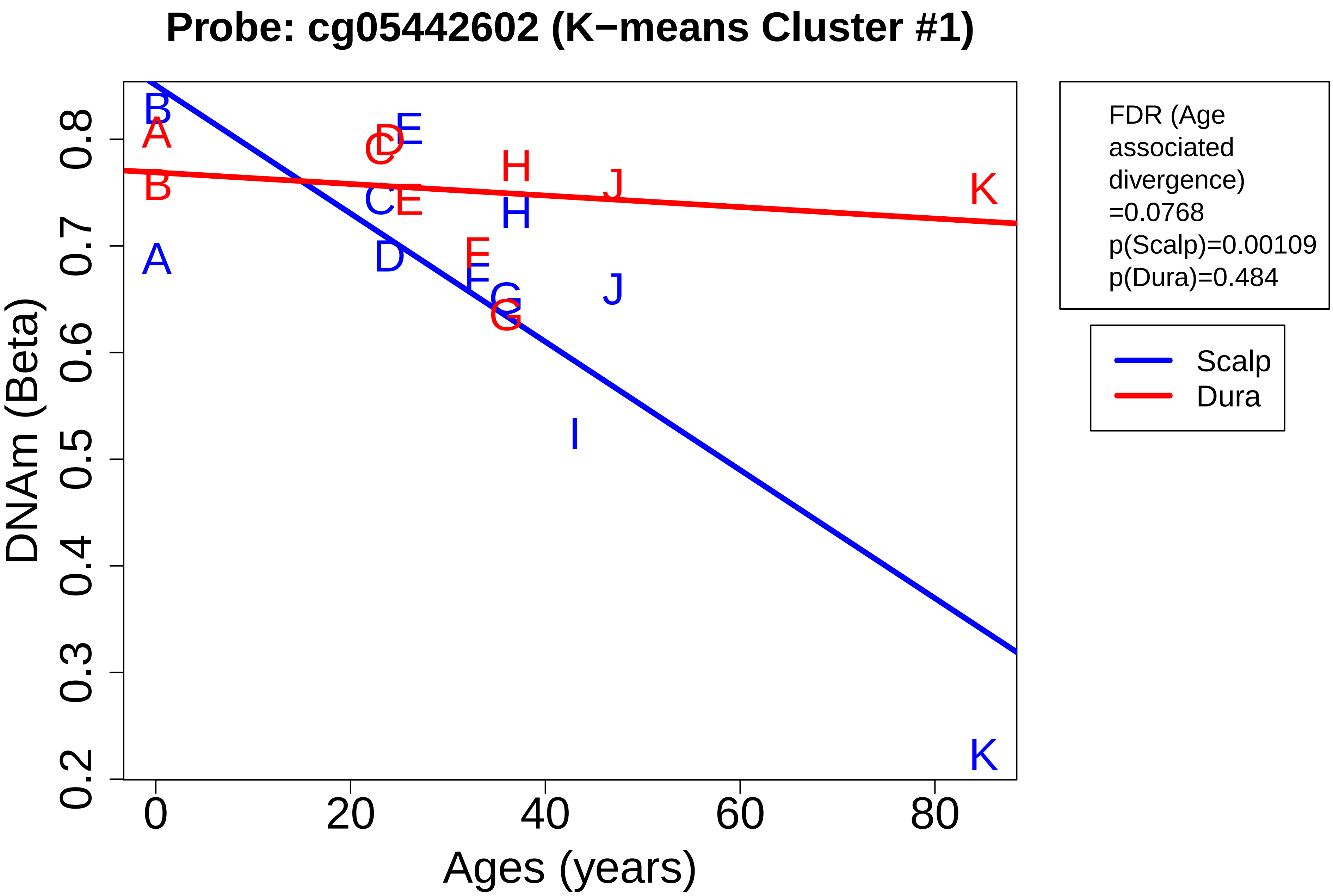
Probe: cg05442602 (K−means Cluster #1)
DNAm (Beta)
Ages (years)
0.2
0.3
0.4
0.5
0.6
0.7
0.8
0
20
40
60
80
FDR (Age associated divergence) =0.0768
p(Scalp)=0.00109
p(Dura)=0.484
Scalp
Dura

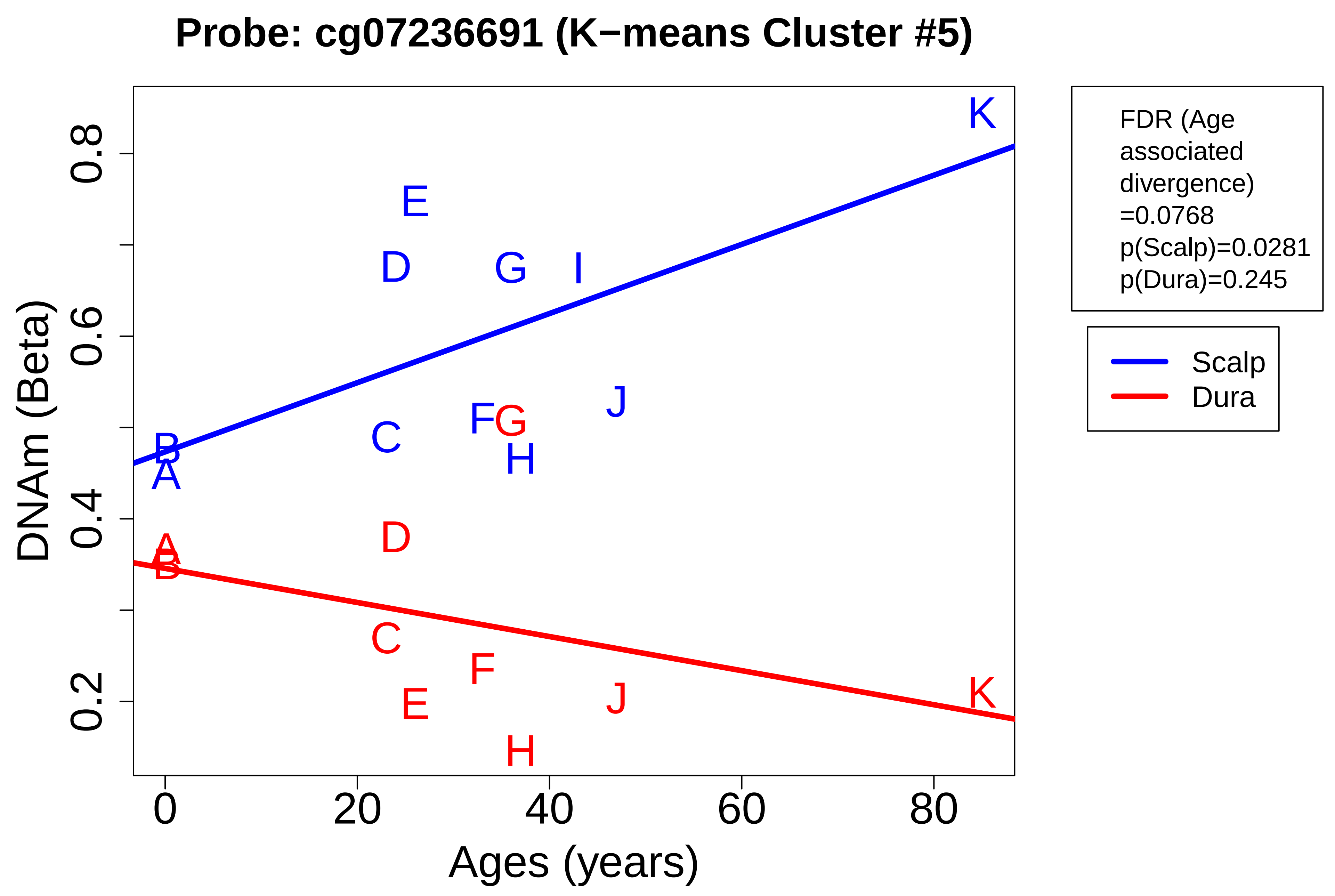

Probe: cg07236691 (K−means Cluster #5)
DNAm (Beta)
0.2
0.4
0.6
0.8
0
20
40
60
80
Ages (years)
A
B
C
D
E
F
G
H
I
J
K
FDR (Age associated divergence) =0.0768
p(Scalp)=0.0281
p(Dura)=0.245
Scalp
Dura

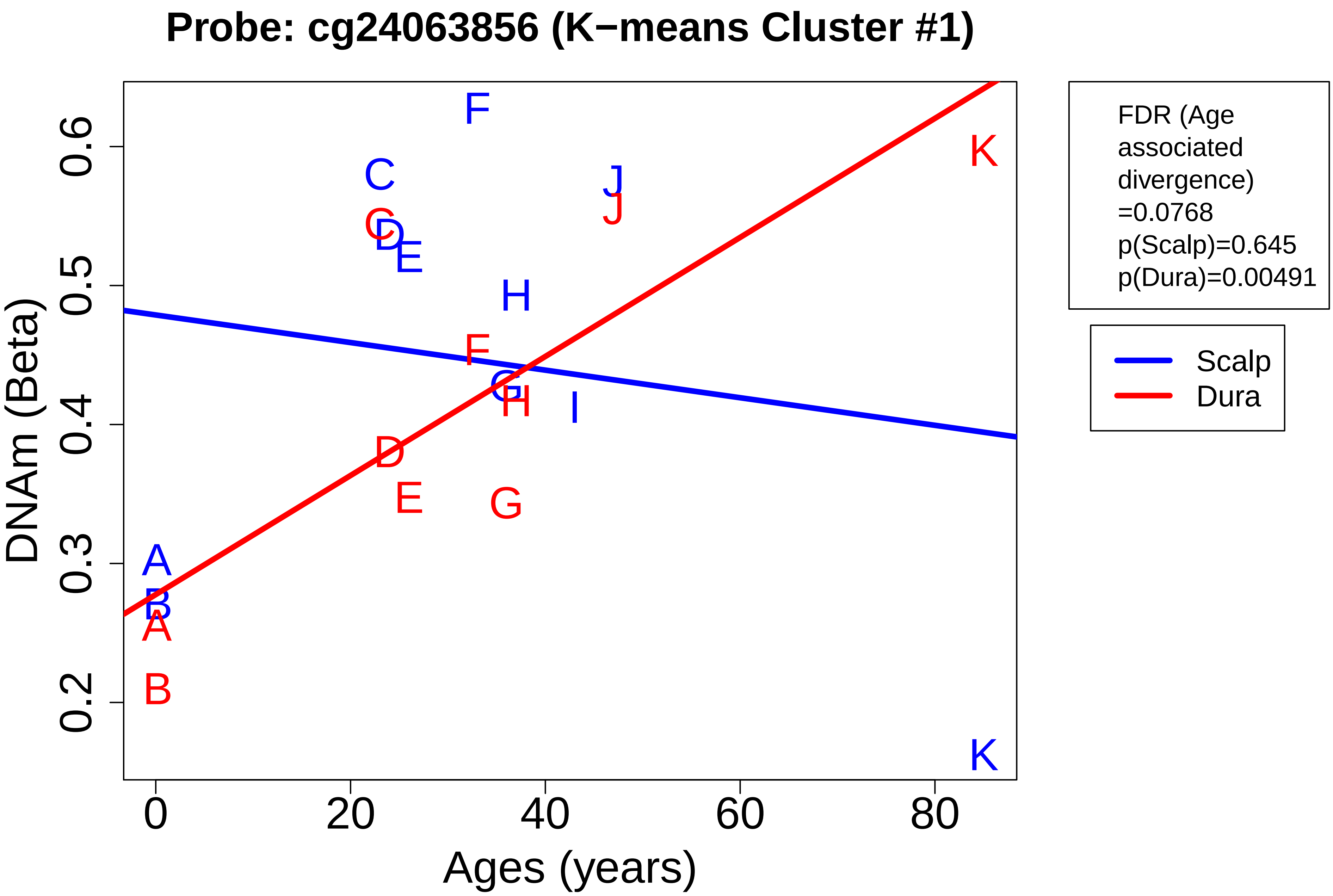

Probe: cg24063856 (K−means Cluster #1)
FDR (Age associated divergence) =0.0768
p(Scalp)=0.645
p(Dura)=0.00491
Scalp
Dura
DNAm (Beta)
Ages (years)
0.2
0.3
0.4
0.5
0.6
0
20
40
60
80

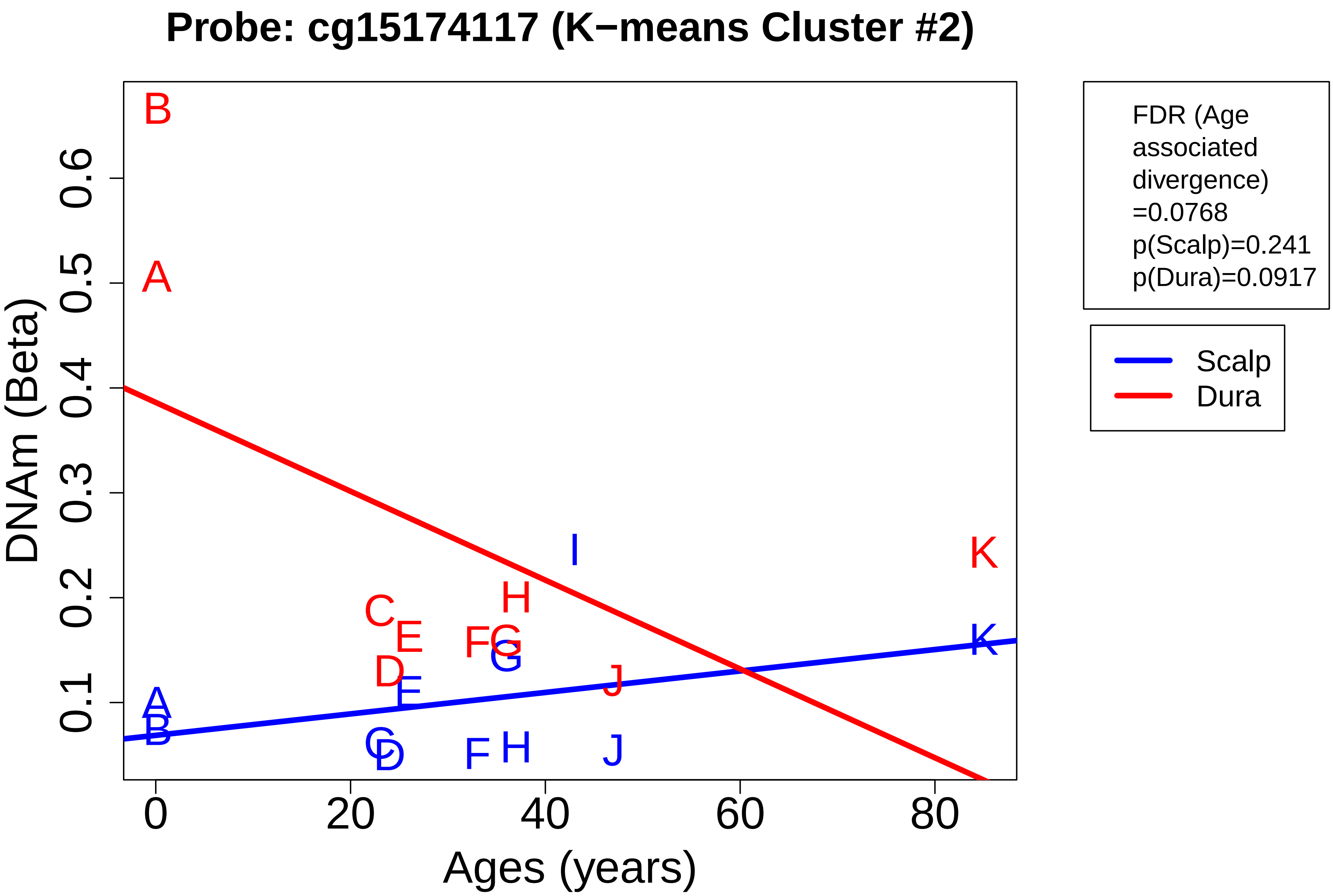
Probe: cg15174117 (K−means Cluster #2)
DNAm (Beta)
0.1
0.2
0.3
0.4
0.5
0.6
0
20
40
60
80
Ages (years)
A
B
C
D
E
F
G
H
I
J
K
FDR (Age associated divergence) =0.0768
p(Scalp)=0.241
p(Dura)=0.0917
Scalp
Dura

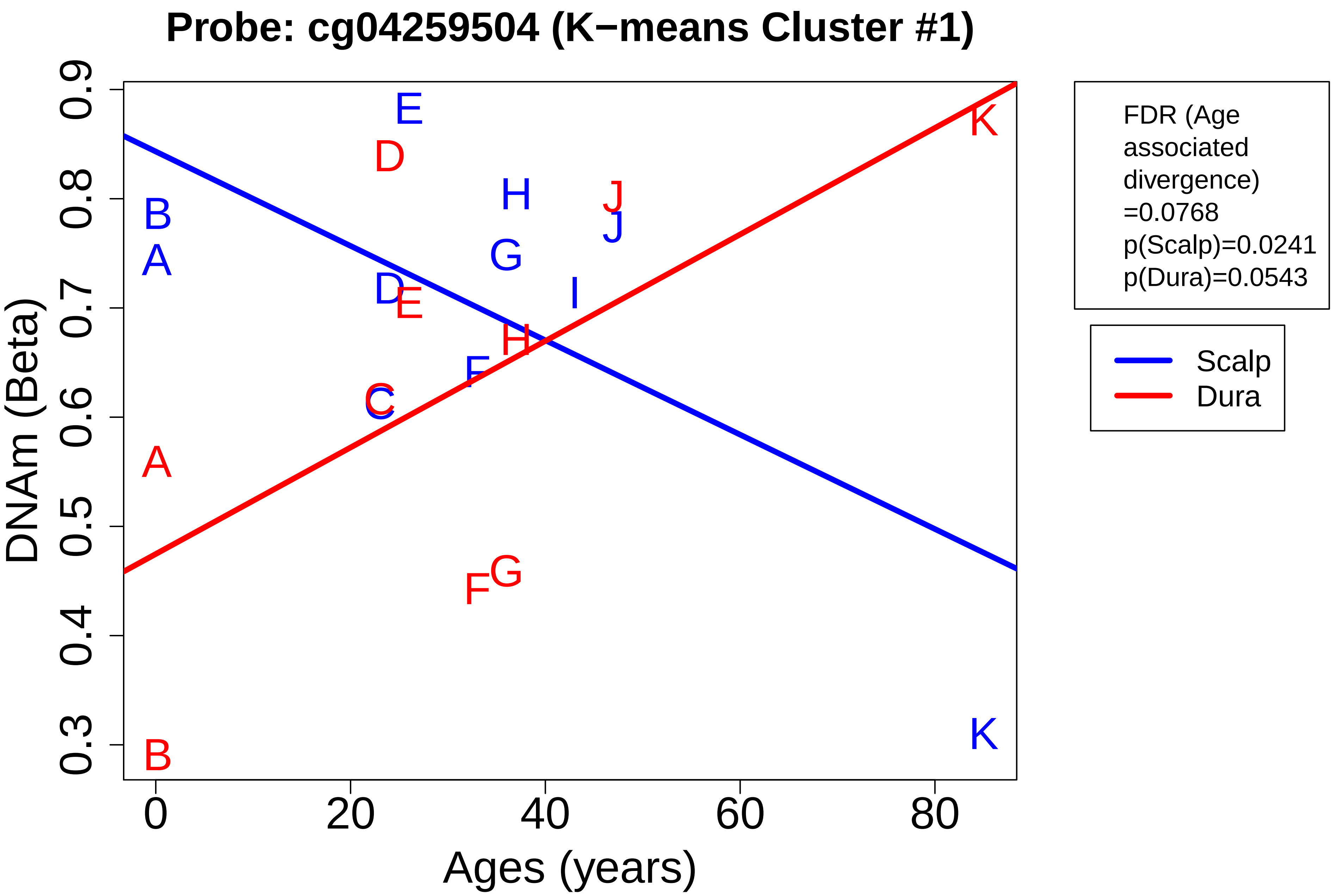

Probe: cg04259504 (K−means Cluster #1)
DNAm (Beta)
0.3
0.4
0.5
0.6
0.7
0.8
0.9
Ages (years)
0
20
40
60
80
FDR (Age associated divergence) =0.0768
p(Scalp)=0.0241
p(Dura)=0.0543
Scalp
Dura
A
B
C
D
E
F
G
H
I
J
K

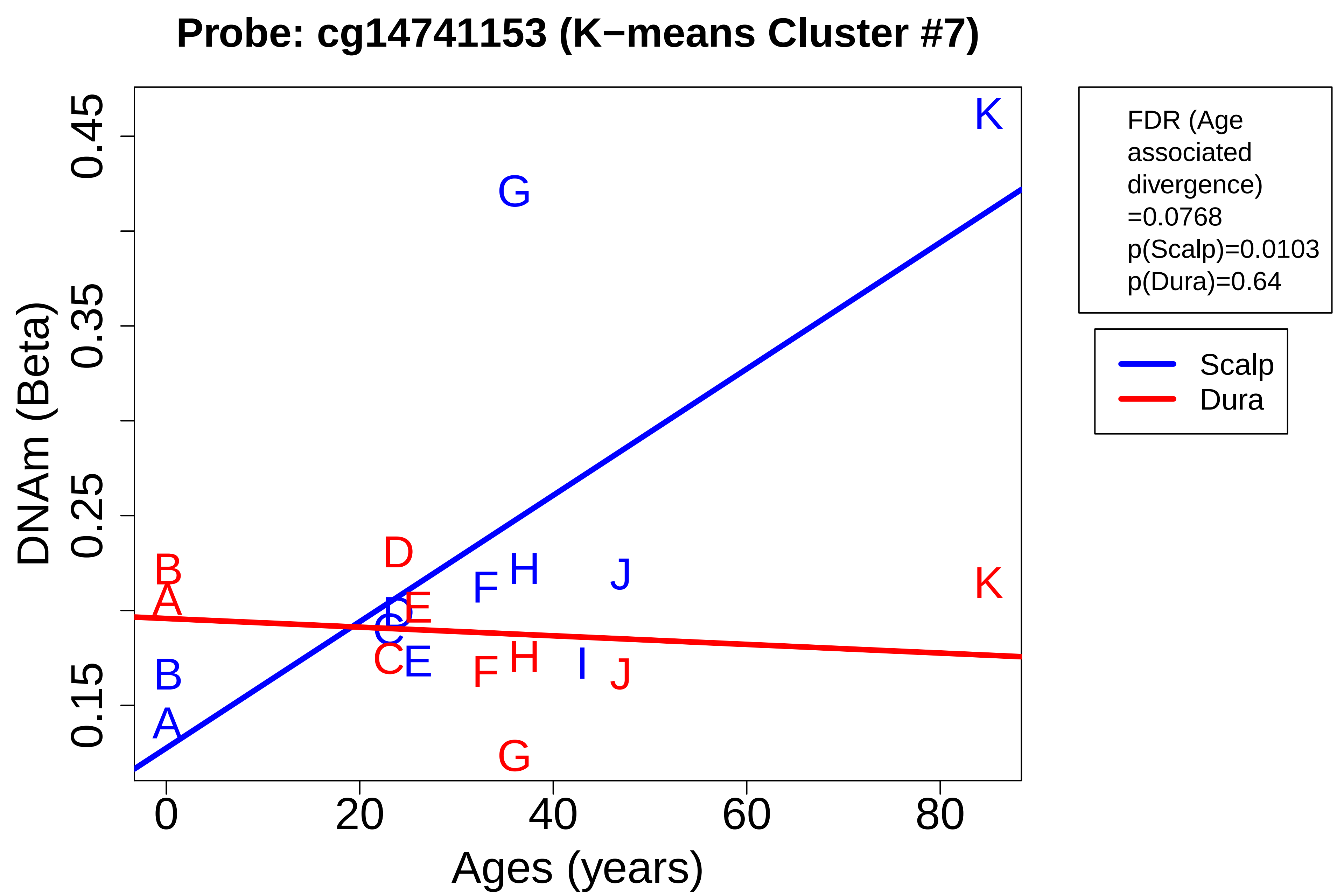

Probe: cg14741153 (K−means Cluster #7)
DNAm (Beta)
0.15
0.25
0.35
0.45
0
20
40
60
80
Ages (years)
FDR (Age associated divergence) =0.0768
p(Scalp)=0.0103
p(Dura)=0.64
Scalp
Dura
A
B
C
D
E
F
G
H
I
J
K

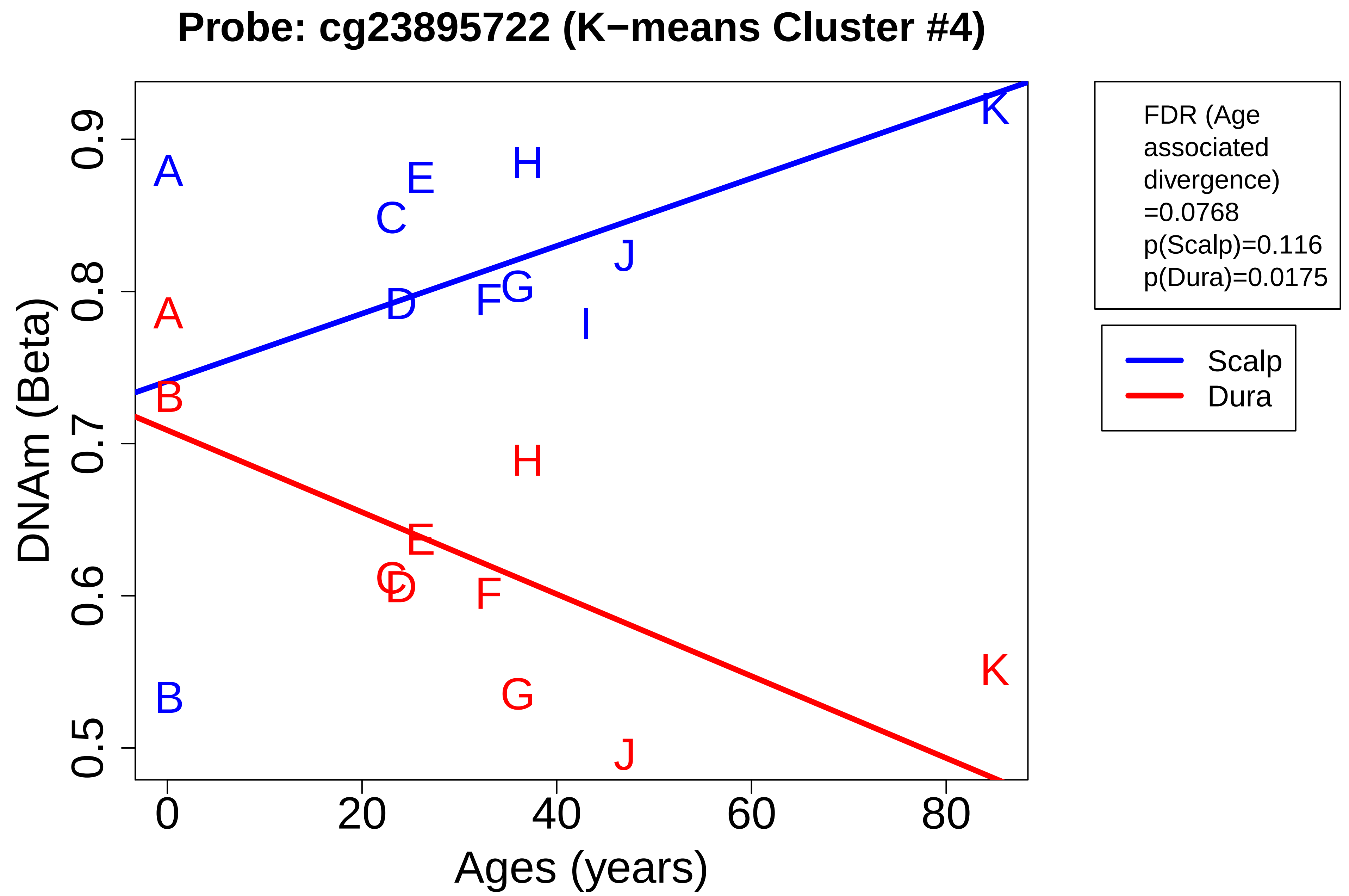

Probe: cg23895722 (K−means Cluster #4)
FDR (Age associated divergence) =0.0768
p(Scalp)=0.116
p(Dura)=0.0175
Scalp
Dura
DNAm (Beta)
0.5
0.6
0.7
0.8
0.9
0
20
40
60
80
Ages (years)
A
B
C
D
E
F
G
H
I
J
K

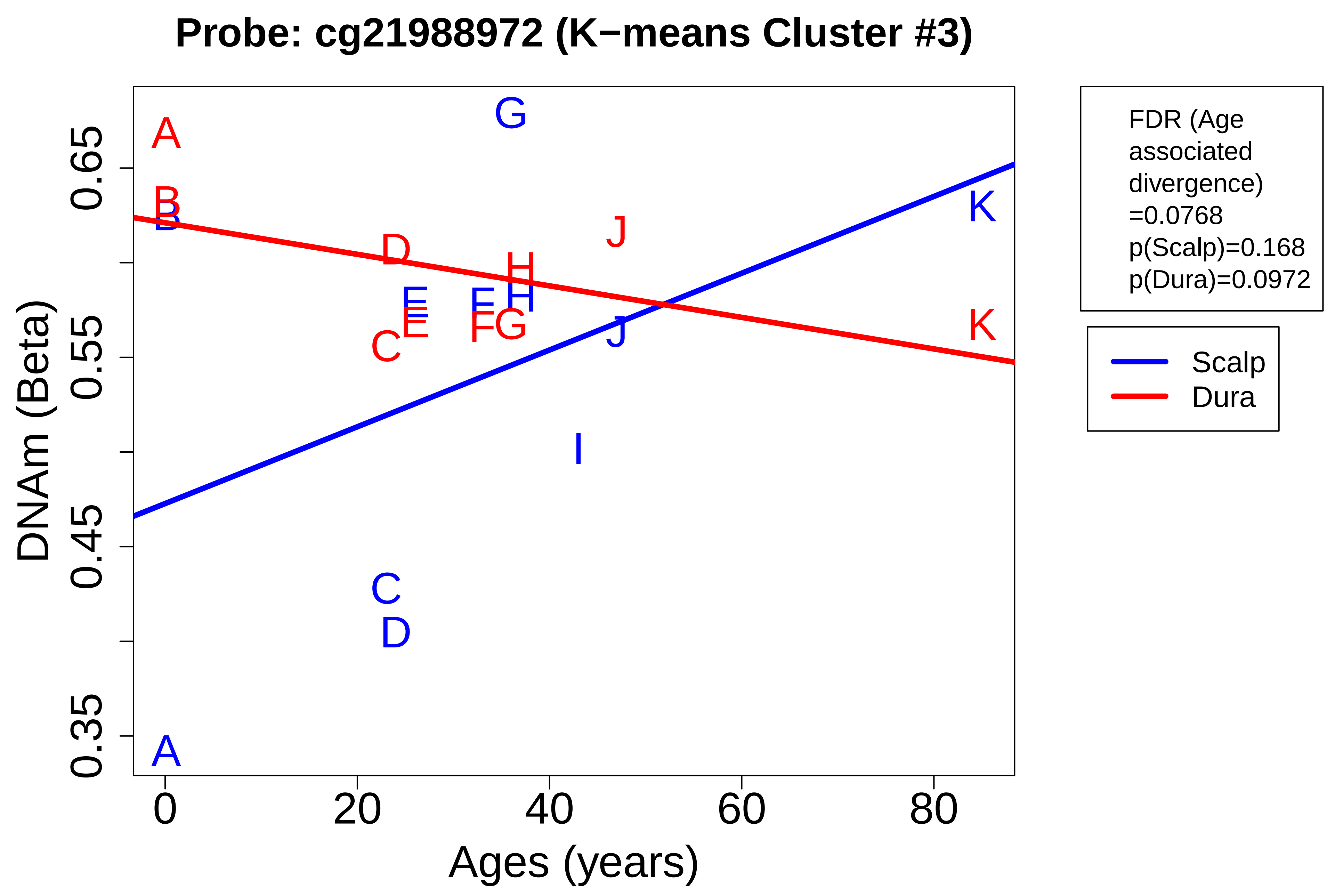

Probe: cg21988972 (K−means Cluster #3)
DNAm (Beta)
0.35
0.45
0.55
0.65
Ages (years)
0
20
40
60
80
A
B
C
D
E
F
G
H
I
J
K
FDR (Age associated divergence) =0.0768
p(Scalp)=0.168
p(Dura)=0.0972
Scalp
Dura

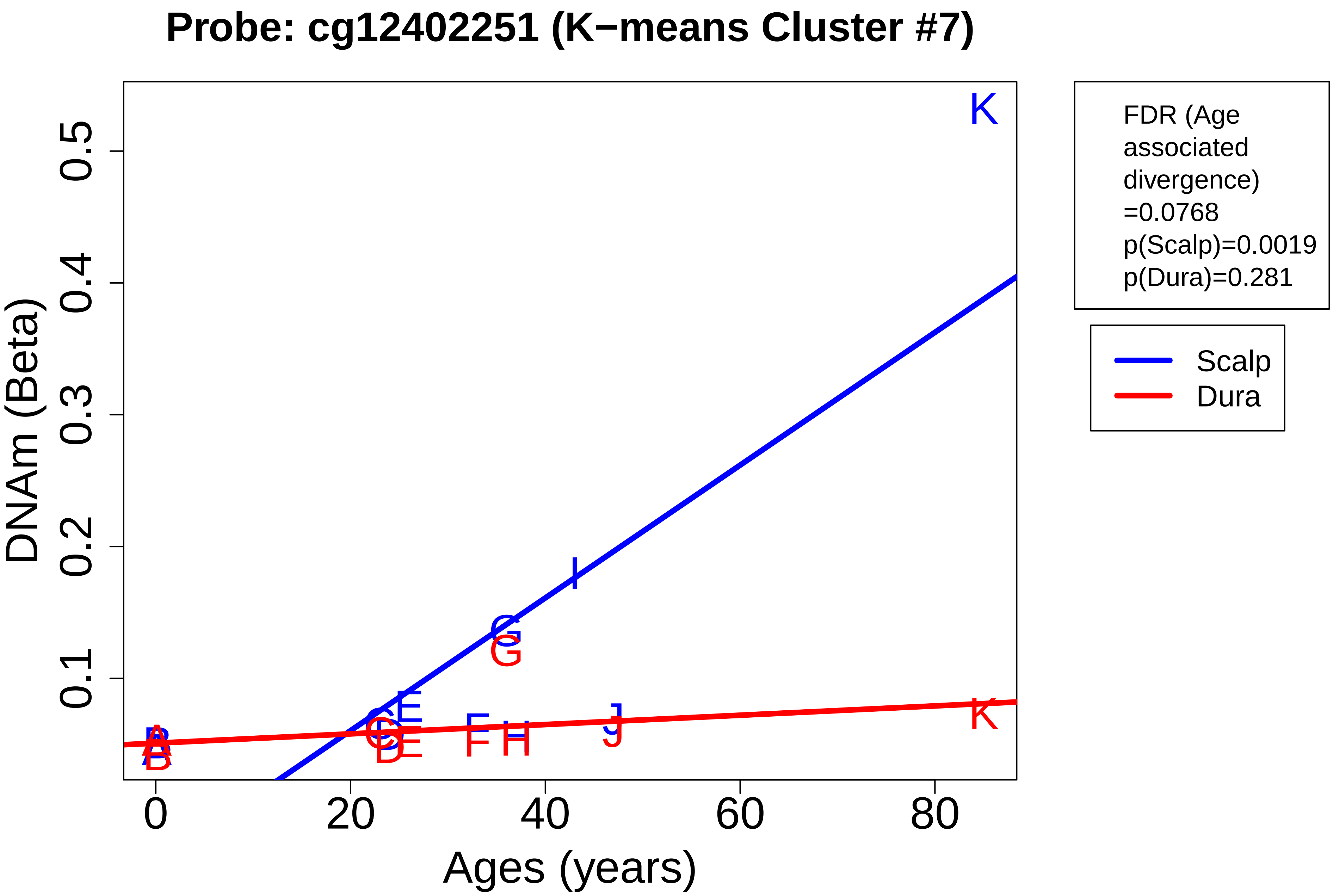

Probe: cg12402251 (K−means Cluster #7)
DNAm (Beta)
0.1
0.2
0.3
0.4
0.5
0
20
40
60
80
Ages (years)
FDR (Age associated divergence) =0.0768
p(Scalp)=0.0019
p(Dura)=0.281
Scalp
Dura

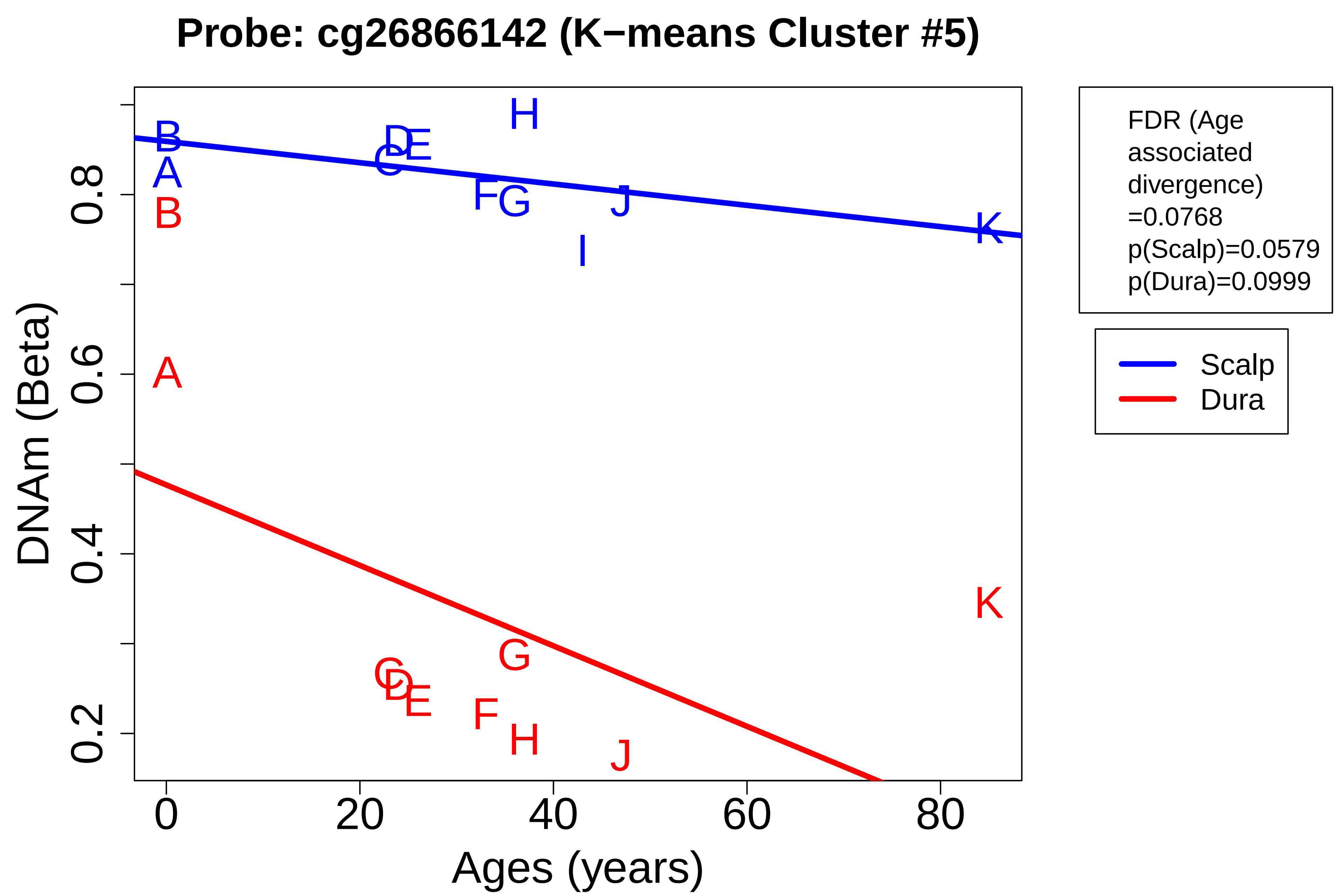

Probe: cg26866142 (K−means Cluster #5)
DNAm (Beta)
0.2
0.4
0.6
0.8
0
20
40
60
80
Ages (years)
A
B
C
D
E
F
G
H
I
J
K
FDR (Age associated divergence) =0.0768
p(Scalp)=0.0579
p(Dura)=0.0999
Scalp
Dura

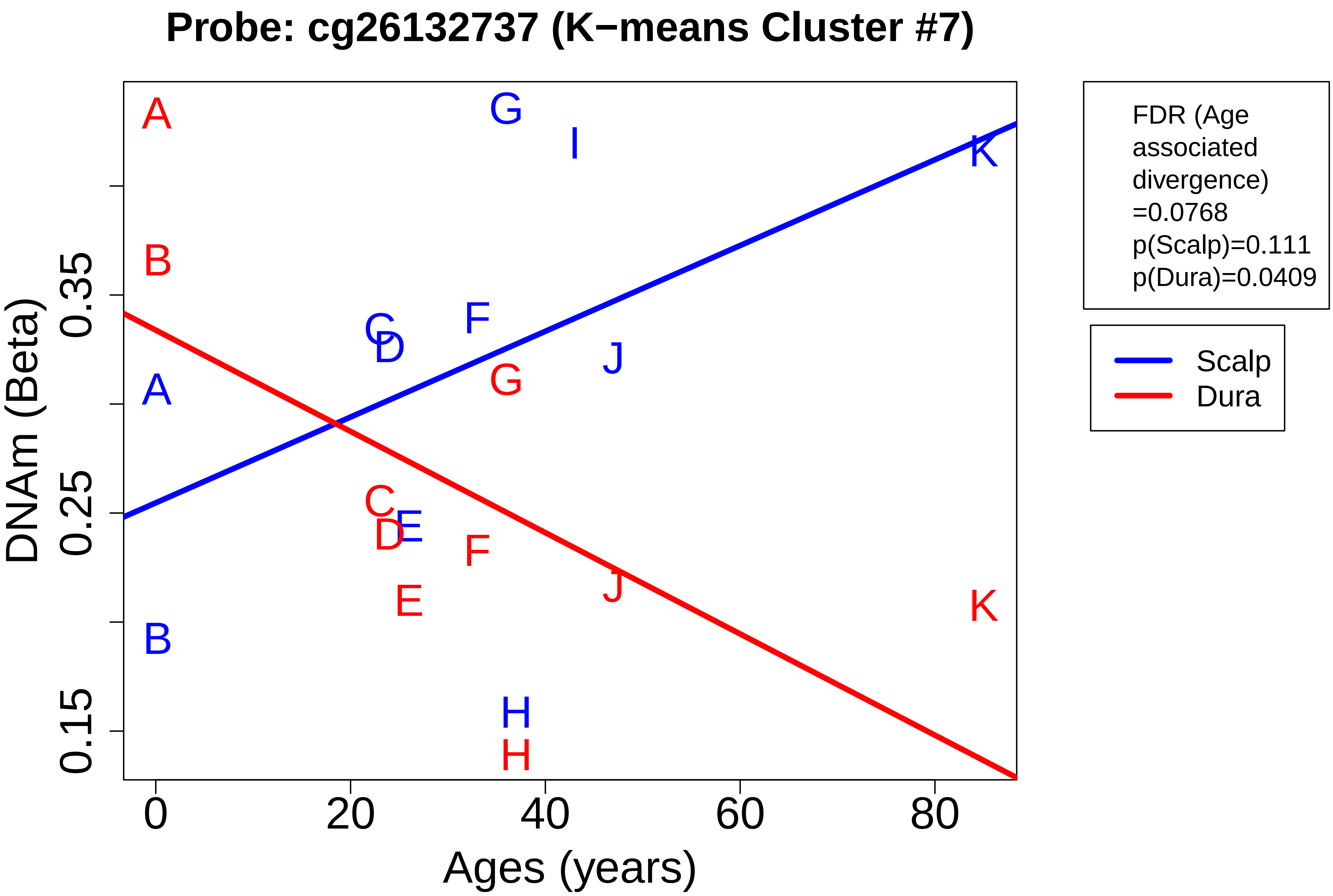

Probe: cg26132737 (K−means Cluster #7)
FDR (Age associated divergence) =0.0768
p(Scalp)=0.111
p(Dura)=0.0409
Scalp
Dura
DNAm (Beta)
0.15
0.25
0.35
0
20
40
60
80
Ages (years)
A
B
C
D
E
F
G
H
I
J
K

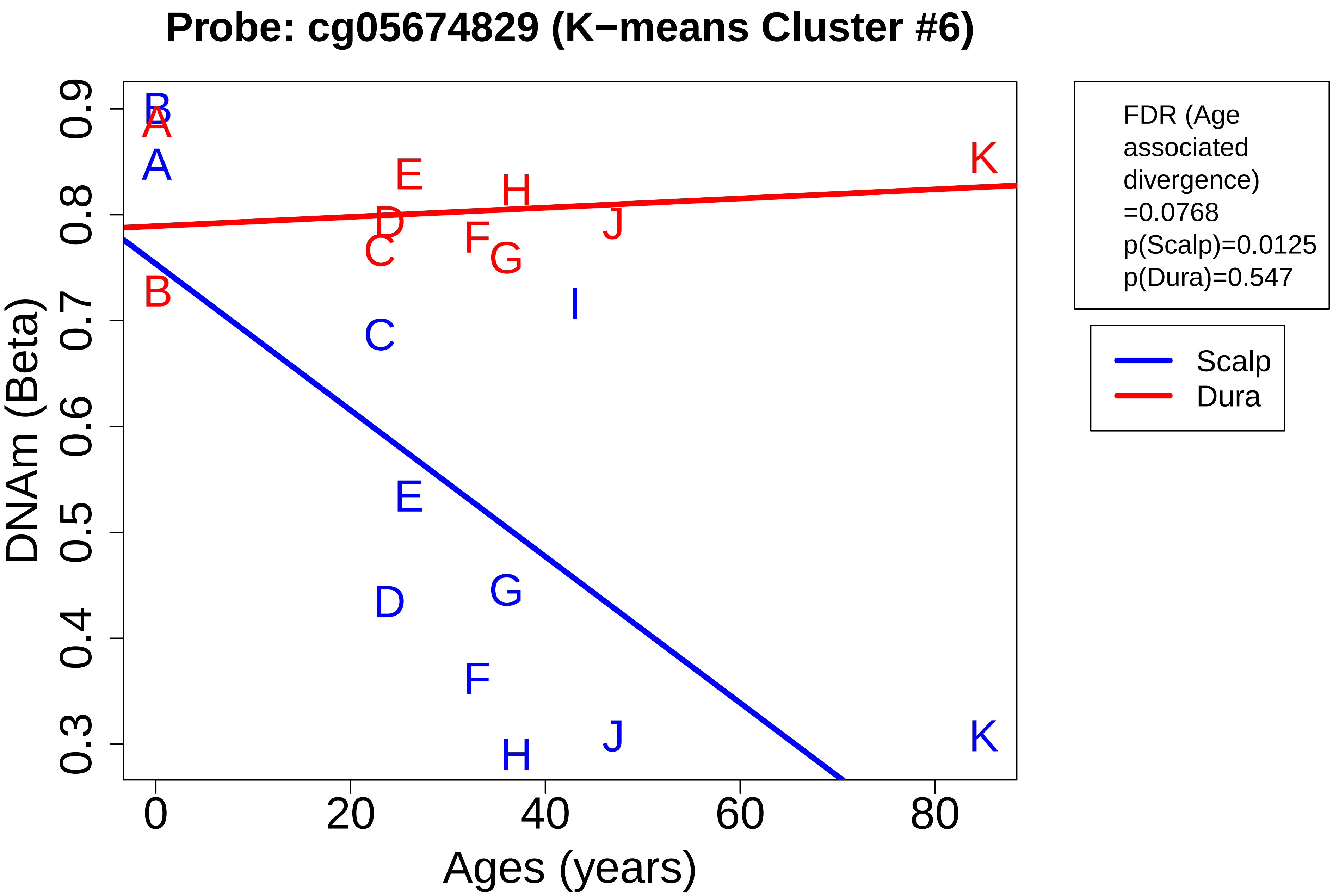

Probe: cg05674829 (K−means Cluster #6)
DNAm (Beta)
0.3
0.4
0.5
0.6
0.7
0.8
0.9
0
20
40
60
80
Ages (years)
A
B
C
D
E
F
G
H
I
J
K
FDR (Age associated divergence) =0.0768
p(Scalp)=0.0125
p(Dura)=0.547
Scalp
Dura

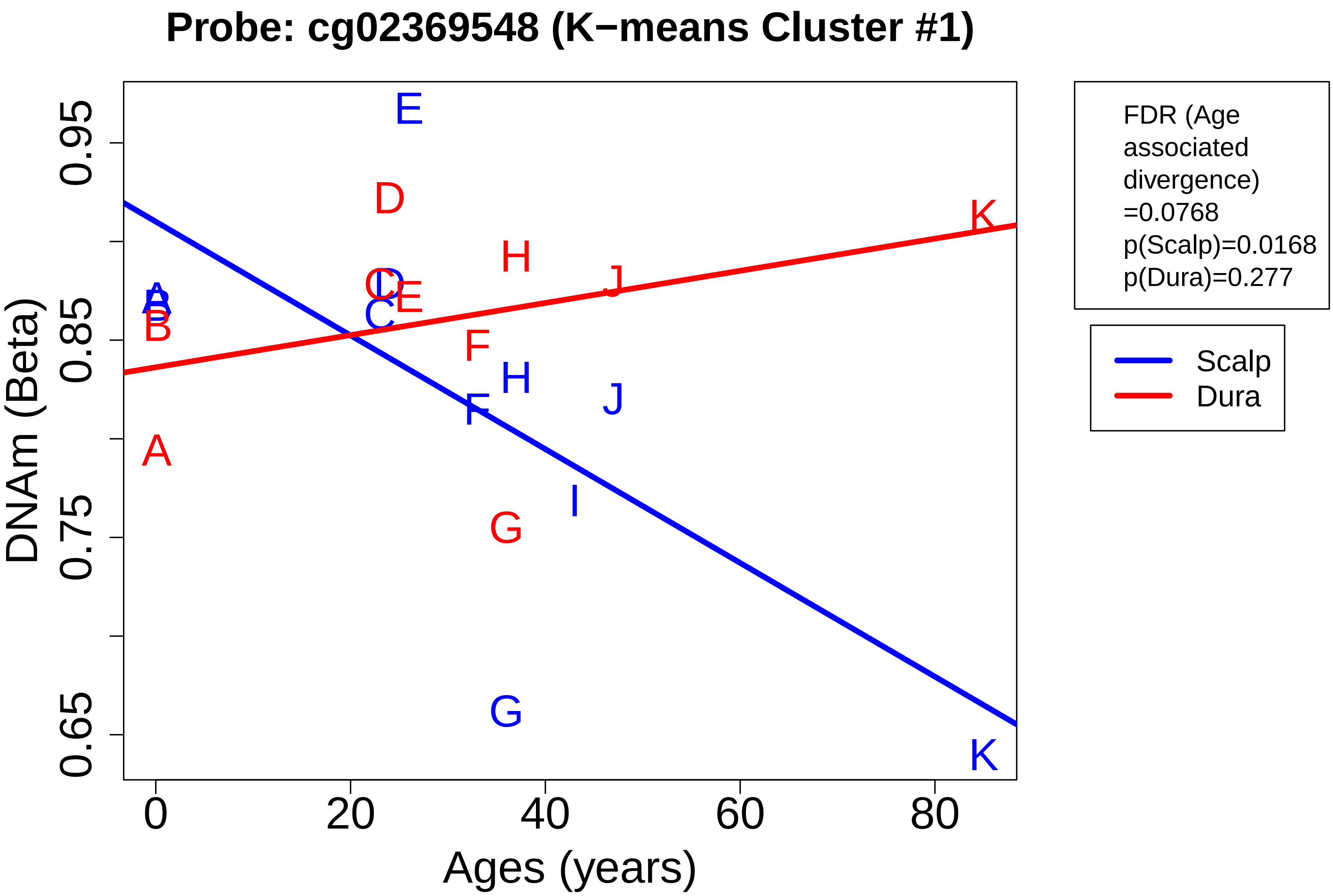

Probe: cg02369548 (K−means Cluster #1)
DNAm (Beta)
0.65
0.75
0.85
0.95
0
20
40
60
80
Ages (years)
FDR (Age associated divergence) =0.0768
p(Scalp)=0.0168
p(Dura)=0.277
Scalp
Dura

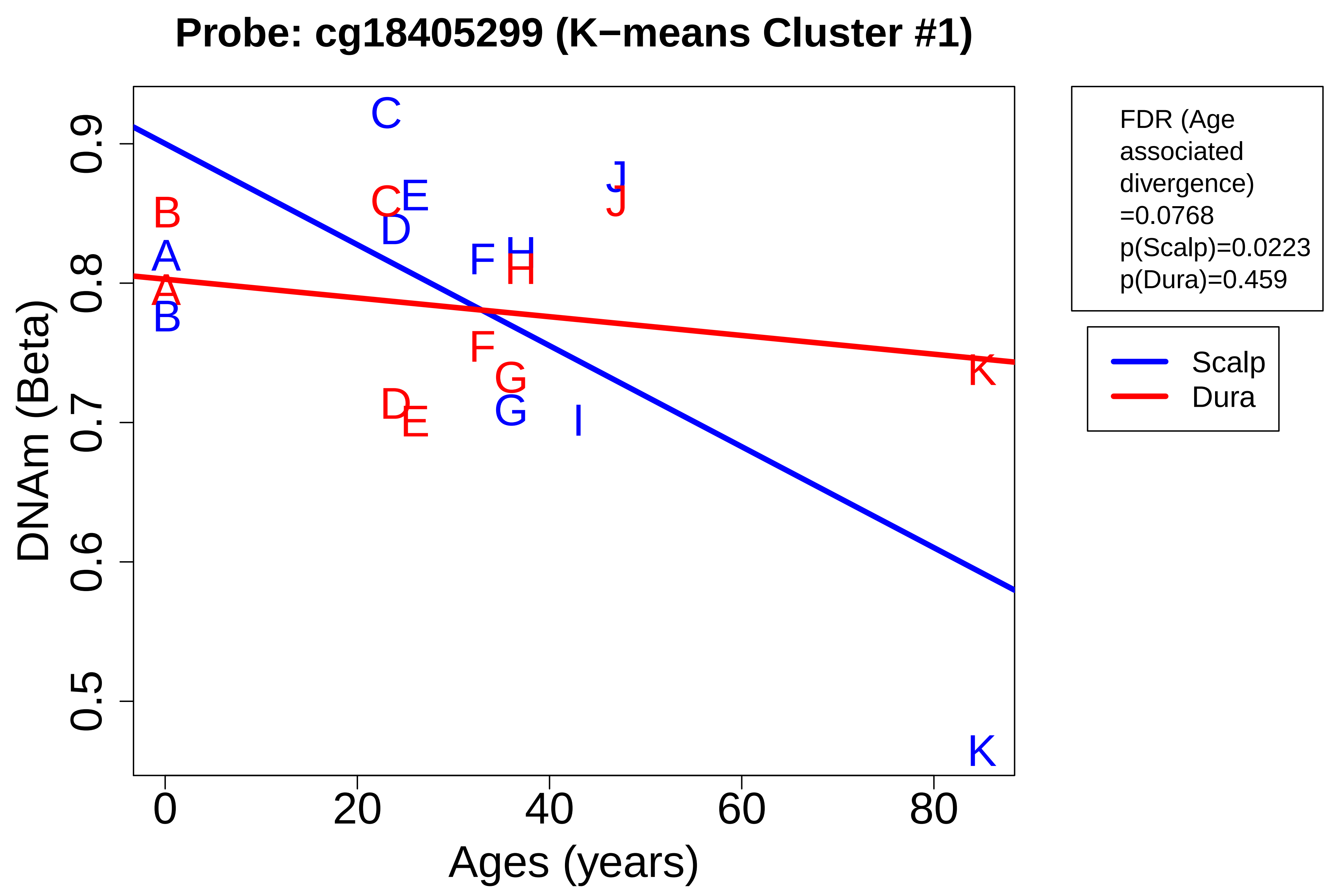

Probe: cg18405299 (K−means Cluster #1)
DNAm (Beta)
0.5
0.6
0.7
0.8
0.9
0
20
40
60
80
Ages (years)
A
B
C
D
E
F
G
H
I
J
K
FDR (Age associated divergence) =0.0768
p(Scalp)=0.0223
p(Dura)=0.459
Scalp
Dura

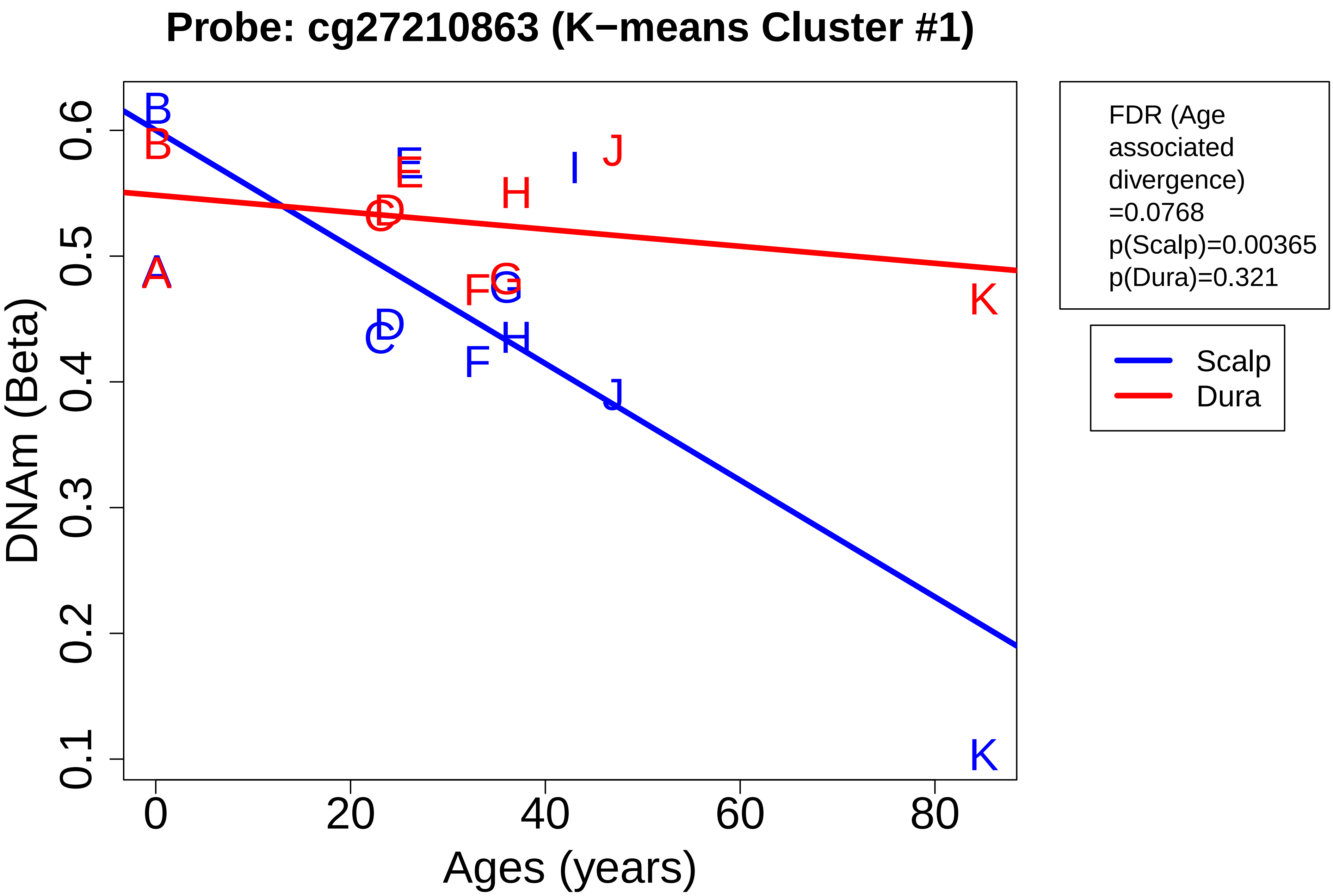

Probe: cg27210863 (K−means Cluster #1)
DNAm (Beta)
Ages (years)
0.1
0.2
0.3
0.4
0.5
0.6
0
20
40
60
80
FDR (Age associated divergence) =0.0768
p(Scalp)=0.00365
p(Dura)=0.321
Scalp
Dura
A
B
C
D
E
F
G
H
I
J
K

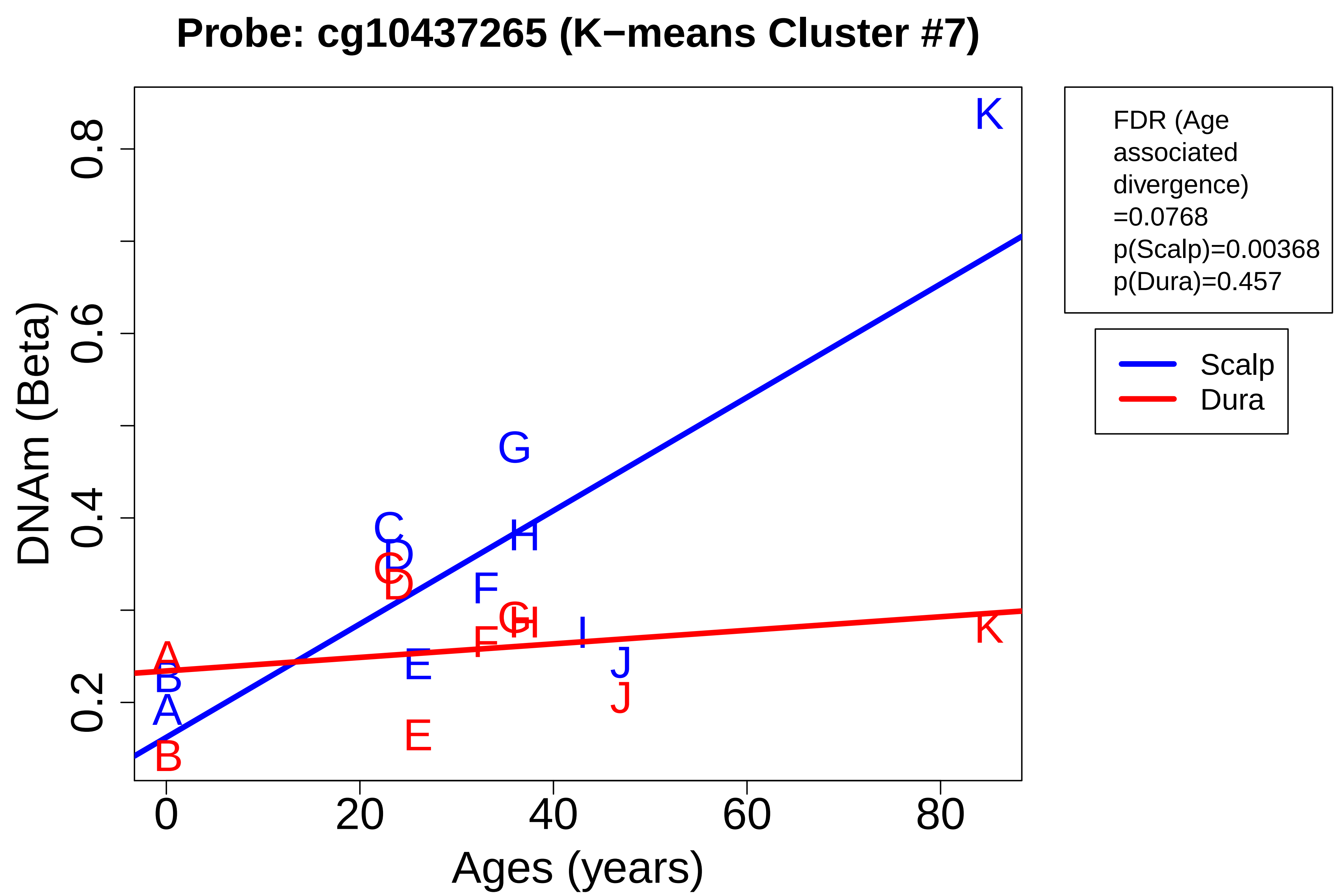

Probe: cg10437265 (K−means Cluster #7)
DNAm (Beta)
0.2
0.4
0.6
0.8
0
20
40
60
80
Ages (years)
FDR (Age associated divergence) =0.0768
p(Scalp)=0.00368
p(Dura)=0.457
Scalp
Dura

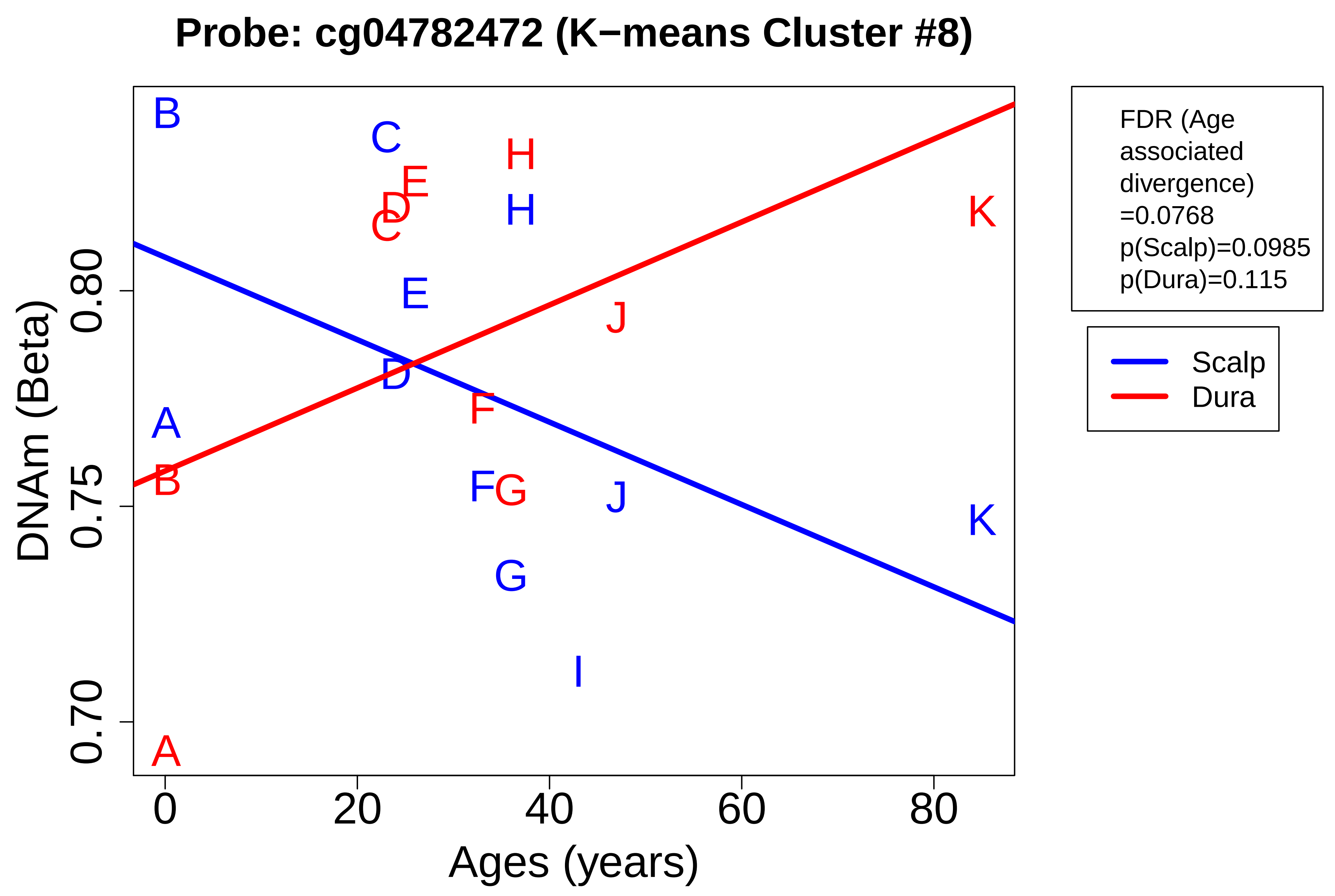

Probe: cg04782472 (K−means Cluster #8)
DNAm (Beta)
0.70
0.75
0.80
Ages (years)
0
20
40
60
80
FDR (Age associated divergence) =0.0768
p(Scalp)=0.0985
p(Dura)=0.115
Scalp
Dura

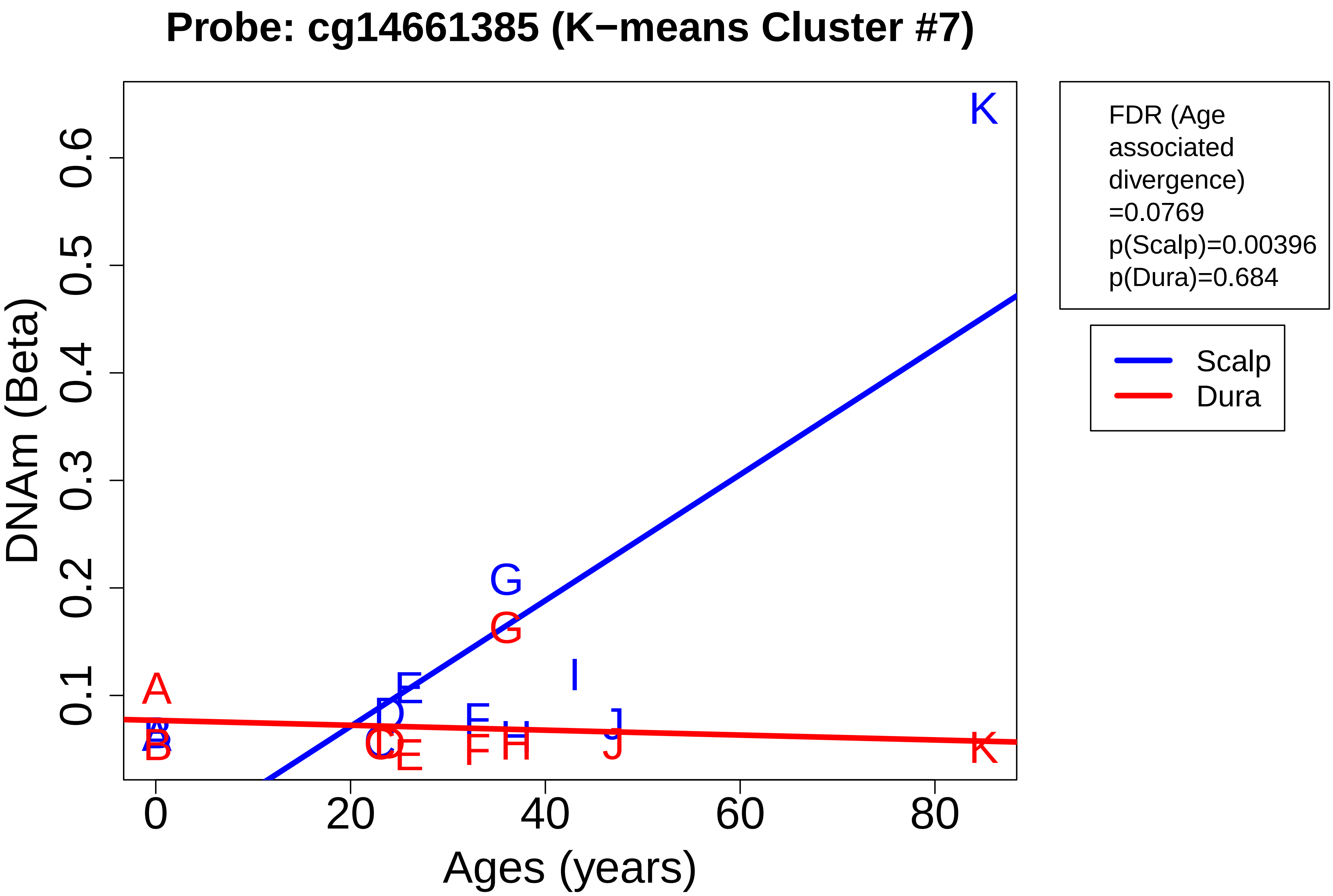

Probe: cg14661385 (K−means Cluster #7)
DNAm (Beta)
0.1
0.2
0.3
0.4
0.5
0.6
0
20
40
60
80
Ages (years)
FDR (Age associated divergence) =0.0769
p(Scalp)=0.00396
p(Dura)=0.684
Scalp
Dura

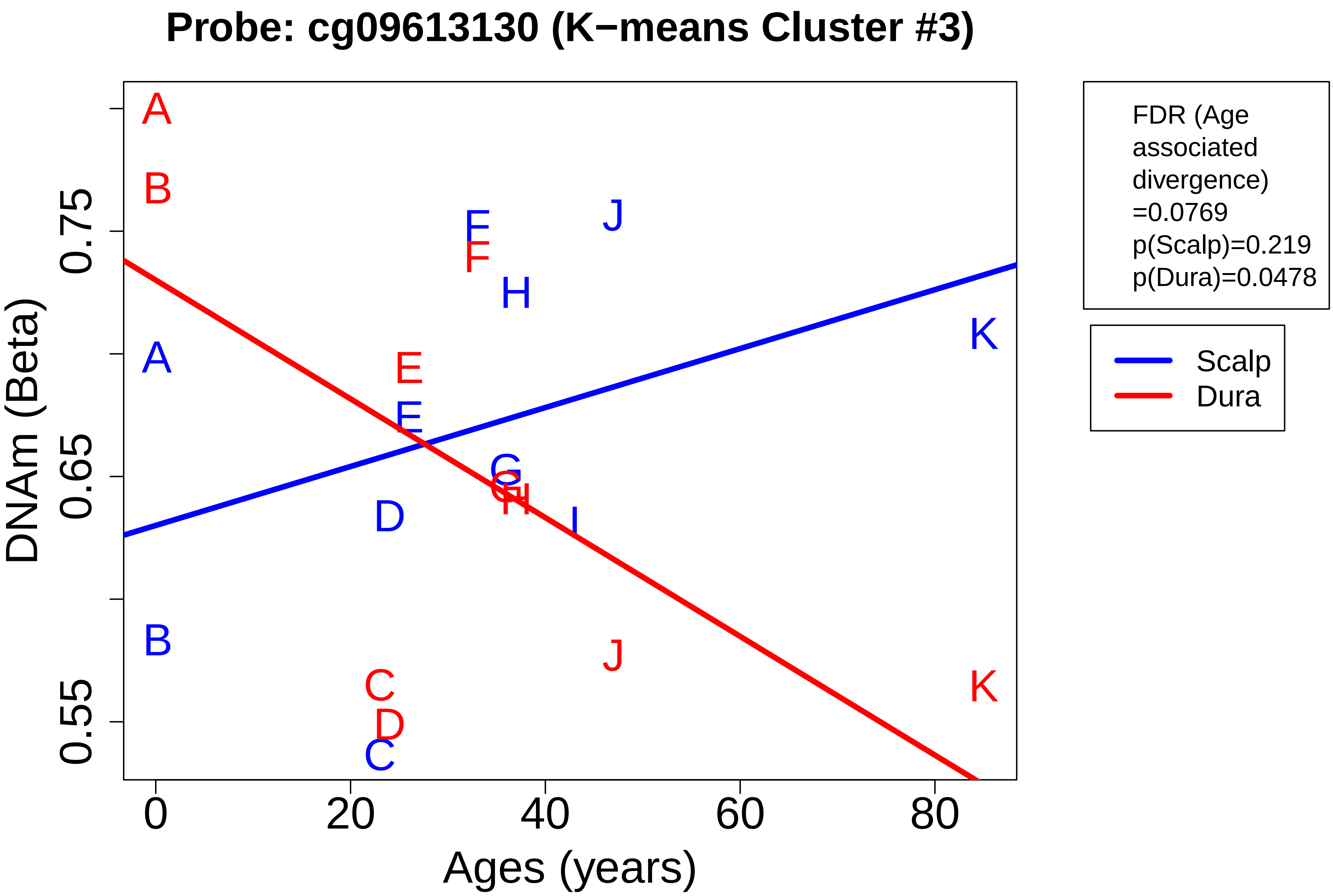

Probe: cg09613130 (K−means Cluster #3)
FDR (Age associated divergence) =0.0769
p(Scalp)=0.219
p(Dura)=0.0478
Scalp
Dura
DNAm (Beta)
0.55
0.65
0.75
Ages (years)
0
20
40
60
80

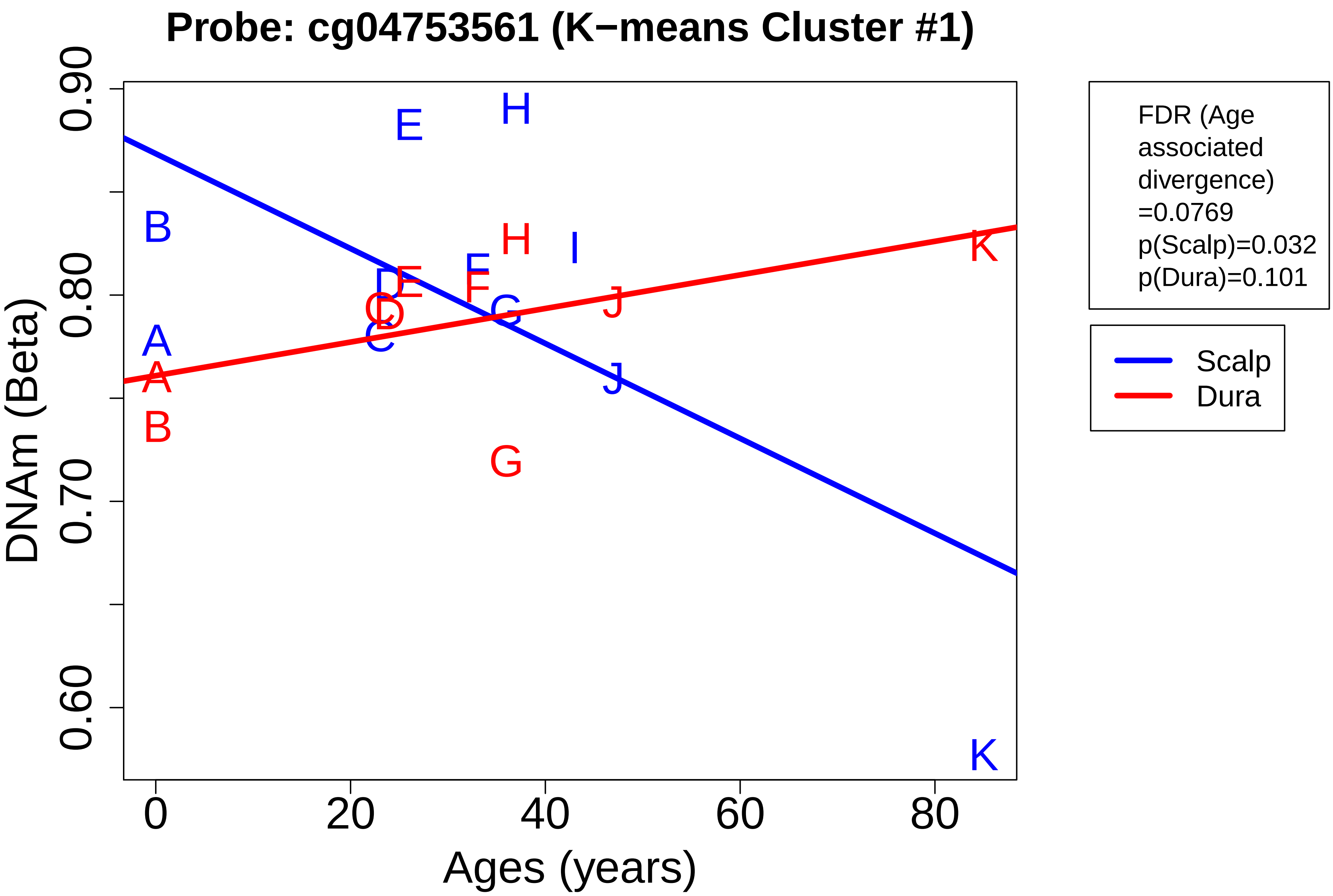

Probe: cg04753561 (K−means Cluster #1)
DNAm (Beta)
0.60
0.70
0.80
0.90
0
20
40
60
80
Ages (years)
FDR (Age associated divergence) =0.0769
p(Scalp)=0.032
p(Dura)=0.101
Scalp
Dura

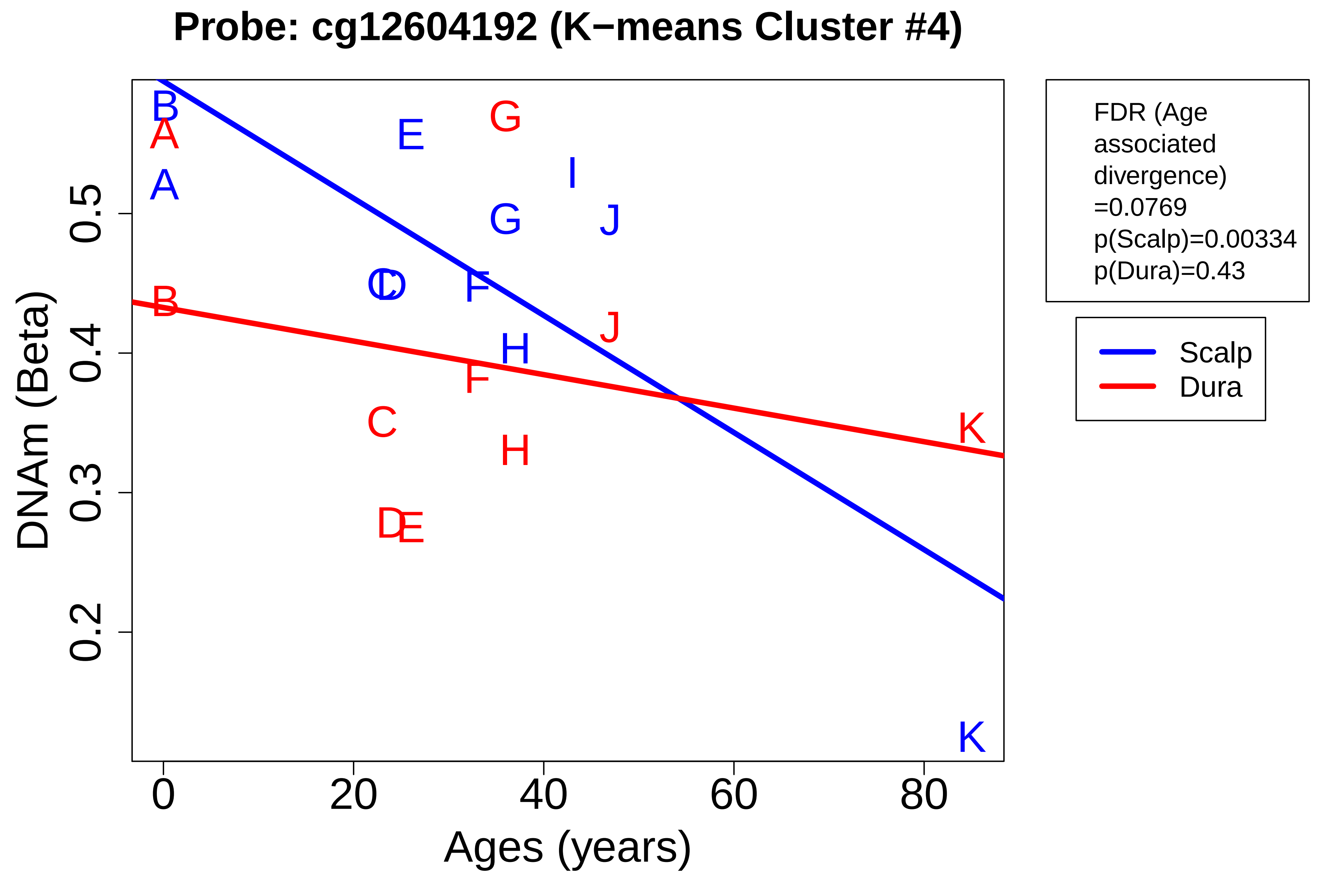

Probe: cg12604192 (K−means Cluster #4)
FDR (Age associated divergence) =0.0769
p(Scalp)=0.00334
p(Dura)=0.43
Scalp
Dura
DNAm (Beta)
Ages (years)
0.2
0.3
0.4
0.5
0
20
40
60
80
A
B
C
D
E
F
G
H
I
J
K

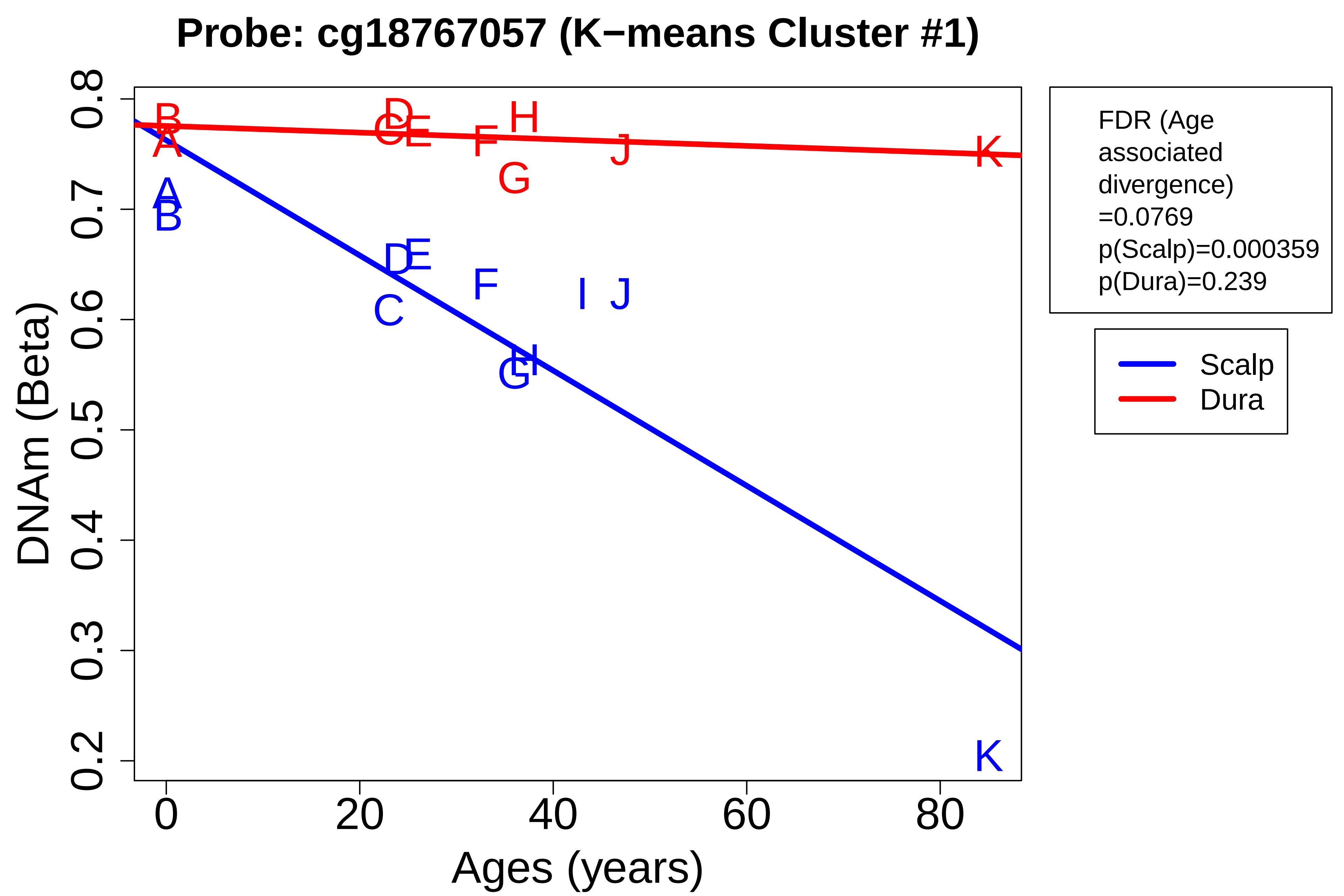

Probe: cg18767057 (K−means Cluster #1)
DNAm (Beta)
0.2
0.3
0.4
0.5
0.6
0.7
0.8
Ages (years)
0
20
40
60
80
FDR (Age associated divergence) =0.0769
p(Scalp)=0.000359
p(Dura)=0.239
Scalp
Dura

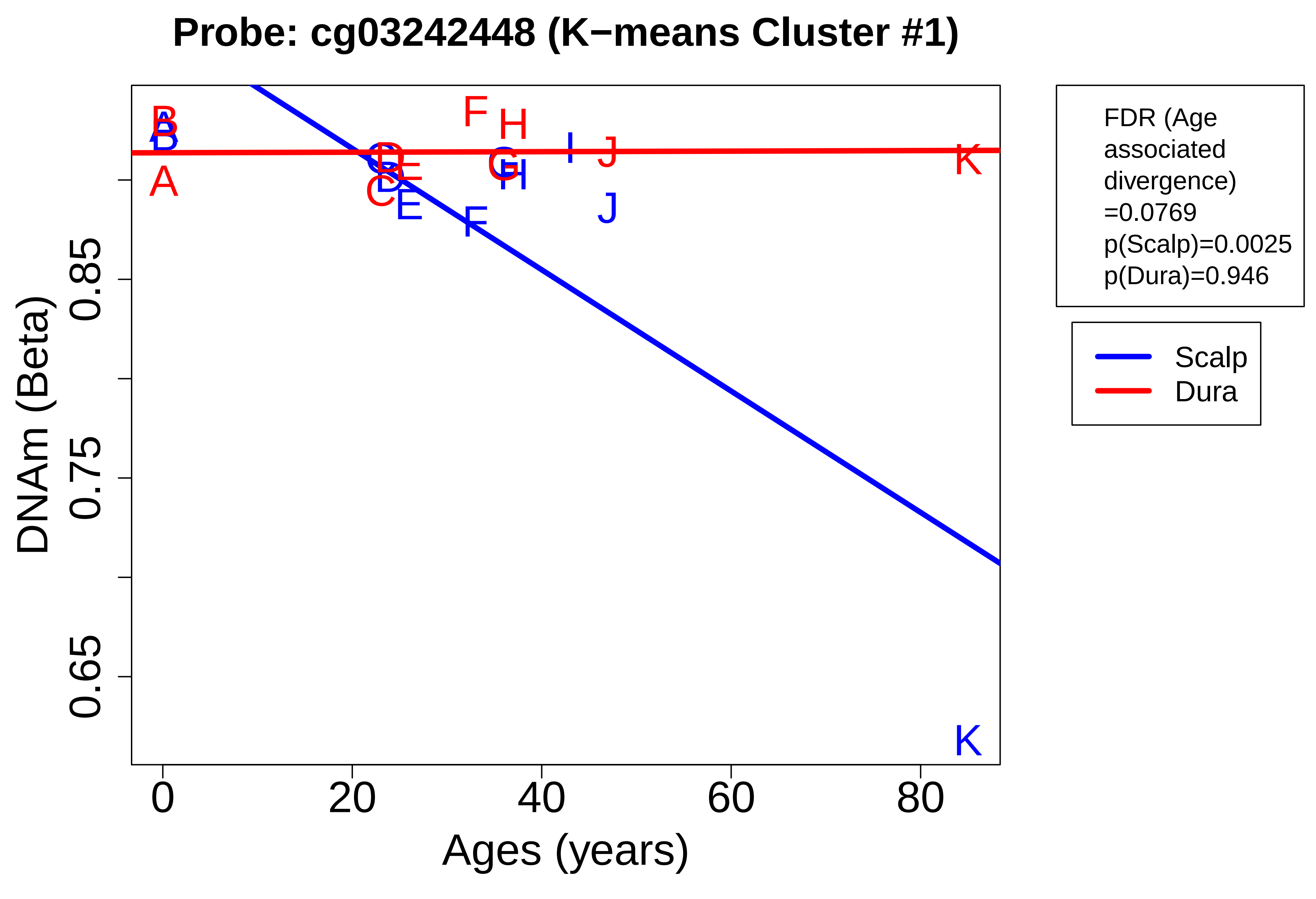

Probe: cg03242448 (K−means Cluster #1)
DNAm (Beta)
0.65
0.75
0.85
Ages (years)
0
20
40
60
80
FDR (Age associated divergence) =0.0769
p(Scalp)=0.0025
p(Dura)=0.946
Scalp
Dura

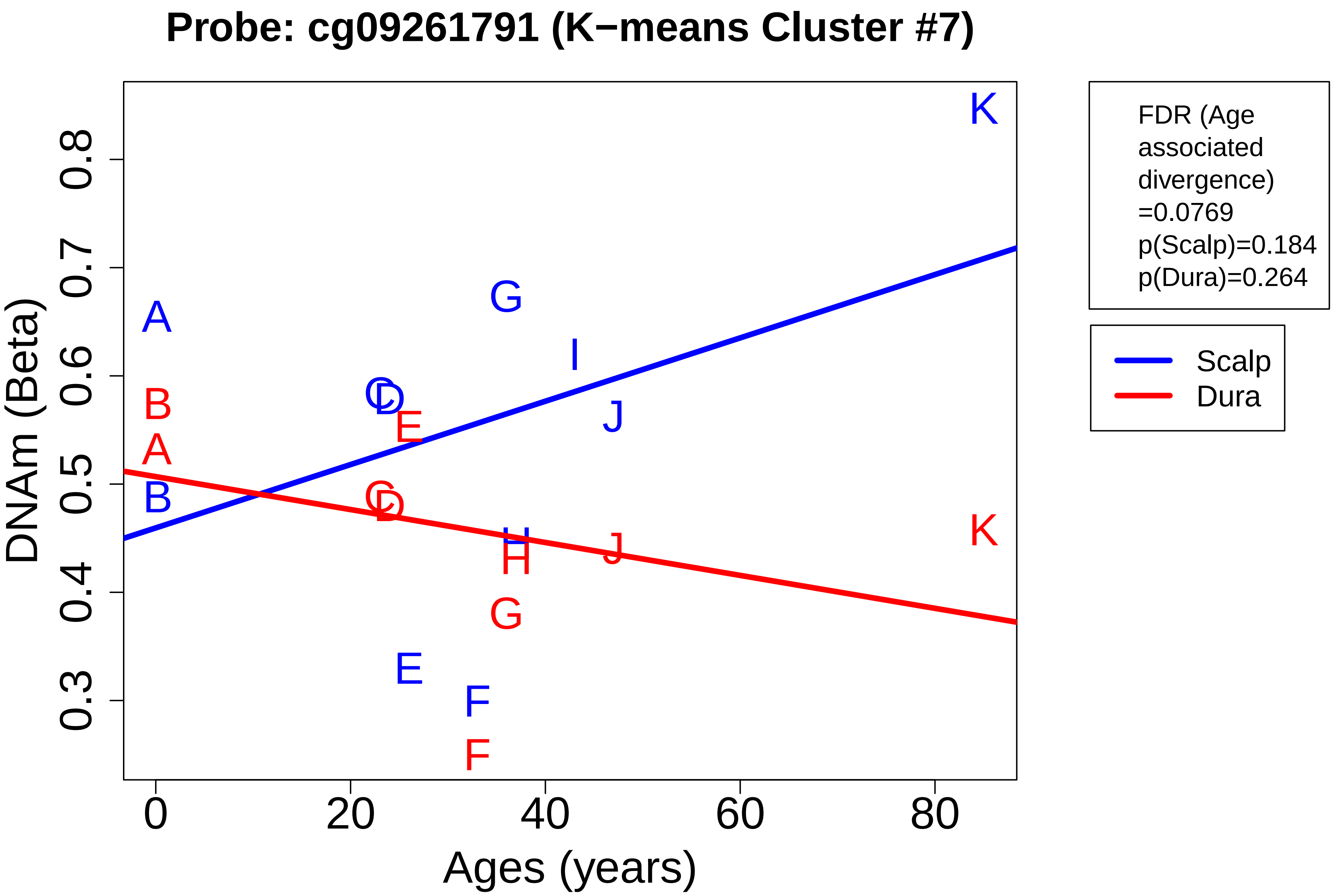

Probe: cg09261791 (K−means Cluster #7)
DNAm (Beta)
0.3
0.4
0.5
0.6
0.7
0.8
0
20
40
60
80
Ages (years)
A
B
C
D
E
F
G
H
I
J
K
FDR (Age associated divergence) =0.0769
p(Scalp)=0.184
p(Dura)=0.264
Scalp
Dura

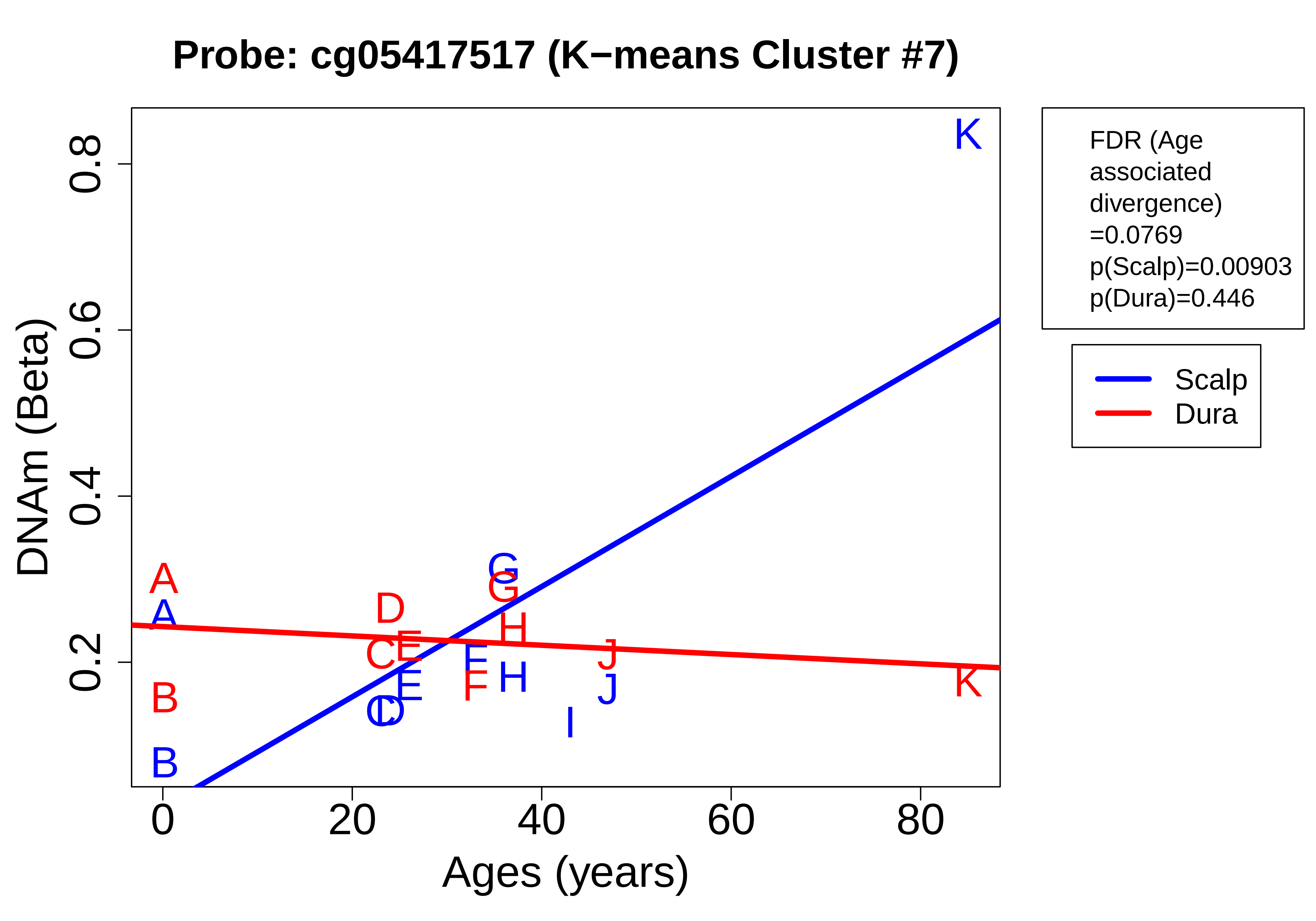
Probe: cg05417517 (K−means Cluster #7)
DNAm (Beta)
0.2
0.4
0.6
0.8
0
20
40
60
80
Ages (years)
FDR (Age associated divergence) =0.0769
p(Scalp)=0.00903
p(Dura)=0.446
Scalp
Dura
A
B
C
D
E
F
G
H
I
J
K

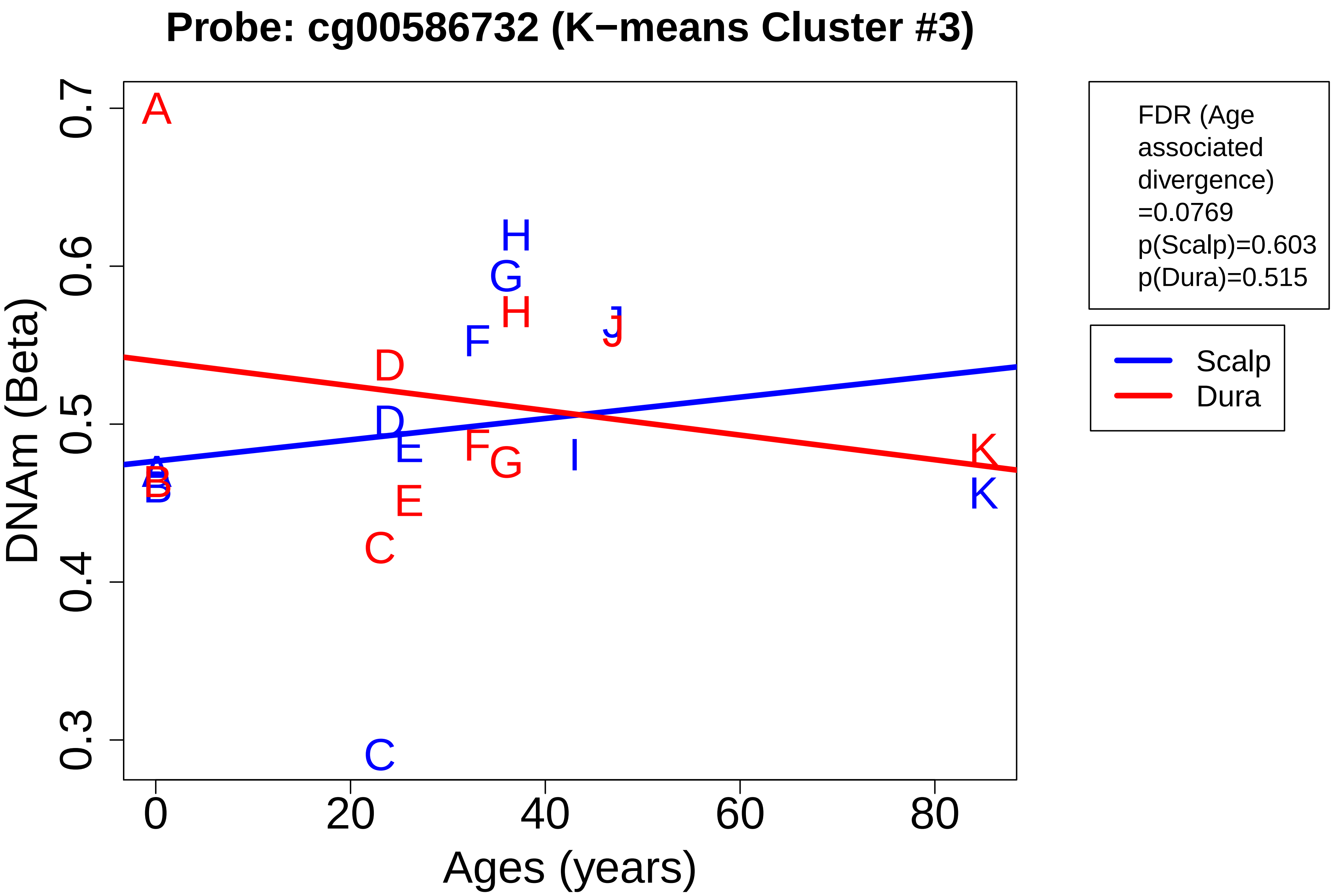

Probe: cg00586732 (K−means Cluster #3)
DNAm (Beta)
0.3
0.4
0.5
0.6
0.7
0
20
40
60
80
Ages (years)
A
B
C
D
E
F
G
H
I
J
K
FDR (Age associated divergence) =0.0769
p(Scalp)=0.603
p(Dura)=0.515
Scalp
Dura

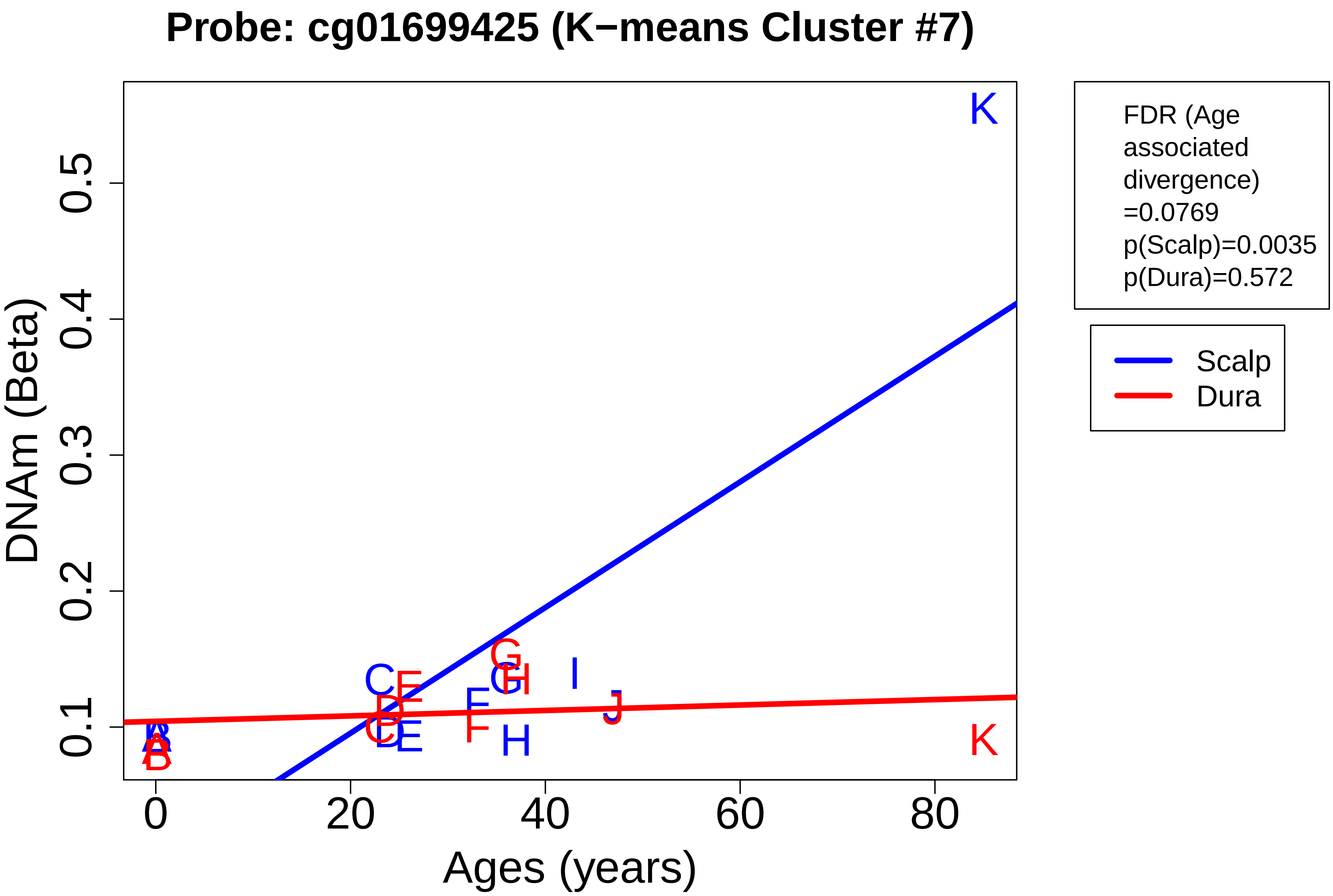

Probe: cg01699425 (K−means Cluster #7)
DNAm (Beta)
0.1
0.2
0.3
0.4
0.5
0
20
40
60
80
Ages (years)
FDR (Age associated divergence) =0.0769
p(Scalp)=0.0035
p(Dura)=0.572
Scalp
Dura

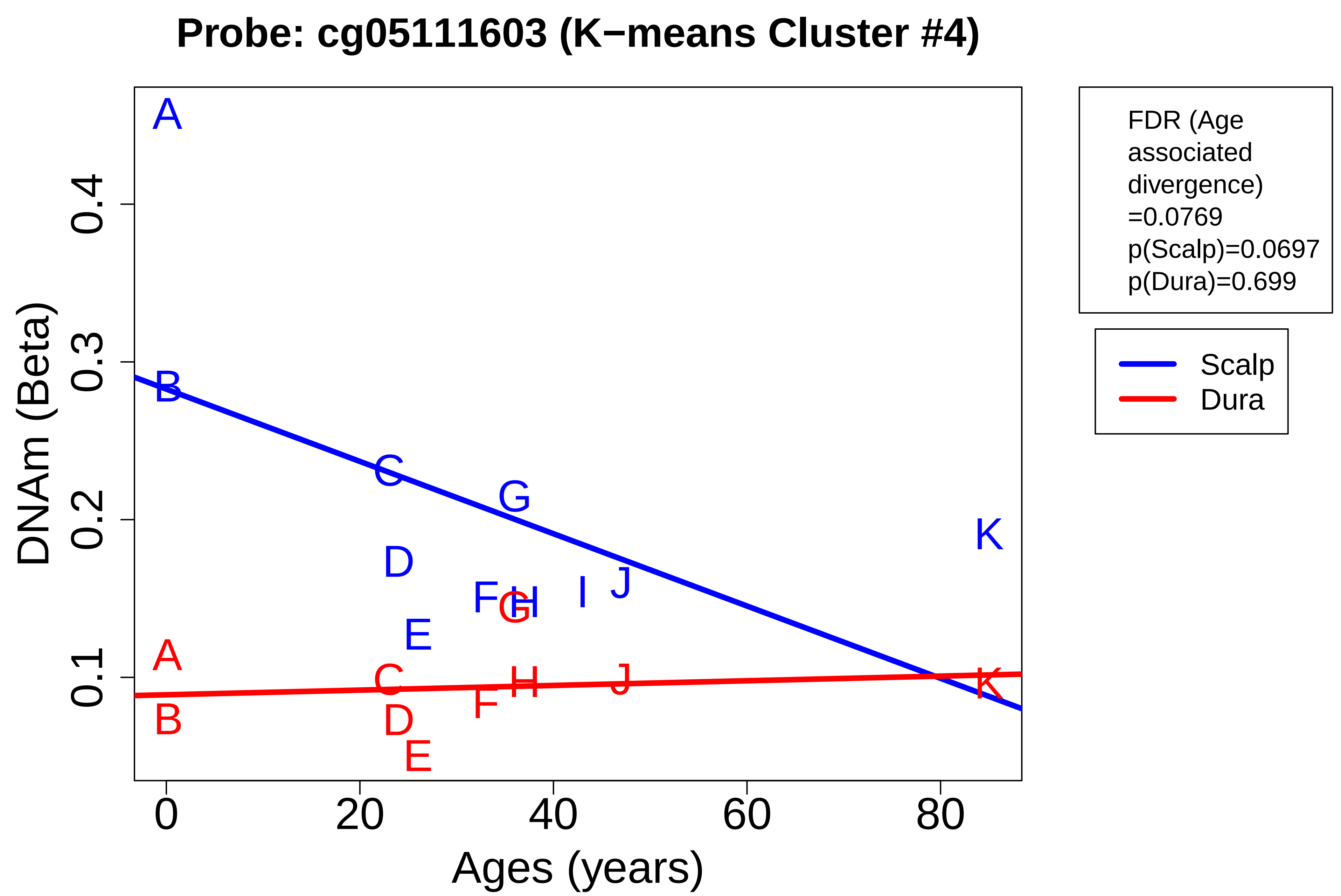
Probe: cg05111603 (K−means Cluster #4)
DNAm (Beta)
0.1
0.2
0.3
0.4
Ages (years)
0
20
40
60
80
FDR (Age associated divergence) =0.0769
p(Scalp)=0.0697
p(Dura)=0.699
Scalp
Dura

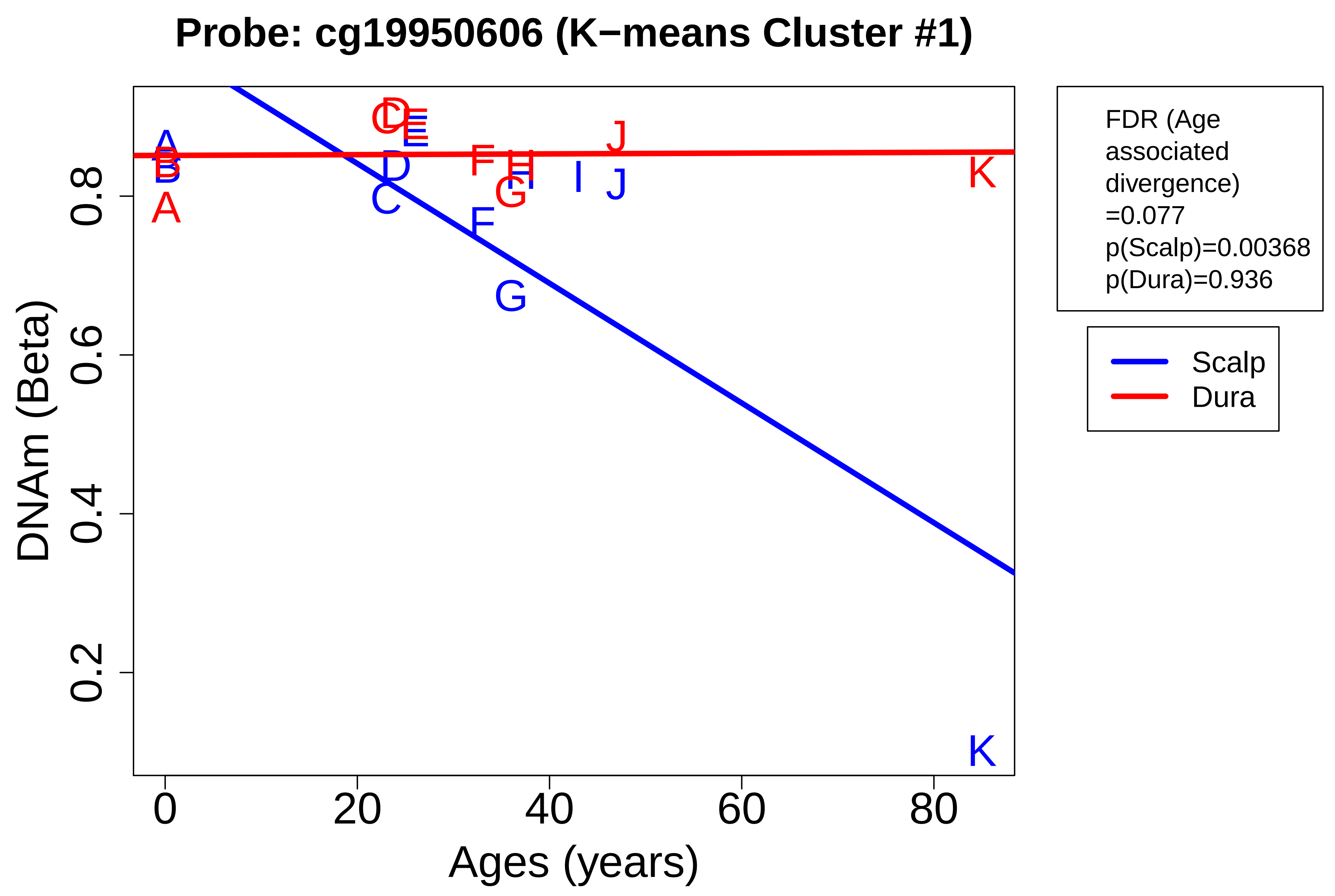

Probe: cg19950606 (K−means Cluster #1)
DNAm (Beta)
0.2
0.4
0.6
0.8
0
20
40
60
80
Ages (years)
FDR (Age associated divergence) =0.077
p(Scalp)=0.00368
p(Dura)=0.936
Scalp
Dura

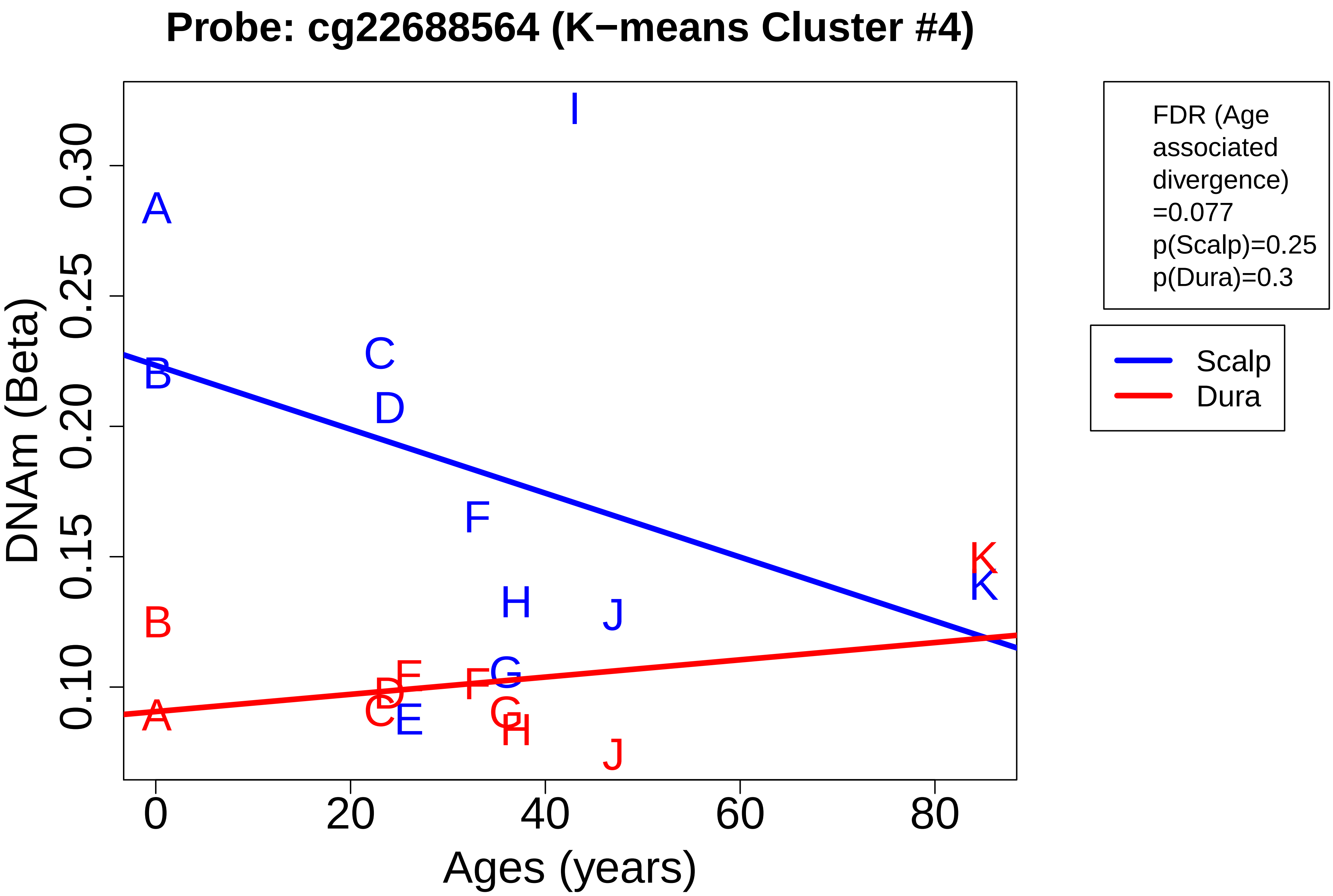

Probe: cg22688564 (K−means Cluster #4)
DNAm (Beta)
0.10
0.15
0.20
0.25
0.30
0
20
40
60
80
Ages (years)
FDR (Age associated divergence) =0.077
p(Scalp)=0.25
p(Dura)=0.3
Scalp
Dura

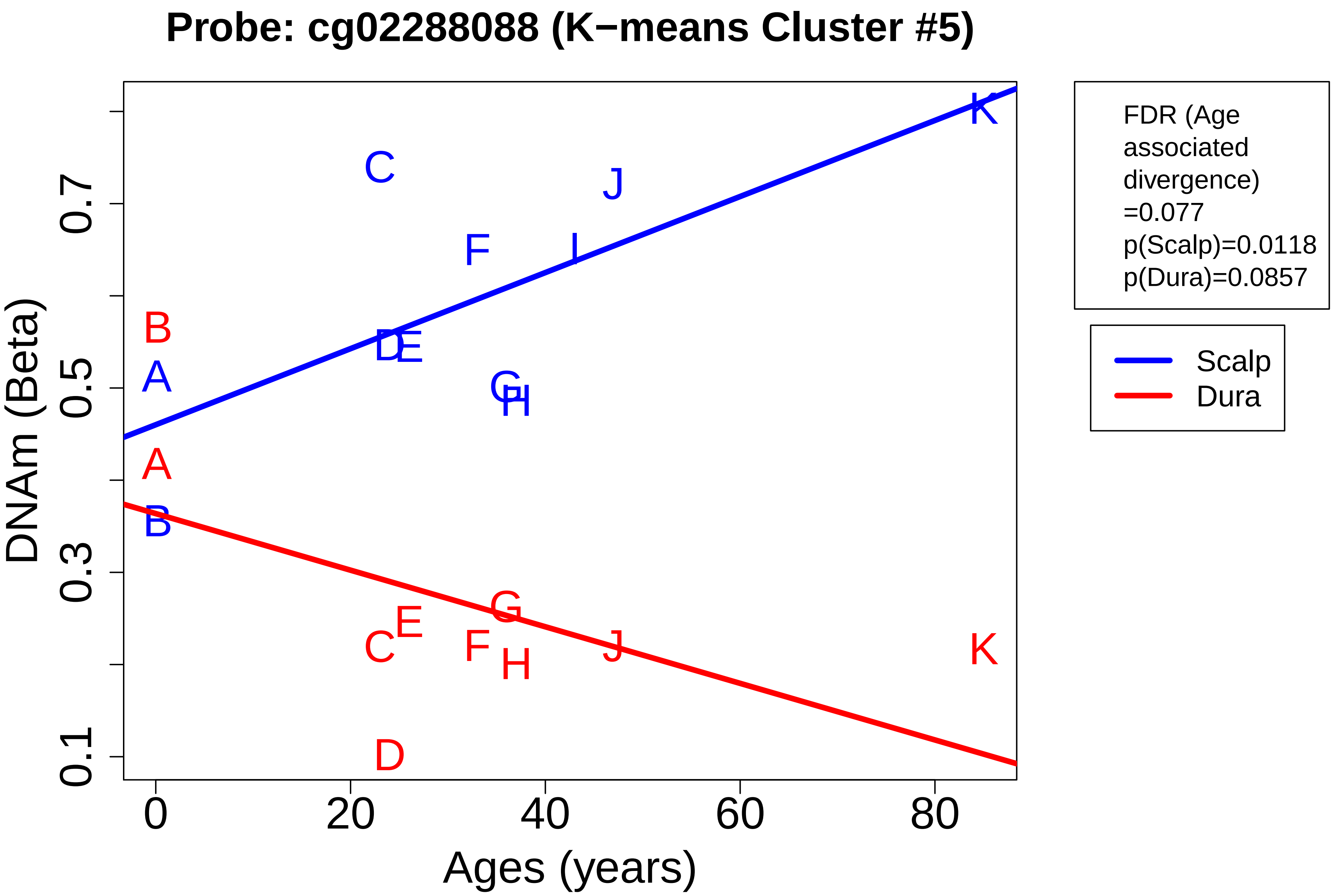
Probe: cg02288088 (K−means Cluster #5)
DNAm (Beta)
Ages (years)
0.1
0.3
0.5
0.7
0
20
40
60
80
FDR (Age associated divergence) =0.077
p(Scalp)=0.0118
p(Dura)=0.0857
Scalp
Dura
A
B
C
D
E
F
G
H
I
J
K

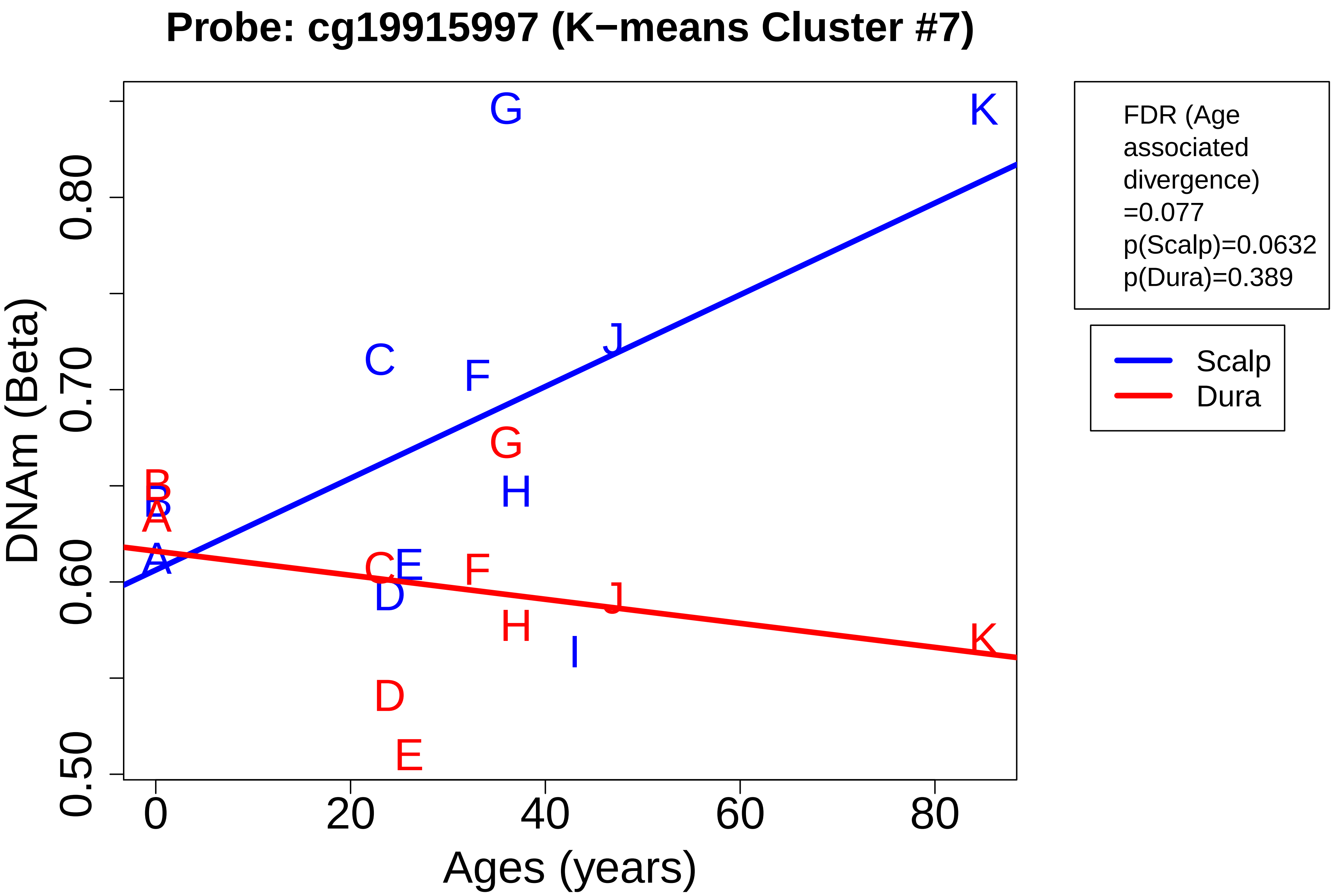
Probe: cg19915997 (K−means Cluster #7)
FDR (Age associated divergence) =0.077
p(Scalp)=0.0632
p(Dura)=0.389
Scalp
Dura
DNAm (Beta)
0.50
0.60
0.70
0.80
0
20
40
60
80
Ages (years)

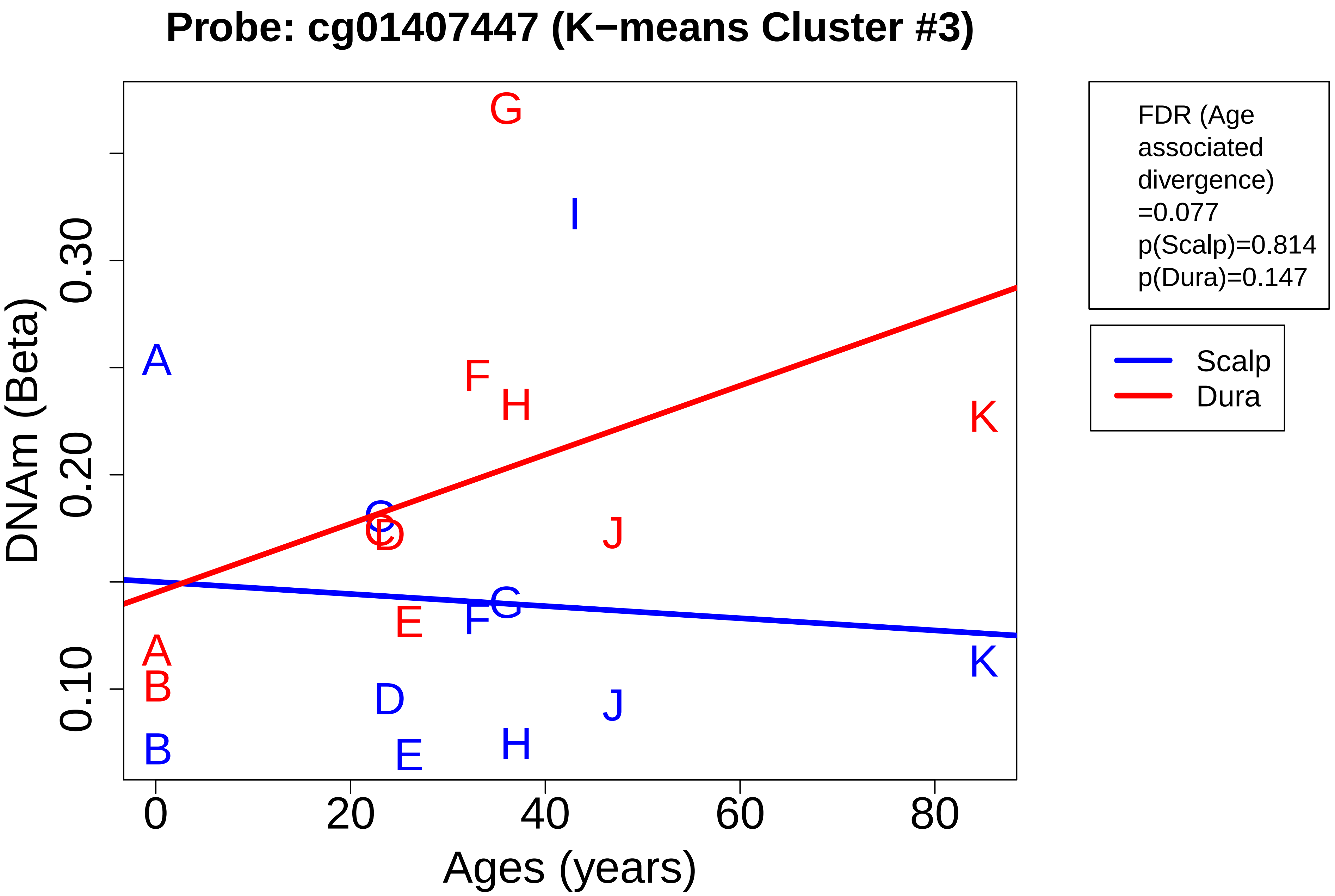

Probe: cg01407447 (K−means Cluster #3)
DNAm (Beta)
0.10
0.20
0.30
0
20
40
60
80
Ages (years)
FDR (Age associated divergence) =0.077
p(Scalp)=0.814
p(Dura)=0.147
Scalp
Dura

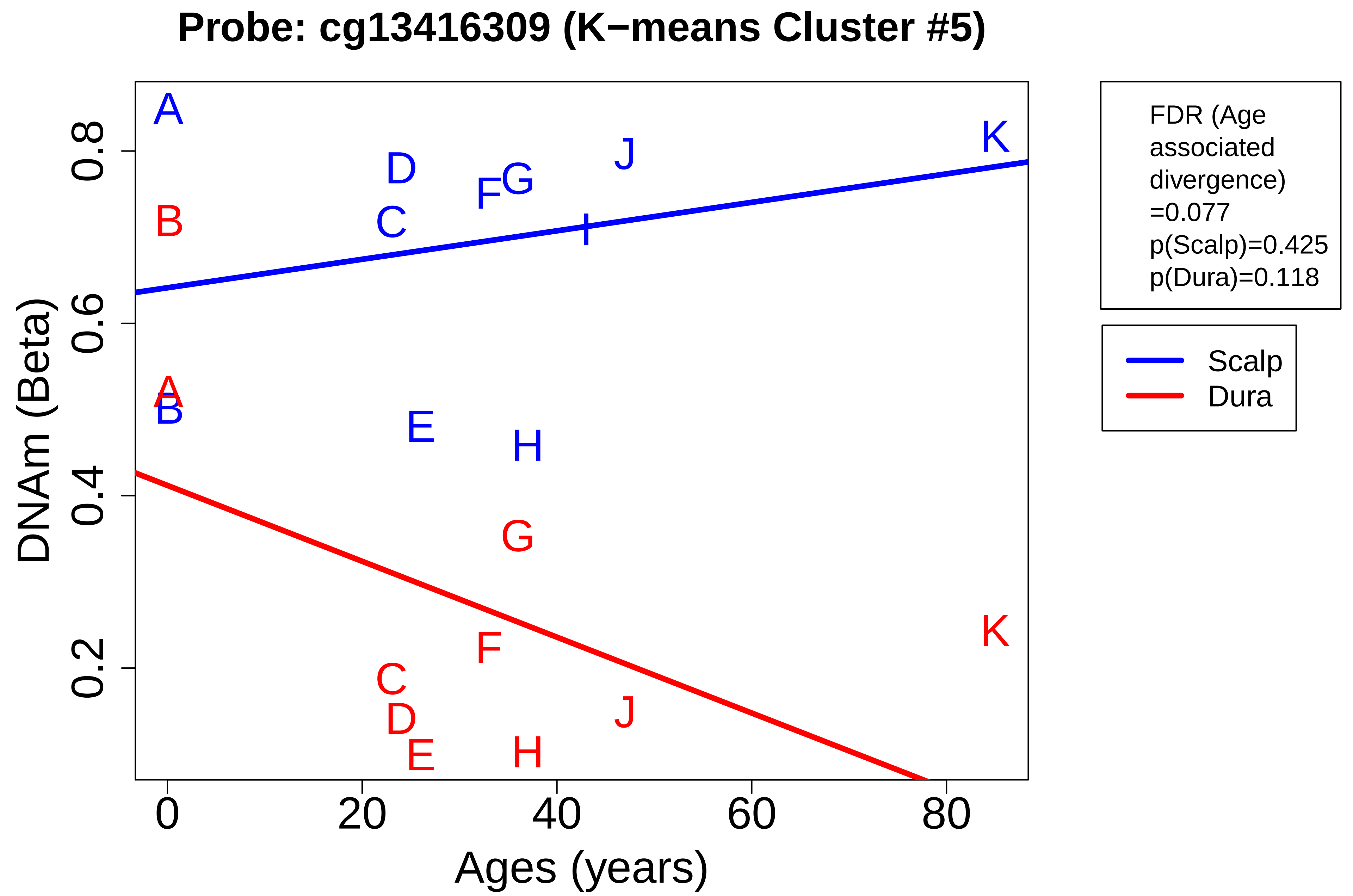

Probe: cg13416309 (K−means Cluster #5)
DNAm (Beta)
0.2
0.4
0.6
0.8
Ages (years)
0
20
40
60
80
A
B
C
D
E
F
G
H
I
J
K
FDR (Age associated divergence) =0.077
p(Scalp)=0.425
p(Dura)=0.118
Scalp
Dura

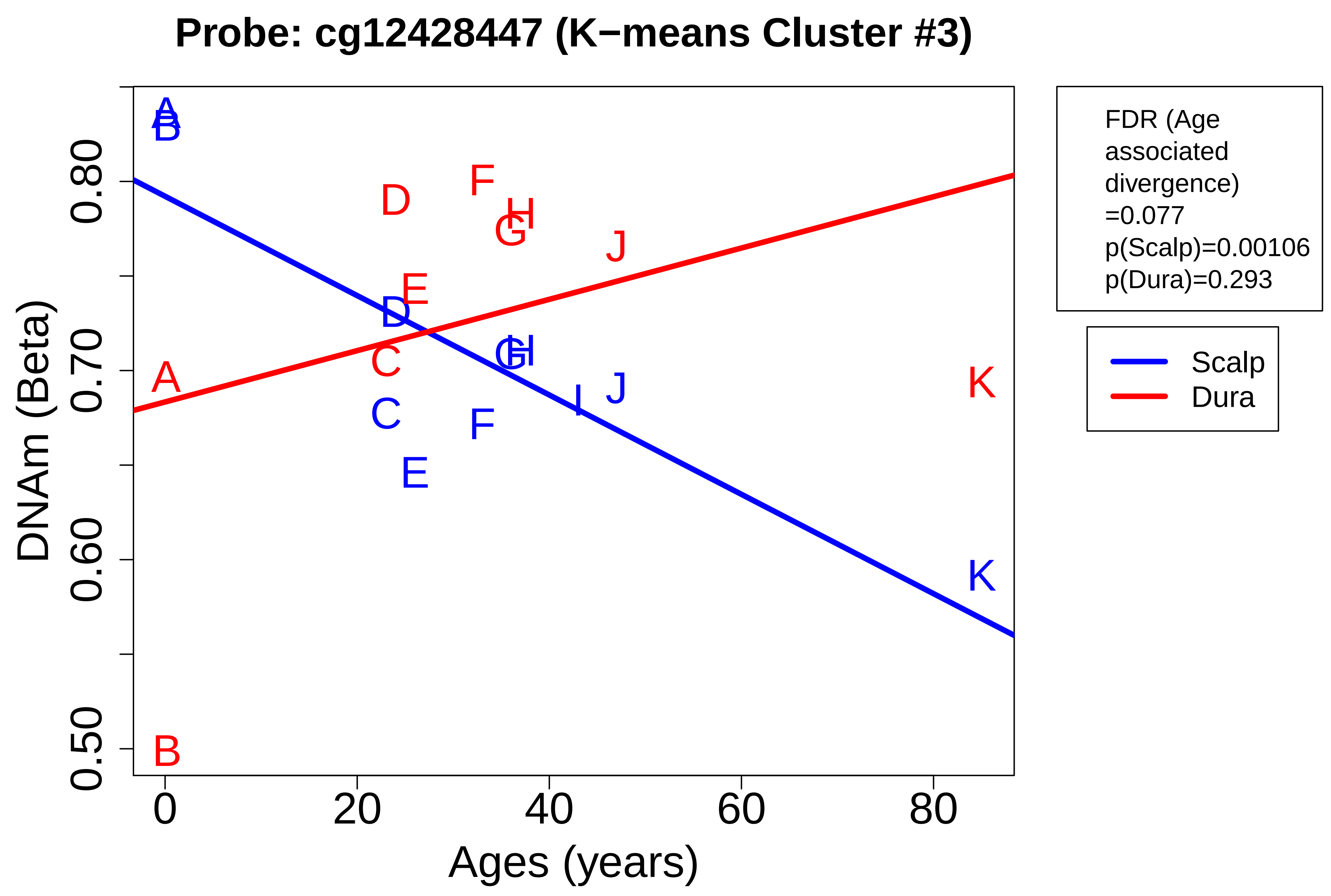
Probe: cg12428447 (K−means Cluster #3)
DNAm (Beta)
0.50
0.60
0.70
0.80
0
20
40
60
80
Ages (years)
A
B
C
D
E
F
G
H
I
J
K
FDR (Age associated divergence) =0.077
p(Scalp)=0.00106
p(Dura)=0.293
Scalp
Dura

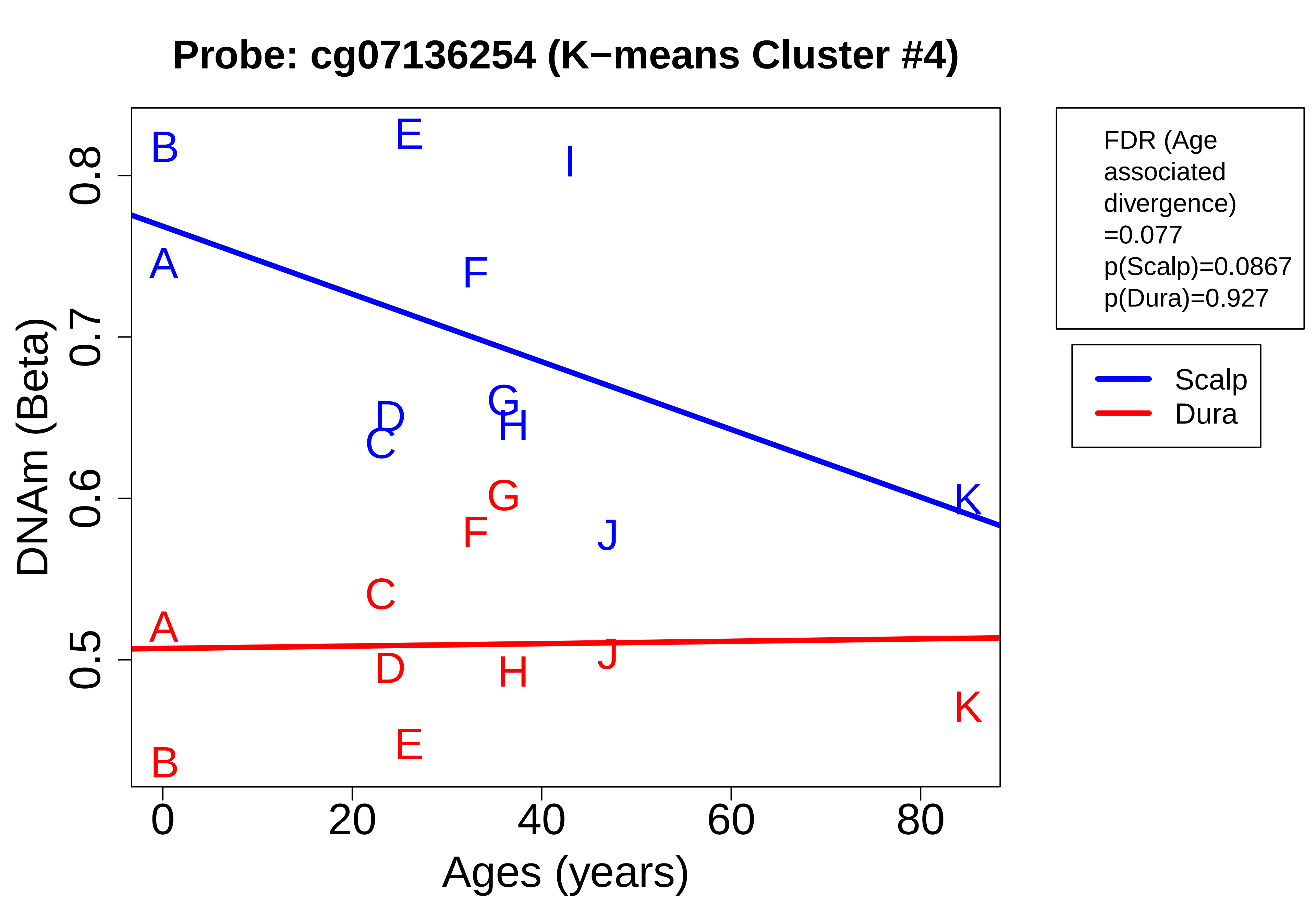

Probe: cg07136254 (K−means Cluster #4)
DNAm (Beta)
0.5
0.6
0.7
0.8
Ages (years)
0
20
40
60
80
FDR (Age associated divergence) =0.077
p(Scalp)=0.0867
p(Dura)=0.927
Scalp
Dura
A
B
C
D
E
F
G
H
I
J
K

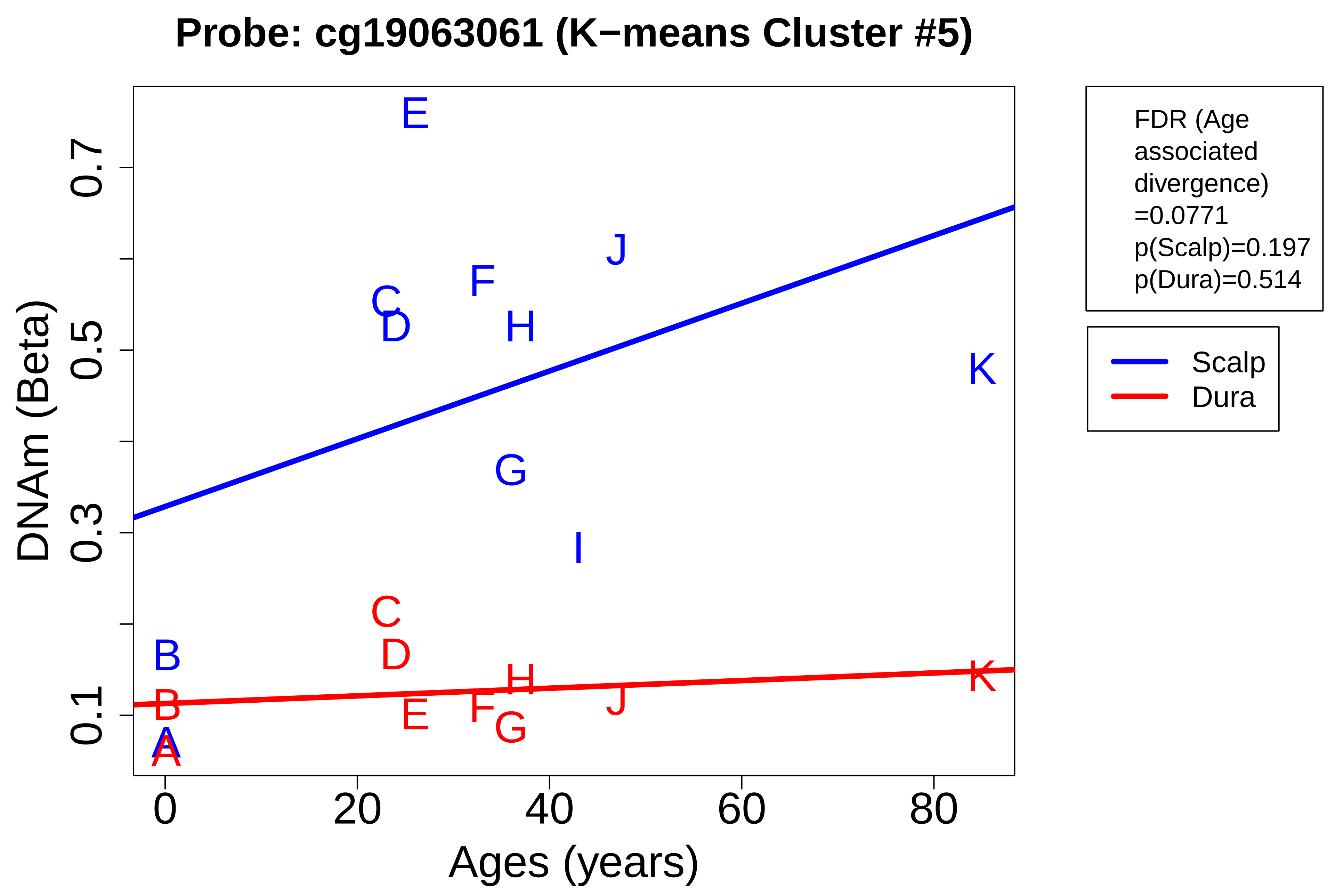

Probe: cg19063061 (K−means Cluster #5)
DNAm (Beta)
0.1
0.3
0.5
0.7
Ages (years)
0
20
40
60
80
A
B
C
D
E
F
G
H
I
J
K
FDR (Age associated divergence) =0.0771
p(Scalp)=0.197
p(Dura)=0.514
Scalp
Dura

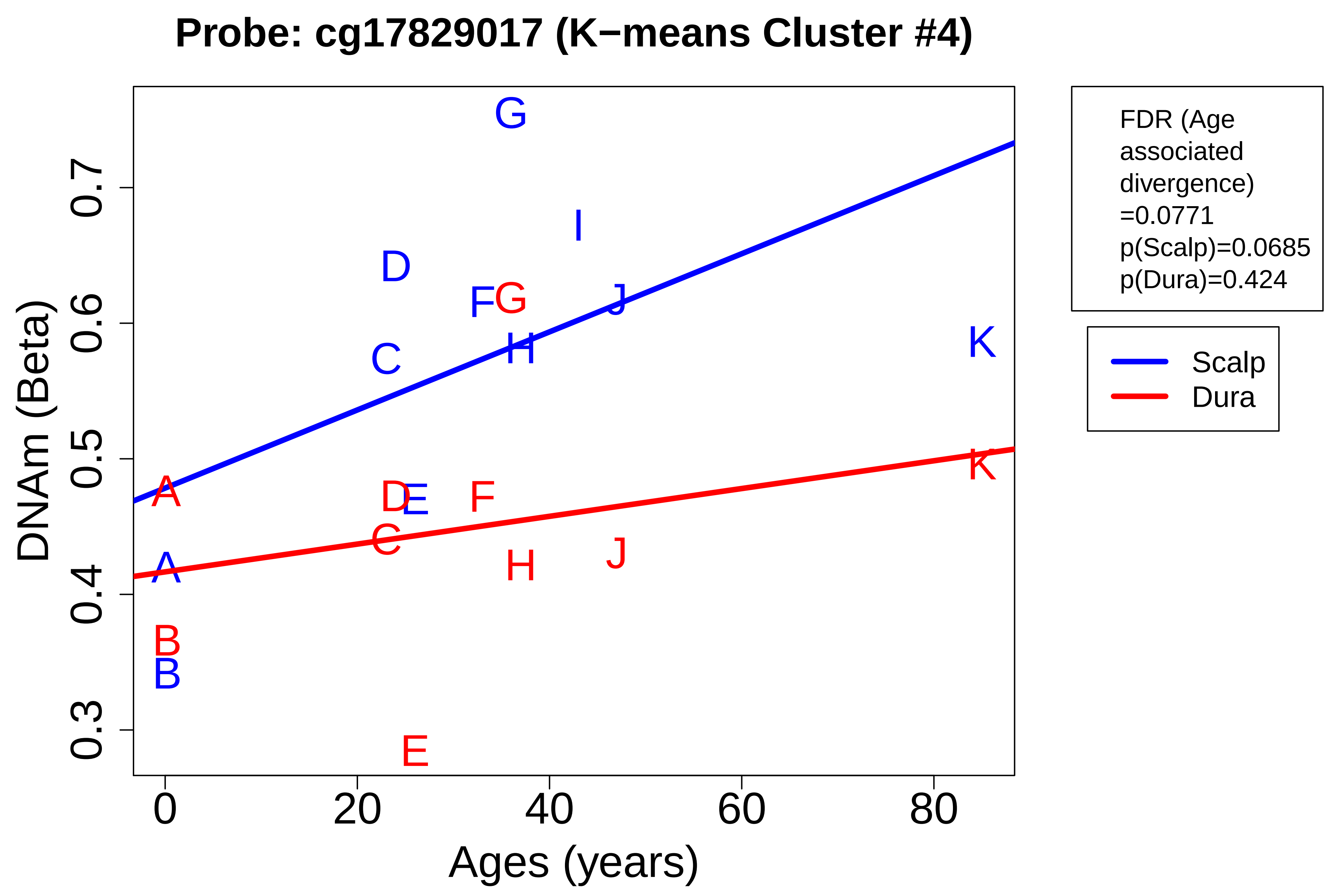

Probe: cg17829017 (K−means Cluster #4)
DNAm (Beta)
0.3
0.4
0.5
0.6
0.7
Ages (years)
0
20
40
60
80
A
B
C
D
E
F
G
H
I
J
K
FDR (Age associated divergence) =0.0771
p(Scalp)=0.0685
p(Dura)=0.424
Scalp
Dura

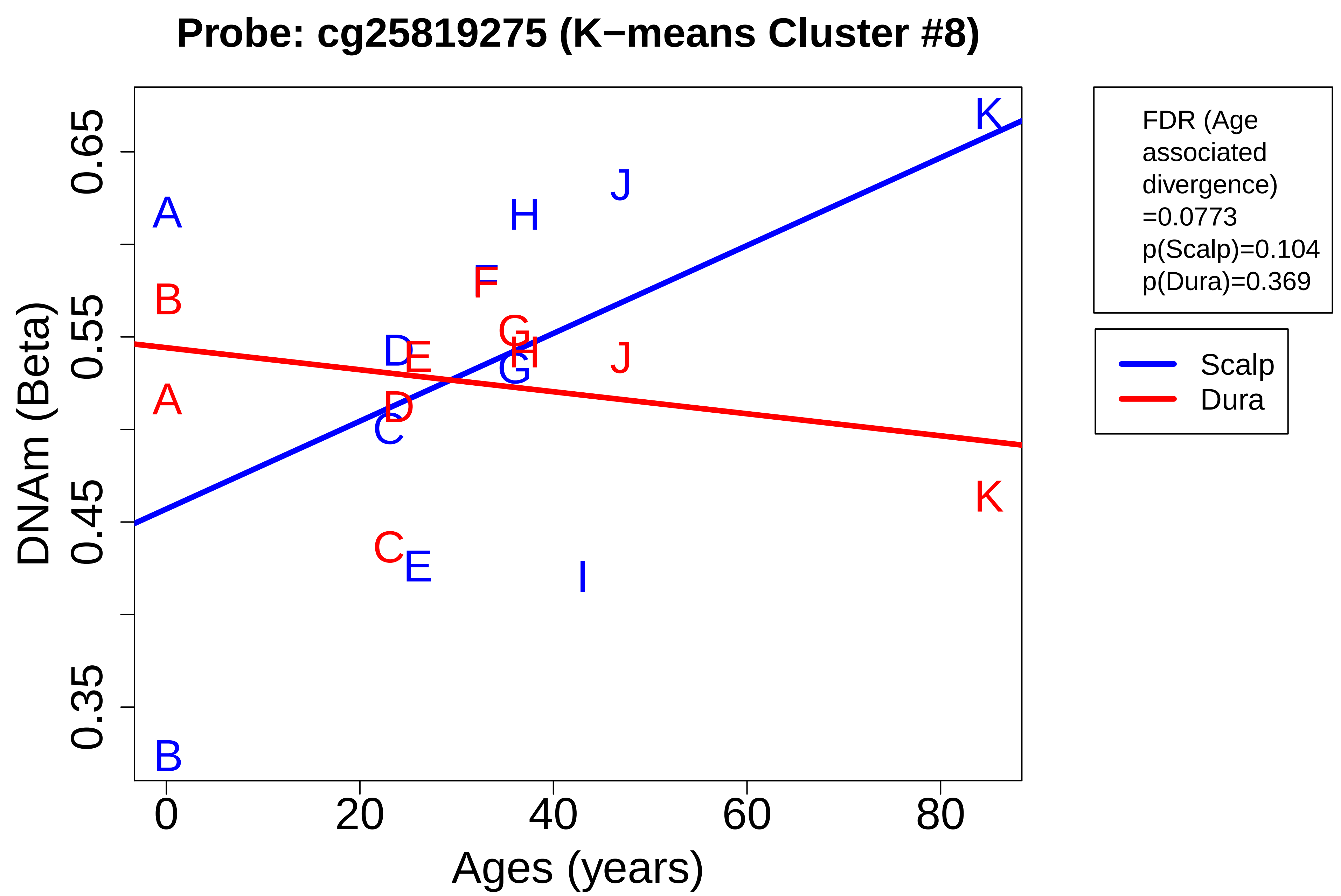
Probe: cg25819275 (K−means Cluster #8)
DNAm (Beta)
0.35
0.45
0.55
0.65
Ages (years)
0
20
40
60
80
A
B
C
D
E
F
G
H
I
J
K
FDR (Age associated divergence) =0.0773
p(Scalp)=0.104
p(Dura)=0.369
Scalp
Dura

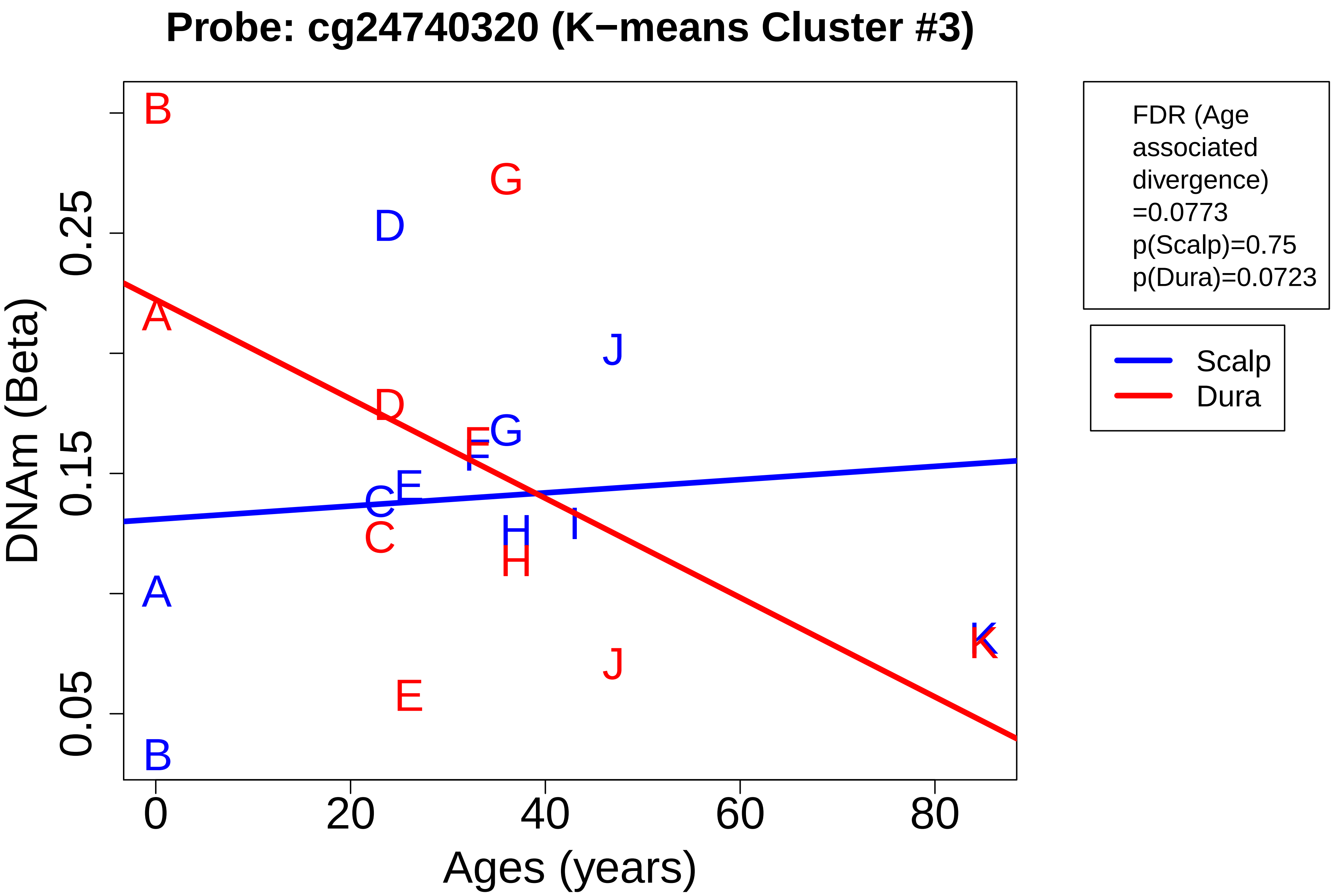
Probe: cg24740320 (K−means Cluster #3)
DNAm (Beta)
0.05
0.15
0.25
0
20
40
60
80
Ages (years)
B
G
D
A
J
D
F
G
E
C
C
H
I
H
A
K
J
E
B
FDR (Age associated divergence) =0.0773
p(Scalp)=0.75
p(Dura)=0.0723
Scalp
Dura

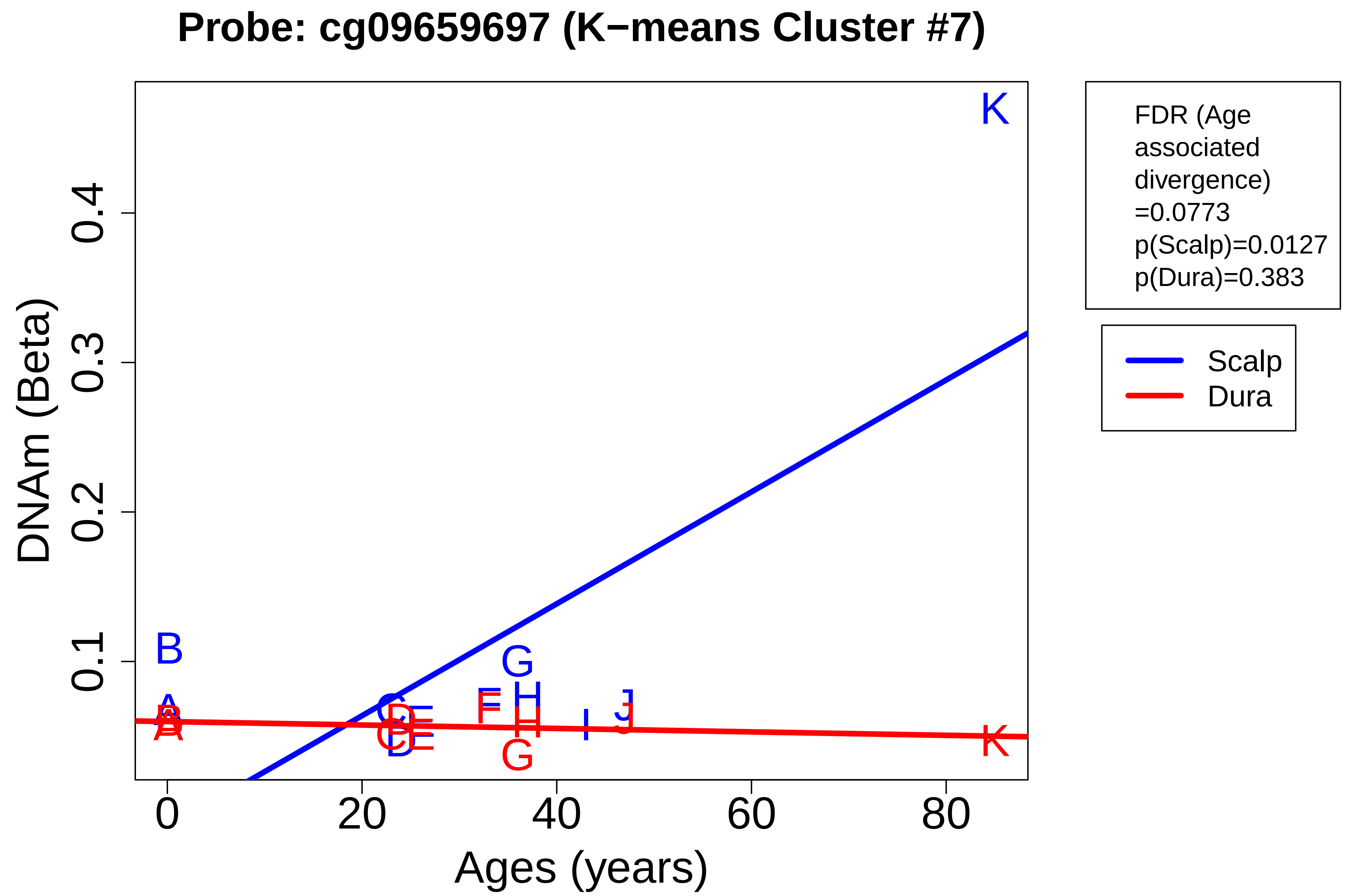

Probe: cg09659697 (K−means Cluster #7)
DNAm (Beta)
0.1
0.2
0.3
0.4
0
20
40
60
80
Ages (years)
FDR (Age associated divergence) =0.0773
p(Scalp)=0.0127
p(Dura)=0.383
Scalp
Dura

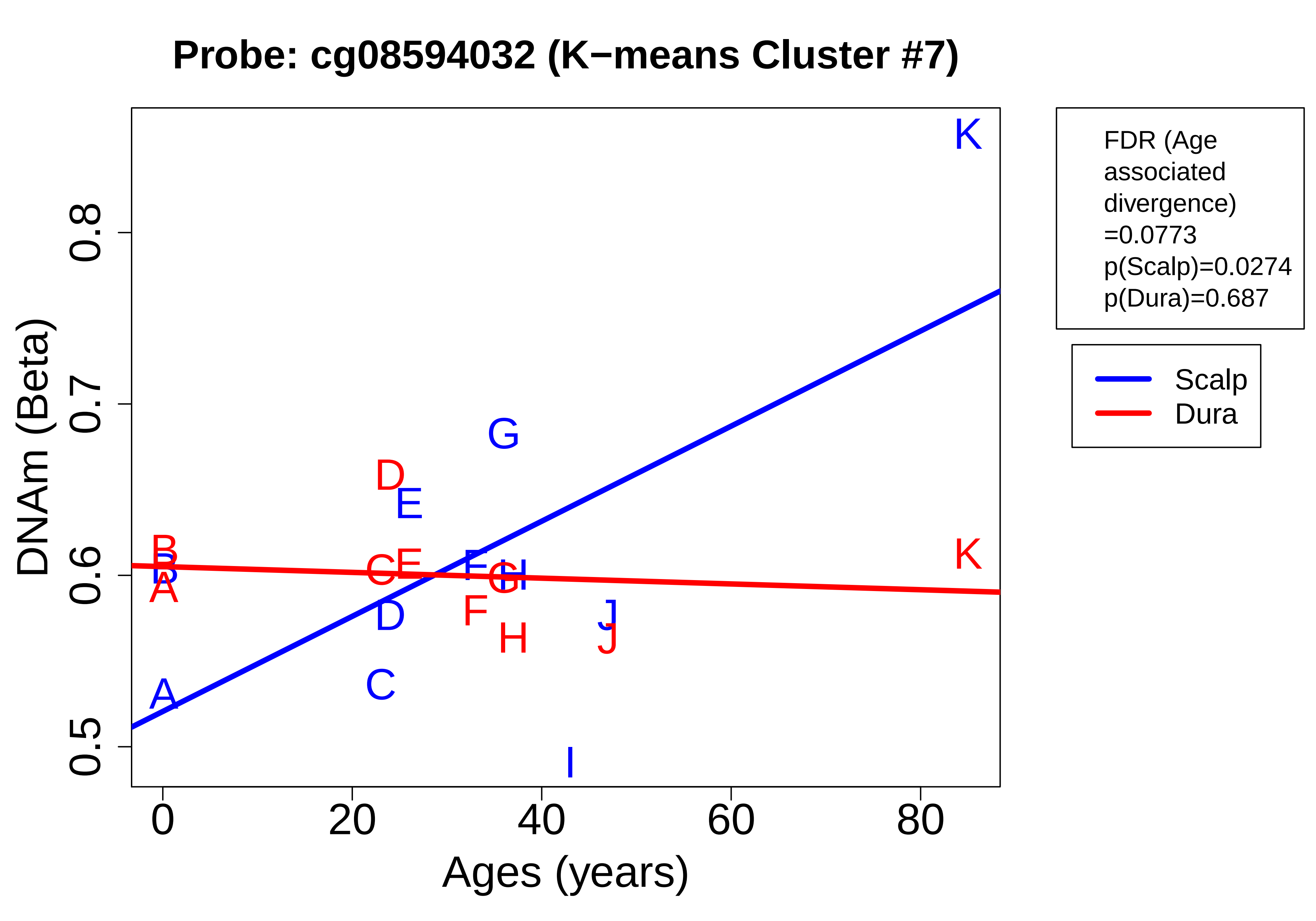

Probe: cg08594032 (K−means Cluster #7)
DNAm (Beta)
0.5
0.6
0.7
0.8
0
20
40
60
80
Ages (years)
FDR (Age associated divergence) =0.0773
p(Scalp)=0.0274
p(Dura)=0.687
Scalp
Dura
A
B
C
D
E
F
G
H
I
J
K

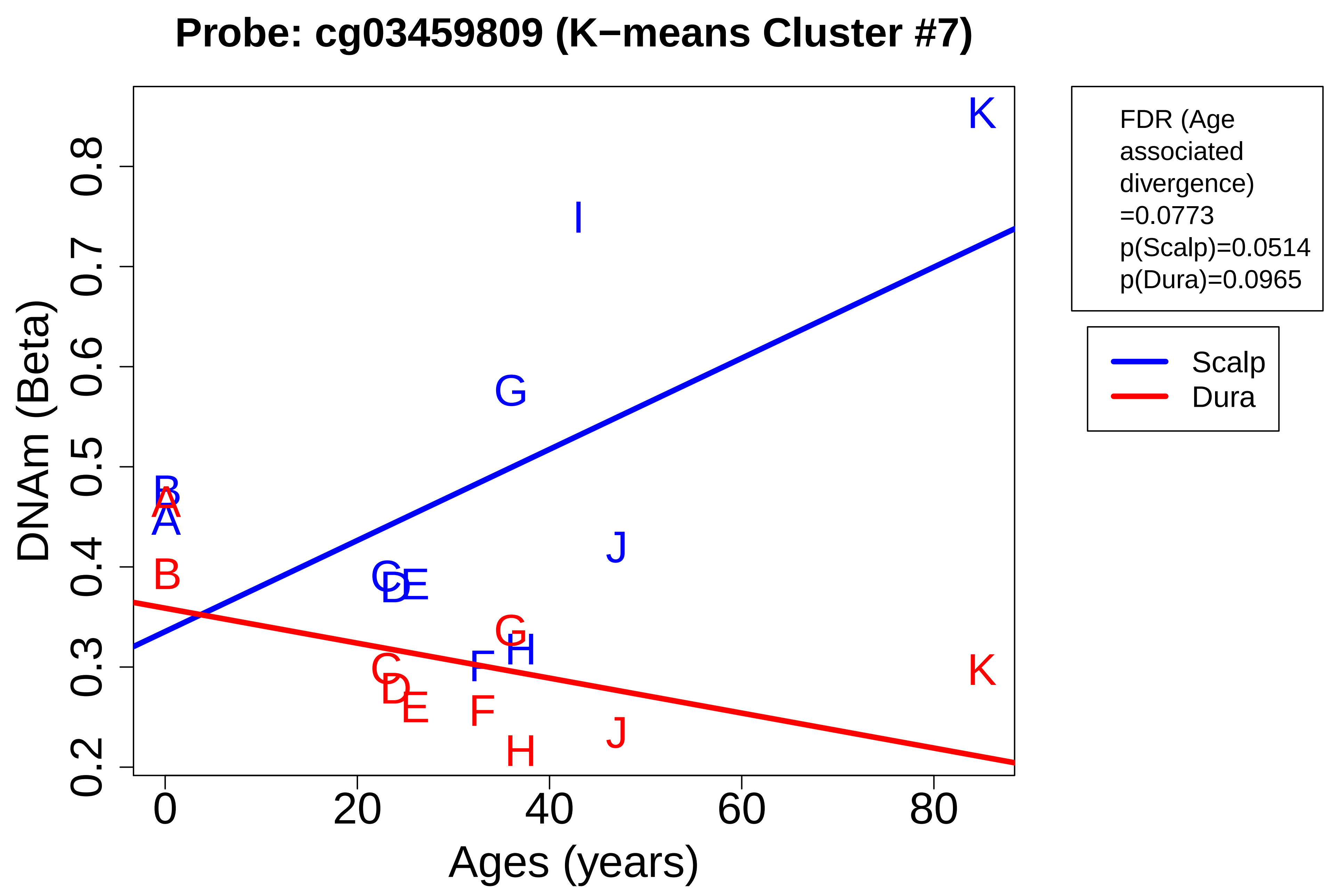
Probe: cg03459809 (K−means Cluster #7)
FDR (Age associated divergence) =0.0773
p(Scalp)=0.0514
p(Dura)=0.0965
Scalp
Dura
DNAm (Beta)
0.2
0.3
0.4
0.5
0.6
0.7
0.8
Ages (years)
0
20
40
60
80
A
B
C
D
E
F
G
H
I
J
K

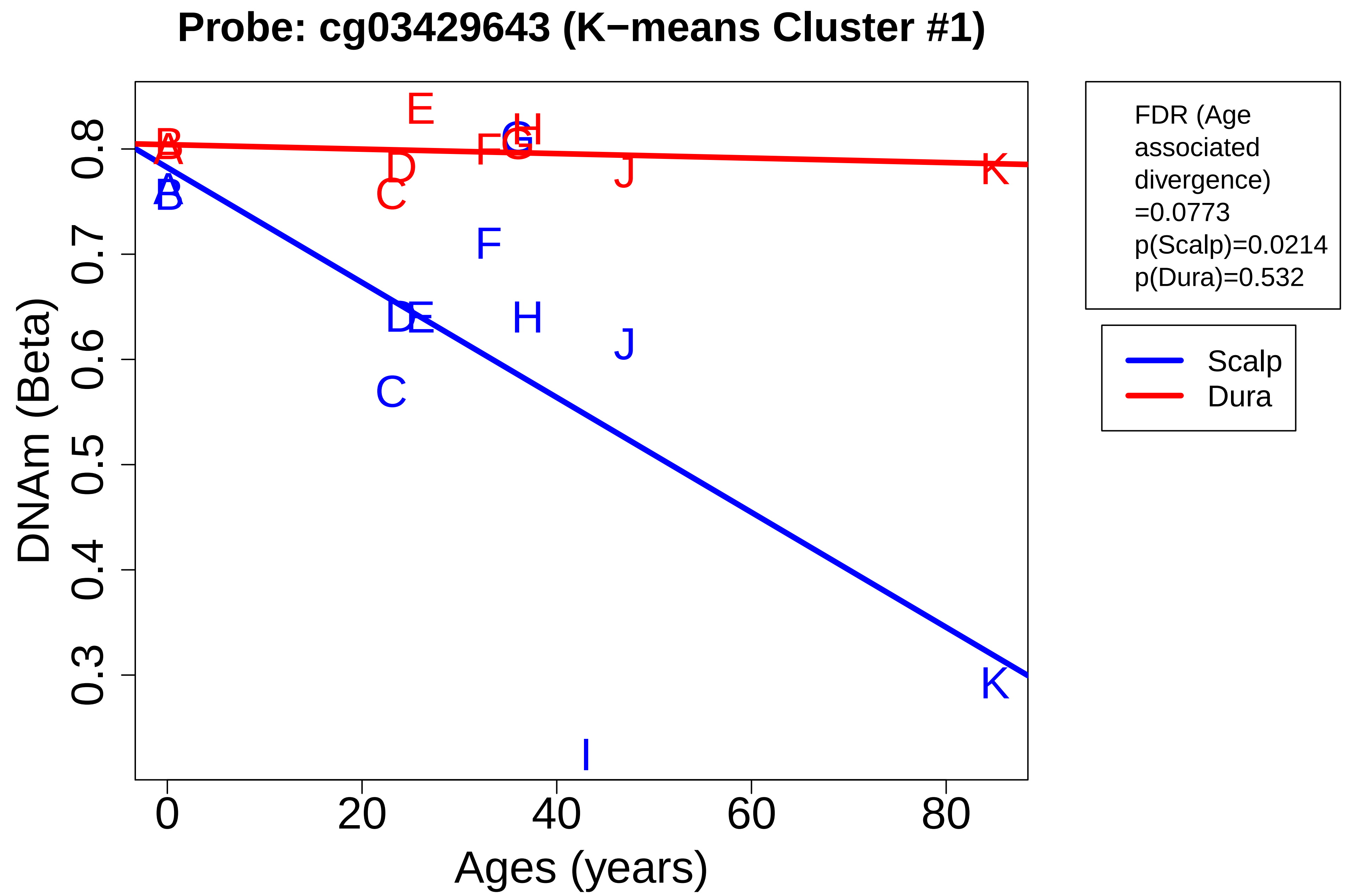
Probe: cg03429643 (K−means Cluster #1)
DNAm (Beta)
0.3
0.4
0.5
0.6
0.7
0.8
0
20
40
60
80
Ages (years)
FDR (Age associated divergence) =0.0773
p(Scalp)=0.0214
p(Dura)=0.532
Scalp
Dura

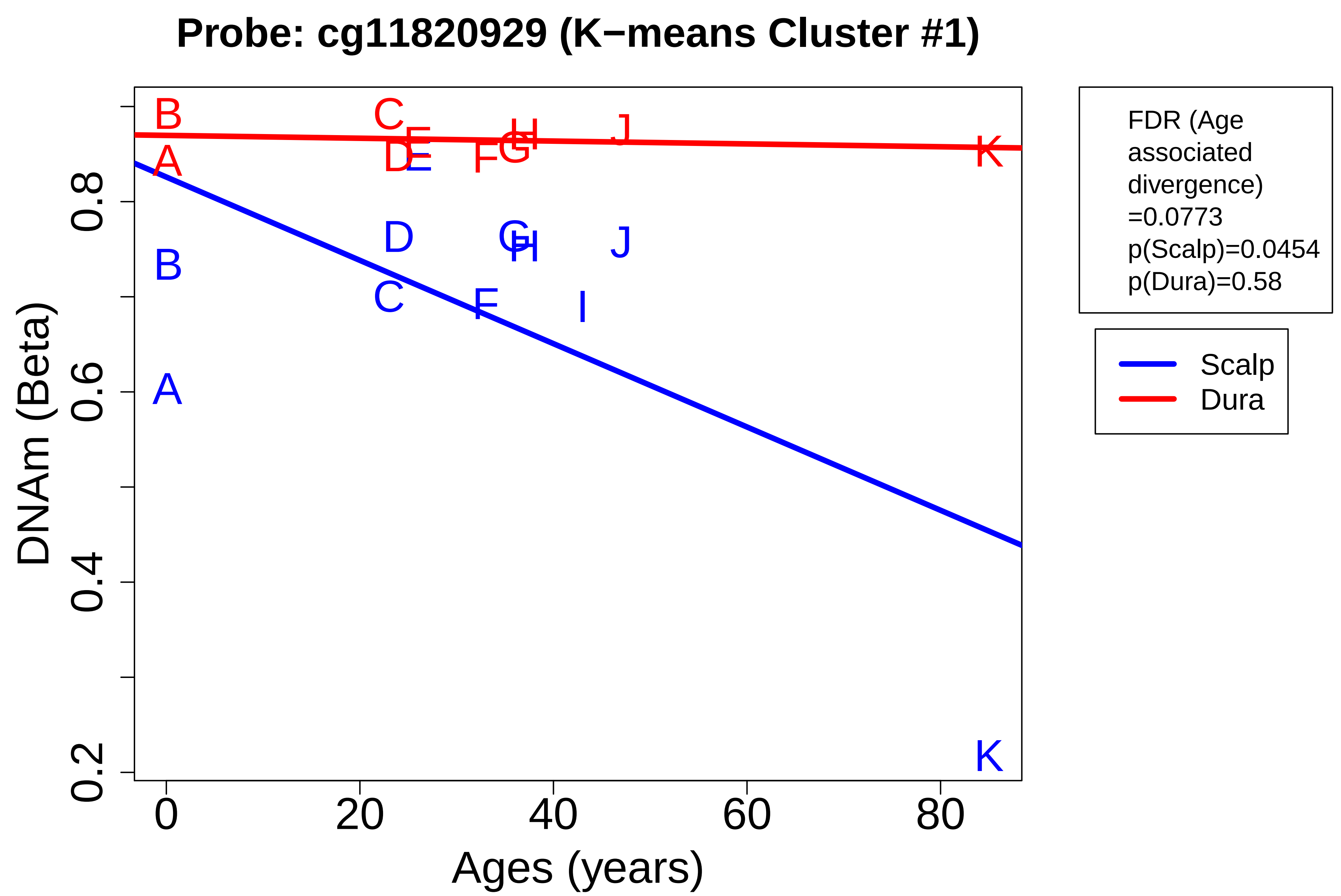

Probe: cg11820929 (K−means Cluster #1)
DNAm (Beta)
0.2
0.4
0.6
0.8
0
20
40
60
80
Ages (years)
A
B
C
D
E
F
G
H
I
J
K
FDR (Age associated divergence) =0.0773
p(Scalp)=0.0454
p(Dura)=0.58
Scalp
Dura

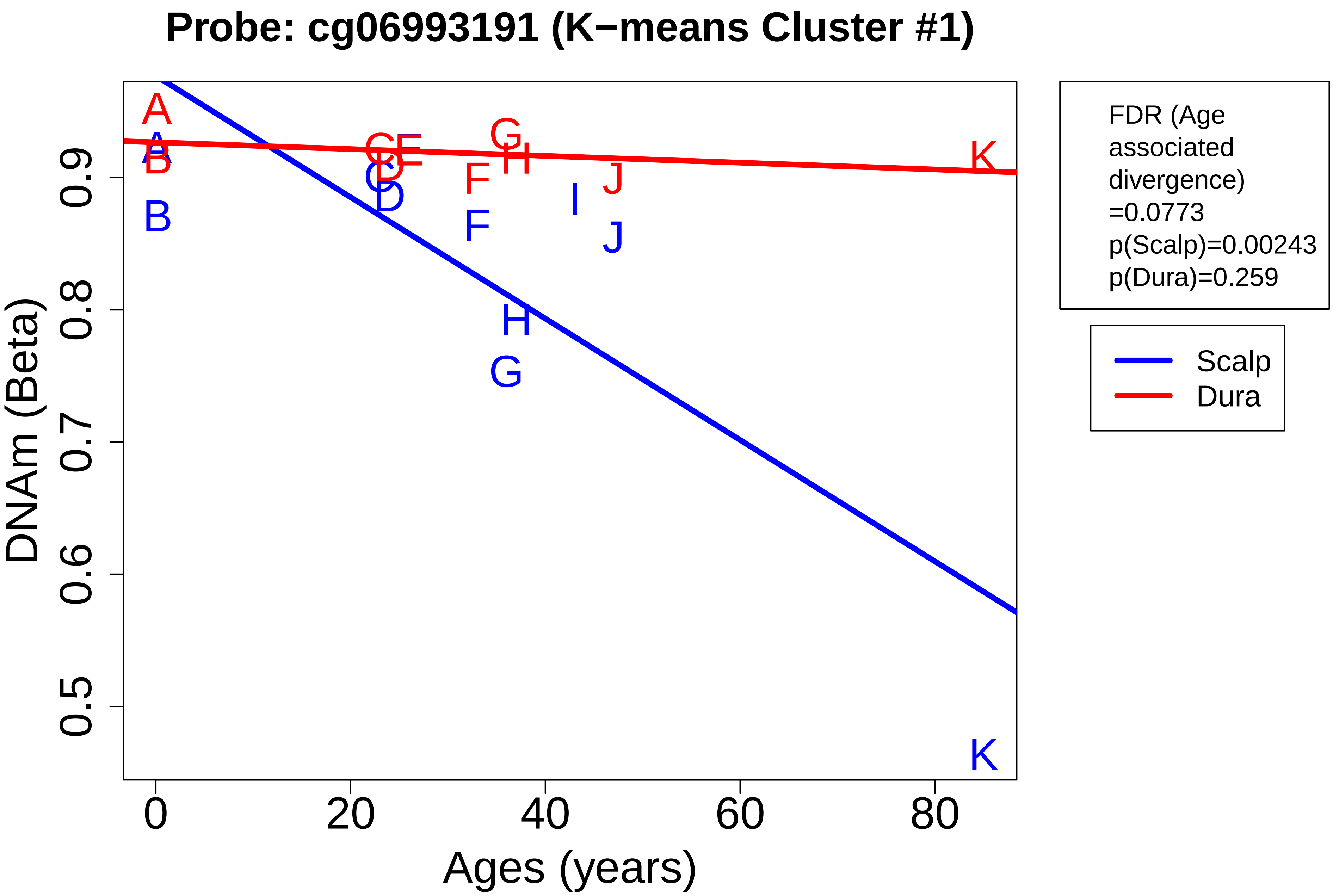

Probe: cg06993191 (K−means Cluster #1)
DNAm (Beta)
0.5
0.6
0.7
0.8
0.9
Ages (years)
0
20
40
60
80
A
B
C
D
E
F
G
H
I
J
K
FDR (Age associated divergence) =0.0773
p(Scalp)=0.00243
p(Dura)=0.259
Scalp
Dura

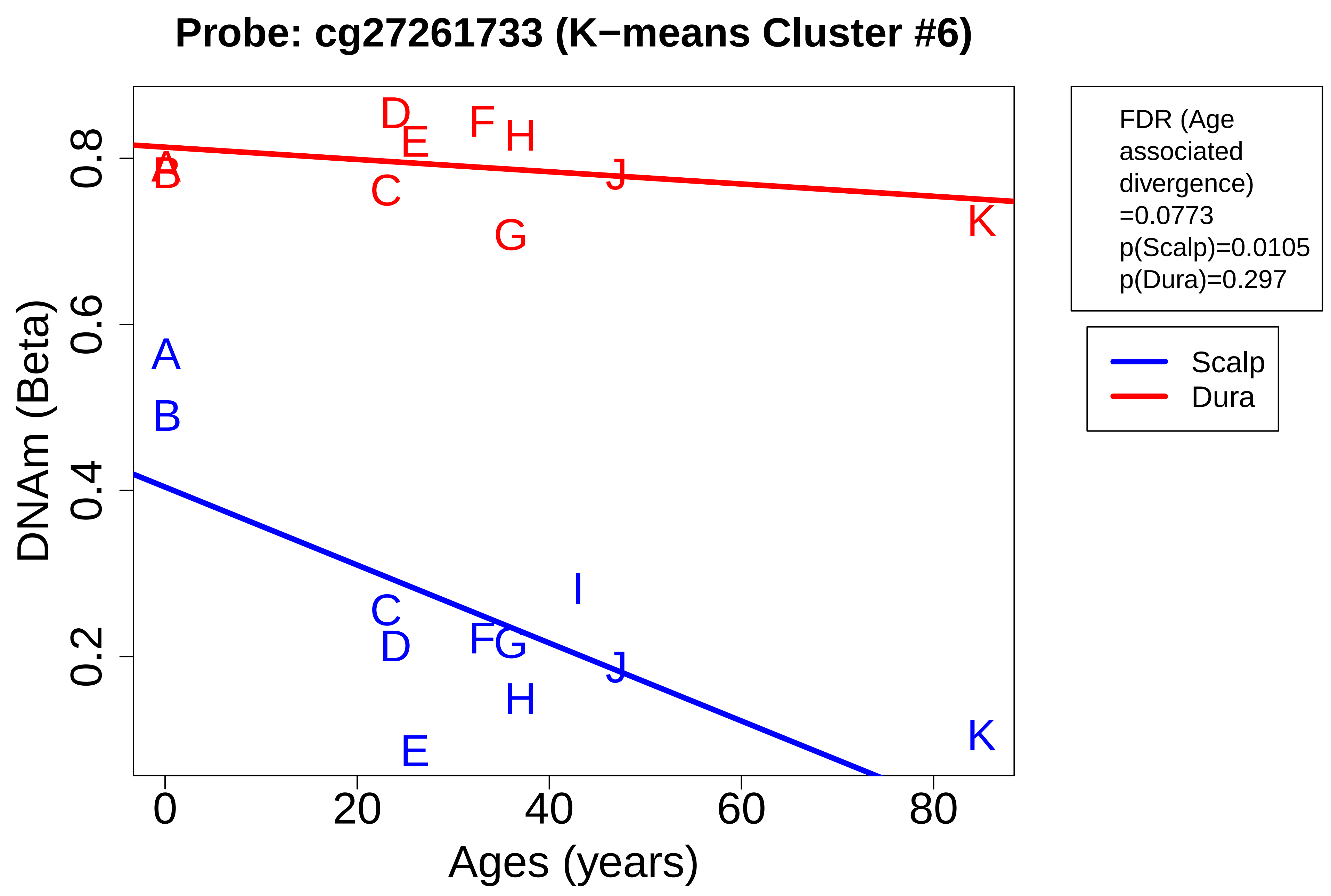
Probe: cg27261733 (K−means Cluster #6)
DNAm (Beta)
Ages (years)
0.2
0.4
0.6
0.8
0
20
40
60
80
FDR (Age associated divergence) =0.0773
p(Scalp)=0.0105
p(Dura)=0.297
Scalp
Dura
A
B
C
D
E
F
G
H
I
J
K

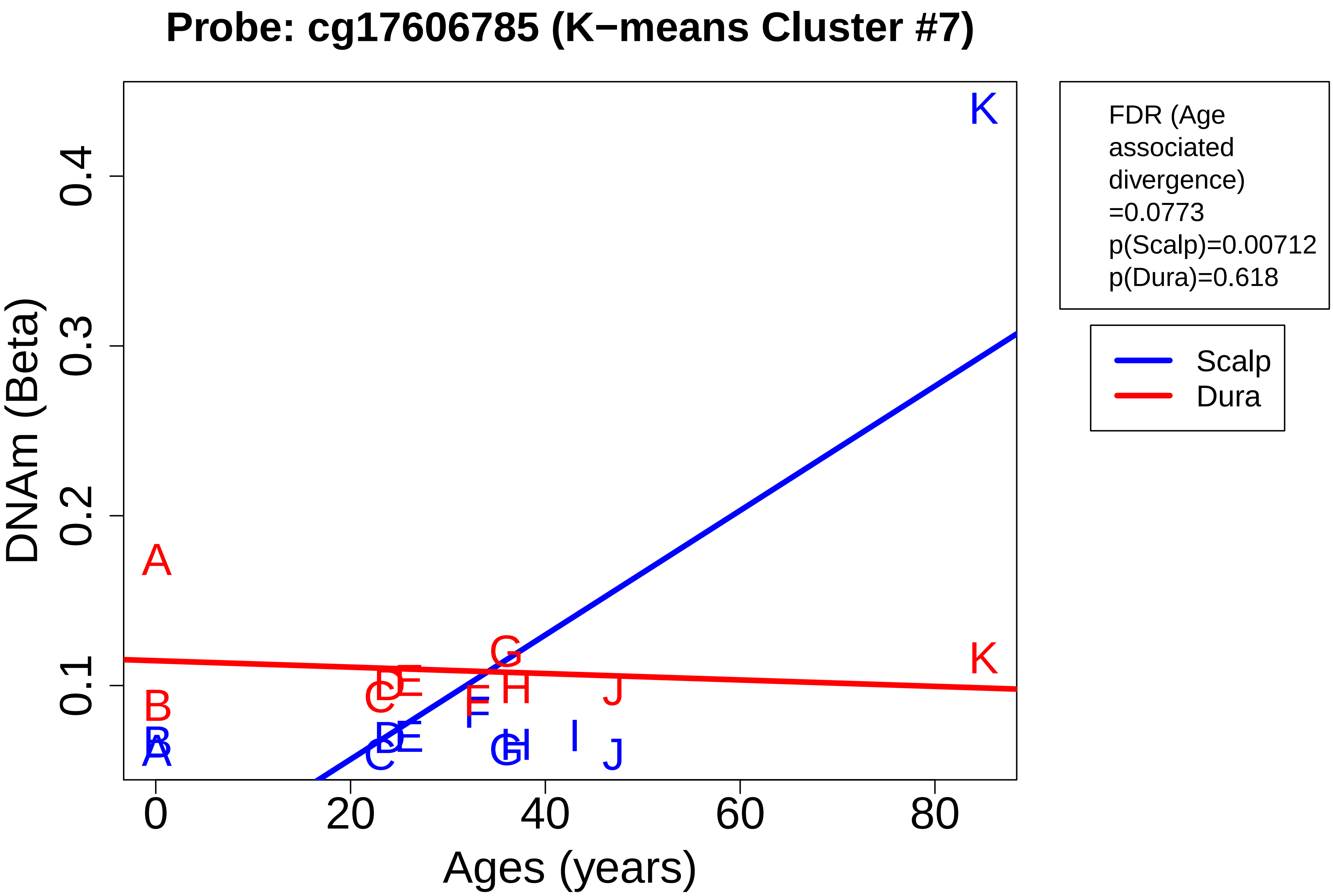

Probe: cg17606785 (K−means Cluster #7)
DNAm (Beta)
0.1
0.2
0.3
0.4
Ages (years)
0
20
40
60
80
A
B
C
D
E
F
G
H
I
J
K
FDR (Age associated divergence) =0.0773
p(Scalp)=0.00712
p(Dura)=0.618
Scalp
Dura

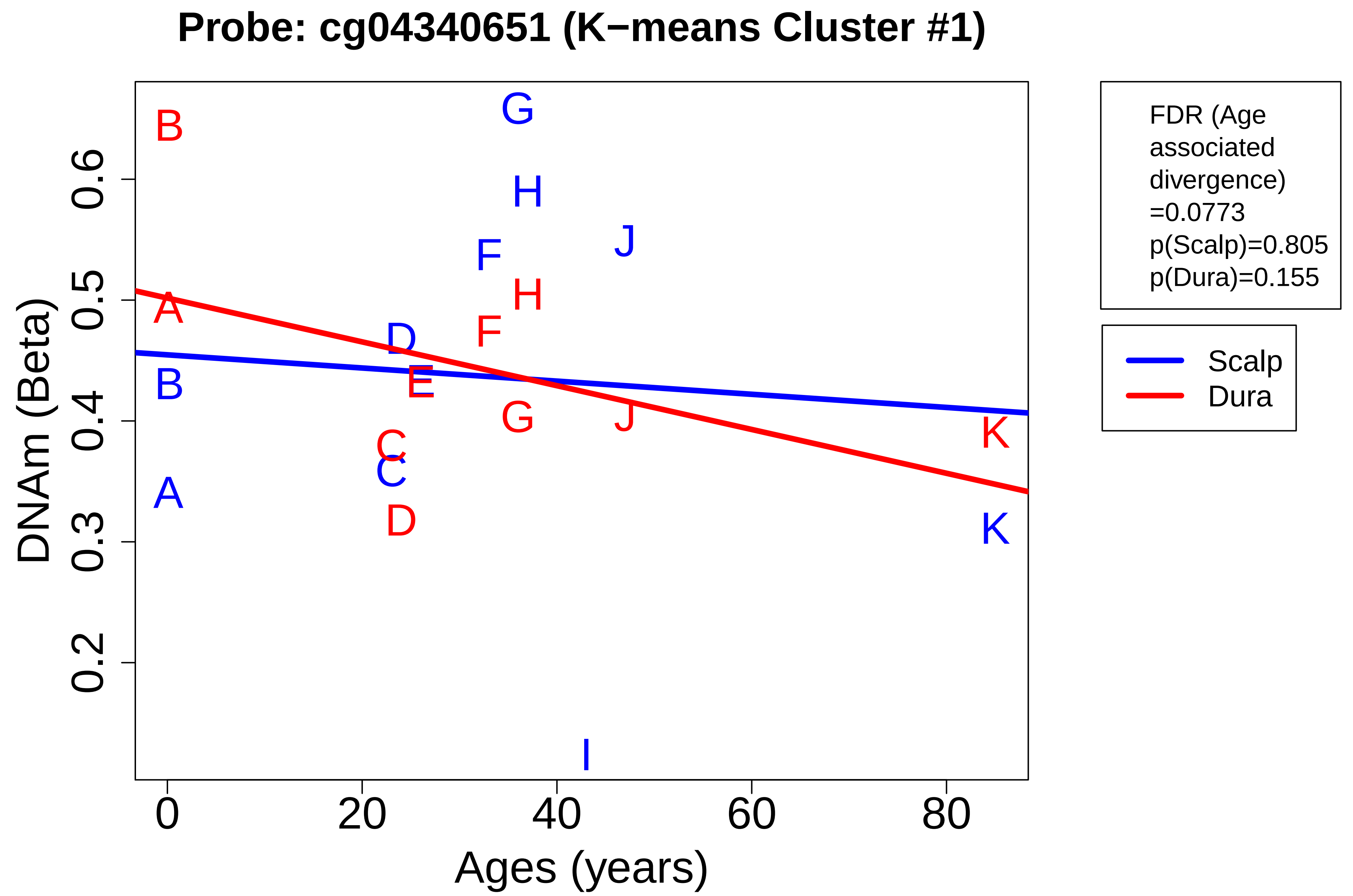

Probe: cg04340651 (K−means Cluster #1)
DNAm (Beta)
0.2
0.3
0.4
0.5
0.6
0
20
40
60
80
Ages (years)
FDR (Age associated divergence) =0.0773
p(Scalp)=0.805
p(Dura)=0.155
Scalp
Dura

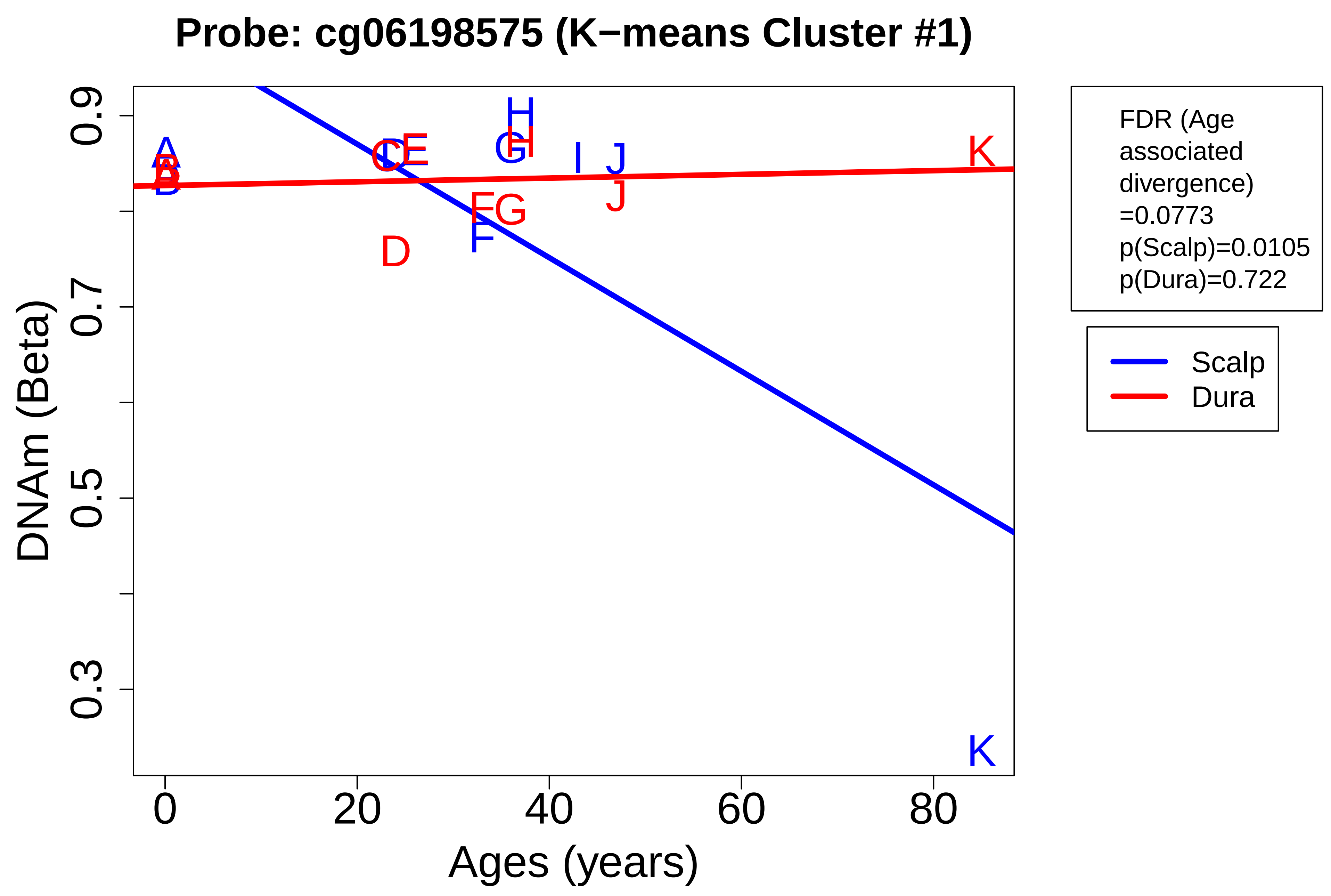

Probe: cg06198575 (K−means Cluster #1)
DNAm (Beta)
0.9
0.7
0.5
0.3
0
20
40
60
80
Ages (years)
FDR (Age associated divergence) =0.0773
p(Scalp)=0.0105
p(Dura)=0.722
Scalp
Dura

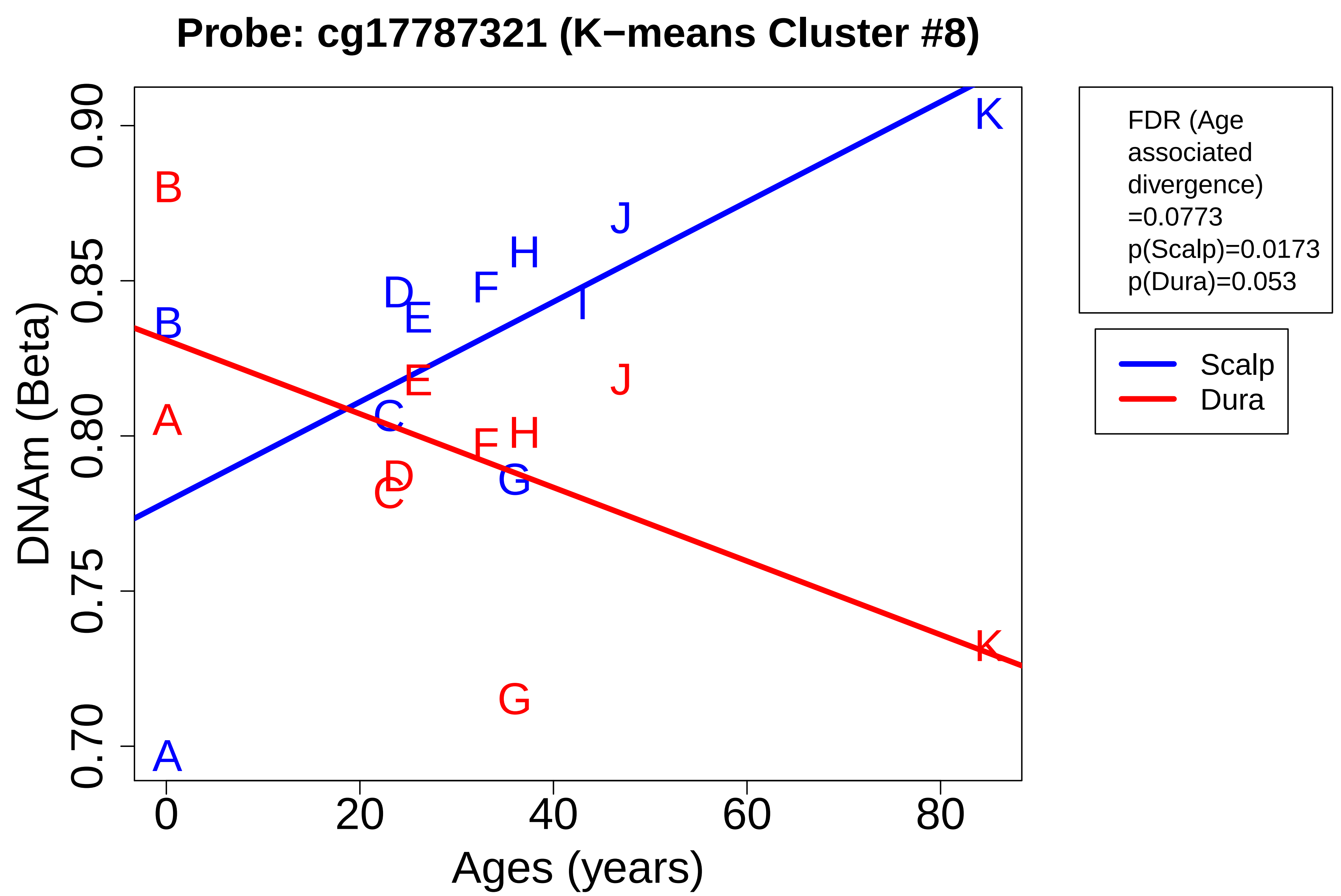

Probe: cg17787321 (K−means Cluster #8)
DNAm (Beta)
0.70
0.75
0.80
0.85
0.90
0
20
40
60
80
Ages (years)
A
B
C
D
E
F
G
H
I
J
K
FDR (Age associated divergence) =0.0773
p(Scalp)=0.0173
p(Dura)=0.053
Scalp
Dura

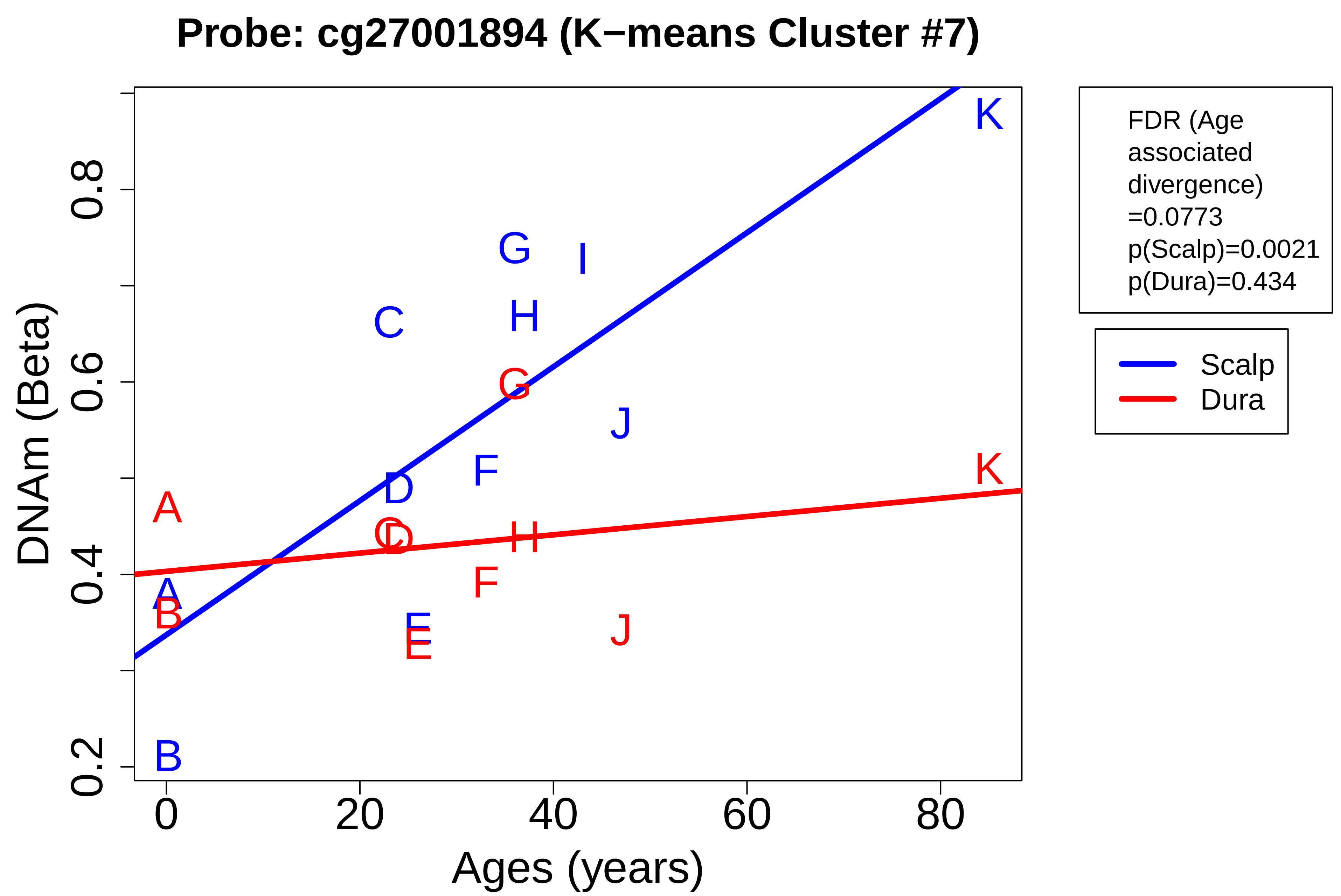

Probe: cg27001894 (K−means Cluster #7)
DNAm (Beta)
0.2
0.4
0.6
0.8
0
20
40
60
80
Ages (years)
FDR (Age associated divergence) =0.0773
p(Scalp)=0.0021
p(Dura)=0.434
Scalp
Dura
A
B
C
D
E
F
G
H
I
J
K

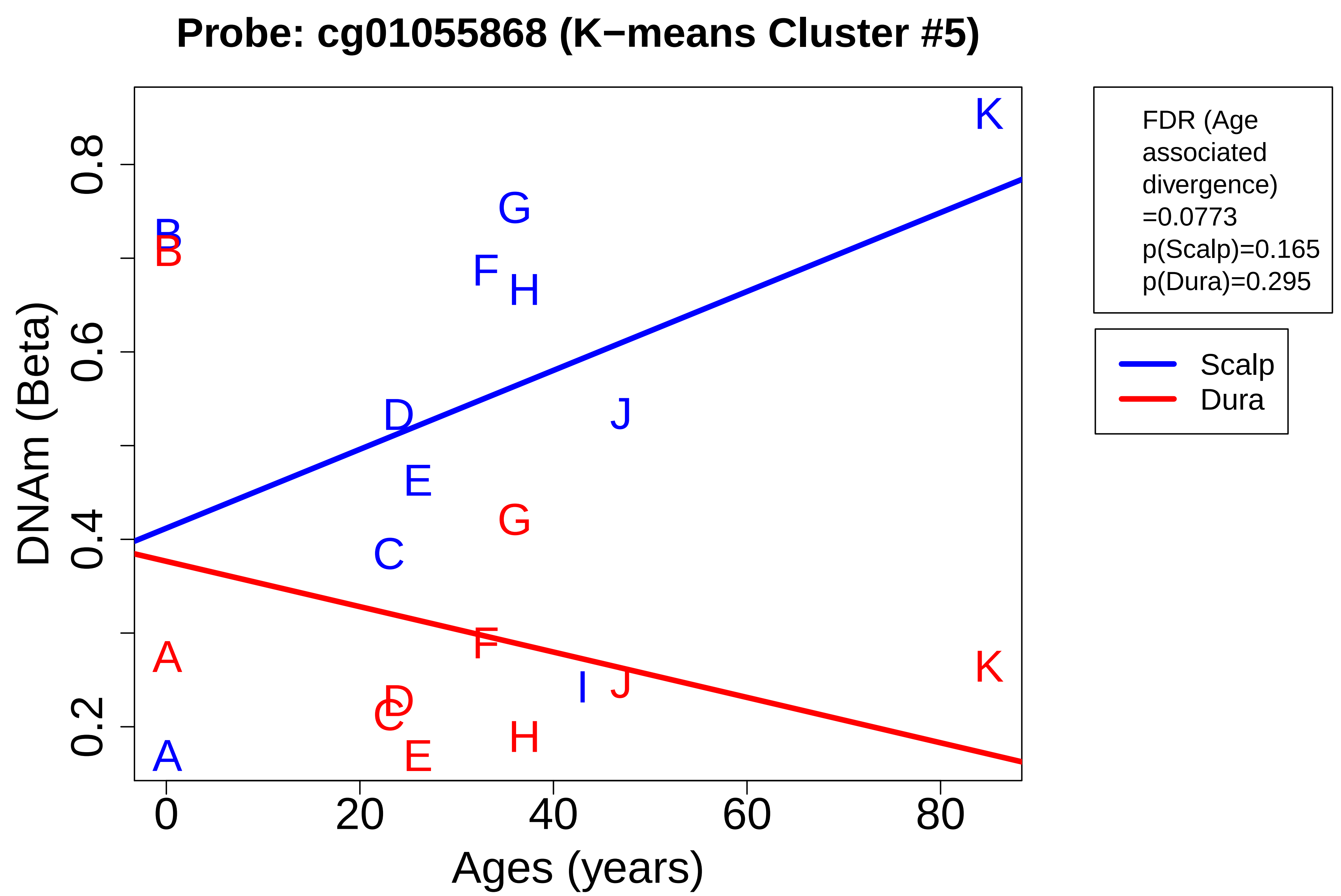

Probe: cg01055868 (K−means Cluster #5)
DNAm (Beta)
0.2
0.4
0.6
0.8
Ages (years)
0
20
40
60
80
FDR (Age associated divergence) =0.0773
p(Scalp)=0.165
p(Dura)=0.295
Scalp
Dura

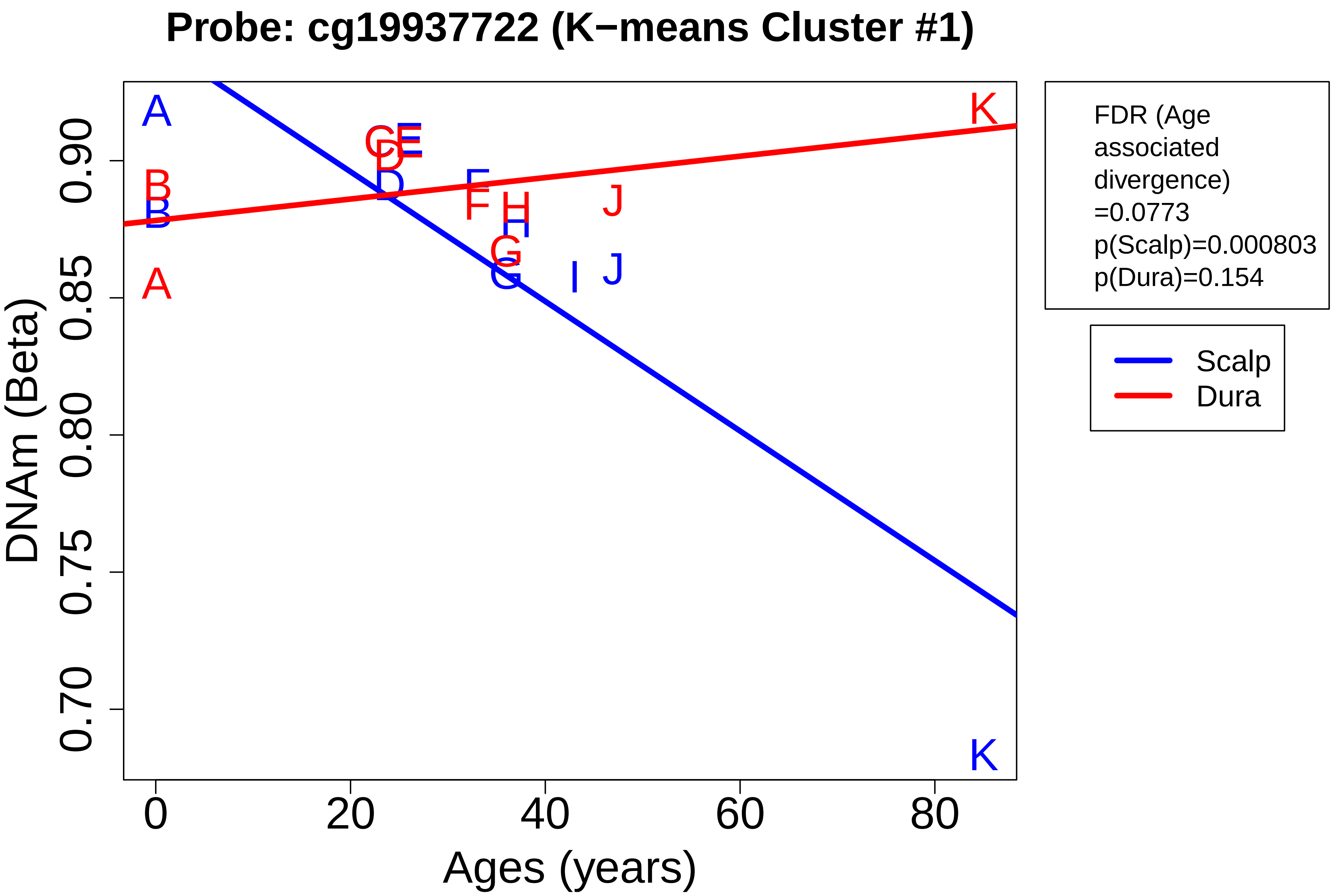

Probe: cg19937722 (K−means Cluster #1)
DNAm (Beta)
0.70
0.75
0.80
0.85
0.90
Ages (years)
0
20
40
60
80
A
B
C
D
E
F
G
H
I
J
K
FDR (Age associated divergence) =0.0773
p(Scalp)=0.000803
p(Dura)=0.154
Scalp
Dura

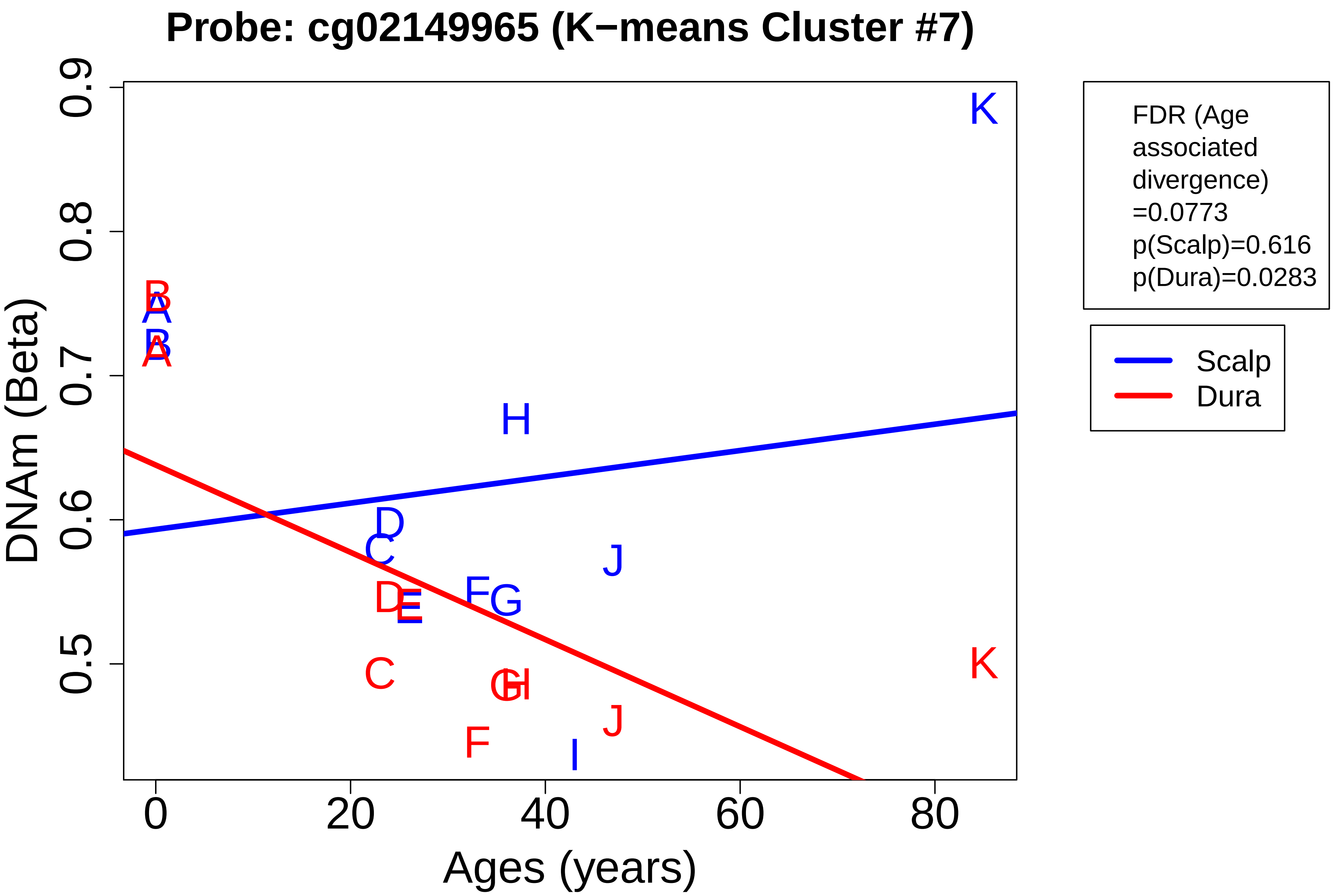

Probe: cg02149965 (K−means Cluster #7)
DNAm (Beta)
0.5
0.6
0.7
0.8
0.9
0
20
40
60
80
Ages (years)
FDR (Age associated divergence) =0.0773
p(Scalp)=0.616
p(Dura)=0.0283
Scalp
Dura

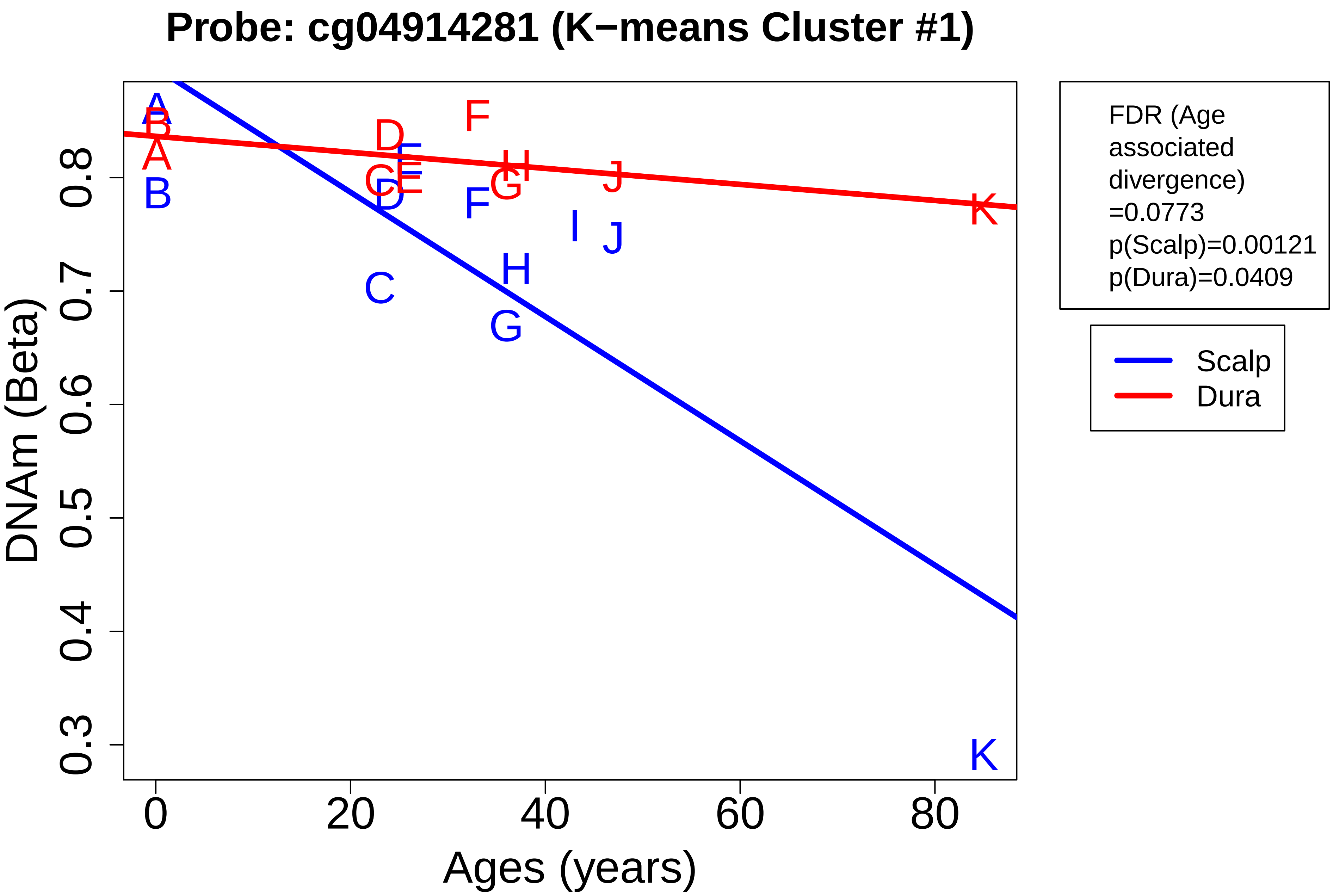

Probe: cg04914281 (K−means Cluster #1)
DNAm (Beta)
0.3
0.4
0.5
0.6
0.7
0.8
0
20
40
60
80
Ages (years)
FDR (Age associated divergence) =0.0773
p(Scalp)=0.00121
p(Dura)=0.0409
Scalp
Dura

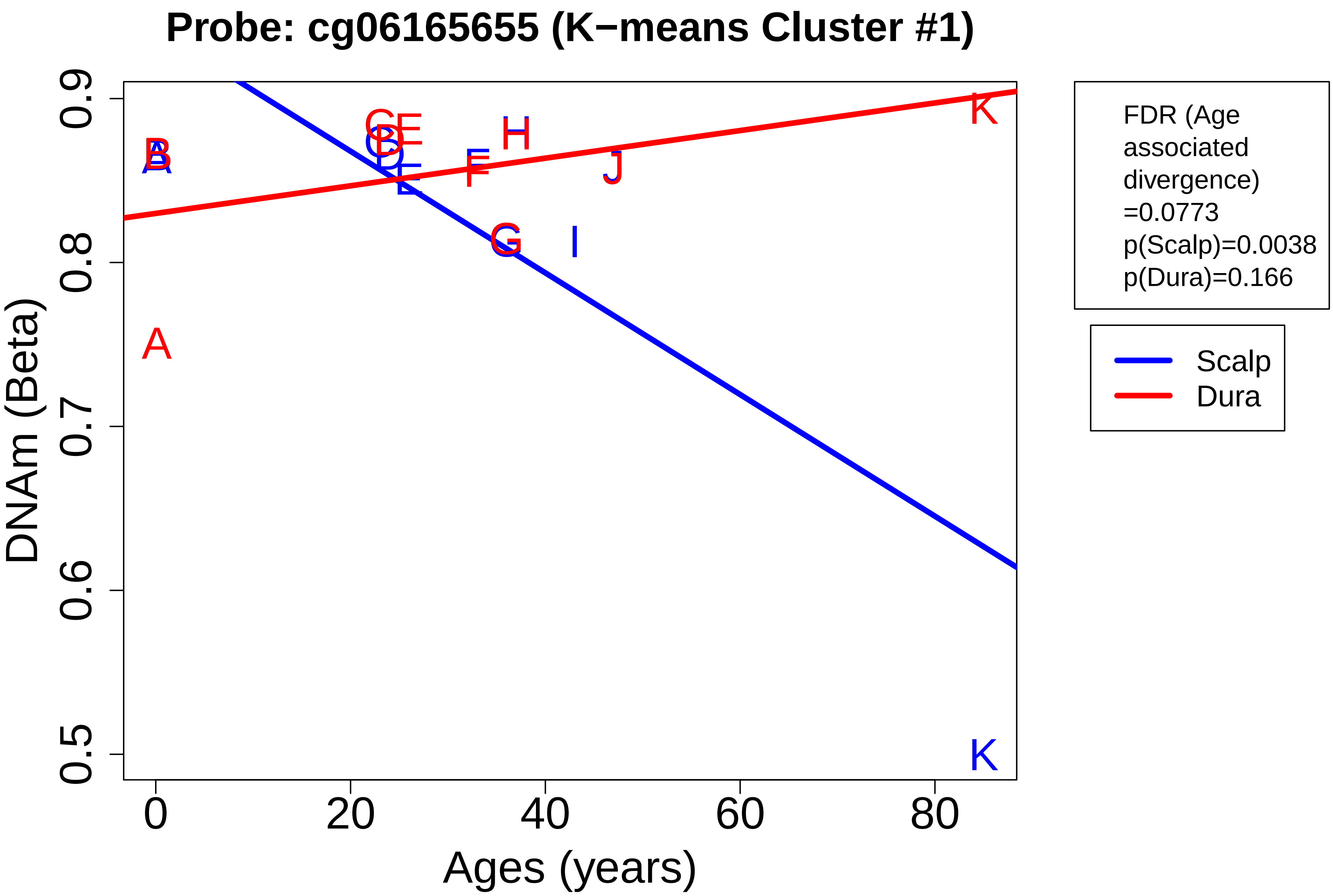

Probe: cg06165655 (K−means Cluster #1)
DNAm (Beta)
0.5
0.6
0.7
0.8
0.9
0
20
40
60
80
Ages (years)
FDR (Age associated divergence) =0.0773
p(Scalp)=0.0038
p(Dura)=0.166
Scalp
Dura

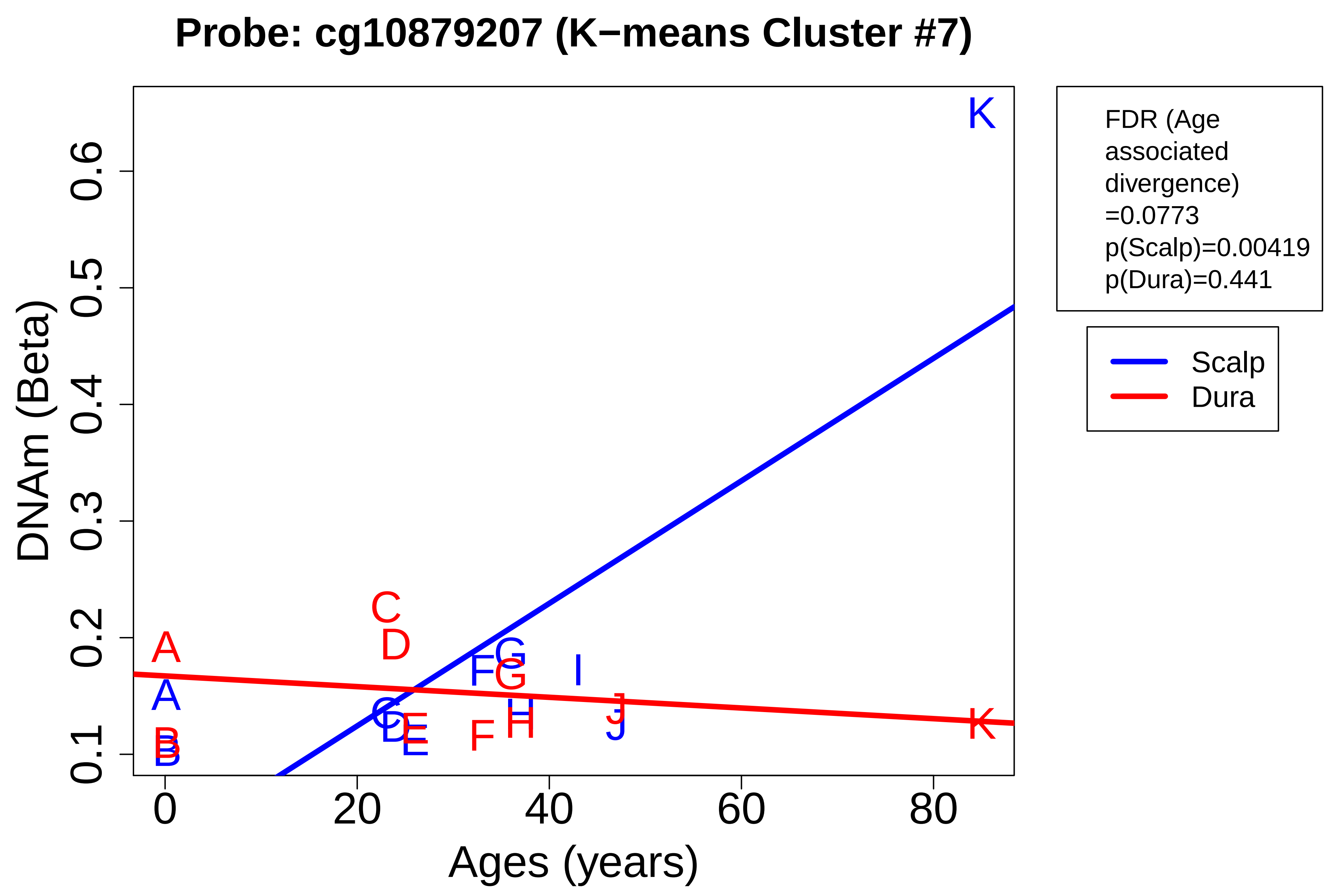

Probe: cg10879207 (K−means Cluster #7)
DNAm (Beta)
0.1
0.2
0.3
0.4
0.5
0.6
0
20
40
60
80
Ages (years)
FDR (Age associated divergence) =0.0773
p(Scalp)=0.00419
p(Dura)=0.441
Scalp
Dura

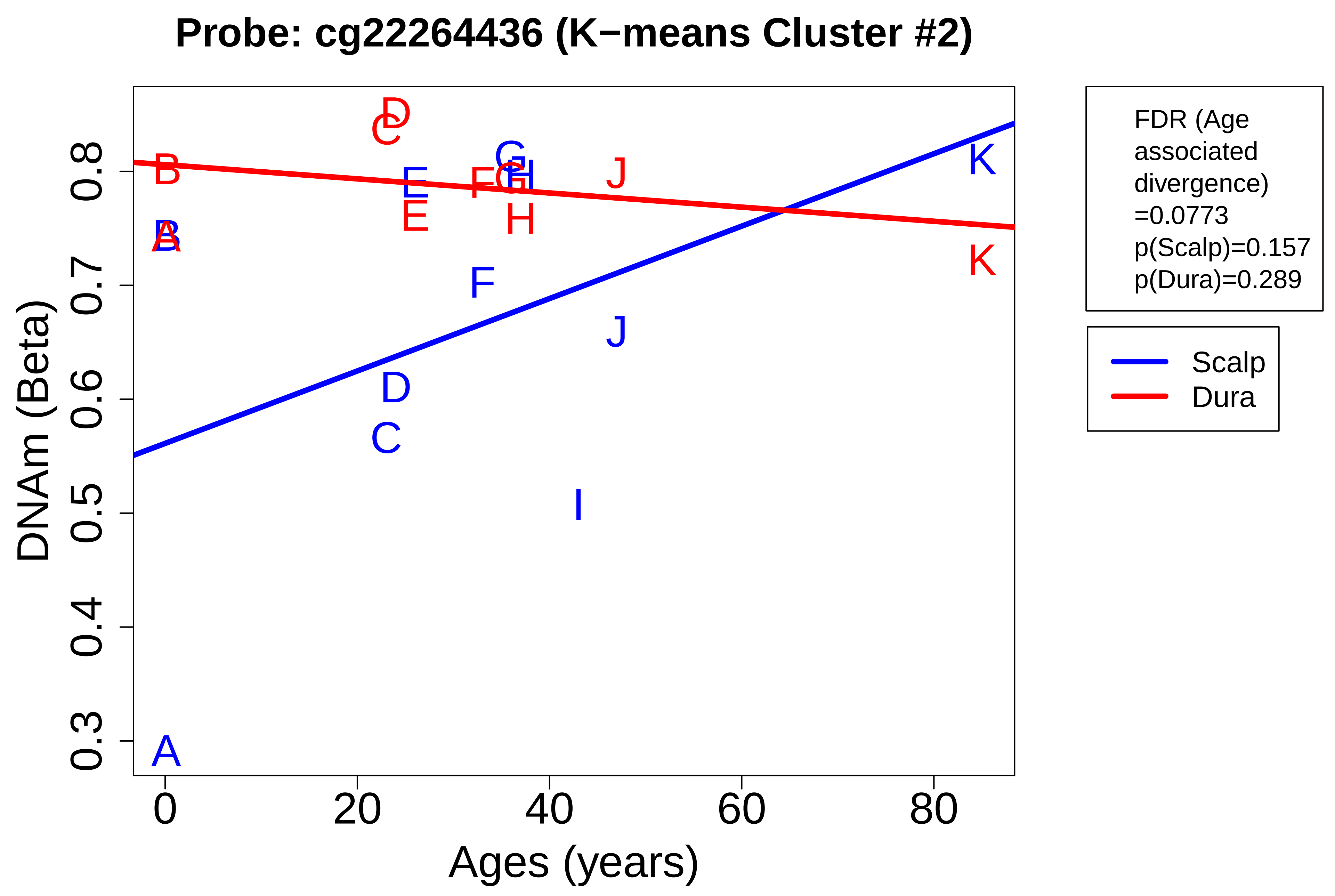

Probe: cg22264436 (K−means Cluster #2)
DNAm (Beta)
0.3
0.4
0.5
0.6
0.7
0.8
0
20
40
60
80
Ages (years)
FDR (Age associated divergence) =0.0773
p(Scalp)=0.157
p(Dura)=0.289
Scalp
Dura
A
B
C
D
E
F
G
H
I
J
K

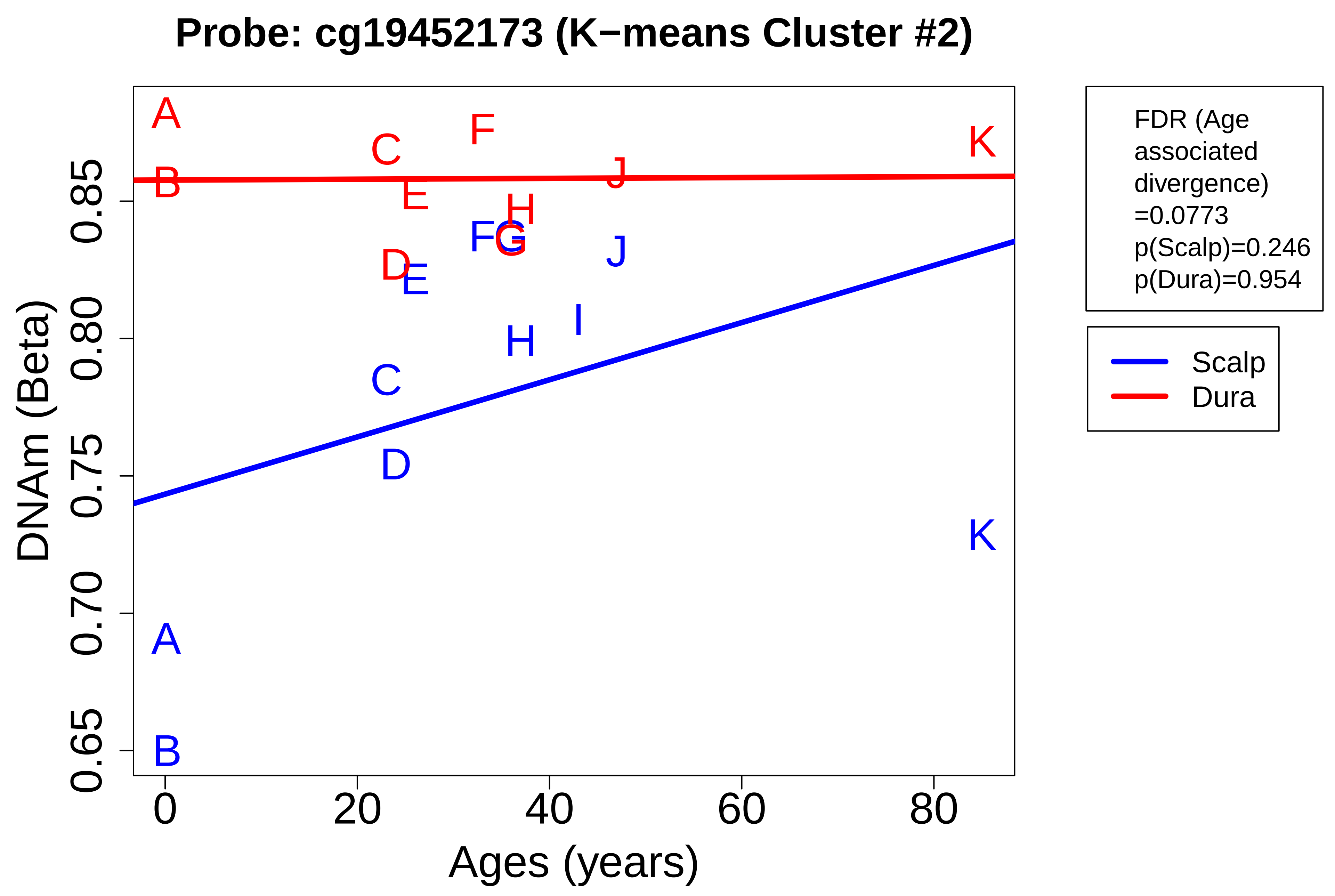

Probe: cg19452173 (K−means Cluster #2)
DNAm (Beta)
0.65
0.70
0.75
0.80
0.85
Ages (years)
0
20
40
60
80
FDR (Age associated divergence) =0.0773
p(Scalp)=0.246
p(Dura)=0.954
Scalp
Dura

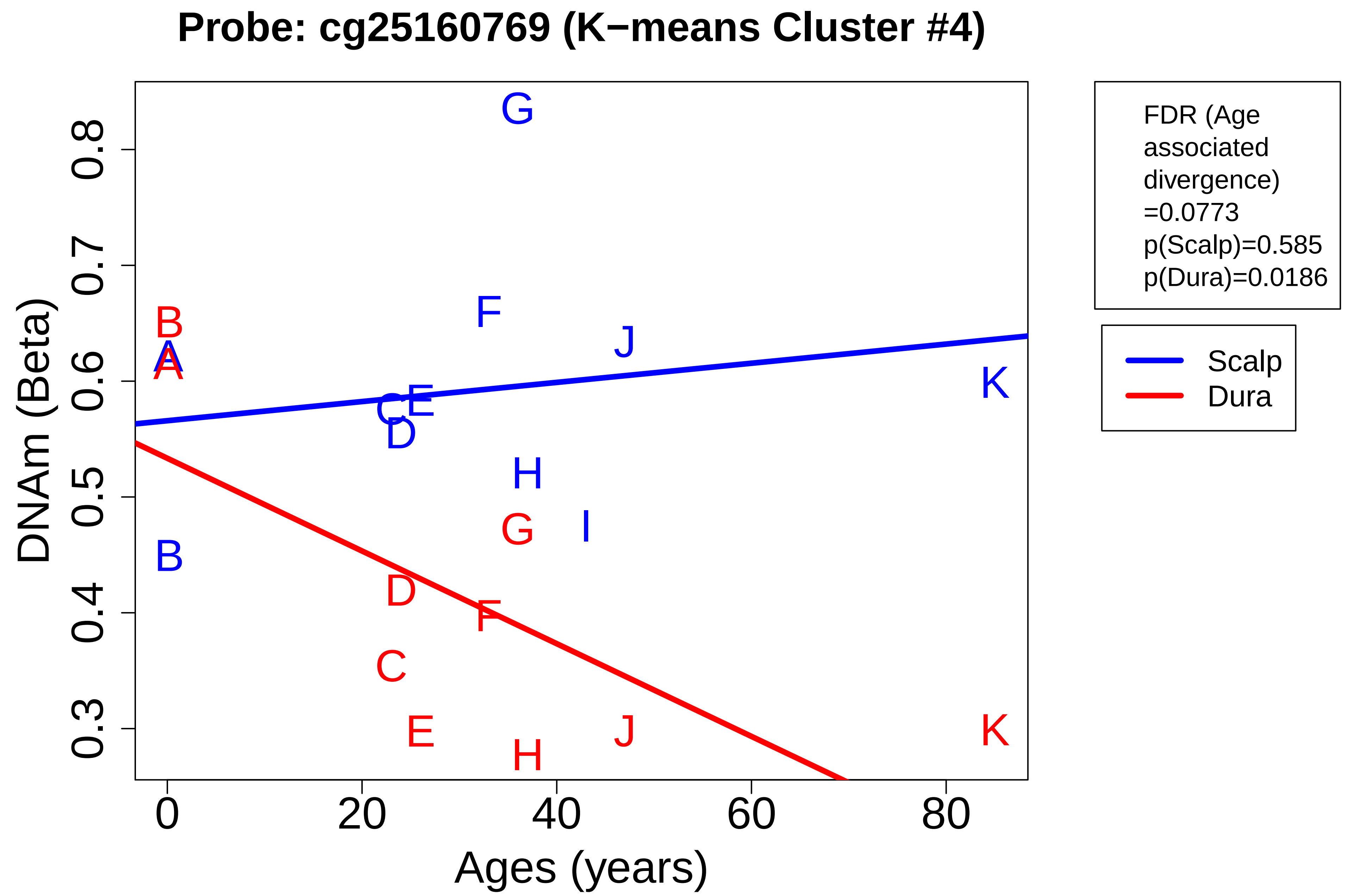

Probe: cg25160769 (K−means Cluster #4)
FDR (Age associated divergence) =0.0773
p(Scalp)=0.585
p(Dura)=0.0186
Scalp
Dura
DNAm (Beta)
0.3
0.4
0.5
0.6
0.7
0.8
0
20
40
60
80
Ages (years)

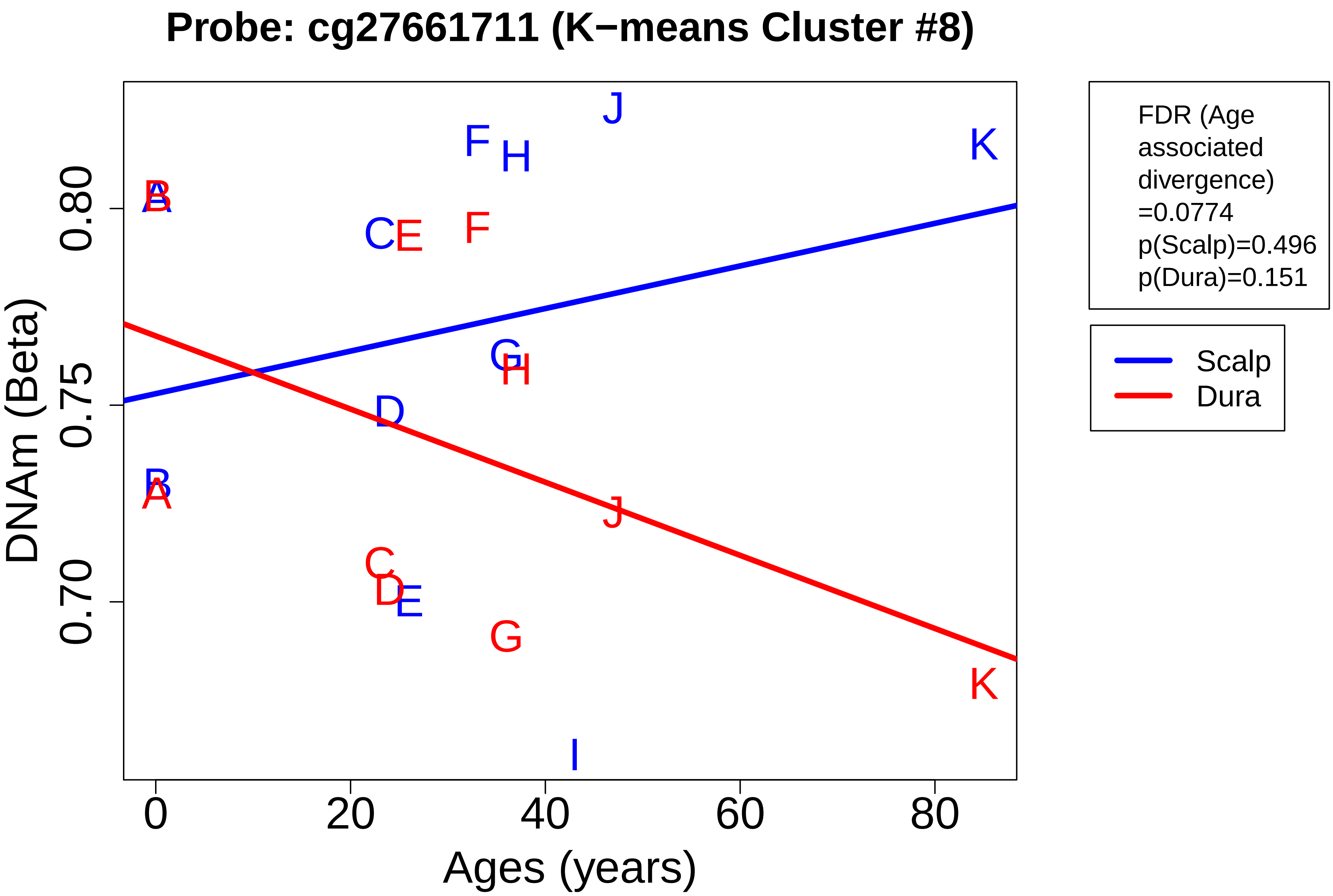

Probe: cg27661711 (K−means Cluster #8)
FDR (Age associated divergence) =0.0774
p(Scalp)=0.496
p(Dura)=0.151
Scalp
Dura
DNAm (Beta)
0.70
0.75
0.80
0
20
40
60
80
Ages (years)
A
B
C
D
E
F
G
H
I
J
K

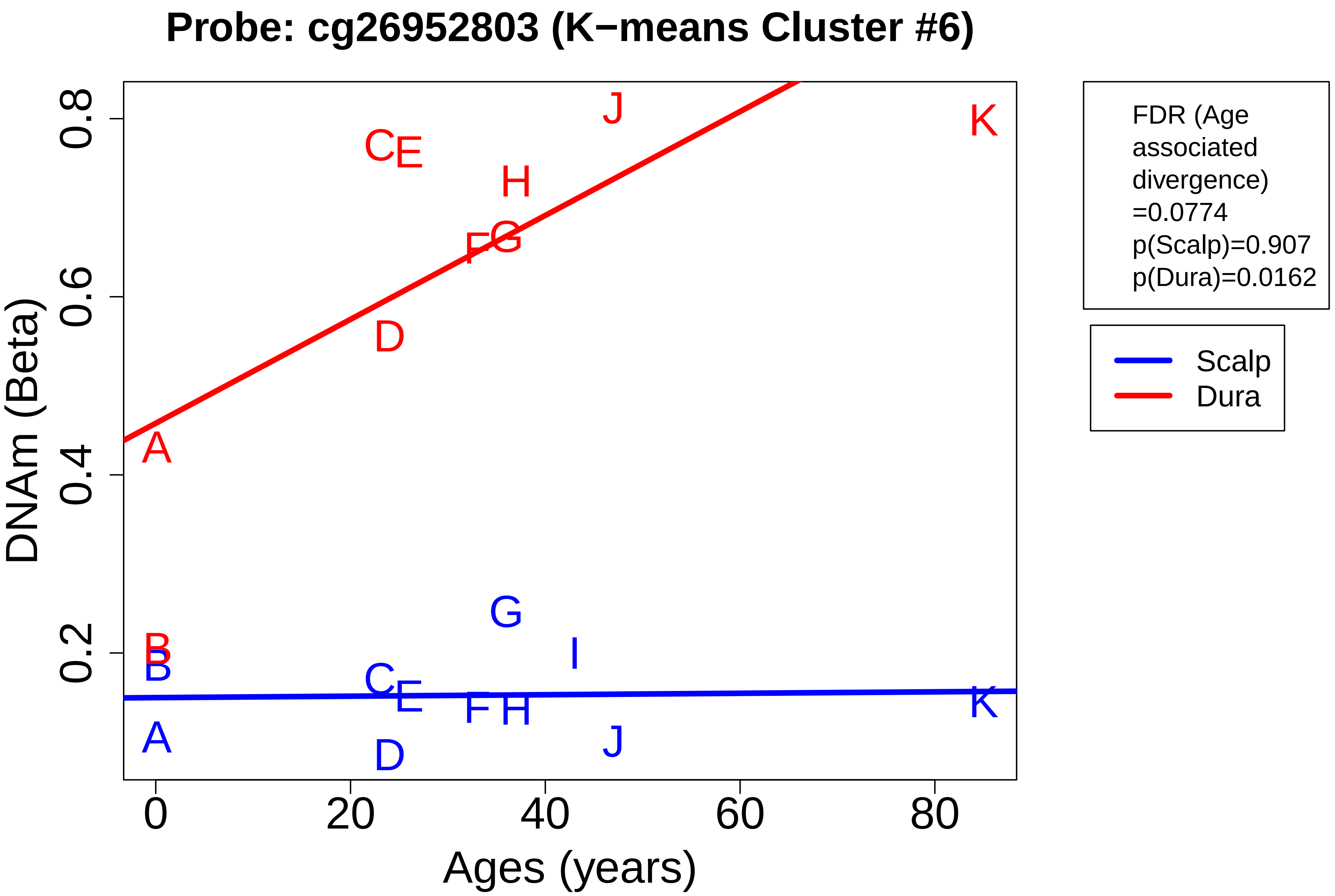
Probe: cg26952803 (K−means Cluster #6)
DNAm (Beta)
Ages (years)
0.2
0.4
0.6
0.8
0
20
40
60
80
FDR (Age associated divergence) =0.0774
p(Scalp)=0.907
p(Dura)=0.0162
Scalp
Dura

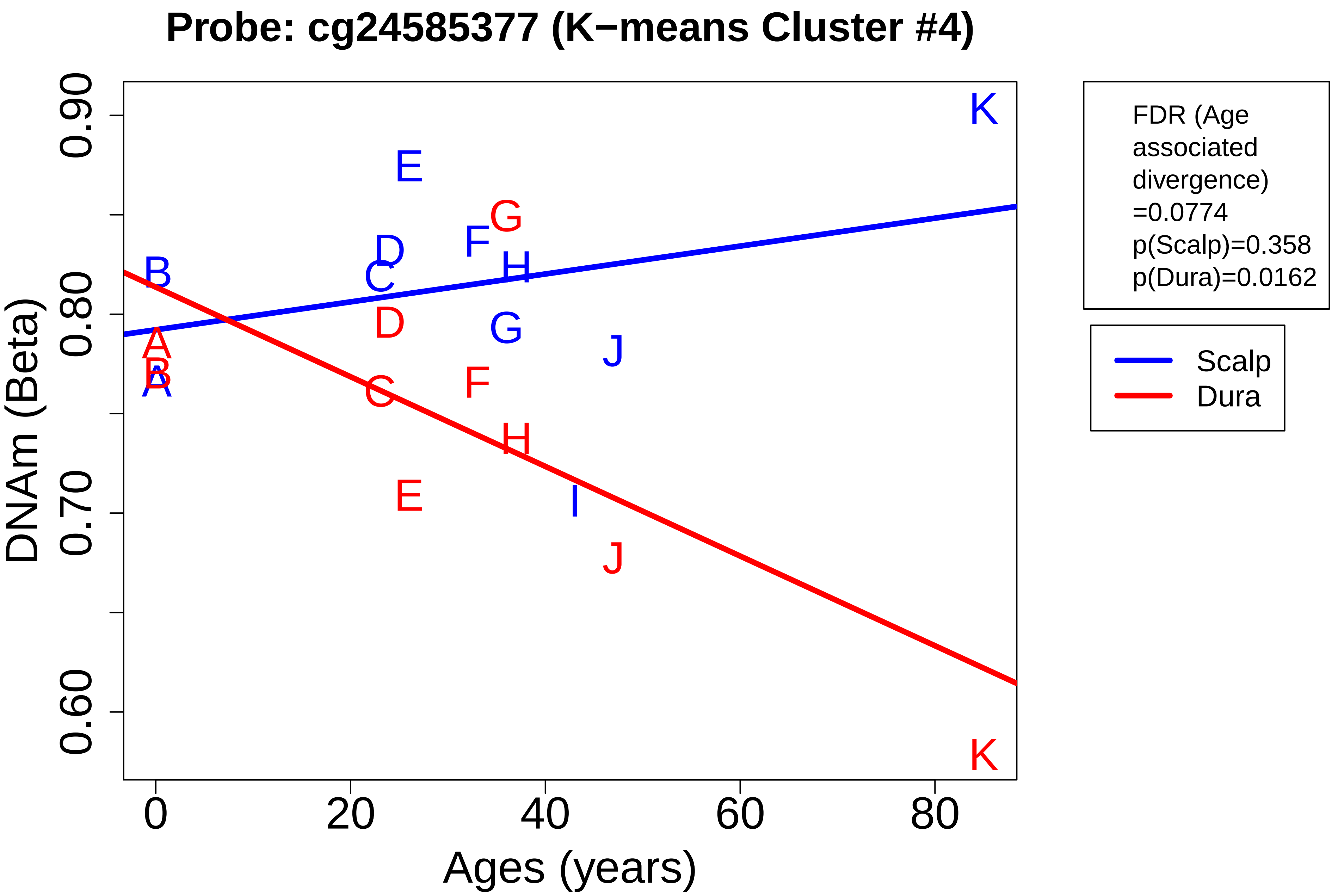

Probe: cg24585377 (K−means Cluster #4)
DNAm (Beta)
0.60
0.70
0.80
0.90
Ages (years)
0
20
40
60
80
FDR (Age associated divergence) =0.0774
p(Scalp)=0.358
p(Dura)=0.0162
Scalp
Dura

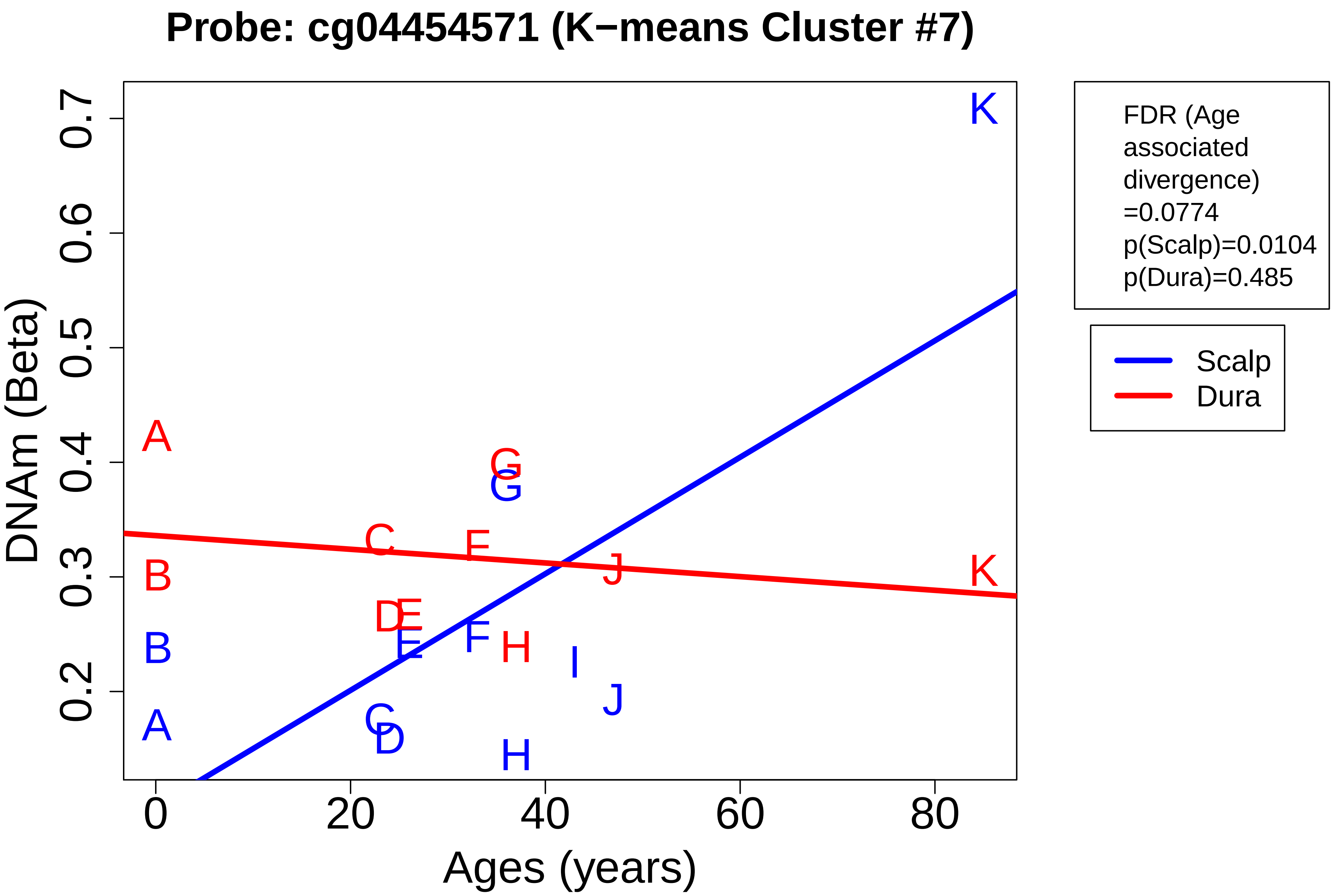

Probe: cg04454571 (K−means Cluster #7)
DNAm (Beta)
0.2
0.3
0.4
0.5
0.6
0.7
Ages (years)
0
20
40
60
80
FDR (Age associated divergence) =0.0774
p(Scalp)=0.0104
p(Dura)=0.485
Scalp
Dura
A
B
C
D
E
F
G
H
I
J
K

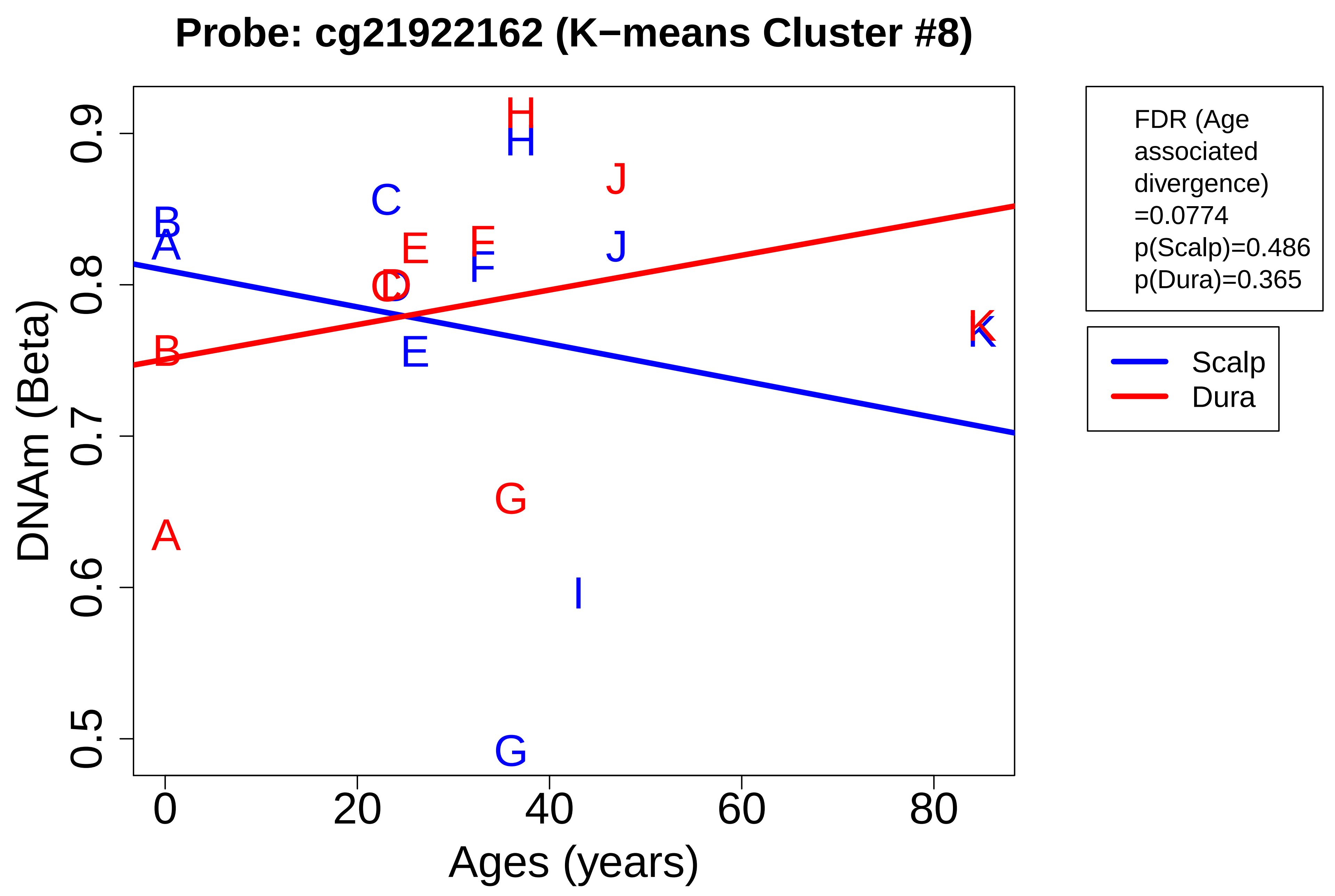

Probe: cg21922162 (K−means Cluster #8)
DNAm (Beta)
0.5
0.6
0.7
0.8
0.9
0
20
40
60
80
Ages (years)
FDR (Age associated divergence) =0.0774
p(Scalp)=0.486
p(Dura)=0.365
Scalp
Dura

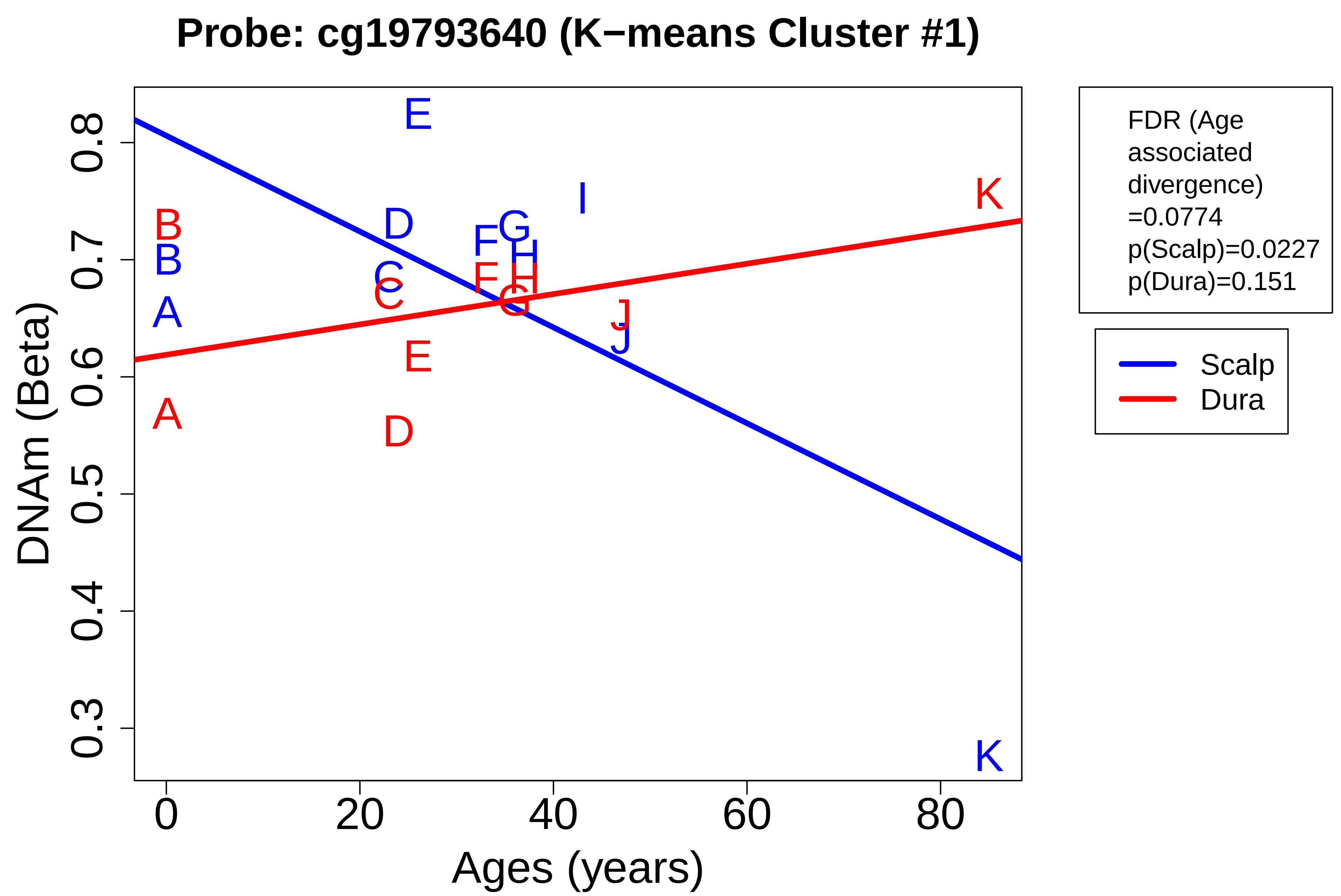

Probe: cg19793640 (K−means Cluster #1)
DNAm (Beta)
0.3
0.4
0.5
0.6
0.7
0.8
0
20
40
60
80
Ages (years)
A
B
C
D
E
F
G
H
I
J
K
FDR (Age associated divergence) =0.0774
p(Scalp)=0.0227
p(Dura)=0.151
Scalp
Dura

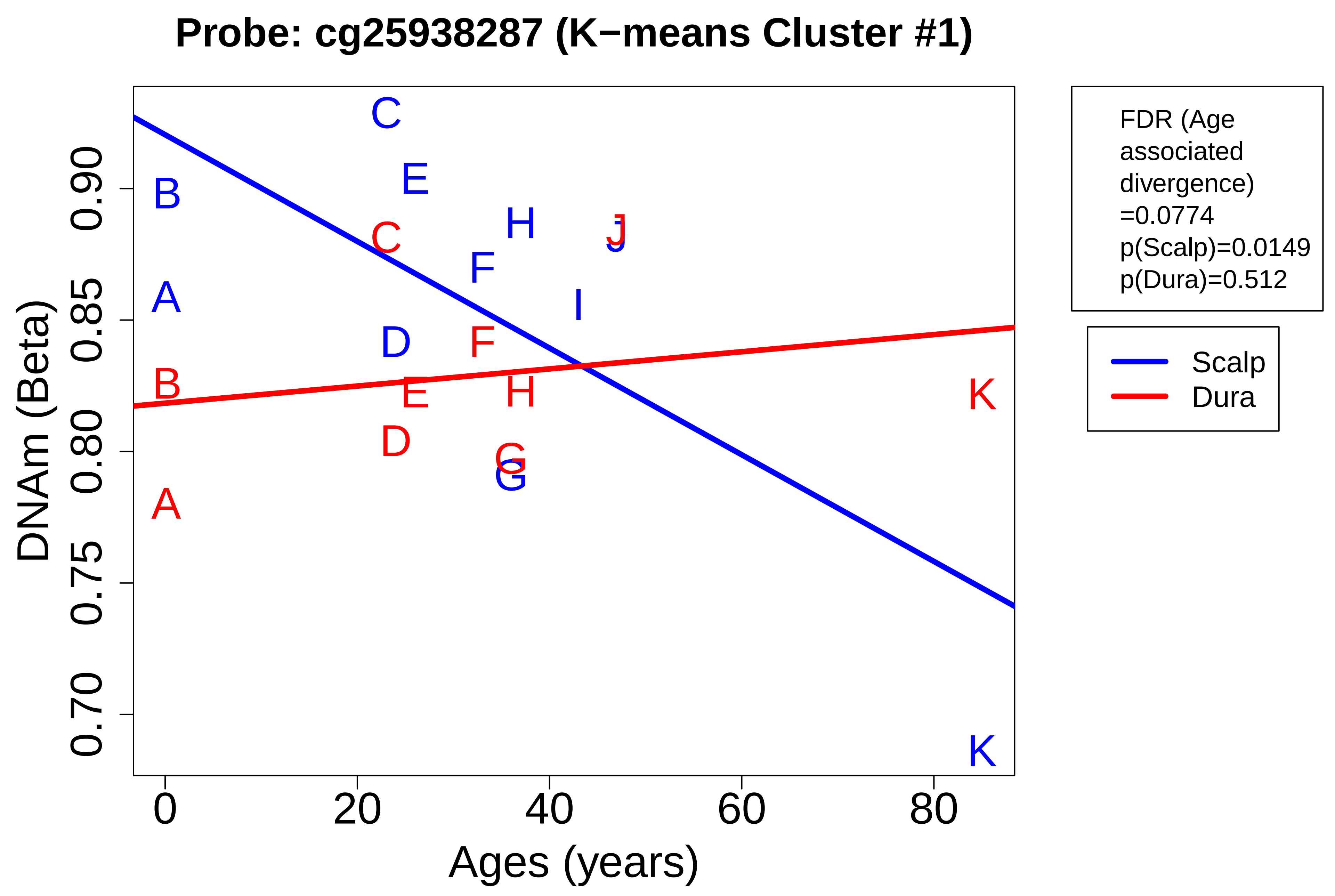

Probe: cg25938287 (K−means Cluster #1)
DNAm (Beta)
0.70
0.75
0.80
0.85
0.90
0
20
40
60
80
Ages (years)
A
B
C
D
E
F
G
H
I
J
K
FDR (Age associated divergence) =0.0774
p(Scalp)=0.0149
p(Dura)=0.512
Scalp
Dura

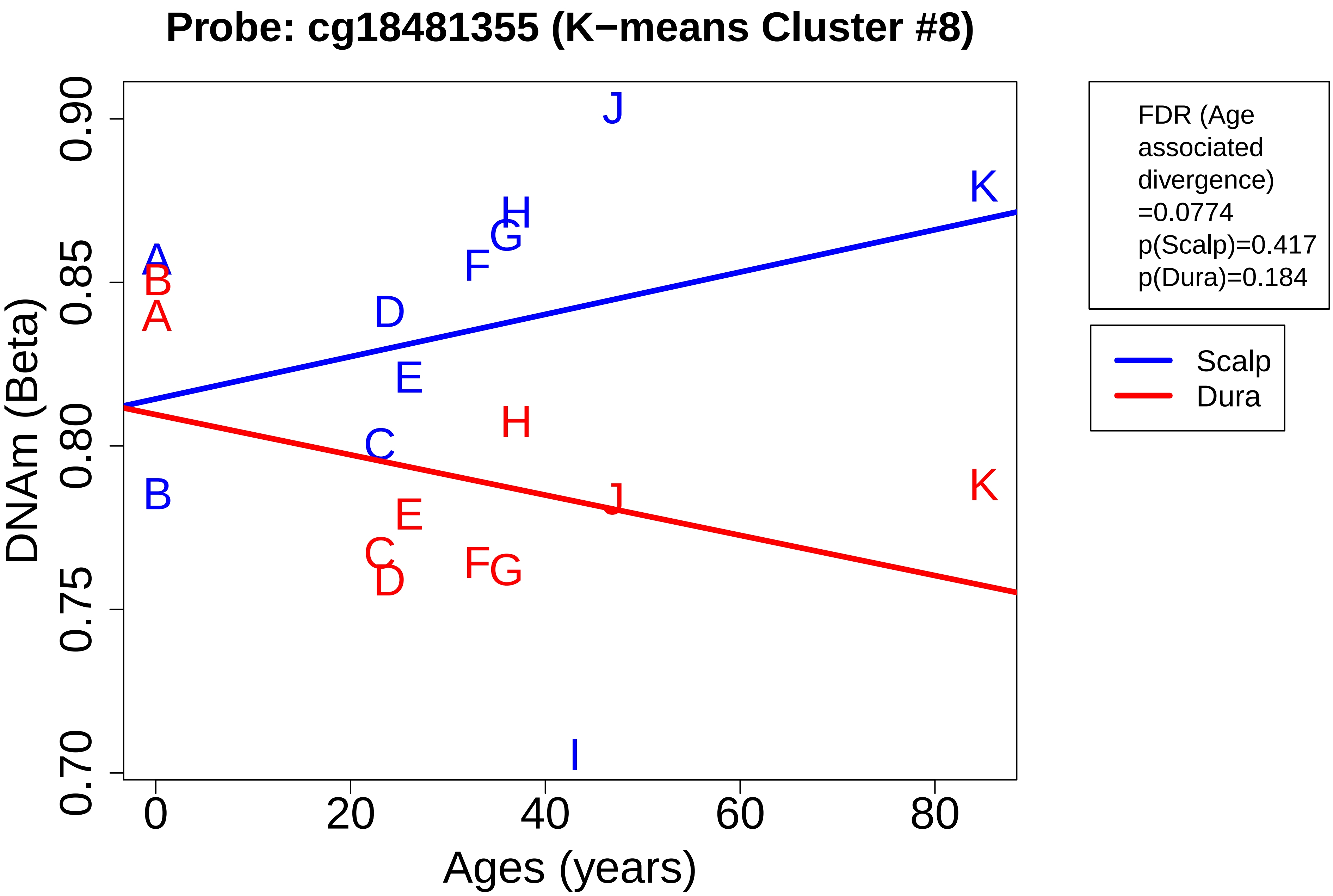

Probe: cg18481355 (K−means Cluster #8)
DNAm (Beta)
0.70
0.75
0.80
0.85
0.90
Ages (years)
0
20
40
60
80
A
B
C
D
E
F
G
H
I
J
K
FDR (Age associated divergence) =0.0774
p(Scalp)=0.417
p(Dura)=0.184
Scalp
Dura

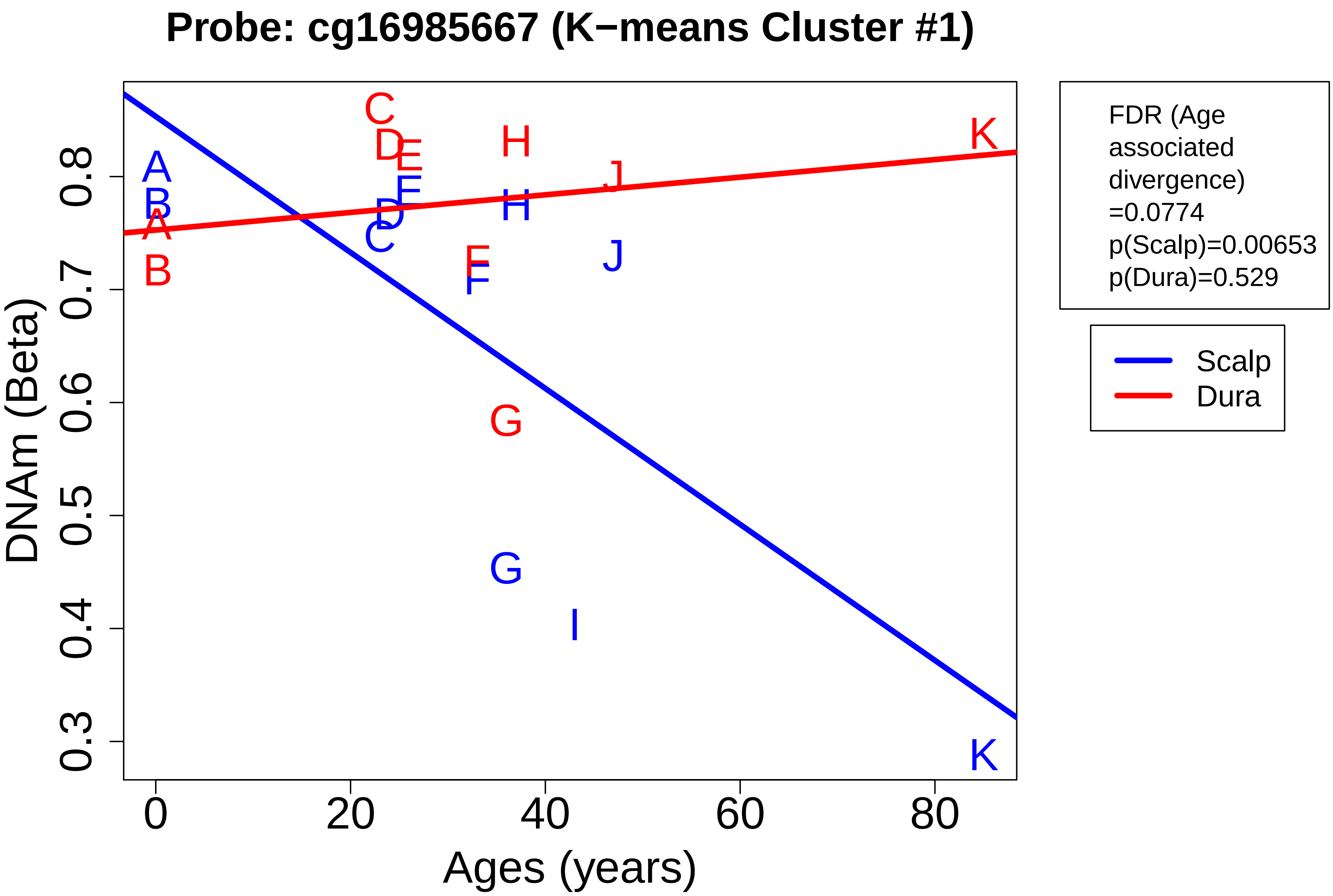

Probe: cg16985667 (K−means Cluster #1)
DNAm (Beta)
0.3
0.4
0.5
0.6
0.7
0.8
0
20
40
60
80
Ages (years)
FDR (Age associated divergence) =0.0774
p(Scalp)=0.00653
p(Dura)=0.529
Scalp
Dura
A
B
C
D
E
F
G
H
I
J
K

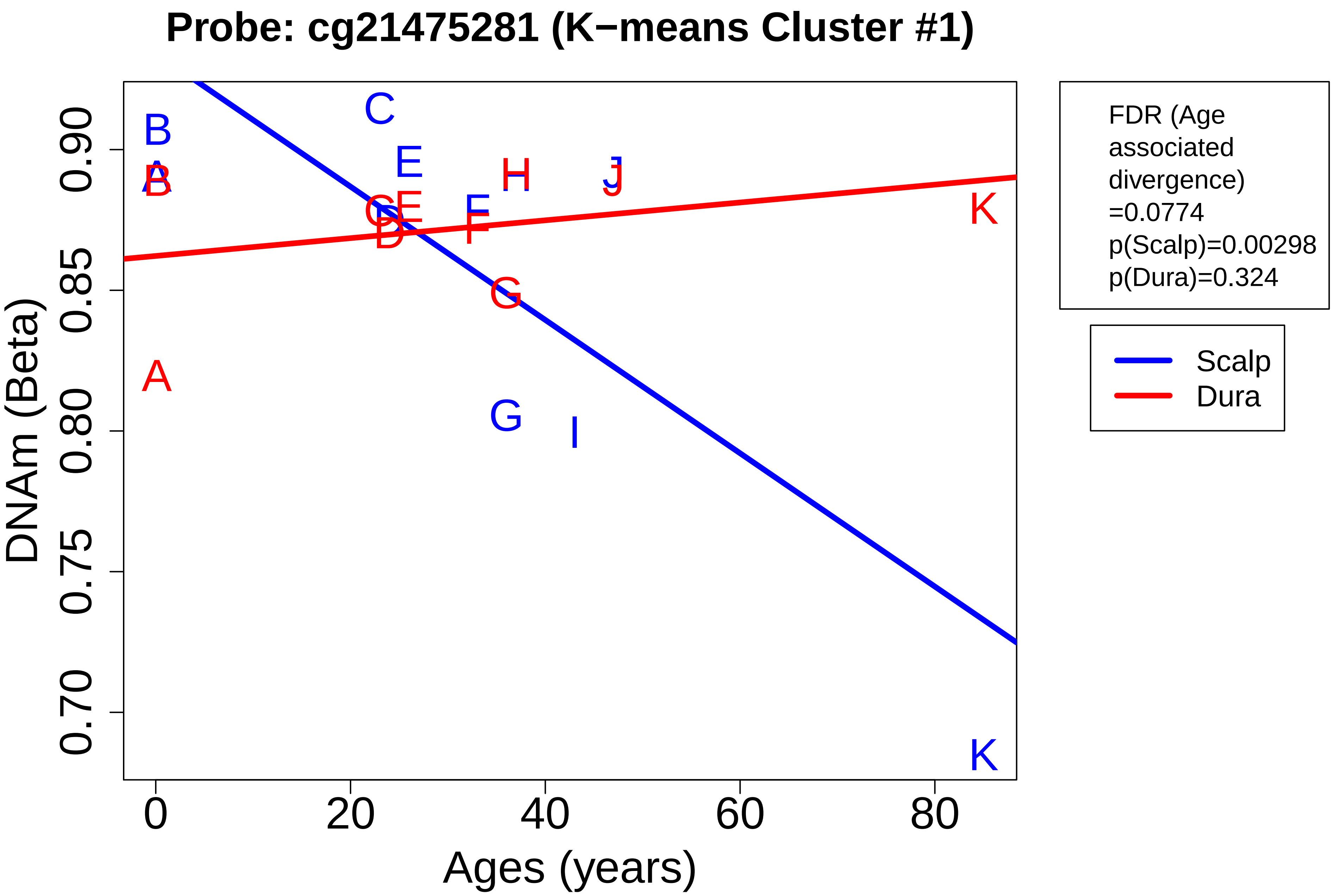

Probe: cg21475281 (K−means Cluster #1)
DNAm (Beta)
0.70
0.75
0.80
0.85
0.90
0
20
40
60
80
Ages (years)
FDR (Age associated divergence) =0.0774
p(Scalp)=0.00298
p(Dura)=0.324
Scalp
Dura

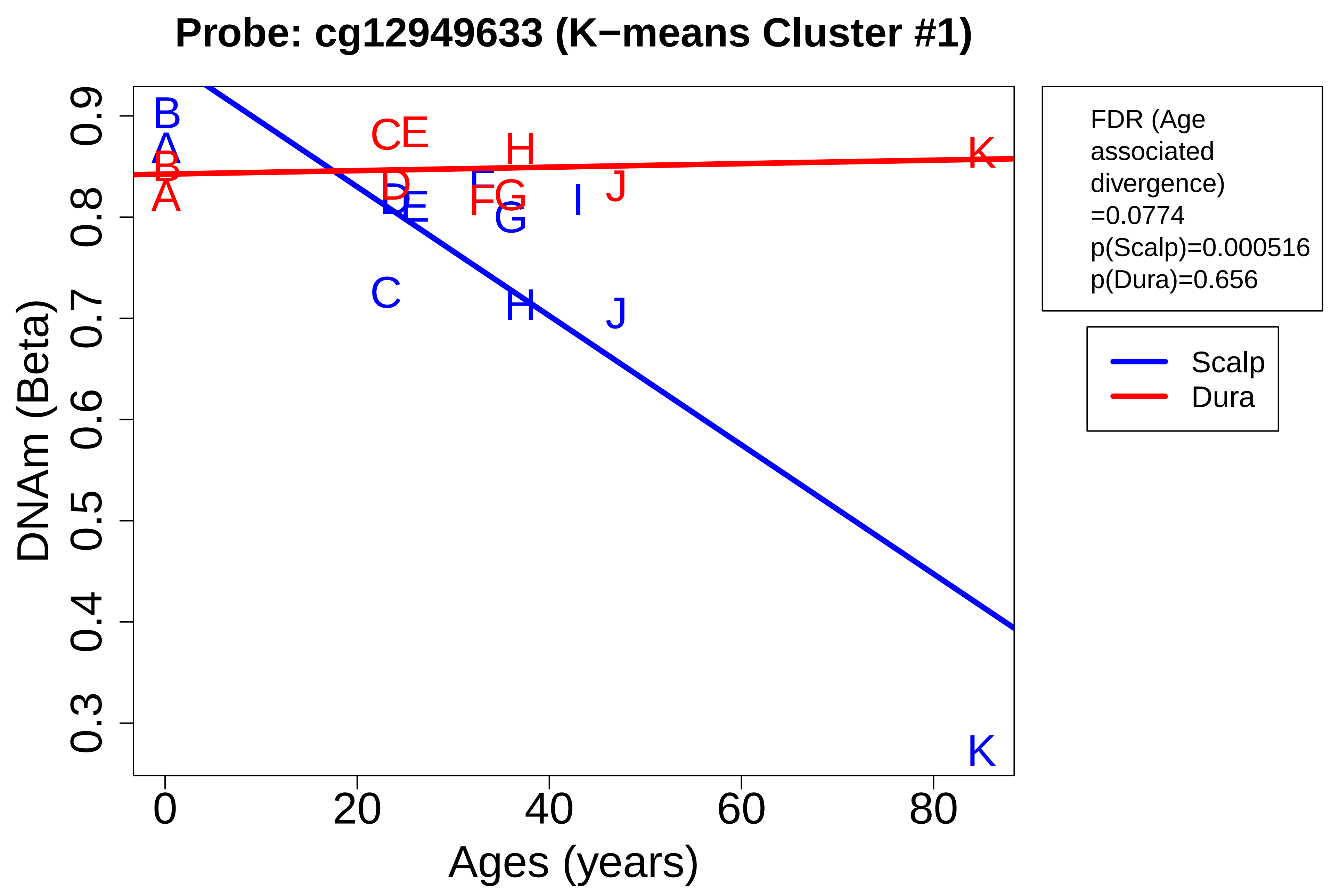

Probe: cg12949633 (K−means Cluster #1)
DNAm (Beta)
0.3
0.4
0.5
0.6
0.7
0.8
0.9
Ages (years)
0
20
40
60
80
B
A
B
A
CE
D
DE
F
FG
G
H
I
J
C
H
J
K
K
FDR (Age associated divergence) =0.0774
p(Scalp)=0.000516
p(Dura)=0.656
Scalp
Dura

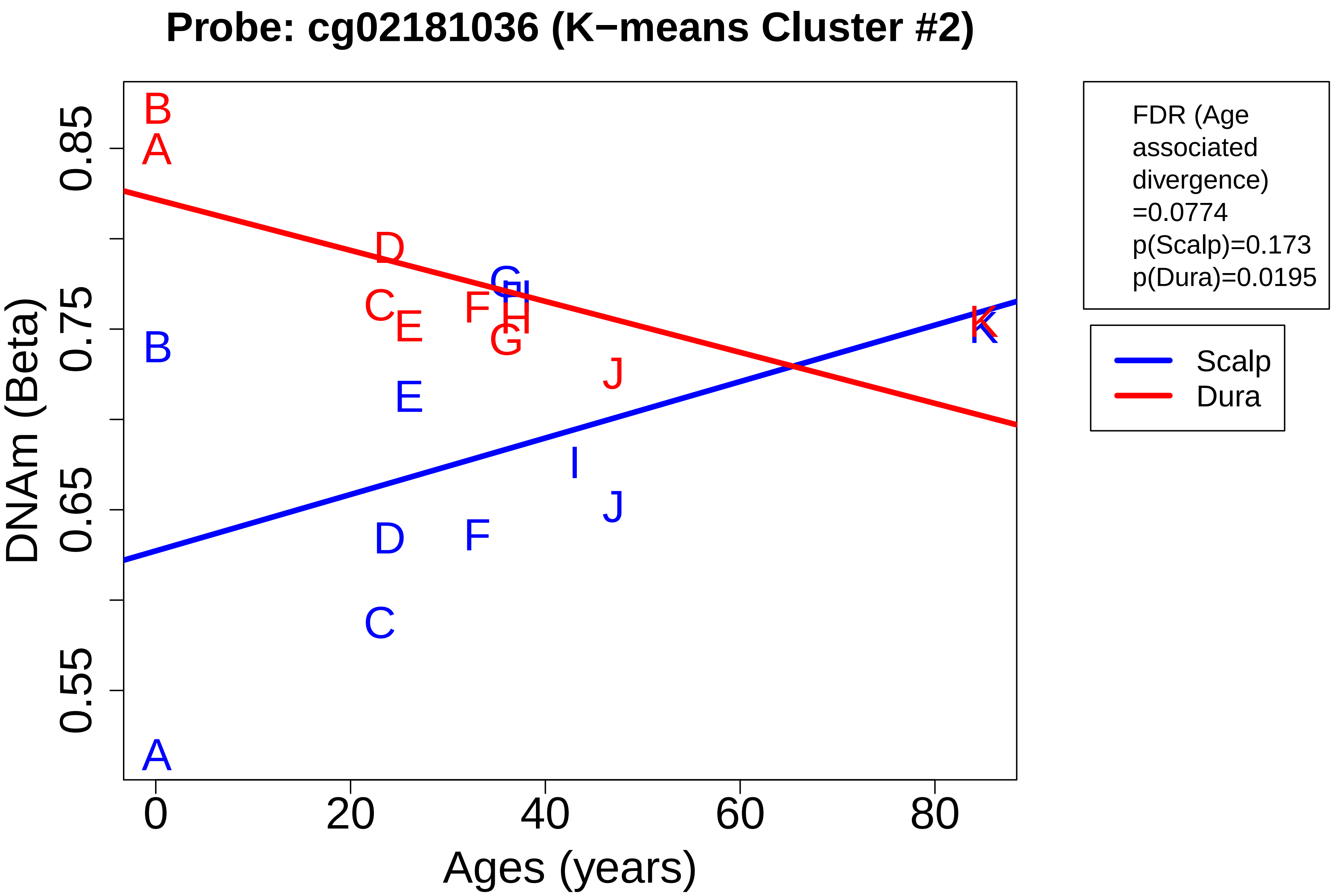

Probe: cg02181036 (K−means Cluster #2)
DNAm (Beta)
0.55
0.65
0.75
0.85
0
20
40
60
80
Ages (years)
FDR (Age associated divergence) =0.0774
p(Scalp)=0.173
p(Dura)=0.0195
Scalp
Dura

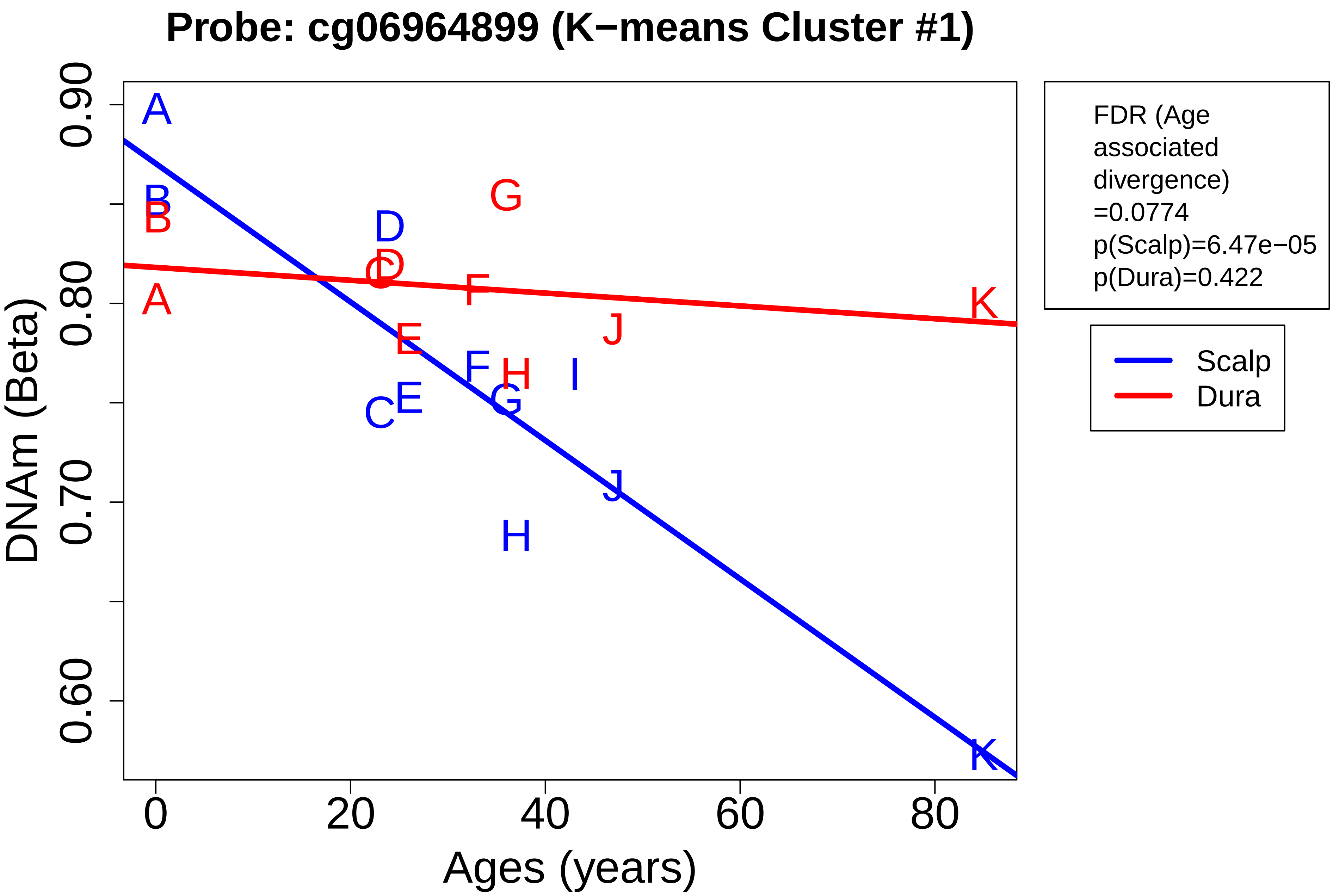

Probe: cg06964899 (K−means Cluster #1)
DNAm (Beta)
0.60
0.70
0.80
0.90
Ages (years)
0
20
40
60
80
A
B
B
A
D
D
C
C
G
F
E
F
H
I
E
G
J
J
H
K
K
FDR (Age associated divergence) =0.0774
p(Scalp)=6.47e−05
p(Dura)=0.422
Scalp
Dura

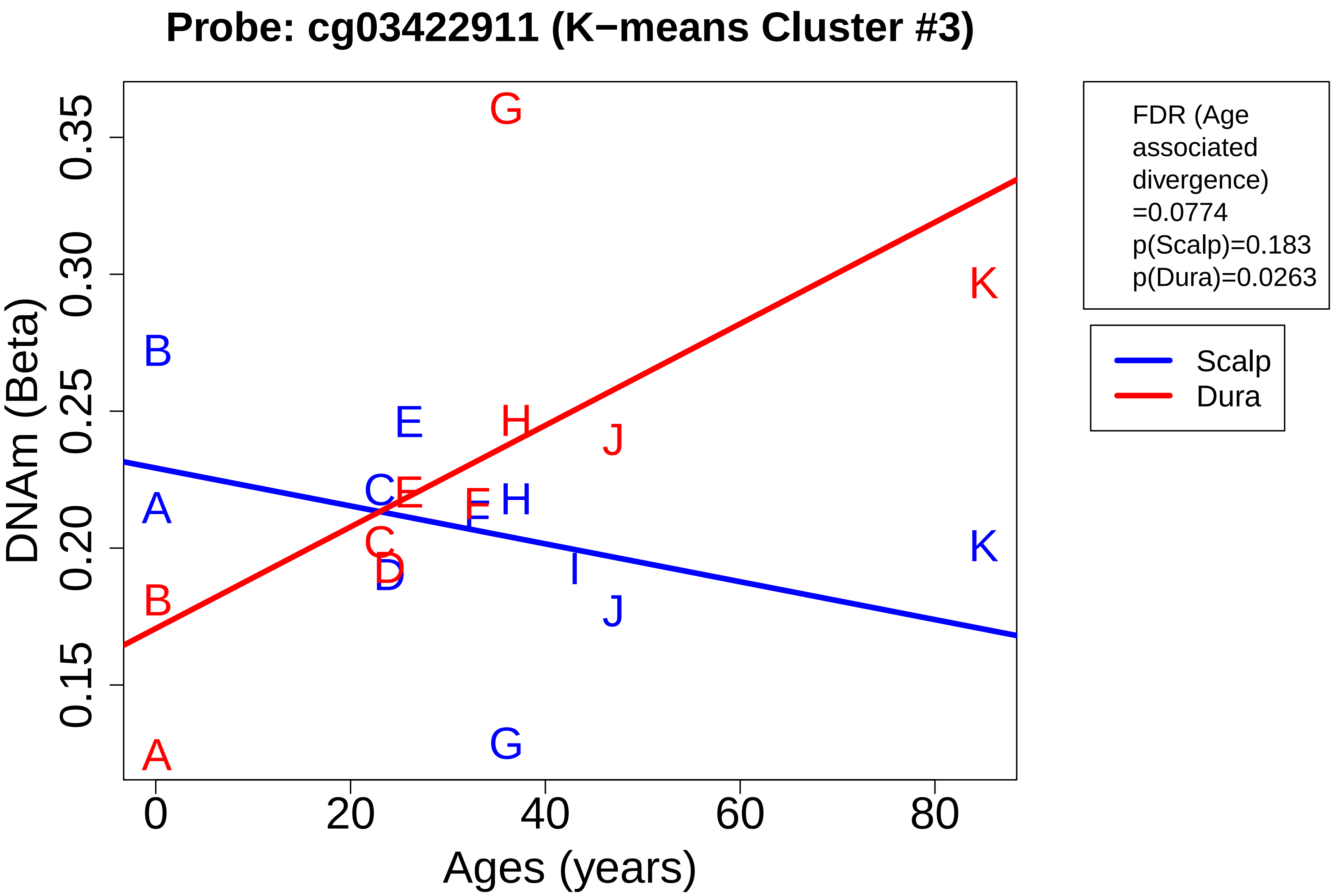

Probe: cg03422911 (K−means Cluster #3)
FDR (Age associated divergence) =0.0774
p(Scalp)=0.183
p(Dura)=0.0263
Scalp
Dura
DNAm (Beta)
0.15
0.20
0.25
0.30
0.35
0
20
40
60
80
Ages (years)

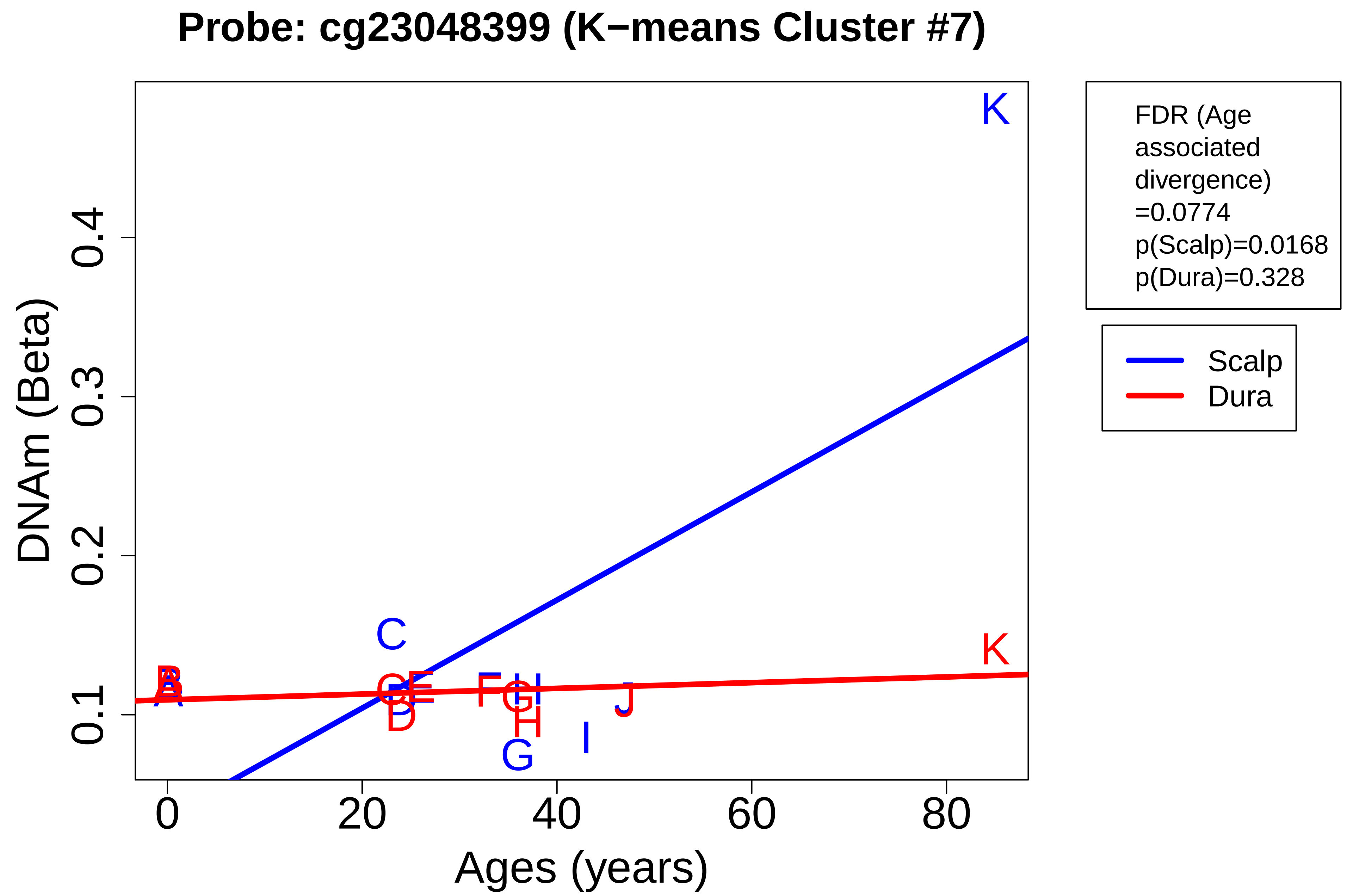
Probe: cg23048399 (K−means Cluster #7)
DNAm (Beta)
0.1
0.2
0.3
0.4
0
20
40
60
80
Ages (years)
FDR (Age associated divergence) =0.0774
p(Scalp)=0.0168
p(Dura)=0.328
Scalp
Dura

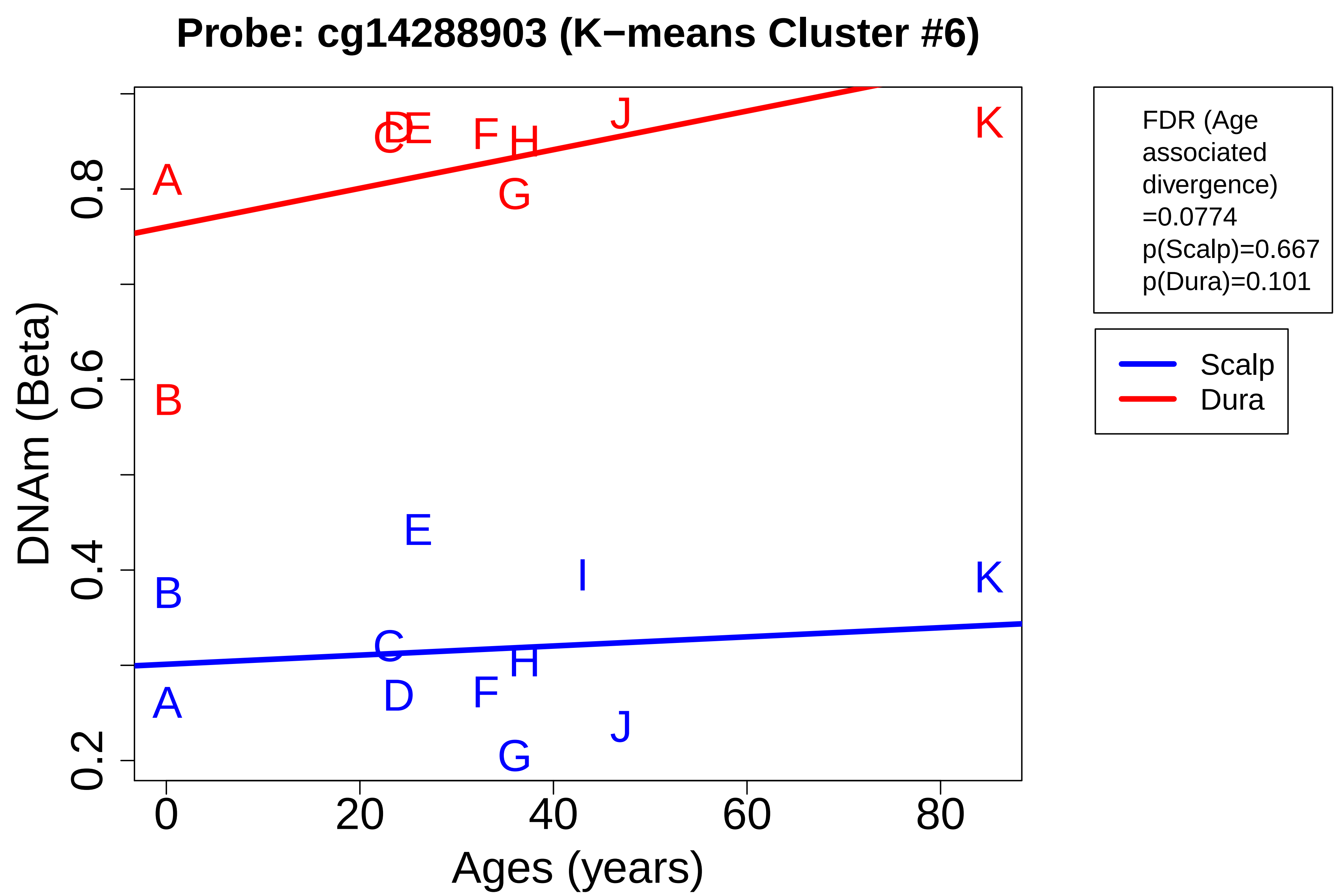

Probe: cg14288903 (K−means Cluster #6)
FDR (Age associated divergence) =0.0774
p(Scalp)=0.667
p(Dura)=0.101
Scalp
Dura
DNAm (Beta)
0.2
0.4
0.6
0.8
0
20
40
60
80
Ages (years)

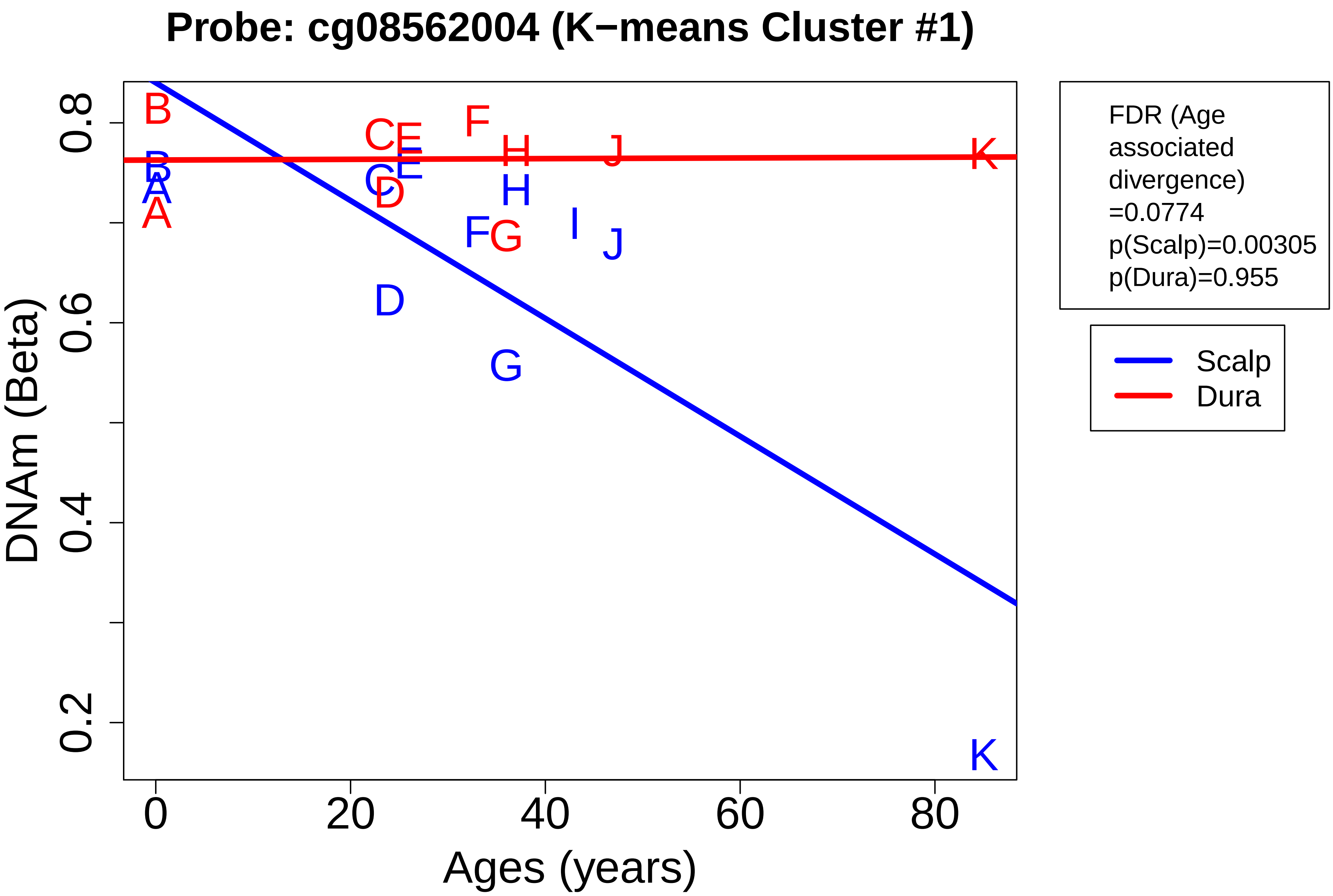

Probe: cg08562004 (K−means Cluster #1)
DNAm (Beta)
0.2
0.4
0.6
0.8
Ages (years)
0
20
40
60
80
FDR (Age associated divergence) =0.0774
p(Scalp)=0.00305
p(Dura)=0.955
Scalp
Dura

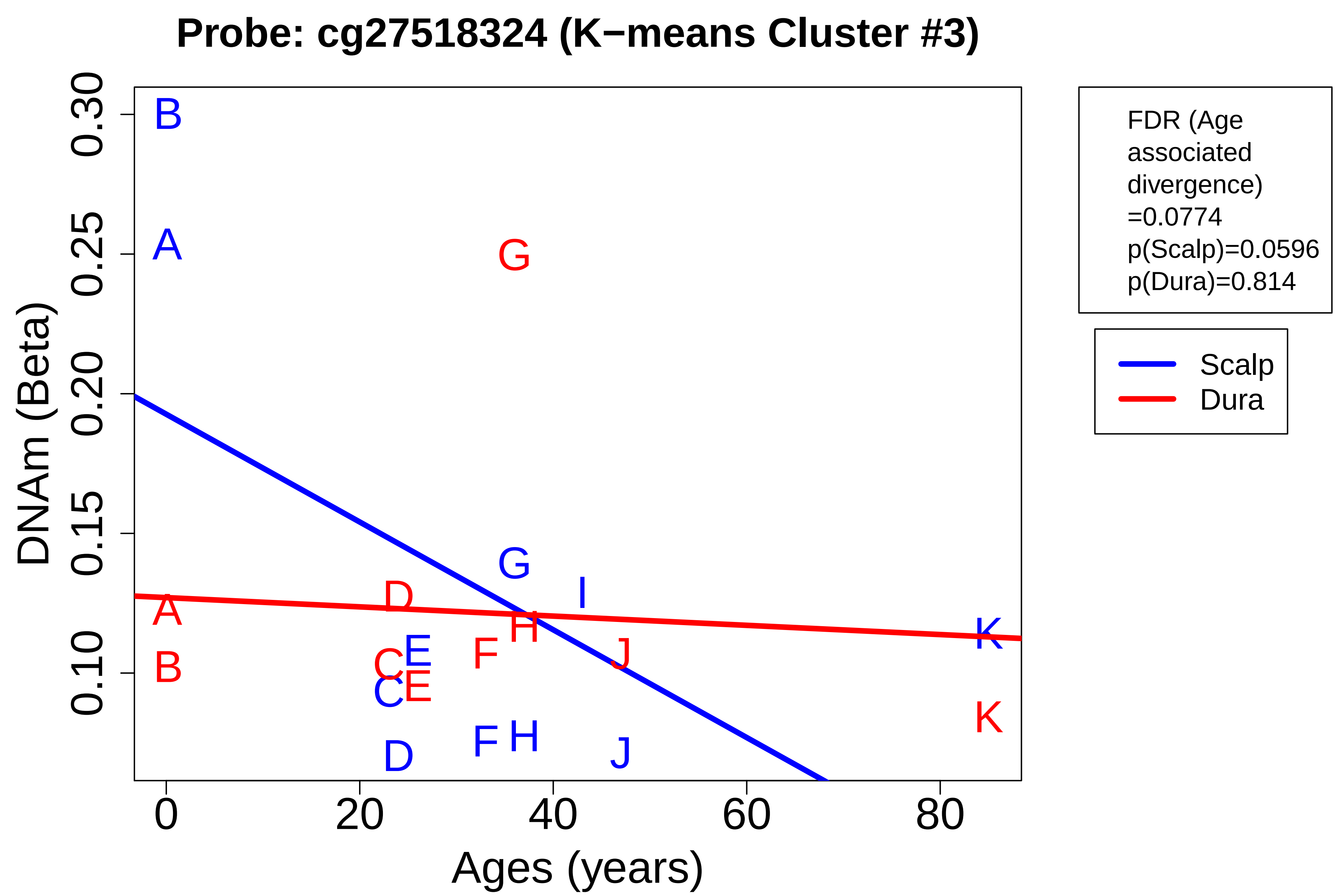

Probe: cg27518324 (K−means Cluster #3)
DNAm (Beta)
0.10
0.15
0.20
0.25
0.30
0
20
40
60
80
Ages (years)
FDR (Age associated divergence) =0.0774
p(Scalp)=0.0596
p(Dura)=0.814
Scalp
Dura
A
B
C
D
E
F
G
H
I
J
K
A
B
C
D
E
F
G
H
J
K

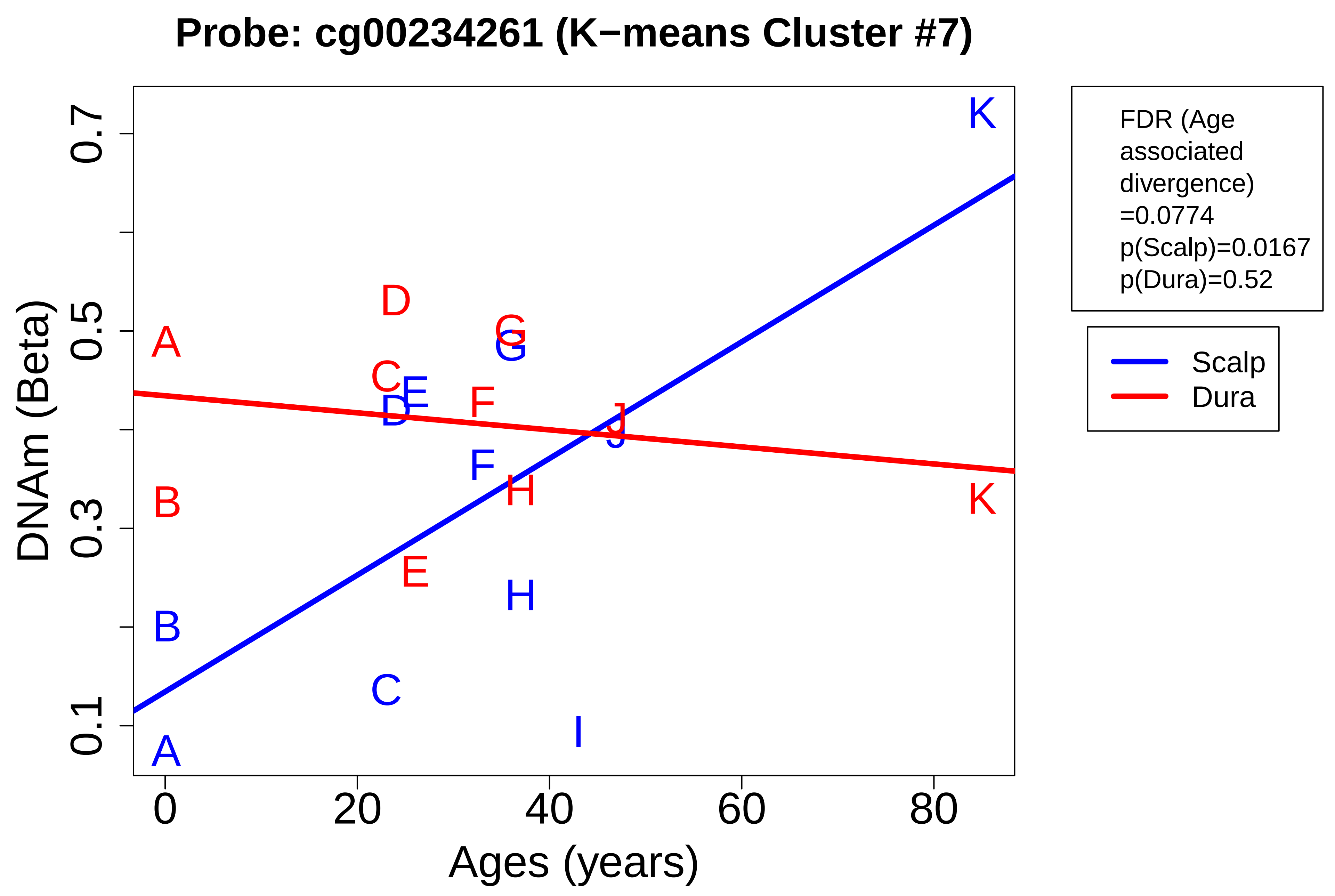

Probe: cg00234261 (K−means Cluster #7)
DNAm (Beta)
0.1
0.3
0.5
0.7
0
20
40
60
80
Ages (years)
A
B
C
D
E
F
G
H
I
J
K
FDR (Age associated divergence) =0.0774
p(Scalp)=0.0167
p(Dura)=0.52
Scalp
Dura

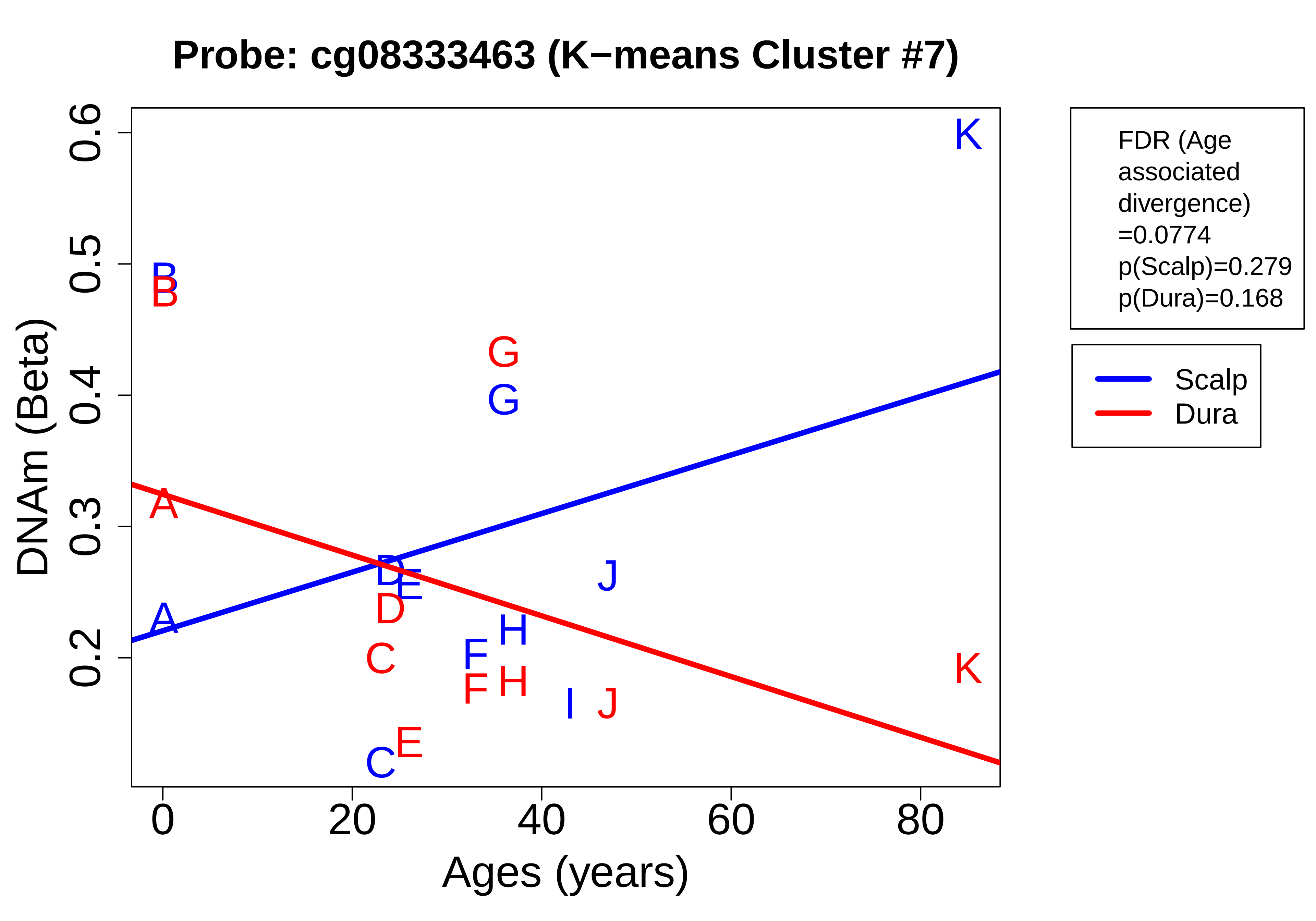

Probe: cg08333463 (K−means Cluster #7)
DNAm (Beta)
0.2
0.3
0.4
0.5
0.6
Ages (years)
0
20
40
60
80
FDR (Age associated divergence) =0.0774
p(Scalp)=0.279
p(Dura)=0.168
Scalp
Dura

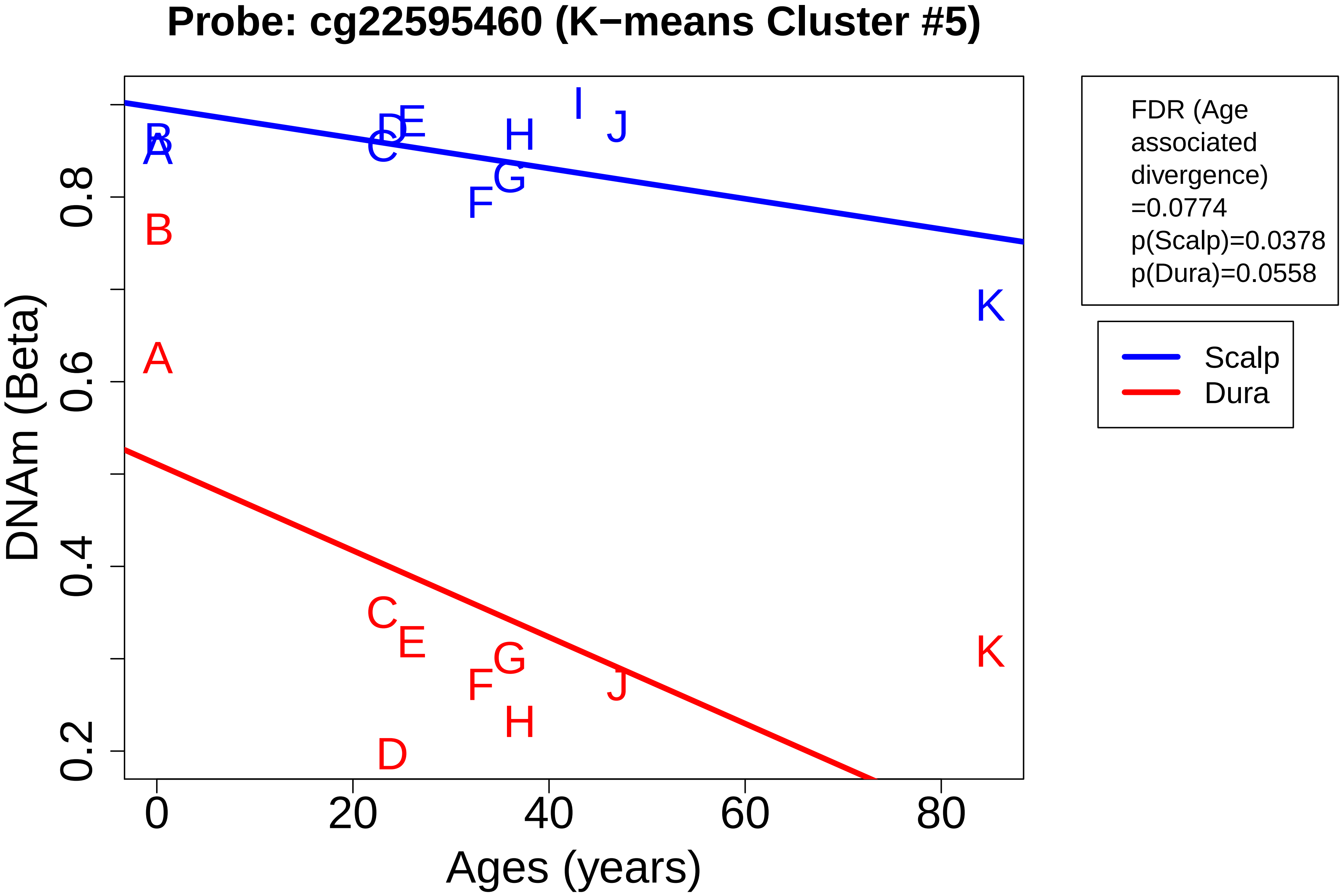

Probe: cg22595460 (K−means Cluster #5)
DNAm (Beta)
Ages (years)
0.2
0.4
0.6
0.8
0
20
40
60
80
FDR (Age associated divergence) =0.0774
p(Scalp)=0.0378
p(Dura)=0.0558
Scalp
Dura
A
B
C
D
E
F
G
H
I
J
K

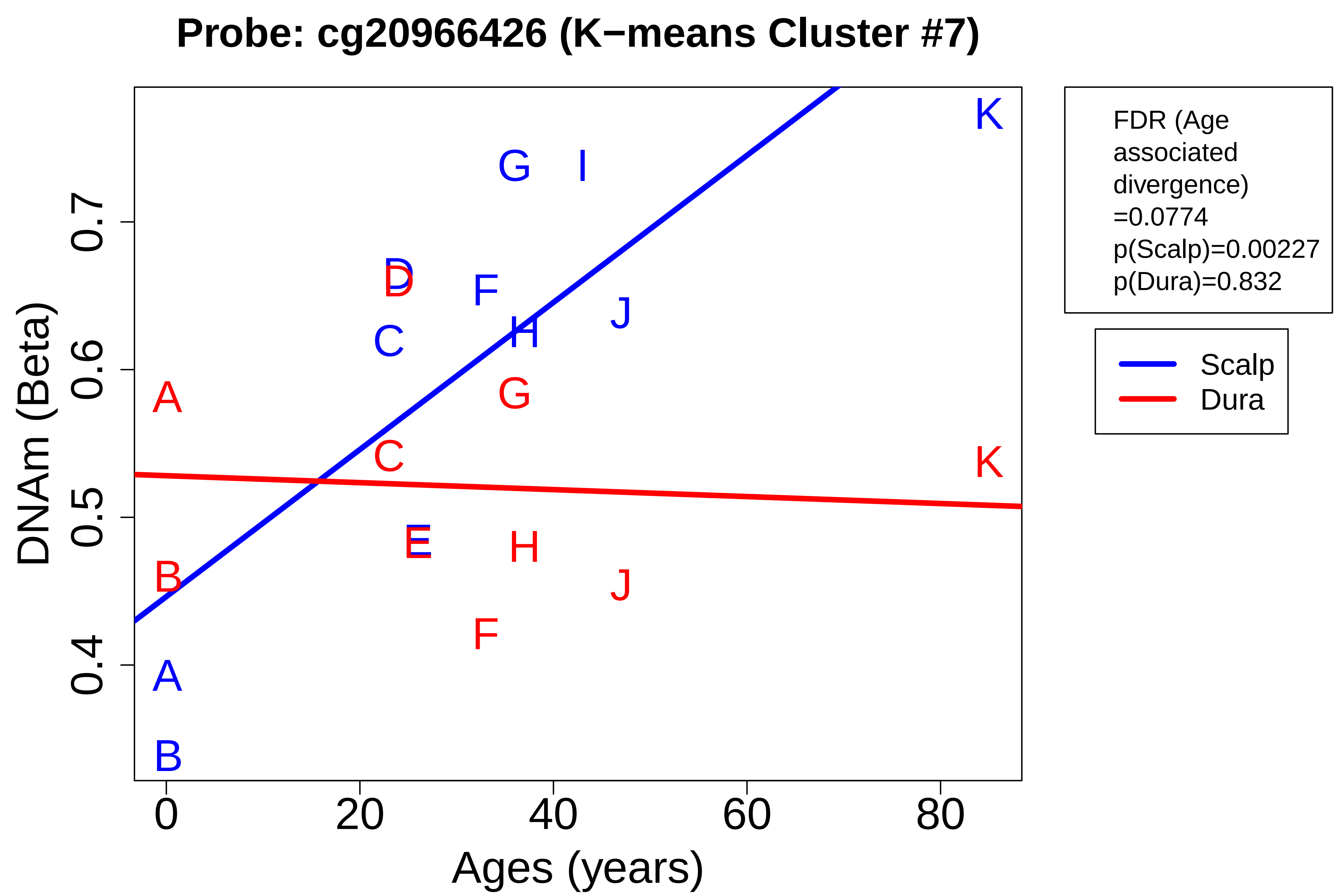

Probe: cg20966426 (K−means Cluster #7)
DNAm (Beta)
0.4
0.5
0.6
0.7
Ages (years)
0
20
40
60
80
A
B
C
D
E
F
G
H
I
J
K
FDR (Age associated divergence) =0.0774
p(Scalp)=0.00227
p(Dura)=0.832
Scalp
Dura

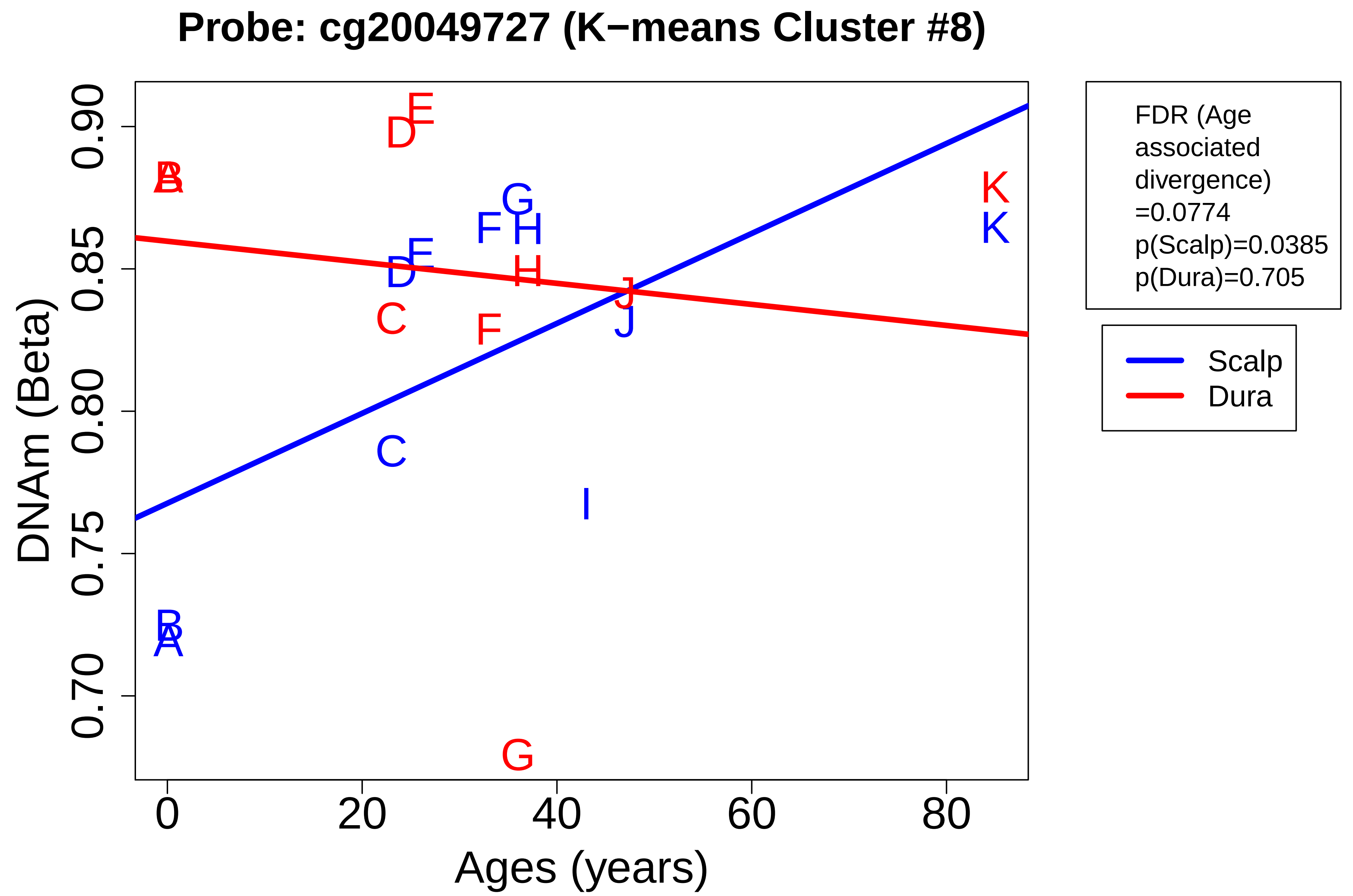

Probe: cg20049727 (K−means Cluster #8)
DNAm (Beta)
0.70
0.75
0.80
0.85
0.90
0
20
40
60
80
Ages (years)
FDR (Age associated divergence) =0.0774
p(Scalp)=0.0385
p(Dura)=0.705
Scalp
Dura

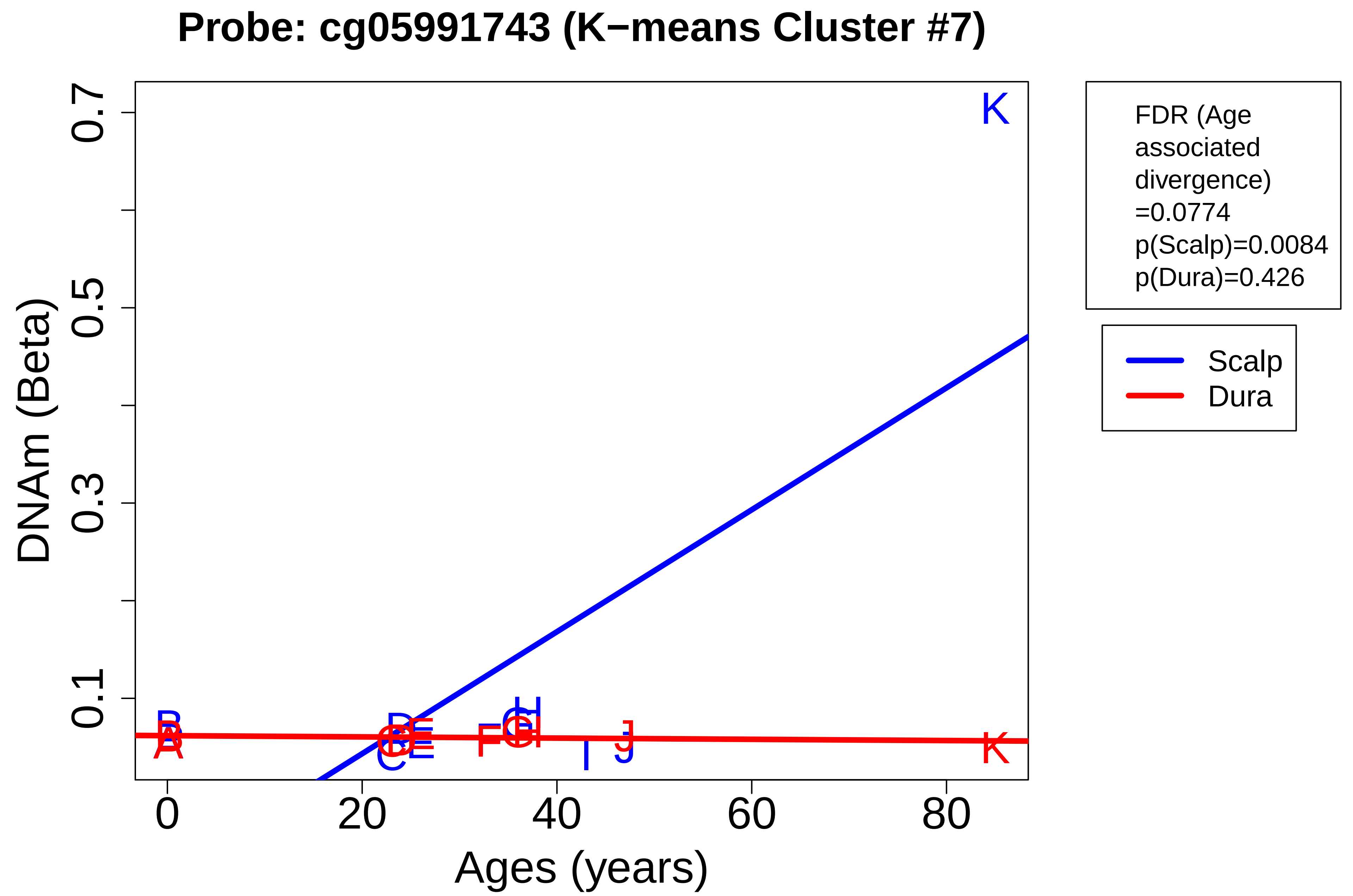

Probe: cg05991743 (K−means Cluster #7)
DNAm (Beta)
0.1
0.3
0.5
0.7
Ages (years)
0
20
40
60
80
FDR (Age associated divergence) =0.0774
p(Scalp)=0.0084
p(Dura)=0.426
Scalp
Dura

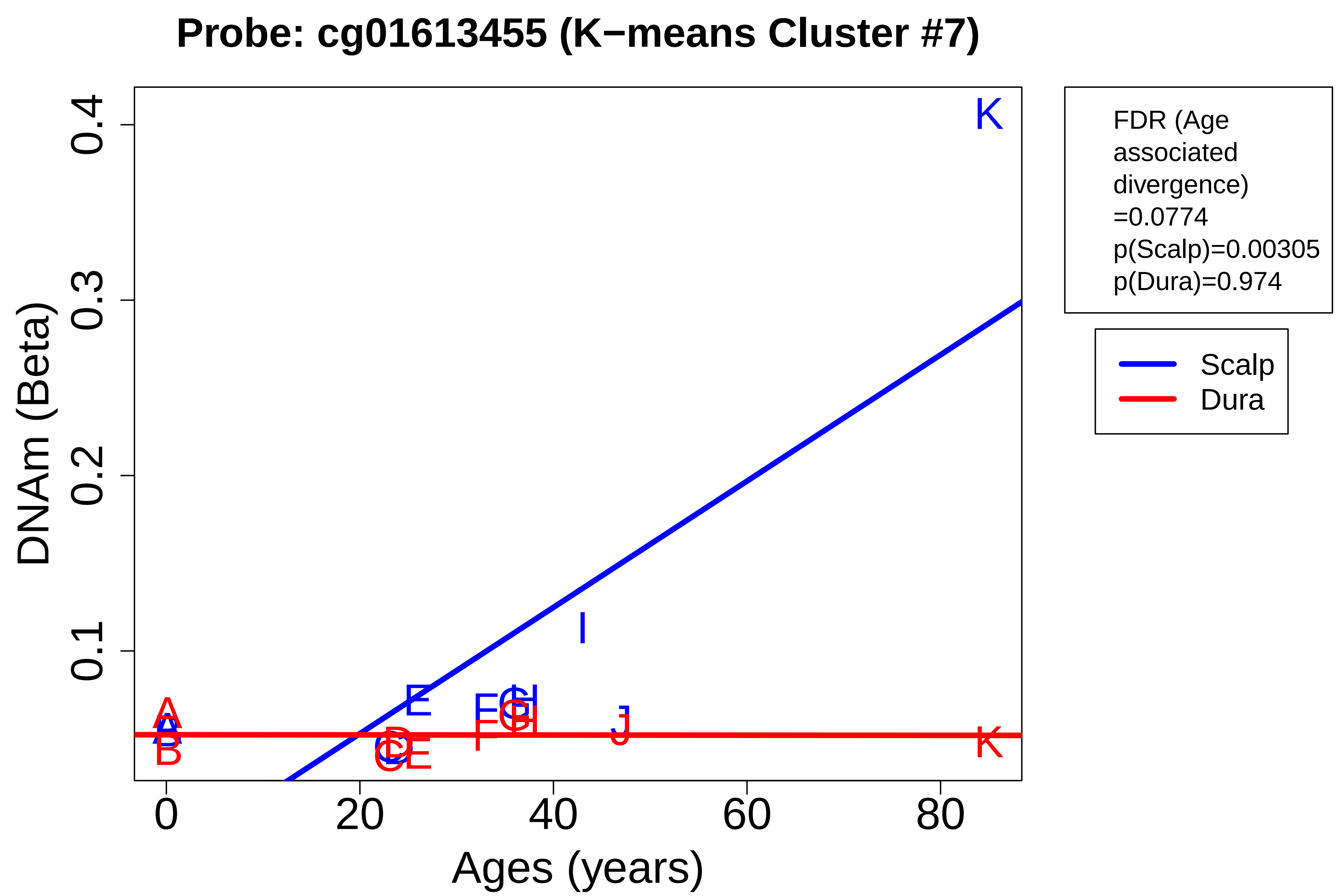

Probe: cg01613455 (K−means Cluster #7)
DNAm (Beta)
Ages (years)
0.1
0.2
0.3
0.4
0
20
40
60
80
FDR (Age associated divergence) =0.0774
p(Scalp)=0.00305
p(Dura)=0.974
Scalp
Dura

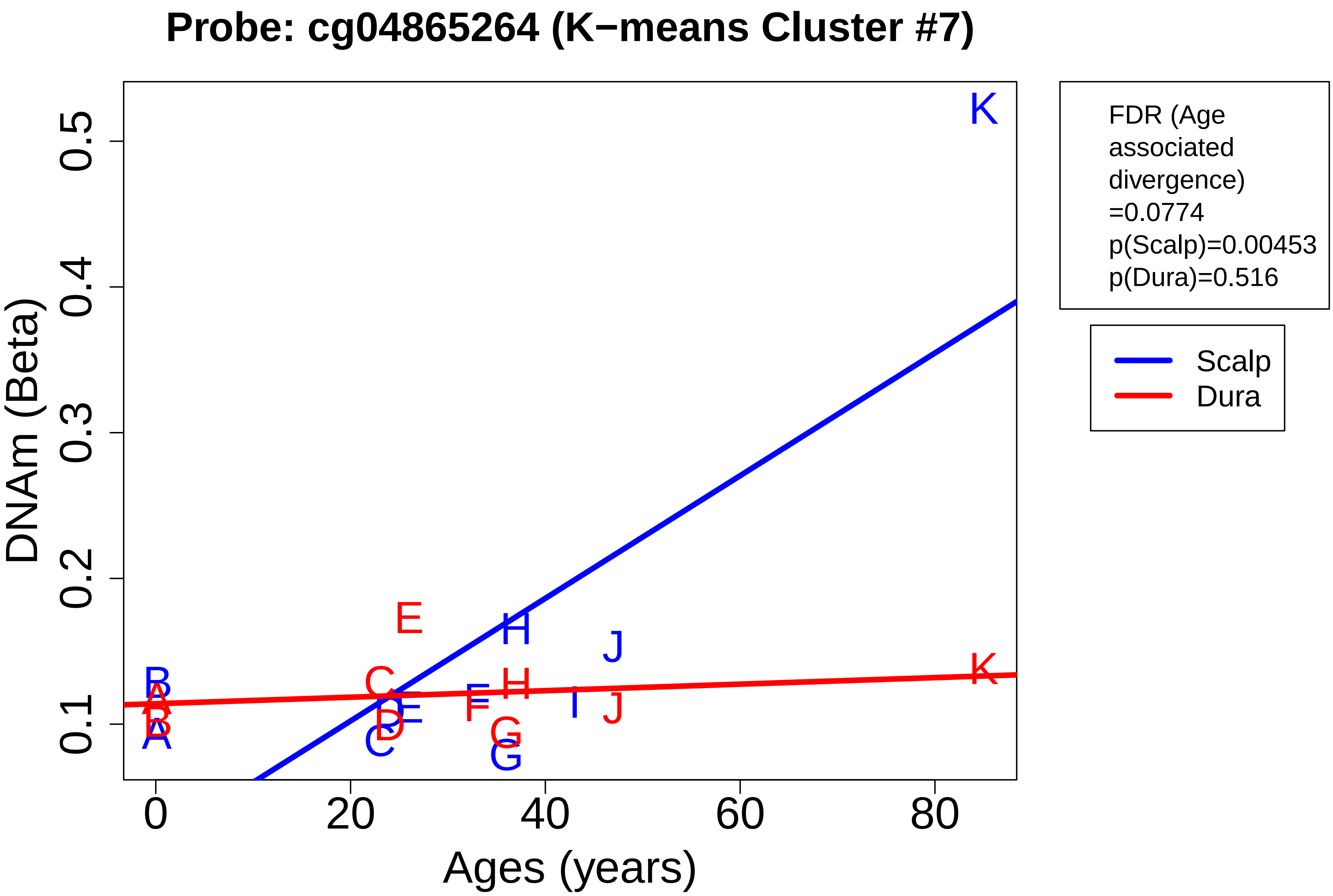

Probe: cg04865264 (K−means Cluster #7)
DNAm (Beta)
Ages (years)
0.1
0.2
0.3
0.4
0.5
0
20
40
60
80
FDR (Age associated divergence) =0.0774
p(Scalp)=0.00453
p(Dura)=0.516
Scalp
Dura

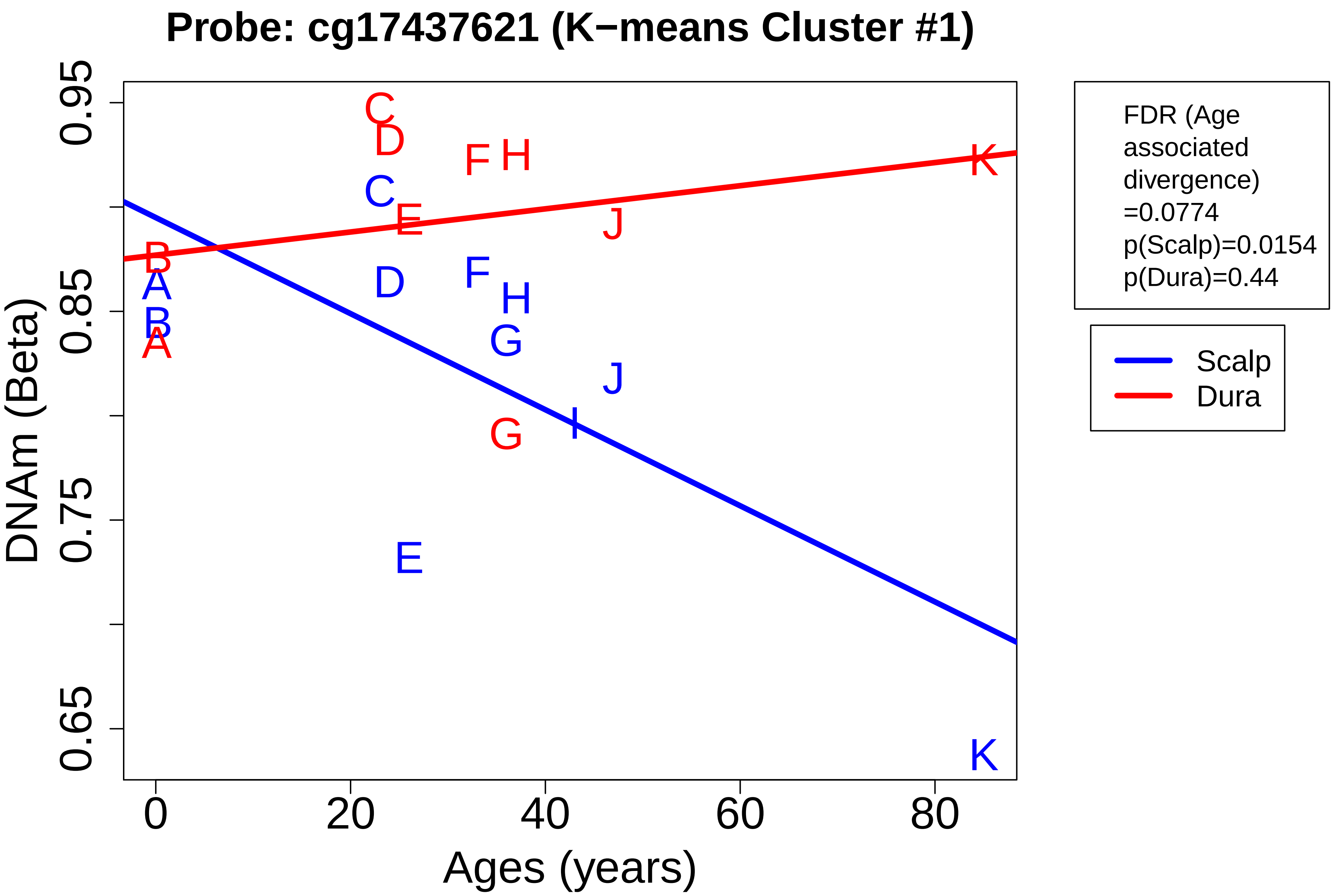

Probe: cg17437621 (K−means Cluster #1)
DNAm (Beta)
0.65
0.75
0.85
0.95
0
20
40
60
80
Ages (years)
FDR (Age associated divergence) =0.0774
p(Scalp)=0.0154
p(Dura)=0.44
Scalp
Dura

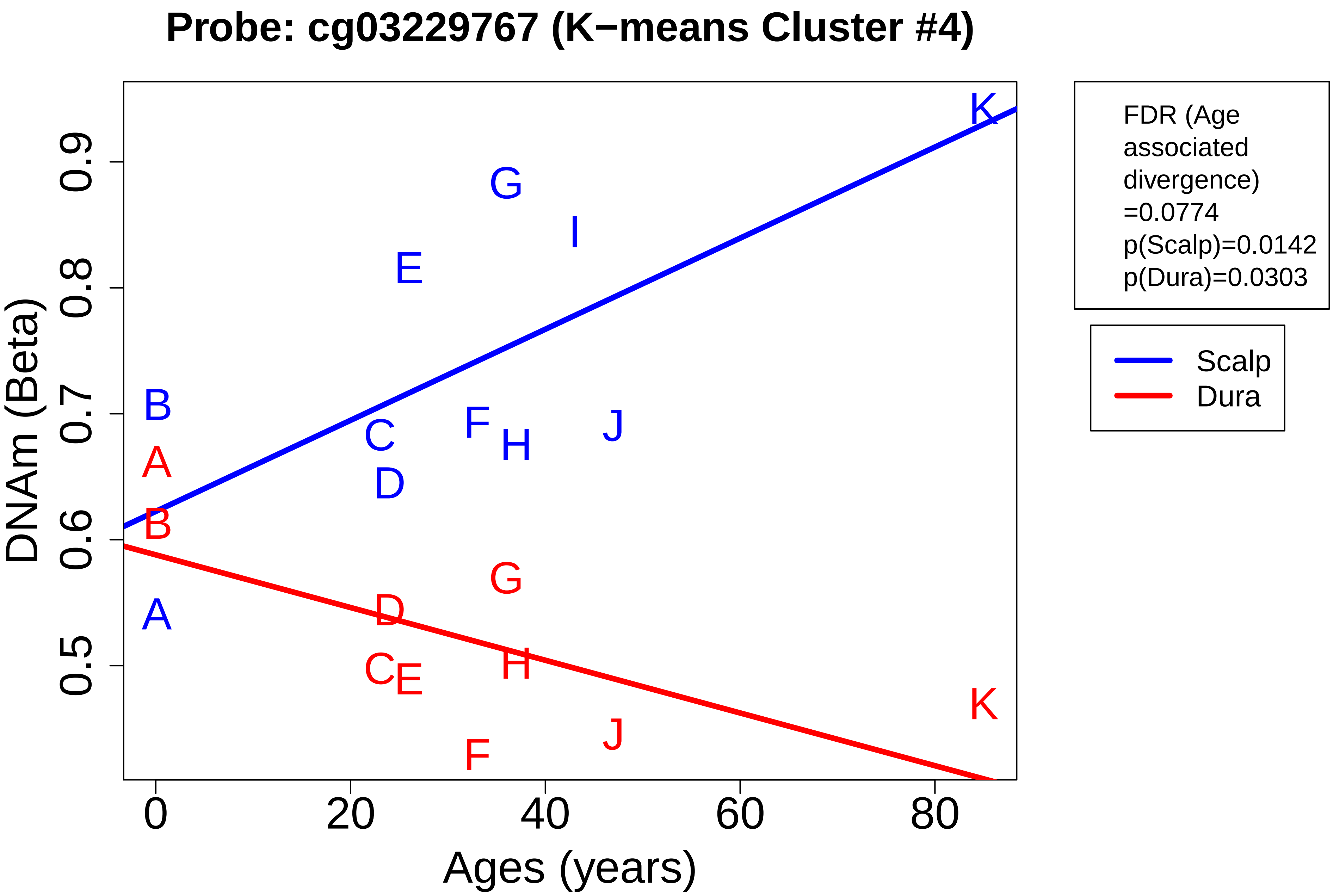

Probe: cg03229767 (K−means Cluster #4)
DNAm (Beta)
0.5
0.6
0.7
0.8
0.9
0
20
40
60
80
Ages (years)
FDR (Age associated divergence) =0.0774
p(Scalp)=0.0142
p(Dura)=0.0303
Scalp
Dura

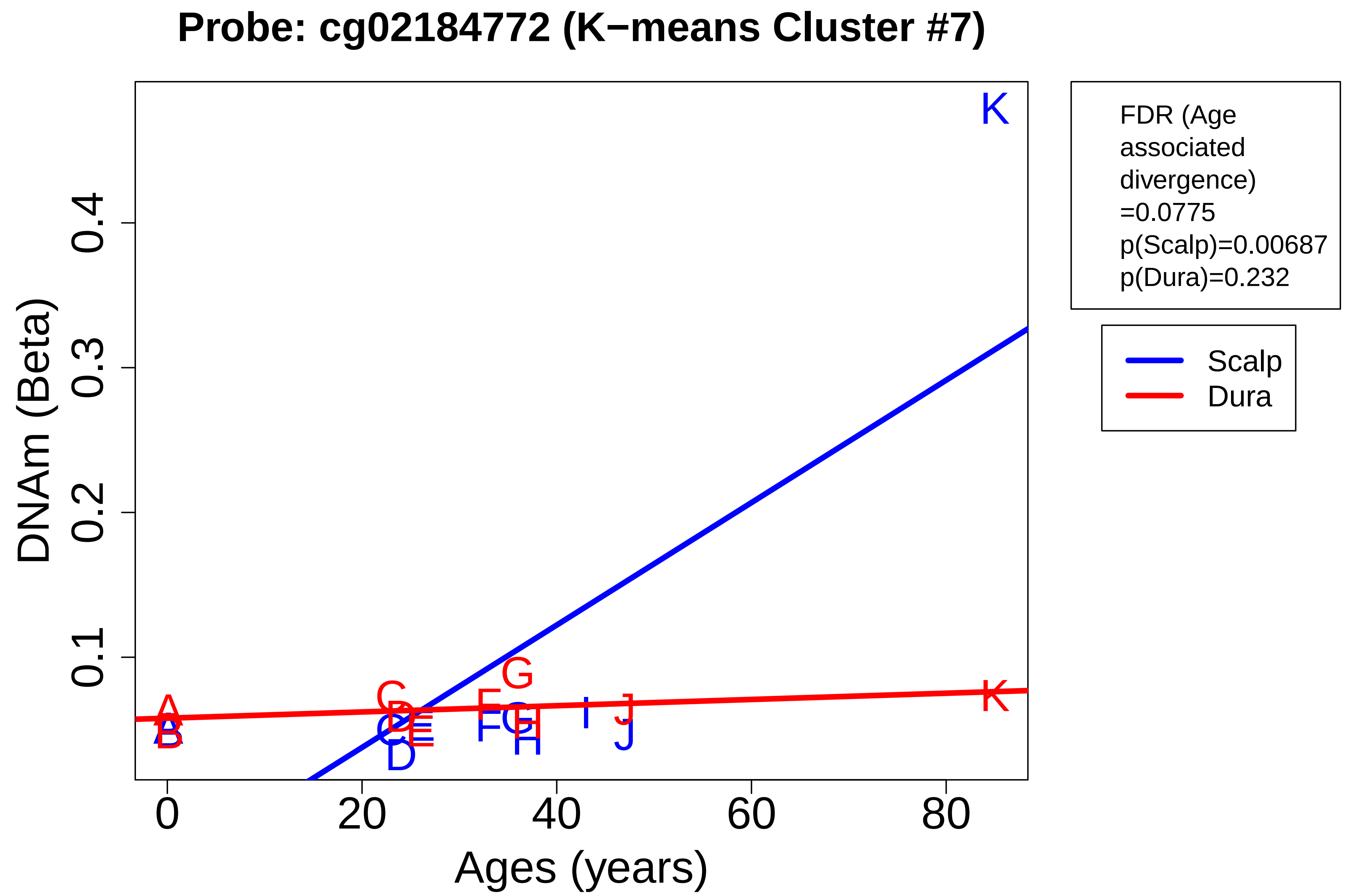

Probe: cg02184772 (K−means Cluster #7)
DNAm (Beta)
0.1
0.2
0.3
0.4
0
20
40
60
80
Ages (years)
FDR (Age associated divergence) =0.0775
p(Scalp)=0.00687
p(Dura)=0.232
Scalp
Dura

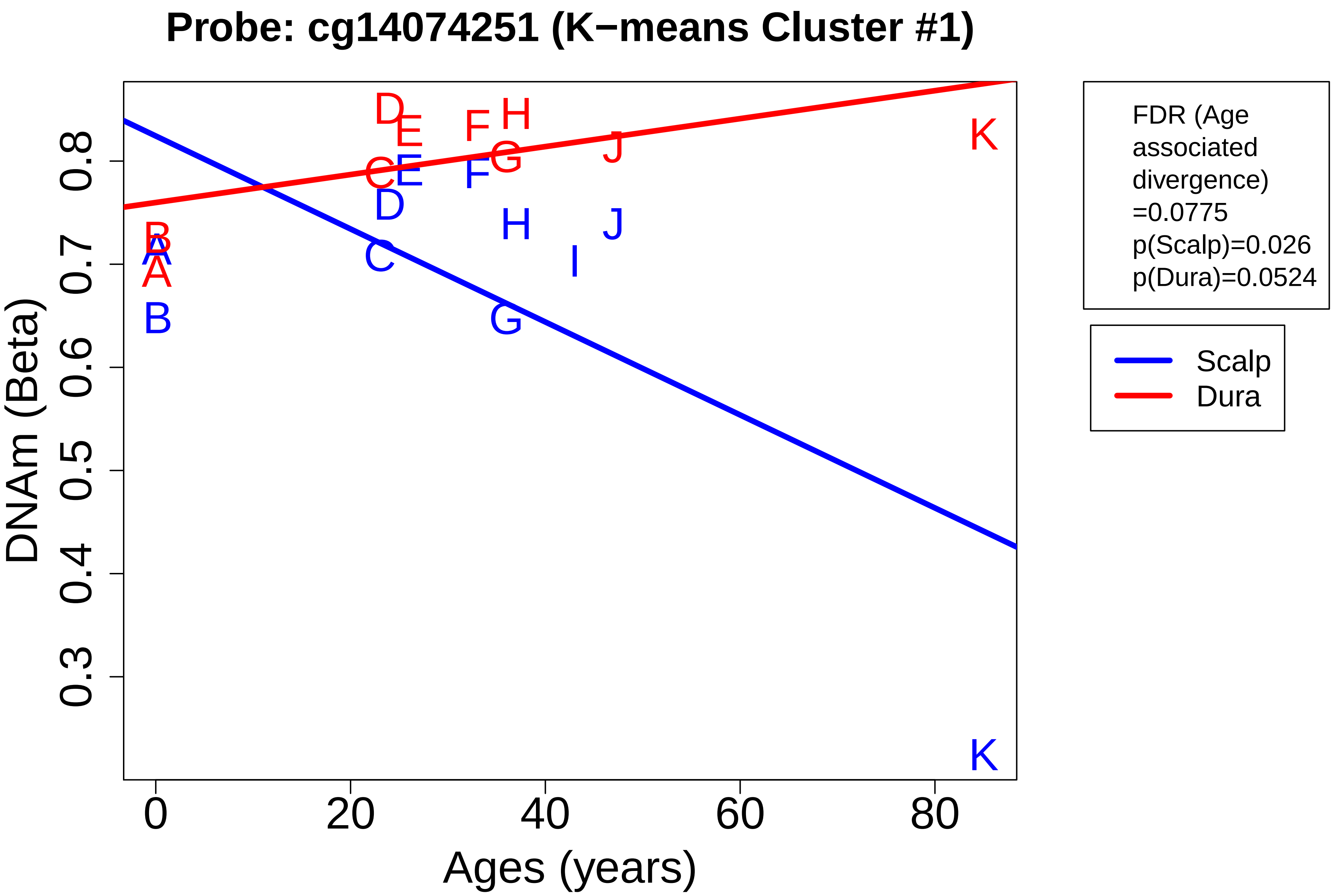
Probe: cg14074251 (K−means Cluster #1)
DNAm (Beta)
0.3
0.4
0.5
0.6
0.7
0.8
Ages (years)
0
20
40
60
80
A B C D E F G H I J K
FDR (Age associated divergence) =0.0775
p(Scalp)=0.026
p(Dura)=0.0524
Scalp
Dura

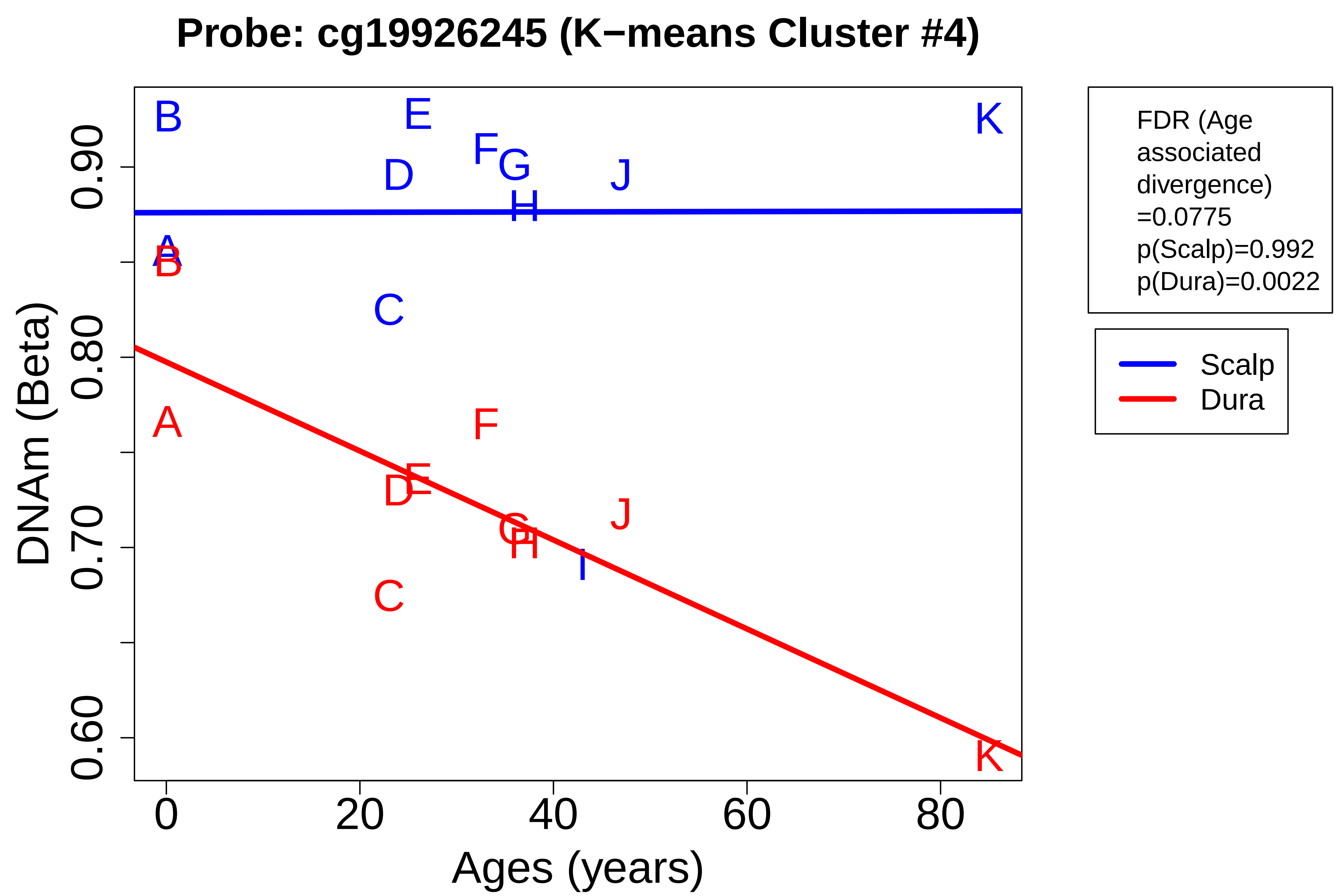
Probe: cg19926245 (K−means Cluster #4)
DNAm (Beta)
0.60
0.70
0.80
0.90
0
20
40
60
80
Ages (years)
B
E
K
F
G
D
J
H
A
B
C
A
F
E
D
J
G
H
I
C
K
FDR (Age associated divergence) =0.0775
p(Scalp)=0.992
p(Dura)=0.0022
Scalp
Dura

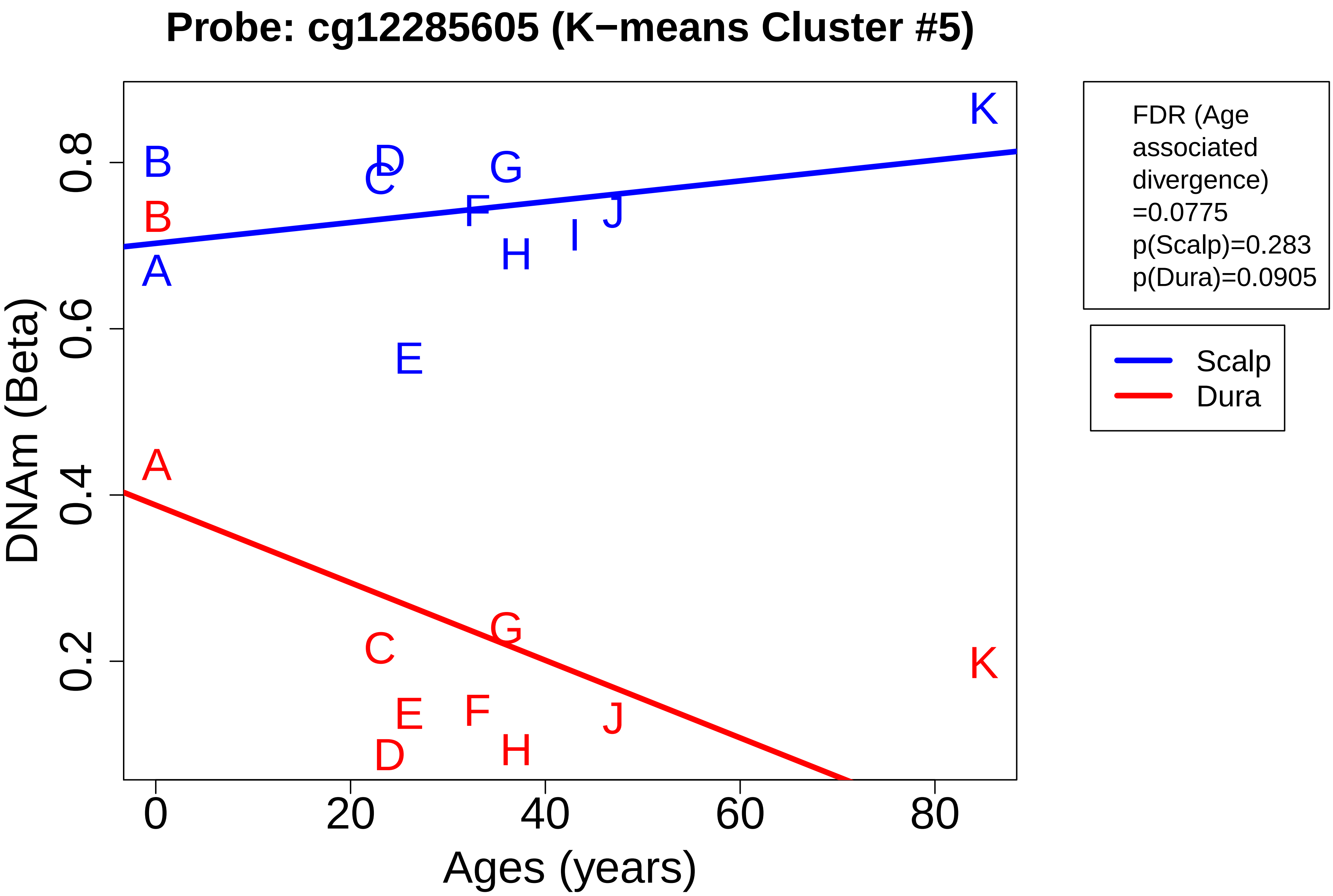

Probe: cg12285605 (K−means Cluster #5)
DNAm (Beta)
0.2
0.4
0.6
0.8
0
20
40
60
80
Ages (years)
FDR (Age associated divergence) =0.0775
p(Scalp)=0.283
p(Dura)=0.0905
Scalp
Dura
A
B
C
D
E
F
G
H
I
J
K

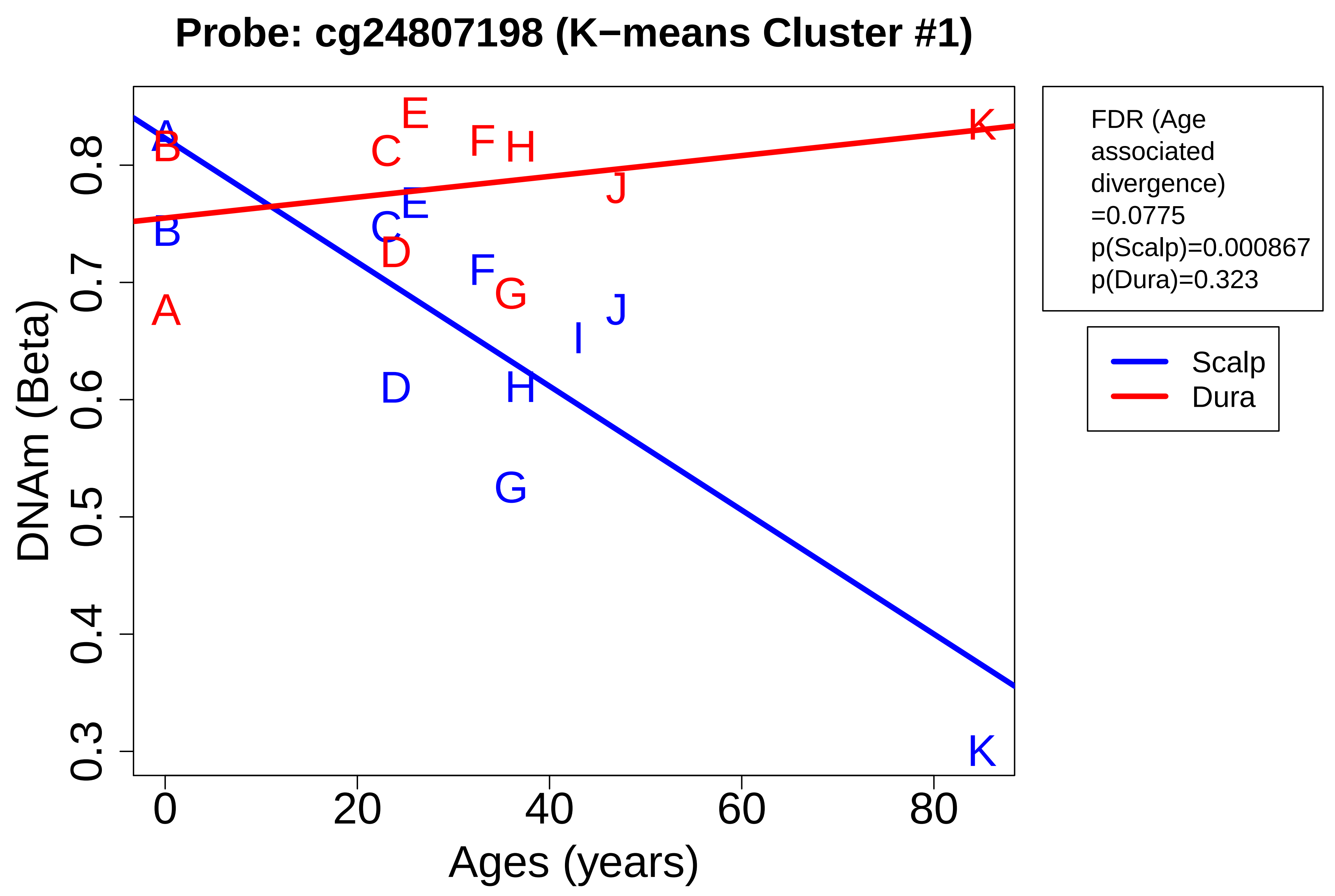

Probe: cg24807198 (K−means Cluster #1)
DNAm (Beta)
0.3
0.4
0.5
0.6
0.7
0.8
0
20
40
60
80
Ages (years)
FDR (Age associated divergence) =0.0775
p(Scalp)=0.000867
p(Dura)=0.323
Scalp
Dura

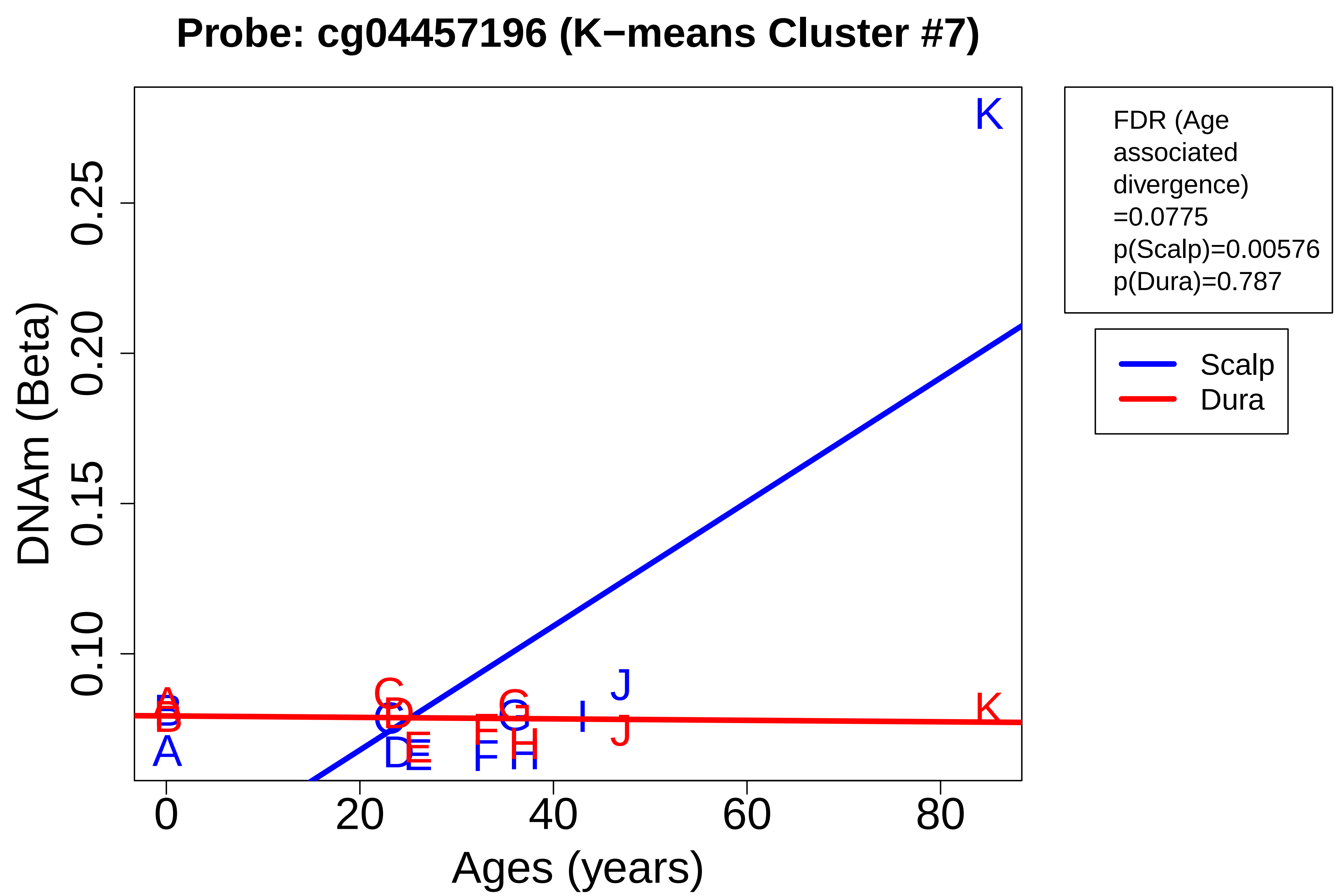

Probe: cg04457196 (K−means Cluster #7)
DNAm (Beta)
0.10
0.15
0.20
0.25
0
20
40
60
80
Ages (years)
FDR (Age associated divergence) =0.0775
p(Scalp)=0.00576
p(Dura)=0.787
Scalp
Dura

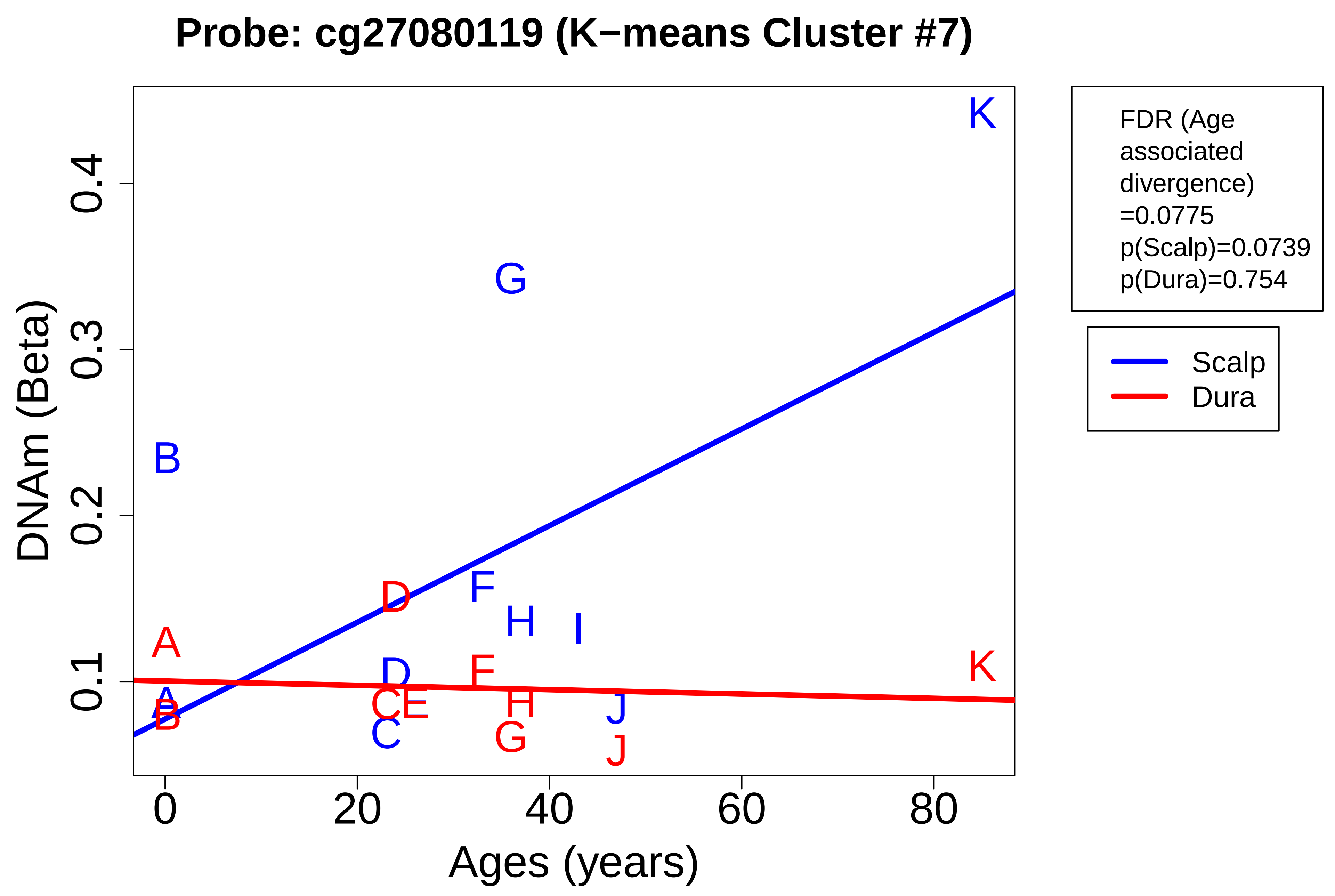

Probe: cg27080119 (K−means Cluster #7)
DNAm (Beta)
0.1
0.2
0.3
0.4
Ages (years)
0
20
40
60
80
A
B
C
D
E
F
G
H
I
J
K
FDR (Age associated divergence) =0.0775
p(Scalp)=0.0739
p(Dura)=0.754
Scalp
Dura

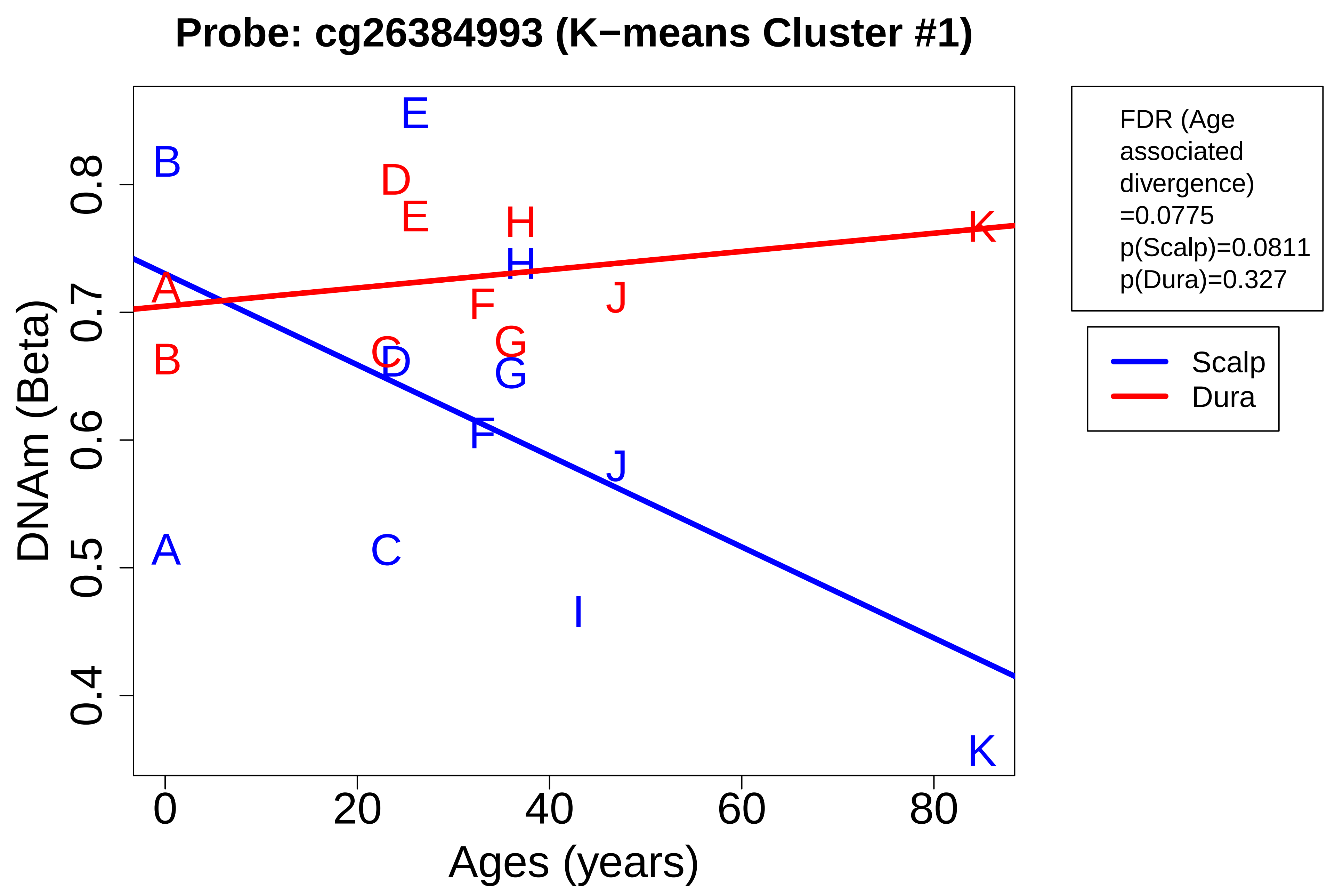
Probe: cg26384993 (K−means Cluster #1)
DNAm (Beta)
0.4
0.5
0.6
0.7
0.8
0
20
40
60
80
Ages (years)
A
B
C
D
E
F
G
H
I
J
K
A
B
C
D
E
F
G
H
J
K
FDR (Age associated divergence) =0.0775
p(Scalp)=0.0811
p(Dura)=0.327
Scalp
Dura

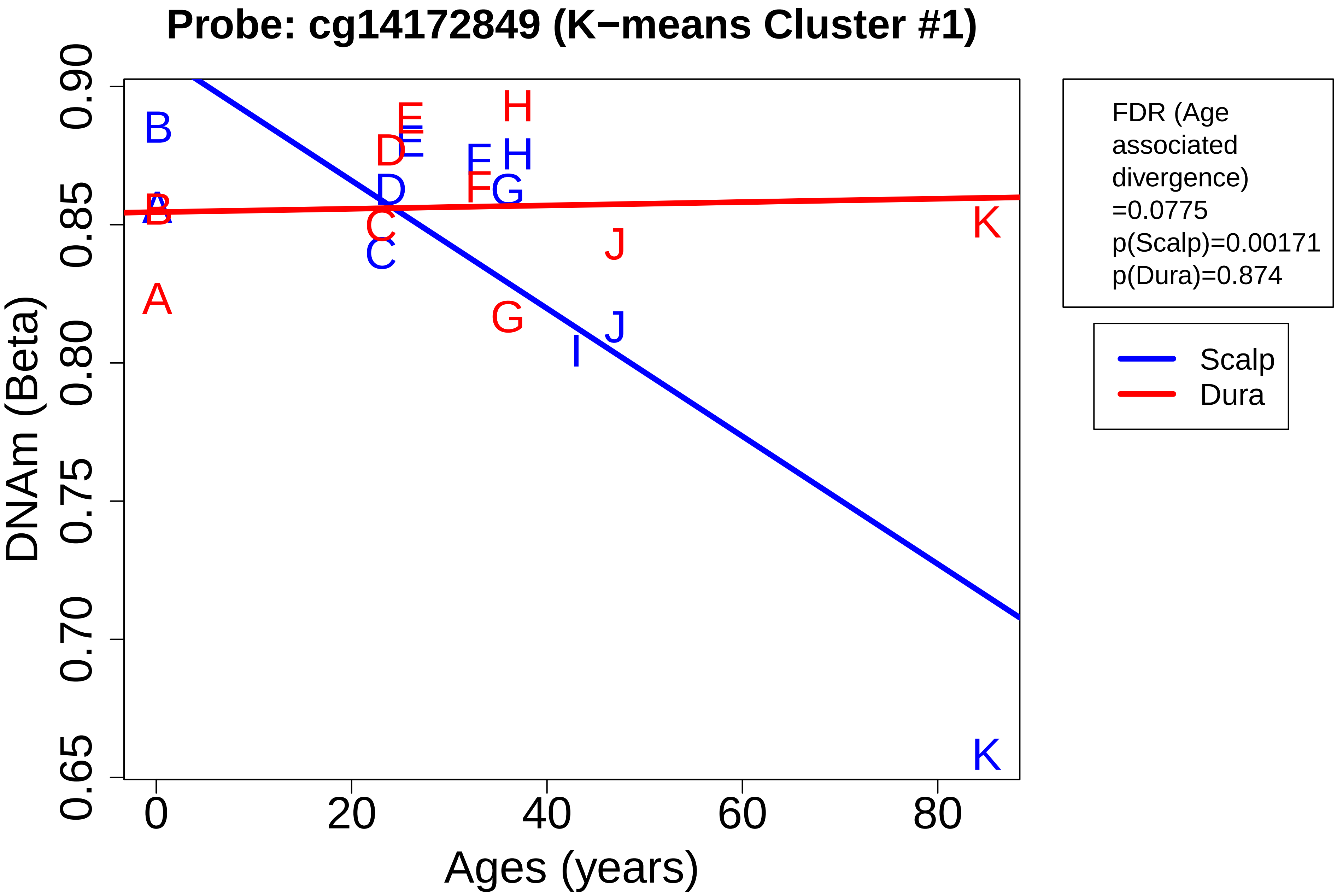
Probe: cg14172849 (K−means Cluster #1)
DNAm (Beta)
0.65
0.70
0.75
0.80
0.85
0.90
0
20
40
60
80
Ages (years)
FDR (Age associated divergence) =0.0775
p(Scalp)=0.00171
p(Dura)=0.874
Scalp
Dura

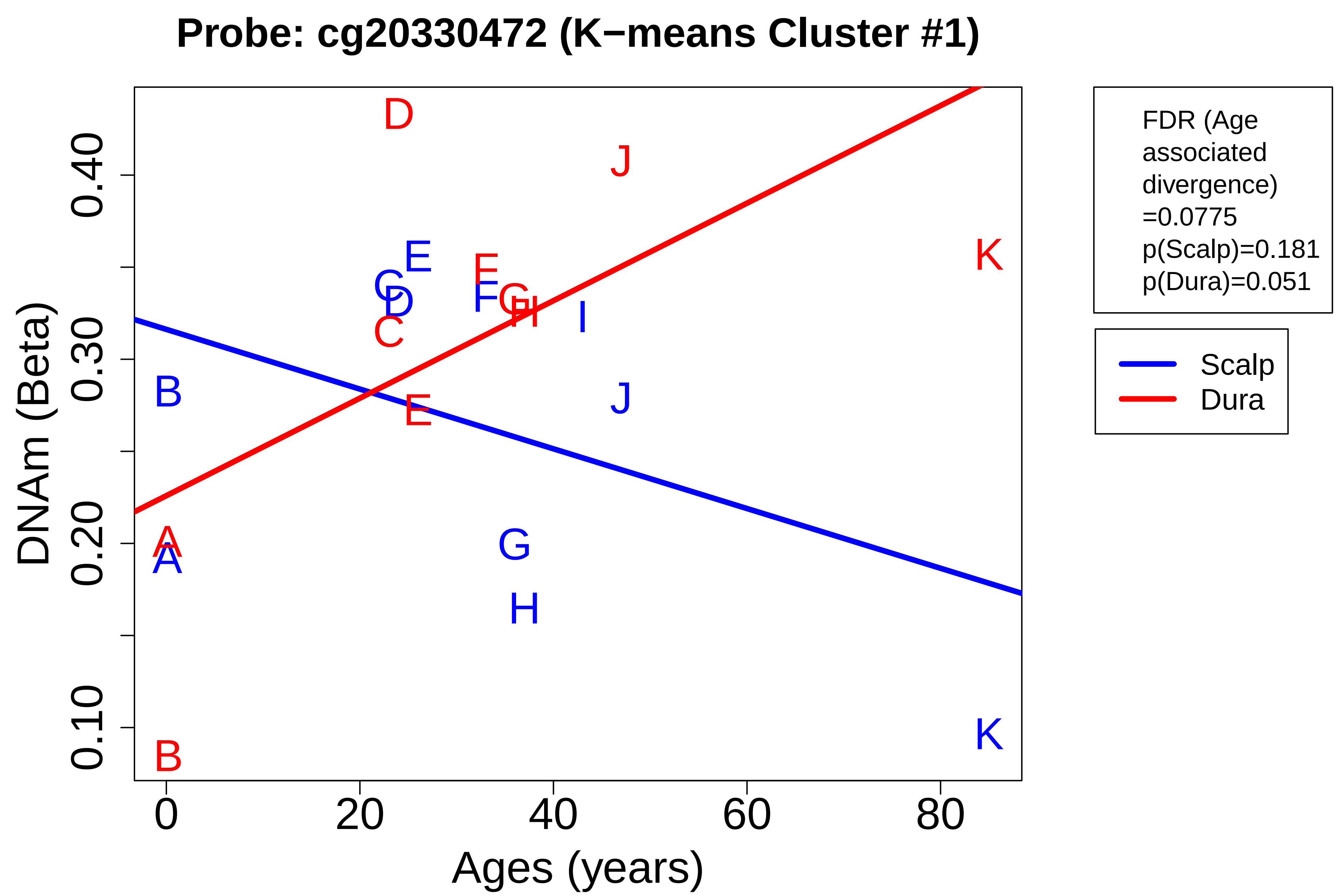
Probe: cg20330472 (K−means Cluster #1)
DNAm (Beta)
Ages (years)
0.10
0.20
0.30
0.40
0
20
40
60
80
FDR (Age associated divergence) =0.0775
p(Scalp)=0.181
p(Dura)=0.051
Scalp
Dura

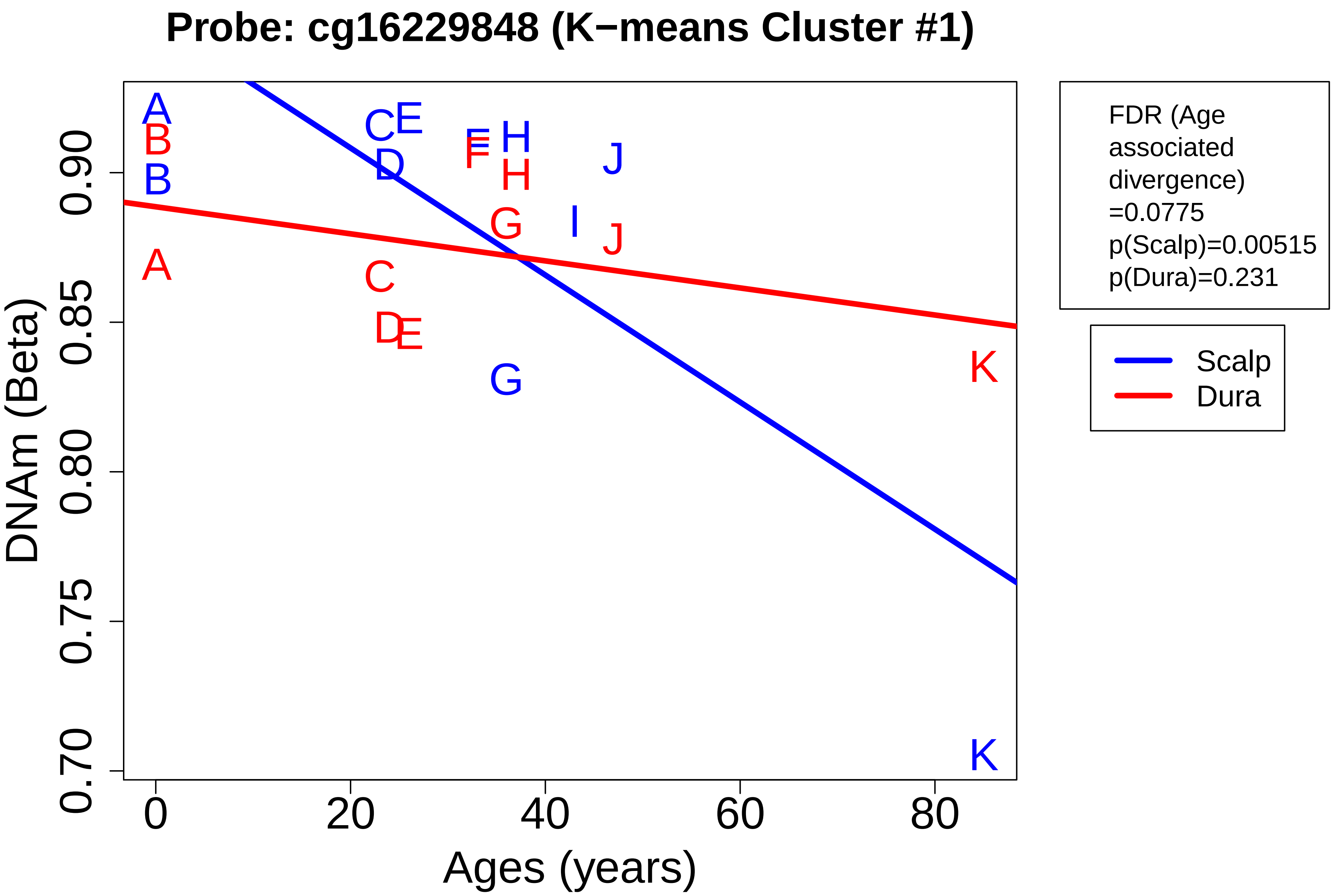
Probe: cg16229848 (K−means Cluster #1)
DNAm (Beta)
0.70
0.75
0.80
0.85
0.90
0
20
40
60
80
Ages (years)
FDR (Age associated divergence) =0.0775
p(Scalp)=0.00515
p(Dura)=0.231
Scalp
Dura

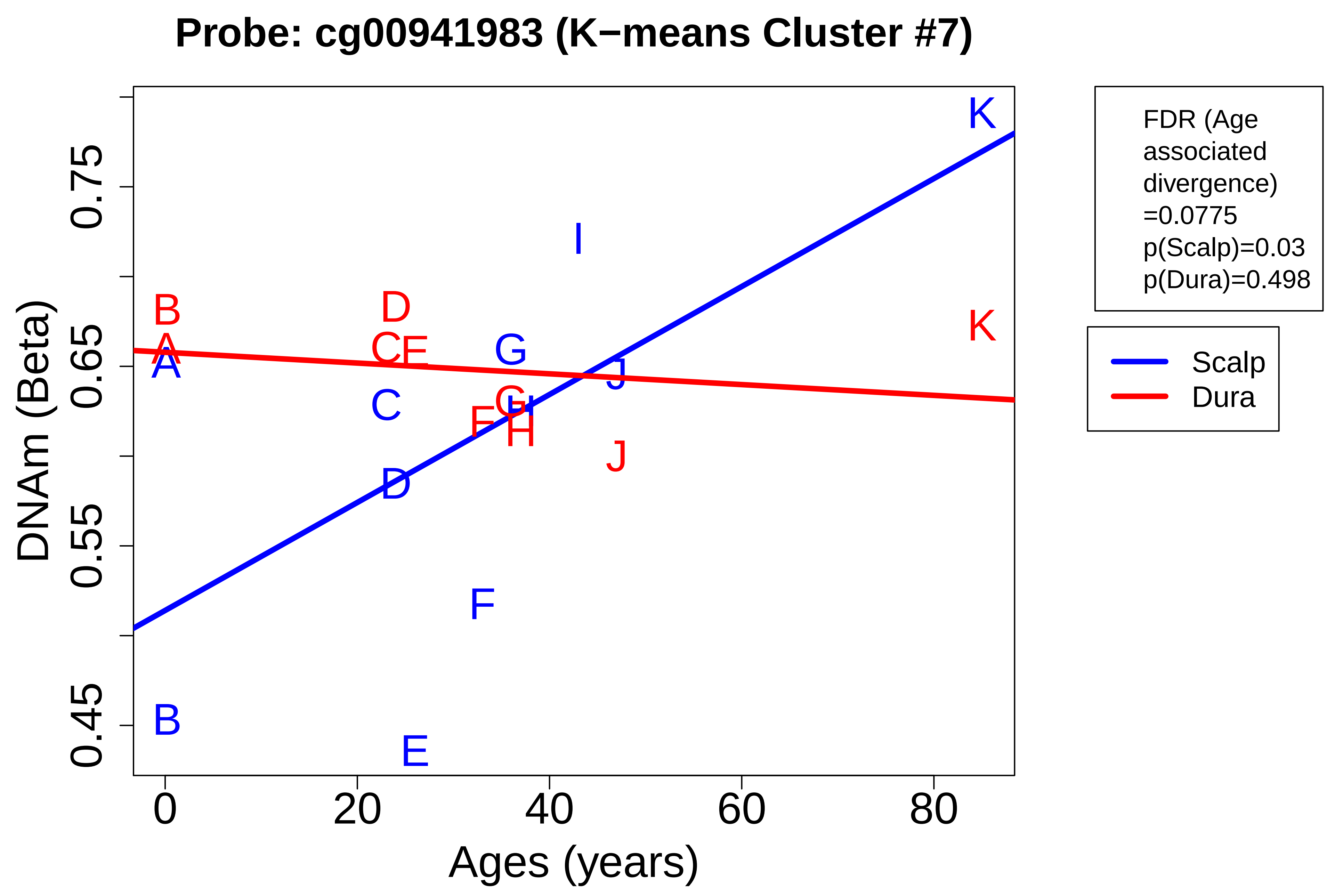

Probe: cg00941983 (K−means Cluster #7)
DNAm (Beta)
0.45
0.55
0.65
0.75
0
20
40
60
80
Ages (years)
A
B
C
D
E
F
G
H
I
J
K
FDR (Age associated divergence) =0.0775
p(Scalp)=0.03
p(Dura)=0.498
Scalp
Dura

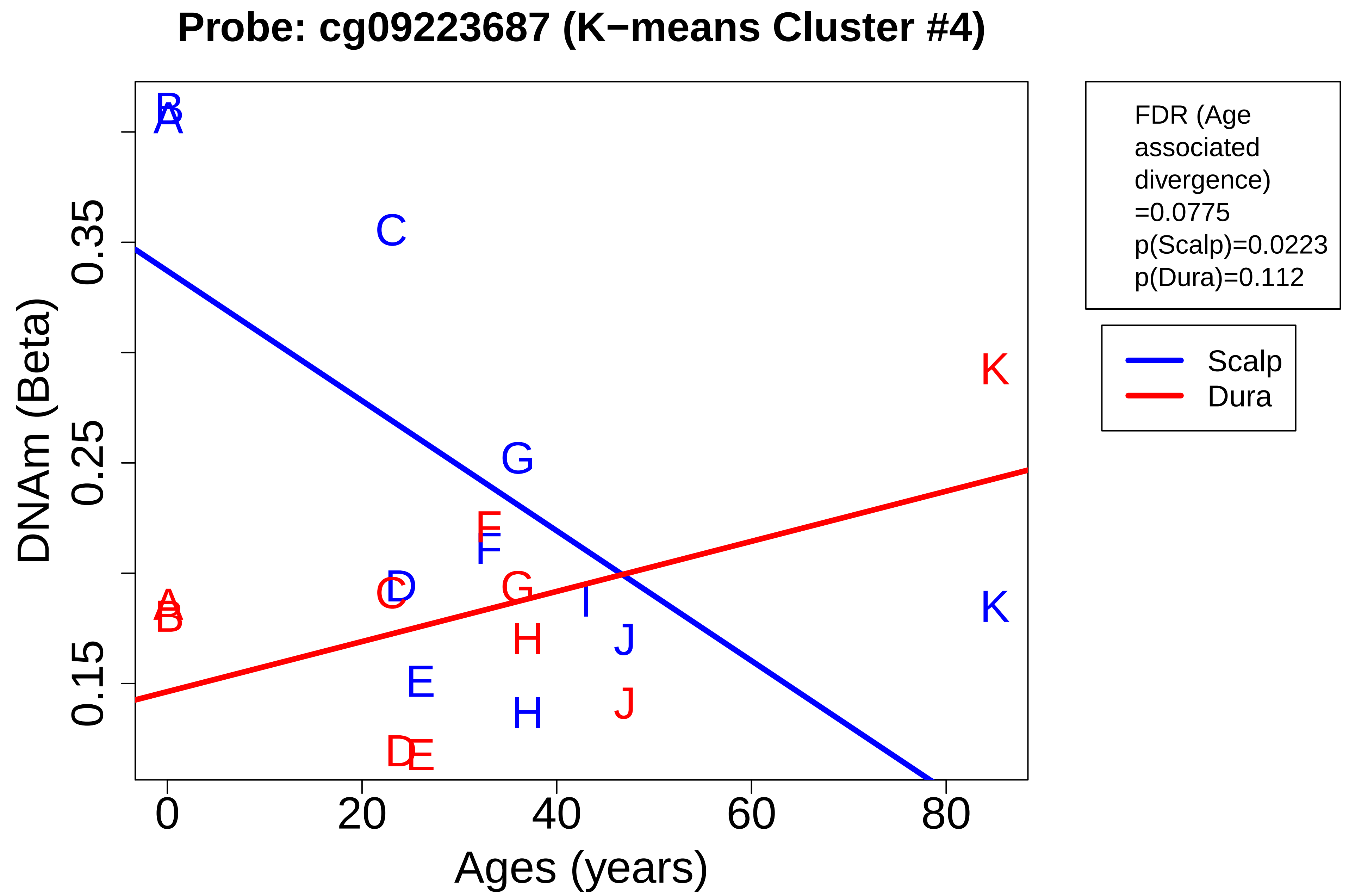
Probe: cg09223687 (K−means Cluster #4)
DNAm (Beta)
0.15
0.25
0.35
Ages (years)
0
20
40
60
80
FDR (Age associated divergence) =0.0775
p(Scalp)=0.0223
p(Dura)=0.112
Scalp
Dura

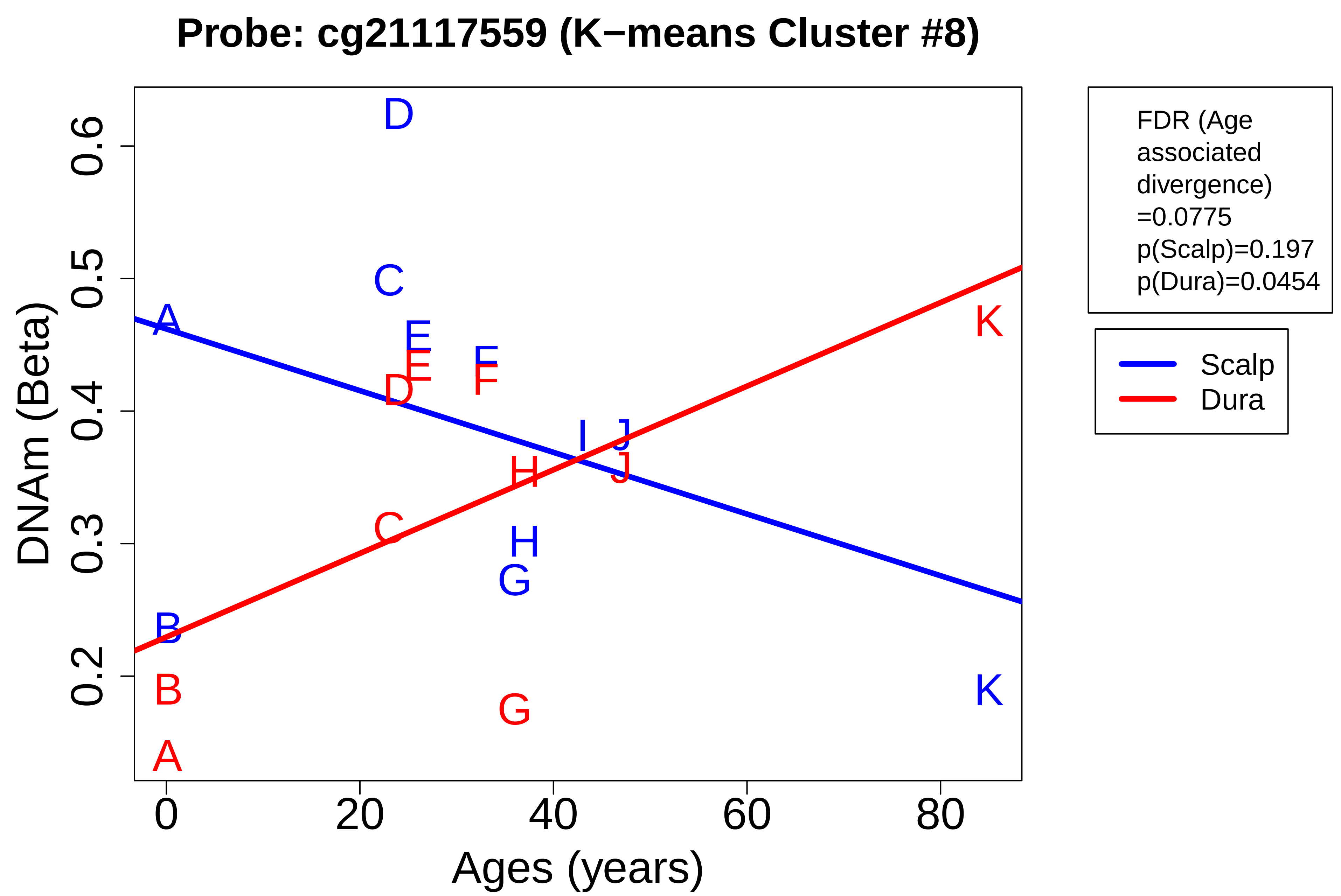

Probe: cg21117559 (K−means Cluster #8)
DNAm (Beta)
0.2
0.3
0.4
0.5
0.6
0
20
40
60
80
Ages (years)
FDR (Age associated divergence) =0.0775
p(Scalp)=0.197
p(Dura)=0.0454
Scalp
Dura

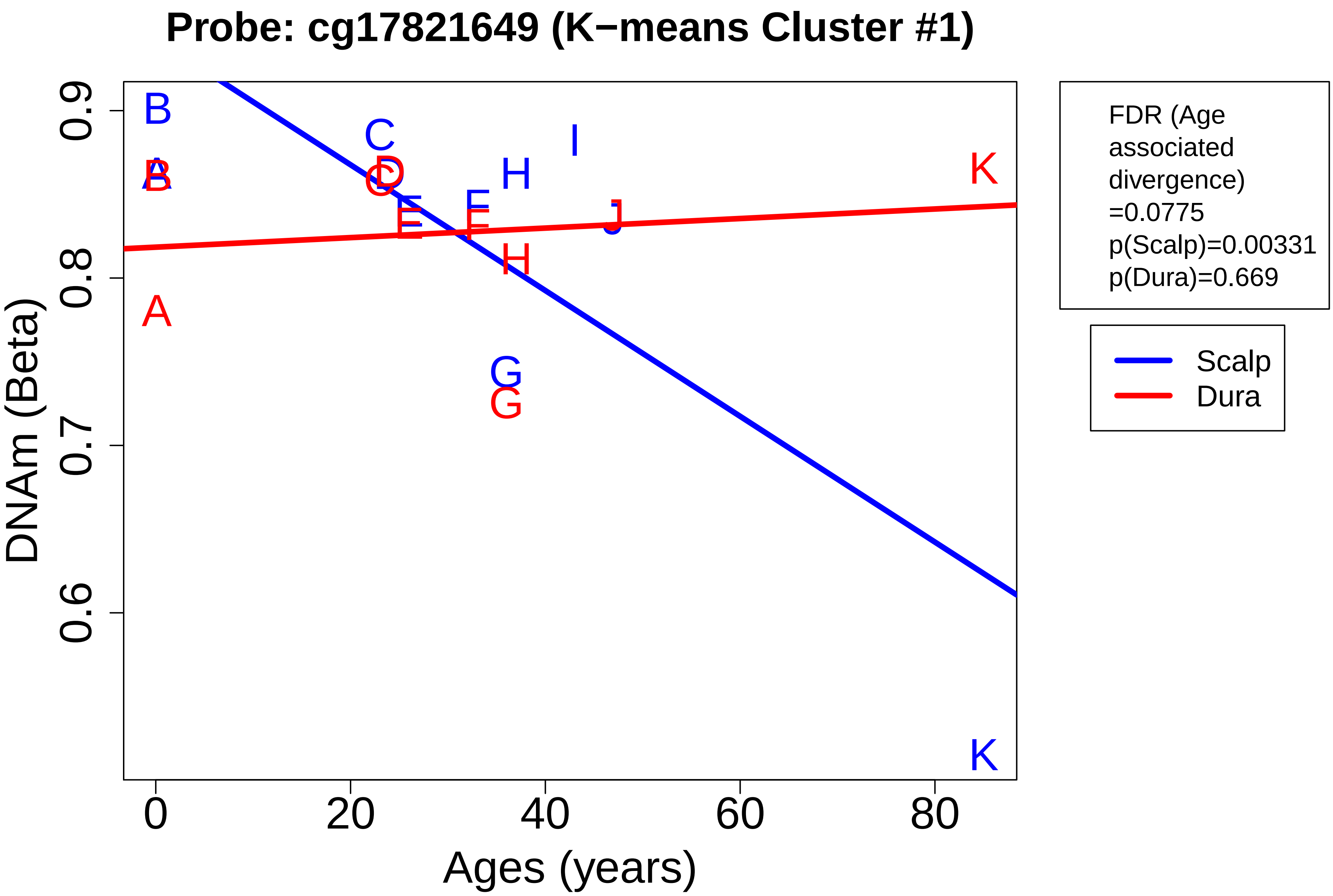

Probe: cg17821649 (K−means Cluster #1)
DNAm (Beta)
Ages (years)
0.6
0.7
0.8
0.9
0
20
40
60
80
FDR (Age associated divergence) =0.0775
p(Scalp)=0.00331
p(Dura)=0.669
Scalp
Dura

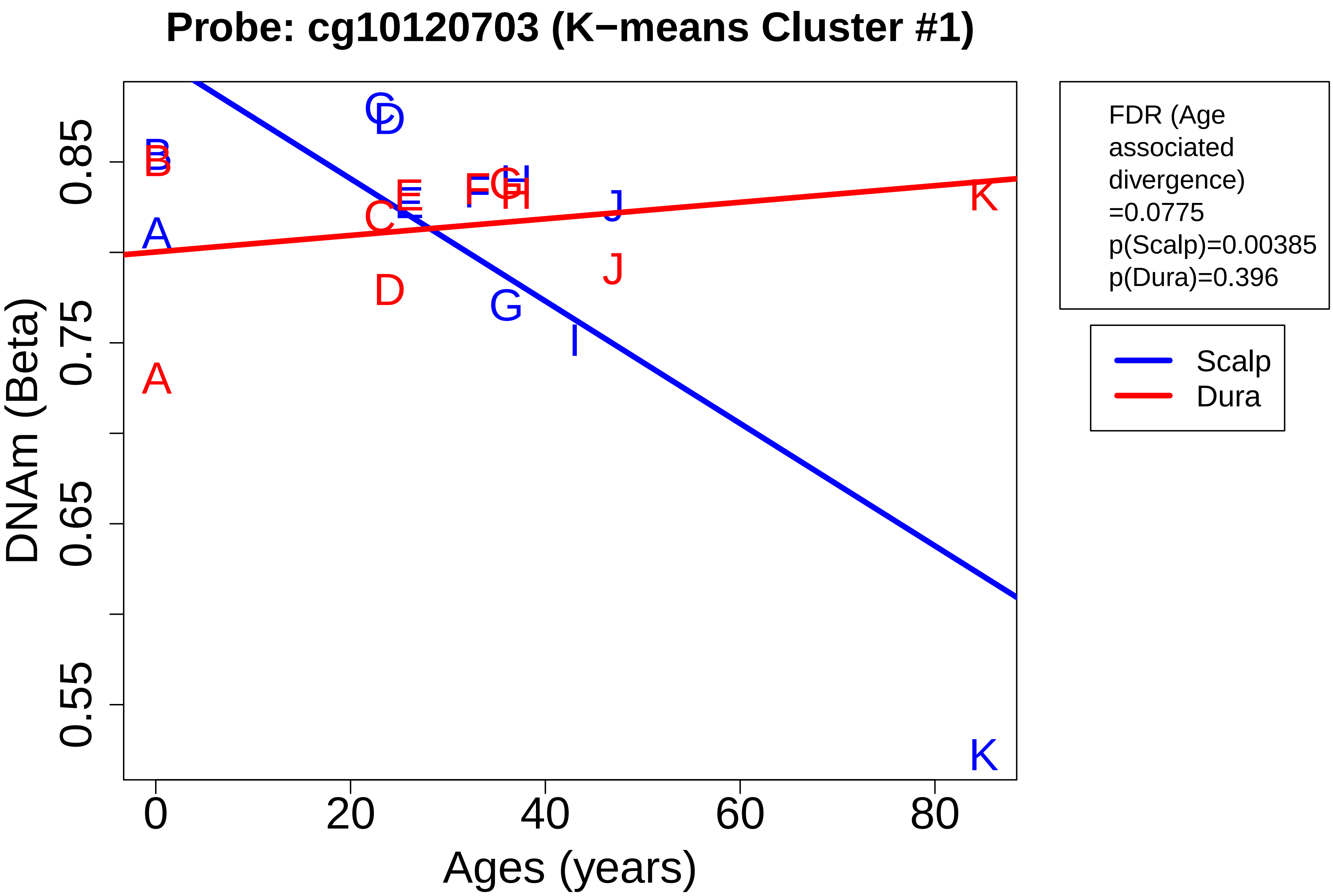

Probe: cg10120703 (K−means Cluster #1)
DNAm (Beta)
0.55
0.65
0.75
0.85
Ages (years)
0
20
40
60
80
A
B
C
D
E
F
G
H
I
J
K
FDR (Age associated divergence) =0.0775
p(Scalp)=0.00385
p(Dura)=0.396
Scalp
Dura

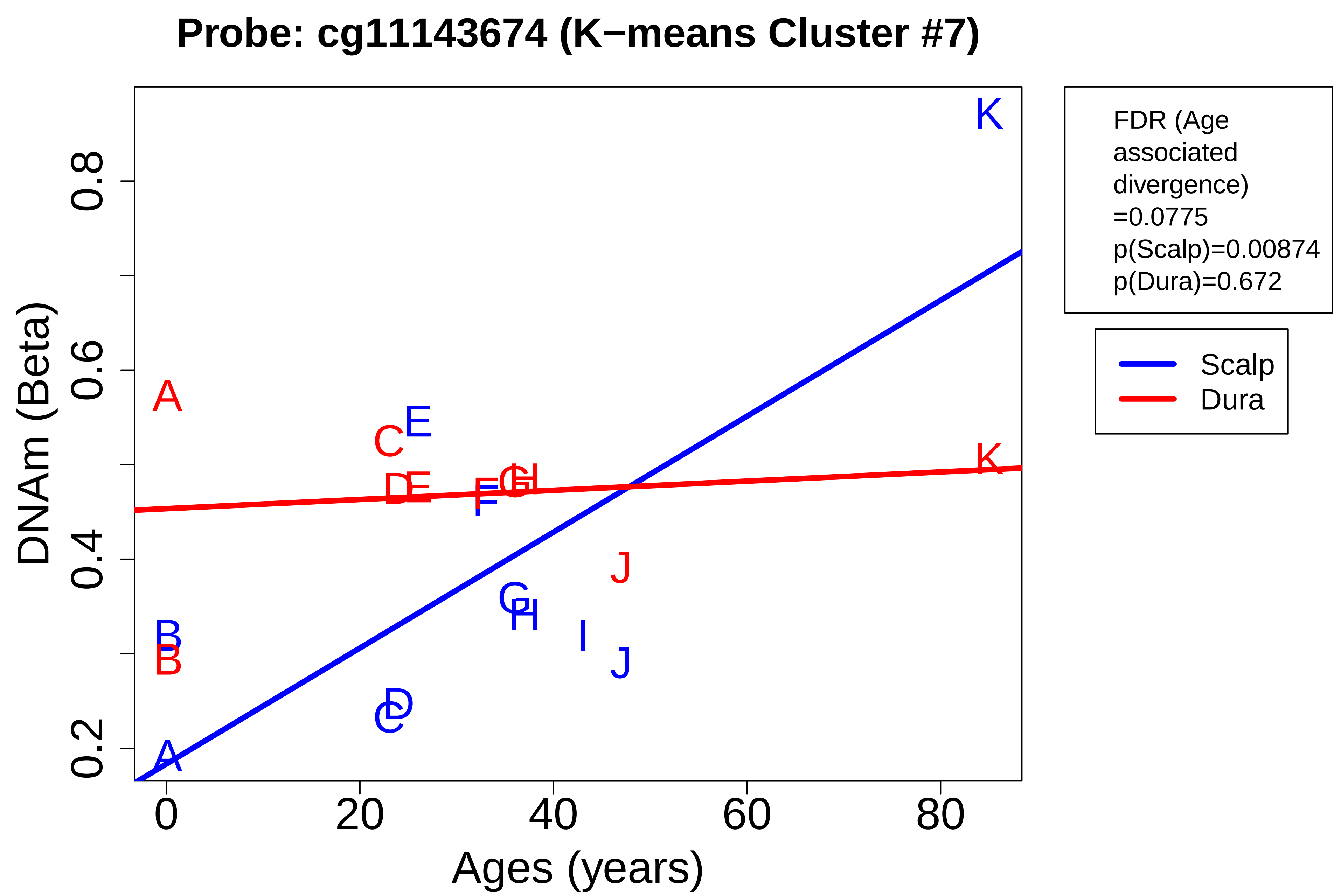

Probe: cg11143674 (K−means Cluster #7)
DNAm (Beta)
0.2
0.4
0.6
0.8
0
20
40
60
80
Ages (years)
A
B
C
D
E
F
G
H
I
J
K
FDR (Age associated divergence) =0.0775
p(Scalp)=0.00874
p(Dura)=0.672
Scalp
Dura

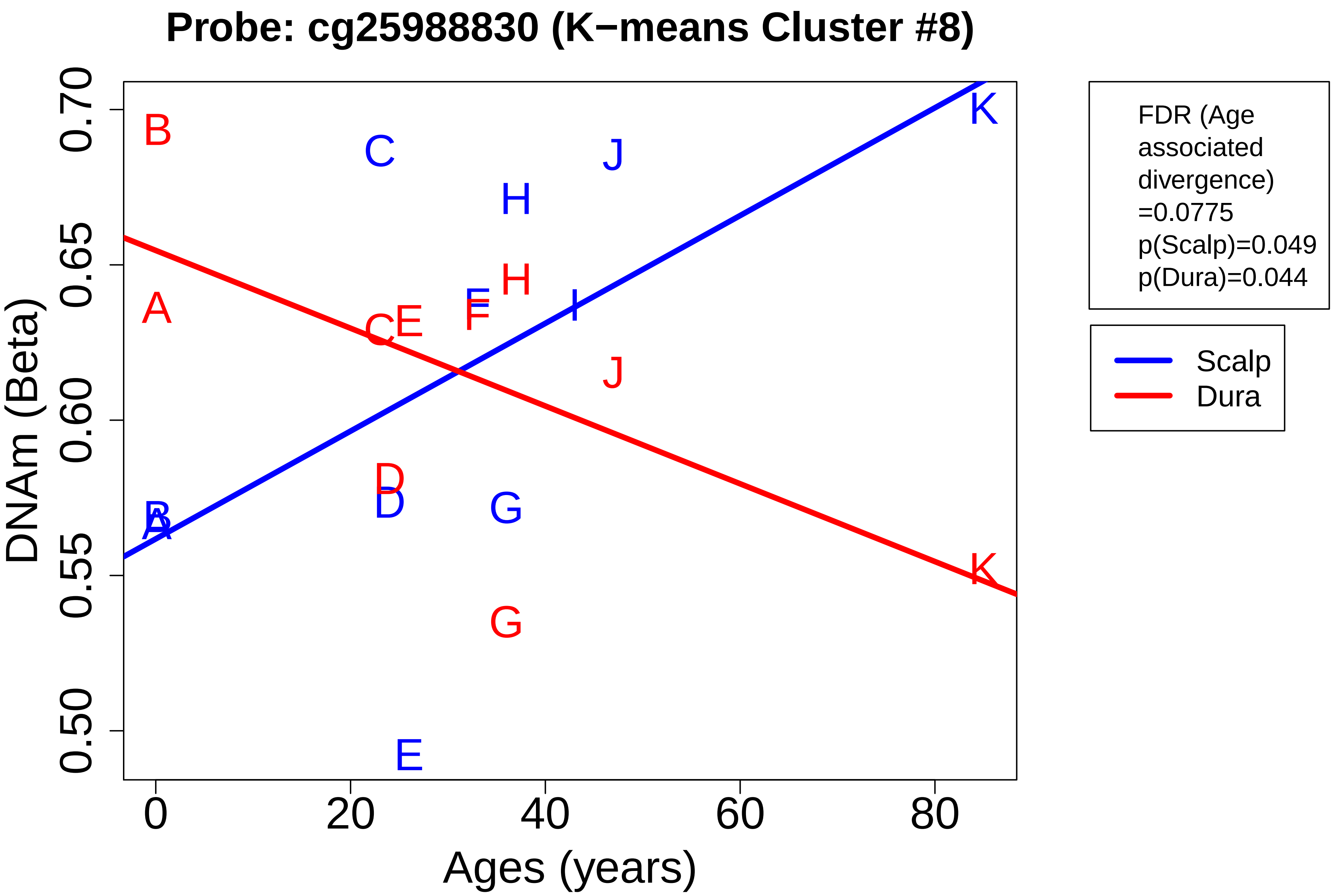
Probe: cg25988830 (K−means Cluster #8)
DNAm (Beta)
0.50
0.55
0.60
0.65
0.70
Ages (years)
0
20
40
60
80
B
A
C
E
D
F
H
G
I
J
K
FDR (Age associated divergence) =0.0775
p(Scalp)=0.049
p(Dura)=0.044
Scalp
Dura

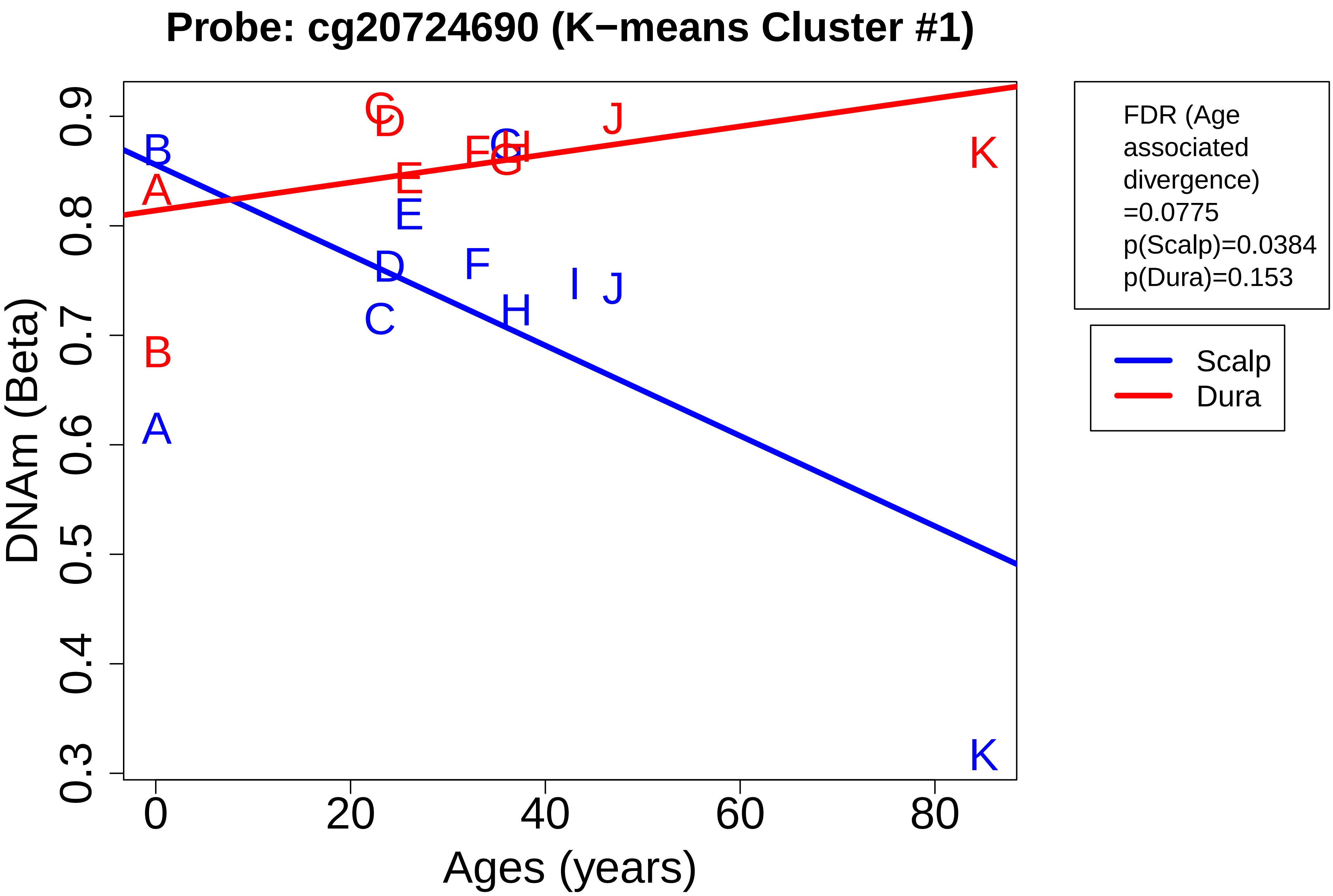
Probe: cg20724690 (K−means Cluster #1)
DNAm (Beta)
0.3
0.4
0.5
0.6
0.7
0.8
0.9
0
20
40
60
80
Ages (years)
A
B
C
D
E
F
G
H
I
J
K
FDR (Age associated divergence) =0.0775
p(Scalp)=0.0384
p(Dura)=0.153
Scalp
Dura

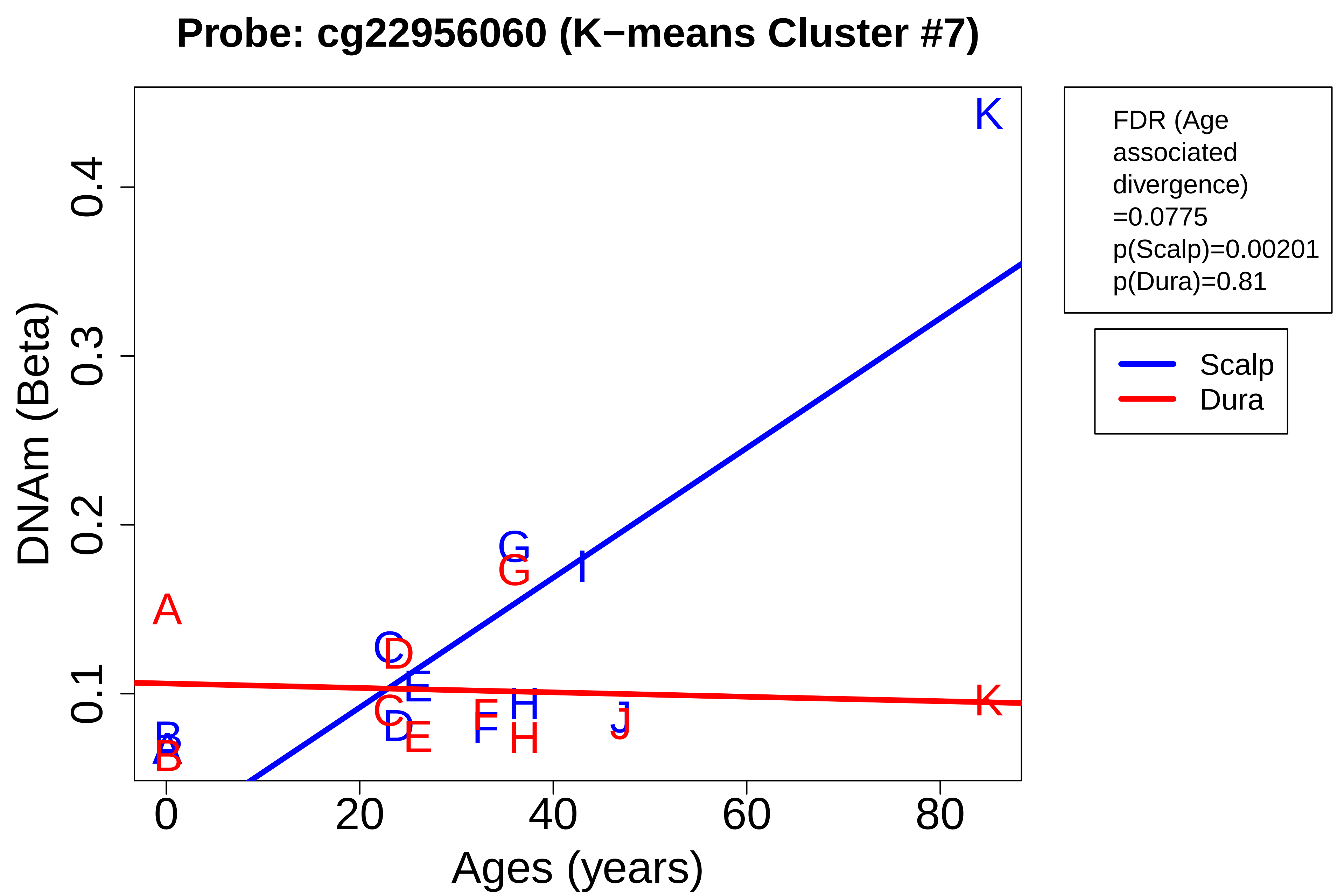

Probe: cg22956060 (K−means Cluster #7)
DNAm (Beta)
0.1
0.2
0.3
0.4
0
20
40
60
80
Ages (years)
FDR (Age associated divergence) =0.0775
p(Scalp)=0.00201
p(Dura)=0.81
Scalp
Dura

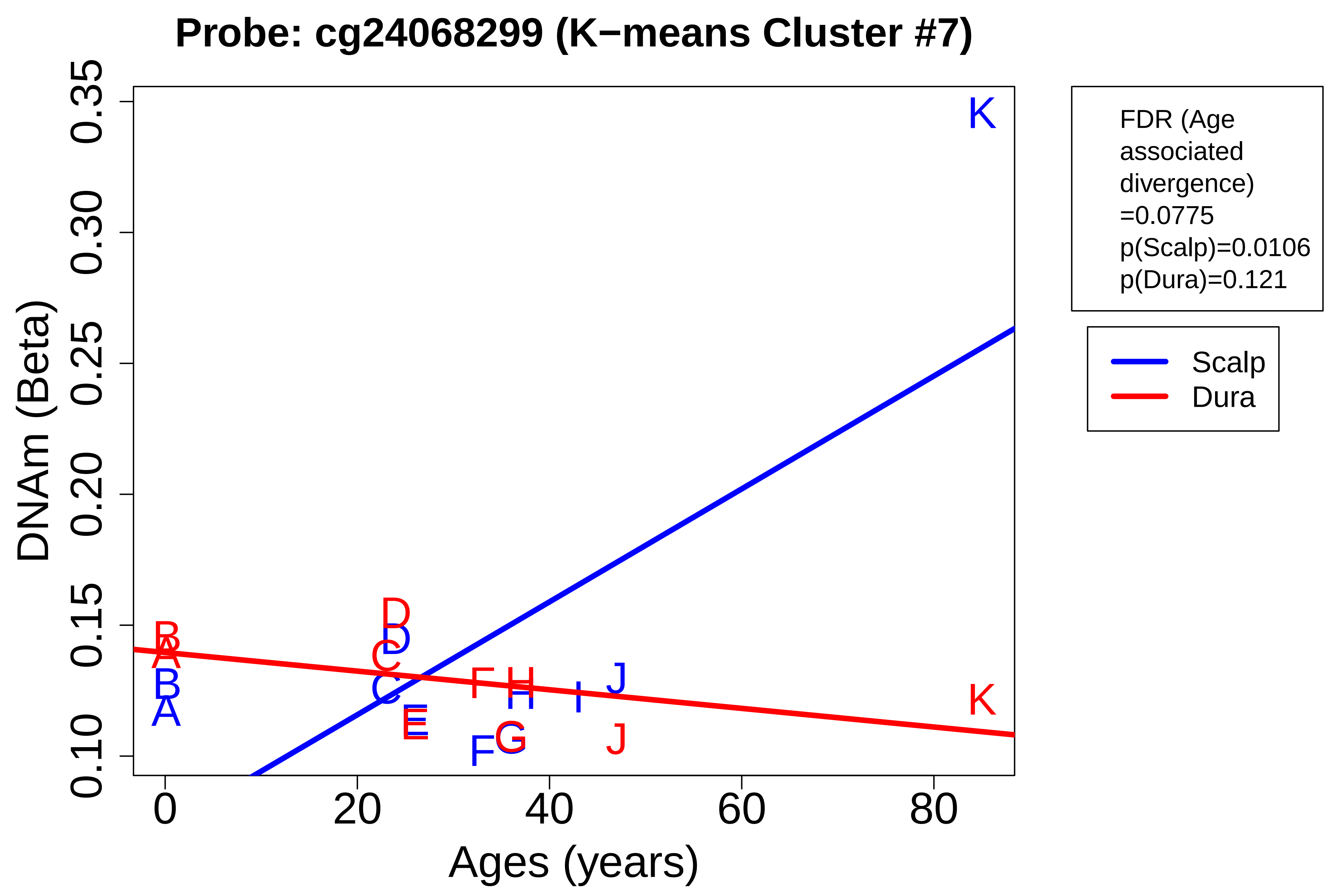

Probe: cg24068299 (K−means Cluster #7)
DNAm (Beta)
0.10
0.15
0.20
0.25
0.30
0.35
Ages (years)
0
20
40
60
80
FDR (Age associated divergence) =0.0775
p(Scalp)=0.0106
p(Dura)=0.121
Scalp
Dura

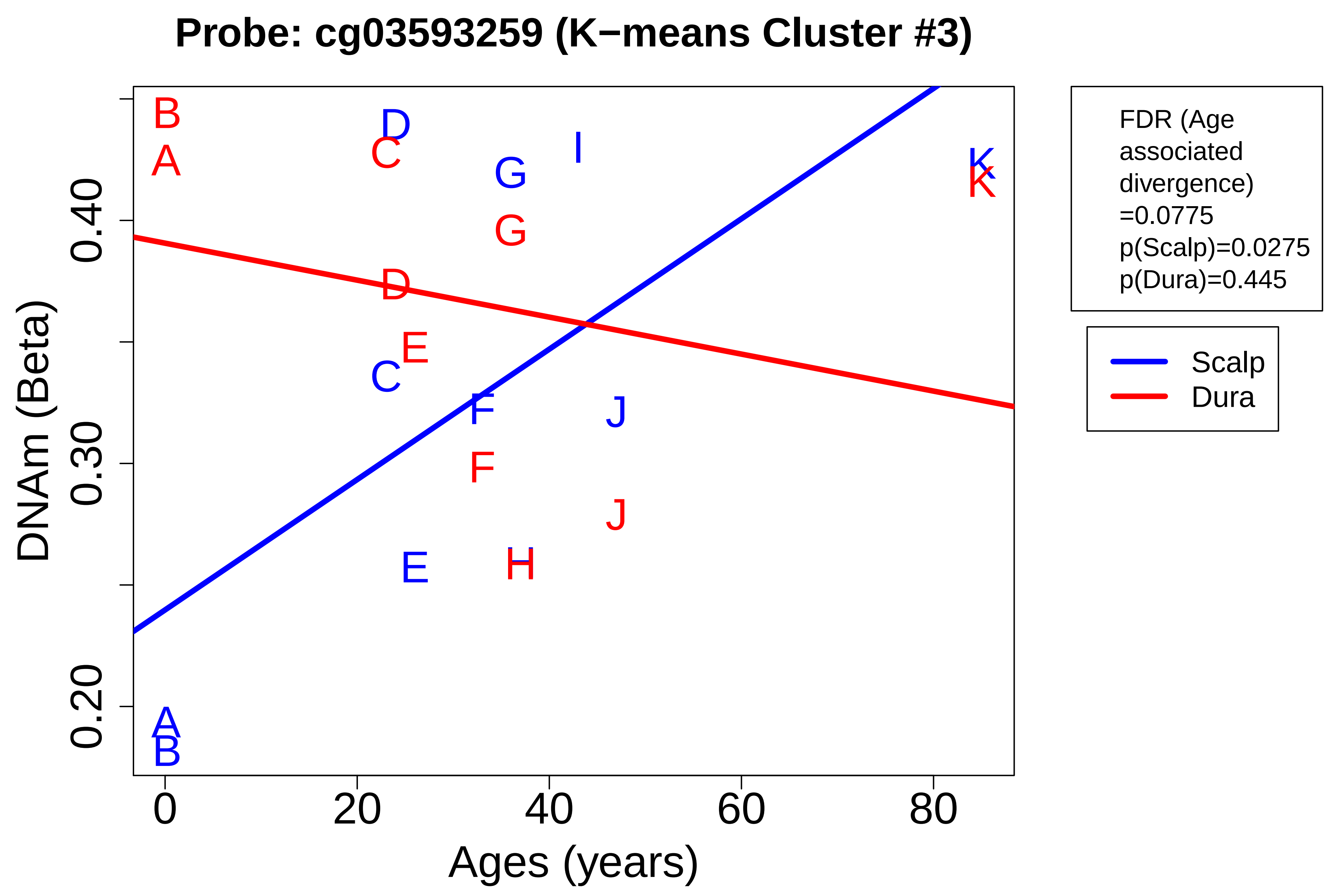

Probe: cg03593259 (K−means Cluster #3)
DNAm (Beta)
0.20
0.30
0.40
0
20
40
60
80
Ages (years)
FDR (Age associated divergence) =0.0775
p(Scalp)=0.0275
p(Dura)=0.445
Scalp
Dura
A
B
C
D
E
F
G
H
I
J
K

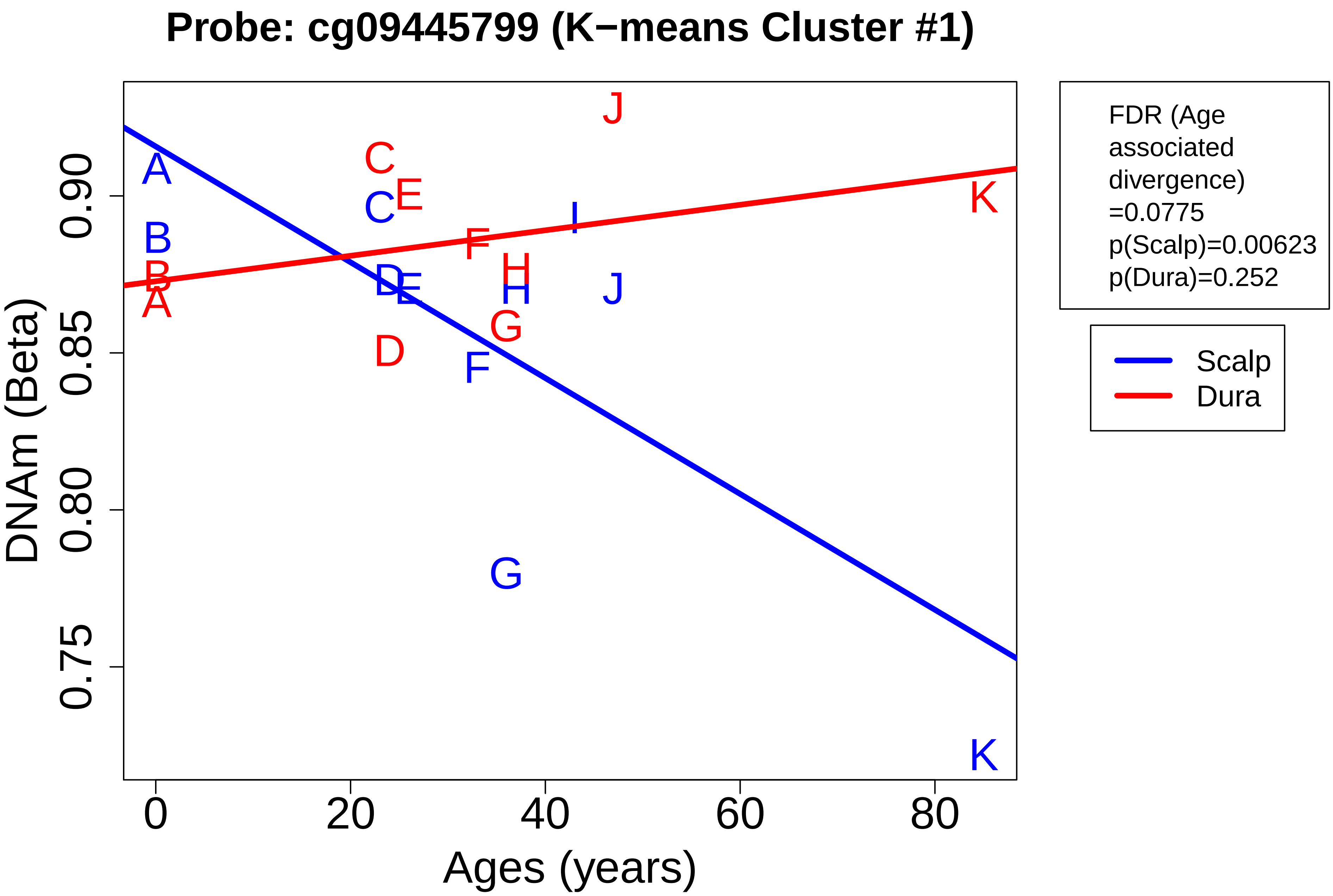

Probe: cg09445799 (K−means Cluster #1)
DNAm (Beta)
0.75
0.80
0.85
0.90
0
20
40
60
80
Ages (years)
FDR (Age associated divergence) =0.0775
p(Scalp)=0.00623
p(Dura)=0.252
Scalp
Dura

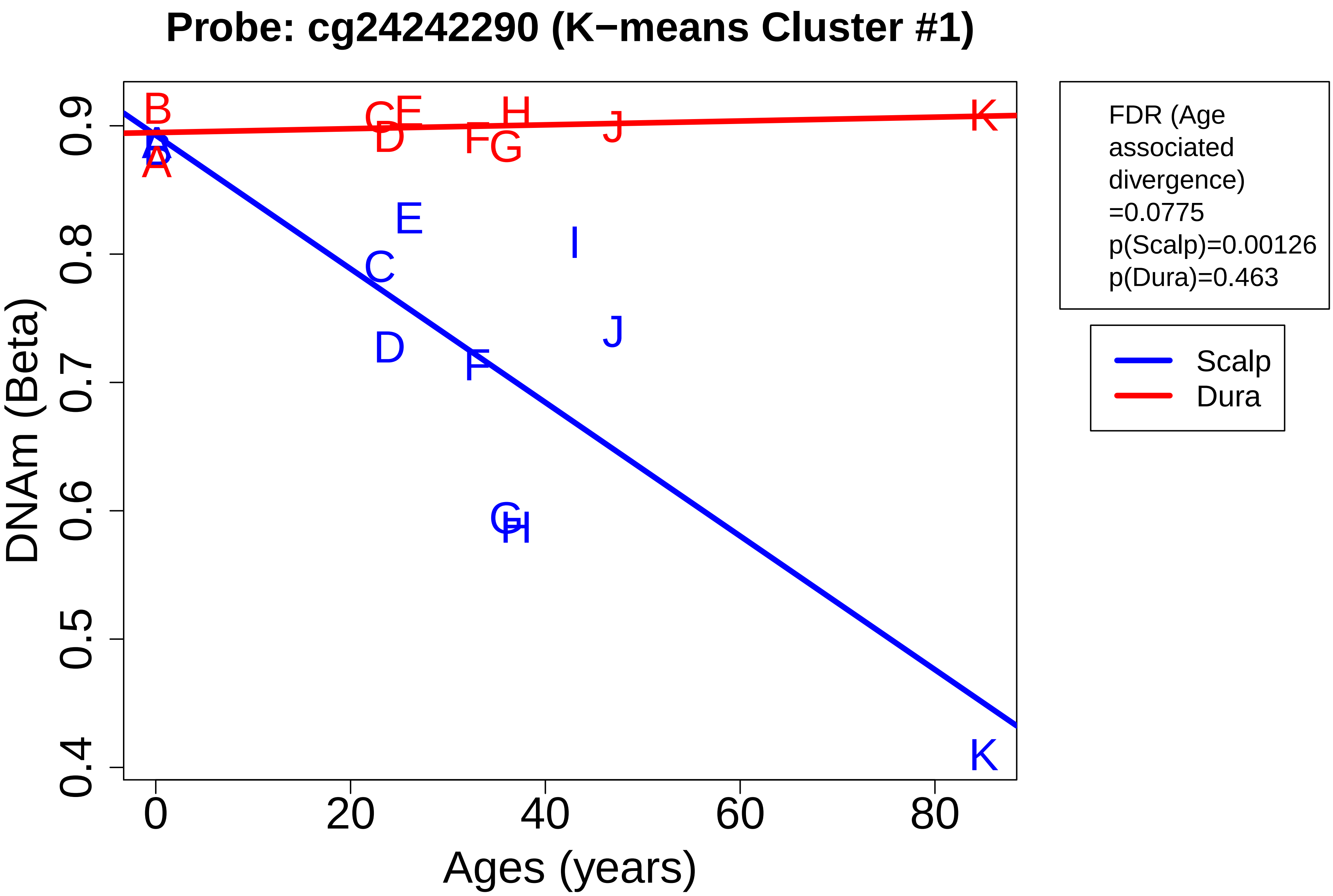

Probe: cg24242290 (K−means Cluster #1)
DNAm (Beta)
0.4
0.5
0.6
0.7
0.8
0.9
0
20
40
60
80
Ages (years)
FDR (Age associated divergence) =0.0775
p(Scalp)=0.00126
p(Dura)=0.463
Scalp
Dura

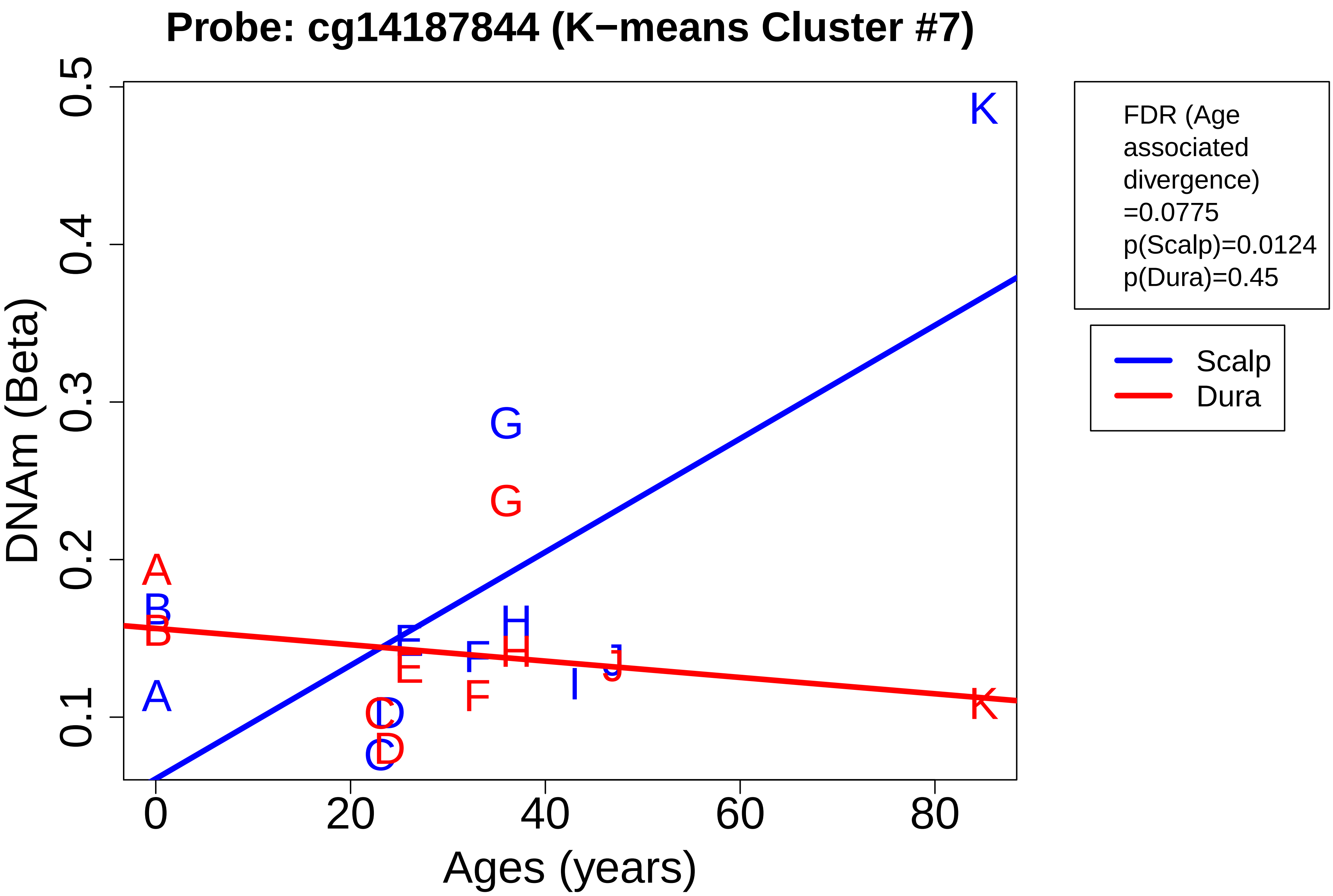

Probe: cg14187844 (K−means Cluster #7)
DNAm (Beta)
0.1
0.2
0.3
0.4
0.5
0
20
40
60
80
Ages (years)
FDR (Age associated divergence) =0.0775
p(Scalp)=0.0124
p(Dura)=0.45
Scalp
Dura
A
B
C
D
E
F
G
H
I
J
K

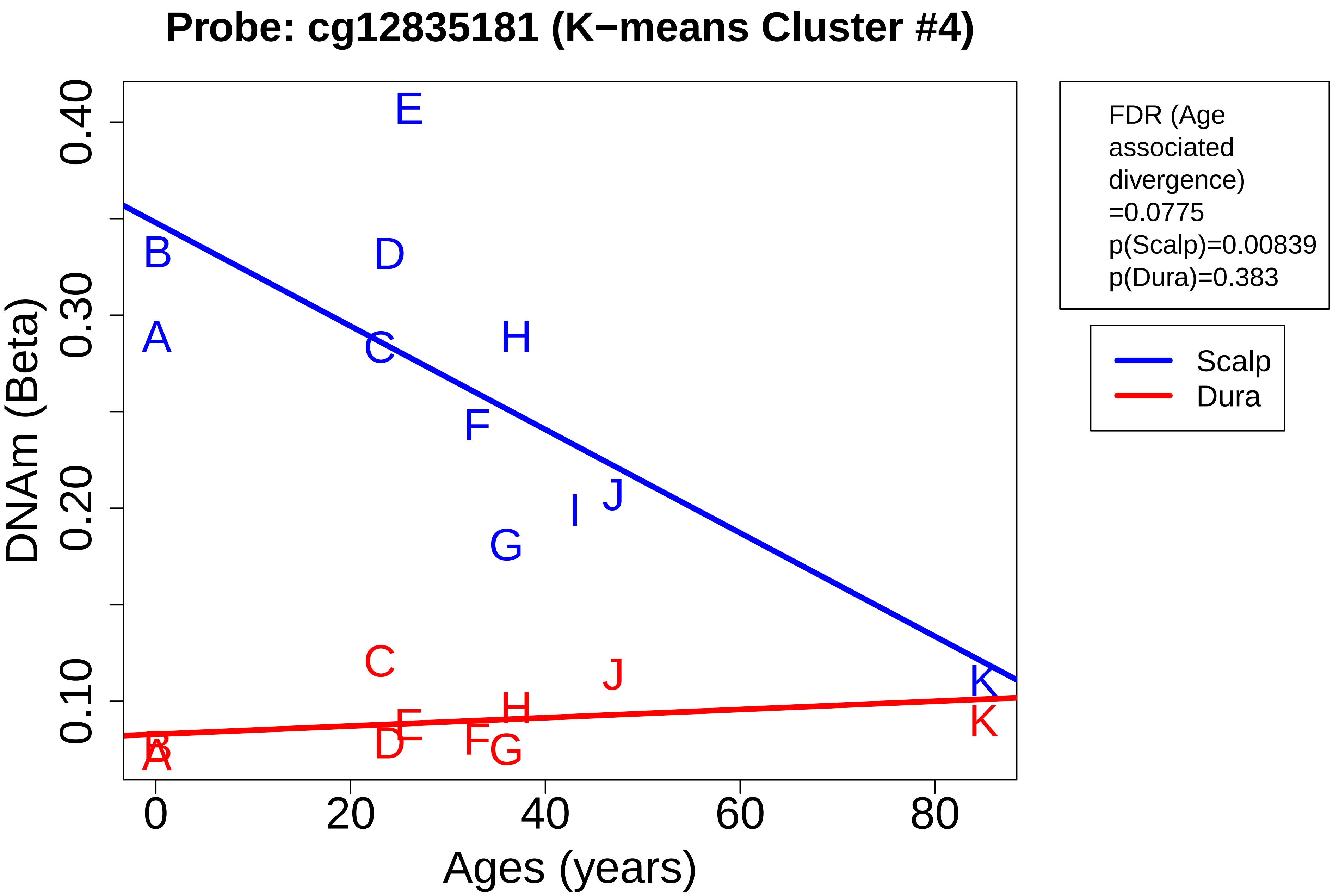

Probe: cg12835181 (K−means Cluster #4)
DNAm (Beta)
0.10
0.20
0.30
0.40
Ages (years)
0
20
40
60
80
A
B
C
D
E
F
G
H
I
J
K
FDR (Age associated divergence) =0.0775
p(Scalp)=0.00839
p(Dura)=0.383
Scalp
Dura

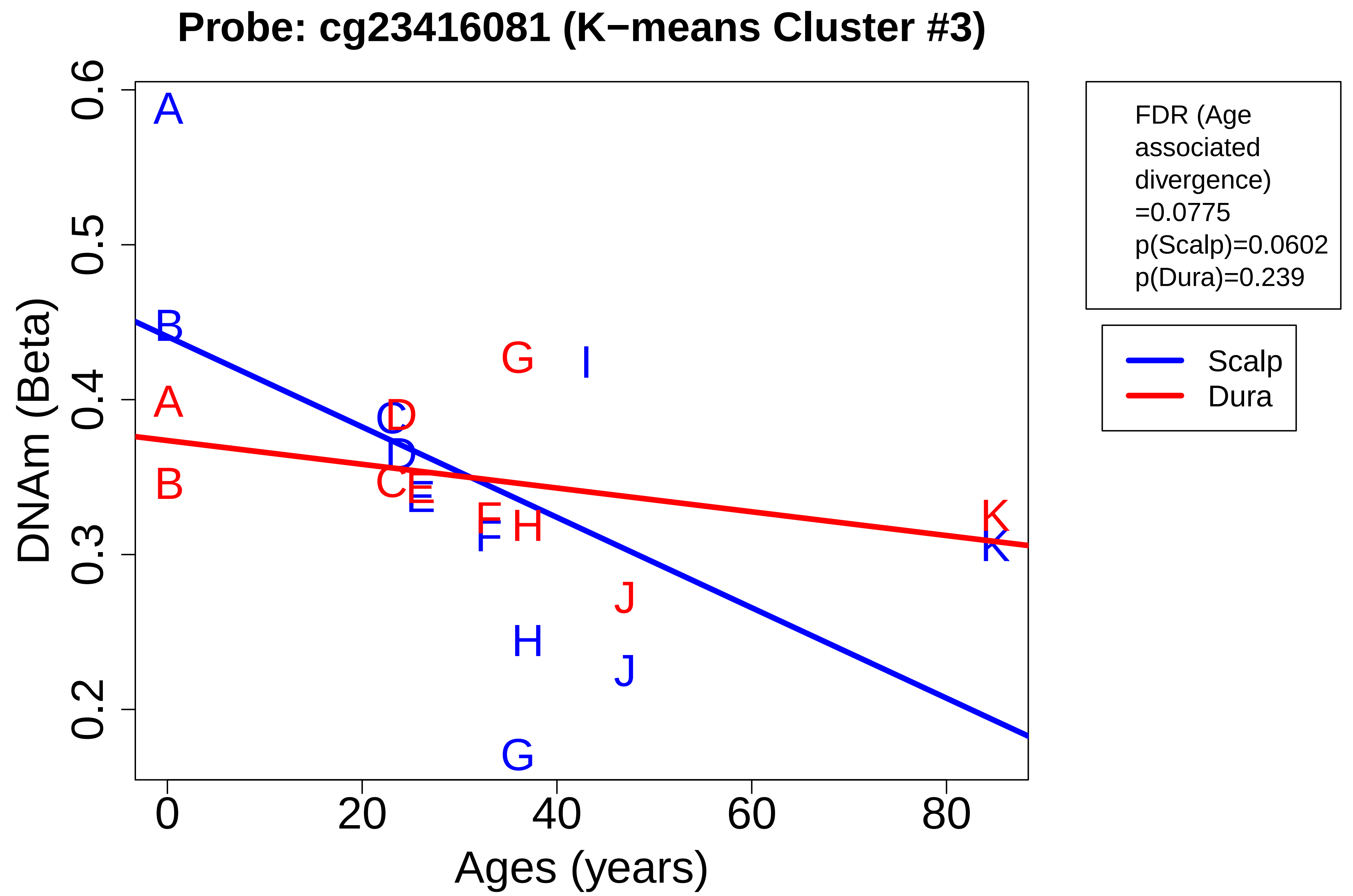

Probe: cg23416081 (K−means Cluster #3)
DNAm (Beta)
0.2
0.3
0.4
0.5
0.6
0
20
40
60
80
Ages (years)
FDR (Age associated divergence) =0.0775
p(Scalp)=0.0602
p(Dura)=0.239
Scalp
Dura

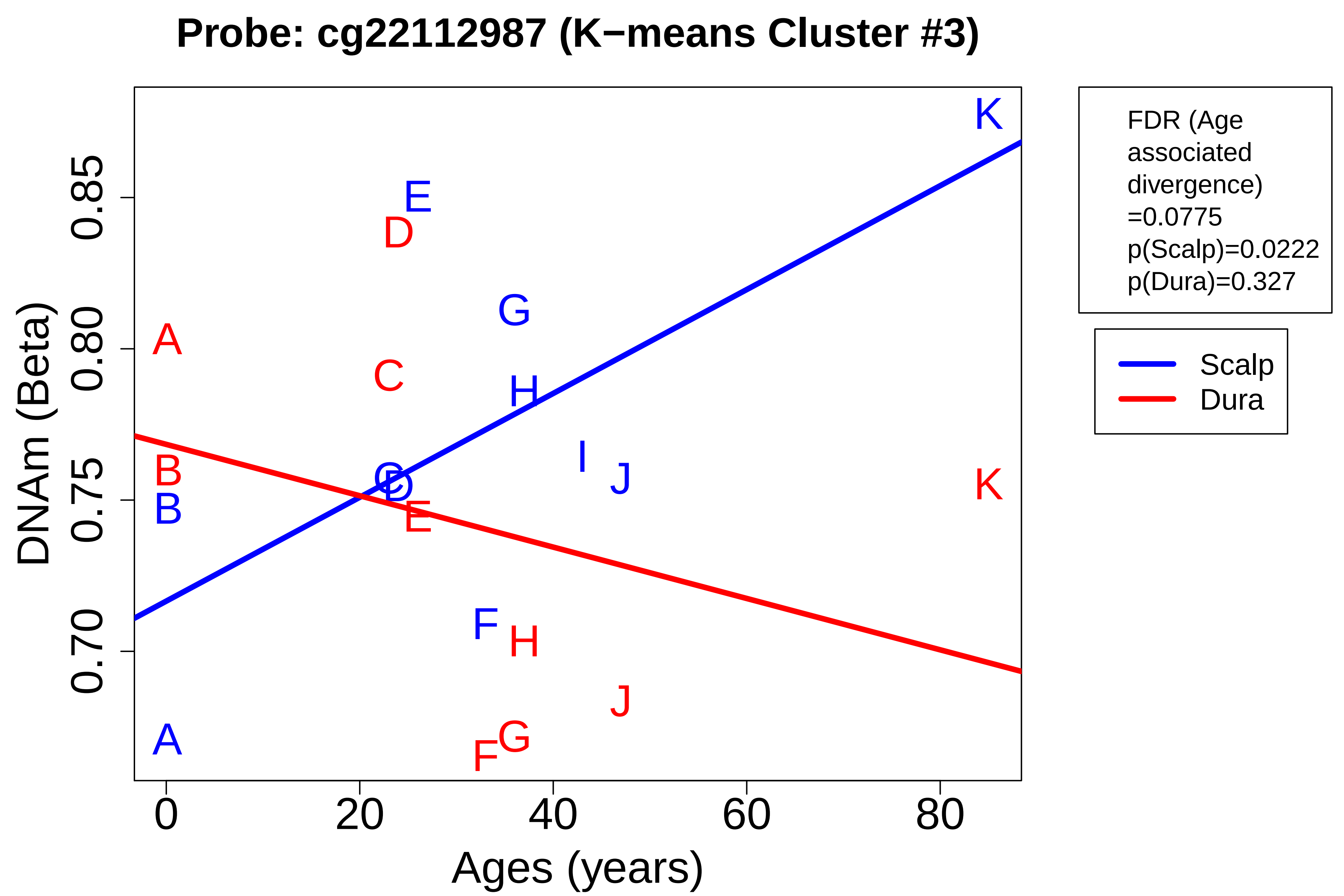

Probe: cg22112987 (K−means Cluster #3)
DNAm (Beta)
0.70
0.75
0.80
0.85
Ages (years)
0
20
40
60
80
A
B
C
D
E
F
G
H
I
J
K
FDR (Age associated divergence) =0.0775
p(Scalp)=0.0222
p(Dura)=0.327
Scalp
Dura

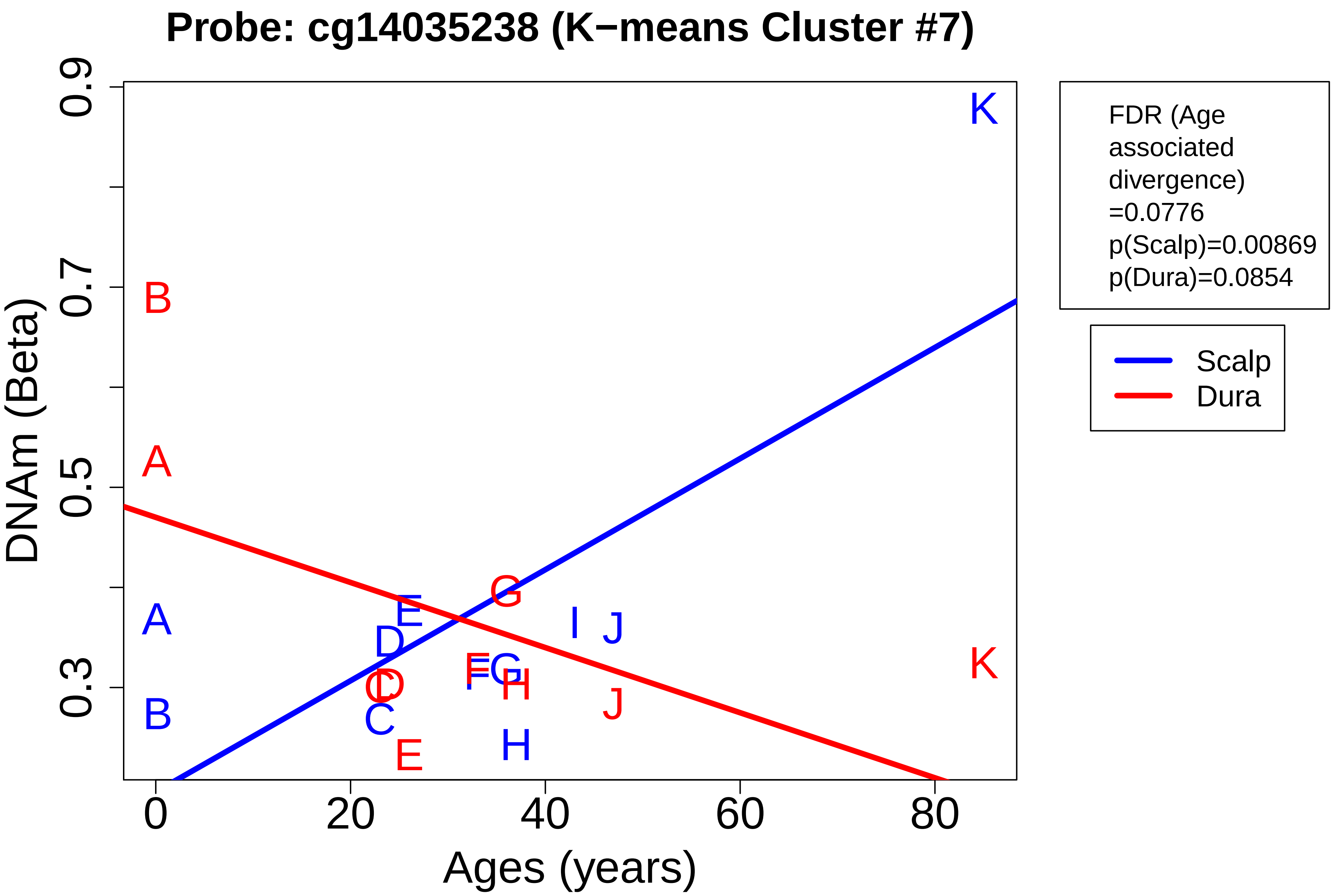

Probe: cg14035238 (K−means Cluster #7)
DNAm (Beta)
0.3
0.5
0.7
0.9
Ages (years)
0
20
40
60
80
FDR (Age associated divergence) =0.0776
p(Scalp)=0.00869
p(Dura)=0.0854
Scalp
Dura

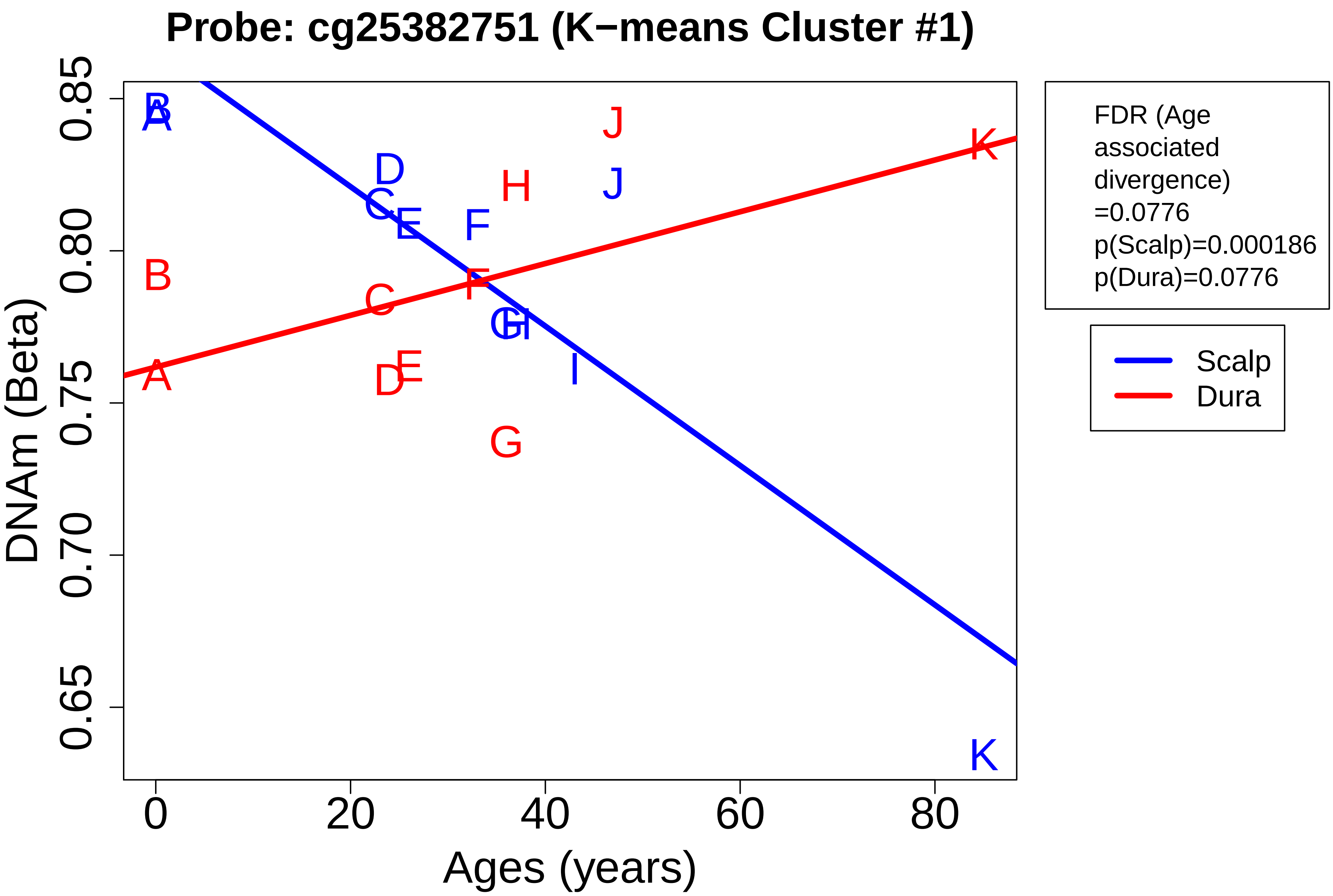
Probe: cg25382751 (K−means Cluster #1)
DNAm (Beta)
Ages (years)
0.65
0.70
0.75
0.80
0.85
0
20
40
60
80
FDR (Age associated divergence) =0.0776
p(Scalp)=0.000186
p(Dura)=0.0776
Scalp
Dura

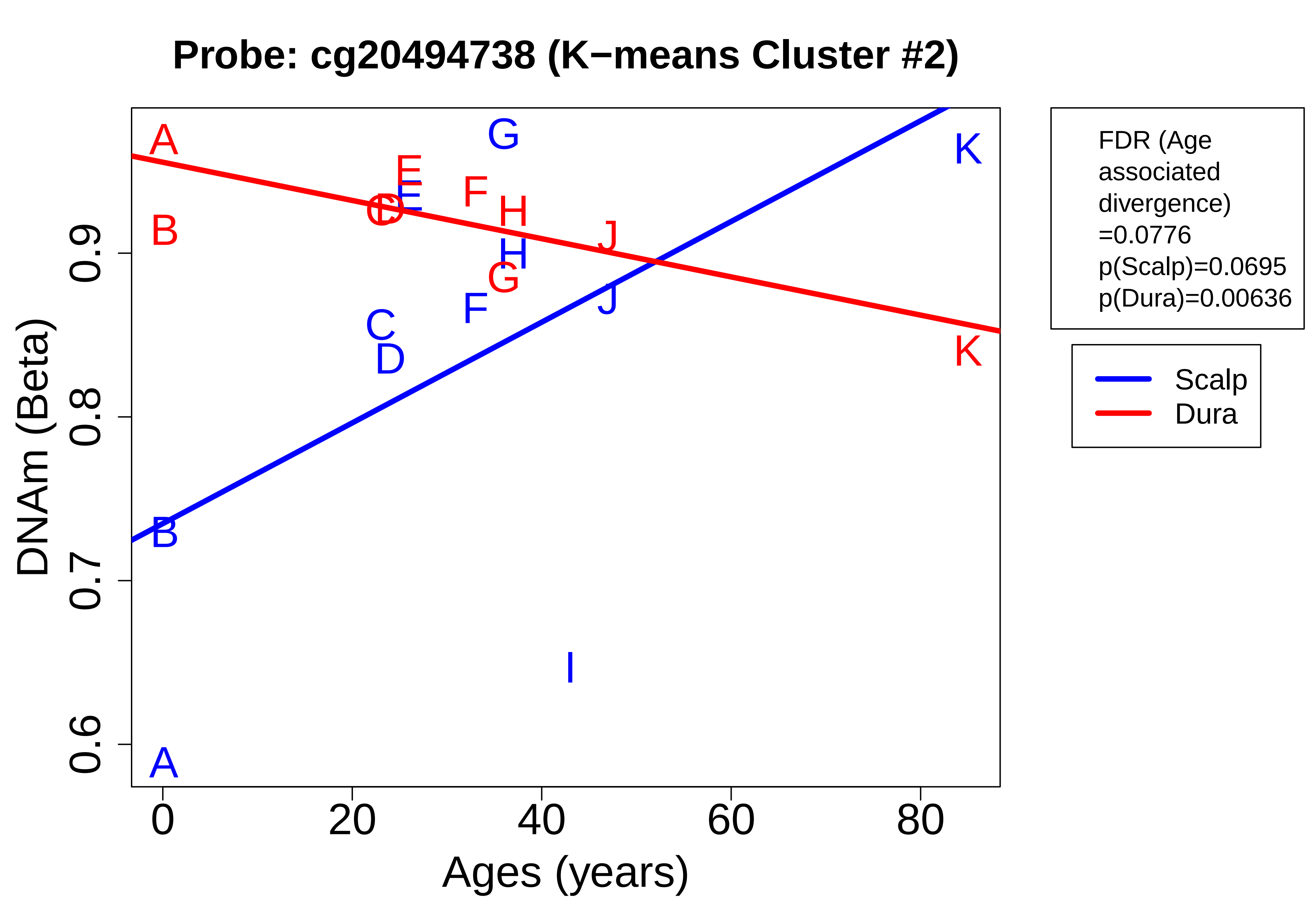

Probe: cg20494738 (K−means Cluster #2)
DNAm (Beta)
Ages (years)
0.6
0.7
0.8
0.9
0
20
40
60
80
FDR (Age associated divergence) =0.0776
p(Scalp)=0.0695
p(Dura)=0.00636
Scalp
Dura

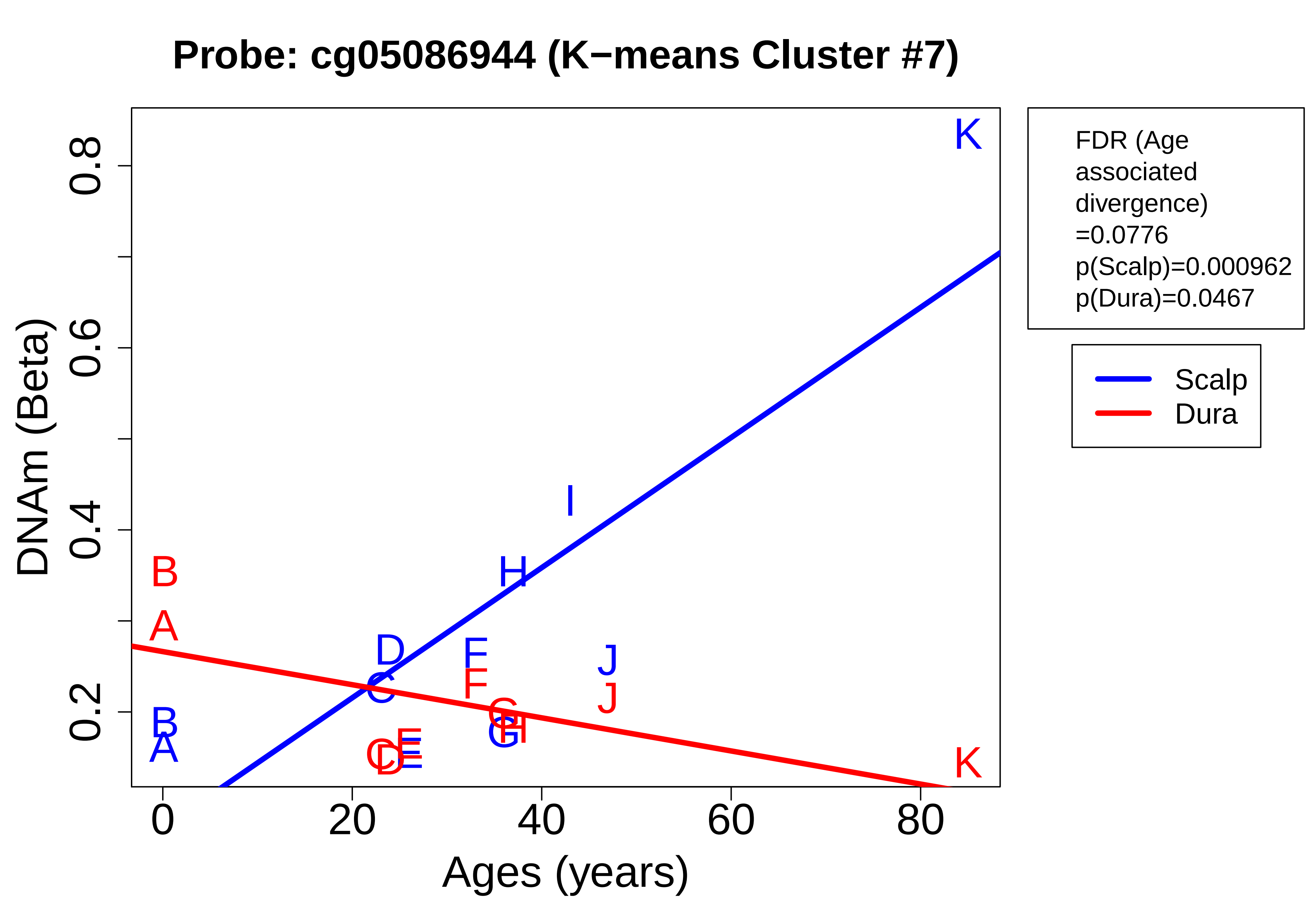

Probe: cg05086944 (K−means Cluster #7)
DNAm (Beta)
0.2
0.4
0.6
0.8
0
20
40
60
80
Ages (years)
FDR (Age associated divergence) =0.0776
p(Scalp)=0.000962
p(Dura)=0.0467
Scalp
Dura
A
B
C
D
E
F
G
H
I
J
K

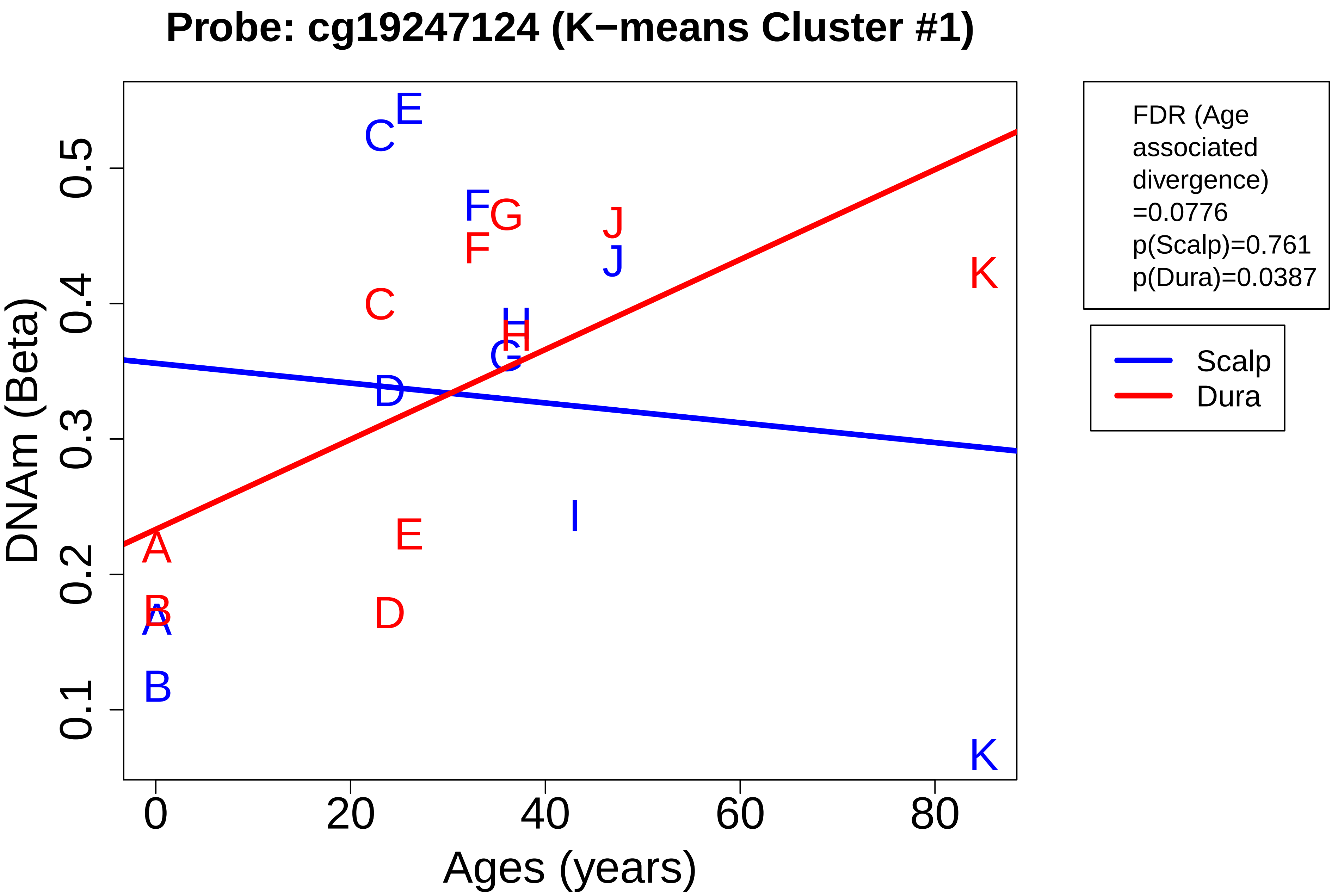

Probe: cg19247124 (K−means Cluster #1)
DNAm (Beta)
0.1
0.2
0.3
0.4
0.5
0
20
40
60
80
Ages (years)
FDR (Age associated divergence) =0.0776
p(Scalp)=0.761
p(Dura)=0.0387
Scalp
Dura

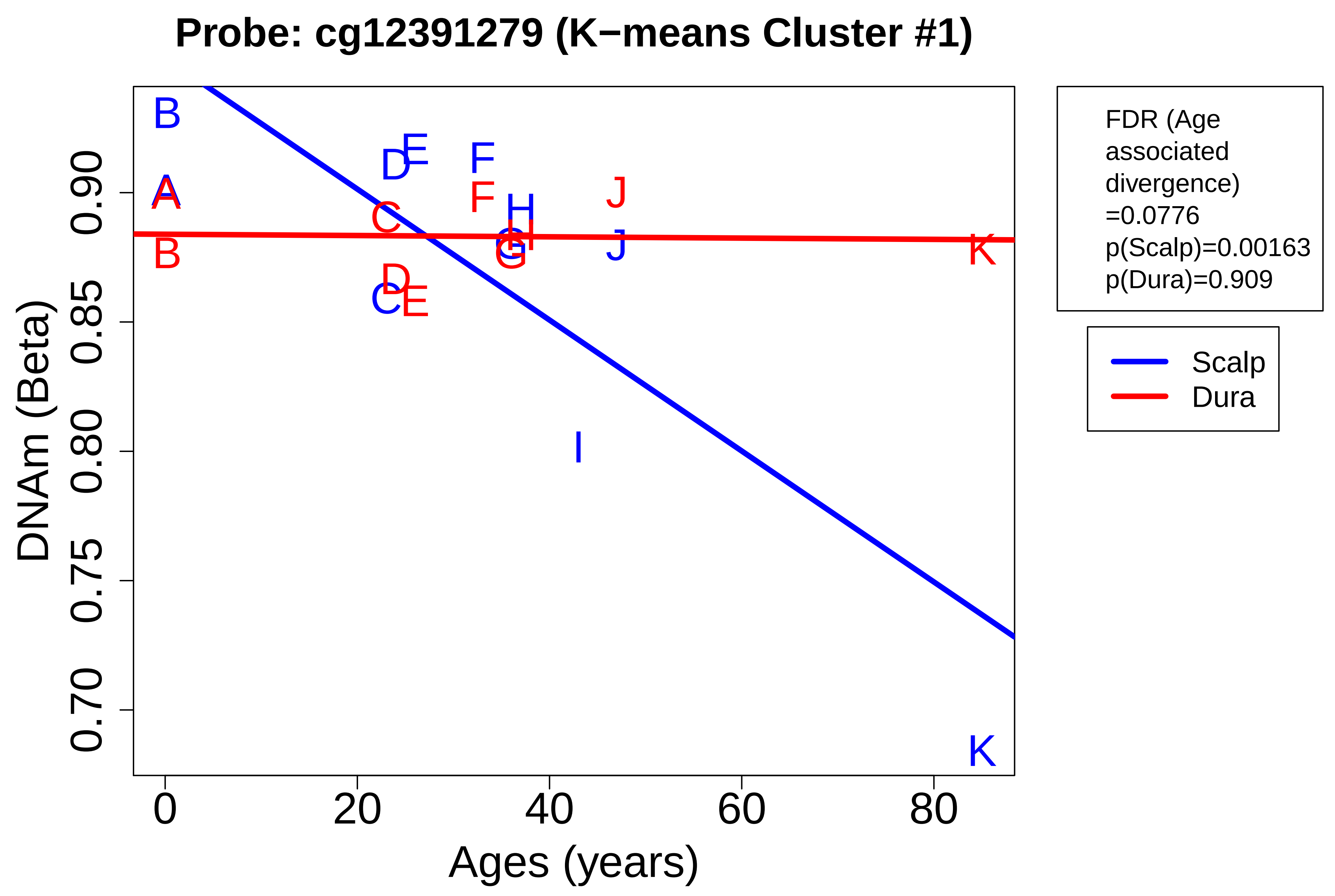

Probe: cg12391279 (K−means Cluster #1)
DNAm (Beta)
0.70
0.75
0.80
0.85
0.90
Ages (years)
0
20
40
60
80
FDR (Age associated divergence) =0.0776
p(Scalp)=0.00163
p(Dura)=0.909
Scalp
Dura

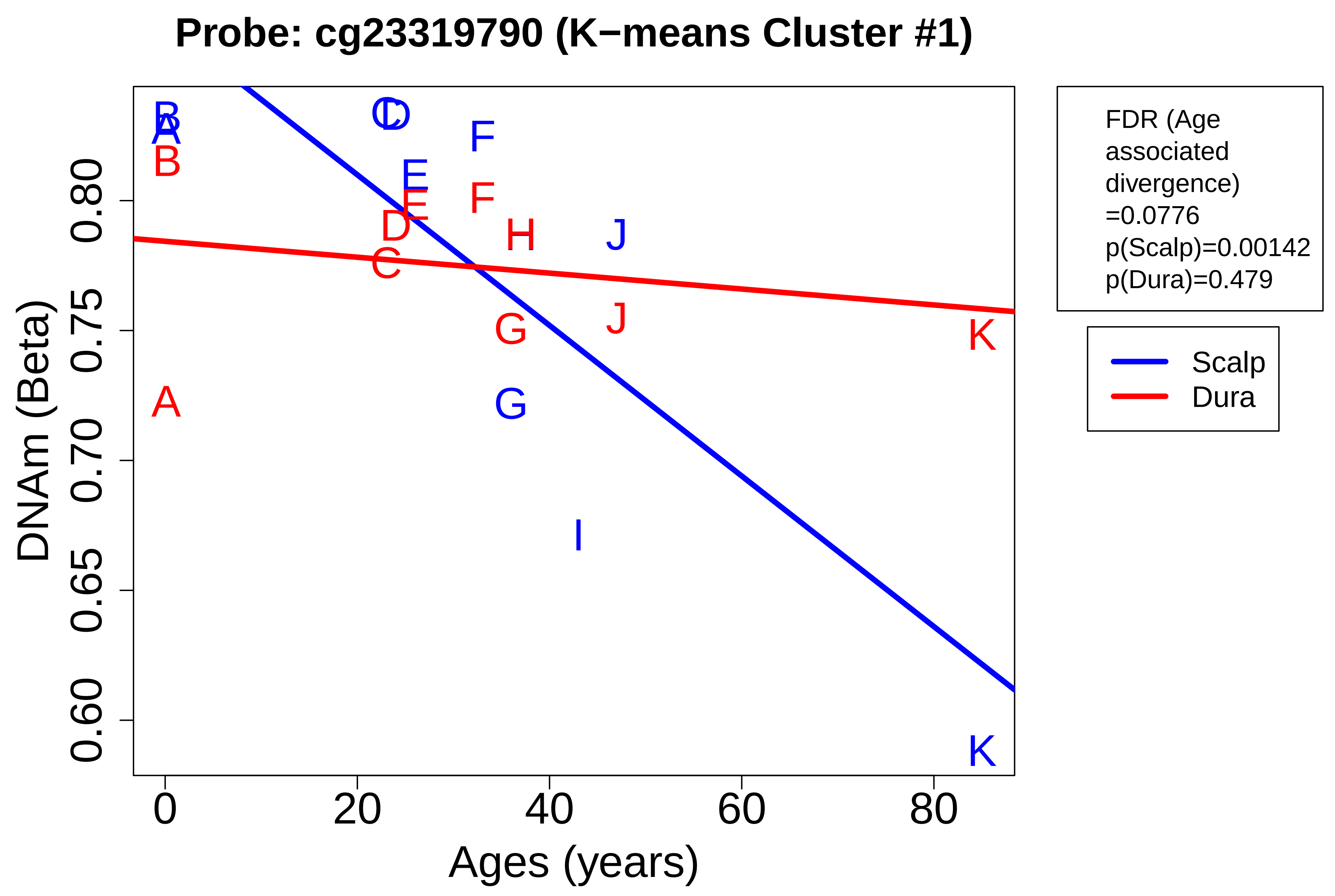

Probe: cg23319790 (K−means Cluster #1)
FDR (Age associated divergence) =0.0776
p(Scalp)=0.00142
p(Dura)=0.479
Scalp
Dura
DNAm (Beta)
0.60
0.65
0.70
0.75
0.80
Ages (years)
0
20
40
60
80
A
B
C
D
E
F
G
H
I
J
K

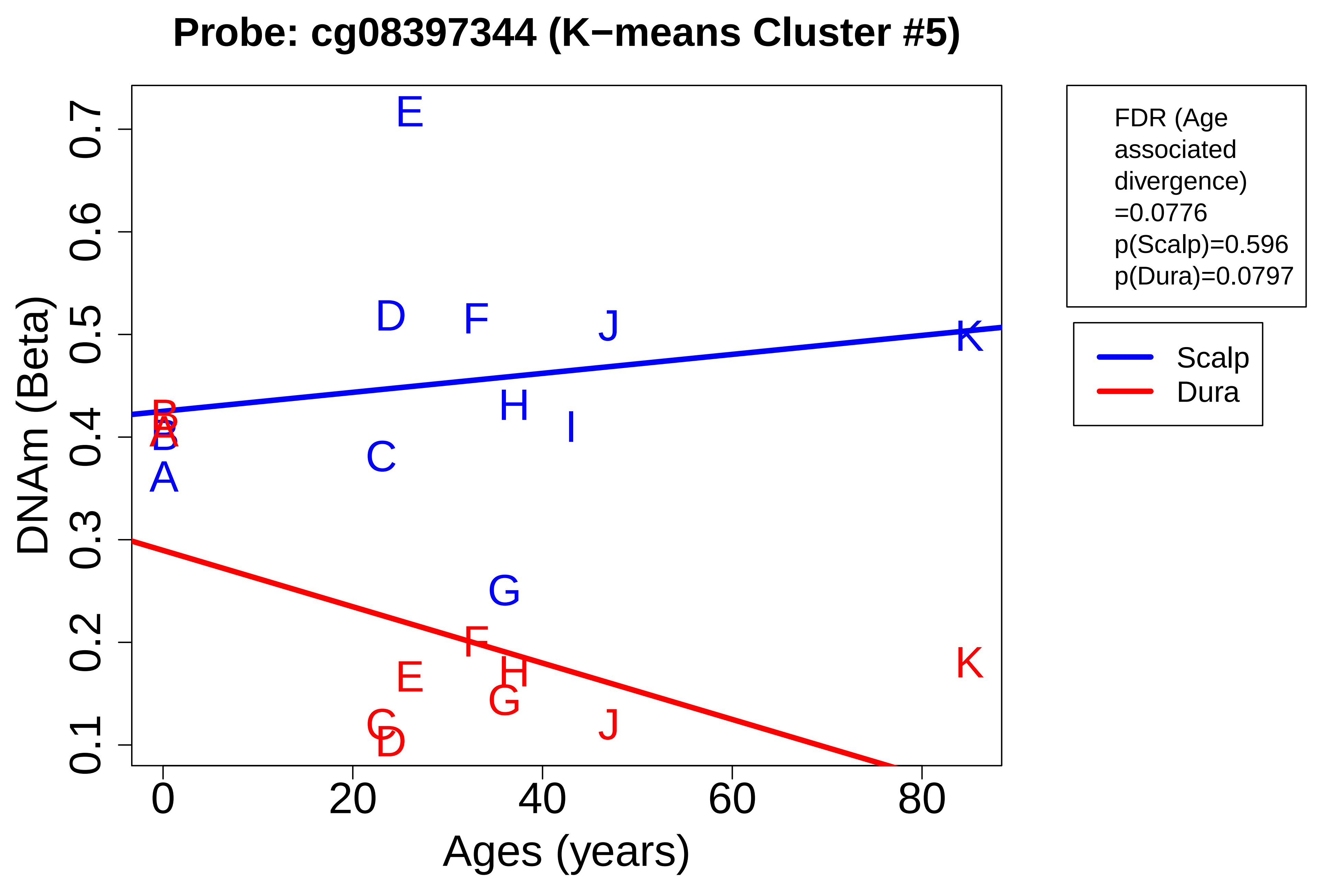
Probe: cg08397344 (K−means Cluster #5)
DNAm (Beta)
Ages (years)
0.1
0.2
0.3
0.4
0.5
0.6
0.7
0
20
40
60
80
FDR (Age associated divergence) =0.0776
p(Scalp)=0.596
p(Dura)=0.0797
Scalp
Dura
A
B
C
D
E
F
G
H
I
J
K

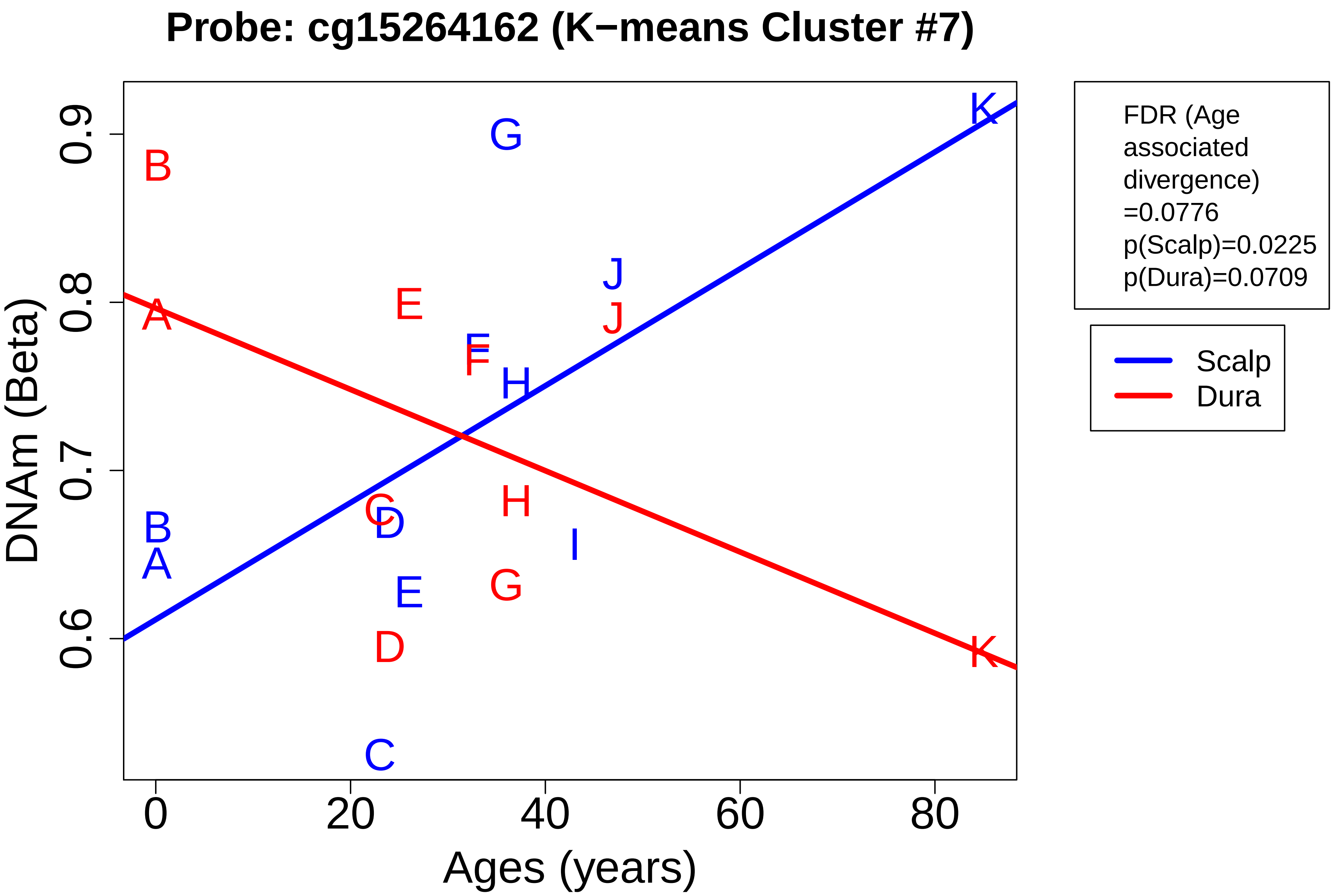

Probe: cg15264162 (K−means Cluster #7)
DNAm (Beta)
0.6
0.7
0.8
0.9
Ages (years)
0
20
40
60
80
FDR (Age associated divergence) =0.0776
p(Scalp)=0.0225
p(Dura)=0.0709
Scalp
Dura
A
B
C
D
E
F
G
H
I
J
K

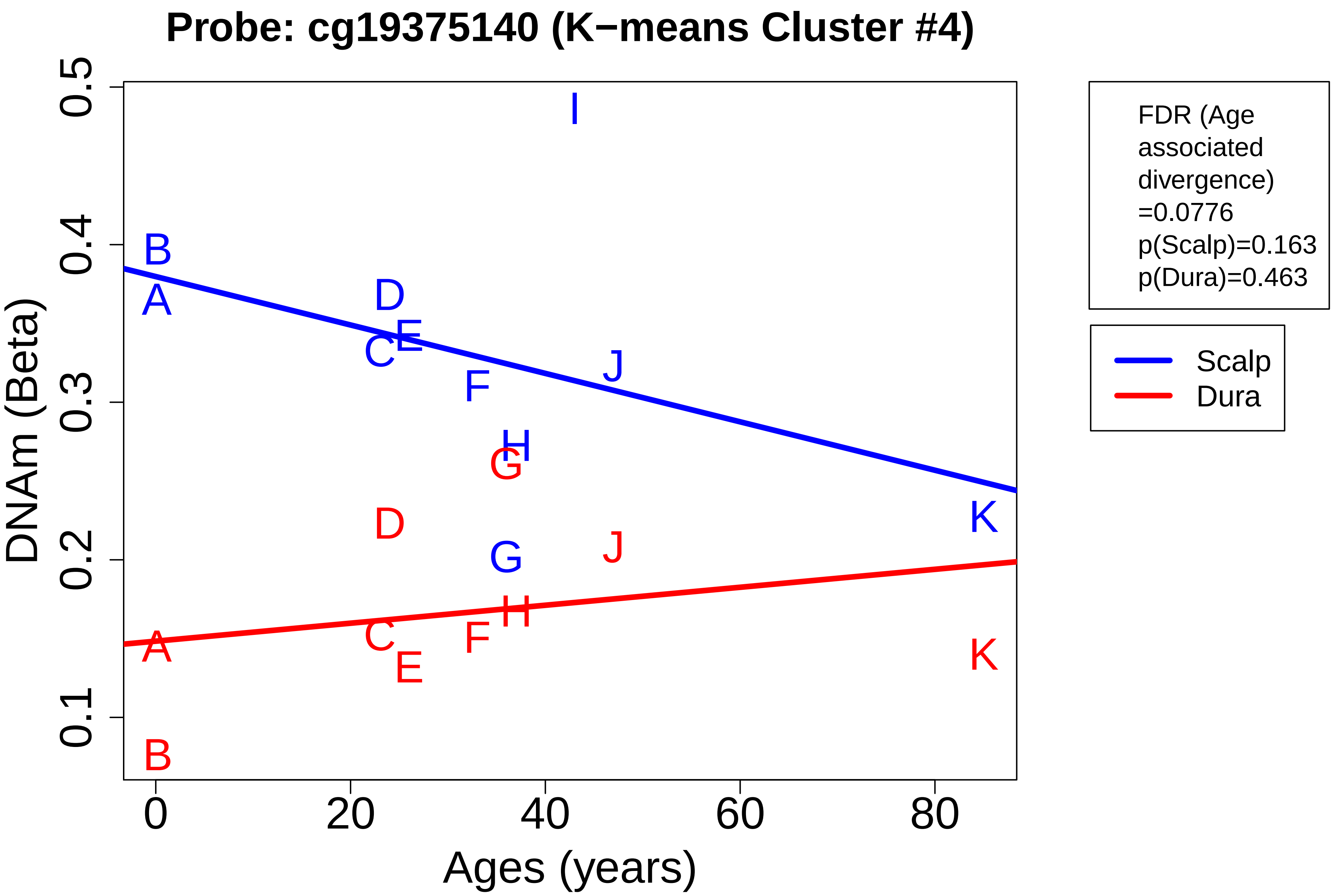

Probe: cg19375140 (K−means Cluster #4)
DNAm (Beta)
0.1
0.2
0.3
0.4
0.5
0
20
40
60
80
Ages (years)
FDR (Age associated divergence) =0.0776
p(Scalp)=0.163
p(Dura)=0.463
Scalp
Dura

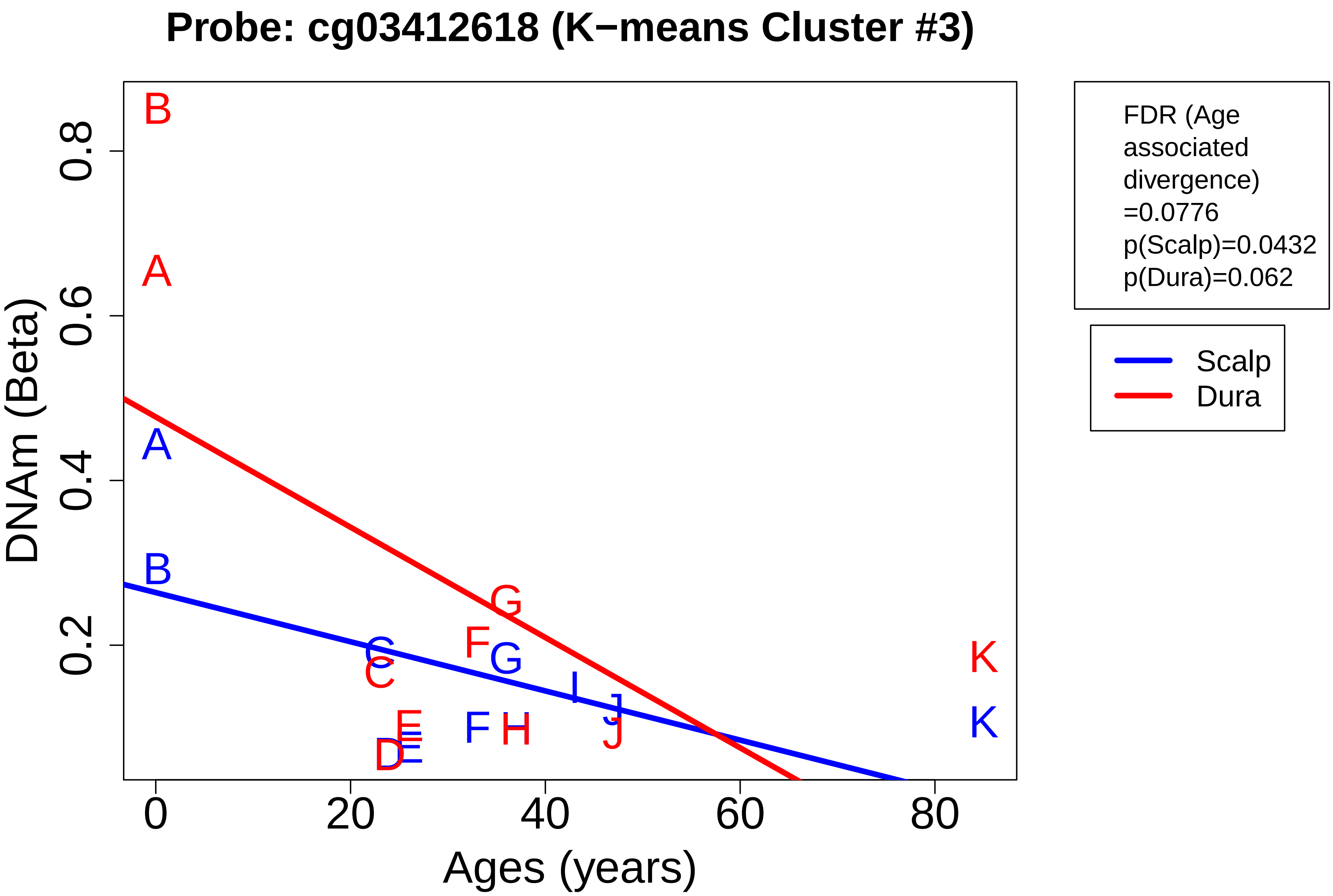

Probe: cg03412618 (K−means Cluster #3)
DNAm (Beta)
0.2
0.4
0.6
0.8
0
20
40
60
80
Ages (years)
FDR (Age associated divergence) =0.0776
p(Scalp)=0.0432
p(Dura)=0.062
Scalp
Dura
A
B
C
D
E
F
G
H
I
J
K

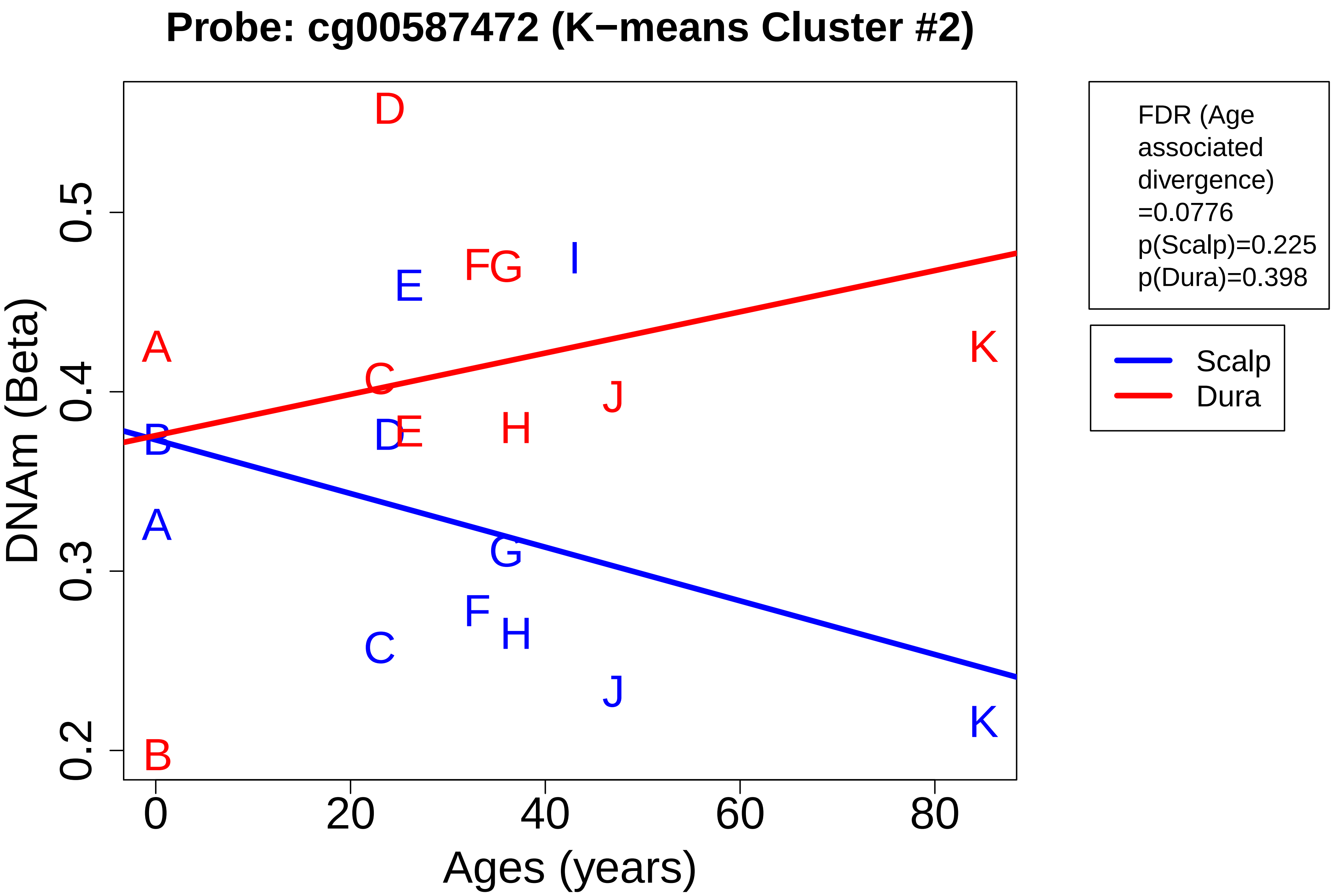
Probe: cg00587472 (K−means Cluster #2)
FDR (Age associated divergence) =0.0776
p(Scalp)=0.225
p(Dura)=0.398
Scalp
Dura
DNAm (Beta)
0.2
0.3
0.4
0.5
0
20
40
60
80
Ages (years)
A B C D E F G H I J K

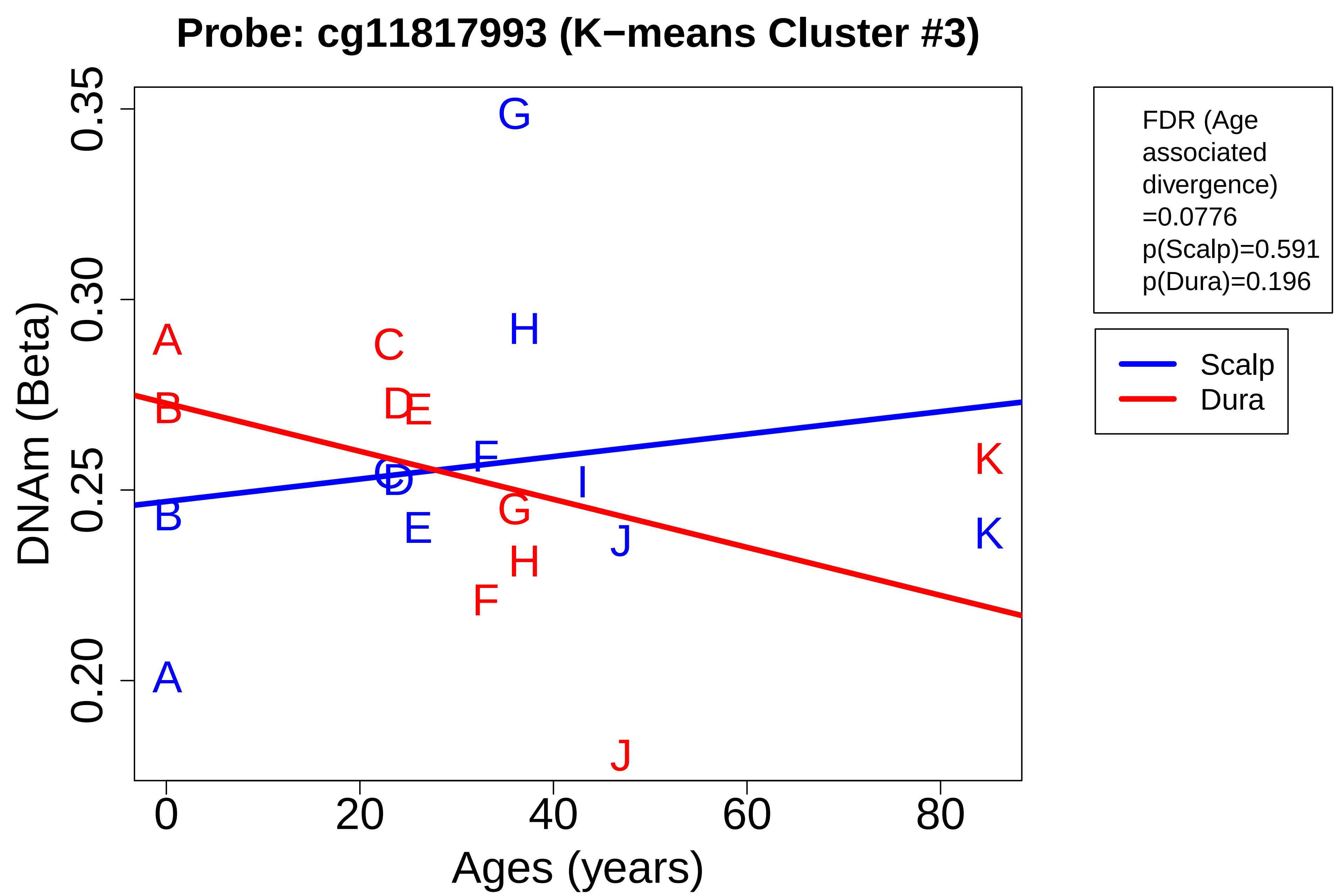

Probe: cg11817993 (K−means Cluster #3)
DNAm (Beta)
0.20
0.25
0.30
0.35
Ages (years)
0
20
40
60
80
FDR (Age associated divergence) =0.0776
p(Scalp)=0.591
p(Dura)=0.196
Scalp
Dura

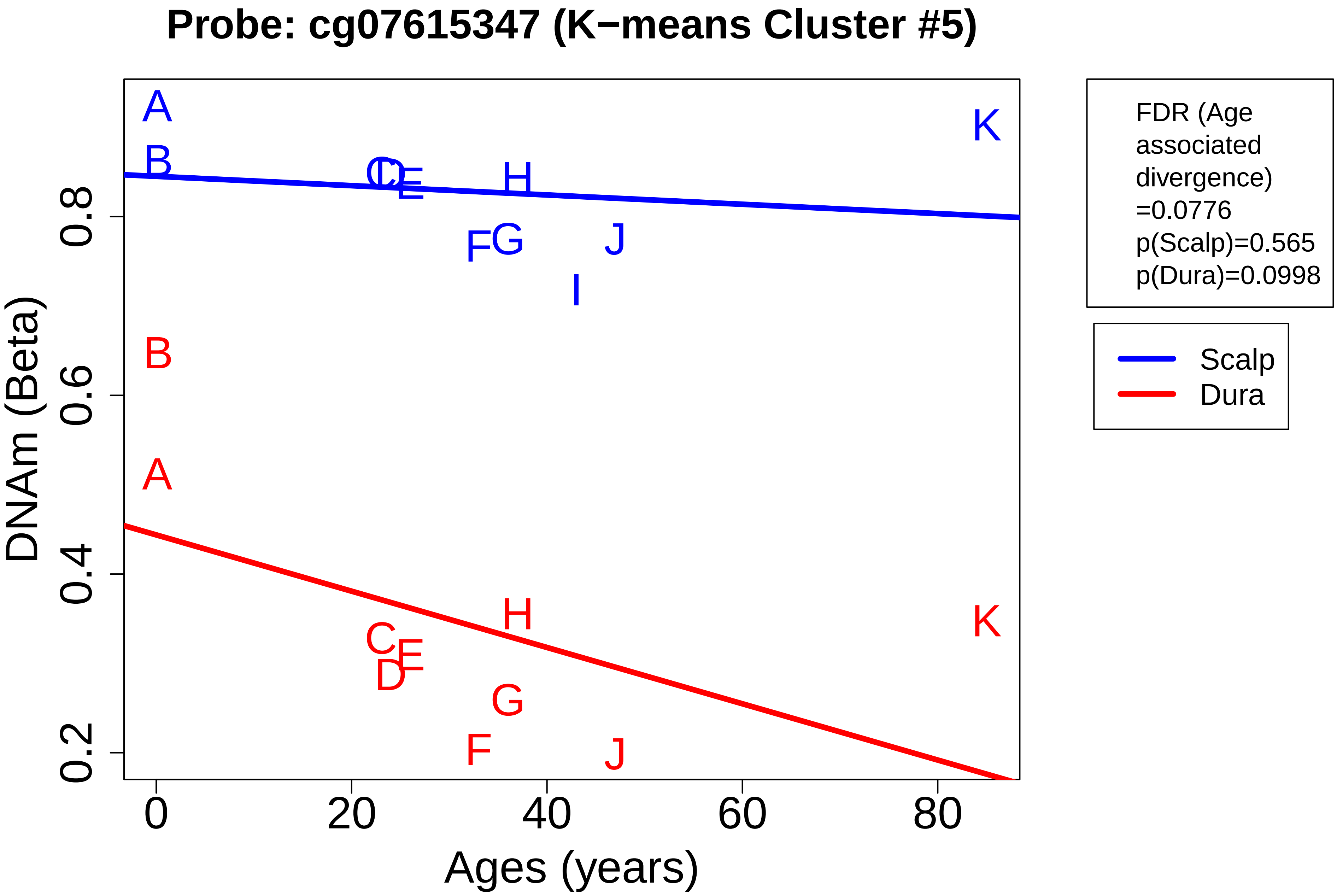

Probe: cg07615347 (K−means Cluster #5)
DNAm (Beta)
0.2
0.4
0.6
0.8
0
20
40
60
80
Ages (years)
A
B
C
D
E
F
G
H
I
J
K
FDR (Age associated divergence) =0.0776
p(Scalp)=0.565
p(Dura)=0.0998
Scalp
Dura

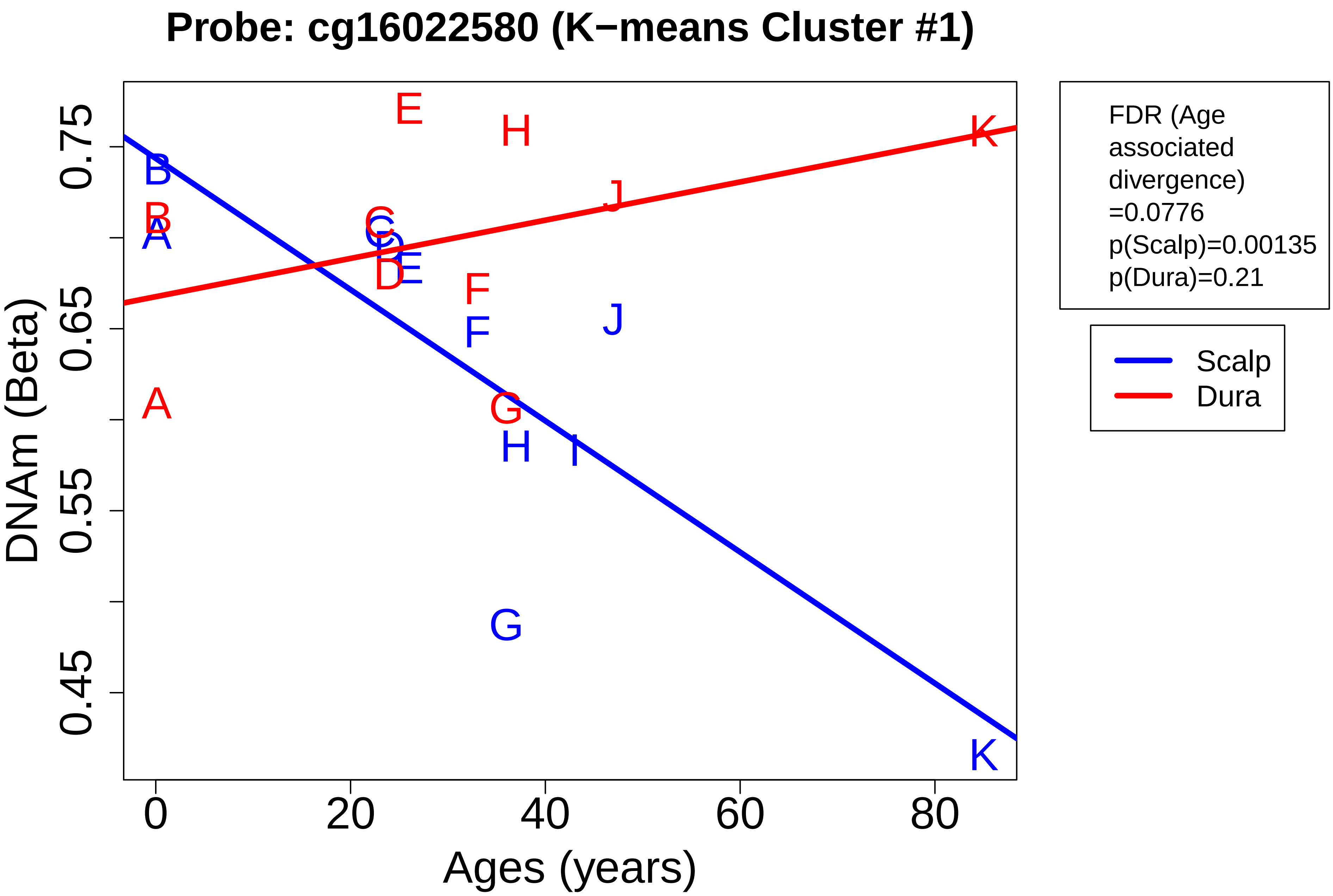

Probe: cg16022580 (K−means Cluster #1)
DNAm (Beta)
0.45
0.55
0.65
0.75
Ages (years)
0
20
40
60
80
FDR (Age associated divergence) =0.0776
p(Scalp)=0.00135
p(Dura)=0.21
Scalp
Dura

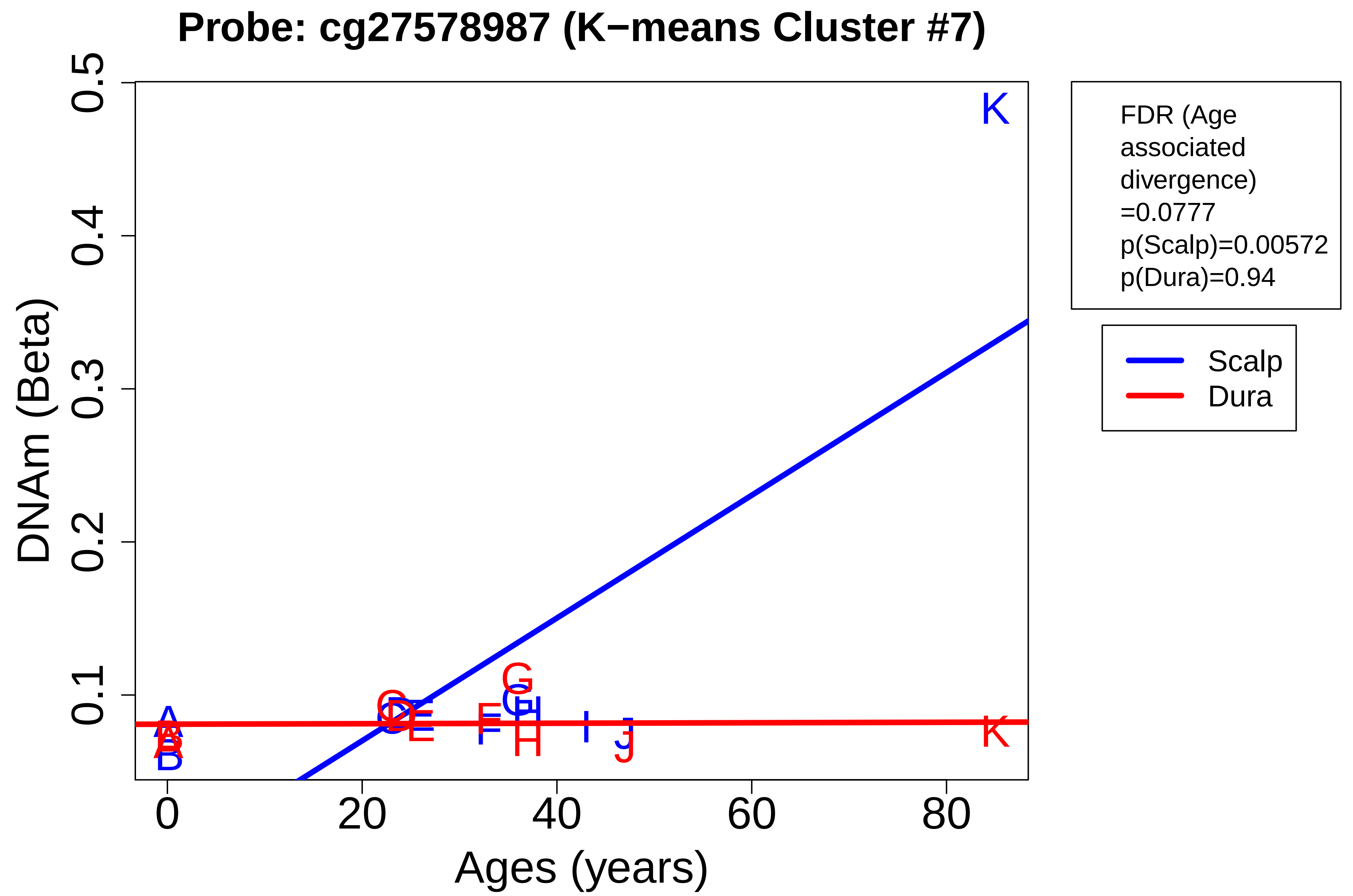

Probe: cg27578987 (K−means Cluster #7)
DNAm (Beta)
0.1
0.2
0.3
0.4
0.5
Ages (years)
0
20
40
60
80
FDR (Age associated divergence) =0.0777
p(Scalp)=0.00572
p(Dura)=0.94
Scalp
Dura

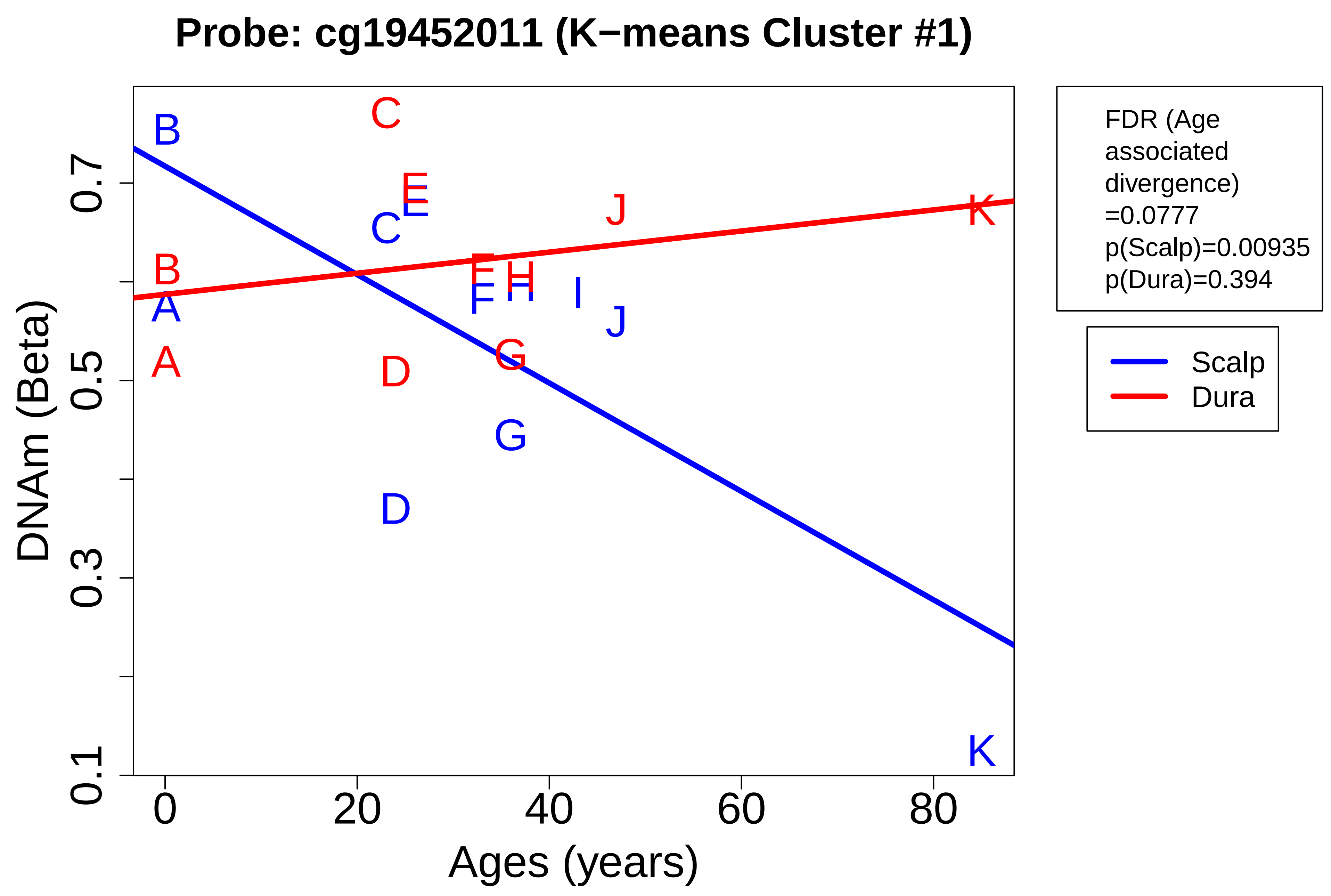

Probe: cg19452011 (K−means Cluster #1)
DNAm (Beta)
0.1
0.3
0.5
0.7
Ages (years)
0
20
40
60
80
B
C
E
C
E
J
K
B
A
F
H
F
H
I
J
G
A
D
G
D
K
FDR (Age associated divergence) =0.0777
p(Scalp)=0.00935
p(Dura)=0.394
Scalp
Dura

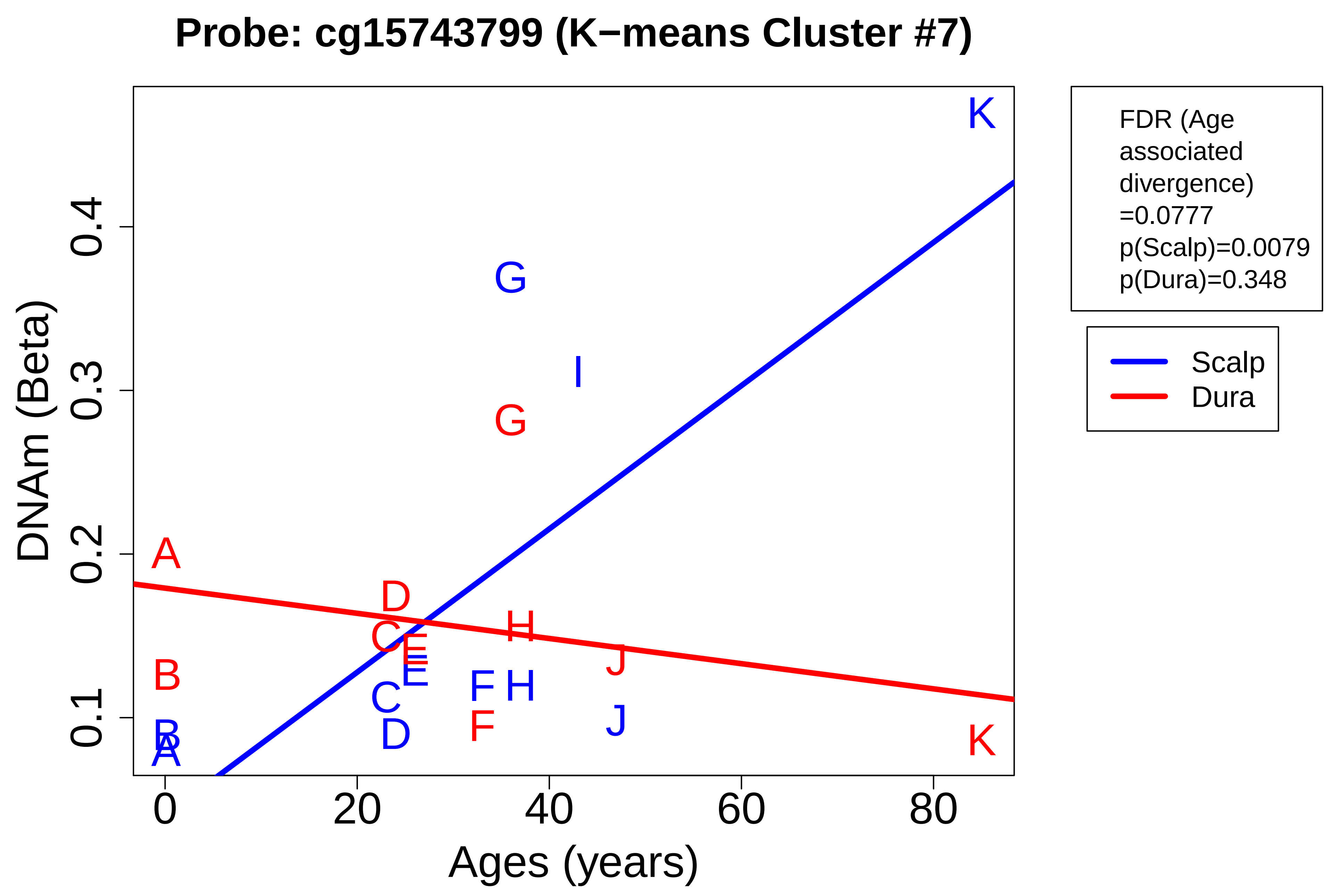

Probe: cg15743799 (K−means Cluster #7)
DNAm (Beta)
0.1
0.2
0.3
0.4
0
20
40
60
80
Ages (years)
FDR (Age associated divergence) =0.0777
p(Scalp)=0.0079
p(Dura)=0.348
Scalp
Dura
A
B
C
D
E
F
G
H
I
J
K

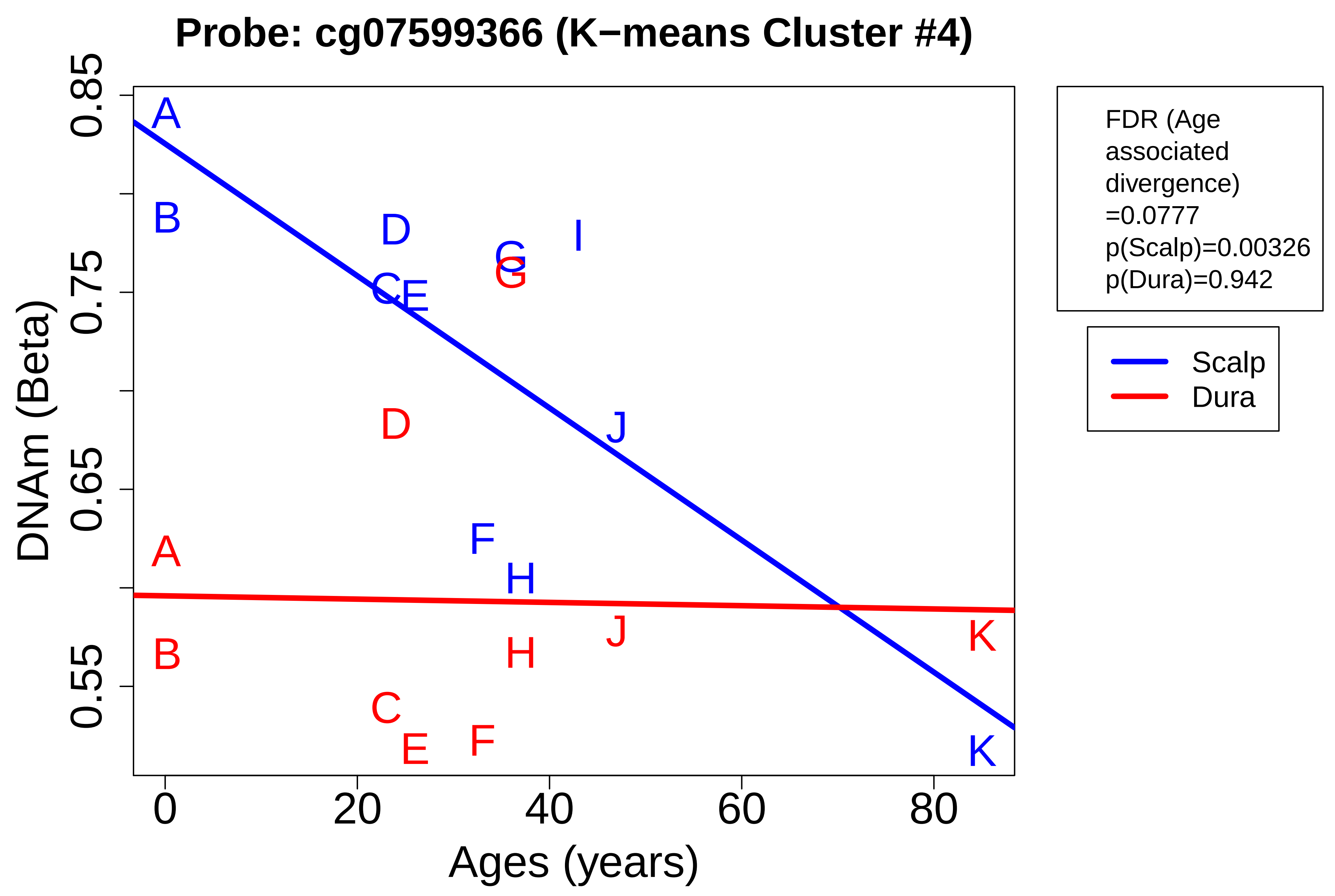
Probe: cg07599366 (K−means Cluster #4)
DNAm (Beta)
0.85
0.75
0.65
0.55
Ages (years)
0
20
40
60
80
A
B
C
D
E
F
G
H
I
J
K
FDR (Age associated divergence) =0.0777
p(Scalp)=0.00326
p(Dura)=0.942
Scalp
Dura

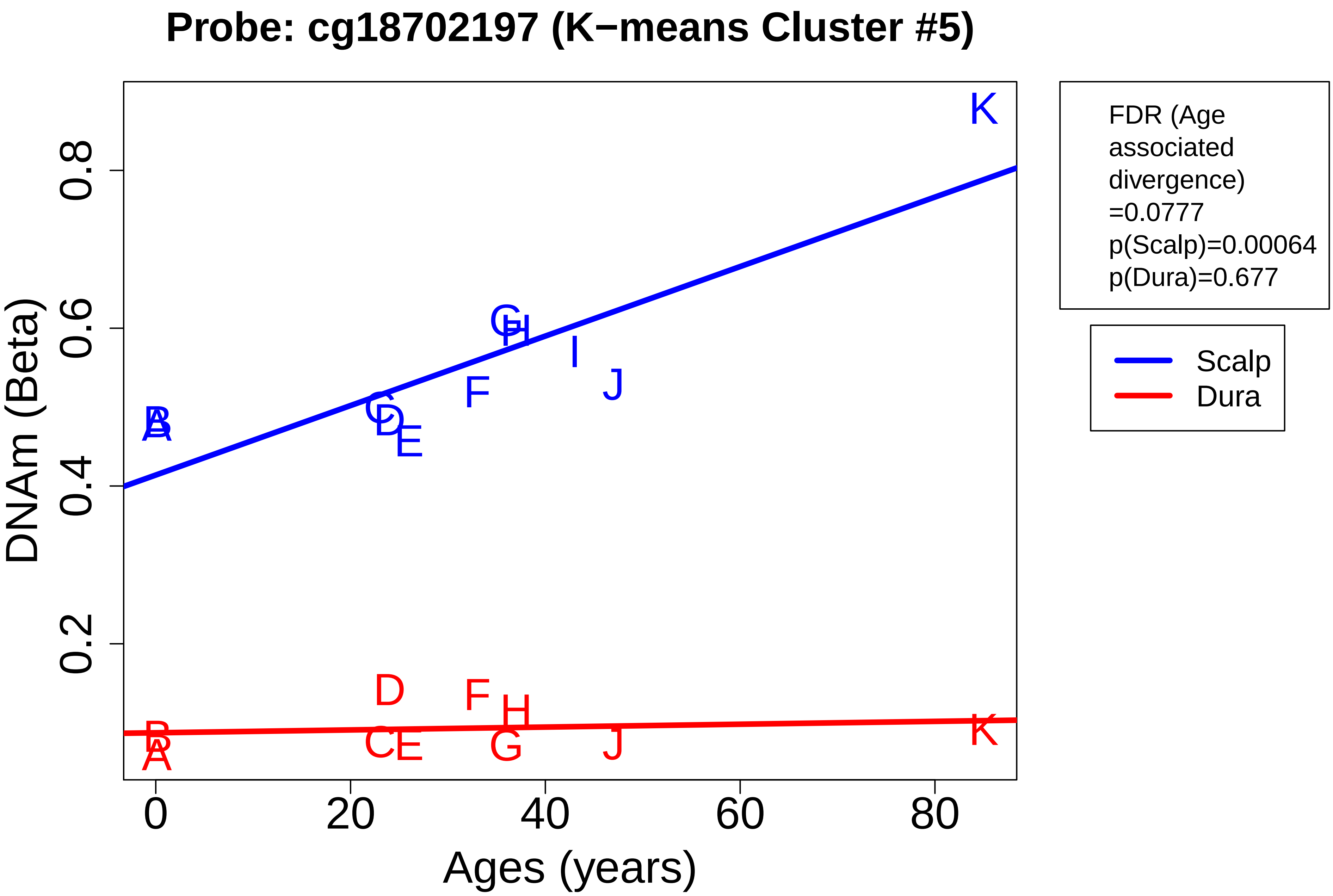

Probe: cg18702197 (K−means Cluster #5)
FDR (Age associated divergence) =0.0777
p(Scalp)=0.00064
p(Dura)=0.677
Scalp
Dura
DNAm (Beta)
0.2
0.4
0.6
0.8
Ages (years)
0
20
40
60
80
A
B
C
D
E
F
G
H
I
J
K

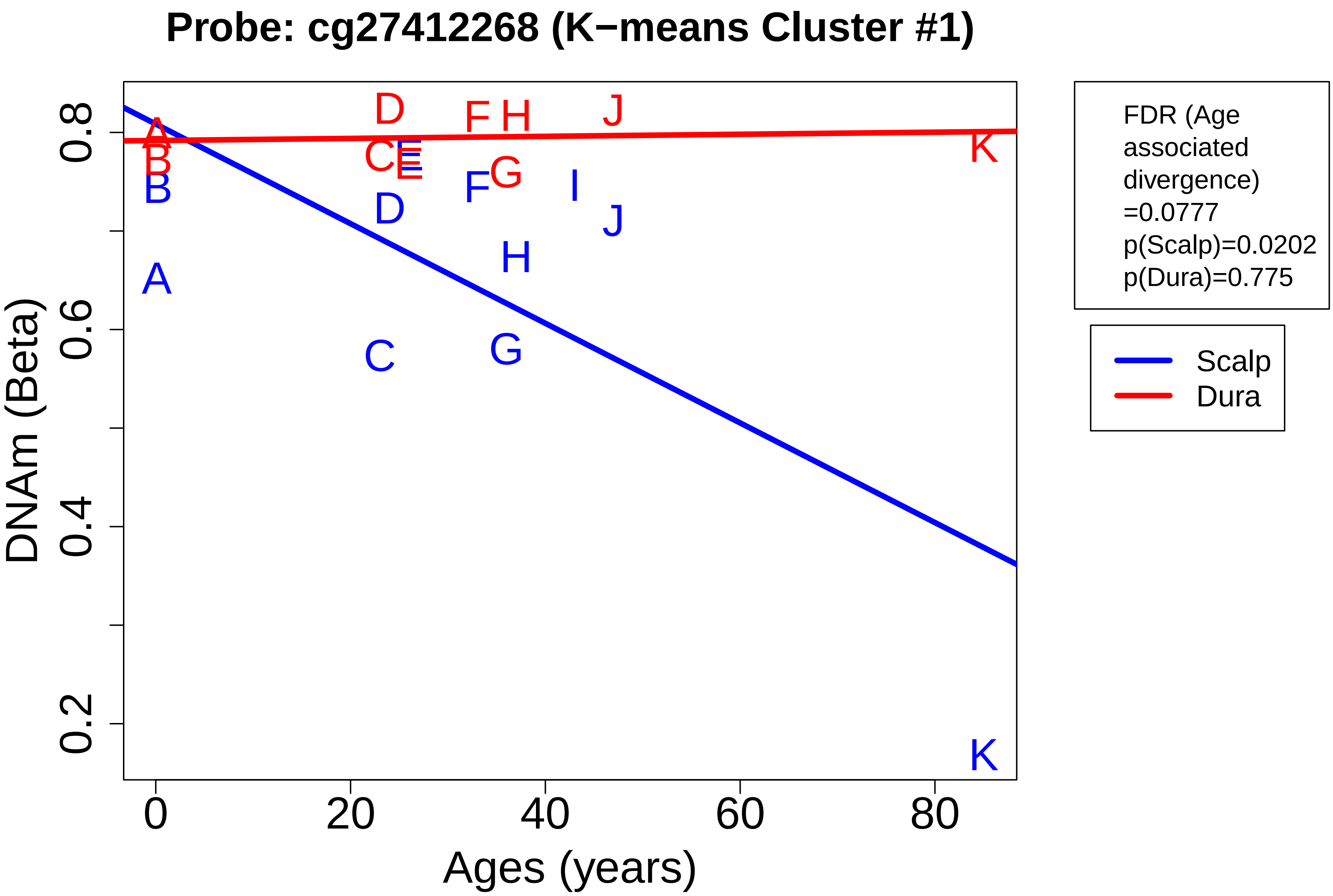

Probe: cg27412268 (K−means Cluster #1)
DNAm (Beta)
0.2
0.4
0.6
0.8
0
20
40
60
80
Ages (years)
FDR (Age associated divergence) =0.0777
p(Scalp)=0.0202
p(Dura)=0.775
Scalp
Dura

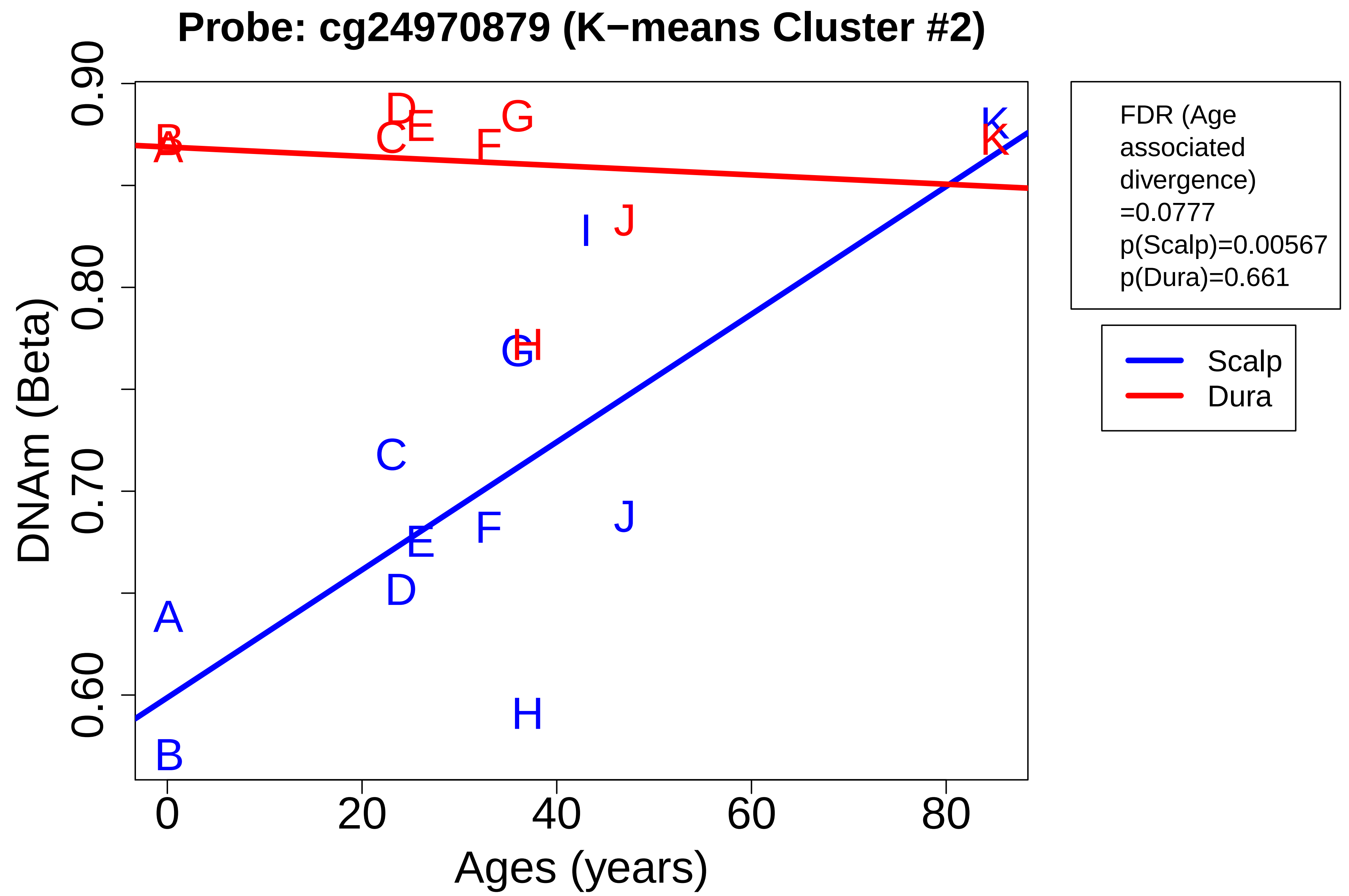

Probe: cg24970879 (K−means Cluster #2)
DNAm (Beta)
0.60
0.70
0.80
0.90
0
20
40
60
80
Ages (years)
FDR (Age associated divergence) =0.0777
p(Scalp)=0.00567
p(Dura)=0.661
Scalp
Dura

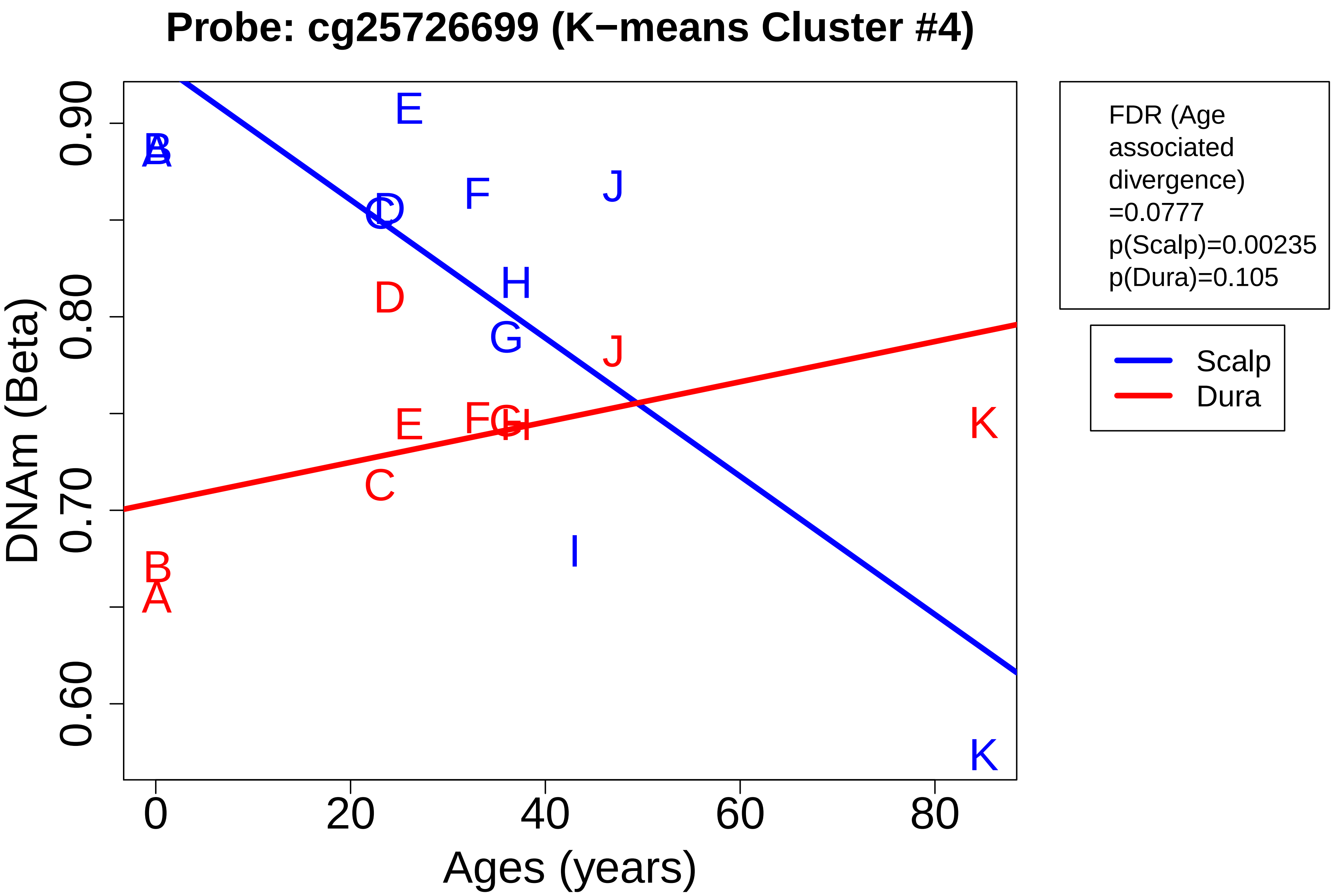

Probe: cg25726699 (K−means Cluster #4)
DNAm (Beta)
0.60
0.70
0.80
0.90
0
20
40
60
80
Ages (years)
FDR (Age associated divergence) =0.0777
p(Scalp)=0.00235
p(Dura)=0.105
Scalp
Dura

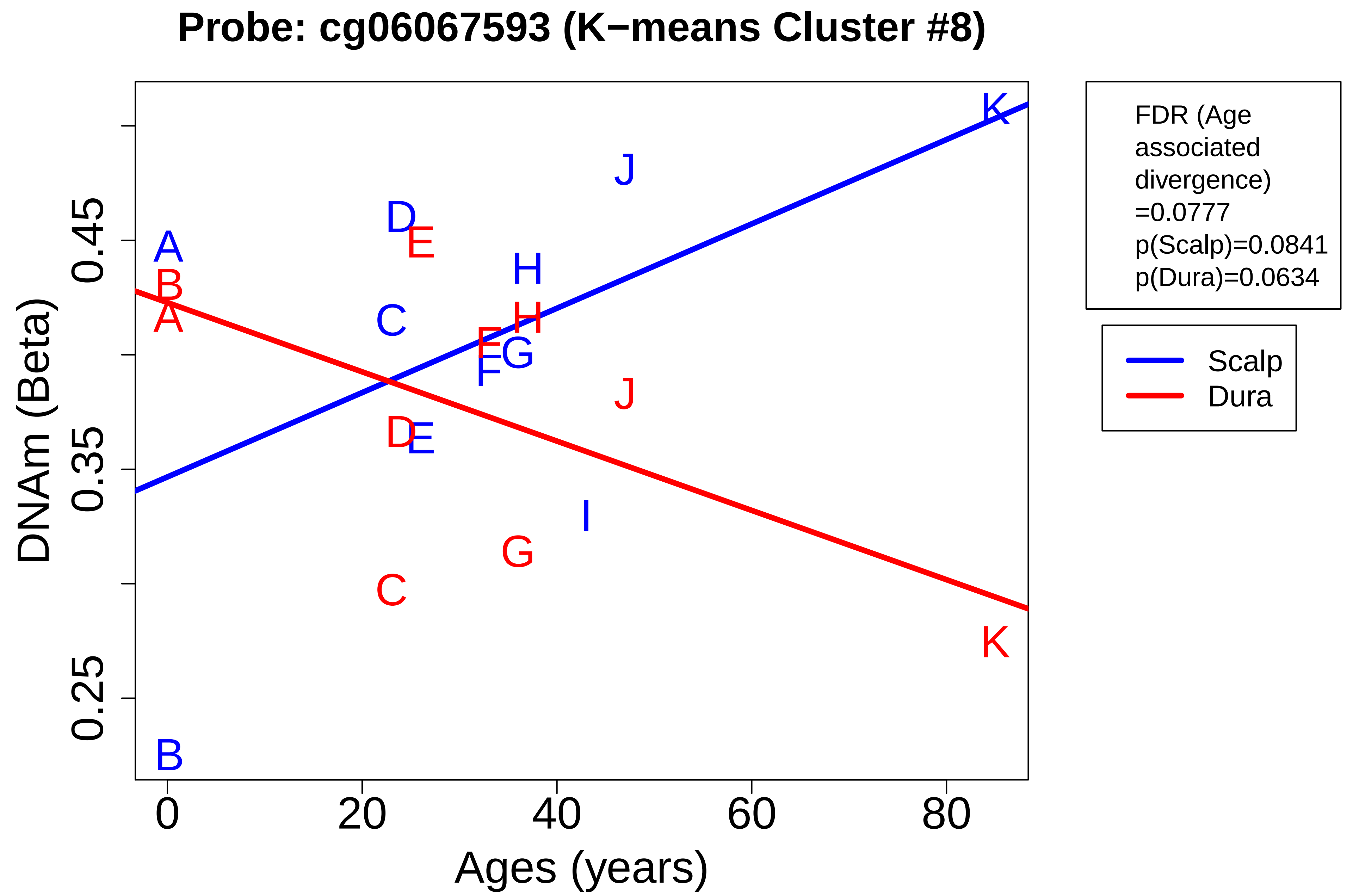

Probe: cg06067593 (K−means Cluster #8)
DNAm (Beta)
0.25
0.35
0.45
Ages (years)
0
20
40
60
80
A
B
C
D
E
F
G
H
I
J
K
FDR (Age associated divergence) =0.0777
p(Scalp)=0.0841
p(Dura)=0.0634
Scalp
Dura

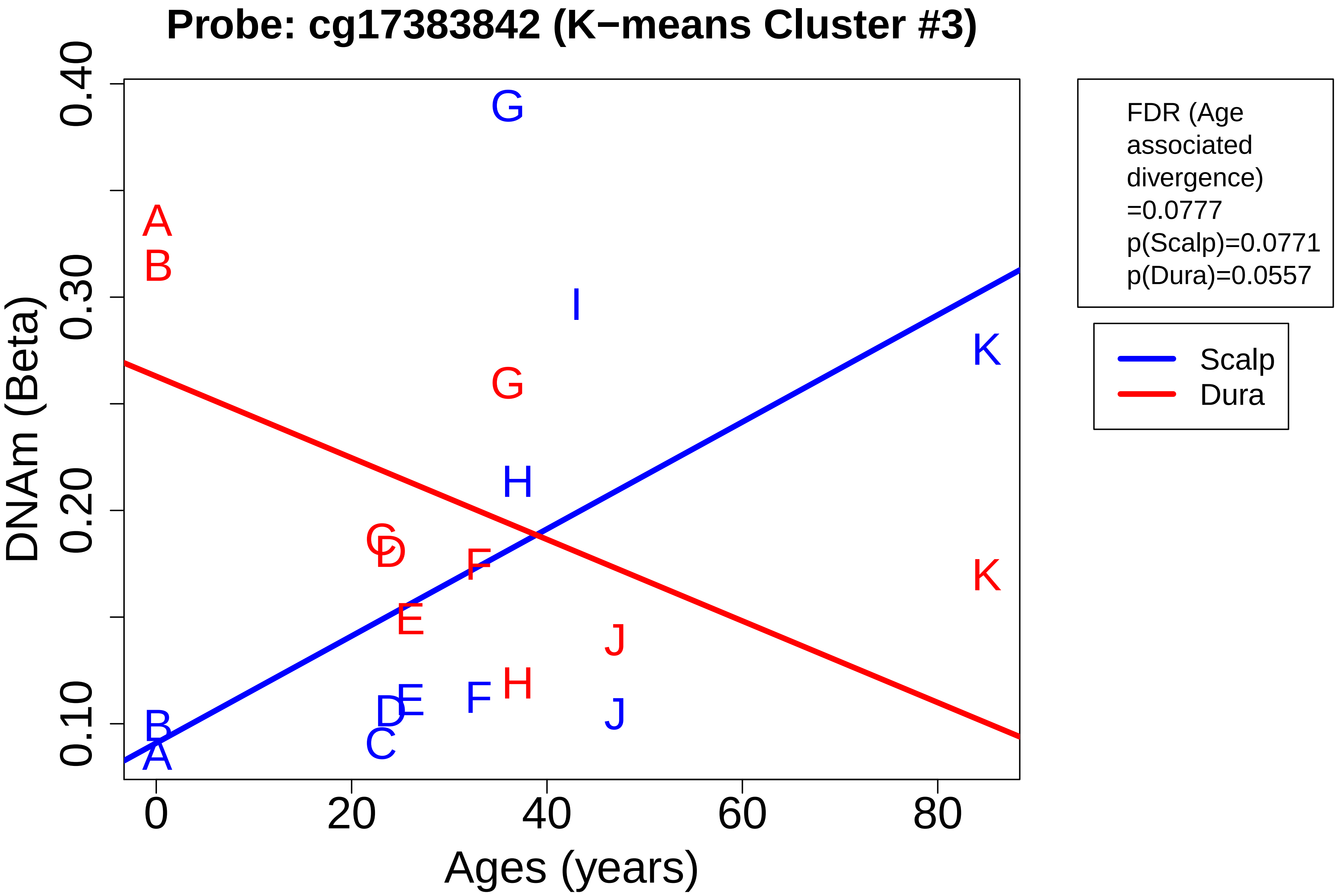

Probe: cg17383842 (K−means Cluster #3)
DNAm (Beta)
0.10
0.20
0.30
0.40
0
20
40
60
80
Ages (years)
FDR (Age associated divergence) =0.0777
p(Scalp)=0.0771
p(Dura)=0.0557
Scalp
Dura

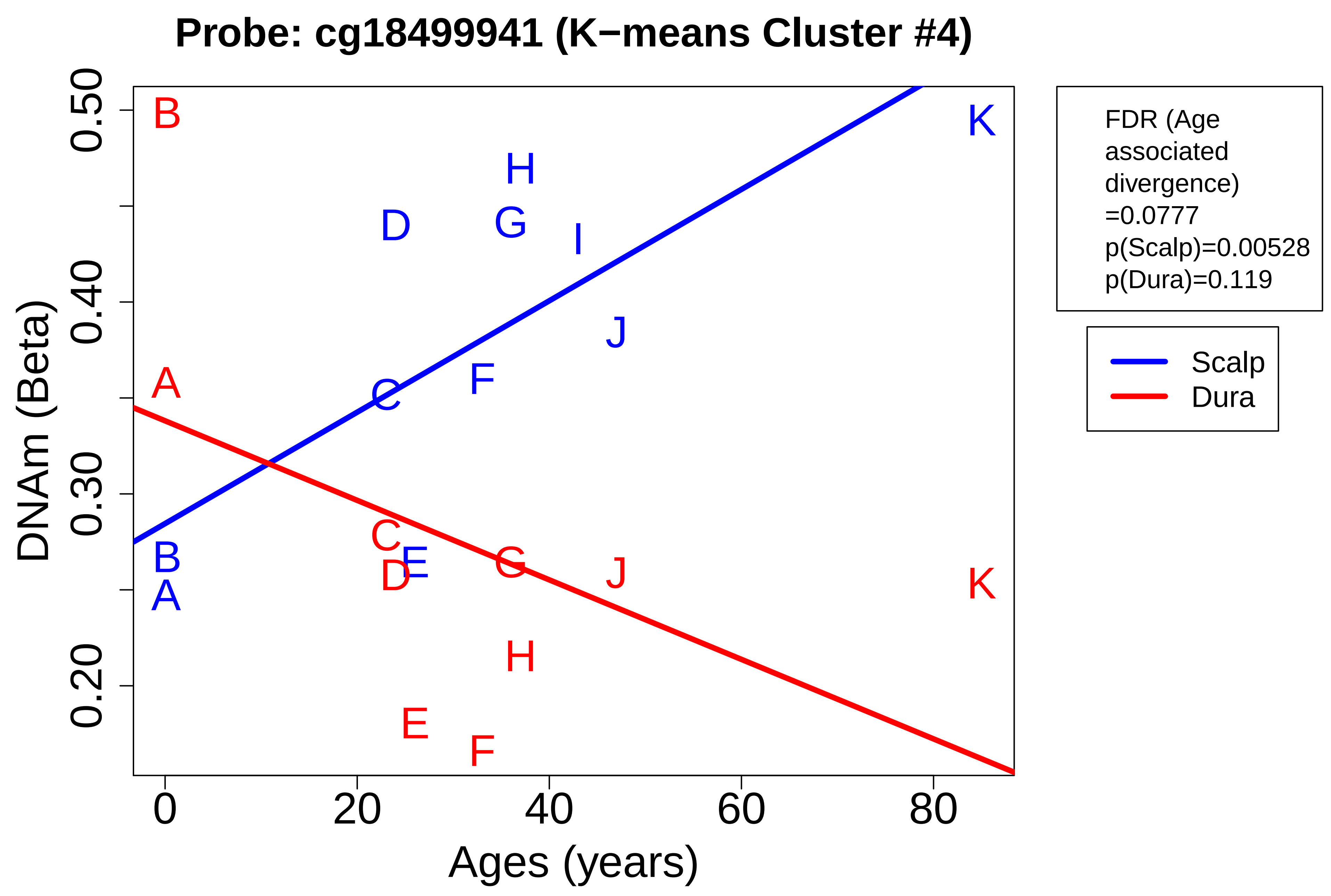
Probe: cg18499941 (K−means Cluster #4)
DNAm (Beta)
0.20
0.30
0.40
0.50
Ages (years)
0
20
40
60
80
A
B
C
D
E
F
G
H
I
J
K
FDR (Age associated divergence) =0.0777
p(Scalp)=0.00528
p(Dura)=0.119
Scalp
Dura

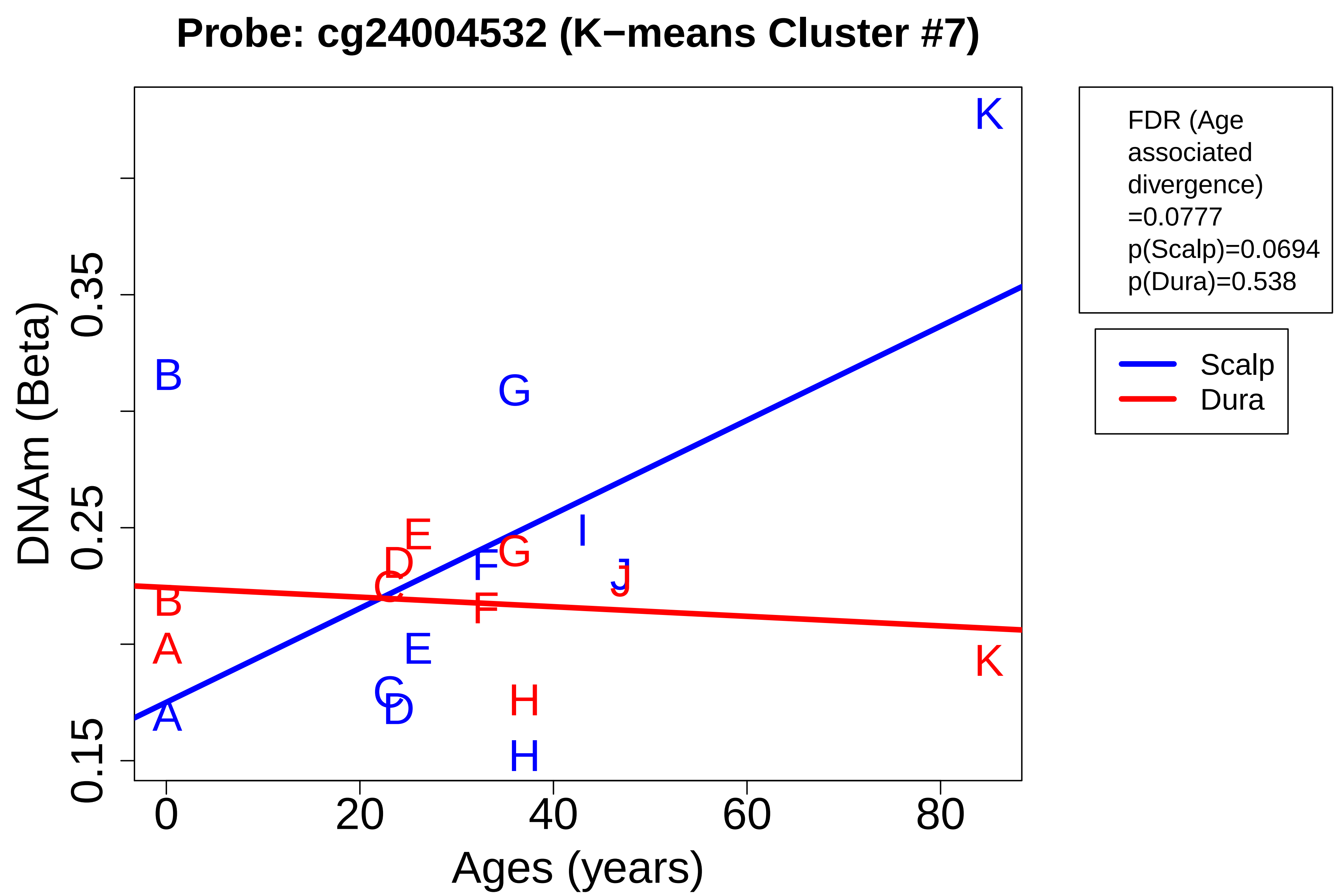
Probe: cg24004532 (K−means Cluster #7)
DNAm (Beta)
0.15
0.25
0.35
Ages (years)
0
20
40
60
80
A
B
C
D
E
F
G
H
I
J
K
FDR (Age associated divergence) =0.0777
p(Scalp)=0.0694
p(Dura)=0.538
Scalp
Dura

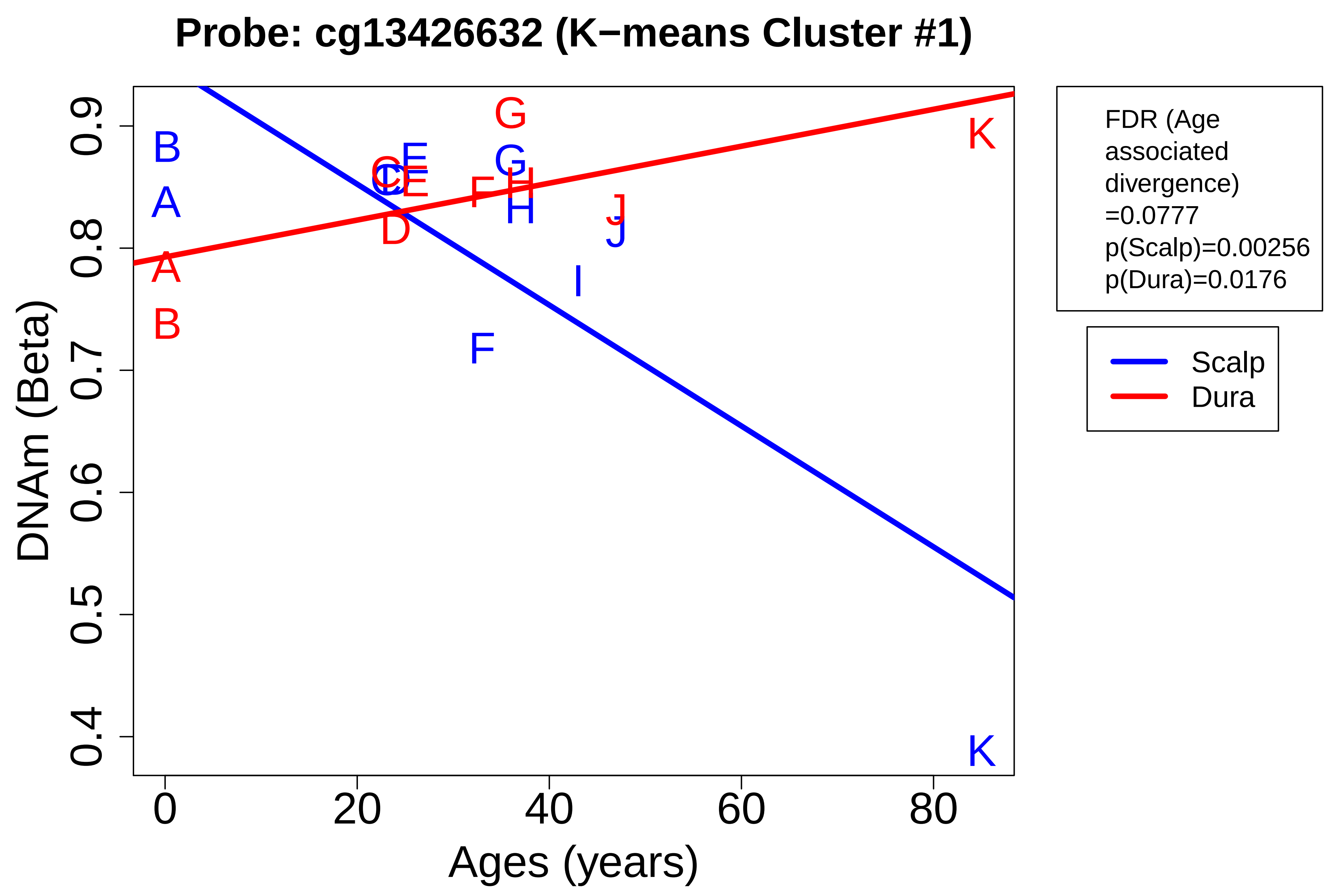

Probe: cg13426632 (K−means Cluster #1)
DNAm (Beta)
0.4
0.5
0.6
0.7
0.8
0.9
Ages (years)
0
20
40
60
80
FDR (Age associated divergence) =0.0777
p(Scalp)=0.00256
p(Dura)=0.0176
Scalp
Dura

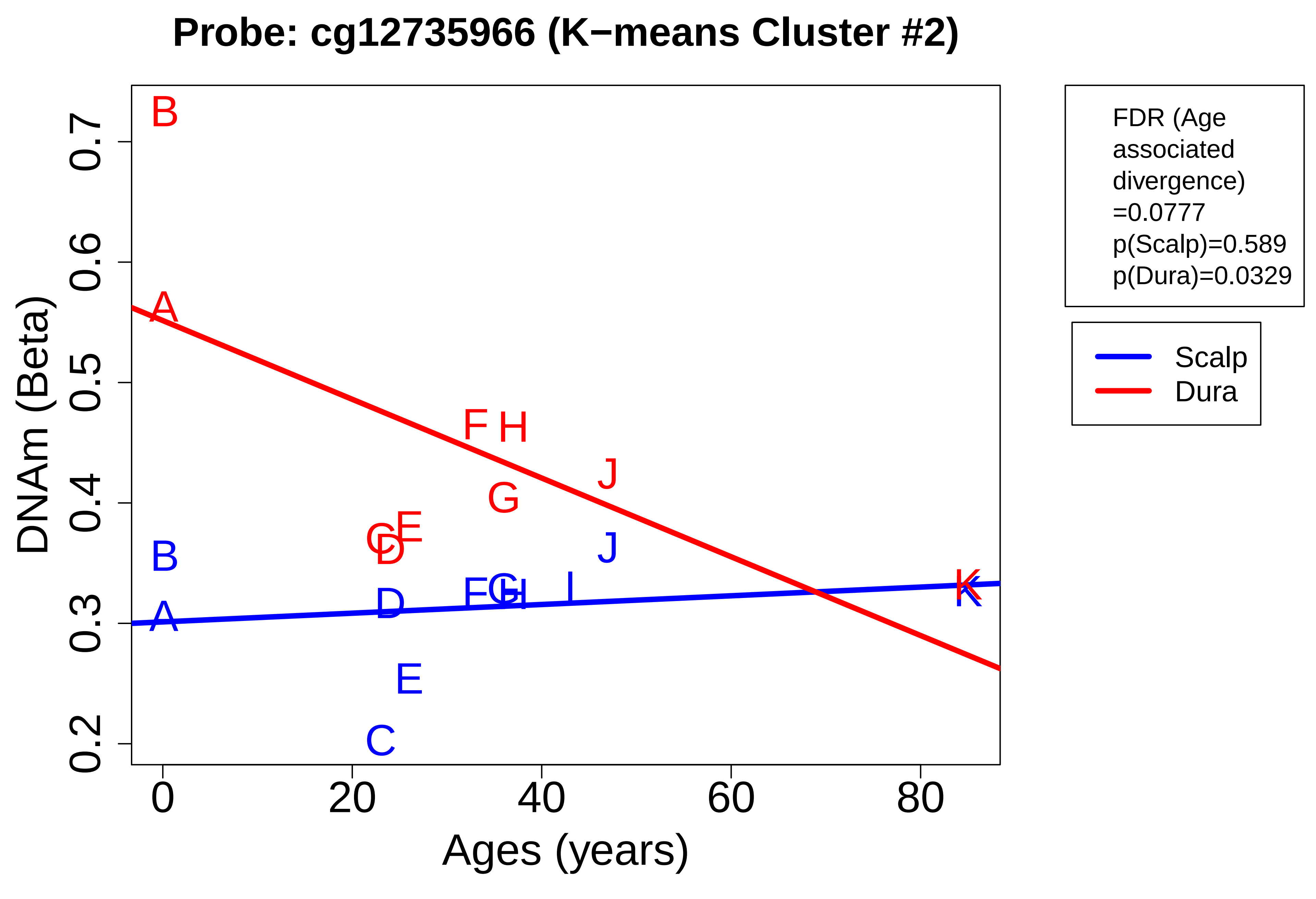

Probe: cg12735966 (K−means Cluster #2)
DNAm (Beta)
0.2
0.3
0.4
0.5
0.6
0.7
0
20
40
60
80
Ages (years)
FDR (Age associated divergence) =0.0777
p(Scalp)=0.589
p(Dura)=0.0329
Scalp
Dura

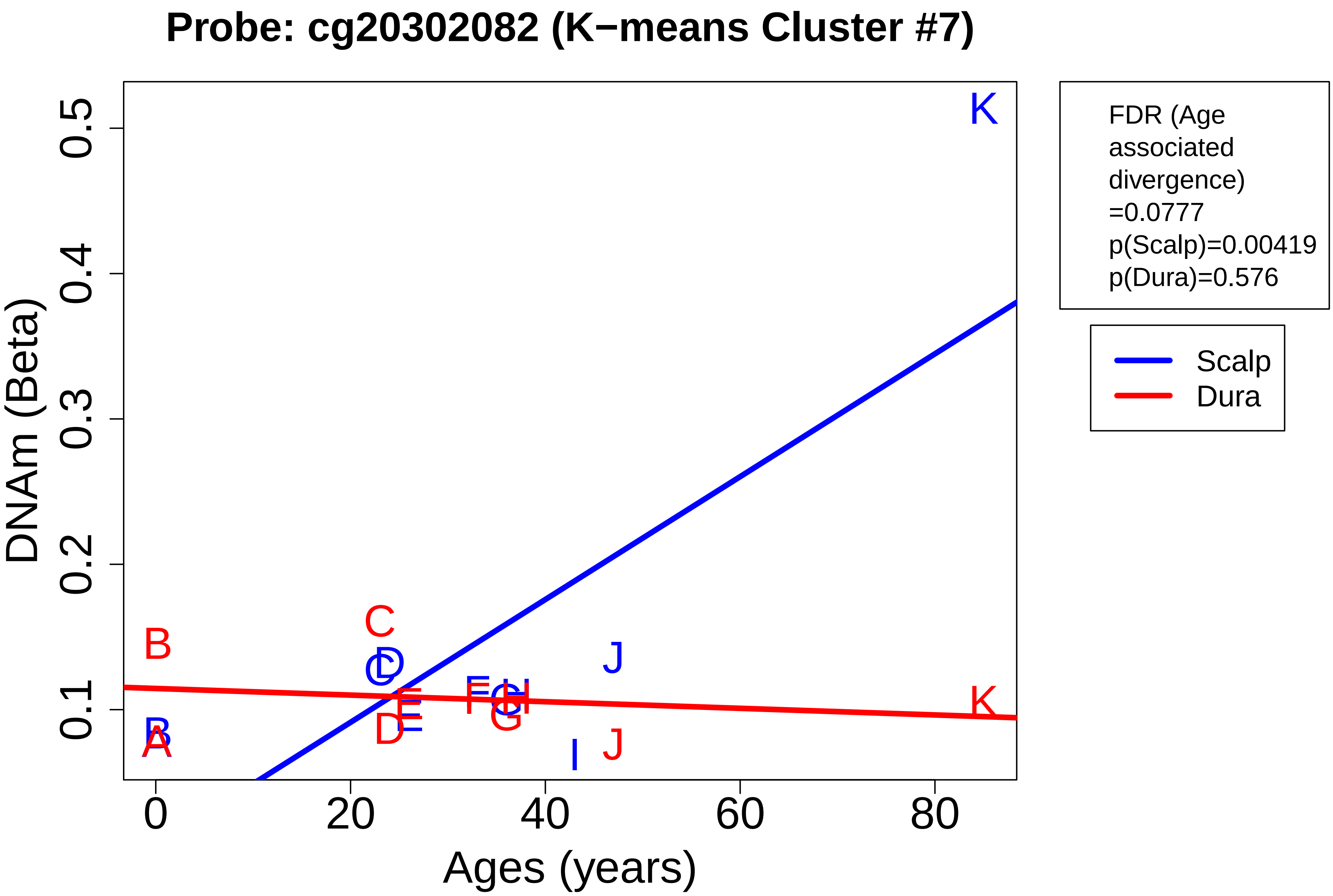

Probe: cg20302082 (K−means Cluster #7)
DNAm (Beta)
Ages (years)
0.1
0.2
0.3
0.4
0.5
0
20
40
60
80
FDR (Age associated divergence) =0.0777
p(Scalp)=0.00419
p(Dura)=0.576
Scalp
Dura
A
B
C
D
E
F
G
H
I
J
K

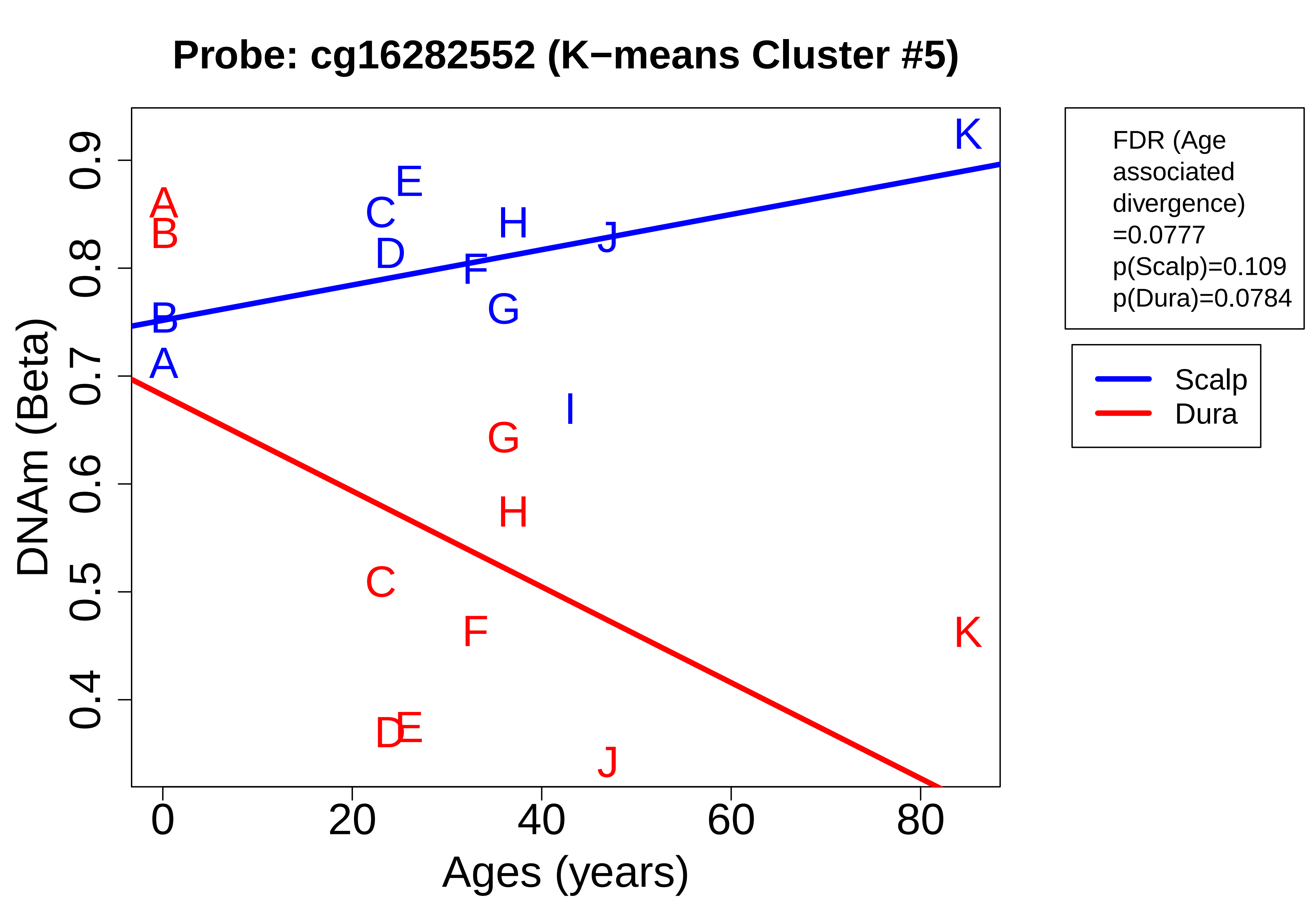
Probe: cg16282552 (K−means Cluster #5)
DNAm (Beta)
Ages (years)
0.4
0.5
0.6
0.7
0.8
0.9
0
20
40
60
80
FDR (Age associated divergence) =0.0777
p(Scalp)=0.109
p(Dura)=0.0784
Scalp
Dura

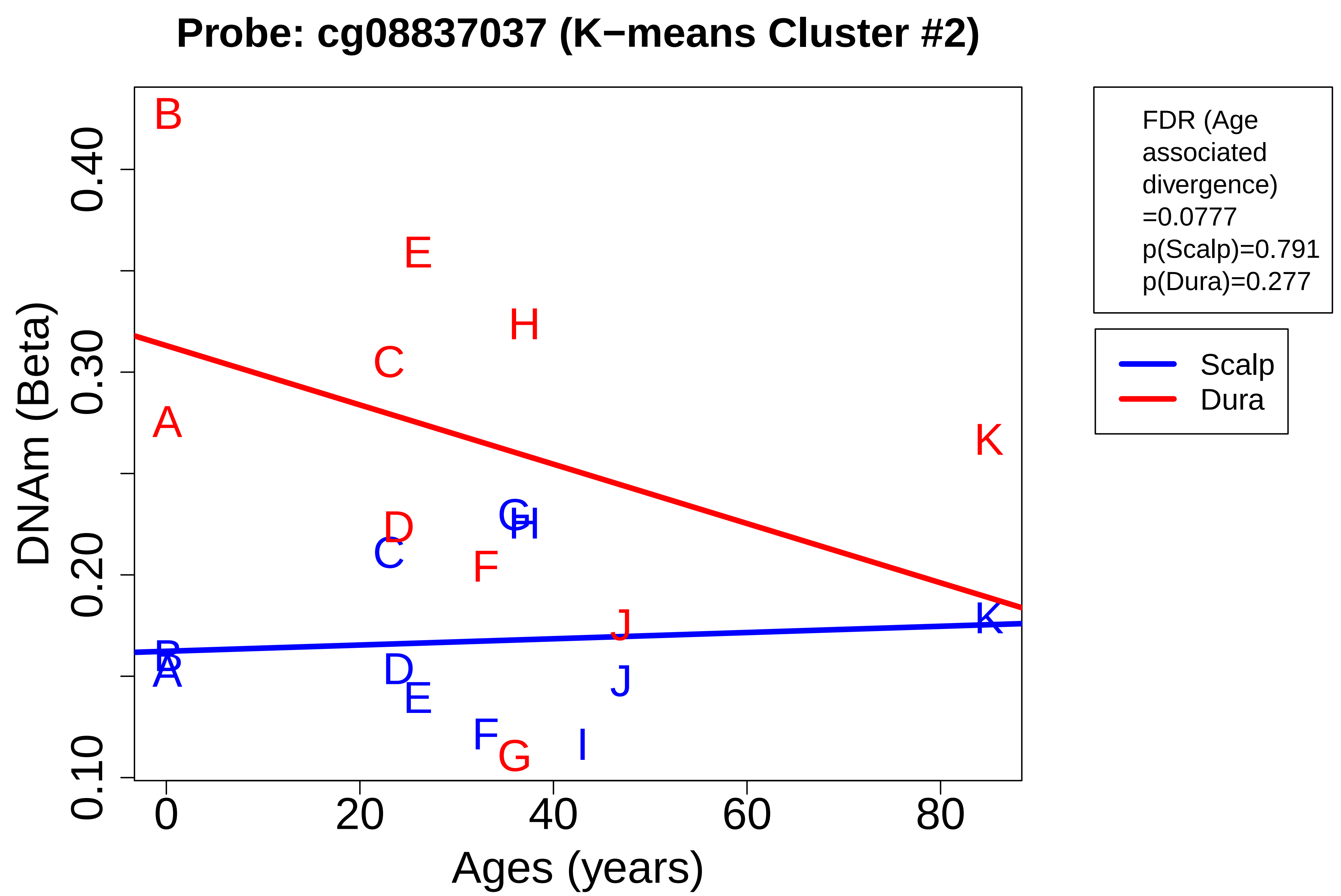

Probe: cg08837037 (K−means Cluster #2)
FDR (Age associated divergence) =0.0777
p(Scalp)=0.791
p(Dura)=0.277
Scalp
Dura
DNAm (Beta)
0.10
0.20
0.30
0.40
0
20
40
60
80
Ages (years)
A
B
C
D
E
F
G
H
I
J
K

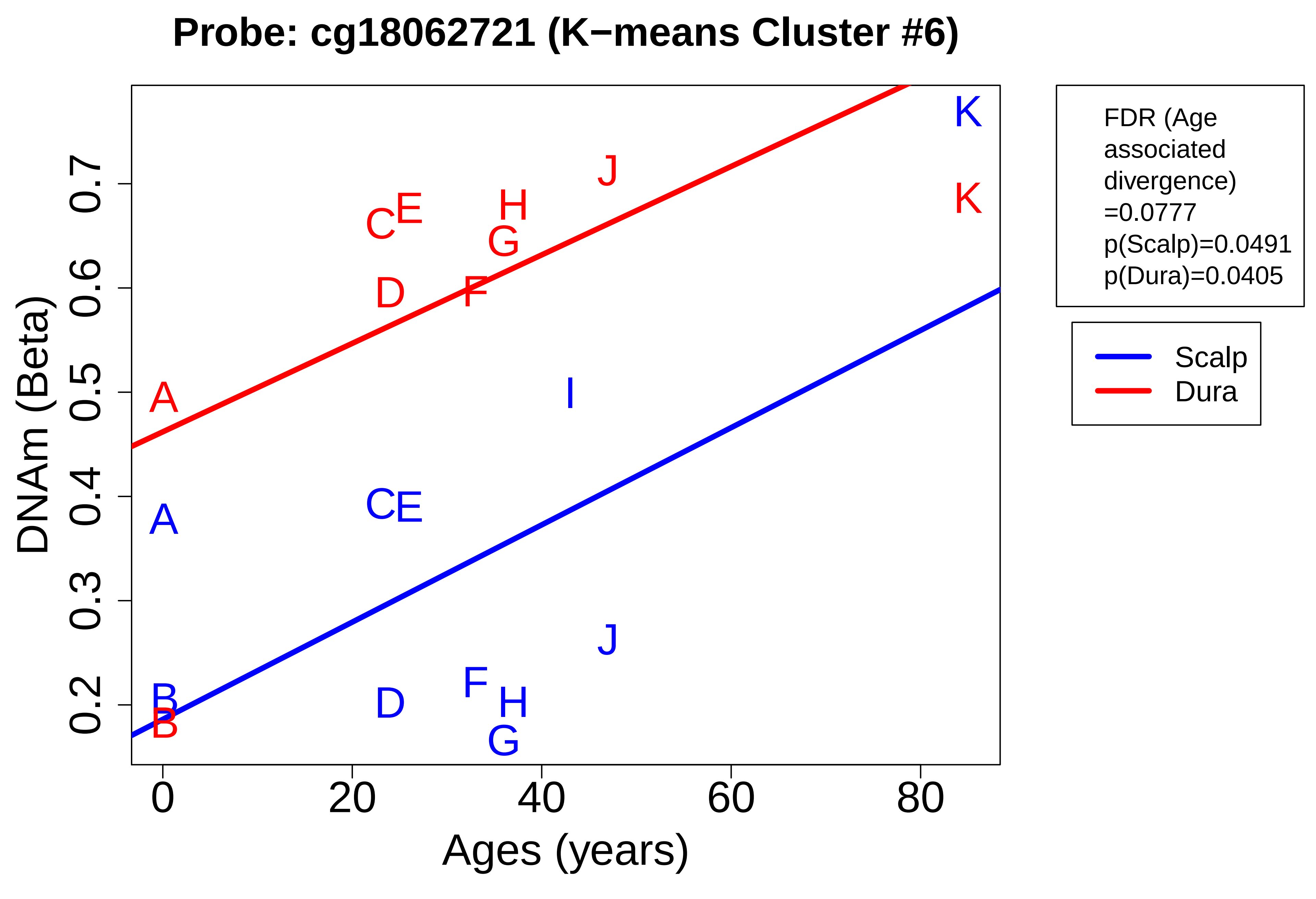

Probe: cg18062721 (K−means Cluster #6)
DNAm (Beta)
0.2
0.3
0.4
0.5
0.6
0.7
0
20
40
60
80
Ages (years)
A
B
C
D
E
F
G
H
I
J
K
FDR (Age associated divergence) =0.0777
p(Scalp)=0.0491
p(Dura)=0.0405
Scalp
Dura

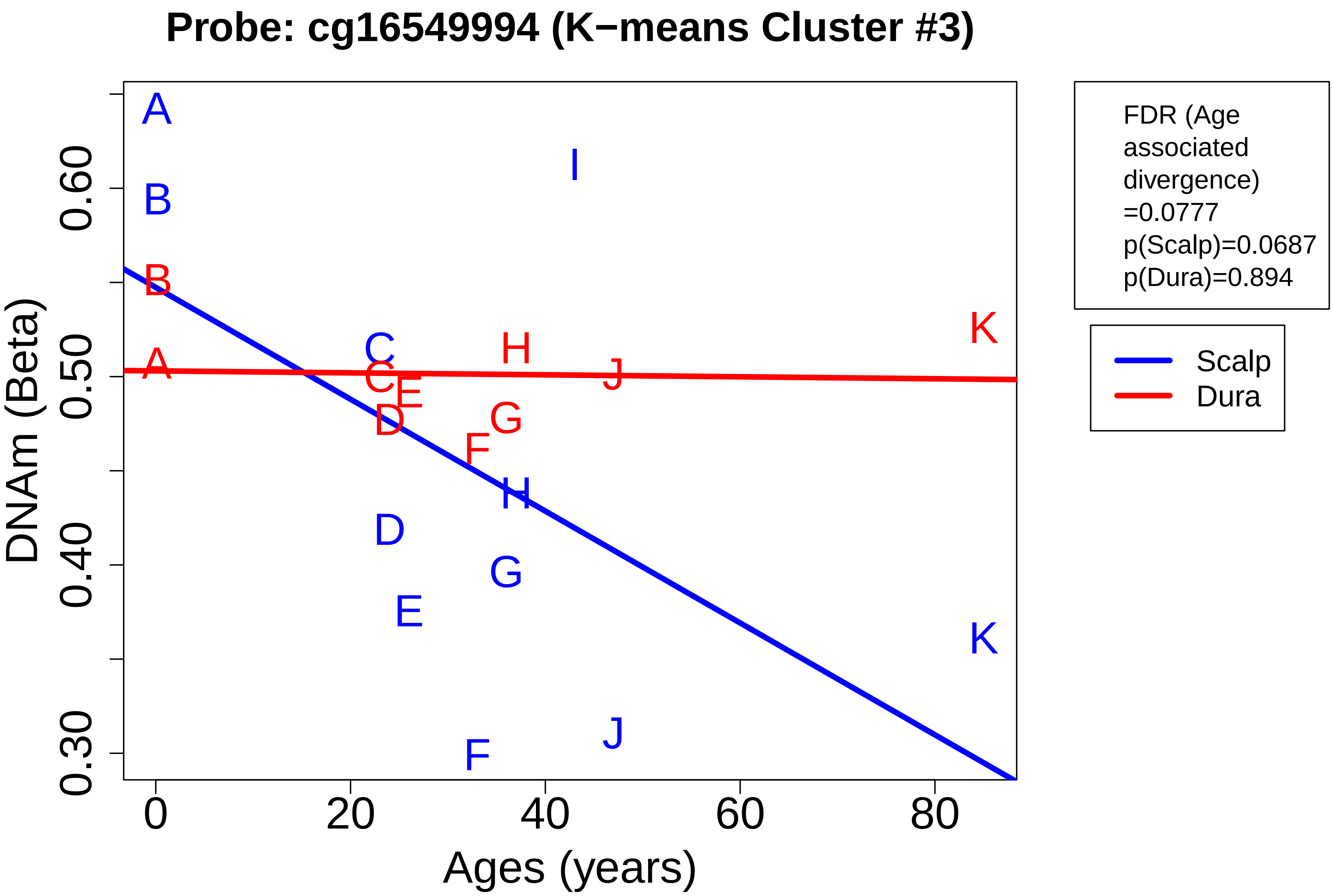

Probe: cg16549994 (K−means Cluster #3)
DNAm (Beta)
0.30
0.40
0.50
0.60
0
20
40
60
80
Ages (years)
A
B
I
B
A
C
C
E
H
J
K
D
G
F
H
D
G
E
K
F
J
FDR (Age associated divergence) =0.0777
p(Scalp)=0.0687
p(Dura)=0.894
Scalp
Dura

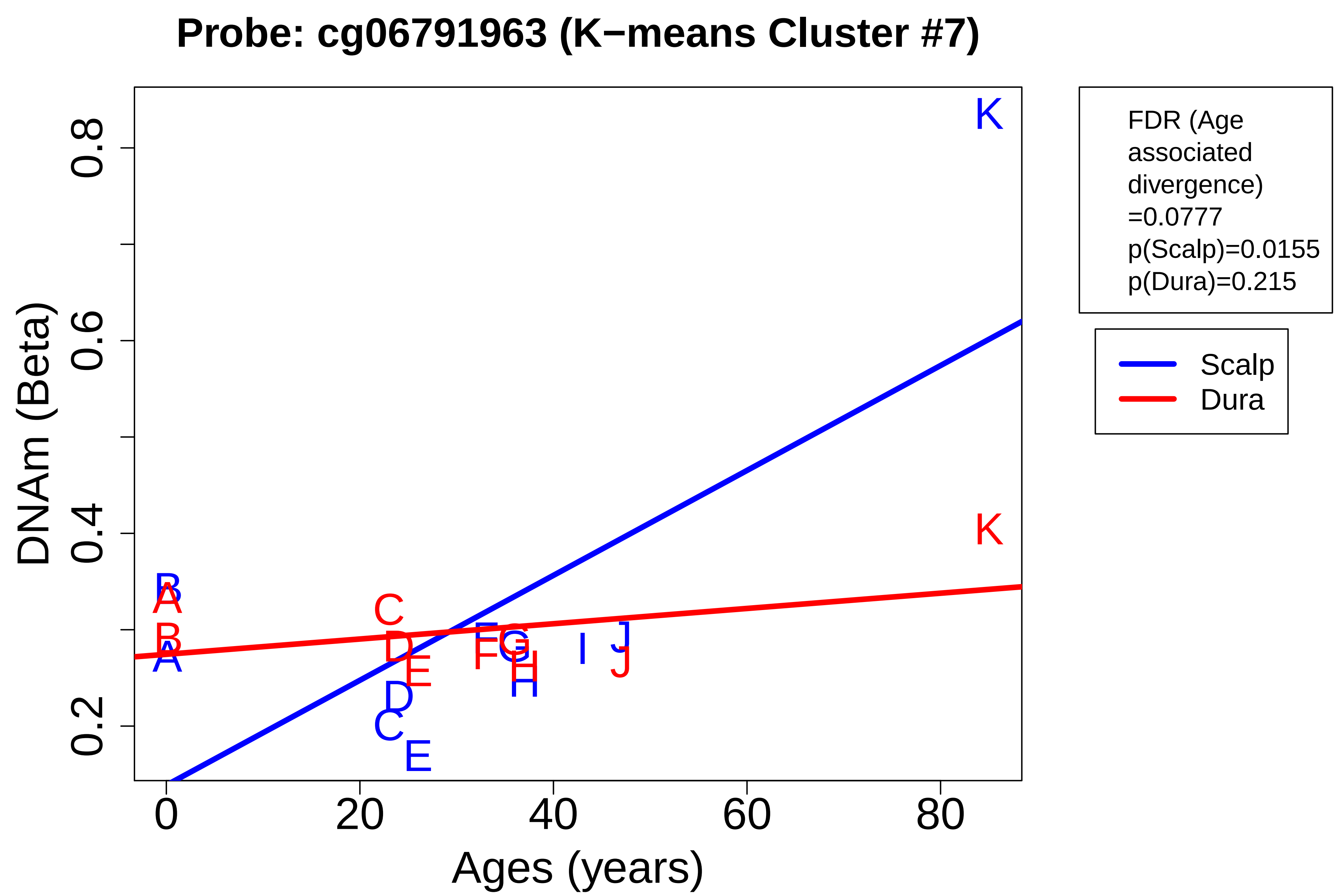

Probe: cg06791963 (K−means Cluster #7)
DNAm (Beta)
0.2
0.4
0.6
0.8
Ages (years)
0
20
40
60
80
K
B
A
C
D
E
F
G
H
I
J
FDR (Age associated divergence) =0.0777
p(Scalp)=0.0155
p(Dura)=0.215
Scalp
Dura

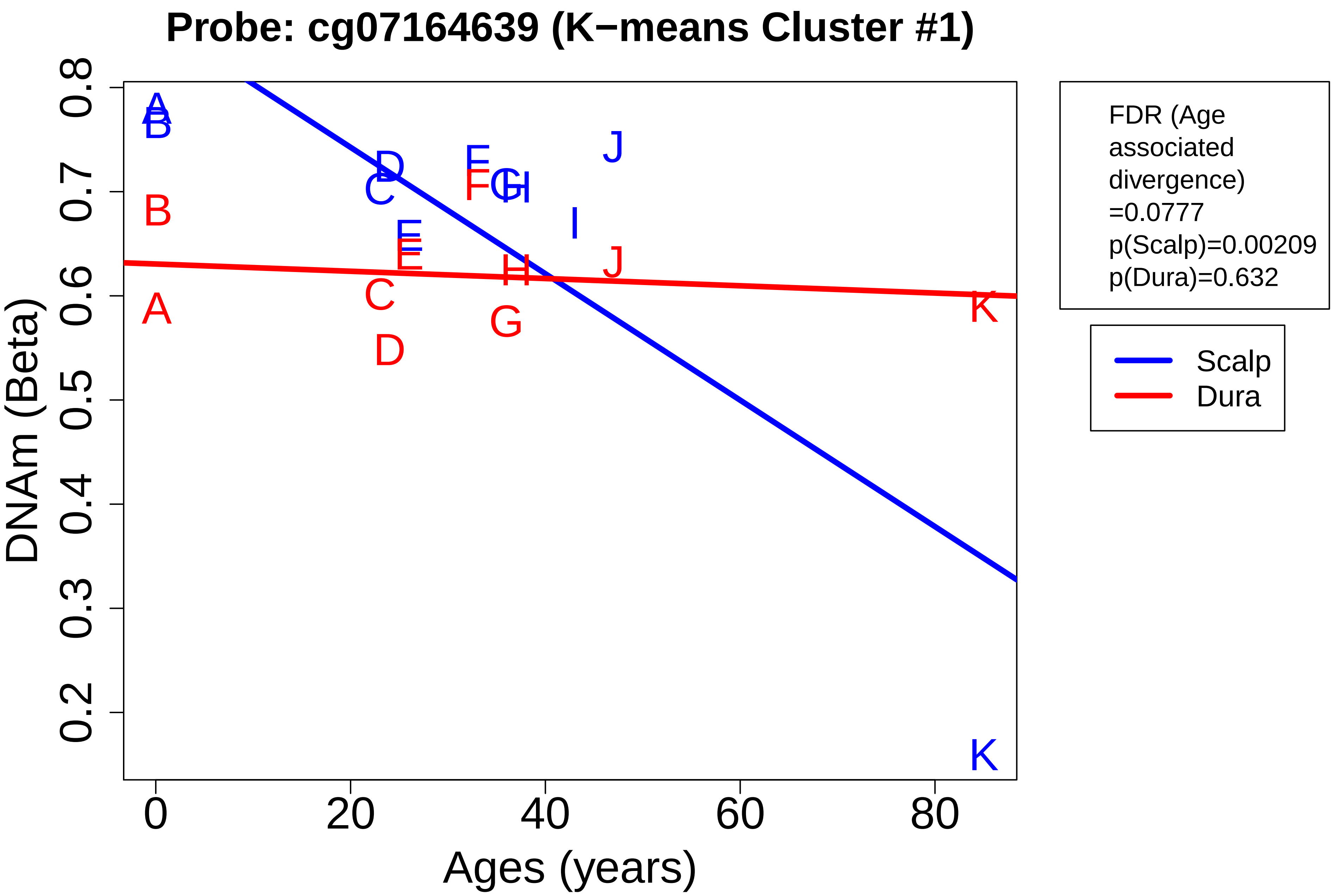
Probe: cg07164639 (K−means Cluster #1)
DNAm (Beta)
0.2
0.3
0.4
0.5
0.6
0.7
0.8
Ages (years)
0
20
40
60
80
A
B
C
D
E
F
G
H
I
J
K
FDR (Age associated divergence) =0.0777
p(Scalp)=0.00209
p(Dura)=0.632
Scalp
Dura

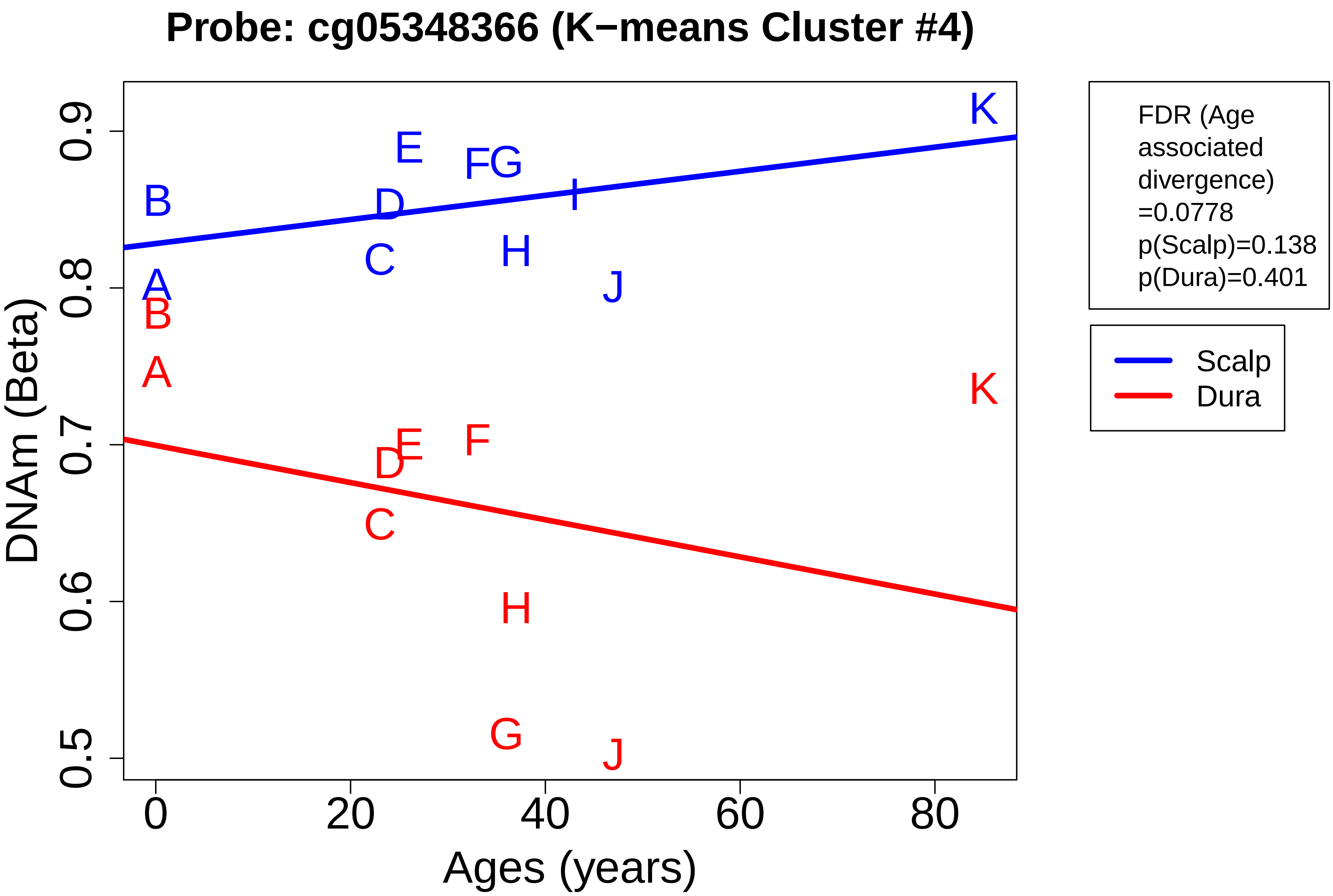

Probe: cg05348366 (K−means Cluster #4)
DNAm (Beta)
0.5
0.6
0.7
0.8
0.9
0
20
40
60
80
Ages (years)
A
B
C
D
E
F
G
H
I
J
K
FDR (Age associated divergence) =0.0778
p(Scalp)=0.138
p(Dura)=0.401
Scalp
Dura

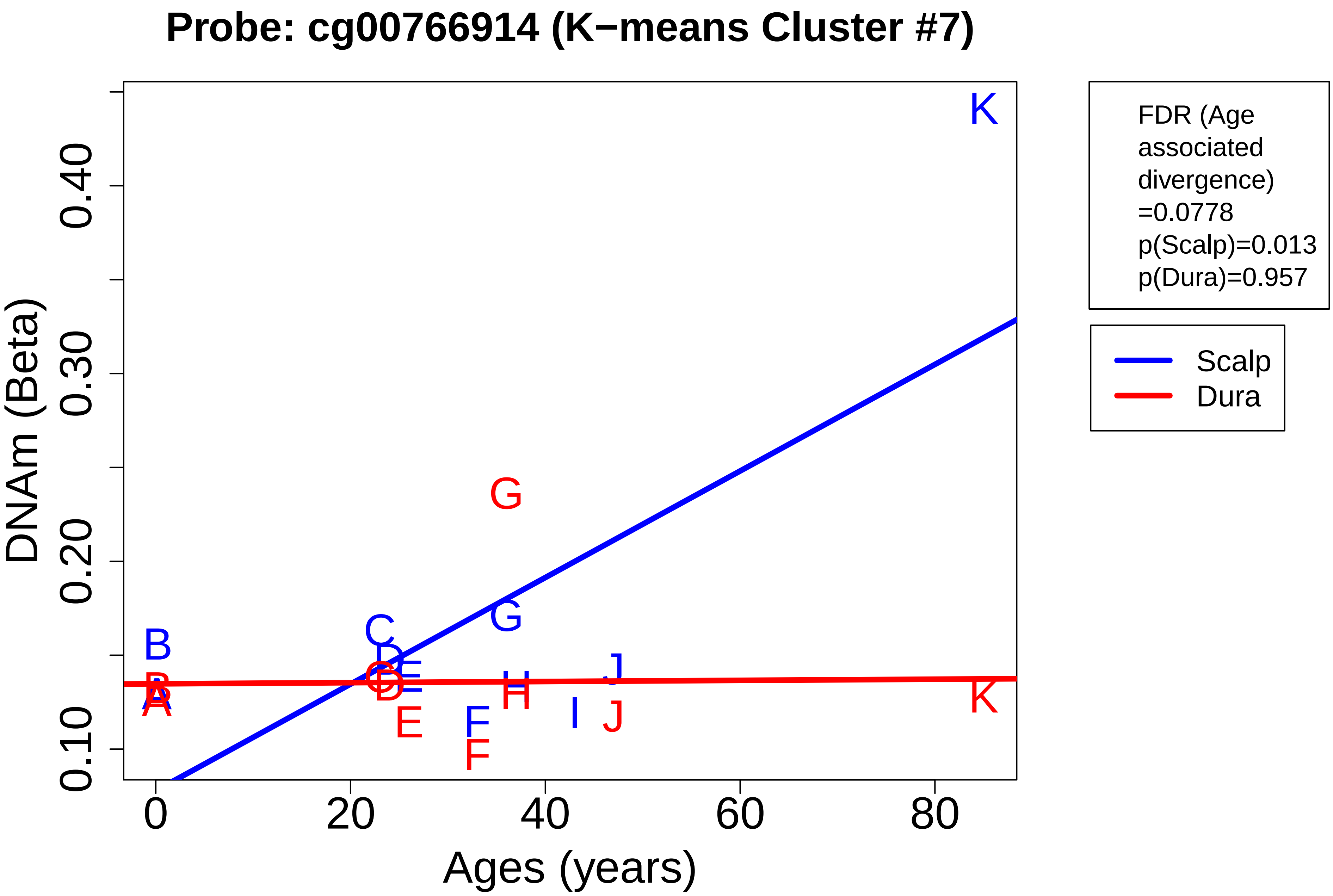
Probe: cg00766914 (K−means Cluster #7)
DNAm (Beta)
0.10
0.20
0.30
0.40
0
20
40
60
80
Ages (years)
FDR (Age associated divergence) =0.0778
p(Scalp)=0.013
p(Dura)=0.957
Scalp
Dura
A
B
C
D
E
F
G
H
I
J
K

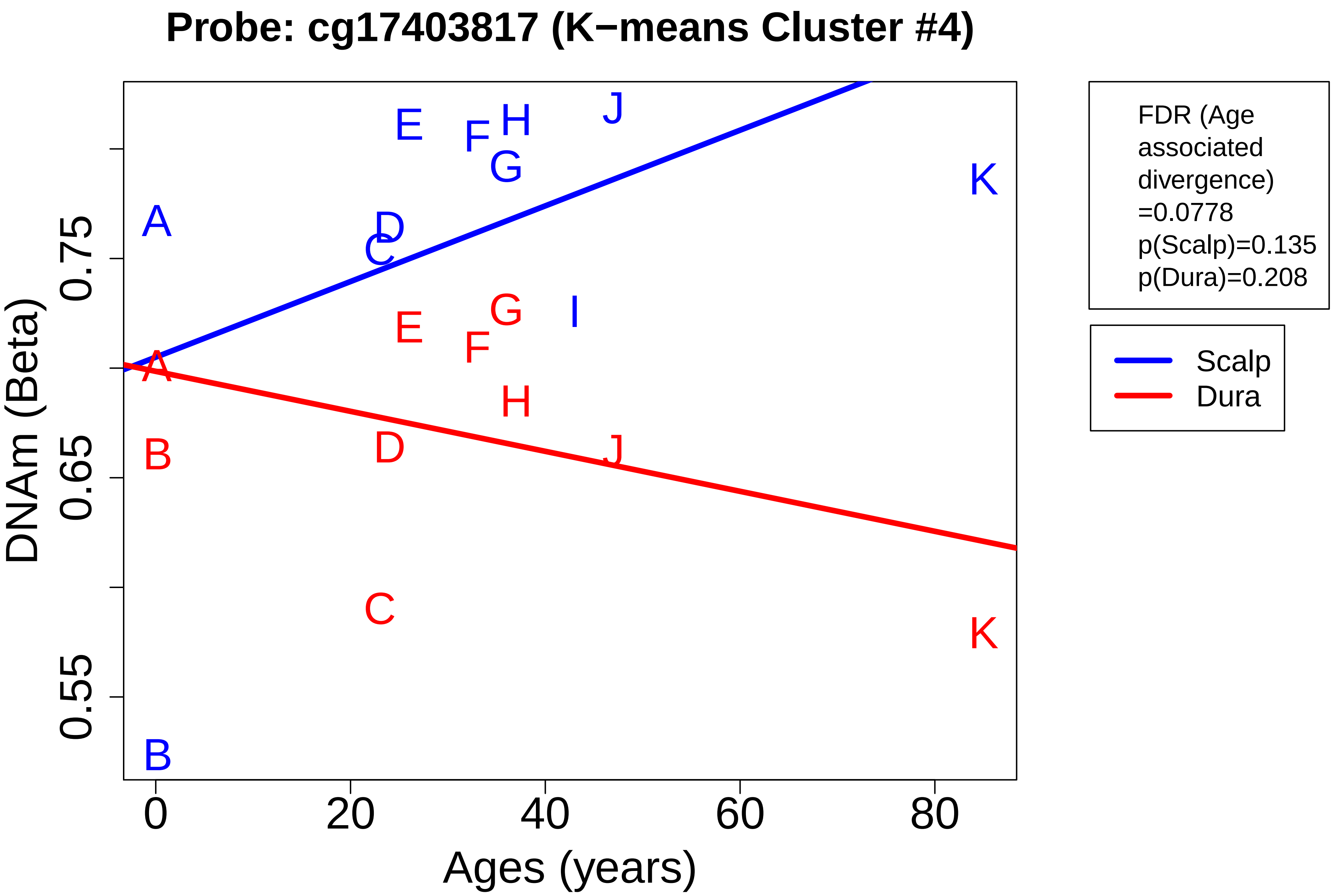
Probe: cg17403817 (K−means Cluster #4)
DNAm (Beta)
0.55
0.65
0.75
Ages (years)
0
20
40
60
80
FDR (Age associated divergence) =0.0778
p(Scalp)=0.135
p(Dura)=0.208
Scalp
Dura
A
B
C
D
E
F
G
H
I
J
K

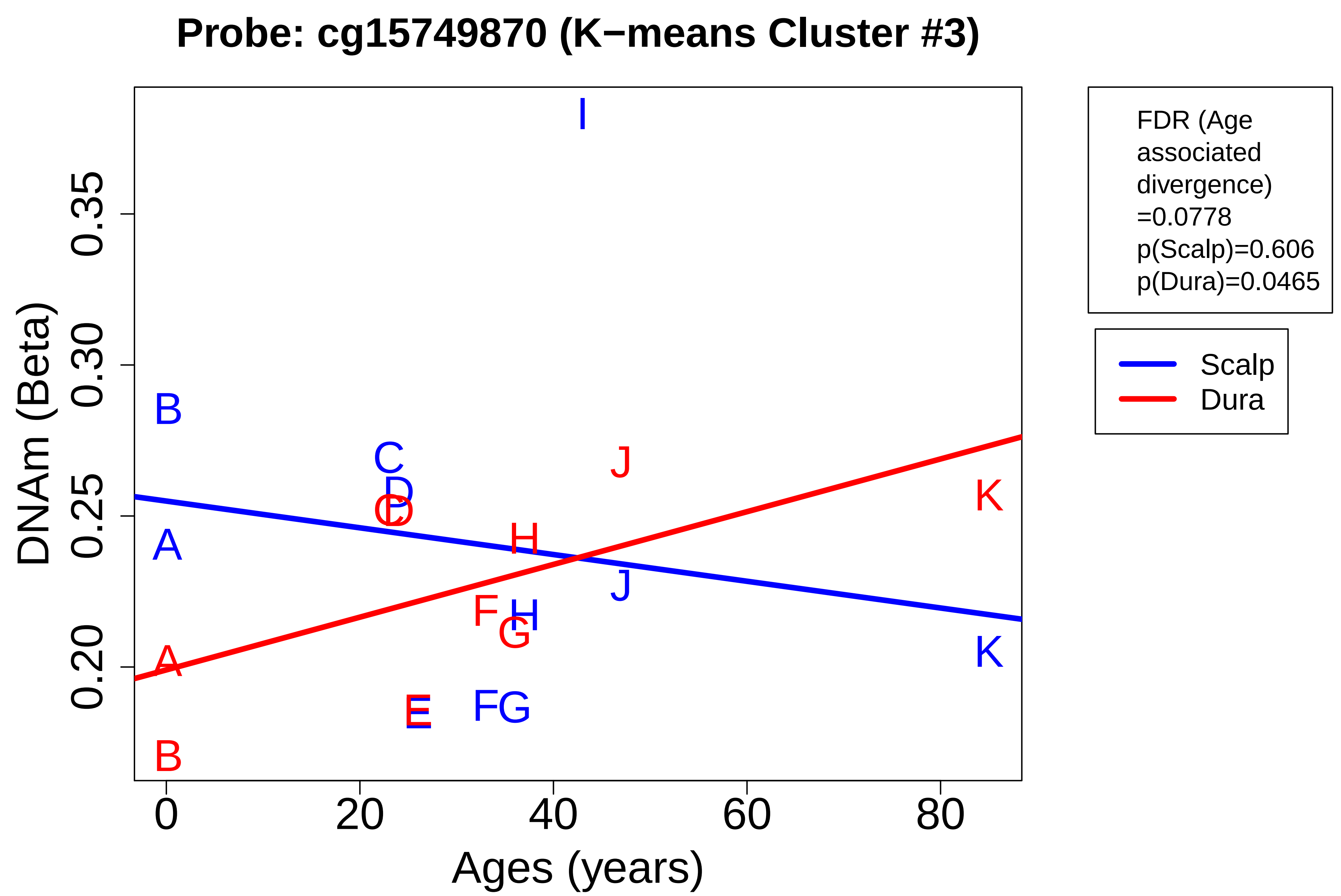

Probe: cg15749870 (K−means Cluster #3)
FDR (Age associated divergence) =0.0778
p(Scalp)=0.606
p(Dura)=0.0465
Scalp
Dura
DNAm (Beta)
0.20
0.25
0.30
0.35
0
20
40
60
80
Ages (years)

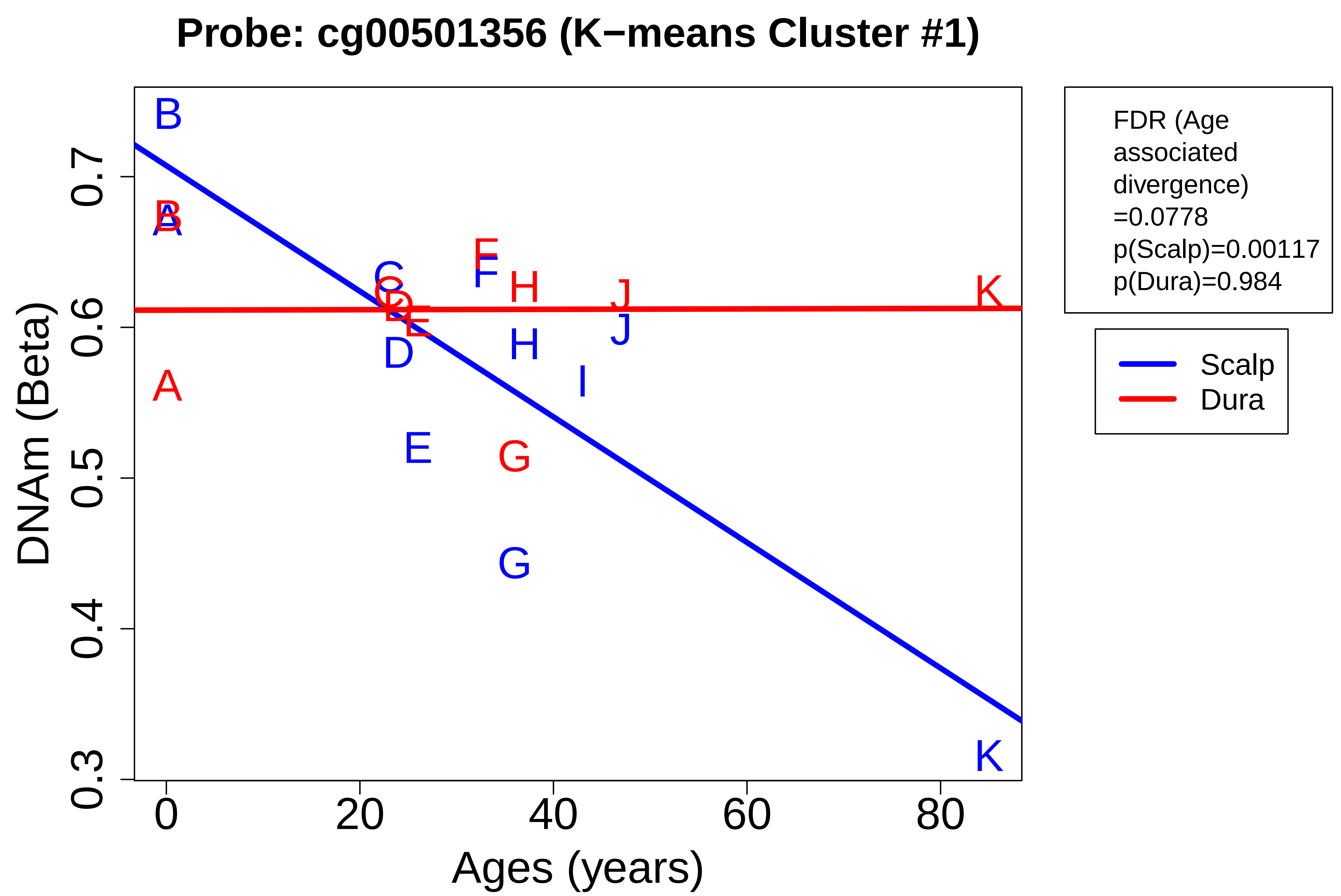

Probe: cg00501356 (K−means Cluster #1)
DNAm (Beta)
0.3
0.4
0.5
0.6
0.7
Ages (years)
0
20
40
60
80
B
A
B
A
C
C
D
D
E
E
F
F
G
G
H
H
I
J
J
K
K
FDR (Age associated divergence) =0.0778
p(Scalp)=0.00117
p(Dura)=0.984
Scalp
Dura

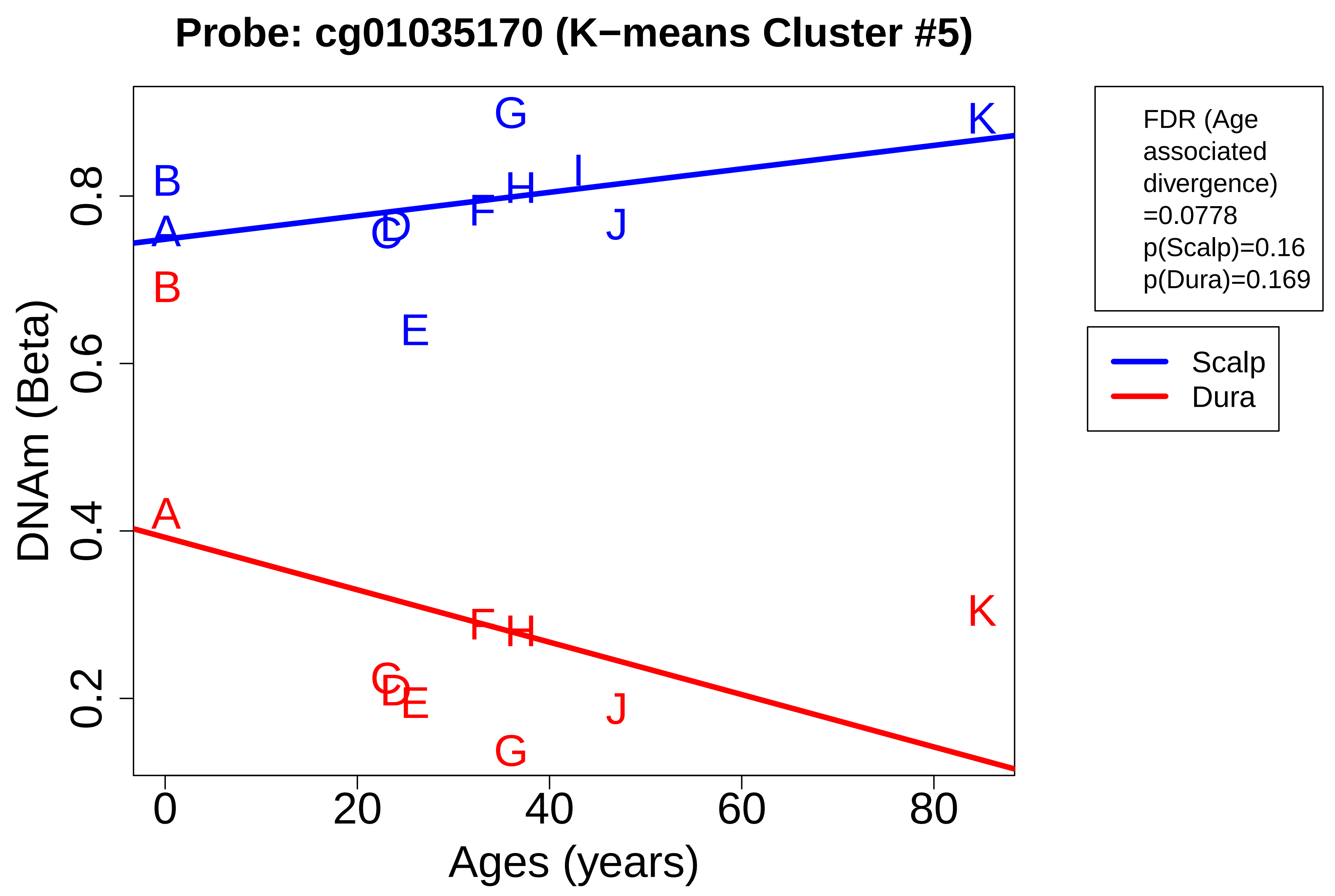

Probe: cg01035170 (K−means Cluster #5)
DNAm (Beta)
0.2
0.4
0.6
0.8
0
20
40
60
80
Ages (years)
A
B
C
D
E
F
G
H
I
J
K
FDR (Age associated divergence) =0.0778
p(Scalp)=0.16
p(Dura)=0.169
Scalp
Dura

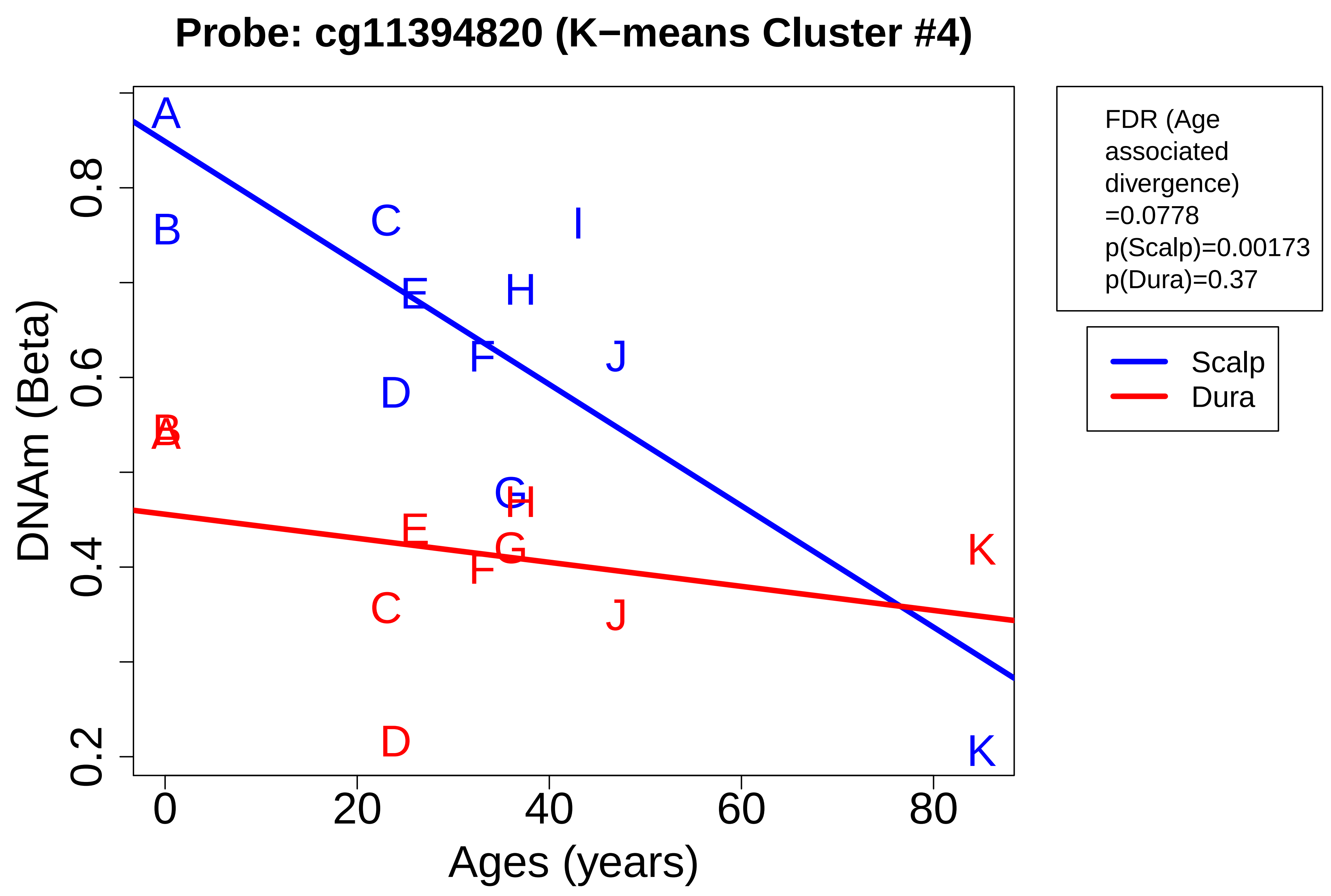

Probe: cg11394820 (K−means Cluster #4)
FDR (Age associated divergence) =0.0778
p(Scalp)=0.00173
p(Dura)=0.37
Scalp
Dura
DNAm (Beta)
0.2
0.4
0.6
0.8
0
20
40
60
80
Ages (years)
A
B
C
D
E
F
G
H
I
J
K

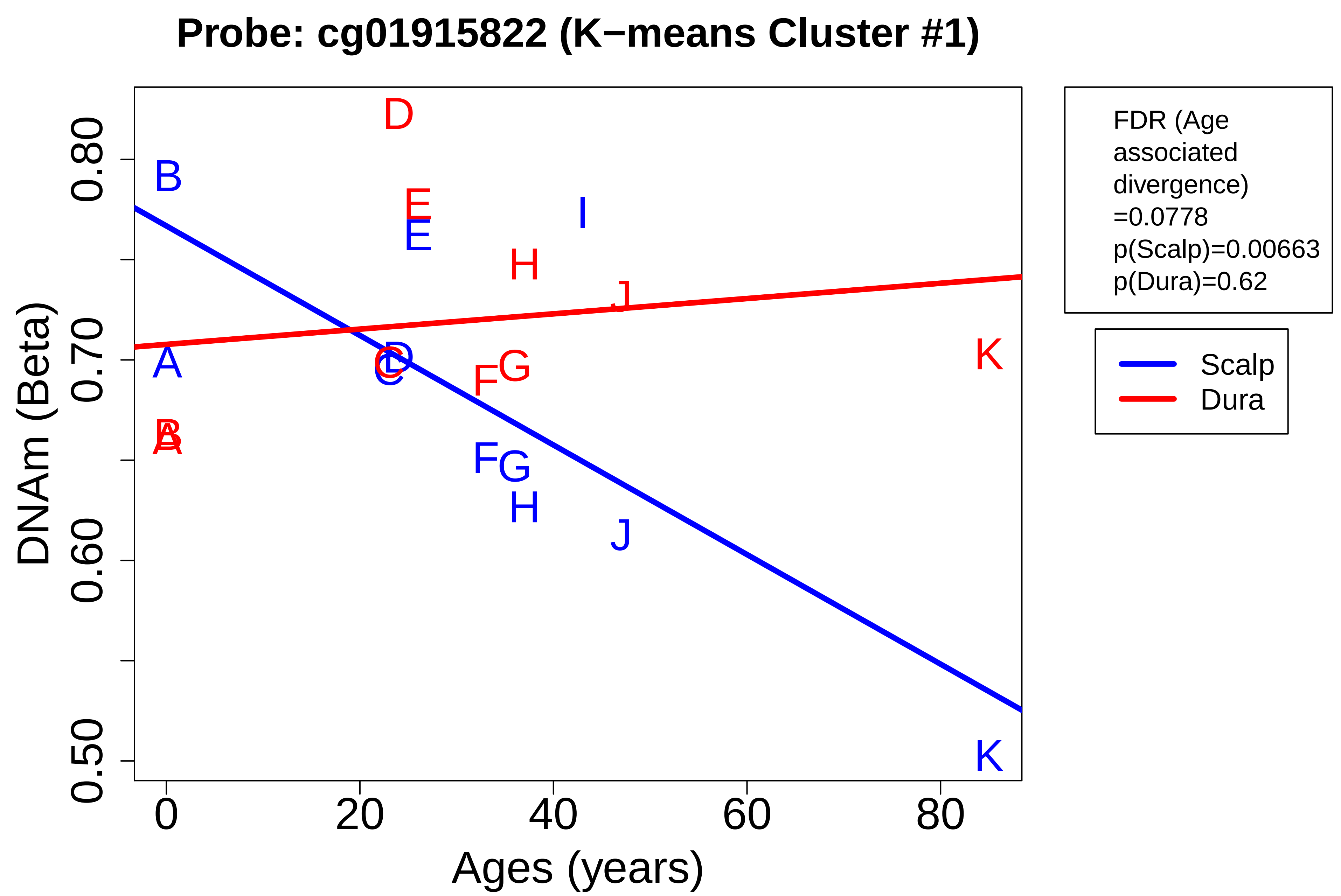

Probe: cg01915822 (K−means Cluster #1)
DNAm (Beta)
0.50
0.60
0.70
0.80
Ages (years)
0
20
40
60
80
FDR (Age associated divergence) =0.0778
p(Scalp)=0.00663
p(Dura)=0.62
Scalp
Dura

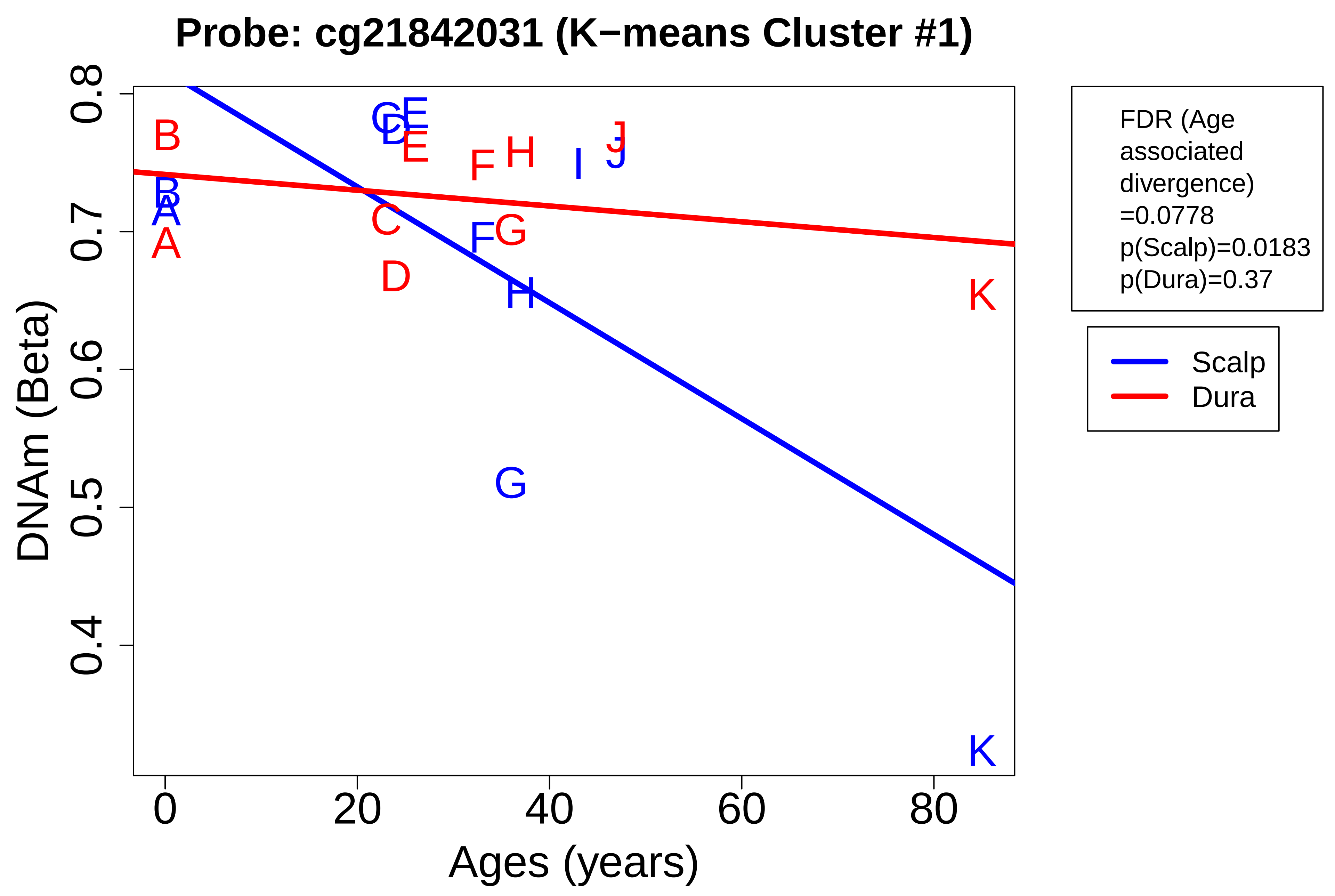

Probe: cg21842031 (K−means Cluster #1)
DNAm (Beta)
0.4
0.5
0.6
0.7
0.8
Ages (years)
0
20
40
60
80
FDR (Age associated divergence) =0.0778
p(Scalp)=0.0183
p(Dura)=0.37
Scalp
Dura

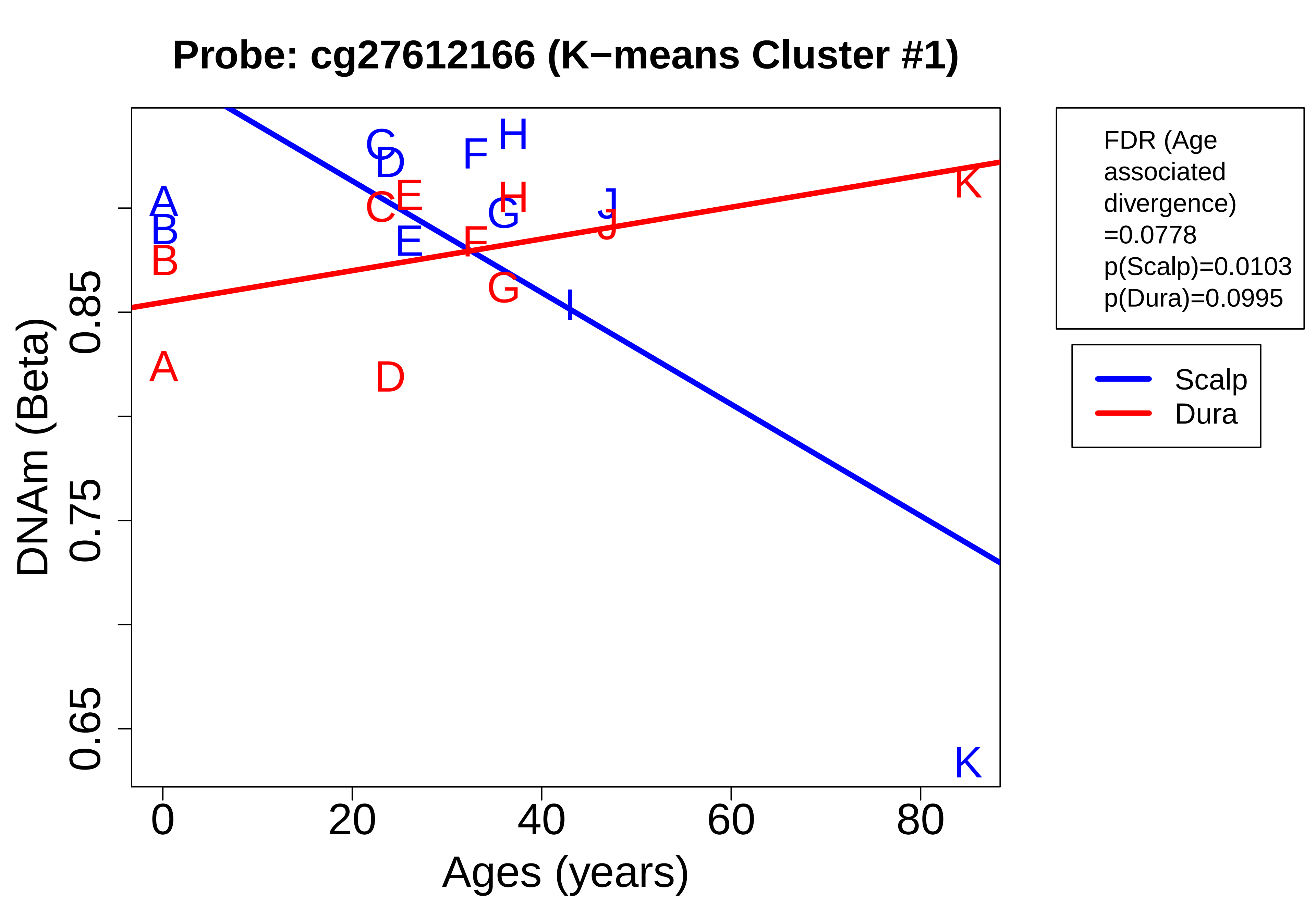

Probe: cg27612166 (K−means Cluster #1)
DNAm (Beta)
0.65
0.75
0.85
Ages (years)
0
20
40
60
80
FDR (Age associated divergence) =0.0778
p(Scalp)=0.0103
p(Dura)=0.0995
Scalp
Dura
A
B
C
D
E
F
G
H
I
J
K

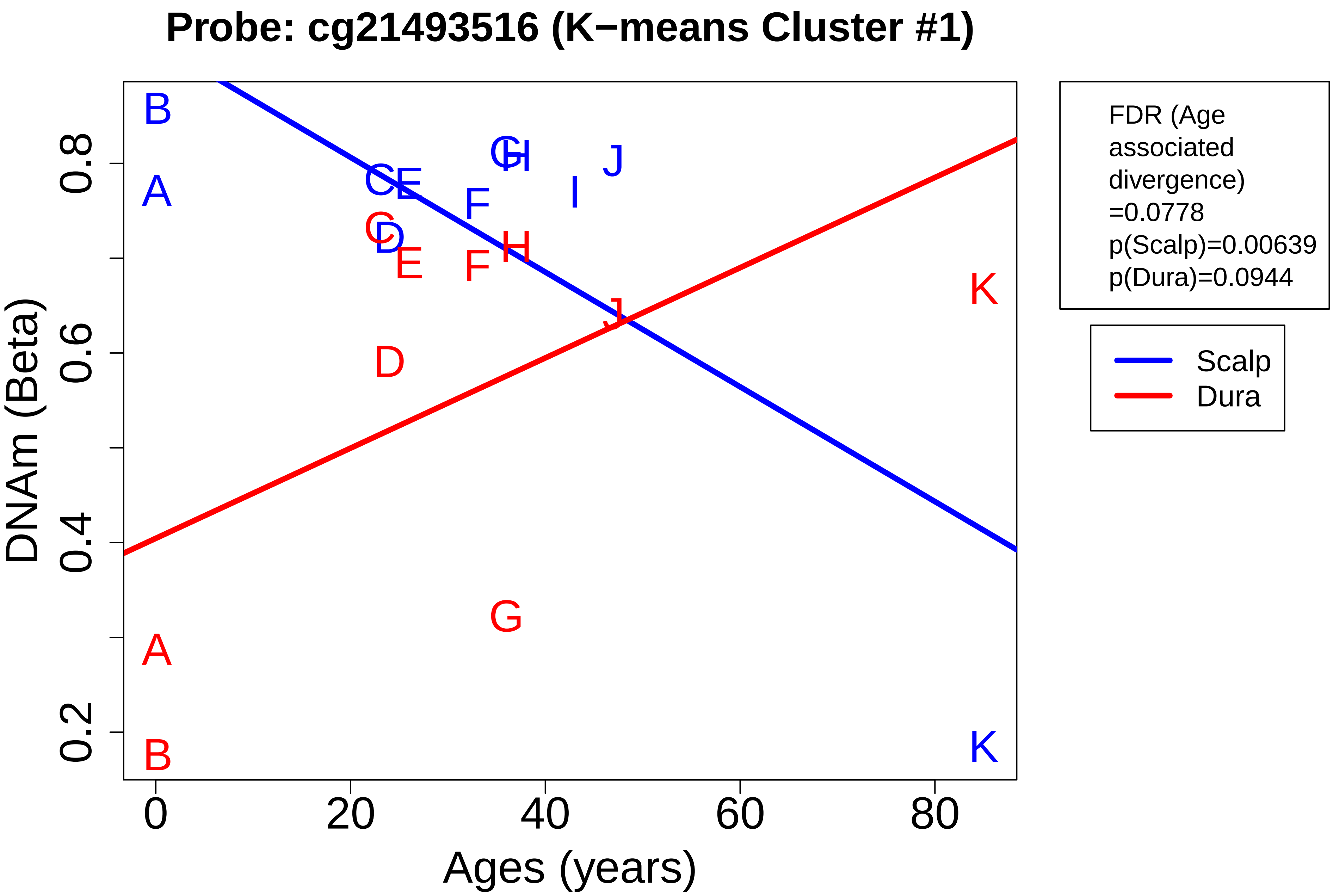

Probe: cg21493516 (K−means Cluster #1)
DNAm (Beta)
0.2
0.4
0.6
0.8
Ages (years)
0
20
40
60
80
FDR (Age associated divergence) =0.0778
p(Scalp)=0.00639
p(Dura)=0.0944
Scalp
Dura

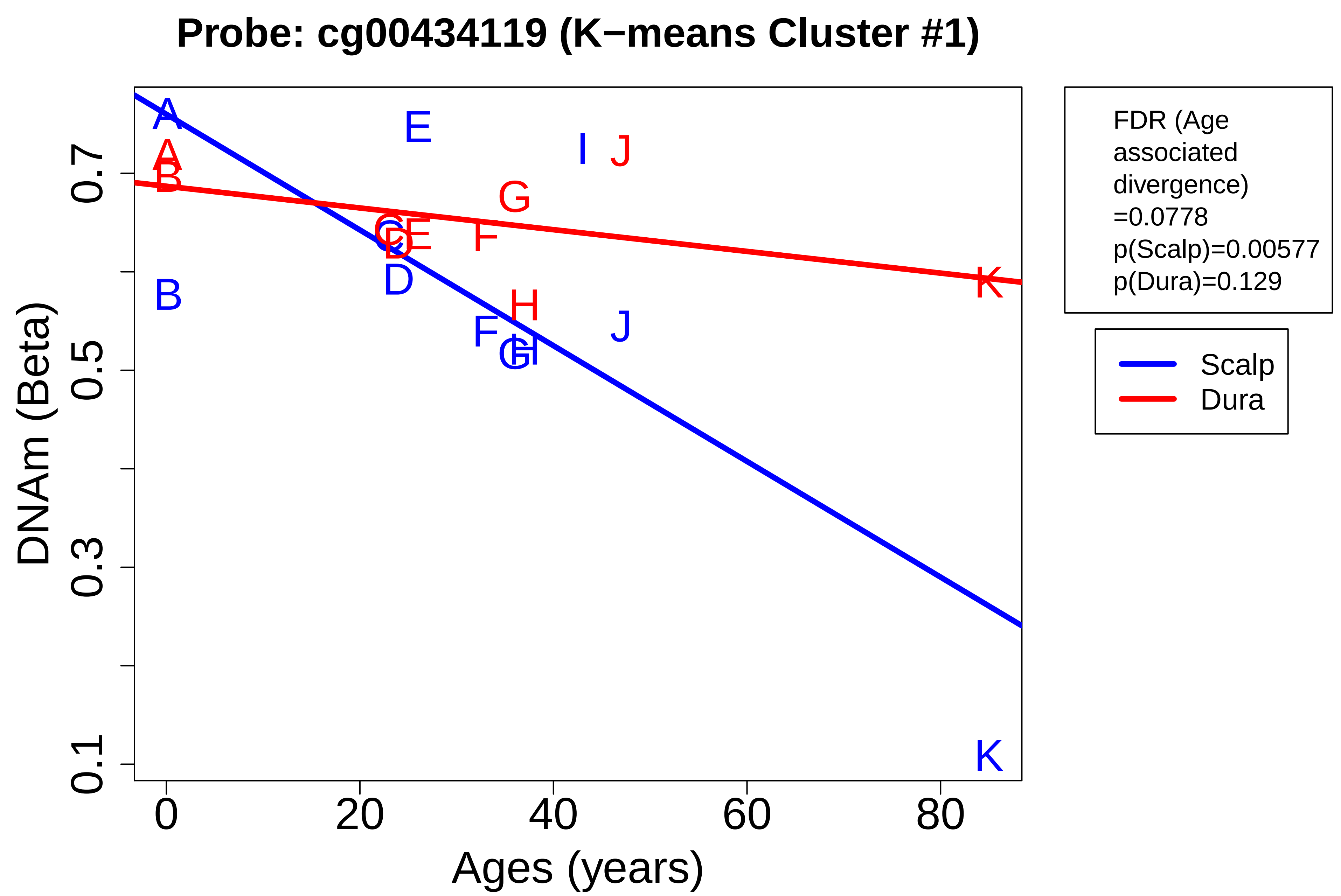

Probe: cg00434119 (K−means Cluster #1)
DNAm (Beta)
0.1
0.3
0.5
0.7
0
20
40
60
80
Ages (years)
A
B
C
D
E
F
G
H
I
J
K
FDR (Age associated divergence) =0.0778
p(Scalp)=0.00577
p(Dura)=0.129
Scalp
Dura

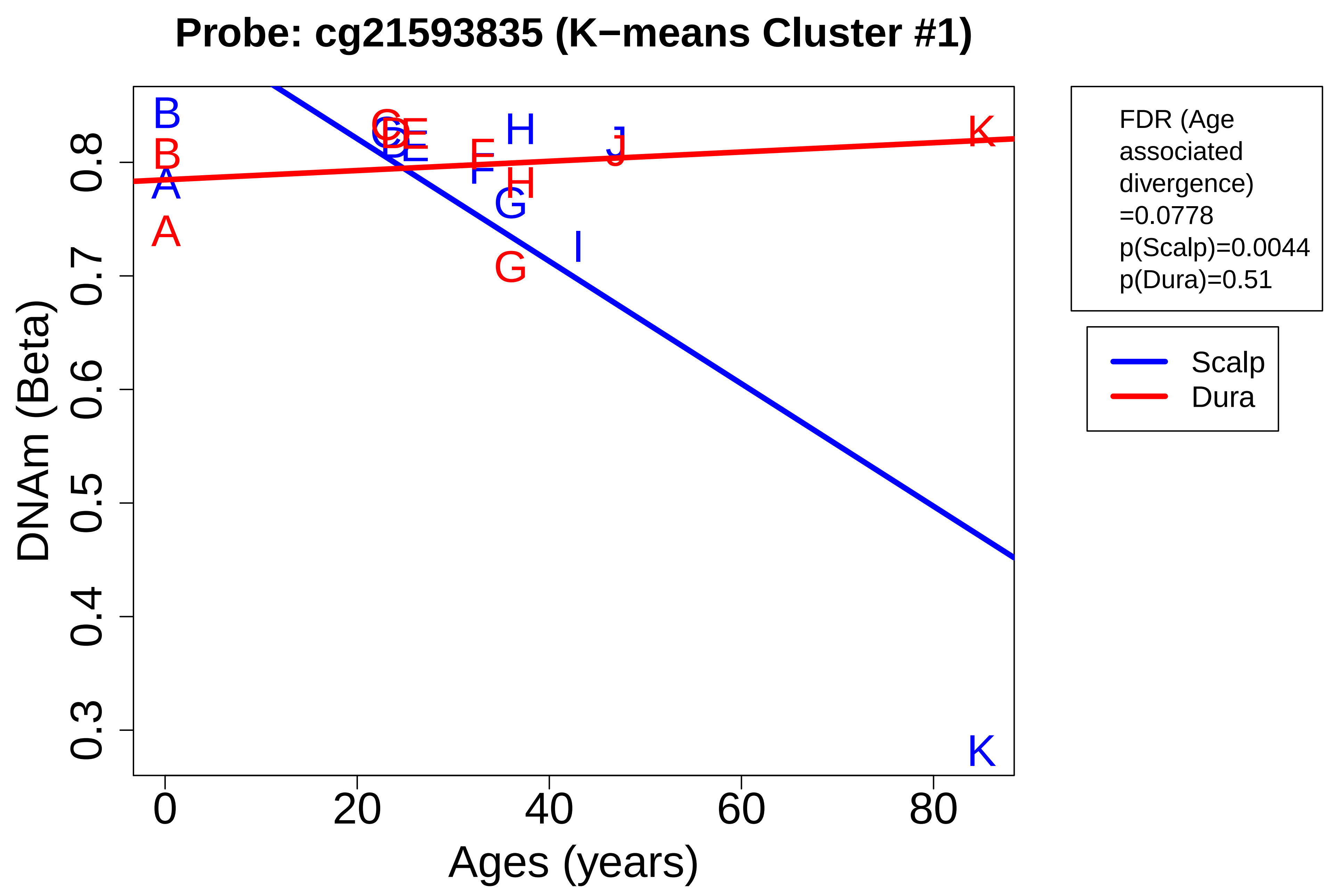

Probe: cg21593835 (K−means Cluster #1)
DNAm (Beta)
0.3
0.4
0.5
0.6
0.7
0.8
0
20
40
60
80
Ages (years)
FDR (Age associated divergence) =0.0778
p(Scalp)=0.0044
p(Dura)=0.51
Scalp
Dura

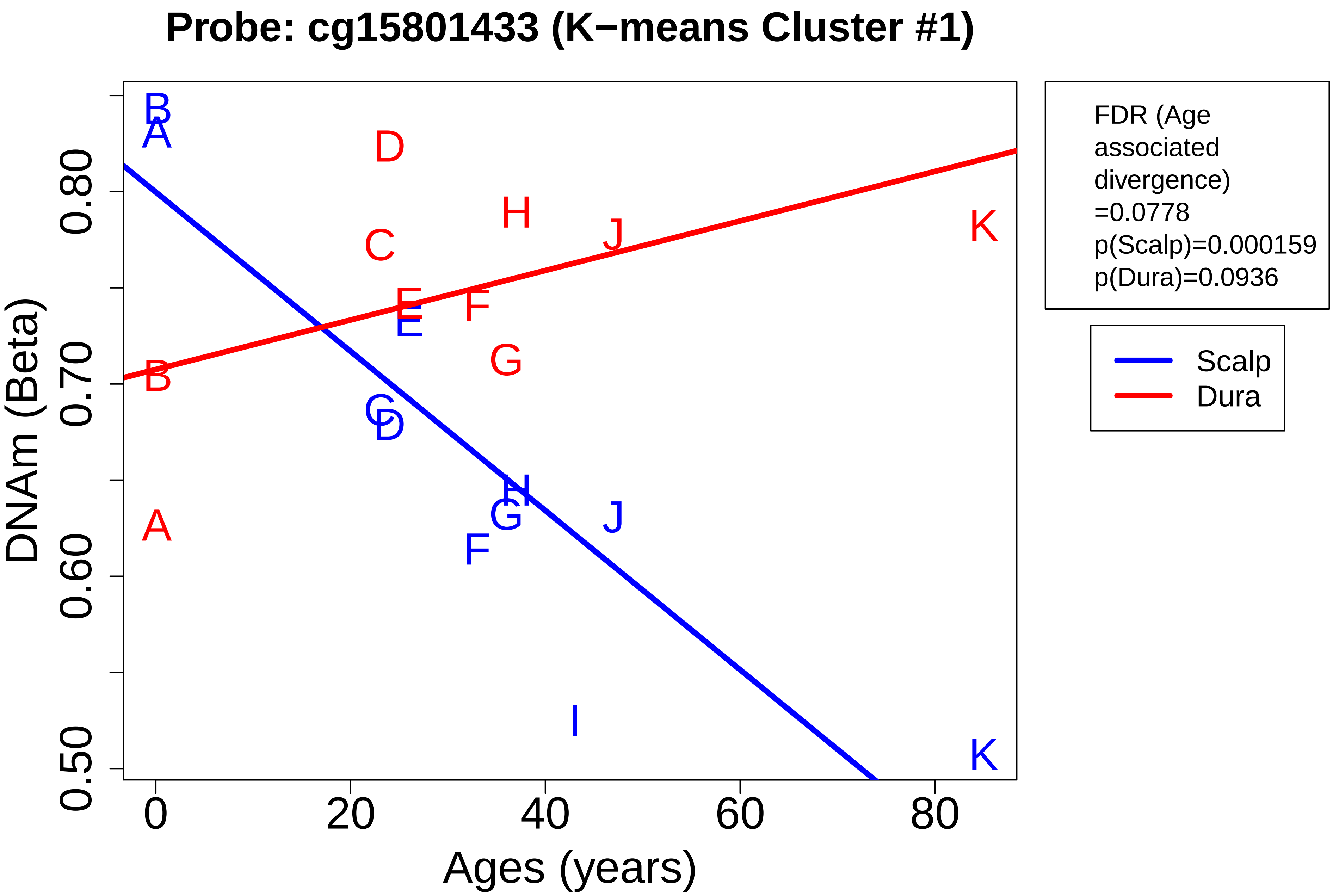

Probe: cg15801433 (K−means Cluster #1)
DNAm (Beta)
0.50
0.60
0.70
0.80
0
20
40
60
80
Ages (years)
B
A
D
C
H
J
K
E
F
G
B
A
C
D
E
H
G
J
F
I
K
FDR (Age associated divergence) =0.0778
p(Scalp)=0.000159
p(Dura)=0.0936
Scalp
Dura

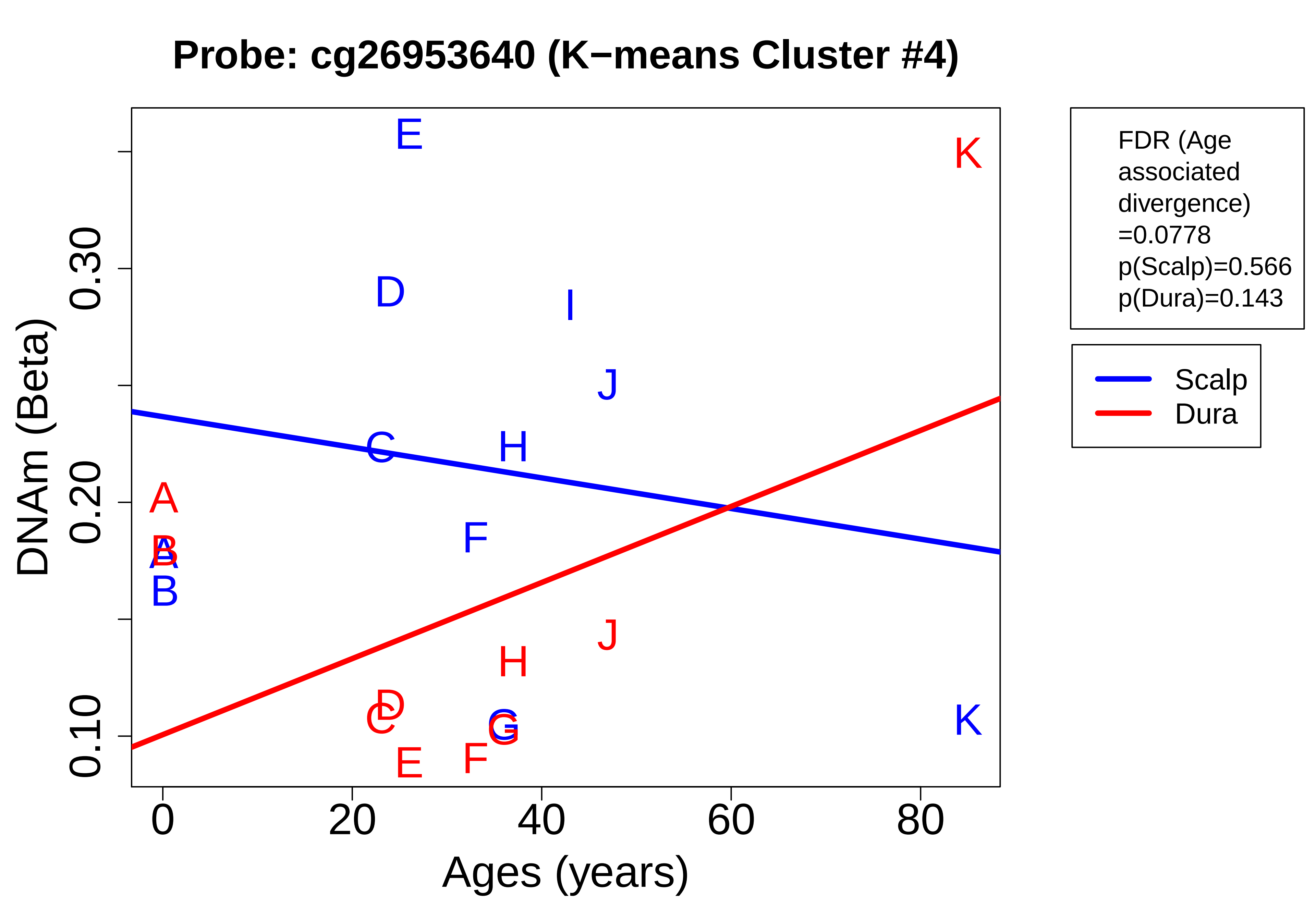

Probe: cg26953640 (K−means Cluster #4)
FDR (Age associated divergence) =0.0778
p(Scalp)=0.566
p(Dura)=0.143
Scalp
Dura
DNAm (Beta)
0.10
0.20
0.30
0
20
40
60
80
Ages (years)

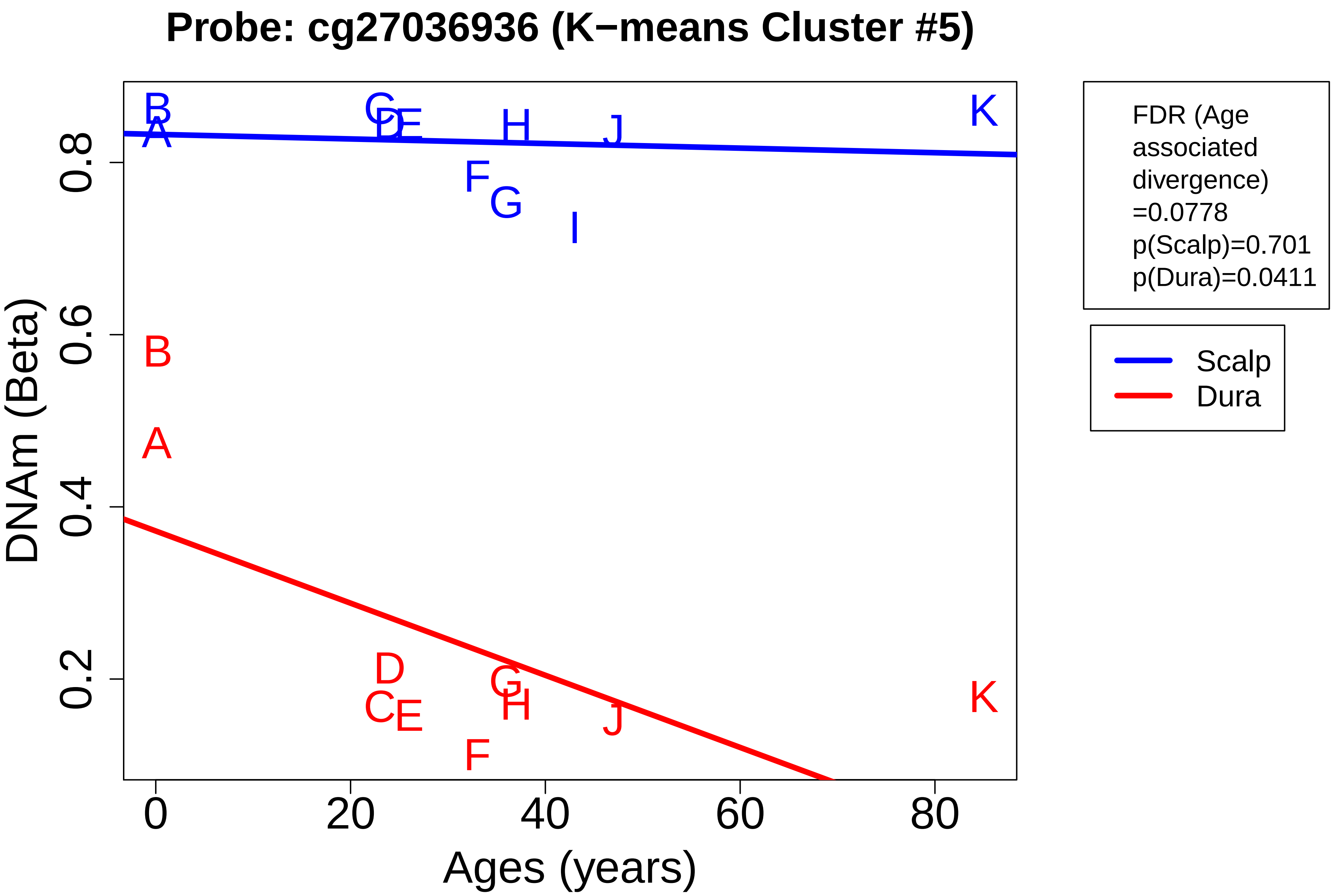
Probe: cg27036936 (K−means Cluster #5)
DNAm (Beta)
0.2
0.4
0.6
0.8
0
20
40
60
80
Ages (years)
B
A
C
D
E
H
J
K
F
G
I
B
A
D
C
E
G
H
J
K
F
FDR (Age associated divergence) =0.0778
p(Scalp)=0.701
p(Dura)=0.0411
Scalp
Dura

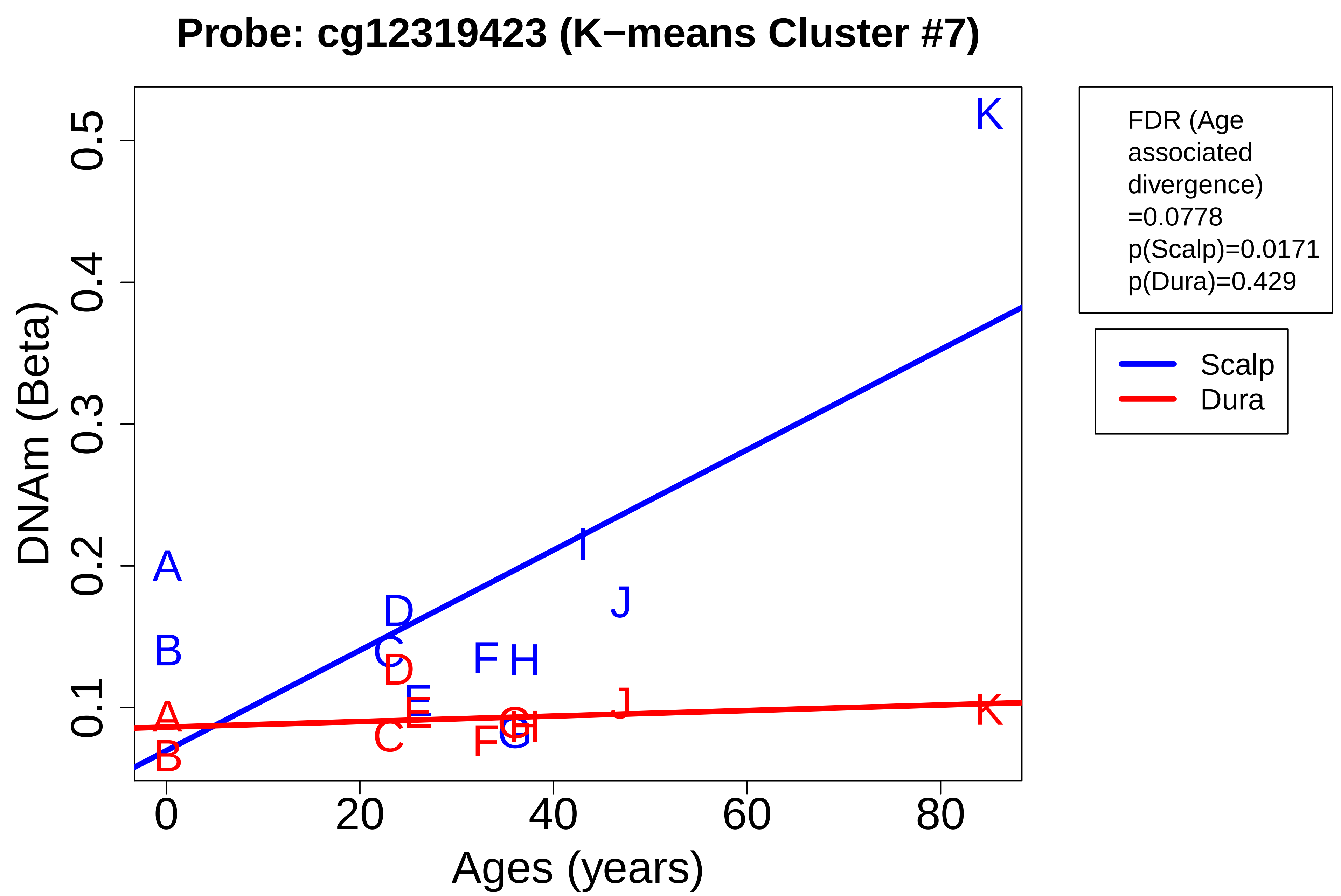

Probe: cg12319423 (K−means Cluster #7)
DNAm (Beta)
0.1
0.2
0.3
0.4
0.5
0
20
40
60
80
Ages (years)
FDR (Age associated divergence) =0.0778
p(Scalp)=0.0171
p(Dura)=0.429
Scalp
Dura

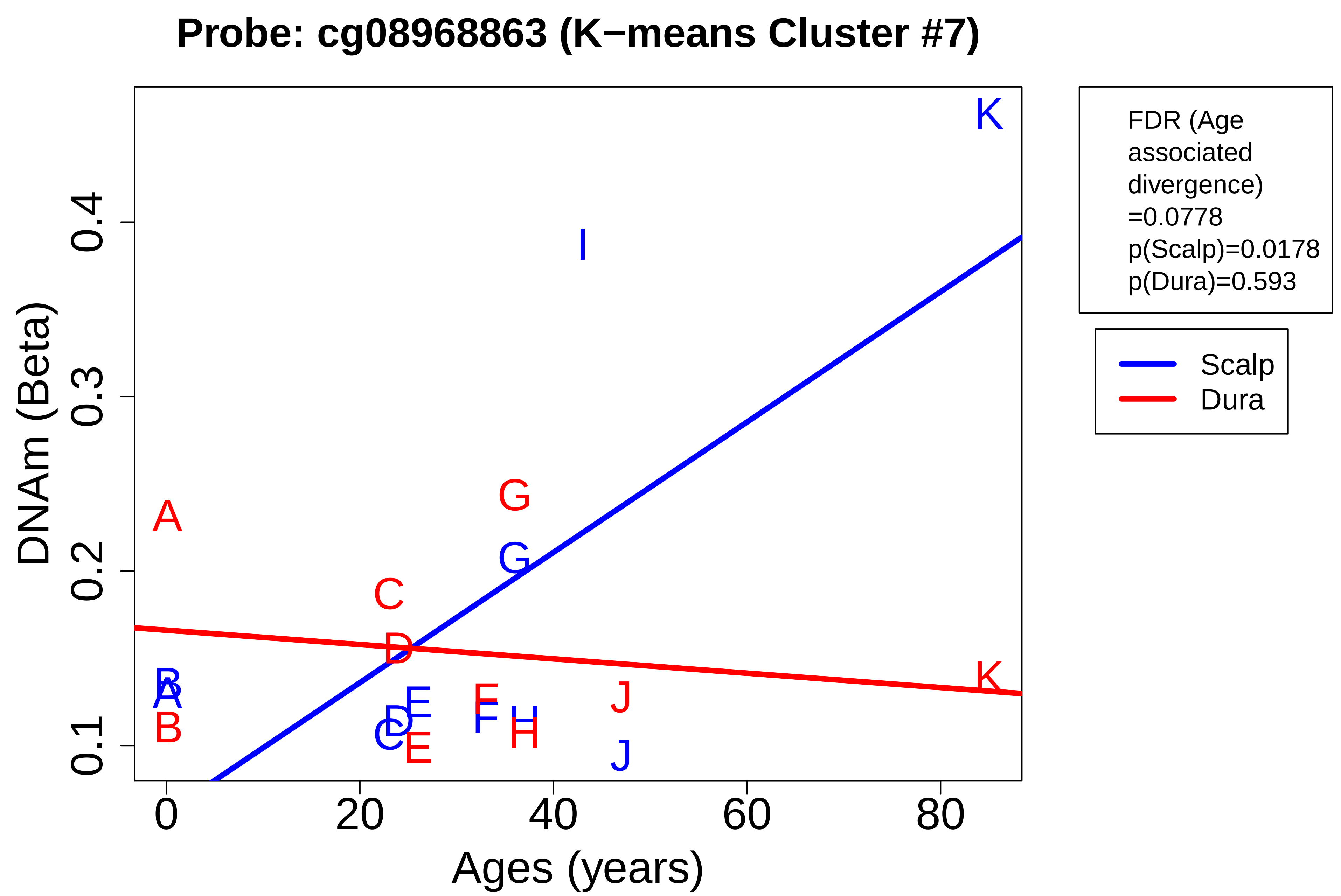

Probe: cg08968863 (K−means Cluster #7)
DNAm (Beta)
0.1
0.2
0.3
0.4
0
20
40
60
80
Ages (years)
FDR (Age associated divergence) =0.0778
p(Scalp)=0.0178
p(Dura)=0.593
Scalp
Dura

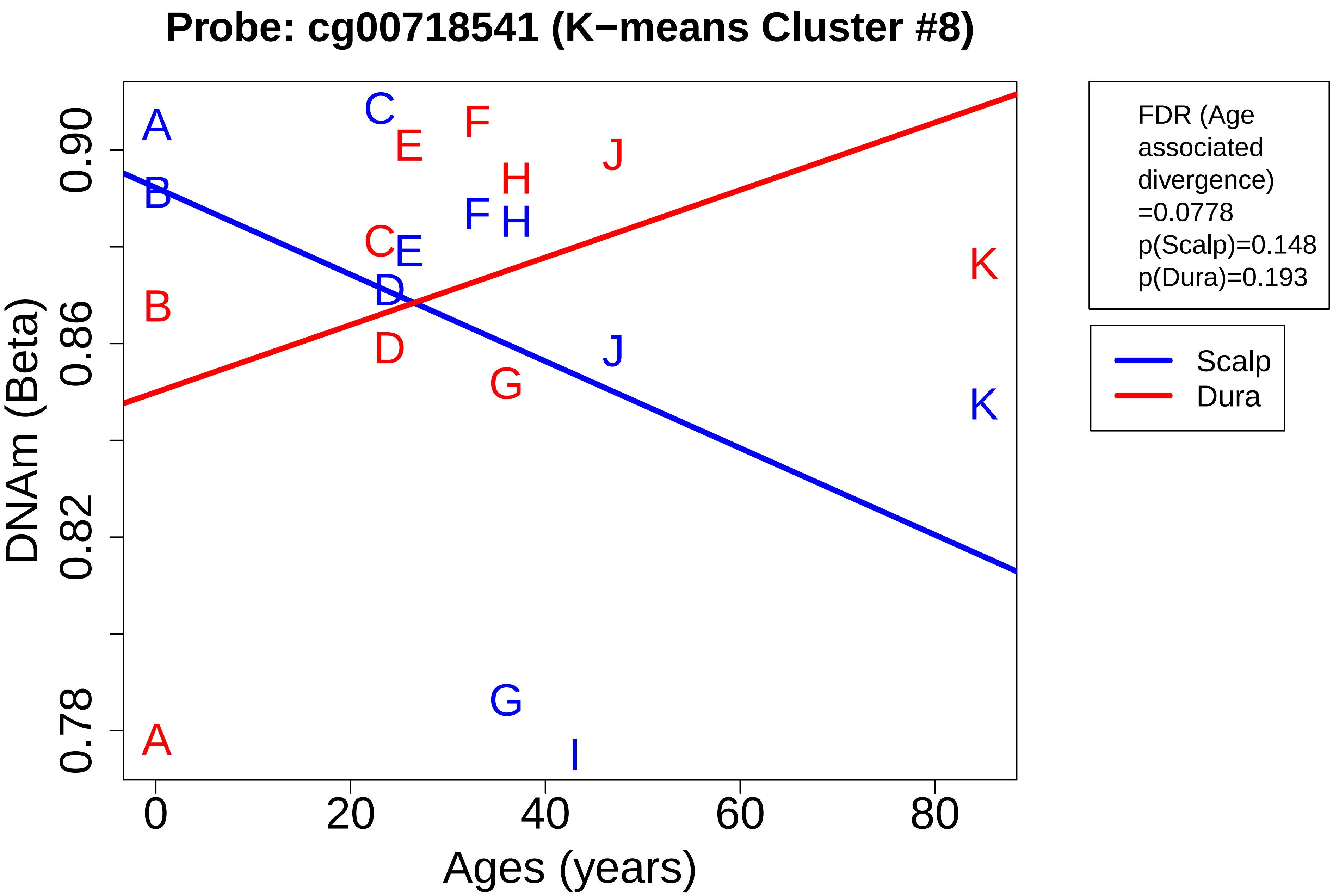

Probe: cg00718541 (K−means Cluster #8)
DNAm (Beta)
0.78
0.82
0.86
0.90
0
20
40
60
80
Ages (years)
A
B
C
D
E
F
G
H
I
J
K
FDR (Age associated divergence) =0.0778
p(Scalp)=0.148
p(Dura)=0.193
Scalp
Dura

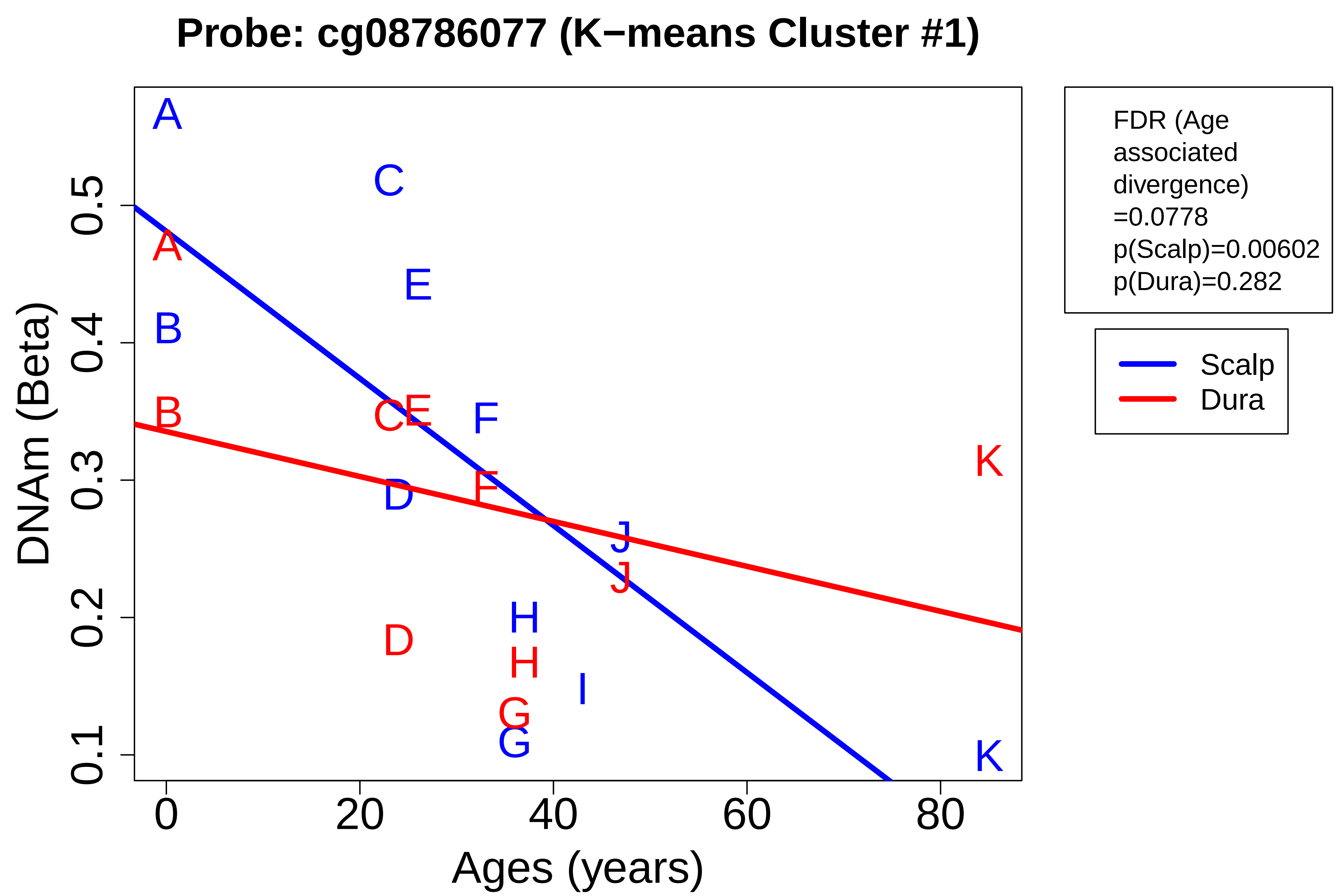
Probe: cg08786077 (K−means Cluster #1)
DNAm (Beta)
Ages (years)
0.1
0.2
0.3
0.4
0.5
0
20
40
60
80
A
B
C
D
E
F
G
H
I
J
K
FDR (Age associated divergence) =0.0778
p(Scalp)=0.00602
p(Dura)=0.282
Scalp
Dura

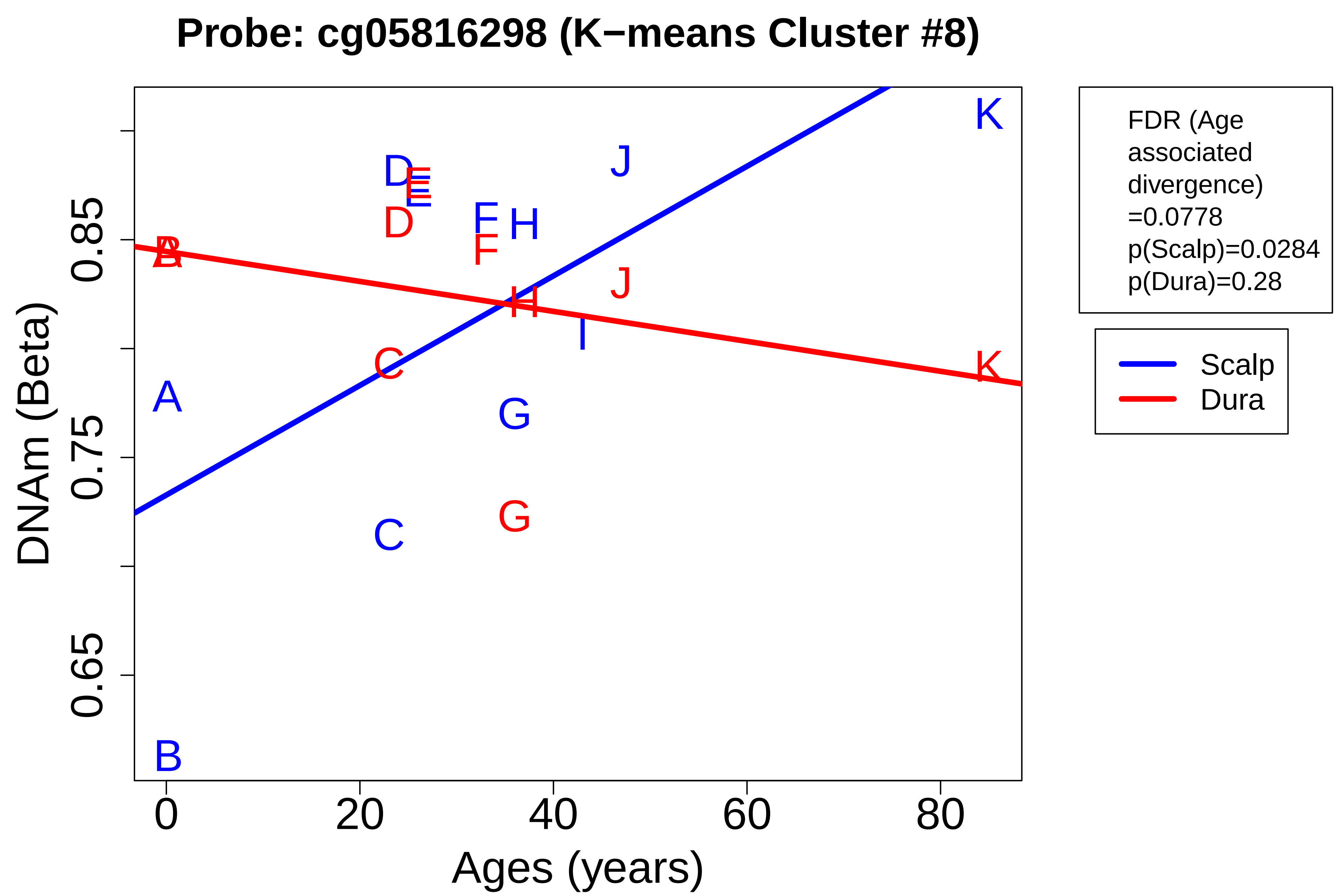

Probe: cg05816298 (K−means Cluster #8)
DNAm (Beta)
0.65
0.75
0.85
Ages (years)
0
20
40
60
80
FDR (Age associated divergence) =0.0778
p(Scalp)=0.0284
p(Dura)=0.28
Scalp
Dura

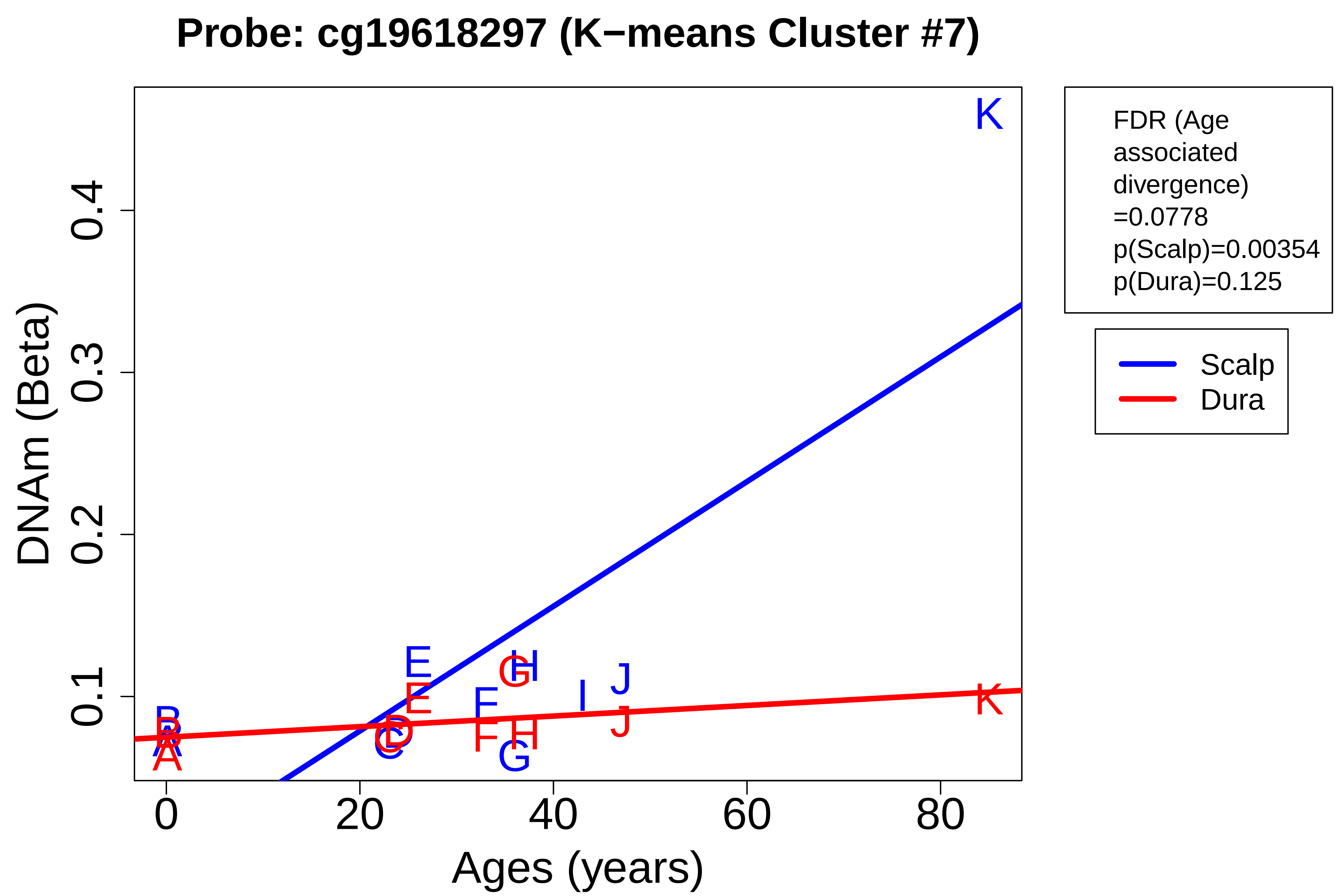
Probe: cg19618297 (K−means Cluster #7)
DNAm (Beta)
0.1
0.2
0.3
0.4
0
20
40
60
80
Ages (years)
A
B
C
D
E
F
G
H
I
J
K
FDR (Age associated divergence) =0.0778
p(Scalp)=0.00354
p(Dura)=0.125
Scalp
Dura

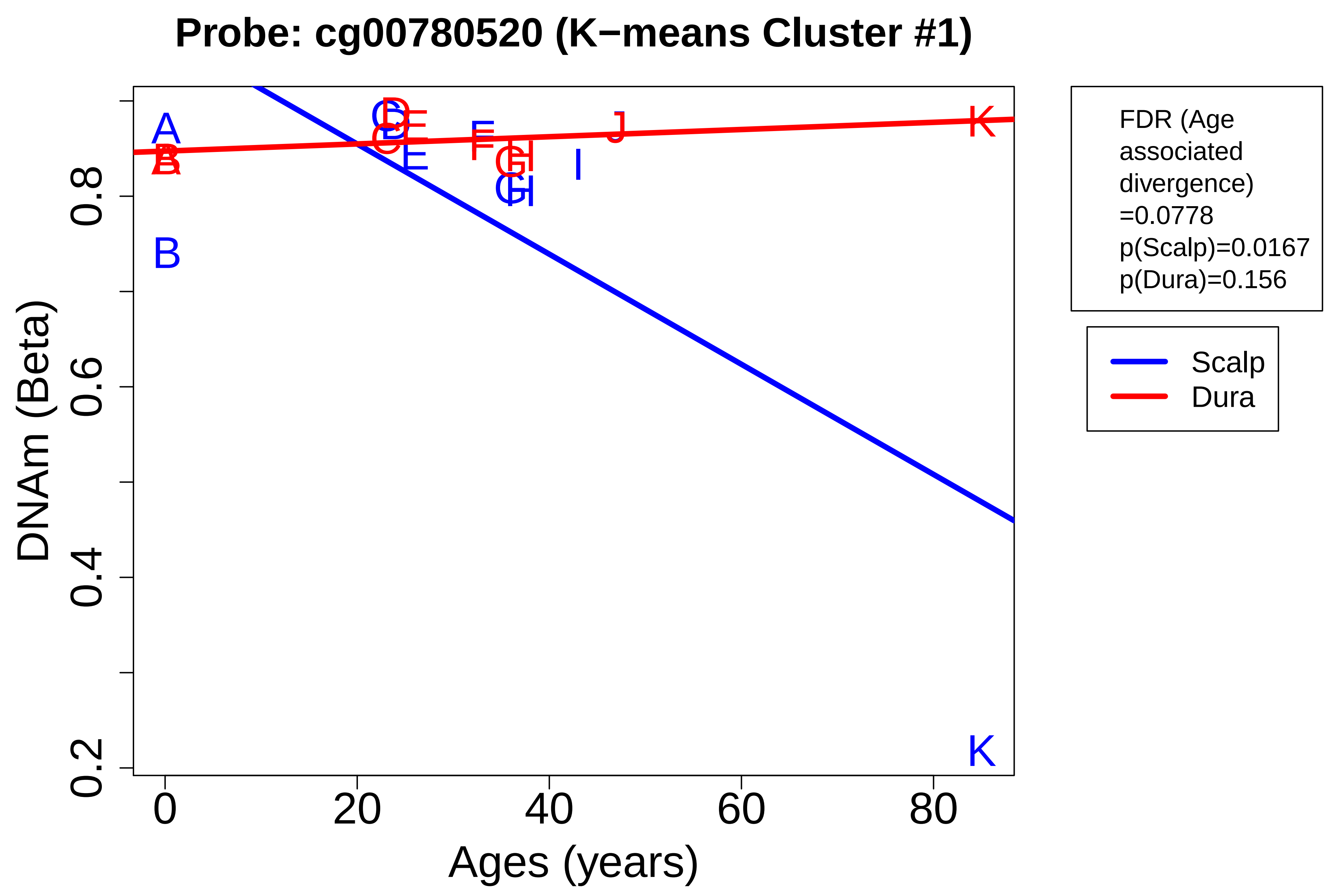
Probe: cg00780520 (K−means Cluster #1)
DNAm (Beta)
0.2
0.4
0.6
0.8
0
20
40
60
80
Ages (years)
FDR (Age associated divergence) =0.0778
p(Scalp)=0.0167
p(Dura)=0.156
Scalp
Dura
A
B
C
D
E
F
G
H
I
J
K

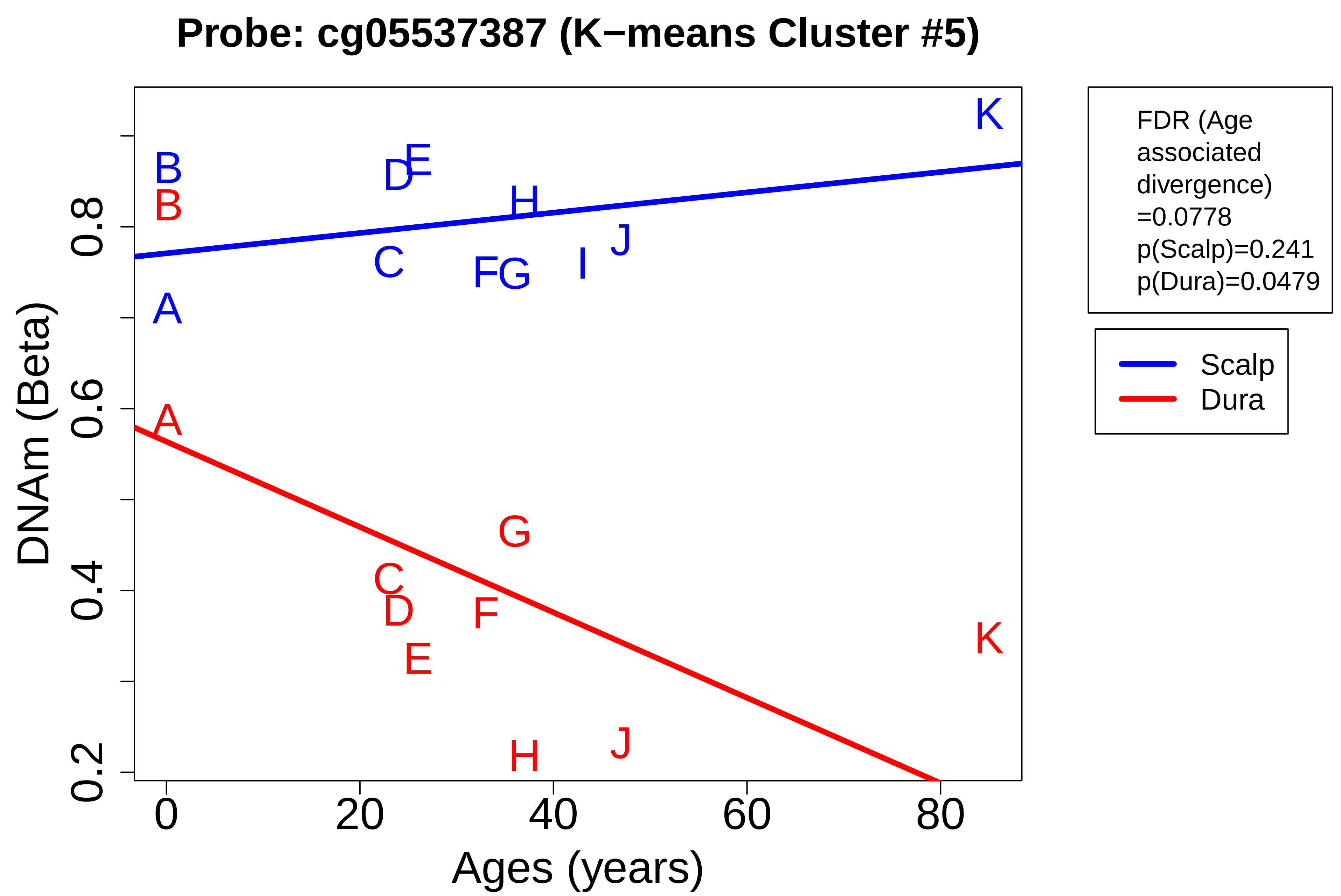

Probe: cg05537387 (K−means Cluster #5)
DNAm (Beta)
0.2
0.4
0.6
0.8
Ages (years)
0
20
40
60
80
A
B
C
D
E
F
G
H
I
J
K
FDR (Age associated divergence) =0.0778
p(Scalp)=0.241
p(Dura)=0.0479
Scalp
Dura

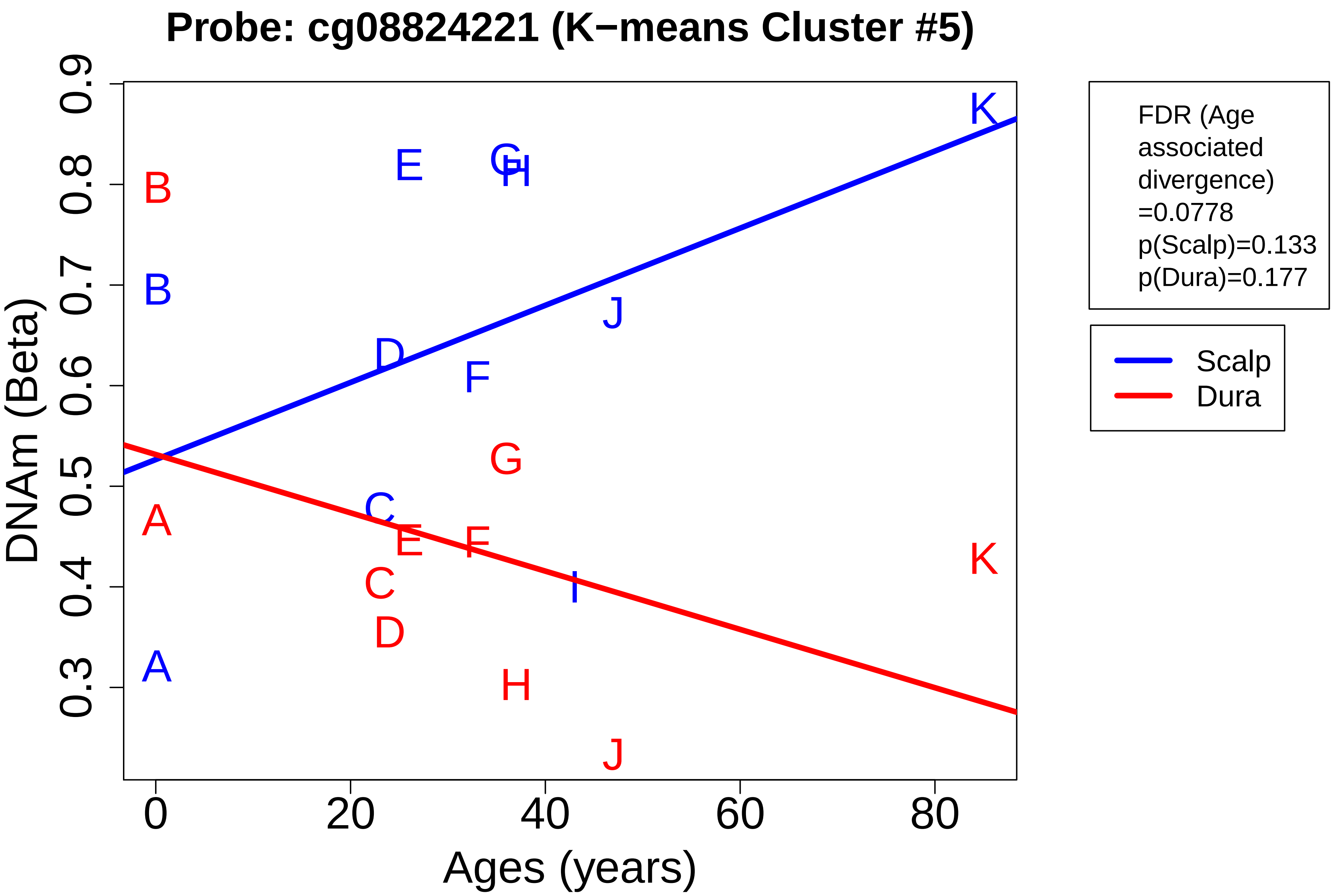
Probe: cg08824221 (K−means Cluster #5)
DNAm (Beta)
0.3
0.4
0.5
0.6
0.7
0.8
0.9
0
20
40
60
80
Ages (years)
FDR (Age associated divergence) =0.0778
p(Scalp)=0.133
p(Dura)=0.177
Scalp
Dura
A
B
C
D
E
F
G
H
I
J
K

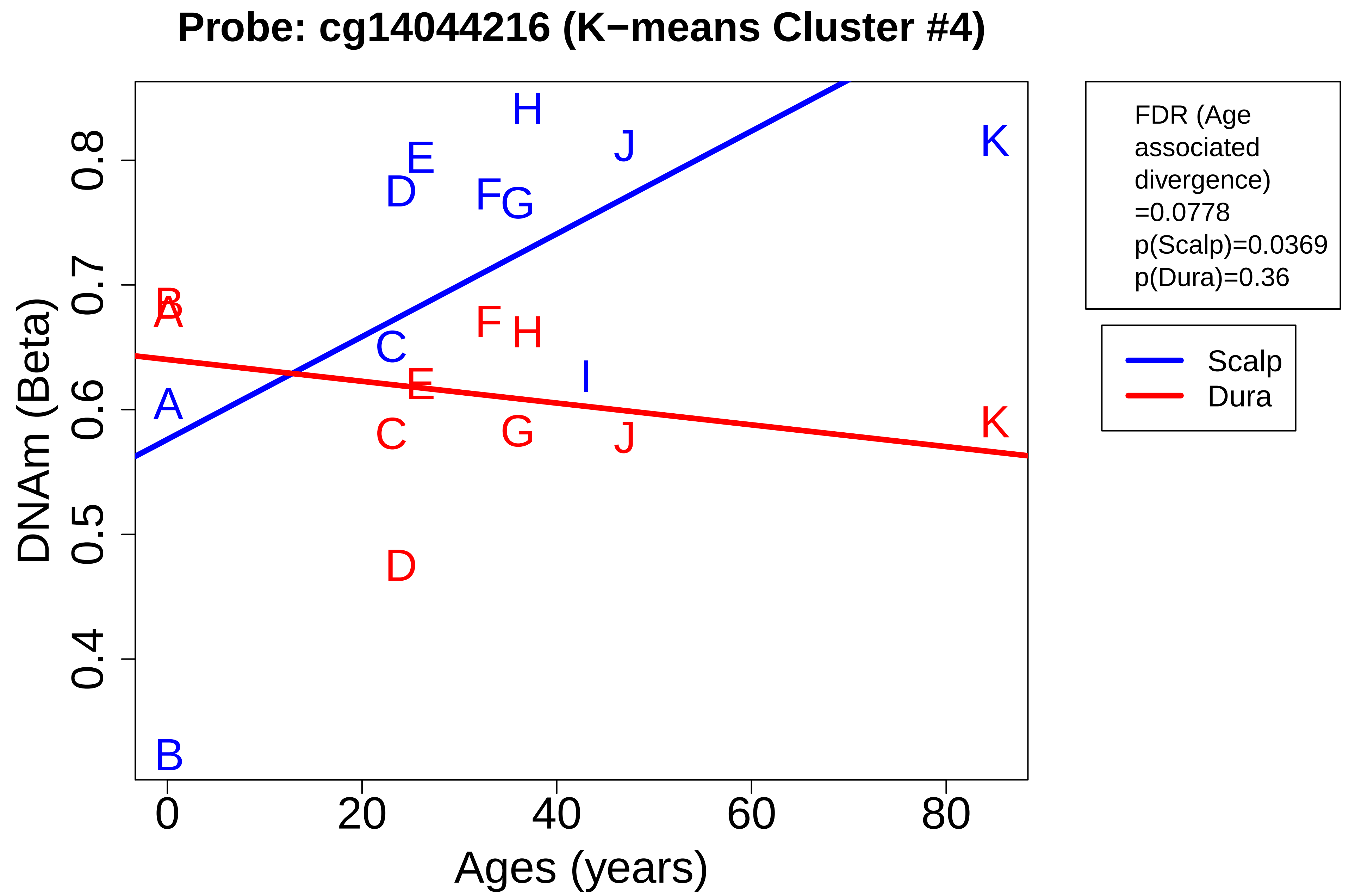

Probe: cg14044216 (K−means Cluster #4)
DNAm (Beta)
0.4
0.5
0.6
0.7
0.8
0
20
40
60
80
Ages (years)
A
B
C
D
E
F
G
H
I
J
K
FDR (Age associated divergence) =0.0778
p(Scalp)=0.0369
p(Dura)=0.36
Scalp
Dura

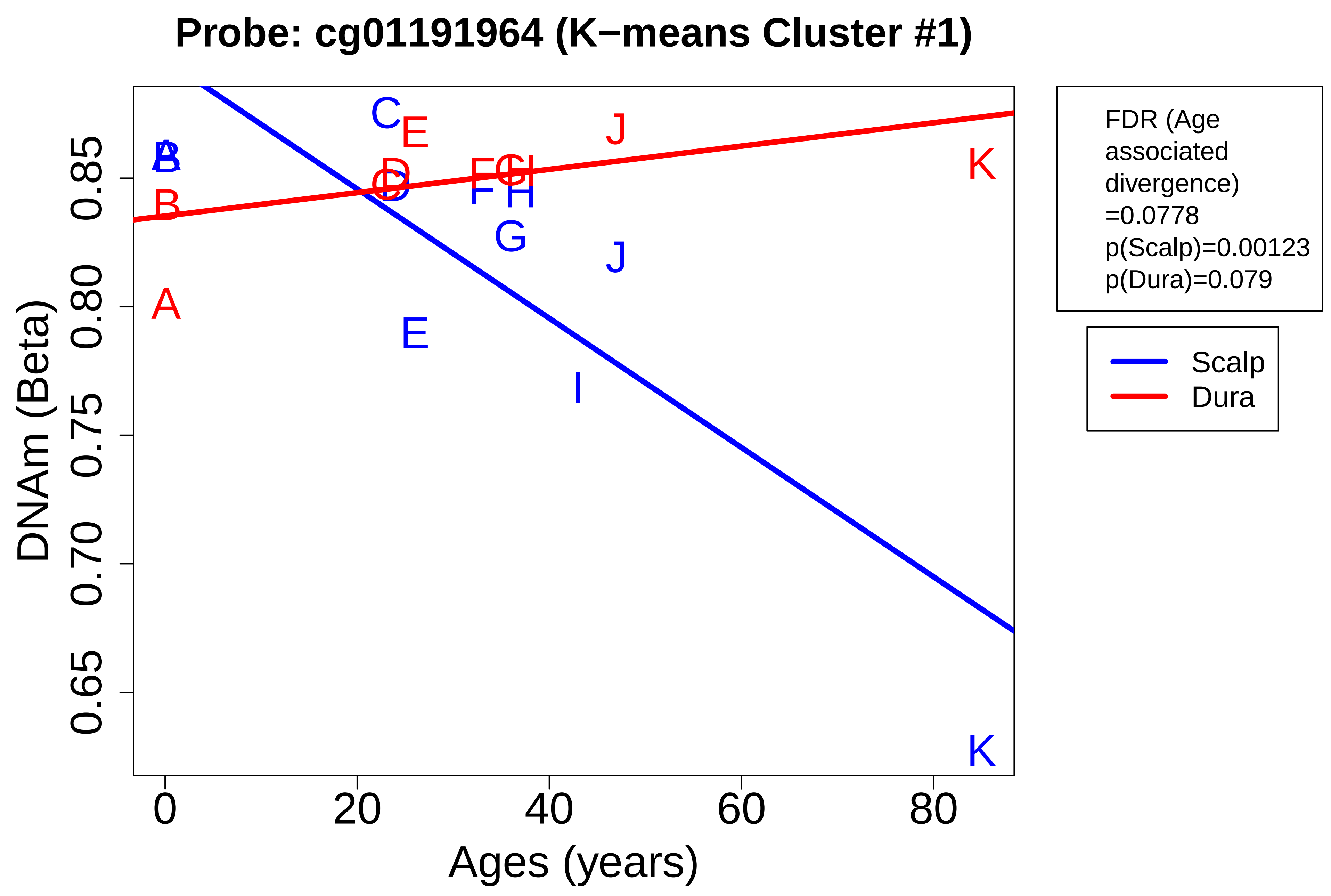

Probe: cg01191964 (K−means Cluster #1)
DNAm (Beta)
0.65
0.70
0.75
0.80
0.85
Ages (years)
0
20
40
60
80
FDR (Age associated divergence) =0.0778
p(Scalp)=0.00123
p(Dura)=0.079
Scalp
Dura
A
B
C
D
E
F
G
H
I
J
K

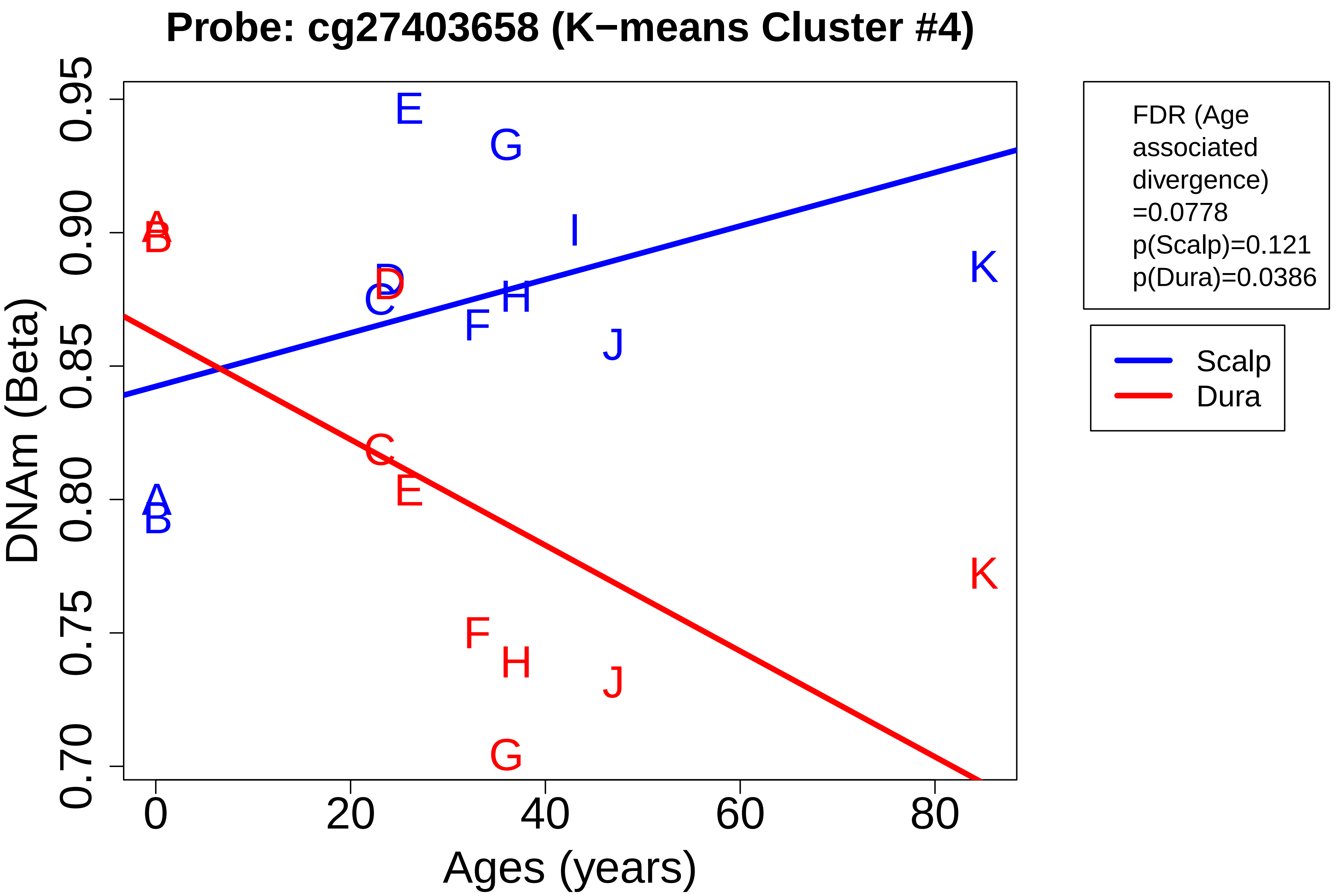

Probe: cg27403658 (K−means Cluster #4)
DNAm (Beta)
0.70
0.75
0.80
0.85
0.90
0.95
Ages (years)
0
20
40
60
80
FDR (Age associated divergence) =0.0778
p(Scalp)=0.121
p(Dura)=0.0386
Scalp
Dura

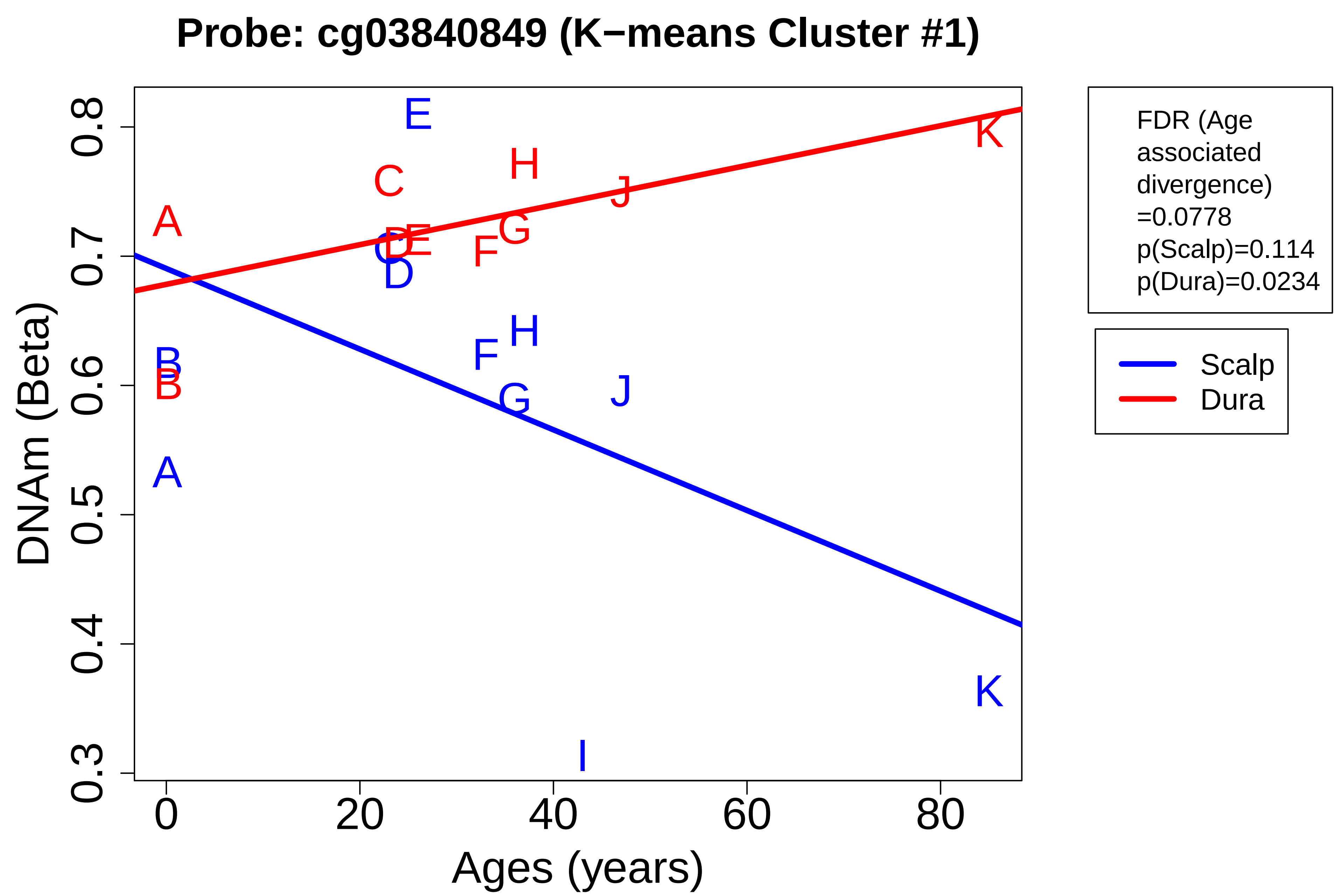

Probe: cg03840849 (K−means Cluster #1)
DNAm (Beta)
0.3
0.4
0.5
0.6
0.7
0.8
0
20
40
60
80
Ages (years)
FDR (Age associated divergence) =0.0778
p(Scalp)=0.114
p(Dura)=0.0234
Scalp
Dura

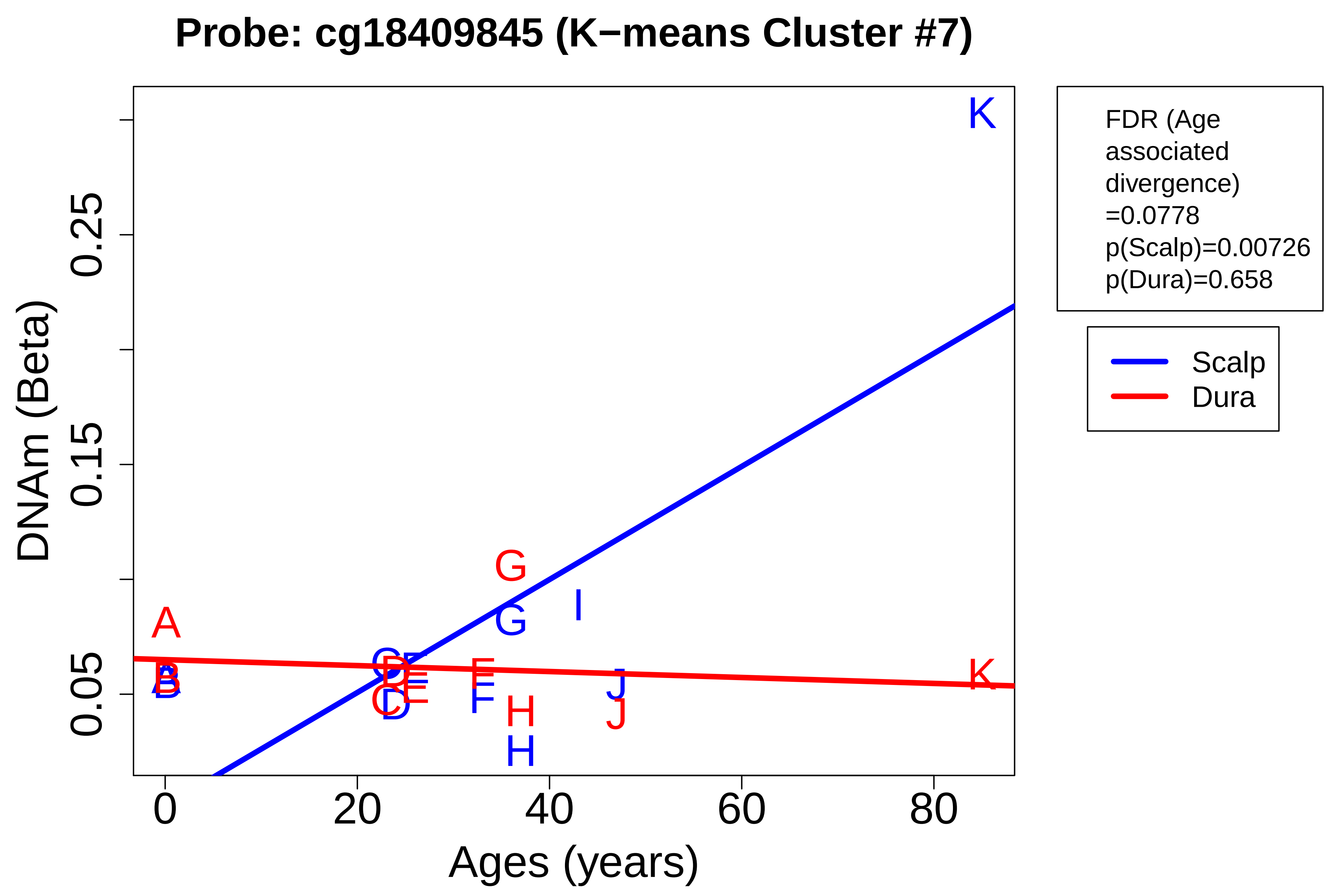

Probe: cg18409845 (K−means Cluster #7)
DNAm (Beta)
0.05
0.15
0.25
0
20
40
60
80
Ages (years)
FDR (Age associated divergence) =0.0778
p(Scalp)=0.00726
p(Dura)=0.658
Scalp
Dura

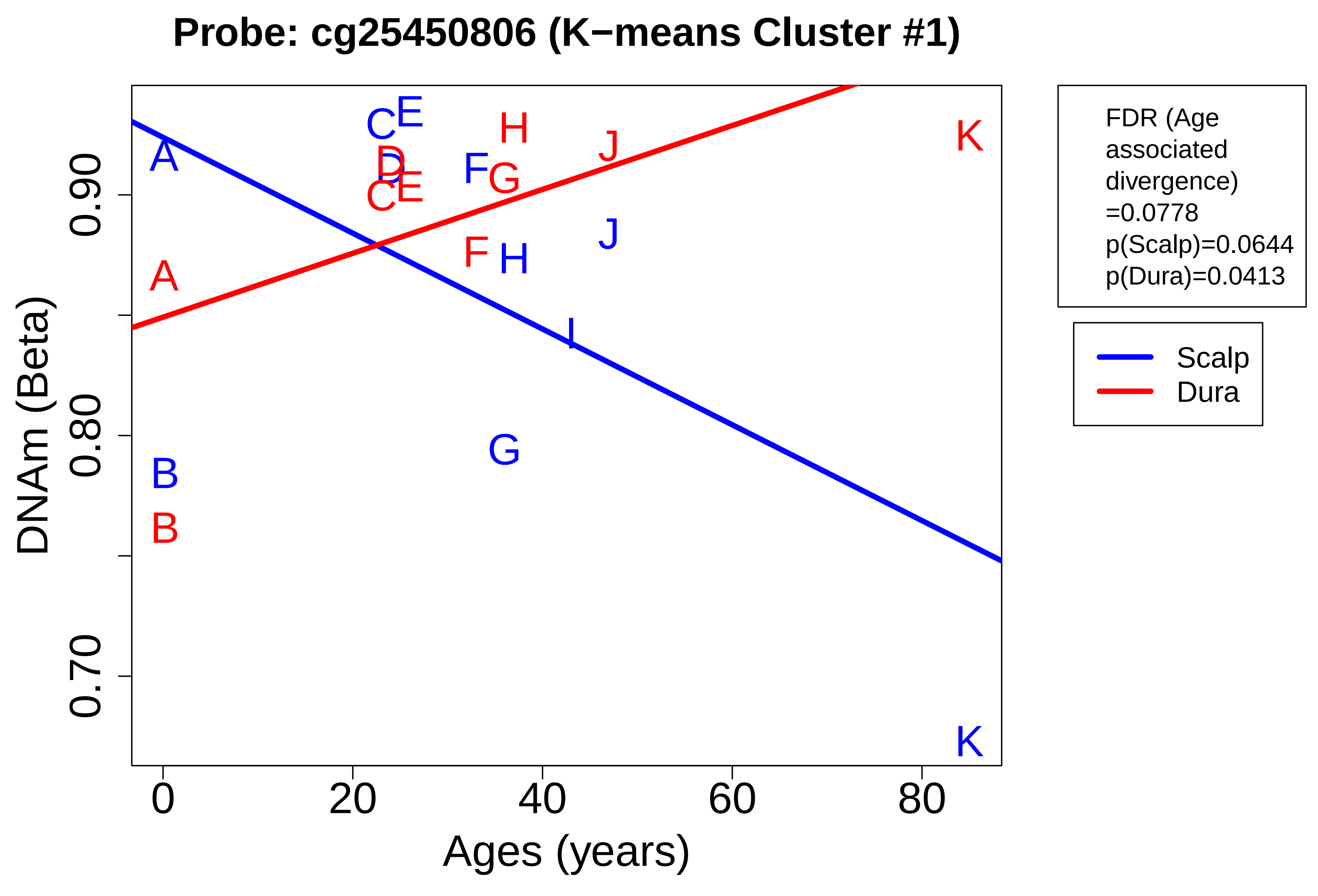

Probe: cg25450806 (K−means Cluster #1)
DNAm (Beta)
0.70
0.80
0.90
0
20
40
60
80
Ages (years)
A
B
C
D
E
F
G
H
I
J
K
FDR (Age associated divergence) =0.0778
p(Scalp)=0.0644
p(Dura)=0.0413
Scalp
Dura

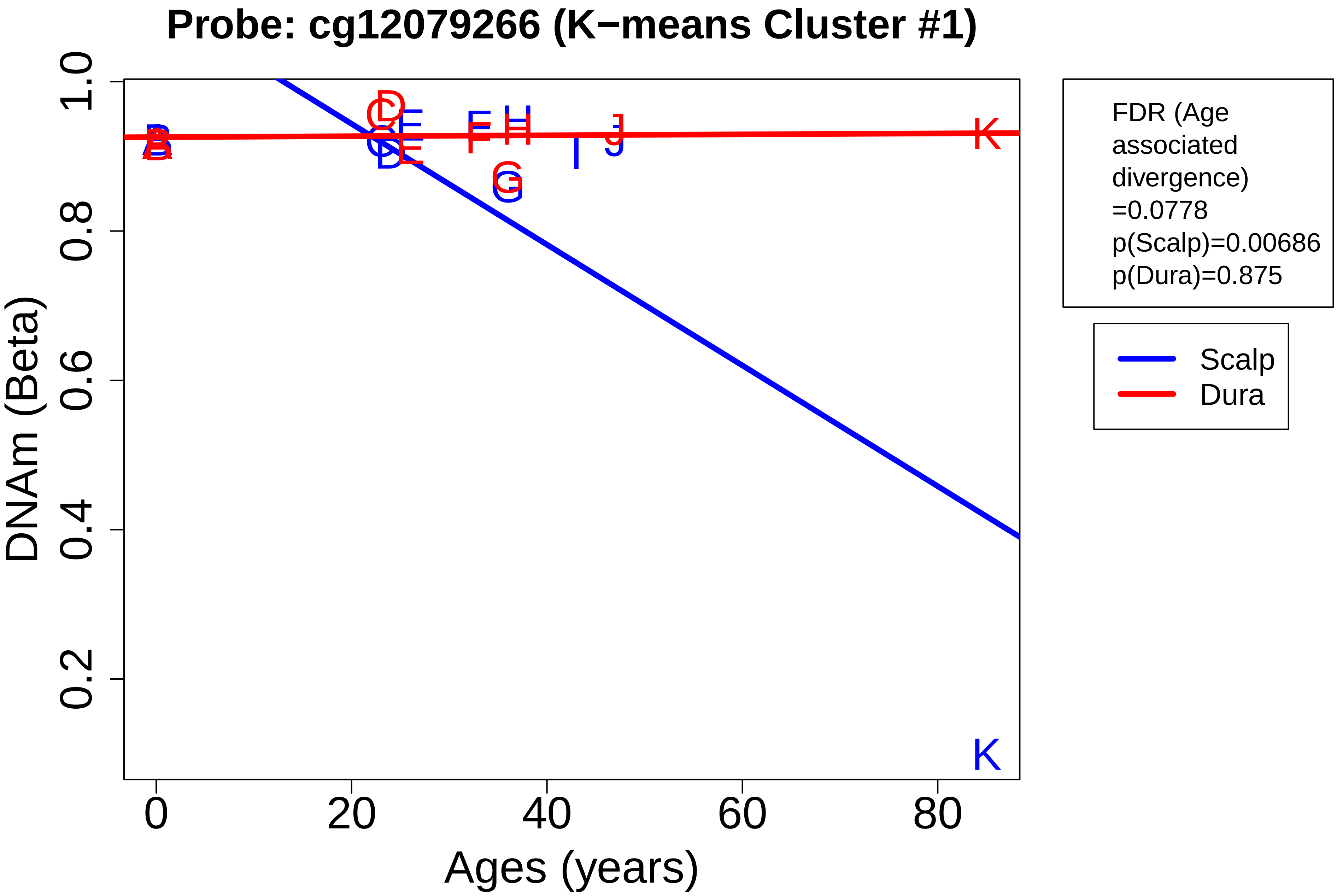

Probe: cg12079266 (K−means Cluster #1)
DNAm (Beta)
1.0
0.8
0.6
0.4
0.2
0
20
40
60
80
Ages (years)
FDR (Age associated divergence) =0.0778
p(Scalp)=0.00686
p(Dura)=0.875
Scalp
Dura

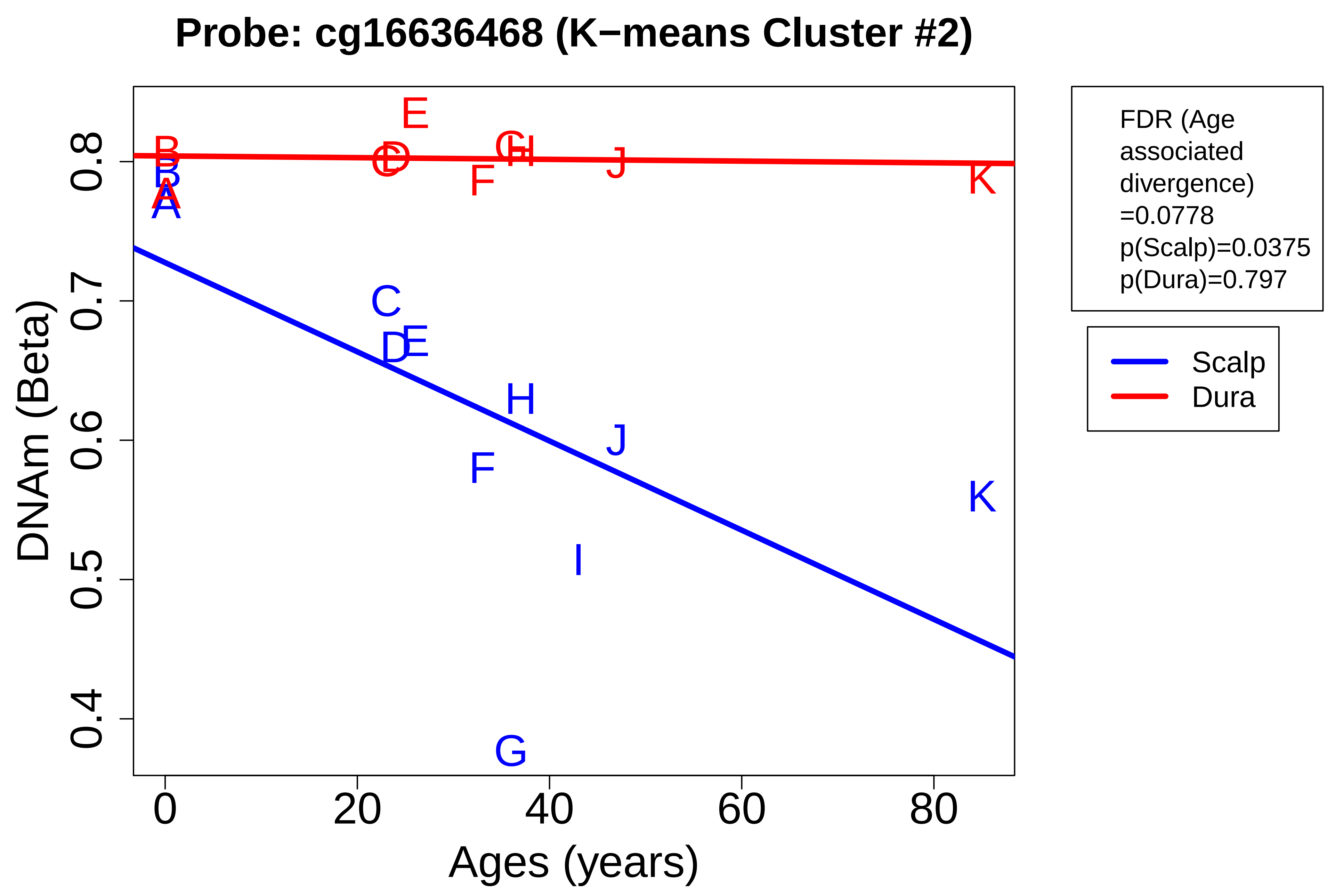

Probe: cg16636468 (K−means Cluster #2)
DNAm (Beta)
Ages (years)
0.4
0.5
0.6
0.7
0.8
0
20
40
60
80
FDR (Age associated divergence) =0.0778
p(Scalp)=0.0375
p(Dura)=0.797
Scalp
Dura

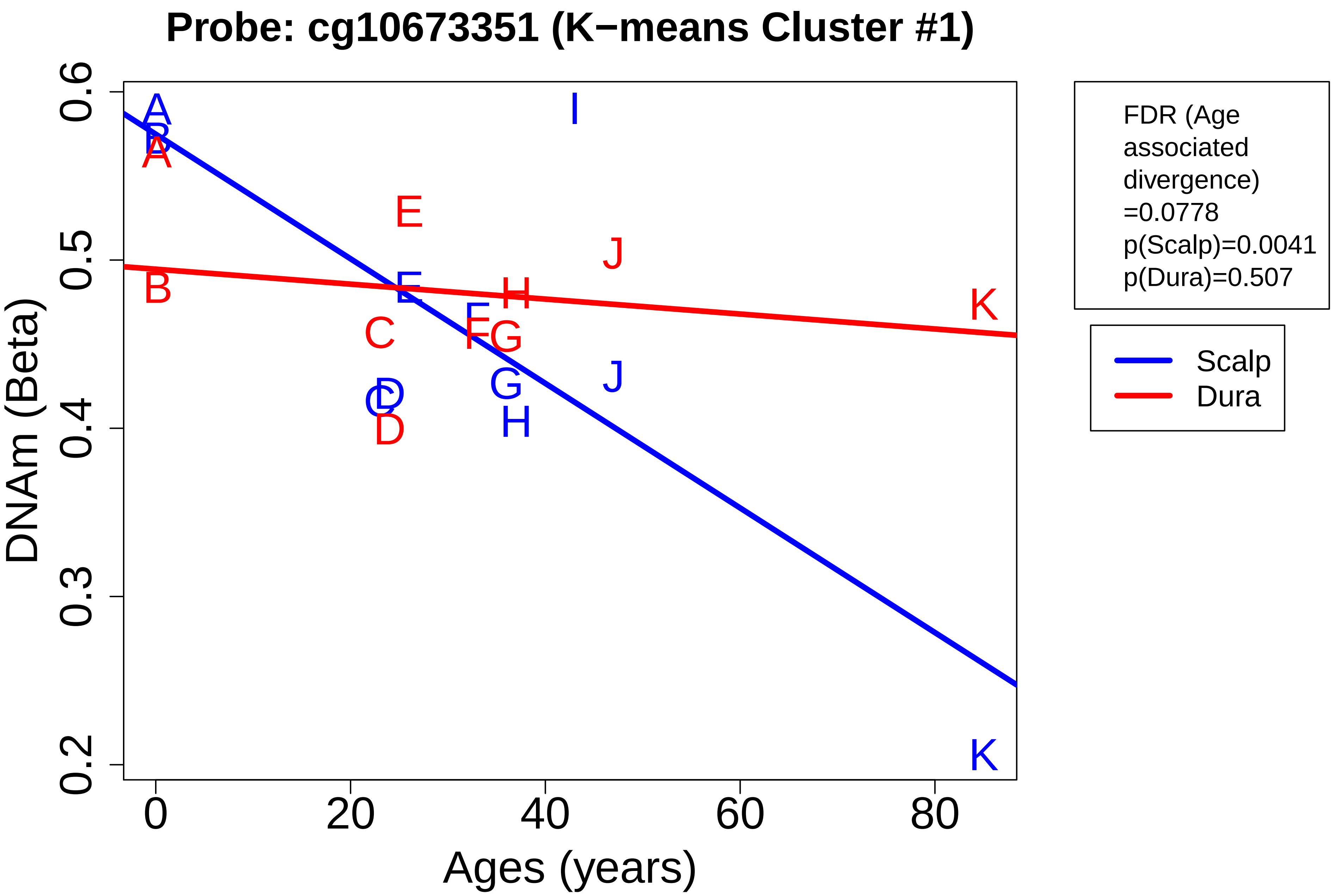

Probe: cg10673351 (K−means Cluster #1)
DNAm (Beta)
0.2
0.3
0.4
0.5
0.6
Ages (years)
0
20
40
60
80
FDR (Age associated divergence) =0.0778
p(Scalp)=0.0041
p(Dura)=0.507
Scalp
Dura
A
B
C
D
E
F
G
H
I
J
K

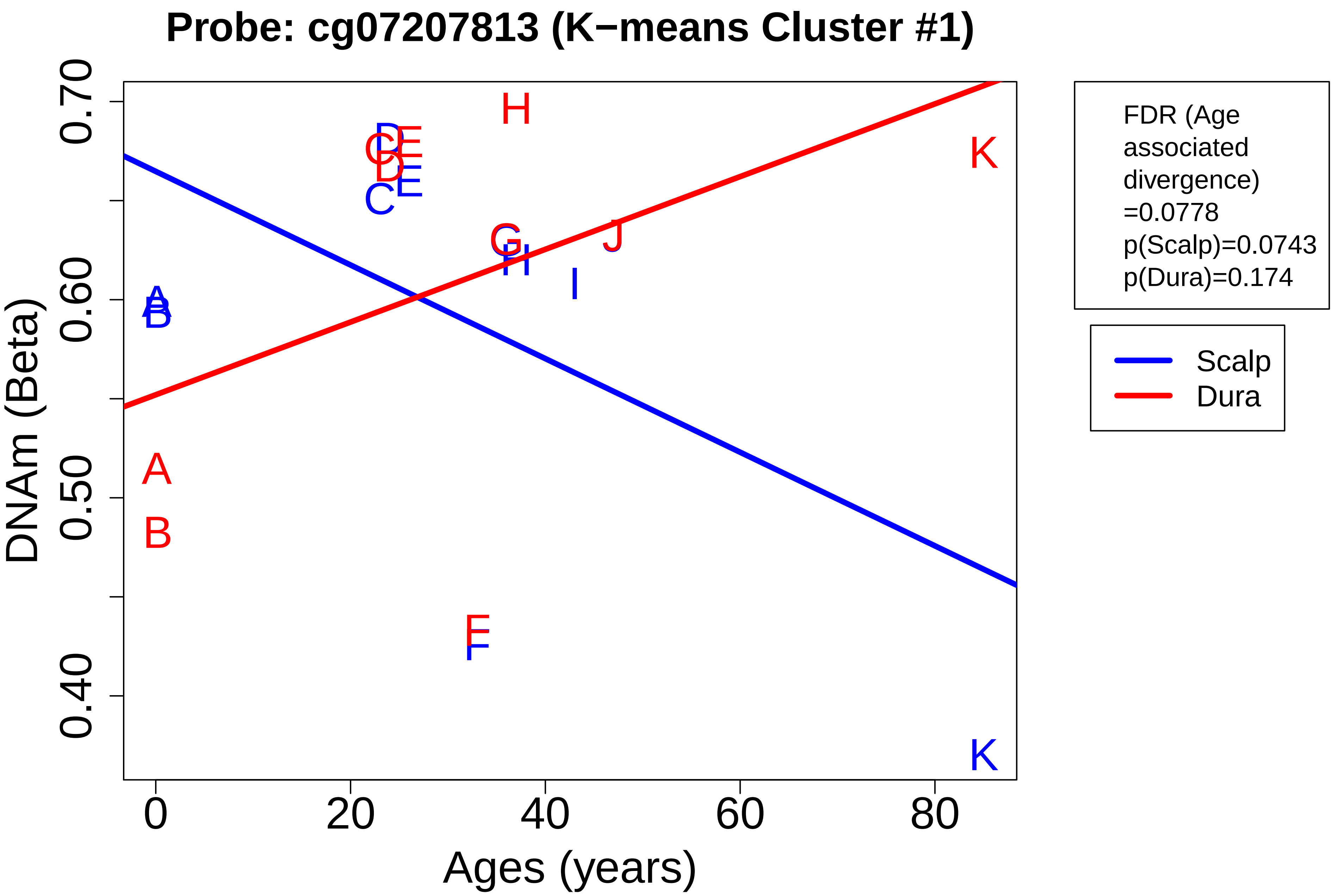

Probe: cg07207813 (K−means Cluster #1)
DNAm (Beta)
0.40
0.50
0.60
0.70
0
20
40
60
80
Ages (years)
FDR (Age associated divergence) =0.0778
p(Scalp)=0.0743
p(Dura)=0.174
Scalp
Dura

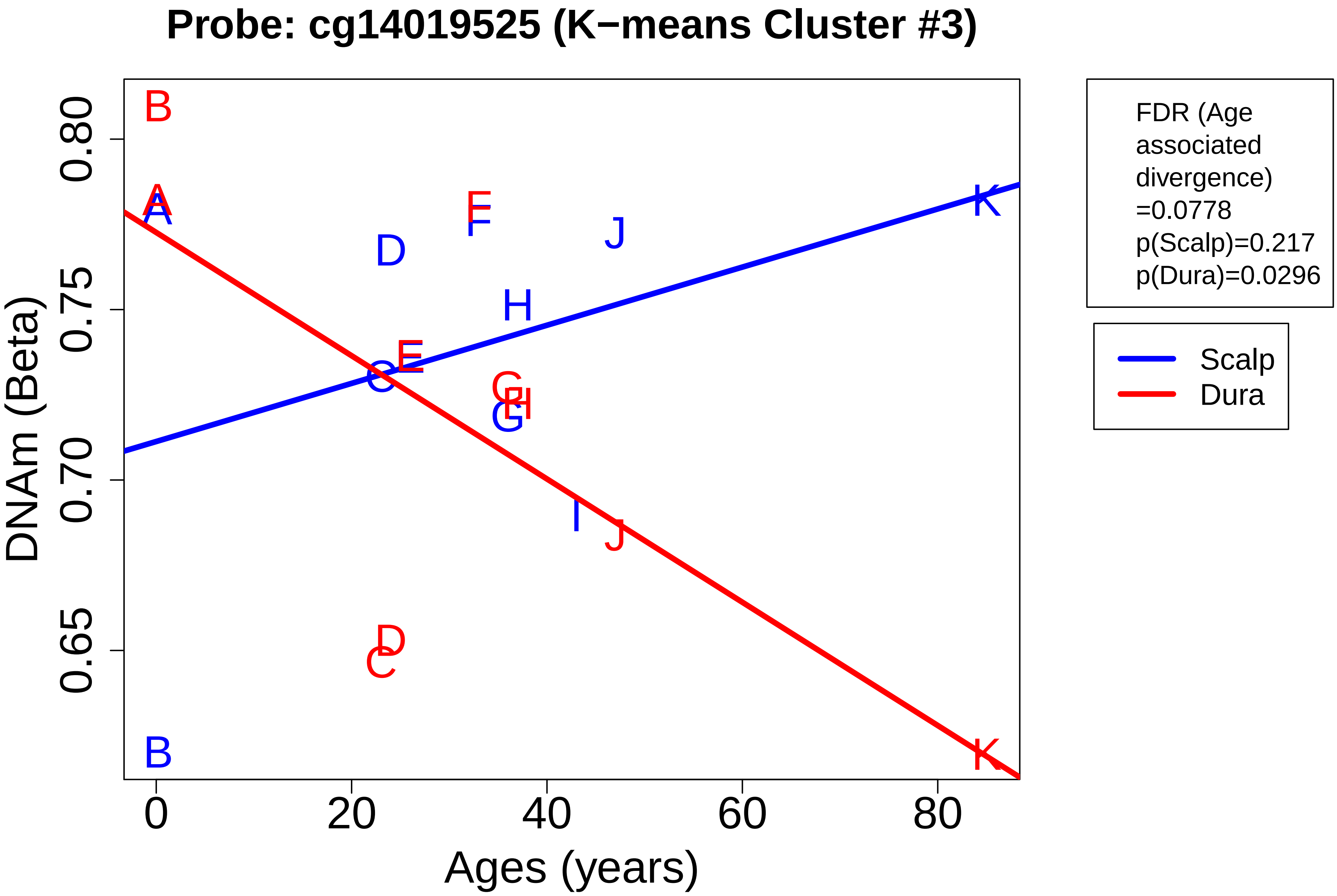
Probe: cg14019525 (K−means Cluster #3)
FDR (Age associated divergence) =0.0778
p(Scalp)=0.217
p(Dura)=0.0296
Scalp
Dura
DNAm (Beta)
0.65
0.70
0.75
0.80
0
20
40
60
80
Ages (years)
A
B
C
D
E
F
G
H
I
J
K

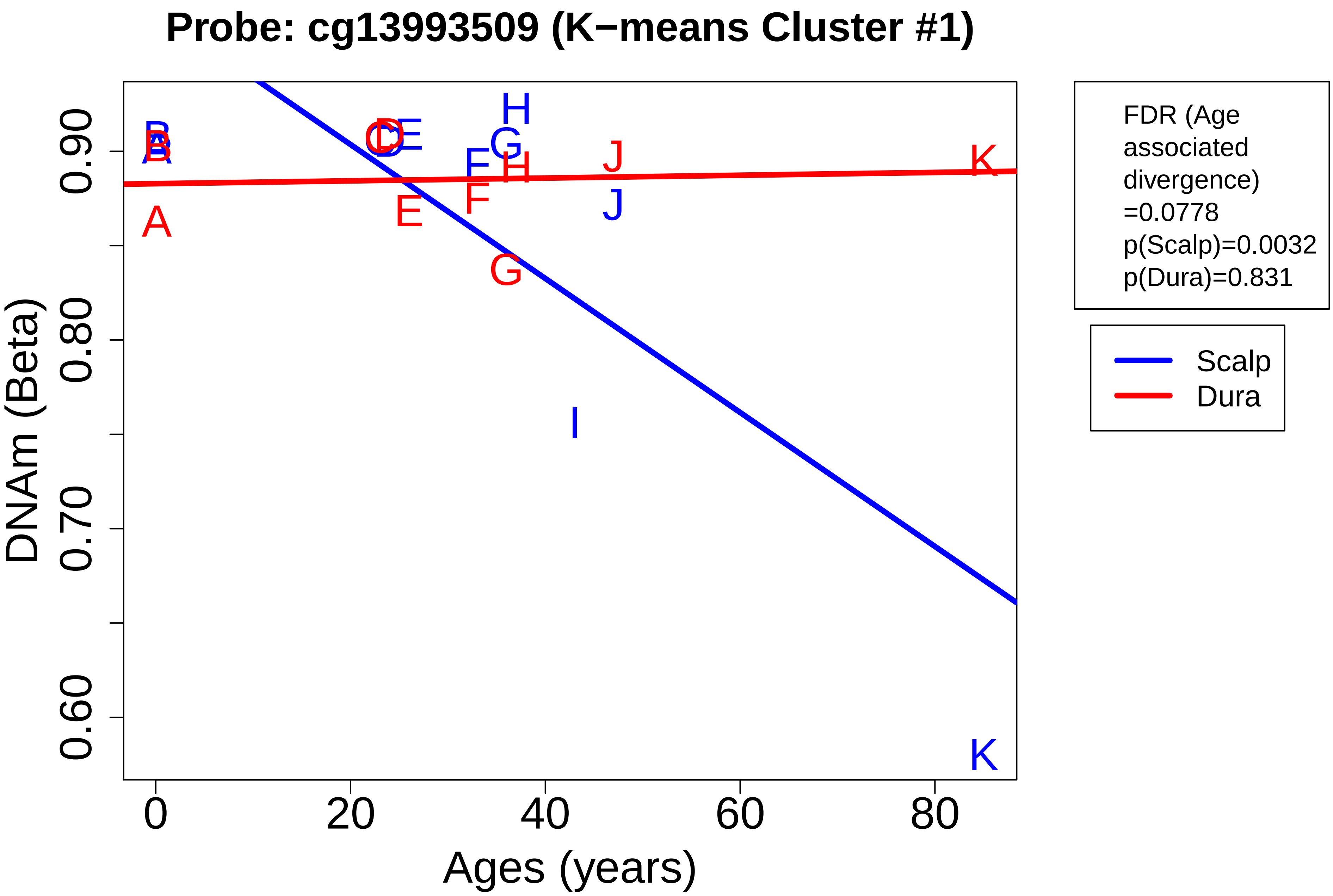

Probe: cg13993509 (K−means Cluster #1)
DNAm (Beta)
0.60
0.70
0.80
0.90
Ages (years)
0
20
40
60
80
FDR (Age associated divergence) =0.0778
p(Scalp)=0.0032
p(Dura)=0.831
Scalp
Dura

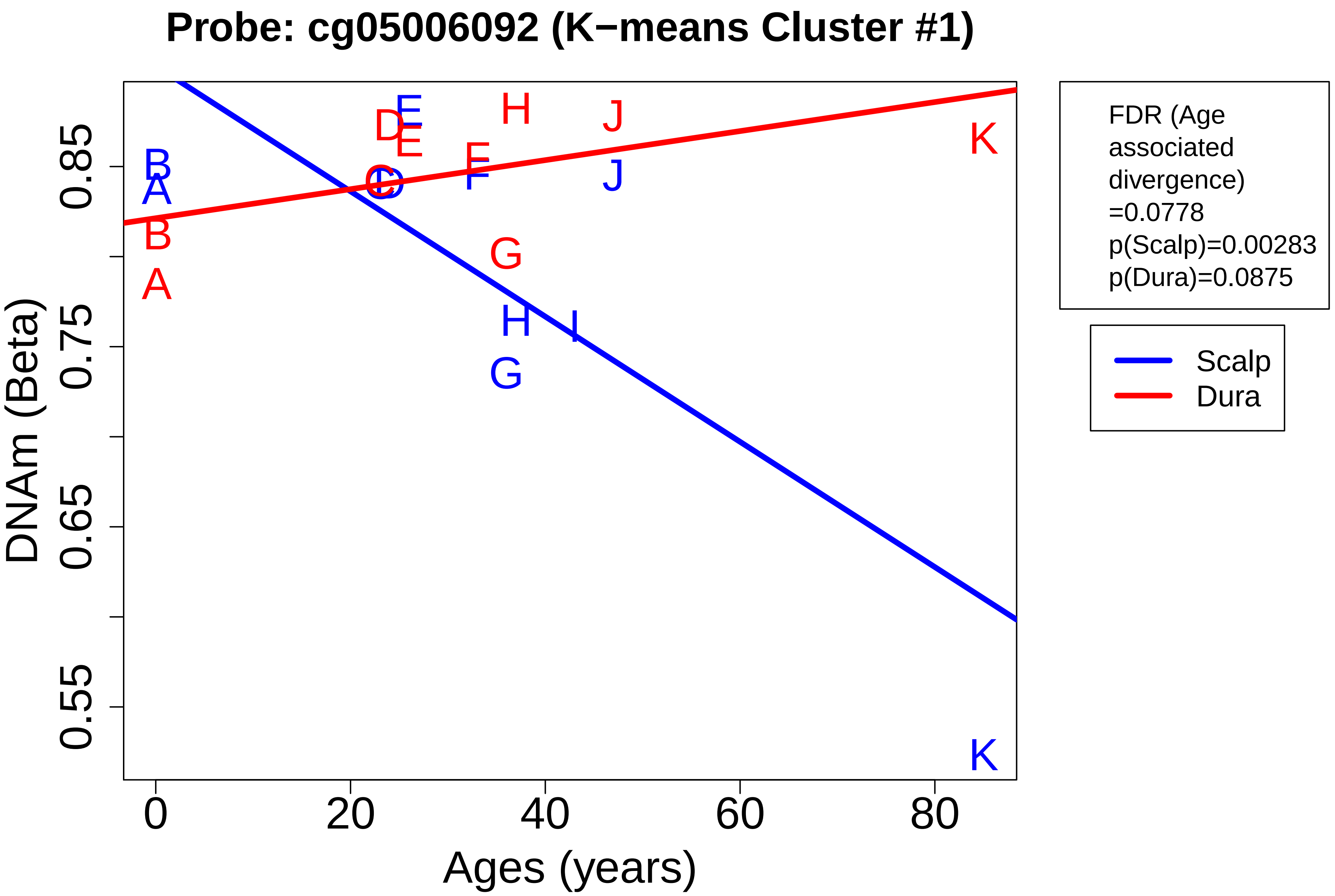

Probe: cg05006092 (K−means Cluster #1)
DNAm (Beta)
0.55
0.65
0.75
0.85
0
20
40
60
80
Ages (years)
FDR (Age associated divergence) =0.0778
p(Scalp)=0.00283
p(Dura)=0.0875
Scalp
Dura

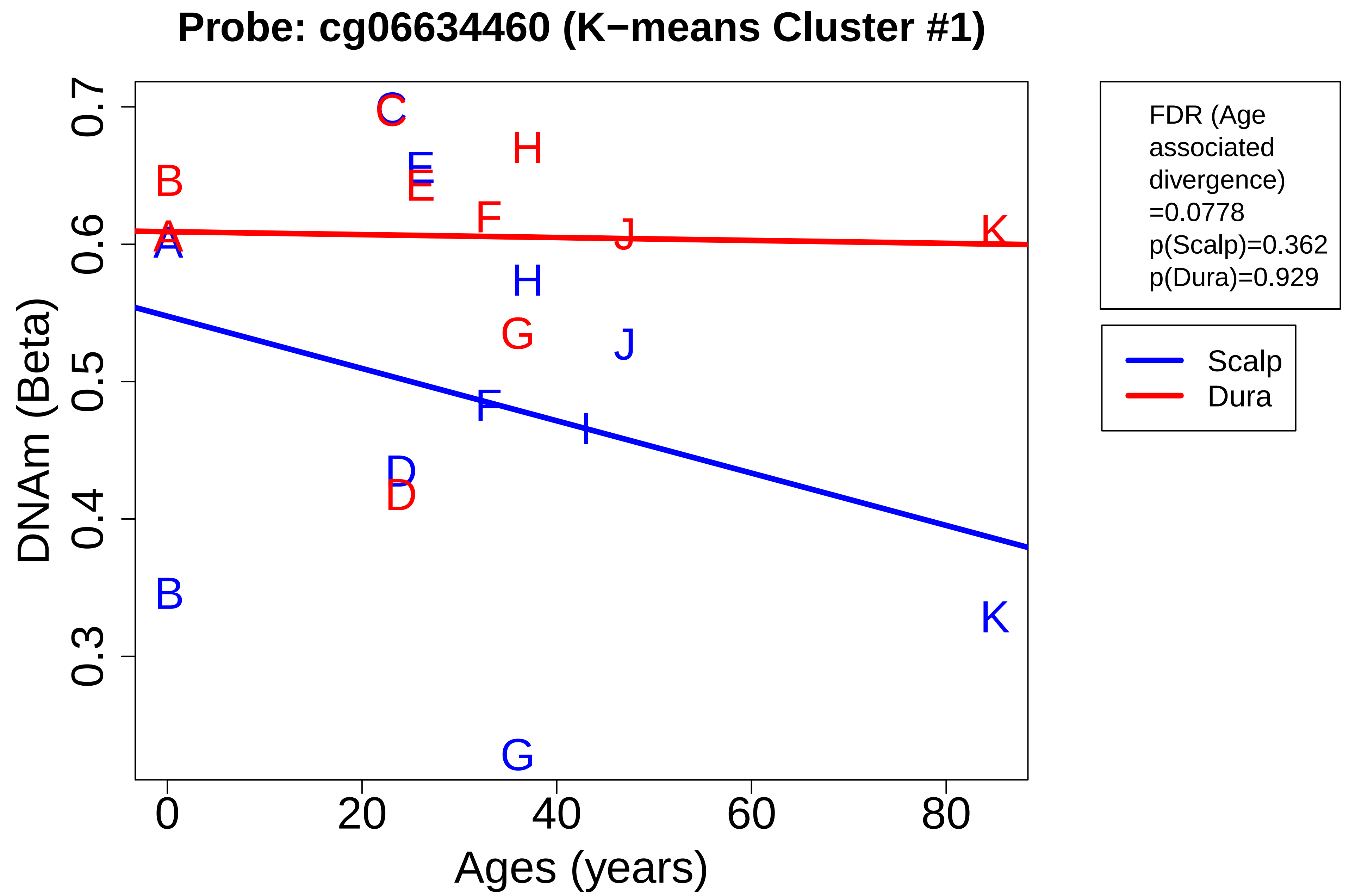

Probe: cg06634460 (K−means Cluster #1)
DNAm (Beta)
0.3
0.4
0.5
0.6
0.7
0
20
40
60
80
Ages (years)
A
B
C
D
E
F
G
H
I
J
K
FDR (Age associated divergence) =0.0778
p(Scalp)=0.362
p(Dura)=0.929
Scalp
Dura

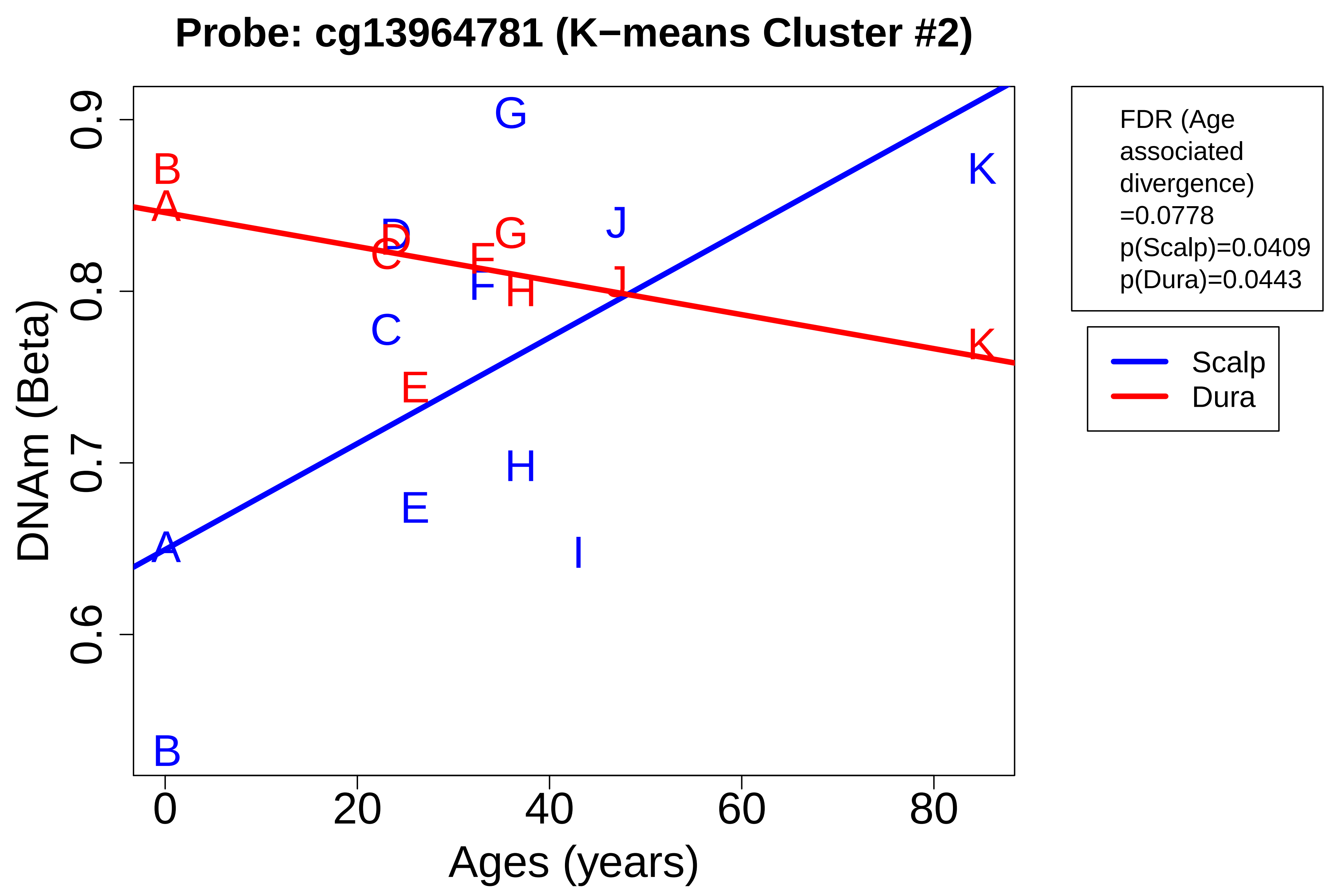

Probe: cg13964781 (K−means Cluster #2)
DNAm (Beta)
0.6
0.7
0.8
0.9
Ages (years)
0
20
40
60
80
FDR (Age associated divergence) =0.0778
p(Scalp)=0.0409
p(Dura)=0.0443
Scalp
Dura

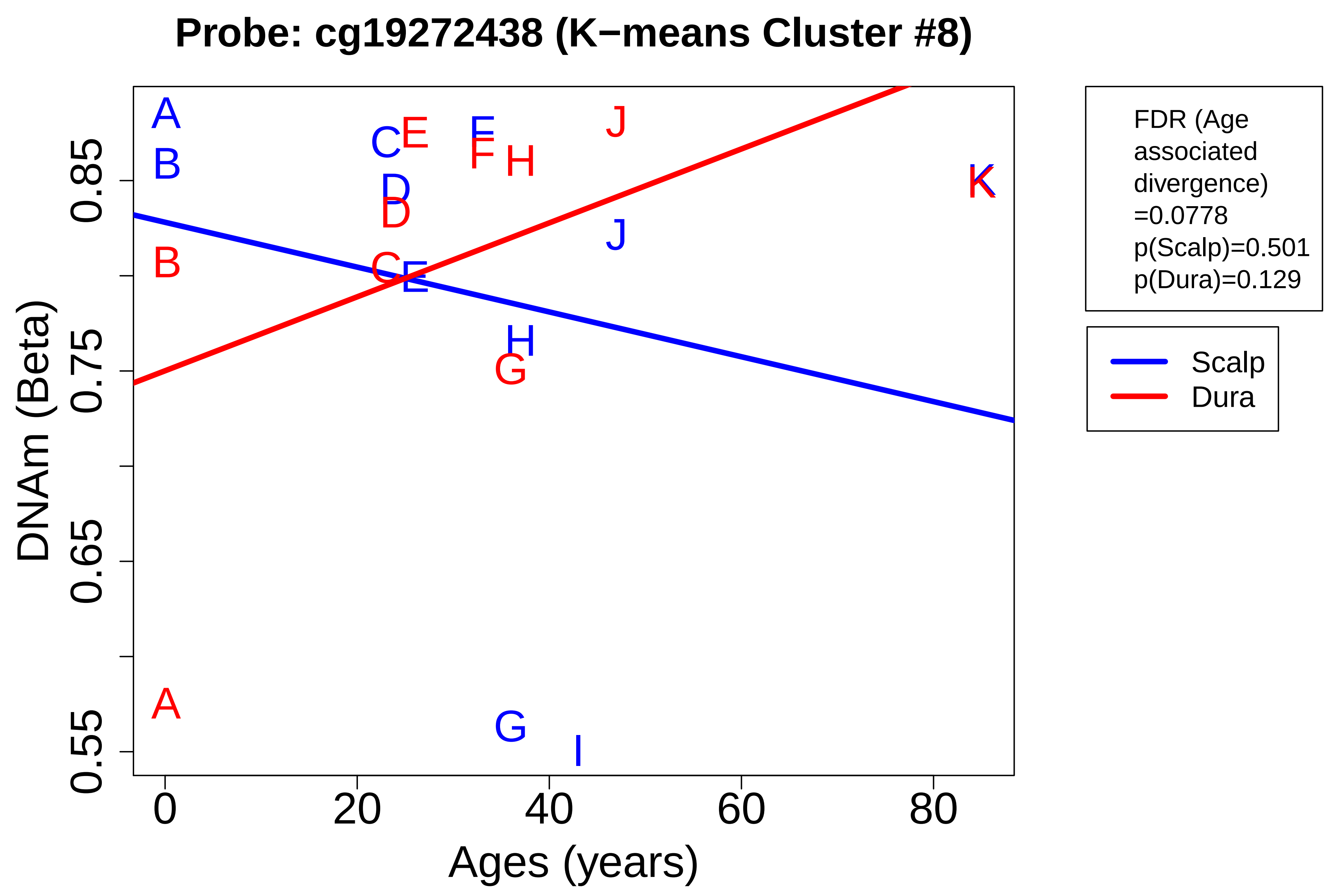

Probe: cg19272438 (K−means Cluster #8)
DNAm (Beta)
0.55
0.65
0.75
0.85
Ages (years)
0
20
40
60
80
A
B
C
D
E
F
G
H
I
J
K
FDR (Age associated divergence) =0.0778
p(Scalp)=0.501
p(Dura)=0.129
Scalp
Dura

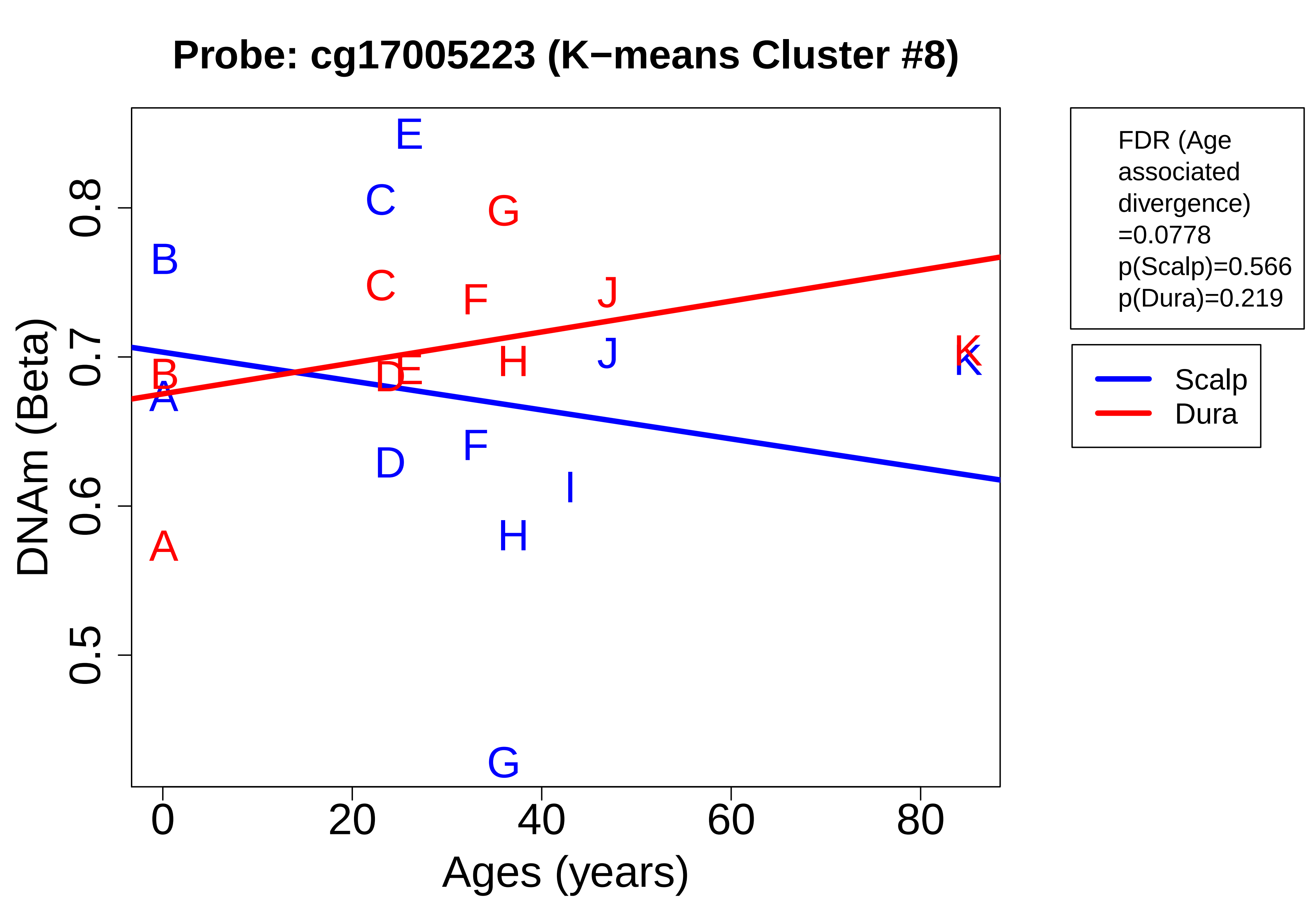

Probe: cg17005223 (K−means Cluster #8)
FDR (Age associated divergence) =0.0778
p(Scalp)=0.566
p(Dura)=0.219
Scalp
Dura
DNAm (Beta)
0.5
0.6
0.7
0.8
0
20
40
60
80
Ages (years)

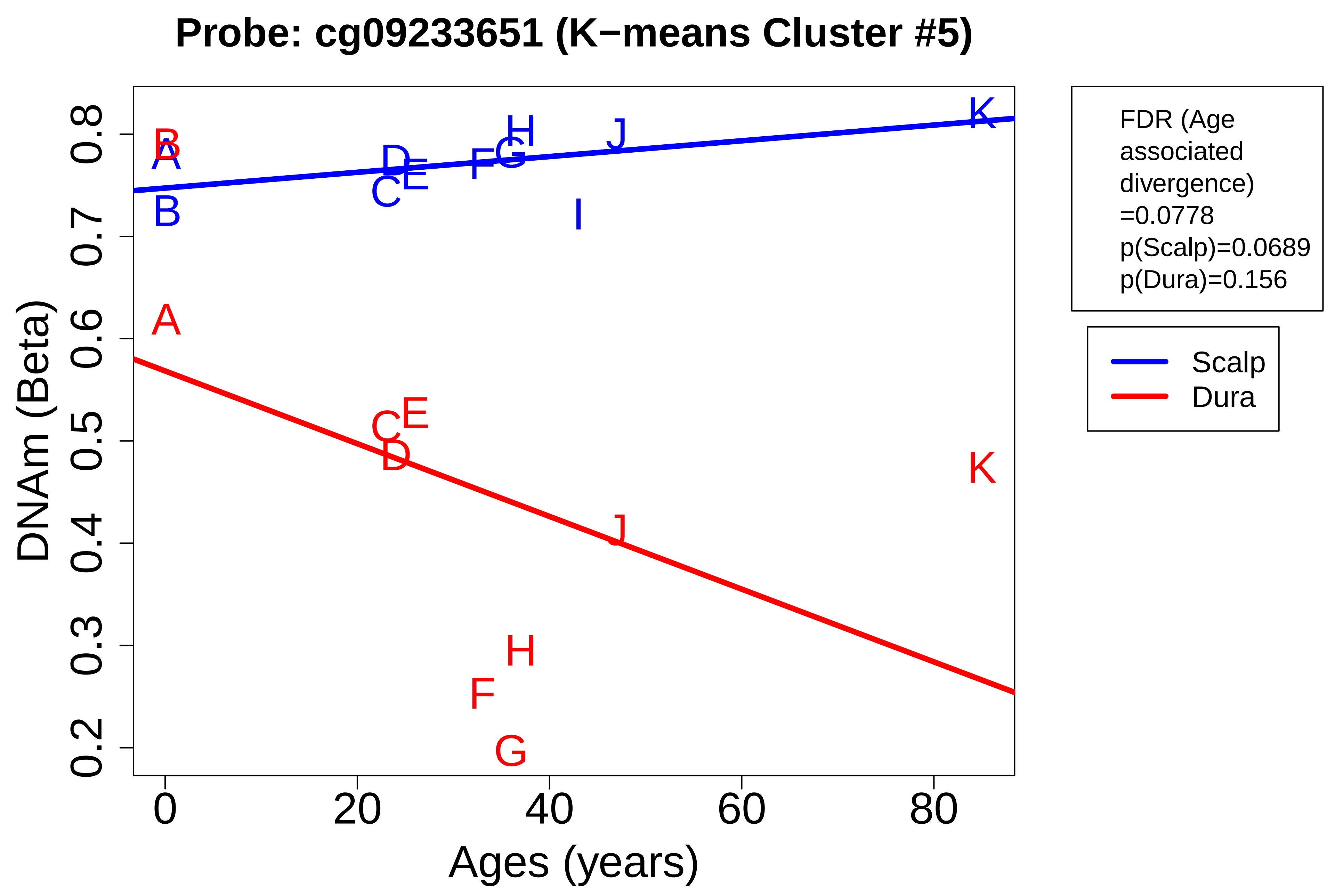

Probe: cg09233651 (K−means Cluster #5)
DNAm (Beta)
0.2
0.3
0.4
0.5
0.6
0.7
0.8
0
20
40
60
80
Ages (years)
FDR (Age associated divergence) =0.0778
p(Scalp)=0.0689
p(Dura)=0.156
Scalp
Dura
A
B
C
D
E
F
G
H
I
J
K

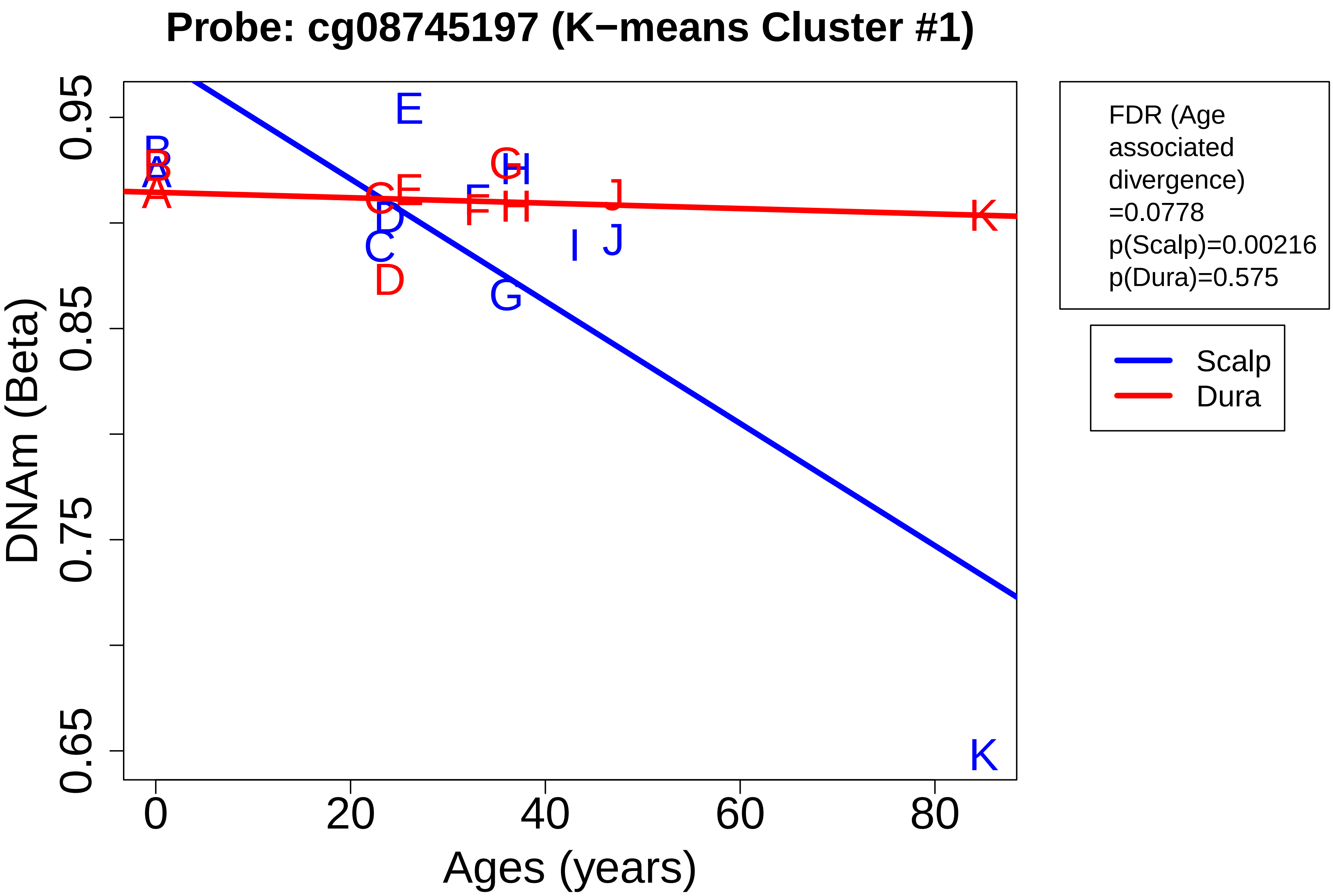

Probe: cg08745197 (K−means Cluster #1)
DNAm (Beta)
0.65
0.75
0.85
0.95
0
20
40
60
80
Ages (years)
FDR (Age associated divergence) =0.0778
p(Scalp)=0.00216
p(Dura)=0.575
Scalp
Dura

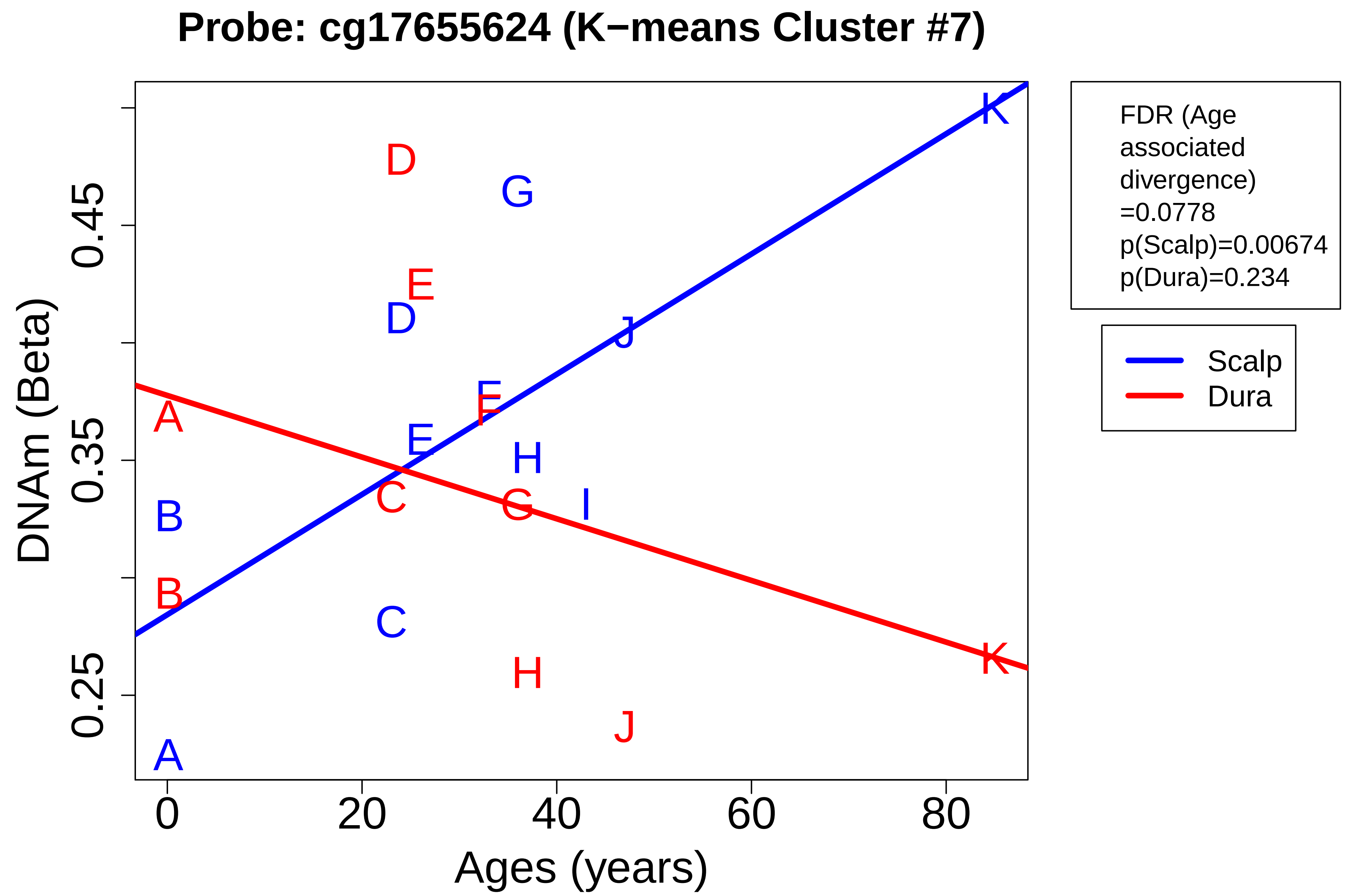

Probe: cg17655624 (K−means Cluster #7)
DNAm (Beta)
0.25
0.35
0.45
0
20
40
60
80
Ages (years)
FDR (Age associated divergence) =0.0778
p(Scalp)=0.00674
p(Dura)=0.234
Scalp
Dura
A
B
C
D
E
F
G
H
I
J
K

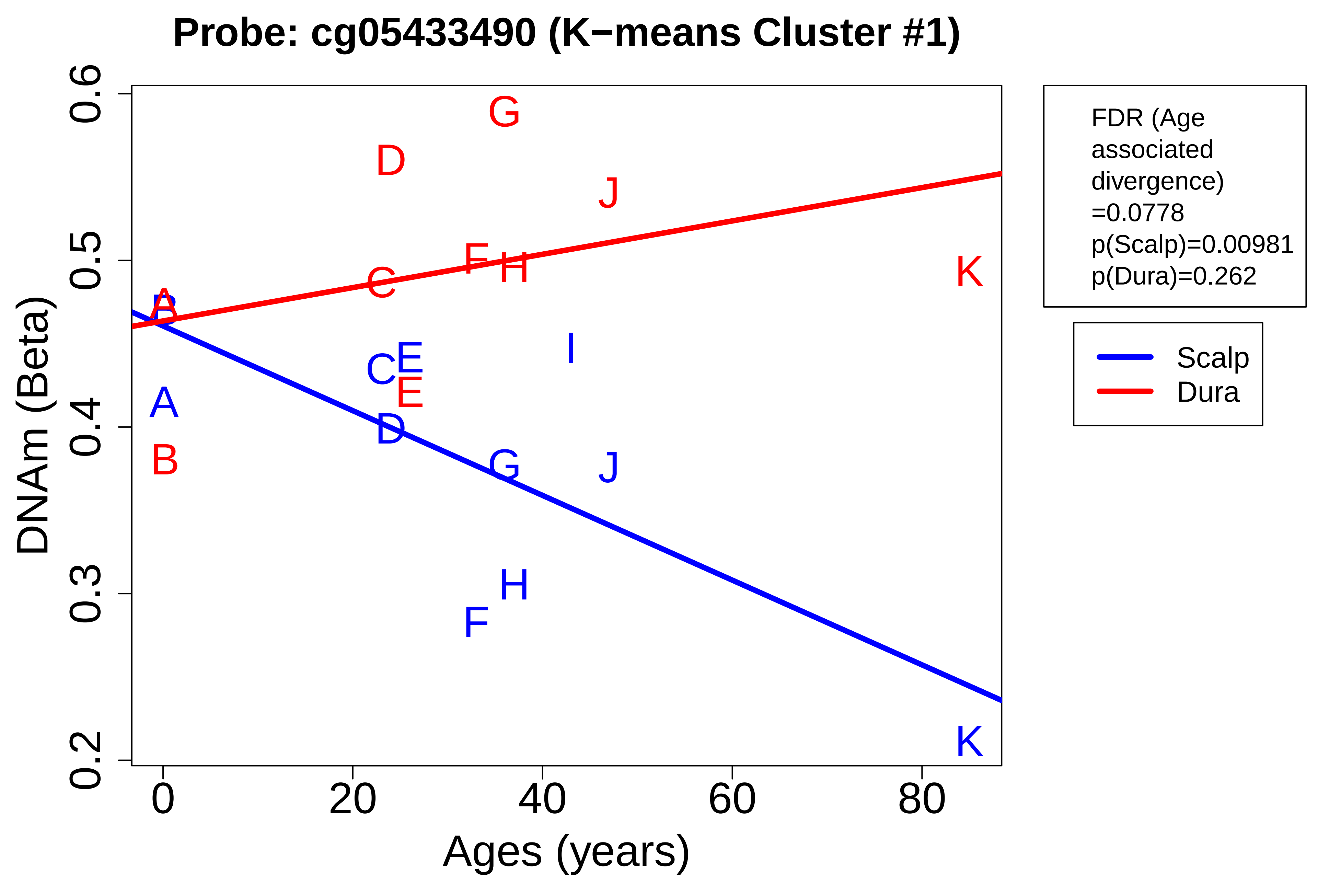
Probe: cg05433490 (K−means Cluster #1)
DNAm (Beta)
Ages (years)
0.2
0.3
0.4
0.5
0.6
0
20
40
60
80
FDR (Age associated divergence) =0.0778
p(Scalp)=0.00981
p(Dura)=0.262
Scalp
Dura
A
B
C
D
E
F
G
H
I
J
K

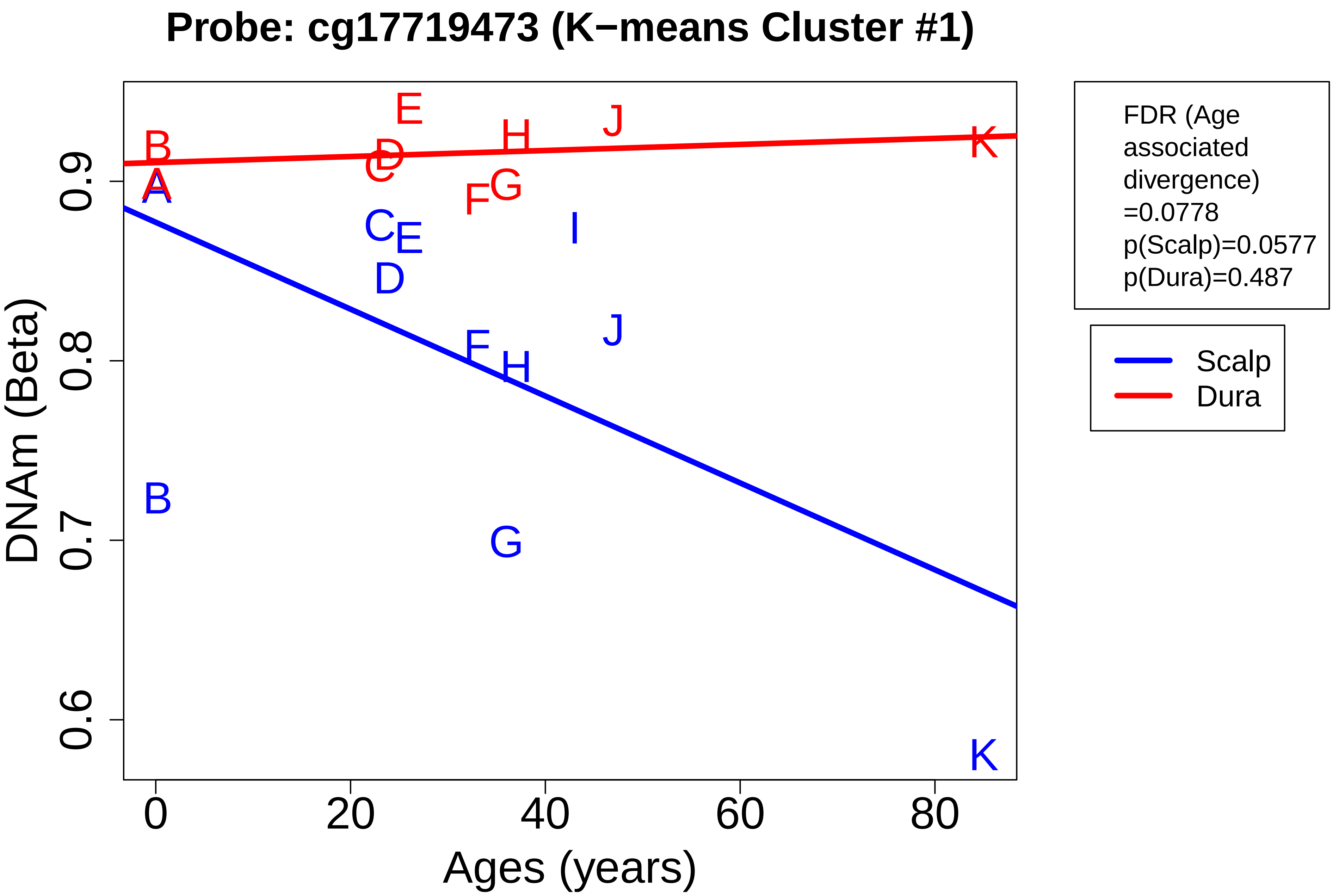

Probe: cg17719473 (K−means Cluster #1)
FDR (Age associated divergence) =0.0778
p(Scalp)=0.0577
p(Dura)=0.487
Scalp
Dura
DNAm (Beta)
0.6
0.7
0.8
0.9
Ages (years)
0
20
40
60
80

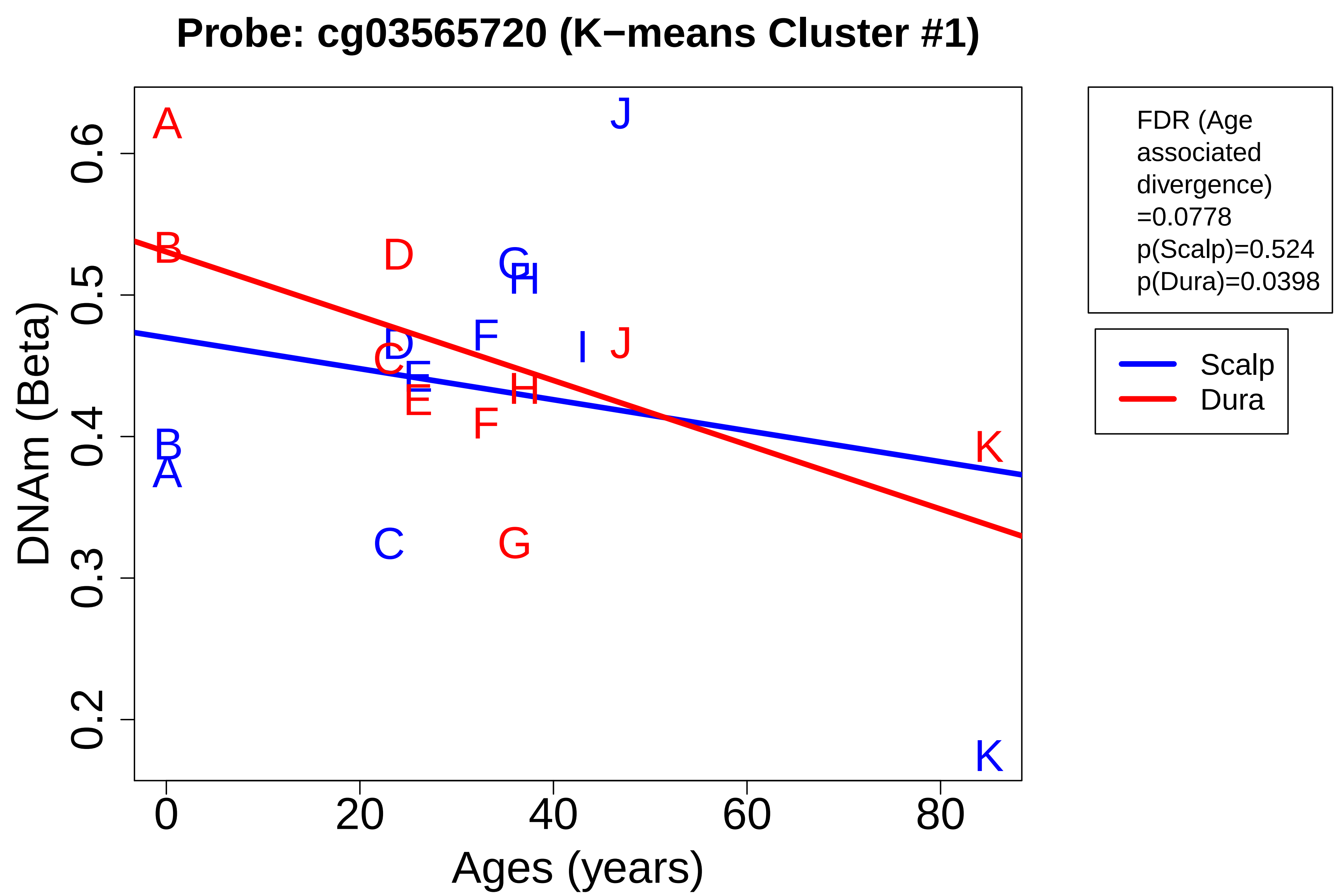

Probe: cg03565720 (K−means Cluster #1)
DNAm (Beta)
0.2
0.3
0.4
0.5
0.6
0
20
40
60
80
Ages (years)
A
B
C
D
E
F
G
H
I
J
K
FDR (Age associated divergence) =0.0778
p(Scalp)=0.524
p(Dura)=0.0398
Scalp
Dura

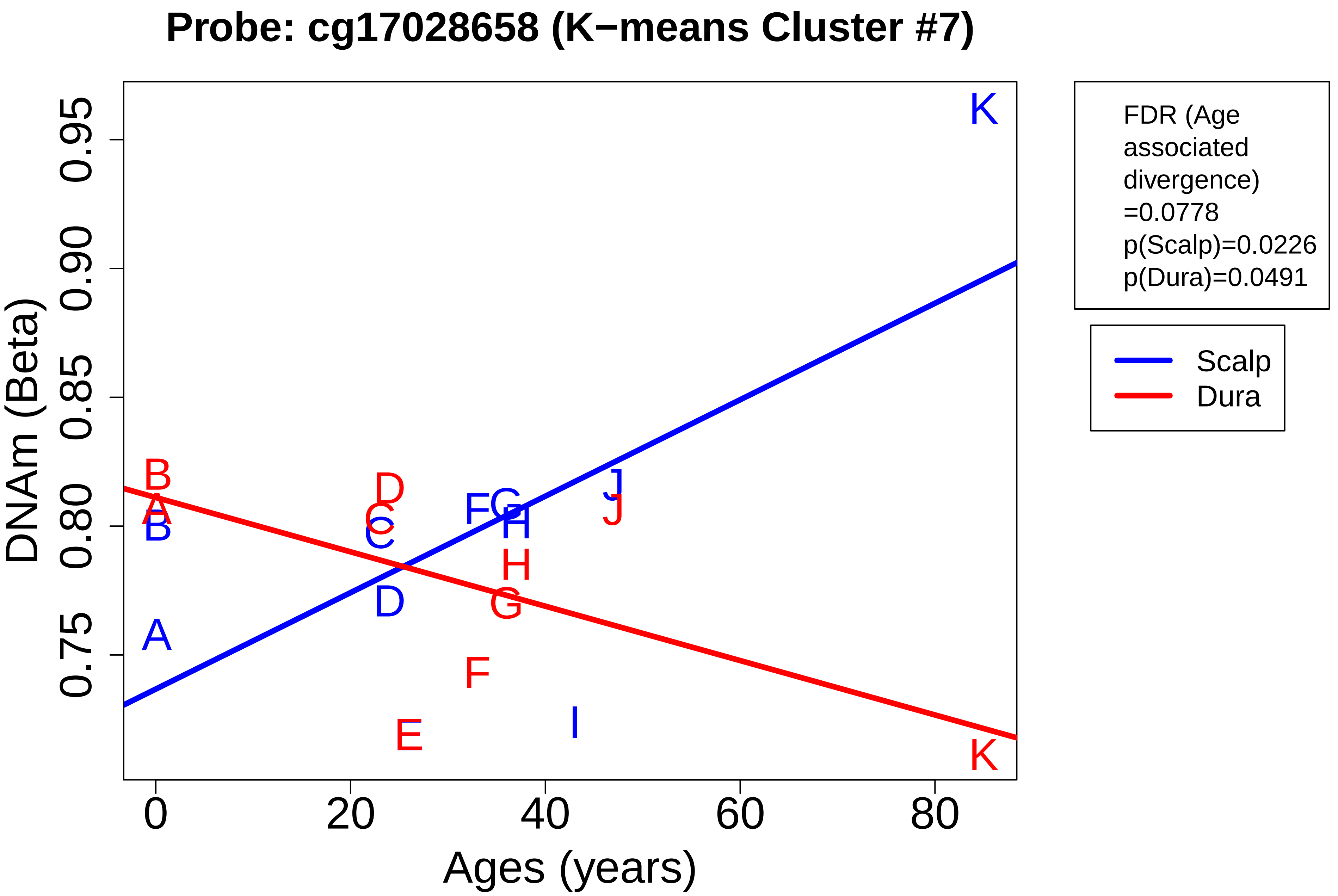

Probe: cg17028658 (K−means Cluster #7)
DNAm (Beta)
0.75
0.80
0.85
0.90
0.95
0
20
40
60
80
Ages (years)
FDR (Age associated divergence) =0.0778
p(Scalp)=0.0226
p(Dura)=0.0491
Scalp
Dura
A
B
C
D
E
F
G
H
I
J
K

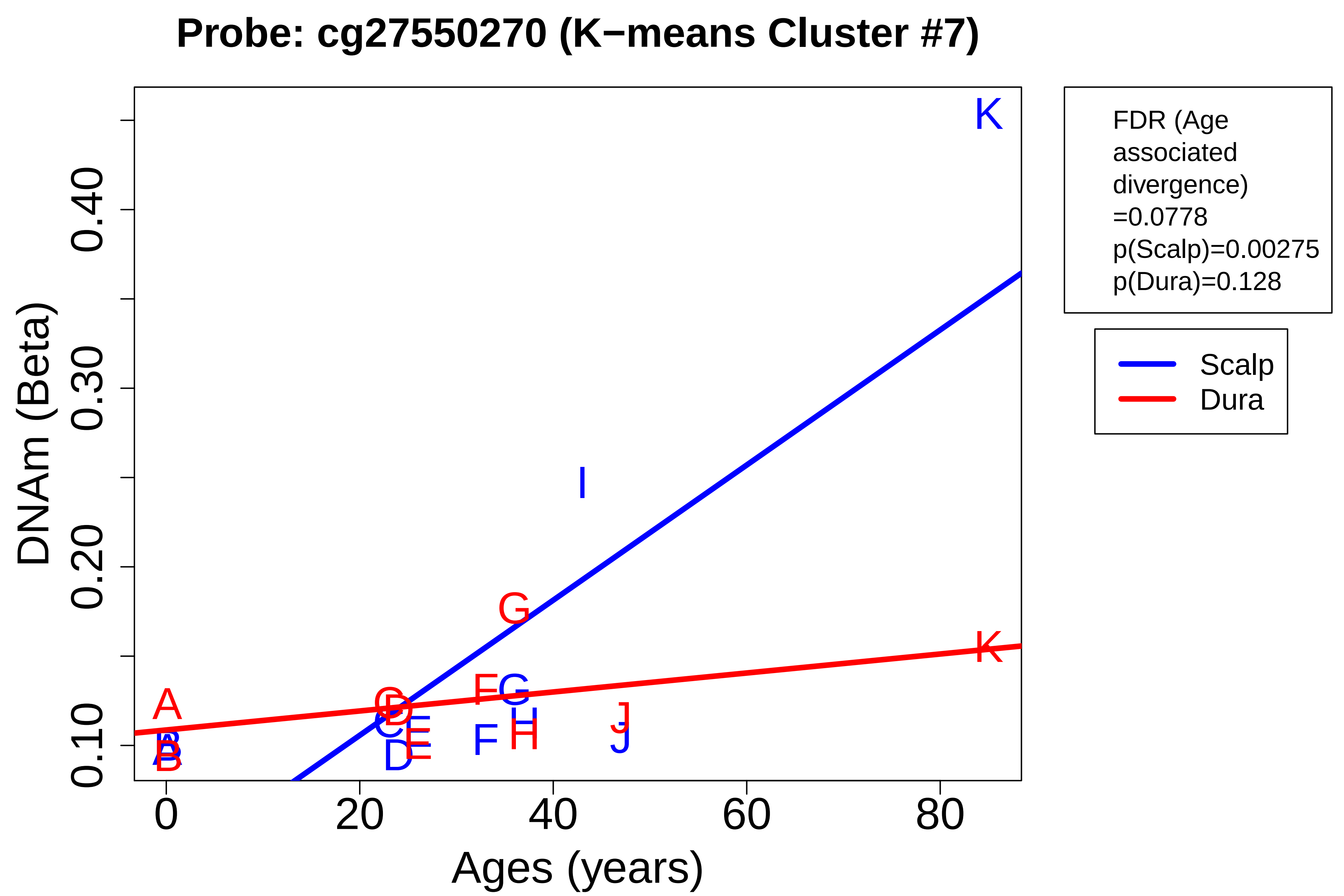

Probe: cg27550270 (K−means Cluster #7)
DNAm (Beta)
0.10
0.20
0.30
0.40
0
20
40
60
80
Ages (years)
FDR (Age associated divergence) =0.0778
p(Scalp)=0.00275
p(Dura)=0.128
Scalp
Dura

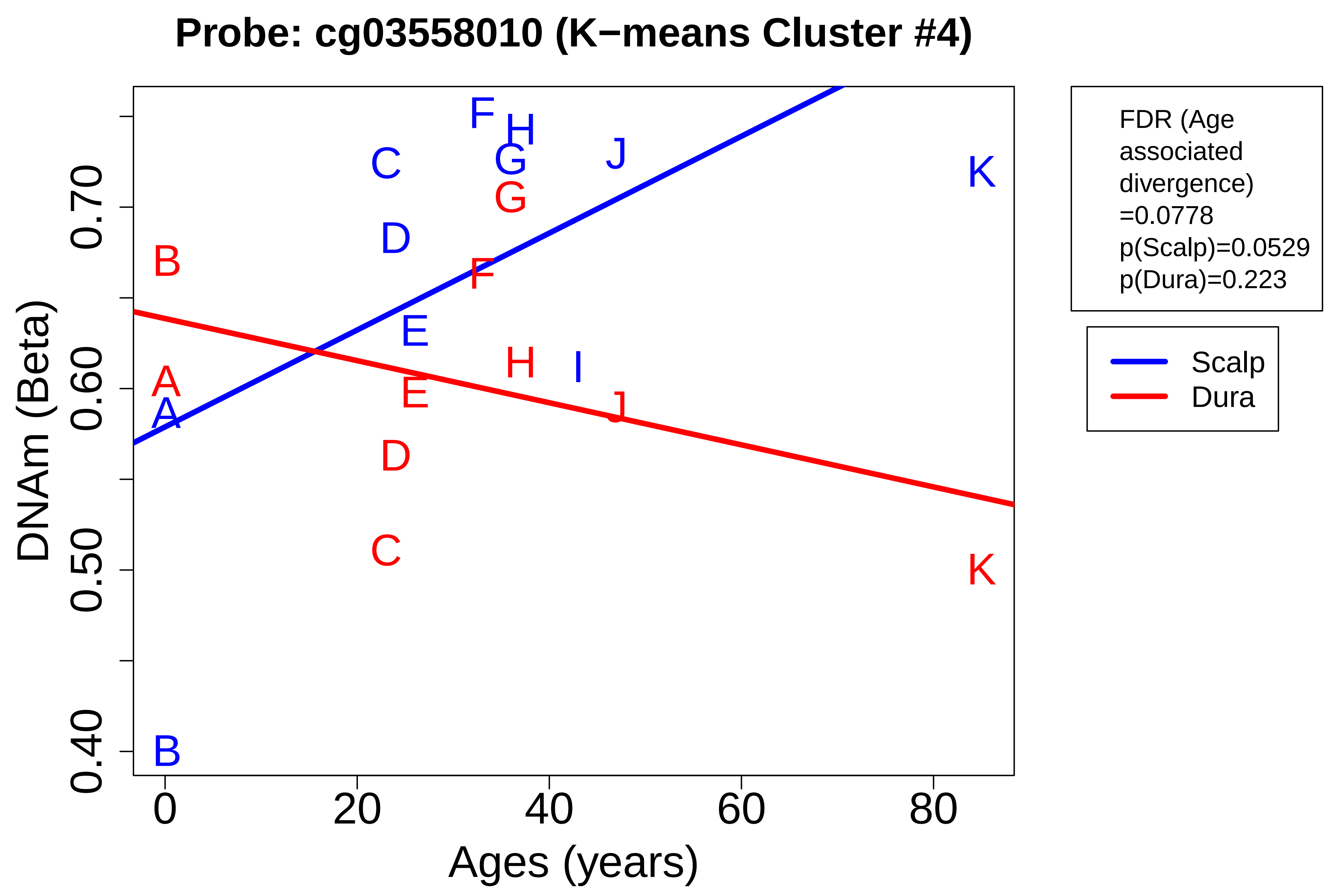
Probe: cg03558010 (K−means Cluster #4)
DNAm (Beta)
0.40
0.50
0.60
0.70
0
20
40
60
80
Ages (years)
FDR (Age associated divergence) =0.0778
p(Scalp)=0.0529
p(Dura)=0.223
Scalp
Dura

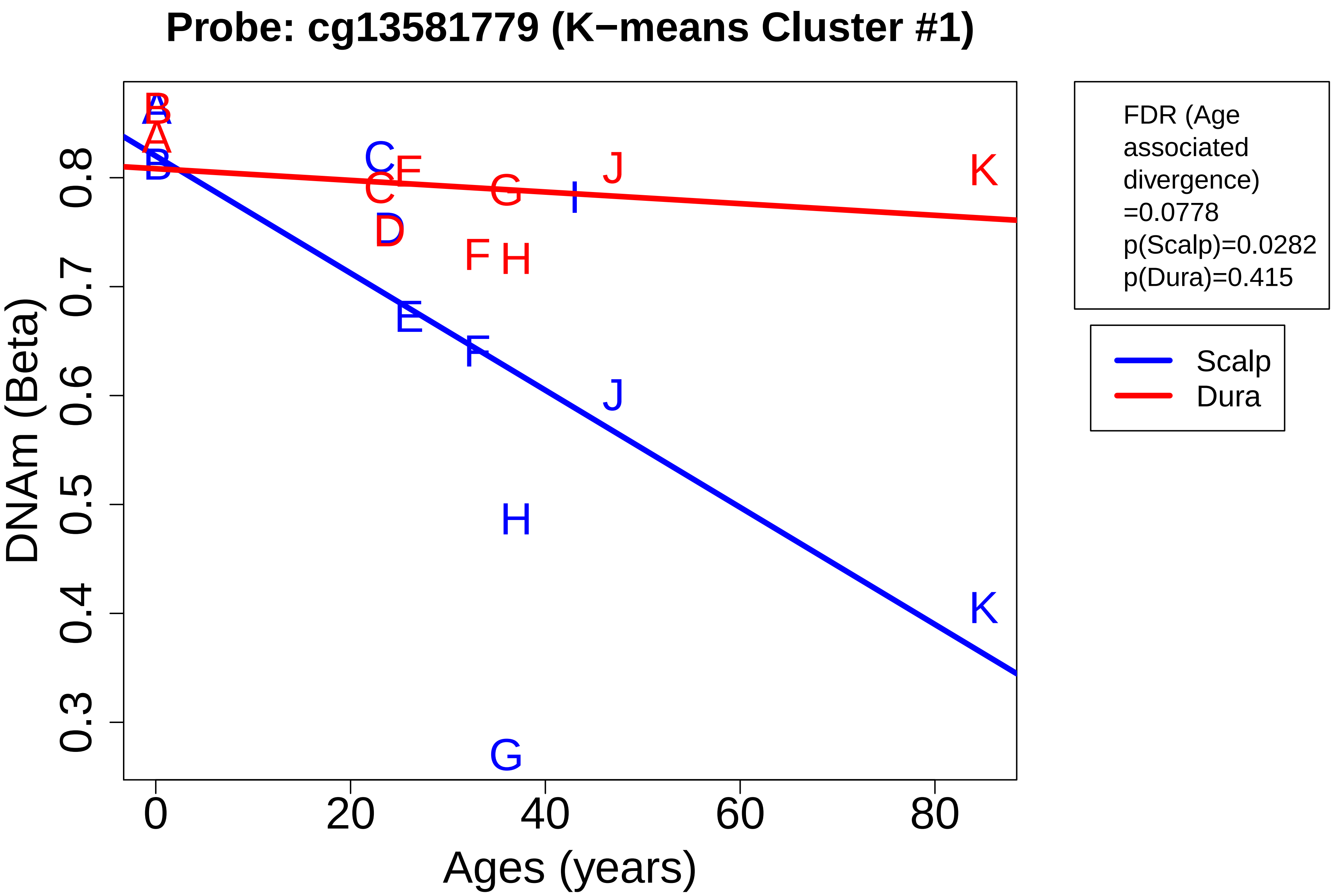
Probe: cg13581779 (K−means Cluster #1)
FDR (Age associated divergence) =0.0778
p(Scalp)=0.0282
p(Dura)=0.415
Scalp
Dura
DNAm (Beta)
0.3
0.4
0.5
0.6
0.7
0.8
0
20
40
60
80
Ages (years)

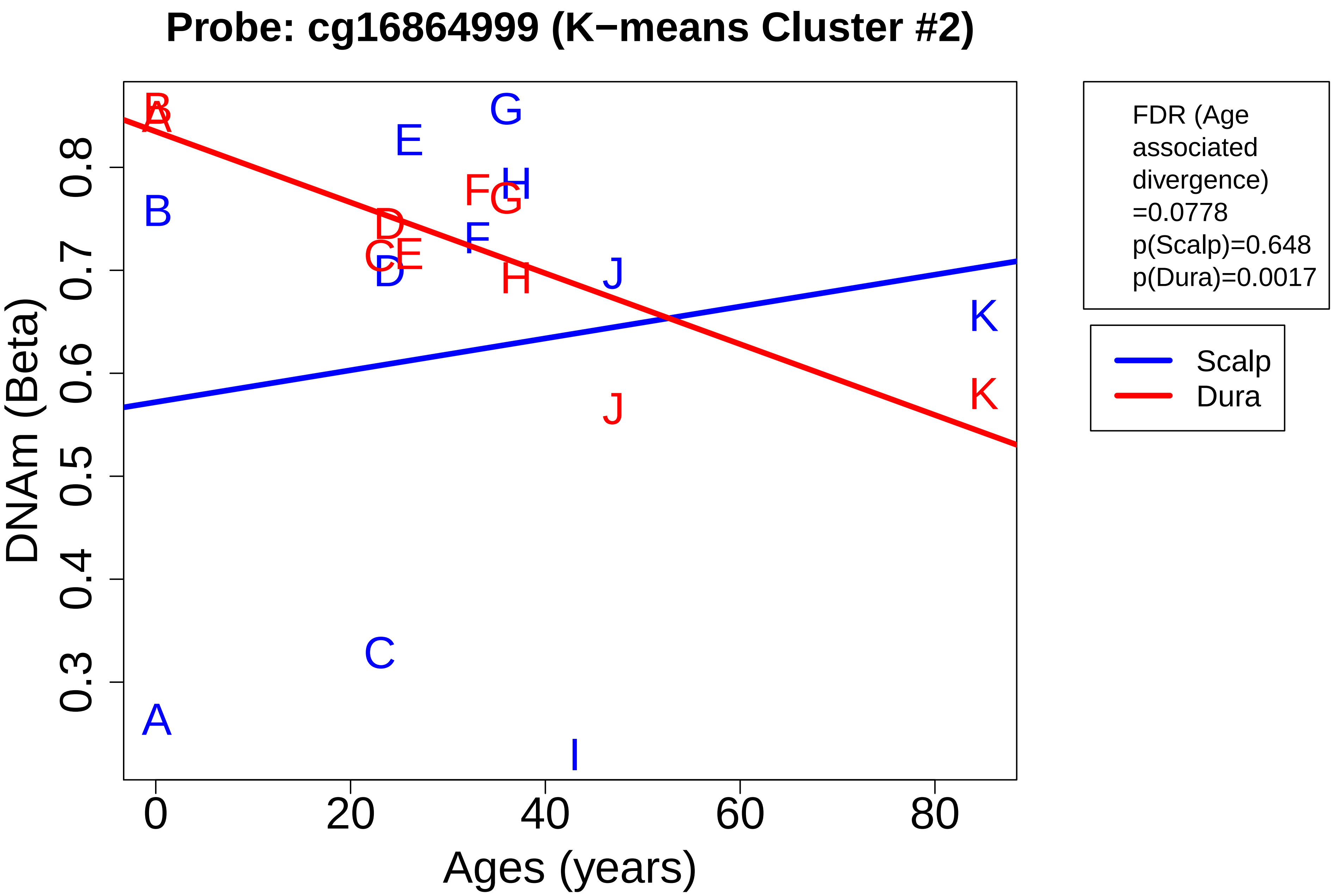

Probe: cg16864999 (K−means Cluster #2)
DNAm (Beta)
0.3
0.4
0.5
0.6
0.7
0.8
0
20
40
60
80
Ages (years)
FDR (Age associated divergence) =0.0778
p(Scalp)=0.648
p(Dura)=0.0017
Scalp
Dura

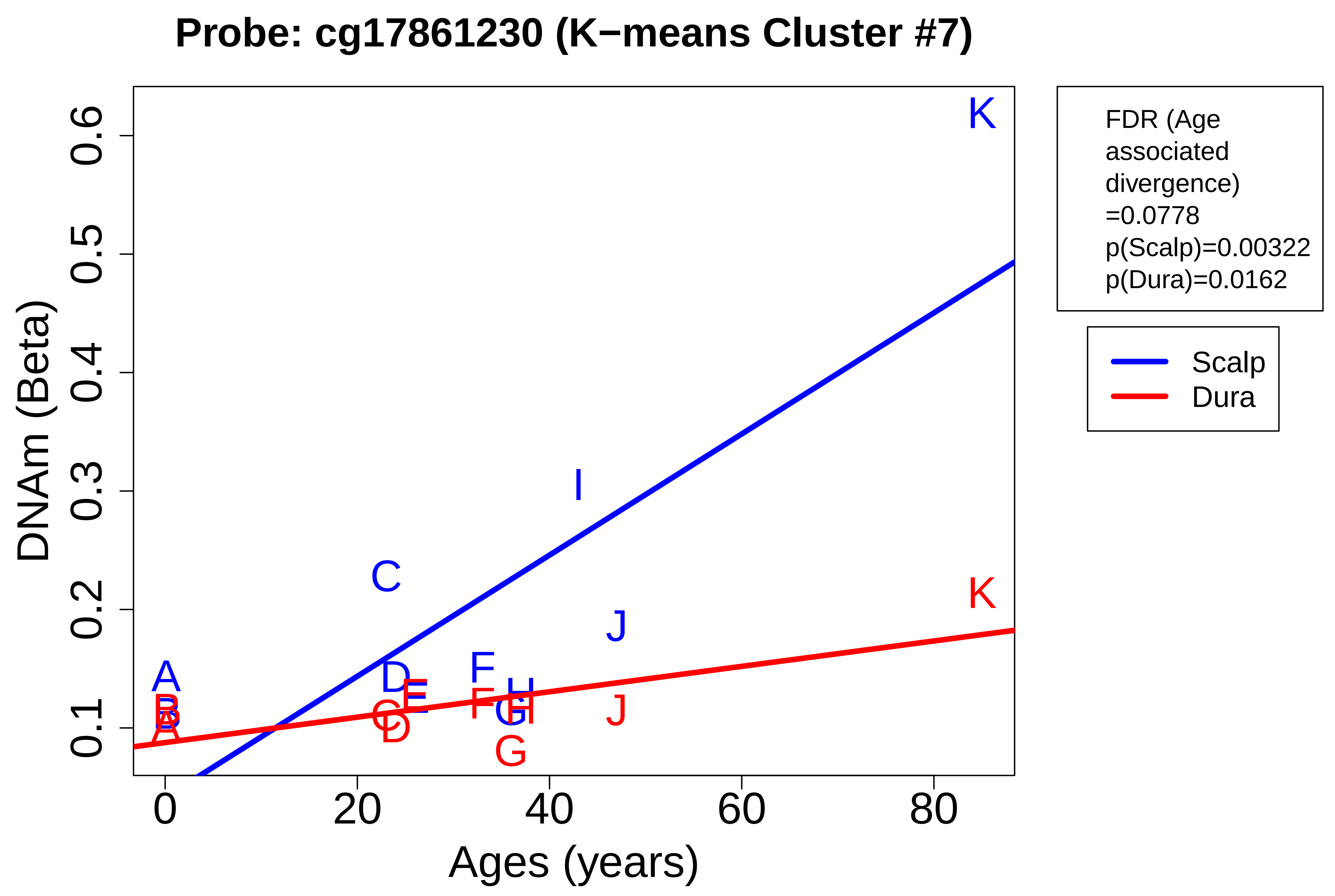

Probe: cg17861230 (K−means Cluster #7)
DNAm (Beta)
0.1
0.2
0.3
0.4
0.5
0.6
Ages (years)
0
20
40
60
80
FDR (Age associated divergence) =0.0778
p(Scalp)=0.00322
p(Dura)=0.0162
Scalp
Dura
A
B
C
D
E
F
G
H
I
J
K

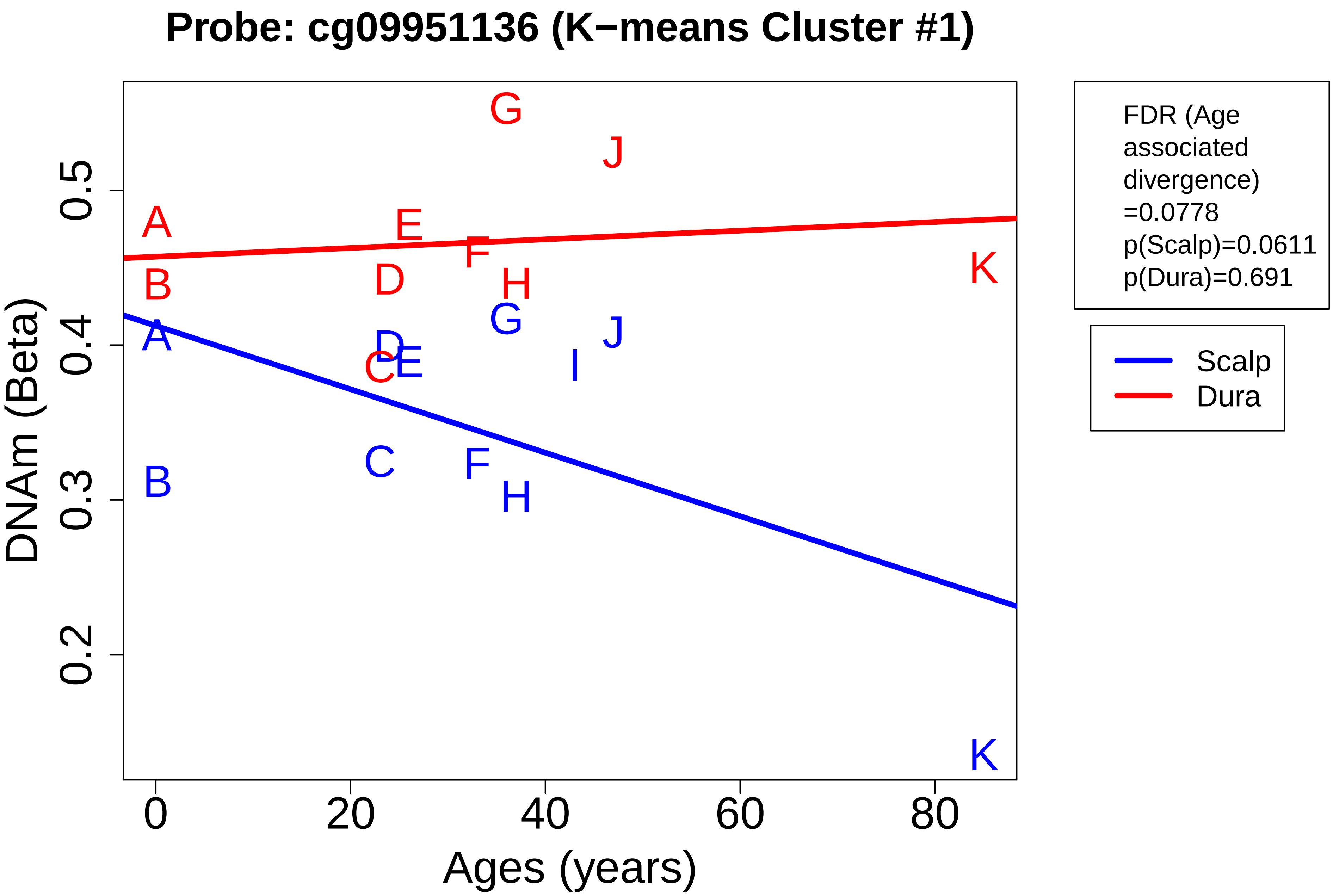

Probe: cg09951136 (K−means Cluster #1)
DNAm (Beta)
0.2
0.3
0.4
0.5
0
20
40
60
80
Ages (years)
A
B
C
D
E
F
G
H
I
J
K
FDR (Age associated divergence) =0.0778
p(Scalp)=0.0611
p(Dura)=0.691
Scalp
Dura

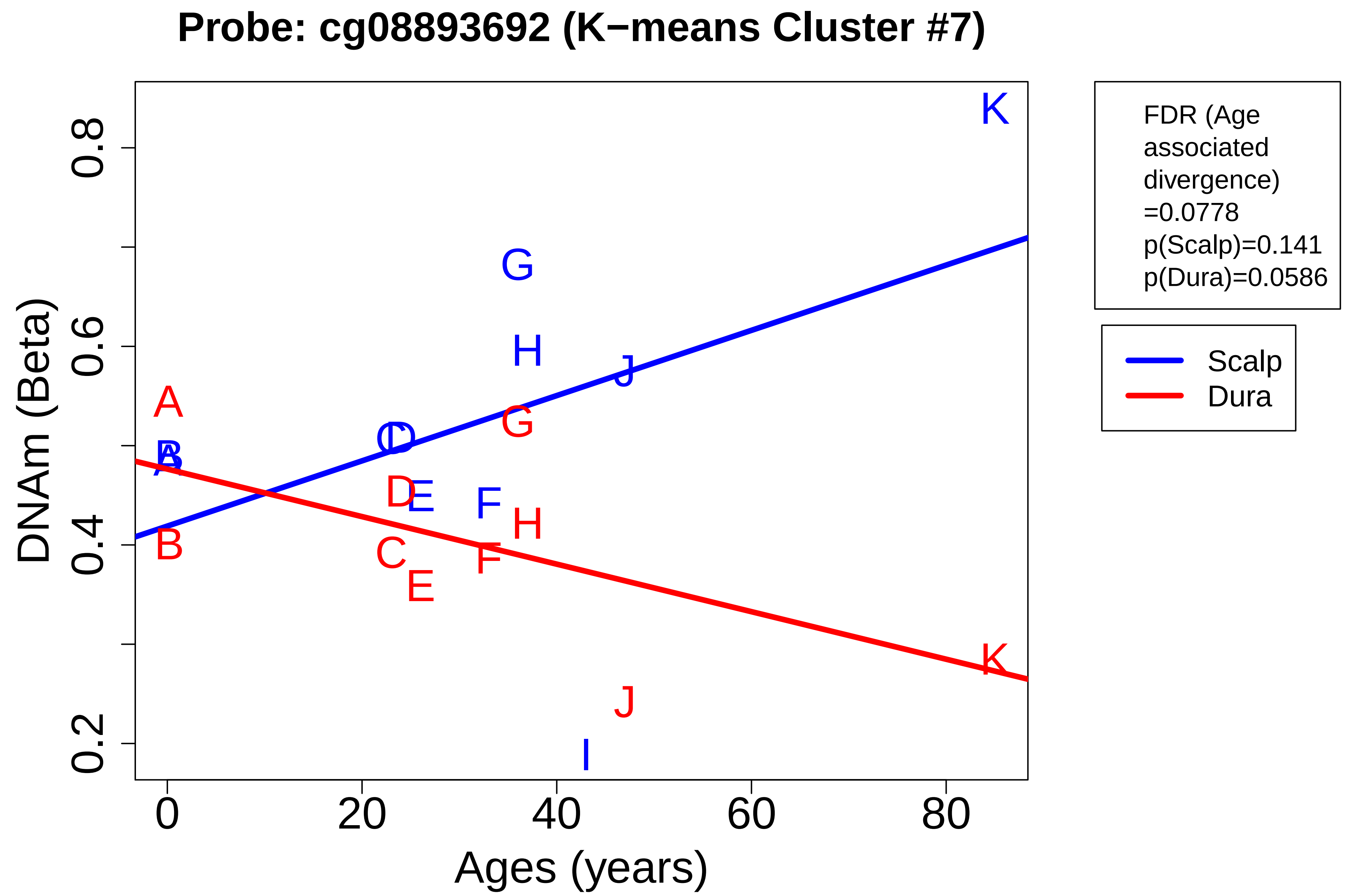

Probe: cg08893692 (K−means Cluster #7)
DNAm (Beta)
0.2
0.4
0.6
0.8
Ages (years)
0
20
40
60
80
A
B
C
D
E
F
G
H
I
J
K
FDR (Age associated divergence) =0.0778
p(Scalp)=0.141
p(Dura)=0.0586
Scalp
Dura

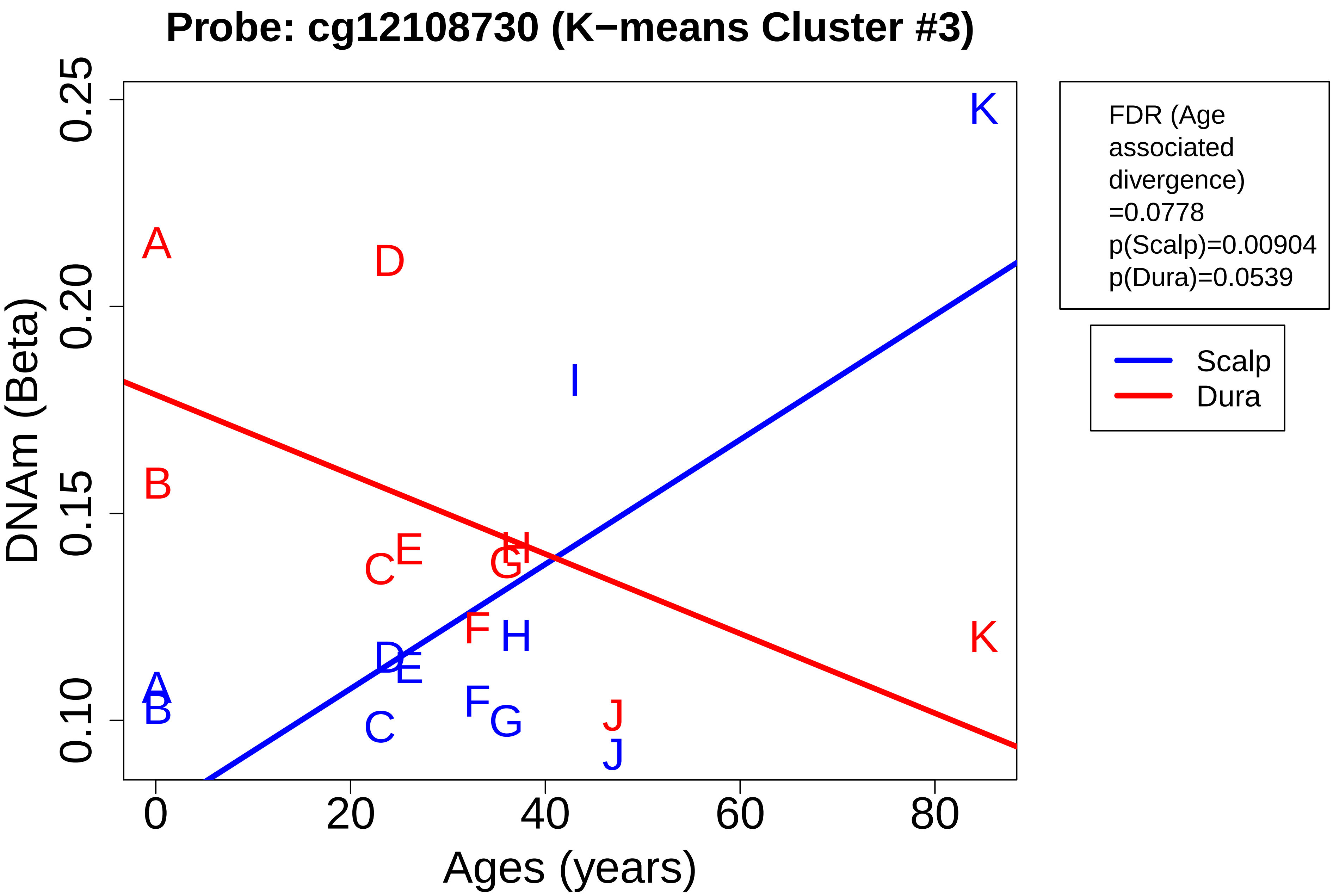
Probe: cg12108730 (K−means Cluster #3)
DNAm (Beta)
0.10
0.15
0.20
0.25
0
20
40
60
80
Ages (years)
A
B
C
D
E
F
G
H
I
J
K
FDR (Age associated divergence) =0.0778
p(Scalp)=0.00904
p(Dura)=0.0539
Scalp
Dura

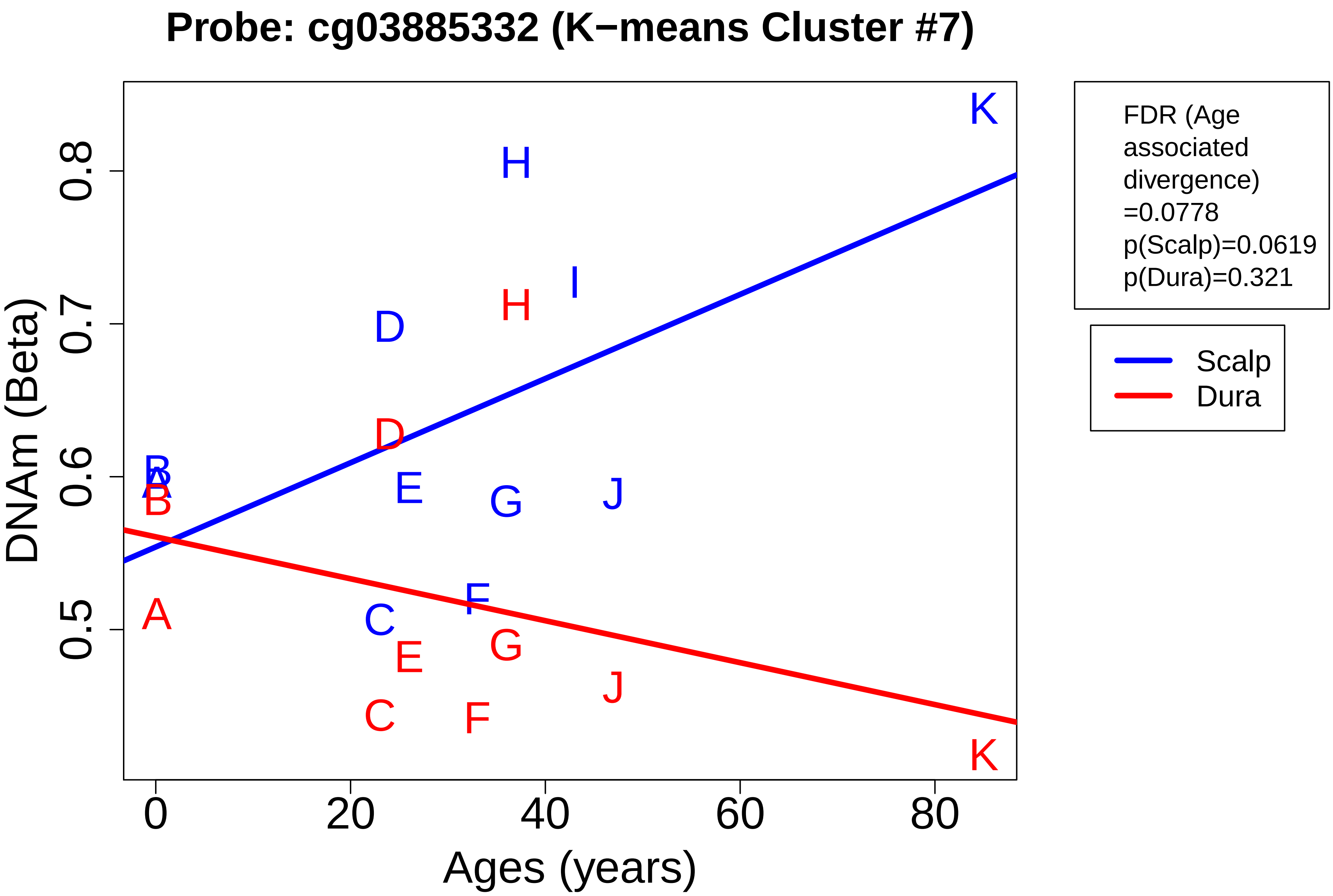

Probe: cg03885332 (K−means Cluster #7)
FDR (Age associated divergence) =0.0778
p(Scalp)=0.0619
p(Dura)=0.321
Scalp
Dura
DNAm (Beta)
0.5
0.6
0.7
0.8
0
20
40
60
80
Ages (years)
A
B
C
D
E
F
G
H
I
J
K

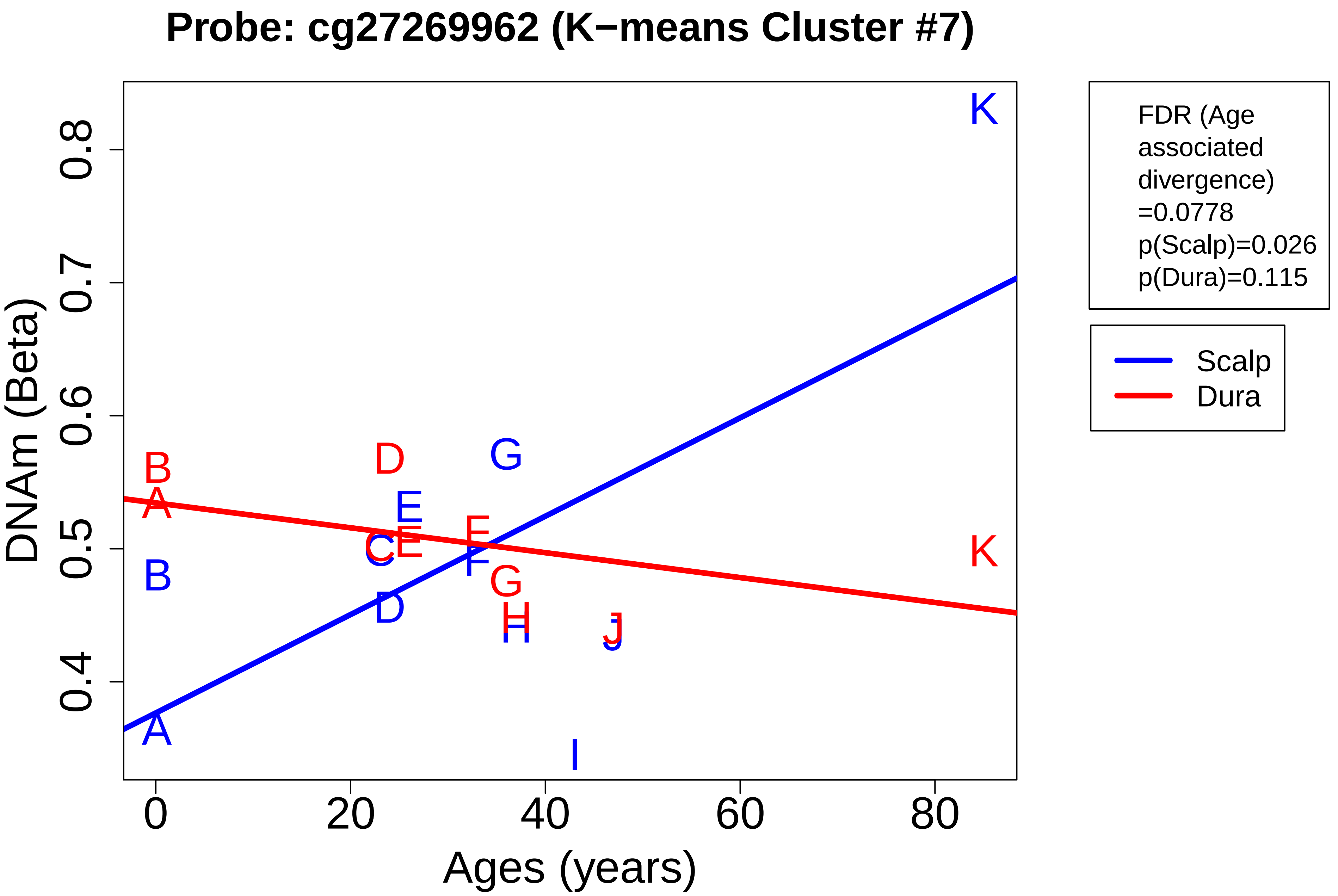

Probe: cg27269962 (K−means Cluster #7)
DNAm (Beta)
0.4
0.5
0.6
0.7
0.8
0
20
40
60
80
Ages (years)
FDR (Age associated divergence) =0.0778
p(Scalp)=0.026
p(Dura)=0.115
Scalp
Dura

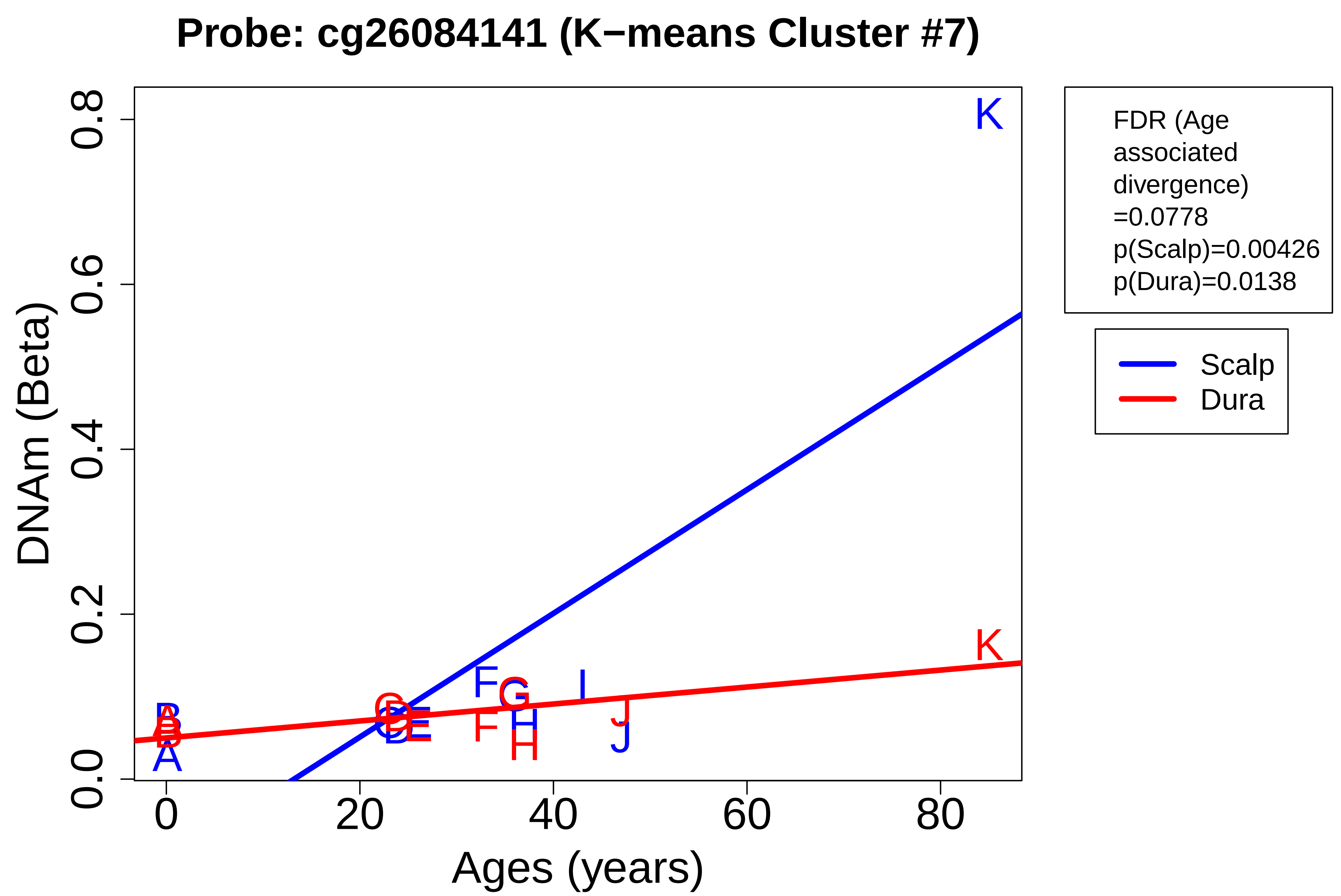

Probe: cg26084141 (K−means Cluster #7)
DNAm (Beta)
0.0
0.2
0.4
0.6
0.8
0
20
40
60
80
Ages (years)
FDR (Age associated divergence) =0.0778
p(Scalp)=0.00426
p(Dura)=0.0138
Scalp
Dura

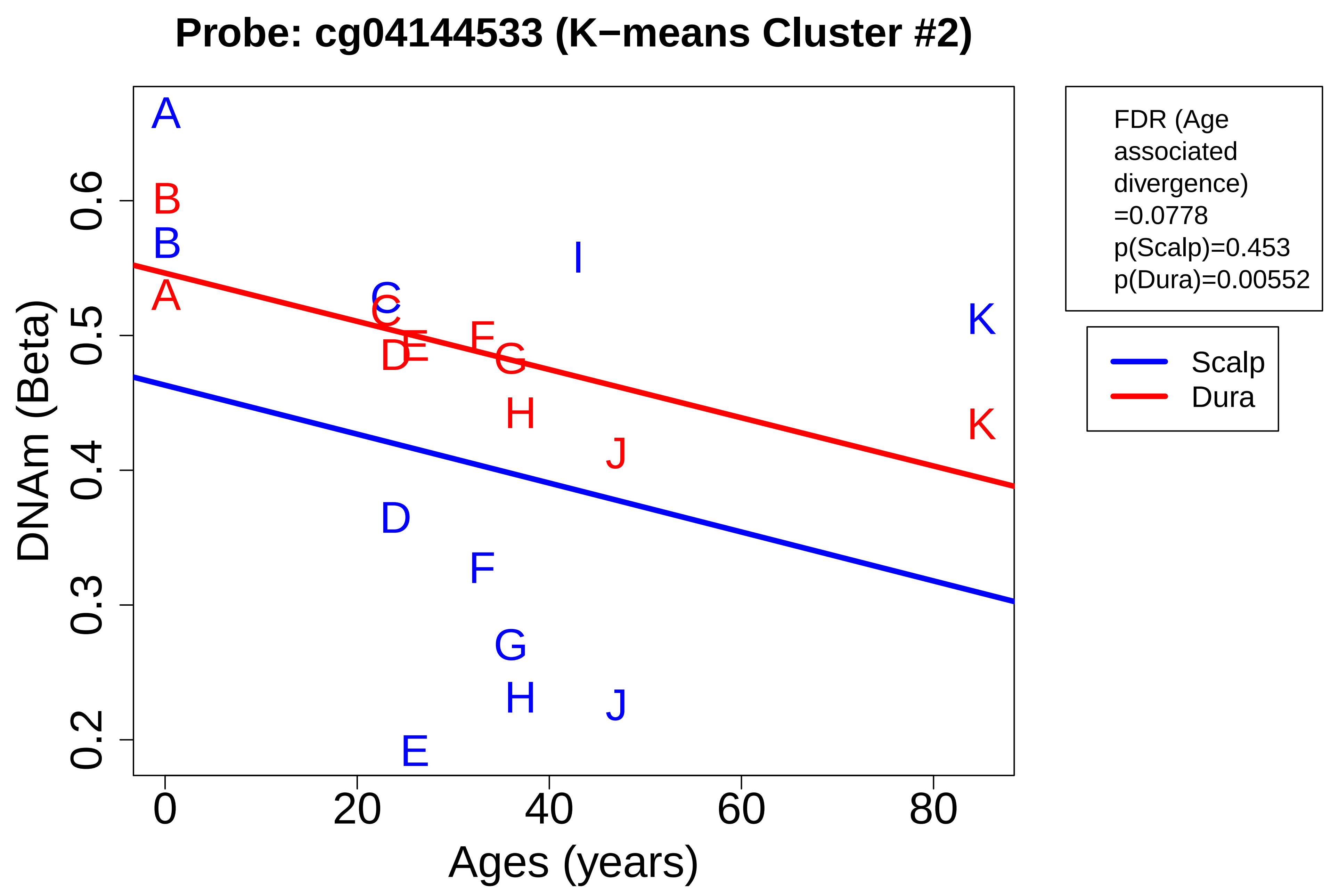

Probe: cg04144533 (K−means Cluster #2)
DNAm (Beta)
0.2
0.3
0.4
0.5
0.6
Ages (years)
0
20
40
60
80
A
B
B
A
C
C
D
E
F
G
H
I
J
K
K
D
F
G
H
J
E
FDR (Age associated divergence) =0.0778
p(Scalp)=0.453
p(Dura)=0.00552
Scalp
Dura

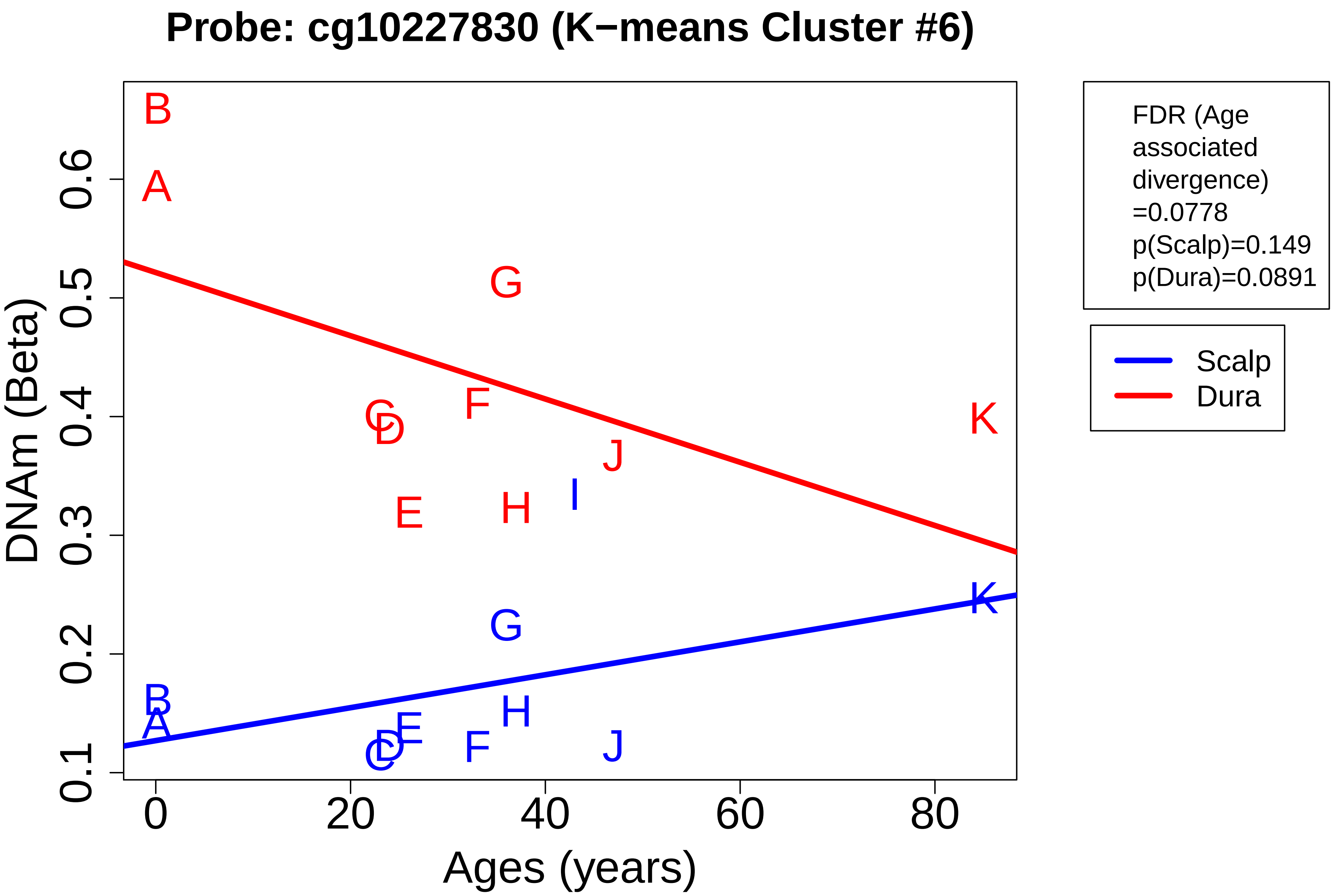

Probe: cg10227830 (K−means Cluster #6)
DNAm (Beta)
Ages (years)
0.1
0.2
0.3
0.4
0.5
0.6
0
20
40
60
80
FDR (Age associated divergence) =0.0778
p(Scalp)=0.149
p(Dura)=0.0891
Scalp
Dura

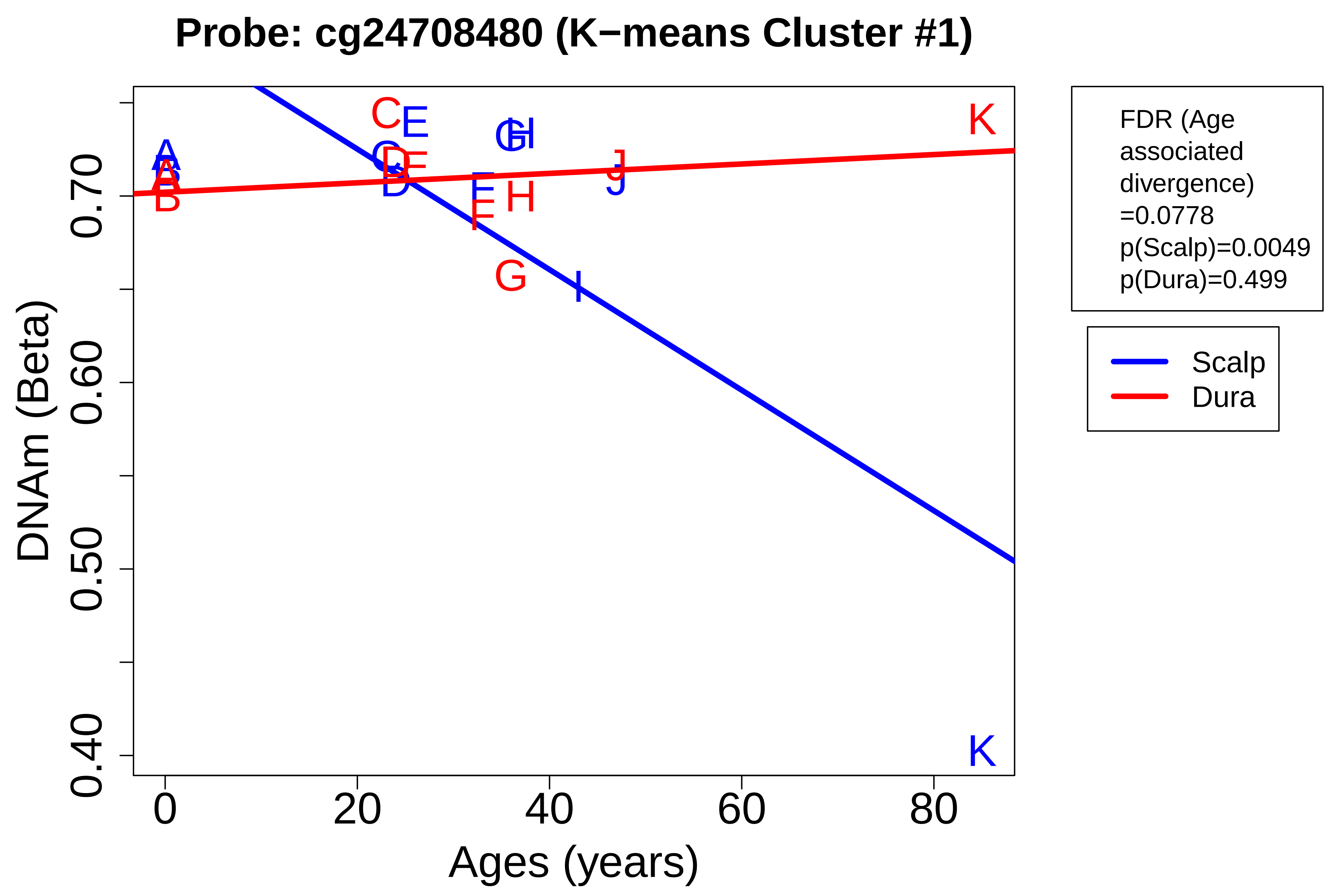

Probe: cg24708480 (K−means Cluster #1)
DNAm (Beta)
0.40
0.50
0.60
0.70
0
20
40
60
80
Ages (years)
FDR (Age associated divergence) =0.0778
p(Scalp)=0.0049
p(Dura)=0.499
Scalp
Dura
A
B
C
D
E
F
G
H
I
J
K

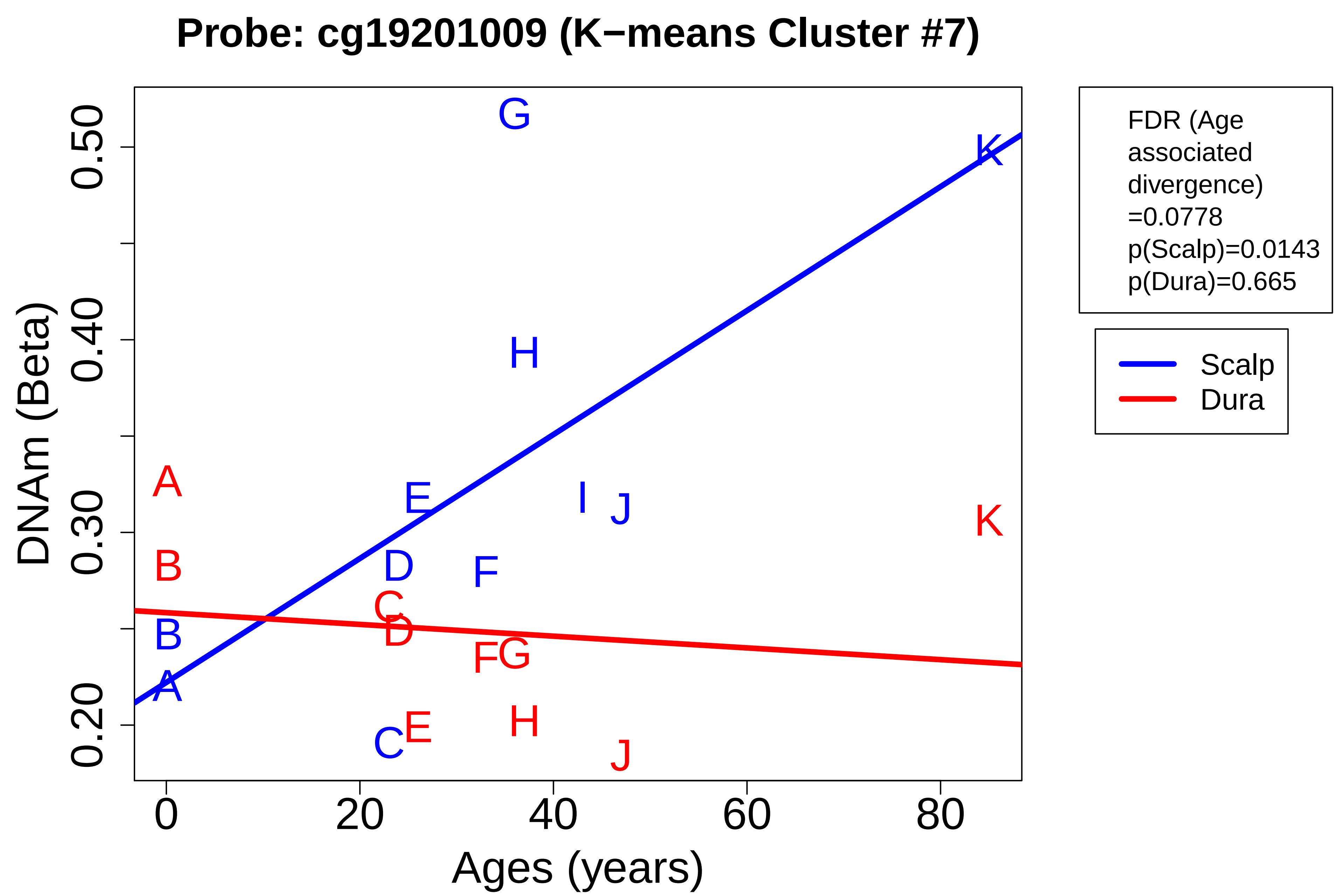
Probe: cg19201009 (K−means Cluster #7)
DNAm (Beta)
Ages (years)
0.20
0.30
0.40
0.50
0
20
40
60
80
FDR (Age associated divergence) =0.0778
p(Scalp)=0.0143
p(Dura)=0.665
Scalp
Dura

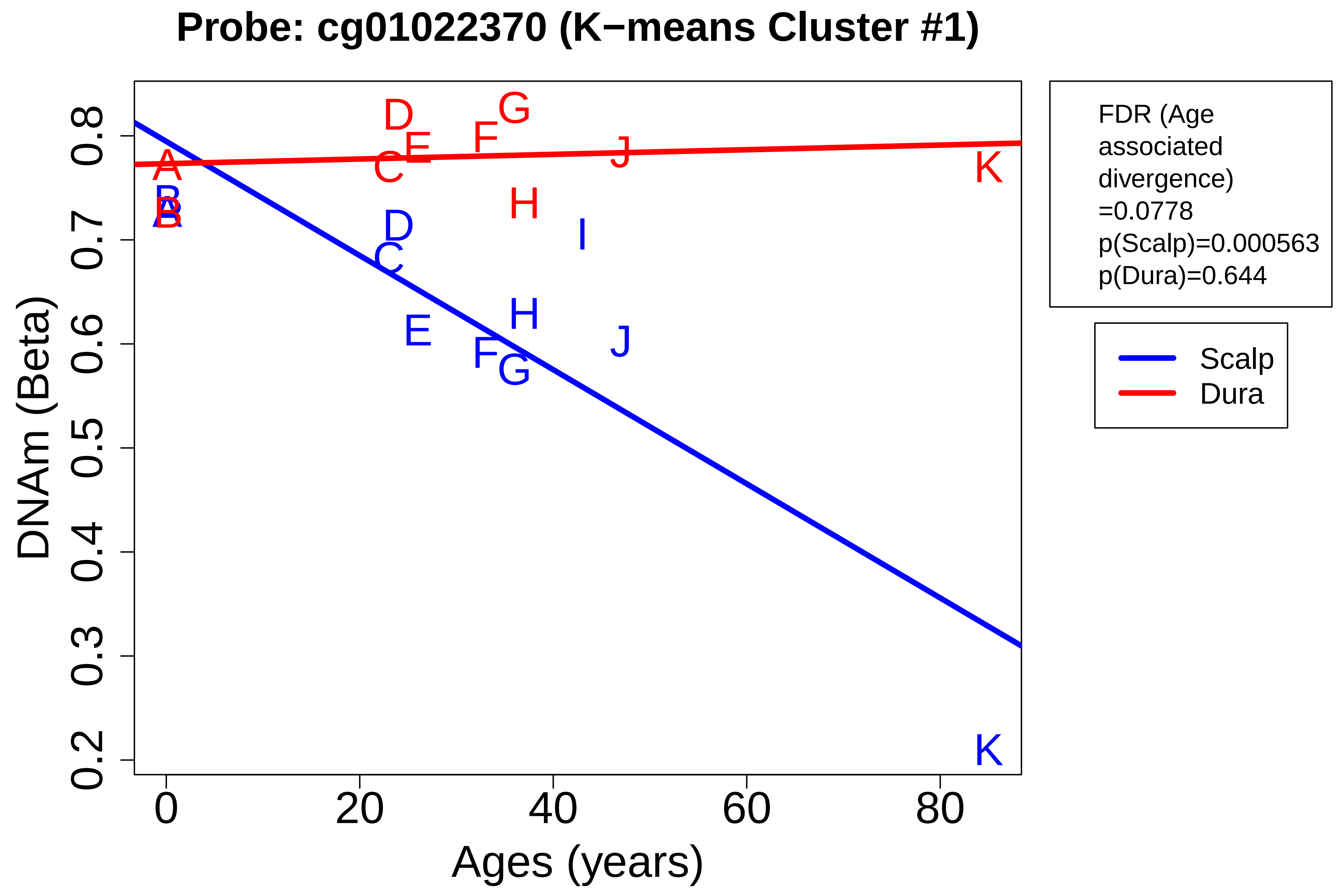

Probe: cg01022370 (K−means Cluster #1)
DNAm (Beta)
0.2
0.3
0.4
0.5
0.6
0.7
0.8
Ages (years)
0
20
40
60
80
FDR (Age associated divergence) =0.0778
p(Scalp)=0.000563
p(Dura)=0.644
Scalp
Dura

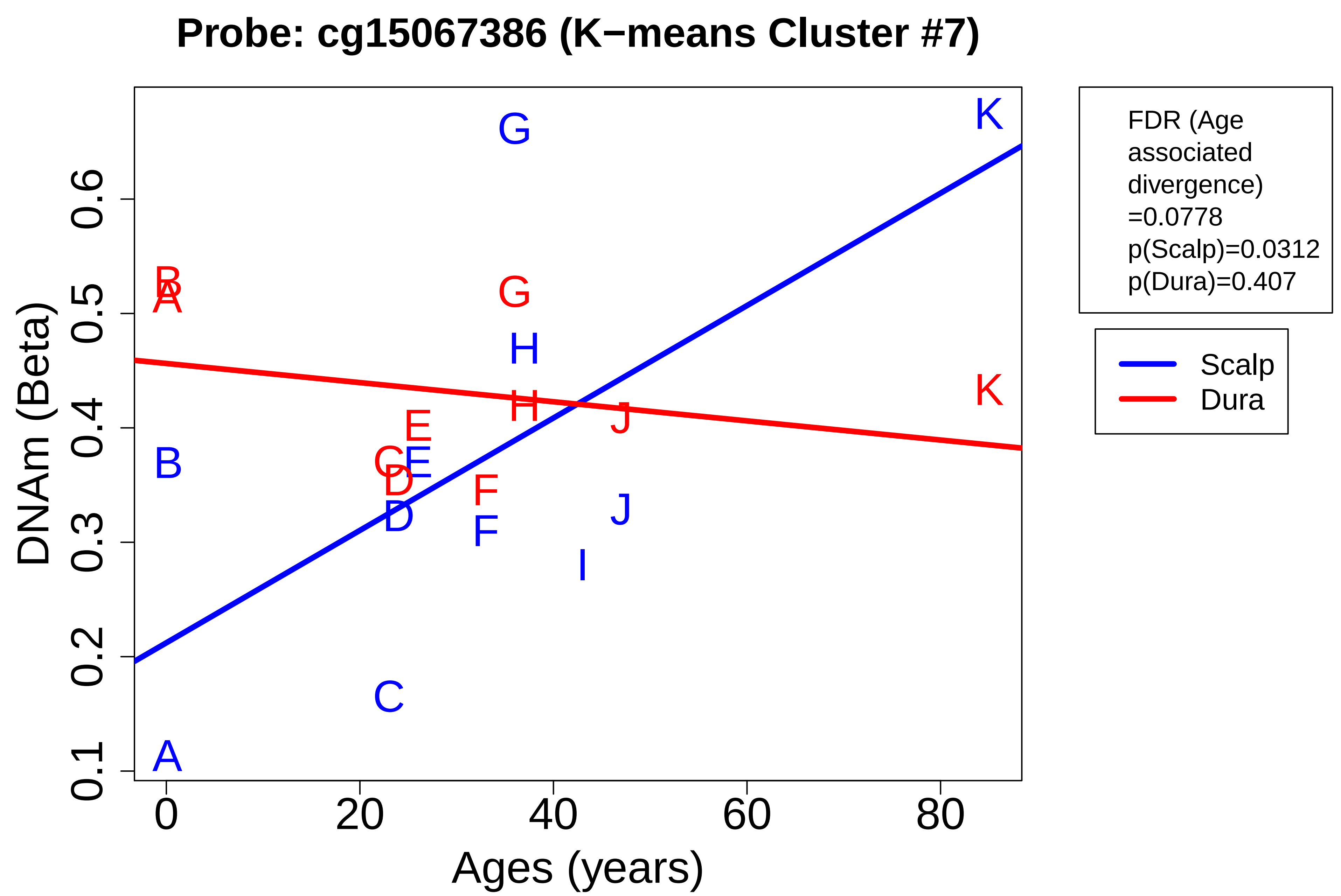

Probe: cg15067386 (K−means Cluster #7)
DNAm (Beta)
0.1
0.2
0.3
0.4
0.5
0.6
0
20
40
60
80
Ages (years)
FDR (Age associated divergence) =0.0778
p(Scalp)=0.0312
p(Dura)=0.407
Scalp
Dura
A
B
C
D
E
F
G
H
I
J
K

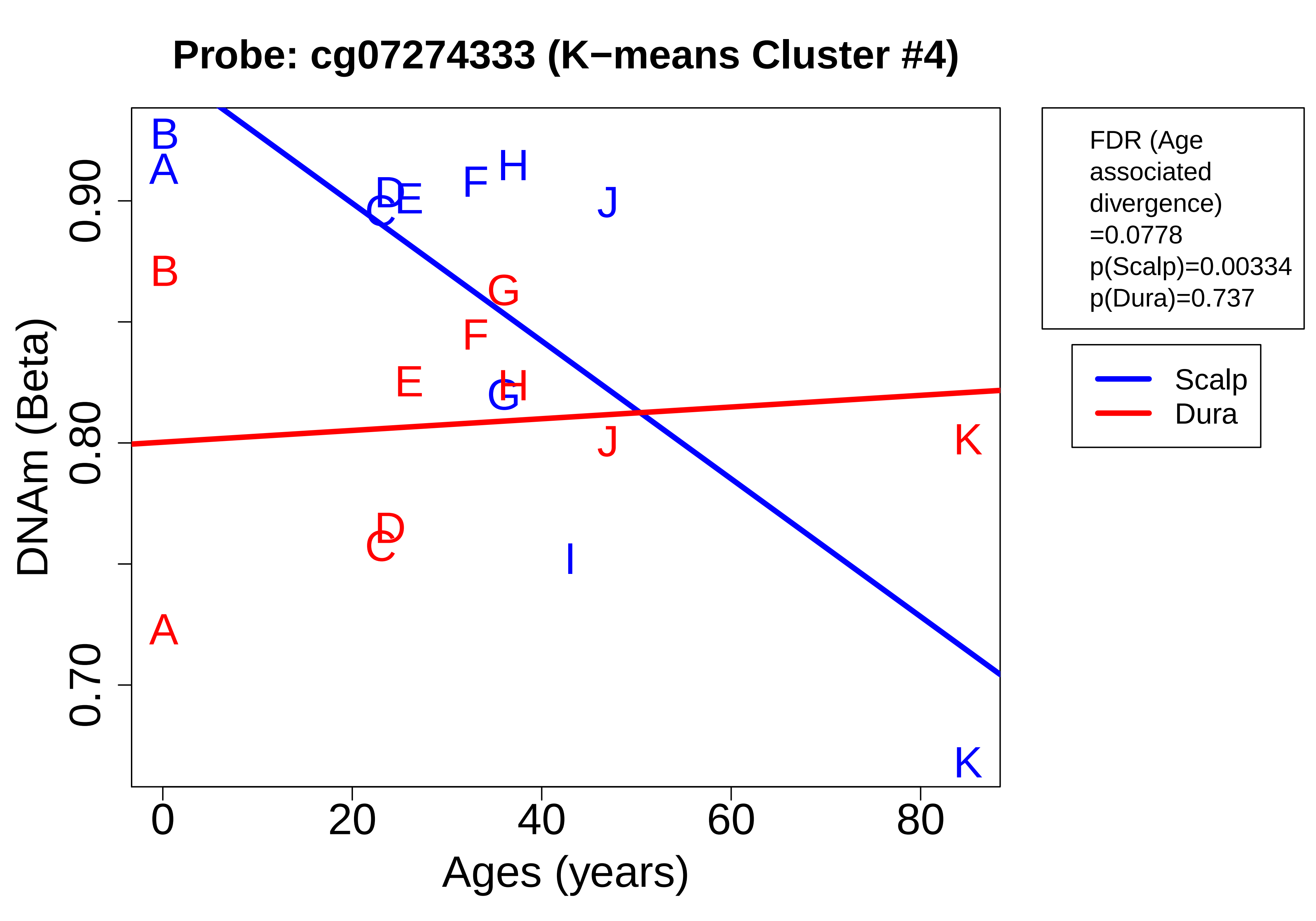
Probe: cg07274333 (K−means Cluster #4)
DNAm (Beta)
0.70
0.80
0.90
Ages (years)
0
20
40
60
80
FDR (Age associated divergence) =0.0778
p(Scalp)=0.00334
p(Dura)=0.737
Scalp
Dura
A
B
C
D
E
F
G
H
I
J
K

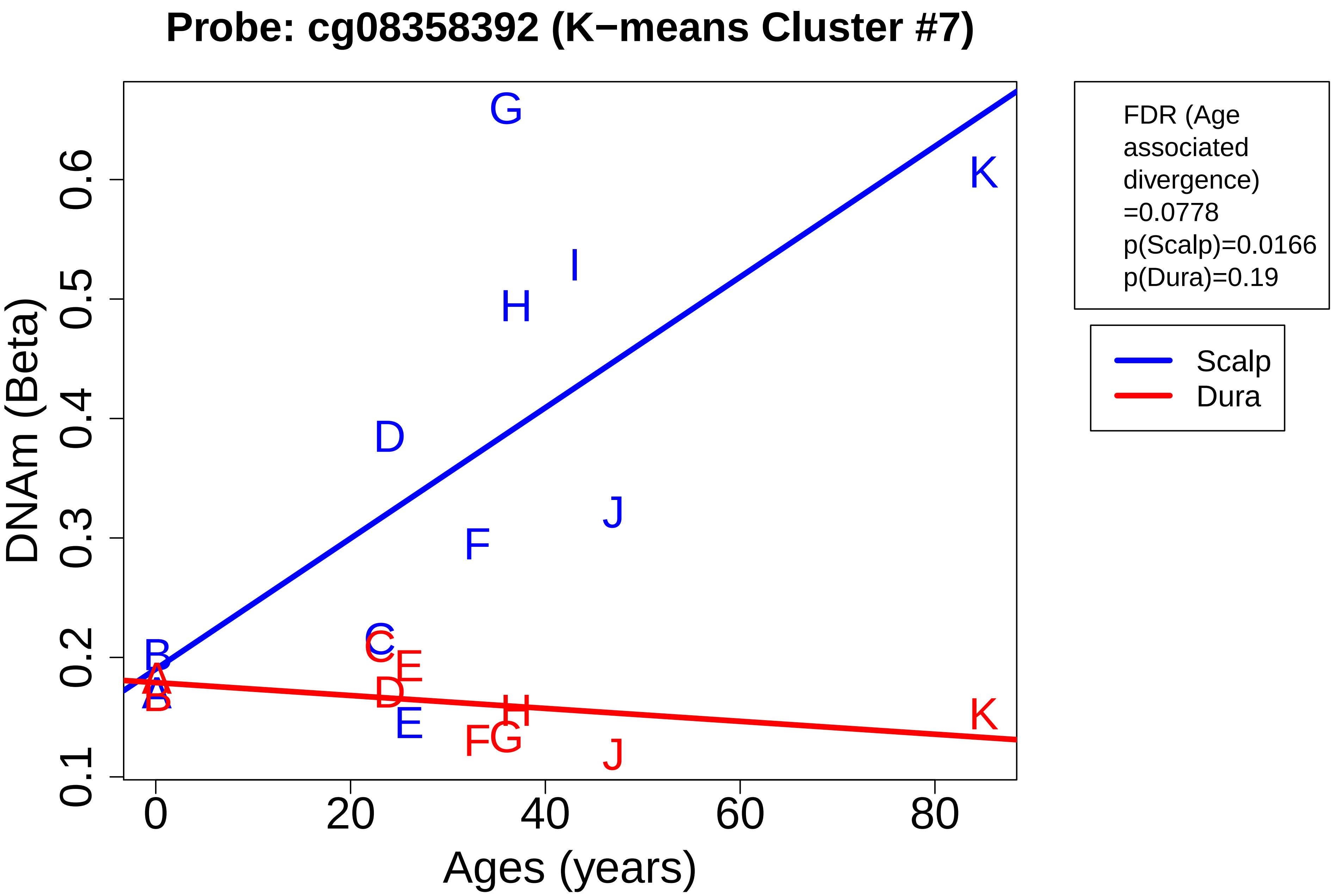
Probe: cg08358392 (K−means Cluster #7)
DNAm (Beta)
0.1
0.2
0.3
0.4
0.5
0.6
Ages (years)
0
20
40
60
80
G
K
I
H
D
J
F
C
B
A
E
FDR (Age associated divergence) =0.0778
p(Scalp)=0.0166
p(Dura)=0.19
Scalp
Dura

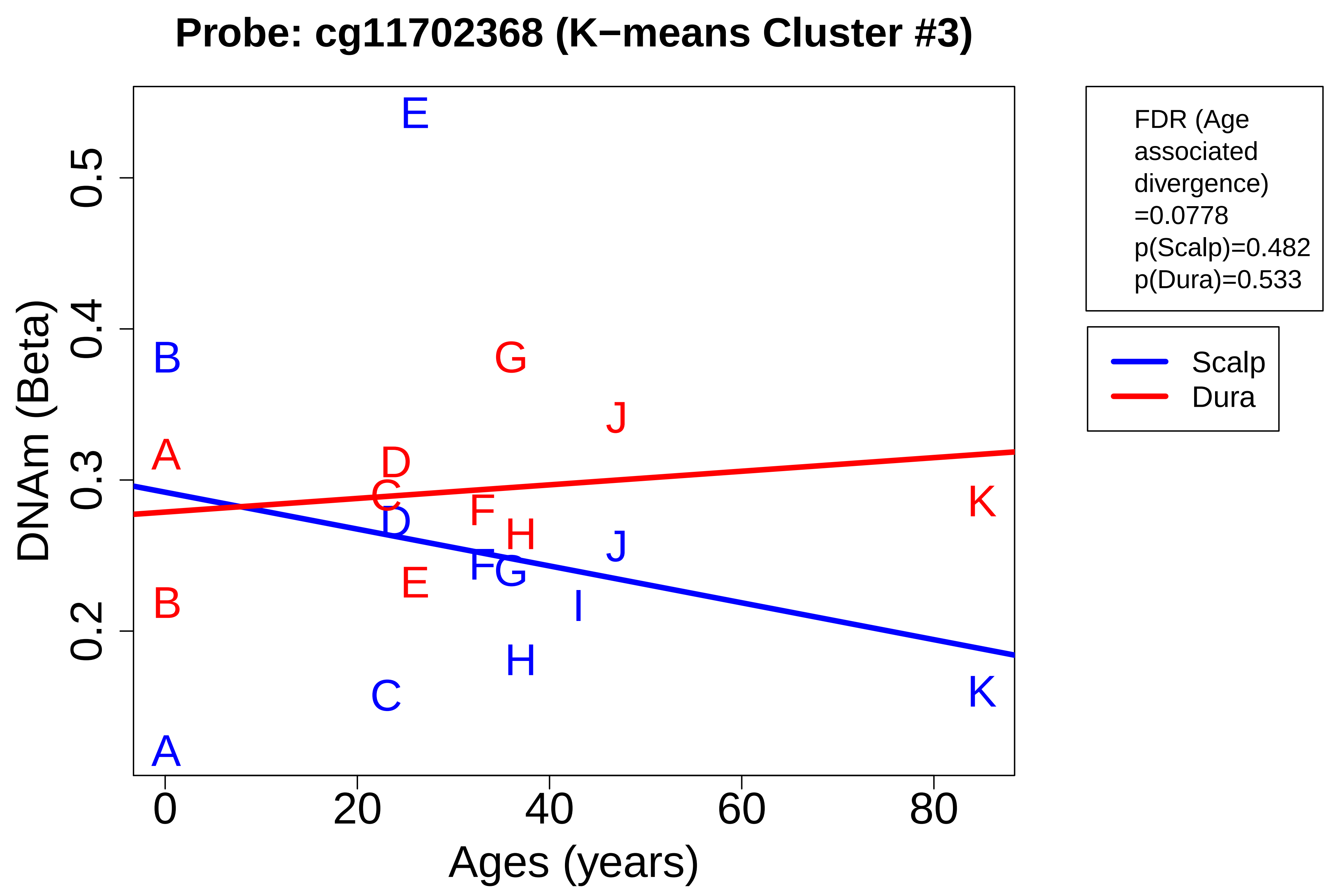

Probe: cg11702368 (K−means Cluster #3)
DNAm (Beta)
Ages (years)
0.2
0.3
0.4
0.5
0
20
40
60
80
FDR (Age associated divergence) =0.0778
p(Scalp)=0.482
p(Dura)=0.533
Scalp
Dura
A
B
C
D
E
F
G
H
I
J
K

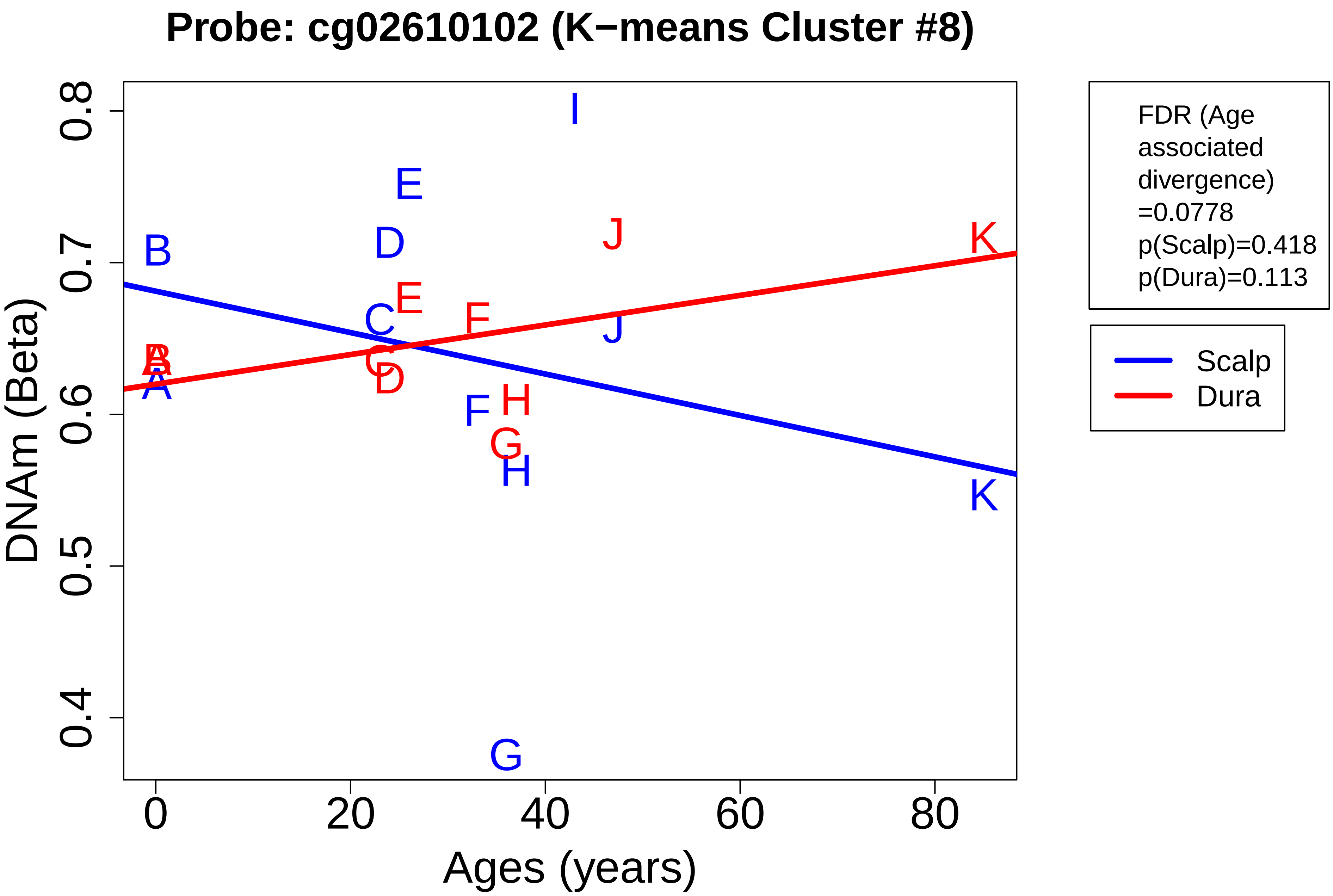

Probe: cg02610102 (K−means Cluster #8)
DNAm (Beta)
0.4
0.5
0.6
0.7
0.8
Ages (years)
0
20
40
60
80
FDR (Age associated divergence) =0.0778
p(Scalp)=0.418
p(Dura)=0.113
Scalp
Dura

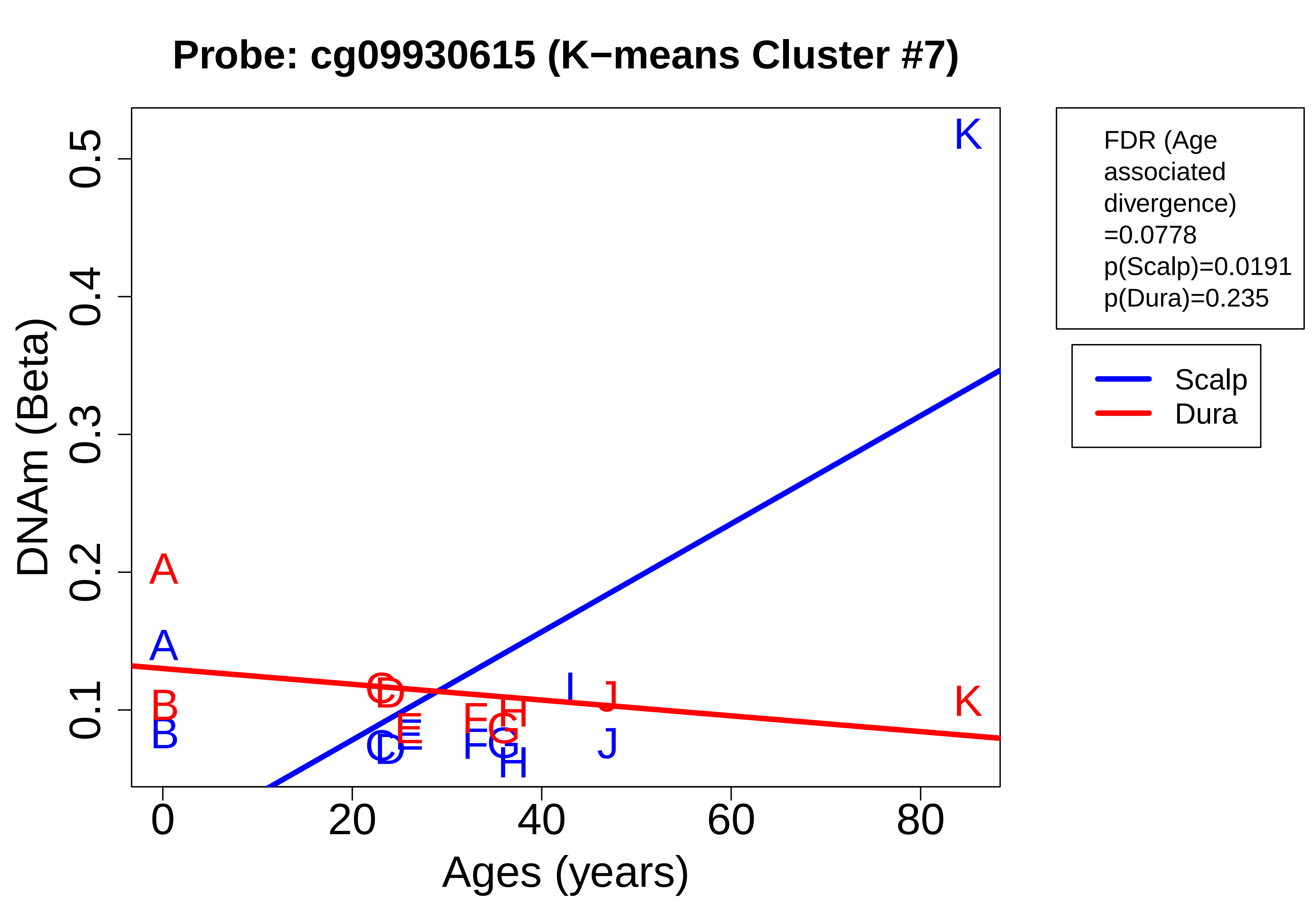

Probe: cg09930615 (K−means Cluster #7)
FDR (Age associated divergence) =0.0778
p(Scalp)=0.0191
p(Dura)=0.235
Scalp
Dura
DNAm (Beta)
0.1
0.2
0.3
0.4
0.5
0
20
40
60
80
Ages (years)
A
B
C
D
E
F
G
H
I
J
K

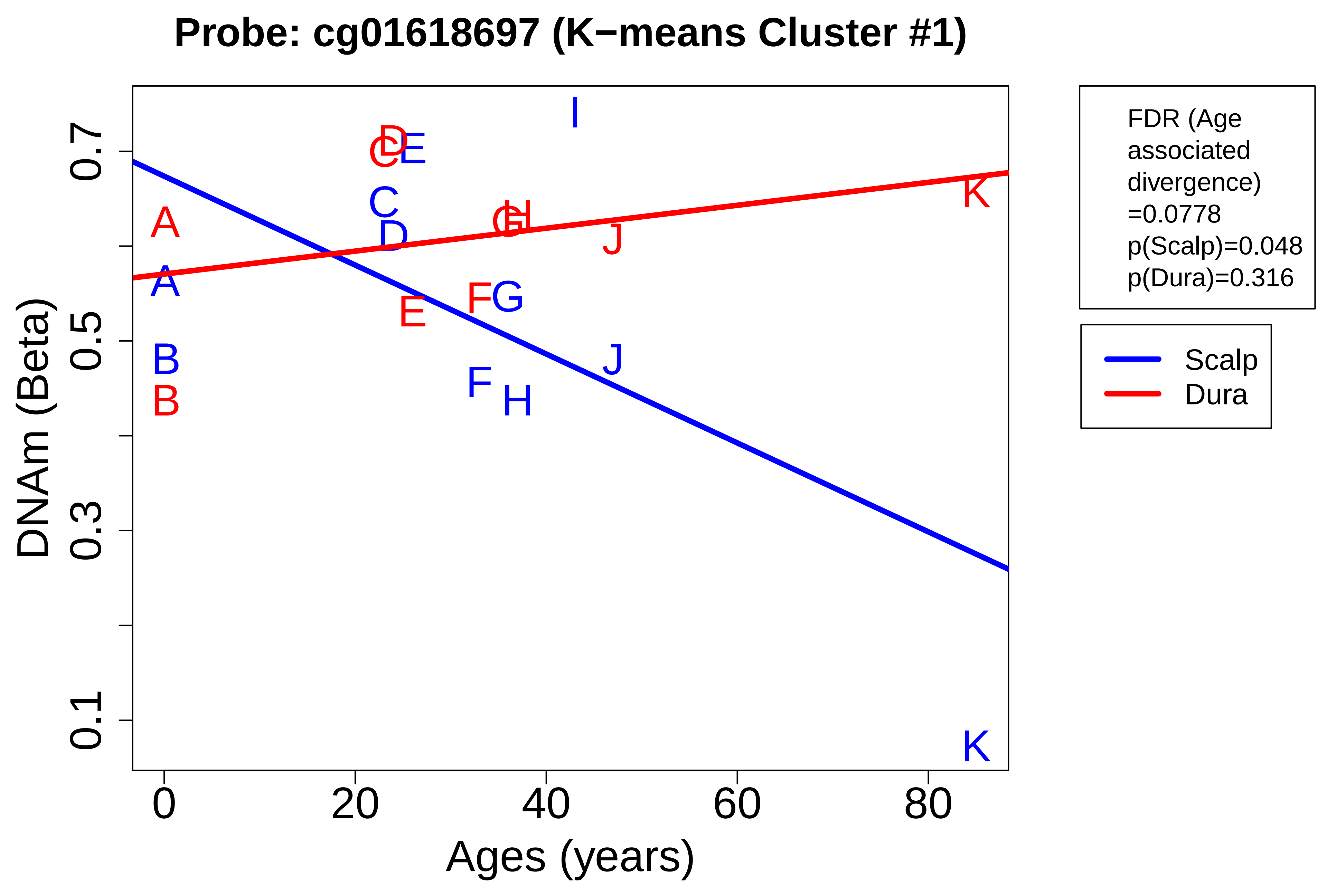

Probe: cg01618697 (K−means Cluster #1)
DNAm (Beta)
0.1
0.3
0.5
0.7
Ages (years)
0
20
40
60
80
A
B
C
D
E
F
G
H
I
J
K
FDR (Age associated divergence) =0.0778
p(Scalp)=0.048
p(Dura)=0.316
Scalp
Dura

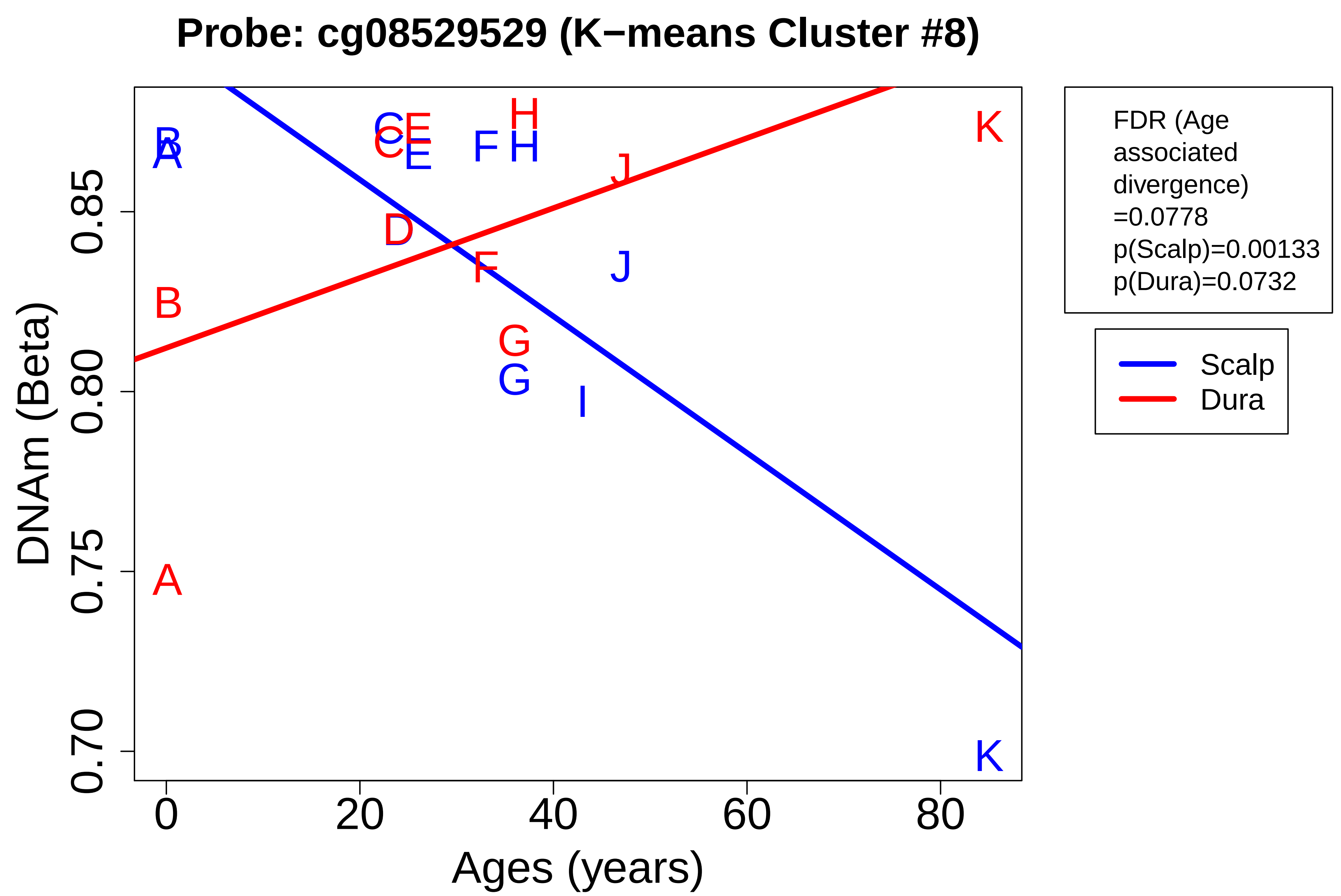

Probe: cg08529529 (K−means Cluster #8)
FDR (Age associated divergence) =0.0778
p(Scalp)=0.00133
p(Dura)=0.0732
Scalp
Dura
DNAm (Beta)
0.70
0.75
0.80
0.85
0
20
40
60
80
Ages (years)
A
B
C
D
E
F
G
H
I
J
K

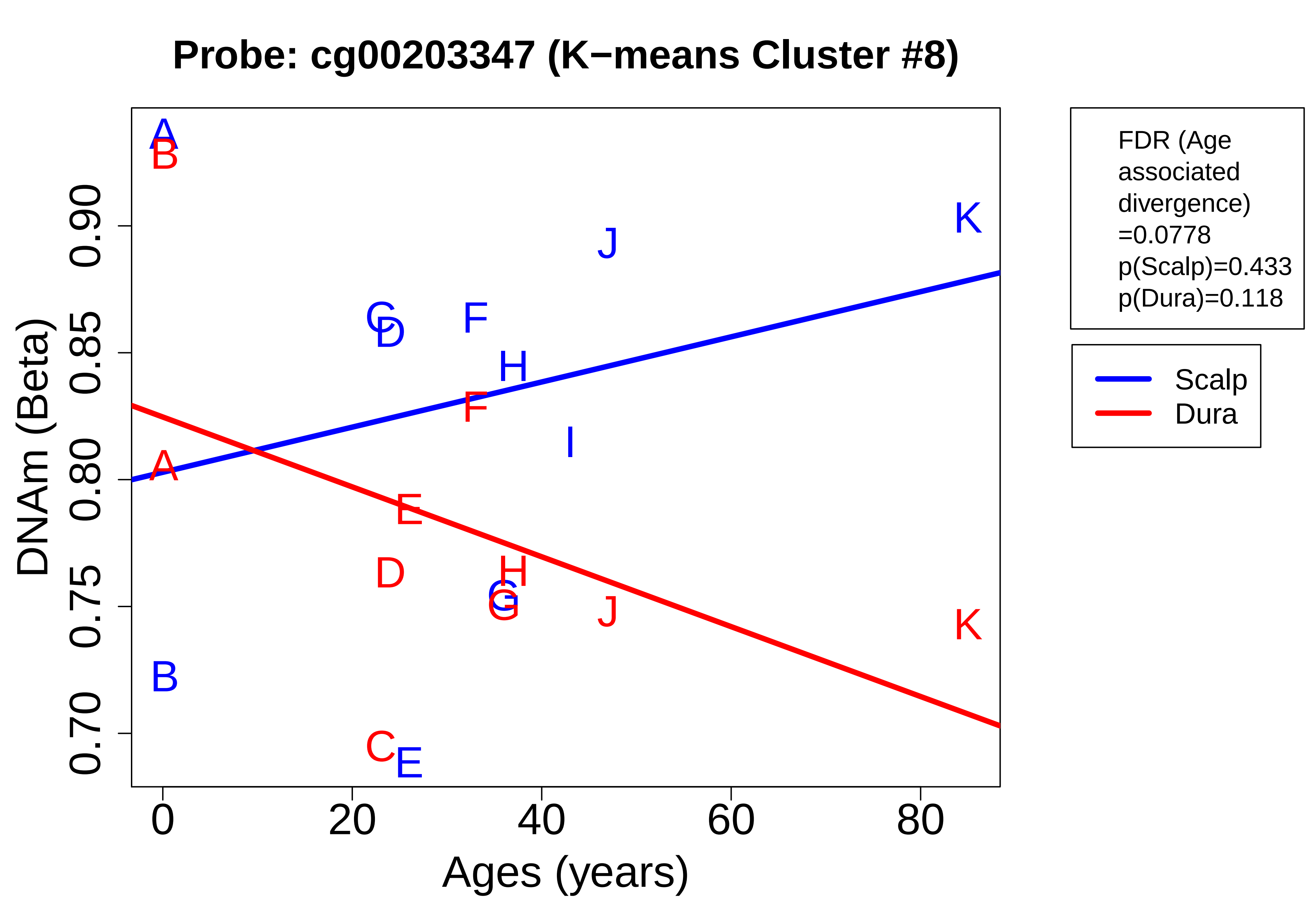

Probe: cg00203347 (K−means Cluster #8)
DNAm (Beta)
0.70
0.75
0.80
0.85
0.90
0
20
40
60
80
Ages (years)
FDR (Age associated divergence) =0.0778
p(Scalp)=0.433
p(Dura)=0.118
Scalp
Dura
A
B
C
D
E
F
G
H
I
J
K

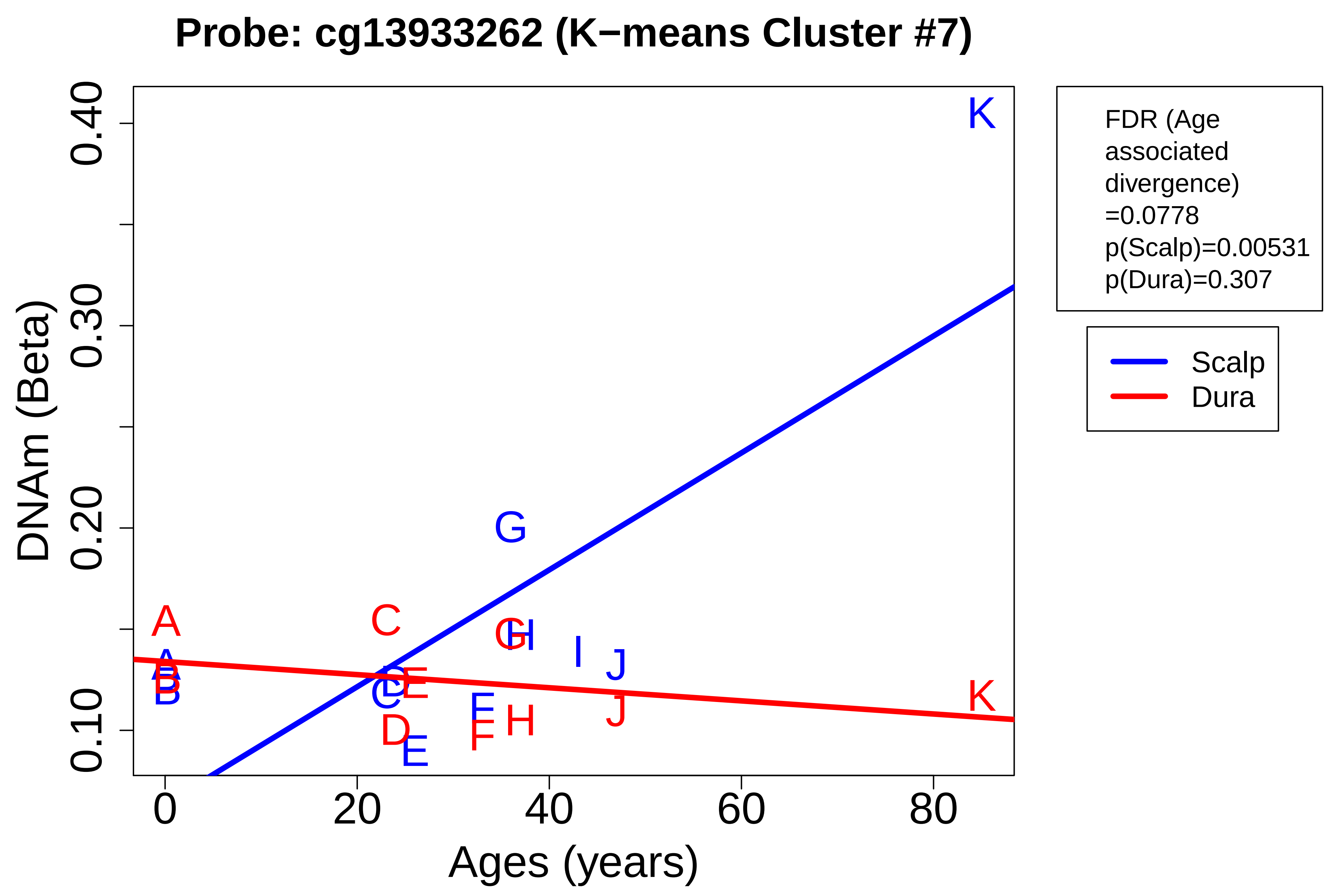

Probe: cg13933262 (K−means Cluster #7)
DNAm (Beta)
0.10
0.20
0.30
0.40
0
20
40
60
80
Ages (years)
FDR (Age associated divergence) =0.0778
p(Scalp)=0.00531
p(Dura)=0.307
Scalp
Dura
A
B
C
D
E
F
G
H
I
J
K

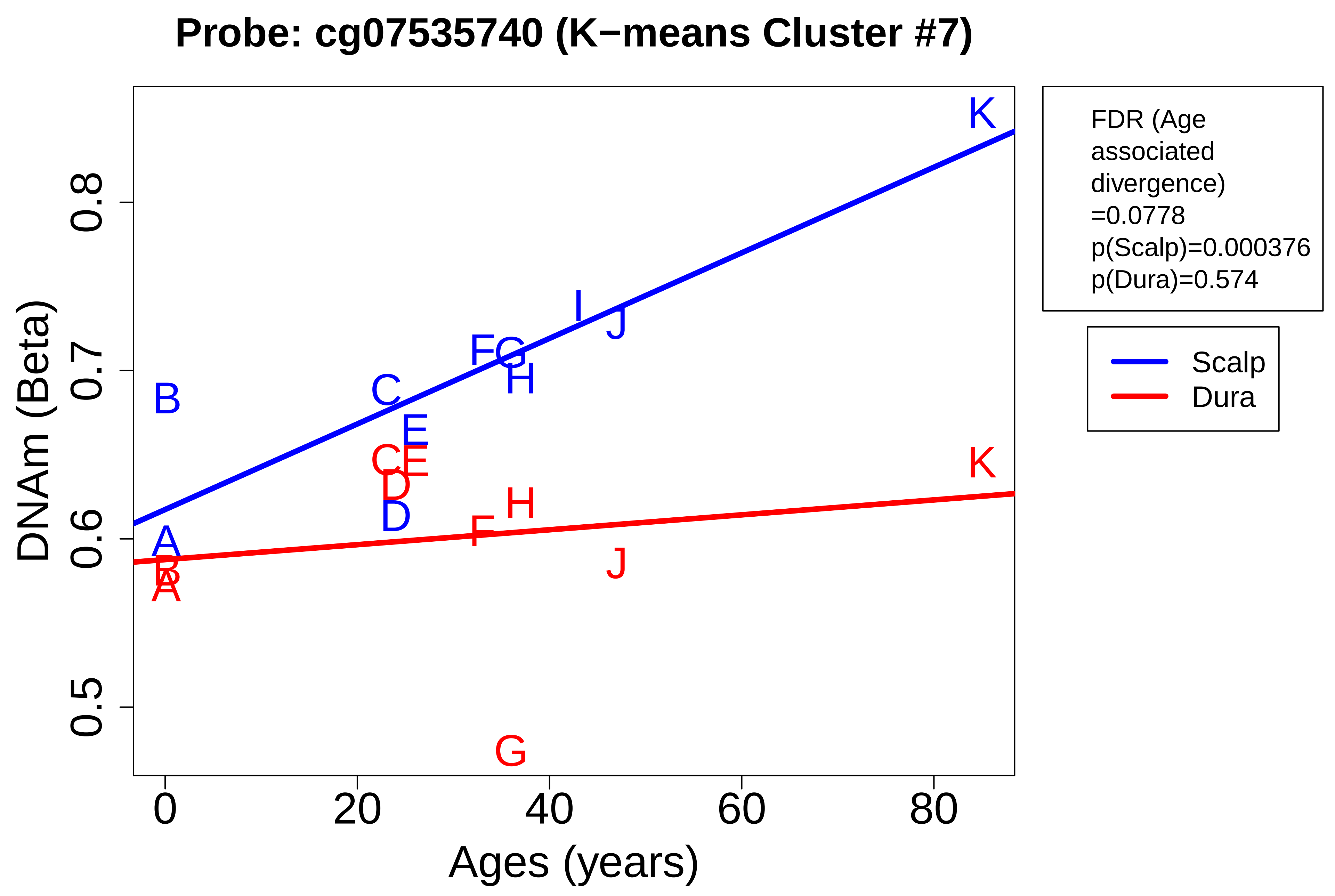

Probe: cg07535740 (K−means Cluster #7)
DNAm (Beta)
0.5
0.6
0.7
0.8
0
20
40
60
80
Ages (years)
FDR (Age associated divergence) =0.0778
p(Scalp)=0.000376
p(Dura)=0.574
Scalp
Dura

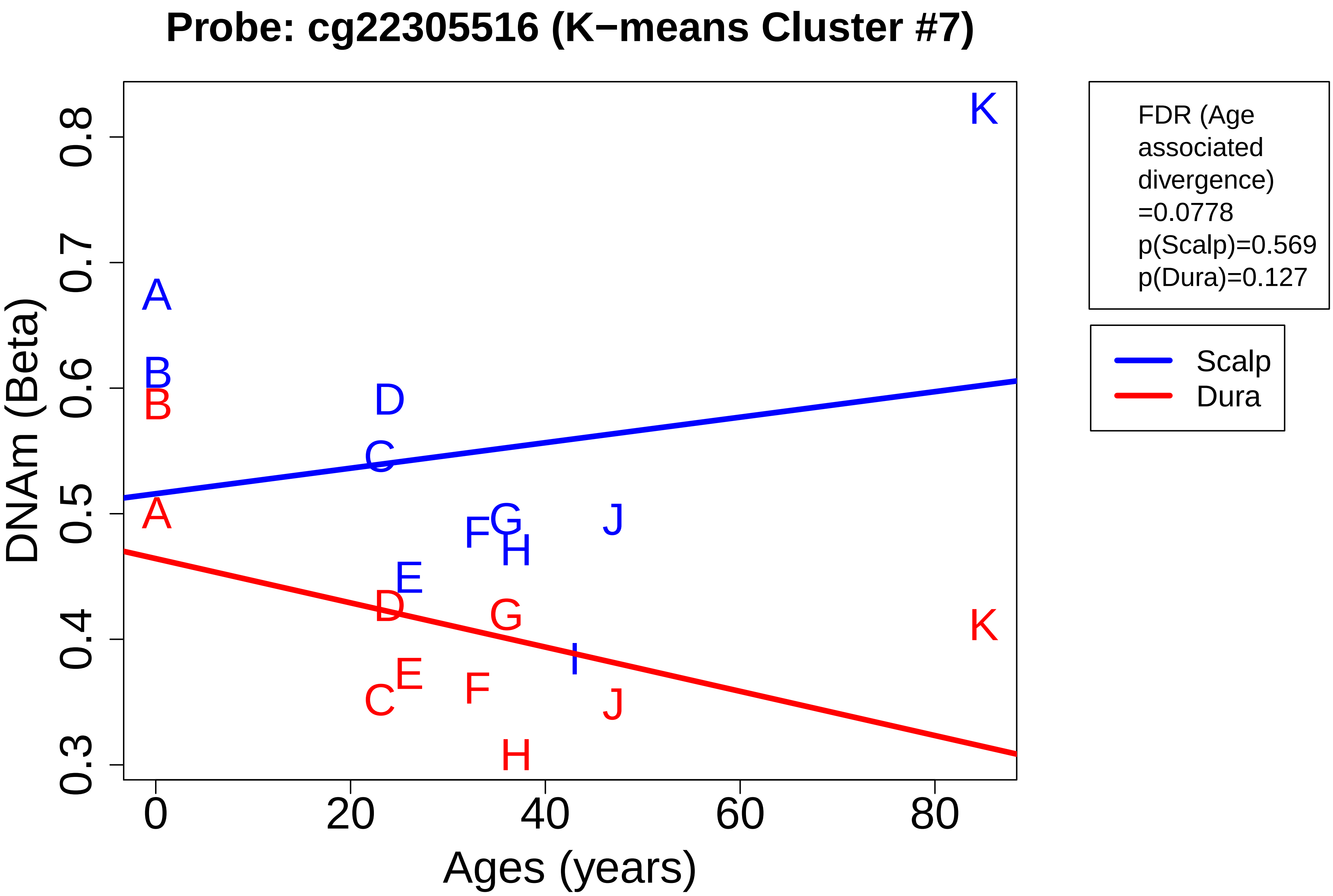

Probe: cg22305516 (K−means Cluster #7)
DNAm (Beta)
0.3
0.4
0.5
0.6
0.7
0.8
0
20
40
60
80
Ages (years)
A B C D E F G H I J K
A B C D E F G H J K
FDR (Age associated divergence) =0.0778
p(Scalp)=0.569
p(Dura)=0.127
Scalp
Dura

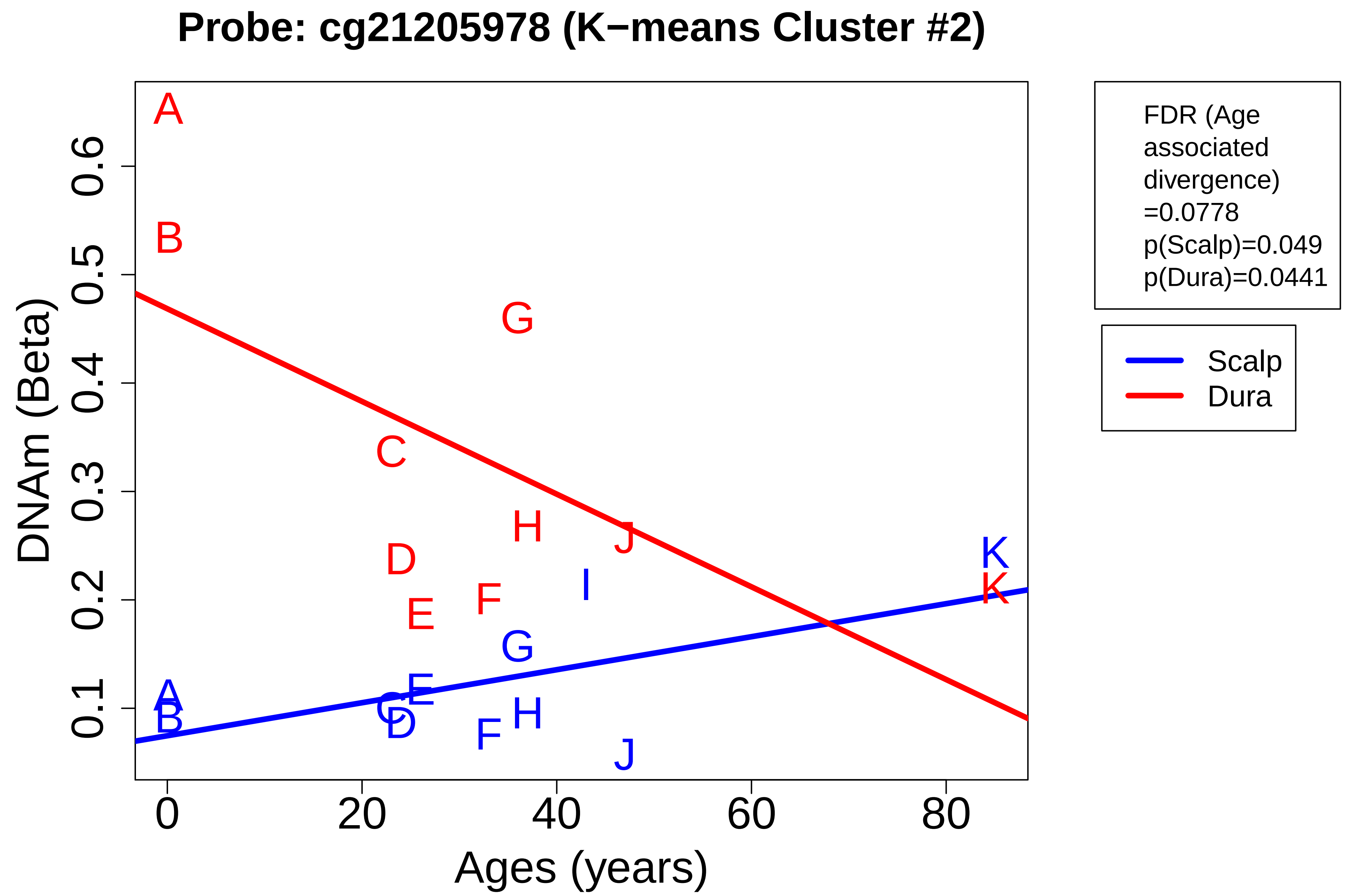

Probe: cg21205978 (K−means Cluster #2)
DNAm (Beta)
0.1
0.2
0.3
0.4
0.5
0.6
Ages (years)
0
20
40
60
80
FDR (Age associated divergence) =0.0778
p(Scalp)=0.049
p(Dura)=0.0441
Scalp
Dura

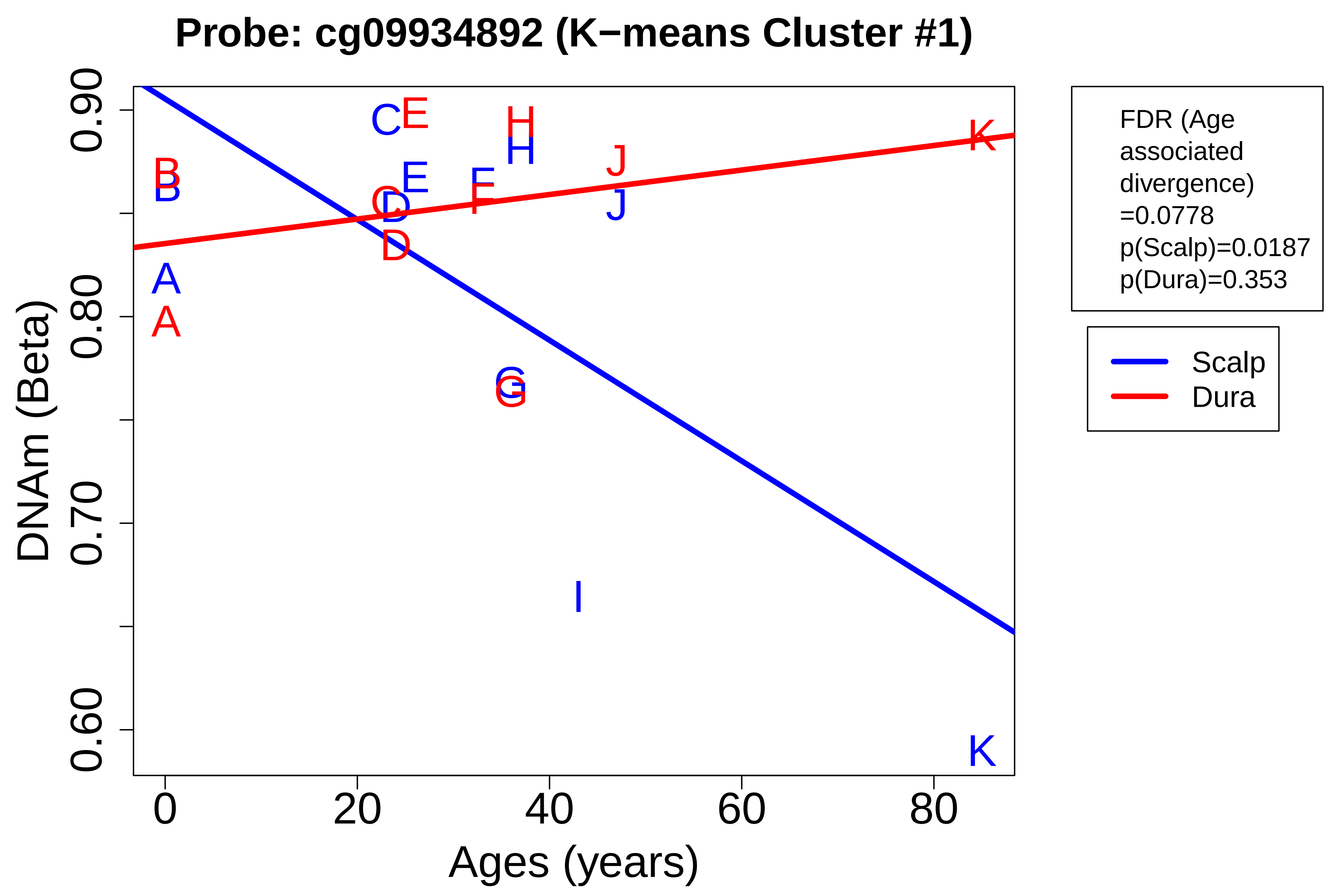

Probe: cg09934892 (K−means Cluster #1)
DNAm (Beta)
0.60
0.70
0.80
0.90
0
20
40
60
80
Ages (years)
A
B
C
D
E
F
G
H
I
J
K
FDR (Age associated divergence) =0.0778
p(Scalp)=0.0187
p(Dura)=0.353
Scalp
Dura

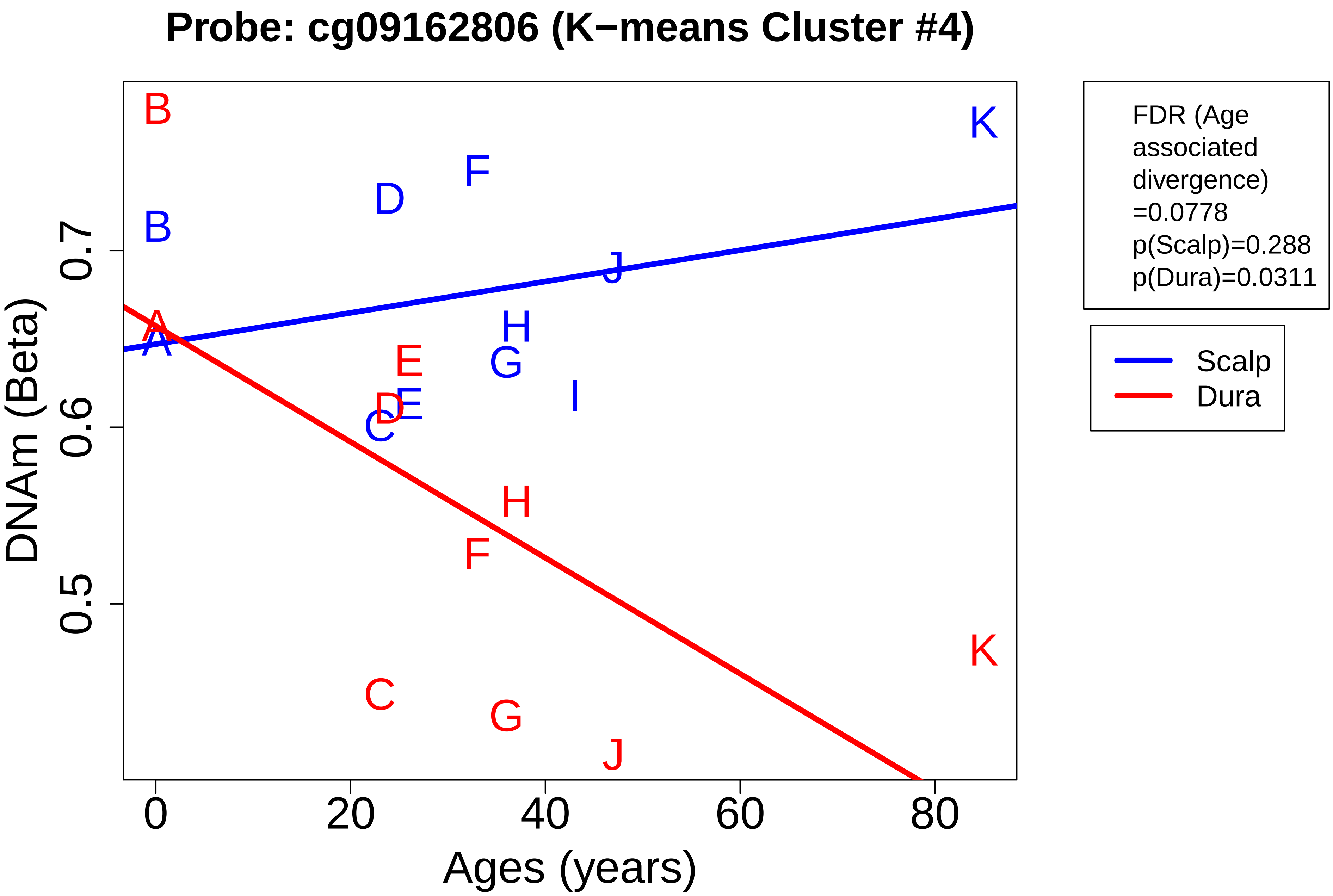

Probe: cg09162806 (K−means Cluster #4)
DNAm (Beta)
0.5
0.6
0.7
Ages (years)
0
20
40
60
80
FDR (Age associated divergence) =0.0778
p(Scalp)=0.288
p(Dura)=0.0311
Scalp
Dura

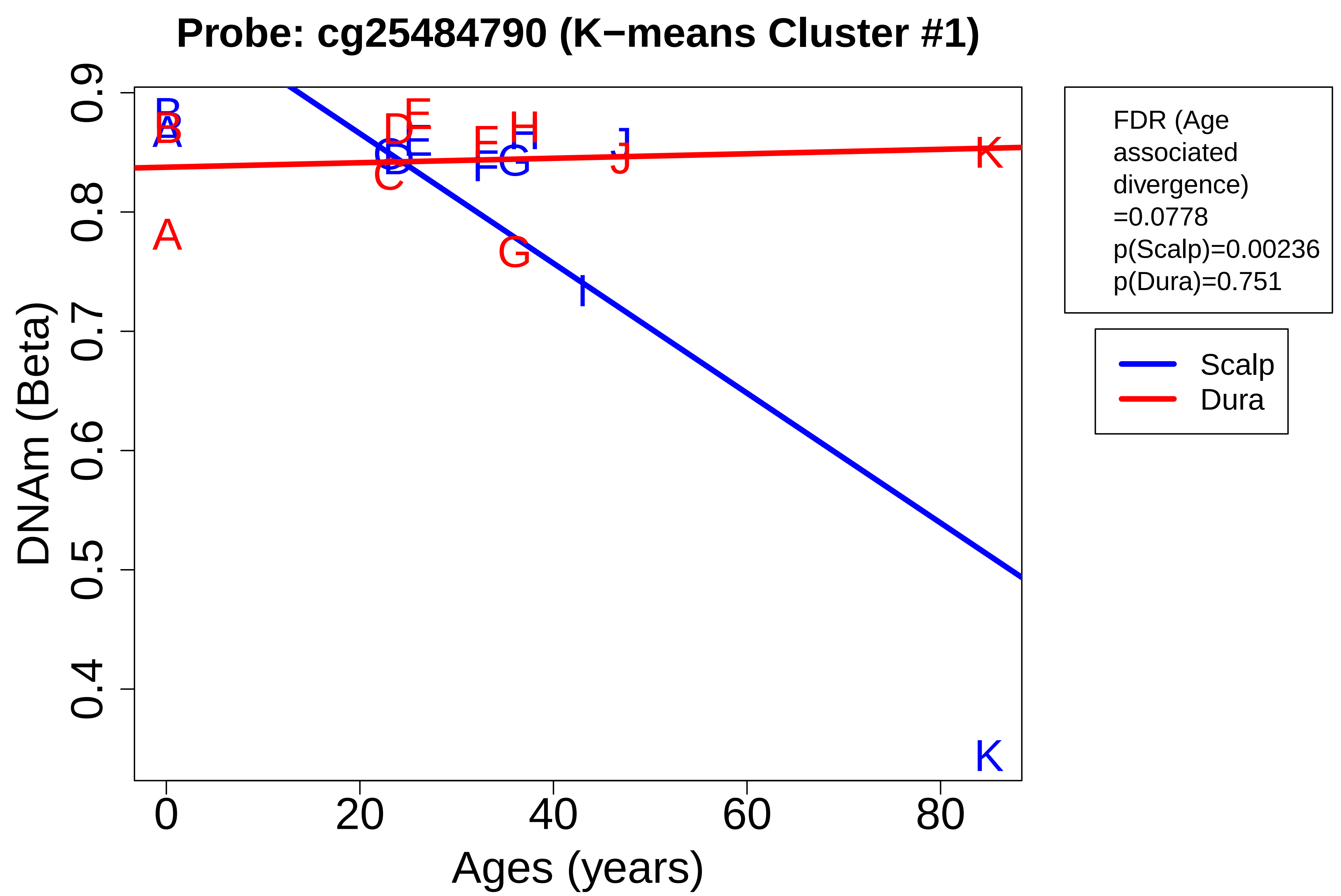

Probe: cg25484790 (K−means Cluster #1)
DNAm (Beta)
0.4
0.5
0.6
0.7
0.8
0.9
0
20
40
60
80
Ages (years)
FDR (Age associated divergence) =0.0778
p(Scalp)=0.00236
p(Dura)=0.751
Scalp
Dura

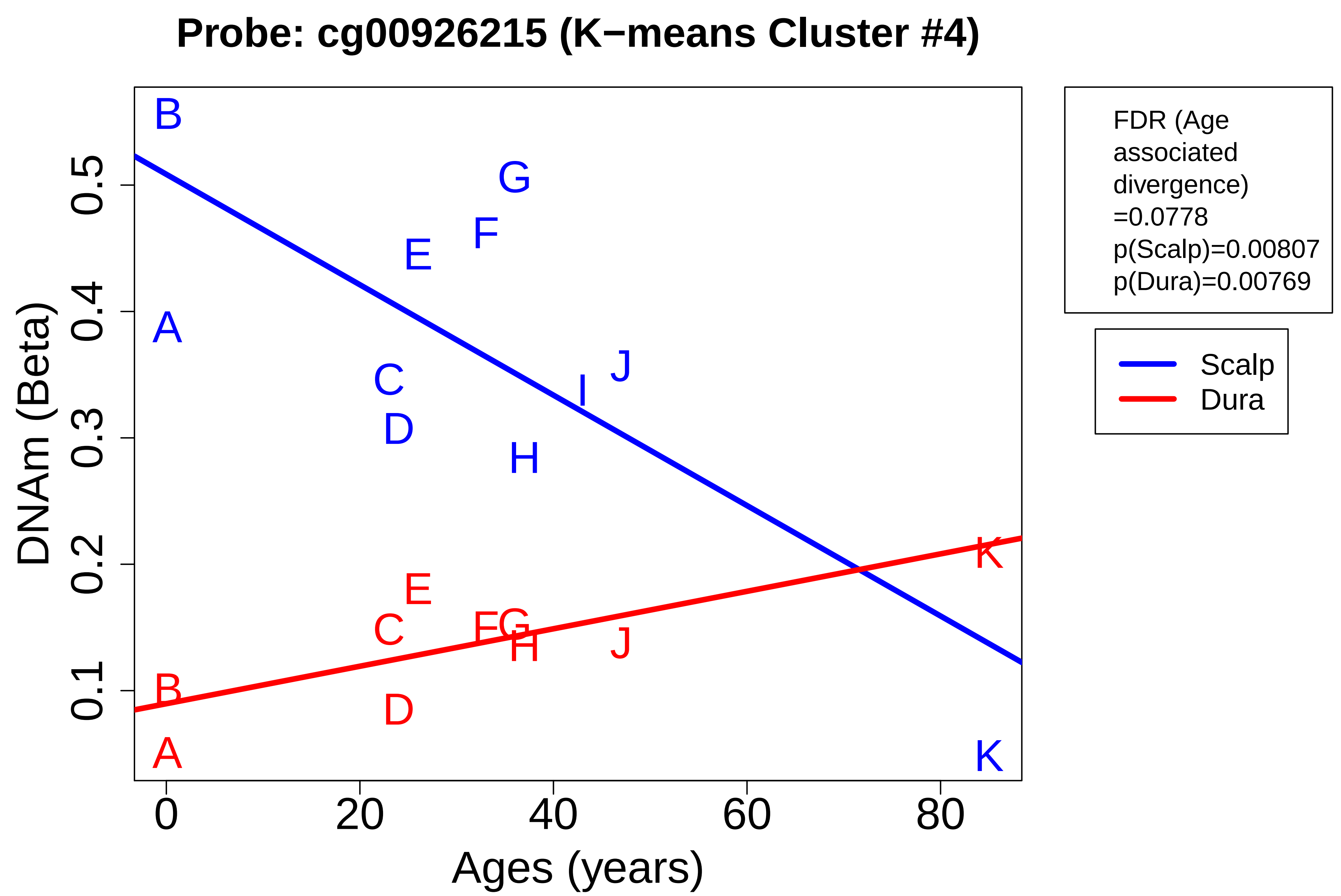

Probe: cg00926215 (K−means Cluster #4)
DNAm (Beta)
0.1
0.2
0.3
0.4
0.5
0
20
40
60
80
Ages (years)
FDR (Age associated divergence) =0.0778
p(Scalp)=0.00807
p(Dura)=0.00769
Scalp
Dura

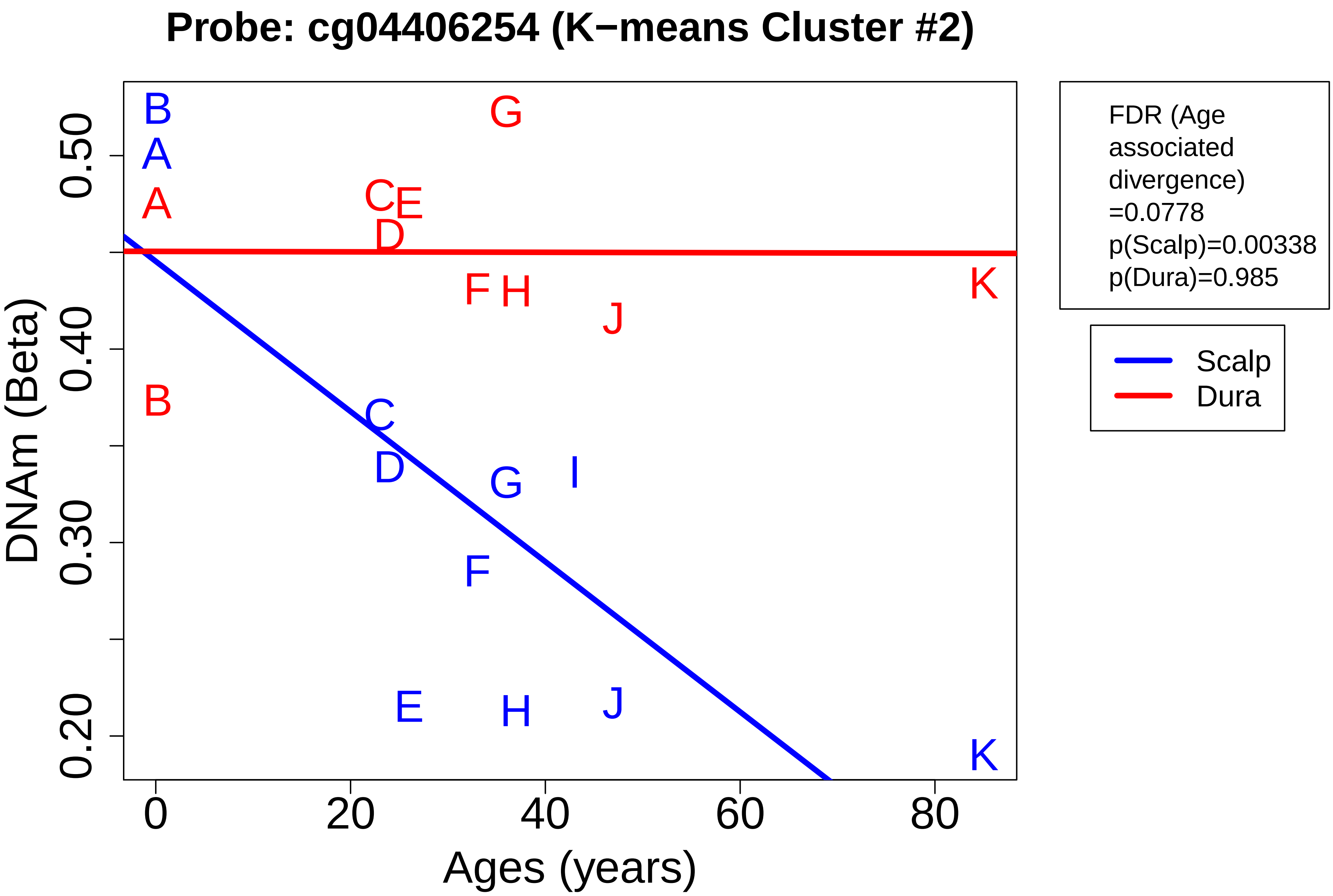

Probe: cg04406254 (K−means Cluster #2)
DNAm (Beta)
0.20
0.30
0.40
0.50
0
20
40
60
80
Ages (years)
B
A
A
G
C
E
D
F
H
K
J
B
C
D
G
I
F
E
H
J
K
FDR (Age associated divergence) =0.0778
p(Scalp)=0.00338
p(Dura)=0.985
Scalp
Dura

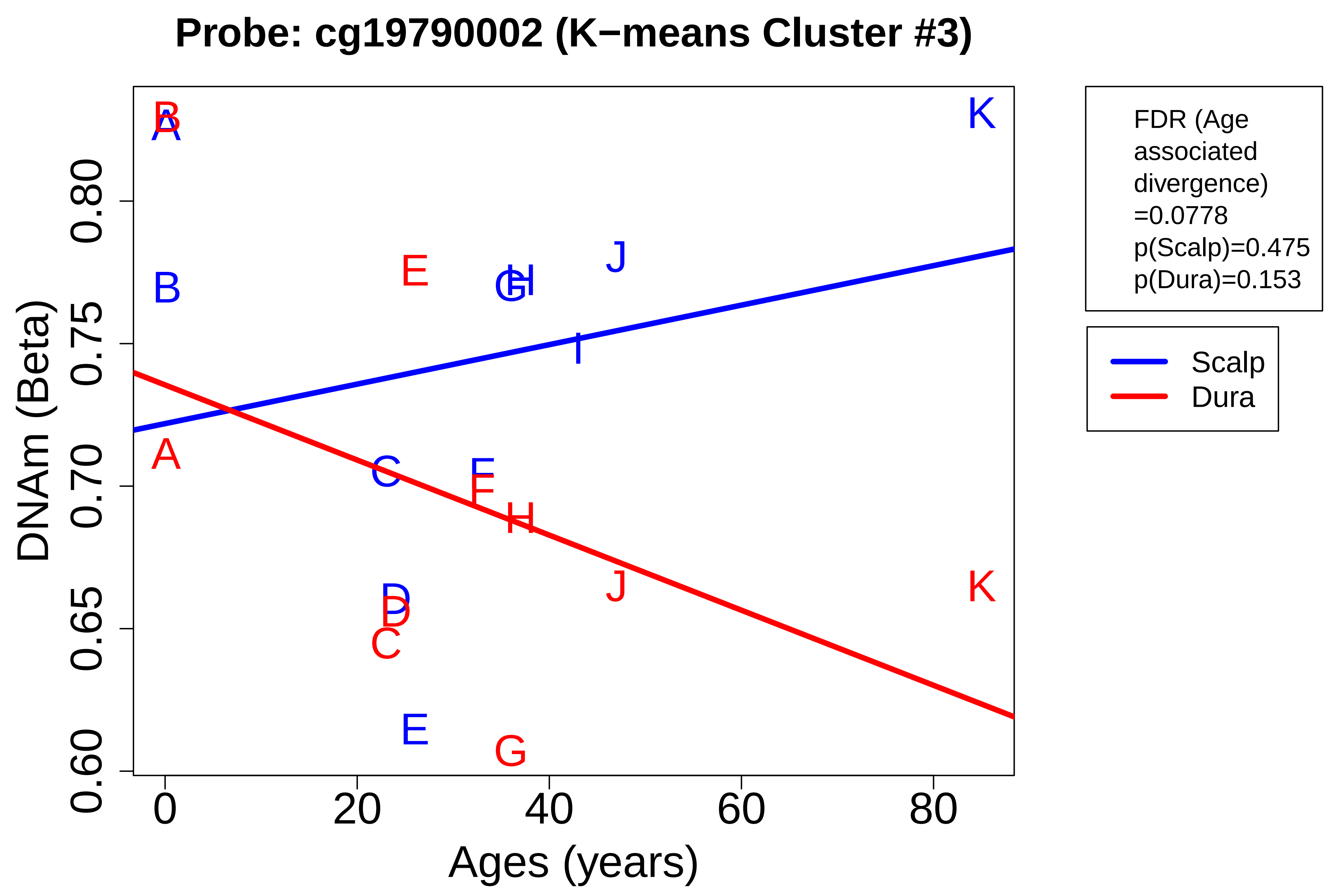

Probe: cg19790002 (K−means Cluster #3)
DNAm (Beta)
0.60
0.65
0.70
0.75
0.80
0
20
40
60
80
Ages (years)
FDR (Age associated divergence) =0.0778
p(Scalp)=0.475
p(Dura)=0.153
Scalp
Dura

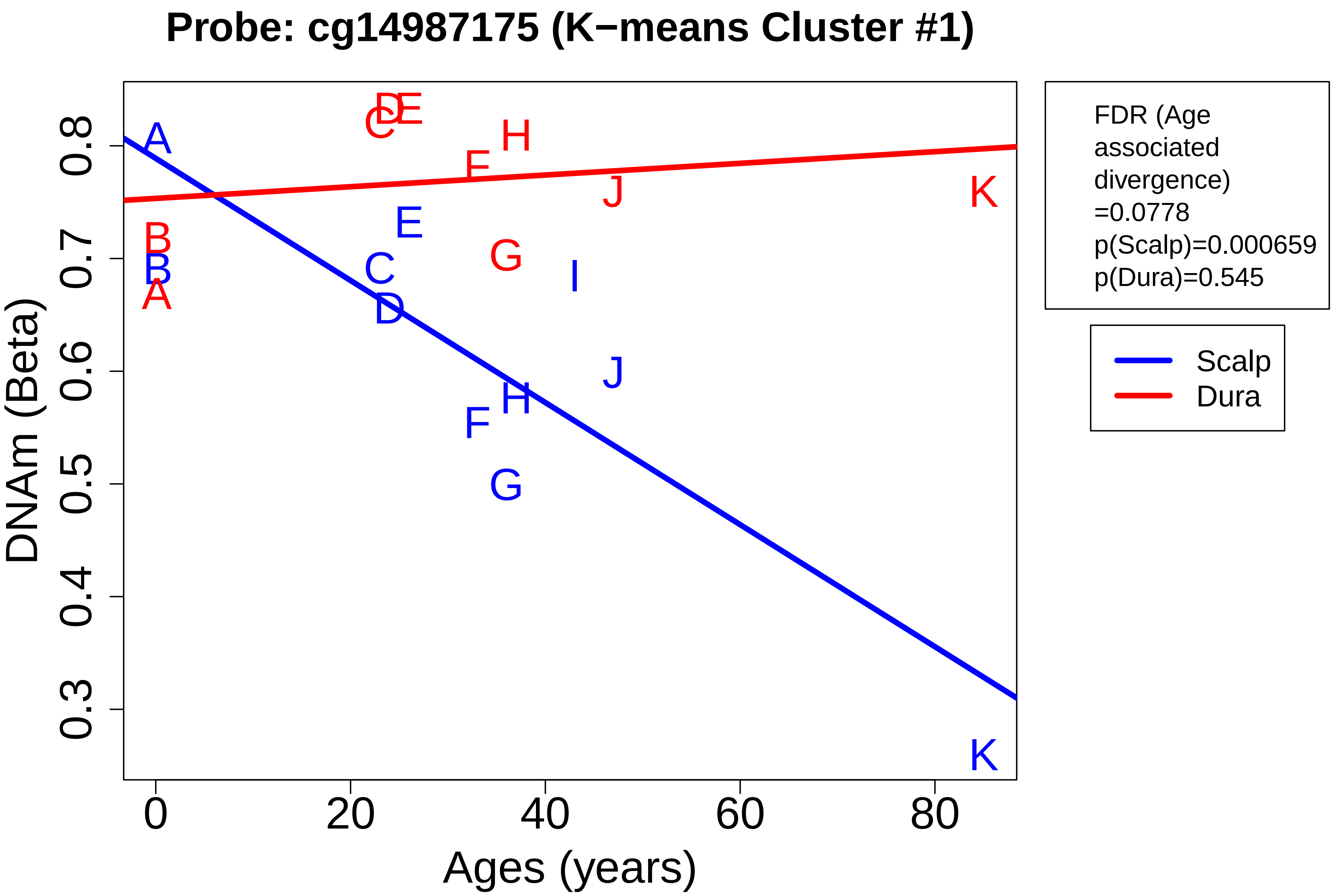

Probe: cg14987175 (K−means Cluster #1)
DNAm (Beta)
0.3
0.4
0.5
0.6
0.7
0.8
0
20
40
60
80
Ages (years)
A
B
C
D
E
F
G
H
I
J
K
FDR (Age associated divergence) =0.0778
p(Scalp)=0.000659
p(Dura)=0.545
Scalp
Dura

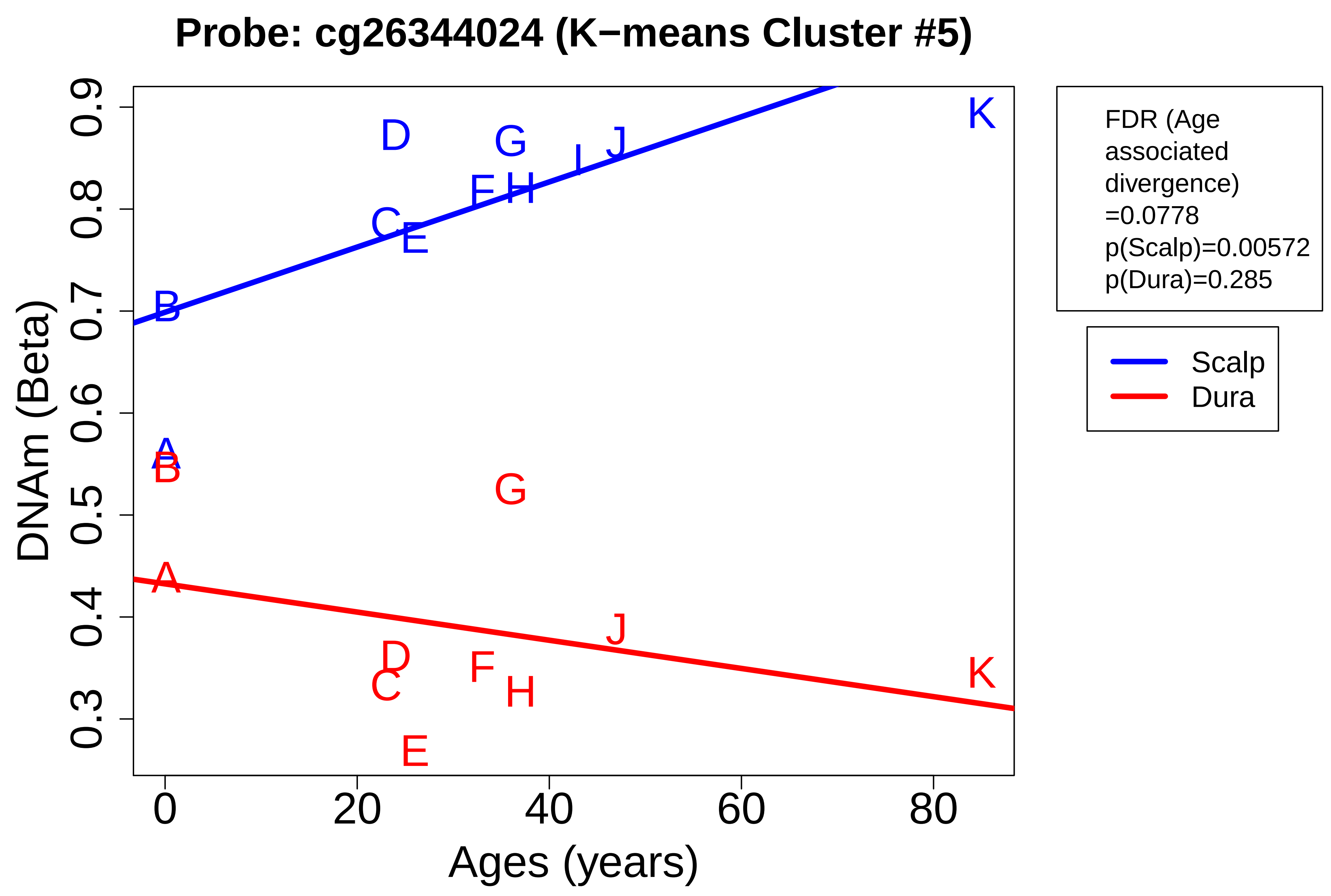
Probe: cg26344024 (K−means Cluster #5)
DNAm (Beta)
0.3
0.4
0.5
0.6
0.7
0.8
0.9
0
20
40
60
80
Ages (years)
A
B
C
D
E
F
G
H
I
J
K
FDR (Age associated divergence) =0.0778
p(Scalp)=0.00572
p(Dura)=0.285
Scalp
Dura

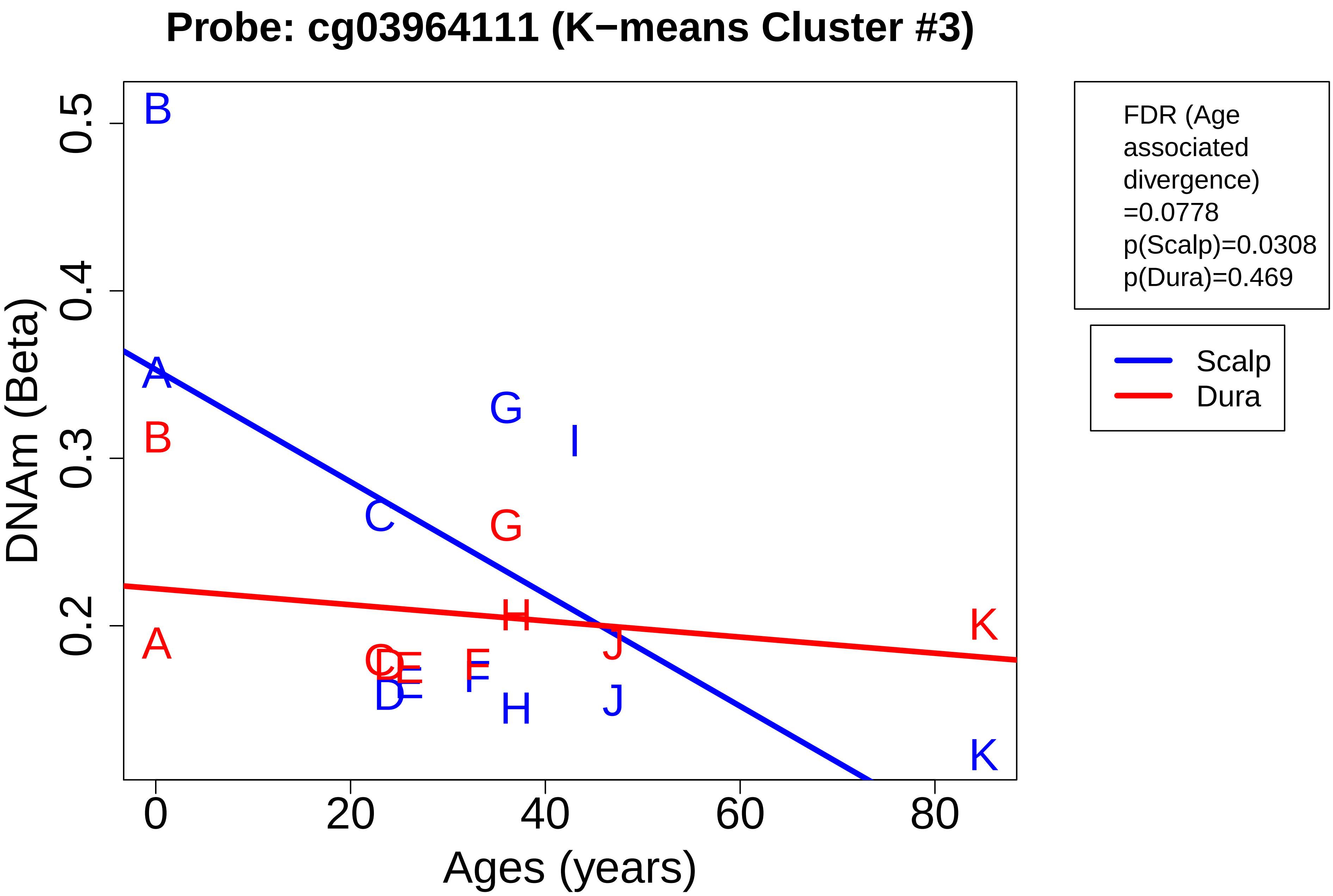

Probe: cg03964111 (K−means Cluster #3)
DNAm (Beta)
0.2
0.3
0.4
0.5
0
20
40
60
80
Ages (years)
FDR (Age associated divergence) =0.0778
p(Scalp)=0.0308
p(Dura)=0.469
Scalp
Dura

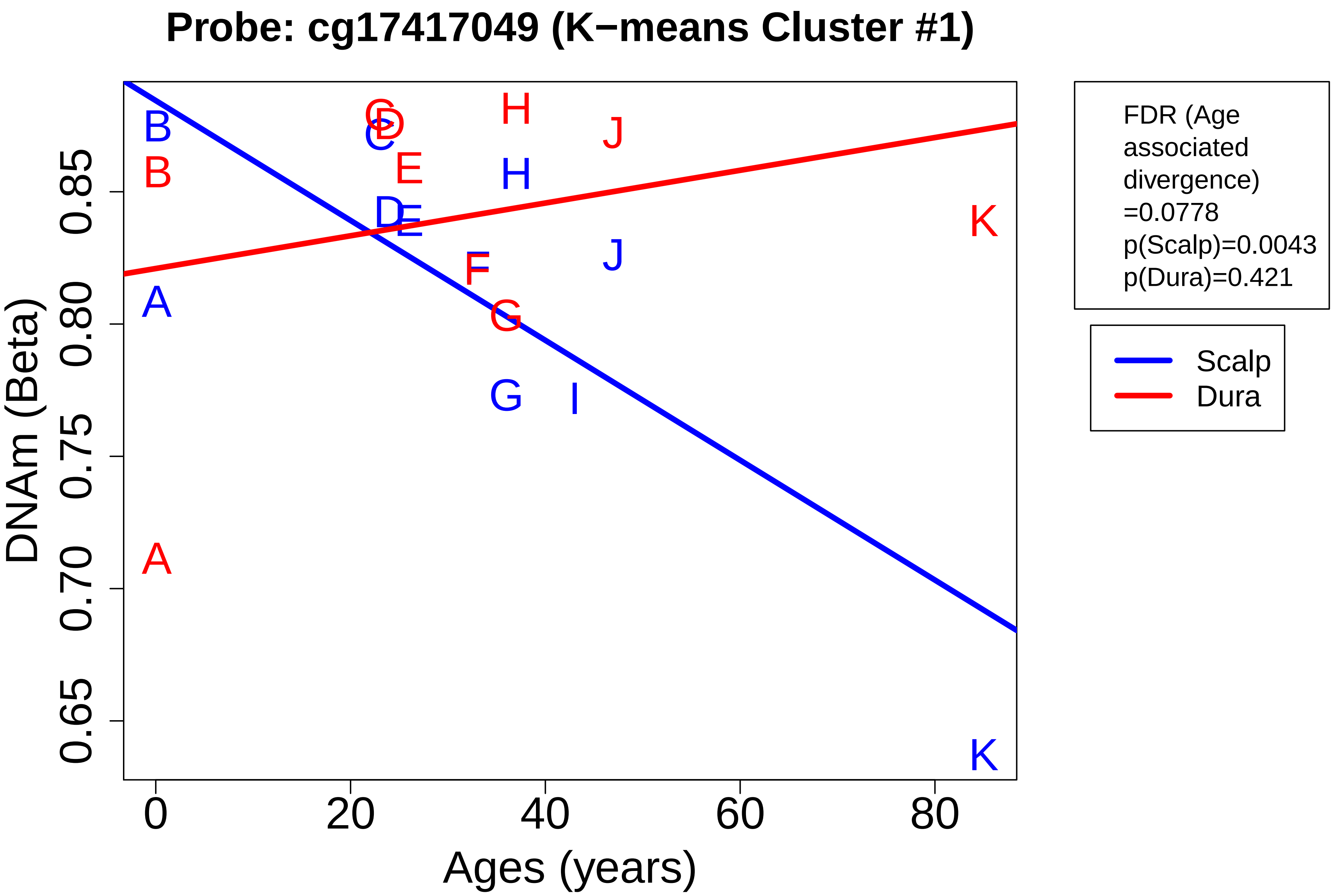

Probe: cg17417049 (K−means Cluster #1)
FDR (Age associated divergence) =0.0778
p(Scalp)=0.0043
p(Dura)=0.421
Scalp
Dura
DNAm (Beta)
0.65
0.70
0.75
0.80
0.85
0
20
40
60
80
Ages (years)
A
B
C
D
E
F
G
H
I
J
K

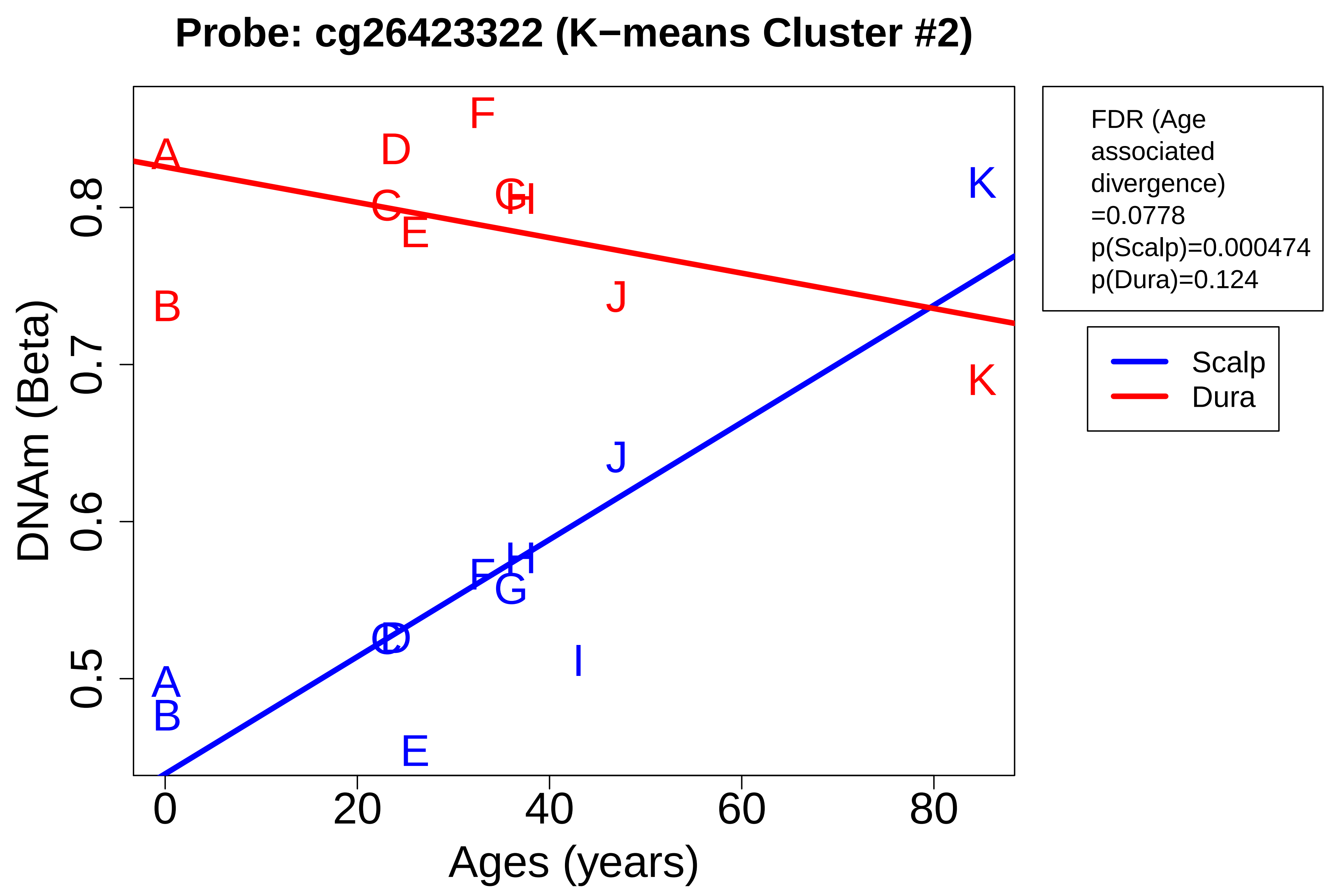

Probe: cg26423322 (K−means Cluster #2)
DNAm (Beta)
0.5
0.6
0.7
0.8
0
20
40
60
80
Ages (years)
FDR (Age associated divergence) =0.0778
p(Scalp)=0.000474
p(Dura)=0.124
Scalp
Dura

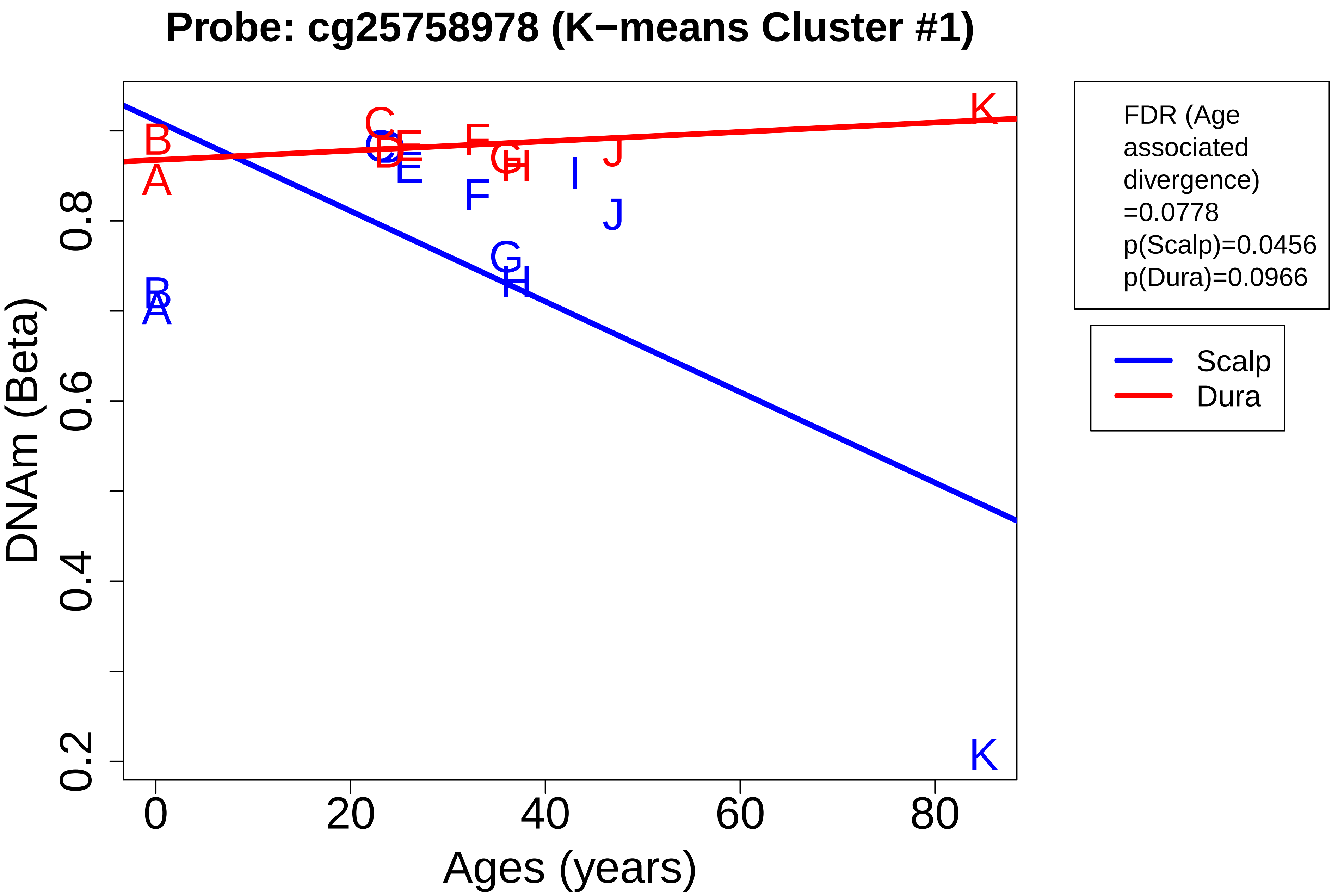

Probe: cg25758978 (K−means Cluster #1)
DNAm (Beta)
0.2
0.4
0.6
0.8
0
20
40
60
80
Ages (years)
FDR (Age associated divergence) =0.0778
p(Scalp)=0.0456
p(Dura)=0.0966
Scalp
Dura

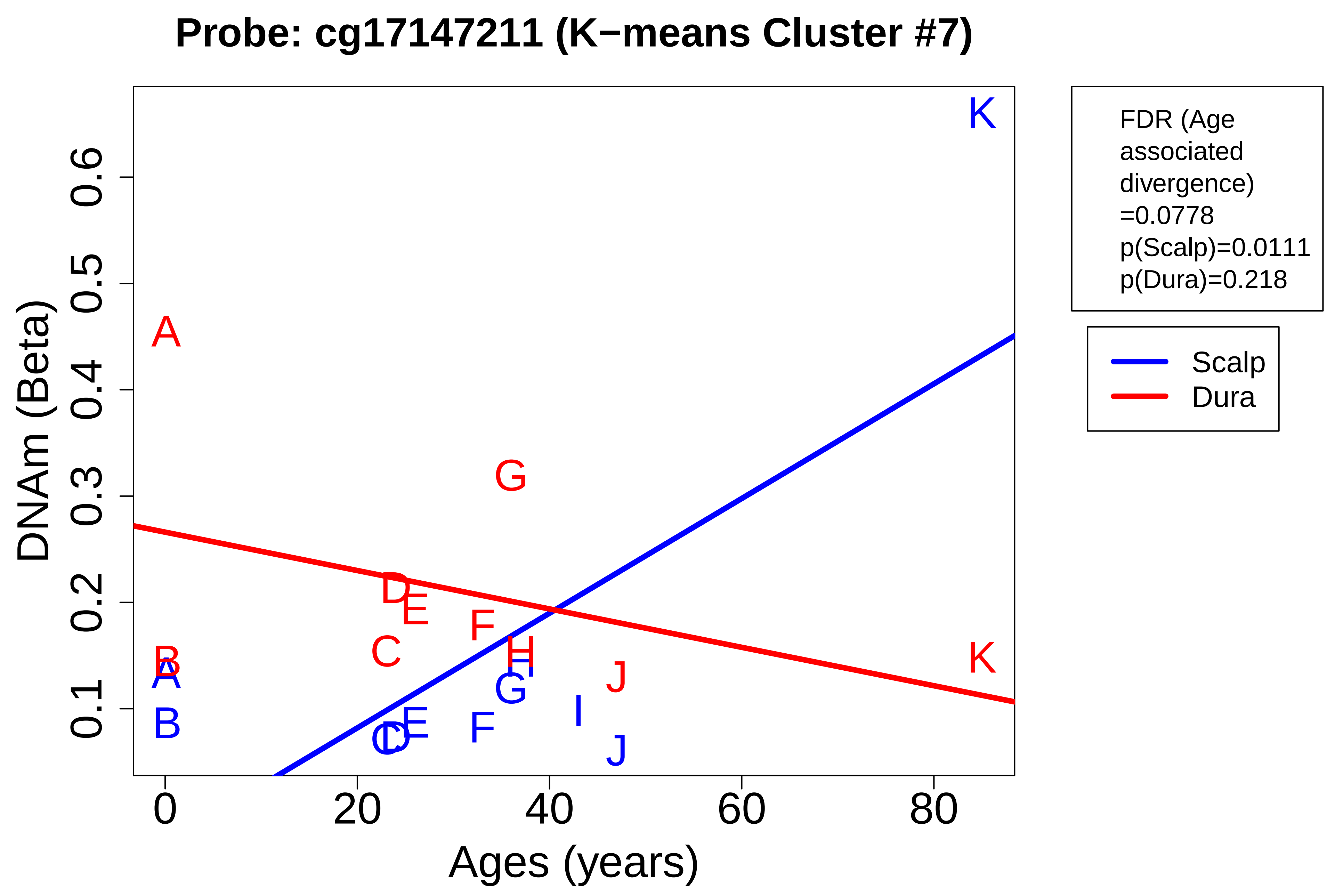

Probe: cg17147211 (K−means Cluster #7)
FDR (Age associated divergence) =0.0778
p(Scalp)=0.0111
p(Dura)=0.218
Scalp
Dura
DNAm (Beta)
Ages (years)
0.1
0.2
0.3
0.4
0.5
0.6
0
20
40
60
80

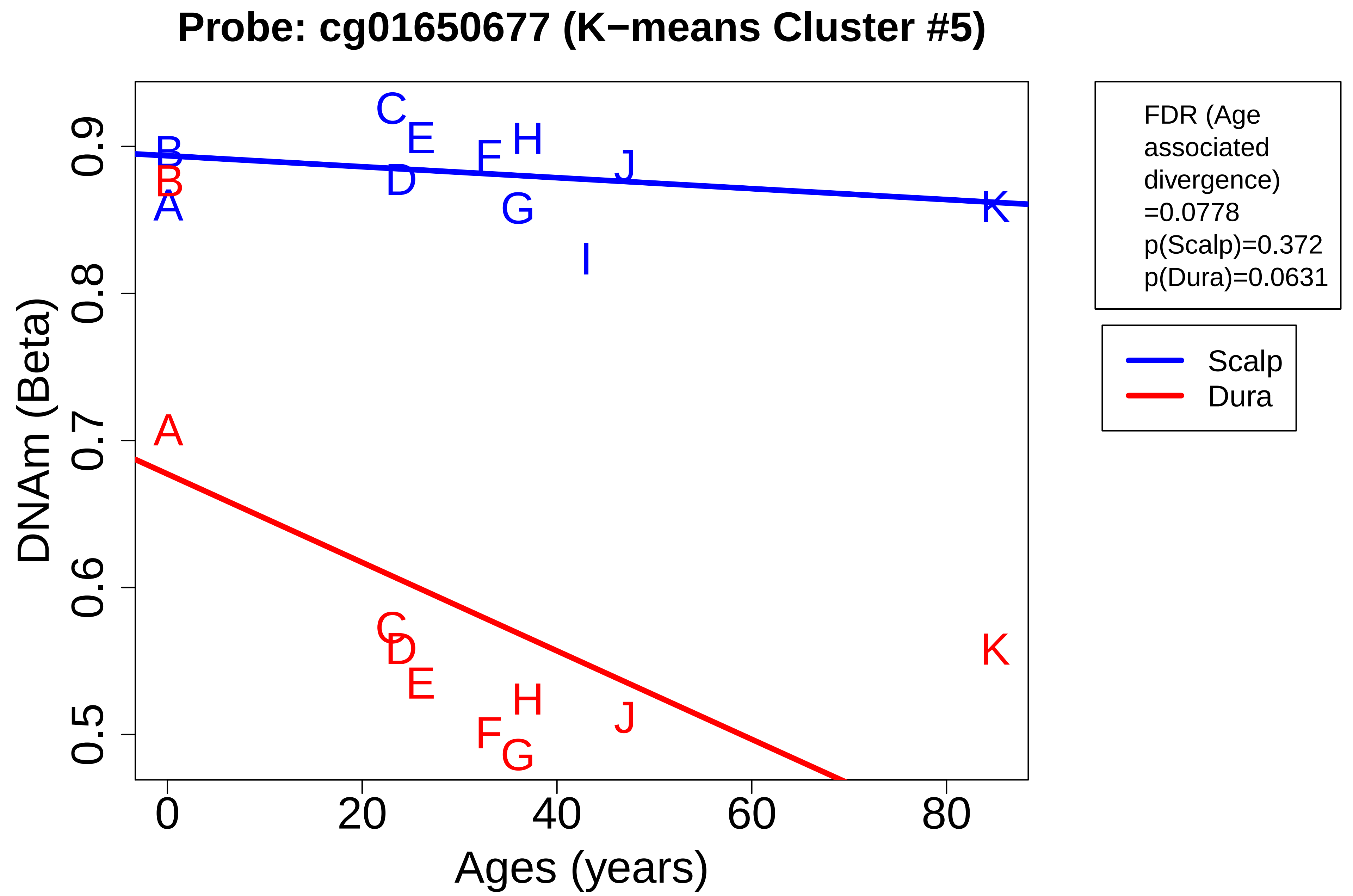

Probe: cg01650677 (K−means Cluster #5)
DNAm (Beta)
0.5
0.6
0.7
0.8
0.9
0
20
40
60
80
Ages (years)
FDR (Age associated divergence) =0.0778
p(Scalp)=0.372
p(Dura)=0.0631
Scalp
Dura
A
B
C
D
E
F
G
H
I
J
K

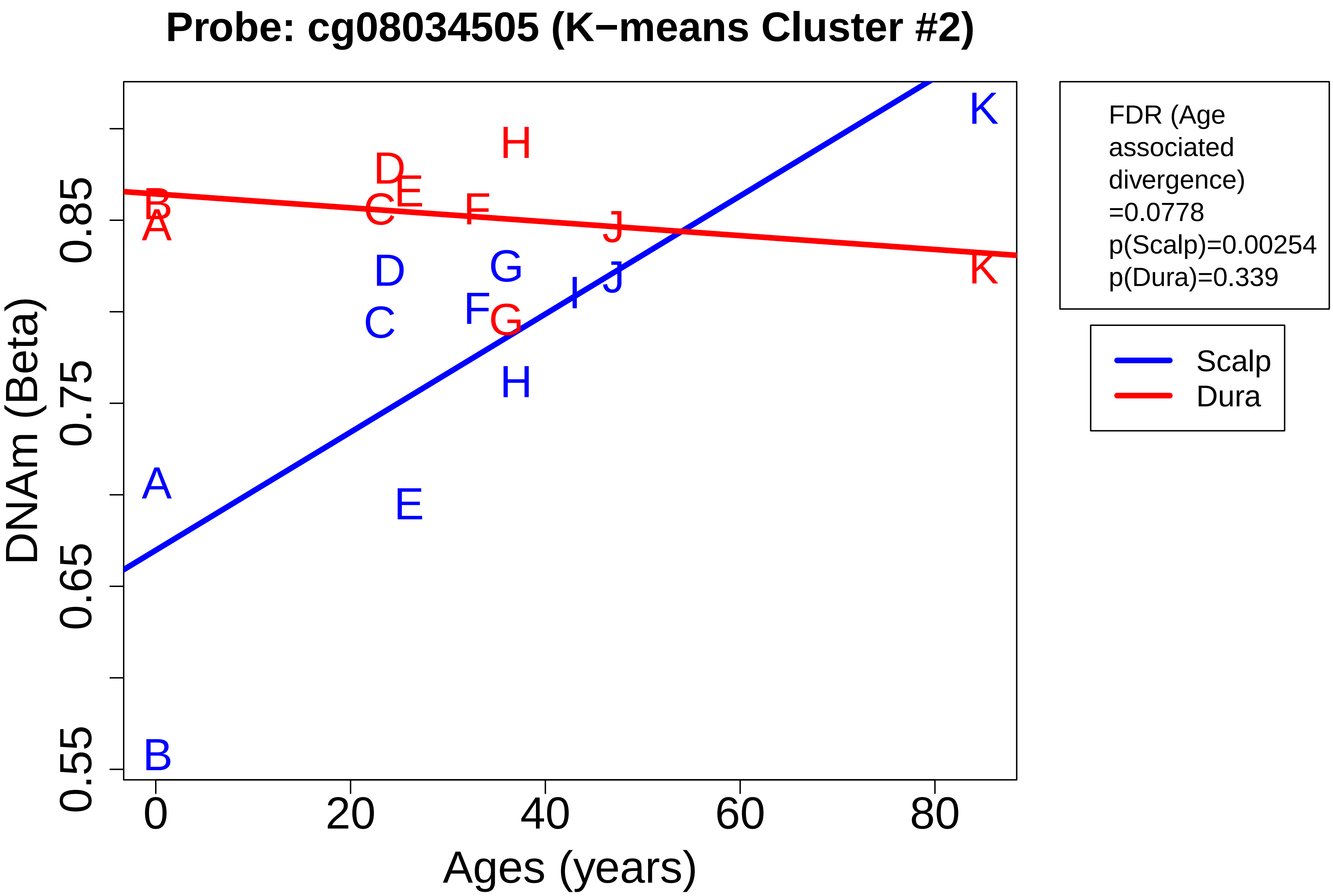

Probe: cg08034505 (K−means Cluster #2)
DNAm (Beta)
0.55
0.65
0.75
0.85
0
20
40
60
80
Ages (years)
FDR (Age associated divergence) =0.0778
p(Scalp)=0.00254
p(Dura)=0.339
Scalp
Dura

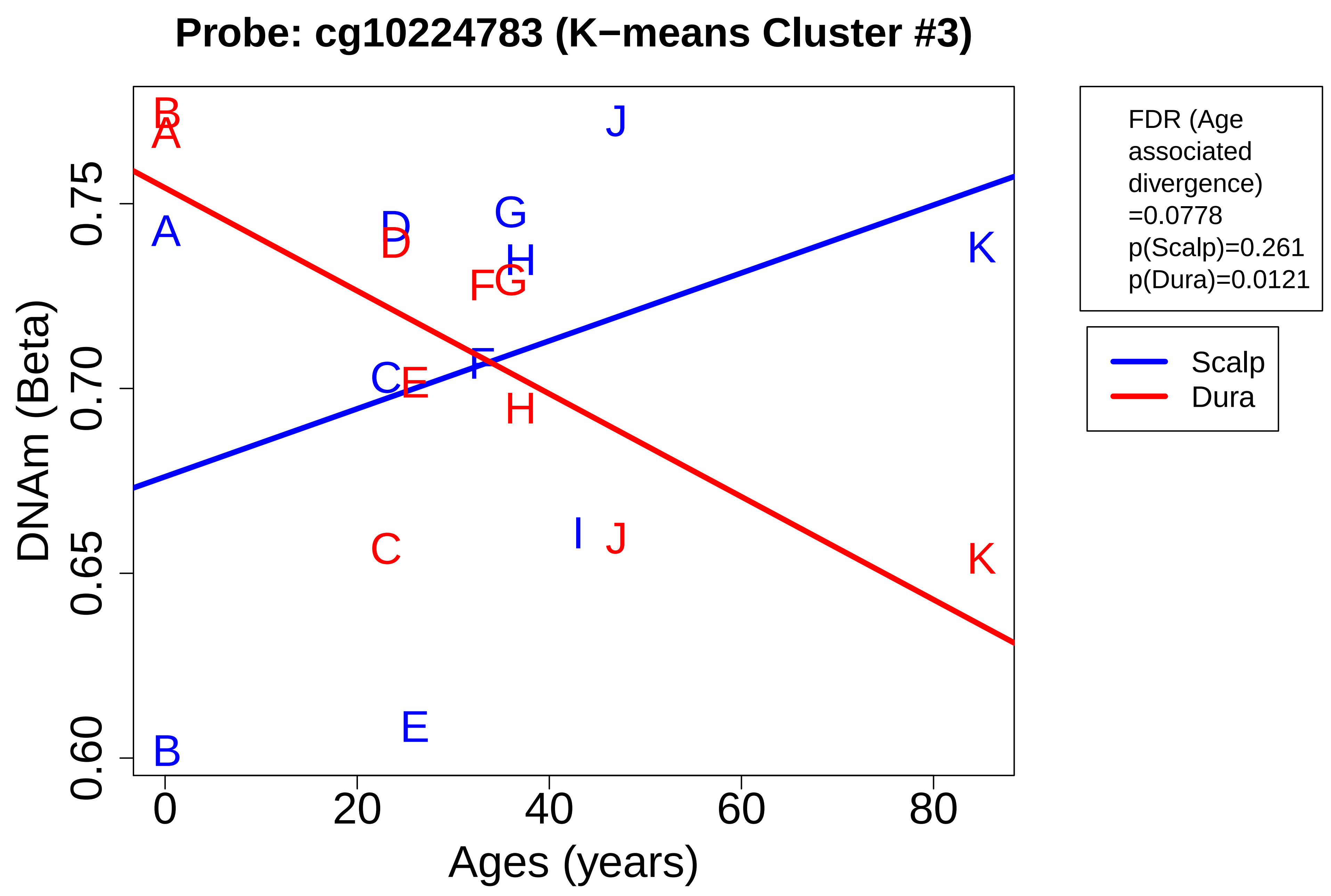
Probe: cg10224783 (K−means Cluster #3)
DNAm (Beta)
Ages (years)
0.60
0.65
0.70
0.75
0
20
40
60
80
FDR (Age associated divergence) =0.0778
p(Scalp)=0.261
p(Dura)=0.0121
Scalp
Dura

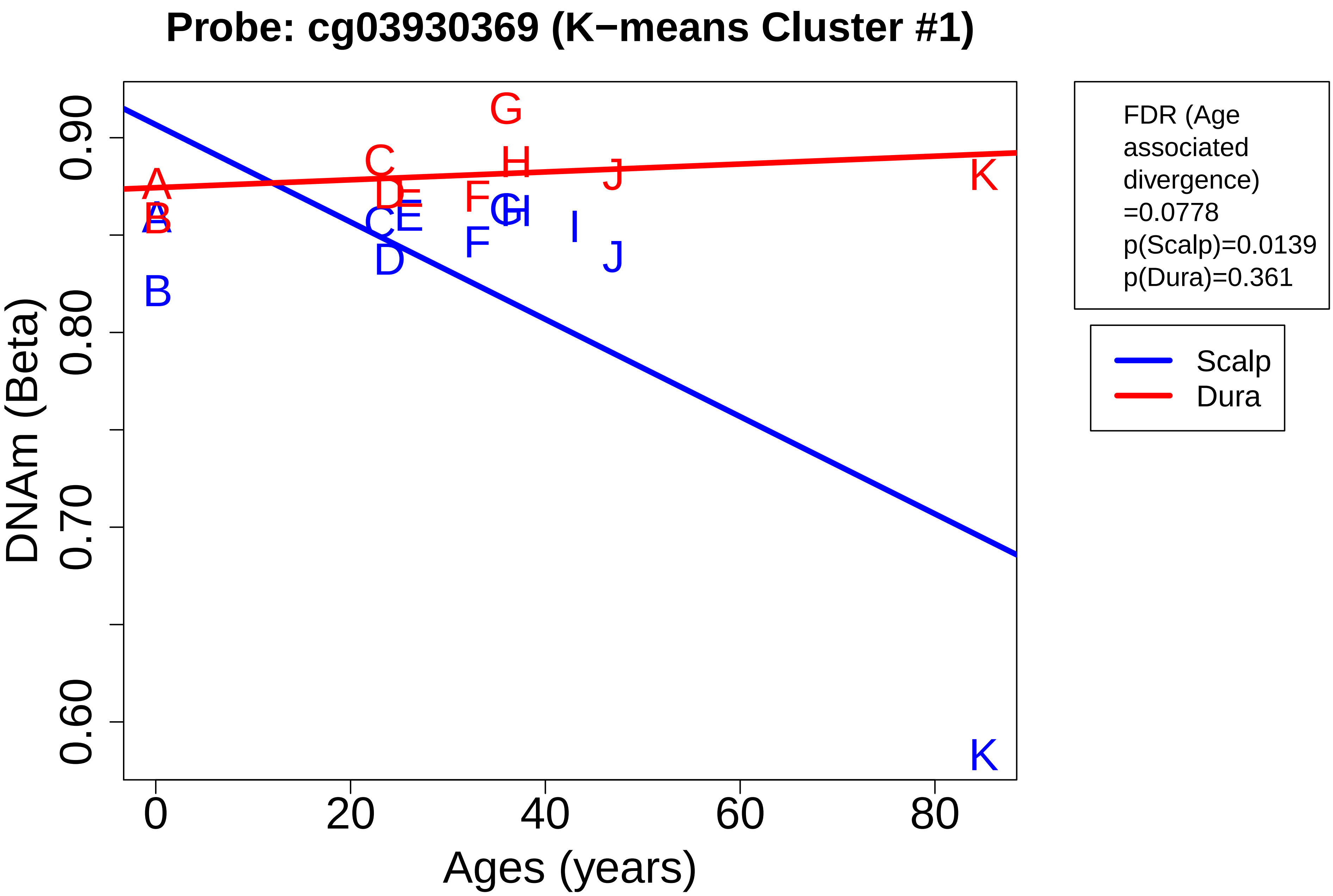

Probe: cg03930369 (K−means Cluster #1)
DNAm (Beta)
0.60
0.70
0.80
0.90
0
20
40
60
80
Ages (years)
FDR (Age associated divergence) =0.0778
p(Scalp)=0.0139
p(Dura)=0.361
Scalp
Dura

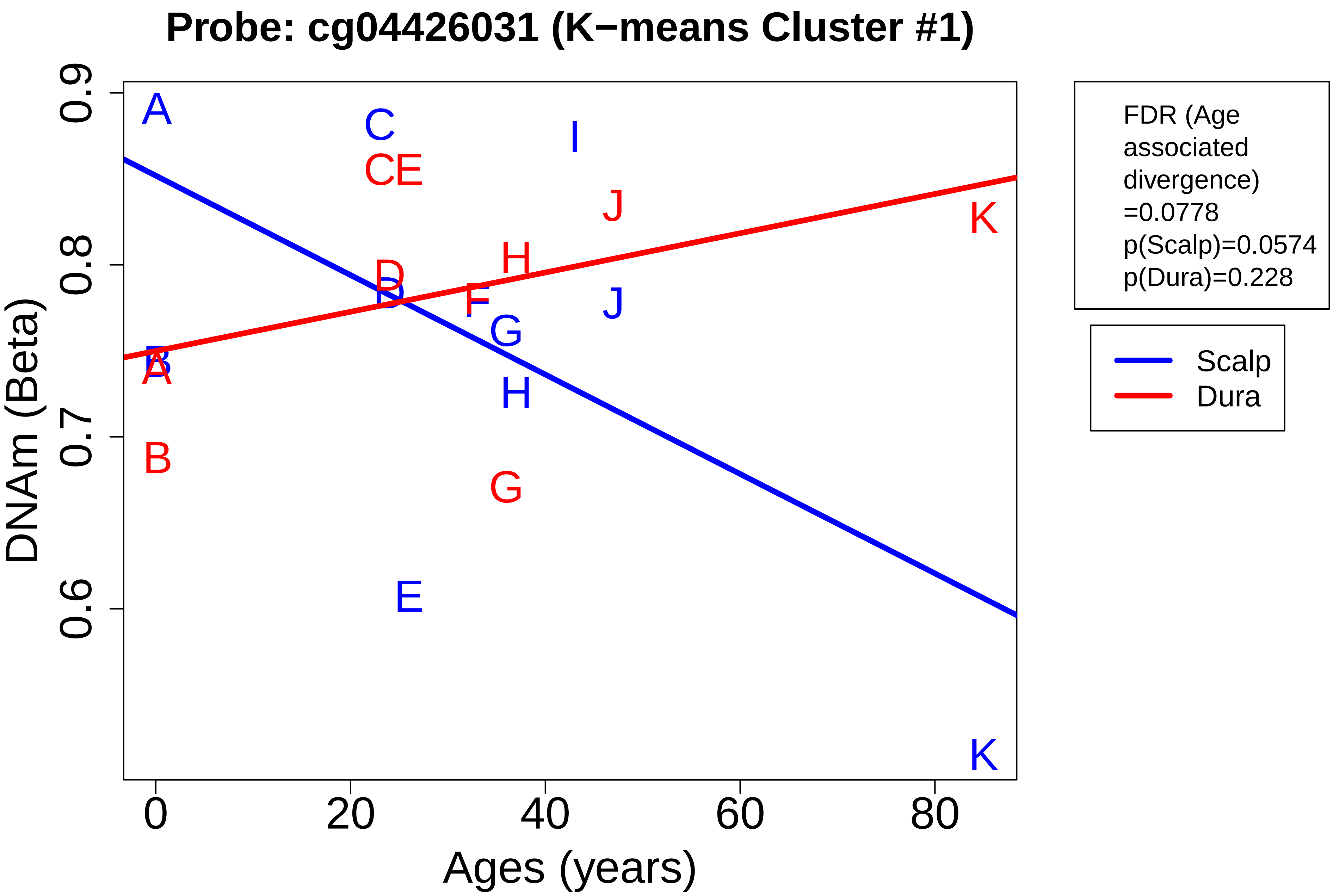

Probe: cg04426031 (K−means Cluster #1)
DNAm (Beta)
0.6
0.7
0.8
0.9
Ages (years)
0
20
40
60
80
FDR (Age associated divergence) =0.0778
p(Scalp)=0.0574
p(Dura)=0.228
Scalp
Dura

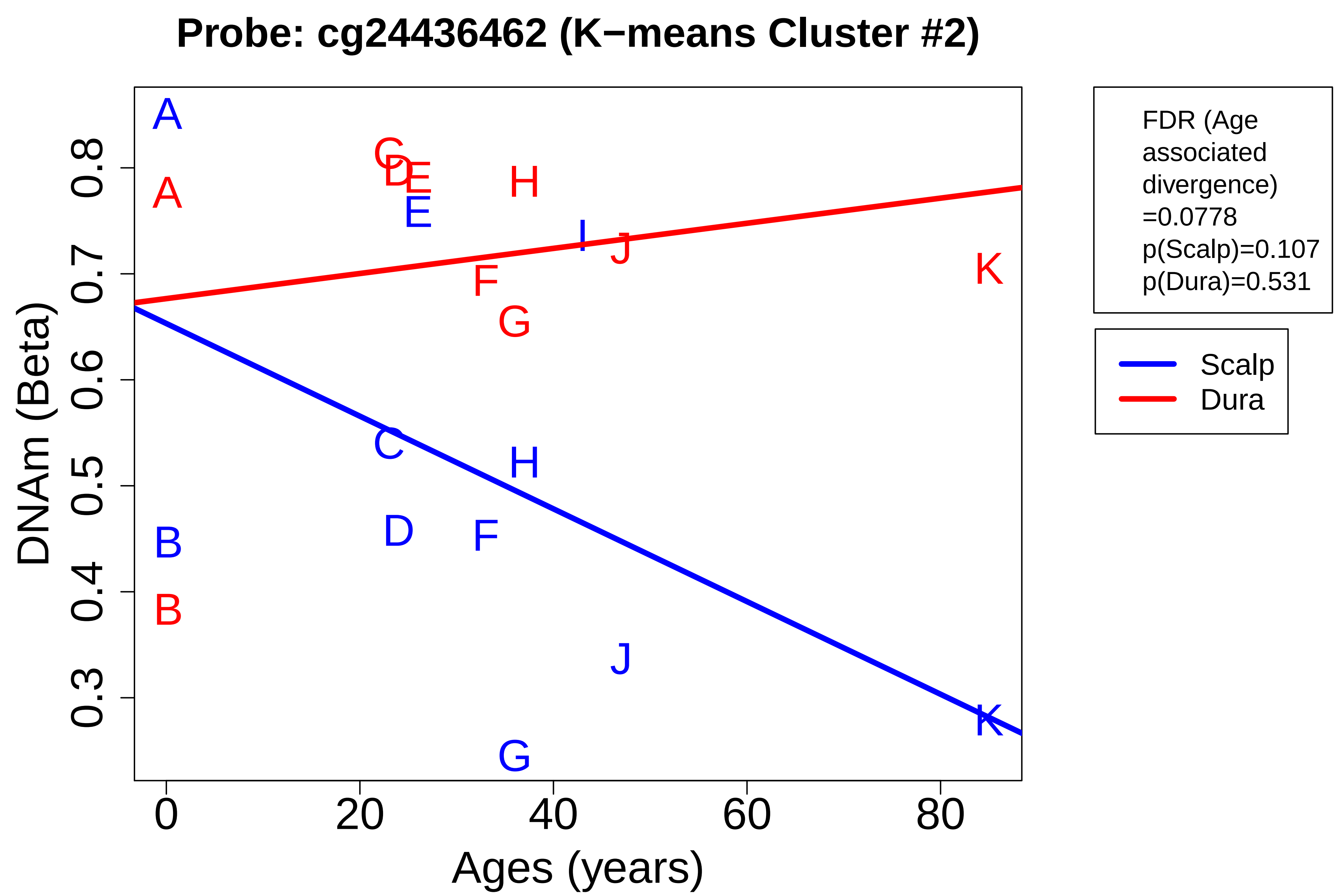
Probe: cg24436462 (K−means Cluster #2)
DNAm (Beta)
Ages (years)
0.3
0.4
0.5
0.6
0.7
0.8
0
20
40
60
80
FDR (Age associated divergence) =0.0778
p(Scalp)=0.107
p(Dura)=0.531
Scalp
Dura

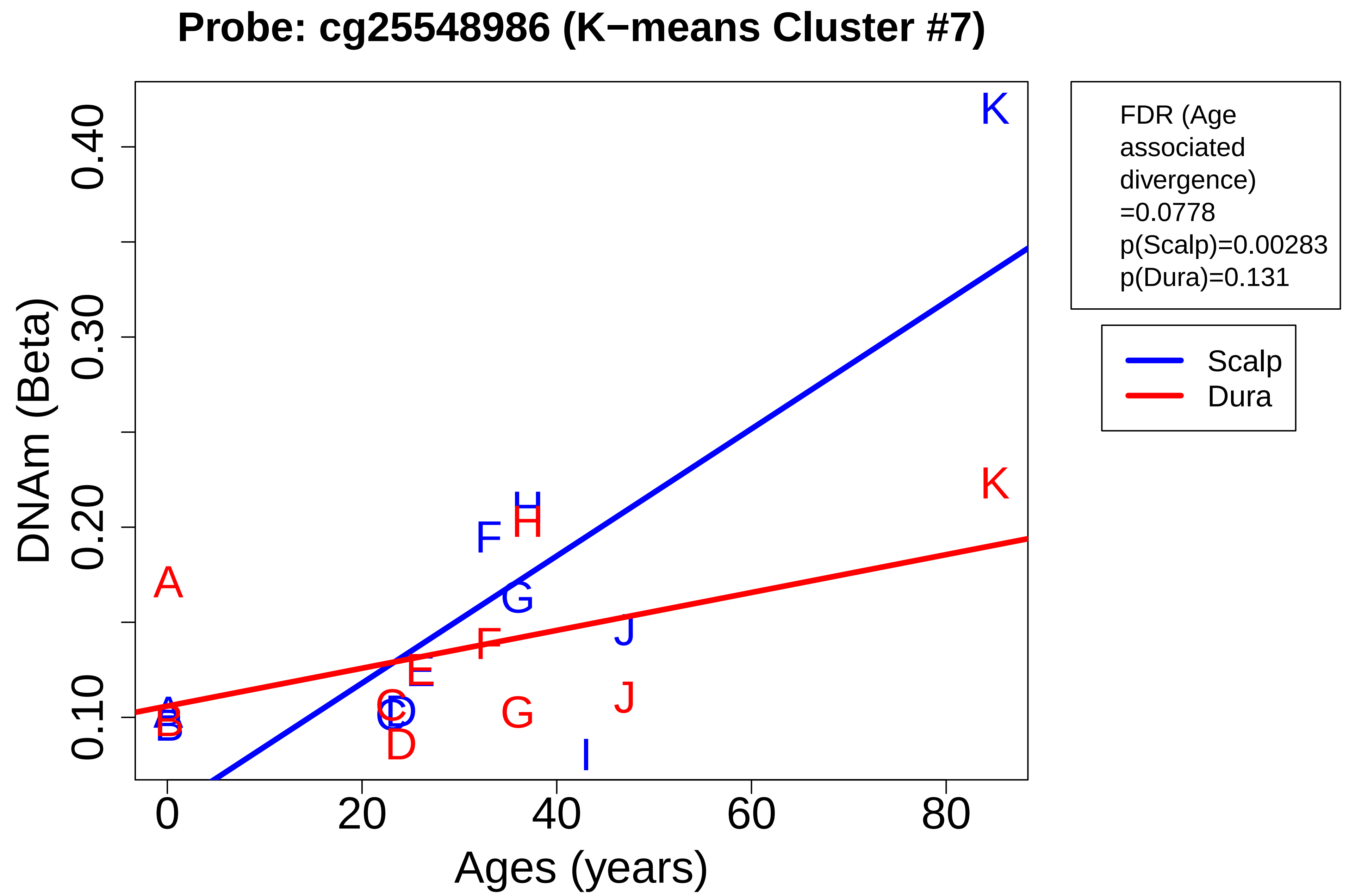

Probe: cg25548986 (K−means Cluster #7)
DNAm (Beta)
0.10
0.20
0.30
0.40
0
20
40
60
80
Ages (years)
FDR (Age associated divergence) =0.0778
p(Scalp)=0.00283
p(Dura)=0.131
Scalp
Dura

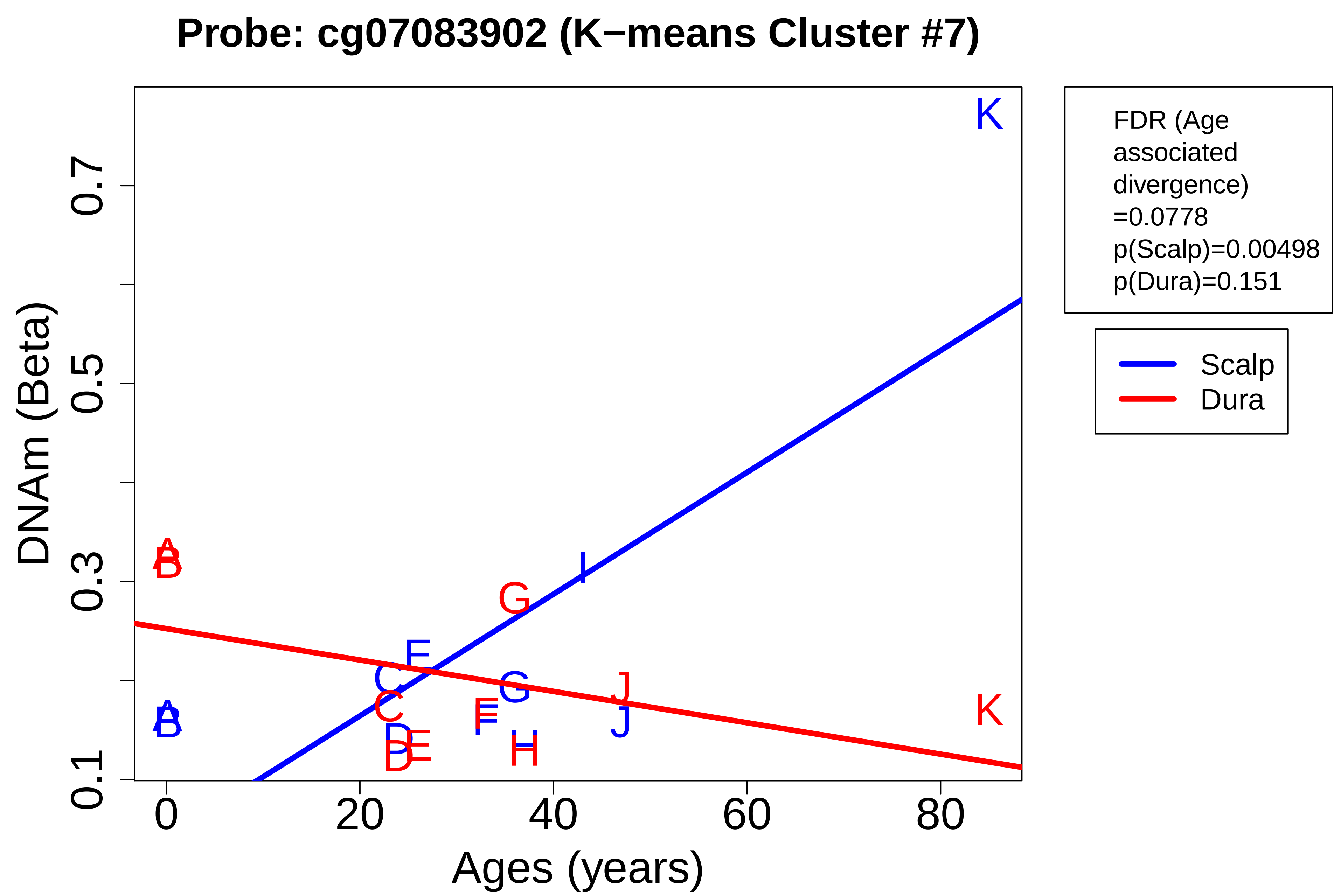

Probe: cg07083902 (K−means Cluster #7)
DNAm (Beta)
0.1
0.3
0.5
0.7
0
20
40
60
80
Ages (years)
FDR (Age associated divergence) =0.0778
p(Scalp)=0.00498
p(Dura)=0.151
Scalp
Dura

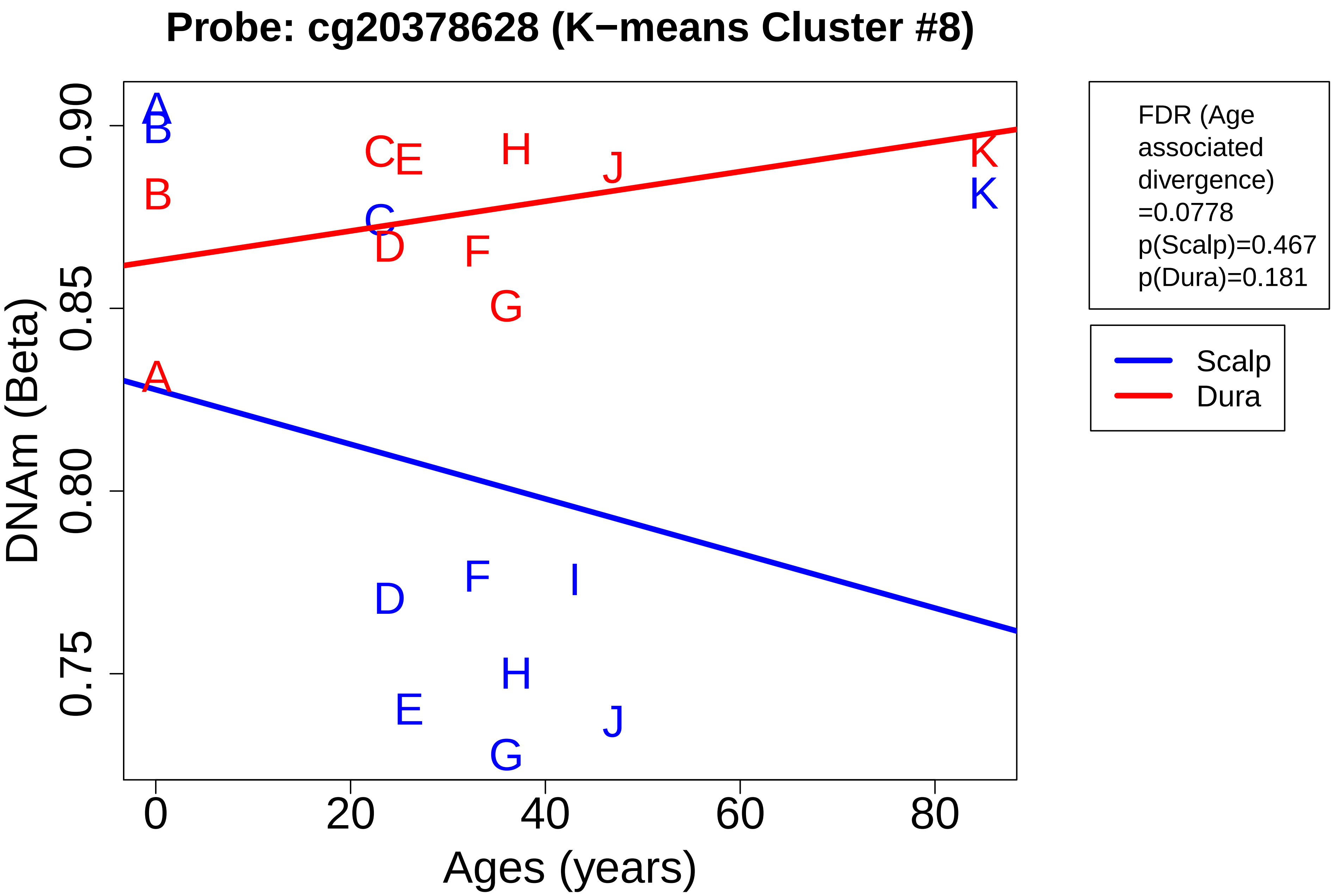
Probe: cg20378628 (K−means Cluster #8)
DNAm (Beta)
0.75
0.80
0.85
0.90
Ages (years)
0
20
40
60
80
FDR (Age associated divergence) =0.0778
p(Scalp)=0.467
p(Dura)=0.181
Scalp
Dura

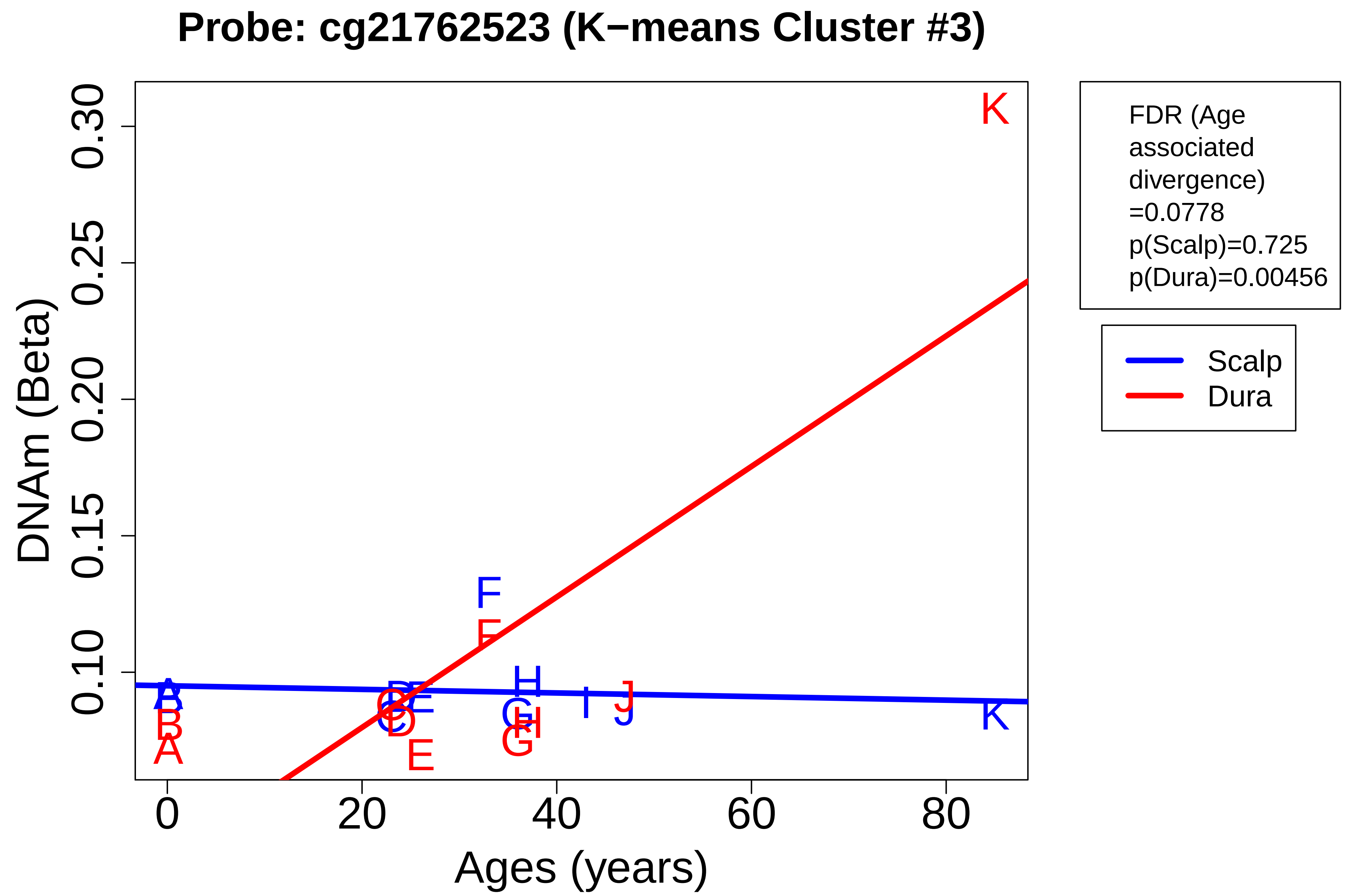

Probe: cg21762523 (K−means Cluster #3)
DNAm (Beta)
0.10
0.15
0.20
0.25
0.30
Ages (years)
0
20
40
60
80
FDR (Age associated divergence) =0.0778
p(Scalp)=0.725
p(Dura)=0.00456
Scalp
Dura

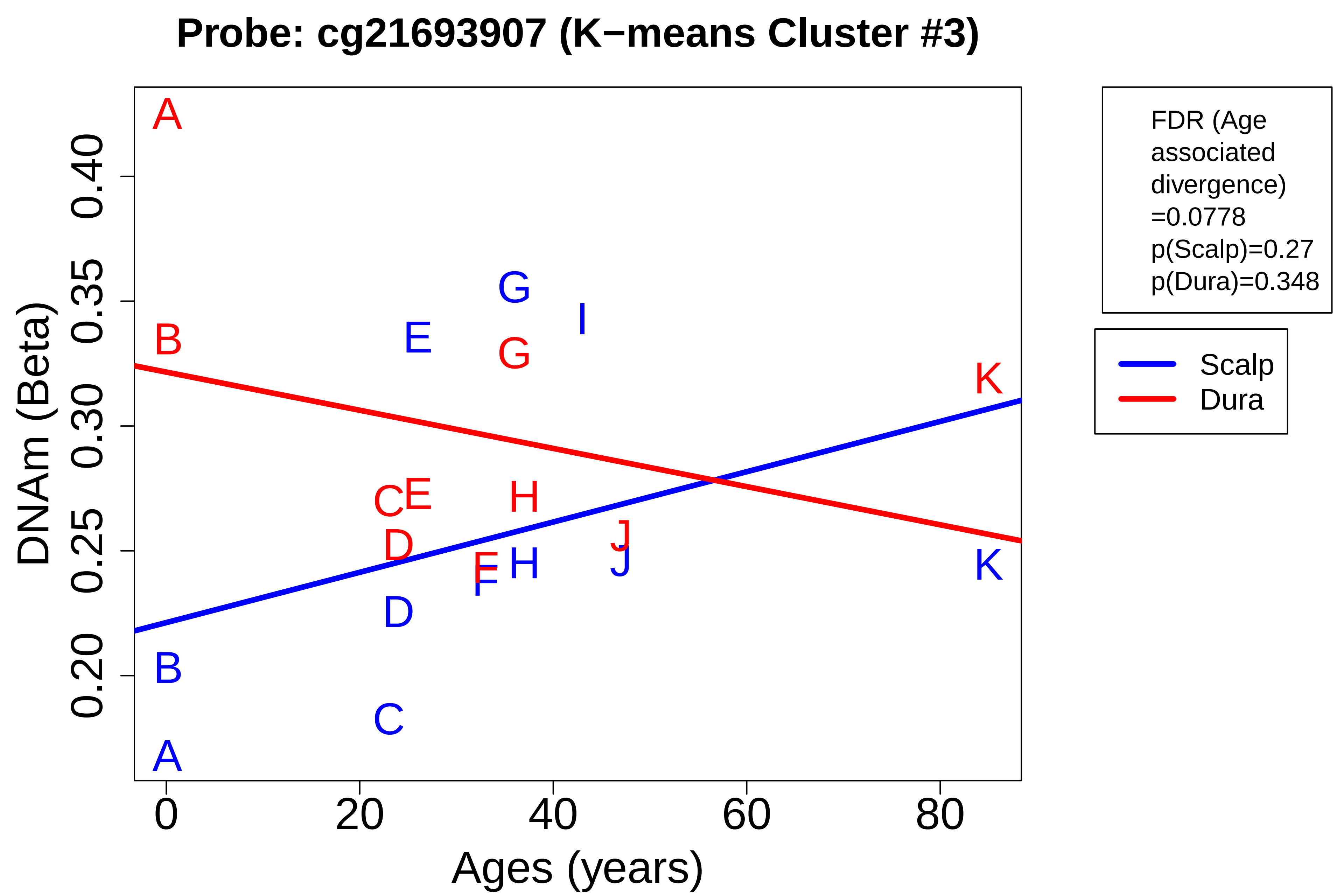

Probe: cg21693907 (K−means Cluster #3)
DNAm (Beta)
0.20
0.25
0.30
0.35
0.40
Ages (years)
0
20
40
60
80
A
B
C
D
E
F
G
H
I
J
K
FDR (Age associated divergence) =0.0778
p(Scalp)=0.27
p(Dura)=0.348
Scalp
Dura

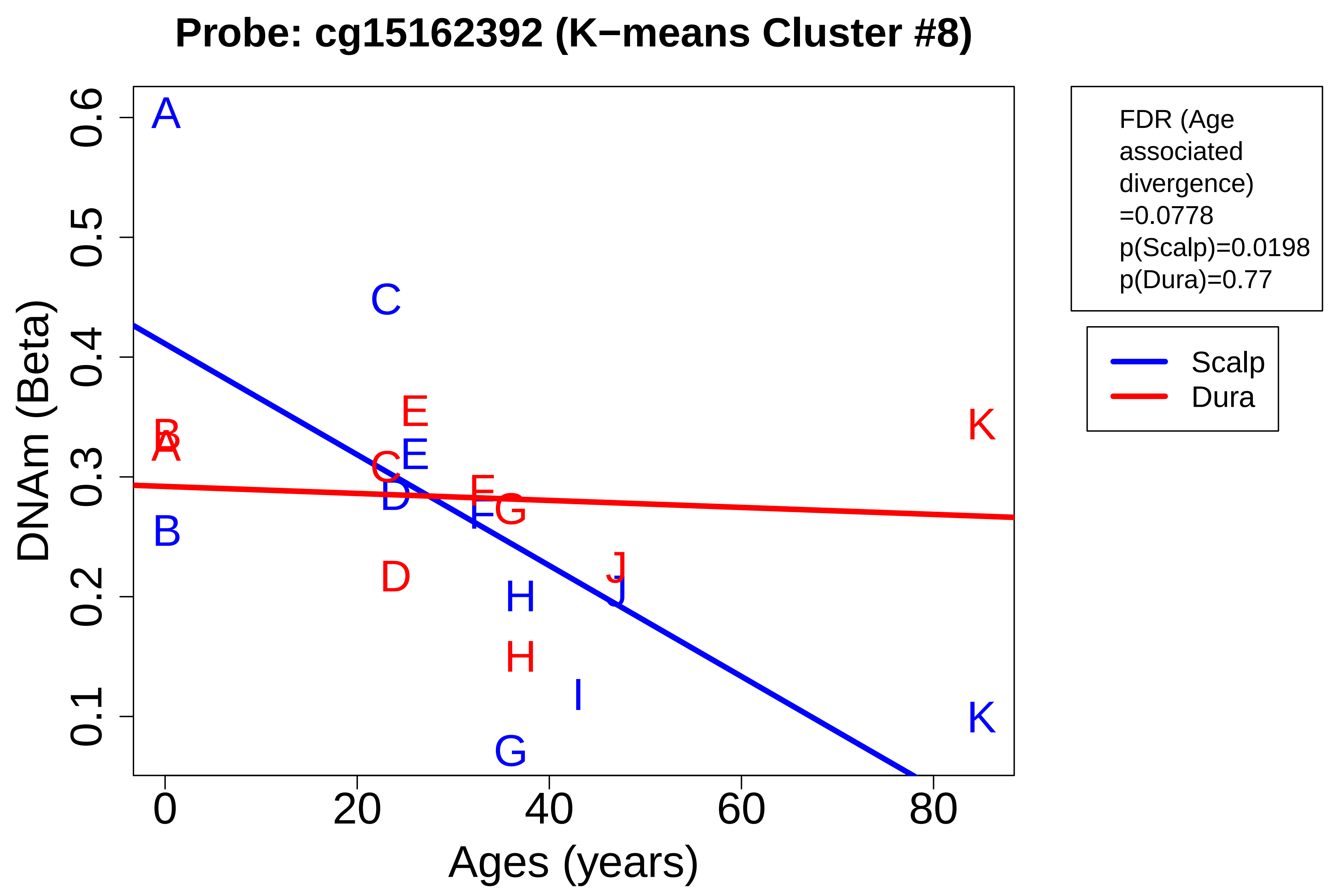

Probe: cg15162392 (K−means Cluster #8)
DNAm (Beta)
Ages (years)
0.1
0.2
0.3
0.4
0.5
0.6
0
20
40
60
80
FDR (Age associated divergence) =0.0778
p(Scalp)=0.0198
p(Dura)=0.77
Scalp
Dura

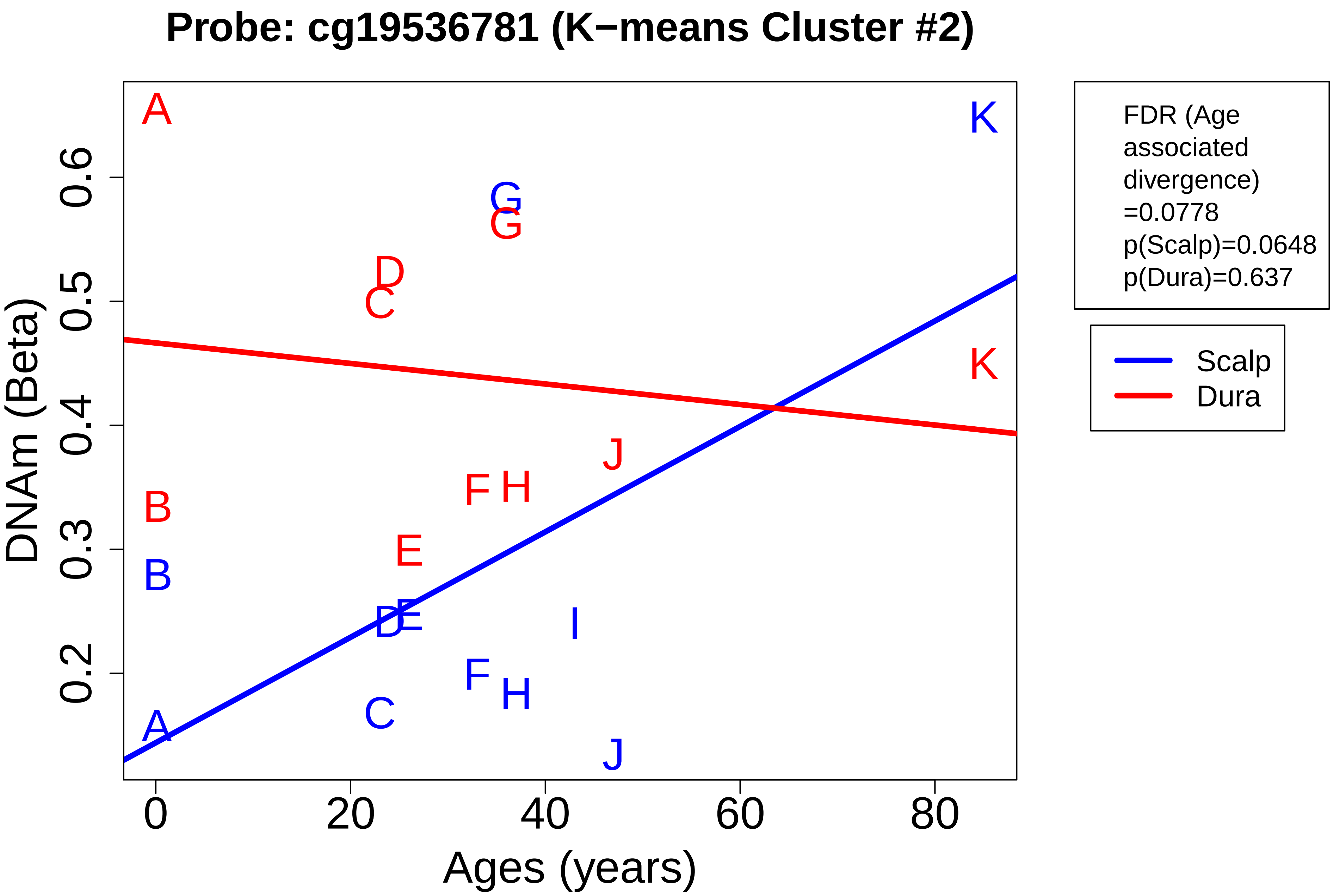

Probe: cg19536781 (K−means Cluster #2)
DNAm (Beta)
Ages (years)
0.2
0.3
0.4
0.5
0.6
0
20
40
60
80
FDR (Age associated divergence) =0.0778
p(Scalp)=0.0648
p(Dura)=0.637
Scalp
Dura

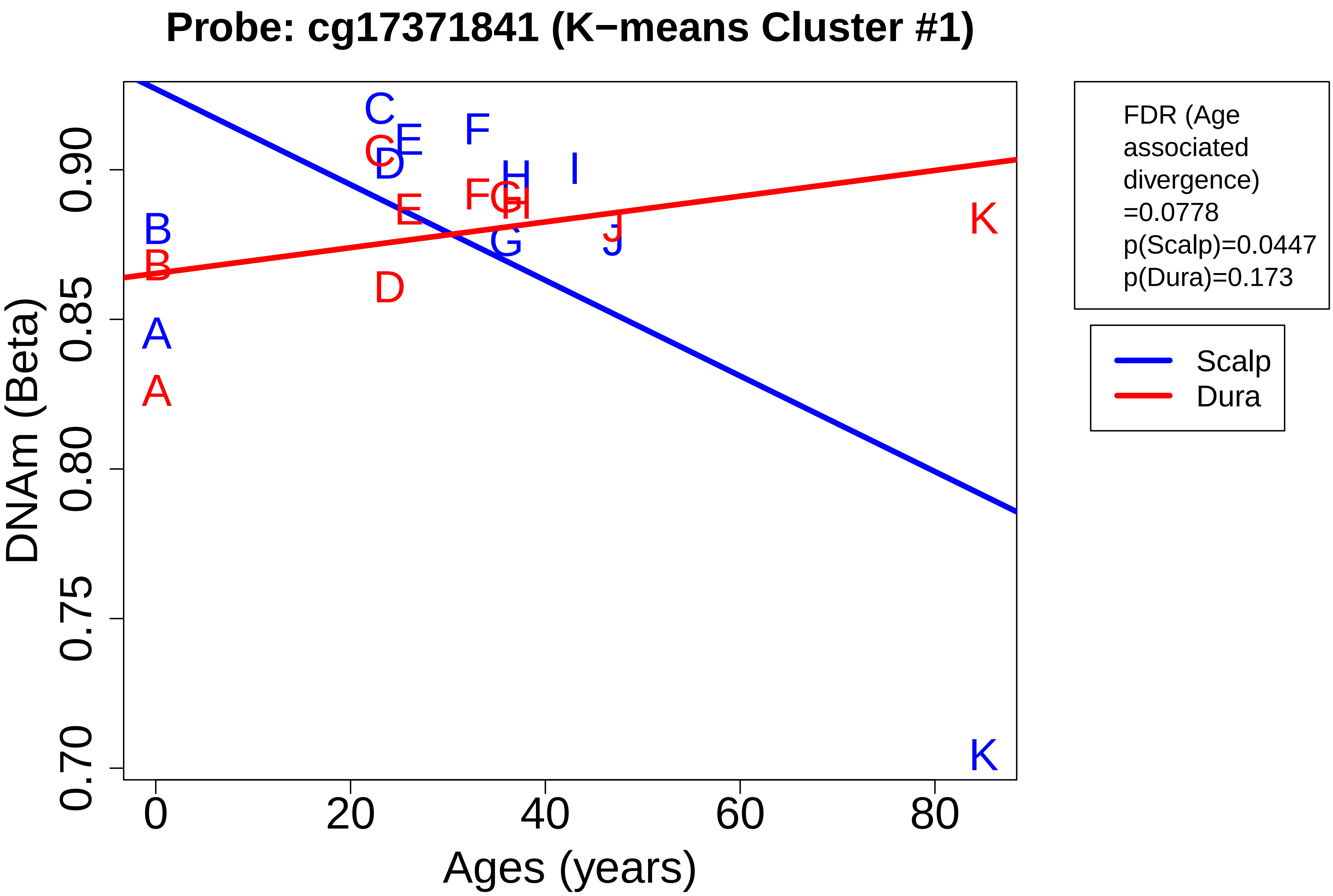

Probe: cg17371841 (K−means Cluster #1)
DNAm (Beta)
0.70
0.75
0.80
0.85
0.90
Ages (years)
0
20
40
60
80
A
B
C
D
E
F
G
H
I
J
K
FDR (Age associated divergence) =0.0778
p(Scalp)=0.0447
p(Dura)=0.173
Scalp
Dura

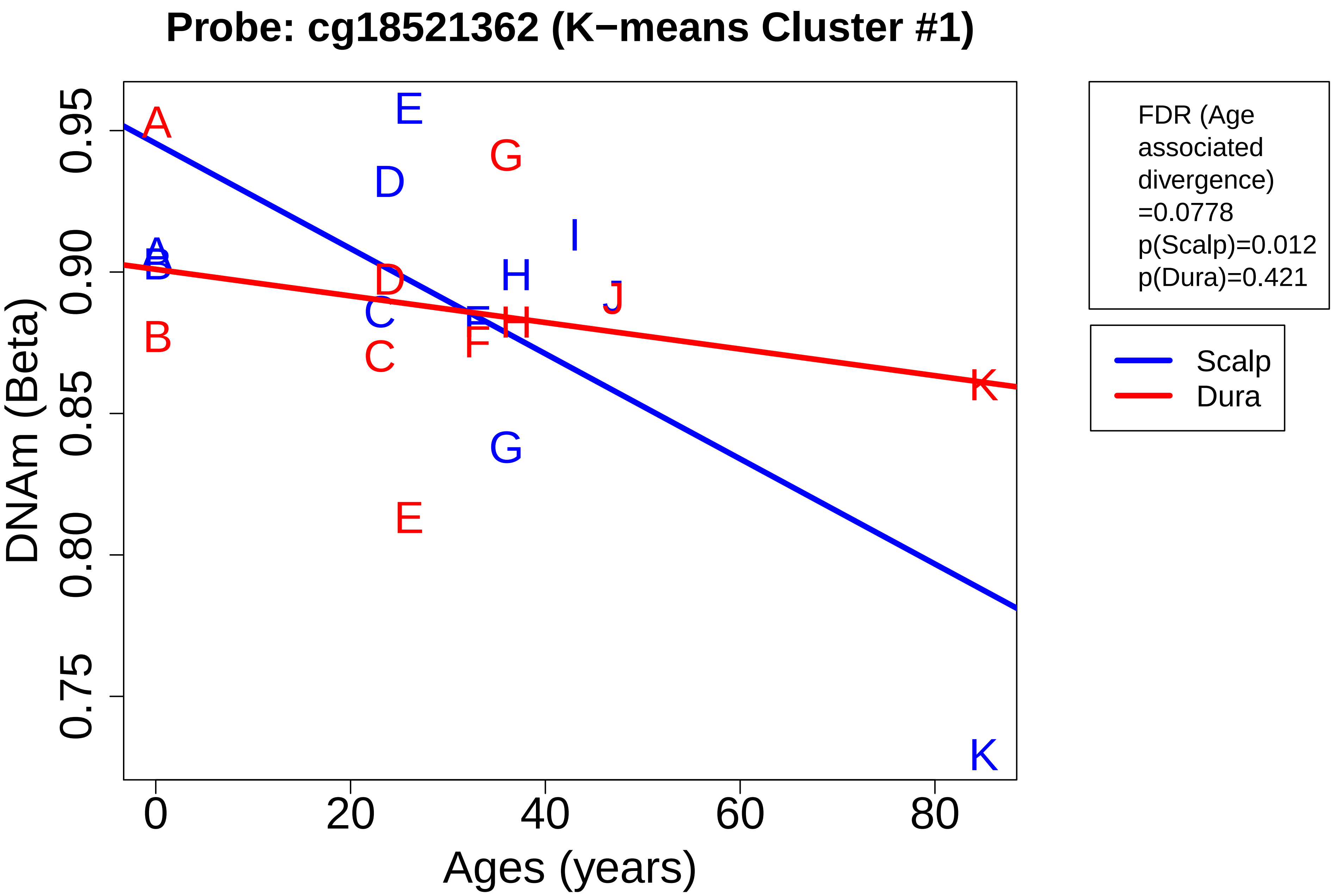

Probe: cg18521362 (K−means Cluster #1)
DNAm (Beta)
0.75
0.80
0.85
0.90
0.95
0
20
40
60
80
Ages (years)
FDR (Age associated divergence) =0.0778
p(Scalp)=0.012
p(Dura)=0.421
Scalp
Dura

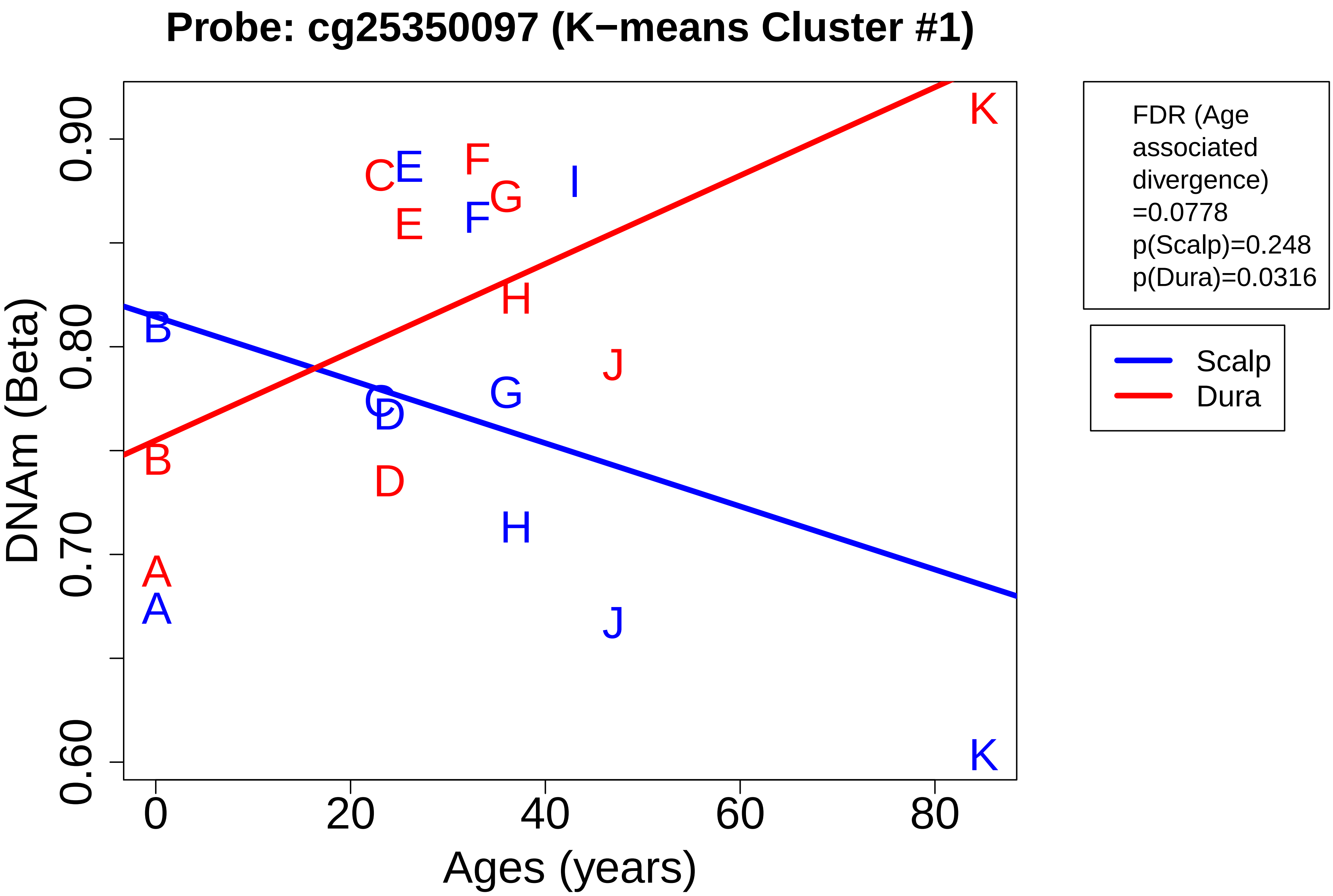

Probe: cg25350097 (K−means Cluster #1)
DNAm (Beta)
0.60
0.70
0.80
0.90
0
20
40
60
80
Ages (years)
FDR (Age associated divergence) =0.0778
p(Scalp)=0.248
p(Dura)=0.0316
Scalp
Dura

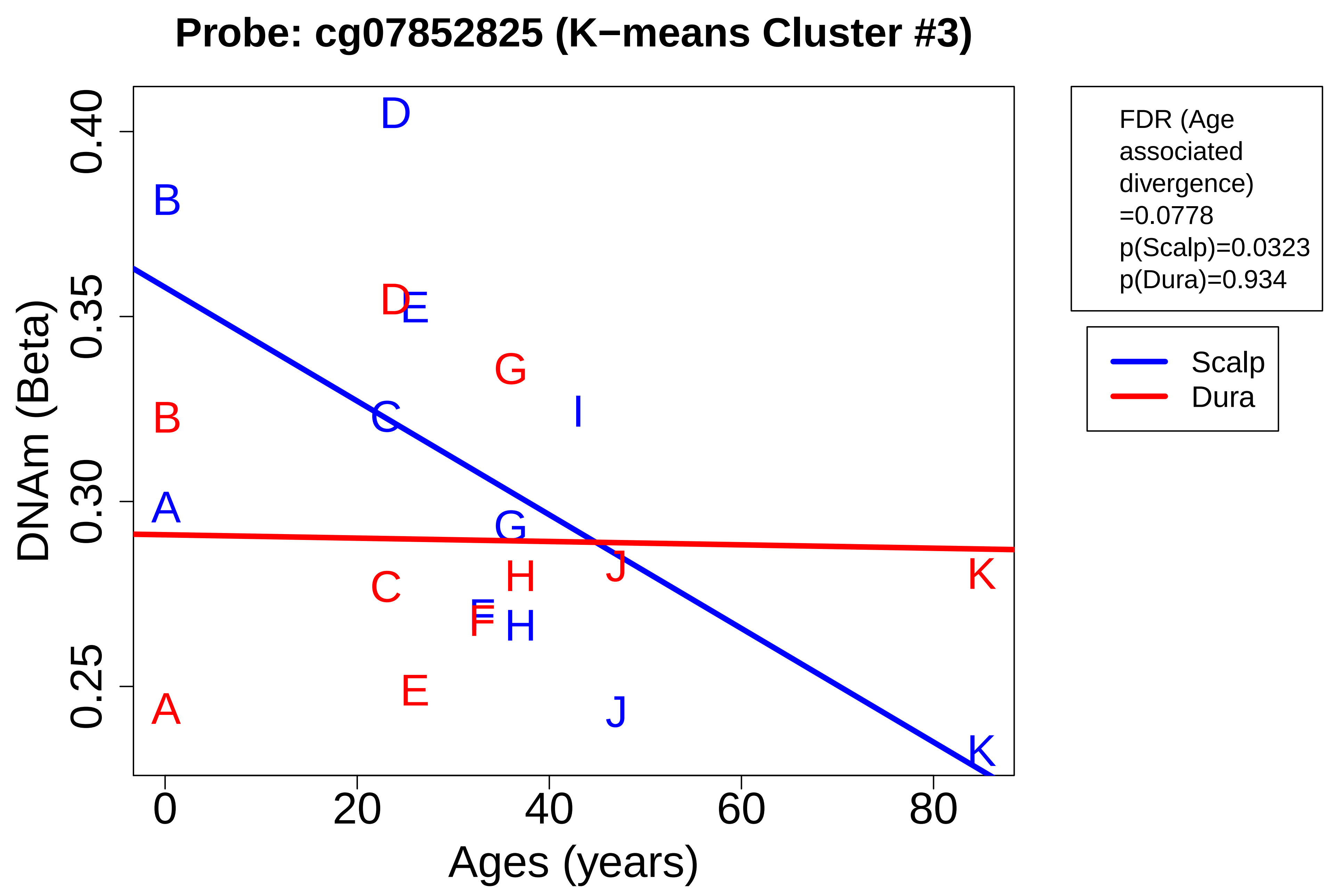

Probe: cg07852825 (K−means Cluster #3)
DNAm (Beta)
0.25
0.30
0.35
0.40
0
20
40
60
80
Ages (years)
FDR (Age associated divergence) =0.0778
p(Scalp)=0.0323
p(Dura)=0.934
Scalp
Dura

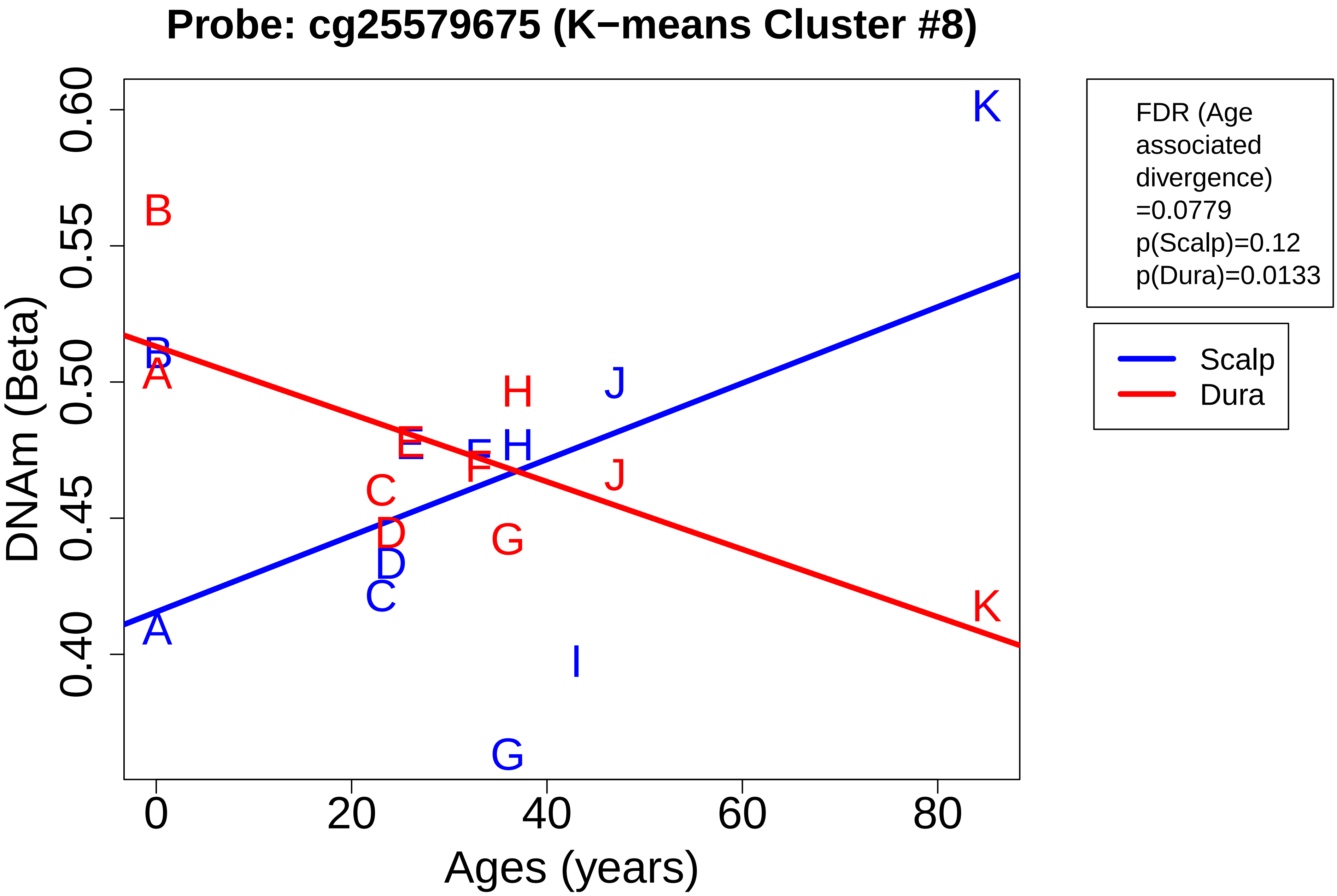

Probe: cg25579675 (K−means Cluster #8)
DNAm (Beta)
0.40
0.45
0.50
0.55
0.60
0
20
40
60
80
Ages (years)
FDR (Age associated divergence) =0.0779
p(Scalp)=0.12
p(Dura)=0.0133
Scalp
Dura

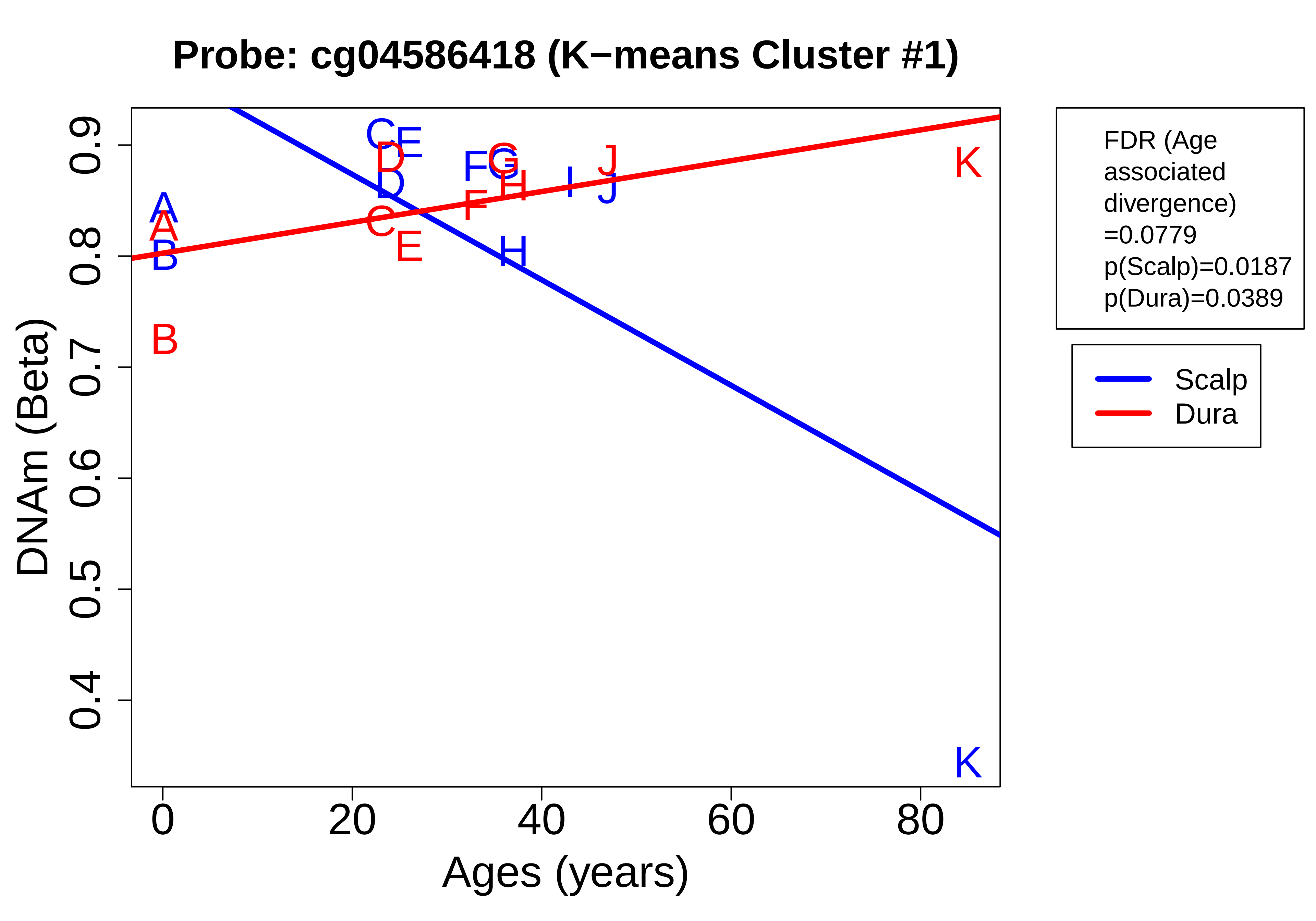

Probe: cg04586418 (K−means Cluster #1)
DNAm (Beta)
0.4
0.5
0.6
0.7
0.8
0.9
0
20
40
60
80
Ages (years)
A
B
C
D
E
F
G
H
I
J
K
FDR (Age associated divergence) =0.0779
p(Scalp)=0.0187
p(Dura)=0.0389
Scalp
Dura

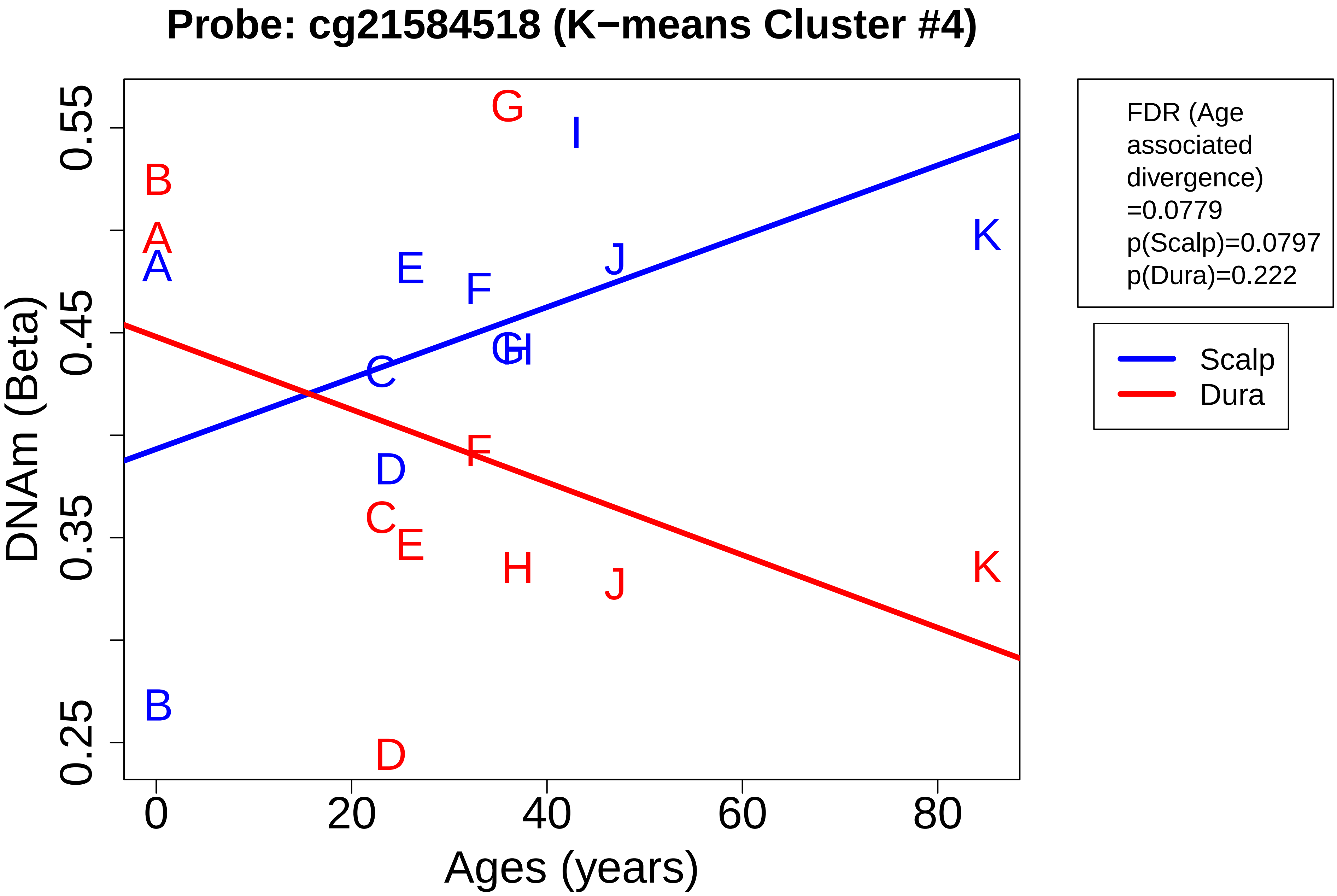
Probe: cg21584518 (K−means Cluster #4)
DNAm (Beta)
0.25
0.35
0.45
0.55
0
20
40
60
80
Ages (years)
A
B
C
D
E
F
G
H
I
J
K
A
B
C
D
E
F
G
H
J
K
FDR (Age associated divergence) =0.0779
p(Scalp)=0.0797
p(Dura)=0.222
Scalp
Dura

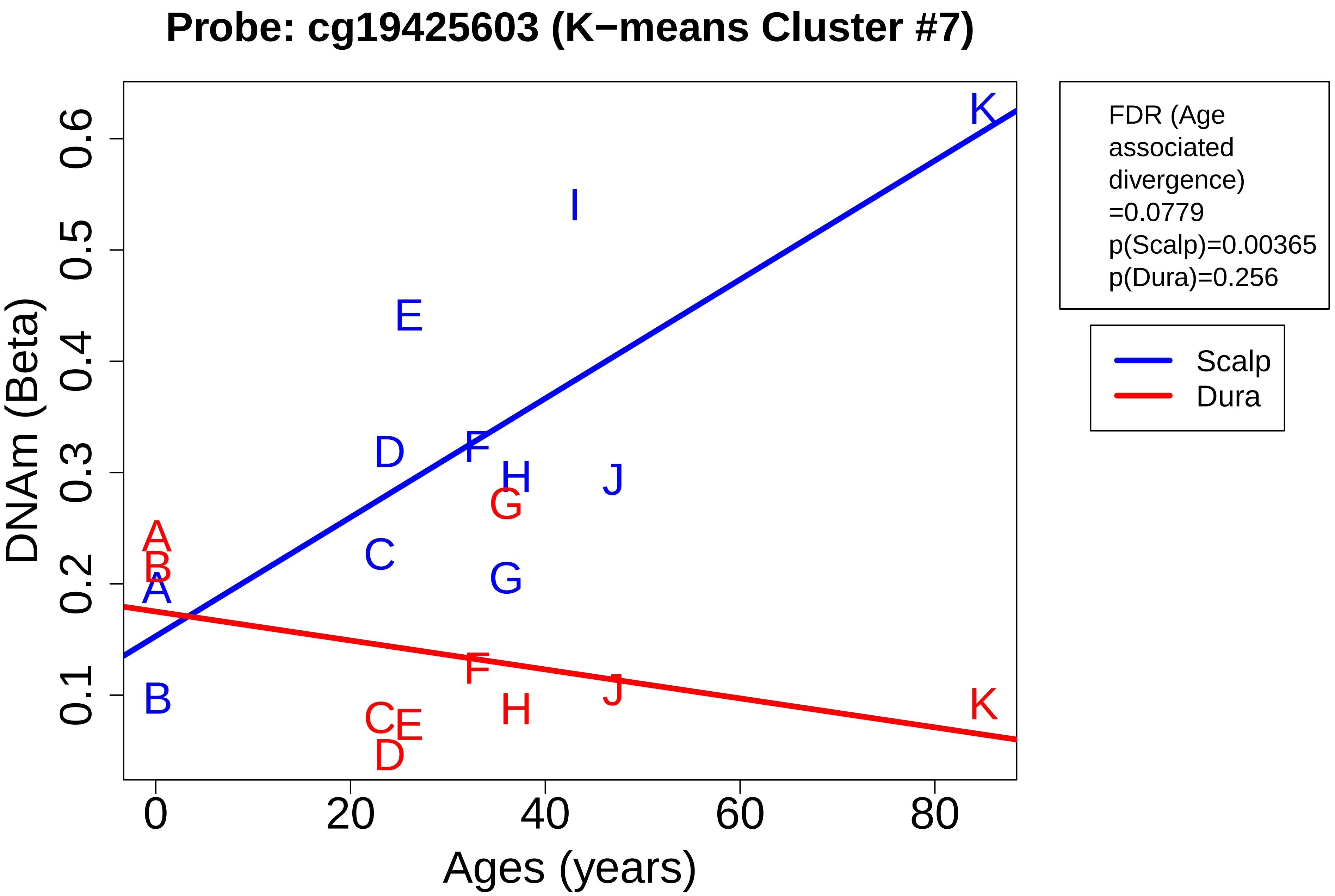

Probe: cg19425603 (K−means Cluster #7)
DNAm (Beta)
0.1
0.2
0.3
0.4
0.5
0.6
0
20
40
60
80
Ages (years)
A
B
C
D
E
F
G
H
I
J
K
FDR (Age associated divergence) =0.0779
p(Scalp)=0.00365
p(Dura)=0.256
Scalp
Dura

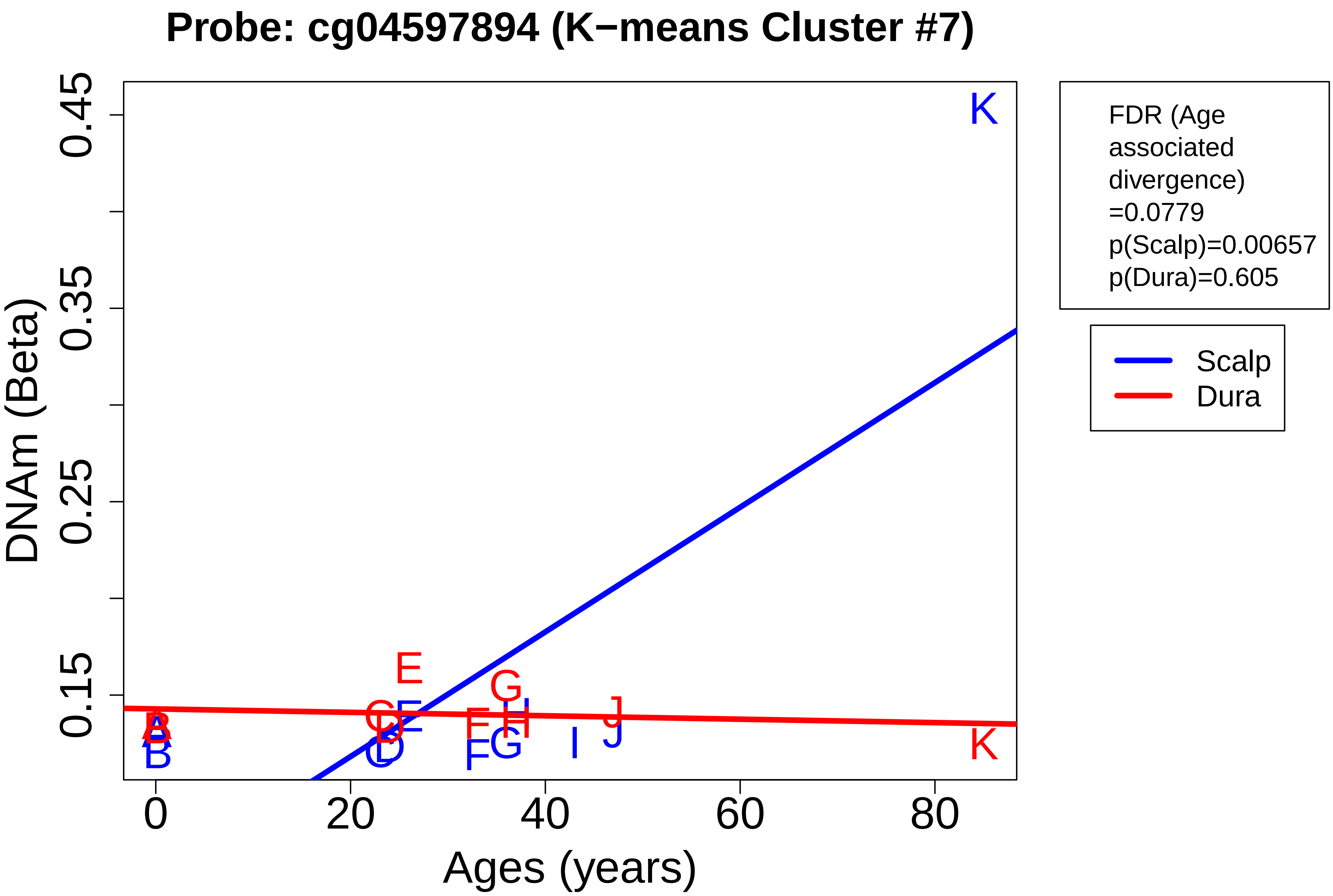

Probe: cg04597894 (K−means Cluster #7)
DNAm (Beta)
0.15
0.25
0.35
0.45
Ages (years)
0
20
40
60
80
FDR (Age associated divergence) =0.0779
p(Scalp)=0.00657
p(Dura)=0.605
Scalp
Dura

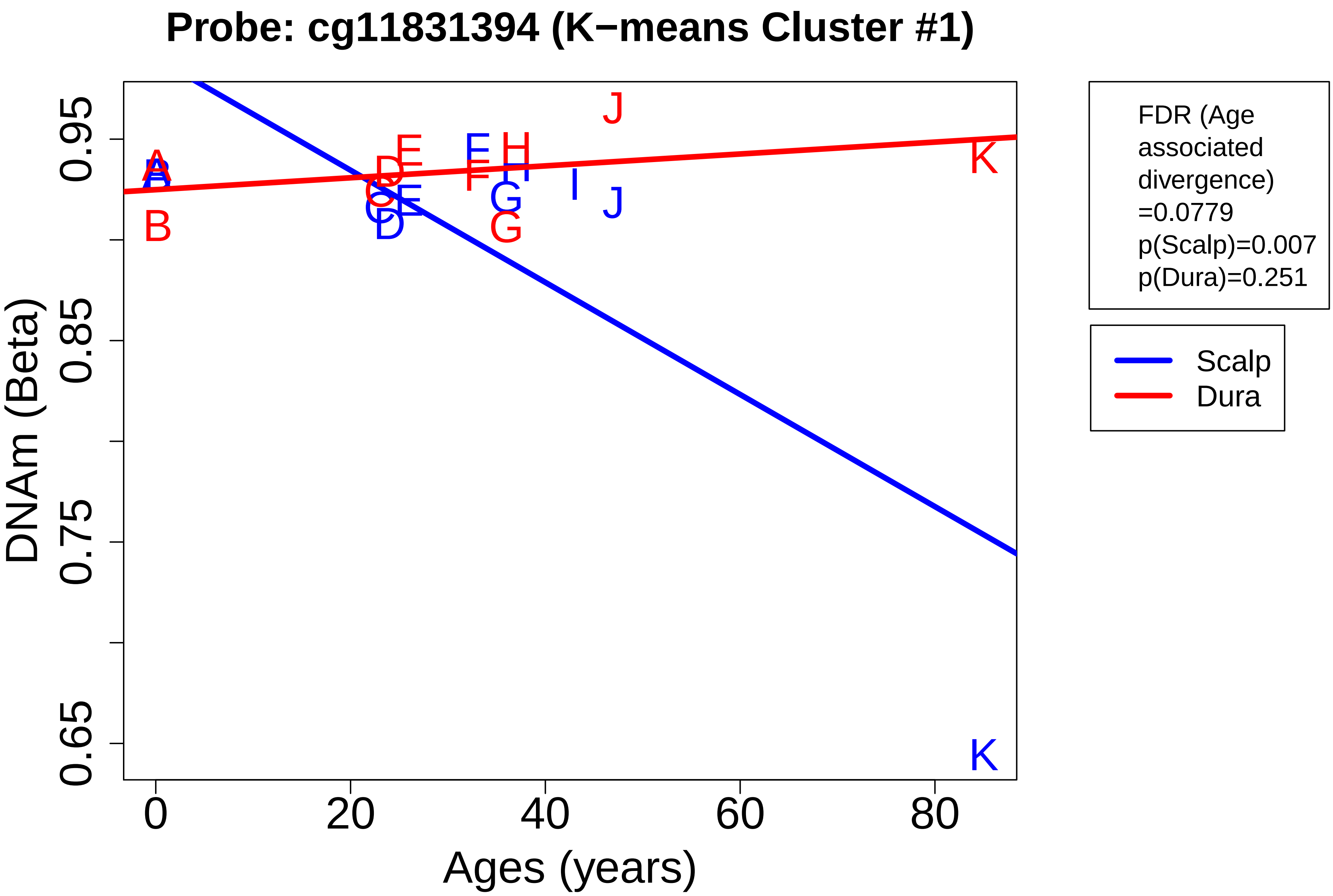
Probe: cg11831394 (K−means Cluster #1)
DNAm (Beta)
0.65
0.75
0.85
0.95
Ages (years)
0
20
40
60
80
FDR (Age associated divergence) =0.0779
p(Scalp)=0.007
p(Dura)=0.251
Scalp
Dura

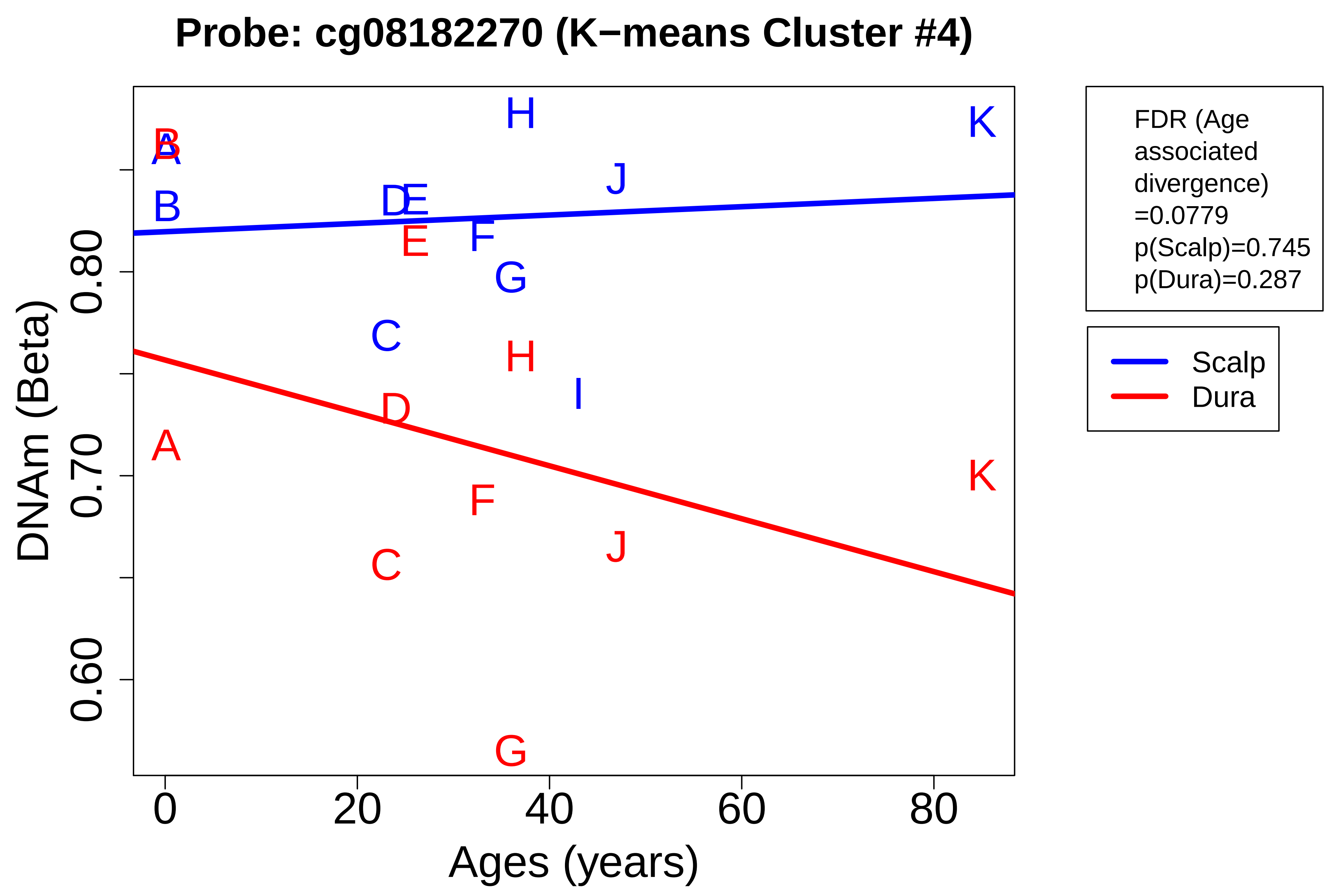

Probe: cg08182270 (K−means Cluster #4)
DNAm (Beta)
0.60
0.70
0.80
Ages (years)
0
20
40
60
80
A
B
C
D
E
F
G
H
I
J
K
FDR (Age associated divergence) =0.0779
p(Scalp)=0.745
p(Dura)=0.287
Scalp
Dura

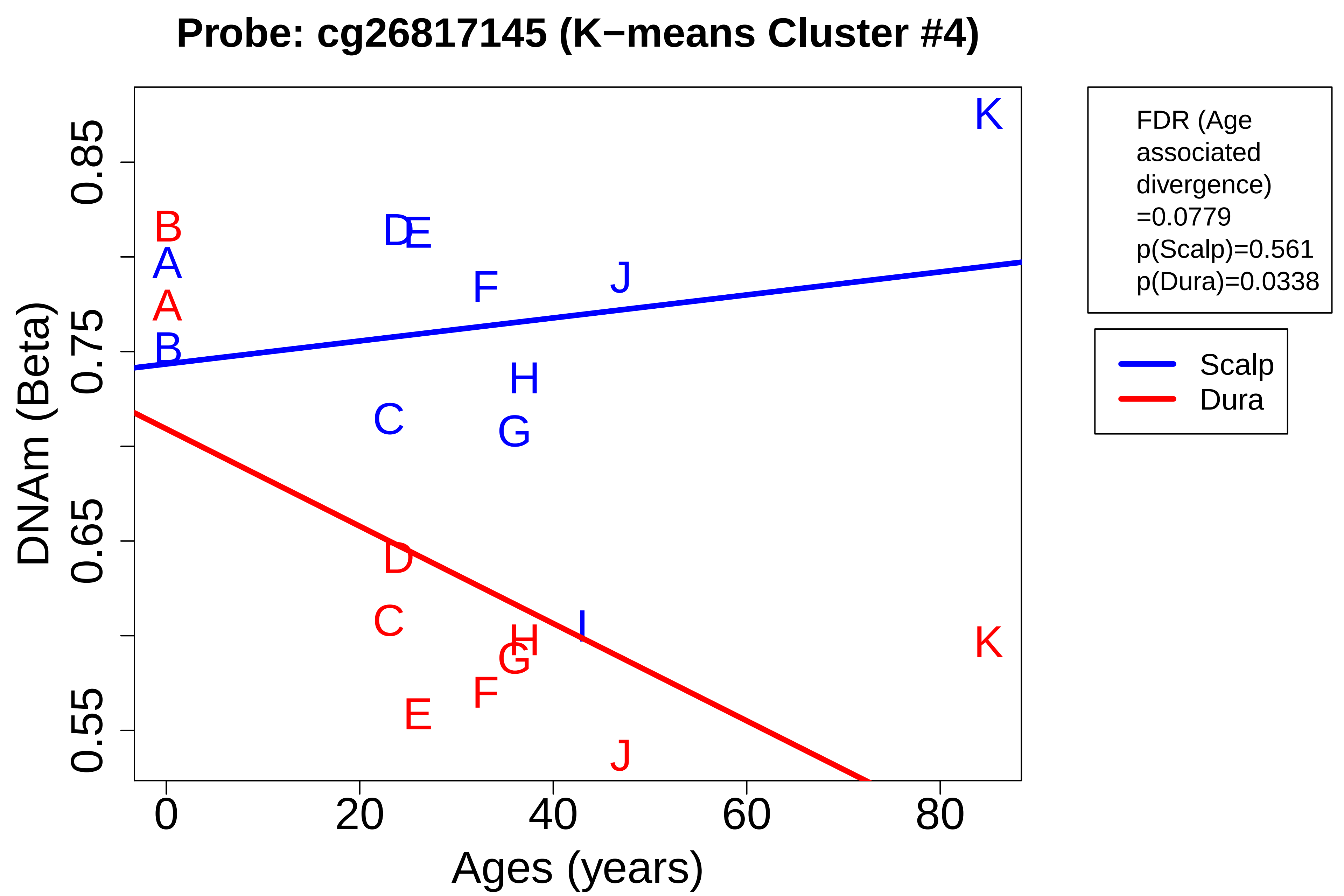

Probe: cg26817145 (K−means Cluster #4)
DNAm (Beta)
0.55
0.65
0.75
0.85
Ages (years)
0
20
40
60
80
FDR (Age associated divergence) =0.0779
p(Scalp)=0.561
p(Dura)=0.0338
Scalp
Dura
A
B
C
D
E
F
G
H
I
J
K

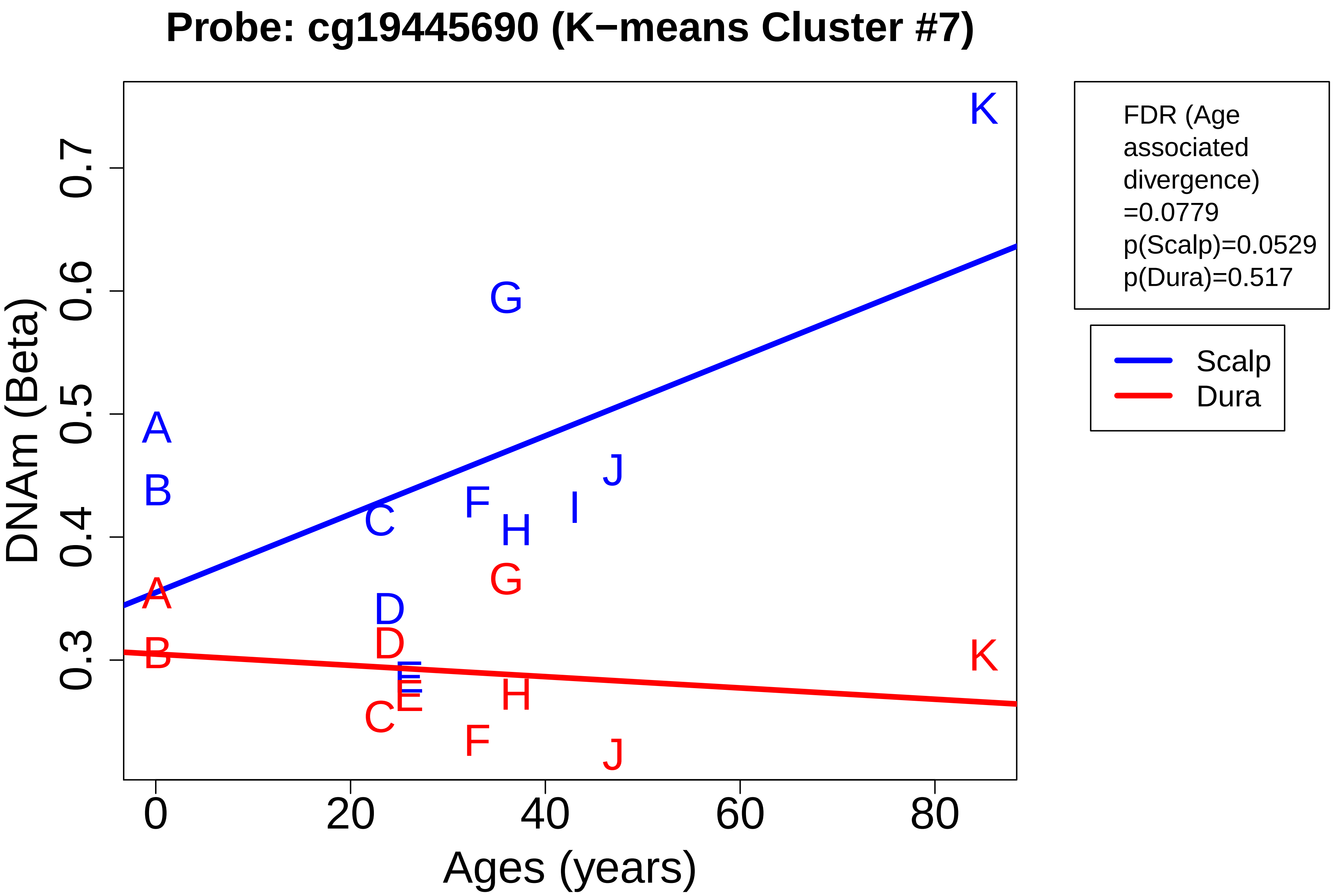

Probe: cg19445690 (K−means Cluster #7)
DNAm (Beta)
0.3
0.4
0.5
0.6
0.7
0
20
40
60
80
Ages (years)
A
B
C
D
E
F
G
H
I
J
K
A
B
C
D
E
F
G
H
J
K
FDR (Age associated divergence) =0.0779
p(Scalp)=0.0529
p(Dura)=0.517
Scalp
Dura

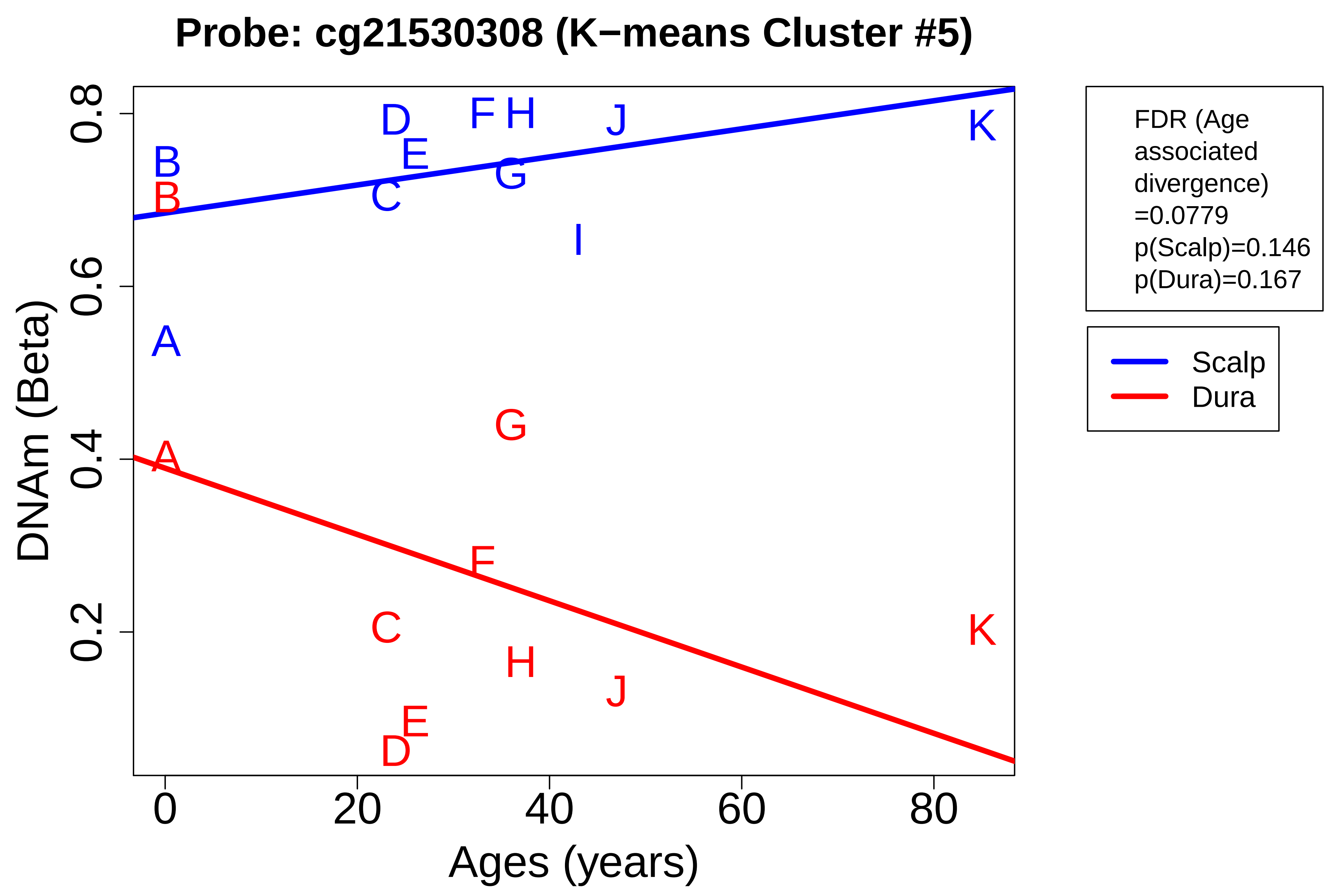
Probe: cg21530308 (K−means Cluster #5)
DNAm (Beta)
Ages (years)
0.2
0.4
0.6
0.8
0
20
40
60
80
A
B
C
D
E
F
G
H
I
J
K
FDR (Age associated divergence) =0.0779
p(Scalp)=0.146
p(Dura)=0.167
Scalp
Dura

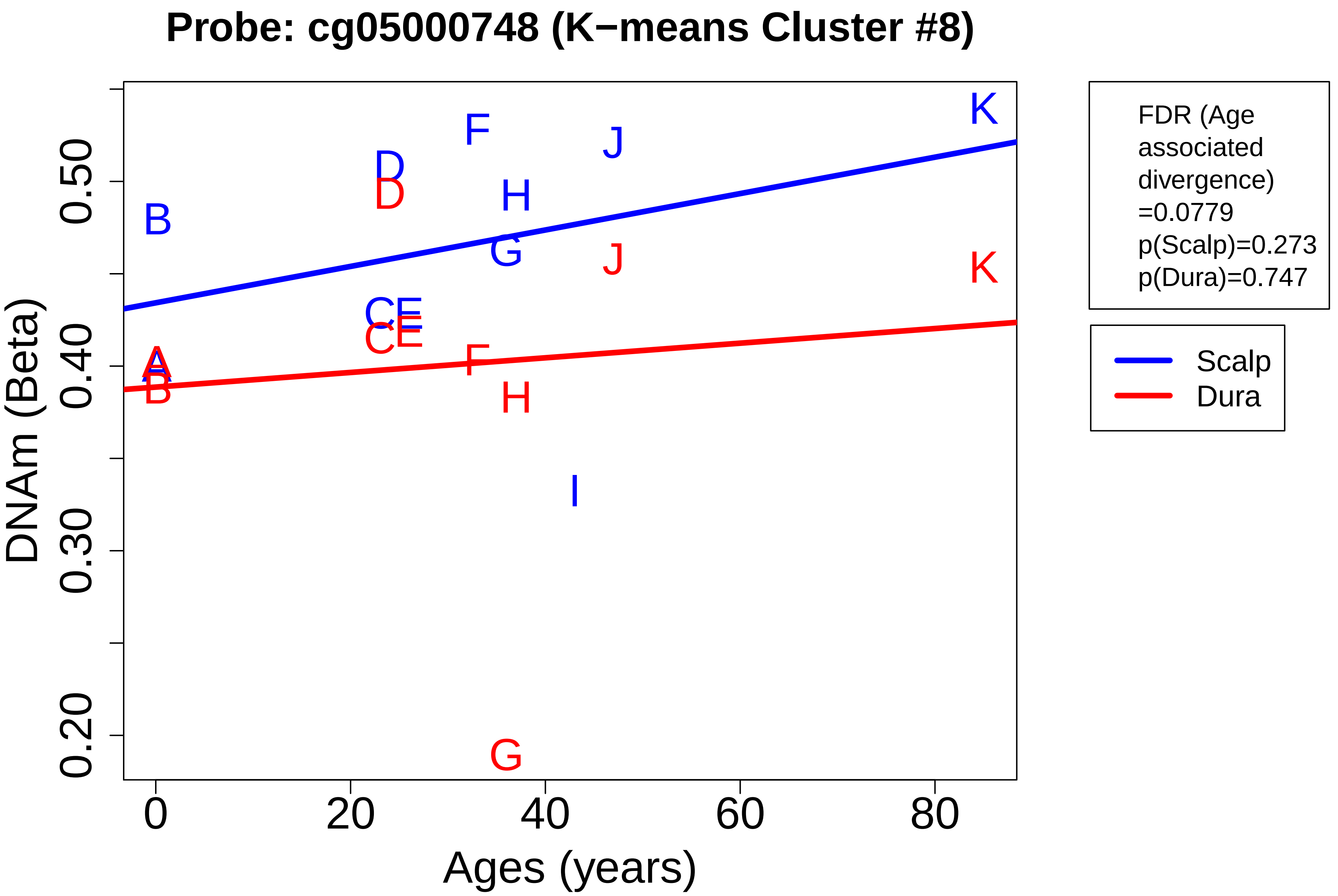

Probe: cg05000748 (K−means Cluster #8)
DNAm (Beta)
0.20
0.30
0.40
0.50
0
20
40
60
80
Ages (years)
FDR (Age associated divergence) =0.0779
p(Scalp)=0.273
p(Dura)=0.747
Scalp
Dura

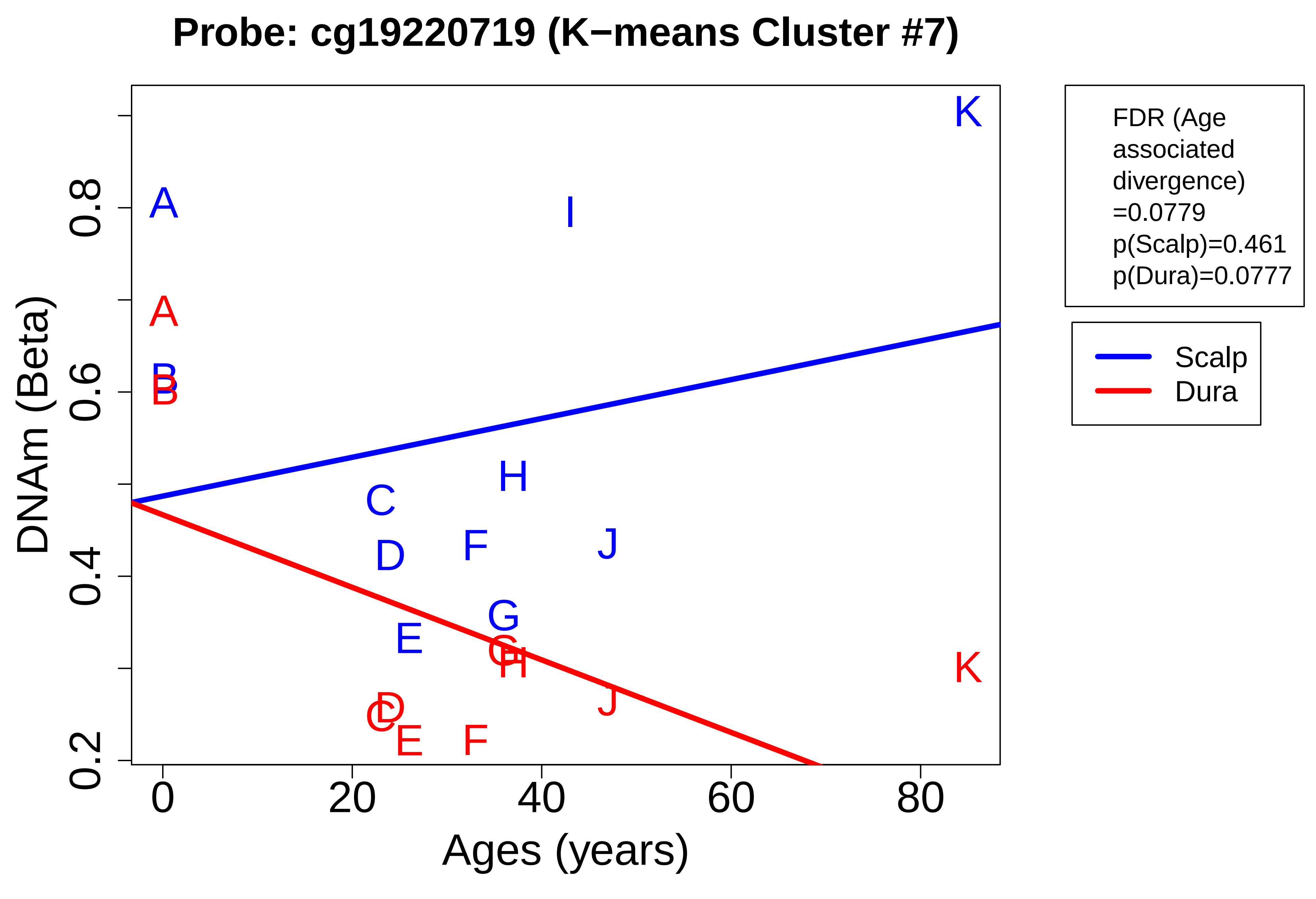

Probe: cg19220719 (K−means Cluster #7)
DNAm (Beta)
0.2
0.4
0.6
0.8
0
20
40
60
80
Ages (years)
A
B
C
D
E
F
G
H
I
J
K
FDR (Age associated divergence) =0.0779
p(Scalp)=0.461
p(Dura)=0.0777
Scalp
Dura

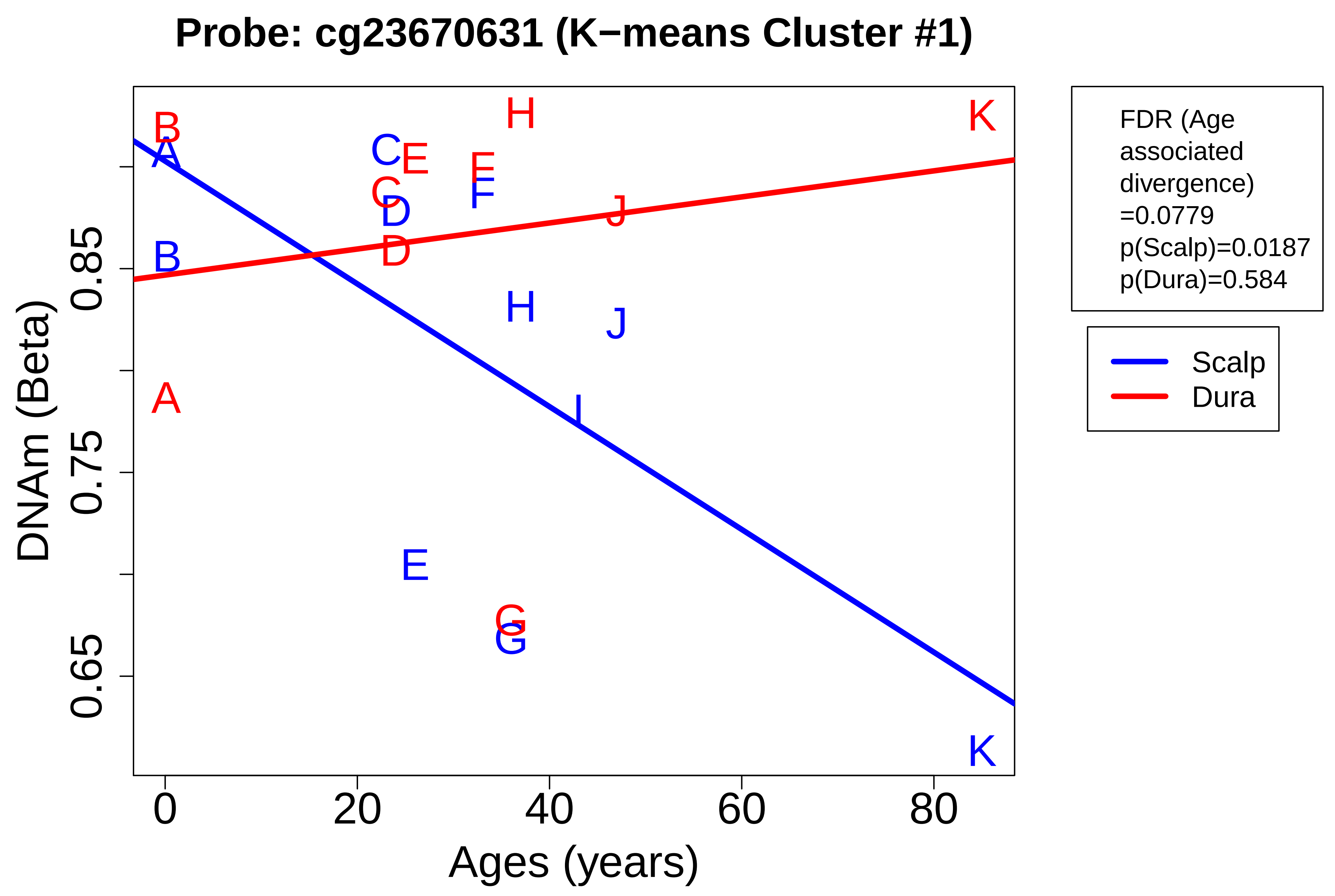

Probe: cg23670631 (K−means Cluster #1)
FDR (Age associated divergence) =0.0779
p(Scalp)=0.0187
p(Dura)=0.584
Scalp
Dura
DNAm (Beta)
0.65
0.75
0.85
0
20
40
60
80
Ages (years)
A
B
C
D
E
F
G
H
I
J
K

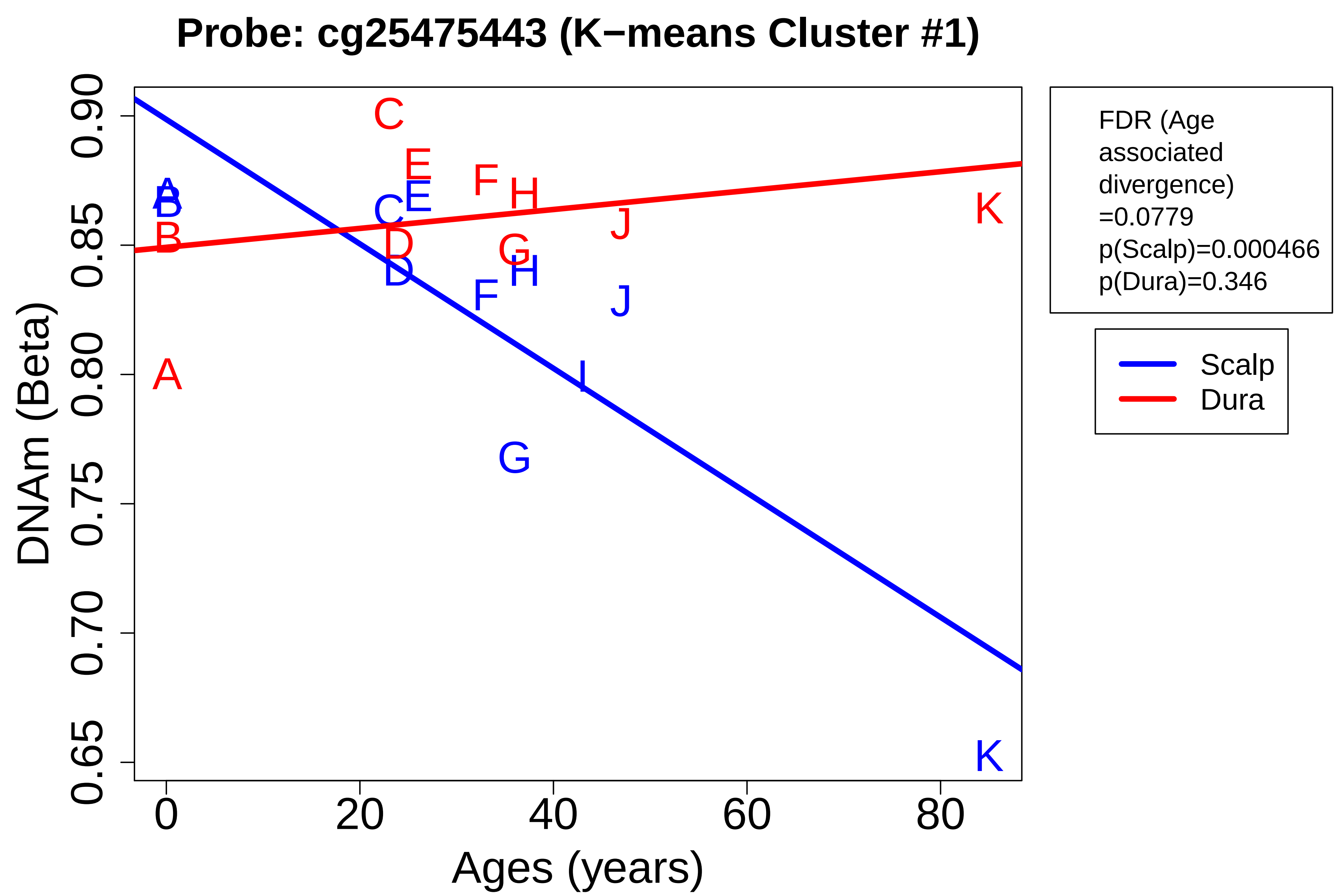
Probe: cg25475443 (K−means Cluster #1)
DNAm (Beta)
0.65
0.70
0.75
0.80
0.85
0.90
0
20
40
60
80
Ages (years)
FDR (Age associated divergence) =0.0779
p(Scalp)=0.000466
p(Dura)=0.346
Scalp
Dura

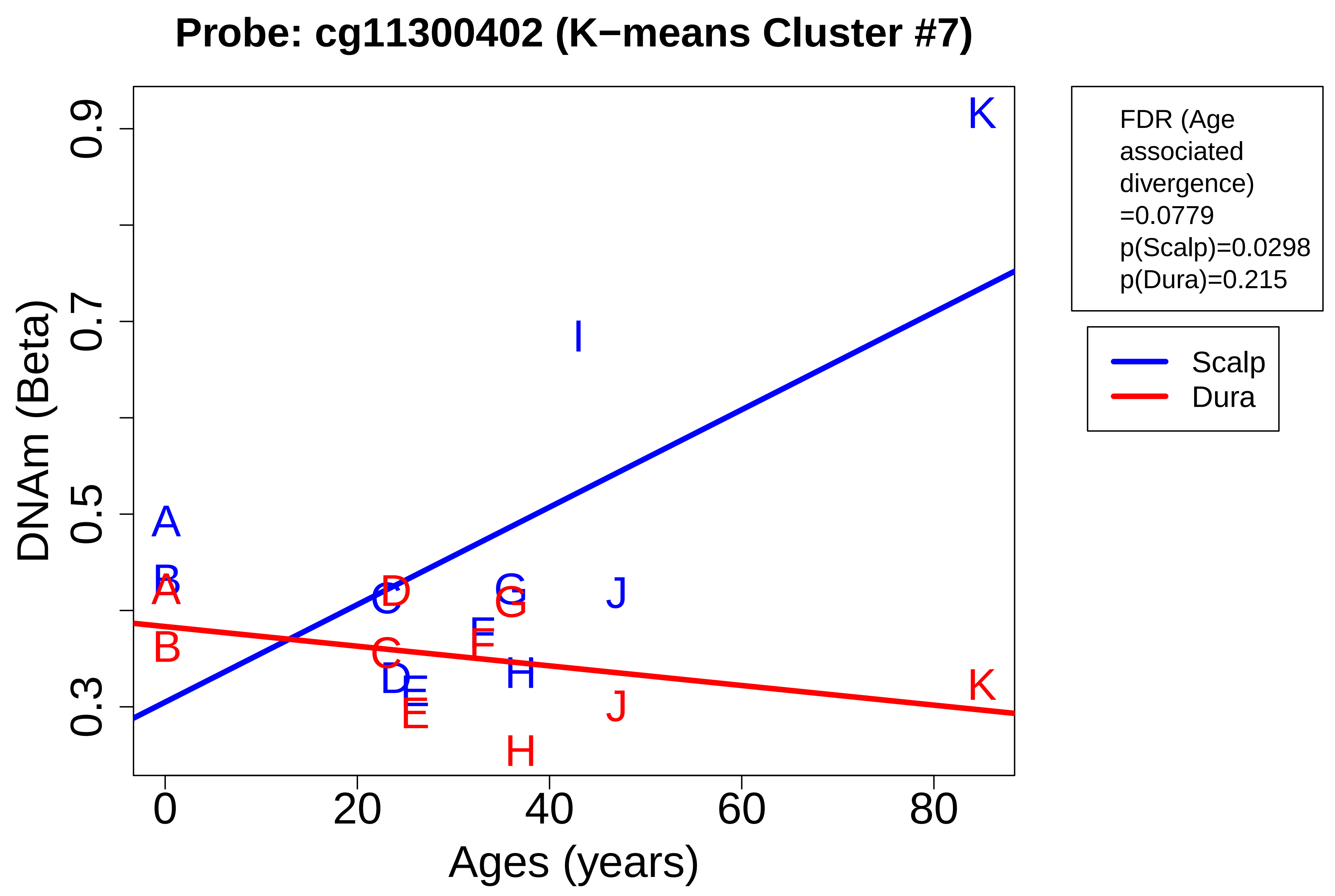

Probe: cg11300402 (K−means Cluster #7)
DNAm (Beta)
0.3
0.5
0.7
0.9
Ages (years)
0
20
40
60
80
FDR (Age associated divergence) =0.0779
p(Scalp)=0.0298
p(Dura)=0.215
Scalp
Dura
A
B
C
D
E
F
G
H
I
J
K

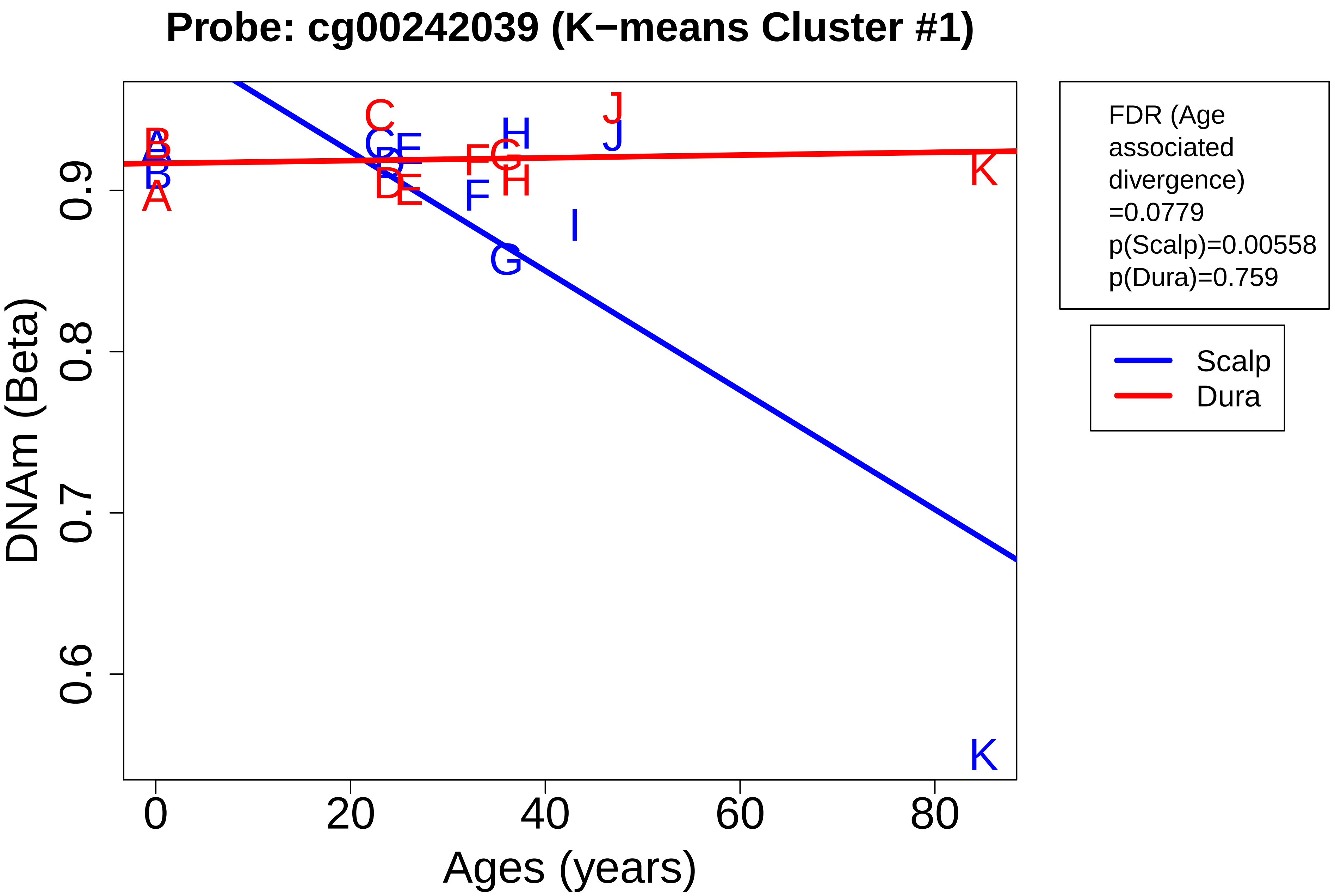

Probe: cg00242039 (K−means Cluster #1)
FDR (Age associated divergence) =0.0779
p(Scalp)=0.00558
p(Dura)=0.759
Scalp
Dura
DNAm (Beta)
0.6
0.7
0.8
0.9
Ages (years)
0
20
40
60
80
A
B
C
D
E
F
G
H
I
J
K

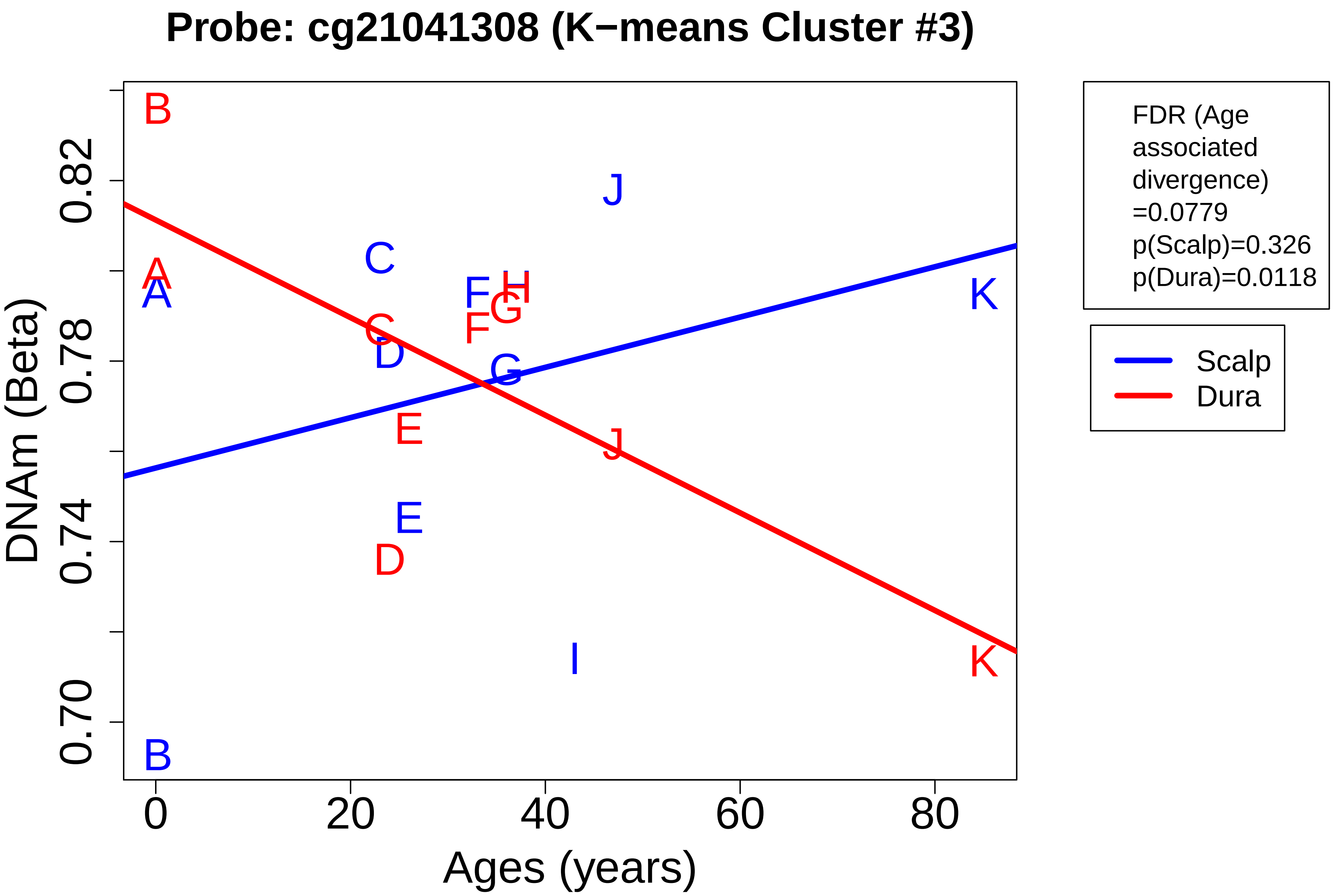

Probe: cg21041308 (K−means Cluster #3)
DNAm (Beta)
0.70
0.74
0.78
0.82
0
20
40
60
80
Ages (years)
FDR (Age associated divergence) =0.0779
p(Scalp)=0.326
p(Dura)=0.0118
Scalp
Dura
A
B
C
D
E
F
G
H
I
J
K

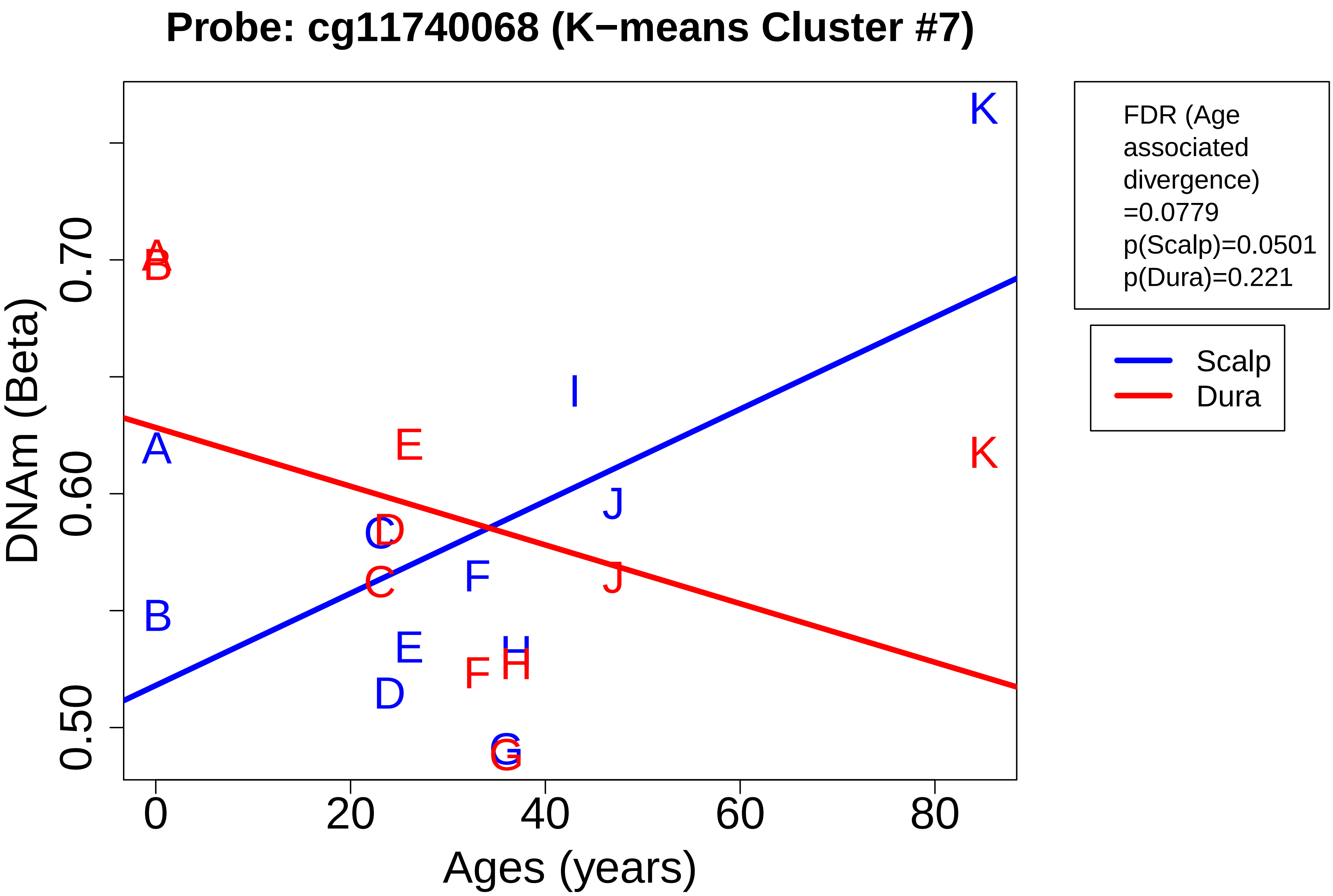

Probe: cg11740068 (K−means Cluster #7)
DNAm (Beta)
0.50
0.60
0.70
Ages (years)
0
20
40
60
80
FDR (Age associated divergence) =0.0779
p(Scalp)=0.0501
p(Dura)=0.221
Scalp
Dura

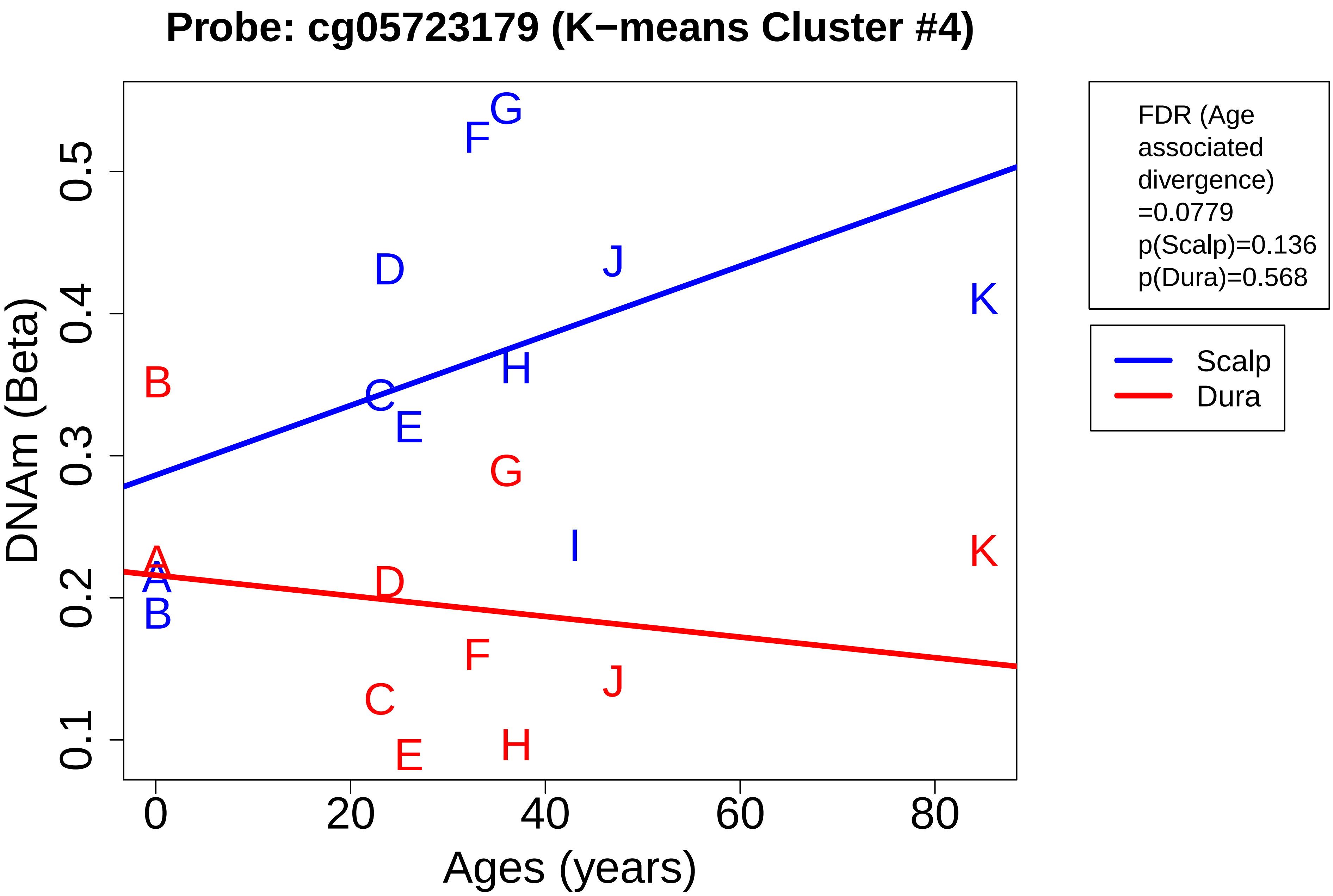

Probe: cg05723179 (K−means Cluster #4)
DNAm (Beta)
0.1
0.2
0.3
0.4
0.5
0
20
40
60
80
Ages (years)
FDR (Age associated divergence) =0.0779
p(Scalp)=0.136
p(Dura)=0.568
Scalp
Dura

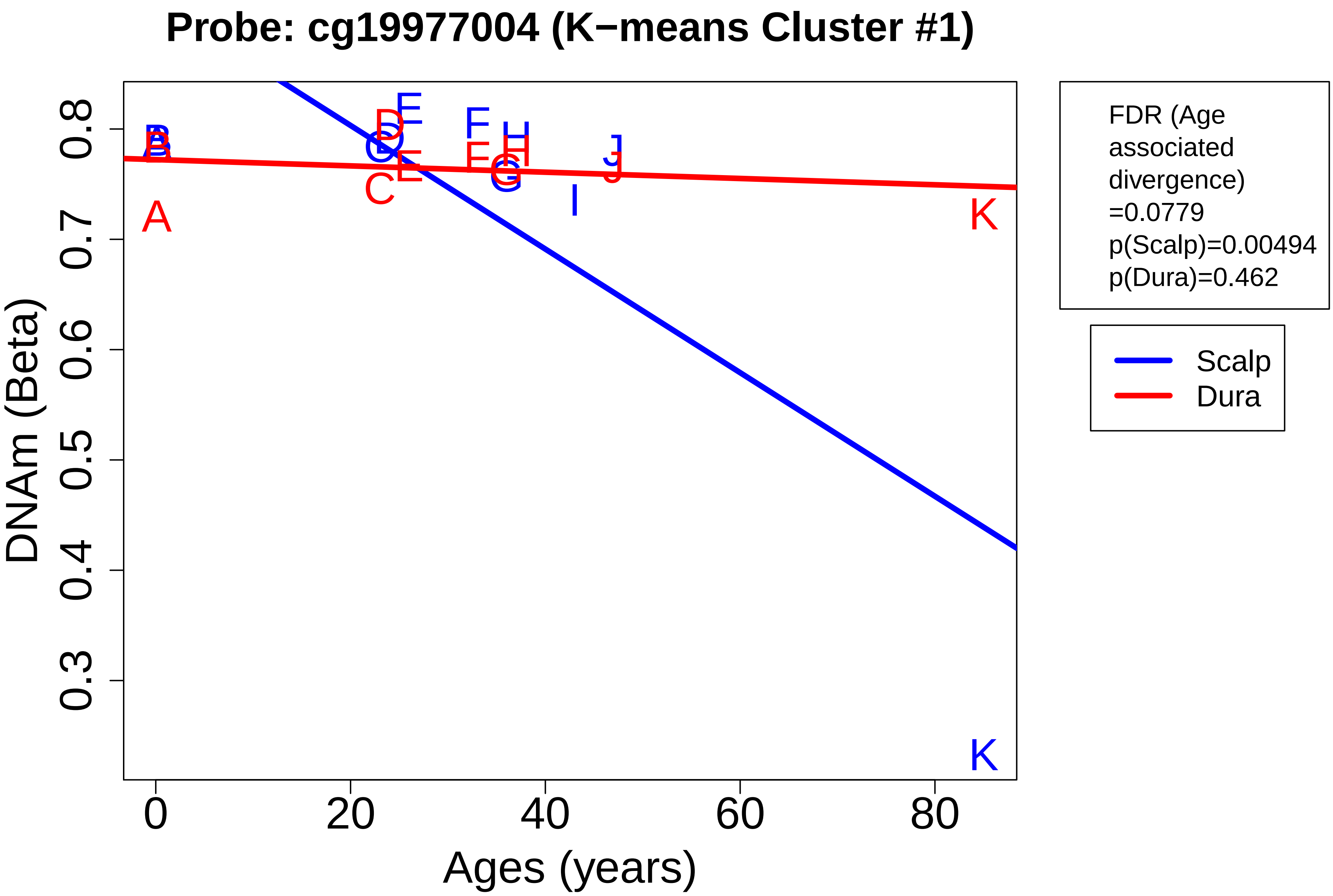

Probe: cg19977004 (K−means Cluster #1)
DNAm (Beta)
0.3
0.4
0.5
0.6
0.7
0.8
Ages (years)
0
20
40
60
80
FDR (Age associated divergence) =0.0779
p(Scalp)=0.00494
p(Dura)=0.462
Scalp
Dura

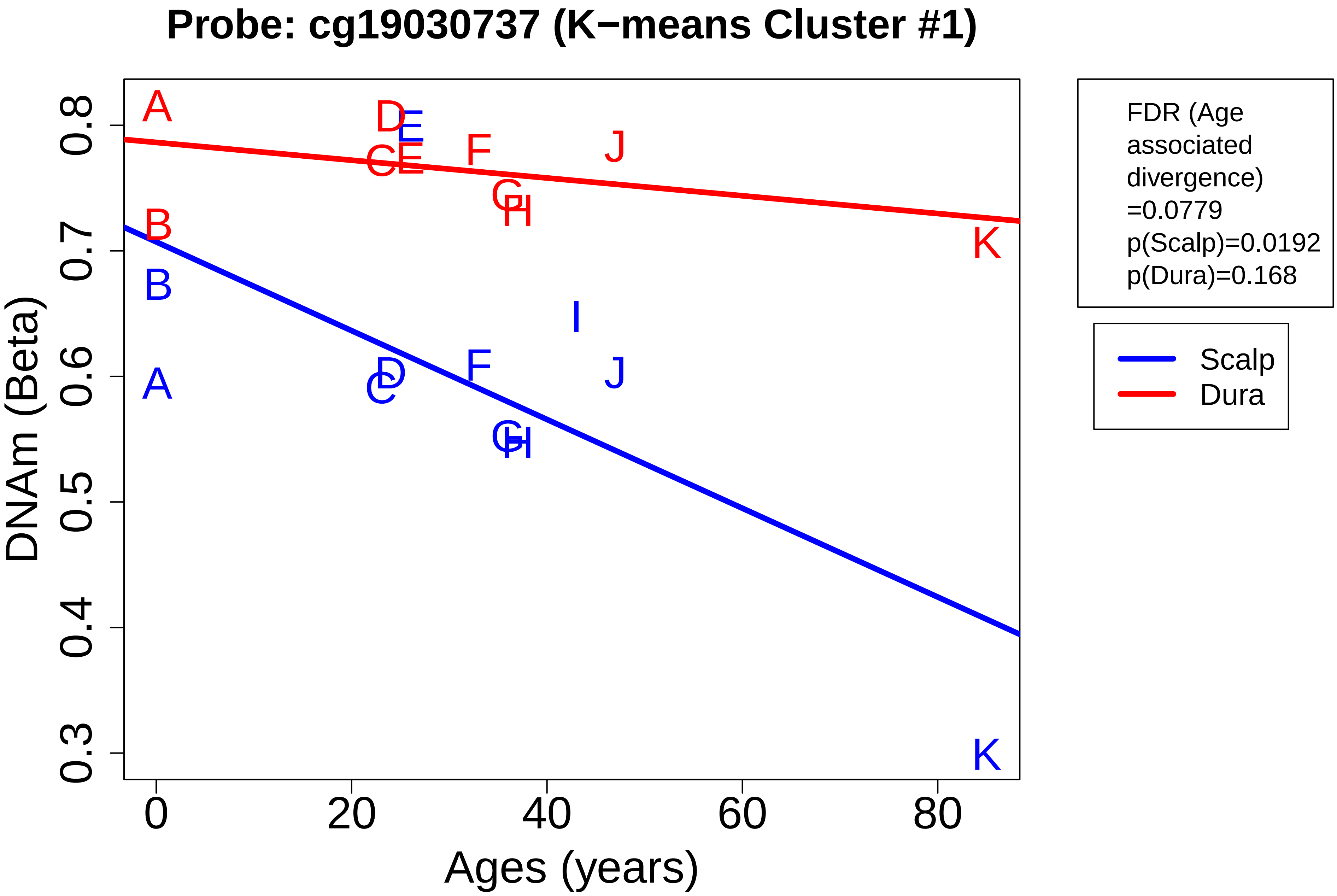

Probe: cg19030737 (K−means Cluster #1)
DNAm (Beta)
0.3
0.4
0.5
0.6
0.7
0.8
0
20
40
60
80
Ages (years)
FDR (Age associated divergence) =0.0779
p(Scalp)=0.0192
p(Dura)=0.168
Scalp
Dura

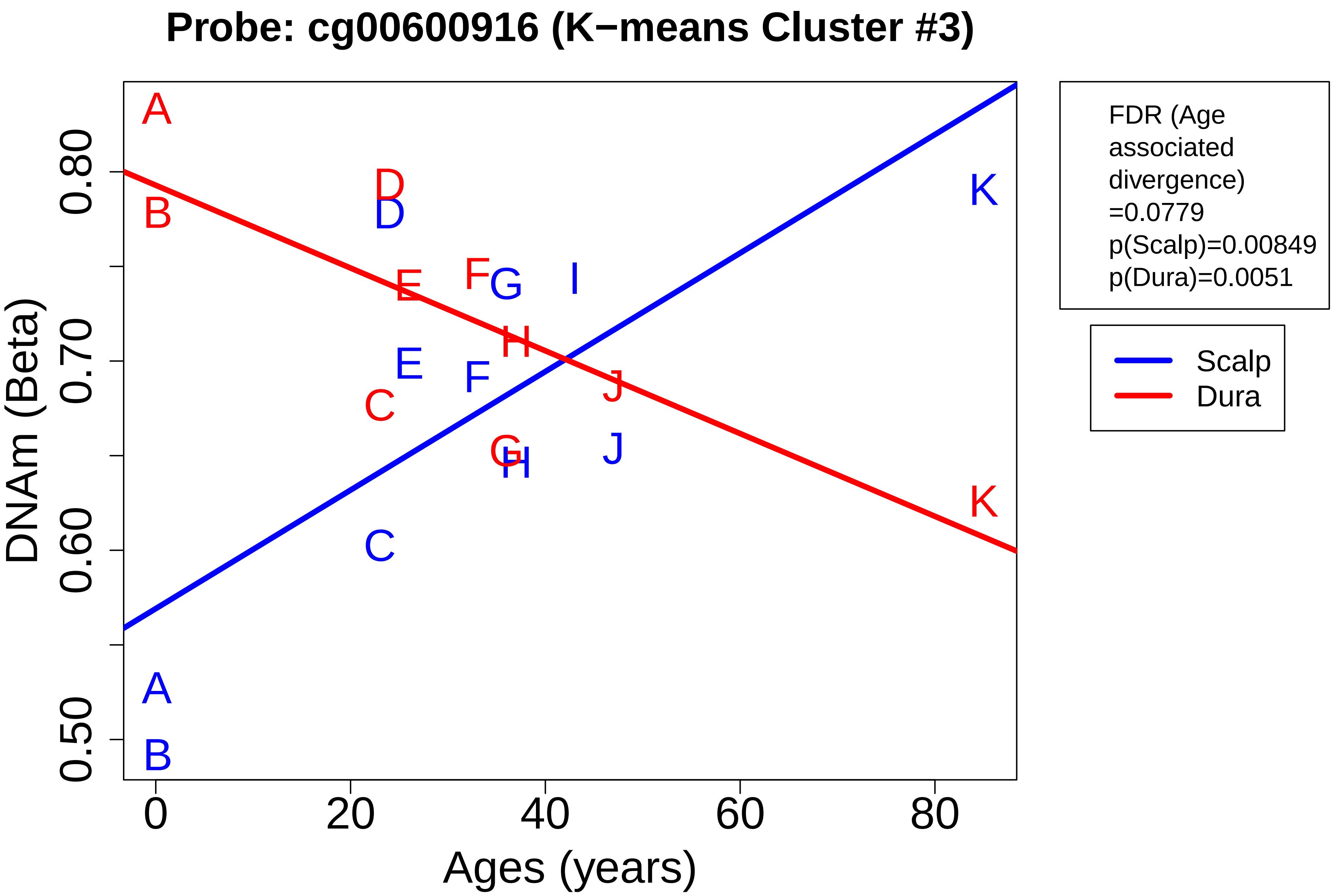

Probe: cg00600916 (K−means Cluster #3)
DNAm (Beta)
0.50
0.60
0.70
0.80
Ages (years)
0
20
40
60
80
A
B
C
D
E
F
G
H
I
J
K
FDR (Age associated divergence) =0.0779
p(Scalp)=0.00849
p(Dura)=0.0051
Scalp
Dura

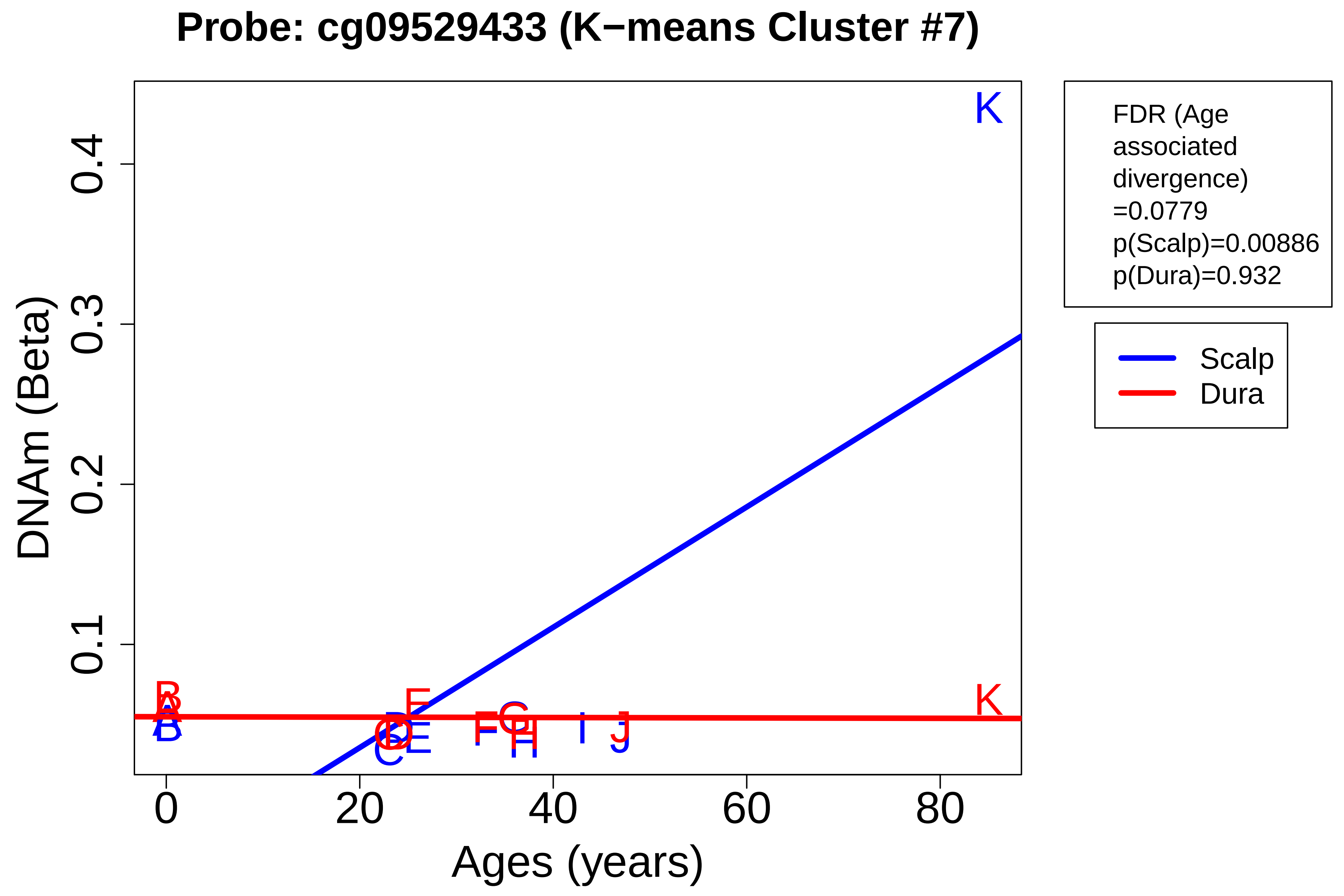

Probe: cg09529433 (K−means Cluster #7)
DNAm (Beta)
Ages (years)
0.1
0.2
0.3
0.4
0
20
40
60
80
FDR (Age associated divergence) =0.0779
p(Scalp)=0.00886
p(Dura)=0.932
Scalp
Dura

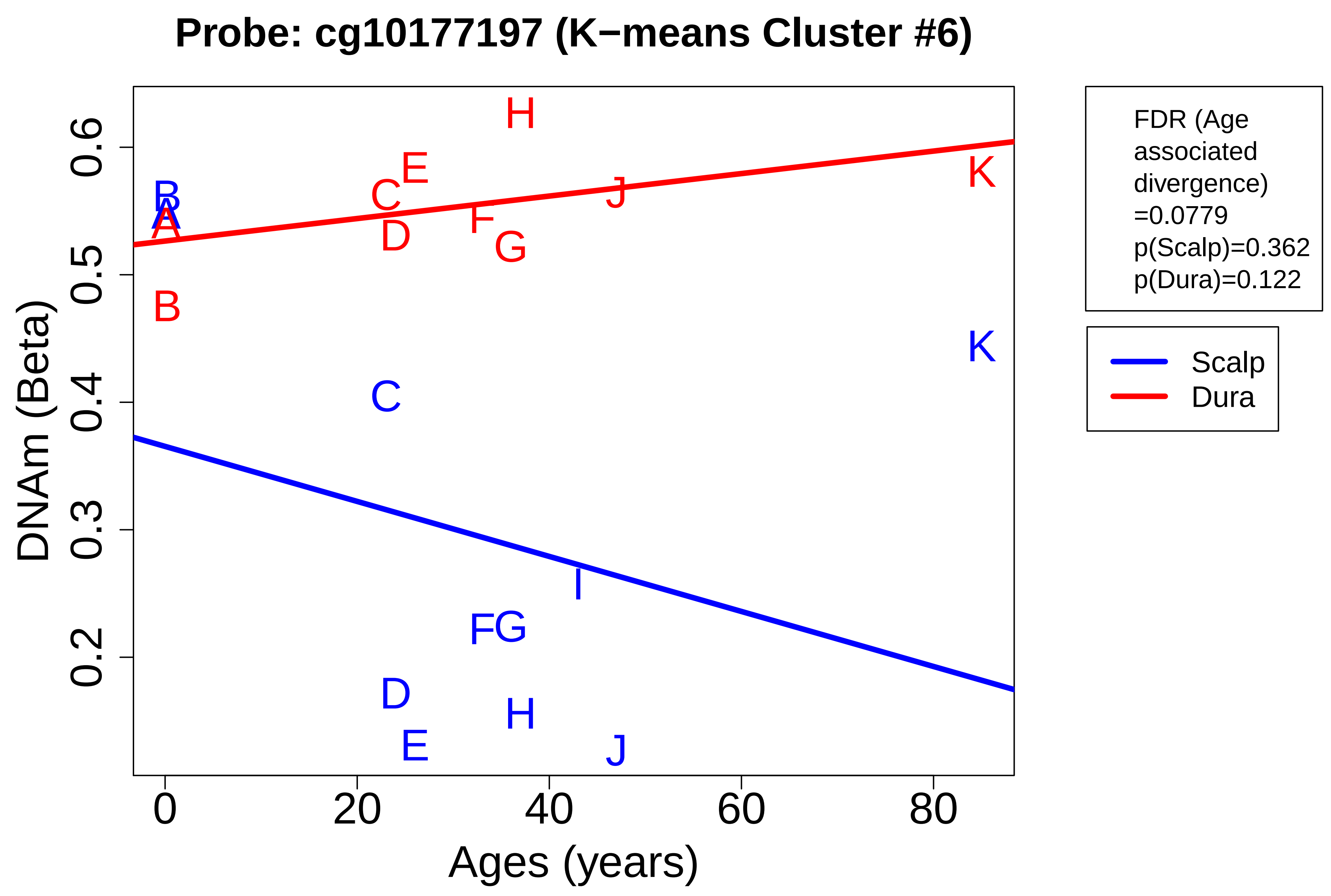

Probe: cg10177197 (K−means Cluster #6)
DNAm (Beta)
0.2
0.3
0.4
0.5
0.6
0
20
40
60
80
Ages (years)
FDR (Age associated divergence) =0.0779
p(Scalp)=0.362
p(Dura)=0.122
Scalp
Dura

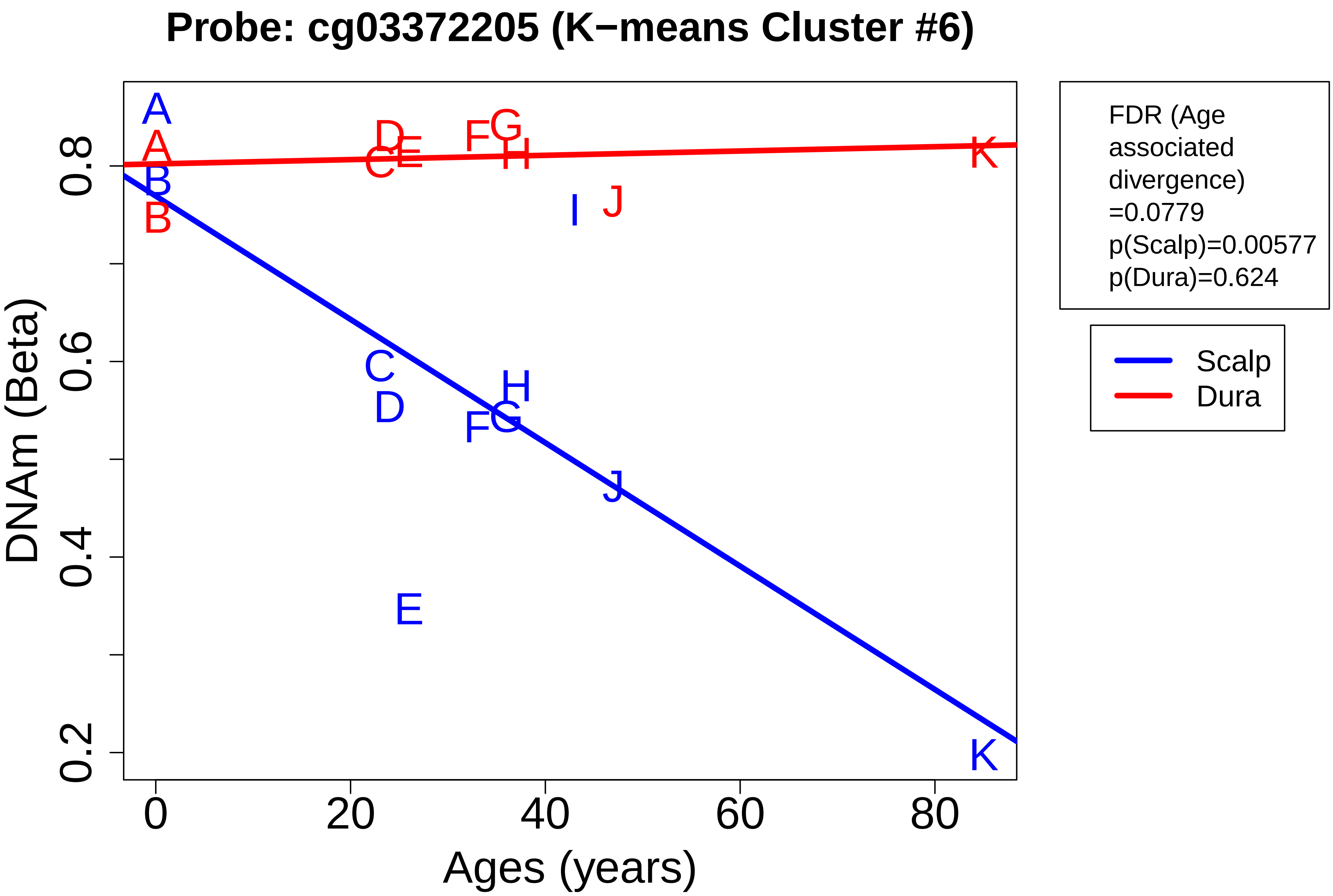

Probe: cg03372205 (K−means Cluster #6)
DNAm (Beta)
0.2
0.4
0.6
0.8
0
20
40
60
80
Ages (years)
A
B
C
D
E
F
G
H
I
J
K
FDR (Age associated divergence) =0.0779
p(Scalp)=0.00577
p(Dura)=0.624
Scalp
Dura

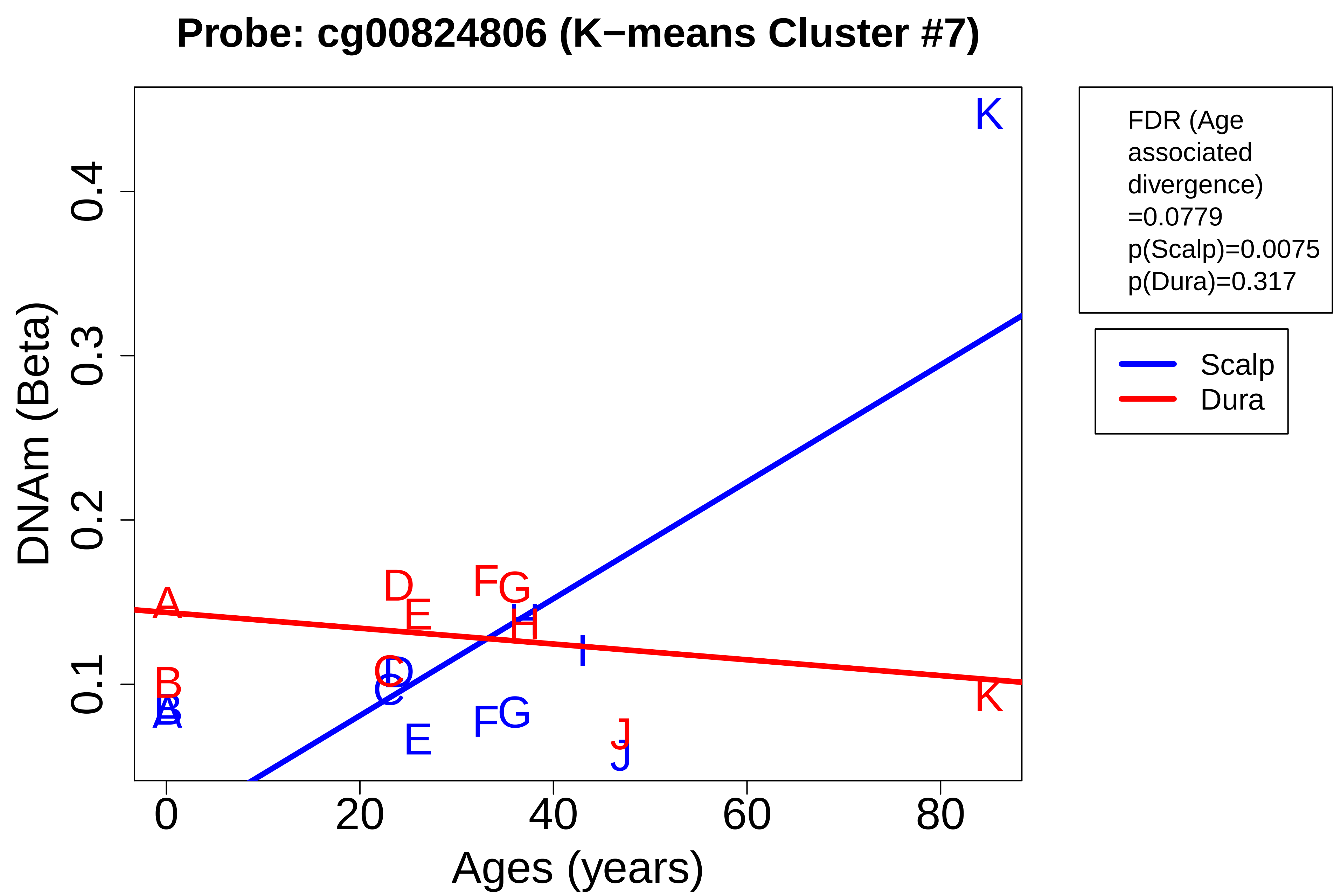
Probe: cg00824806 (K−means Cluster #7)
FDR (Age associated divergence) =0.0779
p(Scalp)=0.0075
p(Dura)=0.317
Scalp
Dura
DNAm (Beta)
0.1
0.2
0.3
0.4
Ages (years)
0
20
40
60
80
A
B
C
D
E
F
G
H
I
J
K

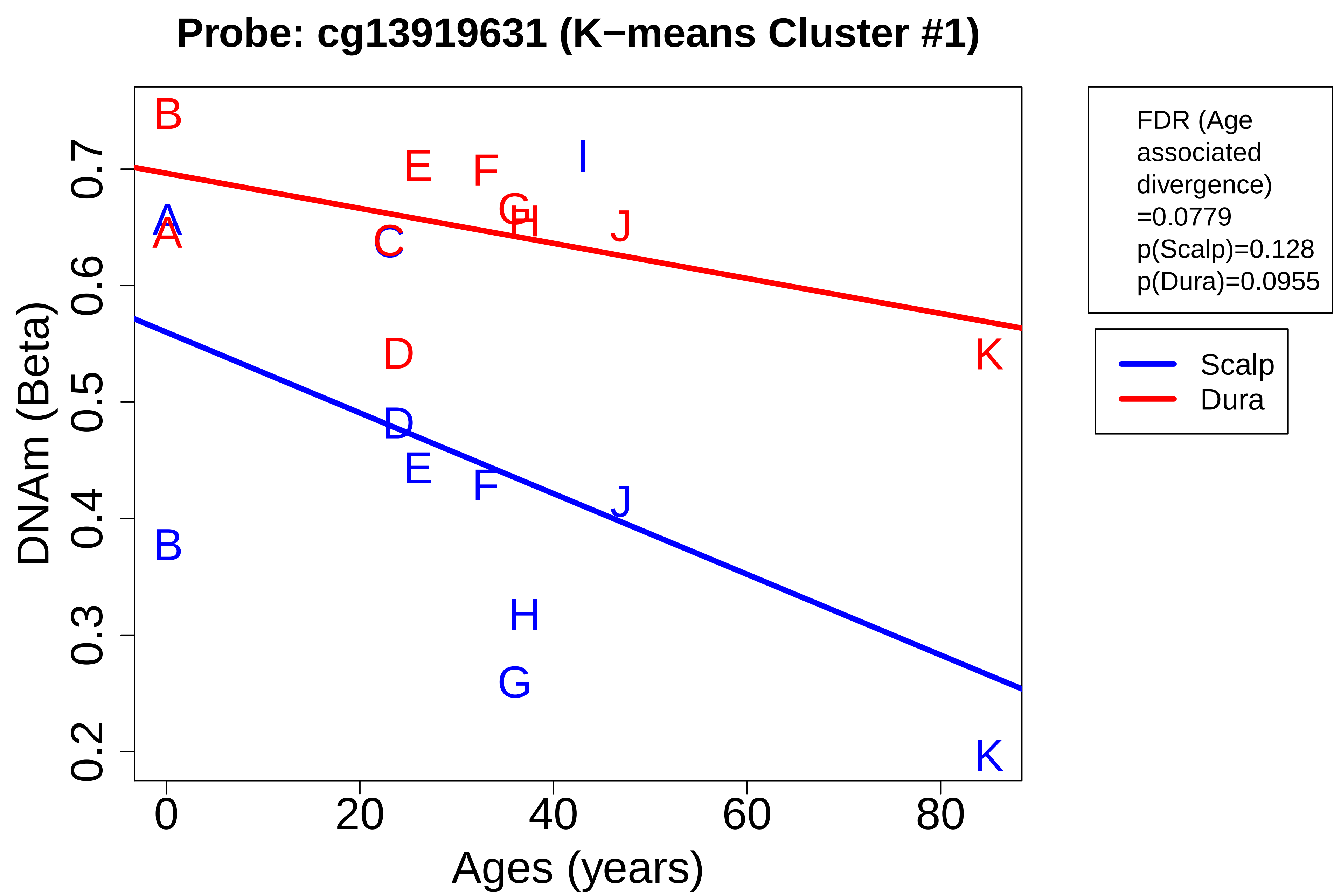

Probe: cg13919631 (K−means Cluster #1)
DNAm (Beta)
0.2
0.3
0.4
0.5
0.6
0.7
Ages (years)
0
20
40
60
80
A
B
C
D
E
F
G
H
I
J
K
FDR (Age associated divergence) =0.0779
p(Scalp)=0.128
p(Dura)=0.0955
Scalp
Dura

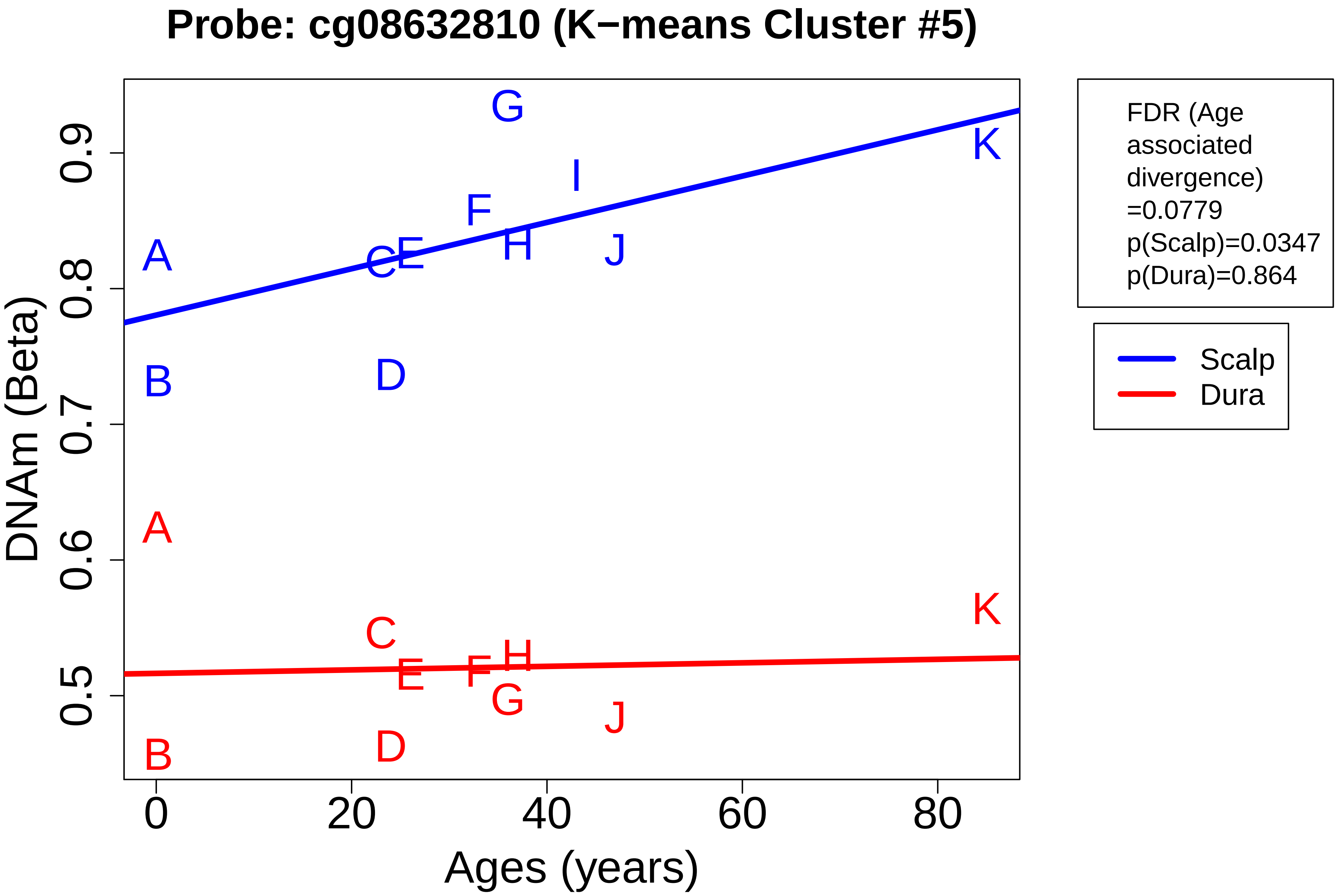

Probe: cg08632810 (K−means Cluster #5)
DNAm (Beta)
0.5
0.6
0.7
0.8
0.9
0
20
40
60
80
Ages (years)
A
B
C
D
E
F
G
H
I
J
K
FDR (Age associated divergence) =0.0779
p(Scalp)=0.0347
p(Dura)=0.864
Scalp
Dura

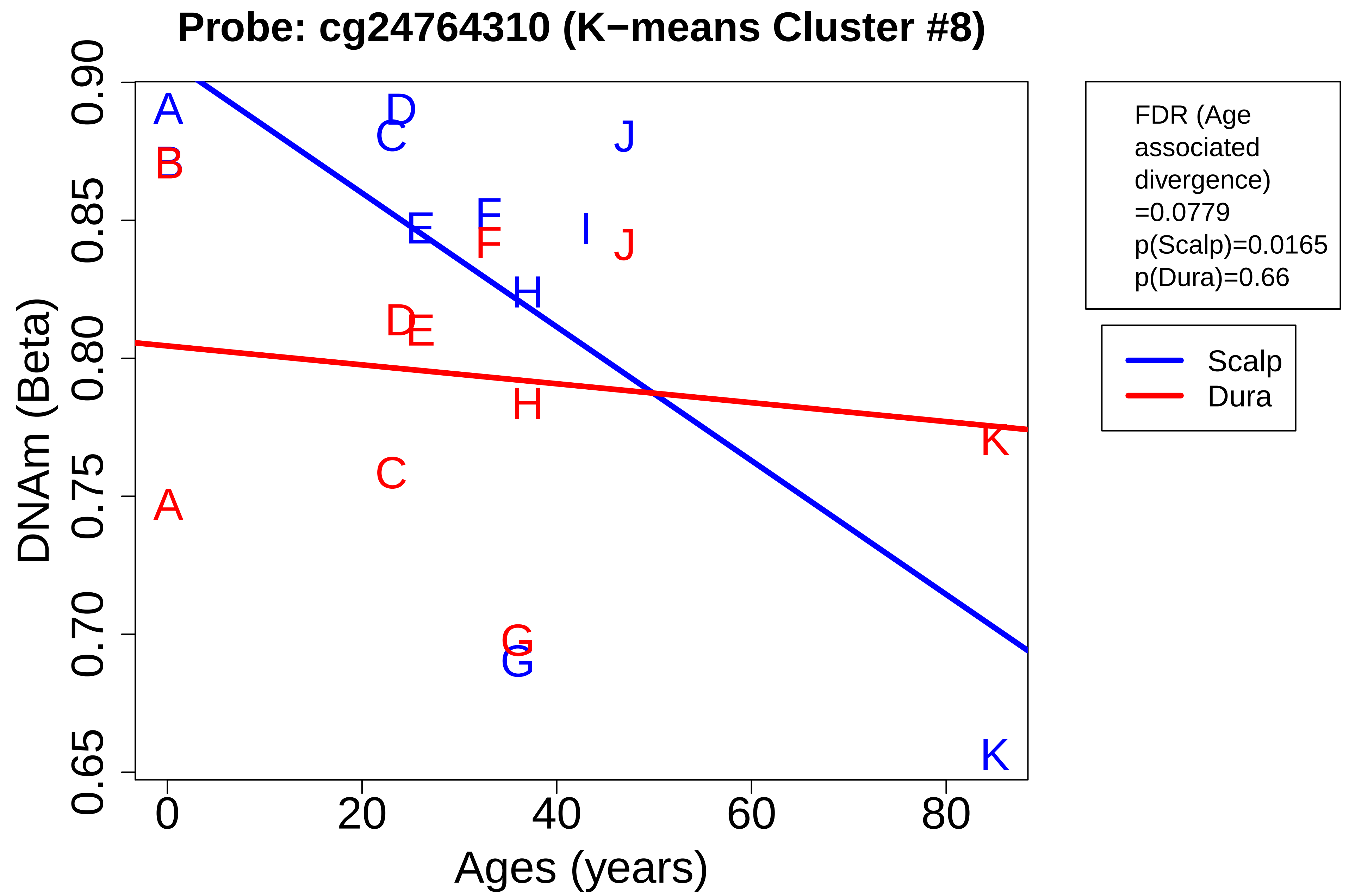

Probe: cg24764310 (K−means Cluster #8)
DNAm (Beta)
0.65
0.70
0.75
0.80
0.85
0.90
0
20
40
60
80
Ages (years)
A
B
C
D
E
F
G
H
I
J
K
FDR (Age associated divergence) =0.0779
p(Scalp)=0.0165
p(Dura)=0.66
Scalp
Dura

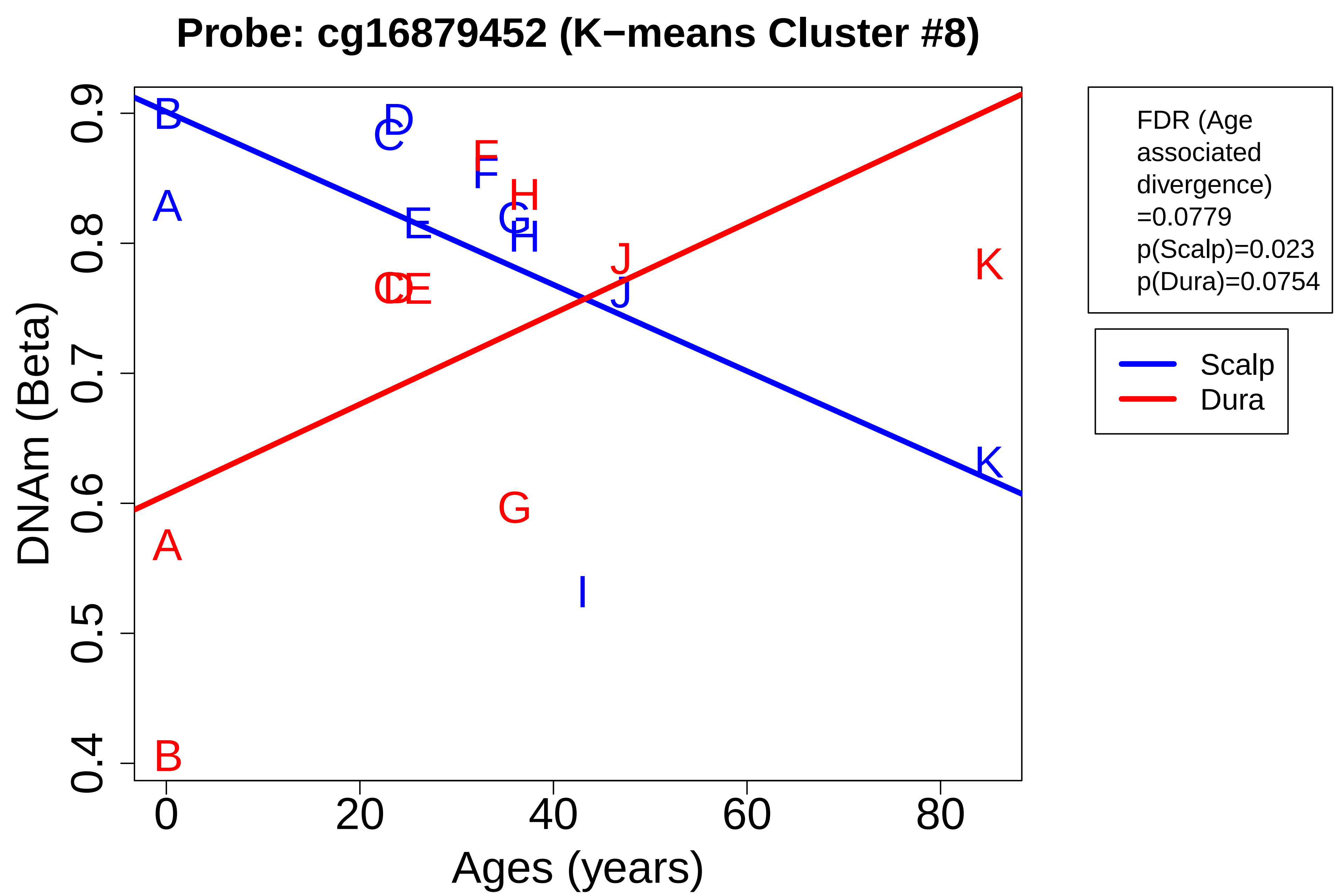

Probe: cg16879452 (K−means Cluster #8)
DNAm (Beta)
0.4
0.5
0.6
0.7
0.8
0.9
0
20
40
60
80
Ages (years)
FDR (Age associated divergence) =0.0779
p(Scalp)=0.023
p(Dura)=0.0754
Scalp
Dura

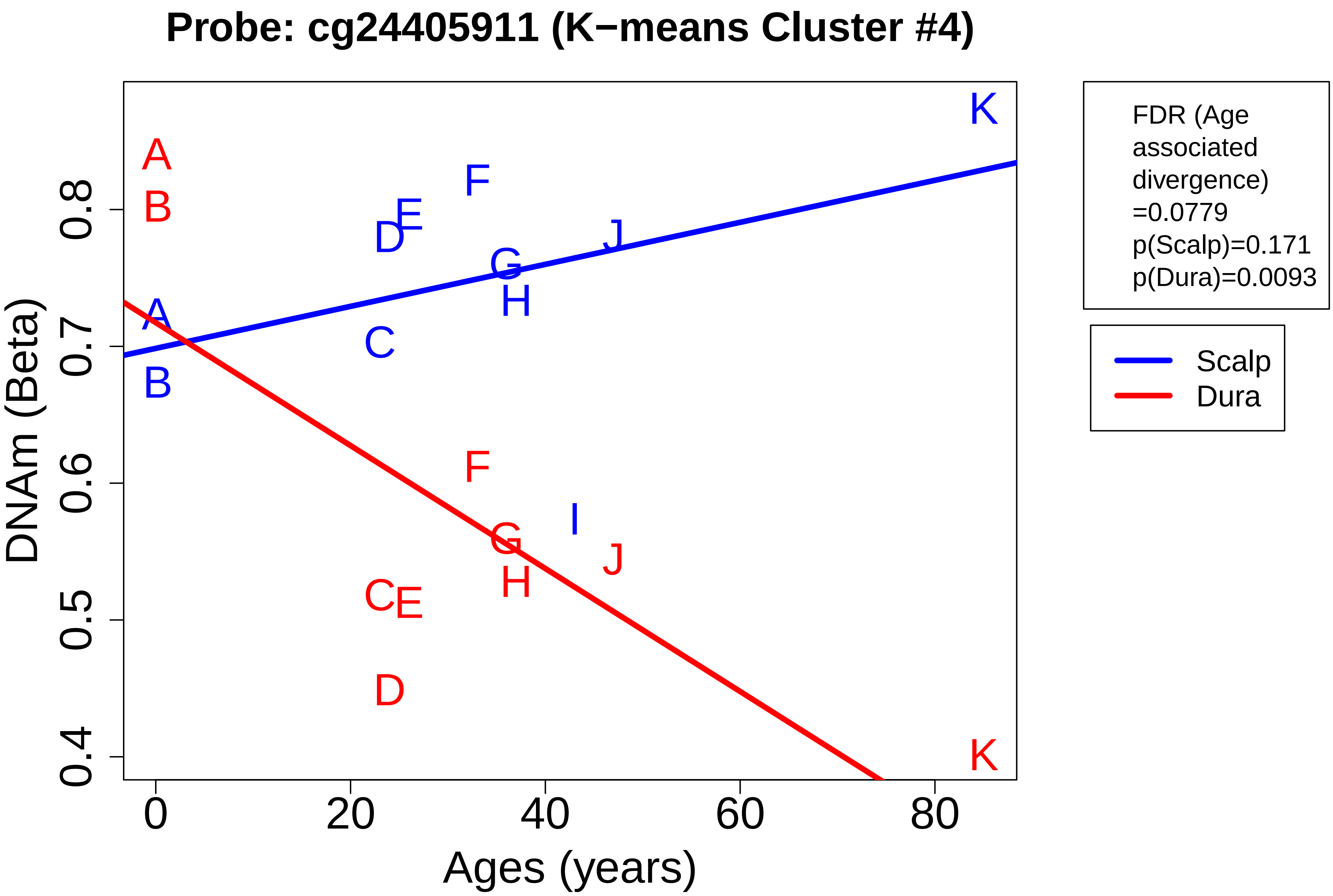
Probe: cg24405911 (K−means Cluster #4)
DNAm (Beta)
0.4
0.5
0.6
0.7
0.8
Ages (years)
0
20
40
60
80
A
B
C
D
E
F
G
H
I
J
K
FDR (Age associated divergence) =0.0779
p(Scalp)=0.171
p(Dura)=0.0093
Scalp
Dura

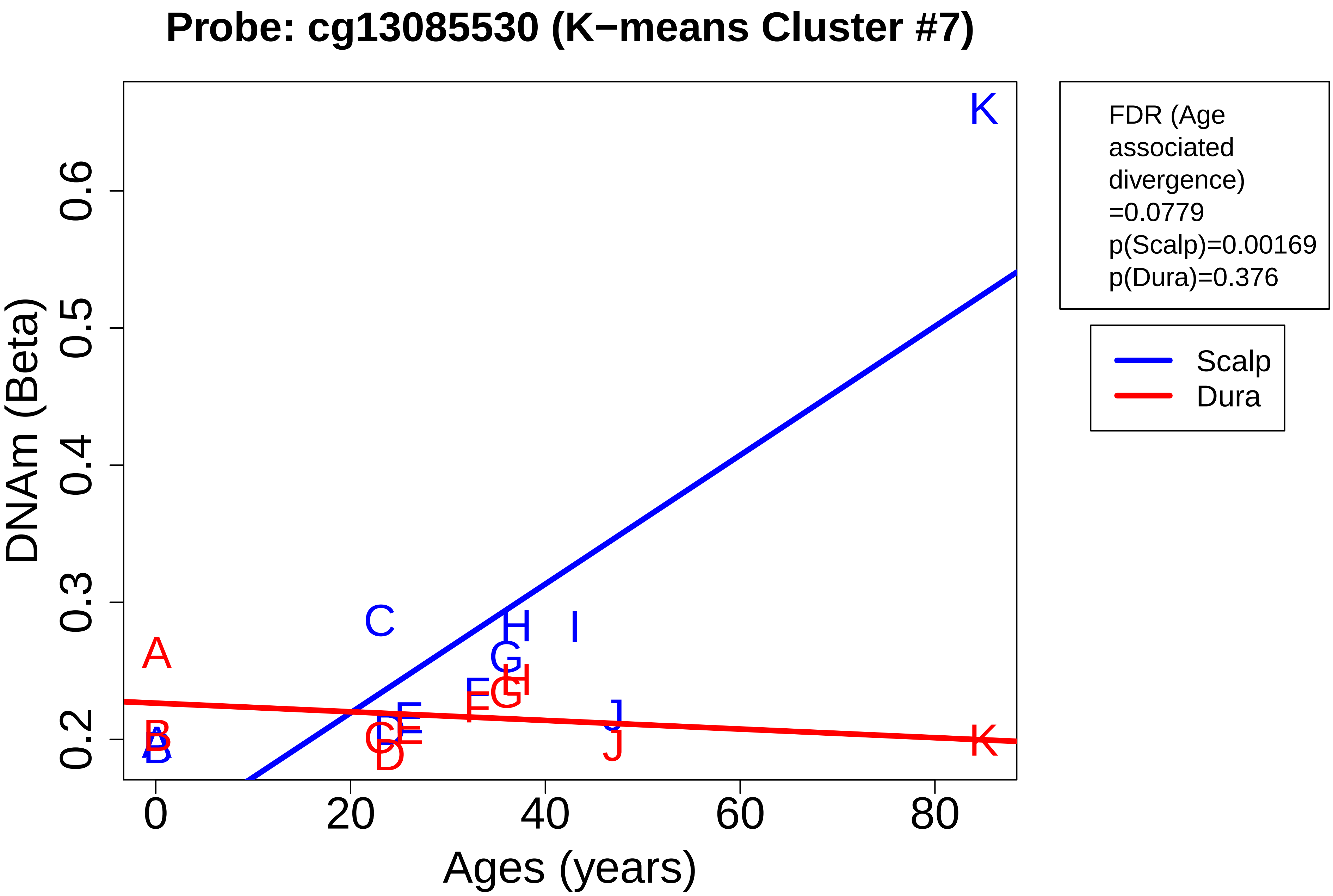

Probe: cg13085530 (K−means Cluster #7)
DNAm (Beta)
0.2
0.3
0.4
0.5
0.6
0
20
40
60
80
Ages (years)
FDR (Age associated divergence) =0.0779
p(Scalp)=0.00169
p(Dura)=0.376
Scalp
Dura

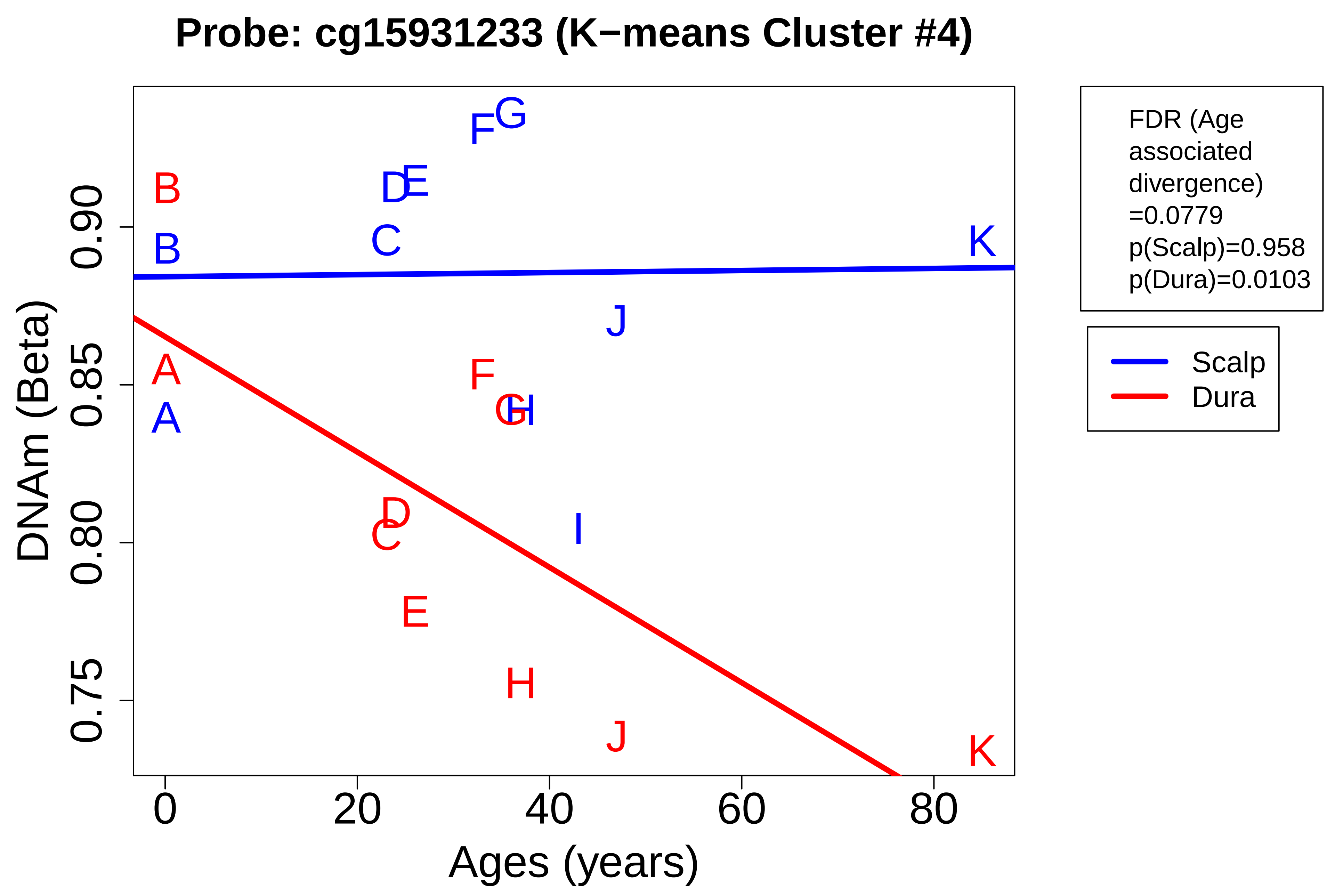

Probe: cg15931233 (K−means Cluster #4)
DNAm (Beta)
0.75
0.80
0.85
0.90
0
20
40
60
80
Ages (years)
A
B
C
D
E
F
G
H
I
J
K
FDR (Age associated divergence) =0.0779
p(Scalp)=0.958
p(Dura)=0.0103
Scalp
Dura

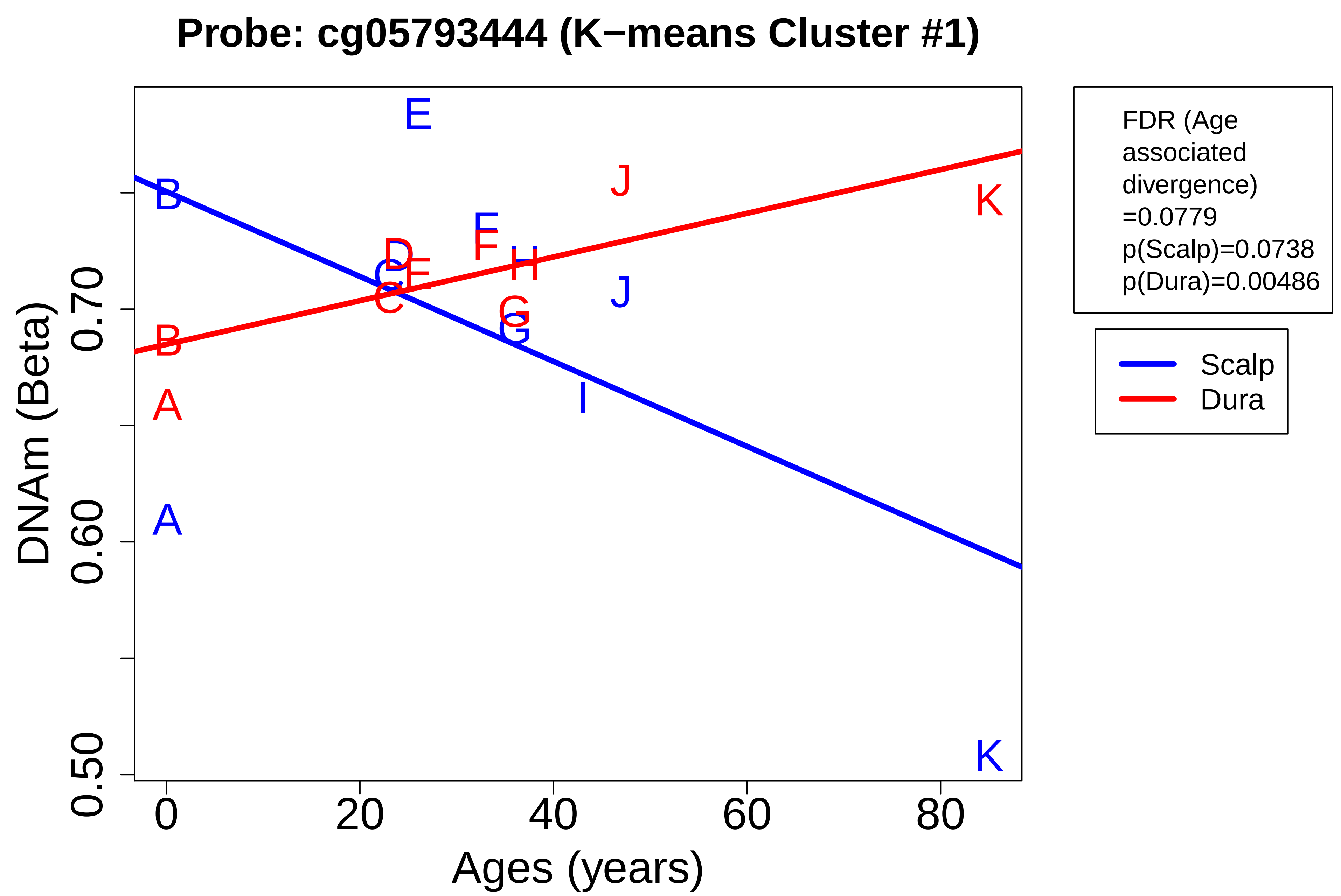
Probe: cg05793444 (K−means Cluster #1)
DNAm (Beta)
0.50
0.60
0.70
0
20
40
60
80
Ages (years)
A
B
C
D
E
F
G
H
I
J
K
FDR (Age associated divergence) =0.0779
p(Scalp)=0.0738
p(Dura)=0.00486
Scalp
Dura

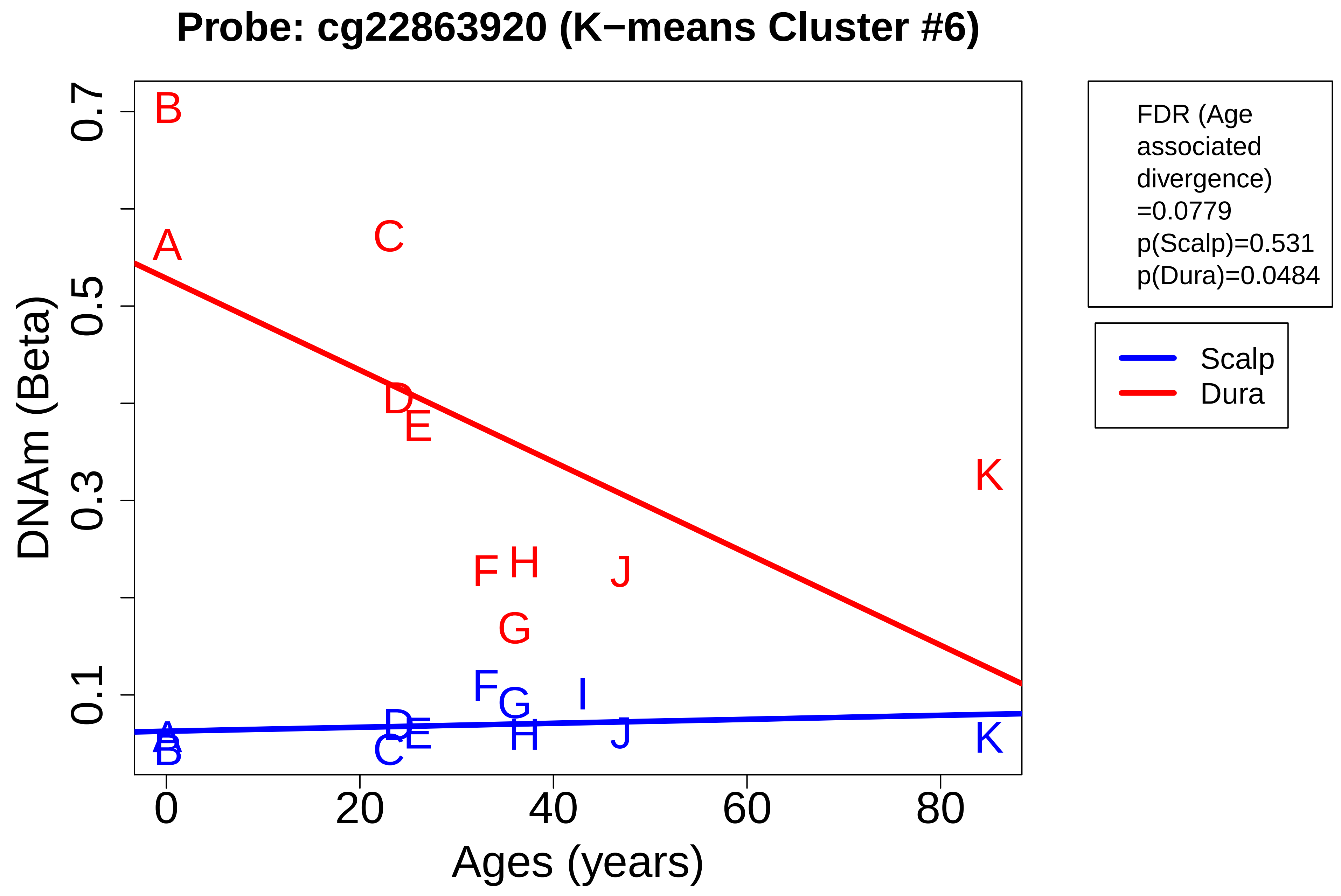

Probe: cg22863920 (K−means Cluster #6)
DNAm (Beta)
0.1
0.3
0.5
0.7
Ages (years)
0
20
40
60
80
FDR (Age associated divergence) =0.0779
p(Scalp)=0.531
p(Dura)=0.0484
Scalp
Dura

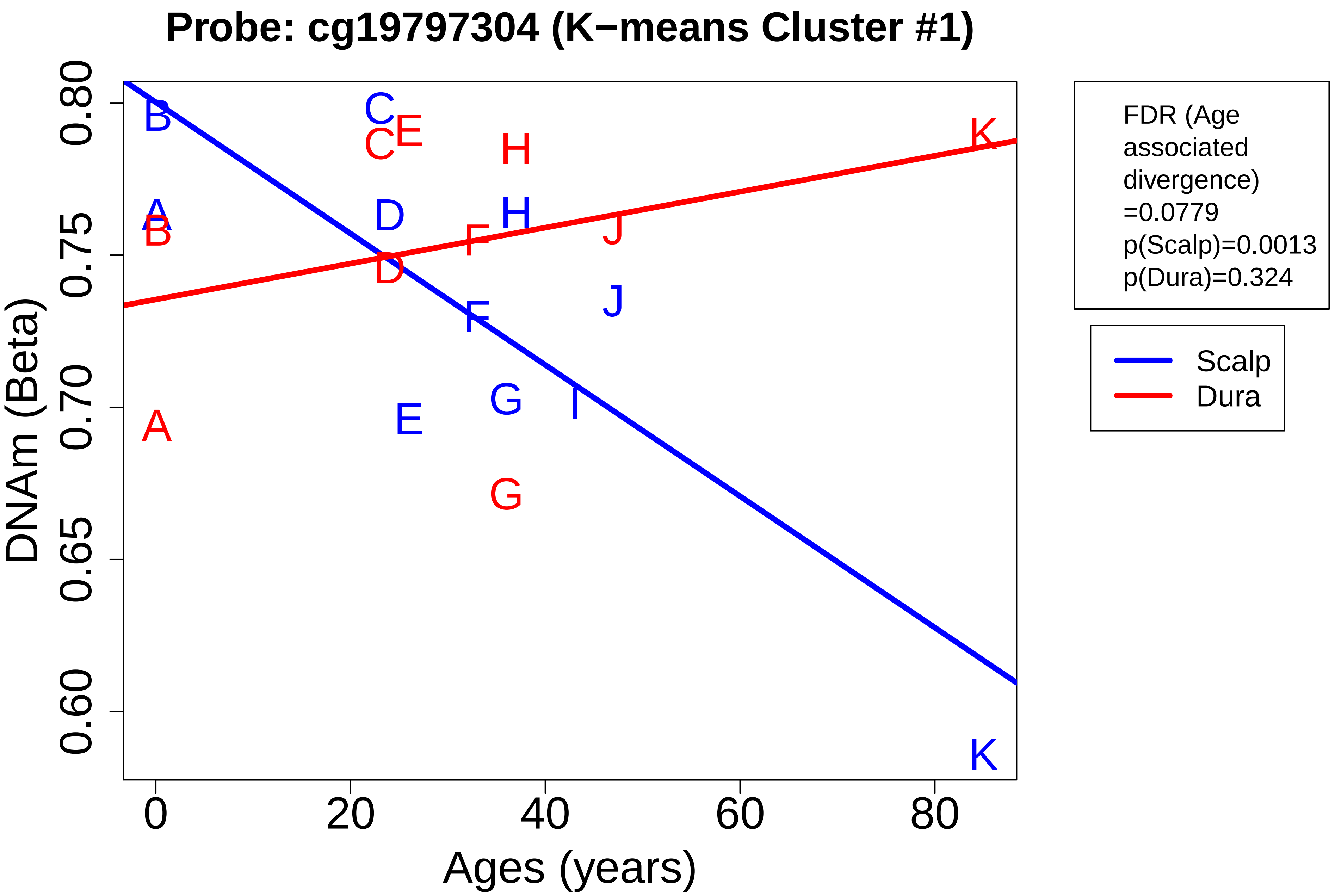
Probe: cg19797304 (K−means Cluster #1)
DNAm (Beta)
0.60
0.65
0.70
0.75
0.80
0
20
40
60
80
Ages (years)
FDR (Age associated divergence) =0.0779
p(Scalp)=0.0013
p(Dura)=0.324
Scalp
Dura

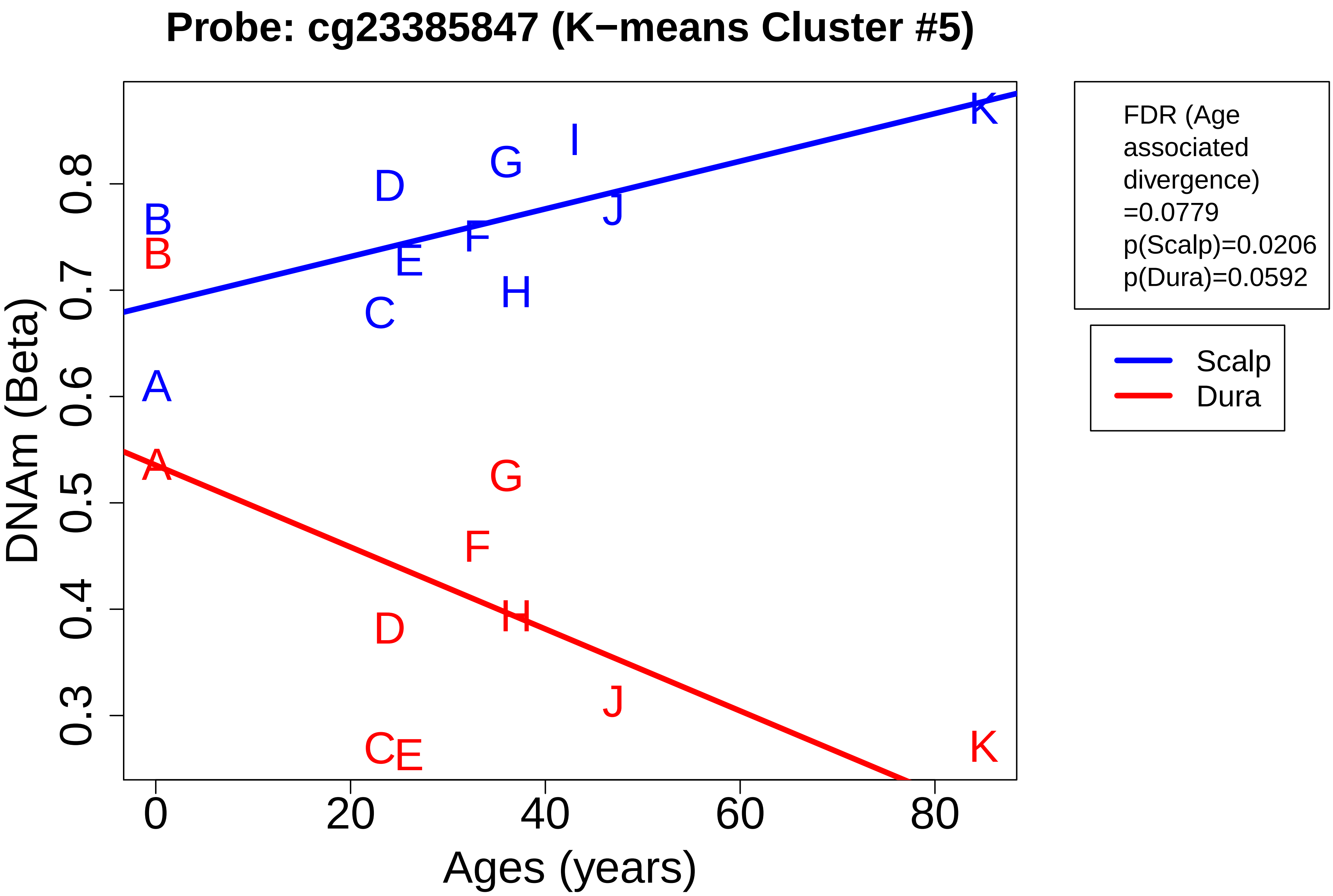

Probe: cg23385847 (K−means Cluster #5)
DNAm (Beta)
Ages (years)
0.3
0.4
0.5
0.6
0.7
0.8
0
20
40
60
80
FDR (Age associated divergence) =0.0779
p(Scalp)=0.0206
p(Dura)=0.0592
Scalp
Dura

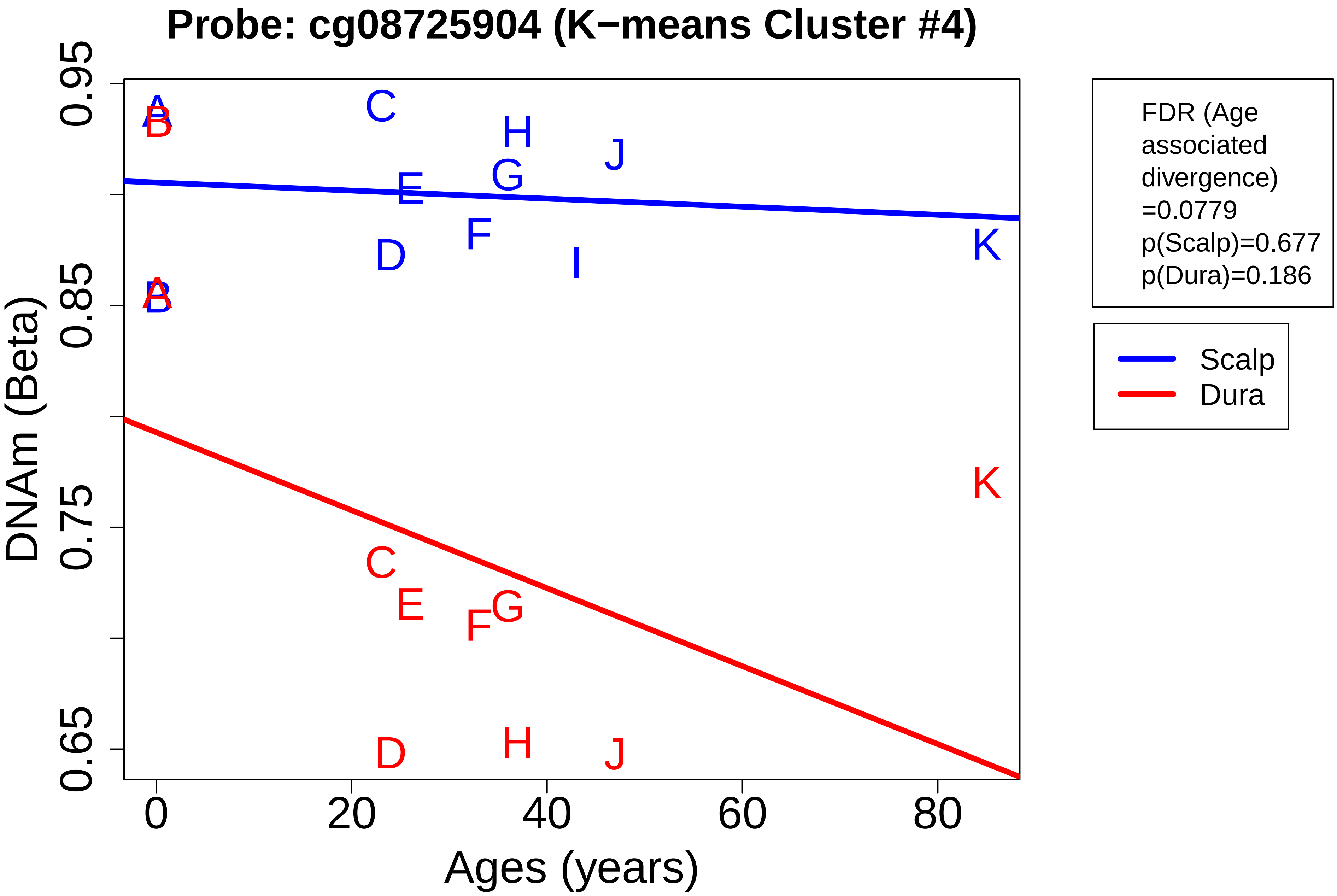

Probe: cg08725904 (K−means Cluster #4)
DNAm (Beta)
0.65
0.75
0.85
0.95
Ages (years)
0
20
40
60
80
FDR (Age associated divergence) =0.0779
p(Scalp)=0.677
p(Dura)=0.186
Scalp
Dura

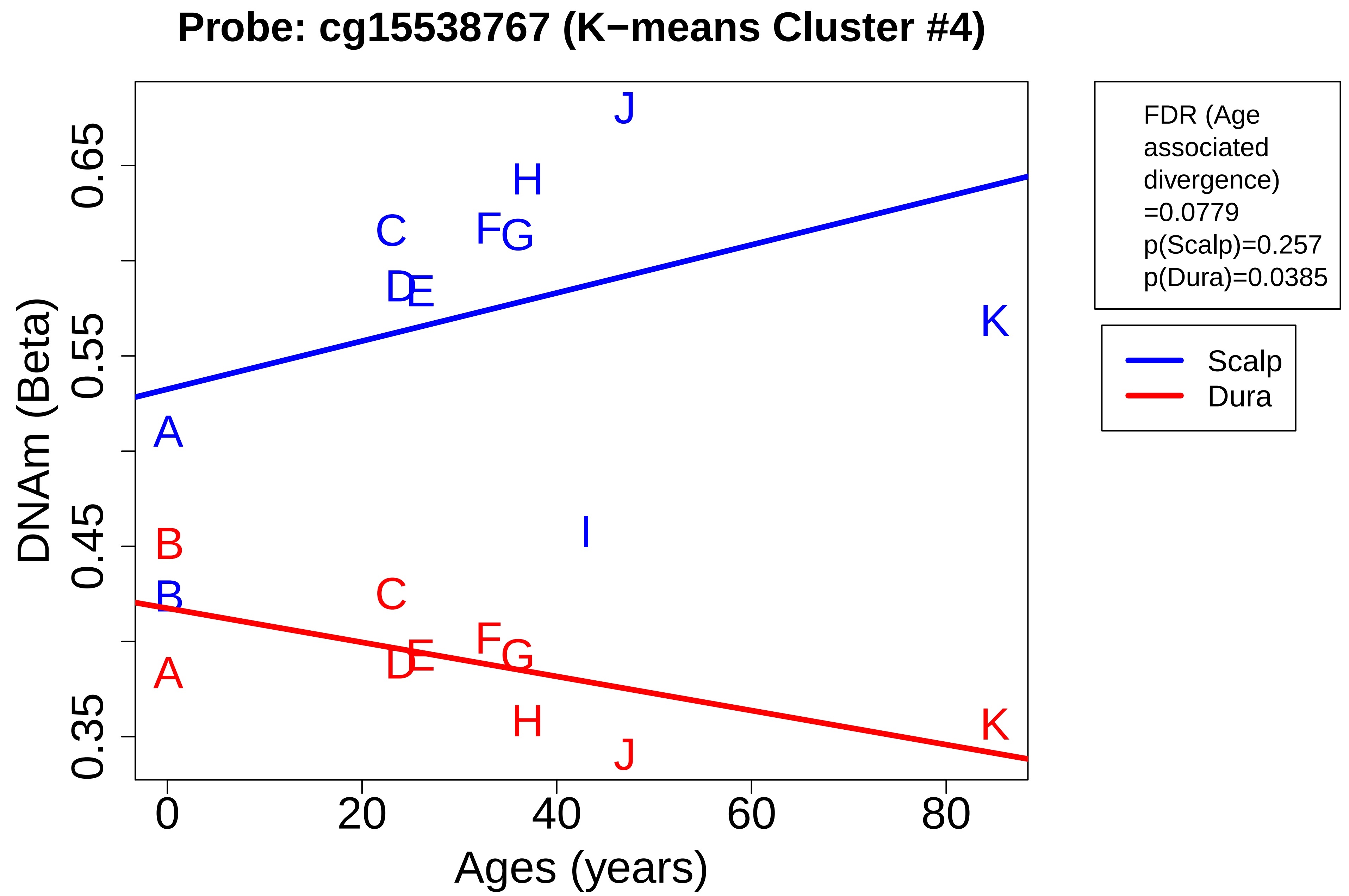

Probe: cg15538767 (K−means Cluster #4)
DNAm (Beta)
0.35
0.45
0.55
0.65
0
20
40
60
80
Ages (years)
J
H
C
F
G
D
E
K
A
I
B
B
C
F
G
E
D
A
H
K
J
FDR (Age associated divergence) =0.0779
p(Scalp)=0.257
p(Dura)=0.0385
Scalp
Dura

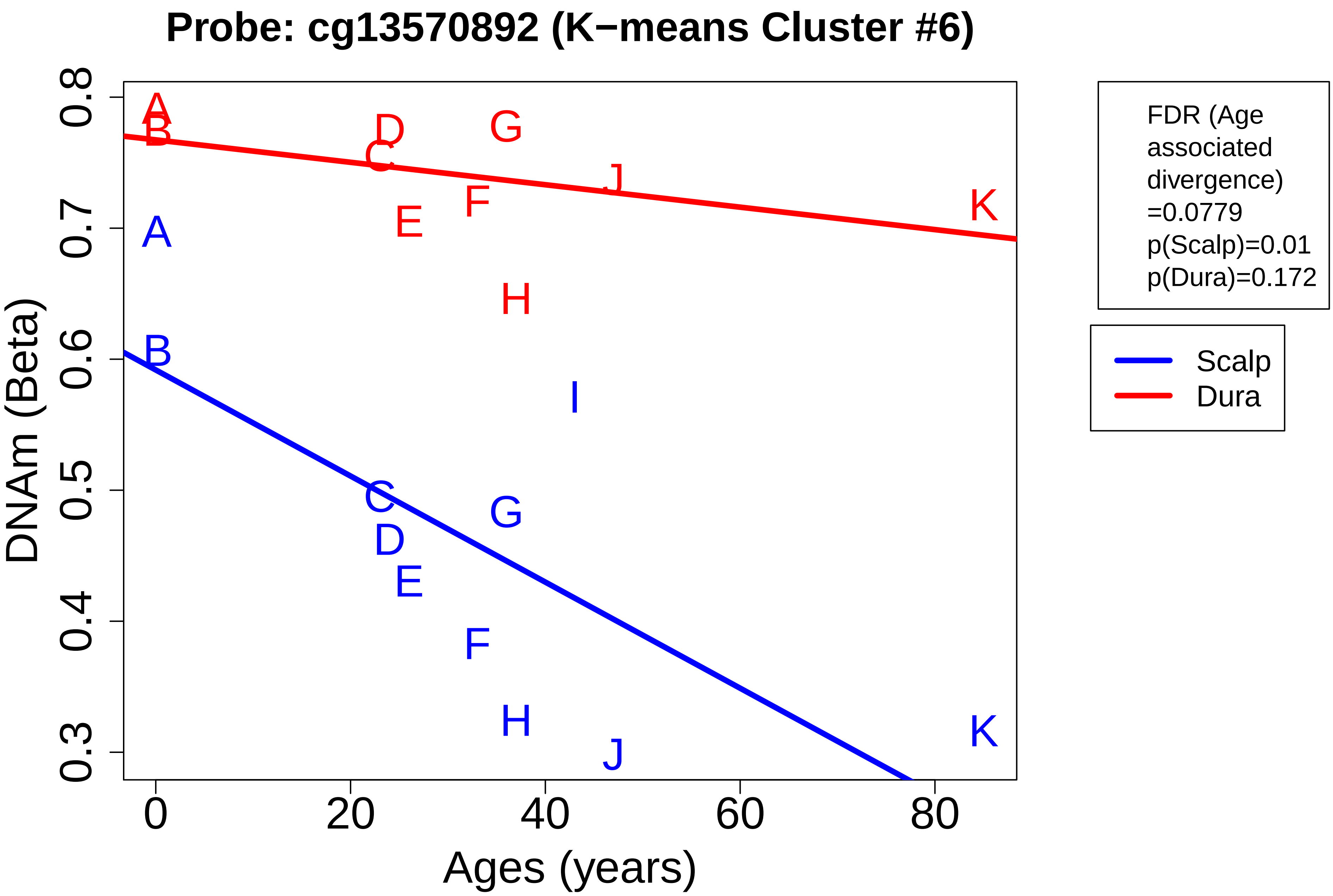
Probe: cg13570892 (K−means Cluster #6)
DNAm (Beta)
0.3
0.4
0.5
0.6
0.7
0.8
0
20
40
60
80
Ages (years)
A
B
C
D
E
F
G
H
I
J
K
FDR (Age associated divergence) =0.0779
p(Scalp)=0.01
p(Dura)=0.172
Scalp
Dura

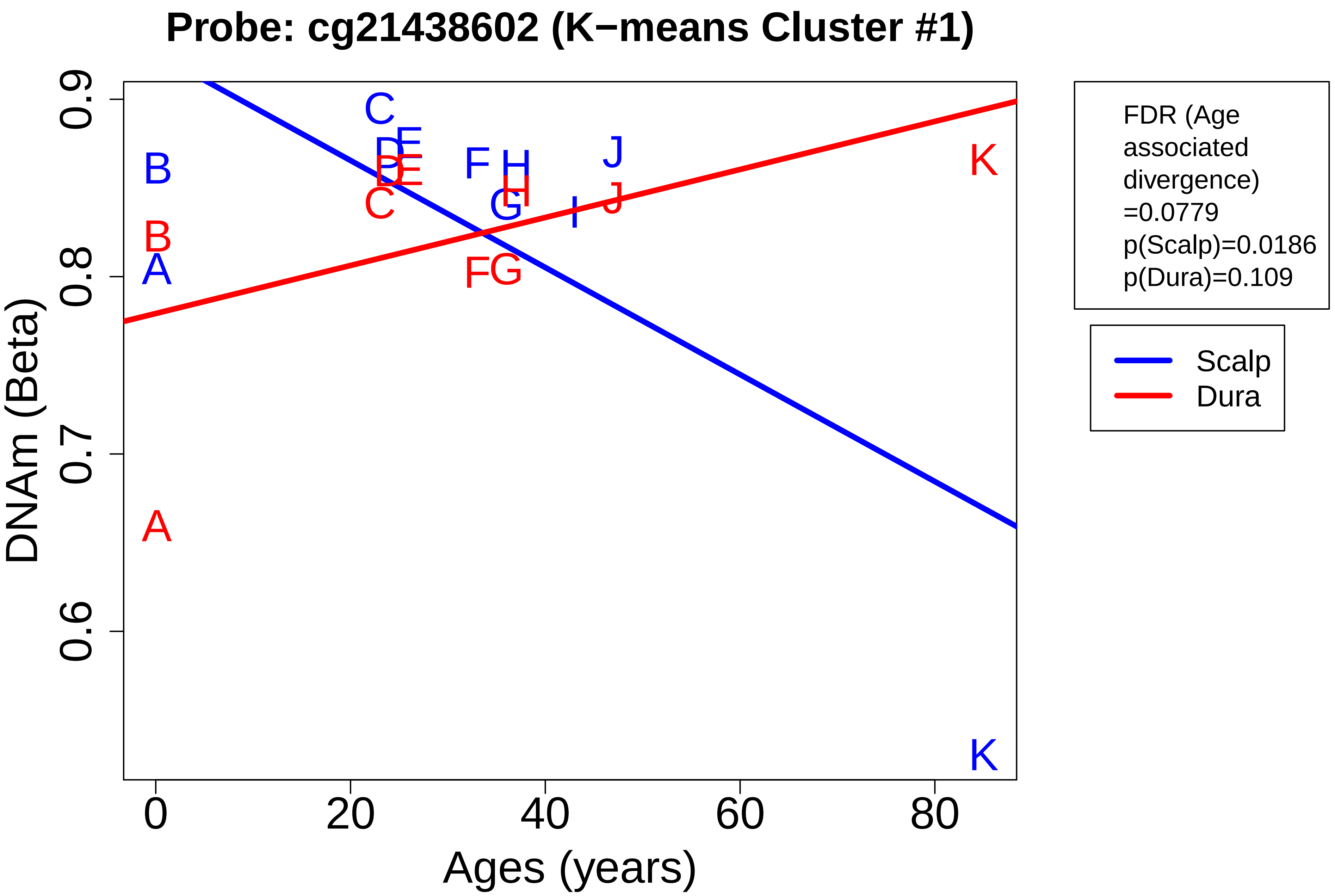

Probe: cg21438602 (K−means Cluster #1)
DNAm (Beta)
0.6
0.7
0.8
0.9
0
20
40
60
80
Ages (years)
FDR (Age associated divergence) =0.0779
p(Scalp)=0.0186
p(Dura)=0.109
Scalp
Dura

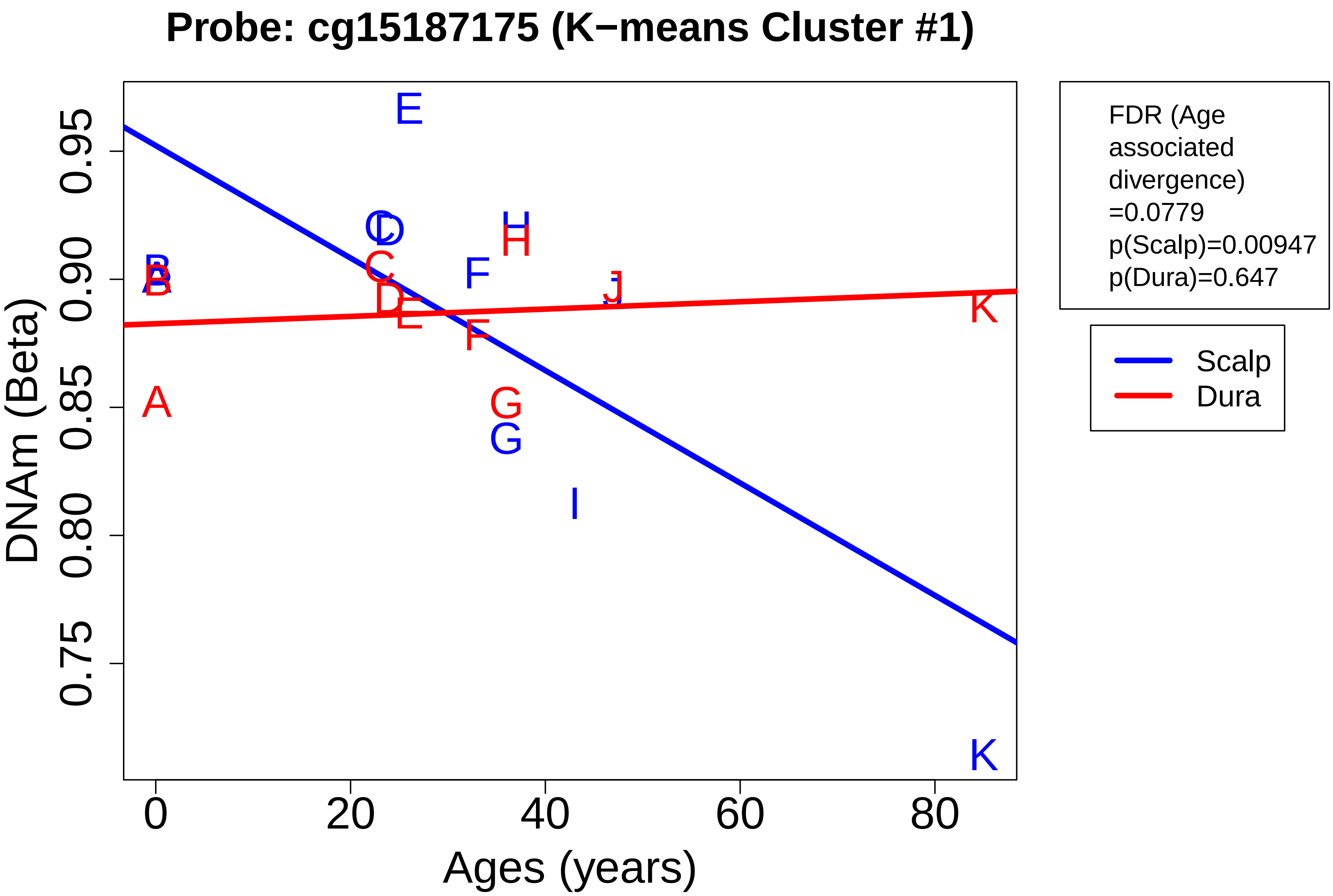

Probe: cg15187175 (K−means Cluster #1)
DNAm (Beta)
0.75
0.80
0.85
0.90
0.95
0
20
40
60
80
Ages (years)
FDR (Age associated divergence) =0.0779
p(Scalp)=0.00947
p(Dura)=0.647
Scalp
Dura

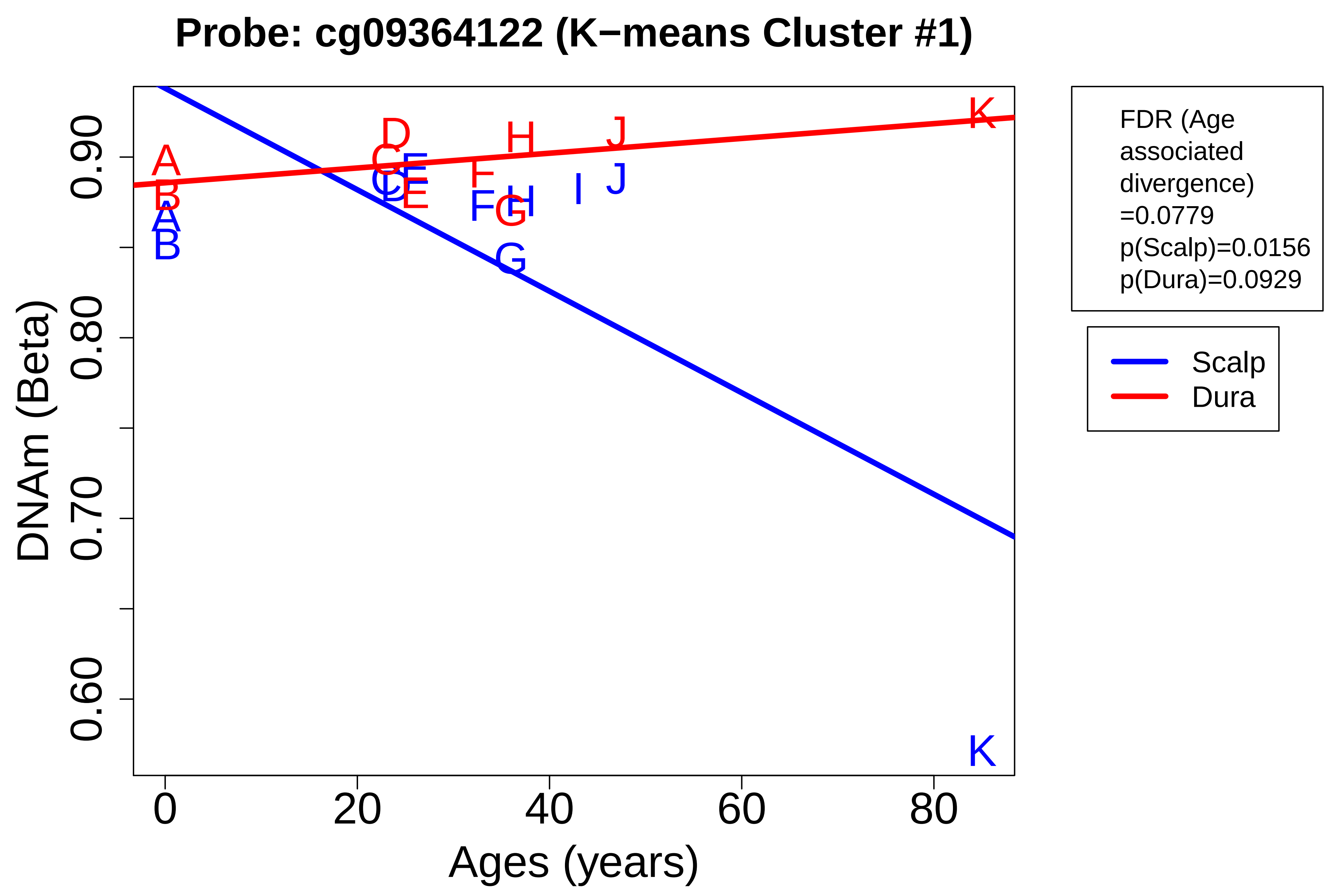

Probe: cg09364122 (K−means Cluster #1)
DNAm (Beta)
0.60
0.70
0.80
0.90
Ages (years)
0
20
40
60
80
A
B
C
D
E
F
G
H
I
J
K
FDR (Age associated divergence) =0.0779
p(Scalp)=0.0156
p(Dura)=0.0929
Scalp
Dura

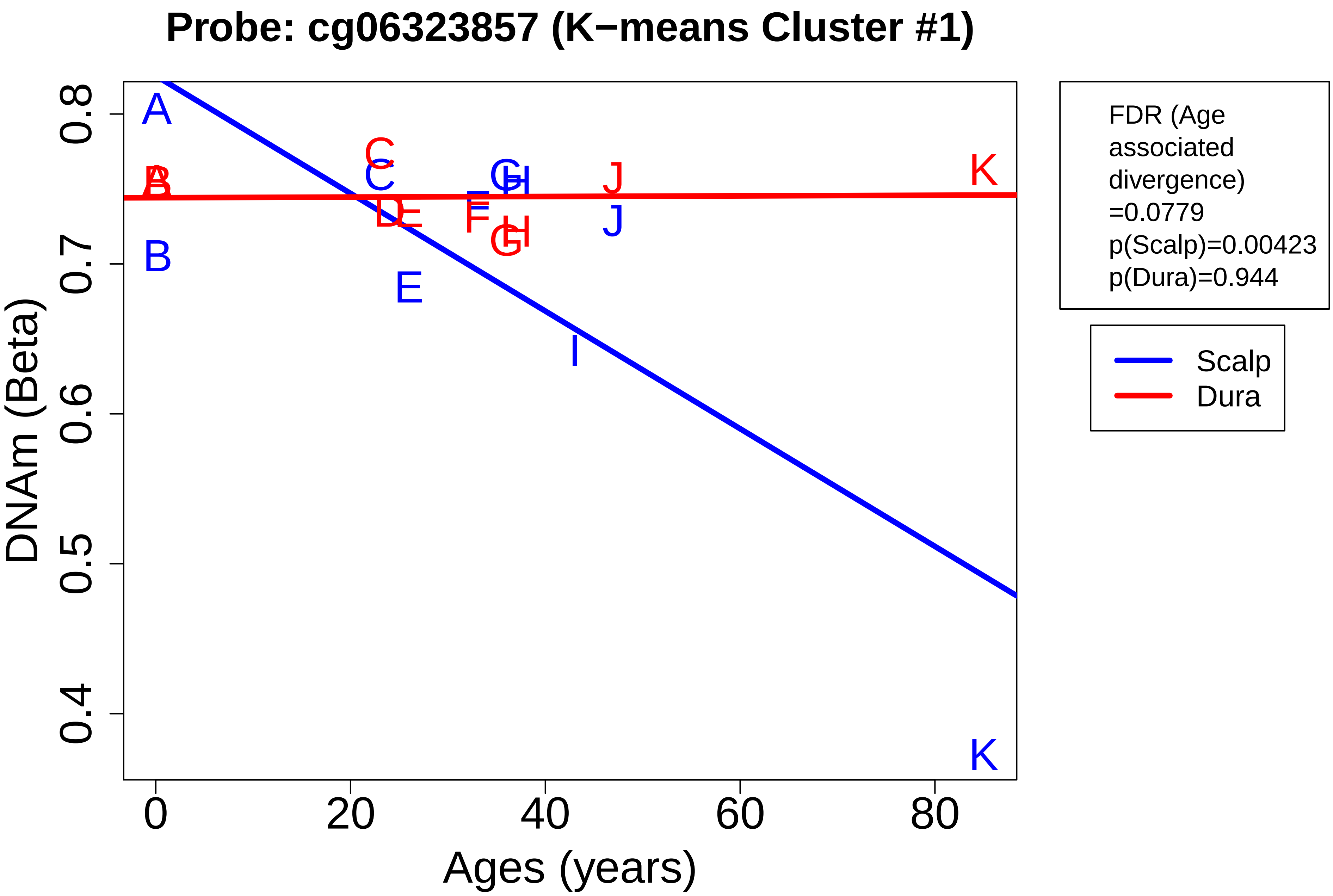

Probe: cg06323857 (K−means Cluster #1)
DNAm (Beta)
Ages (years)
0.4
0.5
0.6
0.7
0.8
0
20
40
60
80
FDR (Age associated divergence) =0.0779
p(Scalp)=0.00423
p(Dura)=0.944
Scalp
Dura

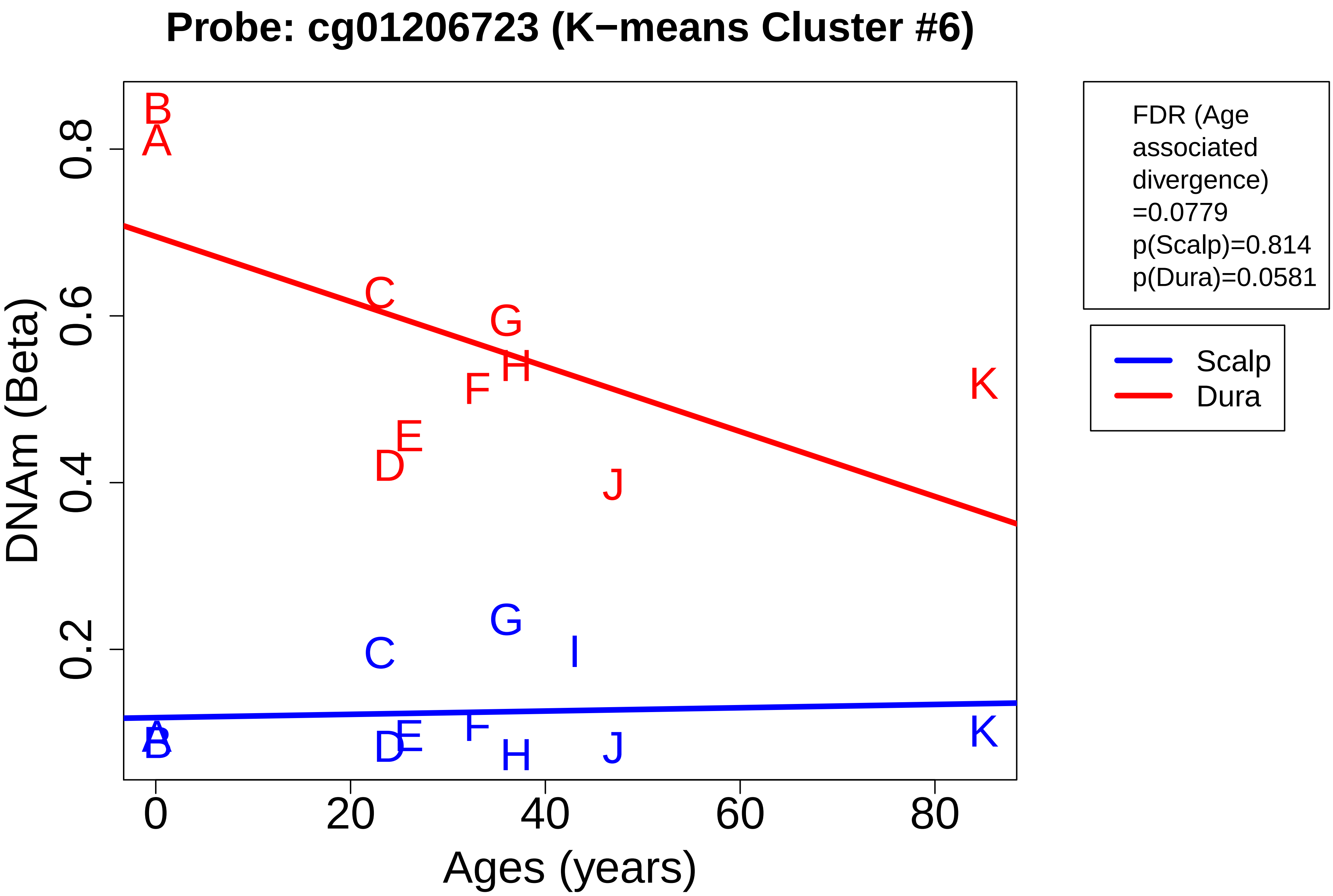

Probe: cg01206723 (K−means Cluster #6)
DNAm (Beta)
0.2
0.4
0.6
0.8
0
20
40
60
80
Ages (years)
FDR (Age associated divergence) =0.0779
p(Scalp)=0.814
p(Dura)=0.0581
Scalp
Dura

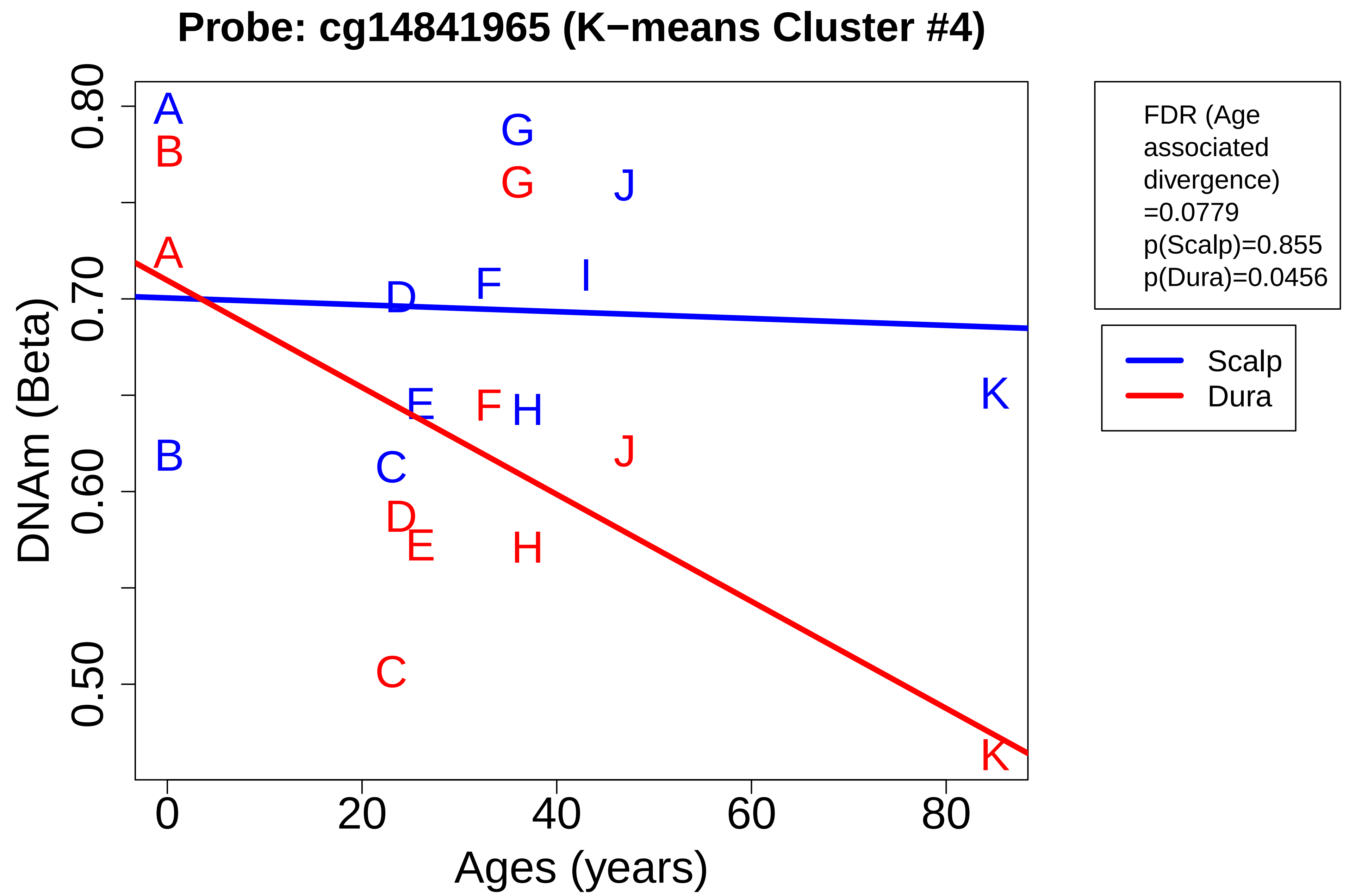

Probe: cg14841965 (K−means Cluster #4)
DNAm (Beta)
0.50
0.60
0.70
0.80
0
20
40
60
80
Ages (years)
A
B
A
B
C
C
D
D
E
E
F
F
G
G
H
H
I
J
J
K
K
FDR (Age associated divergence) =0.0779
p(Scalp)=0.855
p(Dura)=0.0456
Scalp
Dura

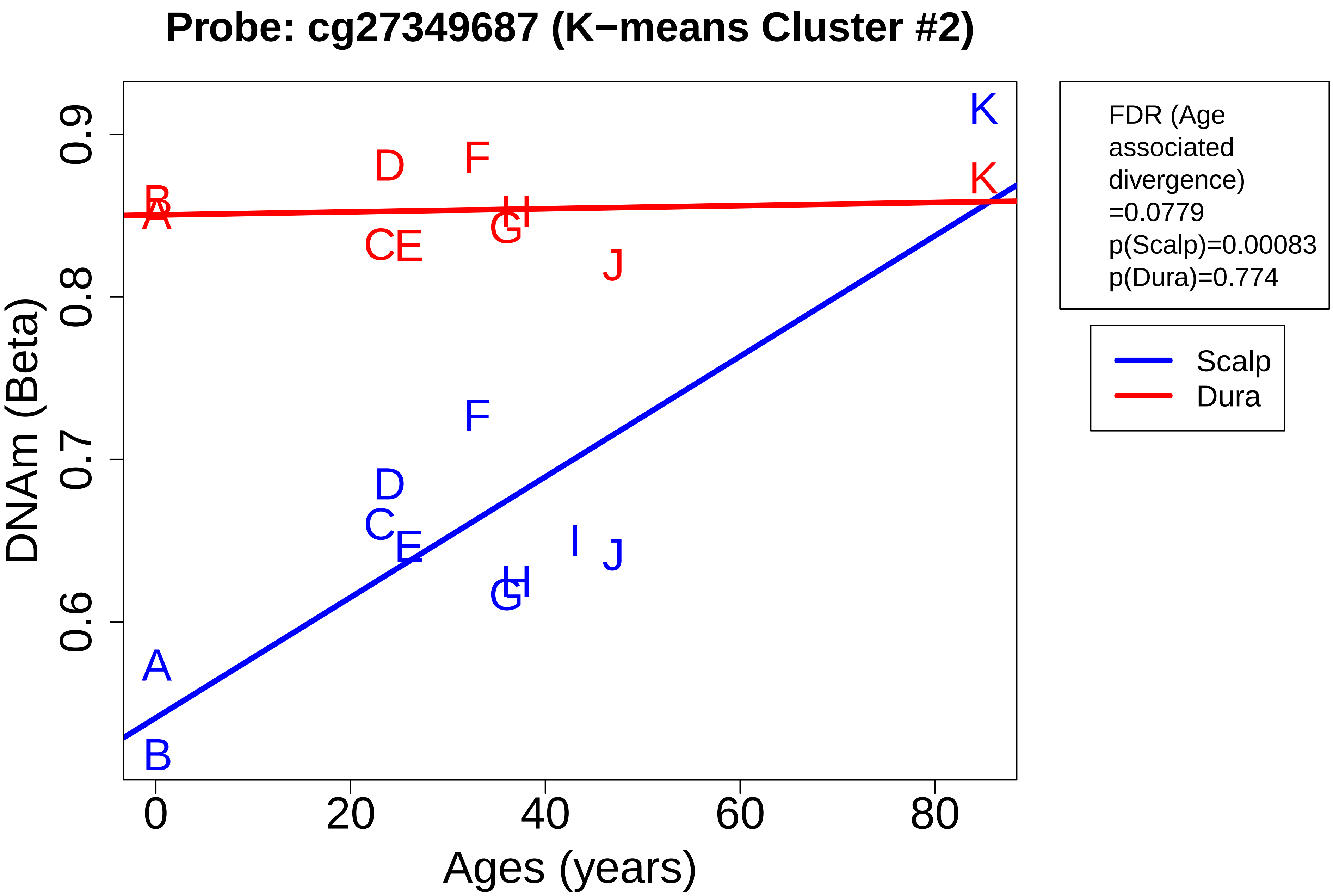

Probe: cg27349687 (K−means Cluster #2)
DNAm (Beta)
0.6
0.7
0.8
0.9
0
20
40
60
80
Ages (years)
FDR (Age associated divergence) =0.0779
p(Scalp)=0.00083
p(Dura)=0.774
Scalp
Dura

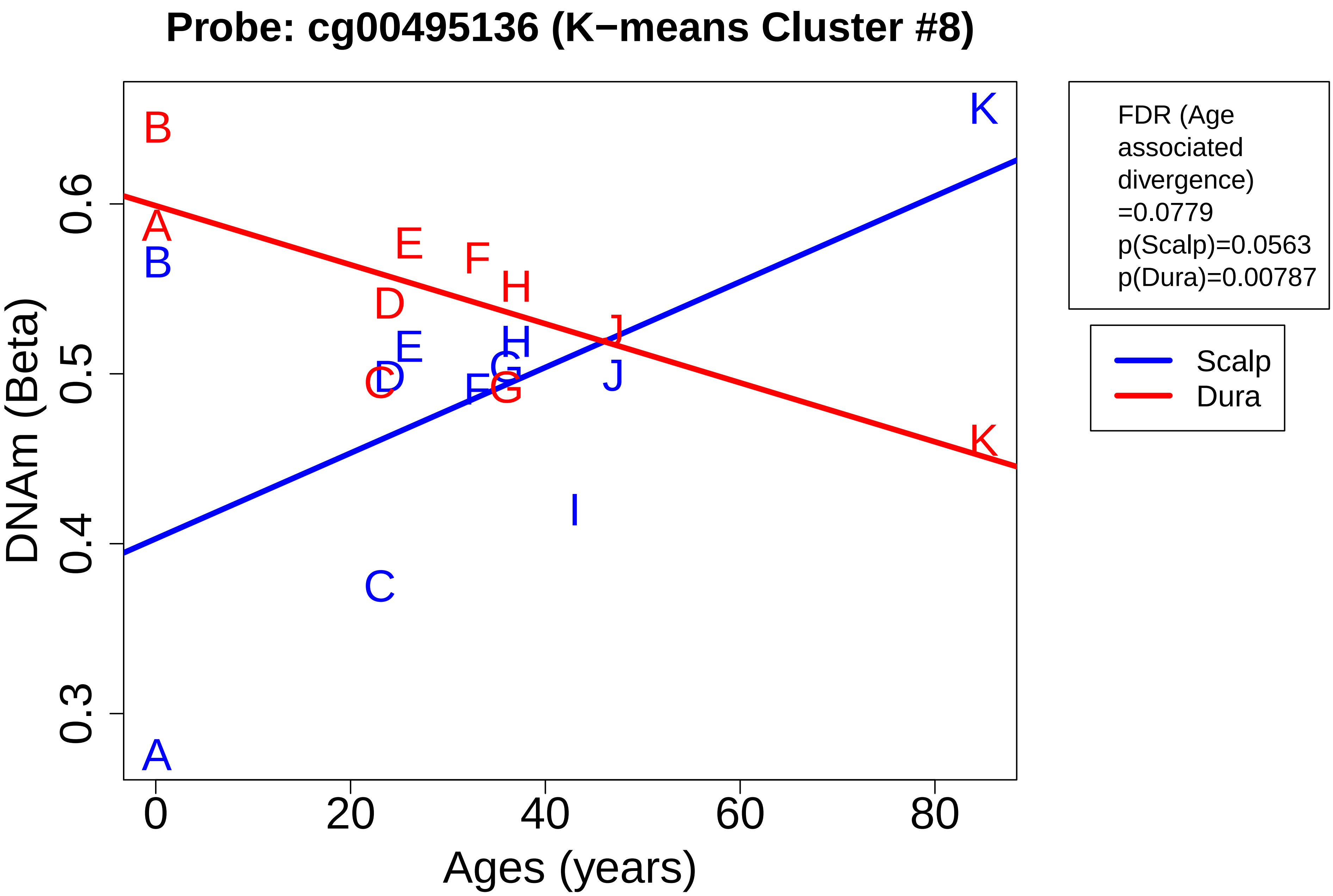

Probe: cg00495136 (K−means Cluster #8)
DNAm (Beta)
0.3
0.4
0.5
0.6
Ages (years)
0
20
40
60
80
FDR (Age associated divergence) =0.0779
p(Scalp)=0.0563
p(Dura)=0.00787
Scalp
Dura

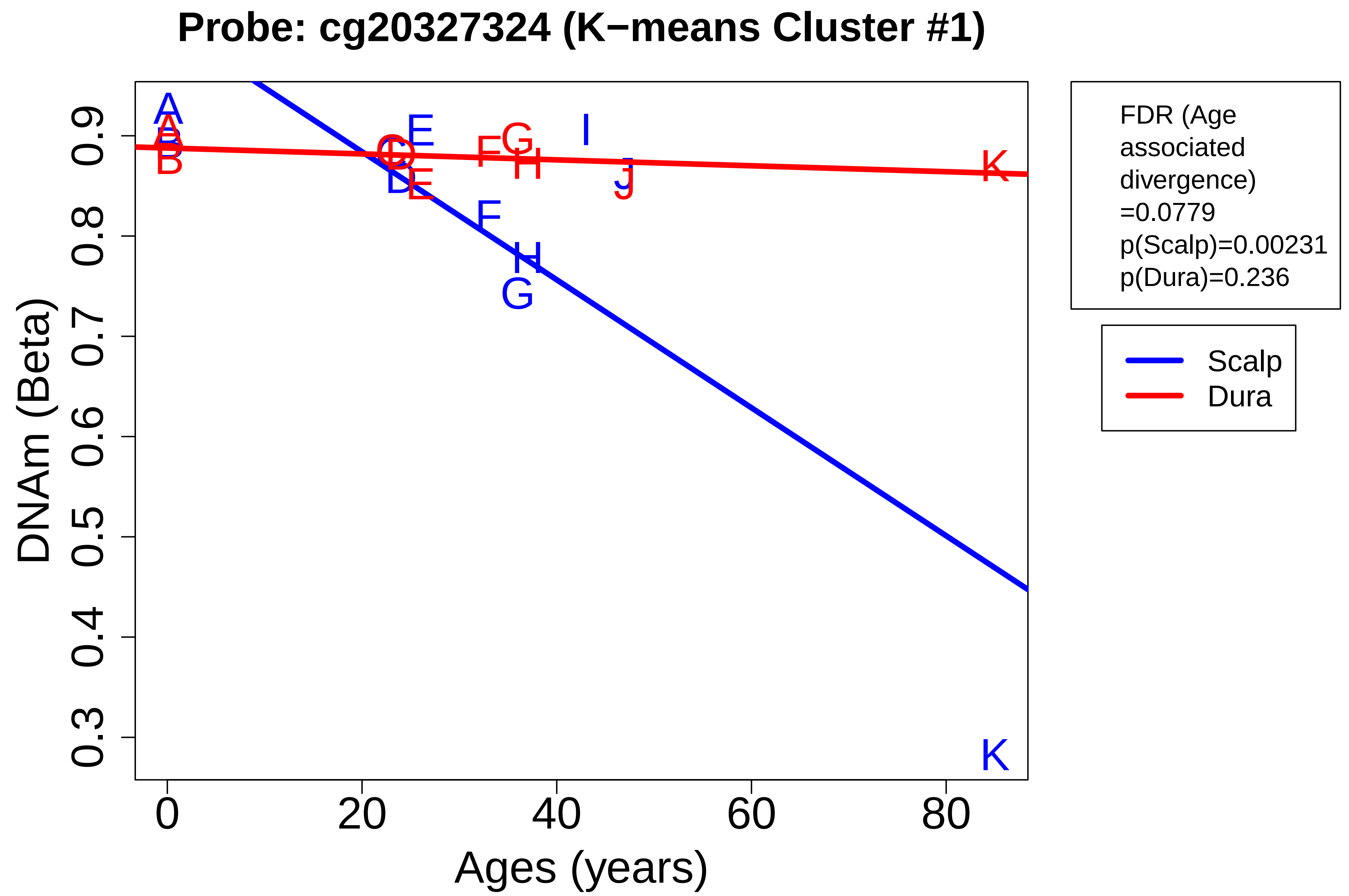

Probe: cg20327324 (K−means Cluster #1)
DNAm (Beta)
0.3
0.4
0.5
0.6
0.7
0.8
0.9
0
20
40
60
80
Ages (years)
A
B
C
D
E
F
G
H
I
J
K
FDR (Age associated divergence) =0.0779
p(Scalp)=0.00231
p(Dura)=0.236
Scalp
Dura

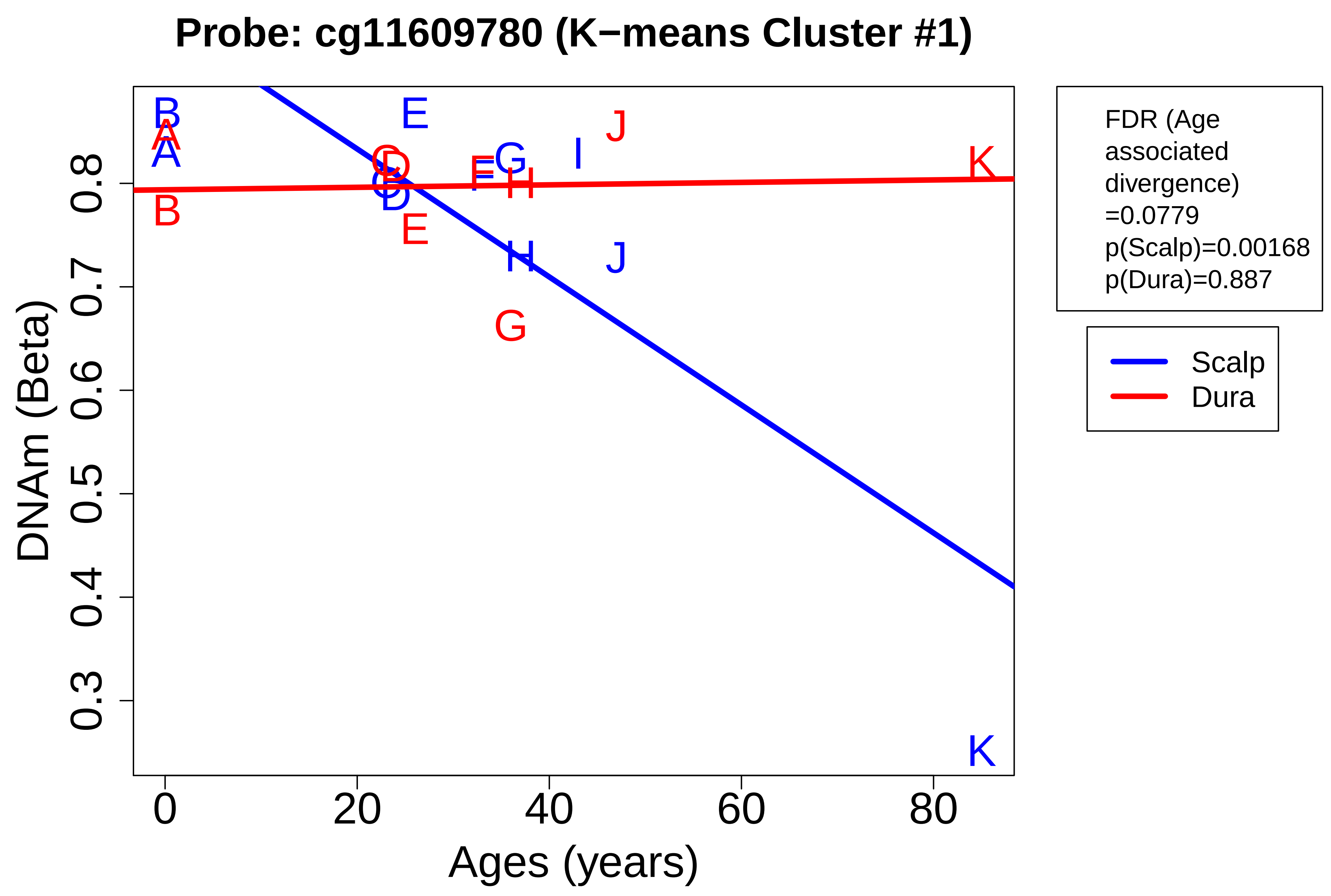
Probe: cg11609780 (K−means Cluster #1)
DNAm (Beta)
Ages (years)
0.3
0.4
0.5
0.6
0.7
0.8
0
20
40
60
80
FDR (Age associated divergence) =0.0779
p(Scalp)=0.00168
p(Dura)=0.887
Scalp
Dura

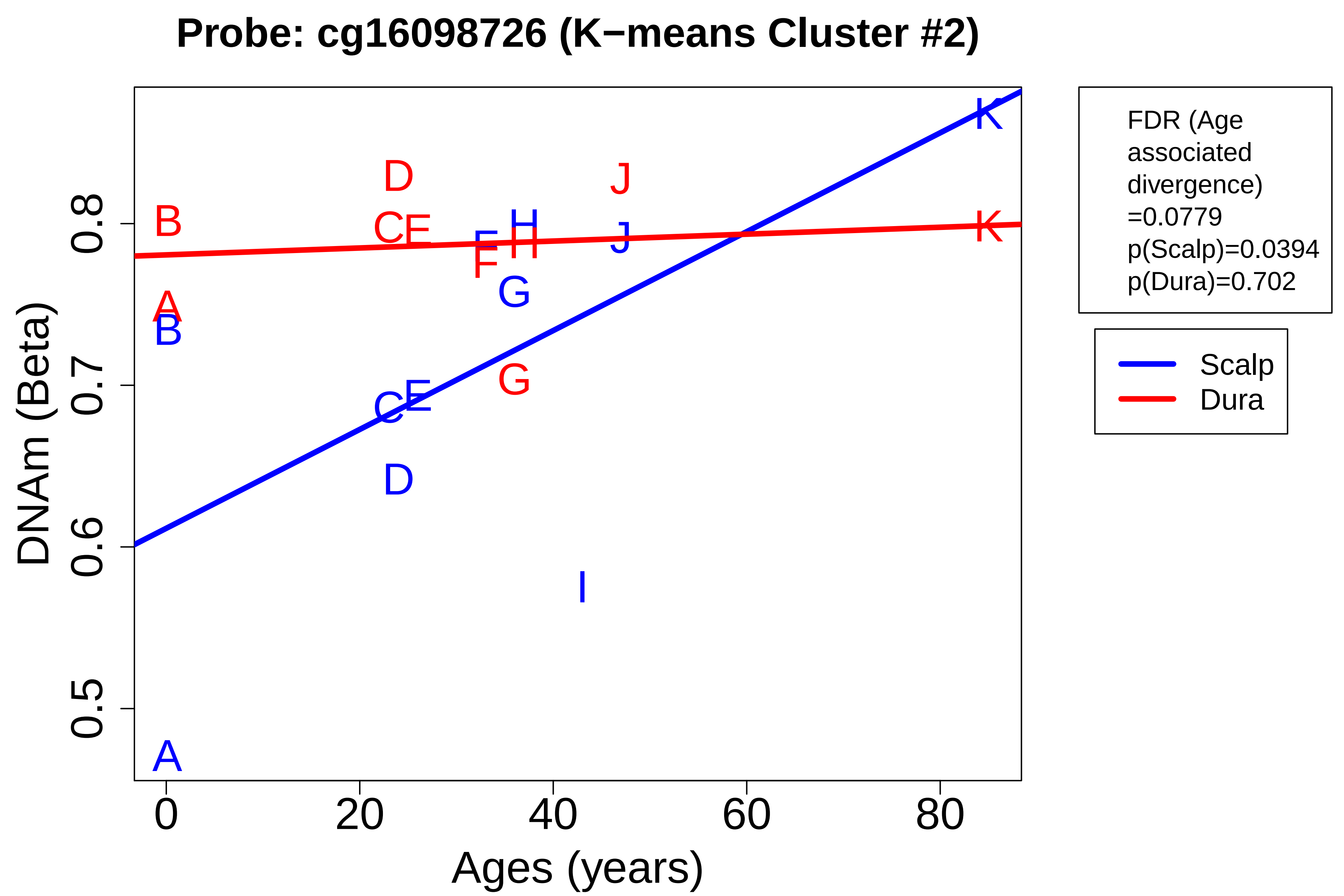

Probe: cg16098726 (K−means Cluster #2)
DNAm (Beta)
0.5
0.6
0.7
0.8
Ages (years)
0
20
40
60
80
A
B
C
D
E
F
G
H
I
J
K
FDR (Age associated divergence) =0.0779
p(Scalp)=0.0394
p(Dura)=0.702
Scalp
Dura

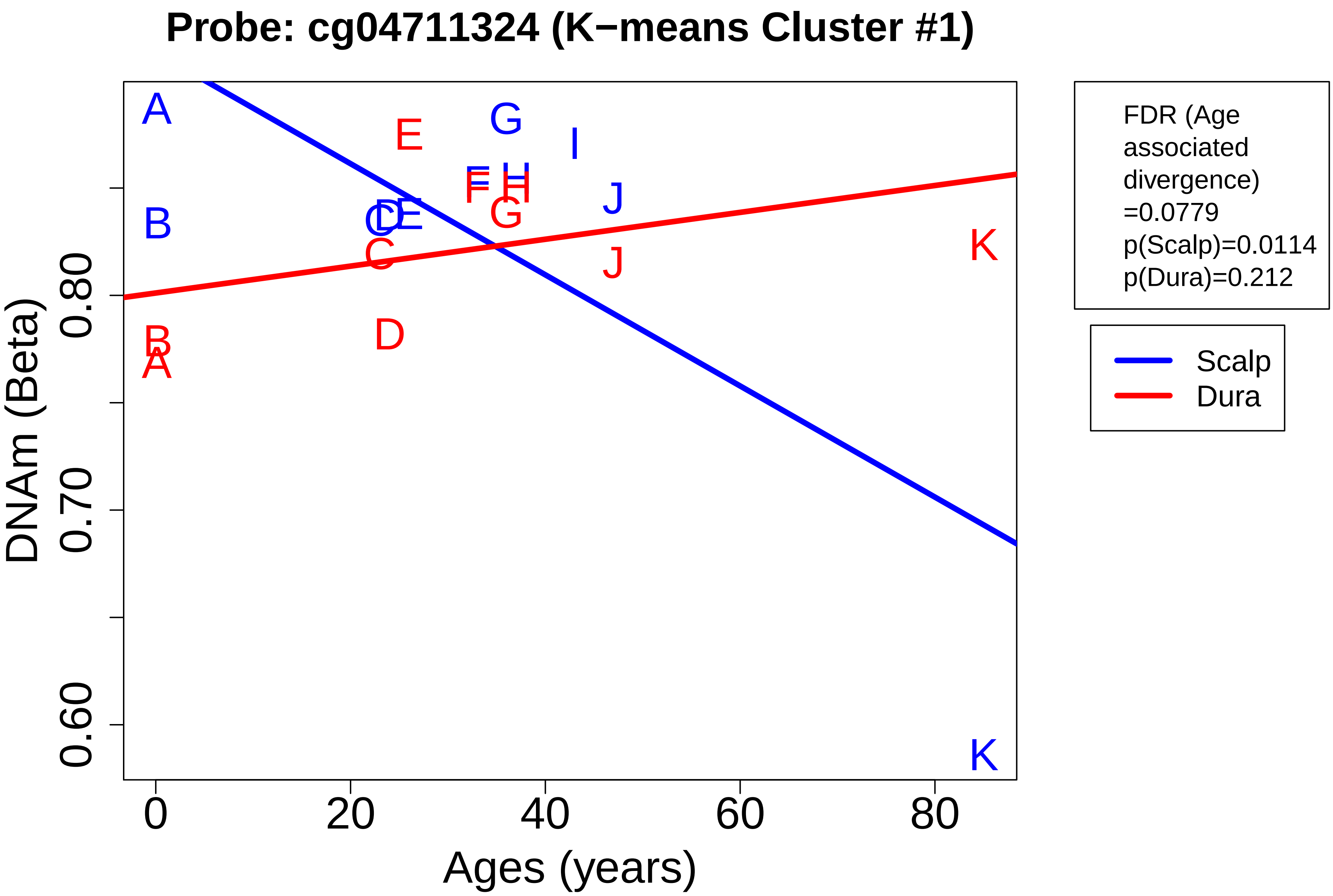
Probe: cg04711324 (K−means Cluster #1)
DNAm (Beta)
0.60
0.70
0.80
0
20
40
60
80
Ages (years)
FDR (Age associated divergence) =0.0779
p(Scalp)=0.0114
p(Dura)=0.212
Scalp
Dura

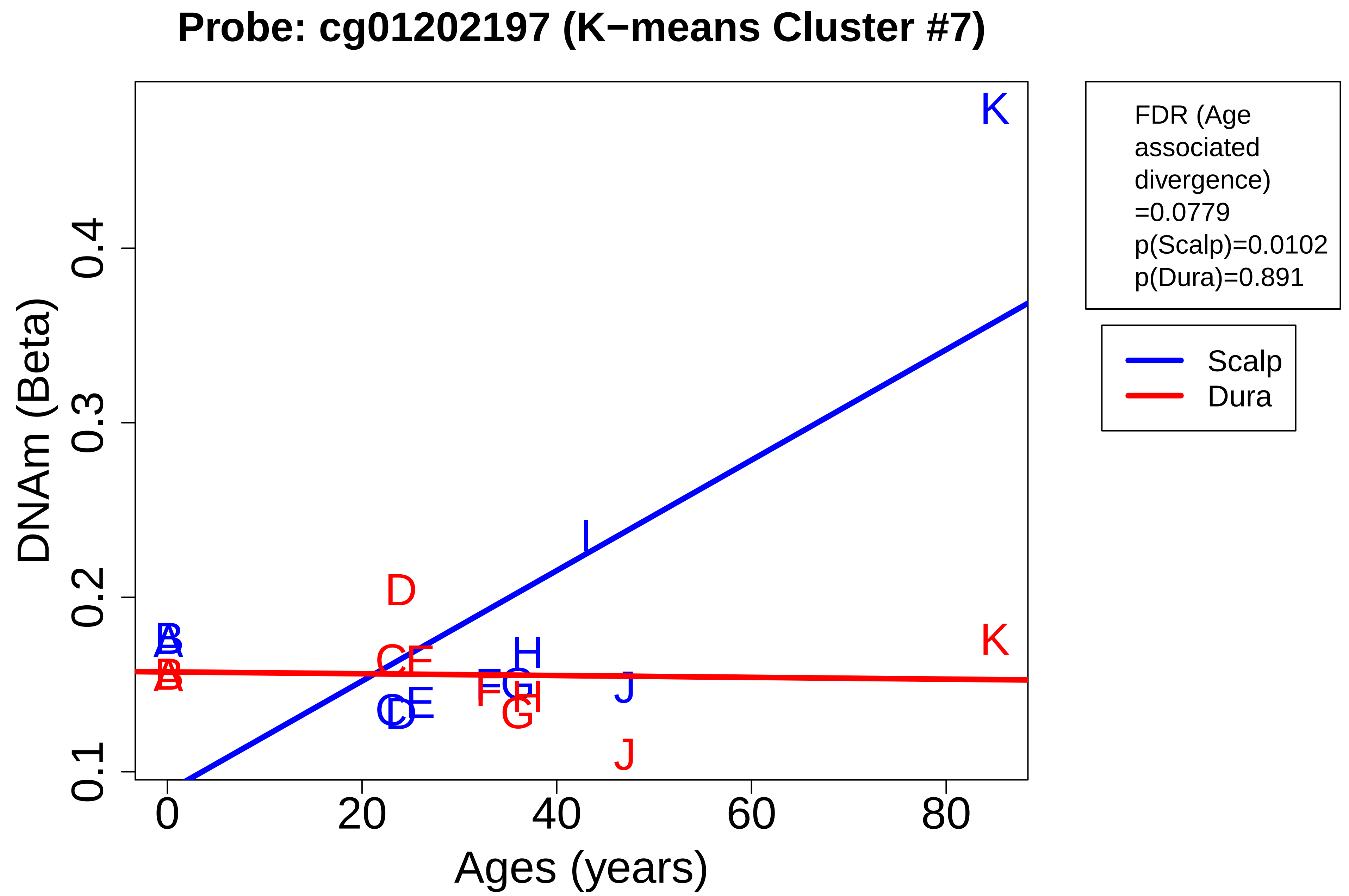

Probe: cg01202197 (K−means Cluster #7)
FDR (Age associated divergence) =0.0779
p(Scalp)=0.0102
p(Dura)=0.891
Scalp
Dura
DNAm (Beta)
0.1
0.2
0.3
0.4
0
20
40
60
80
Ages (years)

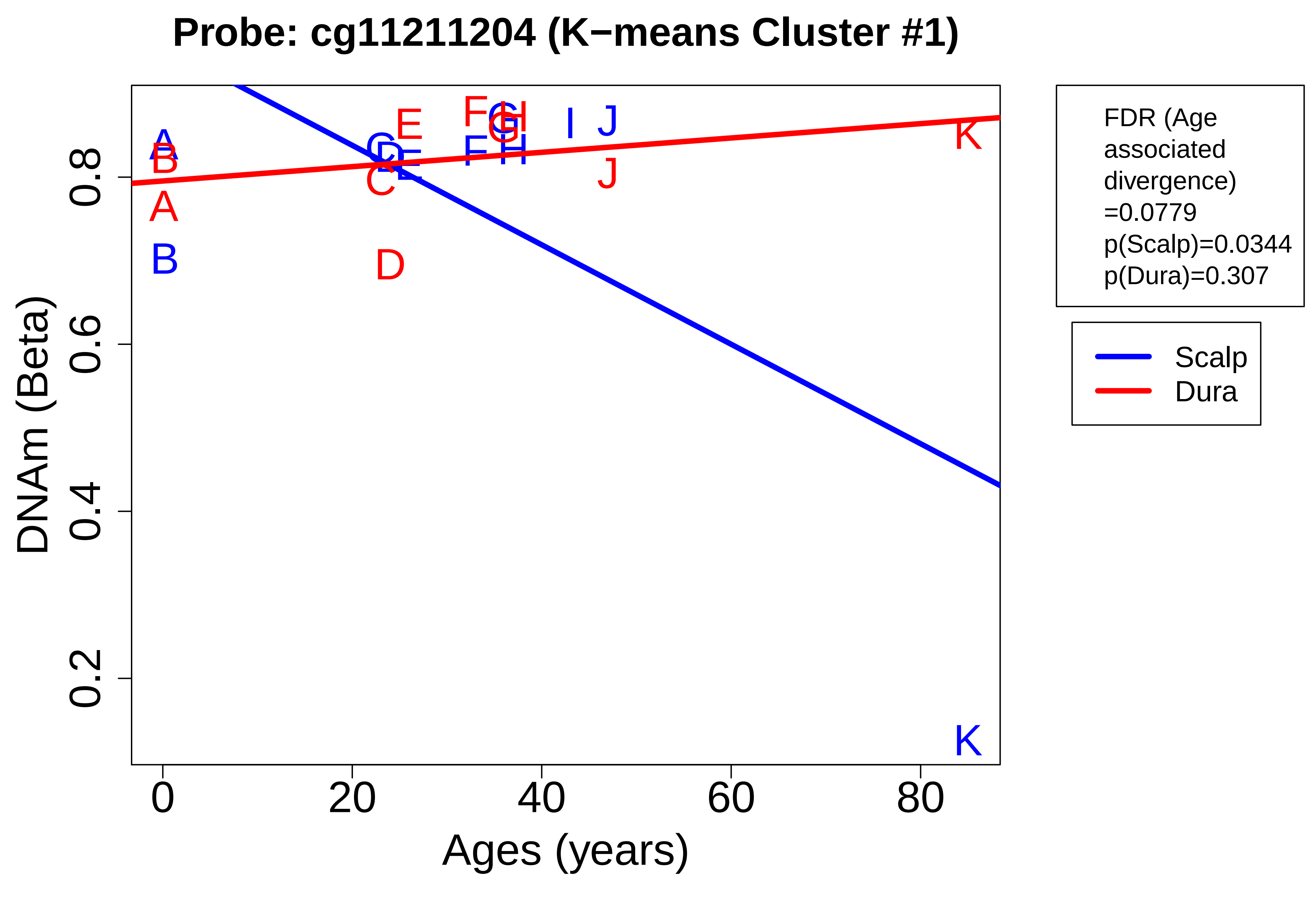

Probe: cg11211204 (K−means Cluster #1)
DNAm (Beta)
0.2
0.4
0.6
0.8
0
20
40
60
80
Ages (years)
FDR (Age associated divergence) =0.0779
p(Scalp)=0.0344
p(Dura)=0.307
Scalp
Dura

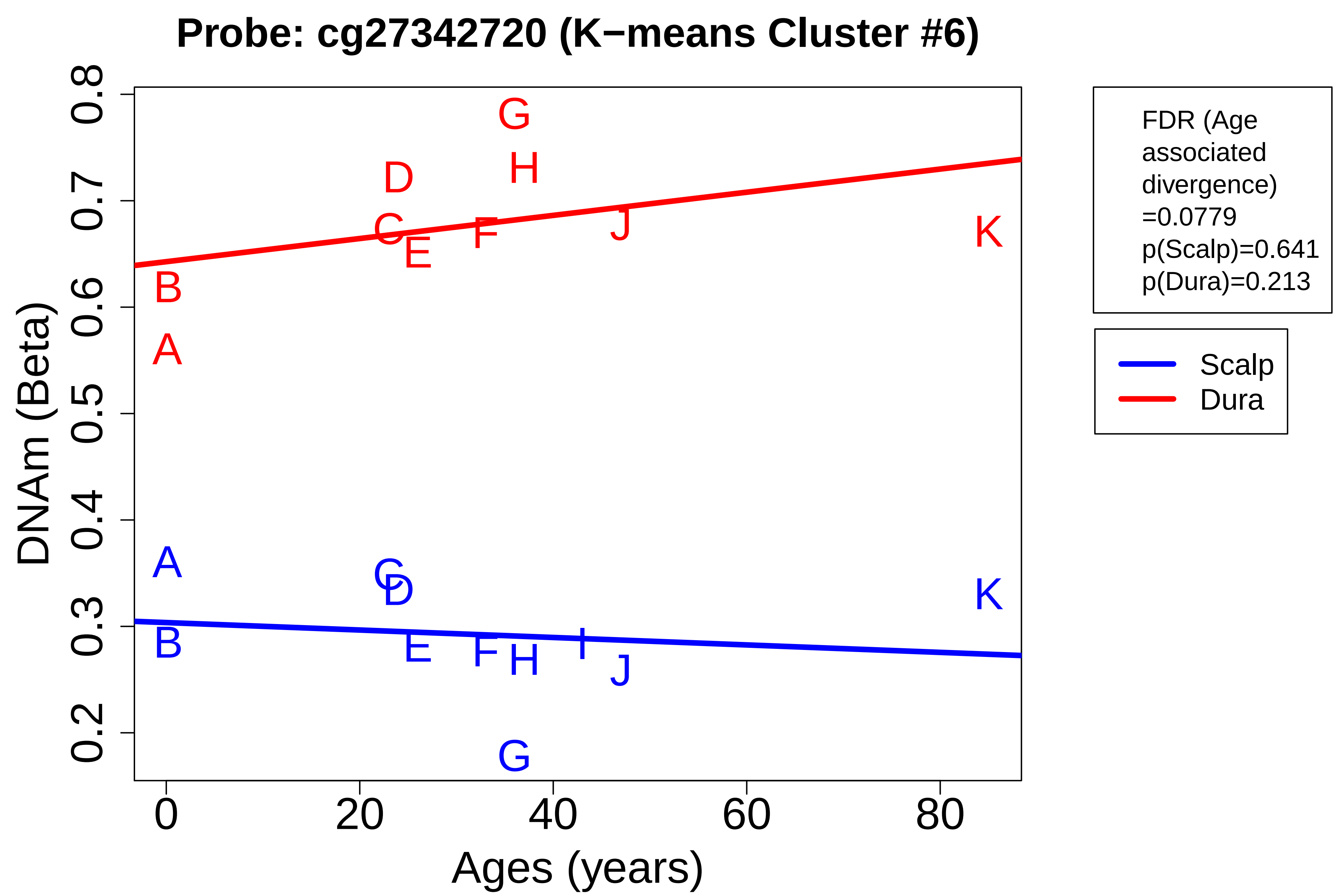

Probe: cg27342720 (K−means Cluster #6)
DNAm (Beta)
0.2
0.3
0.4
0.5
0.6
0.7
0.8
Ages (years)
0
20
40
60
80
FDR (Age associated divergence) =0.0779
p(Scalp)=0.641
p(Dura)=0.213
Scalp
Dura

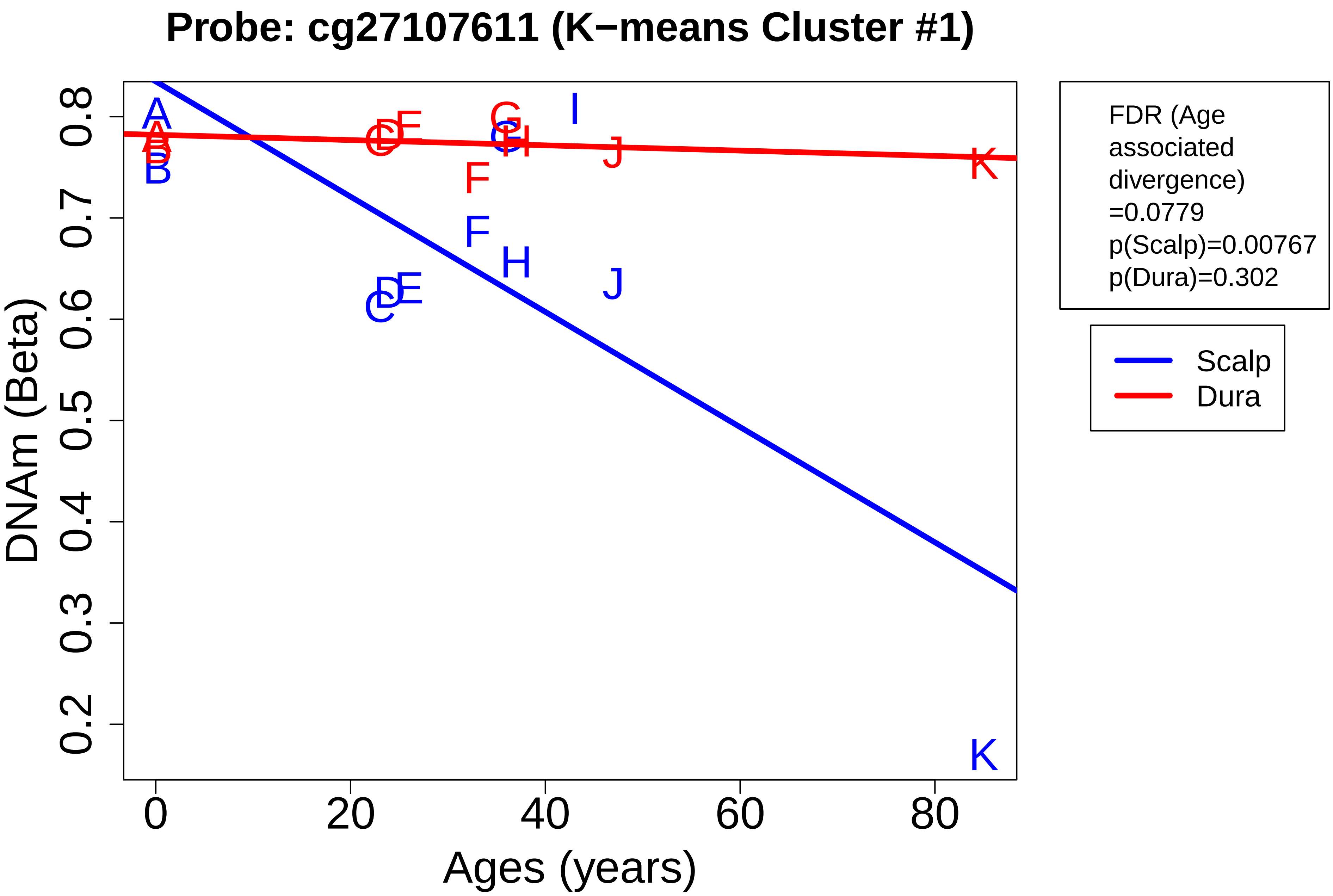
Probe: cg27107611 (K−means Cluster #1)
DNAm (Beta)
Ages (years)
0.2
0.3
0.4
0.5
0.6
0.7
0.8
0
20
40
60
80
FDR (Age associated divergence) =0.0779
p(Scalp)=0.00767
p(Dura)=0.302
Scalp
Dura

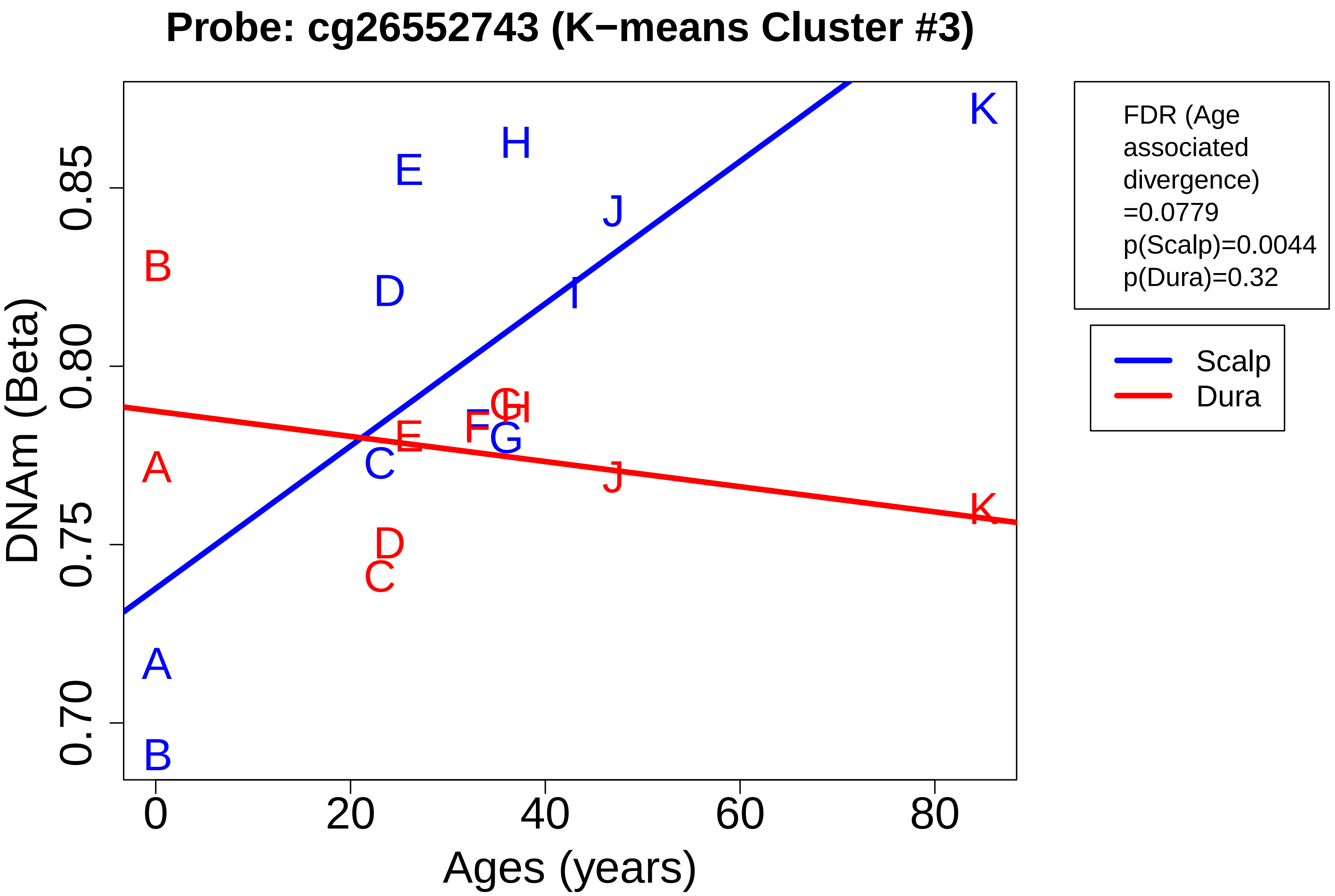

Probe: cg26552743 (K−means Cluster #3)
DNAm (Beta)
0.70
0.75
0.80
0.85
0
20
40
60
80
Ages (years)
FDR (Age associated divergence) =0.0779
p(Scalp)=0.0044
p(Dura)=0.32
Scalp
Dura

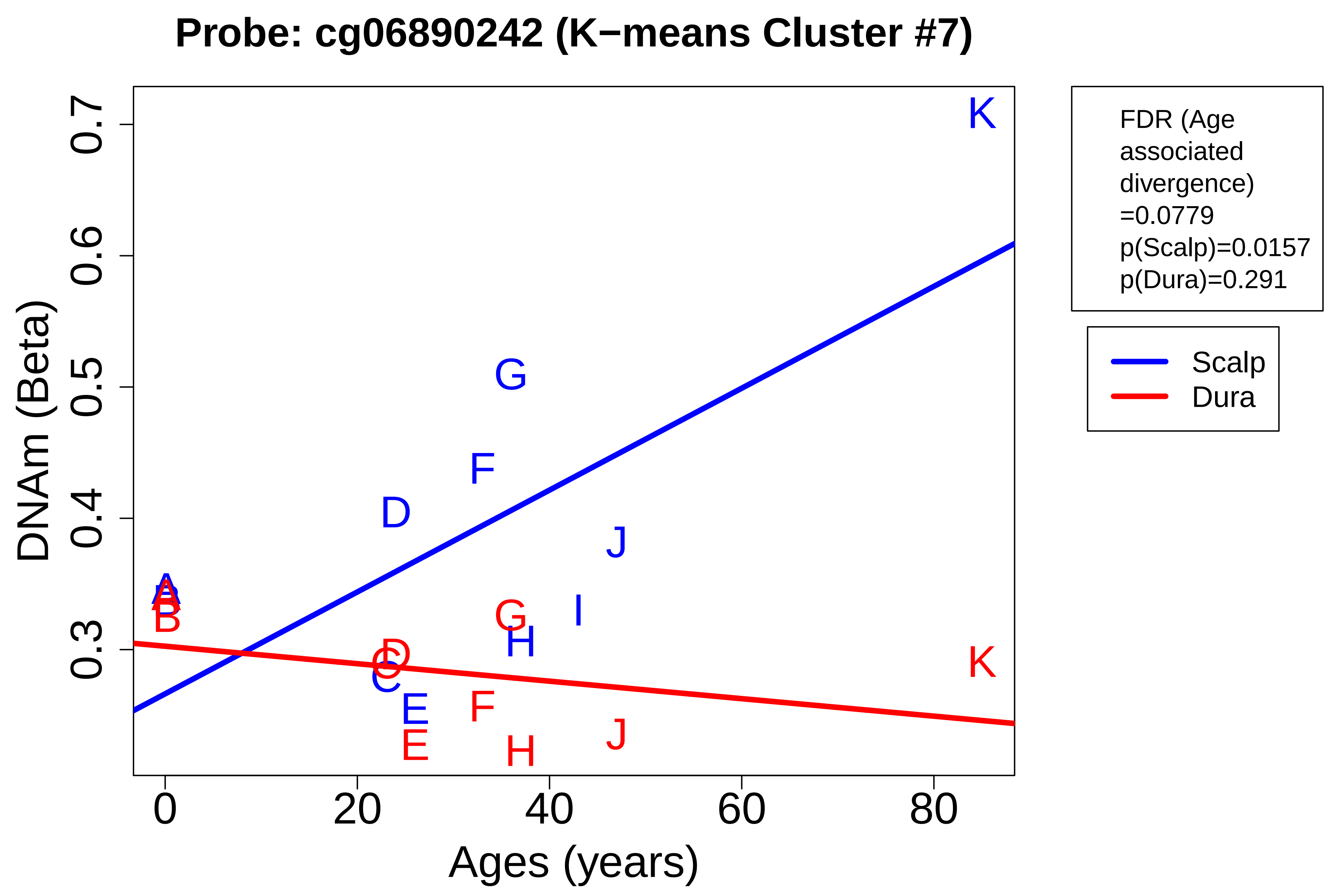
Probe: cg06890242 (K−means Cluster #7)
DNAm (Beta)
0.3
0.4
0.5
0.6
0.7
0
20
40
60
80
Ages (years)
FDR (Age associated divergence) =0.0779
p(Scalp)=0.0157
p(Dura)=0.291
Scalp
Dura
A B C D E F G H I J K

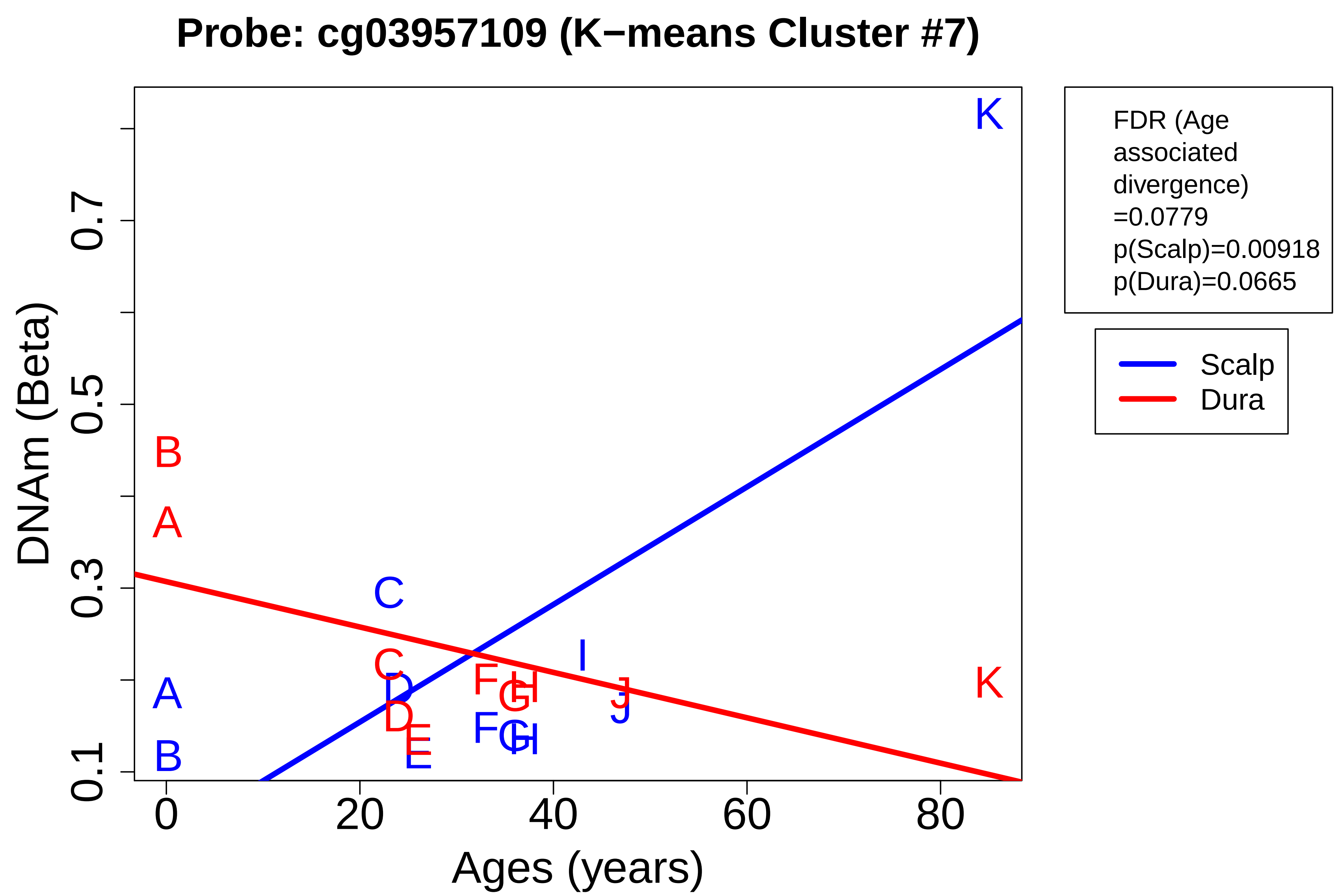

Probe: cg03957109 (K−means Cluster #7)
DNAm (Beta)
0.1
0.3
0.5
0.7
0
20
40
60
80
Ages (years)
A
B
C
D
E
F
G
H
I
J
K
FDR (Age associated divergence) =0.0779
p(Scalp)=0.00918
p(Dura)=0.0665
Scalp
Dura

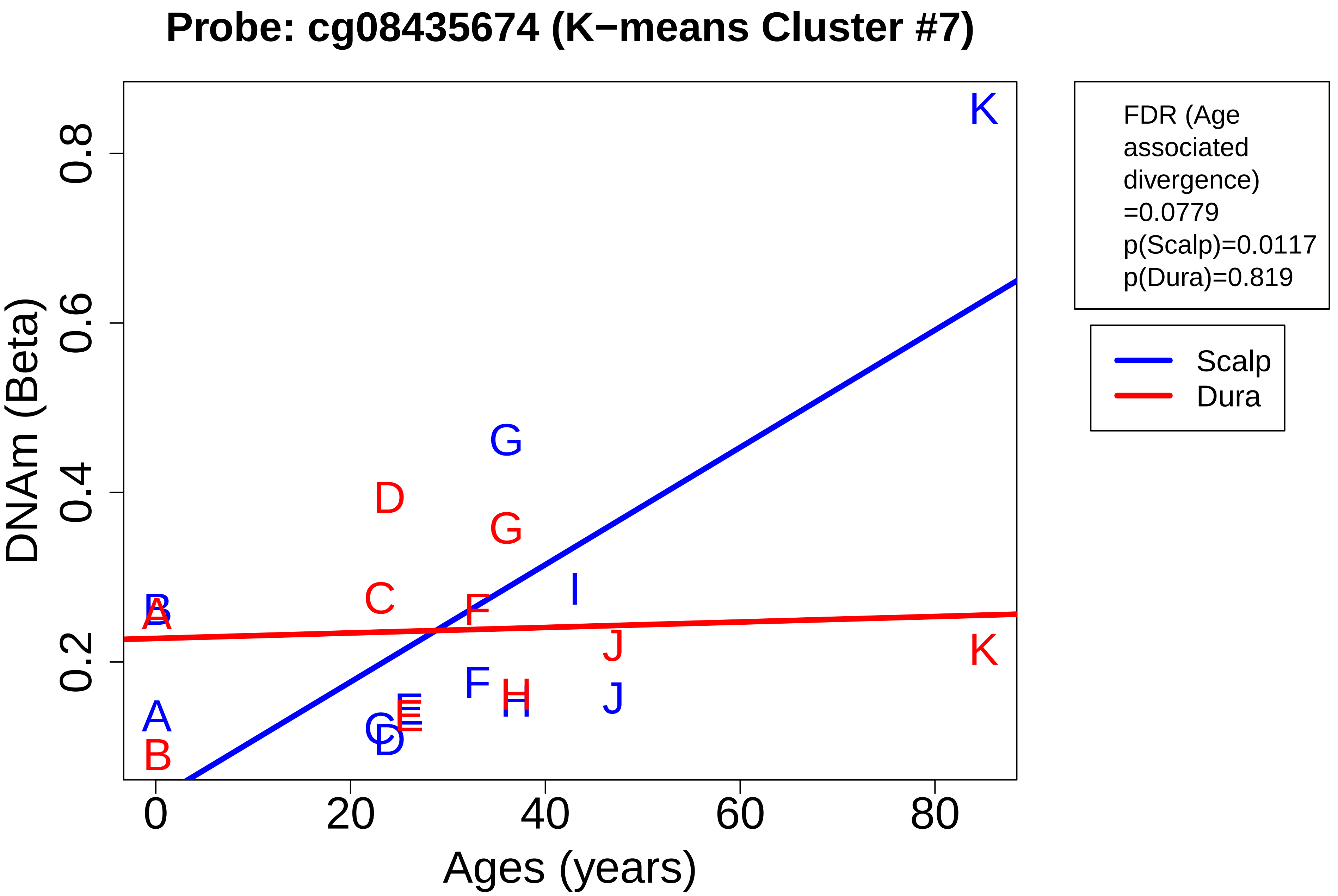

Probe: cg08435674 (K−means Cluster #7)
DNAm (Beta)
0.2
0.4
0.6
0.8
Ages (years)
0
20
40
60
80
FDR (Age associated divergence) =0.0779
p(Scalp)=0.0117
p(Dura)=0.819
Scalp
Dura
A
B
C
D
E
F
G
H
I
J
K

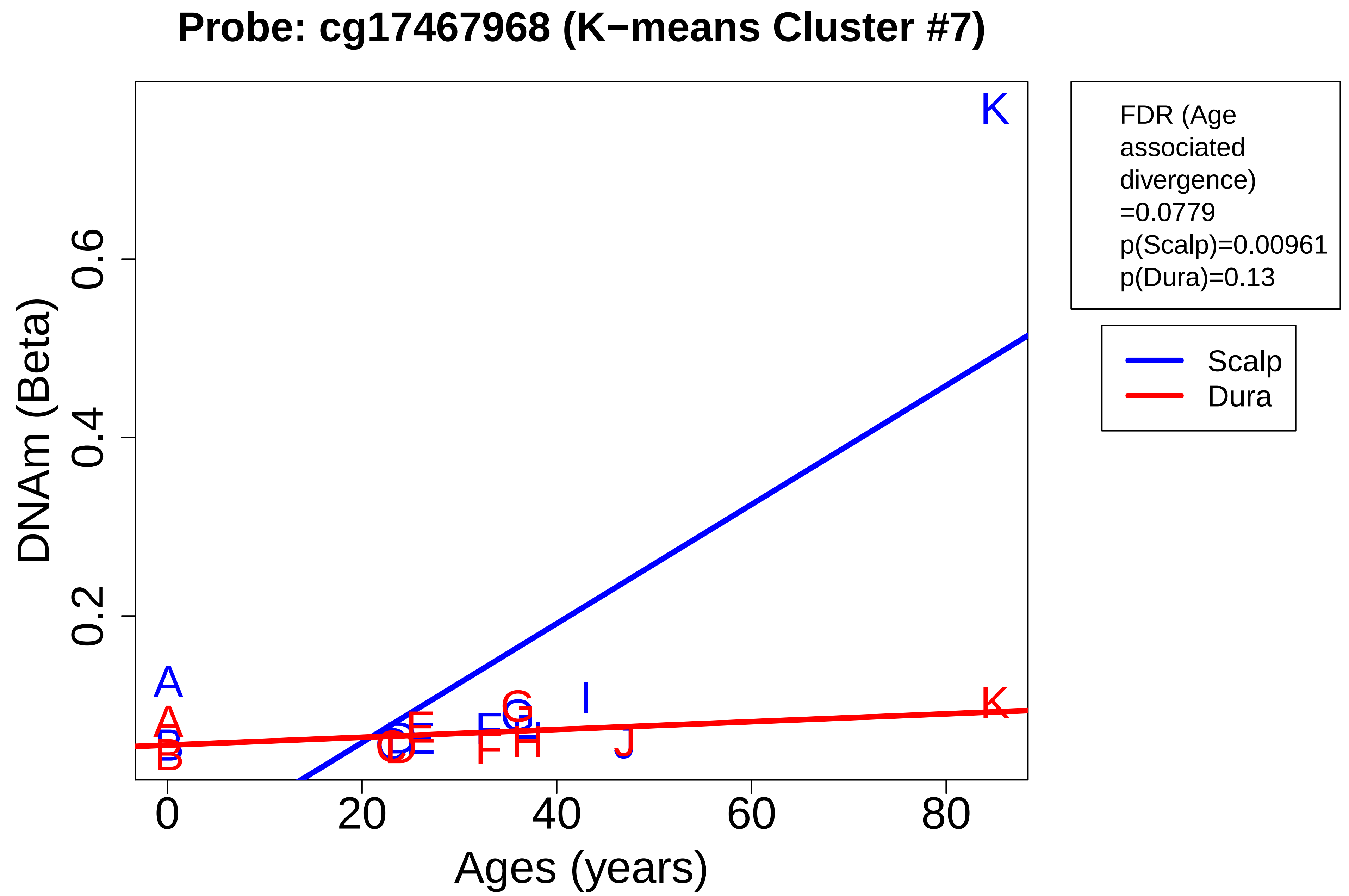
Probe: cg17467968 (K−means Cluster #7)
DNAm (Beta)
0.2
0.4
0.6
Ages (years)
0
20
40
60
80
FDR (Age associated divergence) =0.0779
p(Scalp)=0.00961
p(Dura)=0.13
Scalp
Dura
A
B
C
D
E
F
G
H
I
J
K

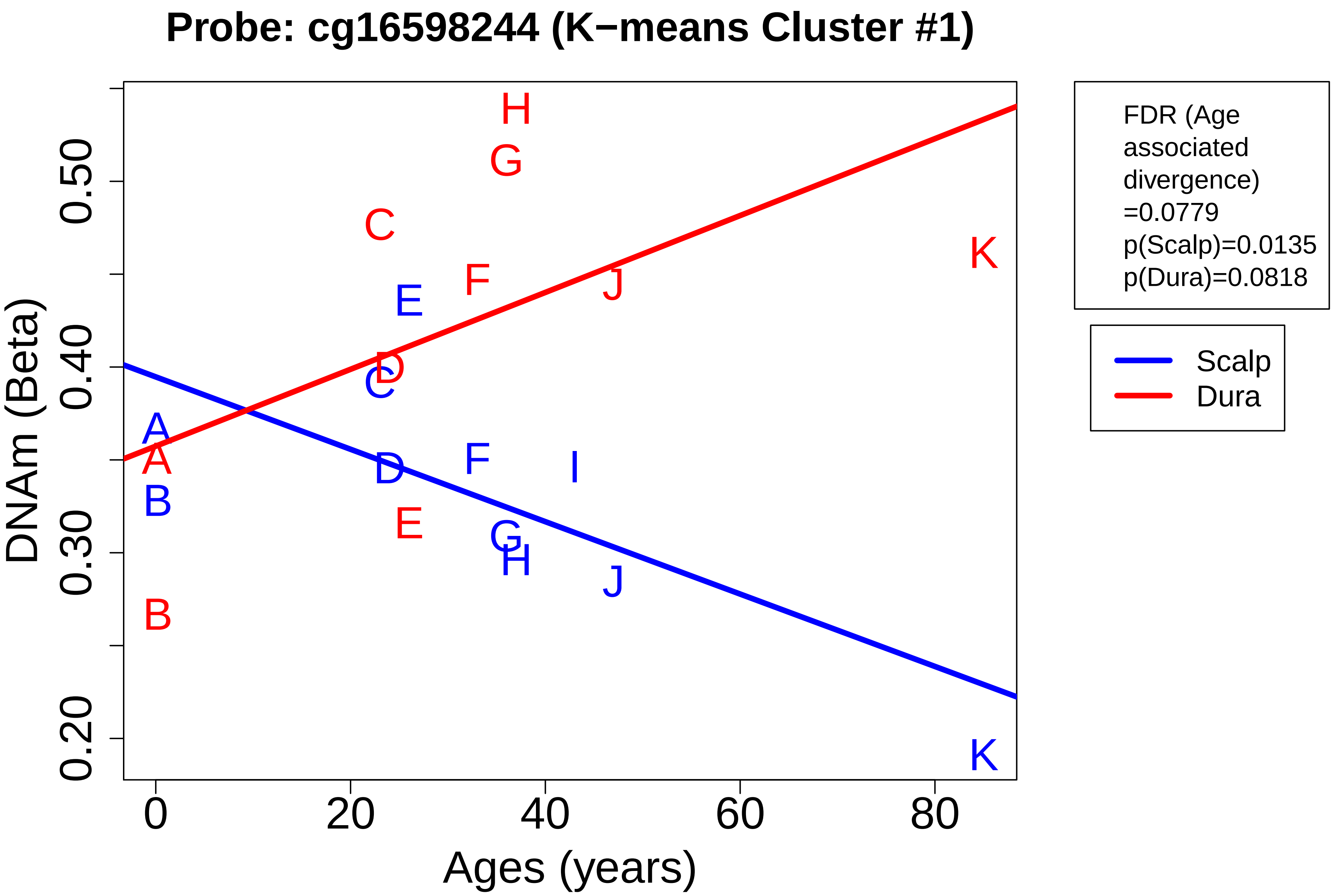

Probe: cg16598244 (K−means Cluster #1)
DNAm (Beta)
0.20
0.30
0.40
0.50
0
20
40
60
80
Ages (years)
FDR (Age associated divergence) =0.0779
p(Scalp)=0.0135
p(Dura)=0.0818
Scalp
Dura

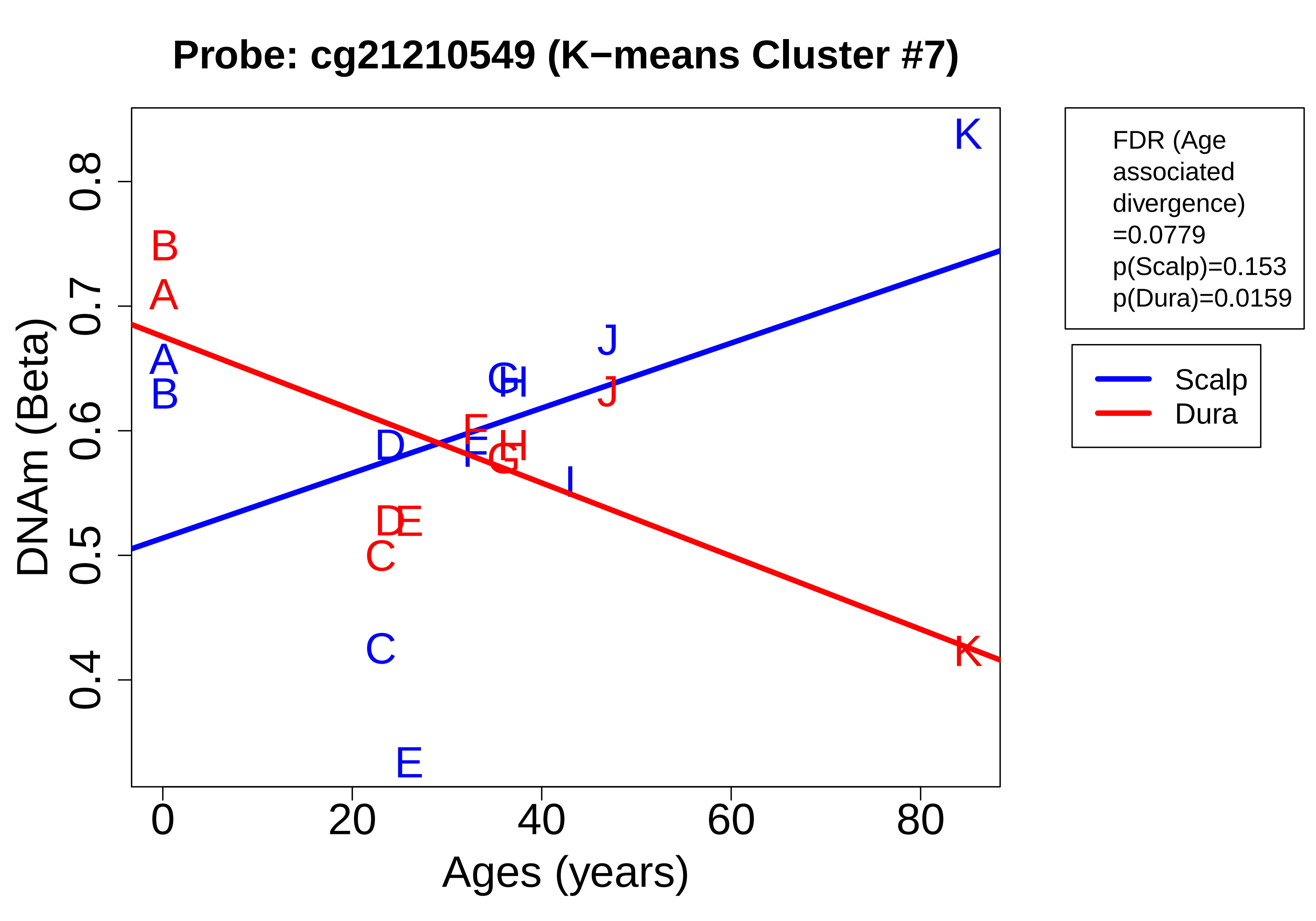
Probe: cg21210549 (K−means Cluster #7)
DNAm (Beta)
0.4
0.5
0.6
0.7
0.8
Ages (years)
0
20
40
60
80
FDR (Age associated divergence) =0.0779
p(Scalp)=0.153
p(Dura)=0.0159
Scalp
Dura

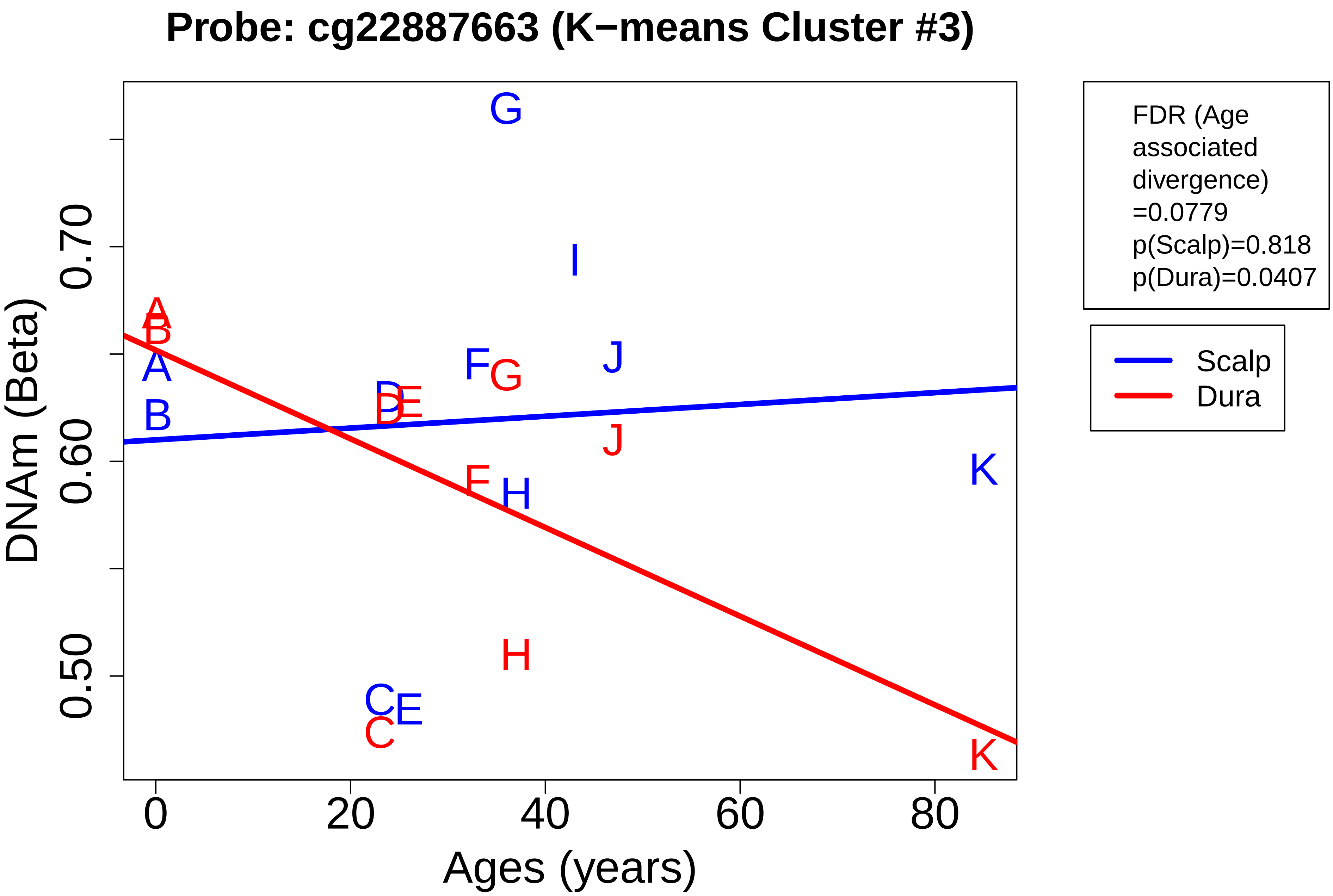

Probe: cg22887663 (K−means Cluster #3)
FDR (Age associated divergence) =0.0779
p(Scalp)=0.818
p(Dura)=0.0407
Scalp
Dura
DNAm (Beta)
0.50
0.60
0.70
0
20
40
60
80
Ages (years)

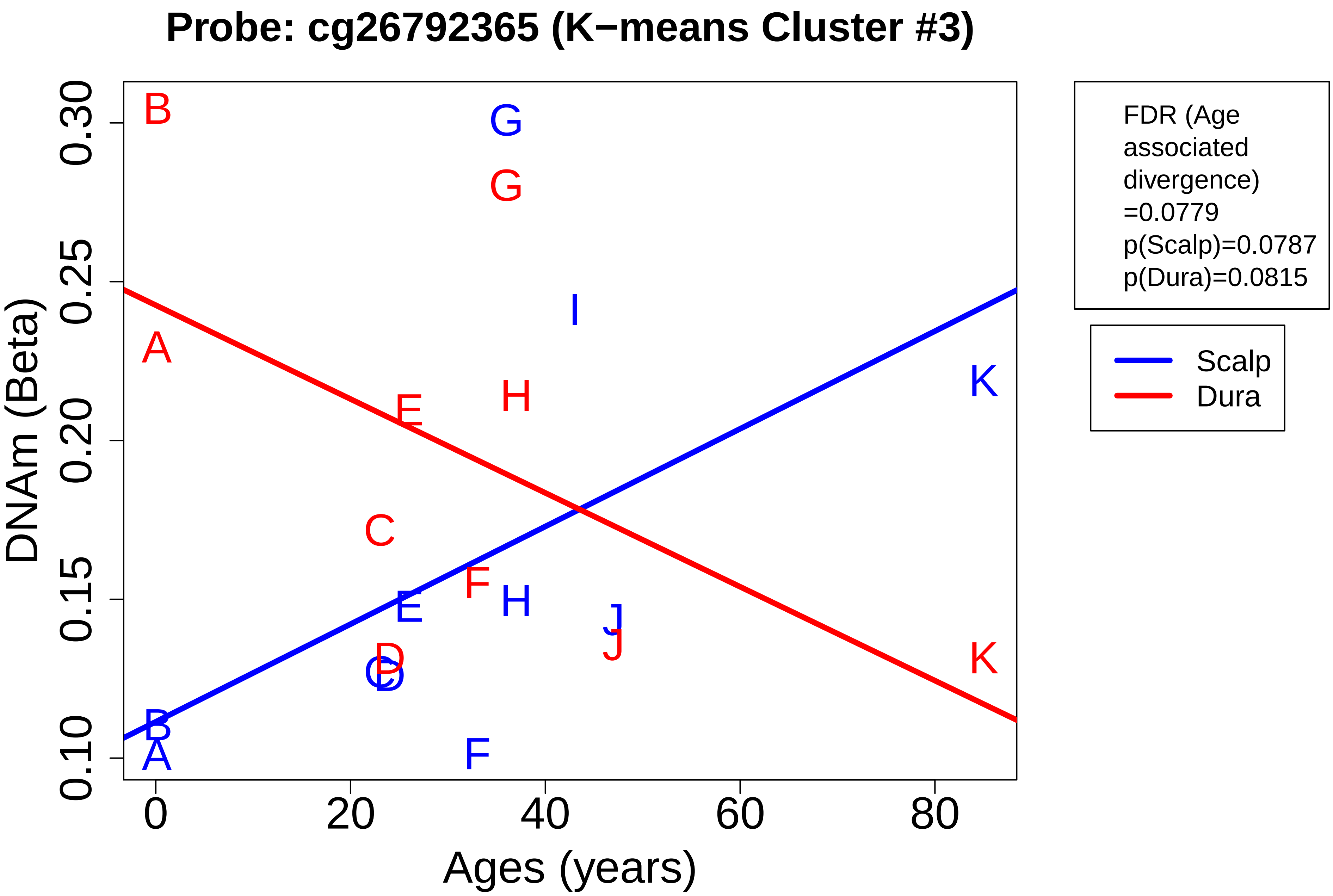

Probe: cg26792365 (K−means Cluster #3)
DNAm (Beta)
0.10
0.15
0.20
0.25
0.30
Ages (years)
0
20
40
60
80
A
B
C
D
E
F
G
H
I
J
K
FDR (Age associated divergence) =0.0779
p(Scalp)=0.0787
p(Dura)=0.0815
Scalp
Dura

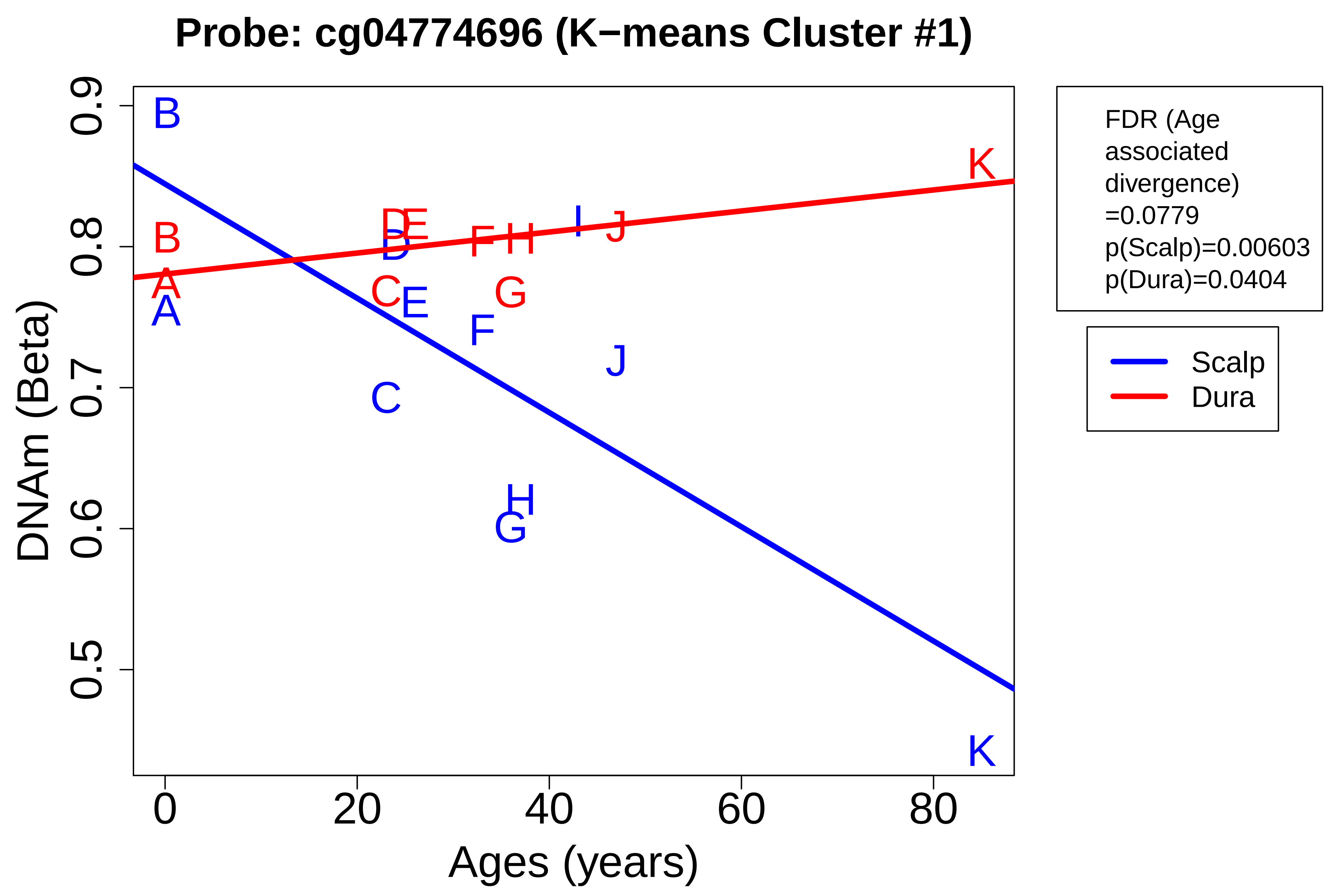

Probe: cg04774696 (K−means Cluster #1)
DNAm (Beta)
0.5
0.6
0.7
0.8
0.9
0
20
40
60
80
Ages (years)
FDR (Age associated divergence) =0.0779
p(Scalp)=0.00603
p(Dura)=0.0404
Scalp
Dura

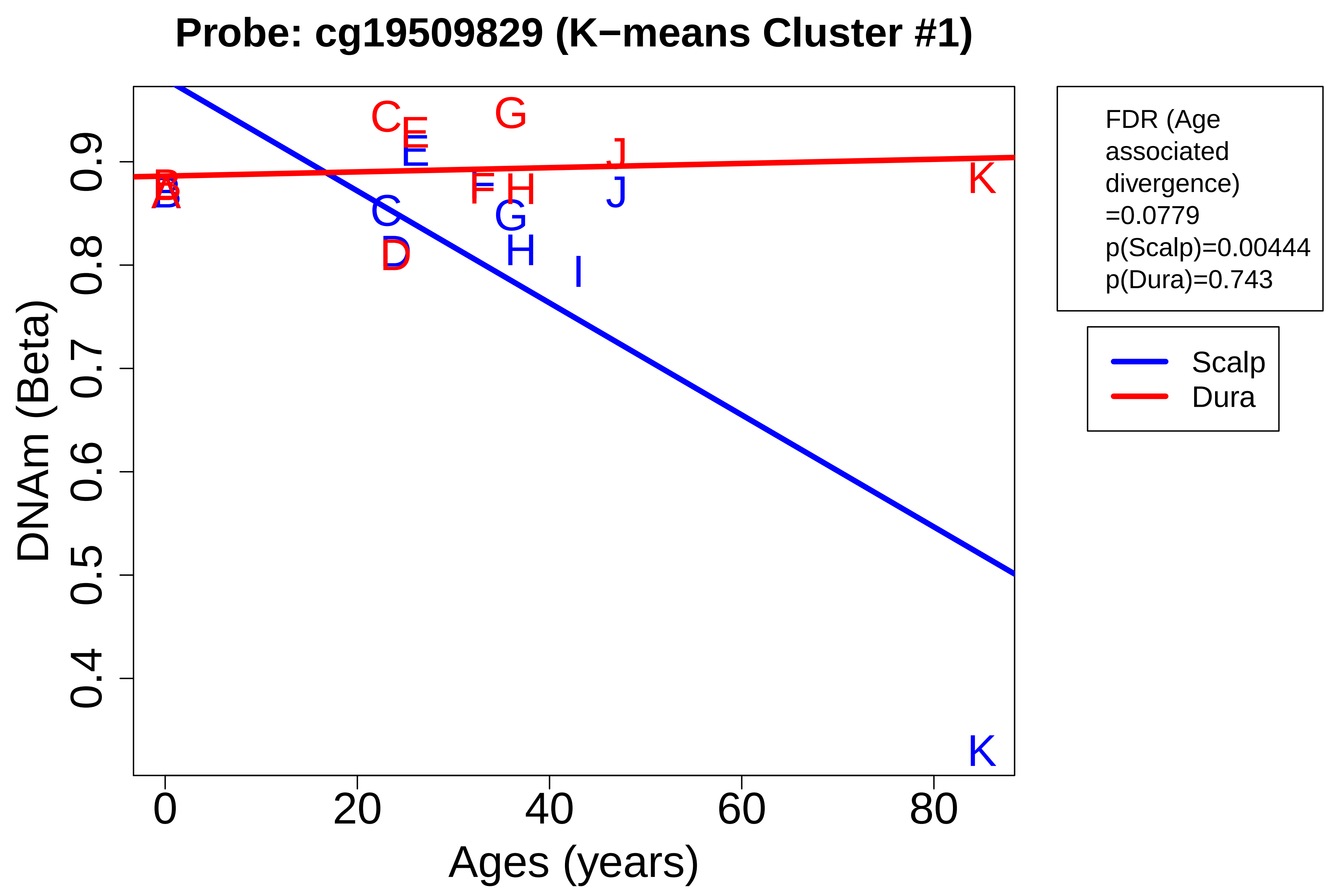
Probe: cg19509829 (K−means Cluster #1)
DNAm (Beta)
Ages (years)
0.4
0.5
0.6
0.7
0.8
0.9
0
20
40
60
80
FDR (Age associated divergence) =0.0779
p(Scalp)=0.00444
p(Dura)=0.743
Scalp
Dura
A
B
C
D
E
F
G
H
I
J
K

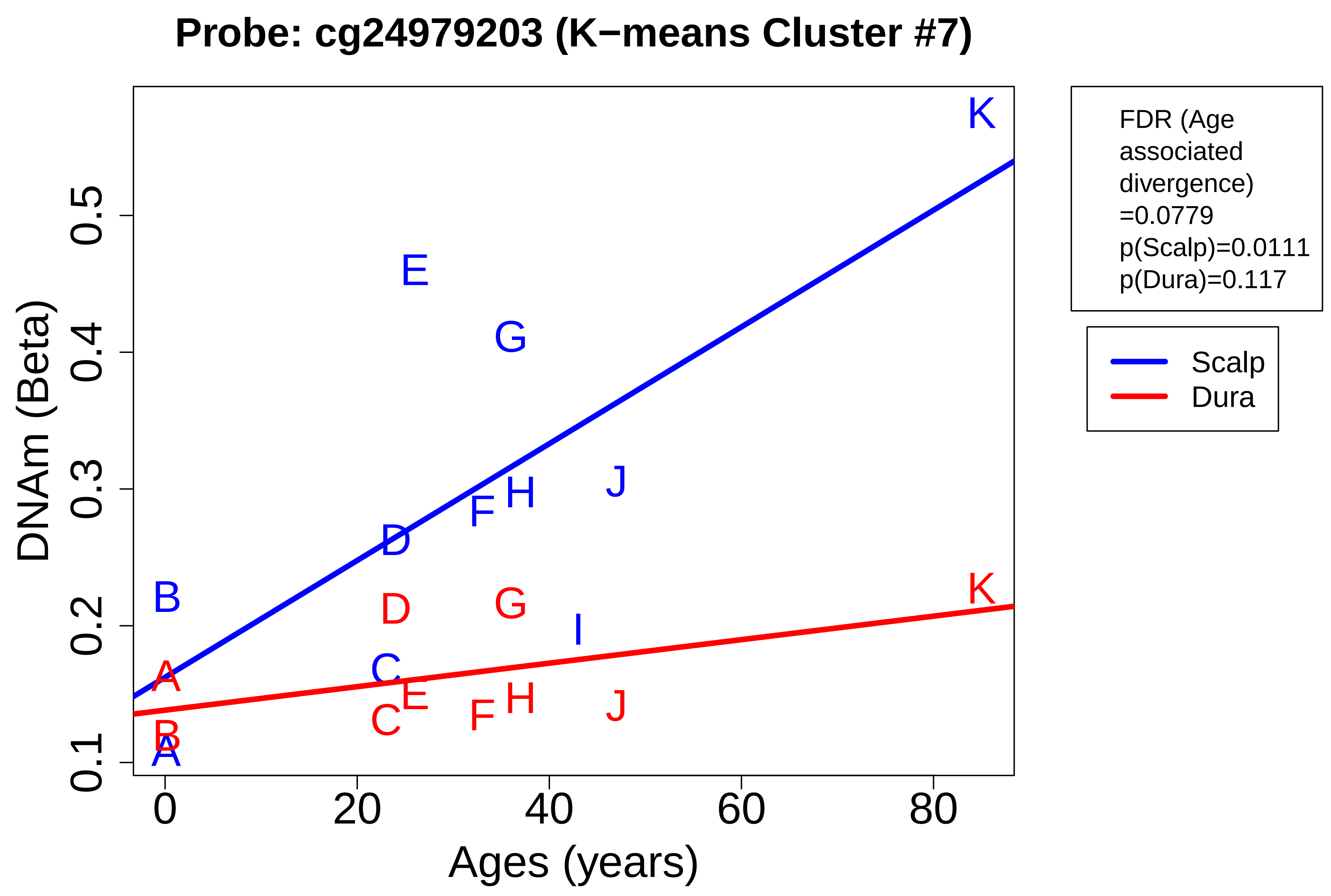

Probe: cg24979203 (K−means Cluster #7)
DNAm (Beta)
0.1
0.2
0.3
0.4
0.5
Ages (years)
0
20
40
60
80
A
B
C
D
E
F
G
H
I
J
K
FDR (Age associated divergence) =0.0779
p(Scalp)=0.0111
p(Dura)=0.117
Scalp
Dura

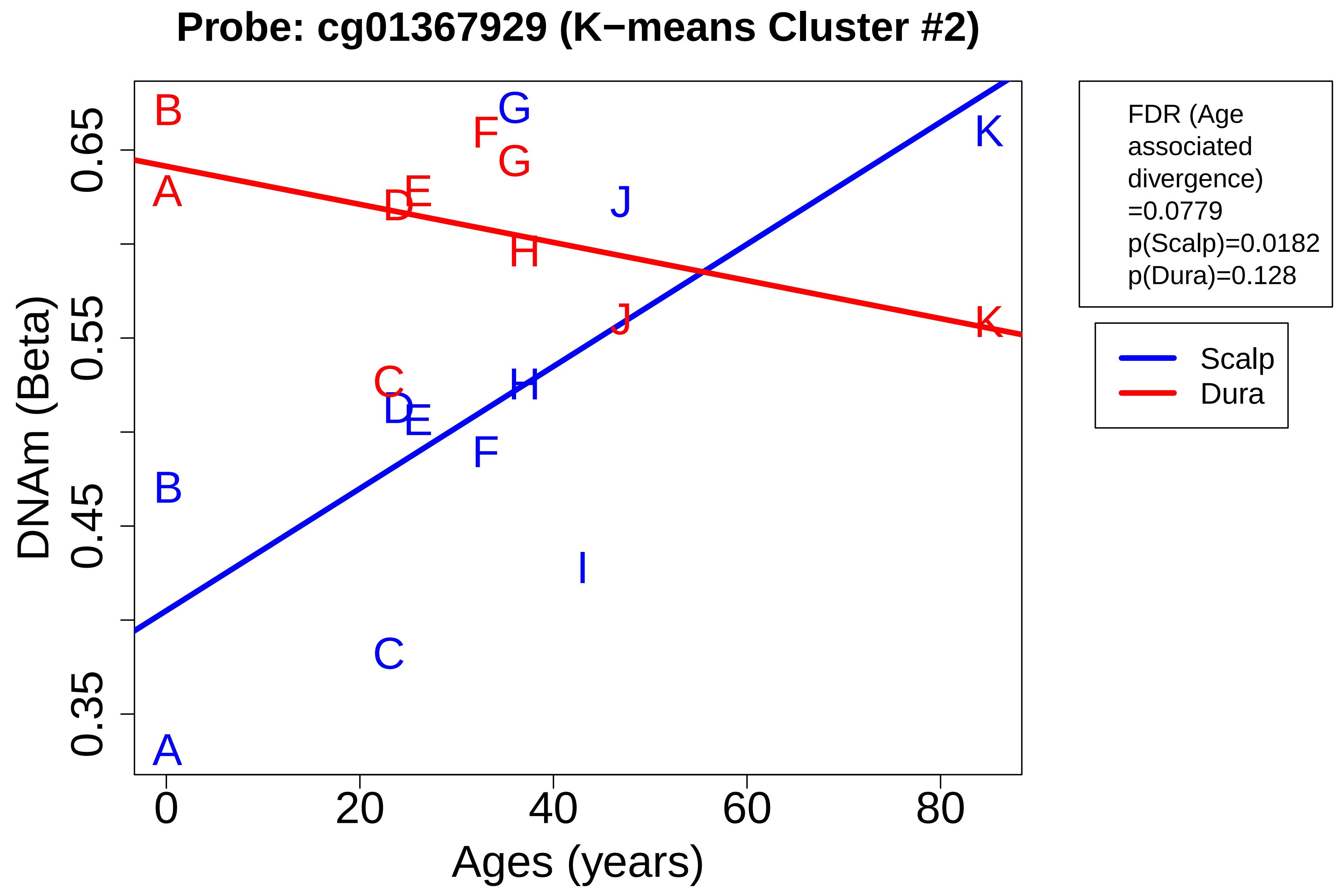

Probe: cg01367929 (K−means Cluster #2)
DNAm (Beta)
0.35
0.45
0.55
0.65
Ages (years)
0
20
40
60
80
FDR (Age associated divergence) =0.0779
p(Scalp)=0.0182
p(Dura)=0.128
Scalp
Dura

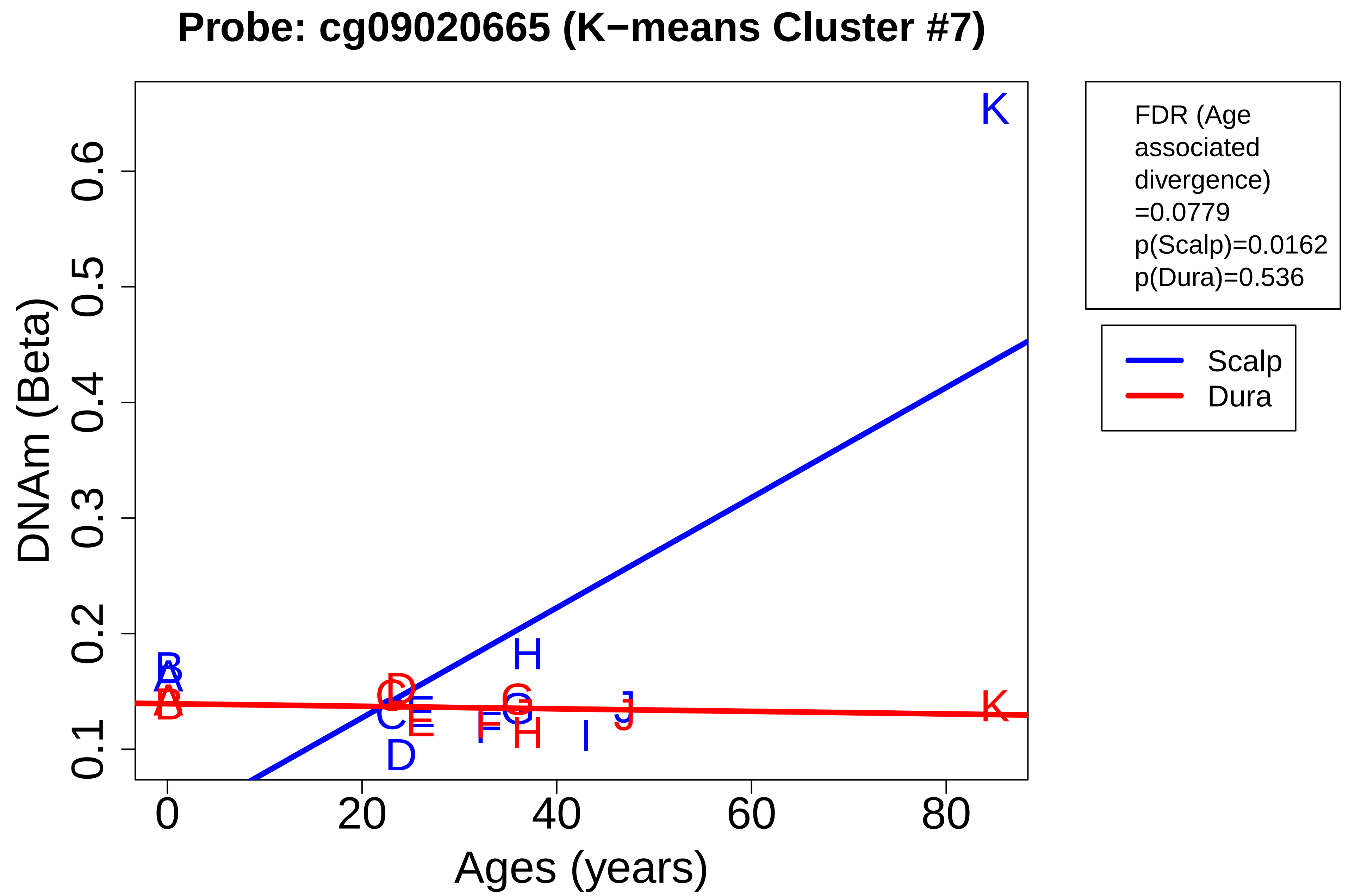

Probe: cg09020665 (K−means Cluster #7)
DNAm (Beta)
Ages (years)
0.1
0.2
0.3
0.4
0.5
0.6
0
20
40
60
80
FDR (Age associated divergence) =0.0779
p(Scalp)=0.0162
p(Dura)=0.536
Scalp
Dura

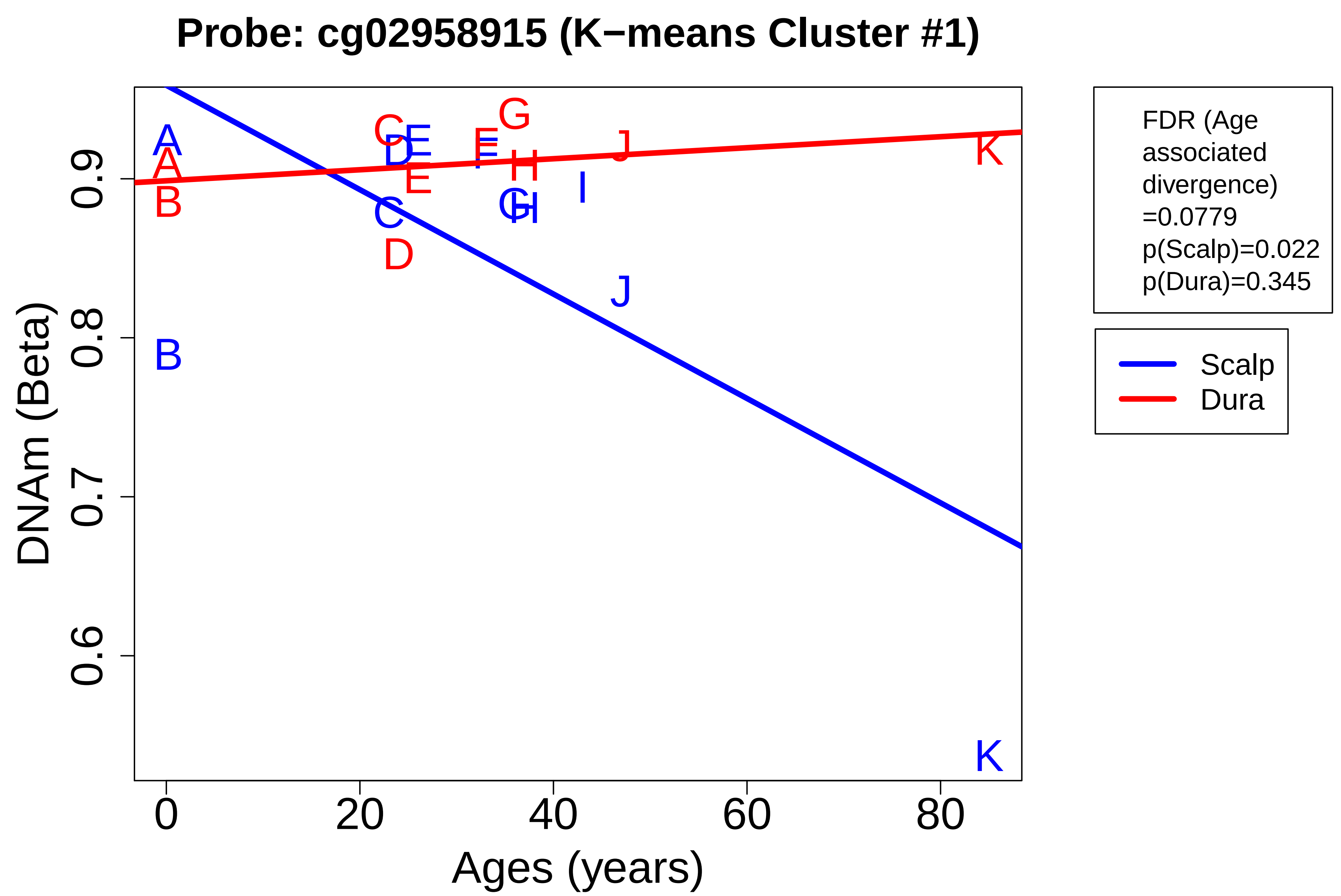

Probe: cg02958915 (K−means Cluster #1)
DNAm (Beta)
0.6
0.7
0.8
0.9
Ages (years)
0
20
40
60
80
FDR (Age associated divergence) =0.0779
p(Scalp)=0.022
p(Dura)=0.345
Scalp
Dura

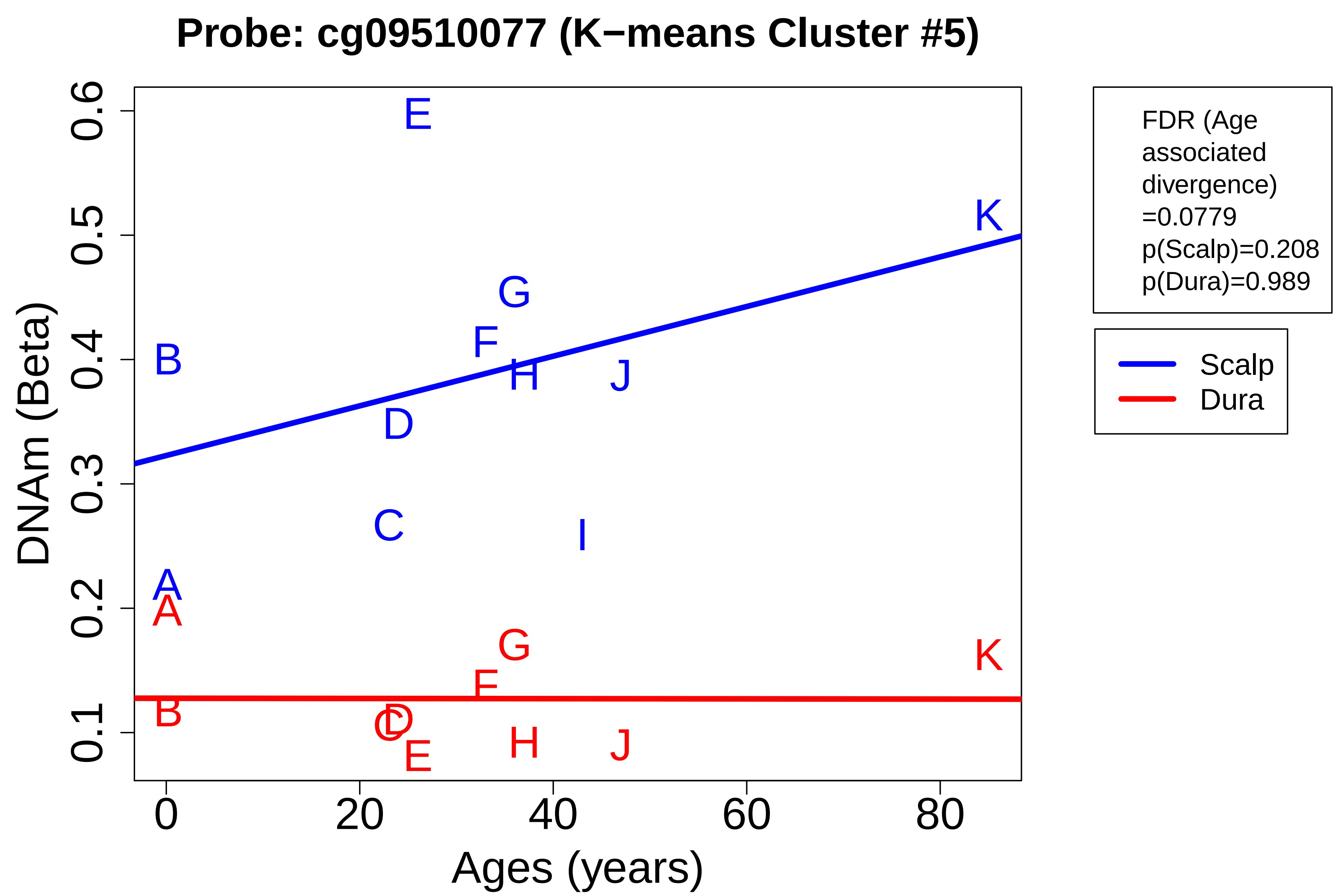

Probe: cg09510077 (K−means Cluster #5)
DNAm (Beta)
0.1
0.2
0.3
0.4
0.5
0.6
0
20
40
60
80
Ages (years)
E
K
G
F
B
H
J
D
C
I
A
A
G
K
F
B
D
C
E
H
J
FDR (Age associated divergence) =0.0779
p(Scalp)=0.208
p(Dura)=0.989
Scalp
Dura

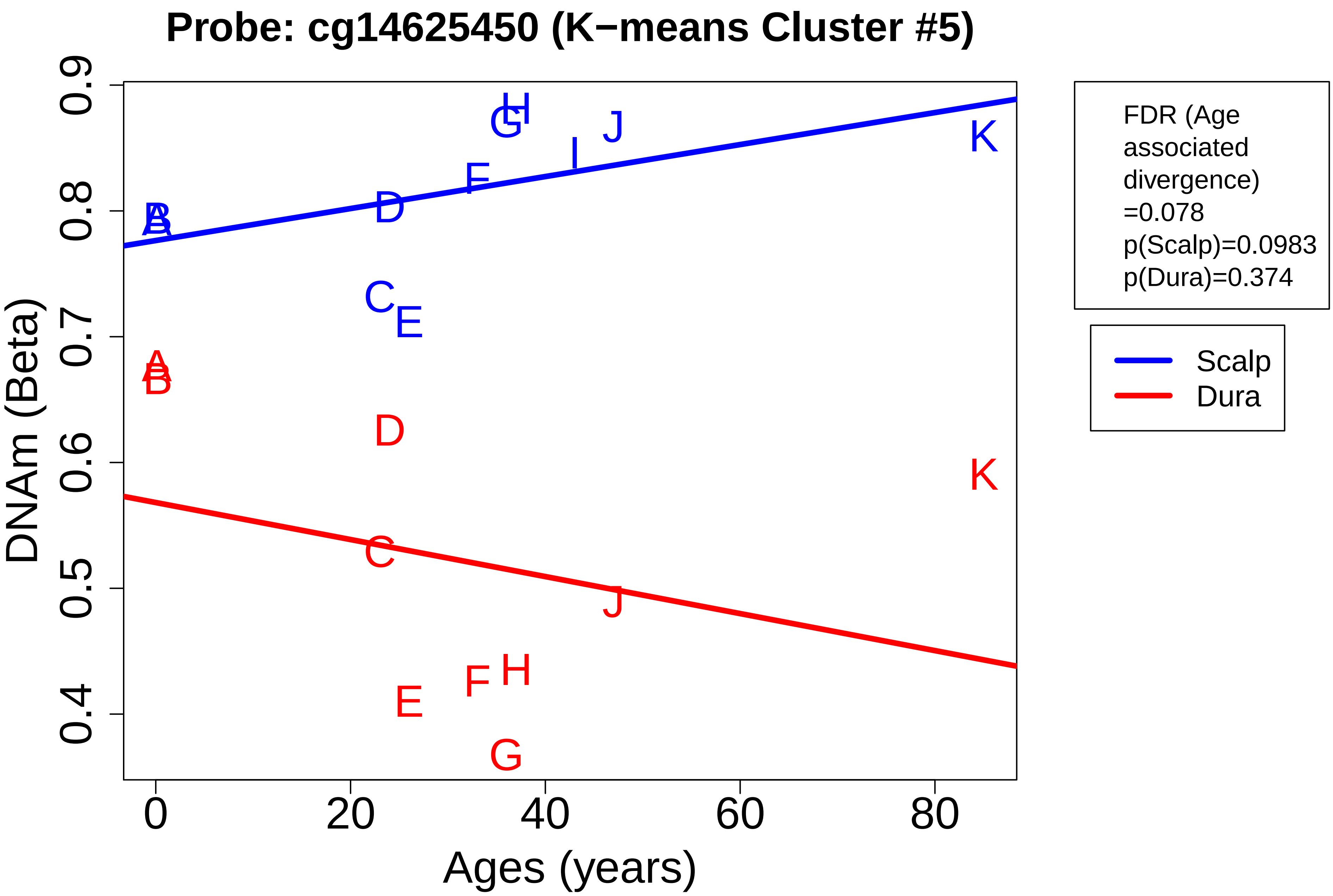

Probe: cg14625450 (K−means Cluster #5)
DNAm (Beta)
Ages (years)
0.4
0.5
0.6
0.7
0.8
0.9
0
20
40
60
80
FDR (Age associated divergence) =0.078
p(Scalp)=0.0983
p(Dura)=0.374
Scalp
Dura

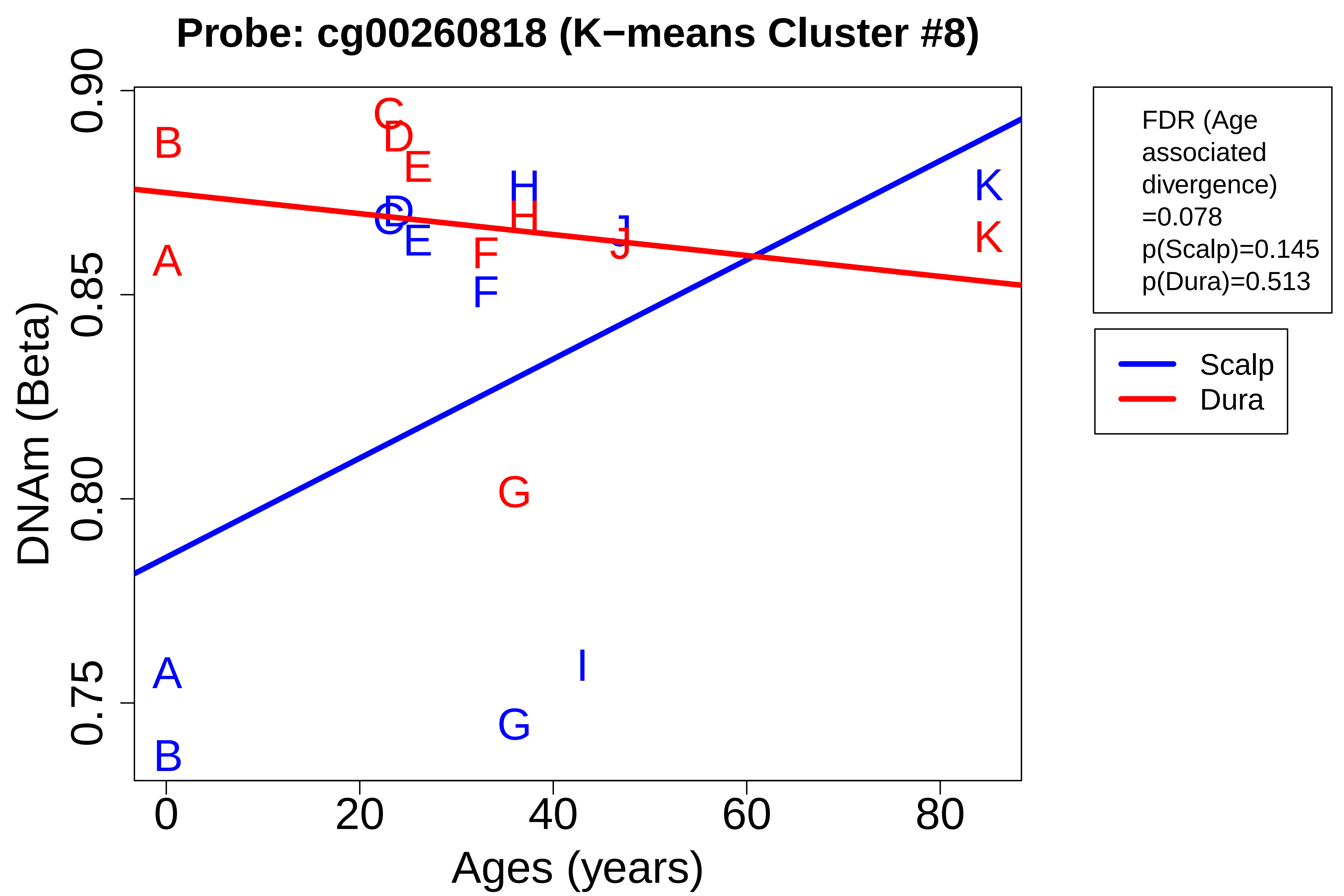

Probe: cg00260818 (K−means Cluster #8)
DNAm (Beta)
0.75
0.80
0.85
0.90
Ages (years)
0
20
40
60
80
FDR (Age associated divergence) =0.078
p(Scalp)=0.145
p(Dura)=0.513
Scalp
Dura

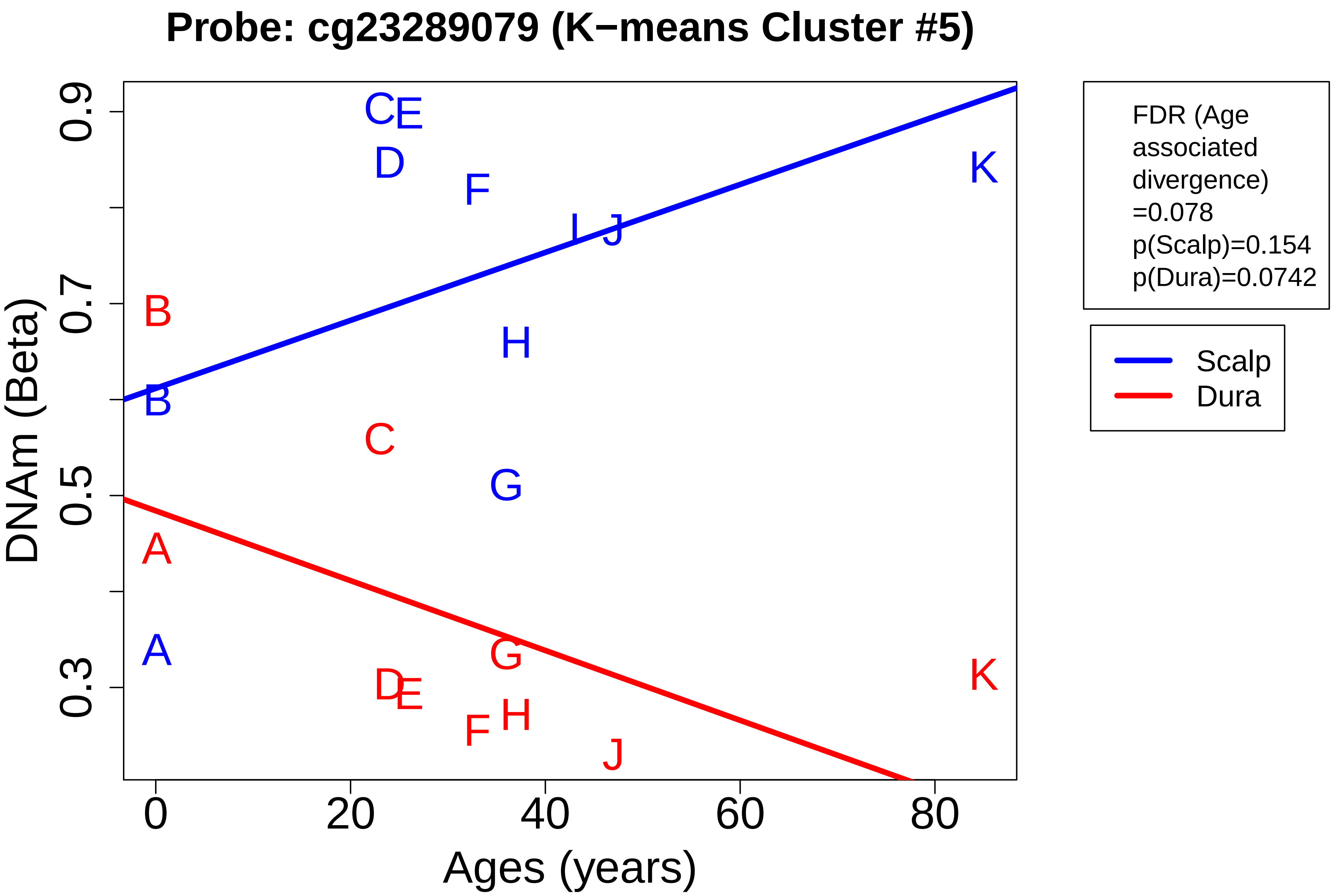
Probe: cg23289079 (K−means Cluster #5)
DNAm (Beta)
0.3
0.5
0.7
0.9
0
20
40
60
80
Ages (years)
FDR (Age associated divergence) =0.078
p(Scalp)=0.154
p(Dura)=0.0742
Scalp
Dura

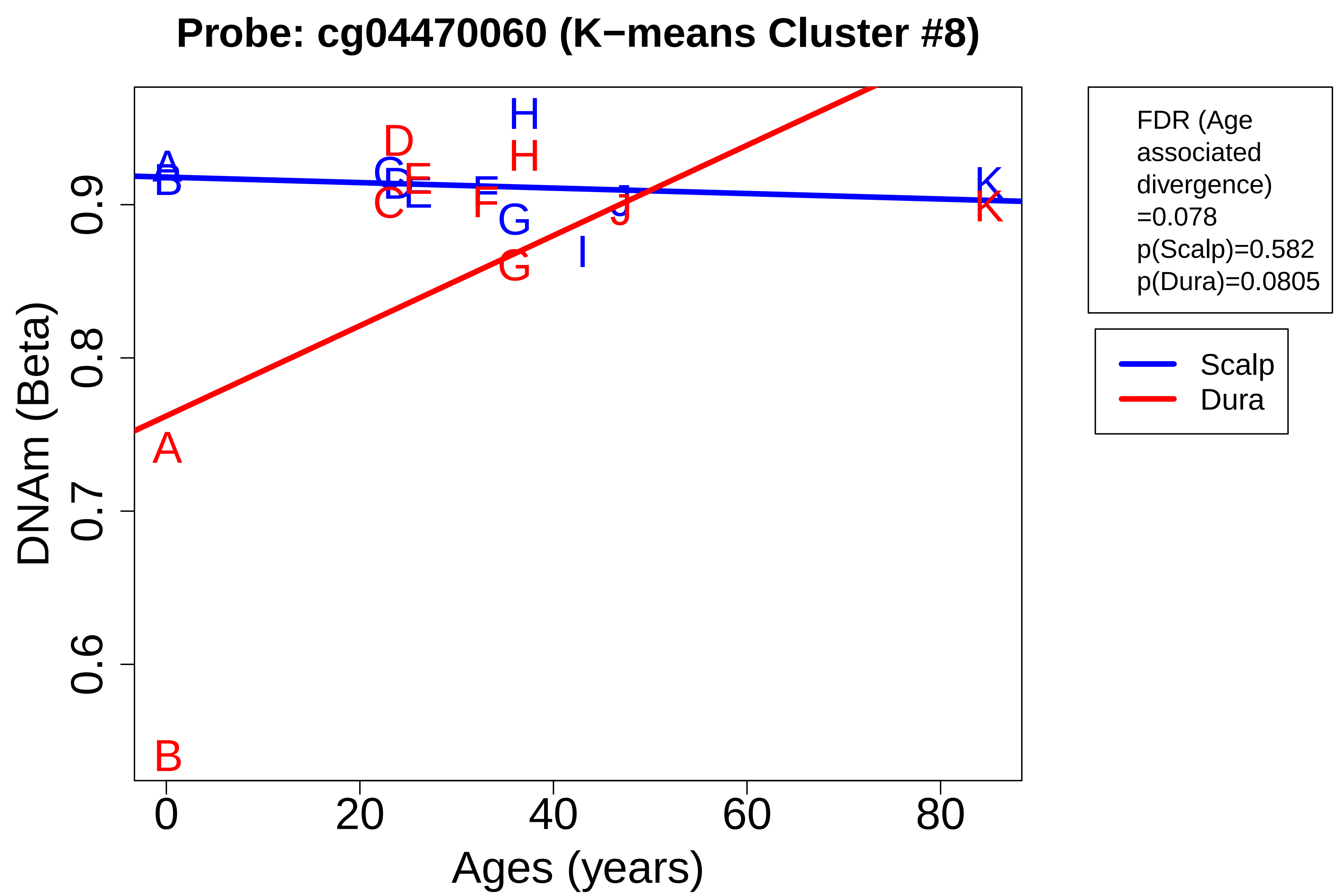

Probe: cg04470060 (K−means Cluster #8)
DNAm (Beta)
Ages (years)
0.6
0.7
0.8
0.9
0
20
40
60
80
FDR (Age associated divergence) =0.078
p(Scalp)=0.582
p(Dura)=0.0805
Scalp
Dura

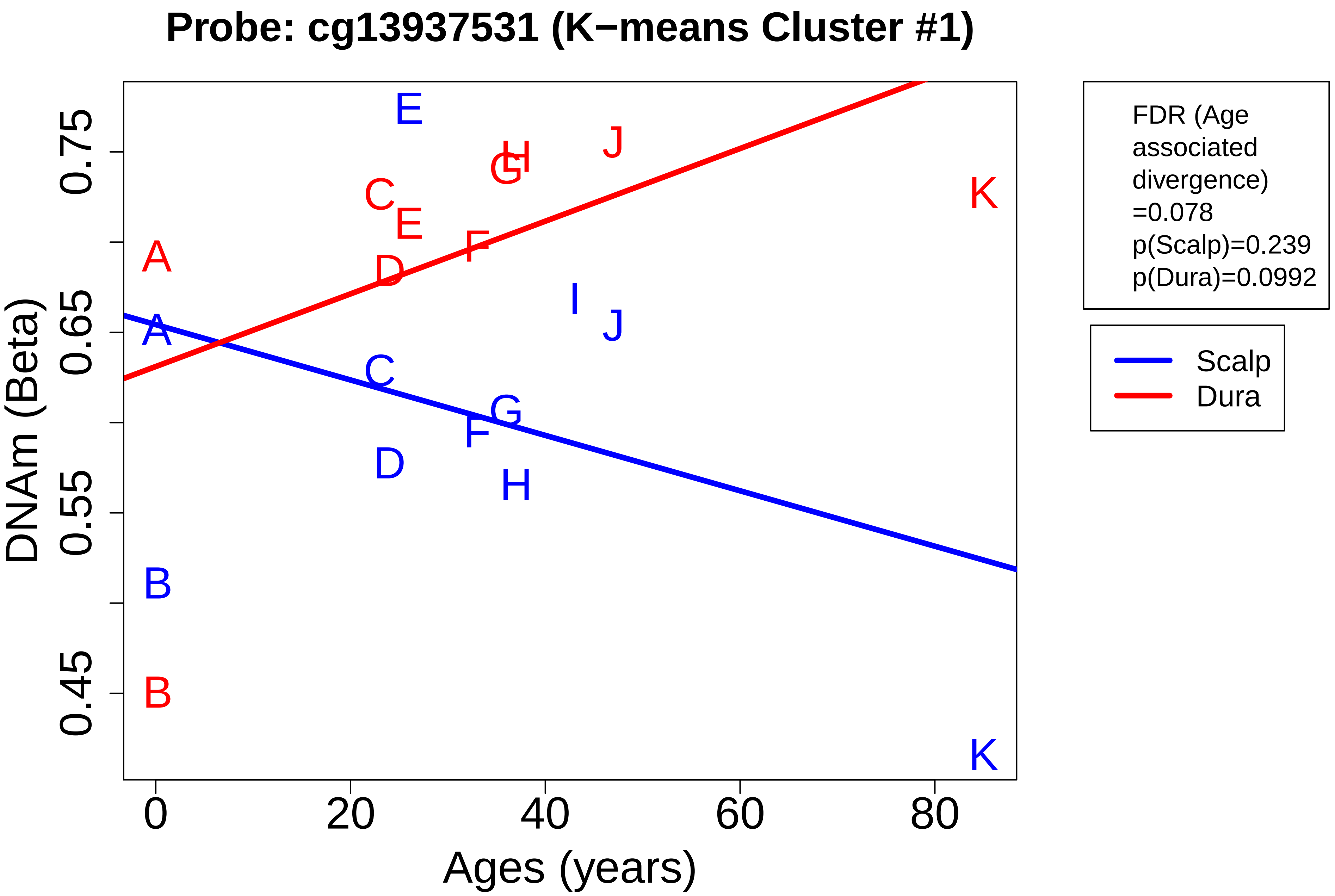
Probe: cg13937531 (K−means Cluster #1)
DNAm (Beta)
0.45
0.55
0.65
0.75
Ages (years)
0
20
40
60
80
FDR (Age associated divergence) =0.078
p(Scalp)=0.239
p(Dura)=0.0992
Scalp
Dura

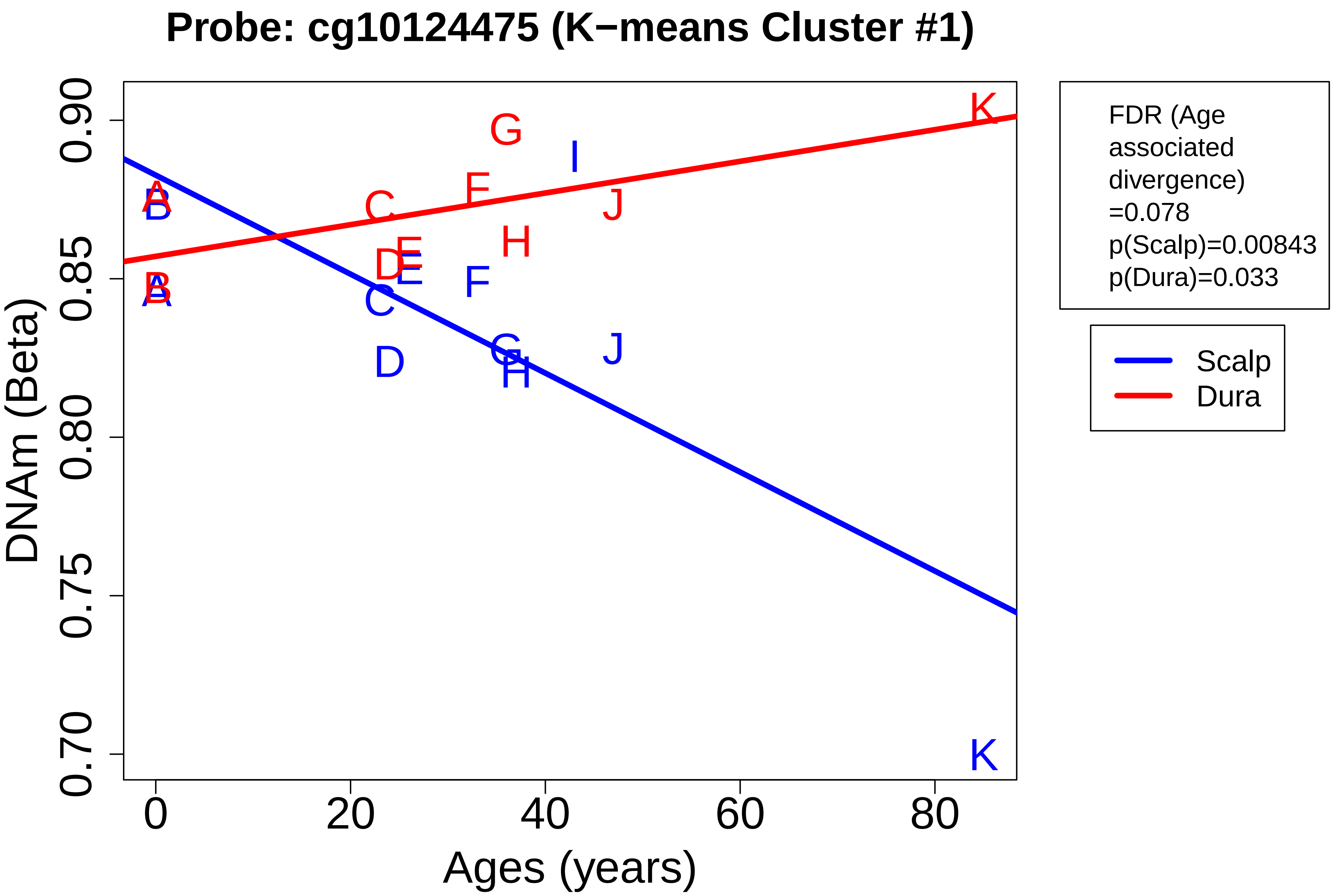

Probe: cg10124475 (K−means Cluster #1)
DNAm (Beta)
0.70
0.75
0.80
0.85
0.90
0
20
40
60
80
Ages (years)
FDR (Age associated divergence) =0.078
p(Scalp)=0.00843
p(Dura)=0.033
Scalp
Dura

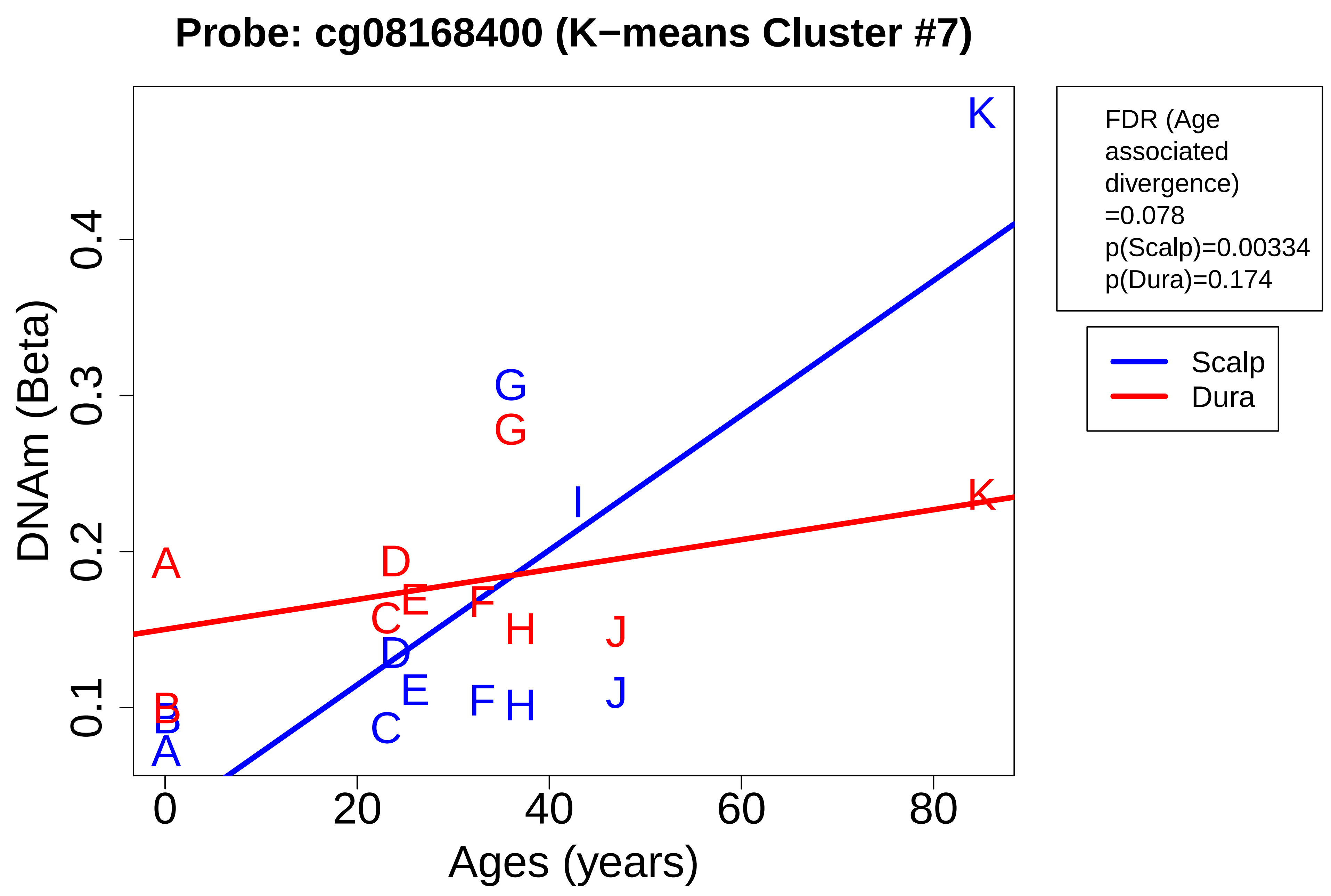
Probe: cg08168400 (K−means Cluster #7)
DNAm (Beta)
0.1
0.2
0.3
0.4
0
20
40
60
80
Ages (years)
A
B
C
D
E
F
G
H
I
J
K
FDR (Age associated divergence) =0.078
p(Scalp)=0.00334
p(Dura)=0.174
Scalp
Dura

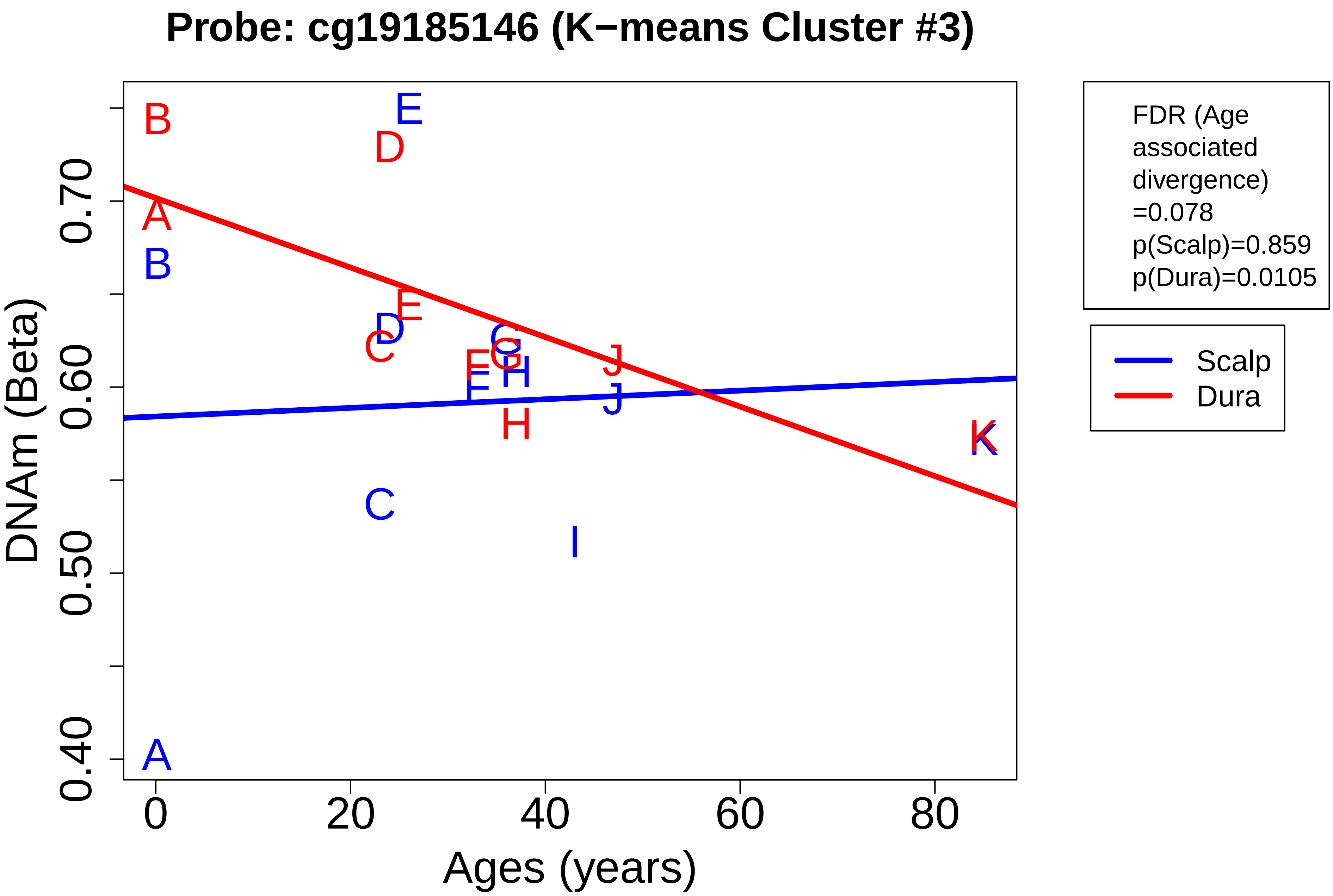

Probe: cg19185146 (K−means Cluster #3)
DNAm (Beta)
0.40
0.50
0.60
0.70
0
20
40
60
80
Ages (years)
FDR (Age associated divergence) =0.078
p(Scalp)=0.859
p(Dura)=0.0105
Scalp
Dura

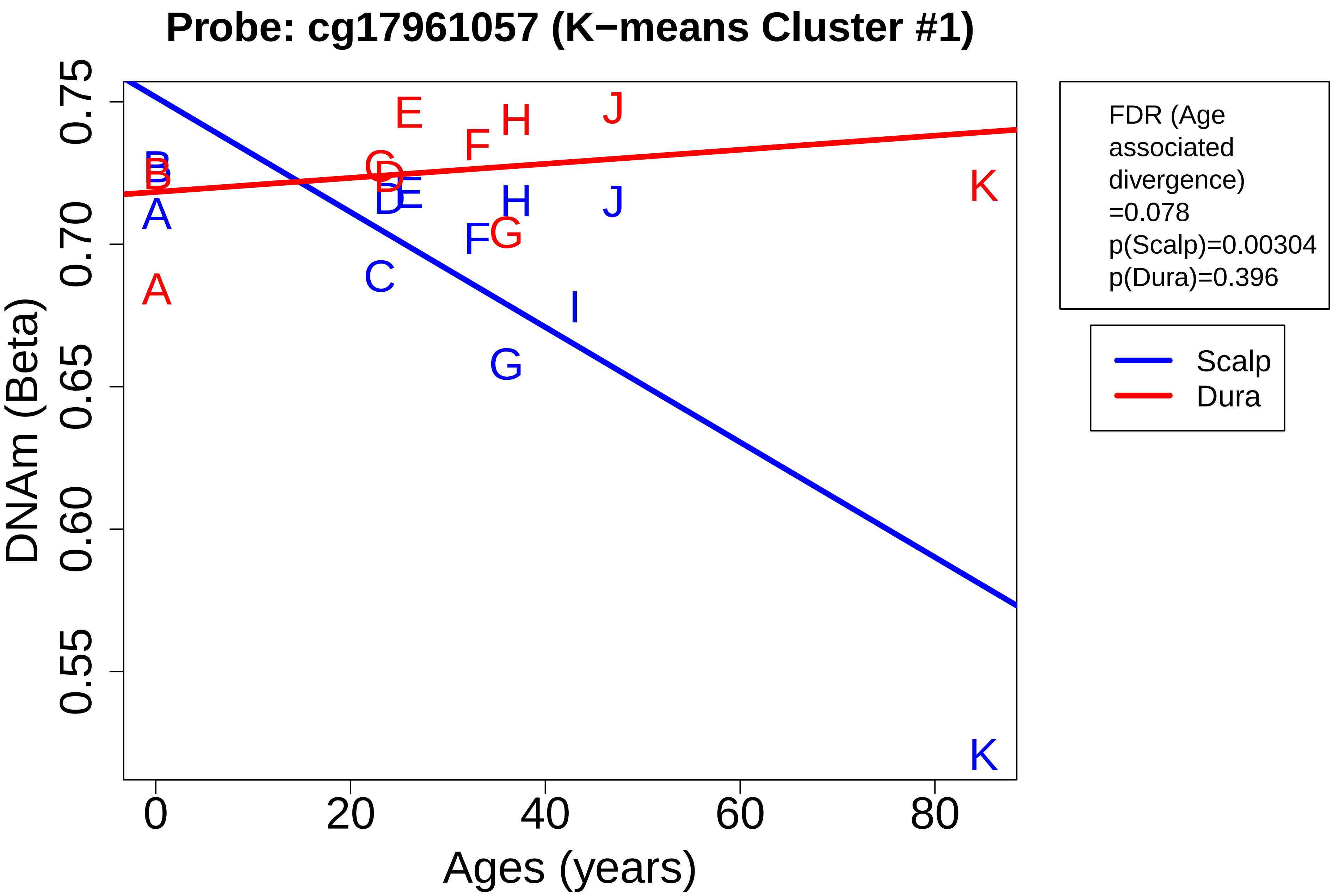

Probe: cg17961057 (K−means Cluster #1)
DNAm (Beta)
0.55
0.60
0.65
0.70
0.75
Ages (years)
0
20
40
60
80
FDR (Age associated divergence) =0.078
p(Scalp)=0.00304
p(Dura)=0.396
Scalp
Dura

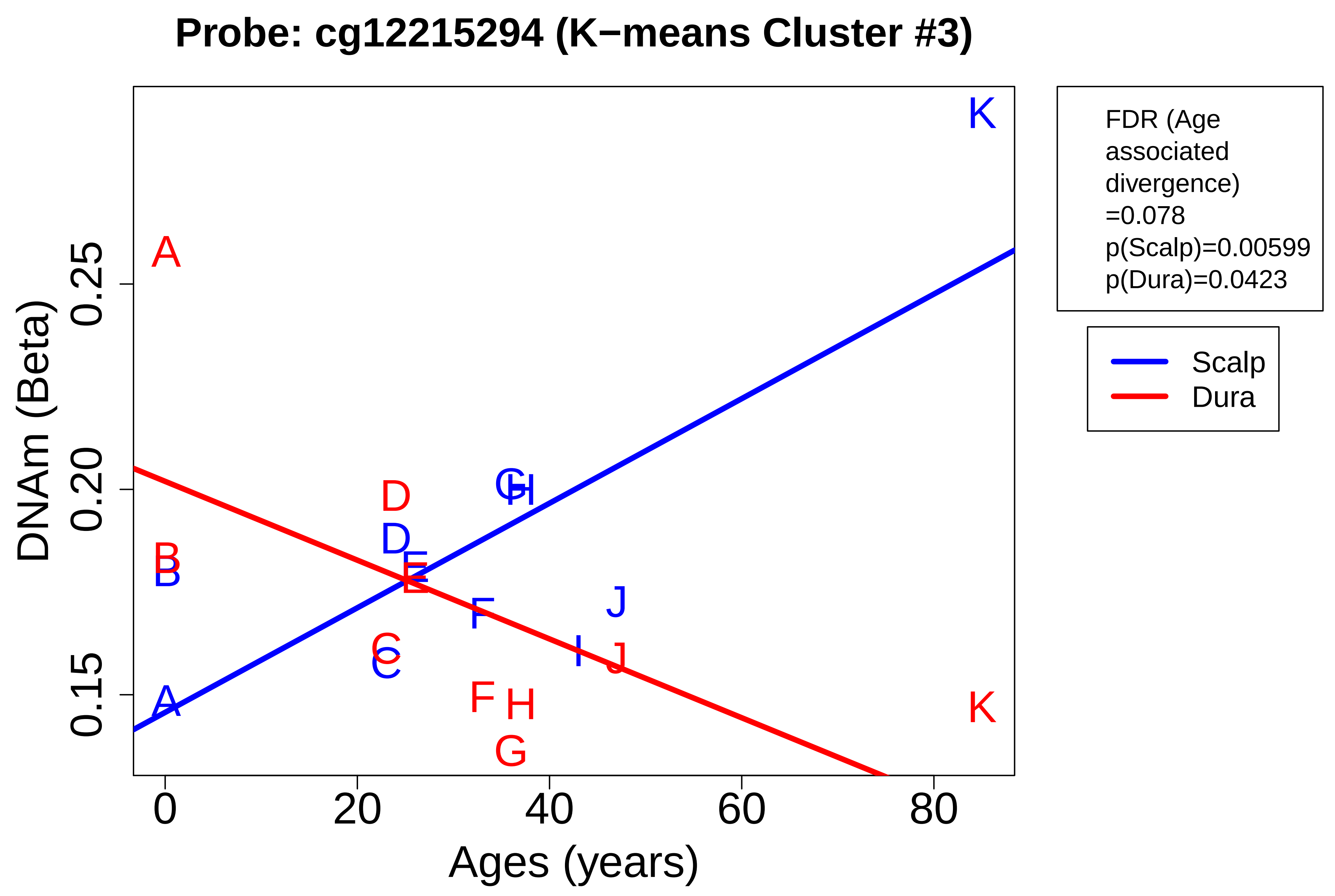

Probe: cg12215294 (K−means Cluster #3)
DNAm (Beta)
0.15
0.20
0.25
Ages (years)
0
20
40
60
80
A
B
C
D
E
F
G
H
I
J
K
FDR (Age associated divergence) =0.078
p(Scalp)=0.00599
p(Dura)=0.0423
Scalp
Dura

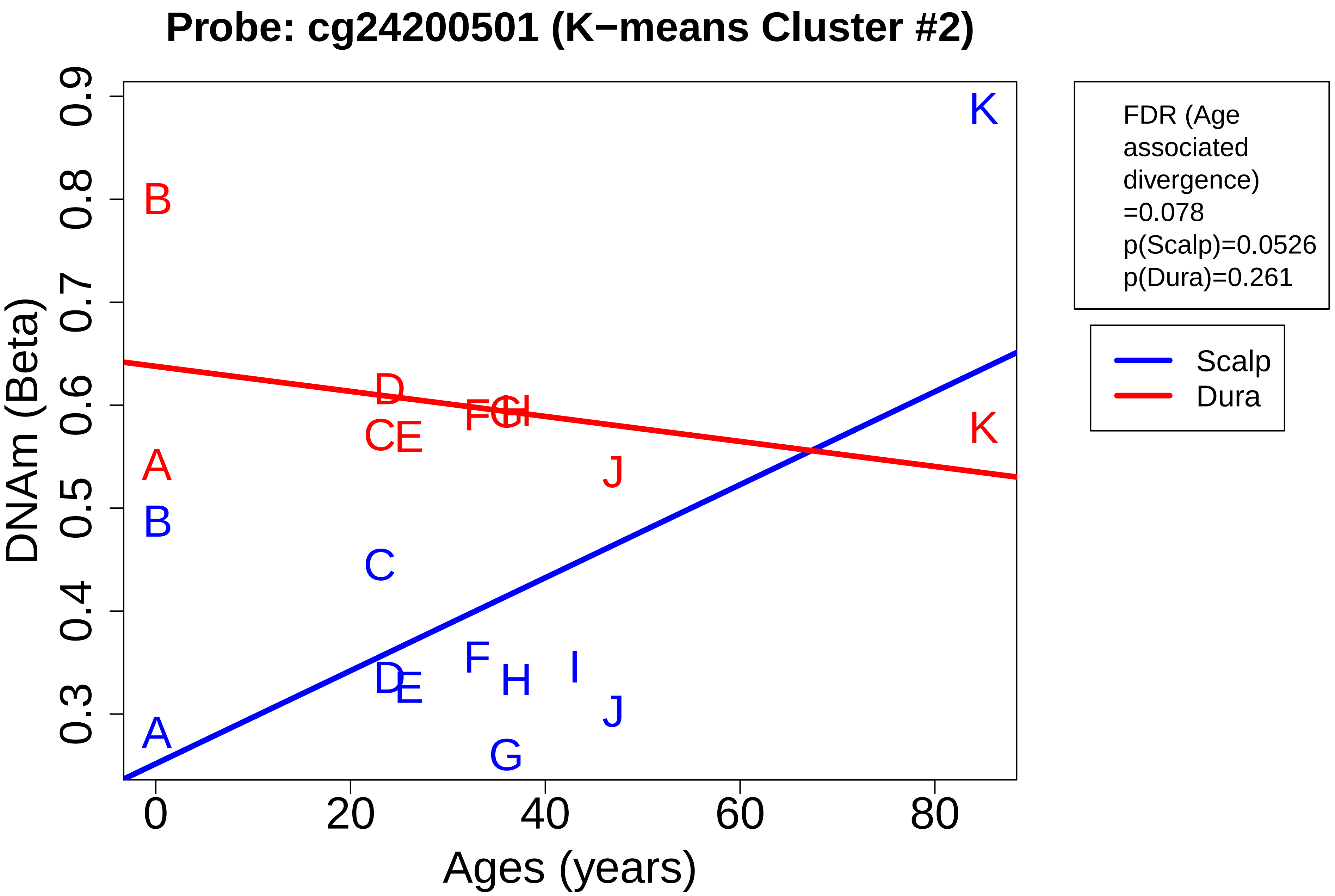

Probe: cg24200501 (K−means Cluster #2)
DNAm (Beta)
0.3
0.4
0.5
0.6
0.7
0.8
0.9
0
20
40
60
80
Ages (years)
FDR (Age associated divergence) =0.078
p(Scalp)=0.0526
p(Dura)=0.261
Scalp
Dura

Probe: cg17176676 (K−means Cluster #4)

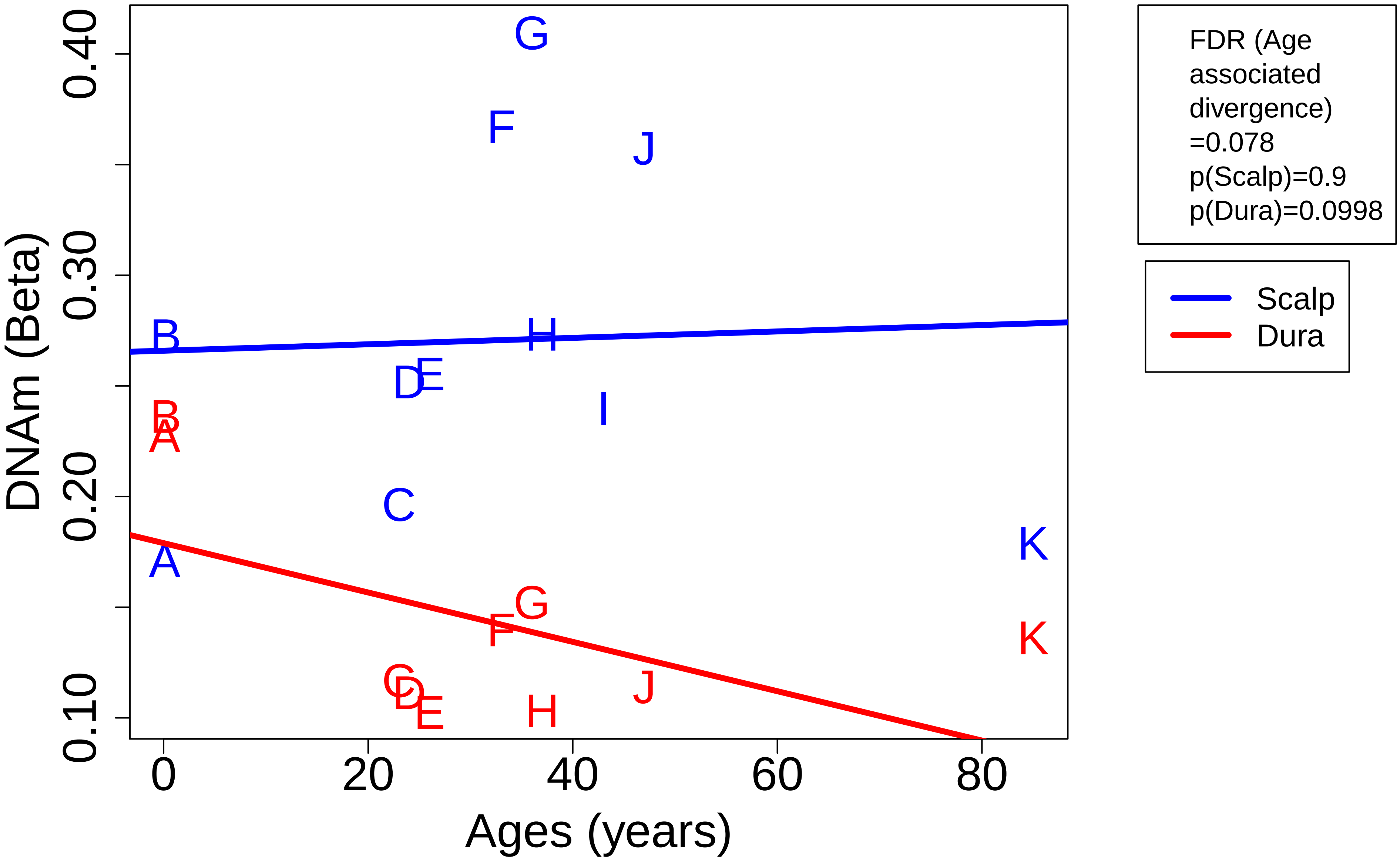

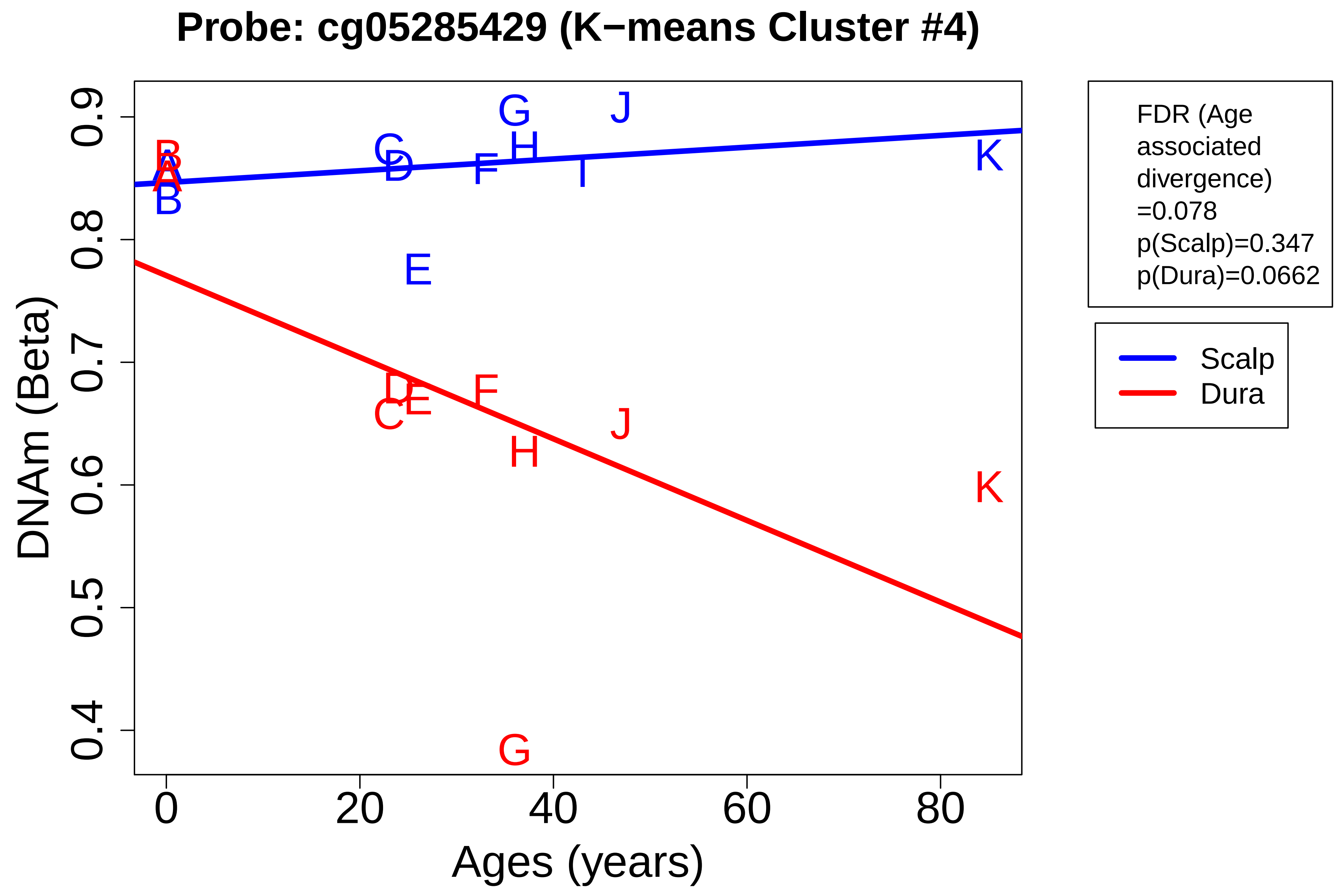

Probe: cg05285429 (K−means Cluster #4)
DNAm (Beta)
0.4
0.5
0.6
0.7
0.8
0.9
0
20
40
60
80
Ages (years)
FDR (Age associated divergence) =0.078
p(Scalp)=0.347
p(Dura)=0.0662
Scalp
Dura

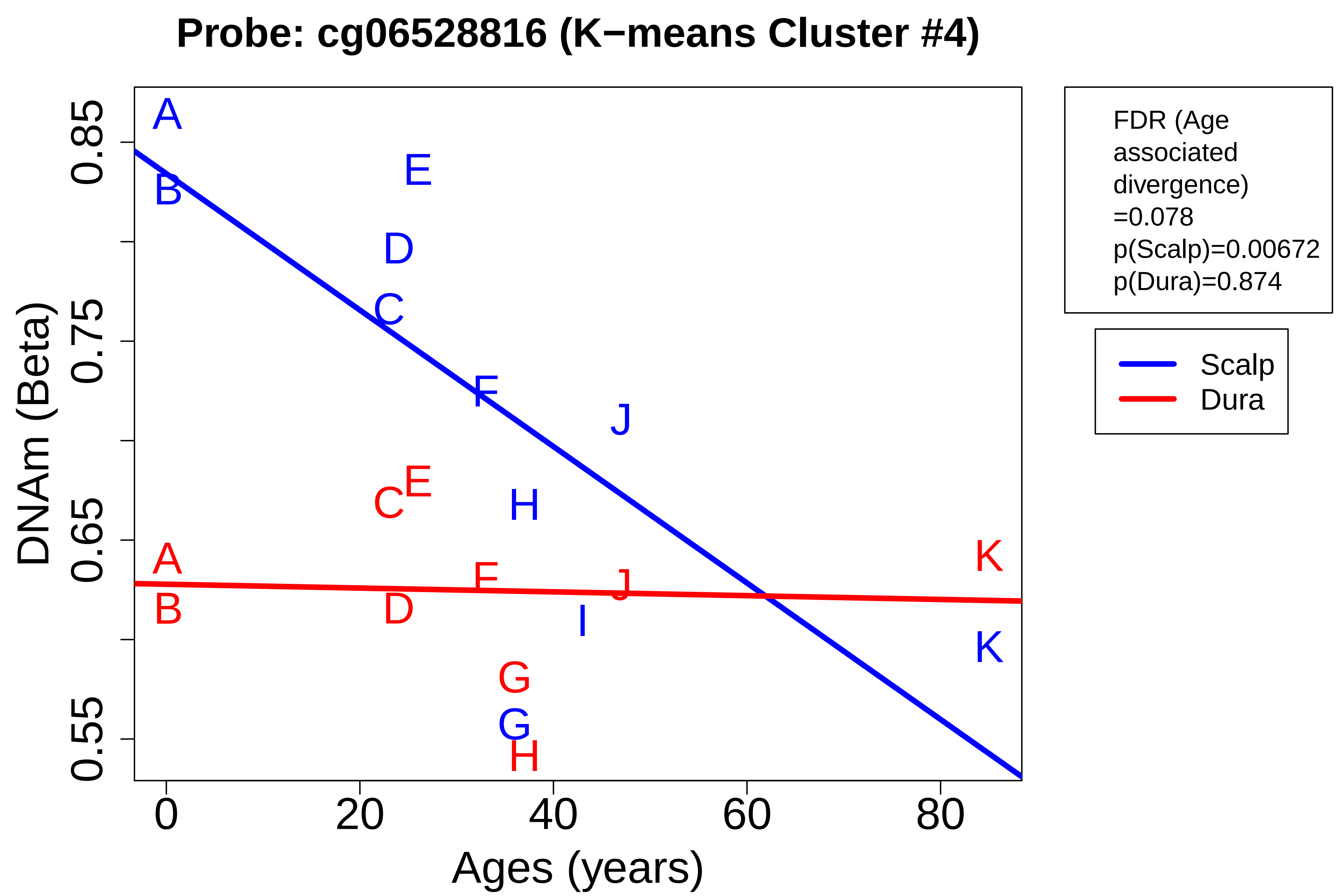

Probe: cg06528816 (K−means Cluster #4)
DNAm (Beta)
0.55
0.65
0.75
0.85
0
20
40
60
80
Ages (years)
A
B
C
D
E
F
G
H
I
J
K
FDR (Age associated divergence) =0.078
p(Scalp)=0.00672
p(Dura)=0.874
Scalp
Dura

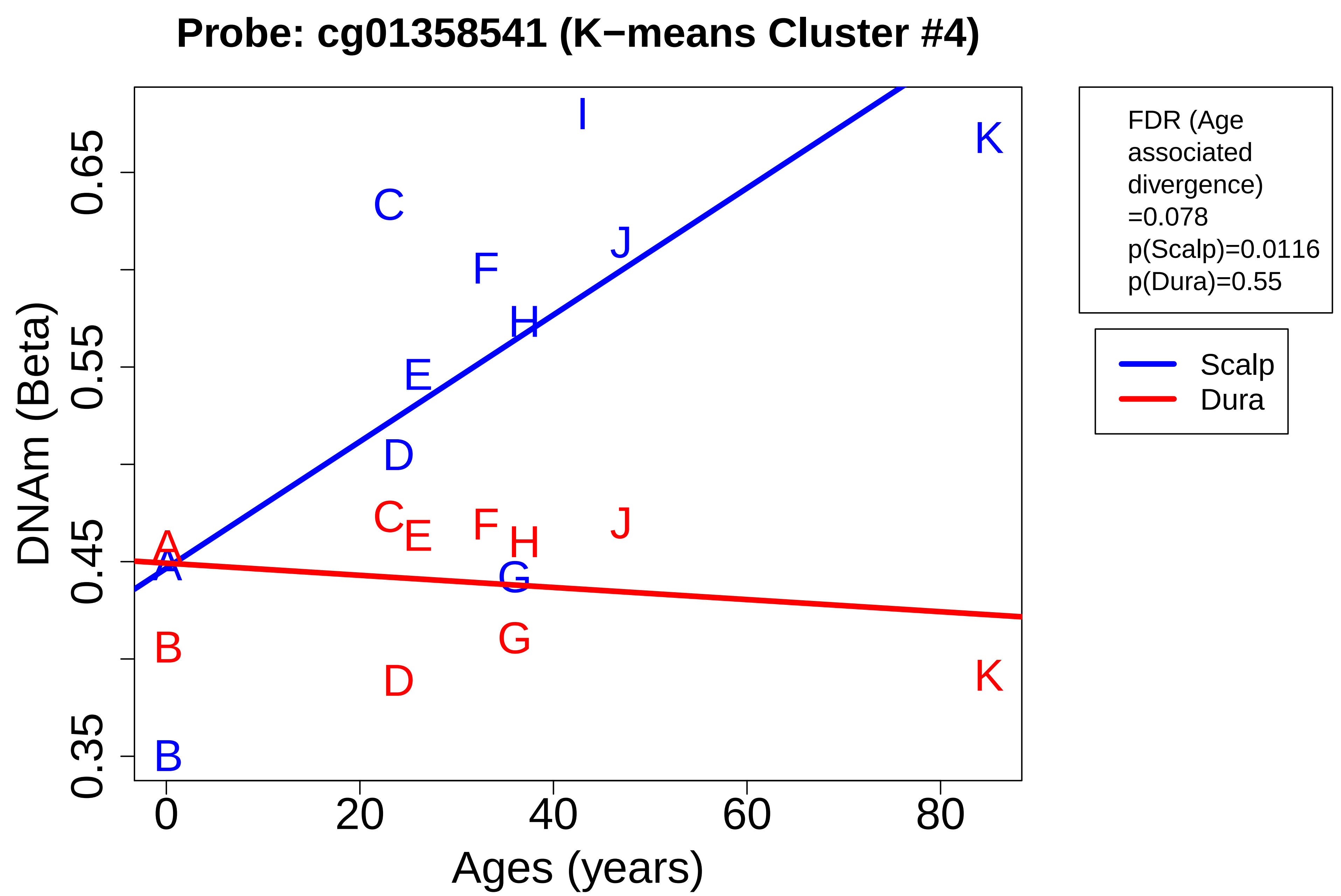
Probe: cg01358541 (K−means Cluster #4)
DNAm (Beta)
0.35
0.45
0.55
0.65
Ages (years)
0
20
40
60
80
FDR (Age associated divergence) =0.078
p(Scalp)=0.0116
p(Dura)=0.55
Scalp
Dura

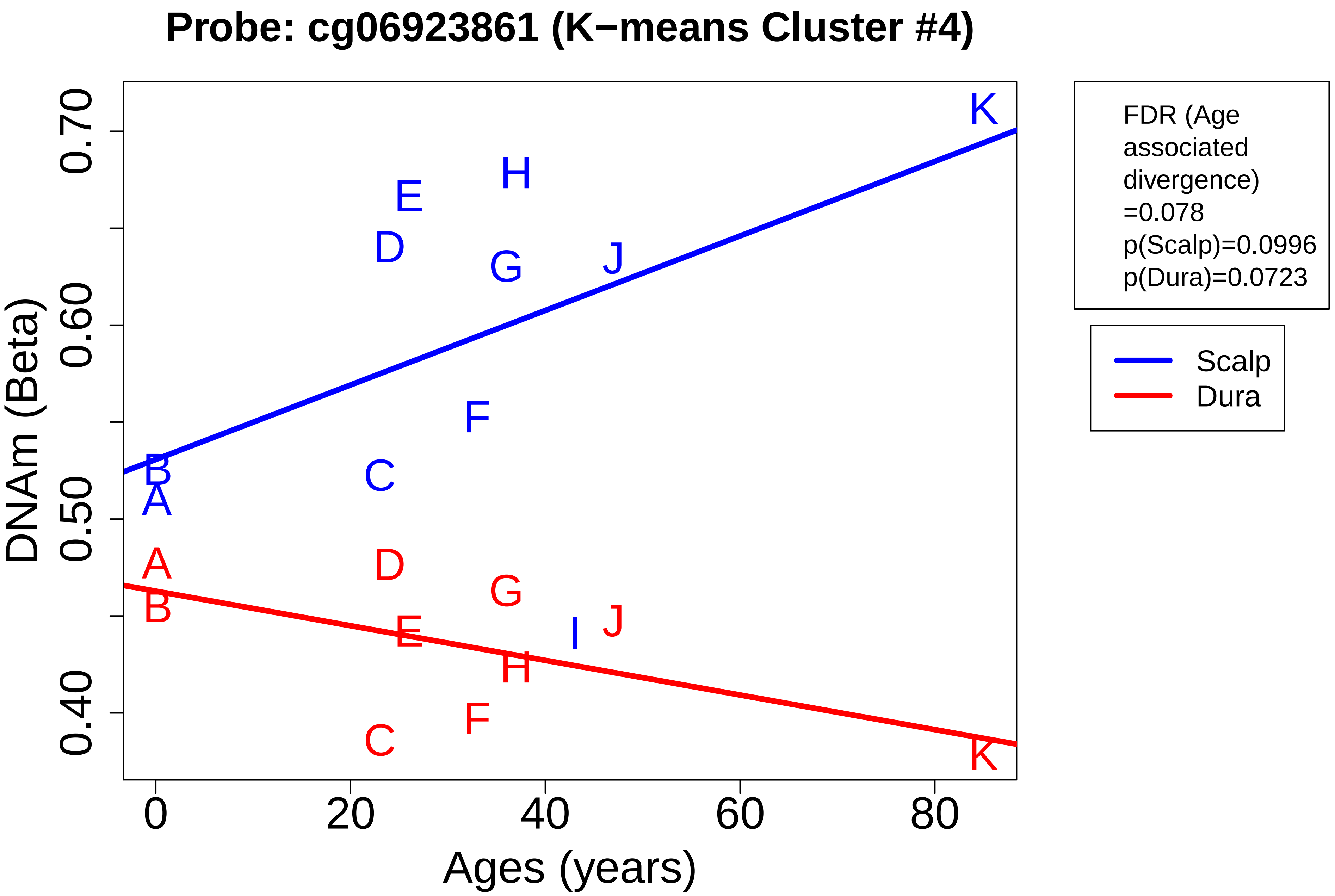

Probe: cg06923861 (K−means Cluster #4)
DNAm (Beta)
0.40
0.50
0.60
0.70
0
20
40
60
80
Ages (years)
FDR (Age associated divergence) =0.078
p(Scalp)=0.0996
p(Dura)=0.0723
Scalp
Dura
A
B
C
D
E
F
G
H
I
J
K

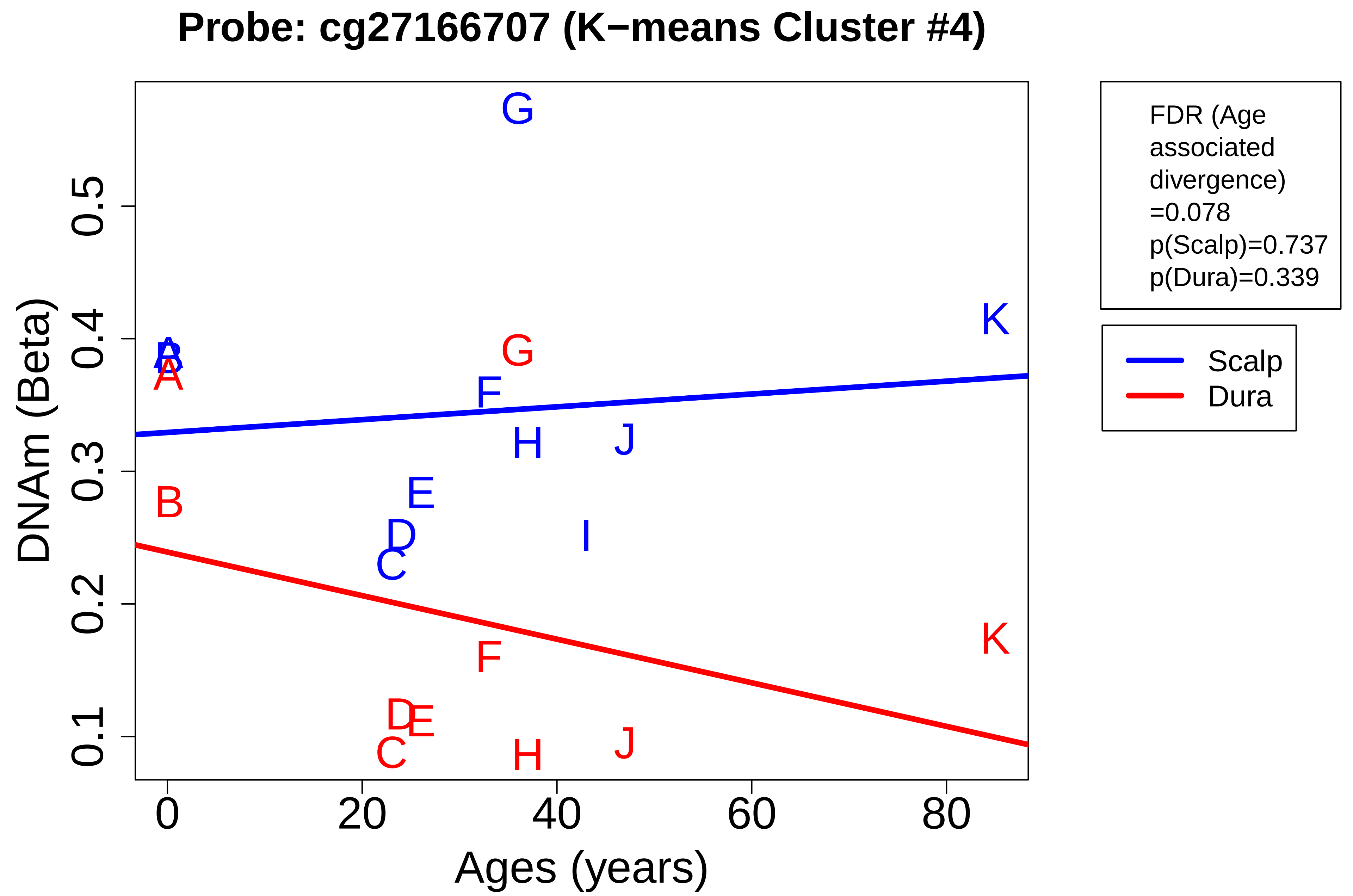
Probe: cg27166707 (K−means Cluster #4)
FDR (Age associated divergence) =0.078
p(Scalp)=0.737
p(Dura)=0.339
Scalp
Dura
DNAm (Beta)
0.1
0.2
0.3
0.4
0.5
0
20
40
60
80
Ages (years)
A
B
C
D
E
F
G
H
I
J
K

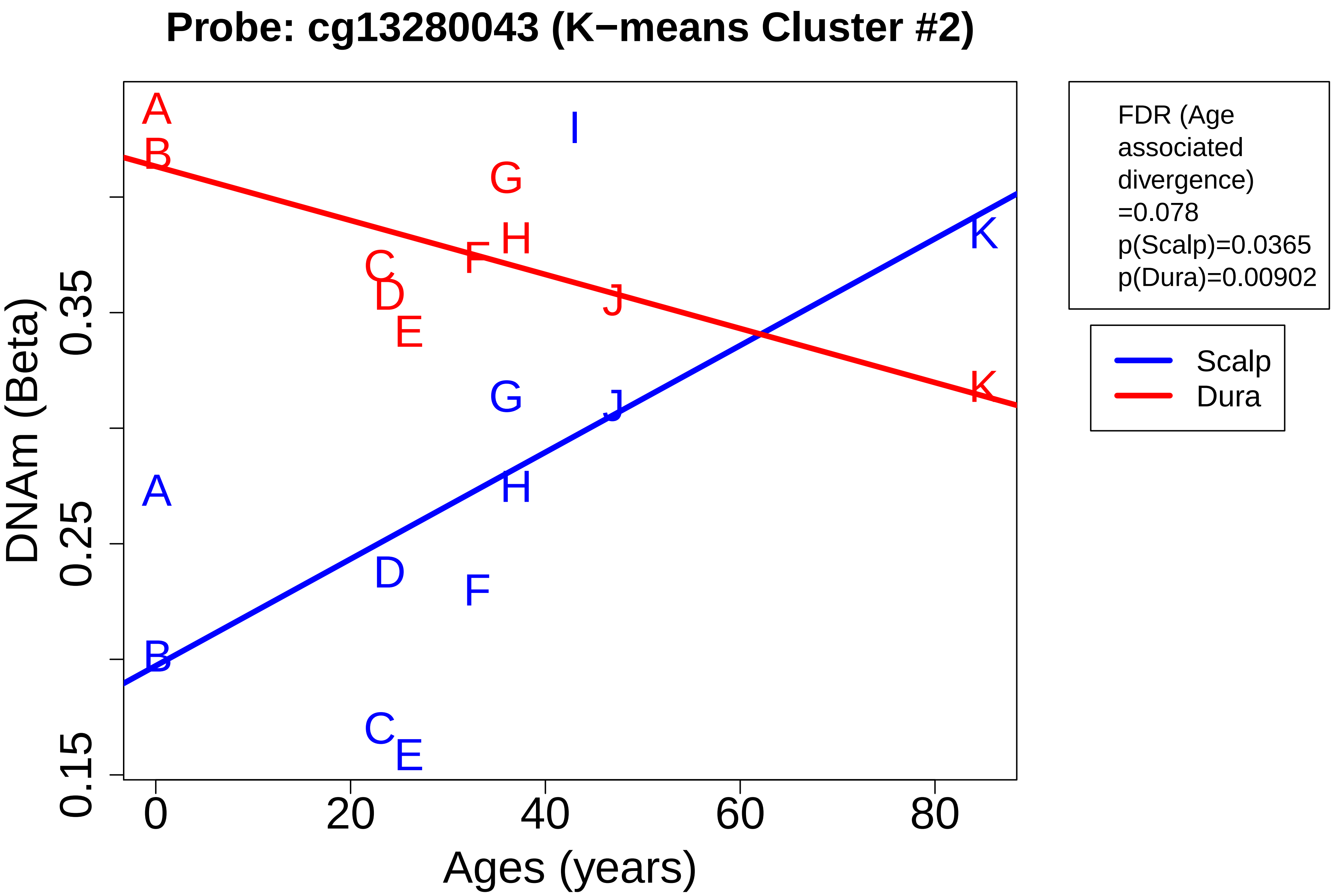

Probe: cg13280043 (K−means Cluster #2)
DNAm (Beta)
0.15
0.25
0.35
0
20
40
60
80
Ages (years)
A
B
C
D
E
F
G
H
I
J
K
FDR (Age associated divergence) =0.078
p(Scalp)=0.0365
p(Dura)=0.00902
Scalp
Dura

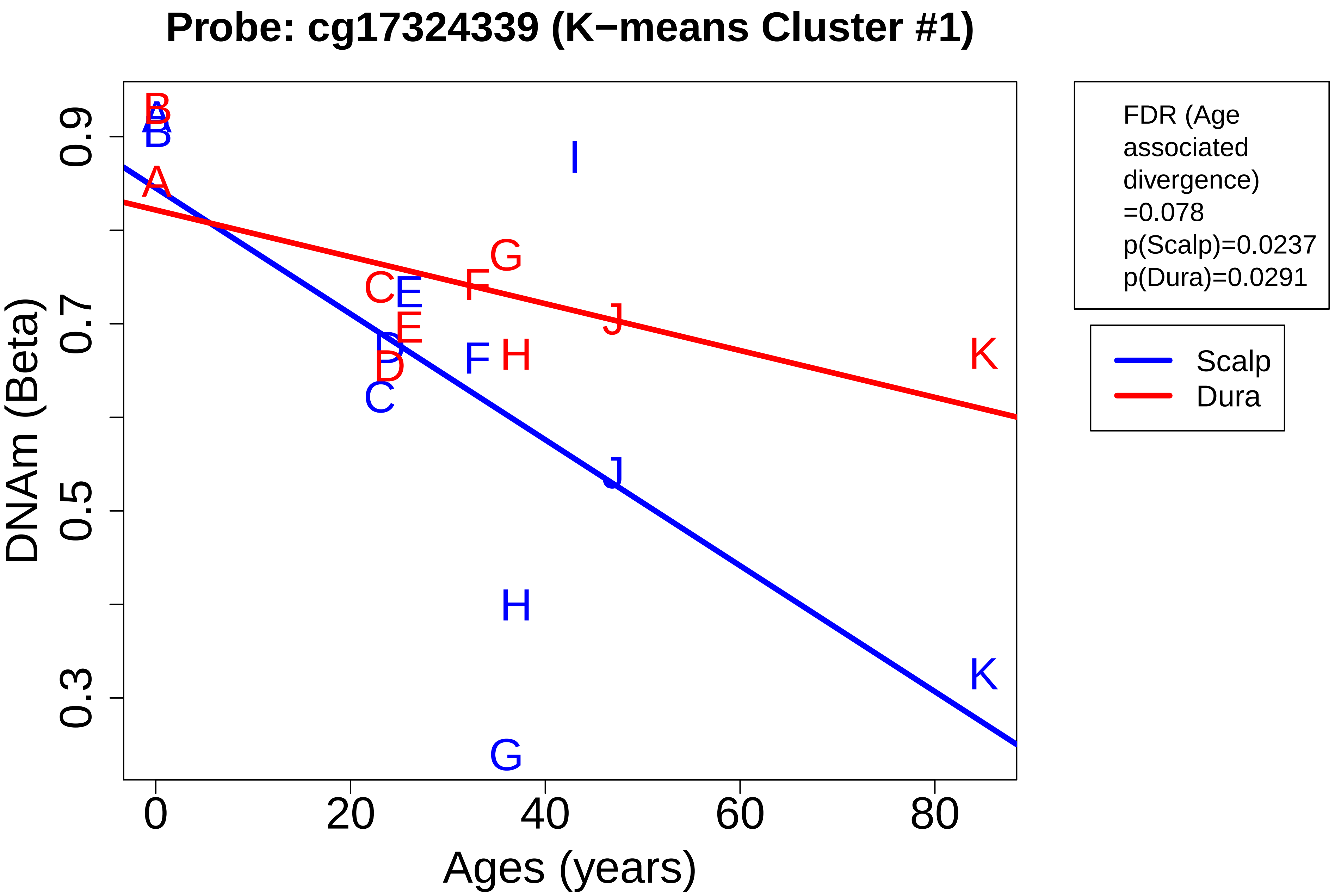

Probe: cg17324339 (K−means Cluster #1)
DNAm (Beta)
0.3
0.5
0.7
0.9
Ages (years)
0
20
40
60
80
FDR (Age associated divergence) =0.078
p(Scalp)=0.0237
p(Dura)=0.0291
Scalp
Dura

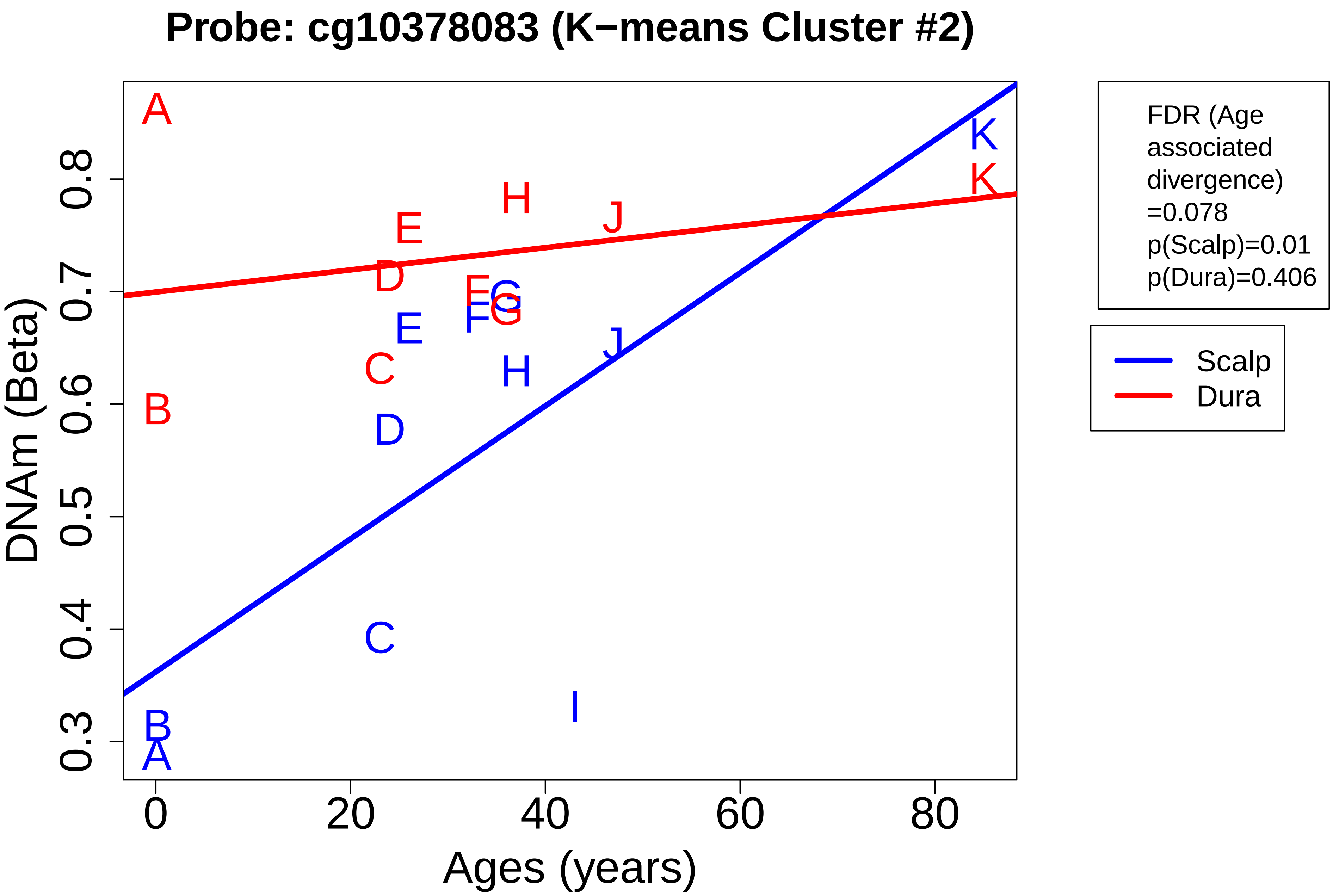

Probe: cg10378083 (K−means Cluster #2)
FDR (Age associated divergence) =0.078
p(Scalp)=0.01
p(Dura)=0.406
Scalp
Dura
DNAm (Beta)
Ages (years)
0.3
0.4
0.5
0.6
0.7
0.8
0
20
40
60
80

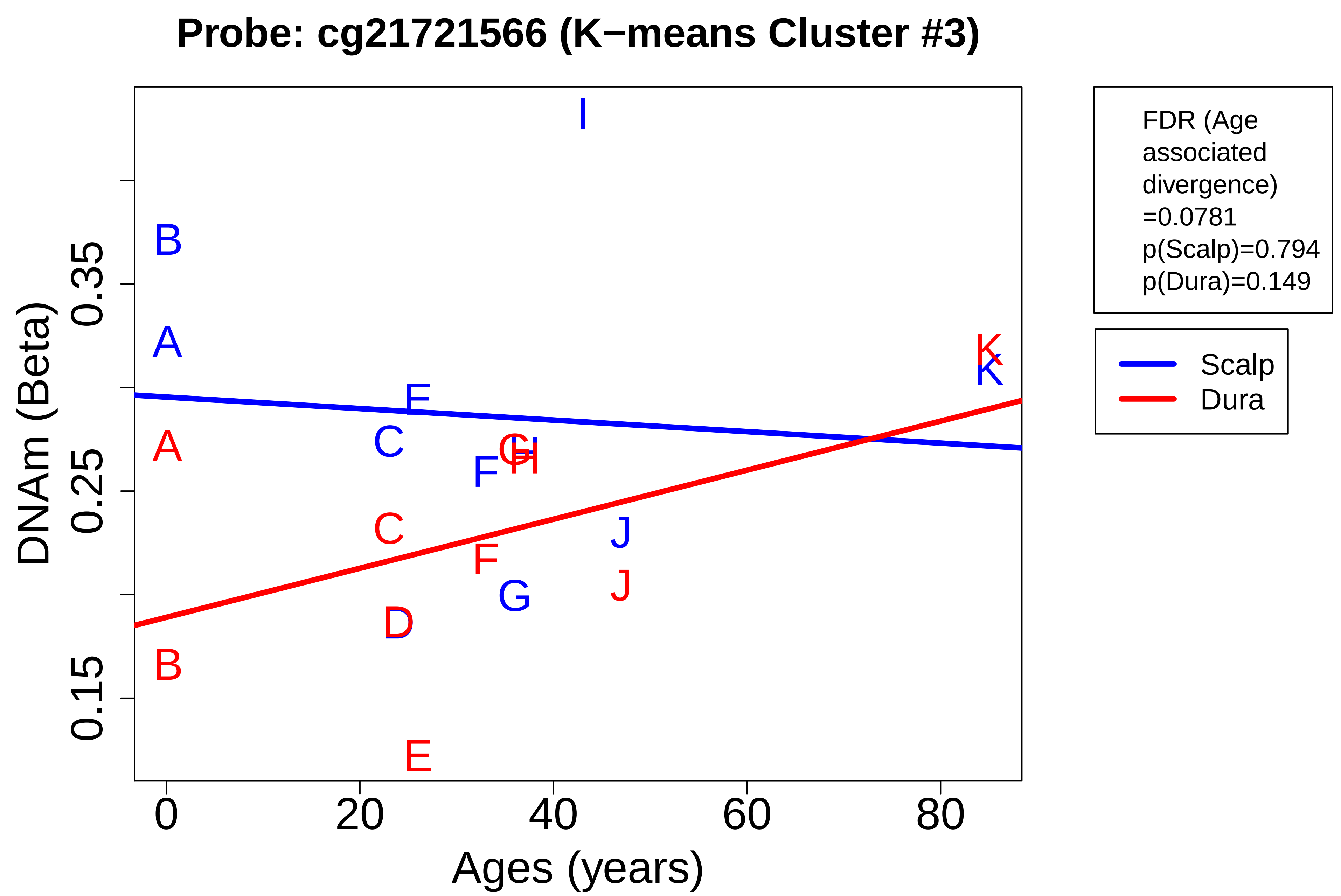

Probe: cg21721566 (K−means Cluster #3)
DNAm (Beta)
0.15
0.25
0.35
0
20
40
60
80
Ages (years)
FDR (Age associated divergence) =0.0781
p(Scalp)=0.794
p(Dura)=0.149
Scalp
Dura
A B C D E F G H I J K

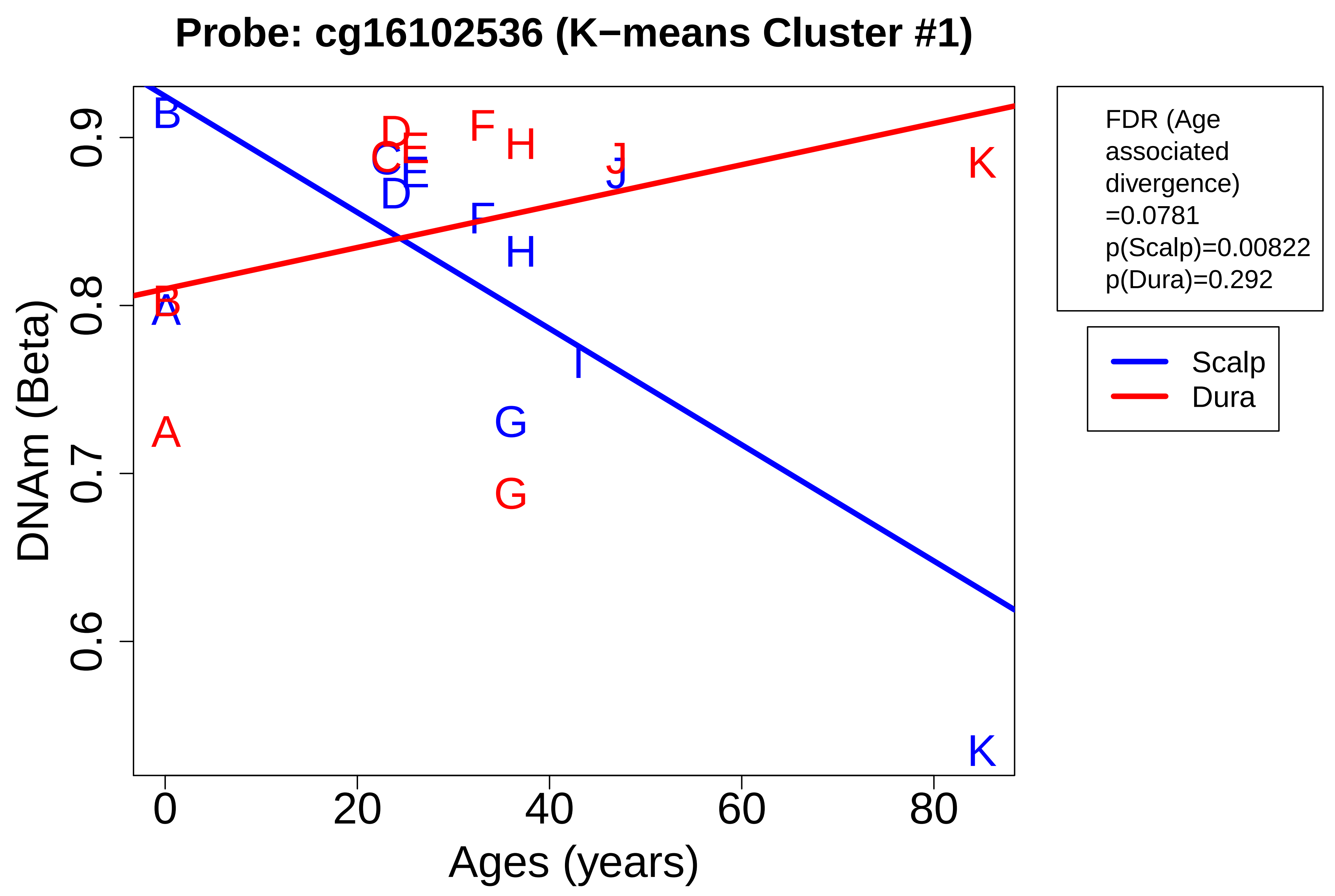

Probe: cg16102536 (K−means Cluster #1)
DNAm (Beta)
0.6
0.7
0.8
0.9
Ages (years)
0
20
40
60
80
FDR (Age associated divergence) =0.0781
p(Scalp)=0.00822
p(Dura)=0.292
Scalp
Dura

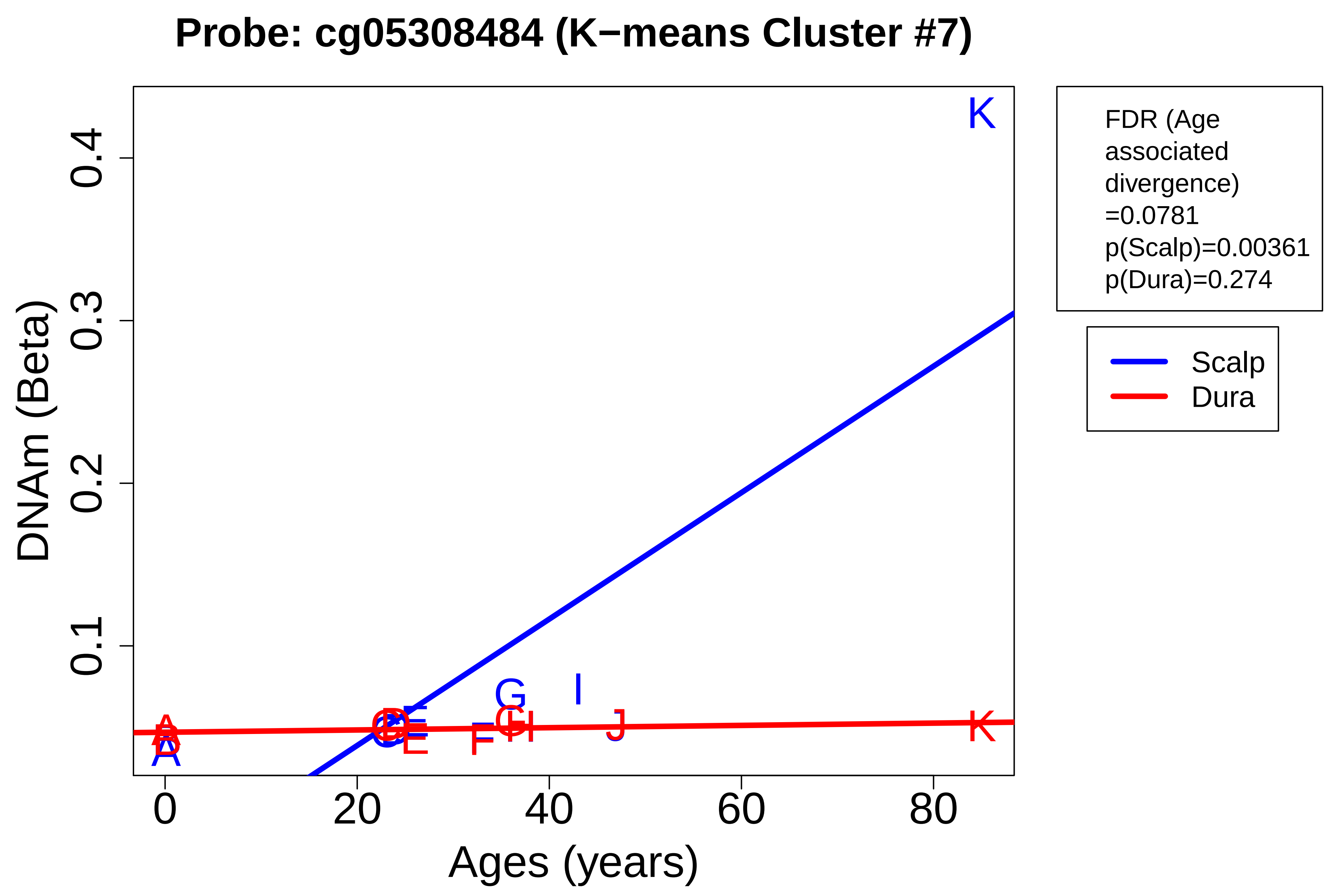

Probe: cg05308484 (K−means Cluster #7)
DNAm (Beta)
0.1
0.2
0.3
0.4
0
20
40
60
80
Ages (years)
A
B
C
D
E
F
G
H
I
J
K
FDR (Age associated divergence) =0.0781
p(Scalp)=0.00361
p(Dura)=0.274
Scalp
Dura

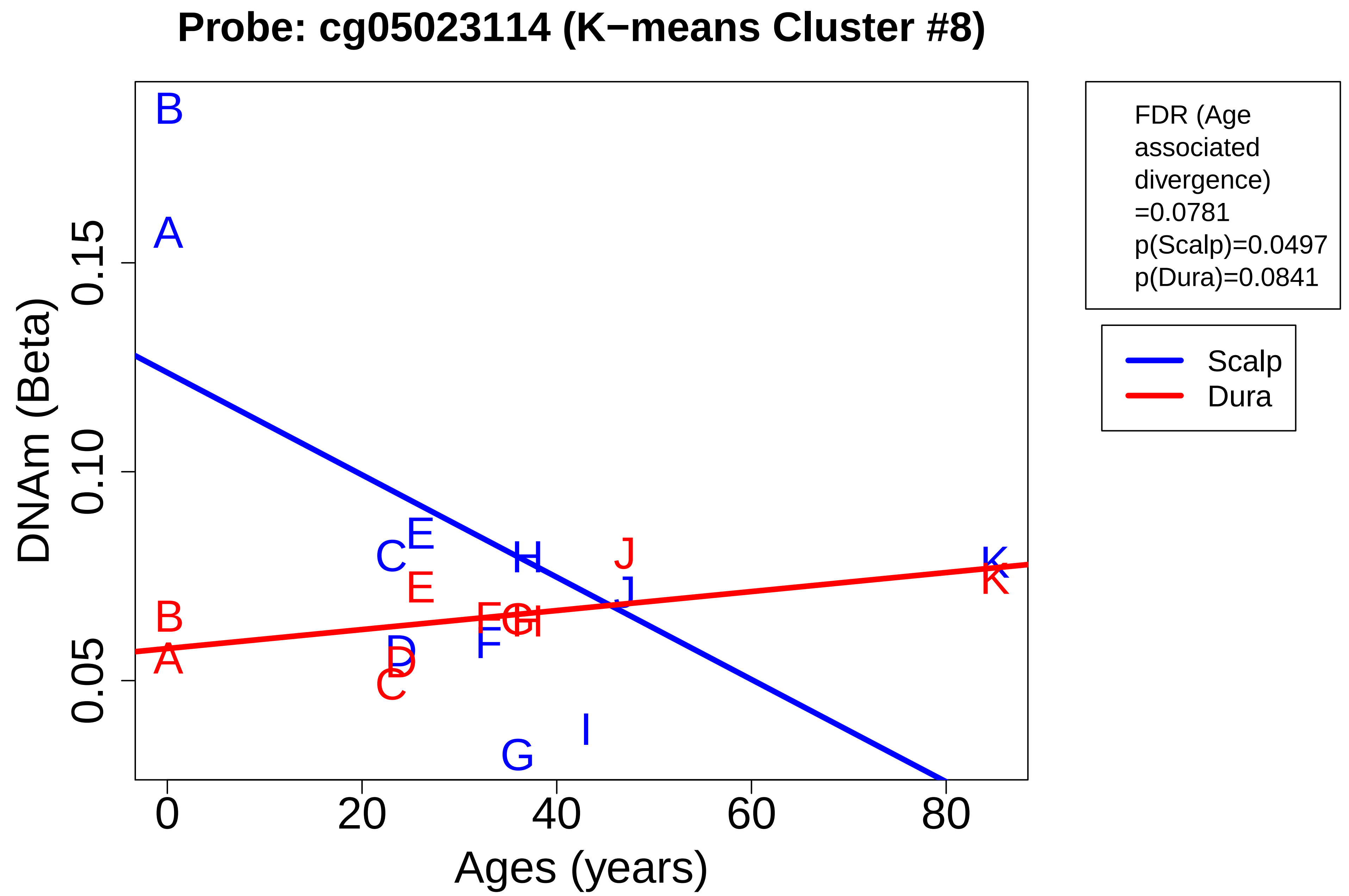

Probe: cg05023114 (K−means Cluster #8)
DNAm (Beta)
0.05
0.10
0.15
Ages (years)
0
20
40
60
80
FDR (Age associated divergence) =0.0781
p(Scalp)=0.0497
p(Dura)=0.0841
Scalp
Dura

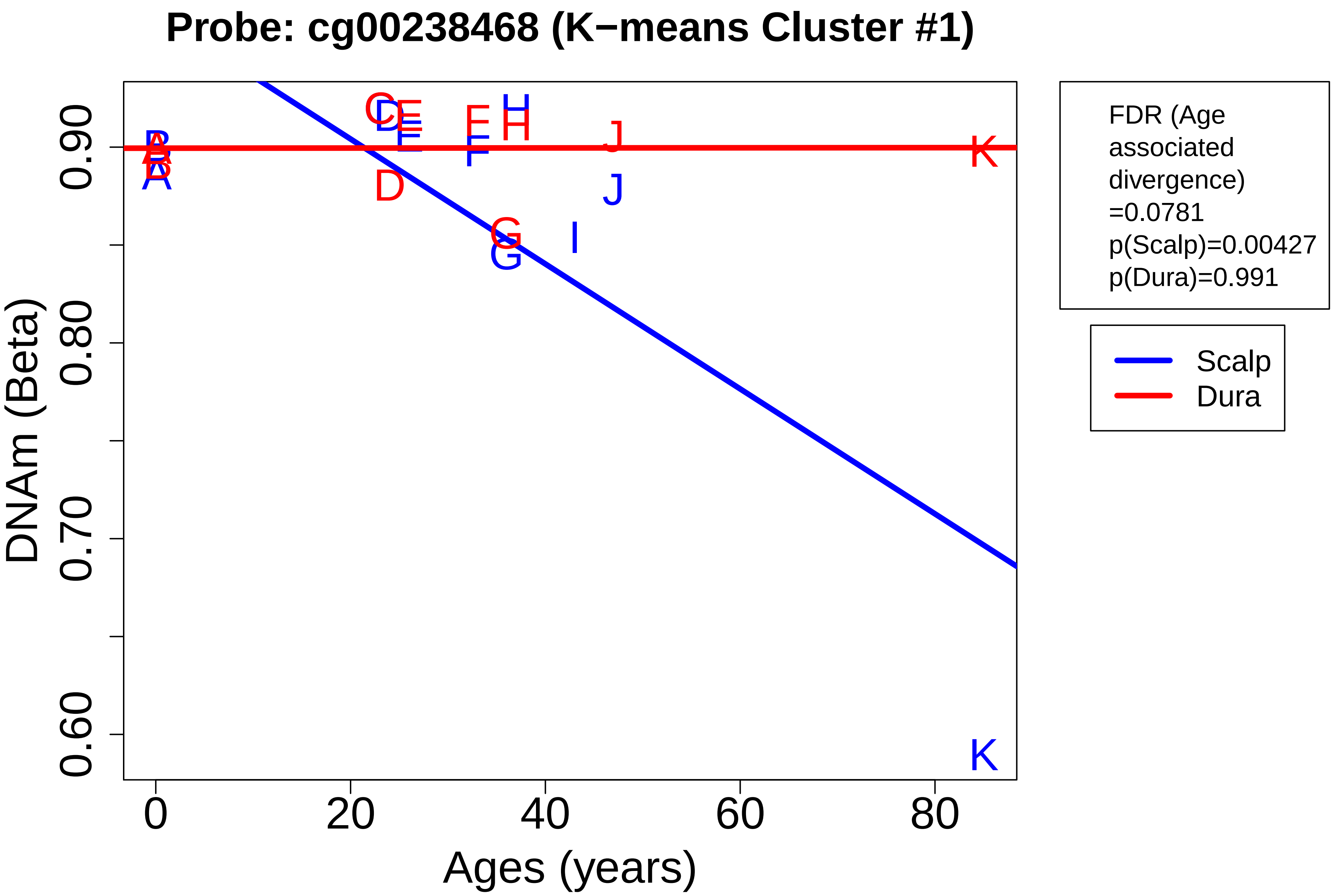
Probe: cg00238468 (K−means Cluster #1)
DNAm (Beta)
0.60
0.70
0.80
0.90
0
20
40
60
80
Ages (years)
FDR (Age associated divergence) =0.0781
p(Scalp)=0.00427
p(Dura)=0.991
Scalp
Dura
A
B
C
D
E
F
G
H
I
J
K

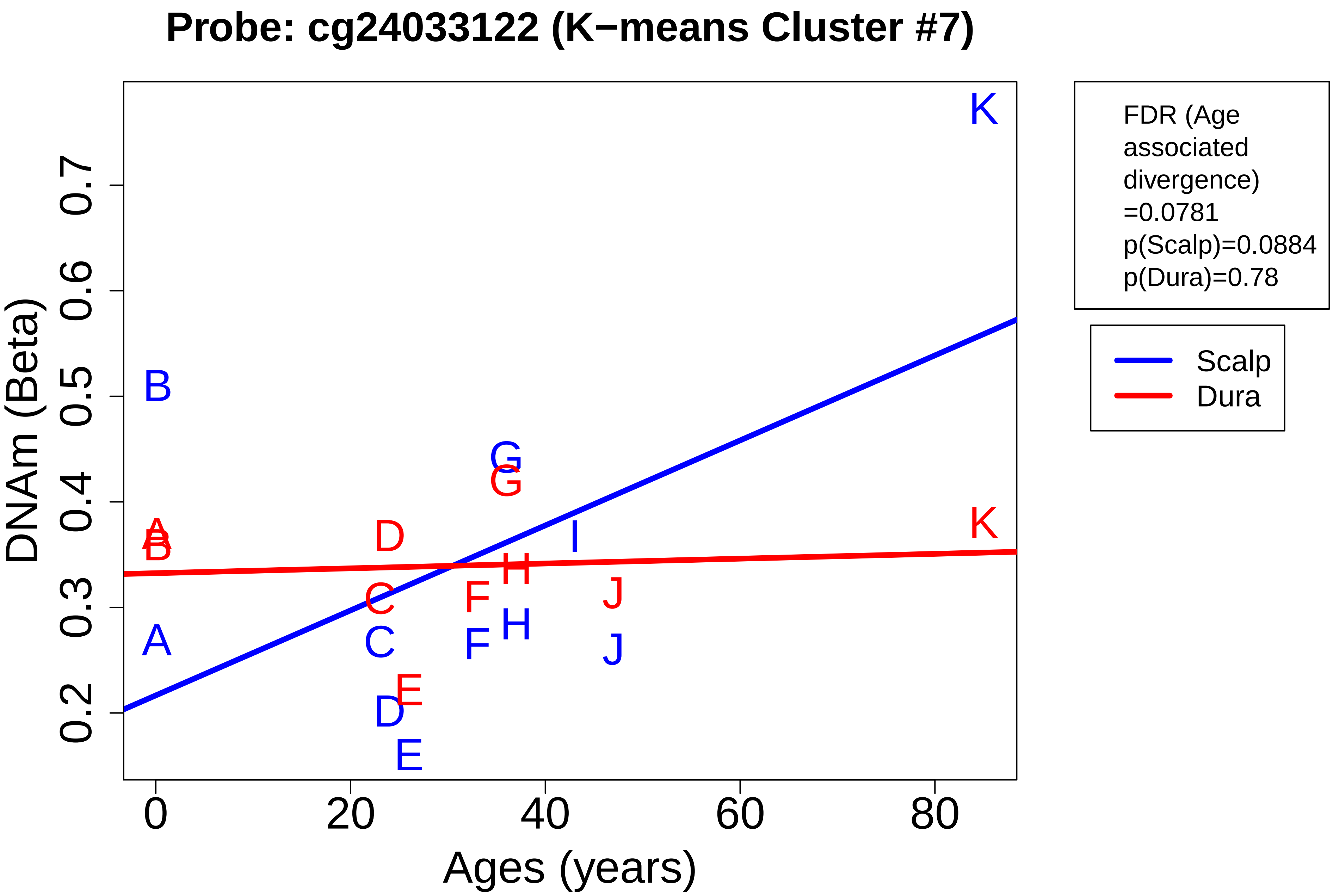
Probe: cg24033122 (K−means Cluster #7)
DNAm (Beta)
Ages (years)
0.2
0.3
0.4
0.5
0.6
0.7
0
20
40
60
80
FDR (Age associated divergence) =0.0781
p(Scalp)=0.0884
p(Dura)=0.78
Scalp
Dura
A
B
C
D
E
F
G
H
I
J
K

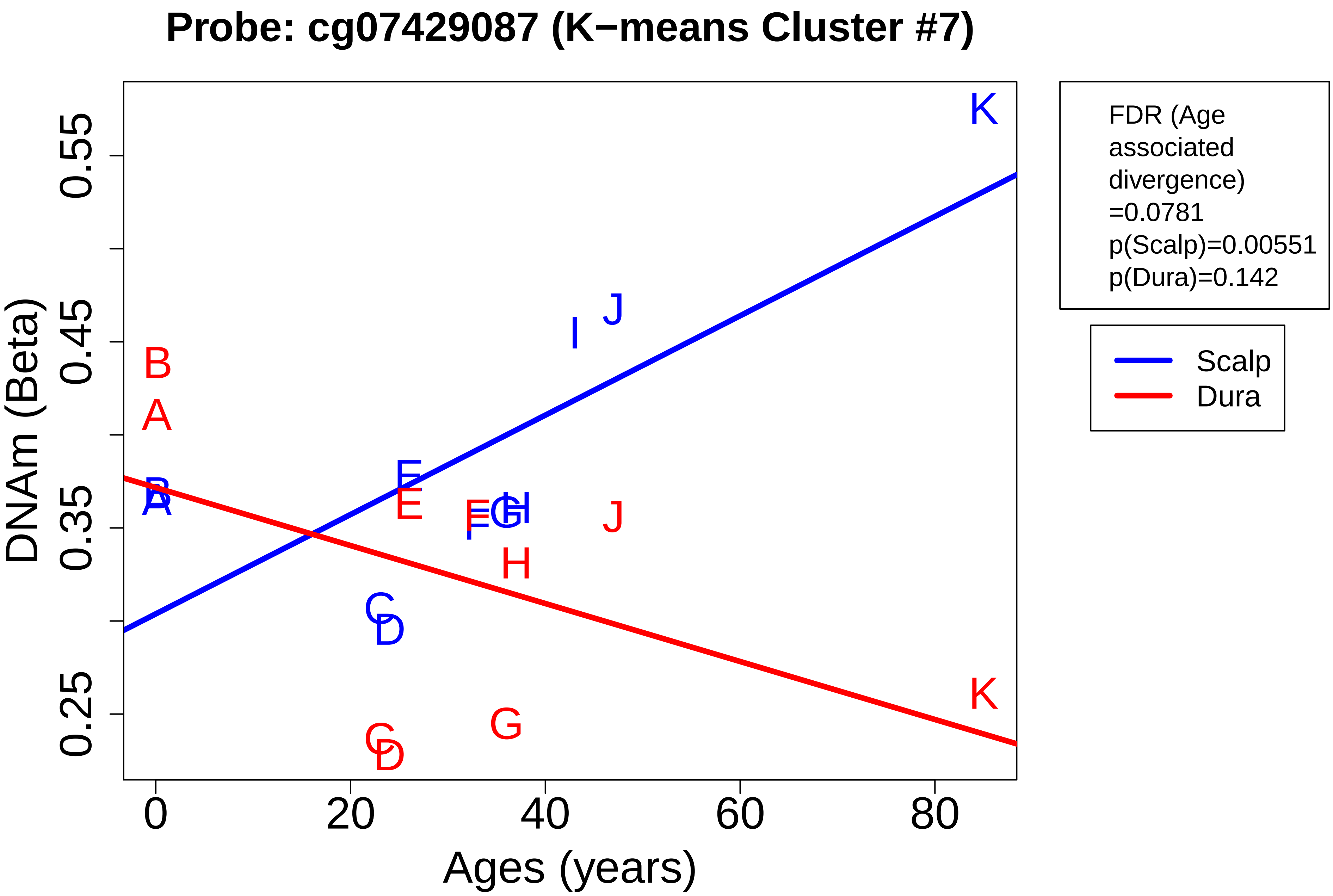
Probe: cg07429087 (K−means Cluster #7)
DNAm (Beta)
0.25
0.35
0.45
0.55
Ages (years)
0
20
40
60
80
FDR (Age associated divergence) =0.0781
p(Scalp)=0.00551
p(Dura)=0.142
Scalp
Dura
A
B
C
D
E
F
G
H
I
J
K

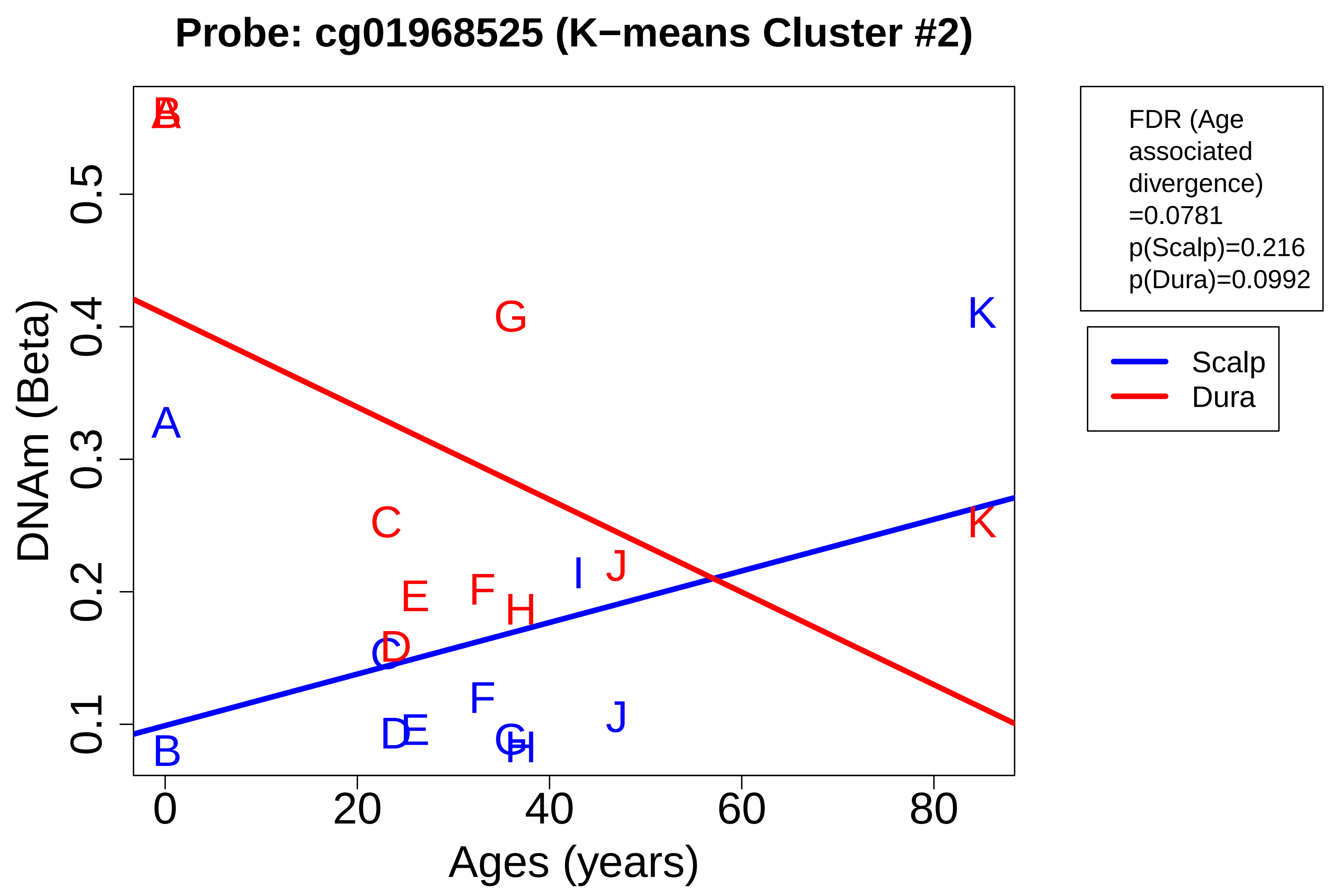

Probe: cg01968525 (K−means Cluster #2)
DNAm (Beta)
0.1
0.2
0.3
0.4
0.5
Ages (years)
0
20
40
60
80
FDR (Age associated divergence) =0.0781
p(Scalp)=0.216
p(Dura)=0.0992
Scalp
Dura

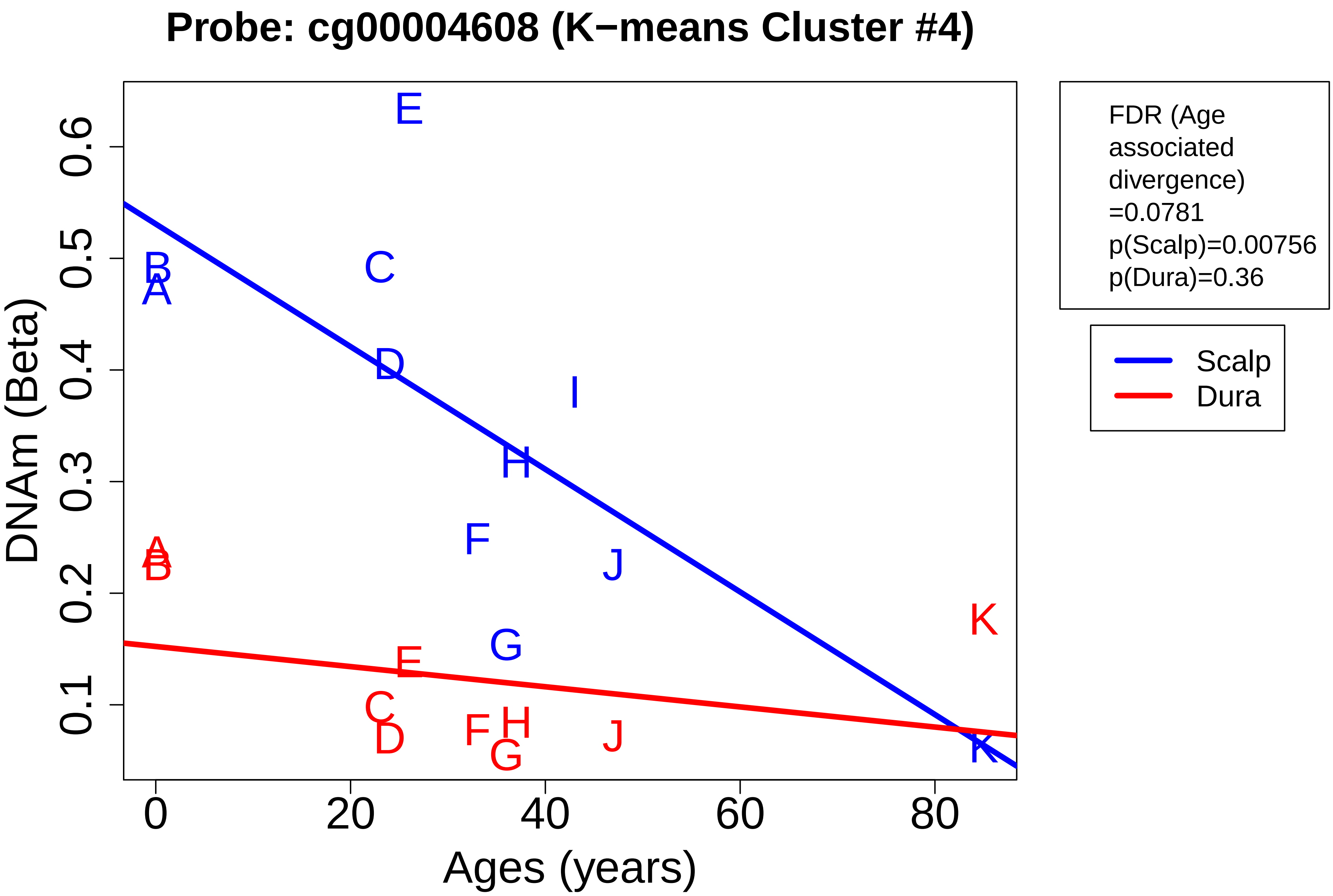

Probe: cg00004608 (K−means Cluster #4)
DNAm (Beta)
0.1
0.2
0.3
0.4
0.5
0.6
0
20
40
60
80
Ages (years)
E
B
A
C
D
I
H
F
J
A
B
K
G
E
C
D
F
H
G
J
K
FDR (Age associated divergence) =0.0781
p(Scalp)=0.00756
p(Dura)=0.36
Scalp
Dura

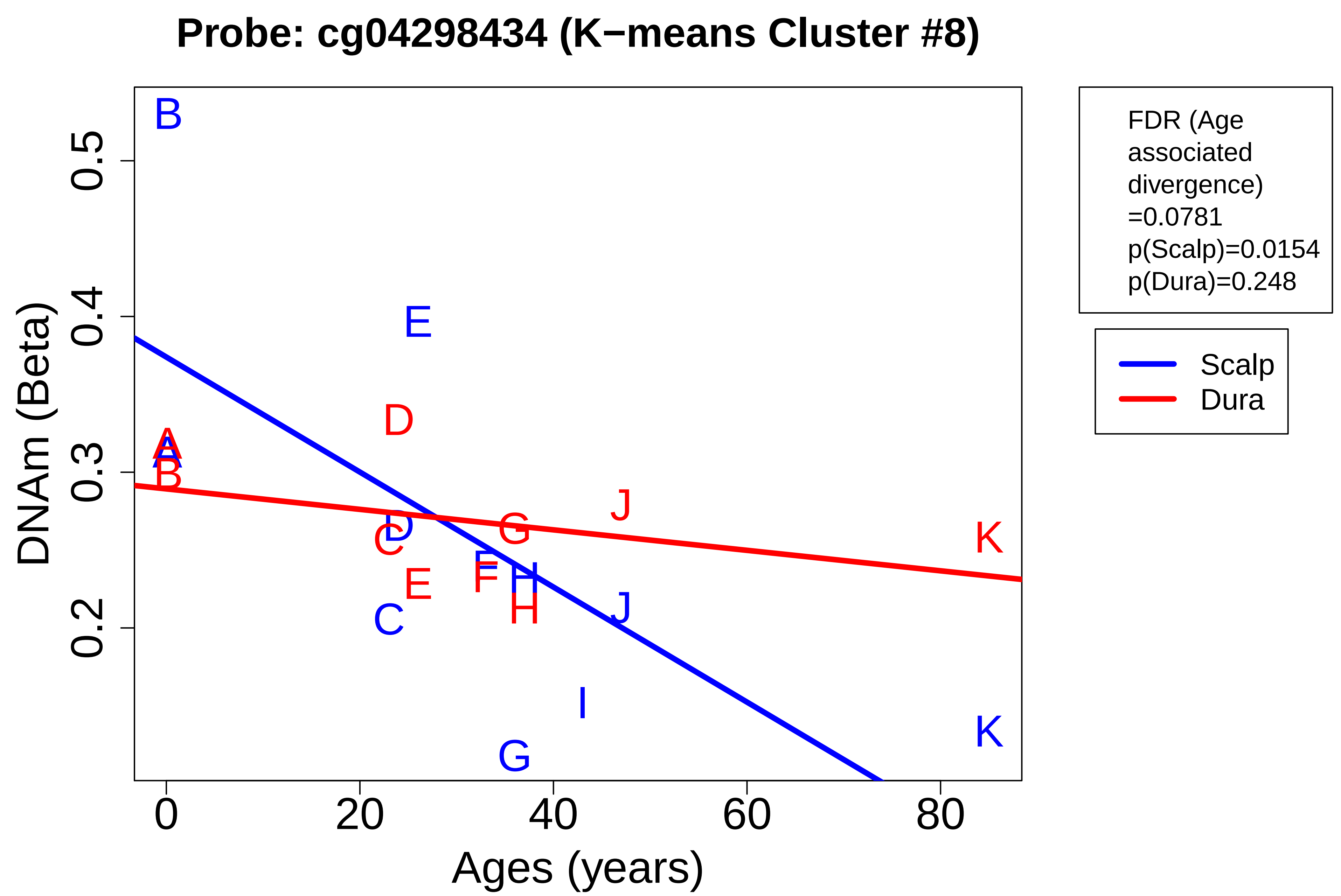

Probe: cg04298434 (K−means Cluster #8)
DNAm (Beta)
0.2
0.3
0.4
0.5
Ages (years)
0
20
40
60
80
FDR (Age associated divergence) =0.0781
p(Scalp)=0.0154
p(Dura)=0.248
Scalp
Dura

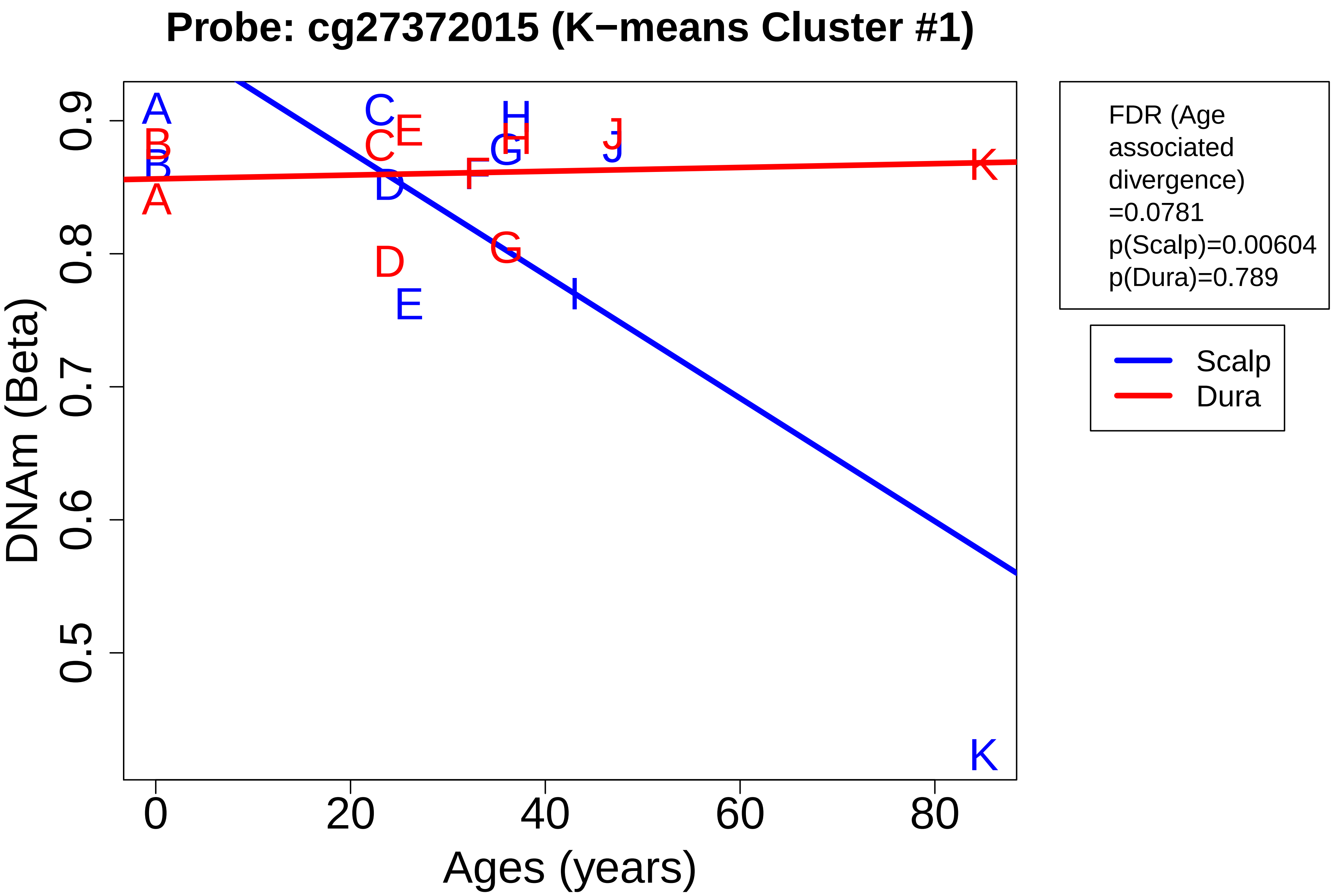

Probe: cg27372015 (K−means Cluster #1)
DNAm (Beta)
0.5
0.6
0.7
0.8
0.9
0
20
40
60
80
Ages (years)
A
B
C
D
E
F
G
H
I
J
K
FDR (Age associated divergence) =0.0781
p(Scalp)=0.00604
p(Dura)=0.789
Scalp
Dura

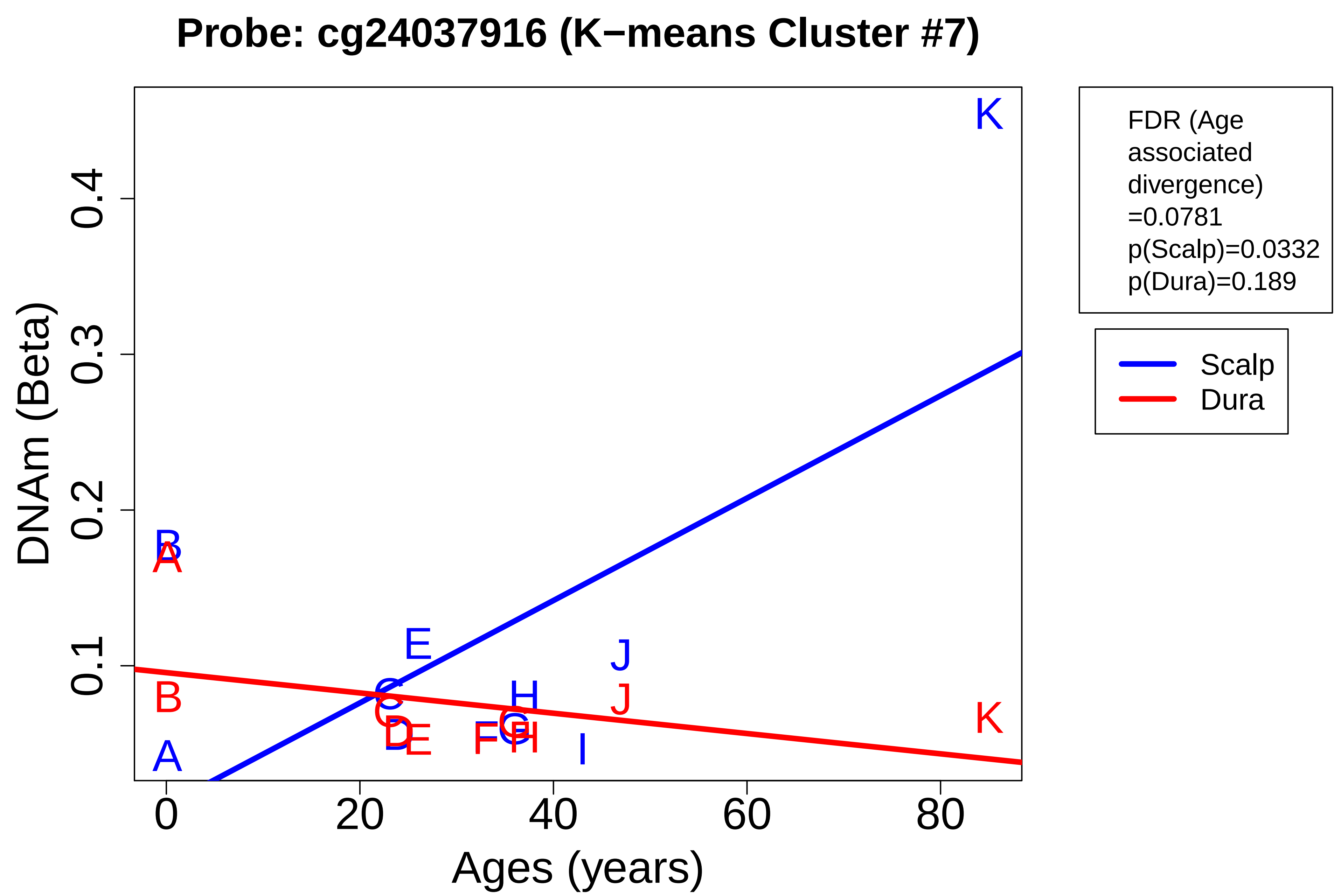

Probe: cg24037916 (K−means Cluster #7)
FDR (Age associated divergence) =0.0781
p(Scalp)=0.0332
p(Dura)=0.189
Scalp
Dura
DNAm (Beta)
0.1
0.2
0.3
0.4
0
20
40
60
80
Ages (years)

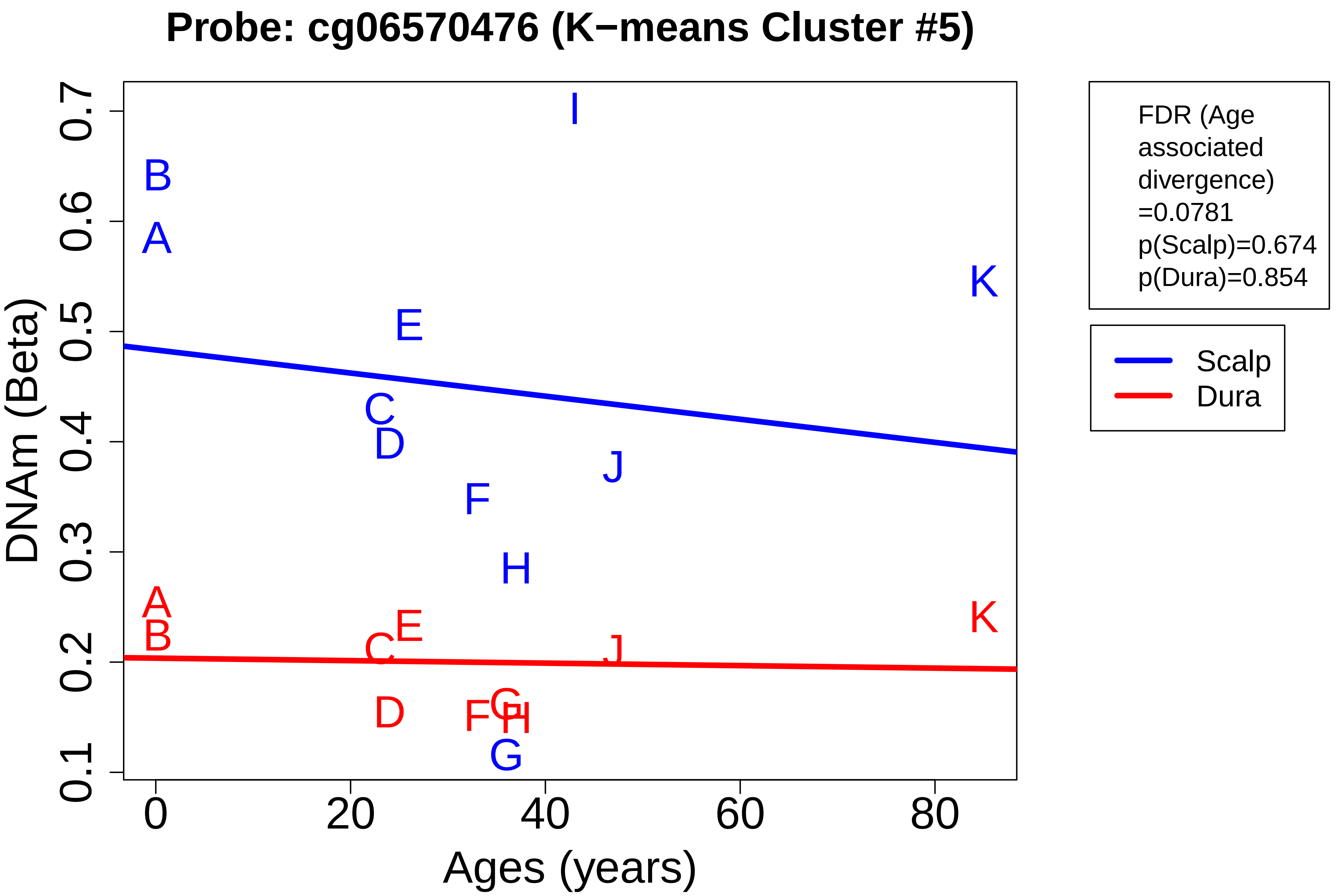

Probe: cg06570476 (K−means Cluster #5)
DNAm (Beta)
Ages (years)
0.1
0.2
0.3
0.4
0.5
0.6
0.7
0
20
40
60
80
FDR (Age associated divergence) =0.0781
p(Scalp)=0.674
p(Dura)=0.854
Scalp
Dura

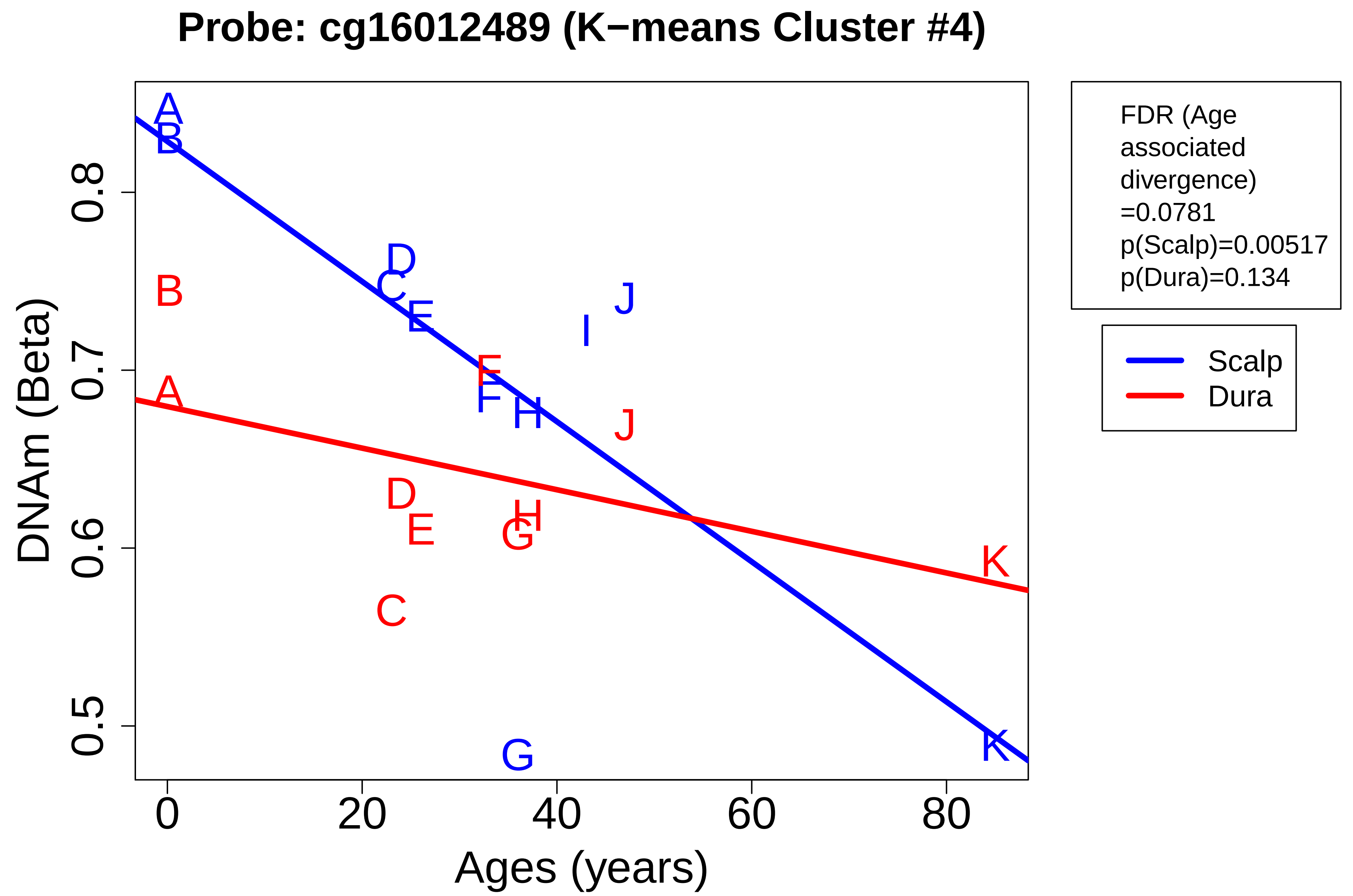

Probe: cg16012489 (K−means Cluster #4)
DNAm (Beta)
0.5
0.6
0.7
0.8
0
20
40
60
80
Ages (years)
A
B
C
D
E
F
G
H
I
J
K
FDR (Age associated divergence) =0.0781
p(Scalp)=0.00517
p(Dura)=0.134
Scalp
Dura

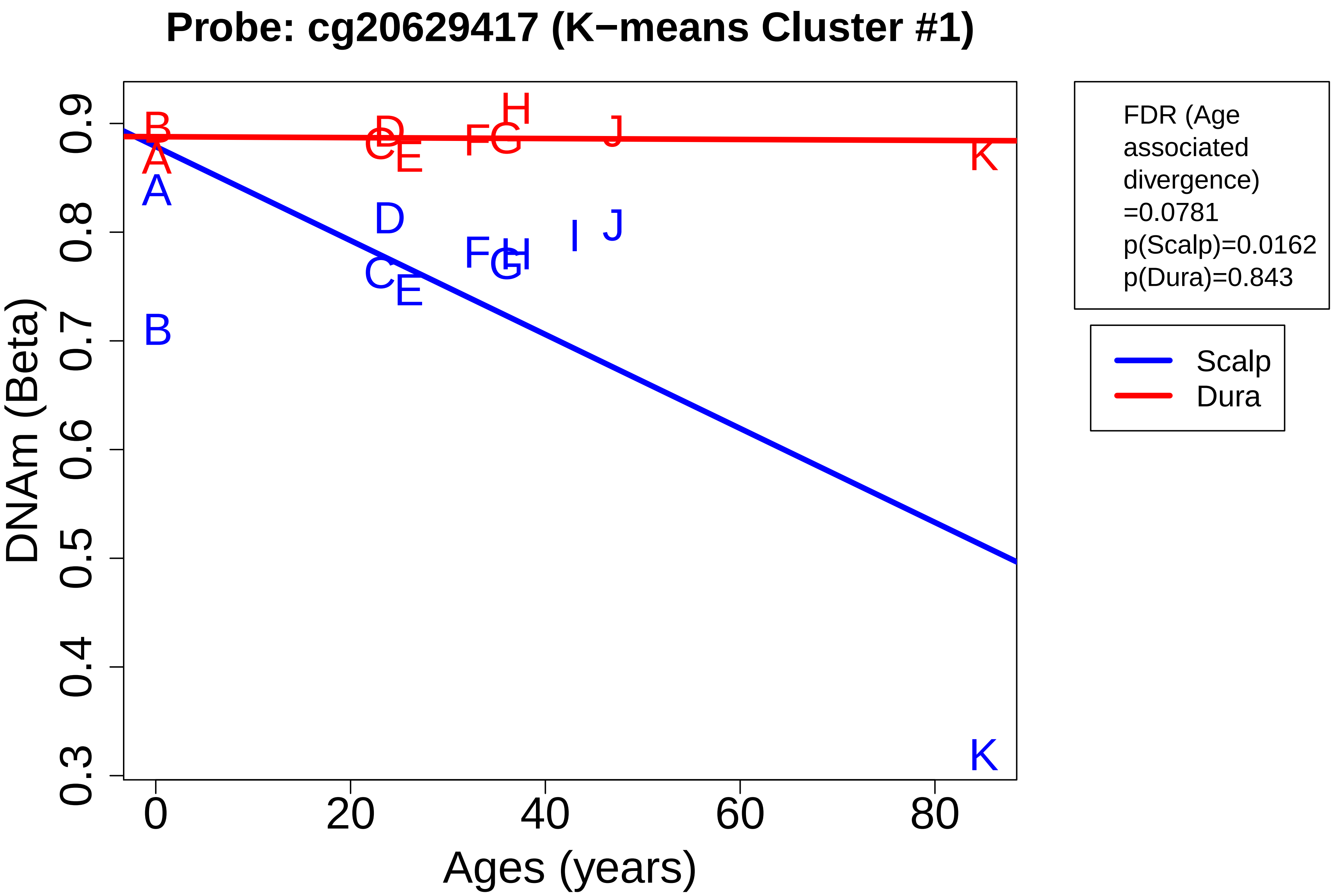

Probe: cg20629417 (K−means Cluster #1)
DNAm (Beta)
0.3
0.4
0.5
0.6
0.7
0.8
0.9
0
20
40
60
80
Ages (years)
FDR (Age associated divergence) =0.0781
p(Scalp)=0.0162
p(Dura)=0.843
Scalp
Dura

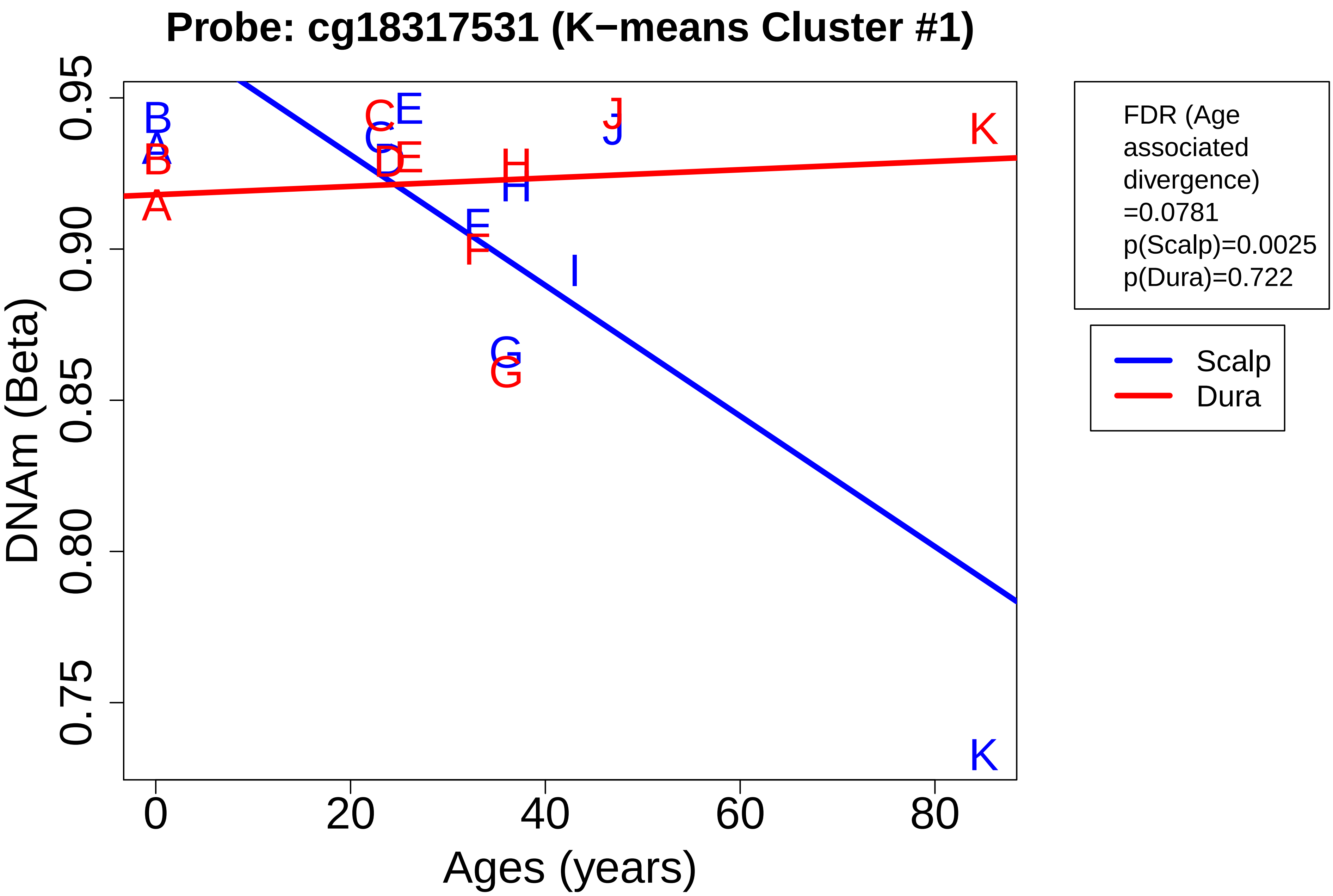

Probe: cg18317531 (K−means Cluster #1)
DNAm (Beta)
0.75
0.80
0.85
0.90
0.95
0
20
40
60
80
Ages (years)
FDR (Age associated divergence) =0.0781
p(Scalp)=0.0025
p(Dura)=0.722
Scalp
Dura

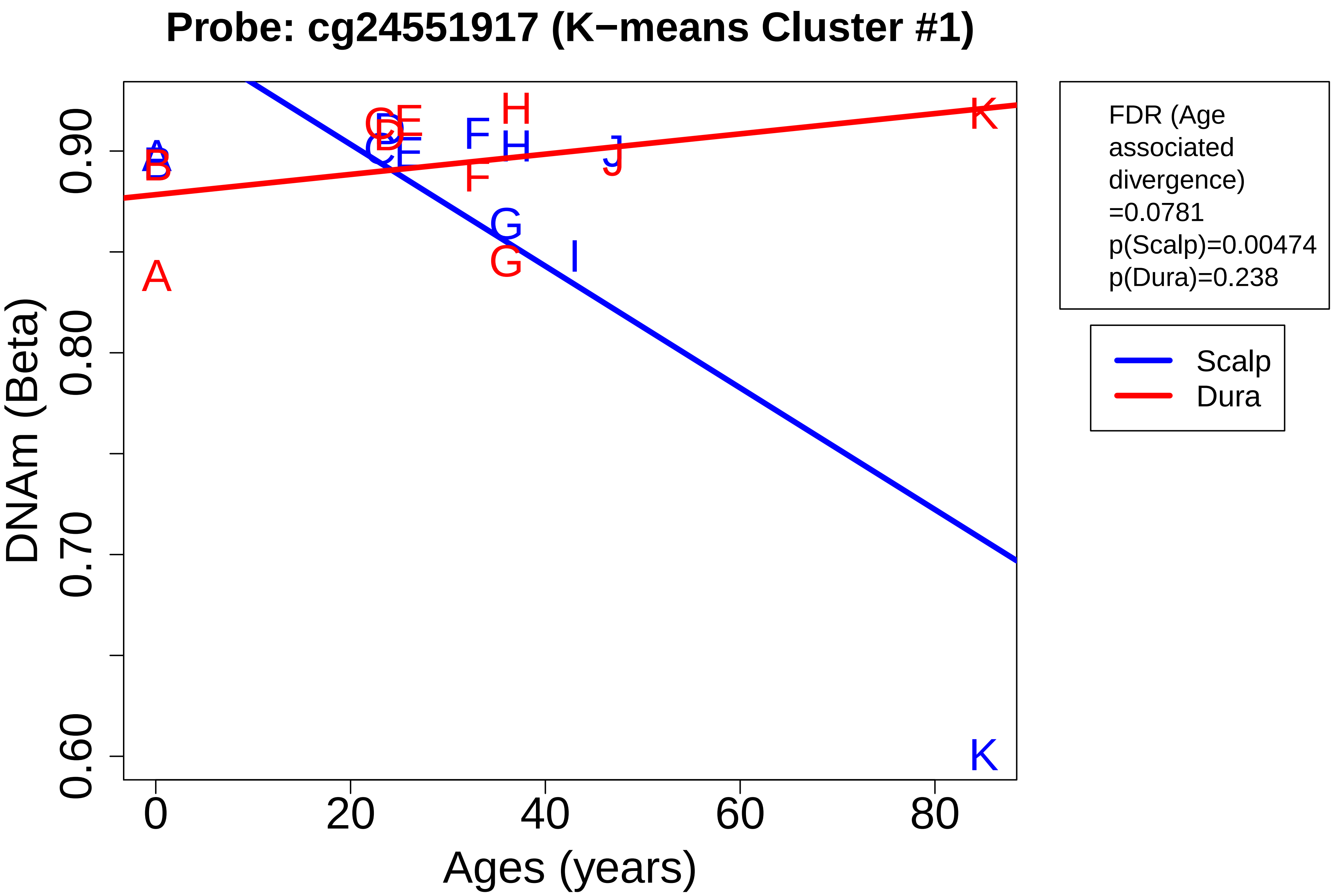

Probe: cg24551917 (K−means Cluster #1)
DNAm (Beta)
0.60
0.70
0.80
0.90
Ages (years)
0
20
40
60
80
FDR (Age associated divergence) =0.0781
p(Scalp)=0.00474
p(Dura)=0.238
Scalp
Dura

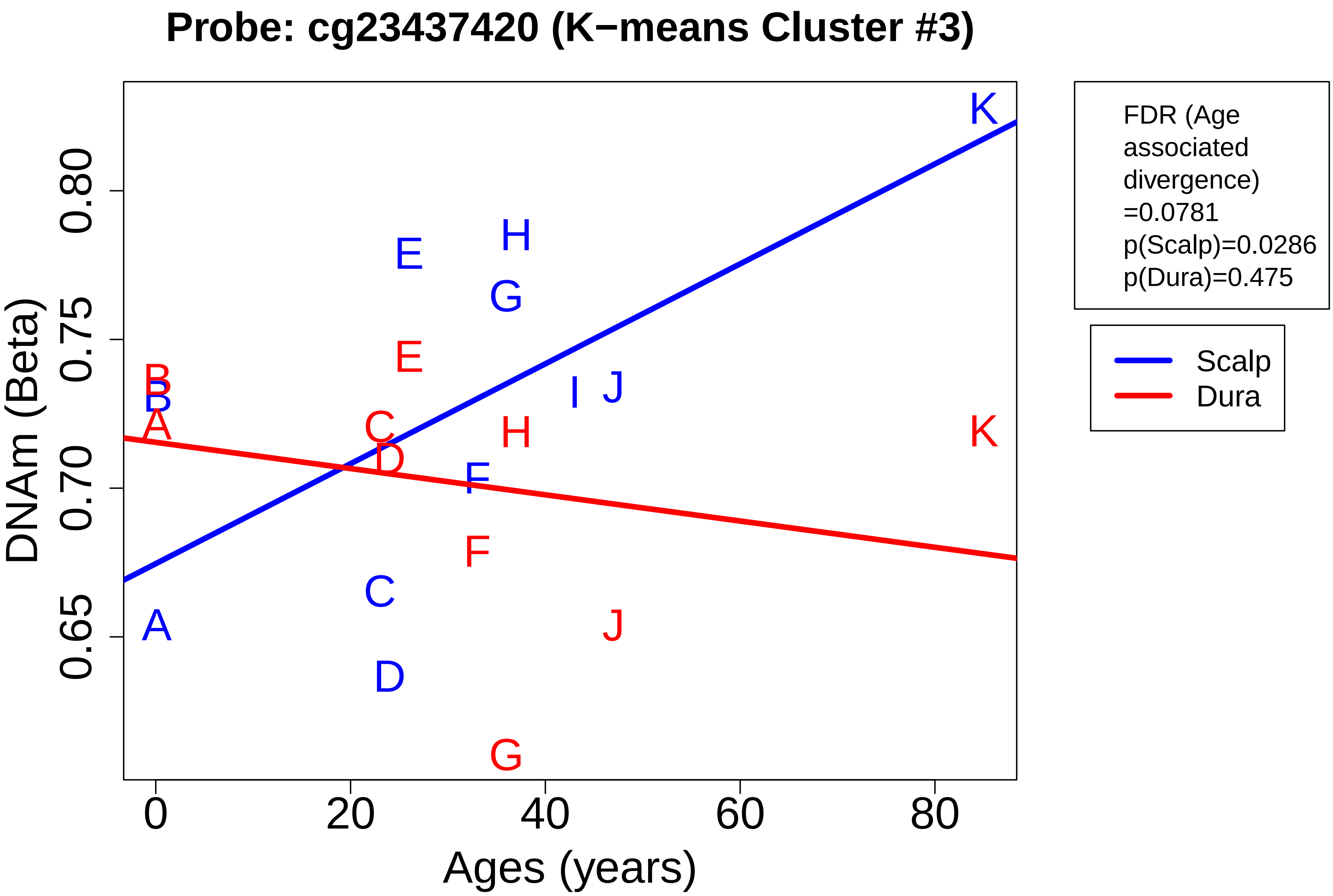

Probe: cg23437420 (K−means Cluster #3)
DNAm (Beta)
0.65
0.70
0.75
0.80
0
20
40
60
80
Ages (years)
FDR (Age associated divergence) =0.0781
p(Scalp)=0.0286
p(Dura)=0.475
Scalp
Dura

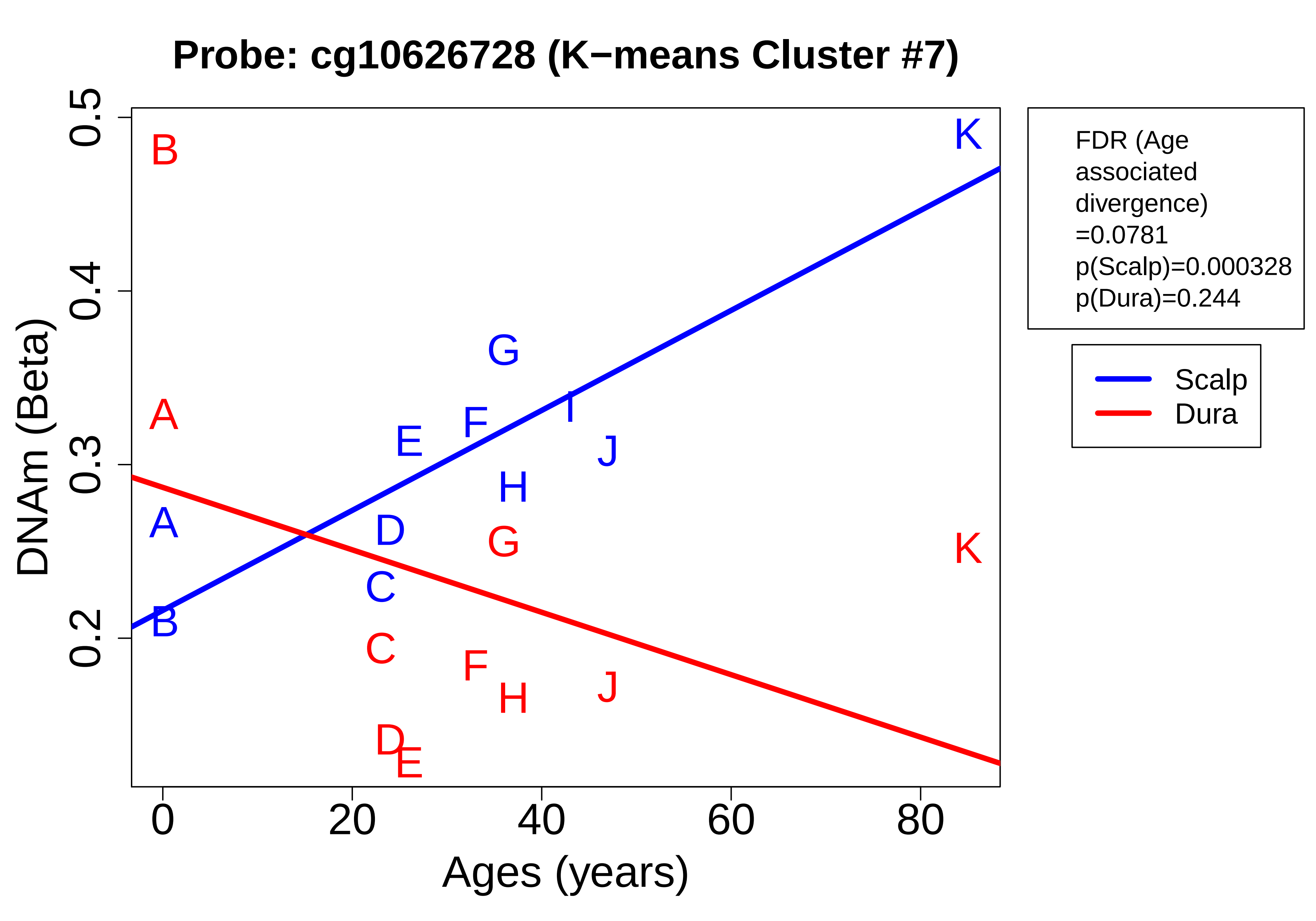
Probe: cg10626728 (K−means Cluster #7)
DNAm (Beta)
0.2
0.3
0.4
0.5
0
20
40
60
80
Ages (years)
FDR (Age associated divergence) =0.0781
p(Scalp)=0.000328
p(Dura)=0.244
Scalp
Dura
A
B
C
D
E
F
G
H
I
J
K

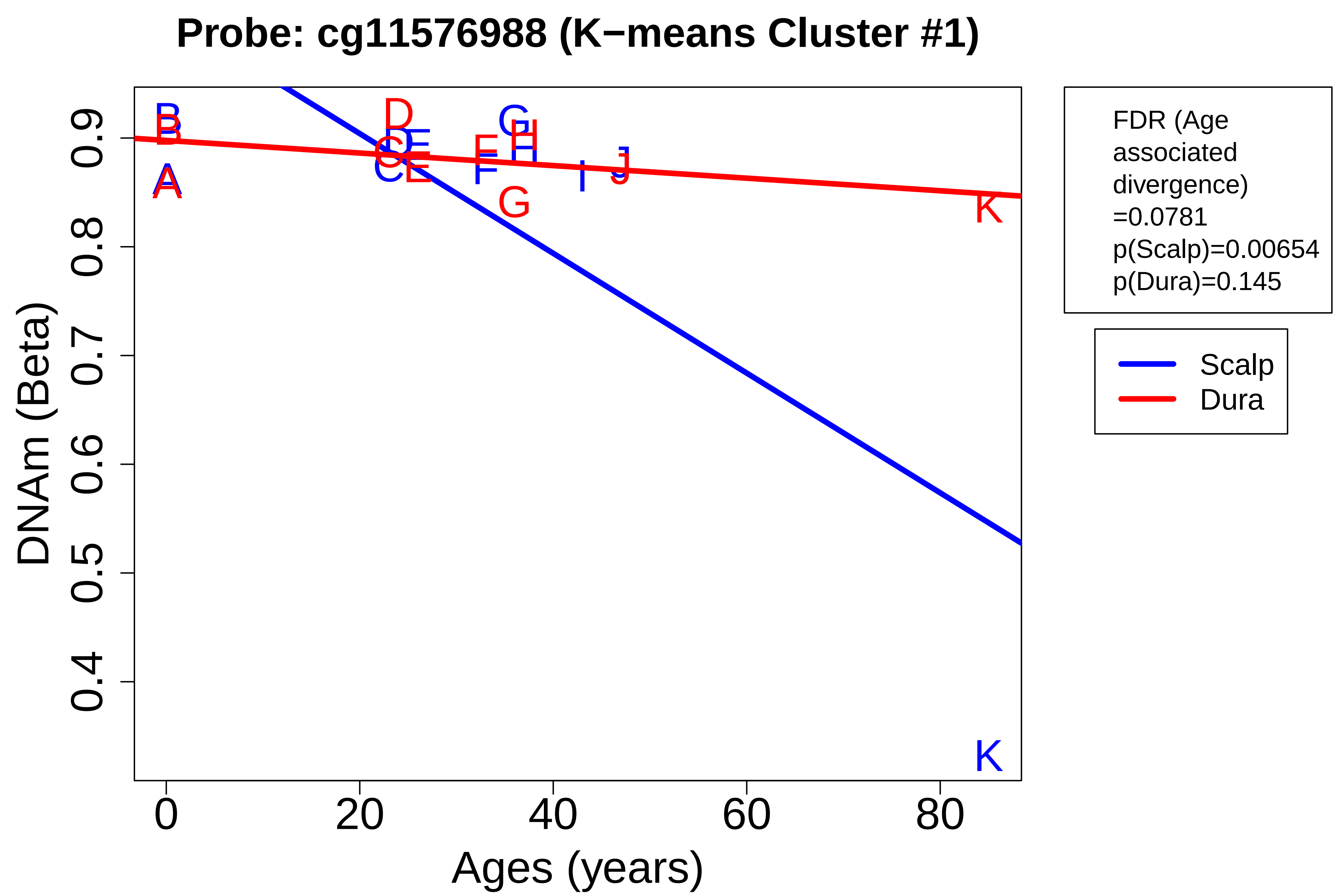

Probe: cg11576988 (K−means Cluster #1)
DNAm (Beta)
0.4
0.5
0.6
0.7
0.8
0.9
0
20
40
60
80
Ages (years)
FDR (Age associated divergence) =0.0781
p(Scalp)=0.00654
p(Dura)=0.145
Scalp
Dura

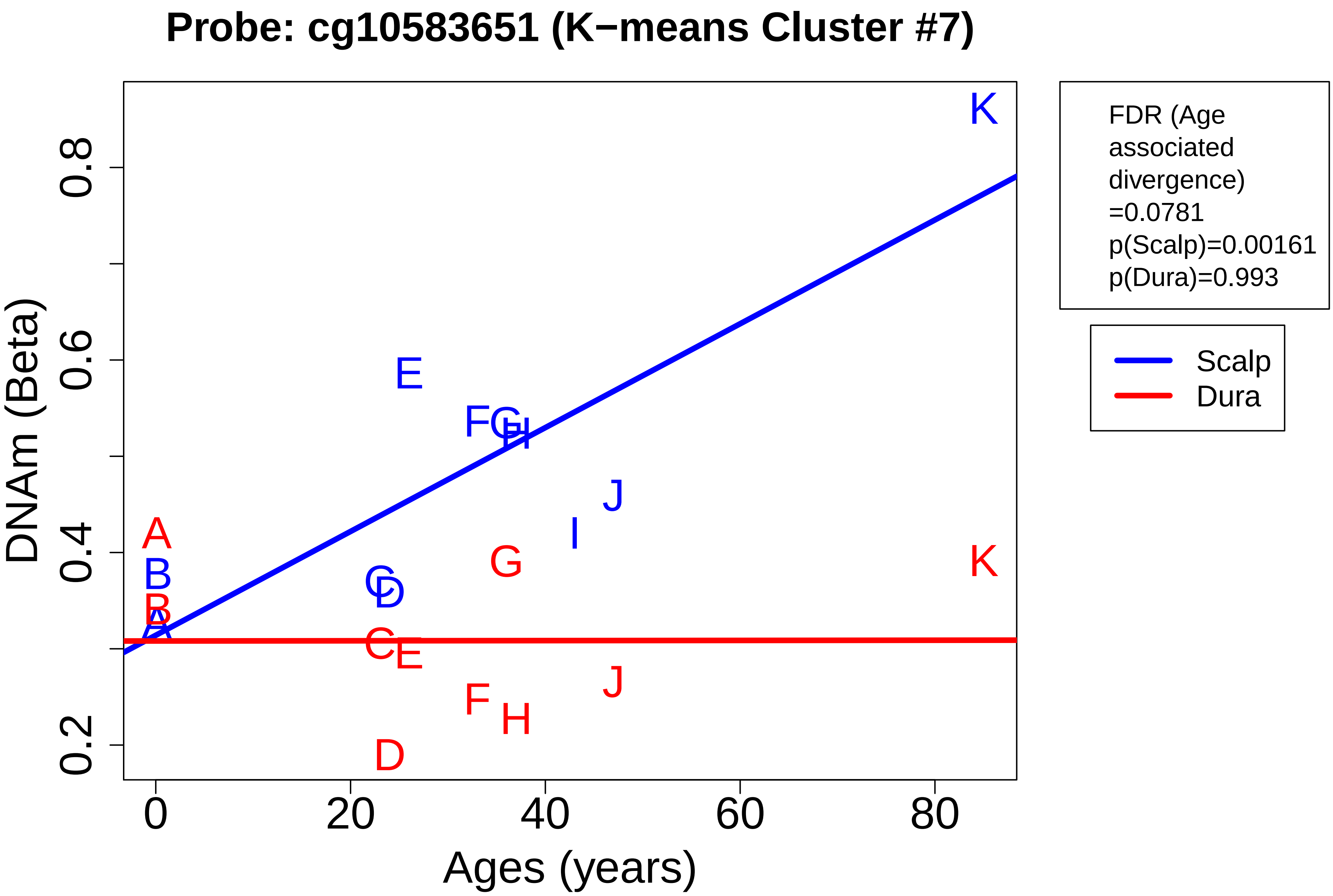

Probe: cg10583651 (K−means Cluster #7)
DNAm (Beta)
0.2
0.4
0.6
0.8
0
20
40
60
80
Ages (years)
FDR (Age associated divergence) =0.0781
p(Scalp)=0.00161
p(Dura)=0.993
Scalp
Dura
A
B
C
D
E
F
G
H
I
J
K

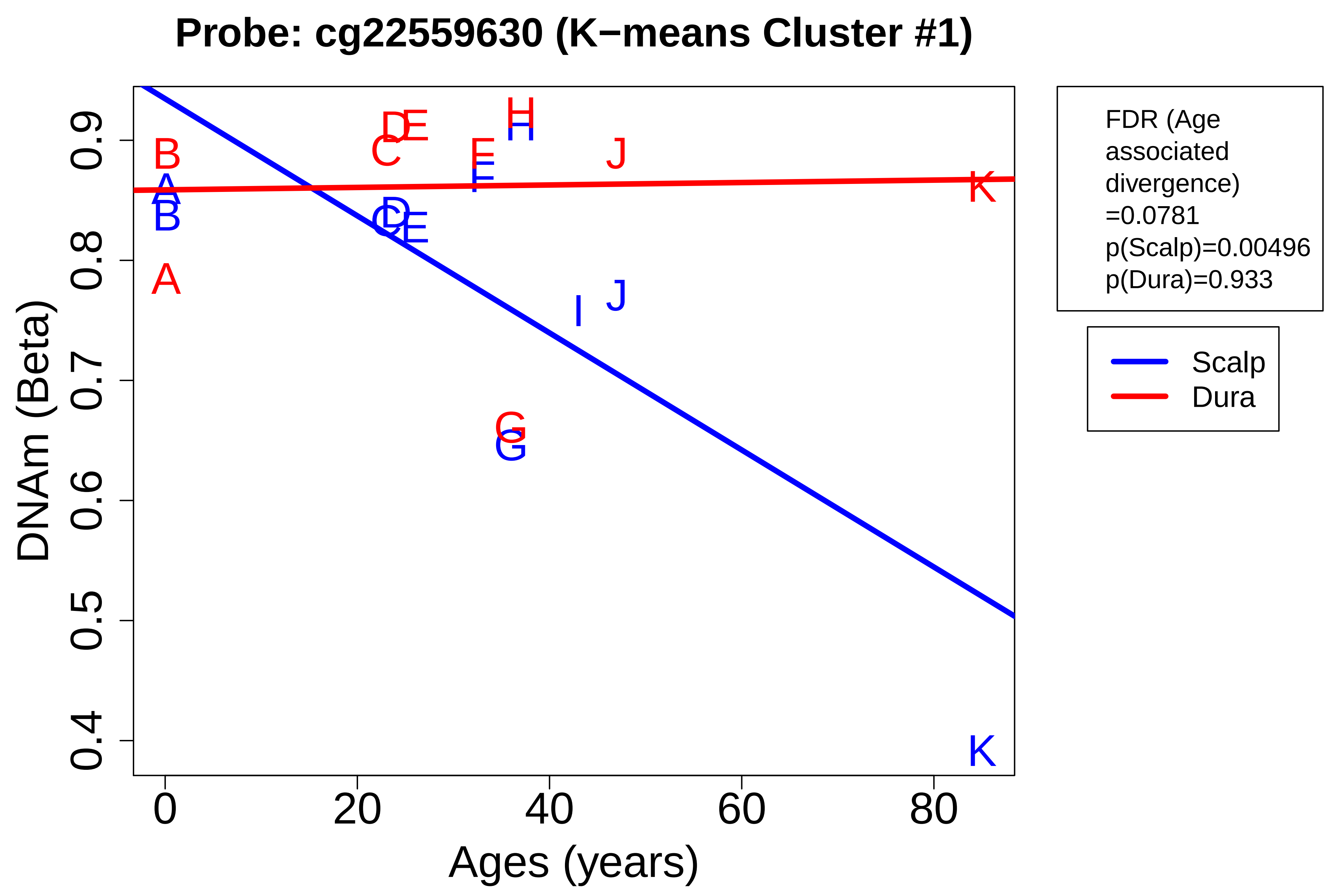

Probe: cg22559630 (K−means Cluster #1)
DNAm (Beta)
0.4
0.5
0.6
0.7
0.8
0.9
0
20
40
60
80
Ages (years)
FDR (Age associated divergence) =0.0781
p(Scalp)=0.00496
p(Dura)=0.933
Scalp
Dura

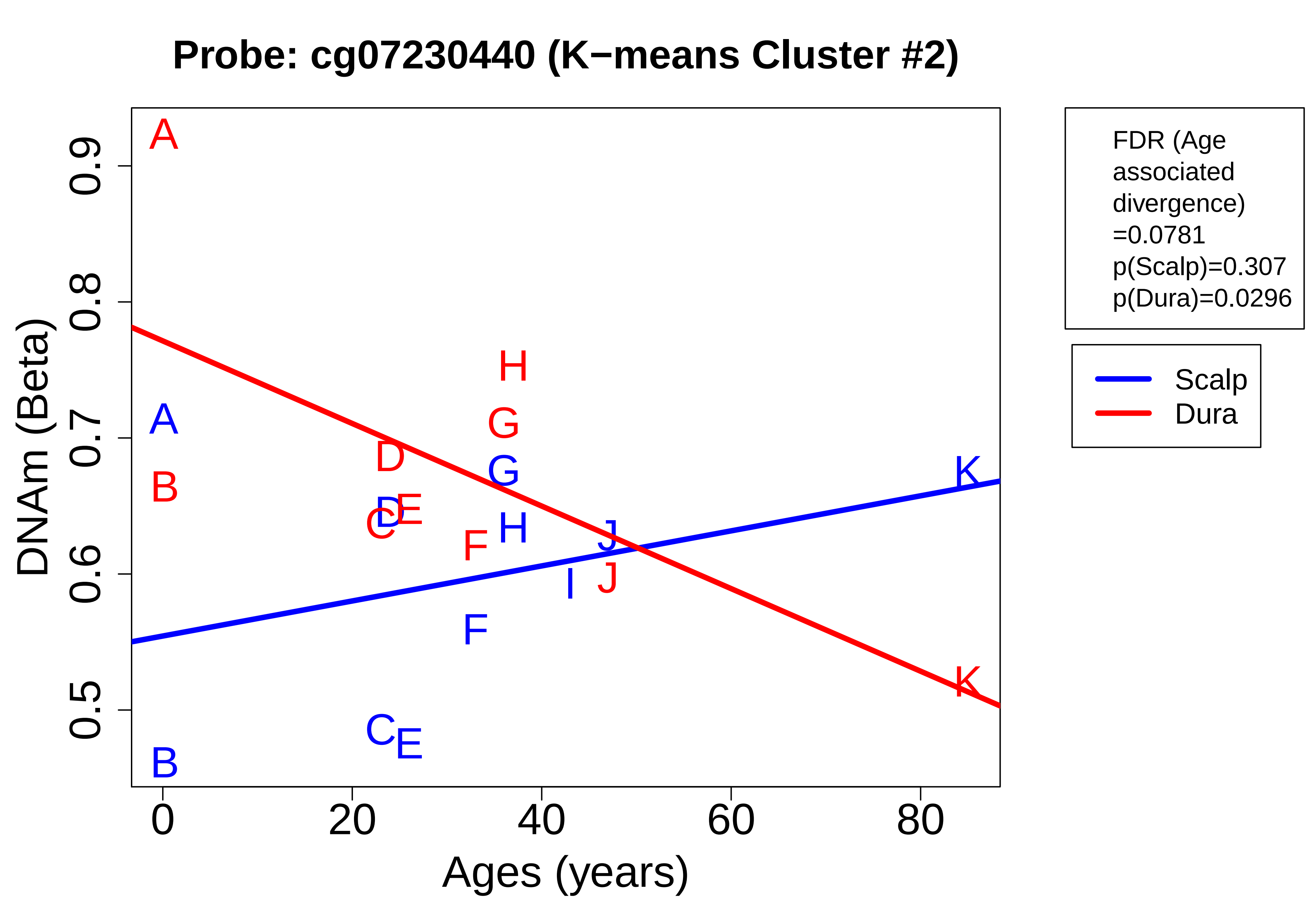

Probe: cg07230440 (K−means Cluster #2)
DNAm (Beta)
0.5
0.6
0.7
0.8
0.9
Ages (years)
0
20
40
60
80
A
B
C
D
E
F
G
H
I
J
K
FDR (Age associated divergence) =0.0781
p(Scalp)=0.307
p(Dura)=0.0296
Scalp
Dura

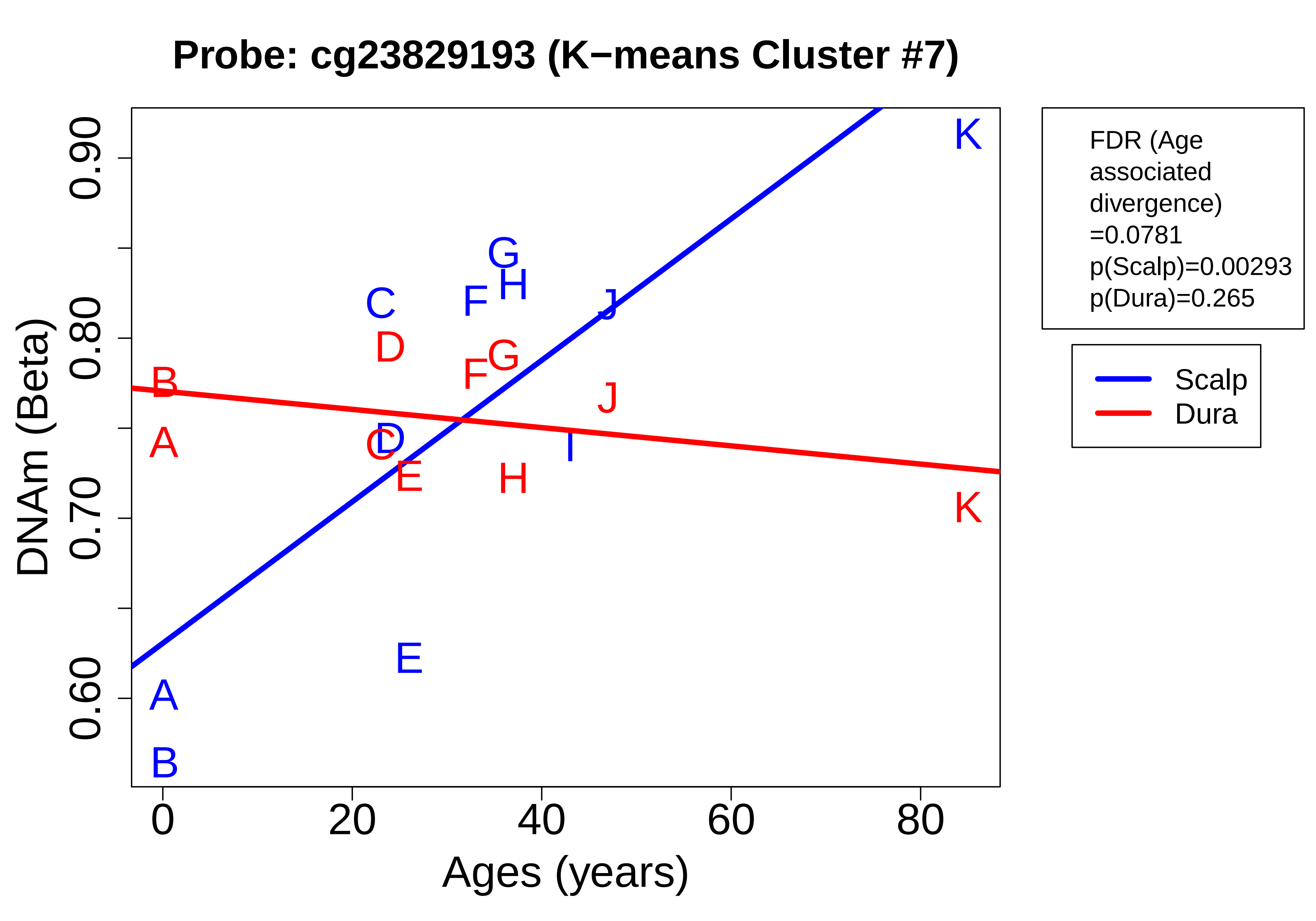

Probe: cg23829193 (K−means Cluster #7)
DNAm (Beta)
0.60
0.70
0.80
0.90
Ages (years)
0
20
40
60
80
A
B
C
D
E
F
G
H
I
J
K
FDR (Age associated divergence) =0.0781
p(Scalp)=0.00293
p(Dura)=0.265
Scalp
Dura

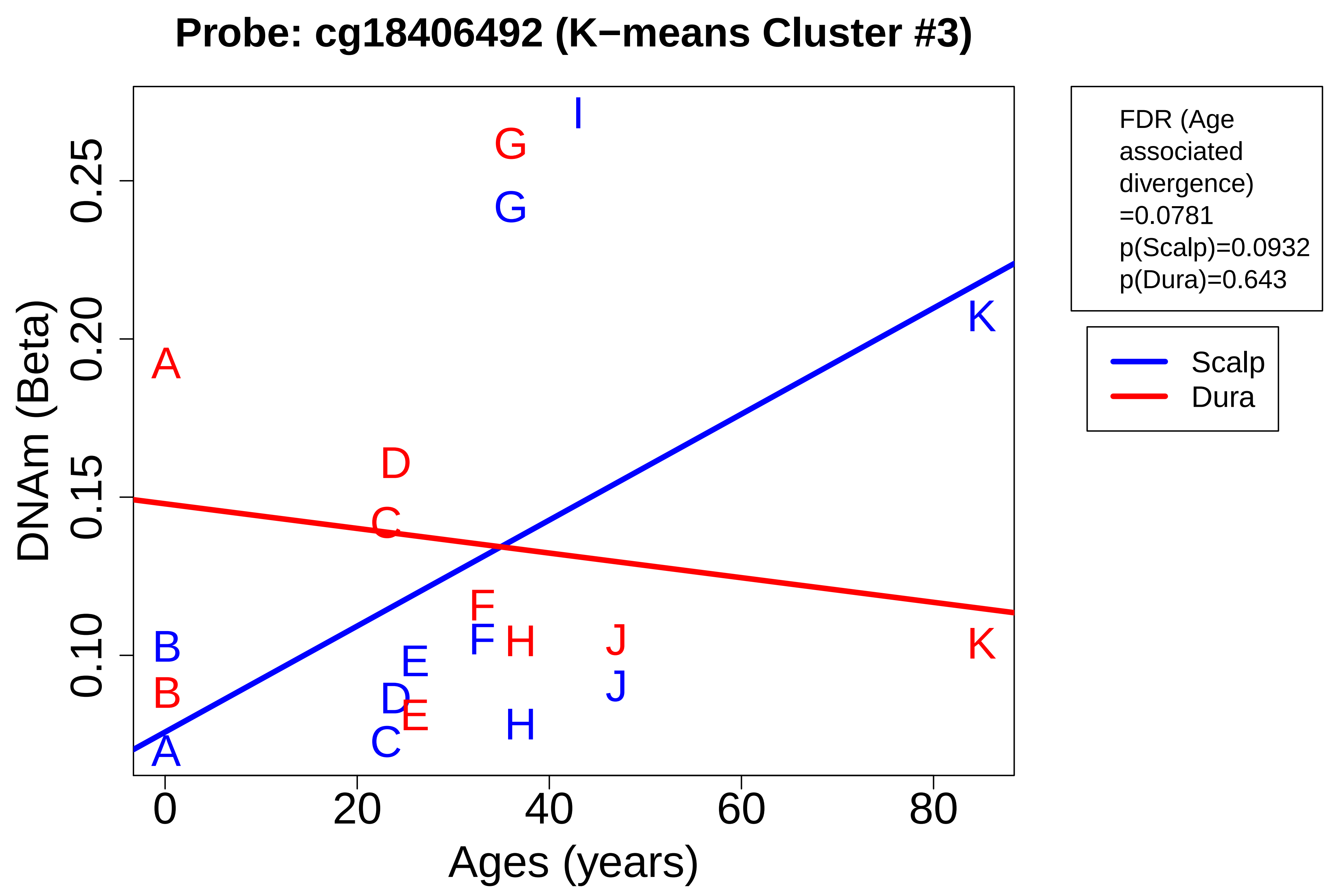
Probe: cg18406492 (K−means Cluster #3)
DNAm (Beta)
0.10
0.15
0.20
0.25
0
20
40
60
80
Ages (years)
A
B
C
D
E
F
G
H
I
J
K
FDR (Age associated divergence) =0.0781
p(Scalp)=0.0932
p(Dura)=0.643
Scalp
Dura

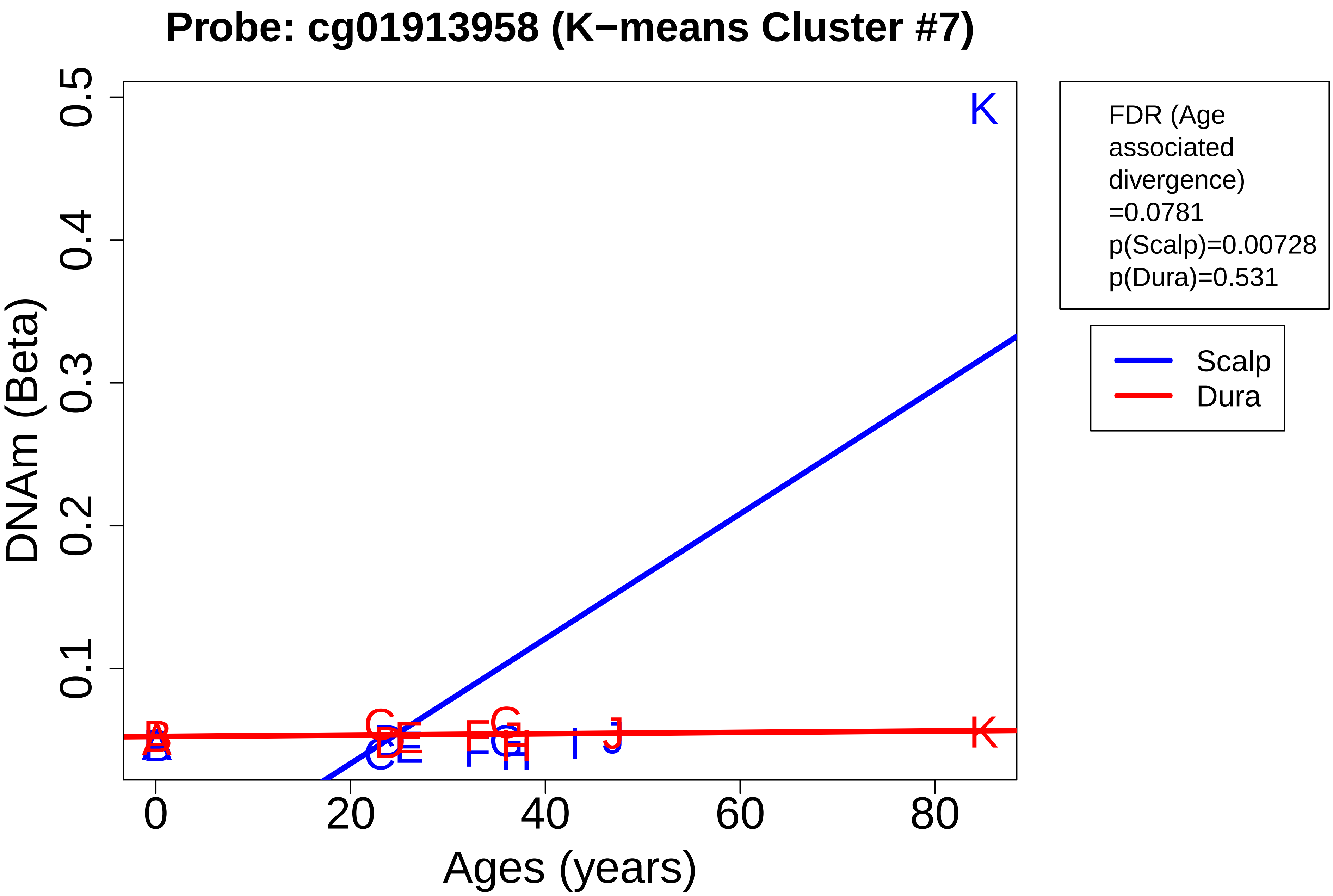

Probe: cg01913958 (K−means Cluster #7)
DNAm (Beta)
0.1
0.2
0.3
0.4
0.5
Ages (years)
0
20
40
60
80
FDR (Age associated divergence) =0.0781
p(Scalp)=0.00728
p(Dura)=0.531
Scalp
Dura

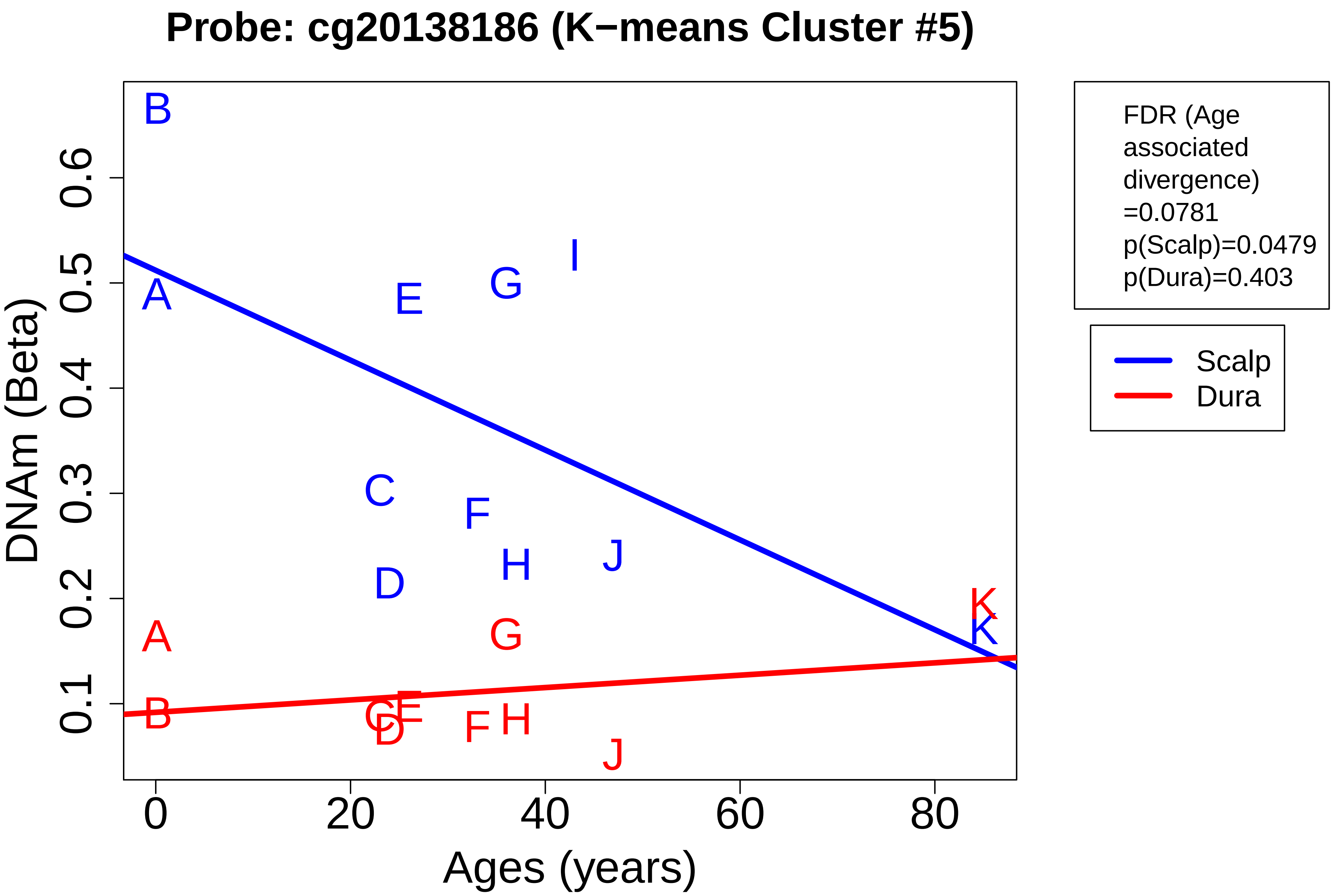

Probe: cg20138186 (K−means Cluster #5)
DNAm (Beta)
0.1
0.2
0.3
0.4
0.5
0.6
0
20
40
60
80
Ages (years)
B
A
E
G
I
C
F
H
J
D
K
A
G
B
C
E
D
F
H
J
K
FDR (Age associated divergence) =0.0781
p(Scalp)=0.0479
p(Dura)=0.403
Scalp
Dura

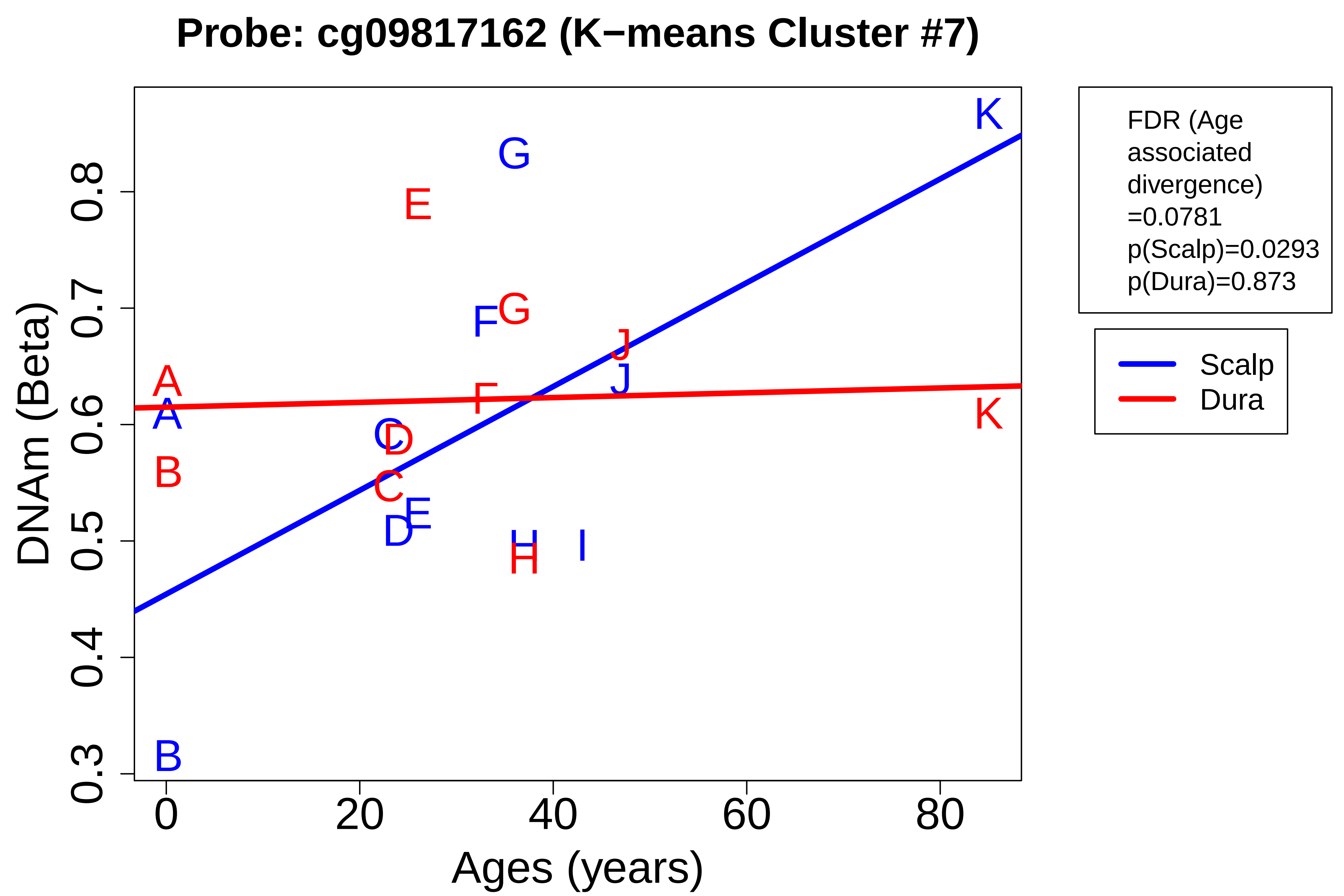

Probe: cg09817162 (K−means Cluster #7)
DNAm (Beta)
0.3
0.4
0.5
0.6
0.7
0.8
0
20
40
60
80
Ages (years)
FDR (Age associated divergence) =0.0781
p(Scalp)=0.0293
p(Dura)=0.873
Scalp
Dura

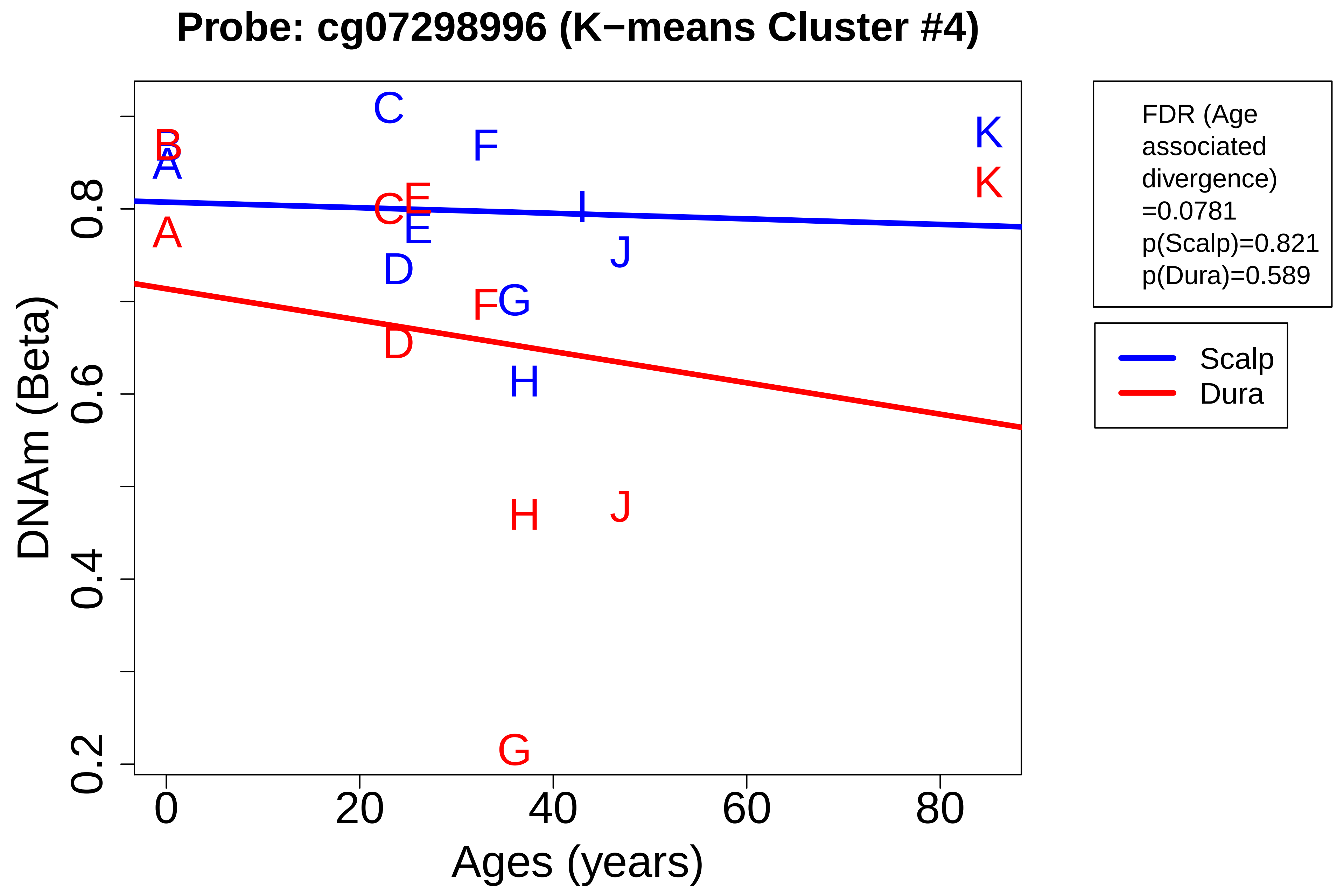

Probe: cg07298996 (K−means Cluster #4)
DNAm (Beta)
0.2
0.4
0.6
0.8
0
20
40
60
80
Ages (years)
FDR (Age associated divergence) =0.0781
p(Scalp)=0.821
p(Dura)=0.589
Scalp
Dura

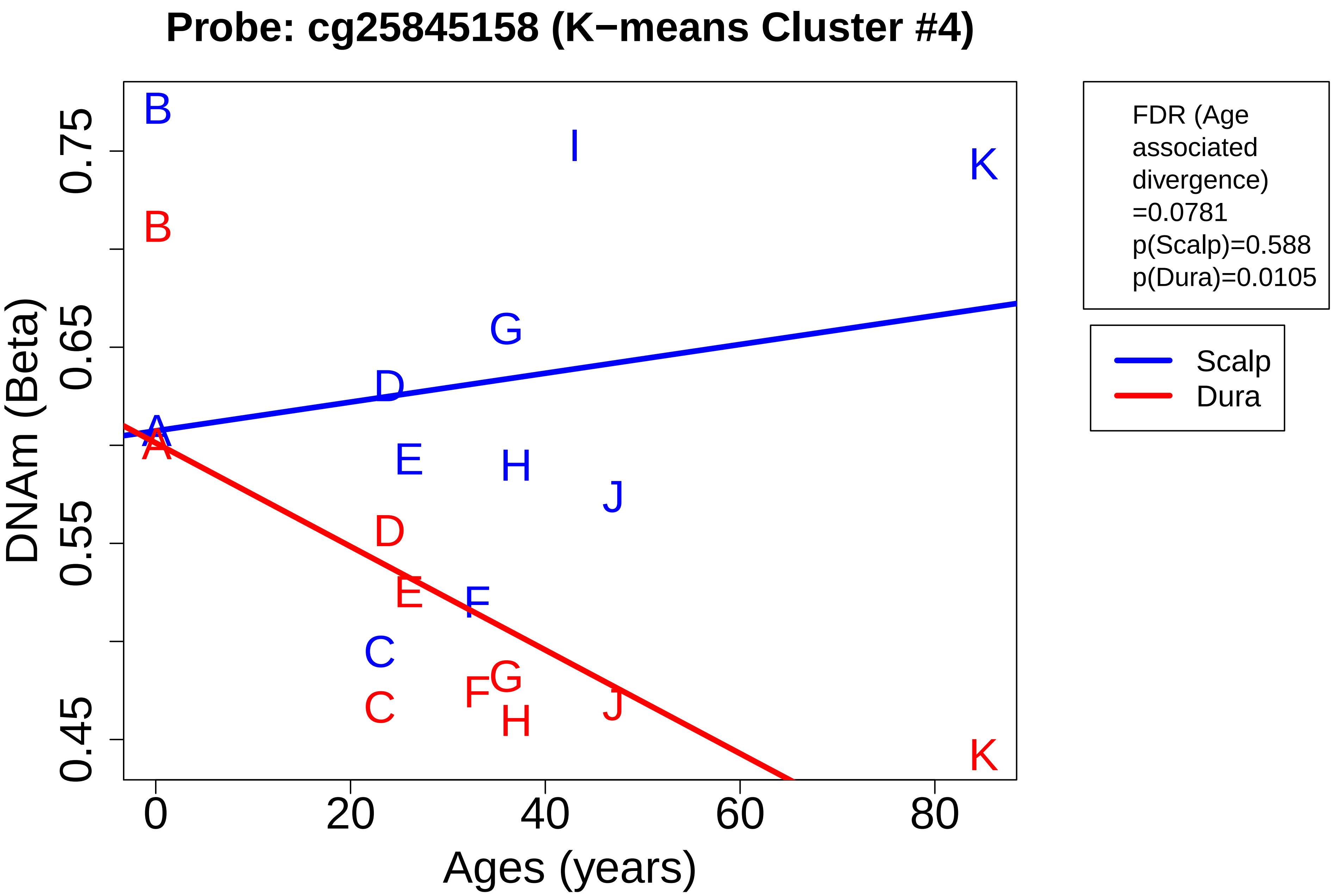

Probe: cg25845158 (K−means Cluster #4)
FDR (Age associated divergence) =0.0781
p(Scalp)=0.588
p(Dura)=0.0105
Scalp
Dura
DNAm (Beta)
0.45
0.55
0.65
0.75
0
20
40
60
80
Ages (years)
A
B
C
D
E
F
G
H
I
J
K

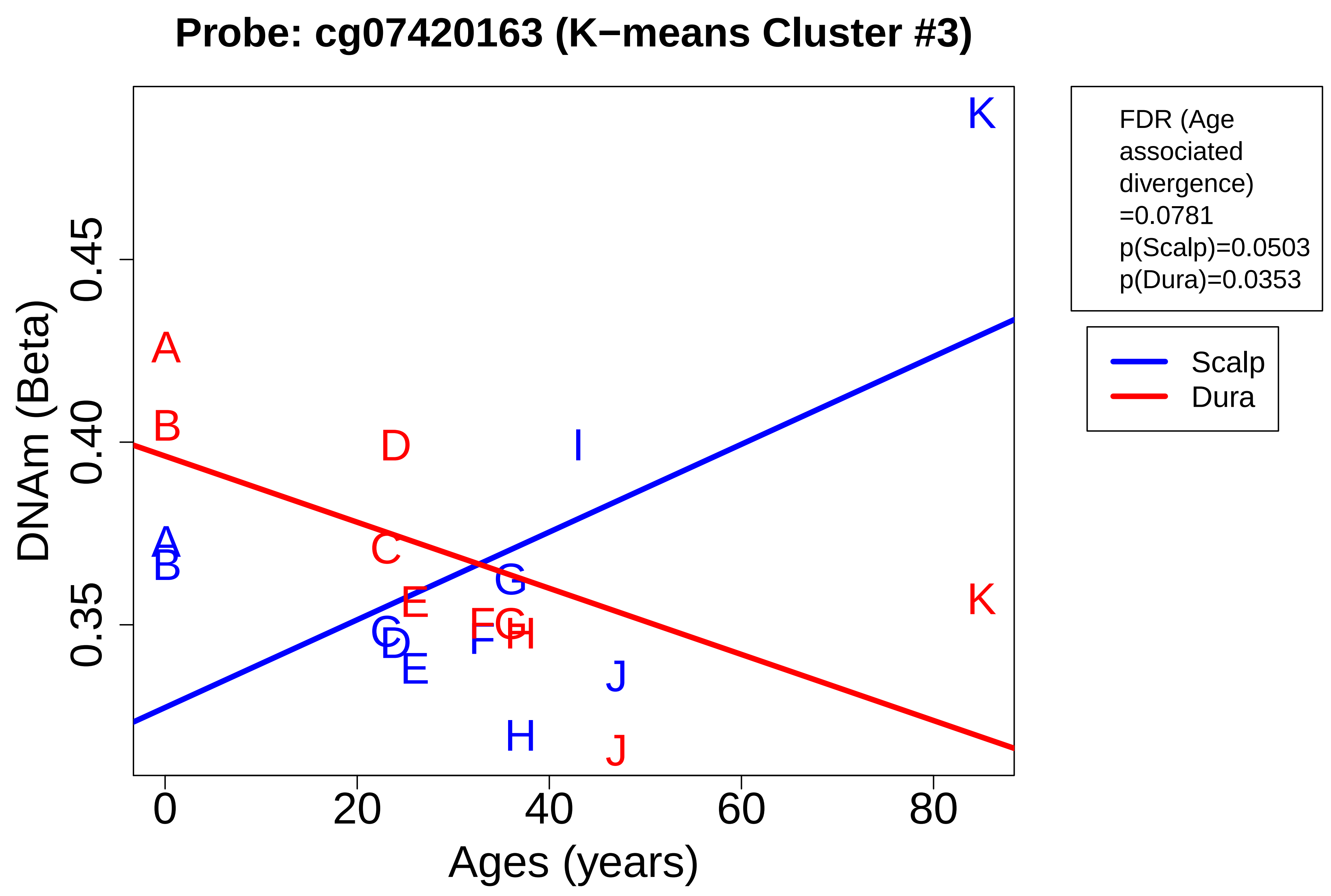

Probe: cg07420163 (K−means Cluster #3)
DNAm (Beta)
0.35
0.40
0.45
0
20
40
60
80
Ages (years)
A
B
C
D
E
F
G
H
I
J
K
FDR (Age associated divergence) =0.0781
p(Scalp)=0.0503
p(Dura)=0.0353
Scalp
Dura

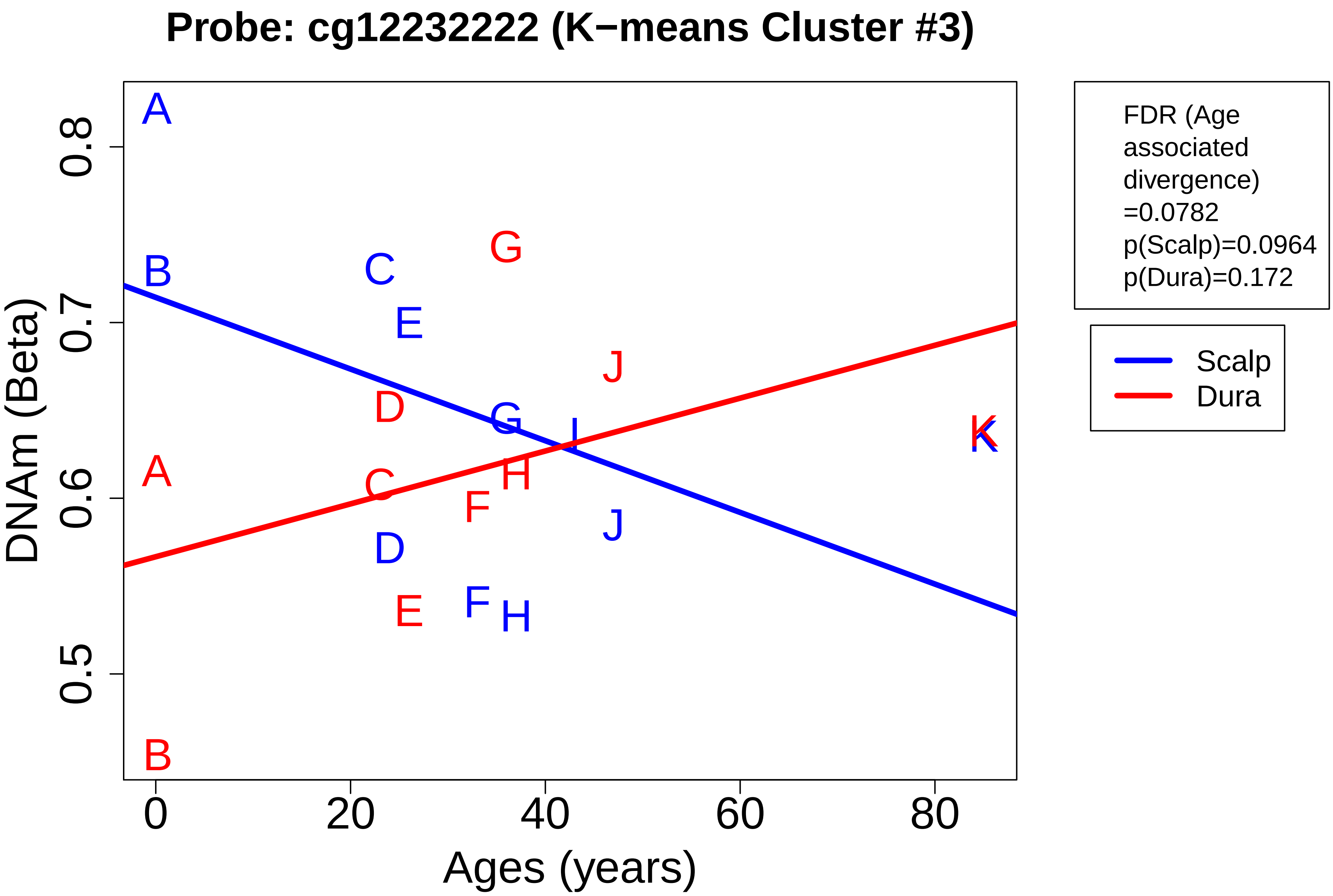
Probe: cg12232222 (K−means Cluster #3)
DNAm (Beta)
0.5
0.6
0.7
0.8
Ages (years)
0
20
40
60
80
A
B
C
D
E
F
G
H
I
J
K
FDR (Age associated divergence) =0.0782
p(Scalp)=0.0964
p(Dura)=0.172
Scalp
Dura

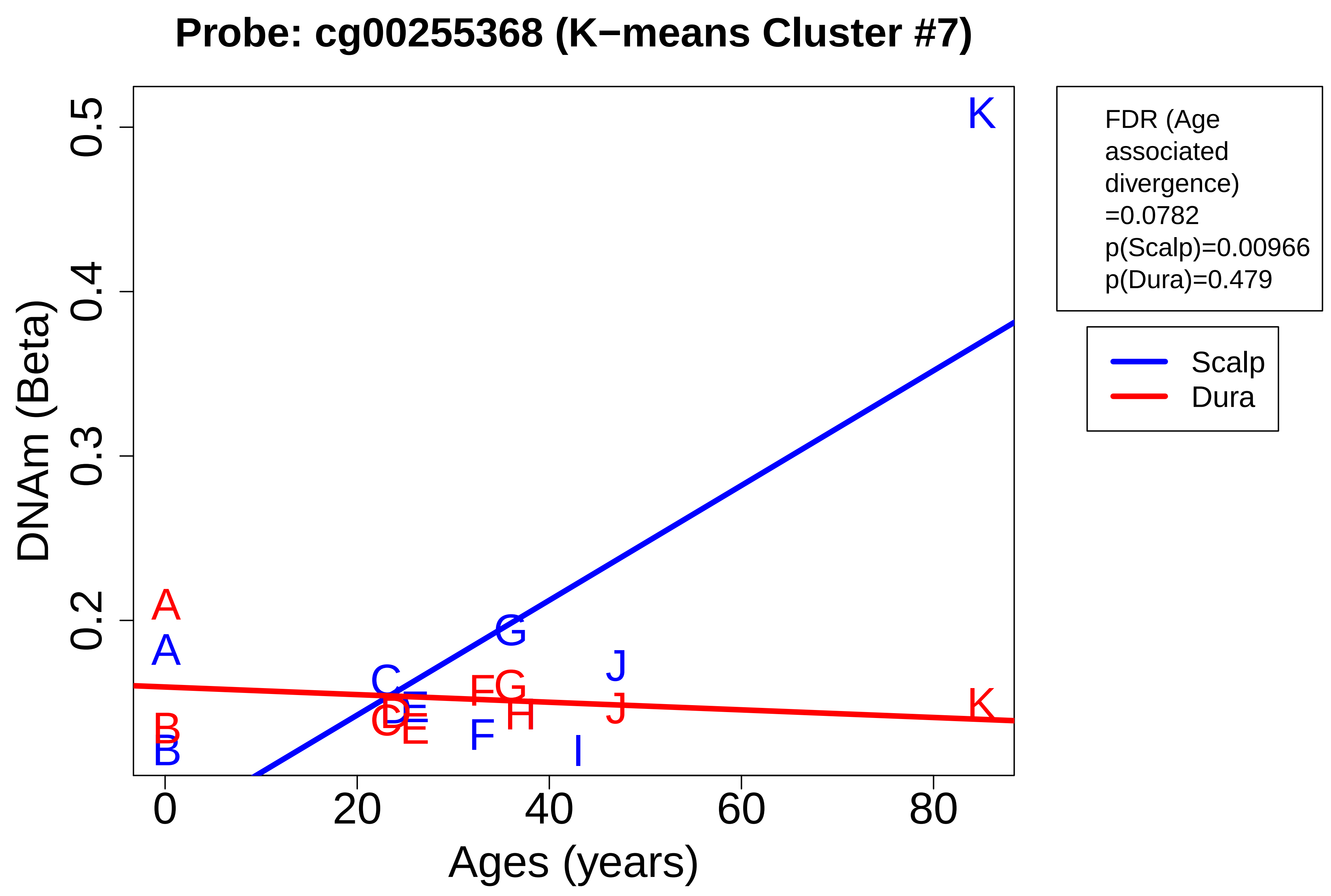

Probe: cg00255368 (K−means Cluster #7)
DNAm (Beta)
0.2
0.3
0.4
0.5
0
20
40
60
80
Ages (years)
FDR (Age associated divergence) =0.0782
p(Scalp)=0.00966
p(Dura)=0.479
Scalp
Dura

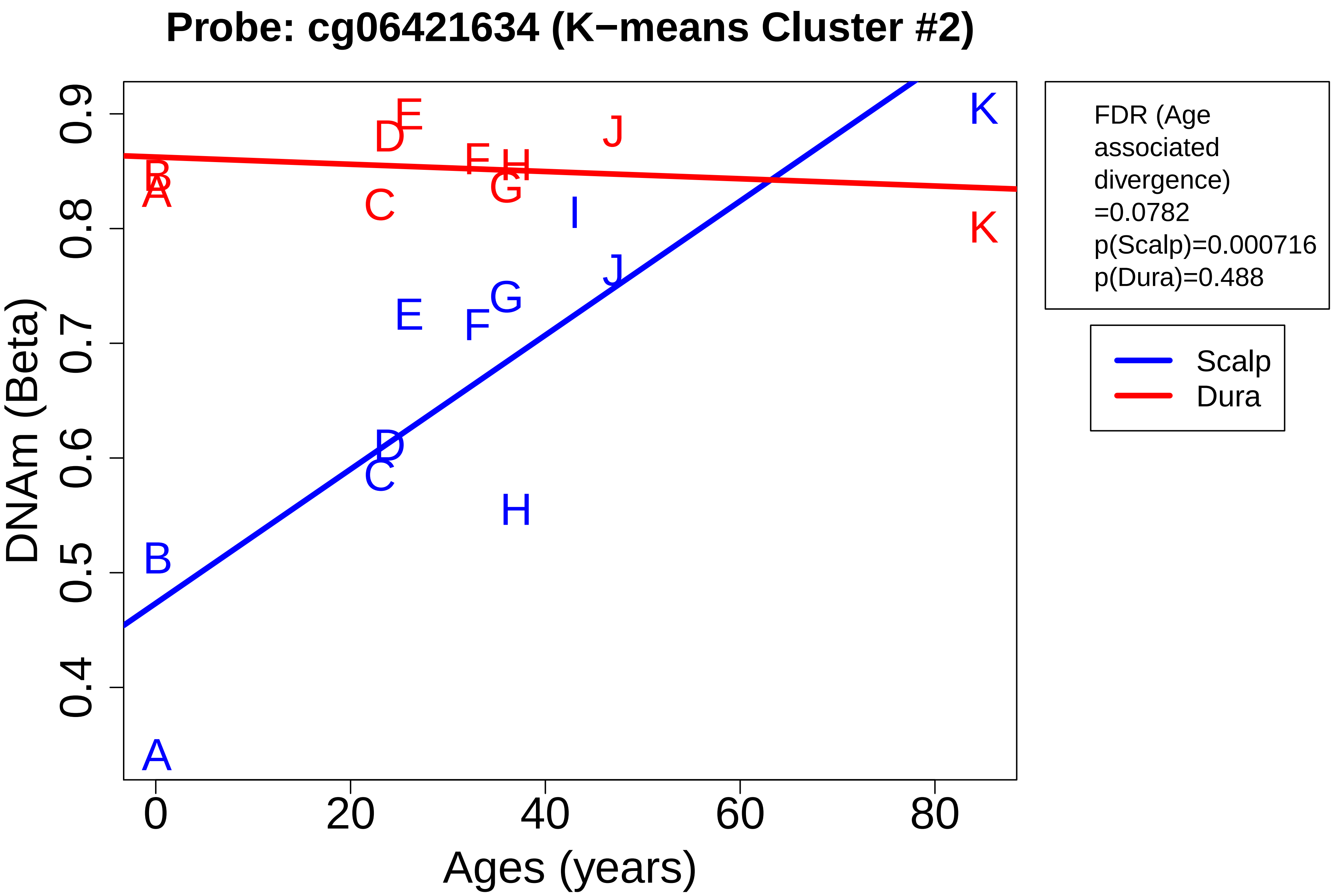

Probe: cg06421634 (K−means Cluster #2)
DNAm (Beta)
0.4
0.5
0.6
0.7
0.8
0.9
0
20
40
60
80
Ages (years)
FDR (Age associated divergence) =0.0782
p(Scalp)=0.000716
p(Dura)=0.488
Scalp
Dura
A
B
C
D
E
F
G
H
I
J
K

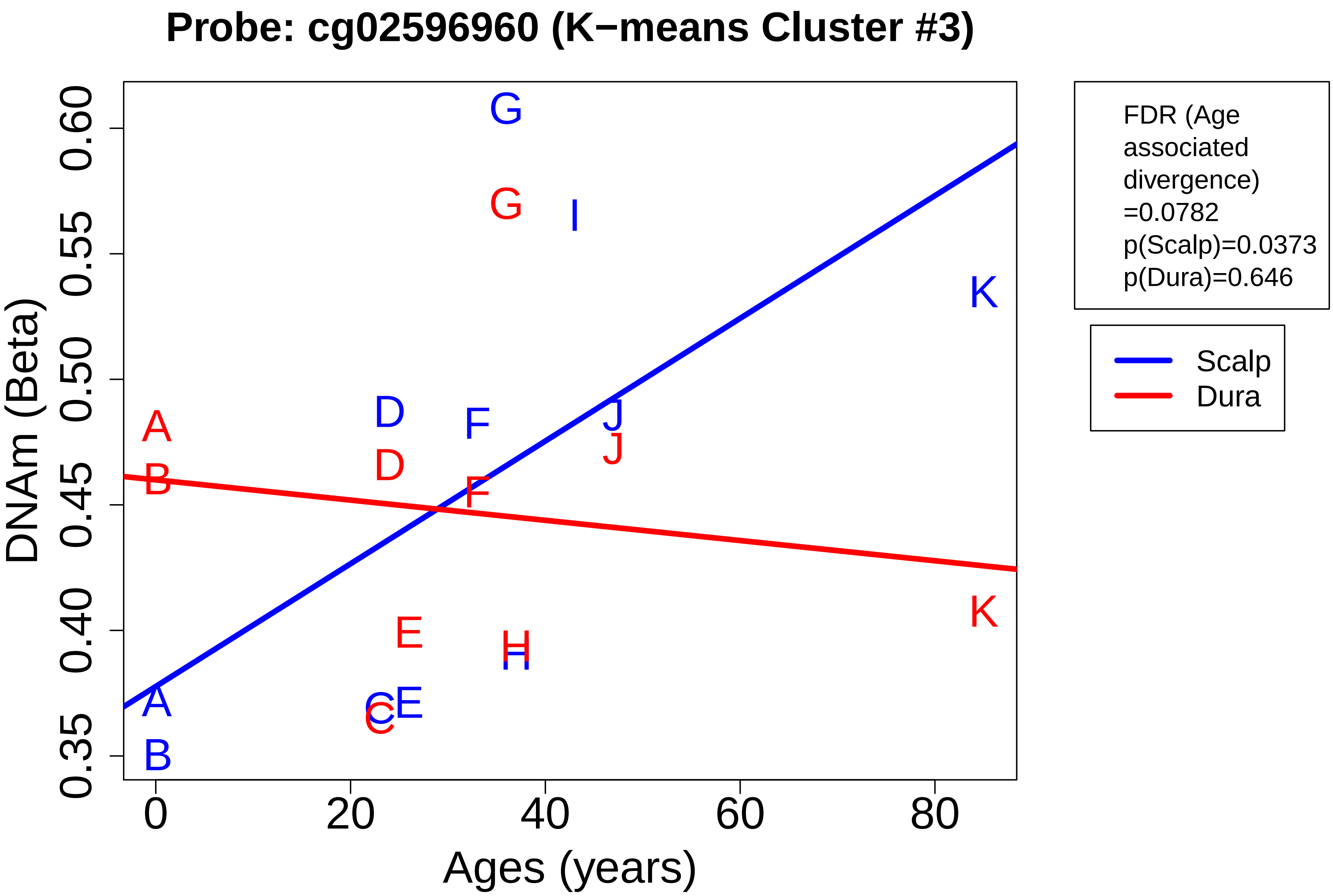

Probe: cg02596960 (K−means Cluster #3)
DNAm (Beta)
0.35
0.40
0.45
0.50
0.55
0.60
Ages (years)
0
20
40
60
80
A
B
C
D
E
F
G
H
I
J
K
FDR (Age associated divergence) =0.0782
p(Scalp)=0.0373
p(Dura)=0.646
Scalp
Dura

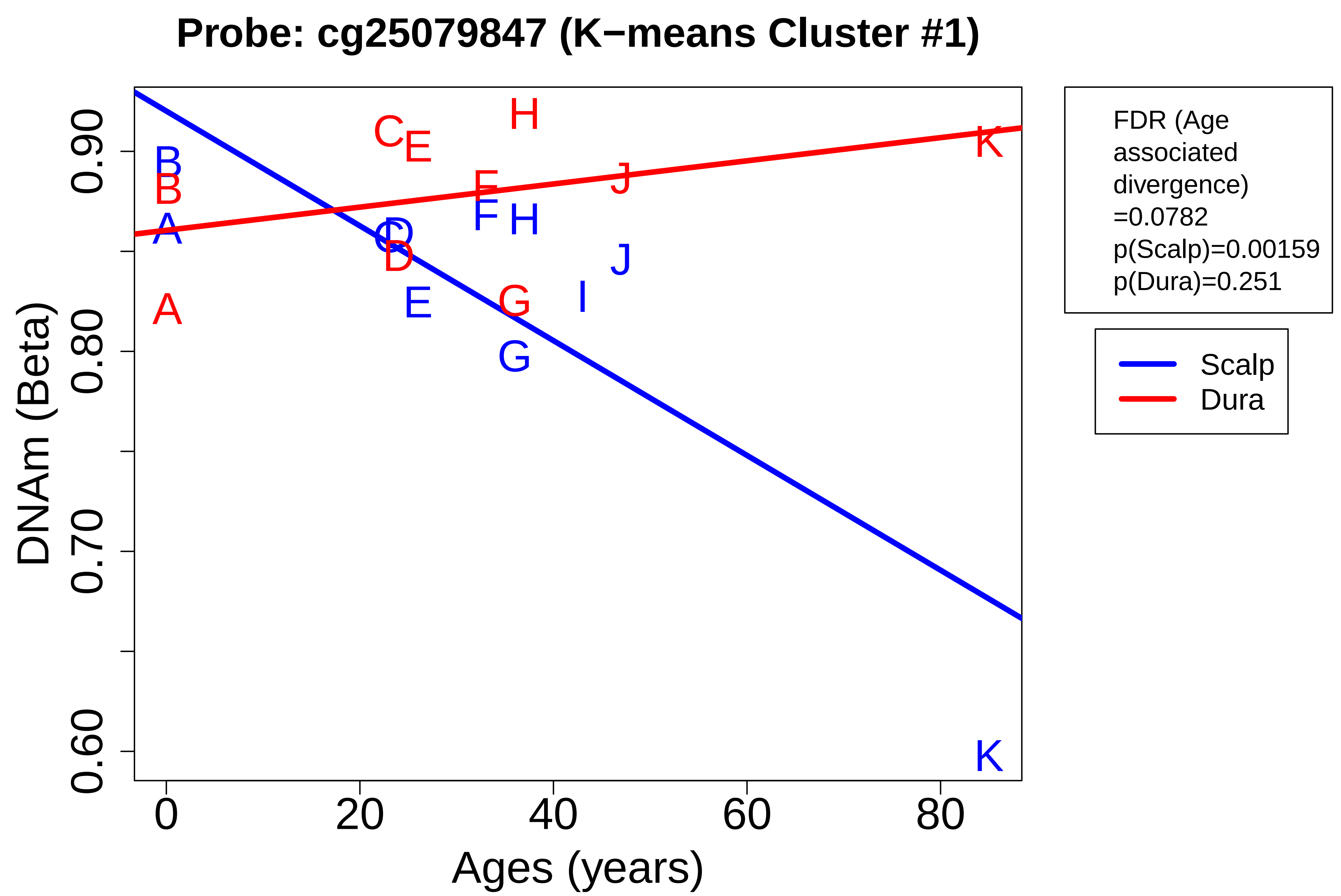

Probe: cg25079847 (K−means Cluster #1)
DNAm (Beta)
0.60
0.70
0.80
0.90
0
20
40
60
80
Ages (years)
FDR (Age associated divergence) =0.0782
p(Scalp)=0.00159
p(Dura)=0.251
Scalp
Dura

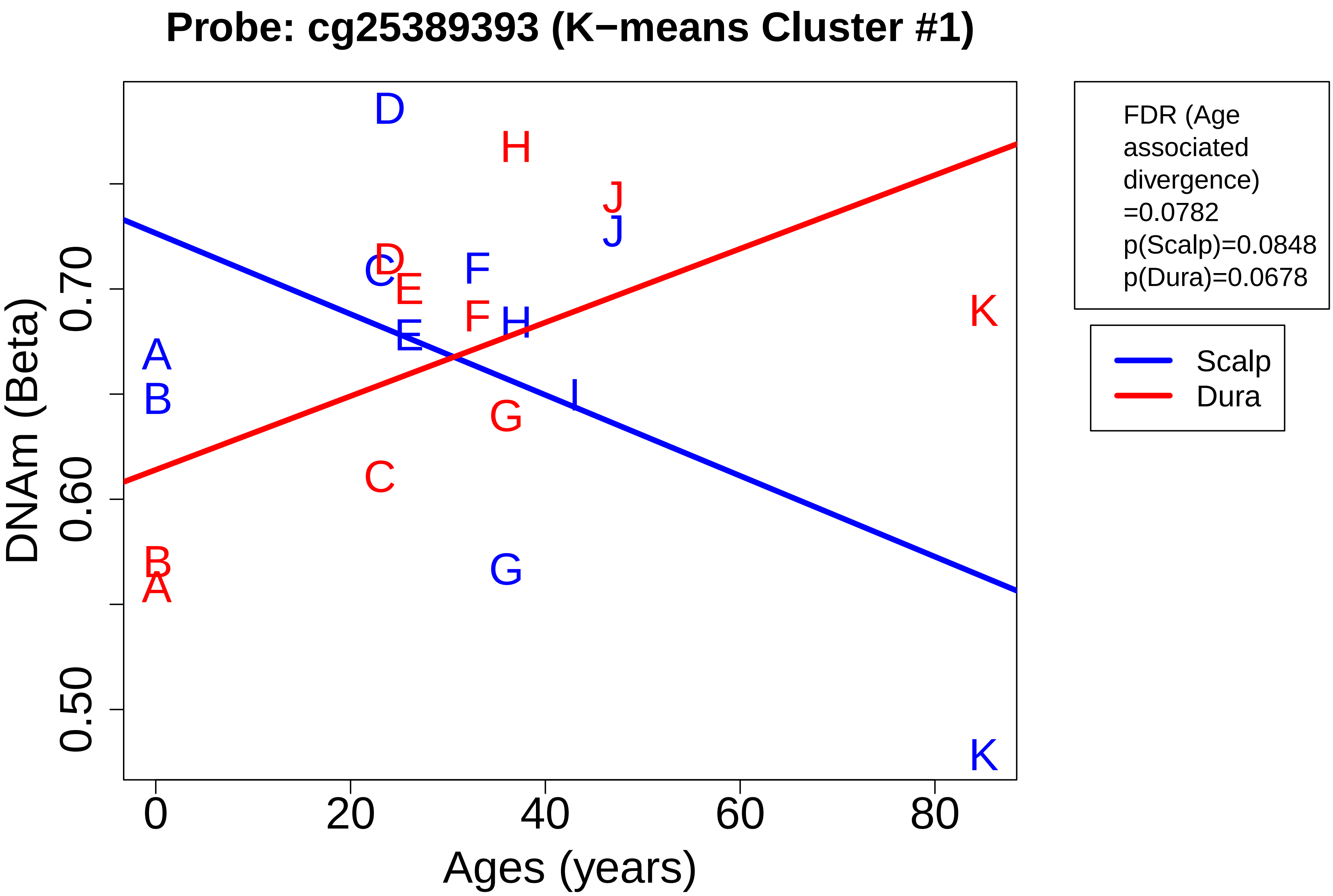

Probe: cg25389393 (K−means Cluster #1)
DNAm (Beta)
0.50
0.60
0.70
0
20
40
60
80
Ages (years)
FDR (Age associated divergence) =0.0782
p(Scalp)=0.0848
p(Dura)=0.0678
Scalp
Dura
A
B
C
D
E
F
G
H
I
J
K

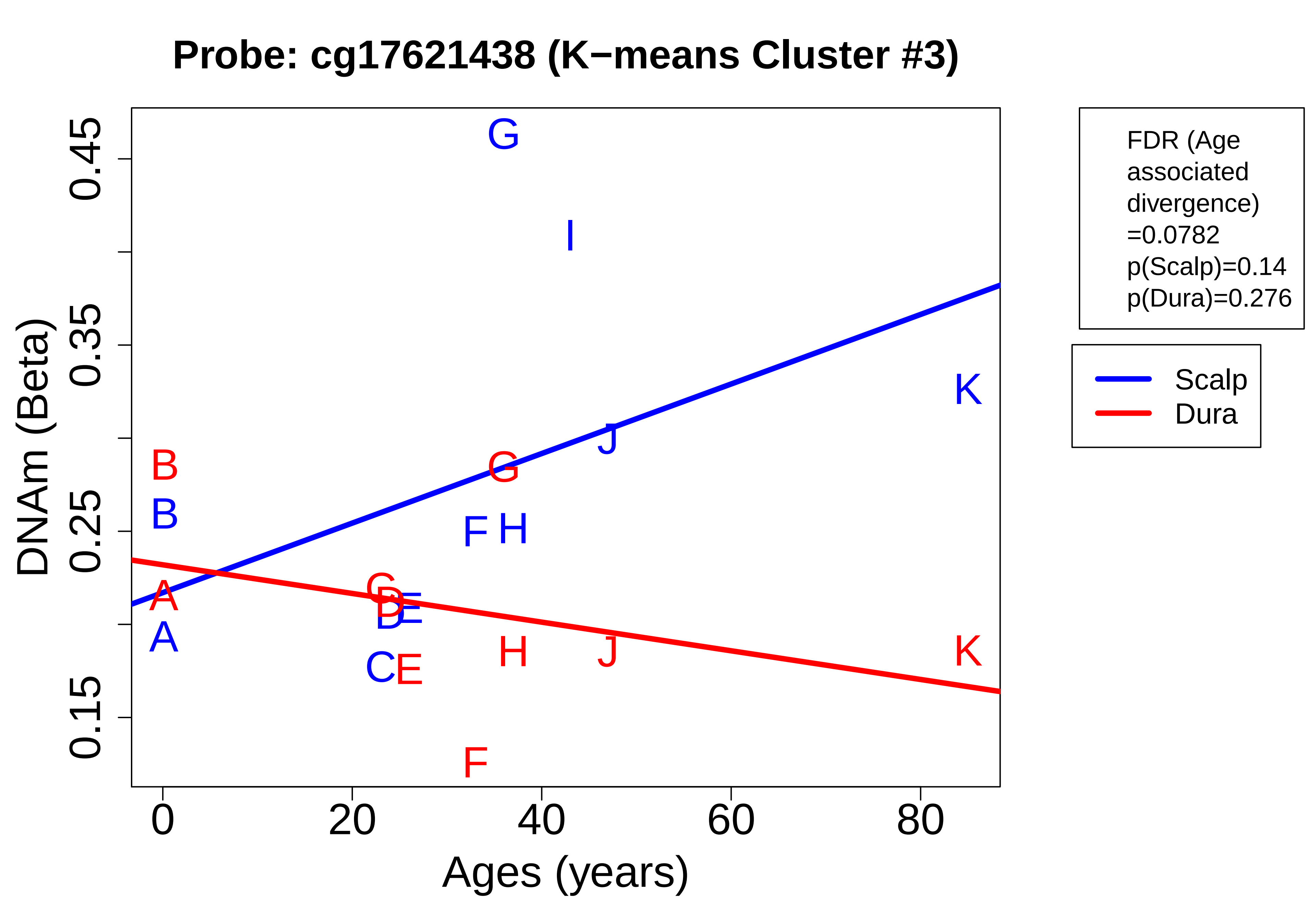

Probe: cg17621438 (K−means Cluster #3)
DNAm (Beta)
0.15
0.25
0.35
0.45
0
20
40
60
80
Ages (years)
FDR (Age associated divergence) =0.0782
p(Scalp)=0.14
p(Dura)=0.276
Scalp
Dura
A
B
C
D
E
F
G
H
I
J
K

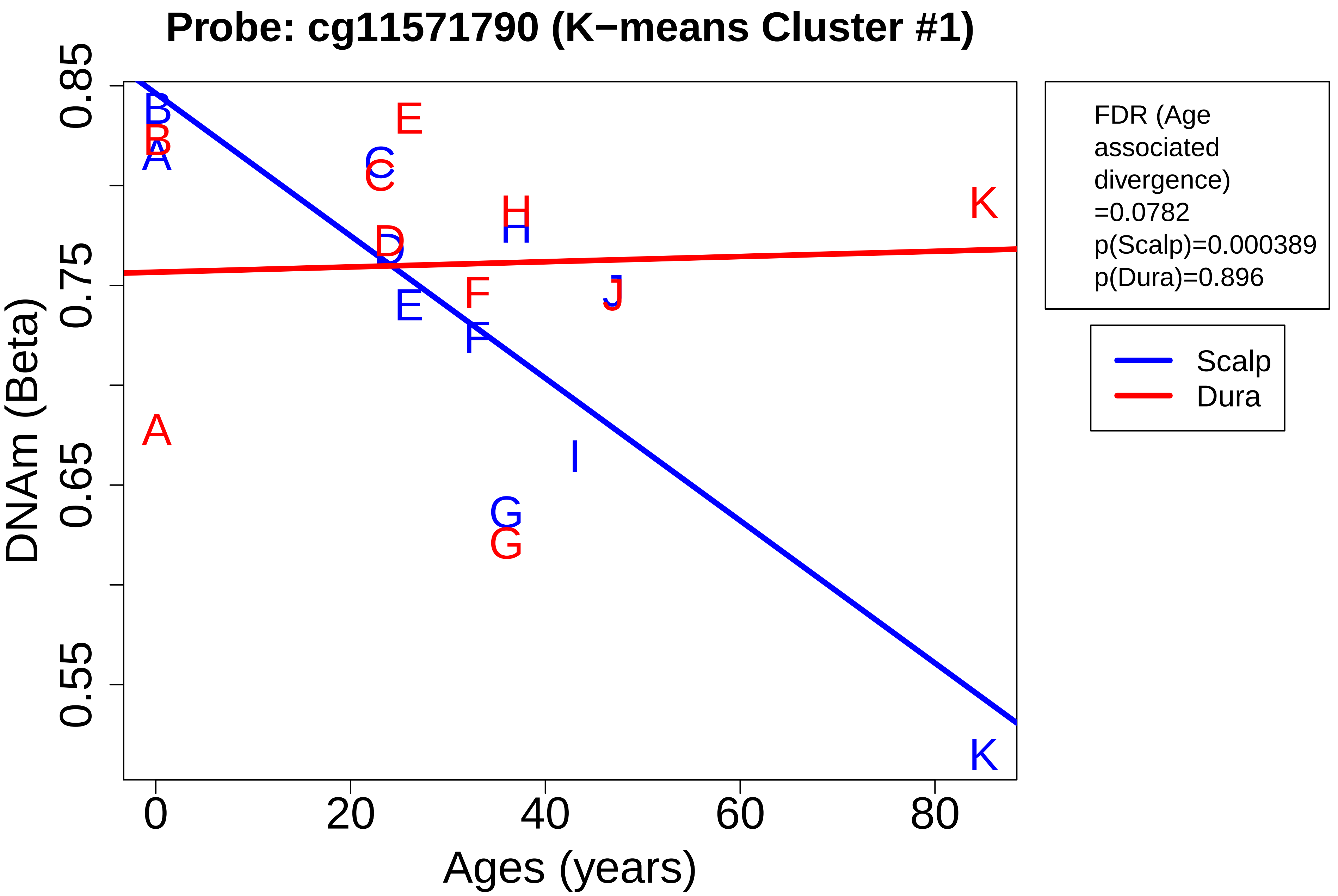

Probe: cg11571790 (K−means Cluster #1)
DNAm (Beta)
0.55
0.65
0.75
0.85
Ages (years)
0
20
40
60
80
FDR (Age associated divergence) =0.0782
p(Scalp)=0.000389
p(Dura)=0.896
Scalp
Dura

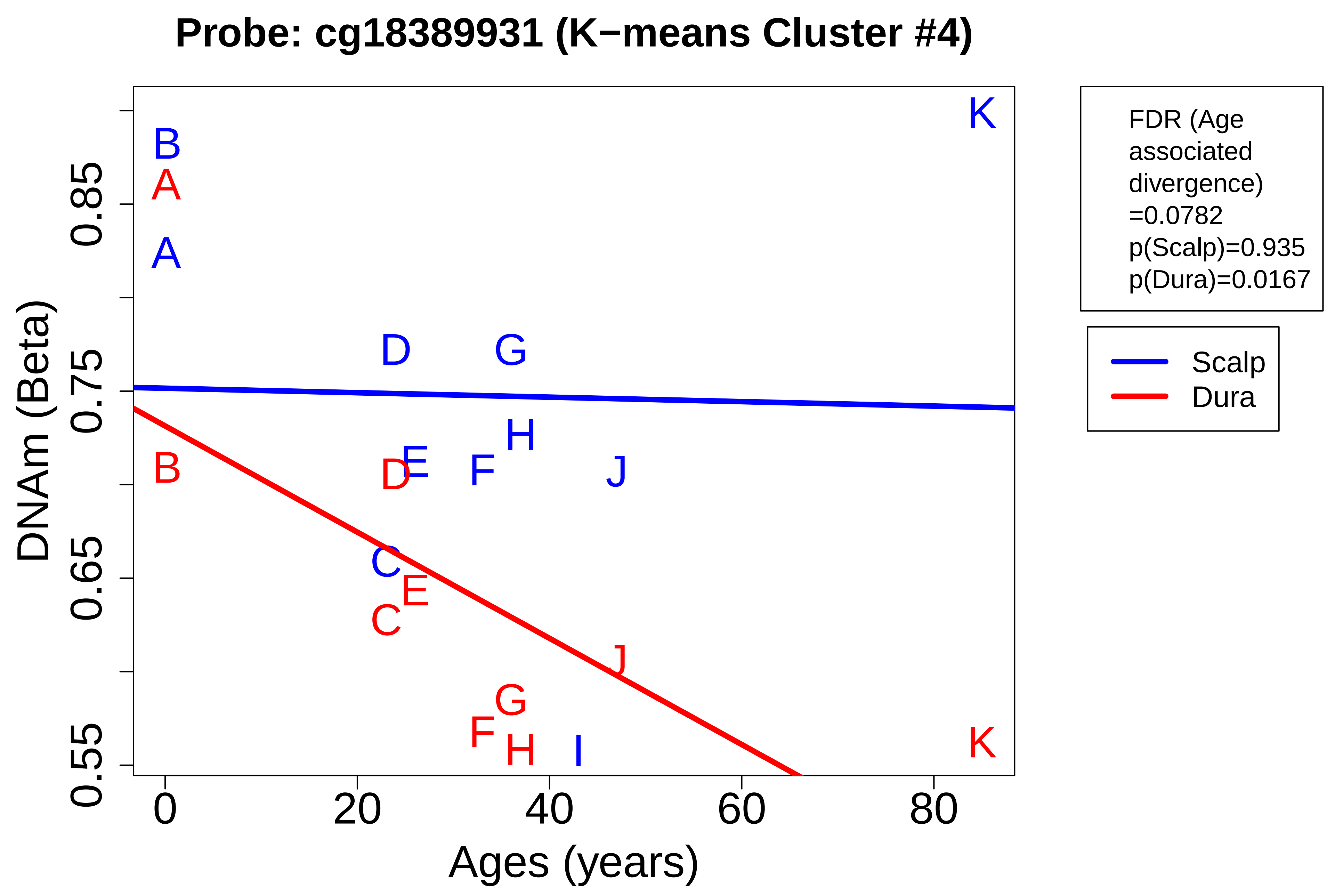

Probe: cg18389931 (K−means Cluster #4)
FDR (Age associated divergence) =0.0782
p(Scalp)=0.935
p(Dura)=0.0167
Scalp
Dura
DNAm (Beta)
0.55
0.65
0.75
0.85
Ages (years)
0
20
40
60
80
A B C D E F G H I J K
A B C D E F G H J K

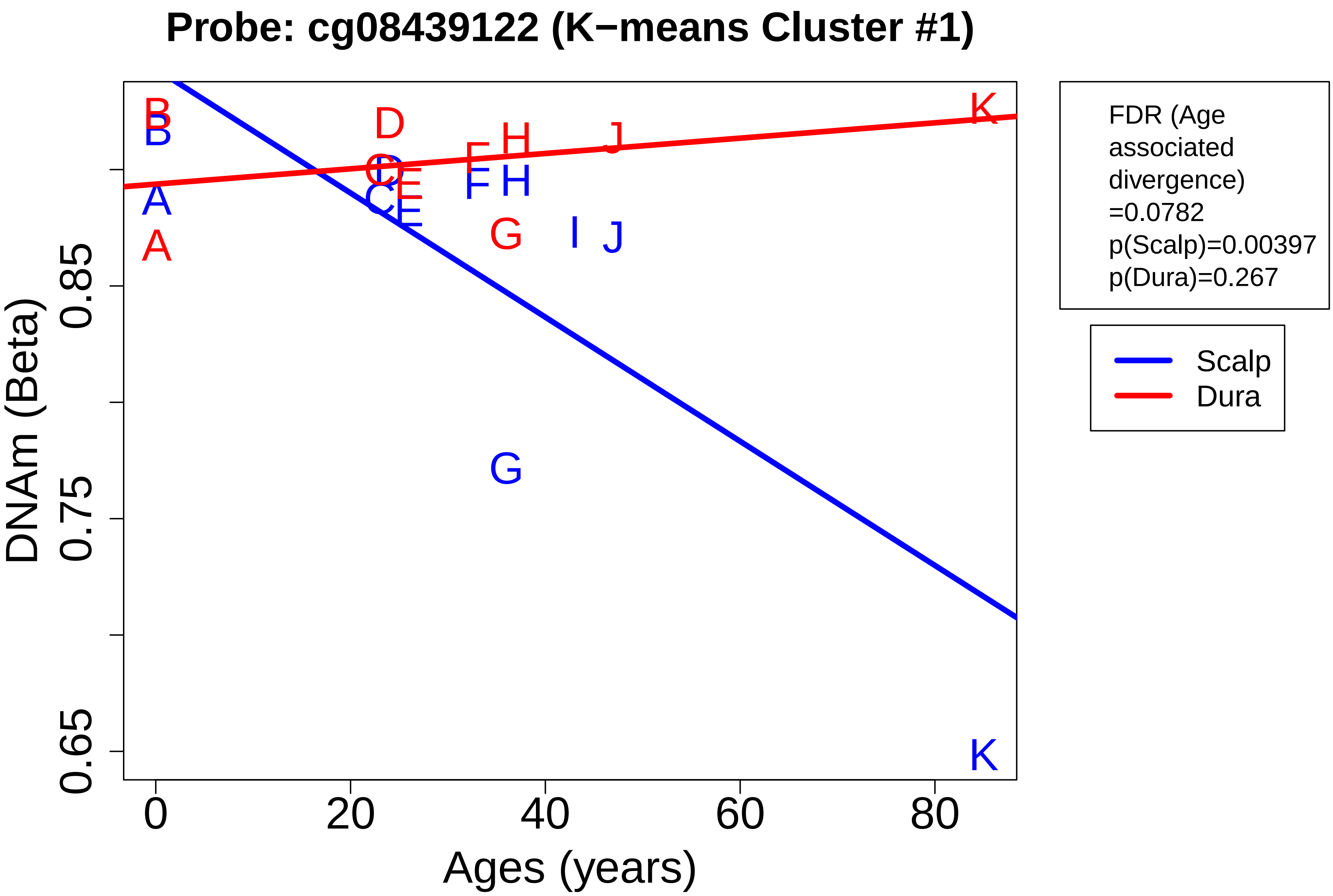

Probe: cg08439122 (K−means Cluster #1)
DNAm (Beta)
0.65
0.75
0.85
Ages (years)
0
20
40
60
80
A
B
C
D
E
F
G
H
I
J
K
FDR (Age associated divergence) =0.0782
p(Scalp)=0.00397
p(Dura)=0.267
Scalp
Dura

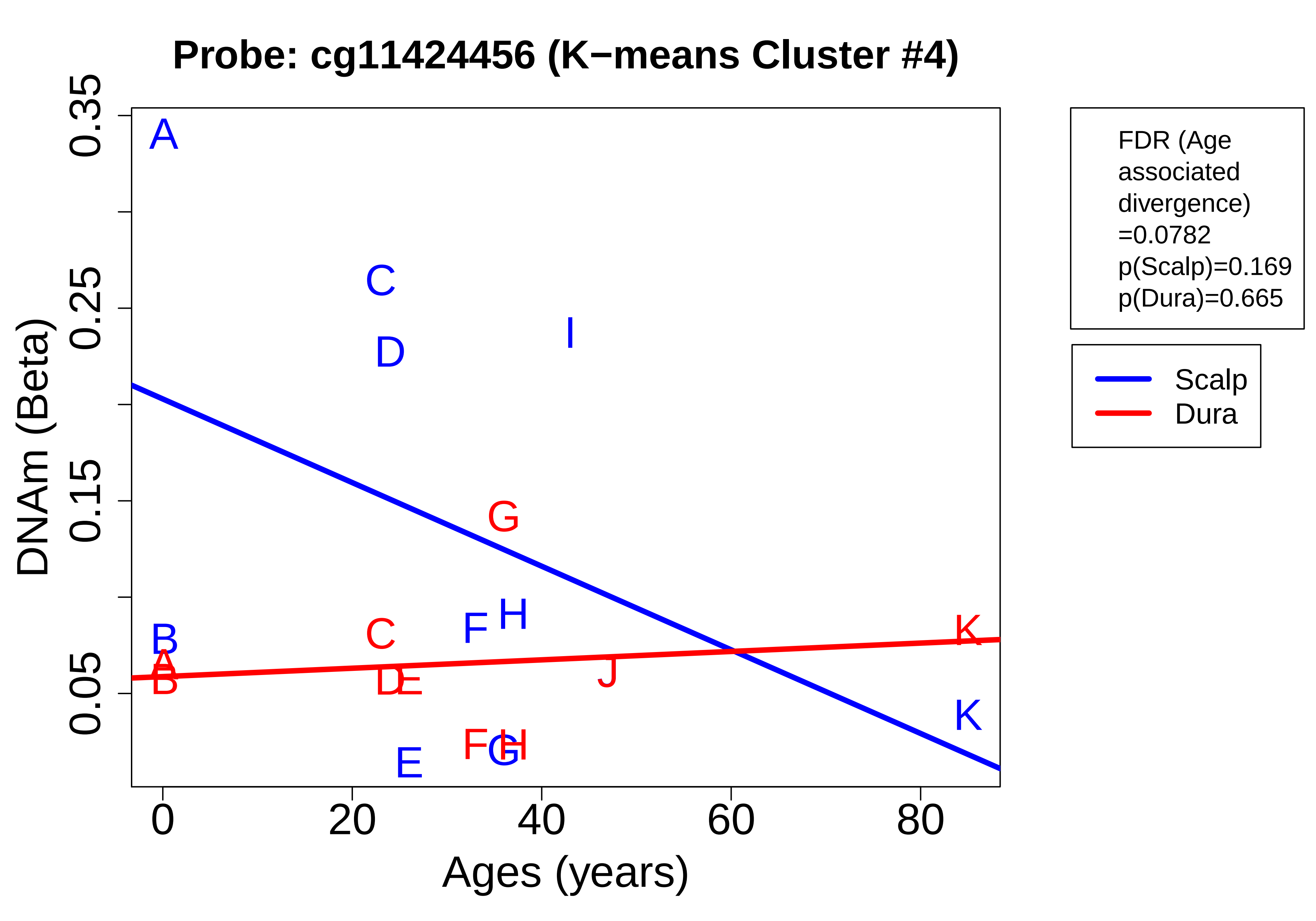

Probe: cg11424456 (K−means Cluster #4)
DNAm (Beta)
0.05
0.15
0.25
0.35
Ages (years)
0
20
40
60
80
A
B
C
D
E
F
G
H
I
J
K
FDR (Age associated divergence) =0.0782
p(Scalp)=0.169
p(Dura)=0.665
Scalp
Dura

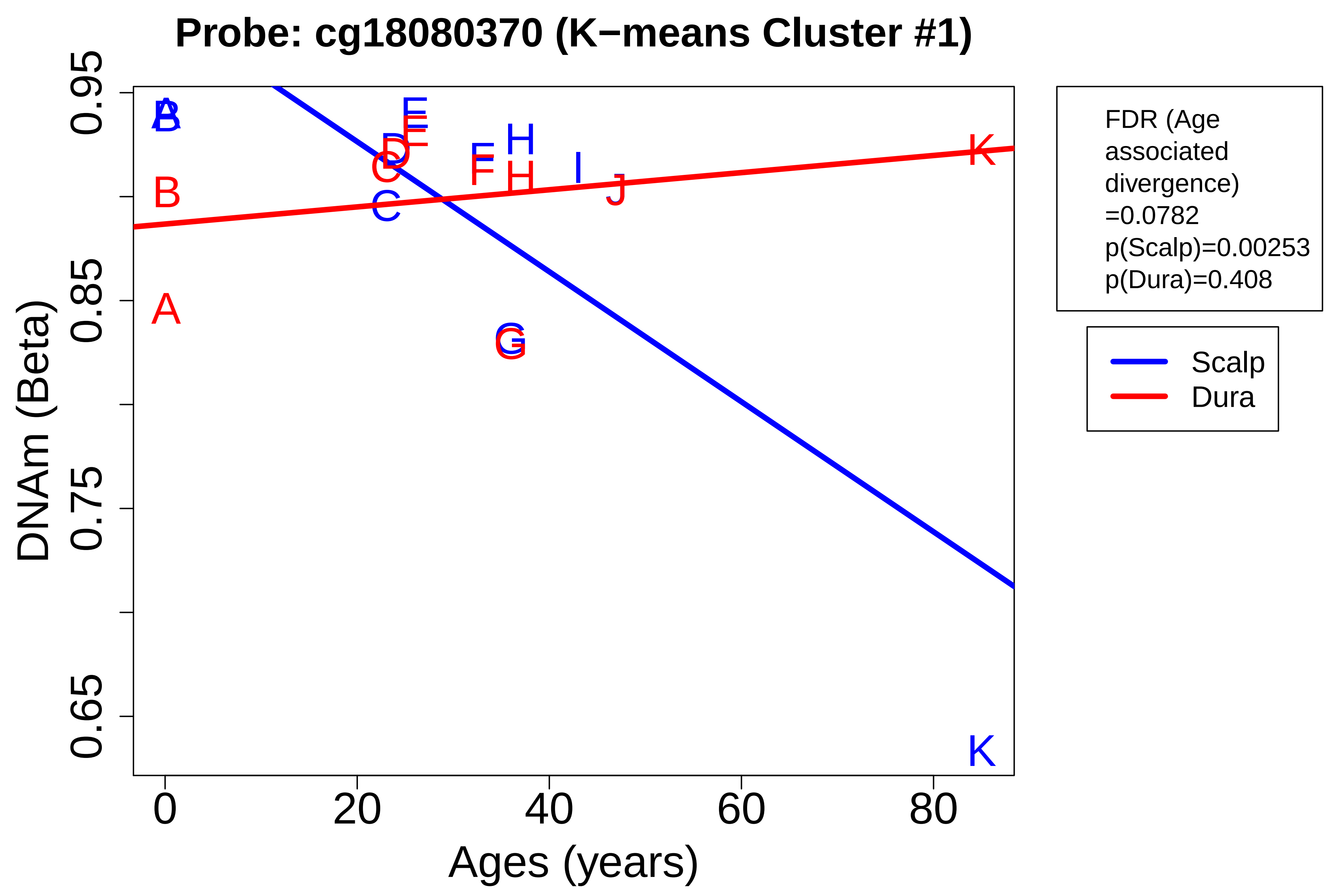

Probe: cg18080370 (K−means Cluster #1)
DNAm (Beta)
0.65
0.75
0.85
0.95
Ages (years)
0
20
40
60
80
A
B
C
D
E
F
G
H
I
J
K
FDR (Age associated divergence) =0.0782
p(Scalp)=0.00253
p(Dura)=0.408
Scalp
Dura

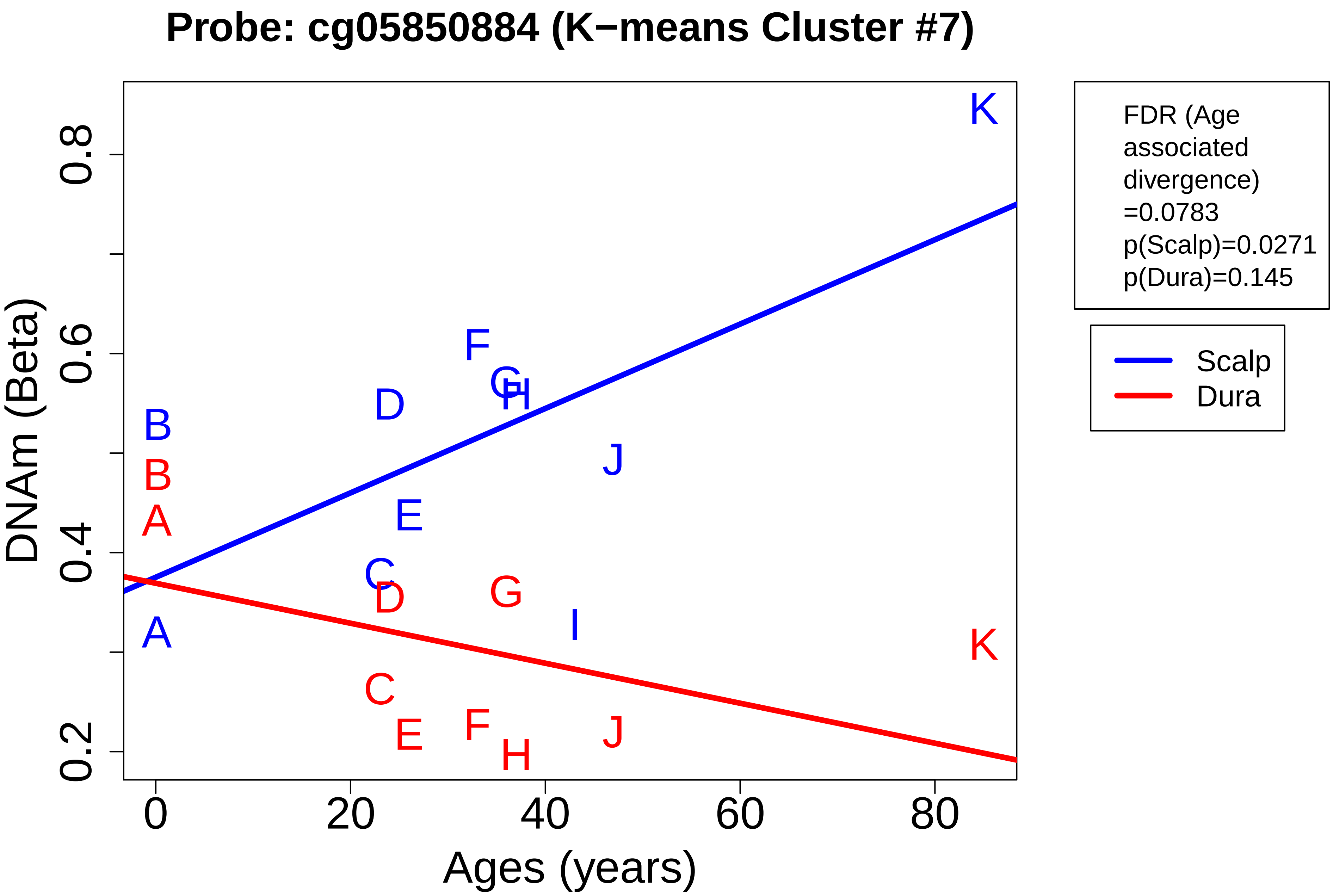
Probe: cg05850884 (K−means Cluster #7)
DNAm (Beta)
0.2
0.4
0.6
0.8
0
20
40
60
80
Ages (years)
FDR (Age associated divergence) =0.0783
p(Scalp)=0.0271
p(Dura)=0.145
Scalp
Dura
A
B
C
D
E
F
G
H
I
J
K

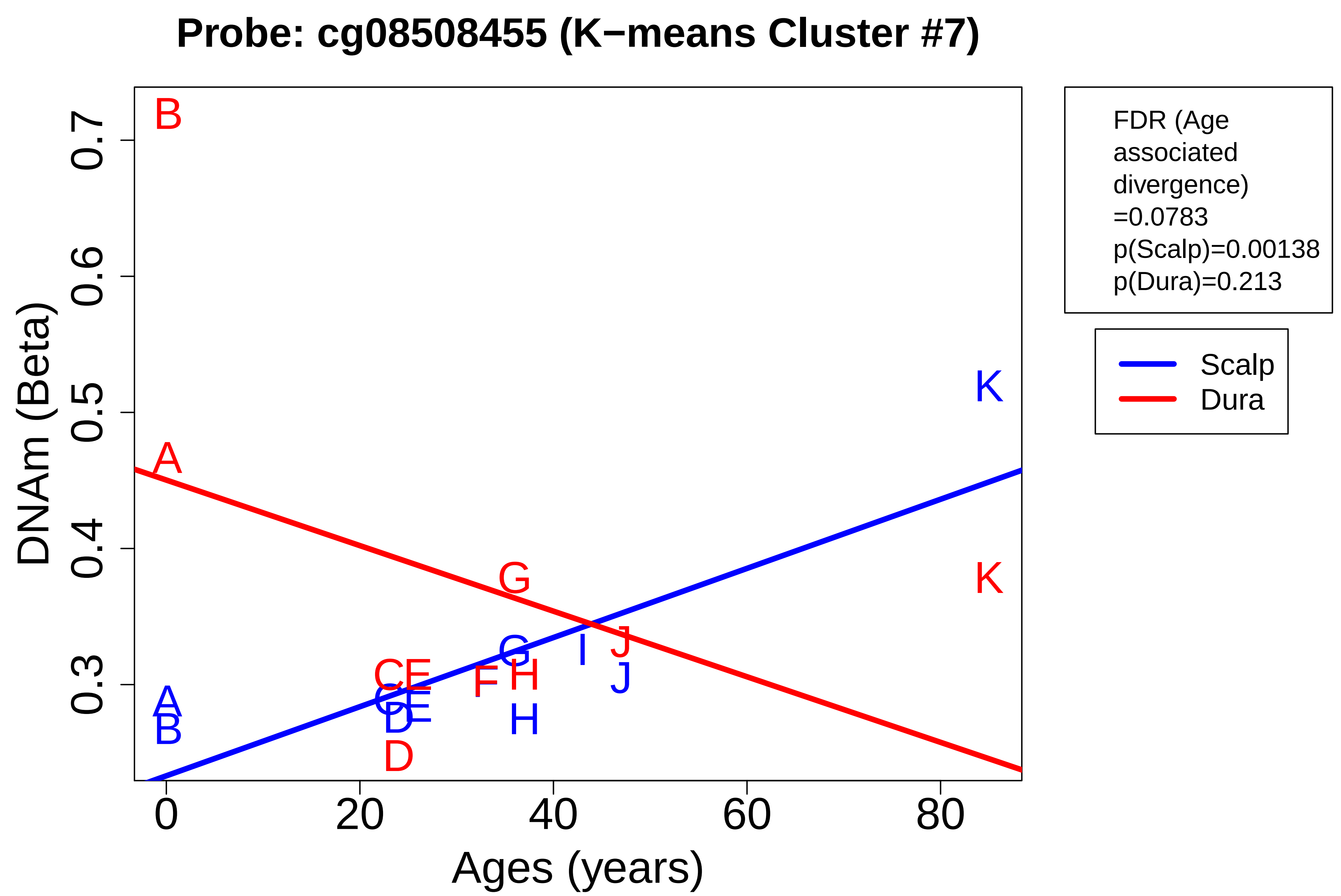

Probe: cg08508455 (K−means Cluster #7)
DNAm (Beta)
0.3
0.4
0.5
0.6
0.7
0
20
40
60
80
Ages (years)
FDR (Age associated divergence) =0.0783
p(Scalp)=0.00138
p(Dura)=0.213
Scalp
Dura

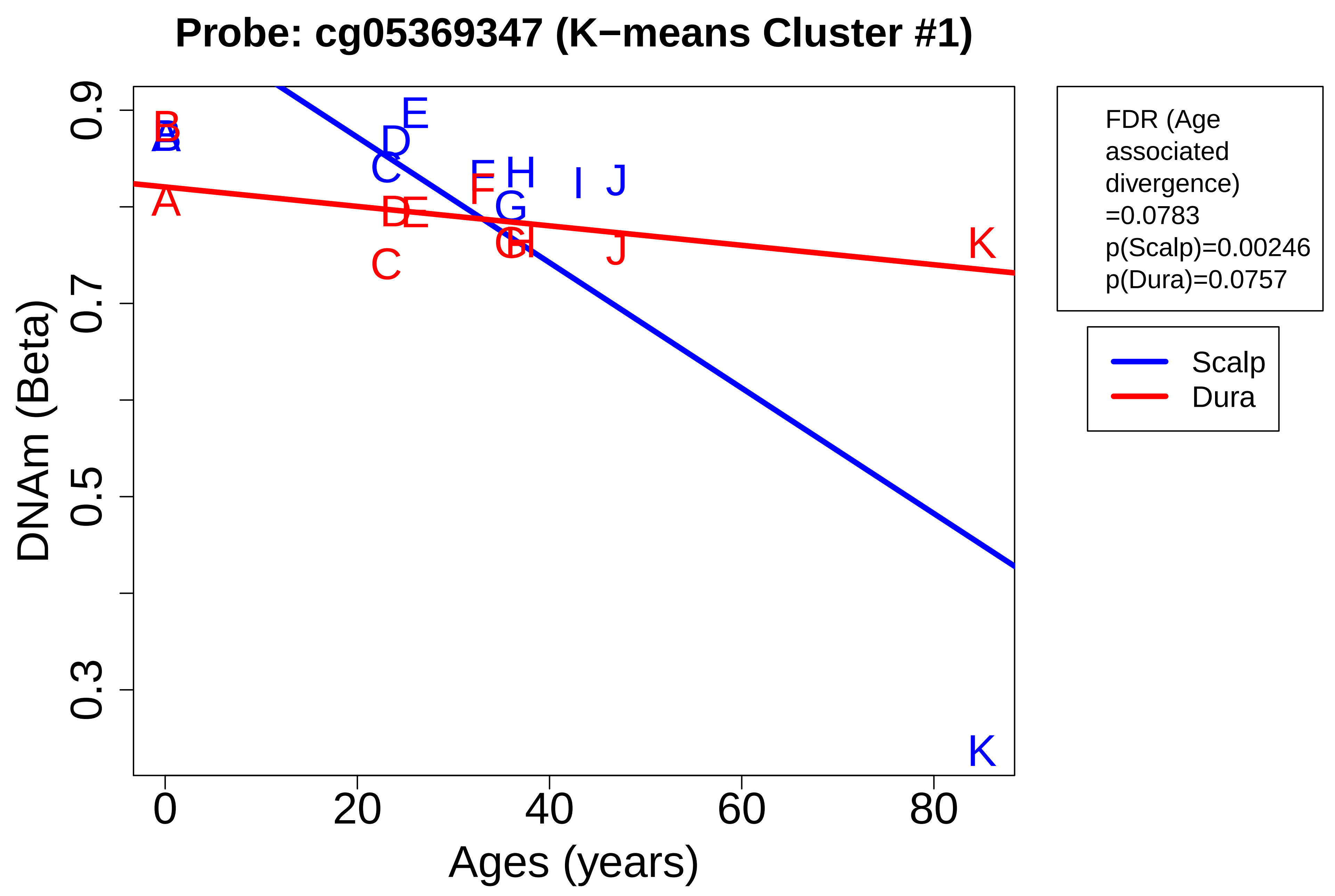

Probe: cg05369347 (K−means Cluster #1)
DNAm (Beta)
Ages (years)
0.3
0.5
0.7
0.9
0
20
40
60
80
FDR (Age associated divergence) =0.0783
p(Scalp)=0.00246
p(Dura)=0.0757
Scalp
Dura
A B C D E F G H I J K

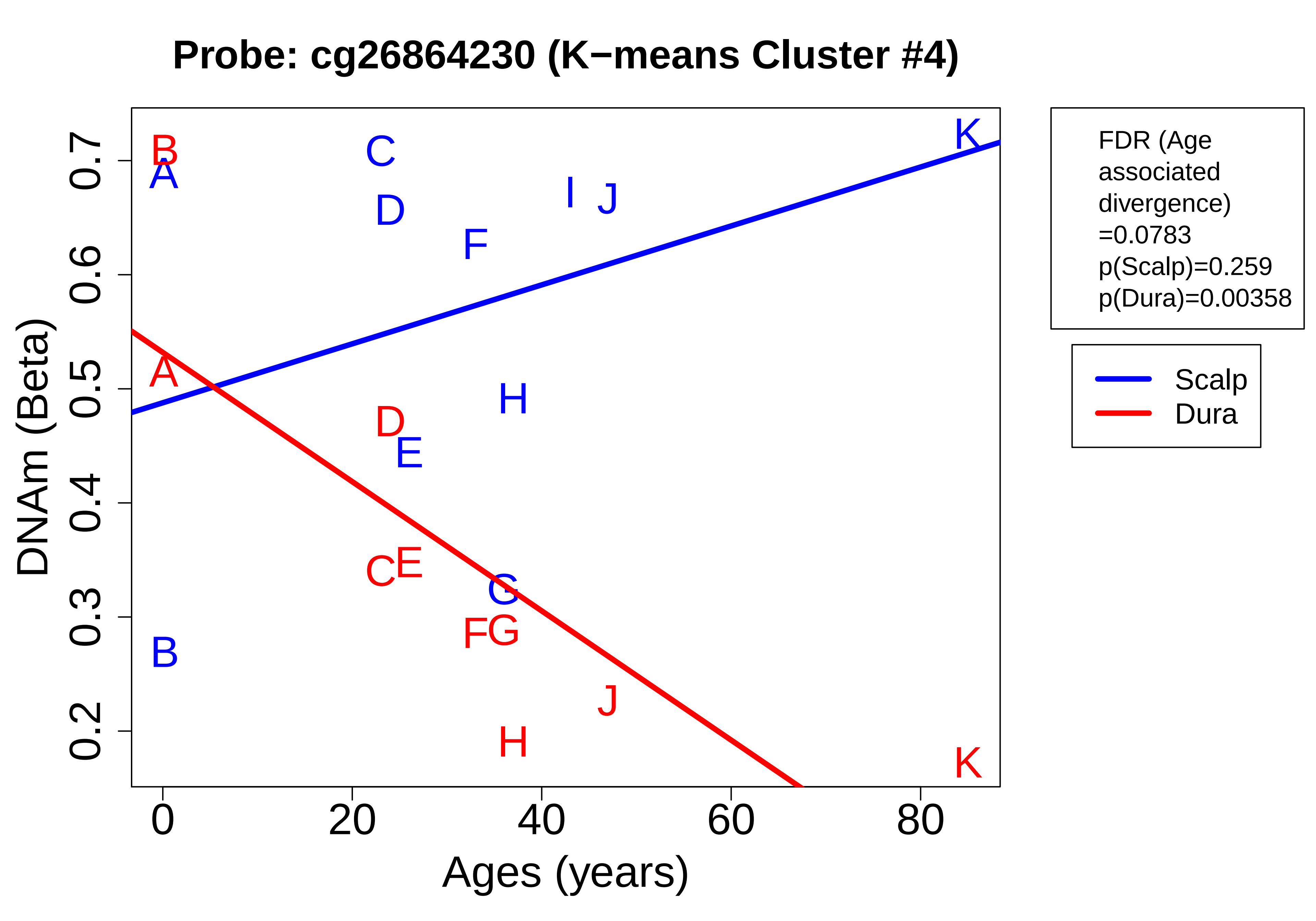

Probe: cg26864230 (K−means Cluster #4)
DNAm (Beta)
0.2
0.3
0.4
0.5
0.6
0.7
Ages (years)
0
20
40
60
80
FDR (Age associated divergence) =0.0783
p(Scalp)=0.259
p(Dura)=0.00358
Scalp
Dura

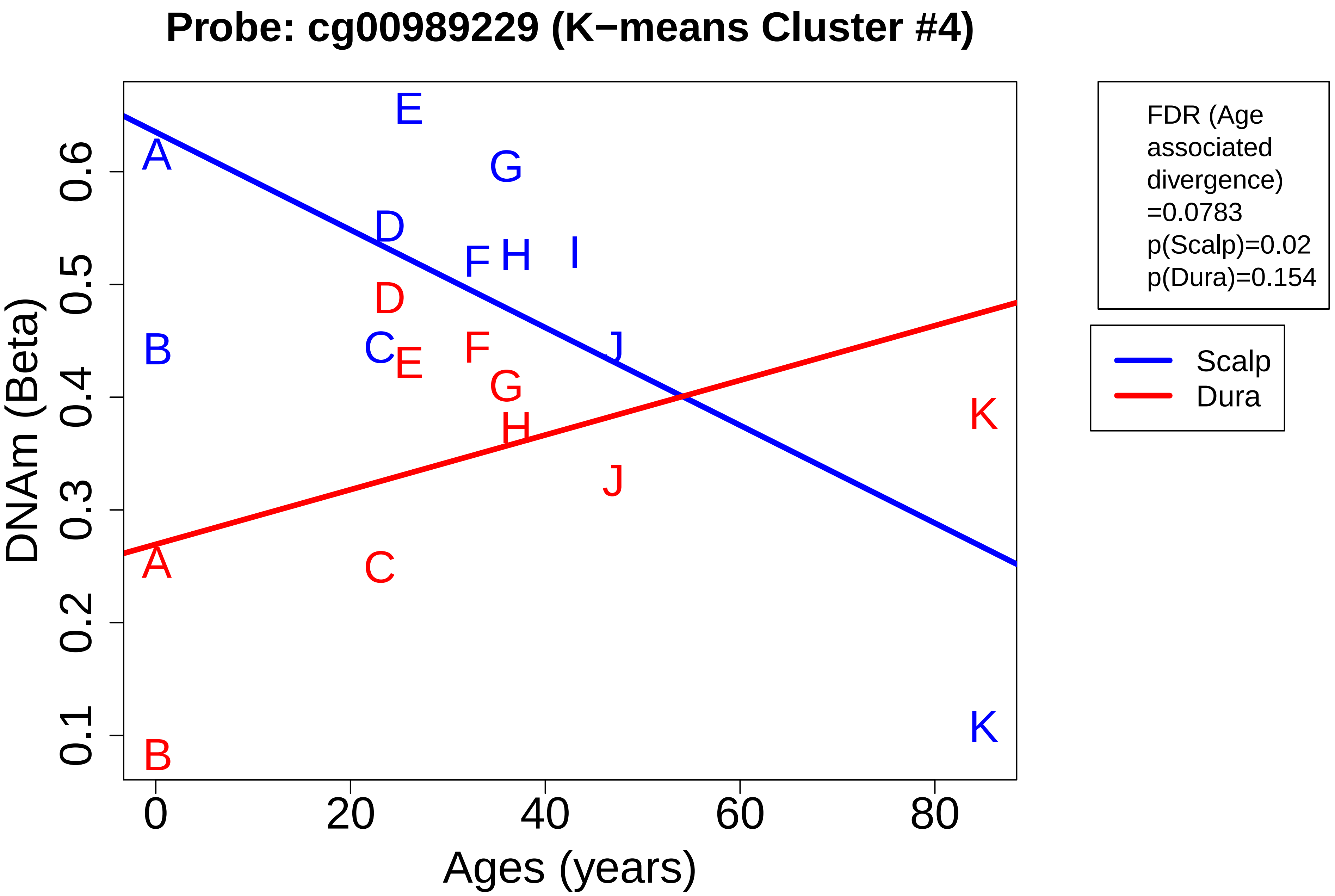

Probe: cg00989229 (K−means Cluster #4)
FDR (Age associated divergence) =0.0783
p(Scalp)=0.02
p(Dura)=0.154
Scalp
Dura
DNAm (Beta)
0.1
0.2
0.3
0.4
0.5
0.6
0
20
40
60
80
Ages (years)
A
B
C
D
E
F
G
H
I
J
K

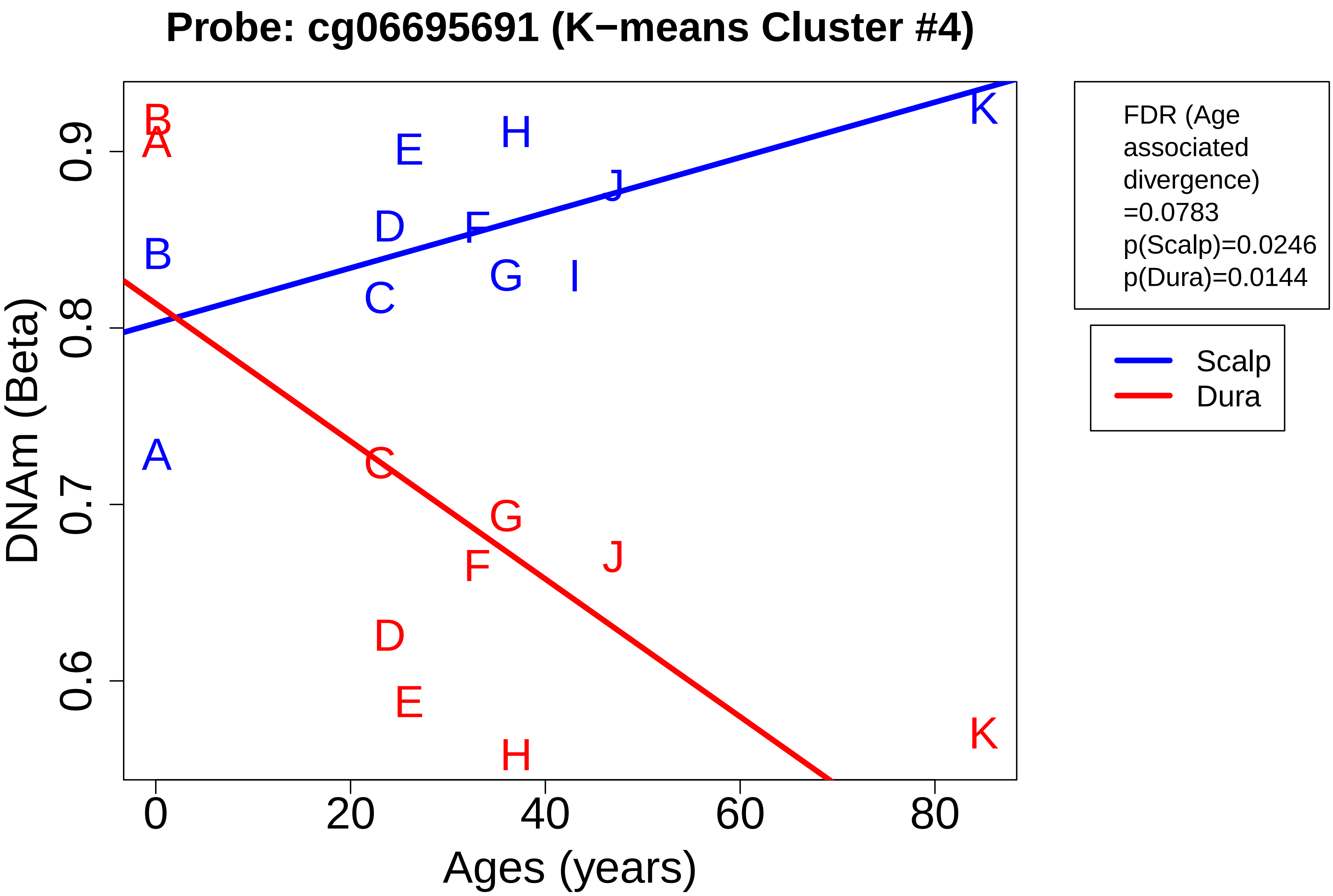
Probe: cg06695691 (K−means Cluster #4)
DNAm (Beta)
Ages (years)
0.6
0.7
0.8
0.9
0
20
40
60
80
FDR (Age associated divergence) =0.0783
p(Scalp)=0.0246
p(Dura)=0.0144
Scalp
Dura

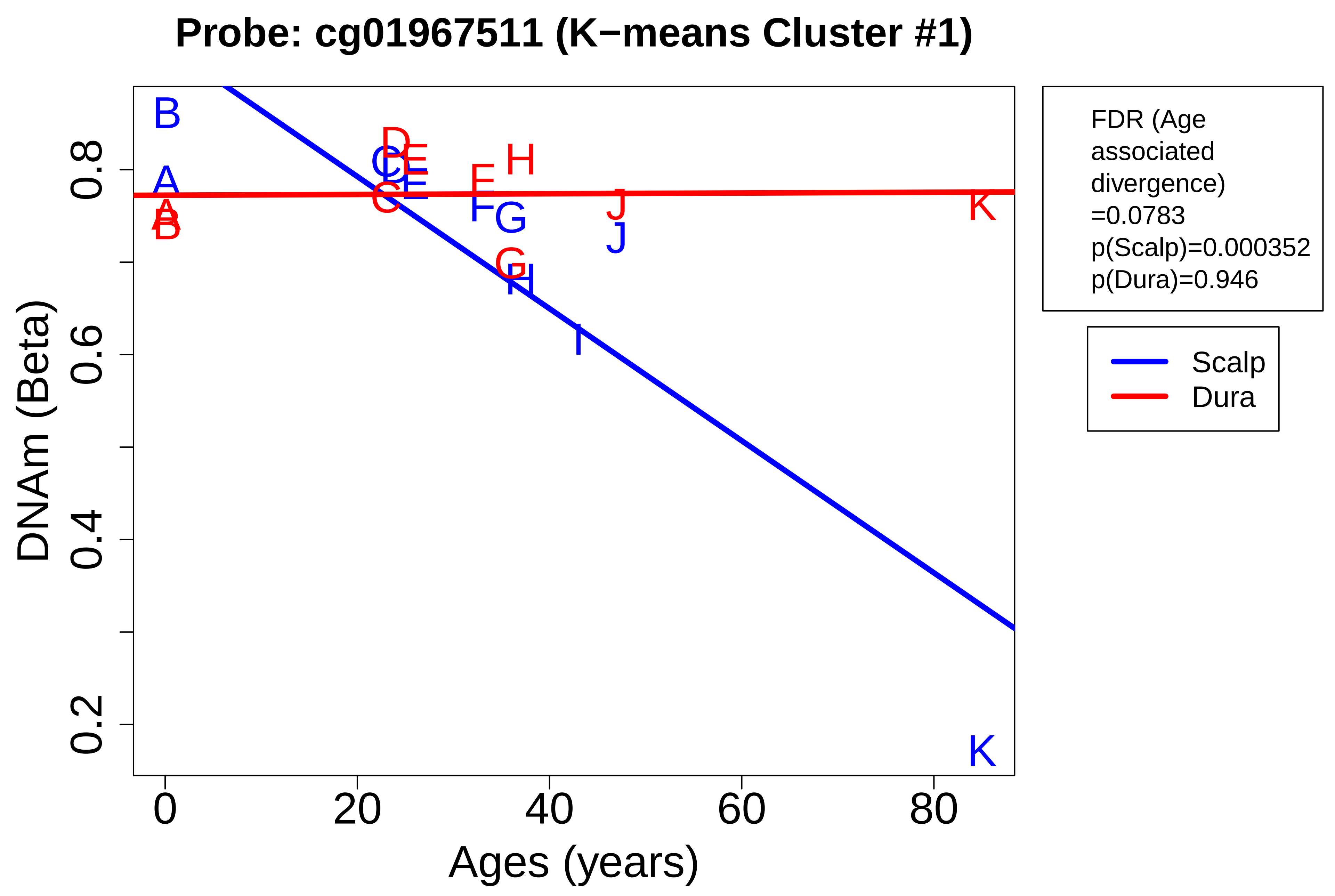

Probe: cg01967511 (K−means Cluster #1)
DNAm (Beta)
0.2
0.4
0.6
0.8
Ages (years)
0
20
40
60
80
FDR (Age associated divergence) =0.0783
p(Scalp)=0.000352
p(Dura)=0.946
Scalp
Dura

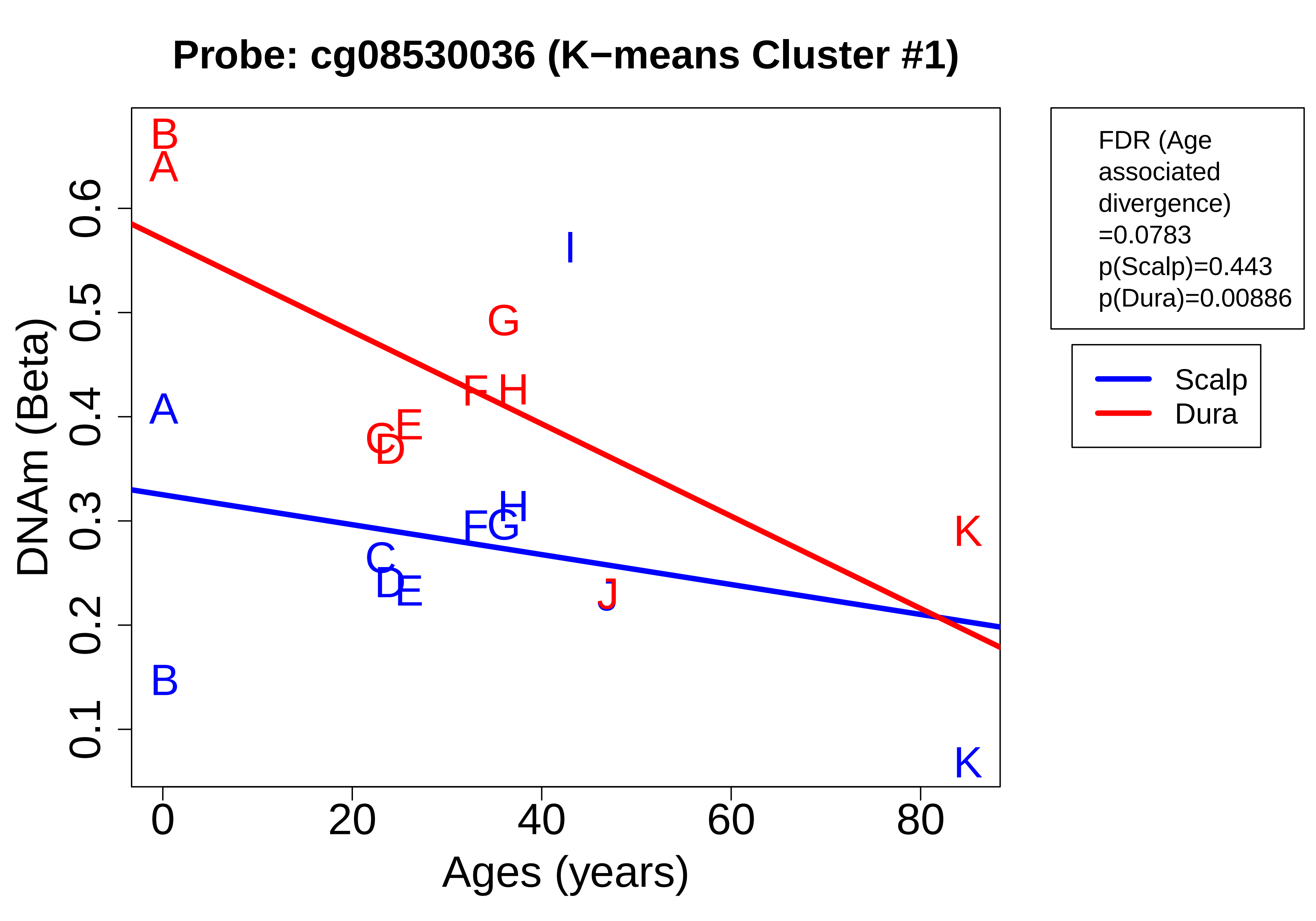

Probe: cg08530036 (K−means Cluster #1)
DNAm (Beta)
0.1
0.2
0.3
0.4
0.5
0.6
0
20
40
60
80
Ages (years)
FDR (Age associated divergence) =0.0783
p(Scalp)=0.443
p(Dura)=0.00886
Scalp
Dura
A
B
C
D
E
F
G
H
I
J
K

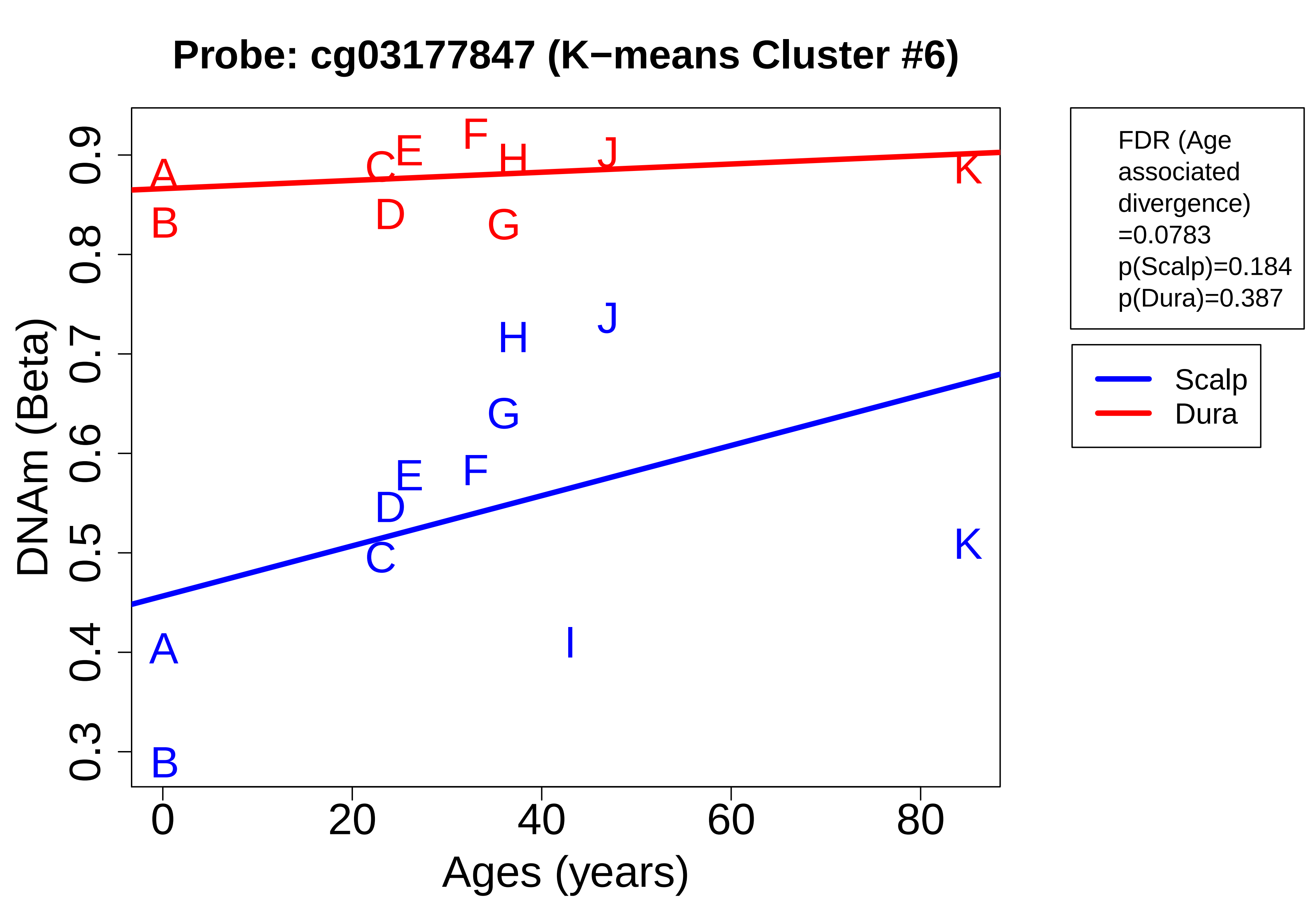
Probe: cg03177847 (K−means Cluster #6)
DNAm (Beta)
0.3
0.4
0.5
0.6
0.7
0.8
0.9
0
20
40
60
80
Ages (years)
A
B
C
D
E
F
G
H
I
J
K
FDR (Age associated divergence) =0.0783
p(Scalp)=0.184
p(Dura)=0.387
Scalp
Dura

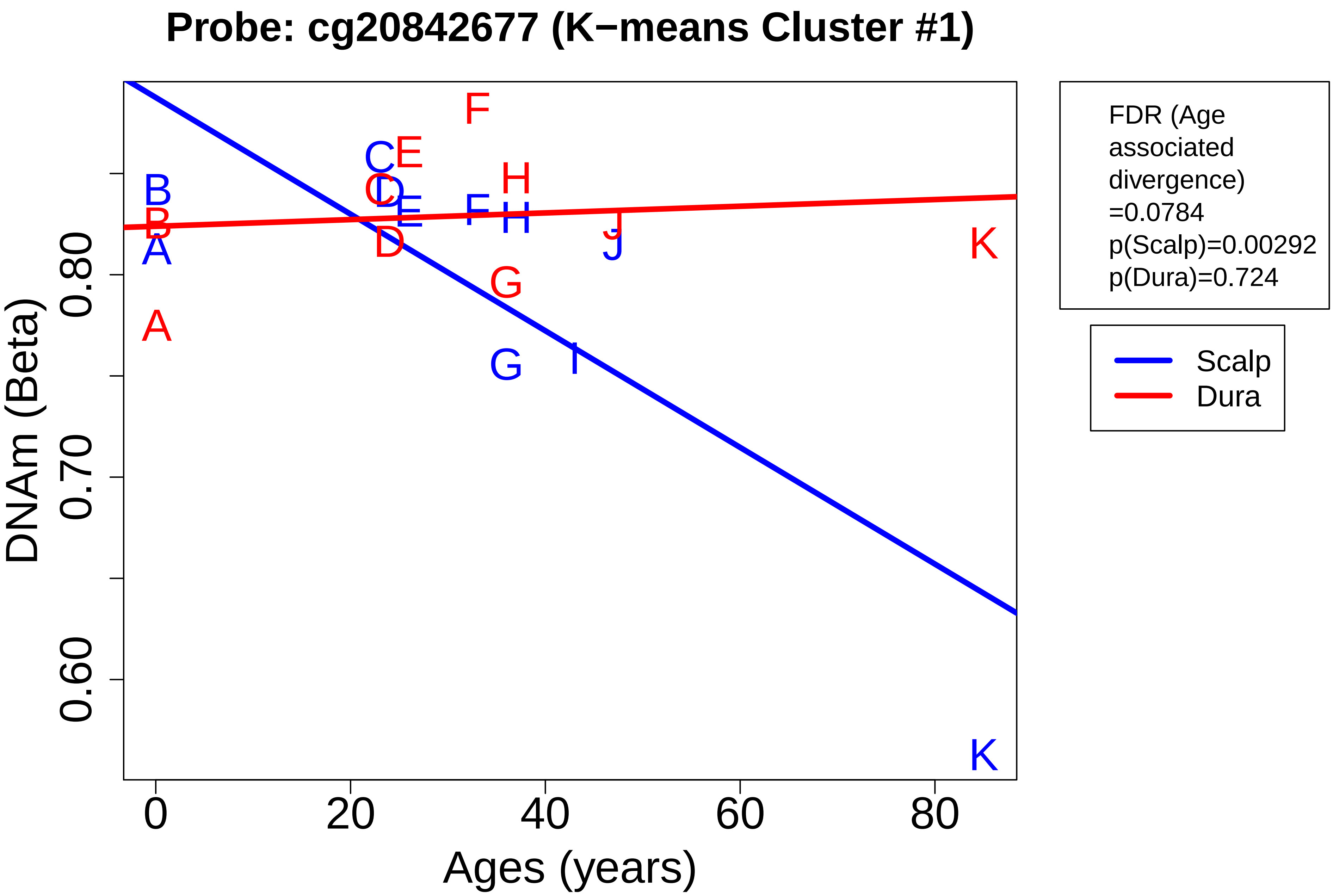

Probe: cg20842677 (K−means Cluster #1)
DNAm (Beta)
0.60
0.70
0.80
0
20
40
60
80
Ages (years)
FDR (Age associated divergence) =0.0784
p(Scalp)=0.00292
p(Dura)=0.724
Scalp
Dura

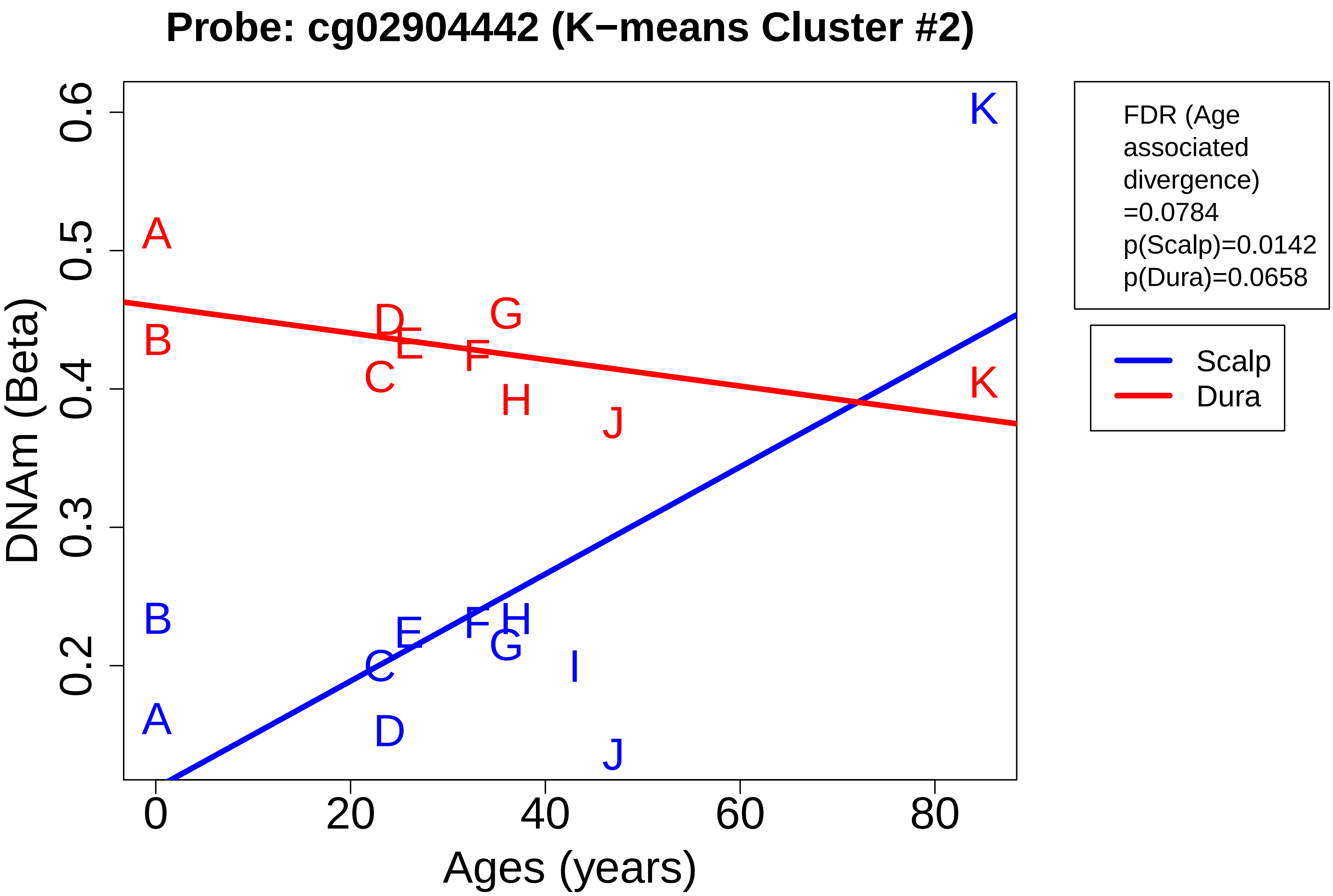

Probe: cg02904442 (K−means Cluster #2)
DNAm (Beta)
0.2
0.3
0.4
0.5
0.6
0
20
40
60
80
Ages (years)
A
B
C
D
E
F
G
H
I
J
K
FDR (Age associated divergence) =0.0784
p(Scalp)=0.0142
p(Dura)=0.0658
Scalp
Dura

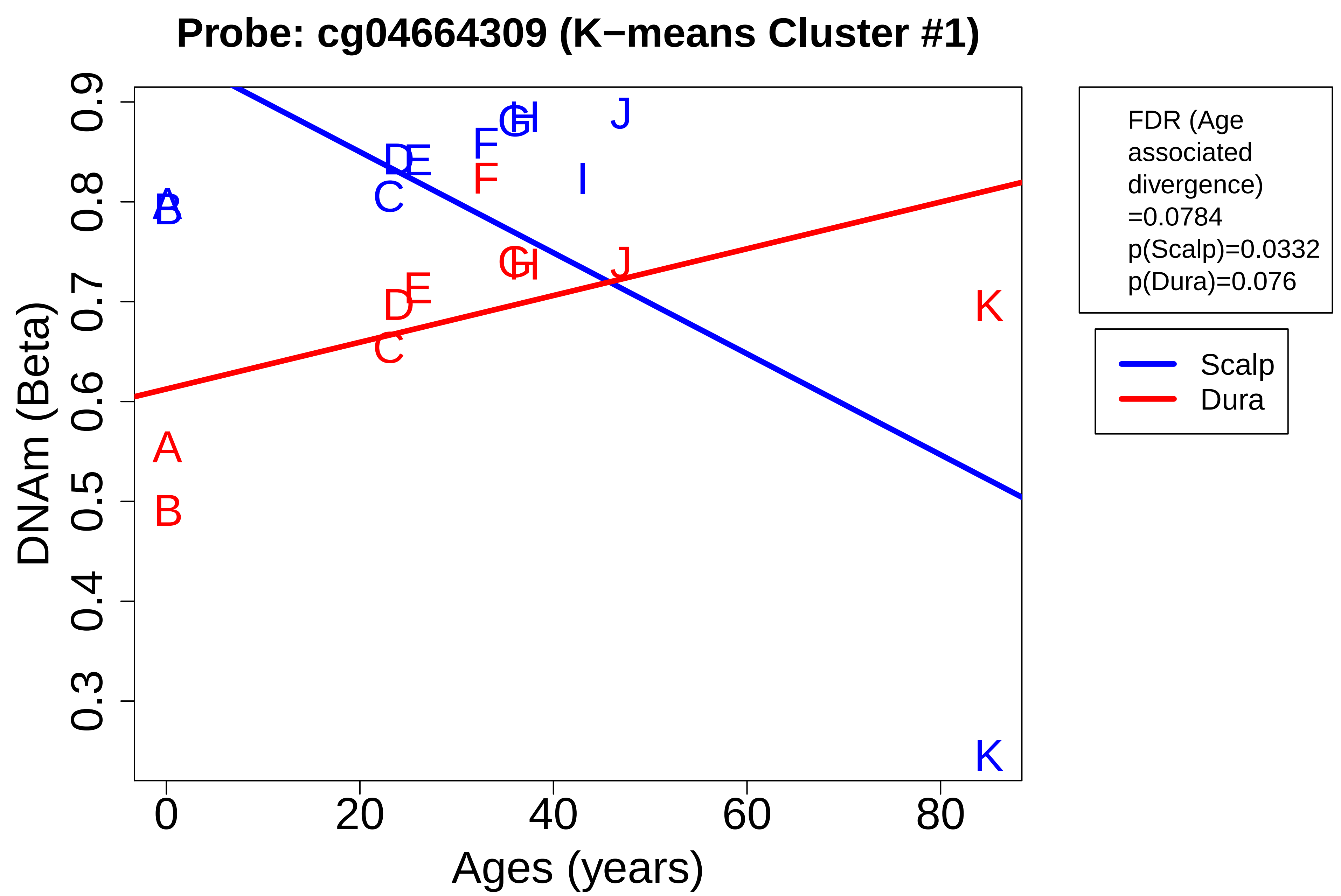
Probe: cg04664309 (K−means Cluster #1)
DNAm (Beta)
0.3
0.4
0.5
0.6
0.7
0.8
0.9
0
20
40
60
80
Ages (years)
FDR (Age associated divergence) =0.0784
p(Scalp)=0.0332
p(Dura)=0.076
Scalp
Dura

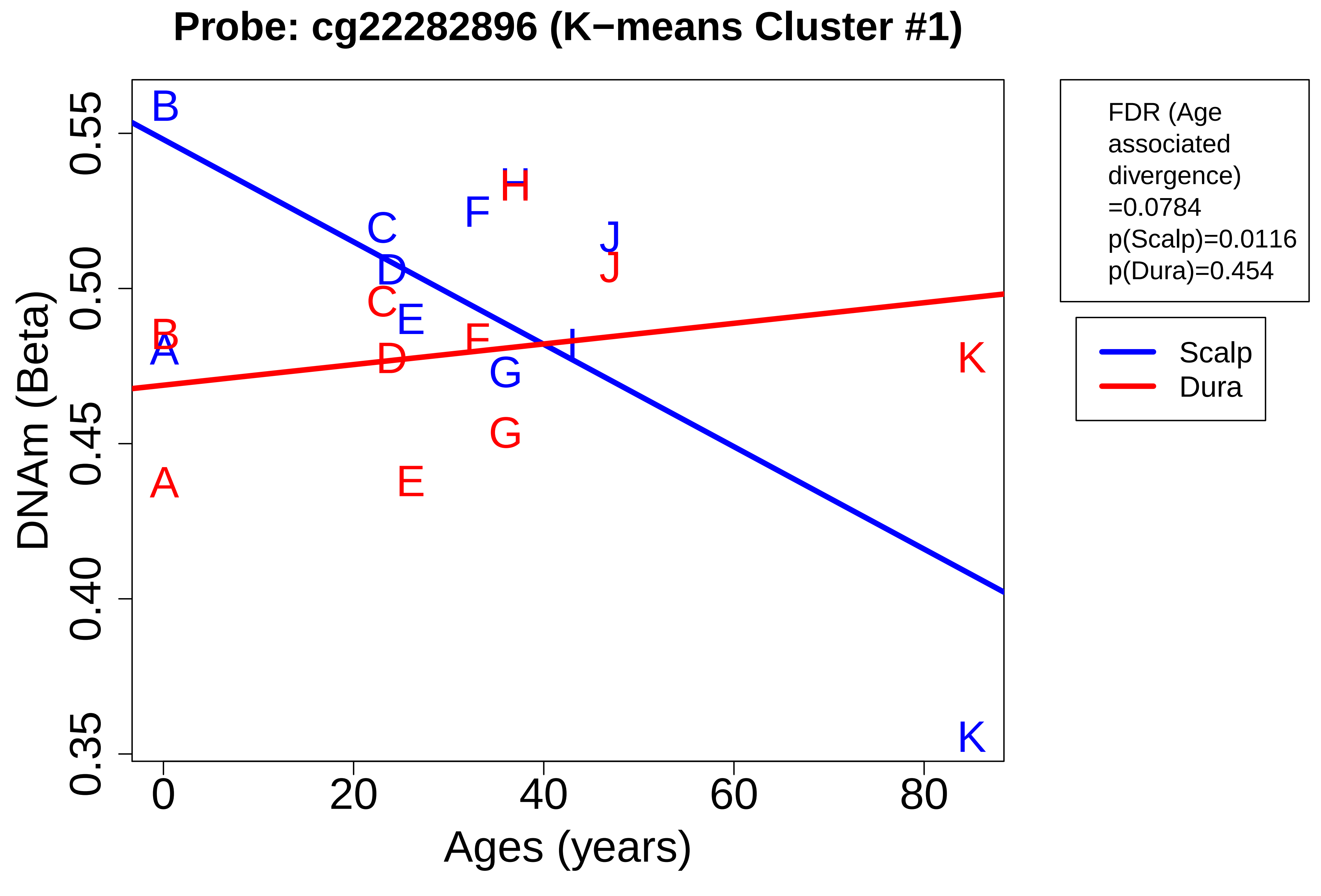

Probe: cg22282896 (K−means Cluster #1)
DNAm (Beta)
Ages (years)
0.35
0.40
0.45
0.50
0.55
0
20
40
60
80
FDR (Age associated divergence) =0.0784
p(Scalp)=0.0116
p(Dura)=0.454
Scalp
Dura

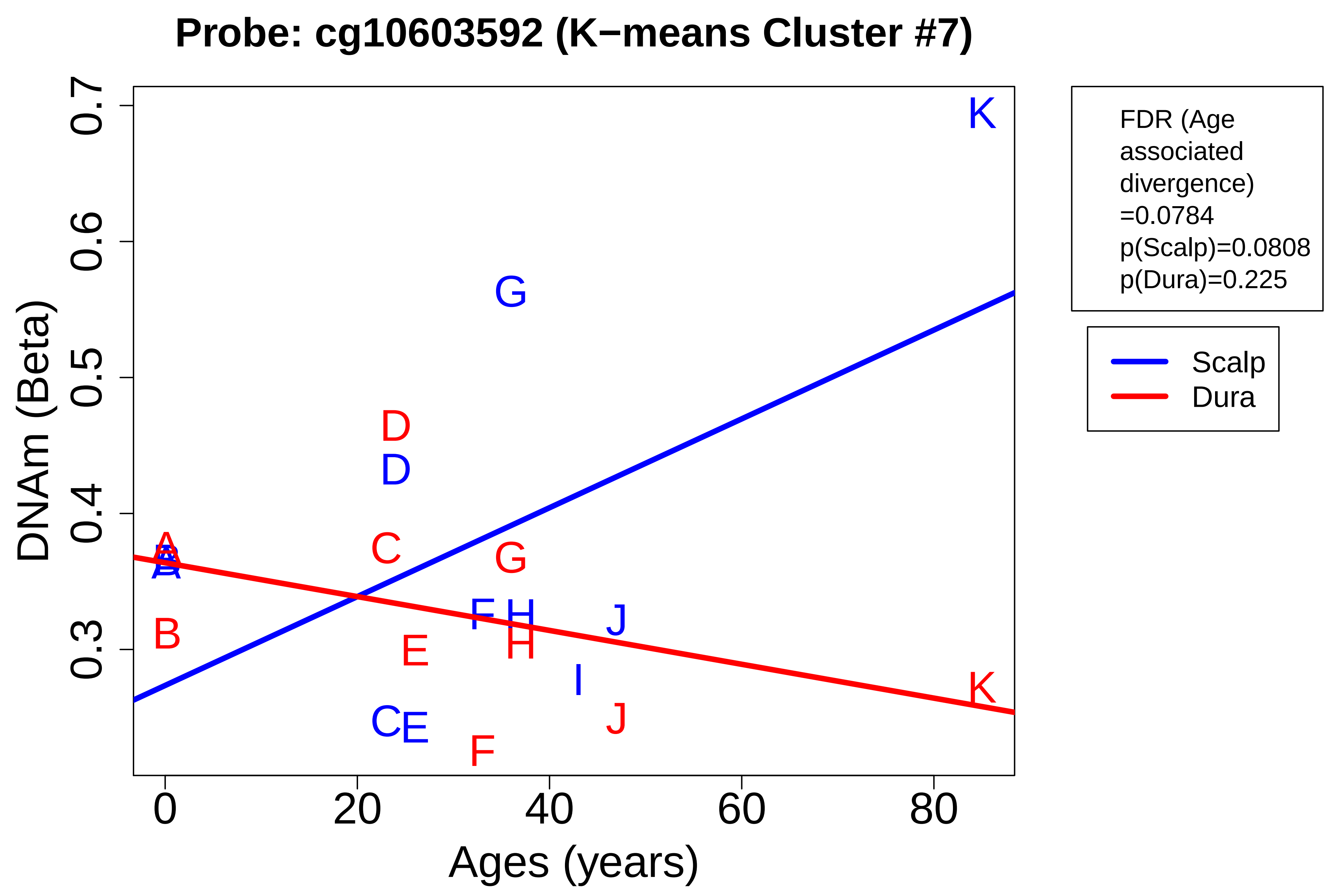

Probe: cg10603592 (K−means Cluster #7)
DNAm (Beta)
0.3
0.4
0.5
0.6
0.7
0
20
40
60
80
Ages (years)
FDR (Age associated divergence) =0.0784
p(Scalp)=0.0808
p(Dura)=0.225
Scalp
Dura

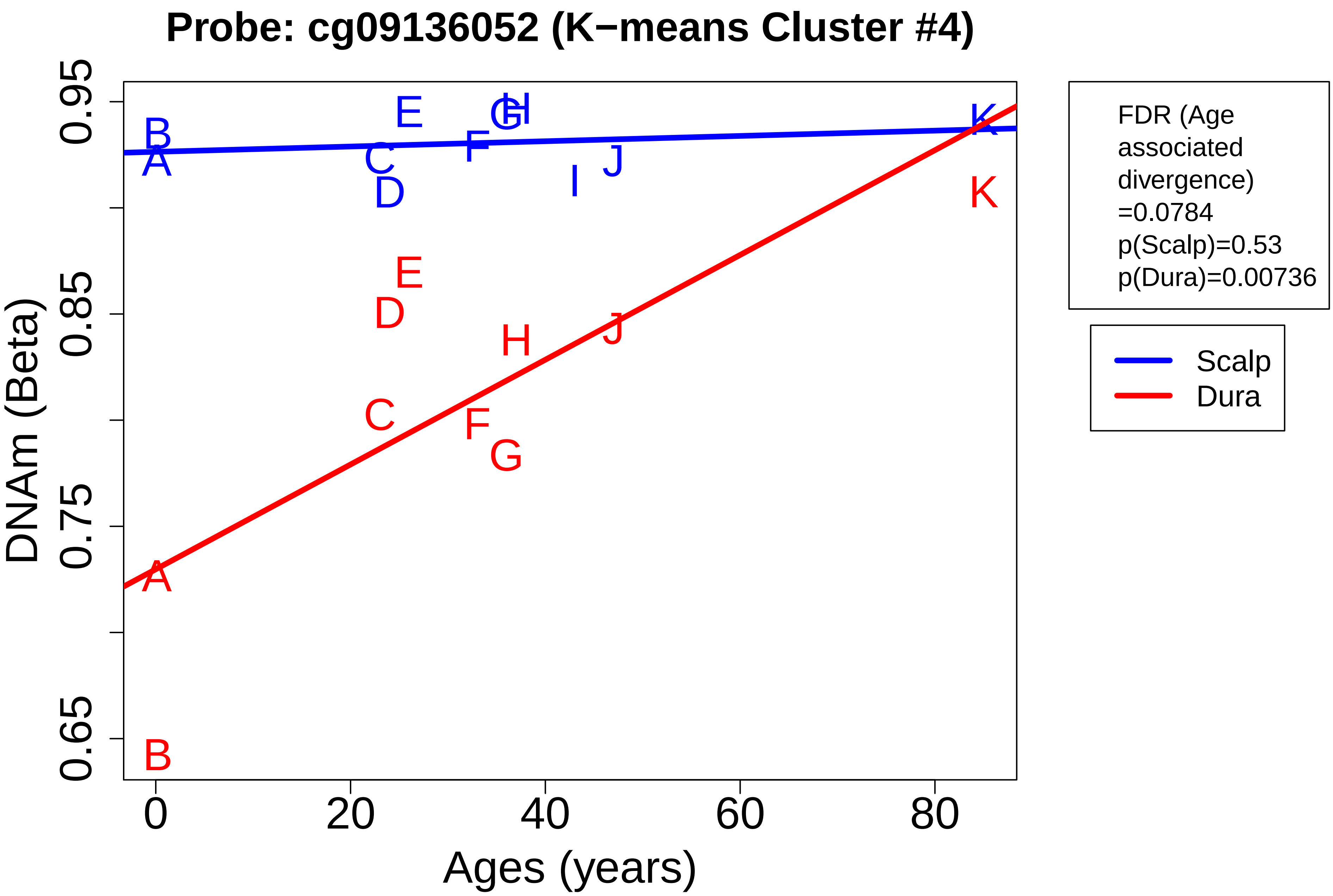

Probe: cg09136052 (K−means Cluster #4)
DNAm (Beta)
0.95
0.85
0.75
0.65
0
20
40
60
80
Ages (years)
FDR (Age associated divergence) =0.0784
p(Scalp)=0.53
p(Dura)=0.00736
Scalp
Dura

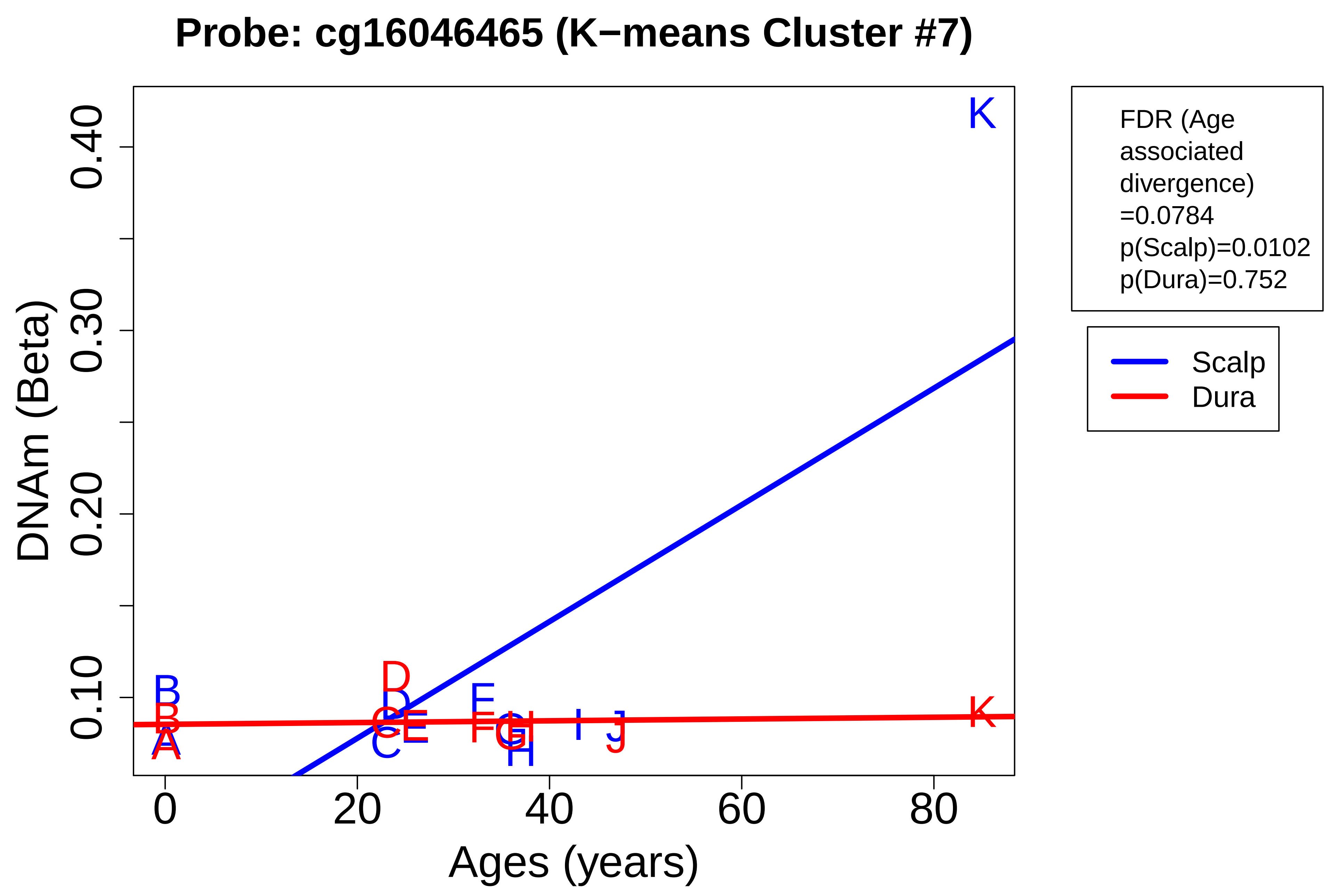

Probe: cg16046465 (K−means Cluster #7)
DNAm (Beta)
0.10
0.20
0.30
0.40
Ages (years)
0
20
40
60
80
A
B
C
D
E
F
G
H
I
J
K
FDR (Age associated divergence) =0.0784
p(Scalp)=0.0102
p(Dura)=0.752
Scalp
Dura

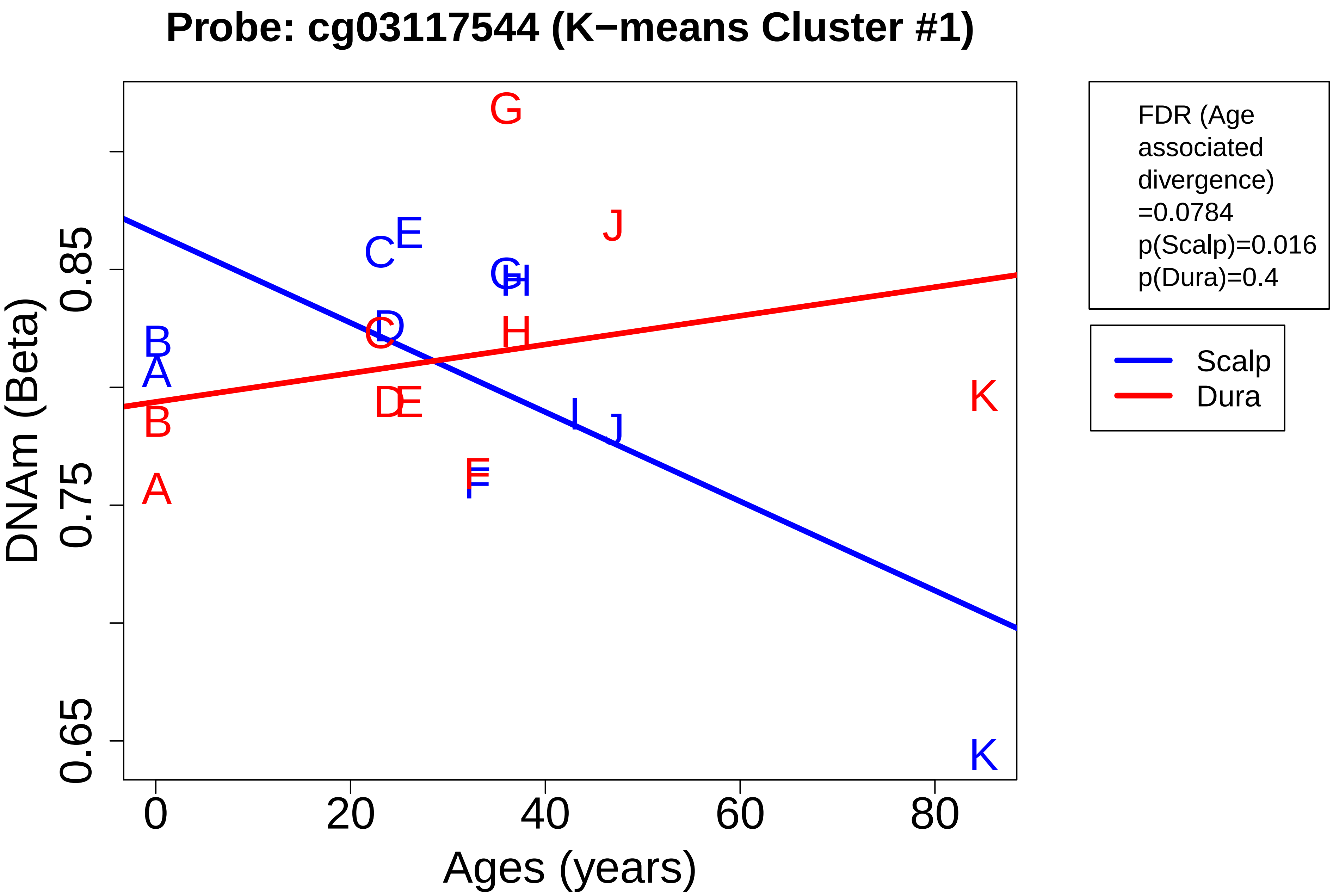

Probe: cg03117544 (K−means Cluster #1)
DNAm (Beta)
0.65
0.75
0.85
0
20
40
60
80
Ages (years)
FDR (Age associated divergence) =0.0784
p(Scalp)=0.016
p(Dura)=0.4
Scalp
Dura
A B C D E F G H I J K

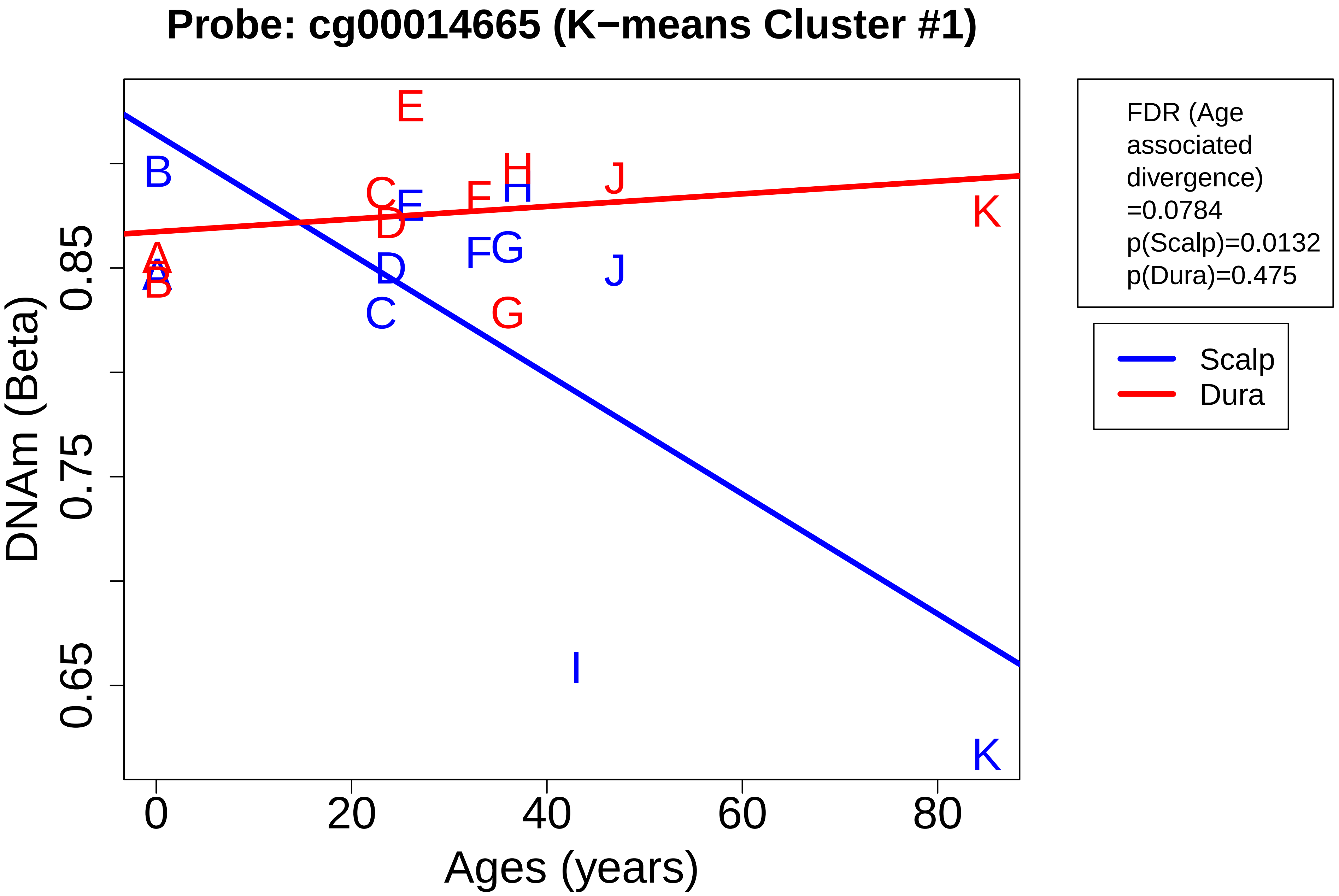

Probe: cg00014665 (K−means Cluster #1)
DNAm (Beta)
0.65
0.75
0.85
0
20
40
60
80
Ages (years)
A
B
C
D
E
F
G
H
I
J
K
FDR (Age associated divergence) =0.0784
p(Scalp)=0.0132
p(Dura)=0.475
Scalp
Dura

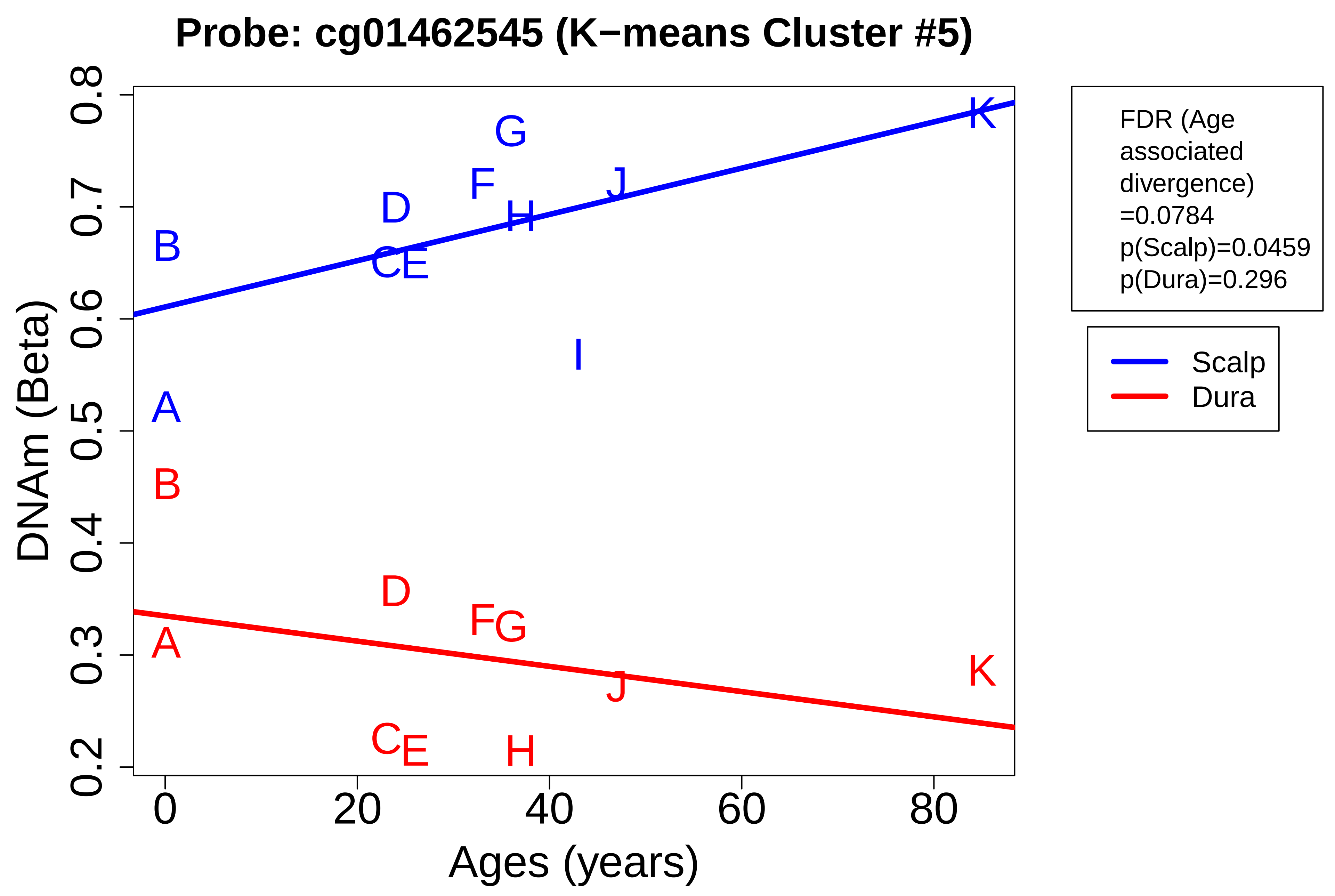

Probe: cg01462545 (K−means Cluster #5)
DNAm (Beta)
0.2
0.3
0.4
0.5
0.6
0.7
0.8
0
20
40
60
80
Ages (years)
A
B
CE
D
F
G
H
I
J
K
A
B
CE
D
FG
H
J
K
FDR (Age associated divergence) =0.0784
p(Scalp)=0.0459
p(Dura)=0.296
Scalp
Dura

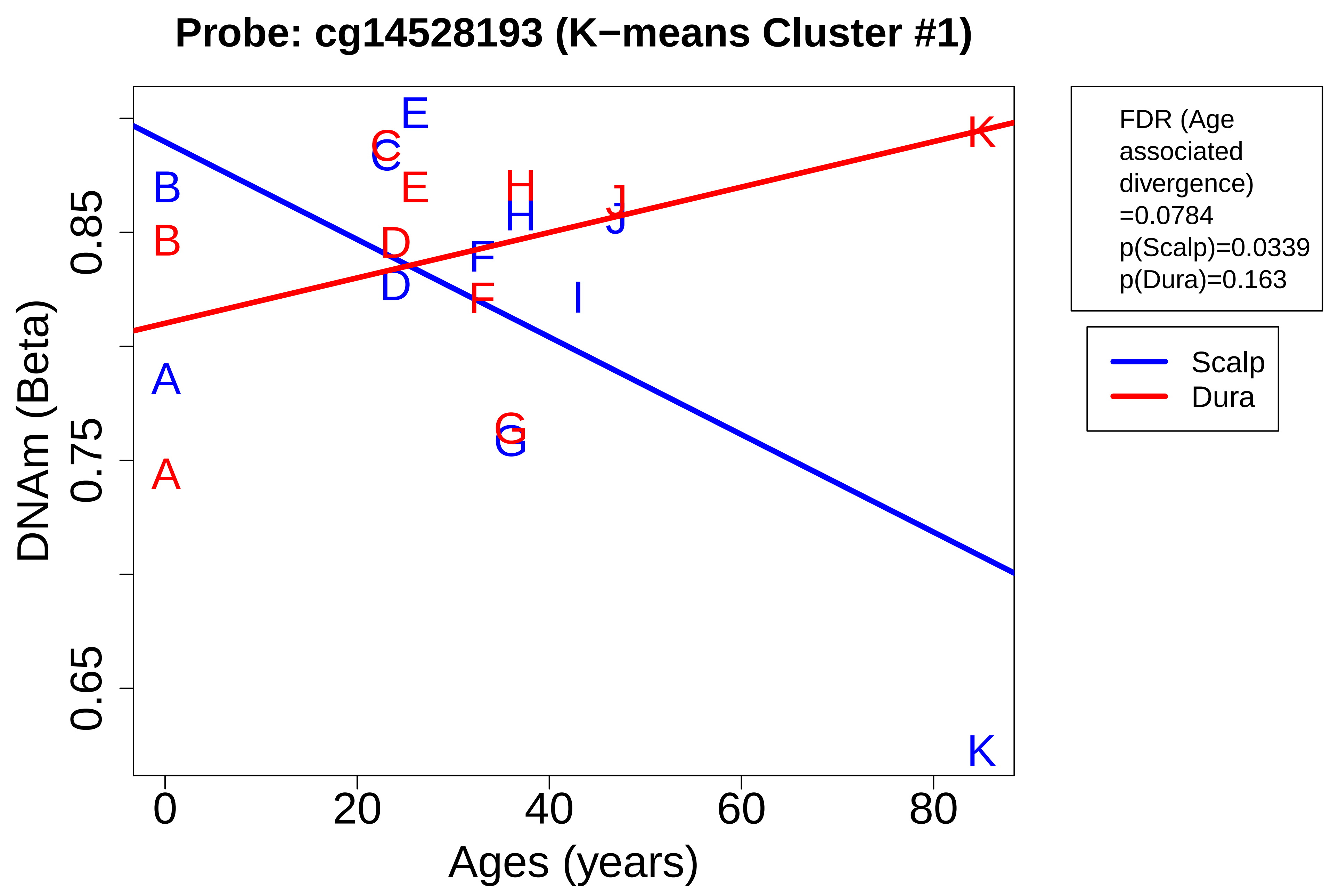
Probe: cg14528193 (K−means Cluster #1)
DNAm (Beta)
0.65
0.75
0.85
Ages (years)
0
20
40
60
80
A
B
C
D
E
F
G
H
I
J
K
FDR (Age associated divergence) =0.0784
p(Scalp)=0.0339
p(Dura)=0.163
Scalp
Dura

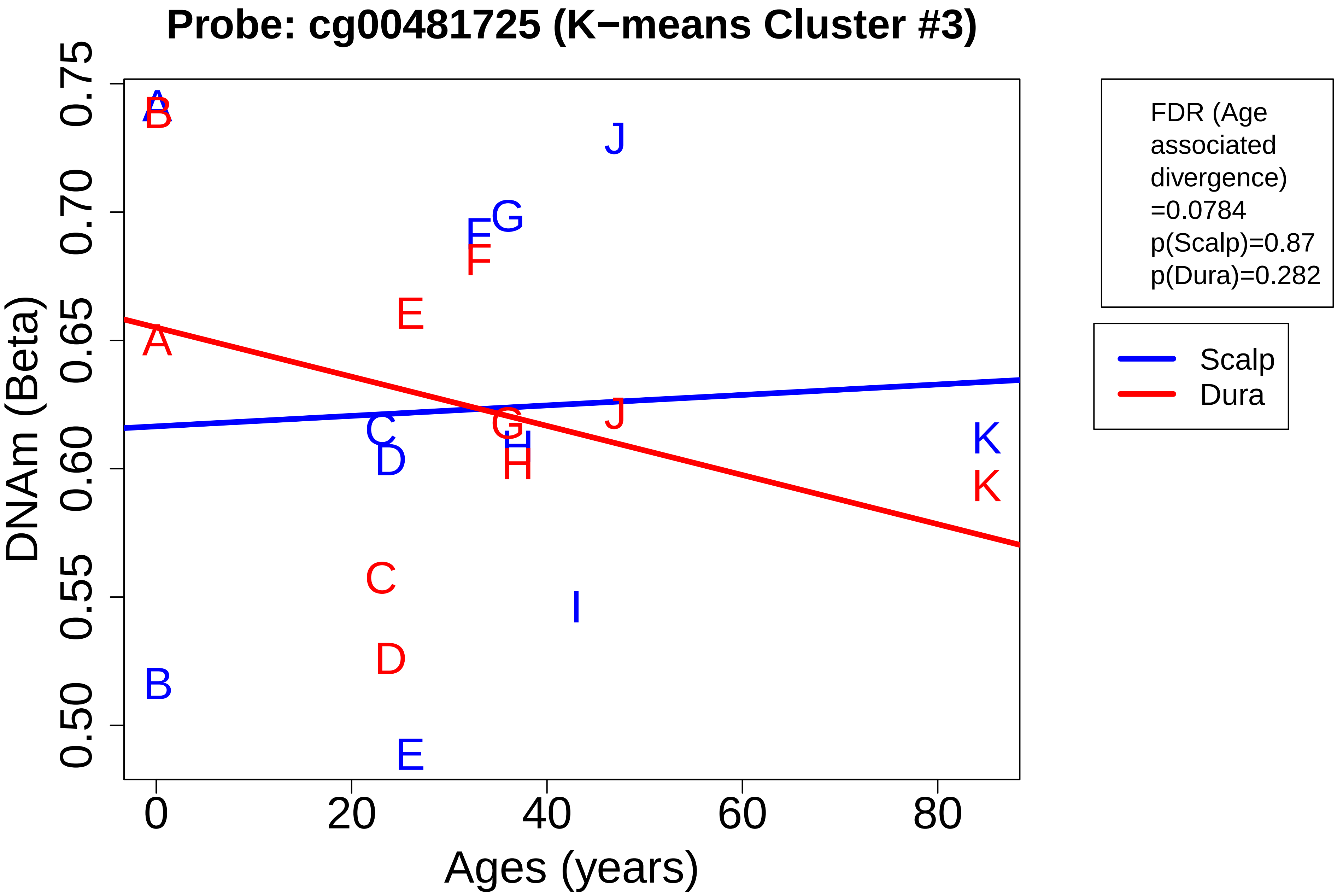

Probe: cg00481725 (K−means Cluster #3)
DNAm (Beta)
0.50
0.55
0.60
0.65
0.70
0.75
0
20
40
60
80
Ages (years)
A
B
C
D
E
F
G
H
I
J
K
FDR (Age associated divergence) =0.0784
p(Scalp)=0.87
p(Dura)=0.282
Scalp
Dura

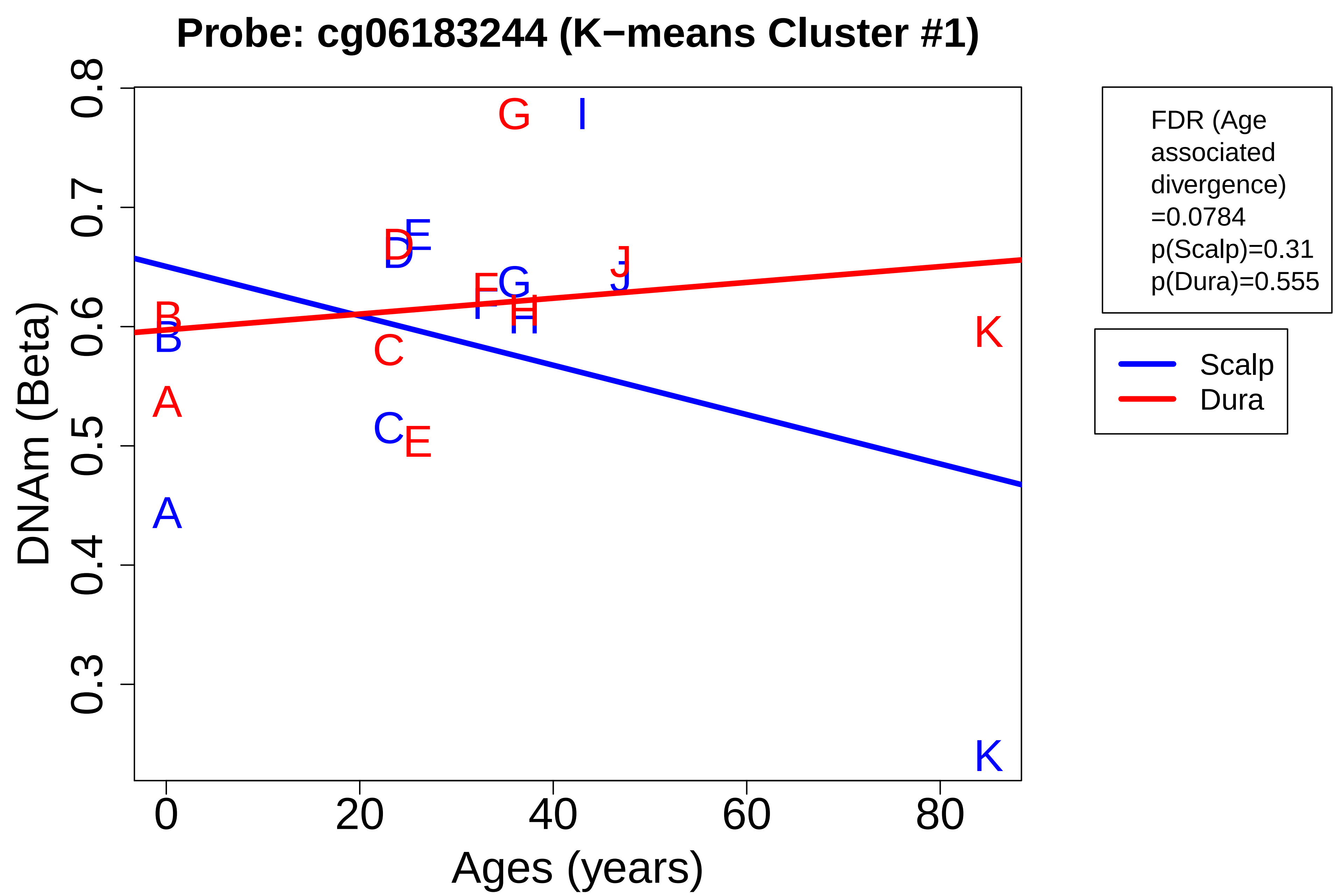
Probe: cg06183244 (K−means Cluster #1)
DNAm (Beta)
0.3
0.4
0.5
0.6
0.7
0.8
Ages (years)
0
20
40
60
80
FDR (Age associated divergence) =0.0784
p(Scalp)=0.31
p(Dura)=0.555
Scalp
Dura
A
B
C
D
E
F
G
H
I
J
K

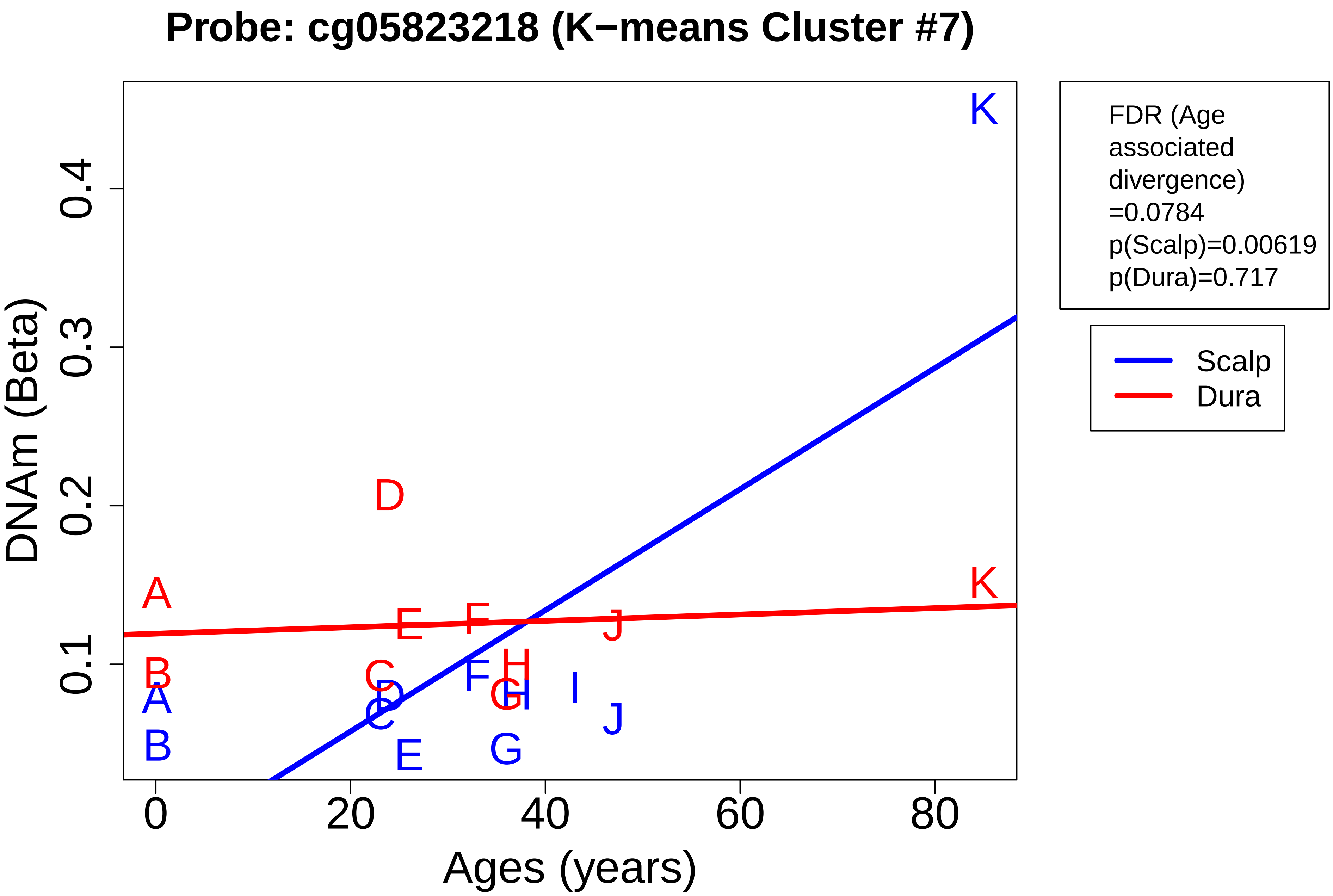

Probe: cg05823218 (K−means Cluster #7)
DNAm (Beta)
0.1
0.2
0.3
0.4
Ages (years)
0
20
40
60
80
FDR (Age associated divergence) =0.0784
p(Scalp)=0.00619
p(Dura)=0.717
Scalp
Dura
A
B
C
D
E
F
G
H
I
J
K

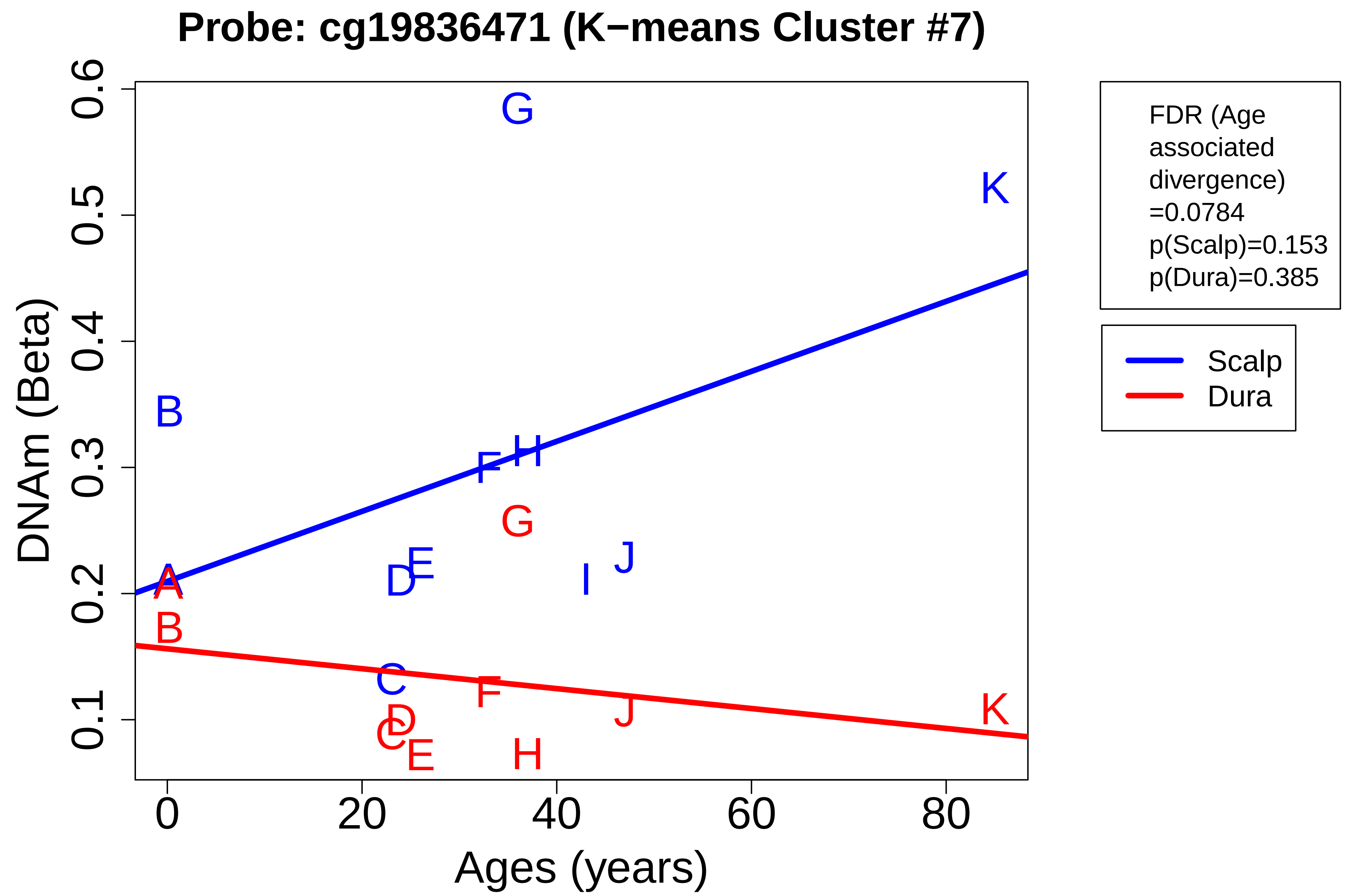

Probe: cg19836471 (K−means Cluster #7)
DNAm (Beta)
0.1
0.2
0.3
0.4
0.5
0.6
Ages (years)
0
20
40
60
80
FDR (Age associated divergence) =0.0784
p(Scalp)=0.153
p(Dura)=0.385
Scalp
Dura

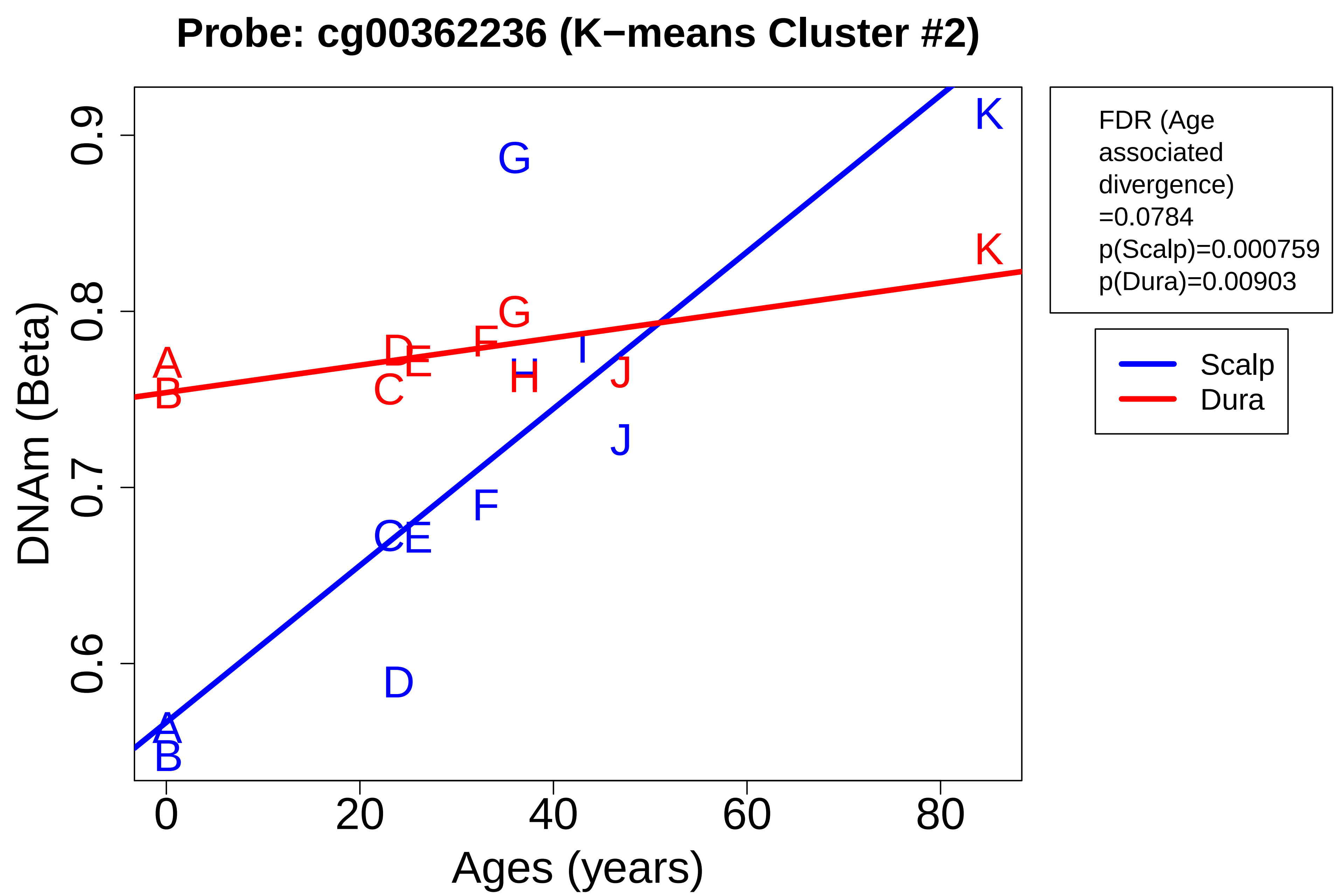

Probe: cg00362236 (K−means Cluster #2)
DNAm (Beta)
0.6
0.7
0.8
0.9
0
20
40
60
80
Ages (years)
A
B
C
D
E
F
G
H
I
J
K
FDR (Age associated divergence) =0.0784
p(Scalp)=0.000759
p(Dura)=0.00903
Scalp
Dura

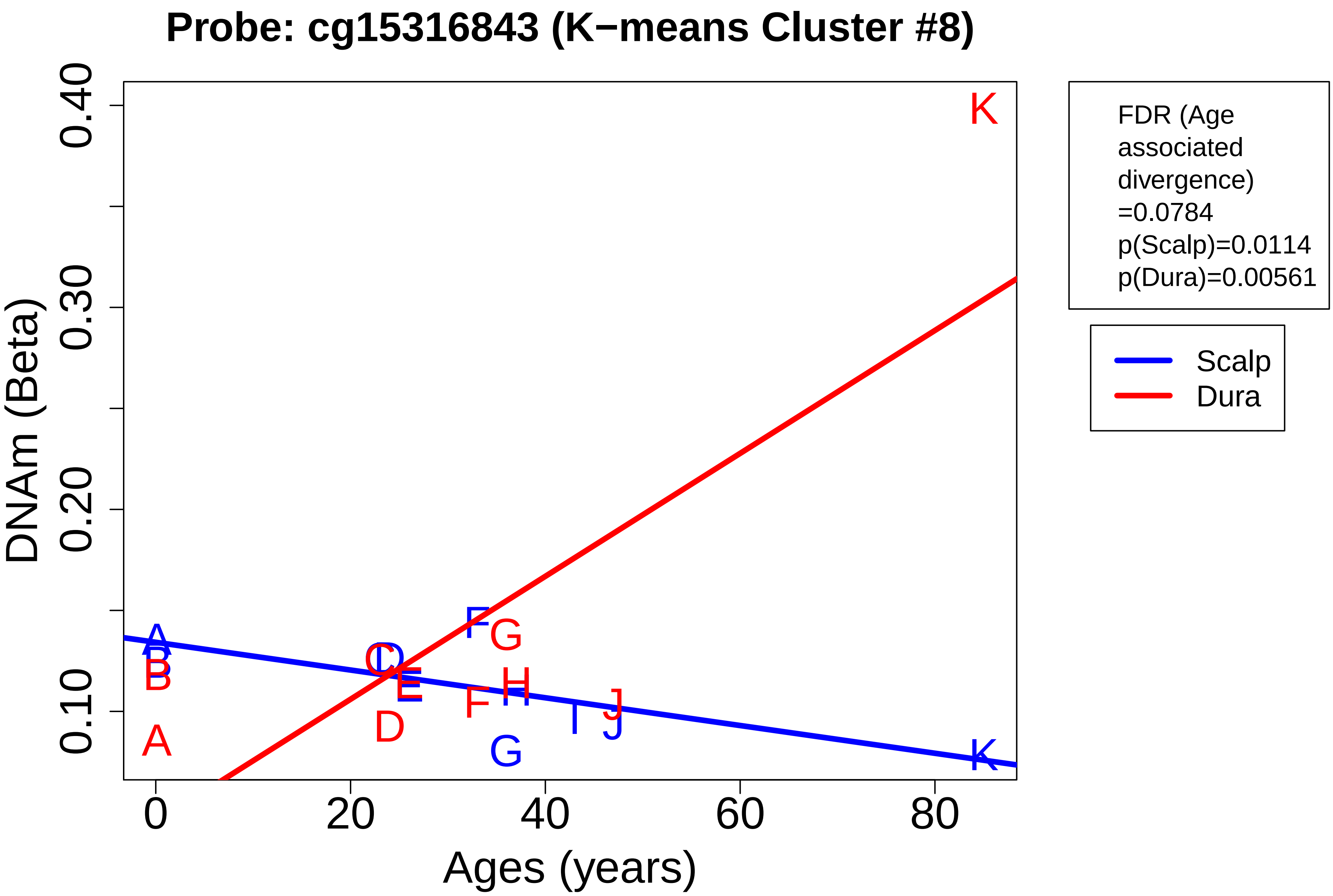

Probe: cg15316843 (K−means Cluster #8)
DNAm (Beta)
0.10
0.20
0.30
0.40
0
20
40
60
80
Ages (years)
FDR (Age associated divergence) =0.0784
p(Scalp)=0.0114
p(Dura)=0.00561
Scalp
Dura

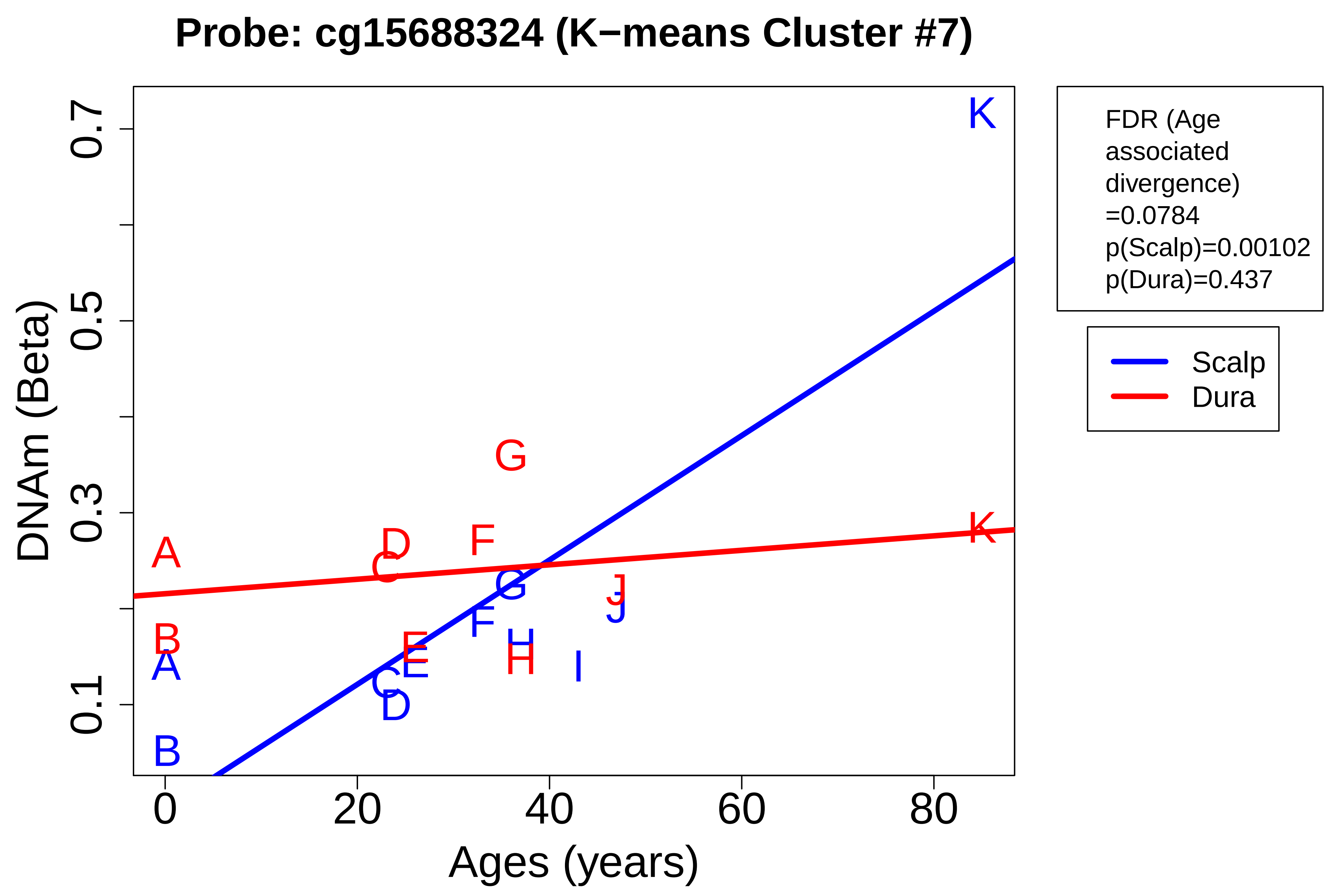

Probe: cg15688324 (K−means Cluster #7)
DNAm (Beta)
0.1
0.3
0.5
0.7
0
20
40
60
80
Ages (years)
A
B
C
D
E
F
G
H
I
J
K
FDR (Age associated divergence) =0.0784
p(Scalp)=0.00102
p(Dura)=0.437
Scalp
Dura

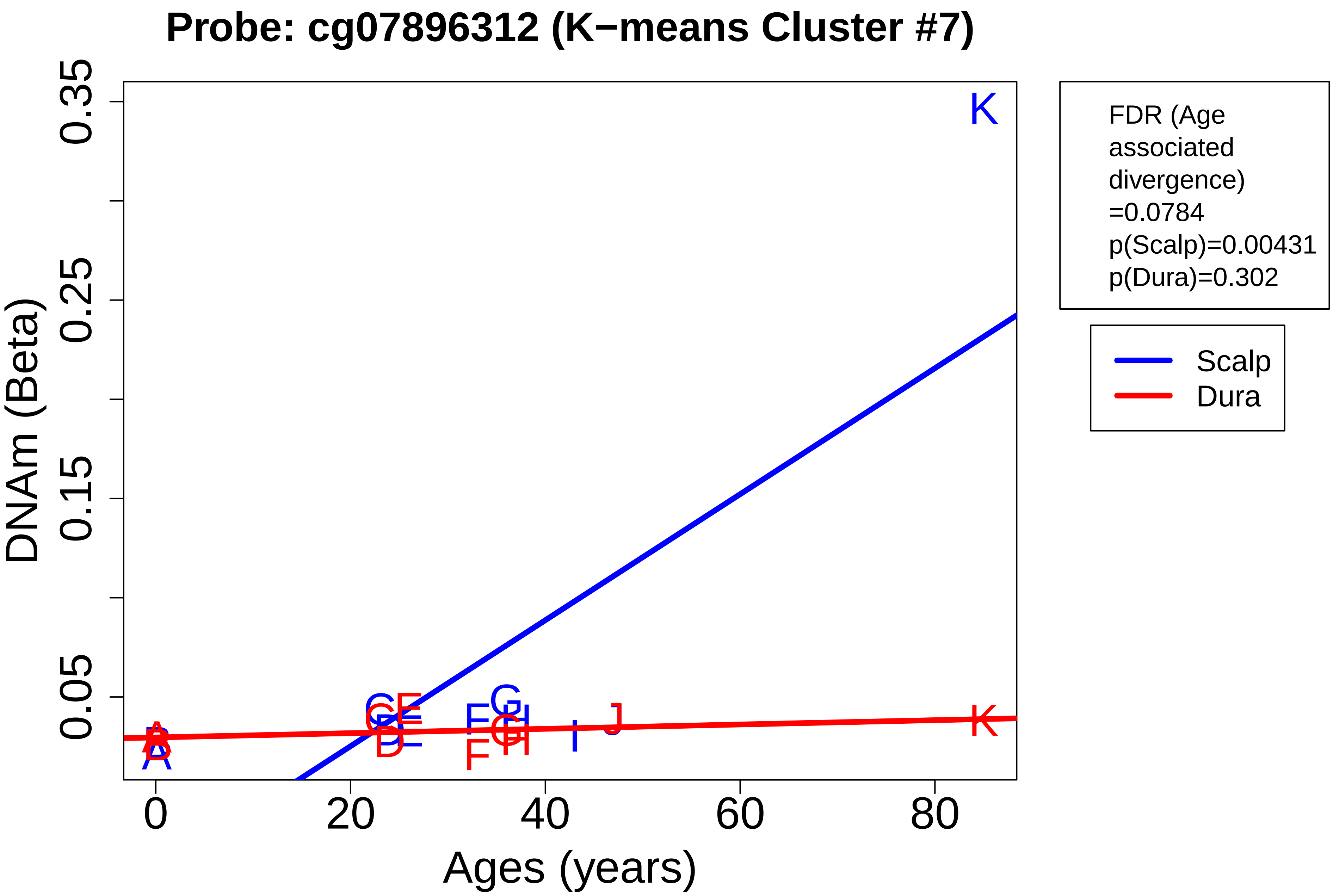

Probe: cg07896312 (K−means Cluster #7)
DNAm (Beta)
0.05
0.15
0.25
0.35
Ages (years)
0
20
40
60
80
FDR (Age associated divergence) =0.0784
p(Scalp)=0.00431
p(Dura)=0.302
Scalp
Dura

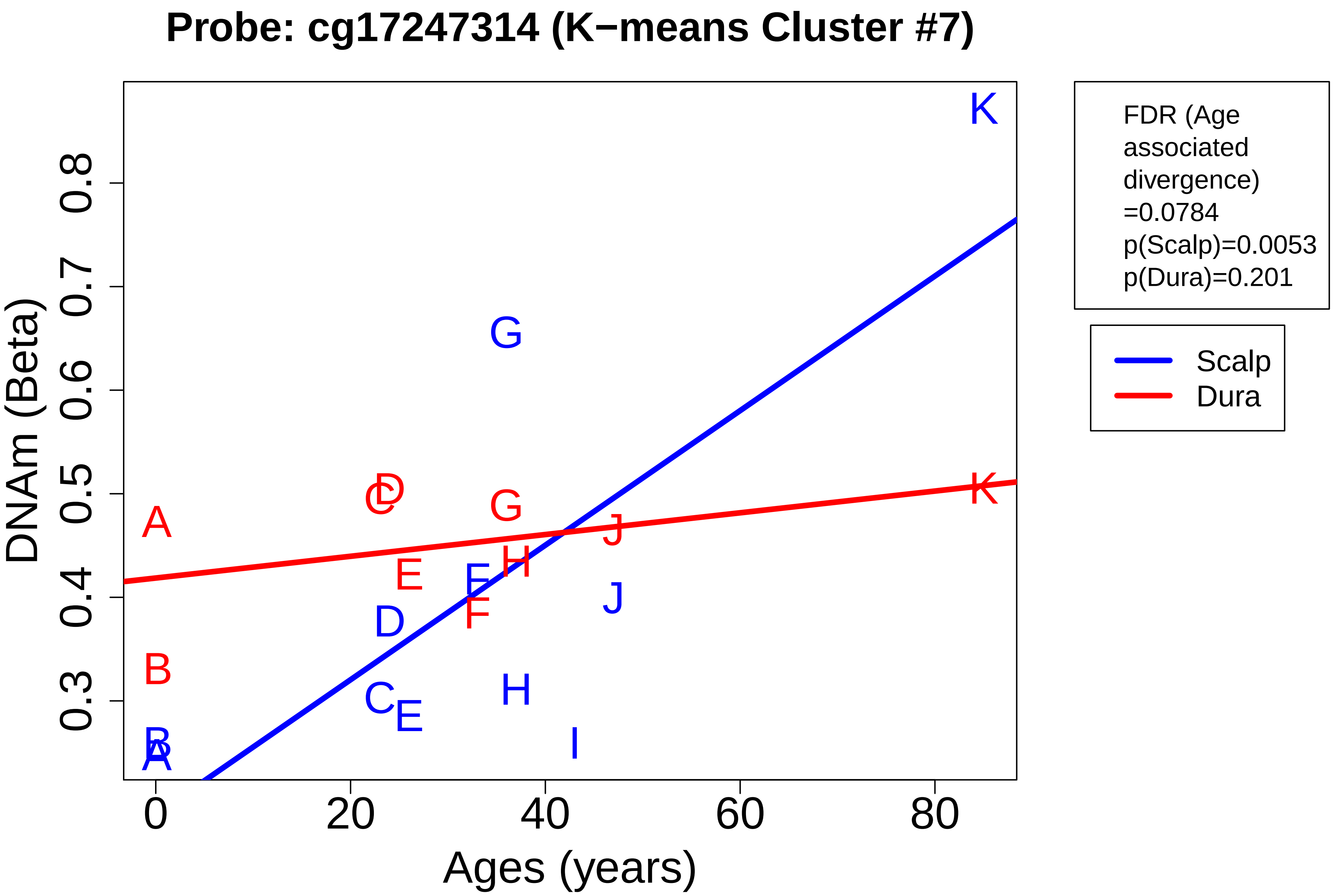

Probe: cg17247314 (K−means Cluster #7)
DNAm (Beta)
0.3
0.4
0.5
0.6
0.7
0.8
0
20
40
60
80
Ages (years)
FDR (Age associated divergence) =0.0784
p(Scalp)=0.0053
p(Dura)=0.201
Scalp
Dura
A
B
C
D
E
F
G
H
I
J
K

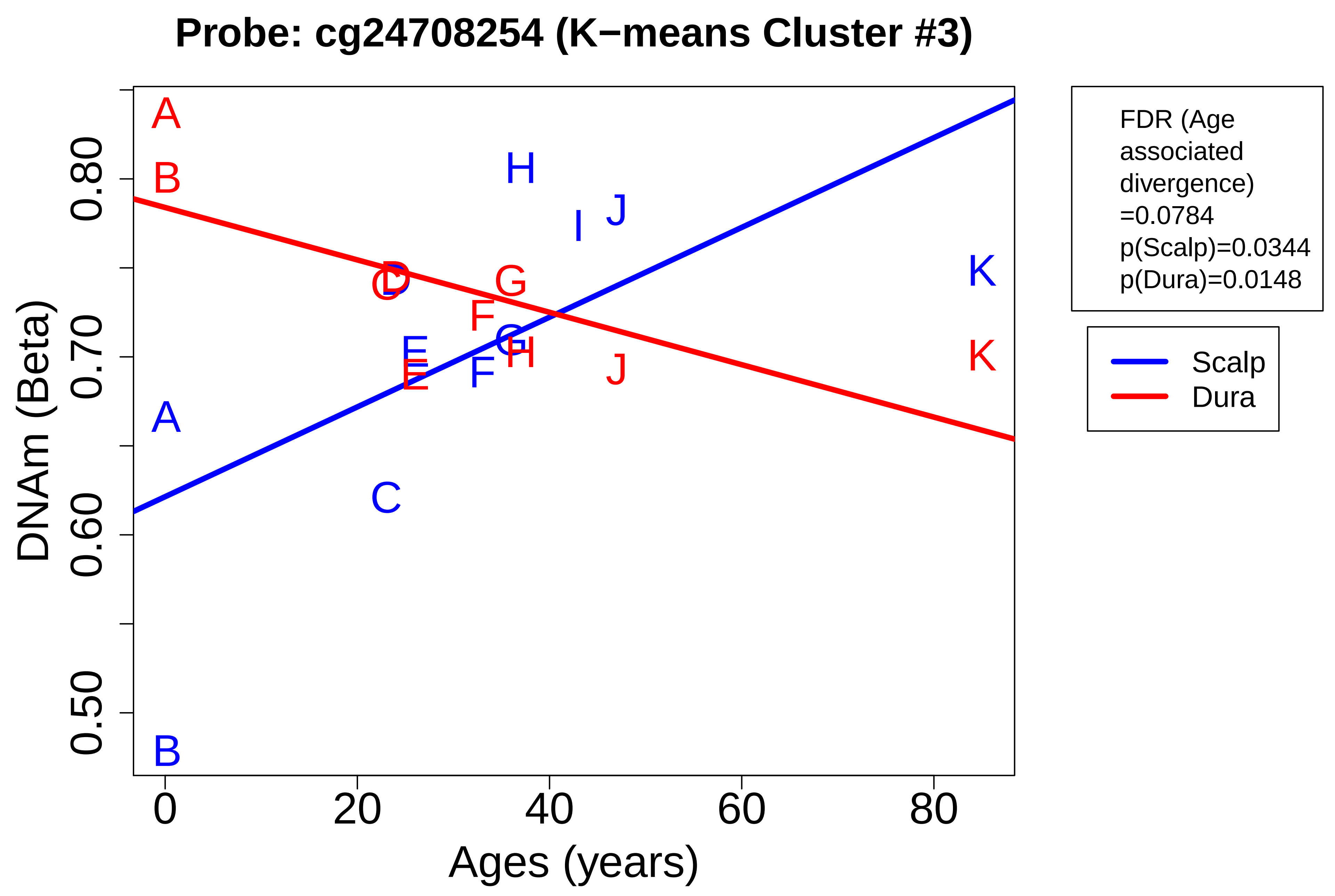

Probe: cg24708254 (K−means Cluster #3)
DNAm (Beta)
0.50
0.60
0.70
0.80
0
20
40
60
80
Ages (years)
FDR (Age associated divergence) =0.0784
p(Scalp)=0.0344
p(Dura)=0.0148
Scalp
Dura

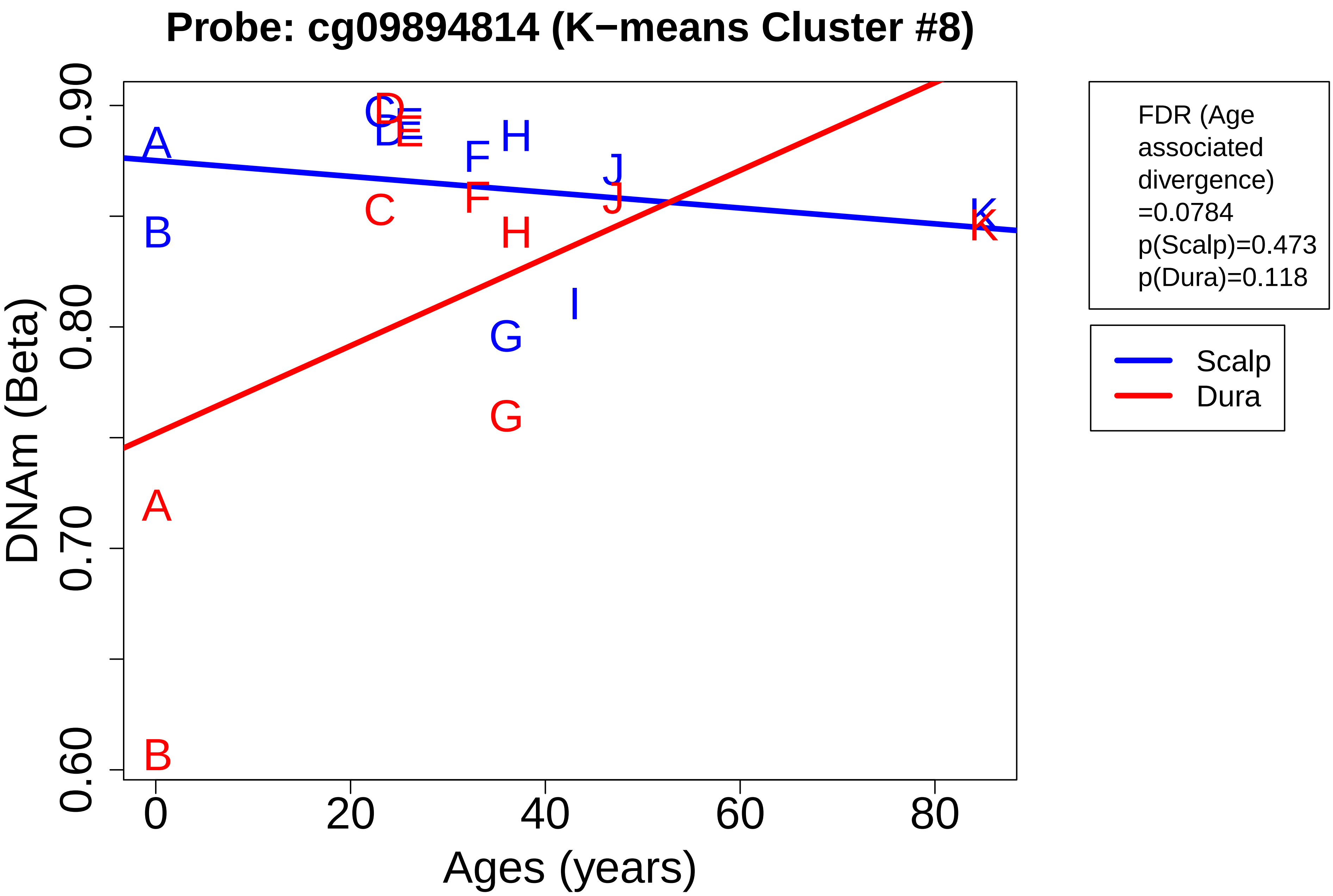

Probe: cg09894814 (K−means Cluster #8)
DNAm (Beta)
0.60
0.70
0.80
0.90
0
20
40
60
80
Ages (years)
FDR (Age associated divergence) =0.0784
p(Scalp)=0.473
p(Dura)=0.118
Scalp
Dura

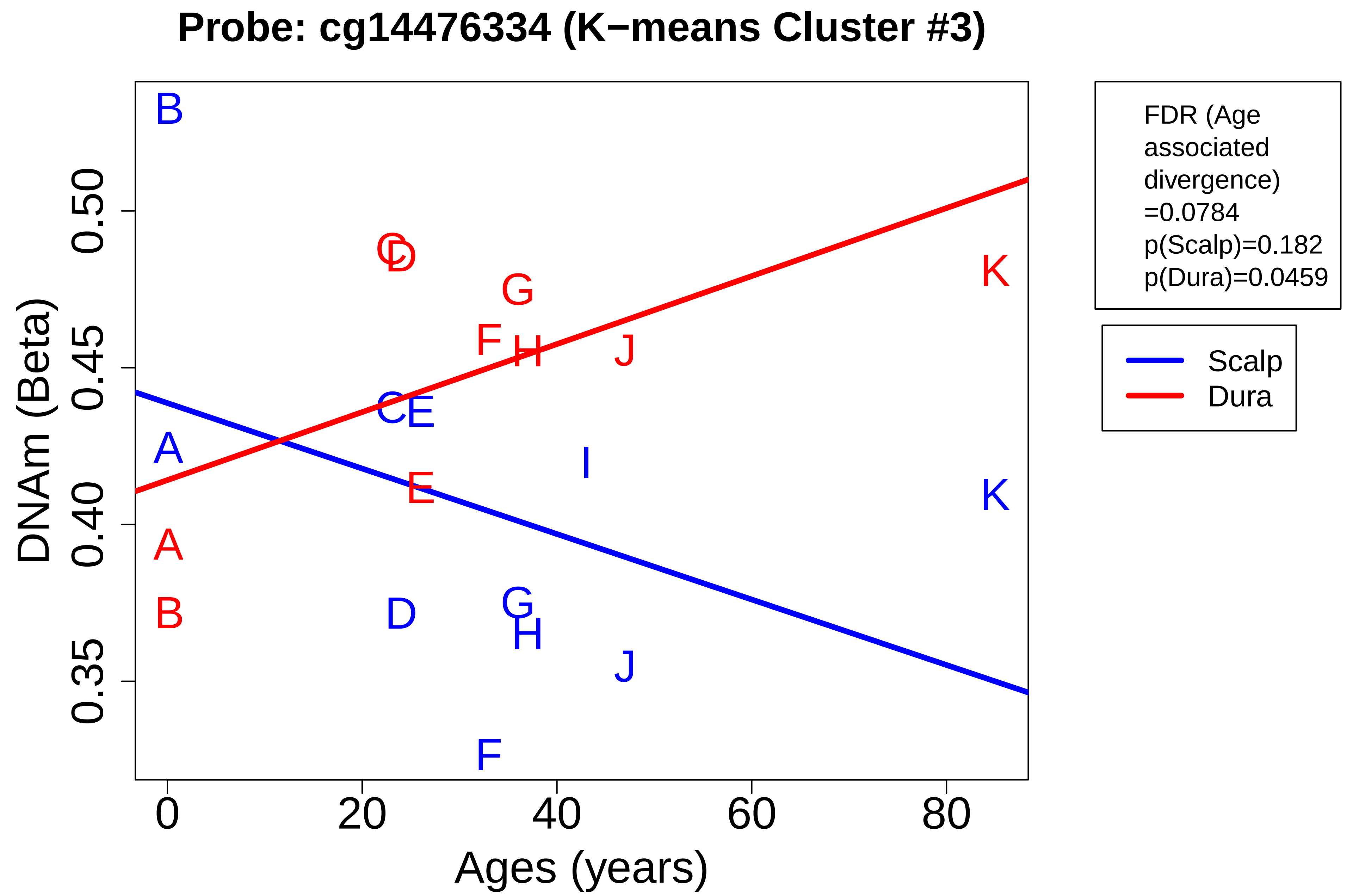

Probe: cg14476334 (K−means Cluster #3)
DNAm (Beta)
0.35
0.40
0.45
0.50
0
20
40
60
80
Ages (years)
B
C
D
G
K
F
H
J
C
E
A
I
E
K
A
B
D
G
H
J
F
FDR (Age associated divergence) =0.0784
p(Scalp)=0.182
p(Dura)=0.0459
Scalp
Dura

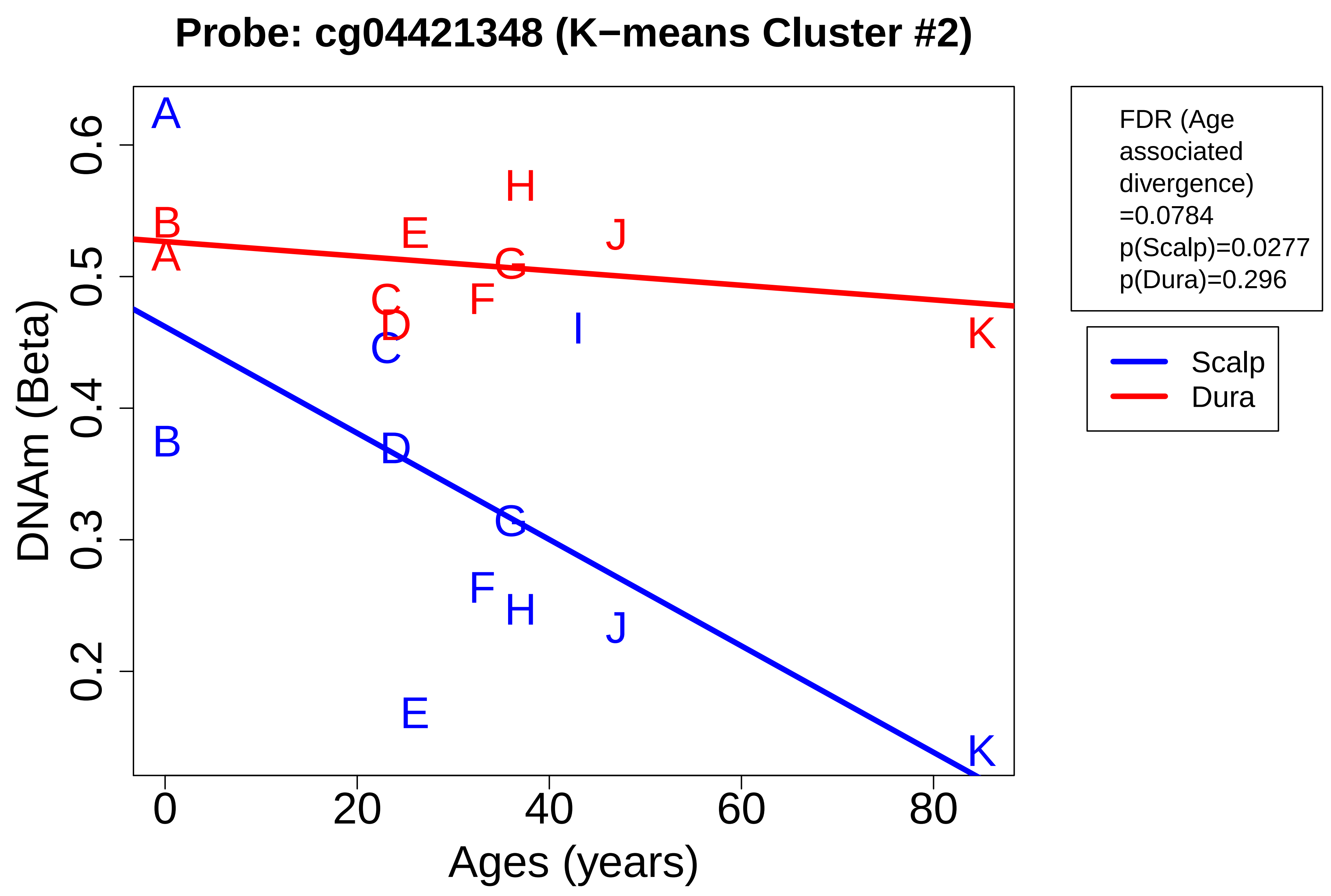

Probe: cg04421348 (K−means Cluster #2)
DNAm (Beta)
0.2
0.3
0.4
0.5
0.6
0
20
40
60
80
Ages (years)
A
B
A
B
E
H
J
G
C
D
F
C
I
K
D
G
F
H
J
E
K
FDR (Age associated divergence) =0.0784
p(Scalp)=0.0277
p(Dura)=0.296
Scalp
Dura

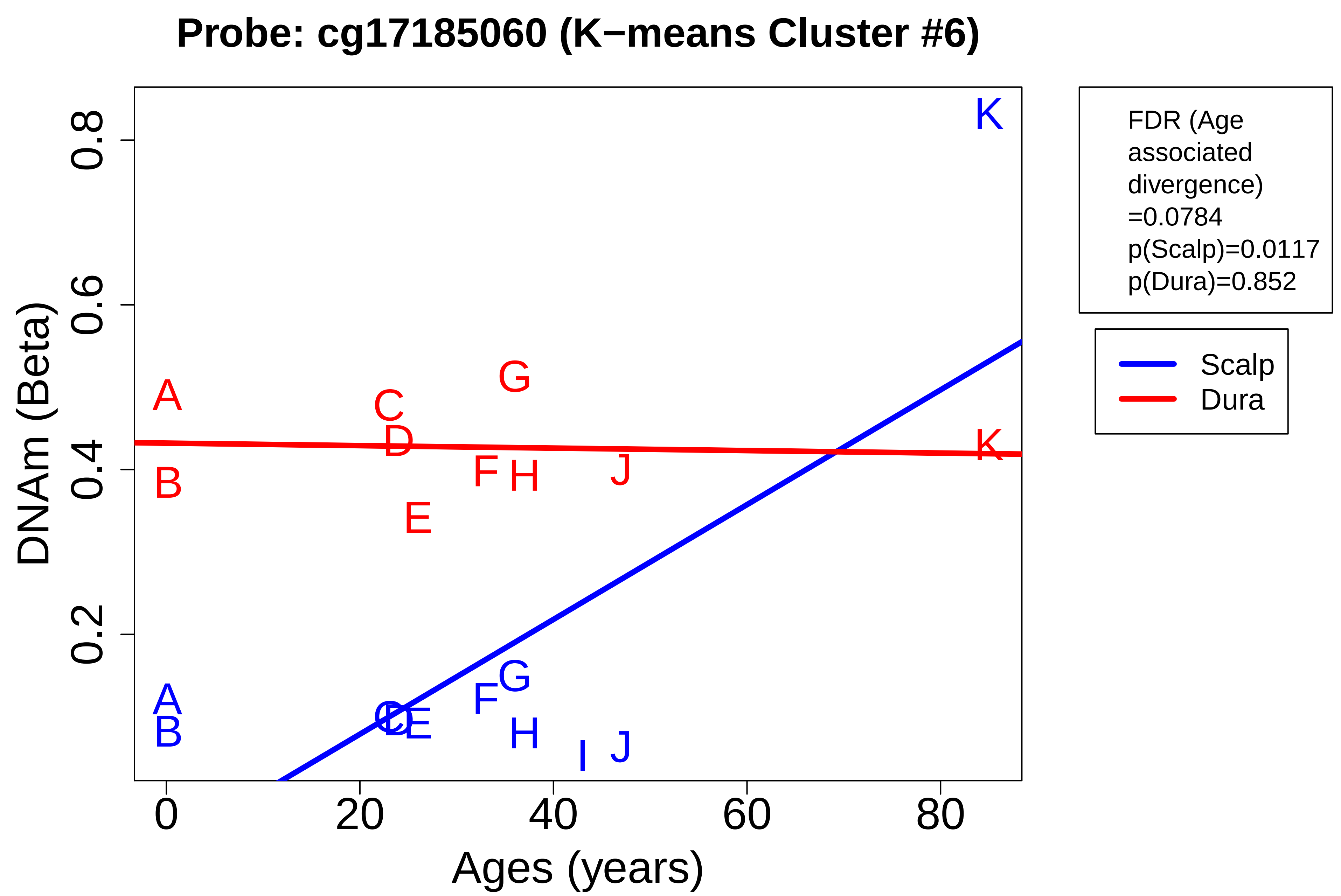

Probe: cg17185060 (K−means Cluster #6)
DNAm (Beta)
0.2
0.4
0.6
0.8
0
20
40
60
80
Ages (years)
A
B
C
D
E
F
G
H
I
J
K
FDR (Age associated divergence) =0.0784
p(Scalp)=0.0117
p(Dura)=0.852
Scalp
Dura

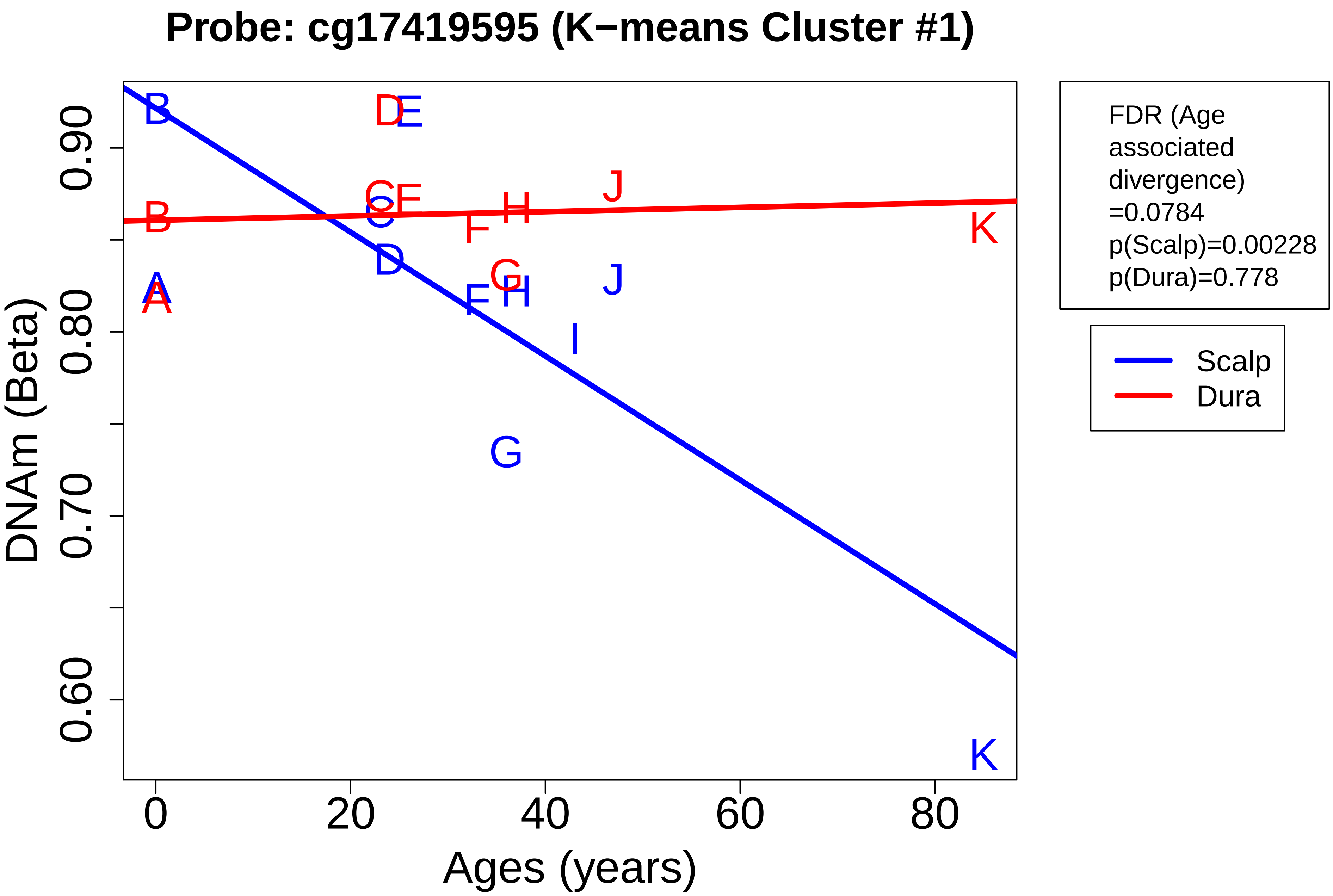
Probe: cg17419595 (K−means Cluster #1)
DNAm (Beta)
0.60
0.70
0.80
0.90
0
20
40
60
80
Ages (years)
FDR (Age associated divergence) =0.0784
p(Scalp)=0.00228
p(Dura)=0.778
Scalp
Dura
A
B
C
D
E
F
G
H
I
J
K

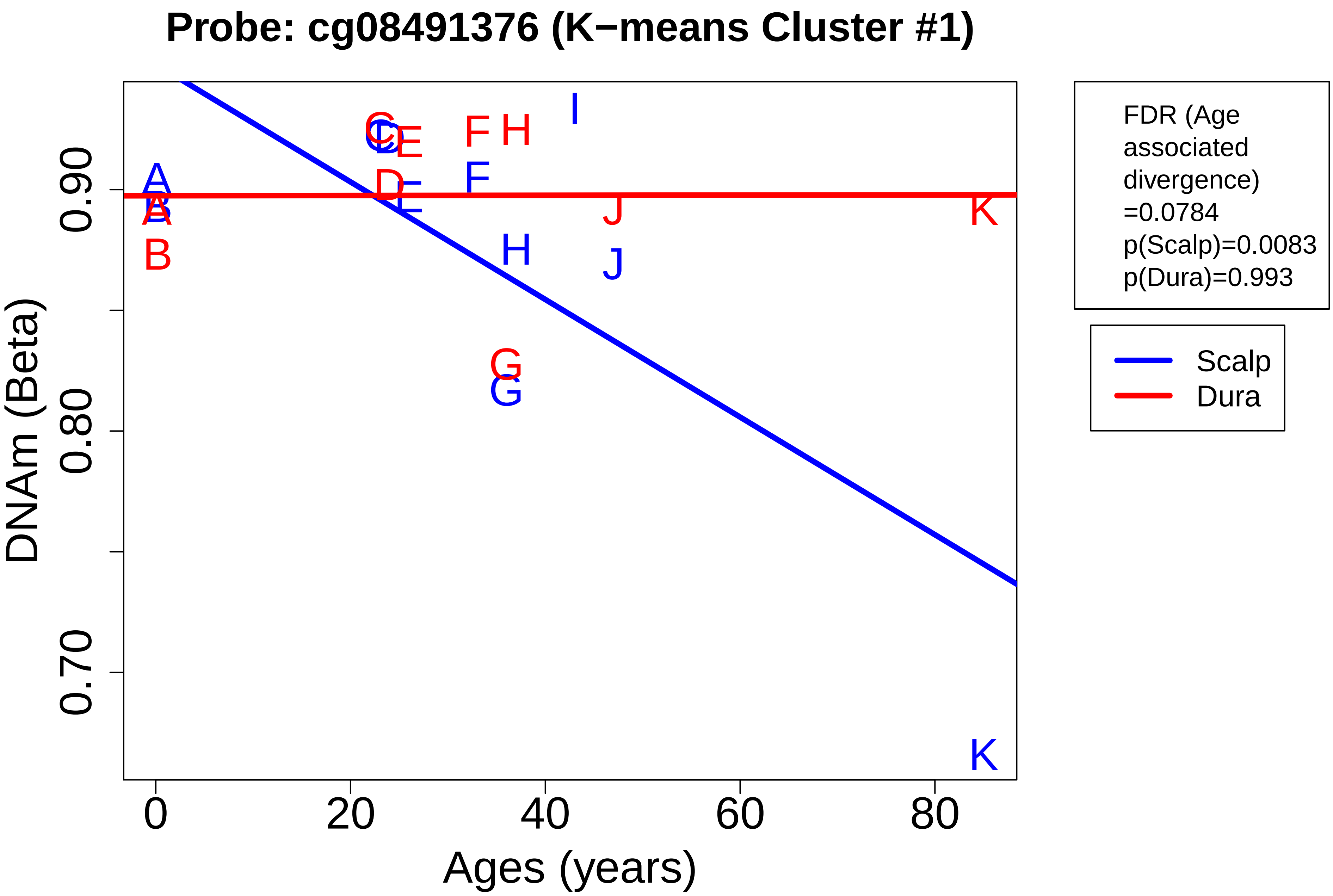

Probe: cg08491376 (K−means Cluster #1)
DNAm (Beta)
0.70
0.80
0.90
Ages (years)
0
20
40
60
80
FDR (Age associated divergence) =0.0784
p(Scalp)=0.0083
p(Dura)=0.993
Scalp
Dura

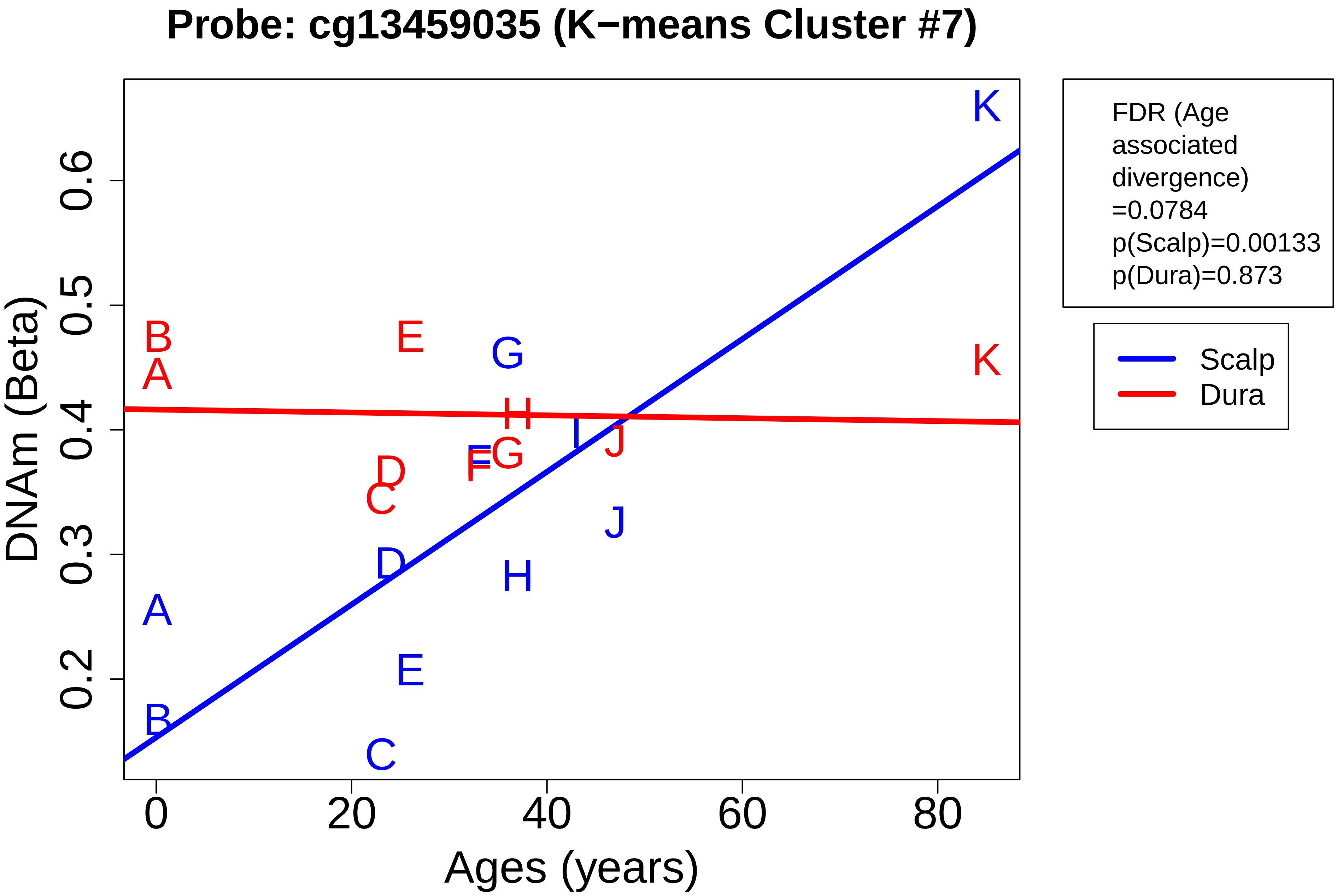

Probe: cg13459035 (K−means Cluster #7)
DNAm (Beta)
0.2
0.3
0.4
0.5
0.6
Ages (years)
0
20
40
60
80
FDR (Age associated divergence) =0.0784
p(Scalp)=0.00133
p(Dura)=0.873
Scalp
Dura

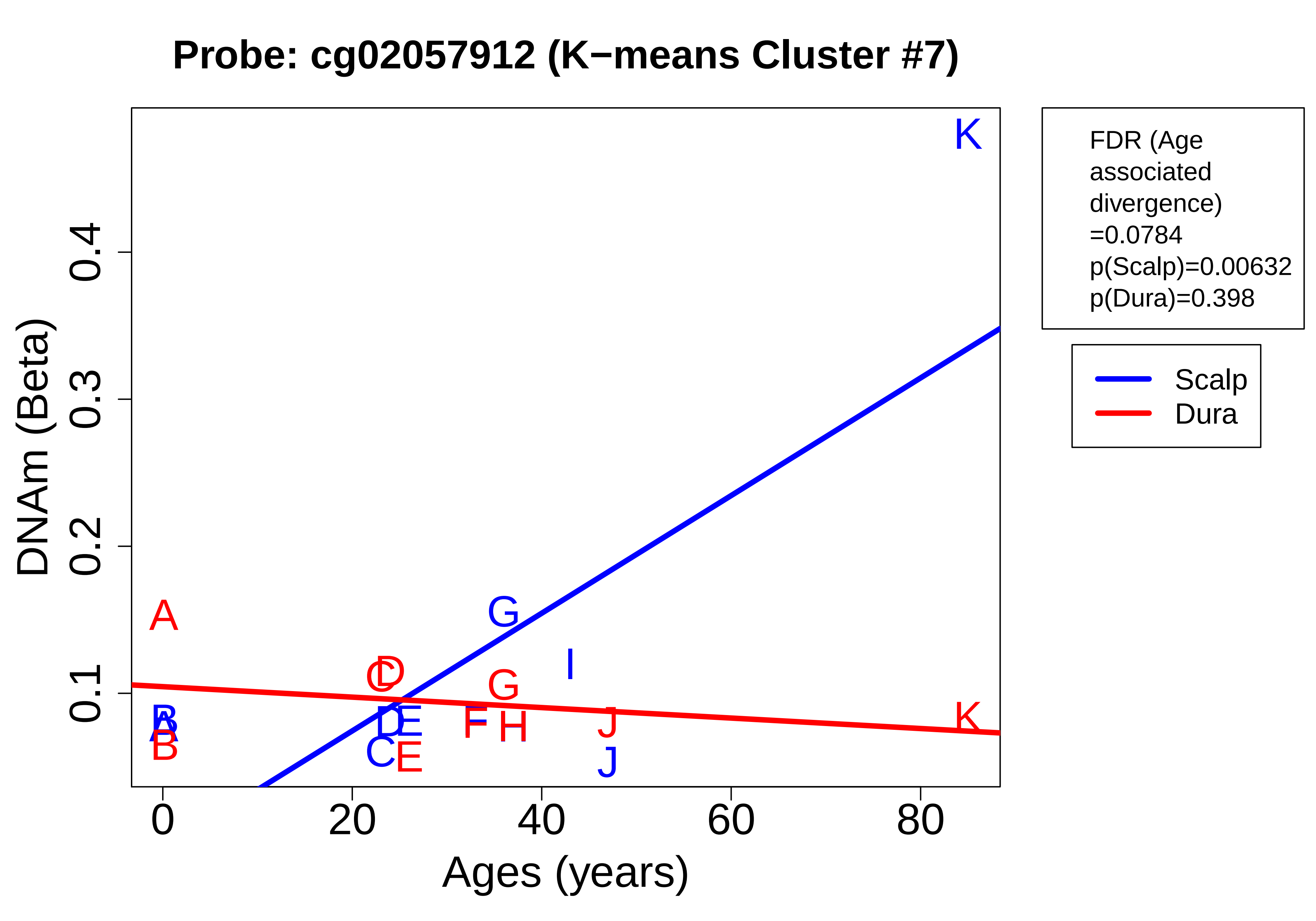

Probe: cg02057912 (K−means Cluster #7)
DNAm (Beta)
0.1
0.2
0.3
0.4
Ages (years)
0
20
40
60
80
A
B
C
D
E
F
G
H
I
J
K
FDR (Age associated divergence) =0.0784
p(Scalp)=0.00632
p(Dura)=0.398
Scalp
Dura

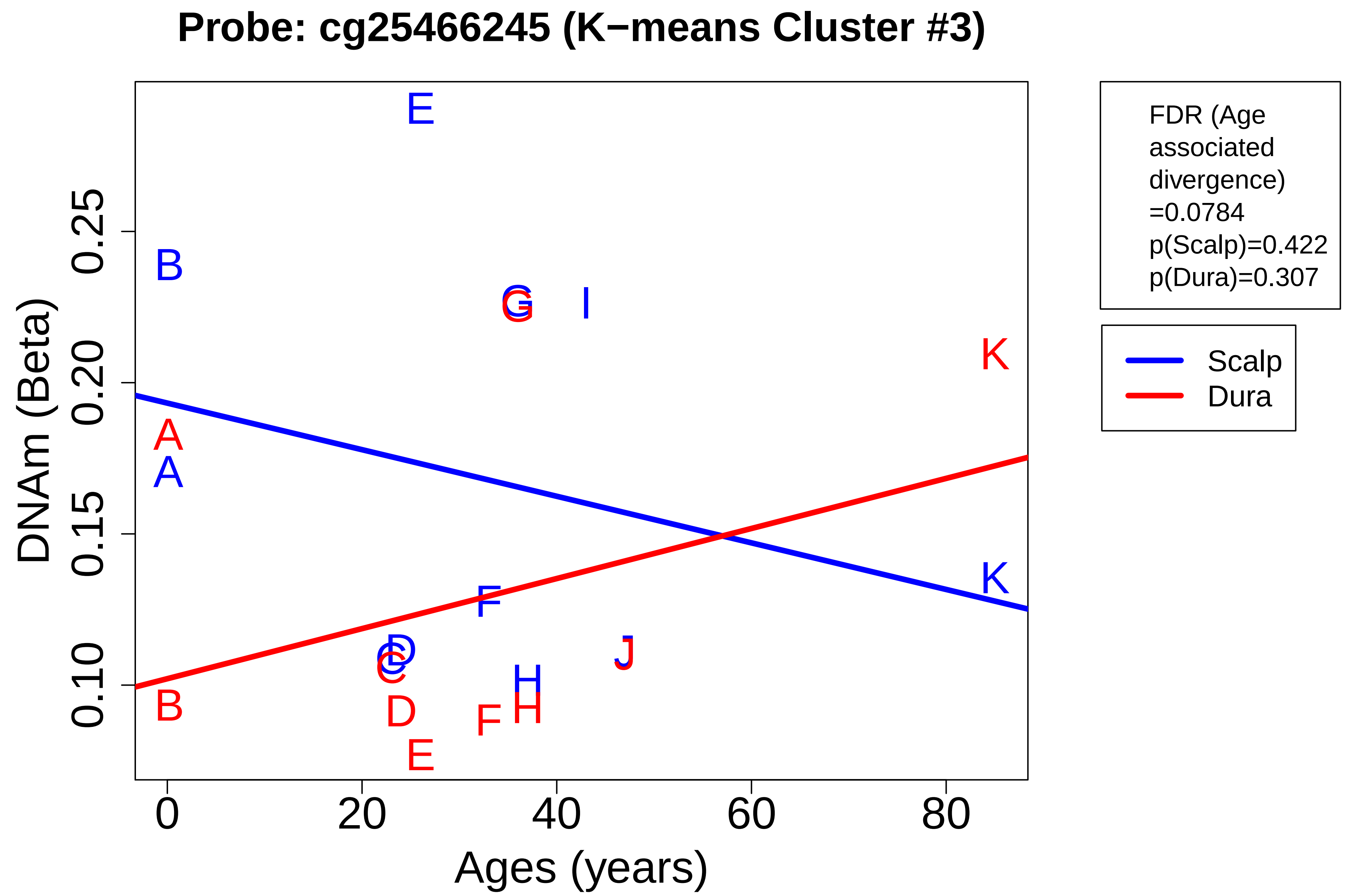

Probe: cg25466245 (K−means Cluster #3)
DNAm (Beta)
0.10
0.15
0.20
0.25
Ages (years)
0
20
40
60
80
FDR (Age associated divergence) =0.0784
p(Scalp)=0.422
p(Dura)=0.307
Scalp
Dura

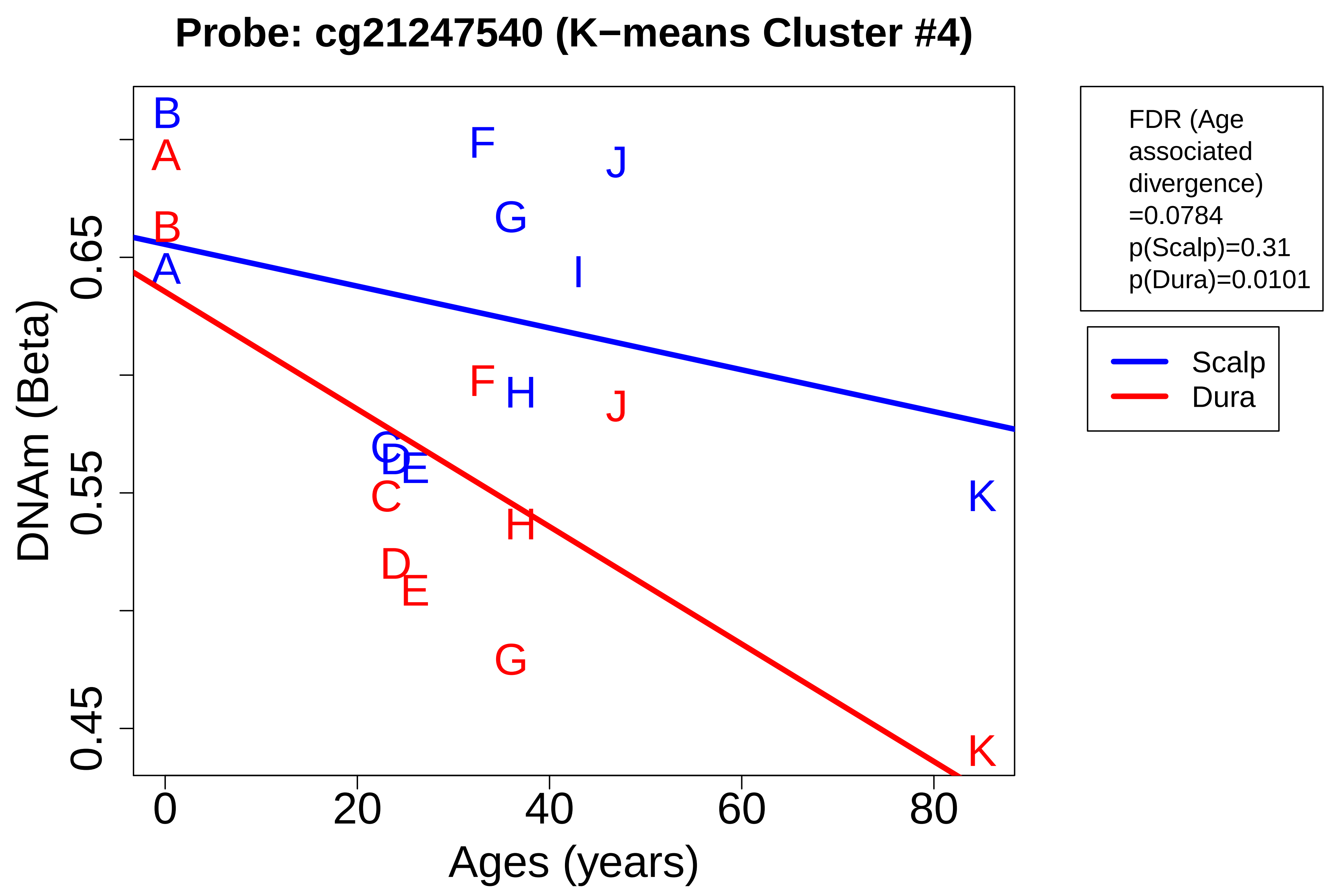

Probe: cg21247540 (K−means Cluster #4)
DNAm (Beta)
0.45
0.55
0.65
0
20
40
60
80
Ages (years)
FDR (Age associated divergence) =0.0784
p(Scalp)=0.31
p(Dura)=0.0101
Scalp
Dura

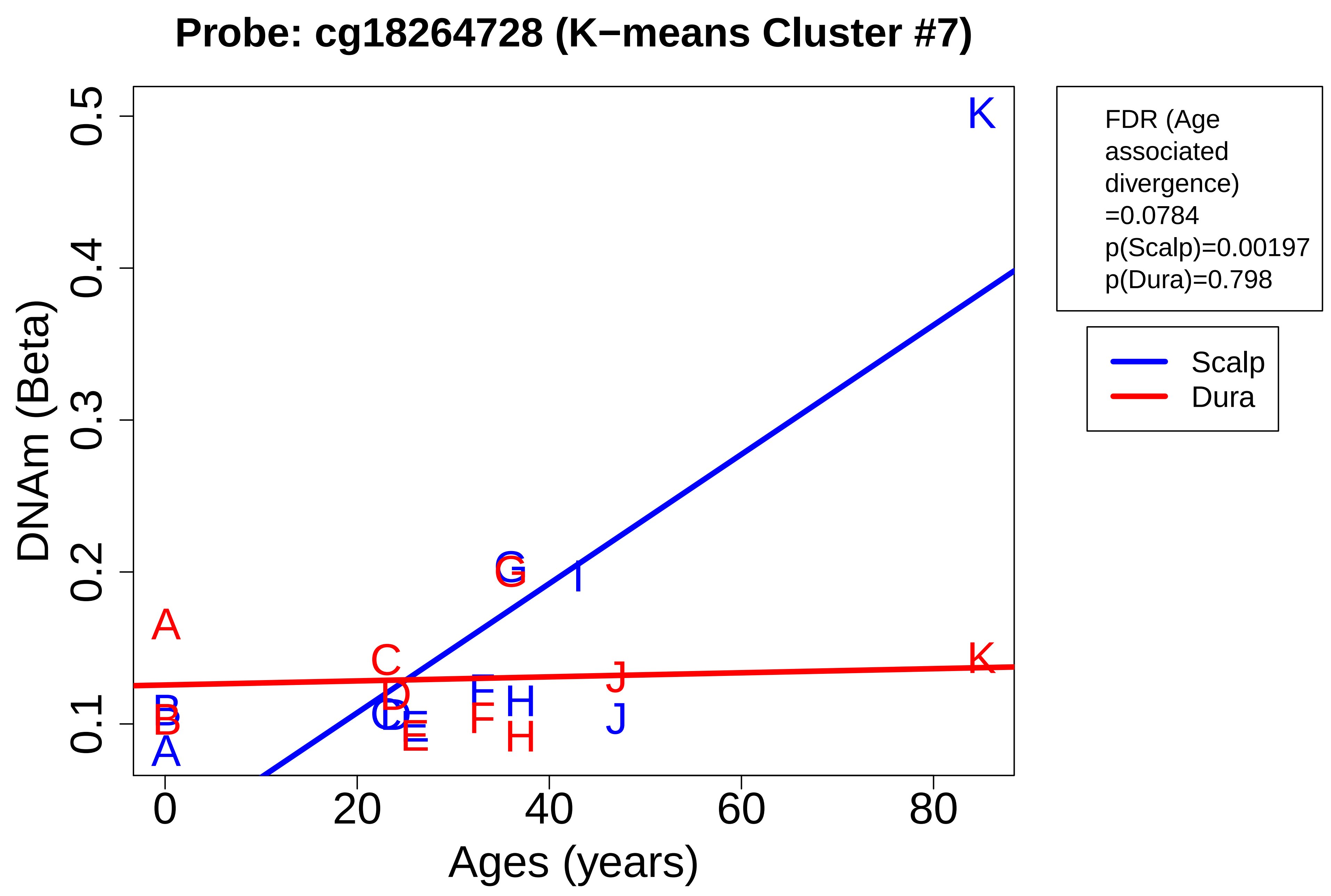

Probe: cg18264728 (K−means Cluster #7)
DNAm (Beta)
0.1
0.2
0.3
0.4
0.5
Ages (years)
0
20
40
60
80
FDR (Age associated divergence) =0.0784
p(Scalp)=0.00197
p(Dura)=0.798
Scalp
Dura

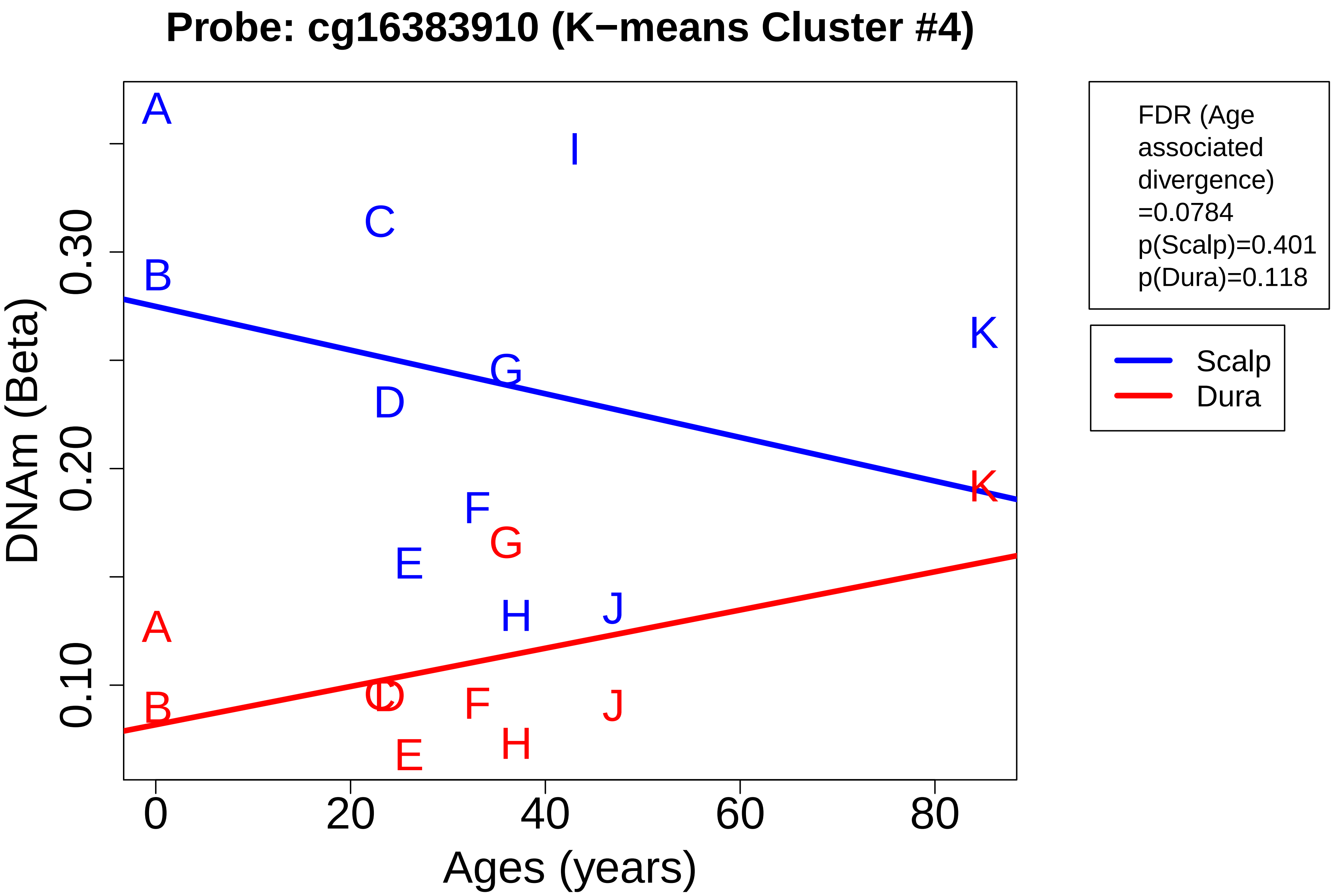

Probe: cg16383910 (K−means Cluster #4)
DNAm (Beta)
0.10
0.20
0.30
0
20
40
60
80
Ages (years)
FDR (Age associated divergence) =0.0784
p(Scalp)=0.401
p(Dura)=0.118
Scalp
Dura
A
B
C
D
E
F
G
H
I
J
K

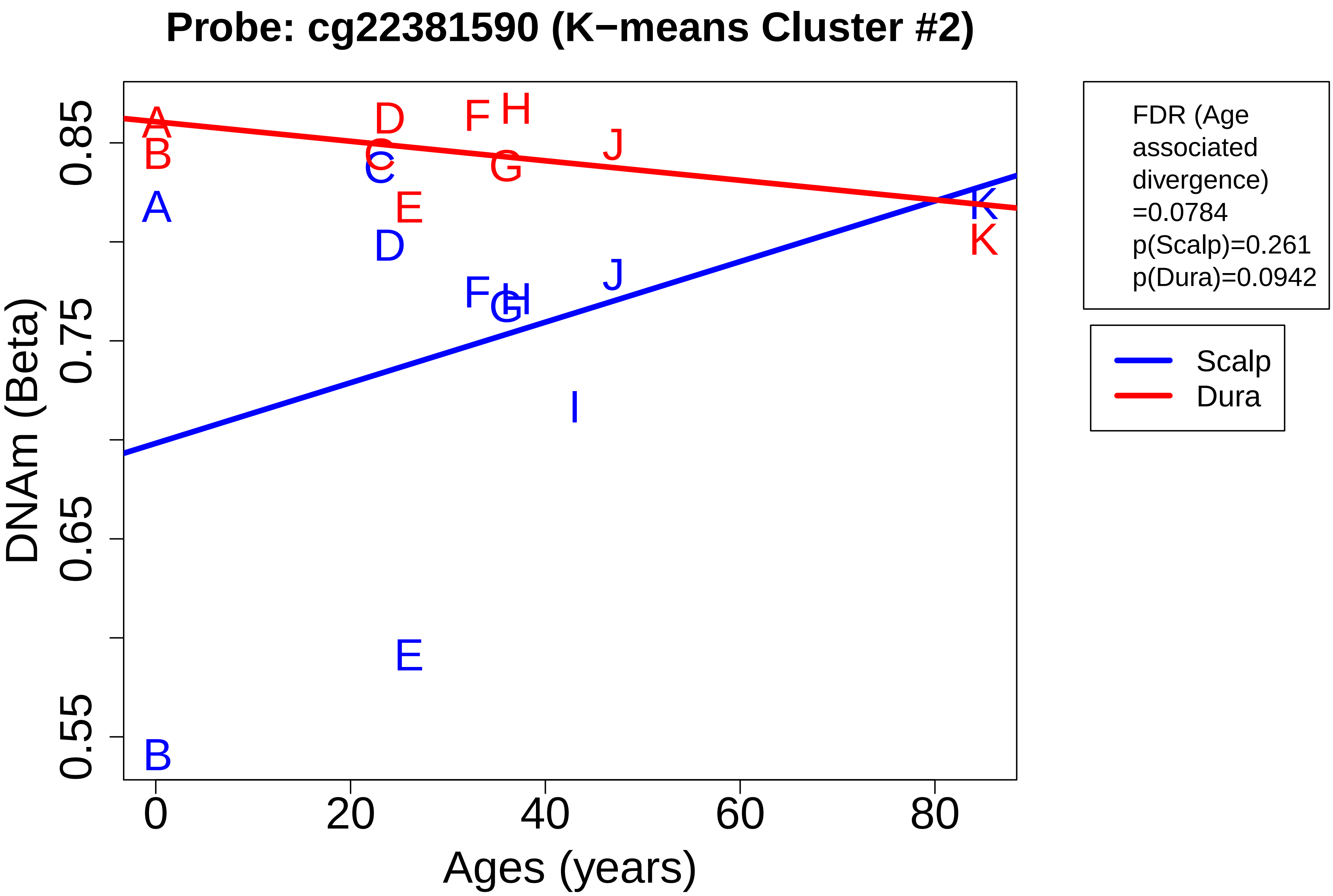
Probe: cg22381590 (K−means Cluster #2)
DNAm (Beta)
0.55
0.65
0.75
0.85
Ages (years)
0
20
40
60
80
A
B
C
D
E
F
G
H
I
J
K
FDR (Age associated divergence) =0.0784
p(Scalp)=0.261
p(Dura)=0.0942
Scalp
Dura

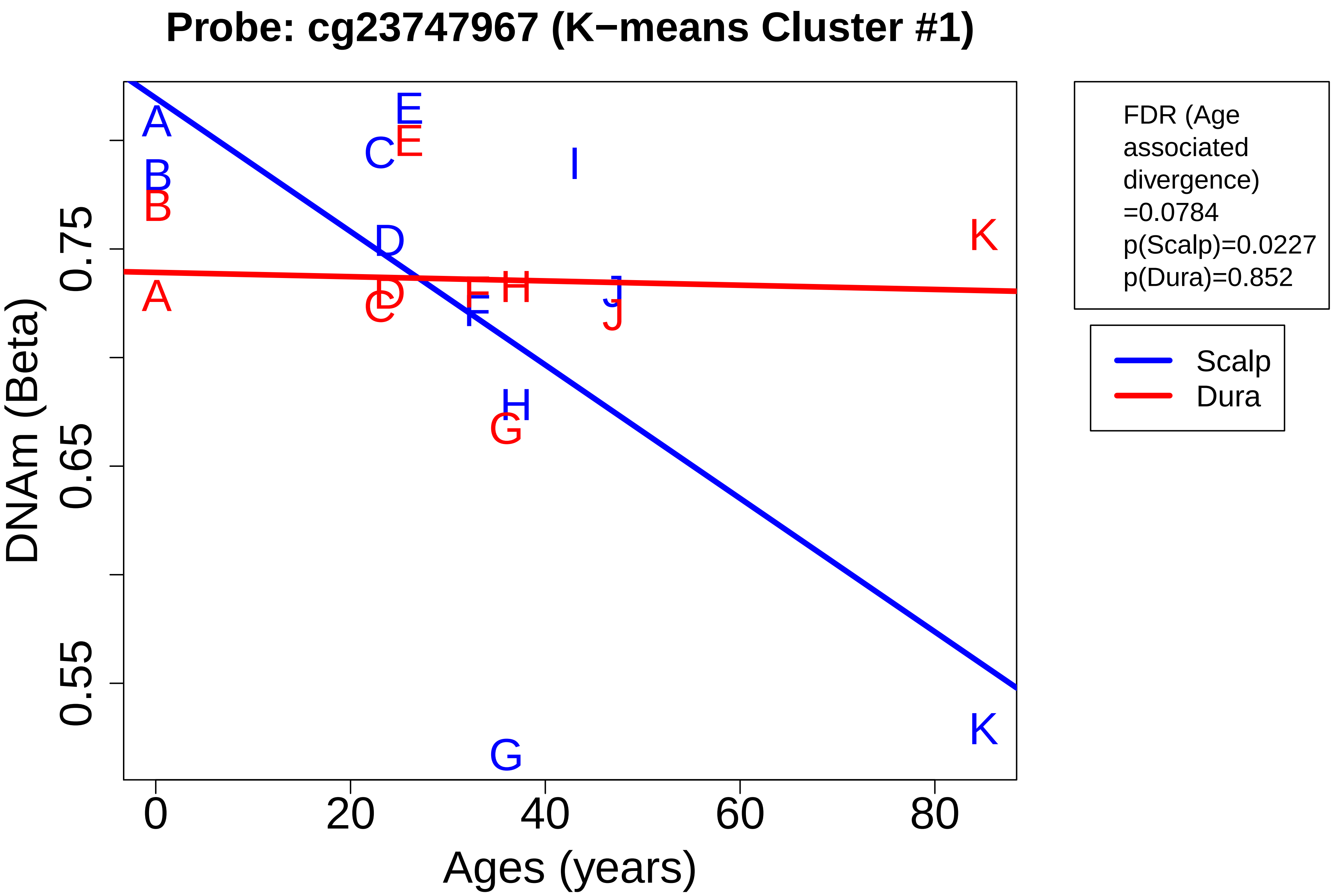

Probe: cg23747967 (K−means Cluster #1)
DNAm (Beta)
0.55
0.65
0.75
Ages (years)
0
20
40
60
80
FDR (Age associated divergence) =0.0784
p(Scalp)=0.0227
p(Dura)=0.852
Scalp
Dura

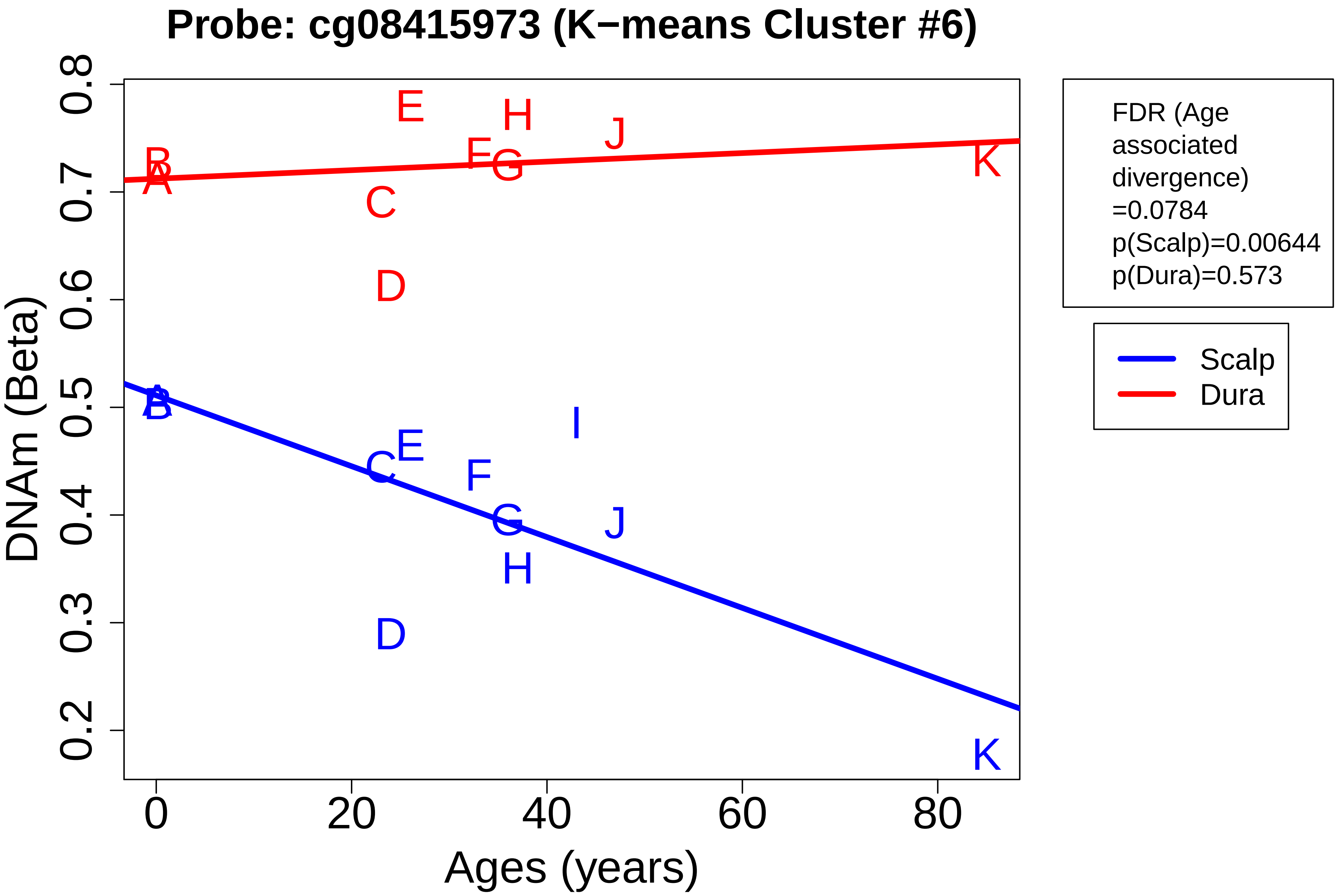

Probe: cg08415973 (K−means Cluster #6)
DNAm (Beta)
0.2
0.3
0.4
0.5
0.6
0.7
0.8
Ages (years)
0
20
40
60
80
FDR (Age associated divergence) =0.0784
p(Scalp)=0.00644
p(Dura)=0.573
Scalp
Dura

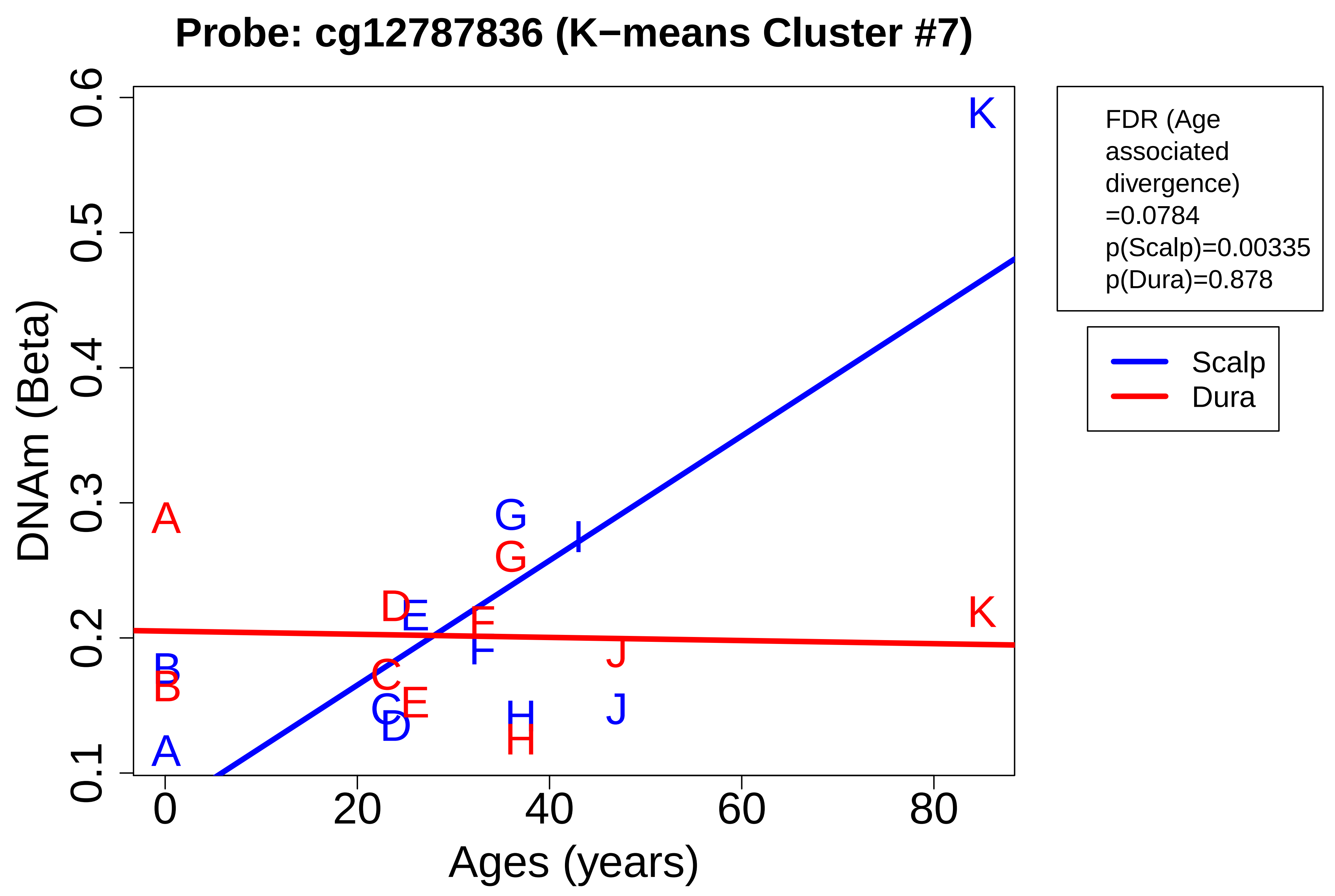

Probe: cg12787836 (K−means Cluster #7)
DNAm (Beta)
0.1
0.2
0.3
0.4
0.5
0.6
0
20
40
60
80
Ages (years)
FDR (Age associated divergence) =0.0784
p(Scalp)=0.00335
p(Dura)=0.878
Scalp
Dura

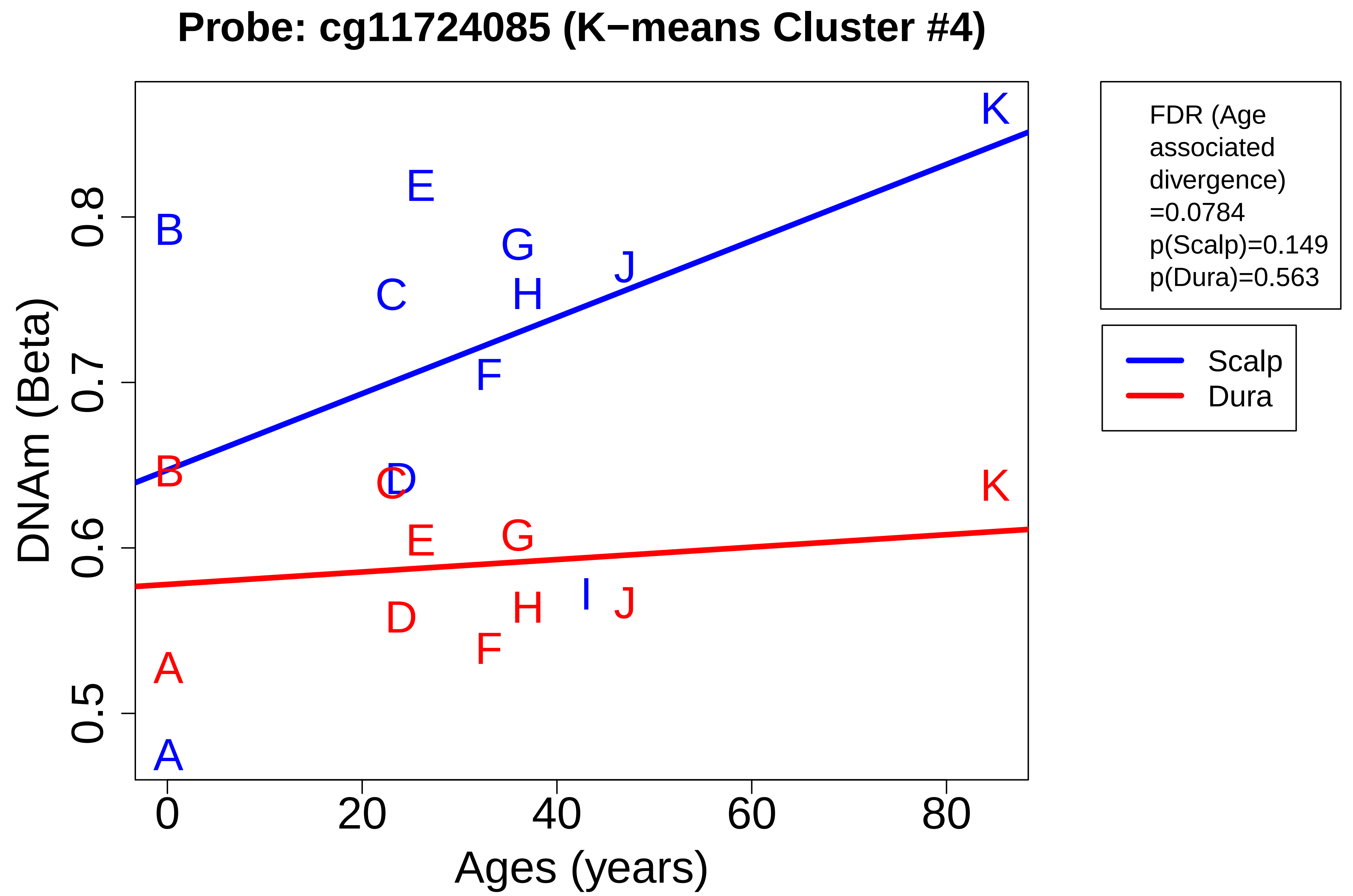

Probe: cg11724085 (K−means Cluster #4)
DNAm (Beta)
0.5
0.6
0.7
0.8
0
20
40
60
80
Ages (years)
A
B
C
D
E
F
G
H
I
J
K
FDR (Age associated divergence) =0.0784
p(Scalp)=0.149
p(Dura)=0.563
Scalp
Dura

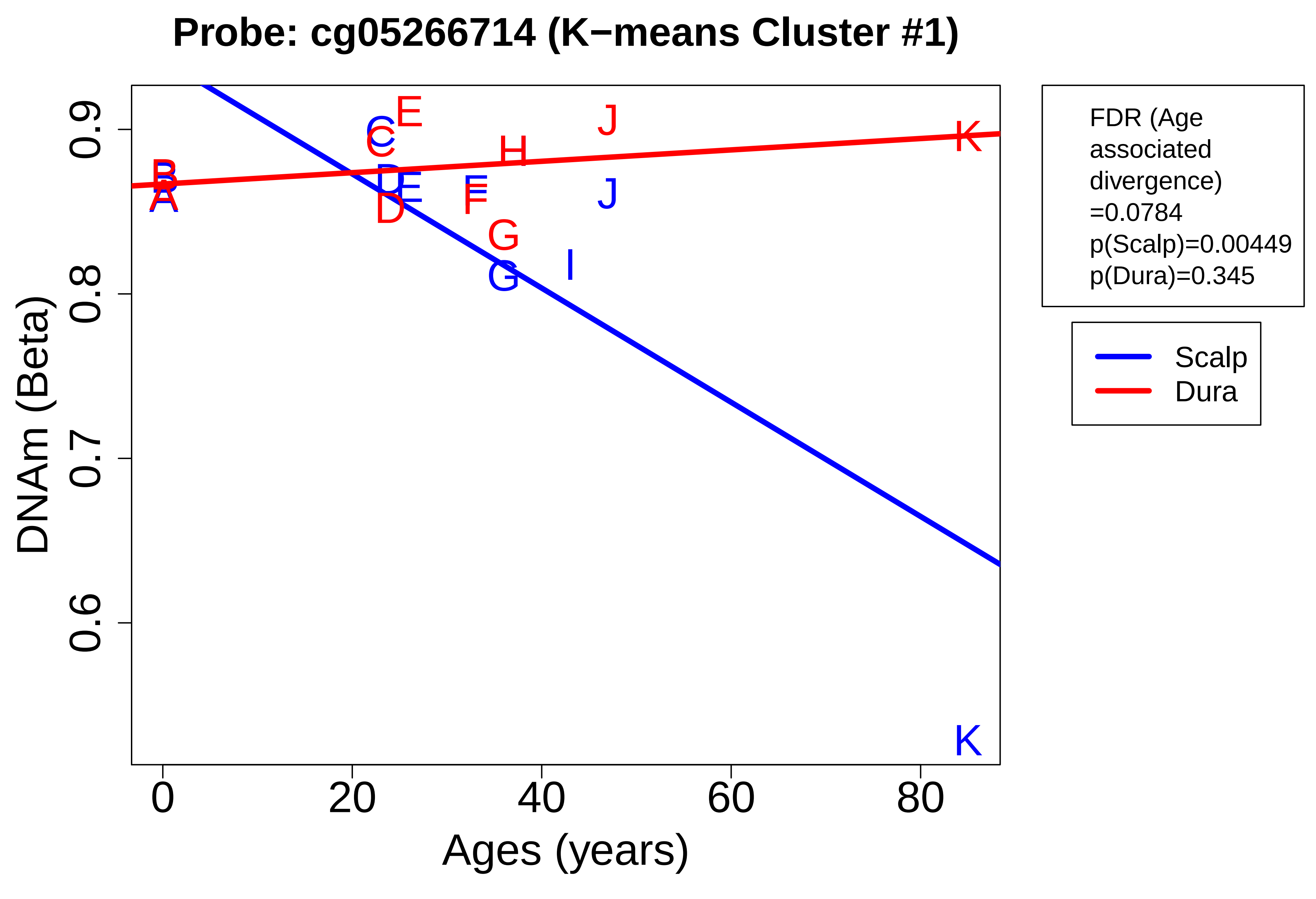

Probe: cg05266714 (K−means Cluster #1)
DNAm (Beta)
0.6
0.7
0.8
0.9
Ages (years)
0
20
40
60
80
FDR (Age associated divergence) =0.0784
p(Scalp)=0.00449
p(Dura)=0.345
Scalp
Dura
A
B
C
D
E
F
G
H
I
J
K

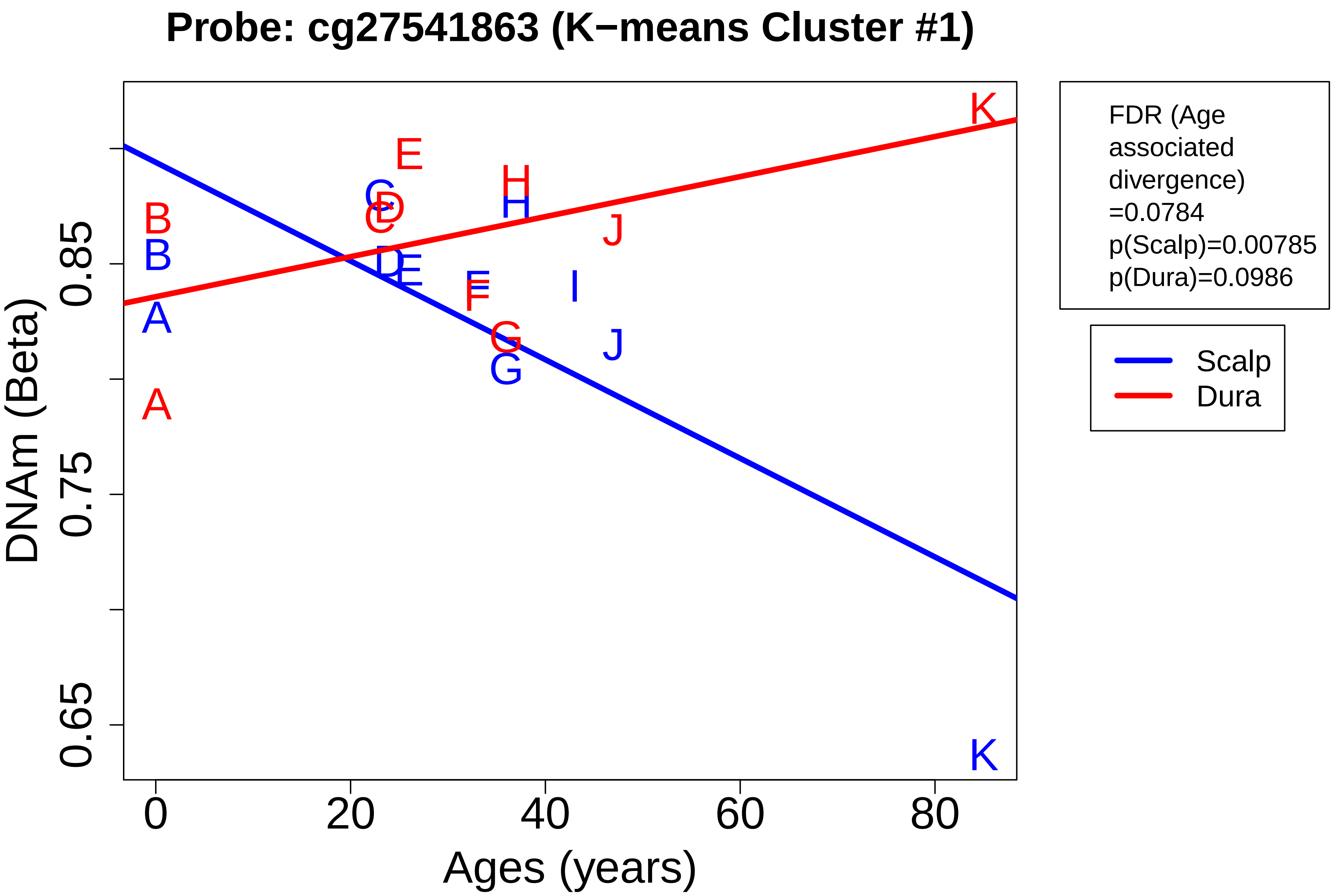

Probe: cg27541863 (K−means Cluster #1)
DNAm (Beta)
0.65
0.75
0.85
Ages (years)
0
20
40
60
80
FDR (Age associated divergence) =0.0784
p(Scalp)=0.00785
p(Dura)=0.0986
Scalp
Dura

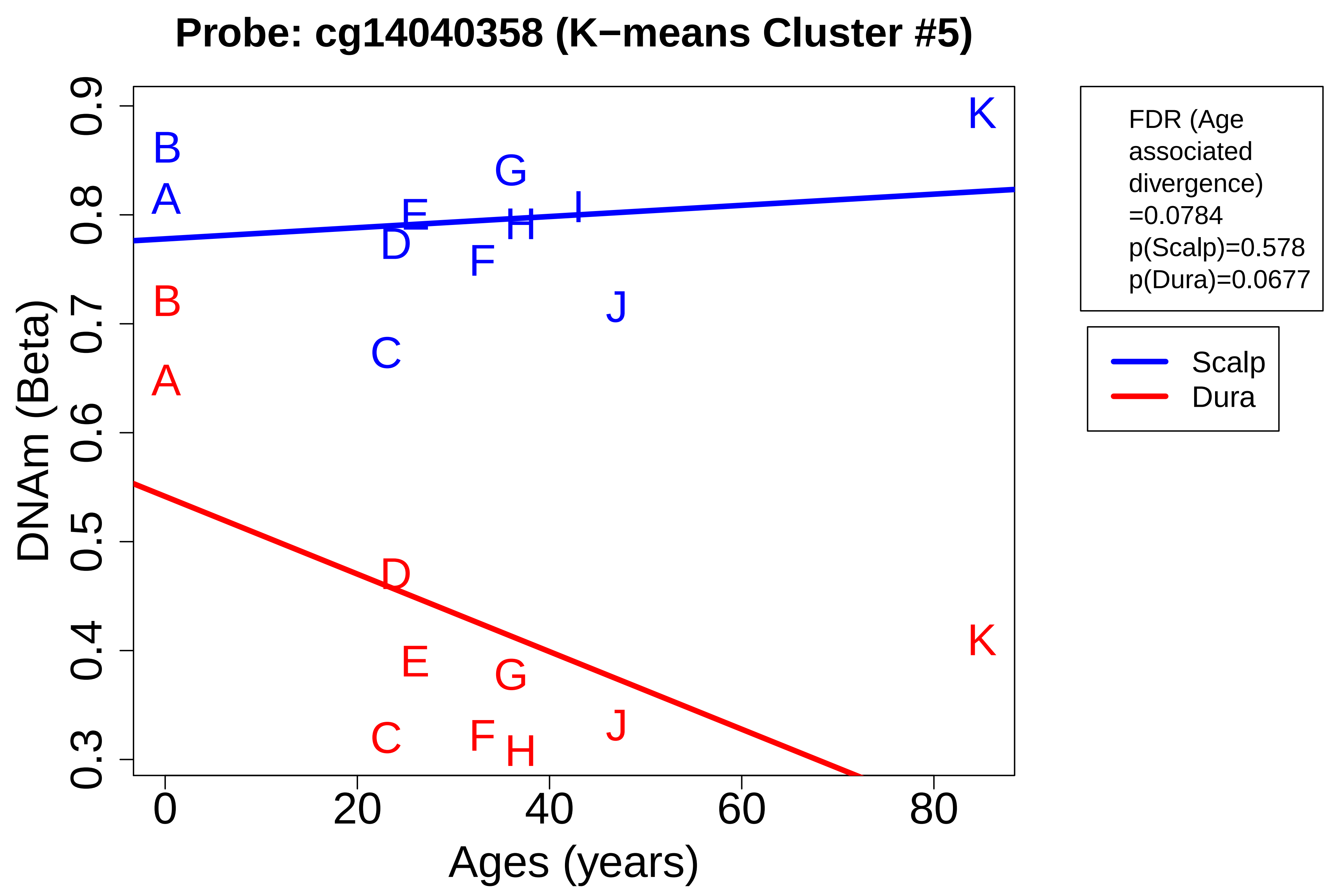
Probe: cg14040358 (K−means Cluster #5)
DNAm (Beta)
0.3
0.4
0.5
0.6
0.7
0.8
0.9
0
20
40
60
80
Ages (years)
A
B
C
D
E
F
G
H
I
J
K
FDR (Age associated divergence) =0.0784
p(Scalp)=0.578
p(Dura)=0.0677
Scalp
Dura

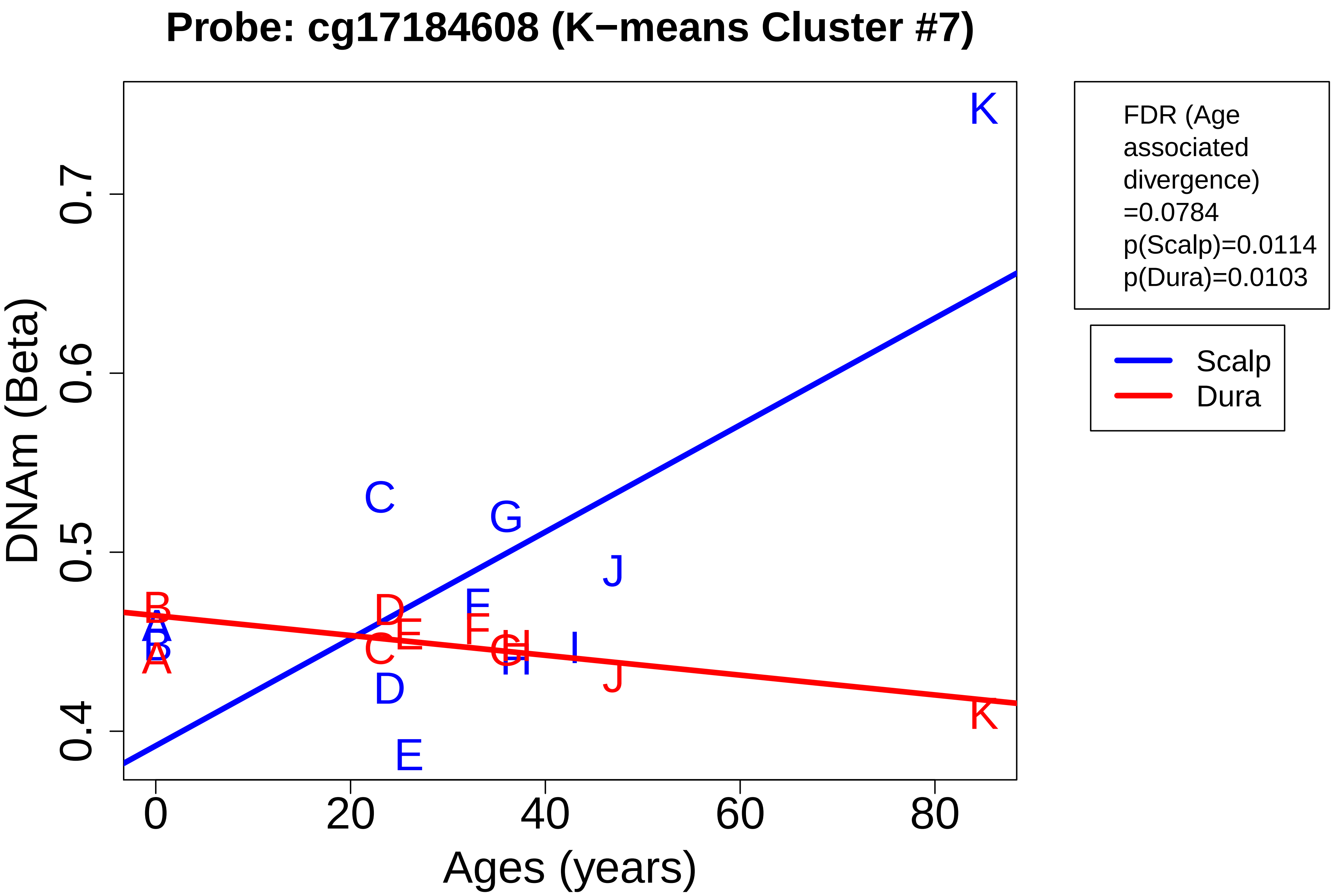

Probe: cg17184608 (K−means Cluster #7)
DNAm (Beta)
0.4
0.5
0.6
0.7
0
20
40
60
80
Ages (years)
FDR (Age associated divergence) =0.0784
p(Scalp)=0.0114
p(Dura)=0.0103
Scalp
Dura

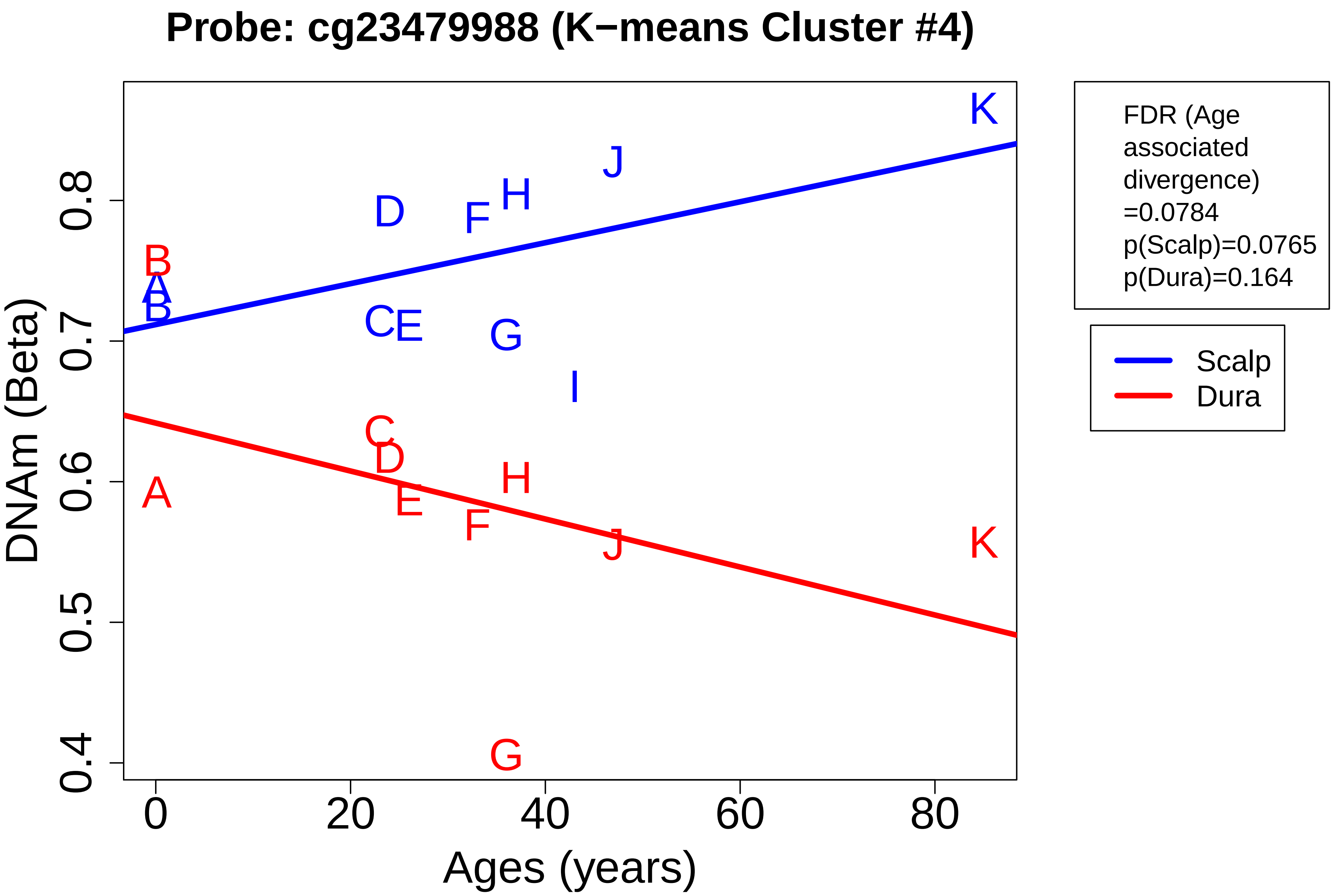

Probe: cg23479988 (K−means Cluster #4)
DNAm (Beta)
0.4
0.5
0.6
0.7
0.8
Ages (years)
0
20
40
60
80
A
B
C
D
E
F
G
H
I
J
K
FDR (Age associated divergence) =0.0784
p(Scalp)=0.0765
p(Dura)=0.164
Scalp
Dura

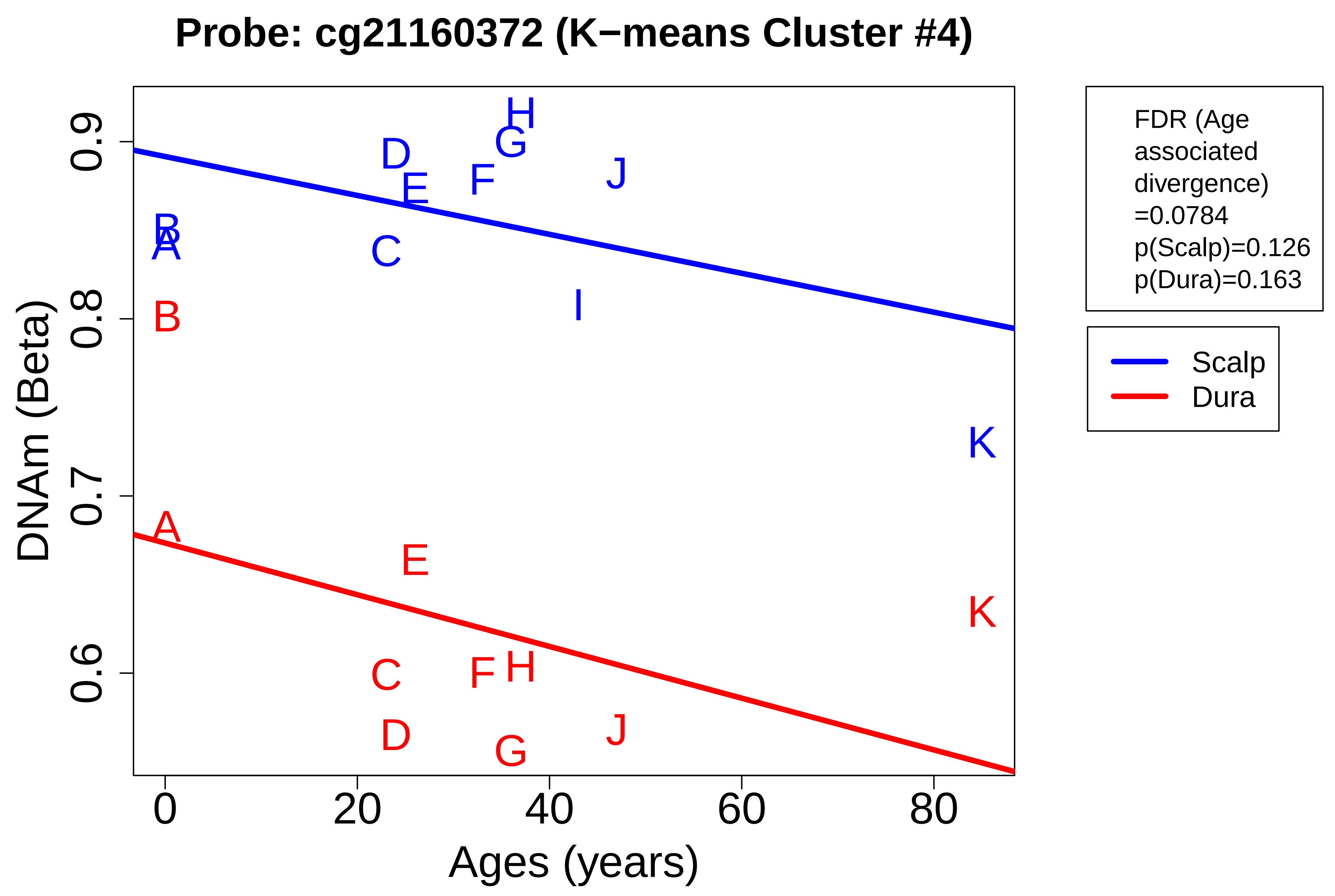
Probe: cg21160372 (K−means Cluster #4)
DNAm (Beta)
Ages (years)
0.6
0.7
0.8
0.9
0
20
40
60
80
FDR (Age associated divergence) =0.0784
p(Scalp)=0.126
p(Dura)=0.163
Scalp
Dura
A
B
C
D
E
F
G
H
I
J
K

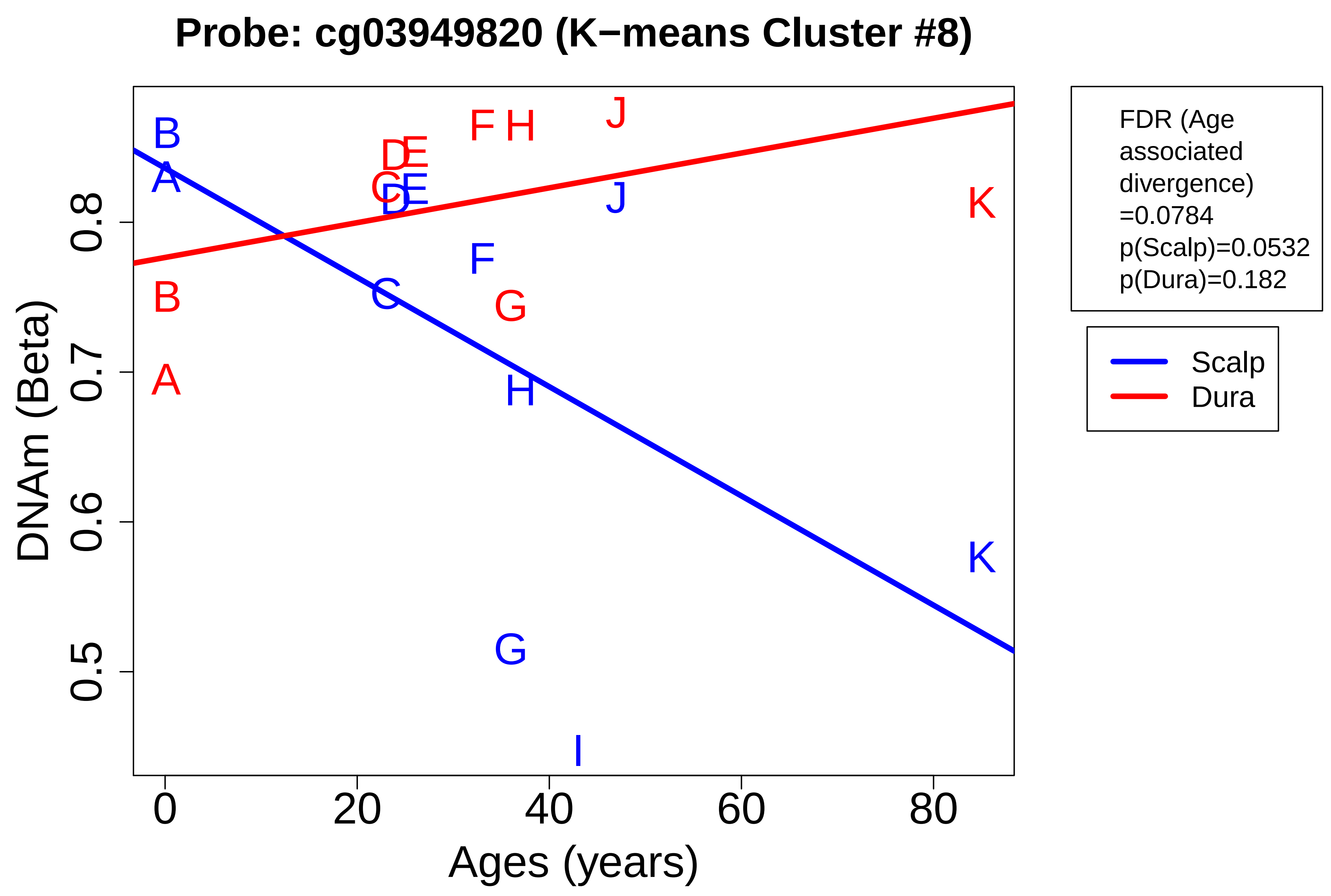

Probe: cg03949820 (K−means Cluster #8)
DNAm (Beta)
Ages (years)
0.5
0.6
0.7
0.8
0
20
40
60
80
FDR (Age associated divergence) =0.0784
p(Scalp)=0.0532
p(Dura)=0.182
Scalp
Dura

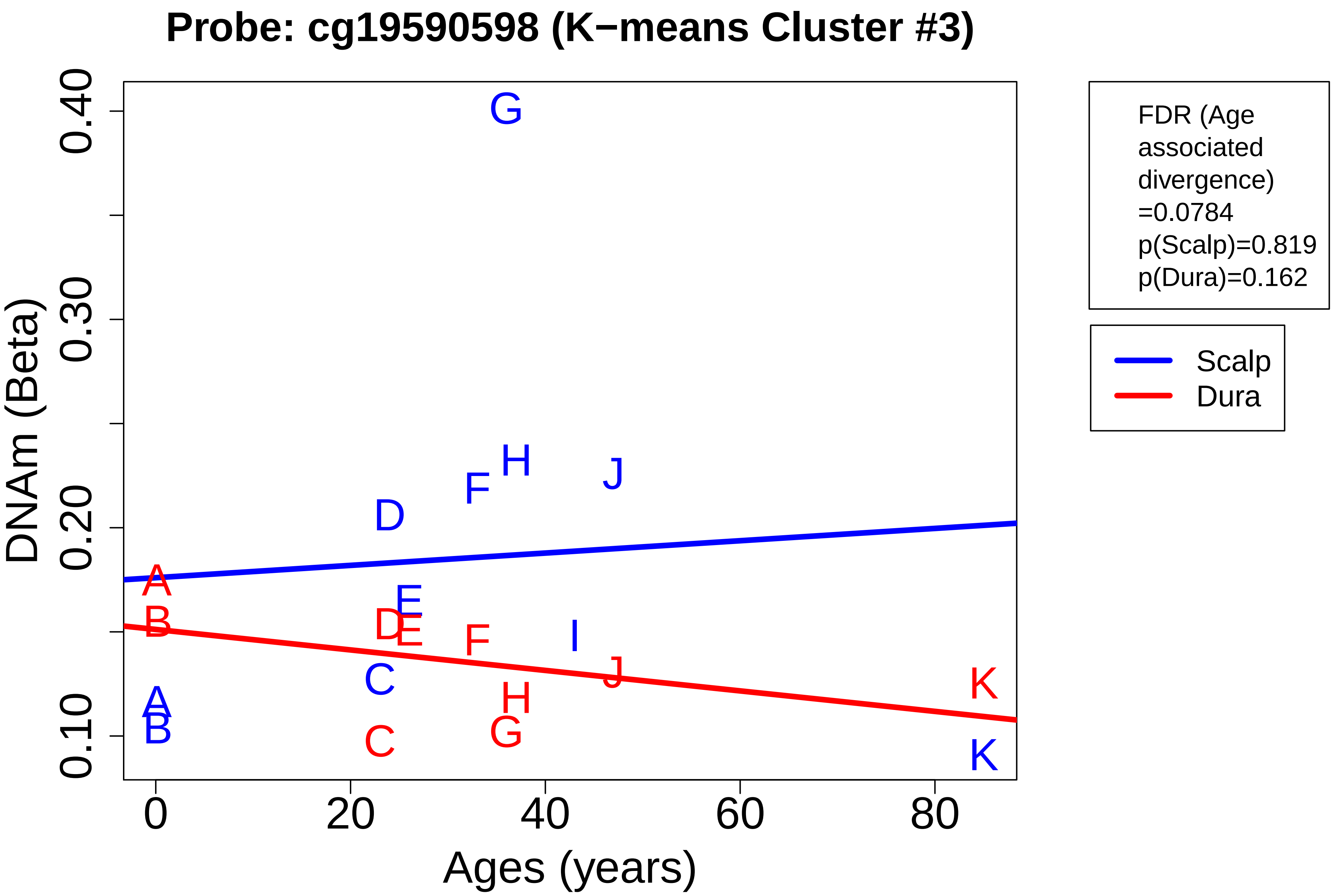

Probe: cg19590598 (K−means Cluster #3)
DNAm (Beta)
0.10
0.20
0.30
0.40
0
20
40
60
80
Ages (years)
FDR (Age associated divergence) =0.0784
p(Scalp)=0.819
p(Dura)=0.162
Scalp
Dura

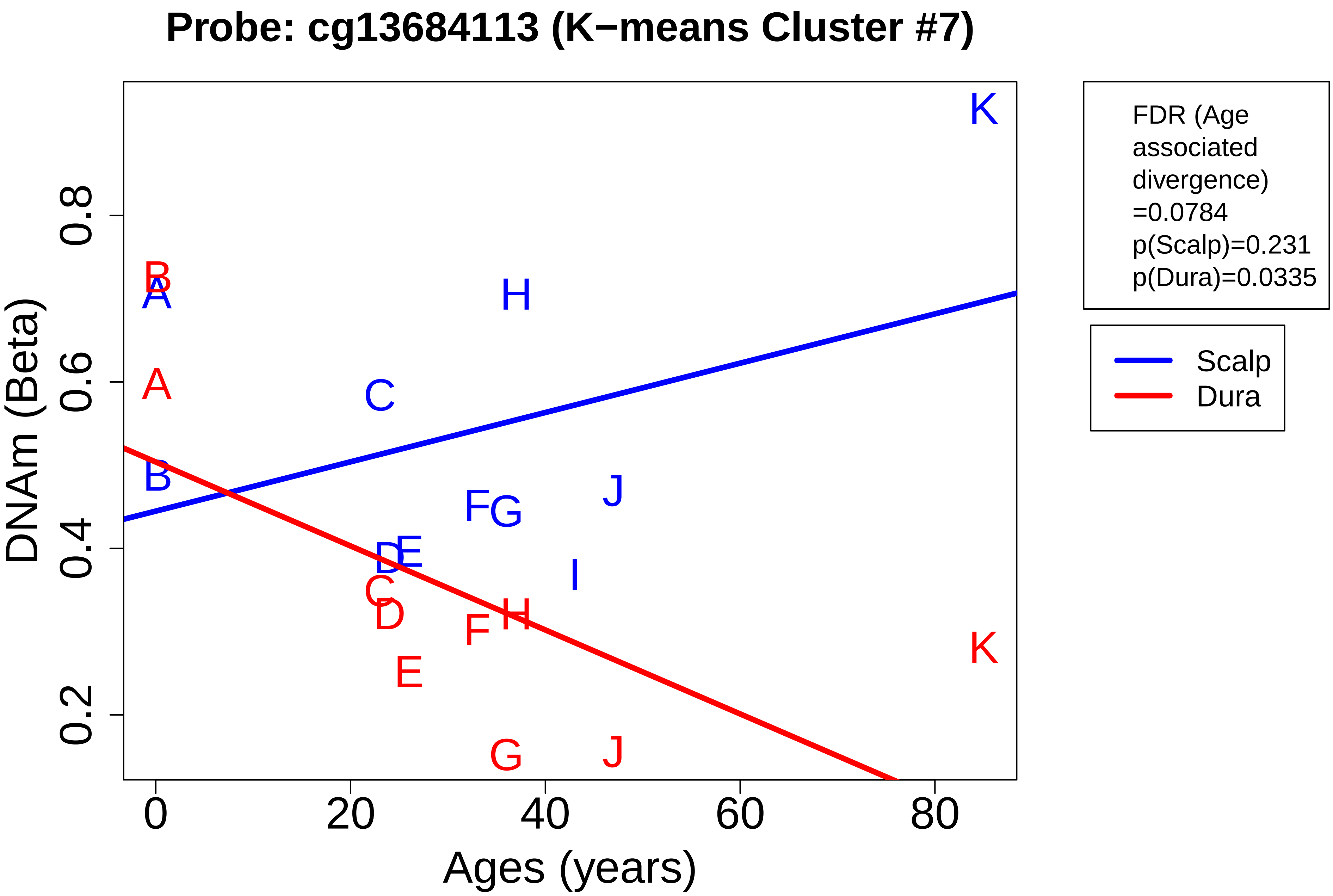

Probe: cg13684113 (K−means Cluster #7)
DNAm (Beta)
0.2
0.4
0.6
0.8
0
20
40
60
80
Ages (years)
FDR (Age associated divergence) =0.0784
p(Scalp)=0.231
p(Dura)=0.0335
Scalp
Dura

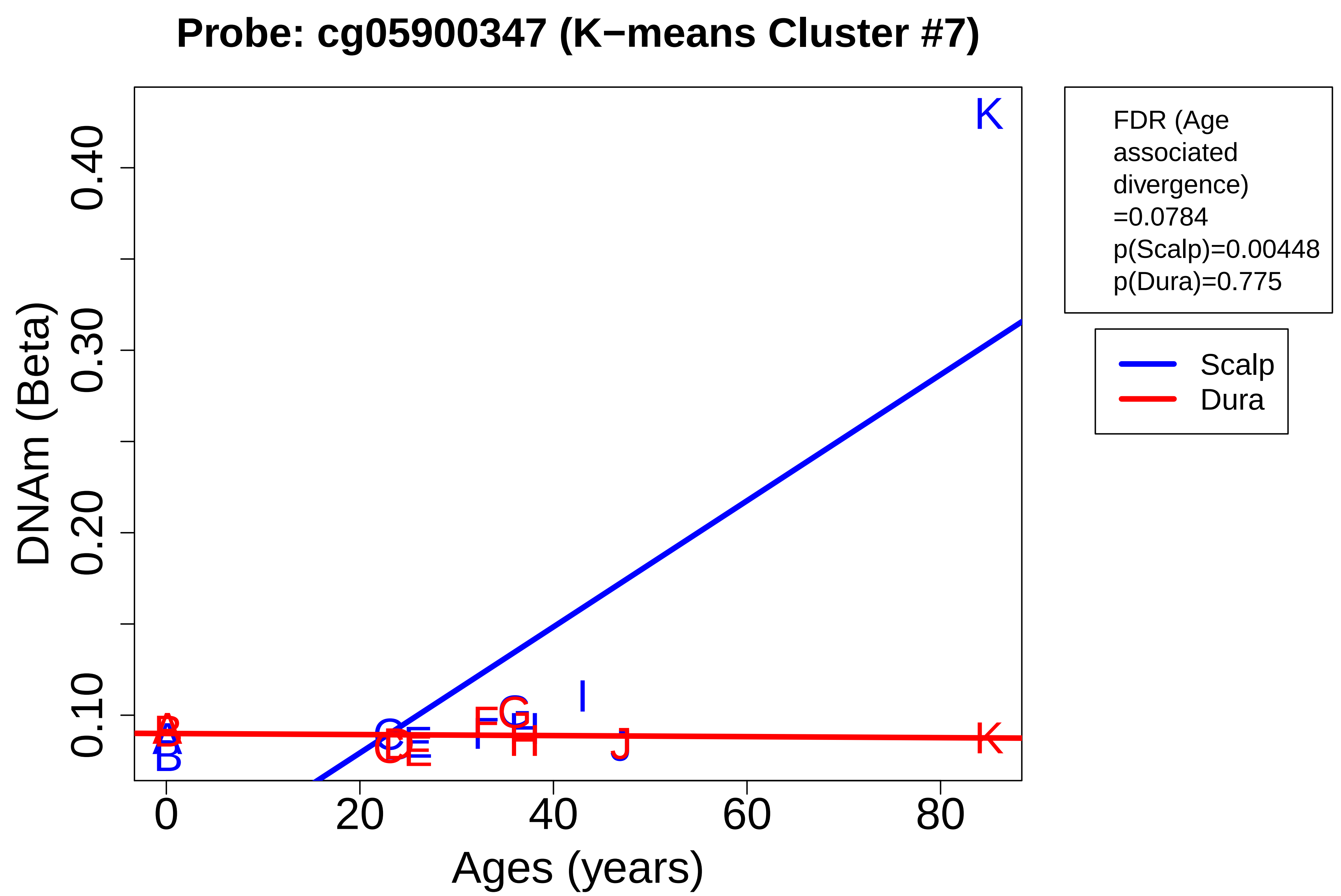

Probe: cg05900347 (K−means Cluster #7)
DNAm (Beta)
0.10
0.20
0.30
0.40
0
20
40
60
80
Ages (years)
FDR (Age associated divergence) =0.0784
p(Scalp)=0.00448
p(Dura)=0.775
Scalp
Dura

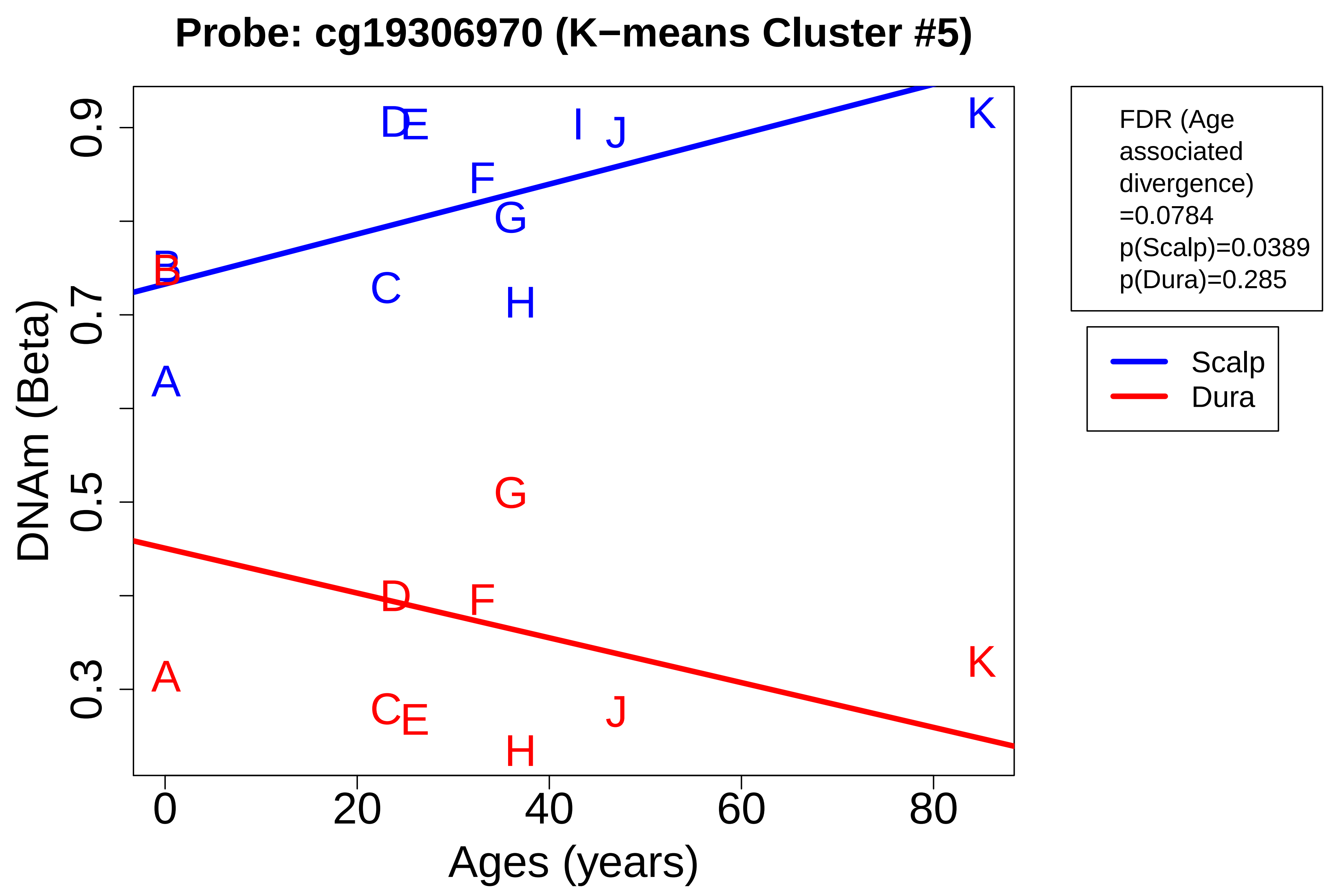

Probe: cg19306970 (K−means Cluster #5)
DNAm (Beta)
0.3
0.5
0.7
0.9
0
20
40
60
80
Ages (years)
A
B
C
D
E
F
G
H
I
J
K
FDR (Age associated divergence) =0.0784
p(Scalp)=0.0389
p(Dura)=0.285
Scalp
Dura

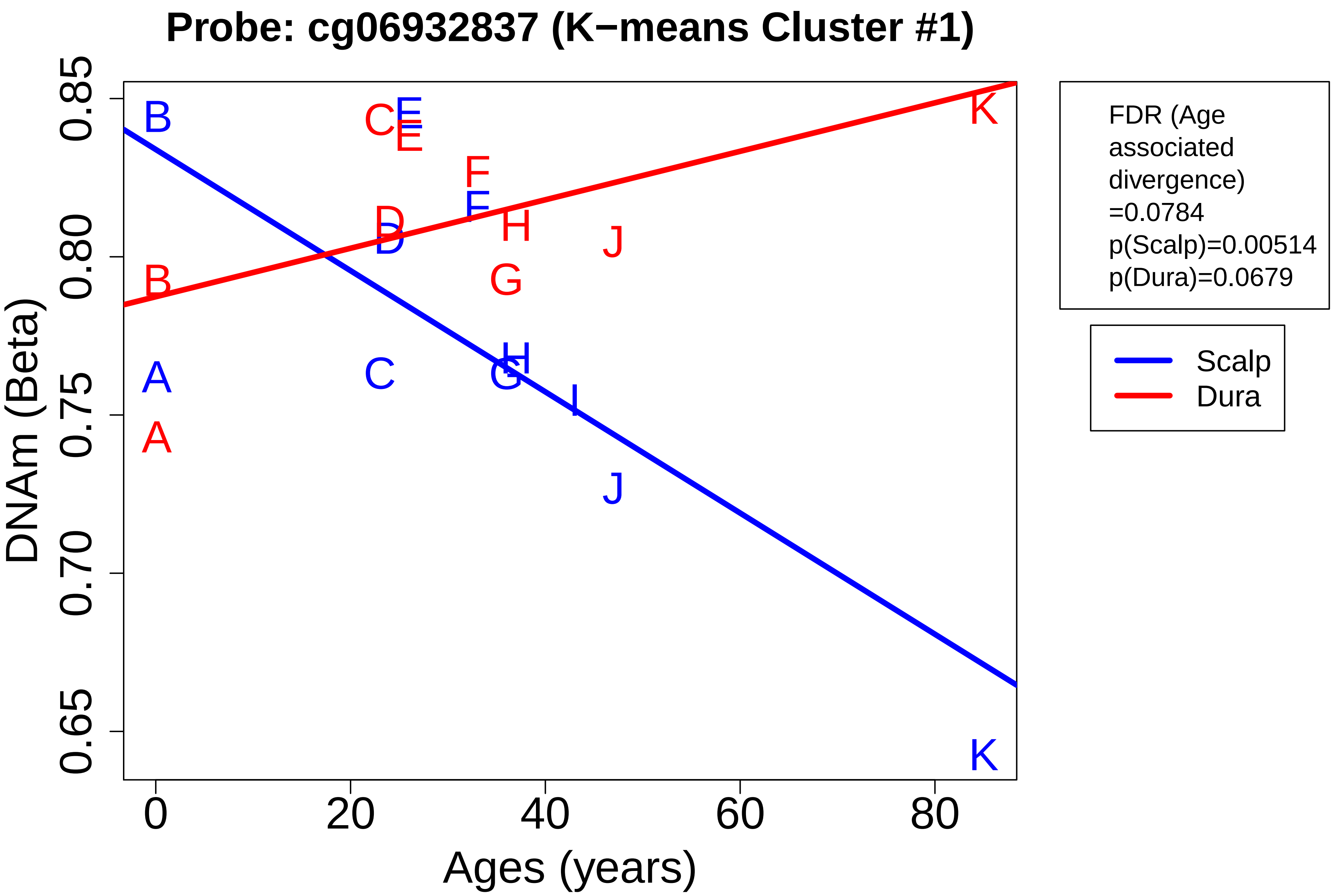

Probe: cg06932837 (K−means Cluster #1)
DNAm (Beta)
0.65
0.70
0.75
0.80
0.85
Ages (years)
0
20
40
60
80
A
B
C
D
E
F
G
H
I
J
K
FDR (Age associated divergence) =0.0784
p(Scalp)=0.00514
p(Dura)=0.0679
Scalp
Dura

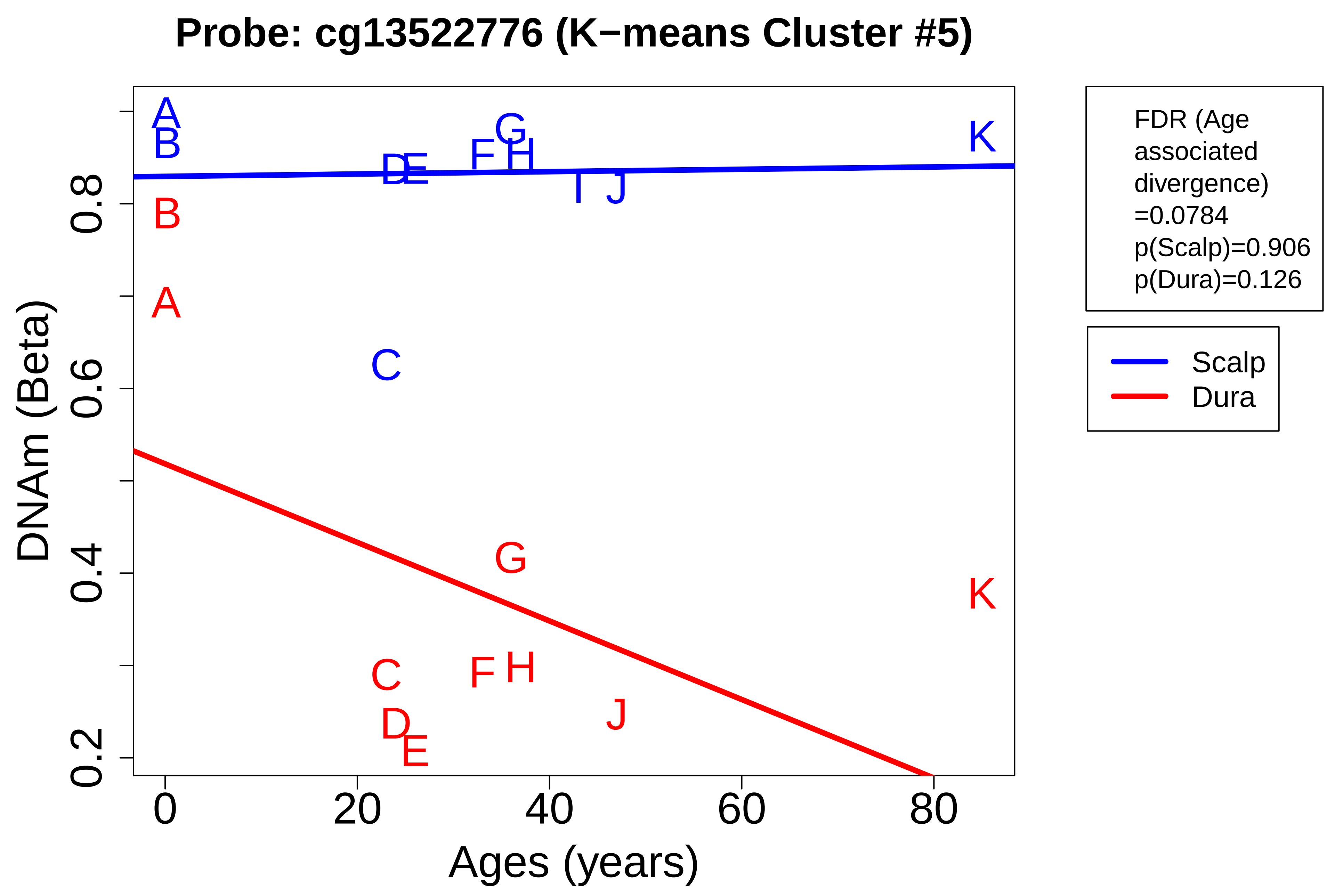
Probe: cg13522776 (K−means Cluster #5)
DNAm (Beta)
0.2
0.4
0.6
0.8
0
20
40
60
80
Ages (years)
A
B
C
D
E
F
G
H
I
J
K
FDR (Age associated divergence) =0.0784
p(Scalp)=0.906
p(Dura)=0.126
Scalp
Dura

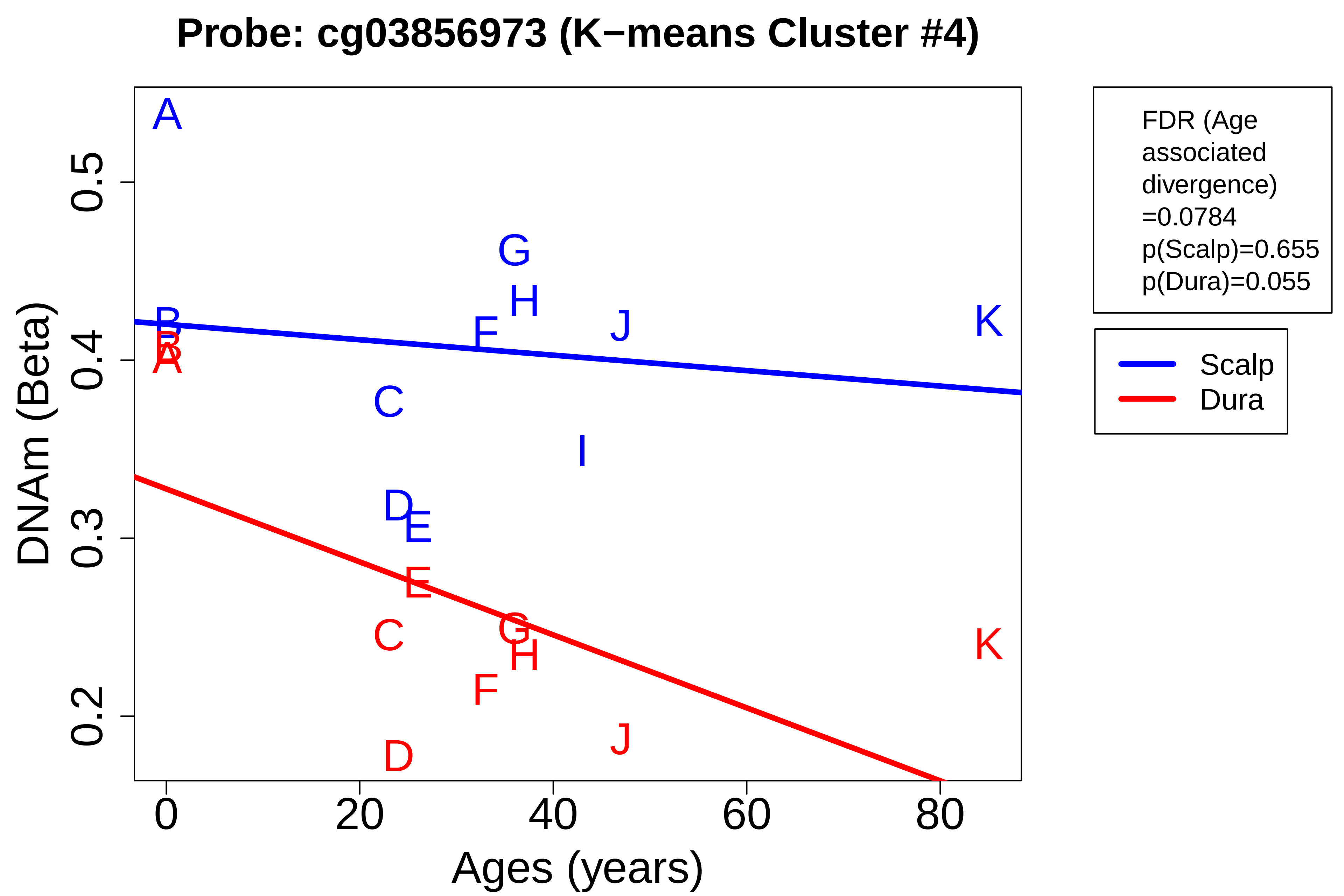

Probe: cg03856973 (K−means Cluster #4)
DNAm (Beta)
0.2
0.3
0.4
0.5
Ages (years)
0
20
40
60
80
A
B
C
D
E
F
G
H
I
J
K
FDR (Age associated divergence) =0.0784
p(Scalp)=0.655
p(Dura)=0.055
Scalp
Dura

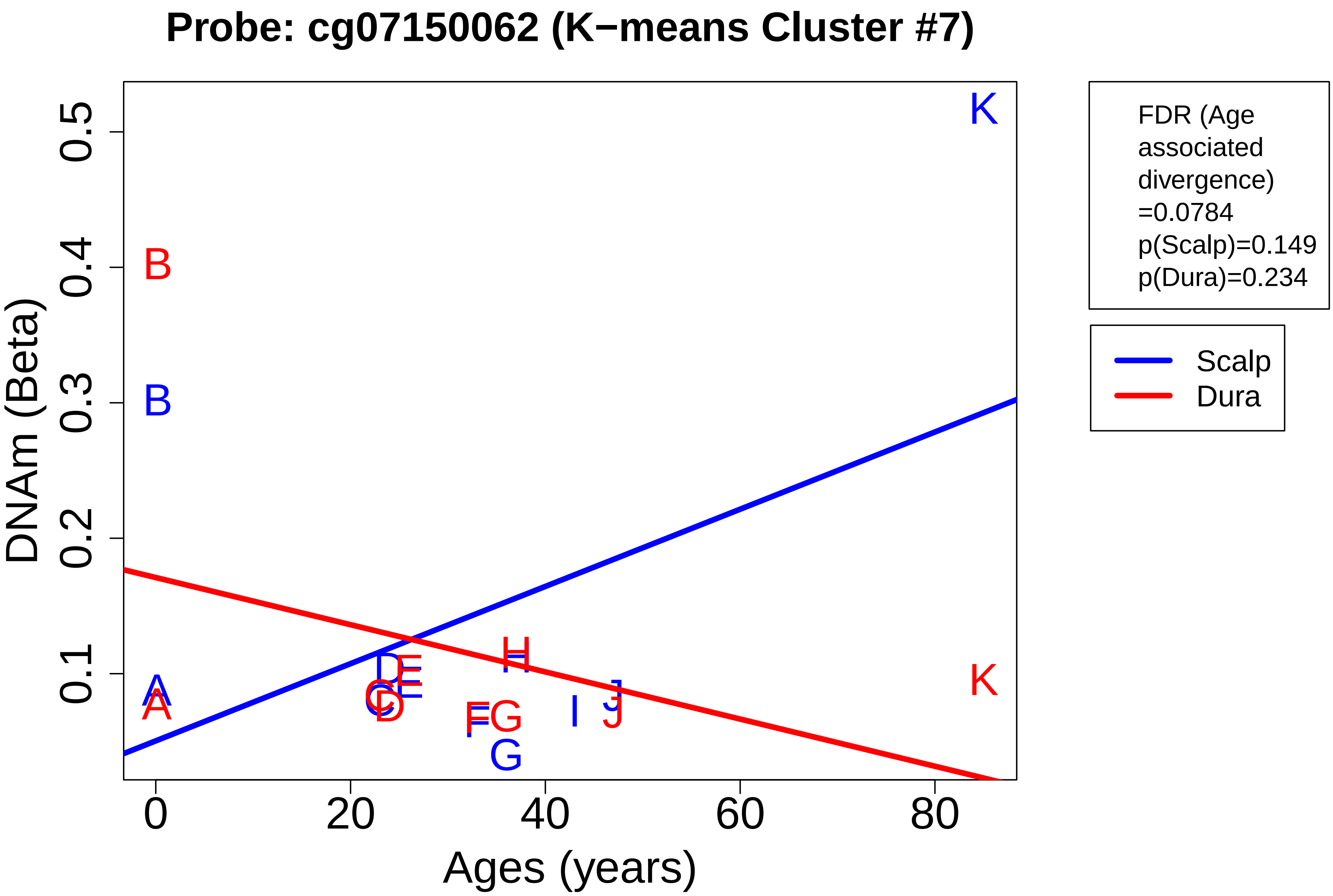

Probe: cg07150062 (K−means Cluster #7)
DNAm (Beta)
0.1
0.2
0.3
0.4
0.5
0
20
40
60
80
Ages (years)
FDR (Age associated divergence) =0.0784
p(Scalp)=0.149
p(Dura)=0.234
Scalp
Dura

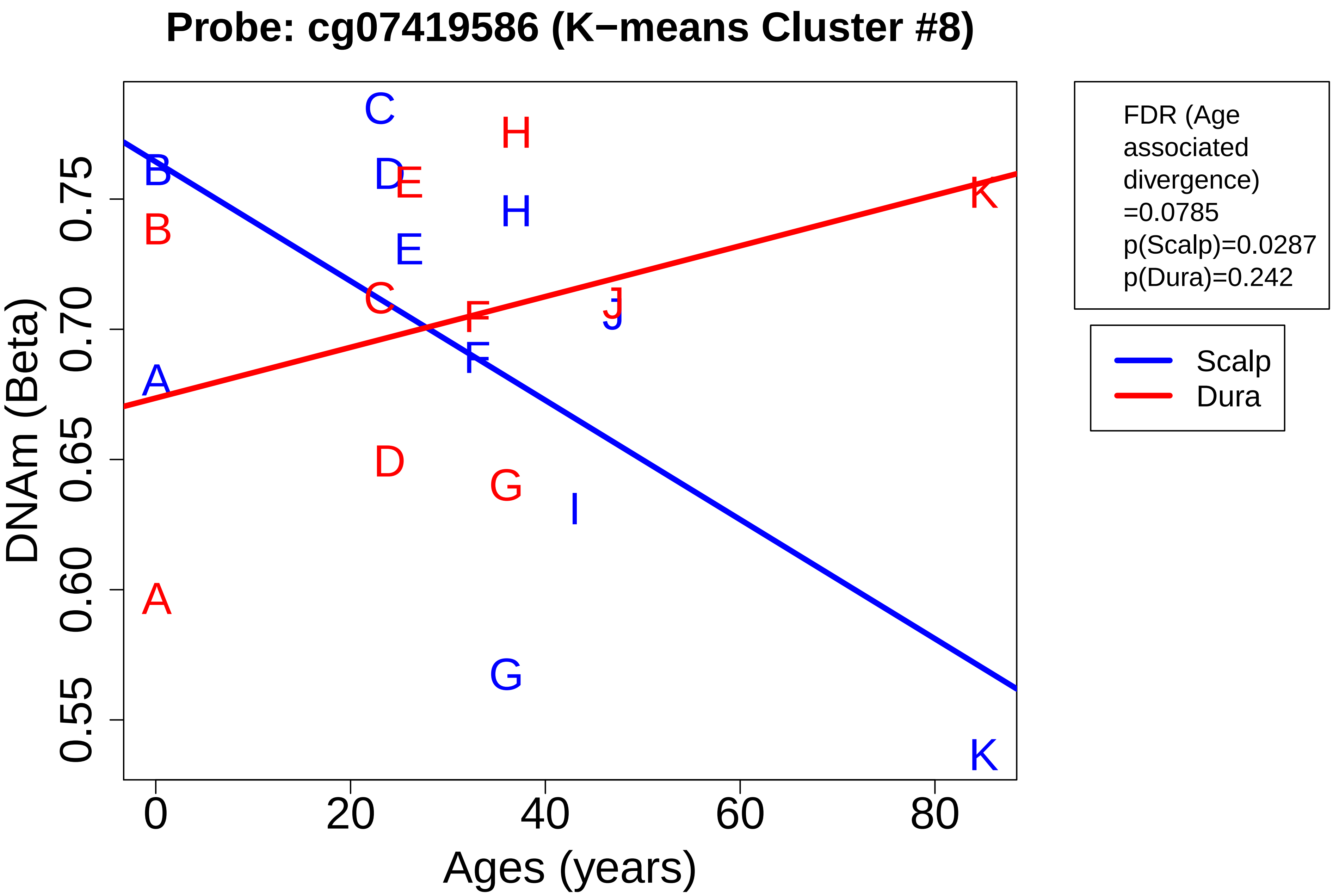

Probe: cg07419586 (K−means Cluster #8)
DNAm (Beta)
0.55
0.60
0.65
0.70
0.75
0
20
40
60
80
Ages (years)
FDR (Age associated divergence) =0.0785
p(Scalp)=0.0287
p(Dura)=0.242
Scalp
Dura

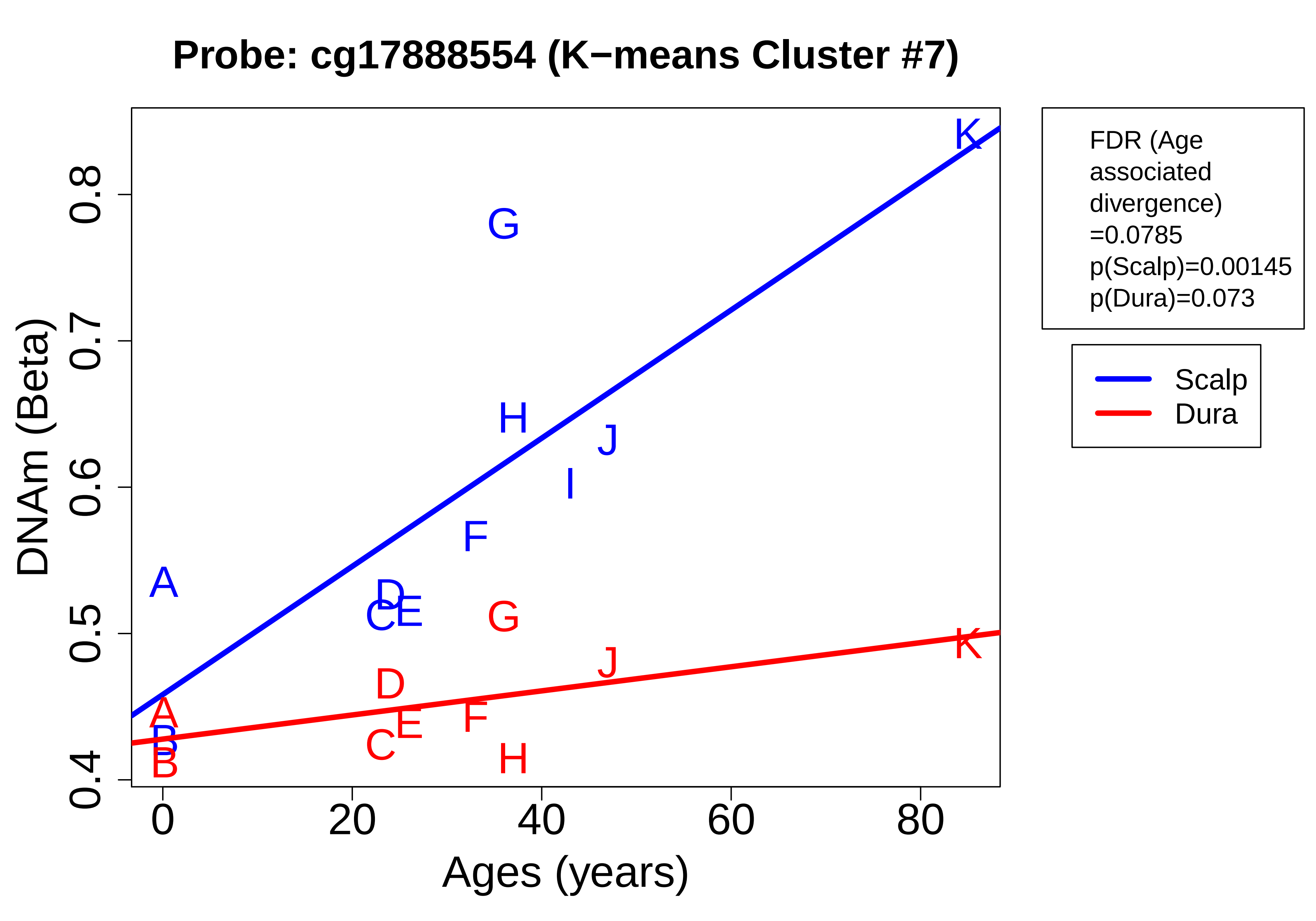

Probe: cg17888554 (K−means Cluster #7)
DNAm (Beta)
Ages (years)
0.4
0.5
0.6
0.7
0.8
0
20
40
60
80
FDR (Age associated divergence) =0.0785
p(Scalp)=0.00145
p(Dura)=0.073
Scalp
Dura
A
B
C
D
E
F
G
H
I
J
K

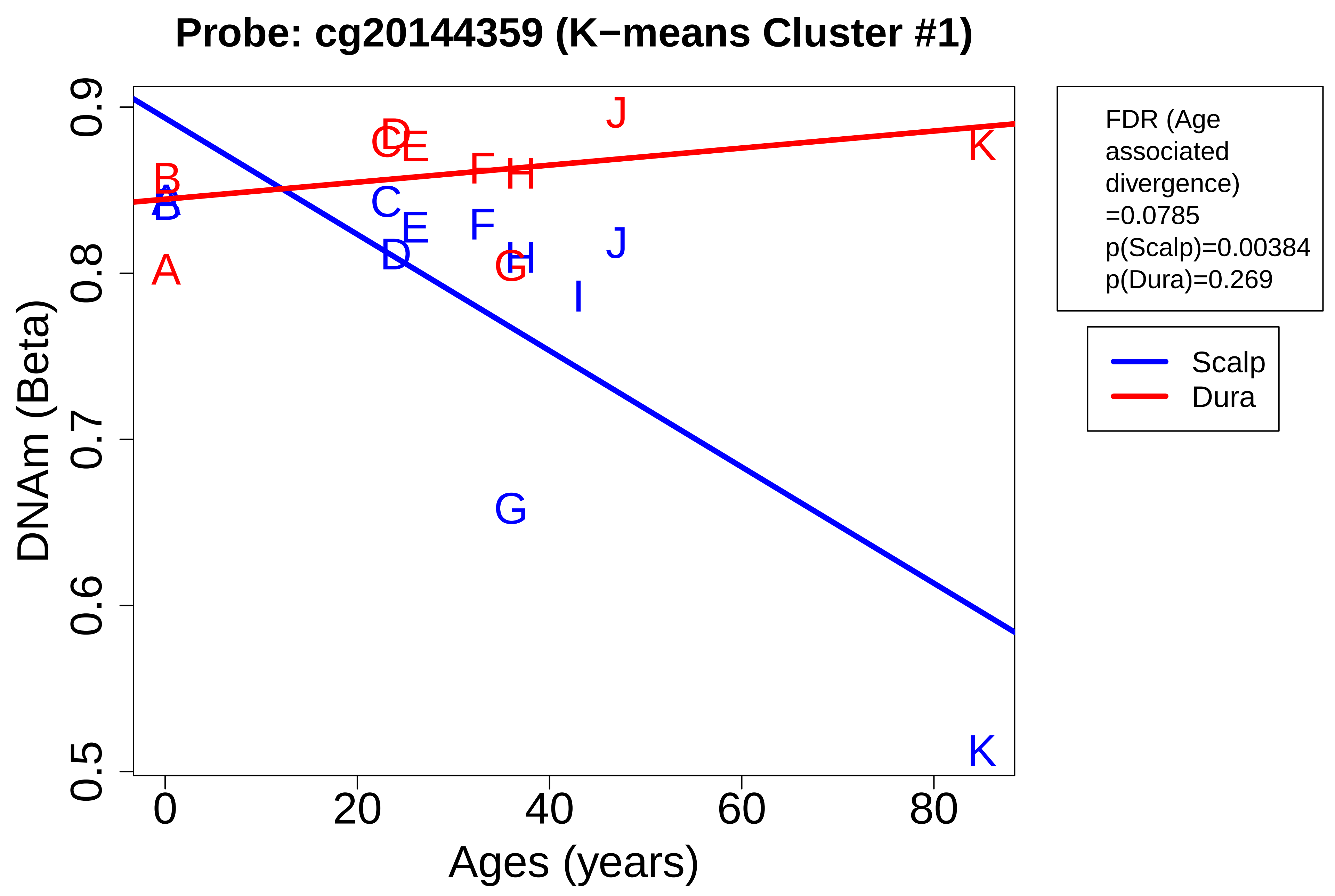

Probe: cg20144359 (K−means Cluster #1)
DNAm (Beta)
0.5
0.6
0.7
0.8
0.9
0
20
40
60
80
Ages (years)
FDR (Age associated divergence) =0.0785
p(Scalp)=0.00384
p(Dura)=0.269
Scalp
Dura

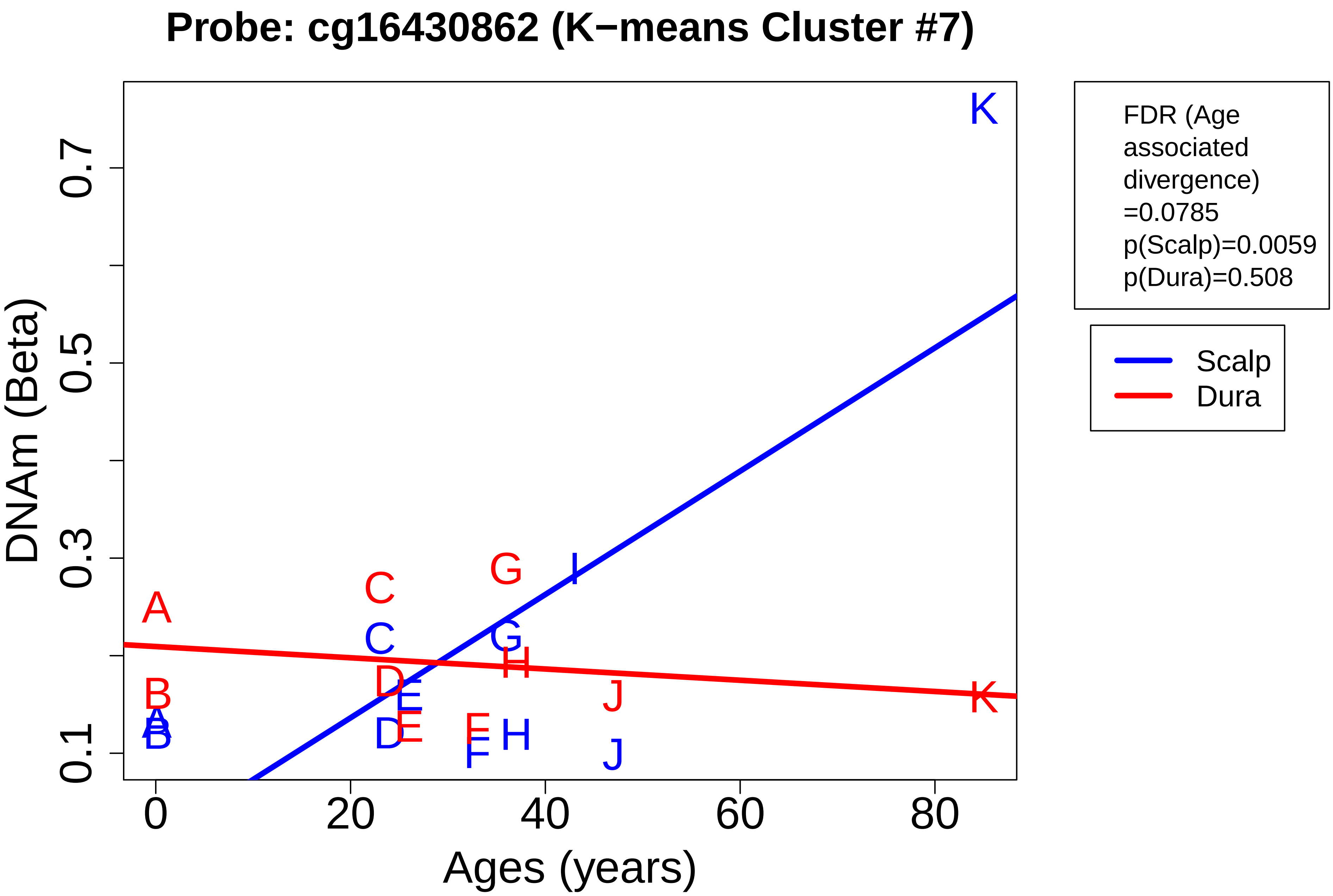

Probe: cg16430862 (K−means Cluster #7)
DNAm (Beta)
0.1
0.3
0.5
0.7
0
20
40
60
80
Ages (years)
FDR (Age associated divergence) =0.0785
p(Scalp)=0.0059
p(Dura)=0.508
Scalp
Dura

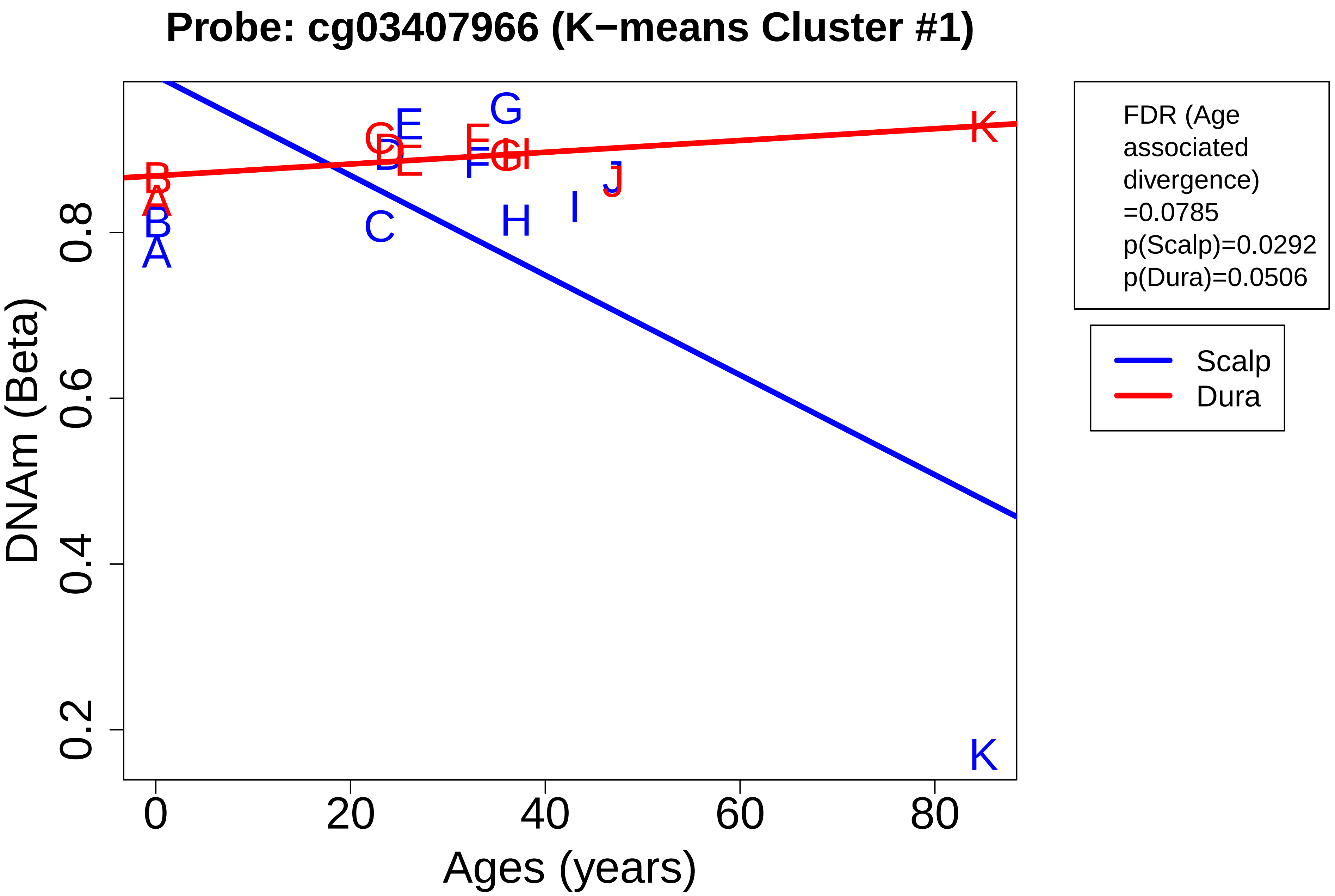

Probe: cg03407966 (K−means Cluster #1)
DNAm (Beta)
0.2
0.4
0.6
0.8
0
20
40
60
80
Ages (years)
FDR (Age associated divergence) =0.0785
p(Scalp)=0.0292
p(Dura)=0.0506
Scalp
Dura

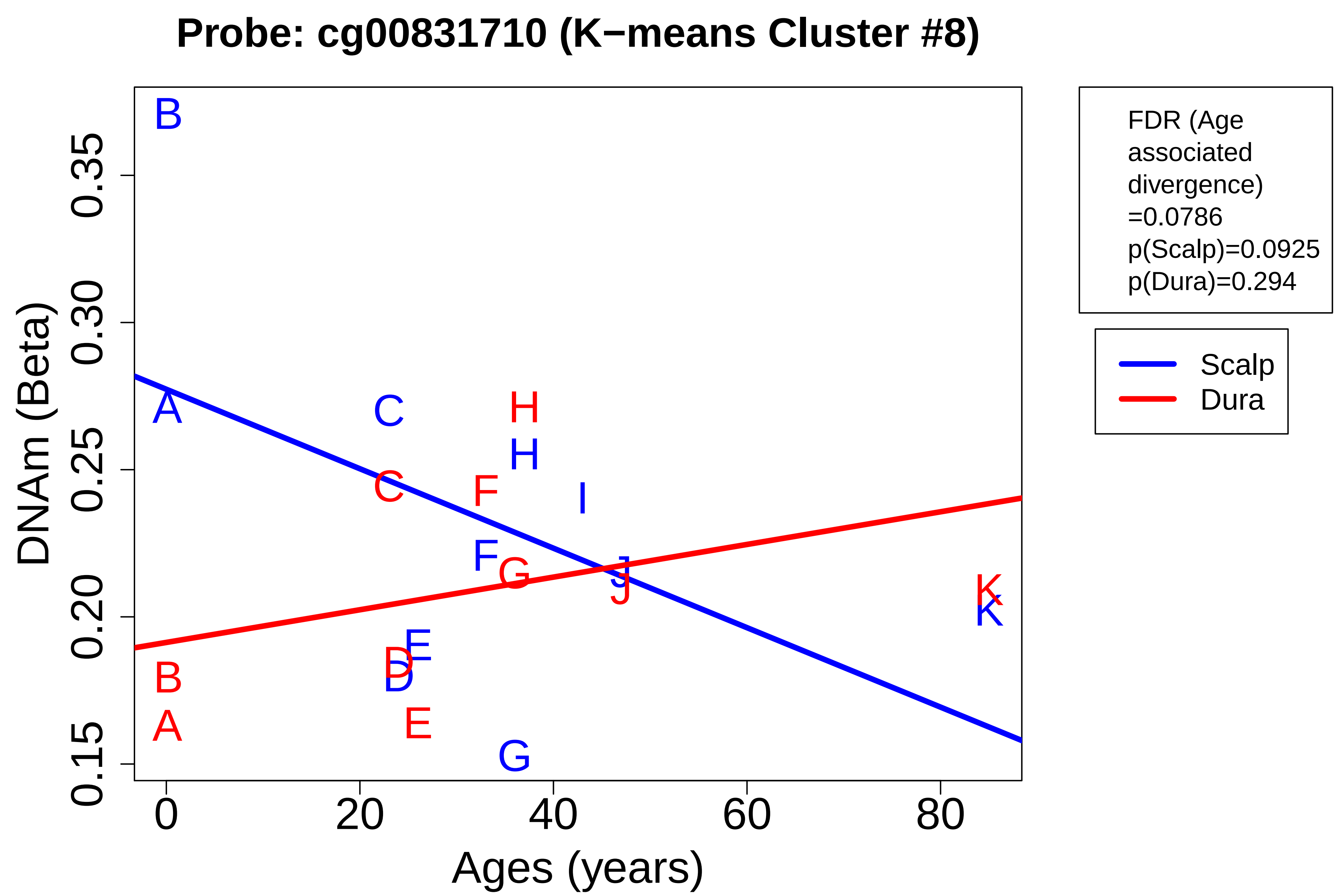

Probe: cg00831710 (K−means Cluster #8)
DNAm (Beta)
0.15
0.20
0.25
0.30
0.35
0
20
40
60
80
Ages (years)
B
A
C
H
H
C
F
I
F
G
J
J
K
K
E
D
D
B
A
E
G
FDR (Age associated divergence) =0.0786
p(Scalp)=0.0925
p(Dura)=0.294
Scalp
Dura

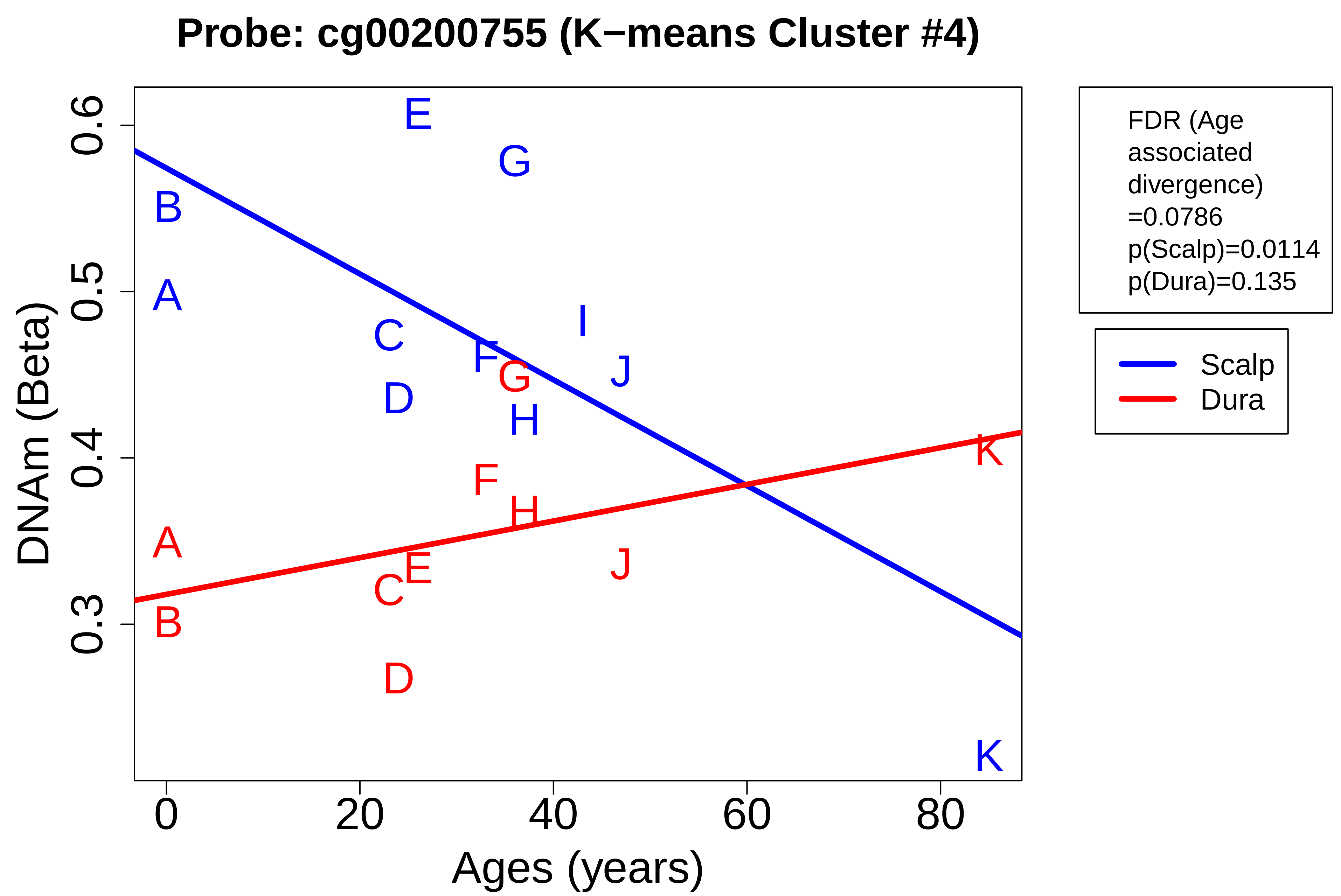

Probe: cg00200755 (K−means Cluster #4)
DNAm (Beta)
0.3
0.4
0.5
0.6
0
20
40
60
80
Ages (years)
A
B
C
D
E
F
G
H
I
J
K
FDR (Age associated divergence) =0.0786
p(Scalp)=0.0114
p(Dura)=0.135
Scalp
Dura

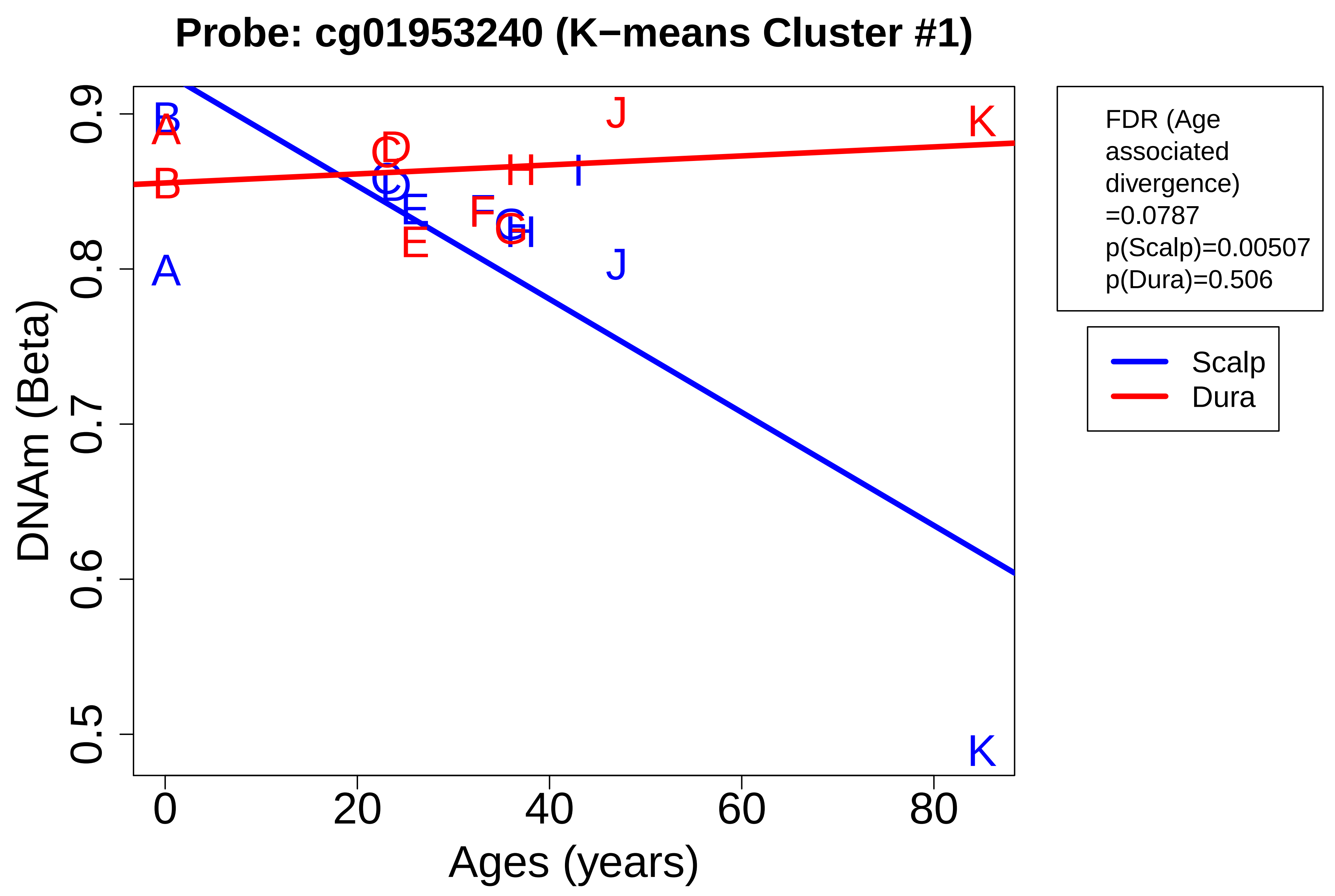

Probe: cg01953240 (K−means Cluster #1)
DNAm (Beta)
0.5
0.6
0.7
0.8
0.9
0
20
40
60
80
Ages (years)
A
B
C
D
E
F
G
H
I
J
K
FDR (Age associated divergence) =0.0787
p(Scalp)=0.00507
p(Dura)=0.506
Scalp
Dura

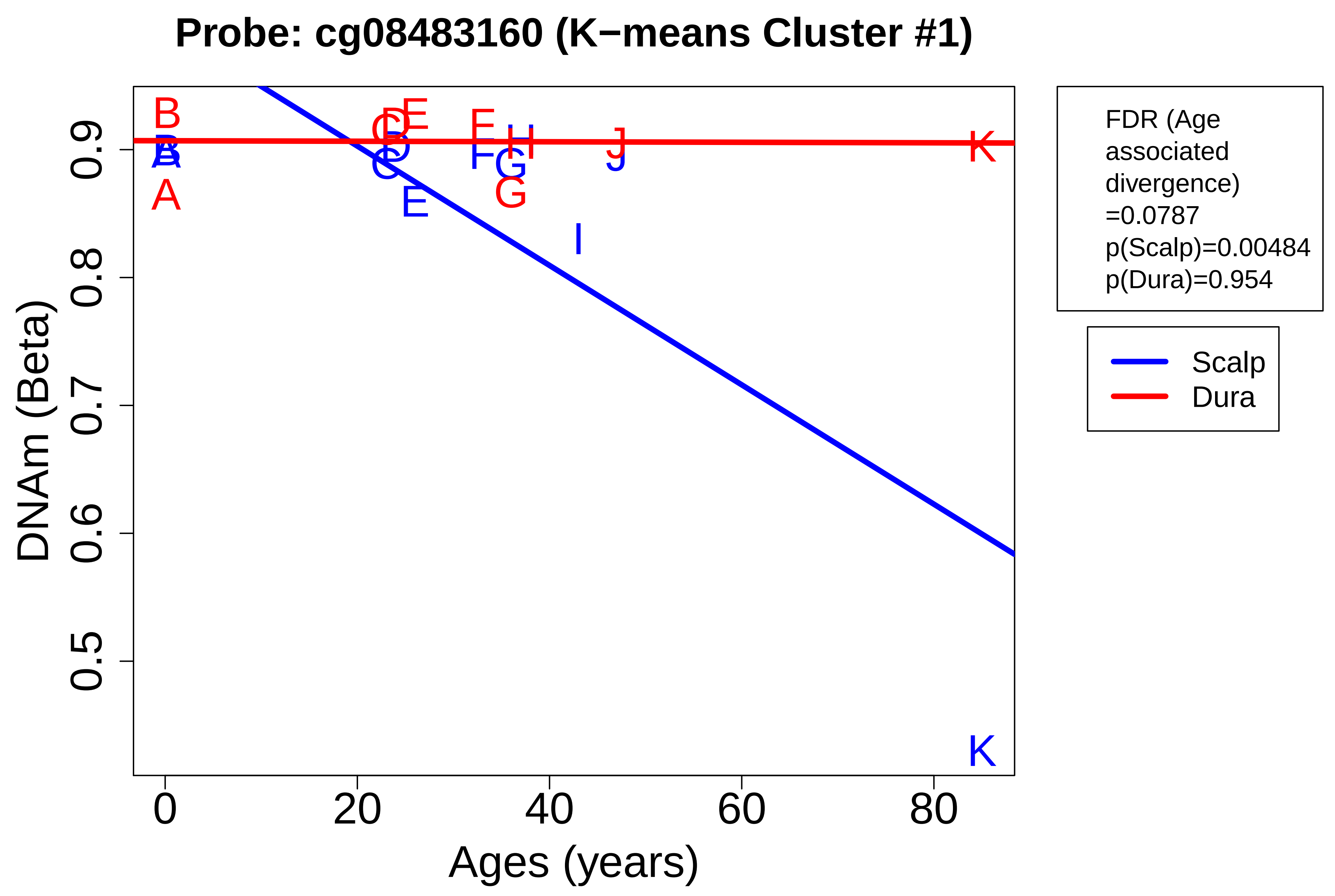

Probe: cg08483160 (K−means Cluster #1)
DNAm (Beta)
0.5
0.6
0.7
0.8
0.9
0
20
40
60
80
Ages (years)
FDR (Age associated divergence) =0.0787
p(Scalp)=0.00484
p(Dura)=0.954
Scalp
Dura

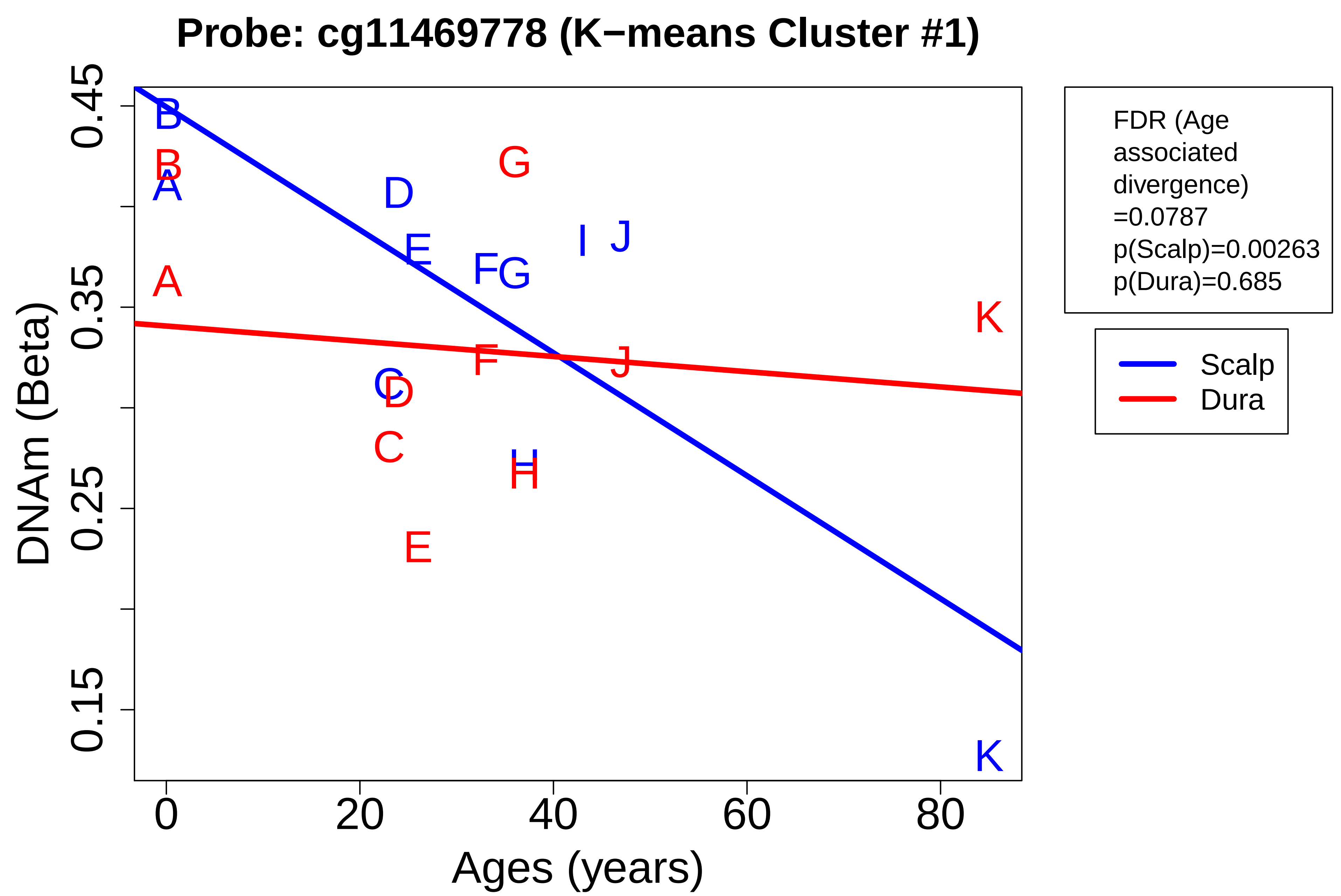

Probe: cg11469778 (K−means Cluster #1)
DNAm (Beta)
0.15
0.25
0.35
0.45
0
20
40
60
80
Ages (years)
A
B
C
D
E
F
G
H
I
J
K
FDR (Age associated divergence) =0.0787
p(Scalp)=0.00263
p(Dura)=0.685
Scalp
Dura

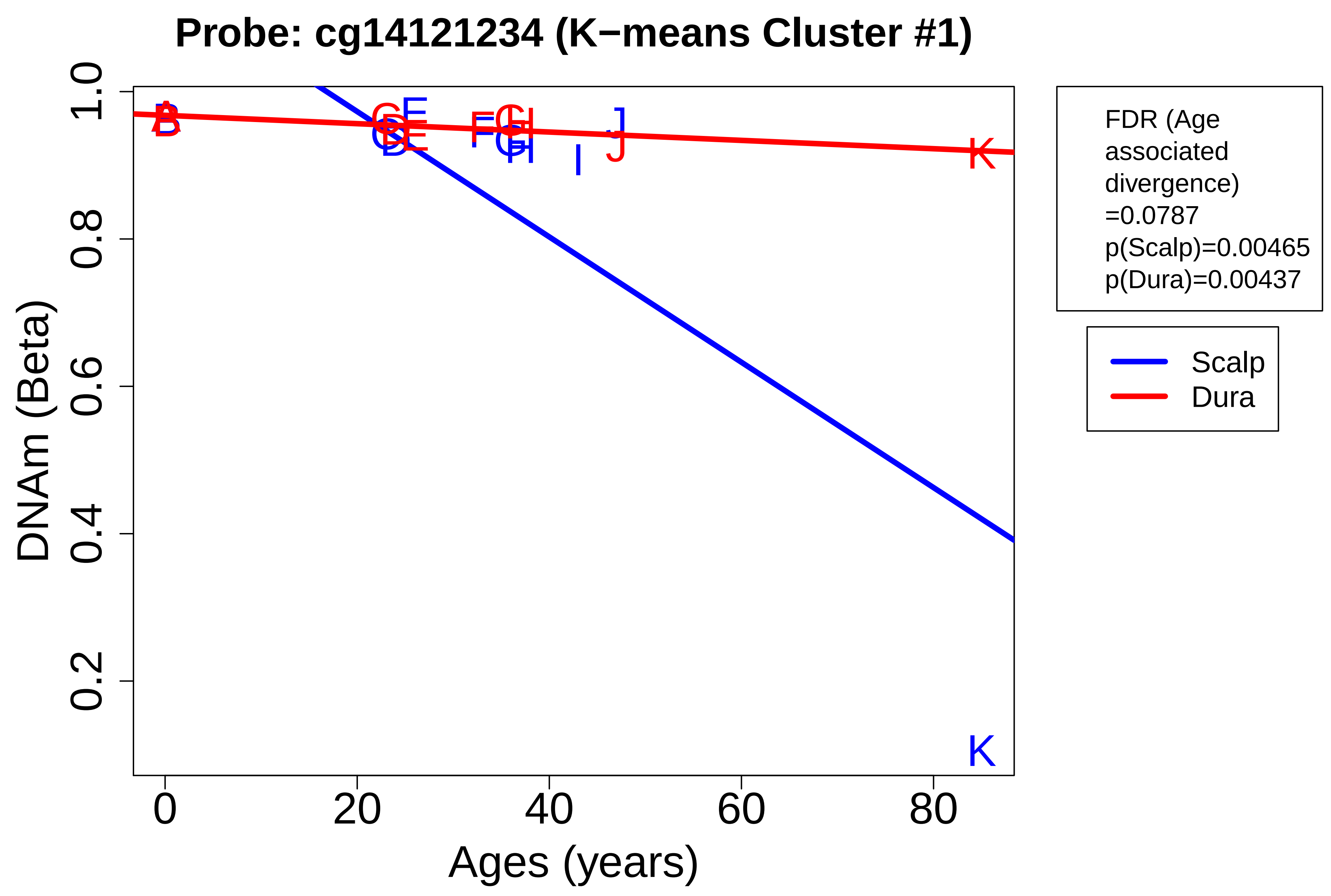

Probe: cg14121234 (K−means Cluster #1)
DNAm (Beta)
Ages (years)
0
20
40
60
80
0.2
0.4
0.6
0.8
1.0
FDR (Age associated divergence) =0.0787
p(Scalp)=0.00465
p(Dura)=0.00437
Scalp
Dura

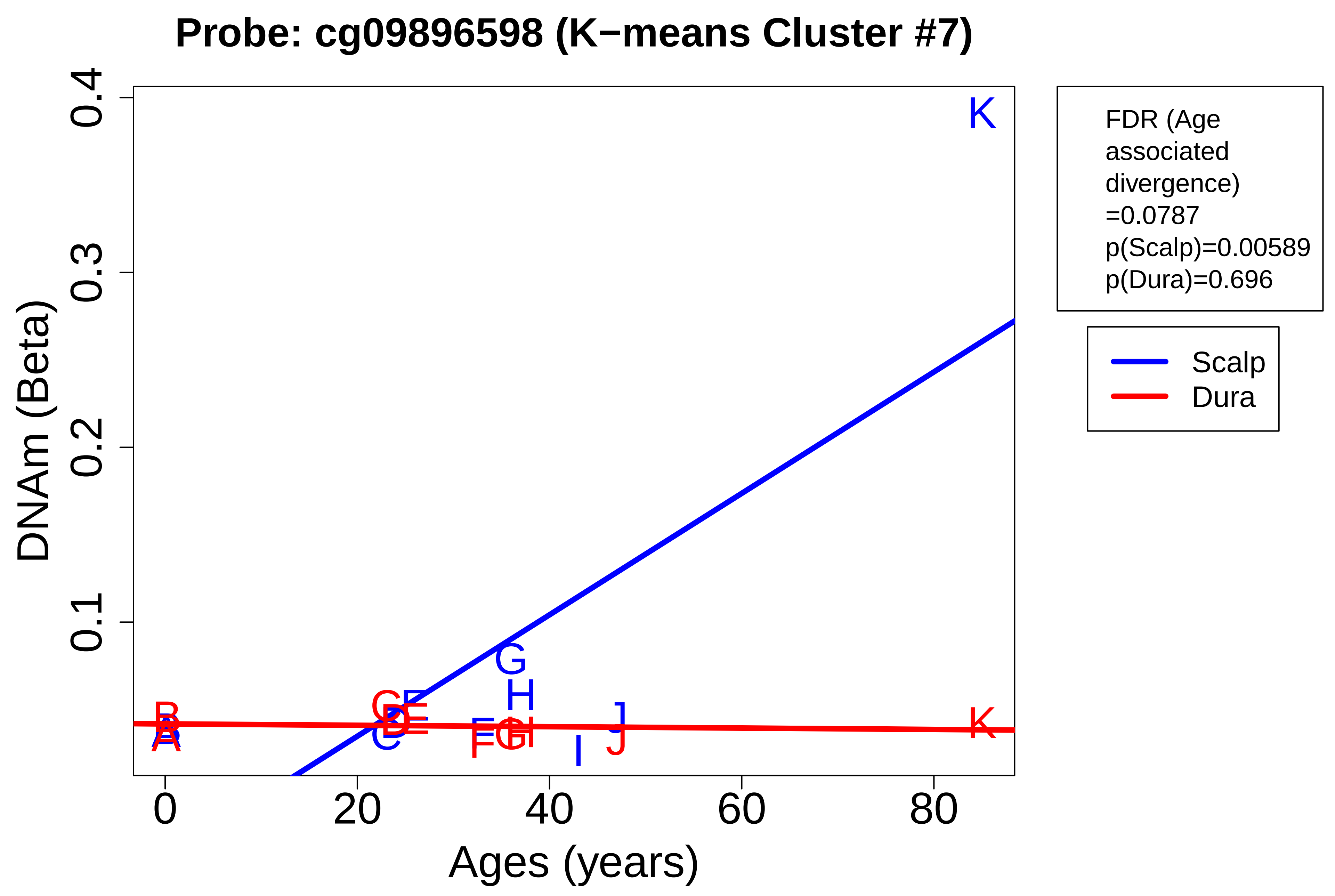

Probe: cg09896598 (K−means Cluster #7)
DNAm (Beta)
0.1
0.2
0.3
0.4
Ages (years)
0
20
40
60
80
FDR (Age associated divergence) =0.0787
p(Scalp)=0.00589
p(Dura)=0.696
Scalp
Dura

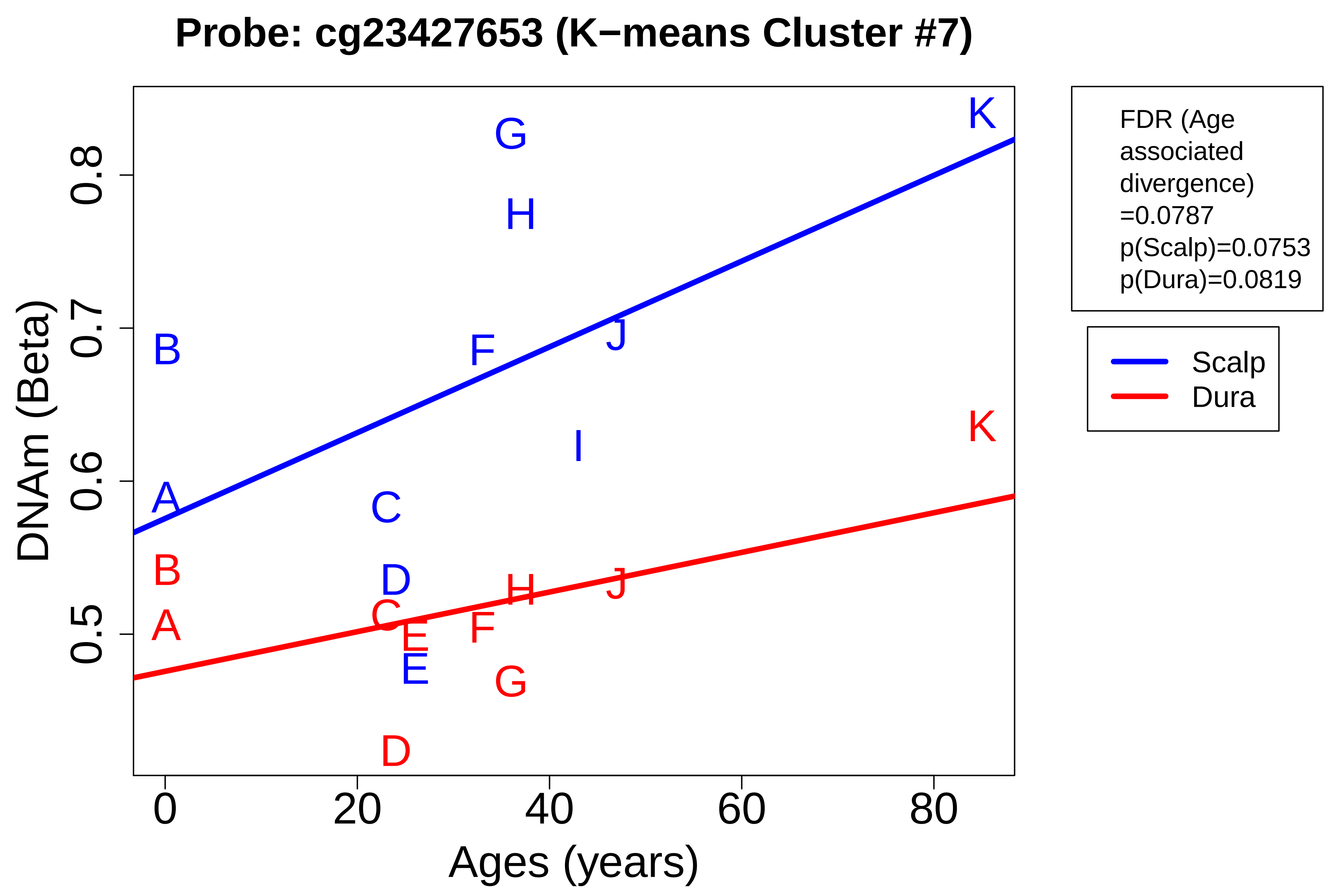

Probe: cg23427653 (K−means Cluster #7)
DNAm (Beta)
Ages (years)
0.5
0.6
0.7
0.8
0
20
40
60
80
FDR (Age associated divergence) =0.0787
p(Scalp)=0.0753
p(Dura)=0.0819
Scalp
Dura

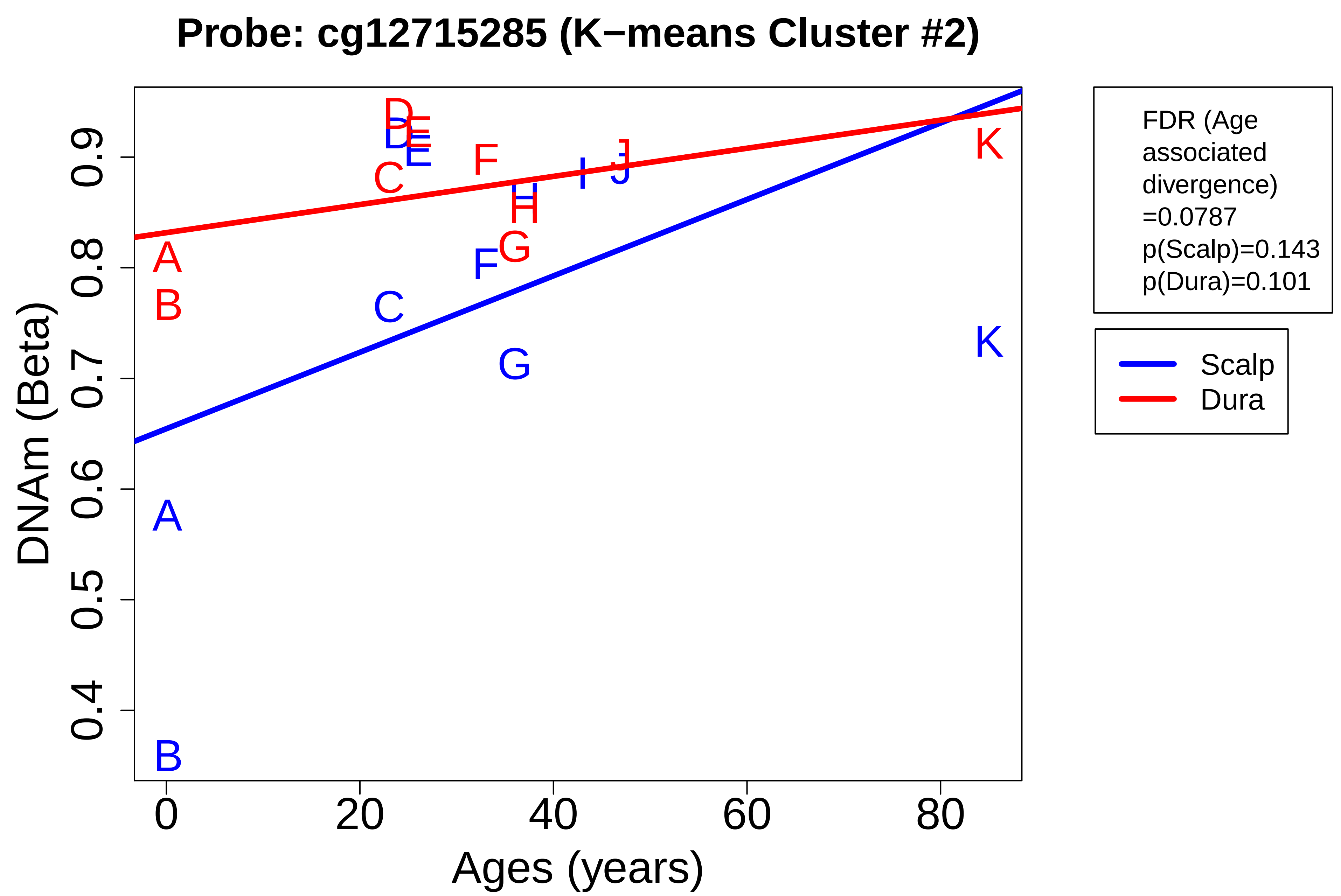

Probe: cg12715285 (K−means Cluster #2)
DNAm (Beta)
0.4
0.5
0.6
0.7
0.8
0.9
0
20
40
60
80
Ages (years)
FDR (Age associated divergence) =0.0787
p(Scalp)=0.143
p(Dura)=0.101
Scalp
Dura
A
B
C
D
E
F
G
H
I
J
K

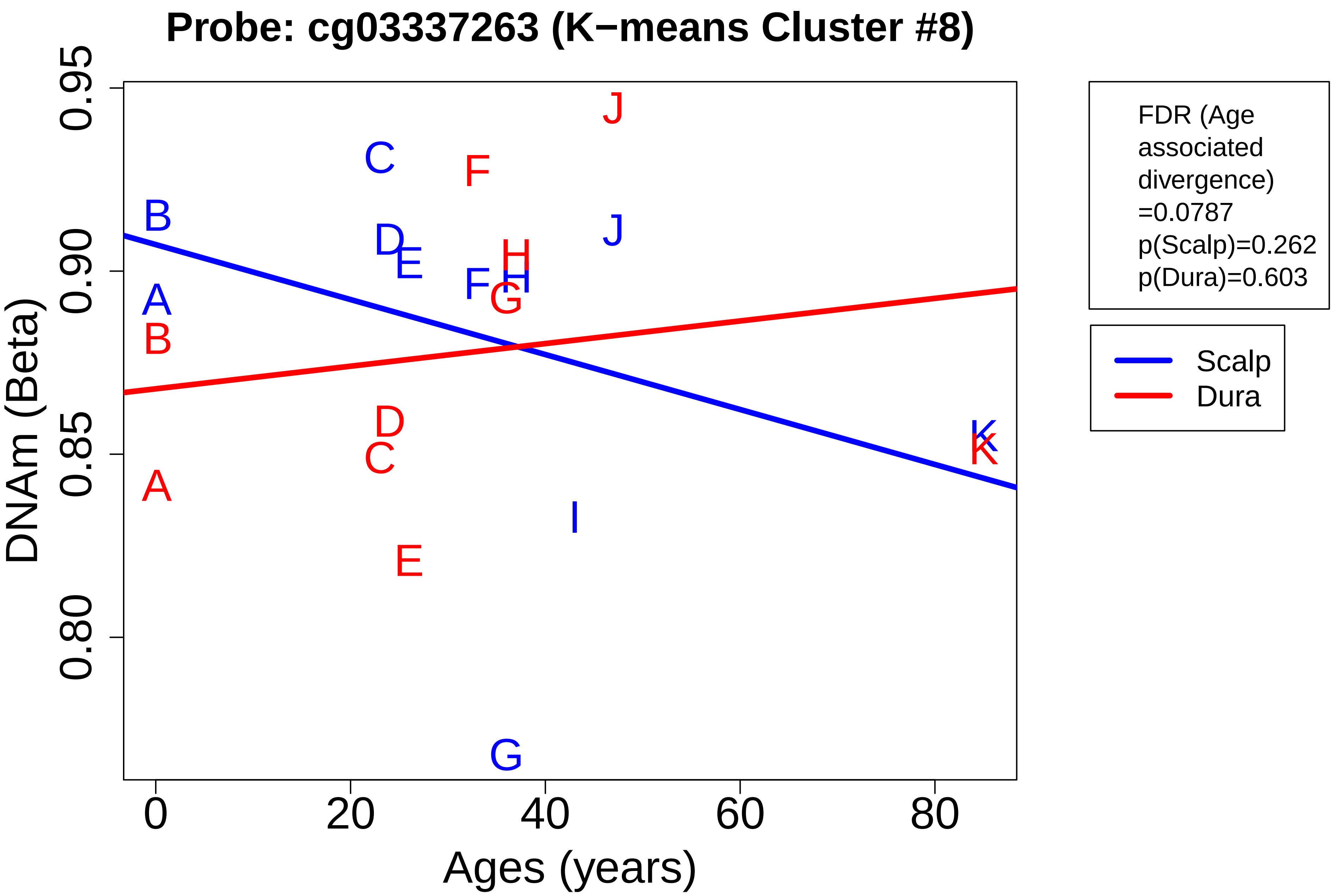

Probe: cg03337263 (K−means Cluster #8)
DNAm (Beta)
0.80
0.85
0.90
0.95
0
20
40
60
80
Ages (years)
FDR (Age associated divergence) =0.0787
p(Scalp)=0.262
p(Dura)=0.603
Scalp
Dura
A
B
C
D
E
F
G
H
I
J
K

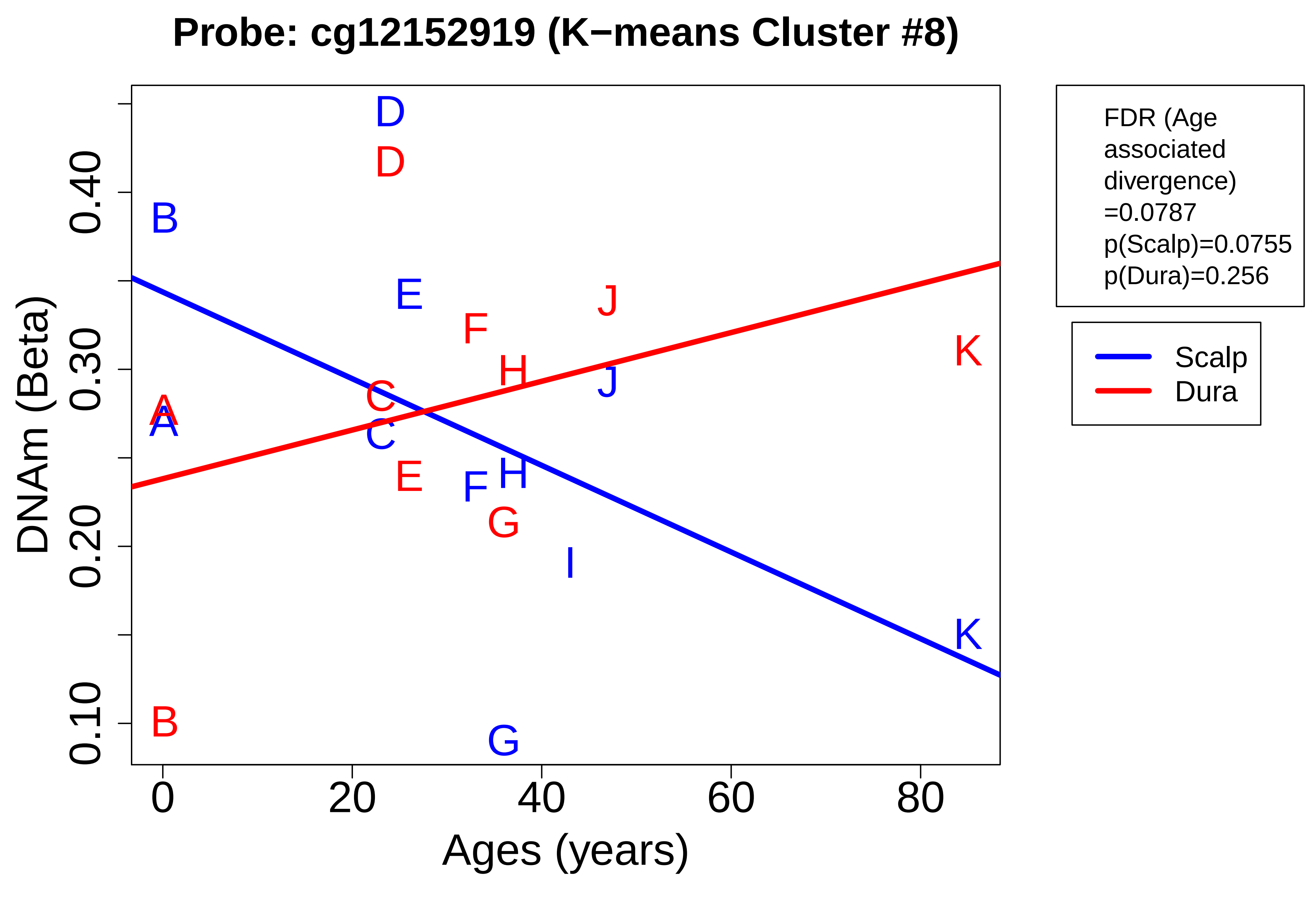

Probe: cg12152919 (K−means Cluster #8)
FDR (Age associated divergence) =0.0787
p(Scalp)=0.0755
p(Dura)=0.256
Scalp
Dura
DNAm (Beta)
0.10
0.20
0.30
0.40
Ages (years)
0
20
40
60
80
A
B
C
D
E
F
G
H
I
J
K

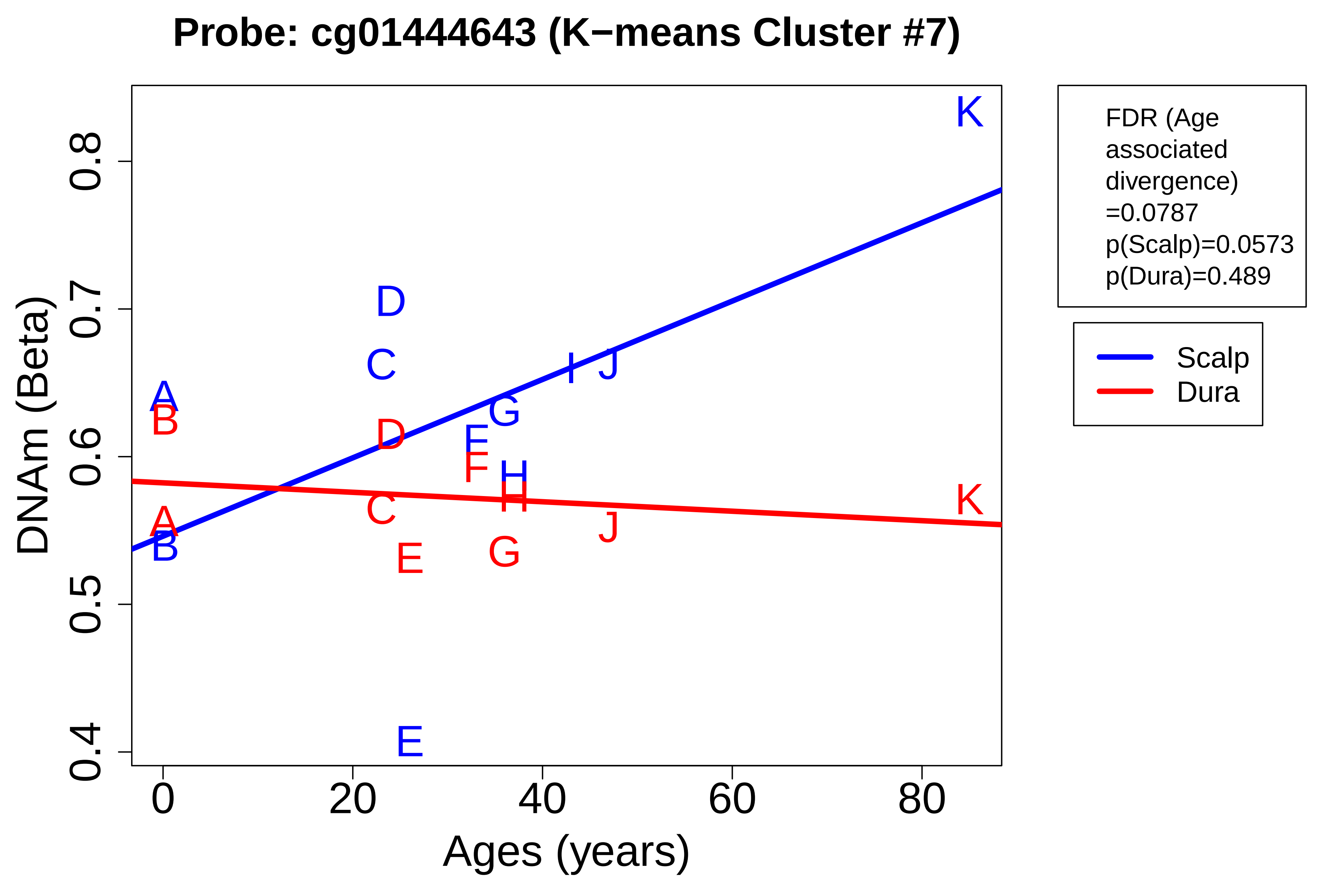

Probe: cg01444643 (K−means Cluster #7)
DNAm (Beta)
0.4
0.5
0.6
0.7
0.8
Ages (years)
0
20
40
60
80
FDR (Age associated divergence) =0.0787
p(Scalp)=0.0573
p(Dura)=0.489
Scalp
Dura
A
B
C
D
E
F
G
H
I
J
K

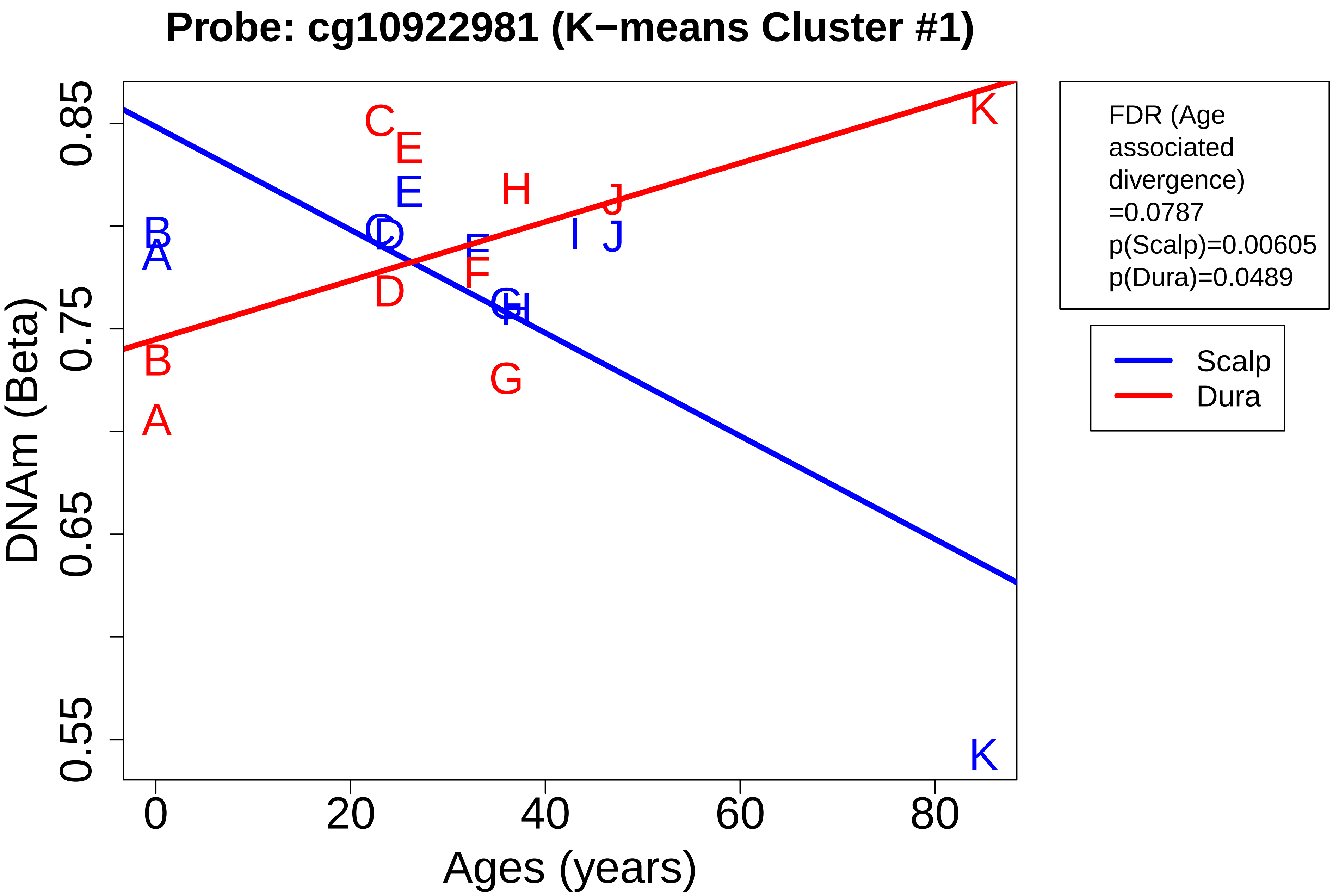

Probe: cg10922981 (K−means Cluster #1)
DNAm (Beta)
0.55
0.65
0.75
0.85
Ages (years)
0
20
40
60
80
FDR (Age associated divergence) =0.0787
p(Scalp)=0.00605
p(Dura)=0.0489
Scalp
Dura

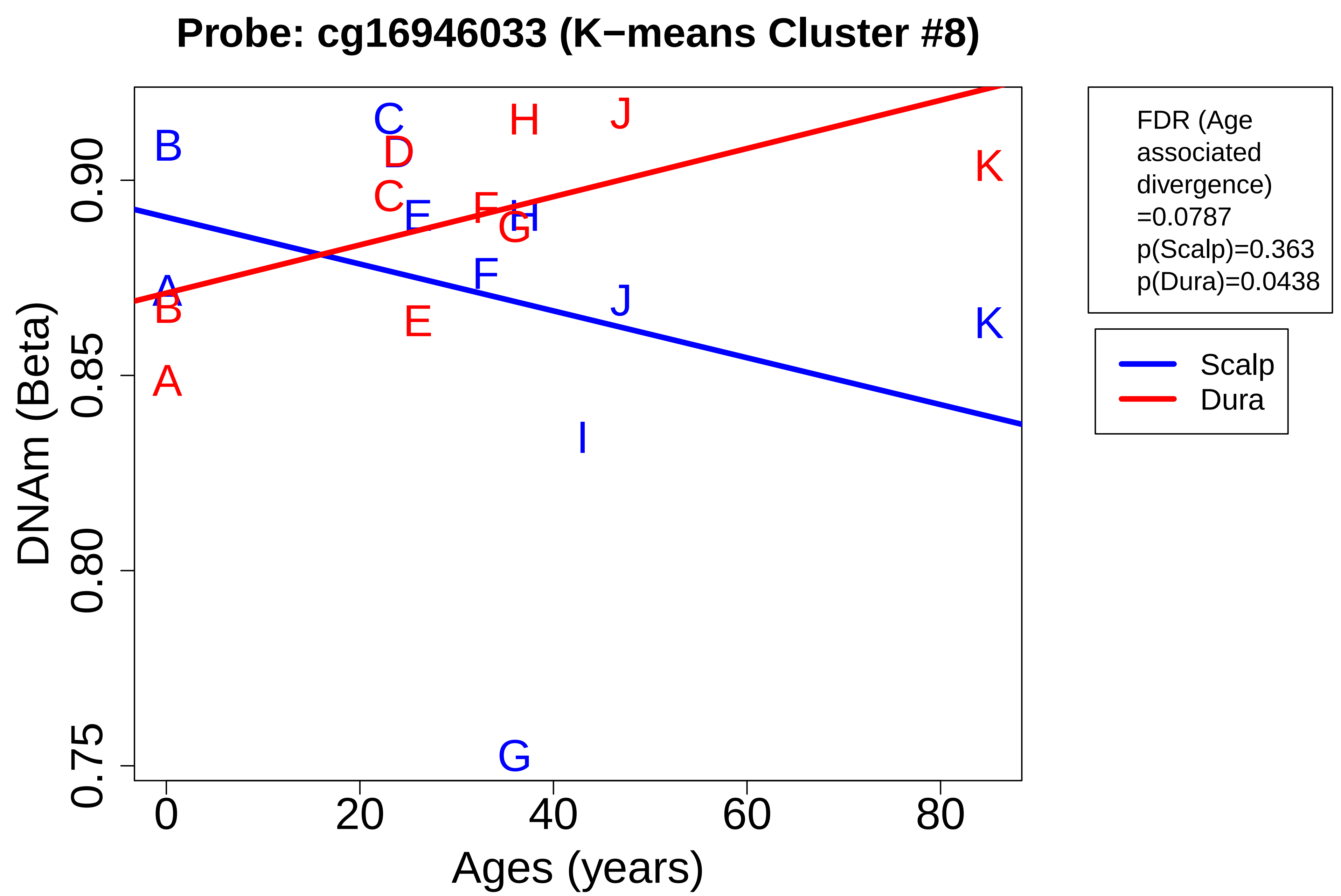

Probe: cg16946033 (K−means Cluster #8)
FDR (Age associated divergence) =0.0787
p(Scalp)=0.363
p(Dura)=0.0438
Scalp
Dura
DNAm (Beta)
0.75
0.80
0.85
0.90
0
20
40
60
80
Ages (years)

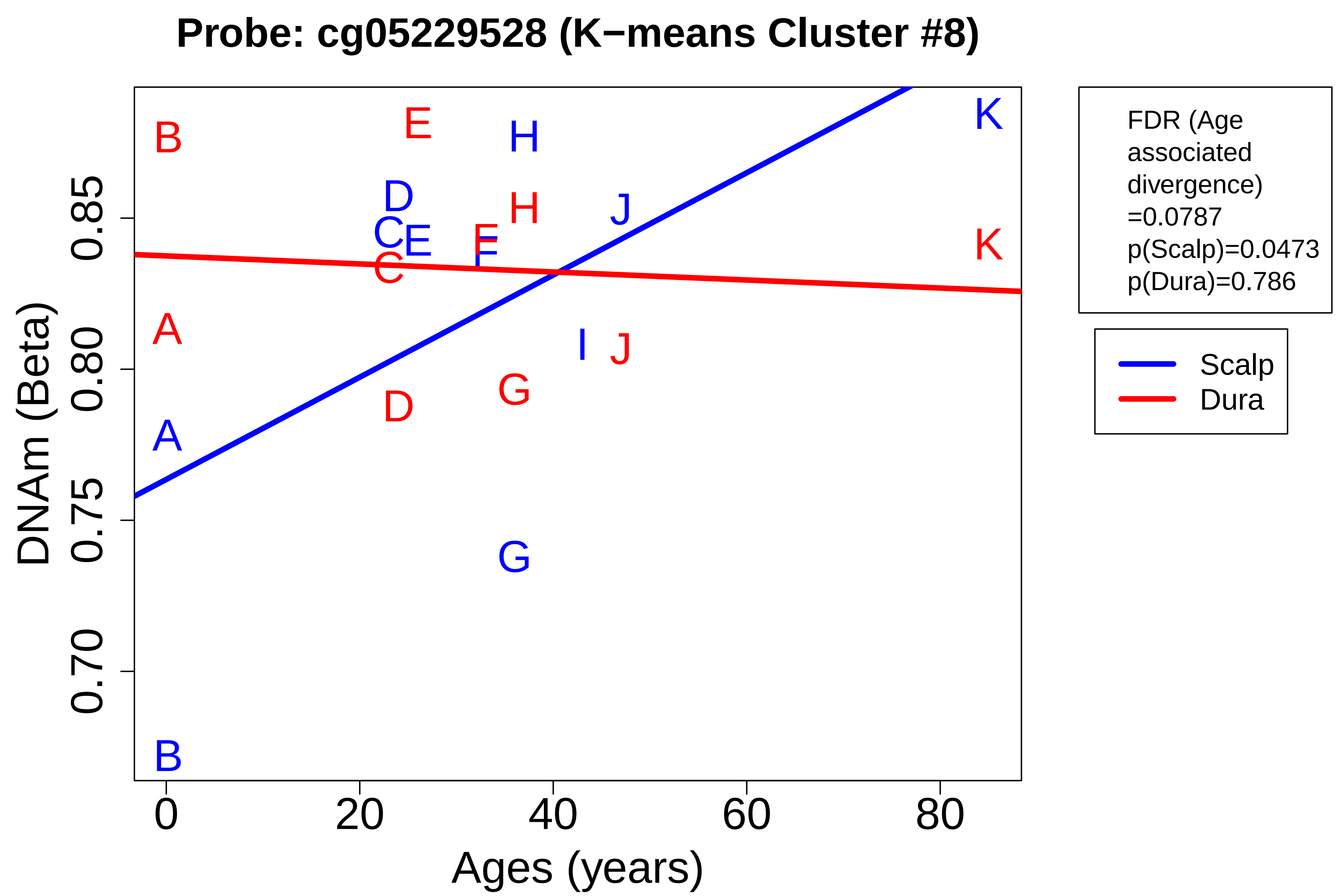

Probe: cg05229528 (K−means Cluster #8)
DNAm (Beta)
0.70
0.75
0.80
0.85
0
20
40
60
80
Ages (years)
FDR (Age associated divergence) =0.0787
p(Scalp)=0.0473
p(Dura)=0.786
Scalp
Dura

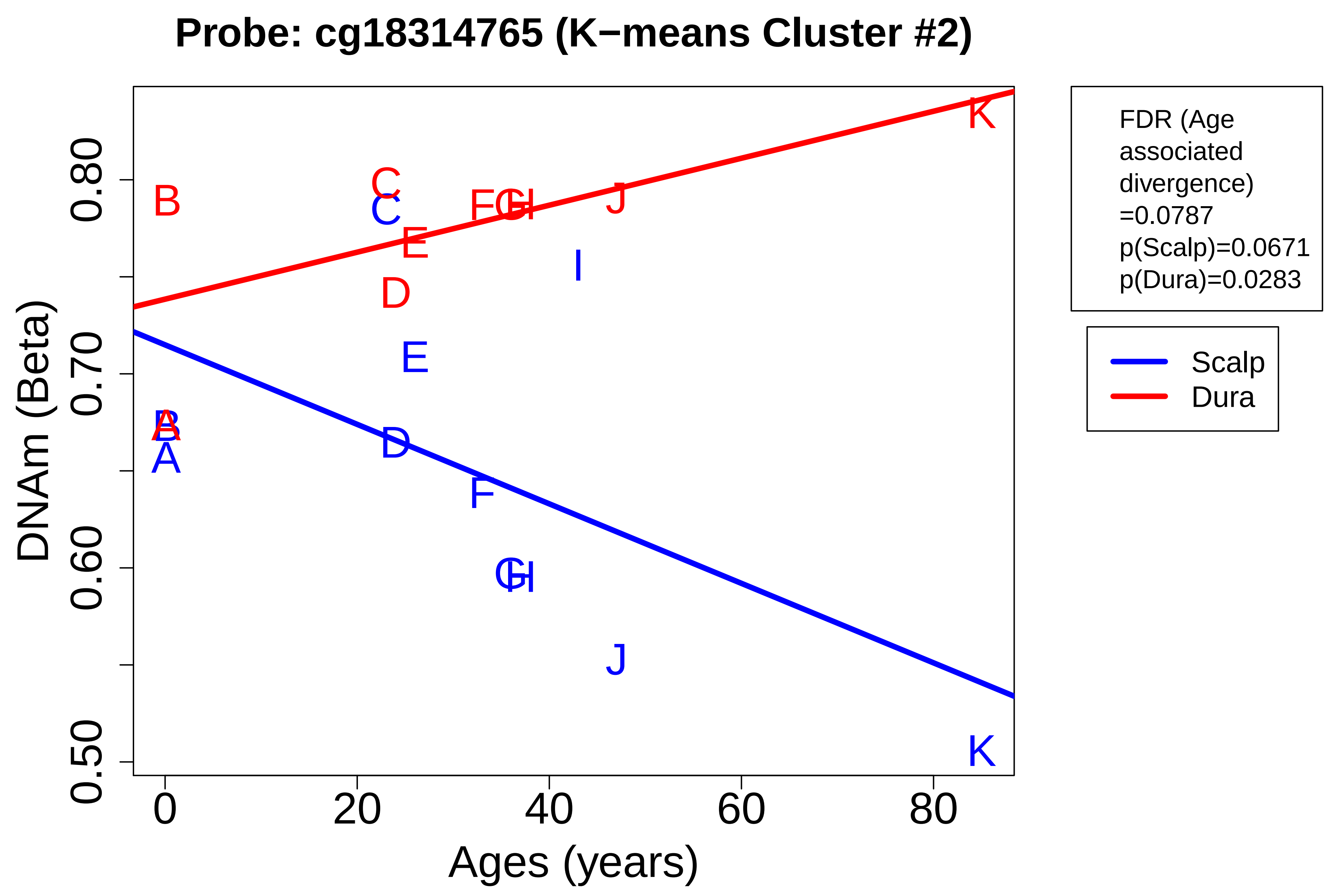

Probe: cg18314765 (K−means Cluster #2)
DNAm (Beta)
0.50
0.60
0.70
0.80
0
20
40
60
80
Ages (years)
FDR (Age associated divergence) =0.0787
p(Scalp)=0.0671
p(Dura)=0.0283
Scalp
Dura

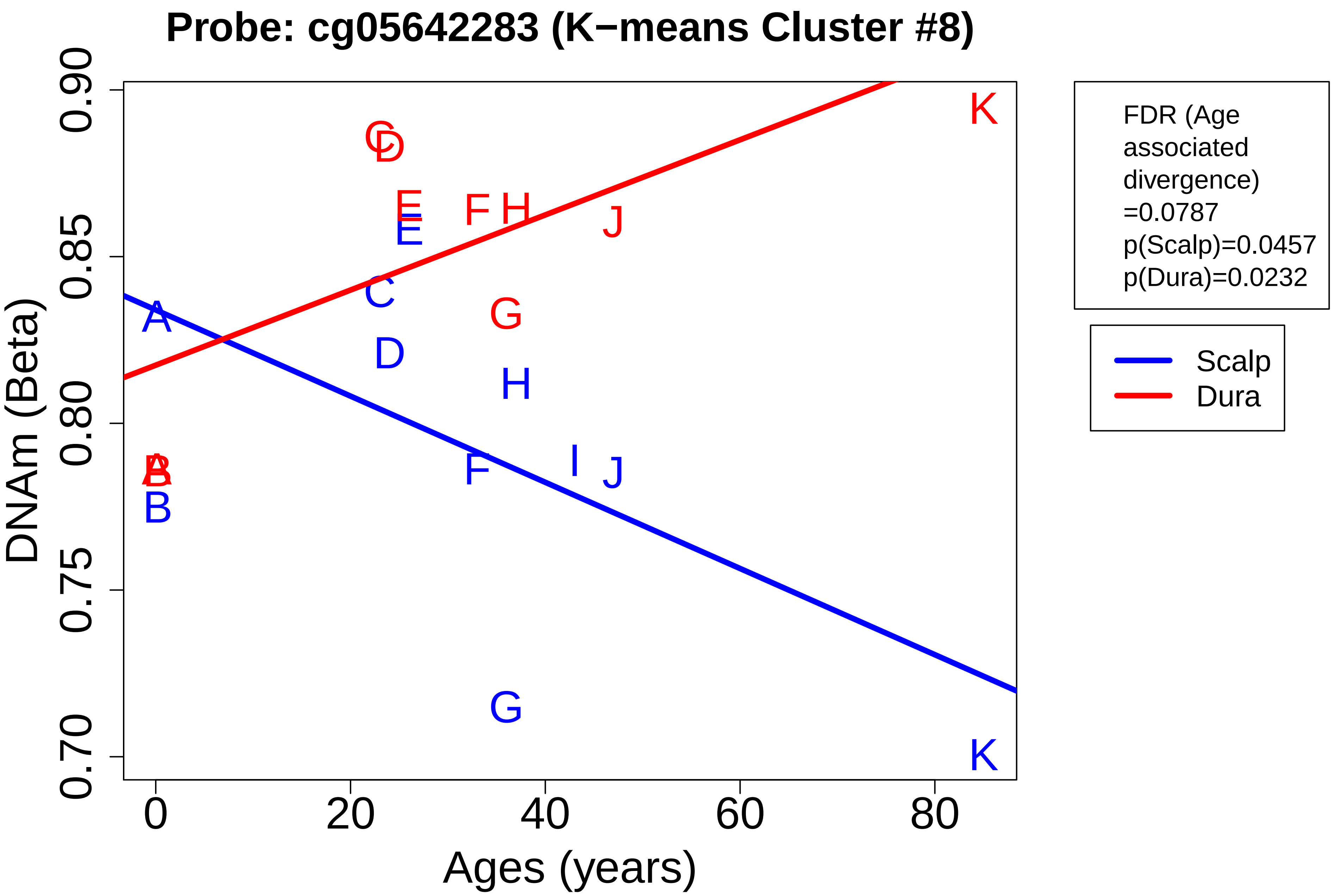

Probe: cg05642283 (K−means Cluster #8)
DNAm (Beta)
0.70
0.75
0.80
0.85
0.90
0
20
40
60
80
Ages (years)
FDR (Age associated divergence) =0.0787
p(Scalp)=0.0457
p(Dura)=0.0232
Scalp
Dura
A
B
C
D
E
F
G
H
I
J
K

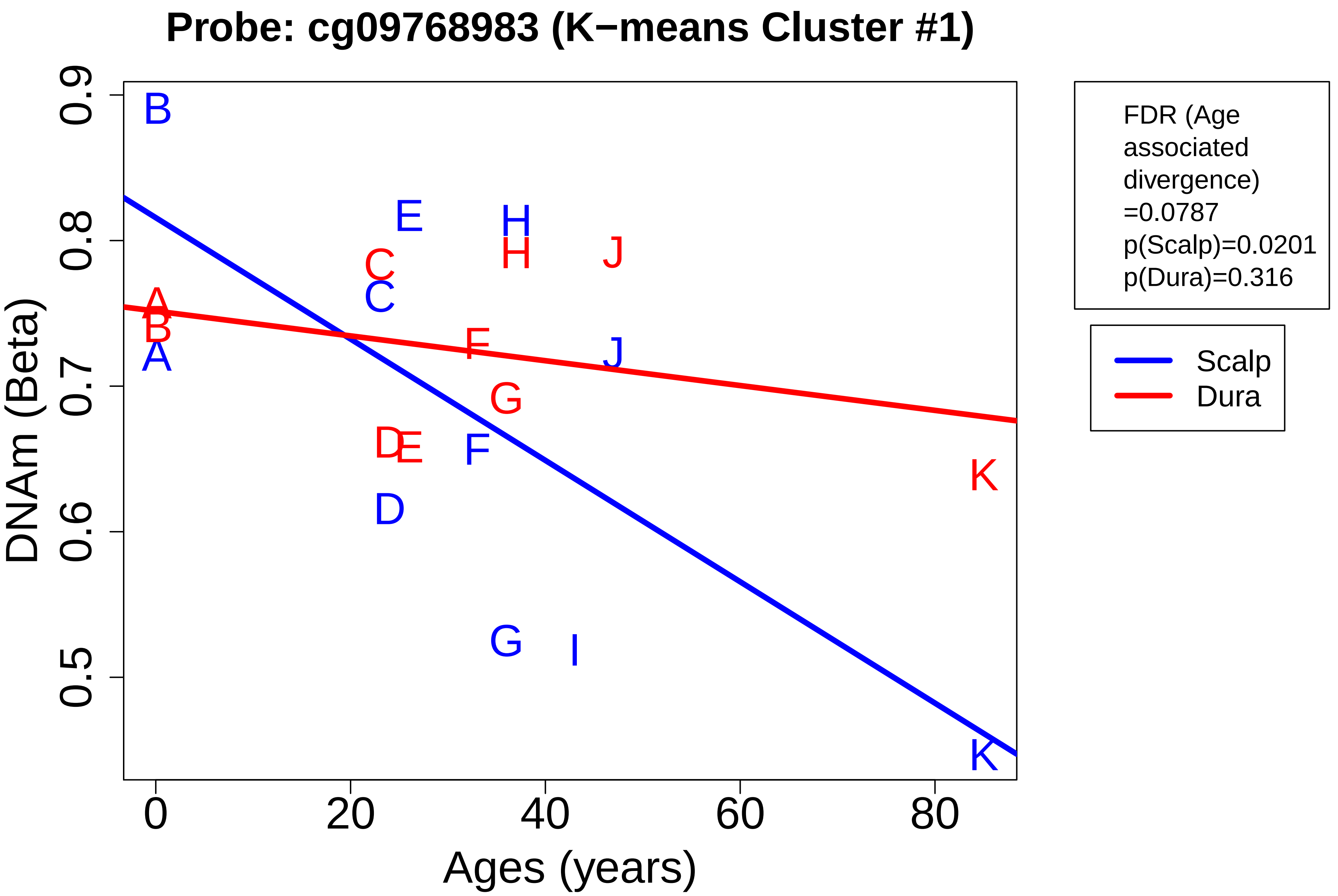

Probe: cg09768983 (K−means Cluster #1)
DNAm (Beta)
0.5
0.6
0.7
0.8
0.9
0
20
40
60
80
Ages (years)
FDR (Age associated divergence) =0.0787
p(Scalp)=0.0201
p(Dura)=0.316
Scalp
Dura
A
B
C
D
E
F
G
H
I
J
K

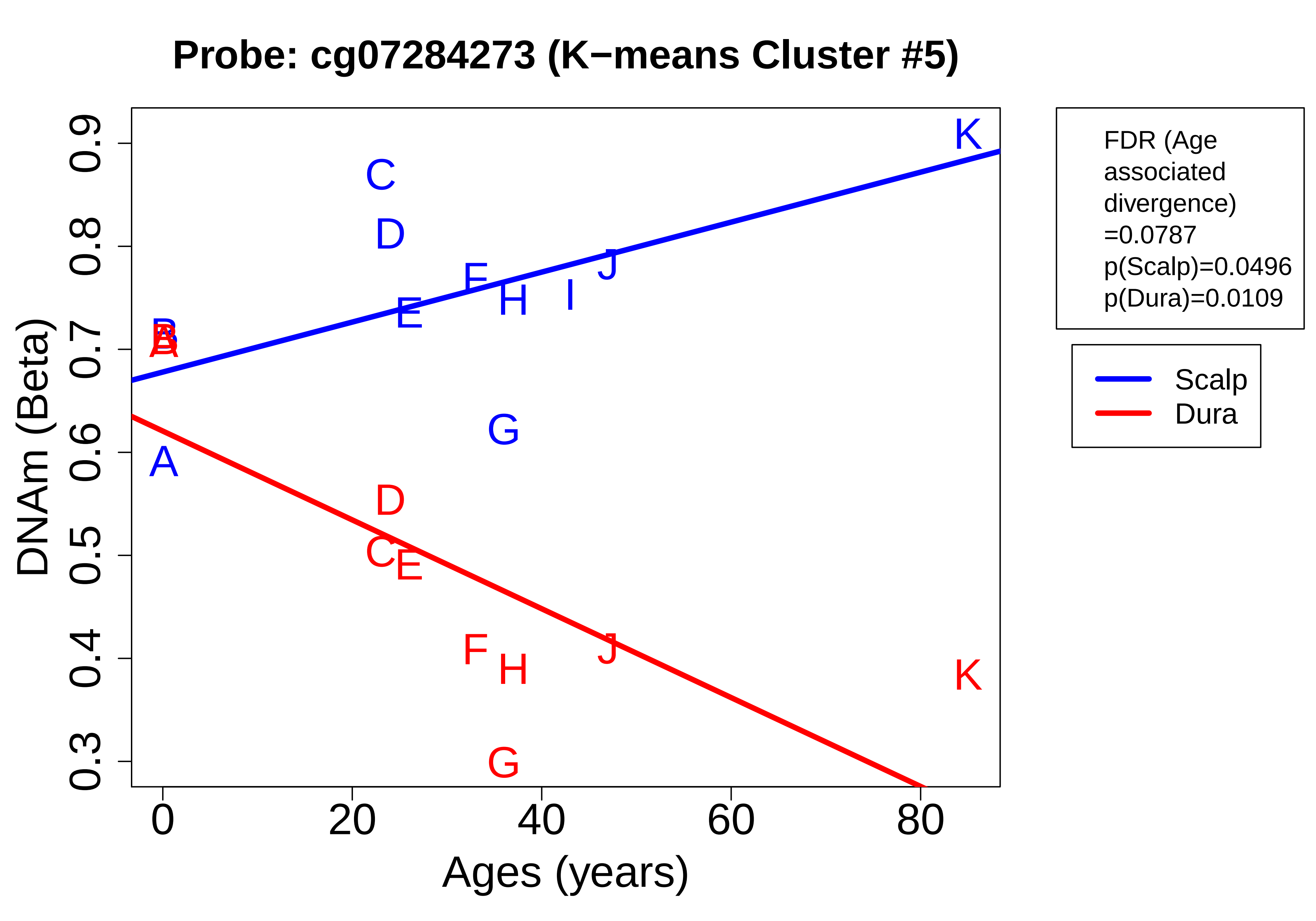

Probe: cg07284273 (K−means Cluster #5)
DNAm (Beta)
Ages (years)
0.3
0.4
0.5
0.6
0.7
0.8
0.9
0
20
40
60
80
FDR (Age associated divergence) =0.0787
p(Scalp)=0.0496
p(Dura)=0.0109
Scalp
Dura
A
B
C
D
E
F
G
H
I
J
K

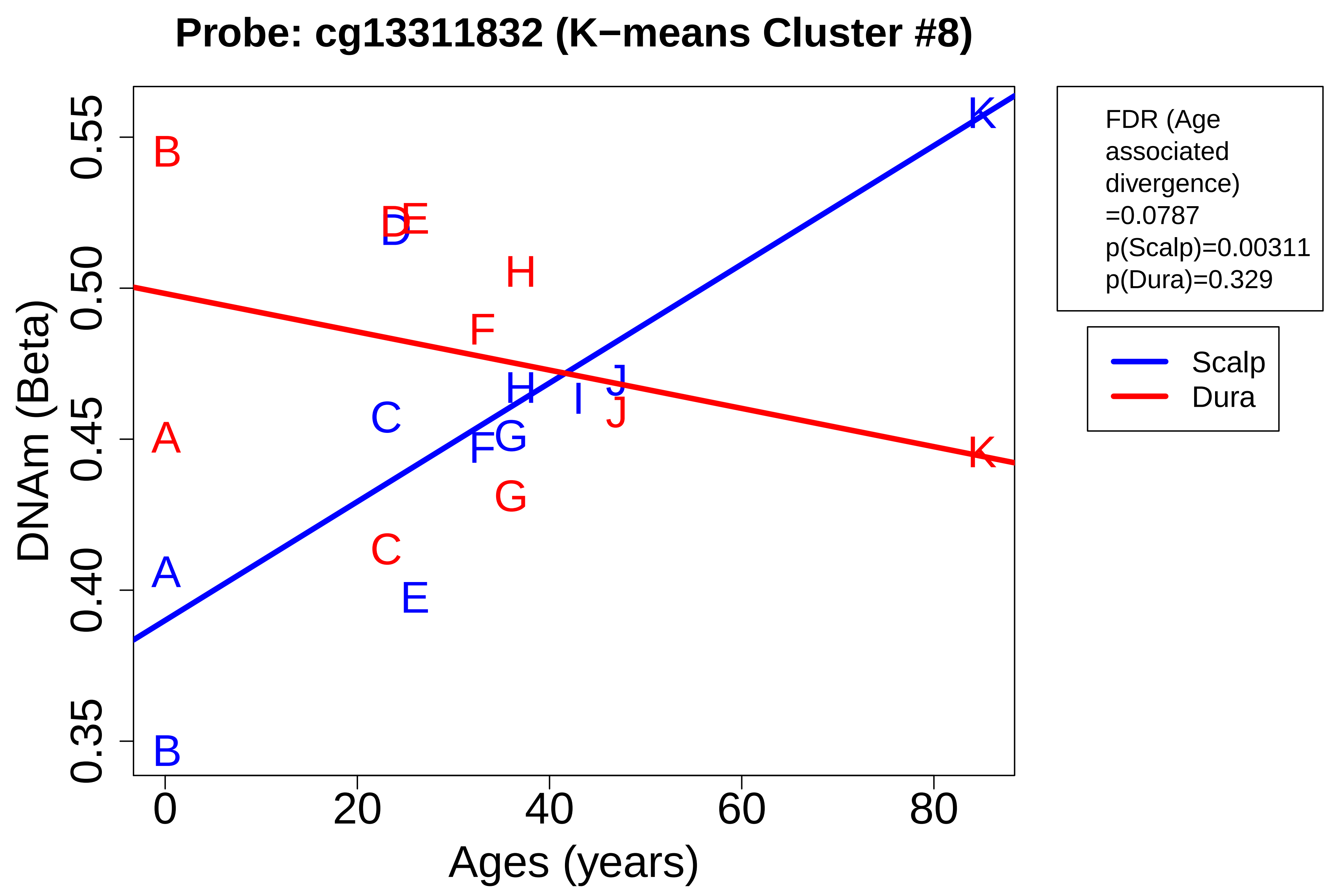

Probe: cg13311832 (K−means Cluster #8)
DNAm (Beta)
Ages (years)
0.35
0.40
0.45
0.50
0.55
0
20
40
60
80
FDR (Age associated divergence) =0.0787
p(Scalp)=0.00311
p(Dura)=0.329
Scalp
Dura

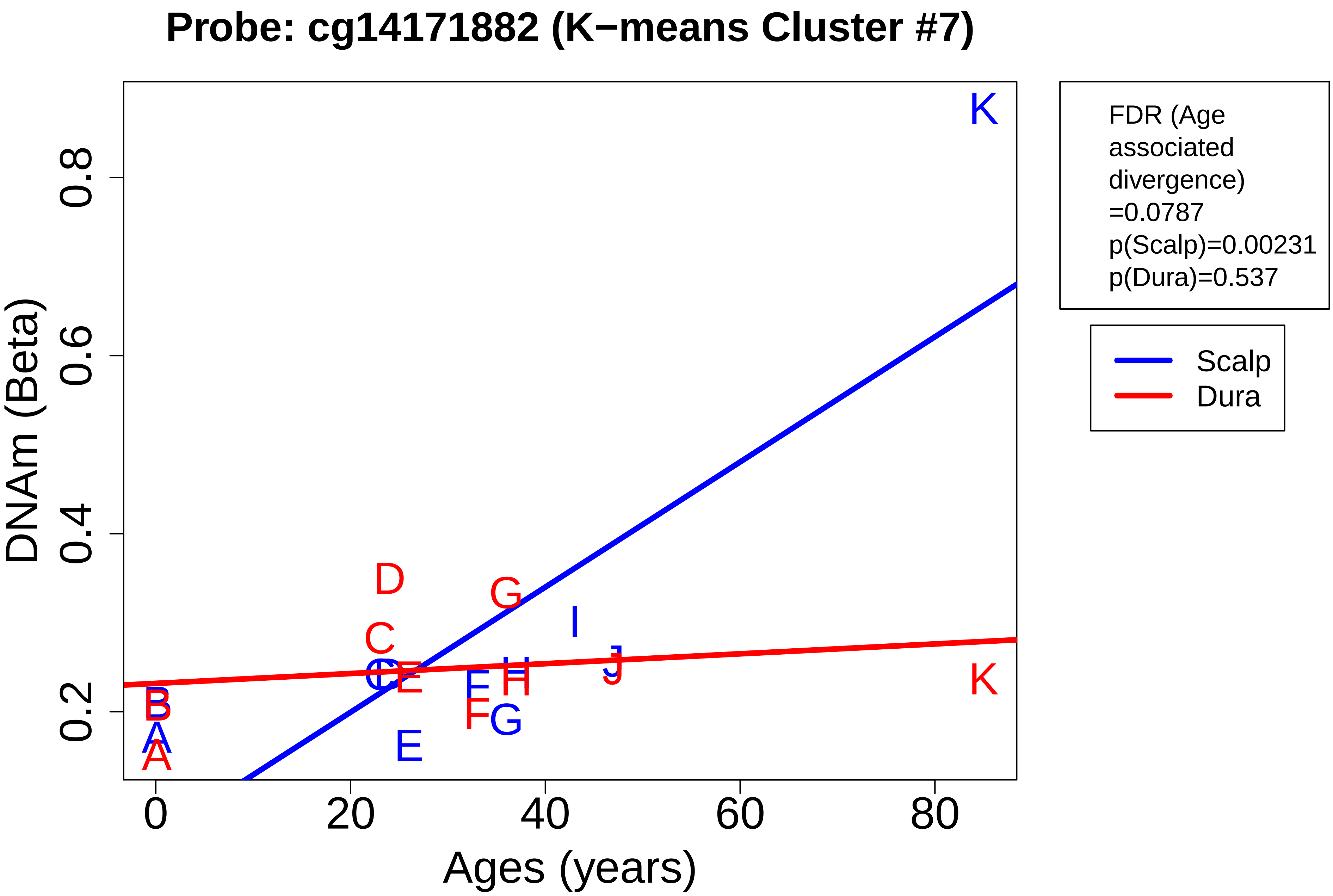

Probe: cg14171882 (K−means Cluster #7)
FDR (Age associated divergence) =0.0787
p(Scalp)=0.00231
p(Dura)=0.537
Scalp
Dura
DNAm (Beta)
0.2
0.4
0.6
0.8
Ages (years)
0
20
40
60
80
A
B
C
D
E
F
G
H
I
J
K

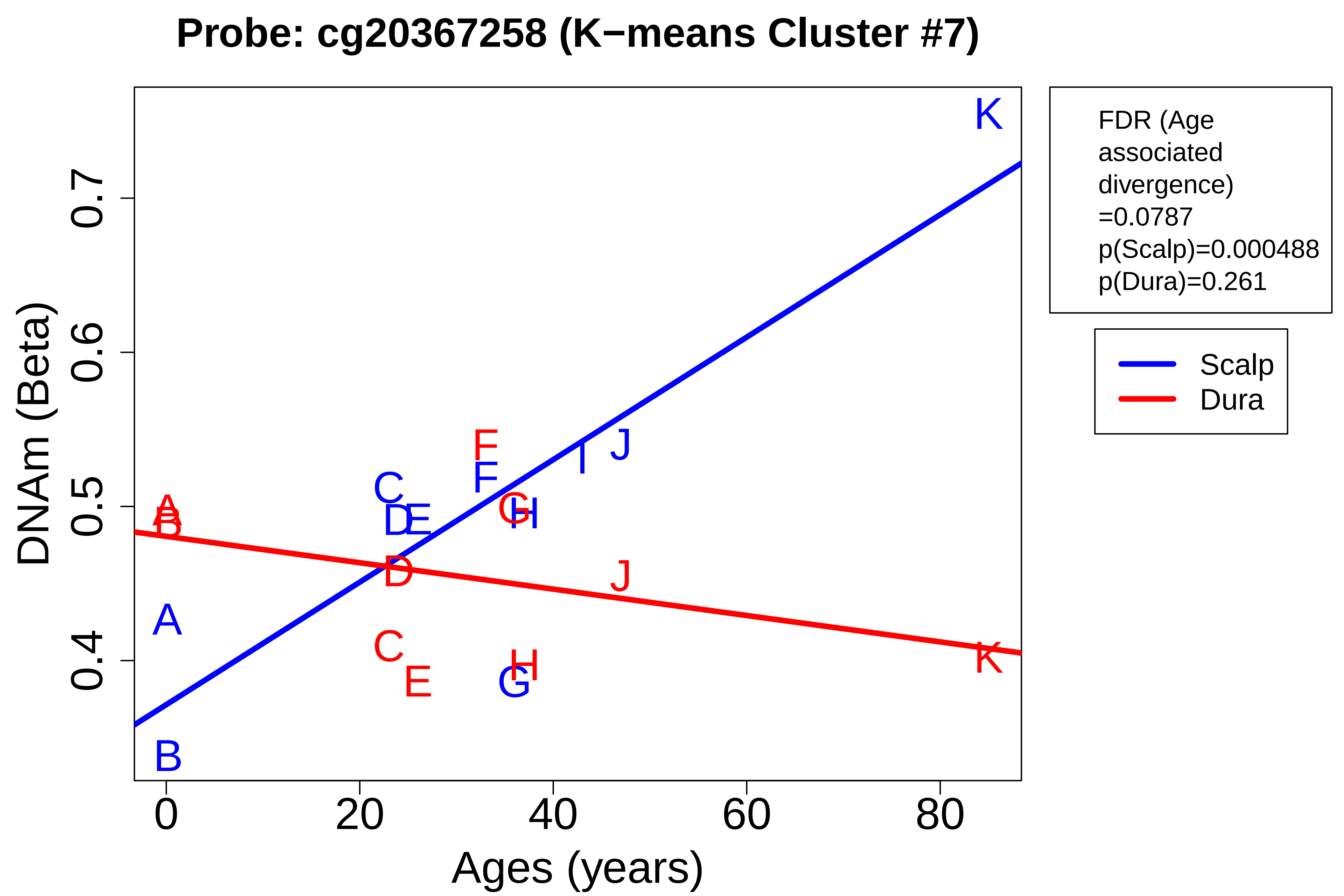

Probe: cg20367258 (K−means Cluster #7)
DNAm (Beta)
0.4
0.5
0.6
0.7
Ages (years)
0
20
40
60
80
FDR (Age associated divergence) =0.0787
p(Scalp)=0.000488
p(Dura)=0.261
Scalp
Dura

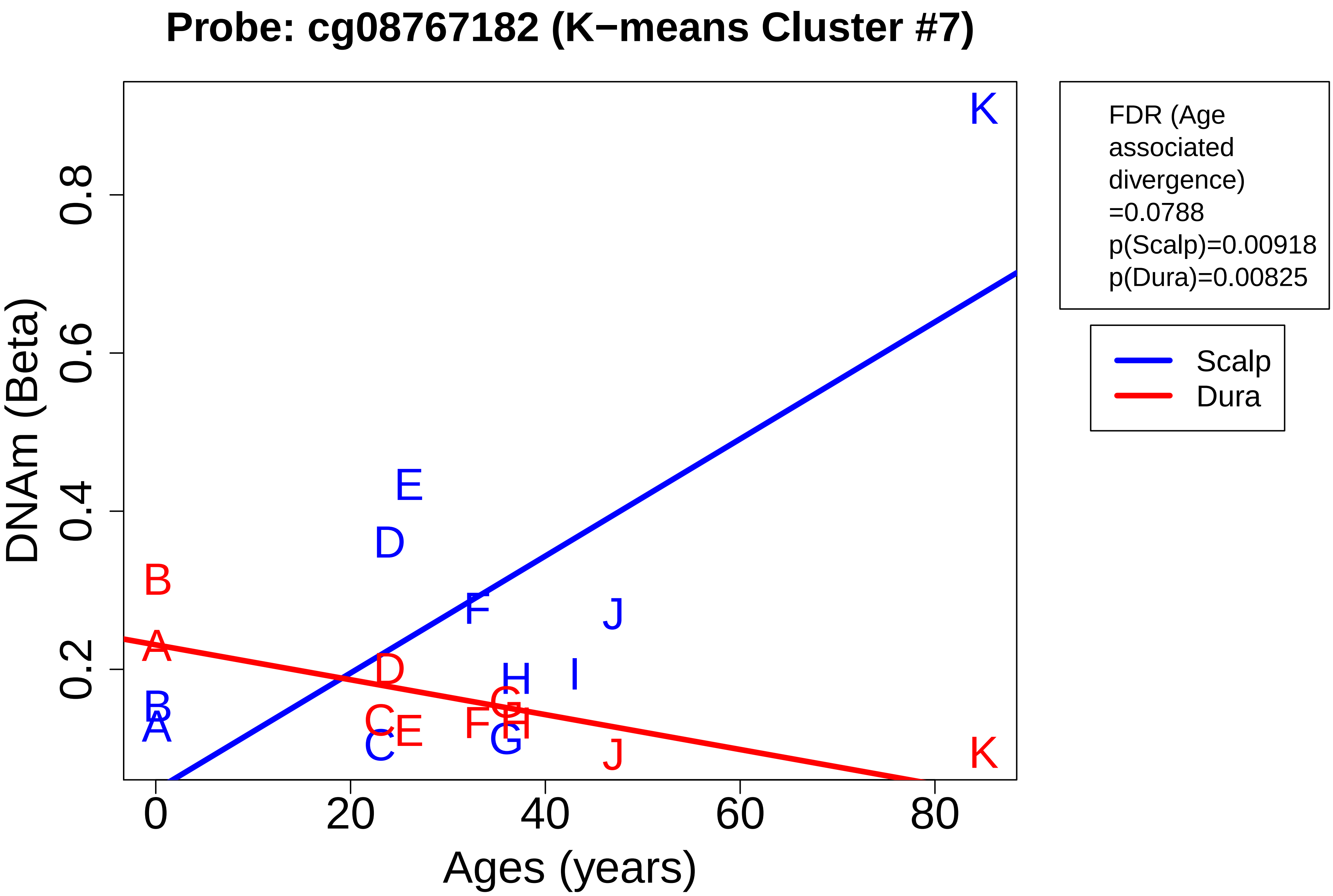

Probe: cg08767182 (K−means Cluster #7)
DNAm (Beta)
0.2
0.4
0.6
0.8
0
20
40
60
80
Ages (years)
FDR (Age associated divergence) =0.0788
p(Scalp)=0.00918
p(Dura)=0.00825
Scalp
Dura
A
B
C
D
E
F
G
H
I
J
K

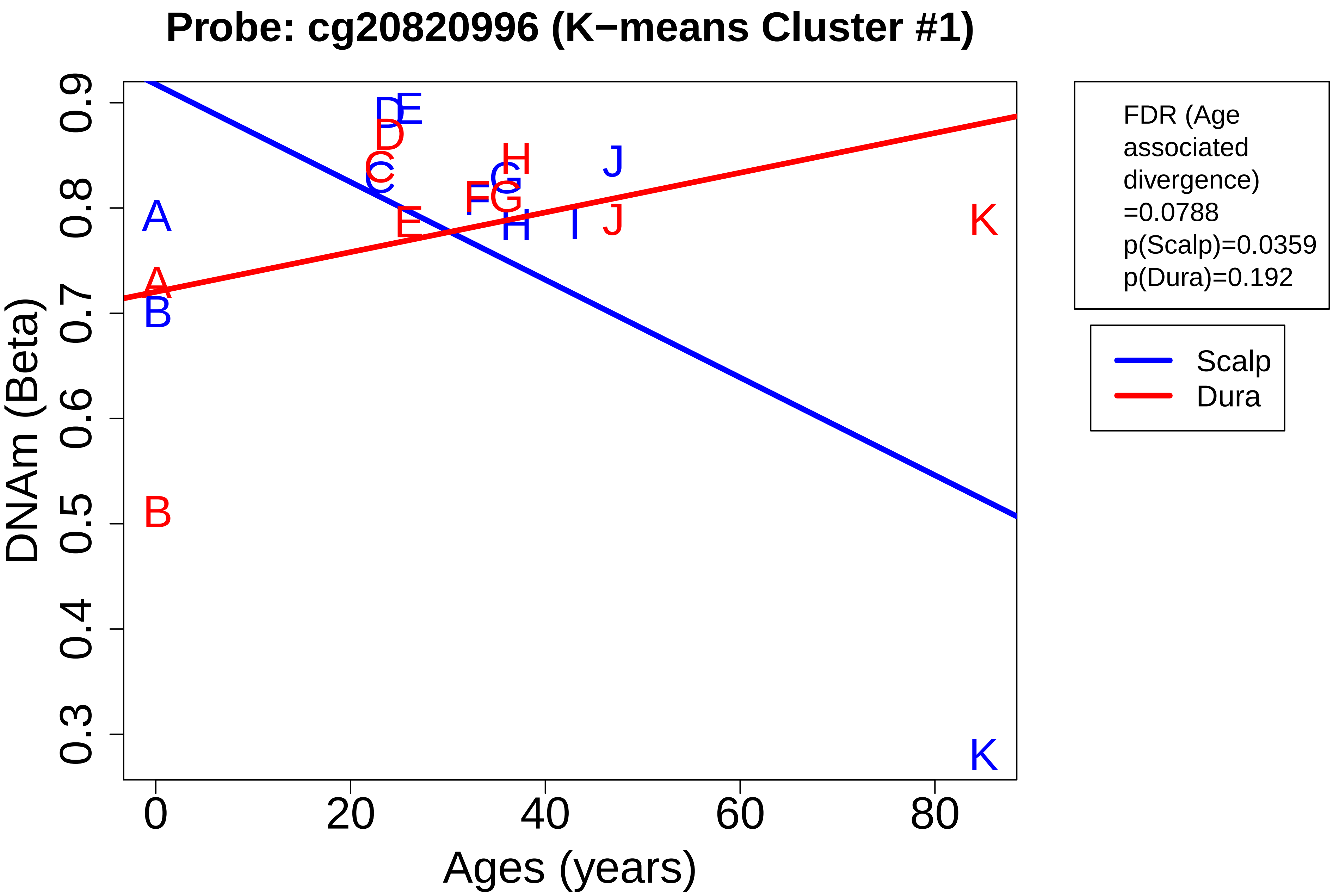

Probe: cg20820996 (K−means Cluster #1)
DNAm (Beta)
0.3
0.4
0.5
0.6
0.7
0.8
0.9
Ages (years)
0
20
40
60
80
FDR (Age associated divergence) =0.0788
p(Scalp)=0.0359
p(Dura)=0.192
Scalp
Dura
A
B
C
D
E
F
G
H
I
J
K

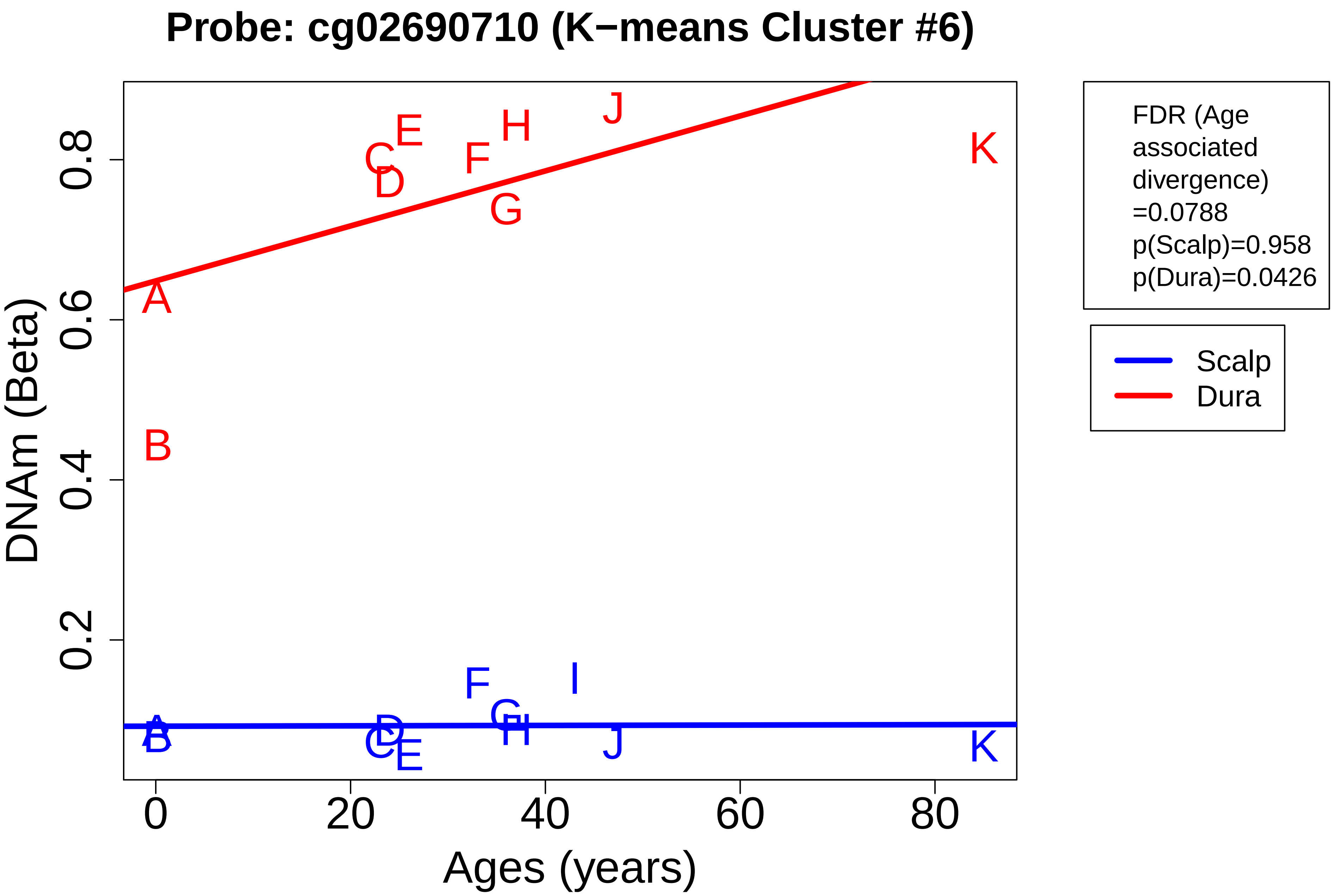

Probe: cg02690710 (K−means Cluster #6)
DNAm (Beta)
0.2
0.4
0.6
0.8
0
20
40
60
80
Ages (years)
FDR (Age associated divergence) =0.0788
p(Scalp)=0.958
p(Dura)=0.0426
Scalp
Dura

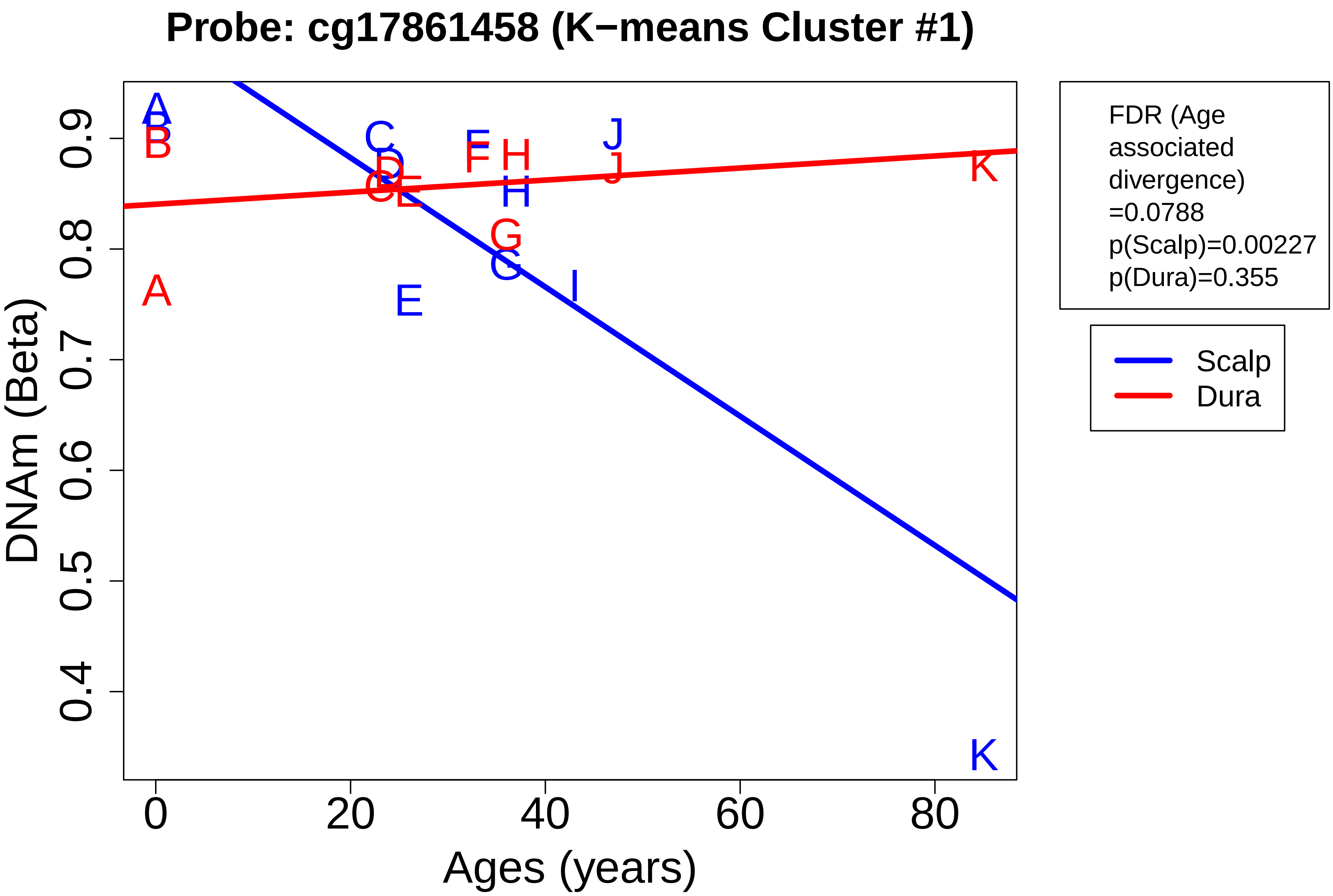

Probe: cg17861458 (K−means Cluster #1)
DNAm (Beta)
0.4
0.5
0.6
0.7
0.8
0.9
Ages (years)
0
20
40
60
80
A
B
C
D
E
F
G
H
I
J
K
FDR (Age associated divergence) =0.0788
p(Scalp)=0.00227
p(Dura)=0.355
Scalp
Dura

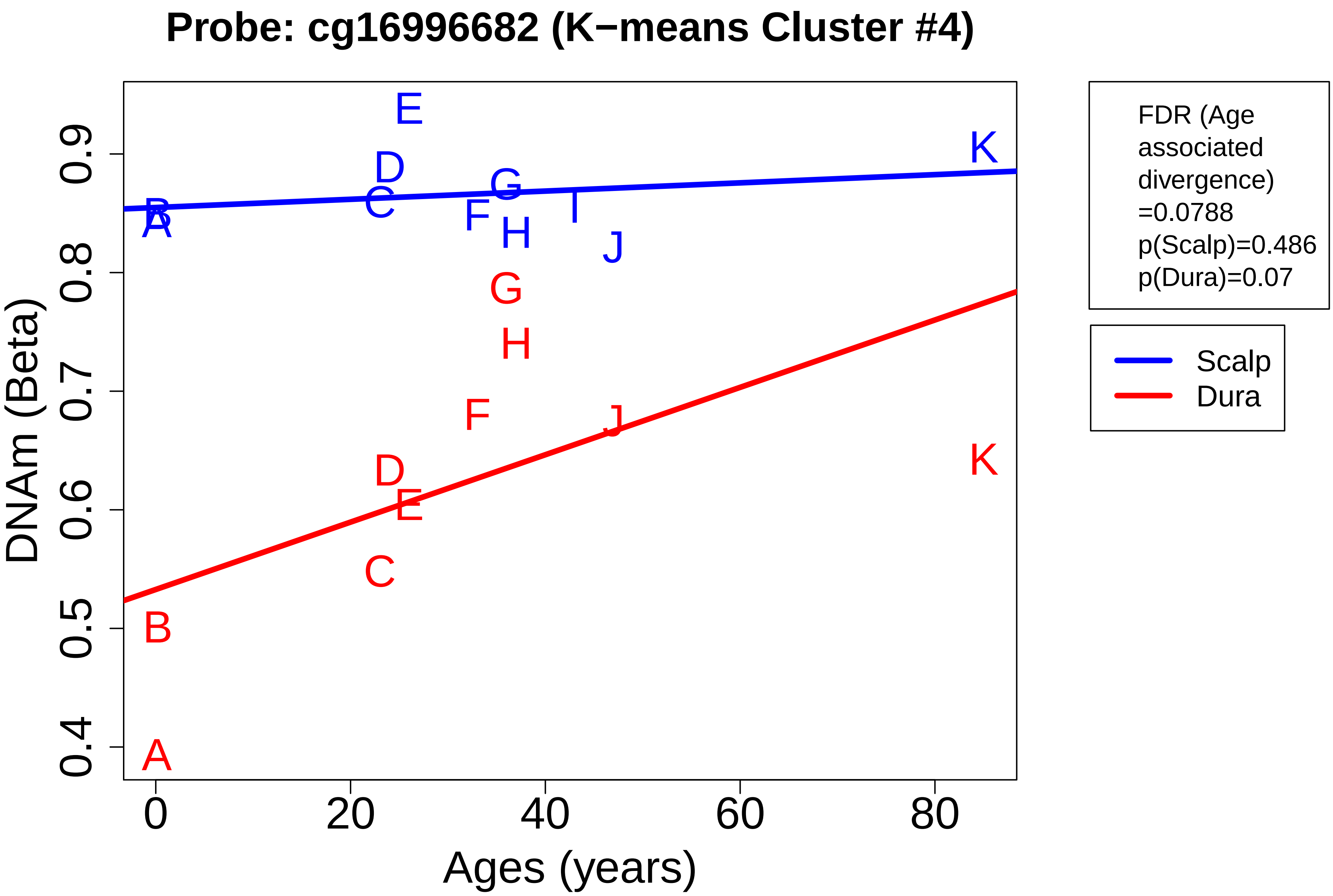

Probe: cg16996682 (K−means Cluster #4)
DNAm (Beta)
0.4
0.5
0.6
0.7
0.8
0.9
0
20
40
60
80
Ages (years)
FDR (Age associated divergence) =0.0788
p(Scalp)=0.486
p(Dura)=0.07
Scalp
Dura

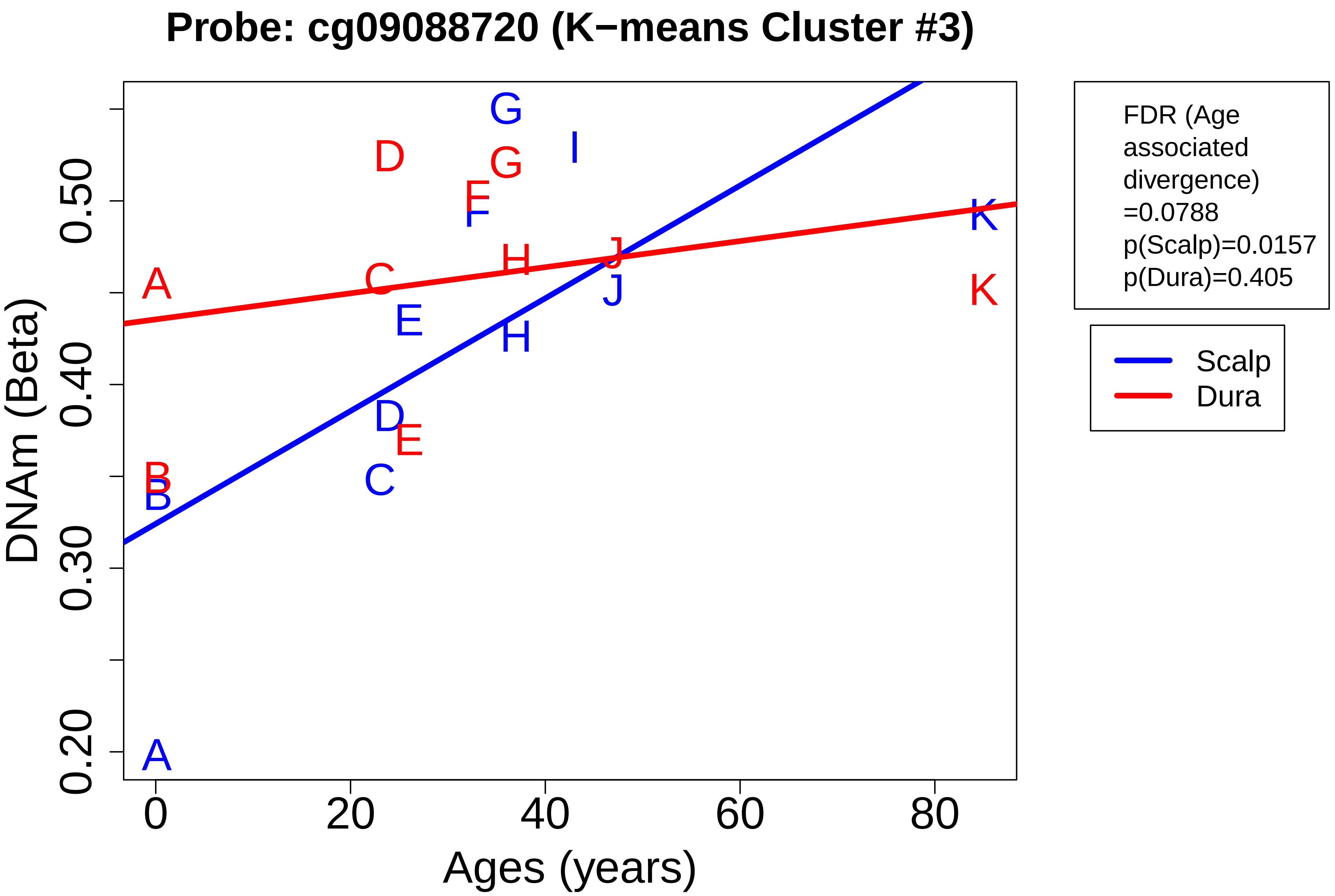

Probe: cg09088720 (K−means Cluster #3)
FDR (Age associated divergence) =0.0788
p(Scalp)=0.0157
p(Dura)=0.405
Scalp
Dura
DNAm (Beta)
0.20
0.30
0.40
0.50
Ages (years)
0
20
40
60
80
A
B
C
D
E
F
G
H
I
J
K

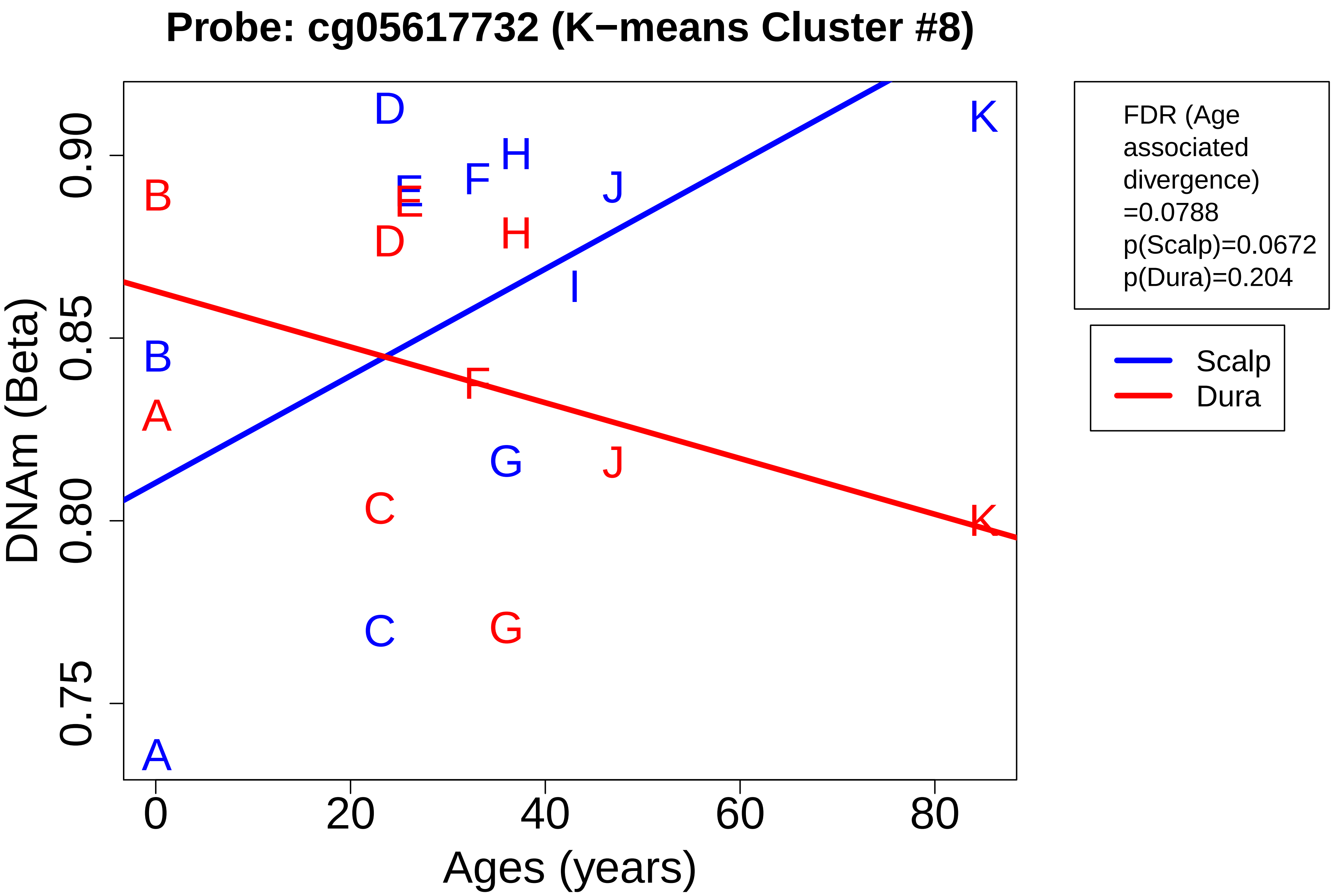

Probe: cg05617732 (K−means Cluster #8)
DNAm (Beta)
0.75
0.80
0.85
0.90
Ages (years)
0
20
40
60
80
A
B
C
D
E
F
G
H
I
J
K
FDR (Age associated divergence) =0.0788
p(Scalp)=0.0672
p(Dura)=0.204
Scalp
Dura

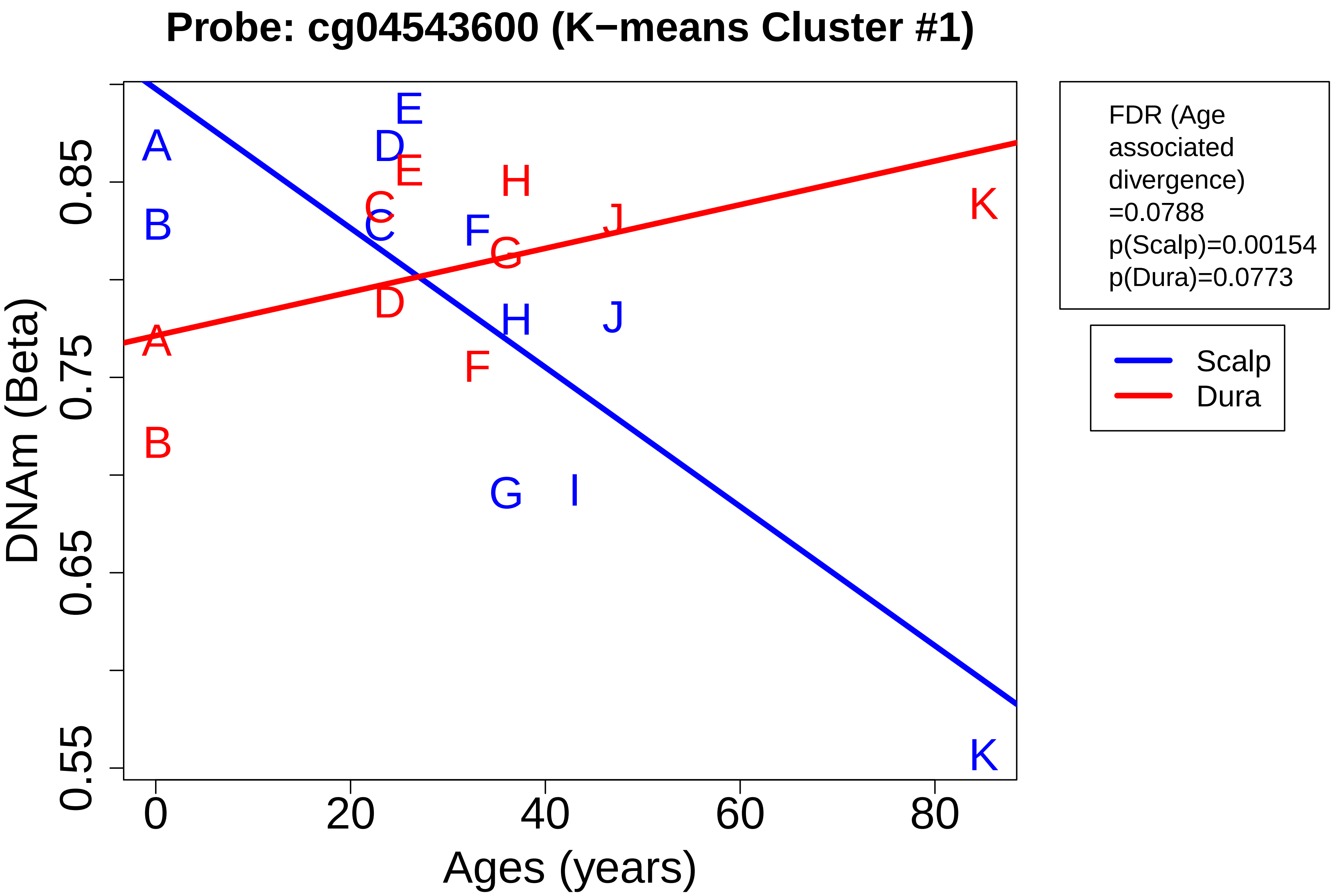

Probe: cg04543600 (K−means Cluster #1)
DNAm (Beta)
0.55
0.65
0.75
0.85
0
20
40
60
80
Ages (years)
FDR (Age associated divergence) =0.0788
p(Scalp)=0.00154
p(Dura)=0.0773
Scalp
Dura
A
B
C
D
E
F
G
H
I
J
K

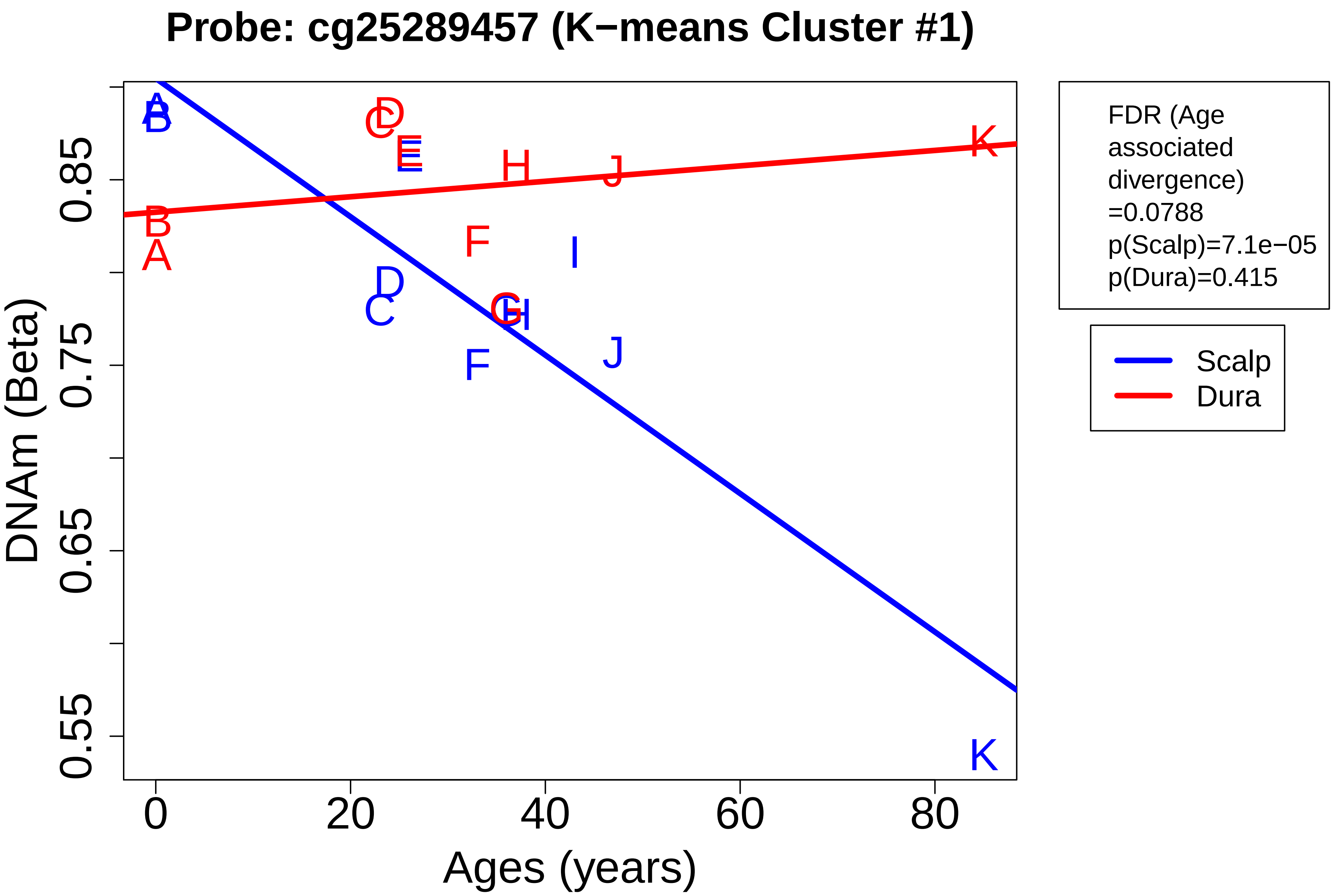

Probe: cg25289457 (K−means Cluster #1)
FDR (Age associated divergence) =0.0788
p(Scalp)=7.1e−05
p(Dura)=0.415
Scalp
Dura
DNAm (Beta)
0.55
0.65
0.75
0.85
Ages (years)
0
20
40
60
80
A
B
C
D
E
F
G
H
I
J
K

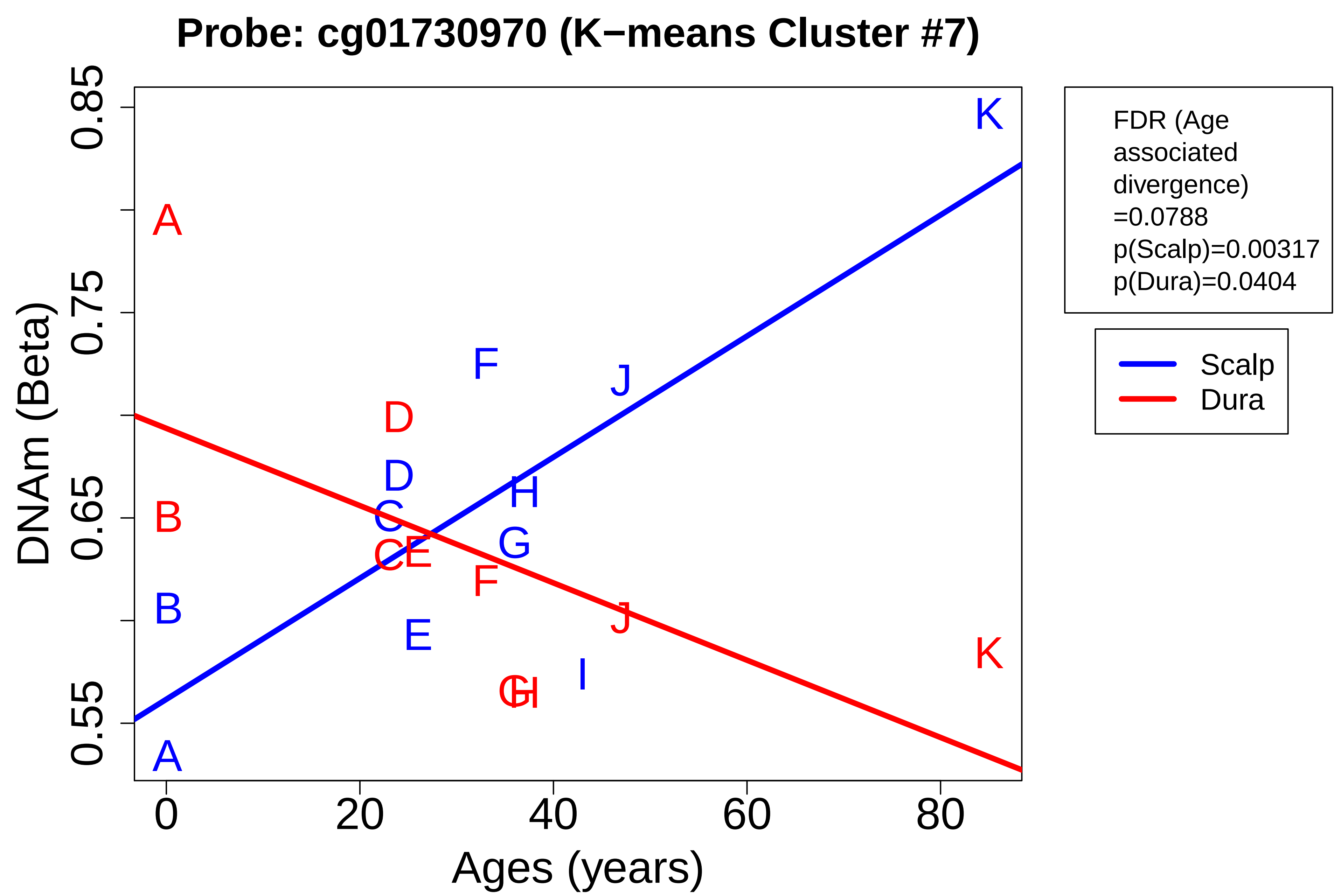

Probe: cg01730970 (K−means Cluster #7)
DNAm (Beta)
0.55
0.65
0.75
0.85
Ages (years)
0
20
40
60
80
A
B
C
D
E
F
G
H
I
J
K
FDR (Age associated divergence) =0.0788
p(Scalp)=0.00317
p(Dura)=0.0404
Scalp
Dura

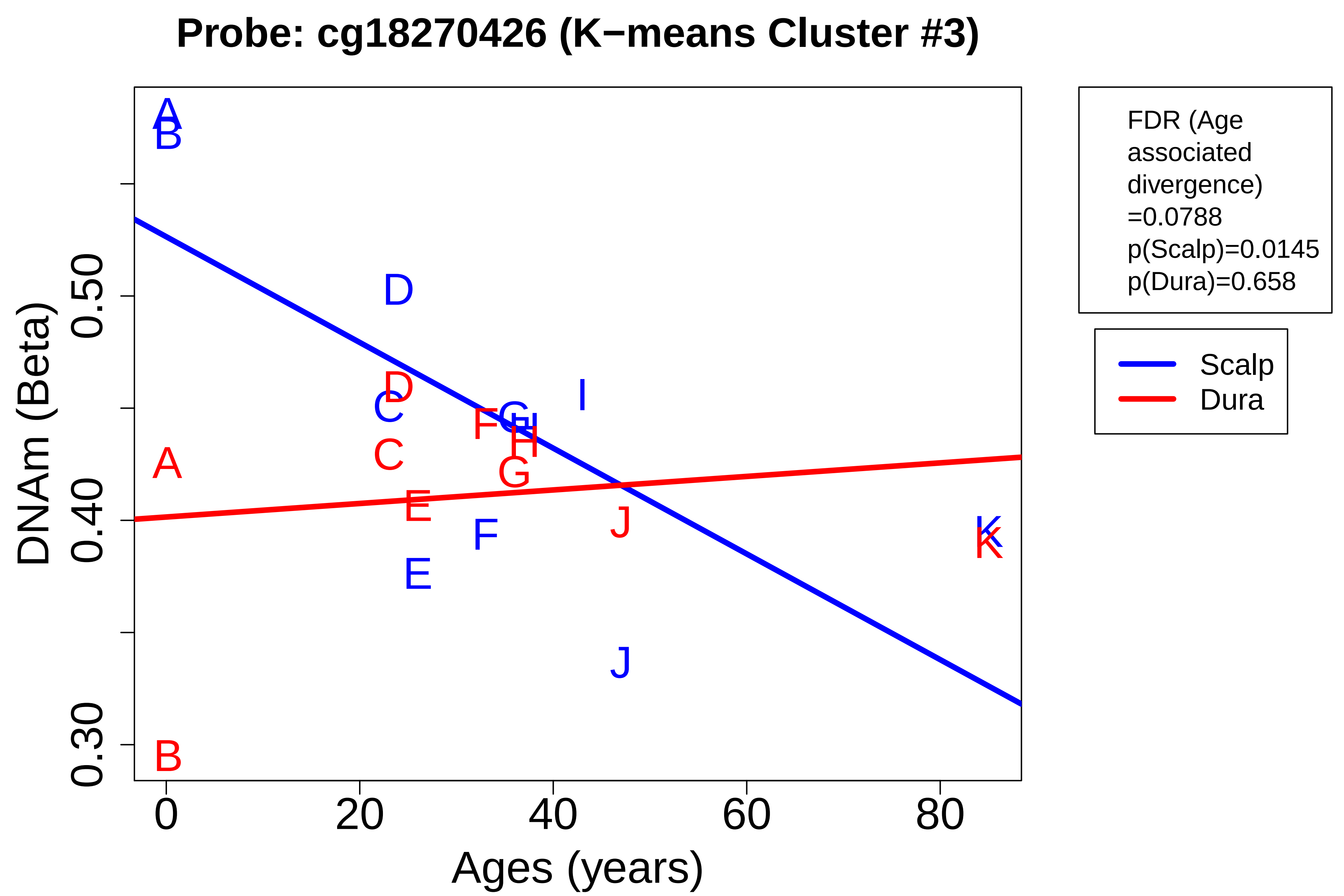
Probe: cg18270426 (K−means Cluster #3)
FDR (Age associated divergence) =0.0788
p(Scalp)=0.0145
p(Dura)=0.658
Scalp
Dura
DNAm (Beta)
0.30
0.40
0.50
0
20
40
60
80
Ages (years)
A
B
C
D
E
F
G
H
I
J
K

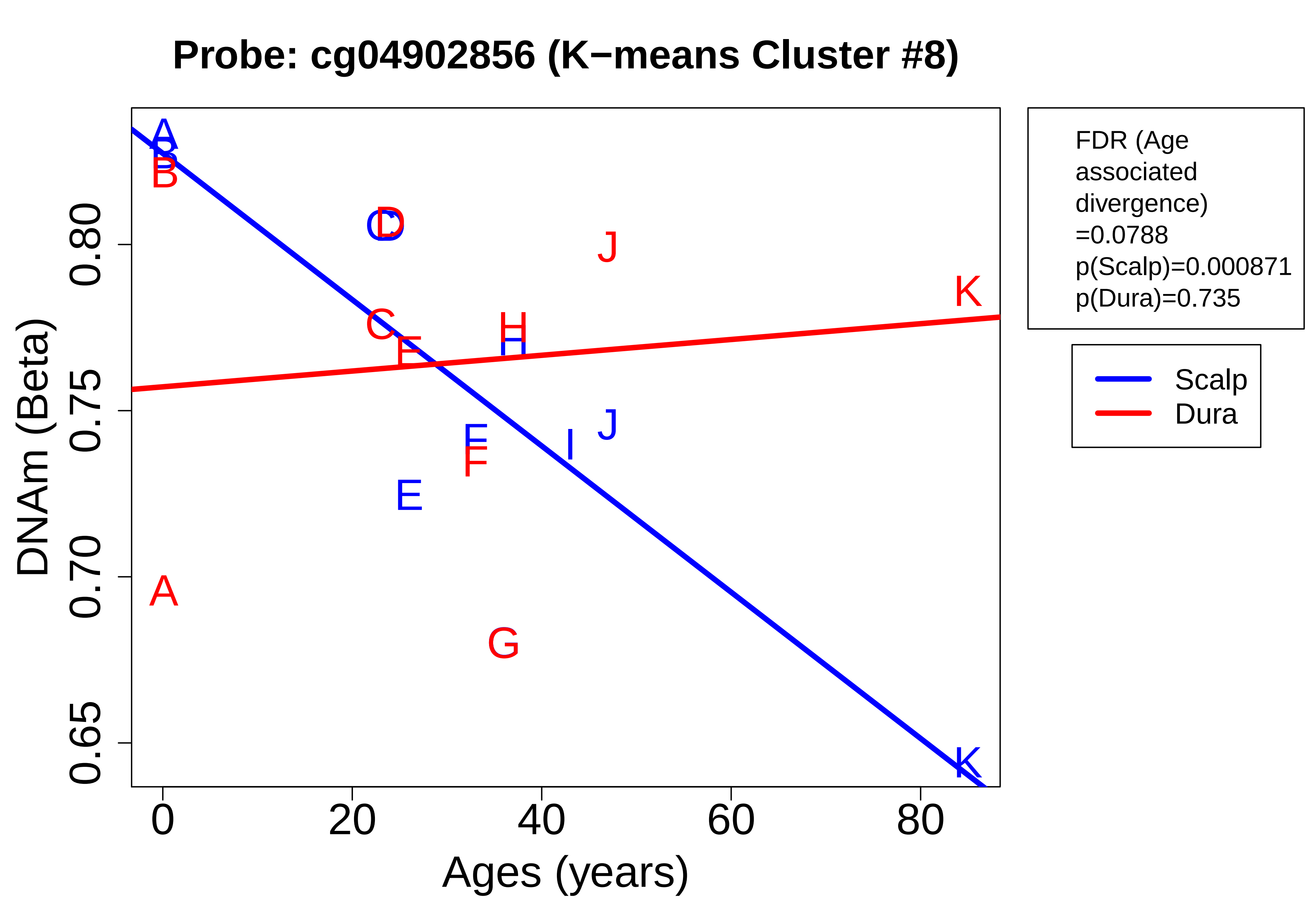

Probe: cg04902856 (K−means Cluster #8)
FDR (Age associated divergence) =0.0788
p(Scalp)=0.000871
p(Dura)=0.735
Scalp
Dura
DNAm (Beta)
0.65
0.70
0.75
0.80
Ages (years)
0
20
40
60
80

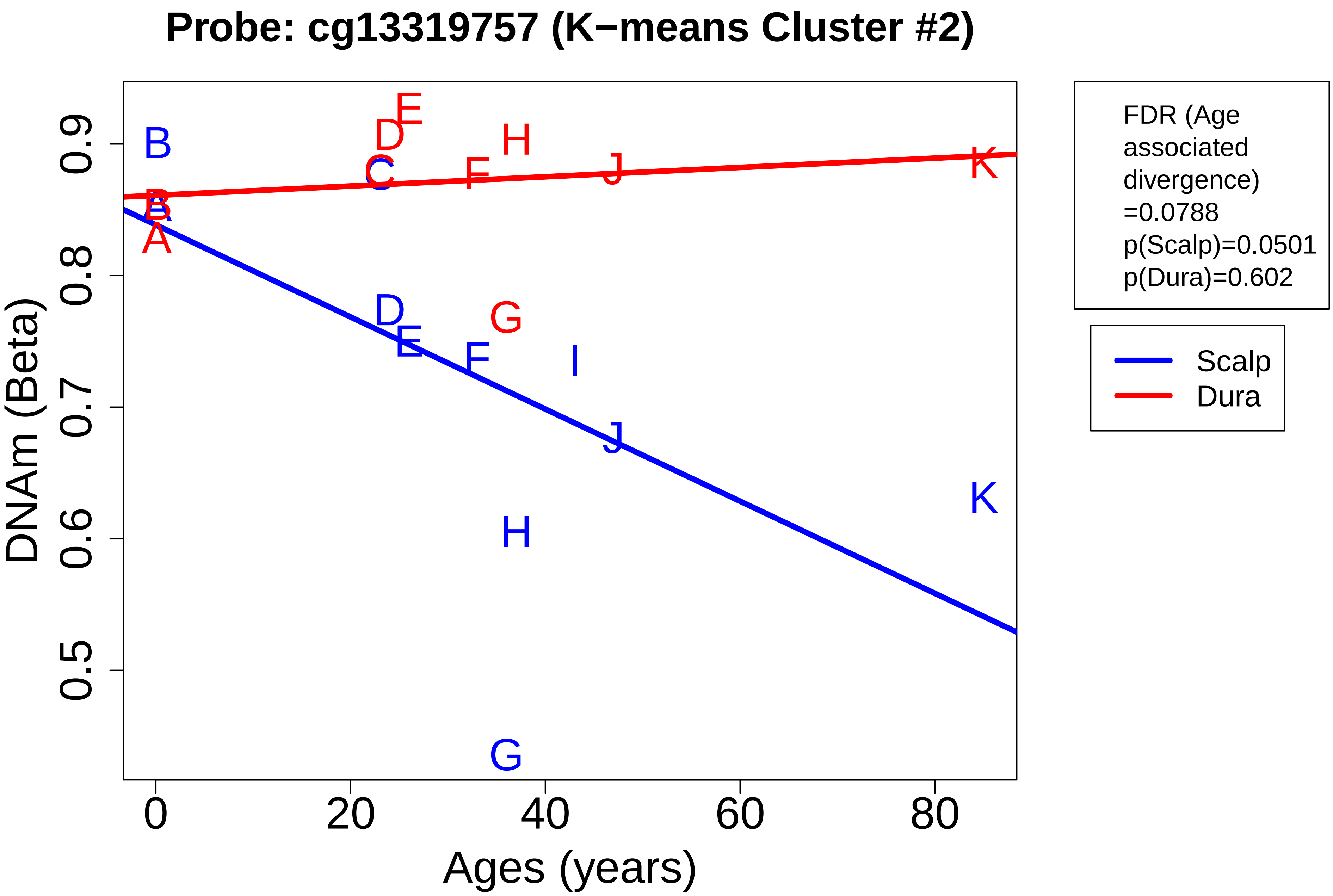

Probe: cg13319757 (K−means Cluster #2)
DNAm (Beta)
0.5
0.6
0.7
0.8
0.9
0
20
40
60
80
Ages (years)
FDR (Age associated divergence) =0.0788
p(Scalp)=0.0501
p(Dura)=0.602
Scalp
Dura

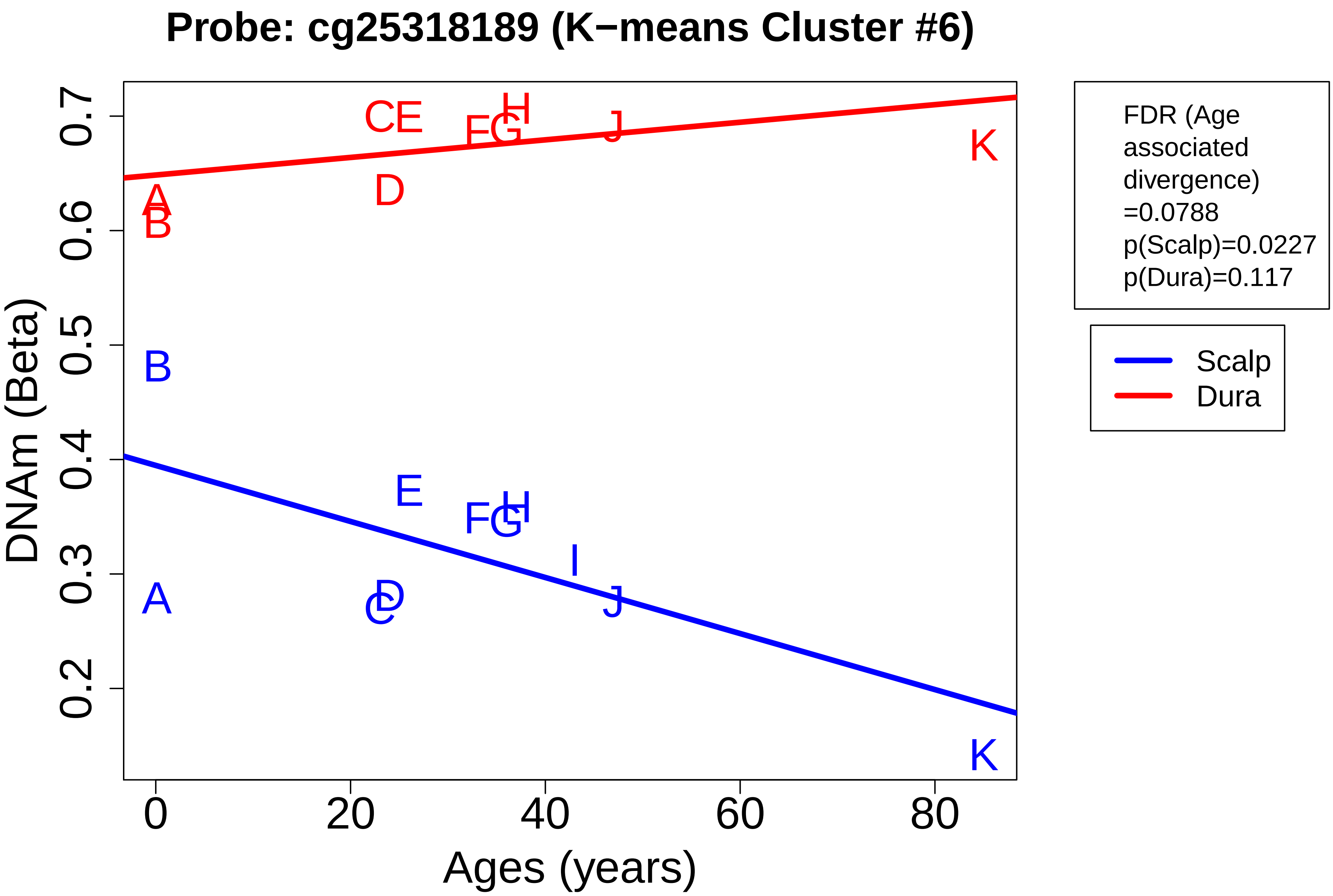

Probe: cg25318189 (K−means Cluster #6)
DNAm (Beta)
0.2
0.3
0.4
0.5
0.6
0.7
0
20
40
60
80
Ages (years)
FDR (Age associated divergence) =0.0788
p(Scalp)=0.0227
p(Dura)=0.117
Scalp
Dura
A
B
CE
D
FG
H
I
J
K

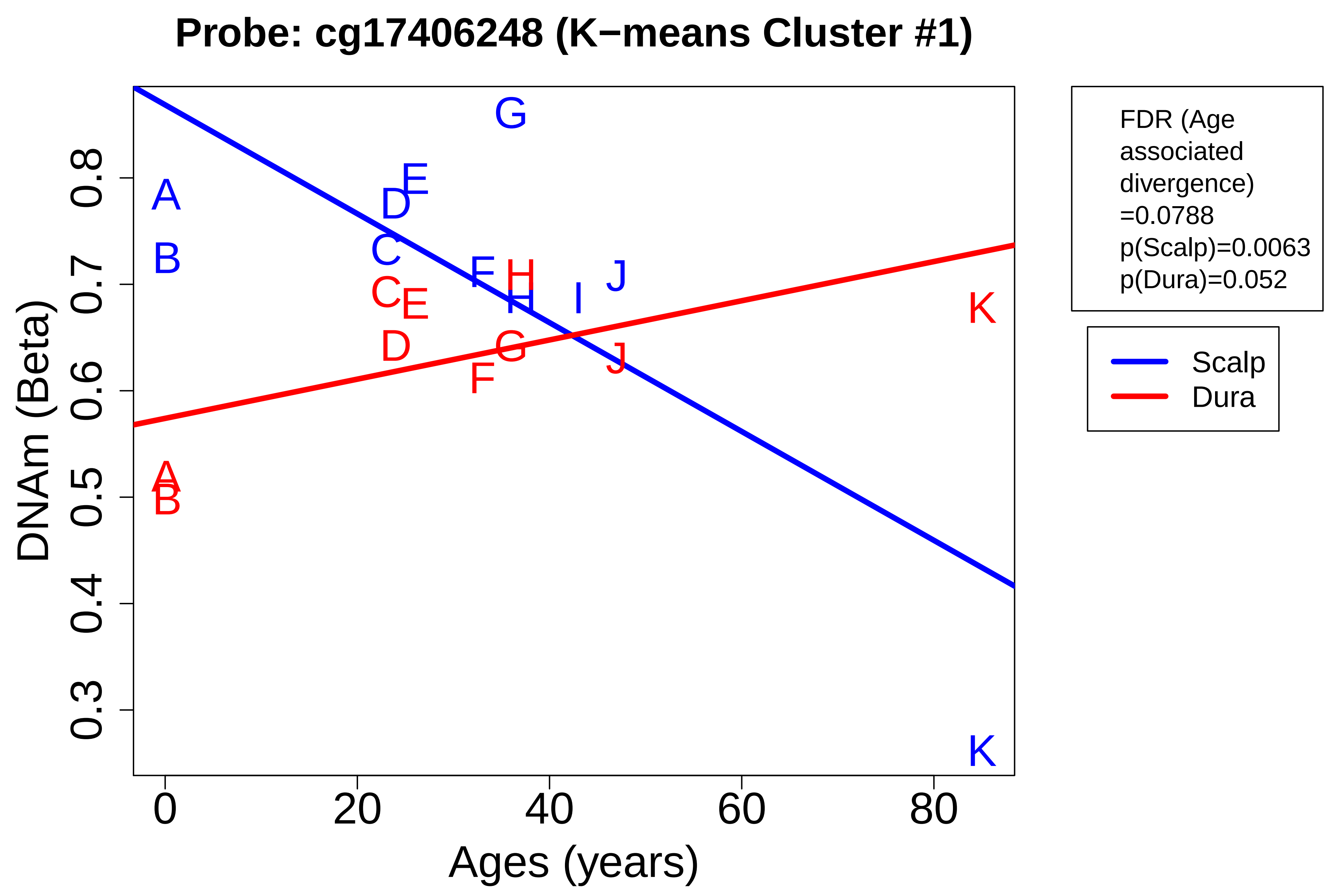

Probe: cg17406248 (K−means Cluster #1)
DNAm (Beta)
0.3
0.4
0.5
0.6
0.7
0.8
0
20
40
60
80
Ages (years)
A
B
C
D
E
F
G
H
I
J
K
FDR (Age associated divergence) =0.0788
p(Scalp)=0.0063
p(Dura)=0.052
Scalp
Dura

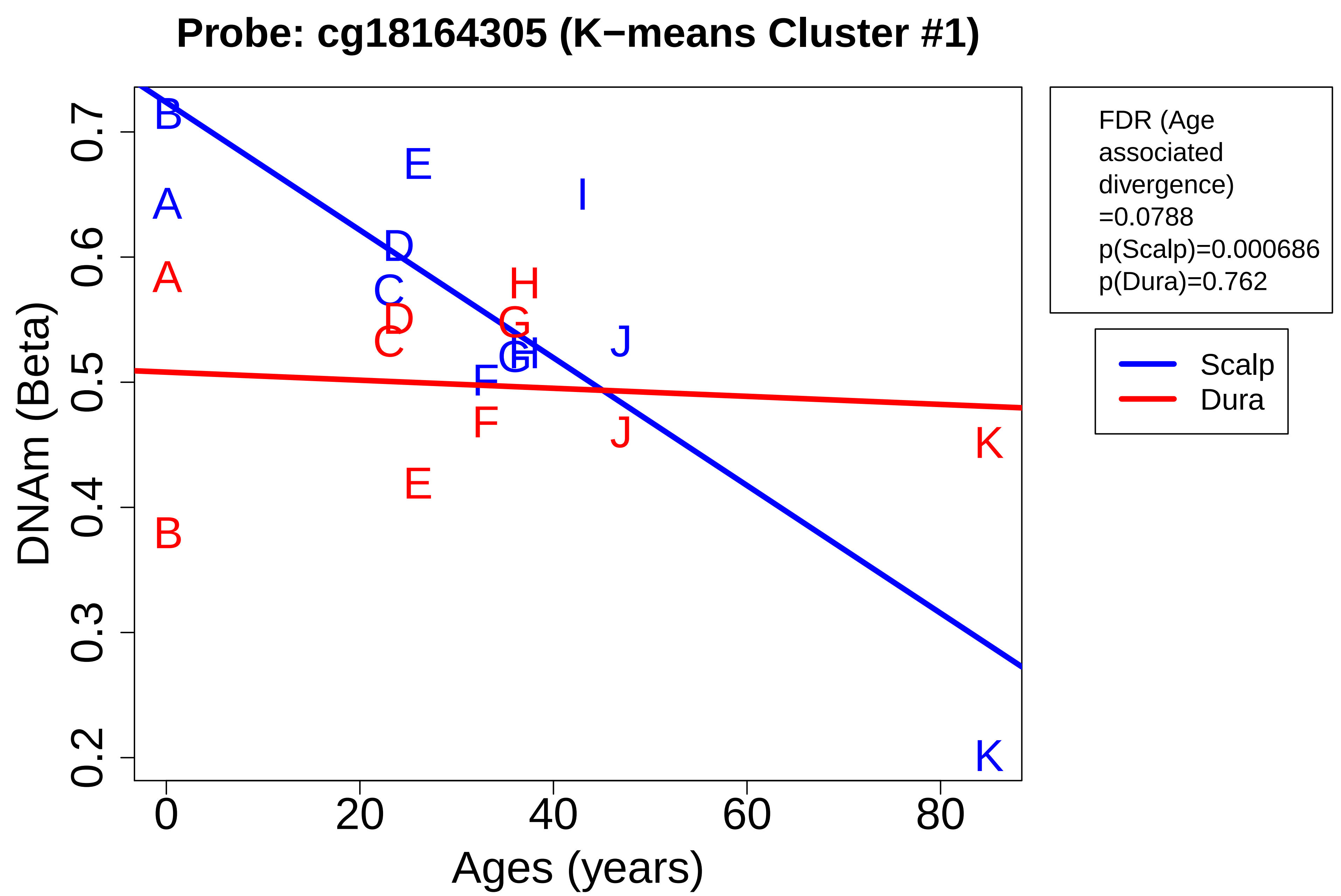
Probe: cg18164305 (K−means Cluster #1)
DNAm (Beta)
0.2
0.3
0.4
0.5
0.6
0.7
0
20
40
60
80
Ages (years)
FDR (Age associated divergence) =0.0788
p(Scalp)=0.000686
p(Dura)=0.762
Scalp
Dura
A
B
C
D
E
F
G
H
I
J
K

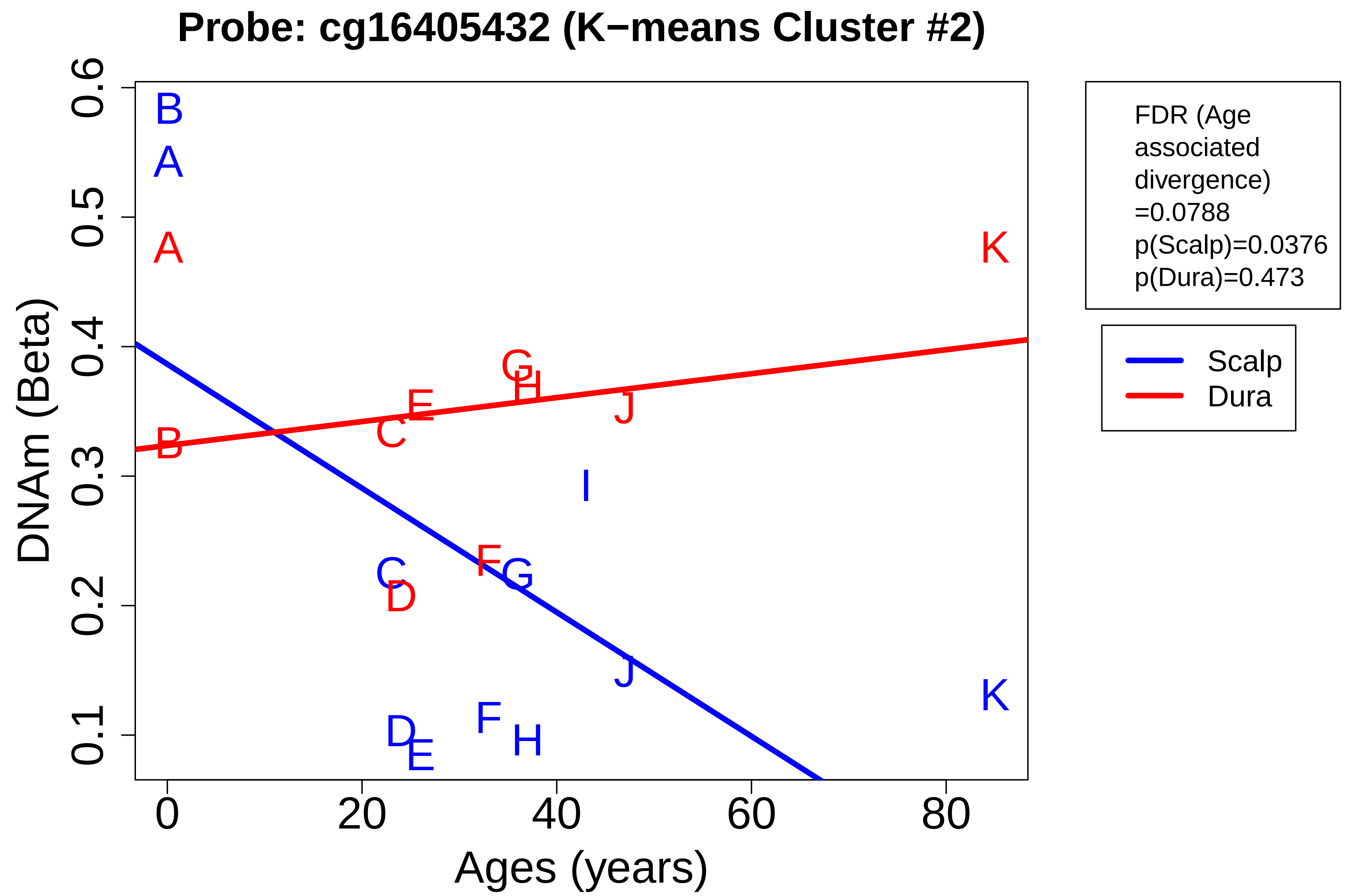

Probe: cg16405432 (K−means Cluster #2)
DNAm (Beta)
Ages (years)
0.1
0.2
0.3
0.4
0.5
0.6
0
20
40
60
80
FDR (Age associated divergence) =0.0788
p(Scalp)=0.0376
p(Dura)=0.473
Scalp
Dura

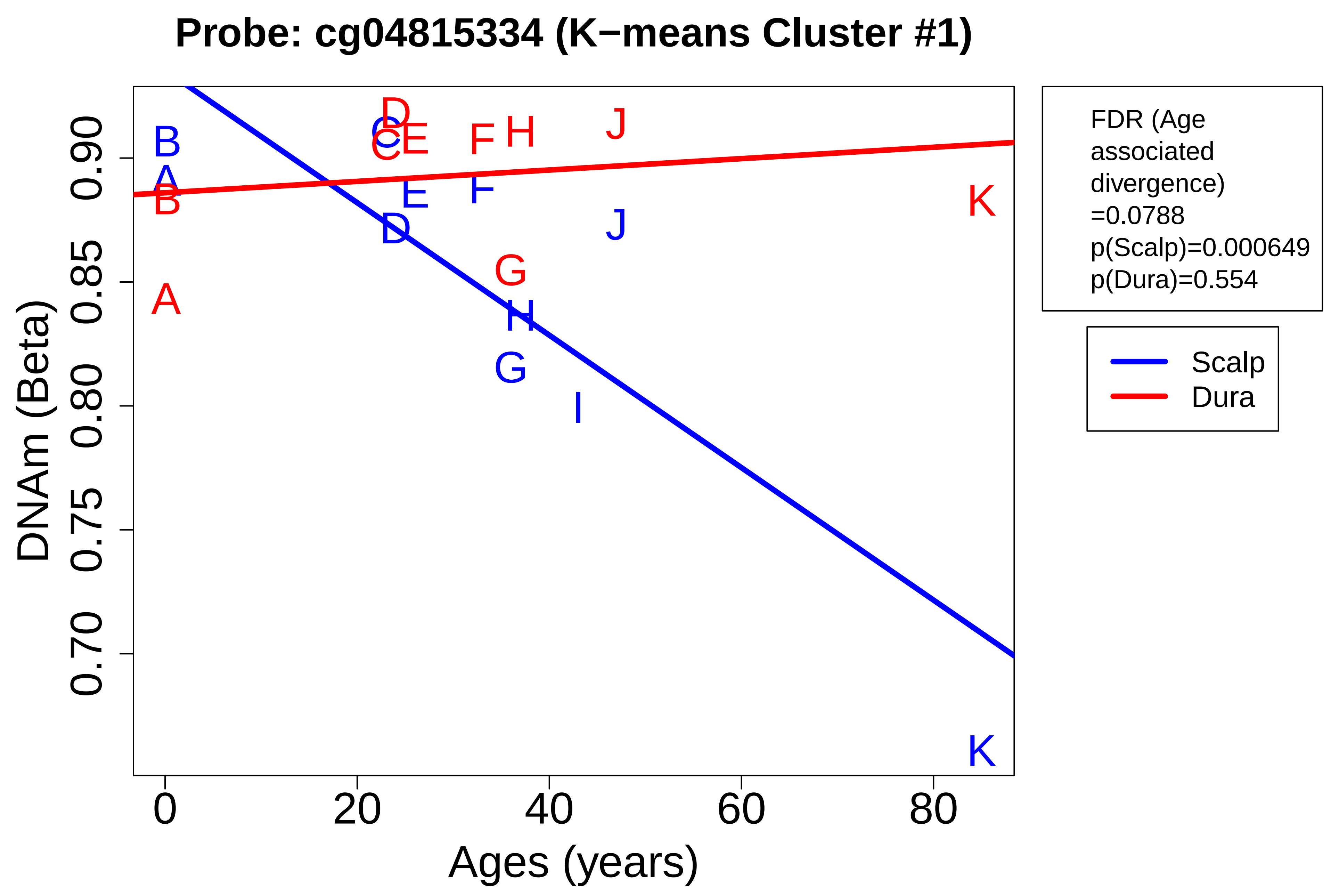

Probe: cg04815334 (K−means Cluster #1)
DNAm (Beta)
0.70
0.75
0.80
0.85
0.90
0
20
40
60
80
Ages (years)
FDR (Age associated divergence) =0.0788
p(Scalp)=0.000649
p(Dura)=0.554
Scalp
Dura

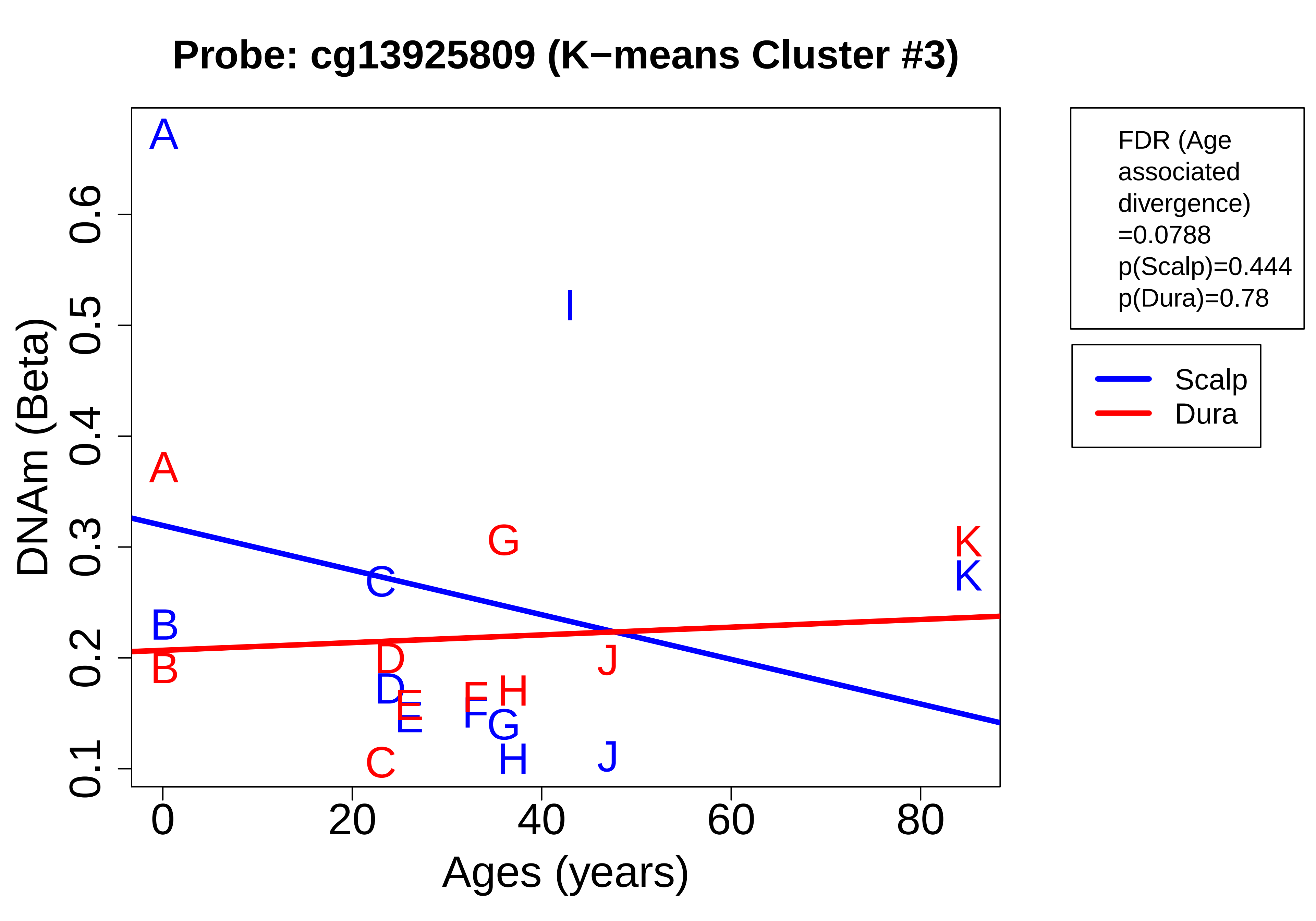

Probe: cg13925809 (K−means Cluster #3)
FDR (Age associated divergence) =0.0788
p(Scalp)=0.444
p(Dura)=0.78
Scalp
Dura
DNAm (Beta)
0.1
0.2
0.3
0.4
0.5
0.6
Ages (years)
0
20
40
60
80
A
B
C
D
E
F
G
H
I
J
K

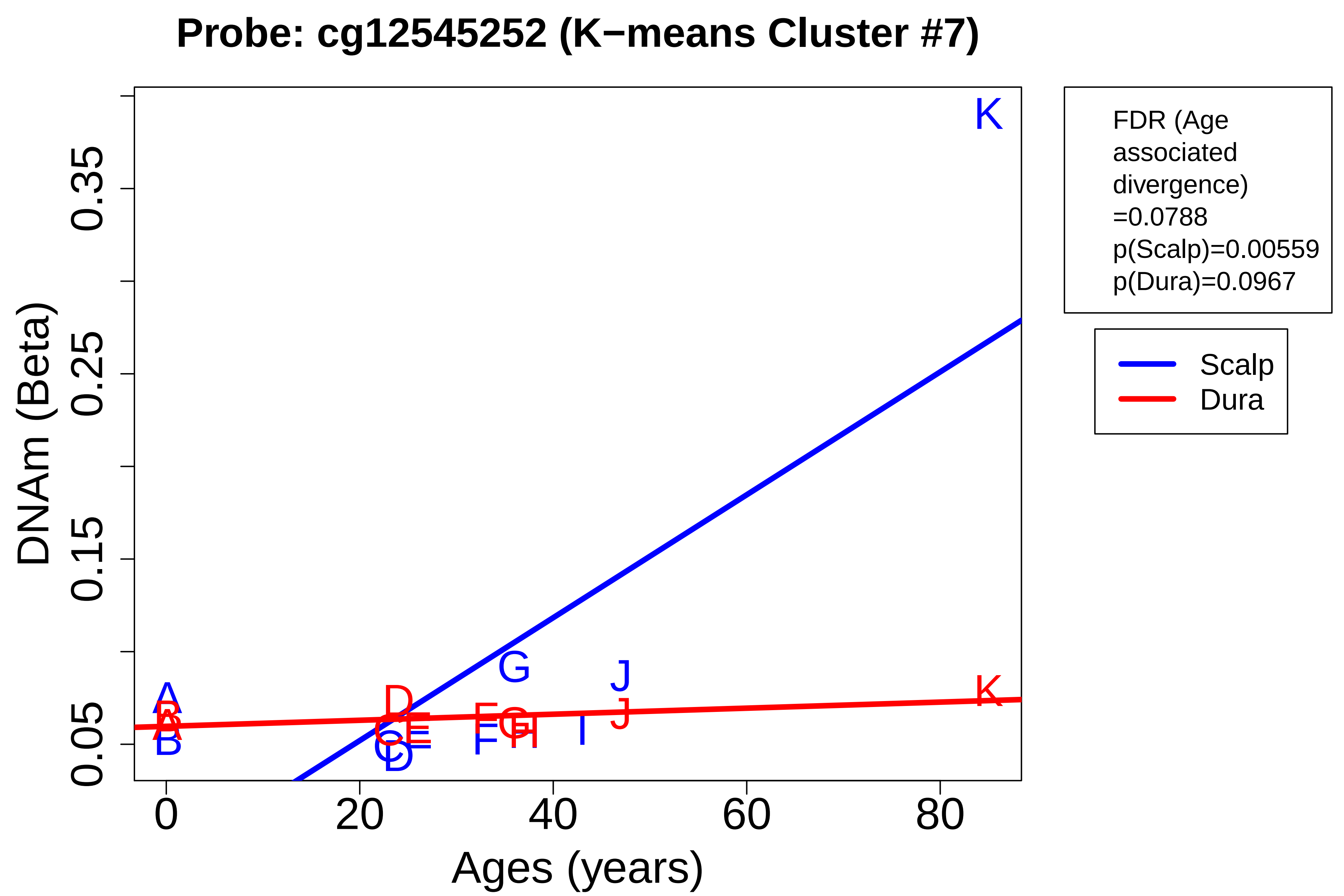

Probe: cg12545252 (K−means Cluster #7)
DNAm (Beta)
0.05
0.15
0.25
0.35
Ages (years)
0
20
40
60
80
FDR (Age associated divergence) =0.0788
p(Scalp)=0.00559
p(Dura)=0.0967
Scalp
Dura

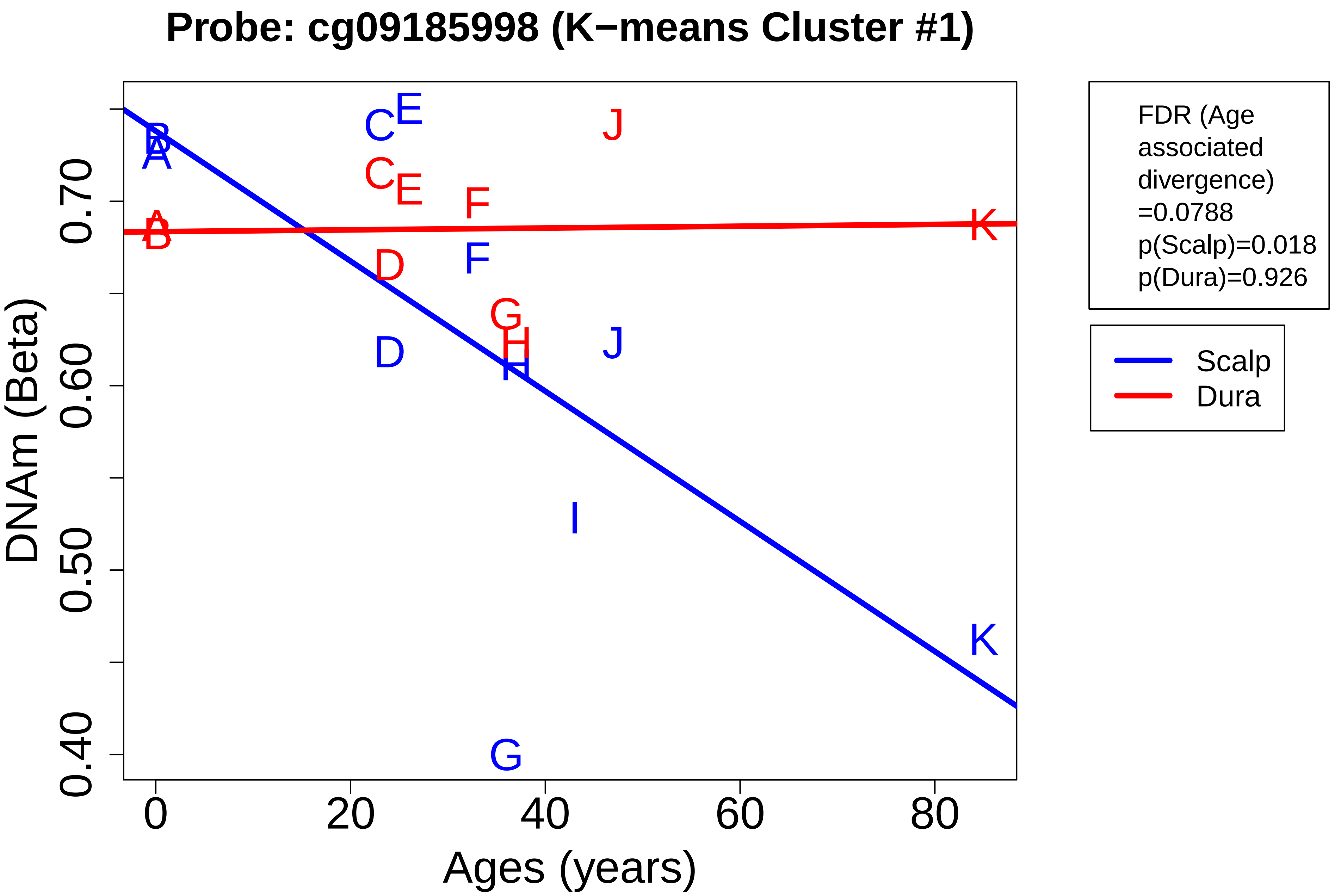

Probe: cg09185998 (K−means Cluster #1)
DNAm (Beta)
0.40
0.50
0.60
0.70
0
20
40
60
80
Ages (years)
FDR (Age associated divergence) =0.0788
p(Scalp)=0.018
p(Dura)=0.926
Scalp
Dura

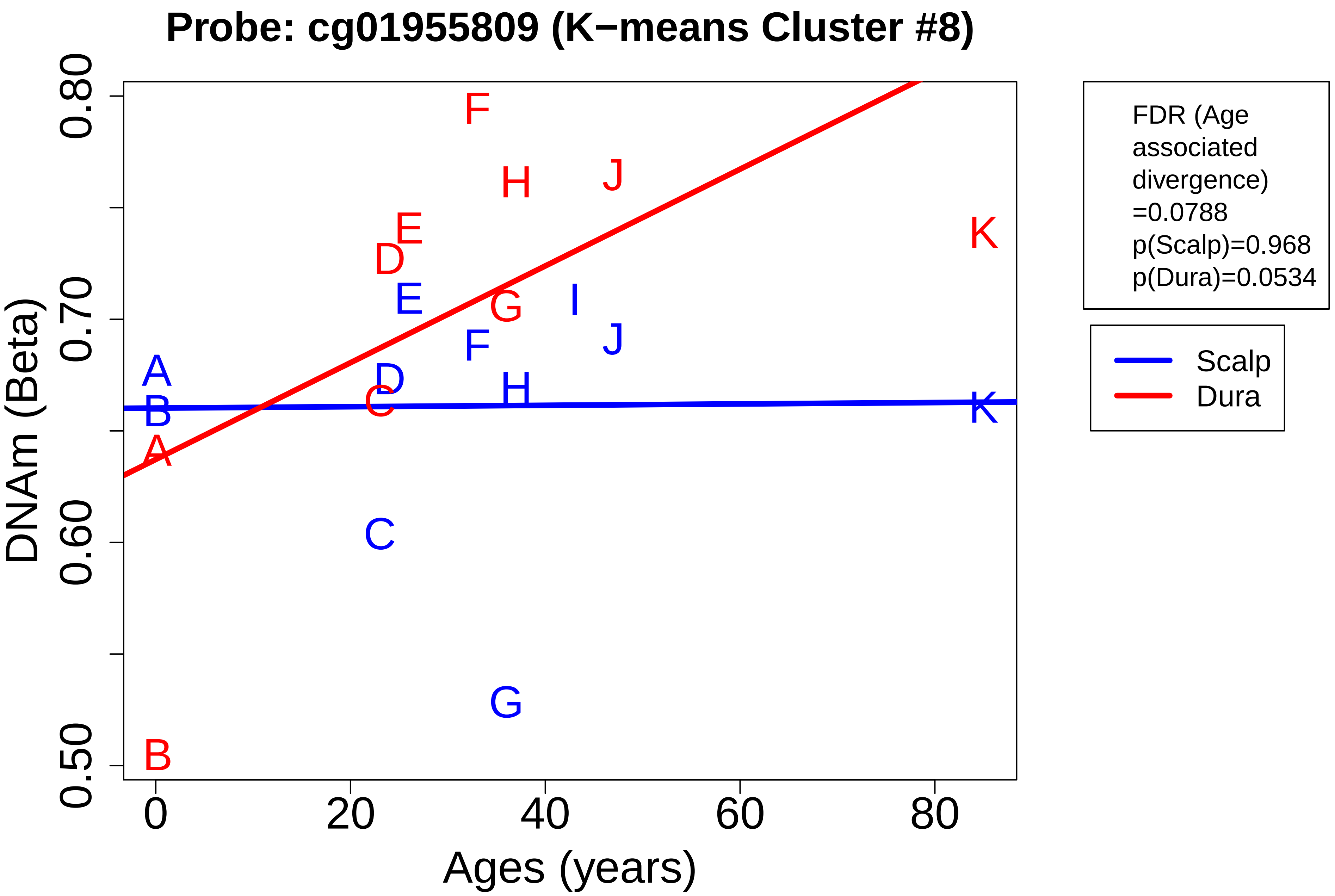
Probe: cg01955809 (K−means Cluster #8)
DNAm (Beta)
0.50
0.60
0.70
0.80
Ages (years)
0
20
40
60
80
FDR (Age associated divergence) =0.0788
p(Scalp)=0.968
p(Dura)=0.0534
Scalp
Dura
A
B
C
D
E
F
G
H
I
J
K

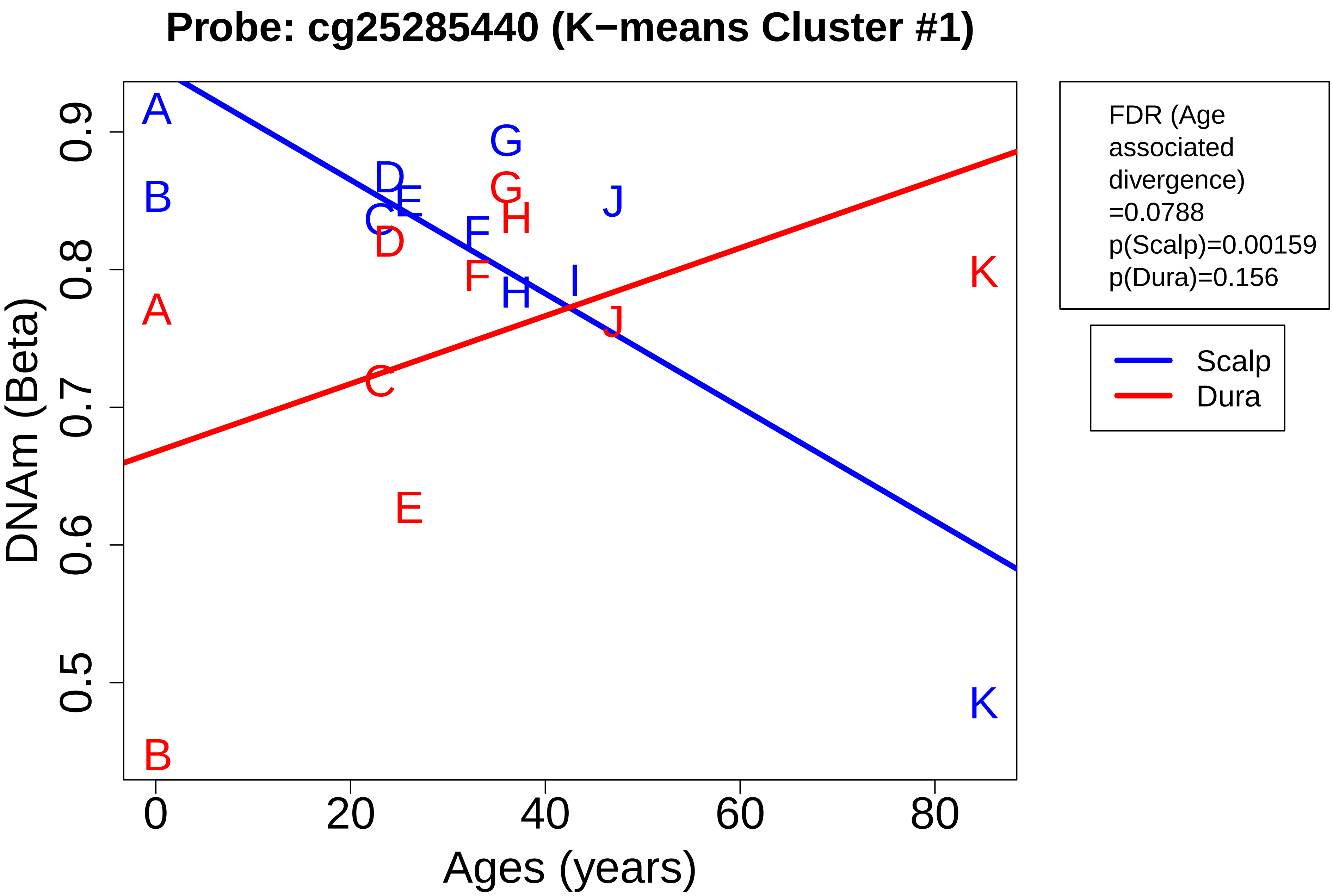

Probe: cg25285440 (K−means Cluster #1)
DNAm (Beta)
0.5
0.6
0.7
0.8
0.9
Ages (years)
0
20
40
60
80
A
B
C
D
E
F
G
H
I
J
K
FDR (Age associated divergence) =0.0788
p(Scalp)=0.00159
p(Dura)=0.156
Scalp
Dura

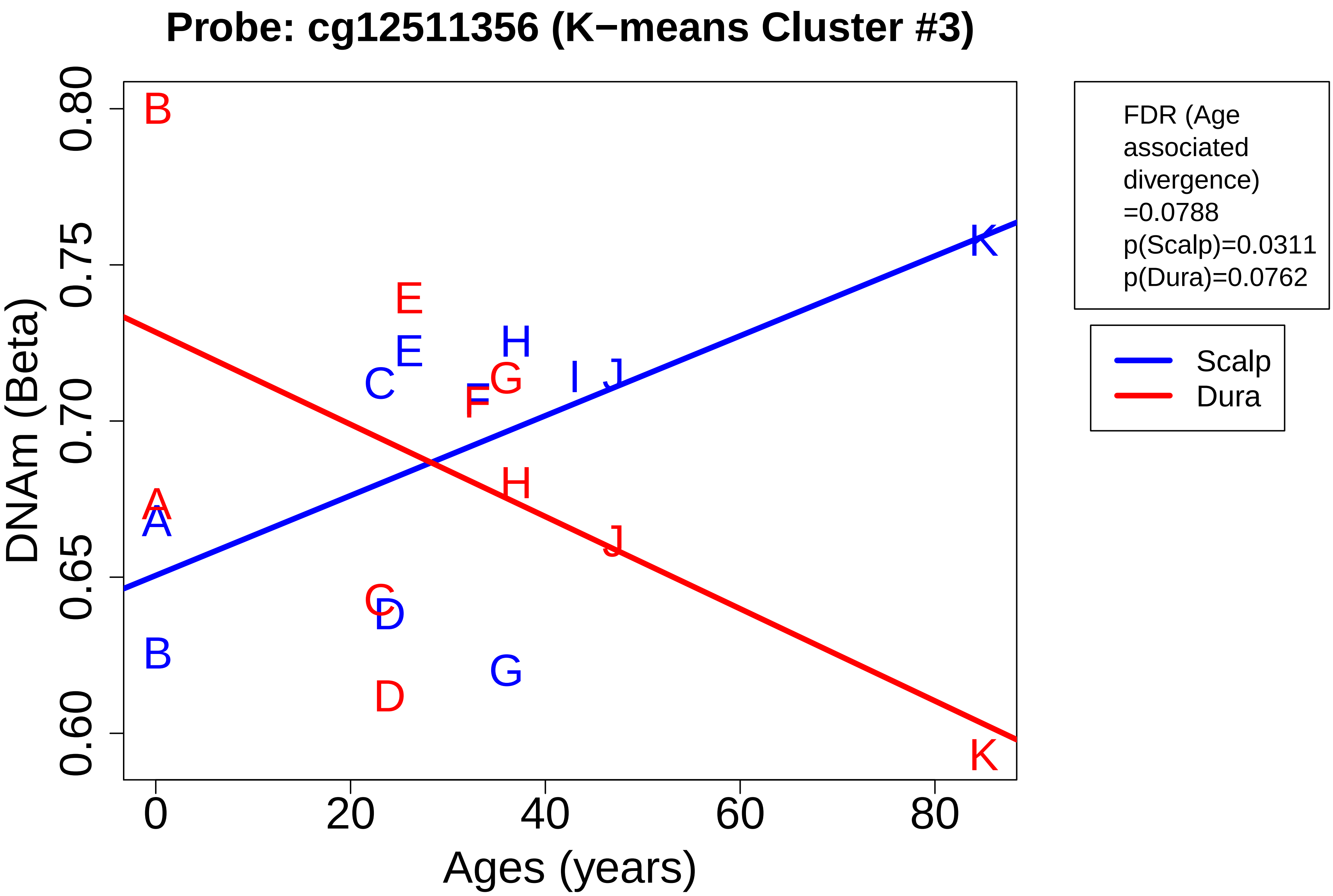
Probe: cg12511356 (K−means Cluster #3)
DNAm (Beta)
Ages (years)
0.60
0.65
0.70
0.75
0.80
0
20
40
60
80
FDR (Age associated divergence) =0.0788
p(Scalp)=0.0311
p(Dura)=0.0762
Scalp
Dura

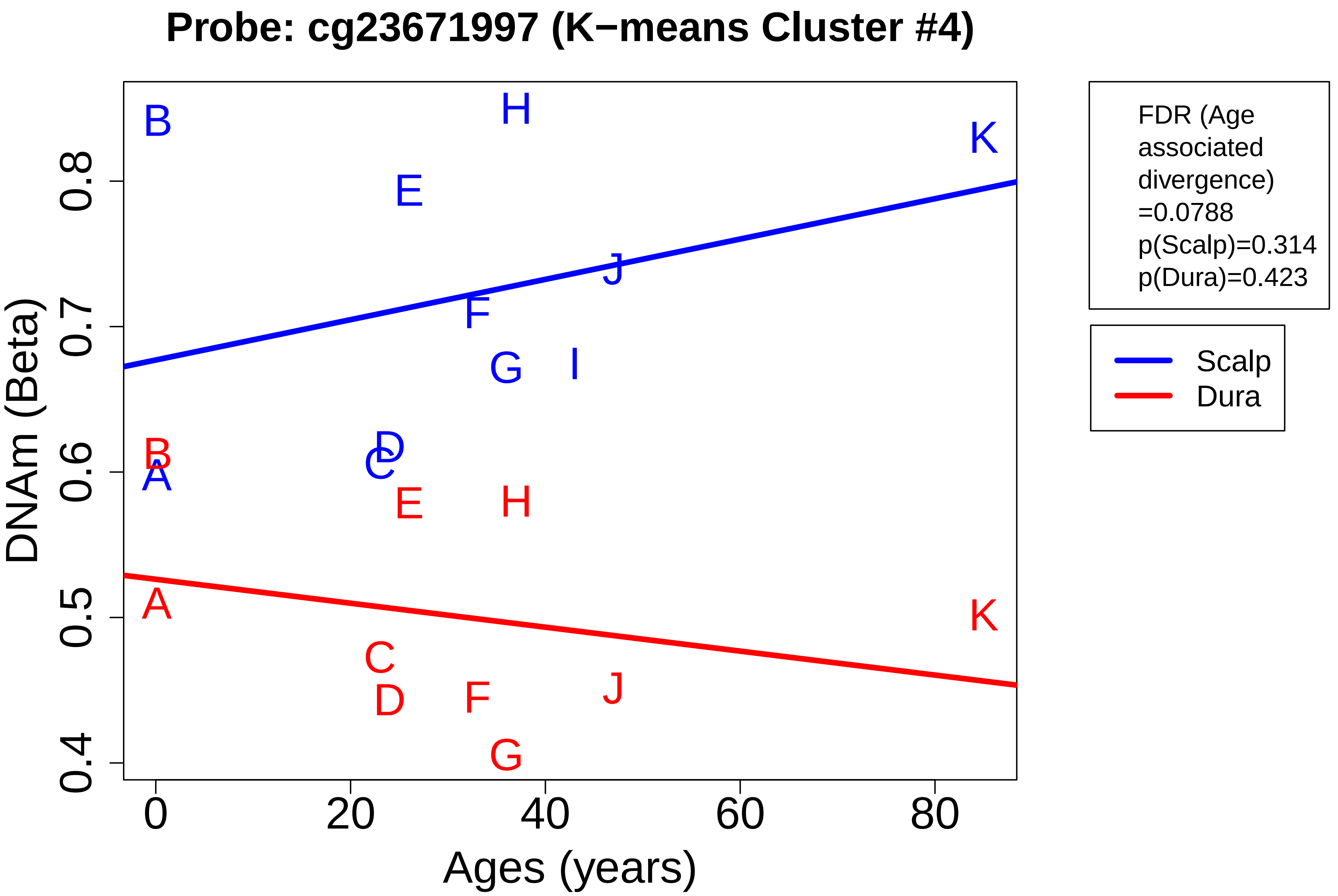

Probe: cg23671997 (K−means Cluster #4)
DNAm (Beta)
0.4
0.5
0.6
0.7
0.8
0
20
40
60
80
Ages (years)
FDR (Age associated divergence) =0.0788
p(Scalp)=0.314
p(Dura)=0.423
Scalp
Dura
A
B
C
D
E
F
G
H
I
J
K
A
B
C
D
E
F
G
H
J
K

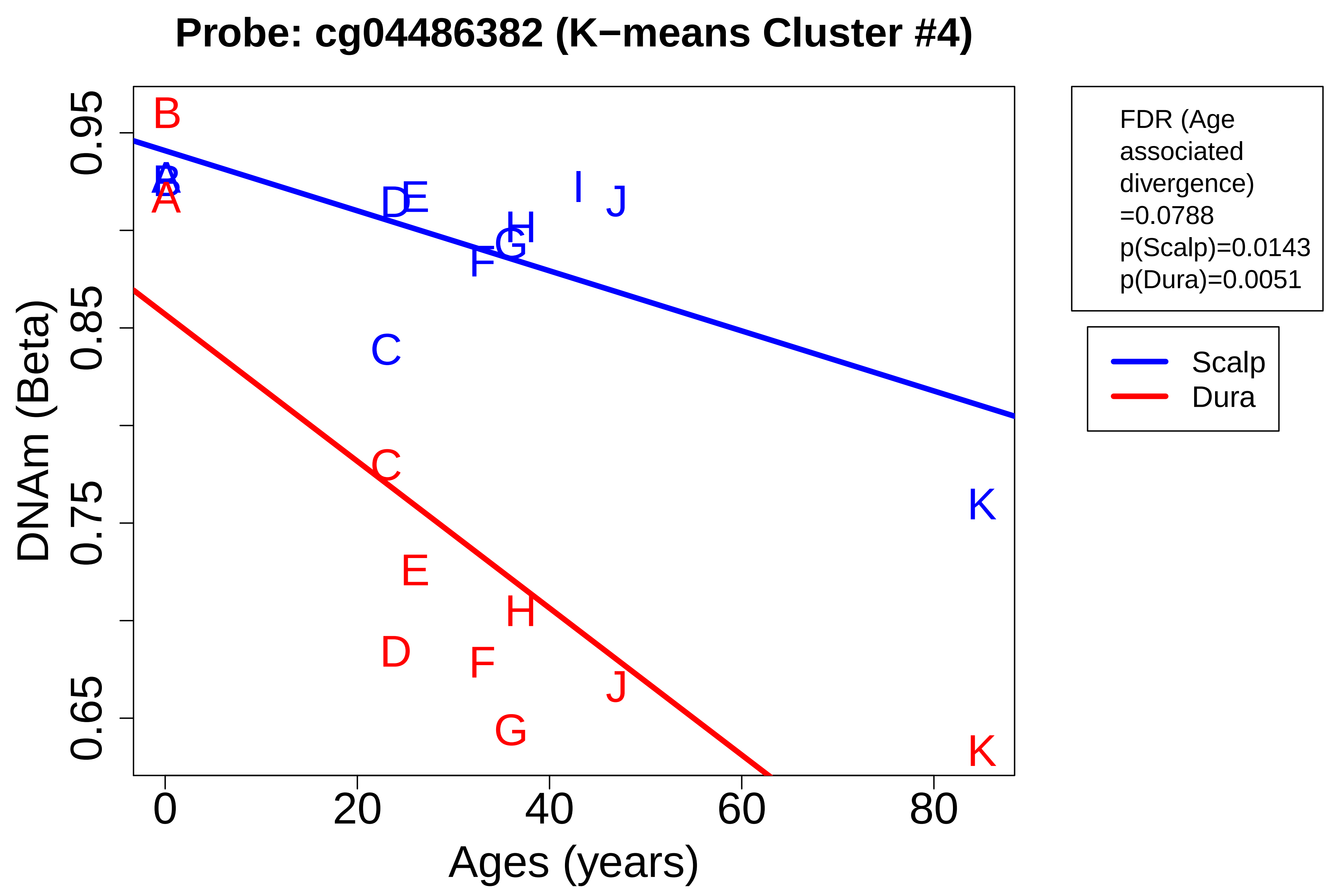

Probe: cg04486382 (K−means Cluster #4)
DNAm (Beta)
0.65
0.75
0.85
0.95
Ages (years)
0
20
40
60
80
A
B
C
D
E
F
G
H
I
J
K
FDR (Age associated divergence) =0.0788
p(Scalp)=0.0143
p(Dura)=0.0051
Scalp
Dura

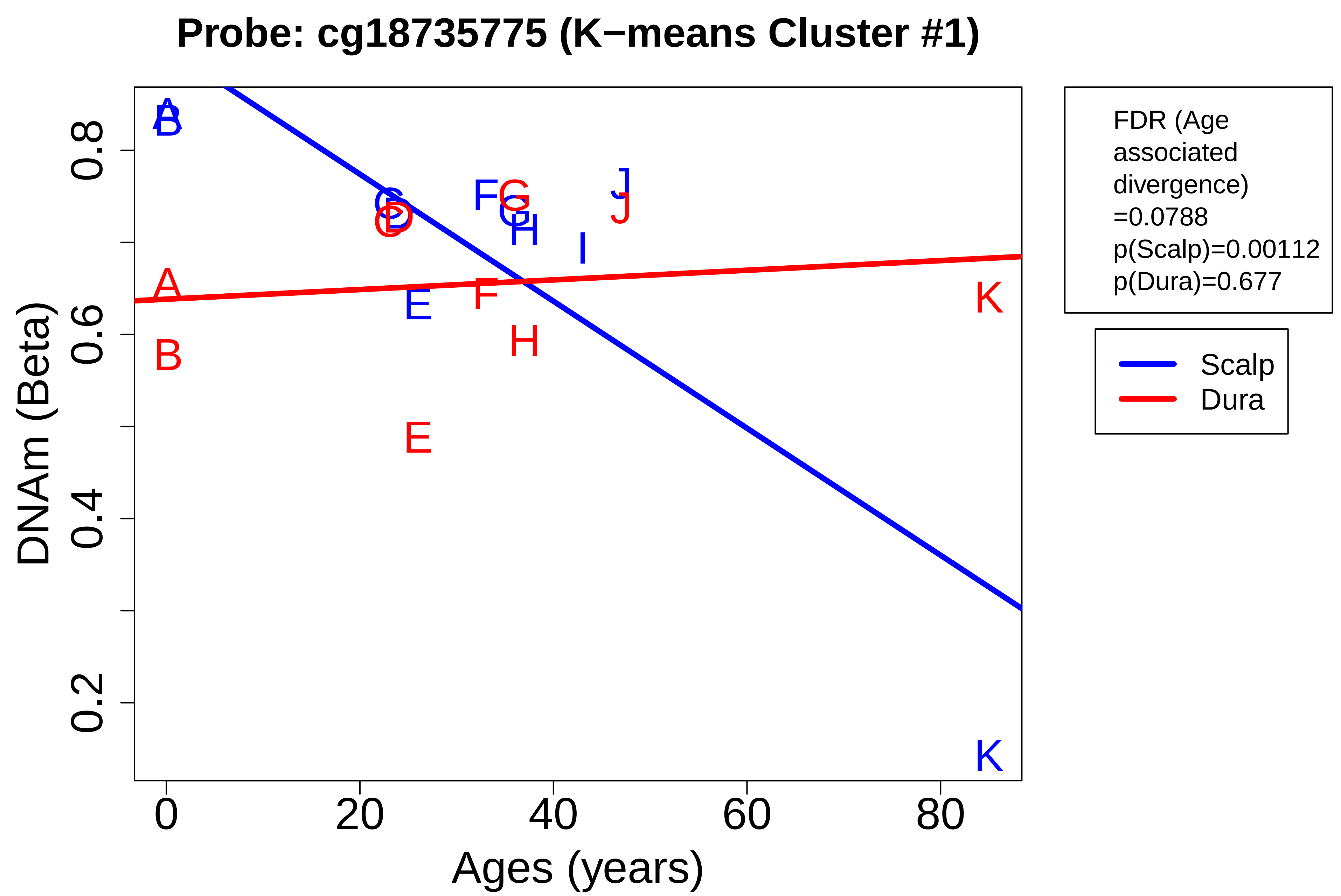

Probe: cg18735775 (K−means Cluster #1)
DNAm (Beta)
0.2
0.4
0.6
0.8
0
20
40
60
80
Ages (years)
A
B
C
D
E
F
G
H
I
J
K
FDR (Age associated divergence) =0.0788
p(Scalp)=0.00112
p(Dura)=0.677
Scalp
Dura

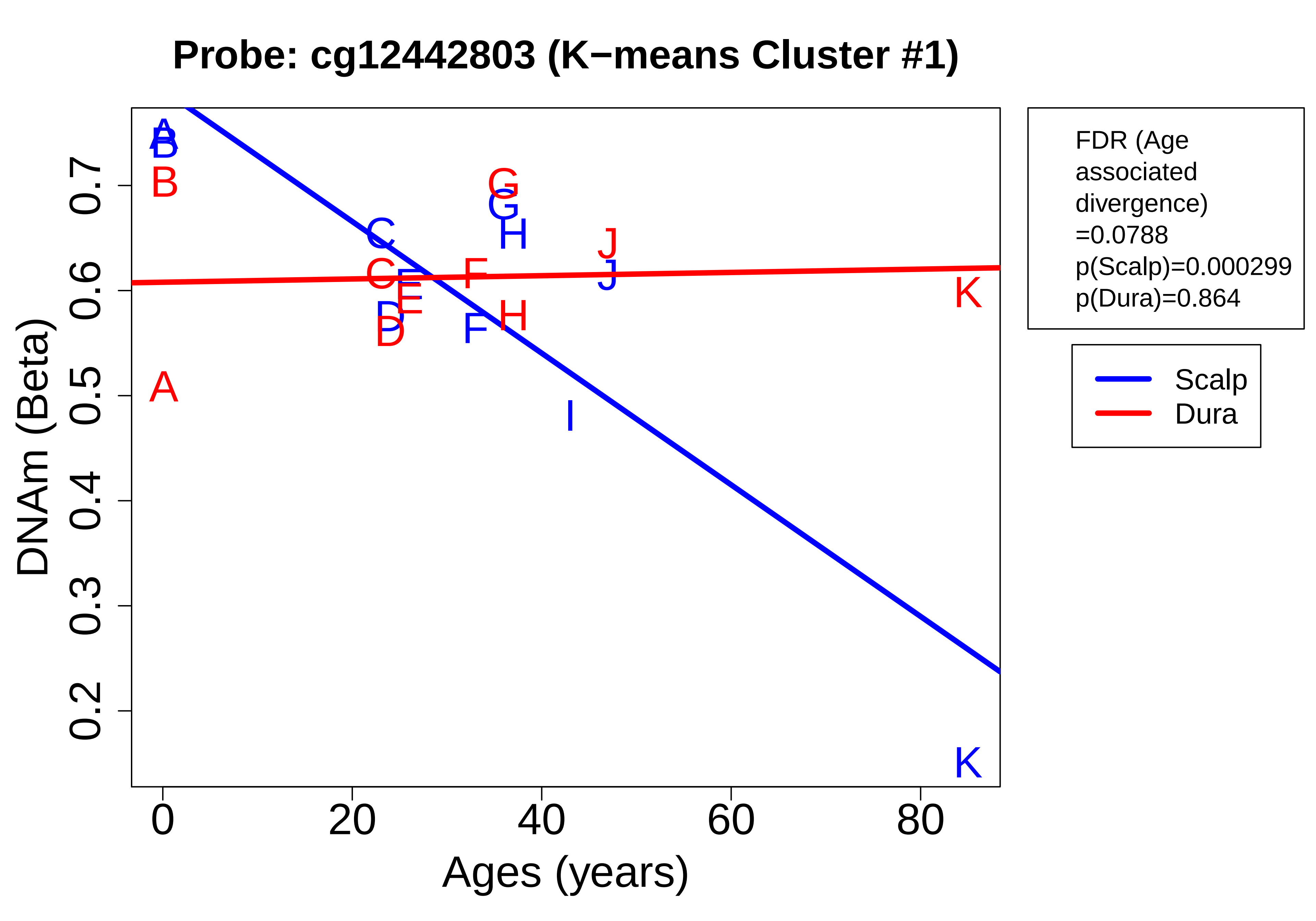

Probe: cg12442803 (K−means Cluster #1)
DNAm (Beta)
0.2
0.3
0.4
0.5
0.6
0.7
Ages (years)
0
20
40
60
80
A
B
C
D
E
F
G
H
I
J
K
FDR (Age associated divergence) =0.0788
p(Scalp)=0.000299
p(Dura)=0.864
Scalp
Dura

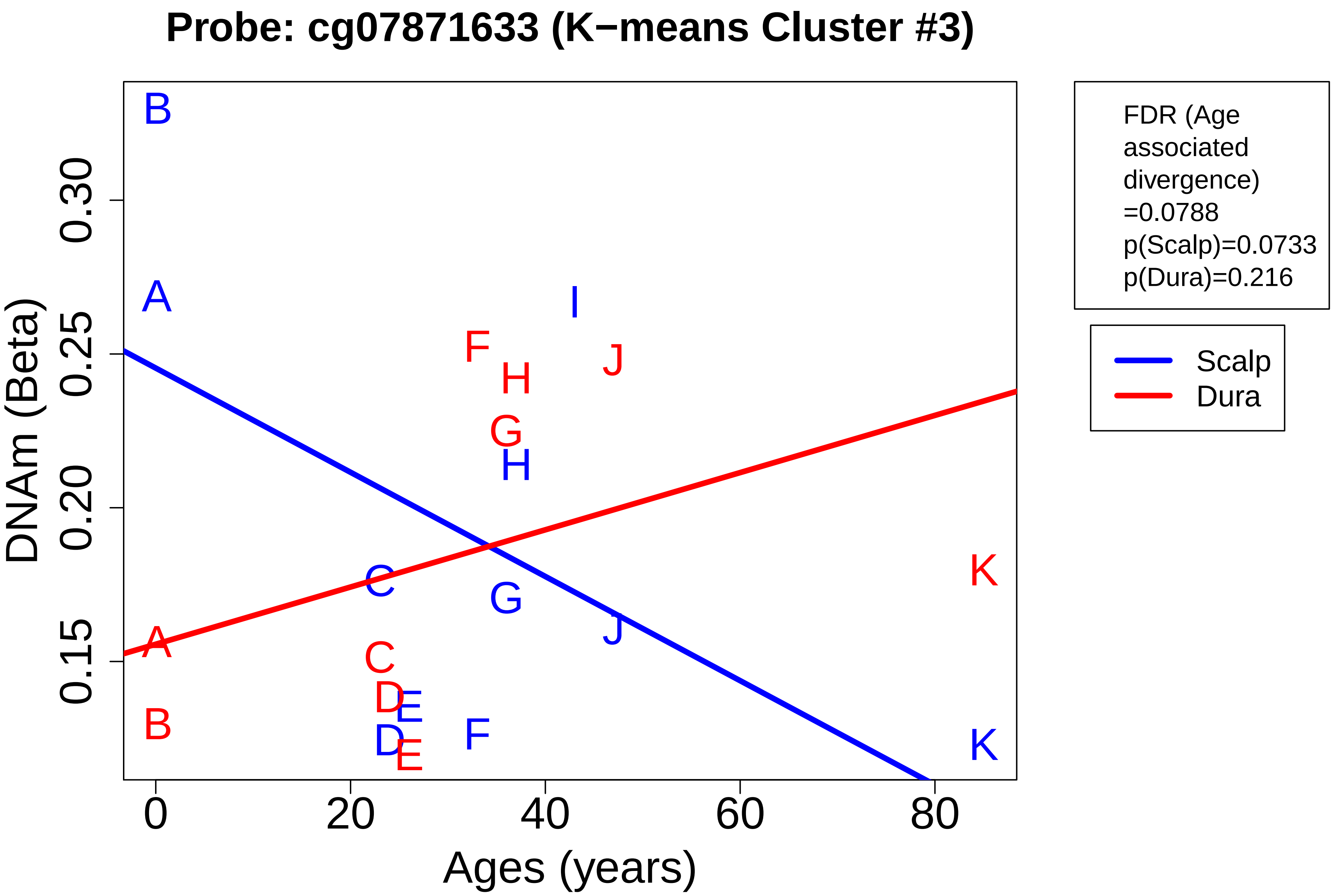

Probe: cg07871633 (K−means Cluster #3)
DNAm (Beta)
0.15
0.20
0.25
0.30
Ages (years)
0
20
40
60
80
A
B
C
D
E
F
G
H
I
J
K
FDR (Age associated divergence) =0.0788
p(Scalp)=0.0733
p(Dura)=0.216
Scalp
Dura

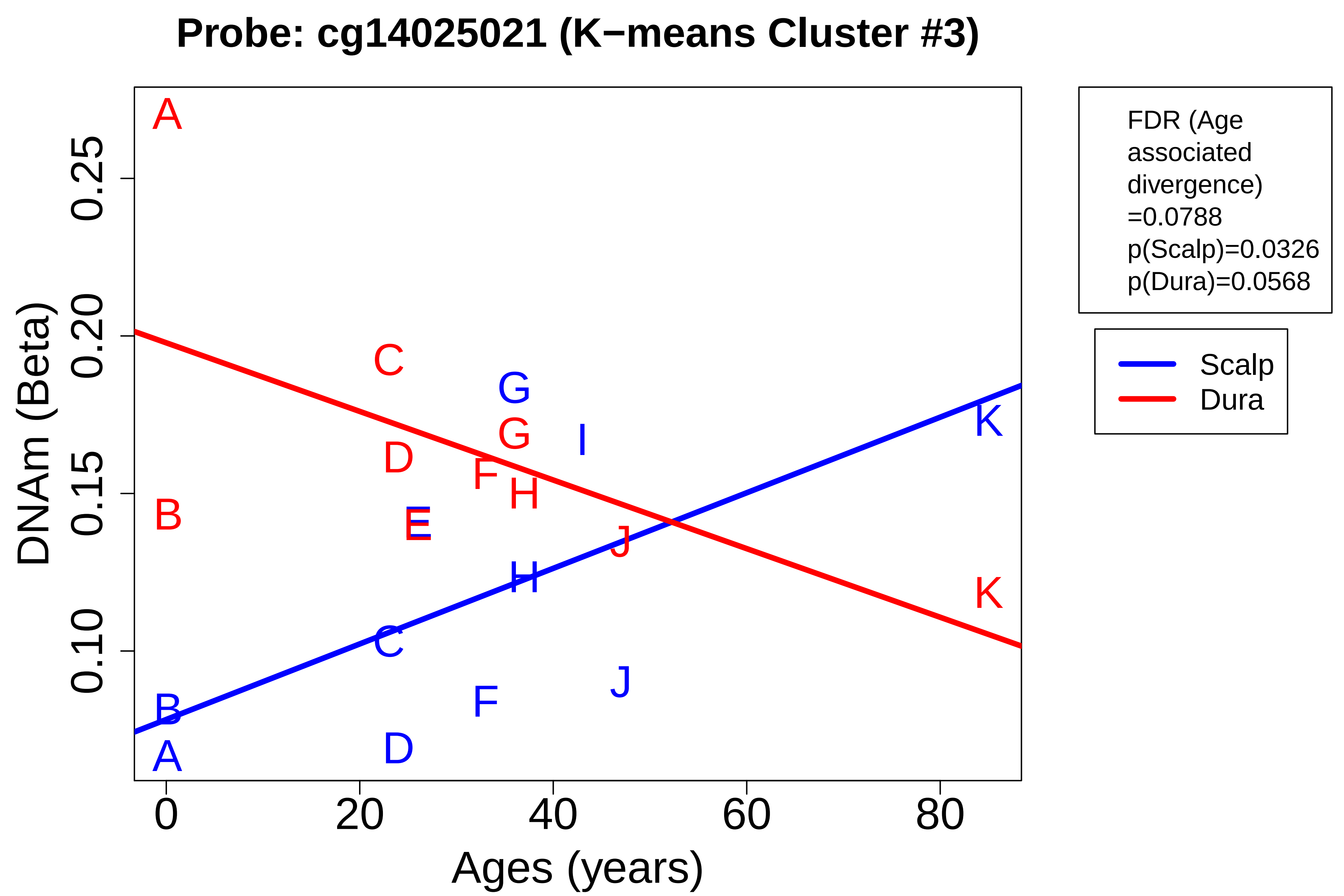

Probe: cg14025021 (K−means Cluster #3)
FDR (Age associated divergence) =0.0788
p(Scalp)=0.0326
p(Dura)=0.0568
Scalp
Dura
DNAm (Beta)
0.10
0.15
0.20
0.25
Ages (years)
0
20
40
60
80
A
B
C
D
E
F
G
H
I
J
K

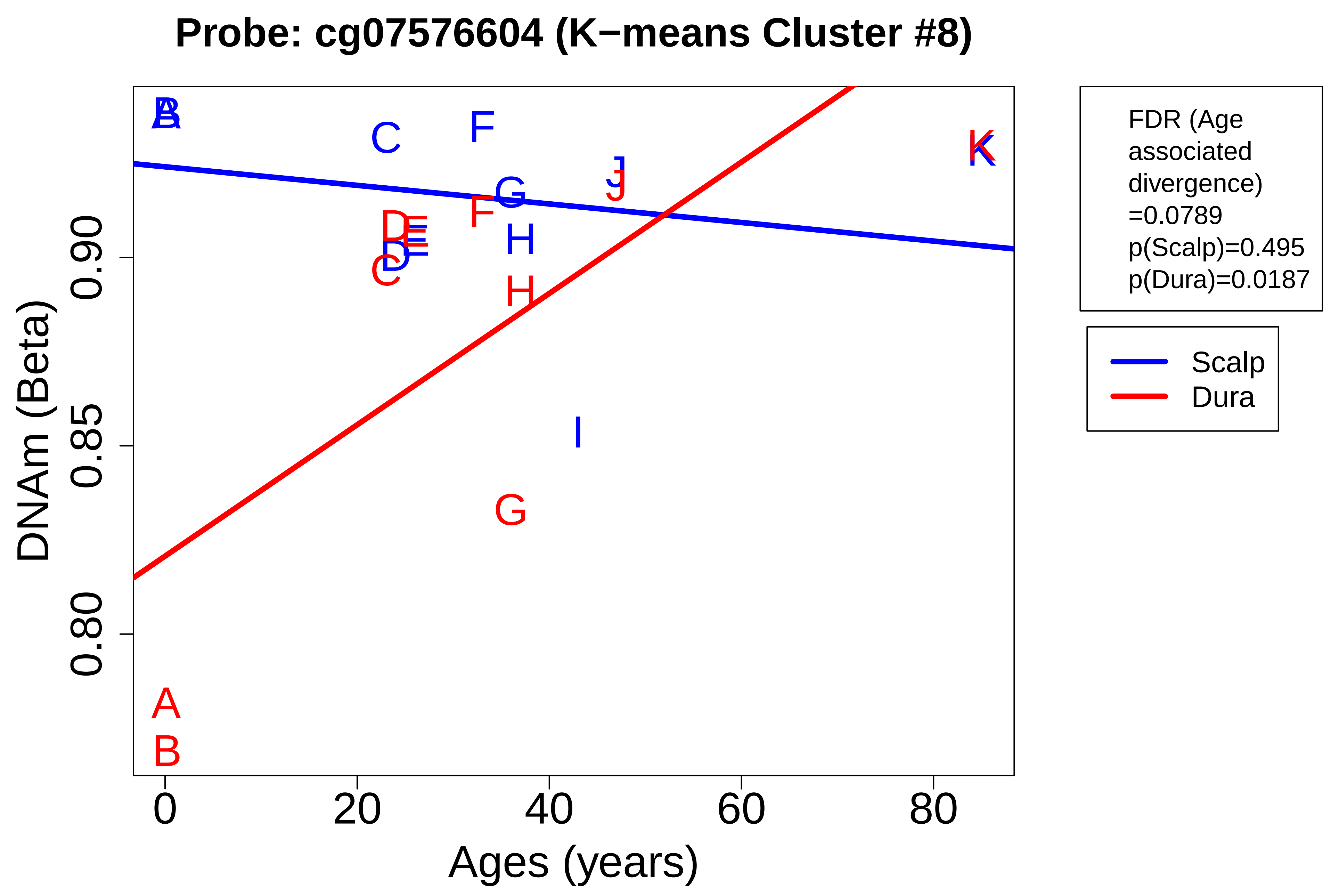

Probe: cg07576604 (K−means Cluster #8)
DNAm (Beta)
0.80
0.85
0.90
0
20
40
60
80
Ages (years)
FDR (Age associated divergence) =0.0789
p(Scalp)=0.495
p(Dura)=0.0187
Scalp
Dura

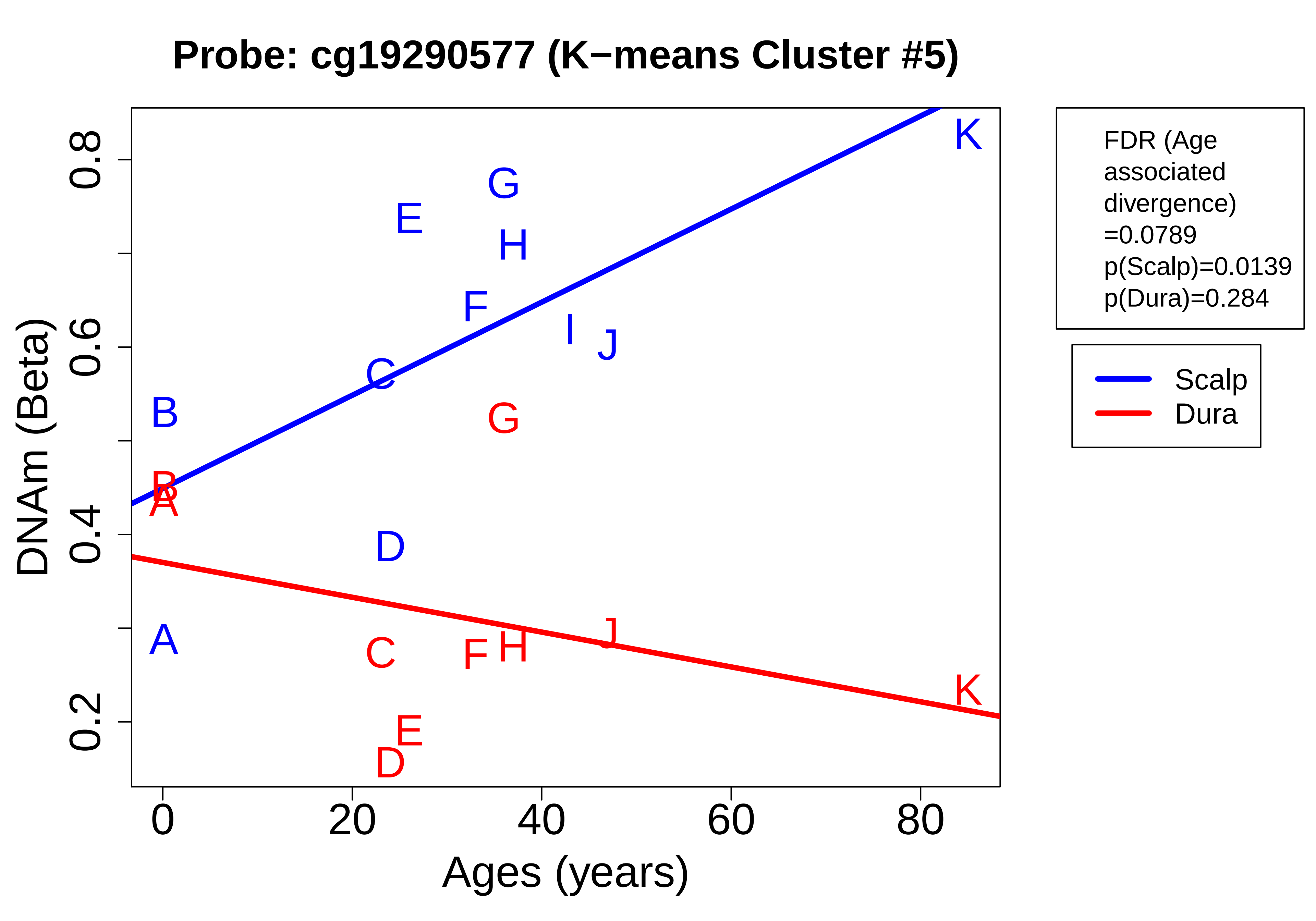
Probe: cg19290577 (K−means Cluster #5)
DNAm (Beta)
0.2
0.4
0.6
0.8
0
20
40
60
80
Ages (years)
FDR (Age associated divergence) =0.0789
p(Scalp)=0.0139
p(Dura)=0.284
Scalp
Dura
A
B
C
D
E
F
G
H
I
J
K

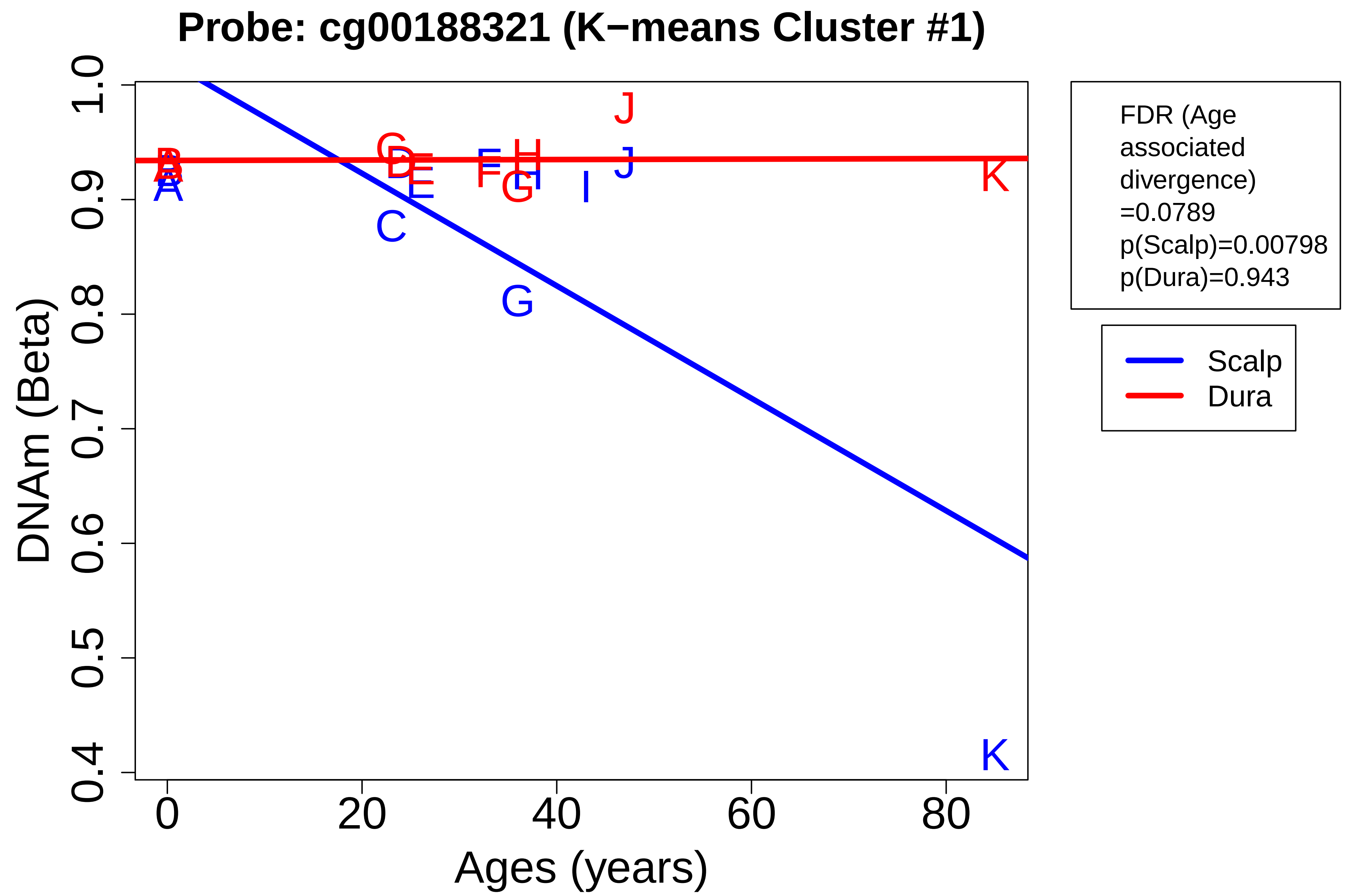

Probe: cg00188321 (K−means Cluster #1)
DNAm (Beta)
1.0
0.9
0.8
0.7
0.6
0.5
0.4
0
20
40
60
80
Ages (years)
A
B
C
D
E
F
G
H
I
J
K
FDR (Age associated divergence) =0.0789
p(Scalp)=0.00798
p(Dura)=0.943
Scalp
Dura

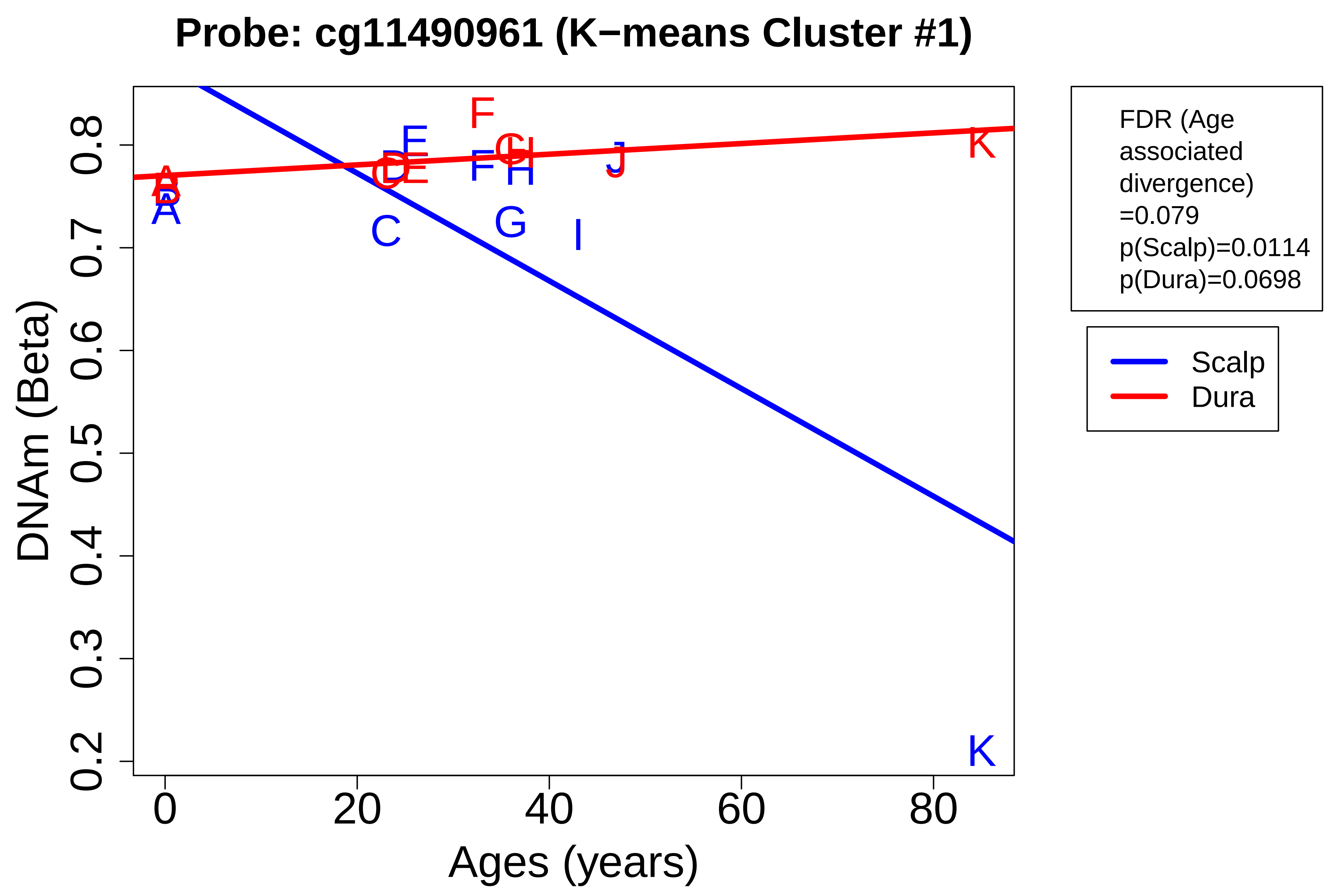
Probe: cg11490961 (K−means Cluster #1)
DNAm (Beta)
0.2
0.3
0.4
0.5
0.6
0.7
0.8
0
20
40
60
80
Ages (years)
A
B
C
D
E
F
G
H
I
J
K
FDR (Age associated divergence) =0.079
p(Scalp)=0.0114
p(Dura)=0.0698
Scalp
Dura

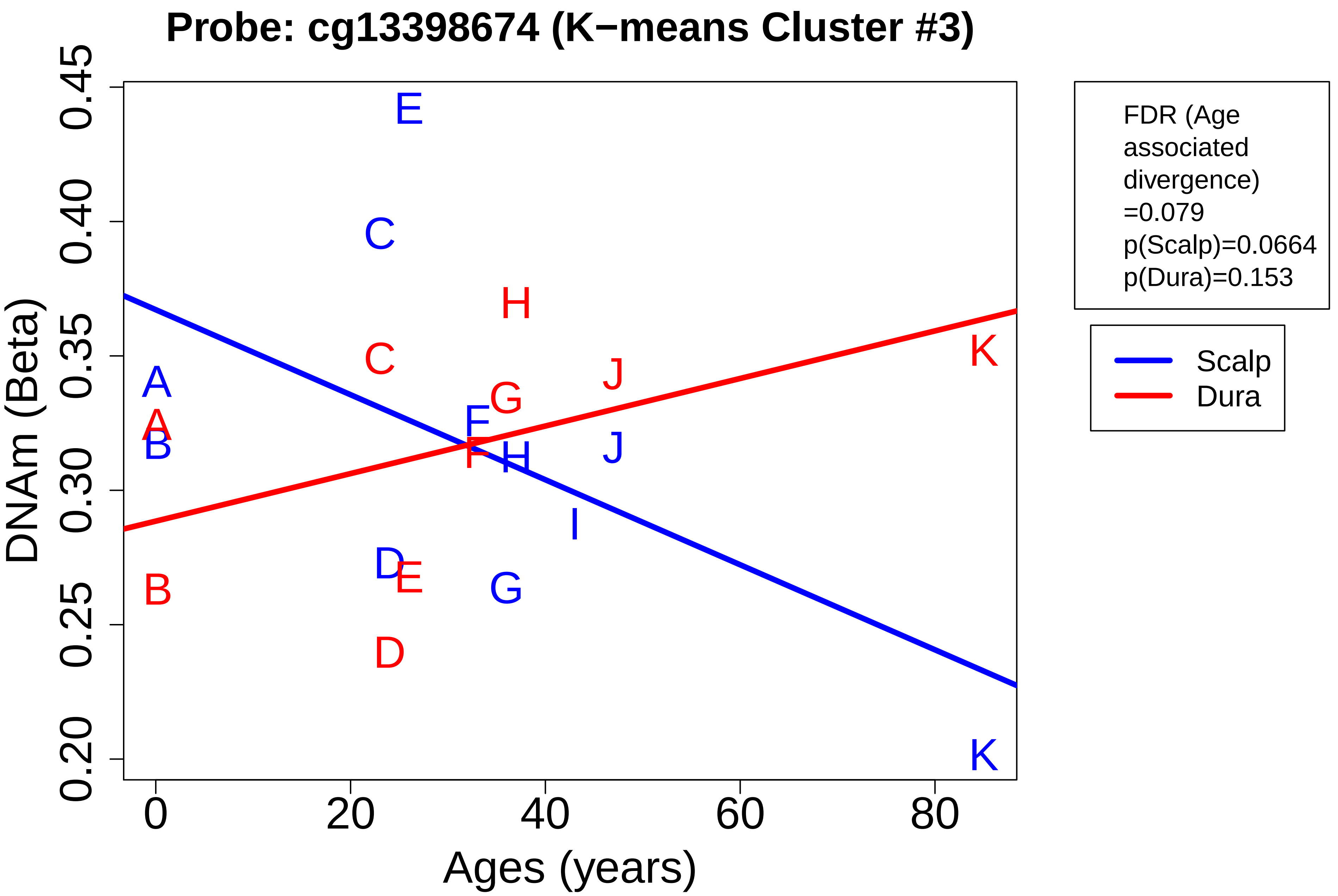

Probe: cg13398674 (K−means Cluster #3)
DNAm (Beta)
0.20
0.25
0.30
0.35
0.40
0.45
0
20
40
60
80
Ages (years)
FDR (Age associated divergence) =0.079
p(Scalp)=0.0664
p(Dura)=0.153
Scalp
Dura

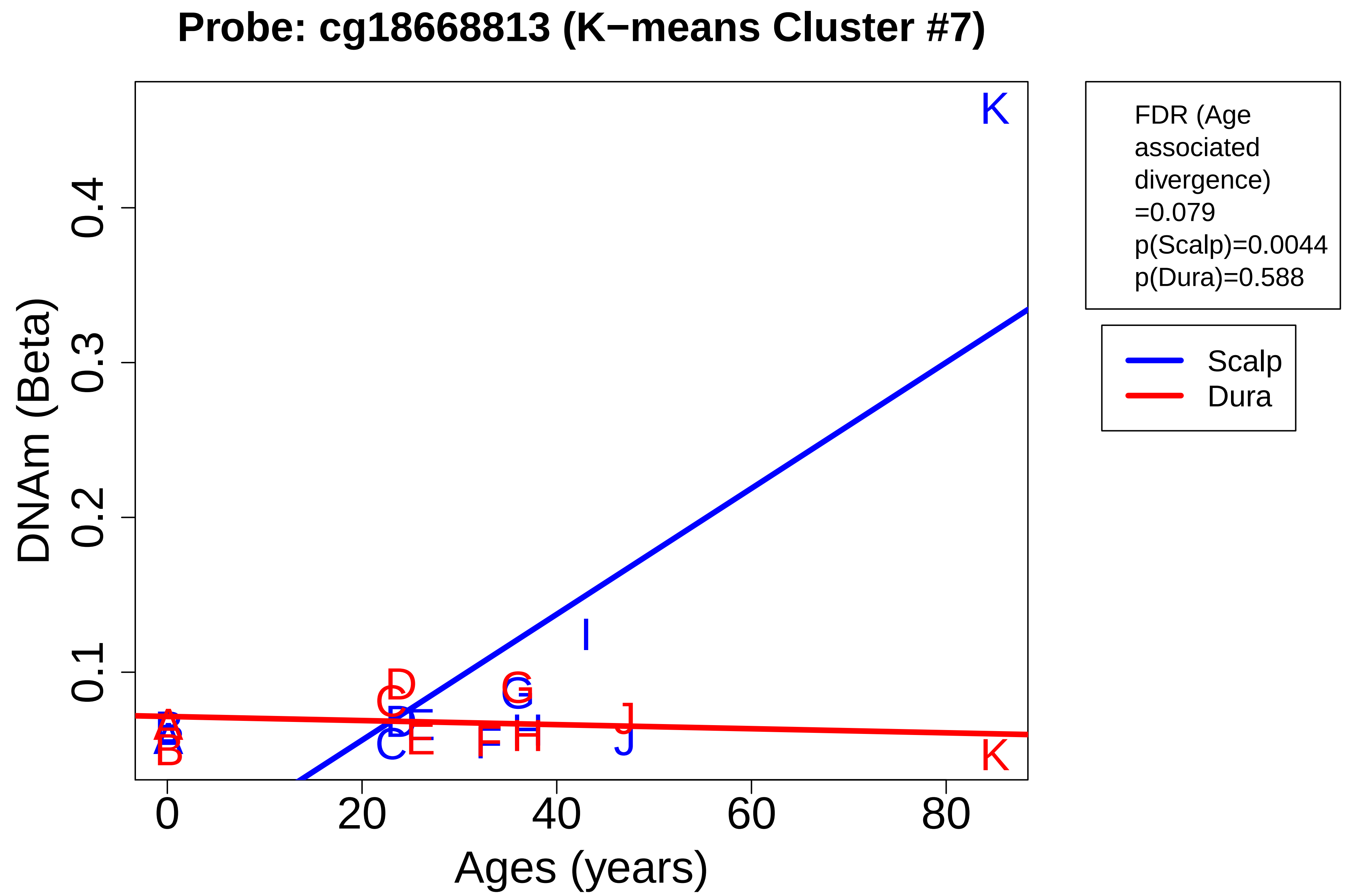

Probe: cg18668813 (K−means Cluster #7)
DNAm (Beta)
0.1
0.2
0.3
0.4
Ages (years)
0
20
40
60
80
FDR (Age associated divergence) =0.079
p(Scalp)=0.0044
p(Dura)=0.588
Scalp
Dura

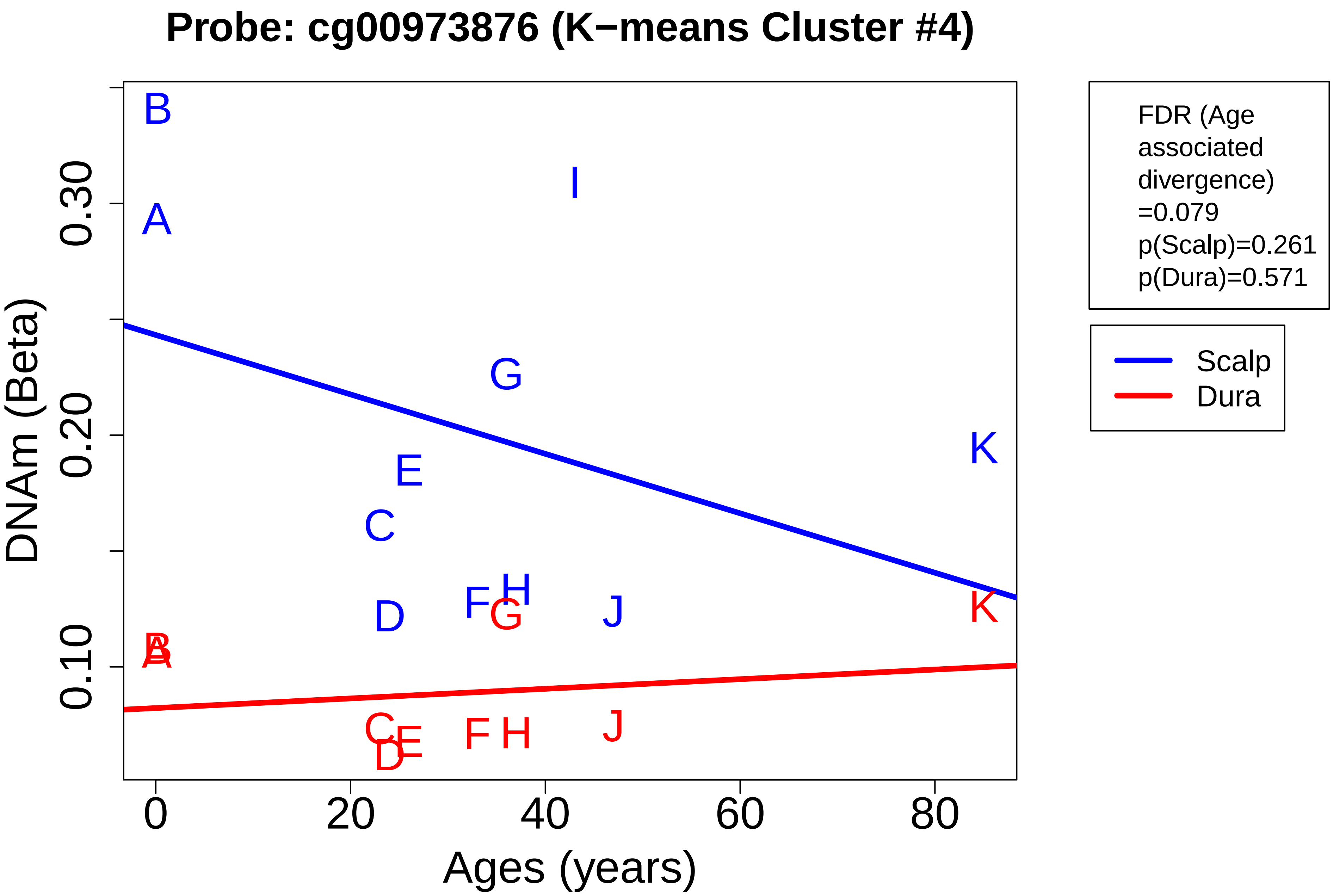

Probe: cg00973876 (K−means Cluster #4)
DNAm (Beta)
0.10
0.20
0.30
0
20
40
60
80
Ages (years)
B
A
I
G
K
E
C
F
H
D
J
B
A
G
K
C
E
D
F
H
J
FDR (Age associated divergence) =0.079
p(Scalp)=0.261
p(Dura)=0.571
Scalp
Dura

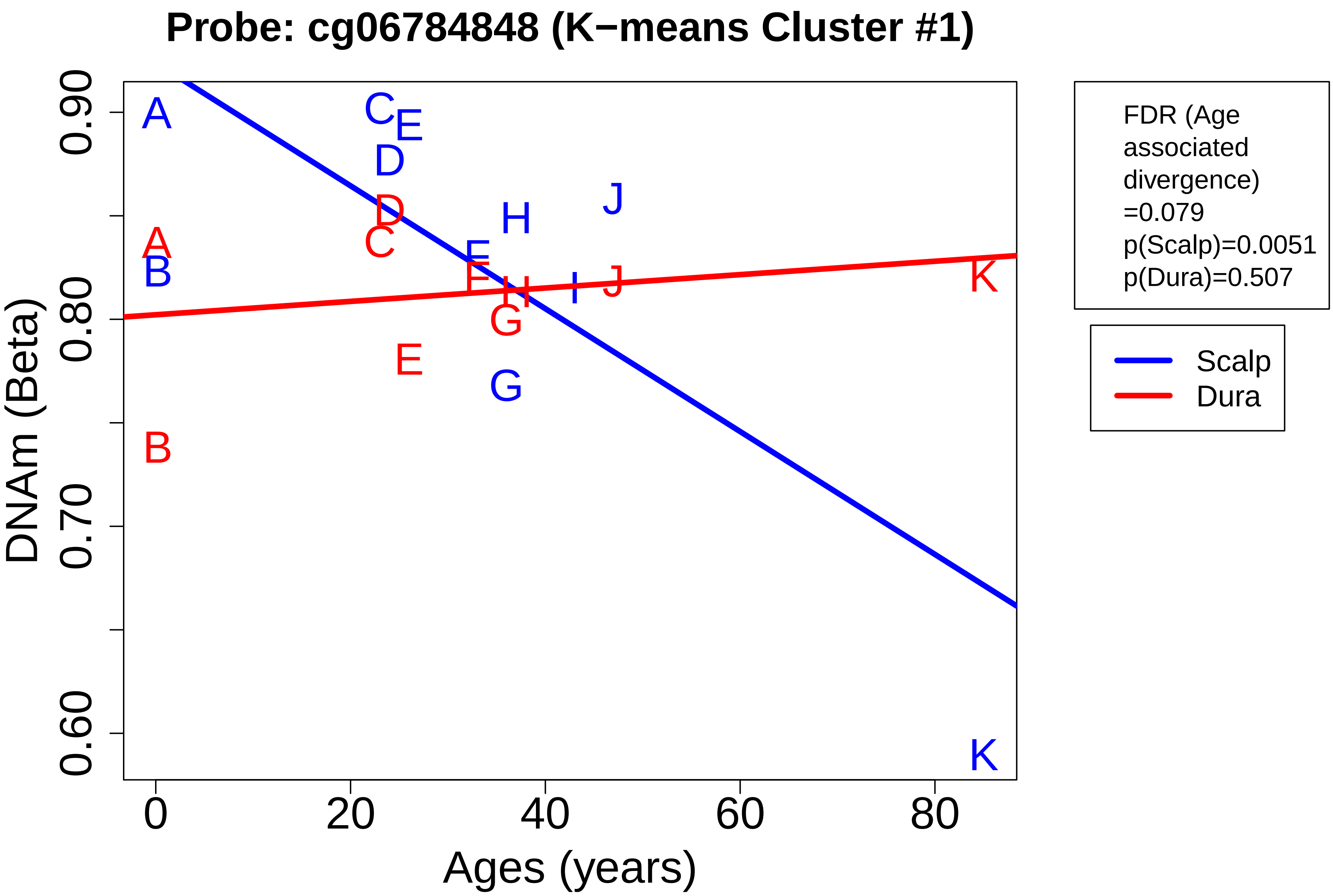
Probe: cg06784848 (K−means Cluster #1)
DNAm (Beta)
0.60
0.70
0.80
0.90
0
20
40
60
80
Ages (years)
FDR (Age associated divergence) =0.079
p(Scalp)=0.0051
p(Dura)=0.507
Scalp
Dura

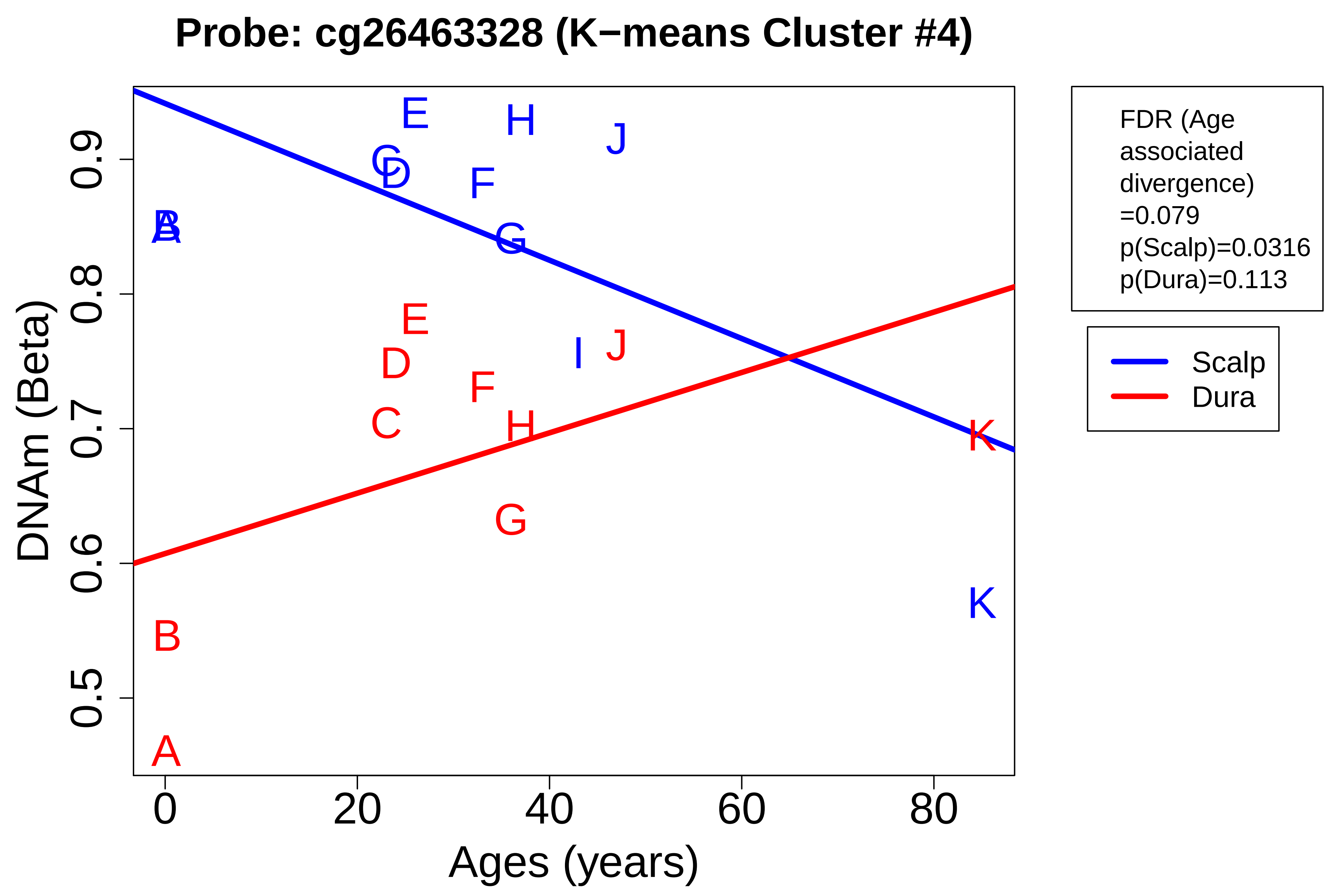

Probe: cg26463328 (K−means Cluster #4)
FDR (Age associated divergence) =0.079
p(Scalp)=0.0316
p(Dura)=0.113
Scalp
Dura
DNAm (Beta)
0.5
0.6
0.7
0.8
0.9
Ages (years)
0
20
40
60
80
A
B
C
D
E
F
G
H
I
J
K

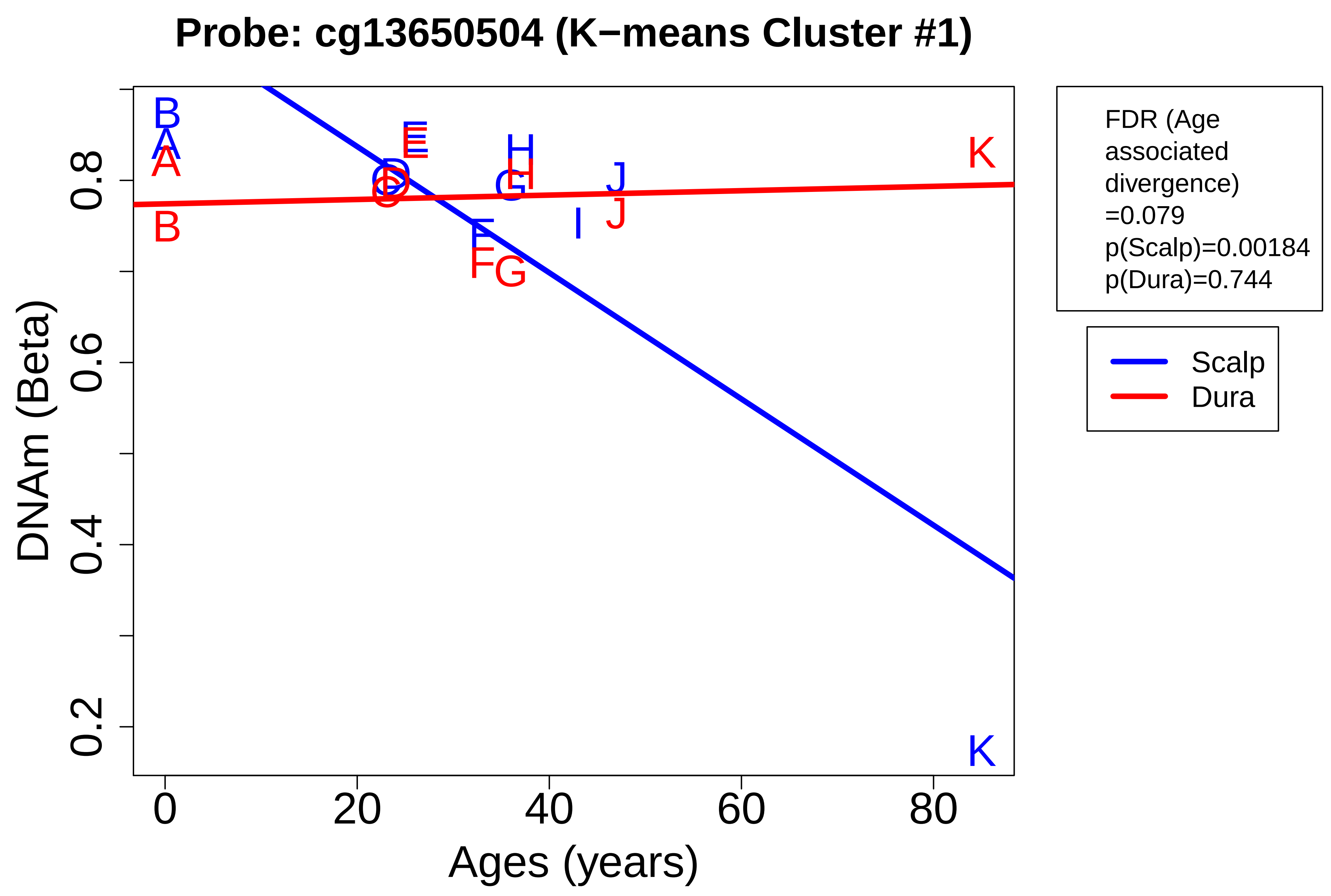
Probe: cg13650504 (K−means Cluster #1)
DNAm (Beta)
0.2
0.4
0.6
0.8
Ages (years)
0
20
40
60
80
FDR (Age associated divergence) =0.079
p(Scalp)=0.00184
p(Dura)=0.744
Scalp
Dura
A
B
C
D
E
F
G
H
I
J
K

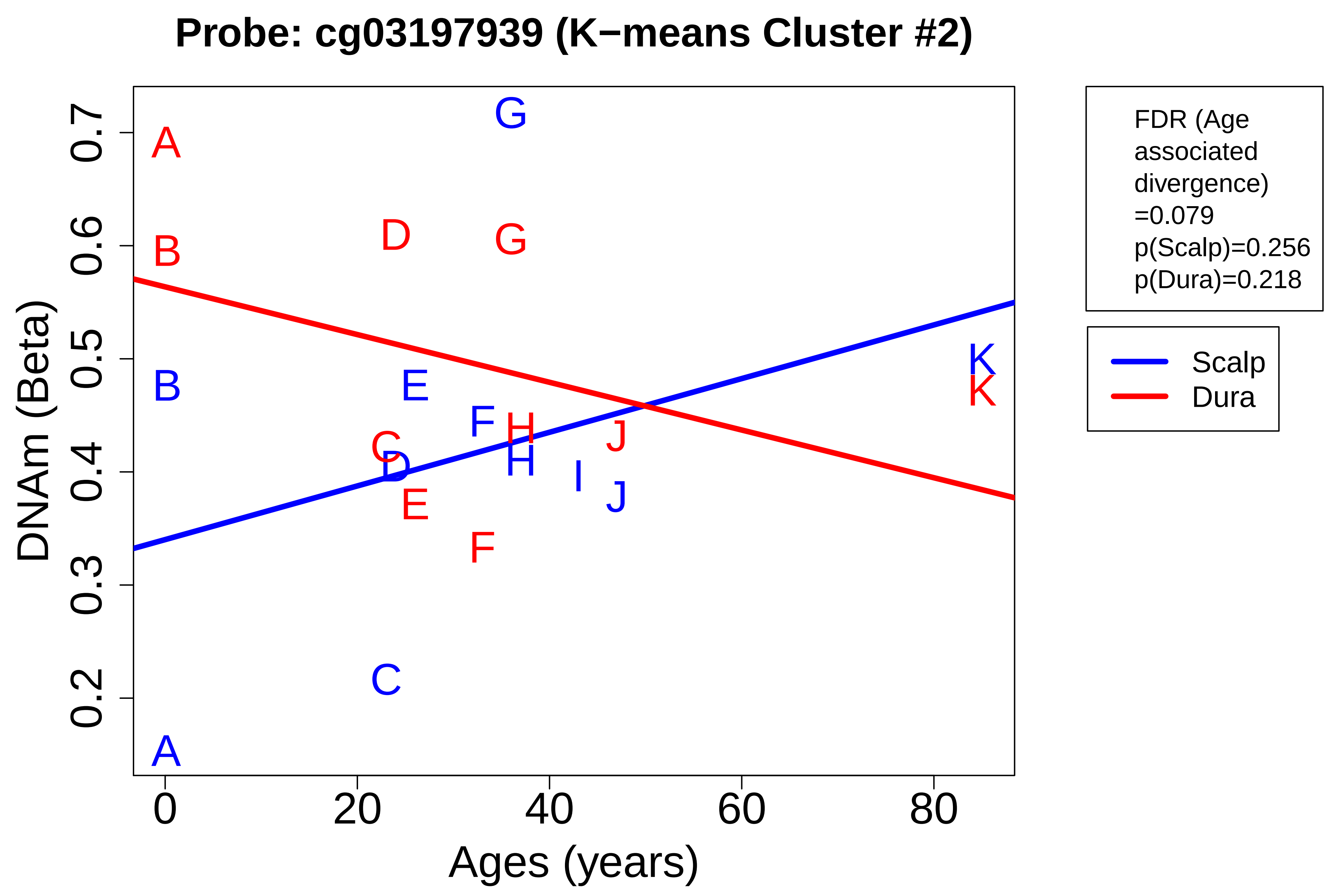

Probe: cg03197939 (K−means Cluster #2)
DNAm (Beta)
0.2
0.3
0.4
0.5
0.6
0.7
Ages (years)
0
20
40
60
80
A
B
C
D
E
F
G
H
I
J
K
FDR (Age associated divergence) =0.079
p(Scalp)=0.256
p(Dura)=0.218
Scalp
Dura

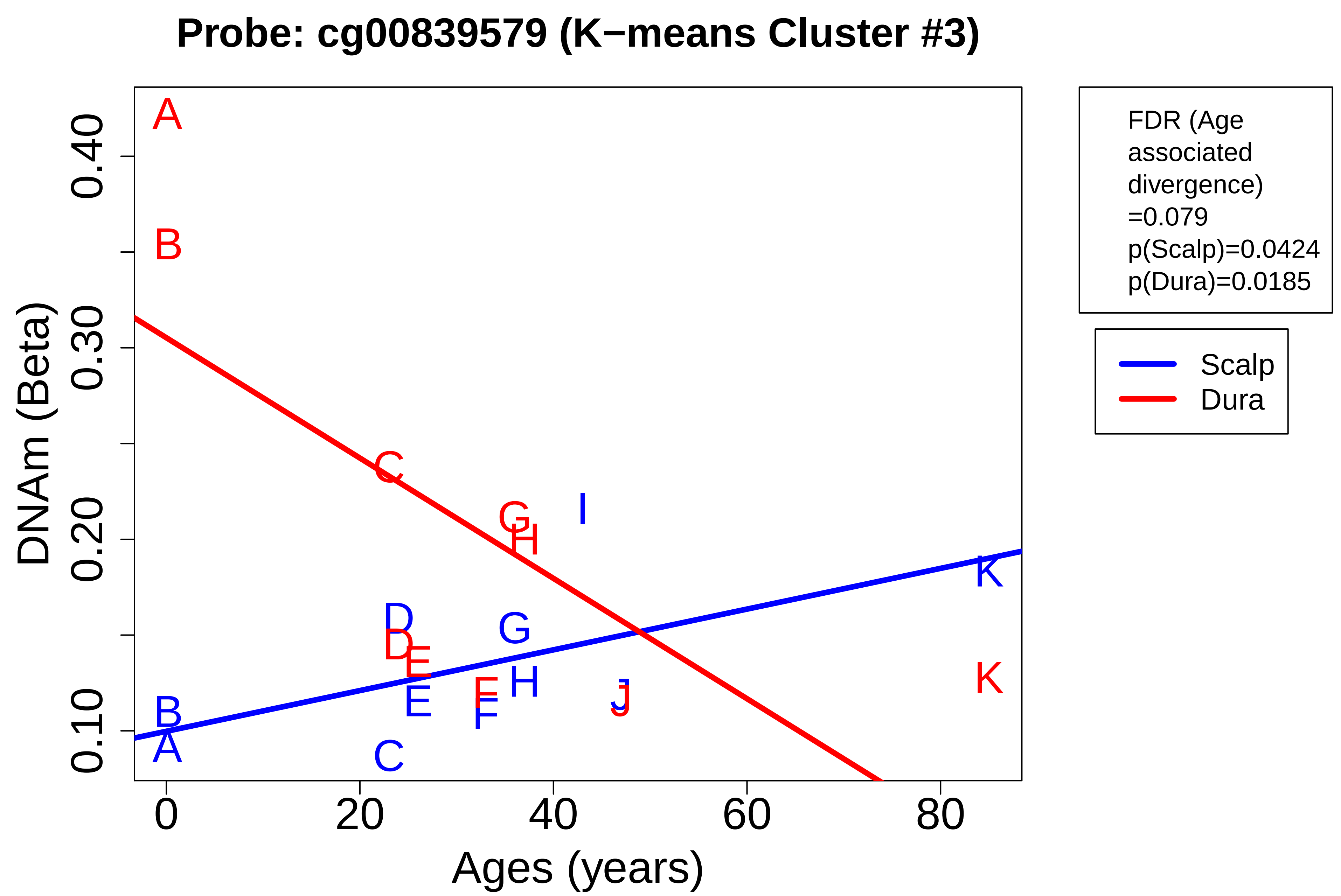
Probe: cg00839579 (K−means Cluster #3)
DNAm (Beta)
0.10
0.20
0.30
0.40
0
20
40
60
80
Ages (years)
A
B
C
D
E
F
G
H
I
J
K
FDR (Age associated divergence) =0.079
p(Scalp)=0.0424
p(Dura)=0.0185
Scalp
Dura

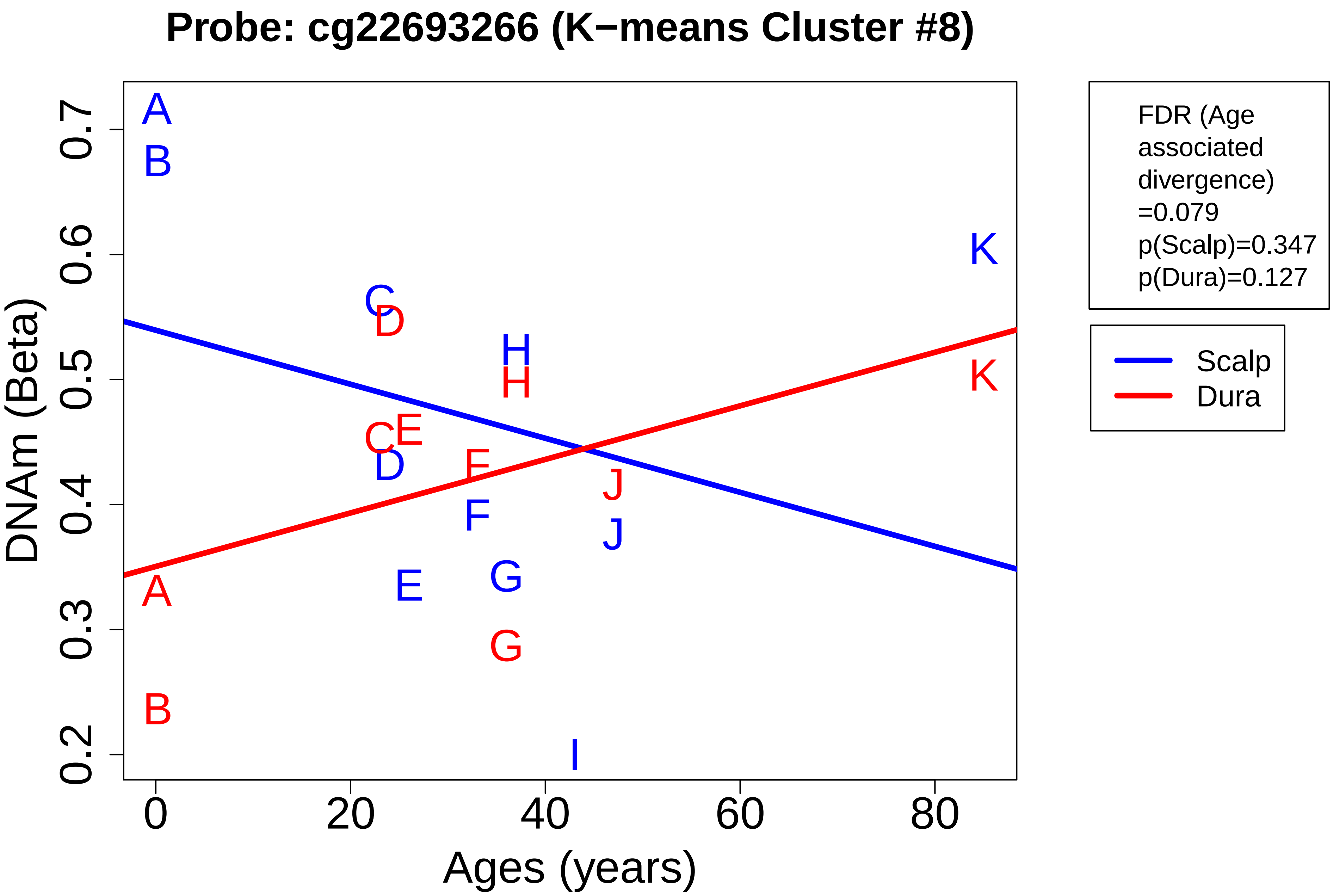

Probe: cg22693266 (K−means Cluster #8)
DNAm (Beta)
Ages (years)
0.2
0.3
0.4
0.5
0.6
0.7
0
20
40
60
80
FDR (Age associated divergence) =0.079
p(Scalp)=0.347
p(Dura)=0.127
Scalp
Dura

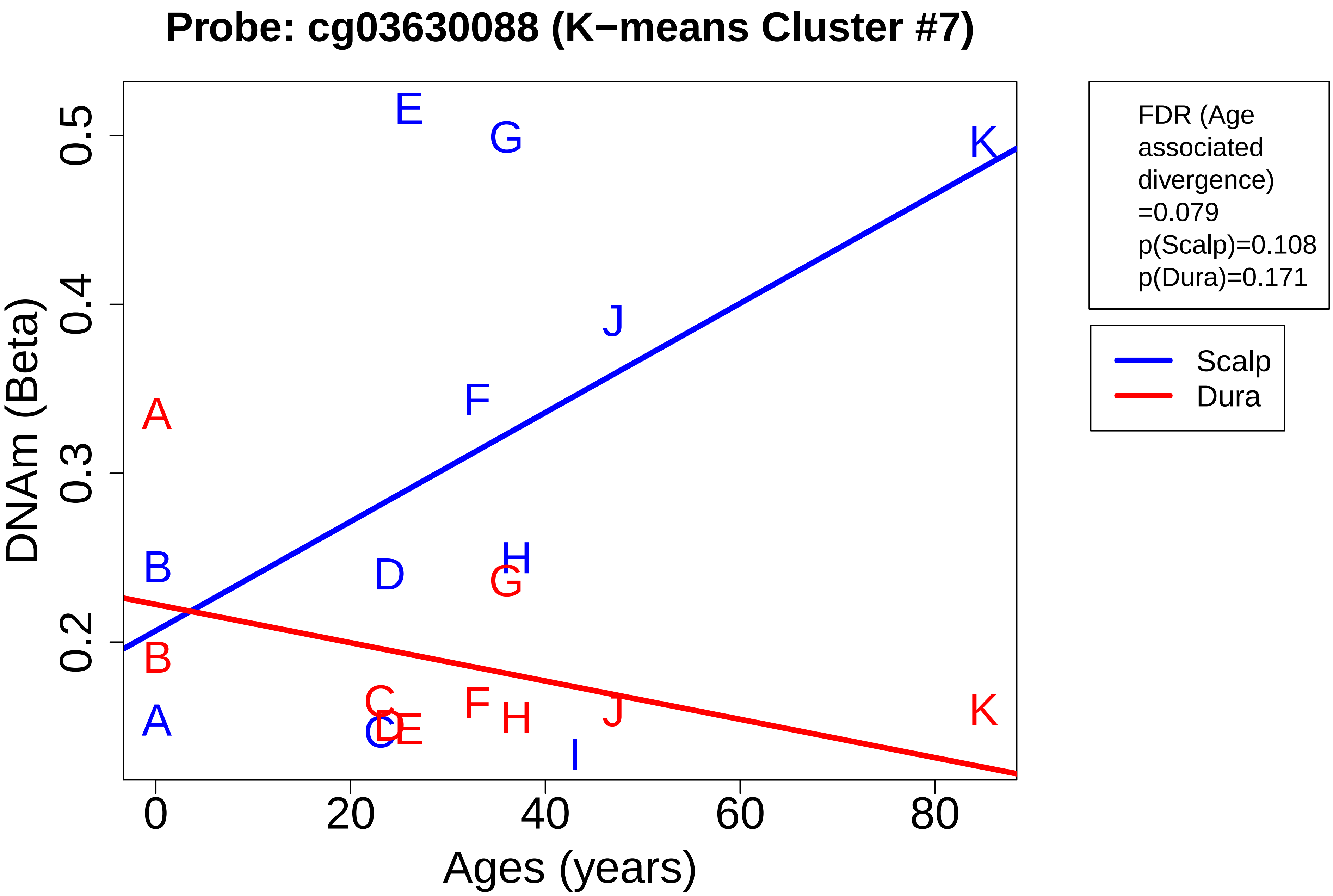

Probe: cg03630088 (K−means Cluster #7)
DNAm (Beta)
0.2
0.3
0.4
0.5
0
20
40
60
80
Ages (years)
FDR (Age associated divergence) =0.079
p(Scalp)=0.108
p(Dura)=0.171
Scalp
Dura

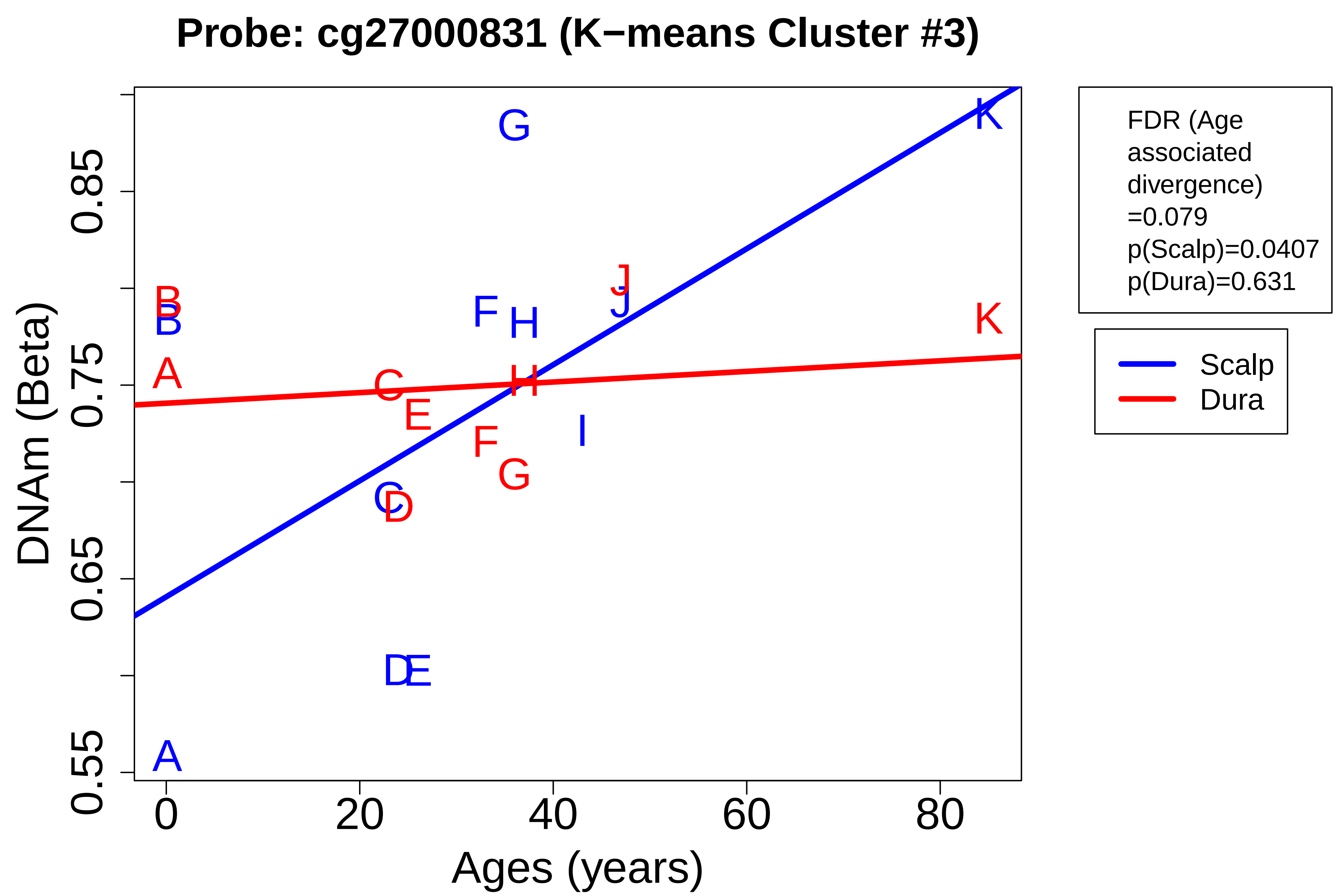

Probe: cg27000831 (K−means Cluster #3)
DNAm (Beta)
0.55
0.65
0.75
0.85
0
20
40
60
80
Ages (years)
FDR (Age associated divergence) =0.079
p(Scalp)=0.0407
p(Dura)=0.631
Scalp
Dura
A
B
C
D
E
F
G
H
I
J
K

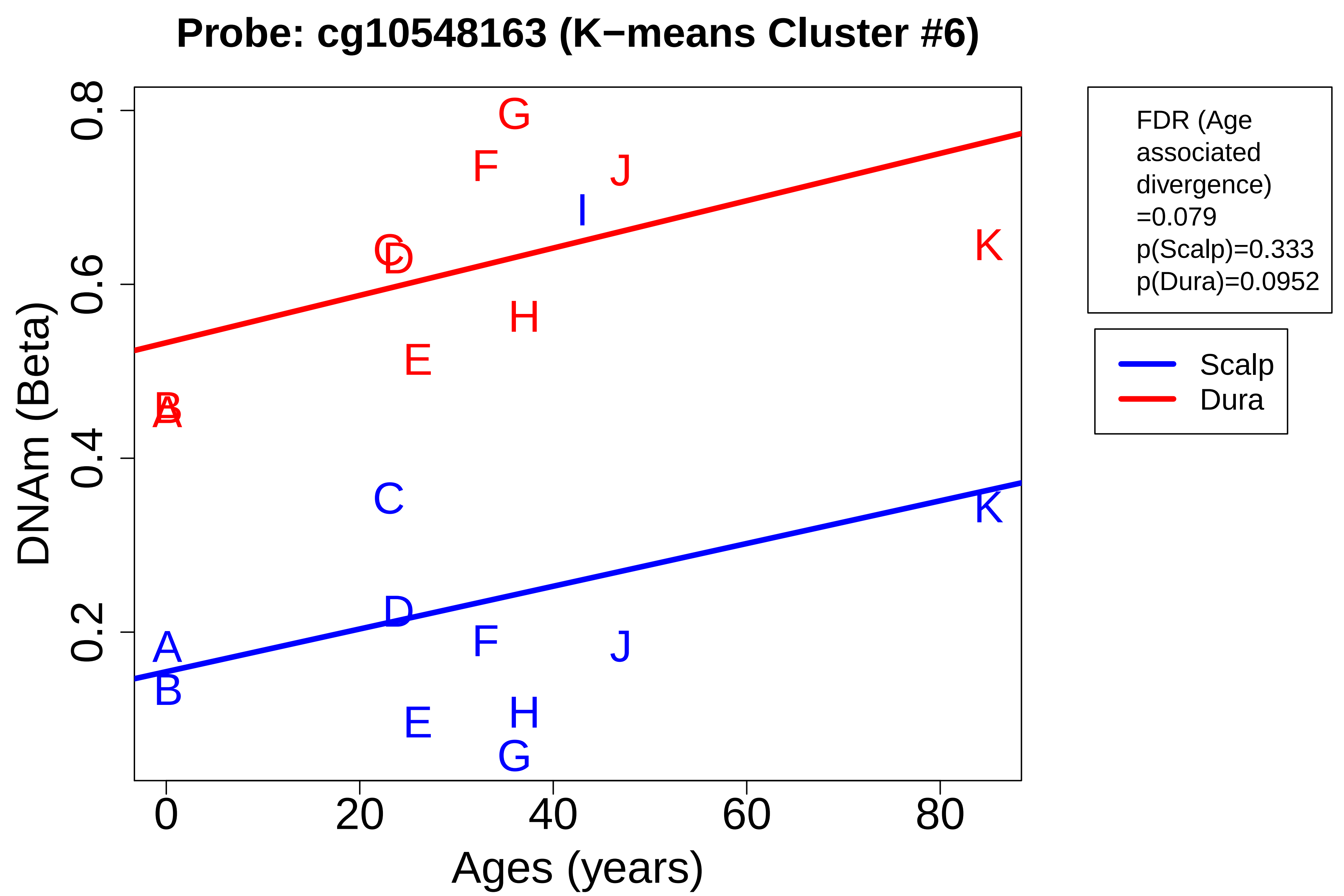

Probe: cg10548163 (K−means Cluster #6)
DNAm (Beta)
0.2
0.4
0.6
0.8
Ages (years)
0
20
40
60
80
FDR (Age associated divergence) =0.079
p(Scalp)=0.333
p(Dura)=0.0952
Scalp
Dura

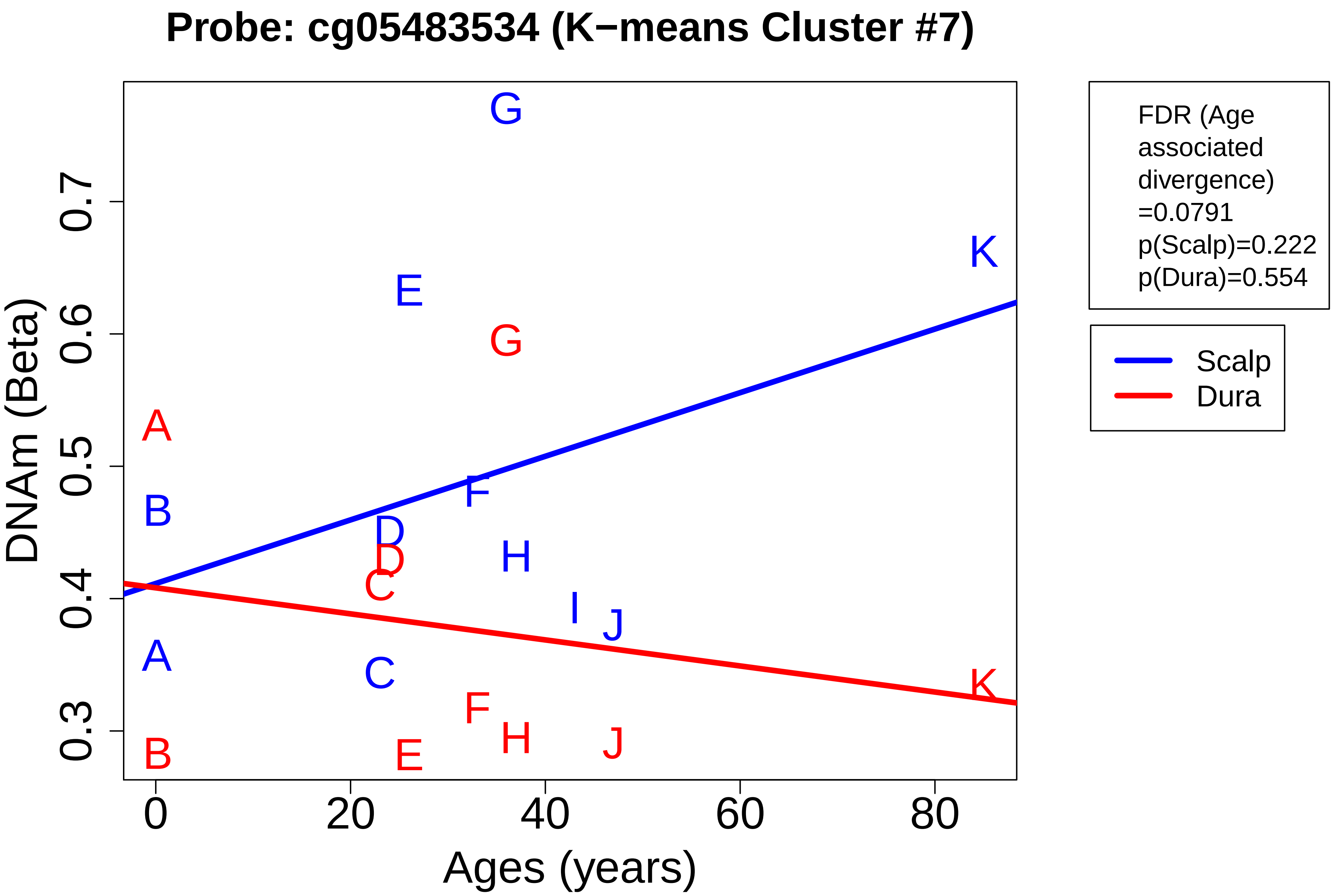
Probe: cg05483534 (K−means Cluster #7)
FDR (Age associated divergence) =0.0791
p(Scalp)=0.222
p(Dura)=0.554
Scalp
Dura
DNAm (Beta)
0.3
0.4
0.5
0.6
0.7
0
20
40
60
80
Ages (years)
A
B
C
D
E
F
G
H
I
J
K

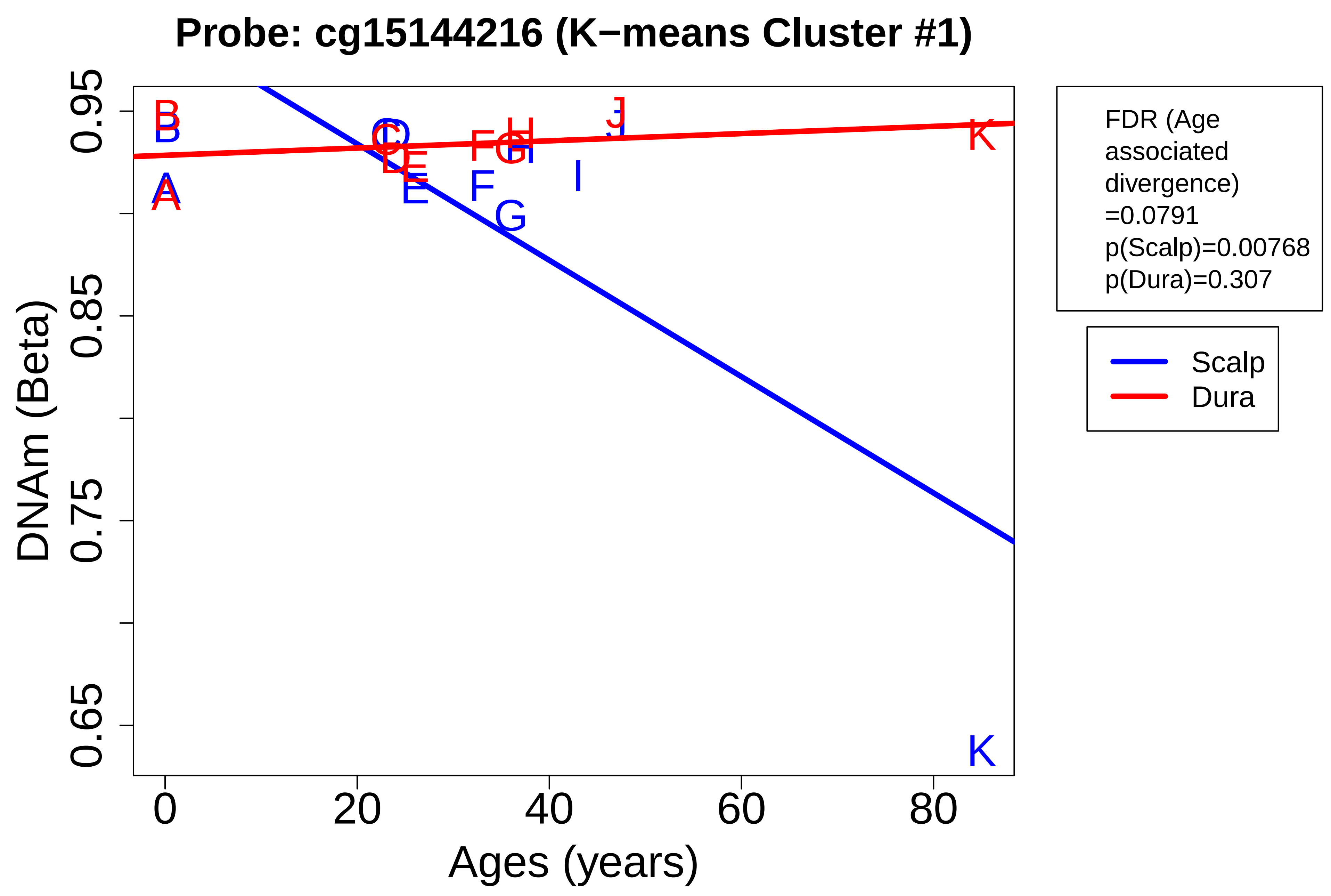

Probe: cg15144216 (K−means Cluster #1)
DNAm (Beta)
0.65
0.75
0.85
0.95
Ages (years)
0
20
40
60
80
FDR (Age associated divergence) =0.0791
p(Scalp)=0.00768
p(Dura)=0.307
Scalp
Dura
A
B
C
D
E
F
G
H
I
J
K

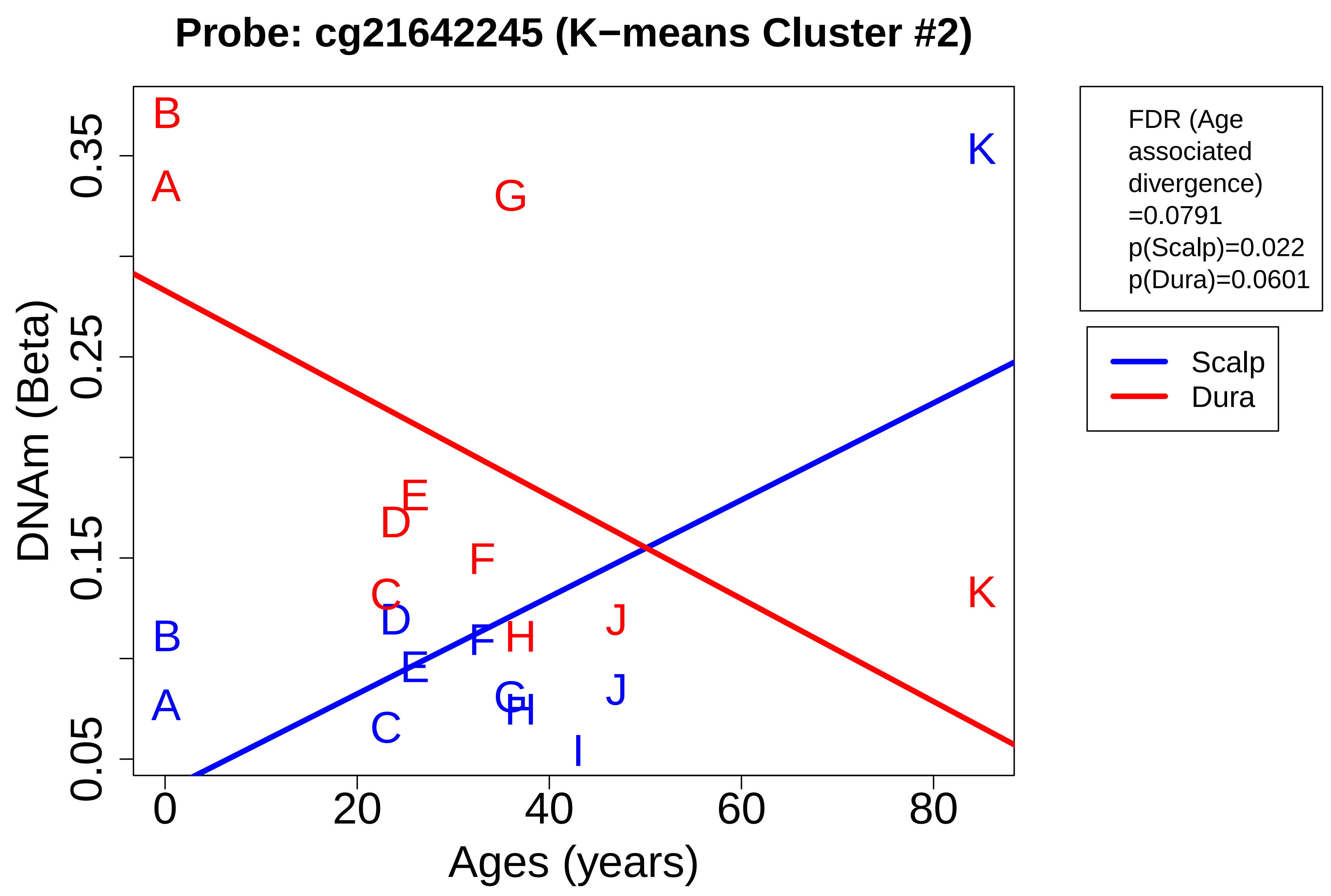

Probe: cg21642245 (K−means Cluster #2)
DNAm (Beta)
0.05
0.15
0.25
0.35
Ages (years)
0
20
40
60
80
FDR (Age associated divergence) =0.0791
p(Scalp)=0.022
p(Dura)=0.0601
Scalp
Dura
A
B
C
D
E
F
G
H
I
J
K

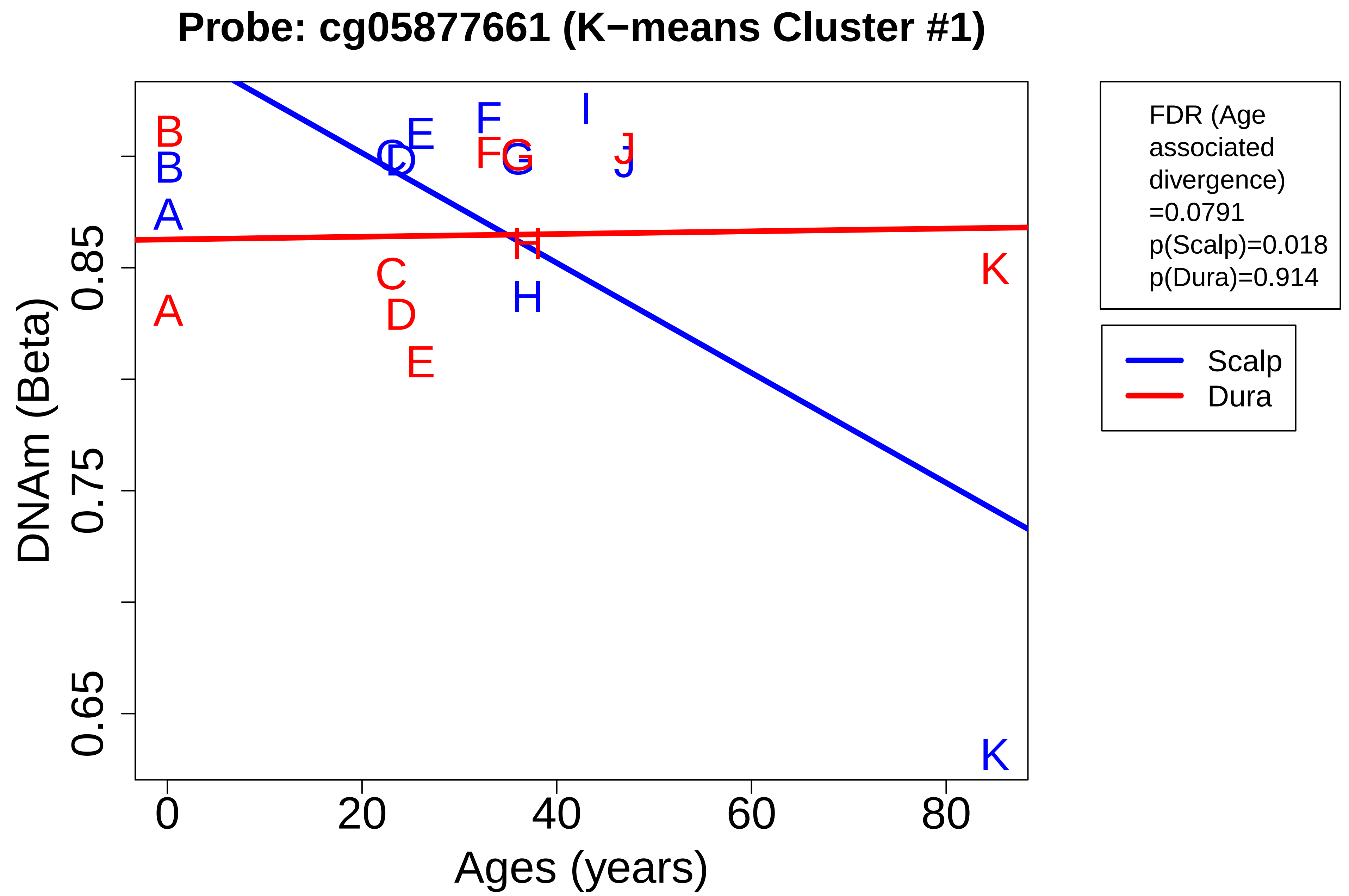

Probe: cg05877661 (K−means Cluster #1)
DNAm (Beta)
0.65
0.75
0.85
Ages (years)
0
20
40
60
80
A
B
C
D
E
F
G
H
I
J
K
FDR (Age associated divergence) =0.0791
p(Scalp)=0.018
p(Dura)=0.914
Scalp
Dura

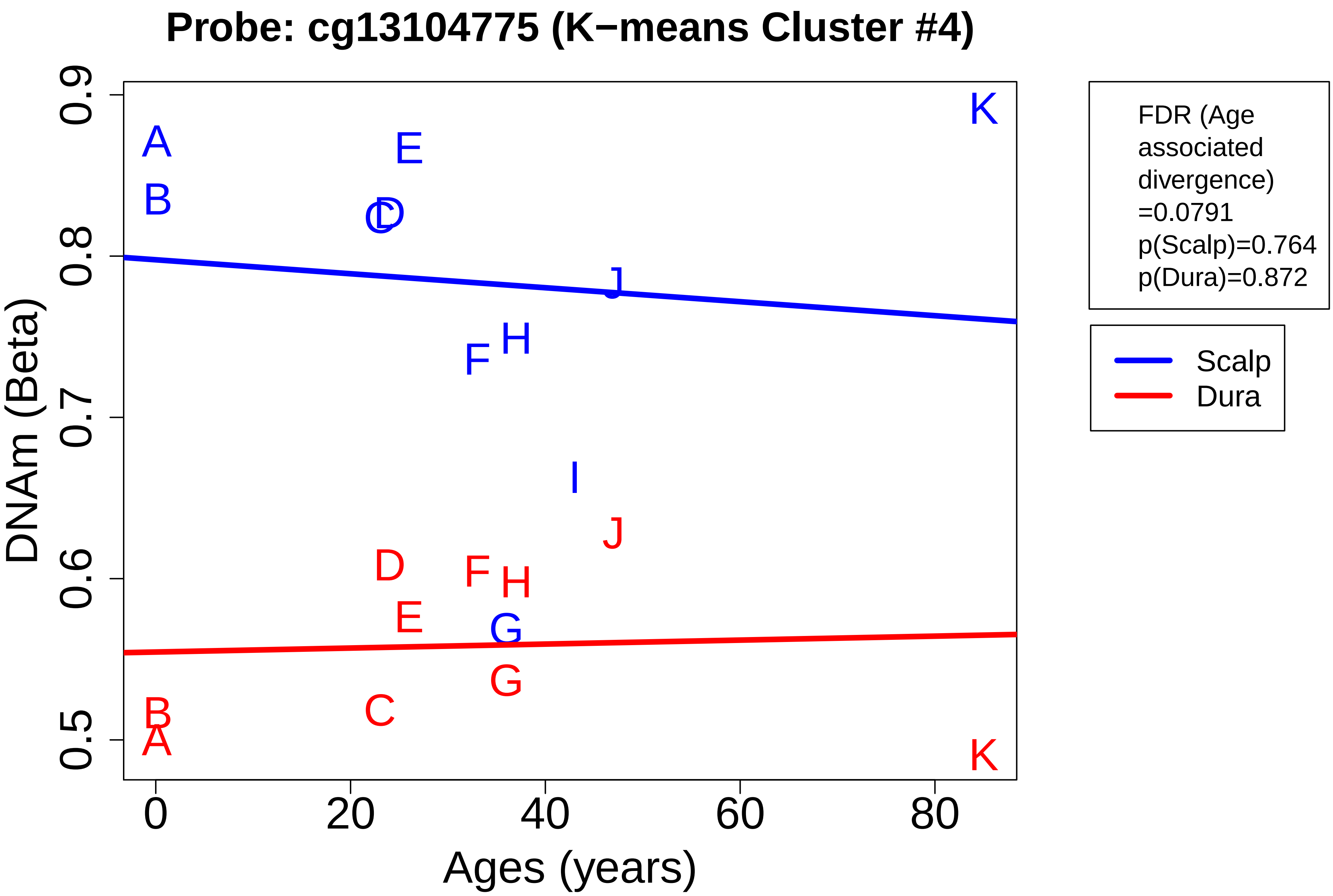

Probe: cg13104775 (K−means Cluster #4)
DNAm (Beta)
Ages (years)
0.5
0.6
0.7
0.8
0.9
0
20
40
60
80
FDR (Age associated divergence) =0.0791
p(Scalp)=0.764
p(Dura)=0.872
Scalp
Dura
A
B
C
D
E
F
G
H
I
J
K

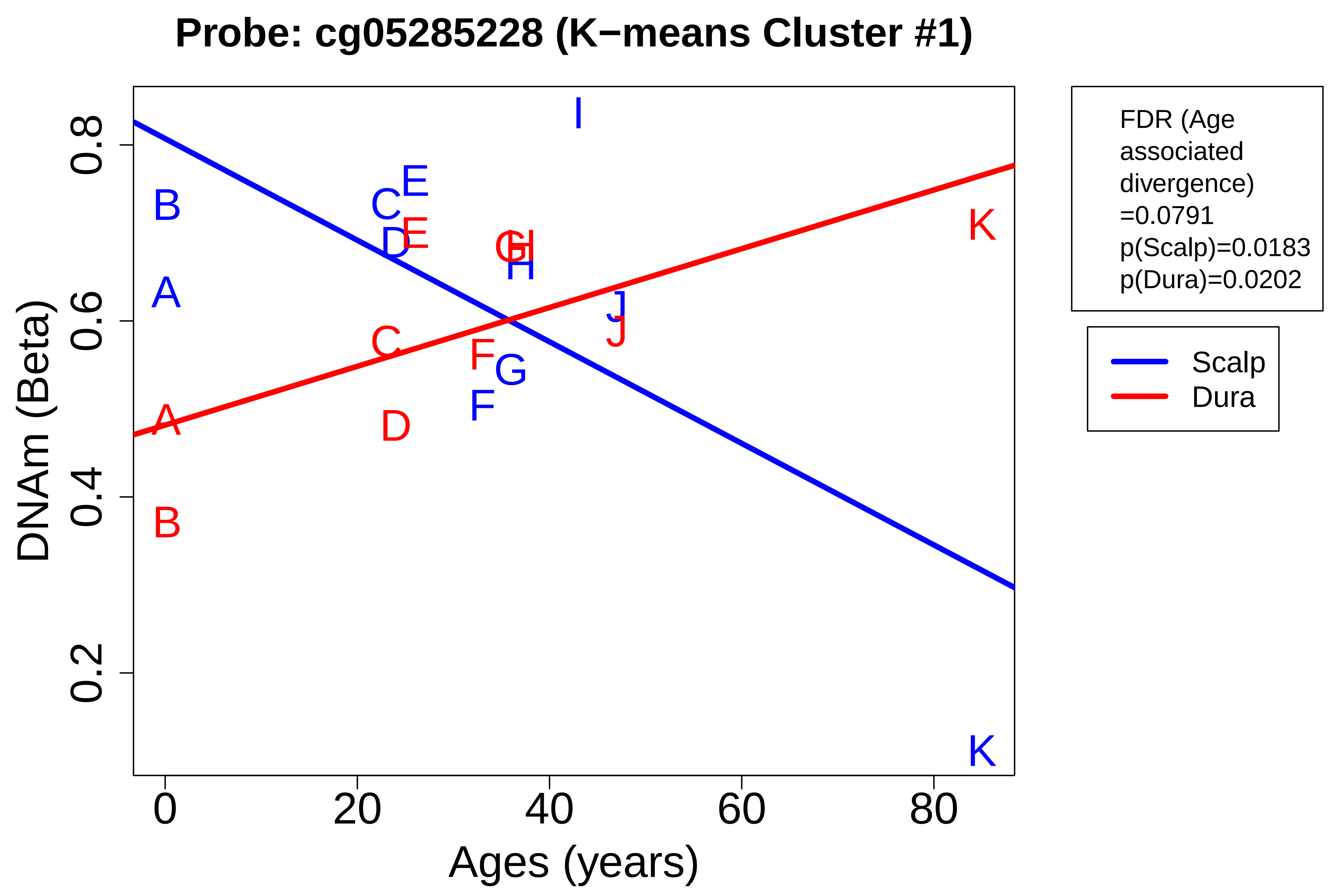
Probe: cg05285228 (K−means Cluster #1)
DNAm (Beta)
0.2
0.4
0.6
0.8
0
20
40
60
80
Ages (years)
FDR (Age associated divergence) =0.0791
p(Scalp)=0.0183
p(Dura)=0.0202
Scalp
Dura
A
B
C
D
E
F
G
H
I
J
K

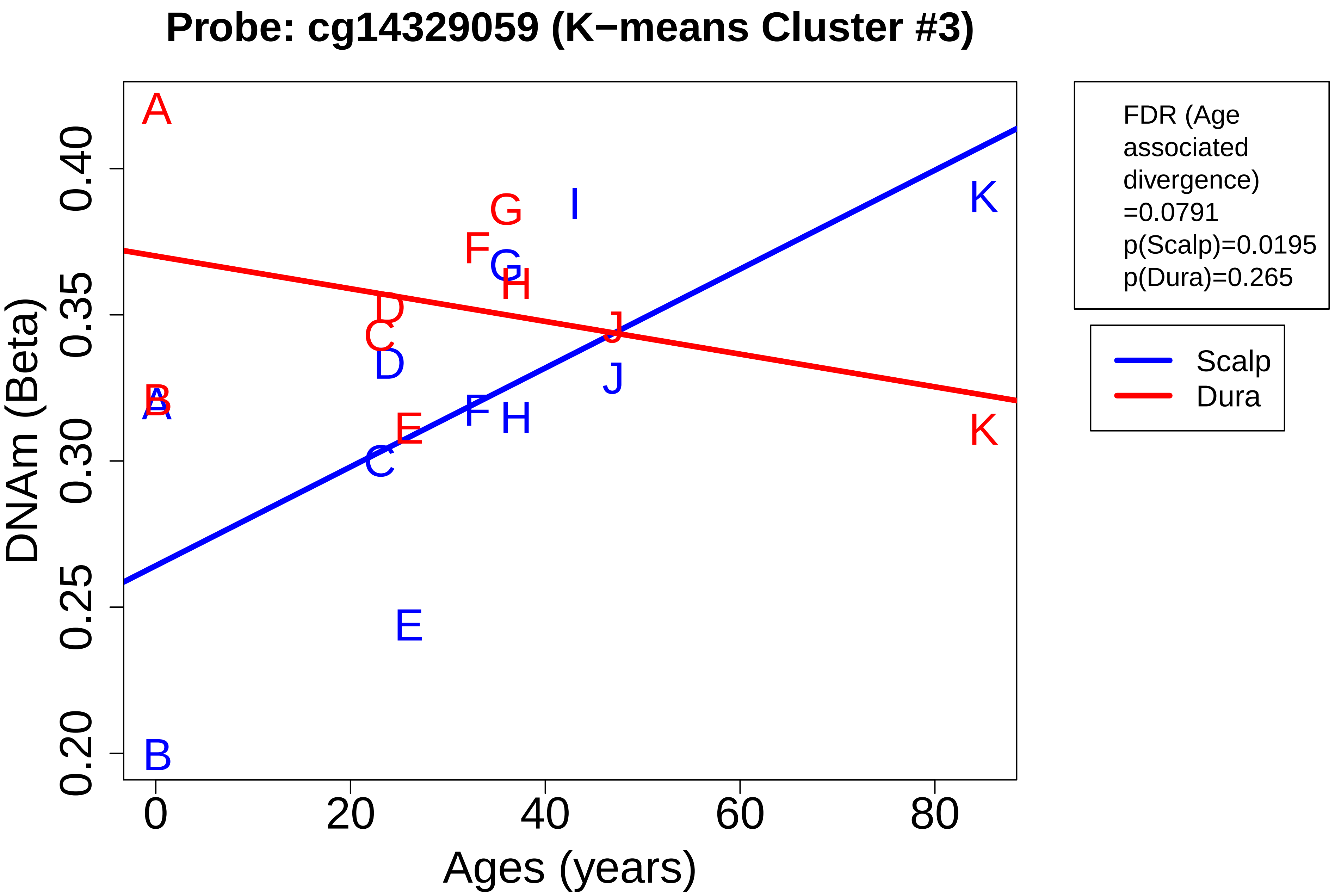

Probe: cg14329059 (K−means Cluster #3)
DNAm (Beta)
0.20
0.25
0.30
0.35
0.40
0
20
40
60
80
Ages (years)
A
B
C
D
E
F
G
H
I
J
K
FDR (Age associated divergence) =0.0791
p(Scalp)=0.0195
p(Dura)=0.265
Scalp
Dura

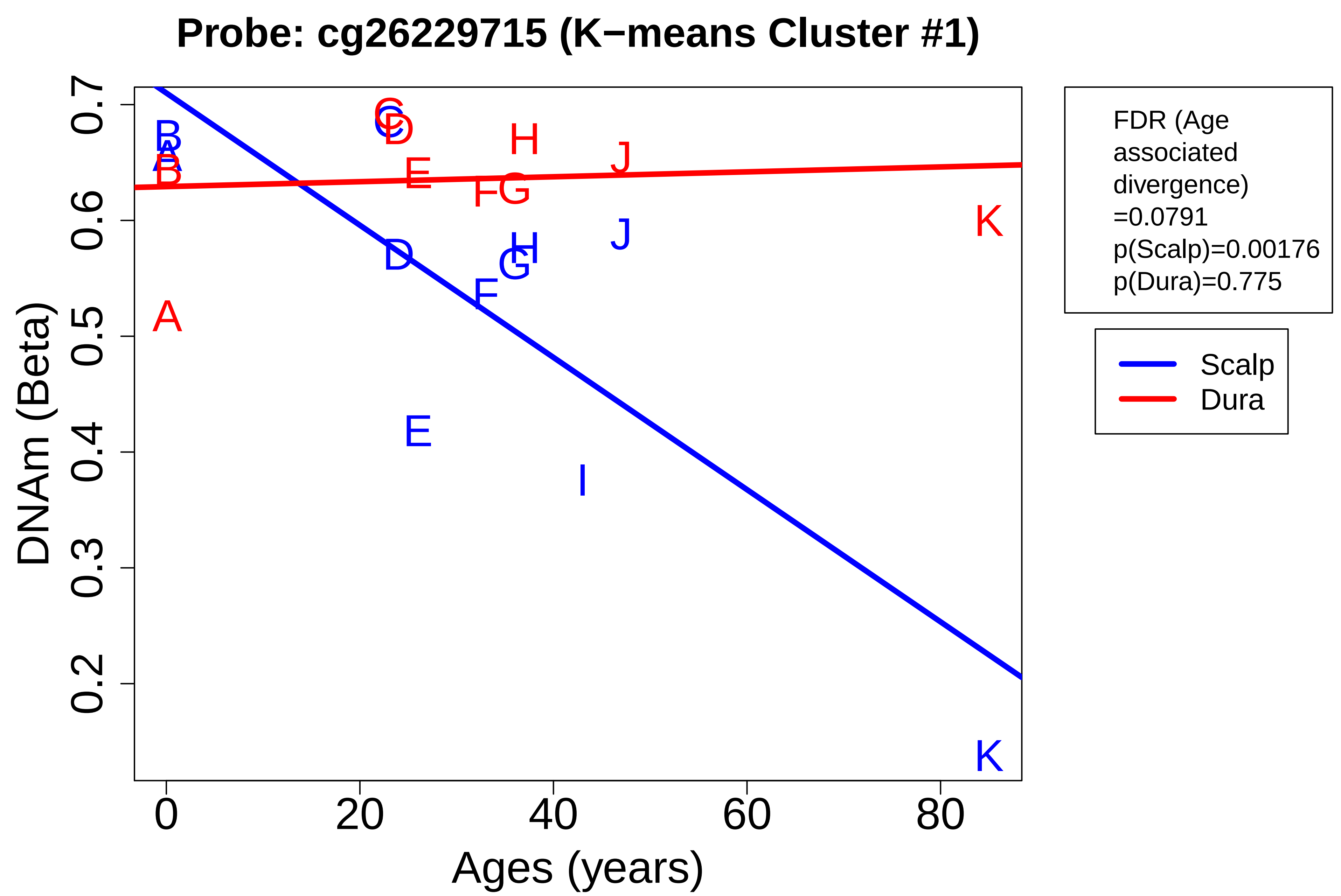

Probe: cg26229715 (K−means Cluster #1)
DNAm (Beta)
0.2
0.3
0.4
0.5
0.6
0.7
Ages (years)
0
20
40
60
80
FDR (Age associated divergence) =0.0791
p(Scalp)=0.00176
p(Dura)=0.775
Scalp
Dura

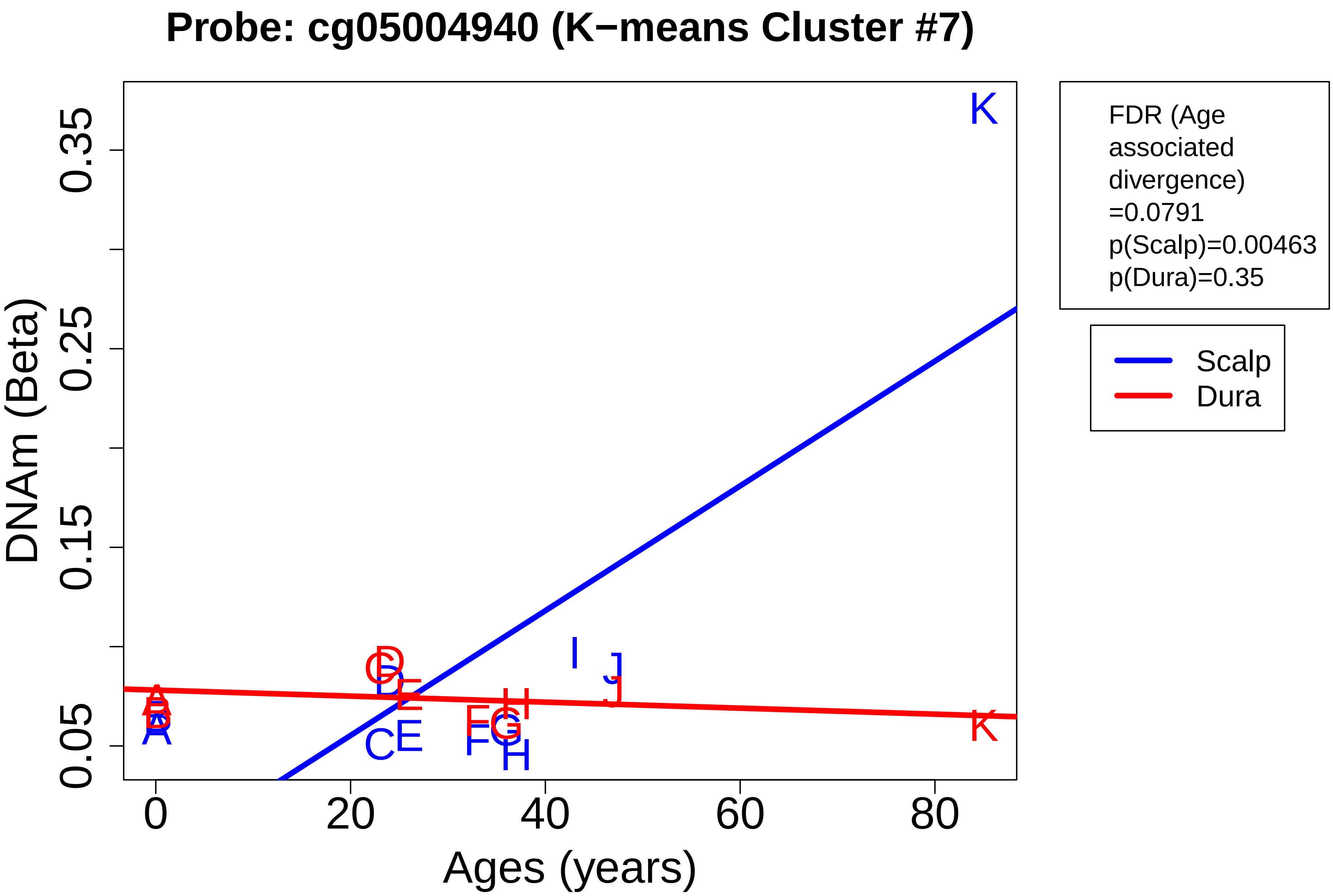

Probe: cg05004940 (K−means Cluster #7)
DNAm (Beta)
0.05
0.15
0.25
0.35
Ages (years)
0
20
40
60
80
FDR (Age associated divergence) =0.0791
p(Scalp)=0.00463
p(Dura)=0.35
Scalp
Dura

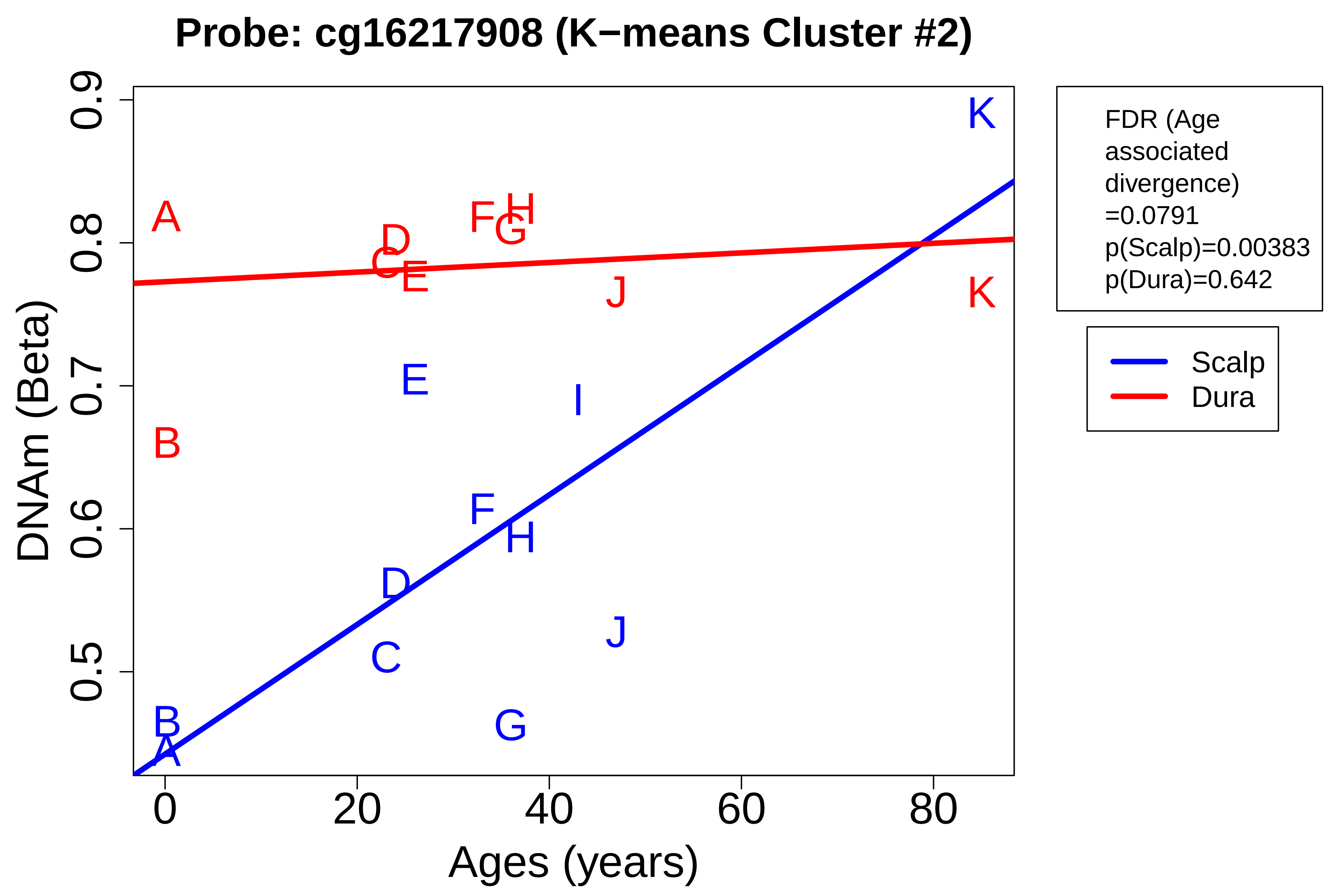

Probe: cg16217908 (K−means Cluster #2)
DNAm (Beta)
0.5
0.6
0.7
0.8
0.9
0
20
40
60
80
Ages (years)
A
B
C
D
E
F
G
H
I
J
K
FDR (Age associated divergence) =0.0791
p(Scalp)=0.00383
p(Dura)=0.642
Scalp
Dura

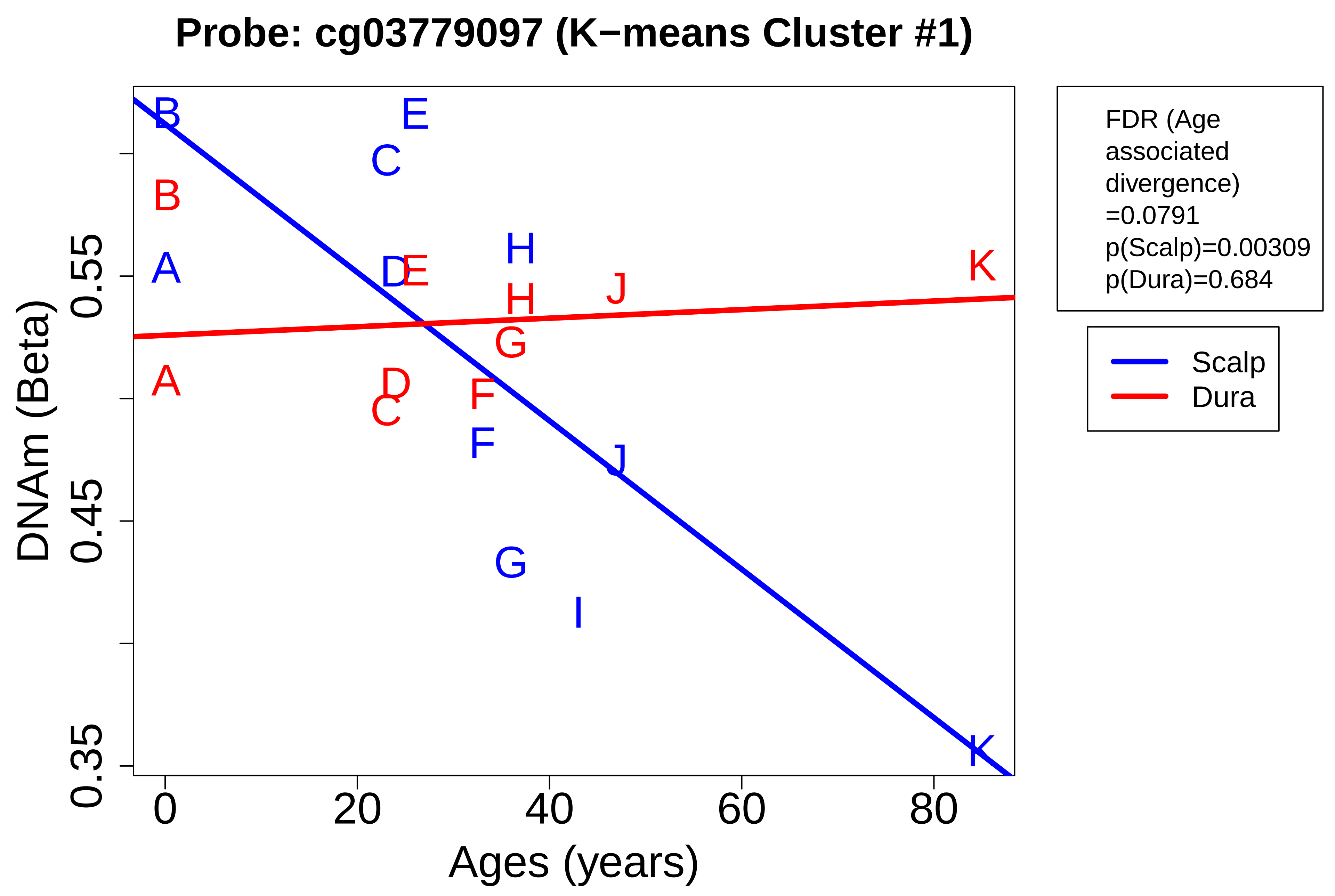

Probe: cg03779097 (K−means Cluster #1)
DNAm (Beta)
0.35
0.45
0.55
Ages (years)
0
20
40
60
80
FDR (Age associated divergence) =0.0791
p(Scalp)=0.00309
p(Dura)=0.684
Scalp
Dura

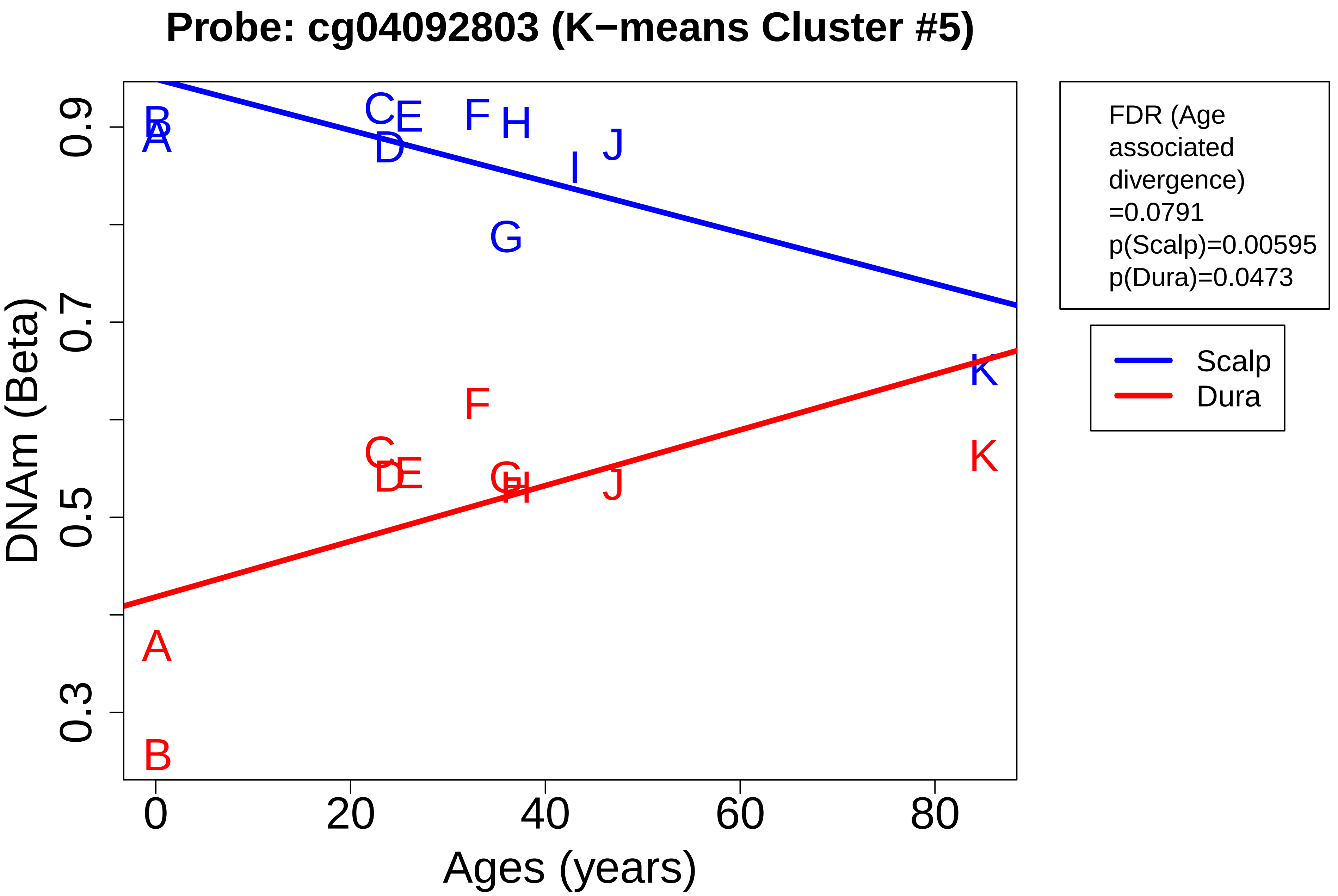

Probe: cg04092803 (K−means Cluster #5)
DNAm (Beta)
0.3
0.5
0.7
0.9
Ages (years)
0
20
40
60
80
FDR (Age associated divergence) =0.0791
p(Scalp)=0.00595
p(Dura)=0.0473
Scalp
Dura
A
B
C
D
E
F
G
H
I
J
K

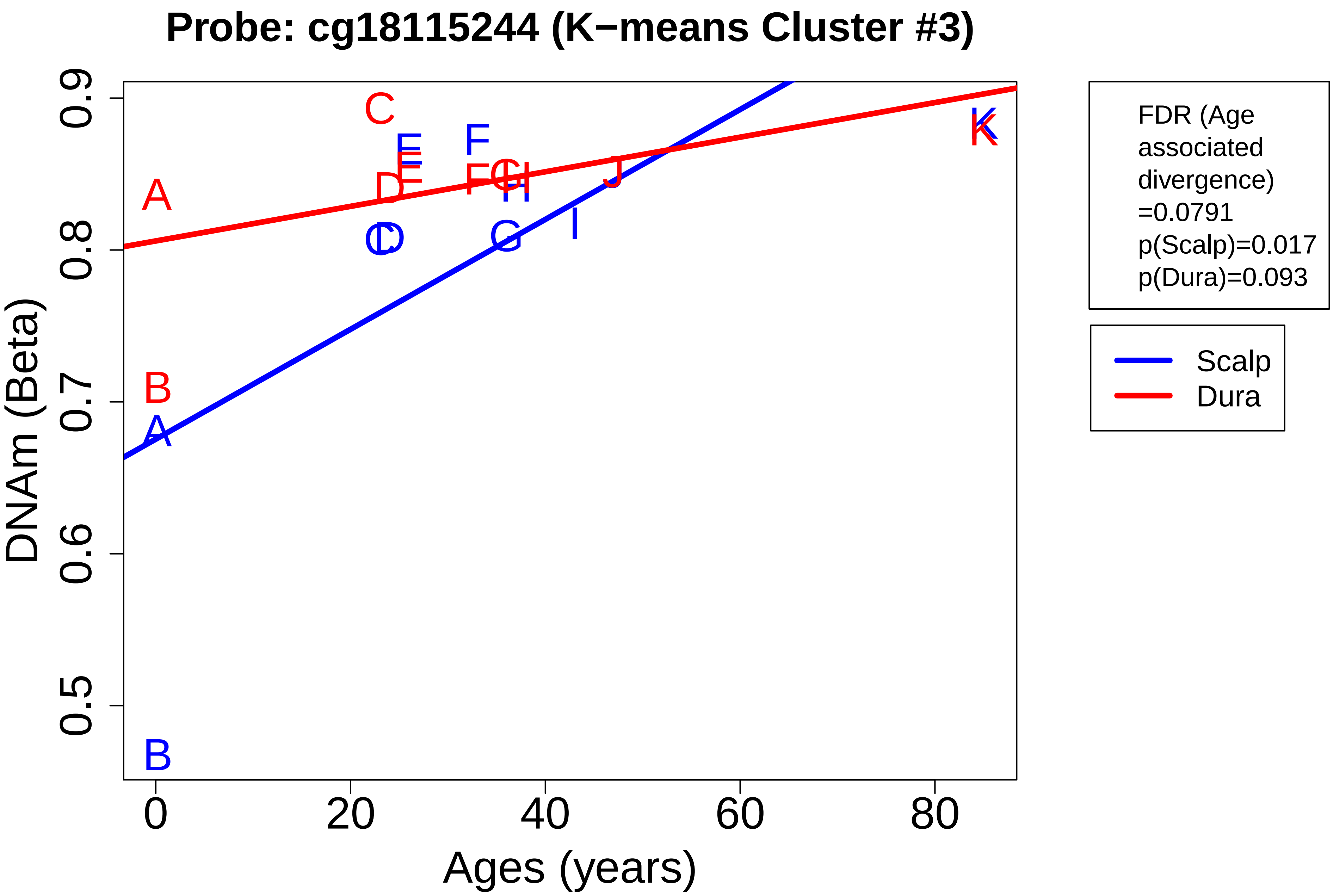

Probe: cg18115244 (K−means Cluster #3)
DNAm (Beta)
Ages (years)
0.5
0.6
0.7
0.8
0.9
0
20
40
60
80
FDR (Age associated divergence) =0.0791
p(Scalp)=0.017
p(Dura)=0.093
Scalp
Dura
A
B
C
D
E
F
G
H
I
J
K

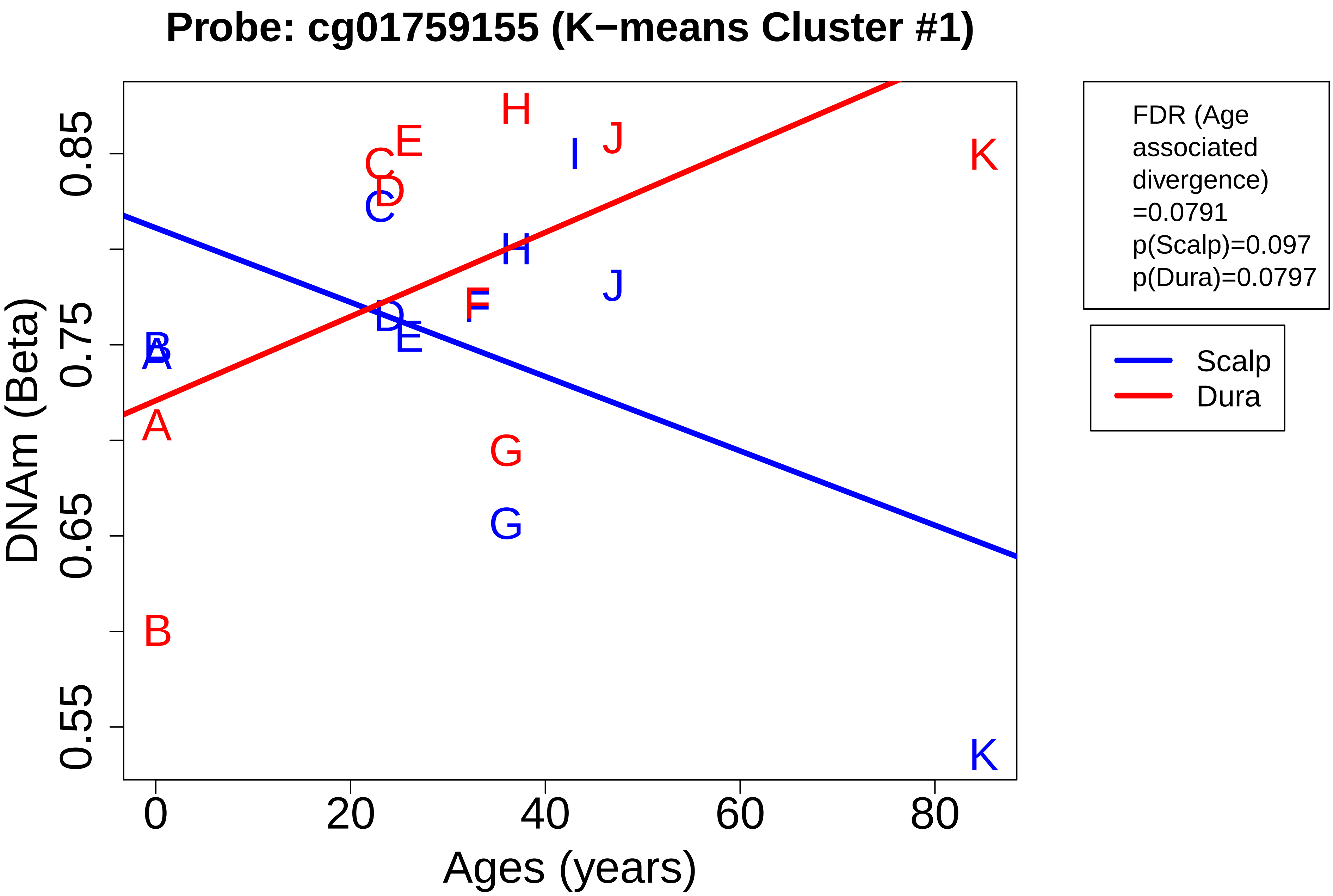

Probe: cg01759155 (K−means Cluster #1)
DNAm (Beta)
0.55
0.65
0.75
0.85
0
20
40
60
80
Ages (years)
FDR (Age associated divergence) =0.0791
p(Scalp)=0.097
p(Dura)=0.0797
Scalp
Dura

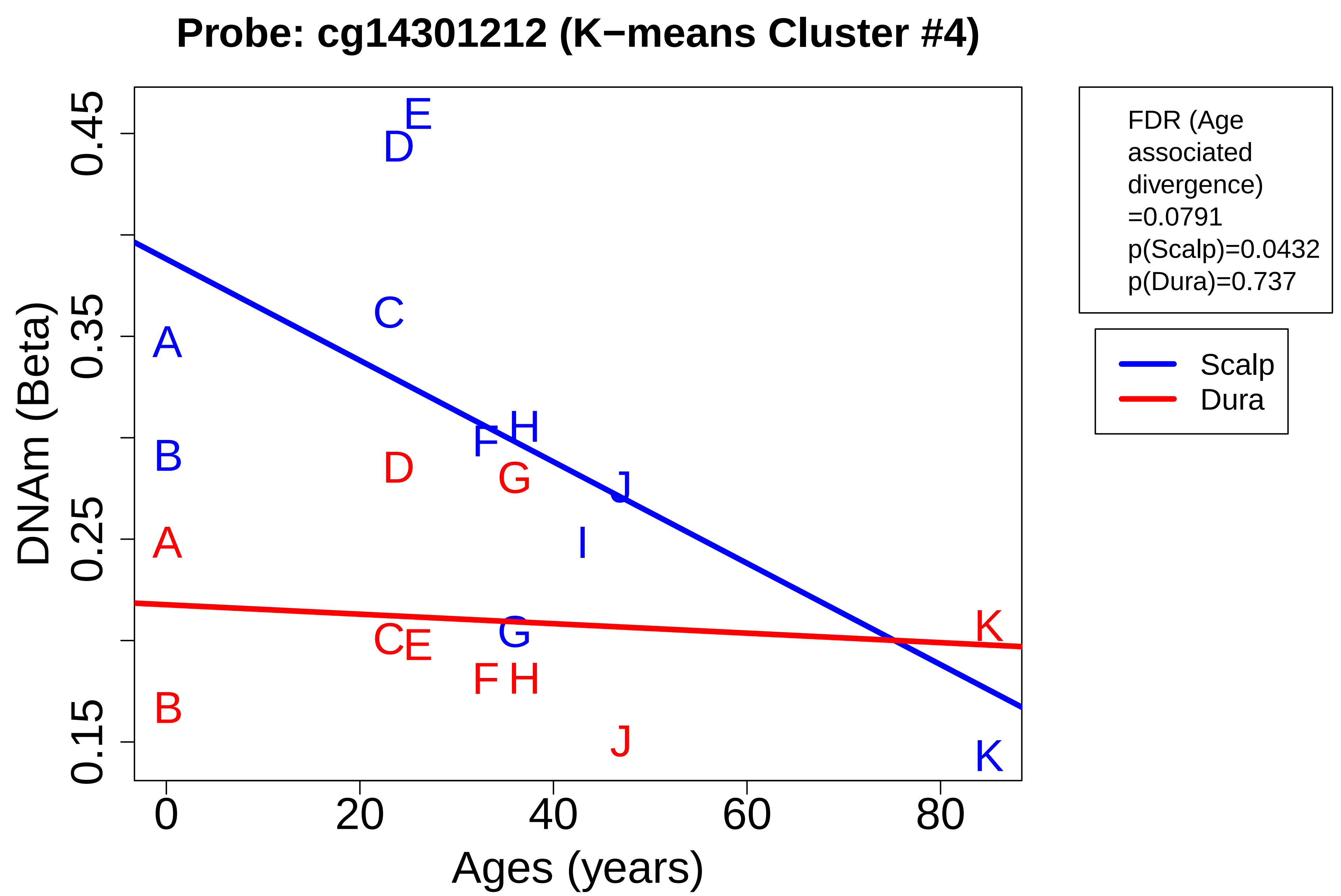

Probe: cg14301212 (K−means Cluster #4)
DNAm (Beta)
0.15
0.25
0.35
0.45
Ages (years)
0
20
40
60
80
A
B
C
D
E
F
G
H
I
J
K
A
B
C
D
E
F
G
H
J
K
FDR (Age associated divergence) =0.0791
p(Scalp)=0.0432
p(Dura)=0.737
Scalp
Dura

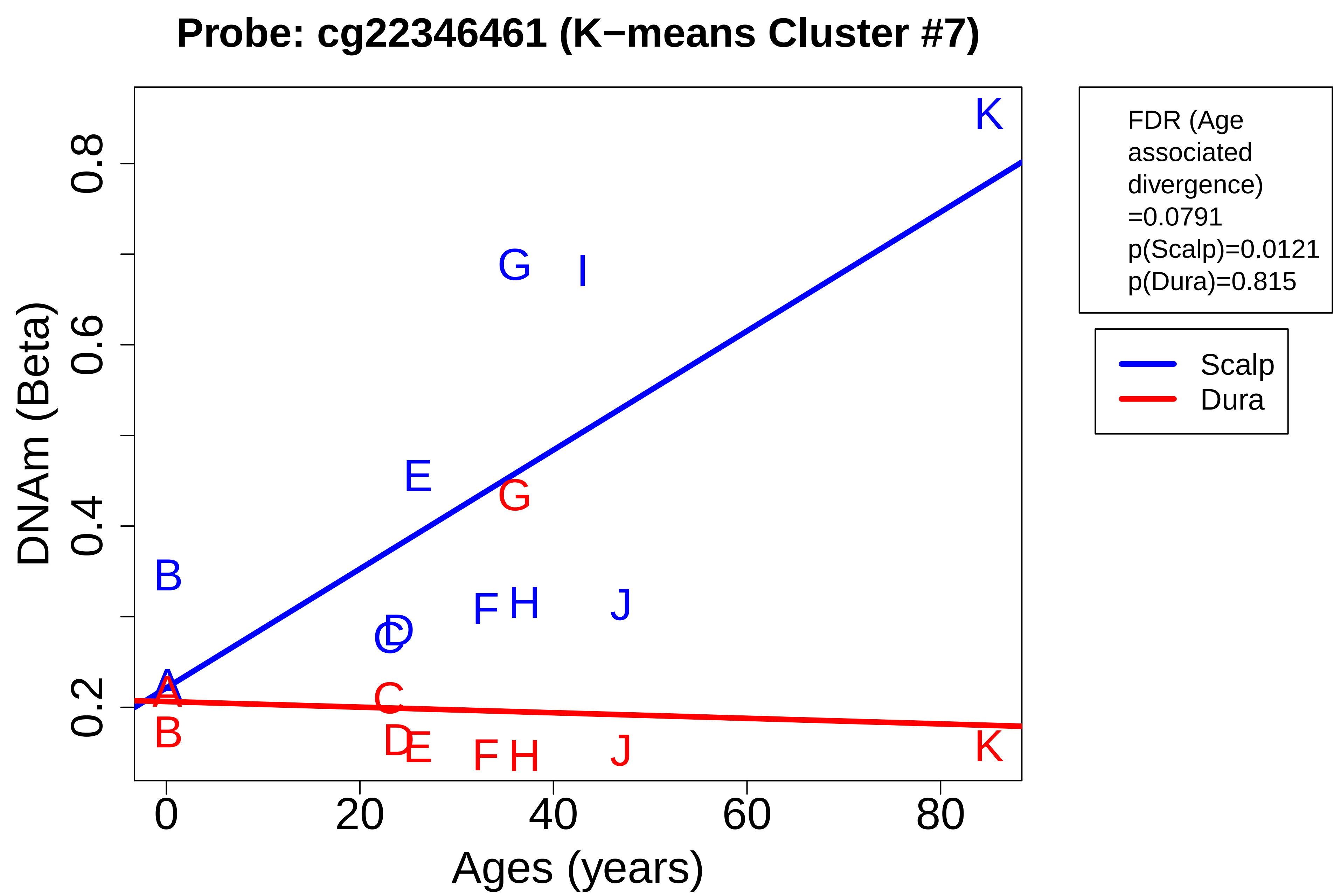

Probe: cg22346461 (K−means Cluster #7)
DNAm (Beta)
0.2
0.4
0.6
0.8
Ages (years)
0
20
40
60
80
FDR (Age associated divergence) =0.0791
p(Scalp)=0.0121
p(Dura)=0.815
Scalp
Dura
A B C D E F G H I J K
A B C D E F G H J K

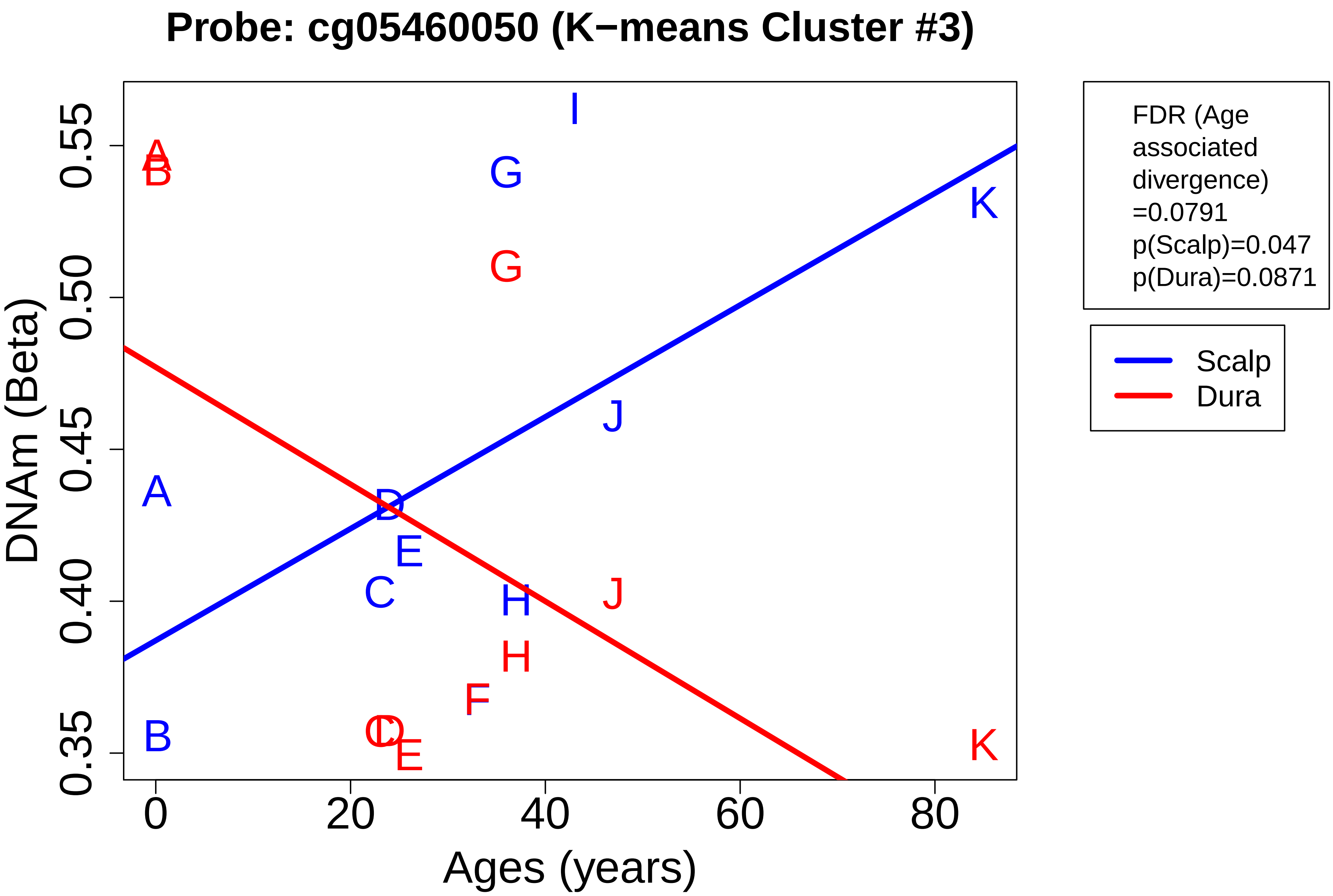

Probe: cg05460050 (K−means Cluster #3)
DNAm (Beta)
0.35
0.40
0.45
0.50
0.55
Ages (years)
0
20
40
60
80
FDR (Age associated divergence) =0.0791
p(Scalp)=0.047
p(Dura)=0.0871
Scalp
Dura

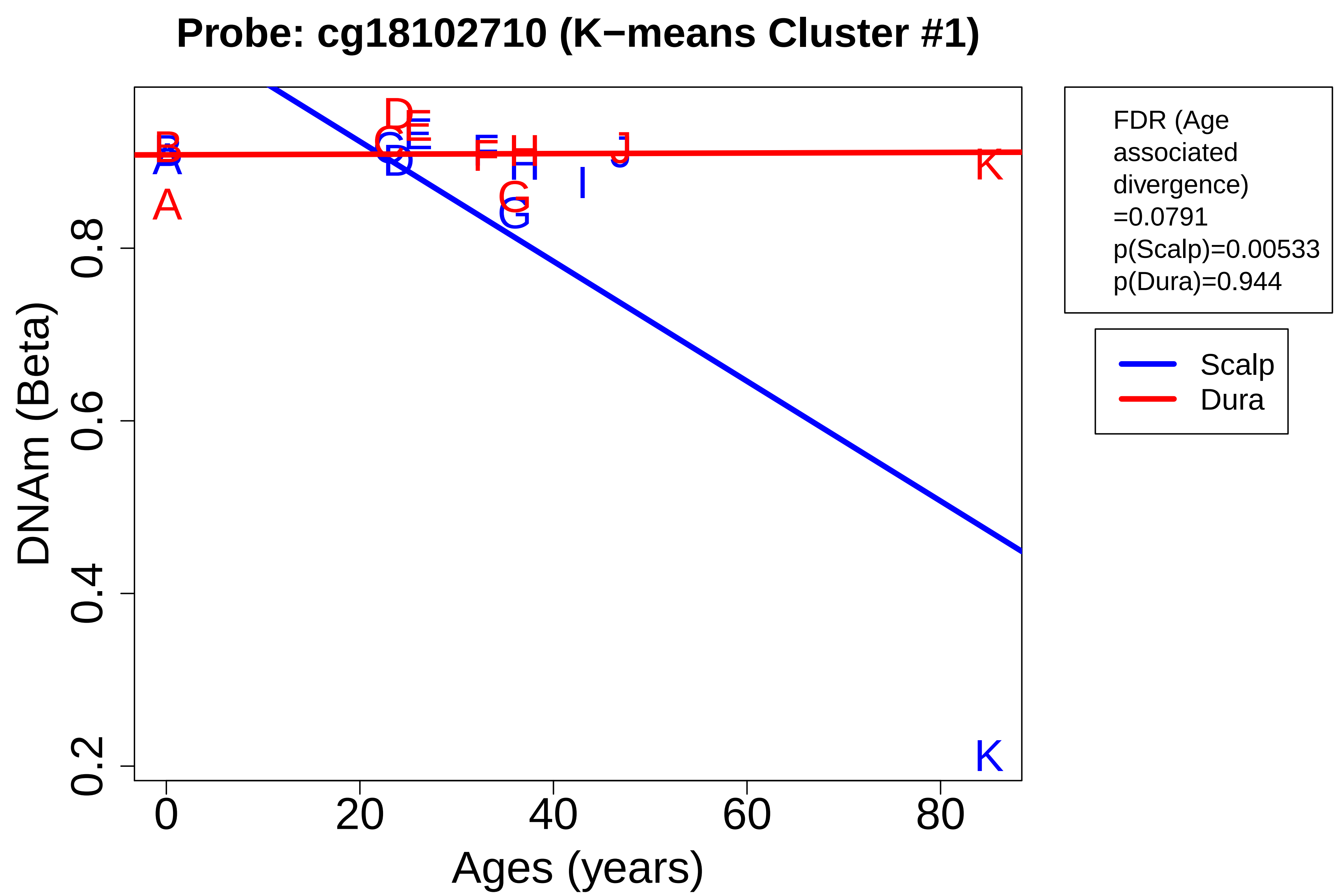

Probe: cg18102710 (K−means Cluster #1)
DNAm (Beta)
0.2
0.4
0.6
0.8
0
20
40
60
80
Ages (years)
A
B
C
D
E
F
G
H
I
J
K
FDR (Age associated divergence) =0.0791
p(Scalp)=0.00533
p(Dura)=0.944
Scalp
Dura

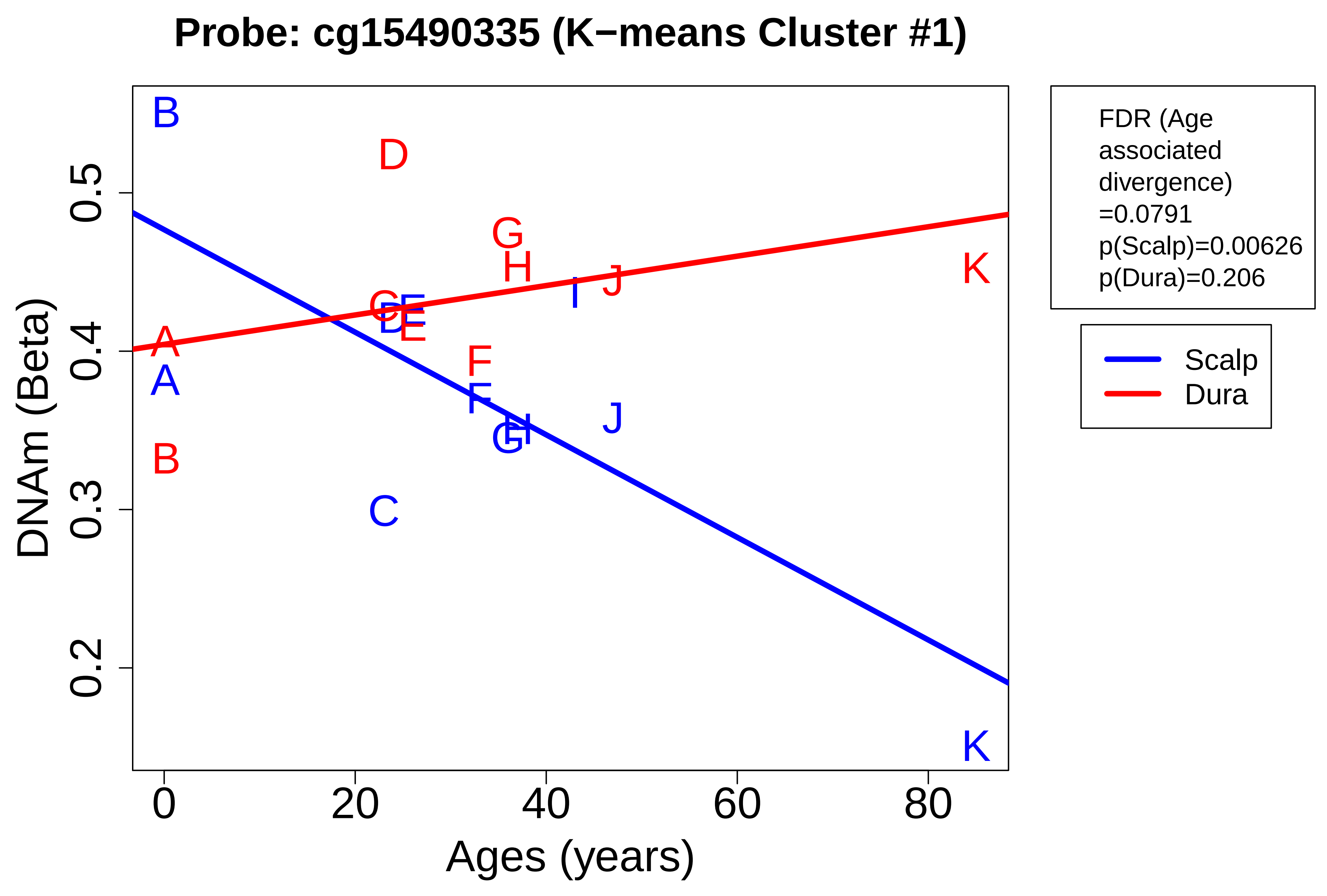

Probe: cg15490335 (K−means Cluster #1)
DNAm (Beta)
0.2
0.3
0.4
0.5
Ages (years)
0
20
40
60
80
FDR (Age associated divergence) =0.0791
p(Scalp)=0.00626
p(Dura)=0.206
Scalp
Dura

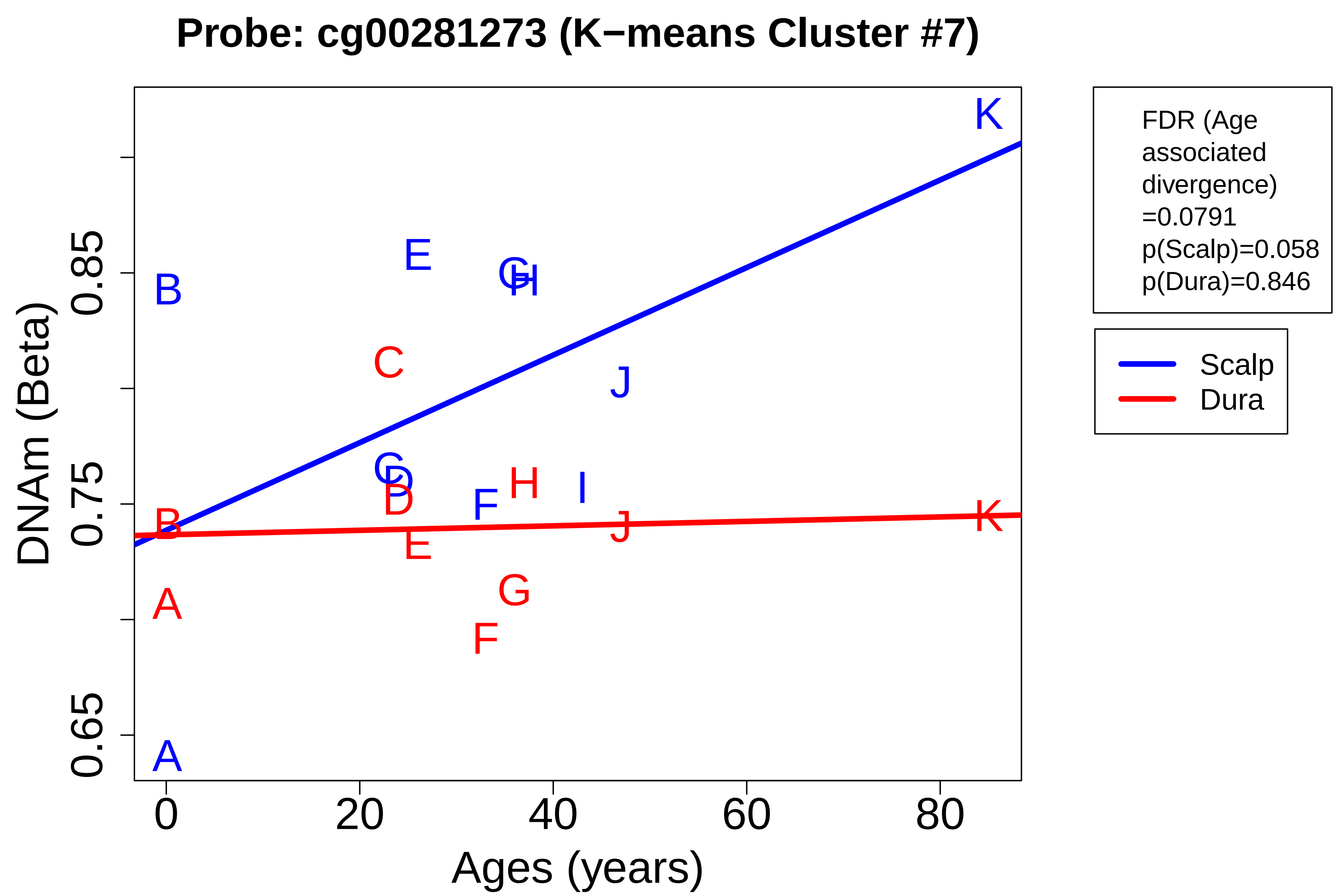

Probe: cg00281273 (K−means Cluster #7)
DNAm (Beta)
0.65
0.75
0.85
0
20
40
60
80
Ages (years)
A
B
C
D
E
F
G
H
I
J
K
FDR (Age associated divergence) =0.0791
p(Scalp)=0.058
p(Dura)=0.846
Scalp
Dura

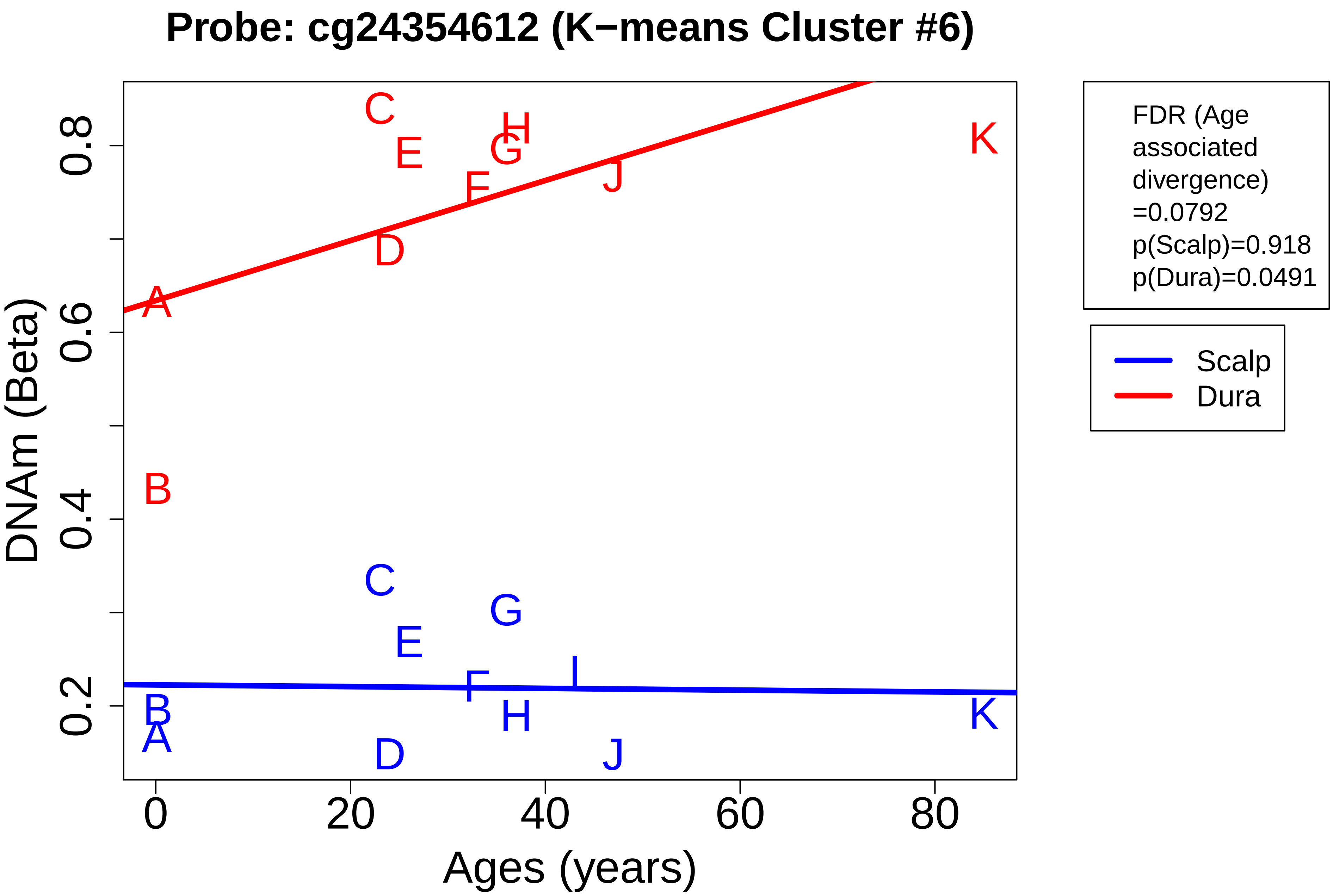

Probe: cg24354612 (K−means Cluster #6)
DNAm (Beta)
0.2
0.4
0.6
0.8
0
20
40
60
80
Ages (years)
FDR (Age associated divergence) =0.0792
p(Scalp)=0.918
p(Dura)=0.0491
Scalp
Dura

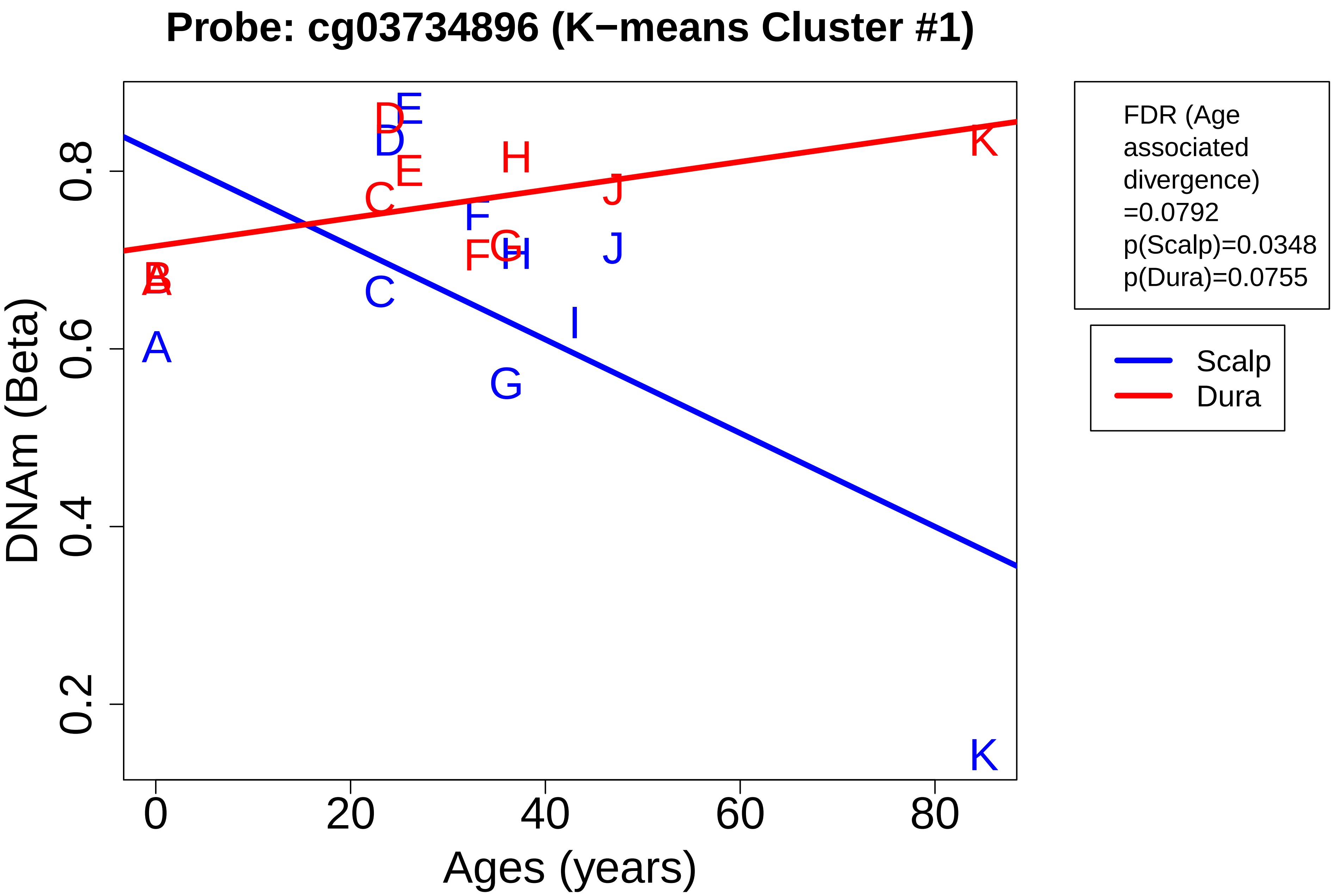
Probe: cg03734896 (K−means Cluster #1)
DNAm (Beta)
0.2
0.4
0.6
0.8
0
20
40
60
80
Ages (years)
A
B
C
D
E
F
G
H
I
J
K
FDR (Age associated divergence) =0.0792
p(Scalp)=0.0348
p(Dura)=0.0755
Scalp
Dura

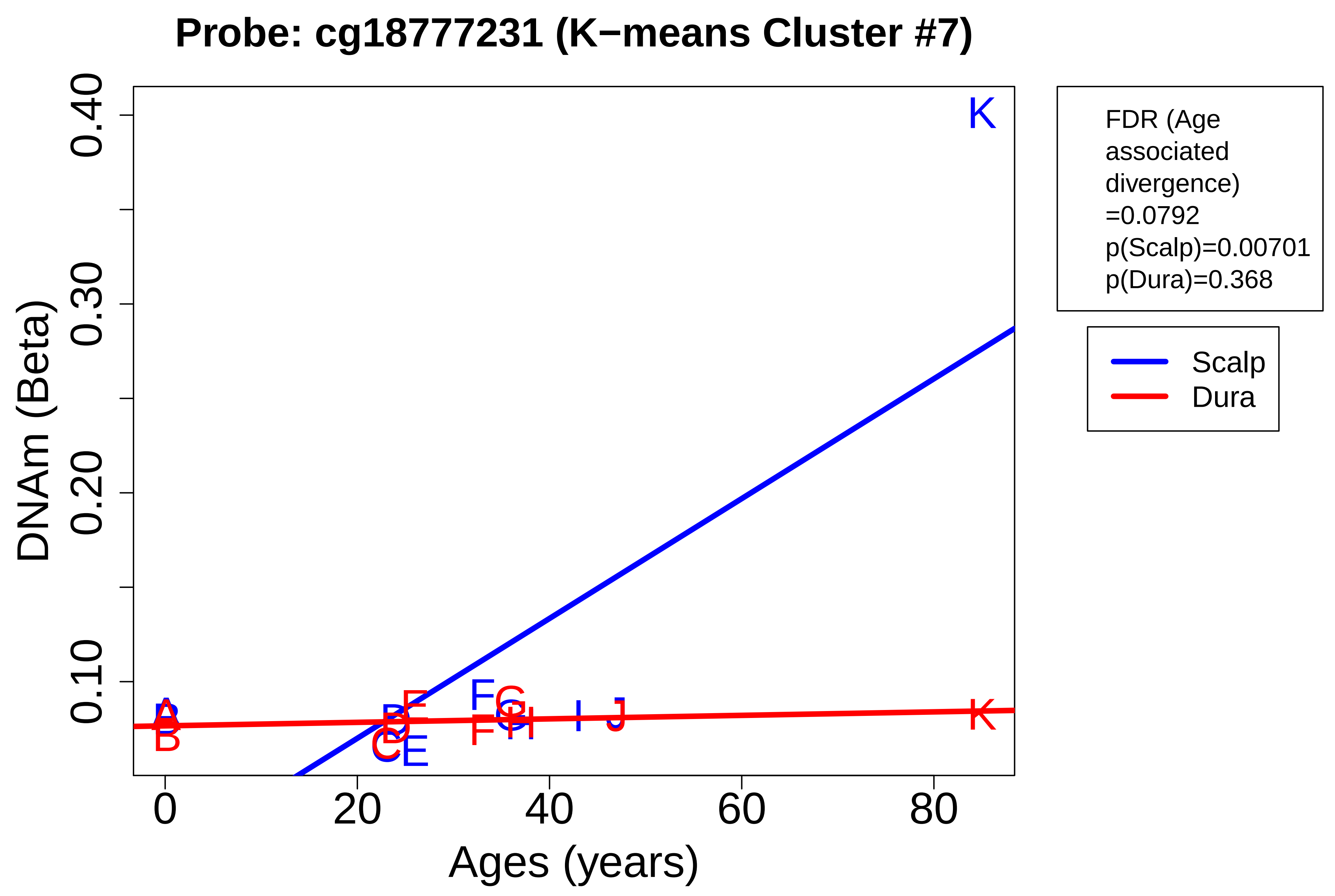

Probe: cg18777231 (K−means Cluster #7)
DNAm (Beta)
0.10
0.20
0.30
0.40
0
20
40
60
80
Ages (years)
FDR (Age associated divergence) =0.0792
p(Scalp)=0.00701
p(Dura)=0.368
Scalp
Dura

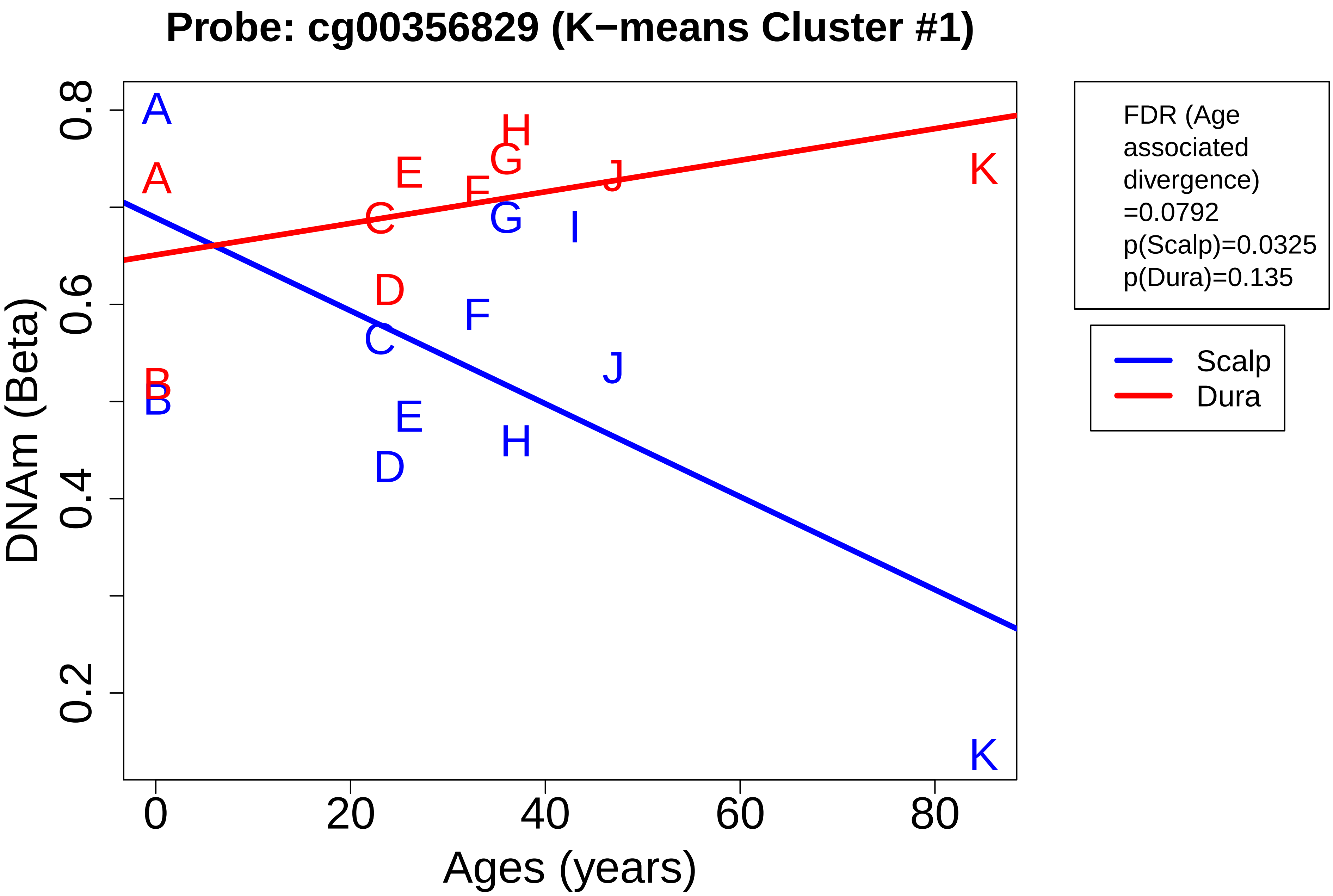

Probe: cg00356829 (K−means Cluster #1)
DNAm (Beta)
0.2
0.4
0.6
0.8
0
20
40
60
80
Ages (years)
A
B
C
D
E
F
G
H
I
J
K
A
B
C
D
E
F
G
H
J
K
FDR (Age associated divergence) =0.0792
p(Scalp)=0.0325
p(Dura)=0.135
Scalp
Dura

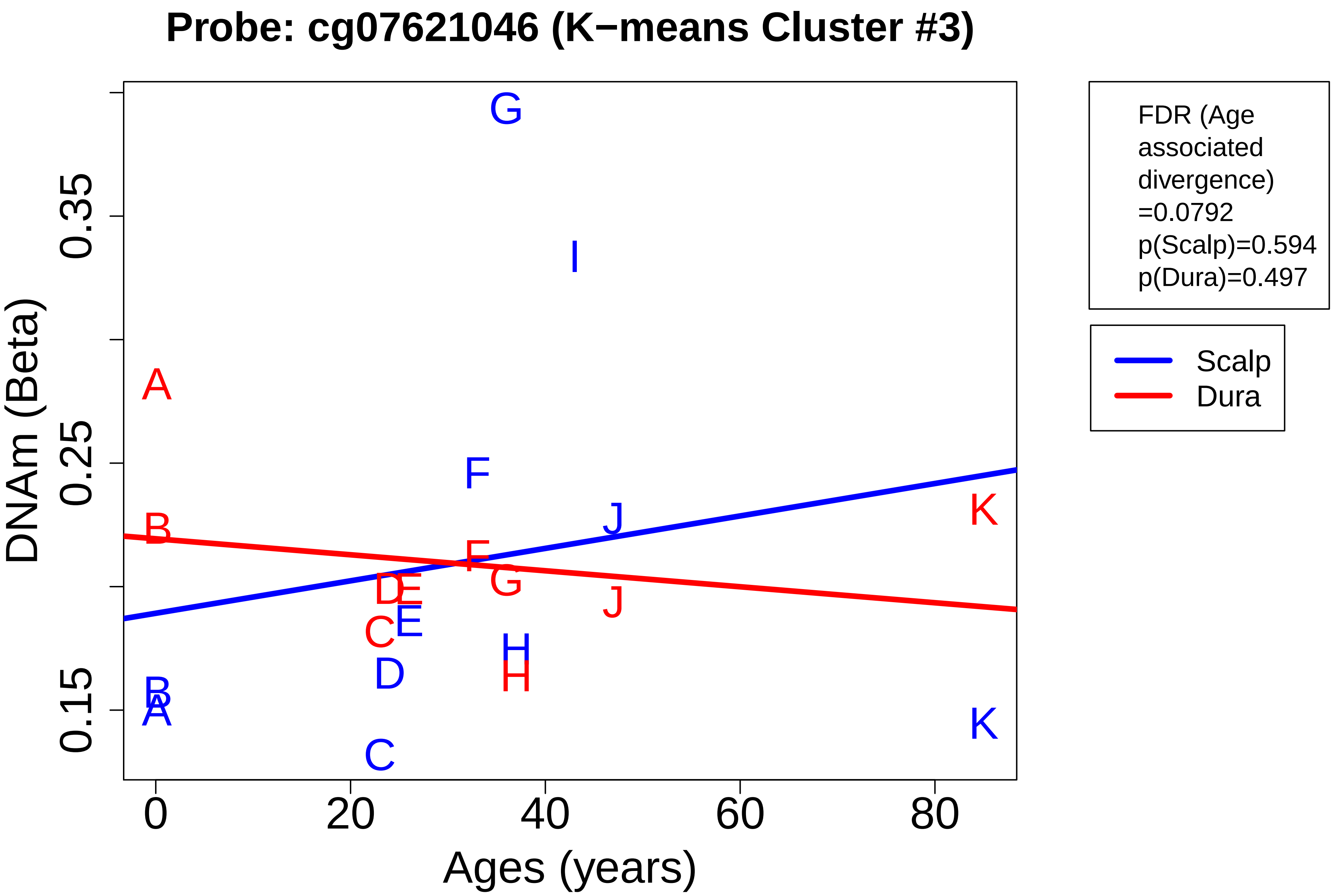

Probe: cg07621046 (K−means Cluster #3)
DNAm (Beta)
0.15
0.25
0.35
Ages (years)
0
20
40
60
80
FDR (Age associated divergence) =0.0792
p(Scalp)=0.594
p(Dura)=0.497
Scalp
Dura
A
B
C
D
E
F
G
H
I
J
K

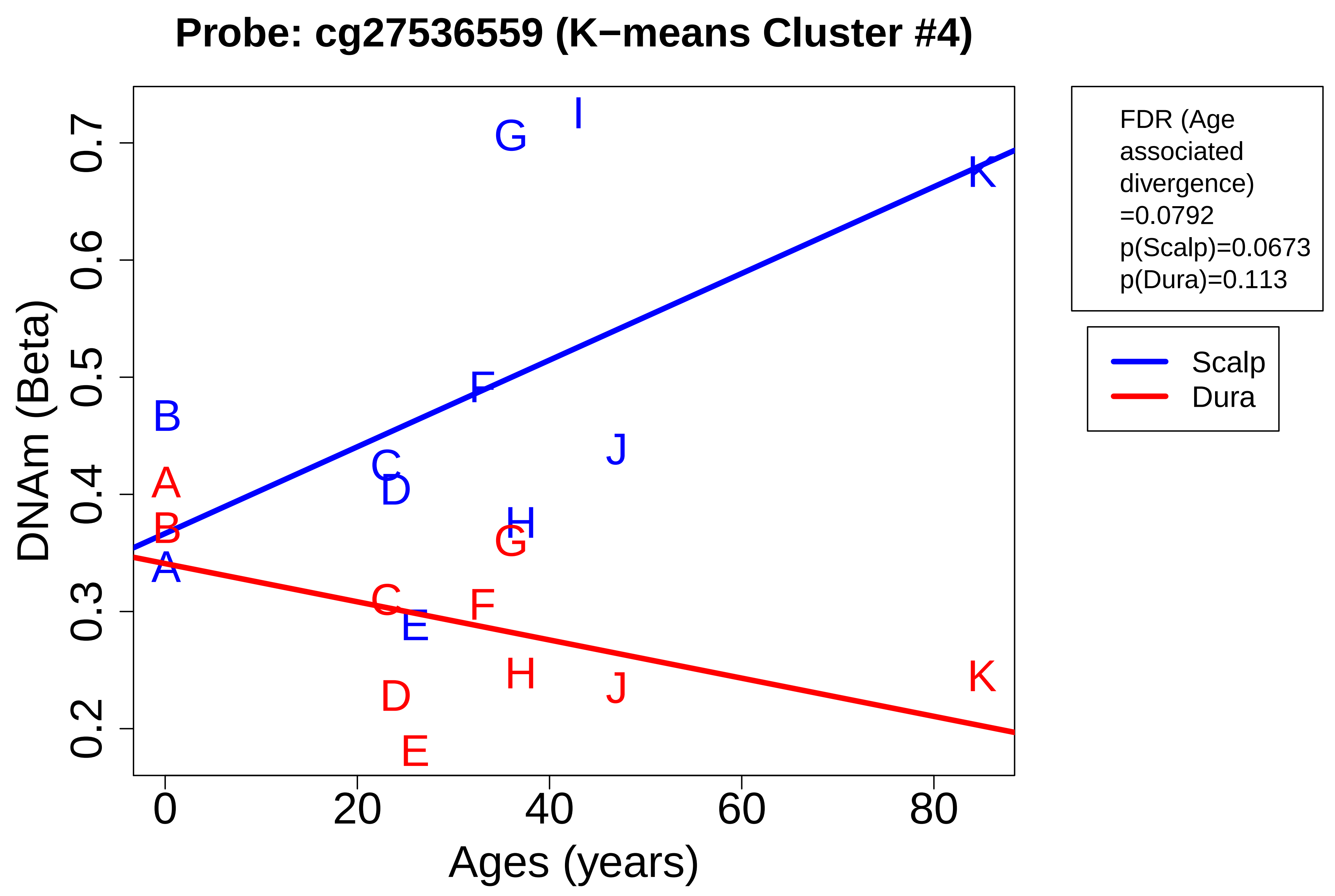
Probe: cg27536559 (K−means Cluster #4)
DNAm (Beta)
0.2
0.3
0.4
0.5
0.6
0.7
0
20
40
60
80
Ages (years)
FDR (Age associated divergence) =0.0792
p(Scalp)=0.0673
p(Dura)=0.113
Scalp
Dura
A B C D E F G H I J K

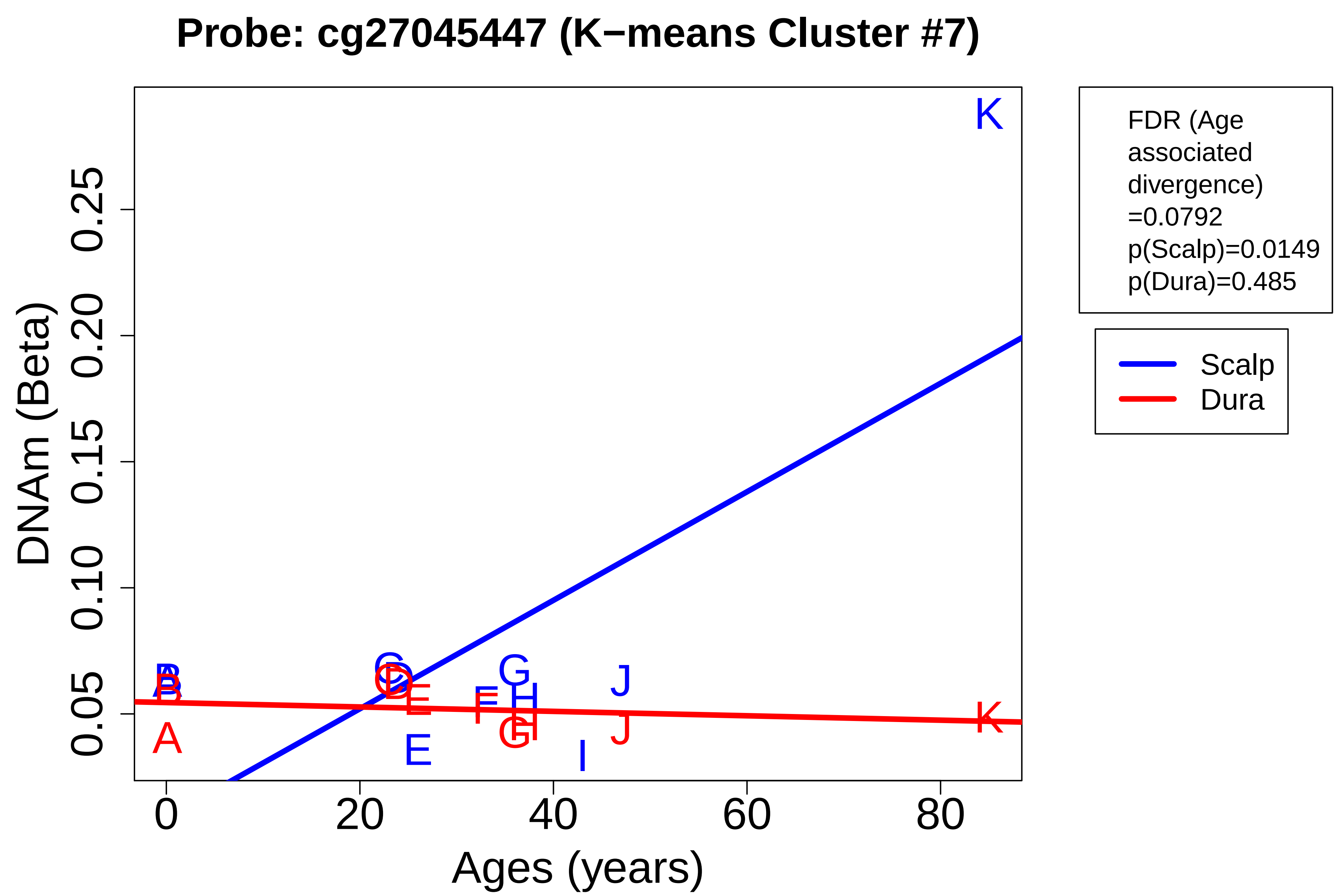

Probe: cg27045447 (K−means Cluster #7)
DNAm (Beta)
0.05
0.10
0.15
0.20
0.25
Ages (years)
0
20
40
60
80
FDR (Age associated divergence) =0.0792
p(Scalp)=0.0149
p(Dura)=0.485
Scalp
Dura

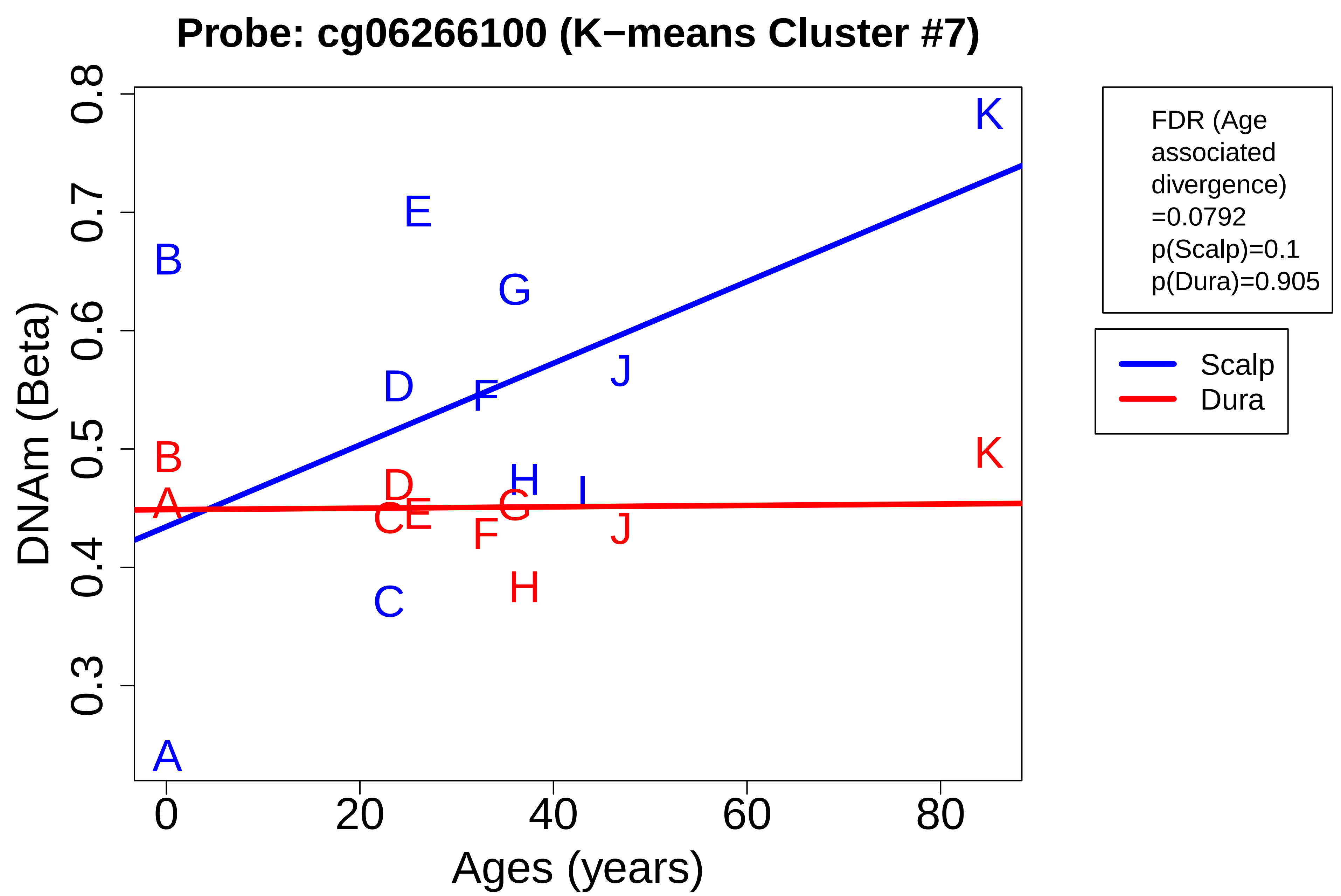
Probe: cg06266100 (K−means Cluster #7)
DNAm (Beta)
0.3
0.4
0.5
0.6
0.7
0.8
0
20
40
60
80
Ages (years)
FDR (Age associated divergence) =0.0792
p(Scalp)=0.1
p(Dura)=0.905
Scalp
Dura
A
B
C
D
E
F
G
H
I
J
K

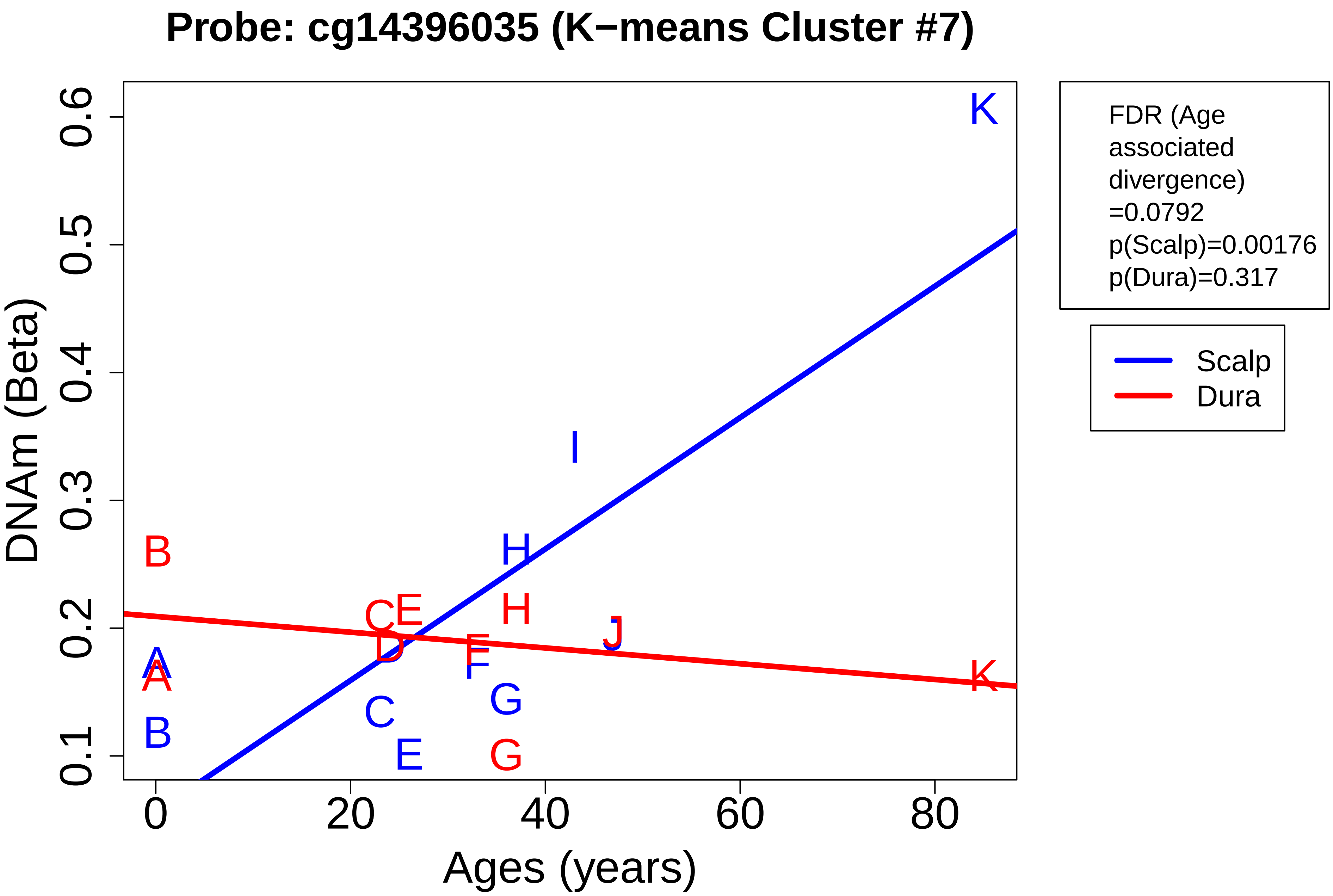

Probe: cg14396035 (K−means Cluster #7)
DNAm (Beta)
0.1
0.2
0.3
0.4
0.5
0.6
Ages (years)
0
20
40
60
80
FDR (Age associated divergence) =0.0792
p(Scalp)=0.00176
p(Dura)=0.317
Scalp
Dura
A
B
C
D
E
F
G
H
I
J
K

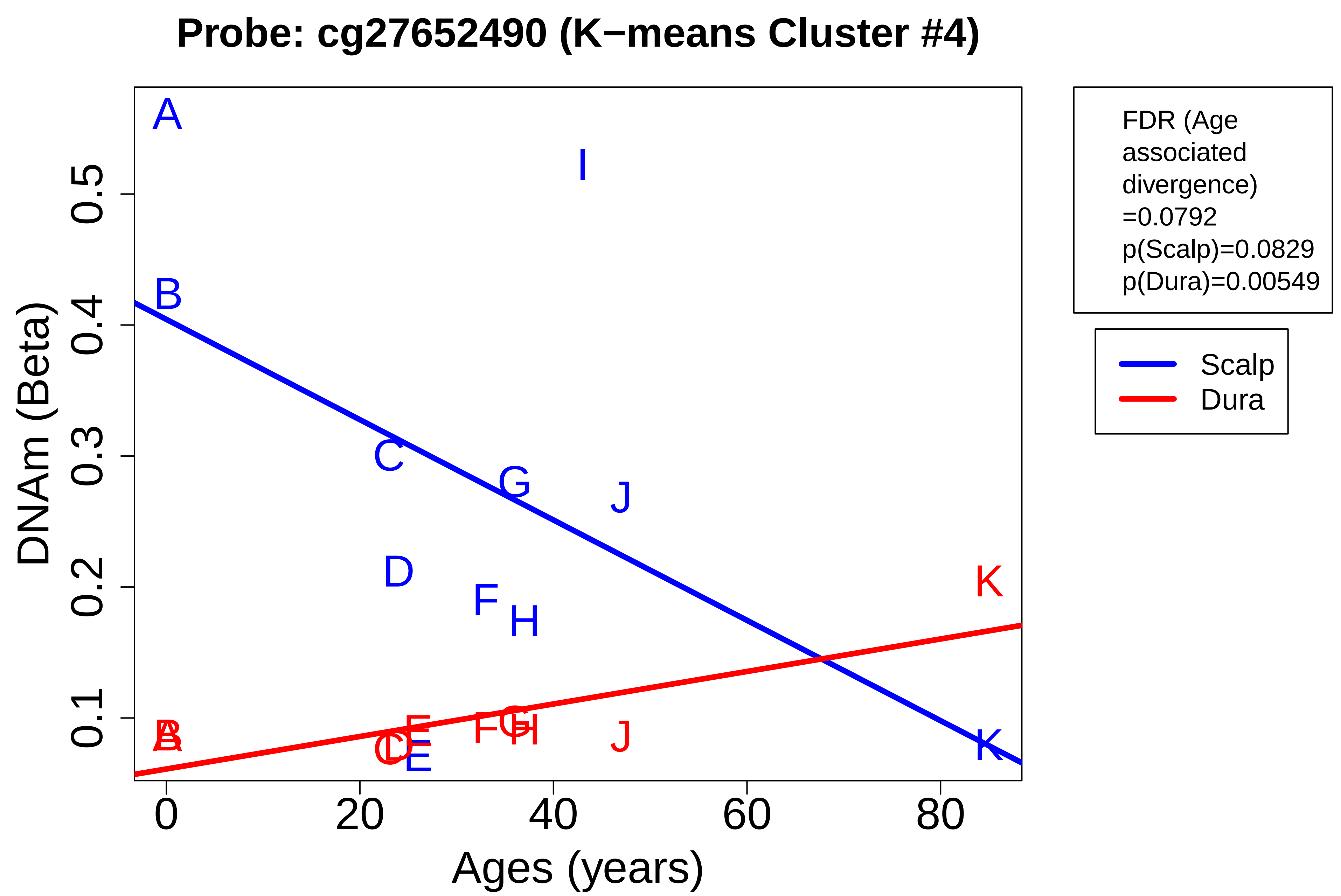

Probe: cg27652490 (K−means Cluster #4)
DNAm (Beta)
0.1
0.2
0.3
0.4
0.5
Ages (years)
0
20
40
60
80
A
B
C
D
E
F
G
H
I
J
K
FDR (Age associated divergence) =0.0792
p(Scalp)=0.0829
p(Dura)=0.00549
Scalp
Dura

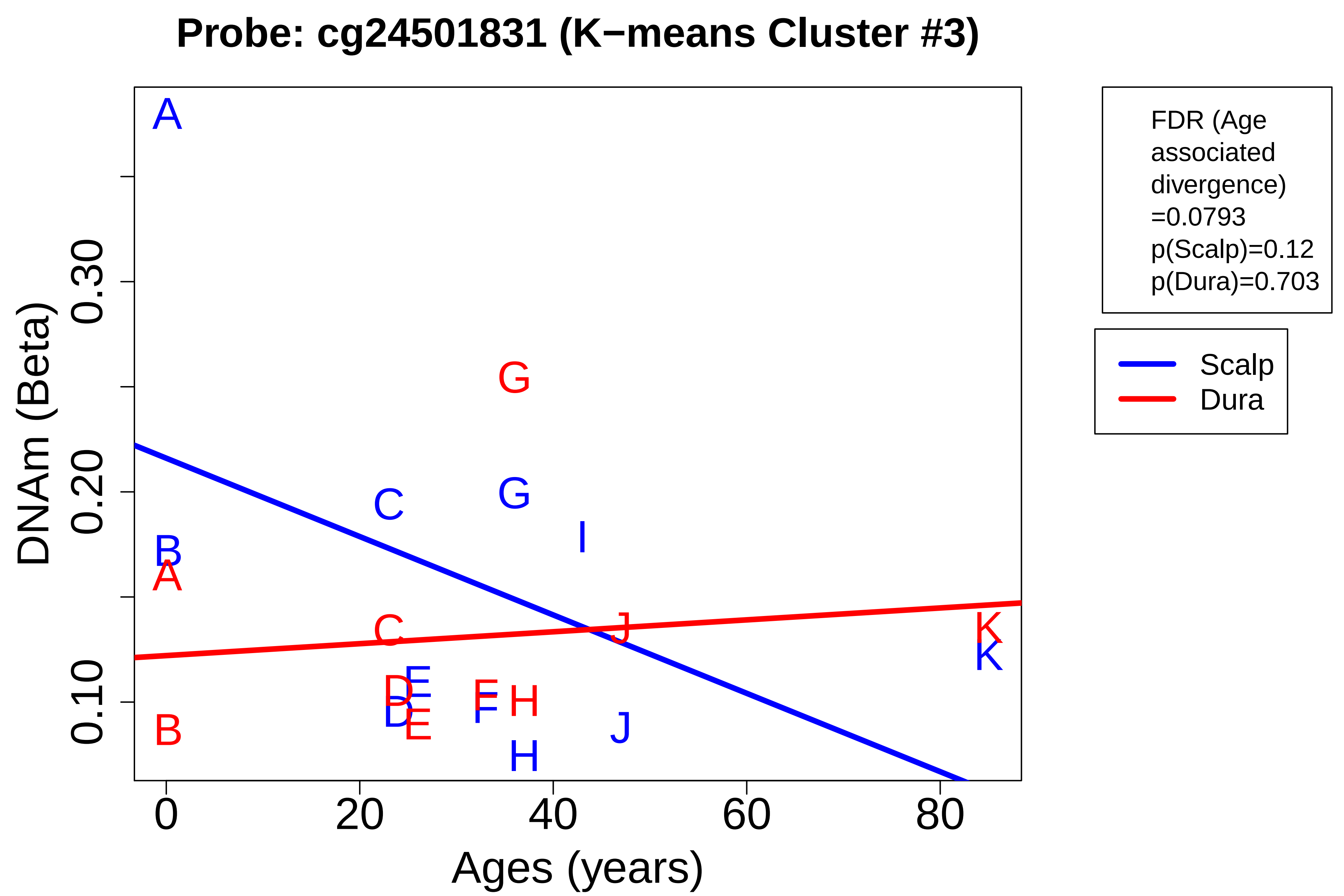
Probe: cg24501831 (K−means Cluster #3)
DNAm (Beta)
0.10
0.20
0.30
Ages (years)
0
20
40
60
80
A
B
C
D
E
F
G
H
I
J
K
FDR (Age associated divergence) =0.0793
p(Scalp)=0.12
p(Dura)=0.703
Scalp
Dura

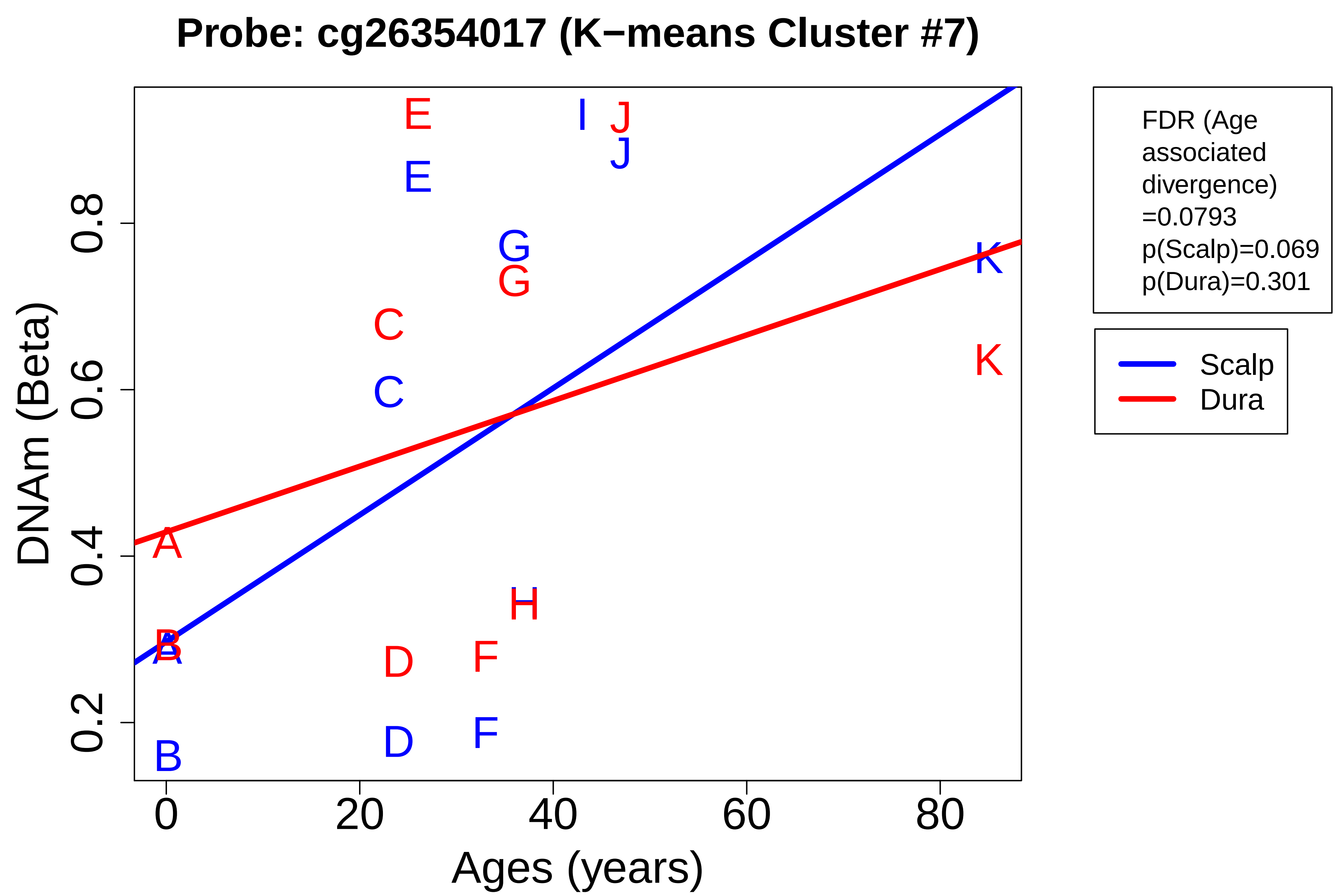

Probe: cg26354017 (K−means Cluster #7)
DNAm (Beta)
0.2
0.4
0.6
0.8
0
20
40
60
80
Ages (years)
FDR (Age associated divergence) =0.0793
p(Scalp)=0.069
p(Dura)=0.301
Scalp
Dura

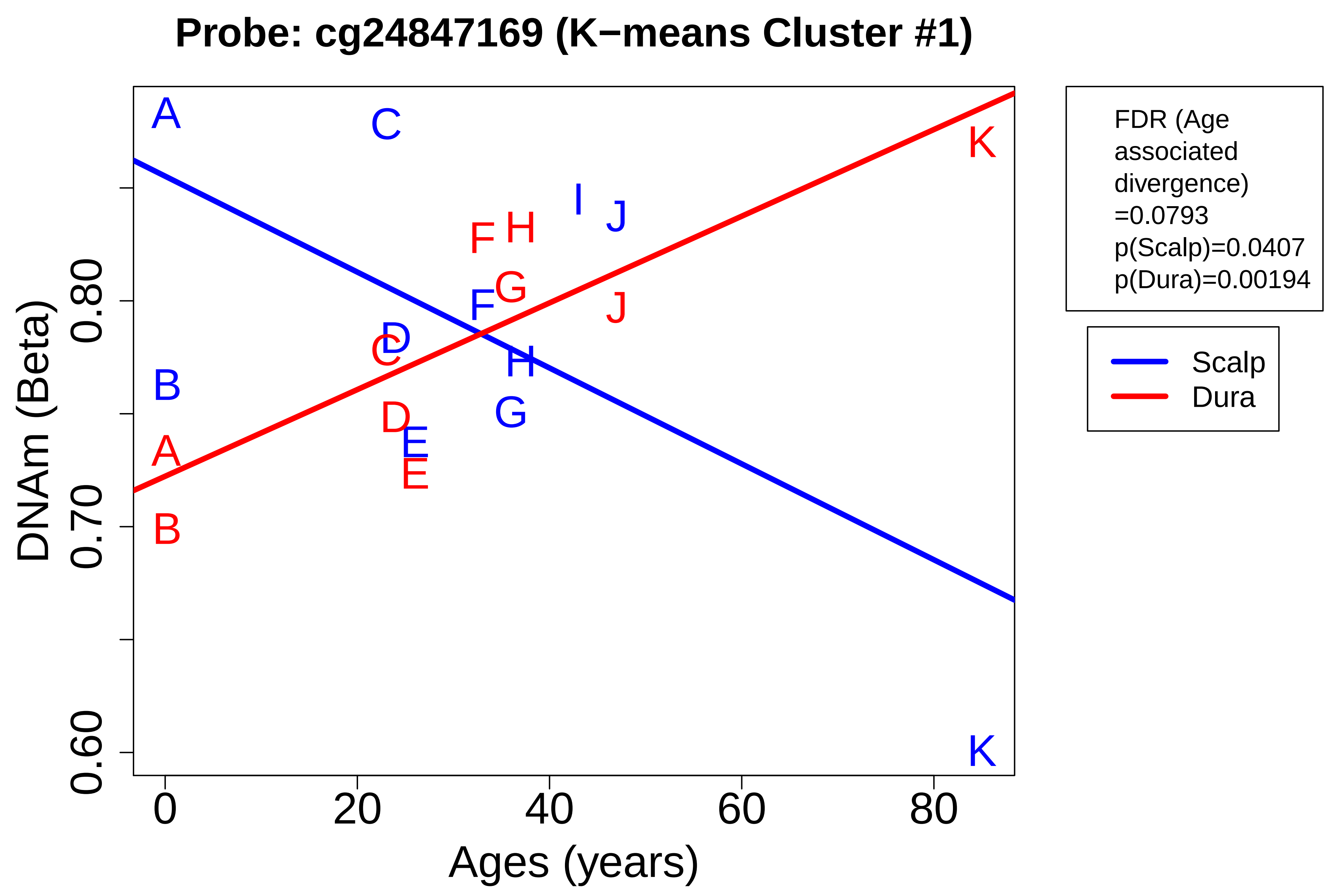

Probe: cg24847169 (K−means Cluster #1)
FDR (Age associated divergence) =0.0793
p(Scalp)=0.0407
p(Dura)=0.00194
Scalp
Dura
DNAm (Beta)
0.60
0.70
0.80
0
20
40
60
80
Ages (years)
A
B
C
D
E
F
G
H
I
J
K

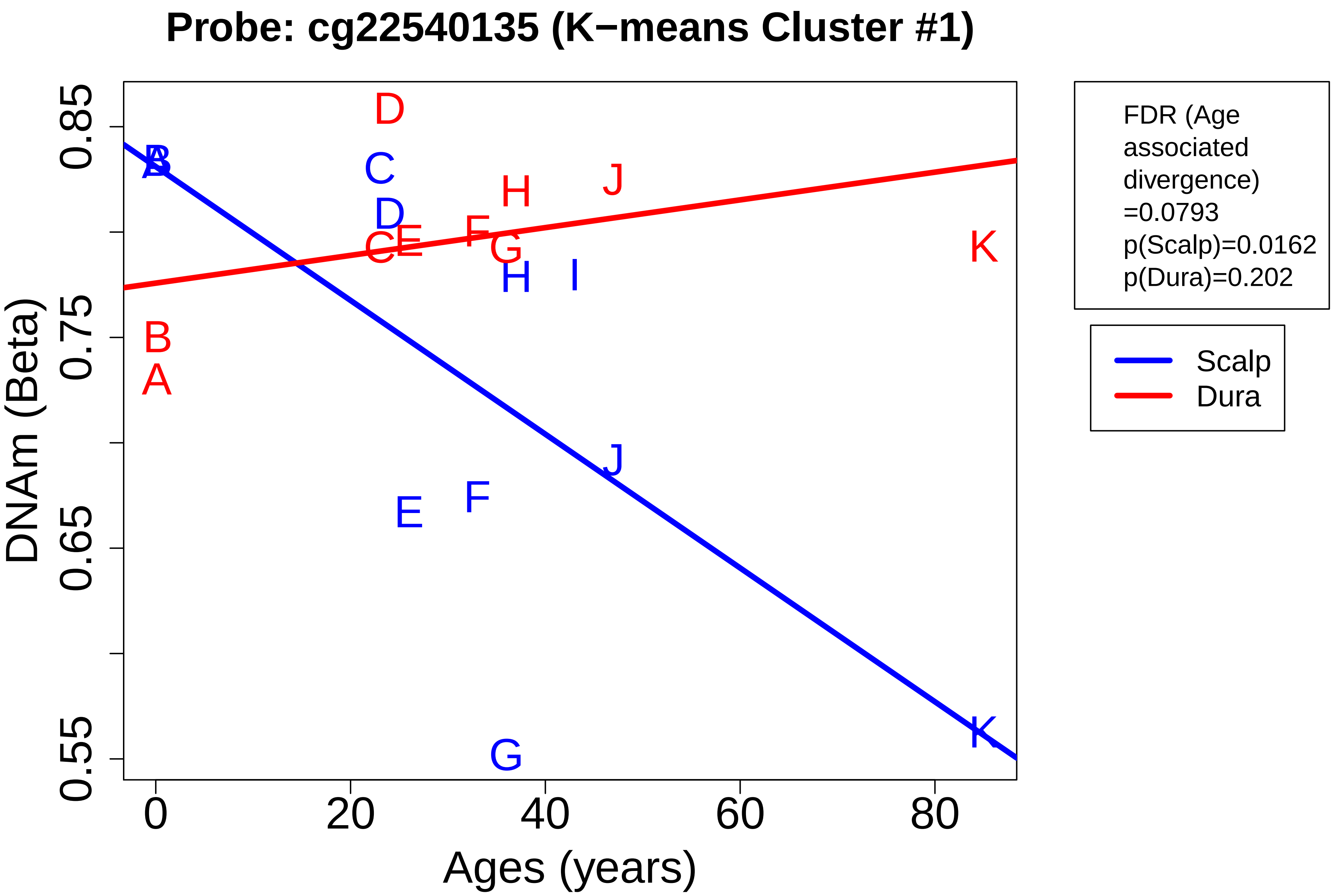
Probe: cg22540135 (K−means Cluster #1)
FDR (Age associated divergence) =0.0793
p(Scalp)=0.0162
p(Dura)=0.202
Scalp
Dura
DNAm (Beta)
0.55
0.65
0.75
0.85
Ages (years)
0
20
40
60
80
A
B
C
D
E
F
G
H
I
J
K

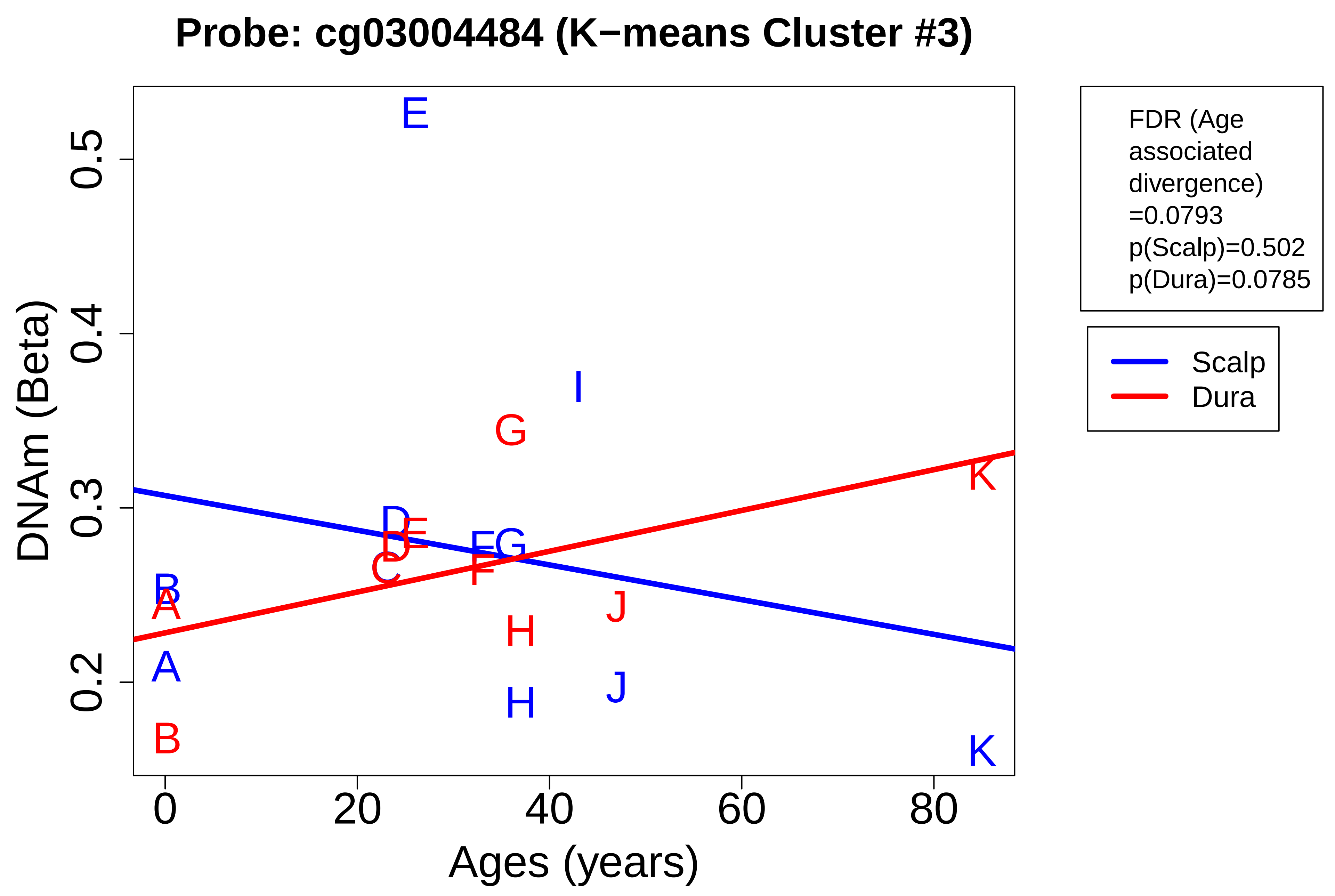

Probe: cg03004484 (K−means Cluster #3)
DNAm (Beta)
0.2
0.3
0.4
0.5
Ages (years)
0
20
40
60
80
FDR (Age associated divergence) =0.0793
p(Scalp)=0.502
p(Dura)=0.0785
Scalp
Dura

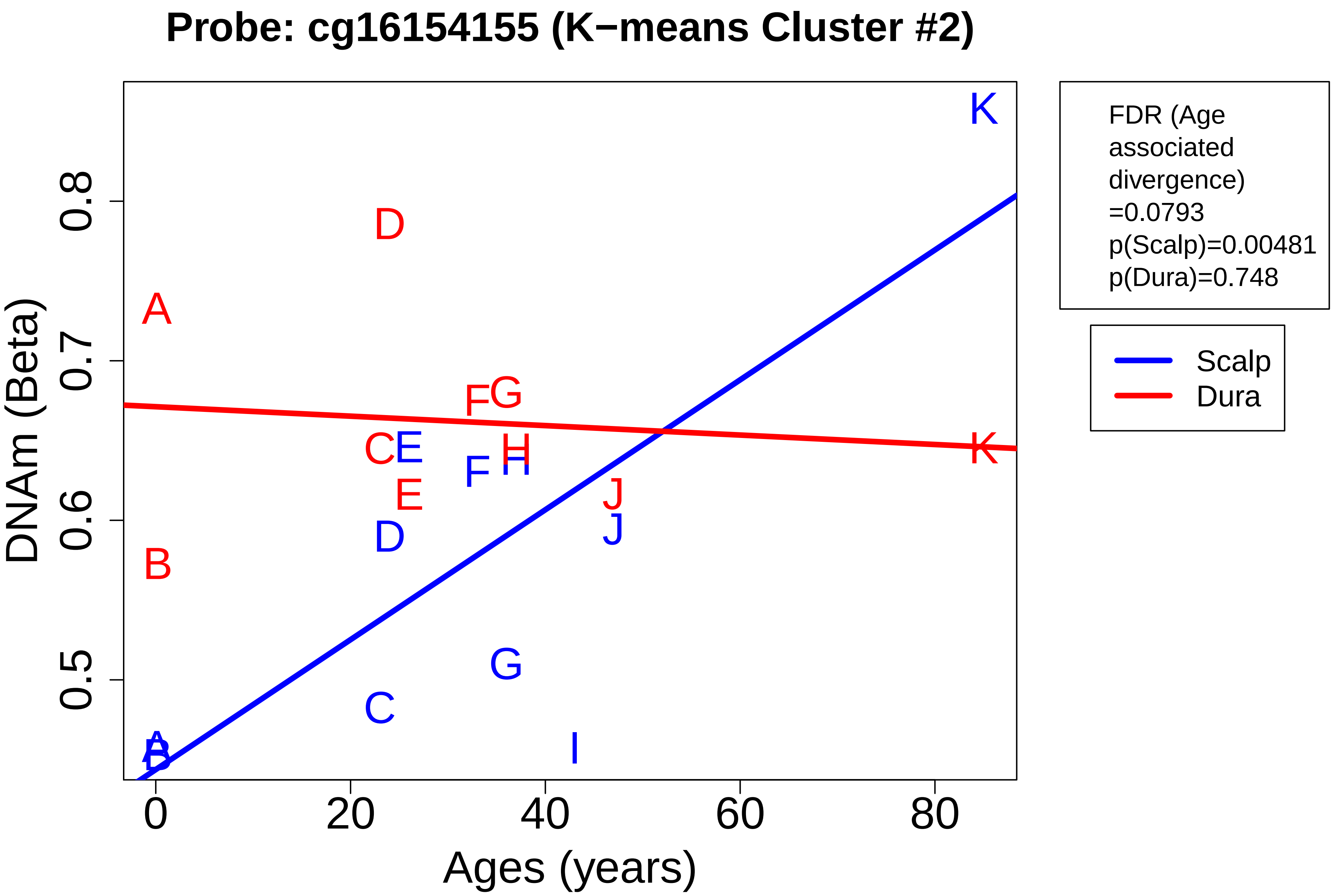
Probe: cg16154155 (K−means Cluster #2)
DNAm (Beta)
0.5
0.6
0.7
0.8
0
20
40
60
80
Ages (years)
FDR (Age associated divergence) =0.0793
p(Scalp)=0.00481
p(Dura)=0.748
Scalp
Dura

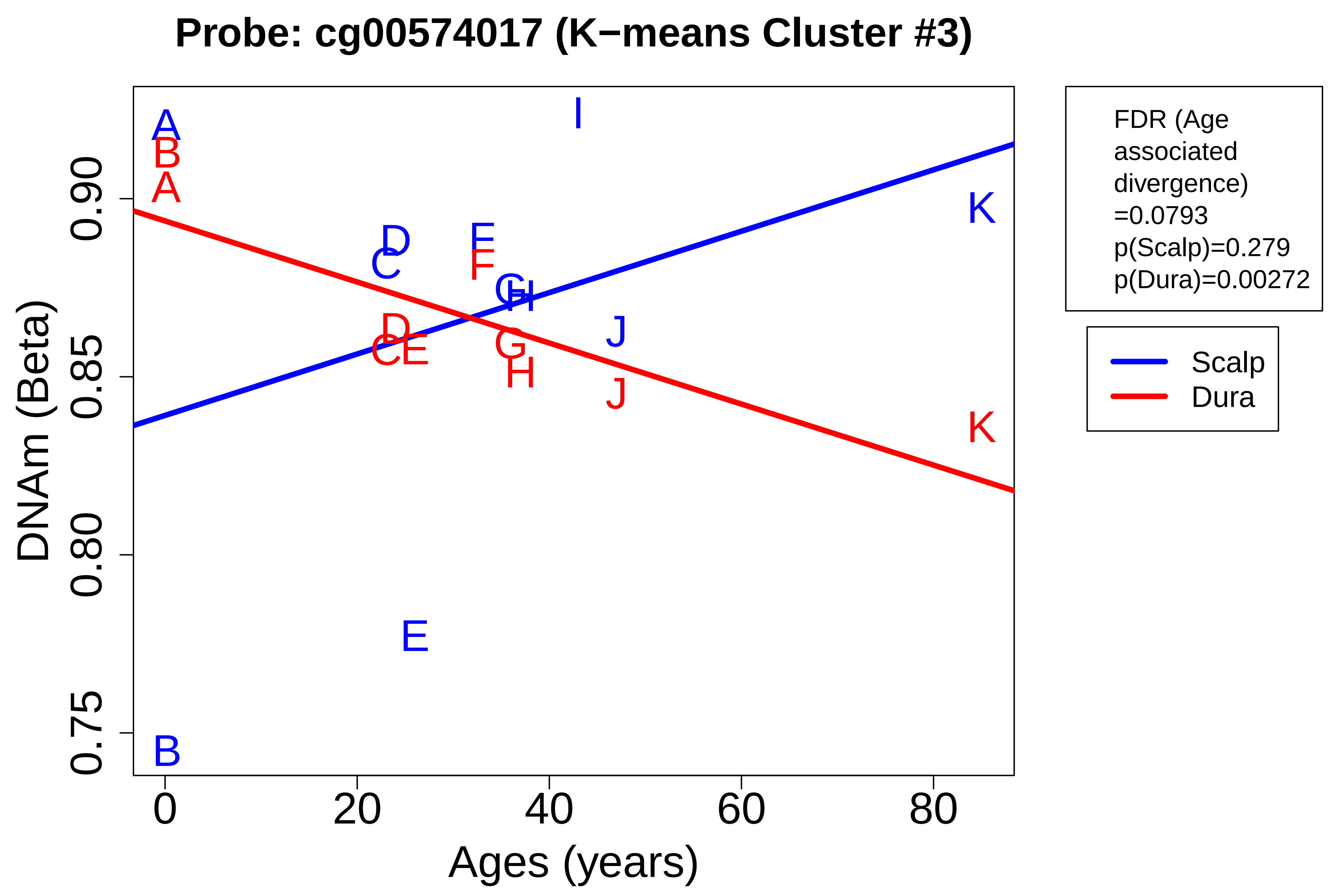

Probe: cg00574017 (K−means Cluster #3)
DNAm (Beta)
0.75
0.80
0.85
0.90
0
20
40
60
80
Ages (years)
A
B
C
D
E
F
G
H
I
J
K
FDR (Age associated divergence) =0.0793
p(Scalp)=0.279
p(Dura)=0.00272
Scalp
Dura

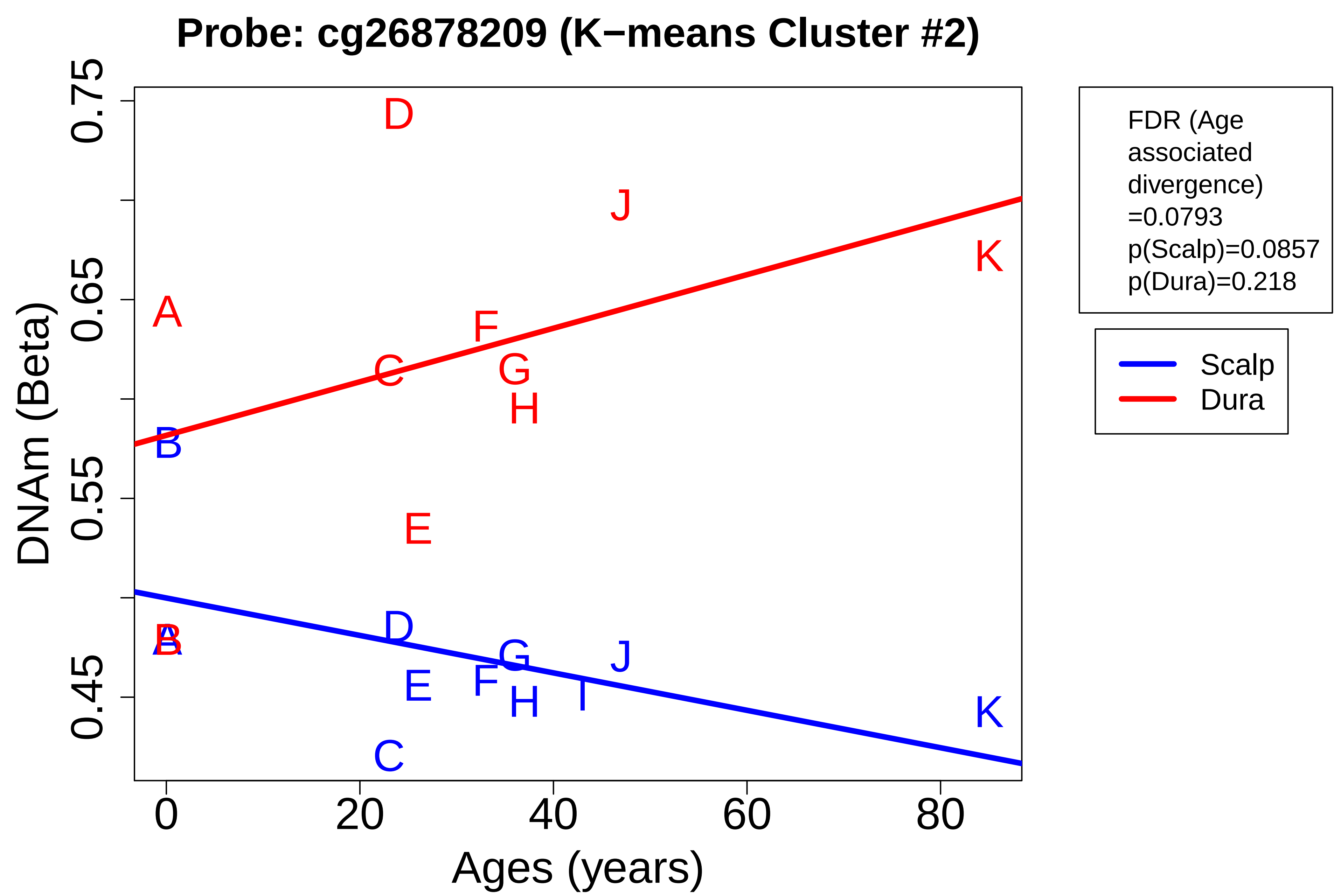
Probe: cg26878209 (K−means Cluster #2)
DNAm (Beta)
0.75
0.65
0.55
0.45
0
20
40
60
80
Ages (years)
FDR (Age associated divergence) =0.0793
p(Scalp)=0.0857
p(Dura)=0.218
Scalp
Dura
A
B
C
D
E
F
G
H
I
J
K

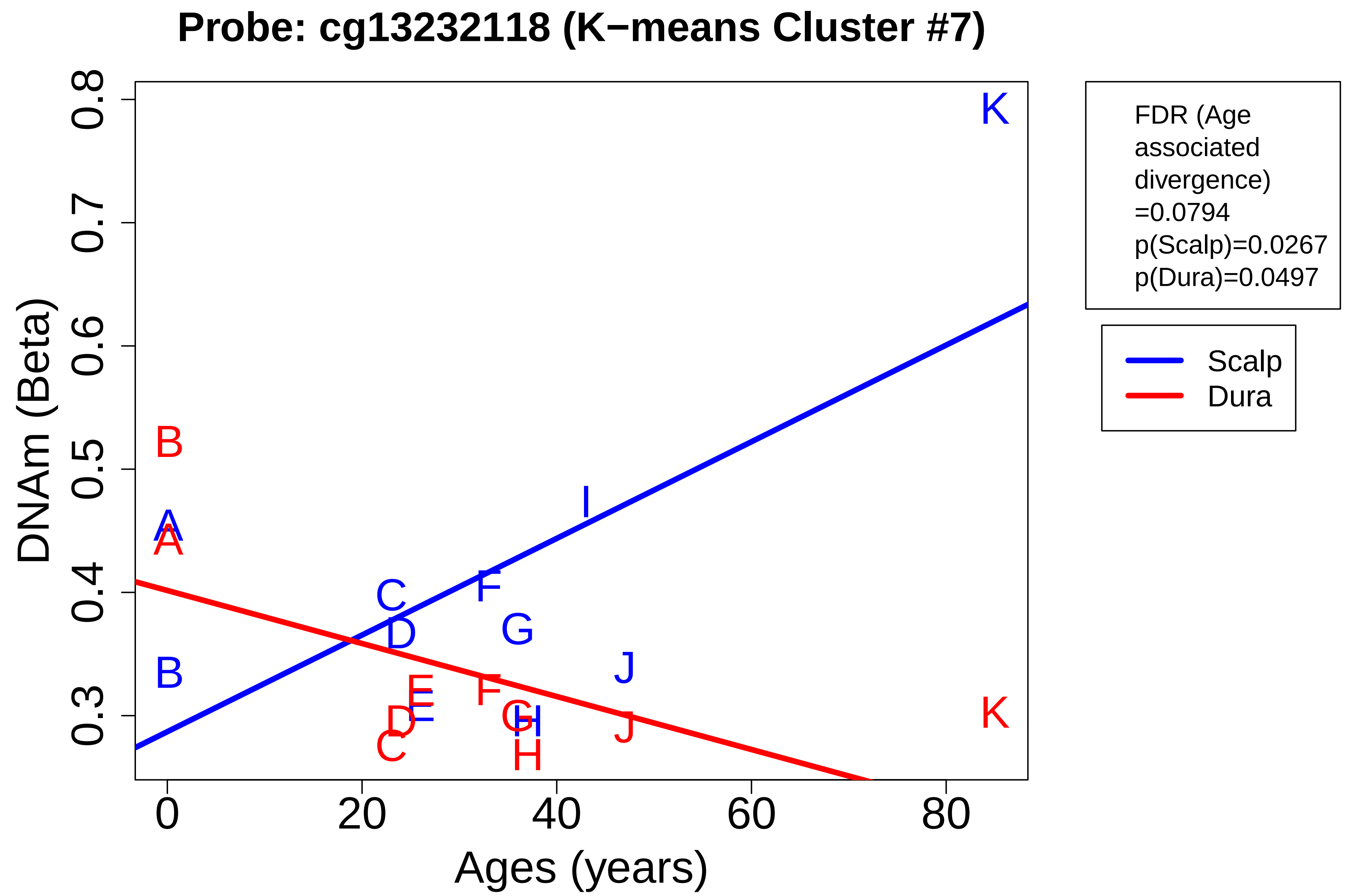

Probe: cg13232118 (K−means Cluster #7)
DNAm (Beta)
0.3
0.4
0.5
0.6
0.7
0.8
0
20
40
60
80
Ages (years)
FDR (Age associated divergence) =0.0794
p(Scalp)=0.0267
p(Dura)=0.0497
Scalp
Dura
A
B
C
D
E
F
G
H
I
J
K

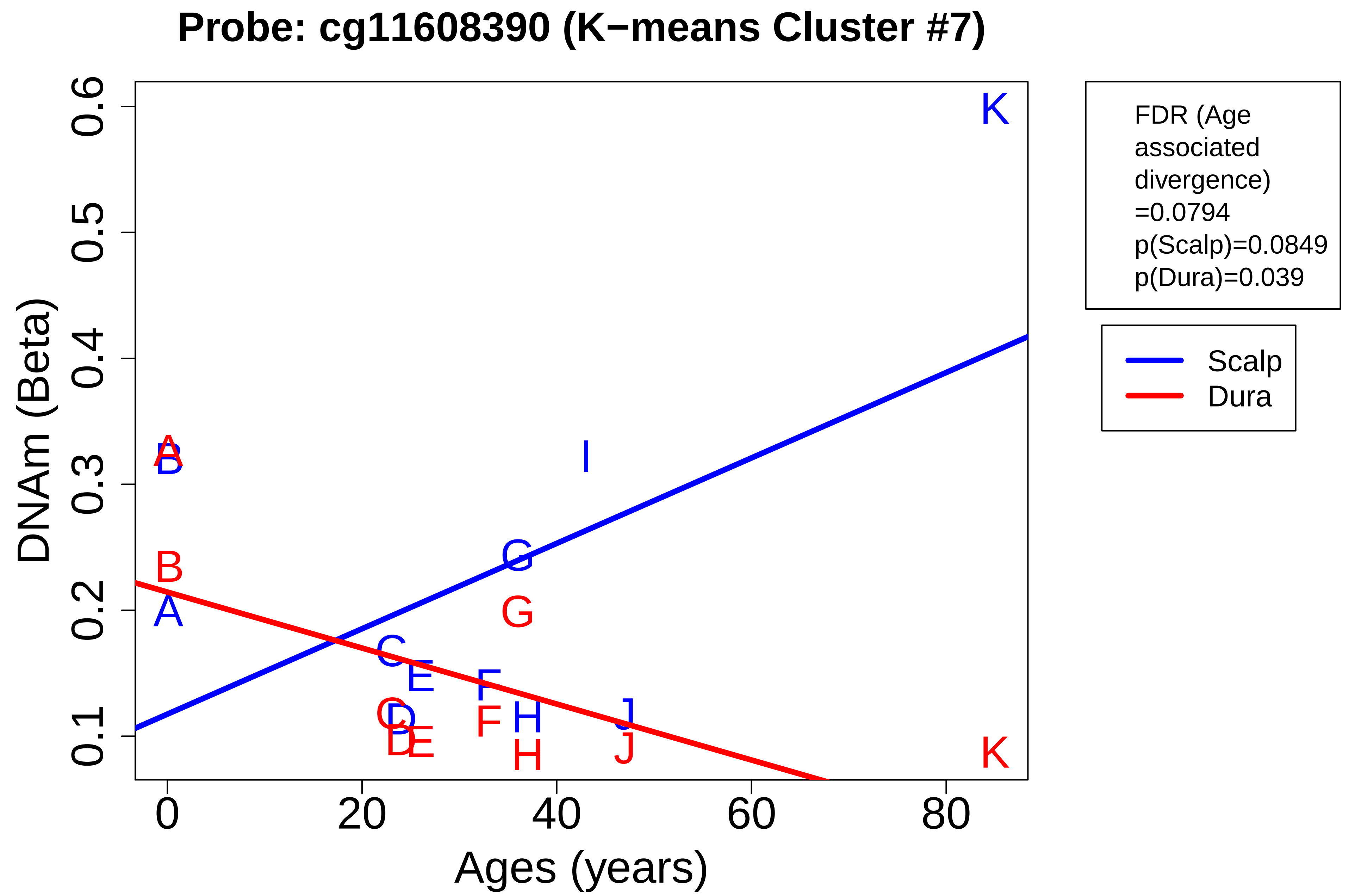

Probe: cg11608390 (K−means Cluster #7)
DNAm (Beta)
0.1
0.2
0.3
0.4
0.5
0.6
0
20
40
60
80
Ages (years)
FDR (Age associated divergence) =0.0794
p(Scalp)=0.0849
p(Dura)=0.039
Scalp
Dura

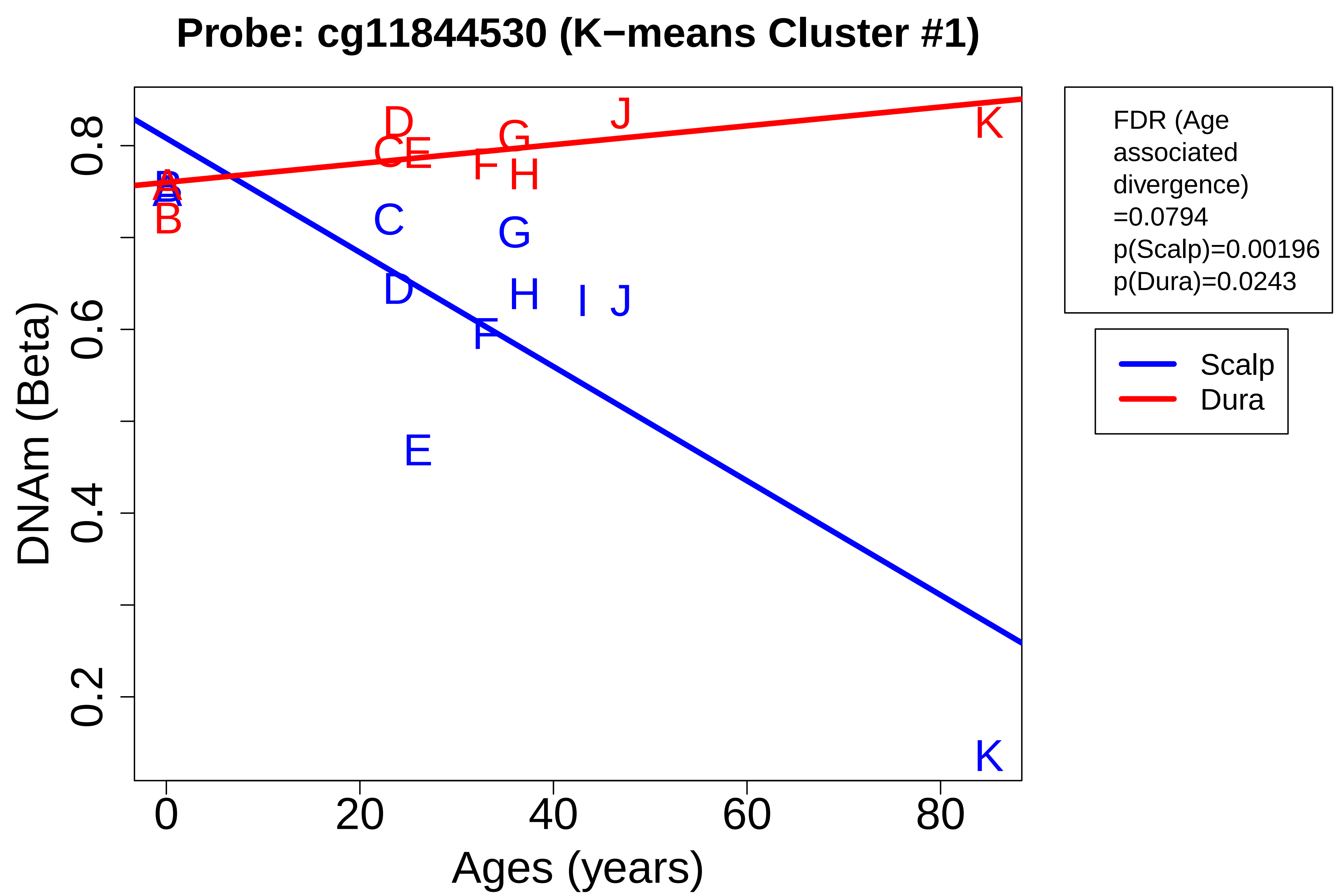

Probe: cg11844530 (K−means Cluster #1)
DNAm (Beta)
0.2
0.4
0.6
0.8
Ages (years)
0
20
40
60
80
A B C D E F G H I J K
FDR (Age associated divergence) =0.0794
p(Scalp)=0.00196
p(Dura)=0.0243
Scalp
Dura

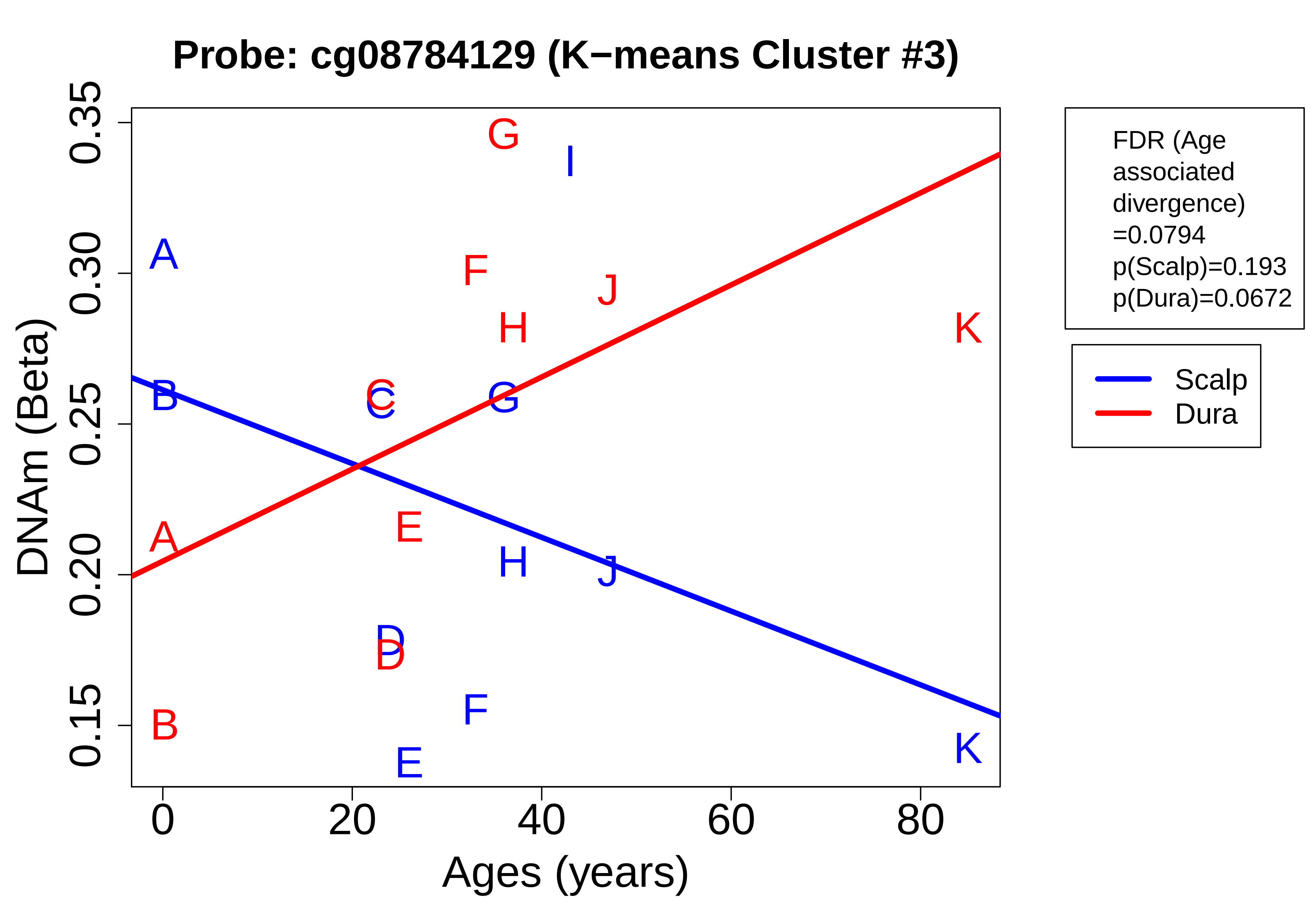
Probe: cg08784129 (K−means Cluster #3)
DNAm (Beta)
Ages (years)
0.15
0.20
0.25
0.30
0.35
0
20
40
60
80
FDR (Age associated divergence) =0.0794
p(Scalp)=0.193
p(Dura)=0.0672
Scalp
Dura

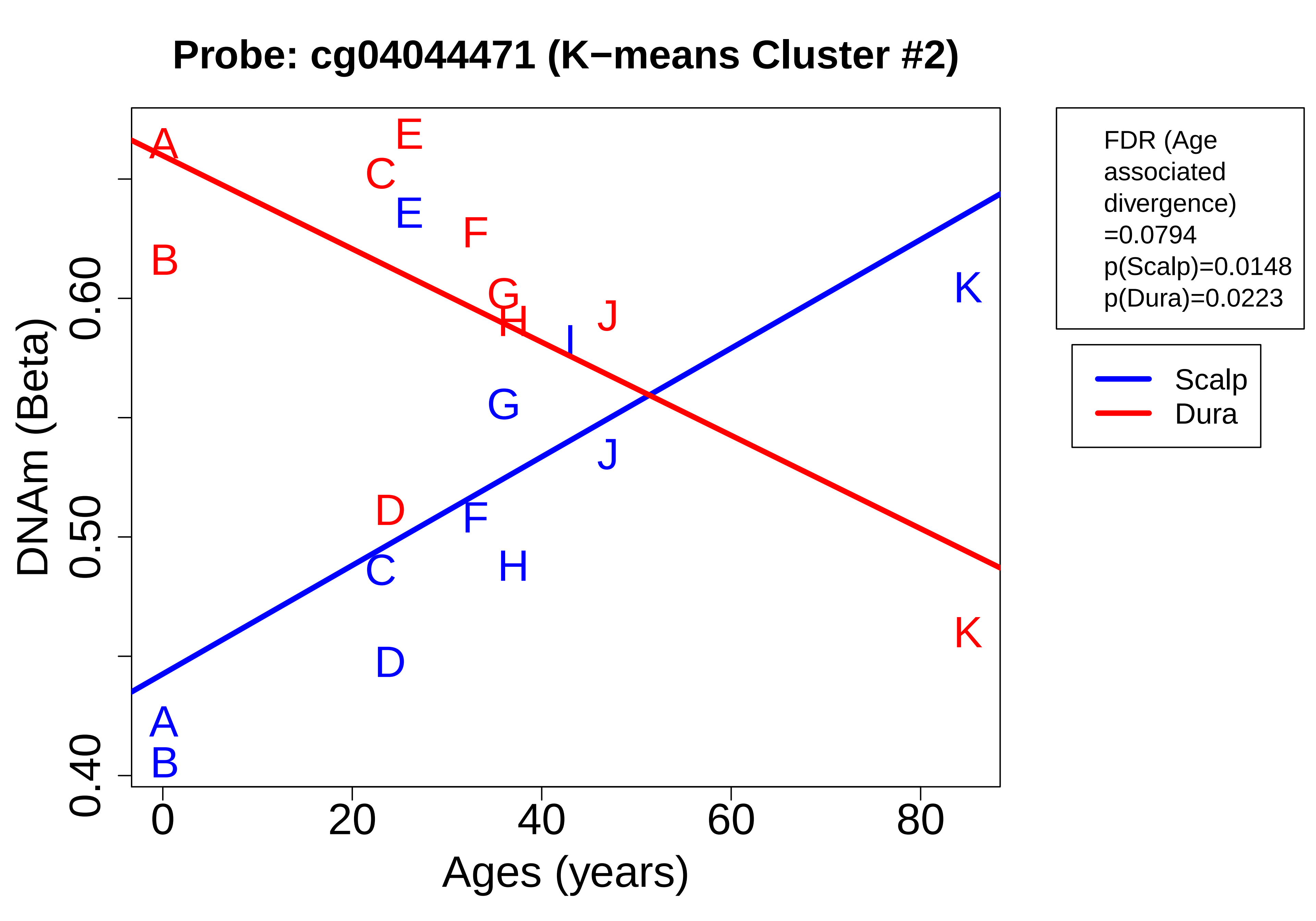

Probe: cg04044471 (K−means Cluster #2)
DNAm (Beta)
0.40
0.50
0.60
Ages (years)
0
20
40
60
80
A
B
C
D
E
F
G
H
I
J
K
FDR (Age associated divergence) =0.0794
p(Scalp)=0.0148
p(Dura)=0.0223
Scalp
Dura

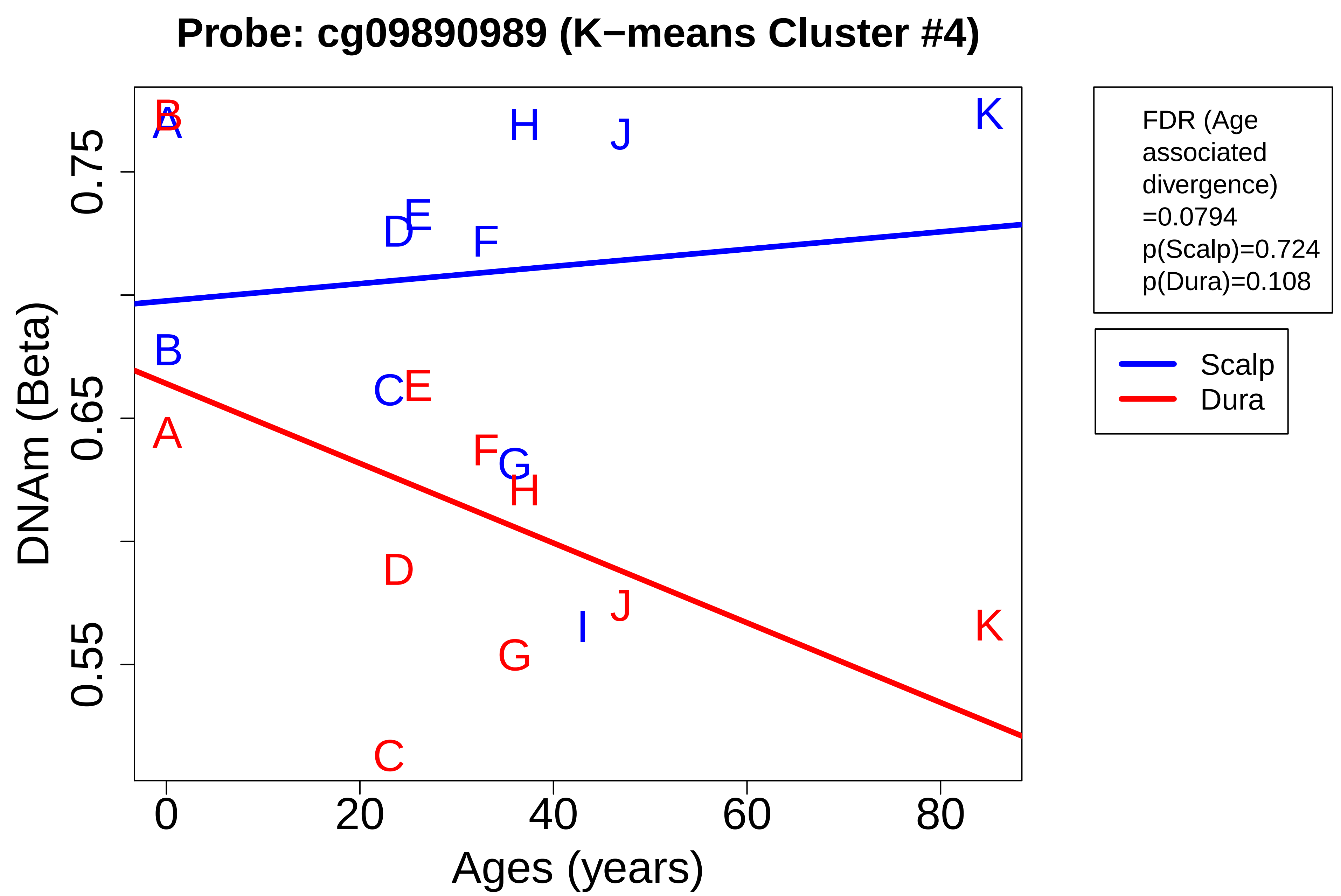
Probe: cg09890989 (K−means Cluster #4)
DNAm (Beta)
0.55
0.65
0.75
Ages (years)
0
20
40
60
80
FDR (Age associated divergence) =0.0794
p(Scalp)=0.724
p(Dura)=0.108
Scalp
Dura
A
B
C
D
E
F
G
H
I
J
K

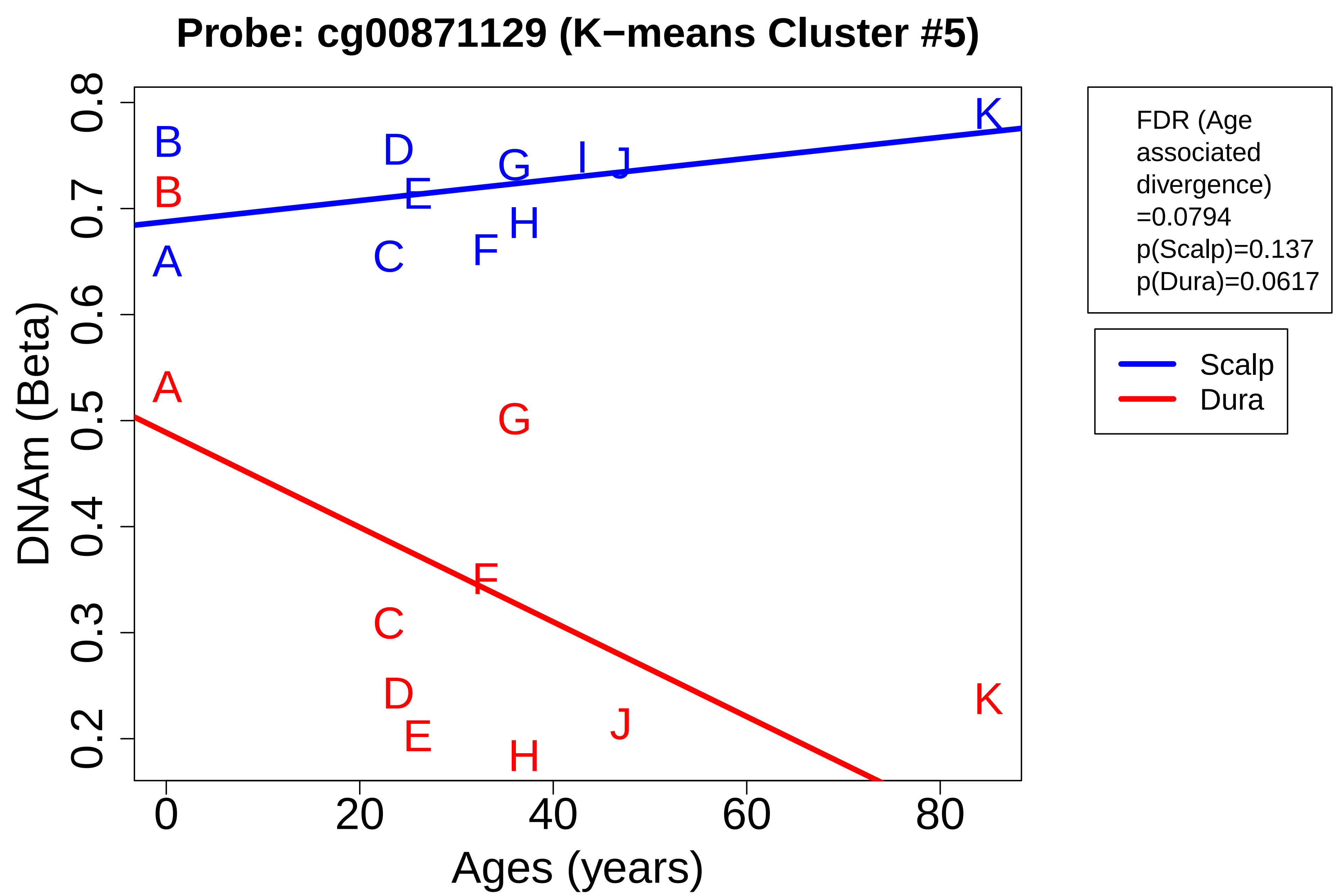
Probe: cg00871129 (K−means Cluster #5)
DNAm (Beta)
0.2
0.3
0.4
0.5
0.6
0.7
0.8
Ages (years)
0
20
40
60
80
FDR (Age associated divergence) =0.0794
p(Scalp)=0.137
p(Dura)=0.0617
Scalp
Dura
A
B
C
D
E
F
G
H
I
J
K

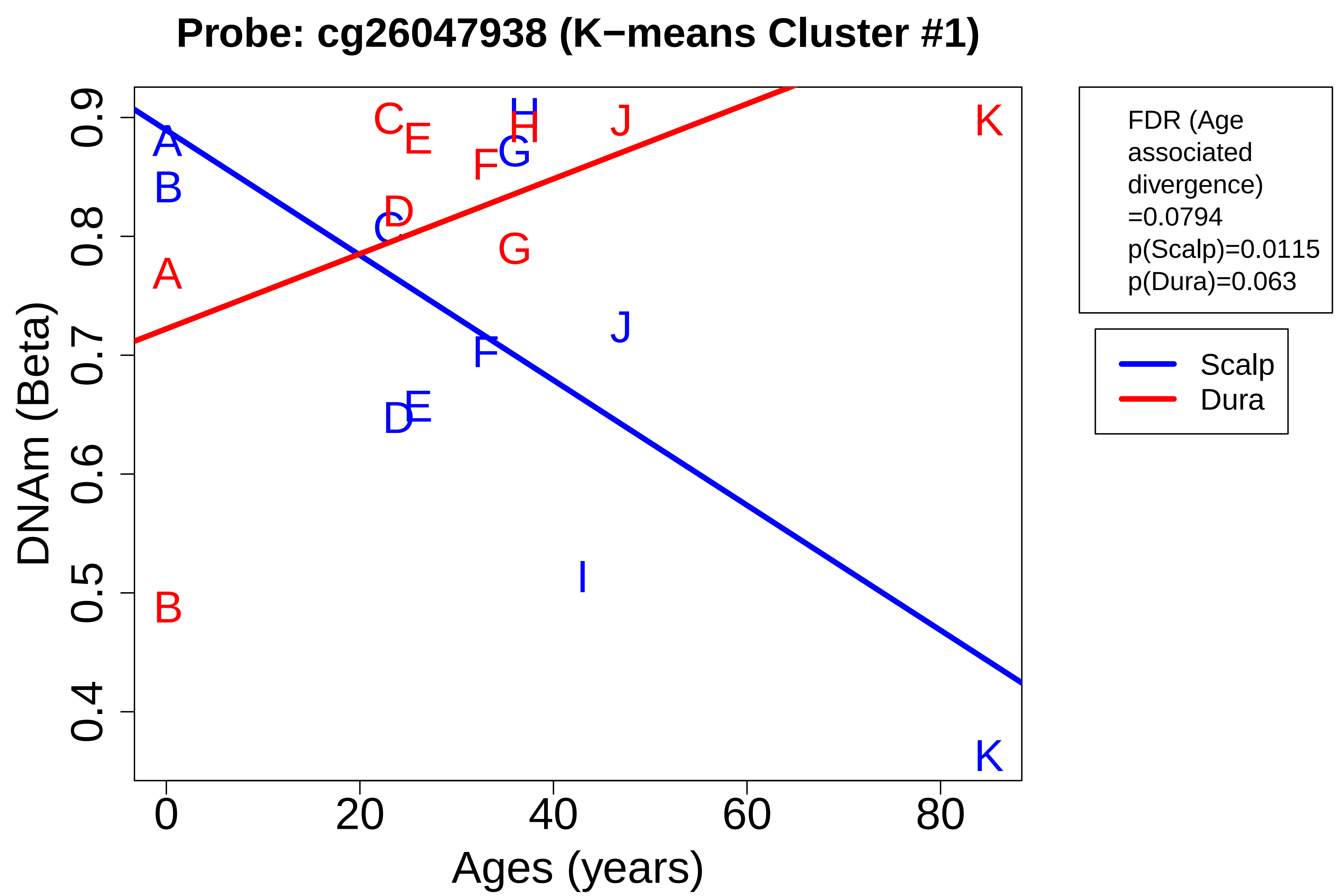
Probe: cg26047938 (K−means Cluster #1)
DNAm (Beta)
Ages (years)
0.4
0.5
0.6
0.7
0.8
0.9
0
20
40
60
80
FDR (Age associated divergence) =0.0794
p(Scalp)=0.0115
p(Dura)=0.063
Scalp
Dura

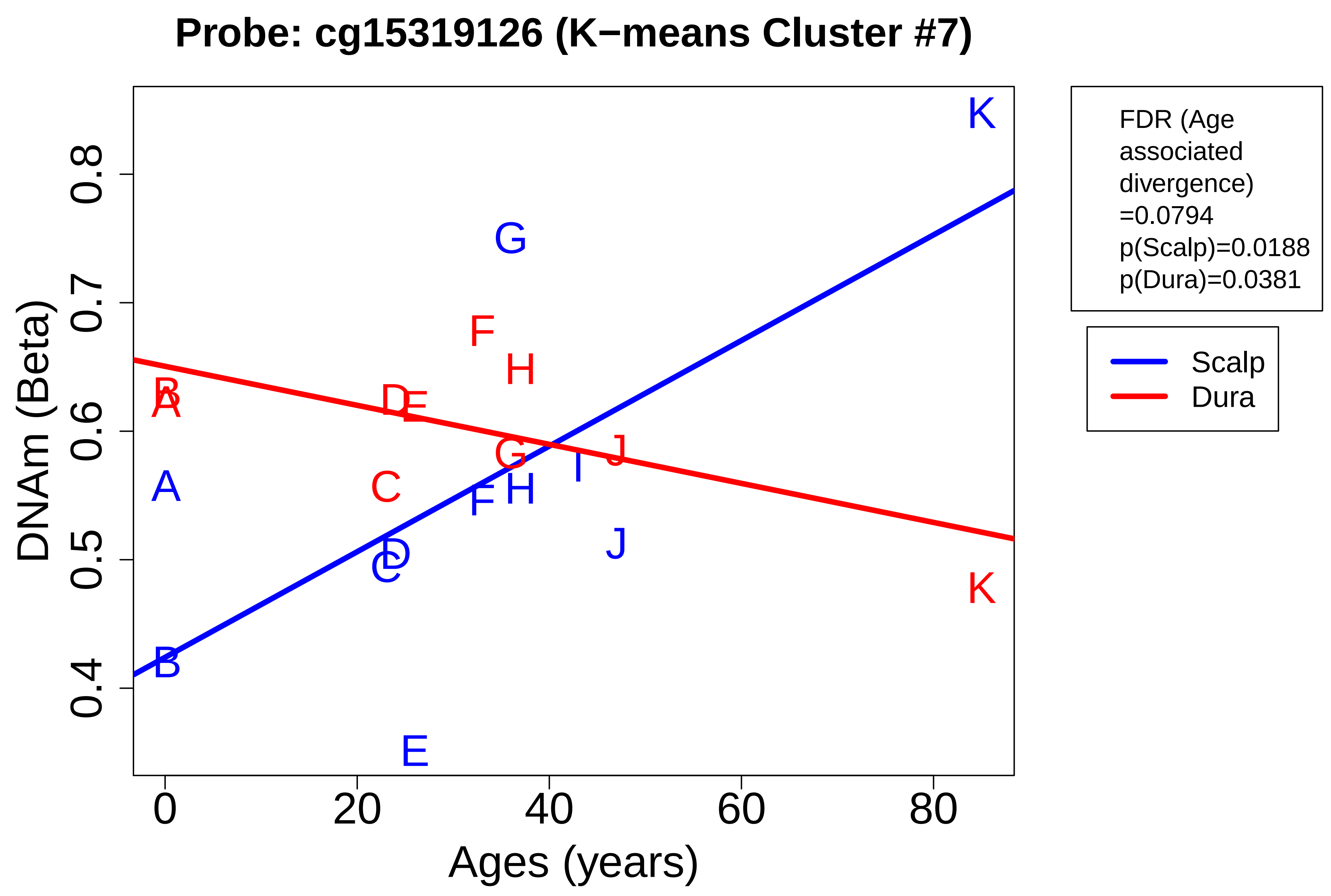

Probe: cg15319126 (K−means Cluster #7)
DNAm (Beta)
0.4
0.5
0.6
0.7
0.8
0
20
40
60
80
Ages (years)
FDR (Age associated divergence) =0.0794
p(Scalp)=0.0188
p(Dura)=0.0381
Scalp
Dura

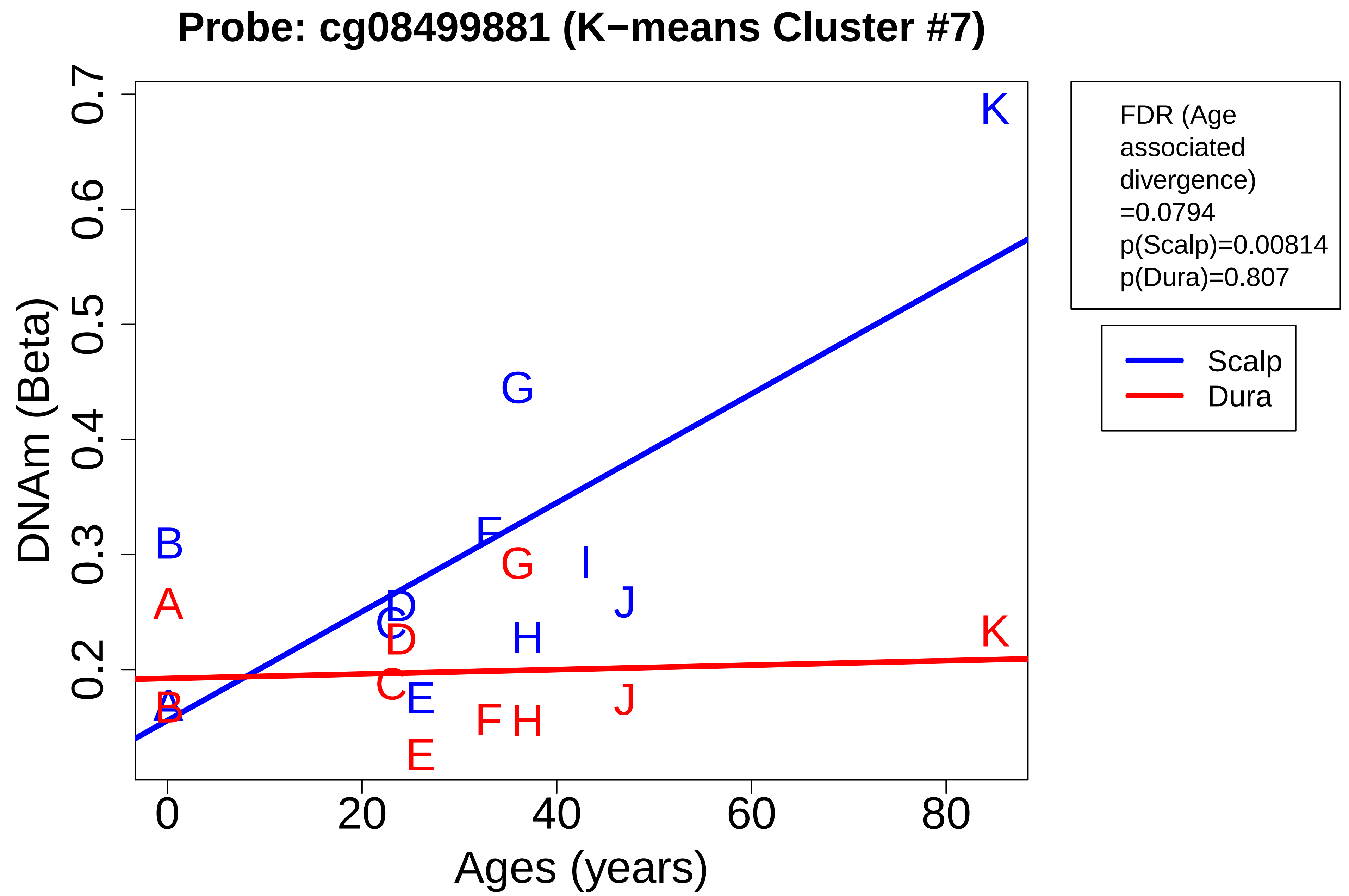

Probe: cg08499881 (K−means Cluster #7)
DNAm (Beta)
0.2
0.3
0.4
0.5
0.6
0.7
Ages (years)
0
20
40
60
80
FDR (Age associated divergence) =0.0794
p(Scalp)=0.00814
p(Dura)=0.807
Scalp
Dura

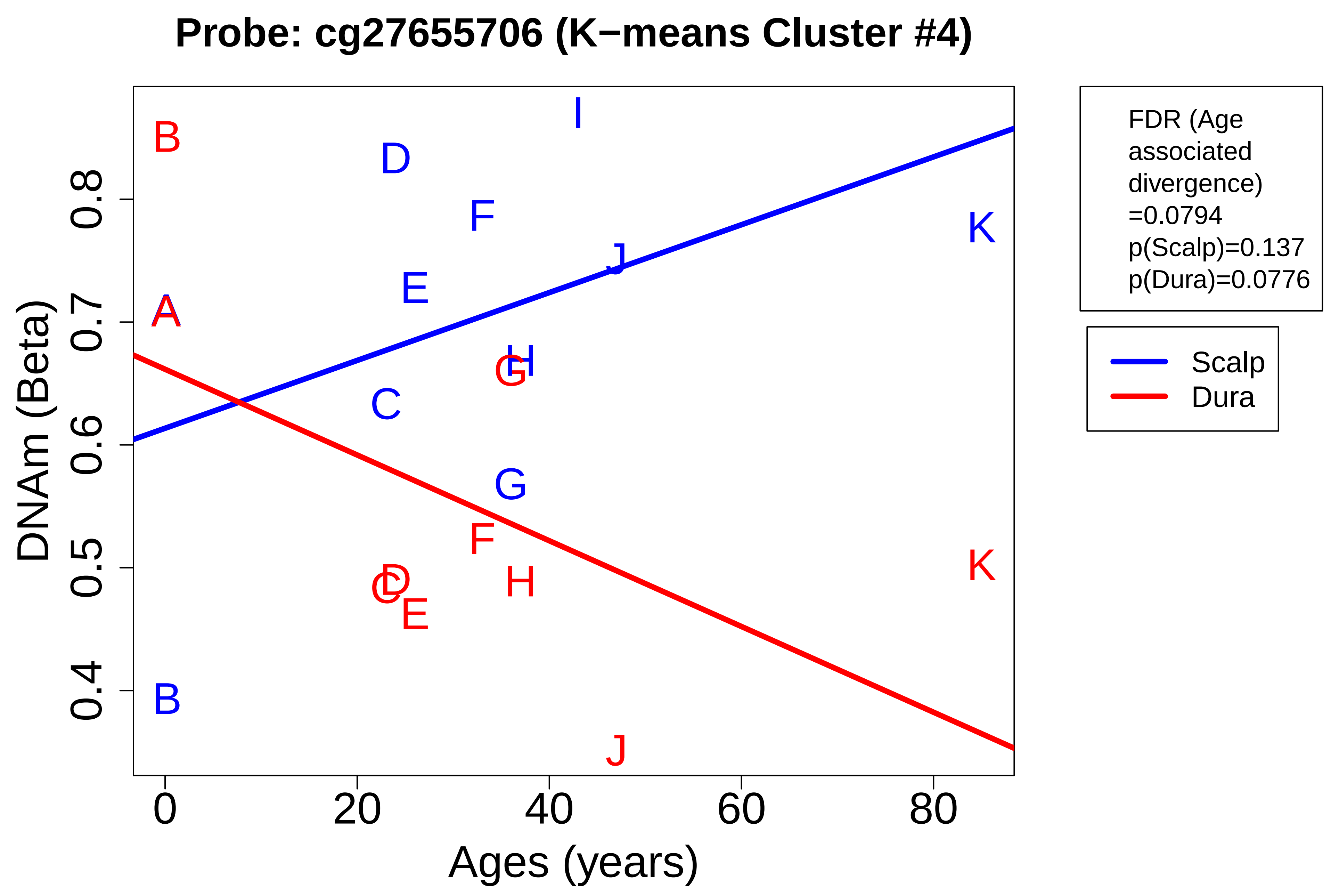

Probe: cg27655706 (K−means Cluster #4)
DNAm (Beta)
0.4
0.5
0.6
0.7
0.8
0
20
40
60
80
Ages (years)
FDR (Age associated divergence) =0.0794
p(Scalp)=0.137
p(Dura)=0.0776
Scalp
Dura
A
B
C
D
E
F
G
H
I
J
K

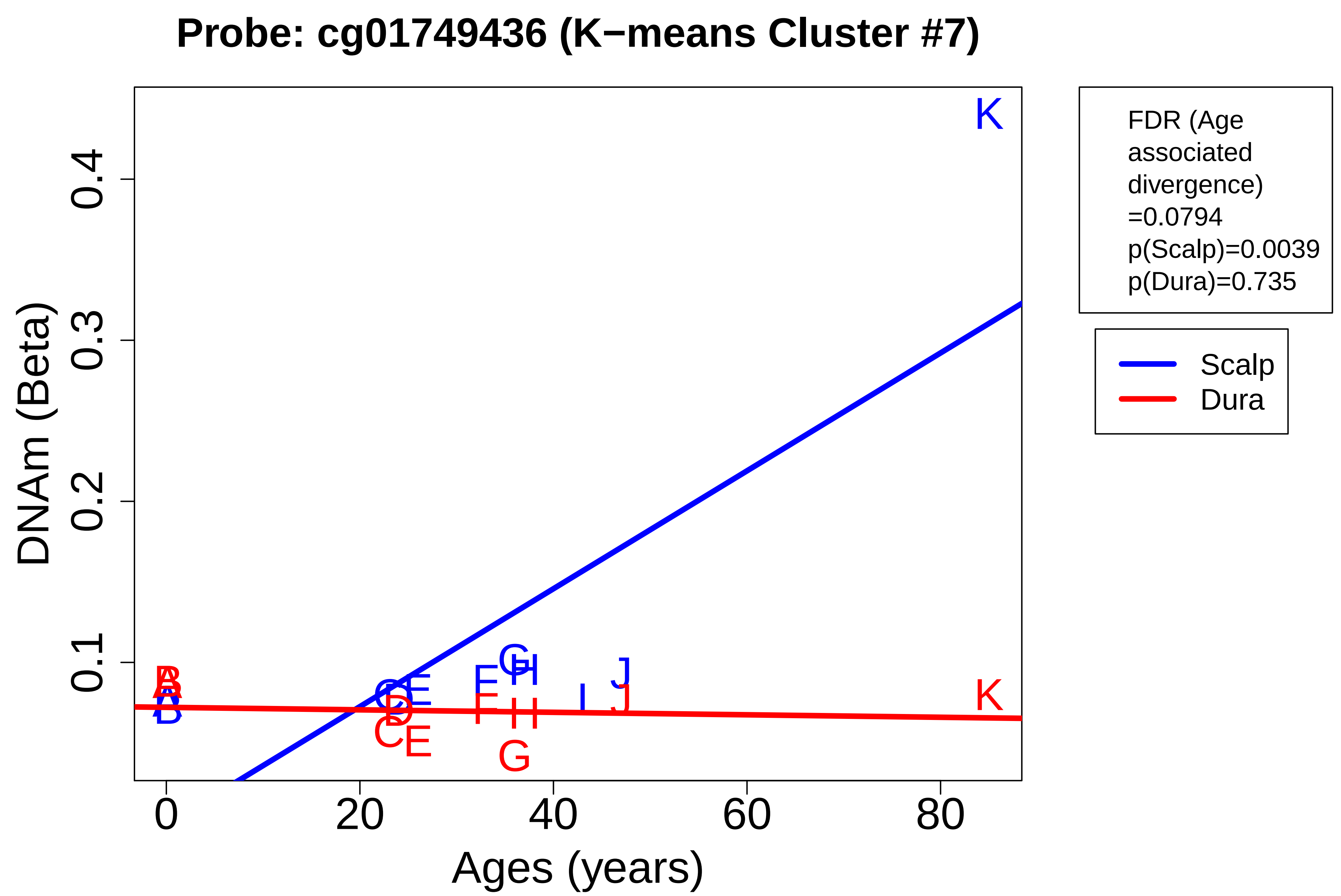
Probe: cg01749436 (K−means Cluster #7)
DNAm (Beta)
0.1
0.2
0.3
0.4
0
20
40
60
80
Ages (years)
FDR (Age associated divergence) =0.0794
p(Scalp)=0.0039
p(Dura)=0.735
Scalp
Dura

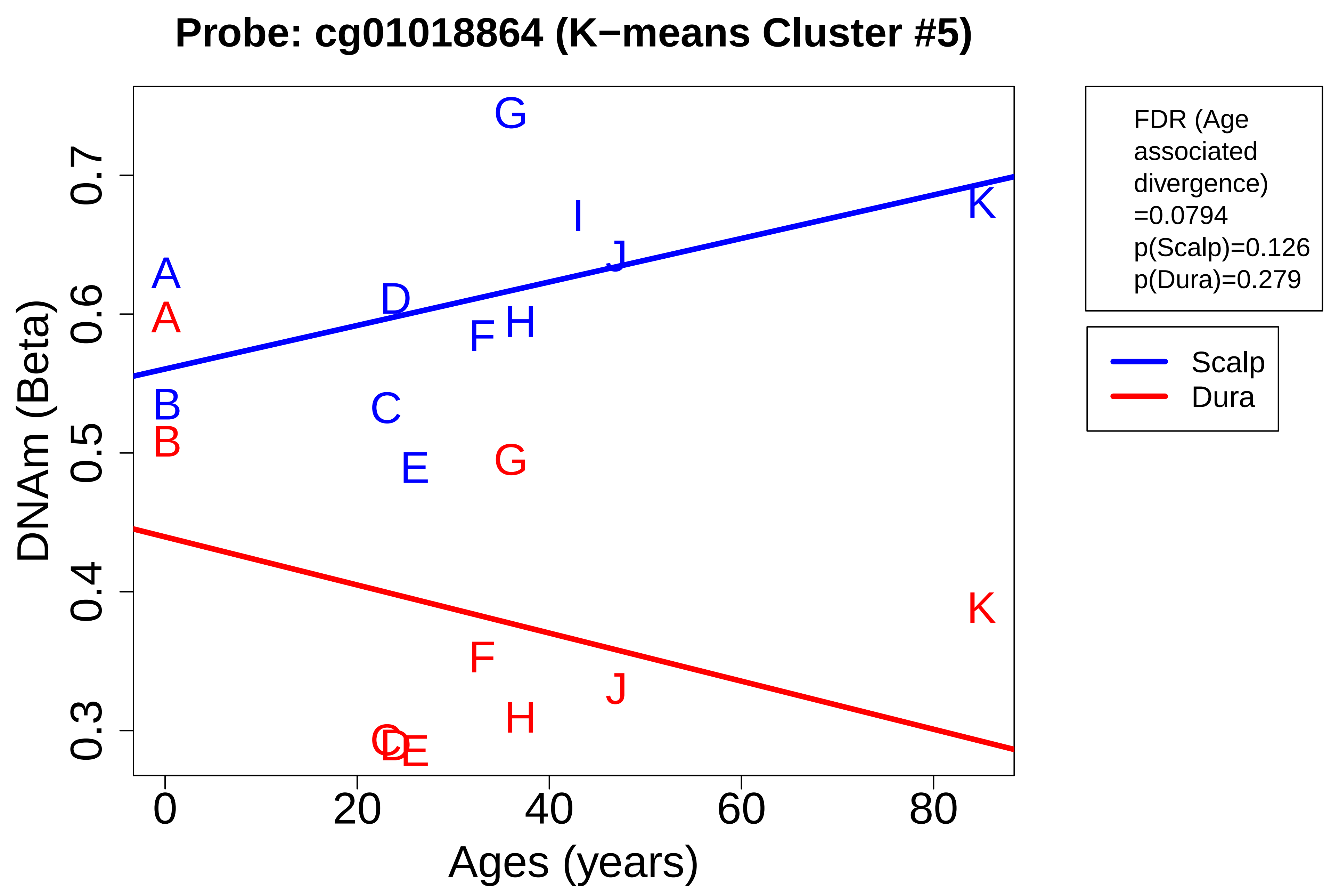

Probe: cg01018864 (K−means Cluster #5)
DNAm (Beta)
Ages (years)
0.3
0.4
0.5
0.6
0.7
0
20
40
60
80
FDR (Age associated divergence) =0.0794
p(Scalp)=0.126
p(Dura)=0.279
Scalp
Dura
A B C D E F G H I J K
A B C D E F G H J K

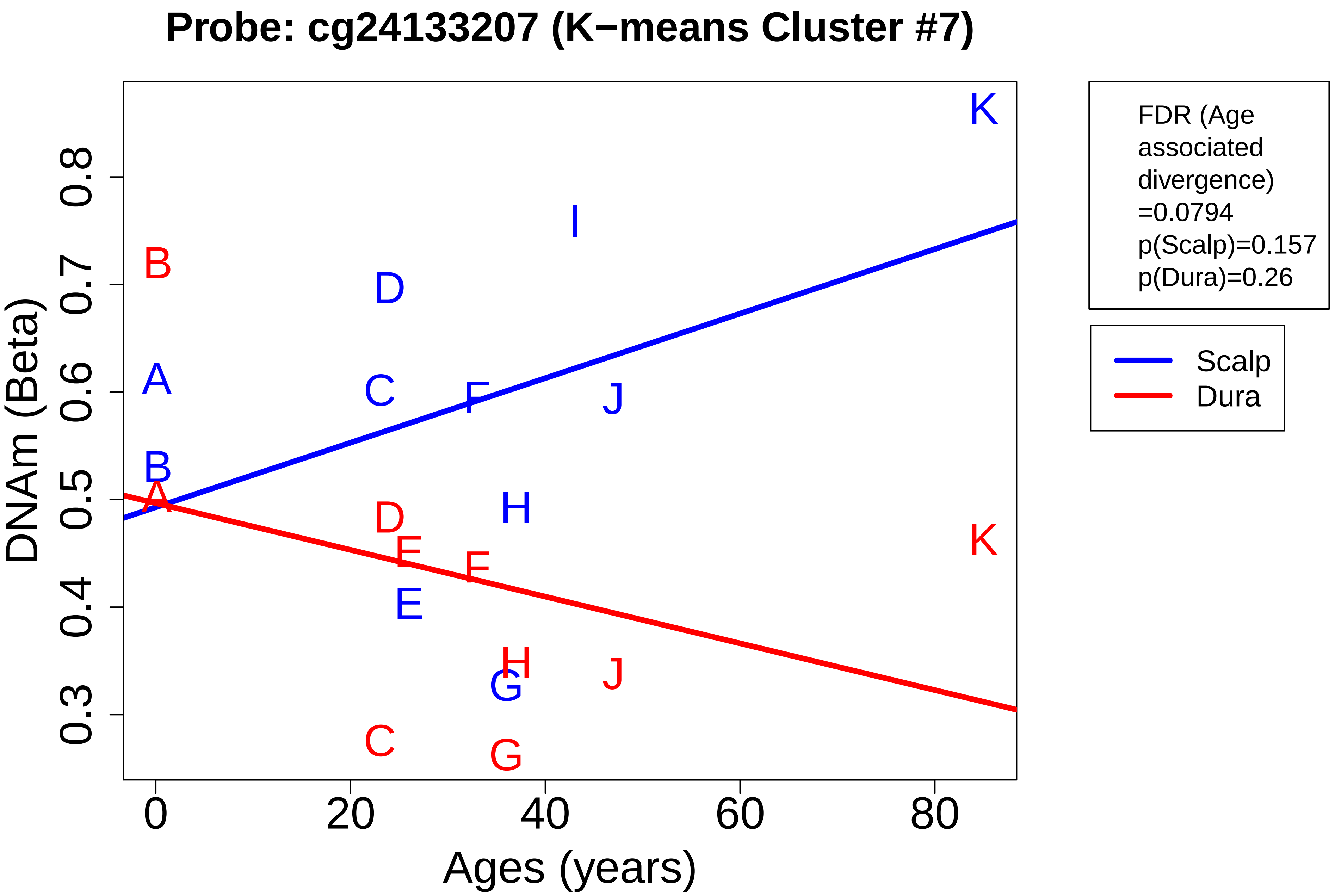
Probe: cg24133207 (K−means Cluster #7)
DNAm (Beta)
Ages (years)
0.3
0.4
0.5
0.6
0.7
0.8
0
20
40
60
80
FDR (Age associated divergence) =0.0794
p(Scalp)=0.157
p(Dura)=0.26
Scalp
Dura
A
B
C
D
E
F
G
H
I
J
K

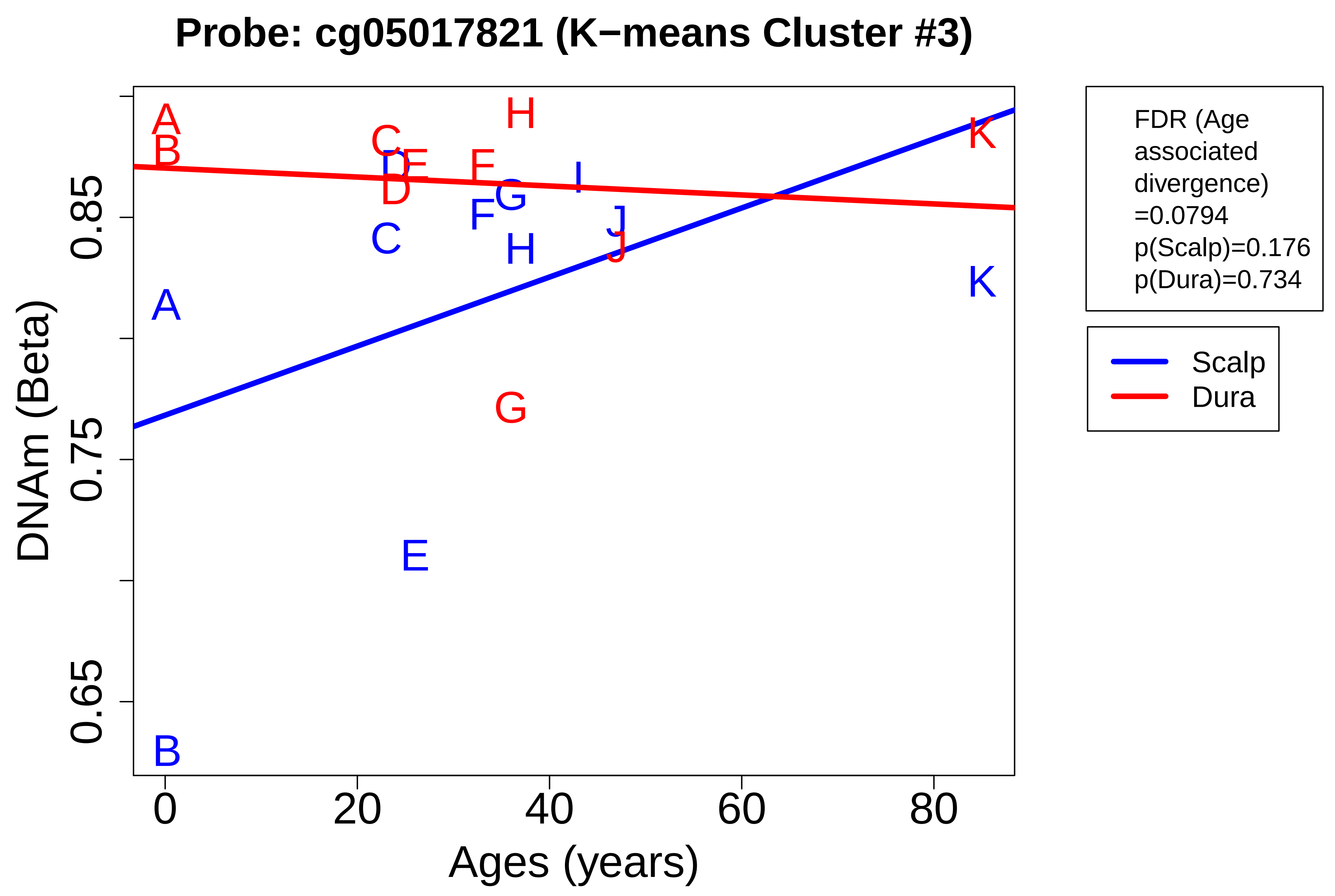

Probe: cg05017821 (K−means Cluster #3)
DNAm (Beta)
0.65
0.75
0.85
Ages (years)
0
20
40
60
80
FDR (Age associated divergence) =0.0794
p(Scalp)=0.176
p(Dura)=0.734
Scalp
Dura

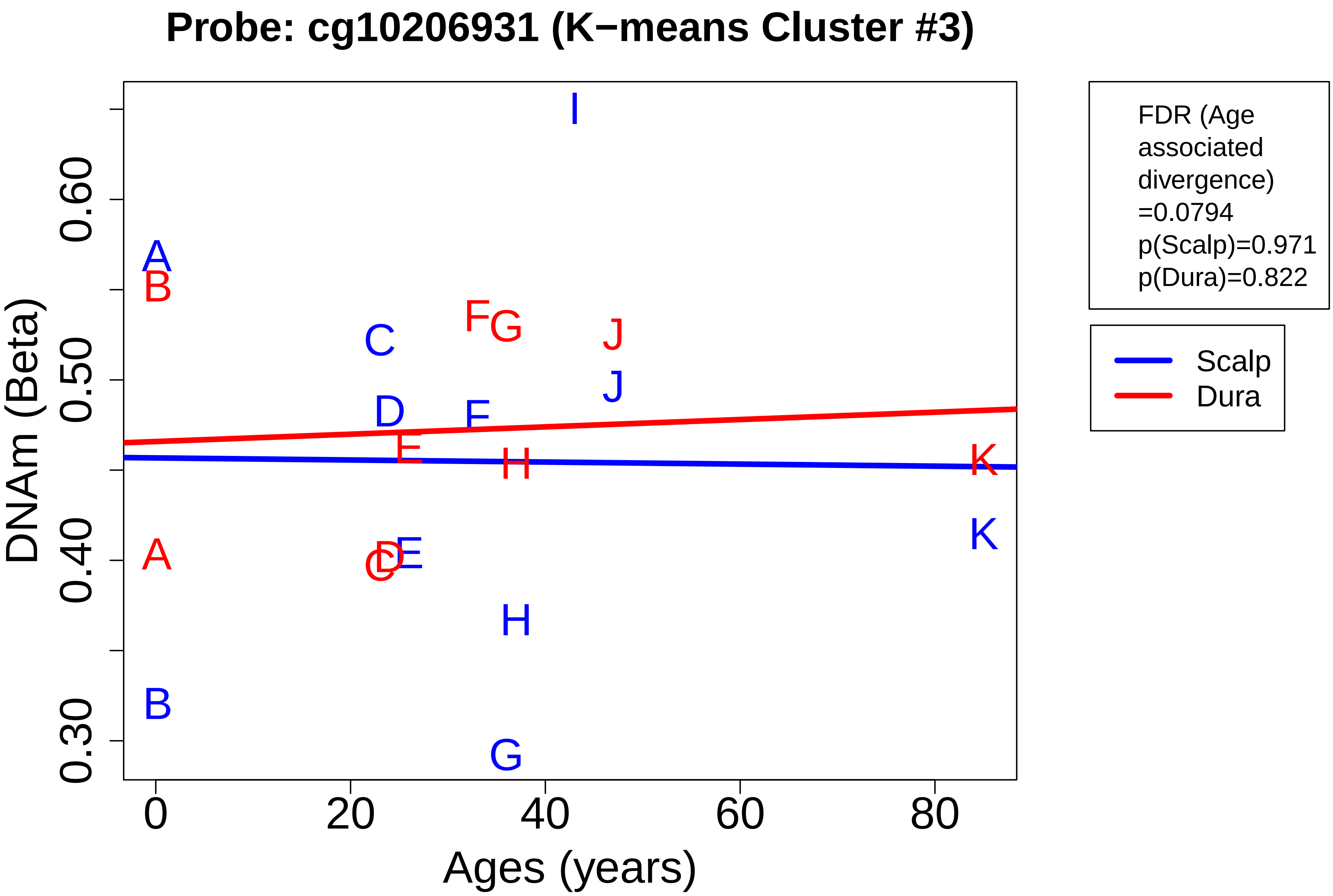
Probe: cg10206931 (K−means Cluster #3)
DNAm (Beta)
0.30
0.40
0.50
0.60
0
20
40
60
80
Ages (years)
FDR (Age associated divergence) =0.0794
p(Scalp)=0.971
p(Dura)=0.822
Scalp
Dura
A
B
C
D
E
F
G
H
I
J
K

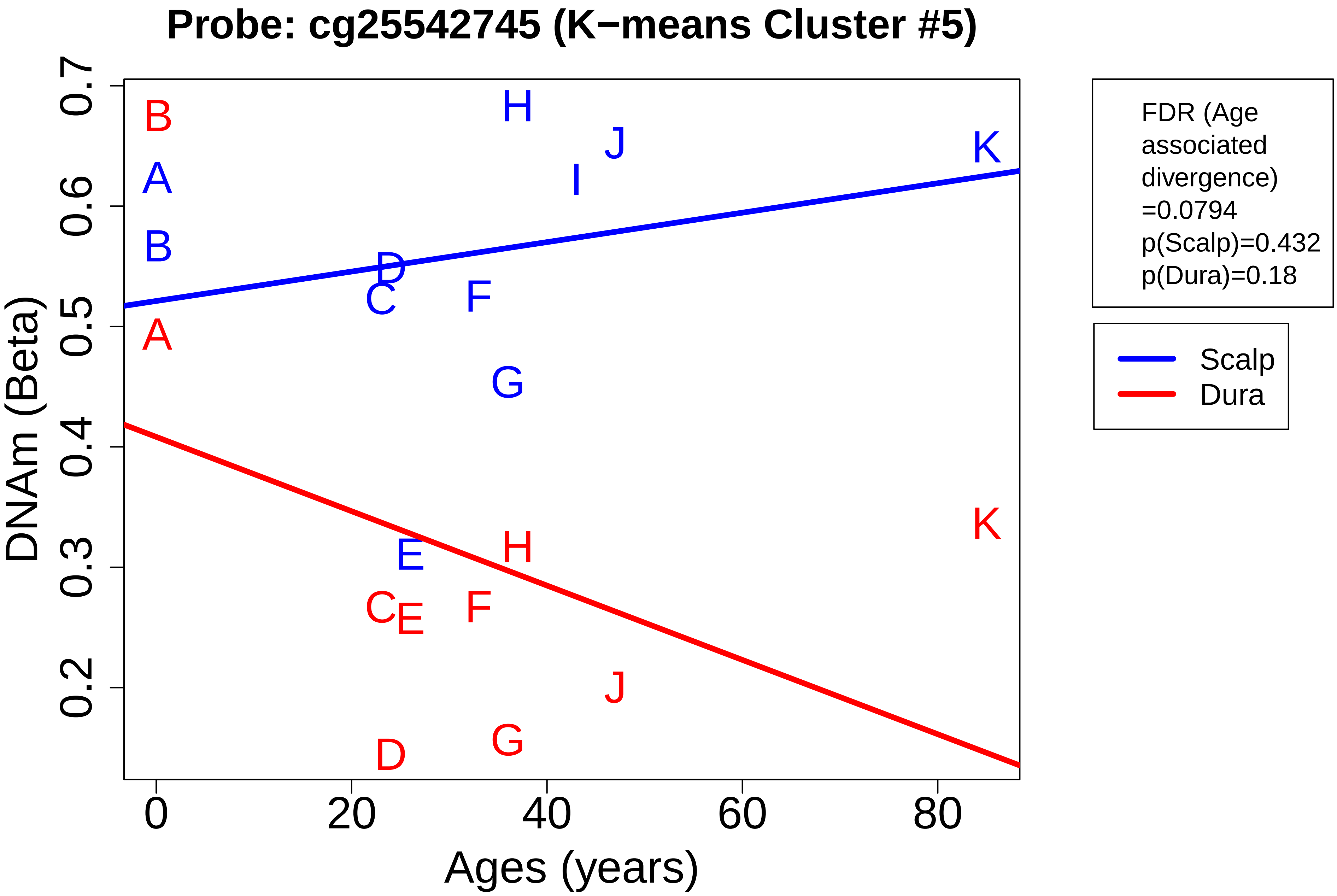
Probe: cg25542745 (K−means Cluster #5)
DNAm (Beta)
0.2
0.3
0.4
0.5
0.6
0.7
Ages (years)
0
20
40
60
80
FDR (Age associated divergence) =0.0794
p(Scalp)=0.432
p(Dura)=0.18
Scalp
Dura
A
B
C
D
E
F
G
H
I
J
K

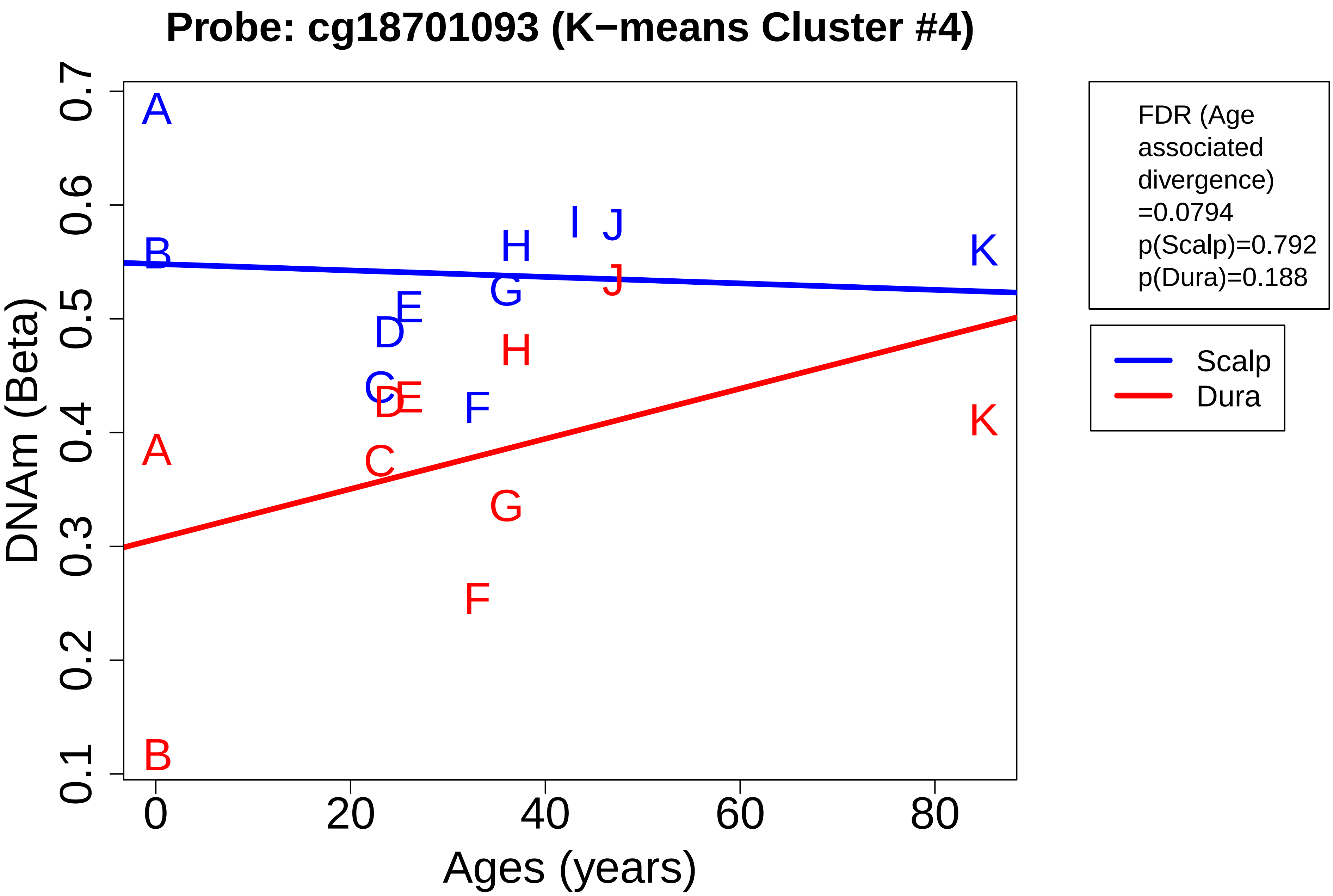

Probe: cg18701093 (K−means Cluster #4)
DNAm (Beta)
0.1
0.2
0.3
0.4
0.5
0.6
0.7
Ages (years)
0
20
40
60
80
FDR (Age associated divergence) =0.0794
p(Scalp)=0.792
p(Dura)=0.188
Scalp
Dura
A
B
C
D
E
F
G
H
I
J
K

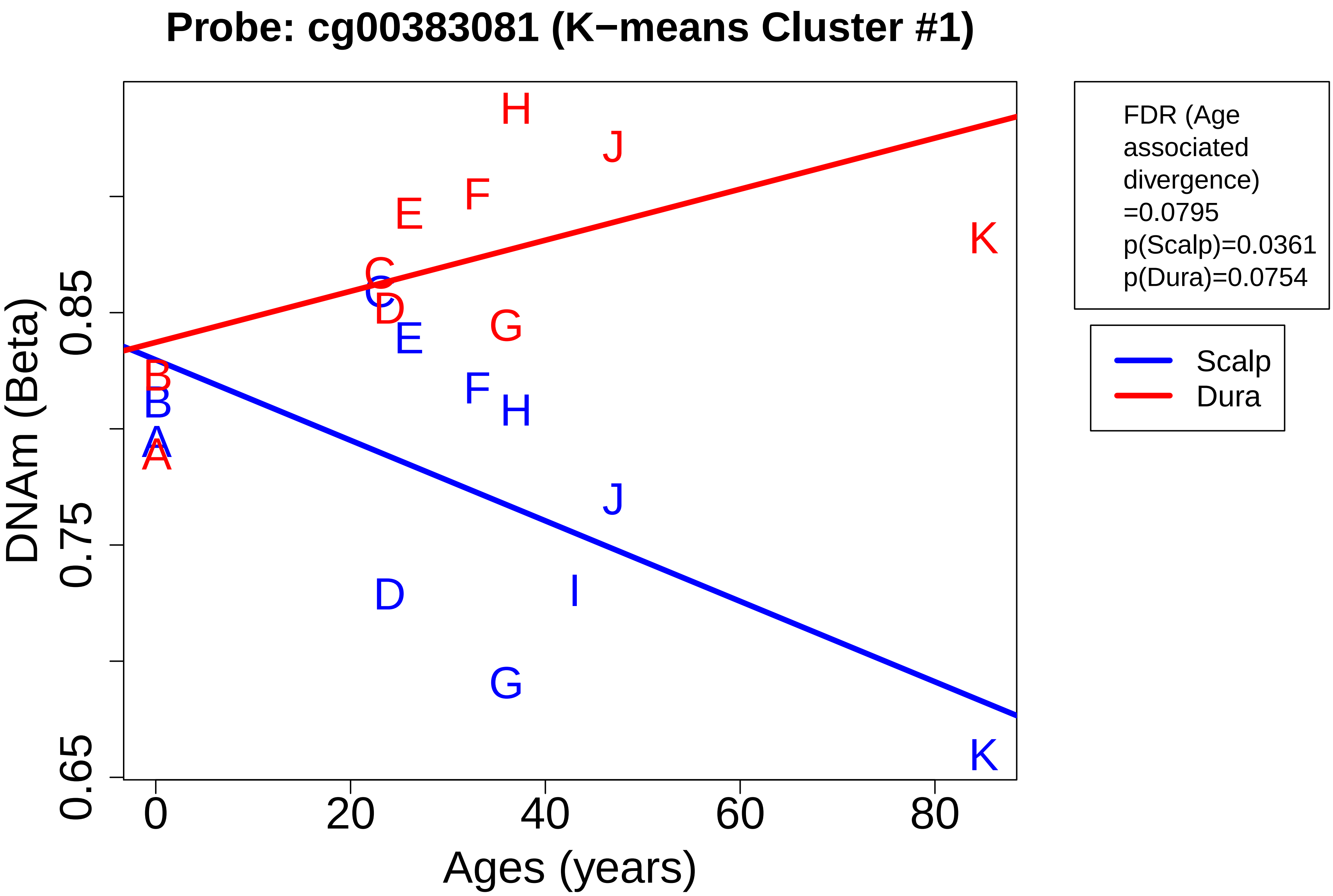

Probe: cg00383081 (K−means Cluster #1)
DNAm (Beta)
0.65
0.75
0.85
Ages (years)
0
20
40
60
80
FDR (Age associated divergence) =0.0795
p(Scalp)=0.0361
p(Dura)=0.0754
Scalp
Dura
A B C D E F G H I J K

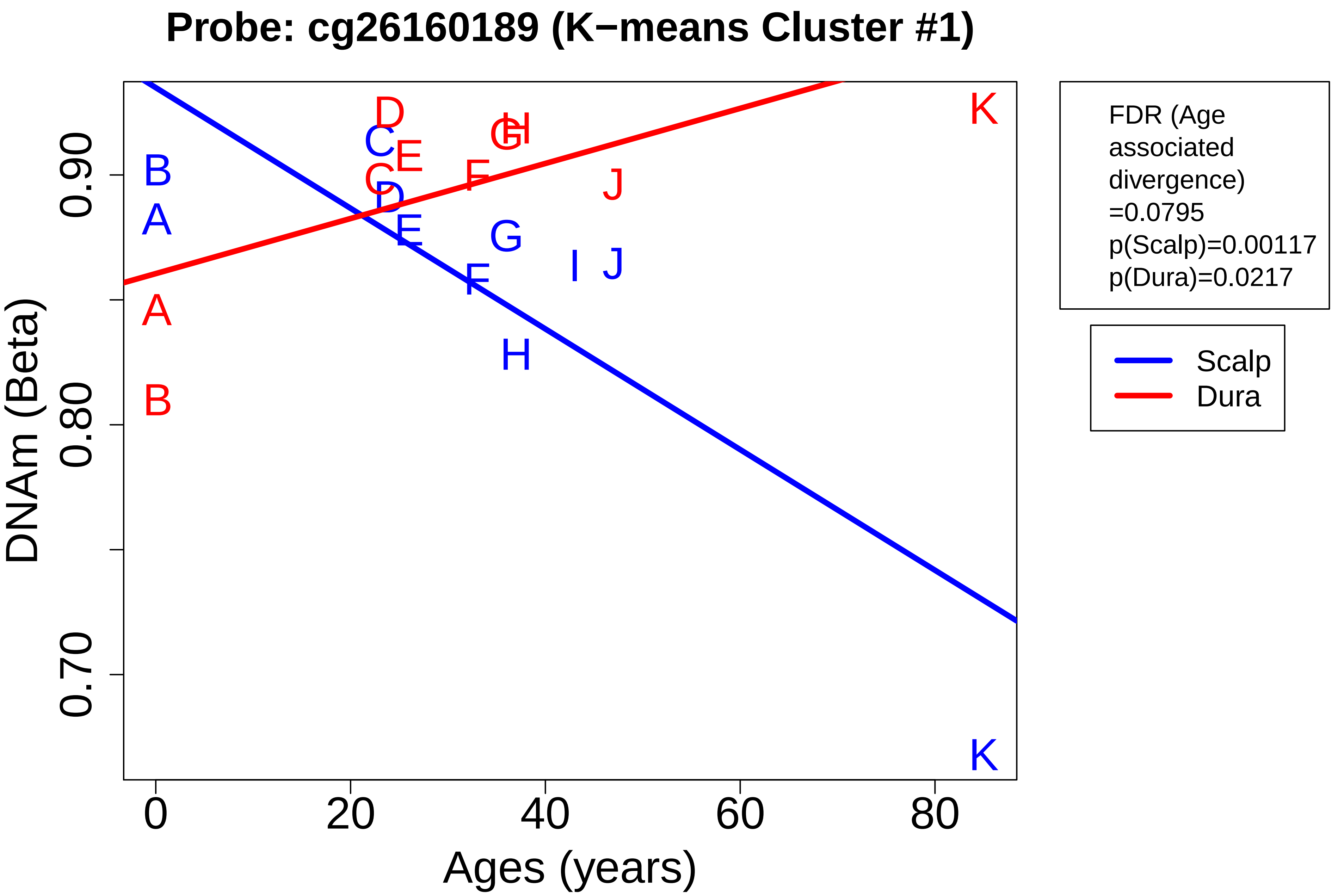

Probe: cg26160189 (K−means Cluster #1)
DNAm (Beta)
0.70
0.80
0.90
Ages (years)
0
20
40
60
80
FDR (Age associated divergence) =0.0795
p(Scalp)=0.00117
p(Dura)=0.0217
Scalp
Dura
A
B
C
D
E
F
G
H
I
J
K

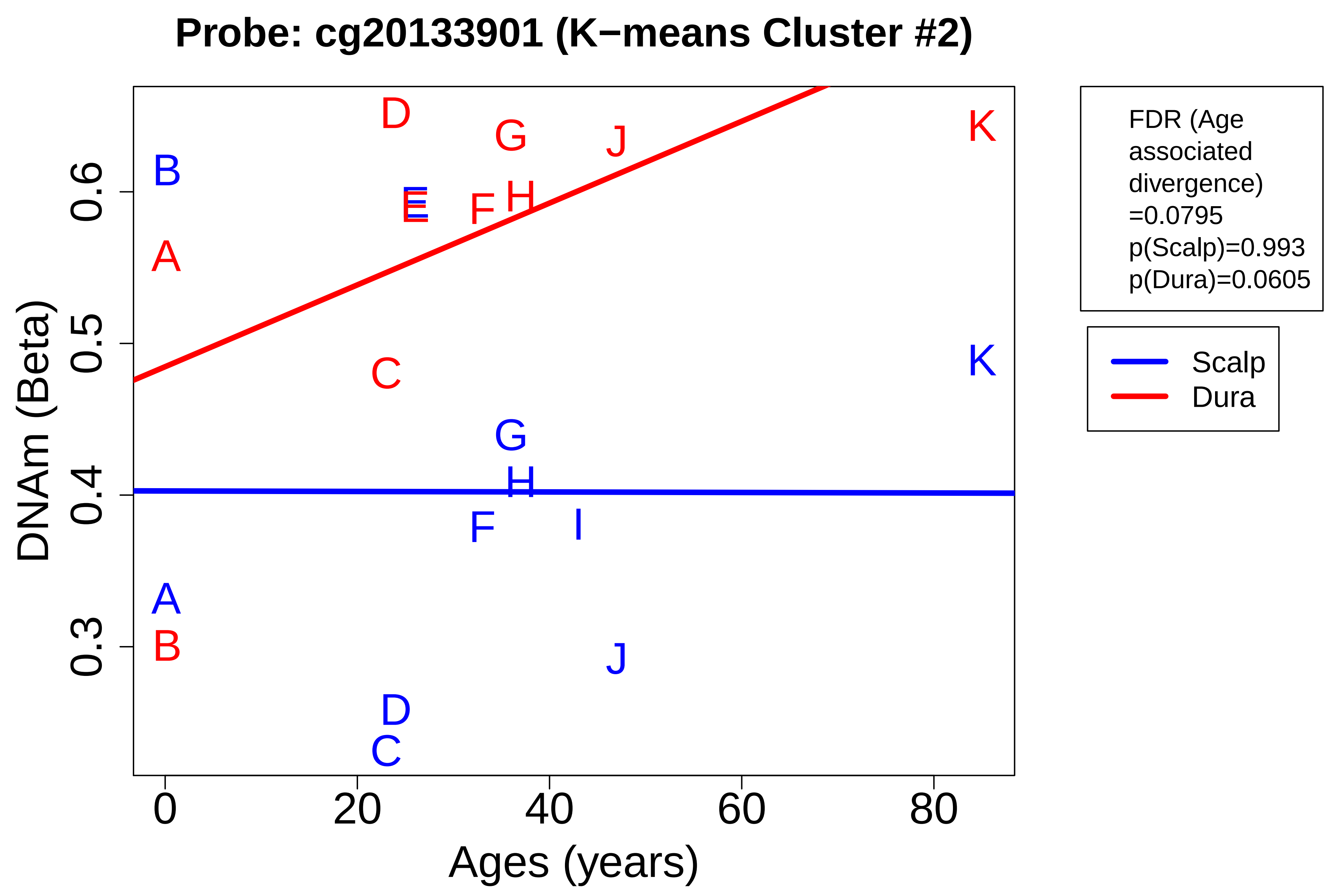
Probe: cg20133901 (K−means Cluster #2)
DNAm (Beta)
0.3
0.4
0.5
0.6
0
20
40
60
80
Ages (years)
FDR (Age associated divergence) =0.0795
p(Scalp)=0.993
p(Dura)=0.0605
Scalp
Dura

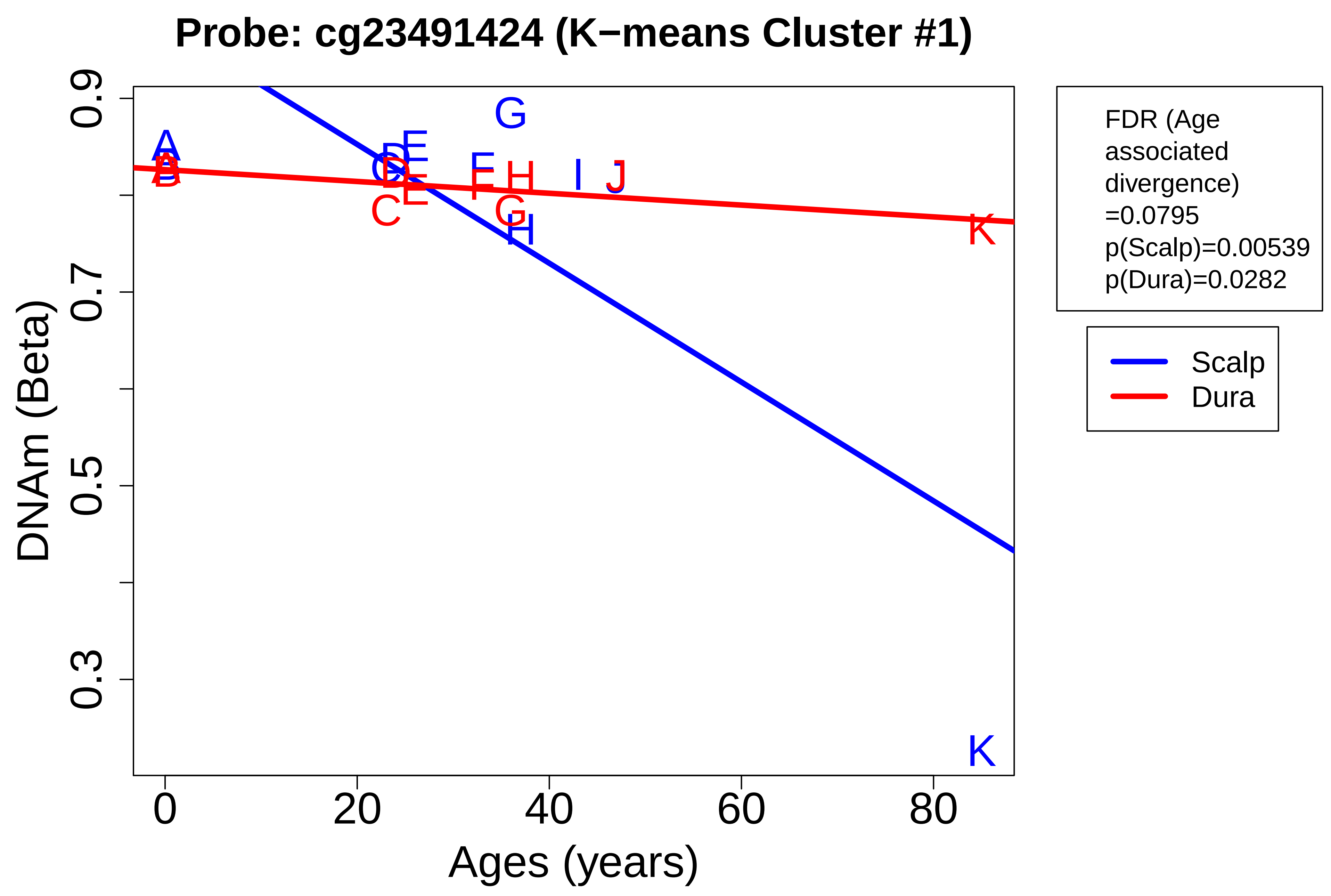

Probe: cg23491424 (K−means Cluster #1)
DNAm (Beta)
0.3
0.5
0.7
0.9
0
20
40
60
80
Ages (years)
FDR (Age associated divergence) =0.0795
p(Scalp)=0.00539
p(Dura)=0.0282
Scalp
Dura

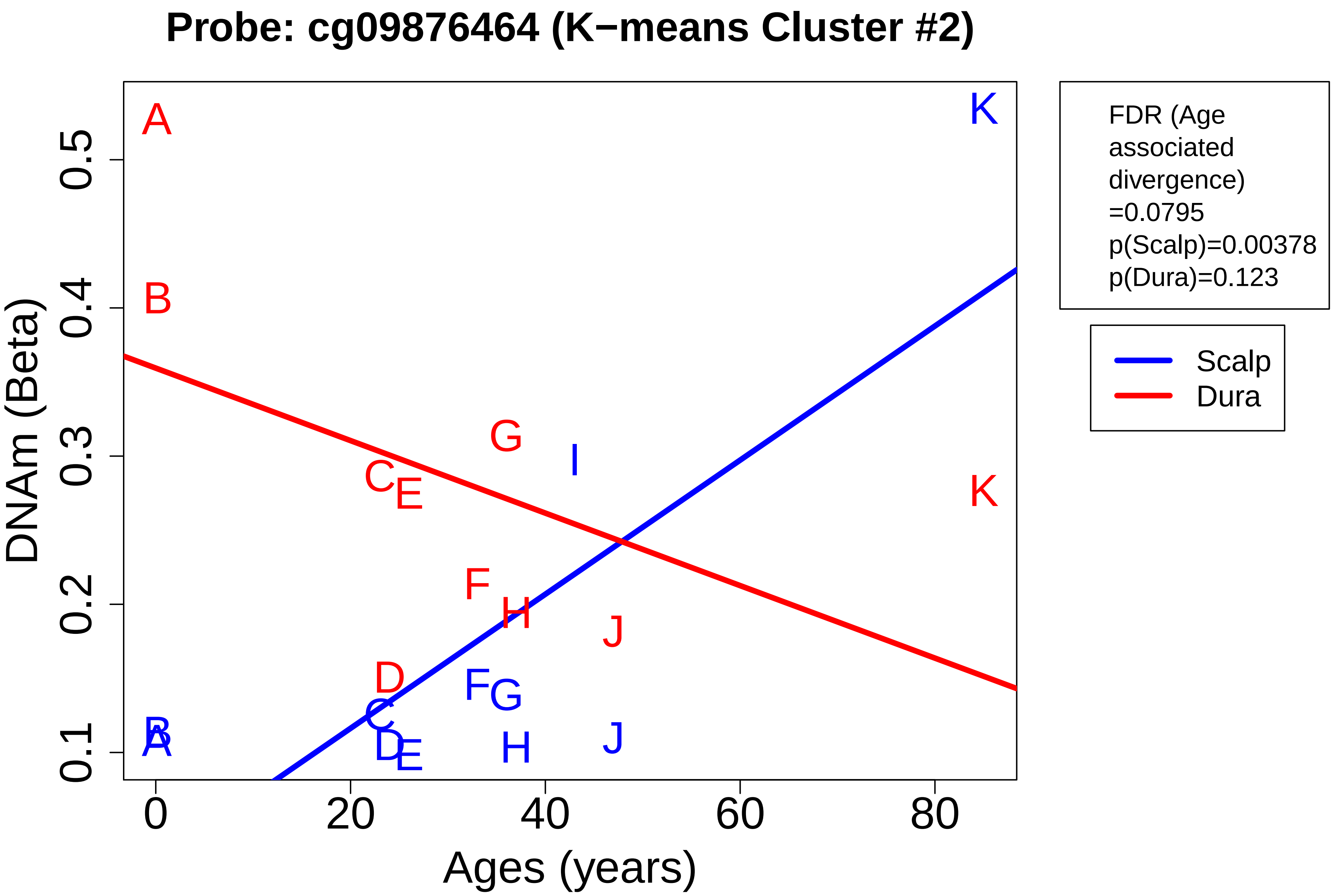
Probe: cg09876464 (K−means Cluster #2)
FDR (Age associated divergence) =0.0795
p(Scalp)=0.00378
p(Dura)=0.123
Scalp
Dura
DNAm (Beta)
Ages (years)
0.1
0.2
0.3
0.4
0.5
0
20
40
60
80
A
B
C
D
E
F
G
H
I
J
K

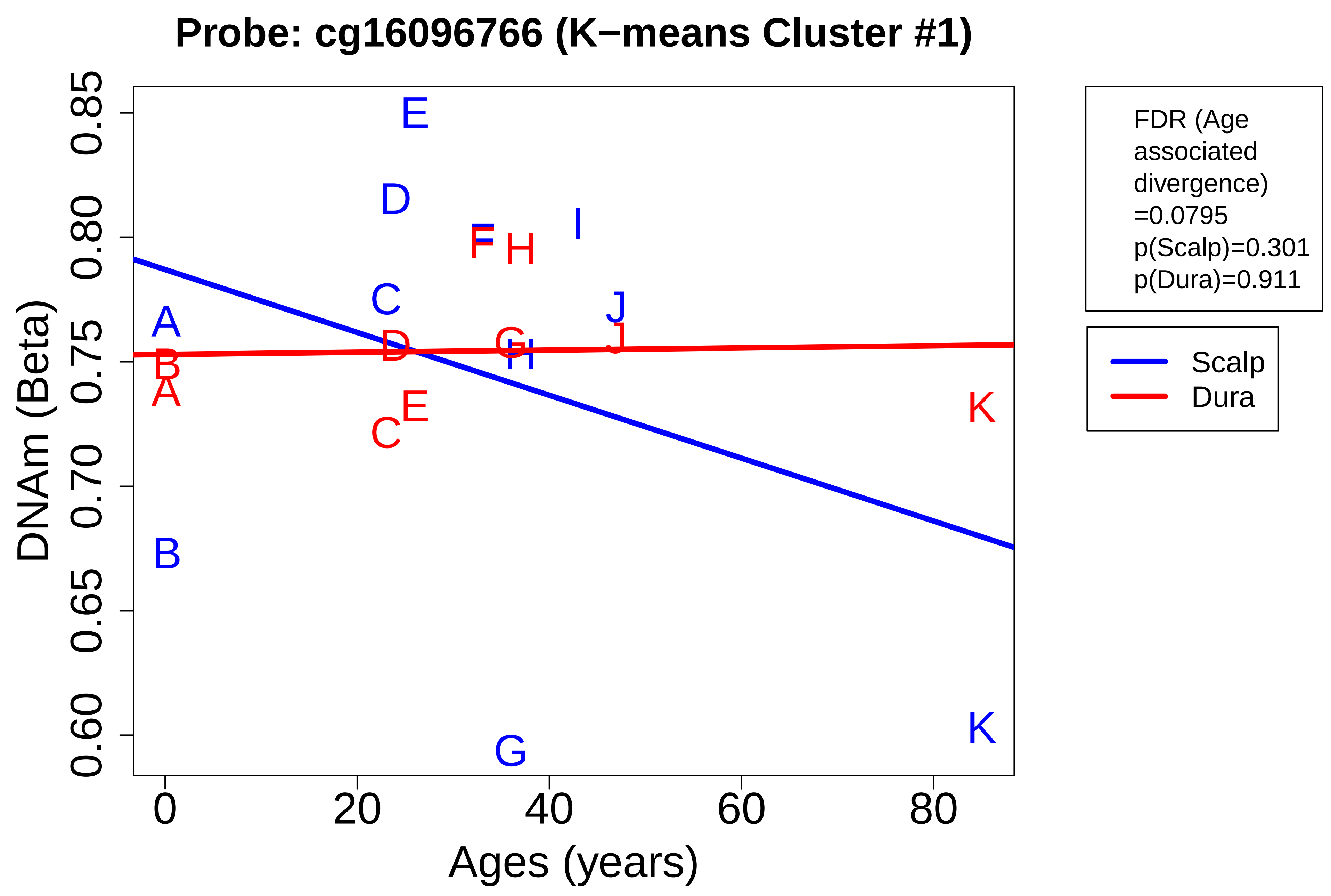

Probe: cg16096766 (K−means Cluster #1)
DNAm (Beta)
0.60
0.65
0.70
0.75
0.80
0.85
0
20
40
60
80
Ages (years)
FDR (Age associated divergence) =0.0795
p(Scalp)=0.301
p(Dura)=0.911
Scalp
Dura

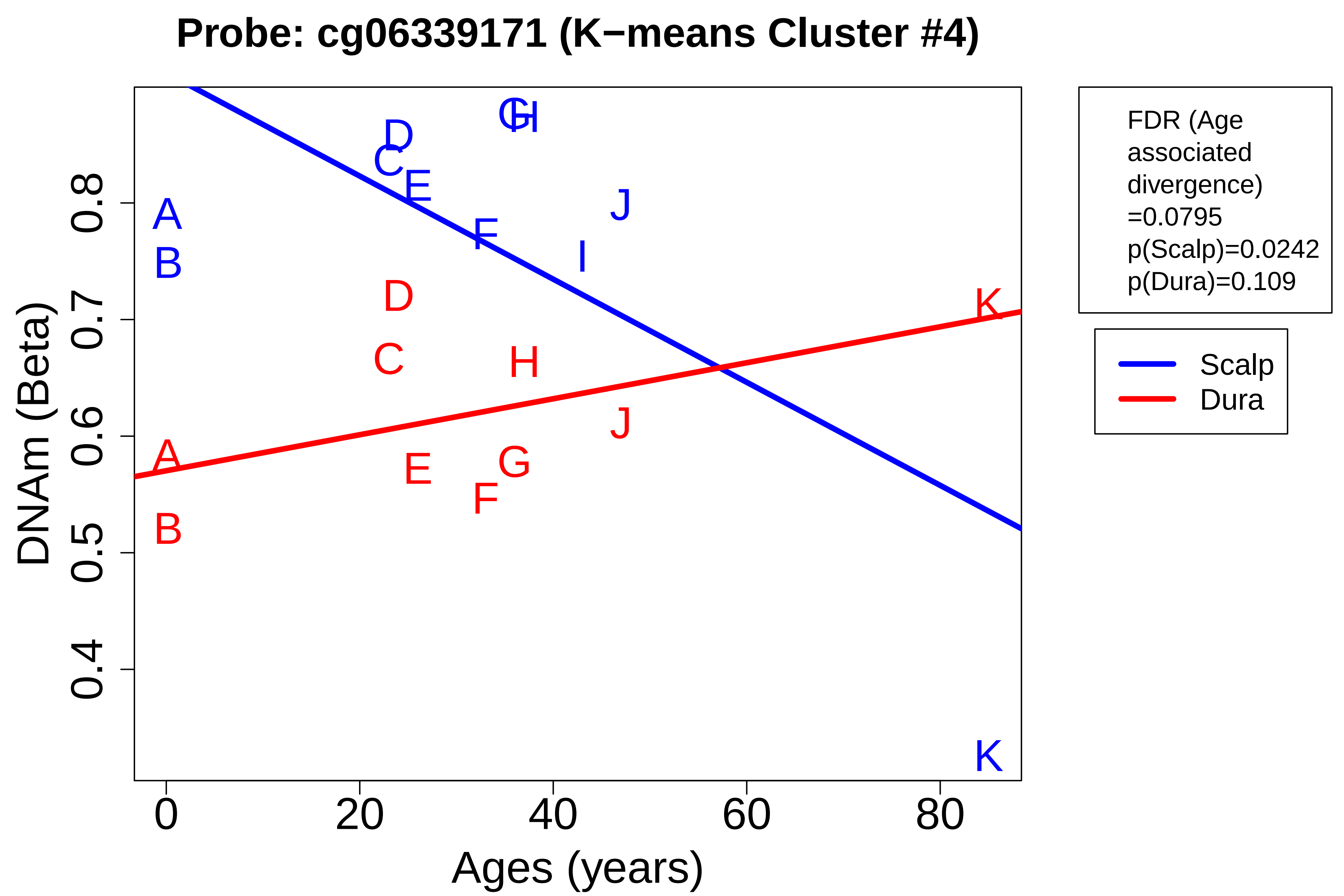

Probe: cg06339171 (K−means Cluster #4)
DNAm (Beta)
0.4
0.5
0.6
0.7
0.8
0
20
40
60
80
Ages (years)
A
B
C
D
E
F
G
H
I
J
K
FDR (Age associated divergence) =0.0795
p(Scalp)=0.0242
p(Dura)=0.109
Scalp
Dura

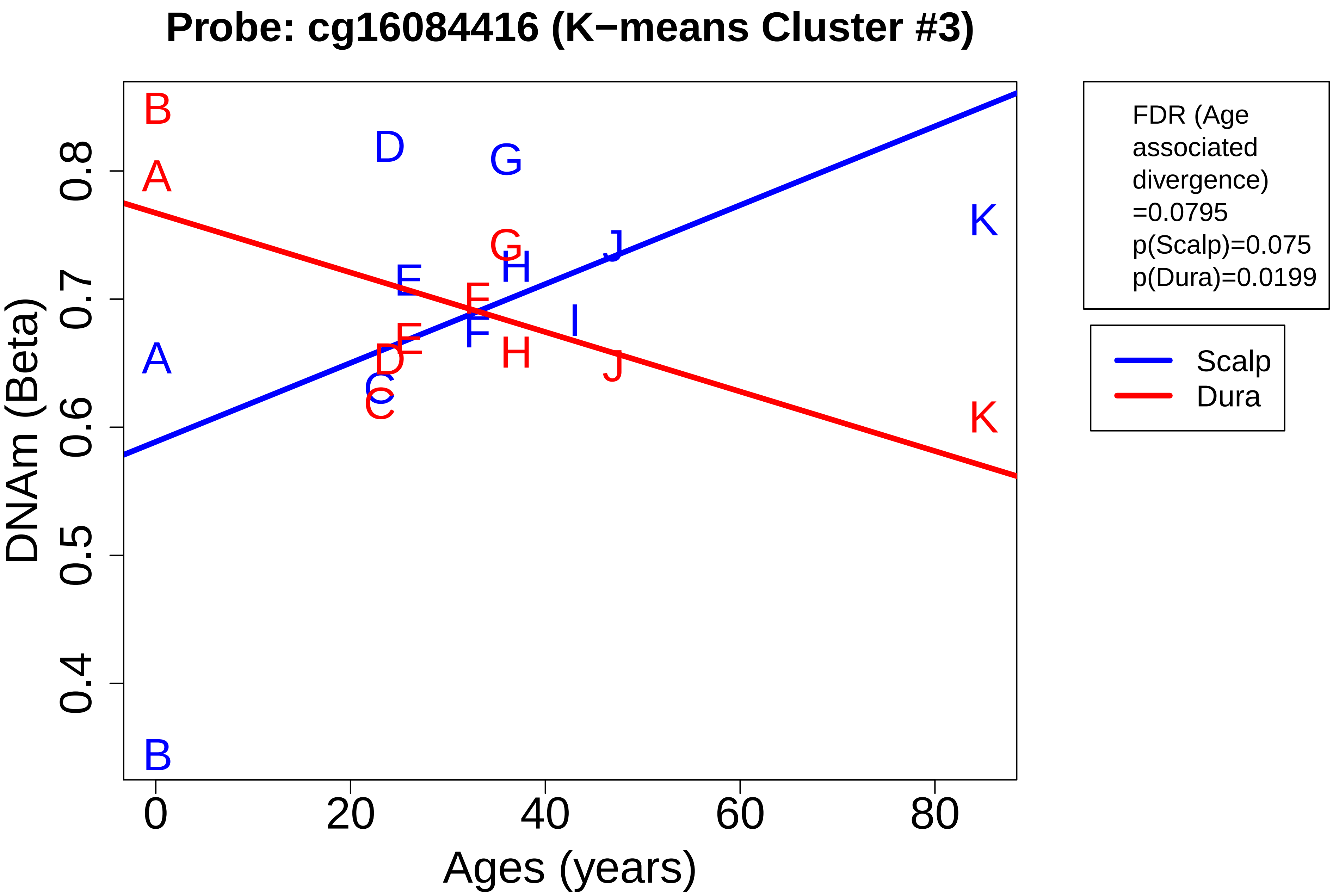

Probe: cg16084416 (K−means Cluster #3)
DNAm (Beta)
0.4
0.5
0.6
0.7
0.8
Ages (years)
0
20
40
60
80
FDR (Age associated divergence) =0.0795
p(Scalp)=0.075
p(Dura)=0.0199
Scalp
Dura

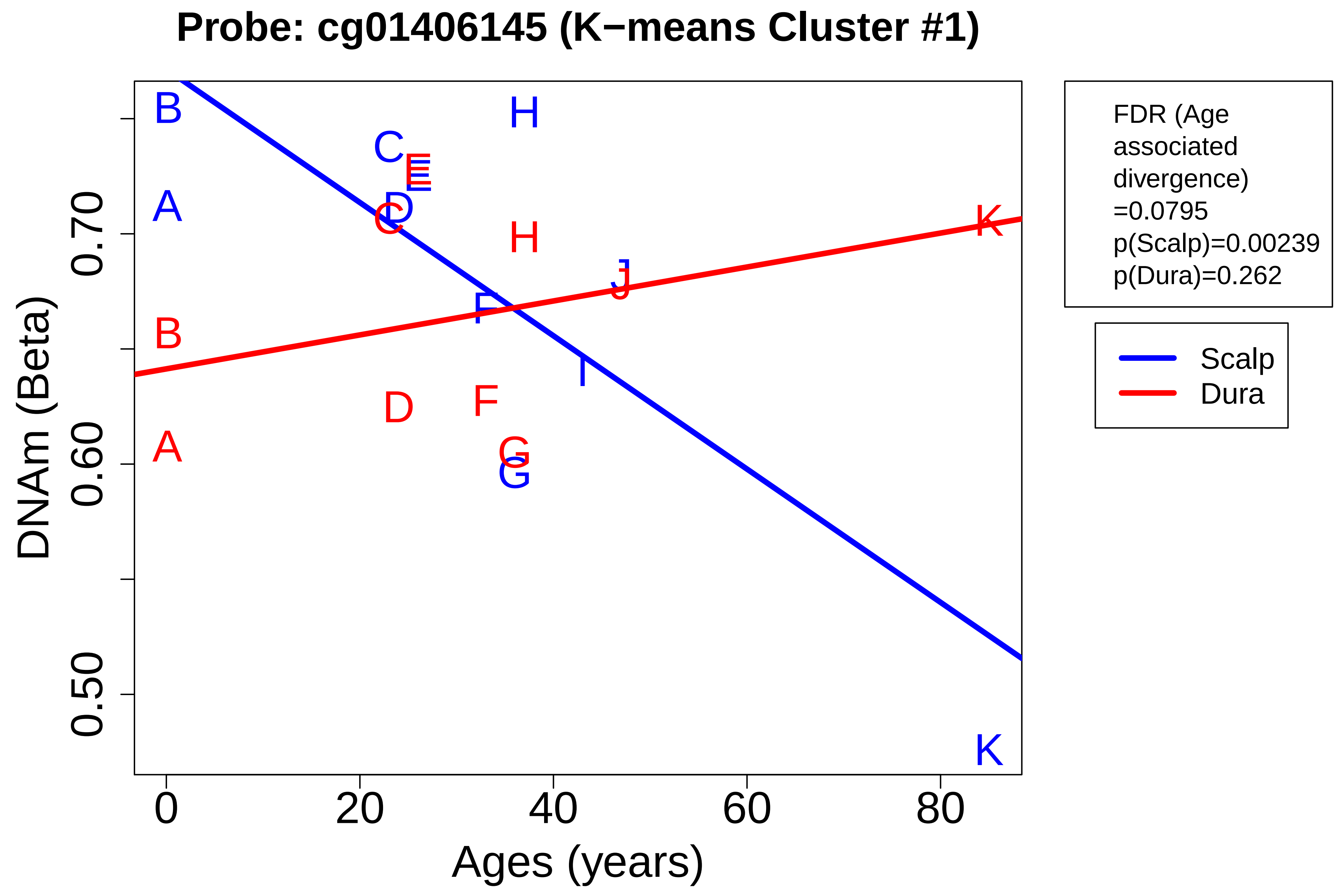

Probe: cg01406145 (K−means Cluster #1)
FDR (Age associated divergence) =0.0795
p(Scalp)=0.00239
p(Dura)=0.262
Scalp
Dura
DNAm (Beta)
0.50
0.60
0.70
Ages (years)
0
20
40
60
80
A
B
C
D
E
F
G
H
I
J
K

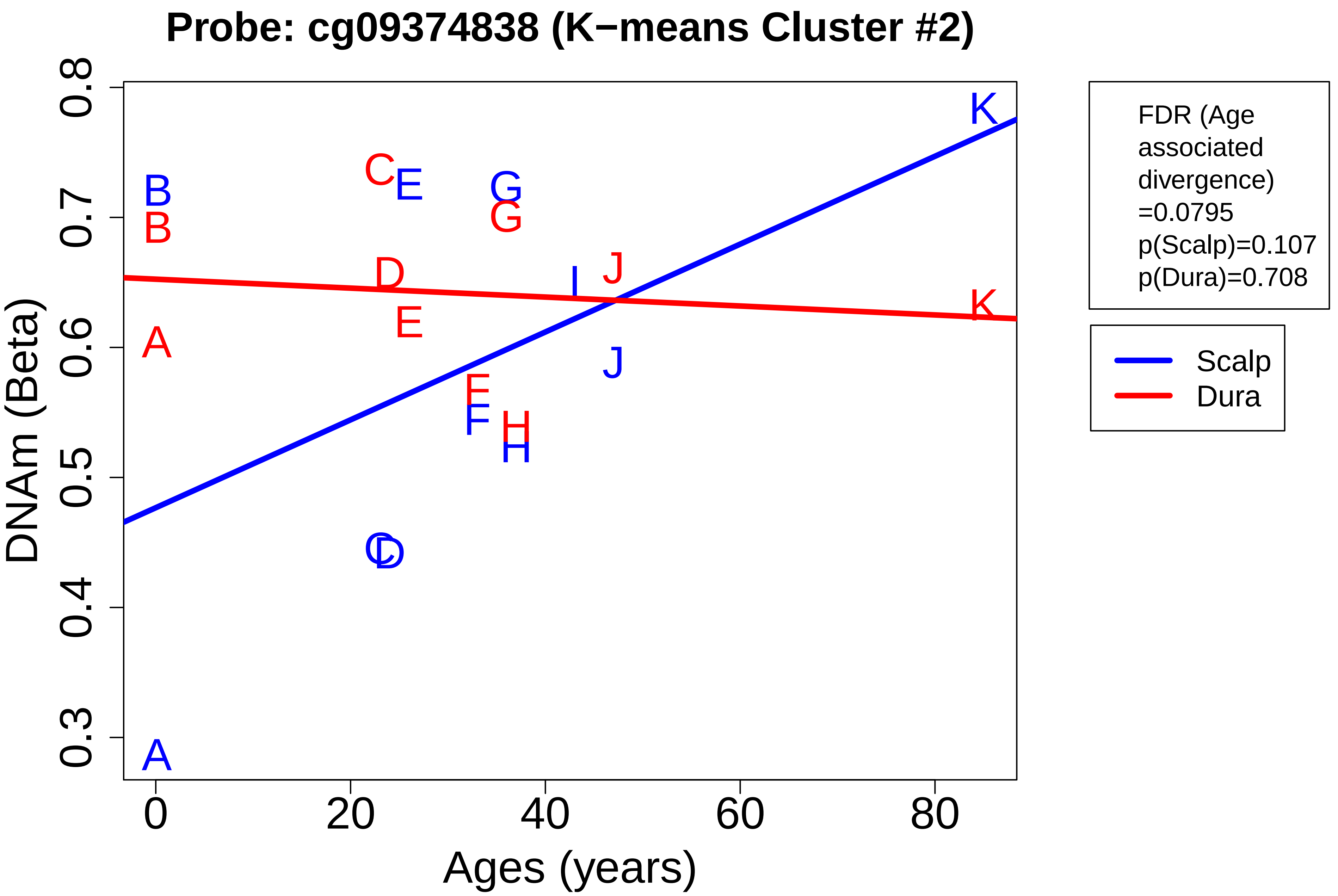

Probe: cg09374838 (K−means Cluster #2)
DNAm (Beta)
0.3
0.4
0.5
0.6
0.7
0.8
0
20
40
60
80
Ages (years)
FDR (Age associated divergence) =0.0795
p(Scalp)=0.107
p(Dura)=0.708
Scalp
Dura

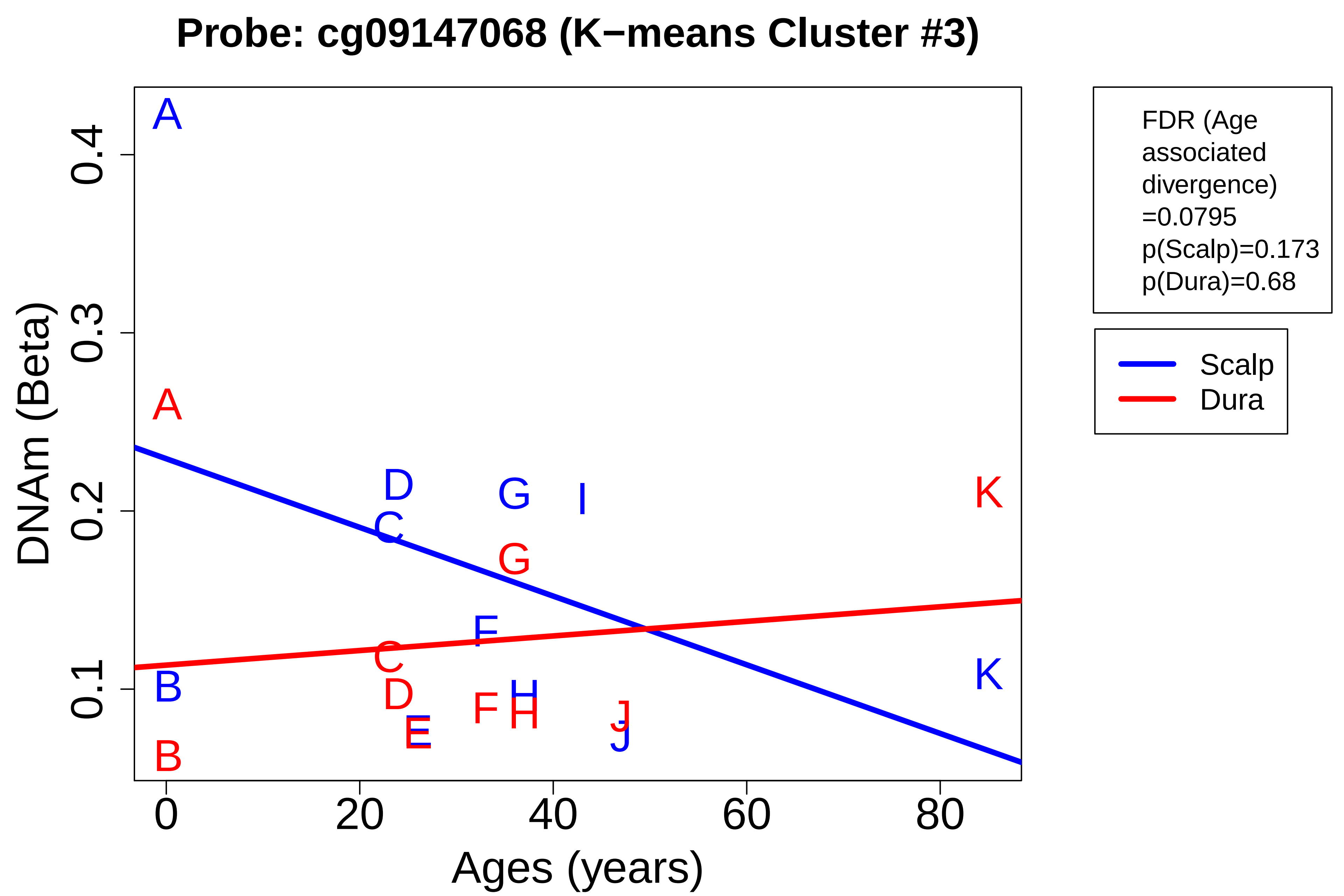

Probe: cg09147068 (K−means Cluster #3)
DNAm (Beta)
0.1
0.2
0.3
0.4
0
20
40
60
80
Ages (years)
A
B
C
D
E
F
G
H
I
J
K
FDR (Age associated divergence) =0.0795
p(Scalp)=0.173
p(Dura)=0.68
Scalp
Dura

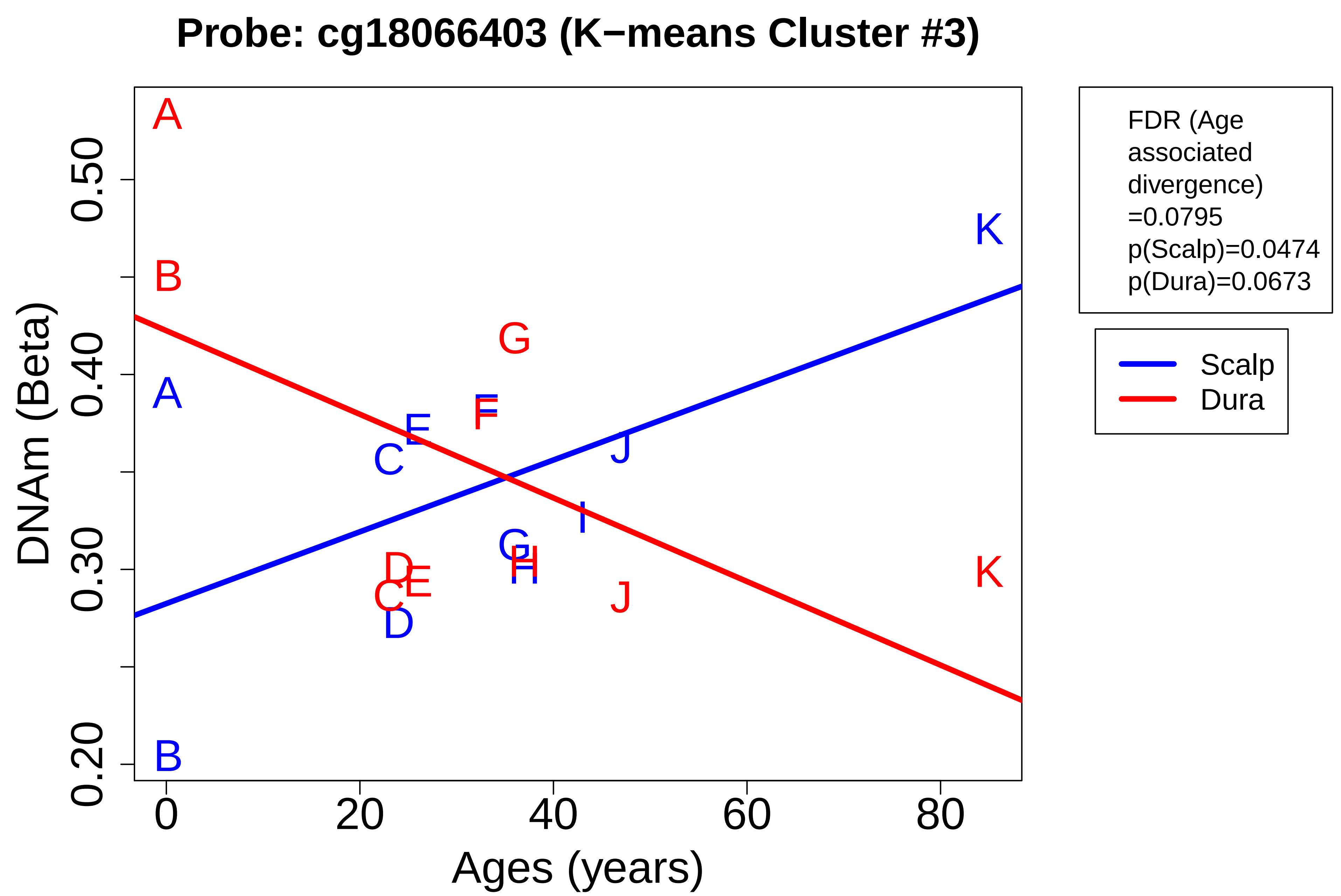

Probe: cg18066403 (K−means Cluster #3)
DNAm (Beta)
0.20
0.30
0.40
0.50
0
20
40
60
80
Ages (years)
A
B
C
D
E
F
G
H
I
J
K
FDR (Age associated divergence) =0.0795
p(Scalp)=0.0474
p(Dura)=0.0673
Scalp
Dura

Probe: cg06084952 (K−means Cluster #7)

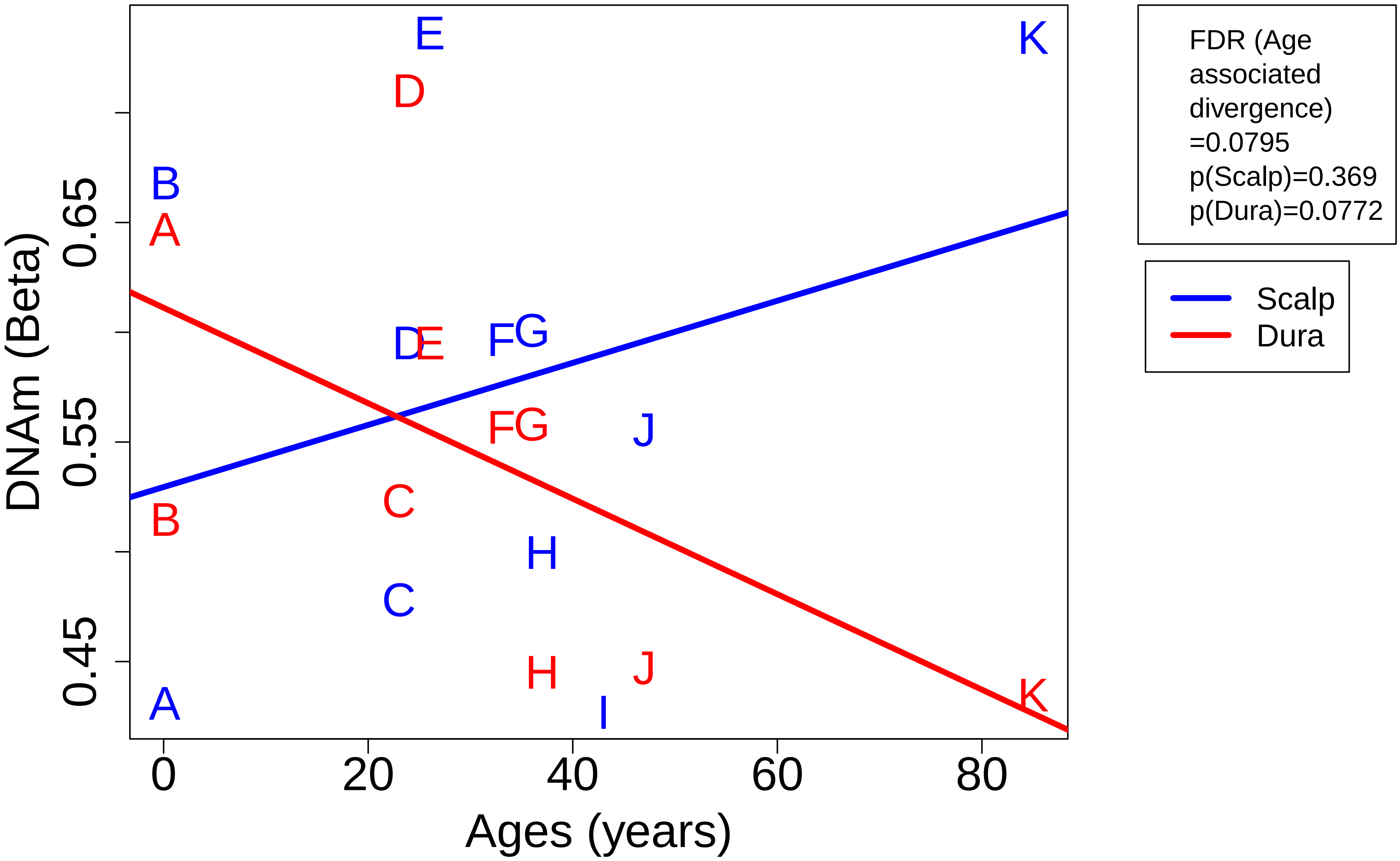

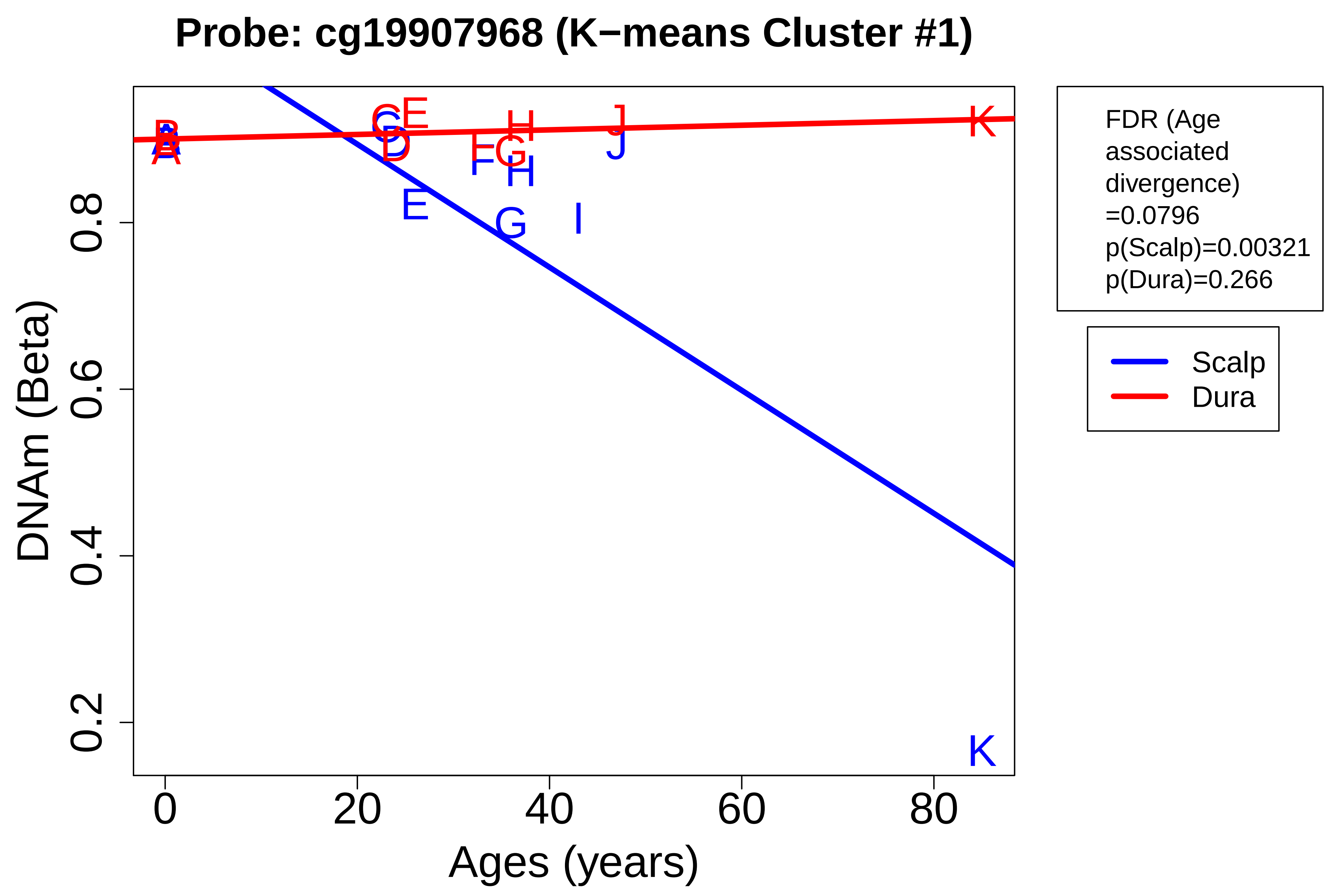

Probe: cg19907968 (K−means Cluster #1)
DNAm (Beta)
0.2
0.4
0.6
0.8
0
20
40
60
80
Ages (years)
FDR (Age associated divergence) =0.0796
p(Scalp)=0.00321
p(Dura)=0.266
Scalp
Dura

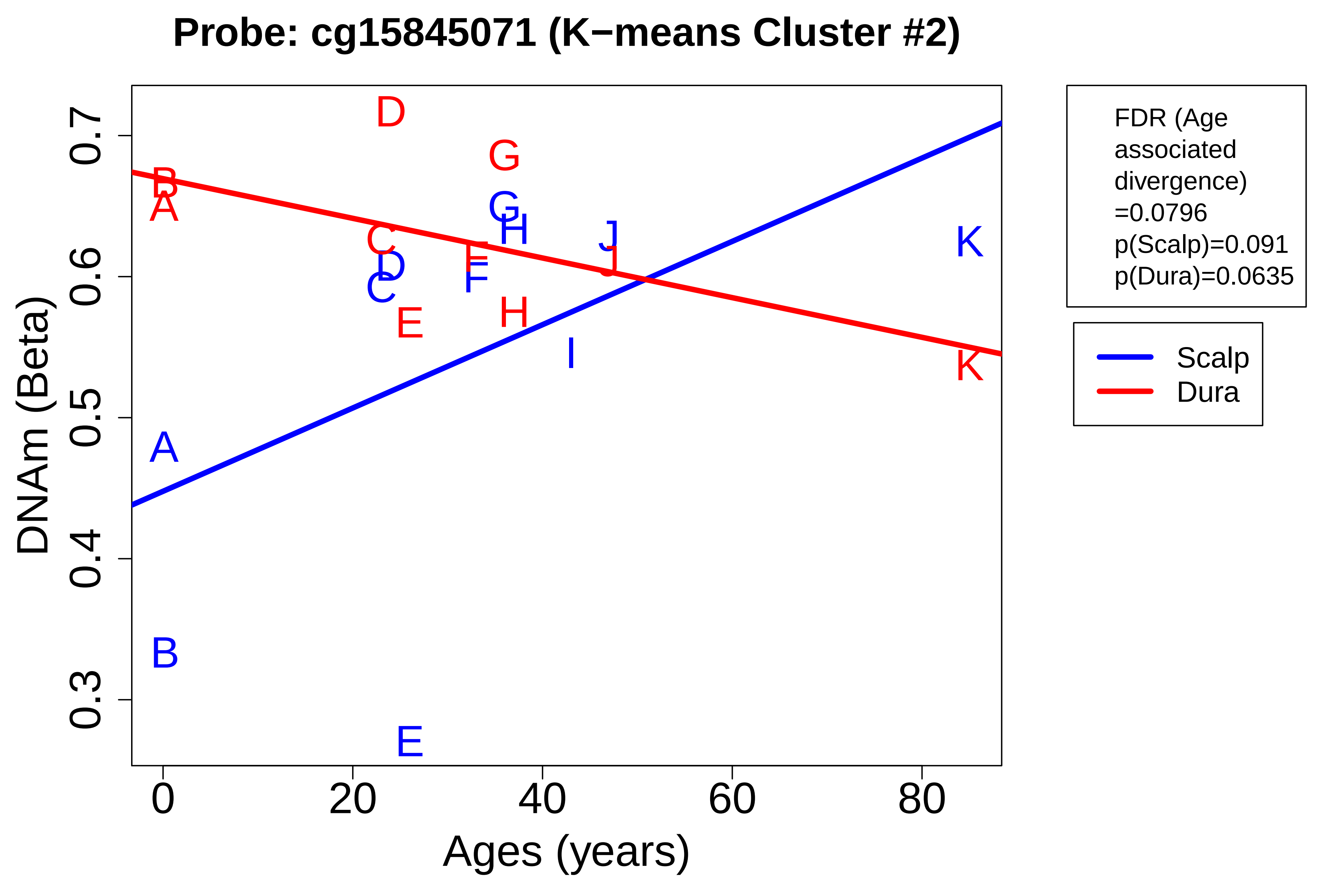

Probe: cg15845071 (K−means Cluster #2)
DNAm (Beta)
0.3
0.4
0.5
0.6
0.7
0
20
40
60
80
Ages (years)
FDR (Age associated divergence) =0.0796
p(Scalp)=0.091
p(Dura)=0.0635
Scalp
Dura

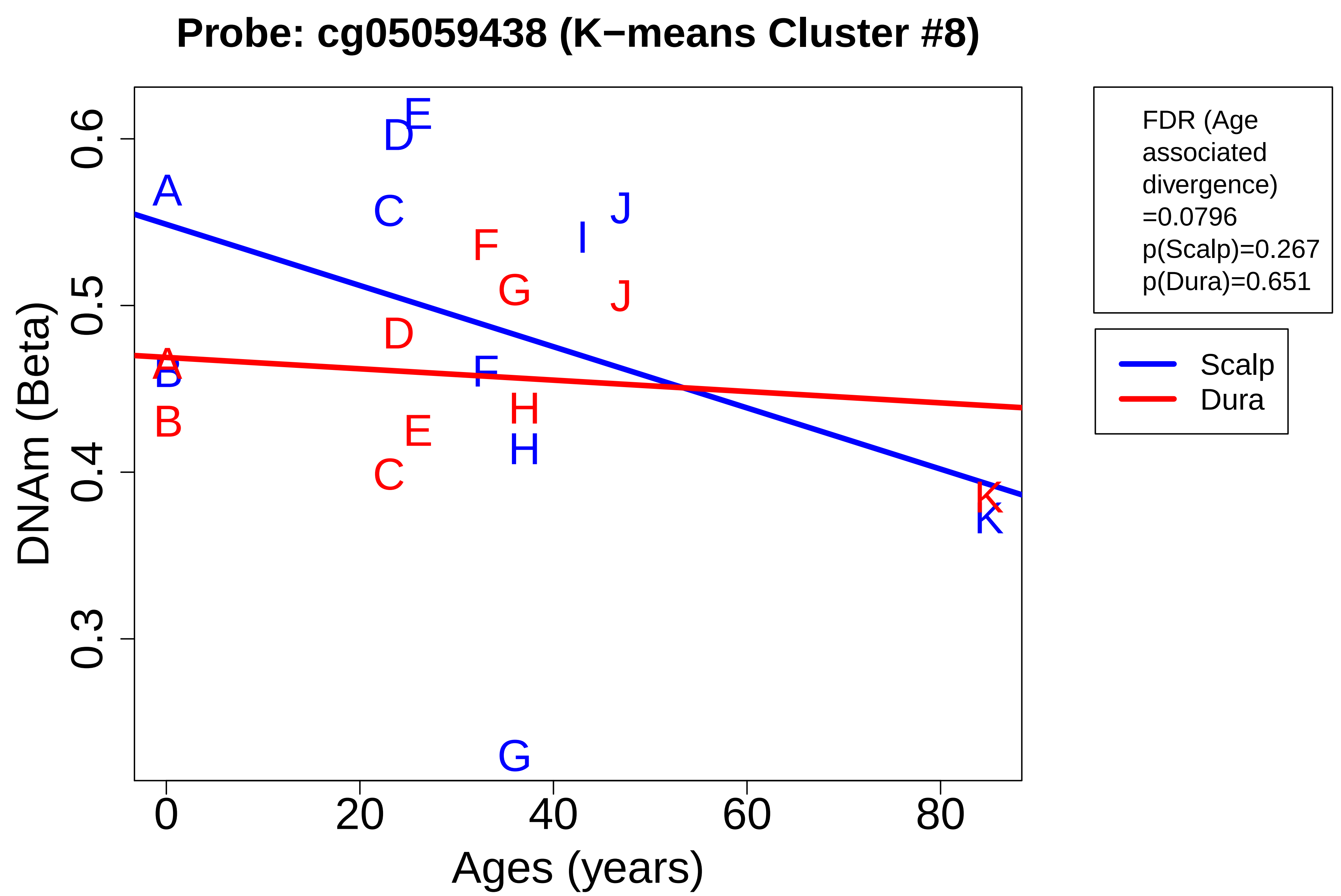

Probe: cg05059438 (K−means Cluster #8)
DNAm (Beta)
0.3
0.4
0.5
0.6
0
20
40
60
80
Ages (years)
A
B
C
D
E
F
G
H
I
J
K
FDR (Age associated divergence) =0.0796
p(Scalp)=0.267
p(Dura)=0.651
Scalp
Dura

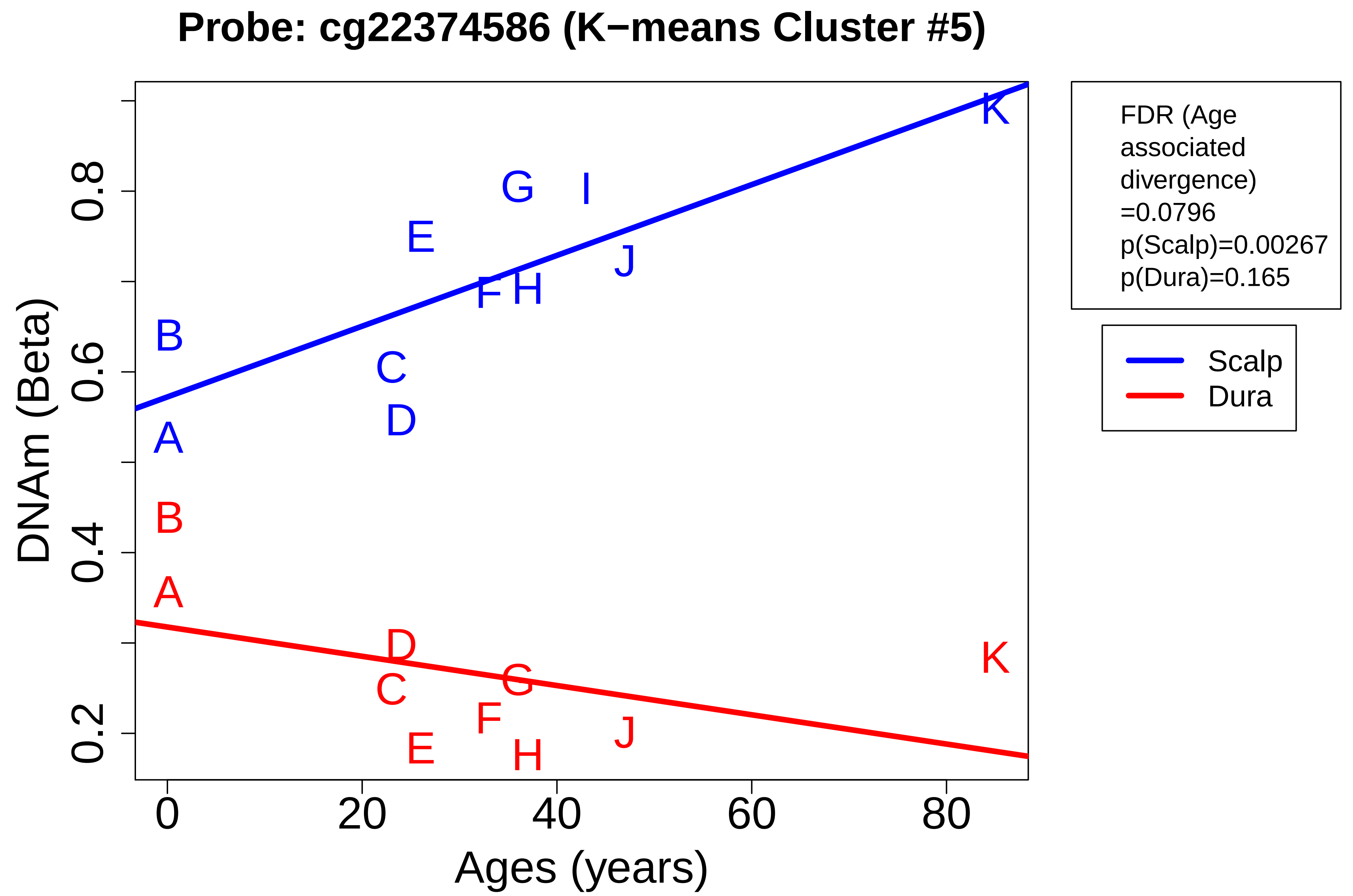

Probe: cg22374586 (K−means Cluster #5)
DNAm (Beta)
0.2
0.4
0.6
0.8
0
20
40
60
80
Ages (years)
FDR (Age associated divergence) =0.0796
p(Scalp)=0.00267
p(Dura)=0.165
Scalp
Dura
A
B
C
D
E
F
G
H
I
J
K

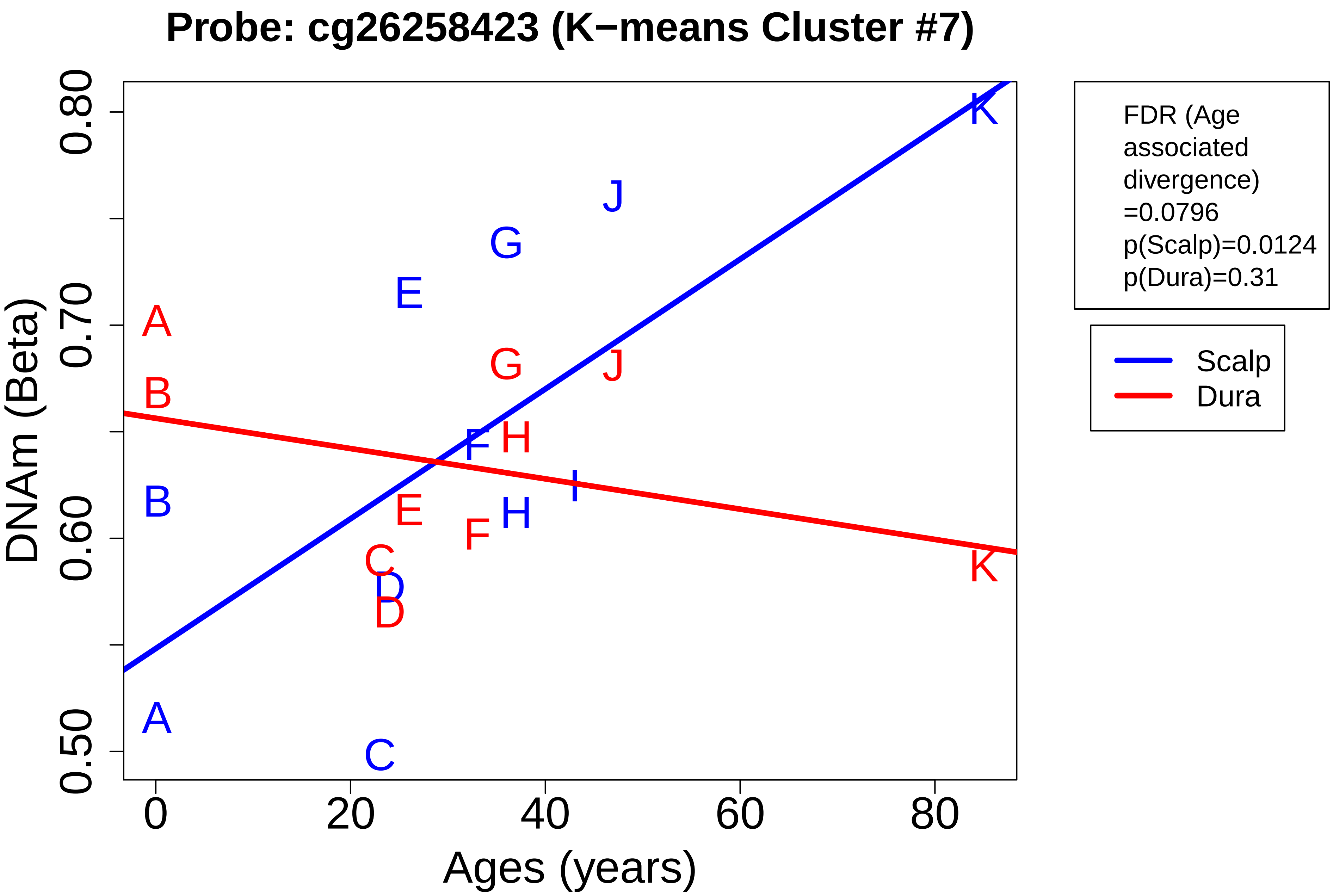
Probe: cg26258423 (K−means Cluster #7)
DNAm (Beta)
0.50
0.60
0.70
0.80
0
20
40
60
80
Ages (years)
FDR (Age associated divergence) =0.0796
p(Scalp)=0.0124
p(Dura)=0.31
Scalp
Dura
A
B
C
D
E
F
G
H
I
J
K

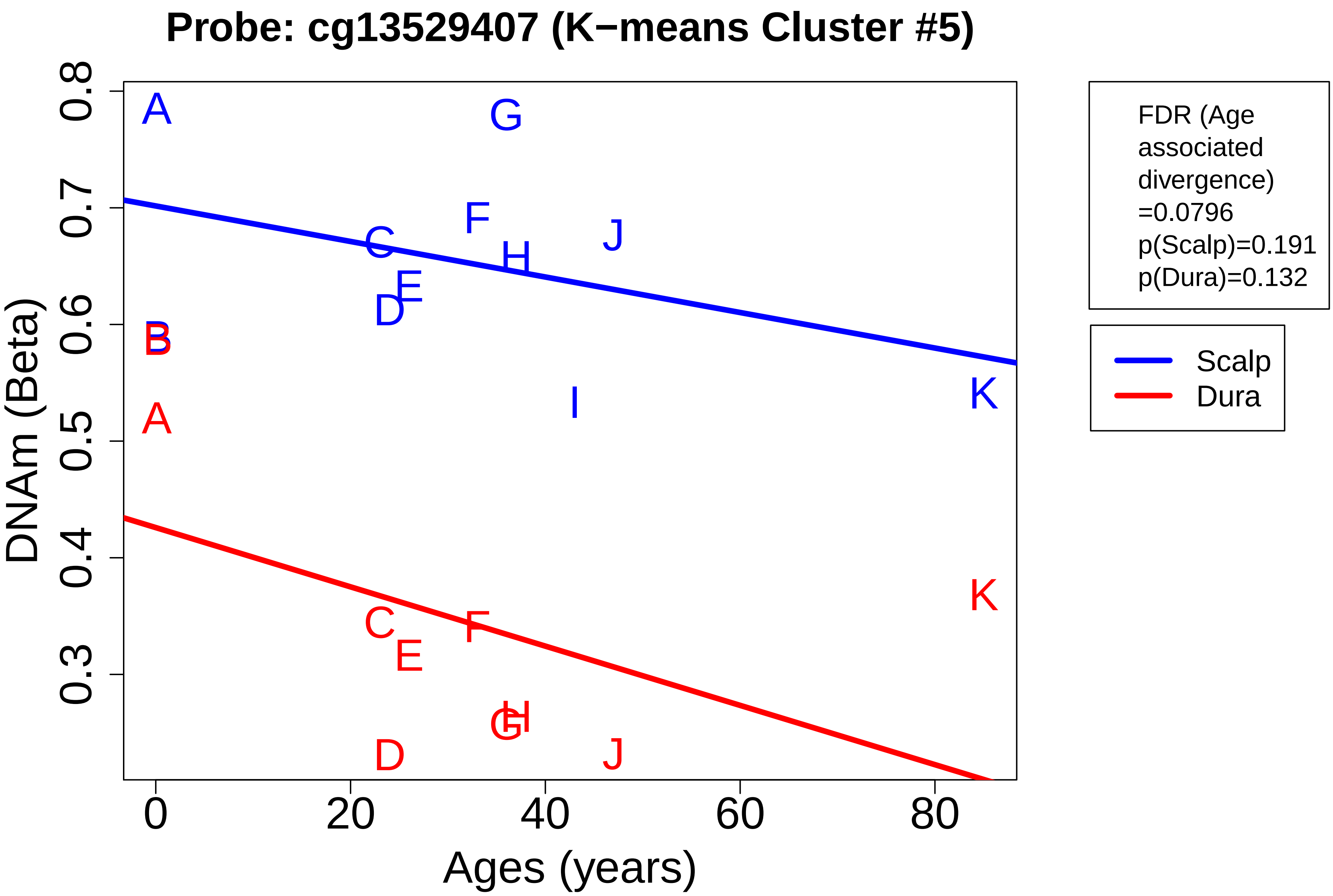

Probe: cg13529407 (K−means Cluster #5)
DNAm (Beta)
Ages (years)
0.8
0.7
0.6
0.5
0.4
0.3
0
20
40
60
80
A
G
F
J
C
H
E
D
B
I
K
A
K
C
F
E
H
G
D
J
FDR (Age associated divergence) =0.0796
p(Scalp)=0.191
p(Dura)=0.132
Scalp
Dura

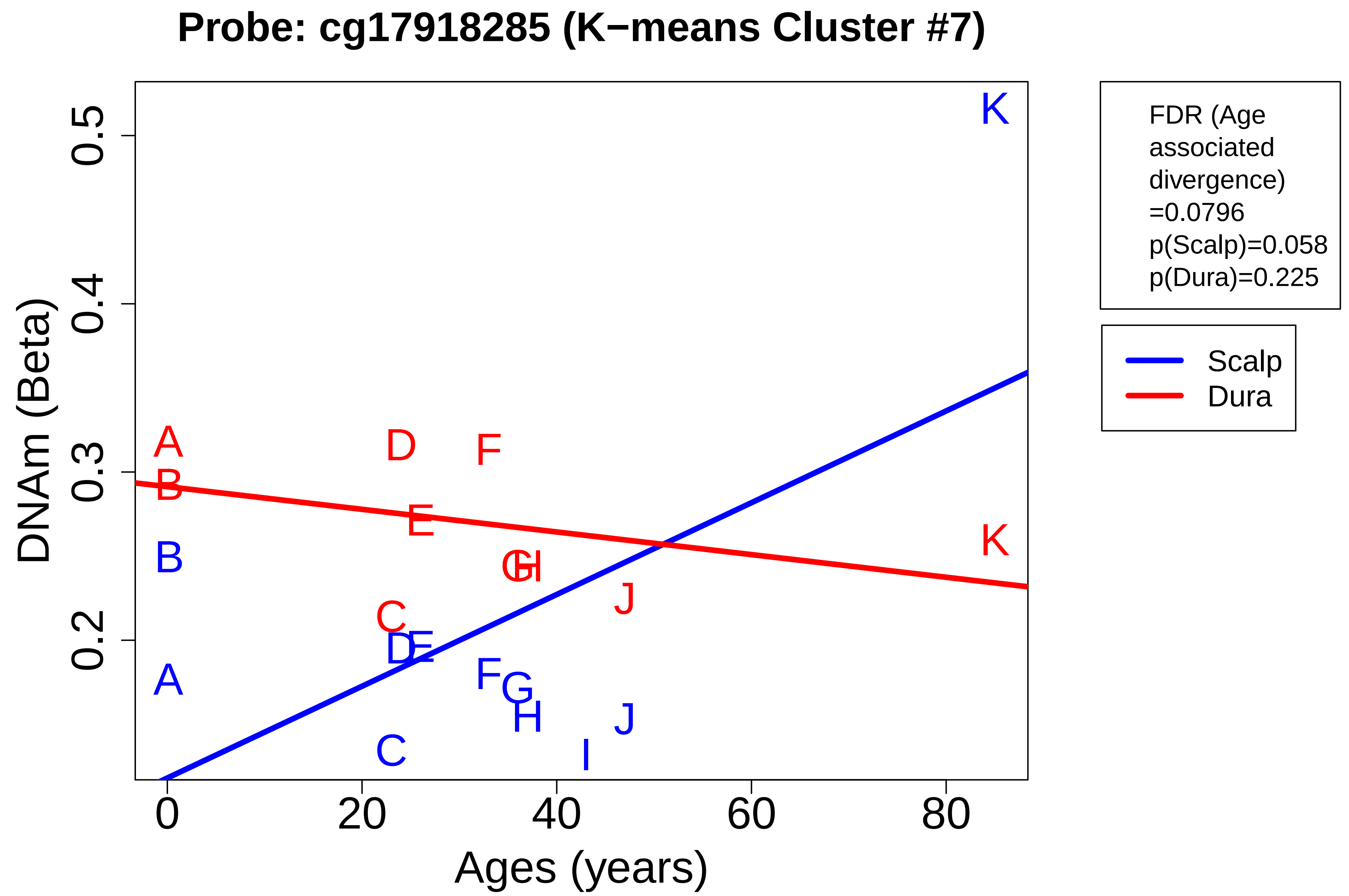
Probe: cg17918285 (K−means Cluster #7)
DNAm (Beta)
0.2
0.3
0.4
0.5
Ages (years)
0
20
40
60
80
FDR (Age associated divergence) =0.0796
p(Scalp)=0.058
p(Dura)=0.225
Scalp
Dura
A
B
C
D
E
F
G
H
I
J
K

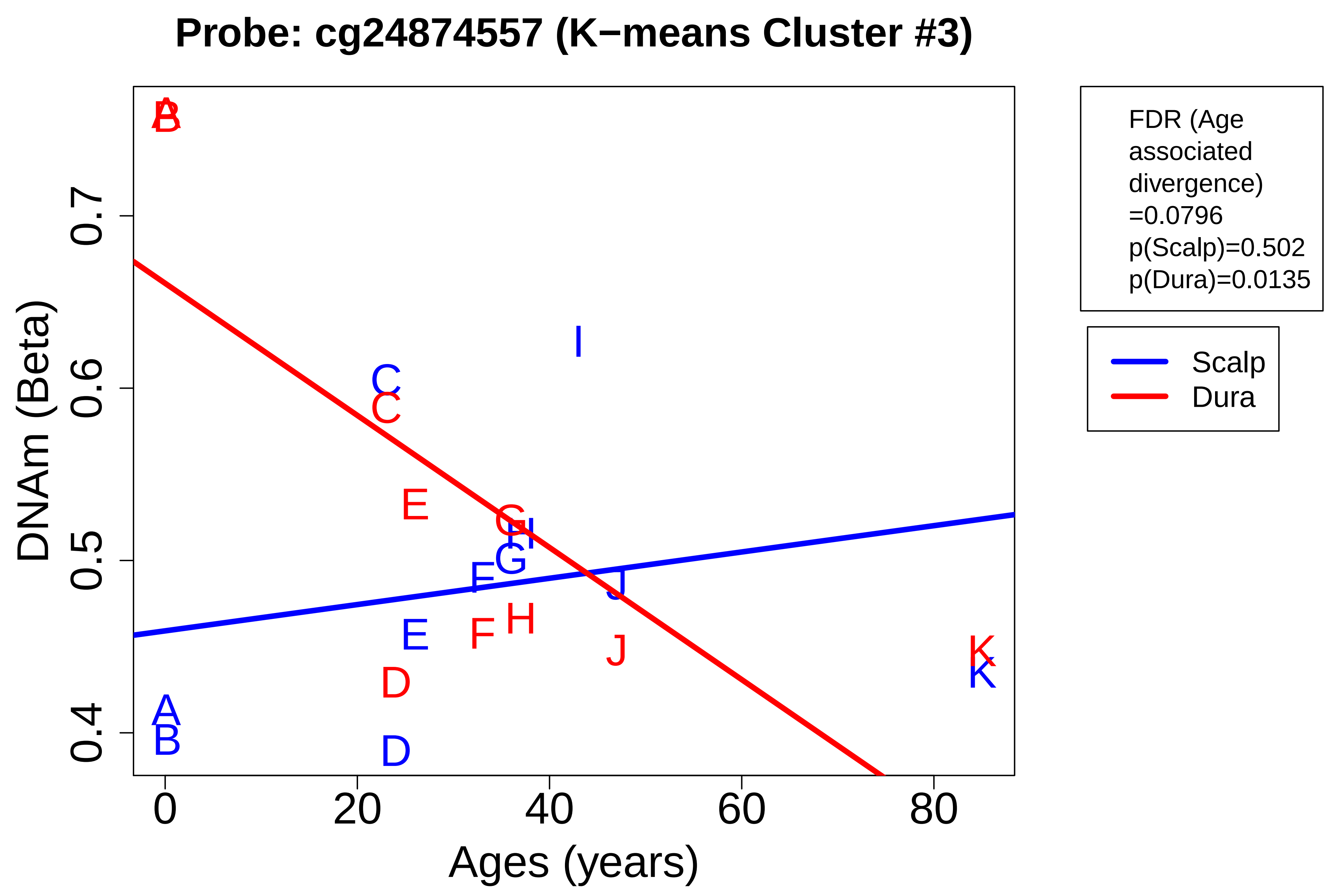

Probe: cg24874557 (K−means Cluster #3)
DNAm (Beta)
0.4
0.5
0.6
0.7
Ages (years)
0
20
40
60
80
FDR (Age associated divergence) =0.0796
p(Scalp)=0.502
p(Dura)=0.0135
Scalp
Dura
A
B
C
D
E
F
G
H
I
J
K

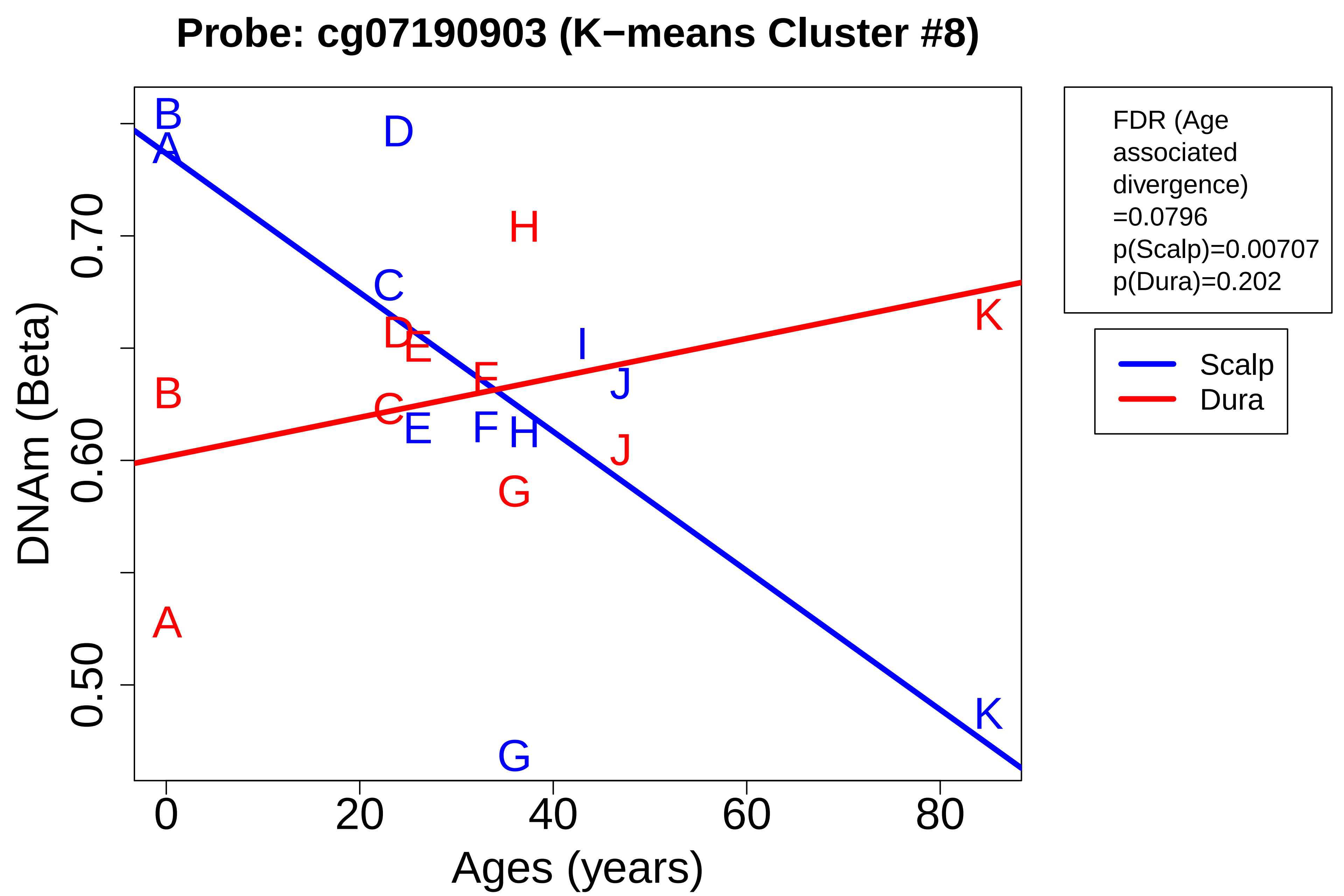
Probe: cg07190903 (K−means Cluster #8)
DNAm (Beta)
0.50
0.60
0.70
0
20
40
60
80
Ages (years)
FDR (Age associated divergence) =0.0796
p(Scalp)=0.00707
p(Dura)=0.202
Scalp
Dura
A
B
C
D
E
F
G
H
I
J
K

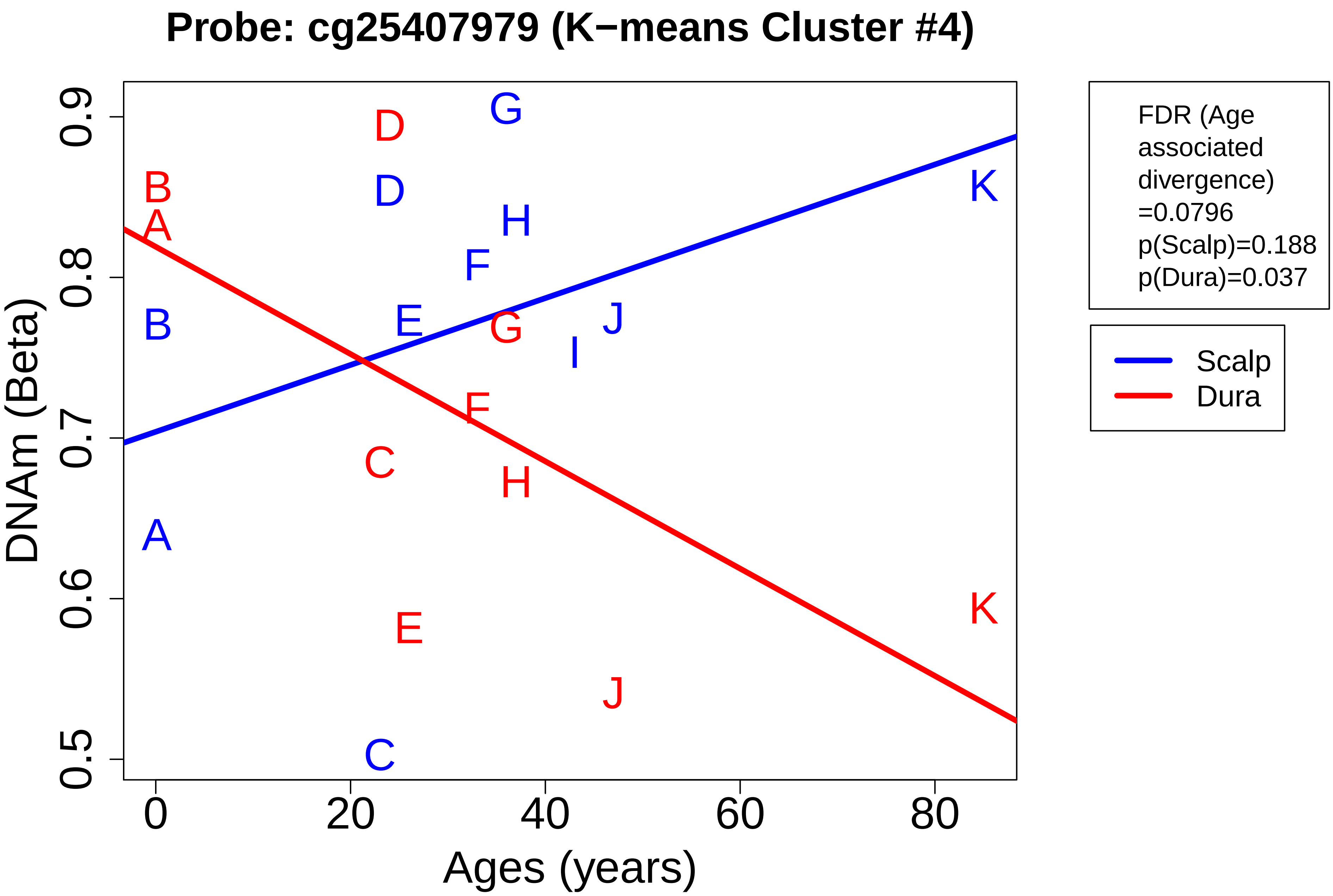

Probe: cg25407979 (K−means Cluster #4)
DNAm (Beta)
0.5
0.6
0.7
0.8
0.9
Ages (years)
0
20
40
60
80
FDR (Age associated divergence) =0.0796
p(Scalp)=0.188
p(Dura)=0.037
Scalp
Dura

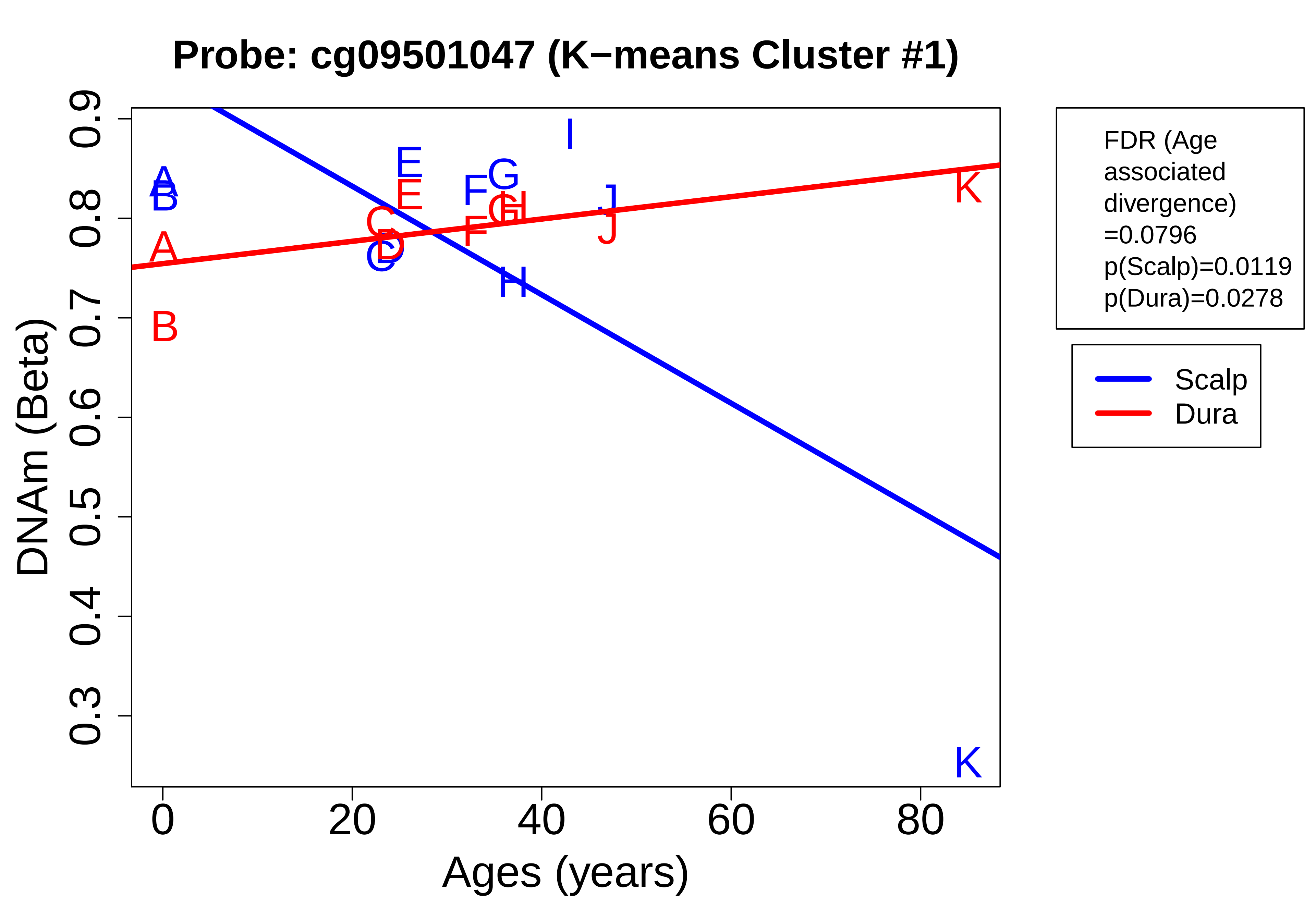

Probe: cg09501047 (K−means Cluster #1)
DNAm (Beta)
0.3
0.4
0.5
0.6
0.7
0.8
0.9
0
20
40
60
80
Ages (years)
A
B
C
D
E
F
G
H
I
J
K
FDR (Age associated divergence) =0.0796
p(Scalp)=0.0119
p(Dura)=0.0278
Scalp
Dura

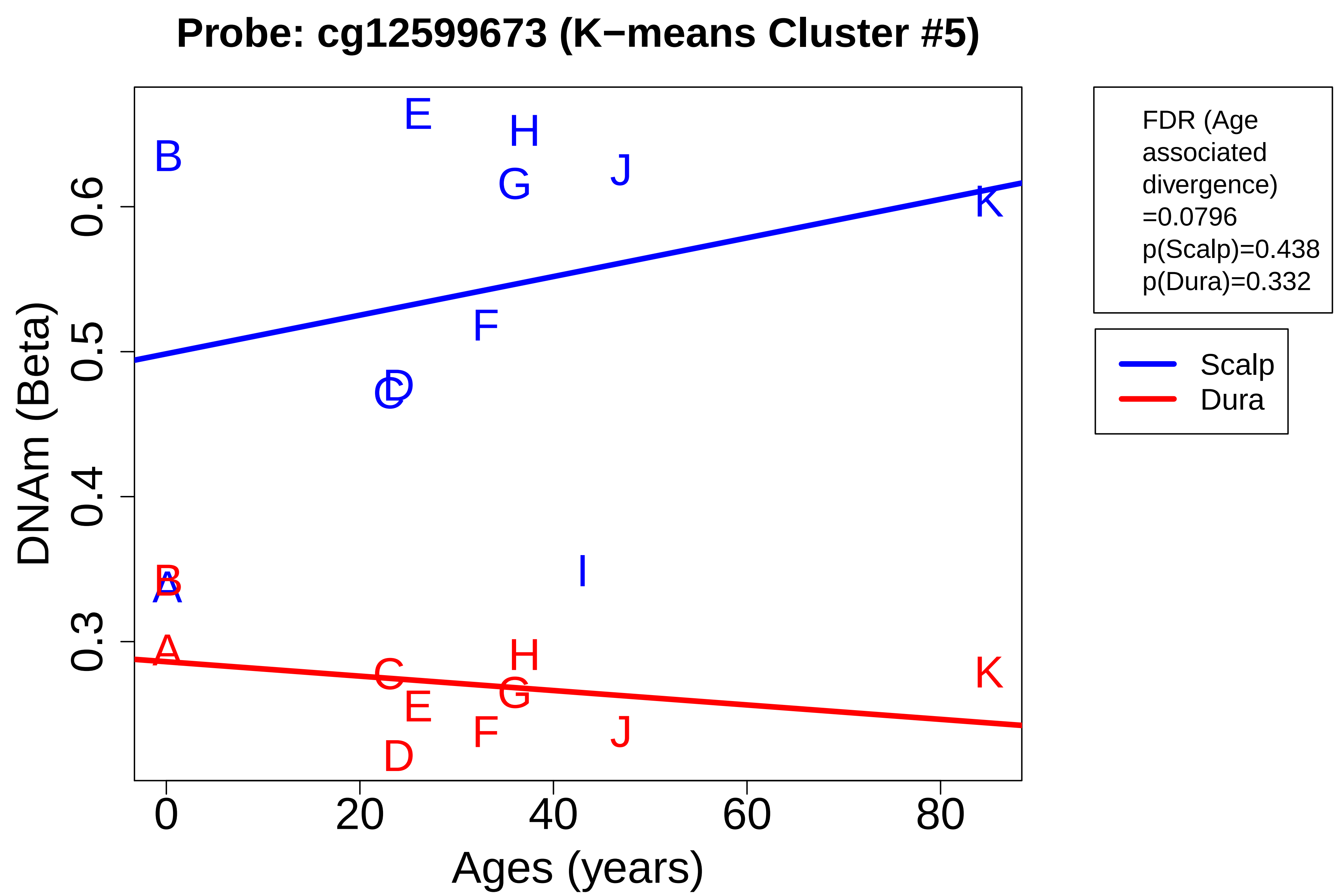

Probe: cg12599673 (K−means Cluster #5)
DNAm (Beta)
0.3
0.4
0.5
0.6
0
20
40
60
80
Ages (years)
A
B
C
D
E
F
G
H
I
J
K
FDR (Age associated divergence) =0.0796
p(Scalp)=0.438
p(Dura)=0.332
Scalp
Dura

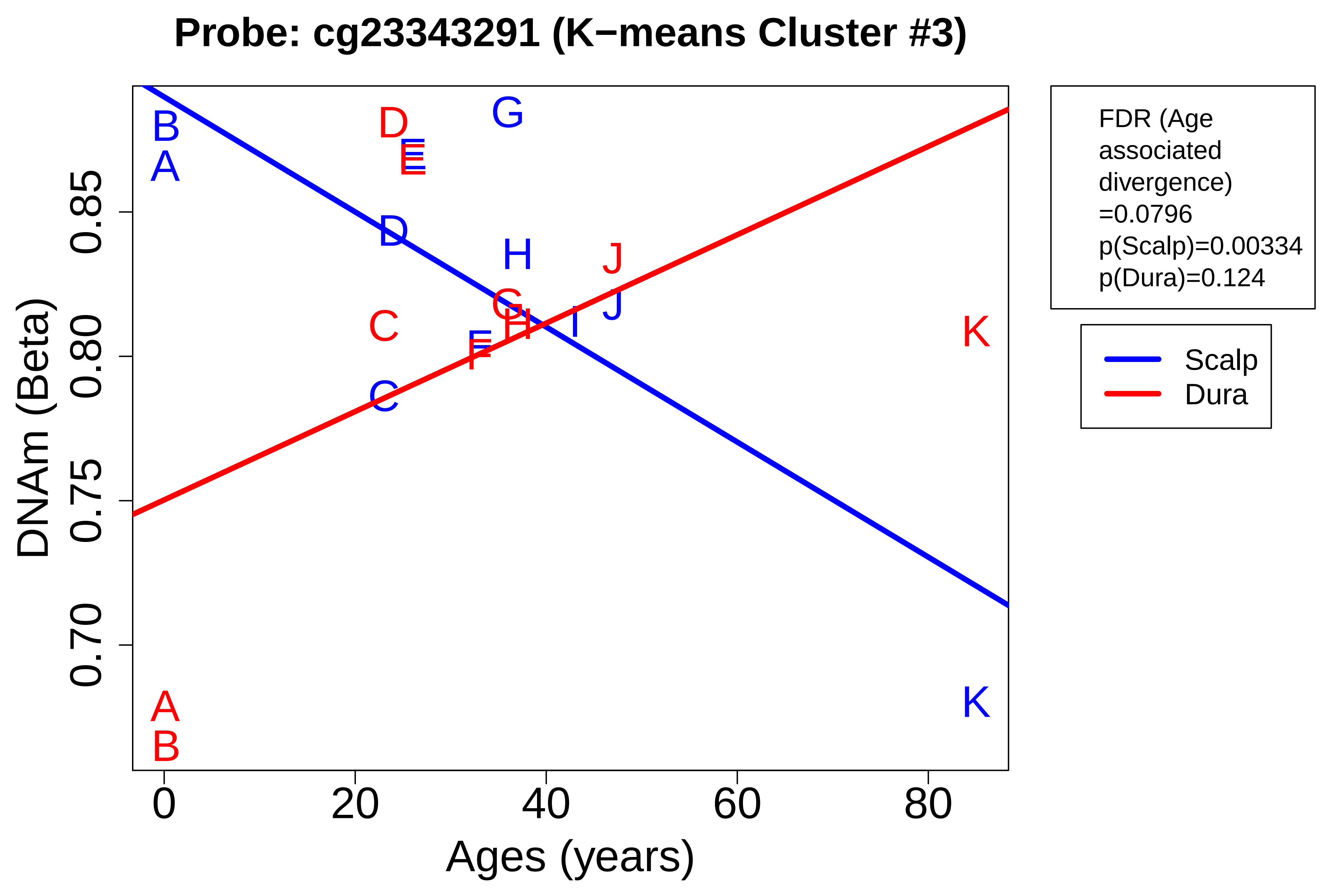

Probe: cg23343291 (K−means Cluster #3)
DNAm (Beta)
0.70
0.75
0.80
0.85
0
20
40
60
80
Ages (years)
A
B
C
D
E
F
G
H
I
J
K
FDR (Age associated divergence) =0.0796
p(Scalp)=0.00334
p(Dura)=0.124
Scalp
Dura

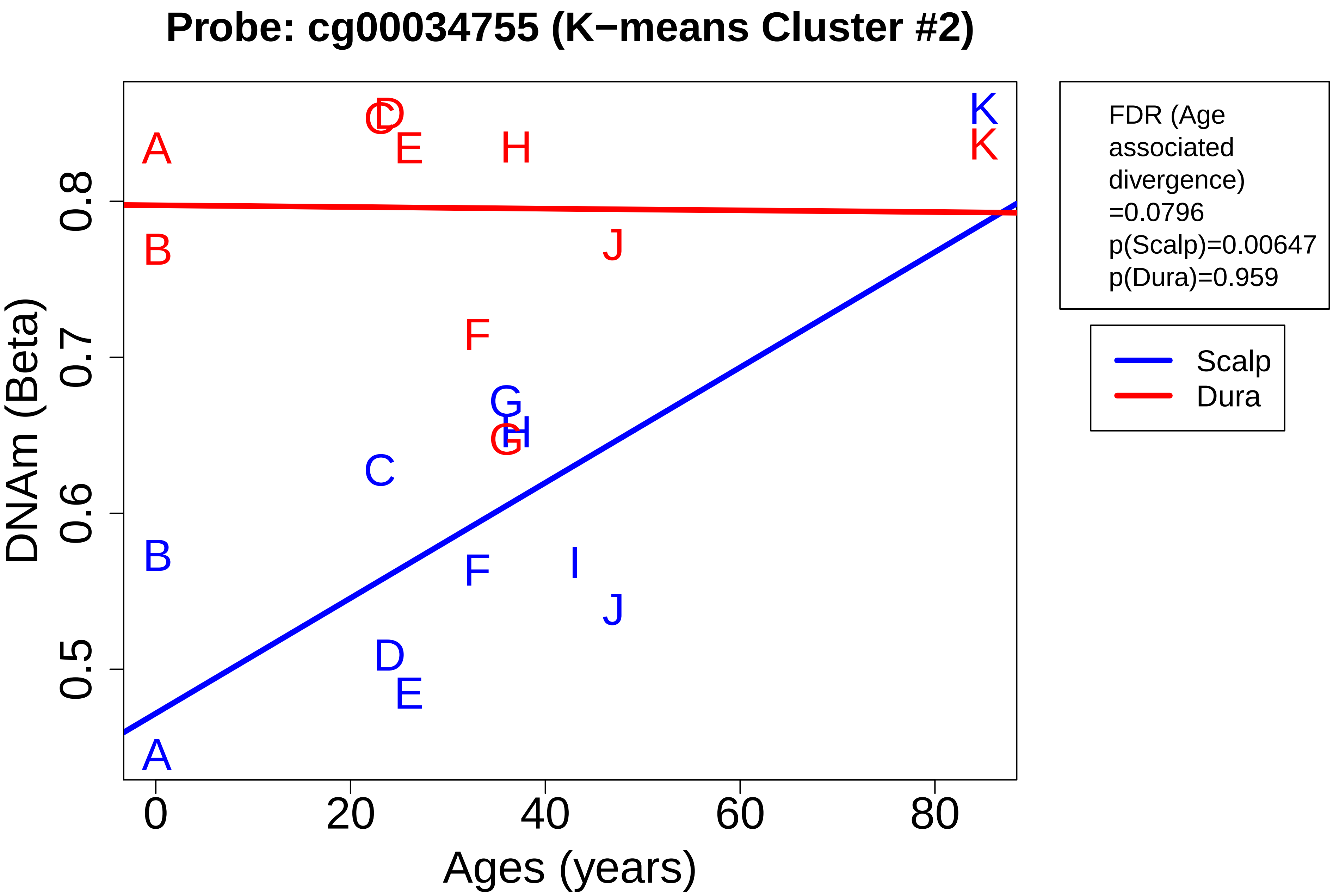

Probe: cg00034755 (K−means Cluster #2)
DNAm (Beta)
0.5
0.6
0.7
0.8
Ages (years)
0
20
40
60
80
A
B
C
D
E
F
G
H
I
J
K
FDR (Age associated divergence) =0.0796
p(Scalp)=0.00647
p(Dura)=0.959
Scalp
Dura

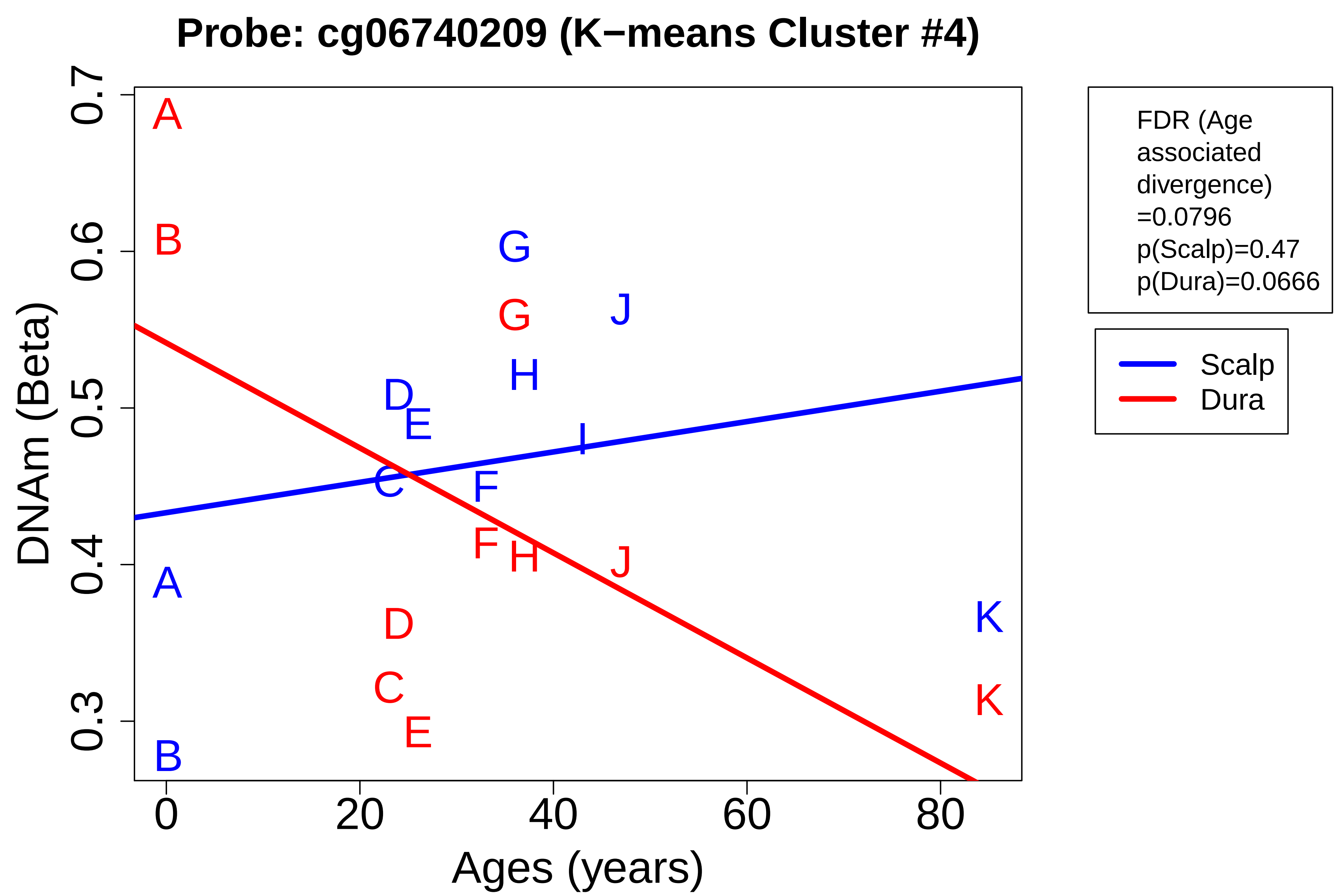

Probe: cg06740209 (K−means Cluster #4)
DNAm (Beta)
0.3
0.4
0.5
0.6
0.7
0
20
40
60
80
Ages (years)
A
B
G
G
J
H
D
E
I
C
F
F
H
J
A
D
K
C
K
E
B
FDR (Age associated divergence) =0.0796
p(Scalp)=0.47
p(Dura)=0.0666
Scalp
Dura

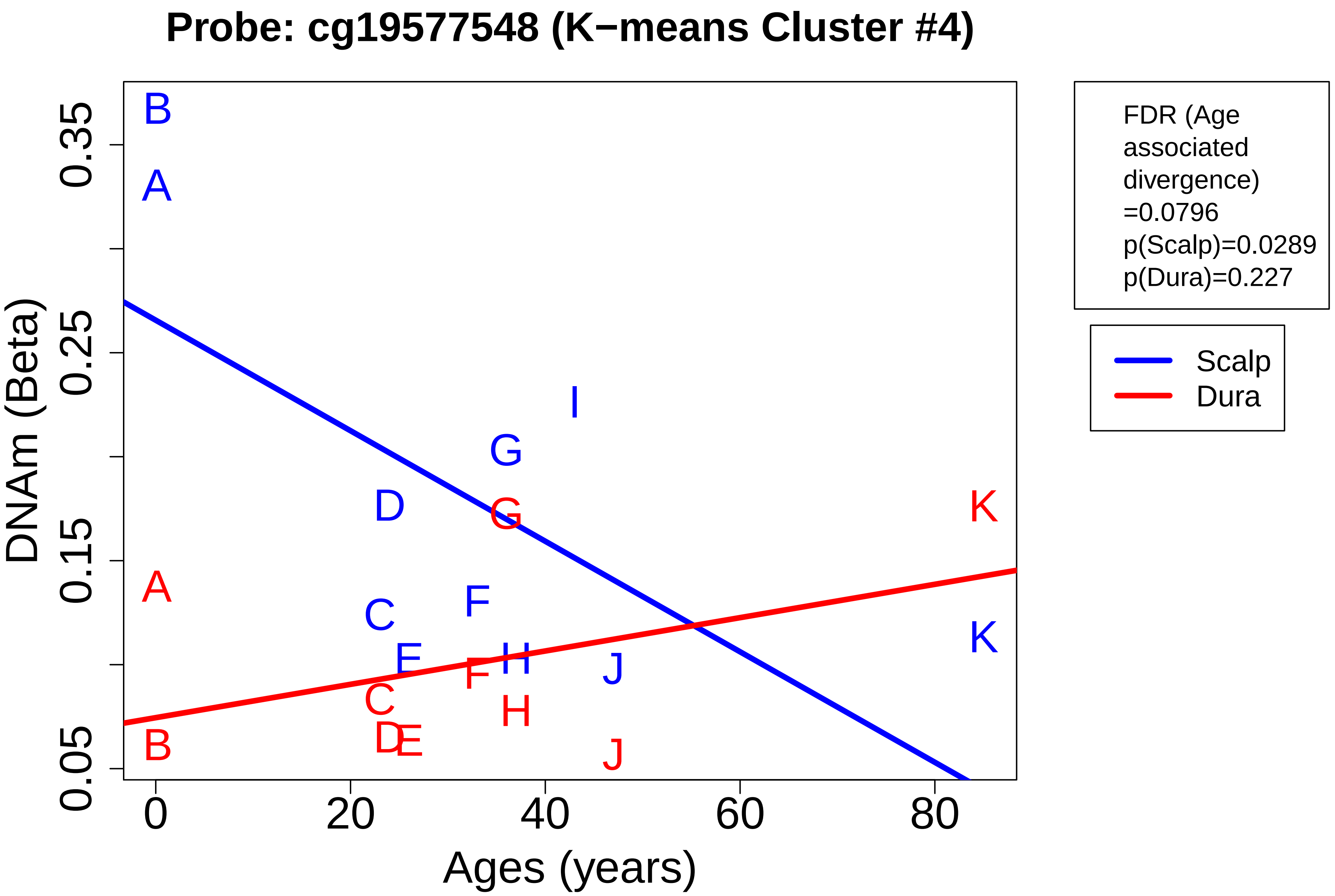
Probe: cg19577548 (K−means Cluster #4)
DNAm (Beta)
0.05
0.15
0.25
0.35
Ages (years)
0
20
40
60
80
A
B
C
D
E
F
G
H
I
J
K
FDR (Age associated divergence) =0.0796
p(Scalp)=0.0289
p(Dura)=0.227
Scalp
Dura

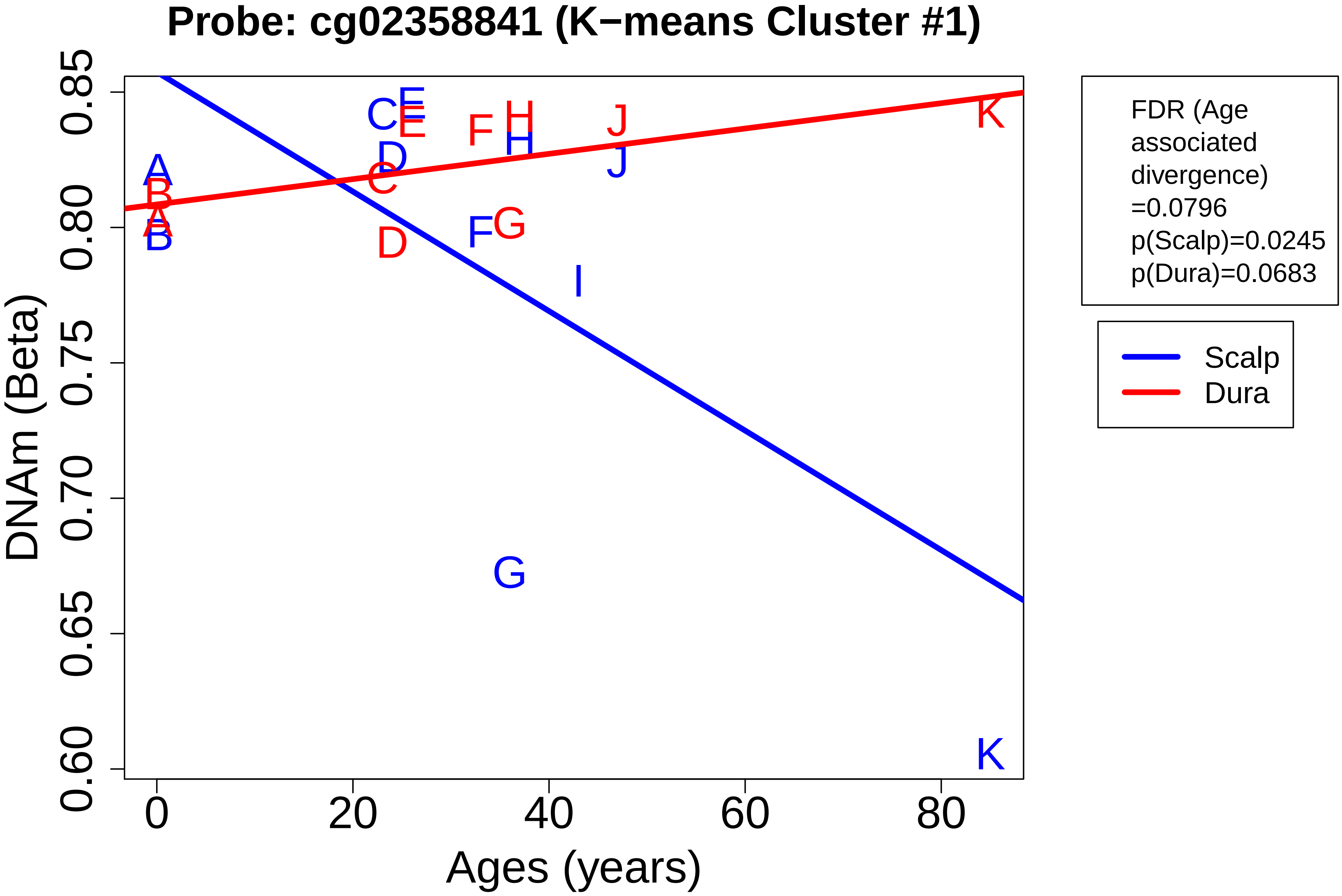

Probe: cg02358841 (K−means Cluster #1)
DNAm (Beta)
0.60
0.65
0.70
0.75
0.80
0.85
0
20
40
60
80
Ages (years)
FDR (Age associated divergence) =0.0796
p(Scalp)=0.0245
p(Dura)=0.0683
Scalp
Dura

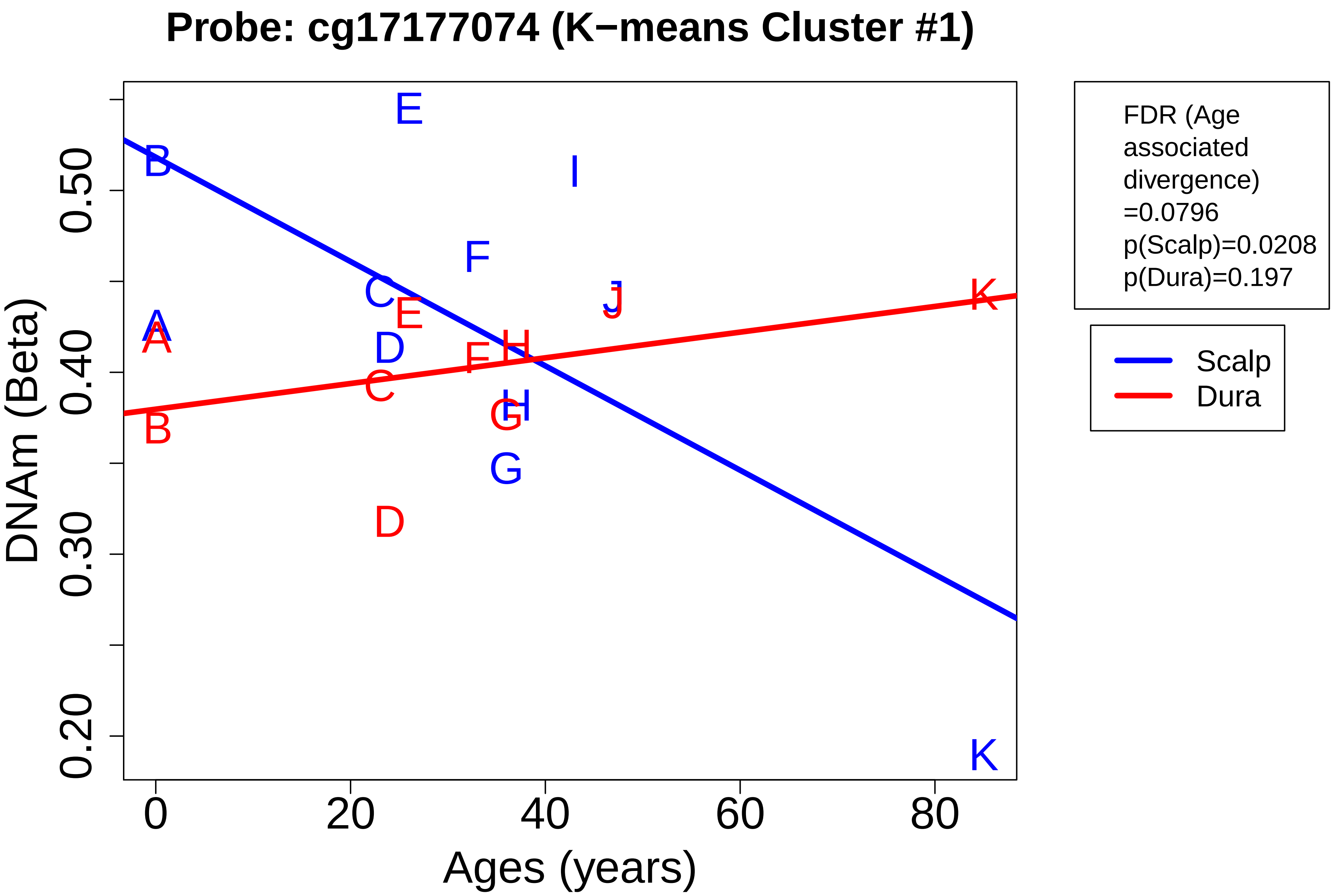

Probe: cg17177074 (K−means Cluster #1)
DNAm (Beta)
0.20
0.30
0.40
0.50
0
20
40
60
80
Ages (years)
FDR (Age associated divergence) =0.0796
p(Scalp)=0.0208
p(Dura)=0.197
Scalp
Dura

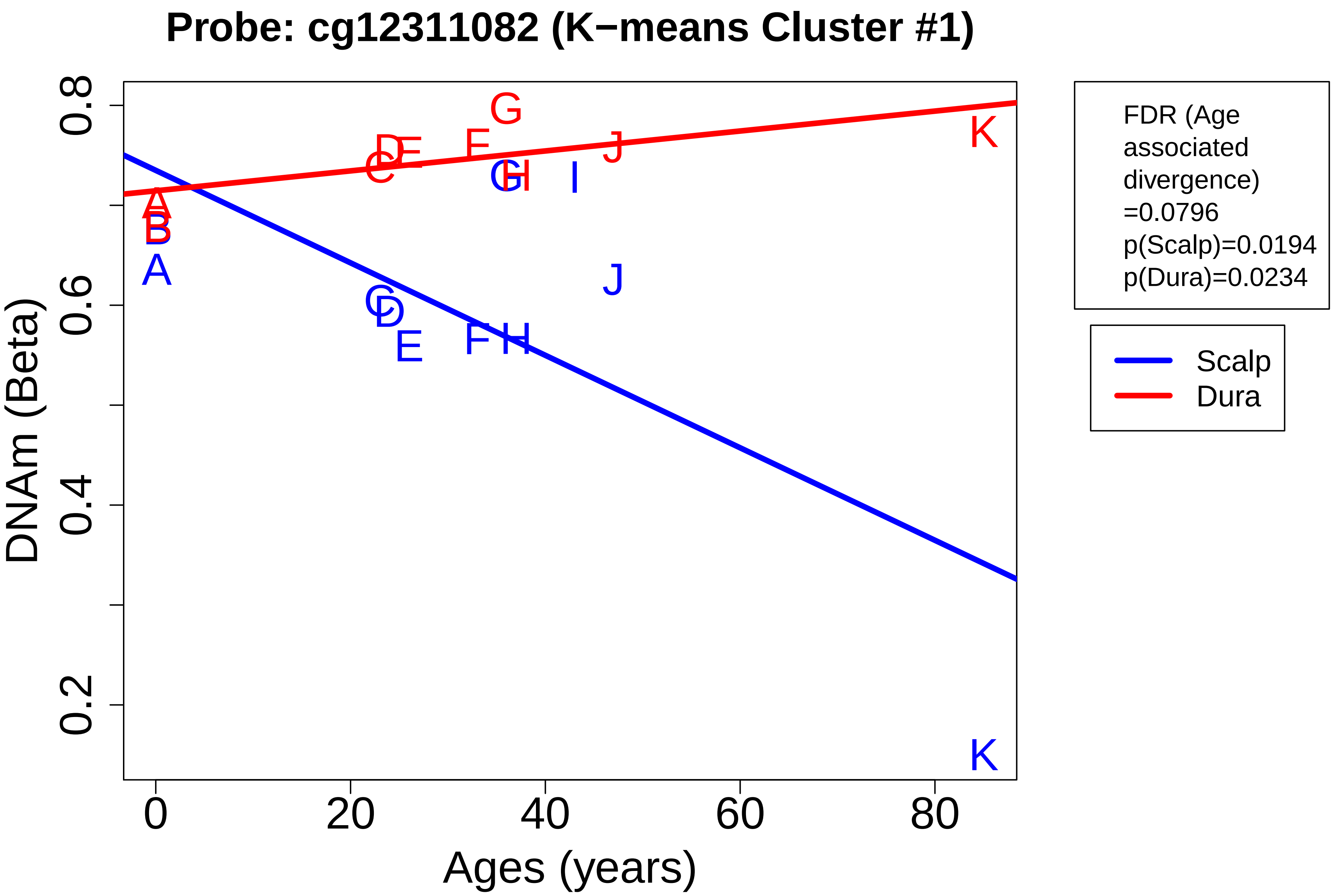

Probe: cg12311082 (K−means Cluster #1)
DNAm (Beta)
0.2
0.4
0.6
0.8
0
20
40
60
80
Ages (years)
FDR (Age associated divergence) =0.0796
p(Scalp)=0.0194
p(Dura)=0.0234
Scalp
Dura

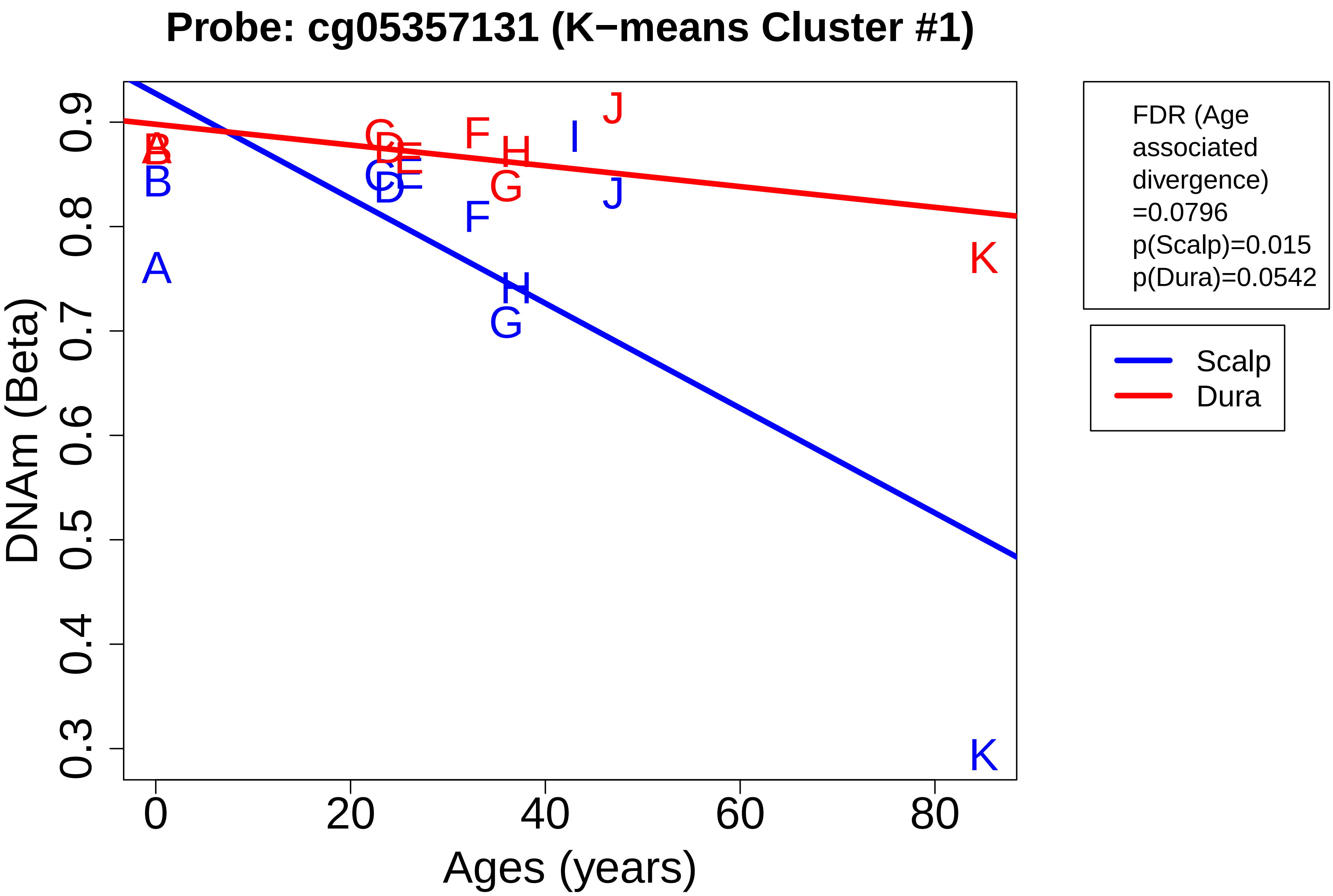

Probe: cg05357131 (K−means Cluster #1)
DNAm (Beta)
0.3
0.4
0.5
0.6
0.7
0.8
0.9
0
20
40
60
80
Ages (years)
A
B
C
D
E
F
G
H
I
J
K
FDR (Age associated divergence) =0.0796
p(Scalp)=0.015
p(Dura)=0.0542
Scalp
Dura

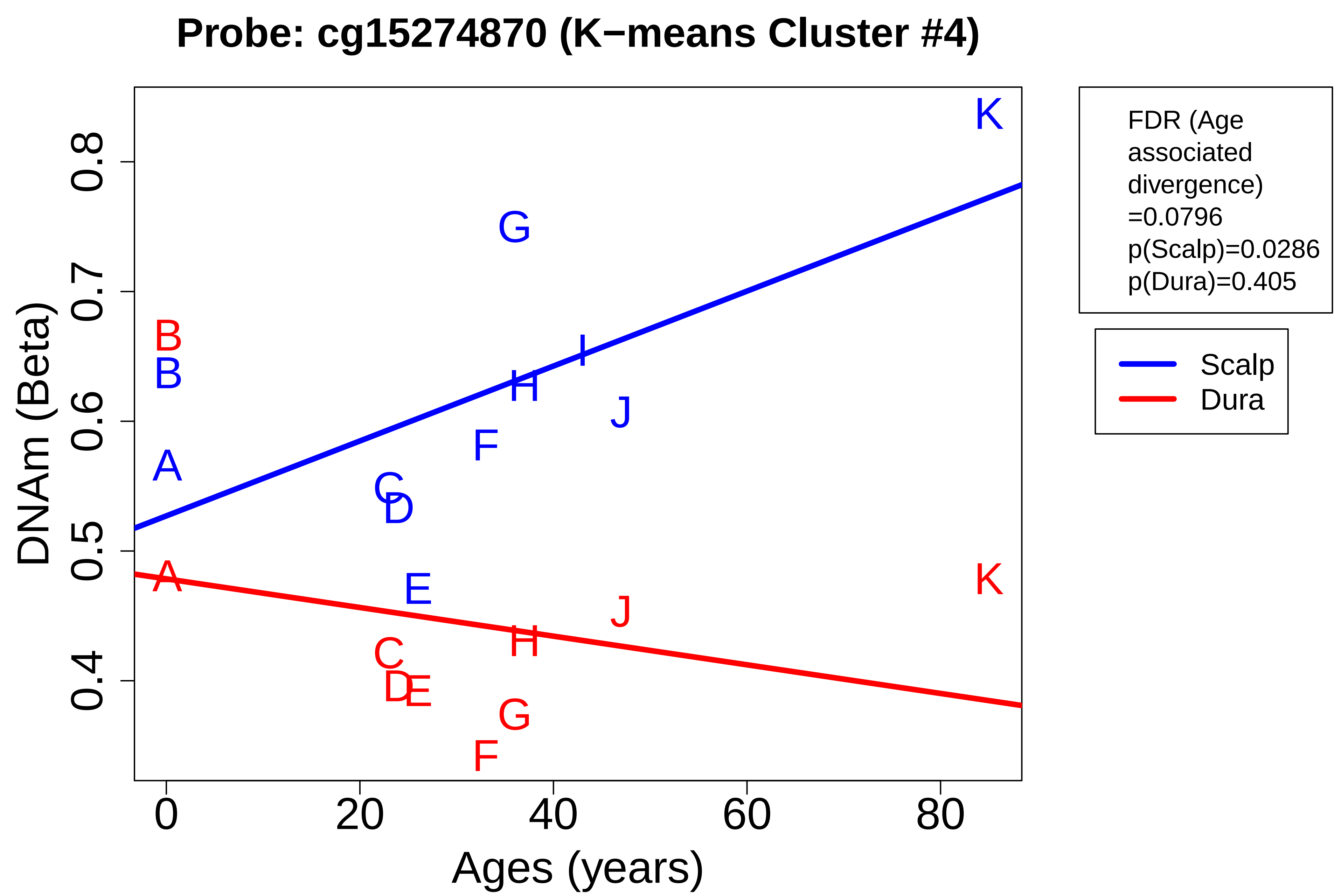

Probe: cg15274870 (K−means Cluster #4)
DNAm (Beta)
0.4
0.5
0.6
0.7
0.8
0
20
40
60
80
Ages (years)
FDR (Age associated divergence) =0.0796
p(Scalp)=0.0286
p(Dura)=0.405
Scalp
Dura
A
B
C
D
E
F
G
H
I
J
K

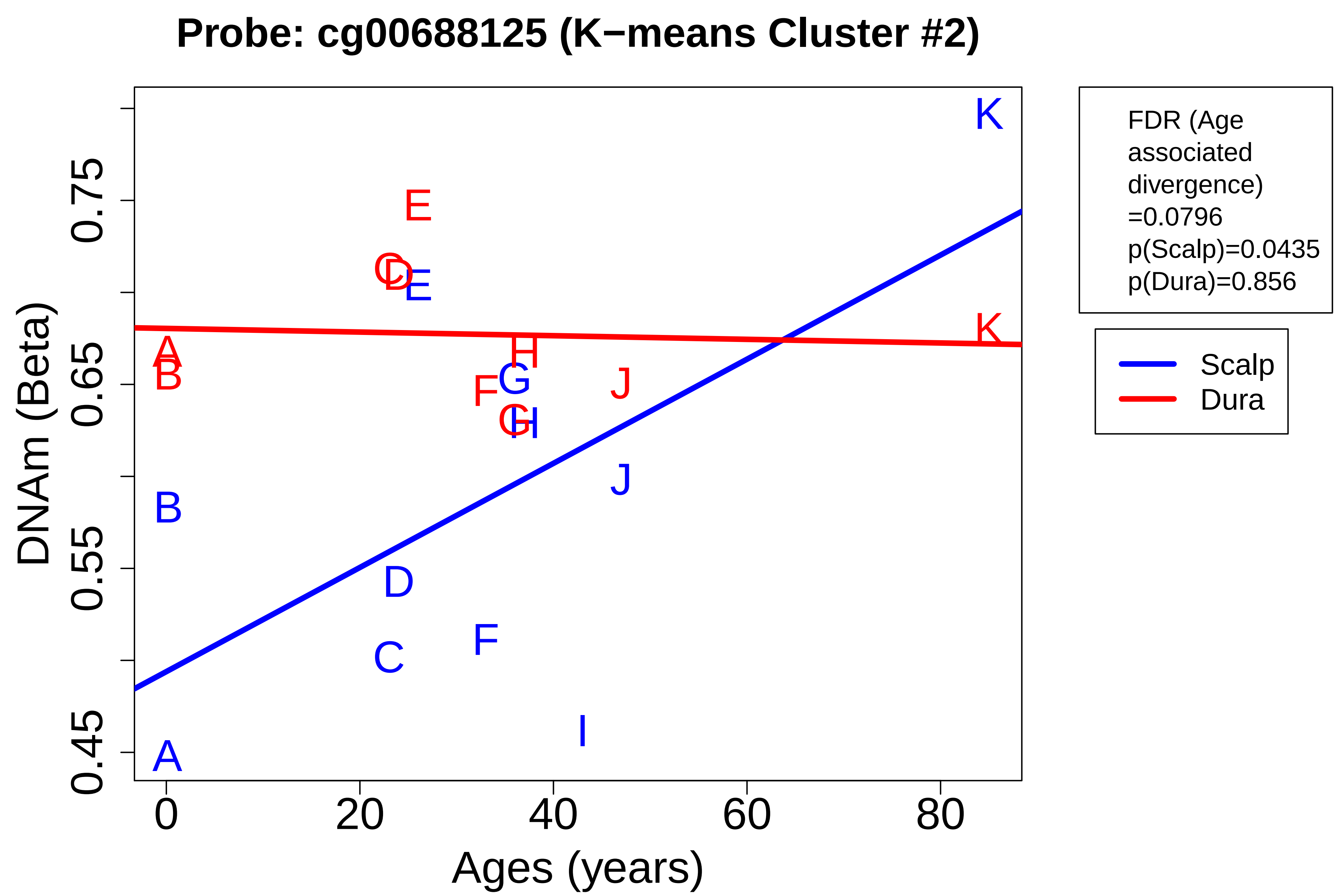

Probe: cg00688125 (K−means Cluster #2)
DNAm (Beta)
0.45
0.55
0.65
0.75
0
20
40
60
80
Ages (years)
A
B
C
D
E
F
G
H
I
J
K
FDR (Age associated divergence) =0.0796
p(Scalp)=0.0435
p(Dura)=0.856
Scalp
Dura

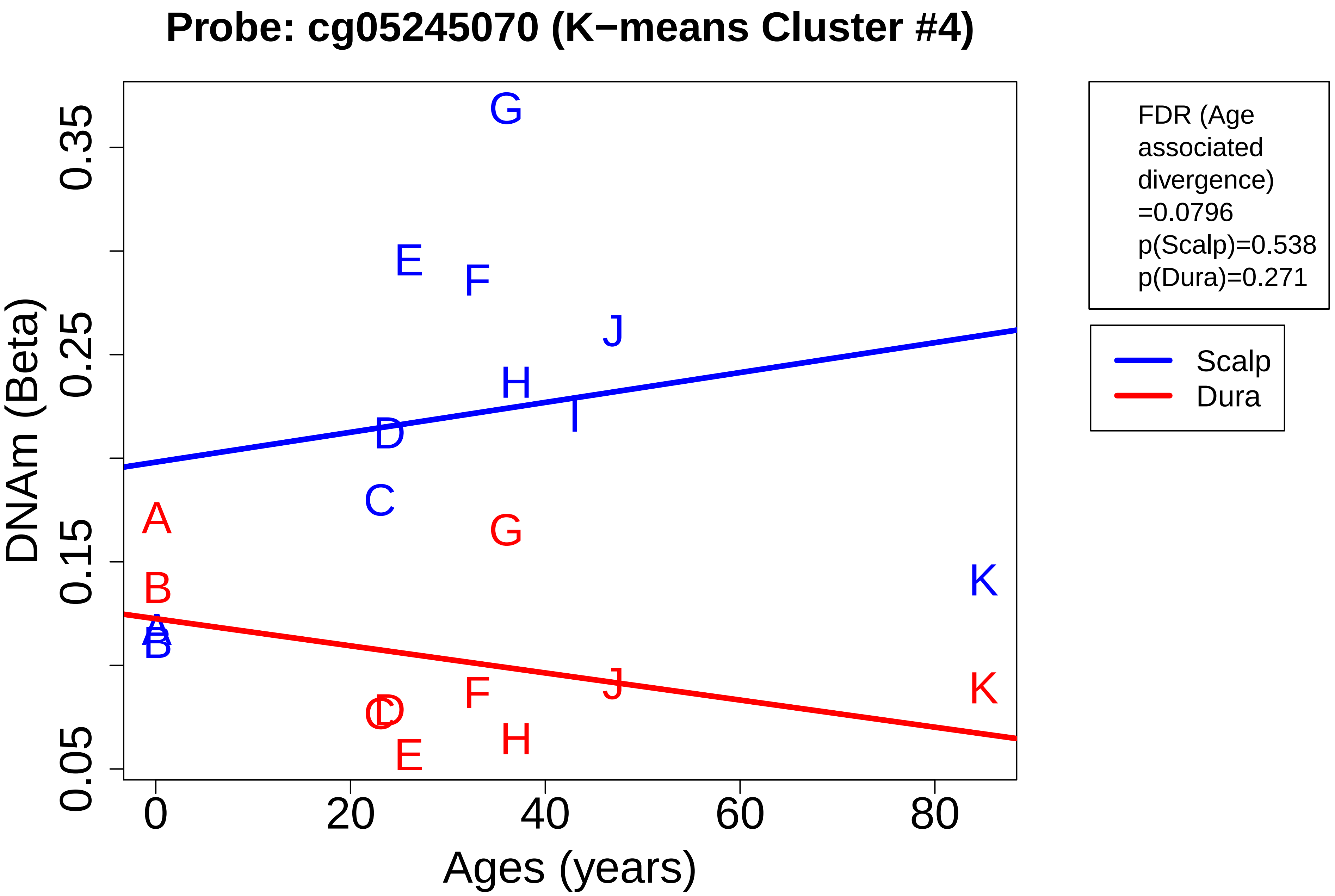

Probe: cg05245070 (K−means Cluster #4)
DNAm (Beta)
0.05
0.15
0.25
0.35
0
20
40
60
80
Ages (years)
FDR (Age associated divergence) =0.0796
p(Scalp)=0.538
p(Dura)=0.271
Scalp
Dura

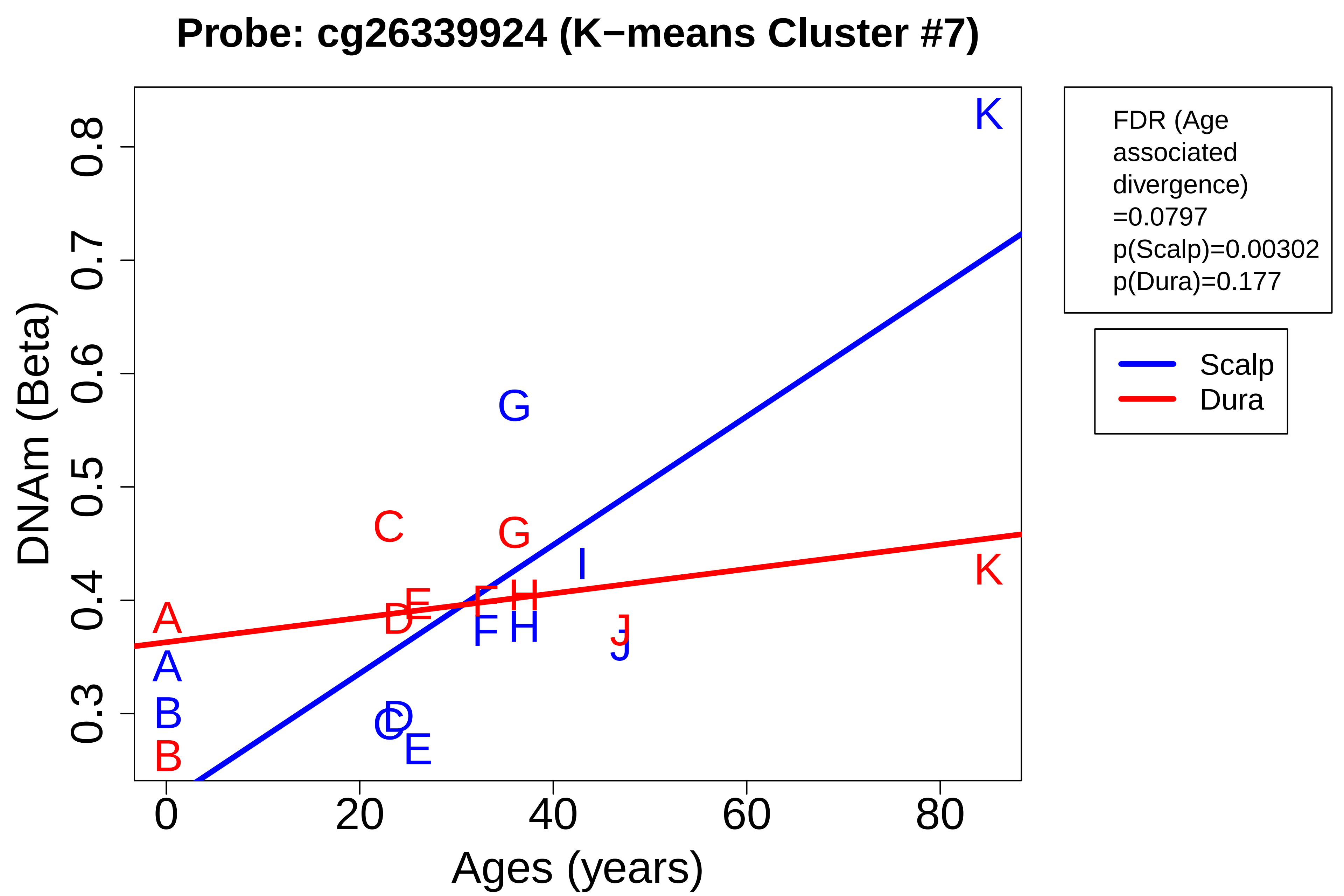
Probe: cg26339924 (K−means Cluster #7)
DNAm (Beta)
0.3
0.4
0.5
0.6
0.7
0.8
0
20
40
60
80
Ages (years)
FDR (Age associated divergence) =0.0797
p(Scalp)=0.00302
p(Dura)=0.177
Scalp
Dura

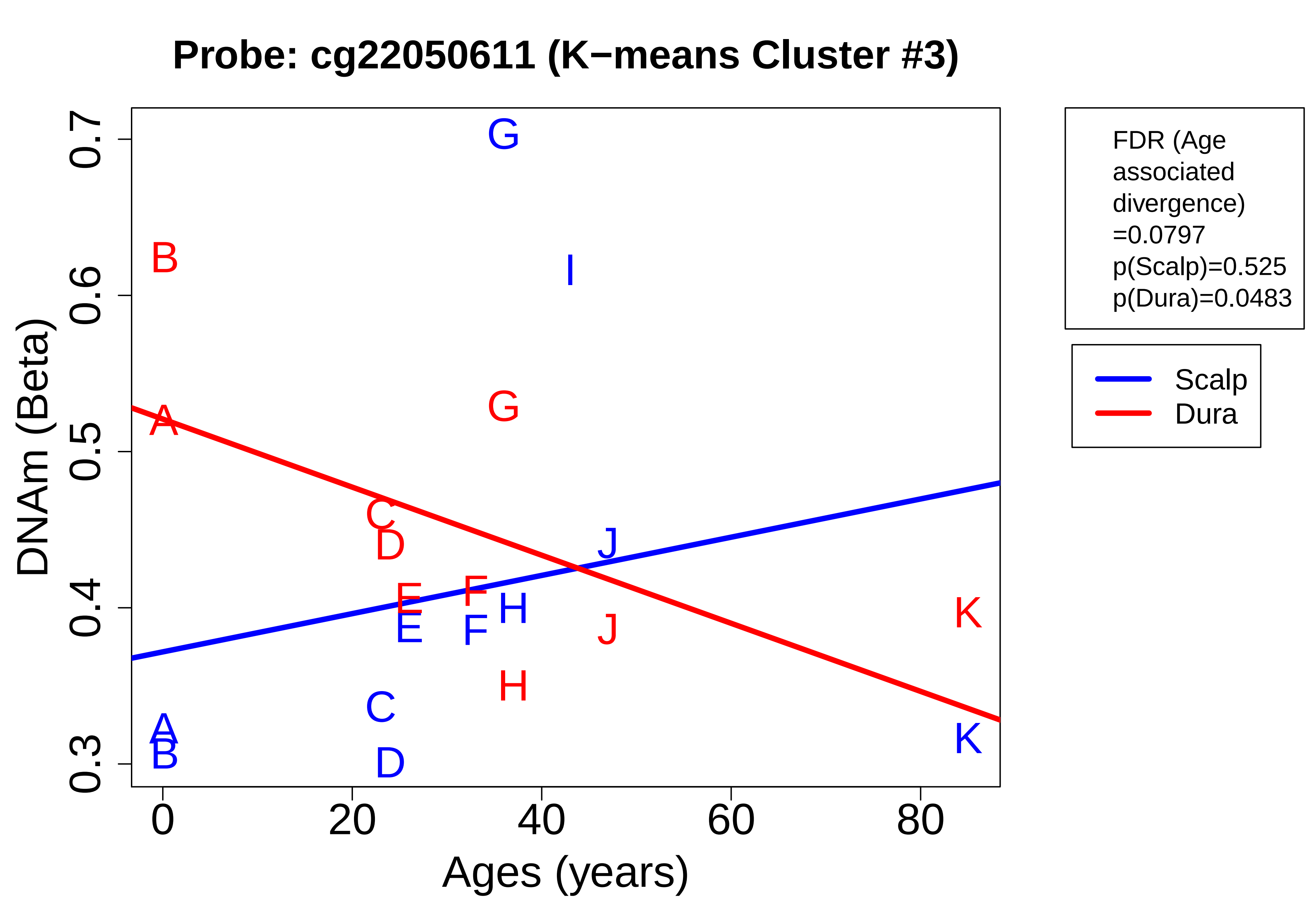
Probe: cg22050611 (K−means Cluster #3)
DNAm (Beta)
0.3
0.4
0.5
0.6
0.7
0
20
40
60
80
Ages (years)
FDR (Age associated divergence) =0.0797
p(Scalp)=0.525
p(Dura)=0.0483
Scalp
Dura

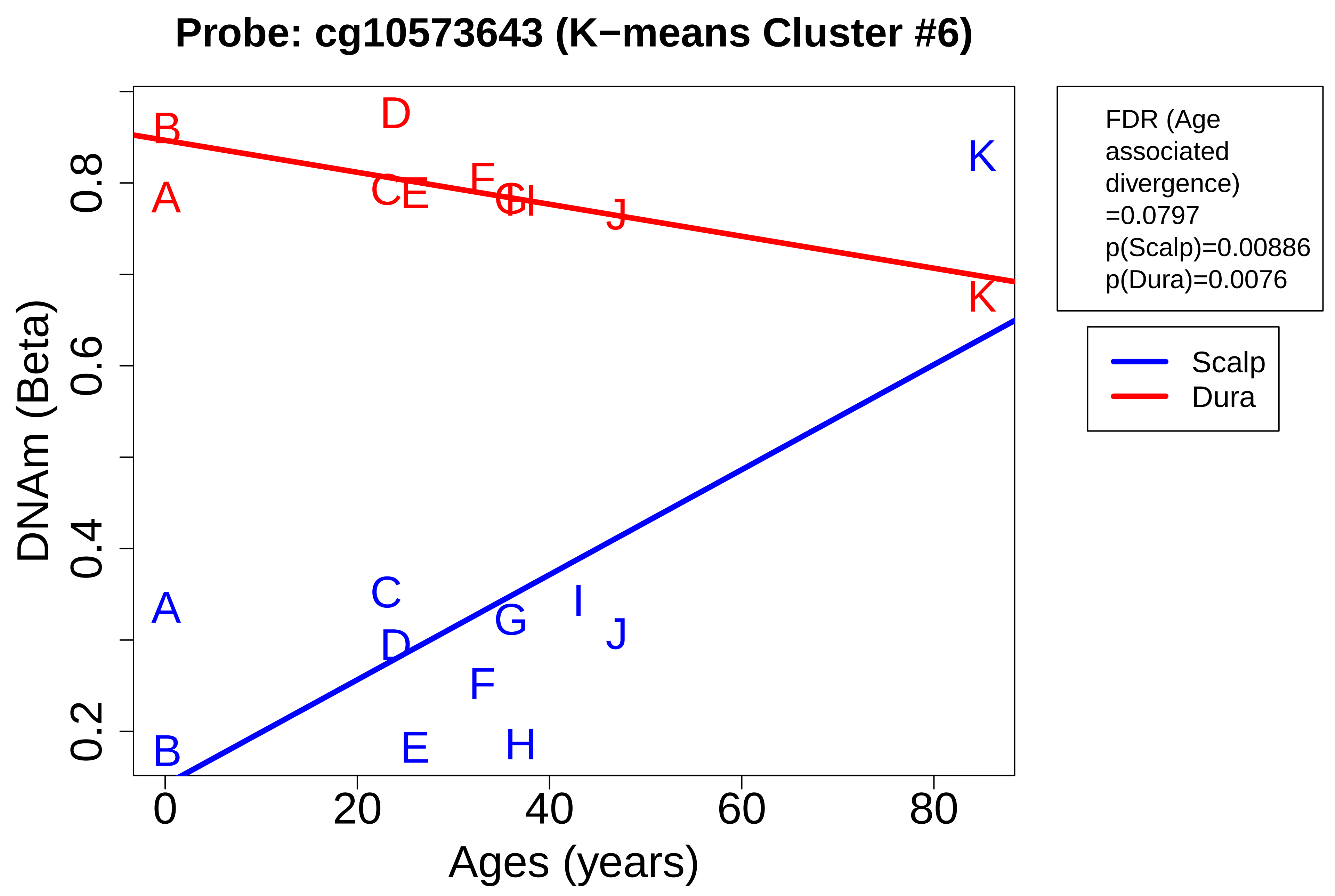
Probe: cg10573643 (K−means Cluster #6)
DNAm (Beta)
0.2
0.4
0.6
0.8
0
20
40
60
80
Ages (years)
FDR (Age associated divergence) =0.0797
p(Scalp)=0.00886
p(Dura)=0.0076
Scalp
Dura
A
B
C
D
E
F
G
H
I
J
K

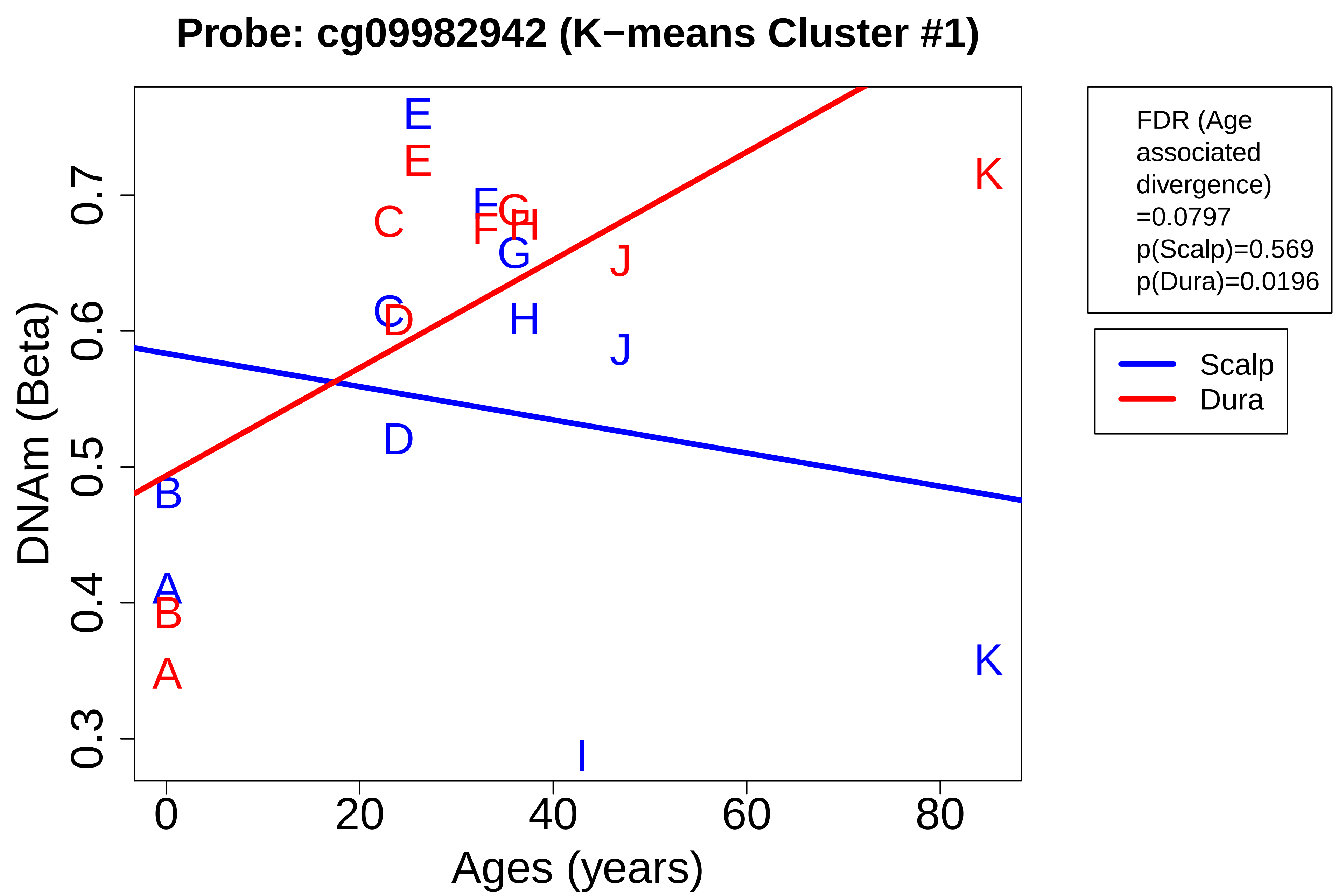

Probe: cg09982942 (K−means Cluster #1)
FDR (Age associated divergence) =0.0797
p(Scalp)=0.569
p(Dura)=0.0196
Scalp
Dura
DNAm (Beta)
Ages (years)
0.3
0.4
0.5
0.6
0.7
0
20
40
60
80
A
B
C
D
E
F
G
H
I
J
K

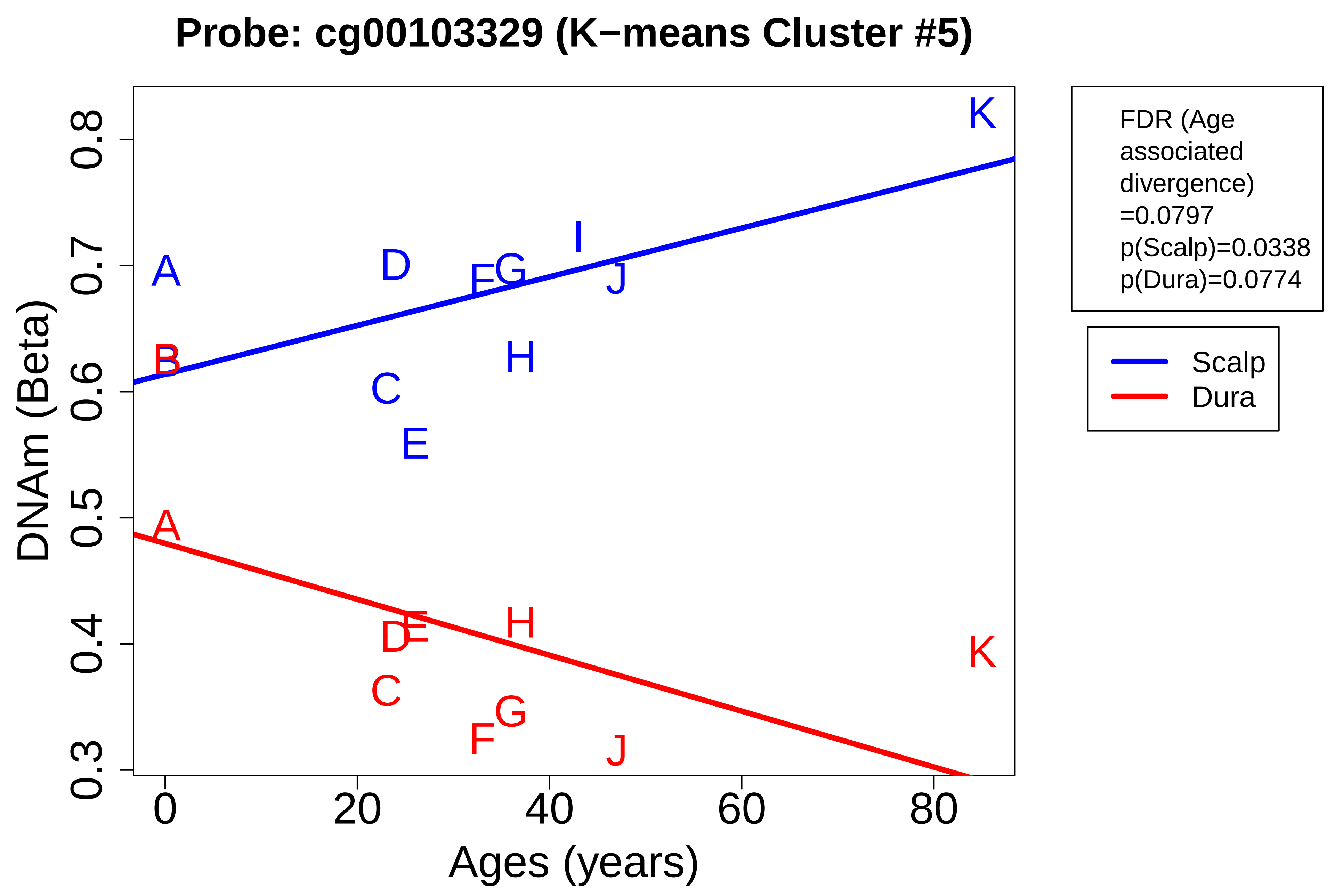

Probe: cg00103329 (K−means Cluster #5)
FDR (Age associated divergence) =0.0797
p(Scalp)=0.0338
p(Dura)=0.0774
Scalp
Dura
DNAm (Beta)
0.3
0.4
0.5
0.6
0.7
0.8
0
20
40
60
80
Ages (years)
A
B
C
D
E
F
G
H
I
J
K

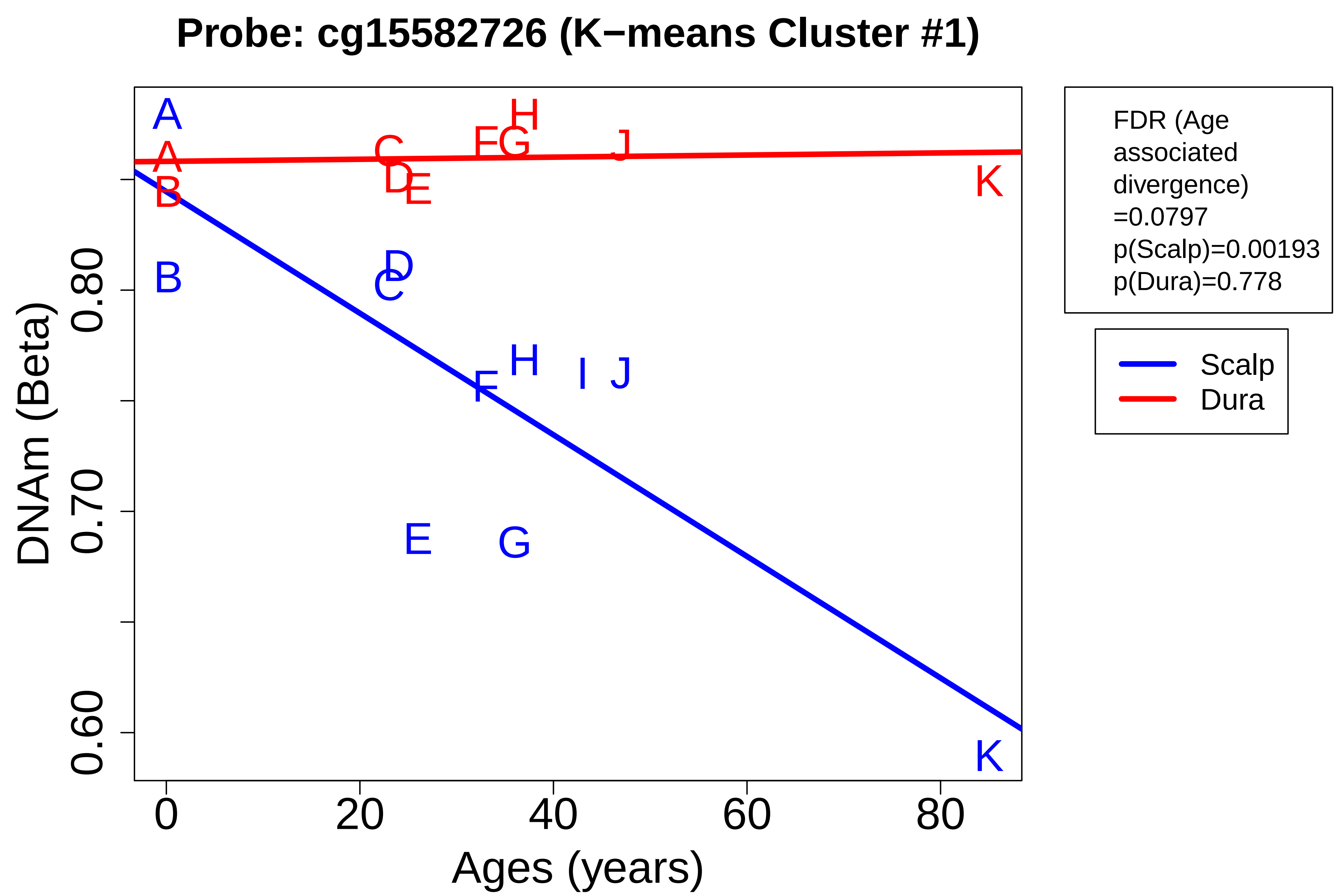
Probe: cg15582726 (K−means Cluster #1)
DNAm (Beta)
0.60
0.70
0.80
Ages (years)
0
20
40
60
80
A
B
C
D
E
F
G
H
I
J
K
FDR (Age associated divergence) =0.0797
p(Scalp)=0.00193
p(Dura)=0.778
Scalp
Dura

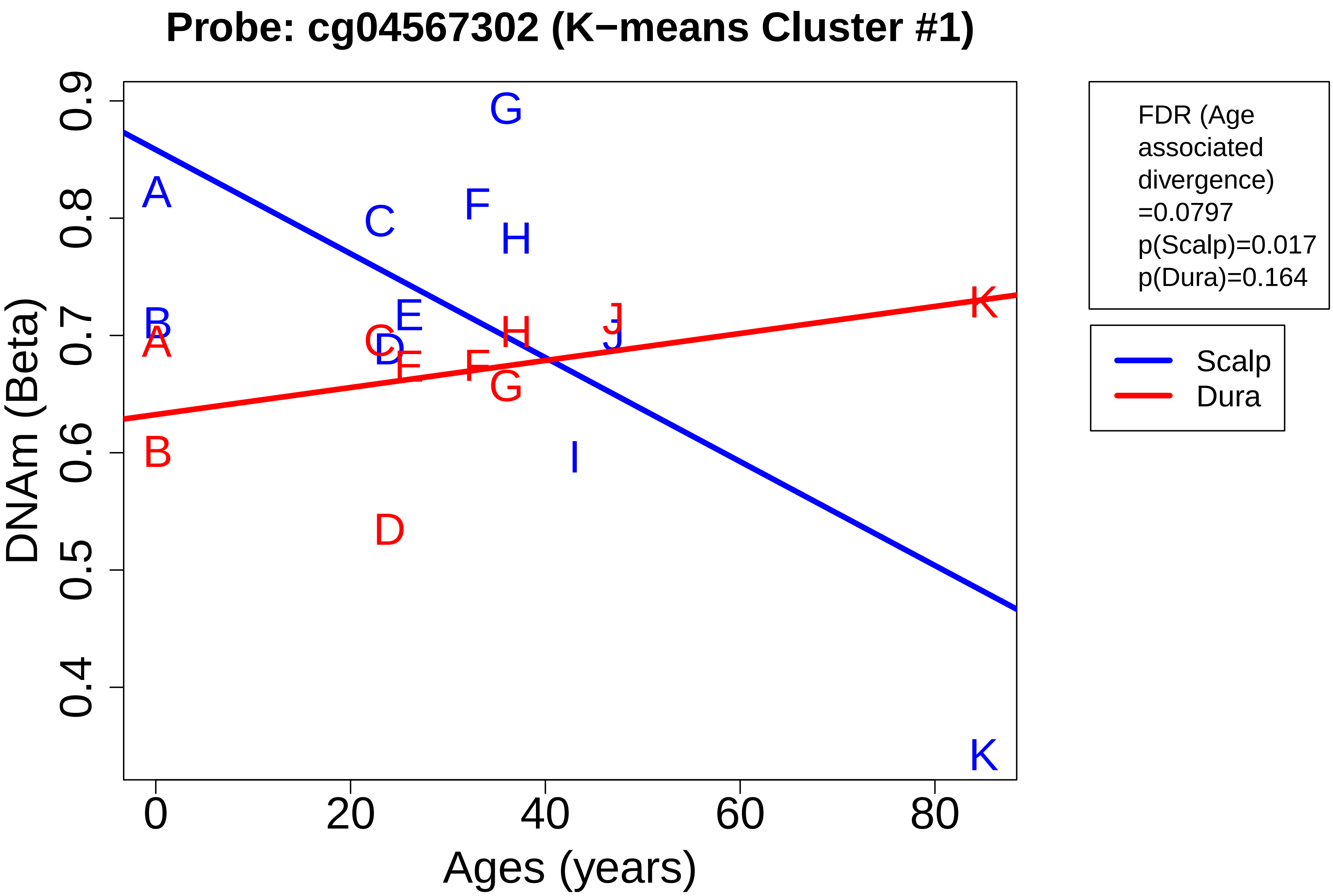

Probe: cg04567302 (K−means Cluster #1)
DNAm (Beta)
0.4
0.5
0.6
0.7
0.8
0.9
0
20
40
60
80
Ages (years)
FDR (Age associated divergence) =0.0797
p(Scalp)=0.017
p(Dura)=0.164
Scalp
Dura

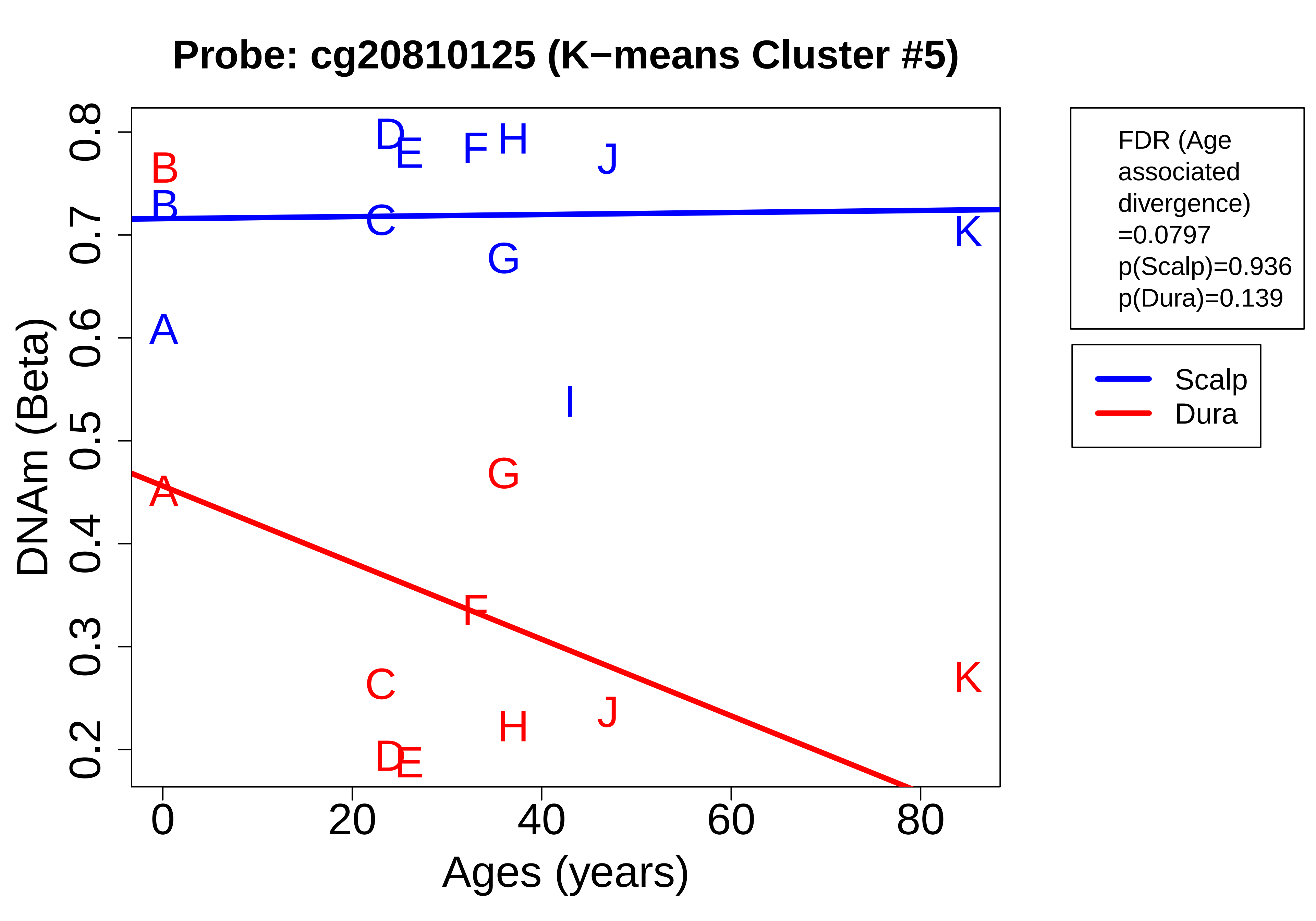

Probe: cg20810125 (K−means Cluster #5)
DNAm (Beta)
0.2
0.3
0.4
0.5
0.6
0.7
0.8
Ages (years)
0
20
40
60
80
A B C D E F G H I J K
FDR (Age associated divergence) =0.0797
p(Scalp)=0.936
p(Dura)=0.139
Scalp
Dura

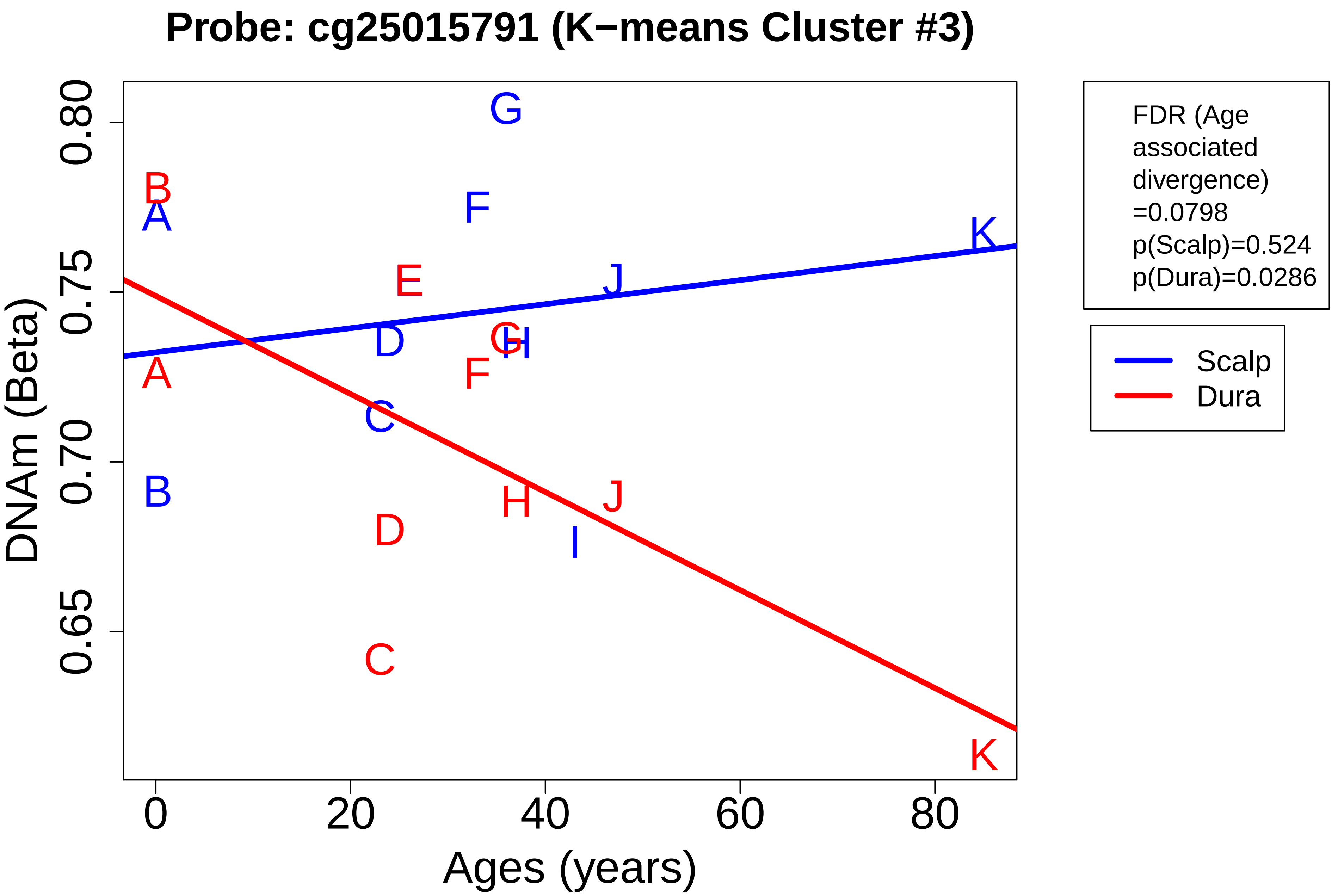

Probe: cg25015791 (K−means Cluster #3)
DNAm (Beta)
Ages (years)
0.65
0.70
0.75
0.80
0
20
40
60
80
A
B
C
D
E
F
G
H
I
J
K
FDR (Age associated divergence) =0.0798
p(Scalp)=0.524
p(Dura)=0.0286
Scalp
Dura

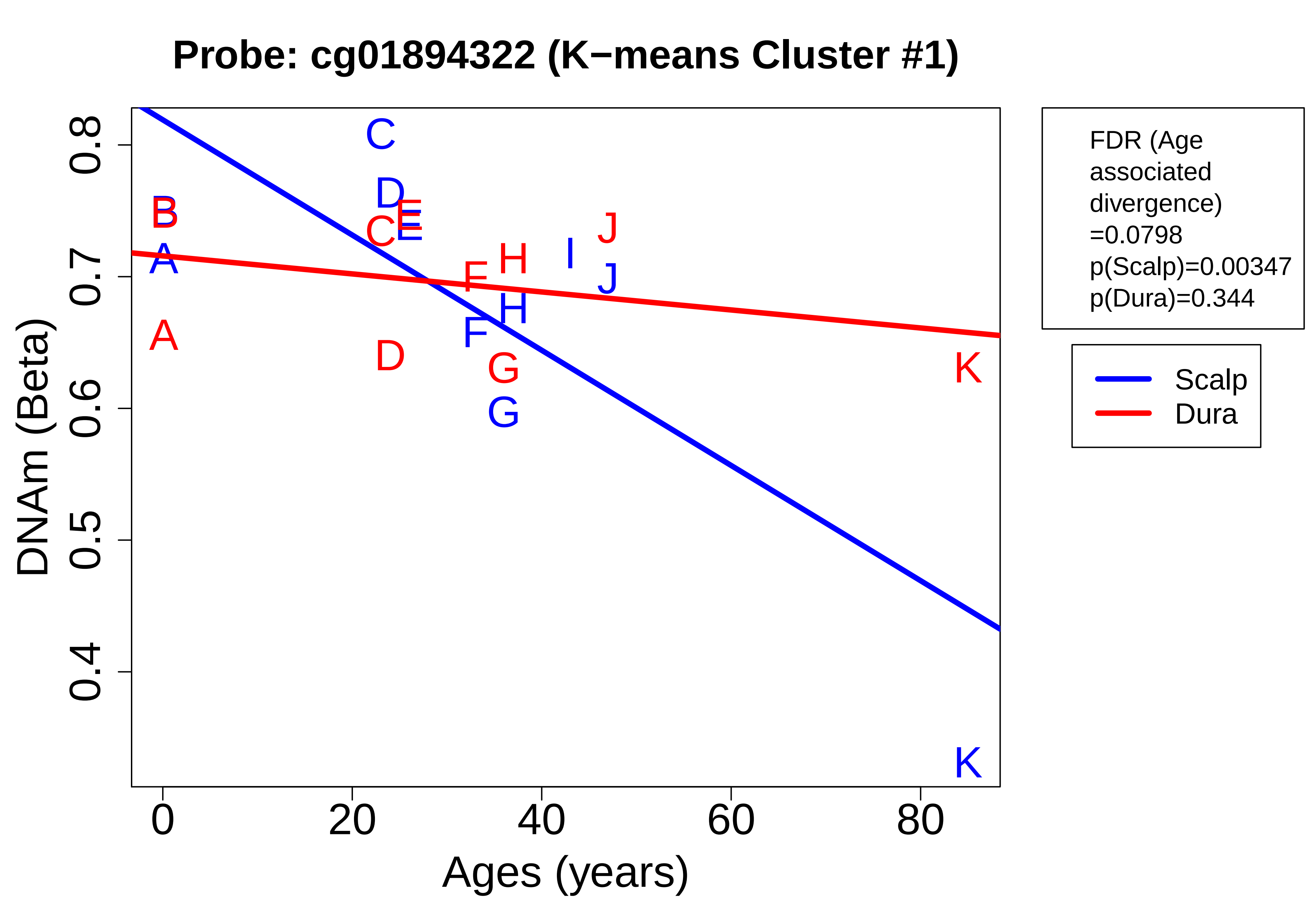

Probe: cg01894322 (K−means Cluster #1)
DNAm (Beta)
0.4
0.5
0.6
0.7
0.8
0
20
40
60
80
Ages (years)
A
B
C
D
E
F
G
H
I
J
K
FDR (Age associated divergence) =0.0798
p(Scalp)=0.00347
p(Dura)=0.344
Scalp
Dura

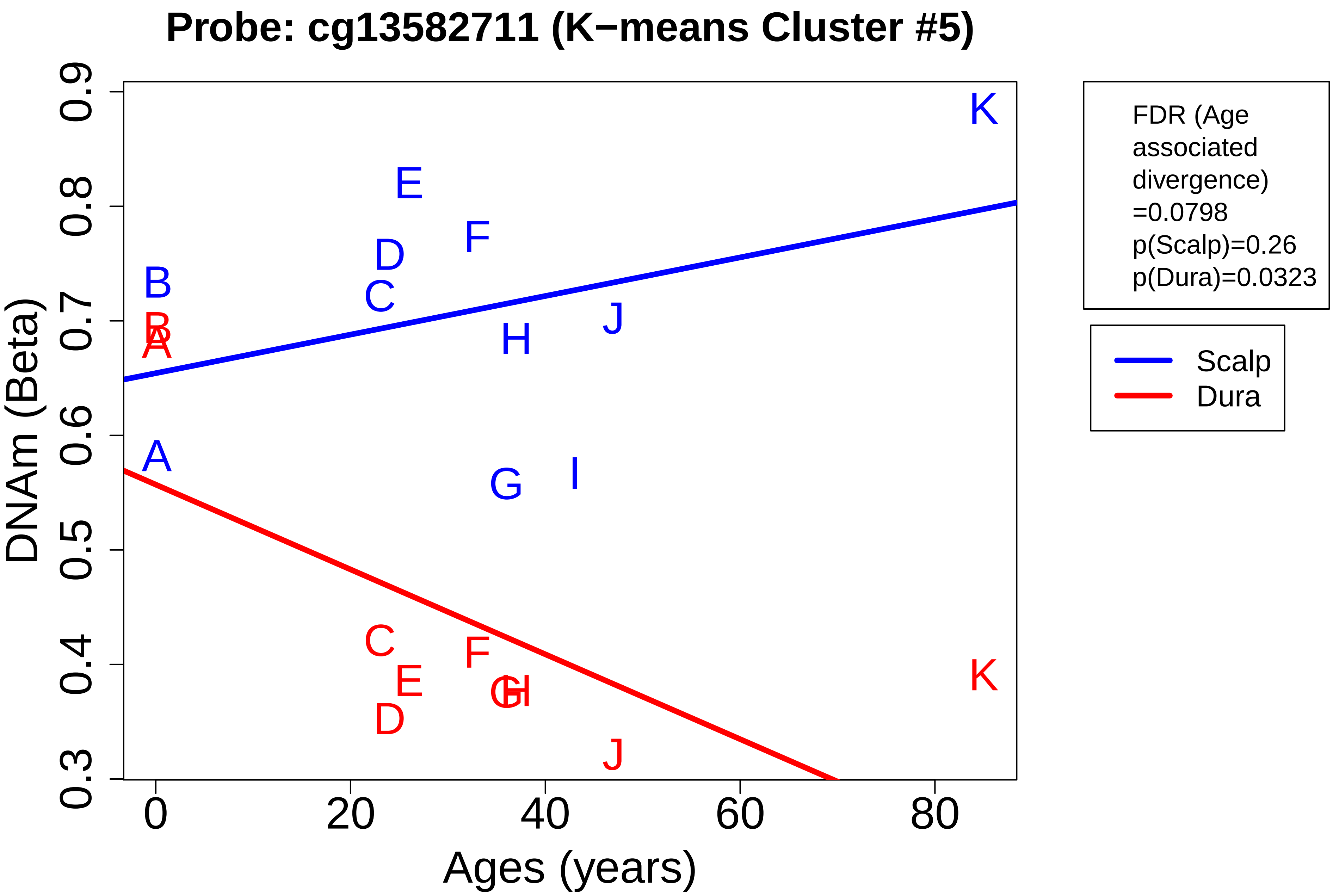

Probe: cg13582711 (K−means Cluster #5)
DNAm (Beta)
0.3
0.4
0.5
0.6
0.7
0.8
0.9
0
20
40
60
80
Ages (years)
K
E
F
D
B
C
J
B
A
H
A
G
I
C
F
E
GH
K
D
J
FDR (Age
associated
divergence)
=0.0798
p(Scalp)=0.26
p(Dura)=0.0323
Scalp
Dura

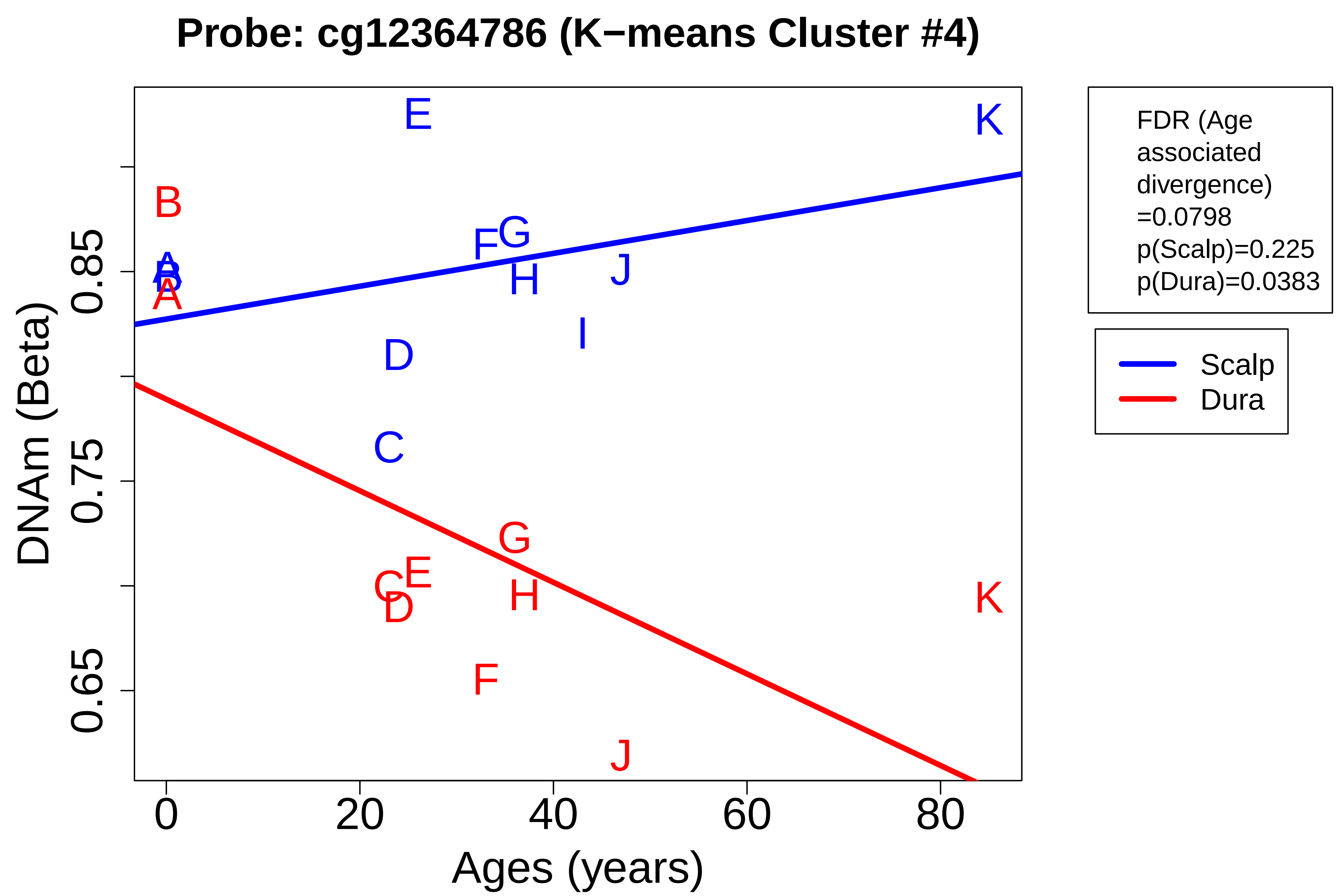

Probe: cg12364786 (K−means Cluster #4)
DNAm (Beta)
0.65
0.75
0.85
Ages (years)
0
20
40
60
80
E
K
B
F
G
A
B
A
H
J
I
D
C
G
C
E
D
H
K
F
J
FDR (Age associated divergence) =0.0798
p(Scalp)=0.225
p(Dura)=0.0383
Scalp
Dura

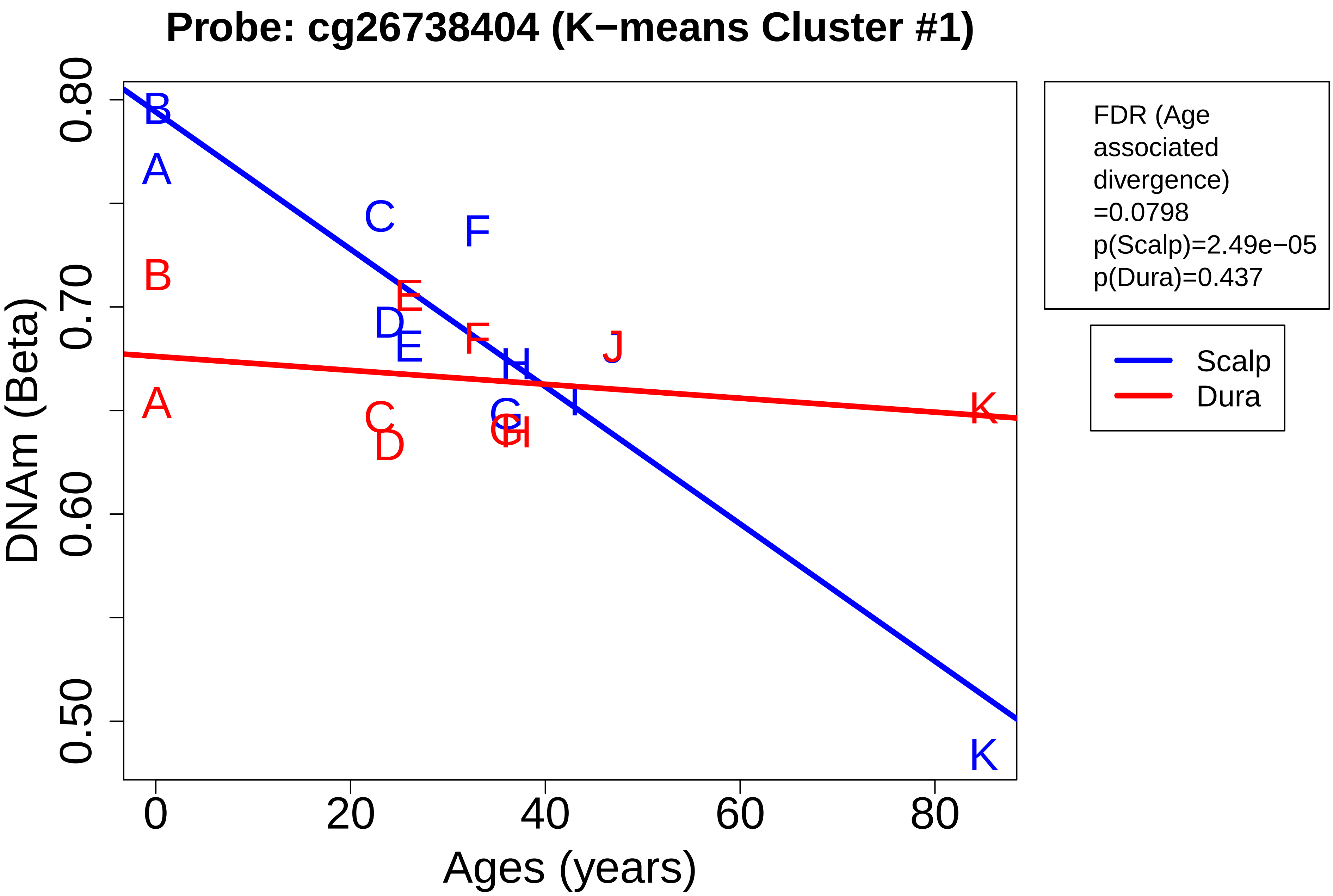

Probe: cg26738404 (K−means Cluster #1)
DNAm (Beta)
0.50
0.60
0.70
0.80
Ages (years)
0
20
40
60
80
FDR (Age associated divergence) =0.0798
p(Scalp)=2.49e−05
p(Dura)=0.437
Scalp
Dura

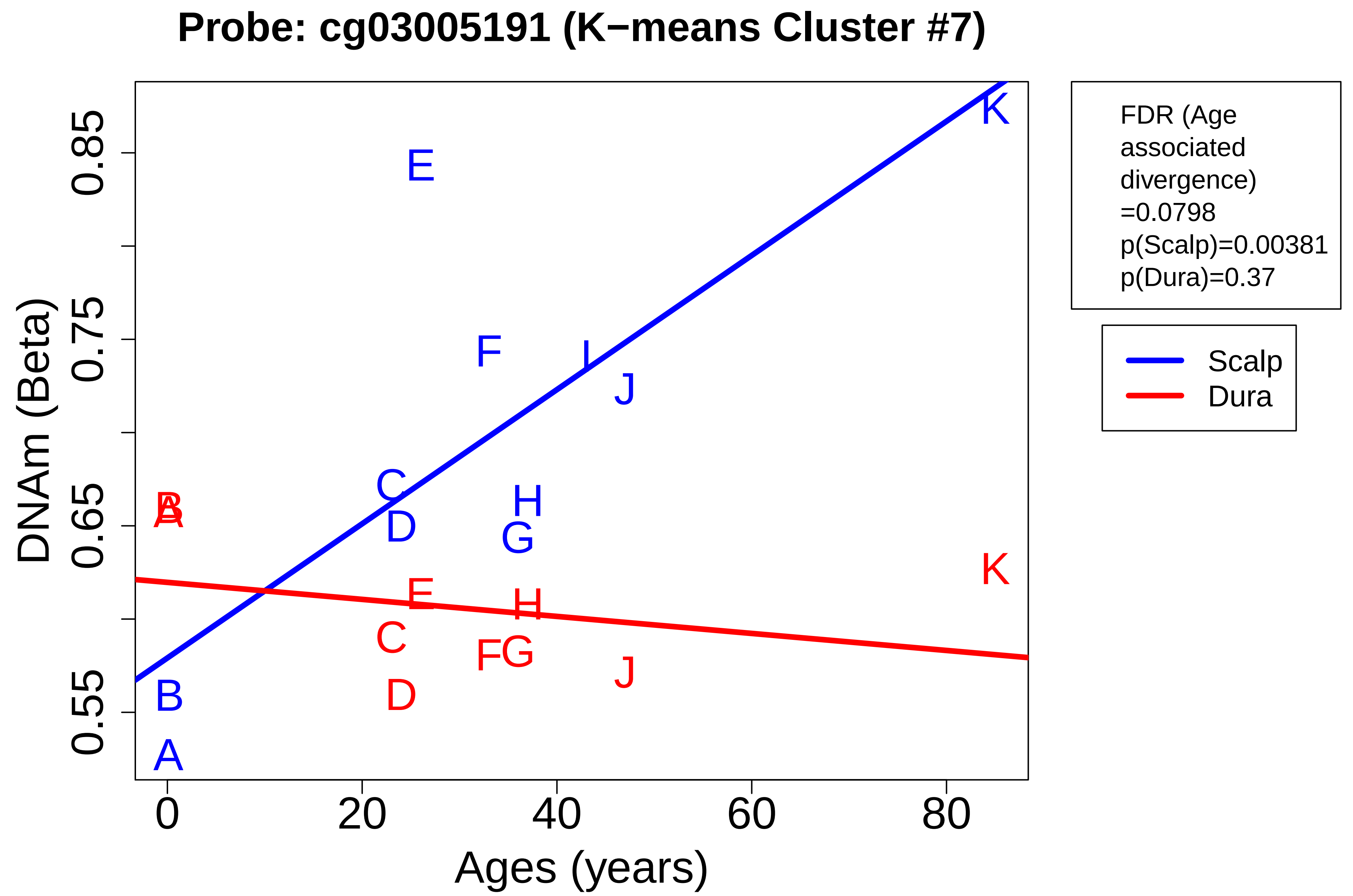

Probe: cg03005191 (K−means Cluster #7)
FDR (Age associated divergence) =0.0798
p(Scalp)=0.00381
p(Dura)=0.37
Scalp
Dura
DNAm (Beta)
0.55
0.65
0.75
0.85
0
20
40
60
80
Ages (years)
A
B
C
D
E
F
G
H
I
J
K

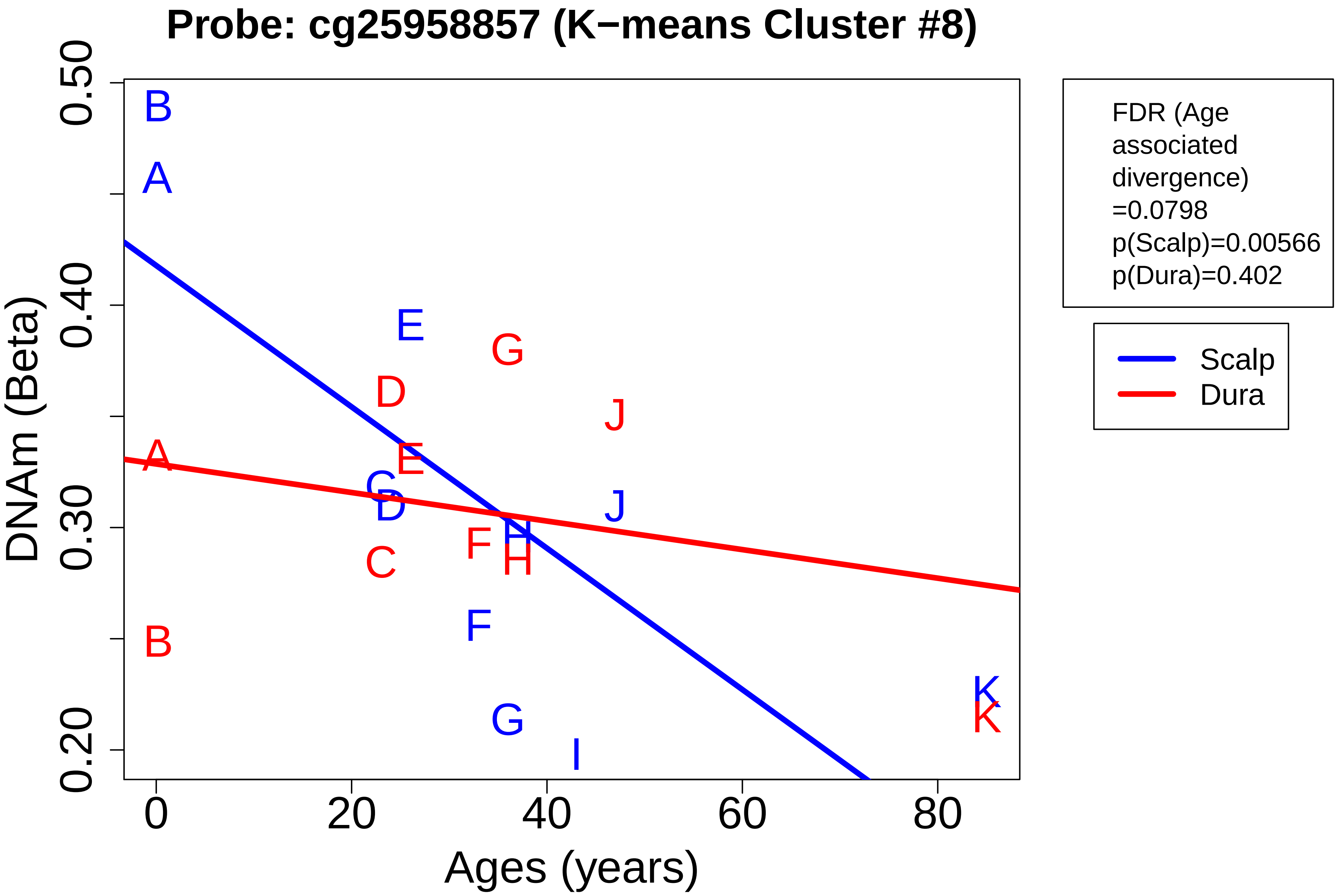

Probe: cg25958857 (K−means Cluster #8)
DNAm (Beta)
0.20
0.30
0.40
0.50
0
20
40
60
80
Ages (years)
FDR (Age associated divergence) =0.0798
p(Scalp)=0.00566
p(Dura)=0.402
Scalp
Dura

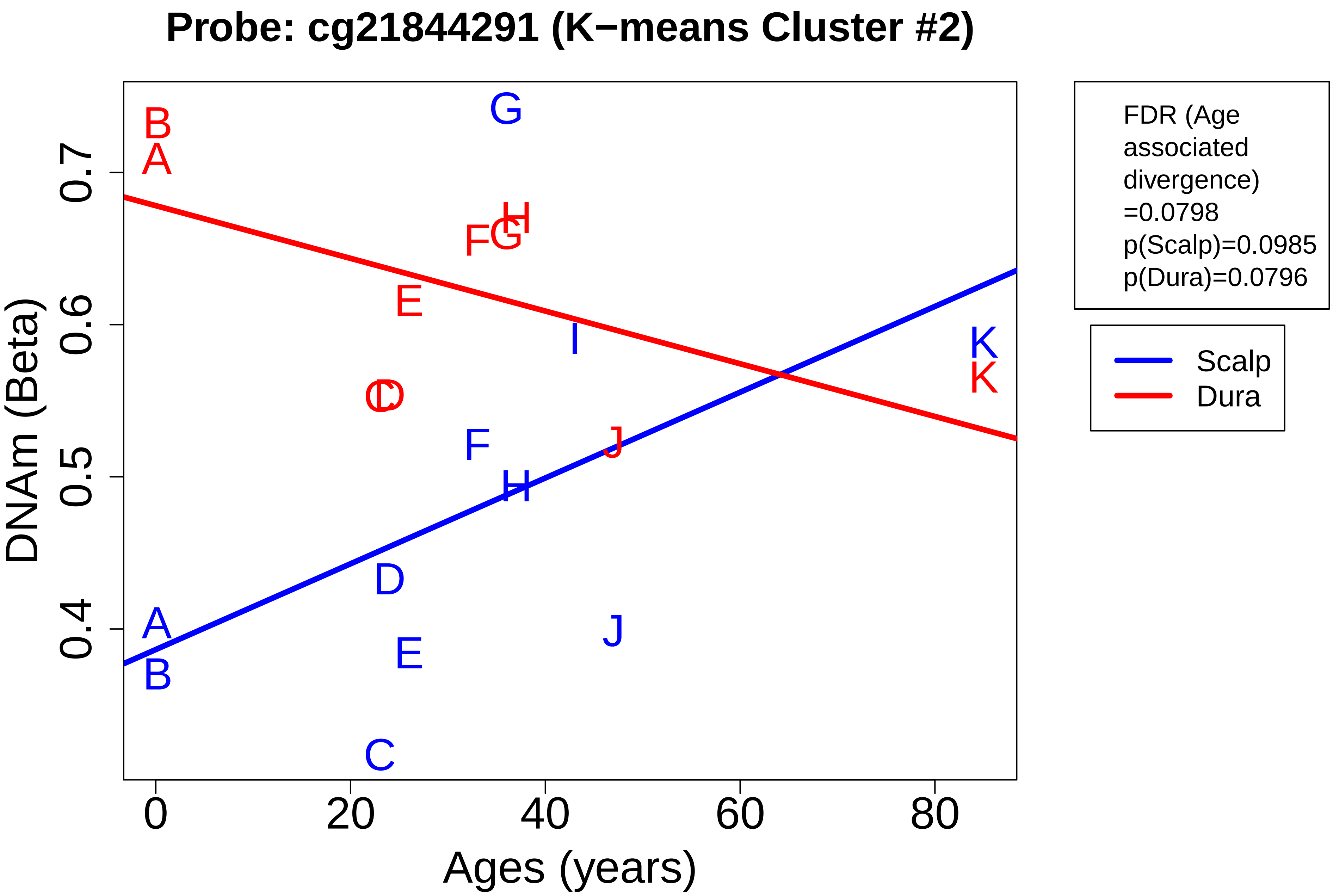
Probe: cg21844291 (K−means Cluster #2)
FDR (Age associated divergence) =0.0798
p(Scalp)=0.0985
p(Dura)=0.0796
Scalp
Dura
DNAm (Beta)
0.4
0.5
0.6
0.7
0
20
40
60
80
Ages (years)

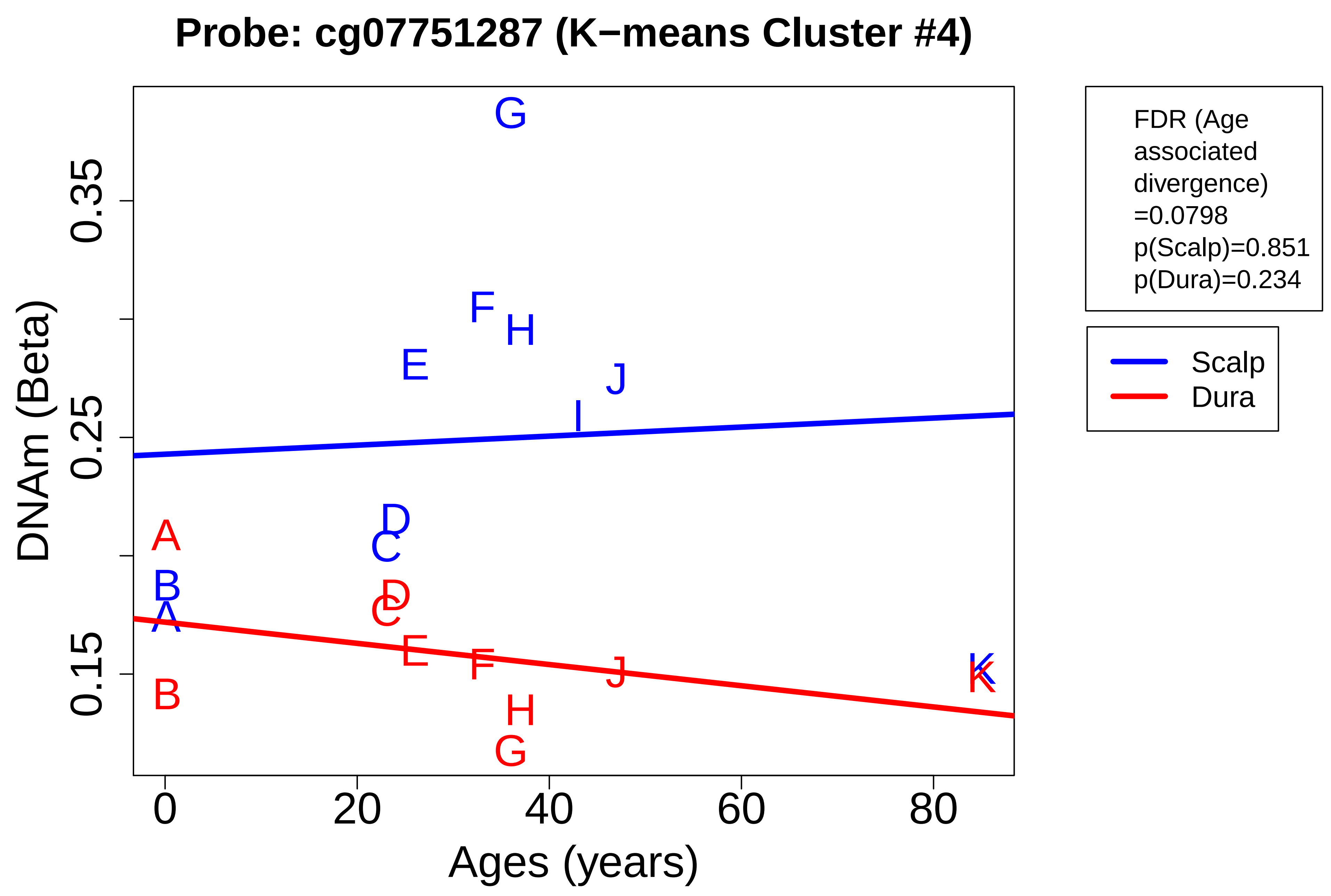

Probe: cg07751287 (K−means Cluster #4)
DNAm (Beta)
0.15
0.25
0.35
Ages (years)
0
20
40
60
80
FDR (Age associated divergence) =0.0798
p(Scalp)=0.851
p(Dura)=0.234
Scalp
Dura
A
B
C
D
E
F
G
H
I
J
K

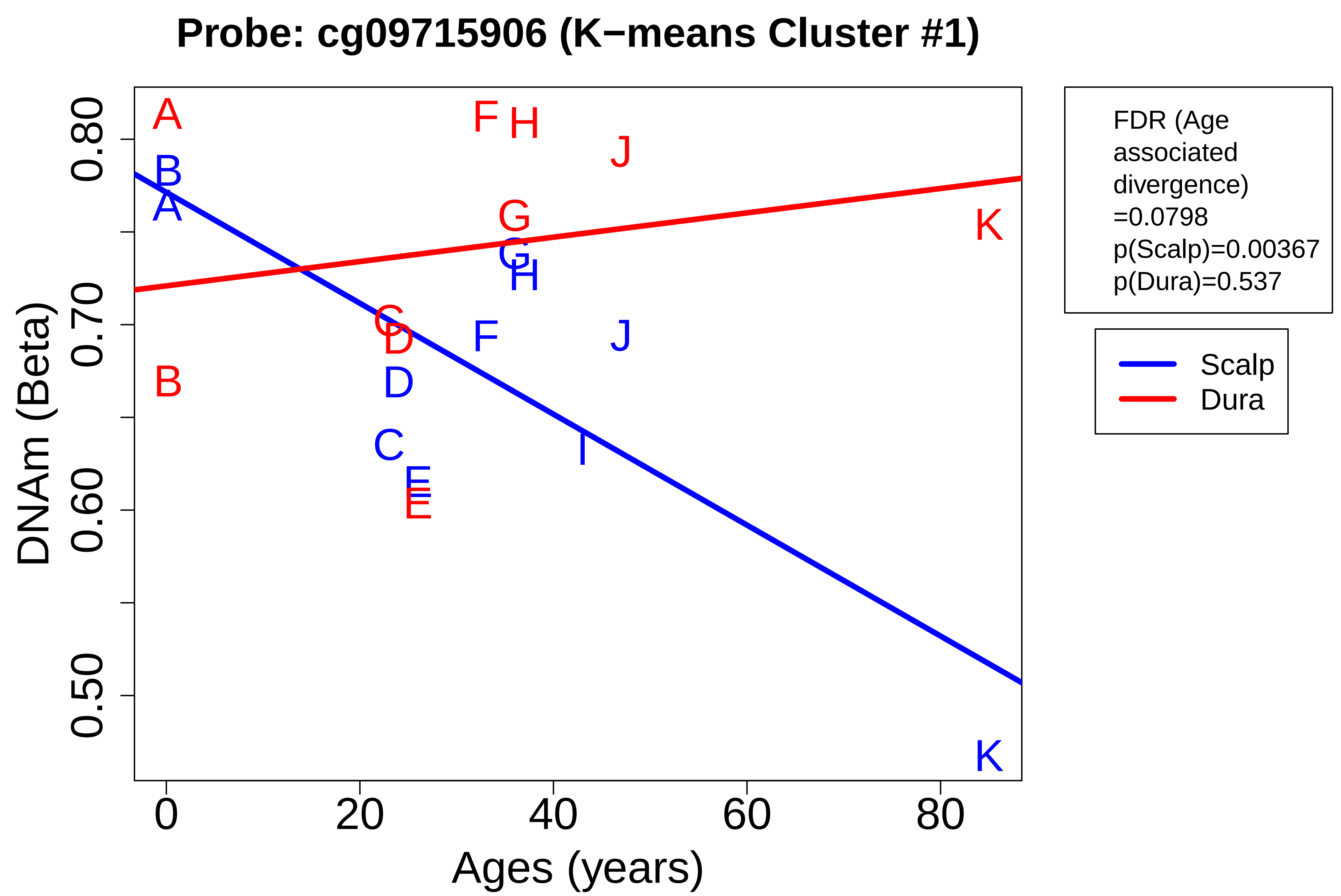
Probe: cg09715906 (K−means Cluster #1)
DNAm (Beta)
0.50
0.60
0.70
0.80
Ages (years)
0
20
40
60
80
A
B
C
D
E
F
G
H
I
J
K
FDR (Age associated divergence) =0.0798
p(Scalp)=0.00367
p(Dura)=0.537
Scalp
Dura

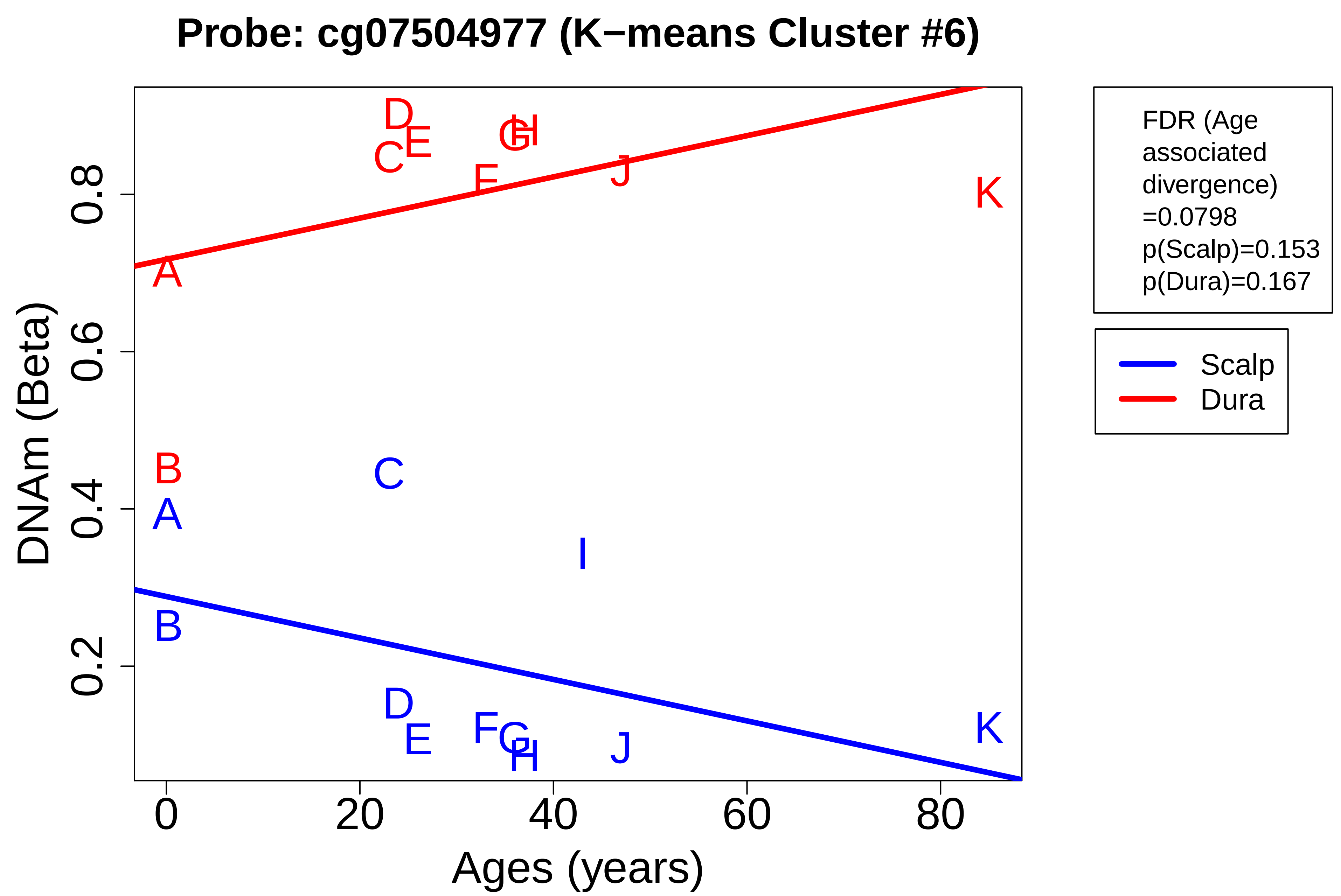

Probe: cg07504977 (K−means Cluster #6)
DNAm (Beta)
0.2
0.4
0.6
0.8
0
20
40
60
80
Ages (years)
FDR (Age associated divergence) =0.0798
p(Scalp)=0.153
p(Dura)=0.167
Scalp
Dura

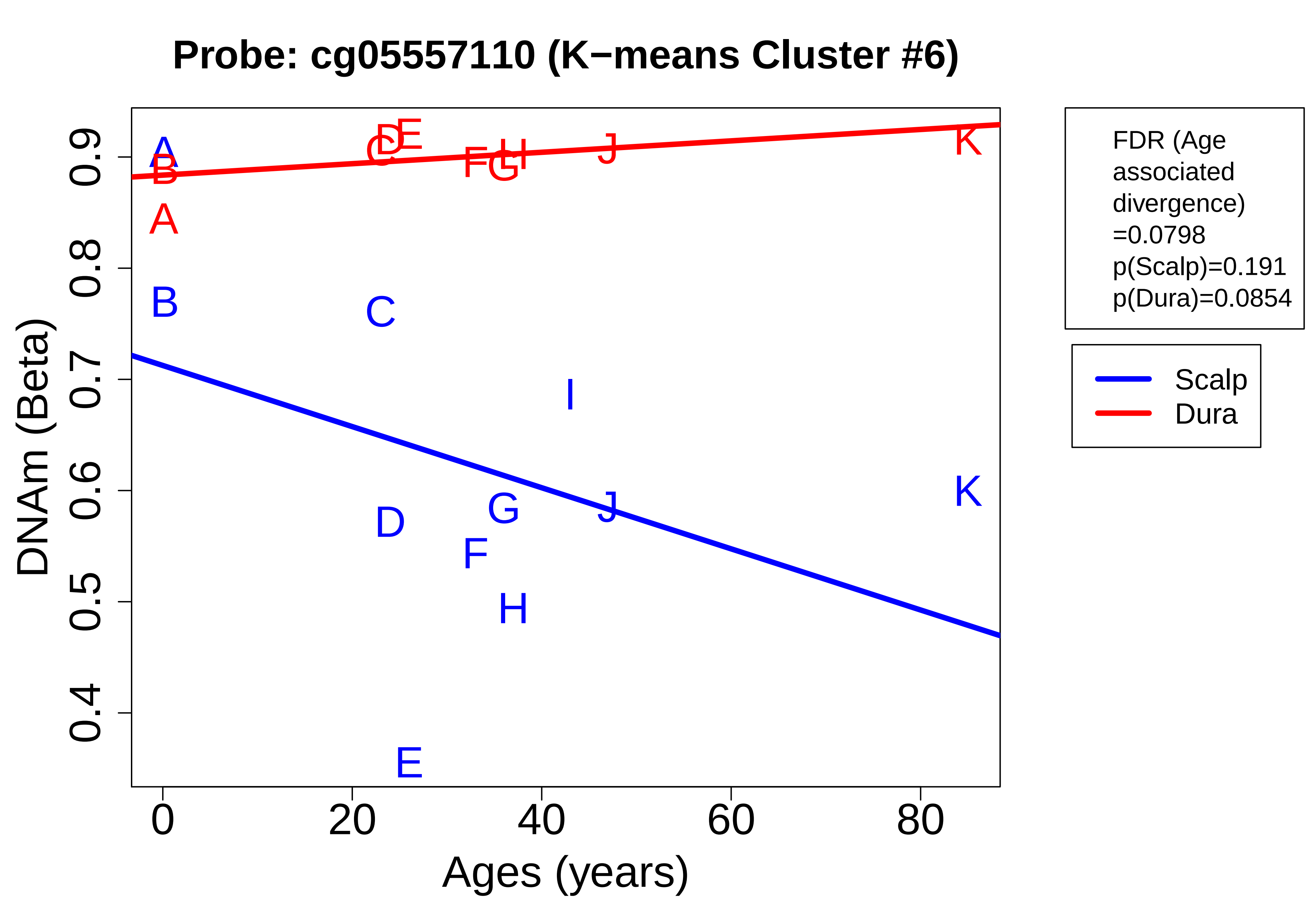

Probe: cg05557110 (K−means Cluster #6)
DNAm (Beta)
0.4
0.5
0.6
0.7
0.8
0.9
0
20
40
60
80
Ages (years)
FDR (Age associated divergence) =0.0798
p(Scalp)=0.191
p(Dura)=0.0854
Scalp
Dura

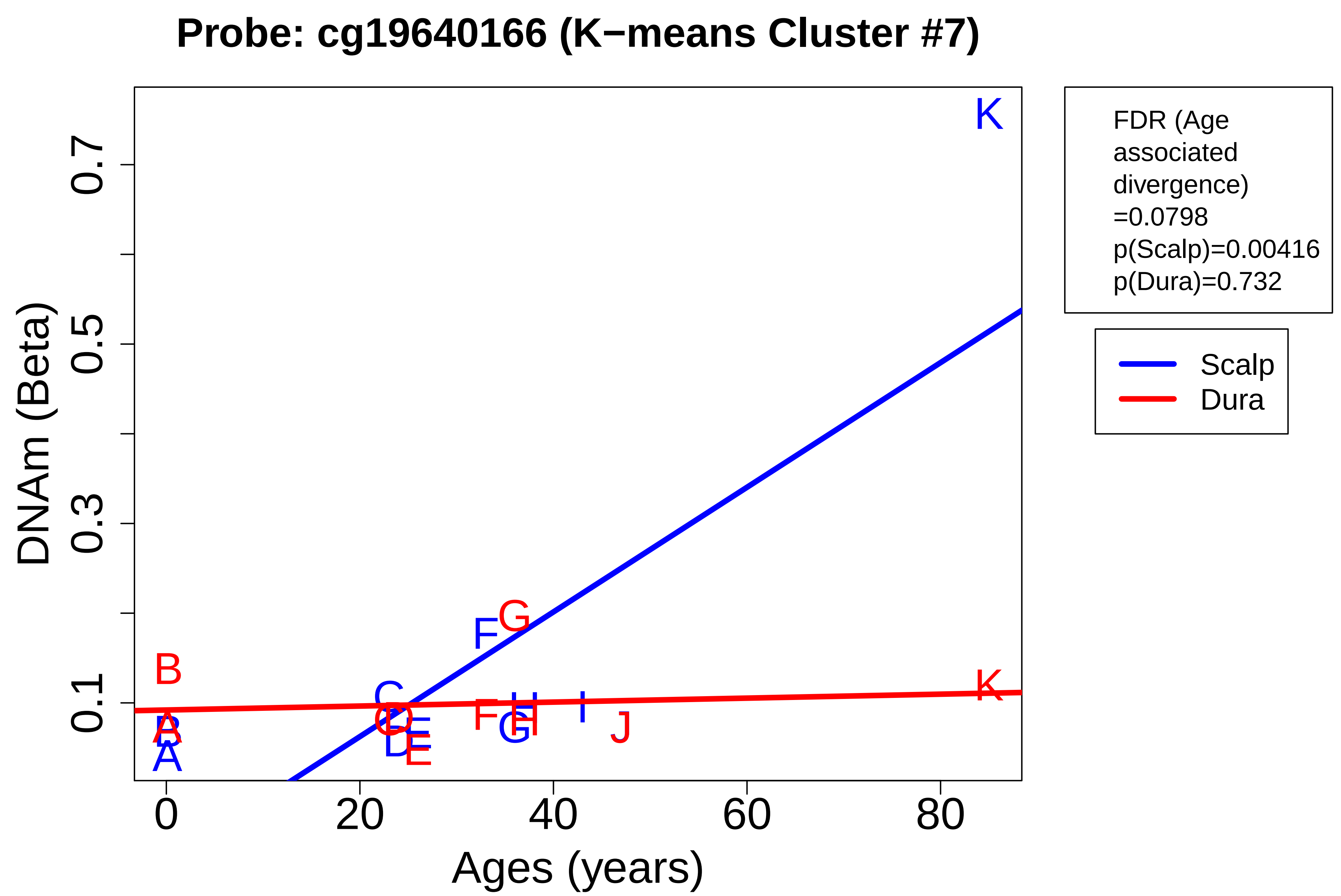

Probe: cg19640166 (K−means Cluster #7)
DNAm (Beta)
0.1
0.3
0.5
0.7
0
20
40
60
80
Ages (years)
FDR (Age associated divergence) =0.0798
p(Scalp)=0.00416
p(Dura)=0.732
Scalp
Dura

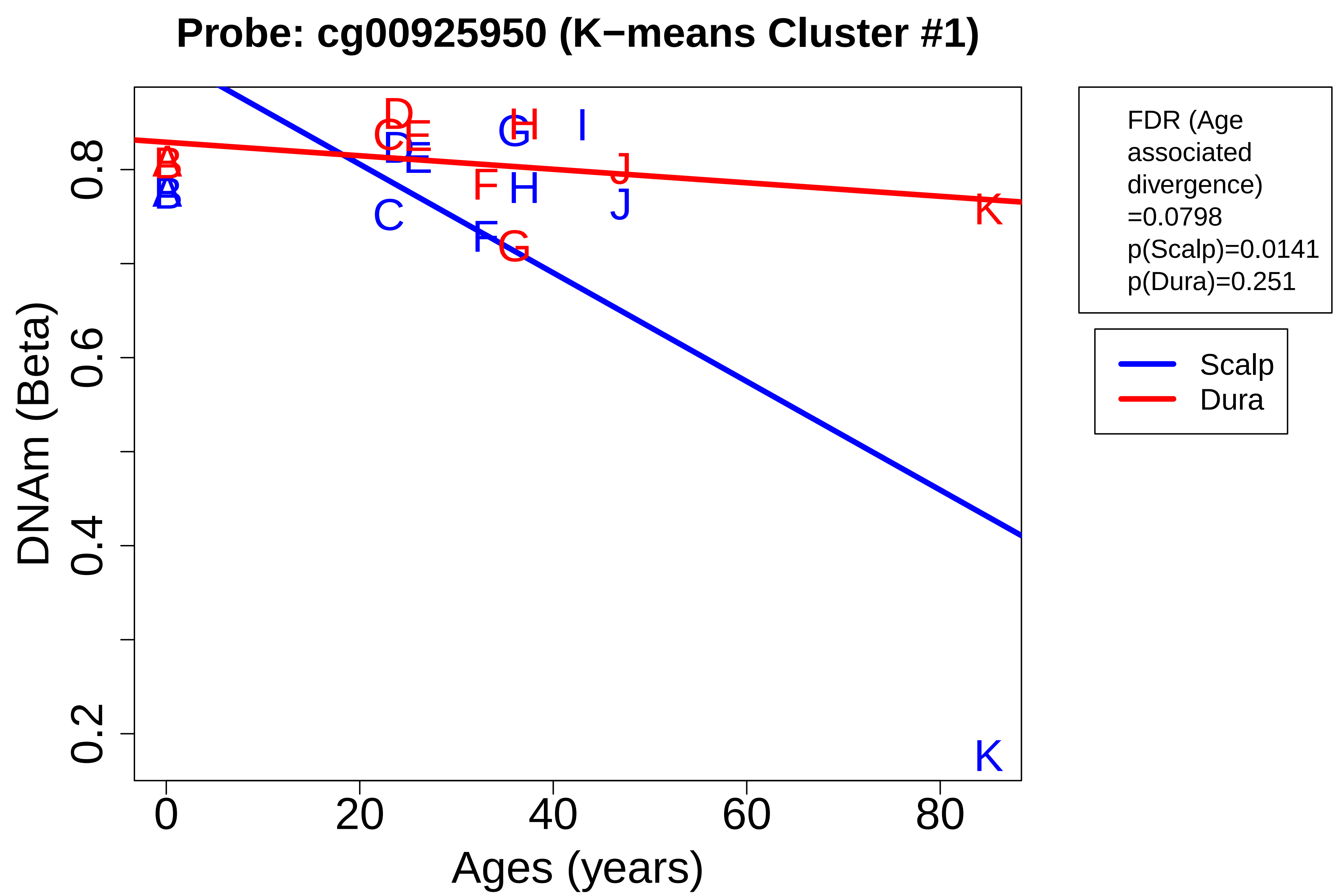

Probe: cg00925950 (K−means Cluster #1)
DNAm (Beta)
0.2
0.4
0.6
0.8
0
20
40
60
80
Ages (years)
FDR (Age associated divergence) =0.0798
p(Scalp)=0.0141
p(Dura)=0.251
Scalp
Dura

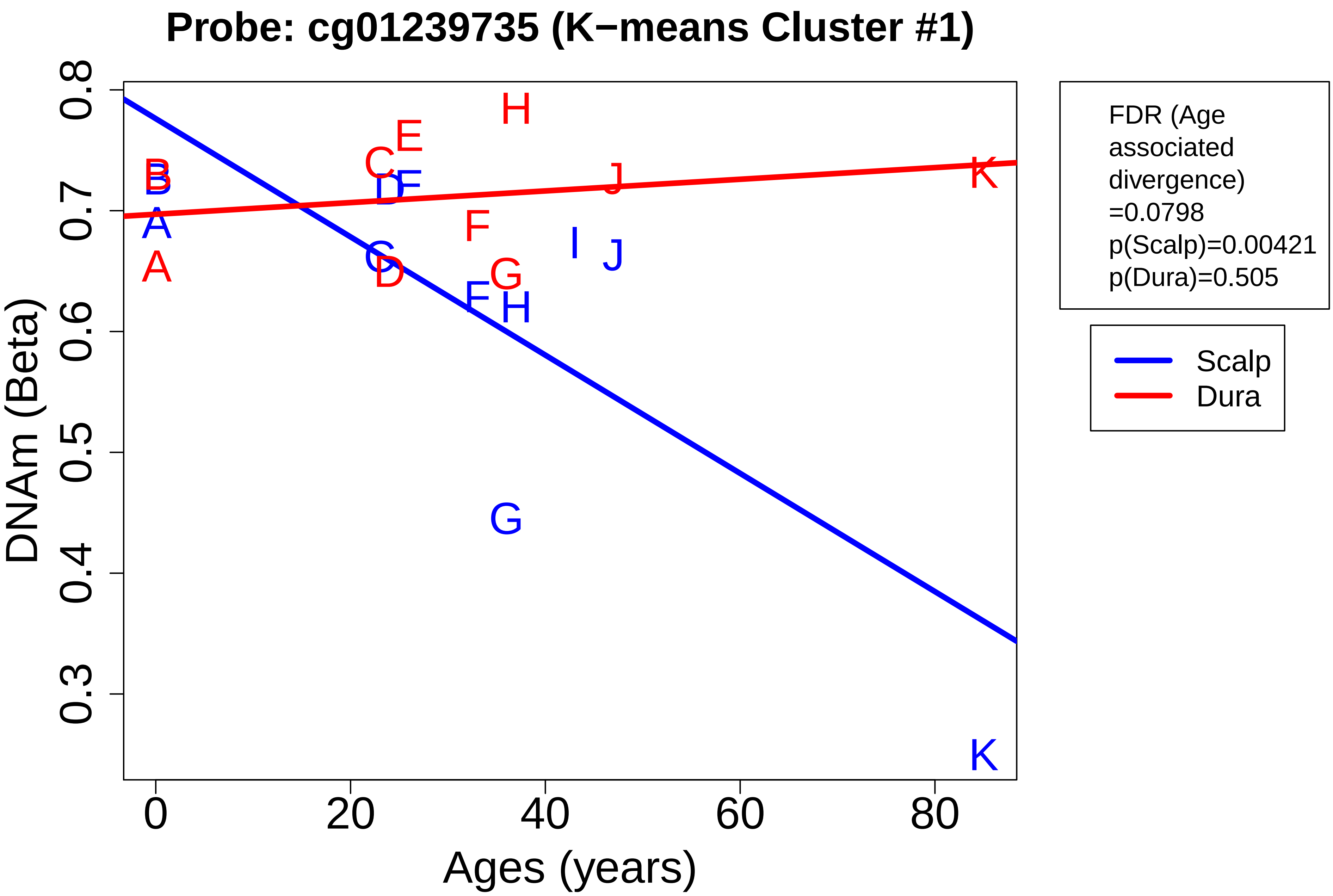

Probe: cg01239735 (K−means Cluster #1)
DNAm (Beta)
0.3
0.4
0.5
0.6
0.7
0.8
0
20
40
60
80
Ages (years)
FDR (Age associated divergence) =0.0798
p(Scalp)=0.00421
p(Dura)=0.505
Scalp
Dura

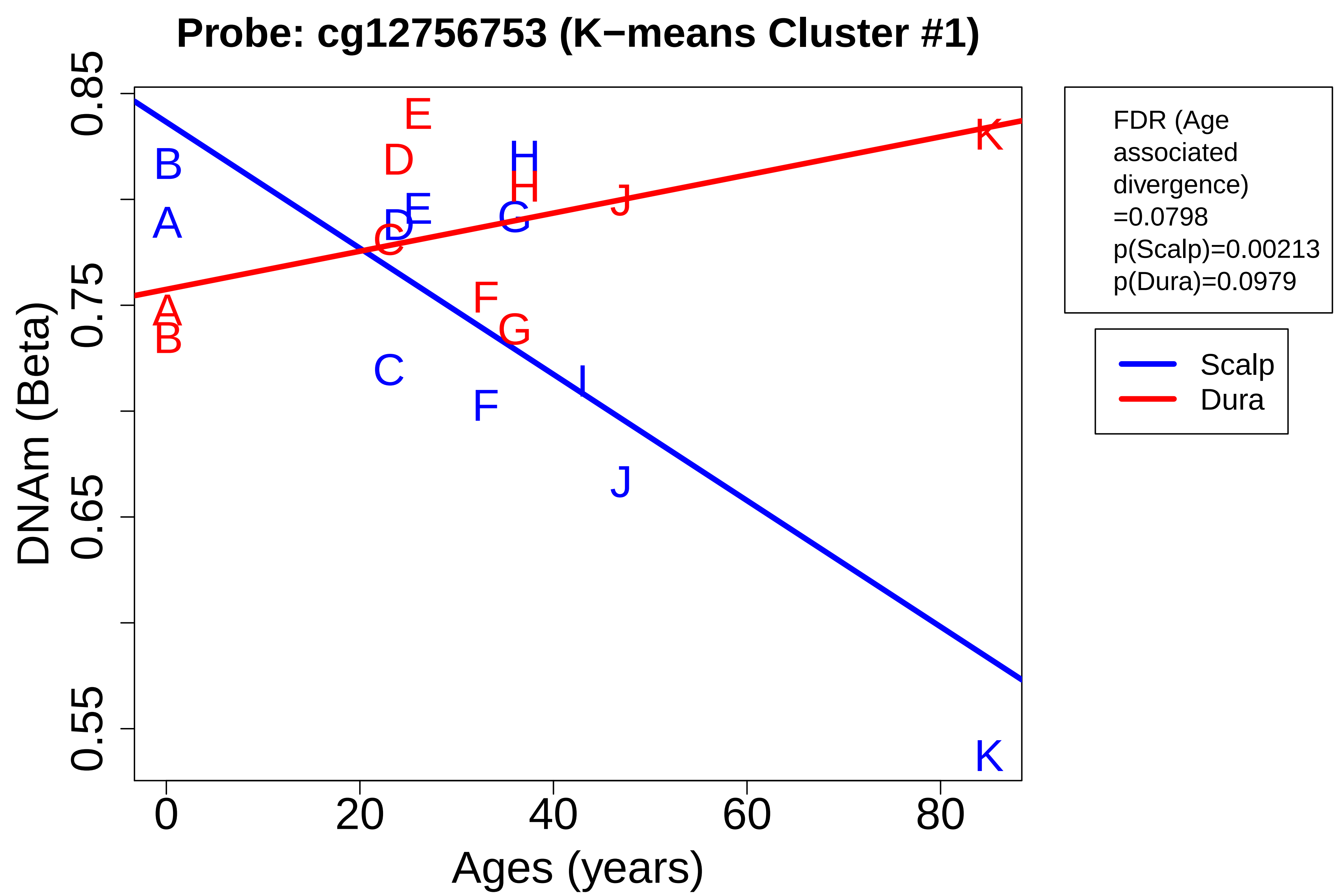

Probe: cg12756753 (K−means Cluster #1)
DNAm (Beta)
0.55
0.65
0.75
0.85
Ages (years)
0
20
40
60
80
FDR (Age associated divergence) =0.0798
p(Scalp)=0.00213
p(Dura)=0.0979
Scalp
Dura
A
B
C
D
E
F
G
H
I
J
K

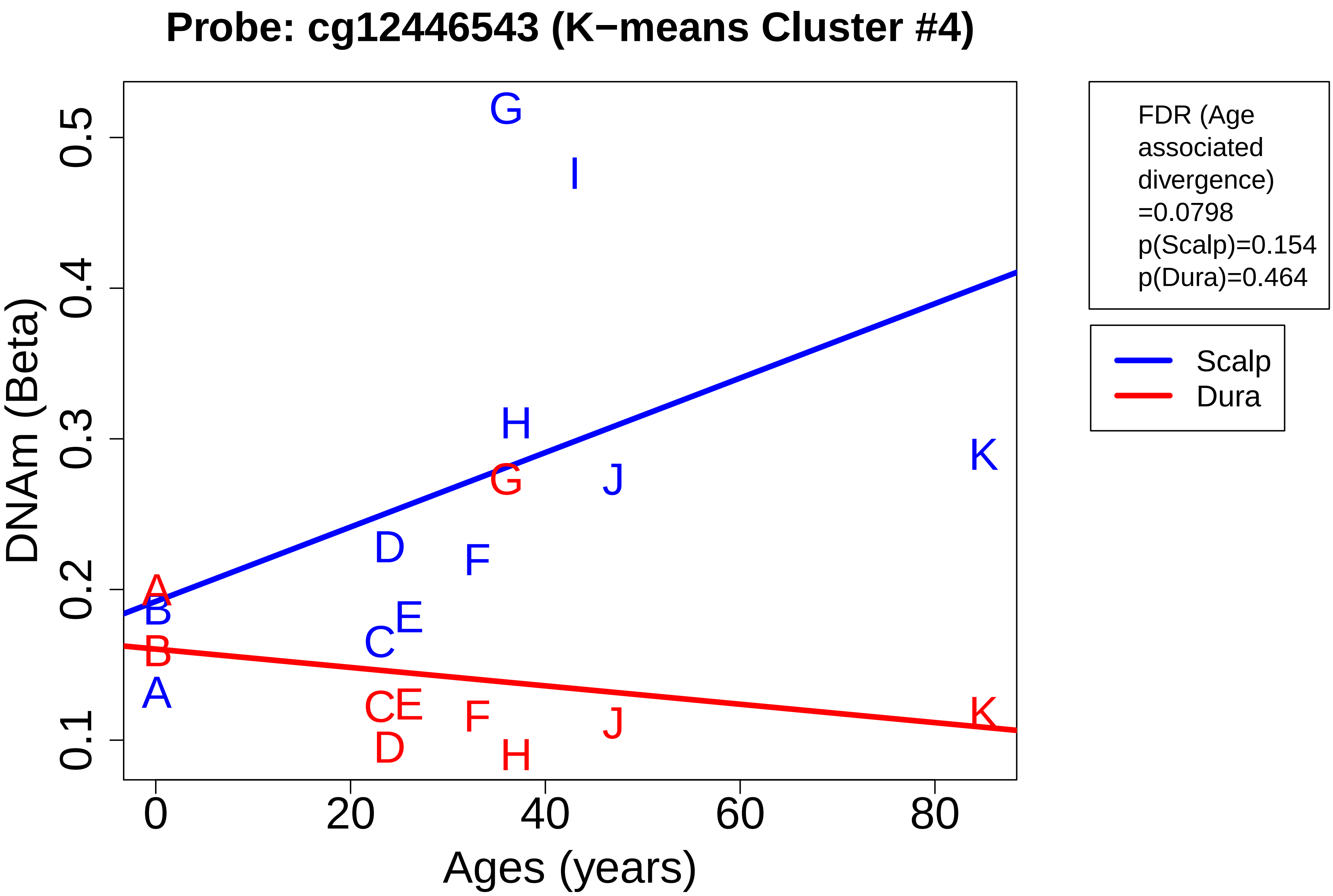

Probe: cg12446543 (K−means Cluster #4)
DNAm (Beta)
0.1
0.2
0.3
0.4
0.5
Ages (years)
0
20
40
60
80
FDR (Age associated divergence) =0.0798
p(Scalp)=0.154
p(Dura)=0.464
Scalp
Dura

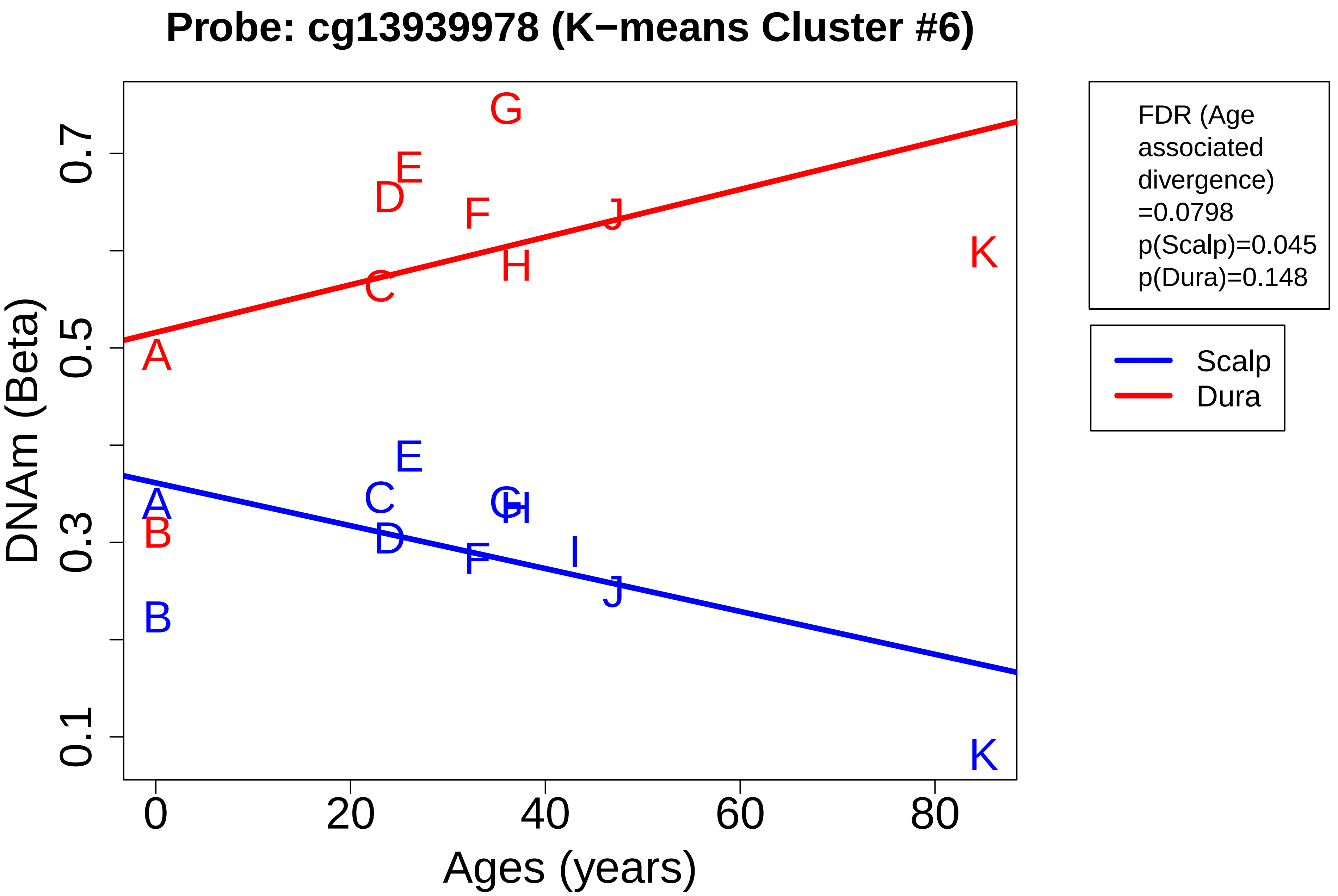
Probe: cg13939978 (K−means Cluster #6)
DNAm (Beta)
0.1
0.3
0.5
0.7
Ages (years)
0
20
40
60
80
FDR (Age associated divergence) =0.0798
p(Scalp)=0.045
p(Dura)=0.148
Scalp
Dura
A
B
C
D
E
F
G
H
I
J
K

Probe: cg15973818 (K−means Cluster #8)

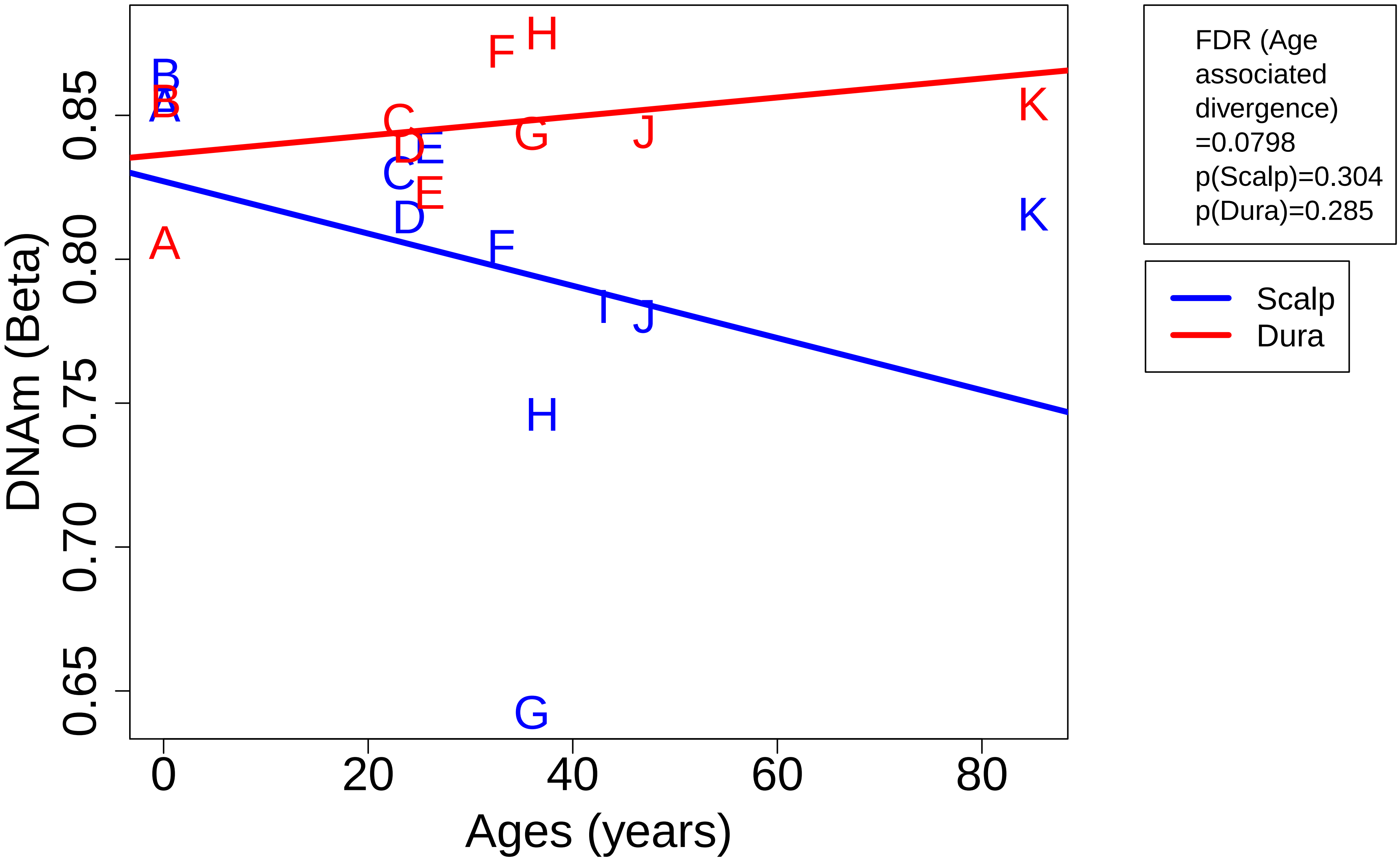

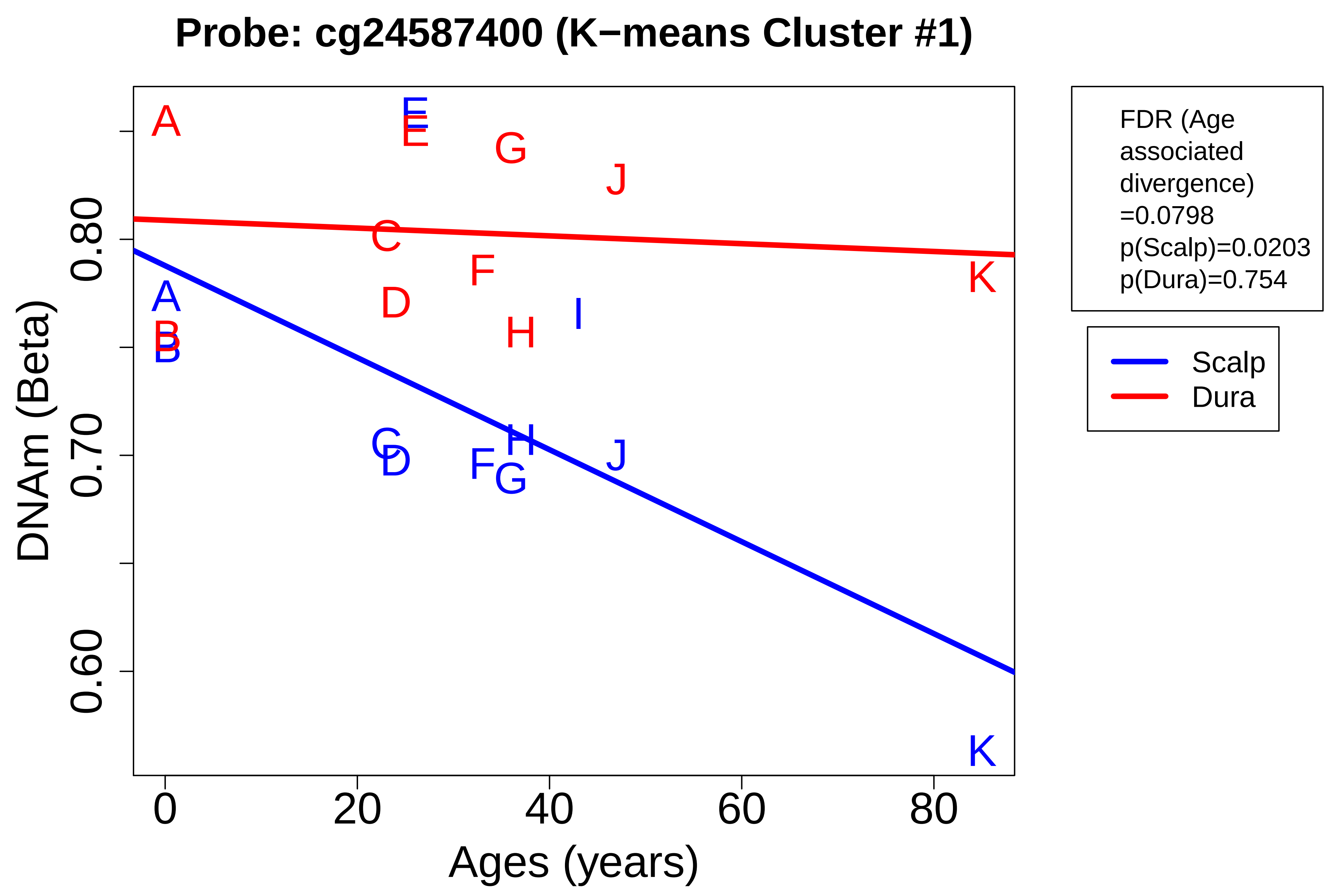

Probe: cg24587400 (K−means Cluster #1)
FDR (Age associated divergence) =0.0798
p(Scalp)=0.0203
p(Dura)=0.754
Scalp
Dura
DNAm (Beta)
0.60
0.70
0.80
0
20
40
60
80
Ages (years)

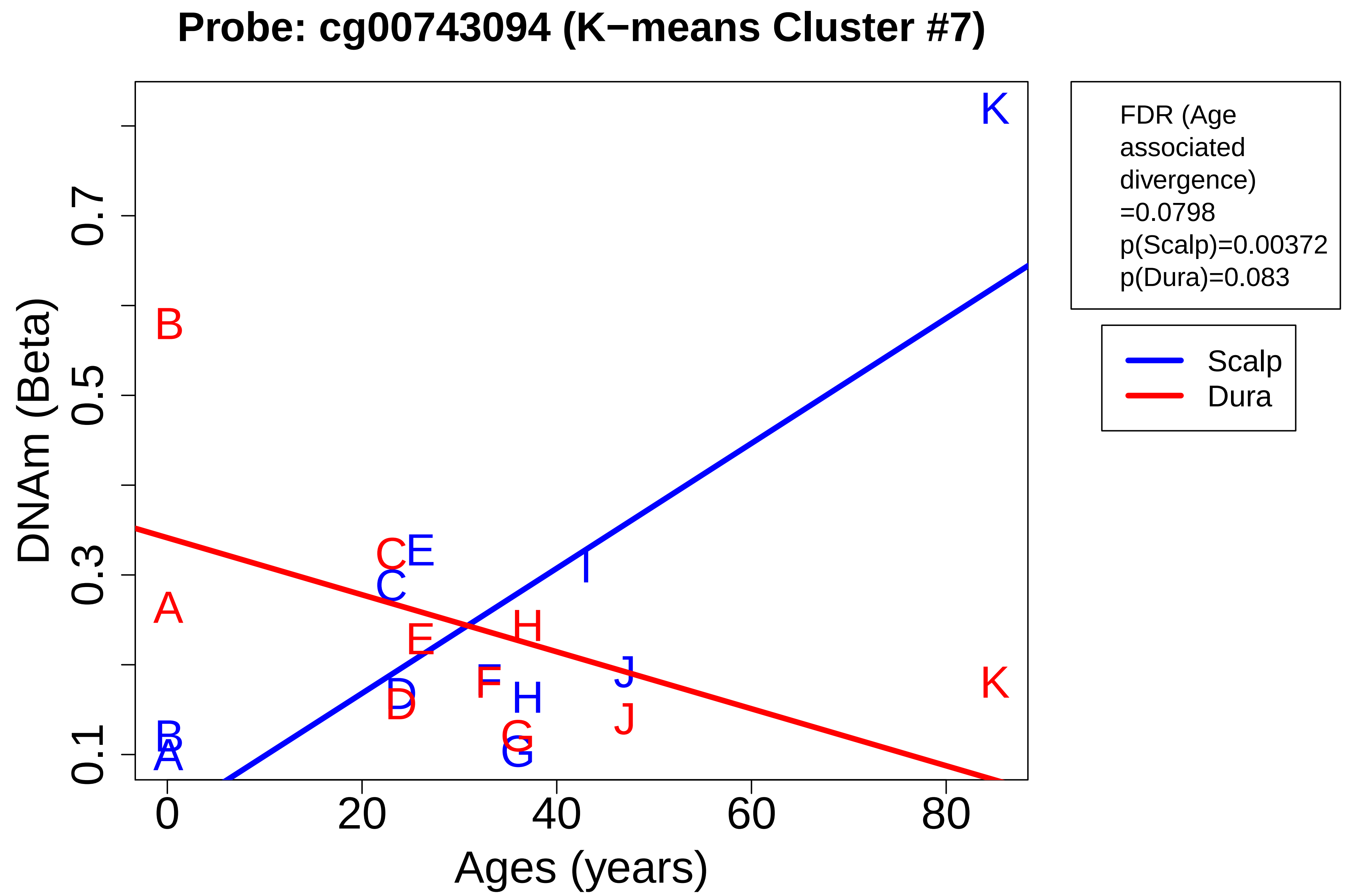

Probe: cg00743094 (K−means Cluster #7)
DNAm (Beta)
Ages (years)
0.1
0.3
0.5
0.7
0
20
40
60
80
FDR (Age associated divergence) =0.0798
p(Scalp)=0.00372
p(Dura)=0.083
Scalp
Dura
A
B
C
D
E
F
G
H
I
J
K

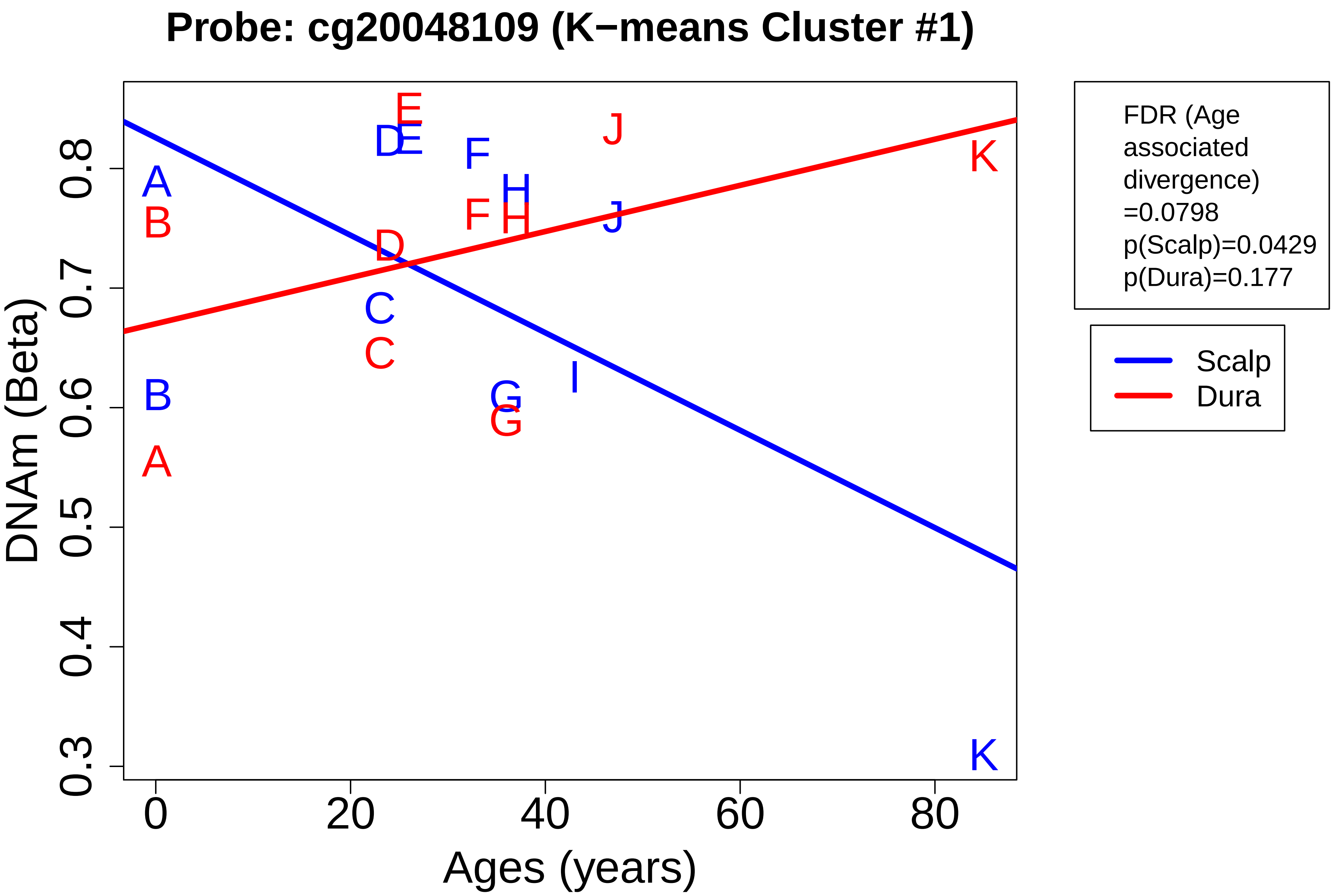

Probe: cg20048109 (K−means Cluster #1)
DNAm (Beta)
0.3
0.4
0.5
0.6
0.7
0.8
0
20
40
60
80
Ages (years)
FDR (Age associated divergence) =0.0798
p(Scalp)=0.0429
p(Dura)=0.177
Scalp
Dura

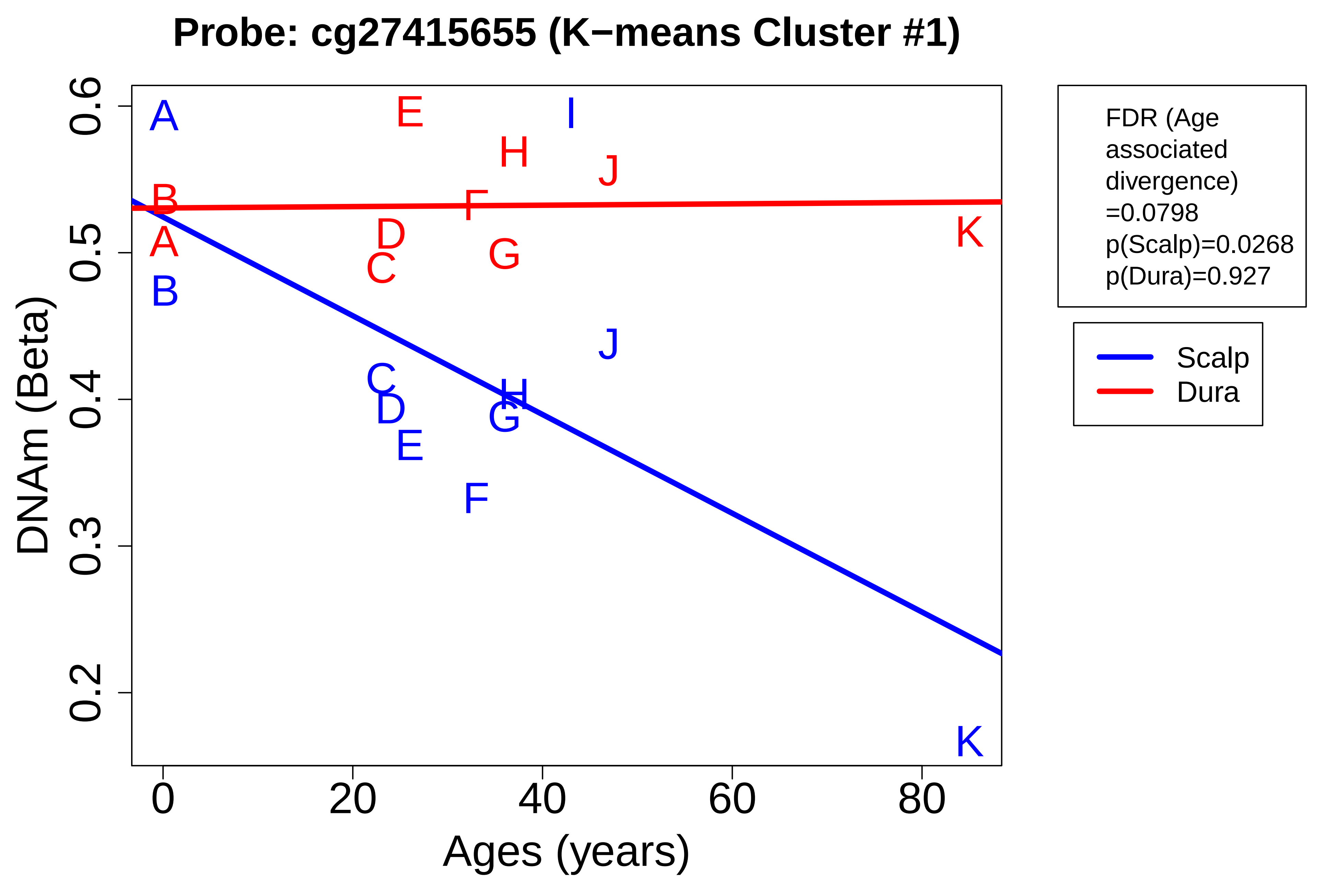

Probe: cg27415655 (K−means Cluster #1)
DNAm (Beta)
0.2
0.3
0.4
0.5
0.6
0
20
40
60
80
Ages (years)
A
B
C
D
E
F
G
H
I
J
K
FDR (Age associated divergence) =0.0798
p(Scalp)=0.0268
p(Dura)=0.927
Scalp
Dura

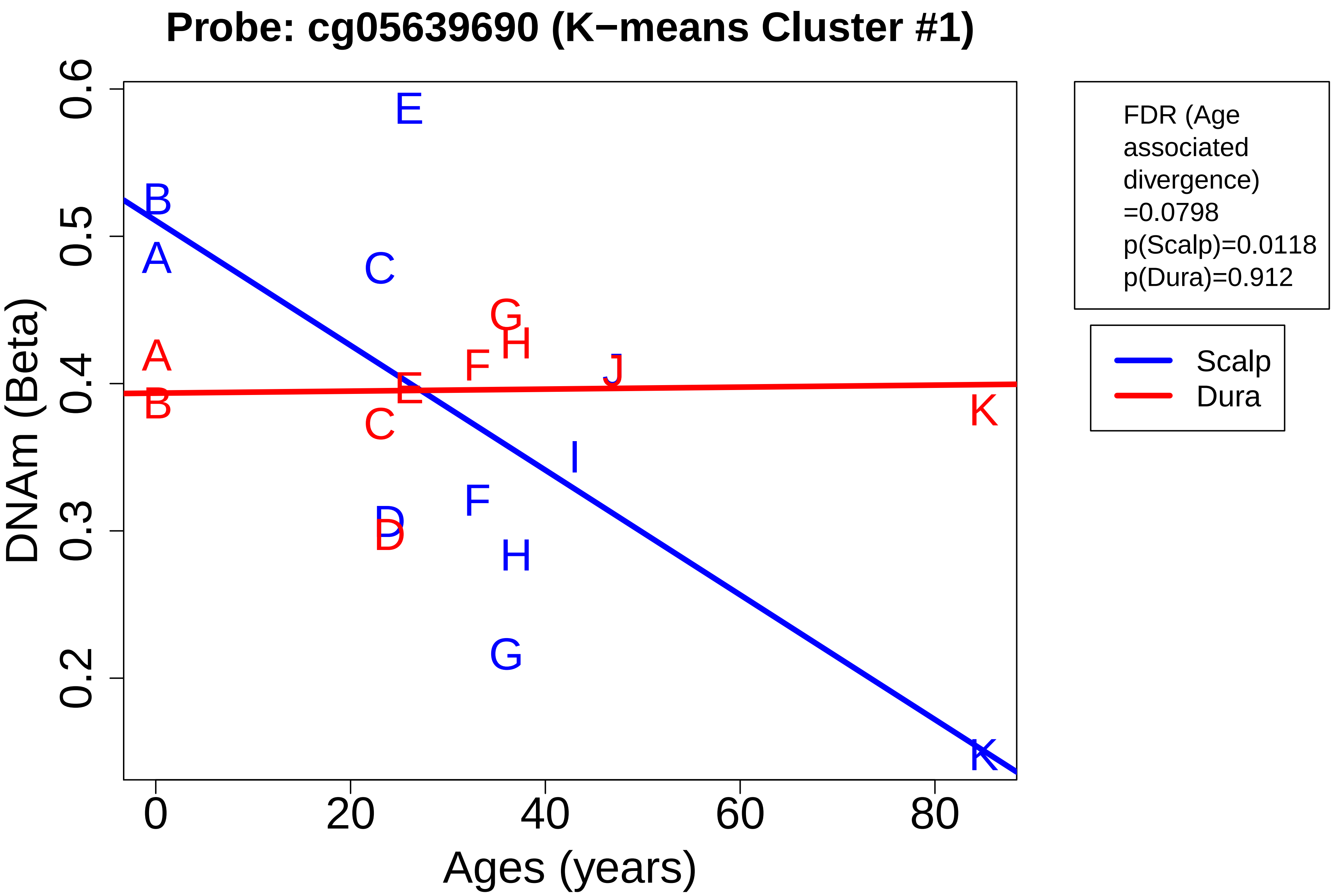

Probe: cg05639690 (K−means Cluster #1)
DNAm (Beta)
0.2
0.3
0.4
0.5
0.6
Ages (years)
0
20
40
60
80
FDR (Age associated divergence) =0.0798
p(Scalp)=0.0118
p(Dura)=0.912
Scalp
Dura

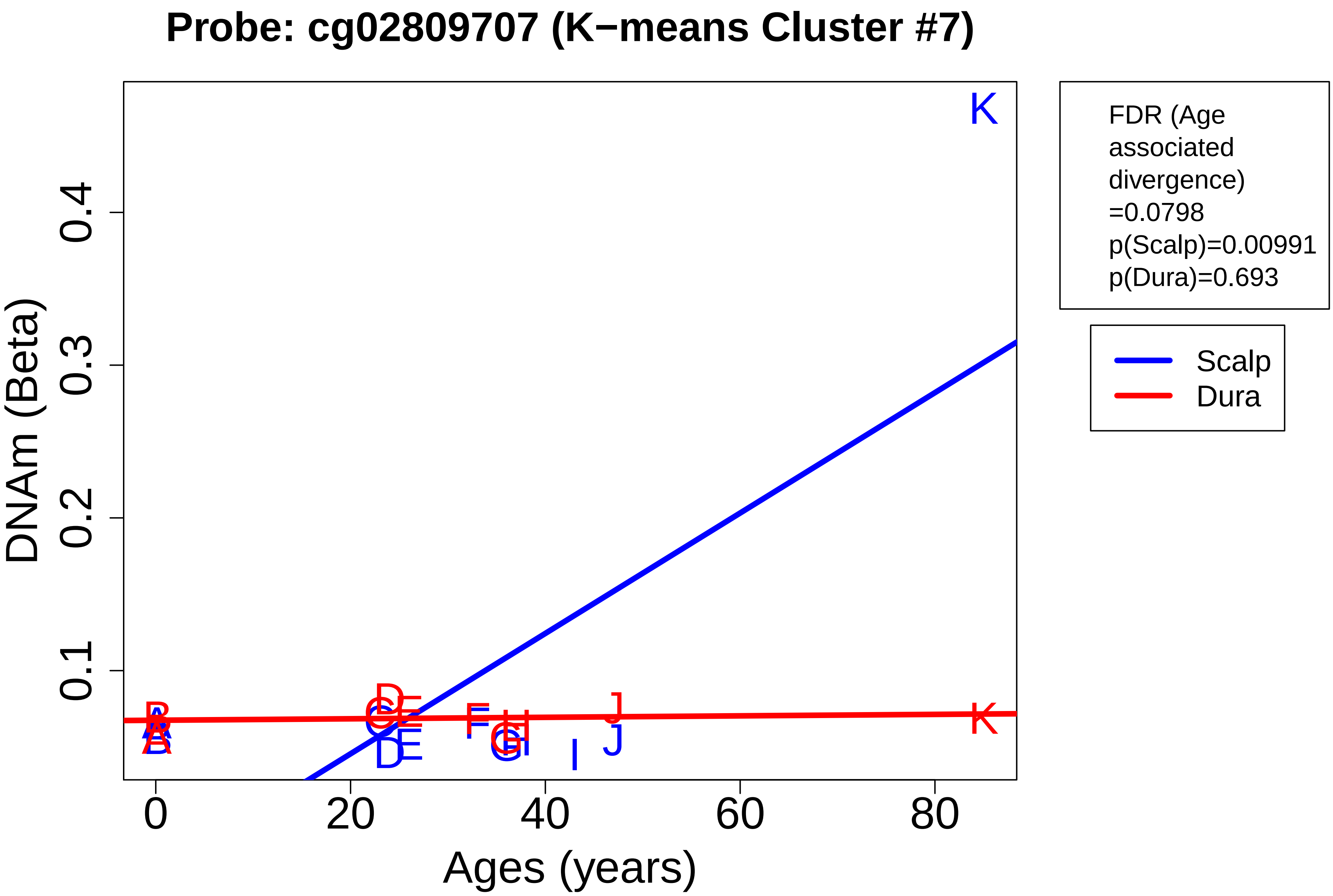

Probe: cg02809707 (K−means Cluster #7)
DNAm (Beta)
0.1
0.2
0.3
0.4
0
20
40
60
80
Ages (years)
FDR (Age associated divergence) =0.0798
p(Scalp)=0.00991
p(Dura)=0.693
Scalp
Dura

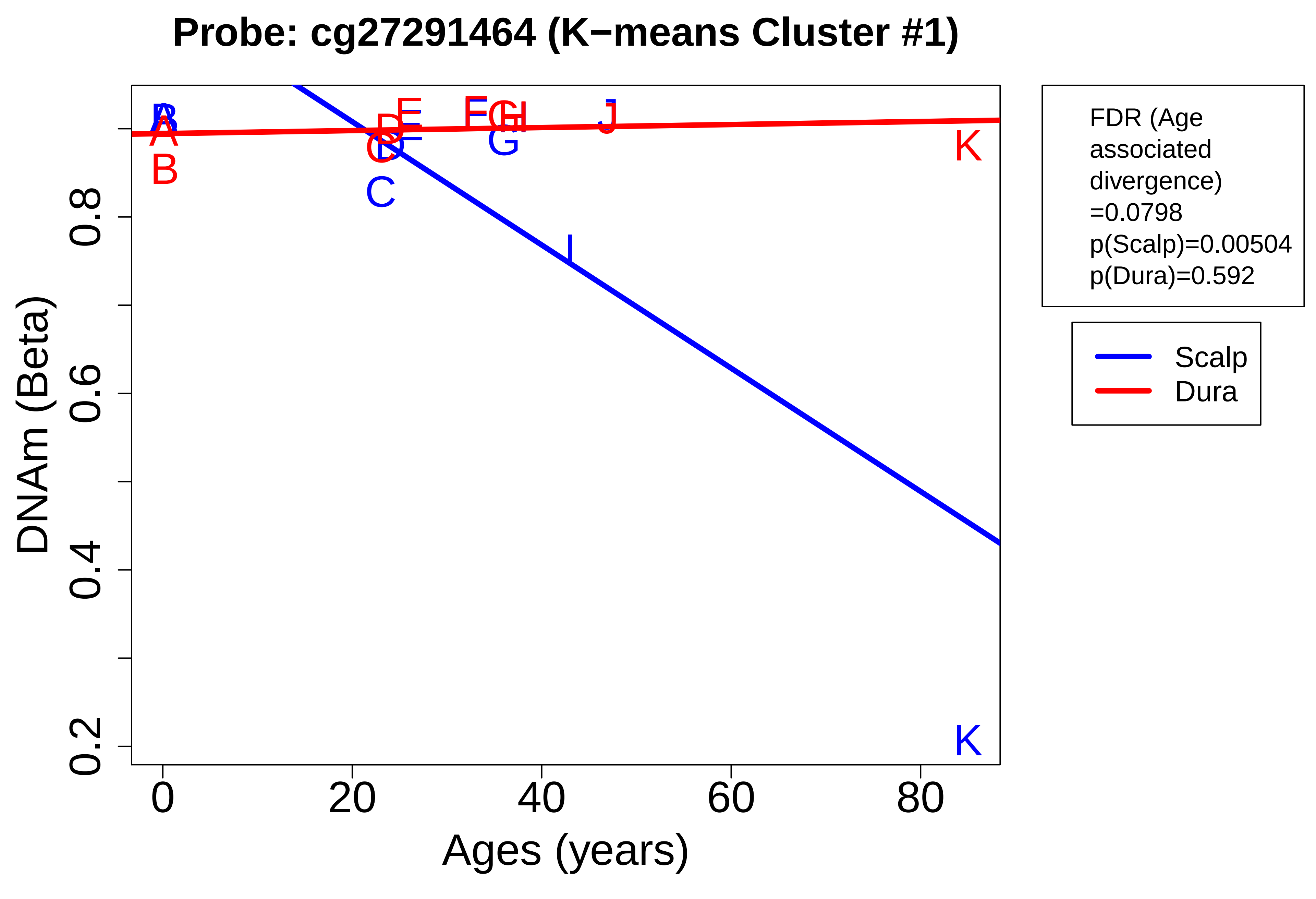

Probe: cg27291464 (K−means Cluster #1)
DNAm (Beta)
0.2
0.4
0.6
0.8
Ages (years)
0
20
40
60
80
FDR (Age associated divergence) =0.0798
p(Scalp)=0.00504
p(Dura)=0.592
Scalp
Dura

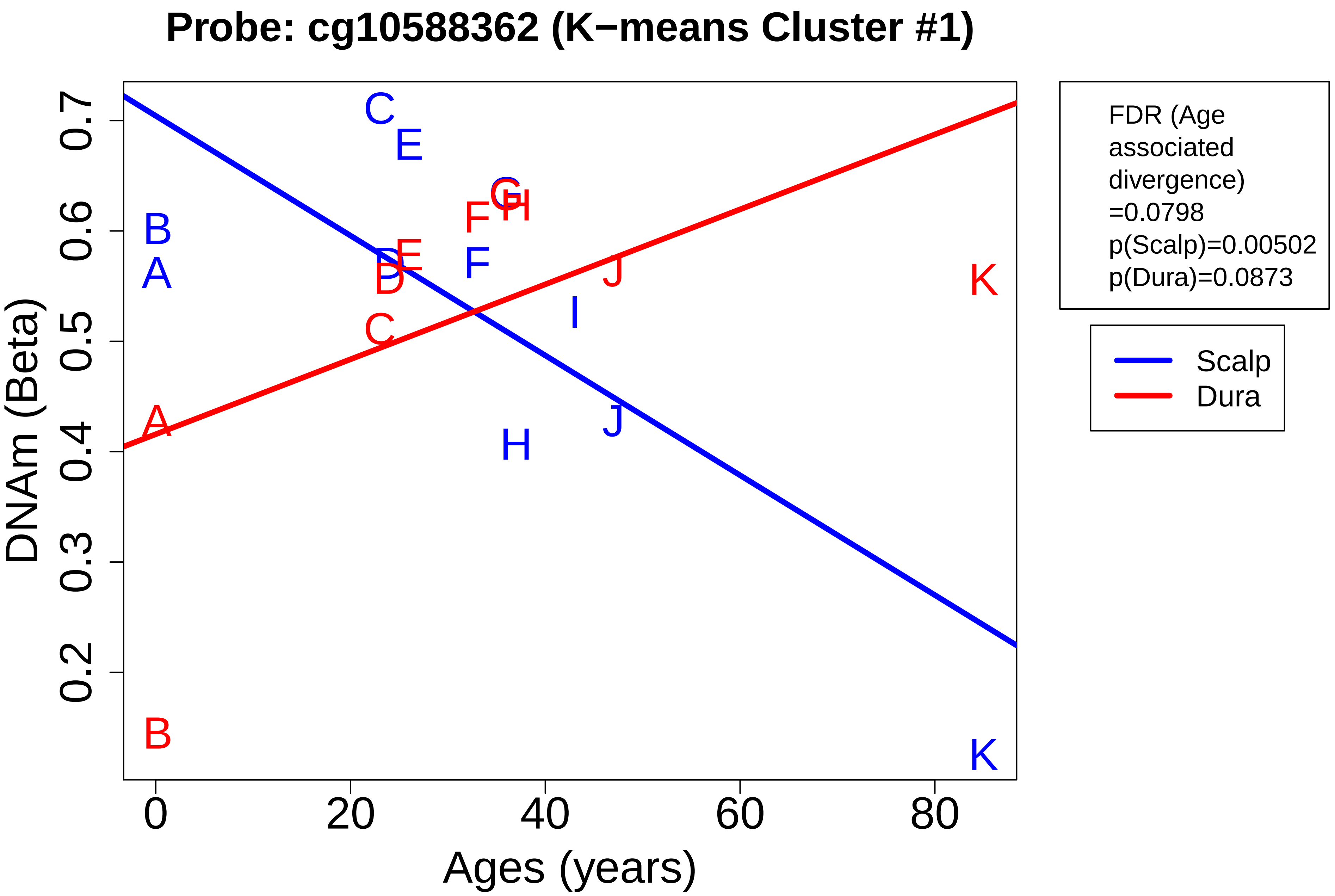

Probe: cg10588362 (K−means Cluster #1)
DNAm (Beta)
0.2
0.3
0.4
0.5
0.6
0.7
Ages (years)
0
20
40
60
80
FDR (Age associated divergence) =0.0798
p(Scalp)=0.00502
p(Dura)=0.0873
Scalp
Dura
A
B
C
D
E
F
G
H
I
J
K

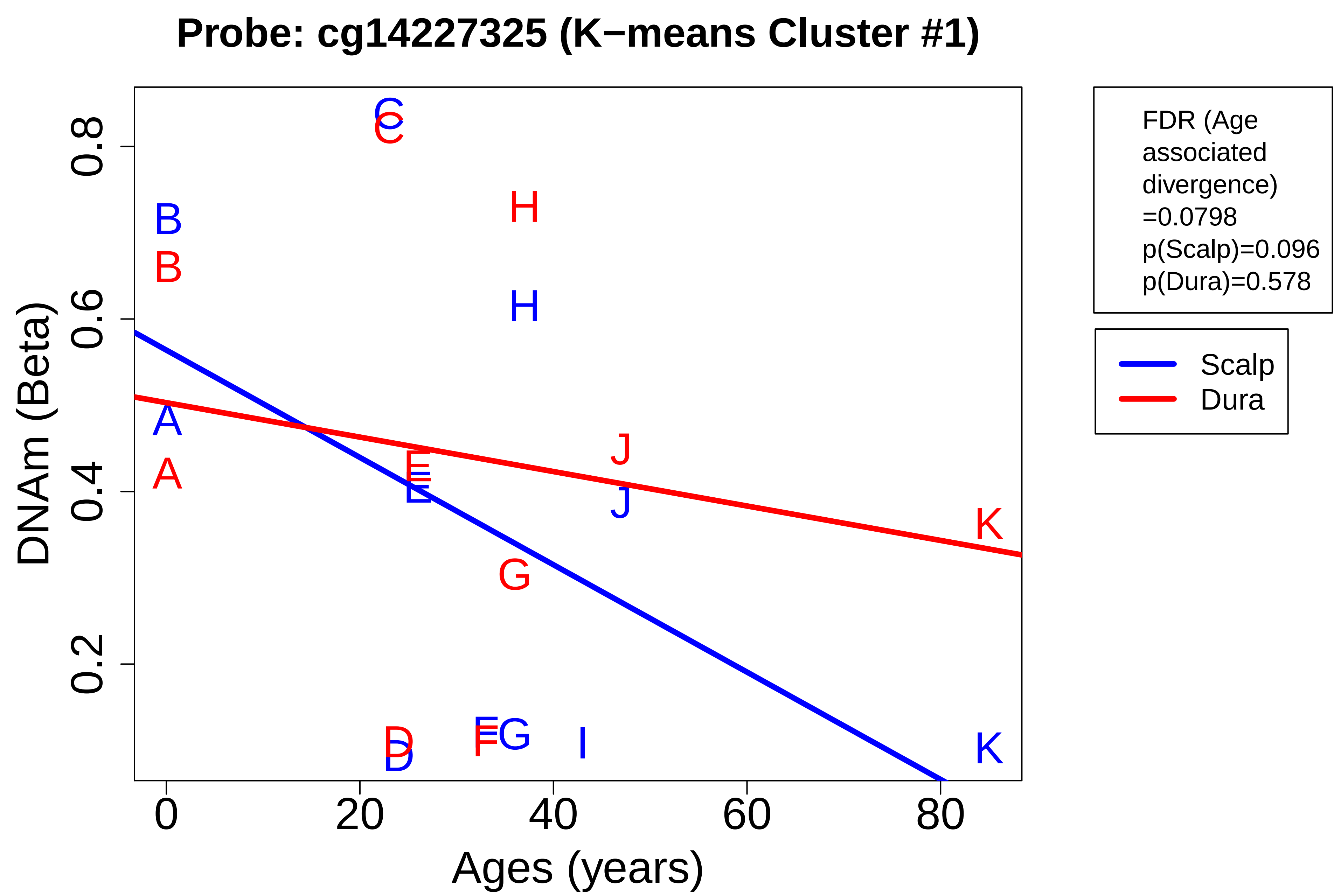

Probe: cg14227325 (K−means Cluster #1)
DNAm (Beta)
0.2
0.4
0.6
0.8
0
20
40
60
80
Ages (years)
A
B
C
D
E
F
G
H
I
J
K
FDR (Age associated divergence) =0.0798
p(Scalp)=0.096
p(Dura)=0.578
Scalp
Dura

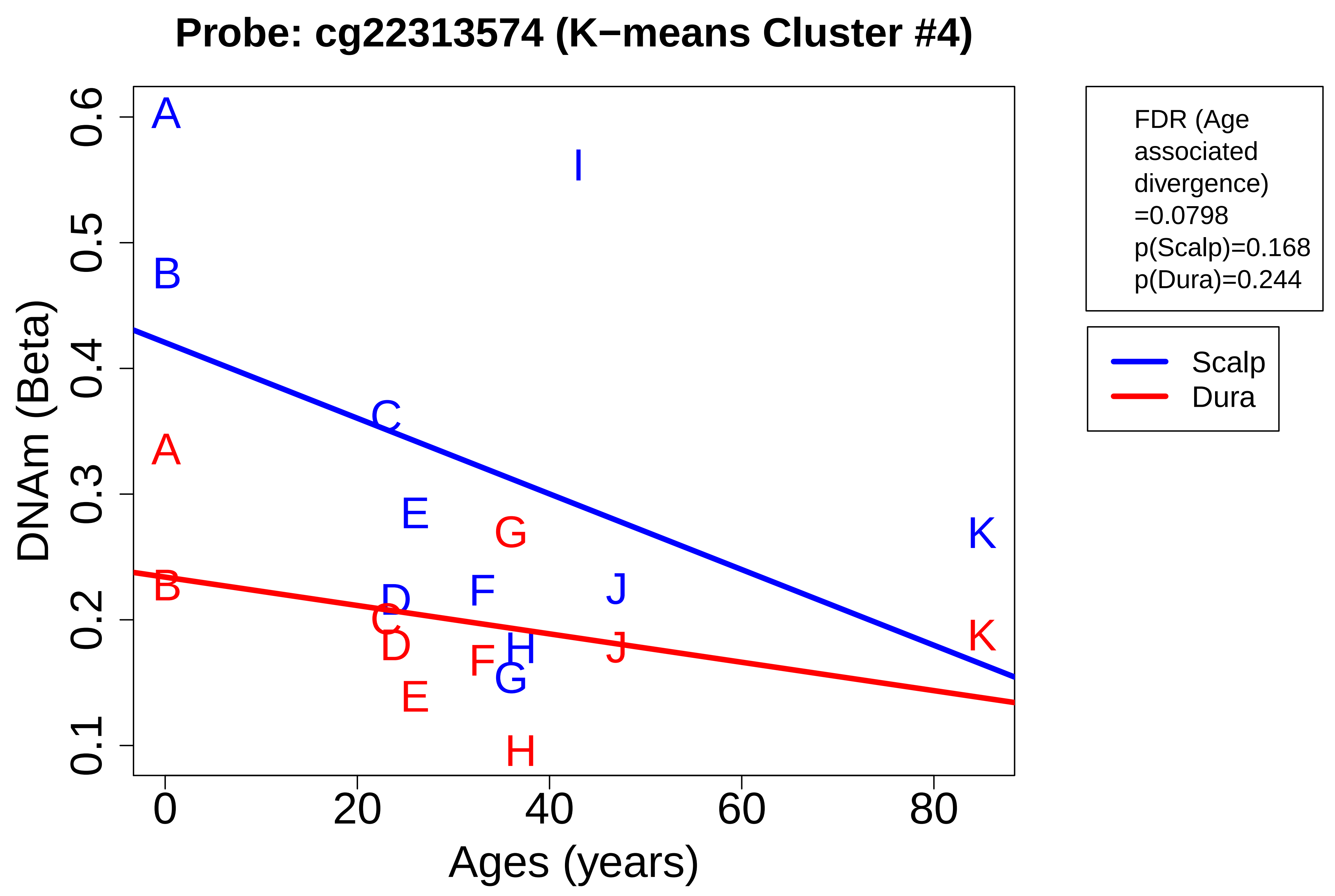

Probe: cg22313574 (K−means Cluster #4)
DNAm (Beta)
Ages (years)
0.1
0.2
0.3
0.4
0.5
0.6
0
20
40
60
80
FDR (Age associated divergence) =0.0798
p(Scalp)=0.168
p(Dura)=0.244
Scalp
Dura

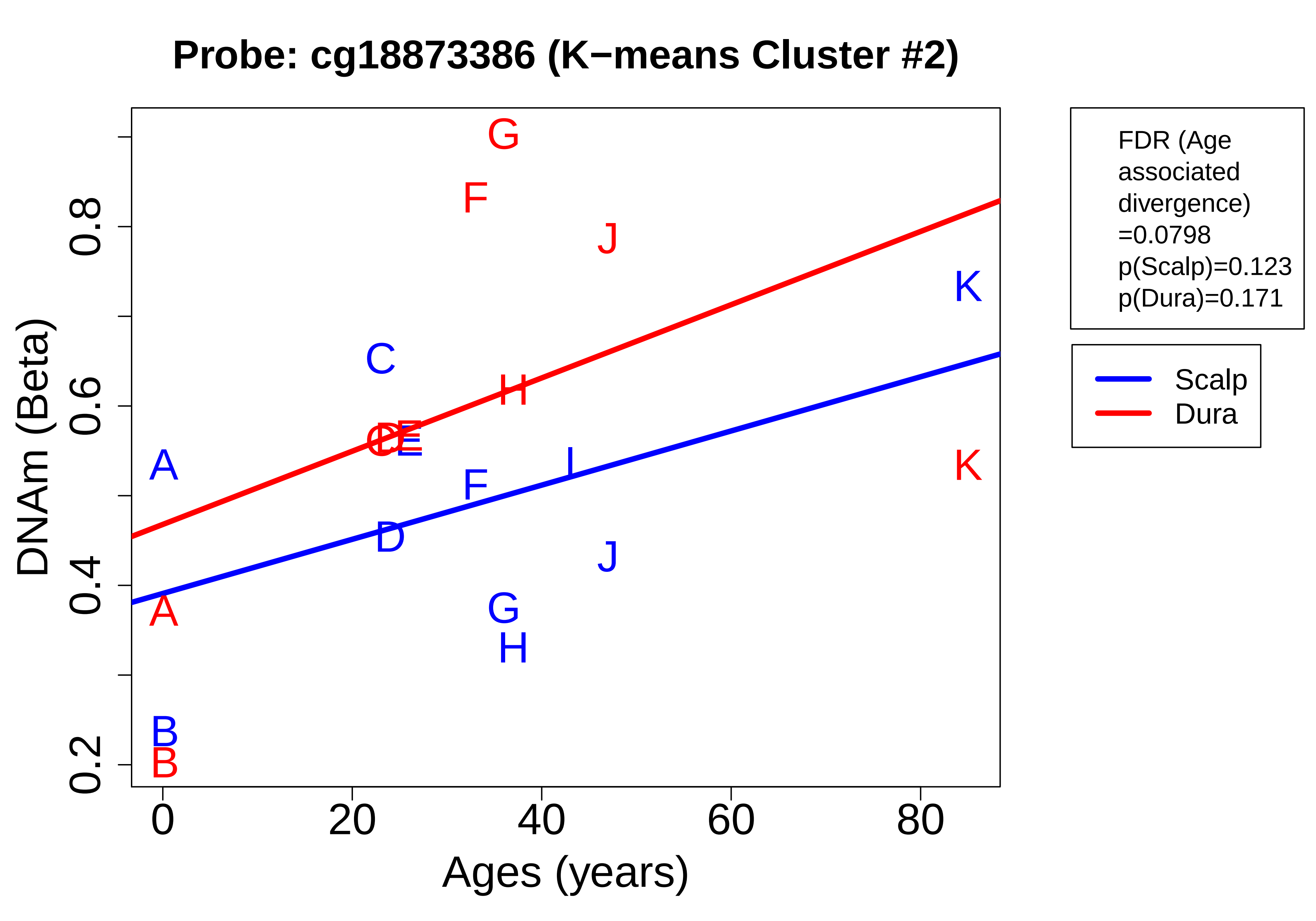

Probe: cg18873386 (K−means Cluster #2)
DNAm (Beta)
0.2
0.4
0.6
0.8
0
20
40
60
80
Ages (years)
FDR (Age associated divergence) =0.0798
p(Scalp)=0.123
p(Dura)=0.171
Scalp
Dura
A
B
C
D
E
F
G
H
I
J
K

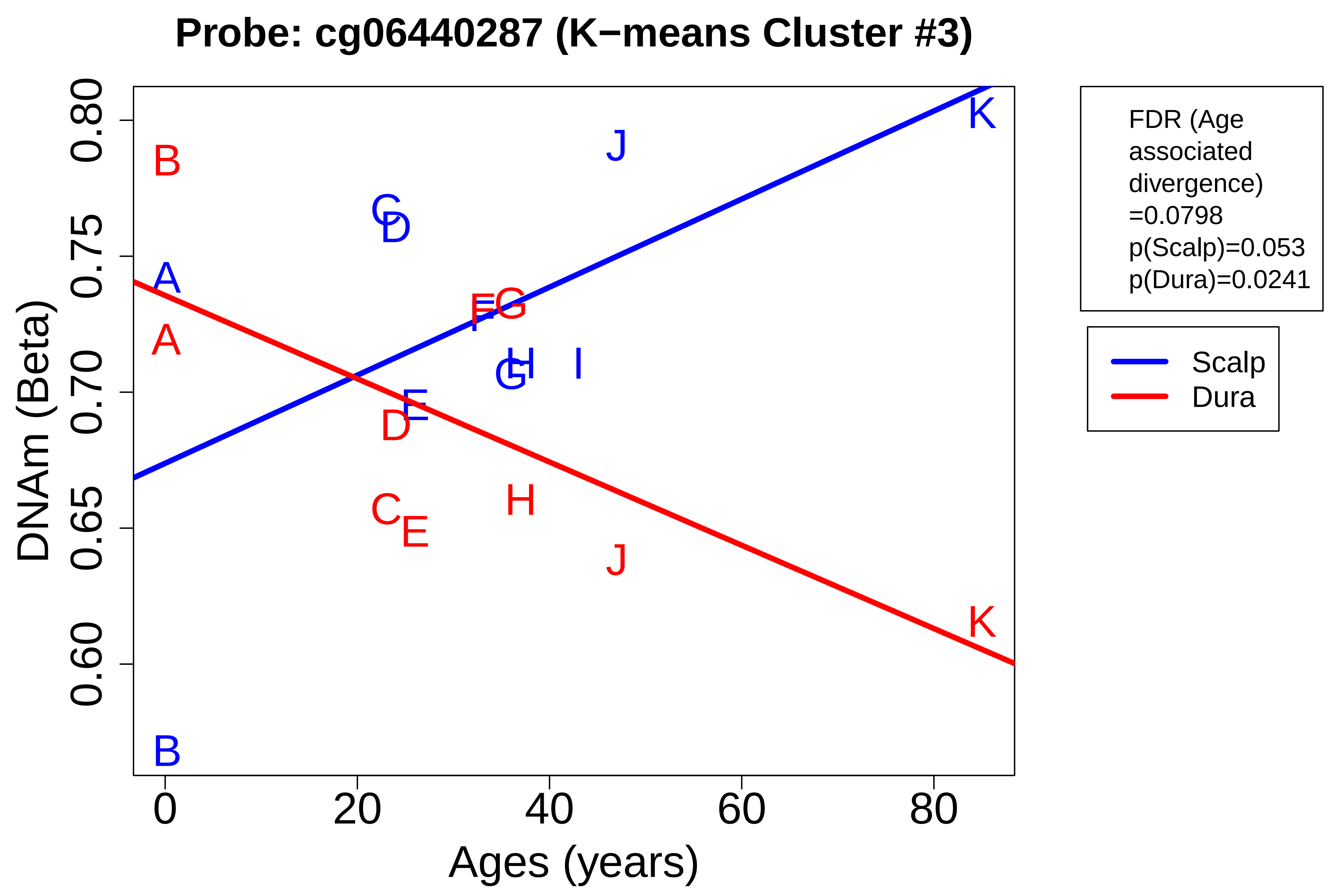

Probe: cg06440287 (K−means Cluster #3)
DNAm (Beta)
Ages (years)
0.60
0.65
0.70
0.75
0.80
0
20
40
60
80
FDR (Age associated divergence) =0.0798
p(Scalp)=0.053
p(Dura)=0.0241
Scalp
Dura

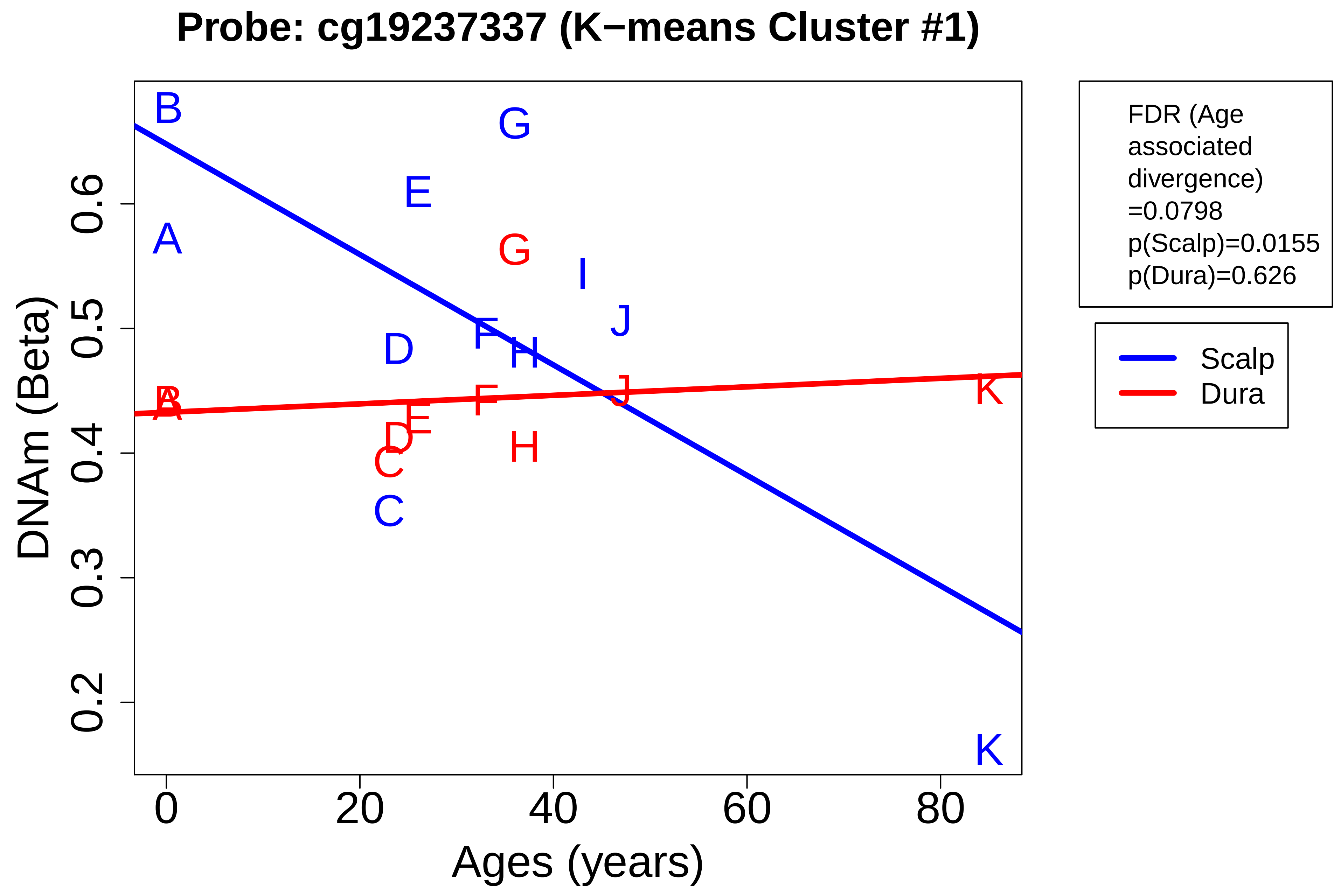

Probe: cg19237337 (K−means Cluster #1)
FDR (Age associated divergence) =0.0798
p(Scalp)=0.0155
p(Dura)=0.626
Scalp
Dura
DNAm (Beta)
0.2
0.3
0.4
0.5
0.6
0
20
40
60
80
Ages (years)
A
B
C
D
E
F
G
H
I
J
K

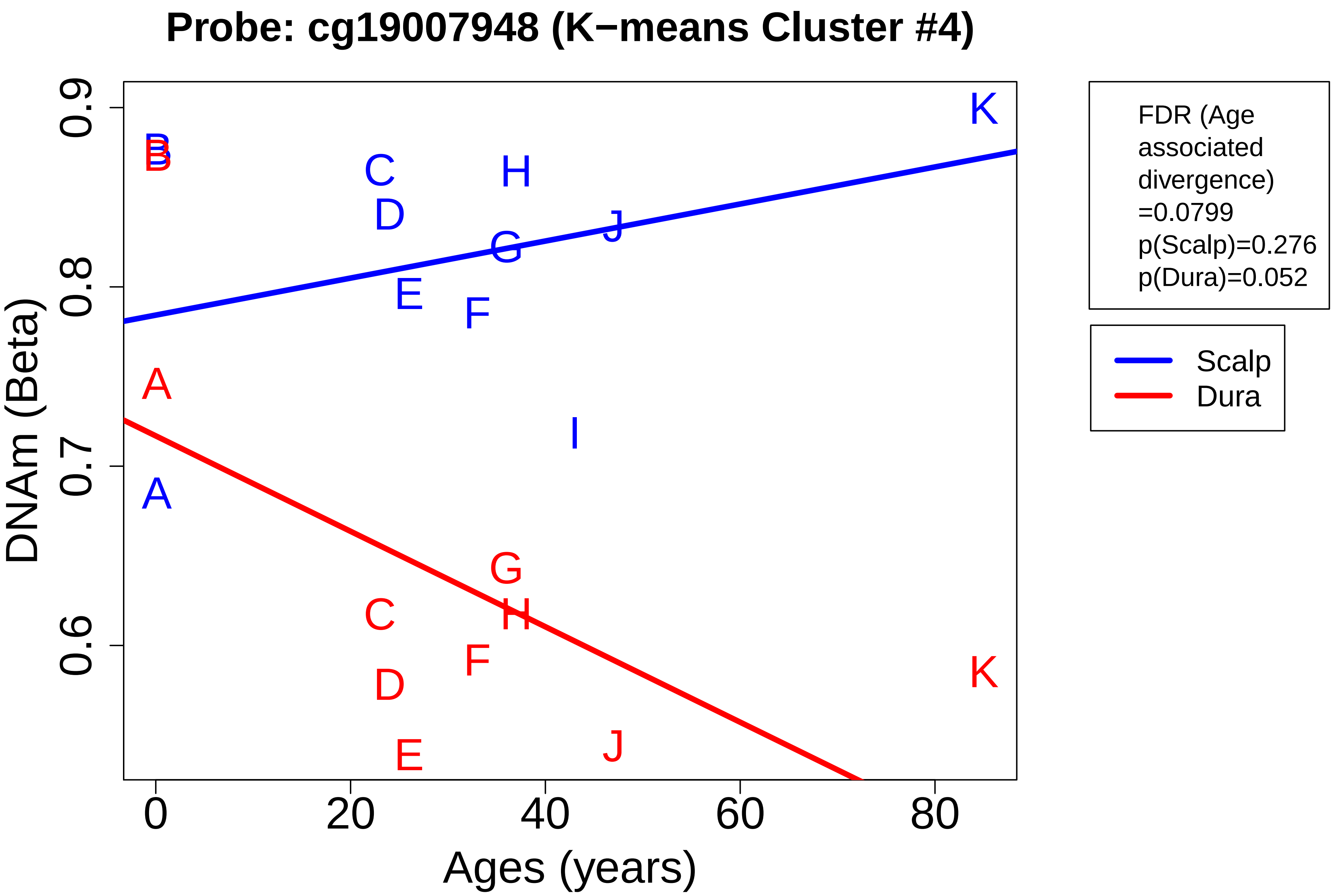

Probe: cg19007948 (K−means Cluster #4)
DNAm (Beta)
0.6
0.7
0.8
0.9
0
20
40
60
80
Ages (years)
FDR (Age associated divergence) =0.0799
p(Scalp)=0.276
p(Dura)=0.052
Scalp
Dura
A
B
C
D
E
F
G
H
I
J
K

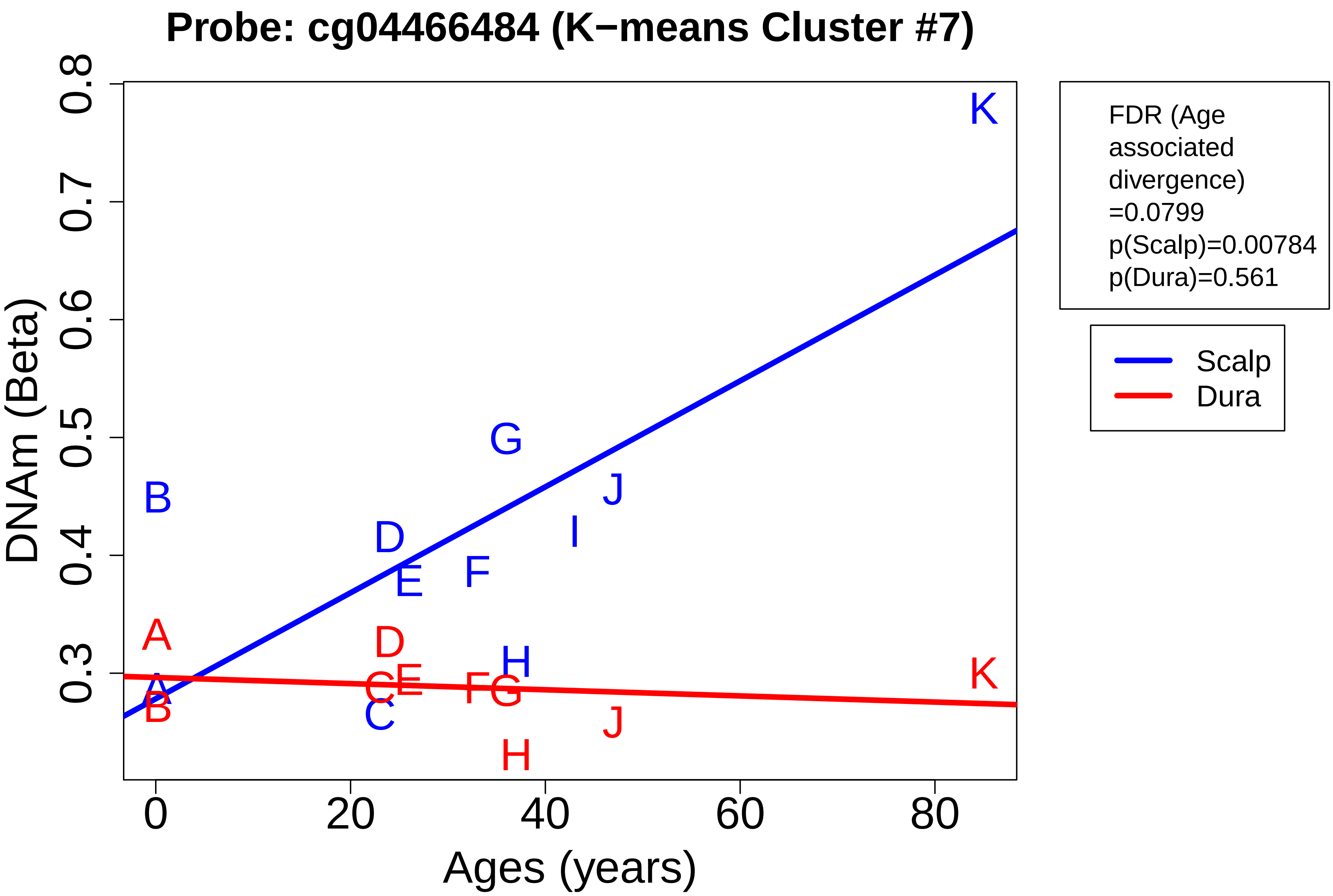

Probe: cg04466484 (K−means Cluster #7)
DNAm (Beta)
0.3
0.4
0.5
0.6
0.7
0.8
0
20
40
60
80
Ages (years)
FDR (Age associated divergence) =0.0799
p(Scalp)=0.00784
p(Dura)=0.561
Scalp
Dura

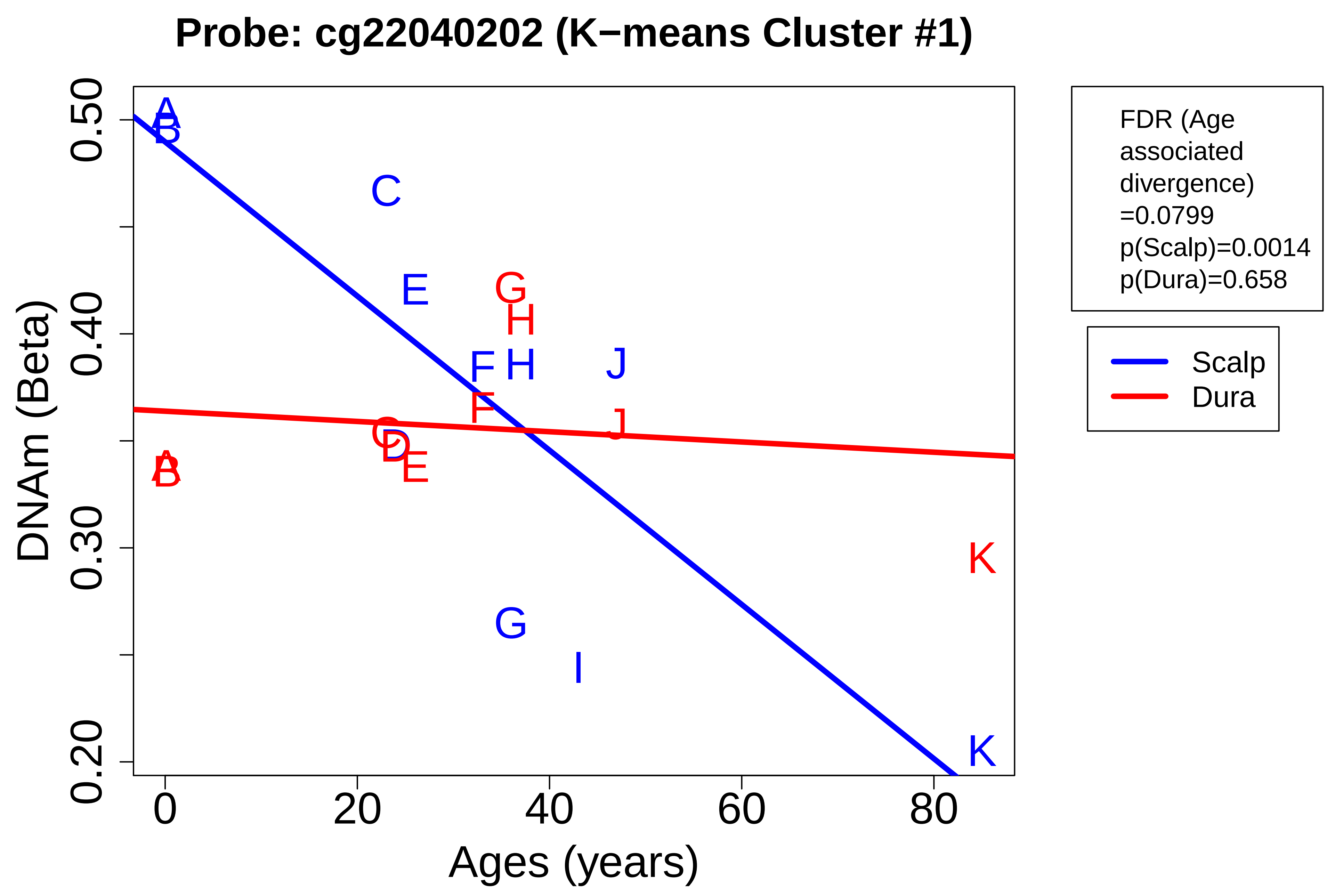

Probe: cg22040202 (K−means Cluster #1)
DNAm (Beta)
0.20
0.30
0.40
0.50
0
20
40
60
80
Ages (years)
A
B
C
D
E
F
G
H
I
J
K
FDR (Age associated divergence) =0.0799
p(Scalp)=0.0014
p(Dura)=0.658
Scalp
Dura

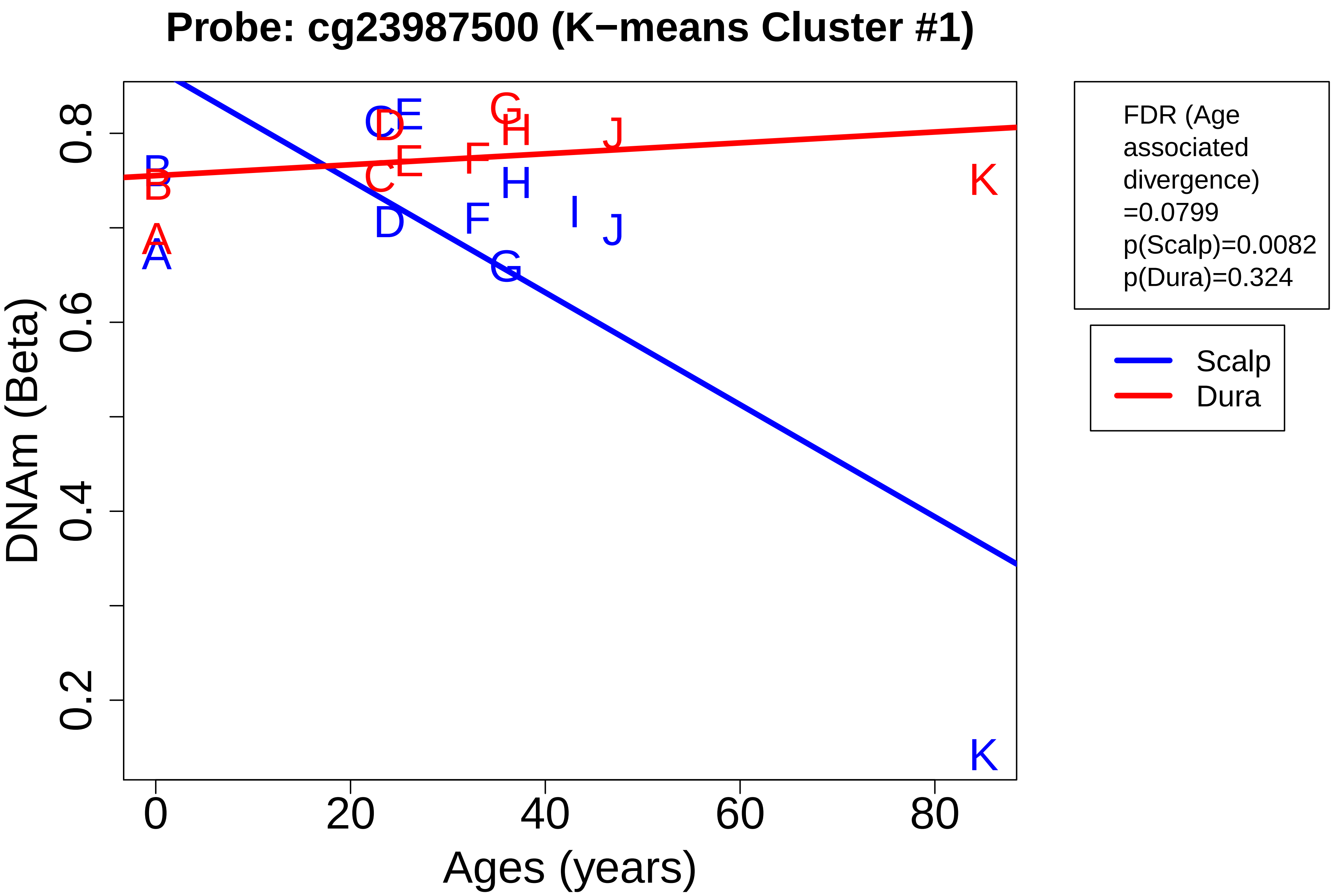
Probe: cg23987500 (K−means Cluster #1)
DNAm (Beta)
0.2
0.4
0.6
0.8
0
20
40
60
80
Ages (years)
A
B
C
D
E
F
G
H
I
J
K
FDR (Age associated divergence) =0.0799
p(Scalp)=0.0082
p(Dura)=0.324
Scalp
Dura

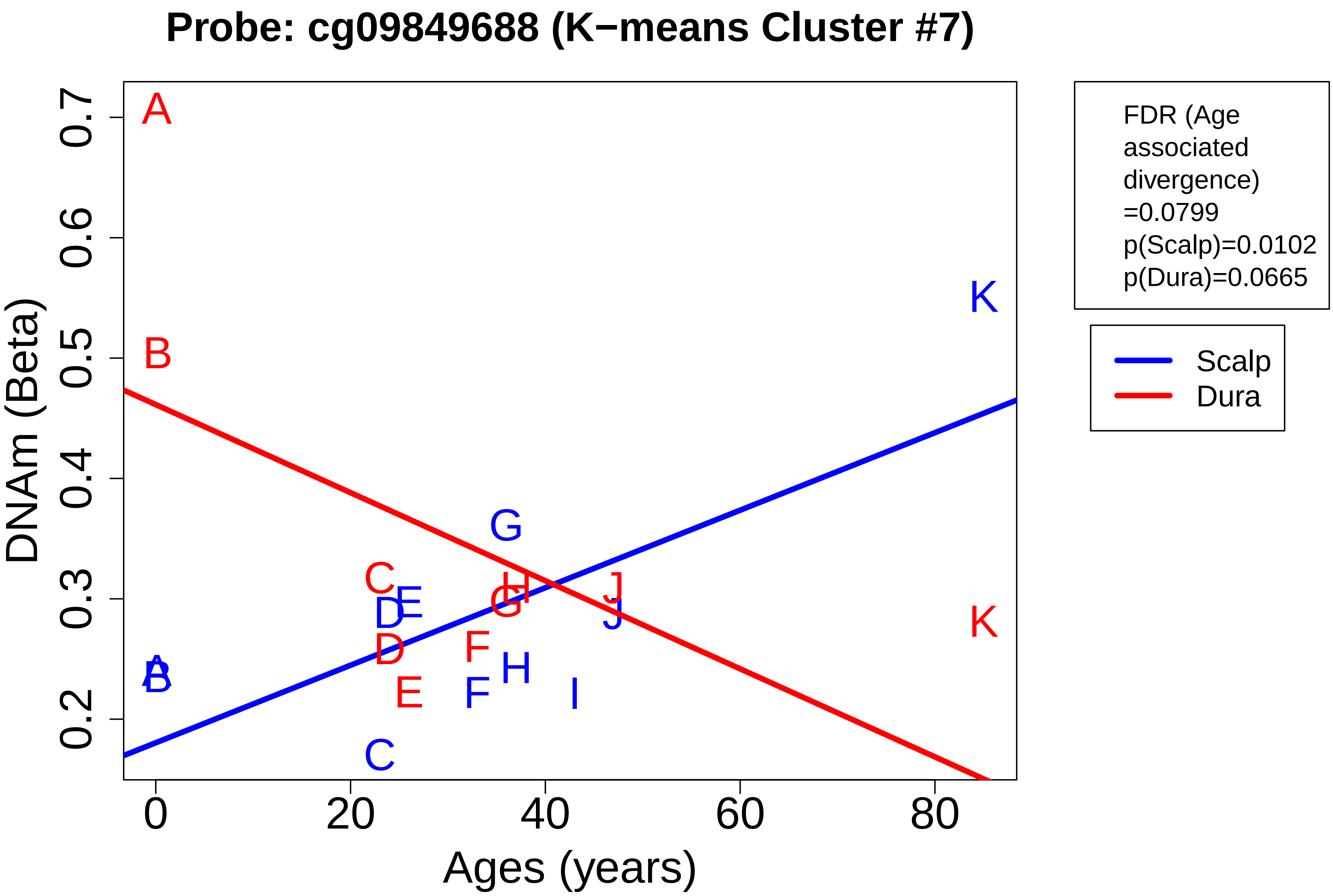

Probe: cg09849688 (K−means Cluster #7)
DNAm (Beta)
Ages (years)
0.2
0.3
0.4
0.5
0.6
0.7
0
20
40
60
80
FDR (Age associated divergence) =0.0799
p(Scalp)=0.0102
p(Dura)=0.0665
Scalp
Dura

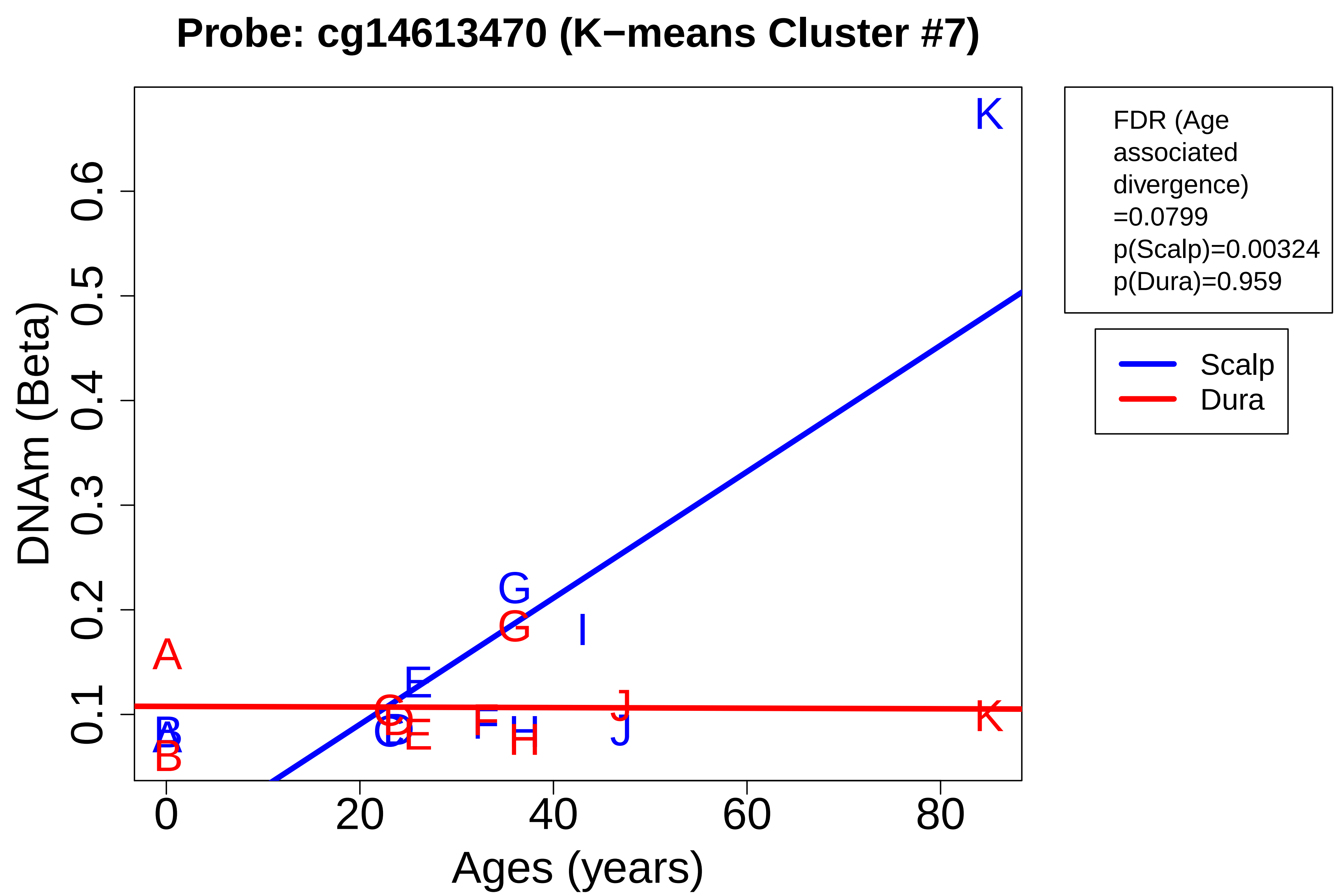

Probe: cg14613470 (K−means Cluster #7)
DNAm (Beta)
0.1
0.2
0.3
0.4
0.5
0.6
Ages (years)
0
20
40
60
80
FDR (Age associated divergence) =0.0799
p(Scalp)=0.00324
p(Dura)=0.959
Scalp
Dura

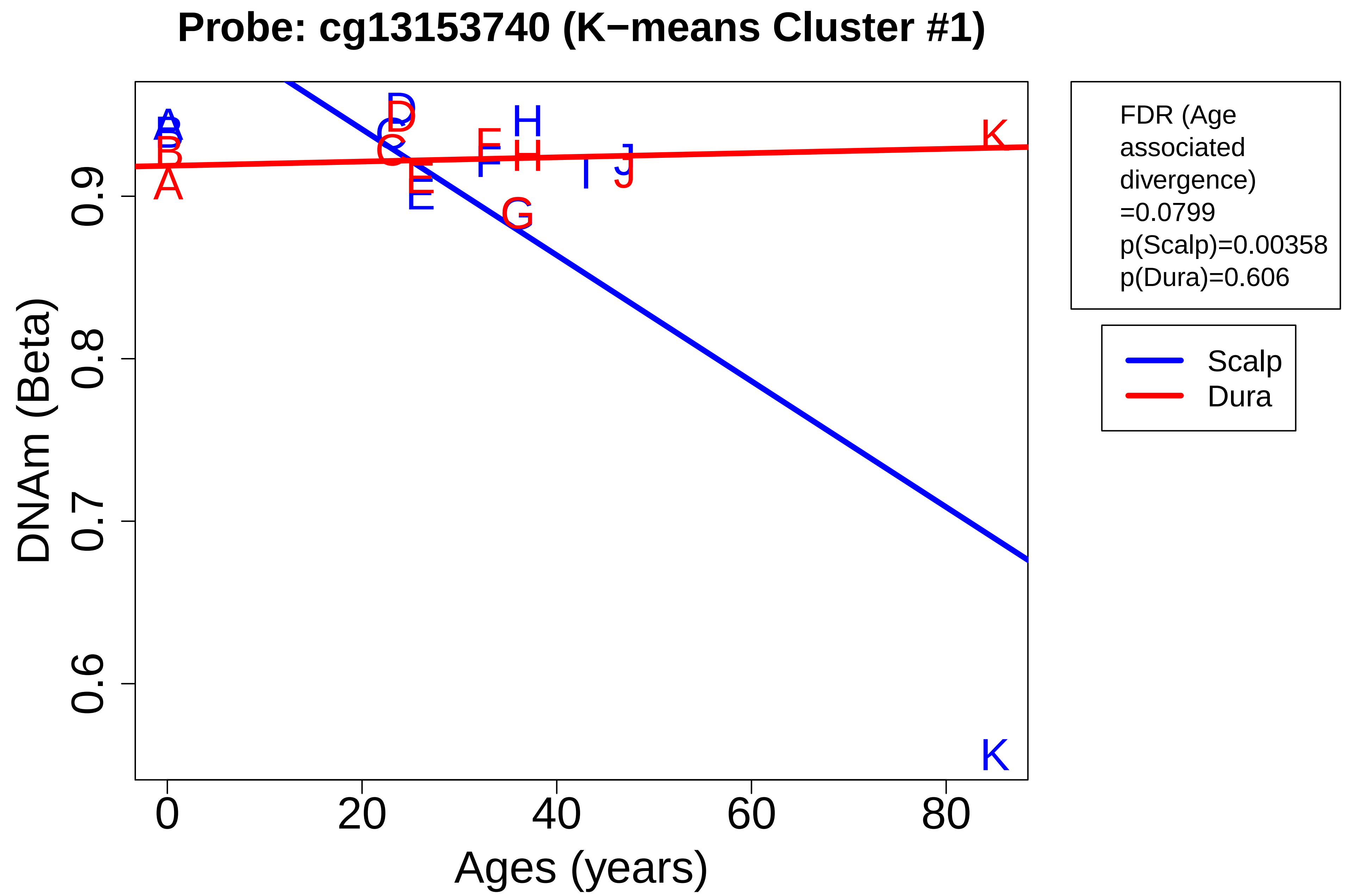

Probe: cg13153740 (K−means Cluster #1)
DNAm (Beta)
0.6
0.7
0.8
0.9
0
20
40
60
80
Ages (years)
FDR (Age associated divergence) =0.0799
p(Scalp)=0.00358
p(Dura)=0.606
Scalp
Dura

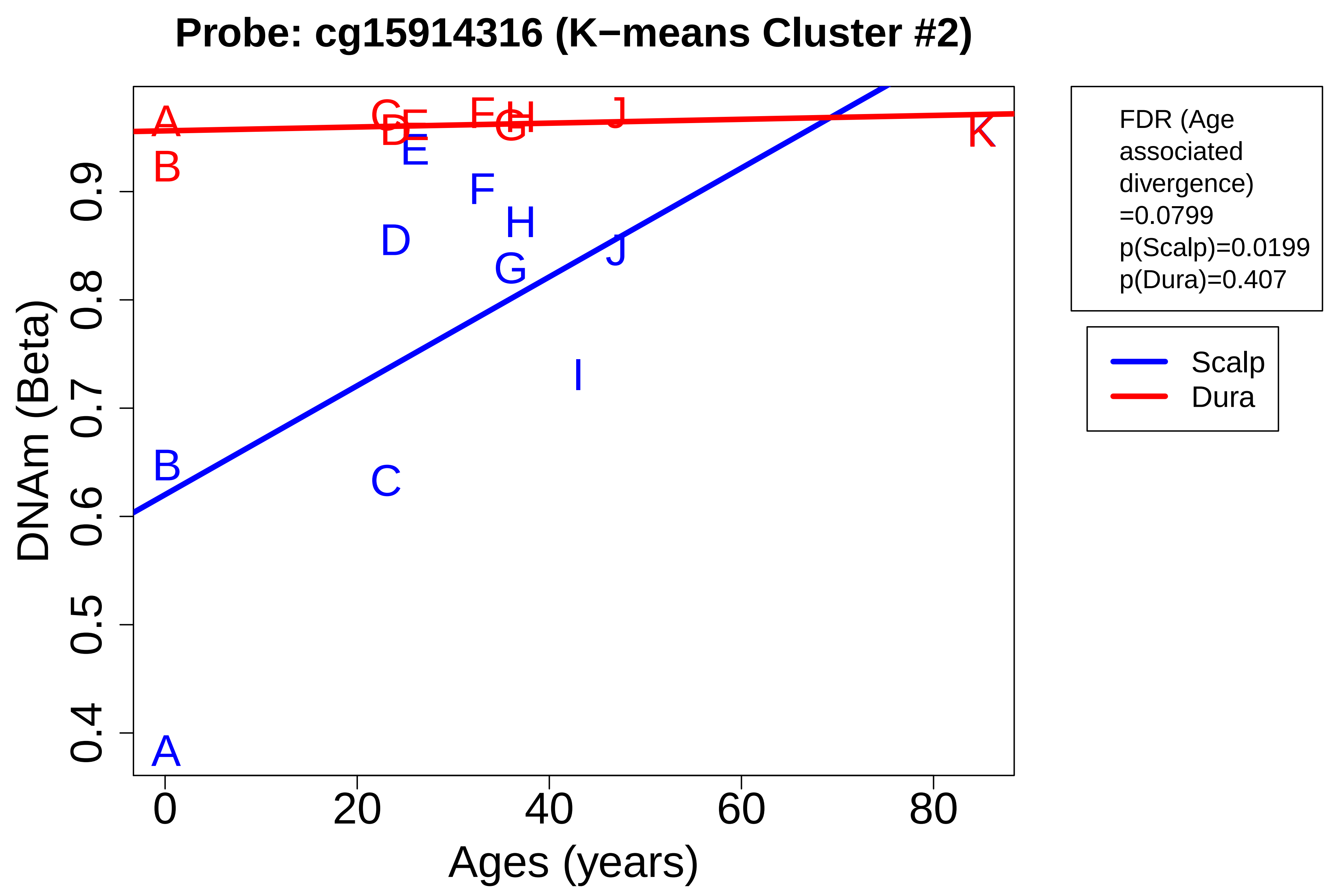
Probe: cg15914316 (K−means Cluster #2)
FDR (Age associated divergence) =0.0799
p(Scalp)=0.0199
p(Dura)=0.407
Scalp
Dura
DNAm (Beta)
0.4
0.5
0.6
0.7
0.8
0.9
0
20
40
60
80
Ages (years)
A
B
C
D
E
F
G
H
I
J
K

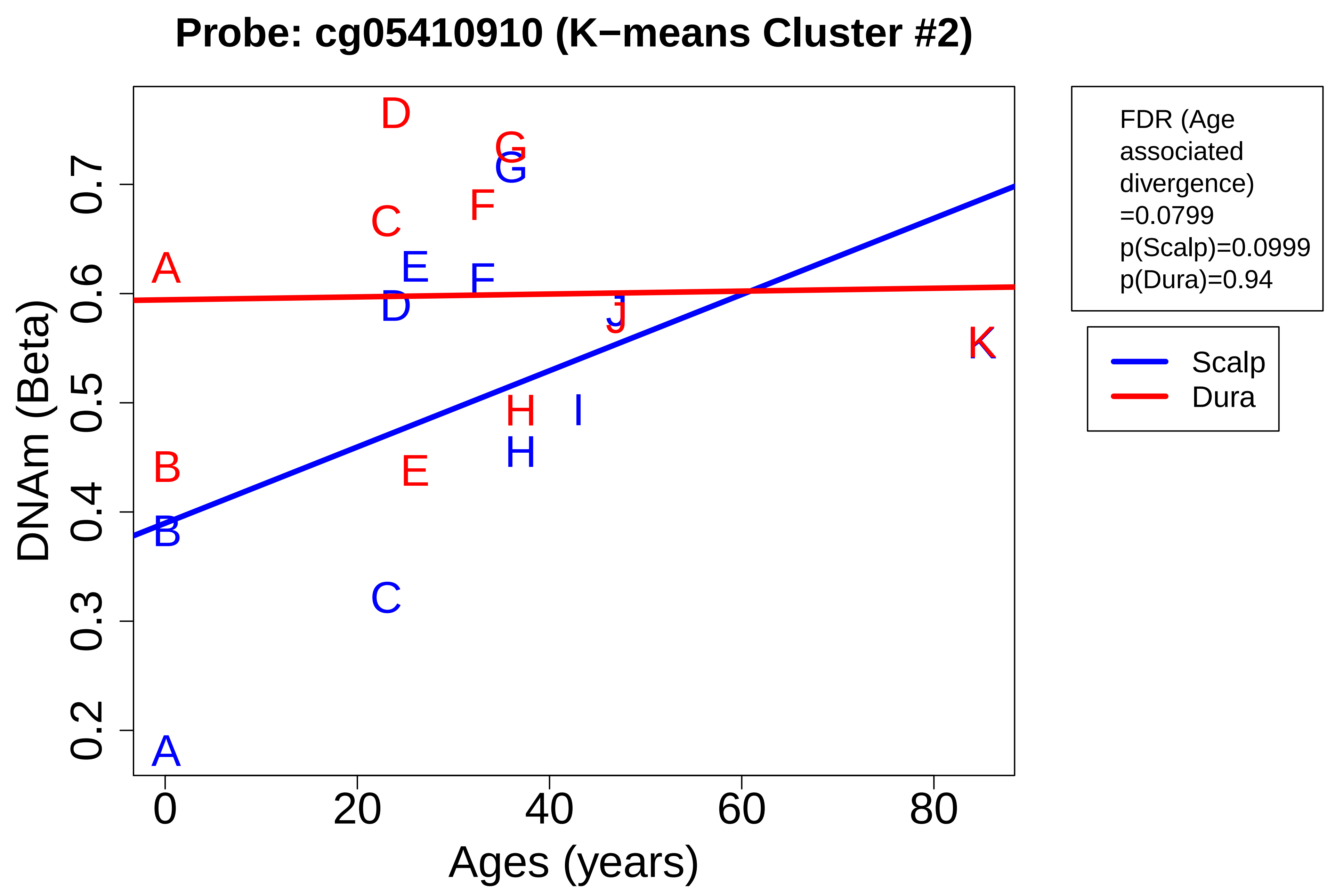

Probe: cg05410910 (K−means Cluster #2)
DNAm (Beta)
Ages (years)
0.2
0.3
0.4
0.5
0.6
0.7
0
20
40
60
80
FDR (Age associated divergence) =0.0799
p(Scalp)=0.0999
p(Dura)=0.94
Scalp
Dura

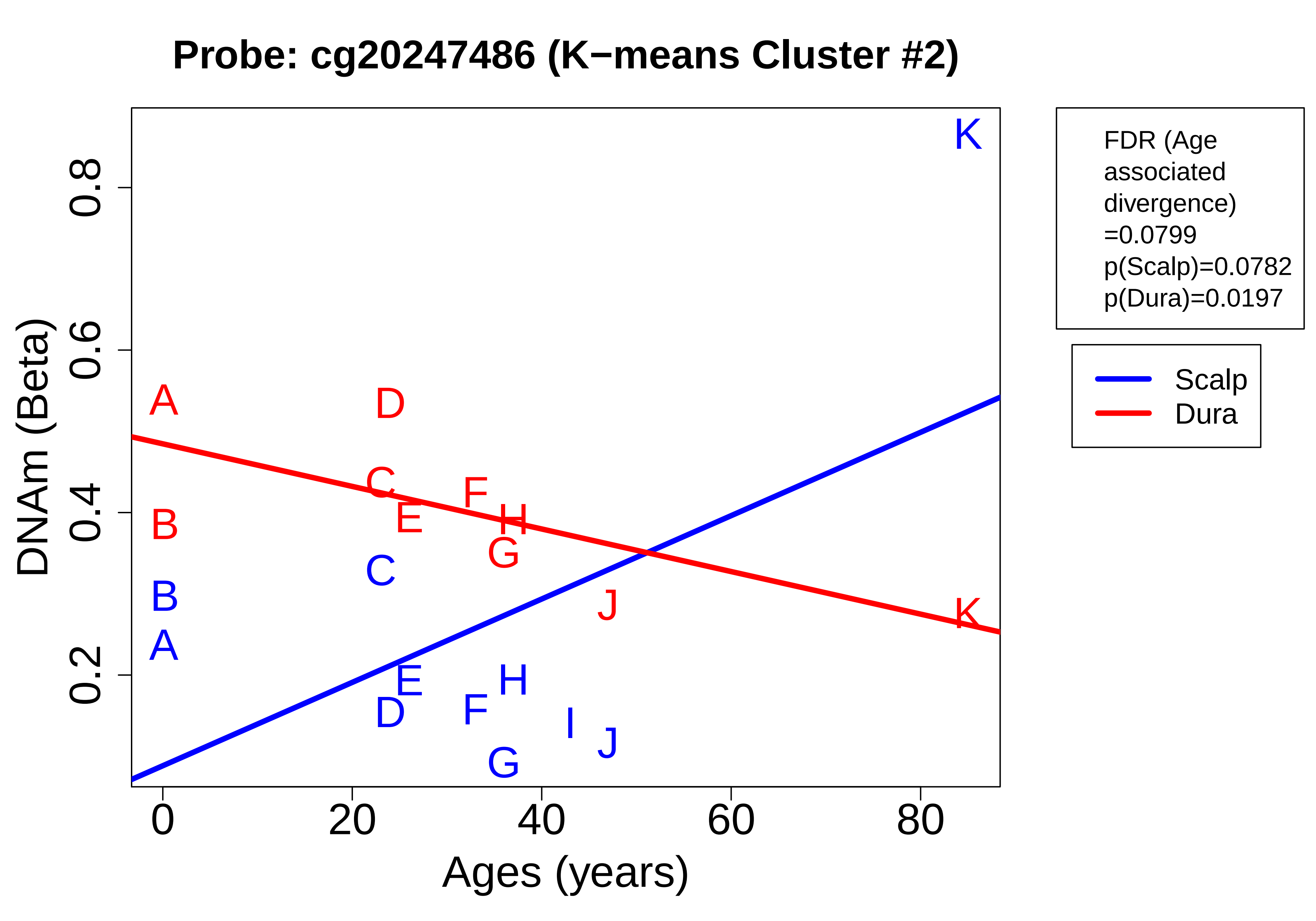

Probe: cg20247486 (K−means Cluster #2)
FDR (Age associated divergence) =0.0799
p(Scalp)=0.0782
p(Dura)=0.0197
Scalp
Dura
DNAm (Beta)
0.2
0.4
0.6
0.8
0
20
40
60
80
Ages (years)
A
B
C
D
E
F
G
H
I
J
K

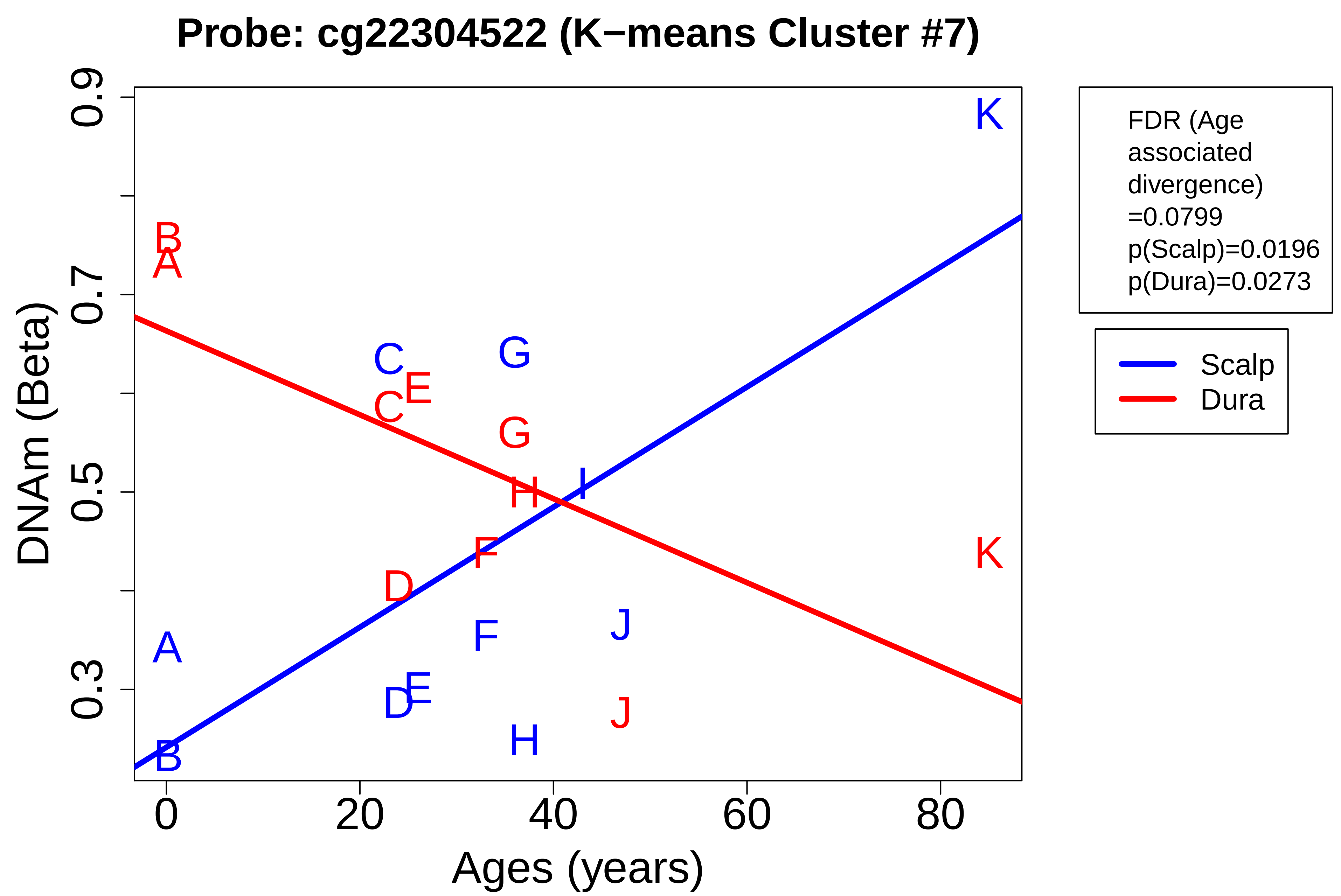
Probe: cg22304522 (K−means Cluster #7)
DNAm (Beta)
0.3
0.5
0.7
0.9
0
20
40
60
80
Ages (years)
FDR (Age associated divergence) =0.0799
p(Scalp)=0.0196
p(Dura)=0.0273
Scalp
Dura
A
B
C
D
E
F
G
H
I
J
K

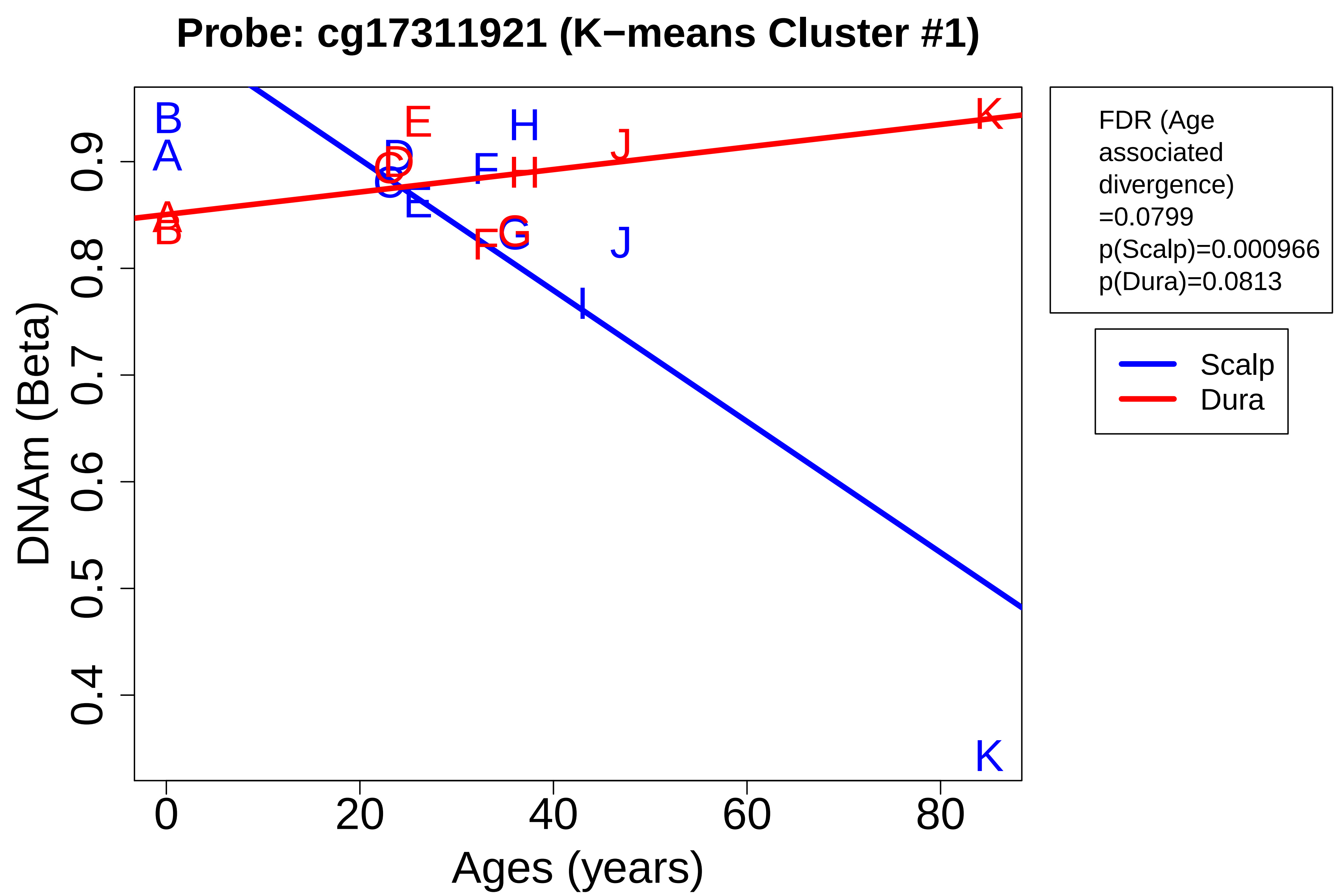

Probe: cg17311921 (K−means Cluster #1)
DNAm (Beta)
0.4
0.5
0.6
0.7
0.8
0.9
0
20
40
60
80
Ages (years)
FDR (Age associated divergence) =0.0799
p(Scalp)=0.000966
p(Dura)=0.0813
Scalp
Dura

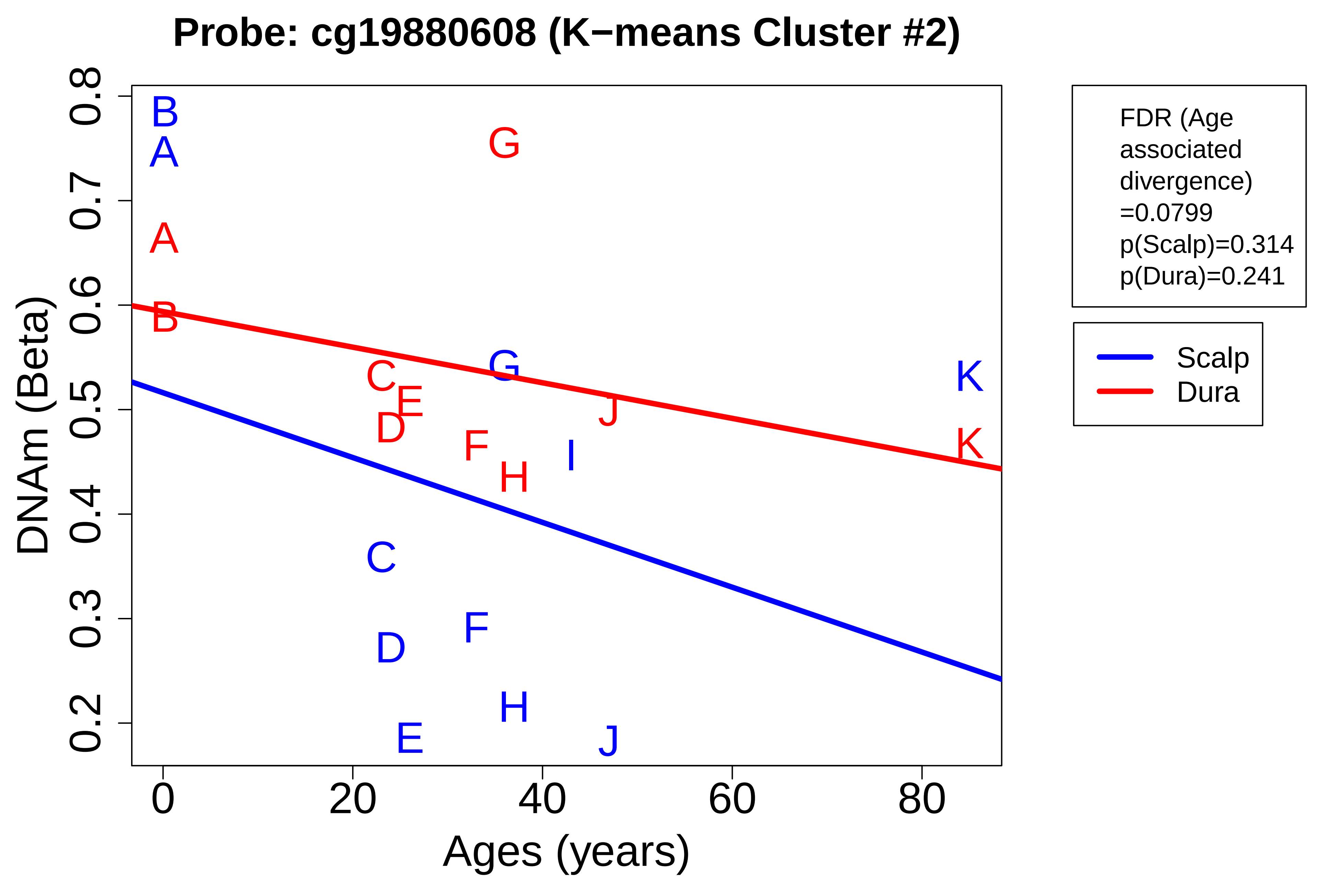

Probe: cg19880608 (K−means Cluster #2)
DNAm (Beta)
Ages (years)
0.2
0.3
0.4
0.5
0.6
0.7
0.8
0
20
40
60
80
FDR (Age associated divergence) =0.0799
p(Scalp)=0.314
p(Dura)=0.241
Scalp
Dura
B
A
G
A
B
G
C
E
D
J
K
F
I
H
K
C
F
D
H
E
J

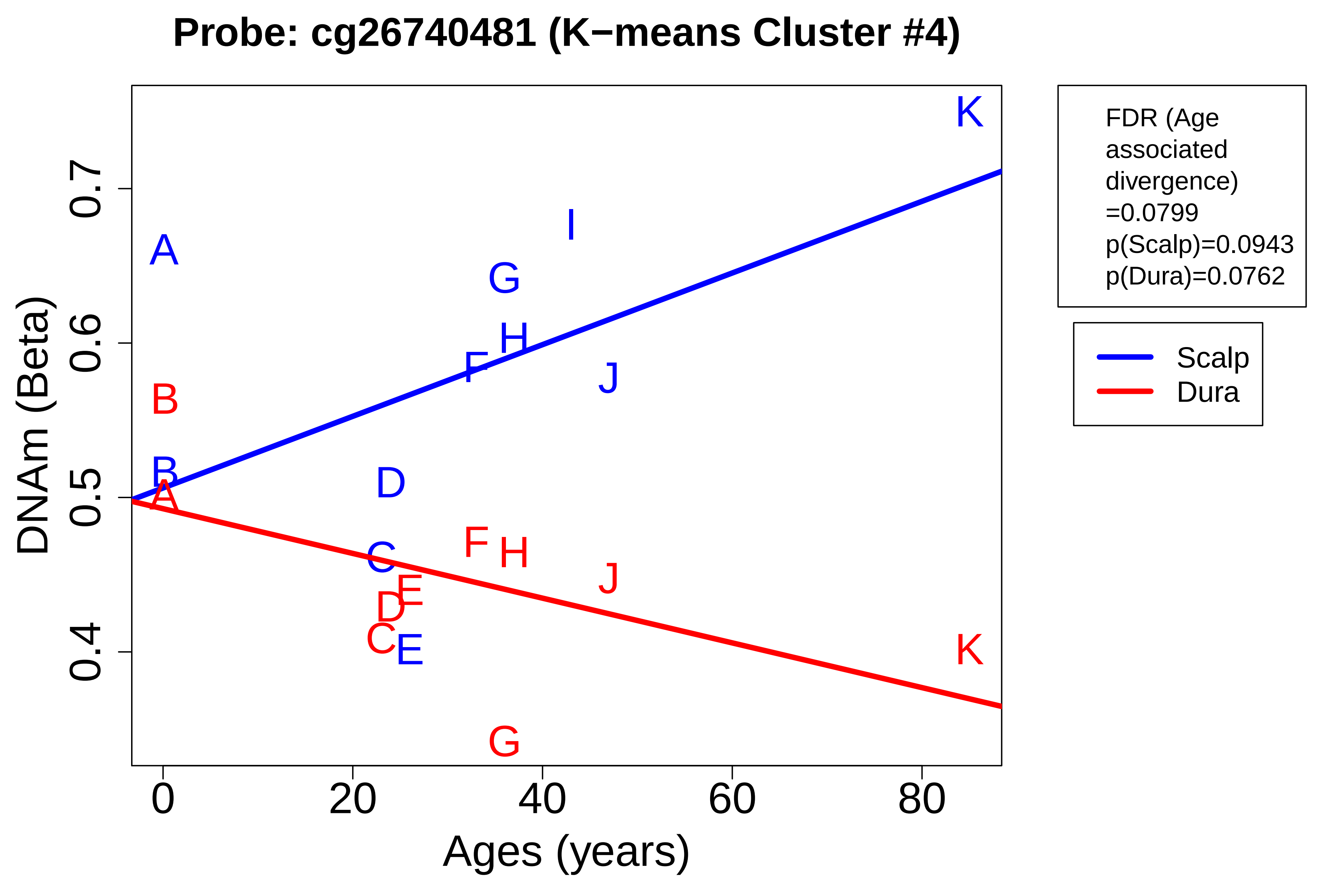
Probe: cg26740481 (K−means Cluster #4)
DNAm (Beta)
0.4
0.5
0.6
0.7
Ages (years)
0
20
40
60
80
A
B
C
D
E
F
G
H
I
J
K
FDR (Age associated divergence) =0.0799
p(Scalp)=0.0943
p(Dura)=0.0762
Scalp
Dura

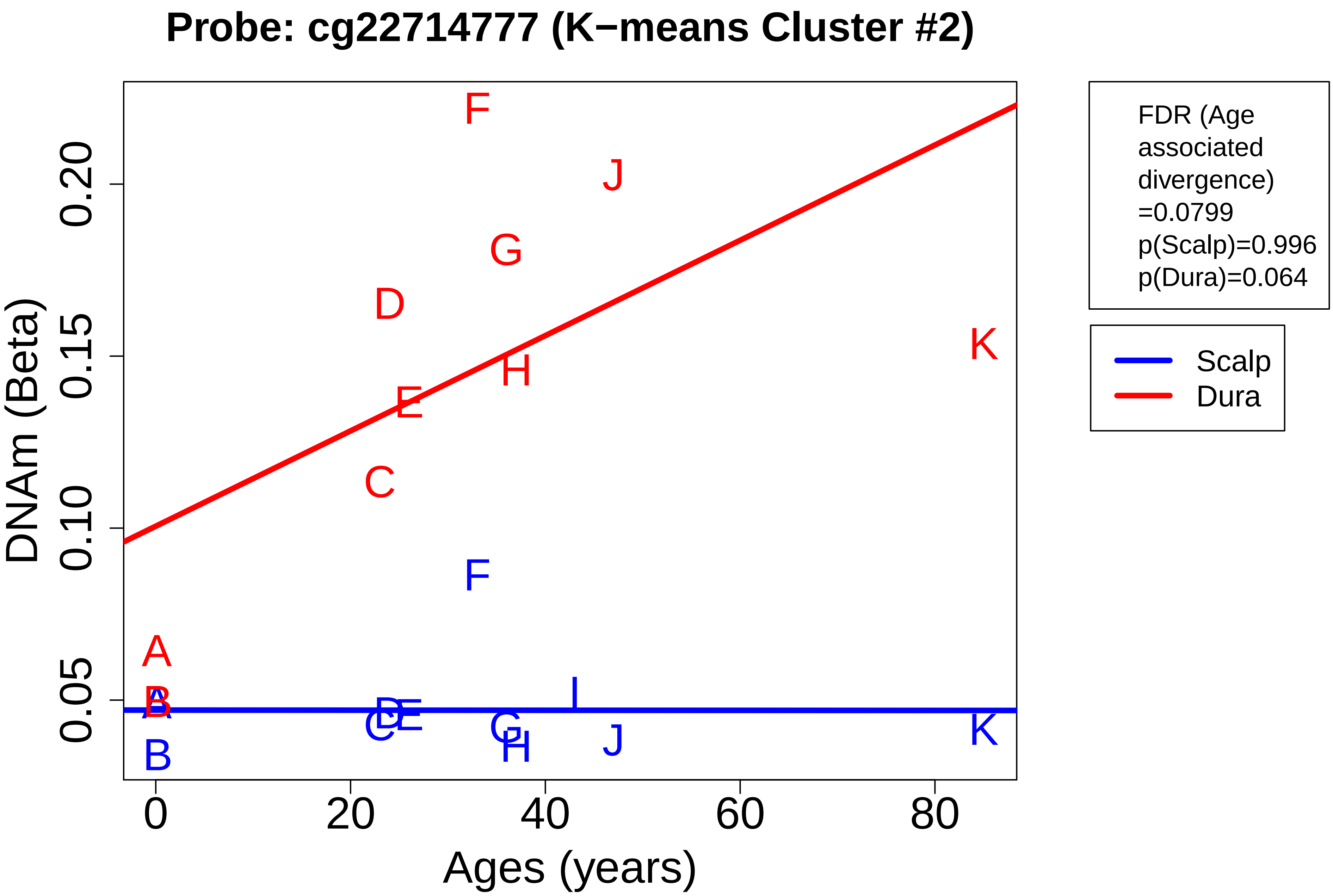

Probe: cg22714777 (K−means Cluster #2)
DNAm (Beta)
0.05
0.10
0.15
0.20
Ages (years)
0
20
40
60
80
FDR (Age associated divergence) =0.0799
p(Scalp)=0.996
p(Dura)=0.064
Scalp
Dura

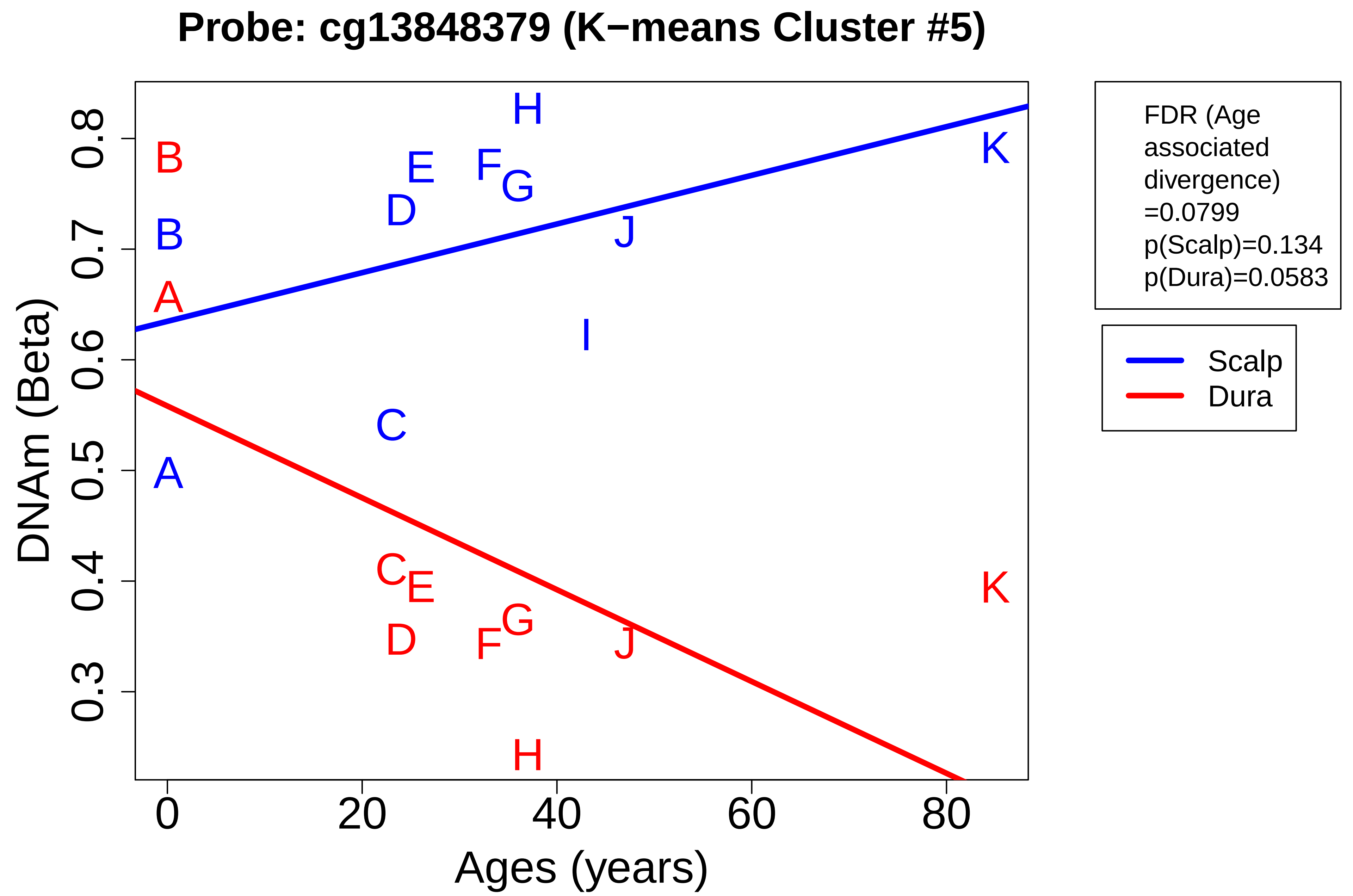

Probe: cg13848379 (K−means Cluster #5)
DNAm (Beta)
0.3
0.4
0.5
0.6
0.7
0.8
0
20
40
60
80
Ages (years)
FDR (Age associated divergence) =0.0799
p(Scalp)=0.134
p(Dura)=0.0583
Scalp
Dura

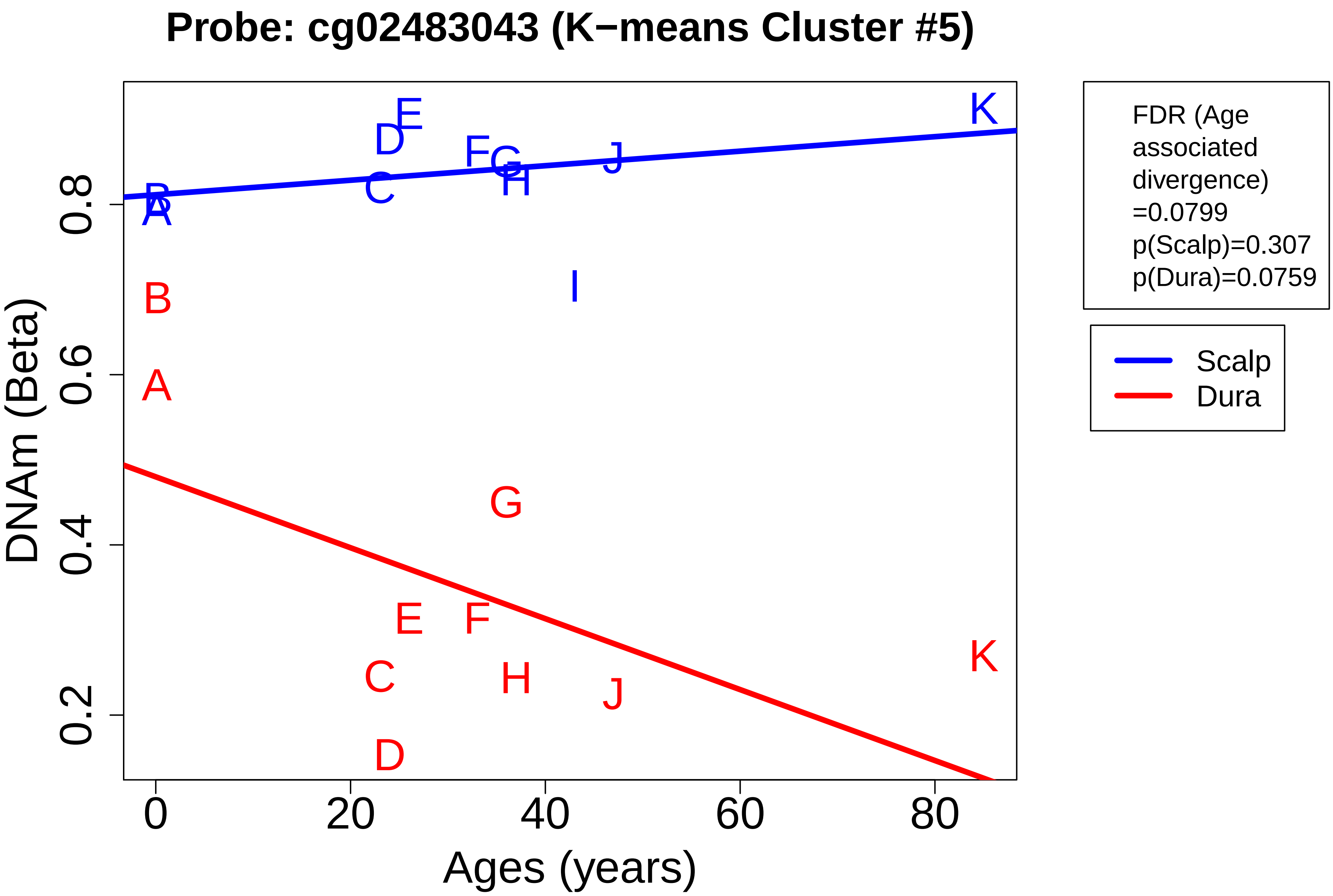
Probe: cg02483043 (K−means Cluster #5)
DNAm (Beta)
Ages (years)
0.2
0.4
0.6
0.8
0
20
40
60
80
A
B
C
D
E
F
G
H
I
J
K
FDR (Age associated divergence) =0.0799
p(Scalp)=0.307
p(Dura)=0.0759
Scalp
Dura

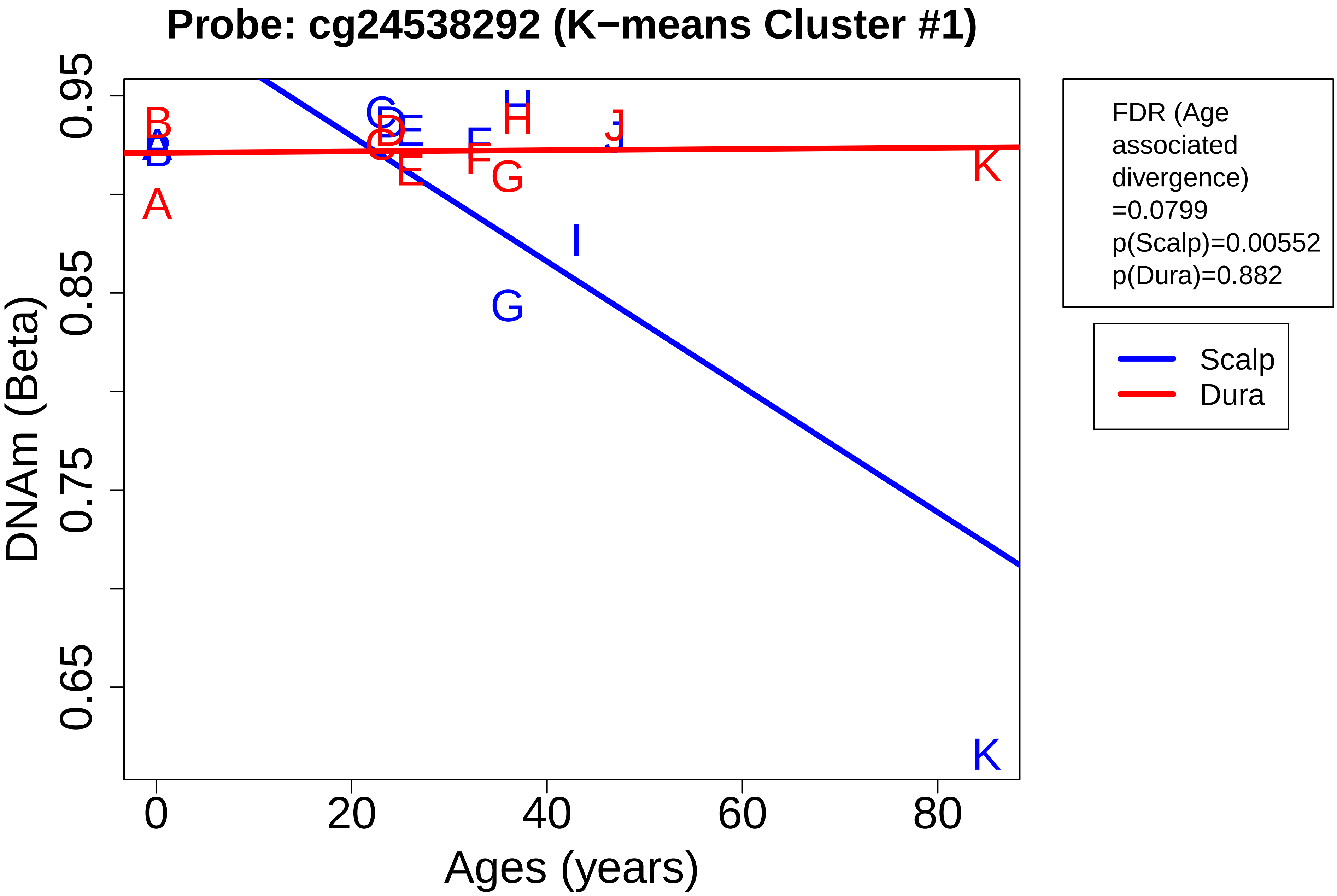

Probe: cg24538292 (K−means Cluster #1)
DNAm (Beta)
0.65
0.75
0.85
0.95
0
20
40
60
80
Ages (years)
FDR (Age associated divergence) =0.0799
p(Scalp)=0.00552
p(Dura)=0.882
Scalp
Dura

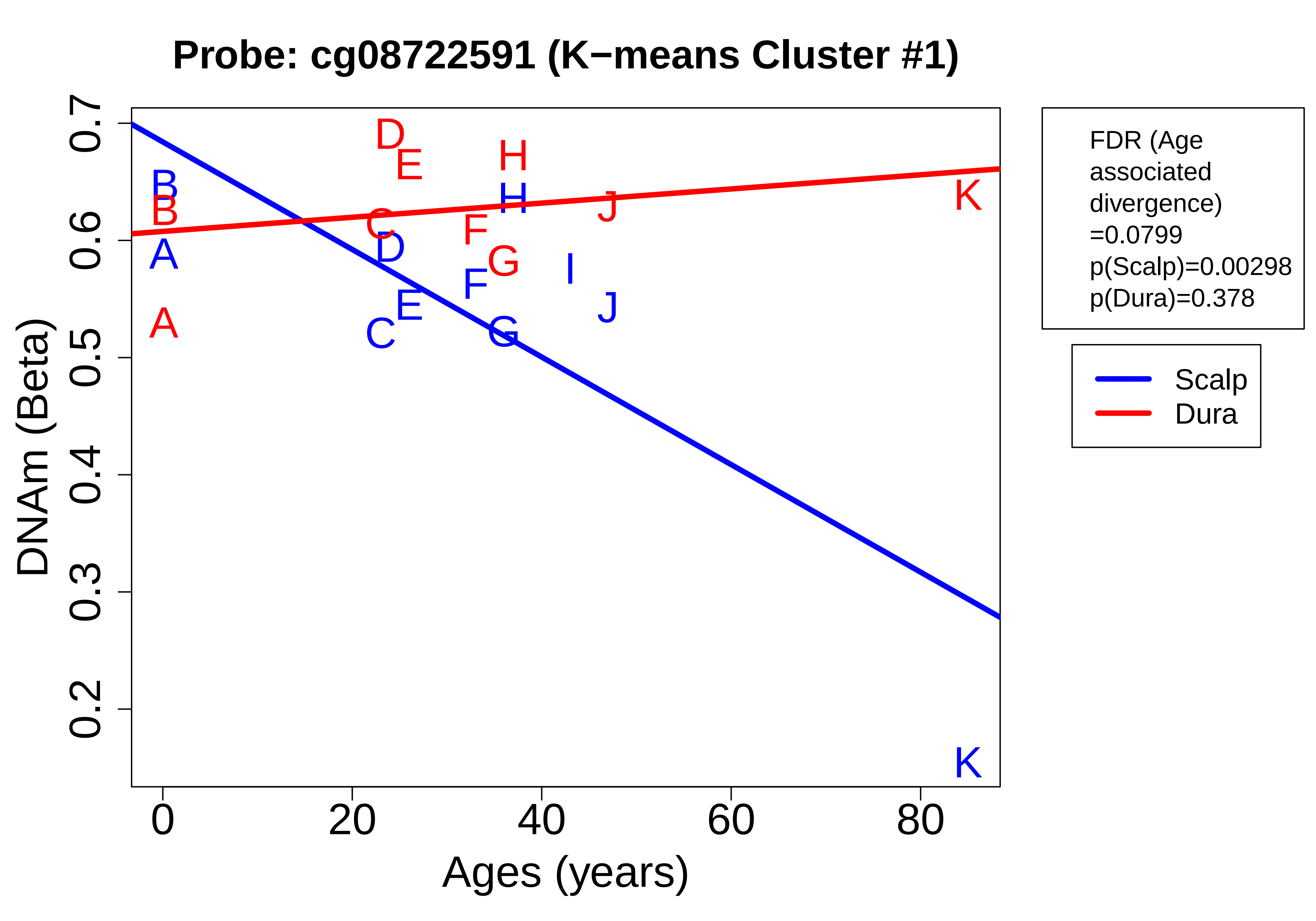

Probe: cg08722591 (K−means Cluster #1)
DNAm (Beta)
0.2
0.3
0.4
0.5
0.6
0.7
Ages (years)
0
20
40
60
80
FDR (Age associated divergence) =0.0799
p(Scalp)=0.00298
p(Dura)=0.378
Scalp
Dura

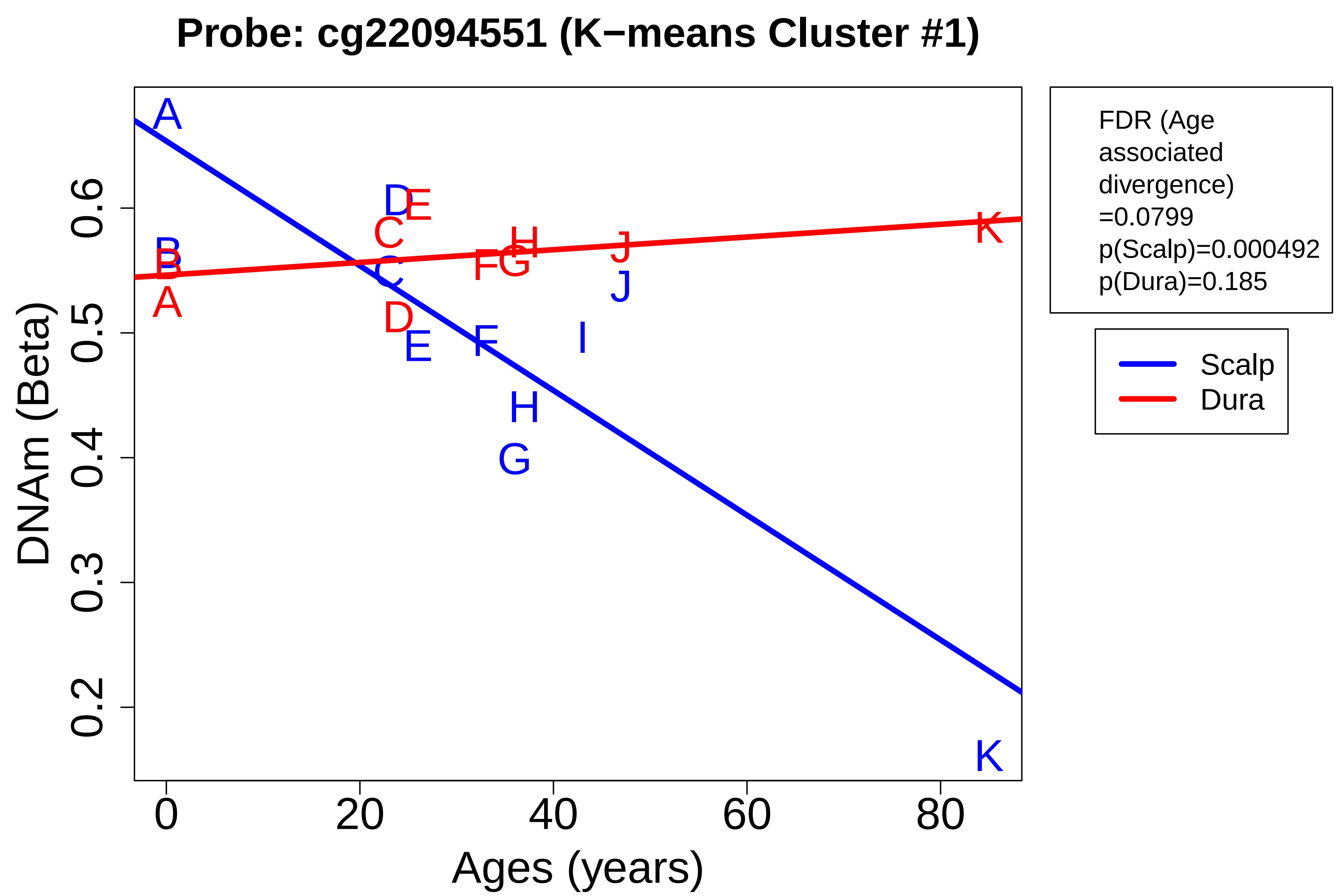

Probe: cg22094551 (K−means Cluster #1)
DNAm (Beta)
0.2
0.3
0.4
0.5
0.6
0
20
40
60
80
Ages (years)
A
B
C
D
E
F
G
H
I
J
K
FDR (Age associated divergence) =0.0799
p(Scalp)=0.000492
p(Dura)=0.185
Scalp
Dura

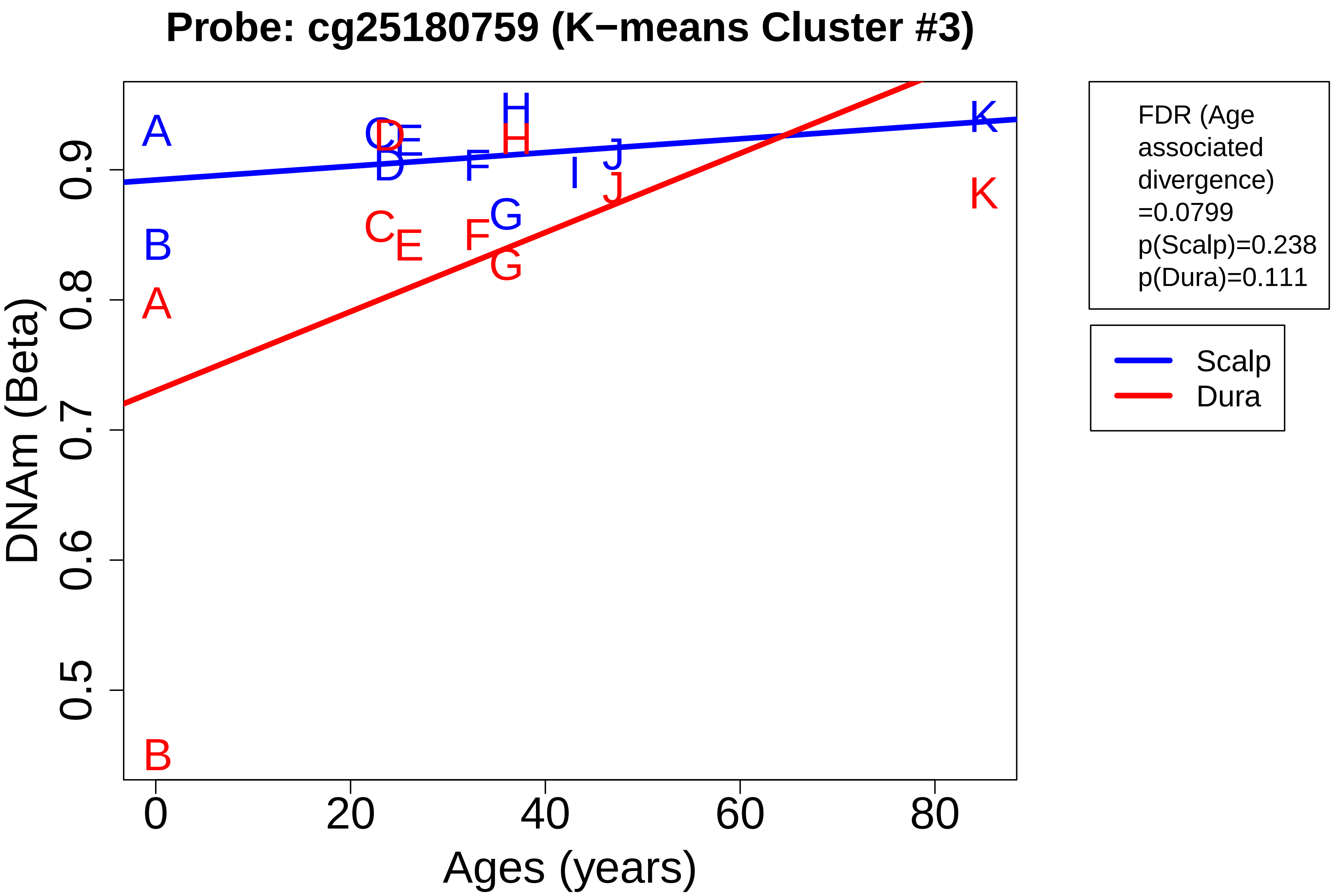

Probe: cg25180759 (K−means Cluster #3)
DNAm (Beta)
0.5
0.6
0.7
0.8
0.9
0
20
40
60
80
Ages (years)
FDR (Age associated divergence) =0.0799
p(Scalp)=0.238
p(Dura)=0.111
Scalp
Dura

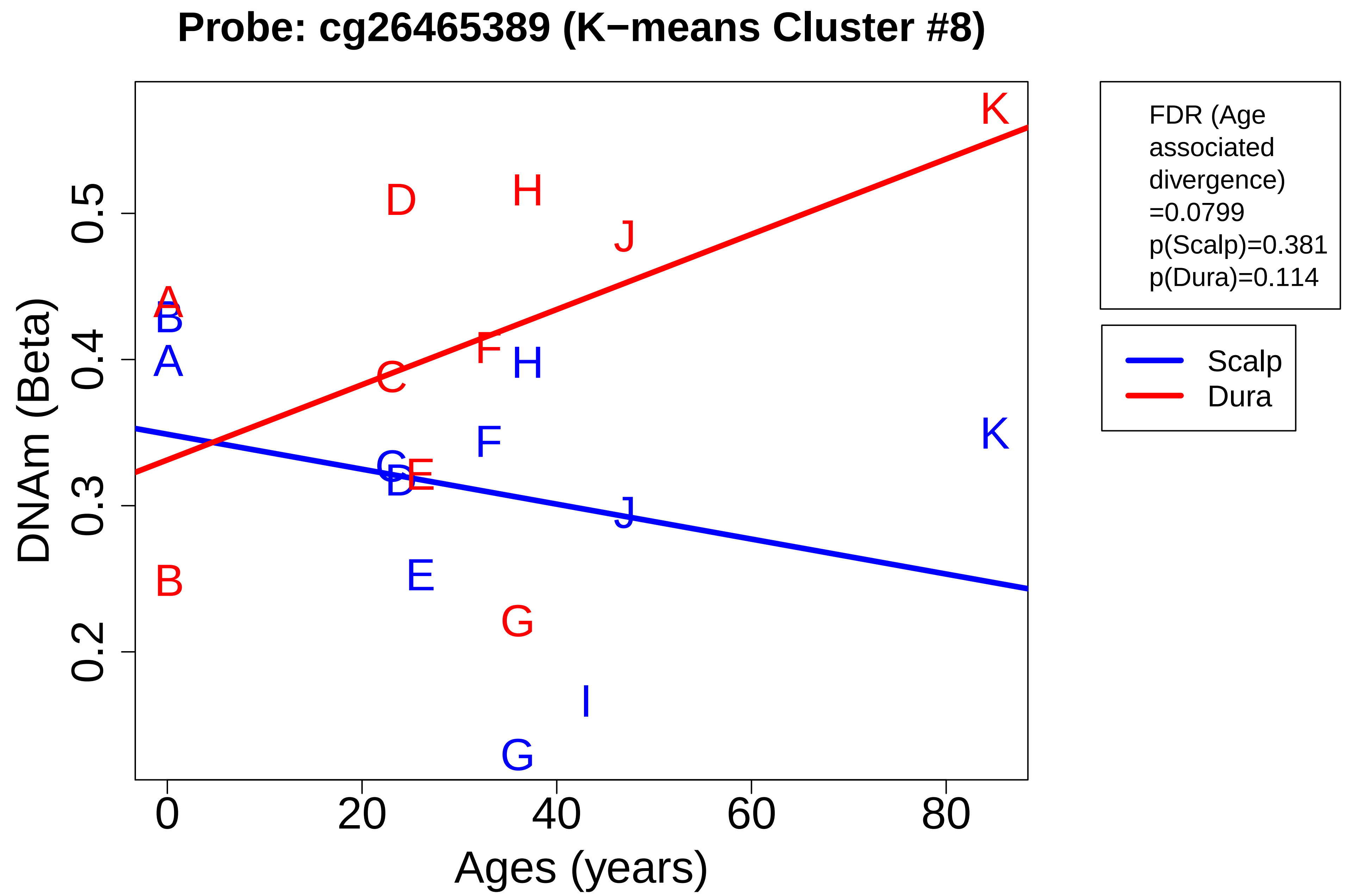

Probe: cg26465389 (K−means Cluster #8)
FDR (Age associated divergence) =0.0799
p(Scalp)=0.381
p(Dura)=0.114
Scalp
Dura
DNAm (Beta)
0.2
0.3
0.4
0.5
0
20
40
60
80
Ages (years)

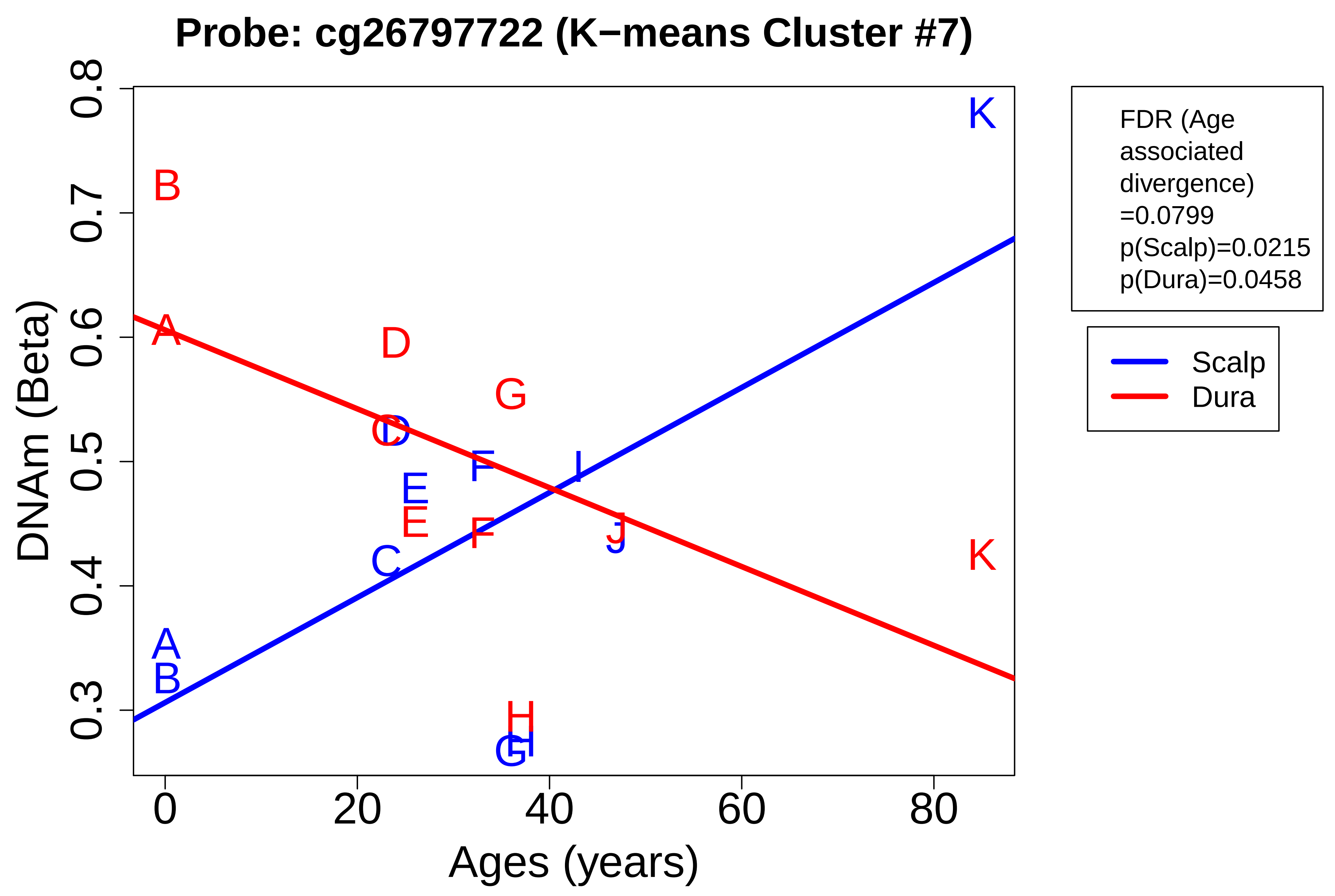

Probe: cg26797722 (K−means Cluster #7)
DNAm (Beta)
0.3
0.4
0.5
0.6
0.7
0.8
0
20
40
60
80
Ages (years)
FDR (Age associated divergence) =0.0799
p(Scalp)=0.0215
p(Dura)=0.0458
Scalp
Dura
A
B
C
D
E
F
G
H
I
J
K

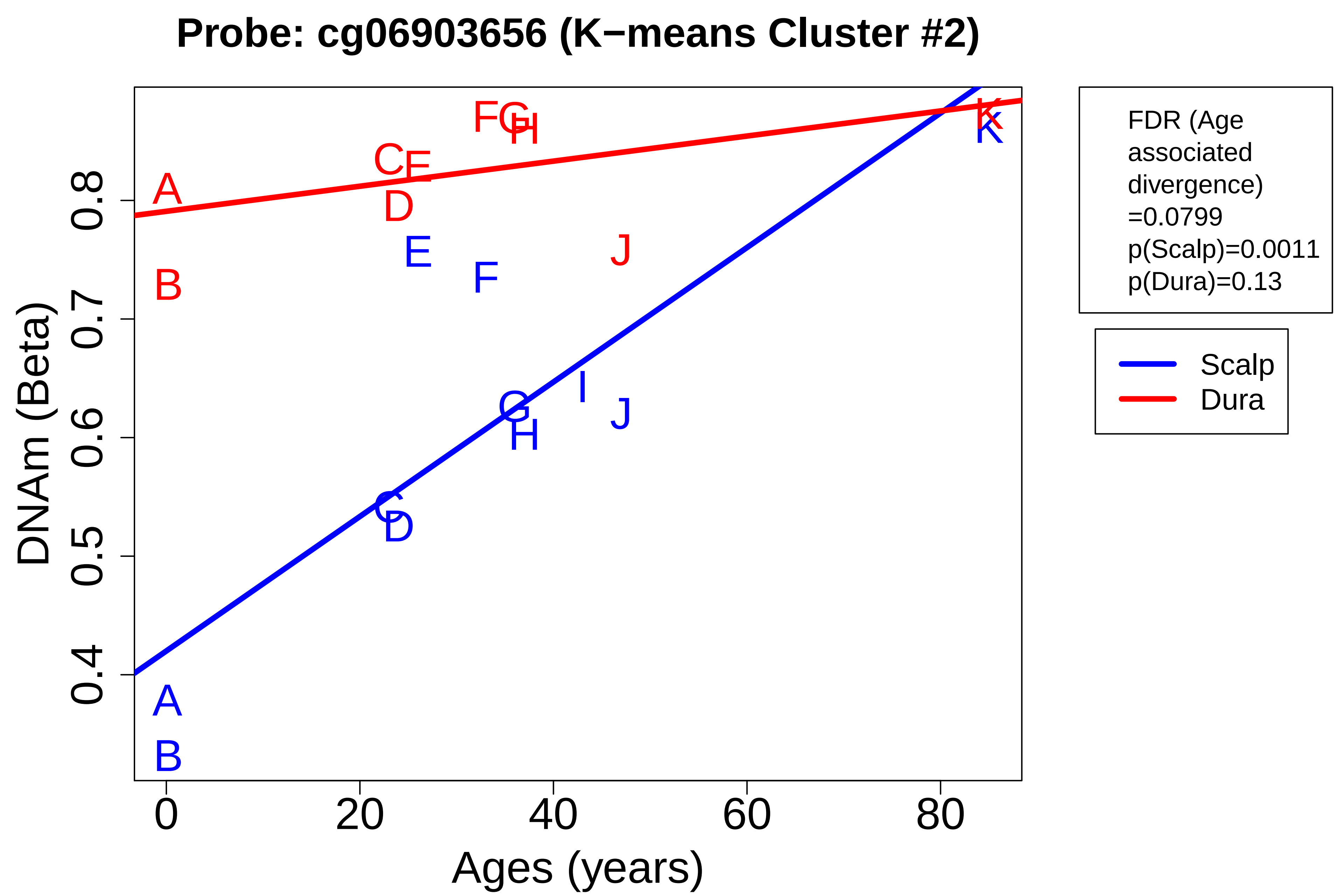
Probe: cg06903656 (K−means Cluster #2)
DNAm (Beta)
0.4
0.5
0.6
0.7
0.8
0
20
40
60
80
Ages (years)
FDR (Age associated divergence) =0.0799
p(Scalp)=0.0011
p(Dura)=0.13
Scalp
Dura

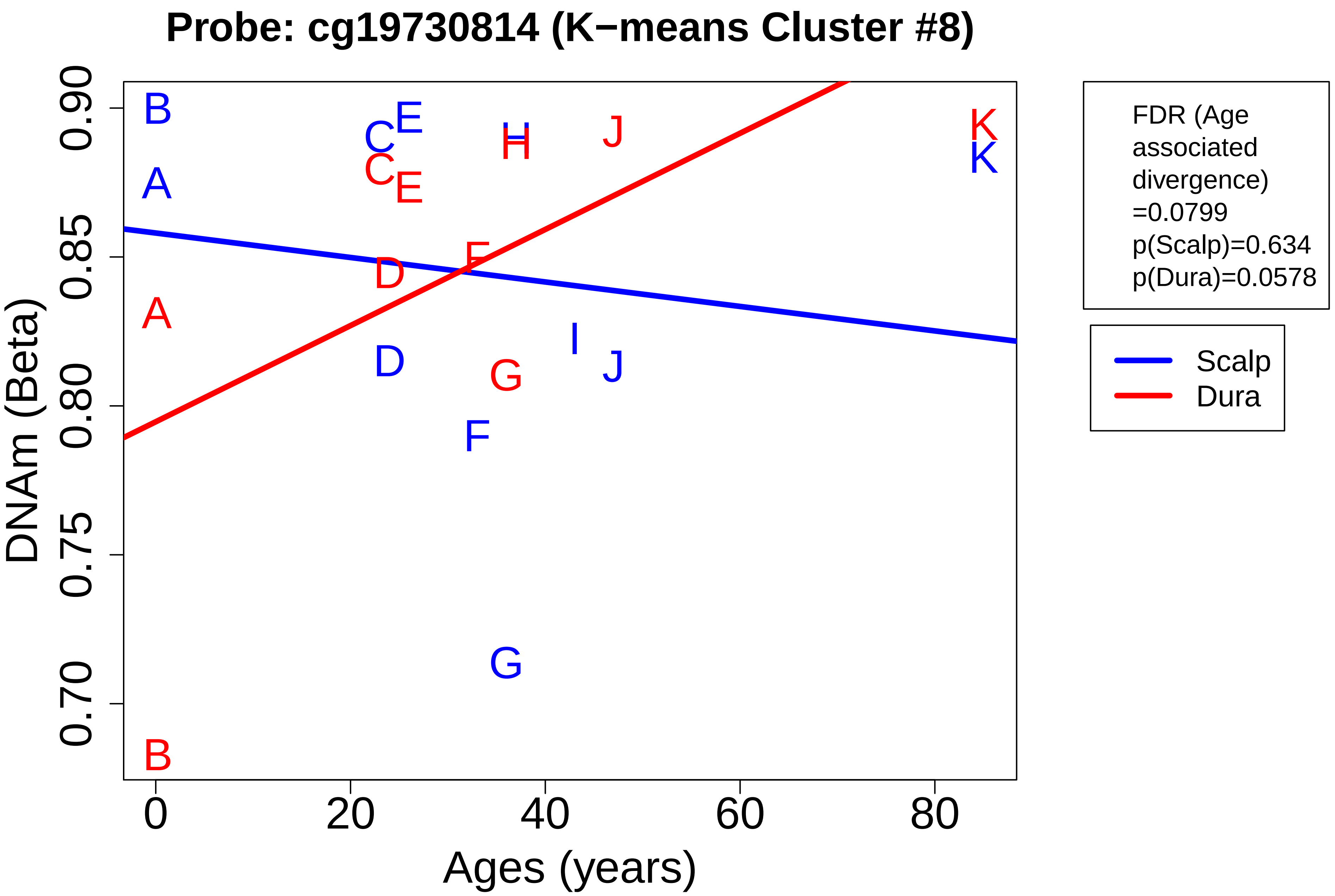

Probe: cg19730814 (K−means Cluster #8)
DNAm (Beta)
0.70
0.75
0.80
0.85
0.90
0
20
40
60
80
Ages (years)
A
B
C
D
E
F
G
H
I
J
K
FDR (Age associated divergence) =0.0799
p(Scalp)=0.634
p(Dura)=0.0578
Scalp
Dura

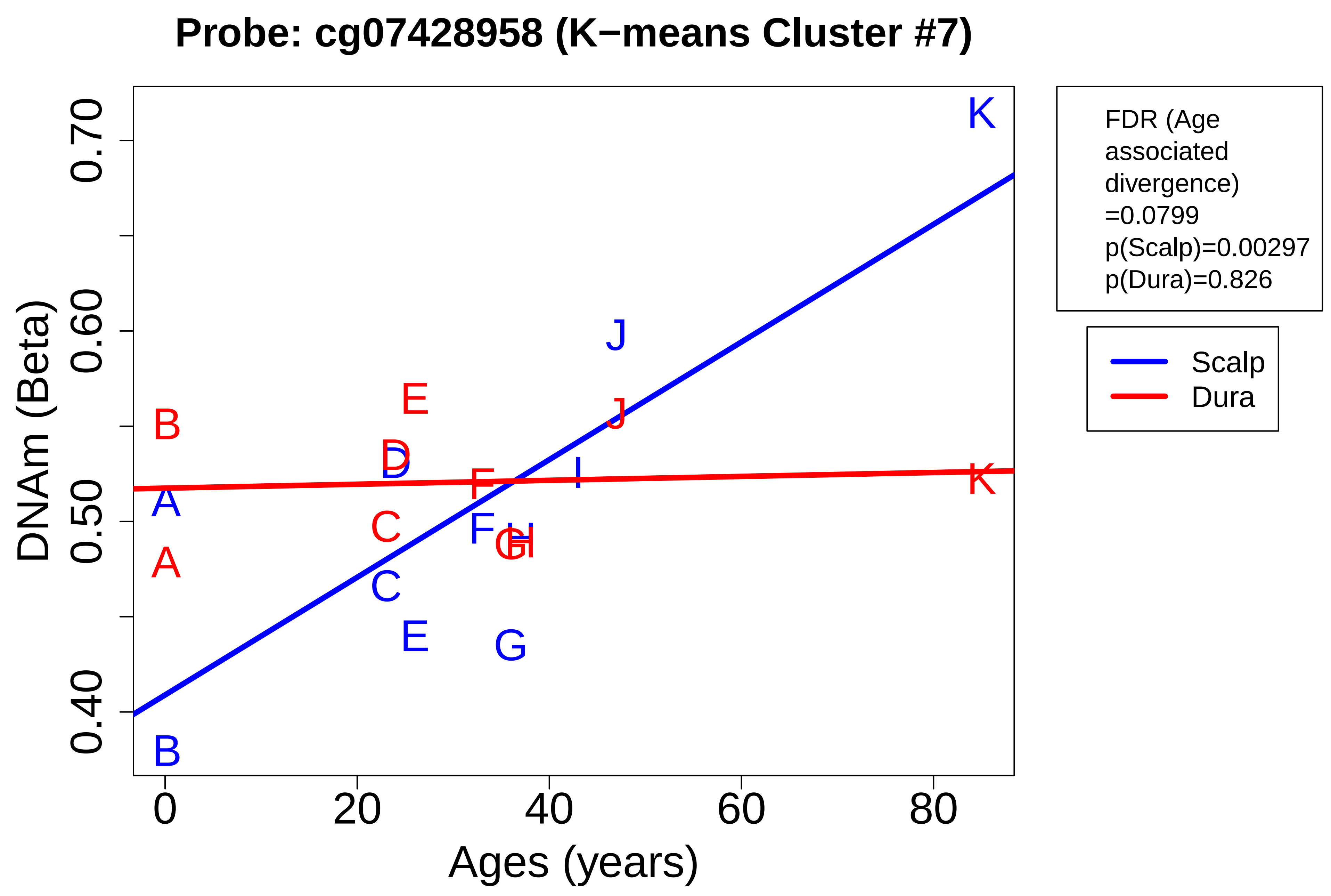
Probe: cg07428958 (K−means Cluster #7)
DNAm (Beta)
0.40
0.50
0.60
0.70
Ages (years)
0
20
40
60
80
FDR (Age associated divergence) =0.0799
p(Scalp)=0.00297
p(Dura)=0.826
Scalp
Dura
A
B
C
D
E
F
G
H
I
J
K

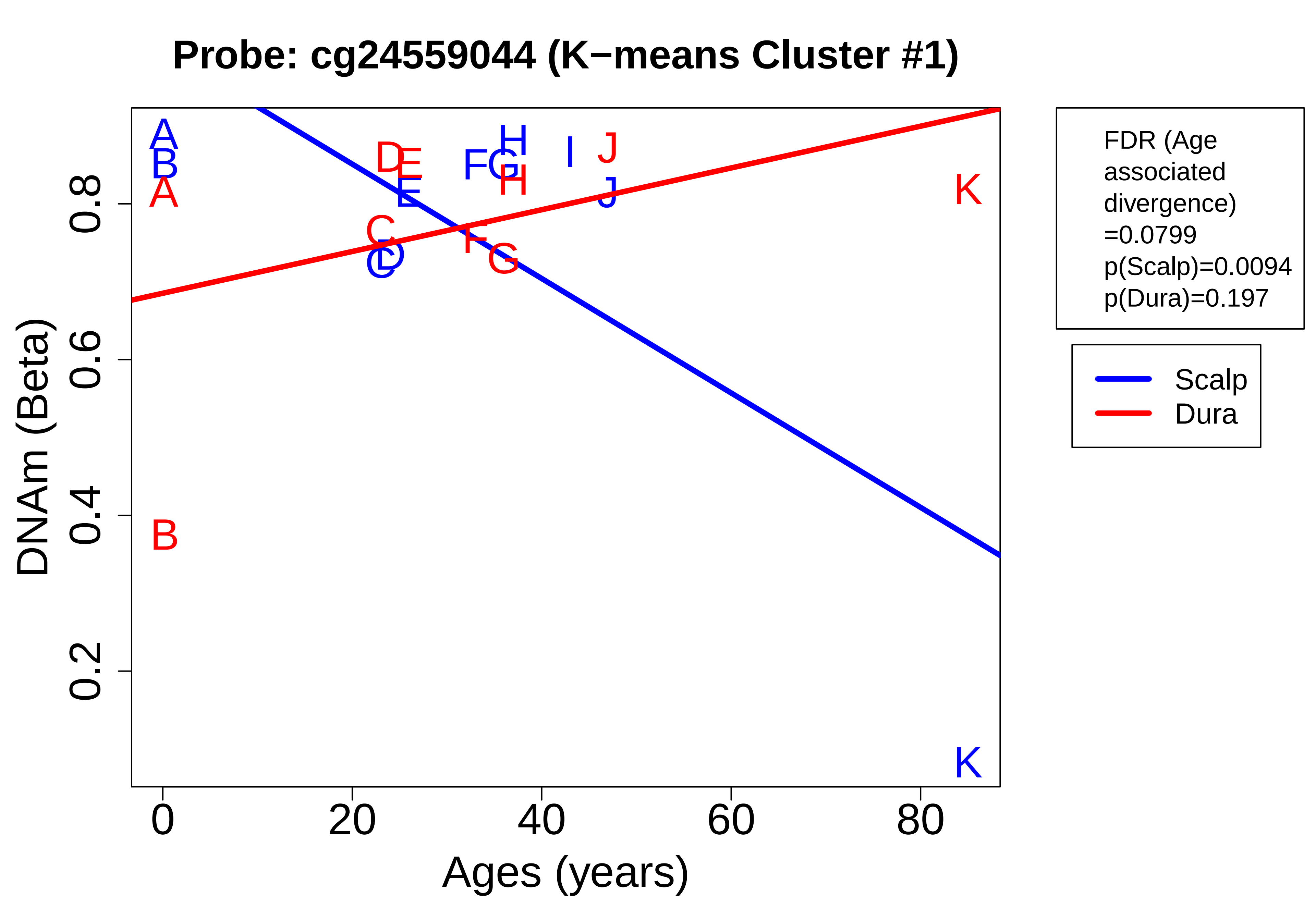

Probe: cg24559044 (K−means Cluster #1)
DNAm (Beta)
0.2
0.4
0.6
0.8
0
20
40
60
80
Ages (years)
A
B
C
D
E
F
G
H
I
J
K
FDR (Age associated divergence) =0.0799
p(Scalp)=0.0094
p(Dura)=0.197
Scalp
Dura

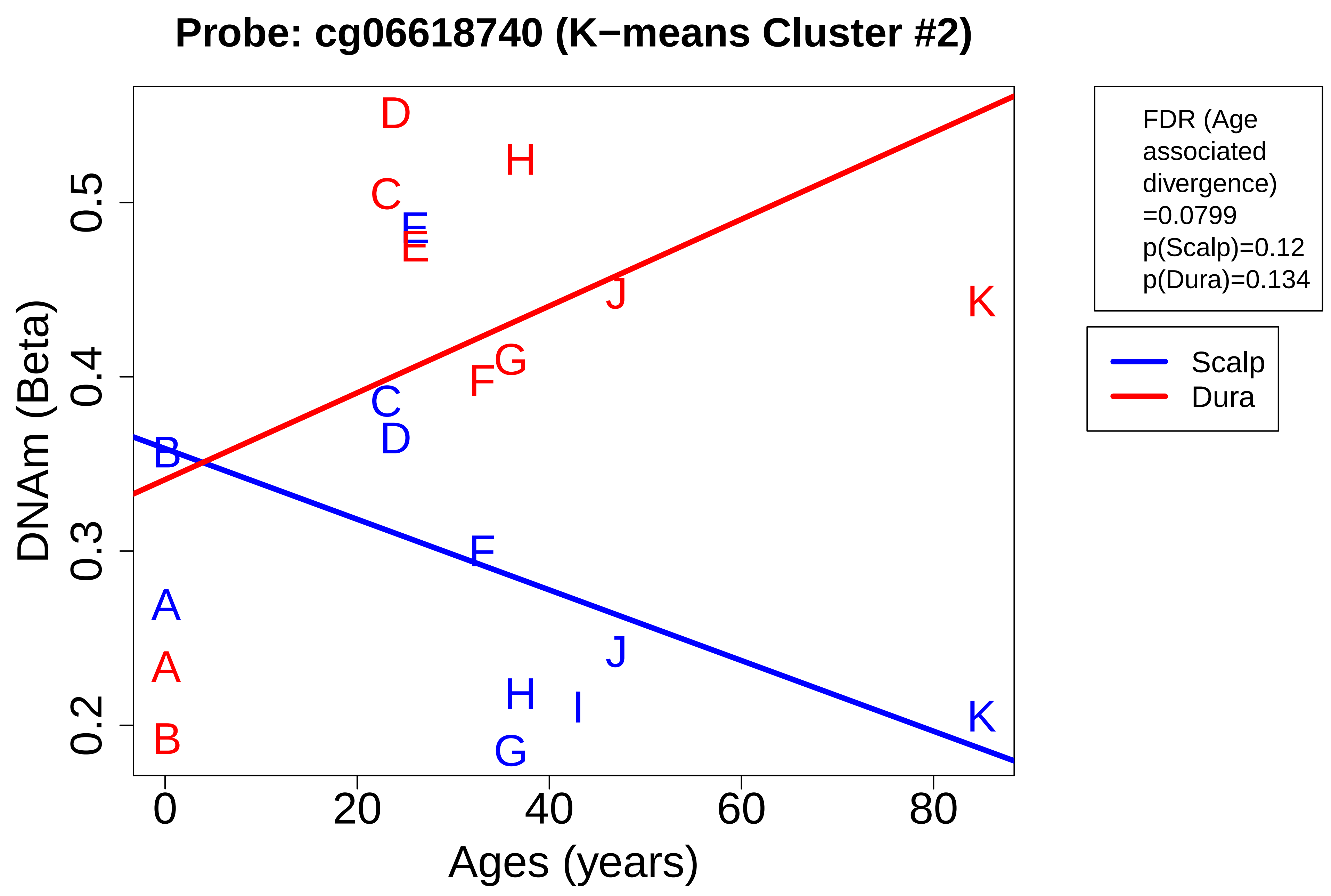

Probe: cg06618740 (K−means Cluster #2)
DNAm (Beta)
Ages (years)
0.2
0.3
0.4
0.5
0
20
40
60
80
FDR (Age associated divergence) =0.0799
p(Scalp)=0.12
p(Dura)=0.134
Scalp
Dura

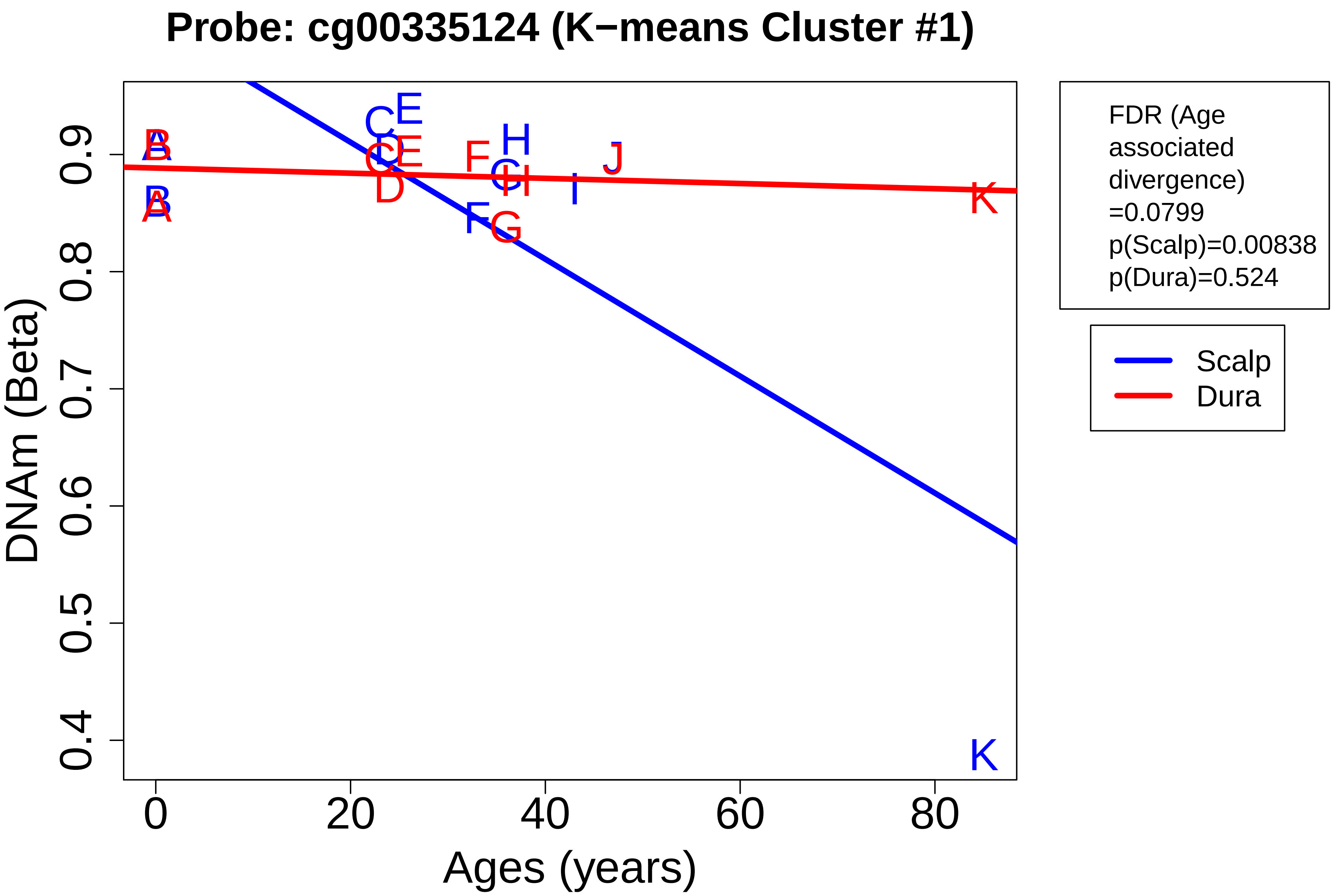
Probe: cg00335124 (K−means Cluster #1)
DNAm (Beta)
0.4
0.5
0.6
0.7
0.8
0.9
0
20
40
60
80
Ages (years)
FDR (Age associated divergence) =0.0799
p(Scalp)=0.00838
p(Dura)=0.524
Scalp
Dura

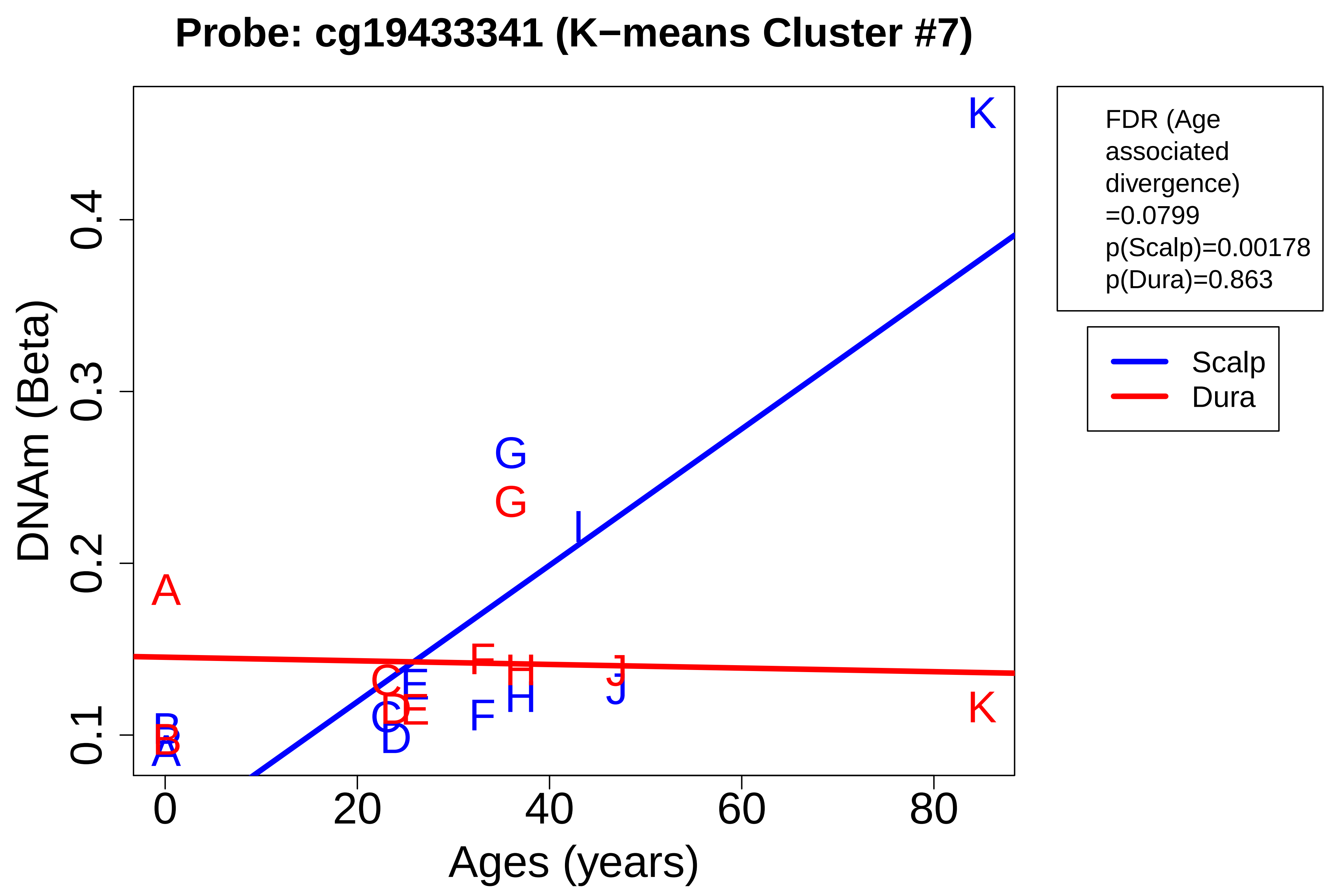
Probe: cg19433341 (K−means Cluster #7)
DNAm (Beta)
0.1
0.2
0.3
0.4
0
20
40
60
80
Ages (years)
FDR (Age associated divergence) =0.0799
p(Scalp)=0.00178
p(Dura)=0.863
Scalp
Dura

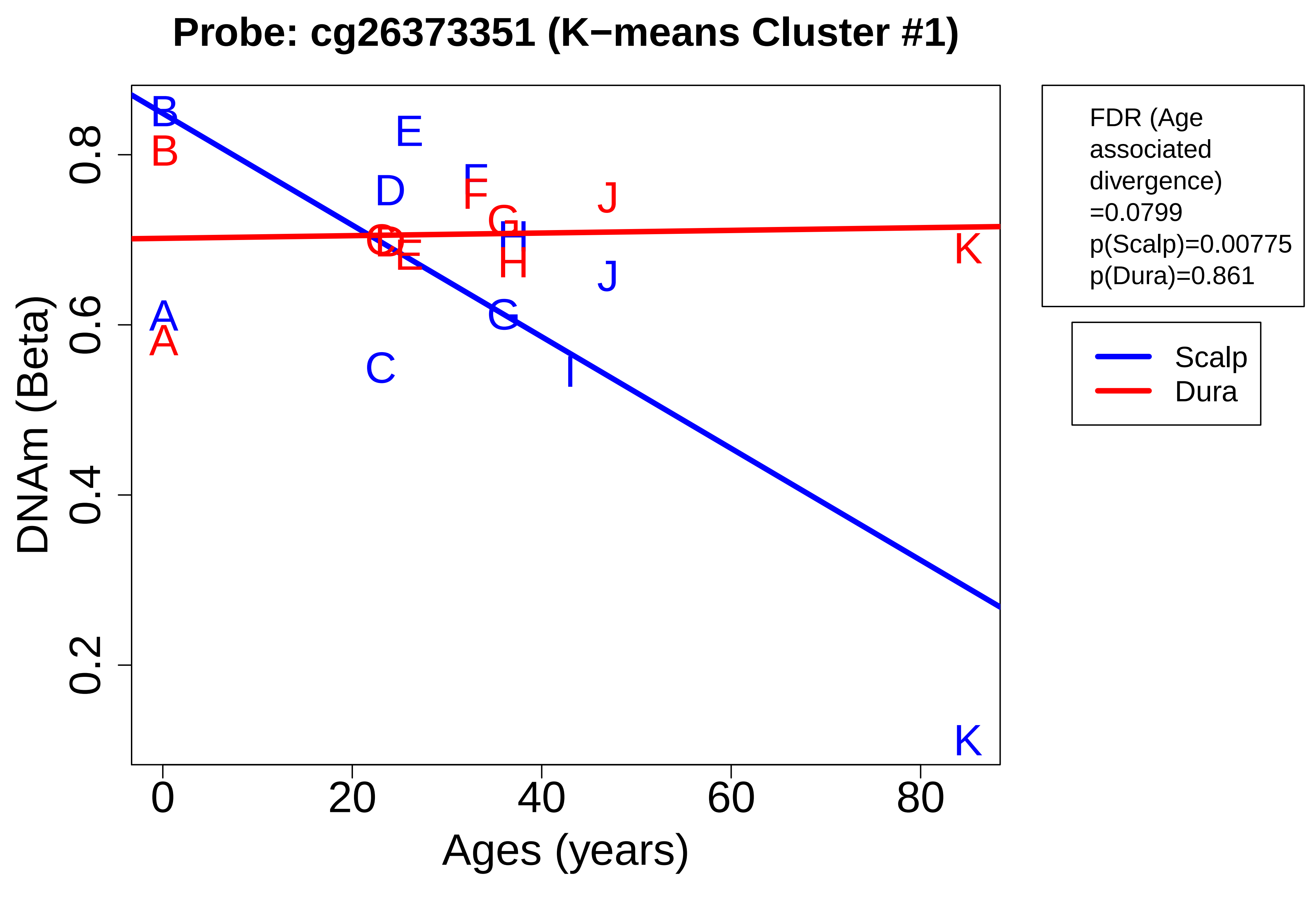

Probe: cg26373351 (K−means Cluster #1)
DNAm (Beta)
0.2
0.4
0.6
0.8
Ages (years)
0
20
40
60
80
FDR (Age associated divergence) =0.0799
p(Scalp)=0.00775
p(Dura)=0.861
Scalp
Dura

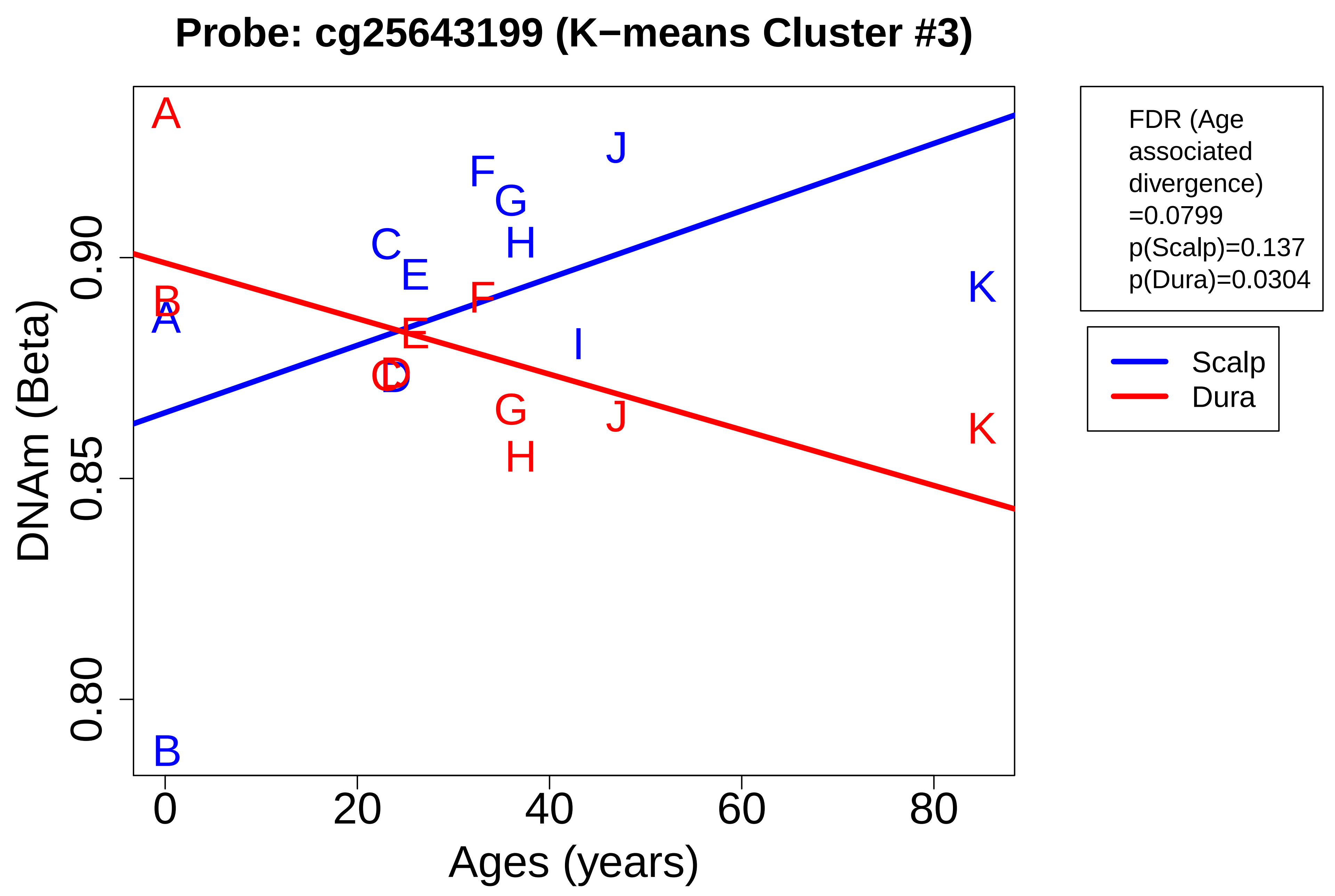

Probe: cg25643199 (K−means Cluster #3)
DNAm (Beta)
Ages (years)
0.80
0.85
0.90
0
20
40
60
80
FDR (Age associated divergence) =0.0799
p(Scalp)=0.137
p(Dura)=0.0304
Scalp
Dura

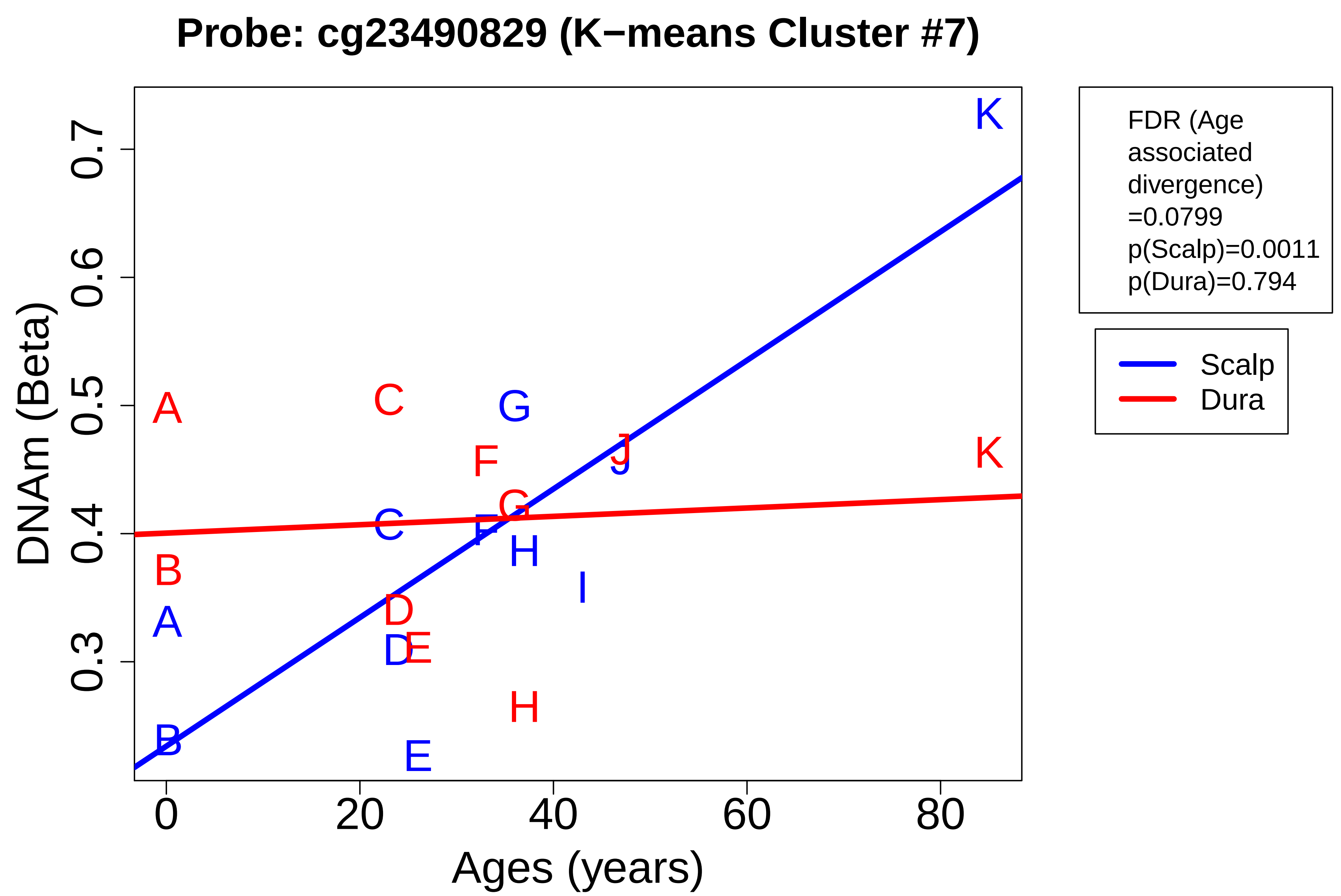

Probe: cg23490829 (K−means Cluster #7)
DNAm (Beta)
0.3
0.4
0.5
0.6
0.7
0
20
40
60
80
Ages (years)
A
B
C
D
E
F
G
H
I
J
K
FDR (Age associated divergence) =0.0799
p(Scalp)=0.0011
p(Dura)=0.794
Scalp
Dura

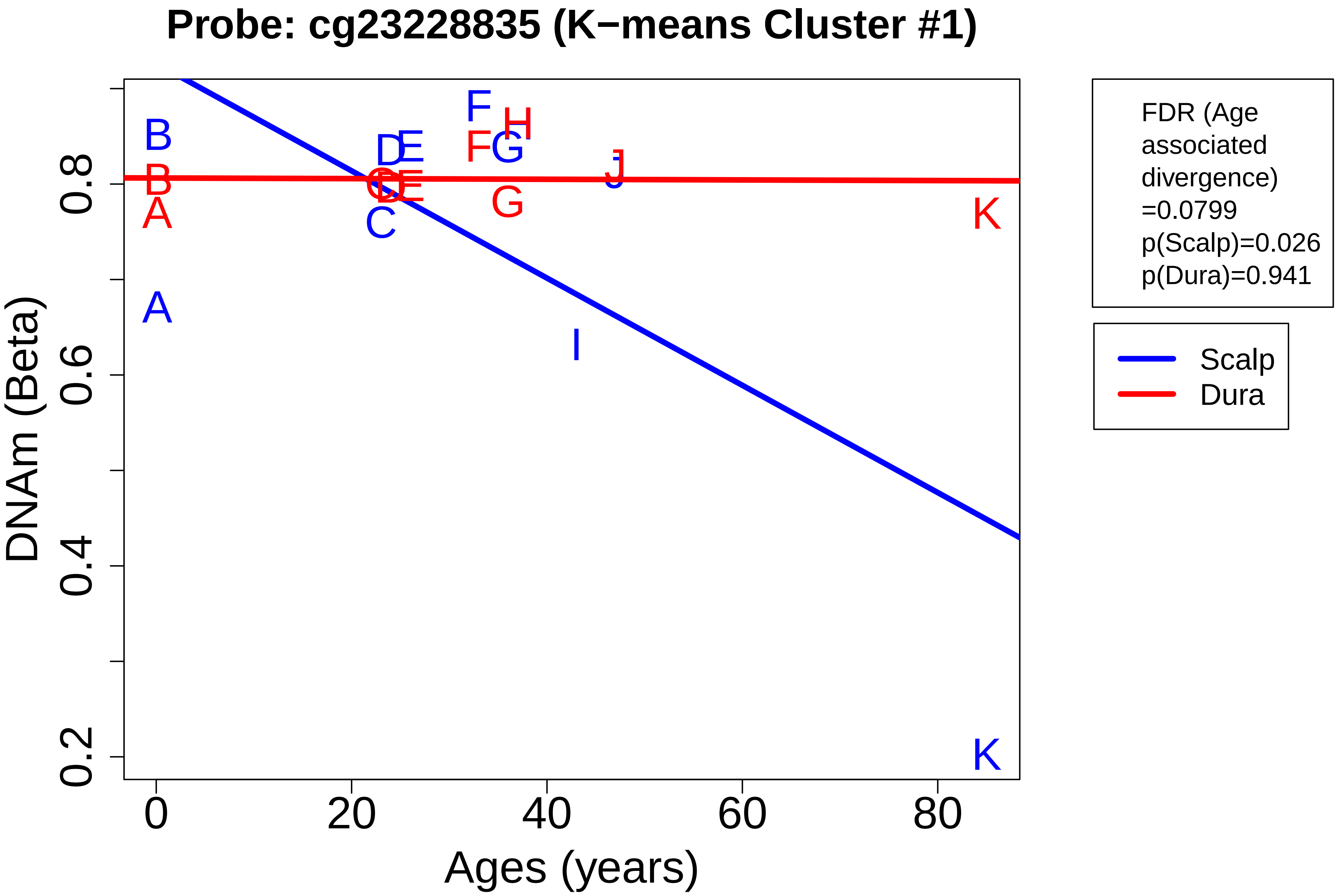

Probe: cg23228835 (K−means Cluster #1)
FDR (Age associated divergence) =0.0799
p(Scalp)=0.026
p(Dura)=0.941
Scalp
Dura
DNAm (Beta)
0.2
0.4
0.6
0.8
Ages (years)
0
20
40
60
80
A
B
C
D
E
F
G
H
I
J
K

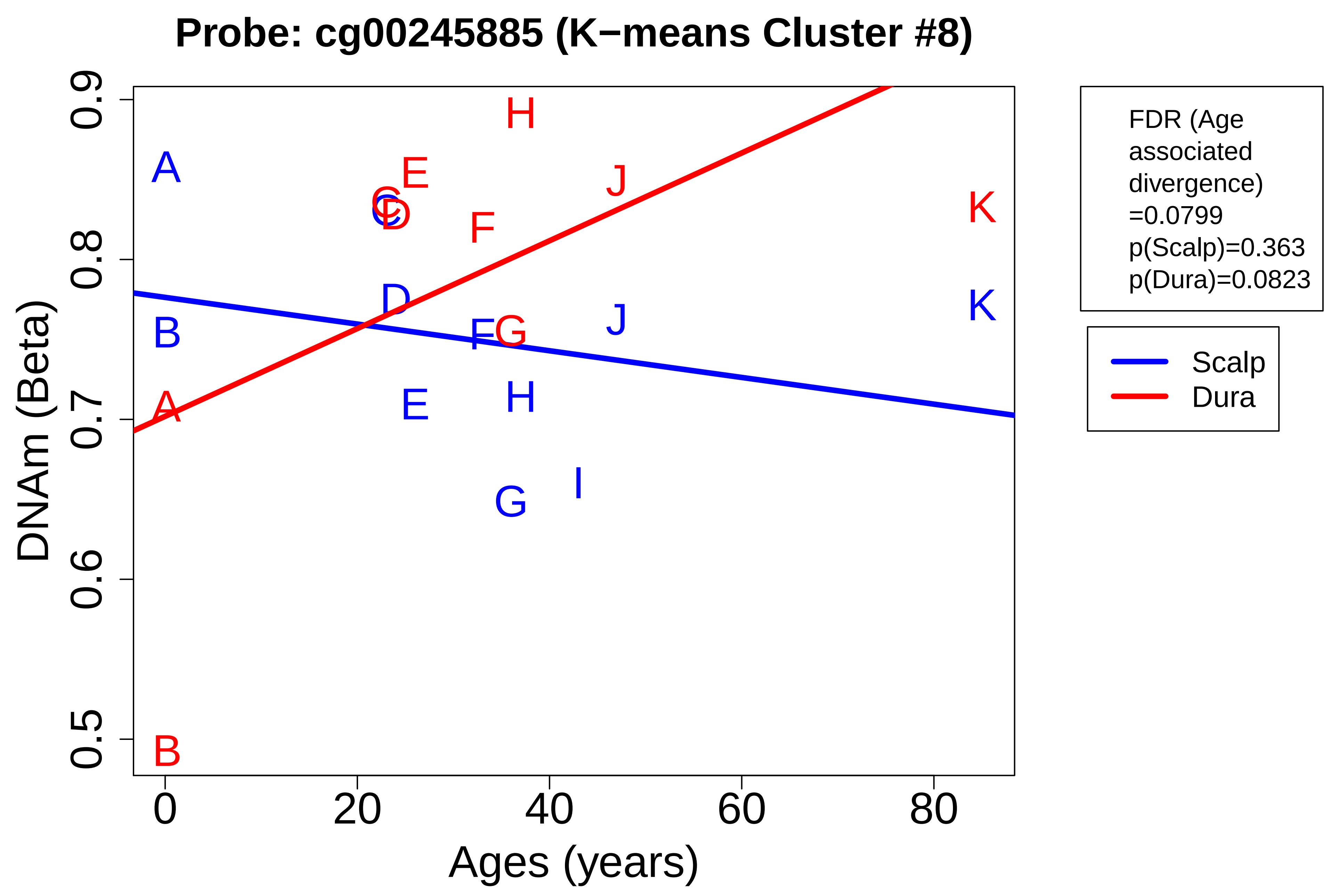

Probe: cg00245885 (K−means Cluster #8)
DNAm (Beta)
0.5
0.6
0.7
0.8
0.9
0
20
40
60
80
Ages (years)
A
B
C
D
E
F
G
H
I
J
K
FDR (Age associated divergence) =0.0799
p(Scalp)=0.363
p(Dura)=0.0823
Scalp
Dura

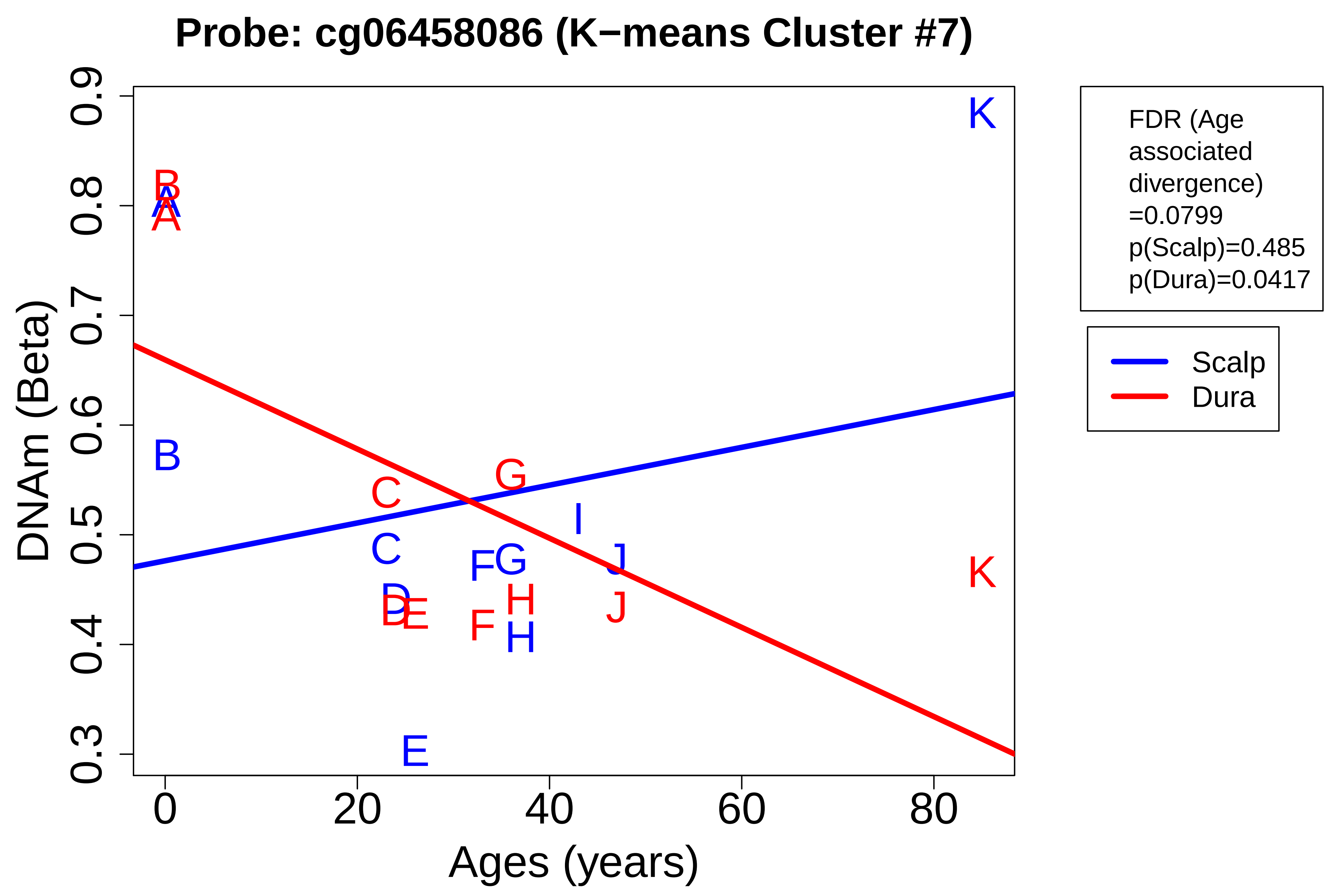

Probe: cg06458086 (K−means Cluster #7)
DNAm (Beta)
0.3
0.4
0.5
0.6
0.7
0.8
0.9
0
20
40
60
80
Ages (years)
A
B
C
D
E
F
G
H
I
J
K
FDR (Age associated divergence) =0.0799
p(Scalp)=0.485
p(Dura)=0.0417
Scalp
Dura

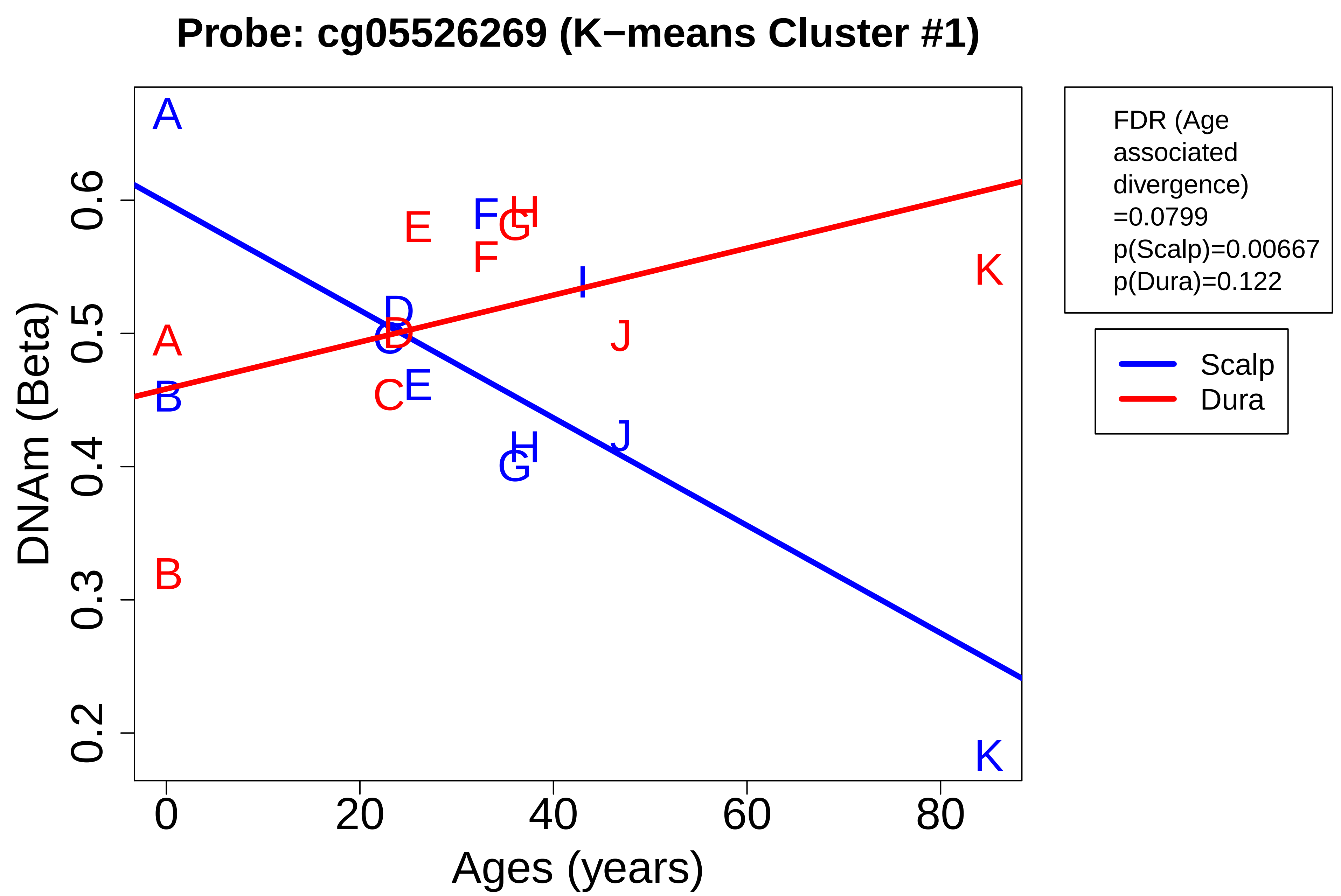

Probe: cg05526269 (K−means Cluster #1)
DNAm (Beta)
Ages (years)
FDR (Age associated divergence) =0.0799
p(Scalp)=0.00667
p(Dura)=0.122
Scalp
Dura
0.2
0.3
0.4
0.5
0.6
0
20
40
60
80

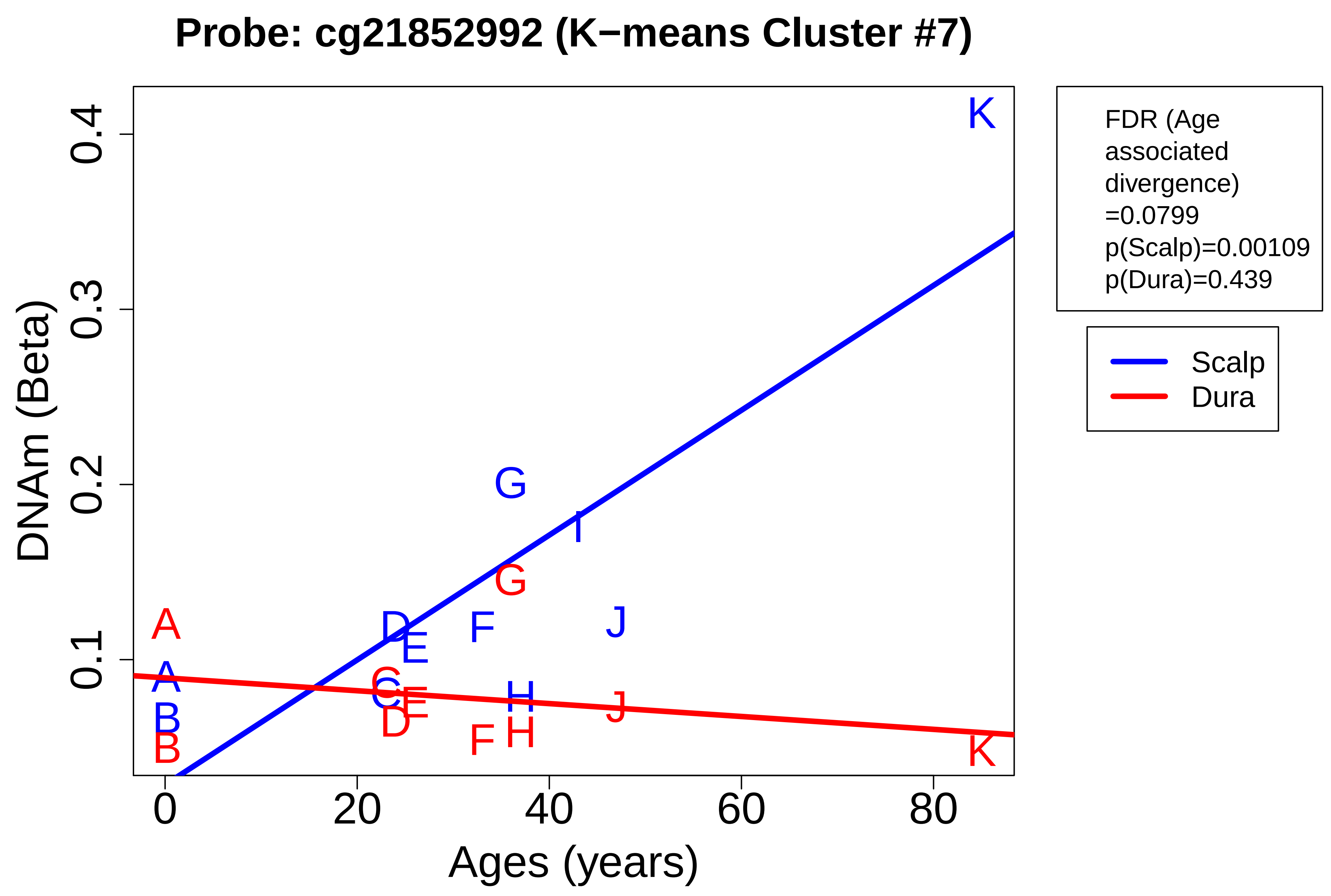
Probe: cg21852992 (K−means Cluster #7)
DNAm (Beta)
0.1
0.2
0.3
0.4
Ages (years)
0
20
40
60
80
A
B
C
D
E
F
G
H
I
J
K
FDR (Age associated divergence) =0.0799
p(Scalp)=0.00109
p(Dura)=0.439
Scalp
Dura

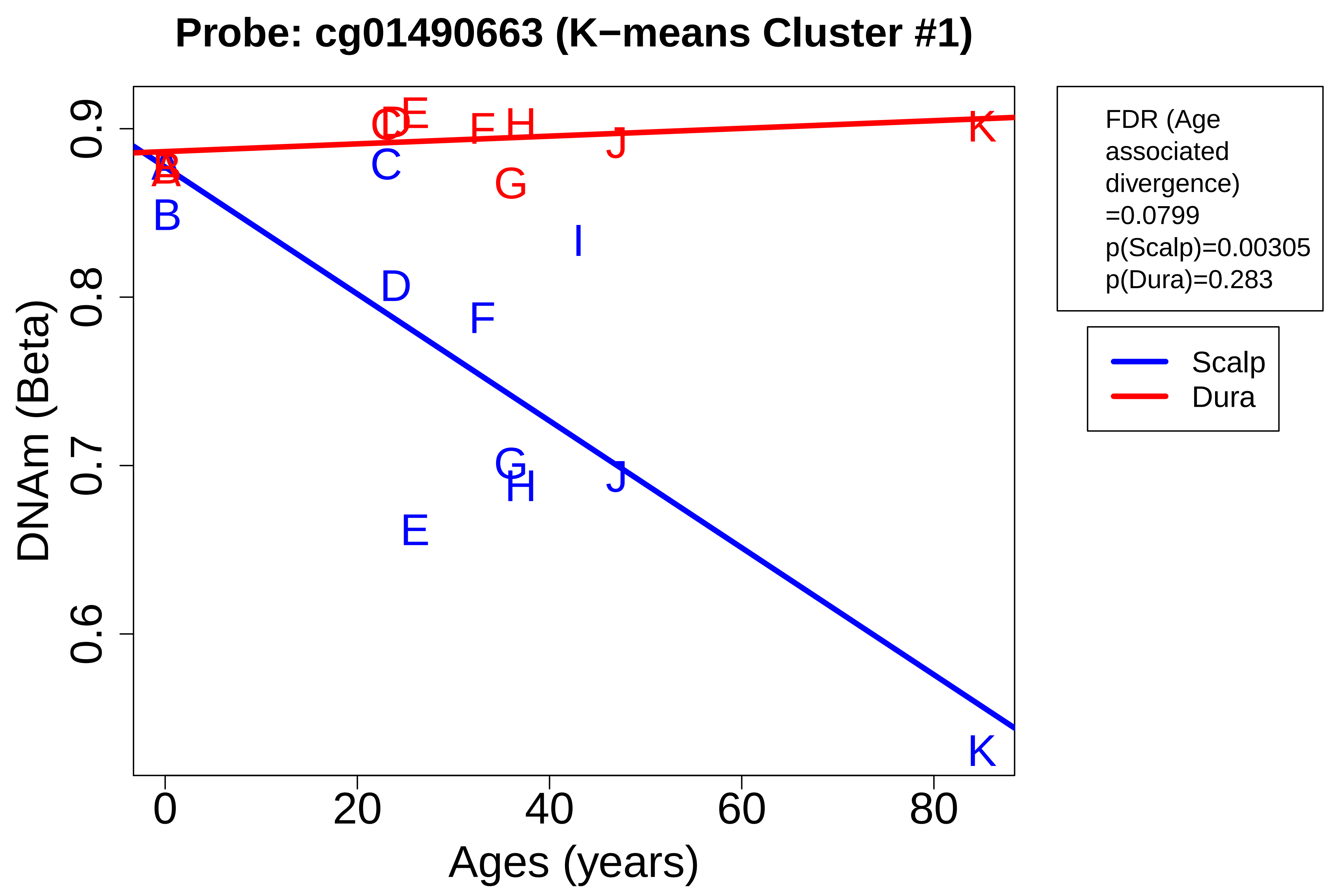

Probe: cg01490663 (K−means Cluster #1)
DNAm (Beta)
0.6
0.7
0.8
0.9
Ages (years)
0
20
40
60
80
FDR (Age associated divergence) =0.0799
p(Scalp)=0.00305
p(Dura)=0.283
Scalp
Dura

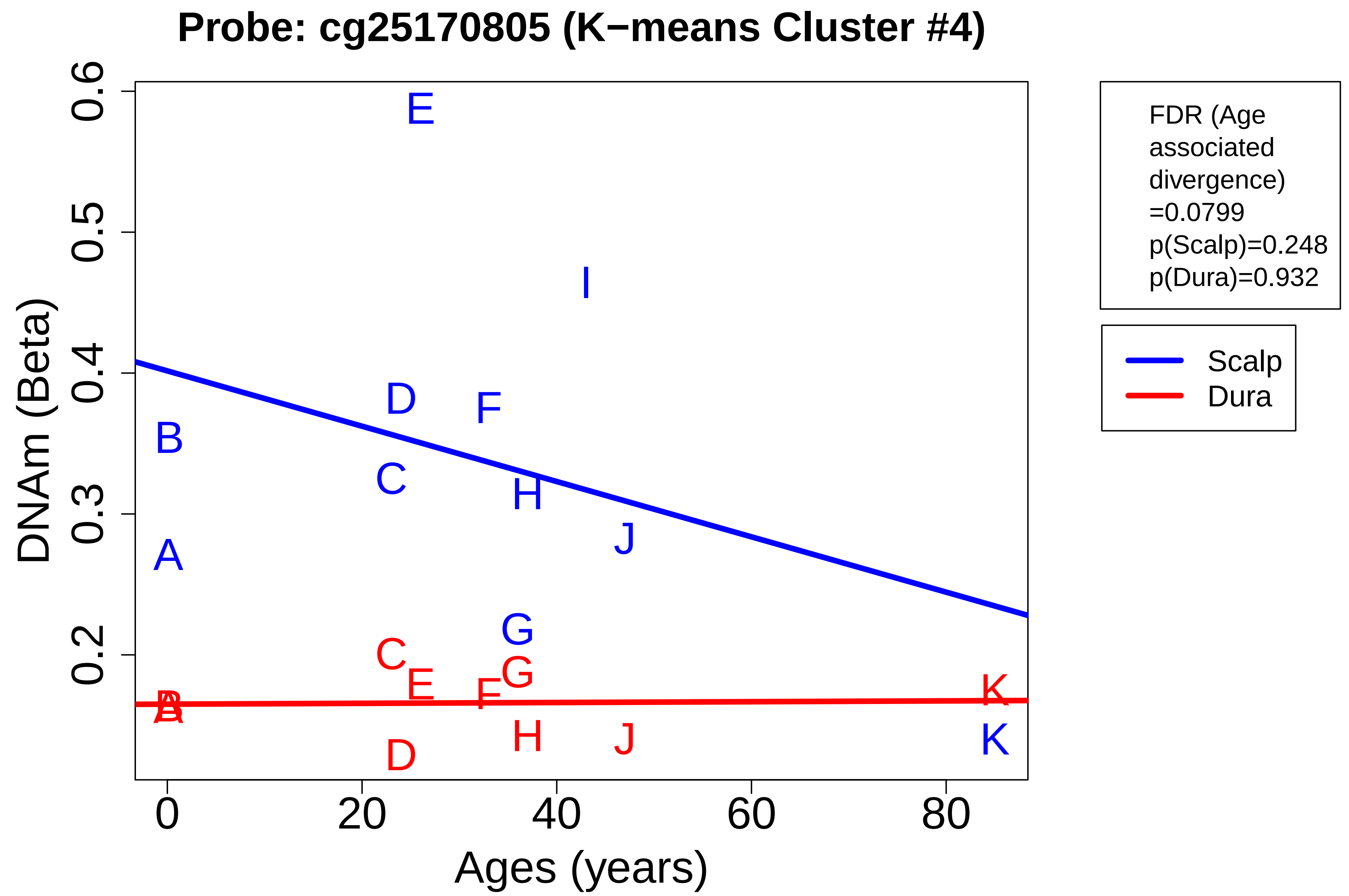

Probe: cg25170805 (K−means Cluster #4)
DNAm (Beta)
0.2
0.3
0.4
0.5
0.6
Ages (years)
0
20
40
60
80
FDR (Age associated divergence) =0.0799
p(Scalp)=0.248
p(Dura)=0.932
Scalp
Dura

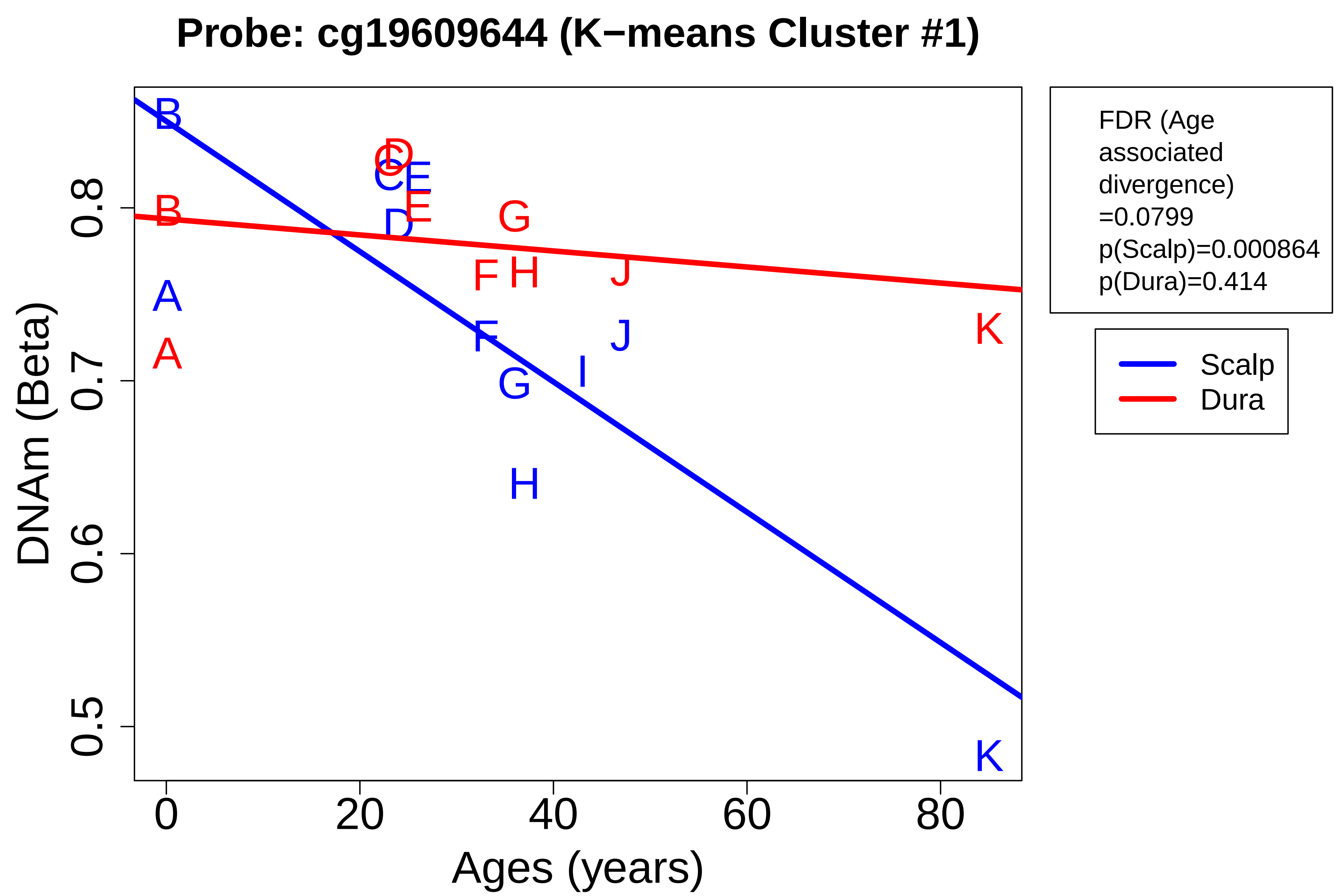

Probe: cg19609644 (K−means Cluster #1)
DNAm (Beta)
0.5
0.6
0.7
0.8
0
20
40
60
80
Ages (years)
A
B
C
D
E
F
G
H
I
J
K
FDR (Age associated divergence) =0.0799
p(Scalp)=0.000864
p(Dura)=0.414
Scalp
Dura

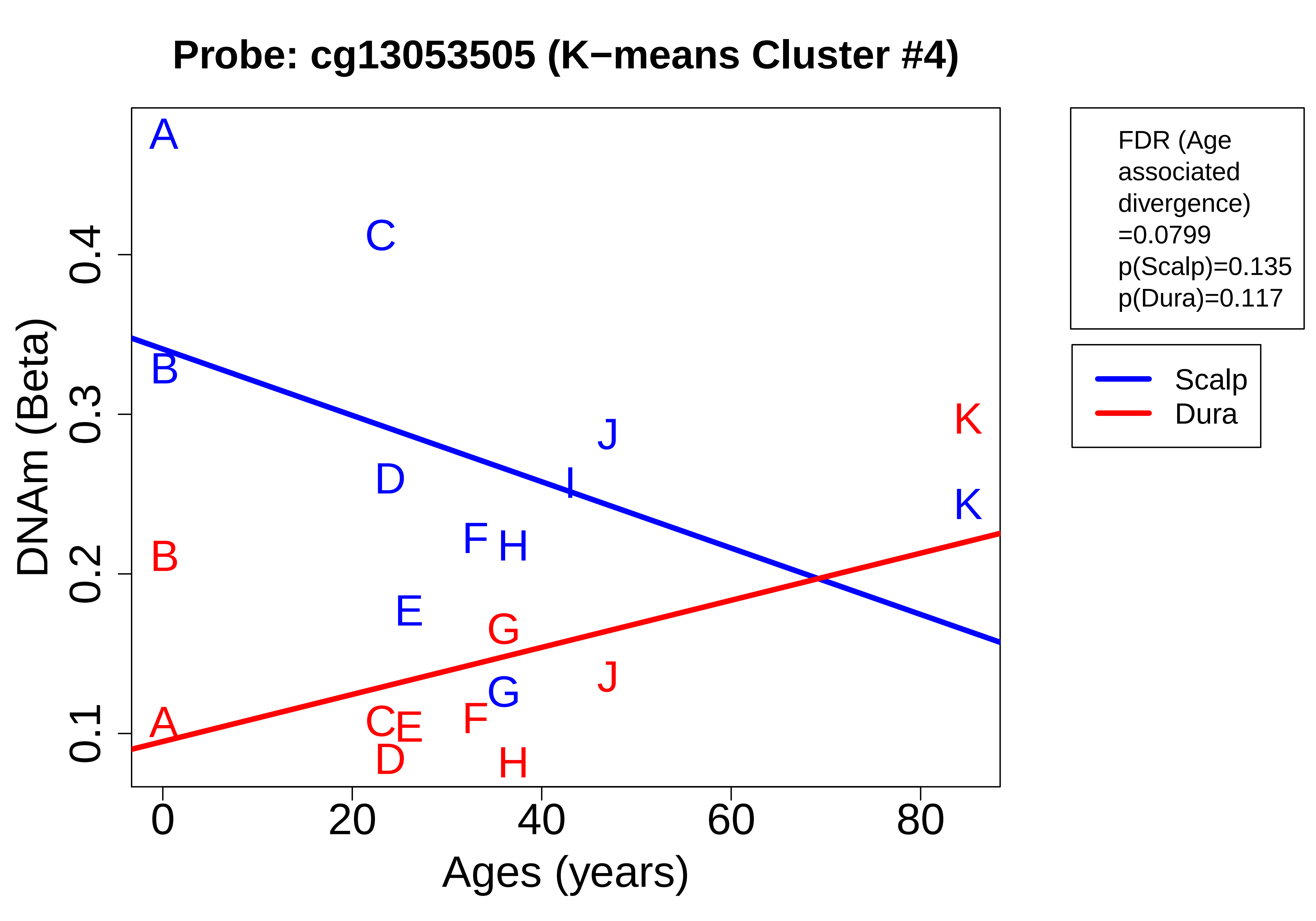

Probe: cg13053505 (K−means Cluster #4)
DNAm (Beta)
0.1
0.2
0.3
0.4
Ages (years)
0
20
40
60
80
A
B
C
D
E
F
G
H
I
J
K
FDR (Age associated divergence) =0.0799
p(Scalp)=0.135
p(Dura)=0.117
Scalp
Dura

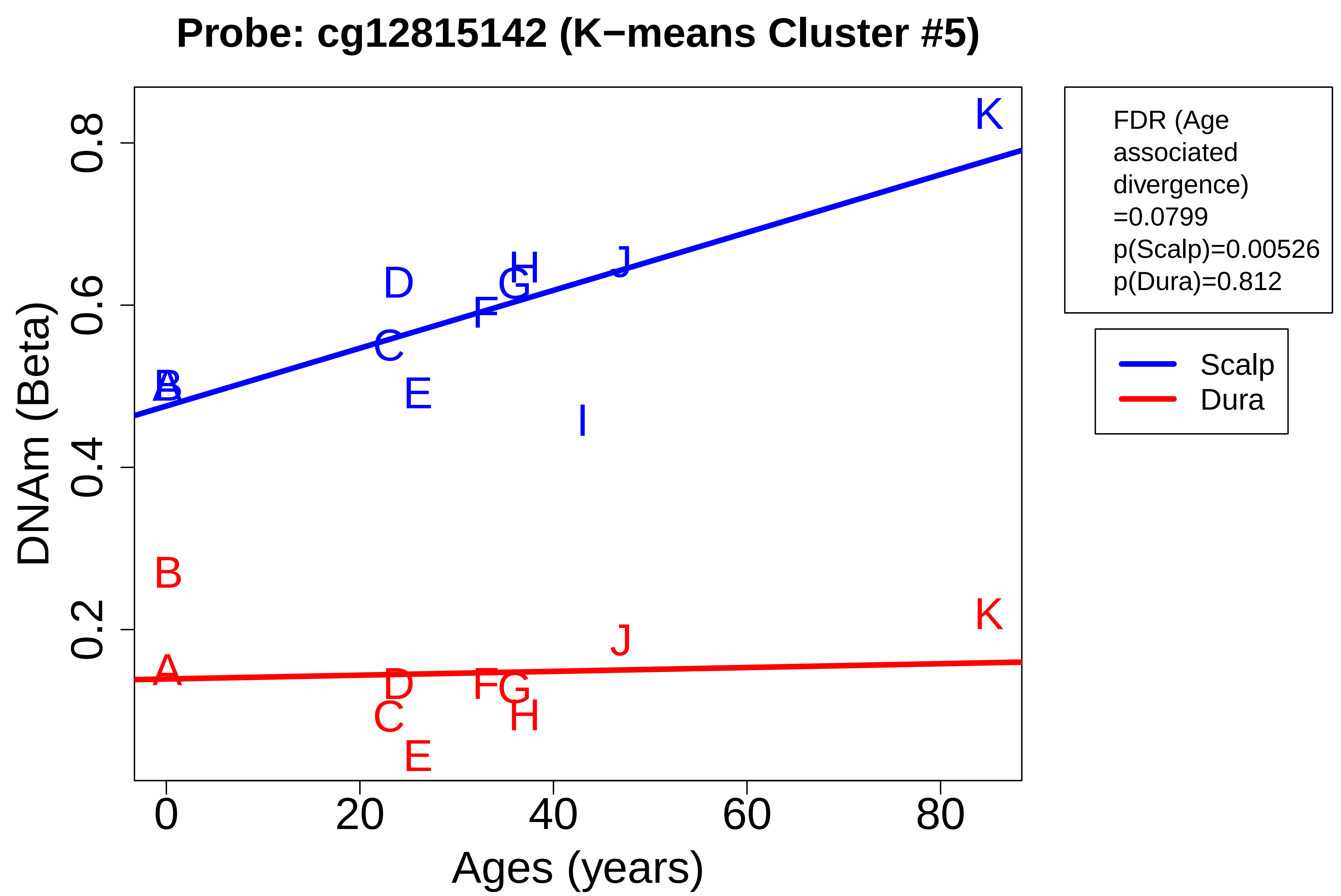

Probe: cg12815142 (K−means Cluster #5)
DNAm (Beta)
0.2
0.4
0.6
0.8
0
20
40
60
80
Ages (years)
FDR (Age associated divergence) =0.0799
p(Scalp)=0.00526
p(Dura)=0.812
Scalp
Dura

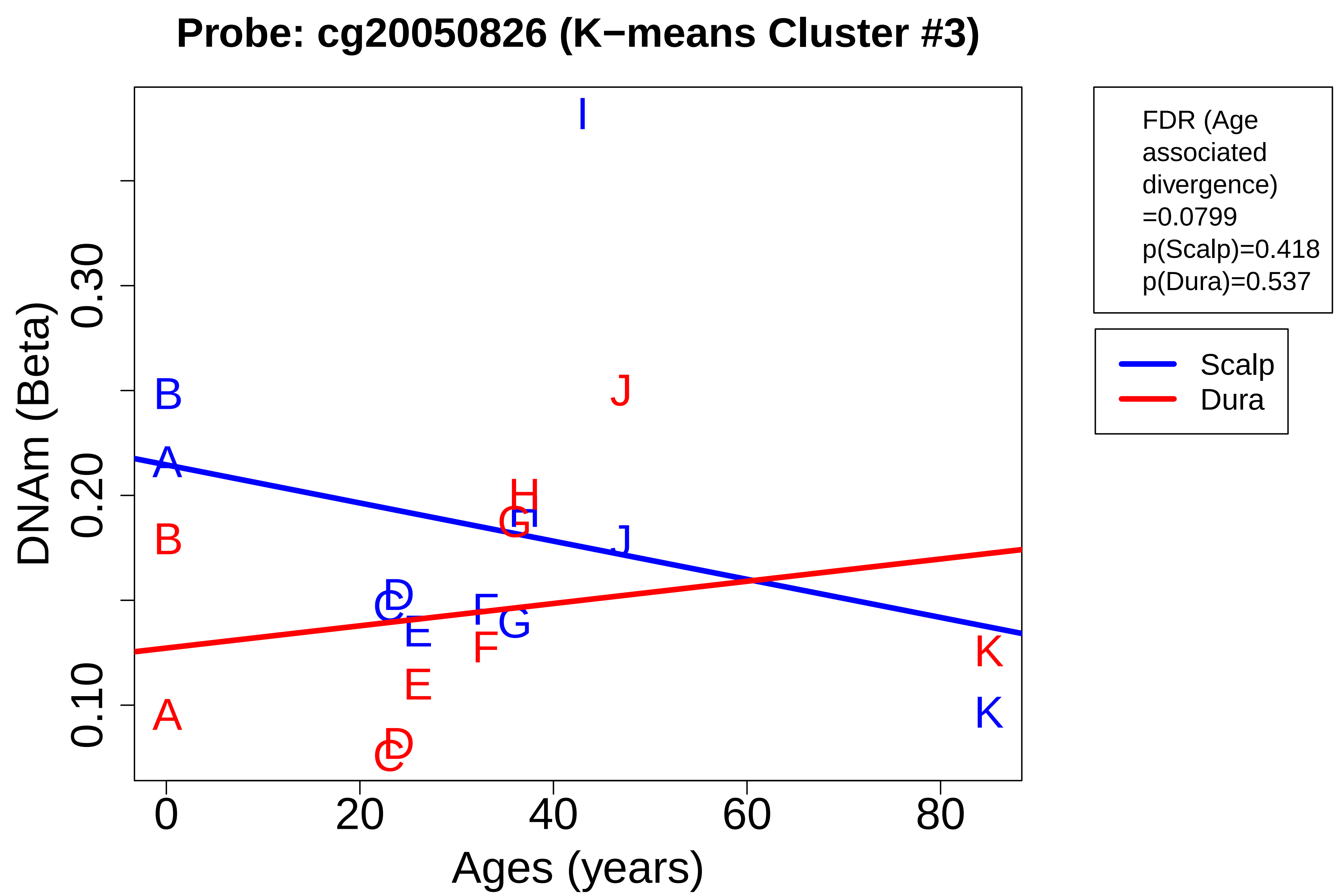

Probe: cg20050826 (K−means Cluster #3)
DNAm (Beta)
0.10
0.20
0.30
Ages (years)
0
20
40
60
80
FDR (Age associated divergence) =0.0799
p(Scalp)=0.418
p(Dura)=0.537
Scalp
Dura
A
B
C
D
E
F
G
H
I
J
K

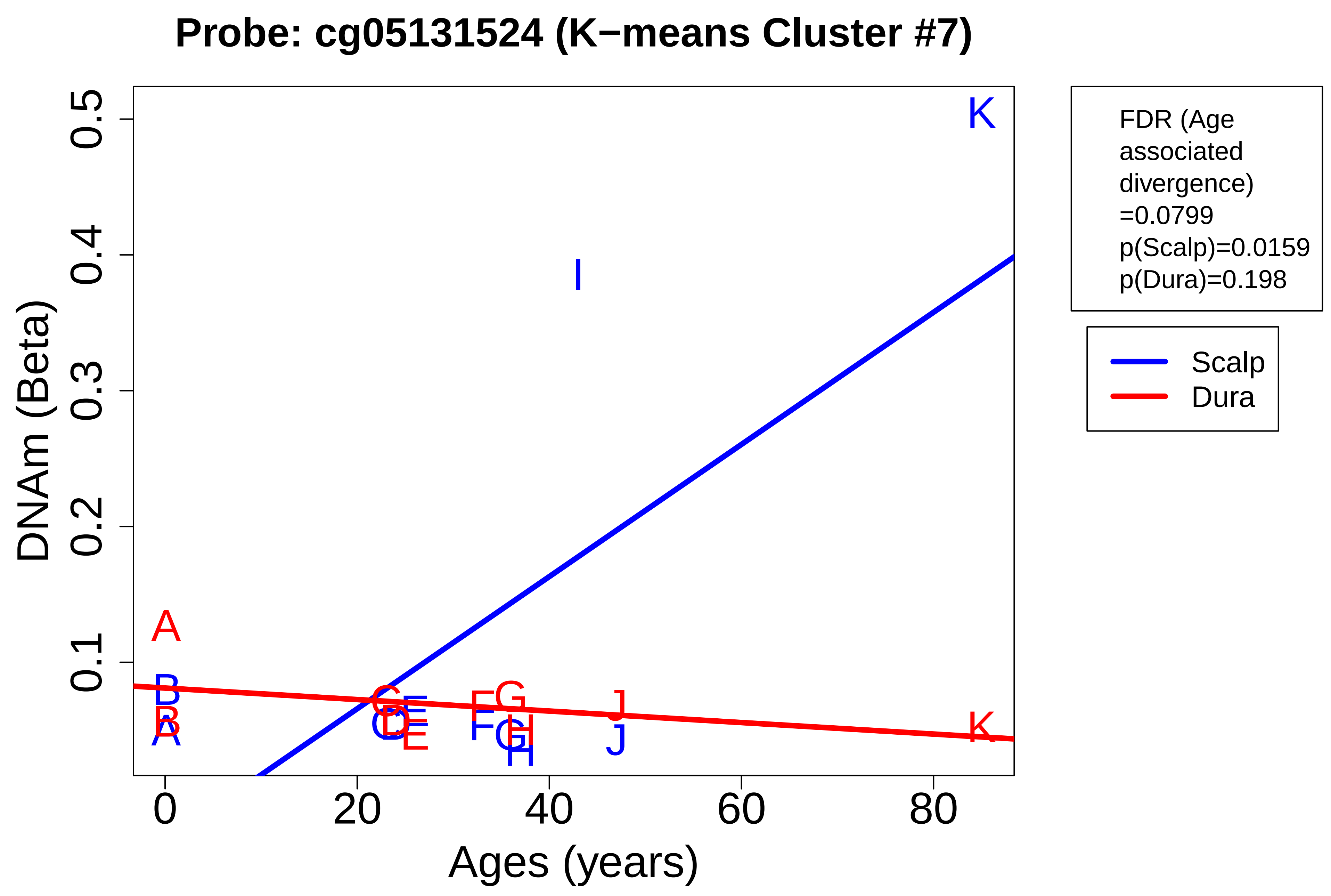

Probe: cg05131524 (K−means Cluster #7)
DNAm (Beta)
Ages (years)
0.1
0.2
0.3
0.4
0.5
0
20
40
60
80
FDR (Age associated divergence) =0.0799
p(Scalp)=0.0159
p(Dura)=0.198
Scalp
Dura

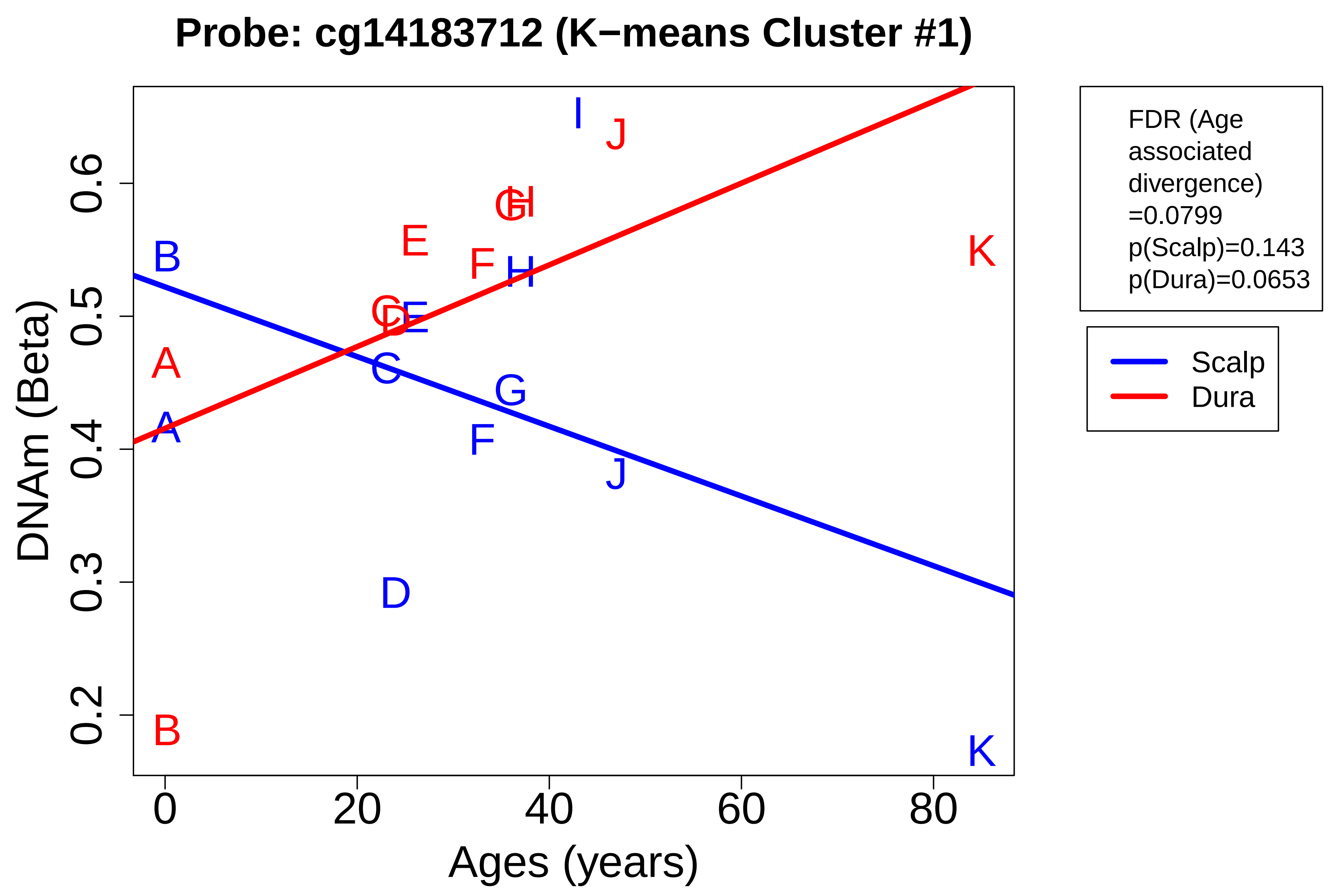

Probe: cg14183712 (K−means Cluster #1)
DNAm (Beta)
0.2
0.3
0.4
0.5
0.6
Ages (years)
0
20
40
60
80
FDR (Age associated divergence) =0.0799
p(Scalp)=0.143
p(Dura)=0.0653
Scalp
Dura

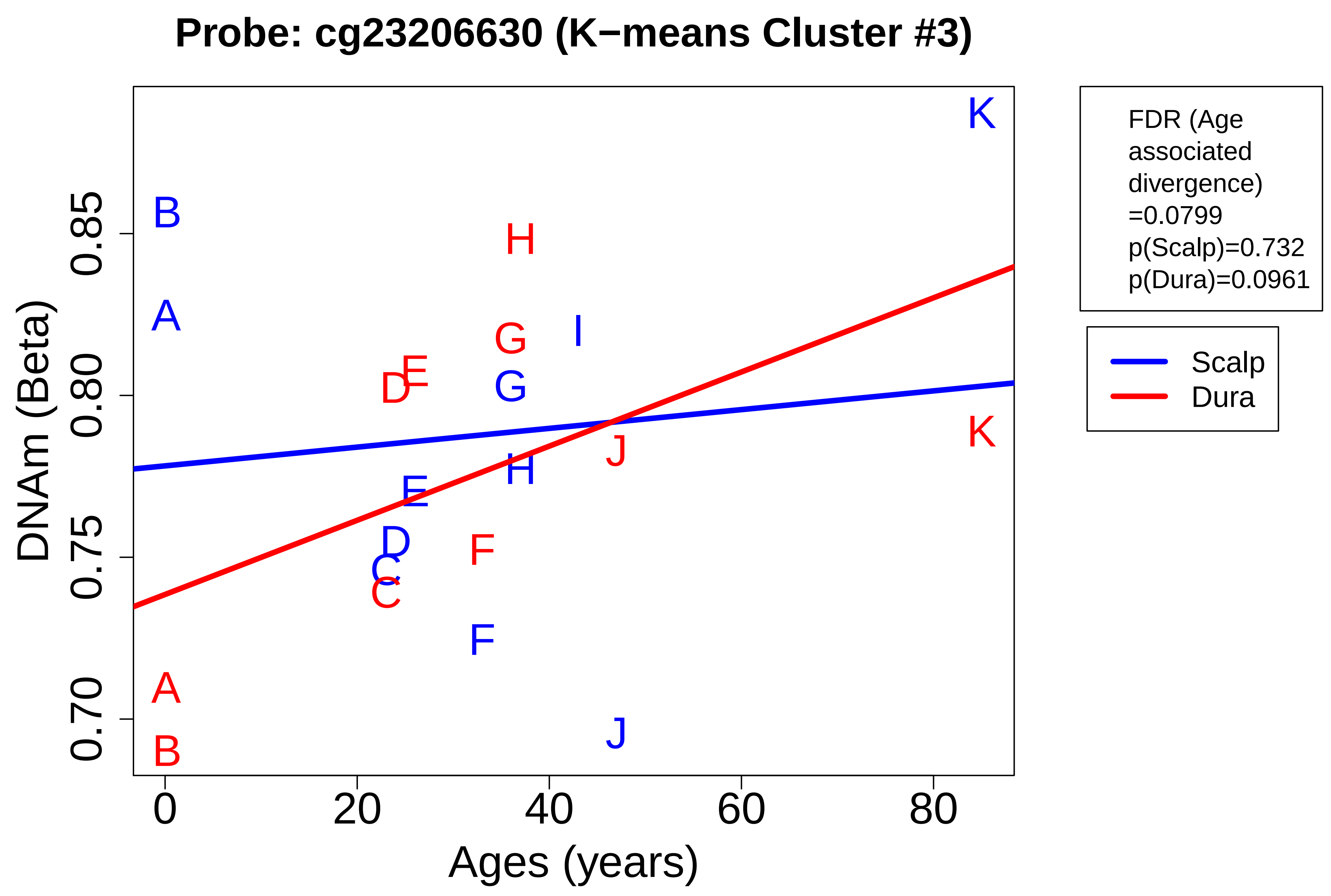
Probe: cg23206630 (K−means Cluster #3)
DNAm (Beta)
0.70
0.75
0.80
0.85
Ages (years)
0
20
40
60
80
FDR (Age associated divergence) =0.0799
p(Scalp)=0.732
p(Dura)=0.0961
Scalp
Dura
A B C D E F G H I J K

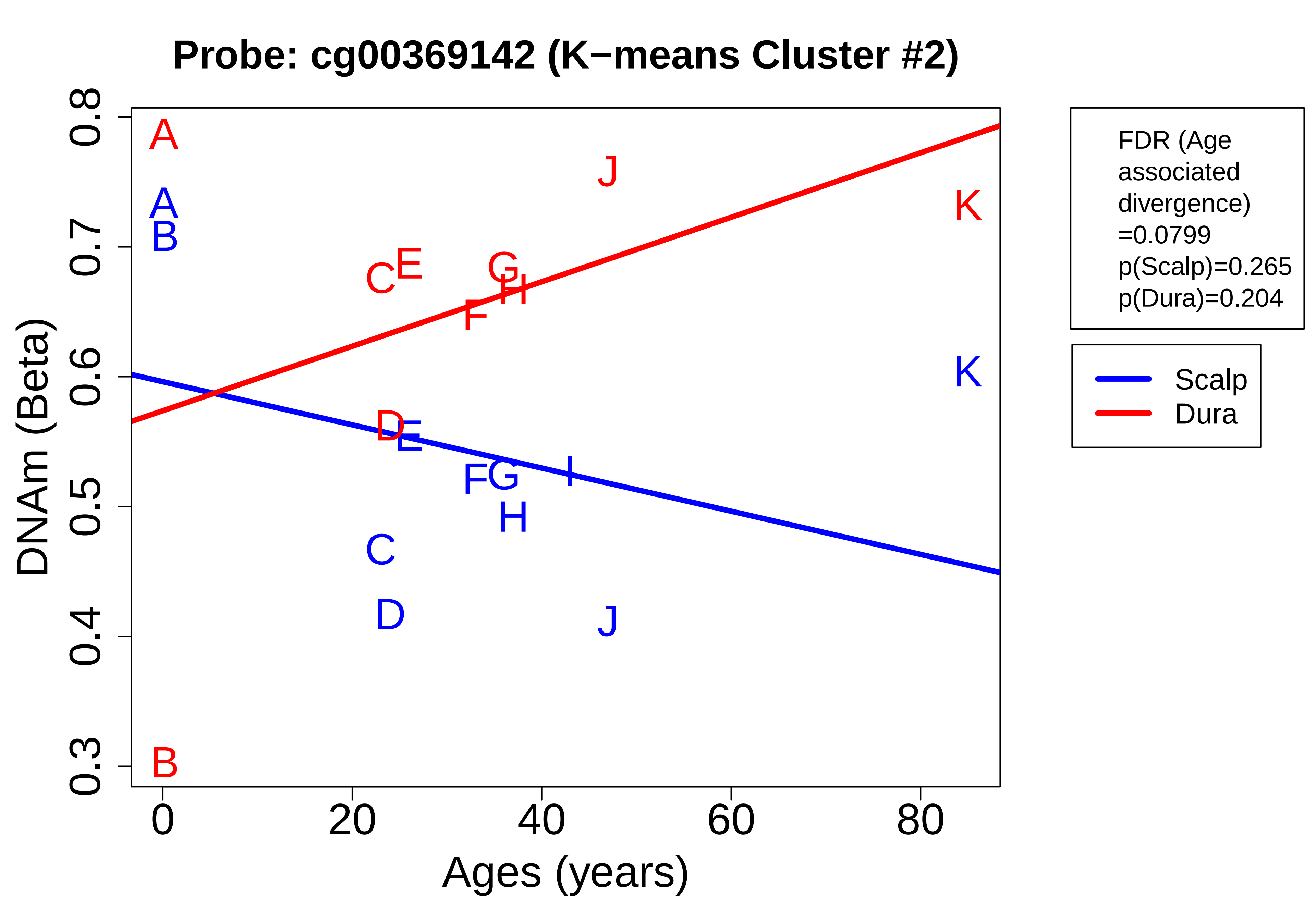

Probe: cg00369142 (K−means Cluster #2)
DNAm (Beta)
Ages (years)
0.3
0.4
0.5
0.6
0.7
0.8
0
20
40
60
80
FDR (Age associated divergence) =0.0799
p(Scalp)=0.265
p(Dura)=0.204
Scalp
Dura

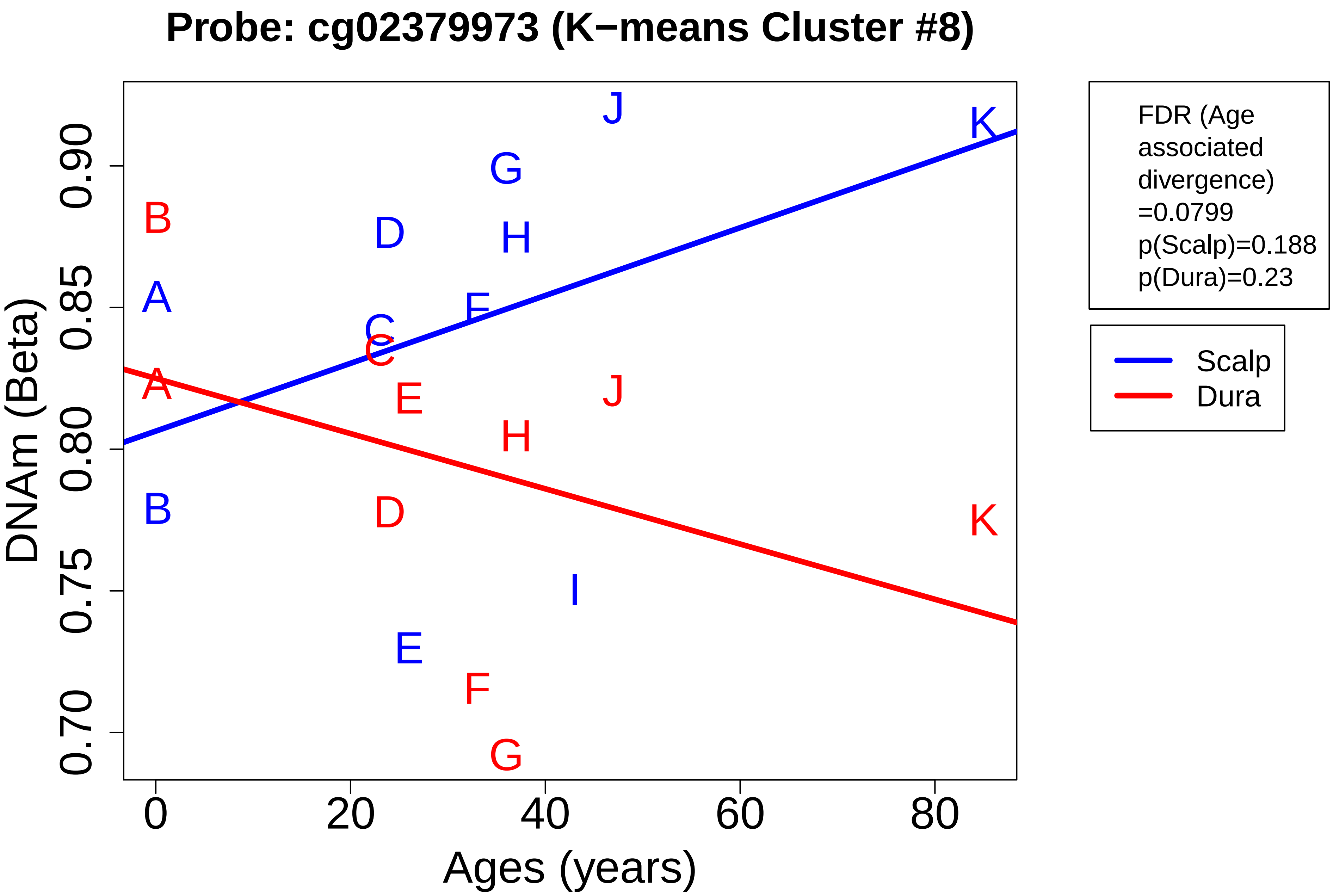

Probe: cg02379973 (K−means Cluster #8)
DNAm (Beta)
0.70
0.75
0.80
0.85
0.90
0
20
40
60
80
Ages (years)
FDR (Age associated divergence) =0.0799
p(Scalp)=0.188
p(Dura)=0.23
Scalp
Dura
A
B
C
D
E
F
G
H
I
J
K

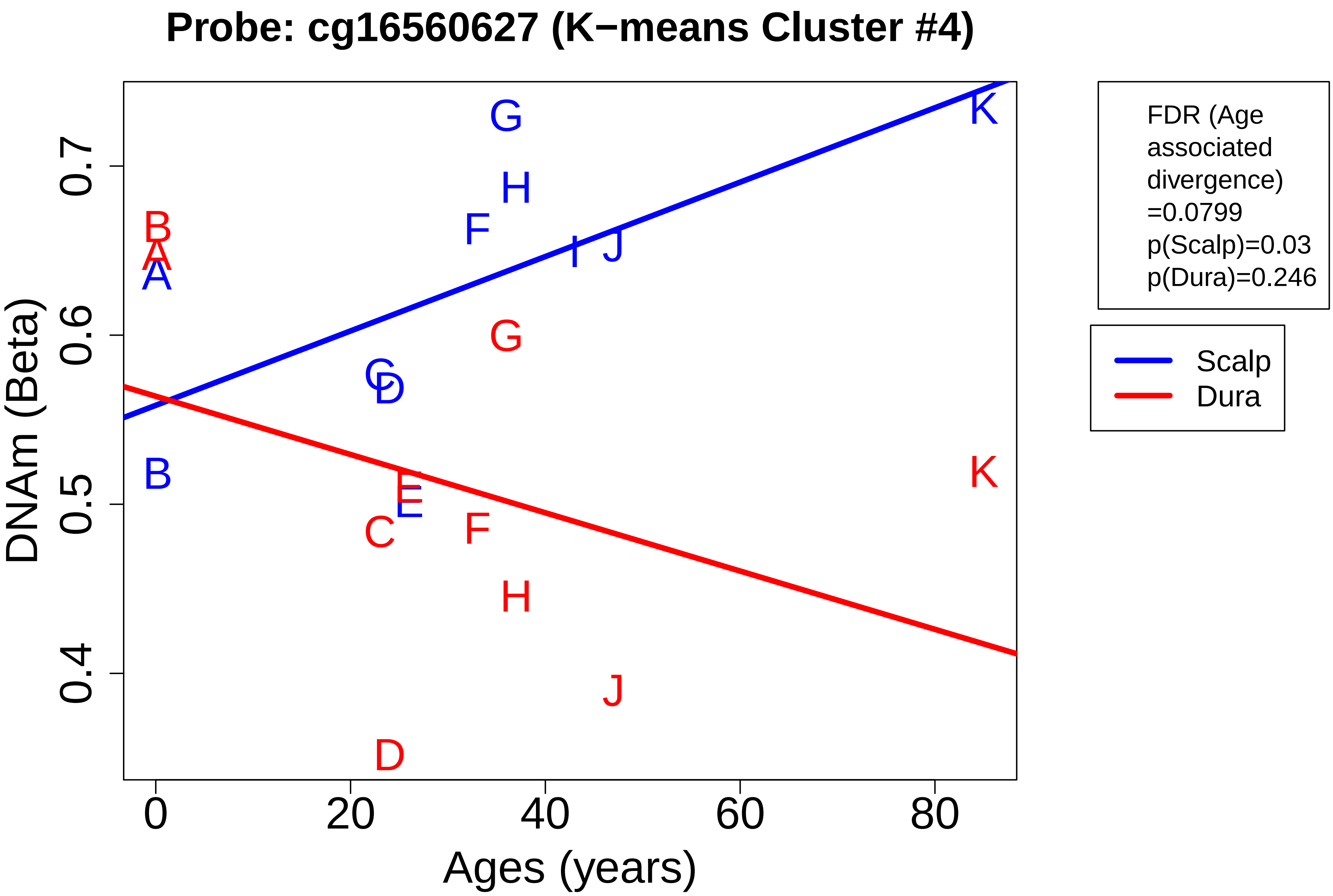
Probe: cg16560627 (K−means Cluster #4)
DNAm (Beta)
0.4
0.5
0.6
0.7
Ages (years)
0
20
40
60
80
A
B
C
D
E
F
G
H
I
J
K
FDR (Age associated divergence) =0.0799
p(Scalp)=0.03
p(Dura)=0.246
Scalp
Dura

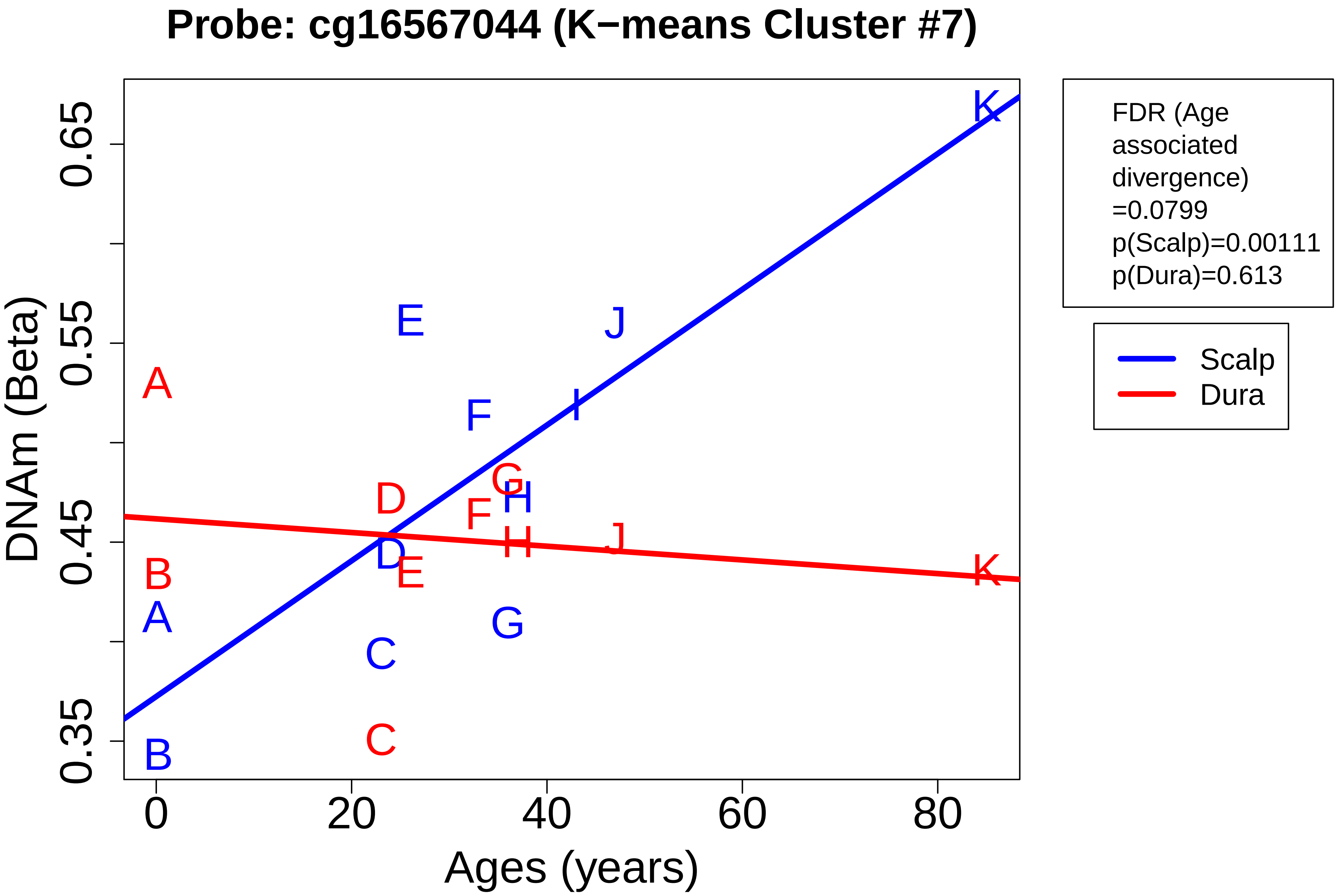

Probe: cg16567044 (K−means Cluster #7)
DNAm (Beta)
0.35
0.45
0.55
0.65
0
20
40
60
80
Ages (years)
FDR (Age associated divergence) =0.0799
p(Scalp)=0.00111
p(Dura)=0.613
Scalp
Dura

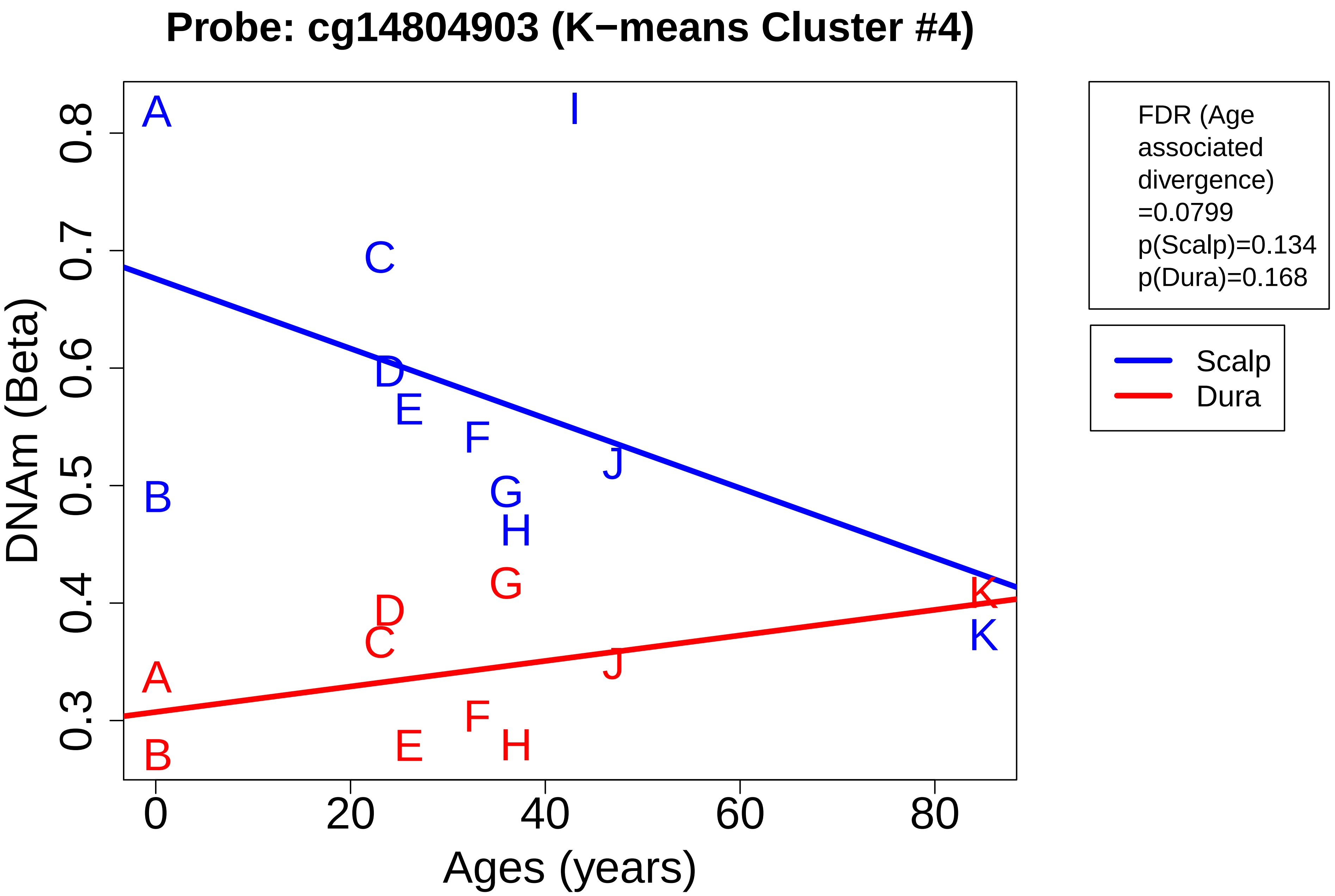

Probe: cg14804903 (K−means Cluster #4)
DNAm (Beta)
0.3
0.4
0.5
0.6
0.7
0.8
0
20
40
60
80
Ages (years)
FDR (Age associated divergence) =0.0799
p(Scalp)=0.134
p(Dura)=0.168
Scalp
Dura
A
B
C
D
E
F
G
H
I
J
K

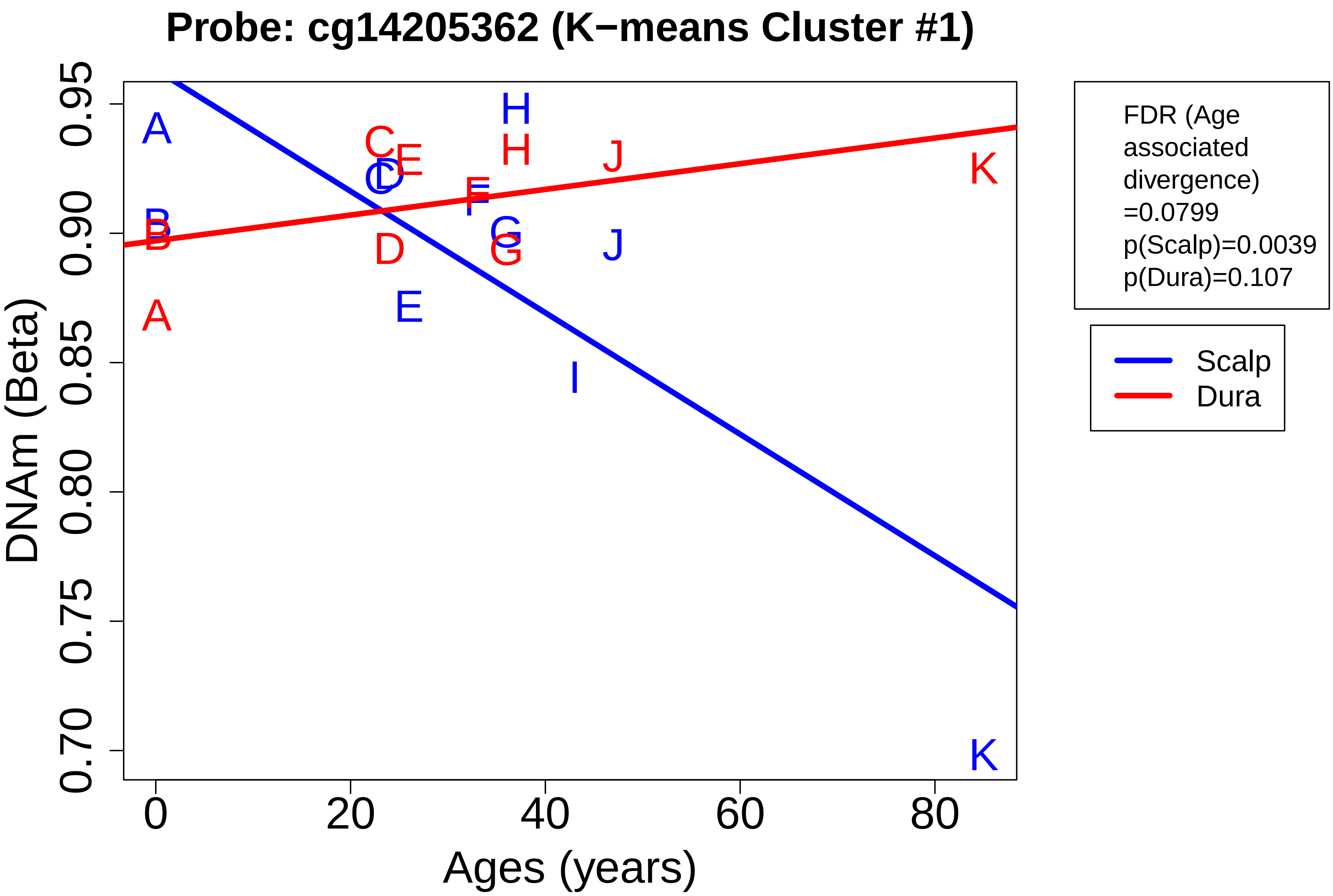

Probe: cg14205362 (K−means Cluster #1)
DNAm (Beta)
0.70
0.75
0.80
0.85
0.90
0.95
Ages (years)
0
20
40
60
80
A
B
C
D
E
F
G
H
I
J
K
FDR (Age
associated
divergence)
=0.0799
p(Scalp)=0.0039
p(Dura)=0.107
Scalp
Dura

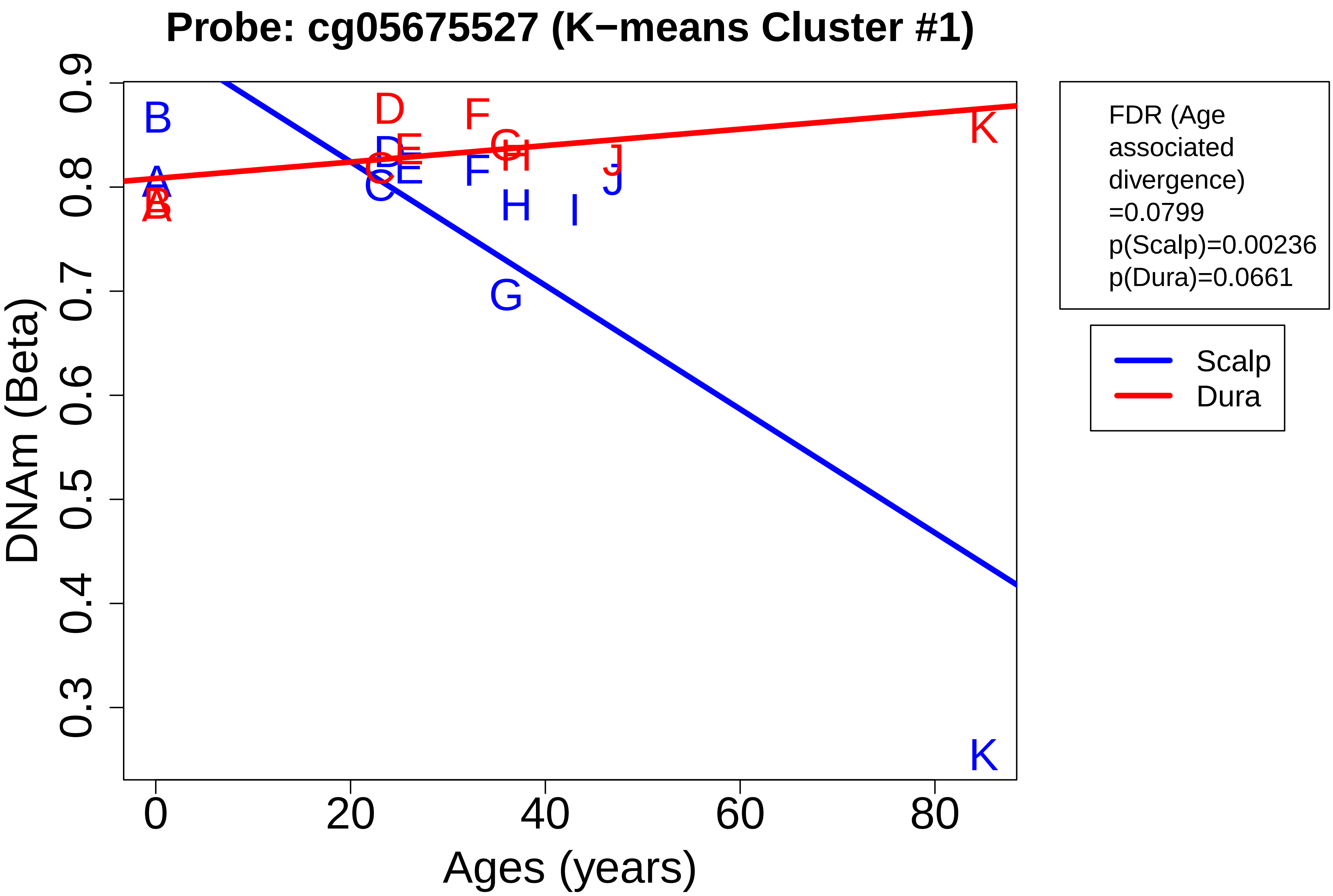
Probe: cg05675527 (K−means Cluster #1)
DNAm (Beta)
Ages (years)
0.3
0.4
0.5
0.6
0.7
0.8
0.9
0
20
40
60
80
FDR (Age associated divergence) =0.0799
p(Scalp)=0.00236
p(Dura)=0.0661
Scalp
Dura

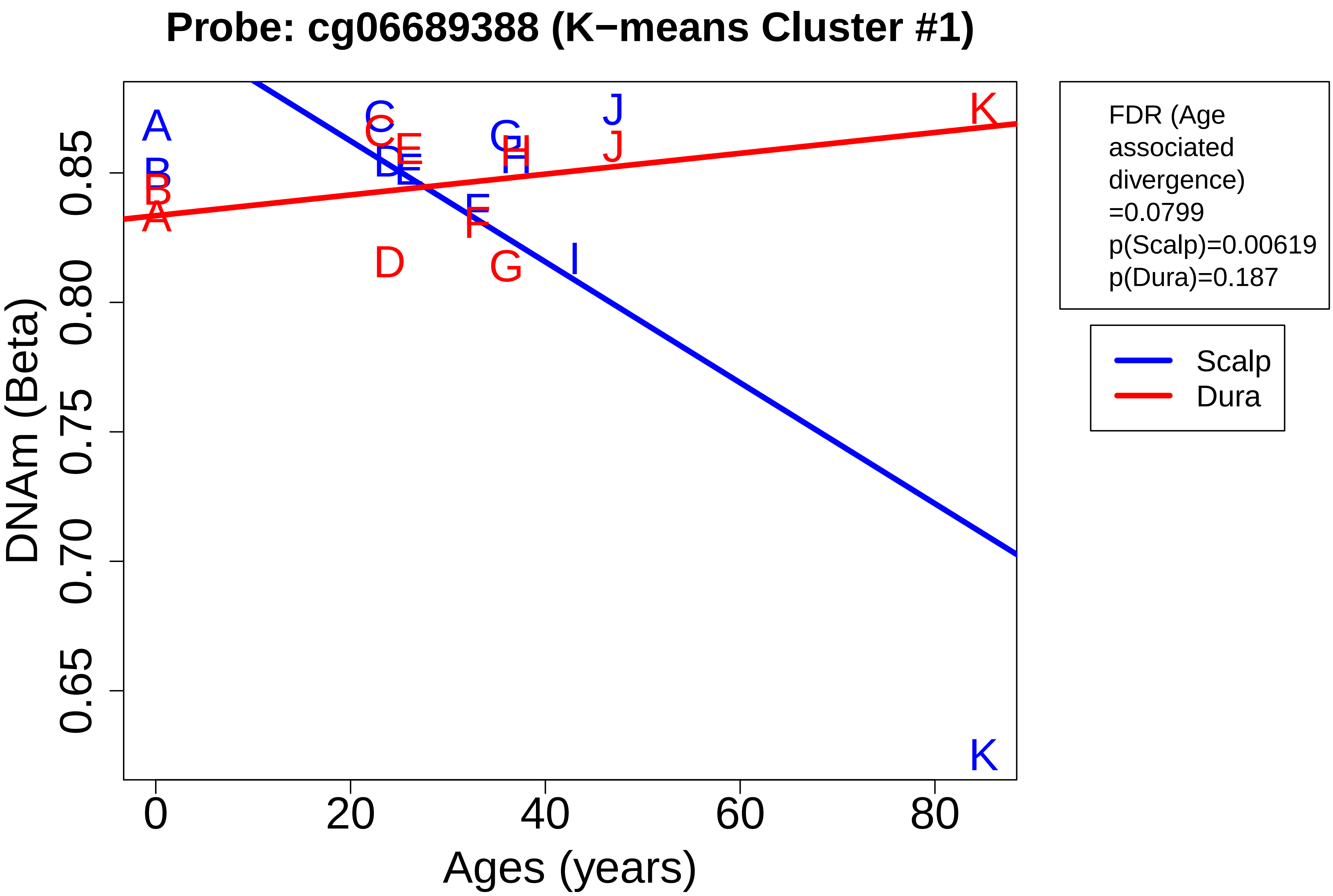

Probe: cg06689388 (K−means Cluster #1)
DNAm (Beta)
0.65
0.70
0.75
0.80
0.85
0
20
40
60
80
Ages (years)
A
B
C
D
E
F
G
H
I
J
K
FDR (Age associated divergence) =0.0799
p(Scalp)=0.00619
p(Dura)=0.187
Scalp
Dura

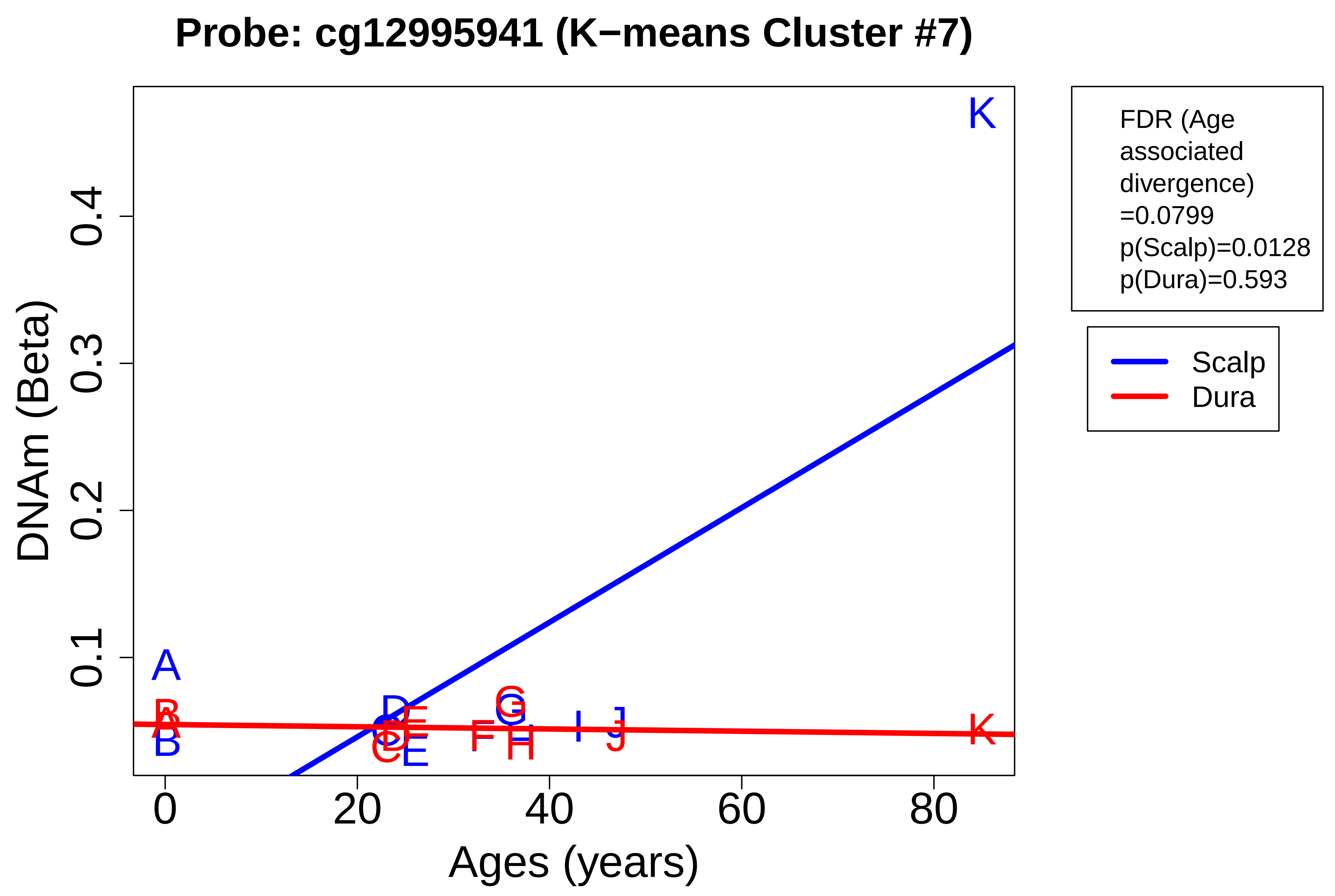

Probe: cg12995941 (K−means Cluster #7)
DNAm (Beta)
0.1
0.2
0.3
0.4
0
20
40
60
80
Ages (years)
FDR (Age associated divergence) =0.0799
p(Scalp)=0.0128
p(Dura)=0.593
Scalp
Dura
A
B
C
D
E
F
G
H
I
J
K

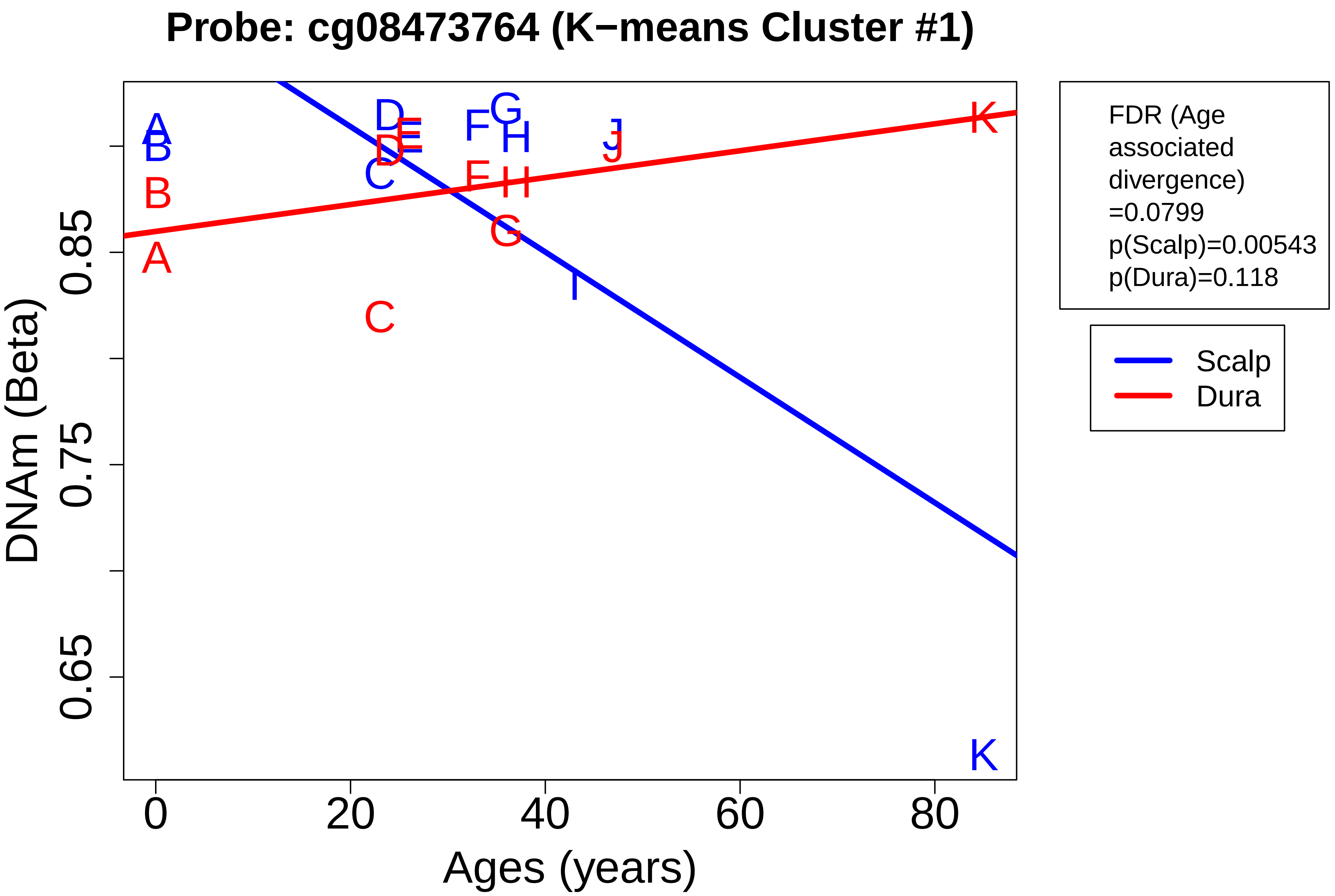
Probe: cg08473764 (K−means Cluster #1)
DNAm (Beta)
0.65
0.75
0.85
Ages (years)
0
20
40
60
80
A
B
C
D
E
F
G
H
I
J
K
FDR (Age associated divergence) =0.0799
p(Scalp)=0.00543
p(Dura)=0.118
Scalp
Dura

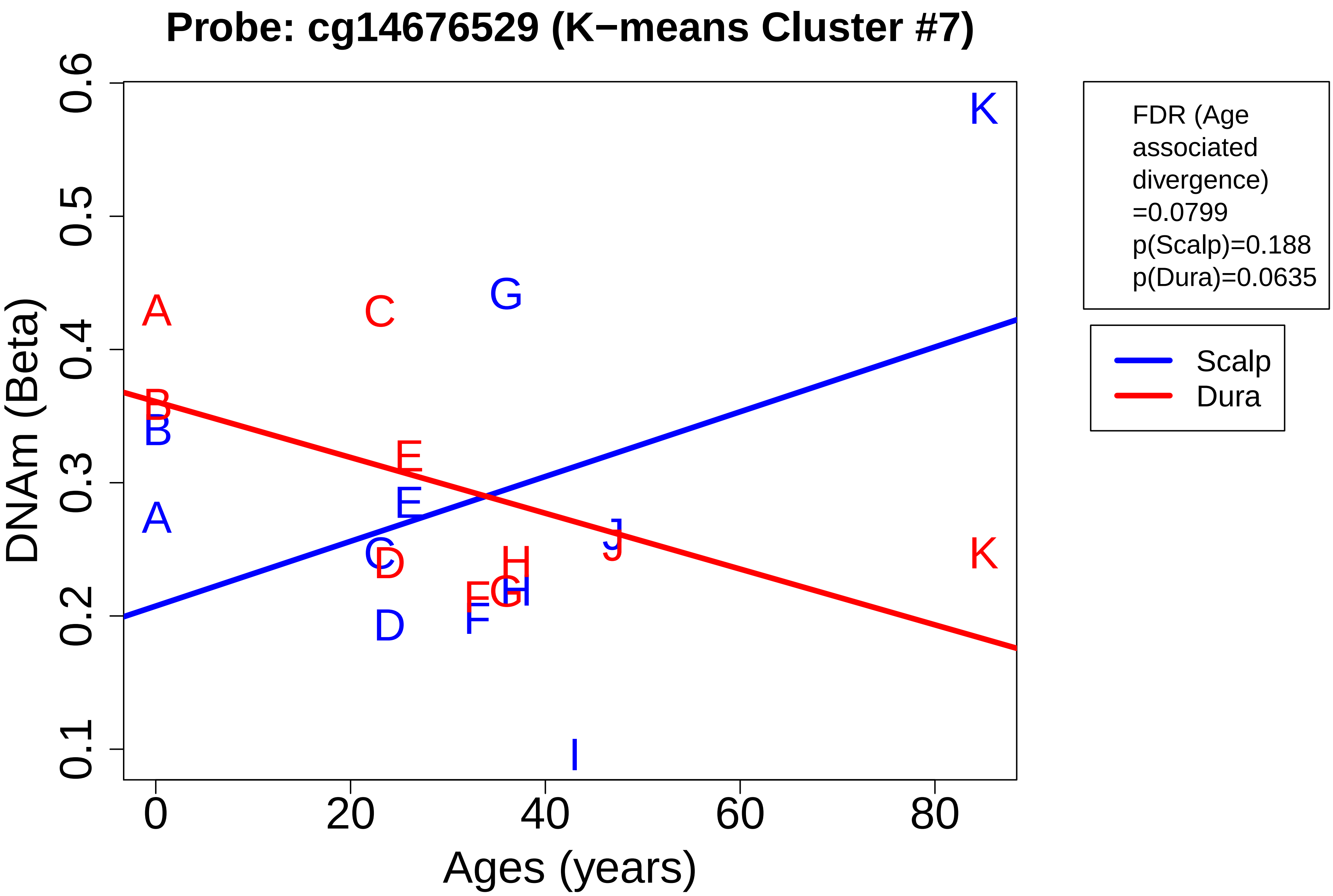

Probe: cg14676529 (K−means Cluster #7)
DNAm (Beta)
0.1
0.2
0.3
0.4
0.5
0.6
Ages (years)
0
20
40
60
80
FDR (Age associated divergence) =0.0799
p(Scalp)=0.188
p(Dura)=0.0635
Scalp
Dura

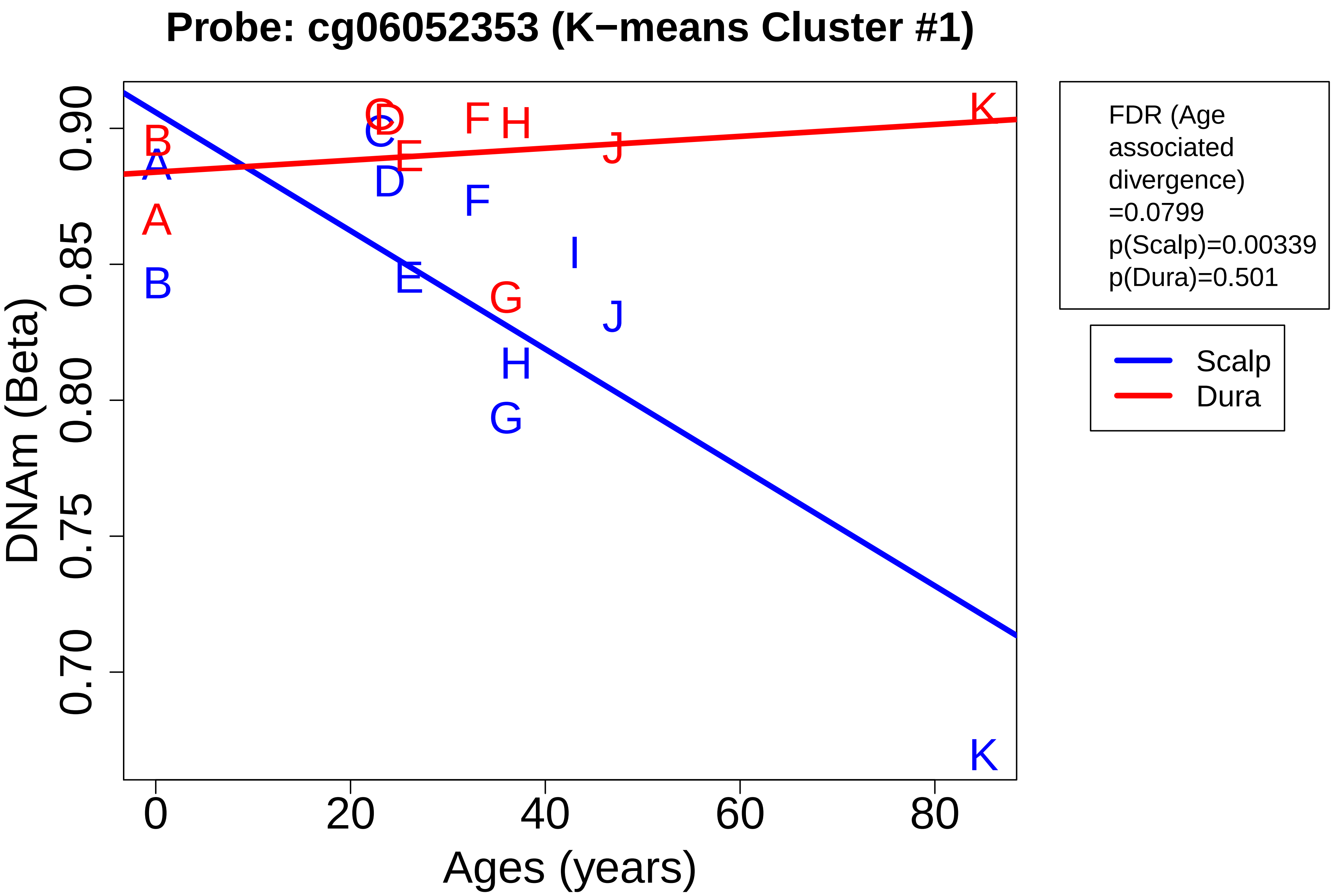

Probe: cg06052353 (K−means Cluster #1)
DNAm (Beta)
0.70
0.75
0.80
0.85
0.90
0
20
40
60
80
Ages (years)
FDR (Age associated divergence) =0.0799
p(Scalp)=0.00339
p(Dura)=0.501
Scalp
Dura

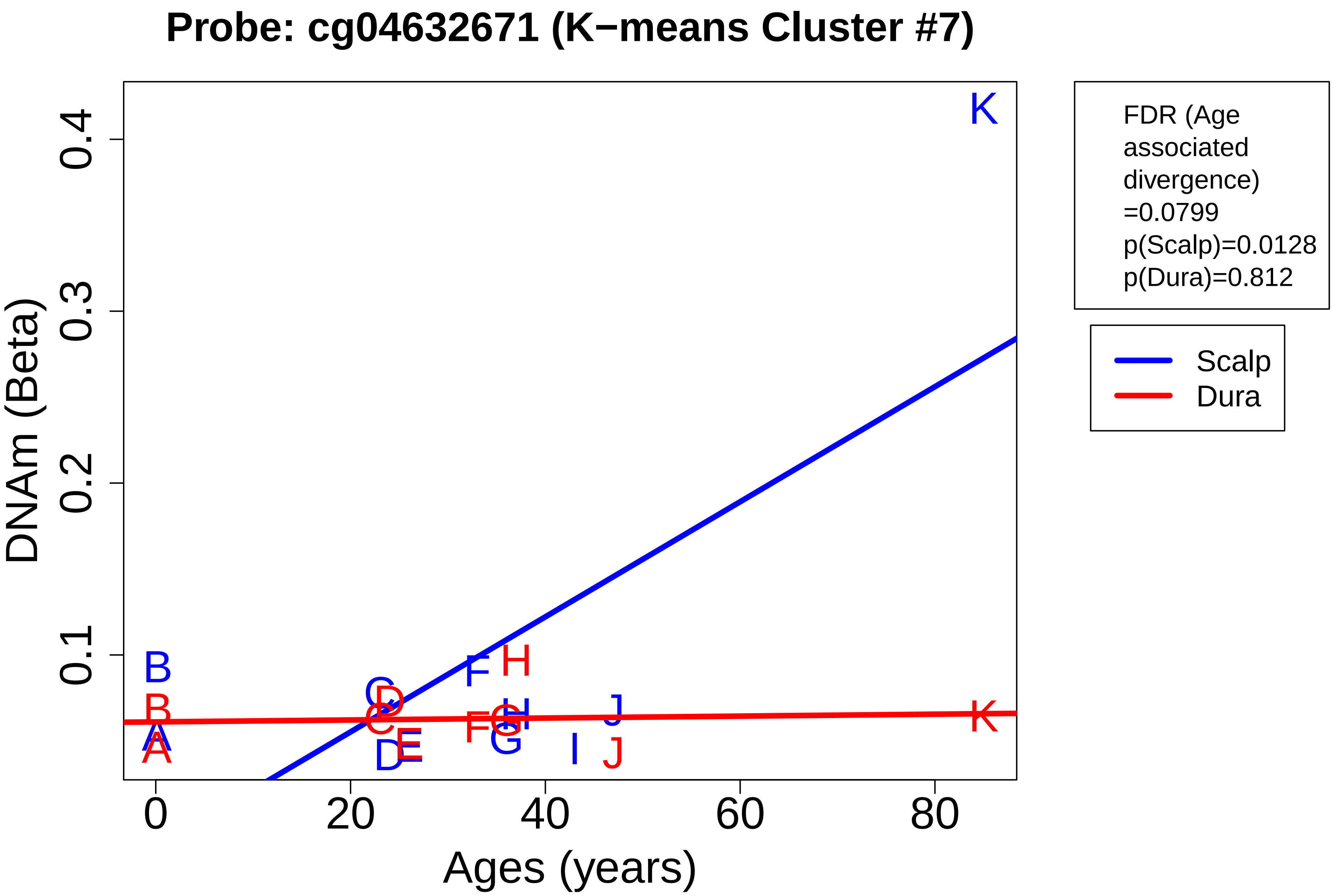

Probe: cg04632671 (K−means Cluster #7)
DNAm (Beta)
0.1
0.2
0.3
0.4
0
20
40
60
80
Ages (years)
FDR (Age associated divergence) =0.0799
p(Scalp)=0.0128
p(Dura)=0.812
Scalp
Dura

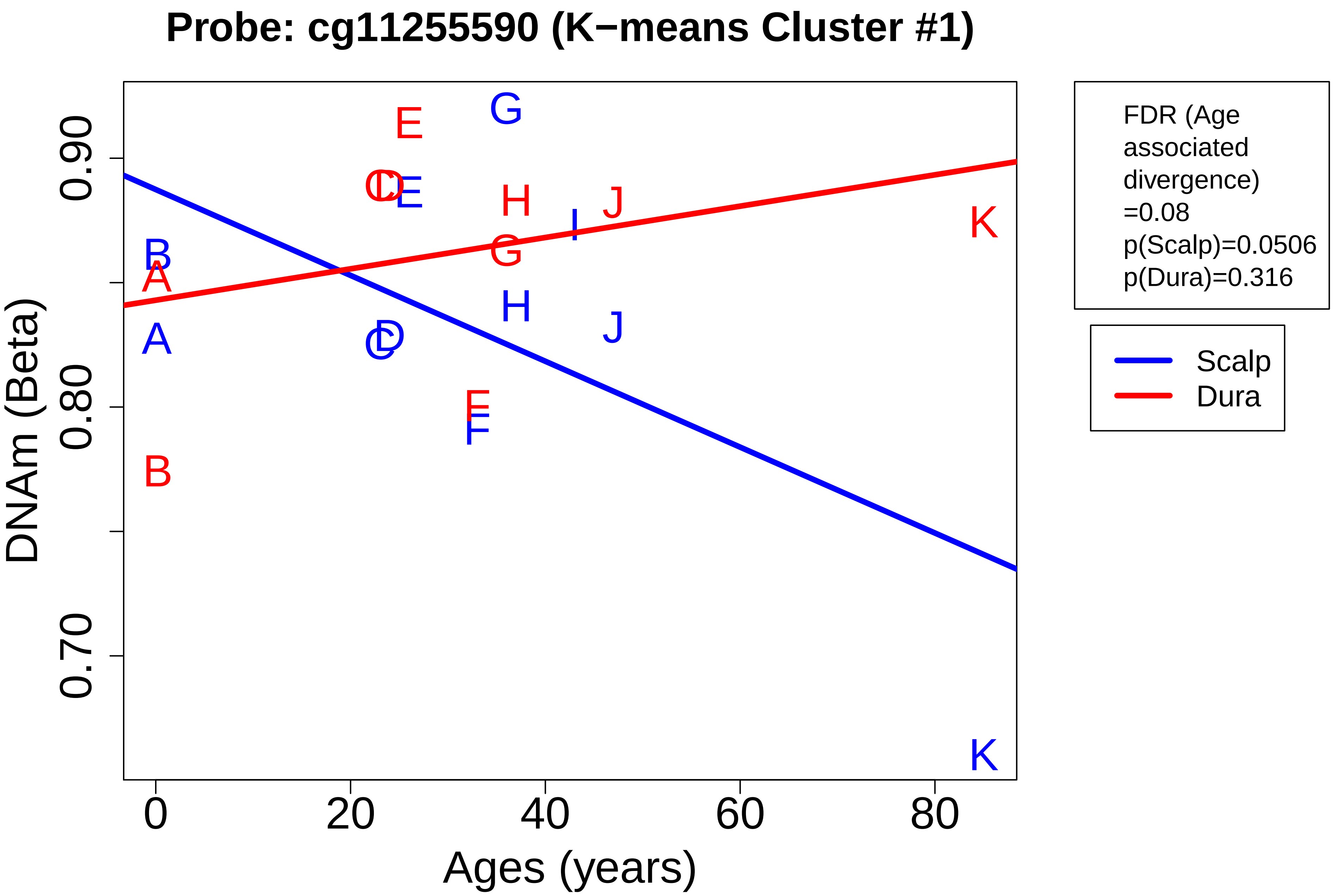

Probe: cg11255590 (K−means Cluster #1)
DNAm (Beta)
0.70
0.80
0.90
0
20
40
60
80
Ages (years)
FDR (Age associated divergence) =0.08
p(Scalp)=0.0506
p(Dura)=0.316
Scalp
Dura

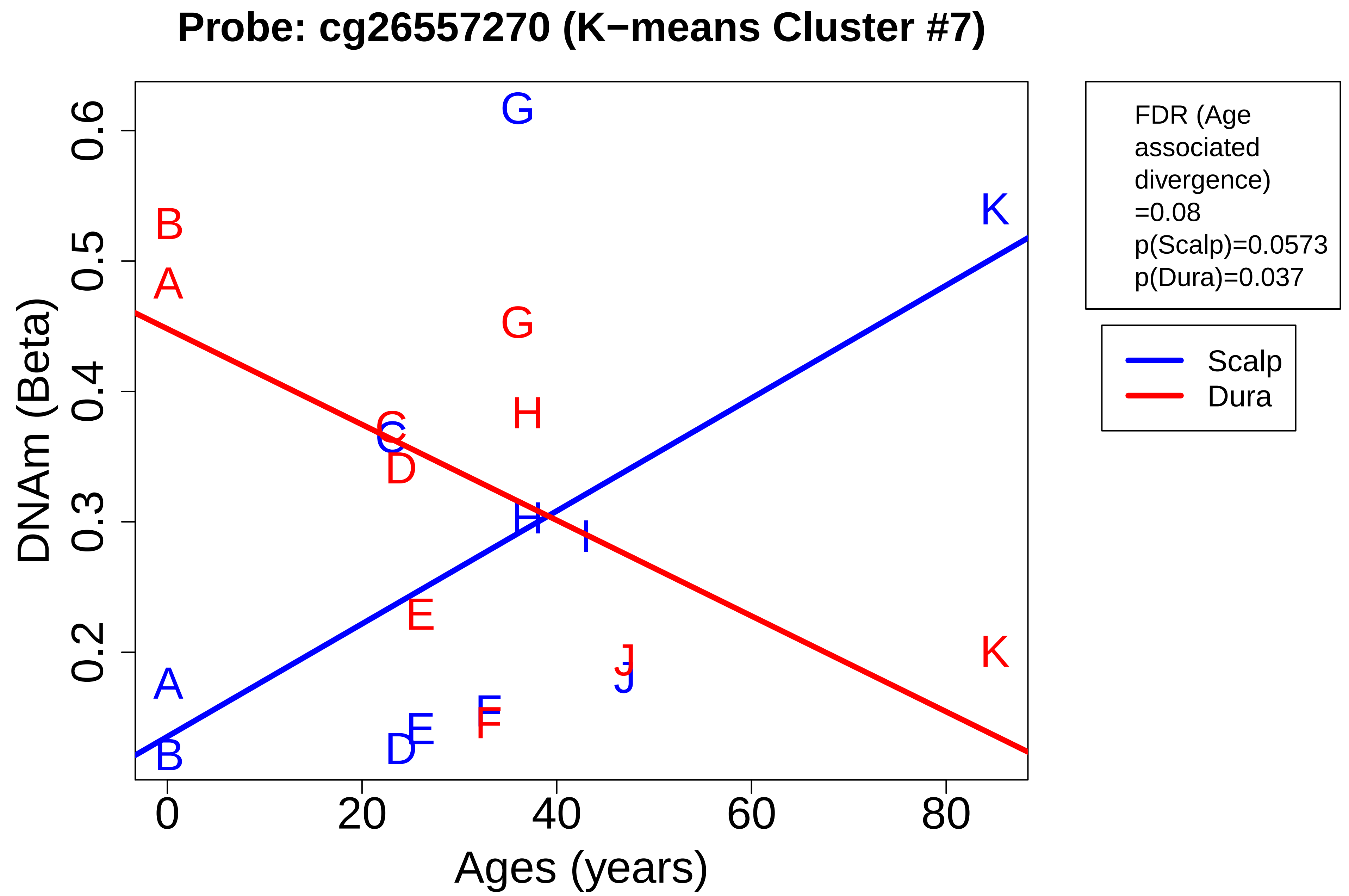

Probe: cg26557270 (K−means Cluster #7)
DNAm (Beta)
Ages (years)
0.2
0.3
0.4
0.5
0.6
0
20
40
60
80
FDR (Age associated divergence) =0.08
p(Scalp)=0.0573
p(Dura)=0.037
Scalp
Dura

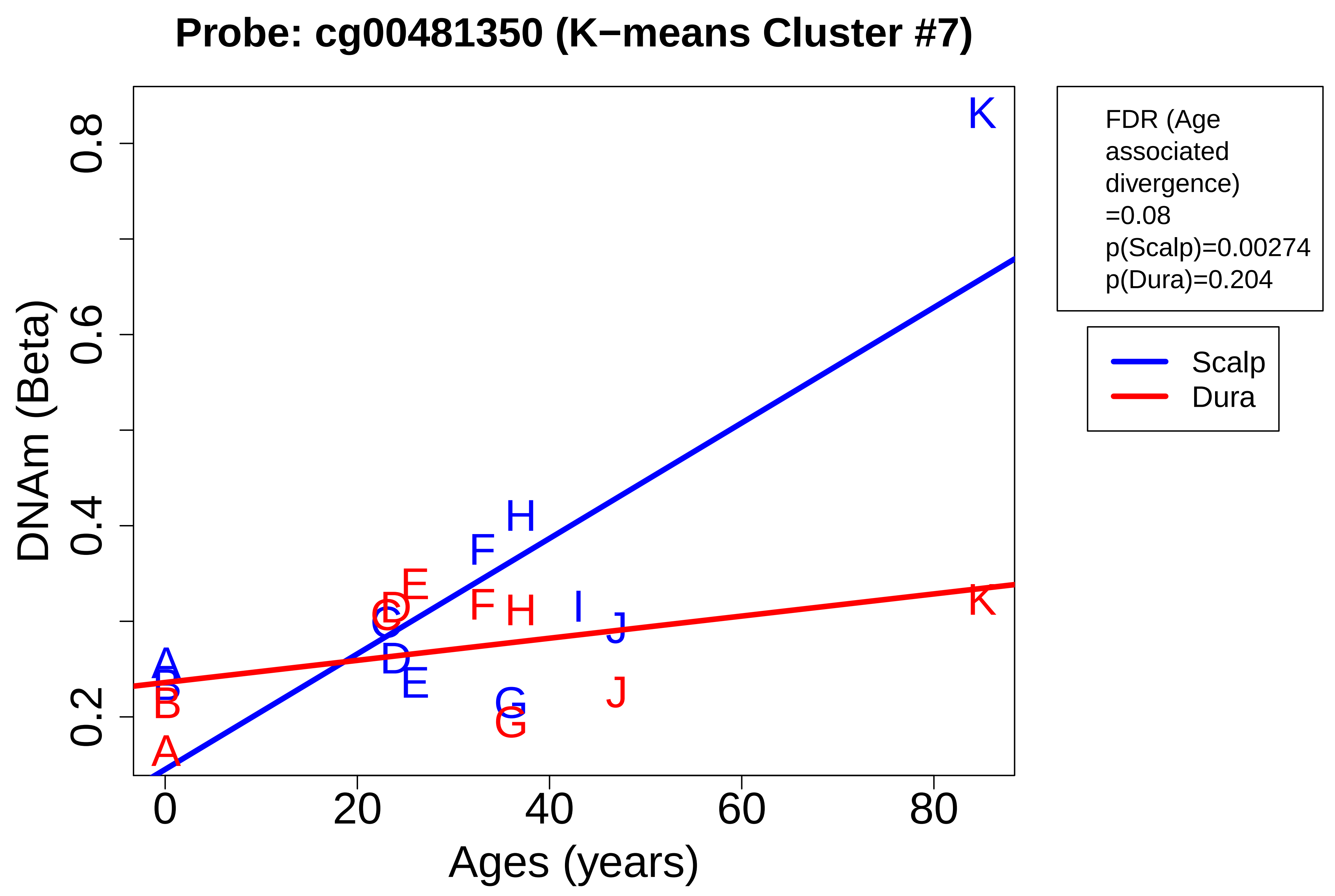

Probe: cg00481350 (K−means Cluster #7)
DNAm (Beta)
0.2
0.4
0.6
0.8
0
20
40
60
80
Ages (years)
FDR (Age associated divergence) =0.08
p(Scalp)=0.00274
p(Dura)=0.204
Scalp
Dura

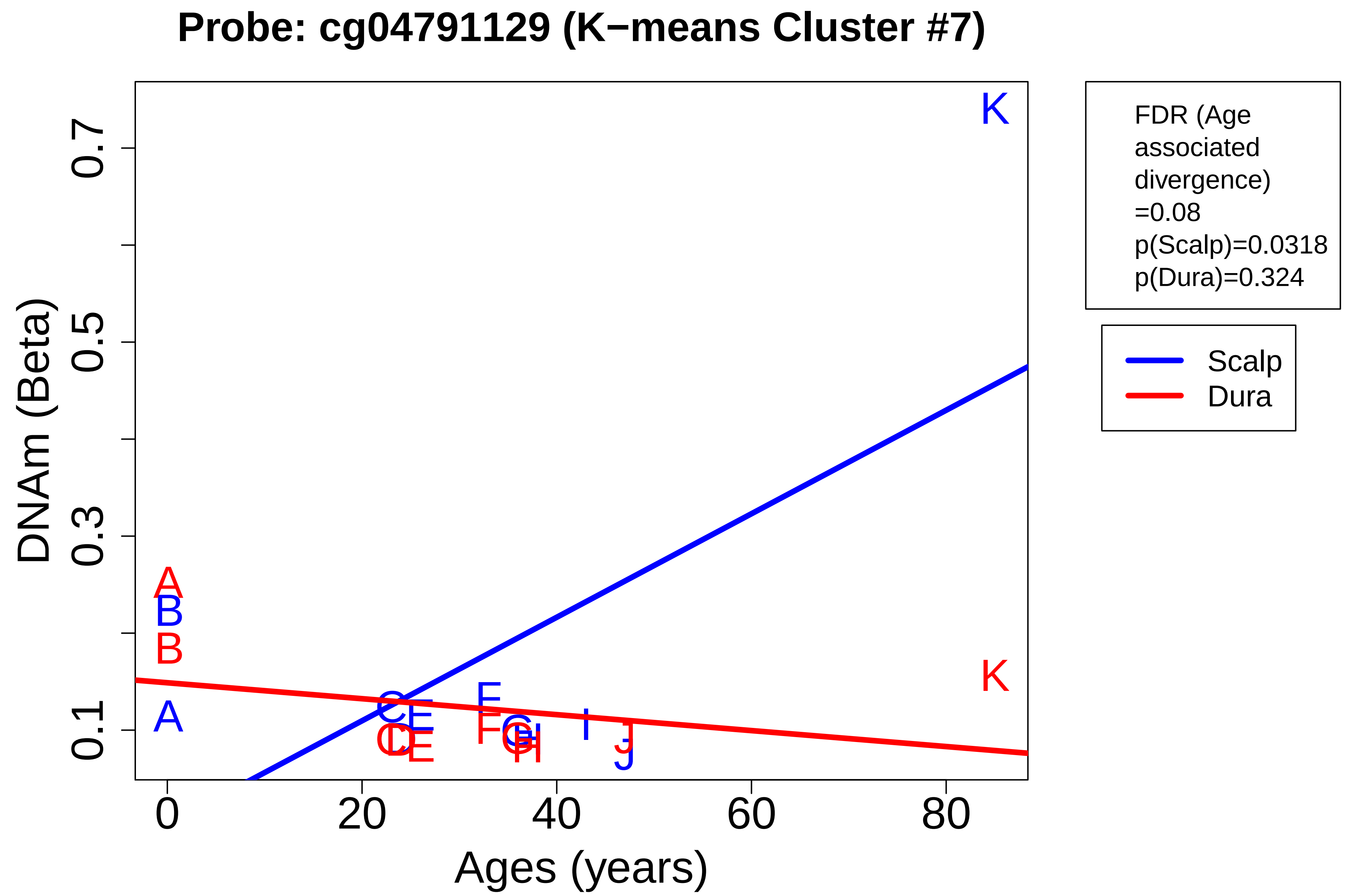

Probe: cg04791129 (K−means Cluster #7)
FDR (Age associated divergence) =0.08
p(Scalp)=0.0318
p(Dura)=0.324
Scalp
Dura
DNAm (Beta)
0.1
0.3
0.5
0.7
Ages (years)
0
20
40
60
80
A
B
C
D
E
F
G
H
I
J
K

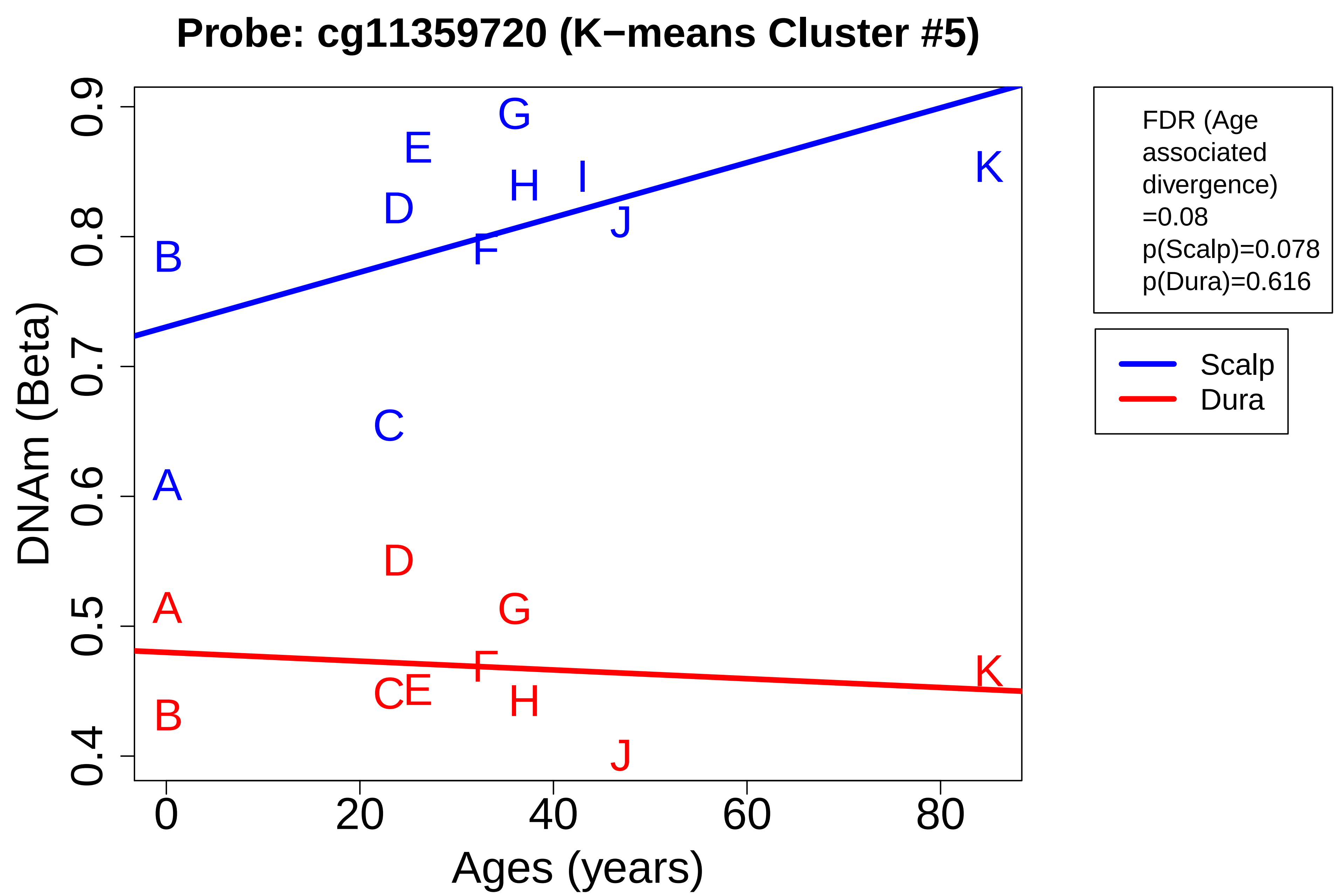

Probe: cg11359720 (K−means Cluster #5)
DNAm (Beta)
0.4
0.5
0.6
0.7
0.8
0.9
0
20
40
60
80
Ages (years)
A
B
C
D
E
F
G
H
I
J
K
A
B
CE
D
F
G
H
J
K
FDR (Age associated divergence) =0.08
p(Scalp)=0.078
p(Dura)=0.616
Scalp
Dura

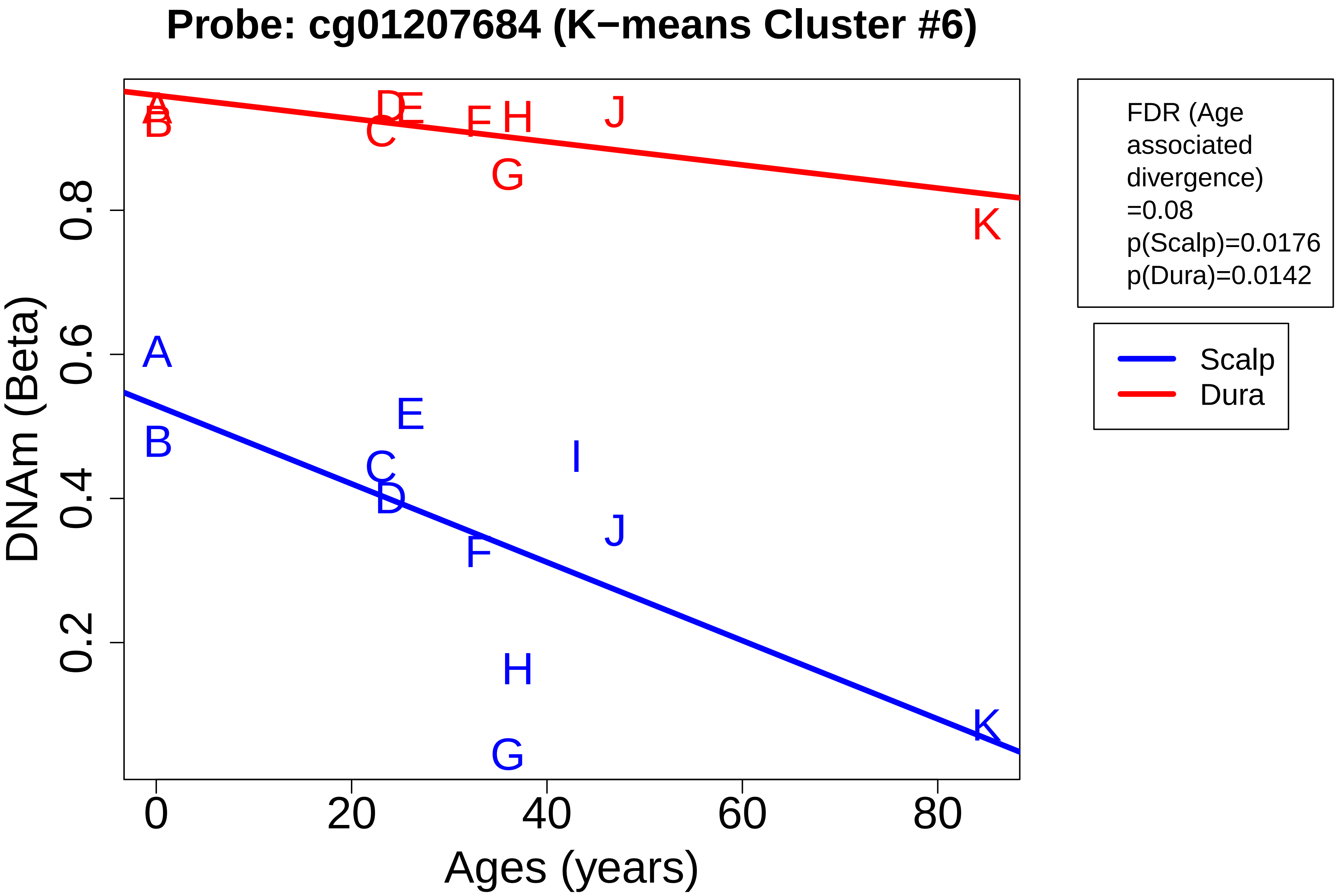
Probe: cg01207684 (K−means Cluster #6)
DNAm (Beta)
0.2
0.4
0.6
0.8
0
20
40
60
80
Ages (years)
FDR (Age associated divergence) =0.08
p(Scalp)=0.0176
p(Dura)=0.0142
Scalp
Dura

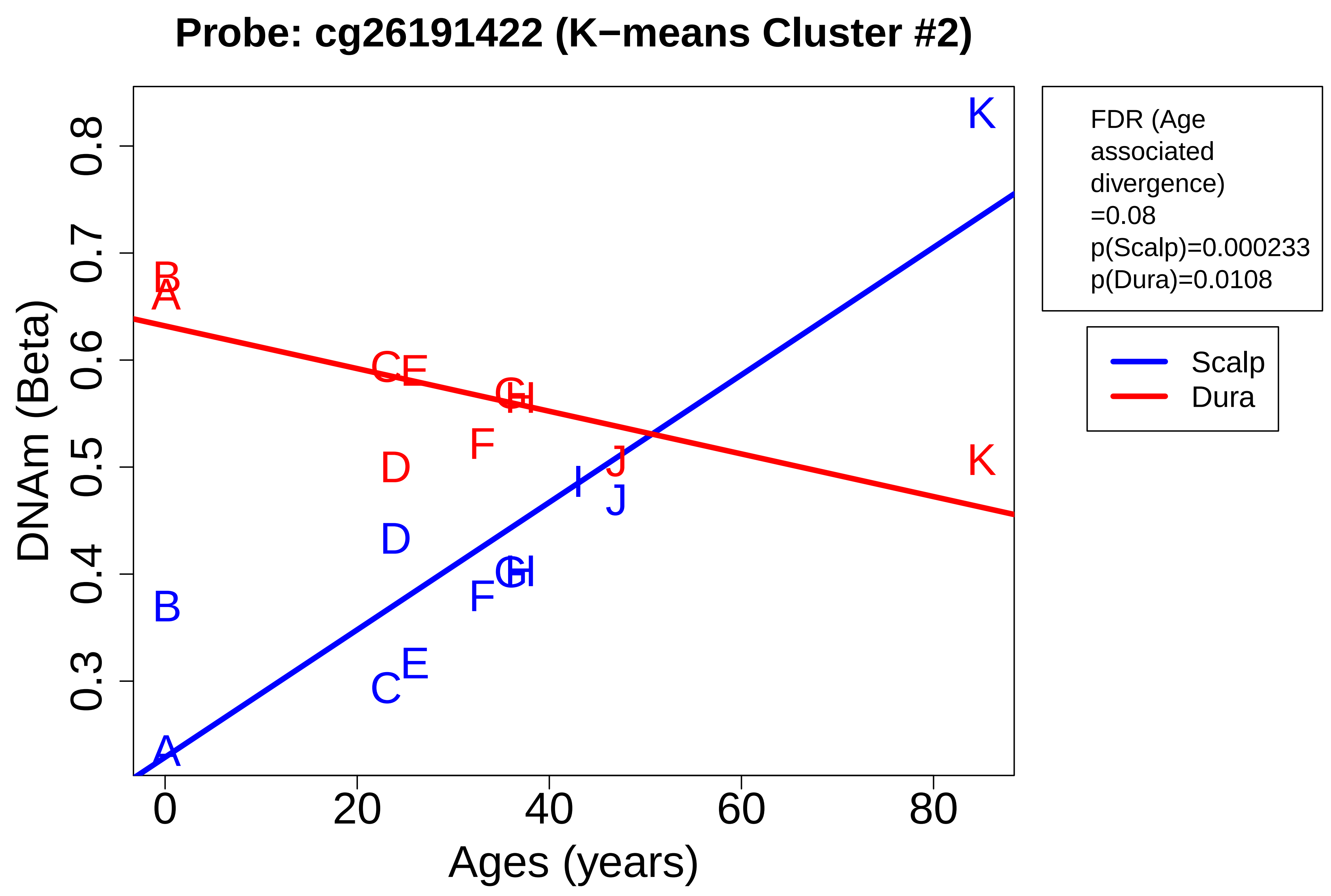

Probe: cg26191422 (K−means Cluster #2)
DNAm (Beta)
0.3
0.4
0.5
0.6
0.7
0.8
Ages (years)
0
20
40
60
80
FDR (Age associated divergence) =0.08
p(Scalp)=0.000233
p(Dura)=0.0108
Scalp
Dura

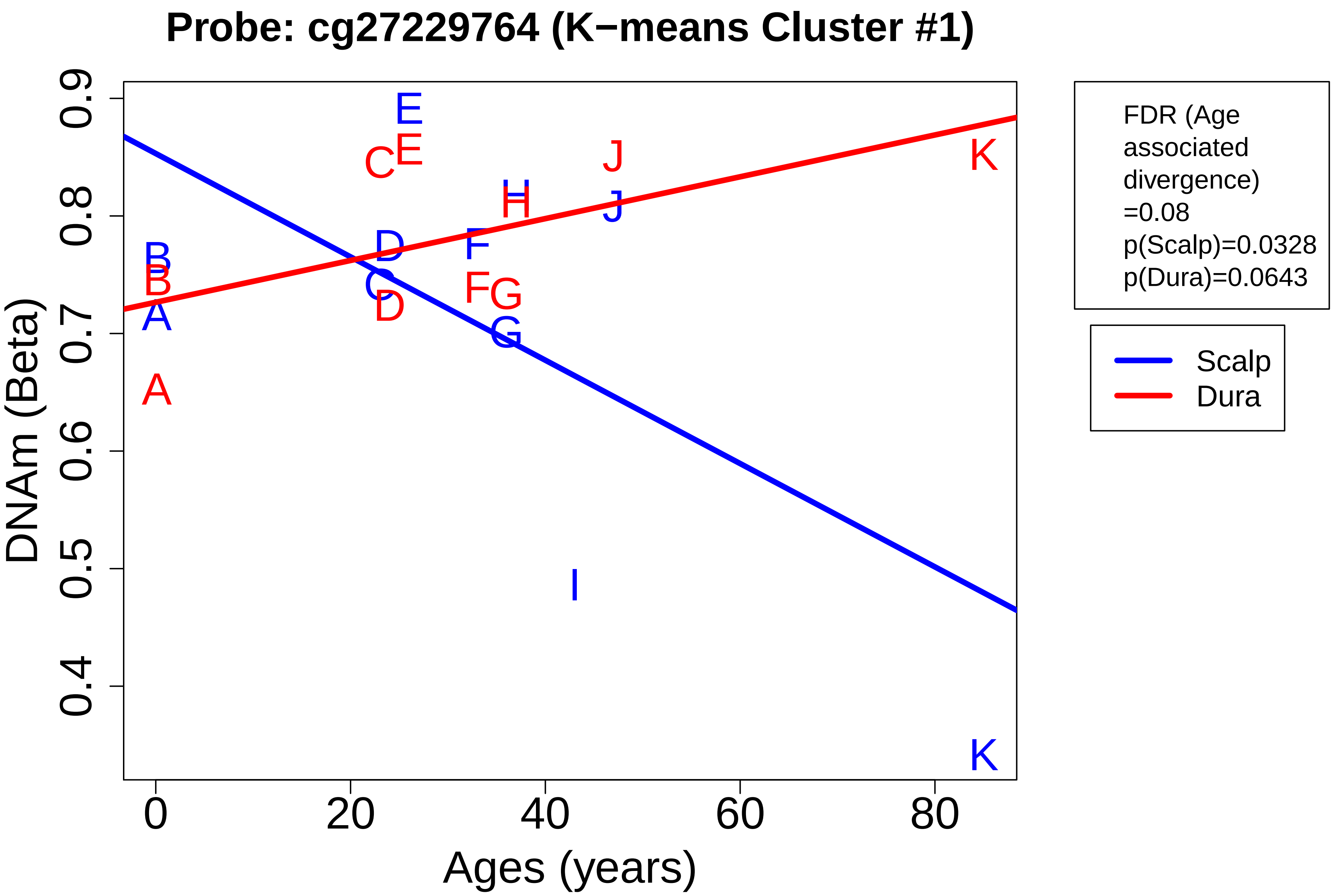

Probe: cg27229764 (K−means Cluster #1)
DNAm (Beta)
0.4
0.5
0.6
0.7
0.8
0.9
0
20
40
60
80
Ages (years)
FDR (Age associated divergence) =0.08
p(Scalp)=0.0328
p(Dura)=0.0643
Scalp
Dura

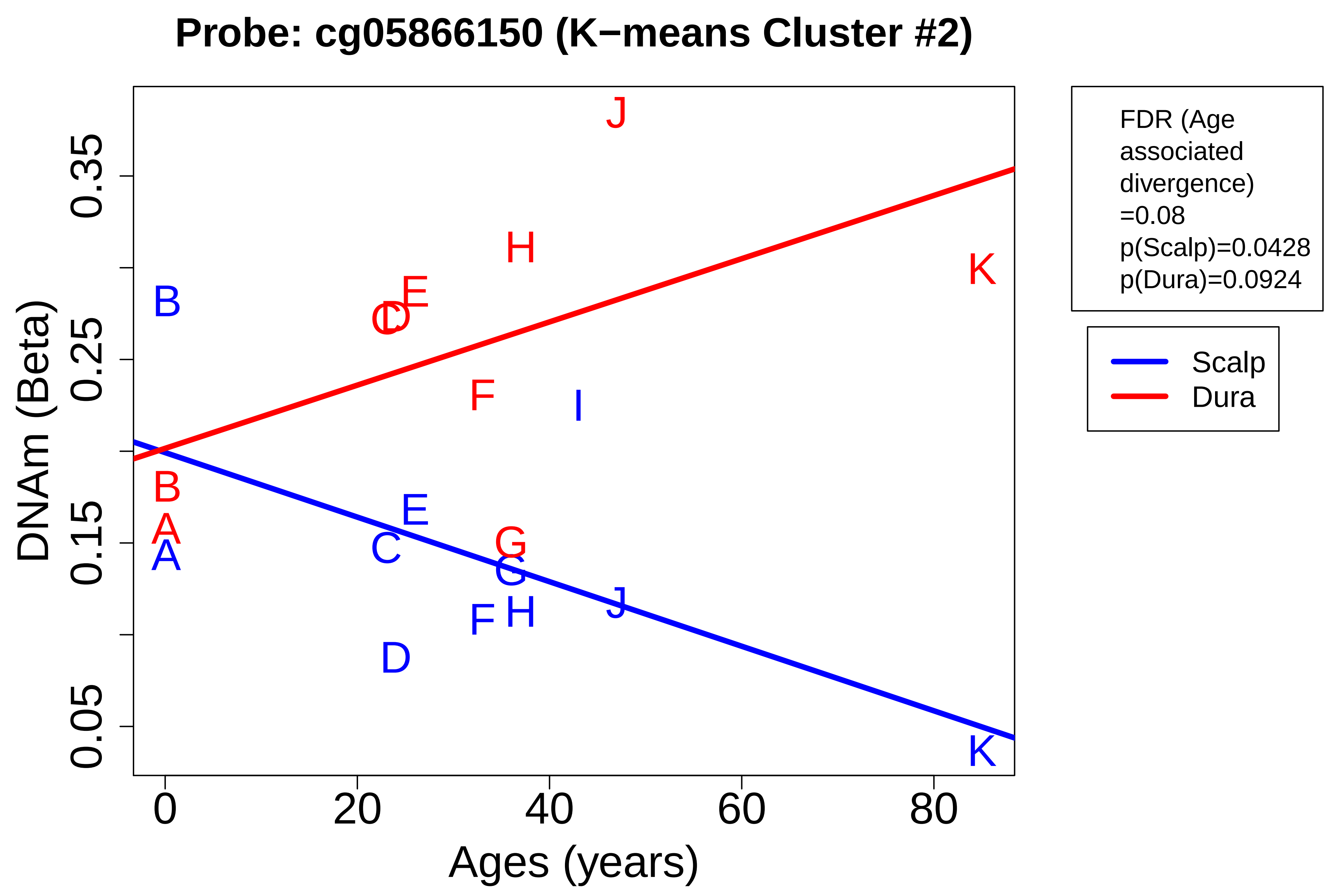
Probe: cg05866150 (K−means Cluster #2)
DNAm (Beta)
0.05
0.15
0.25
0.35
0
20
40
60
80
Ages (years)
FDR (Age associated divergence) =0.08
p(Scalp)=0.0428
p(Dura)=0.0924
Scalp
Dura
A
B
C
D
E
F
G
H
I
J
K

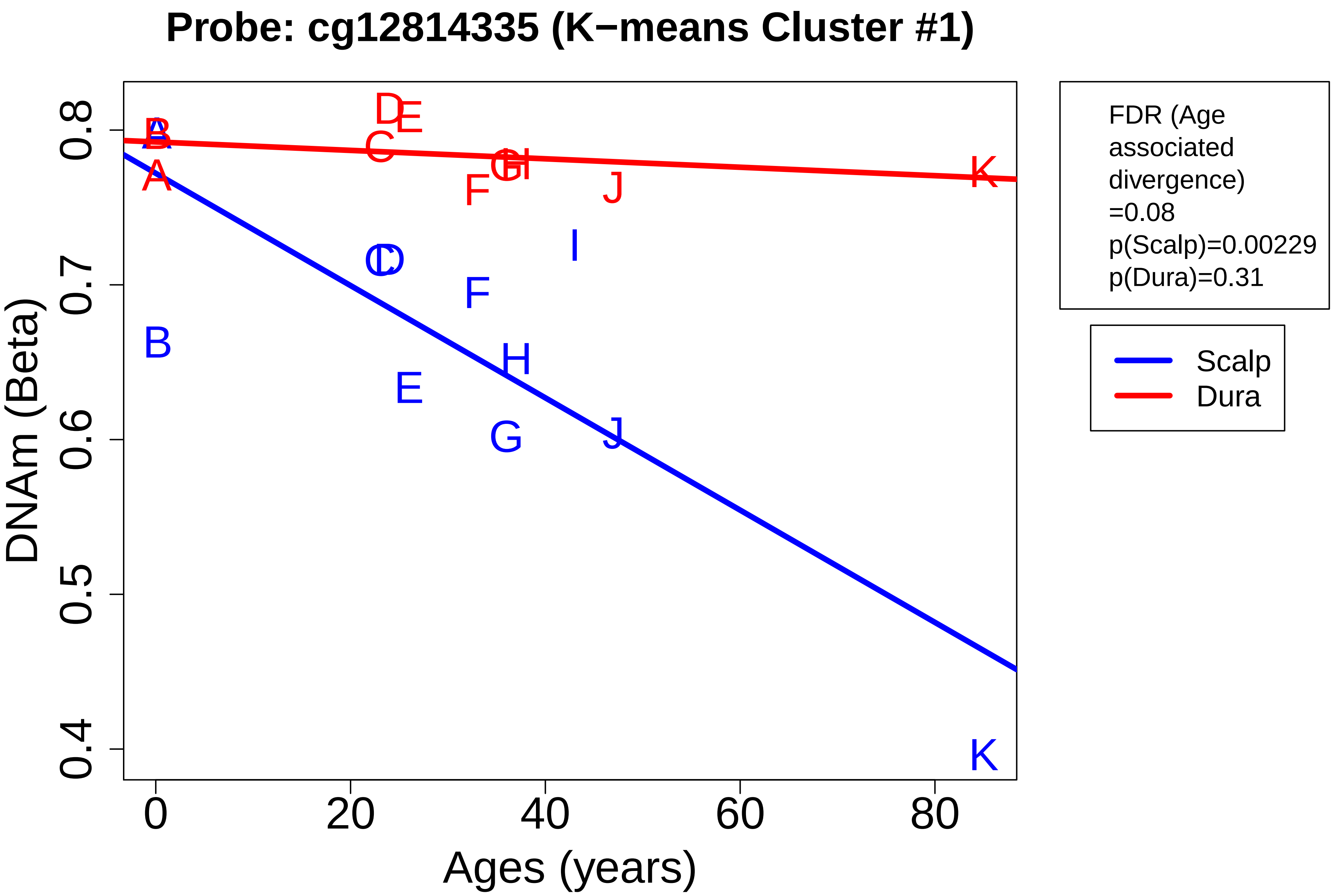

Probe: cg12814335 (K−means Cluster #1)
DNAm (Beta)
0.4
0.5
0.6
0.7
0.8
Ages (years)
0
20
40
60
80
FDR (Age associated divergence) =0.08
p(Scalp)=0.00229
p(Dura)=0.31
Scalp
Dura

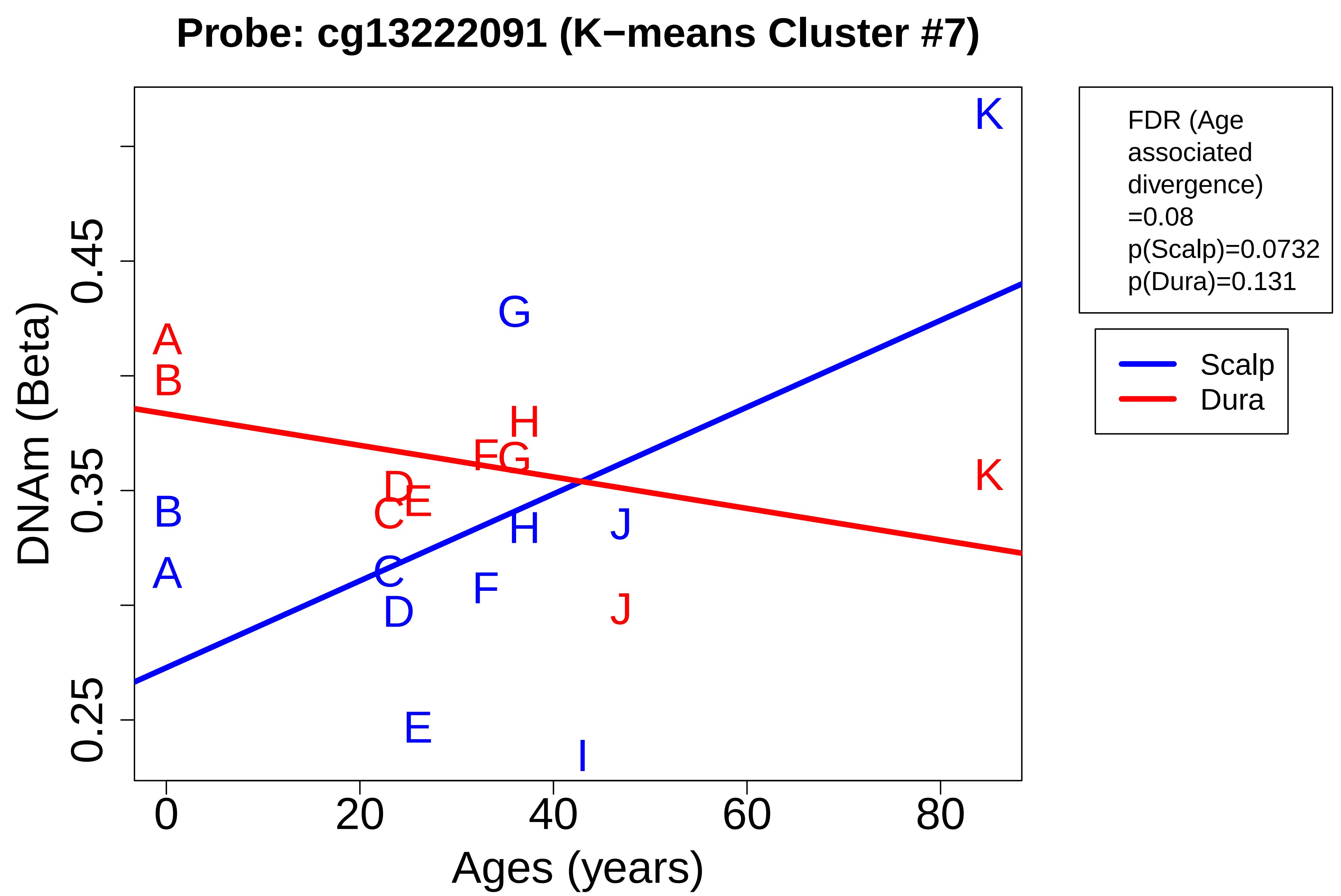
Probe: cg13222091 (K−means Cluster #7)
DNAm (Beta)
0.25
0.35
0.45
Ages (years)
0
20
40
60
80
FDR (Age associated divergence) =0.08
p(Scalp)=0.0732
p(Dura)=0.131
Scalp
Dura

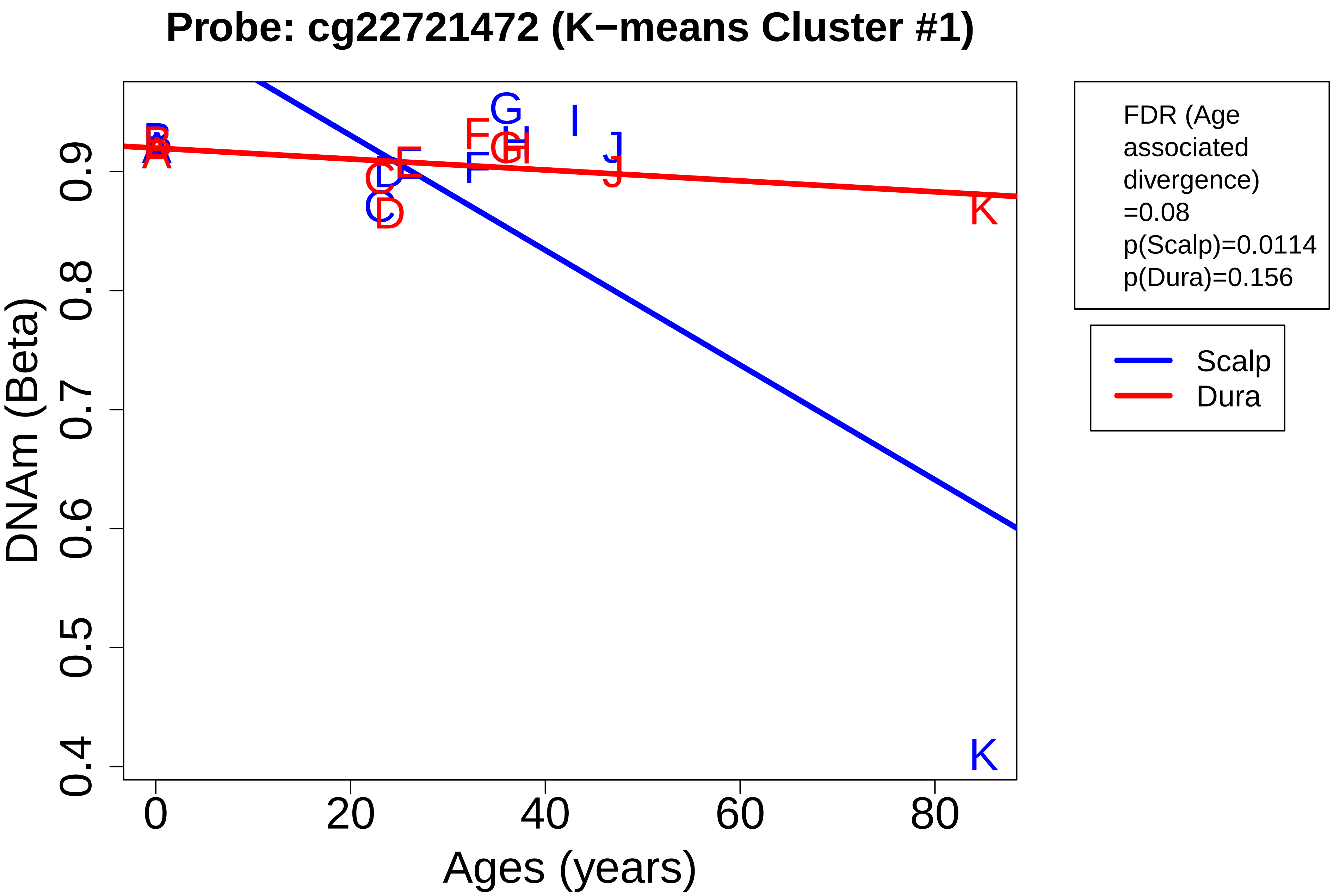

Probe: cg22721472 (K−means Cluster #1)
DNAm (Beta)
0.4
0.5
0.6
0.7
0.8
0.9
0
20
40
60
80
Ages (years)
FDR (Age associated divergence) =0.08
p(Scalp)=0.0114
p(Dura)=0.156
Scalp
Dura

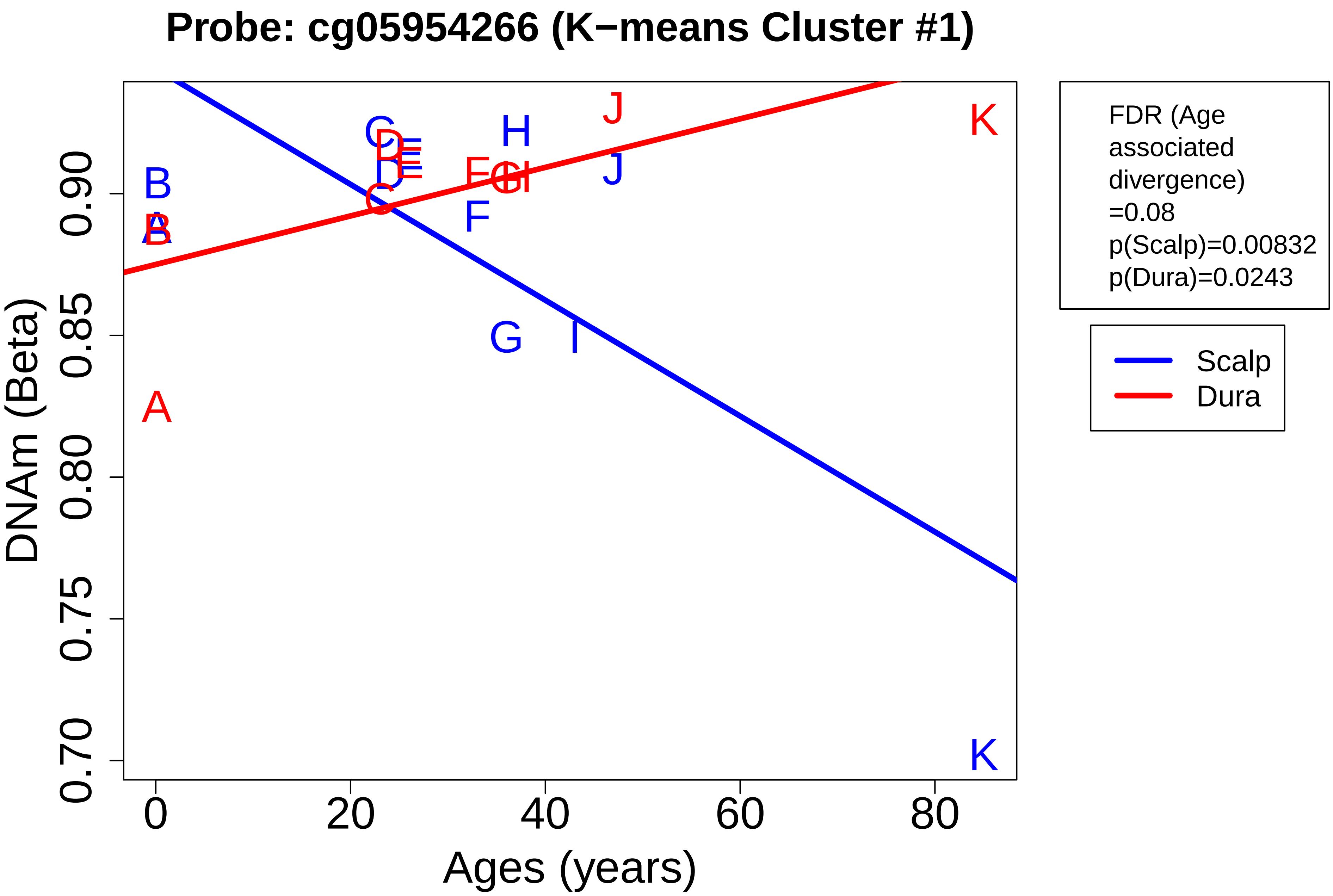

Probe: cg05954266 (K−means Cluster #1)
DNAm (Beta)
Ages (years)
0.70
0.75
0.80
0.85
0.90
0
20
40
60
80
FDR (Age associated divergence) =0.08
p(Scalp)=0.00832
p(Dura)=0.0243
Scalp
Dura

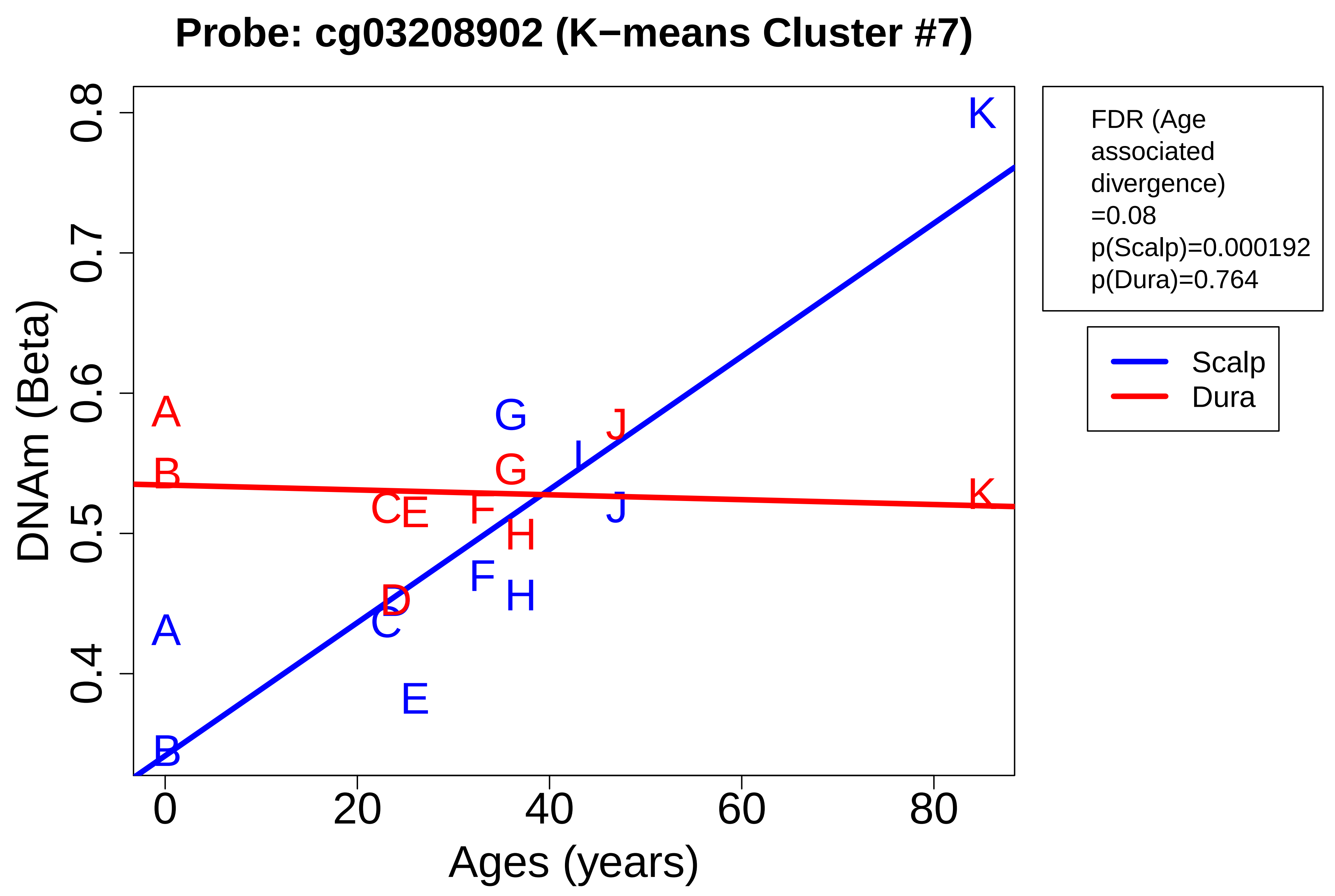

Probe: cg03208902 (K−means Cluster #7)
DNAm (Beta)
0.4
0.5
0.6
0.7
0.8
0
20
40
60
80
Ages (years)
FDR (Age associated divergence) =0.08
p(Scalp)=0.000192
p(Dura)=0.764
Scalp
Dura
A
B
C
D
E
F
G
H
I
J
K

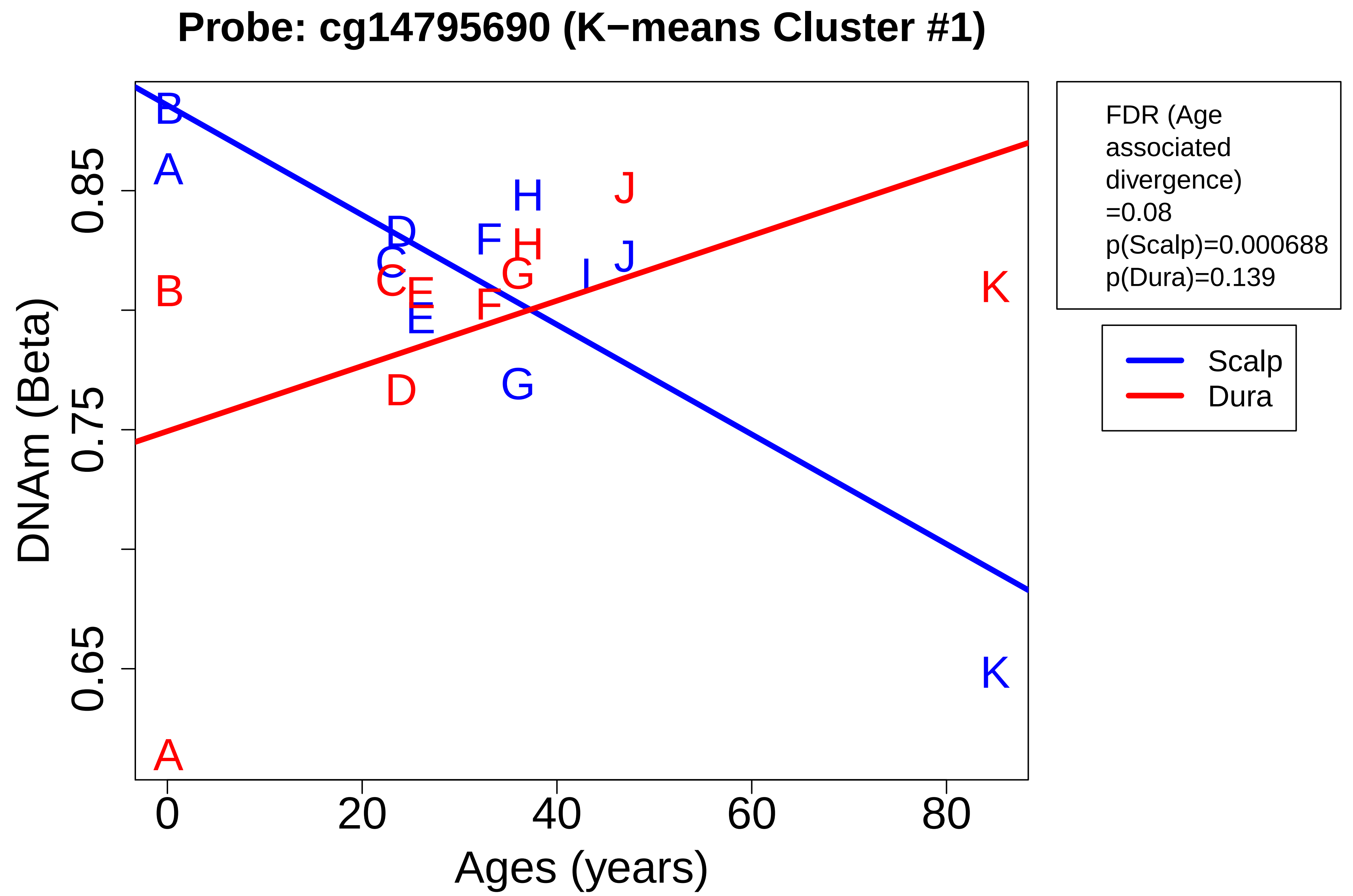

Probe: cg14795690 (K−means Cluster #1)
DNAm (Beta)
0.65
0.75
0.85
Ages (years)
0
20
40
60
80
FDR (Age associated divergence) =0.08
p(Scalp)=0.000688
p(Dura)=0.139
Scalp
Dura

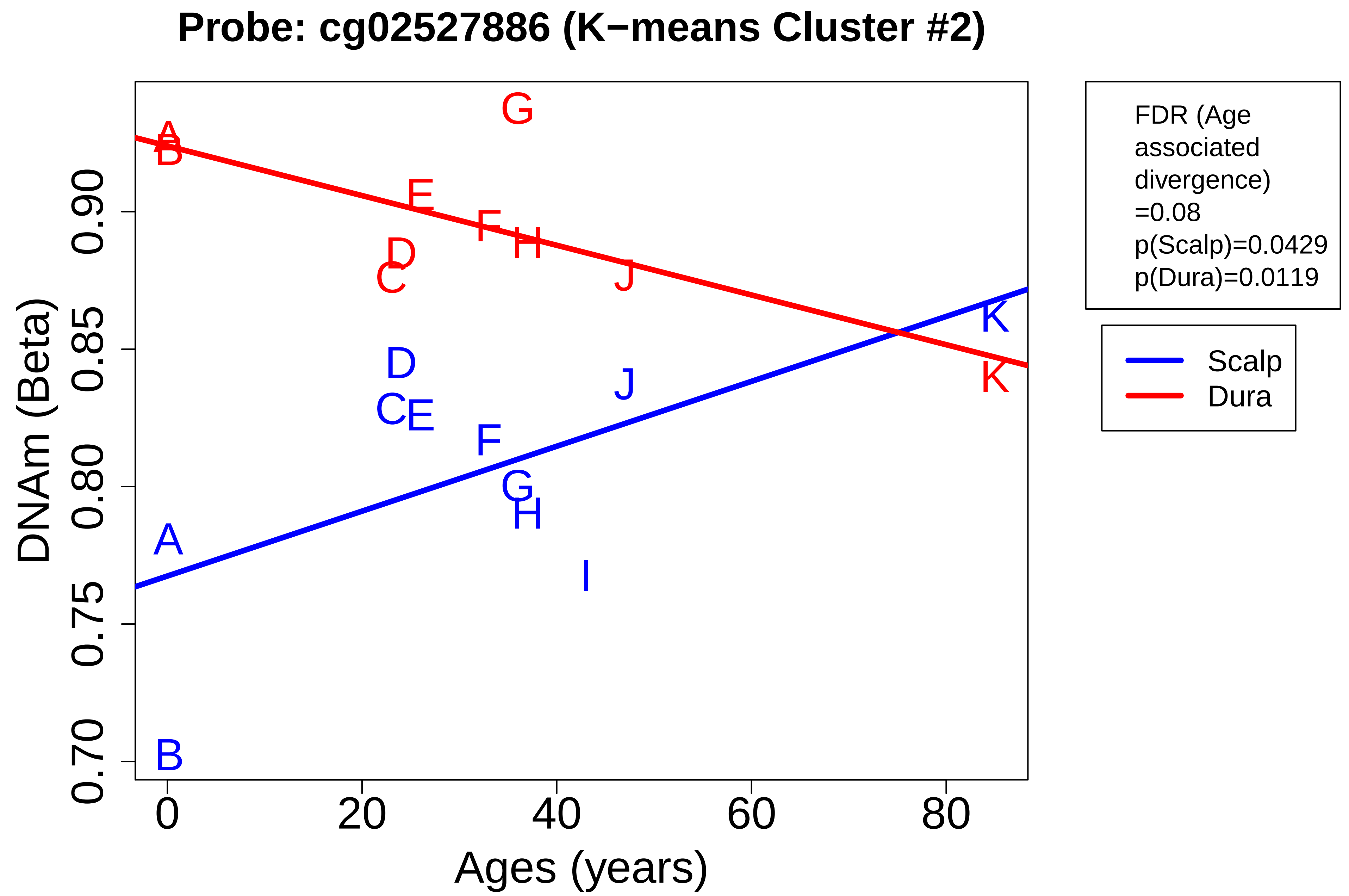

Probe: cg02527886 (K−means Cluster #2)
DNAm (Beta)
0.70
0.75
0.80
0.85
0.90
0
20
40
60
80
Ages (years)
FDR (Age associated divergence) =0.08
p(Scalp)=0.0429
p(Dura)=0.0119
Scalp
Dura

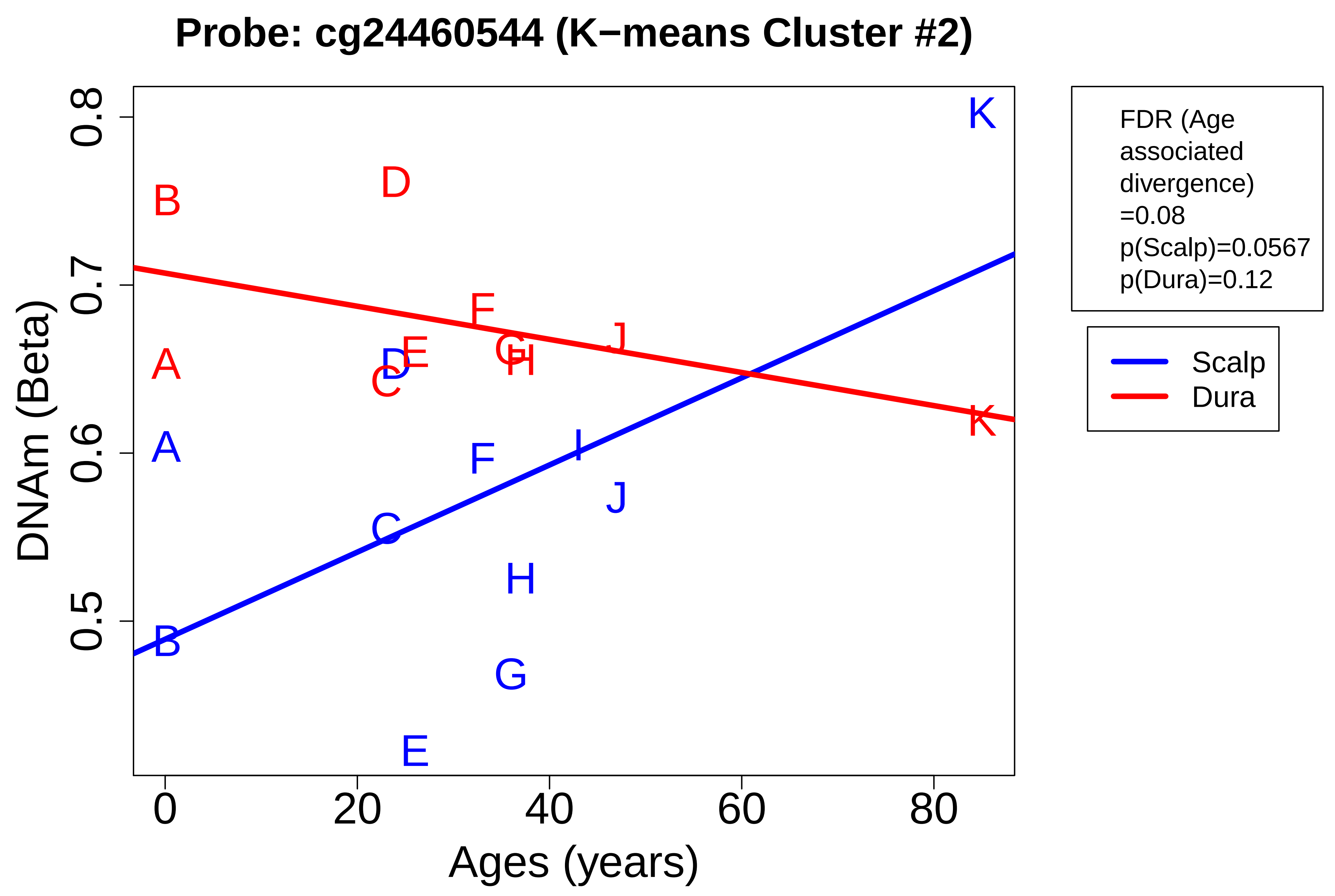

Probe: cg24460544 (K−means Cluster #2)
DNAm (Beta)
0.5
0.6
0.7
0.8
Ages (years)
0
20
40
60
80
FDR (Age associated divergence) =0.08
p(Scalp)=0.0567
p(Dura)=0.12
Scalp
Dura
A
B
C
D
E
F
G
H
I
J
K

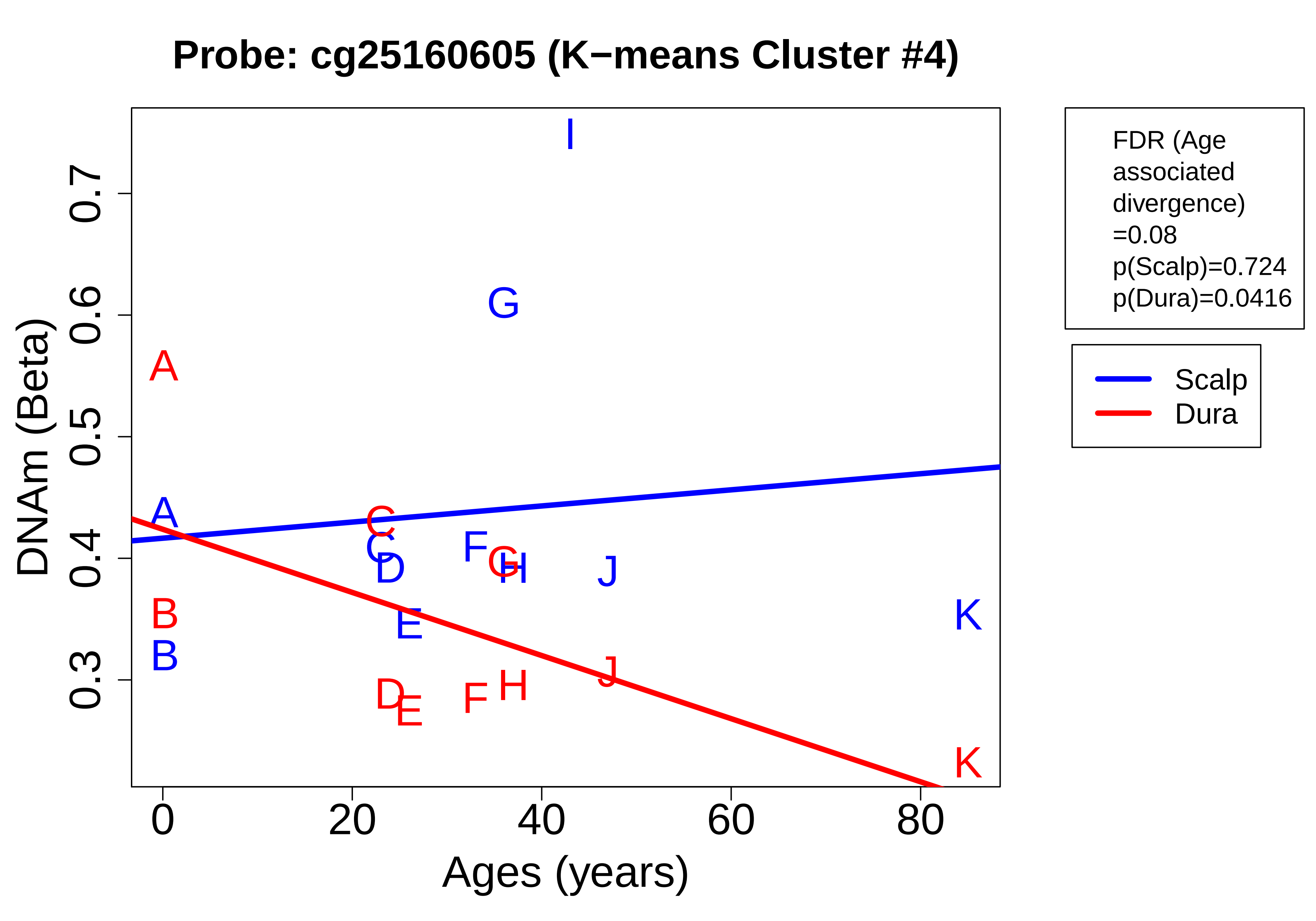

Probe: cg25160605 (K−means Cluster #4)
DNAm (Beta)
0.3
0.4
0.5
0.6
0.7
Ages (years)
0
20
40
60
80
A
B
C
D
E
F
G
H
I
J
K
FDR (Age associated divergence) =0.08
p(Scalp)=0.724
p(Dura)=0.0416
Scalp
Dura

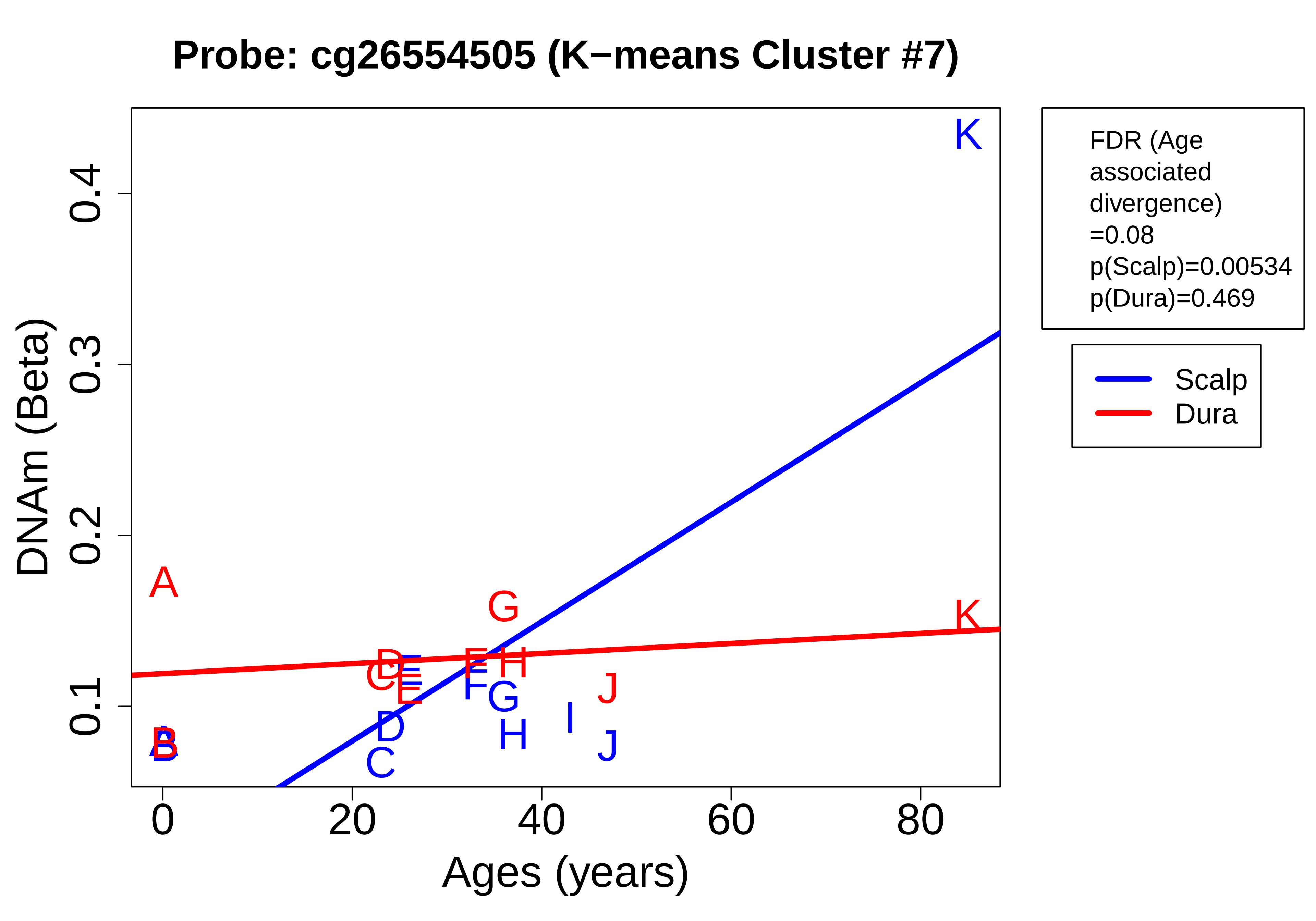
Probe: cg26554505 (K−means Cluster #7)
DNAm (Beta)
Ages (years)
0.1
0.2
0.3
0.4
0
20
40
60
80
FDR (Age associated divergence) =0.08
p(Scalp)=0.00534
p(Dura)=0.469
Scalp
Dura

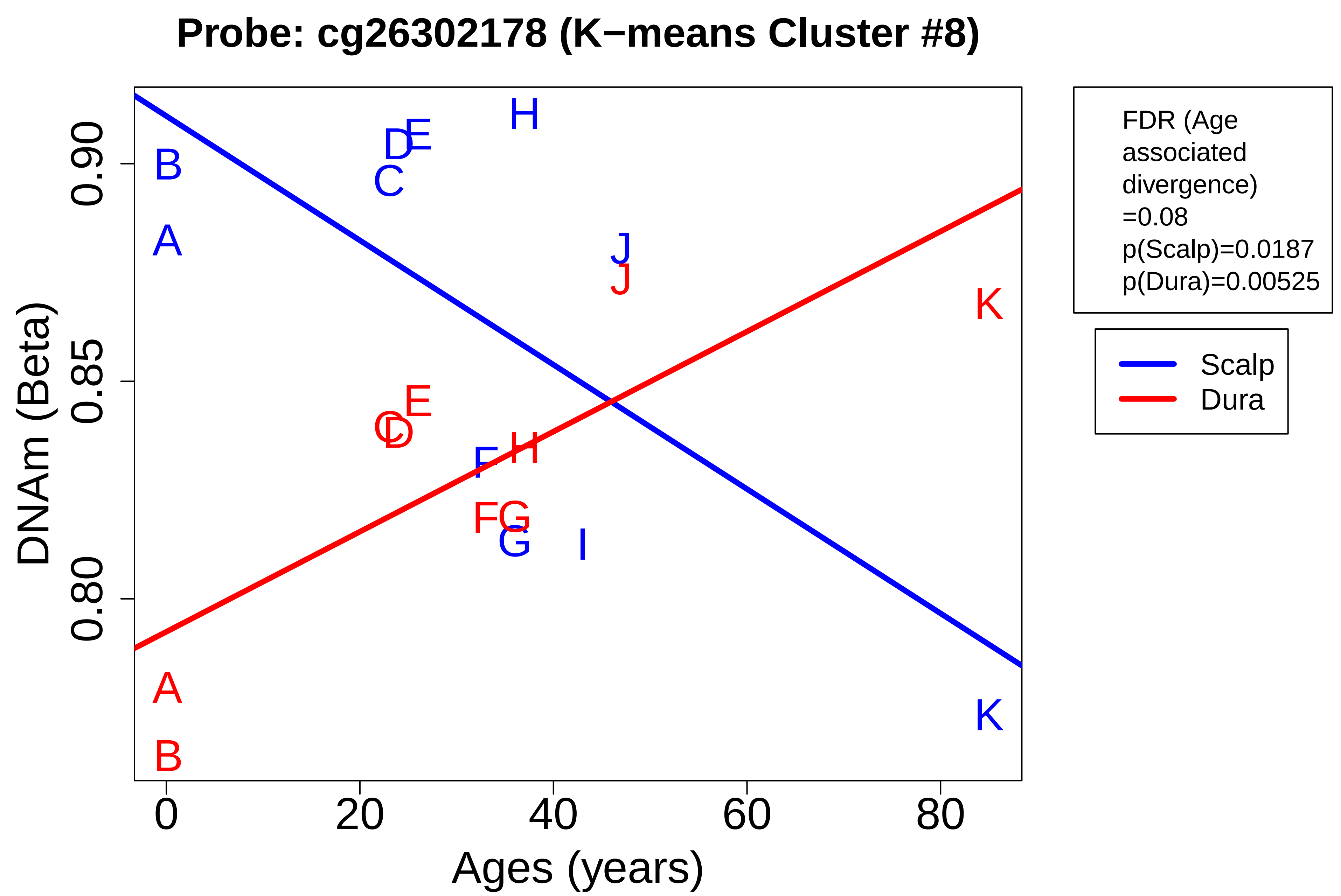

Probe: cg26302178 (K−means Cluster #8)
DNAm (Beta)
0.80
0.85
0.90
Ages (years)
0
20
40
60
80
FDR (Age associated divergence) =0.08
p(Scalp)=0.0187
p(Dura)=0.00525
Scalp
Dura

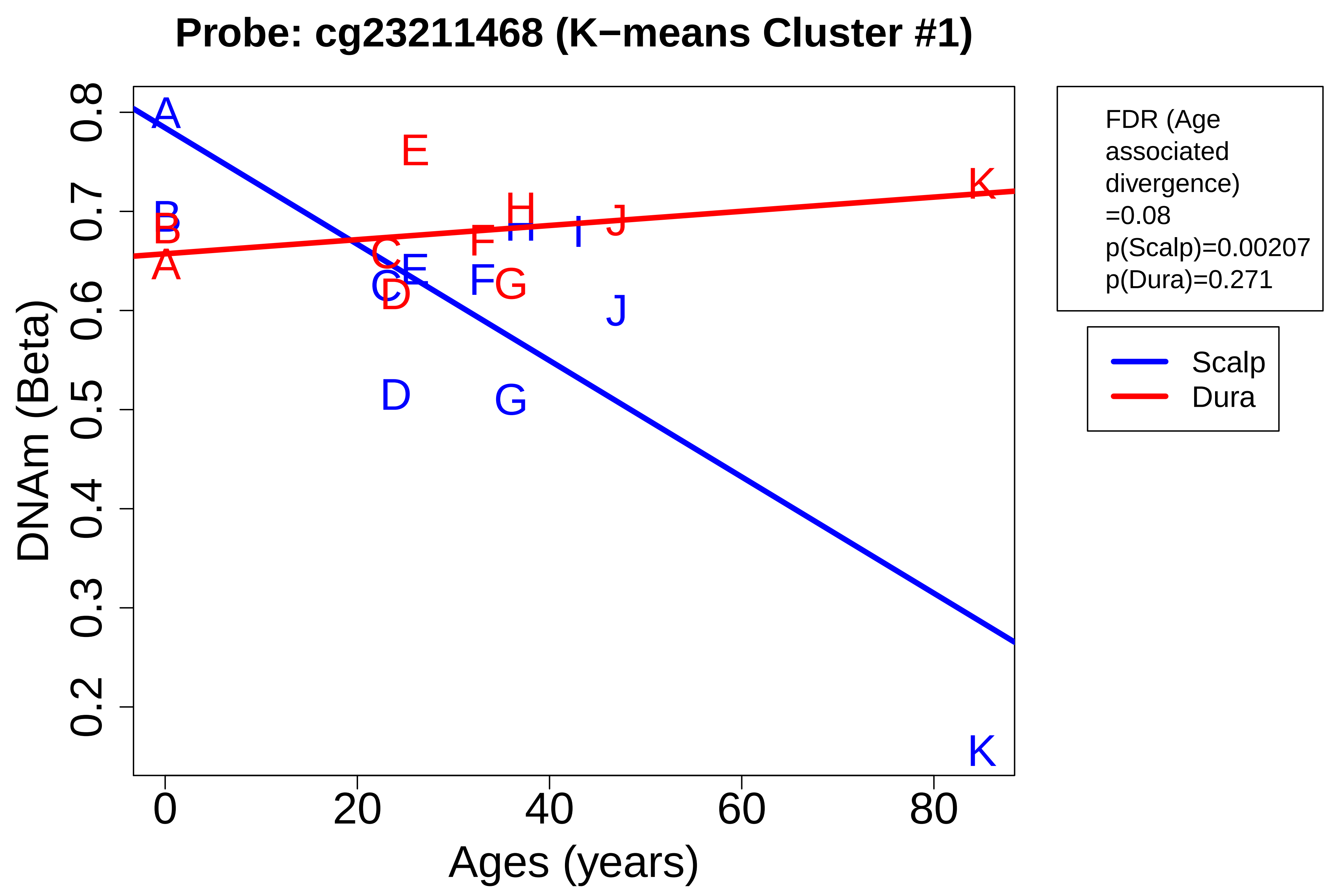

Probe: cg23211468 (K−means Cluster #1)
DNAm (Beta)
0.2
0.3
0.4
0.5
0.6
0.7
0.8
Ages (years)
0
20
40
60
80
FDR (Age associated divergence) =0.08
p(Scalp)=0.00207
p(Dura)=0.271
Scalp
Dura

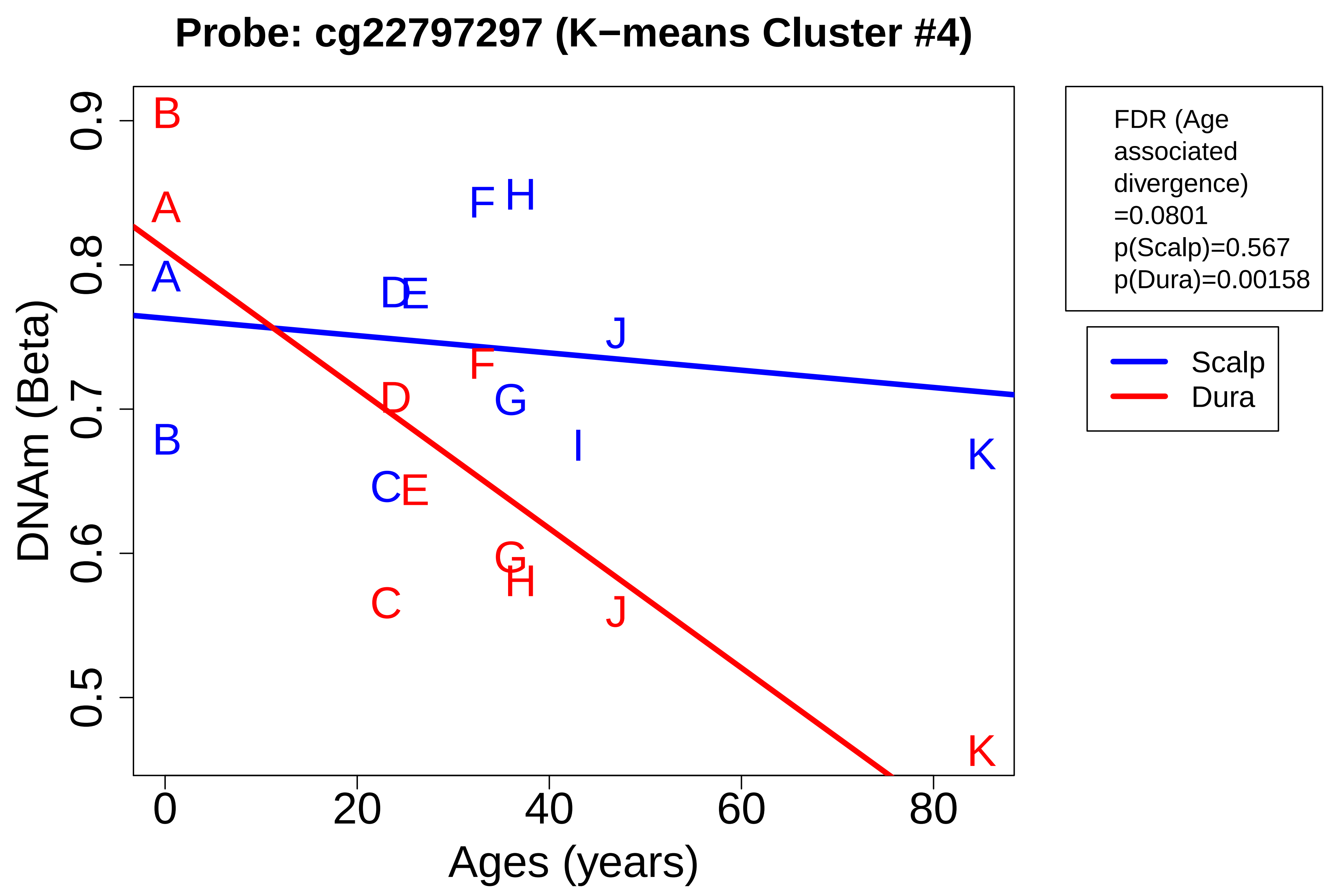

Probe: cg22797297 (K−means Cluster #4)
DNAm (Beta)
0.5
0.6
0.7
0.8
0.9
0
20
40
60
80
Ages (years)
B
A
A
DE
F
H
J
F
D
G
B
I
K
C
E
G
H
C
J
K
FDR (Age associated divergence) =0.0801
p(Scalp)=0.567
p(Dura)=0.00158
Scalp
Dura

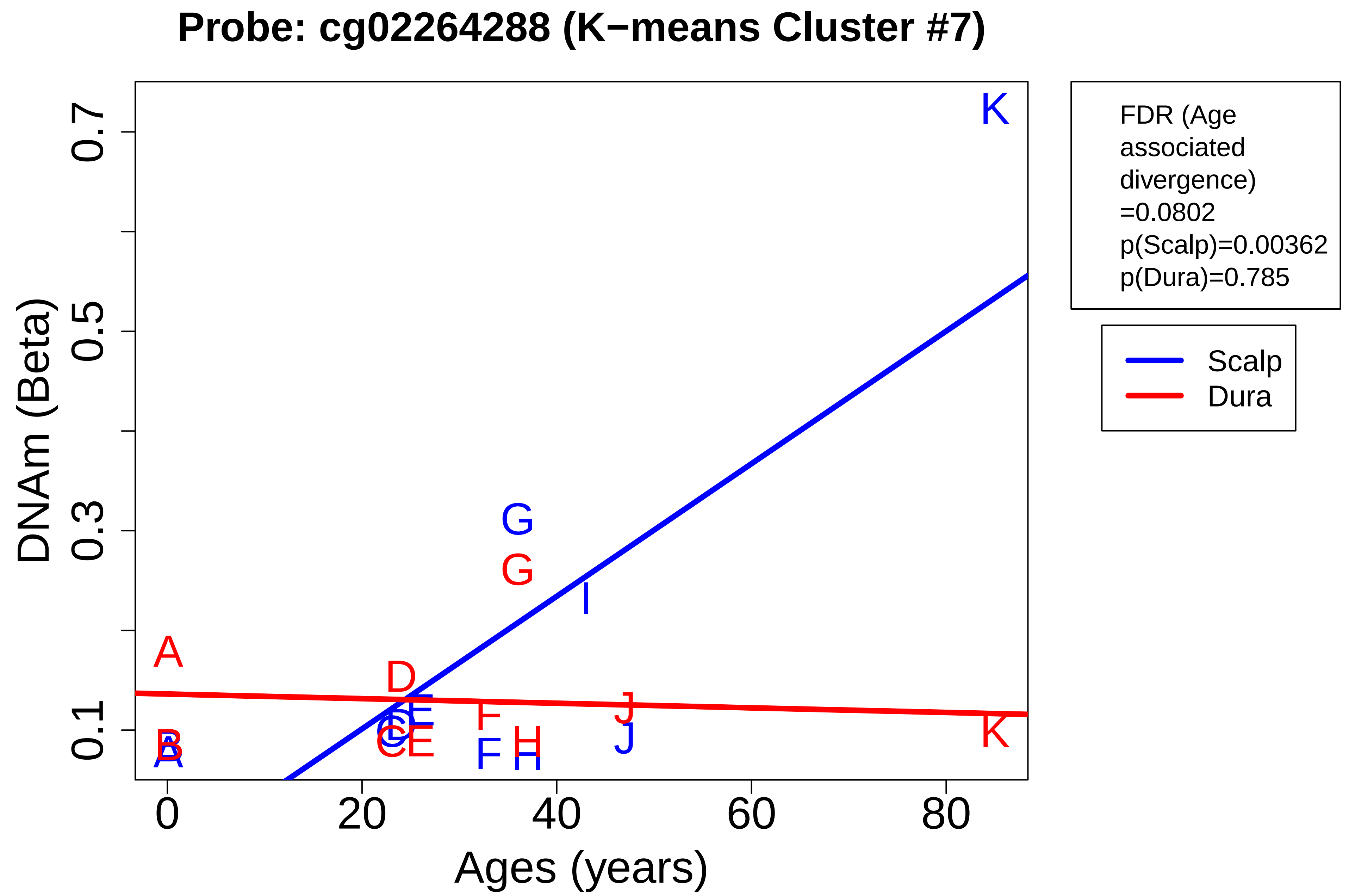

Probe: cg02264288 (K−means Cluster #7)
DNAm (Beta)
0.1
0.3
0.5
0.7
0
20
40
60
80
Ages (years)
FDR (Age associated divergence) =0.0802
p(Scalp)=0.00362
p(Dura)=0.785
Scalp
Dura

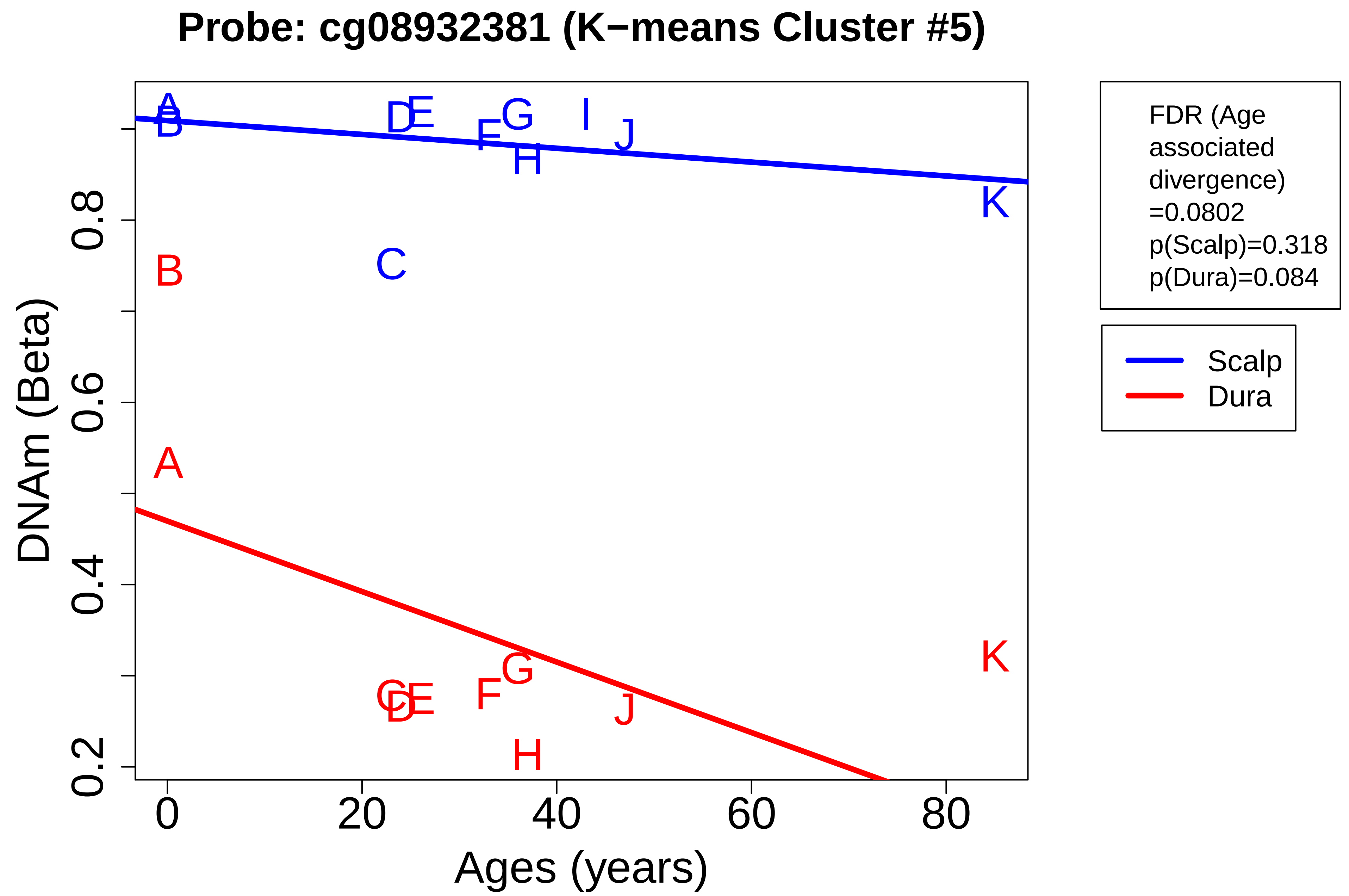

Probe: cg08932381 (K−means Cluster #5)
DNAm (Beta)
0.2
0.4
0.6
0.8
0
20
40
60
80
Ages (years)
FDR (Age associated divergence) =0.0802
p(Scalp)=0.318
p(Dura)=0.084
Scalp
Dura

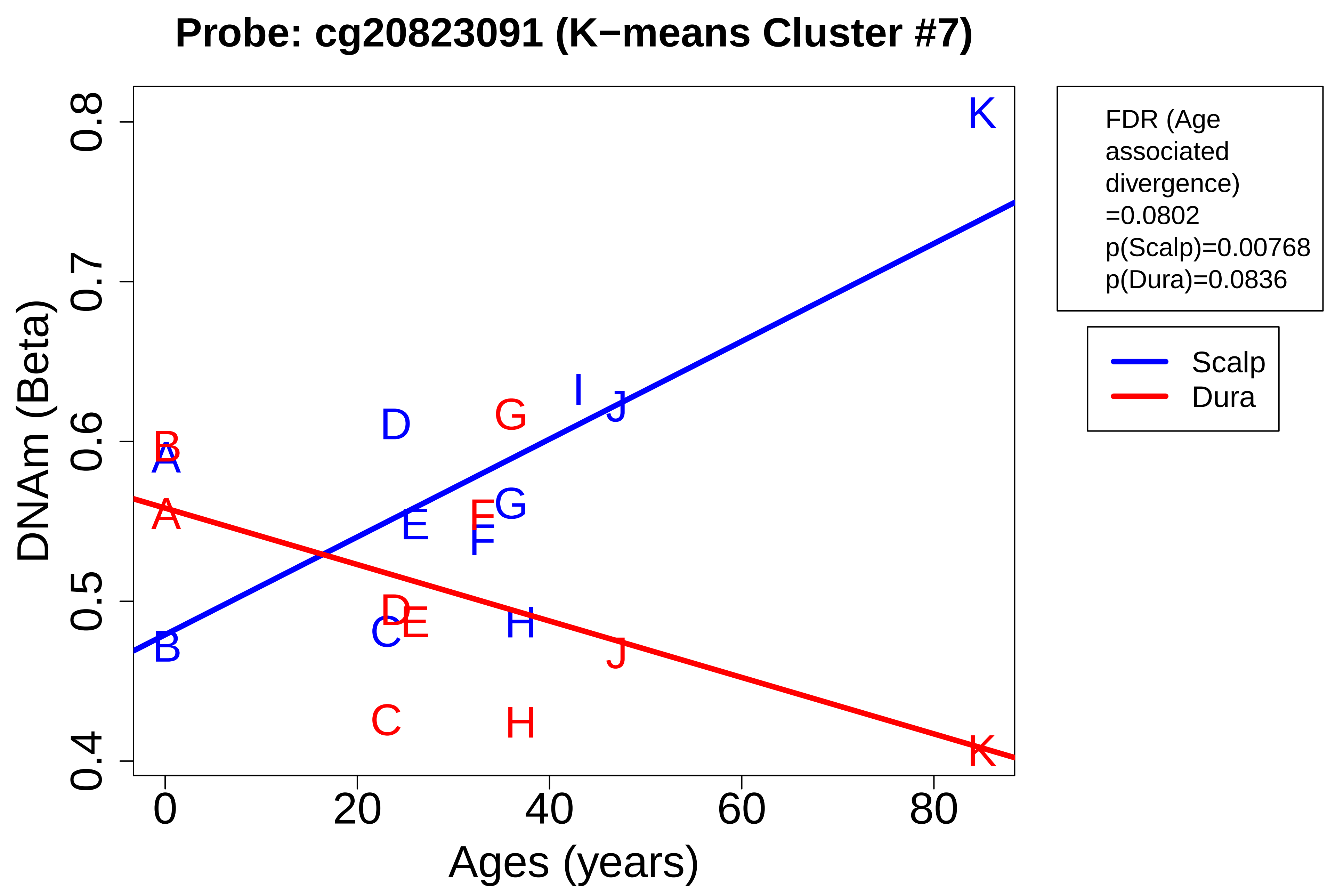
Probe: cg20823091 (K−means Cluster #7)
DNAm (Beta)
Ages (years)
0.4
0.5
0.6
0.7
0.8
0
20
40
60
80
FDR (Age associated divergence) =0.0802
p(Scalp)=0.00768
p(Dura)=0.0836
Scalp
Dura
A
B
C
D
E
F
G
H
I
J
K

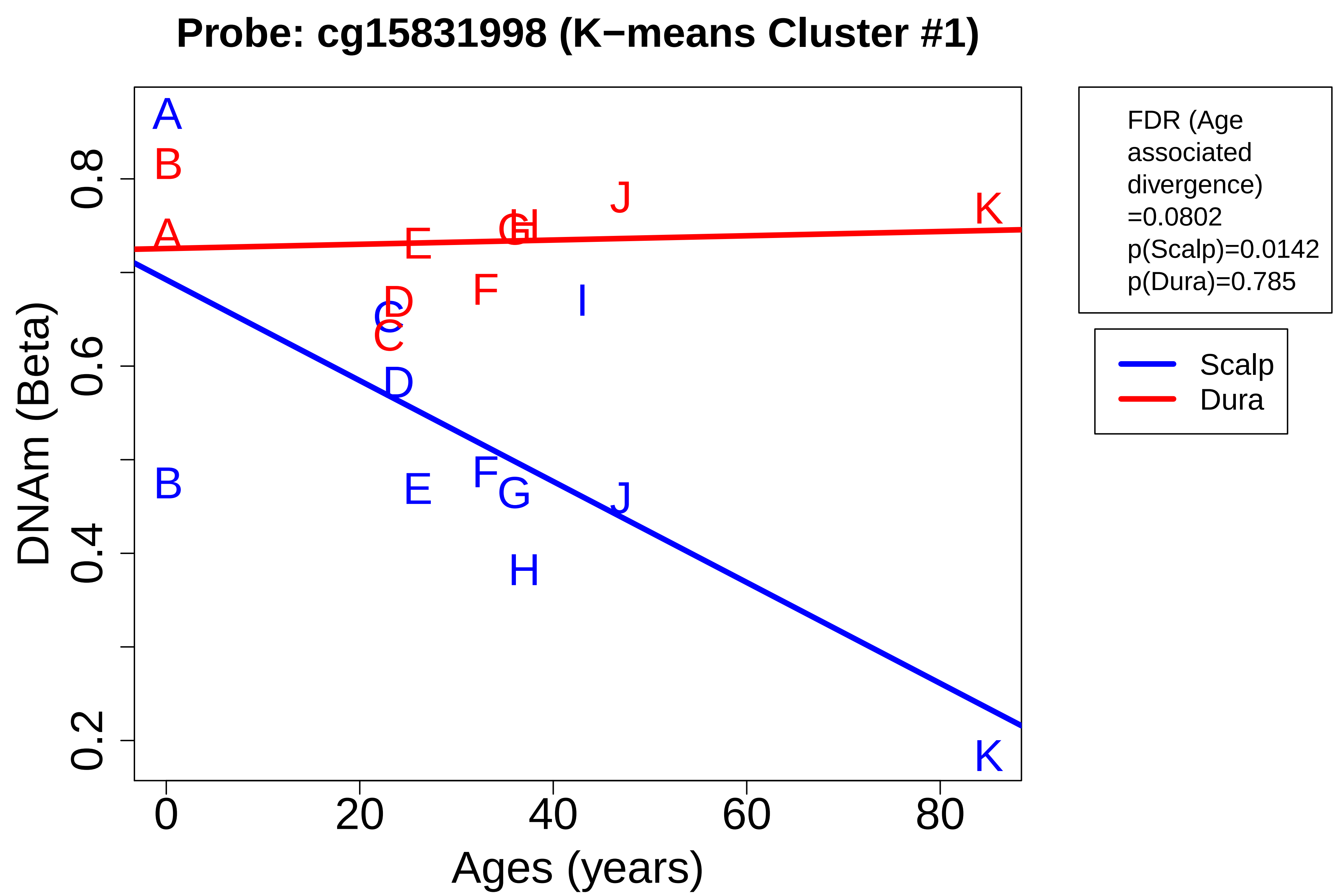

Probe: cg15831998 (K−means Cluster #1)
DNAm (Beta)
0.2
0.4
0.6
0.8
Ages (years)
0
20
40
60
80
FDR (Age associated divergence) =0.0802
p(Scalp)=0.0142
p(Dura)=0.785
Scalp
Dura

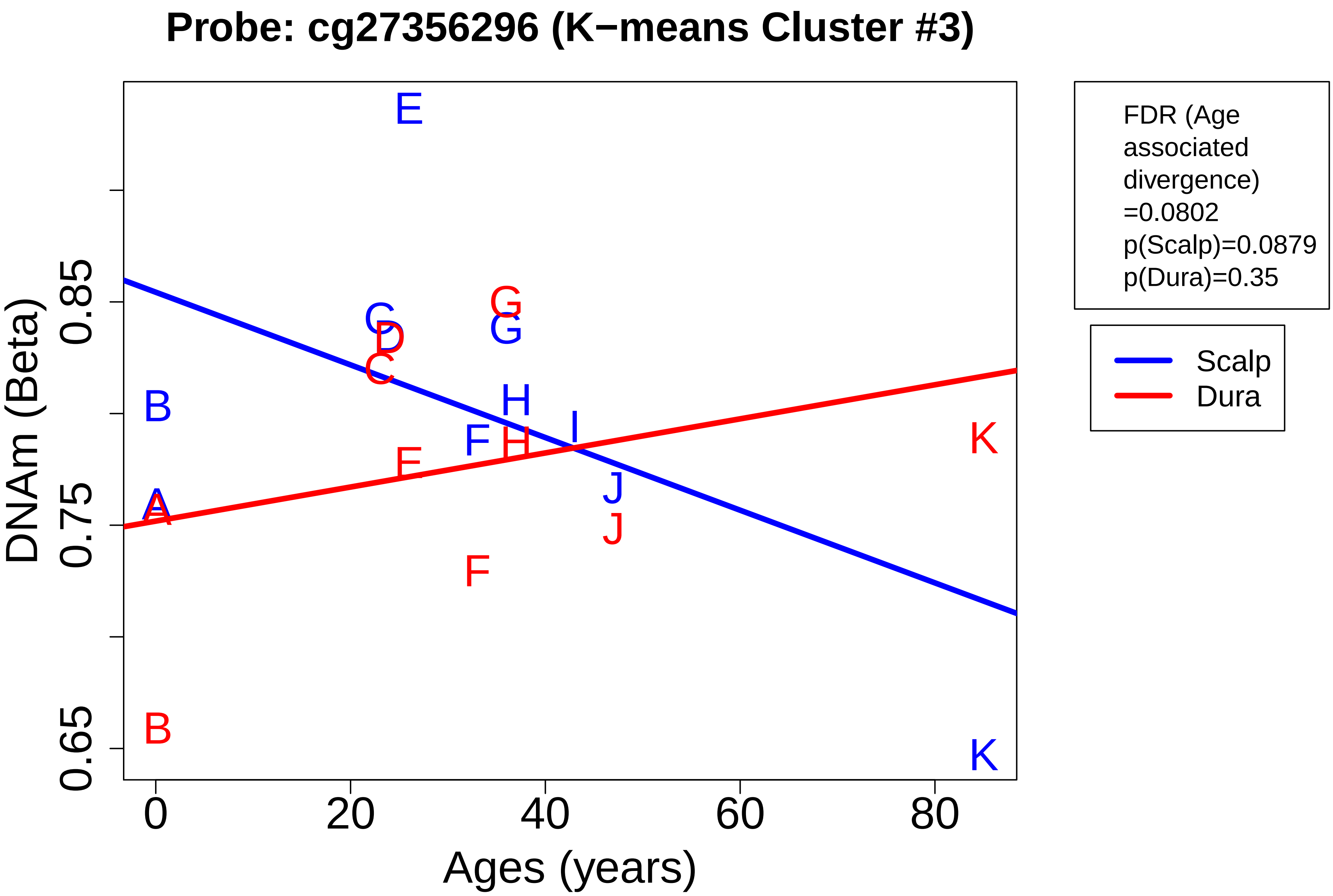
Probe: cg27356296 (K−means Cluster #3)
FDR (Age associated divergence) =0.0802
p(Scalp)=0.0879
p(Dura)=0.35
Scalp
Dura
DNAm (Beta)
0.65
0.75
0.85
0
20
40
60
80
Ages (years)

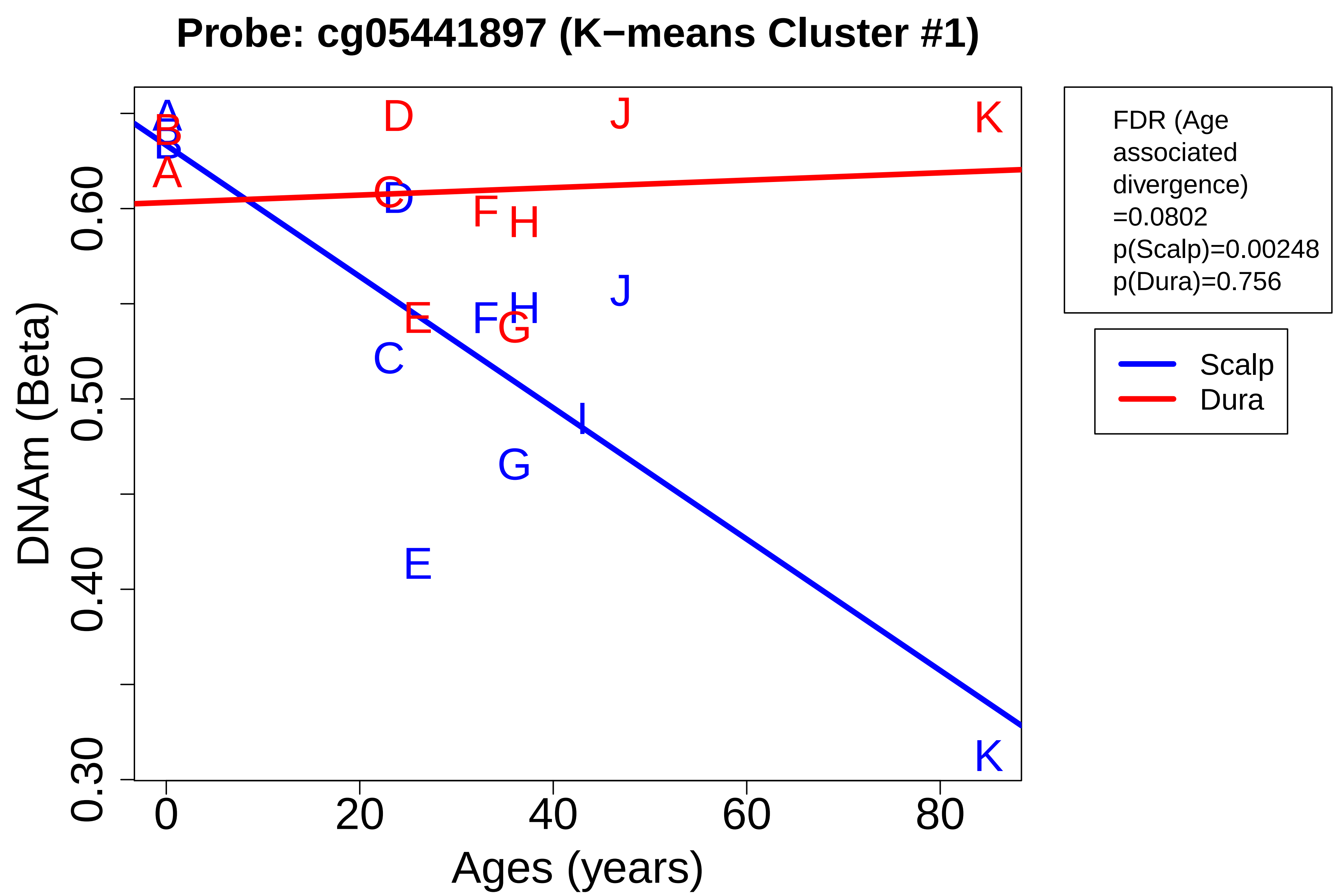
Probe: cg05441897 (K−means Cluster #1)
DNAm (Beta)
Ages (years)
0.30
0.40
0.50
0.60
0
20
40
60
80
FDR (Age associated divergence) =0.0802
p(Scalp)=0.00248
p(Dura)=0.756
Scalp
Dura
A B C D E F G H I J K

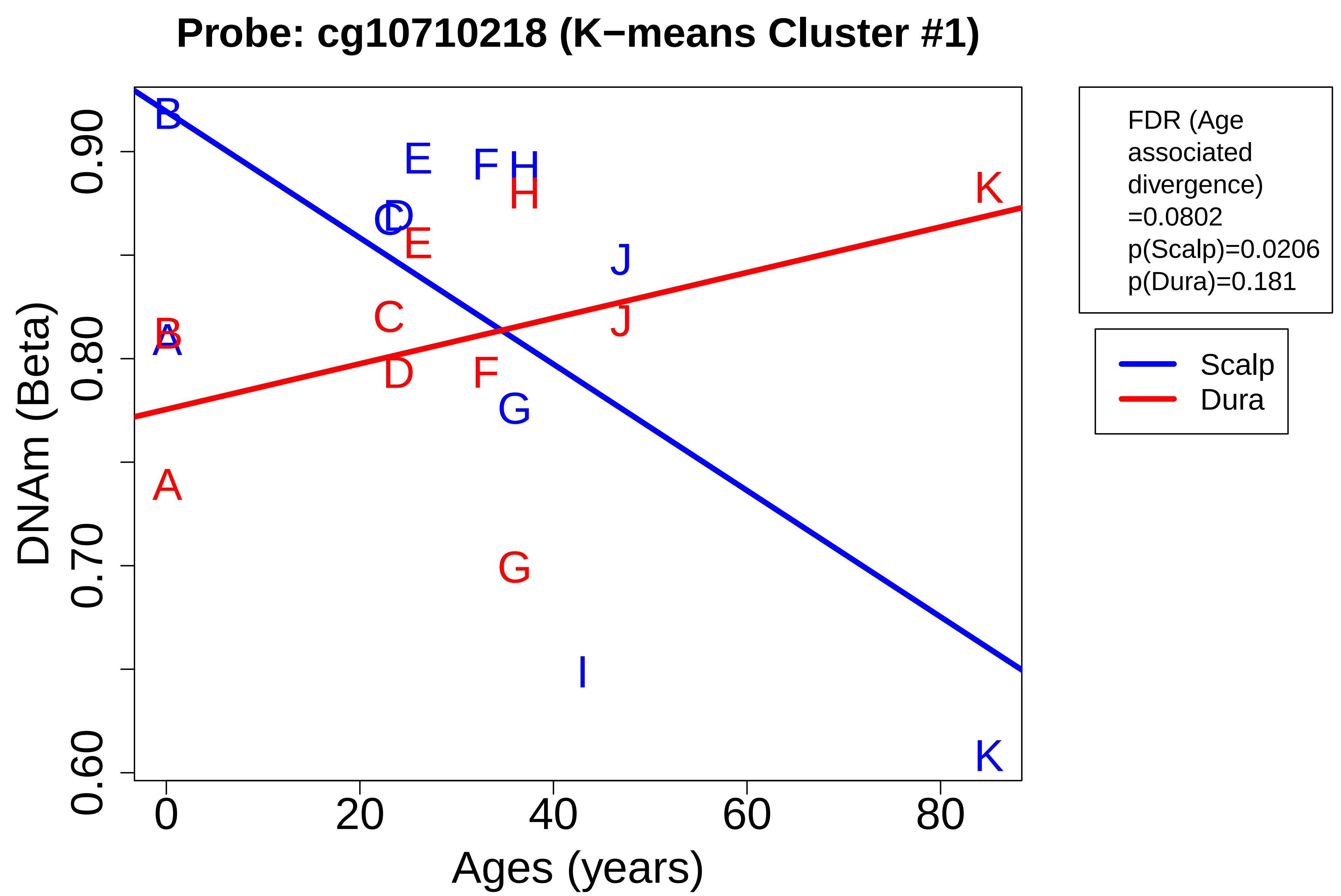

Probe: cg10710218 (K−means Cluster #1)
DNAm (Beta)
0.60
0.70
0.80
0.90
Ages (years)
0
20
40
60
80
A
B
C
D
E
F
G
H
I
J
K
FDR (Age associated divergence) =0.0802
p(Scalp)=0.0206
p(Dura)=0.181
Scalp
Dura

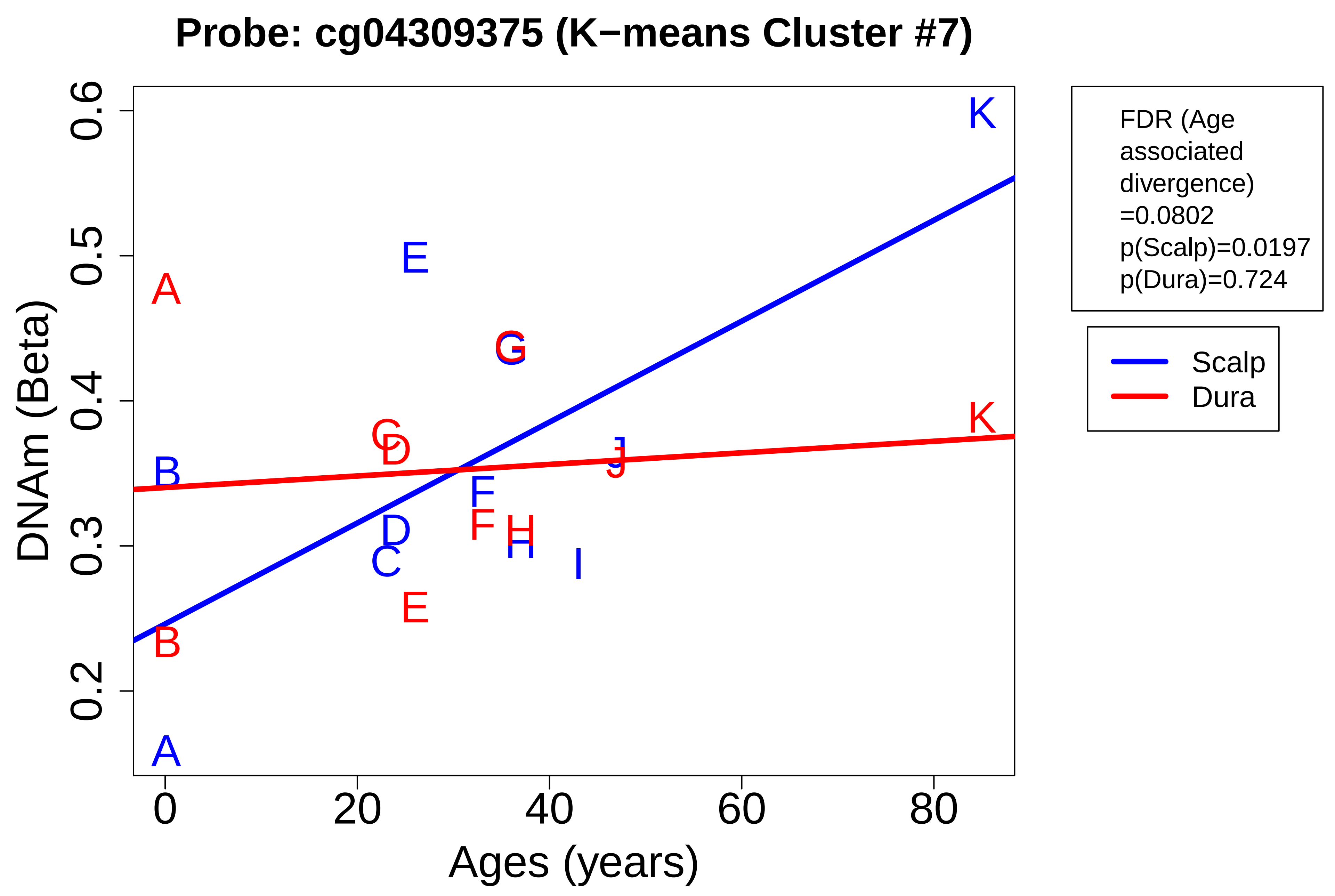
Probe: cg04309375 (K−means Cluster #7)
DNAm (Beta)
Ages (years)
0.2
0.3
0.4
0.5
0.6
0
20
40
60
80
FDR (Age associated divergence) =0.0802
p(Scalp)=0.0197
p(Dura)=0.724
Scalp
Dura
A
B
C
D
E
F
G
H
I
J
K

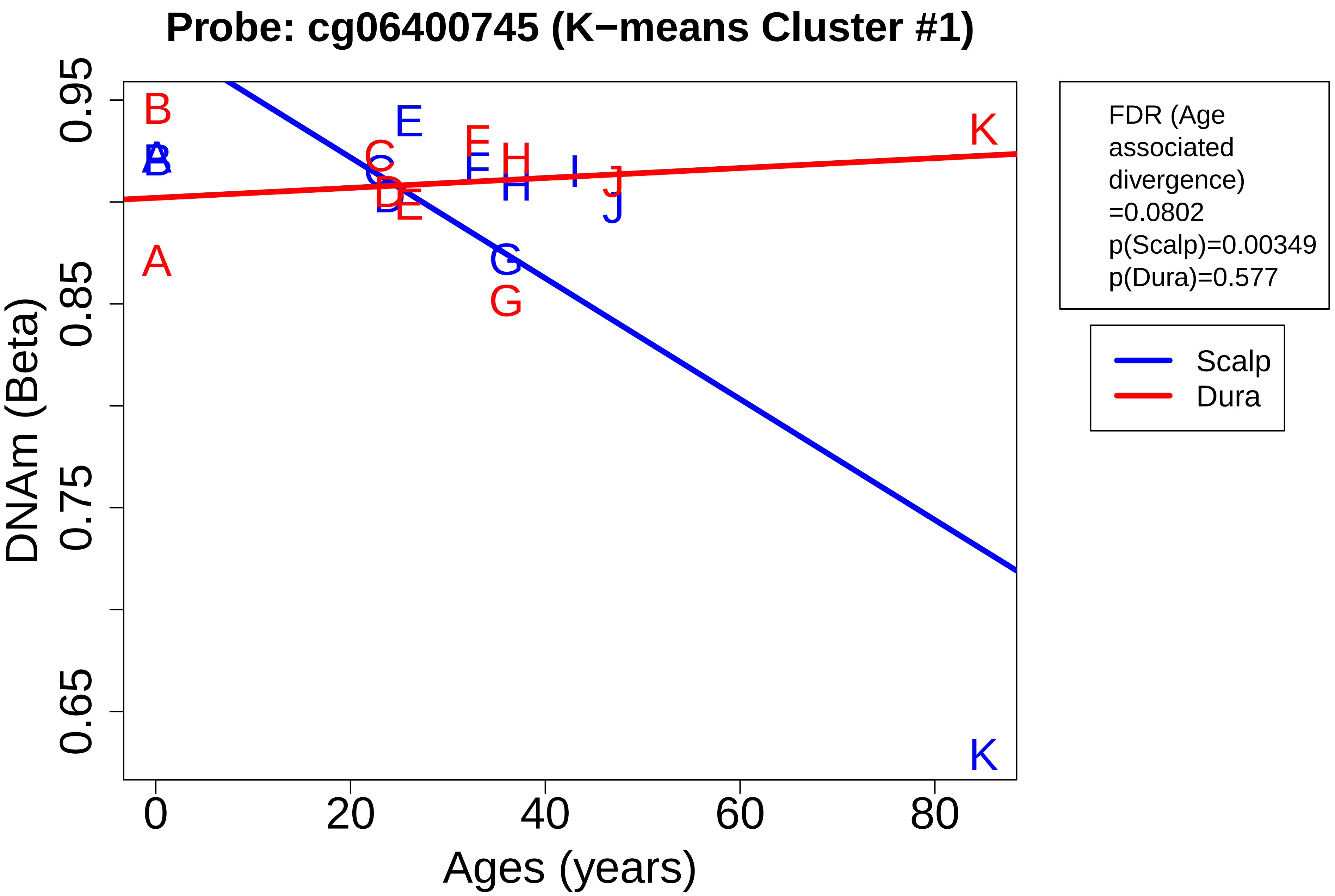

Probe: cg06400745 (K−means Cluster #1)
DNAm (Beta)
0.65
0.75
0.85
0.95
0
20
40
60
80
Ages (years)
FDR (Age associated divergence) =0.0802
p(Scalp)=0.00349
p(Dura)=0.577
Scalp
Dura
A
B
C
D
E
F
G
H
I
J
K

Probe: cg04477101 (K−means Cluster #1)

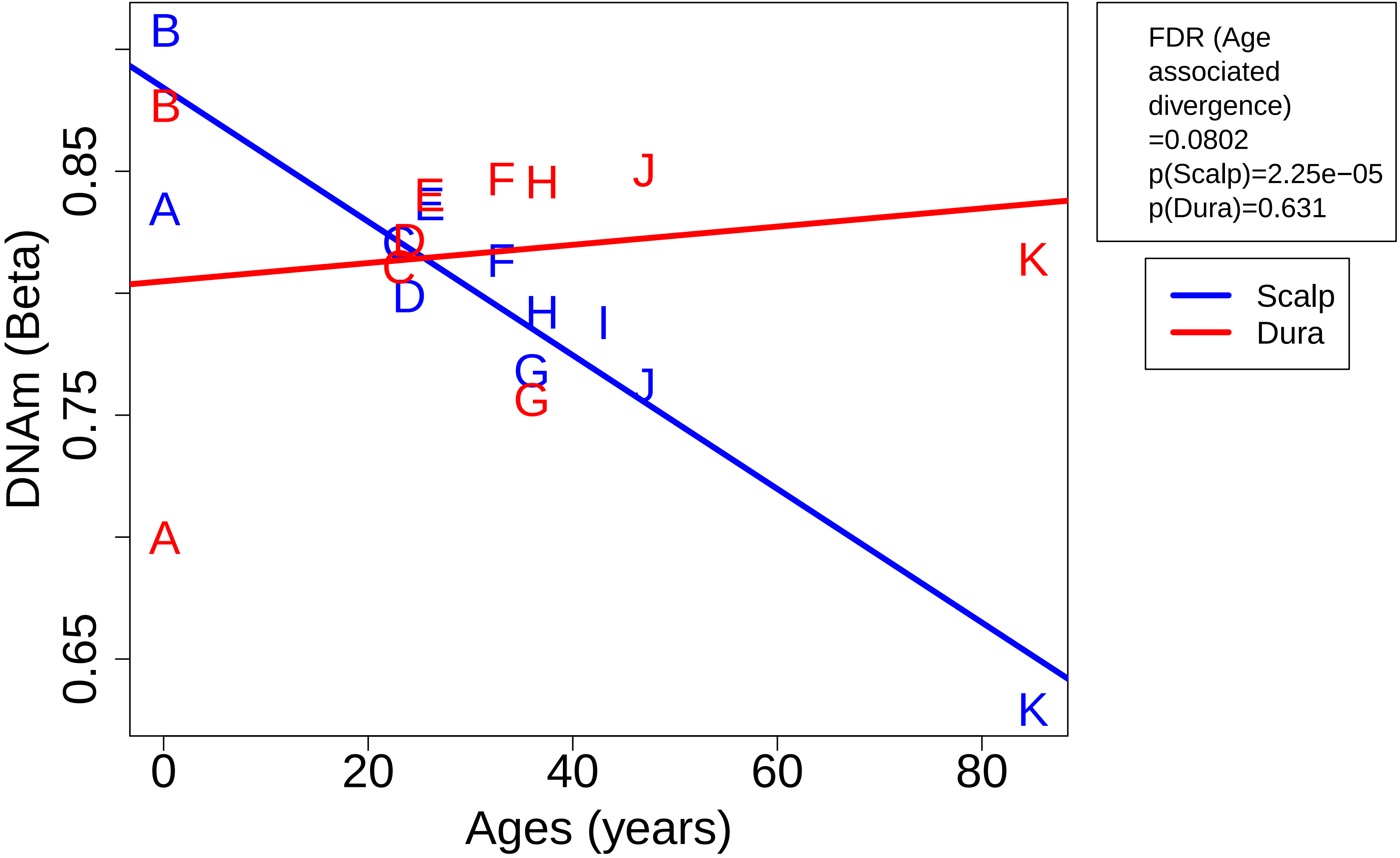

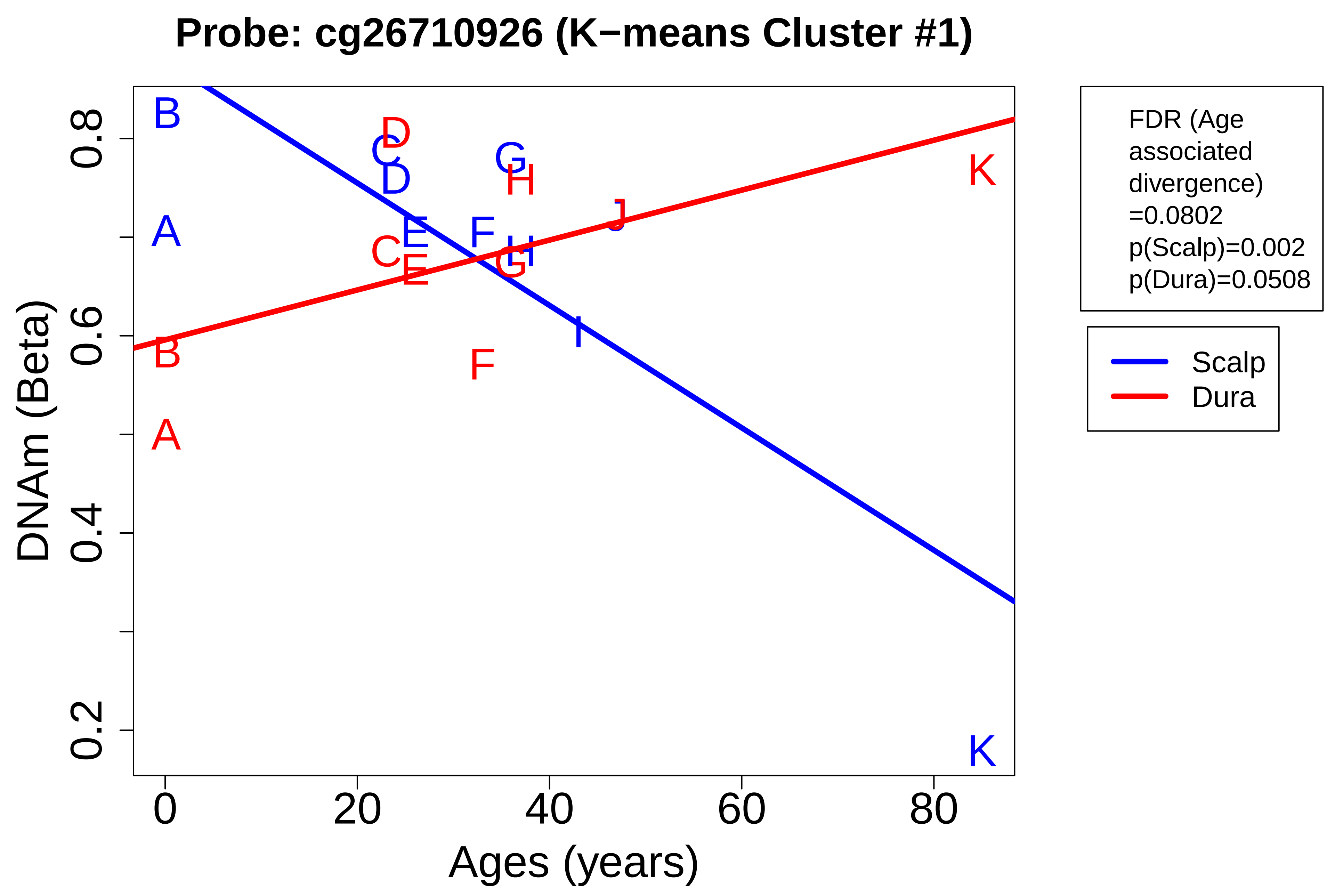
Probe: cg26710926 (K−means Cluster #1)
DNAm (Beta)
0.2
0.4
0.6
0.8
0
20
40
60
80
Ages (years)
A
B
C
D
E
F
G
H
I
J
K
FDR (Age associated divergence) =0.0802
p(Scalp)=0.002
p(Dura)=0.0508
Scalp
Dura

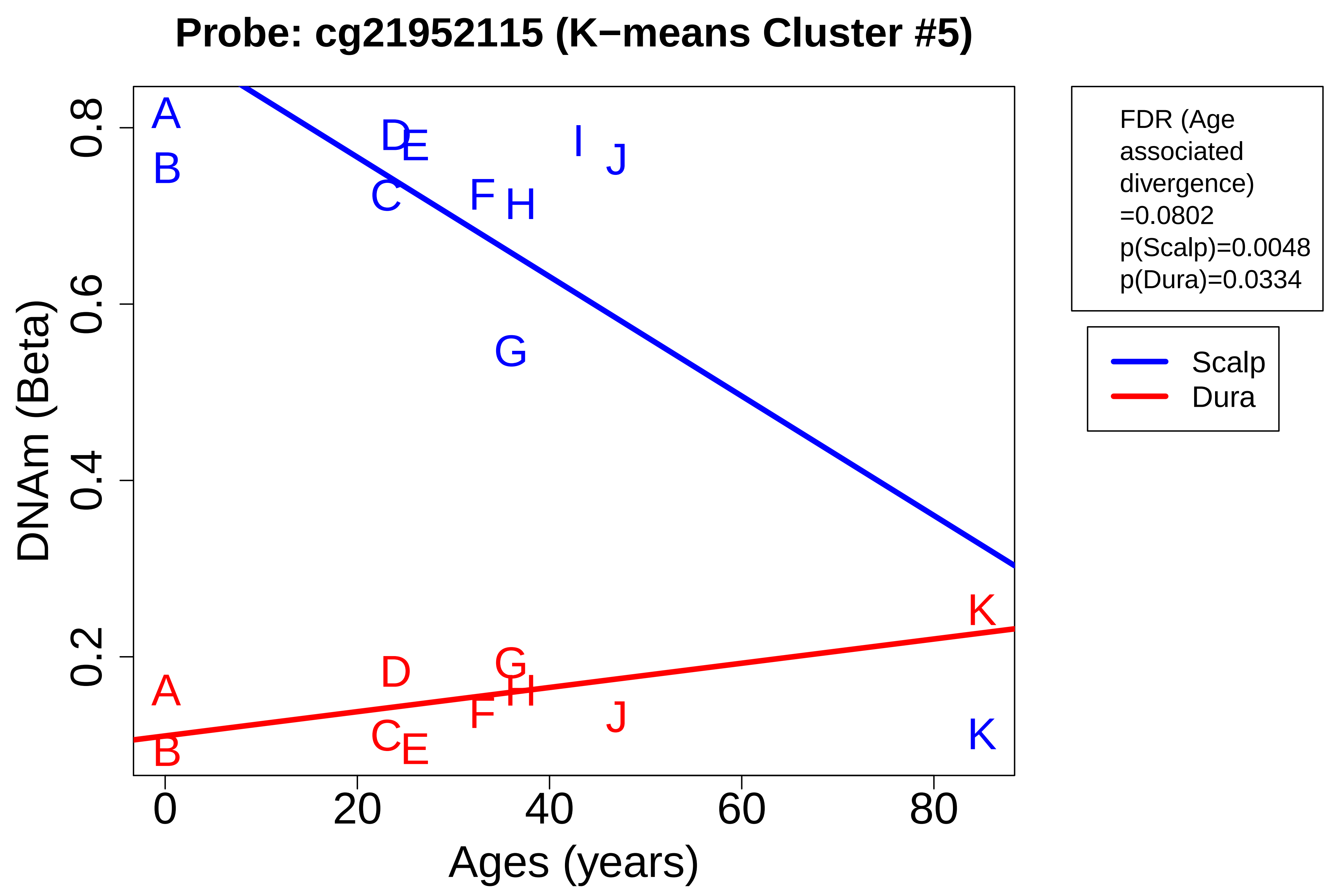

Probe: cg21952115 (K−means Cluster #5)
DNAm (Beta)
0.2
0.4
0.6
0.8
Ages (years)
0
20
40
60
80
A
B
C
D
E
F
G
H
I
J
K
FDR (Age associated divergence) =0.0802
p(Scalp)=0.0048
p(Dura)=0.0334
Scalp
Dura

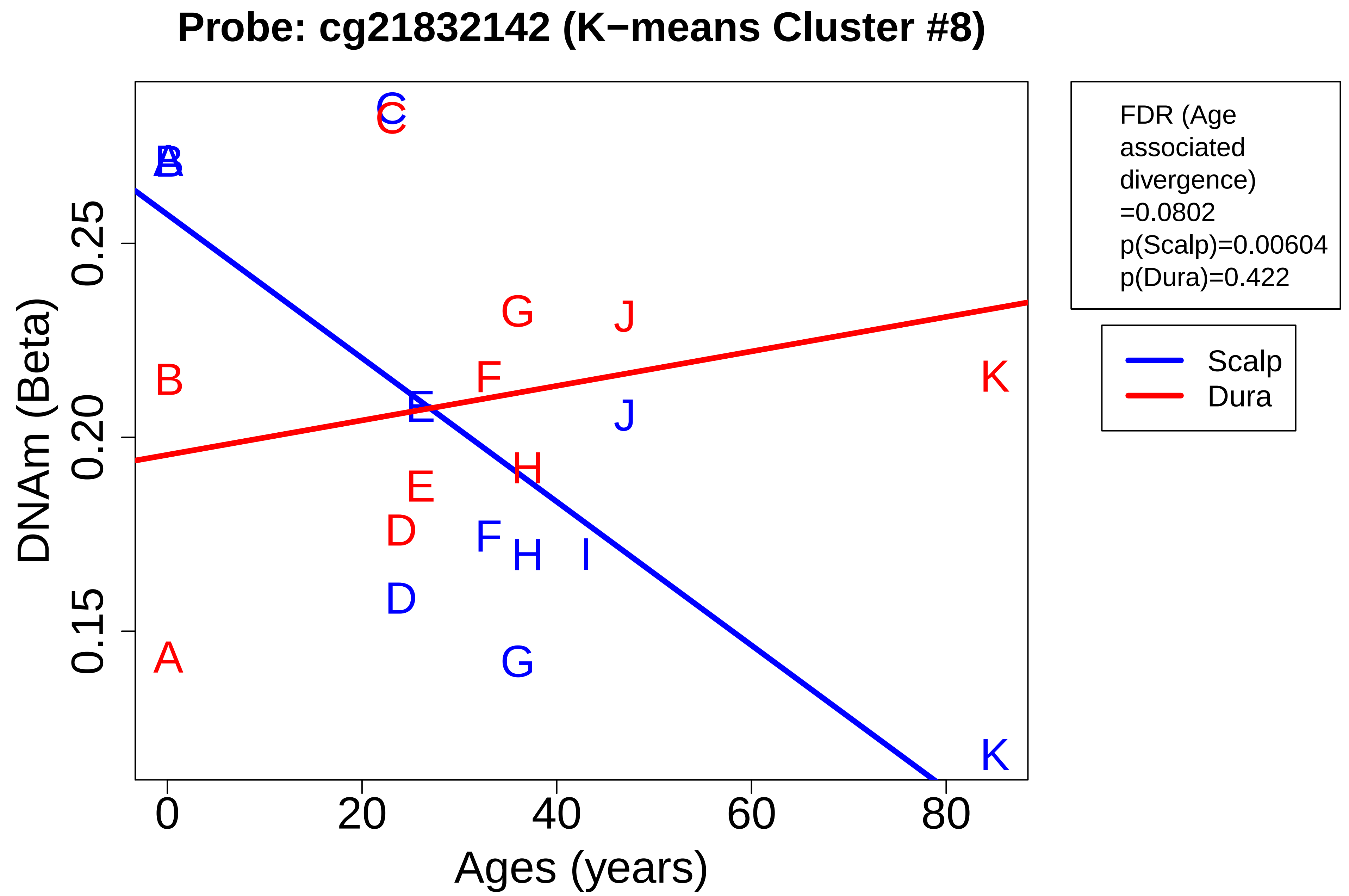

Probe: cg21832142 (K−means Cluster #8)
DNAm (Beta)
0.15
0.20
0.25
Ages (years)
0
20
40
60
80
A
B
C
D
E
F
G
H
I
J
K
FDR (Age associated divergence) =0.0802
p(Scalp)=0.00604
p(Dura)=0.422
Scalp
Dura

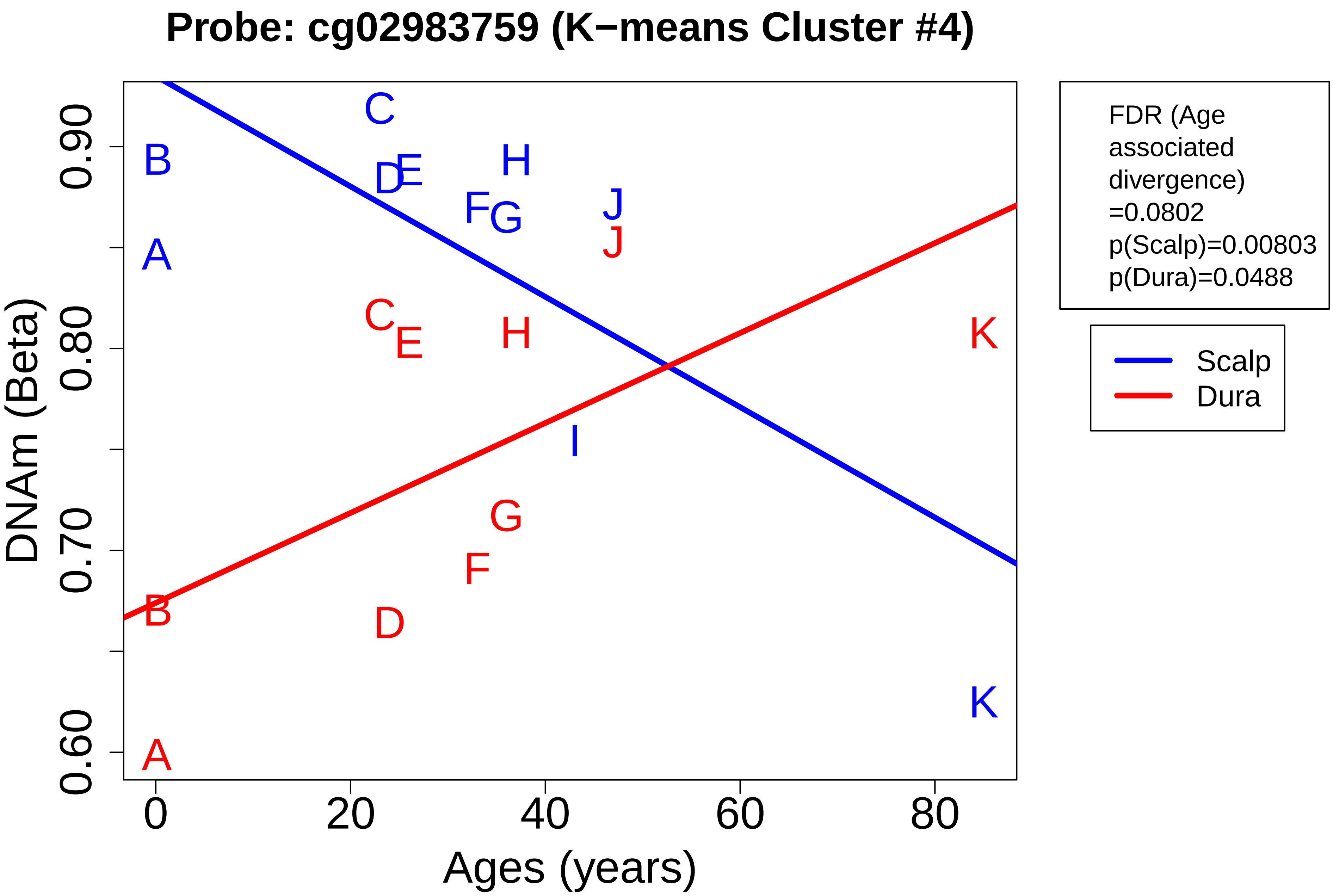

Probe: cg02983759 (K−means Cluster #4)
DNAm (Beta)
0.60
0.70
0.80
0.90
0
20
40
60
80
Ages (years)
A
B
C
D
E
F
G
H
I
J
K
FDR (Age associated divergence) =0.0802
p(Scalp)=0.00803
p(Dura)=0.0488
Scalp
Dura

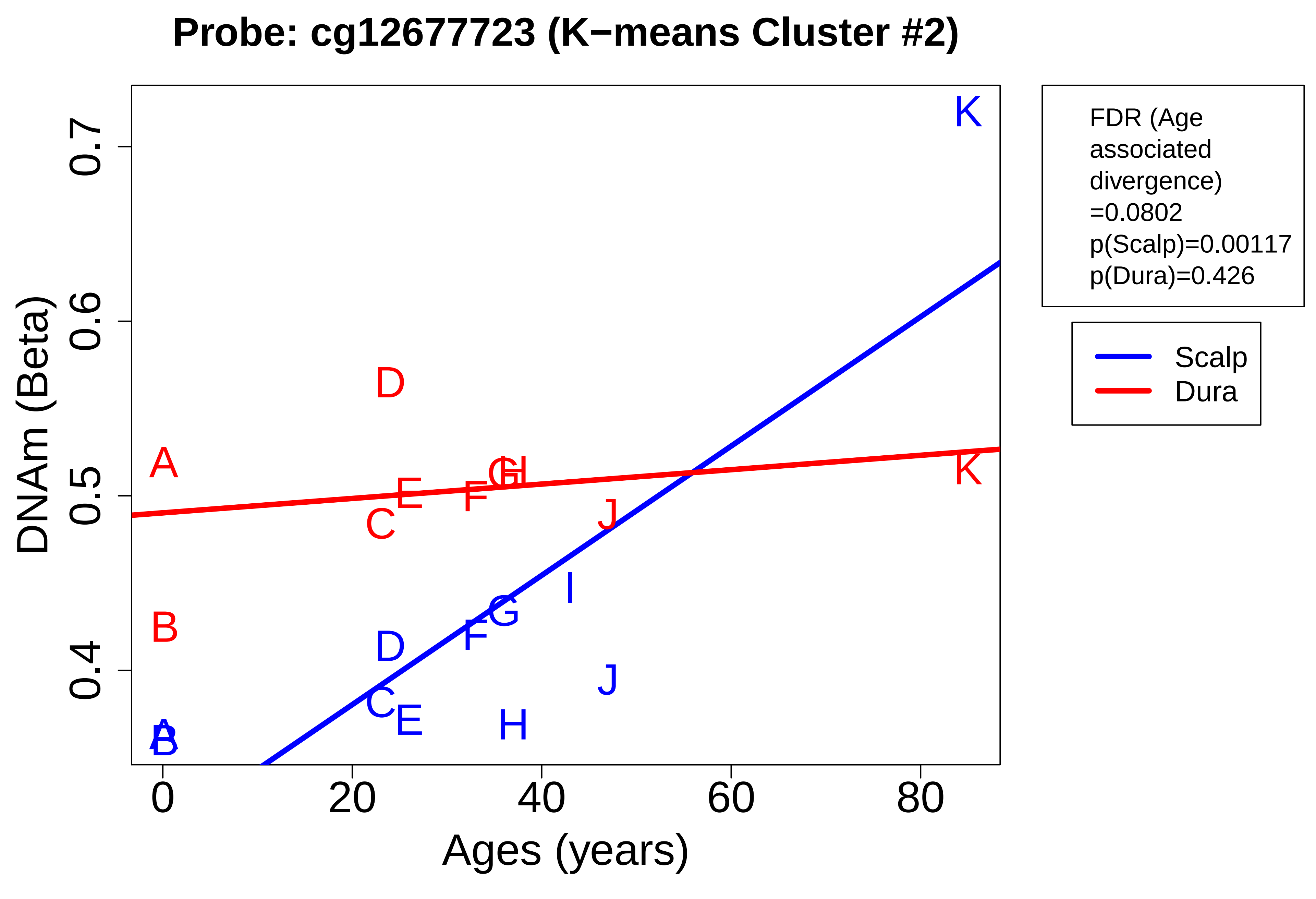
Probe: cg12677723 (K−means Cluster #2)
DNAm (Beta)
0.4
0.5
0.6
0.7
Ages (years)
0
20
40
60
80
FDR (Age associated divergence) =0.0802
p(Scalp)=0.00117
p(Dura)=0.426
Scalp
Dura
A
B
C
D
E
F
G
H
I
J
K

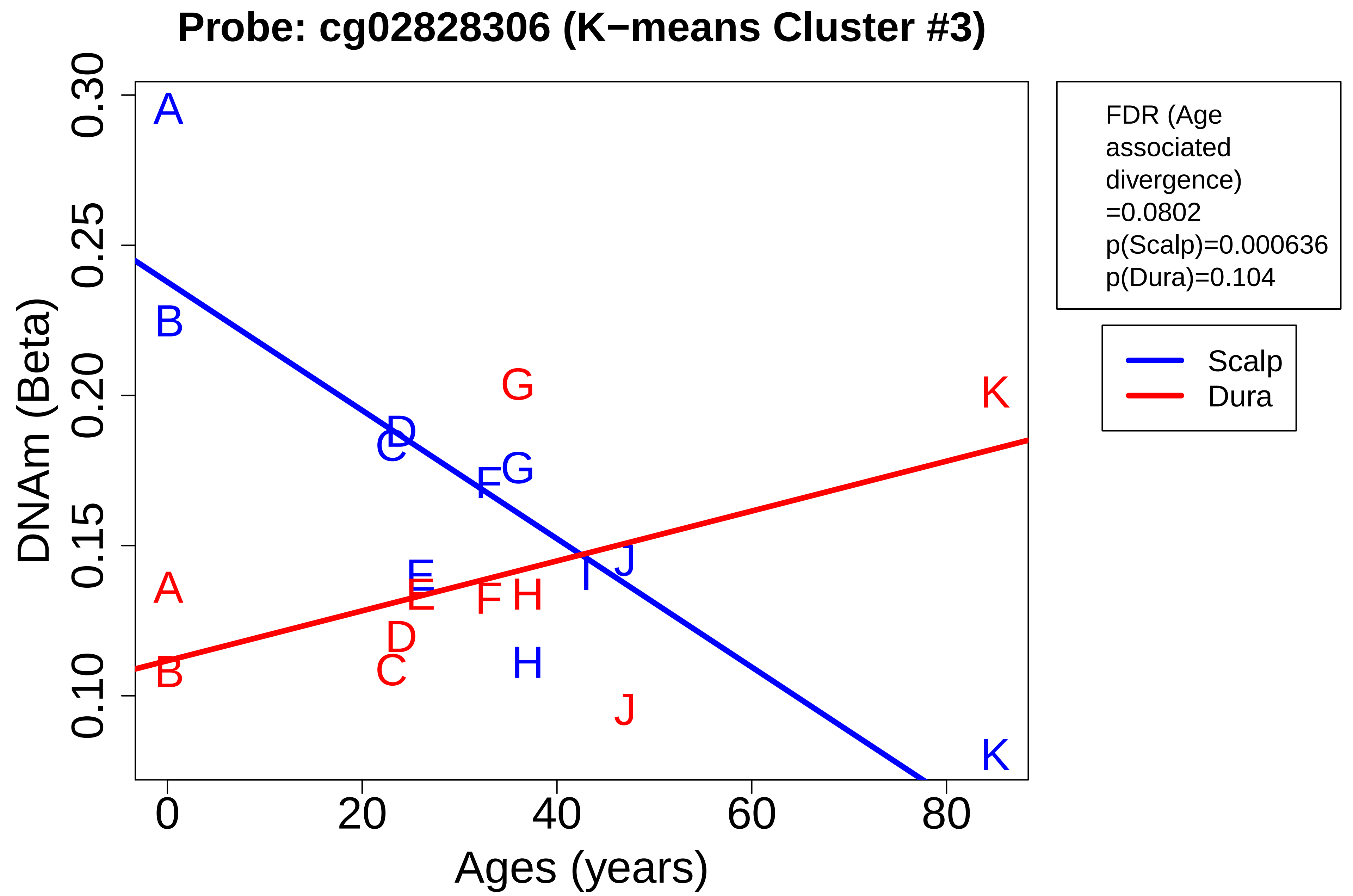

Probe: cg02828306 (K−means Cluster #3)
DNAm (Beta)
0.10
0.15
0.20
0.25
0.30
Ages (years)
0
20
40
60
80
FDR (Age associated divergence) =0.0802
p(Scalp)=0.000636
p(Dura)=0.104
Scalp
Dura
A
B
C
D
E
F
G
H
I
J
K

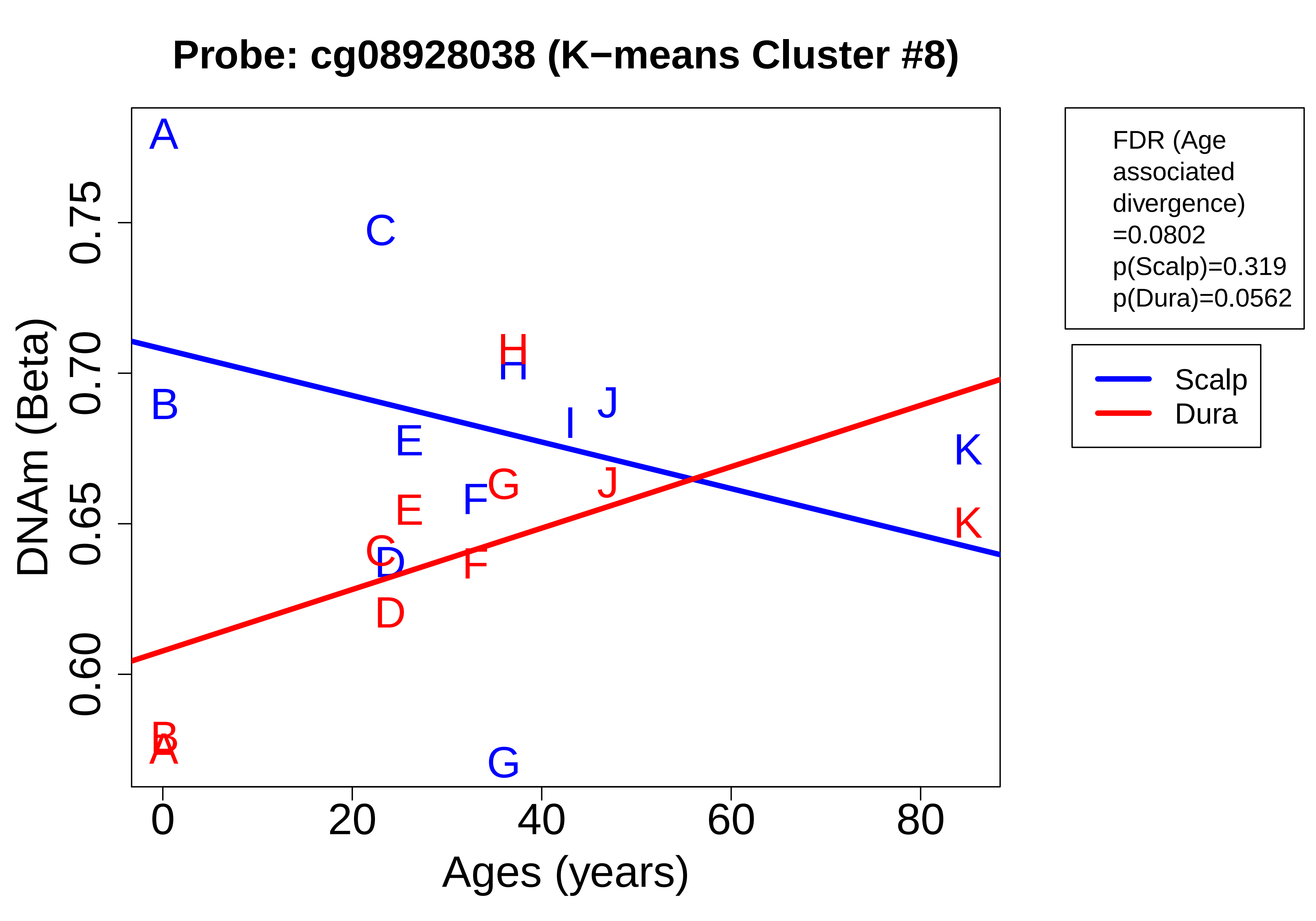
Probe: cg08928038 (K−means Cluster #8)
DNAm (Beta)
0.60
0.65
0.70
0.75
Ages (years)
0
20
40
60
80
A
B
C
D
E
F
G
H
I
J
K
FDR (Age associated divergence) =0.0802
p(Scalp)=0.319
p(Dura)=0.0562
Scalp
Dura

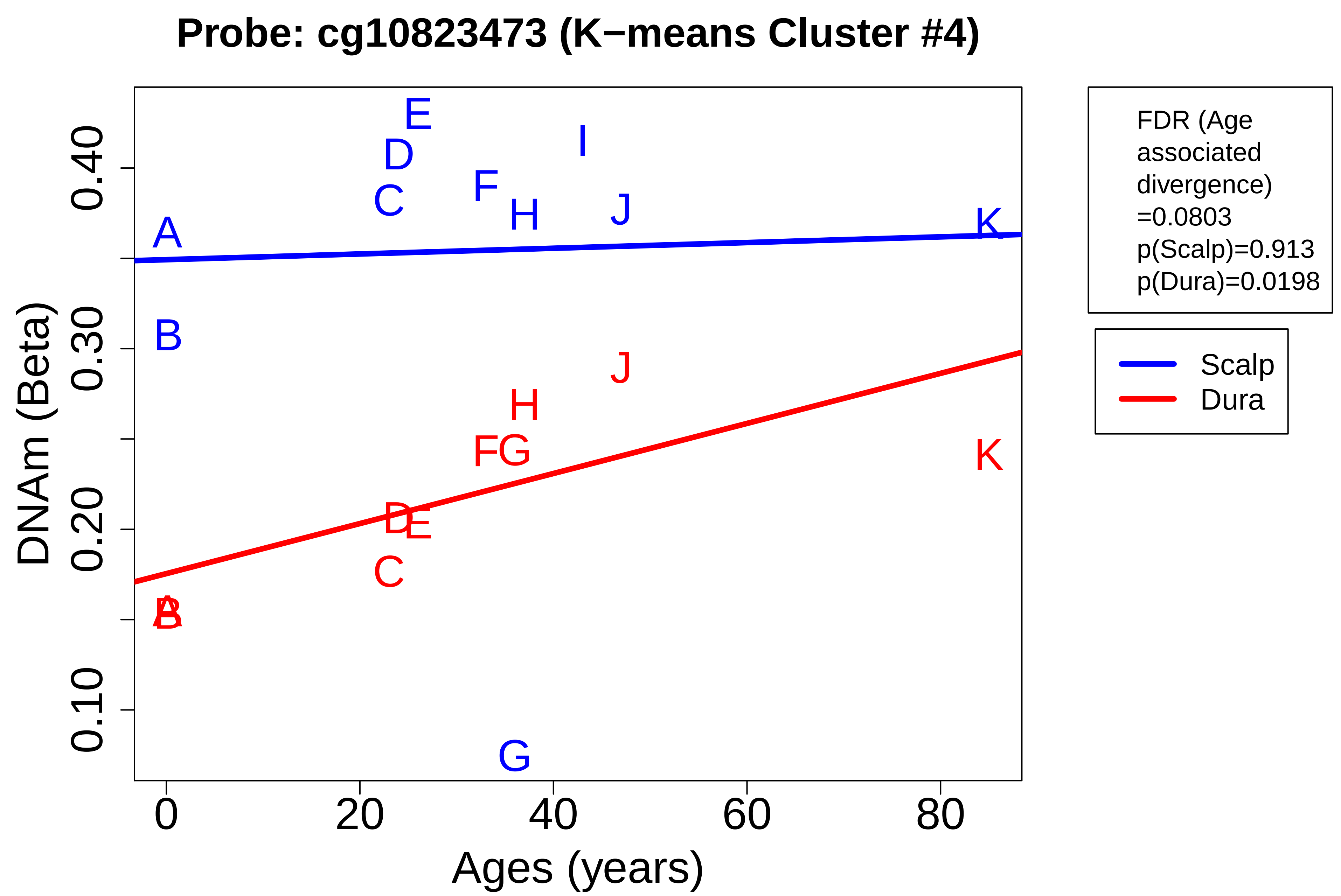

Probe: cg10823473 (K−means Cluster #4)
DNAm (Beta)
0.10
0.20
0.30
0.40
Ages (years)
0
20
40
60
80
FDR (Age associated divergence) =0.0803
p(Scalp)=0.913
p(Dura)=0.0198
Scalp
Dura

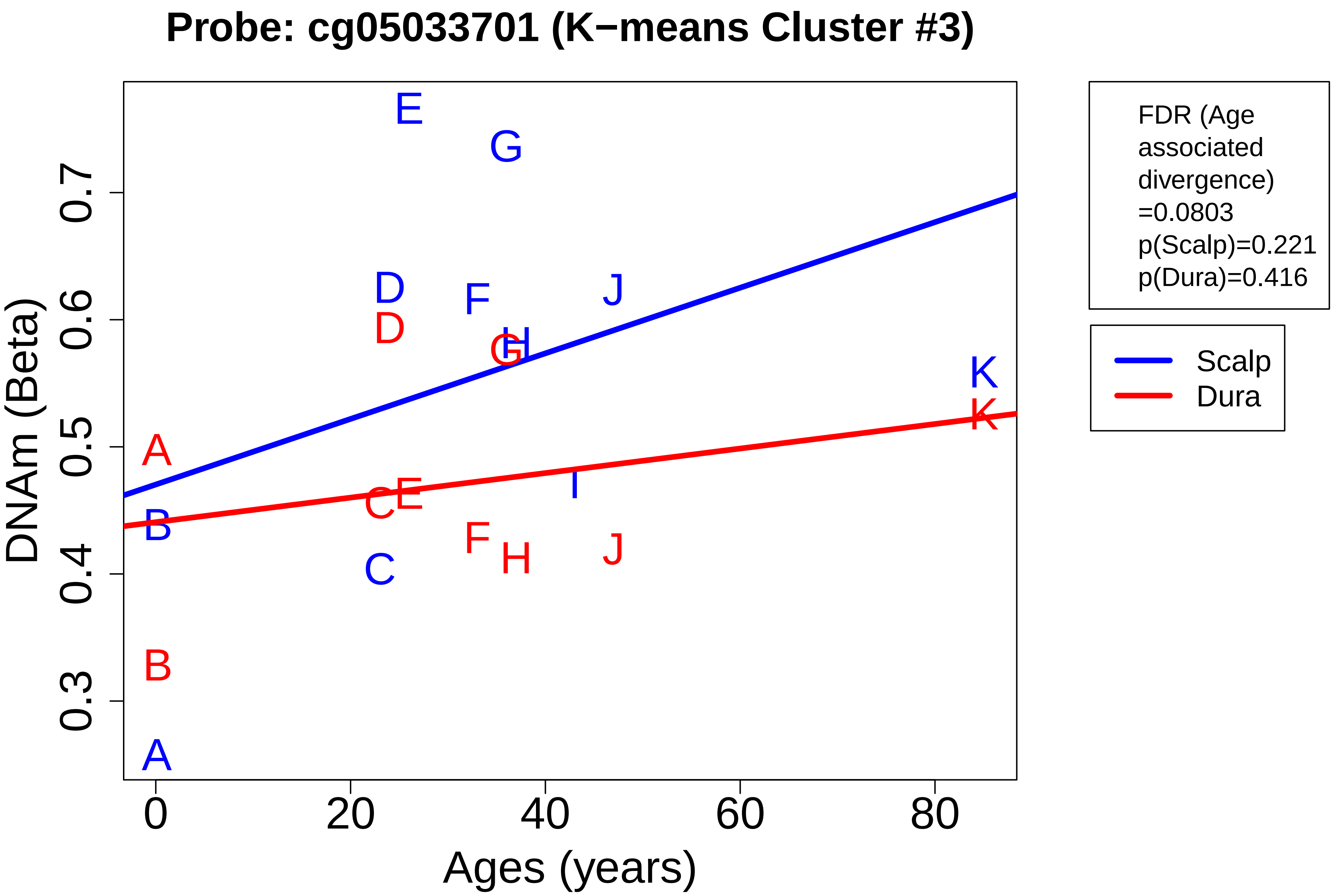
Probe: cg05033701 (K−means Cluster #3)
FDR (Age associated divergence) =0.0803
p(Scalp)=0.221
p(Dura)=0.416
Scalp
Dura
DNAm (Beta)
0.3
0.4
0.5
0.6
0.7
0
20
40
60
80
Ages (years)
A
B
C
D
E
F
G
H
I
J
K

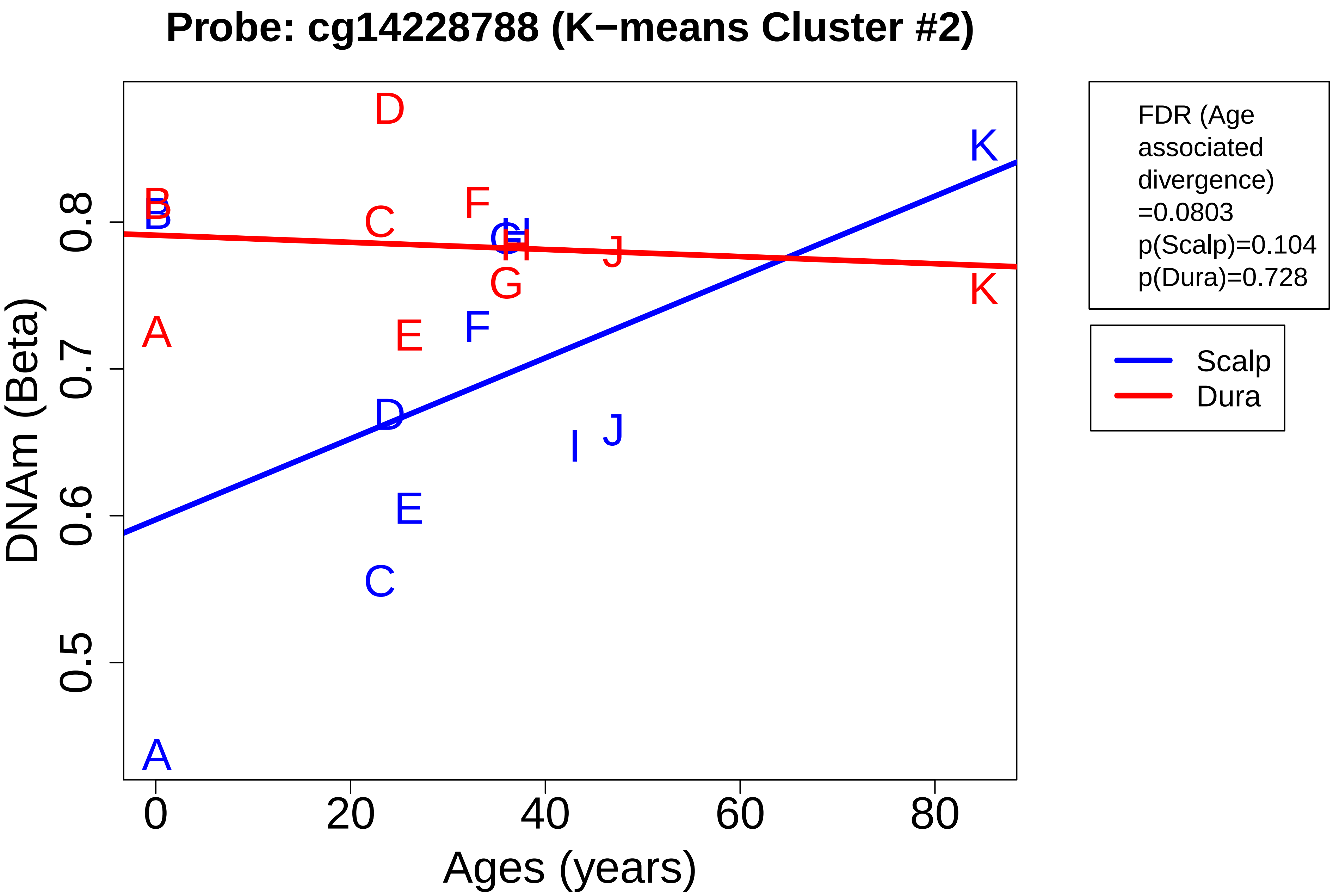

Probe: cg14228788 (K−means Cluster #2)
DNAm (Beta)
0.5
0.6
0.7
0.8
Ages (years)
0
20
40
60
80
FDR (Age associated divergence) =0.0803
p(Scalp)=0.104
p(Dura)=0.728
Scalp
Dura

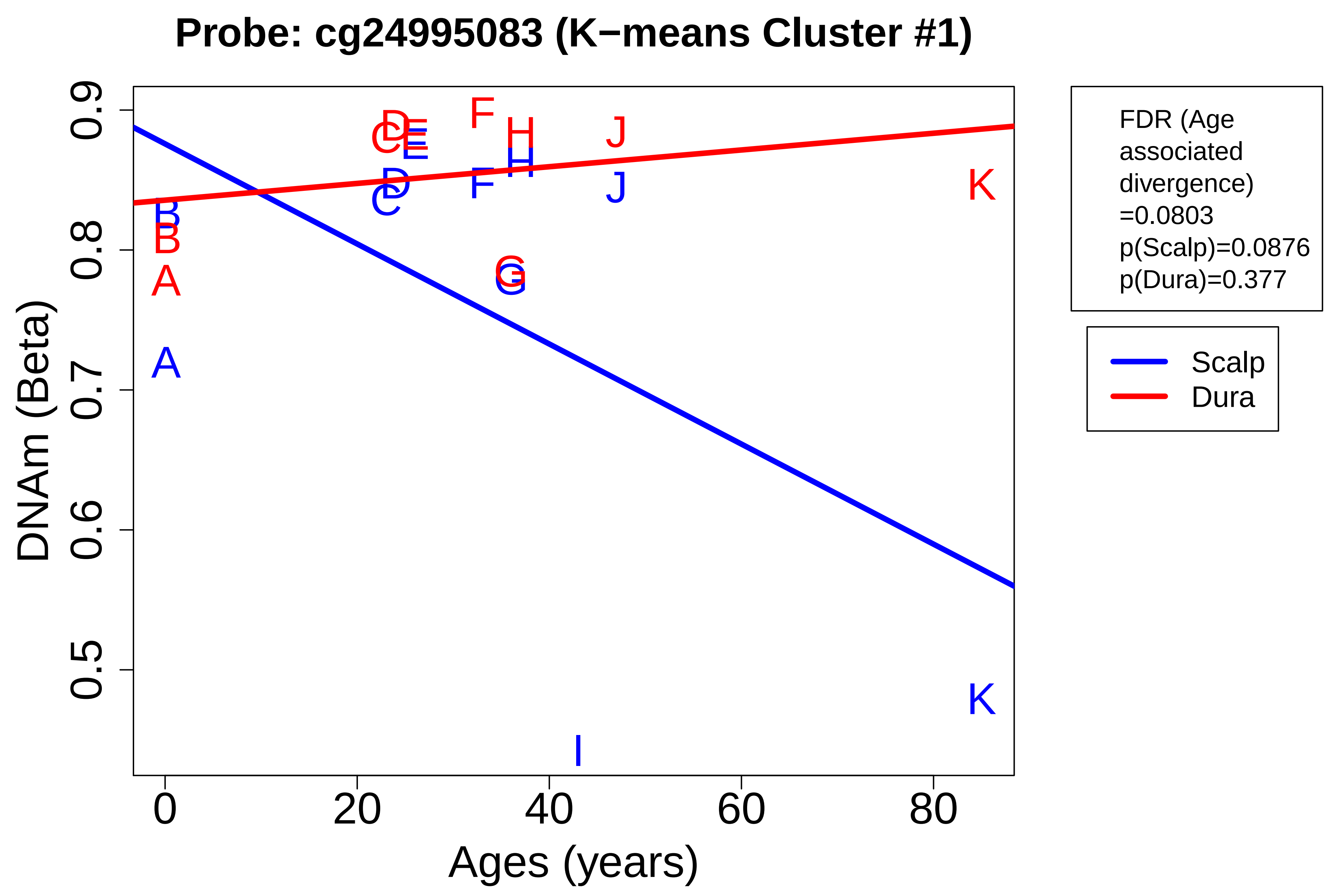

Probe: cg24995083 (K−means Cluster #1)
DNAm (Beta)
0.5
0.6
0.7
0.8
0.9
0
20
40
60
80
Ages (years)
FDR (Age associated divergence) =0.0803
p(Scalp)=0.0876
p(Dura)=0.377
Scalp
Dura

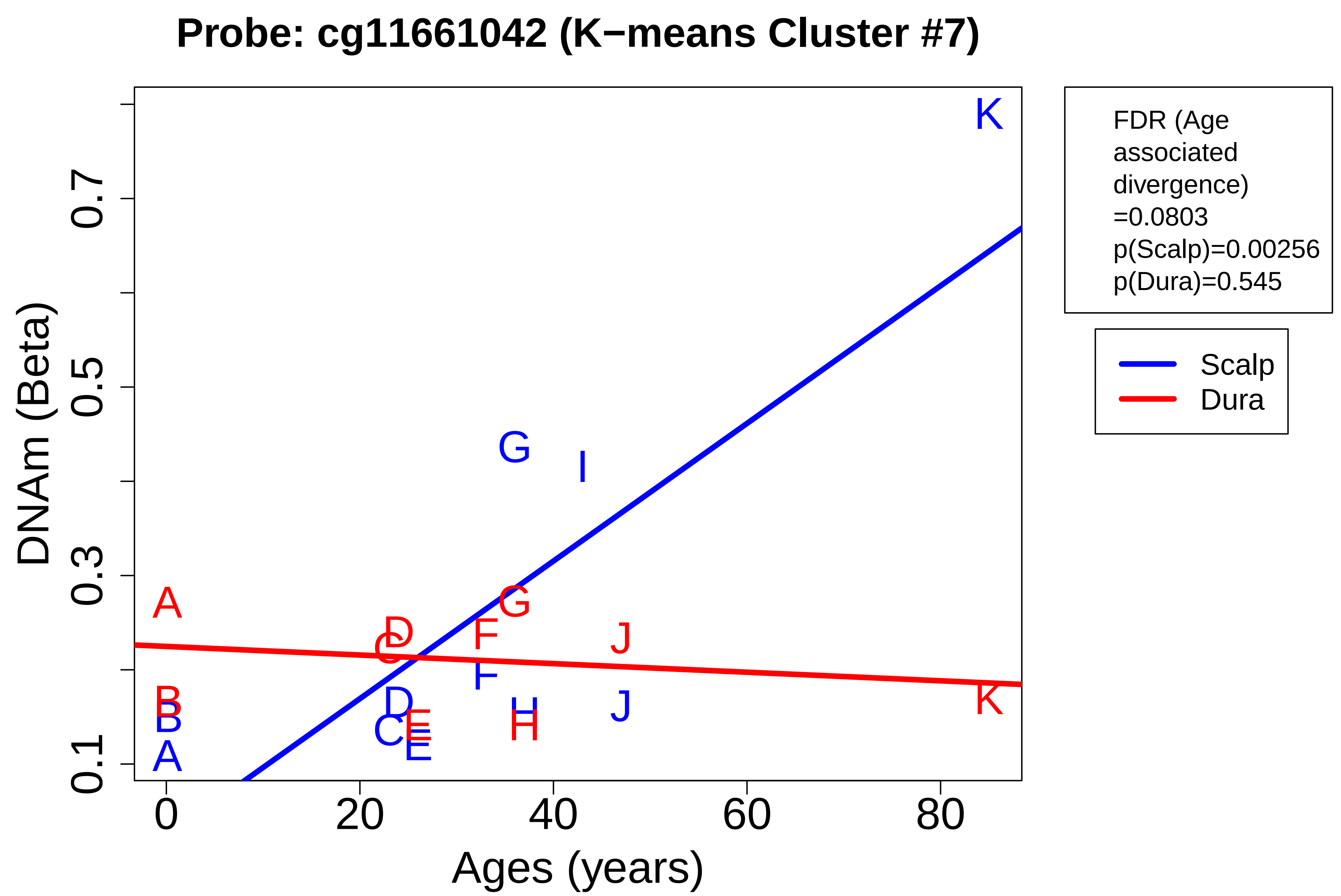

Probe: cg11661042 (K−means Cluster #7)
DNAm (Beta)
0.1
0.3
0.5
0.7
0
20
40
60
80
Ages (years)
FDR (Age associated divergence) =0.0803
p(Scalp)=0.00256
p(Dura)=0.545
Scalp
Dura
A
B
C
D
E
F
G
H
I
J
K

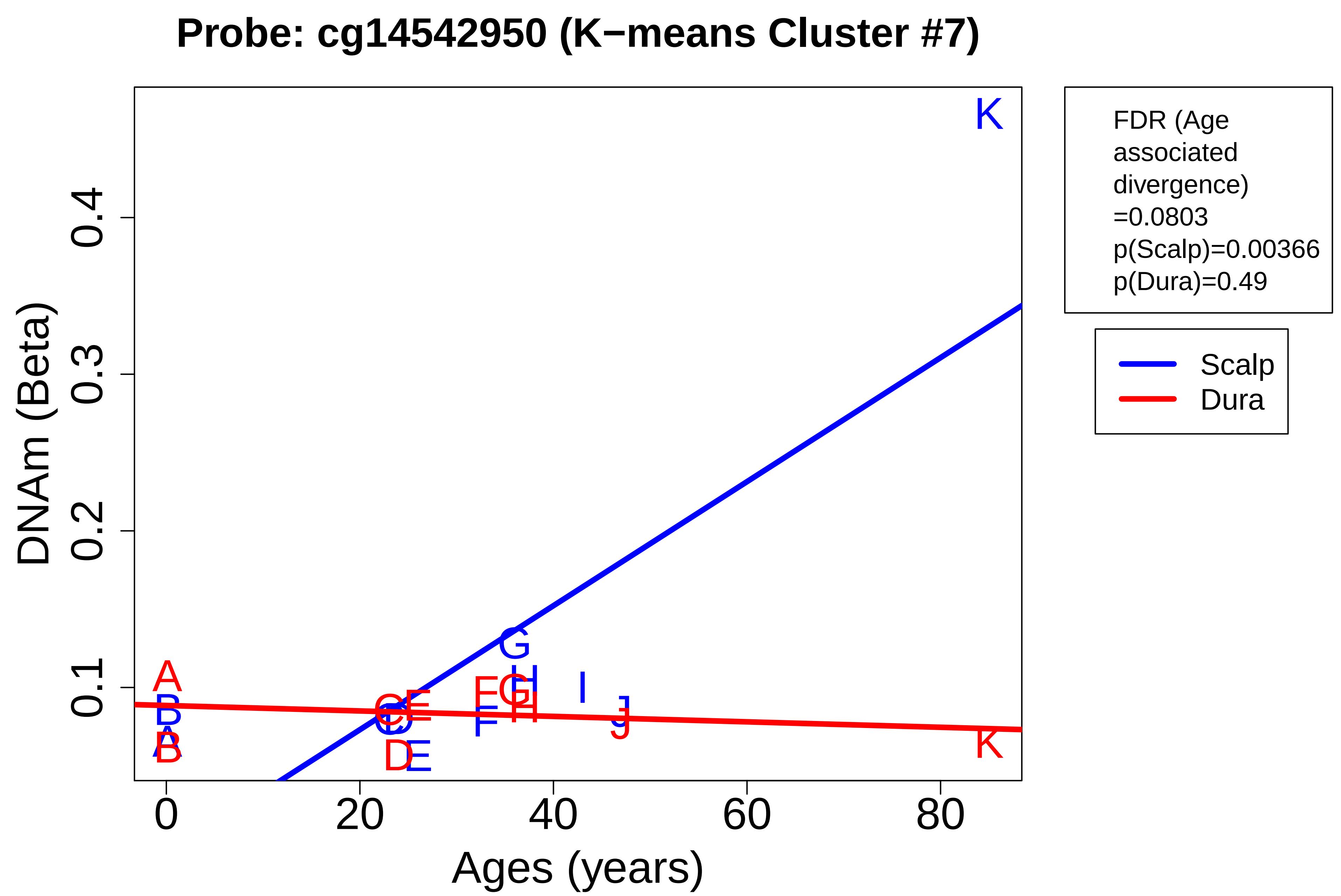

Probe: cg14542950 (K−means Cluster #7)
DNAm (Beta)
0.1
0.2
0.3
0.4
0
20
40
60
80
Ages (years)
FDR (Age associated divergence) =0.0803
p(Scalp)=0.00366
p(Dura)=0.49
Scalp
Dura

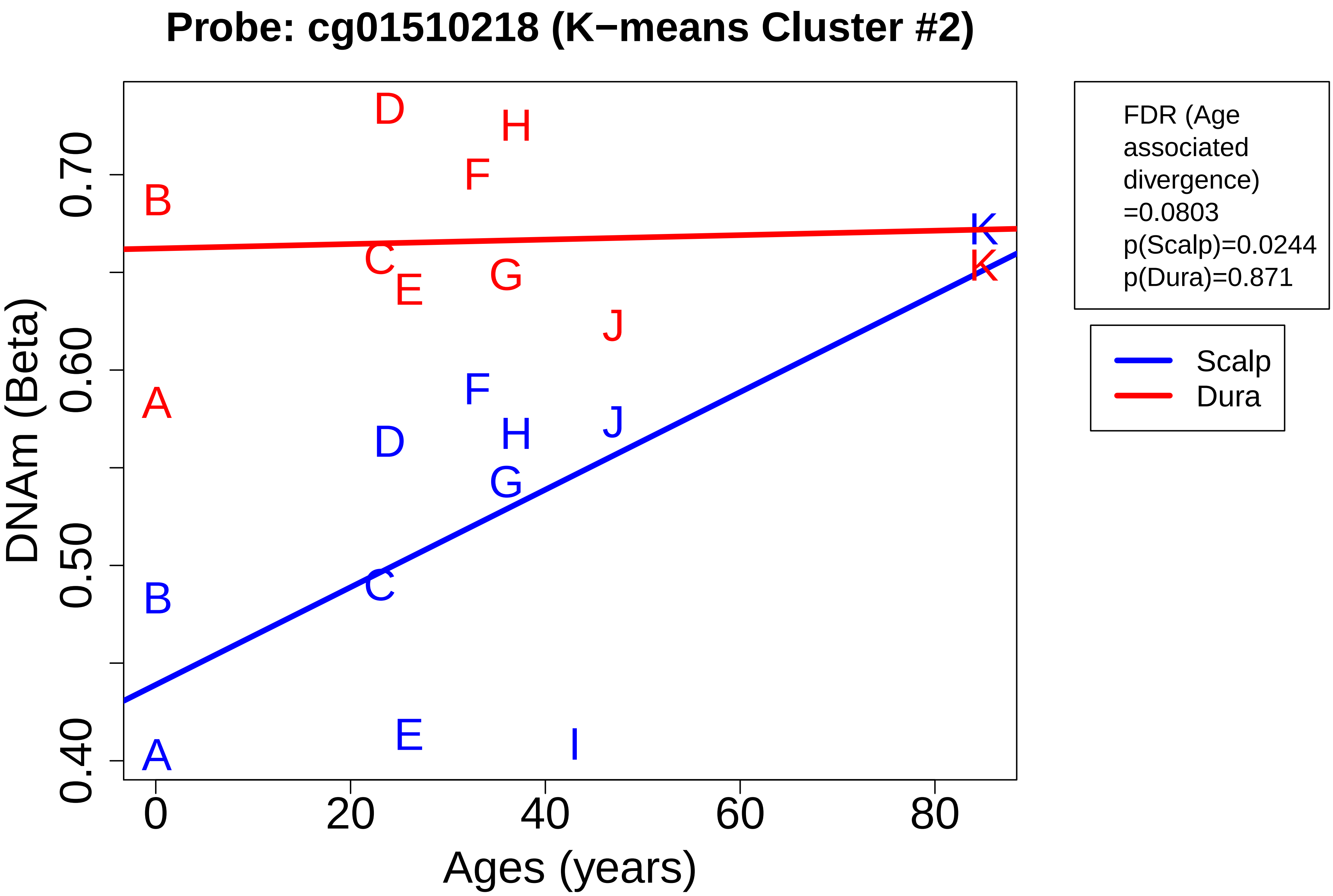
Probe: cg01510218 (K−means Cluster #2)
DNAm (Beta)
0.40
0.50
0.60
0.70
0
20
40
60
80
Ages (years)
A
B
C
D
E
F
G
H
I
J
K
FDR (Age associated divergence) =0.0803
p(Scalp)=0.0244
p(Dura)=0.871
Scalp
Dura

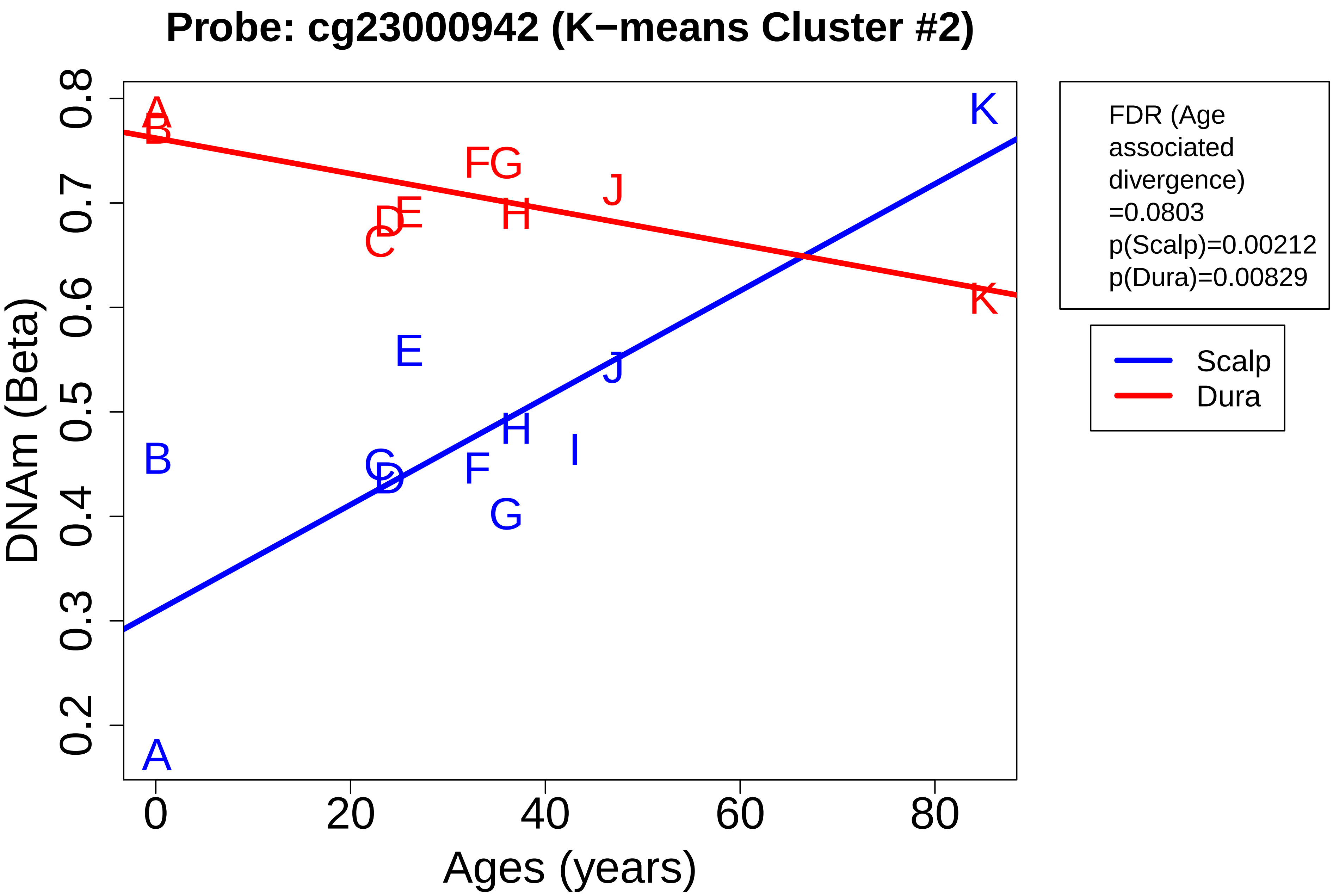

Probe: cg23000942 (K−means Cluster #2)
DNAm (Beta)
0.2
0.3
0.4
0.5
0.6
0.7
0.8
0
20
40
60
80
Ages (years)
A
B
C
D
E
F
G
H
I
J
K
FDR (Age associated divergence) =0.0803
p(Scalp)=0.00212
p(Dura)=0.00829
Scalp
Dura

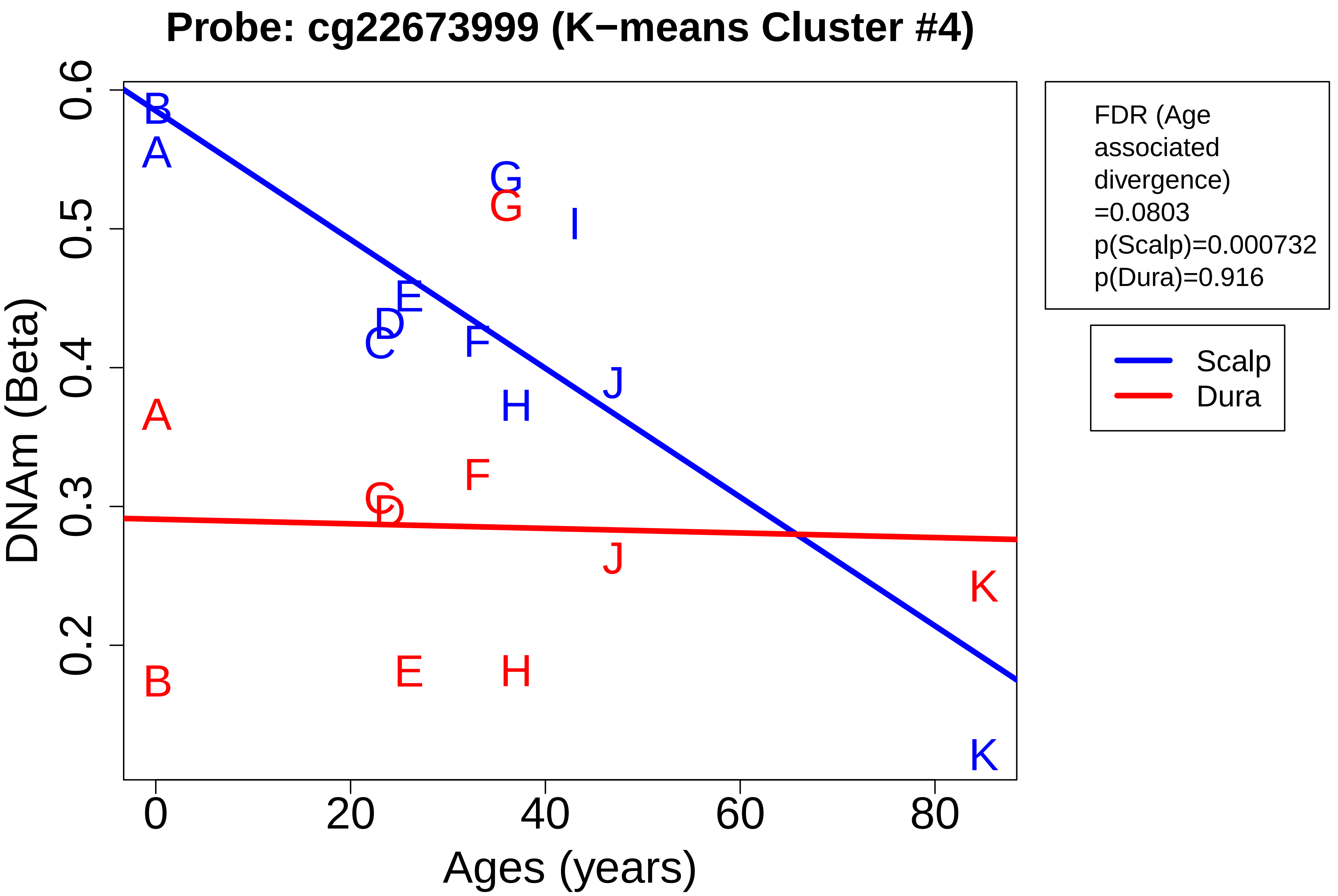

Probe: cg22673999 (K−means Cluster #4)
DNAm (Beta)
Ages (years)
0.2
0.3
0.4
0.5
0.6
0
20
40
60
80
FDR (Age associated divergence) =0.0803
p(Scalp)=0.000732
p(Dura)=0.916
Scalp
Dura

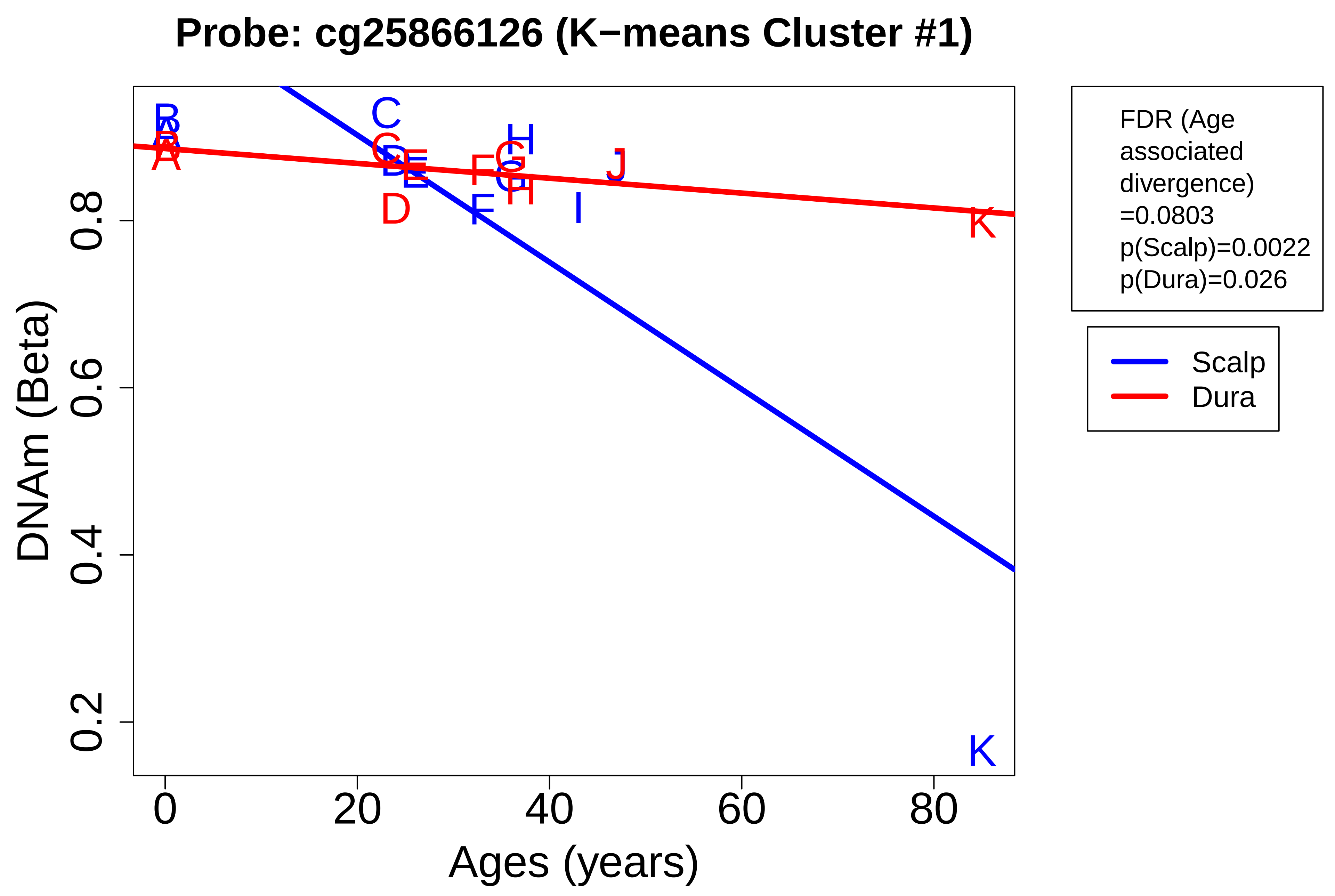

Probe: cg25866126 (K−means Cluster #1)
DNAm (Beta)
0.2
0.4
0.6
0.8
0
20
40
60
80
Ages (years)
FDR (Age associated divergence) =0.0803
p(Scalp)=0.0022
p(Dura)=0.026
Scalp
Dura

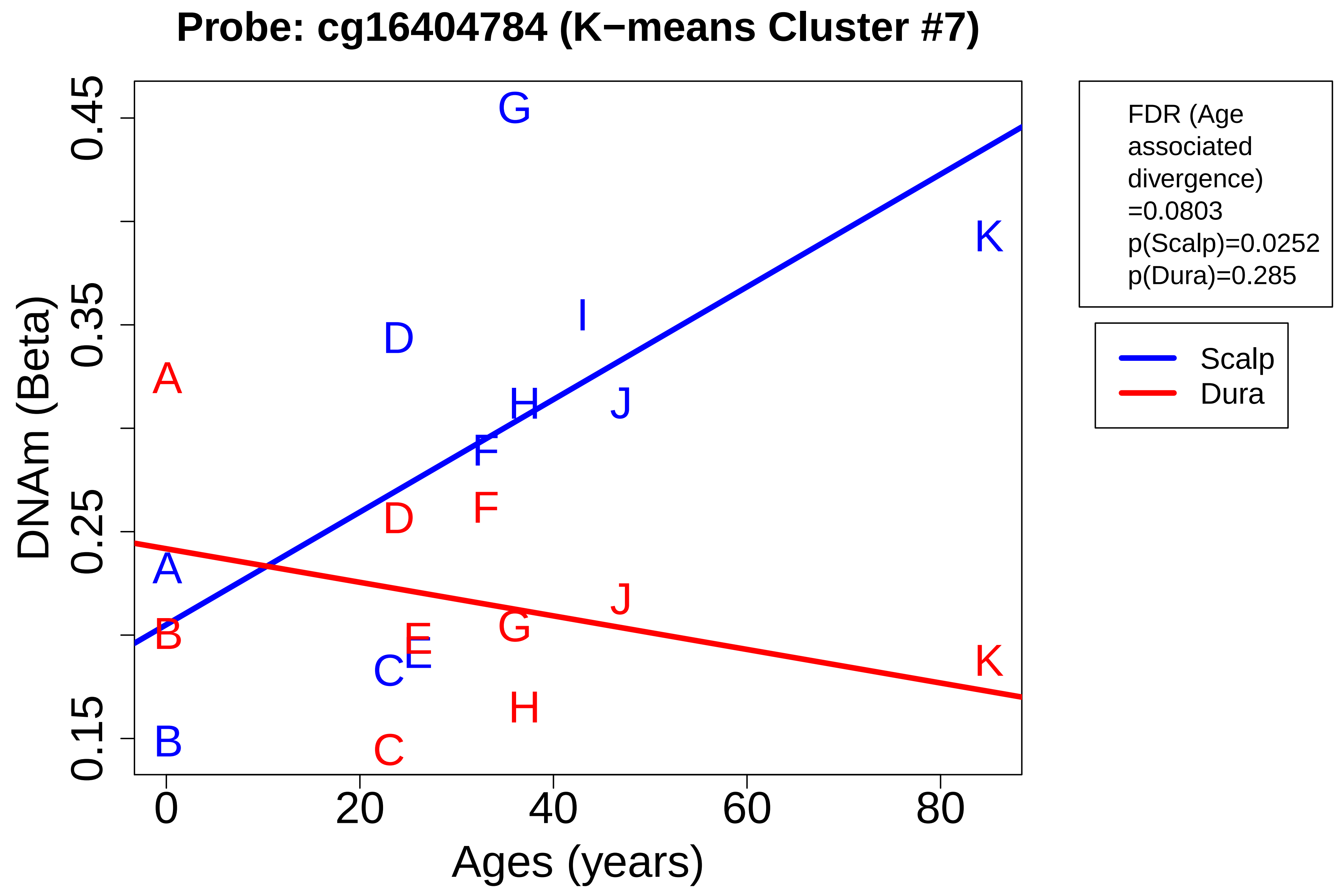
Probe: cg16404784 (K−means Cluster #7)
DNAm (Beta)
Ages (years)
0.15
0.25
0.35
0.45
0
20
40
60
80
FDR (Age associated divergence) =0.0803
p(Scalp)=0.0252
p(Dura)=0.285
Scalp
Dura
A
B
C
D
E
F
G
H
I
J
K

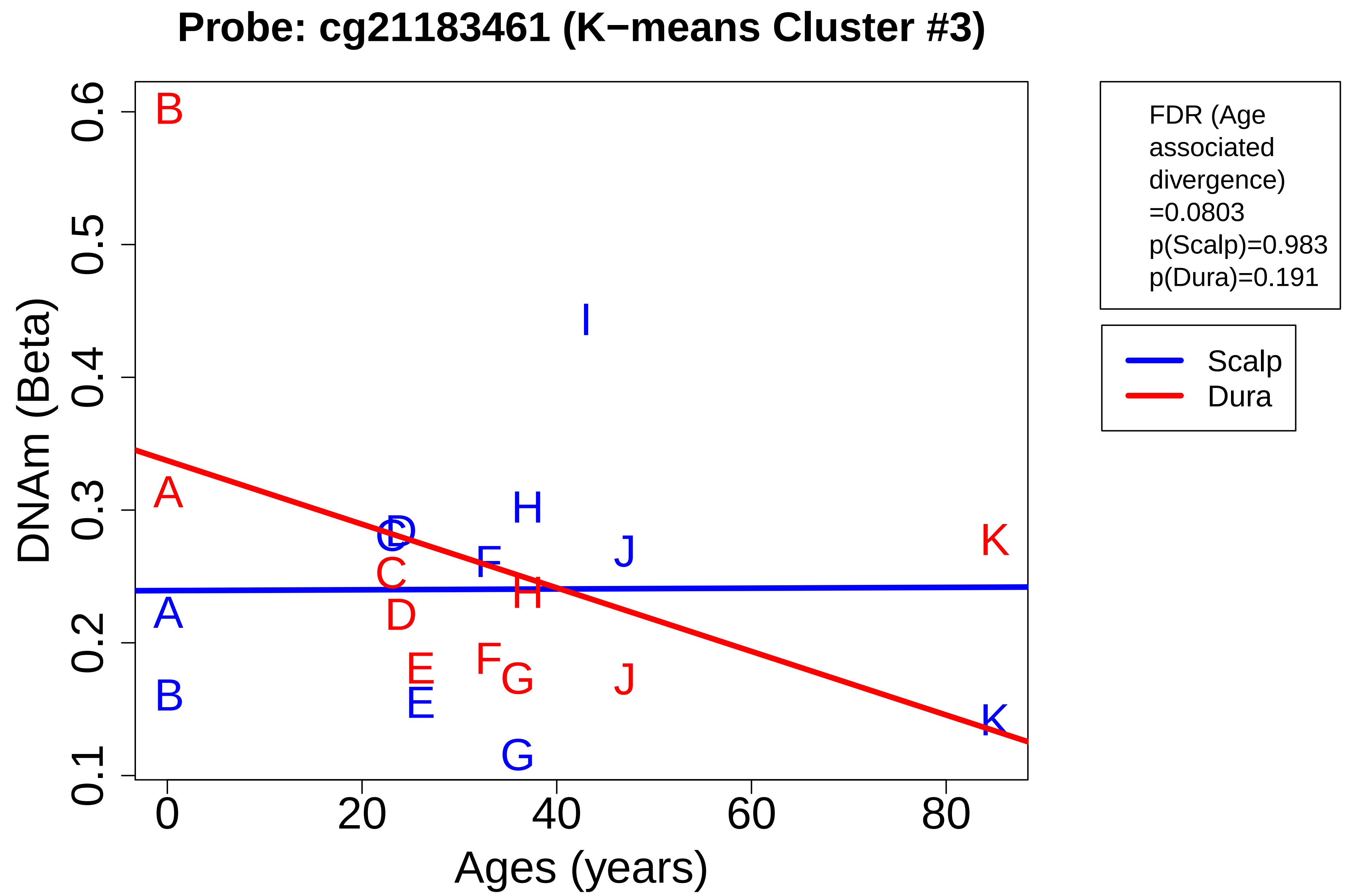

Probe: cg21183461 (K−means Cluster #3)
FDR (Age associated divergence) =0.0803
p(Scalp)=0.983
p(Dura)=0.191
Scalp
Dura
DNAm (Beta)
Ages (years)
0.1
0.2
0.3
0.4
0.5
0.6
0
20
40
60
80

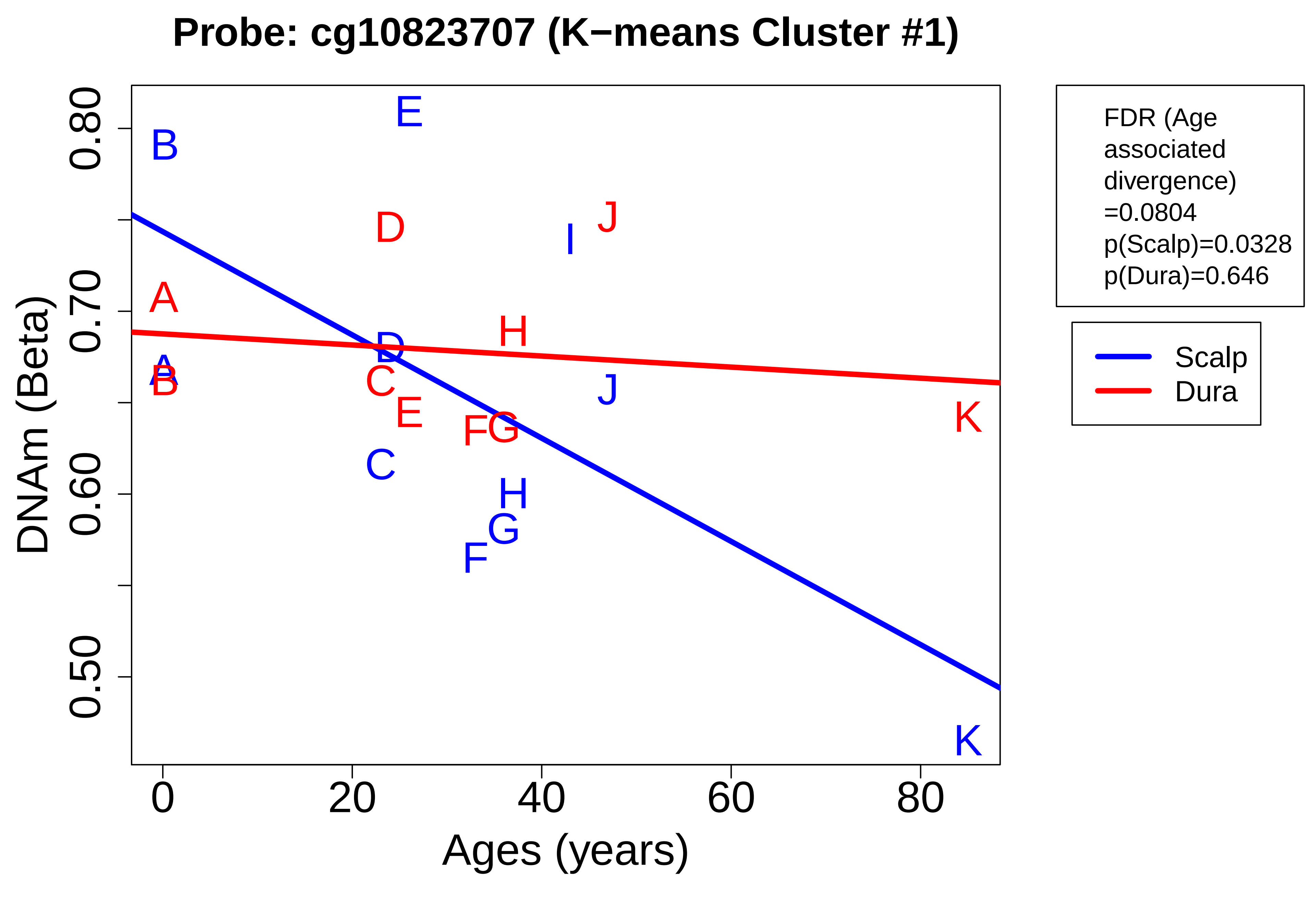
Probe: cg10823707 (K−means Cluster #1)
FDR (Age associated divergence) =0.0804
p(Scalp)=0.0328
p(Dura)=0.646
Scalp
Dura
DNAm (Beta)
0.50
0.60
0.70
0.80
0
20
40
60
80
Ages (years)
A
B
C
D
E
F
G
H
I
J
K

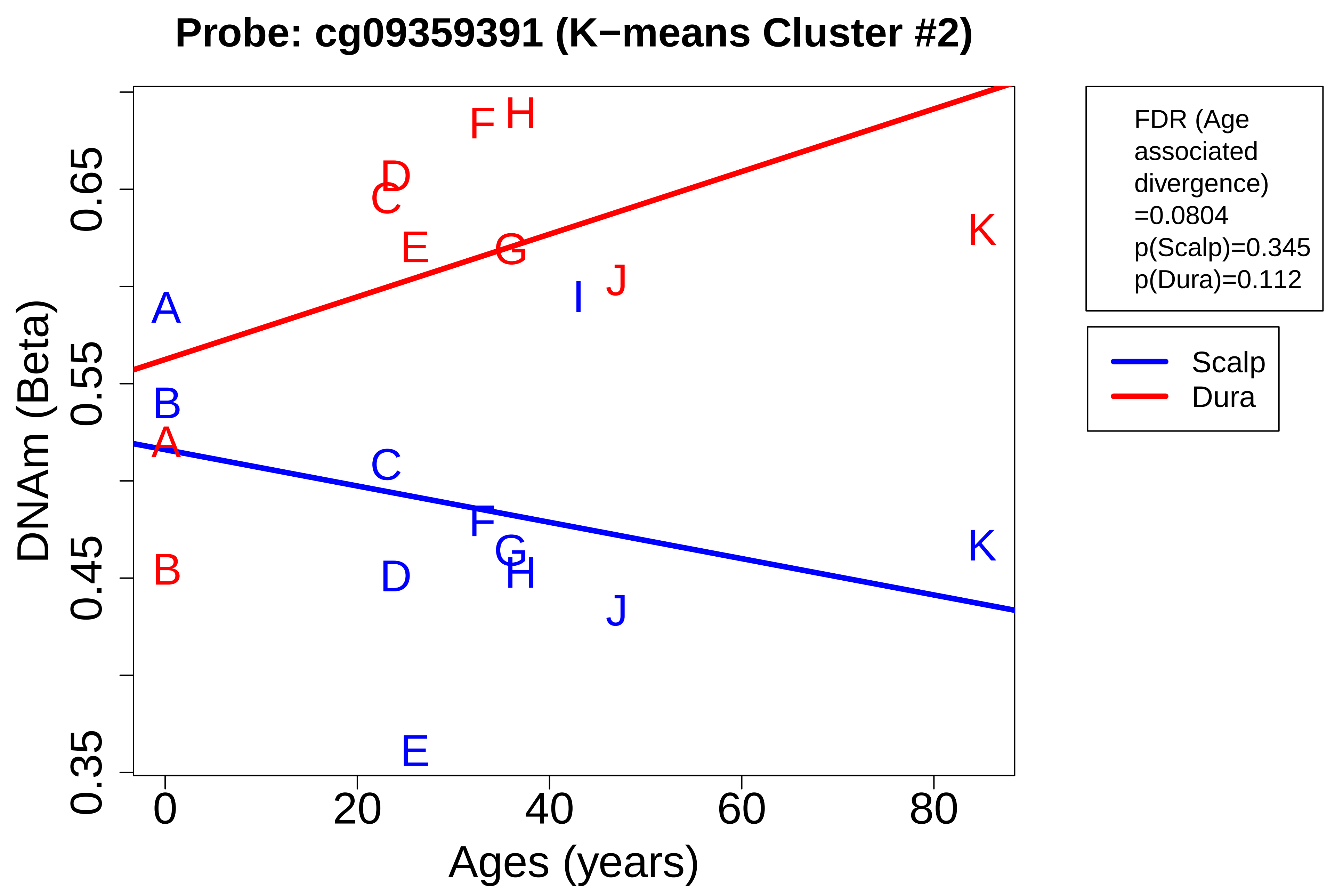

Probe: cg09359391 (K−means Cluster #2)
DNAm (Beta)
0.35
0.45
0.55
0.65
0
20
40
60
80
Ages (years)
A
B
C
D
E
F
G
H
I
J
K
FDR (Age associated divergence) =0.0804
p(Scalp)=0.345
p(Dura)=0.112
Scalp
Dura

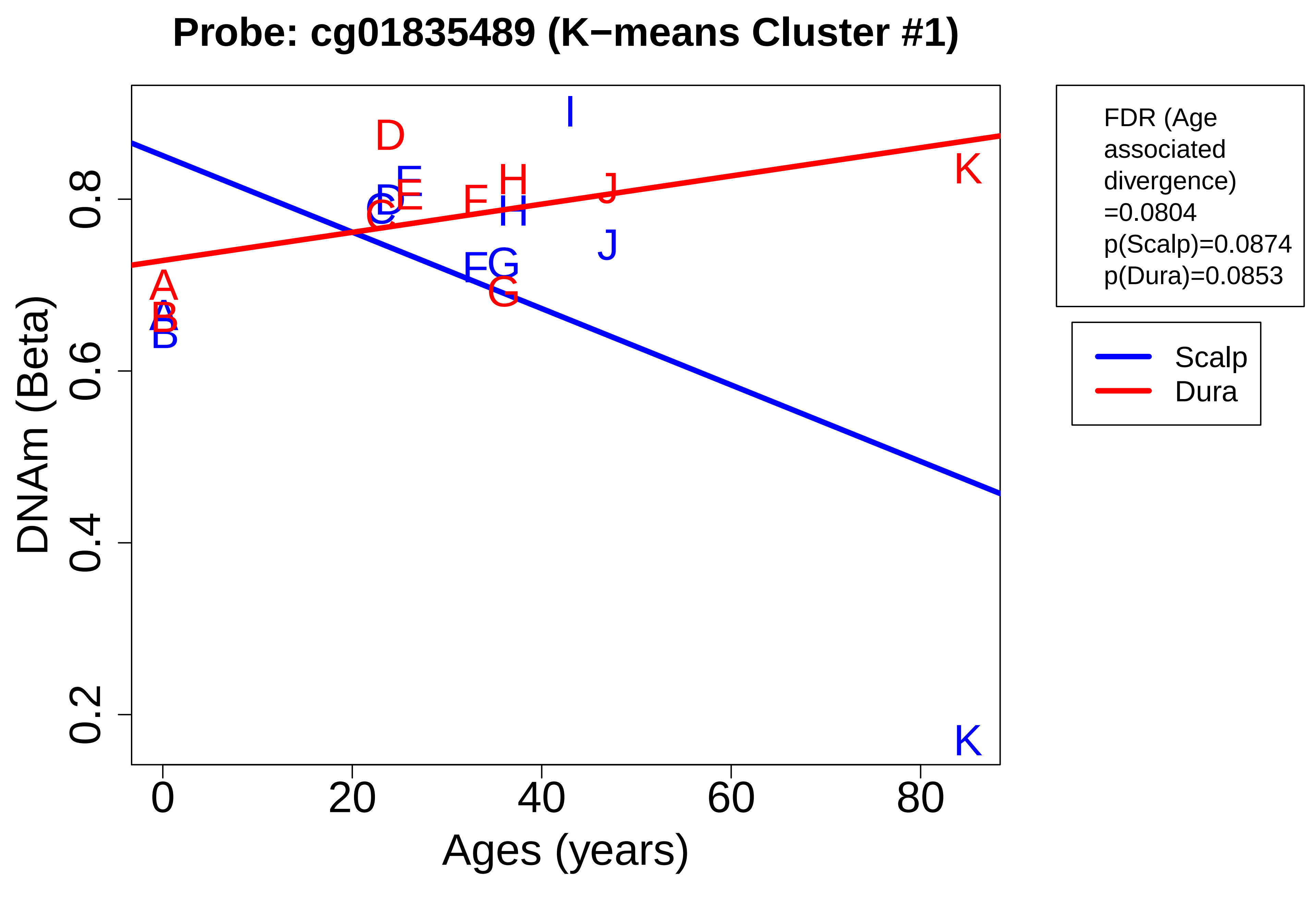
Probe: cg01835489 (K−means Cluster #1)
DNAm (Beta)
0.2
0.4
0.6
0.8
0
20
40
60
80
Ages (years)
A
B
C
D
E
F
G
H
I
J
K
FDR (Age associated divergence) =0.0804
p(Scalp)=0.0874
p(Dura)=0.0853
Scalp
Dura

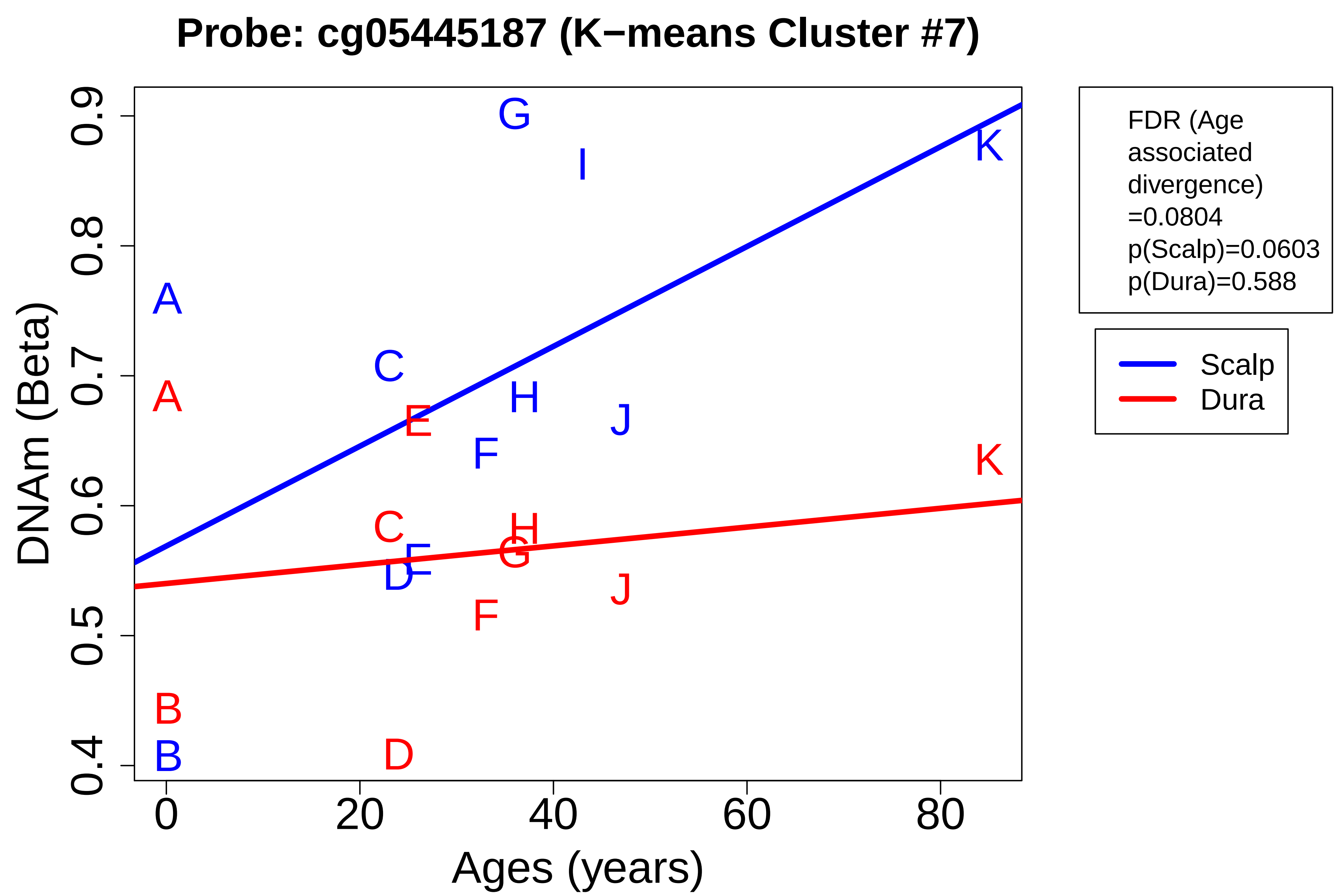

Probe: cg05445187 (K−means Cluster #7)
DNAm (Beta)
0.4
0.5
0.6
0.7
0.8
0.9
0
20
40
60
80
Ages (years)
A
B
C
D
E
F
G
H
I
J
K
A
B
C
D
E
F
G
H
J
K
FDR (Age associated divergence) =0.0804
p(Scalp)=0.0603
p(Dura)=0.588
Scalp
Dura

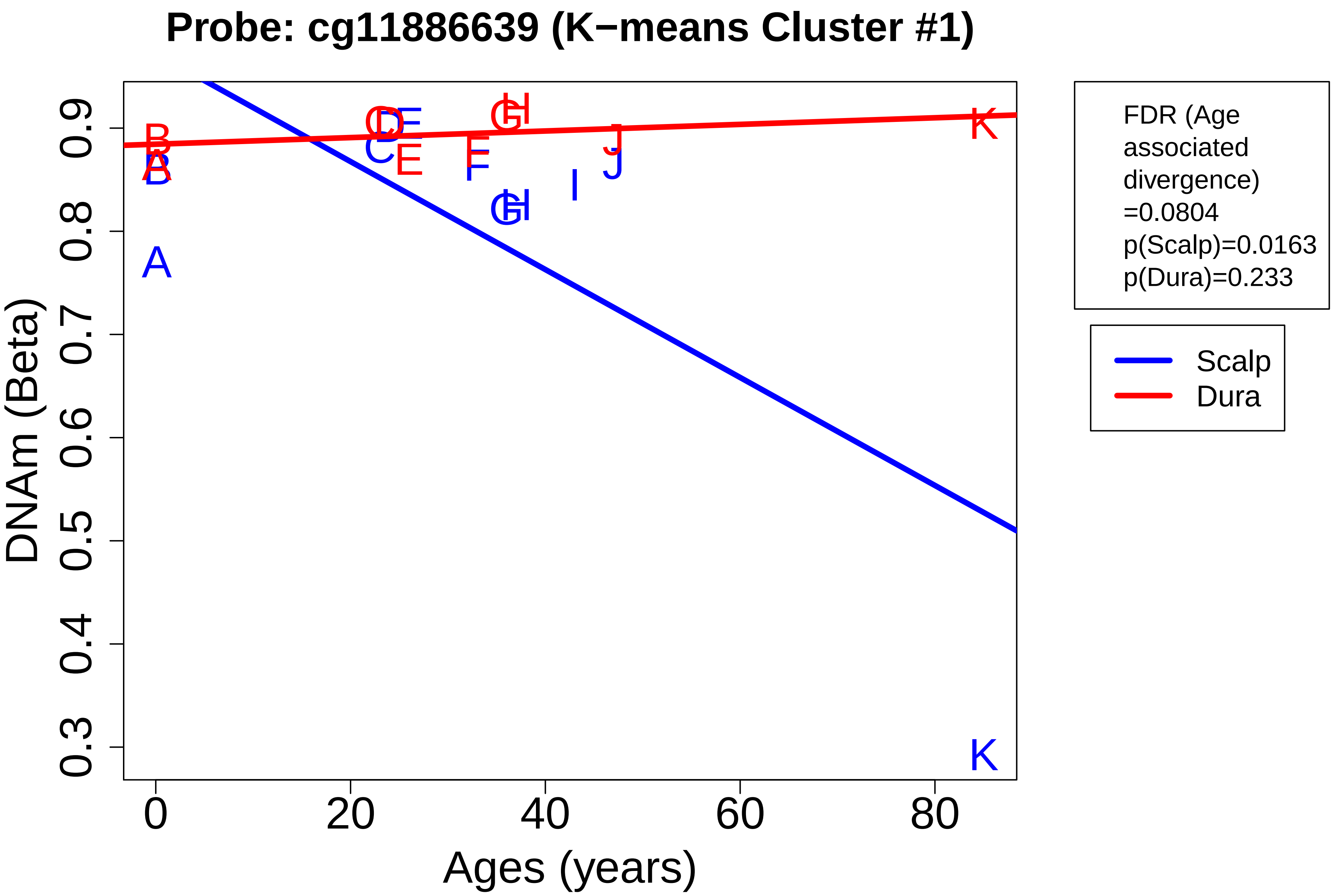
Probe: cg11886639 (K−means Cluster #1)
DNAm (Beta)
0.3
0.4
0.5
0.6
0.7
0.8
0.9
0
20
40
60
80
Ages (years)
A
B
C
D
E
F
G
H
I
J
K
FDR (Age associated divergence) =0.0804
p(Scalp)=0.0163
p(Dura)=0.233
Scalp
Dura

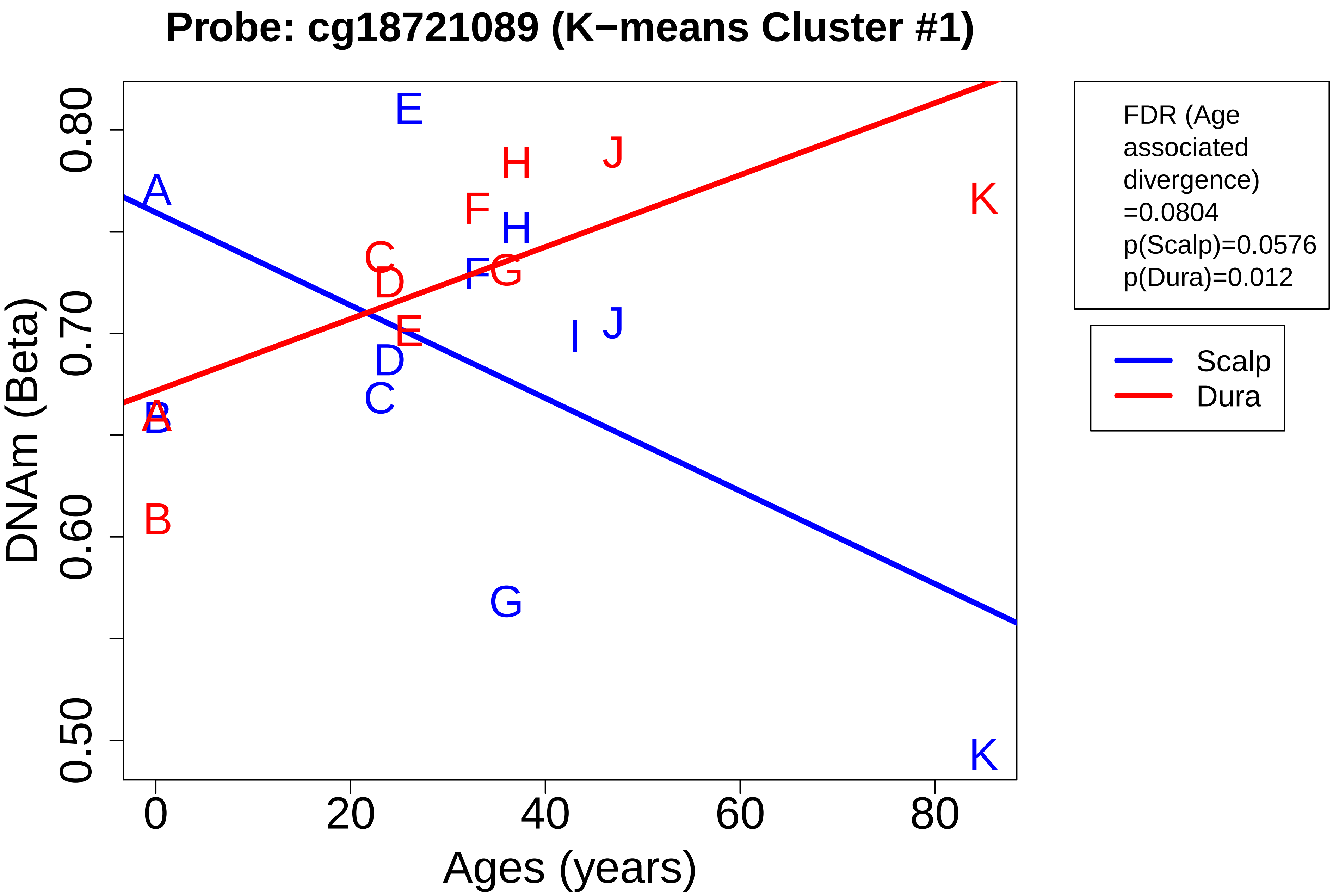

Probe: cg18721089 (K−means Cluster #1)
DNAm (Beta)
0.50
0.60
0.70
0.80
0
20
40
60
80
Ages (years)
FDR (Age associated divergence) =0.0804
p(Scalp)=0.0576
p(Dura)=0.012
Scalp
Dura

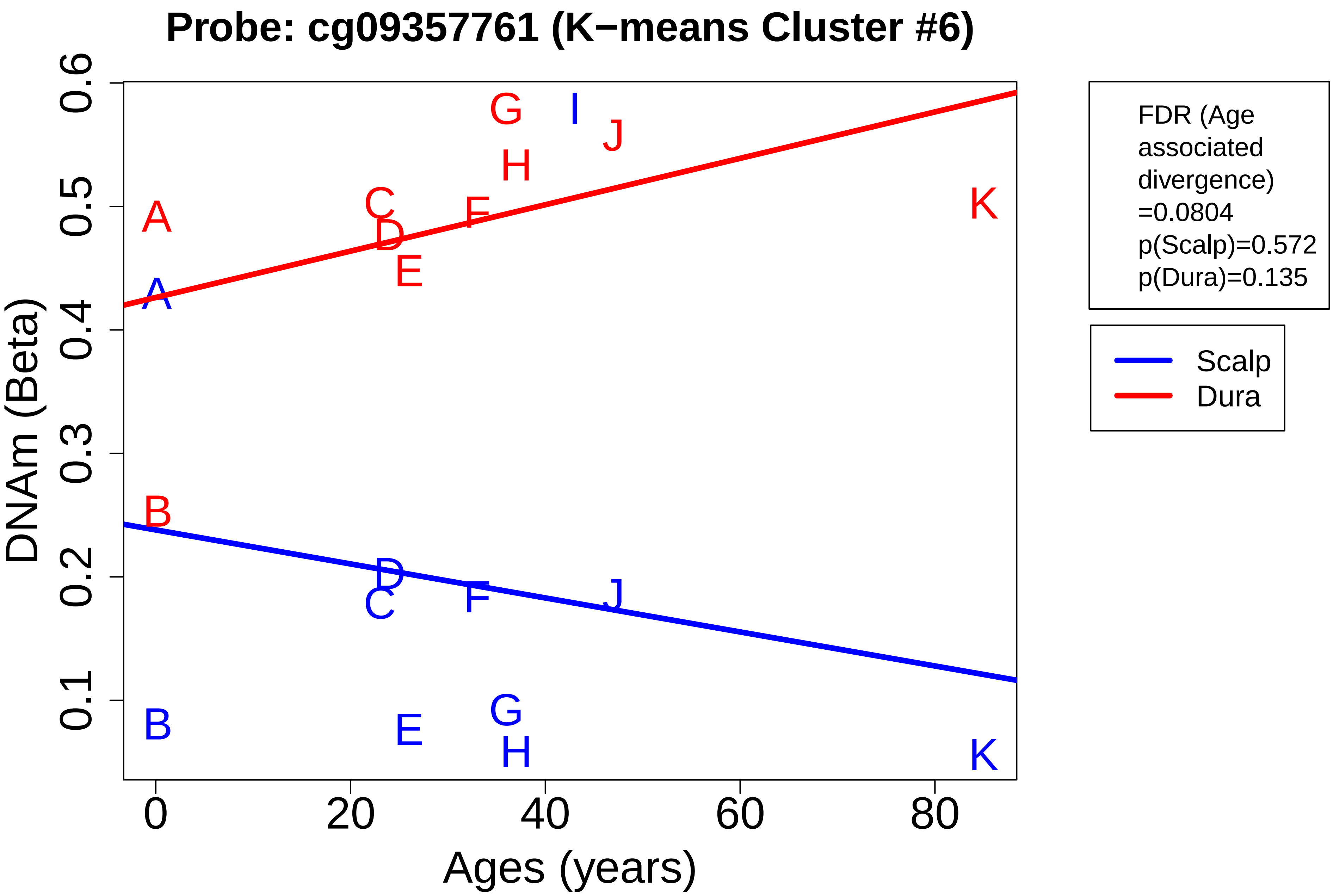

Probe: cg09357761 (K−means Cluster #6)
DNAm (Beta)
0.1
0.2
0.3
0.4
0.5
0.6
Ages (years)
0
20
40
60
80
A
B
C
D
E
F
G
H
I
J
K
FDR (Age associated divergence) =0.0804
p(Scalp)=0.572
p(Dura)=0.135
Scalp
Dura

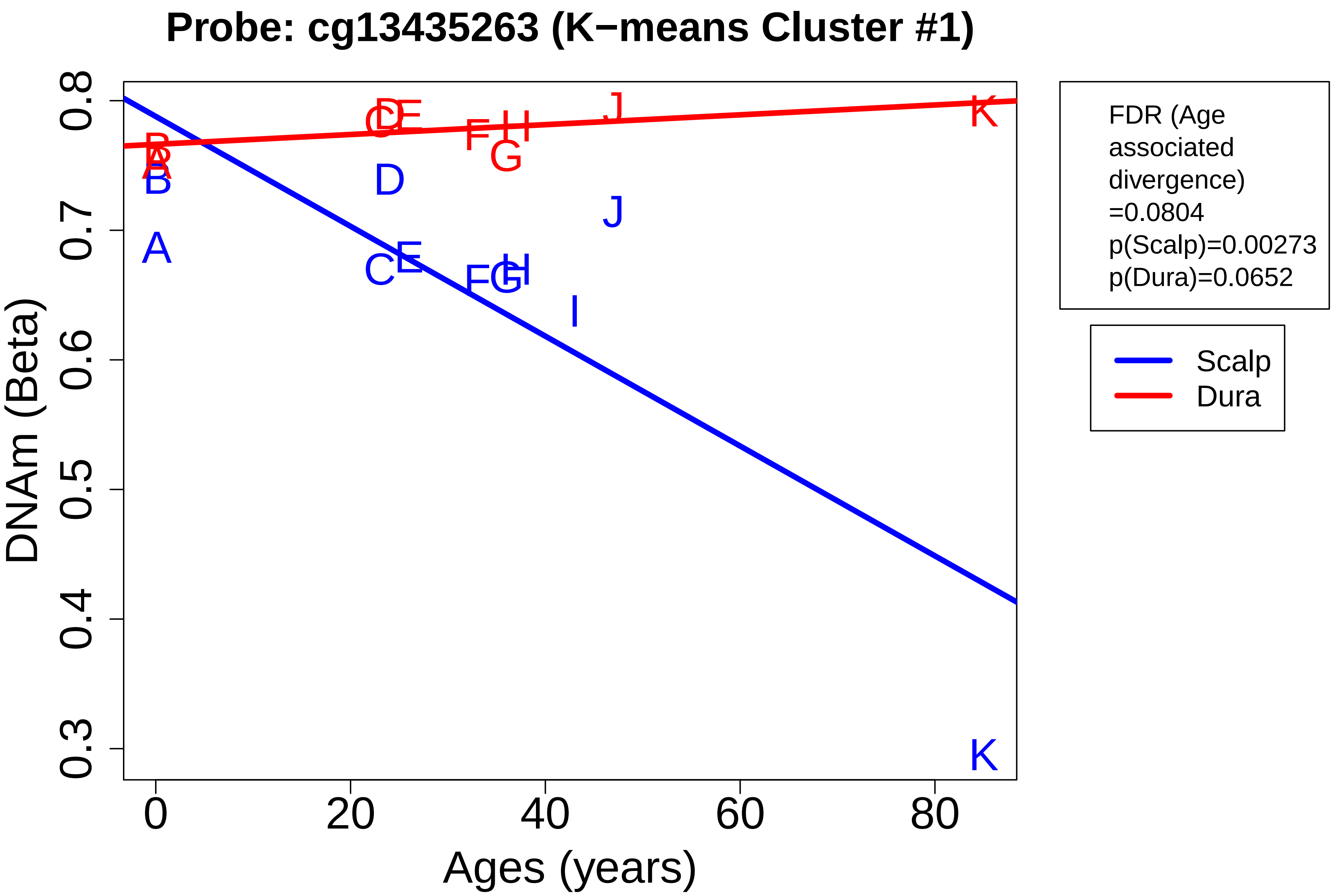

Probe: cg13435263 (K−means Cluster #1)
DNAm (Beta)
0.3
0.4
0.5
0.6
0.7
0.8
0
20
40
60
80
Ages (years)
FDR (Age associated divergence) =0.0804
p(Scalp)=0.00273
p(Dura)=0.0652
Scalp
Dura

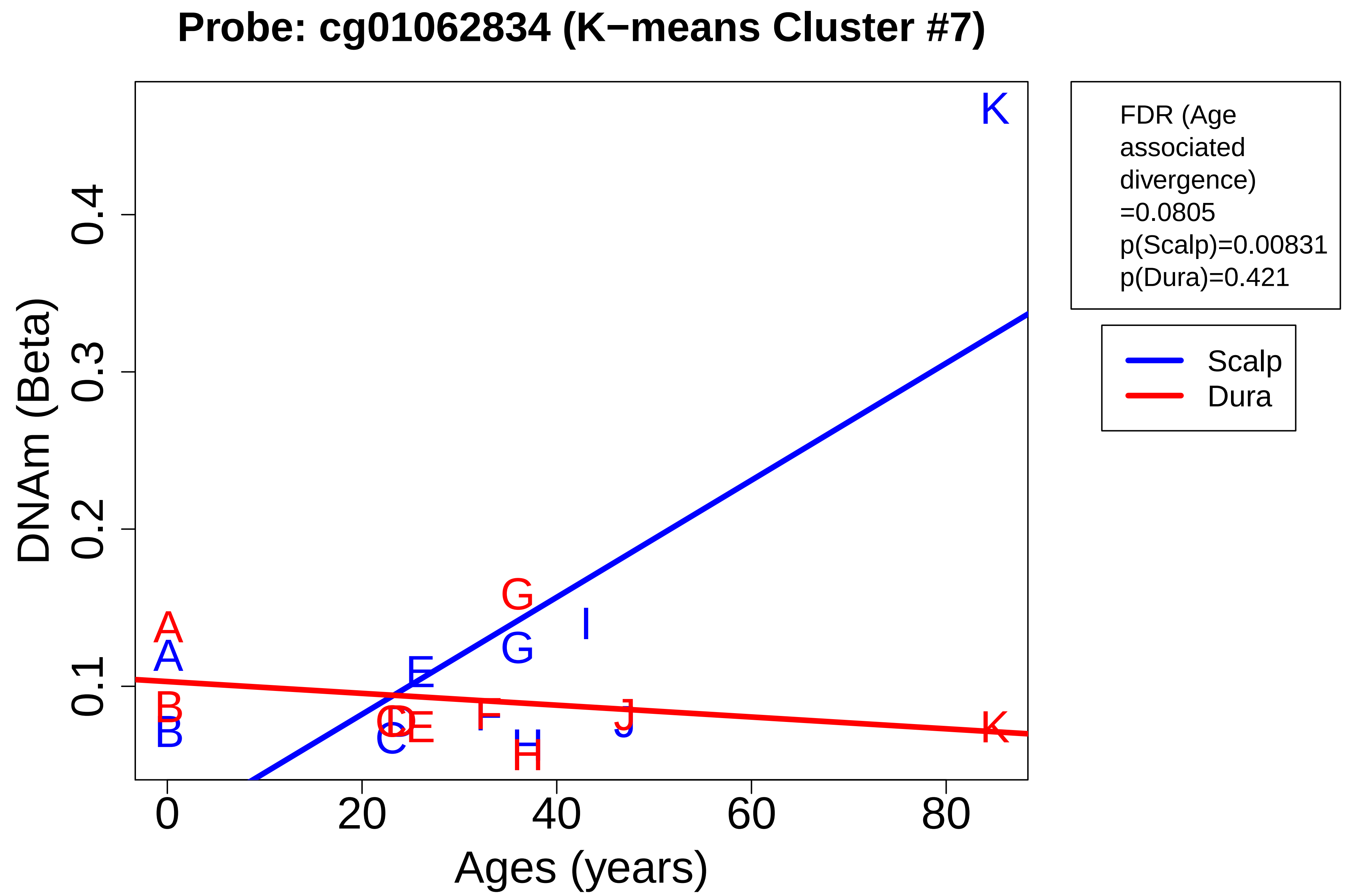

Probe: cg01062834 (K−means Cluster #7)
DNAm (Beta)
0.1
0.2
0.3
0.4
0
20
40
60
80
Ages (years)
FDR (Age associated divergence) =0.0805
p(Scalp)=0.00831
p(Dura)=0.421
Scalp
Dura

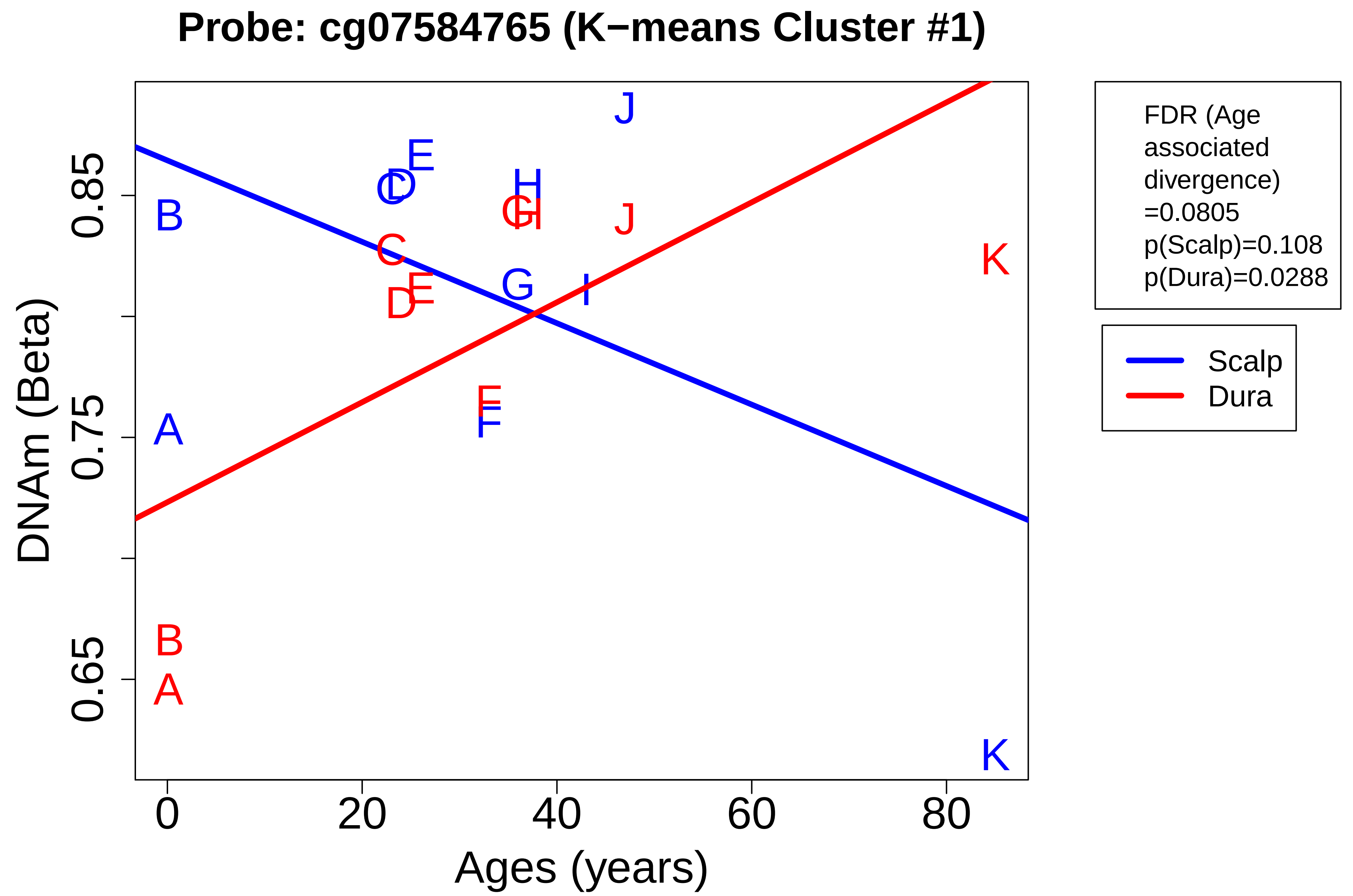

Probe: cg07584765 (K−means Cluster #1)
DNAm (Beta)
0.65
0.75
0.85
Ages (years)
0
20
40
60
80
FDR (Age associated divergence) =0.0805
p(Scalp)=0.108
p(Dura)=0.0288
Scalp
Dura

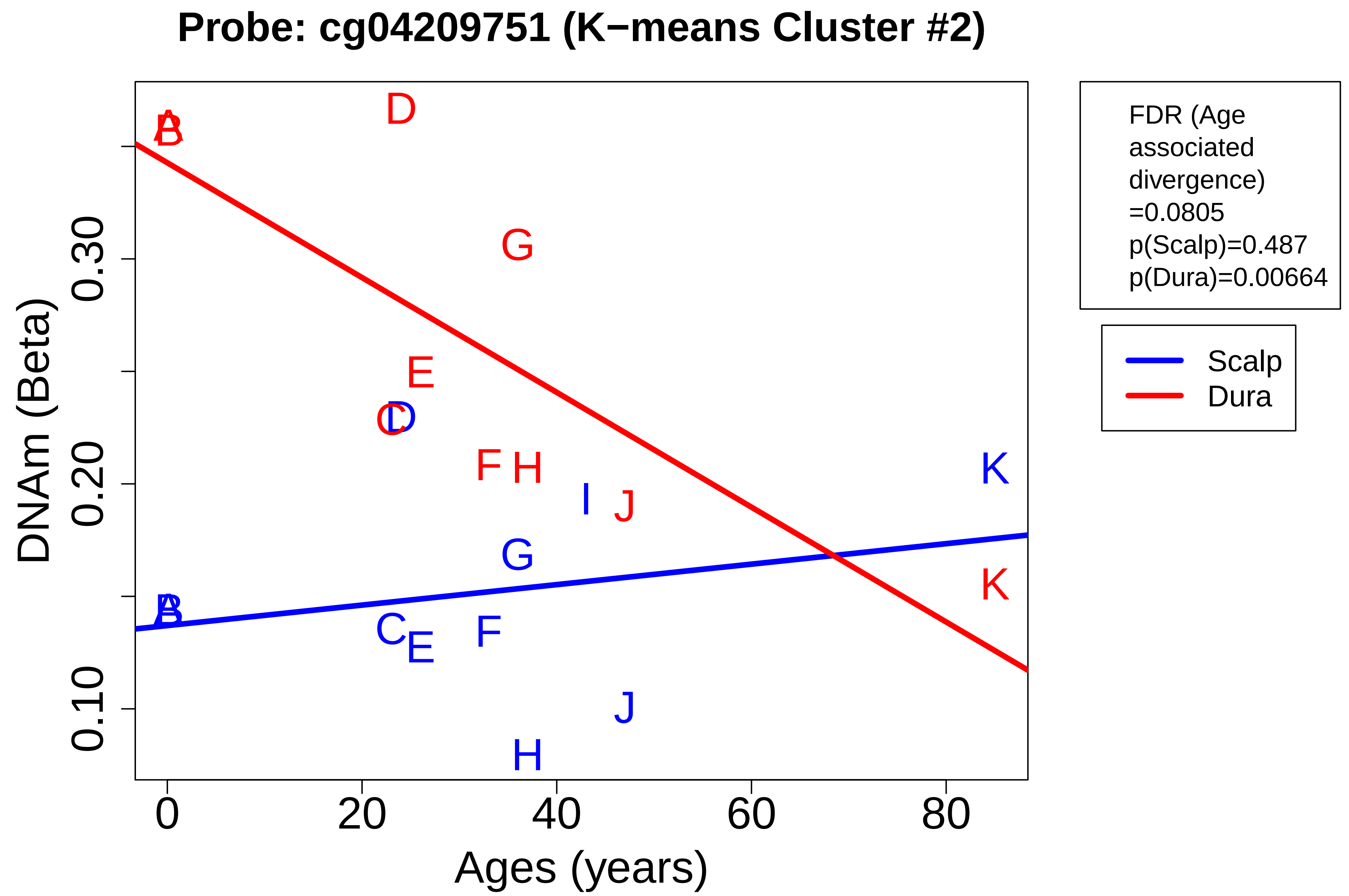

Probe: cg04209751 (K−means Cluster #2)
DNAm (Beta)
0.10
0.20
0.30
Ages (years)
0
20
40
60
80
FDR (Age associated divergence) =0.0805
p(Scalp)=0.487
p(Dura)=0.00664
Scalp
Dura

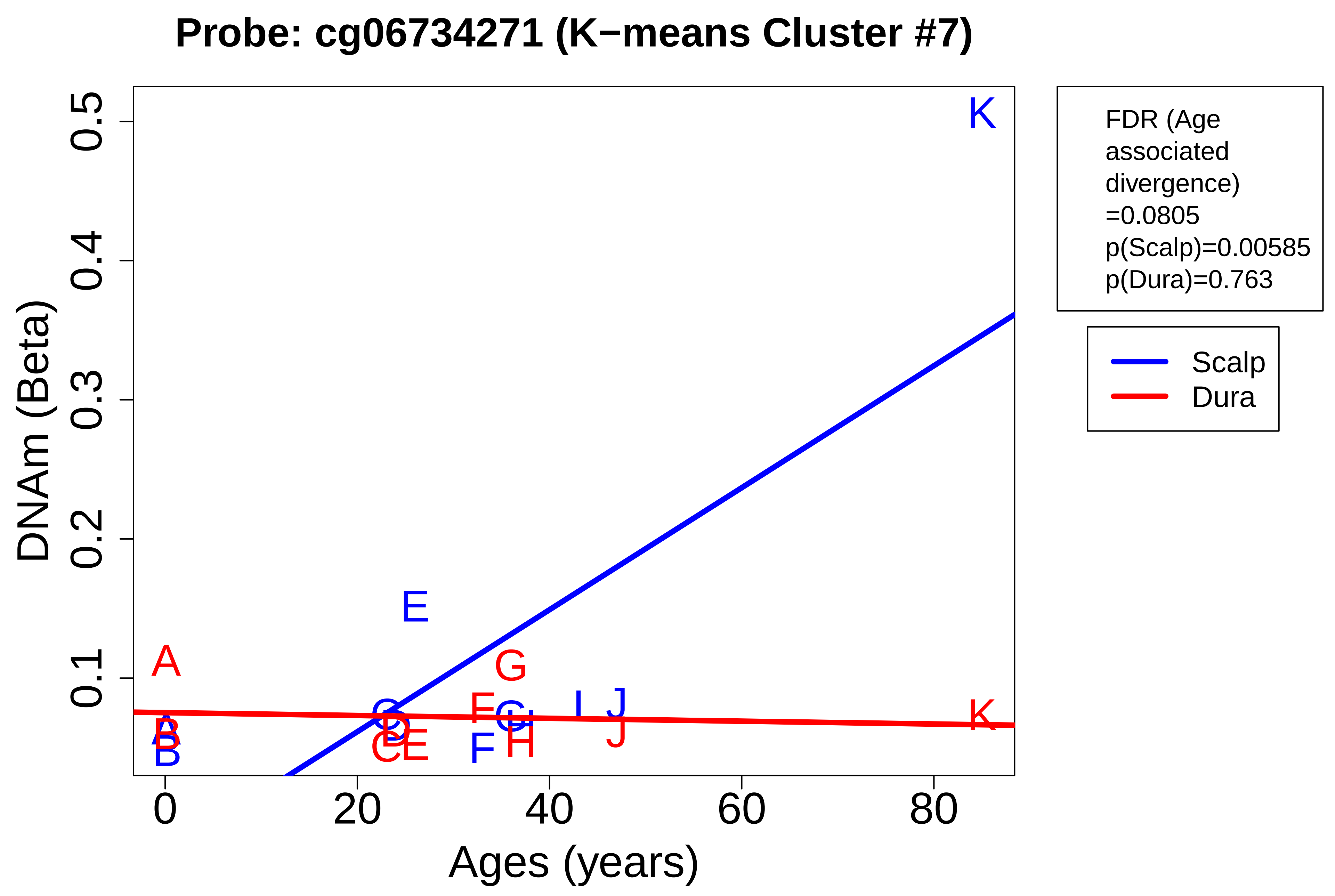

Probe: cg06734271 (K−means Cluster #7)
DNAm (Beta)
0.1
0.2
0.3
0.4
0.5
0
20
40
60
80
Ages (years)
FDR (Age associated divergence) =0.0805
p(Scalp)=0.00585
p(Dura)=0.763
Scalp
Dura

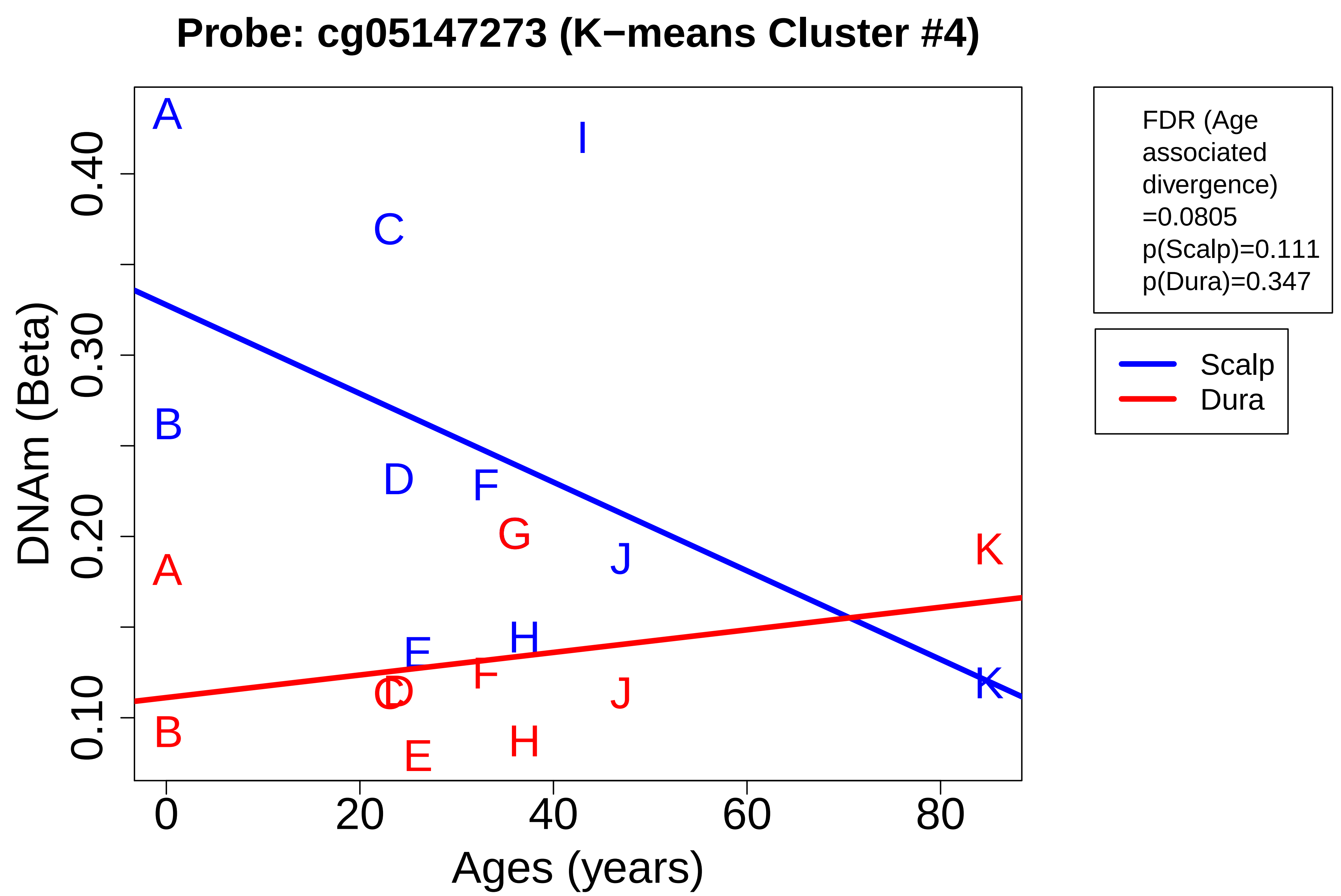

Probe: cg05147273 (K−means Cluster #4)
DNAm (Beta)
0.10
0.20
0.30
0.40
Ages (years)
0
20
40
60
80
A
B
C
D
E
F
G
H
I
J
K
FDR (Age associated divergence) =0.0805
p(Scalp)=0.111
p(Dura)=0.347
Scalp
Dura

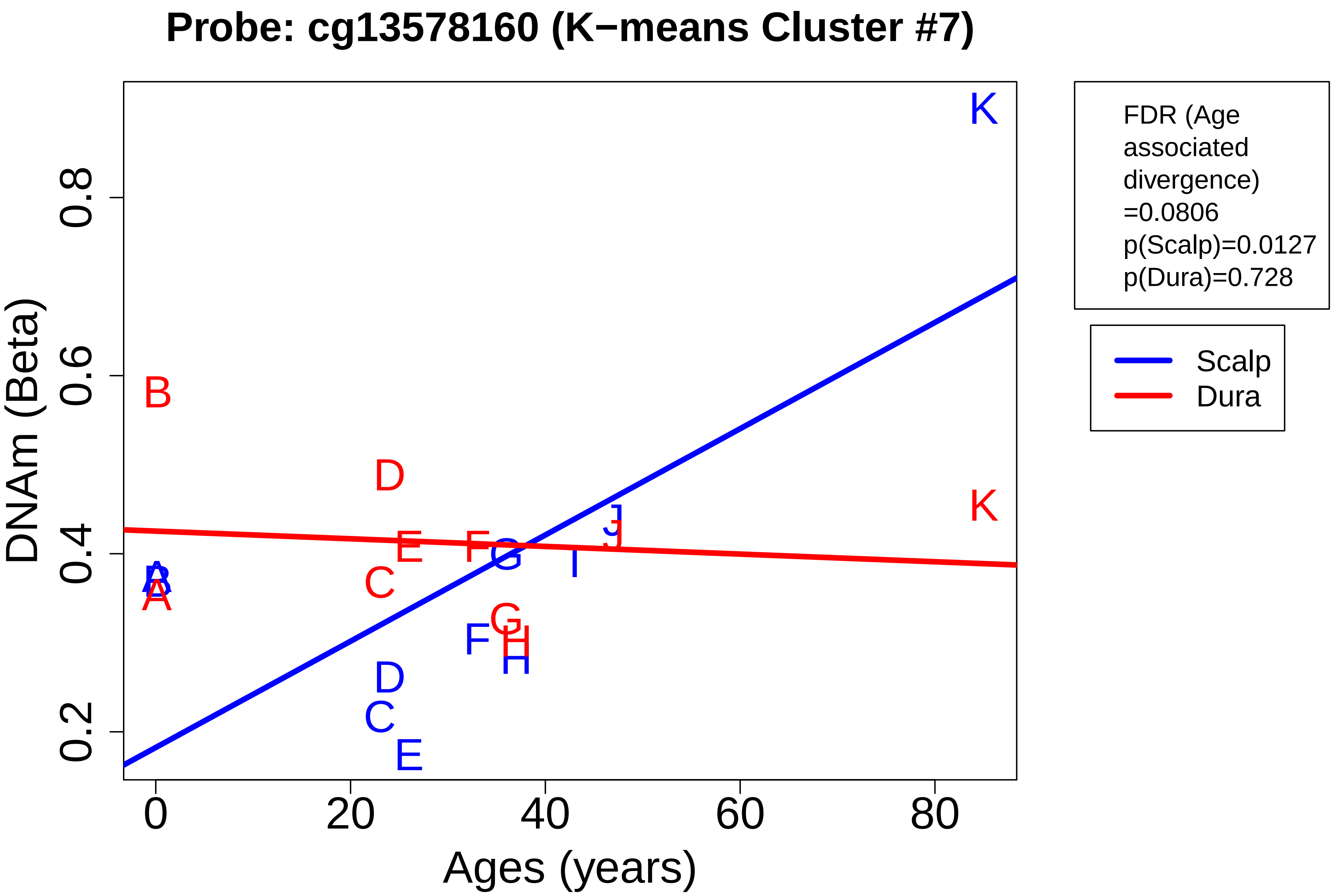

Probe: cg13578160 (K−means Cluster #7)
DNAm (Beta)
0.2
0.4
0.6
0.8
0
20
40
60
80
Ages (years)
FDR (Age associated divergence) =0.0806
p(Scalp)=0.0127
p(Dura)=0.728
Scalp
Dura

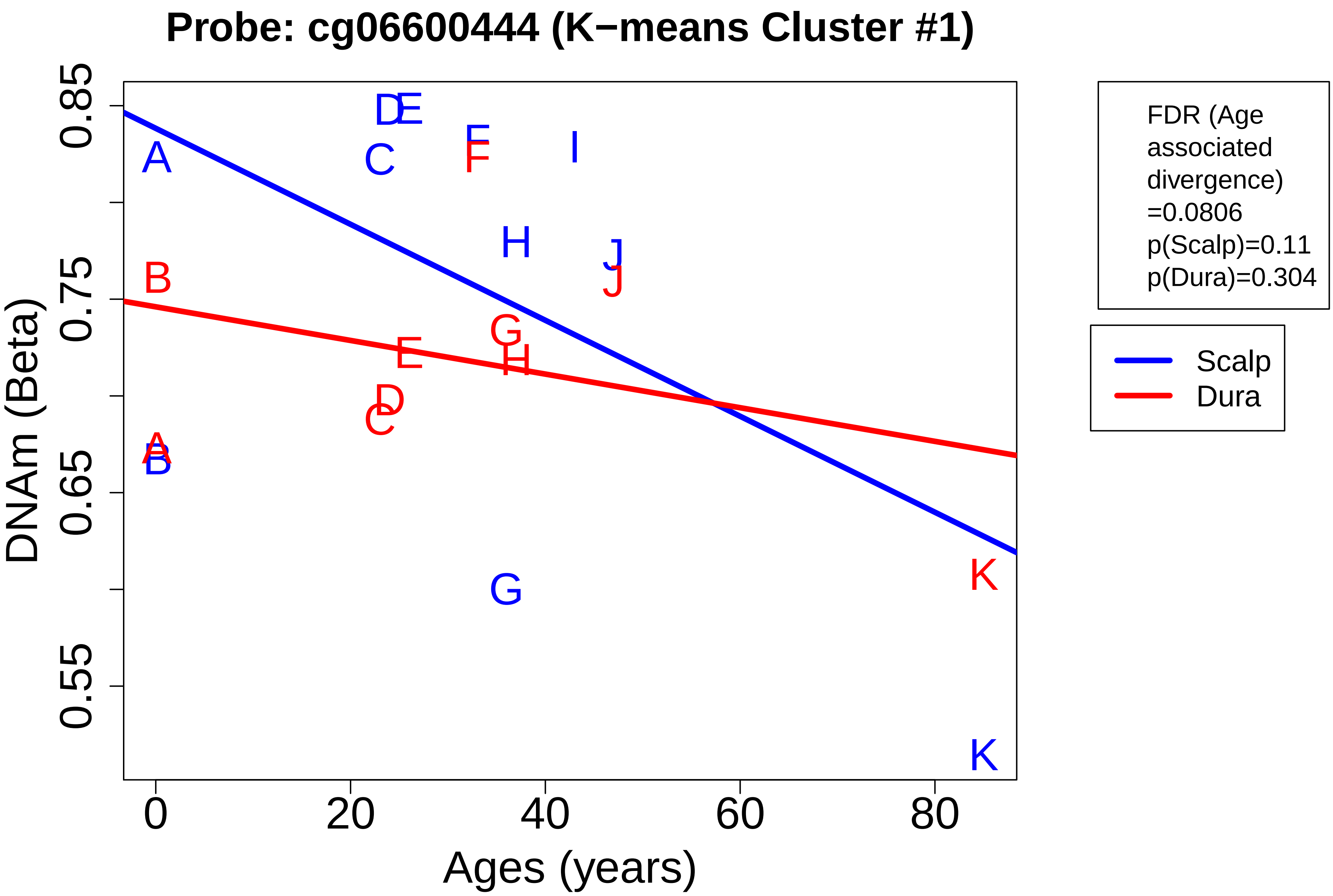

Probe: cg06600444 (K−means Cluster #1)
DNAm (Beta)
0.55
0.65
0.75
0.85
0
20
40
60
80
Ages (years)
FDR (Age associated divergence) =0.0806
p(Scalp)=0.11
p(Dura)=0.304
Scalp
Dura

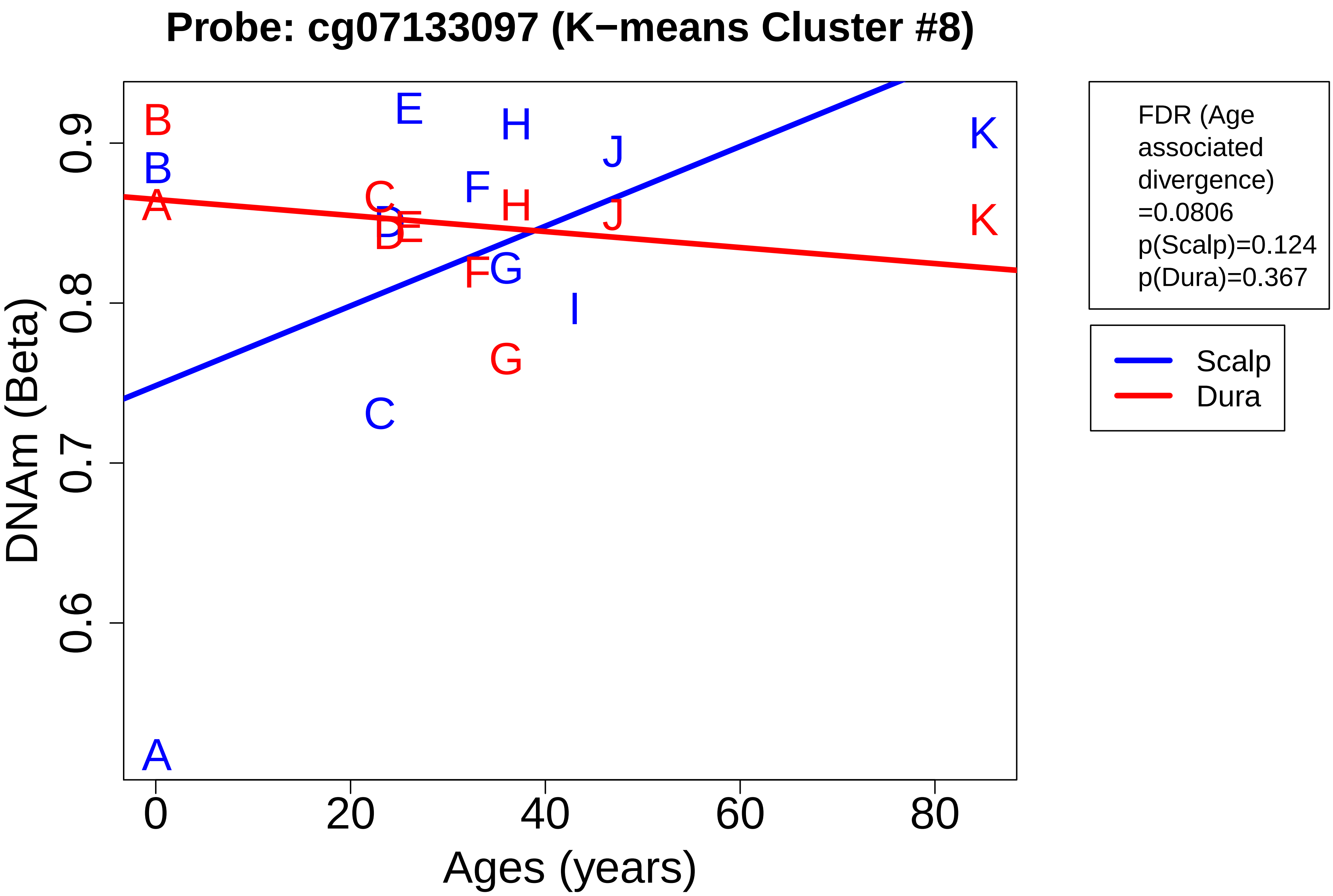

Probe: cg07133097 (K−means Cluster #8)
DNAm (Beta)
0.6
0.7
0.8
0.9
0
20
40
60
80
Ages (years)
FDR (Age associated divergence) =0.0806
p(Scalp)=0.124
p(Dura)=0.367
Scalp
Dura

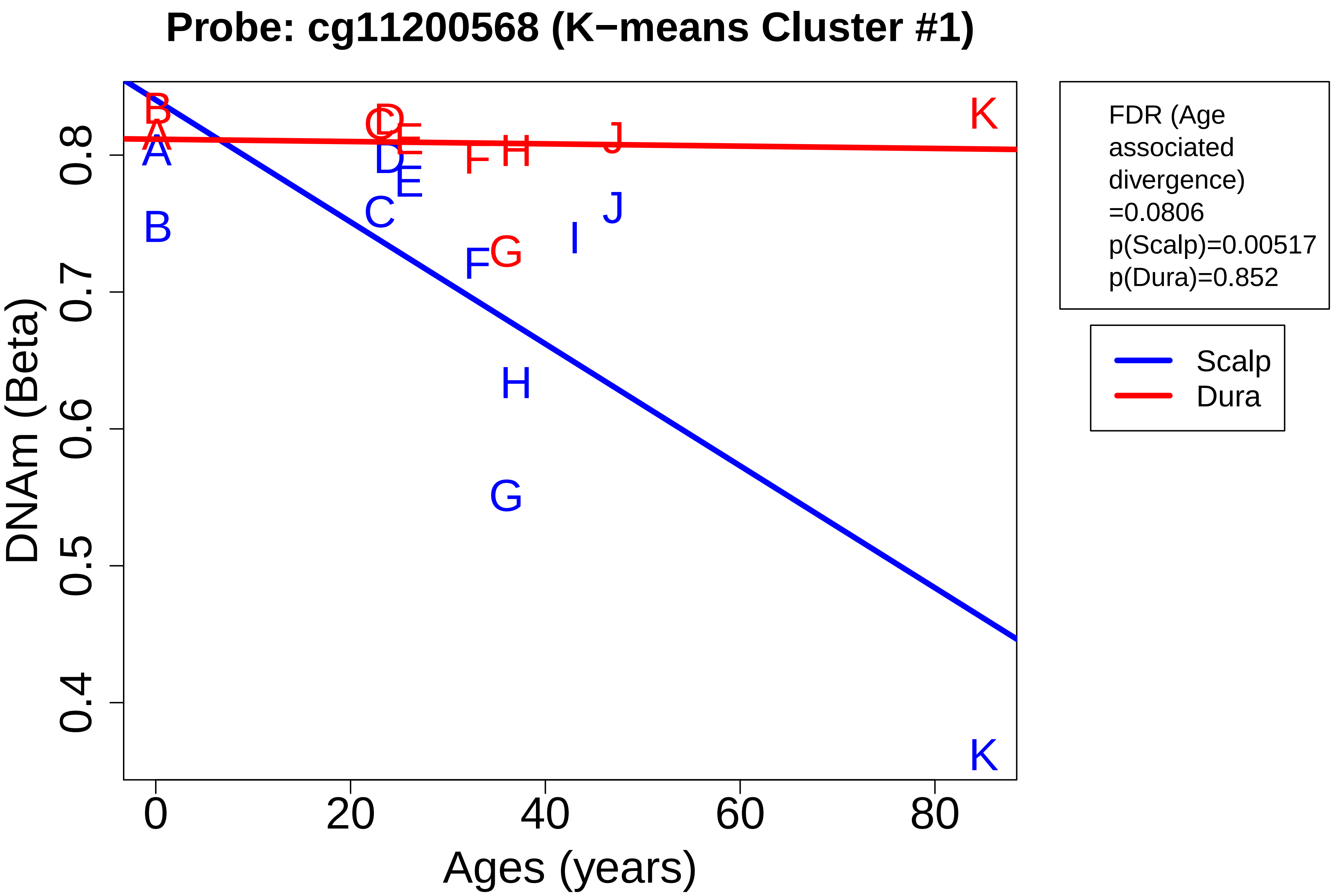

Probe: cg11200568 (K−means Cluster #1)
DNAm (Beta)
0.4
0.5
0.6
0.7
0.8
0
20
40
60
80
Ages (years)
FDR (Age associated divergence) =0.0806
p(Scalp)=0.00517
p(Dura)=0.852
Scalp
Dura

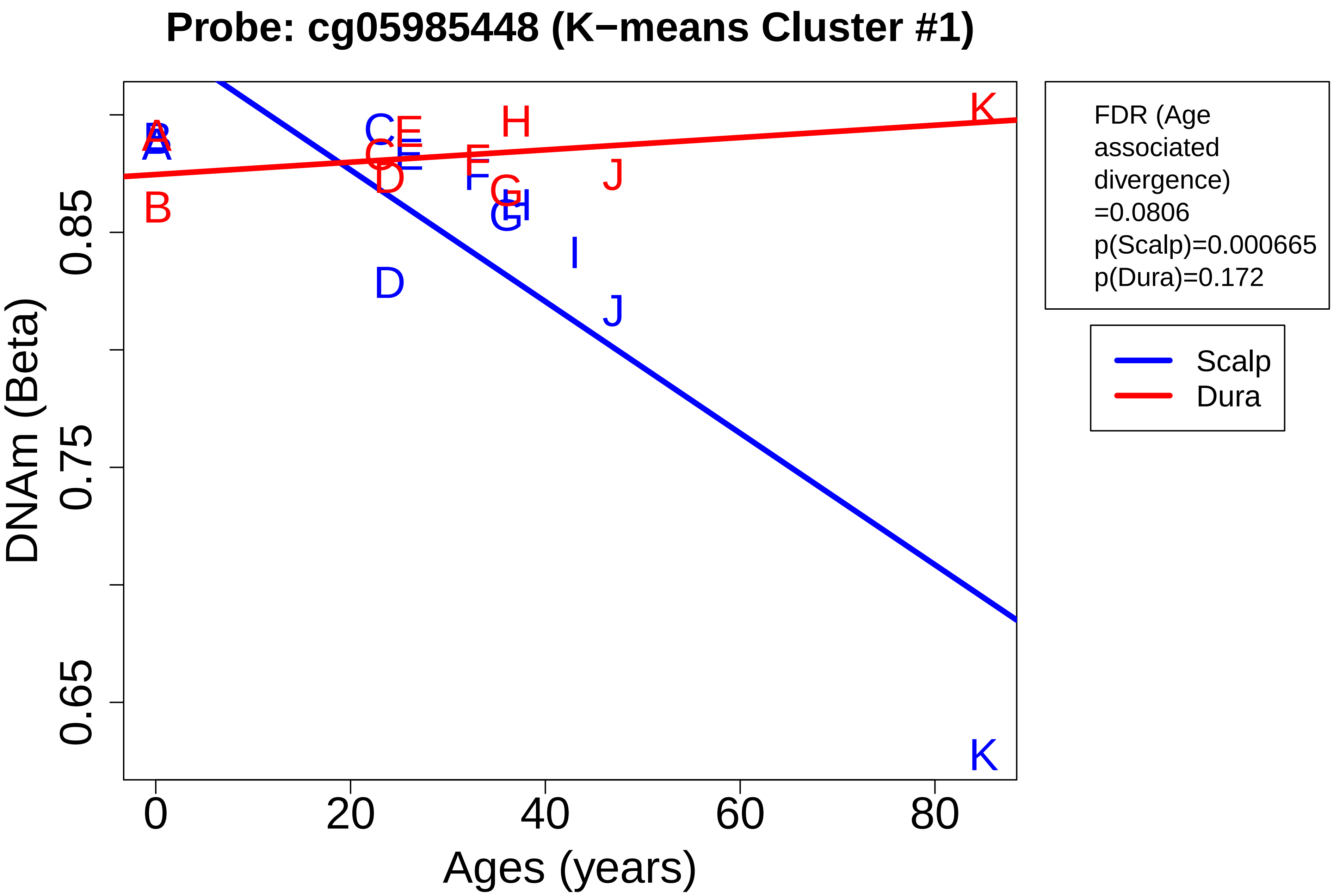

Probe: cg05985448 (K−means Cluster #1)
DNAm (Beta)
0.65
0.75
0.85
Ages (years)
0
20
40
60
80
FDR (Age associated divergence) =0.0806
p(Scalp)=0.000665
p(Dura)=0.172
Scalp
Dura

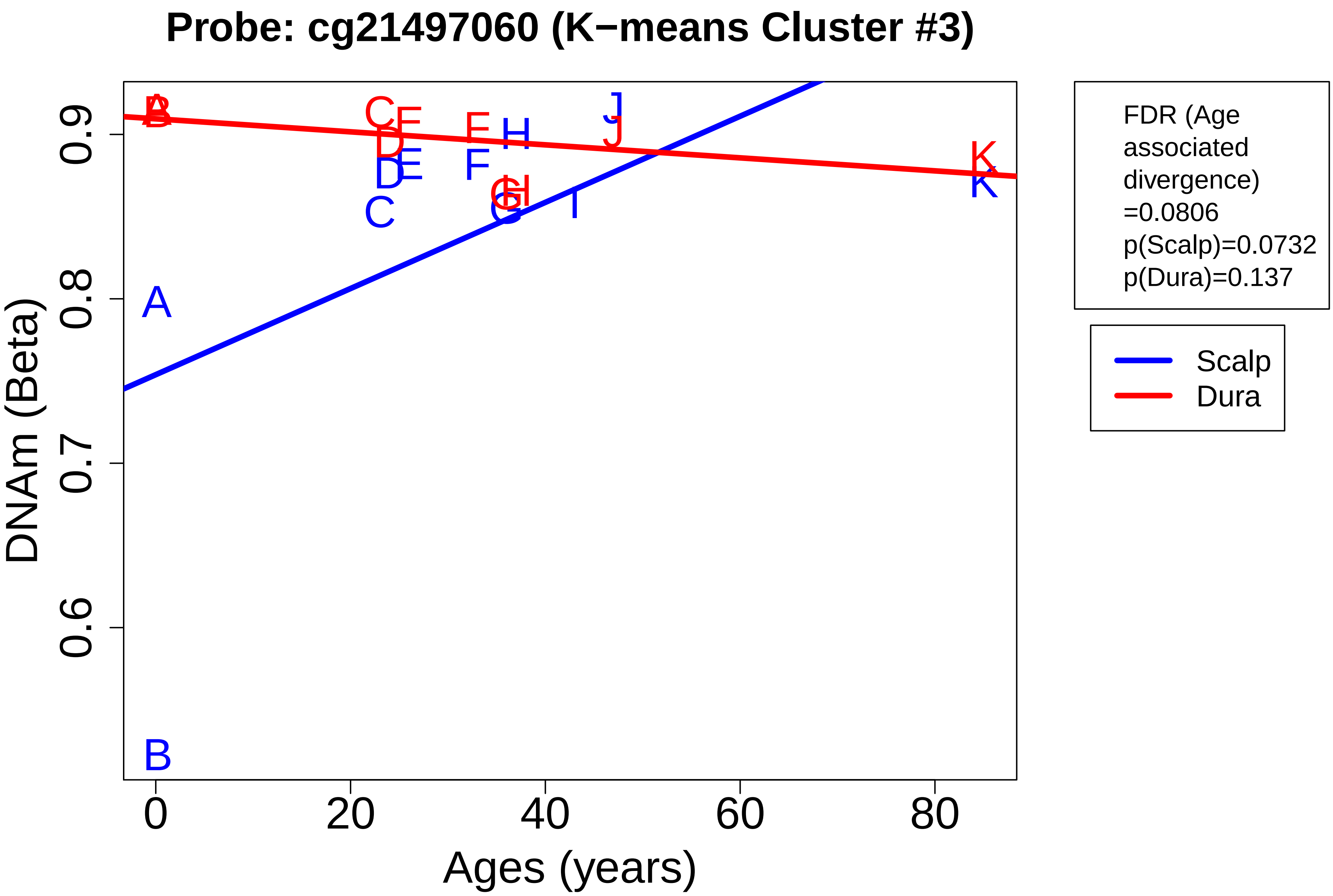

Probe: cg21497060 (K−means Cluster #3)
DNAm (Beta)
0.6
0.7
0.8
0.9
Ages (years)
0
20
40
60
80
FDR (Age associated divergence) =0.0806
p(Scalp)=0.0732
p(Dura)=0.137
Scalp
Dura

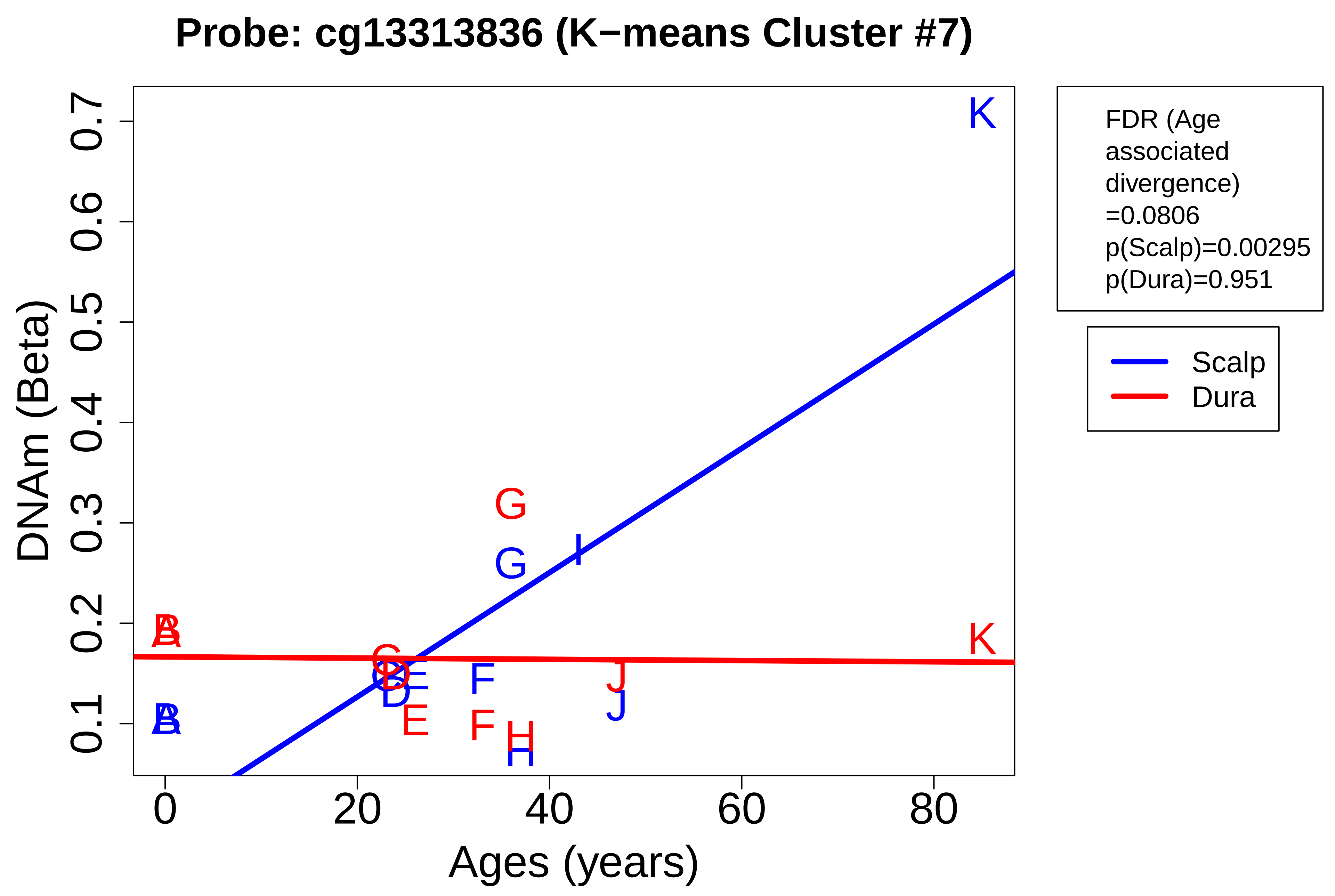
Probe: cg13313836 (K−means Cluster #7)
DNAm (Beta)
Ages (years)
FDR (Age associated divergence) =0.0806
p(Scalp)=0.00295
p(Dura)=0.951
Scalp
Dura
0.1
0.2
0.3
0.4
0.5
0.6
0.7
0
20
40
60
80

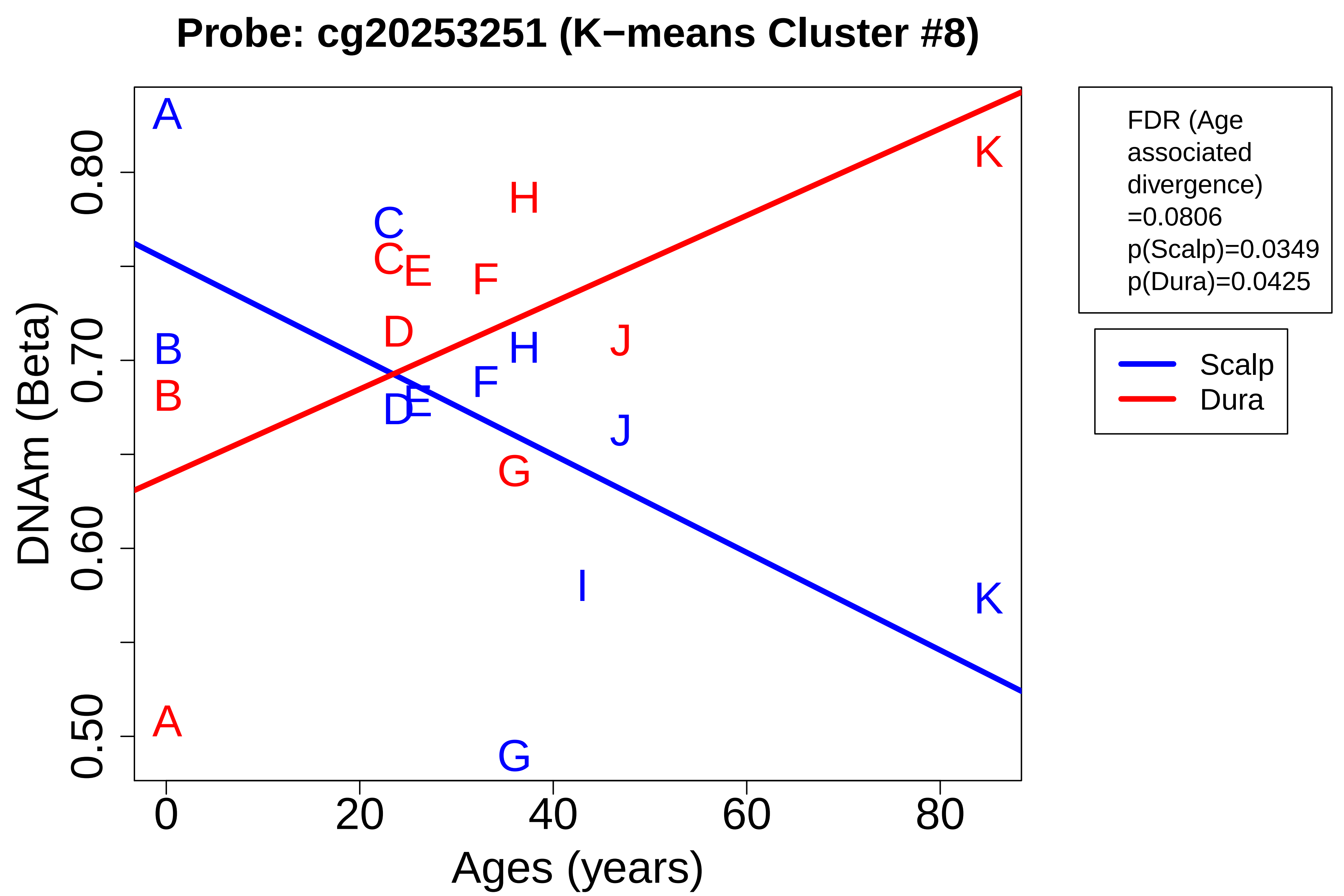

Probe: cg20253251 (K−means Cluster #8)
DNAm (Beta)
0.50
0.60
0.70
0.80
0
20
40
60
80
Ages (years)
A
B
C
D
E
F
G
H
I
J
K
FDR (Age associated divergence) =0.0806
p(Scalp)=0.0349
p(Dura)=0.0425
Scalp
Dura

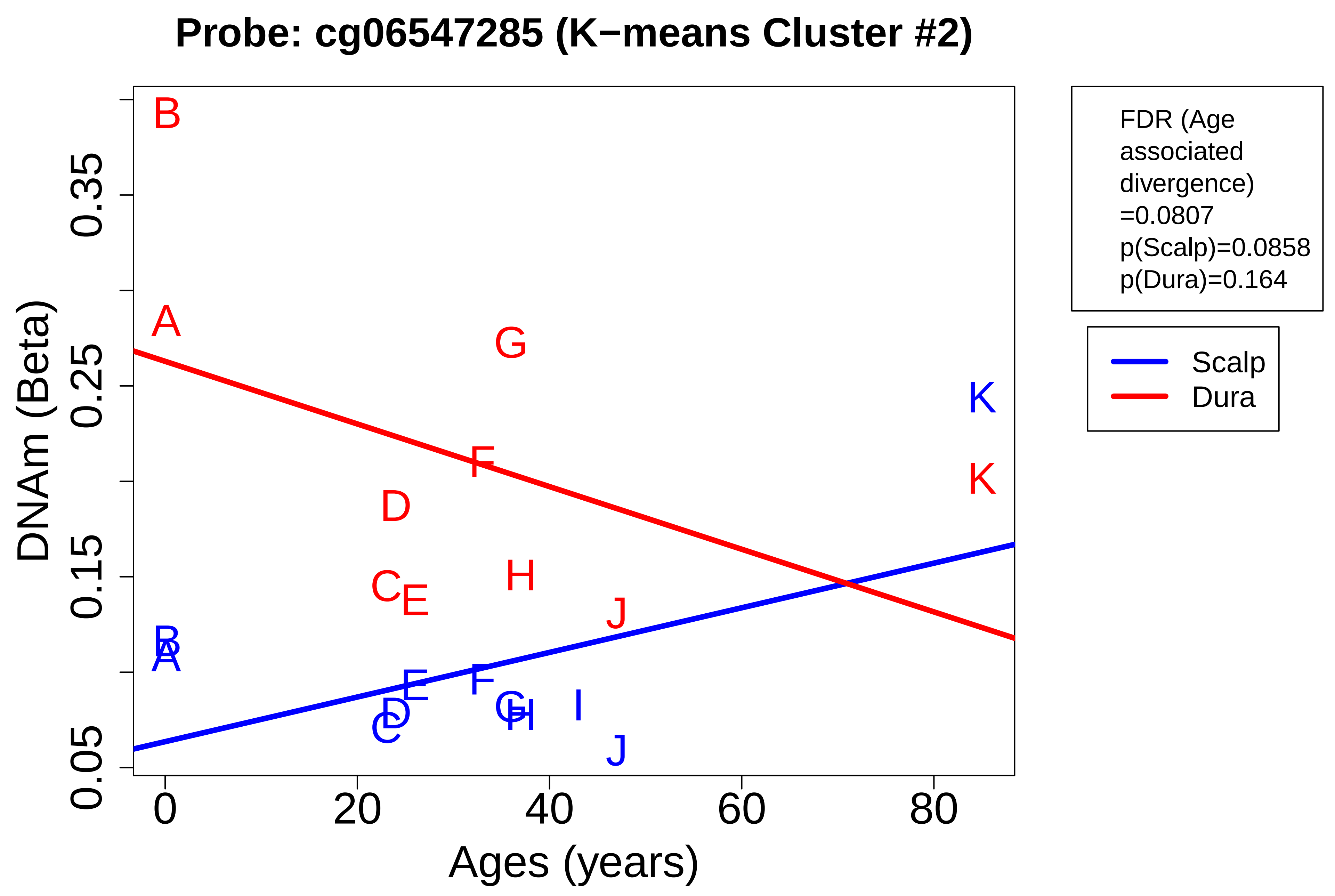

Probe: cg06547285 (K−means Cluster #2)
DNAm (Beta)
0.05
0.15
0.25
0.35
0
20
40
60
80
Ages (years)
FDR (Age associated divergence) =0.0807
p(Scalp)=0.0858
p(Dura)=0.164
Scalp
Dura
A
B
C
D
E
F
G
H
I
J
K

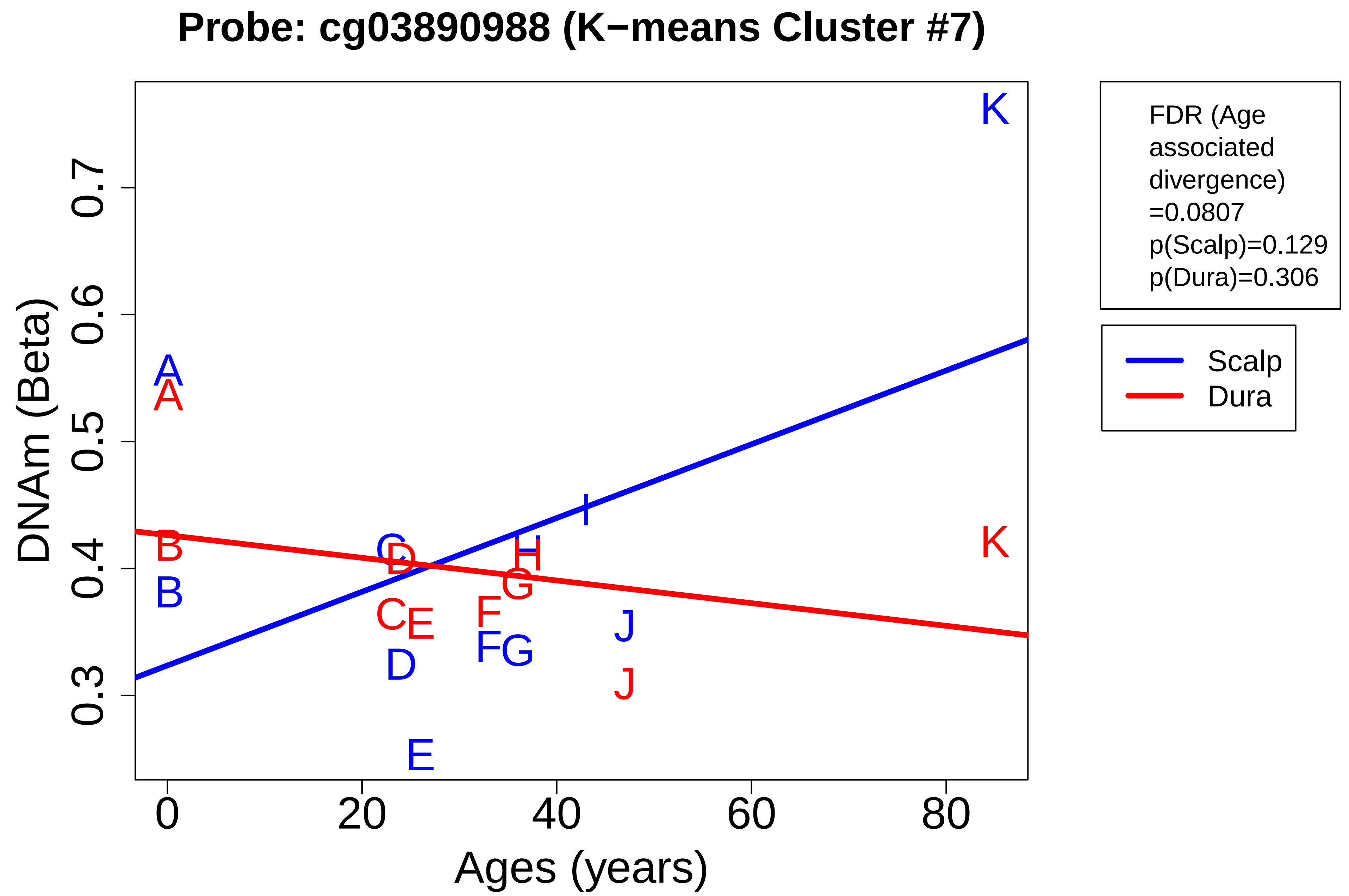

Probe: cg03890988 (K−means Cluster #7)
FDR (Age associated divergence) =0.0807
p(Scalp)=0.129
p(Dura)=0.306
Scalp
Dura
DNAm (Beta)
0.3
0.4
0.5
0.6
0.7
0
20
40
60
80
Ages (years)
A B C D E F G H I J K

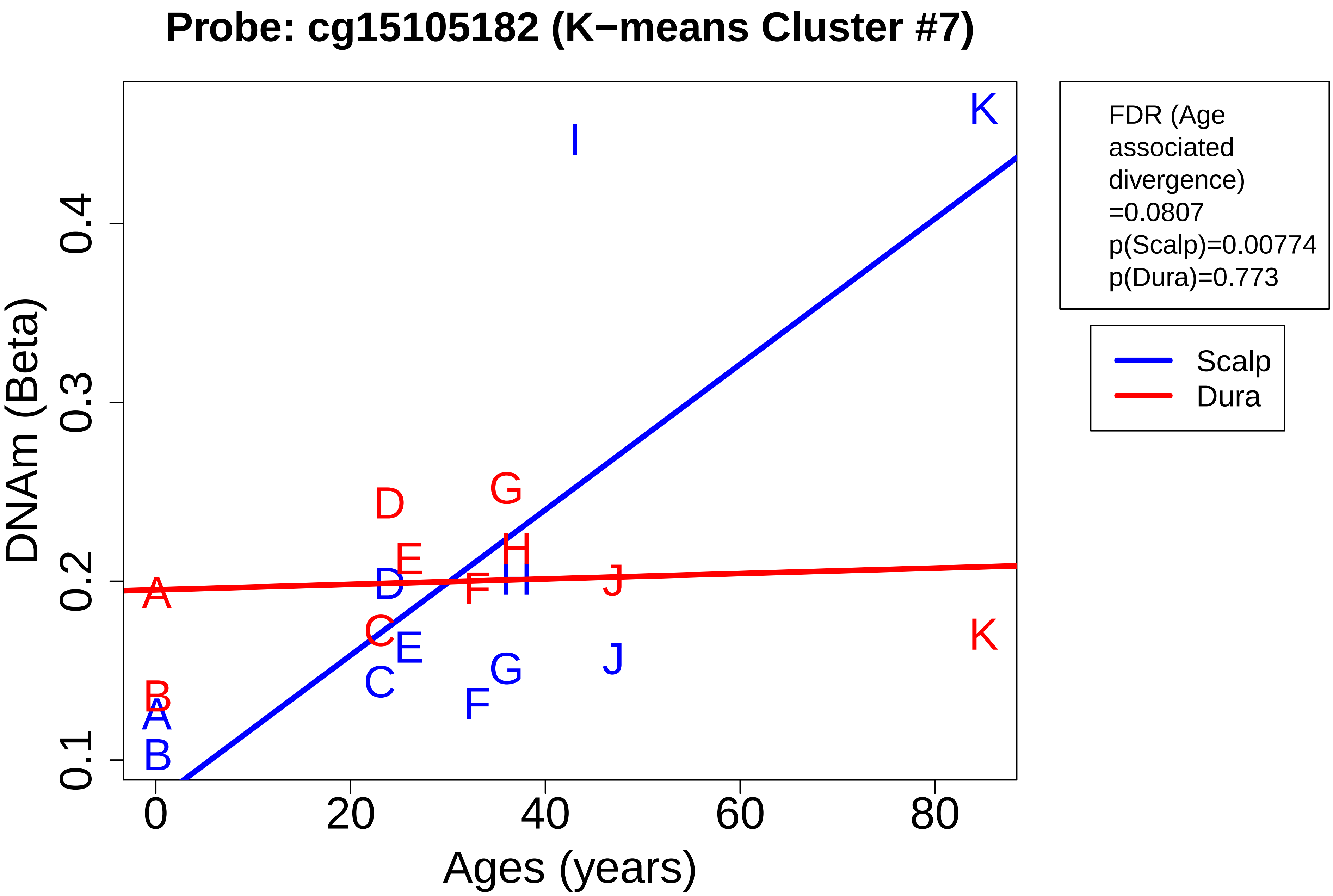
Probe: cg15105182 (K−means Cluster #7)
FDR (Age associated divergence) =0.0807
p(Scalp)=0.00774
p(Dura)=0.773
Scalp
Dura
DNAm (Beta)
0.1
0.2
0.3
0.4
Ages (years)
0
20
40
60
80
A
B
C
D
E
F
G
H
I
J
K

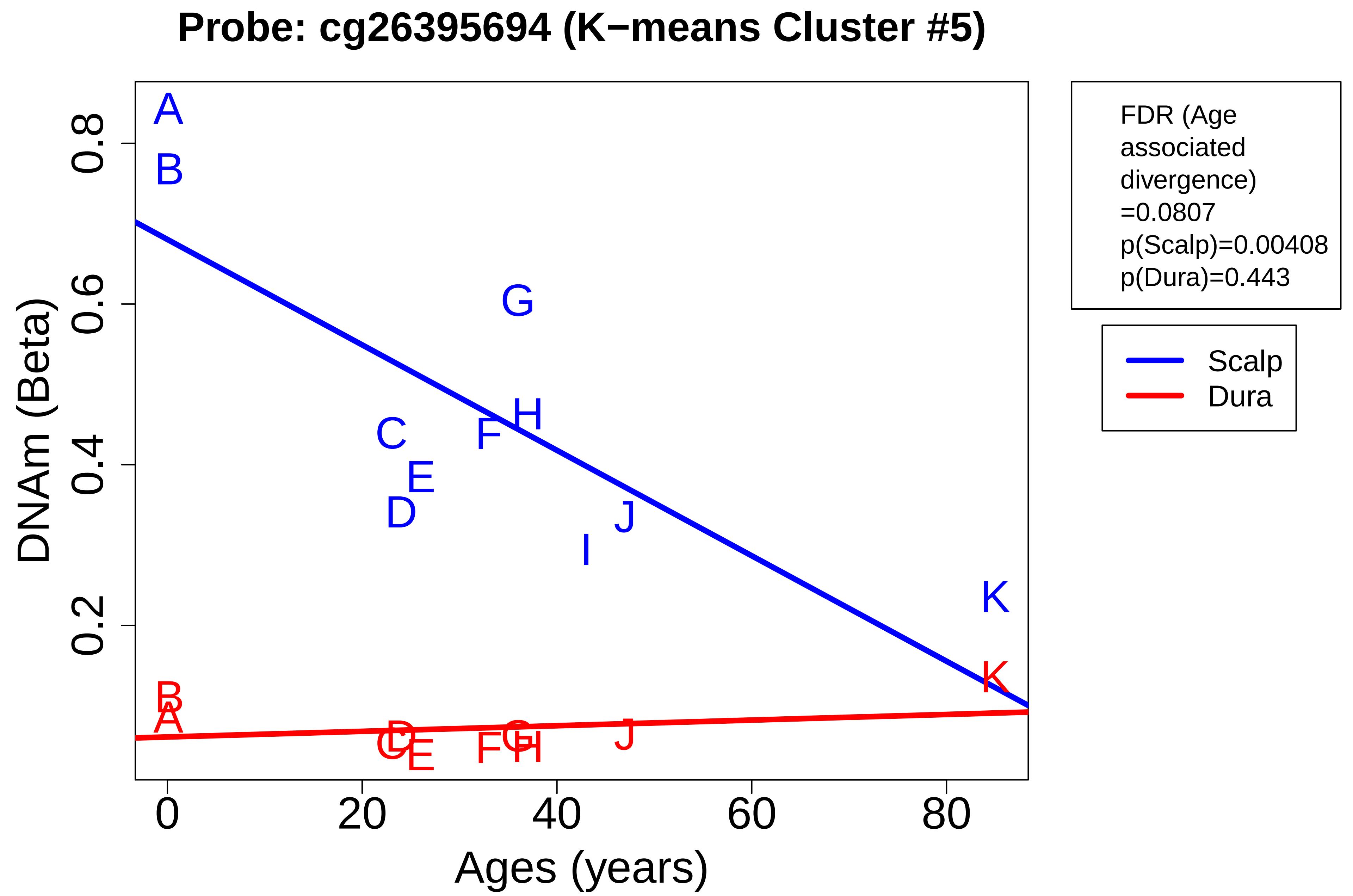

Probe: cg26395694 (K−means Cluster #5)
DNAm (Beta)
0.2
0.4
0.6
0.8
Ages (years)
0
20
40
60
80
FDR (Age associated divergence) =0.0807
p(Scalp)=0.00408
p(Dura)=0.443
Scalp
Dura
A
B
C
D
E
F
G
H
I
J
K

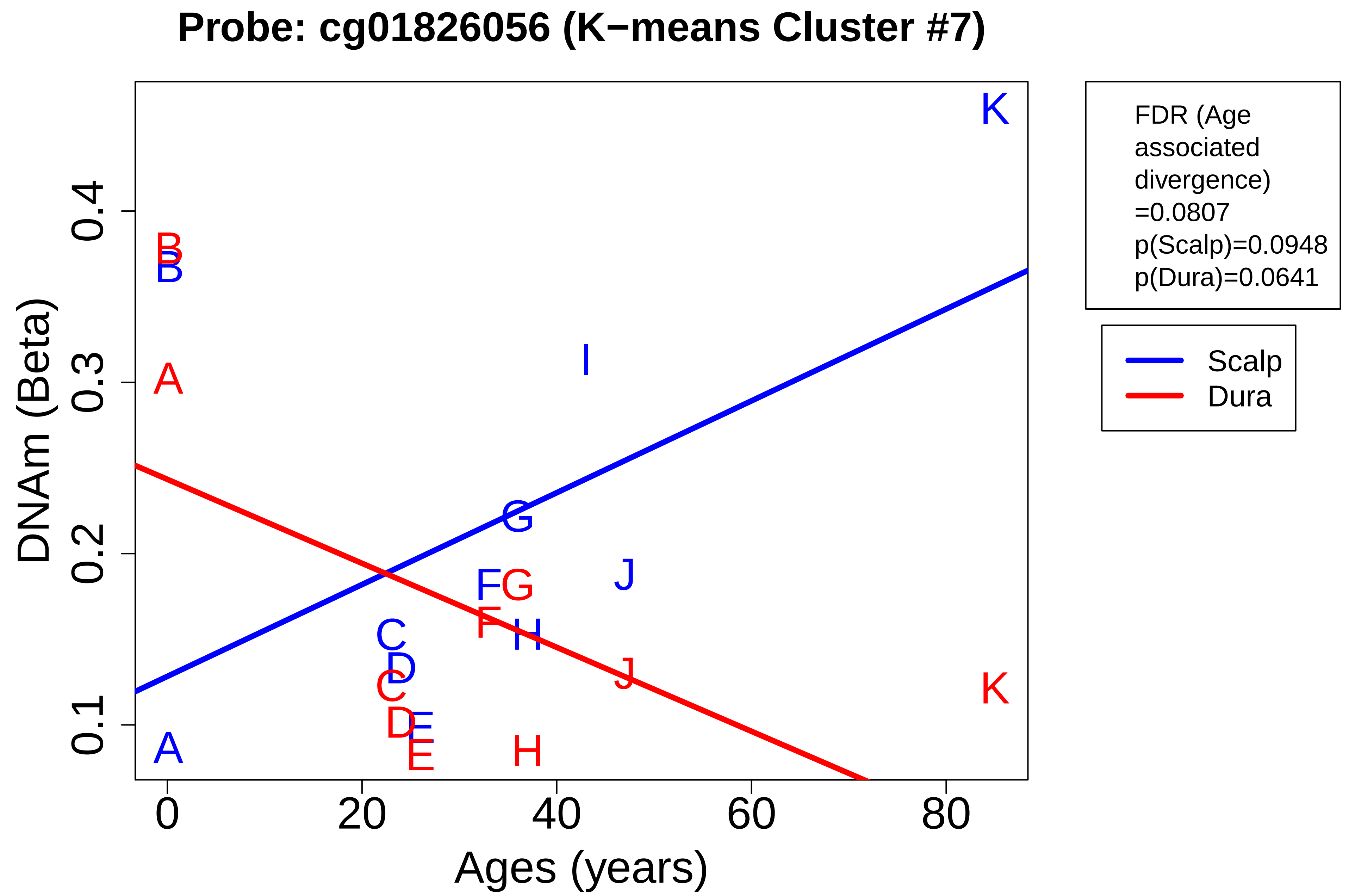

Probe: cg01826056 (K−means Cluster #7)
DNAm (Beta)
0.1
0.2
0.3
0.4
0
20
40
60
80
Ages (years)
FDR (Age associated divergence) =0.0807
p(Scalp)=0.0948
p(Dura)=0.0641
Scalp
Dura

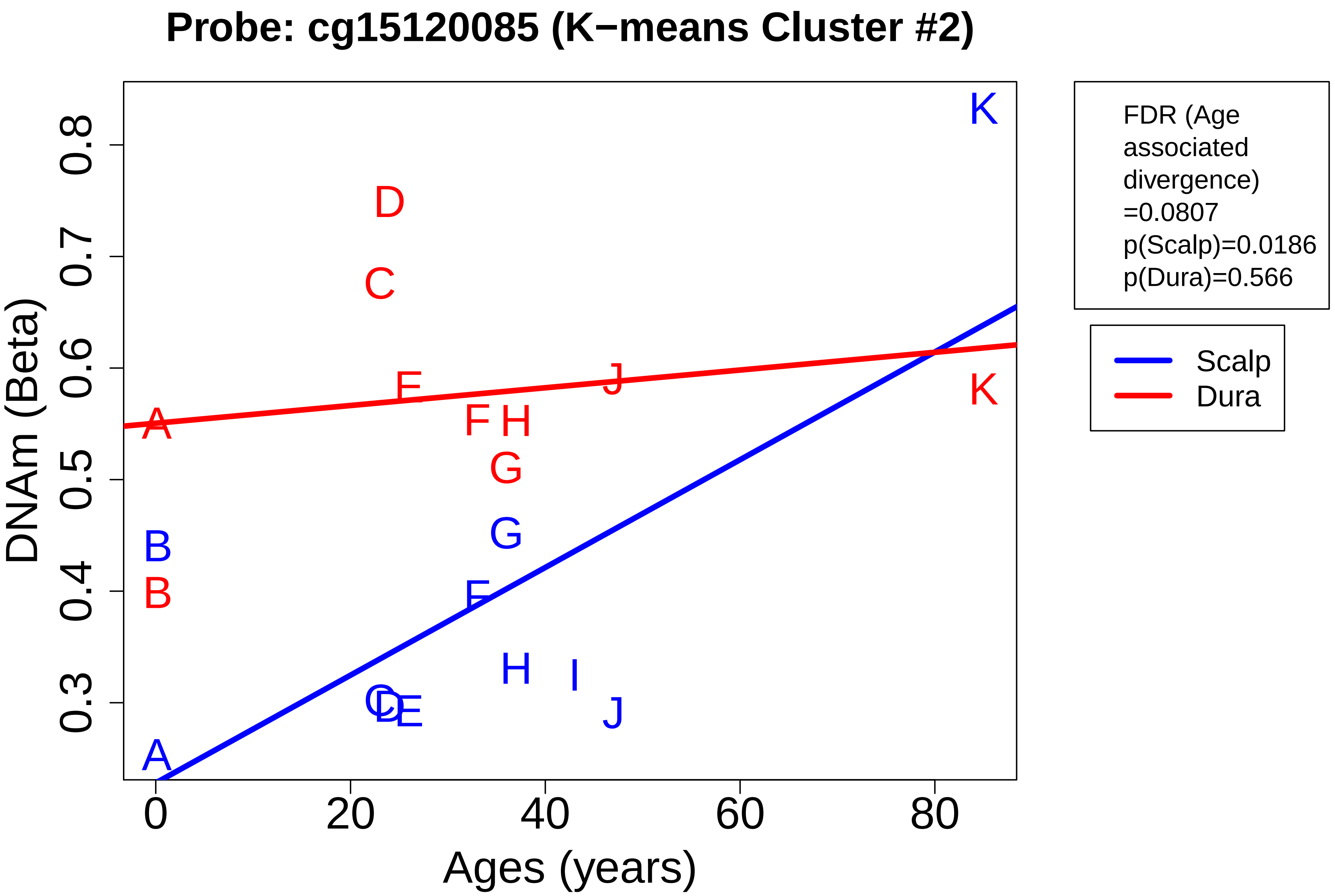

Probe: cg15120085 (K−means Cluster #2)
DNAm (Beta)
0.3
0.4
0.5
0.6
0.7
0.8
Ages (years)
0
20
40
60
80
A
B
C
D
E
F
G
H
I
J
K
FDR (Age associated divergence) =0.0807
p(Scalp)=0.0186
p(Dura)=0.566
Scalp
Dura

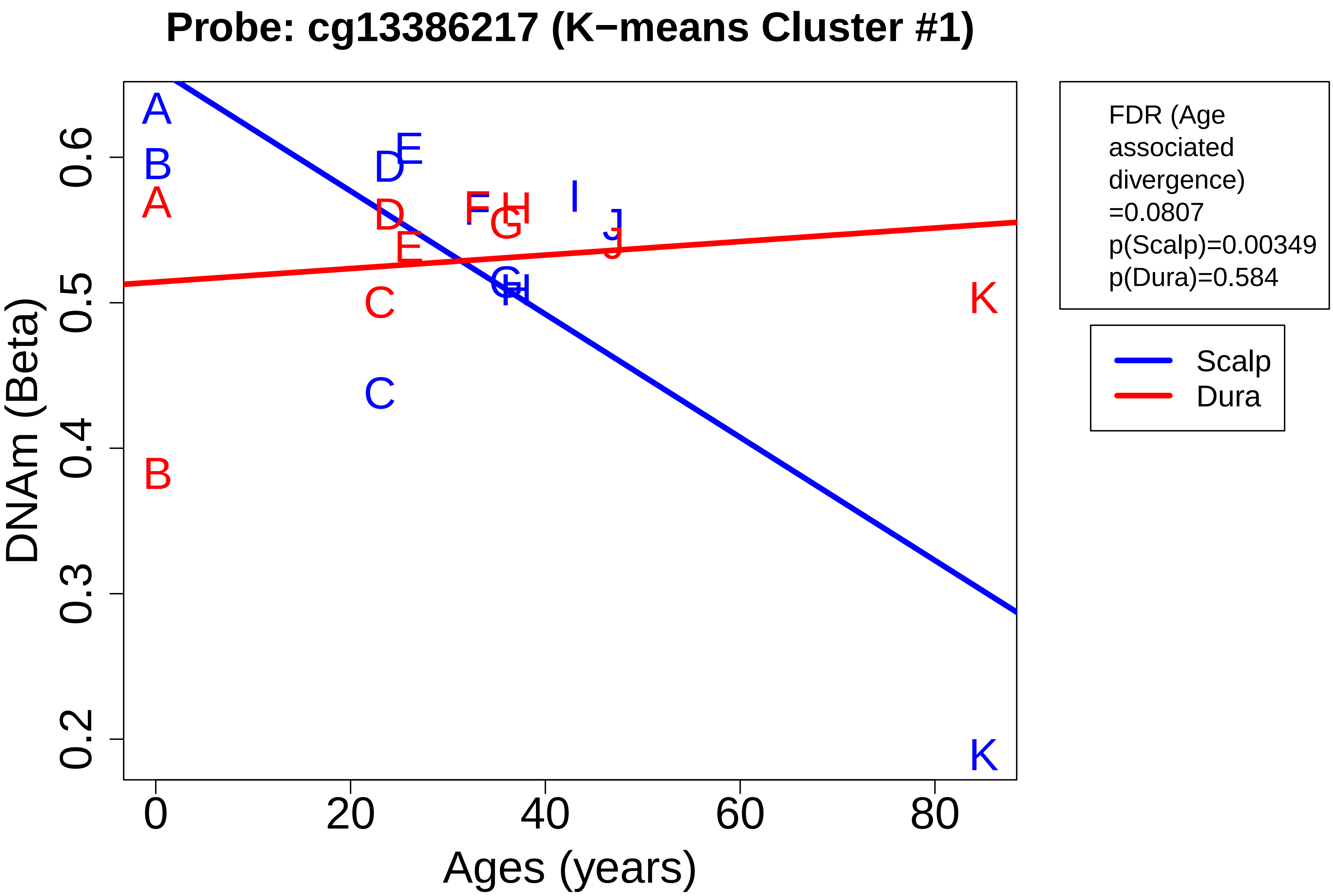

Probe: cg13386217 (K−means Cluster #1)
DNAm (Beta)
0.2
0.3
0.4
0.5
0.6
Ages (years)
0
20
40
60
80
A
B
C
D
E
F
G
H
I
J
K
FDR (Age associated divergence) =0.0807
p(Scalp)=0.00349
p(Dura)=0.584
Scalp
Dura

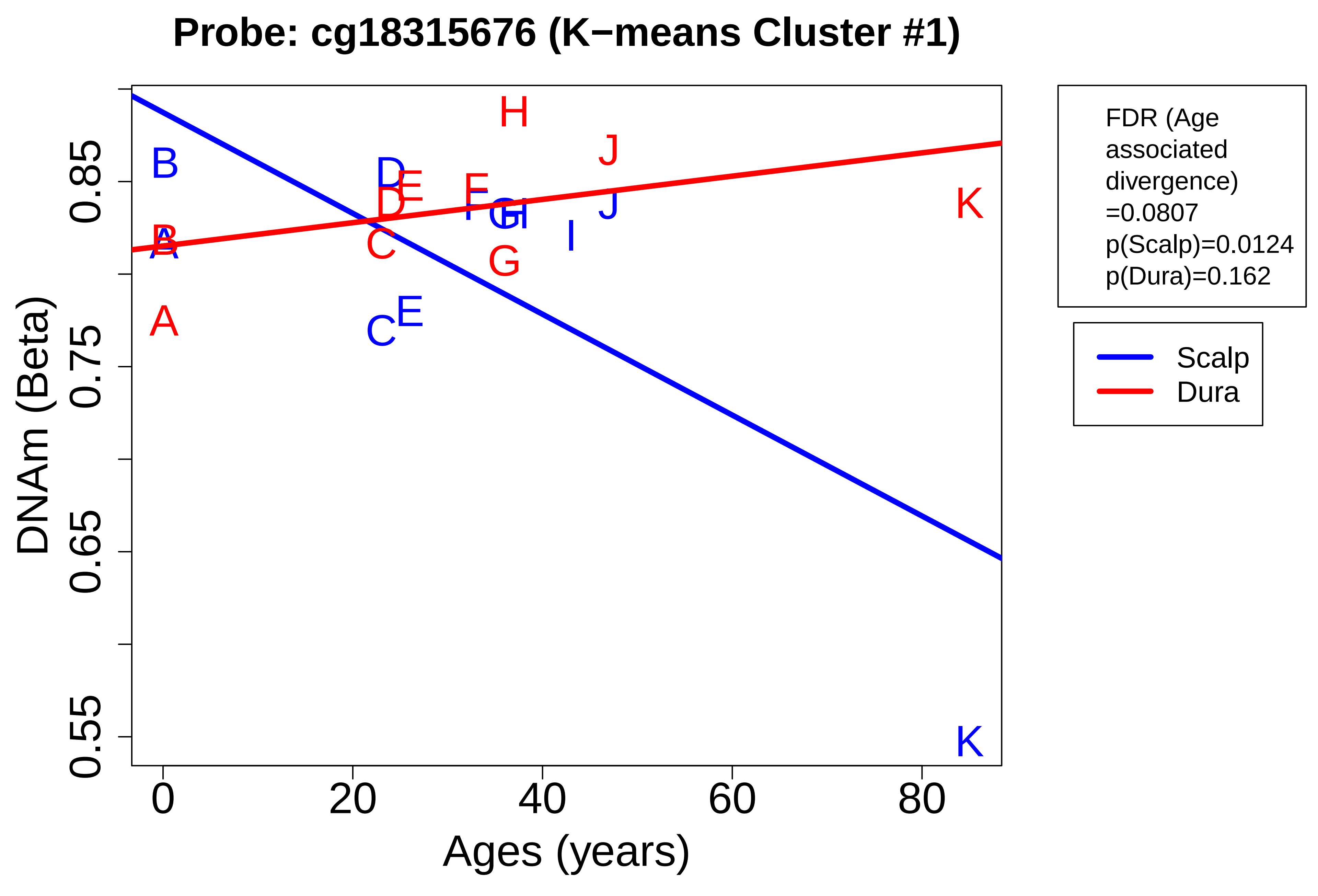

Probe: cg18315676 (K−means Cluster #1)
DNAm (Beta)
0.55
0.65
0.75
0.85
Ages (years)
0
20
40
60
80
FDR (Age associated divergence) =0.0807
p(Scalp)=0.0124
p(Dura)=0.162
Scalp
Dura

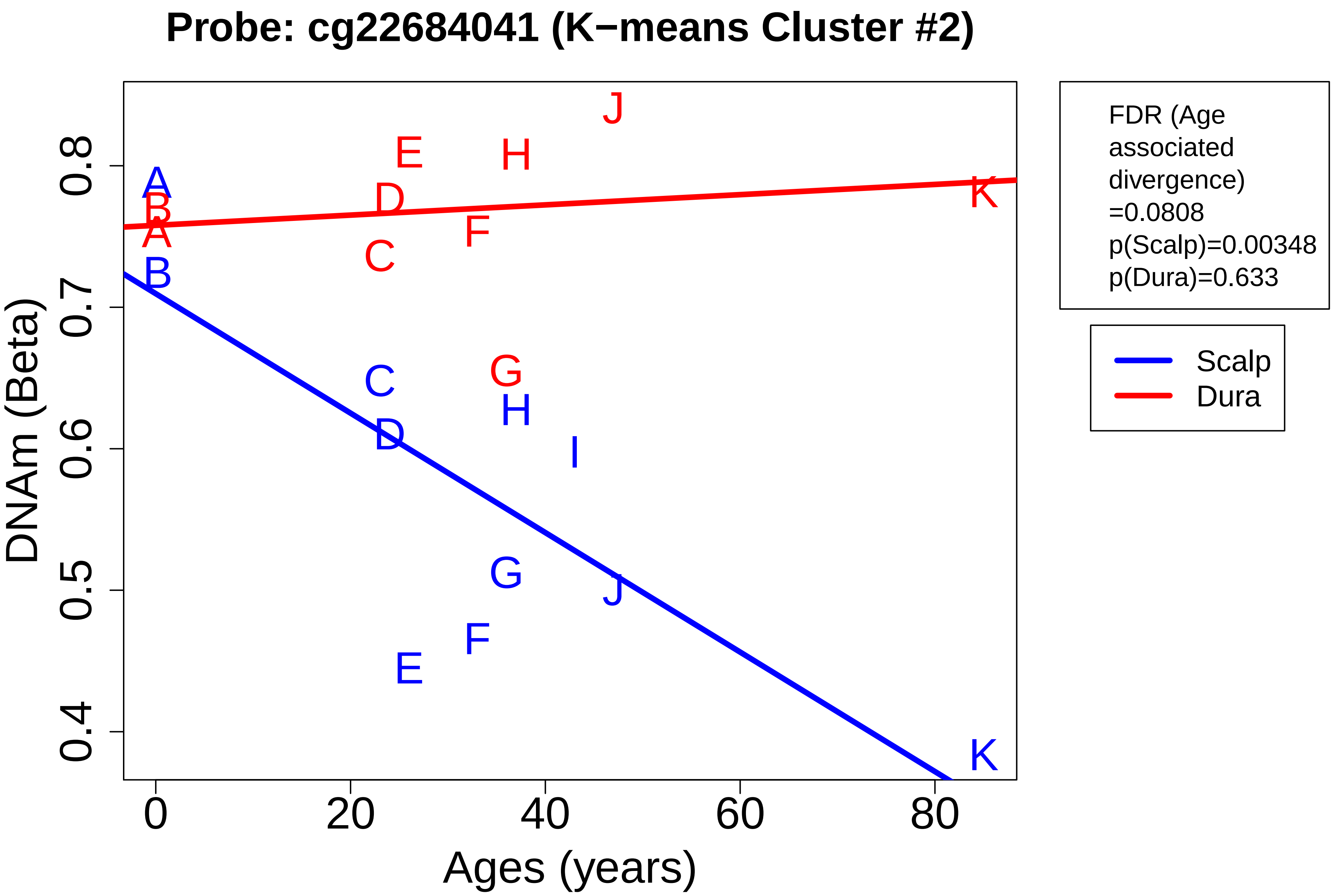

Probe: cg22684041 (K−means Cluster #2)
DNAm (Beta)
0.4
0.5
0.6
0.7
0.8
Ages (years)
0
20
40
60
80
A
B
C
D
E
F
G
H
I
J
K
FDR (Age associated divergence) =0.0808
p(Scalp)=0.00348
p(Dura)=0.633
Scalp
Dura

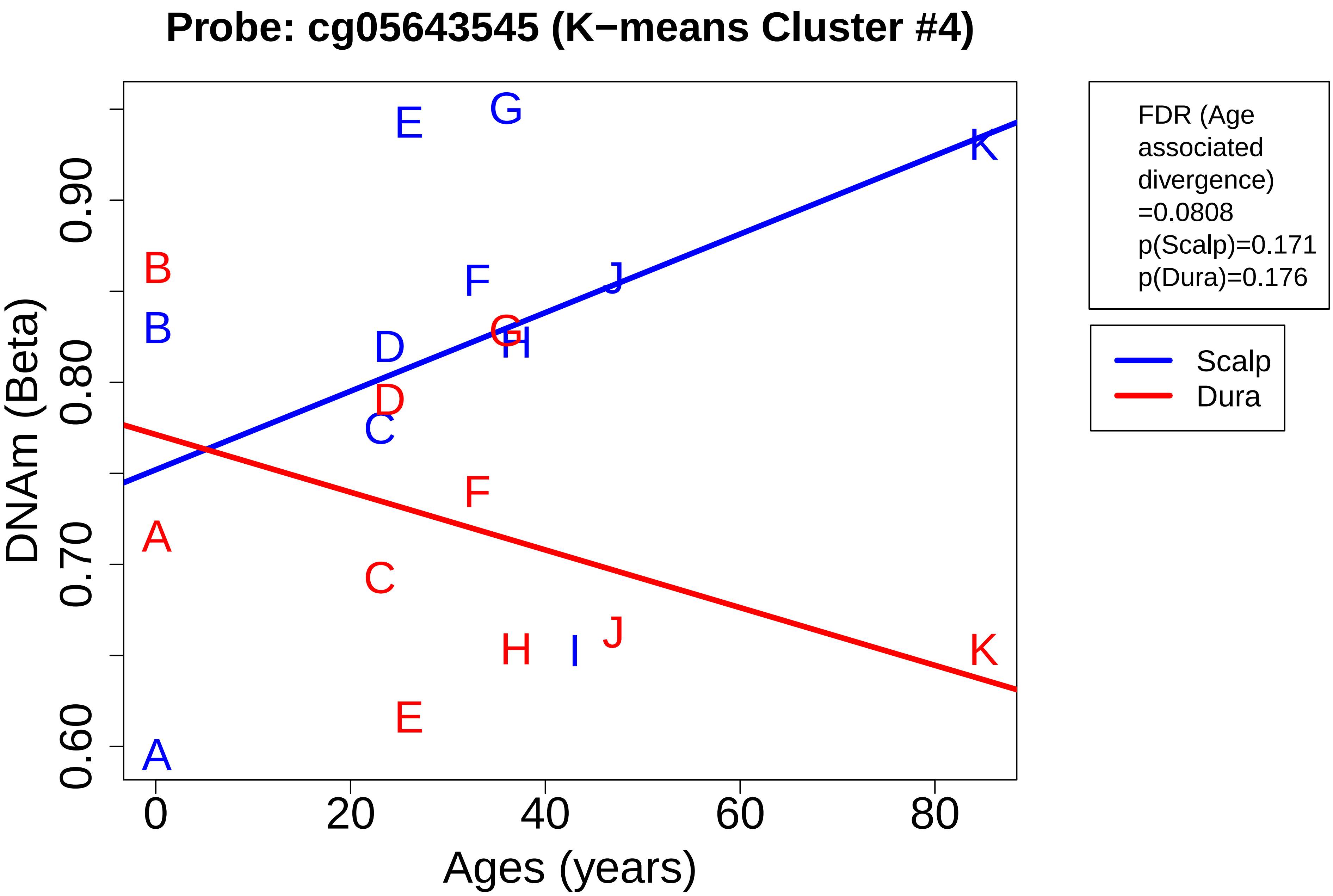

Probe: cg05643545 (K−means Cluster #4)
DNAm (Beta)
0.60
0.70
0.80
0.90
0
20
40
60
80
Ages (years)
FDR (Age associated divergence) =0.0808
p(Scalp)=0.171
p(Dura)=0.176
Scalp
Dura

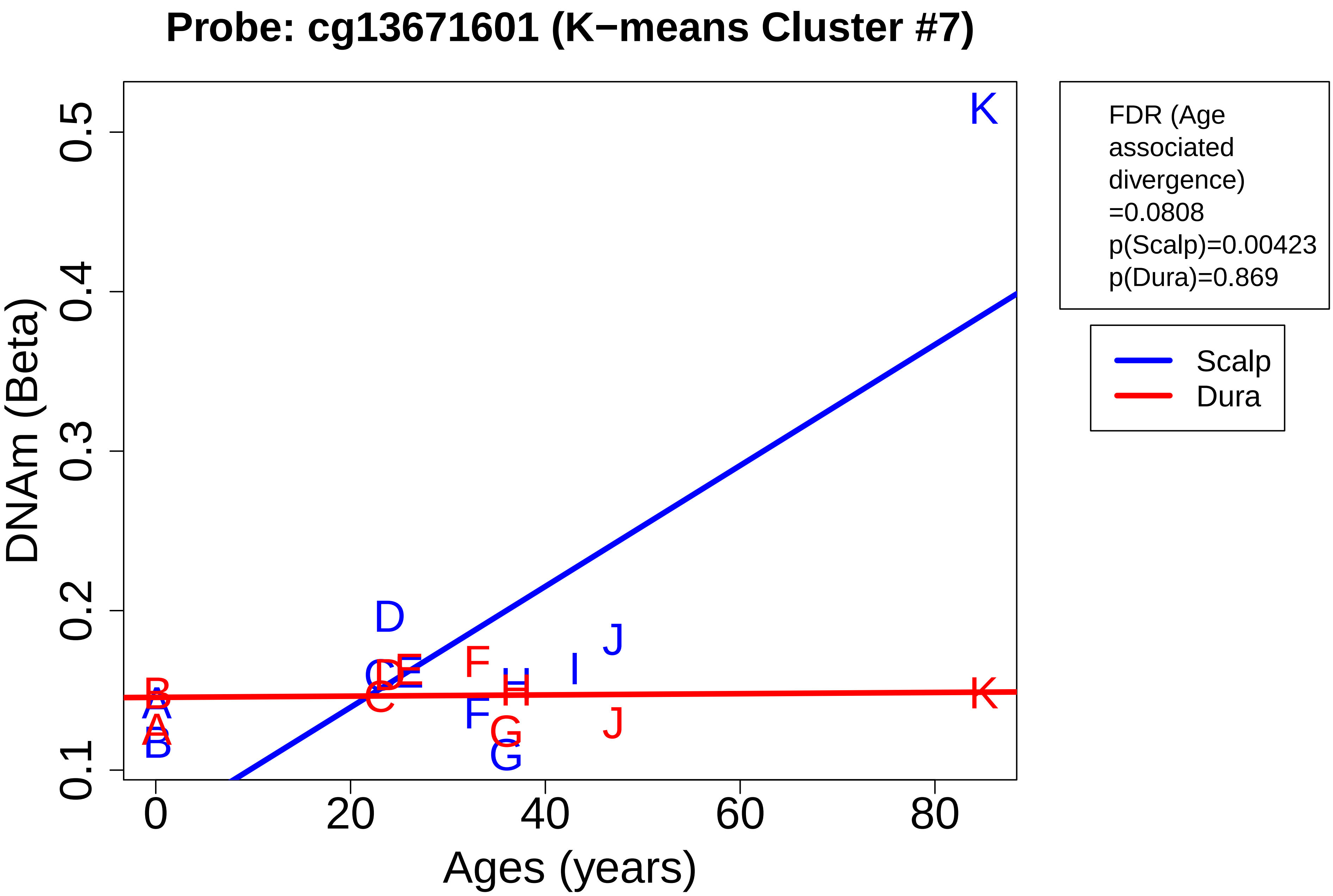

Probe: cg13671601 (K−means Cluster #7)
DNAm (Beta)
0.1
0.2
0.3
0.4
0.5
0
20
40
60
80
Ages (years)
FDR (Age associated divergence) =0.0808
p(Scalp)=0.00423
p(Dura)=0.869
Scalp
Dura
A
B
C
D
E
F
G
H
I
J
K

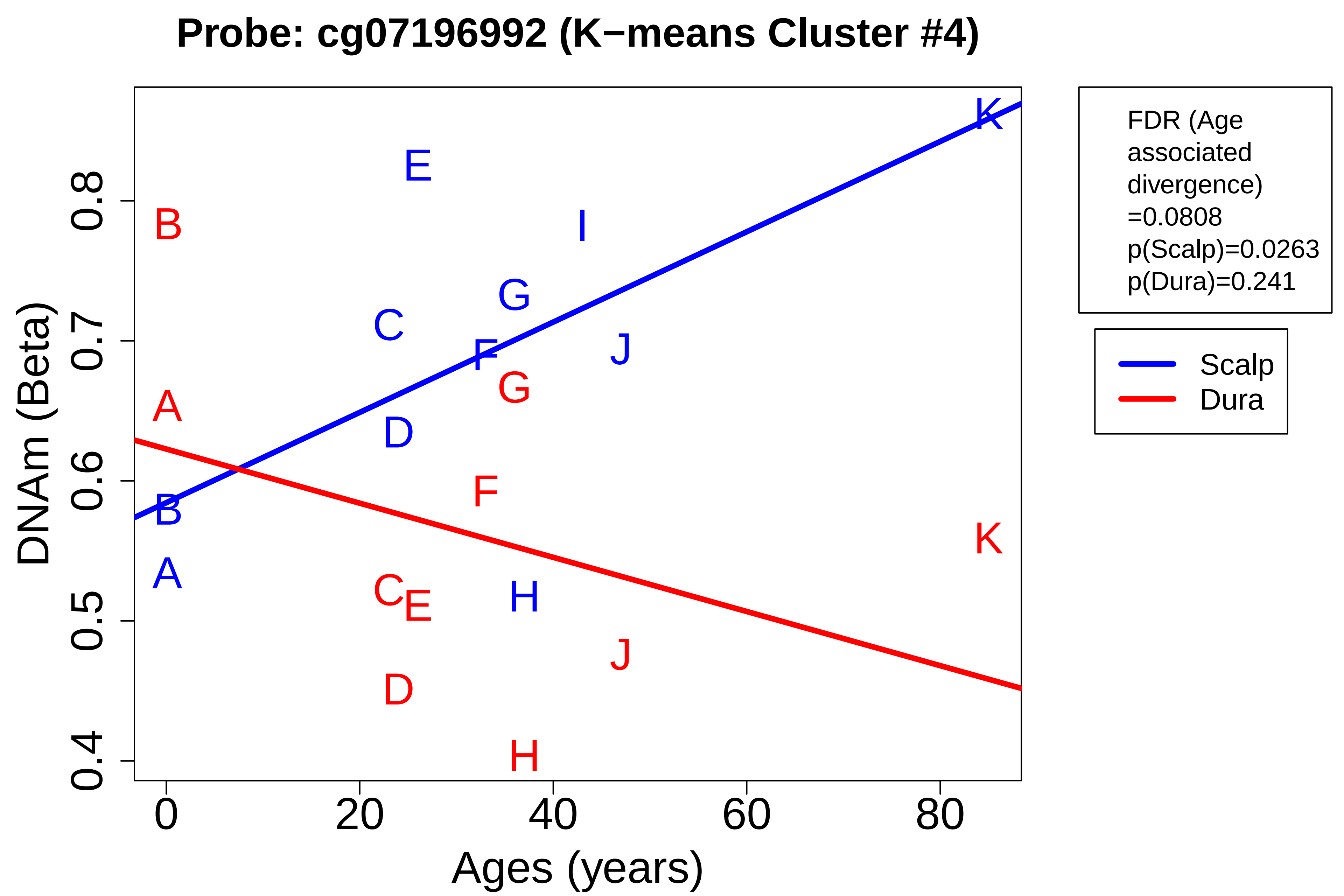

Probe: cg07196992 (K−means Cluster #4)
DNAm (Beta)
0.4
0.5
0.6
0.7
0.8
Ages (years)
0
20
40
60
80
FDR (Age associated divergence) =0.0808
p(Scalp)=0.0263
p(Dura)=0.241
Scalp
Dura
A
B
C
D
E
F
G
H
I
J
K

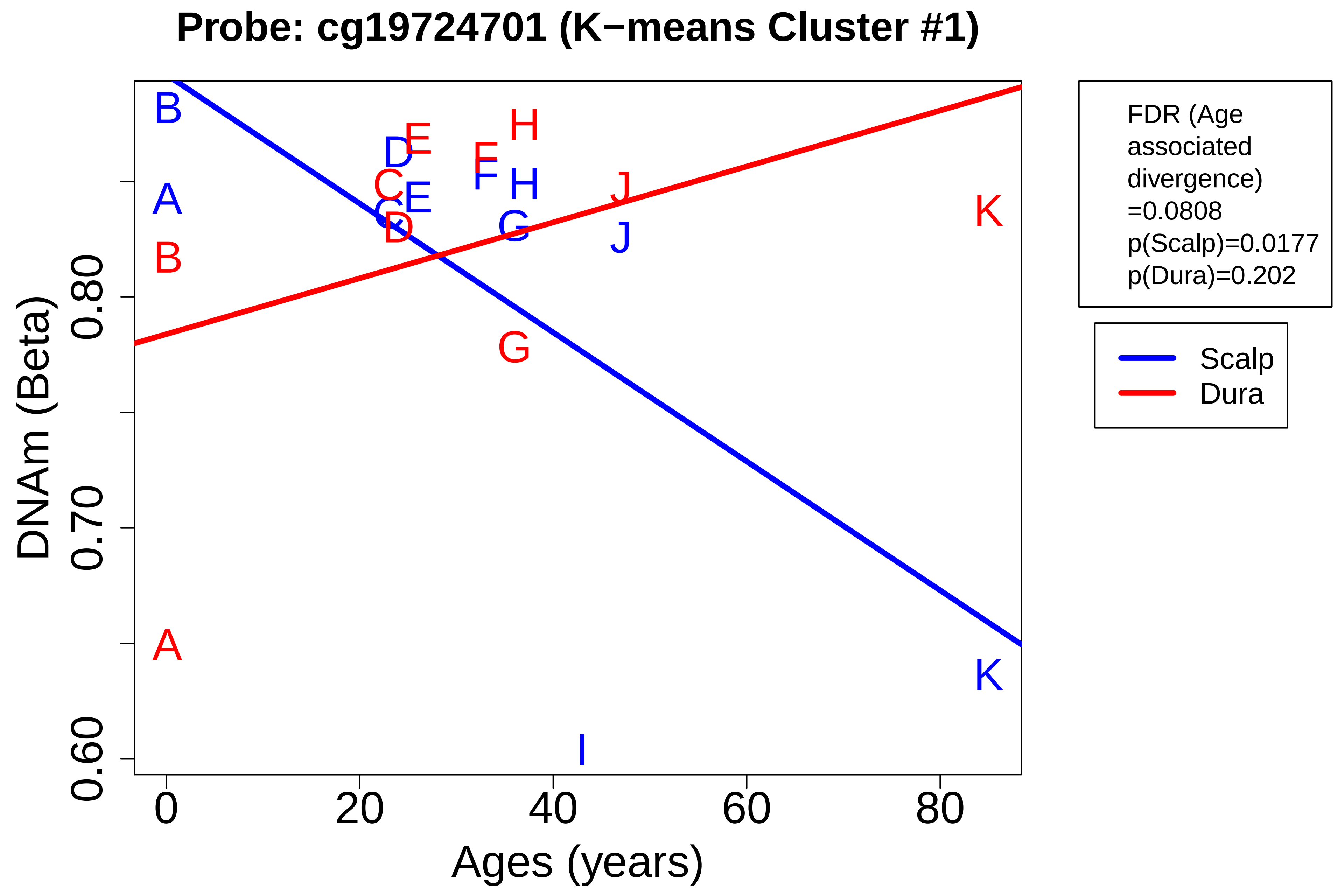

Probe: cg19724701 (K−means Cluster #1)
DNAm (Beta)
0.60
0.70
0.80
Ages (years)
0
20
40
60
80
FDR (Age associated divergence) =0.0808
p(Scalp)=0.0177
p(Dura)=0.202
Scalp
Dura

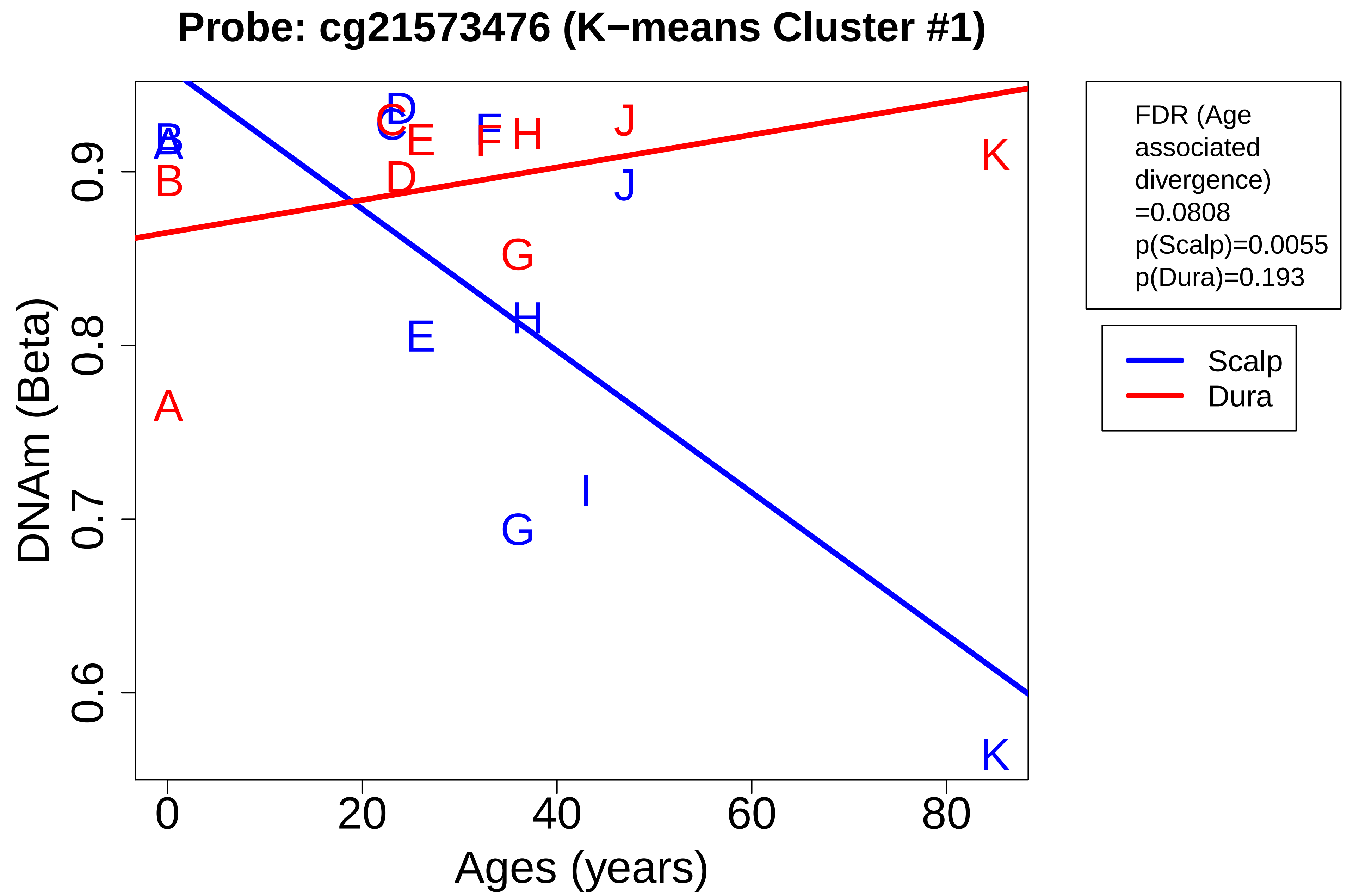

Probe: cg21573476 (K−means Cluster #1)
DNAm (Beta)
0.6
0.7
0.8
0.9
Ages (years)
0
20
40
60
80
A
B
C
D
E
F
G
H
I
J
K
FDR (Age associated divergence) =0.0808
p(Scalp)=0.0055
p(Dura)=0.193
Scalp
Dura

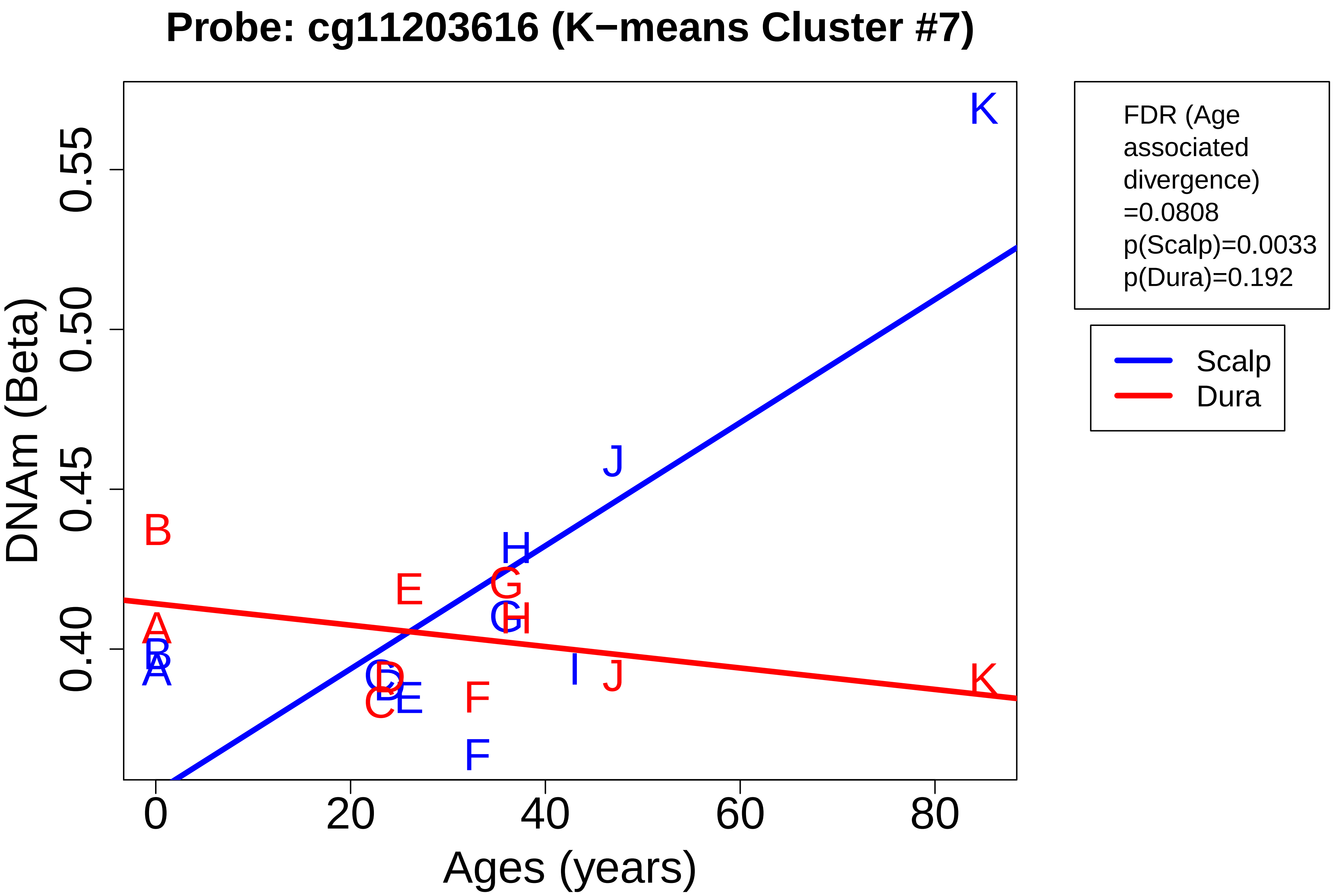

Probe: cg11203616 (K−means Cluster #7)
DNAm (Beta)
0.40
0.45
0.50
0.55
0
20
40
60
80
Ages (years)
FDR (Age associated divergence) =0.0808
p(Scalp)=0.0033
p(Dura)=0.192
Scalp
Dura
A
B
C
D
E
F
G
H
I
J
K

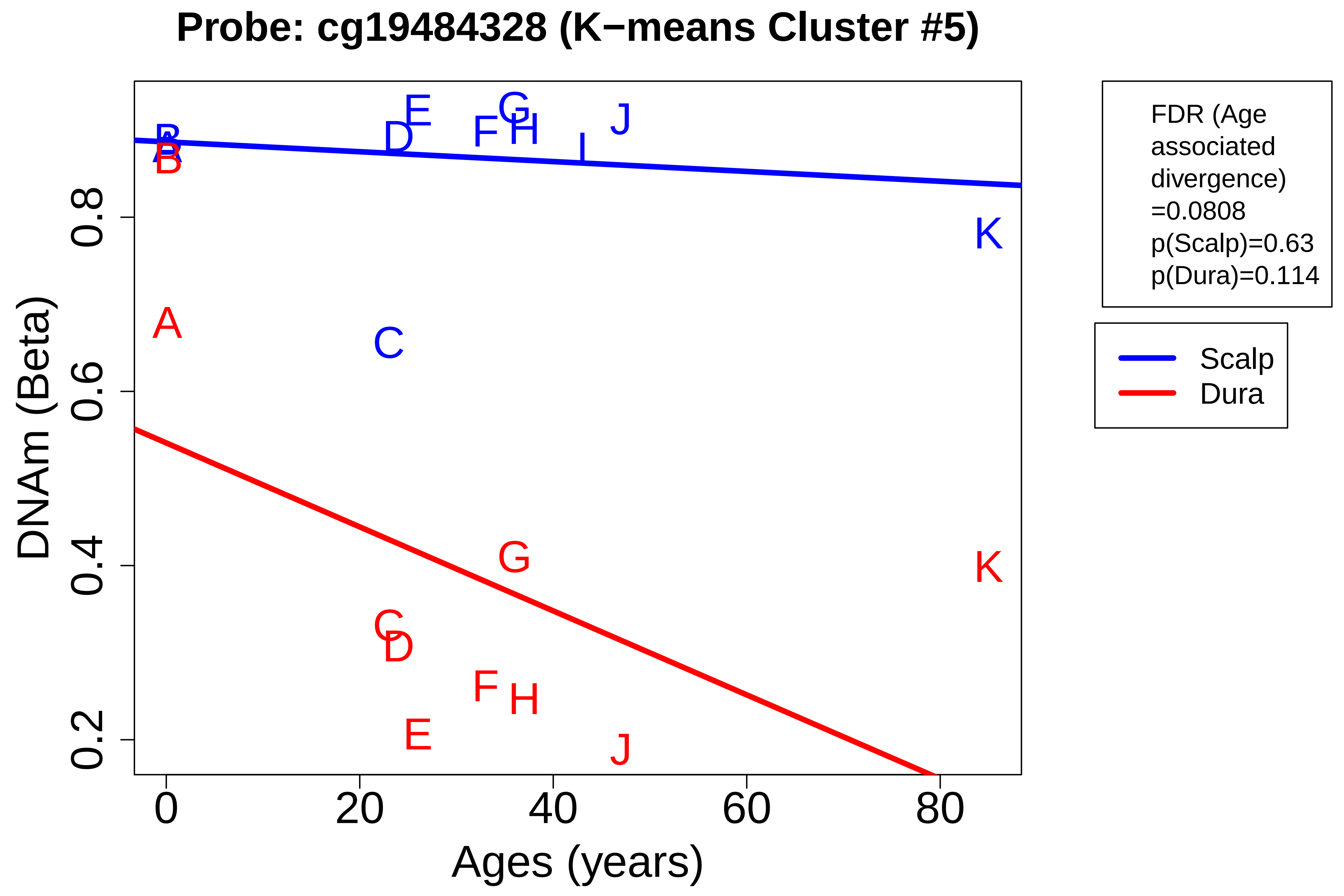

Probe: cg19484328 (K−means Cluster #5)
FDR (Age associated divergence) =0.0808
p(Scalp)=0.63
p(Dura)=0.114
Scalp
Dura
DNAm (Beta)
0.2
0.4
0.6
0.8
0
20
40
60
80
Ages (years)

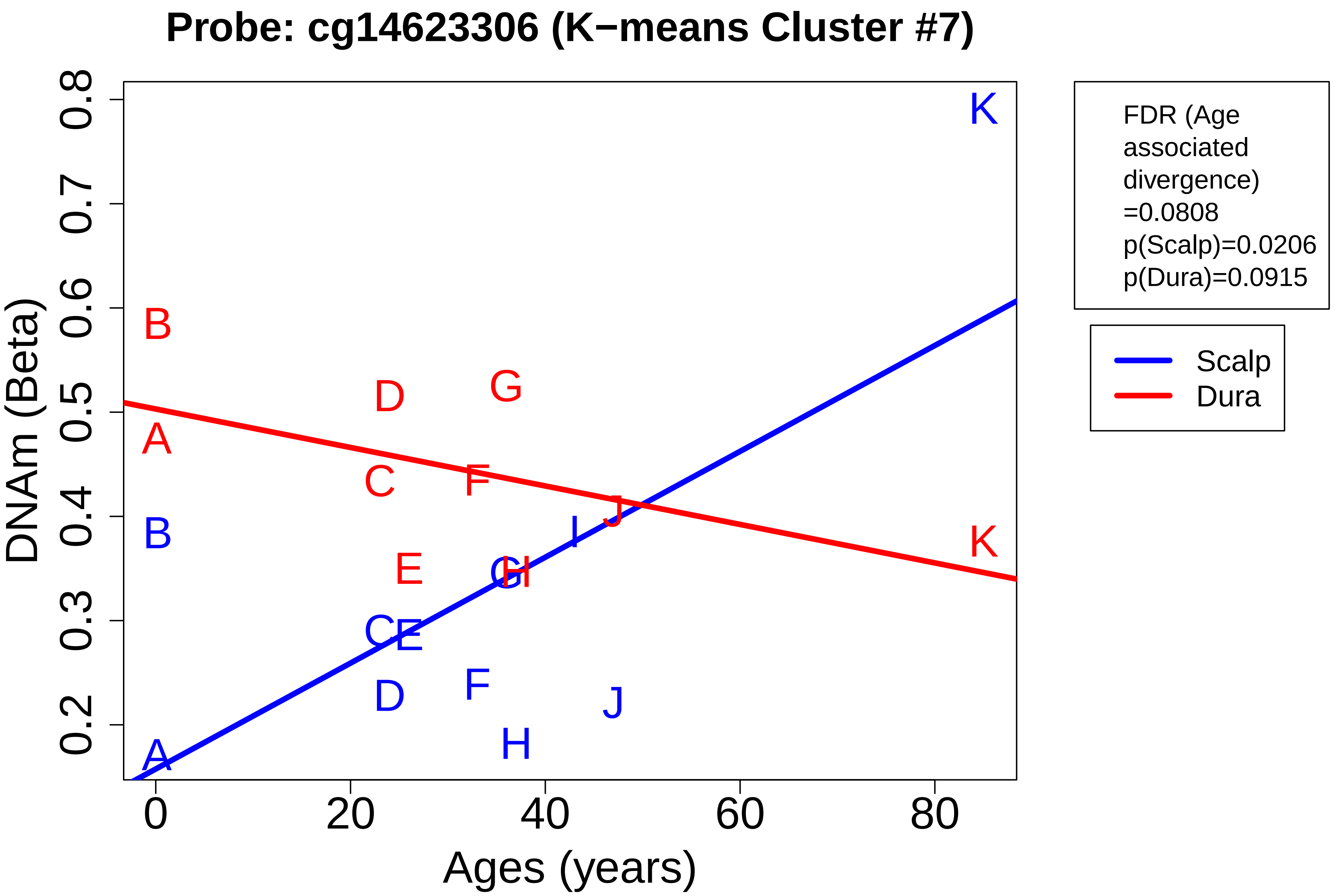

Probe: cg14623306 (K−means Cluster #7)
DNAm (Beta)
0.2
0.3
0.4
0.5
0.6
0.7
0.8
Ages (years)
0
20
40
60
80
FDR (Age associated divergence) =0.0808
p(Scalp)=0.0206
p(Dura)=0.0915
Scalp
Dura
A
B
C
D
E
F
G
H
I
J
K

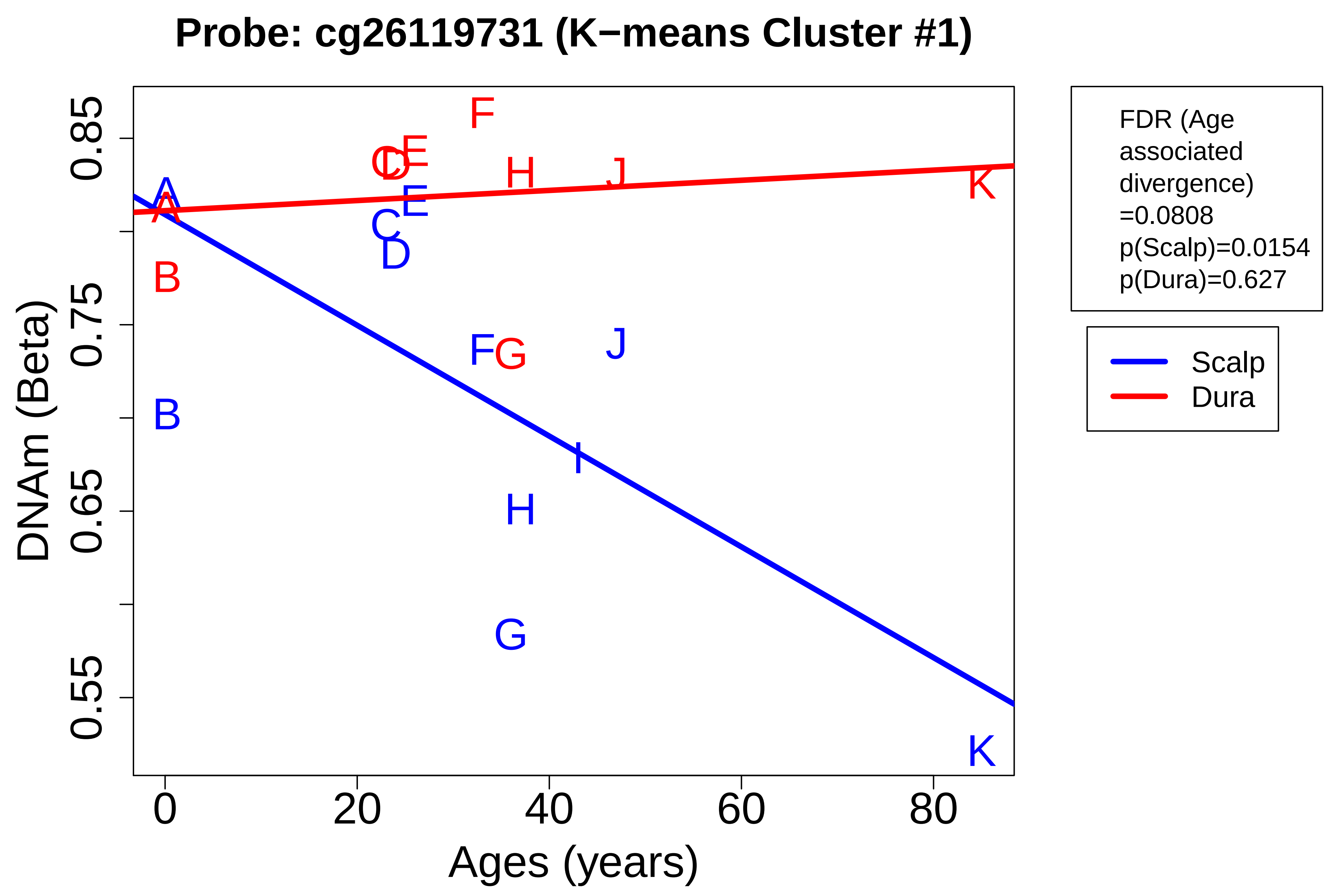
Probe: cg26119731 (K−means Cluster #1)
DNAm (Beta)
0.55
0.65
0.75
0.85
0
20
40
60
80
Ages (years)
FDR (Age associated divergence) =0.0808
p(Scalp)=0.0154
p(Dura)=0.627
Scalp
Dura

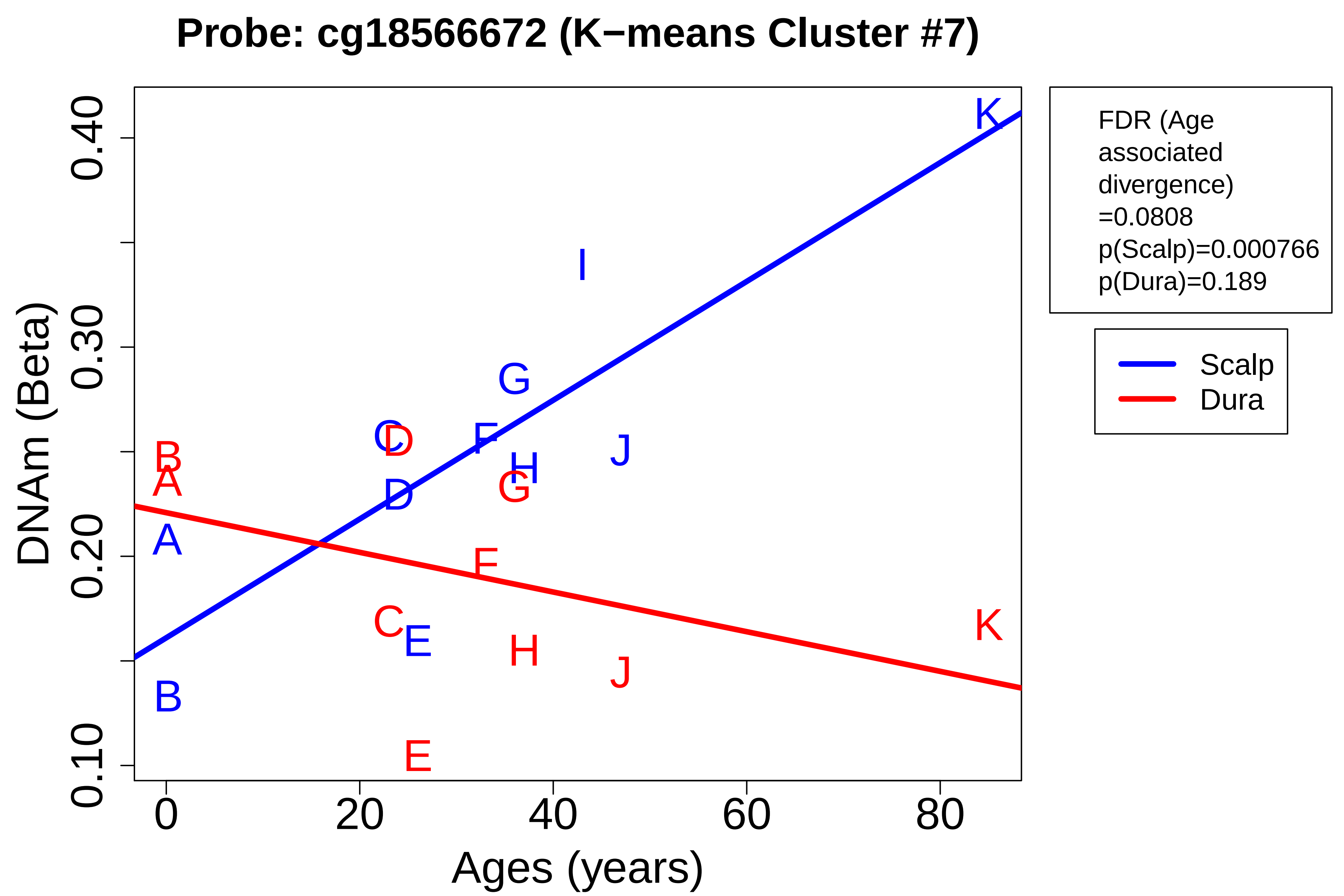

Probe: cg18566672 (K−means Cluster #7)
DNAm (Beta)
0.10
0.20
0.30
0.40
Ages (years)
0
20
40
60
80
A
B
C
D
E
F
G
H
I
J
K
FDR (Age associated divergence) =0.0808
p(Scalp)=0.000766
p(Dura)=0.189
Scalp
Dura

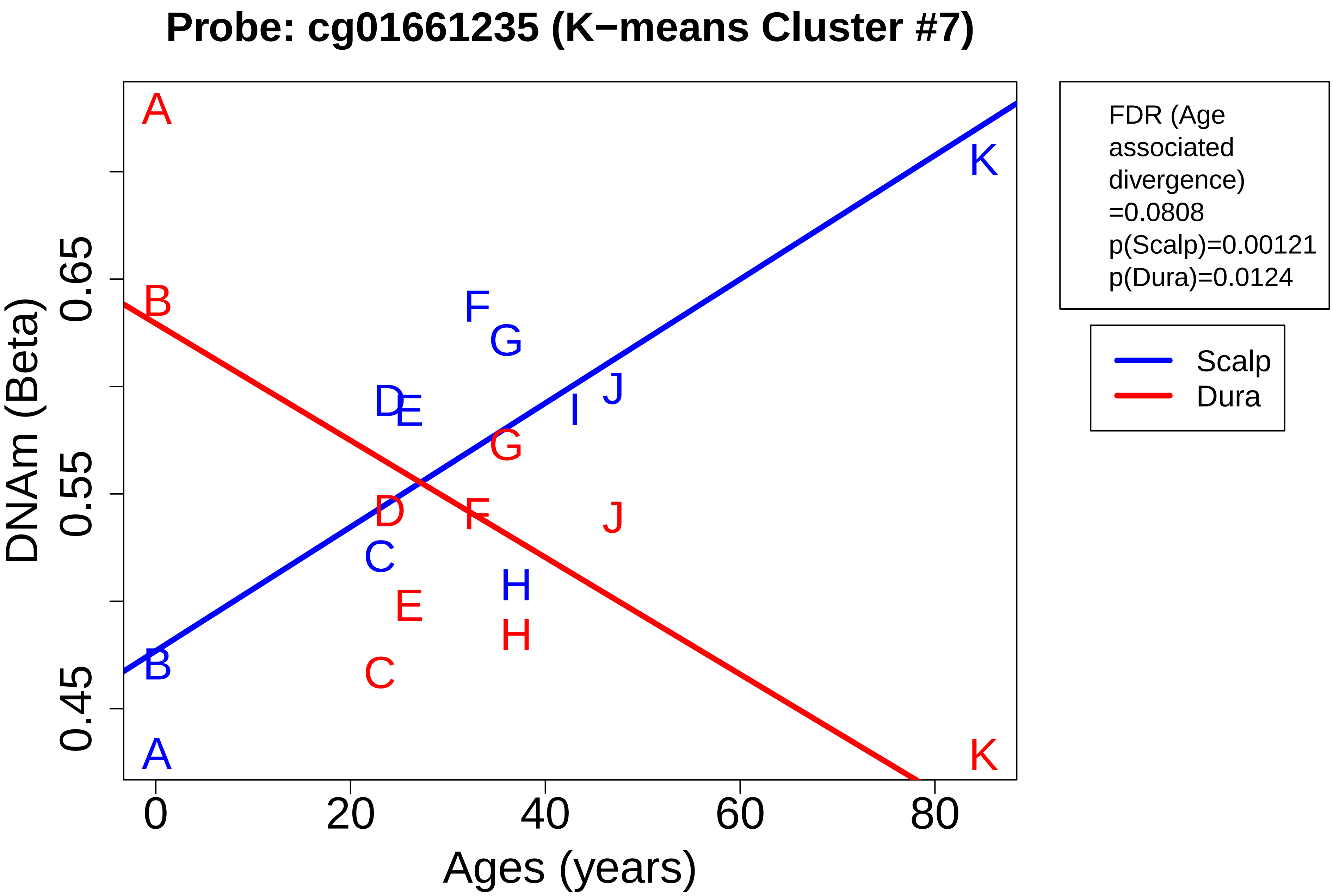

Probe: cg01661235 (K−means Cluster #7)
FDR (Age associated divergence) =0.0808
p(Scalp)=0.00121
p(Dura)=0.0124
Scalp
Dura
DNAm (Beta)
0.45
0.55
0.65
Ages (years)
0
20
40
60
80
A
B
C
D
E
F
G
H
I
J
K

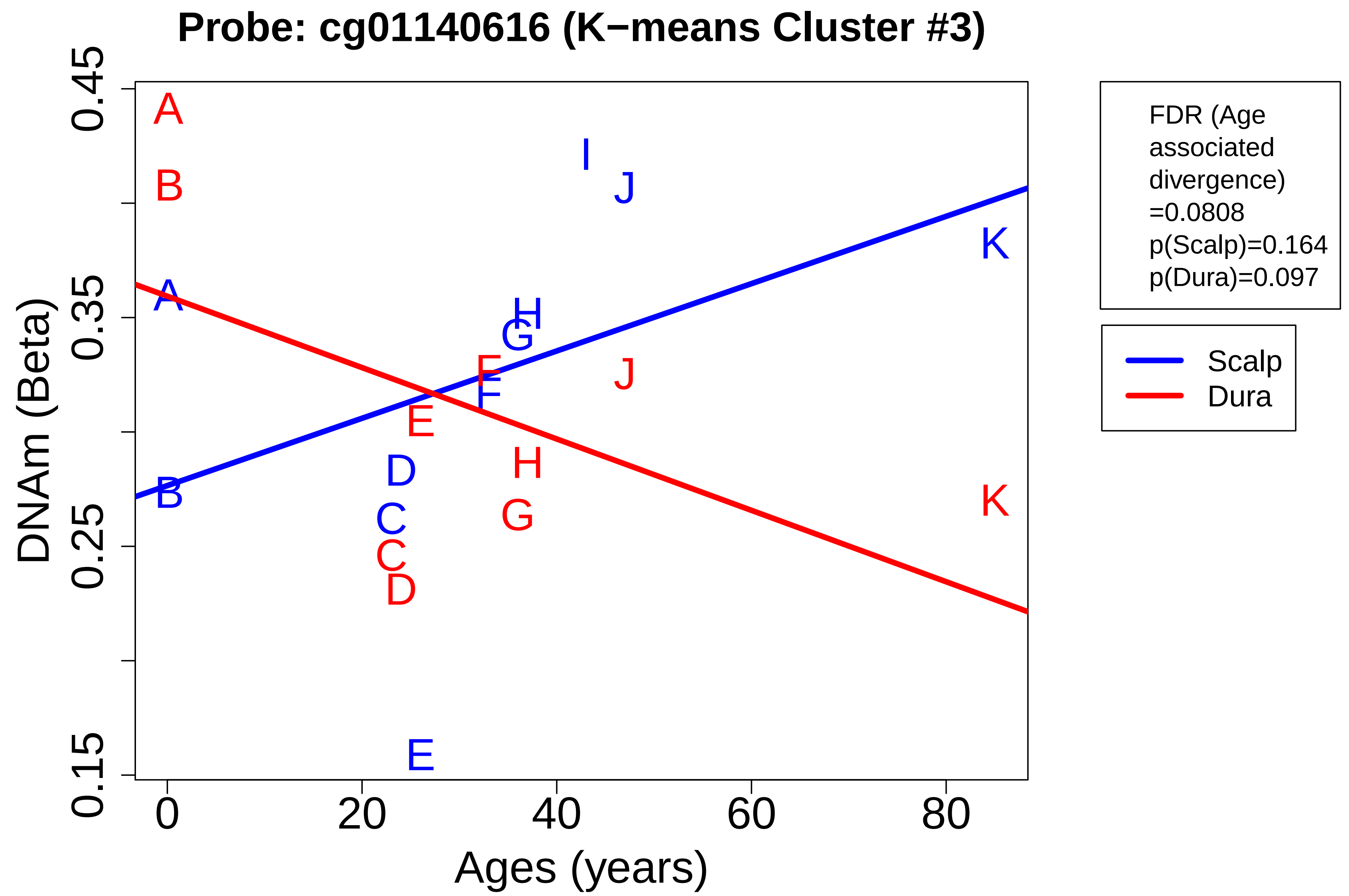

Probe: cg01140616 (K−means Cluster #3)
DNAm (Beta)
0.15
0.25
0.35
0.45
Ages (years)
0
20
40
60
80
A
B
A
H
G
F
J
I
J
K
E
F
D
H
B
C
G
K
C
D
E
FDR (Age associated divergence) =0.0808
p(Scalp)=0.164
p(Dura)=0.097
Scalp
Dura

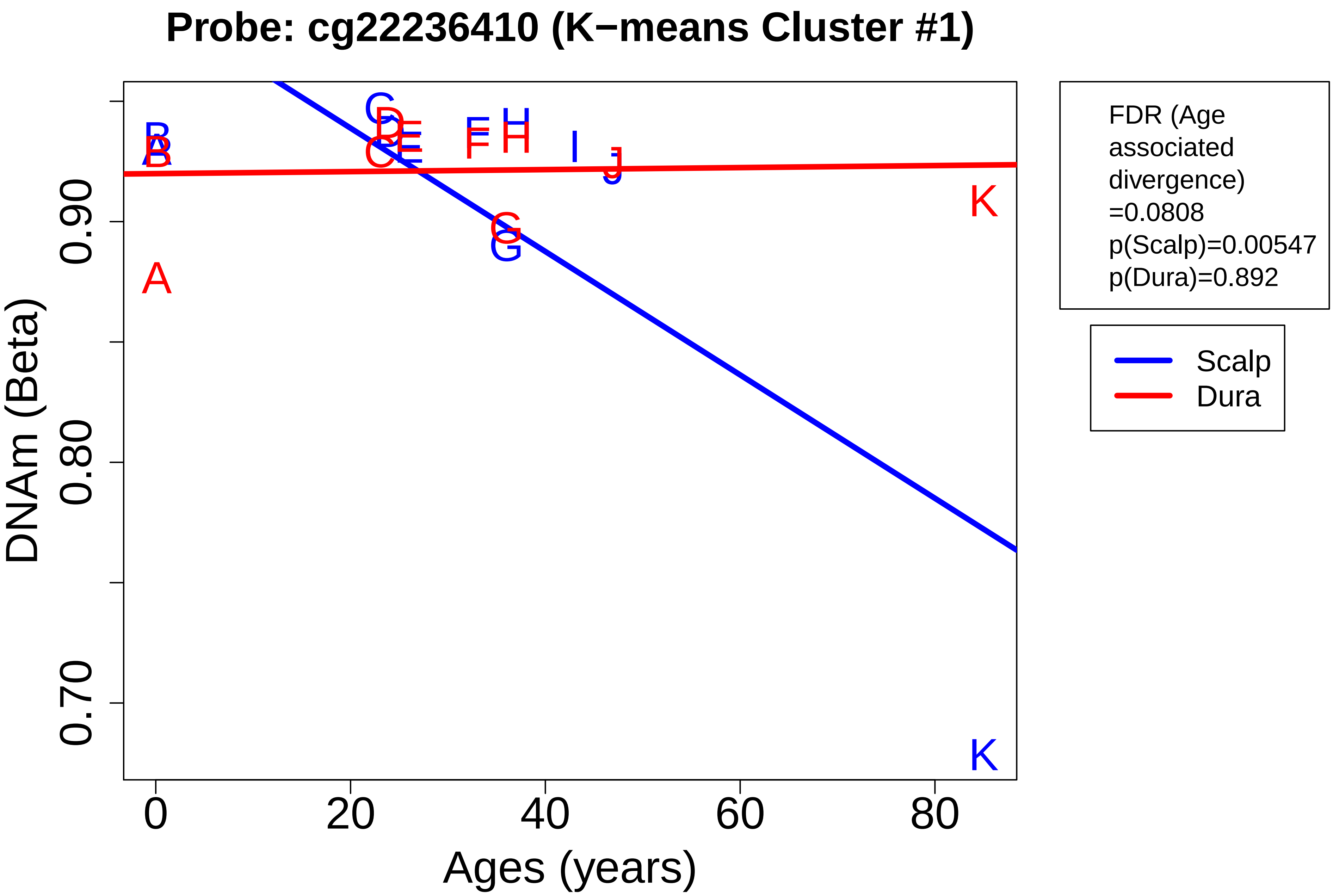

Probe: cg22236410 (K−means Cluster #1)
DNAm (Beta)
0.70
0.80
0.90
Ages (years)
0
20
40
60
80
FDR (Age associated divergence) =0.0808
p(Scalp)=0.00547
p(Dura)=0.892
Scalp
Dura

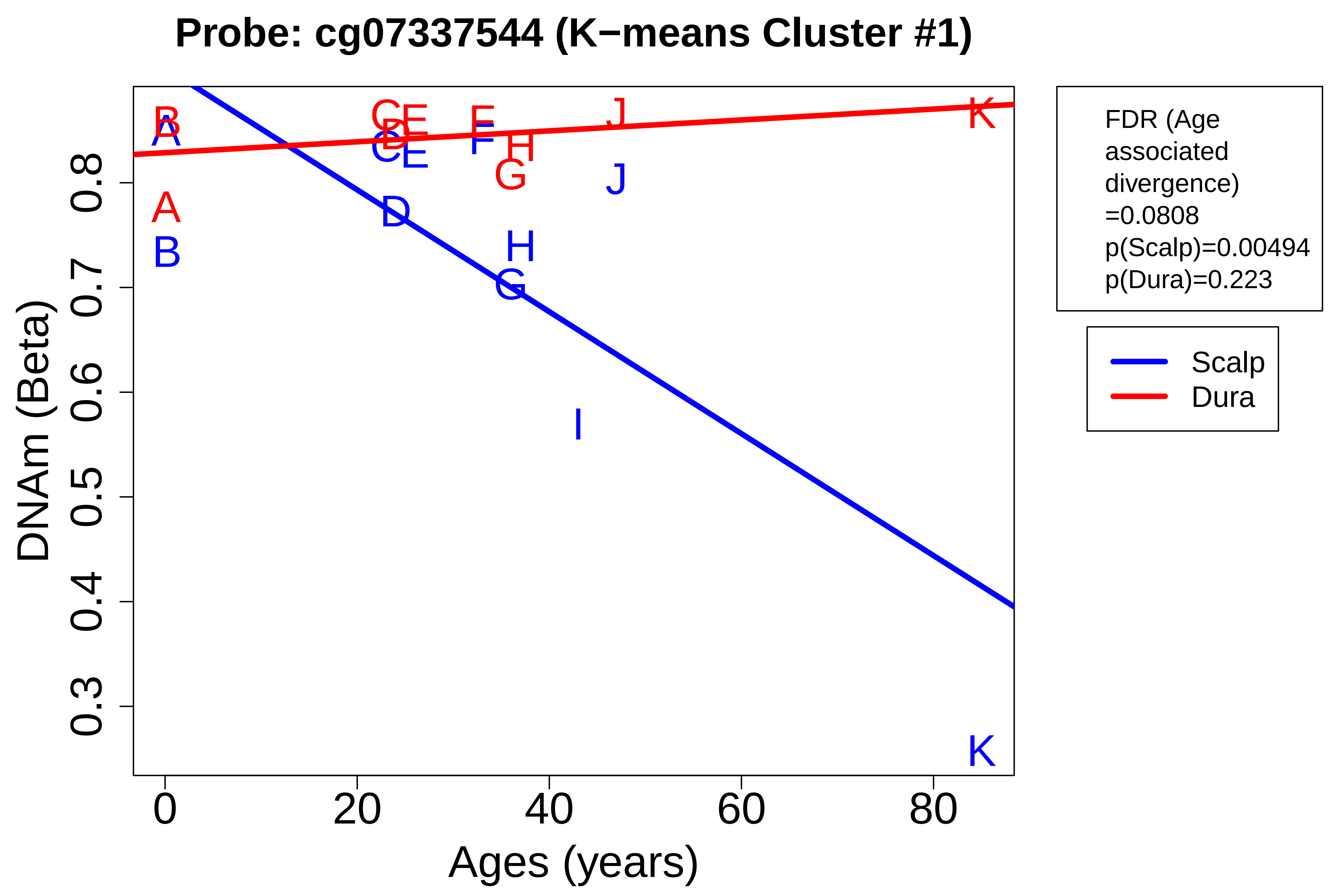

Probe: cg07337544 (K−means Cluster #1)
DNAm (Beta)
0.3
0.4
0.5
0.6
0.7
0.8
0
20
40
60
80
Ages (years)
A
B
C
D
E
F
G
H
I
J
K
FDR (Age associated divergence) =0.0808
p(Scalp)=0.00494
p(Dura)=0.223
Scalp
Dura

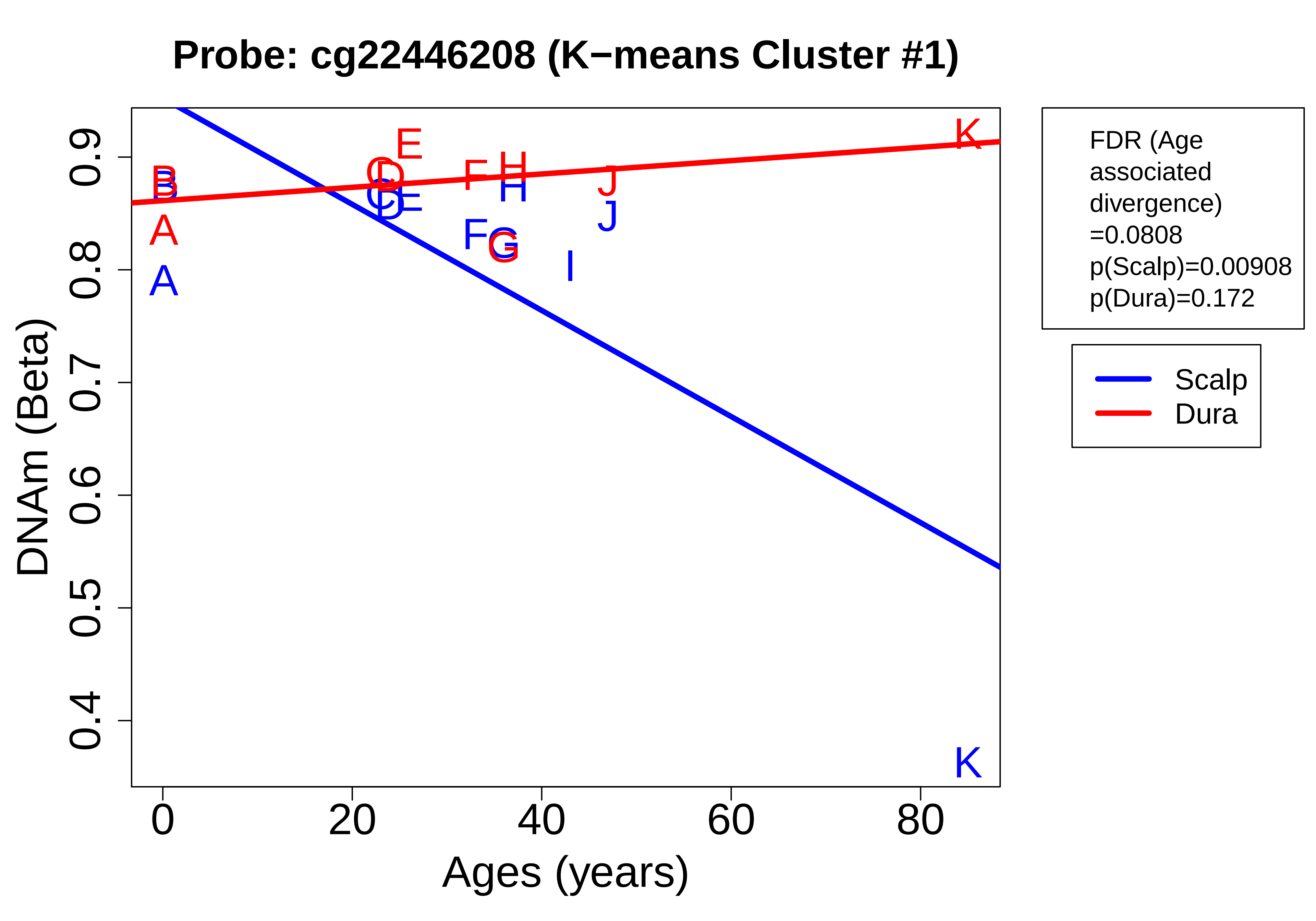

Probe: cg22446208 (K−means Cluster #1)
DNAm (Beta)
Ages (years)
0.4
0.5
0.6
0.7
0.8
0.9
0
20
40
60
80
A
B
C
D
E
F
G
H
I
J
K
FDR (Age associated divergence) =0.0808
p(Scalp)=0.00908
p(Dura)=0.172
Scalp
Dura

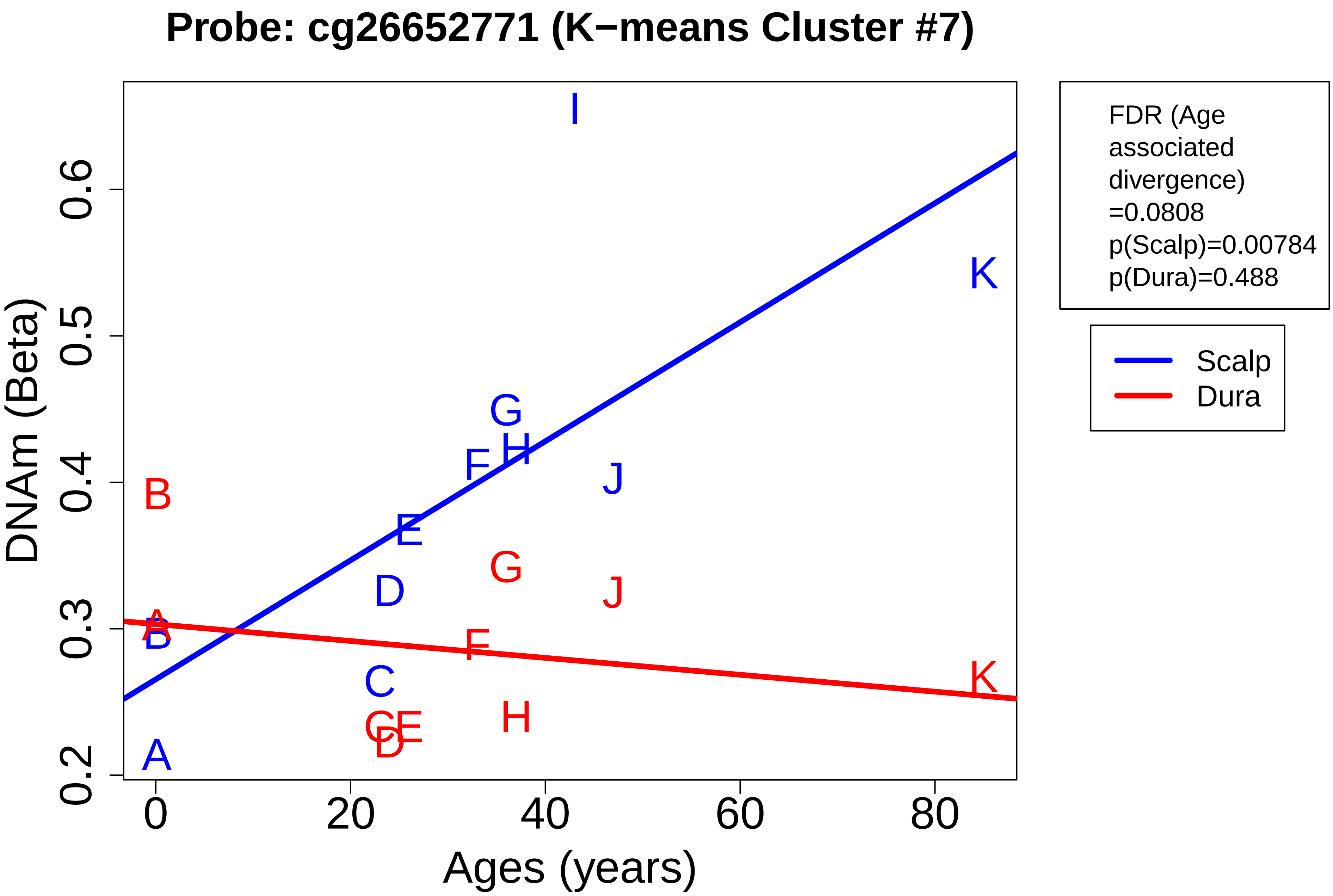
Probe: cg26652771 (K−means Cluster #7)
DNAm (Beta)
0.2
0.3
0.4
0.5
0.6
0
20
40
60
80
Ages (years)
I
K
G
H
F
J
B
E
G
D
J
A
B
F
C
K
H
C
E
D
A
FDR (Age associated divergence) =0.0808
p(Scalp)=0.00784
p(Dura)=0.488
Scalp
Dura

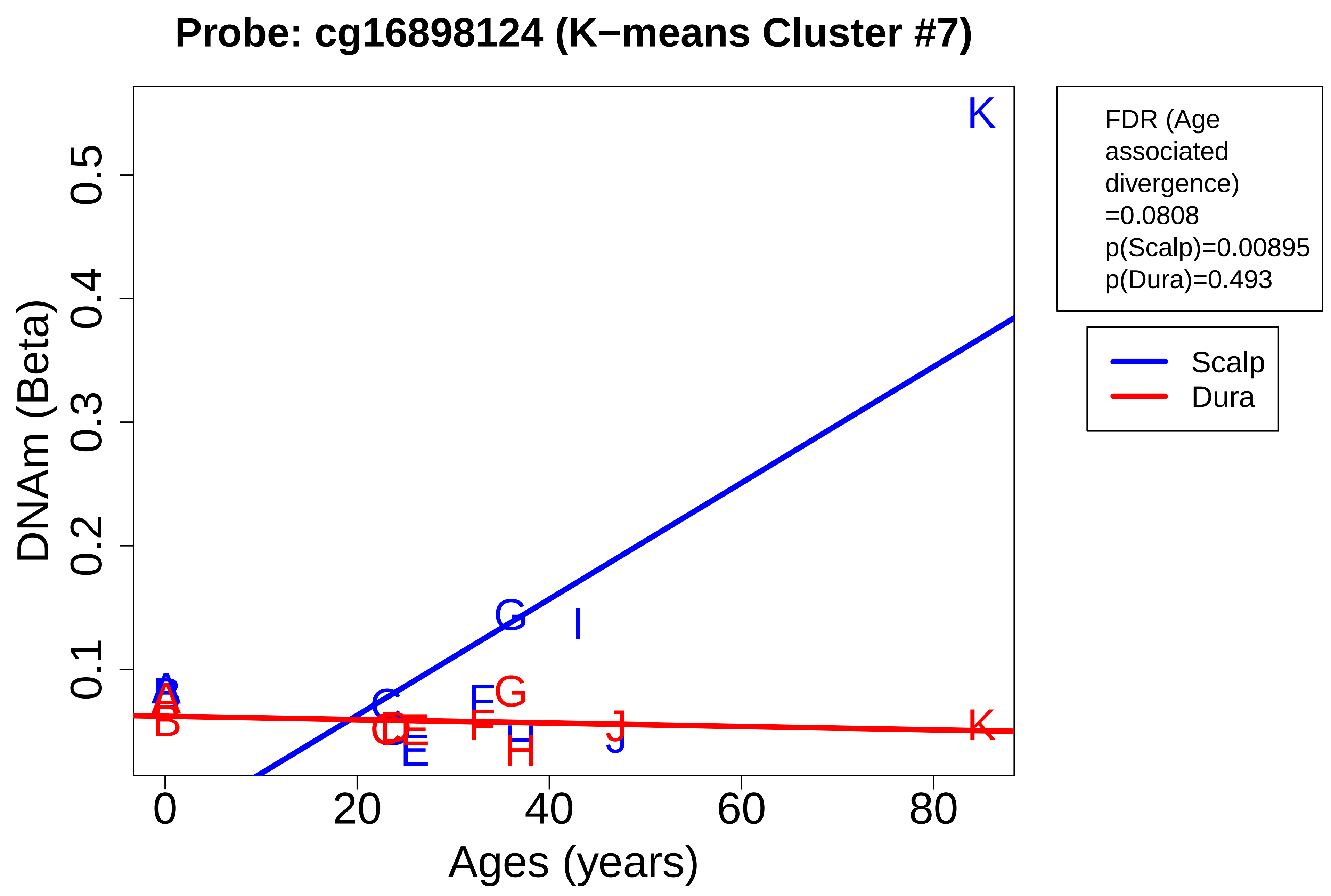

Probe: cg16898124 (K−means Cluster #7)
DNAm (Beta)
0.1
0.2
0.3
0.4
0.5
Ages (years)
0
20
40
60
80
FDR (Age associated divergence) =0.0808
p(Scalp)=0.00895
p(Dura)=0.493
Scalp
Dura

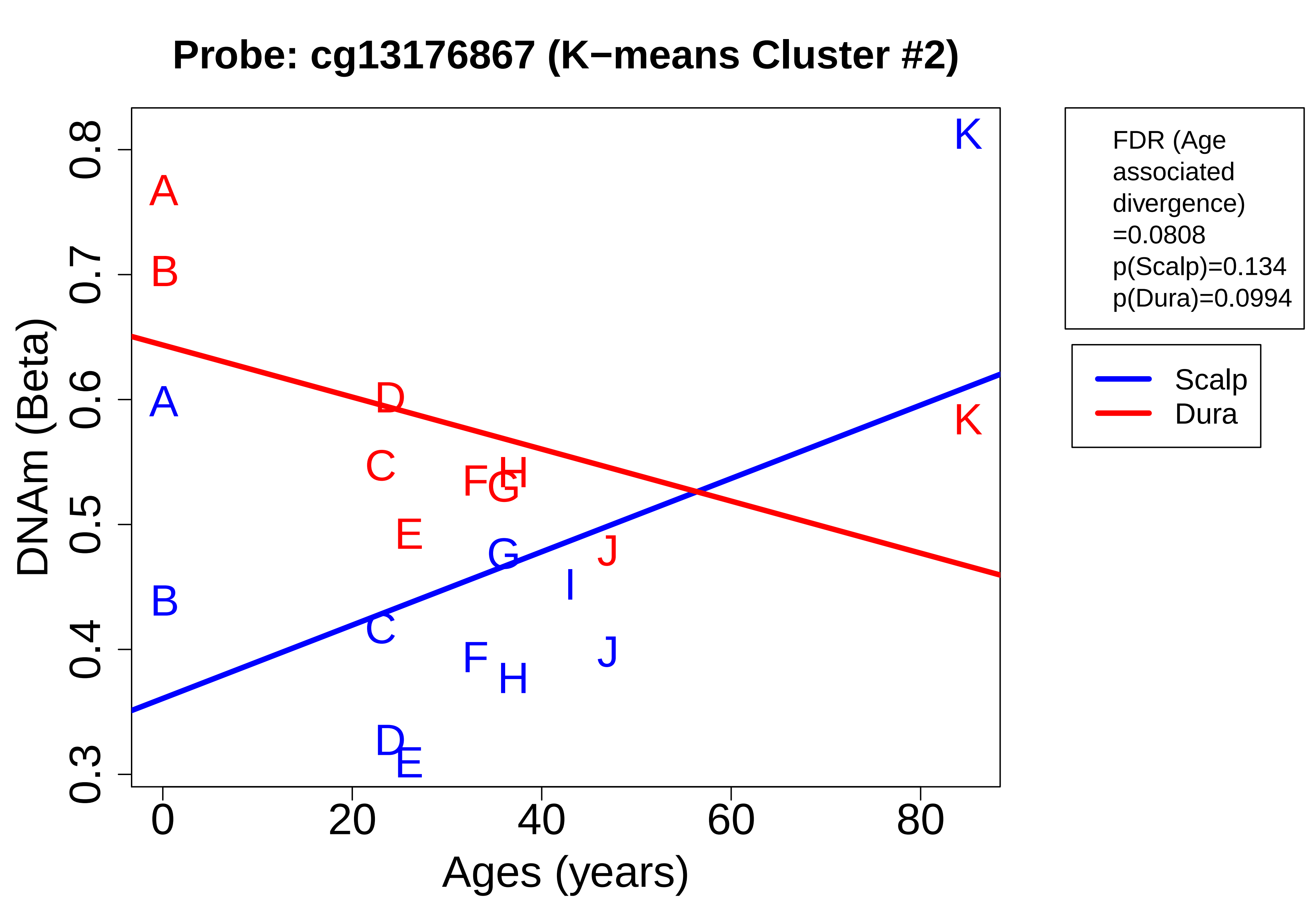
Probe: cg13176867 (K−means Cluster #2)
DNAm (Beta)
0.3
0.4
0.5
0.6
0.7
0.8
0
20
40
60
80
Ages (years)
A
B
C
D
E
F
G
H
I
J
K
FDR (Age associated divergence) =0.0808
p(Scalp)=0.134
p(Dura)=0.0994
Scalp
Dura

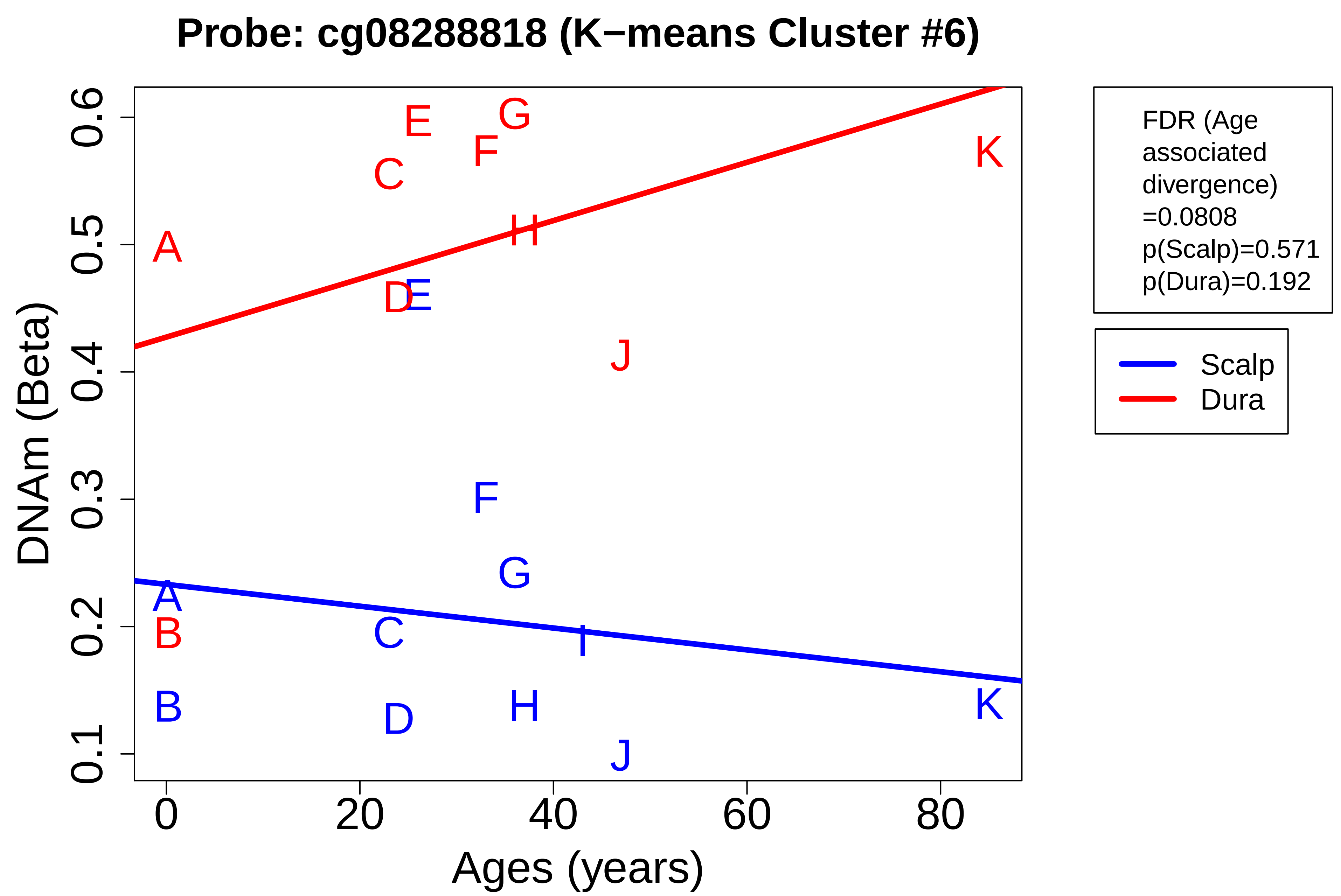

Probe: cg08288818 (K−means Cluster #6)
DNAm (Beta)
0.1
0.2
0.3
0.4
0.5
0.6
Ages (years)
0
20
40
60
80
A
B
C
D
E
F
G
H
I
J
K
FDR (Age associated divergence) =0.0808
p(Scalp)=0.571
p(Dura)=0.192
Scalp
Dura

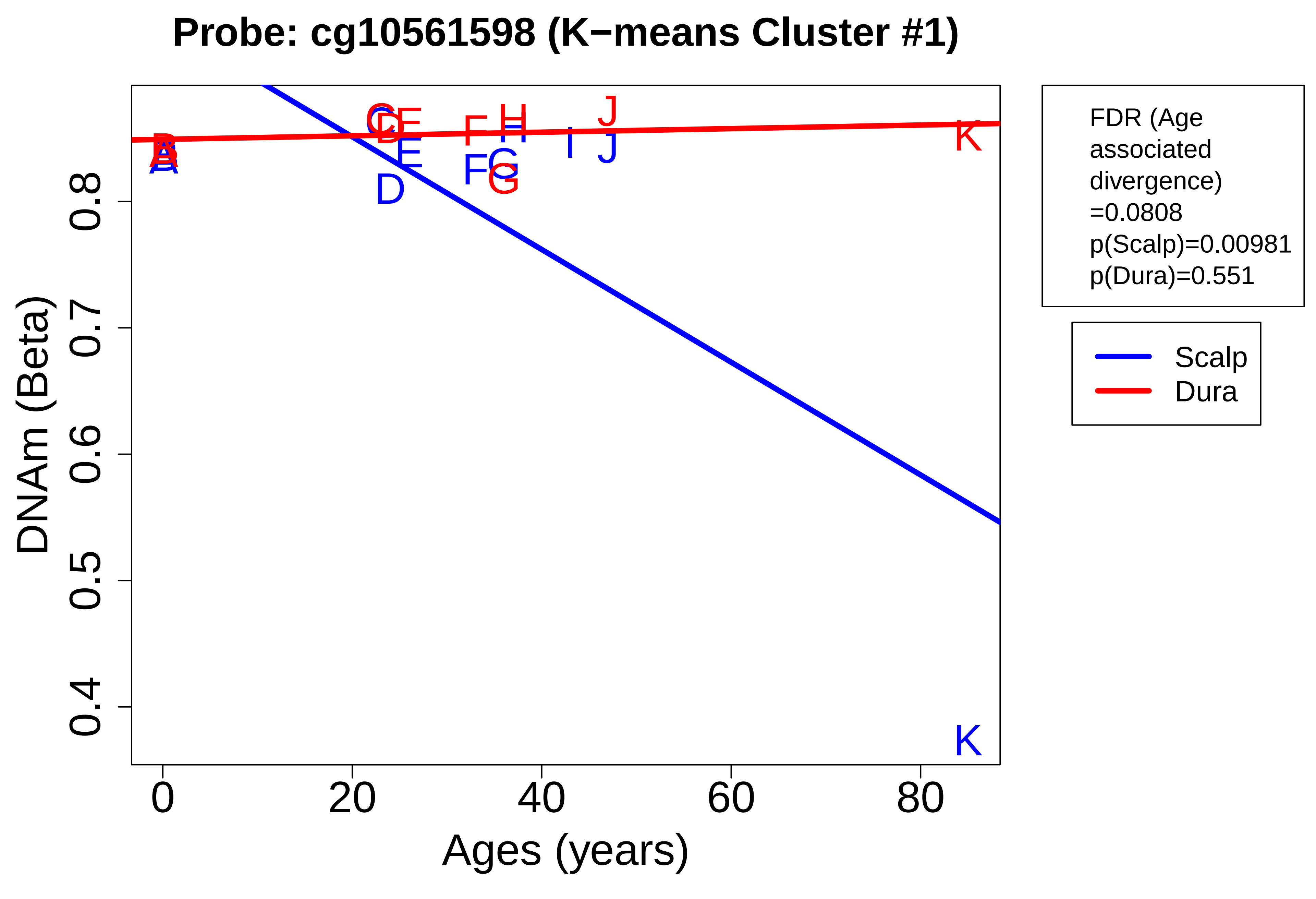

Probe: cg10561598 (K−means Cluster #1)
DNAm (Beta)
0.4
0.5
0.6
0.7
0.8
Ages (years)
0
20
40
60
80
A
B
C
D
E
F
G
H
I
J
K
FDR (Age associated divergence) =0.0808
p(Scalp)=0.00981
p(Dura)=0.551
Scalp
Dura

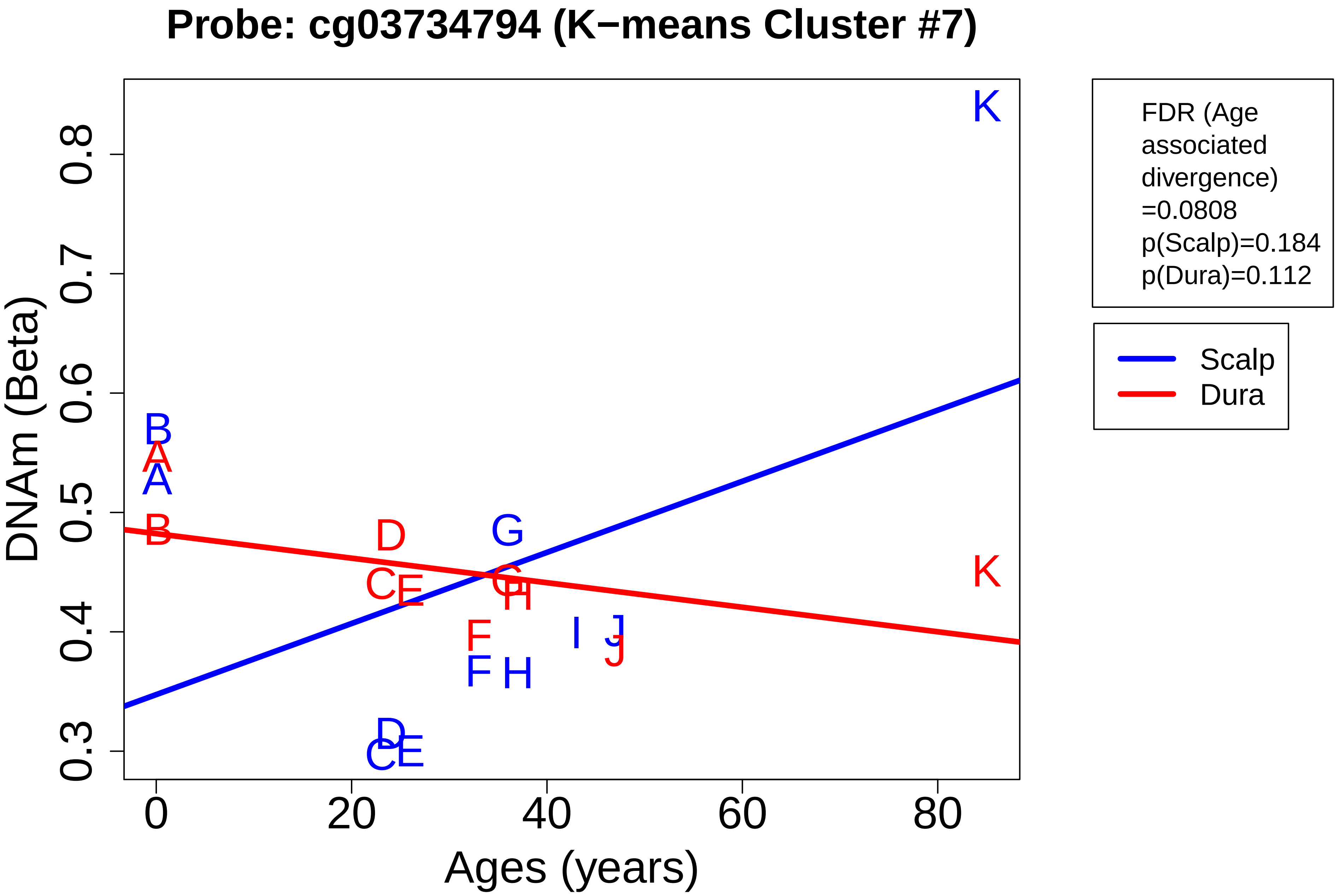

Probe: cg03734794 (K−means Cluster #7)
DNAm (Beta)
0.3
0.4
0.5
0.6
0.7
0.8
Ages (years)
0
20
40
60
80
FDR (Age associated divergence) =0.0808
p(Scalp)=0.184
p(Dura)=0.112
Scalp
Dura

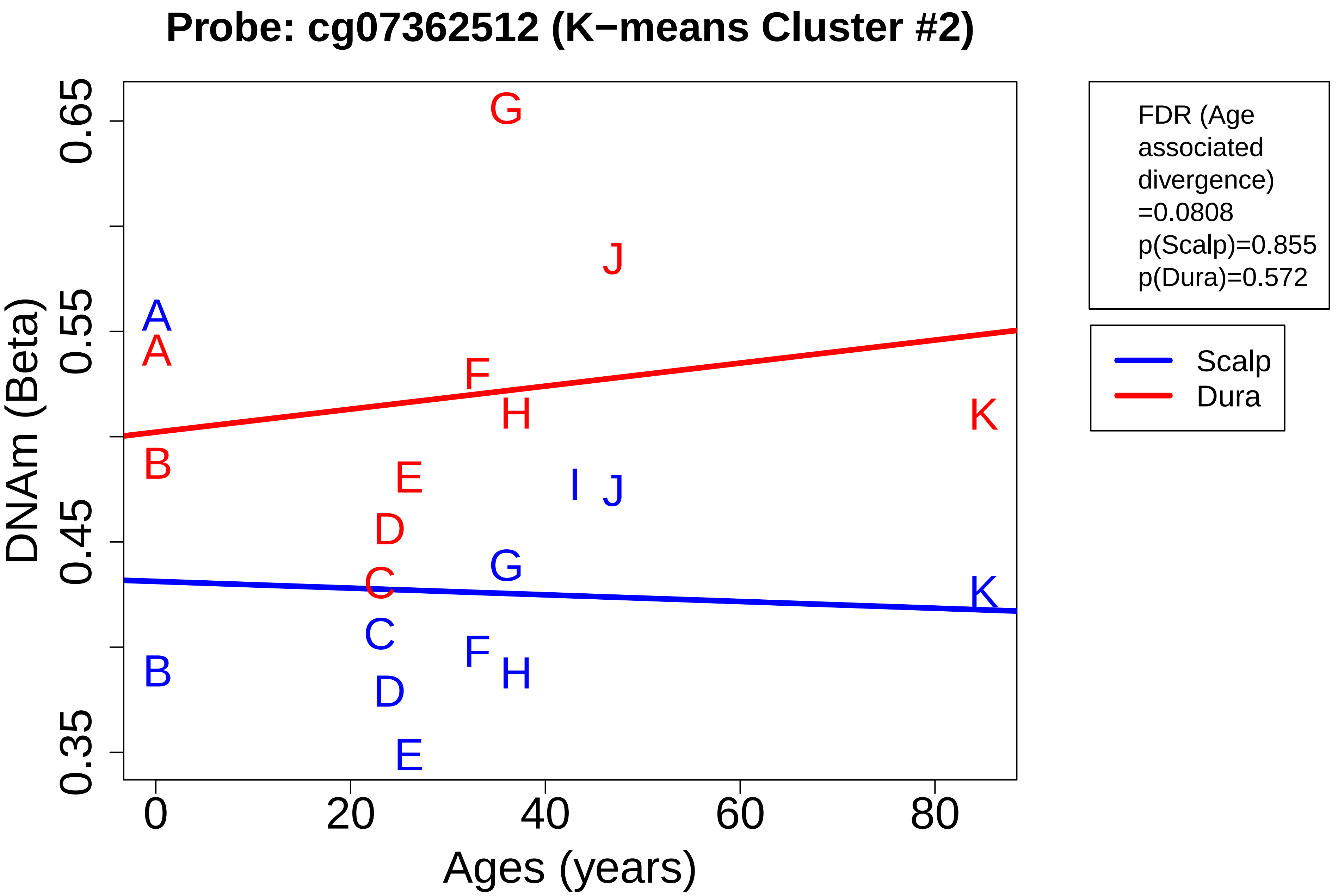

Probe: cg07362512 (K−means Cluster #2)
DNAm (Beta)
0.35
0.45
0.55
0.65
Ages (years)
0
20
40
60
80
A
A
B
B
C
C
D
D
E
E
F
F
G
G
H
H
I
J
J
K
K
FDR (Age associated divergence) =0.0808
p(Scalp)=0.855
p(Dura)=0.572
Scalp
Dura

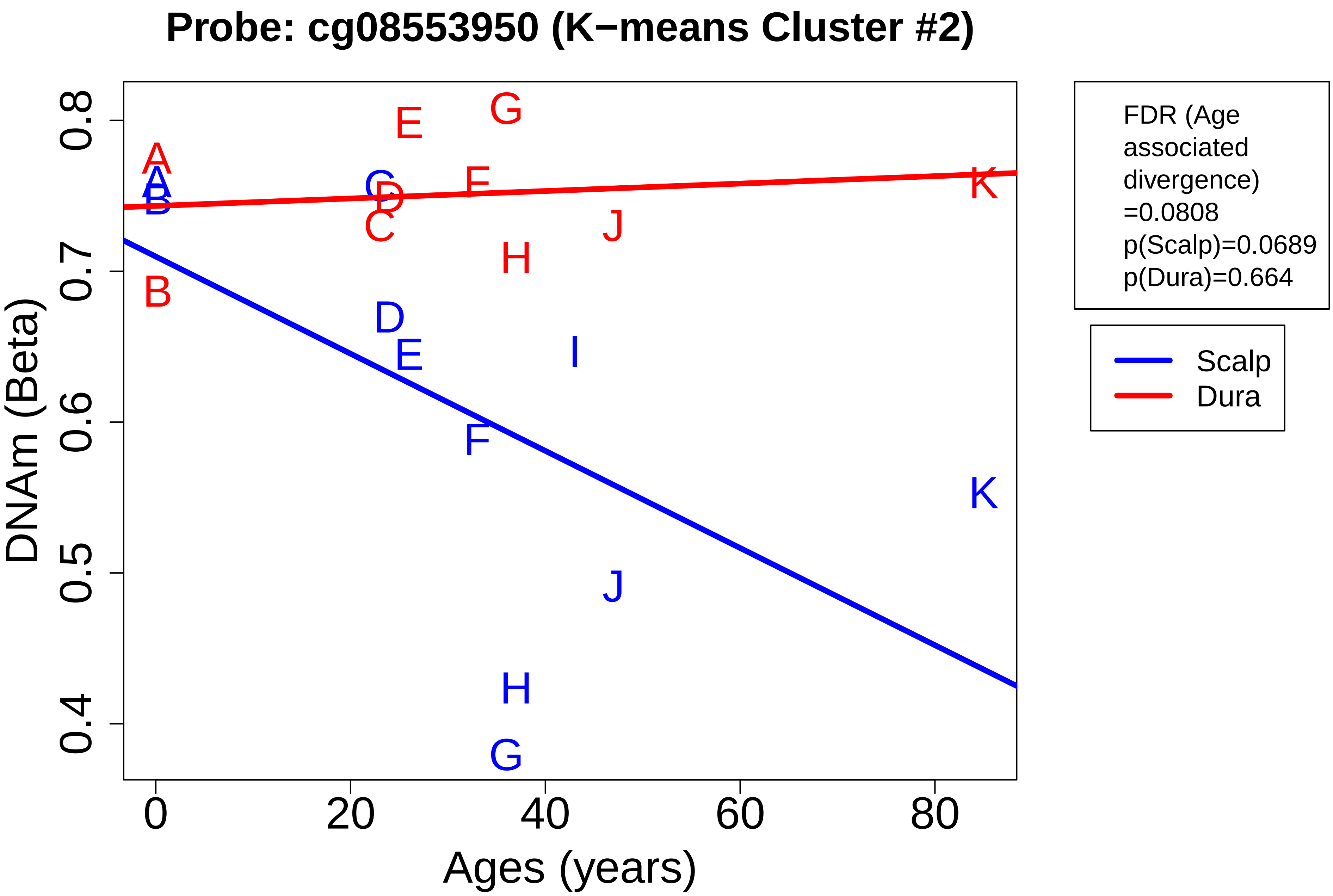

Probe: cg08553950 (K−means Cluster #2)
DNAm (Beta)
0.4
0.5
0.6
0.7
0.8
0
20
40
60
80
Ages (years)
A
B
C
D
E
F
G
H
I
J
K
FDR (Age associated divergence) =0.0808
p(Scalp)=0.0689
p(Dura)=0.664
Scalp
Dura

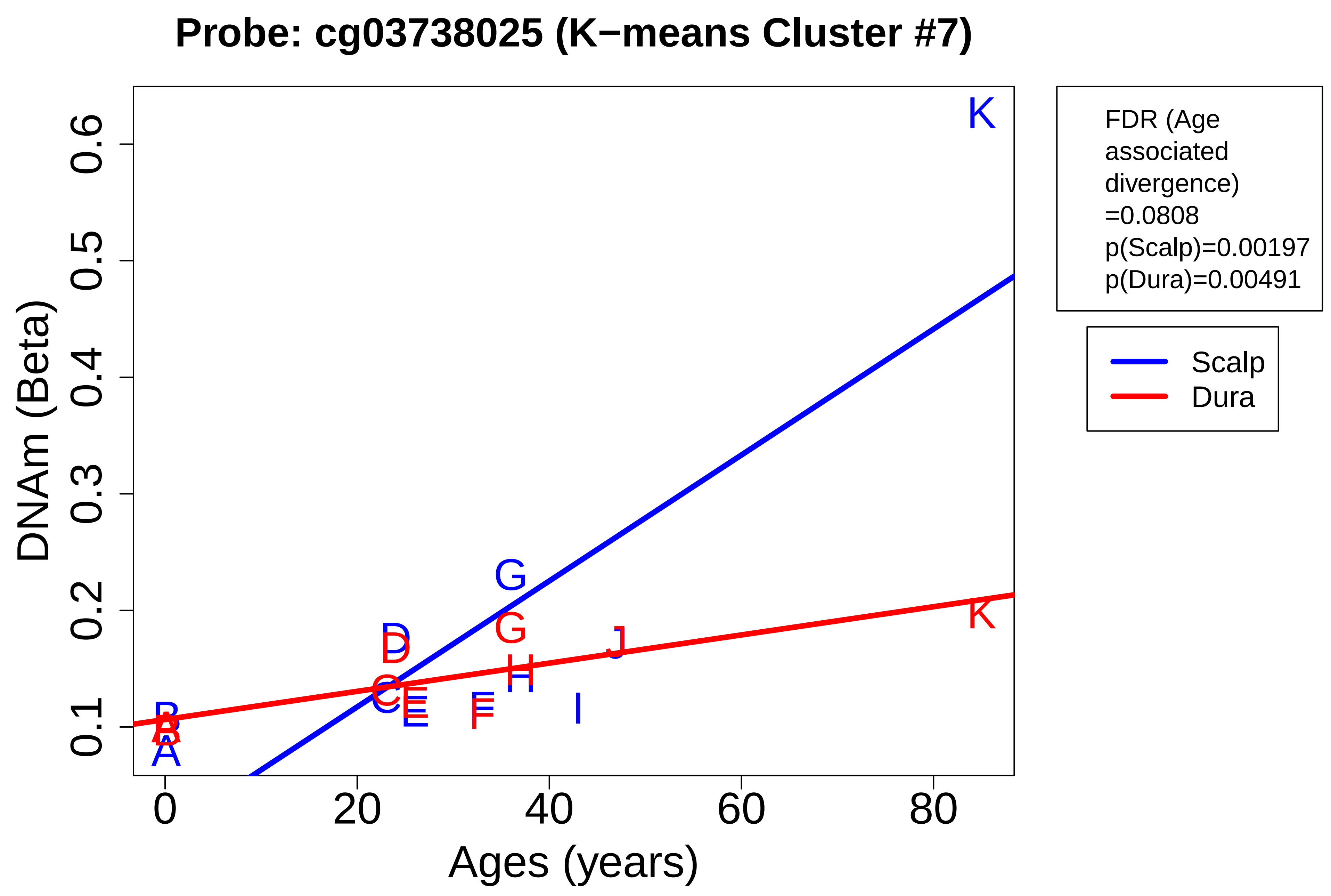

Probe: cg03738025 (K−means Cluster #7)
DNAm (Beta)
0.1
0.2
0.3
0.4
0.5
0.6
Ages (years)
0
20
40
60
80
A
B
C
D
E
F
G
H
I
J
K
FDR (Age associated divergence) =0.0808
p(Scalp)=0.00197
p(Dura)=0.00491
Scalp
Dura

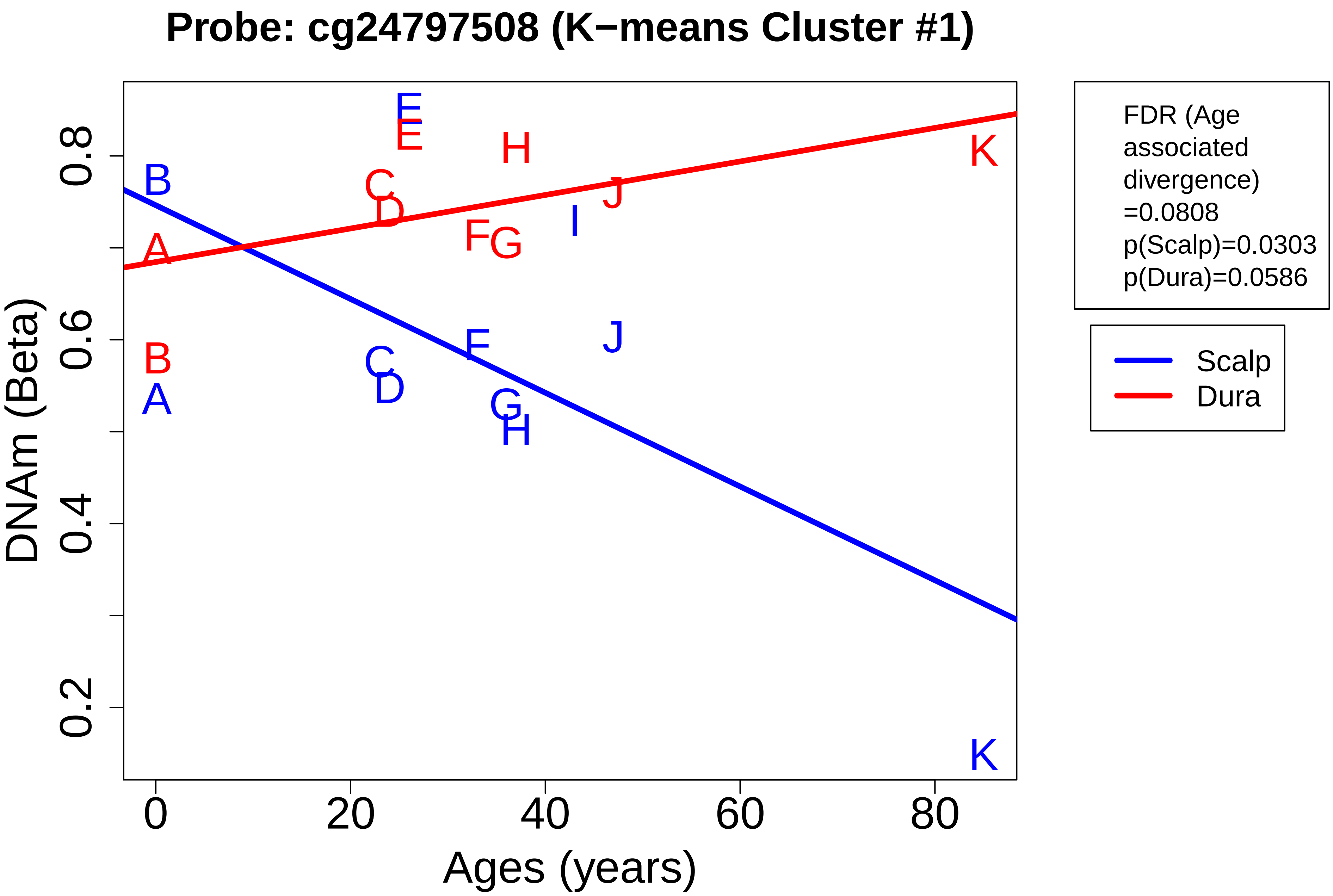

Probe: cg24797508 (K−means Cluster #1)
DNAm (Beta)
0.2
0.4
0.6
0.8
0
20
40
60
80
Ages (years)
A
B
C
D
E
F
G
H
I
J
K
FDR (Age associated divergence) =0.0808
p(Scalp)=0.0303
p(Dura)=0.0586
Scalp
Dura

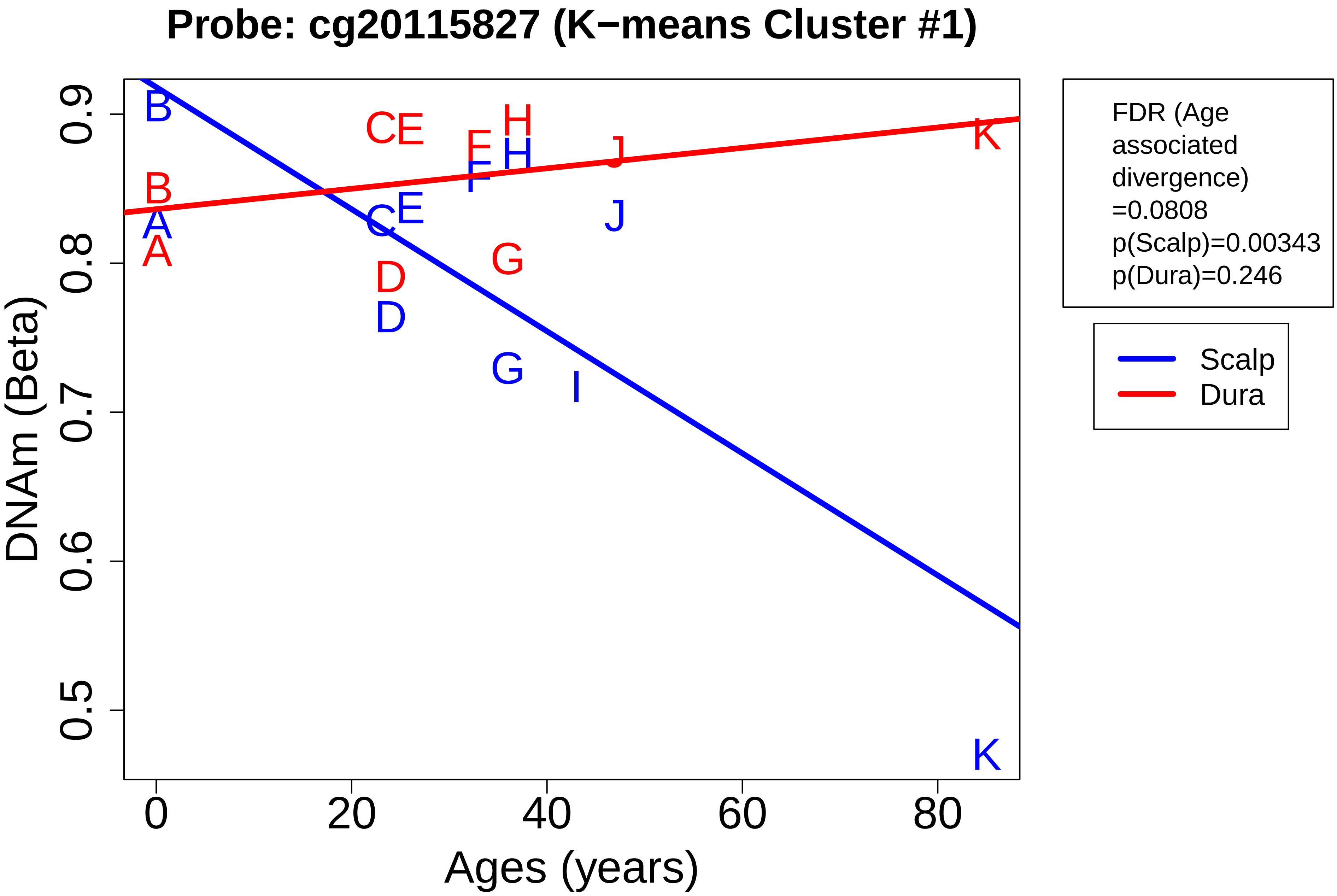

Probe: cg20115827 (K−means Cluster #1)
DNAm (Beta)
Ages (years)
0.5
0.6
0.7
0.8
0.9
0
20
40
60
80
FDR (Age associated divergence) =0.0808
p(Scalp)=0.00343
p(Dura)=0.246
Scalp
Dura

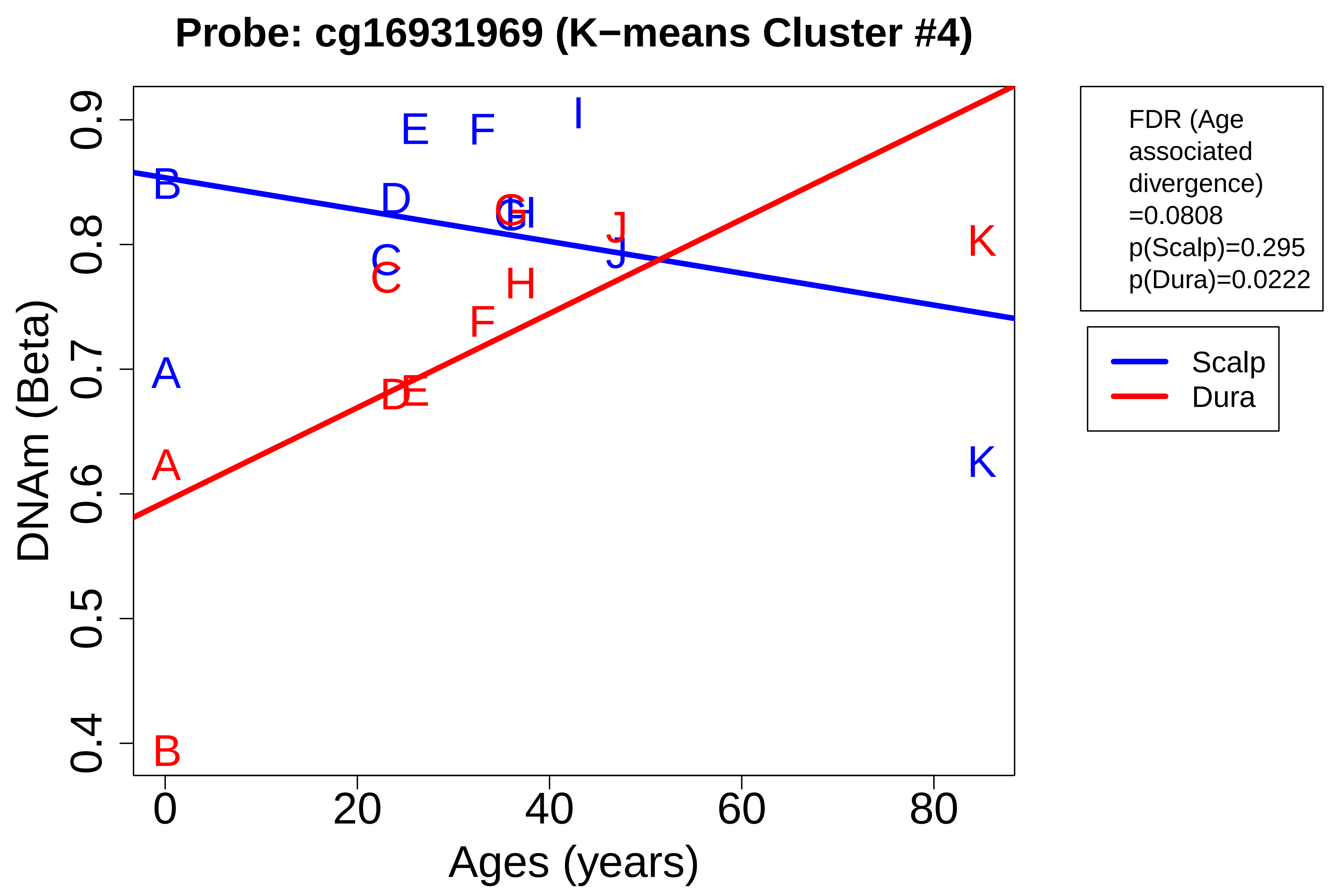
Probe: cg16931969 (K−means Cluster #4)
DNAm (Beta)
Ages (years)
0.4
0.5
0.6
0.7
0.8
0.9
0
20
40
60
80
FDR (Age associated divergence) =0.0808
p(Scalp)=0.295
p(Dura)=0.0222
Scalp
Dura

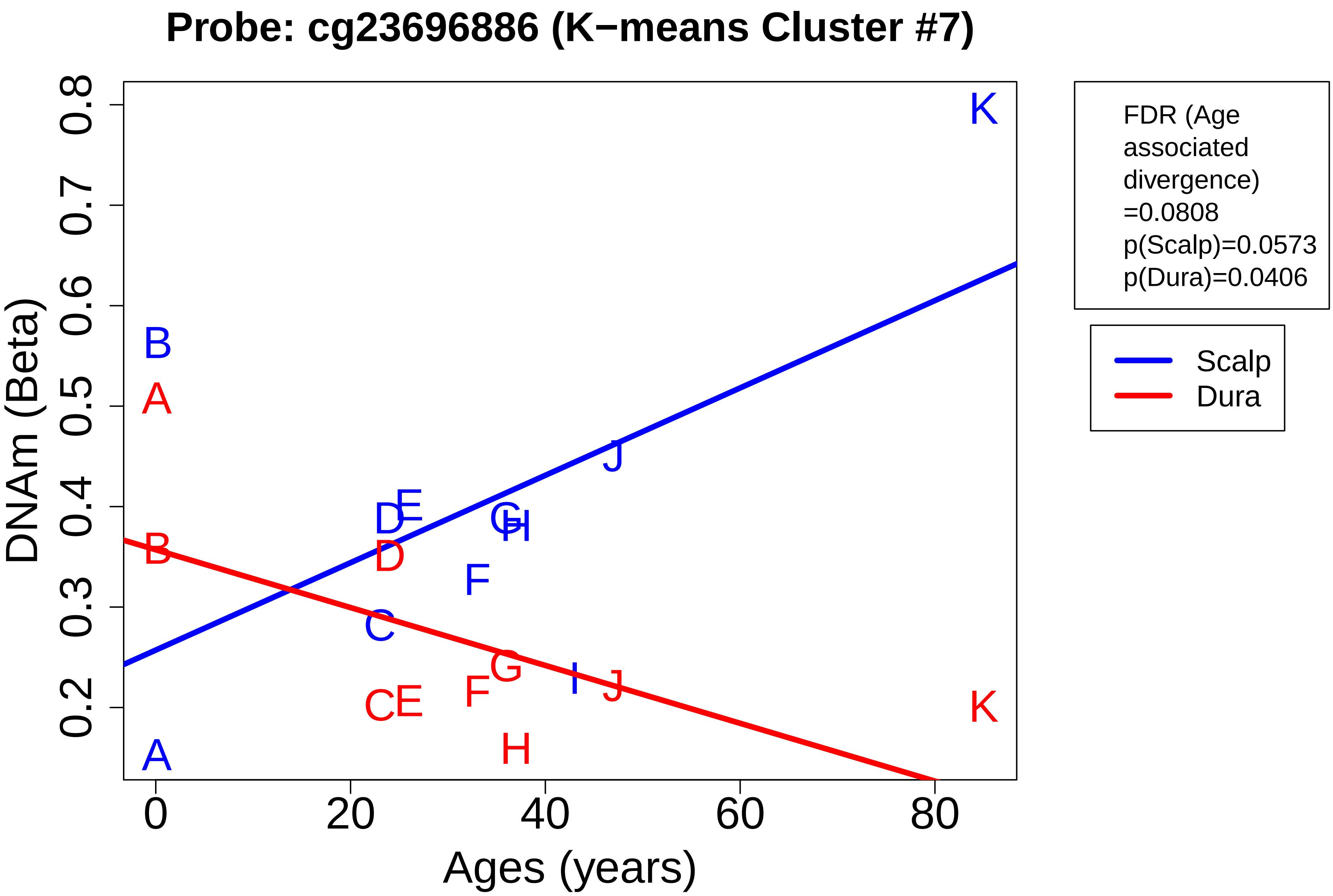

Probe: cg23696886 (K−means Cluster #7)
DNAm (Beta)
0.2
0.3
0.4
0.5
0.6
0.7
0.8
Ages (years)
0
20
40
60
80
FDR (Age associated divergence) =0.0808
p(Scalp)=0.0573
p(Dura)=0.0406
Scalp
Dura

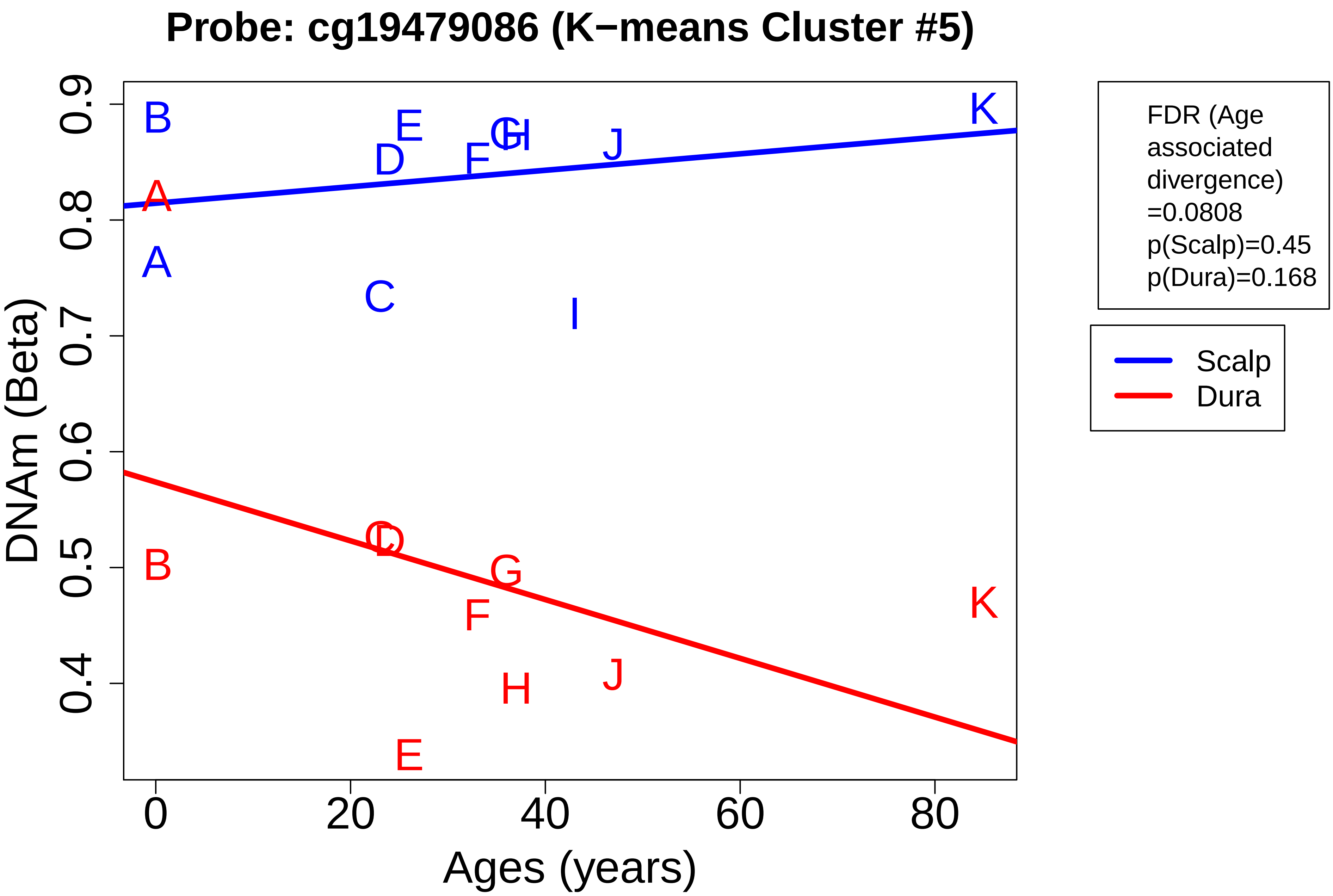

Probe: cg19479086 (K−means Cluster #5)
DNAm (Beta)
0.4
0.5
0.6
0.7
0.8
0.9
0
20
40
60
80
Ages (years)
FDR (Age associated divergence) =0.0808
p(Scalp)=0.45
p(Dura)=0.168
Scalp
Dura

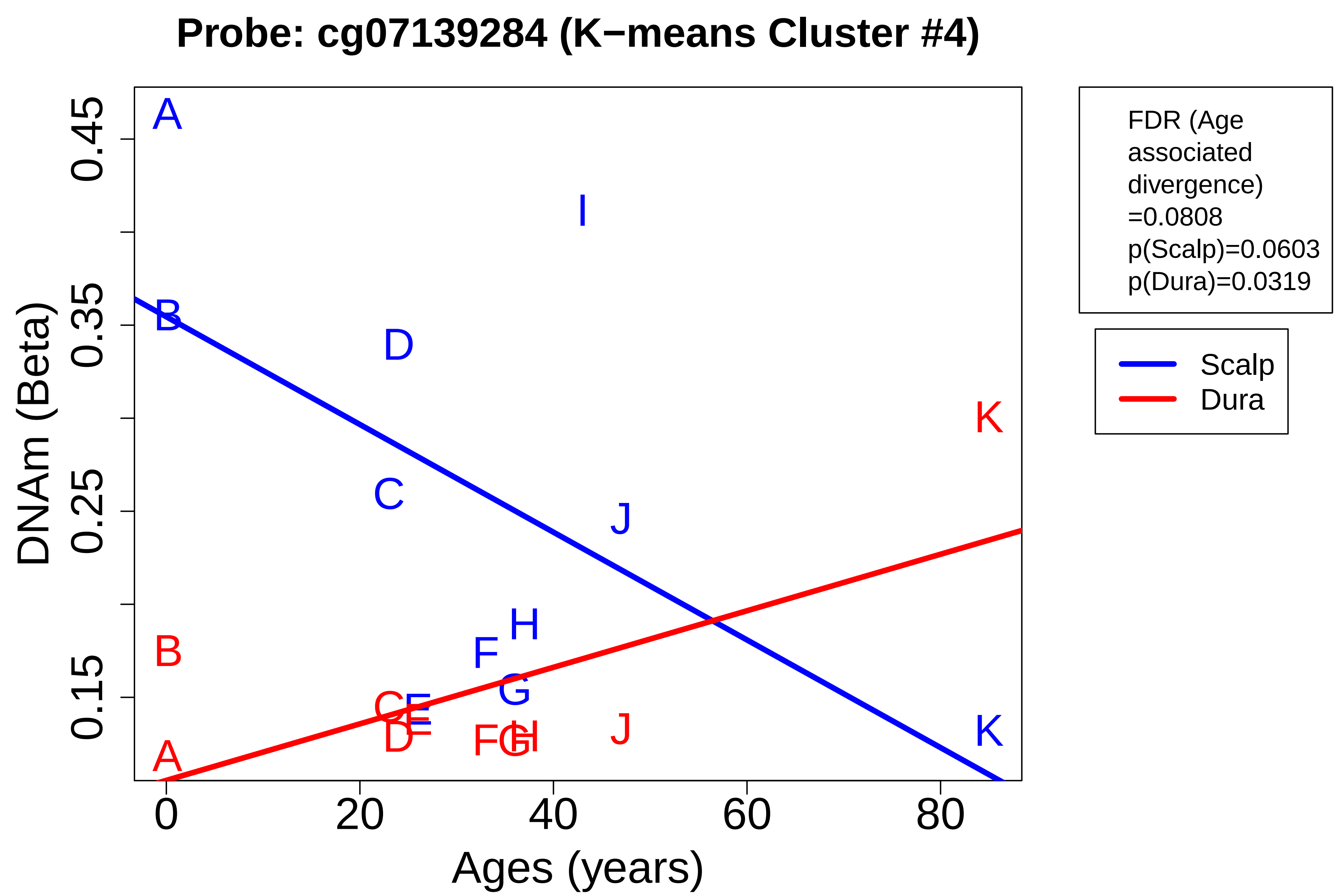

Probe: cg07139284 (K−means Cluster #4)
DNAm (Beta)
0.15
0.25
0.35
0.45
0
20
40
60
80
Ages (years)
A
B
C
D
E
F
G
H
I
J
K
FDR (Age associated divergence) =0.0808
p(Scalp)=0.0603
p(Dura)=0.0319
Scalp
Dura

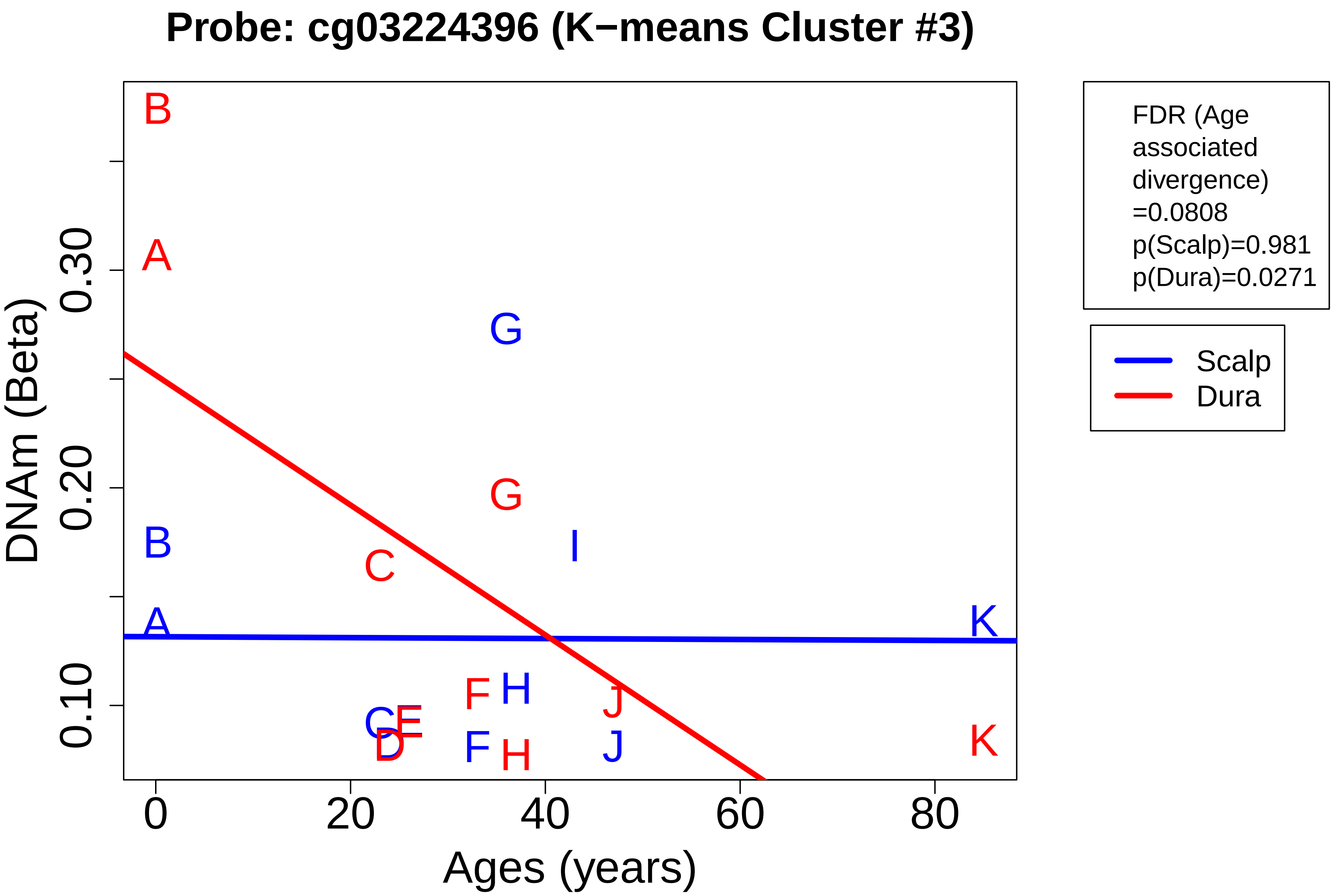

Probe: cg03224396 (K−means Cluster #3)
FDR (Age associated divergence) =0.0808
p(Scalp)=0.981
p(Dura)=0.0271
Scalp
Dura
DNAm (Beta)
0.10
0.20
0.30
0
20
40
60
80
Ages (years)

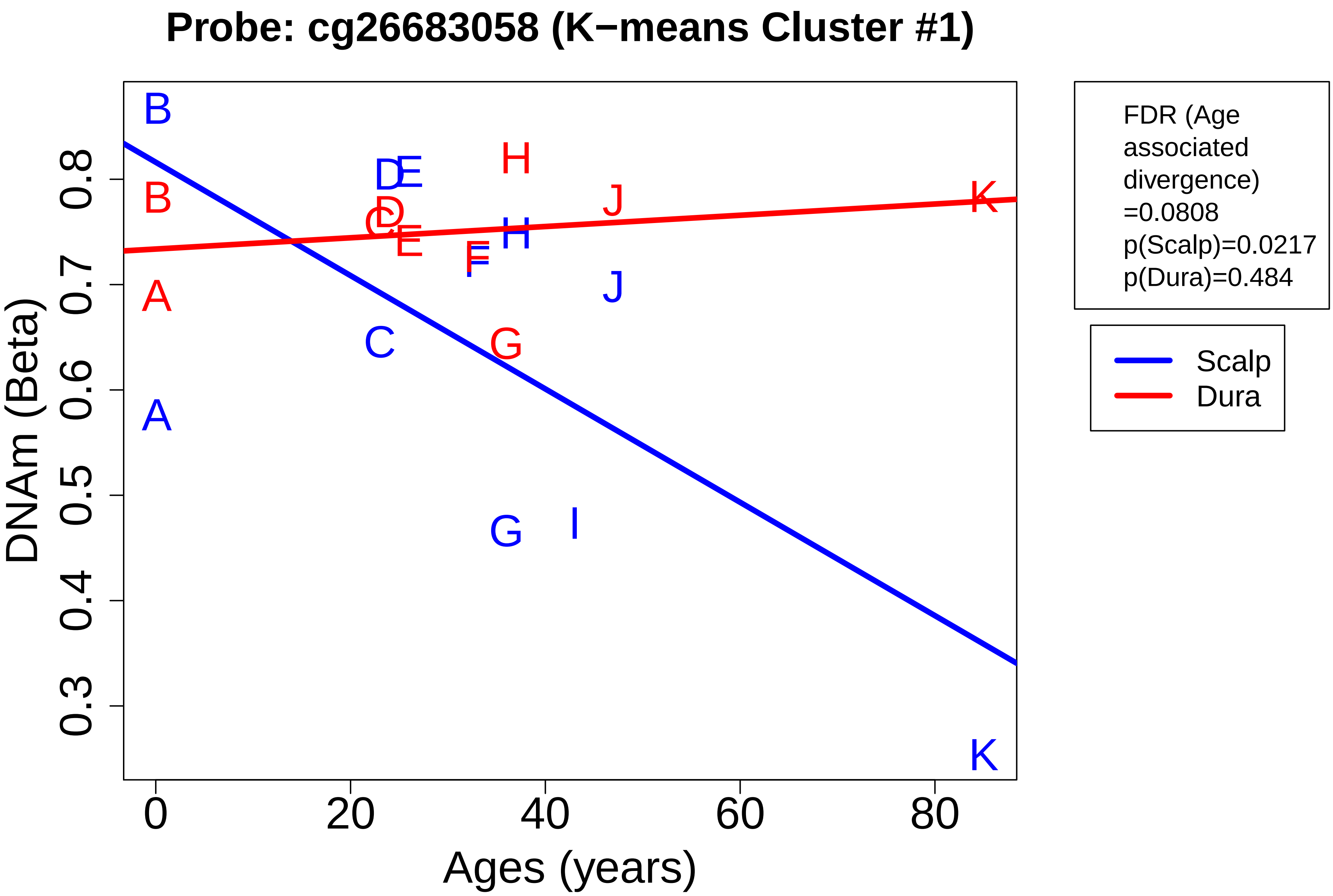

Probe: cg26683058 (K−means Cluster #1)
DNAm (Beta)
0.3
0.4
0.5
0.6
0.7
0.8
Ages (years)
0
20
40
60
80
A
B
C
D
E
F
G
H
I
J
K
FDR (Age associated divergence) =0.0808
p(Scalp)=0.0217
p(Dura)=0.484
Scalp
Dura

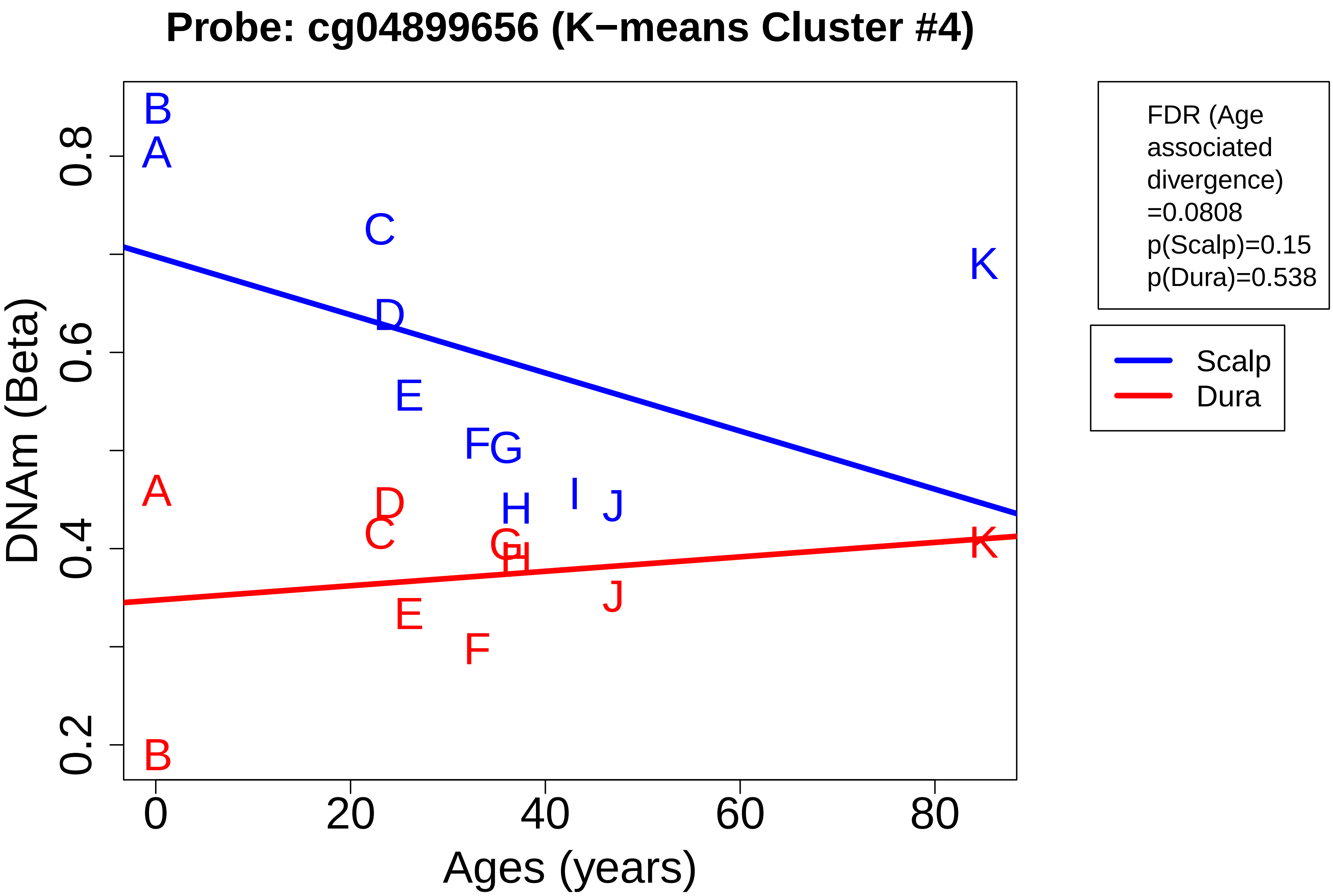

Probe: cg04899656 (K−means Cluster #4)
DNAm (Beta)
0.2
0.4
0.6
0.8
Ages (years)
0
20
40
60
80
A
B
C
D
E
F
G
H
I
J
K
FDR (Age associated divergence) =0.0808
p(Scalp)=0.15
p(Dura)=0.538
Scalp
Dura

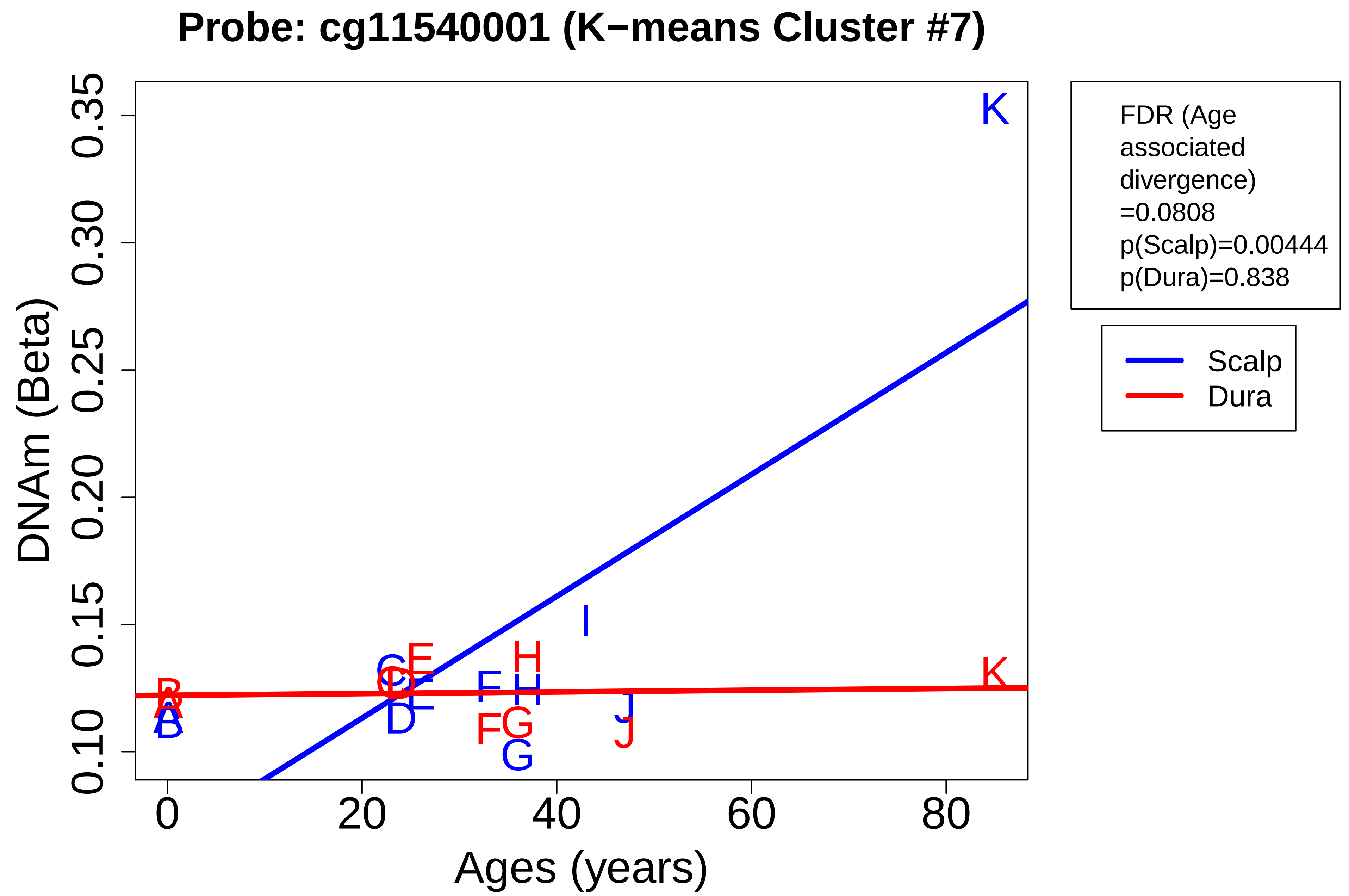
Probe: cg11540001 (K−means Cluster #7)
DNAm (Beta)
0.10
0.15
0.20
0.25
0.30
0.35
0
20
40
60
80
Ages (years)
FDR (Age associated divergence) =0.0808
p(Scalp)=0.00444
p(Dura)=0.838
Scalp
Dura
A
B
C
D
E
F
G
H
I
J
K

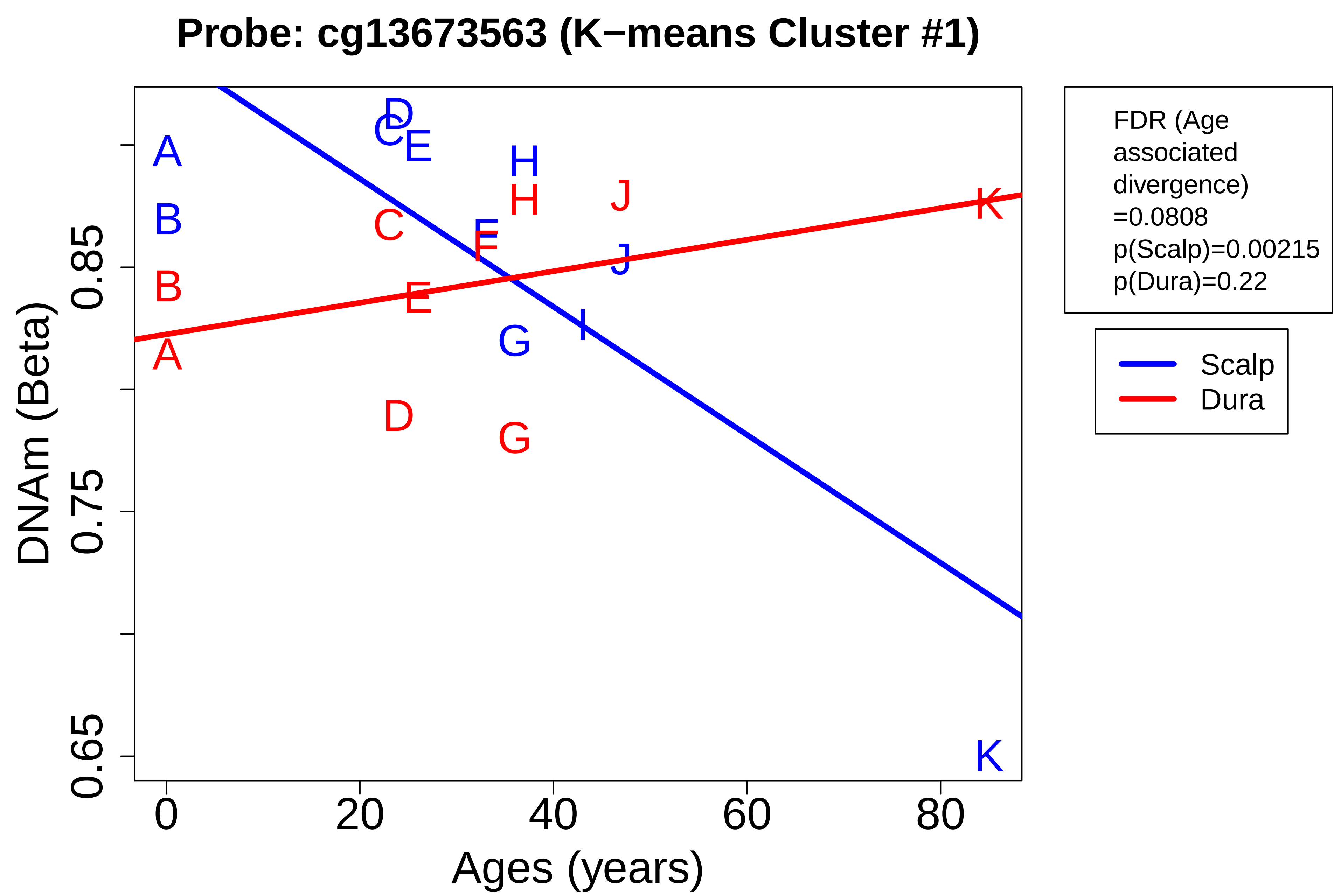
Probe: cg13673563 (K−means Cluster #1)
DNAm (Beta)
0.65
0.75
0.85
Ages (years)
0
20
40
60
80
A
B
C
D
E
F
G
H
I
J
K
A
B
C
D
E
F
G
H
J
K
FDR (Age associated divergence) =0.0808
p(Scalp)=0.00215
p(Dura)=0.22
Scalp
Dura

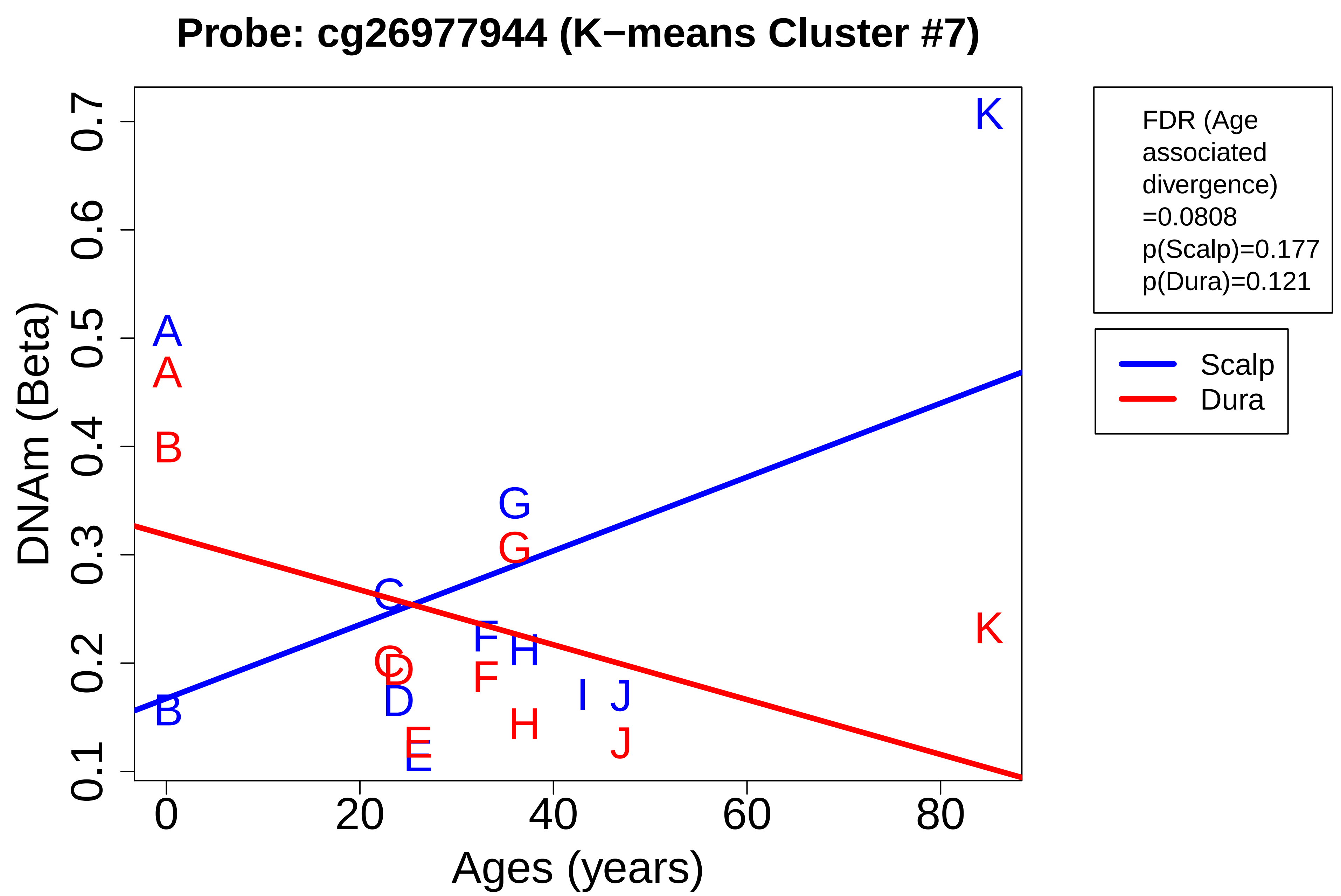

Probe: cg26977944 (K−means Cluster #7)
DNAm (Beta)
Ages (years)
0.1
0.2
0.3
0.4
0.5
0.6
0.7
0
20
40
60
80
FDR (Age associated divergence) =0.0808
p(Scalp)=0.177
p(Dura)=0.121
Scalp
Dura

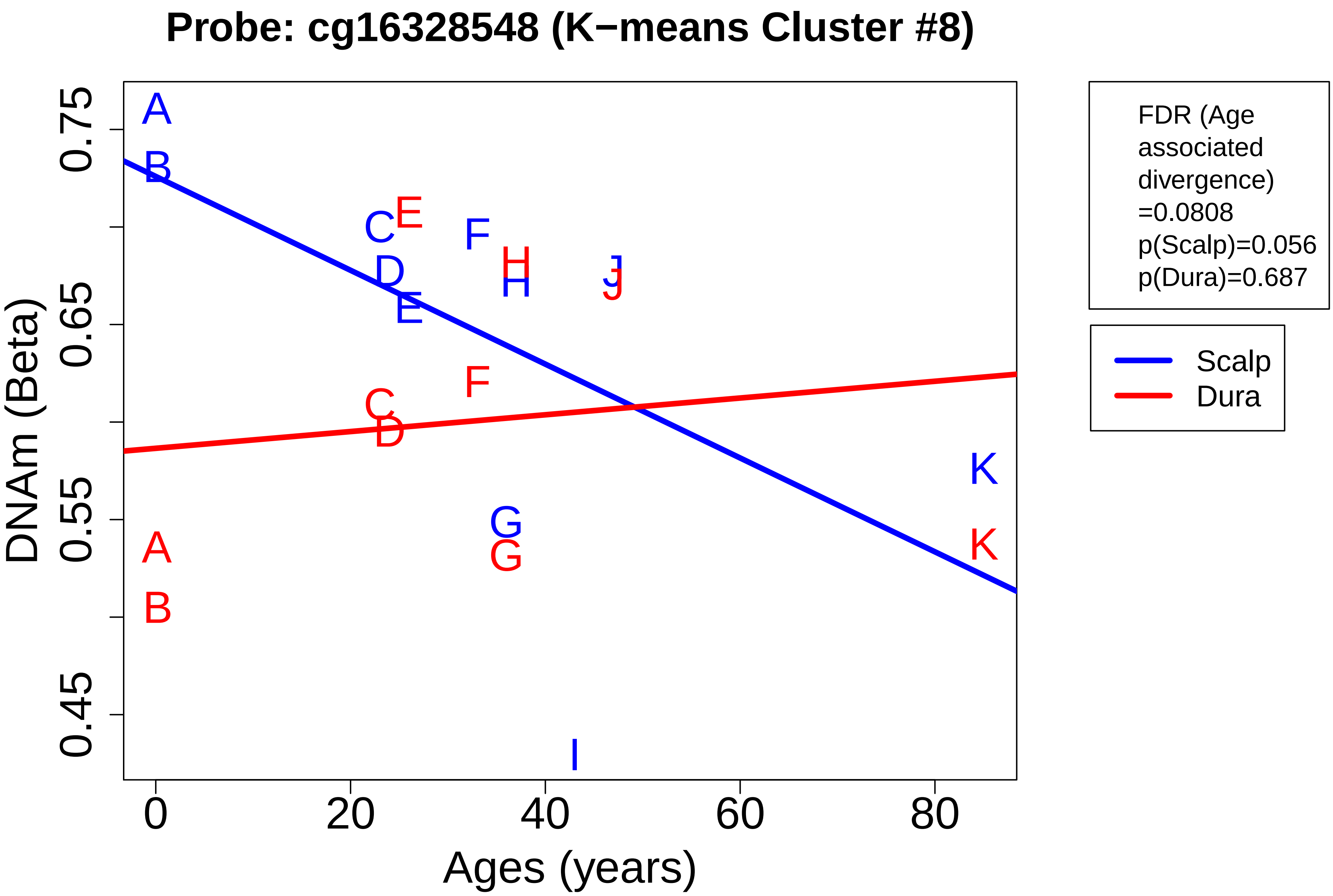
Probe: cg16328548 (K−means Cluster #8)
FDR (Age associated divergence) =0.0808
p(Scalp)=0.056
p(Dura)=0.687
Scalp
Dura
DNAm (Beta)
0.45
0.55
0.65
0.75
Ages (years)
0
20
40
60
80
A
B
C
D
E
F
G
H
I
J
K

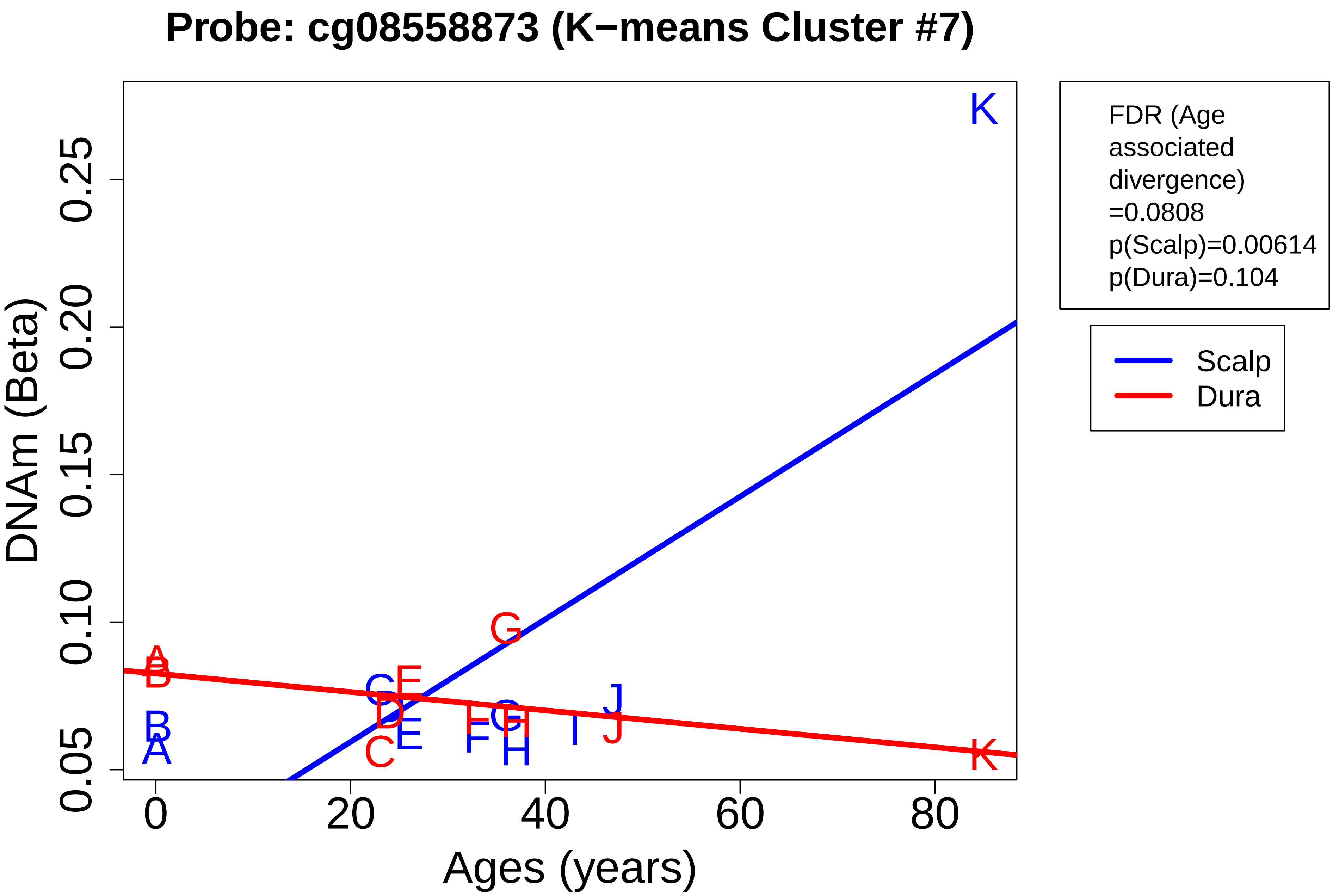

Probe: cg08558873 (K−means Cluster #7)
DNAm (Beta)
Ages (years)
0.05
0.10
0.15
0.20
0.25
0
20
40
60
80
FDR (Age associated divergence) =0.0808
p(Scalp)=0.00614
p(Dura)=0.104
Scalp
Dura

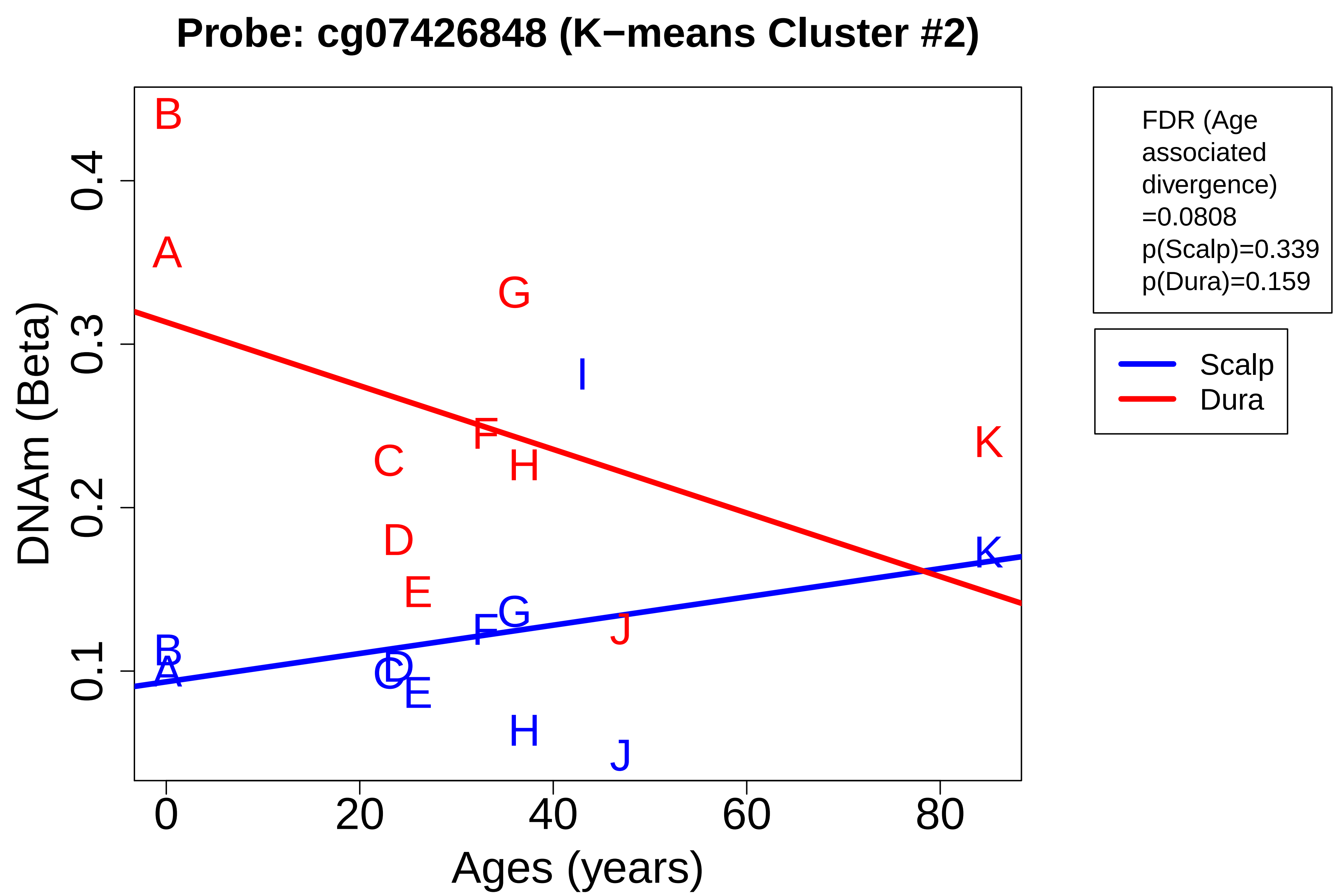

Probe: cg07426848 (K−means Cluster #2)
DNAm (Beta)
0.1
0.2
0.3
0.4
0
20
40
60
80
Ages (years)
FDR (Age associated divergence) =0.0808
p(Scalp)=0.339
p(Dura)=0.159
Scalp
Dura

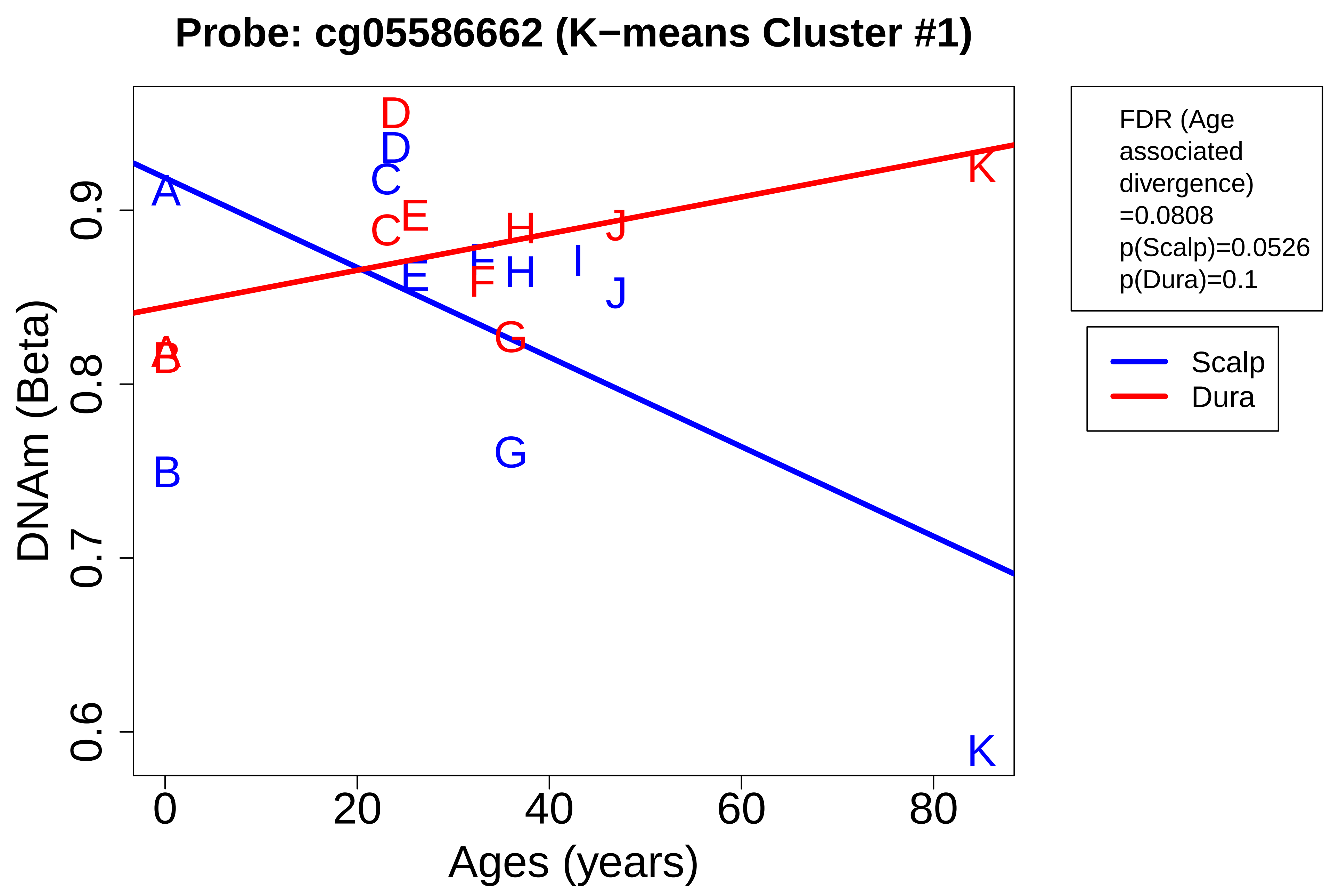

Probe: cg05586662 (K−means Cluster #1)
DNAm (Beta)
Ages (years)
0.6
0.7
0.8
0.9
0
20
40
60
80
FDR (Age associated divergence) =0.0808
p(Scalp)=0.0526
p(Dura)=0.1
Scalp
Dura

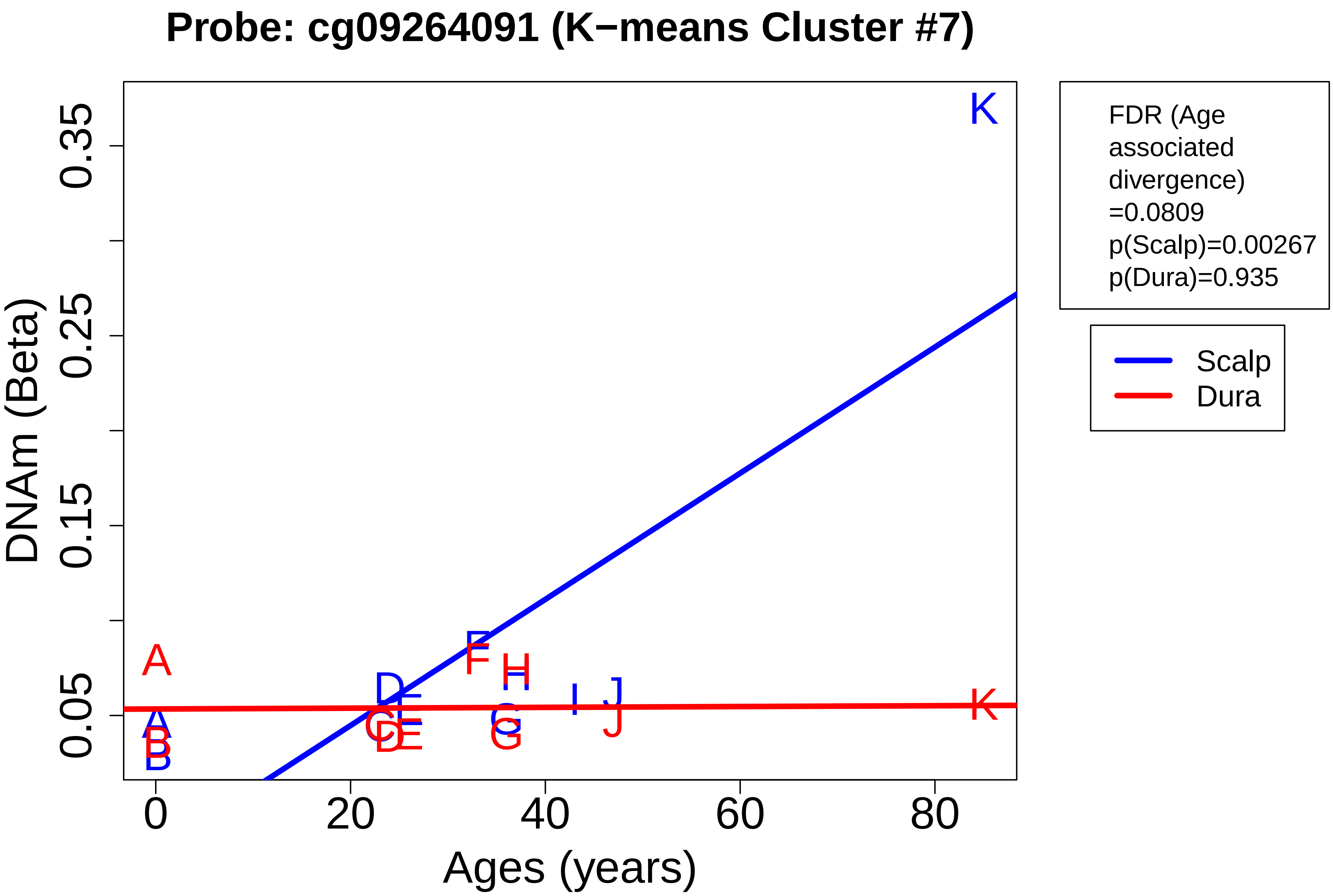

Probe: cg09264091 (K−means Cluster #7)
FDR (Age associated divergence) =0.0809
p(Scalp)=0.00267
p(Dura)=0.935
Scalp
Dura
DNAm (Beta)
0.05
0.15
0.25
0.35
Ages (years)
0
20
40
60
80
A
B
C
D
E
F
G
H
I
J
K

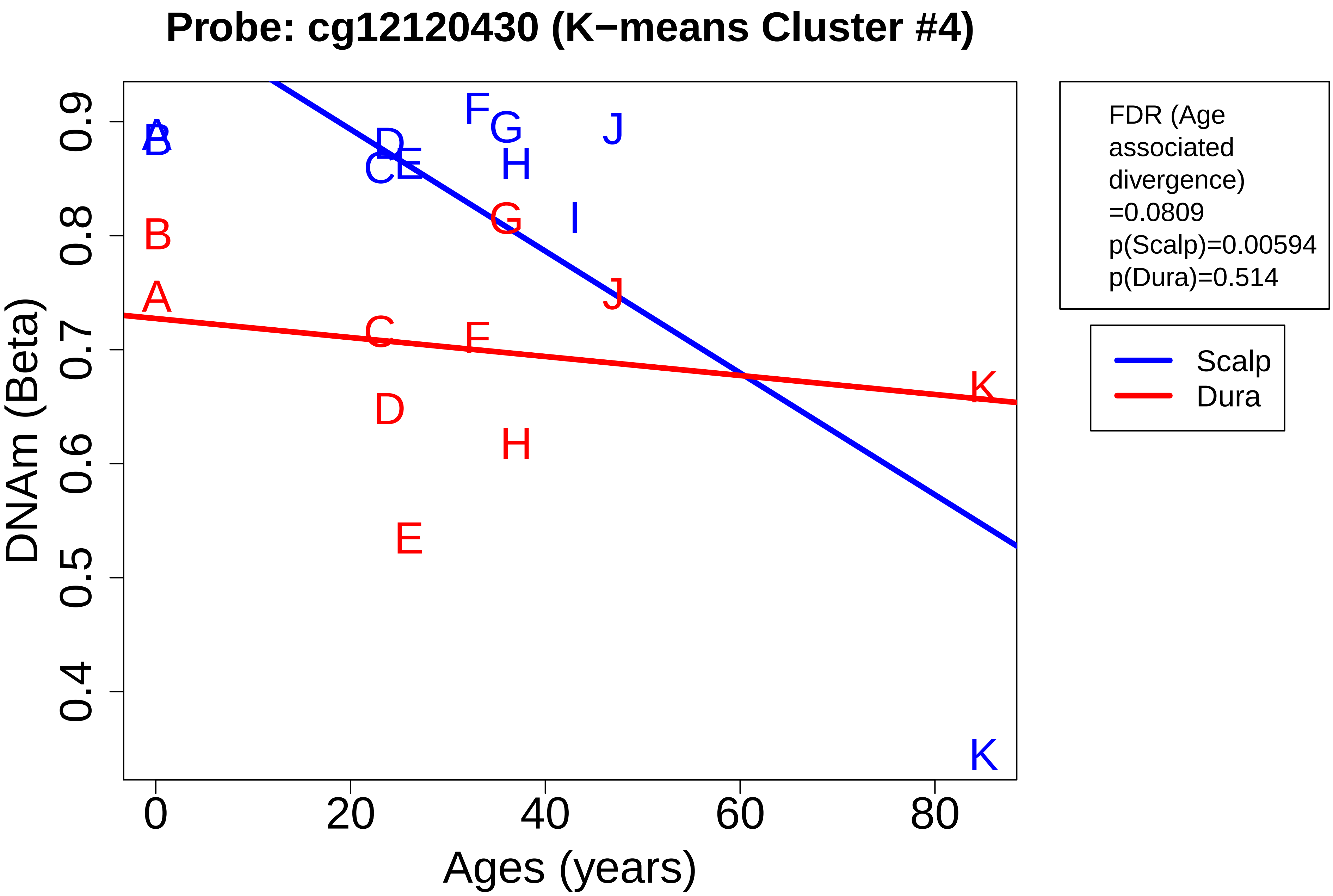

Probe: cg12120430 (K−means Cluster #4)
DNAm (Beta)
0.4
0.5
0.6
0.7
0.8
0.9
0
20
40
60
80
Ages (years)
FDR (Age associated divergence) =0.0809
p(Scalp)=0.00594
p(Dura)=0.514
Scalp
Dura

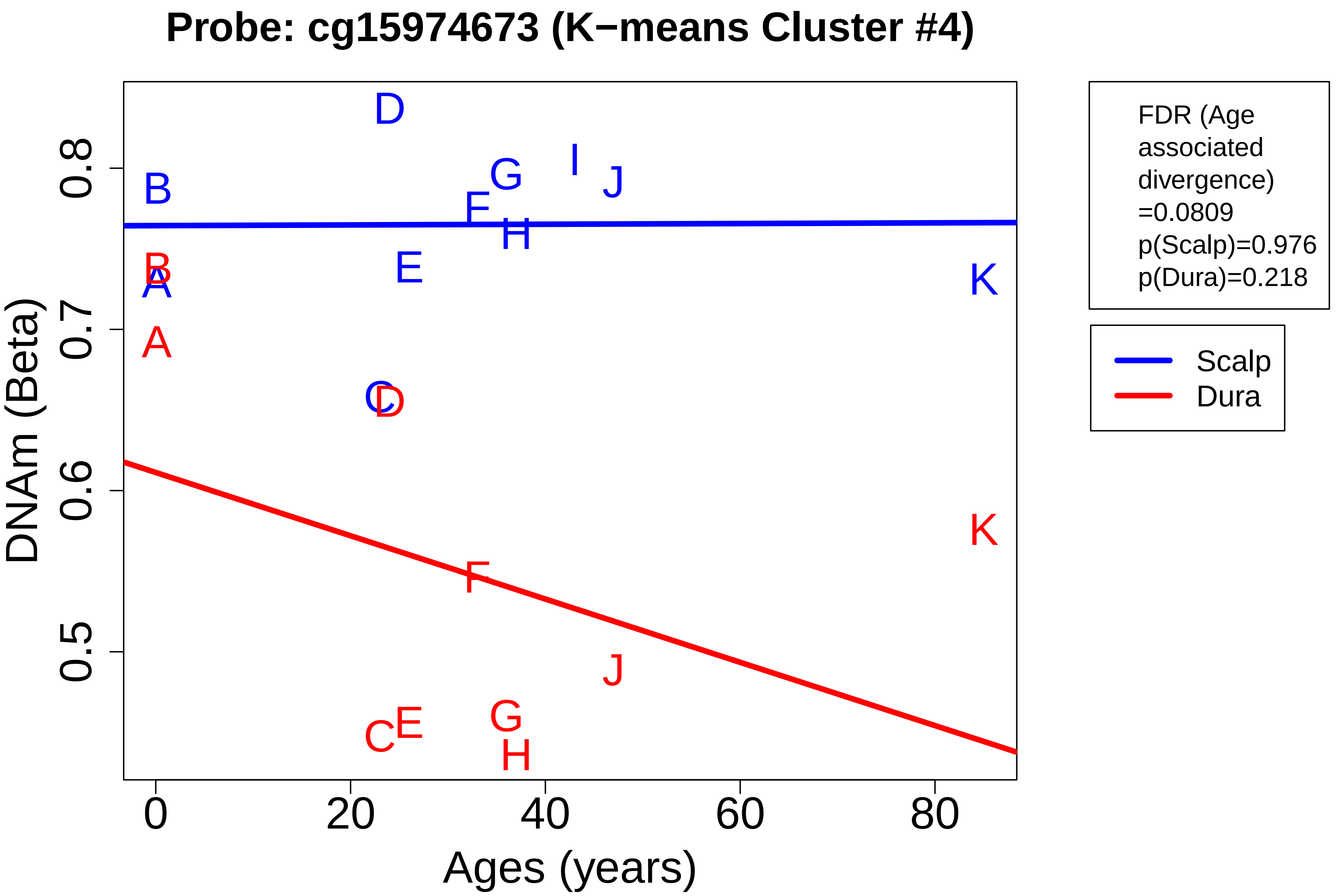

Probe: cg15974673 (K−means Cluster #4)
DNAm (Beta)
0.5
0.6
0.7
0.8
0
20
40
60
80
Ages (years)
FDR (Age associated divergence) =0.0809
p(Scalp)=0.976
p(Dura)=0.218
Scalp
Dura
A
B
C
D
E
F
G
H
I
J
K

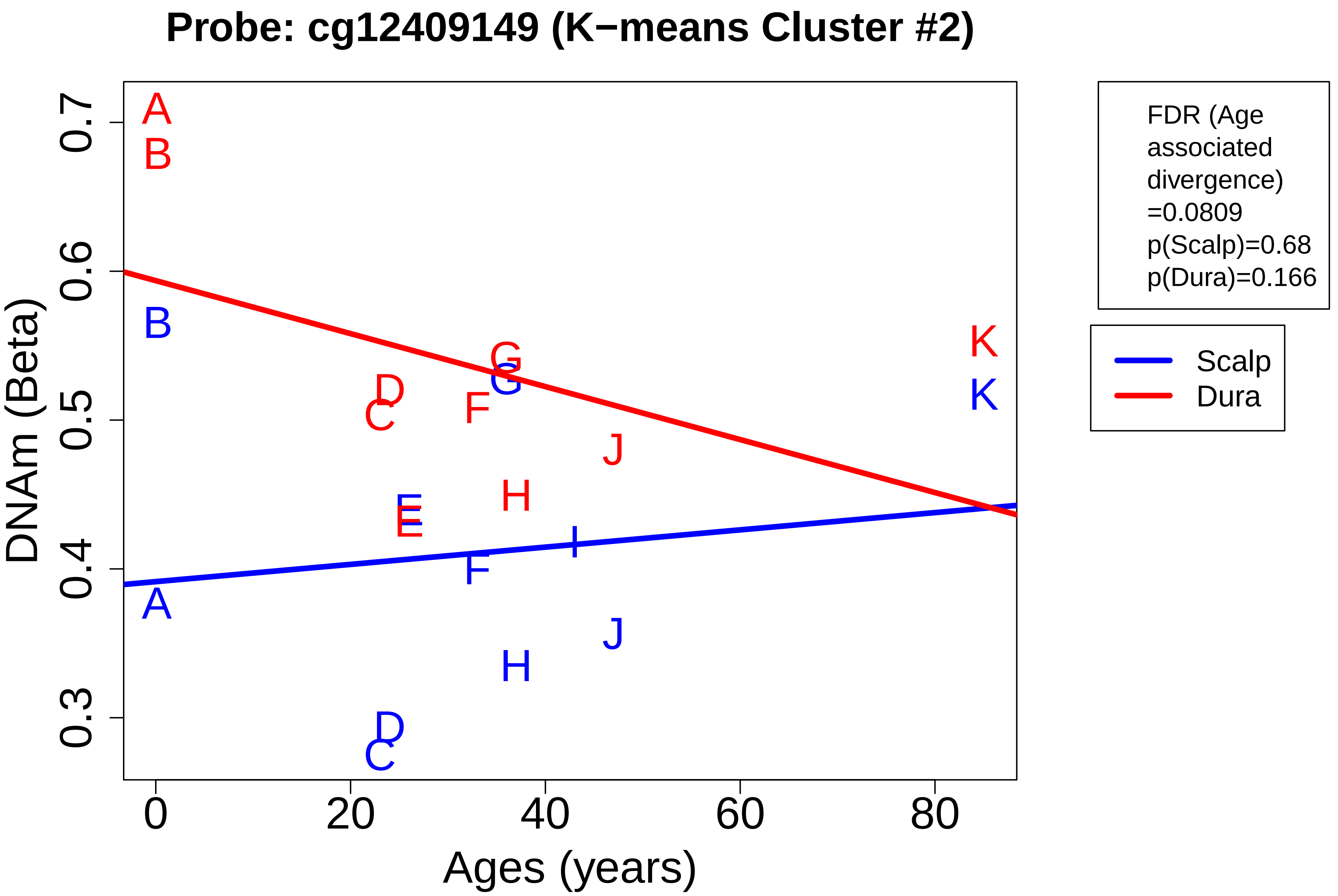

Probe: cg12409149 (K−means Cluster #2)
DNAm (Beta)
0.3
0.4
0.5
0.6
0.7
0
20
40
60
80
Ages (years)
FDR (Age associated divergence) =0.0809
p(Scalp)=0.68
p(Dura)=0.166
Scalp
Dura

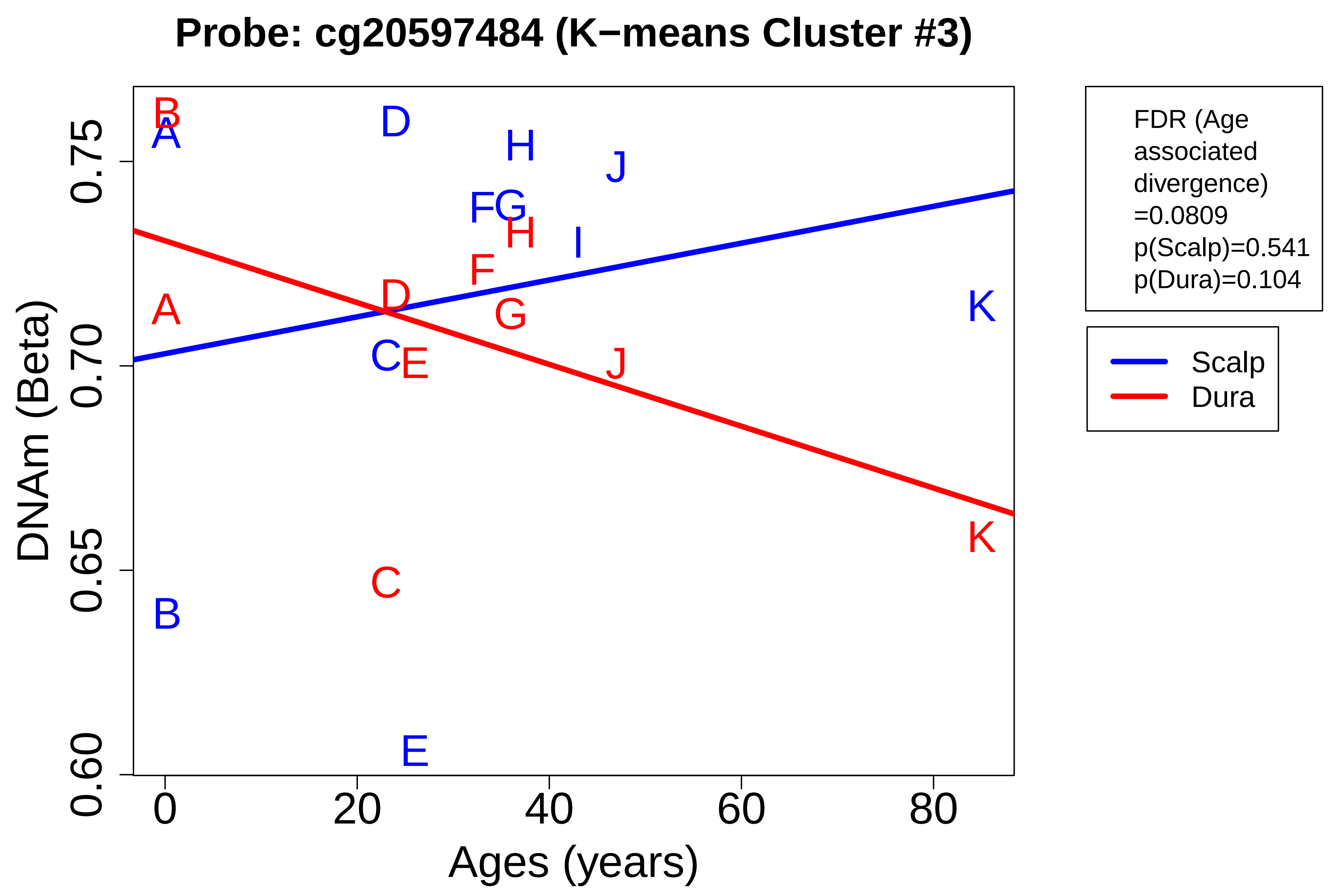

Probe: cg20597484 (K−means Cluster #3)
DNAm (Beta)
0.60
0.65
0.70
0.75
0
20
40
60
80
Ages (years)
FDR (Age associated divergence) =0.0809
p(Scalp)=0.541
p(Dura)=0.104
Scalp
Dura

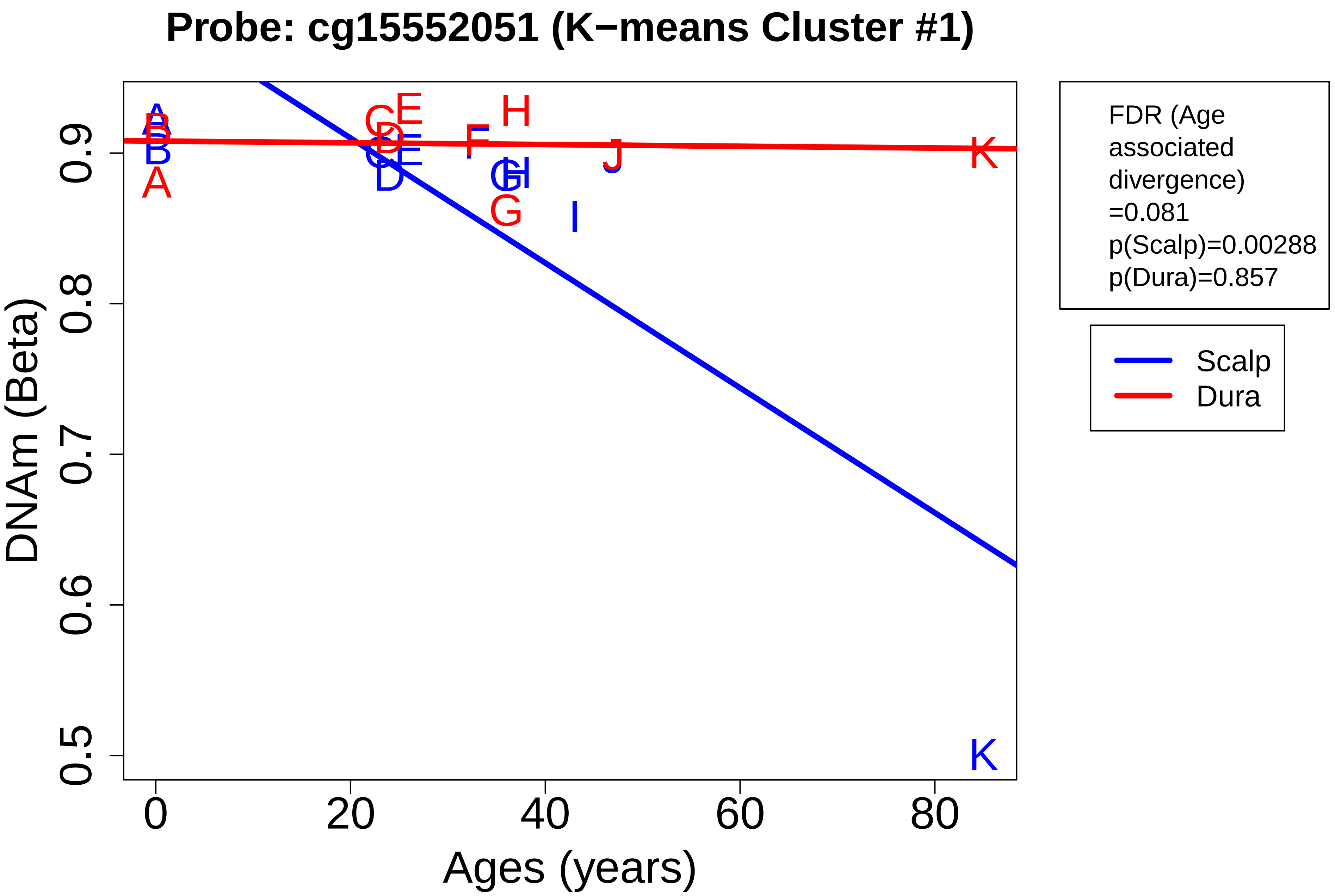

Probe: cg15552051 (K−means Cluster #1)
DNAm (Beta)
0.5
0.6
0.7
0.8
0.9
0
20
40
60
80
Ages (years)
A
B
C
D
E
F
G
H
I
J
K
FDR (Age associated divergence) =0.081
p(Scalp)=0.00288
p(Dura)=0.857
Scalp
Dura

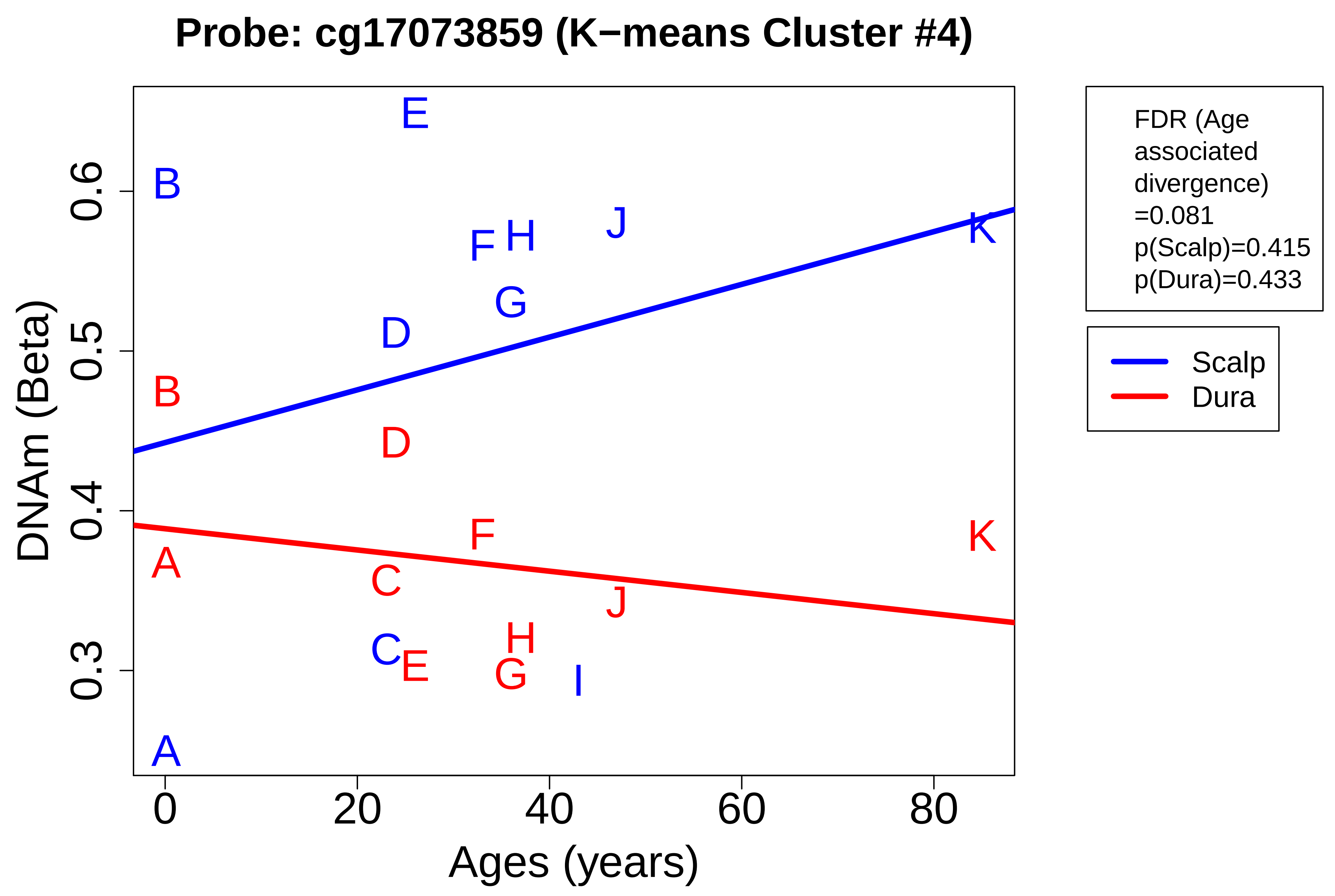

Probe: cg17073859 (K−means Cluster #4)
DNAm (Beta)
0.3
0.4
0.5
0.6
0
20
40
60
80
Ages (years)
A
B
C
D
E
F
G
H
I
J
K
FDR (Age associated divergence) =0.081
p(Scalp)=0.415
p(Dura)=0.433
Scalp
Dura

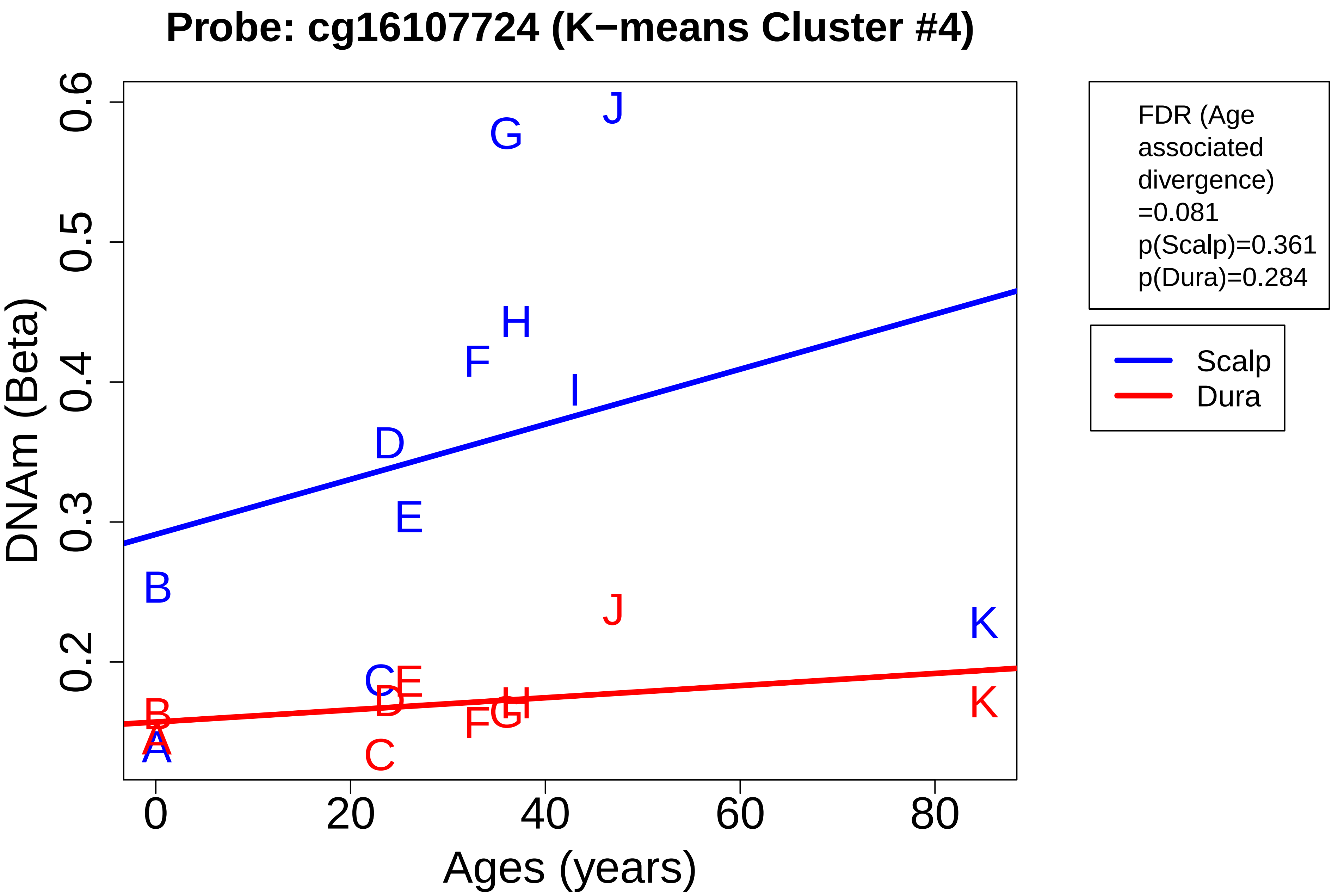
Probe: cg16107724 (K−means Cluster #4)
DNAm (Beta)
0.2
0.3
0.4
0.5
0.6
0
20
40
60
80
Ages (years)
FDR (Age associated divergence) =0.081
p(Scalp)=0.361
p(Dura)=0.284
Scalp
Dura

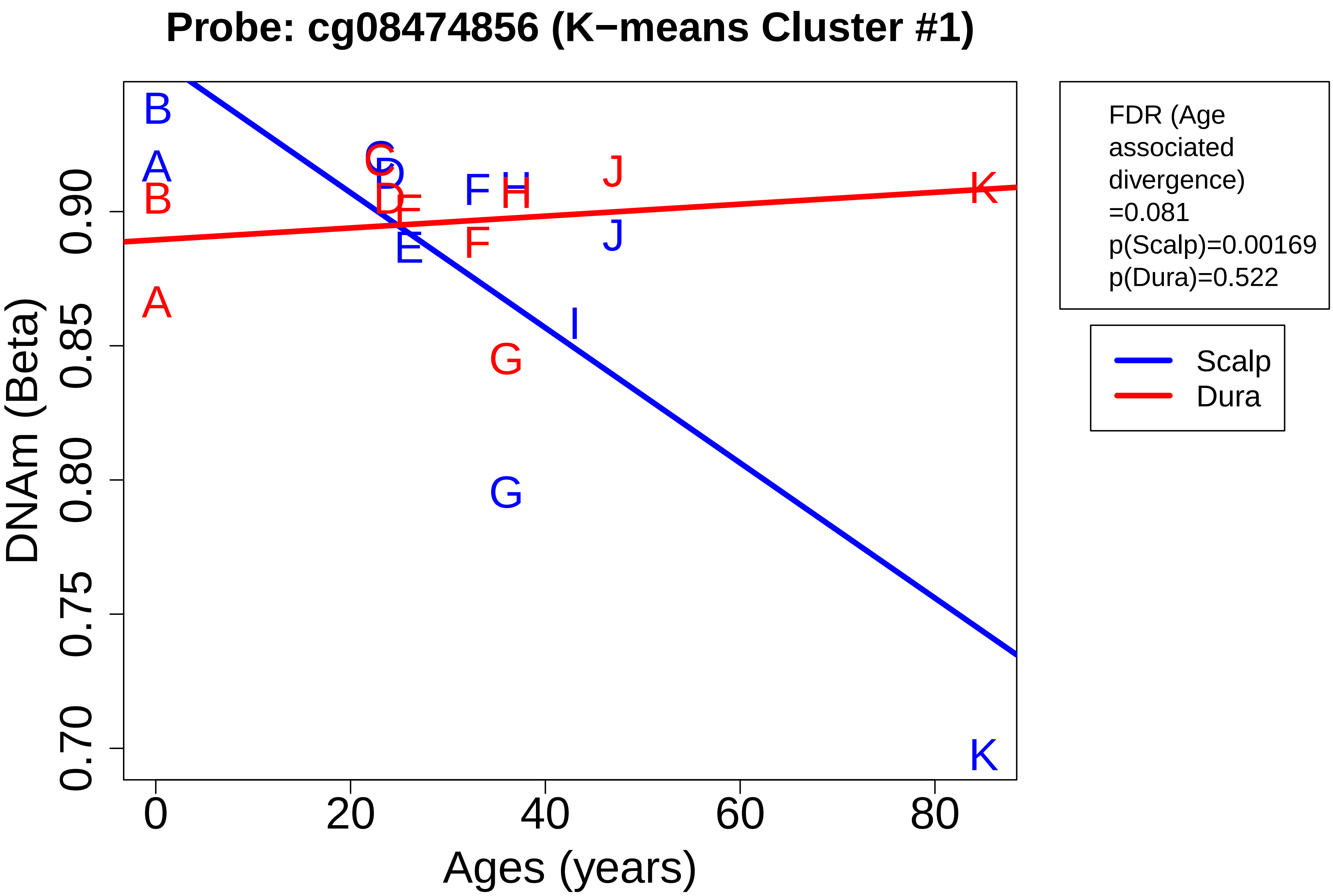

Probe: cg08474856 (K−means Cluster #1)
DNAm (Beta)
0.70
0.75
0.80
0.85
0.90
0
20
40
60
80
Ages (years)
FDR (Age associated divergence) =0.081
p(Scalp)=0.00169
p(Dura)=0.522
Scalp
Dura

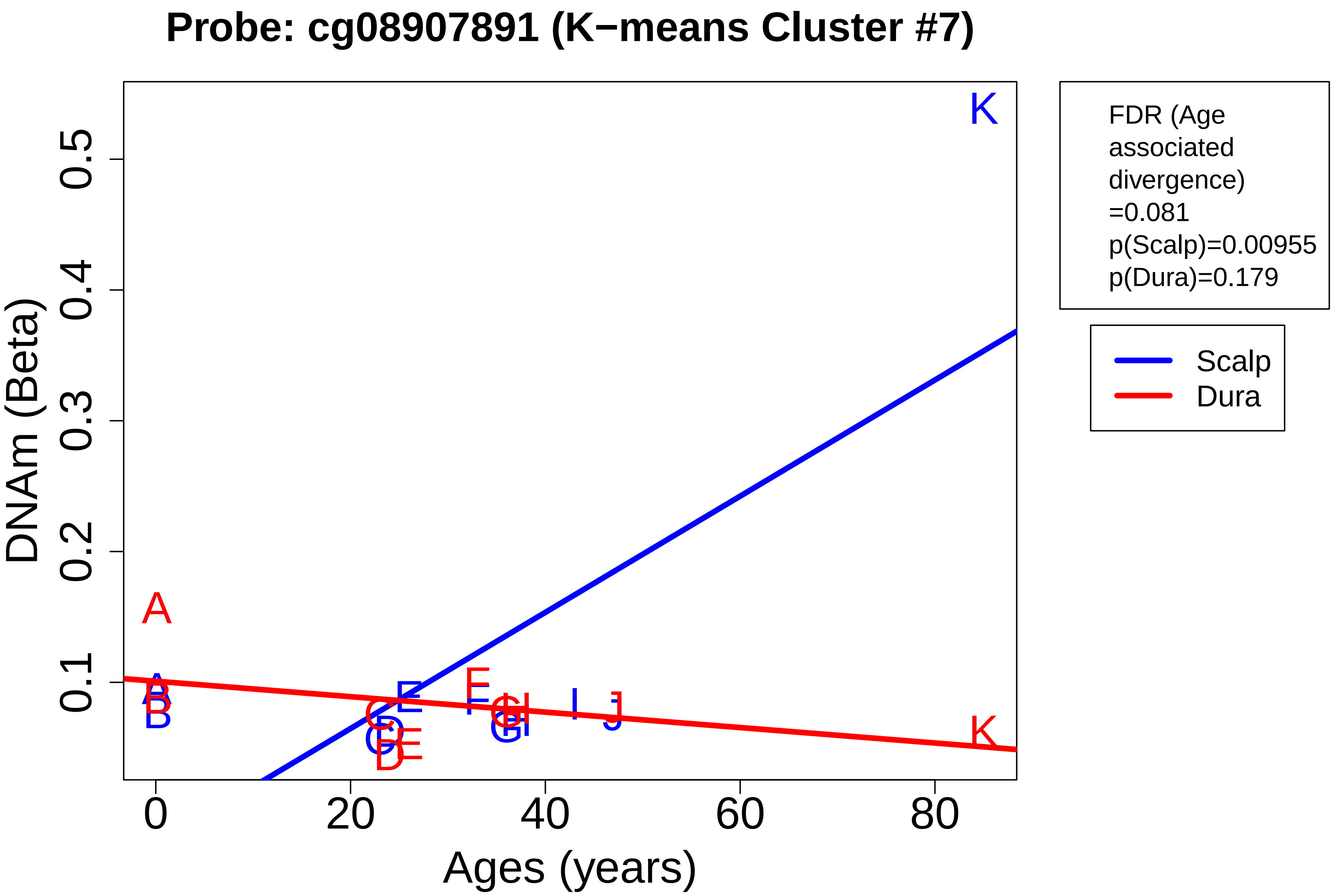

Probe: cg08907891 (K−means Cluster #7)
DNAm (Beta)
0.1
0.2
0.3
0.4
0.5
0
20
40
60
80
Ages (years)
FDR (Age associated divergence) =0.081
p(Scalp)=0.00955
p(Dura)=0.179
Scalp
Dura

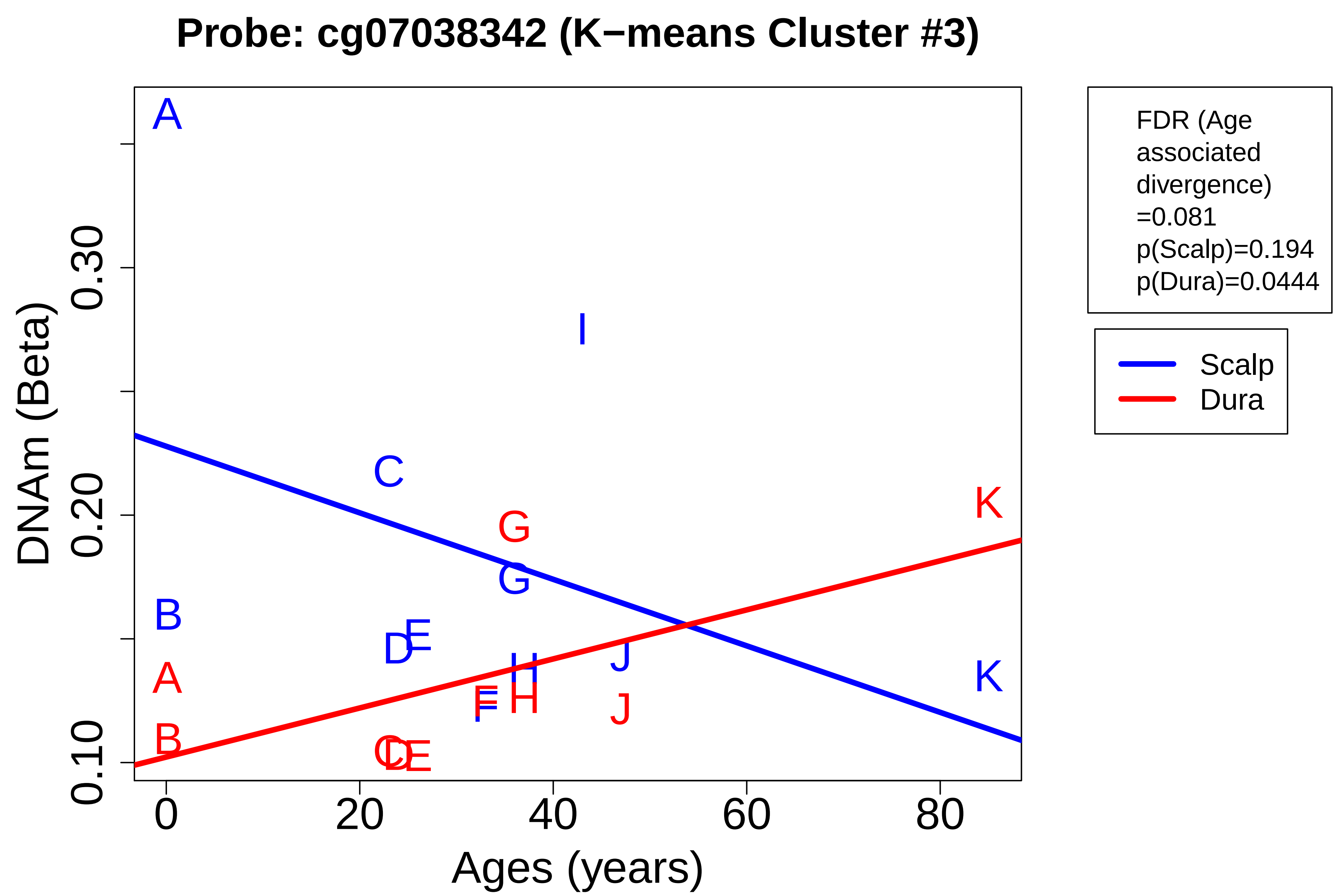

Probe: cg07038342 (K−means Cluster #3)
FDR (Age associated divergence) =0.081
p(Scalp)=0.194
p(Dura)=0.0444
Scalp
Dura
DNAm (Beta)
0.10
0.20
0.30
0
20
40
60
80
Ages (years)
A
B
C
D
E
F
G
H
I
J
K

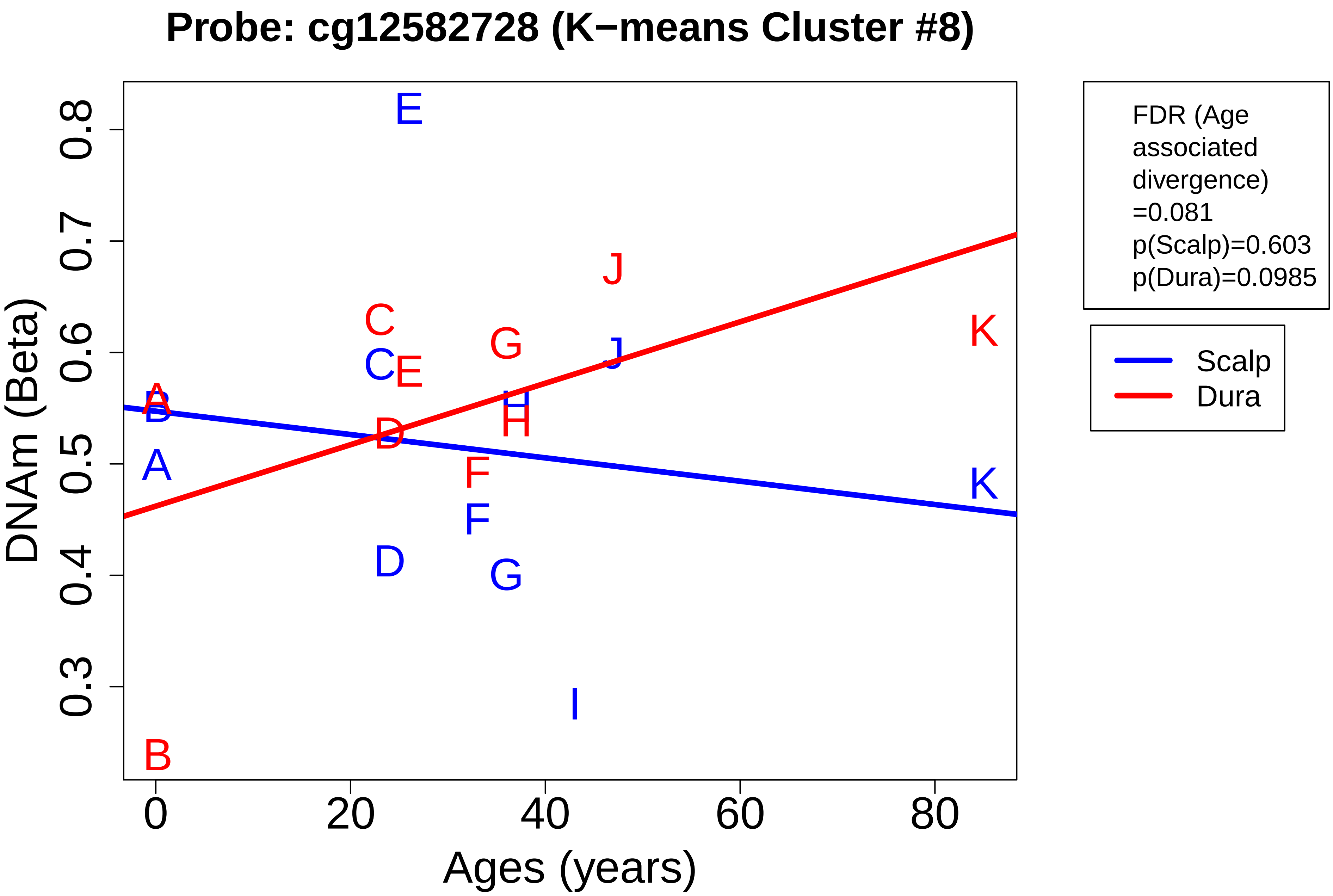

Probe: cg12582728 (K−means Cluster #8)
DNAm (Beta)
Ages (years)
0.3
0.4
0.5
0.6
0.7
0.8
0
20
40
60
80
FDR (Age associated divergence) =0.081
p(Scalp)=0.603
p(Dura)=0.0985
Scalp
Dura

Probe: cg02927519 (K−means Cluster #7)

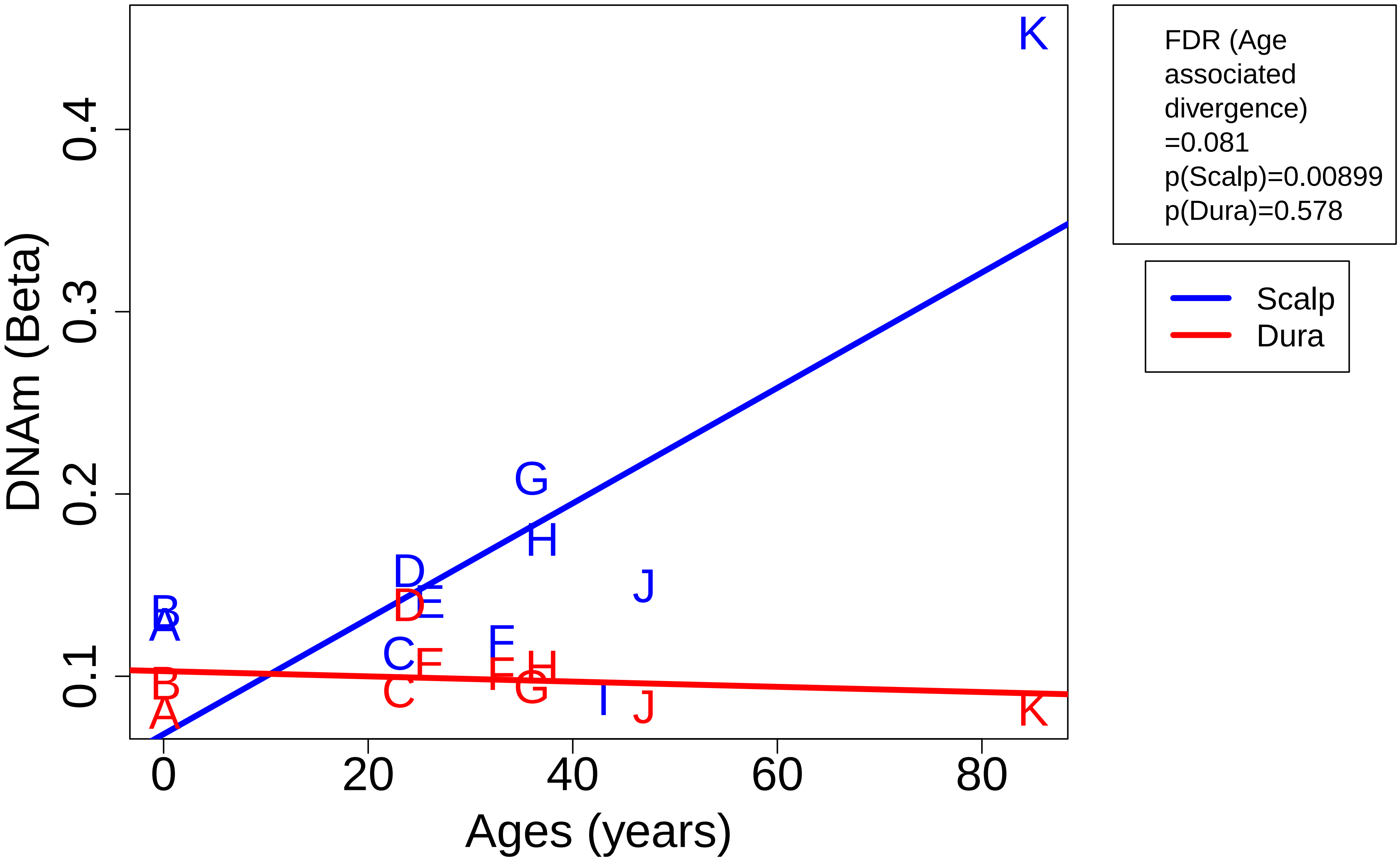

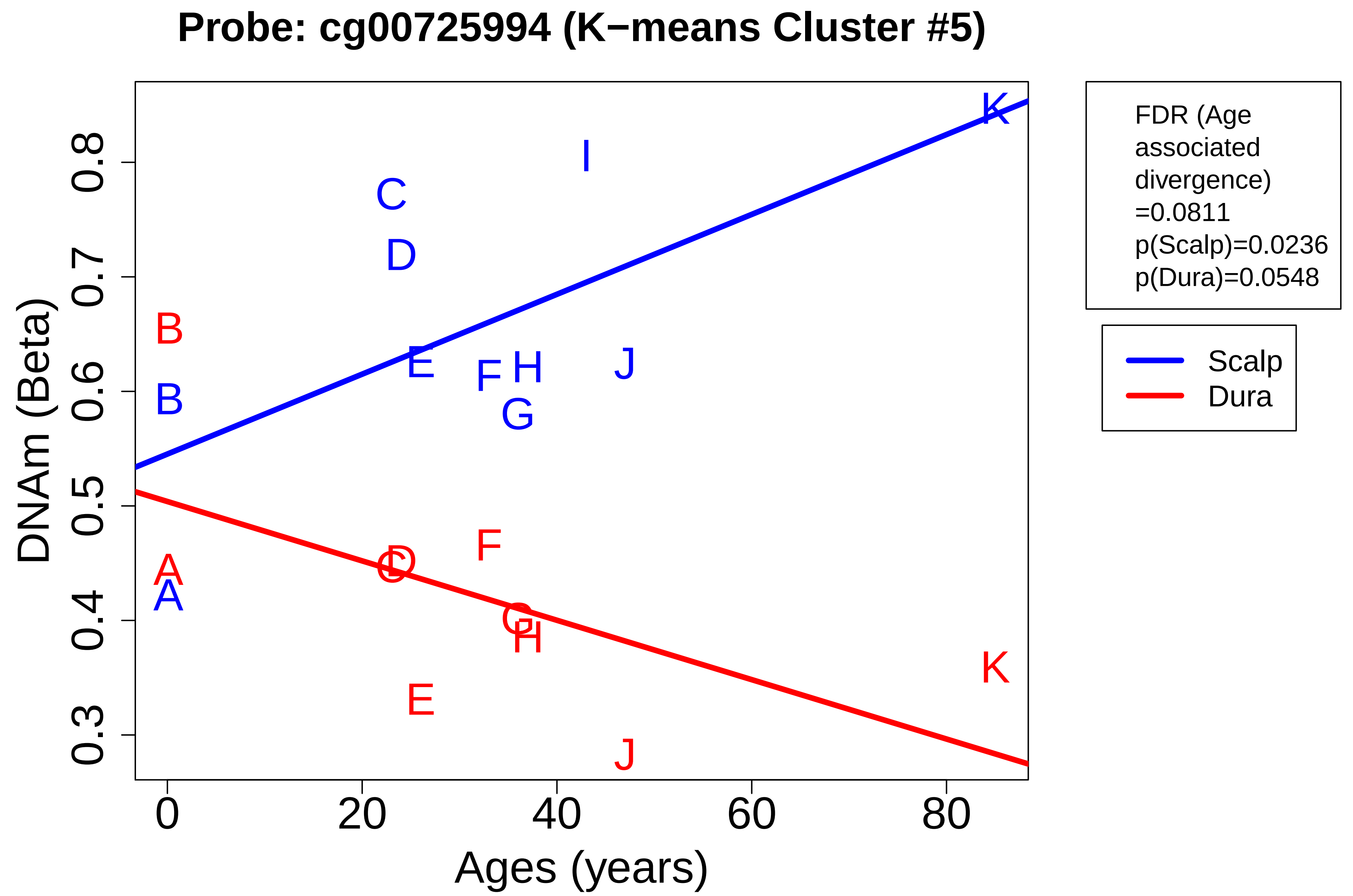

Probe: cg00725994 (K−means Cluster #5)
DNAm (Beta)
0.3
0.4
0.5
0.6
0.7
0.8
Ages (years)
0
20
40
60
80
FDR (Age associated divergence) =0.0811
p(Scalp)=0.0236
p(Dura)=0.0548
Scalp
Dura

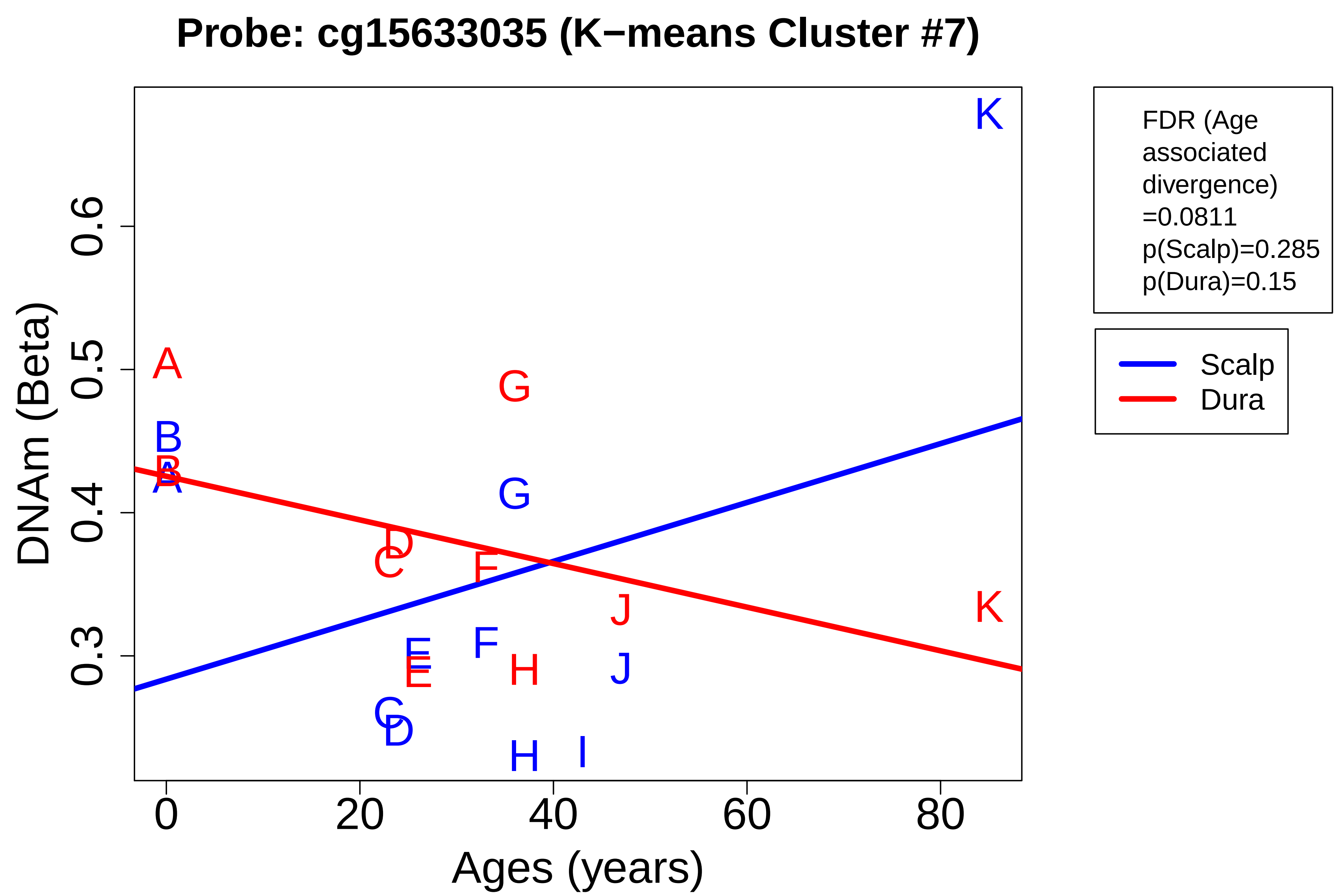

Probe: cg15633035 (K−means Cluster #7)
DNAm (Beta)
0.3
0.4
0.5
0.6
0
20
40
60
80
Ages (years)
FDR (Age associated divergence) =0.0811
p(Scalp)=0.285
p(Dura)=0.15
Scalp
Dura

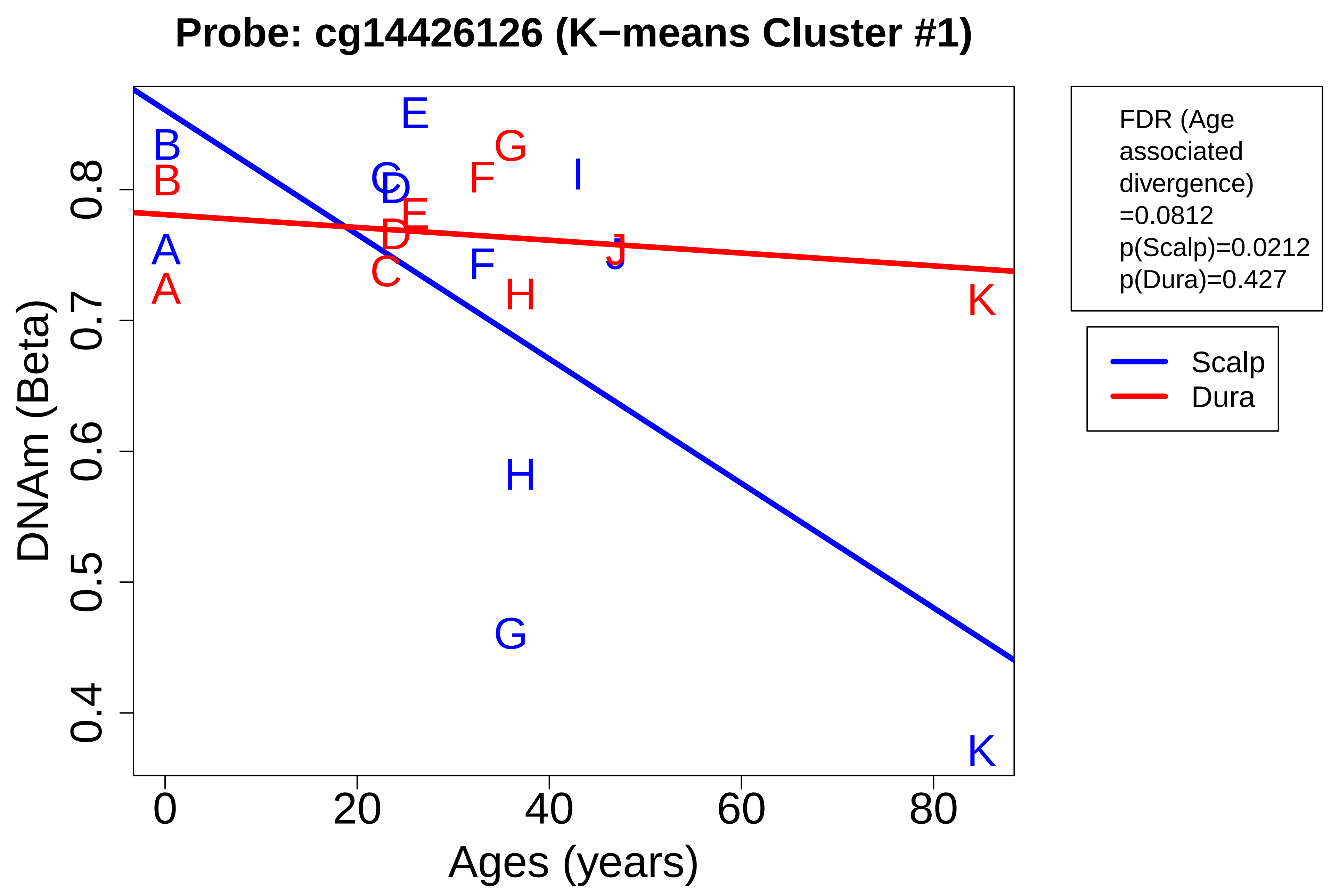

Probe: cg14426126 (K−means Cluster #1)
DNAm (Beta)
0.4
0.5
0.6
0.7
0.8
Ages (years)
0
20
40
60
80
FDR (Age associated divergence) =0.0812
p(Scalp)=0.0212
p(Dura)=0.427
Scalp
Dura
A
B
C
D
E
F
G
H
I
J
K

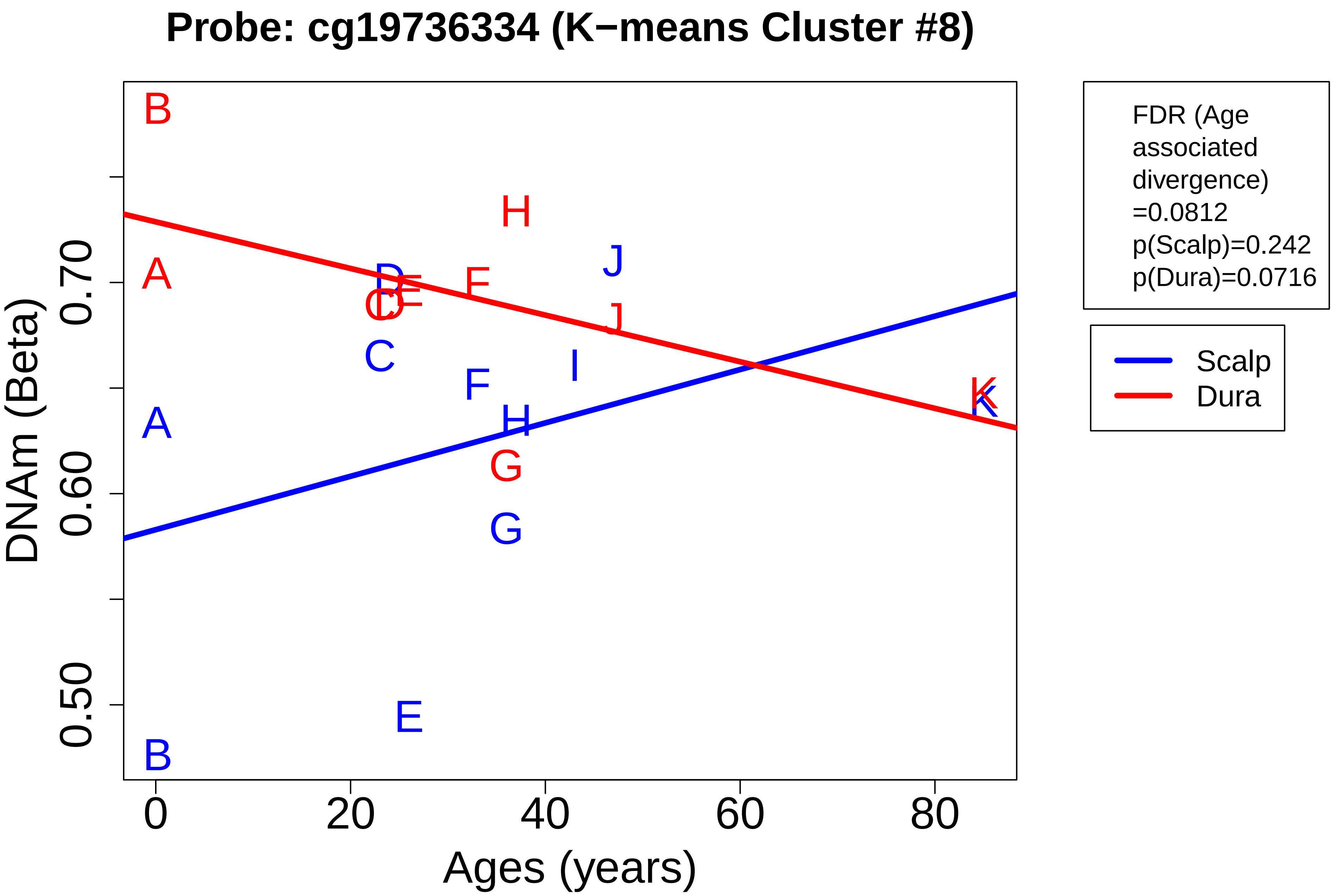

Probe: cg19736334 (K−means Cluster #8)
DNAm (Beta)
0.50
0.60
0.70
0
20
40
60
80
Ages (years)
FDR (Age associated divergence) =0.0812
p(Scalp)=0.242
p(Dura)=0.0716
Scalp
Dura

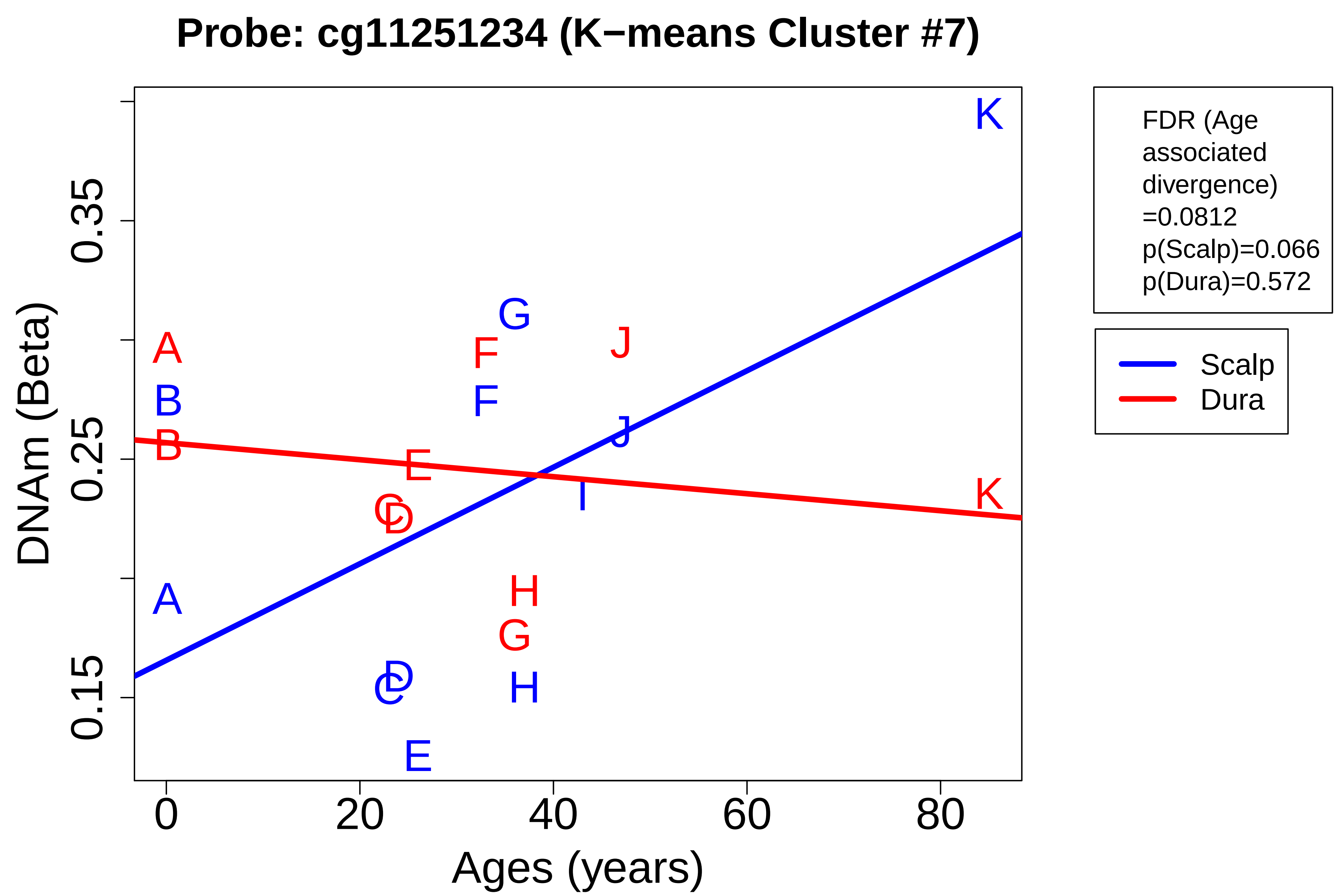
Probe: cg11251234 (K−means Cluster #7)
DNAm (Beta)
0.15
0.25
0.35
Ages (years)
0
20
40
60
80
FDR (Age associated divergence) =0.0812
p(Scalp)=0.066
p(Dura)=0.572
Scalp
Dura
A
B
C
D
E
F
G
H
I
J
K

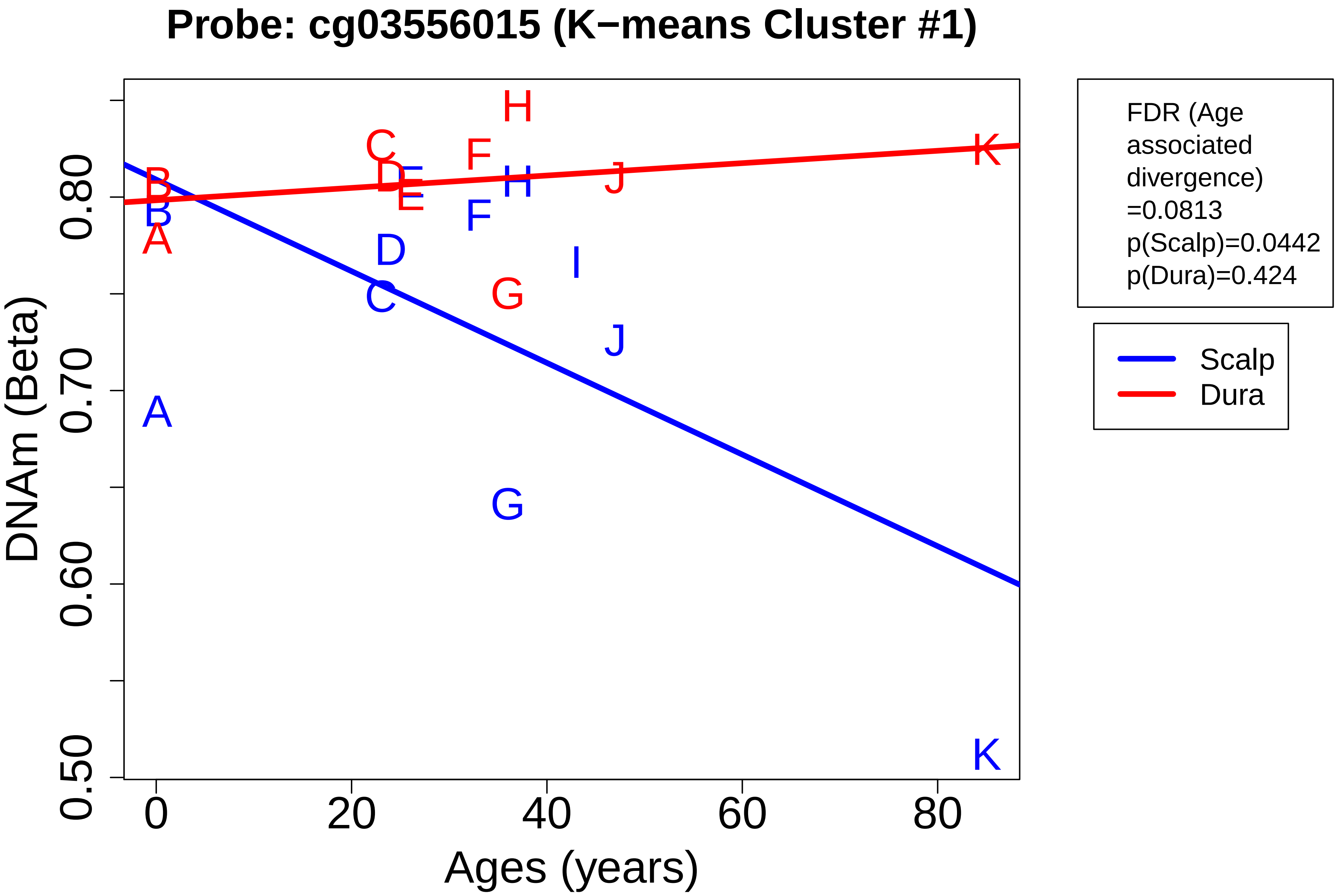

Probe: cg03556015 (K−means Cluster #1)
DNAm (Beta)
0.50
0.60
0.70
0.80
0
20
40
60
80
Ages (years)
FDR (Age associated divergence) =0.0813
p(Scalp)=0.0442
p(Dura)=0.424
Scalp
Dura

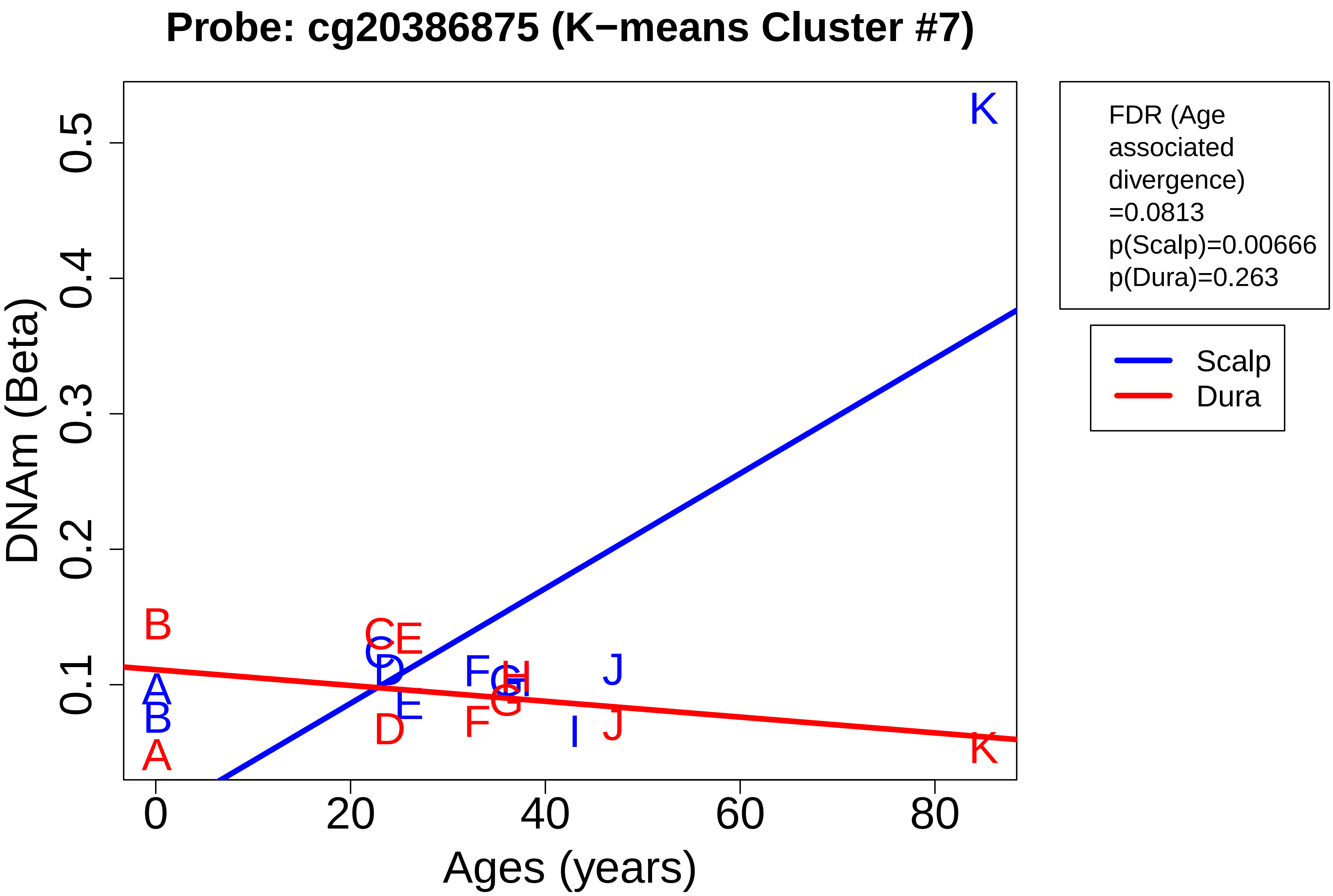

Probe: cg20386875 (K−means Cluster #7)
DNAm (Beta)
0.1
0.2
0.3
0.4
0.5
0
20
40
60
80
Ages (years)
FDR (Age associated divergence) =0.0813
p(Scalp)=0.00666
p(Dura)=0.263
Scalp
Dura

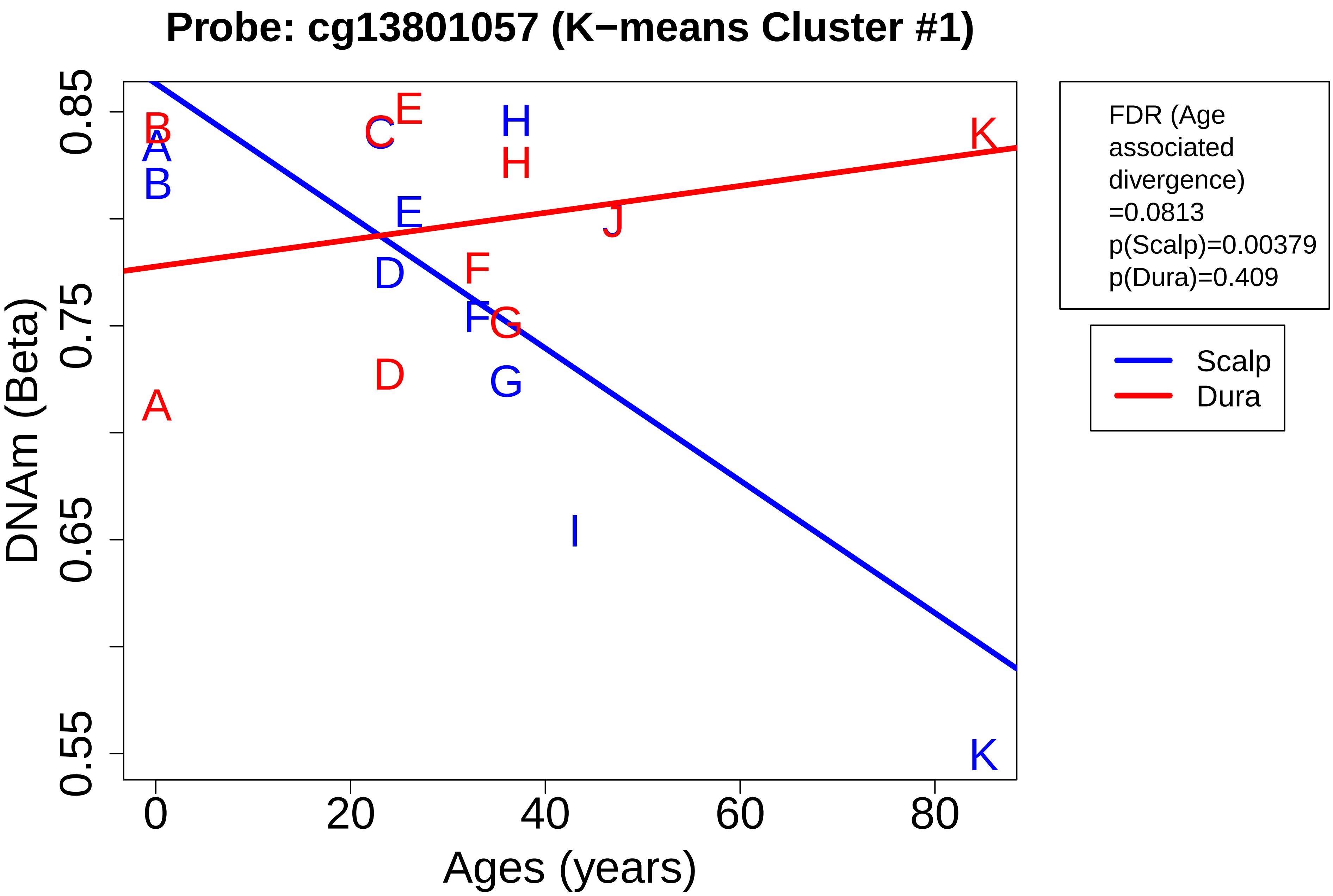

Probe: cg13801057 (K−means Cluster #1)
DNAm (Beta)
0.55
0.65
0.75
0.85
Ages (years)
0
20
40
60
80
A
B
C
D
E
F
G
H
I
J
K
FDR (Age associated divergence) =0.0813
p(Scalp)=0.00379
p(Dura)=0.409
Scalp
Dura

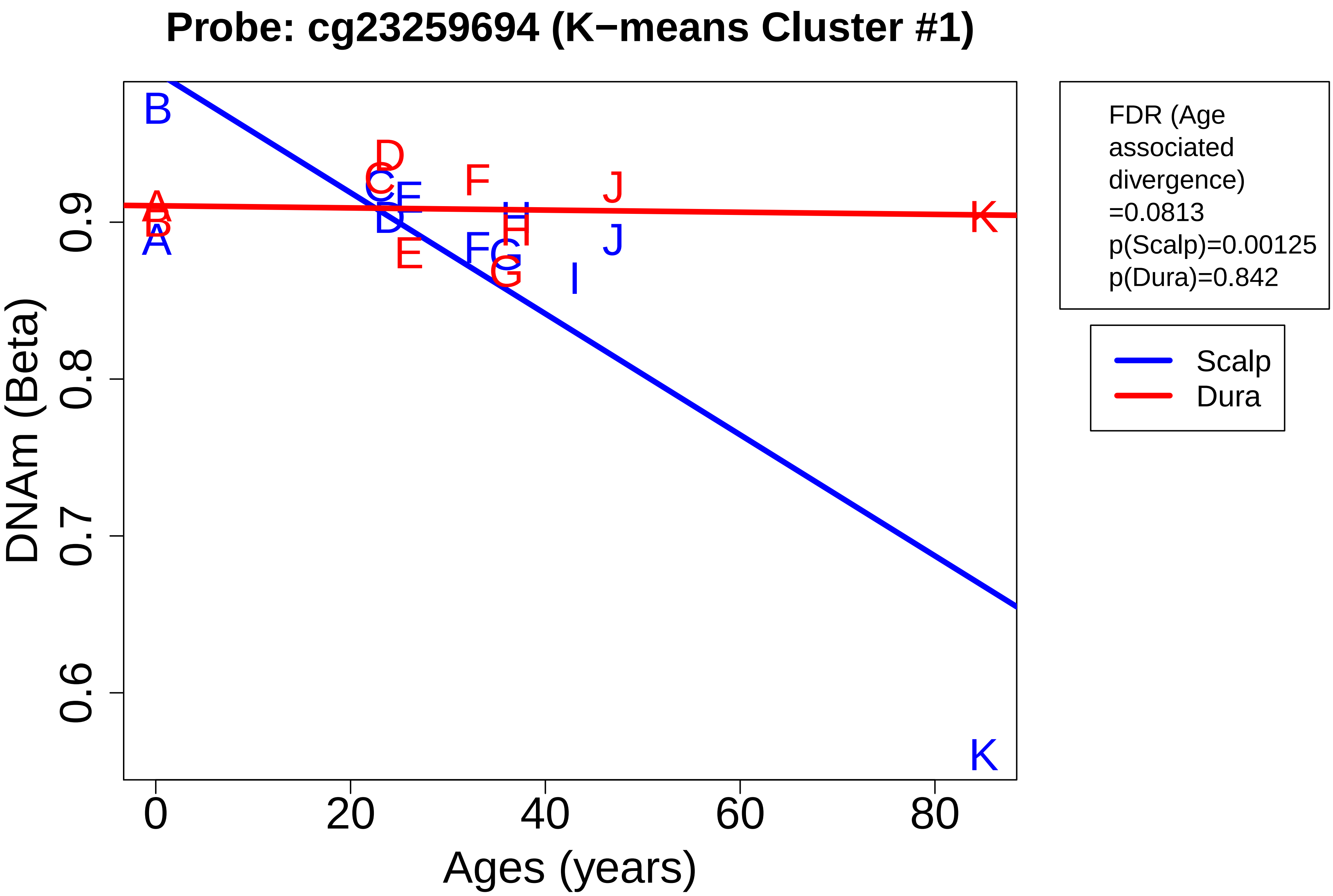

Probe: cg23259694 (K−means Cluster #1)
FDR (Age associated divergence) =0.0813
p(Scalp)=0.00125
p(Dura)=0.842
Scalp
Dura
DNAm (Beta)
0.6
0.7
0.8
0.9
Ages (years)
0
20
40
60
80
A
B
C
D
E
F
G
H
I
J
K

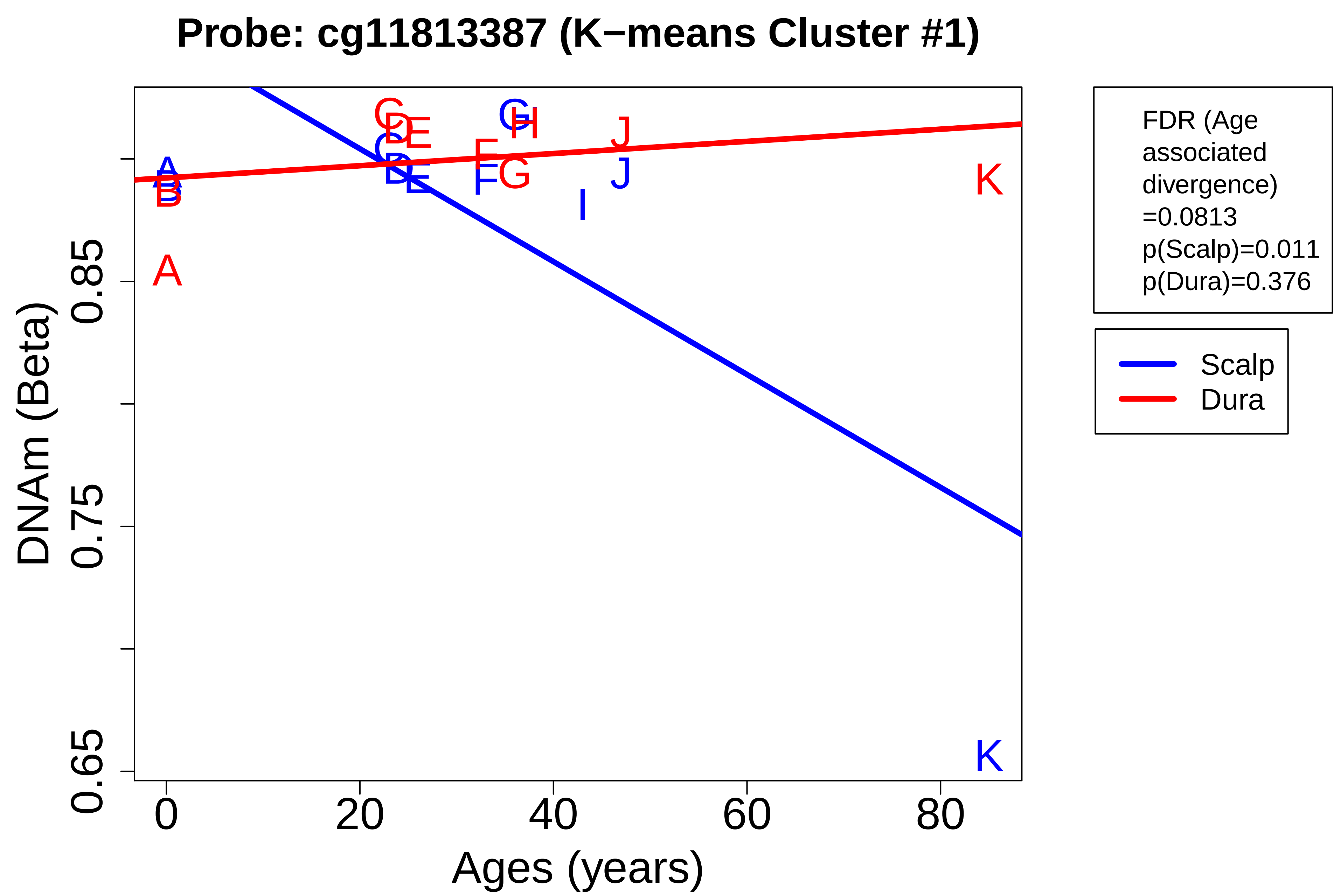

Probe: cg11813387 (K−means Cluster #1)
DNAm (Beta)
0.65
0.75
0.85
Ages (years)
0
20
40
60
80
A
B
C
D
E
F
G
H
I
J
K
FDR (Age associated divergence) =0.0813
p(Scalp)=0.011
p(Dura)=0.376
Scalp
Dura

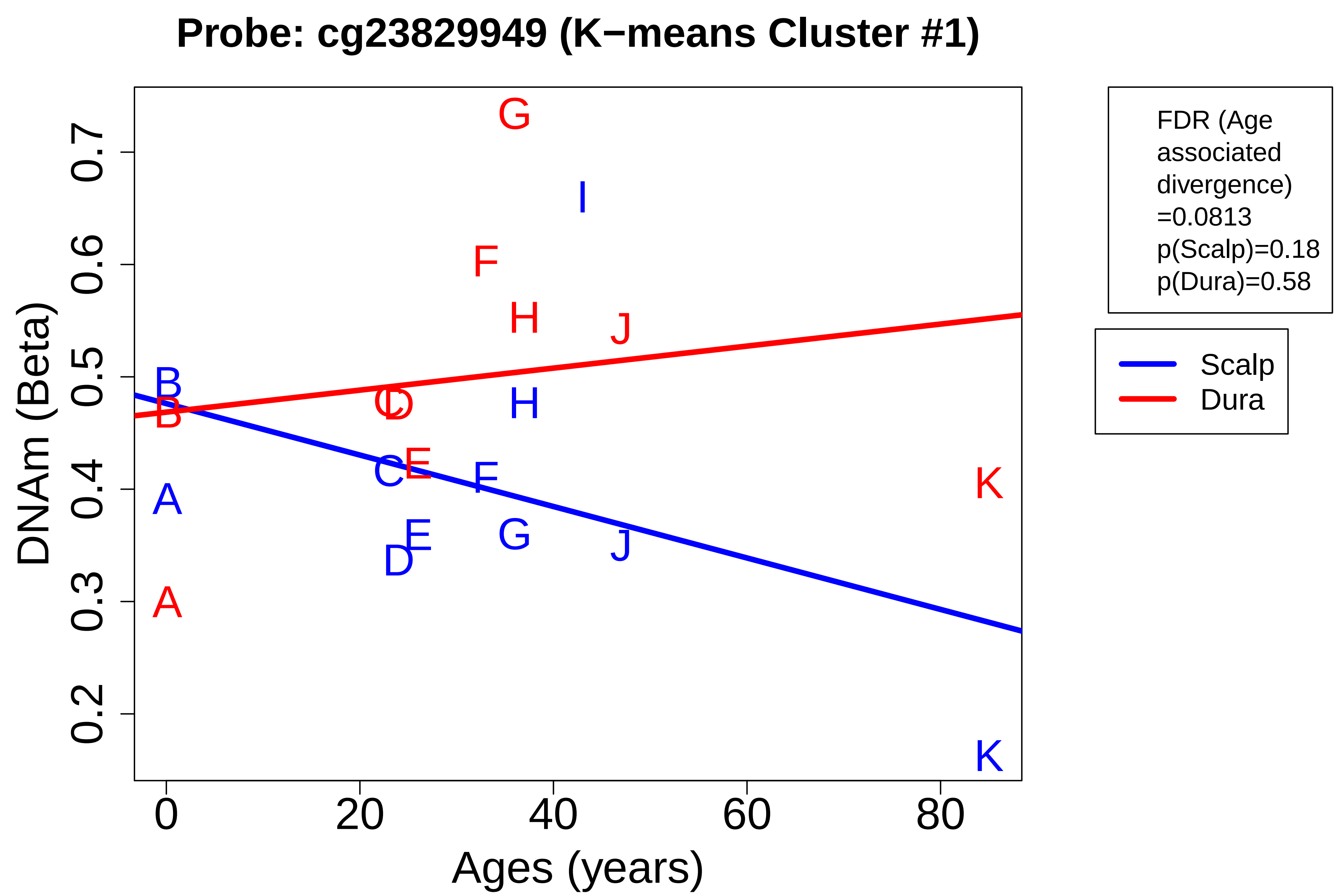
Probe: cg23829949 (K−means Cluster #1)
DNAm (Beta)
0.2
0.3
0.4
0.5
0.6
0.7
Ages (years)
0
20
40
60
80
A
B
C
D
E
F
G
H
I
J
K
FDR (Age associated divergence) =0.0813
p(Scalp)=0.18
p(Dura)=0.58
Scalp
Dura

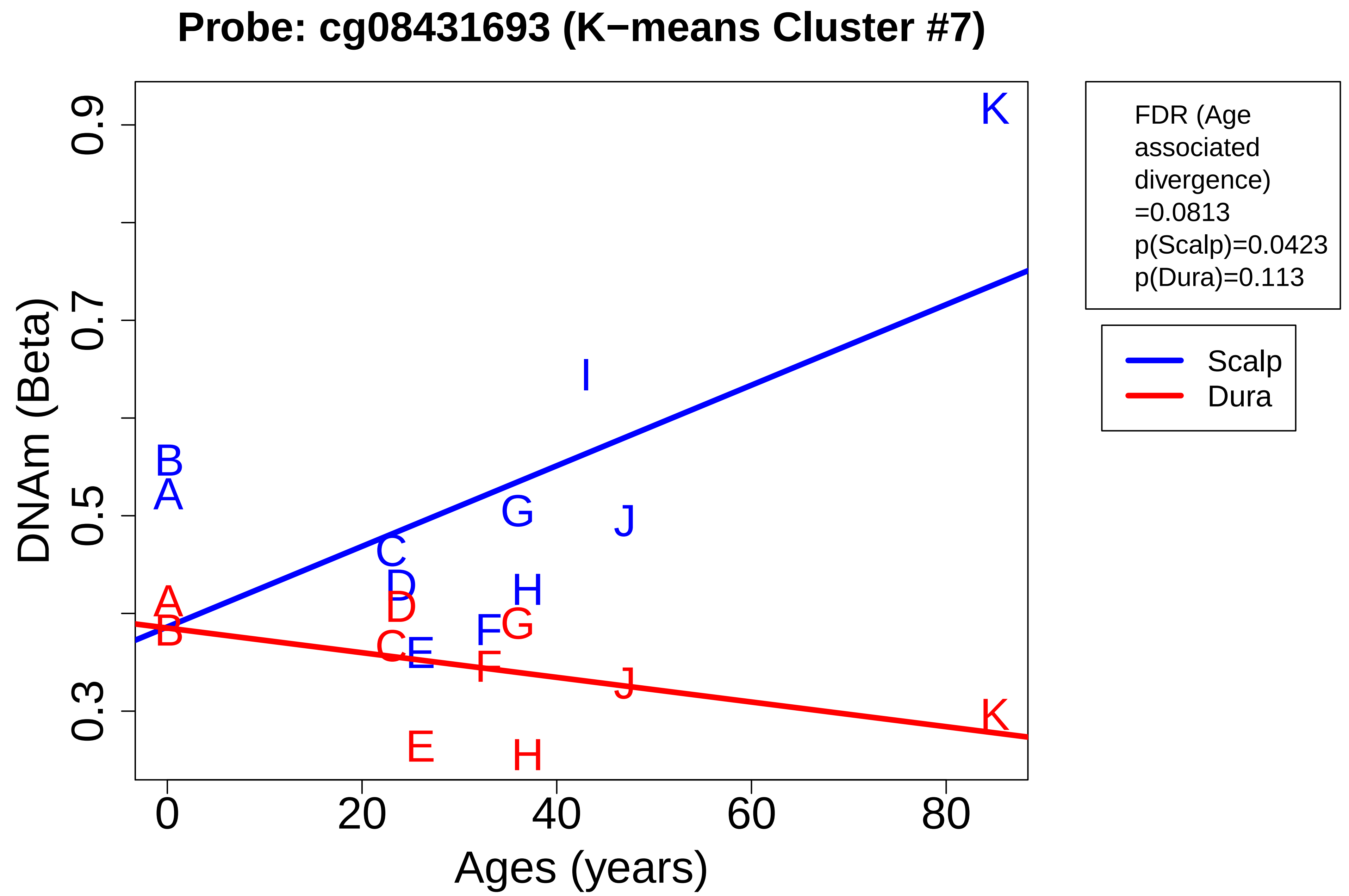

Probe: cg08431693 (K−means Cluster #7)
DNAm (Beta)
0.3
0.5
0.7
0.9
0
20
40
60
80
Ages (years)
FDR (Age associated divergence) =0.0813
p(Scalp)=0.0423
p(Dura)=0.113
Scalp
Dura

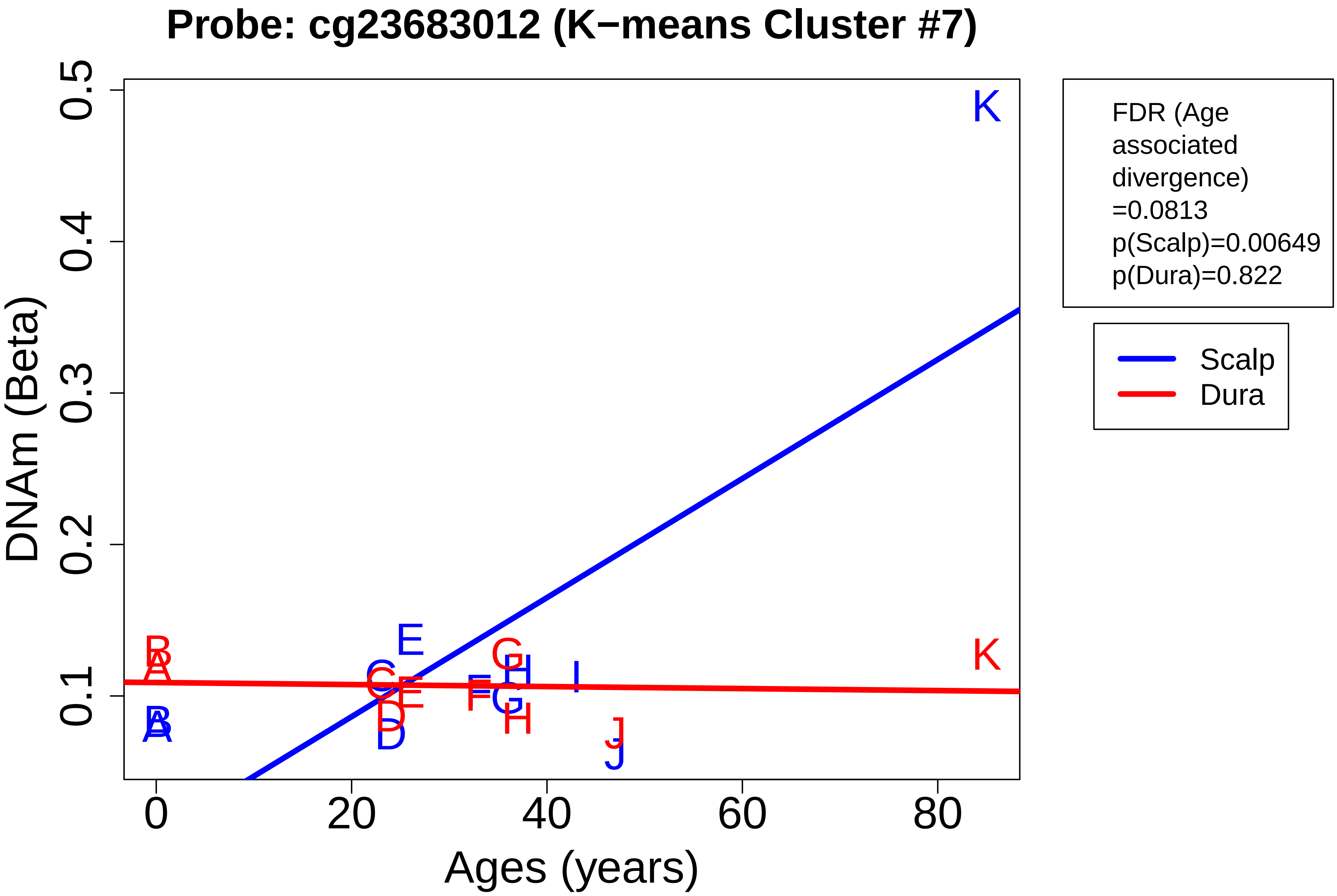
Probe: cg23683012 (K−means Cluster #7)
DNAm (Beta)
0.1
0.2
0.3
0.4
0.5
0
20
40
60
80
Ages (years)
A
B
C
D
E
F
G
H
I
J
K
FDR (Age associated divergence) =0.0813
p(Scalp)=0.00649
p(Dura)=0.822
Scalp
Dura

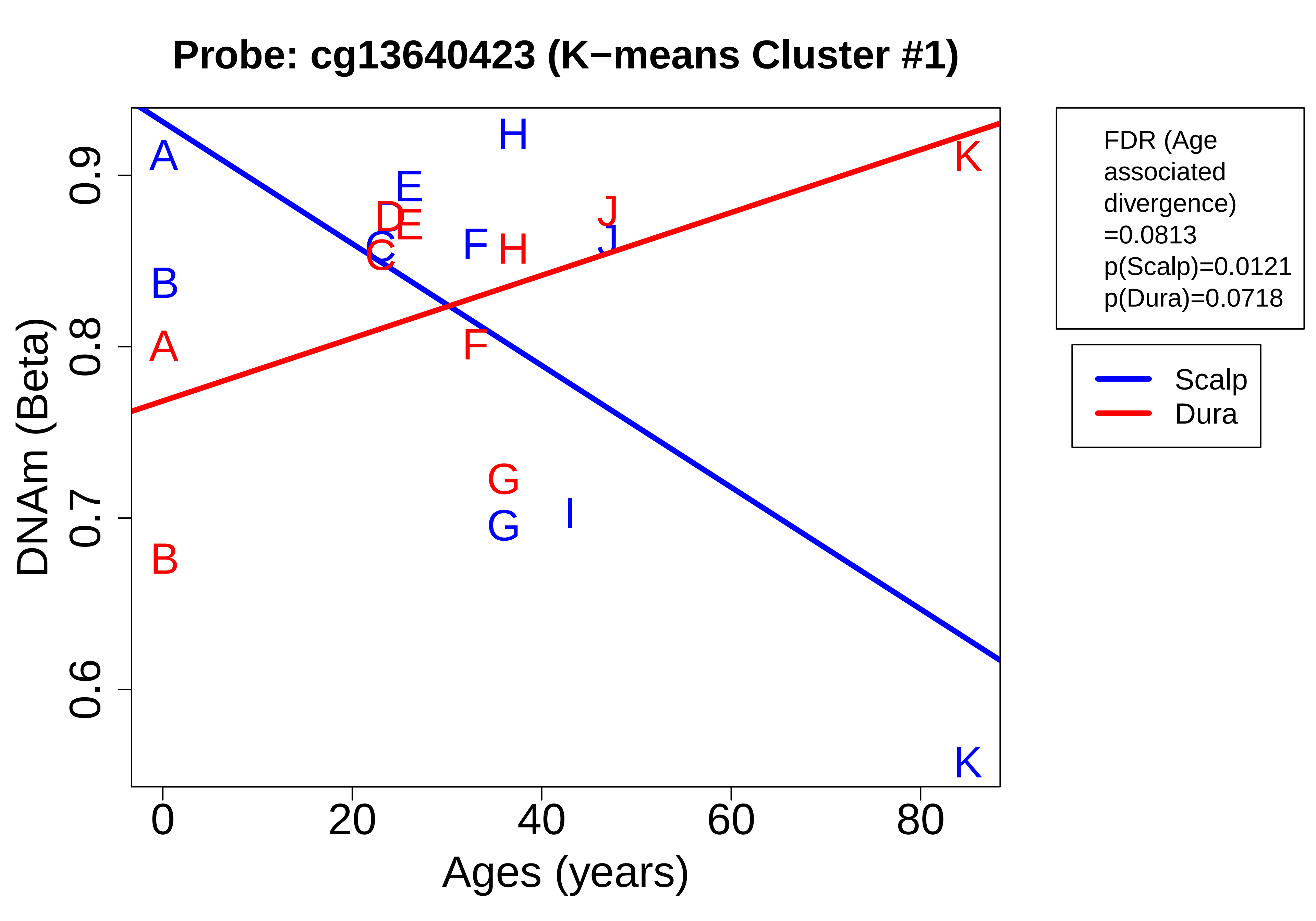
Probe: cg13640423 (K−means Cluster #1)
DNAm (Beta)
0.6
0.7
0.8
0.9
0
20
40
60
80
Ages (years)
A
B
C
D
E
F
G
H
I
J
K
FDR (Age associated divergence) =0.0813
p(Scalp)=0.0121
p(Dura)=0.0718
Scalp
Dura

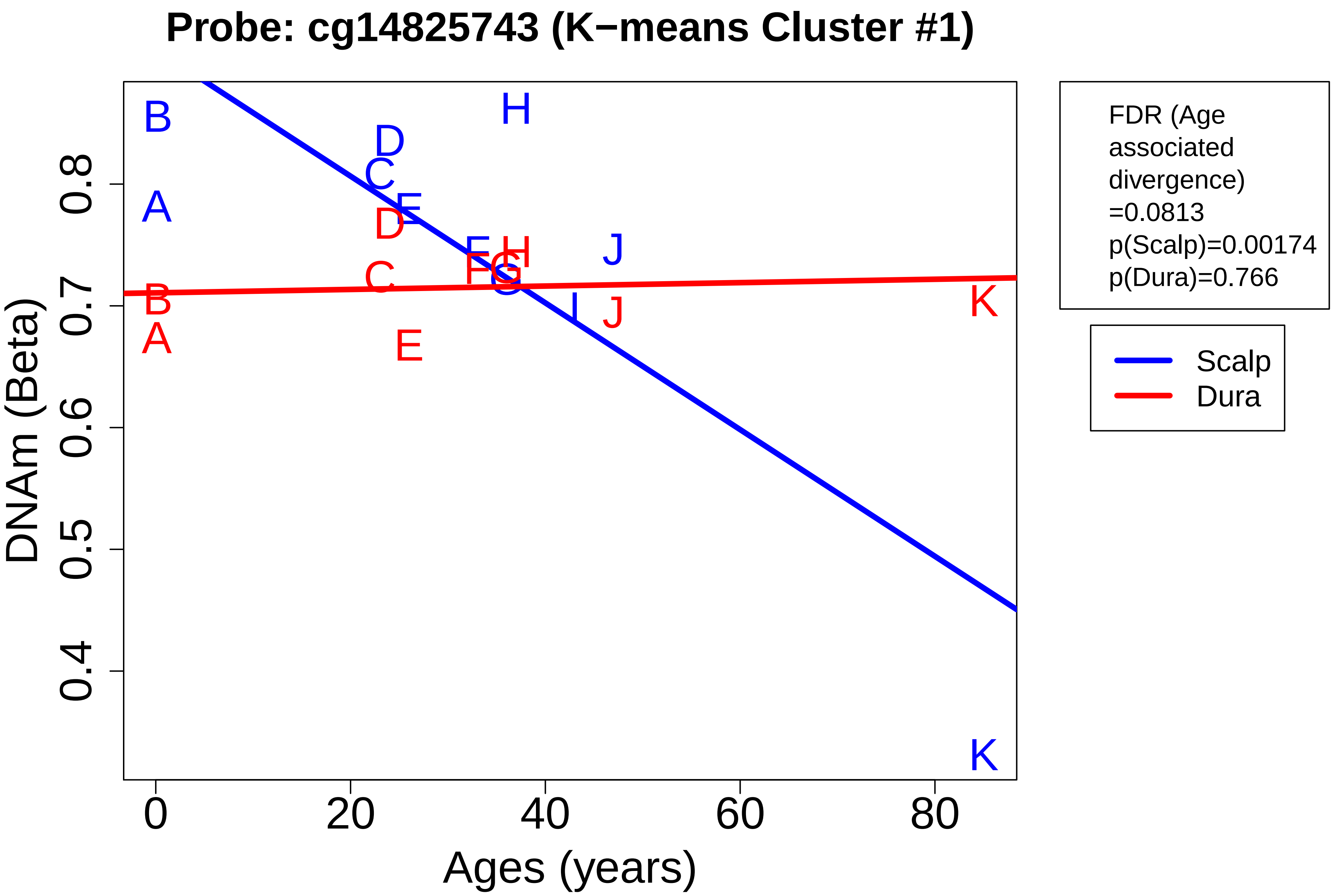

Probe: cg14825743 (K−means Cluster #1)
DNAm (Beta)
0.4
0.5
0.6
0.7
0.8
0
20
40
60
80
Ages (years)
FDR (Age associated divergence) =0.0813
p(Scalp)=0.00174
p(Dura)=0.766
Scalp
Dura

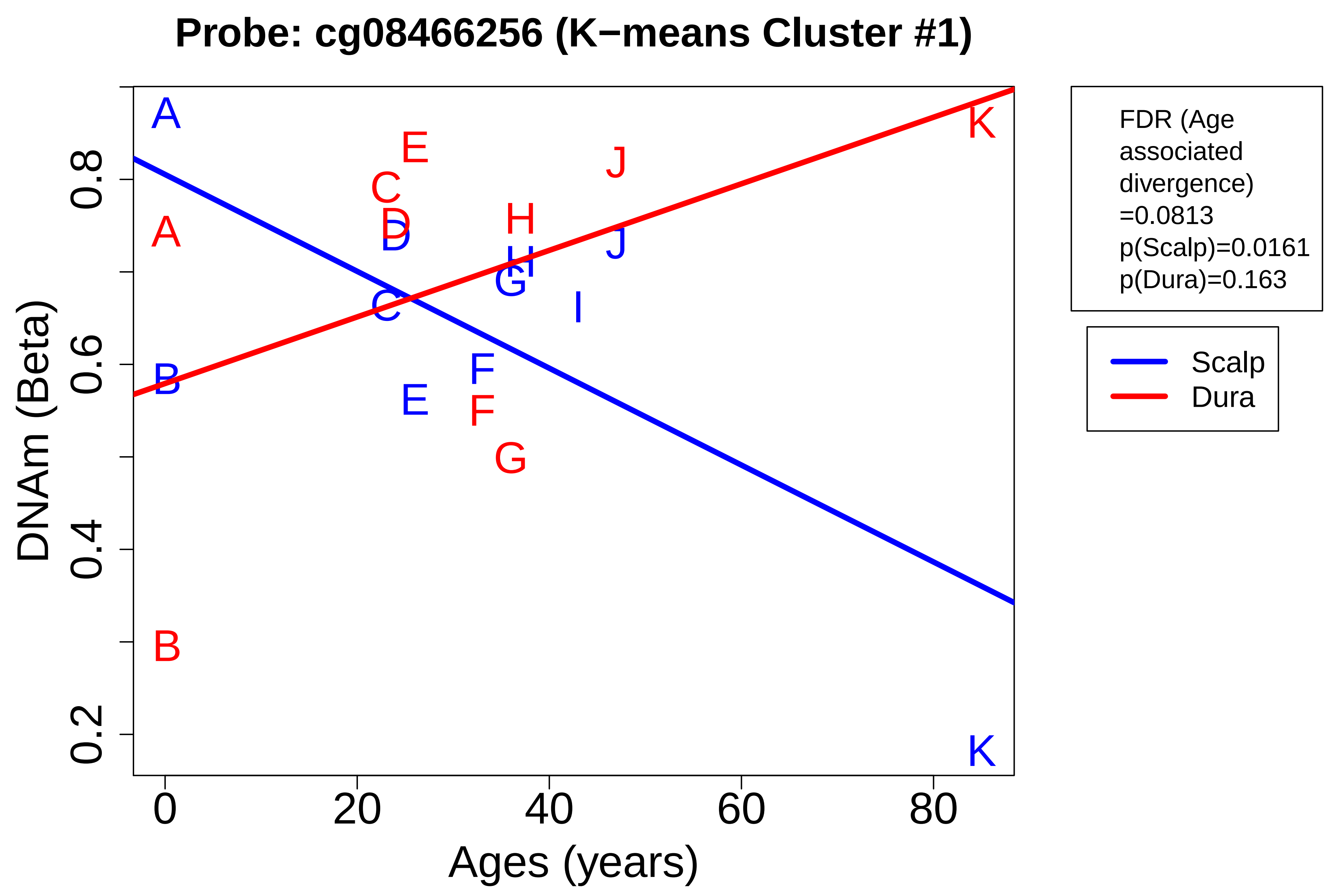

Probe: cg08466256 (K−means Cluster #1)
DNAm (Beta)
0.2
0.4
0.6
0.8
0
20
40
60
80
Ages (years)
A
B
C
D
E
F
G
H
I
J
K
FDR (Age associated divergence) =0.0813
p(Scalp)=0.0161
p(Dura)=0.163
Scalp
Dura

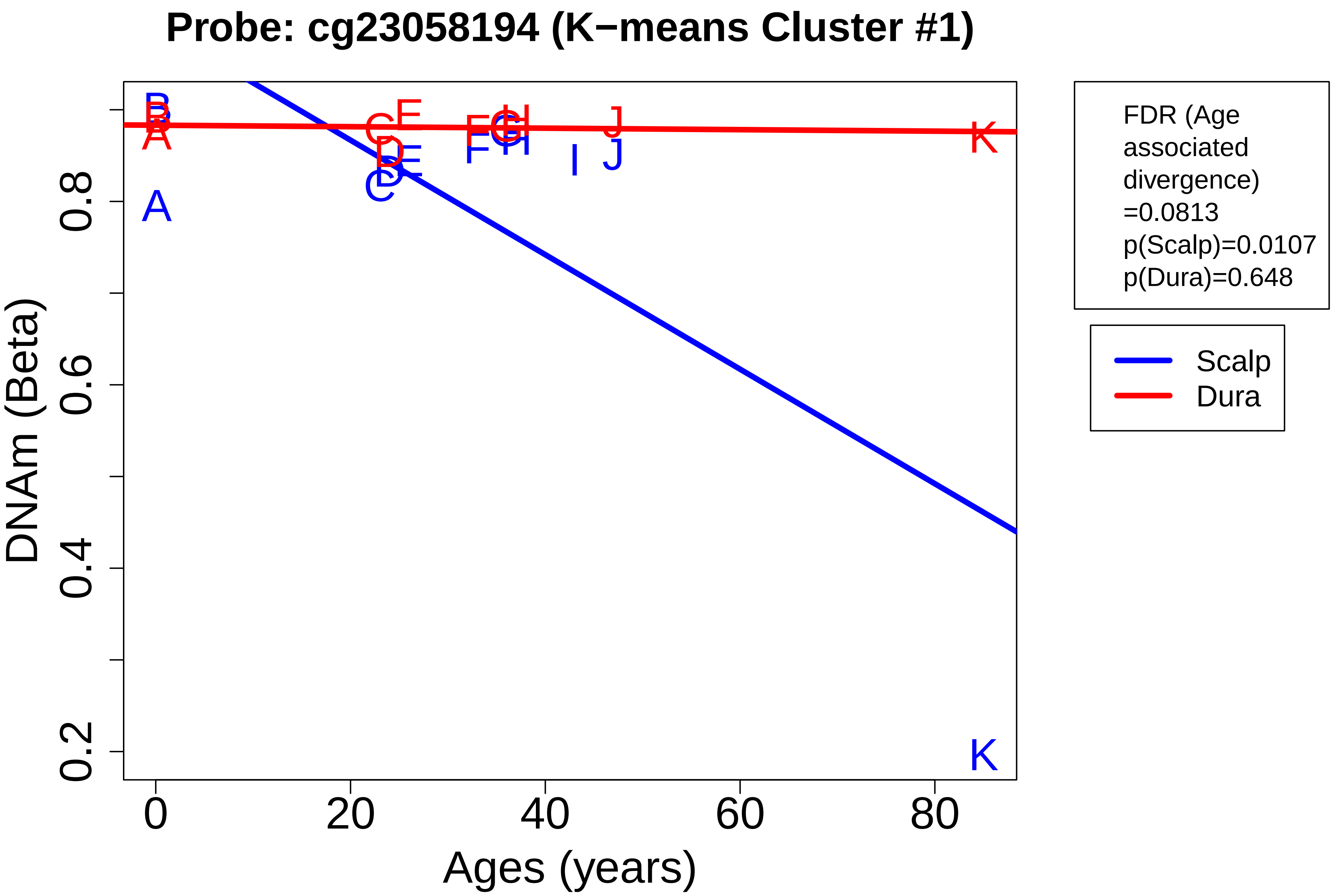

Probe: cg23058194 (K−means Cluster #1)
DNAm (Beta)
0.2
0.4
0.6
0.8
Ages (years)
0
20
40
60
80
FDR (Age associated divergence) =0.0813
p(Scalp)=0.0107
p(Dura)=0.648
Scalp
Dura
A
B
C
D
E
F
G
H
I
J
K

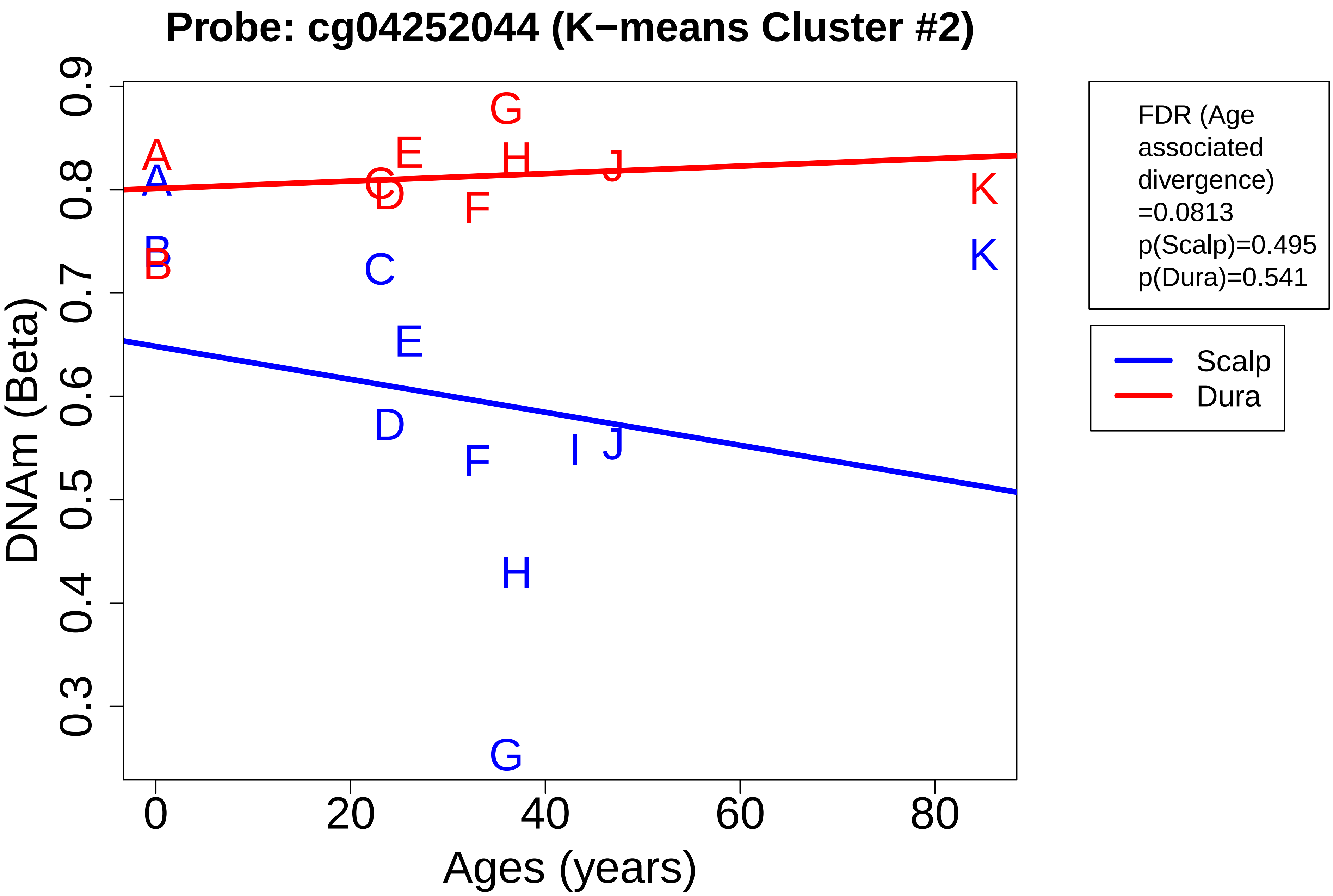

Probe: cg04252044 (K−means Cluster #2)
DNAm (Beta)
0.3
0.4
0.5
0.6
0.7
0.8
0.9
0
20
40
60
80
Ages (years)
FDR (Age associated divergence) =0.0813
p(Scalp)=0.495
p(Dura)=0.541
Scalp
Dura

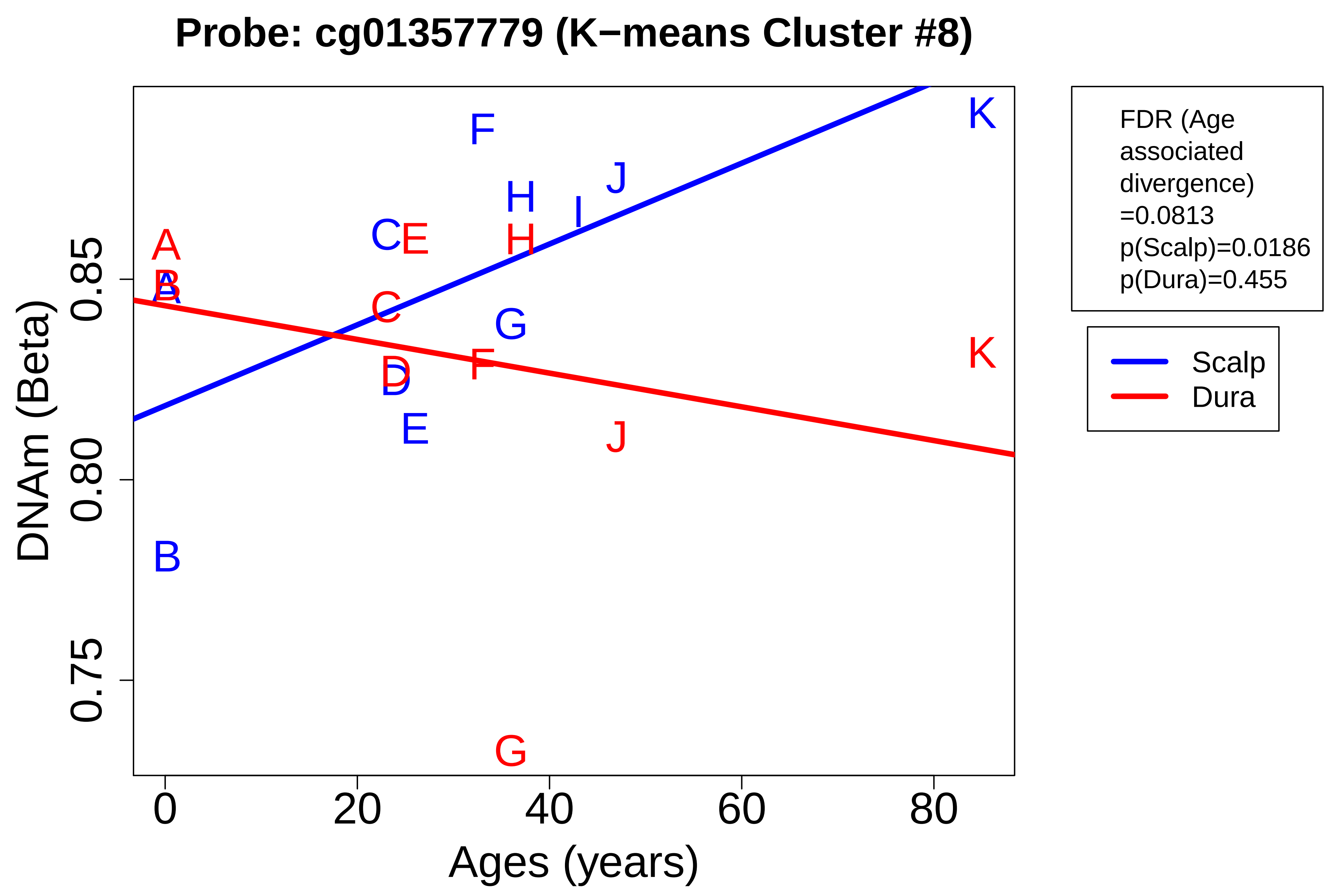

Probe: cg01357779 (K−means Cluster #8)
DNAm (Beta)
0.75
0.80
0.85
Ages (years)
0
20
40
60
80
FDR (Age associated divergence) =0.0813
p(Scalp)=0.0186
p(Dura)=0.455
Scalp
Dura

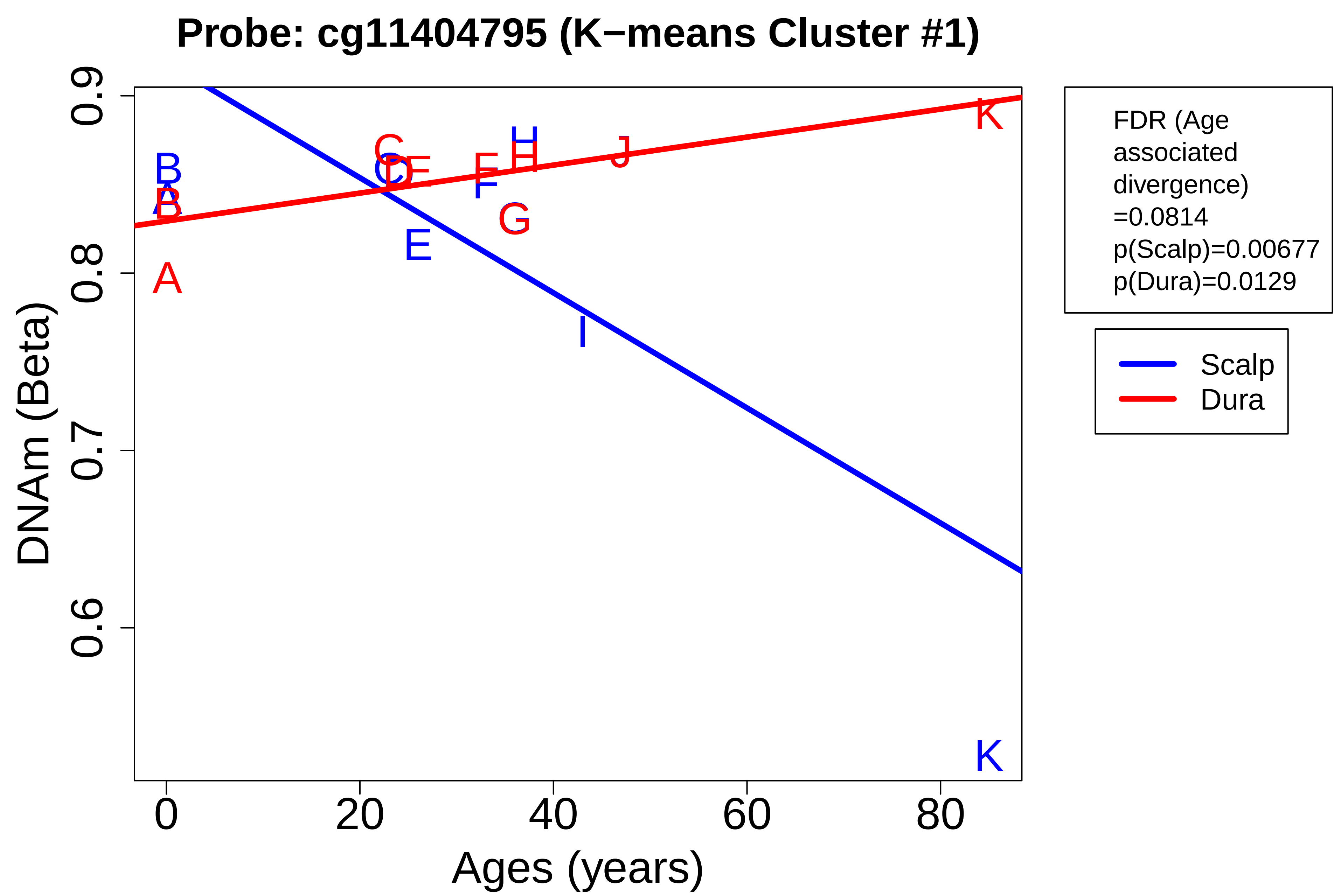

Probe: cg11404795 (K−means Cluster #1)
DNAm (Beta)
0.6
0.7
0.8
0.9
Ages (years)
0
20
40
60
80
FDR (Age associated divergence) =0.0814
p(Scalp)=0.00677
p(Dura)=0.0129
Scalp
Dura
A
B
C
D
E
F
G
H
I
J
K

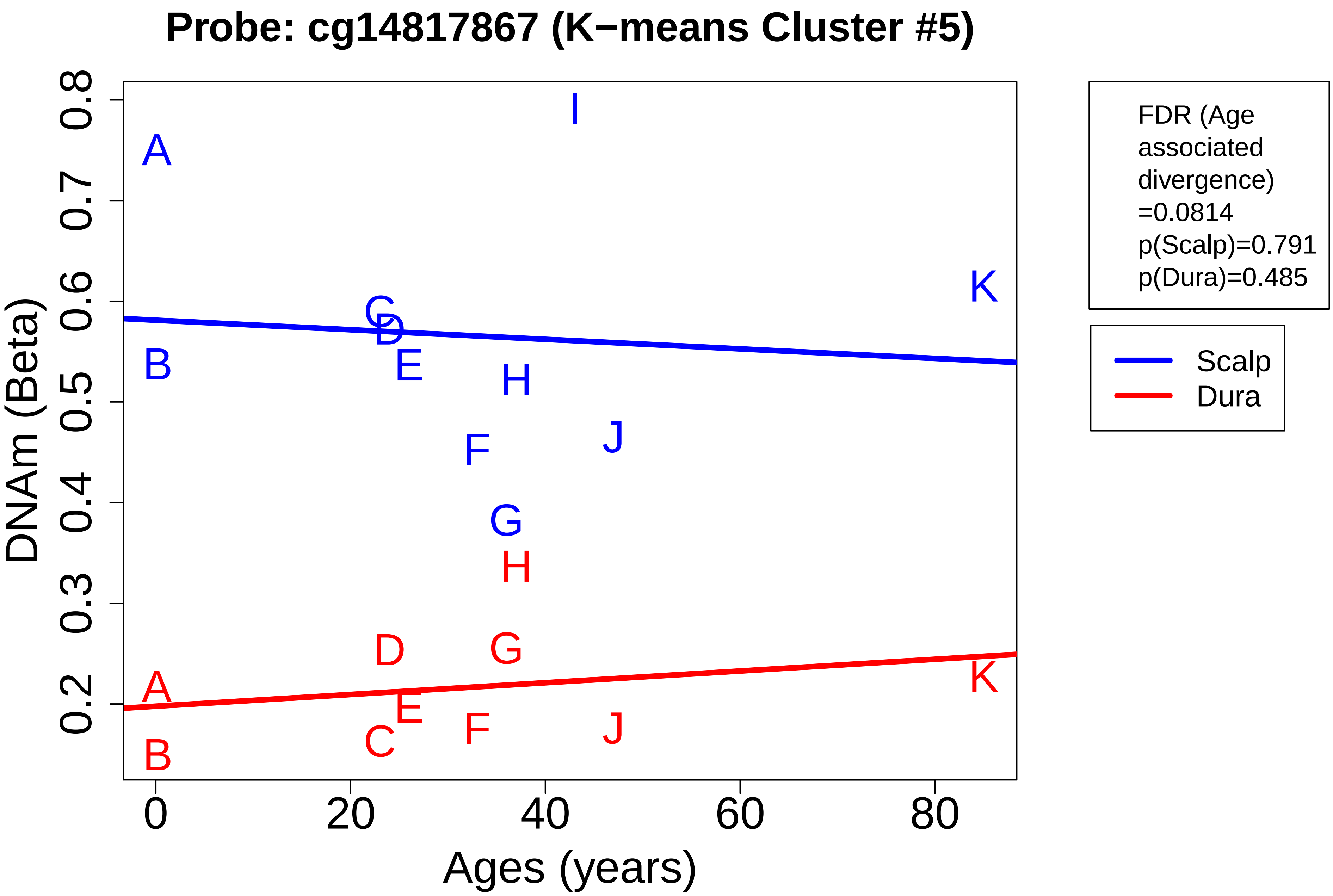

Probe: cg14817867 (K−means Cluster #5)
DNAm (Beta)
0.2
0.3
0.4
0.5
0.6
0.7
0.8
0
20
40
60
80
Ages (years)
A
B
C
D
E
F
G
H
I
J
K
A
B
C
D
E
F
G
H
J
K
FDR (Age associated divergence) =0.0814
p(Scalp)=0.791
p(Dura)=0.485
Scalp
Dura

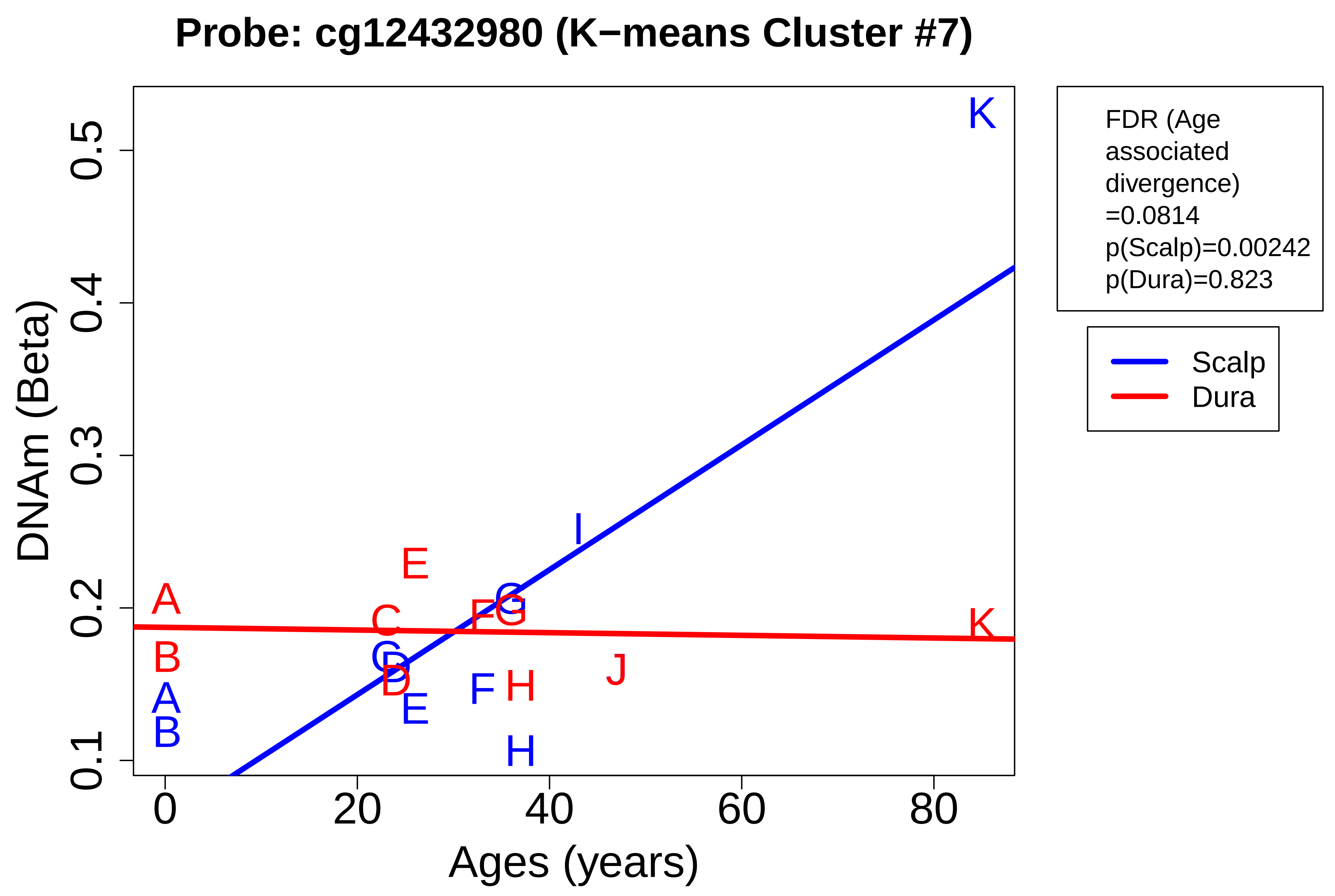

Probe: cg12432980 (K−means Cluster #7)
DNAm (Beta)
0.1
0.2
0.3
0.4
0.5
0
20
40
60
80
Ages (years)
FDR (Age associated divergence) =0.0814
p(Scalp)=0.00242
p(Dura)=0.823
Scalp
Dura
A
B
C
D
E
F
G
H
I
J
K

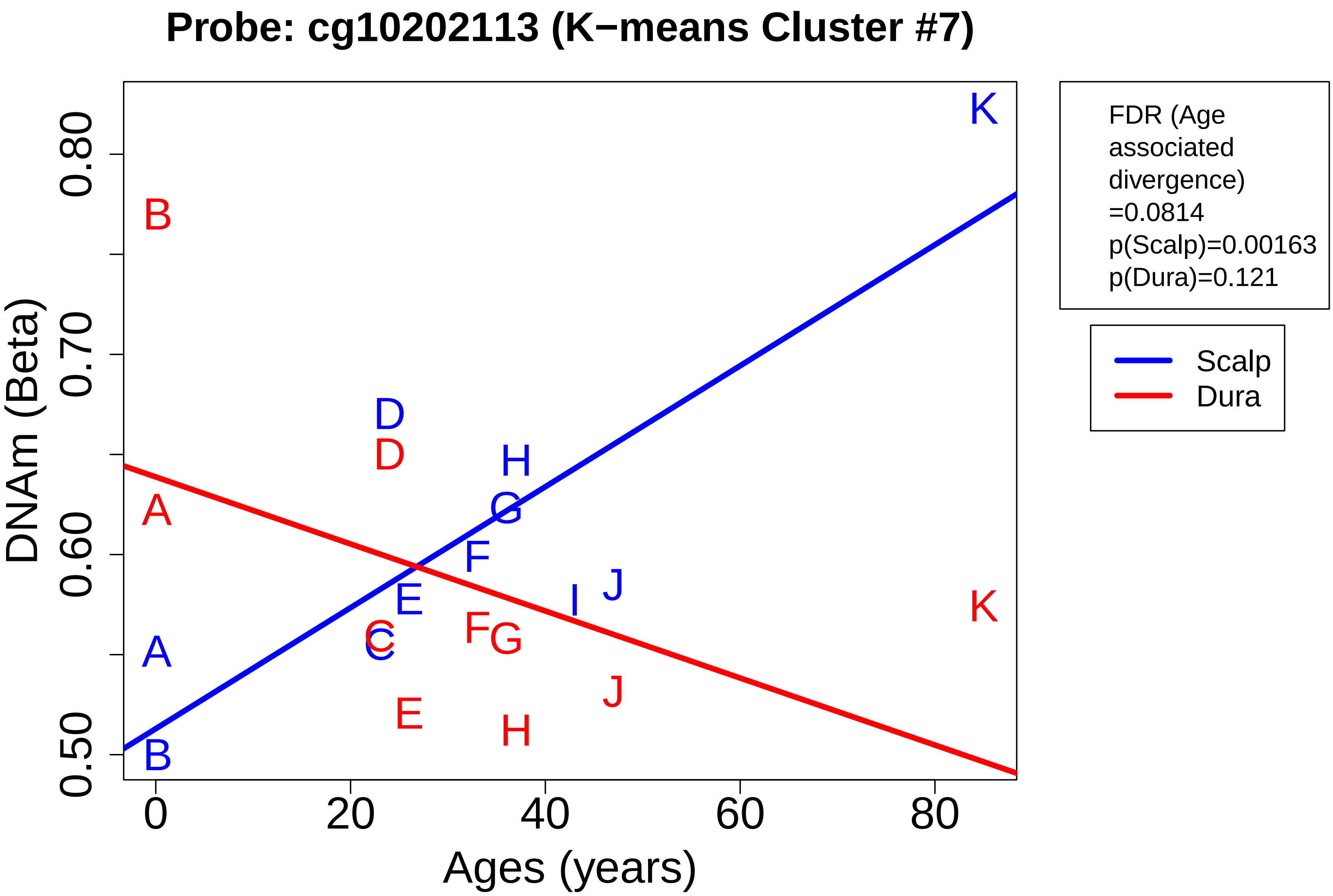

Probe: cg10202113 (K−means Cluster #7)
DNAm (Beta)
0.50
0.60
0.70
0.80
0
20
40
60
80
Ages (years)
FDR (Age associated divergence) =0.0814
p(Scalp)=0.00163
p(Dura)=0.121
Scalp
Dura
A
B
C
D
E
F
G
H
I
J
K

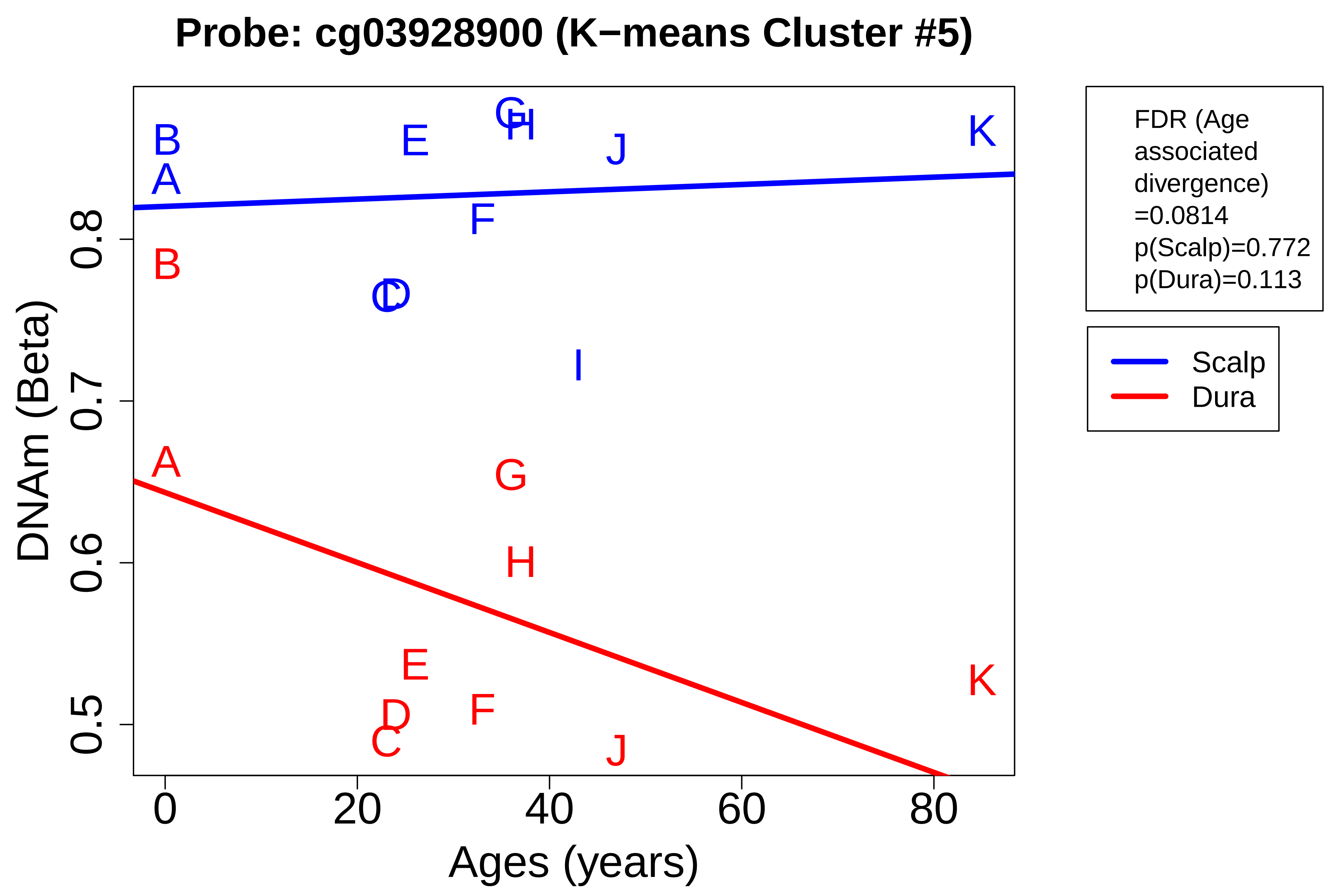

Probe: cg03928900 (K−means Cluster #5)
DNAm (Beta)
0.5
0.6
0.7
0.8
0
20
40
60
80
Ages (years)
A
B
C
D
E
F
G
H
I
J
K
A
B
C
D
E
F
G
H
J
K
FDR (Age associated divergence) =0.0814
p(Scalp)=0.772
p(Dura)=0.113
Scalp
Dura

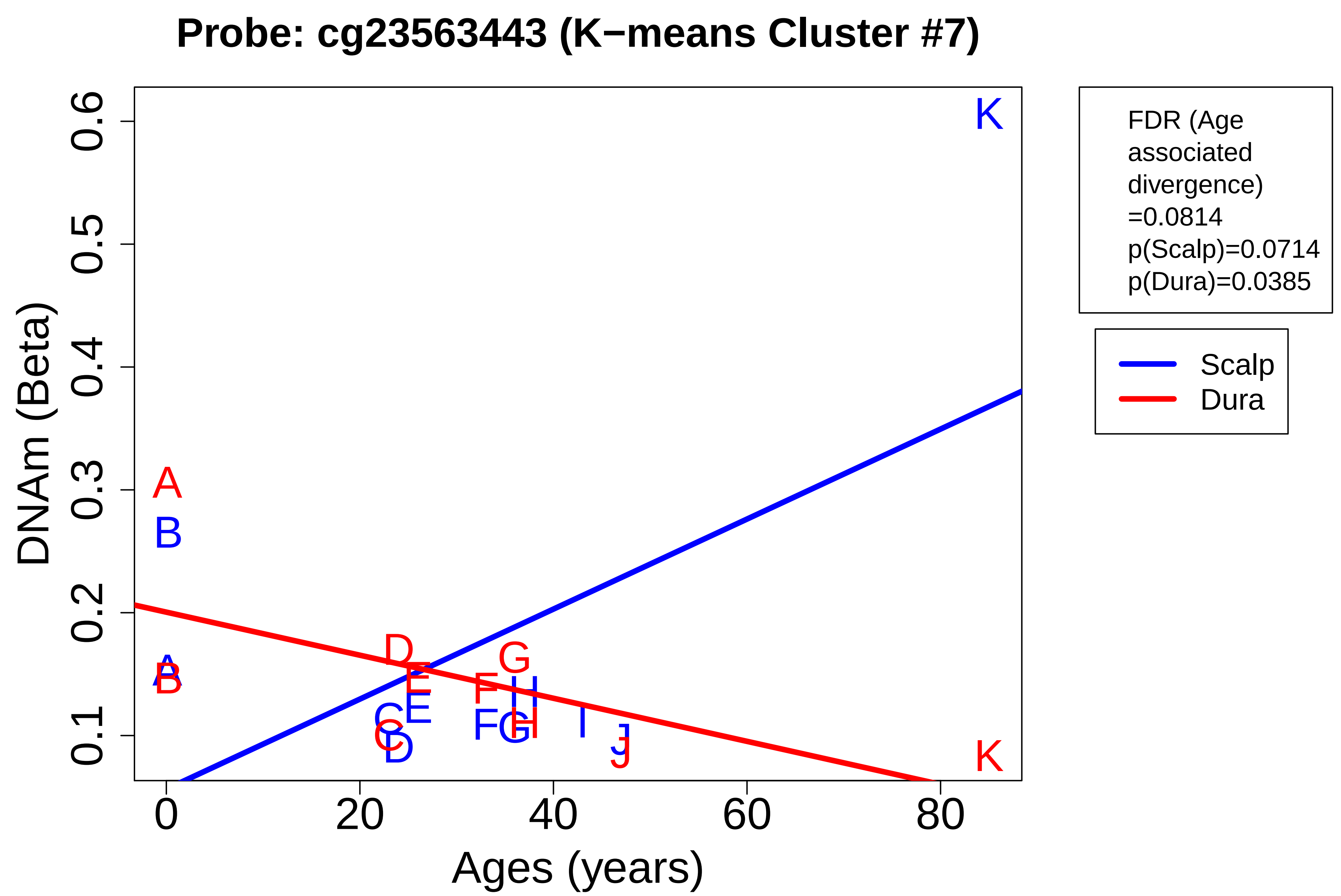
Probe: cg23563443 (K−means Cluster #7)
DNAm (Beta)
0.1
0.2
0.3
0.4
0.5
0.6
Ages (years)
0
20
40
60
80
A
B
C
D
E
F
G
H
I
J
K
FDR (Age associated divergence) =0.0814
p(Scalp)=0.0714
p(Dura)=0.0385
Scalp
Dura

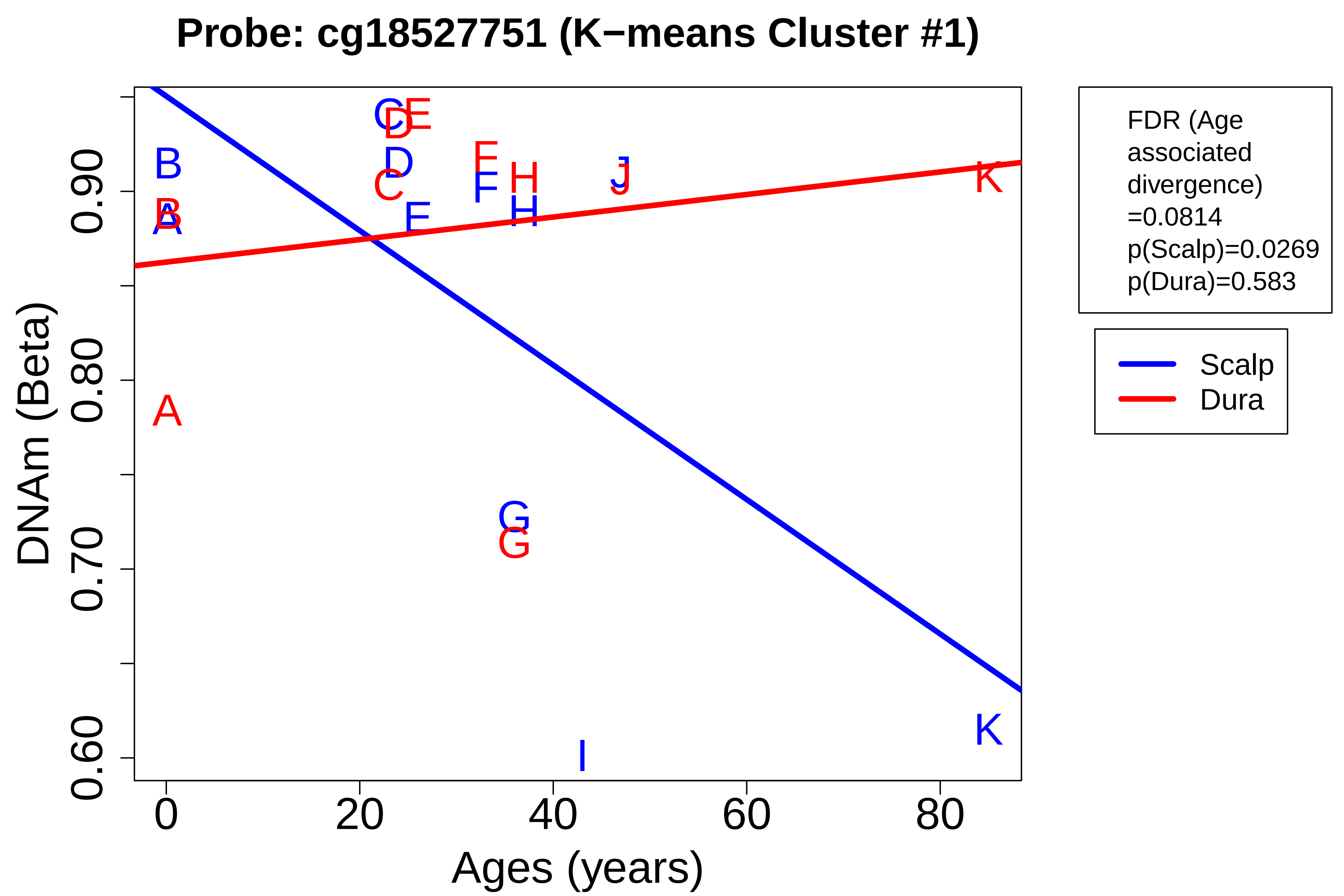

Probe: cg18527751 (K−means Cluster #1)
DNAm (Beta)
0.60
0.70
0.80
0.90
0
20
40
60
80
Ages (years)
A
B
C
D
E
F
G
H
I
J
K
FDR (Age associated divergence) =0.0814
p(Scalp)=0.0269
p(Dura)=0.583
Scalp
Dura

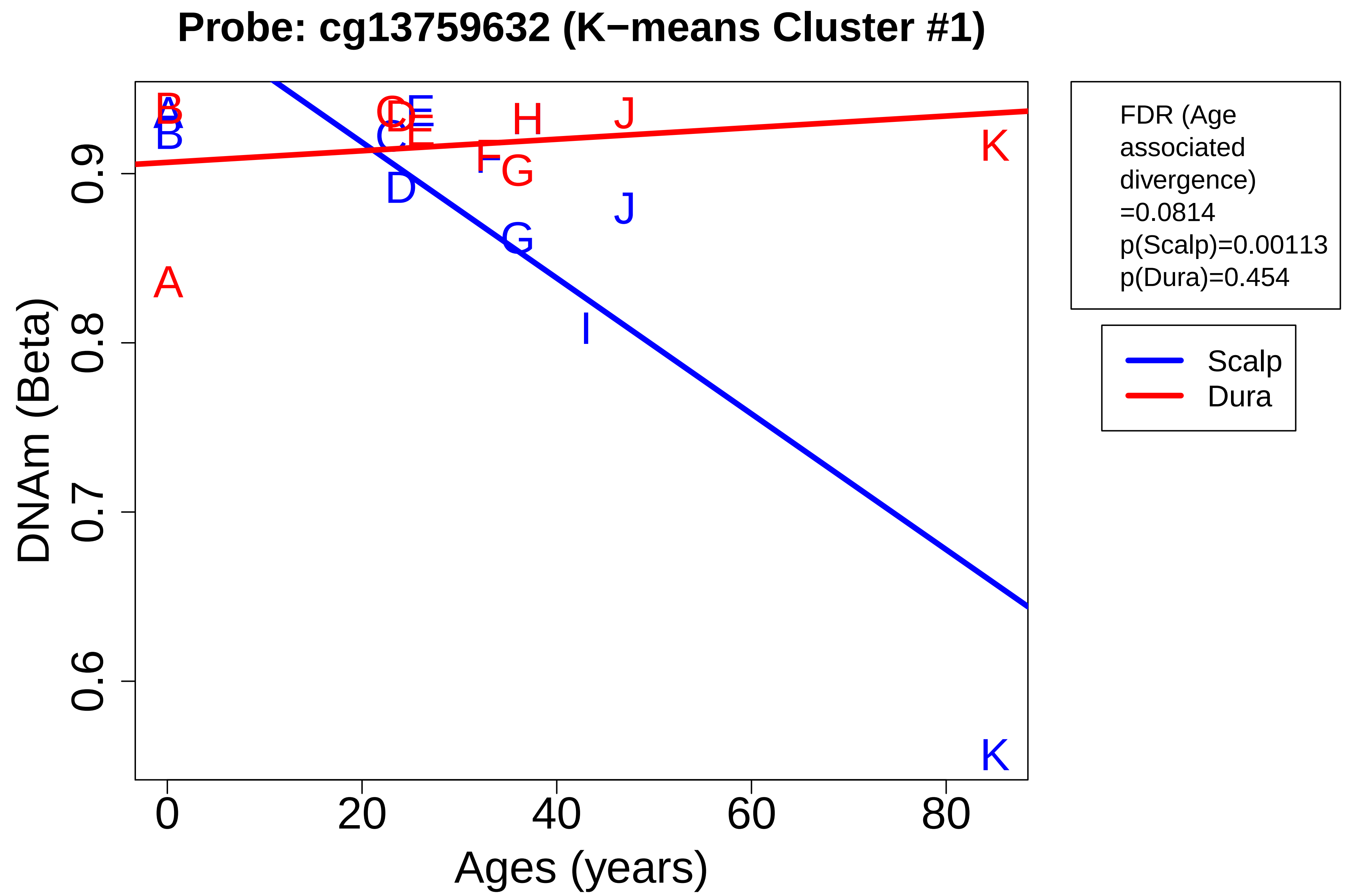
Probe: cg13759632 (K−means Cluster #1)
DNAm (Beta)
0.6
0.7
0.8
0.9
0
20
40
60
80
Ages (years)
FDR (Age associated divergence) =0.0814
p(Scalp)=0.00113
p(Dura)=0.454
Scalp
Dura
A
B
C
D
E
F
G
H
I
J
K

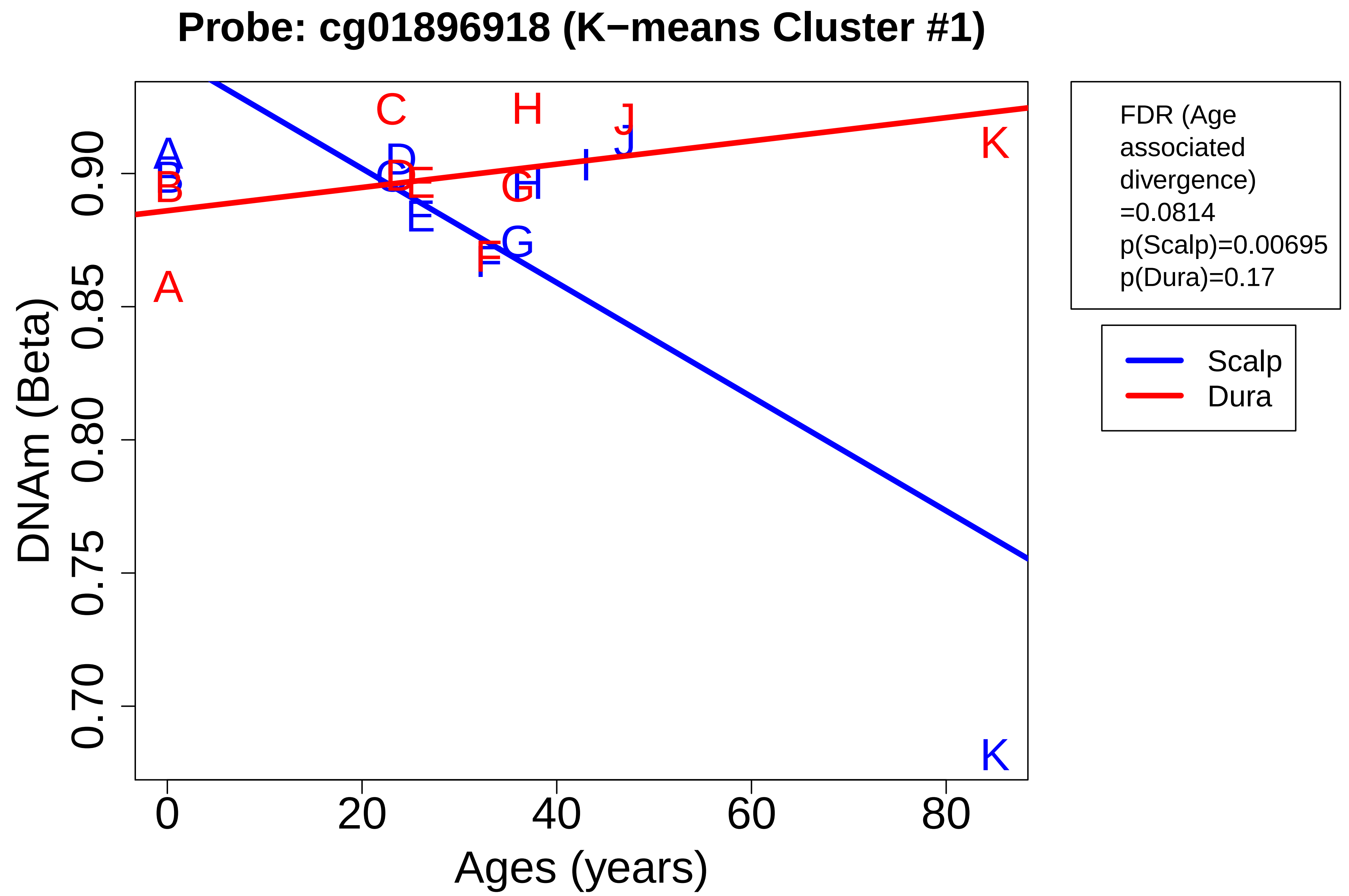

Probe: cg01896918 (K−means Cluster #1)
DNAm (Beta)
0.70
0.75
0.80
0.85
0.90
0
20
40
60
80
Ages (years)
A
B
C
D
E
F
G
H
I
J
K
FDR (Age associated divergence) =0.0814
p(Scalp)=0.00695
p(Dura)=0.17
Scalp
Dura

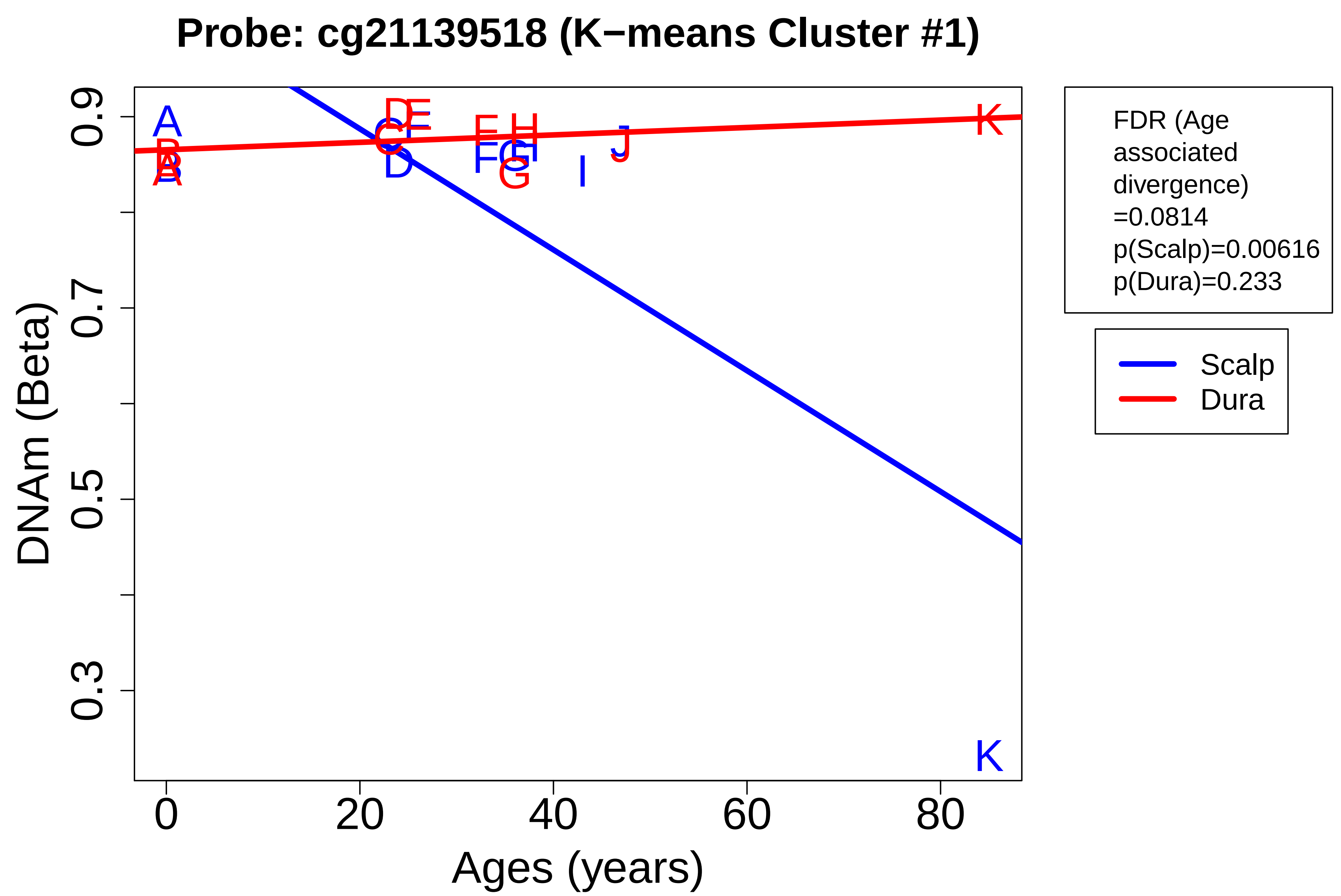
Probe: cg21139518 (K−means Cluster #1)
DNAm (Beta)
0.3
0.5
0.7
0.9
Ages (years)
0
20
40
60
80
A
B
C
D
E
F
G
H
I
J
K
FDR (Age associated divergence) =0.0814
p(Scalp)=0.00616
p(Dura)=0.233
Scalp
Dura

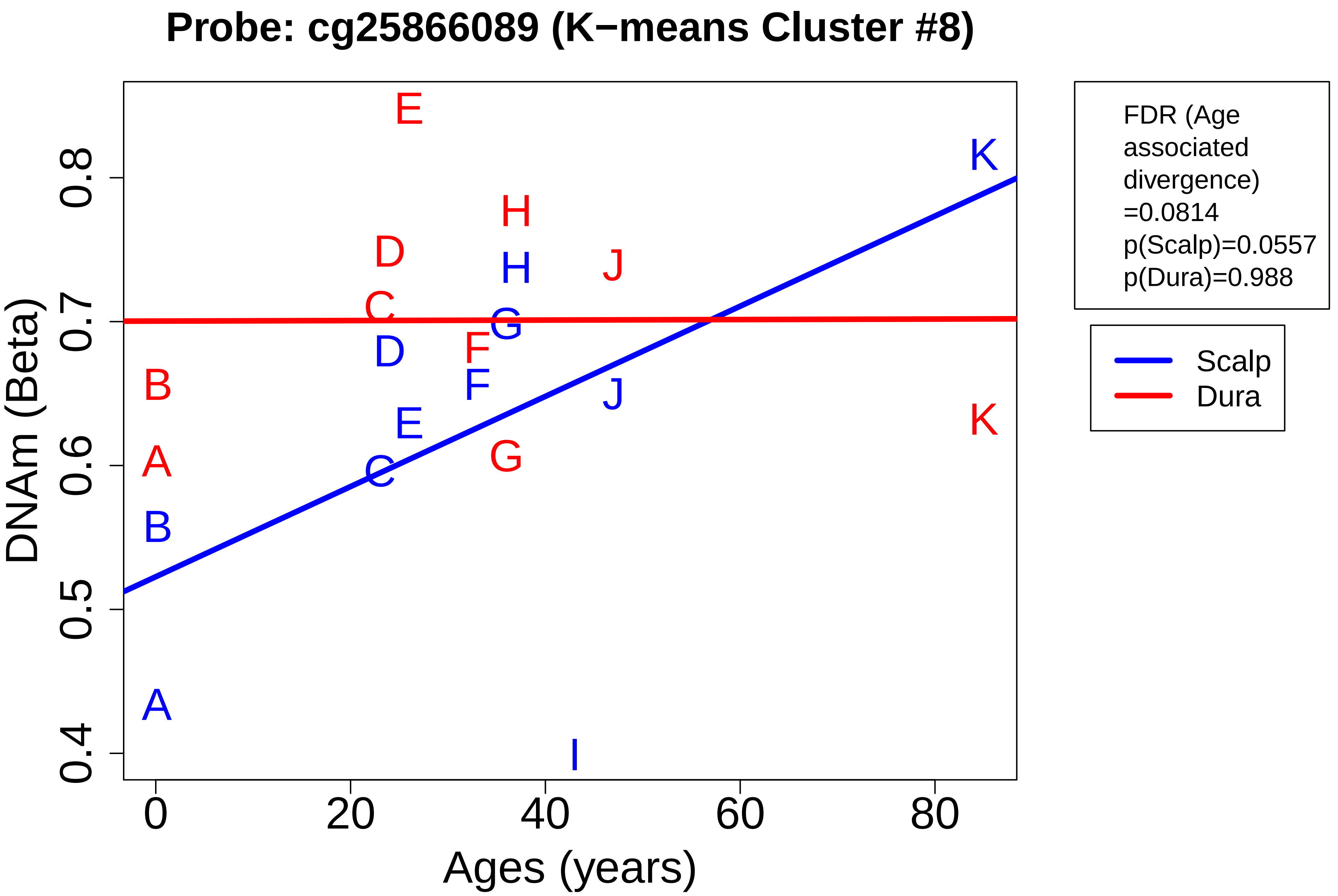

Probe: cg25866089 (K−means Cluster #8)
FDR (Age associated divergence) =0.0814
p(Scalp)=0.0557
p(Dura)=0.988
Scalp
Dura
DNAm (Beta)
0.4
0.5
0.6
0.7
0.8
0
20
40
60
80
Ages (years)

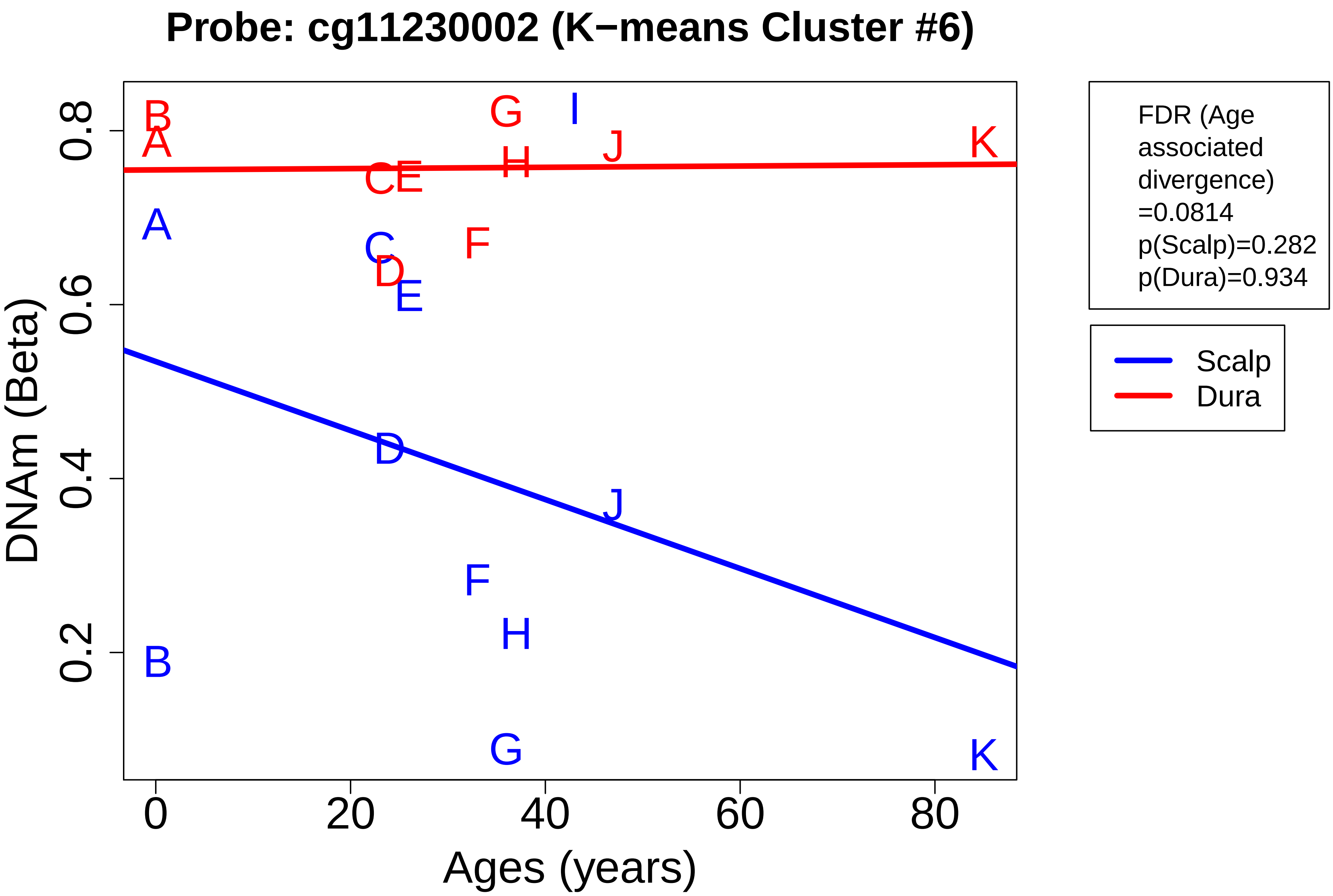

Probe: cg11230002 (K−means Cluster #6)
DNAm (Beta)
0.2
0.4
0.6
0.8
Ages (years)
0
20
40
60
80
FDR (Age associated divergence) =0.0814
p(Scalp)=0.282
p(Dura)=0.934
Scalp
Dura

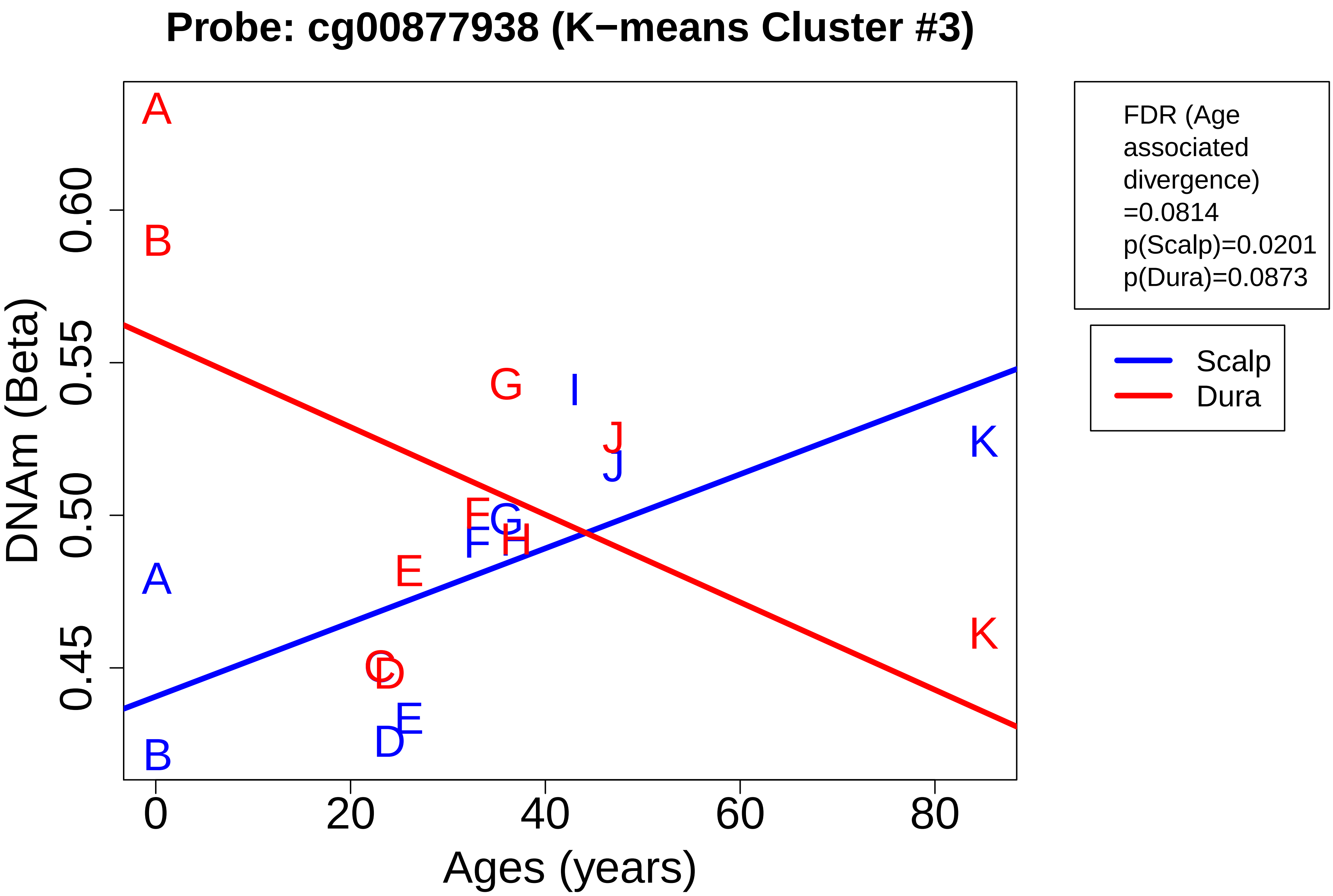

Probe: cg00877938 (K−means Cluster #3)
DNAm (Beta)
0.45
0.50
0.55
0.60
Ages (years)
0
20
40
60
80
A
B
C
D
E
F
G
H
I
J
K
FDR (Age associated divergence) =0.0814
p(Scalp)=0.0201
p(Dura)=0.0873
Scalp
Dura

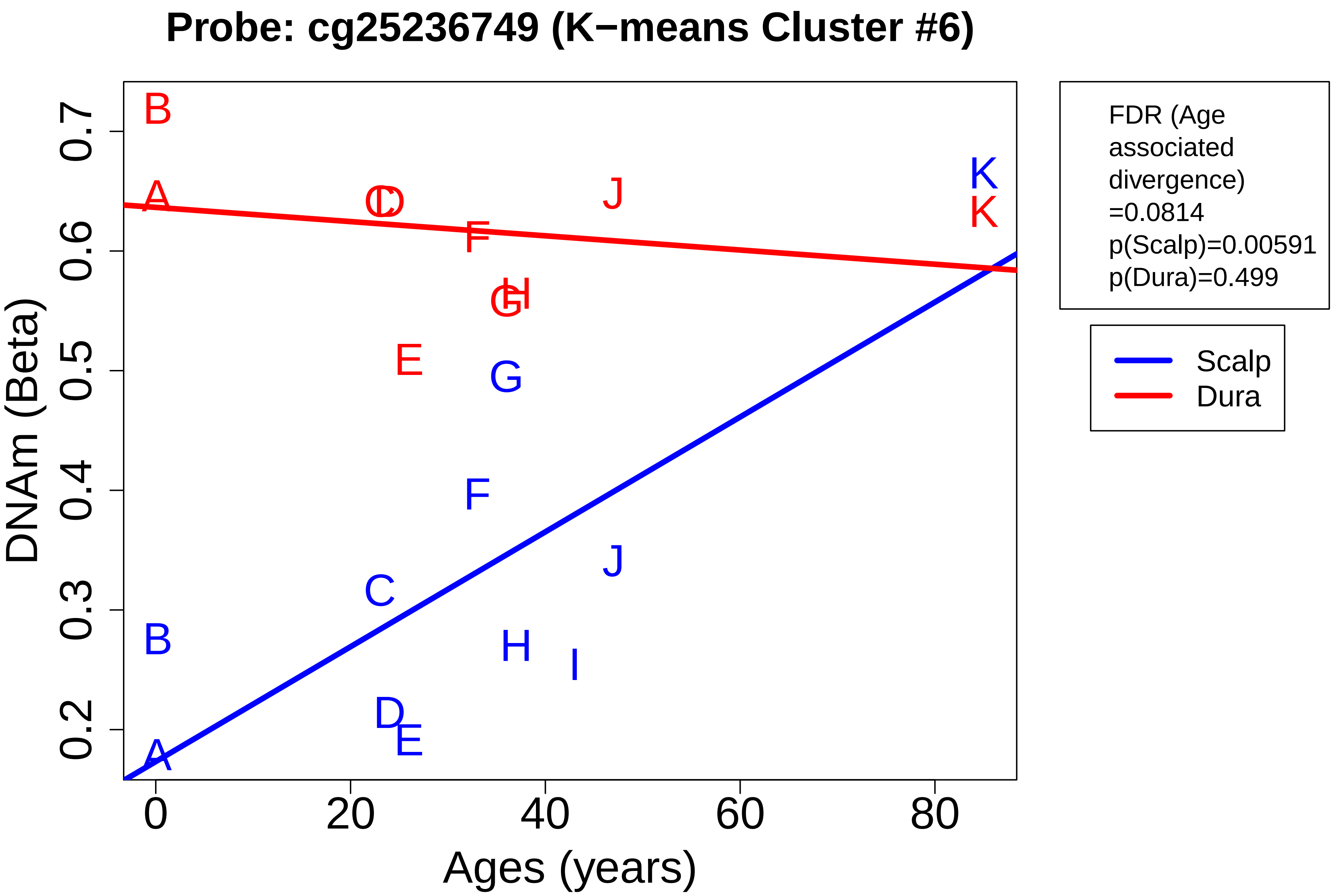

Probe: cg25236749 (K−means Cluster #6)
DNAm (Beta)
0.2
0.3
0.4
0.5
0.6
0.7
0
20
40
60
80
Ages (years)
FDR (Age associated divergence) =0.0814
p(Scalp)=0.00591
p(Dura)=0.499
Scalp
Dura

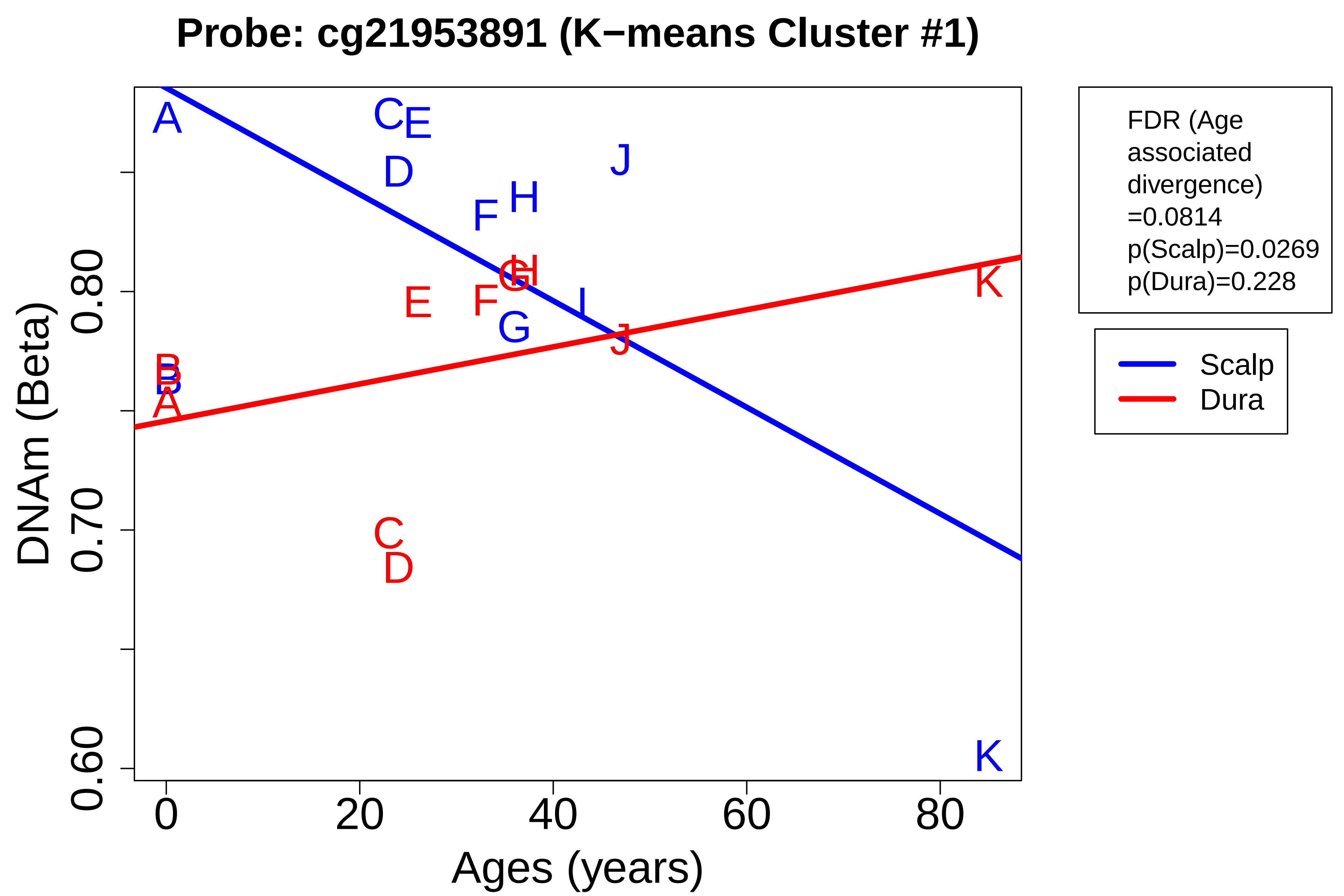

Probe: cg21953891 (K−means Cluster #1)
DNAm (Beta)
0.60
0.70
0.80
0
20
40
60
80
Ages (years)
FDR (Age associated divergence) =0.0814
p(Scalp)=0.0269
p(Dura)=0.228
Scalp
Dura
A
B
C
D
E
F
G
H
I
J
K

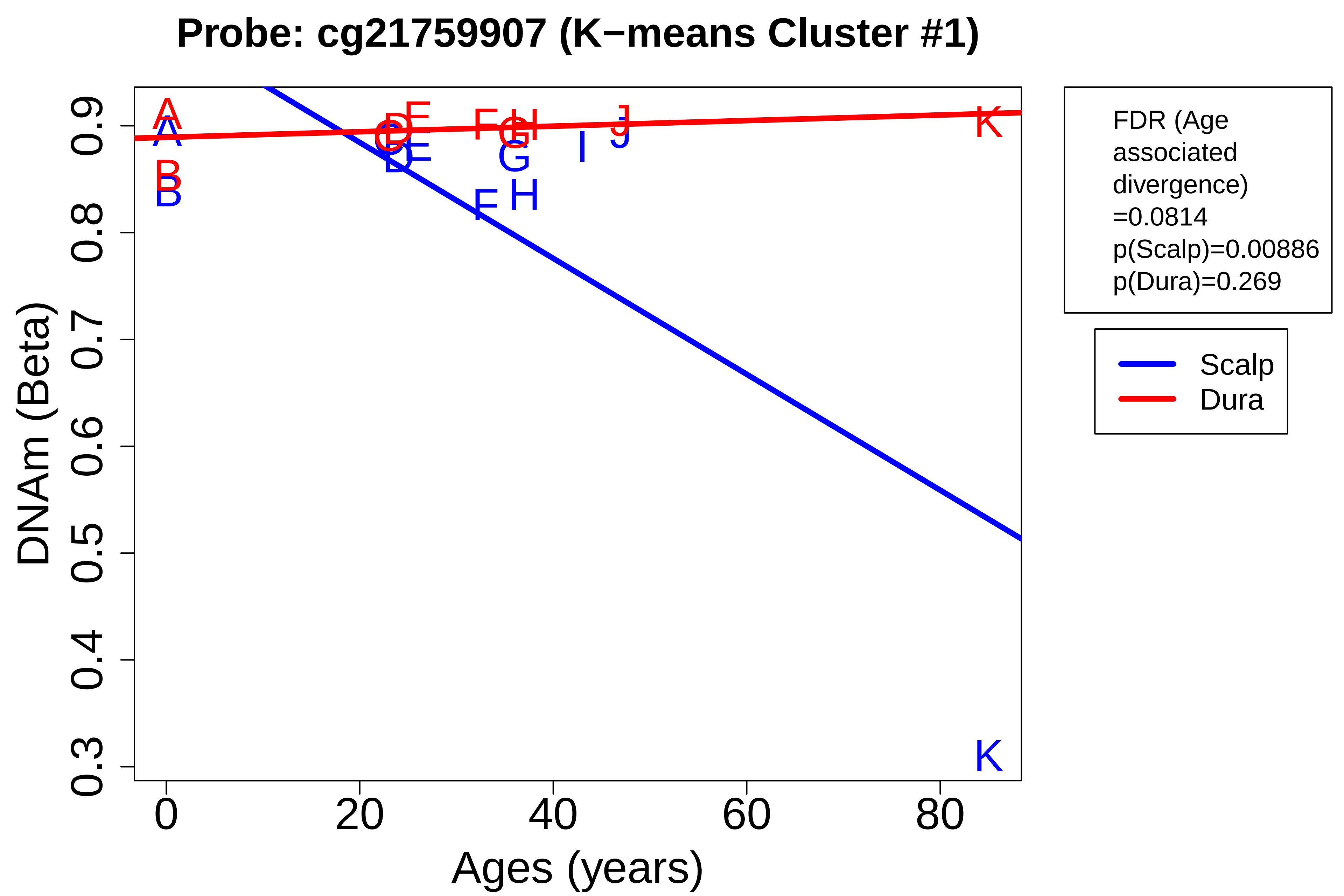

Probe: cg21759907 (K−means Cluster #1)
DNAm (Beta)
Ages (years)
0.3
0.4
0.5
0.6
0.7
0.8
0.9
0
20
40
60
80
A
B
C
D
E
F
G
H
I
J
K
FDR (Age associated divergence) =0.0814
p(Scalp)=0.00886
p(Dura)=0.269
Scalp
Dura

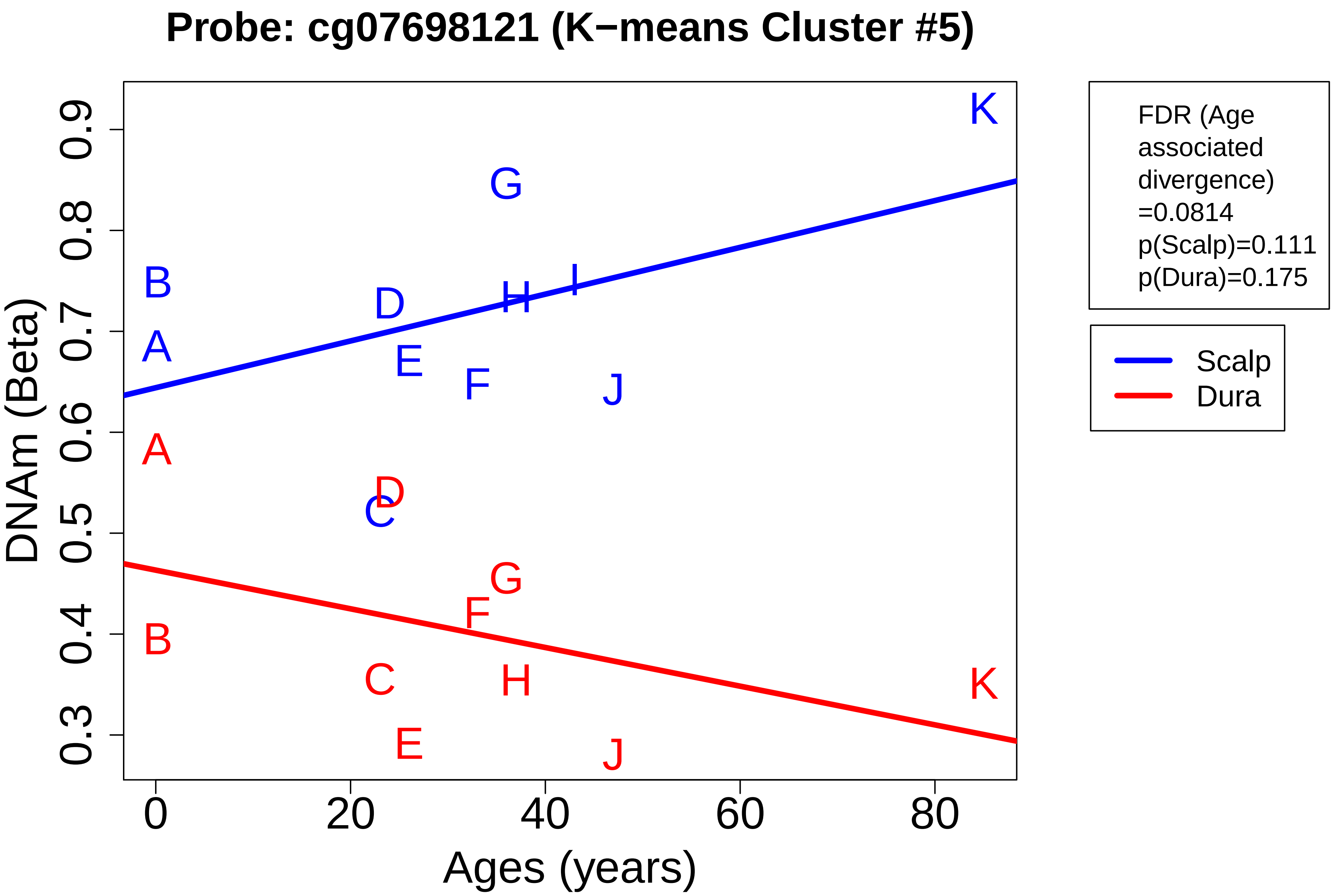

Probe: cg07698121 (K−means Cluster #5)
DNAm (Beta)
0.3
0.4
0.5
0.6
0.7
0.8
0.9
Ages (years)
0
20
40
60
80
A B C D E F G H I J K
FDR (Age associated divergence) =0.0814
p(Scalp)=0.111
p(Dura)=0.175
Scalp
Dura

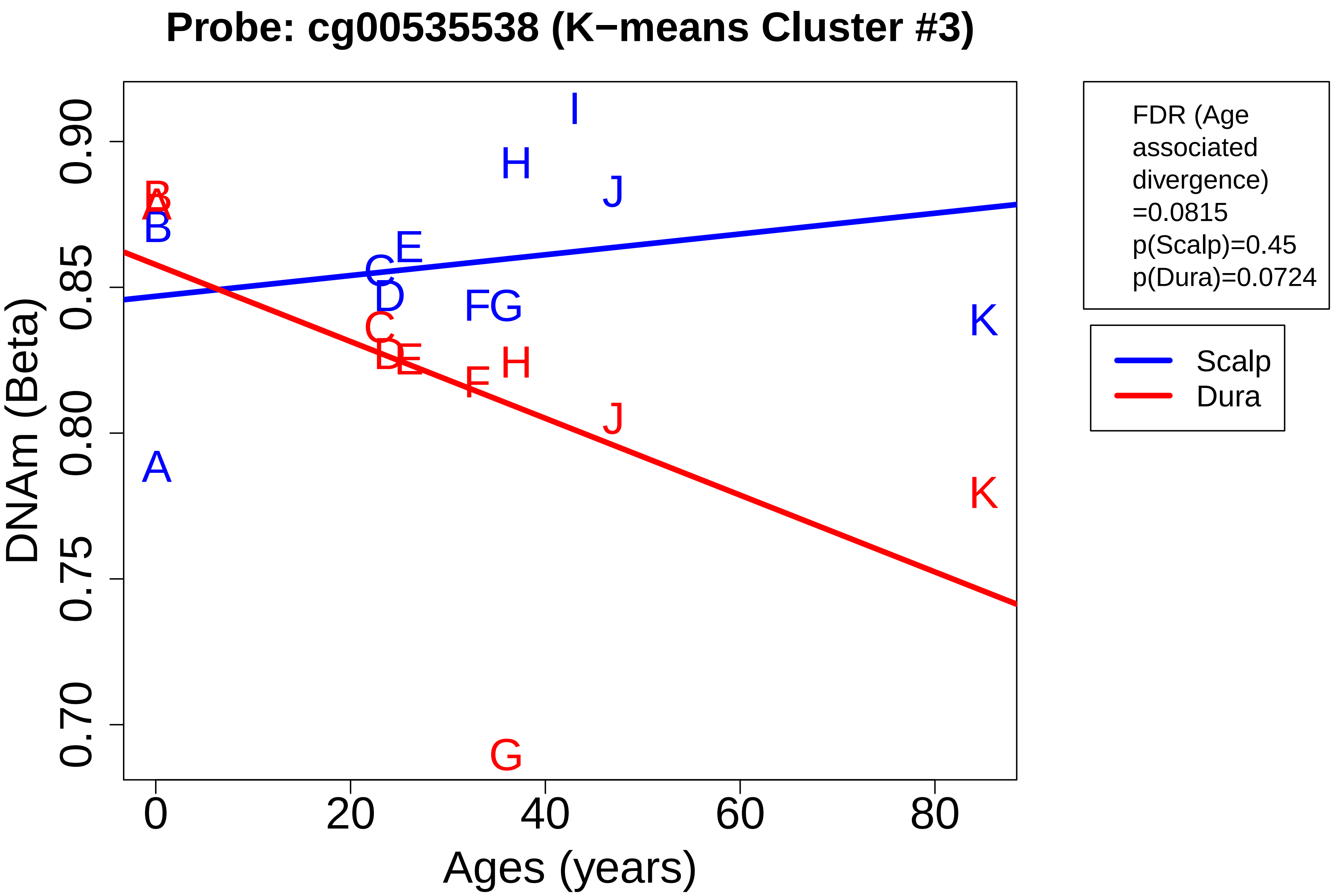

Probe: cg00535538 (K−means Cluster #3)
DNAm (Beta)
0.70
0.75
0.80
0.85
0.90
Ages (years)
0
20
40
60
80
FDR (Age associated divergence) =0.0815
p(Scalp)=0.45
p(Dura)=0.0724
Scalp
Dura

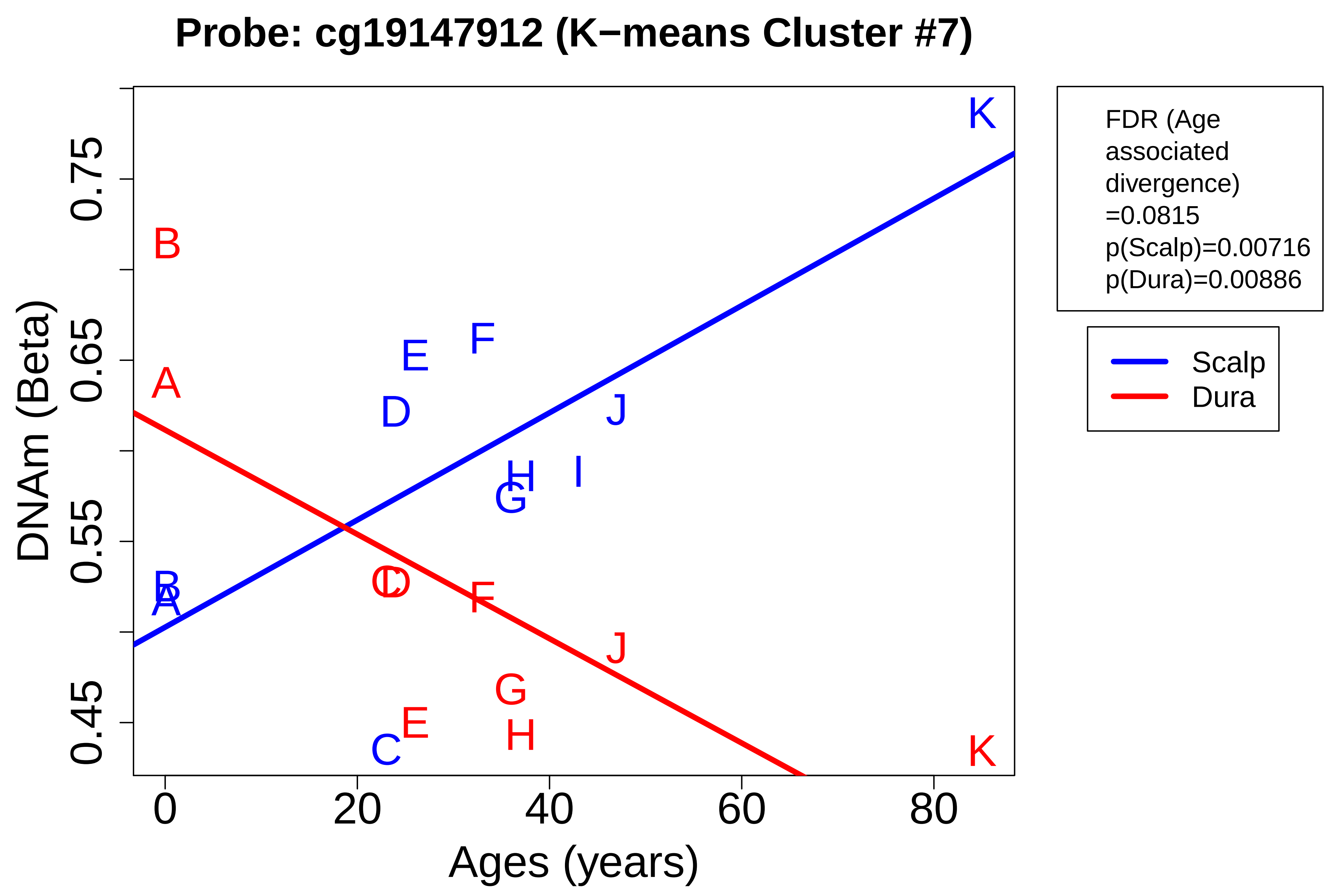

Probe: cg19147912 (K−means Cluster #7)
FDR (Age associated divergence) =0.0815
p(Scalp)=0.00716
p(Dura)=0.00886
Scalp
Dura
DNAm (Beta)
0.45
0.55
0.65
0.75
0
20
40
60
80
Ages (years)

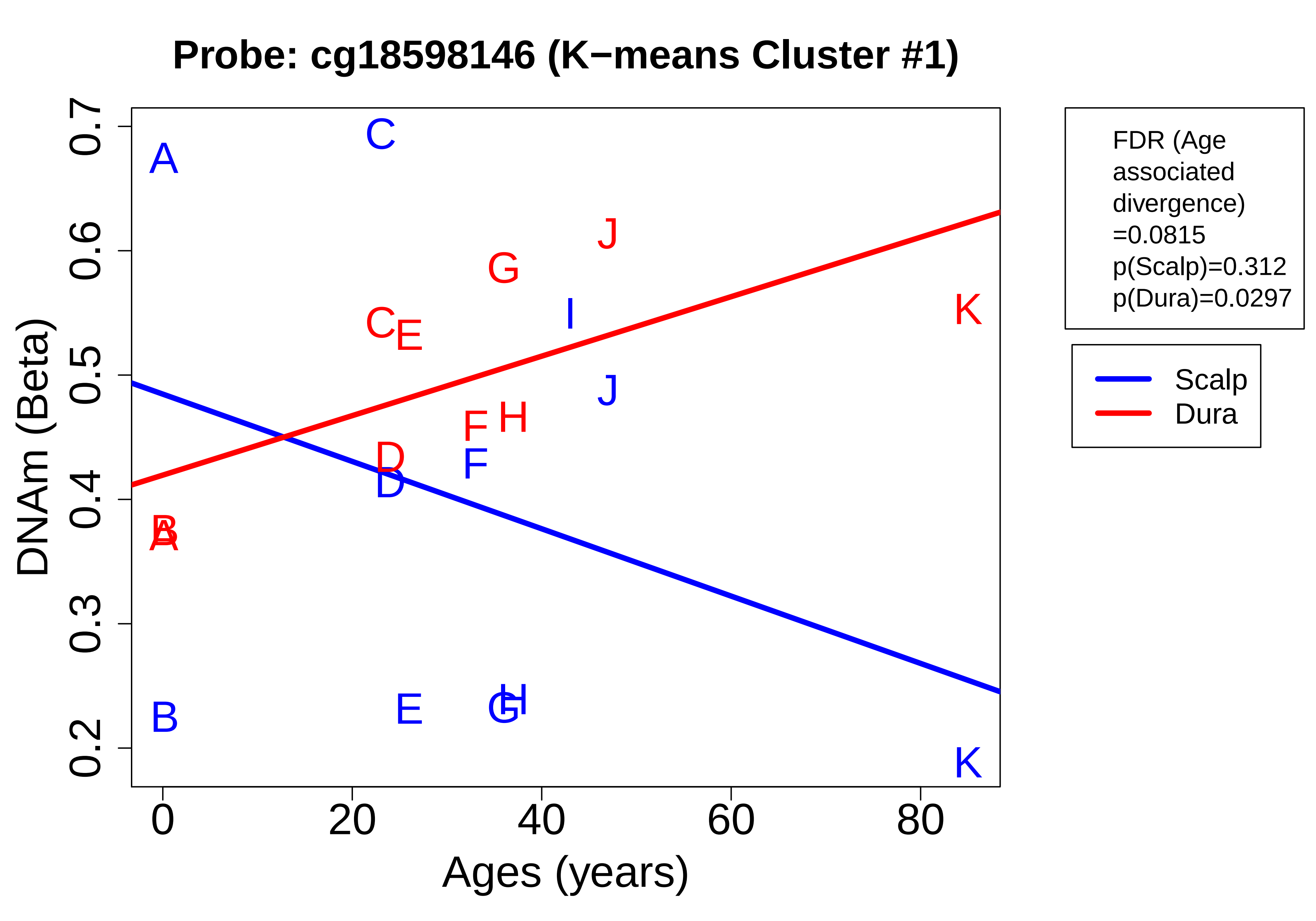

Probe: cg18598146 (K−means Cluster #1)
DNAm (Beta)
Ages (years)
0.2
0.3
0.4
0.5
0.6
0.7
0
20
40
60
80
A
B
C
D
E
F
G
H
I
J
K
FDR (Age associated divergence) =0.0815
p(Scalp)=0.312
p(Dura)=0.0297
Scalp
Dura

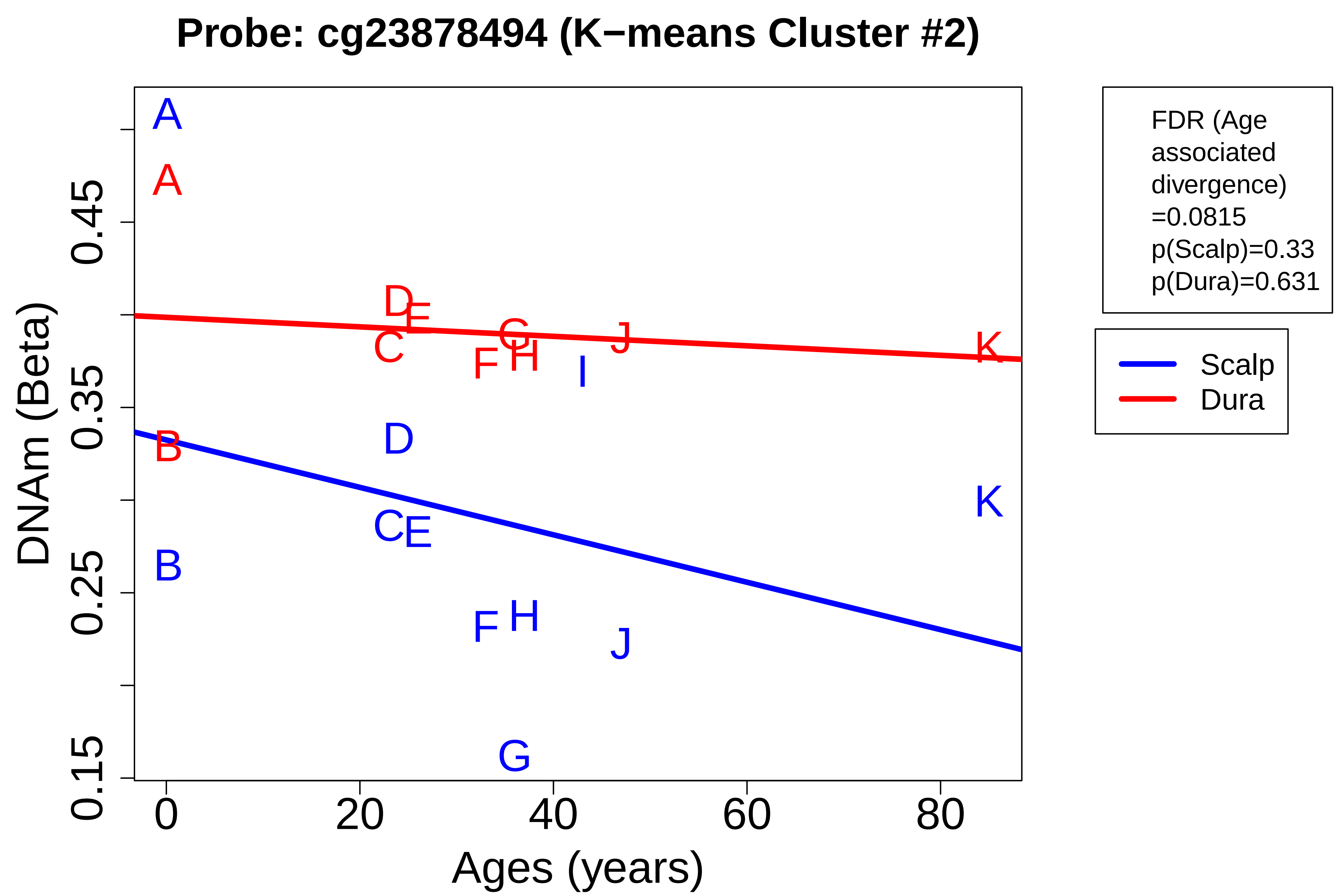

Probe: cg23878494 (K−means Cluster #2)
DNAm (Beta)
0.15
0.25
0.35
0.45
0
20
40
60
80
Ages (years)
A
A
D
E
C
G
H
F
J
I
K
B
D
K
C
E
B
F
H
J
G
FDR (Age associated divergence) =0.0815
p(Scalp)=0.33
p(Dura)=0.631
Scalp
Dura

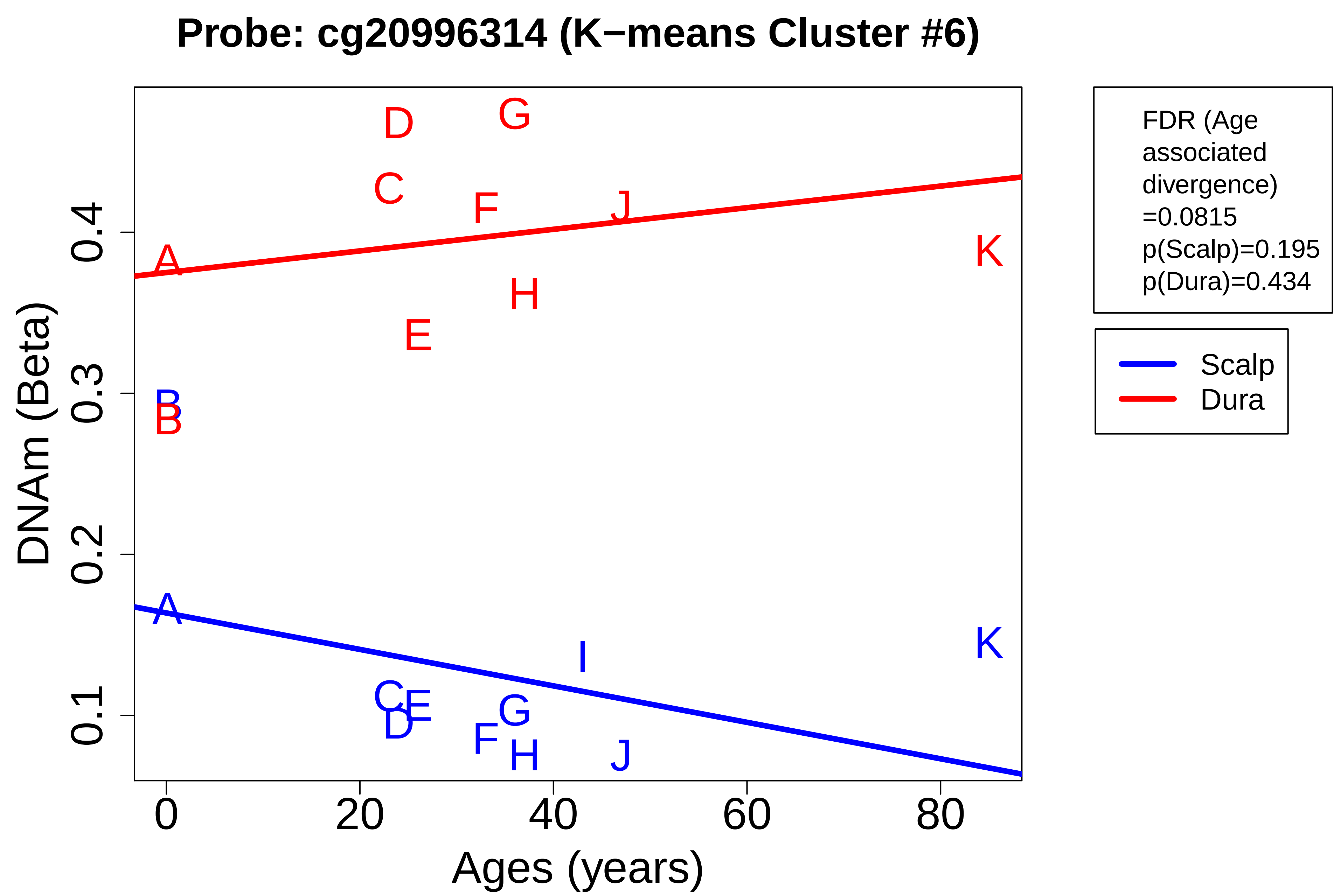

Probe: cg20996314 (K−means Cluster #6)
FDR (Age associated divergence) =0.0815
p(Scalp)=0.195
p(Dura)=0.434
Scalp
Dura
DNAm (Beta)
Ages (years)
0.1
0.2
0.3
0.4
0
20
40
60
80

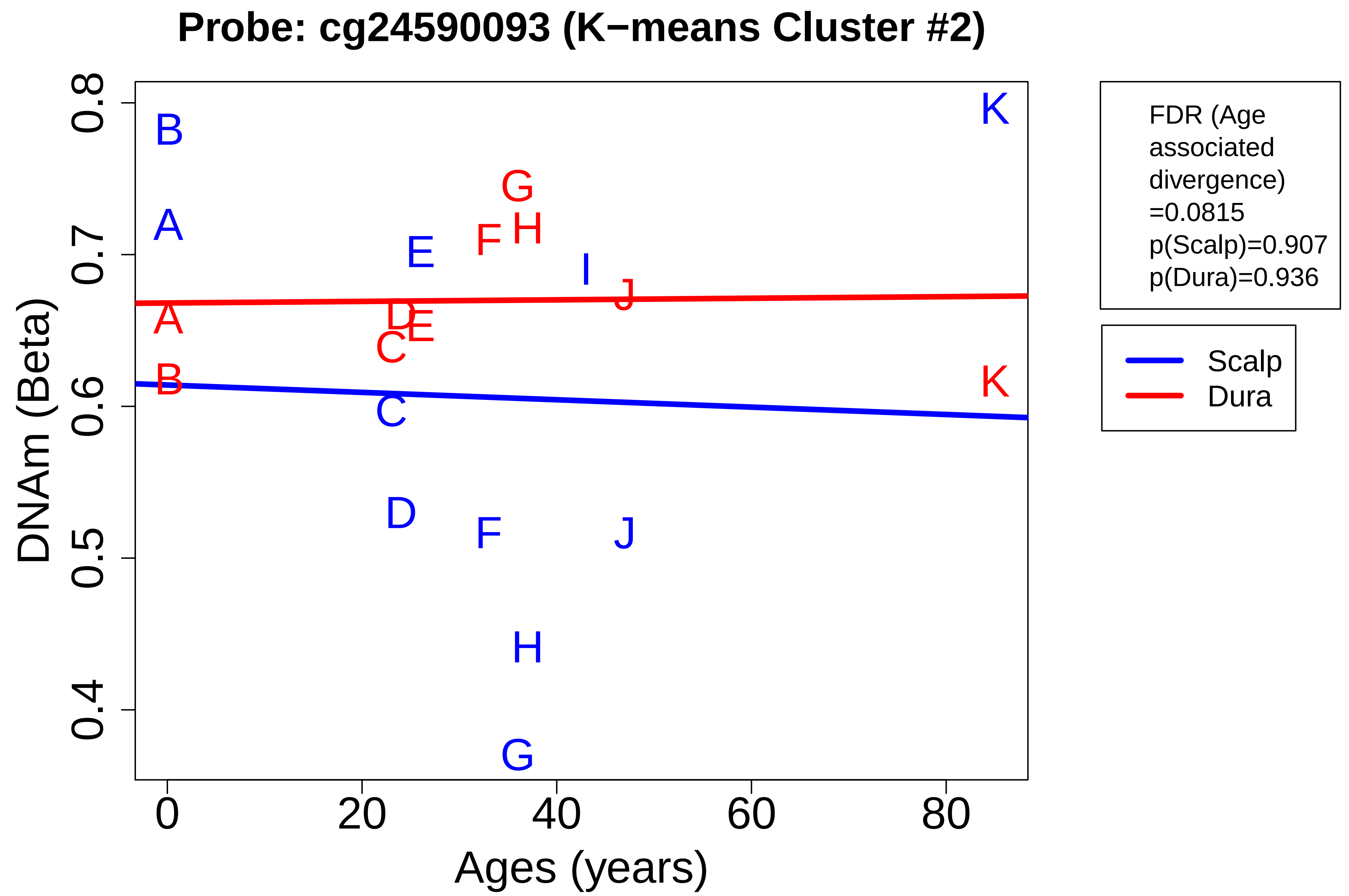

Probe: cg24590093 (K−means Cluster #2)
DNAm (Beta)
0.4
0.5
0.6
0.7
0.8
0
20
40
60
80
Ages (years)
FDR (Age associated divergence) =0.0815
p(Scalp)=0.907
p(Dura)=0.936
Scalp
Dura

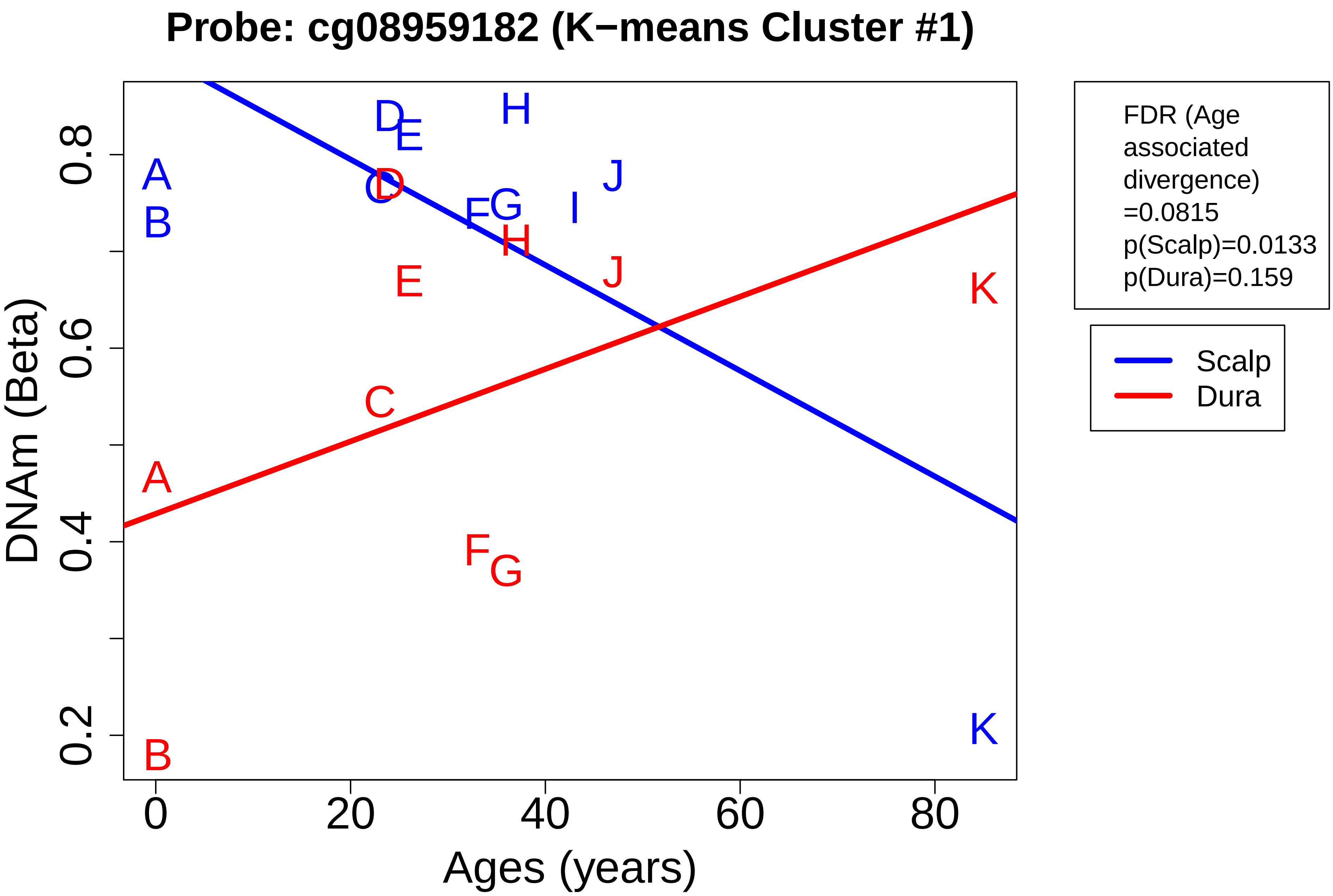
Probe: cg08959182 (K−means Cluster #1)
DNAm (Beta)
0.2
0.4
0.6
0.8
0
20
40
60
80
Ages (years)
A
B
C
D
E
F
G
H
I
J
K
A
B
C
D
E
F
G
H
J
K
FDR (Age associated divergence) =0.0815
p(Scalp)=0.0133
p(Dura)=0.159
Scalp
Dura

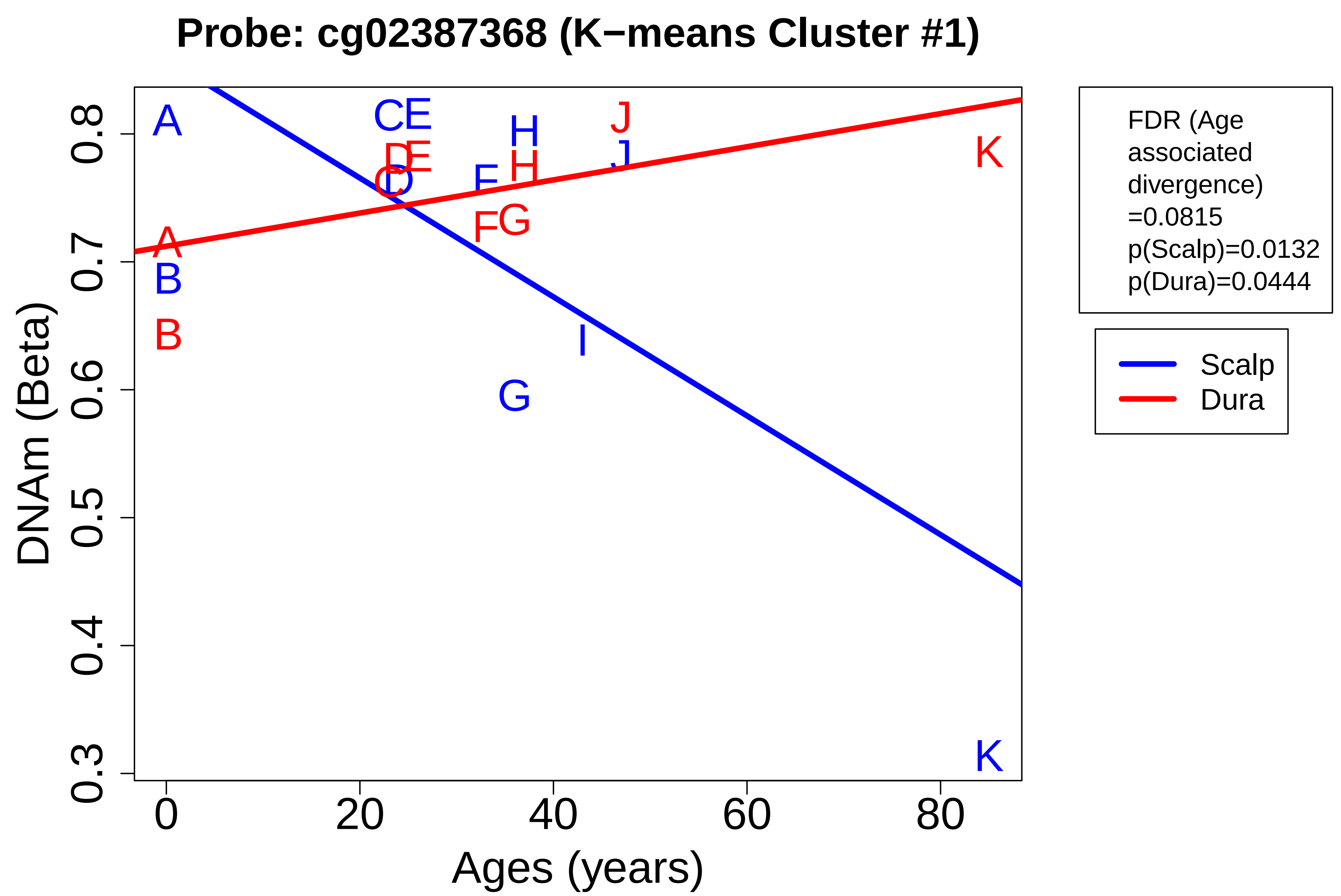

Probe: cg02387368 (K−means Cluster #1)
DNAm (Beta)
0.3
0.4
0.5
0.6
0.7
0.8
0
20
40
60
80
Ages (years)
FDR (Age associated divergence) =0.0815
p(Scalp)=0.0132
p(Dura)=0.0444
Scalp
Dura

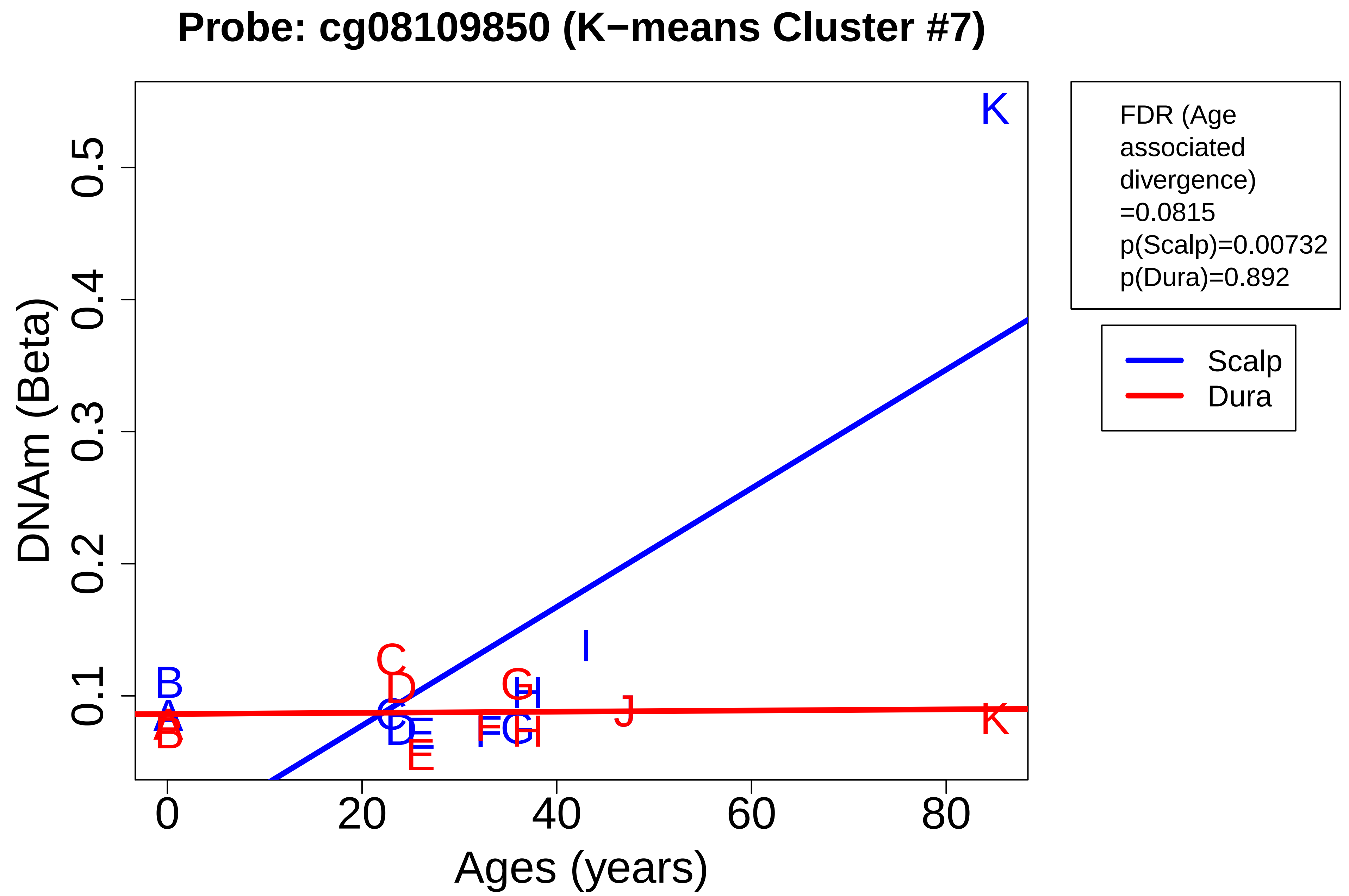

Probe: cg08109850 (K−means Cluster #7)
DNAm (Beta)
0.1
0.2
0.3
0.4
0.5
0
20
40
60
80
Ages (years)
FDR (Age associated divergence) =0.0815
p(Scalp)=0.00732
p(Dura)=0.892
Scalp
Dura

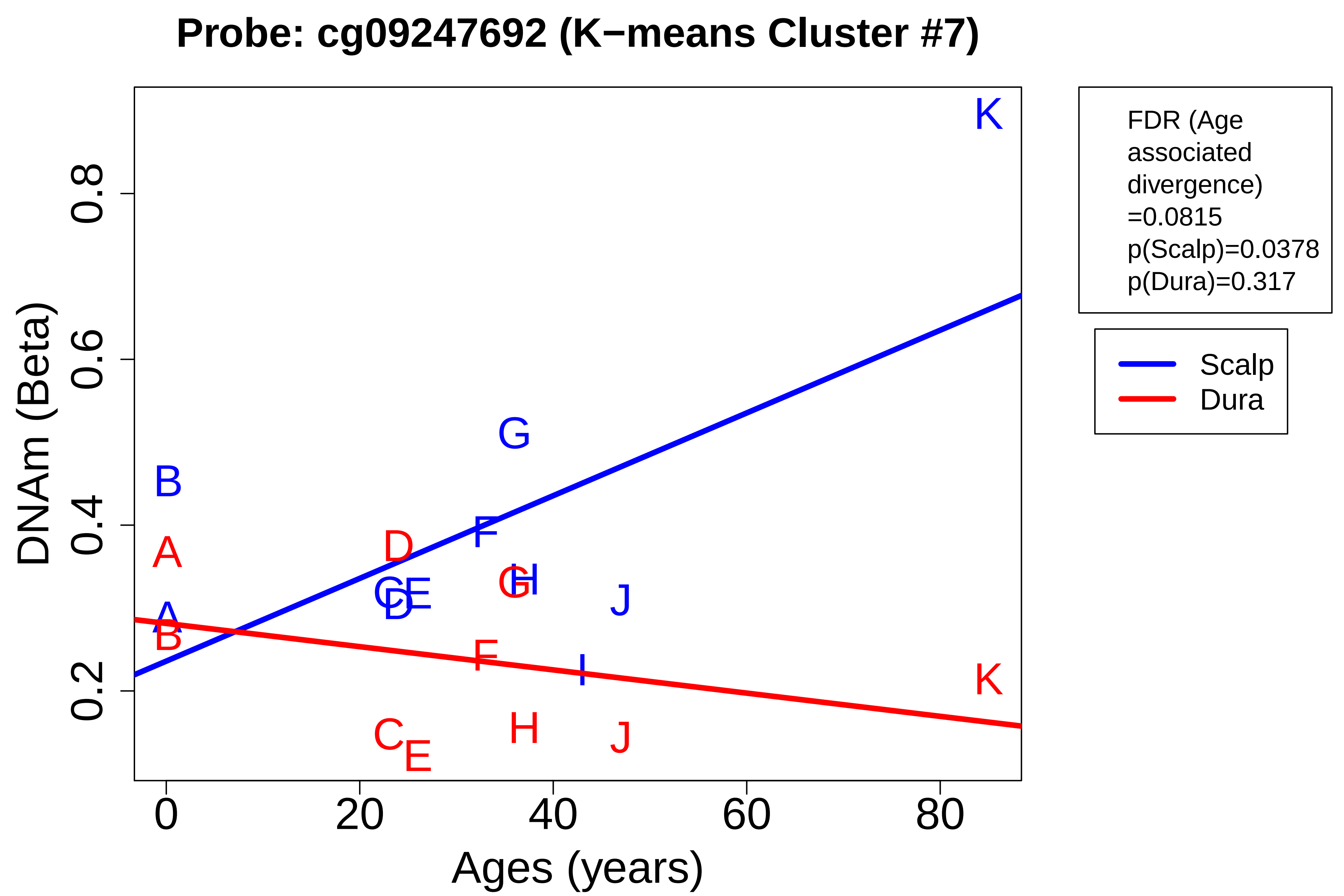
Probe: cg09247692 (K−means Cluster #7)
DNAm (Beta)
0.2
0.4
0.6
0.8
Ages (years)
0
20
40
60
80
FDR (Age associated divergence) =0.0815
p(Scalp)=0.0378
p(Dura)=0.317
Scalp
Dura

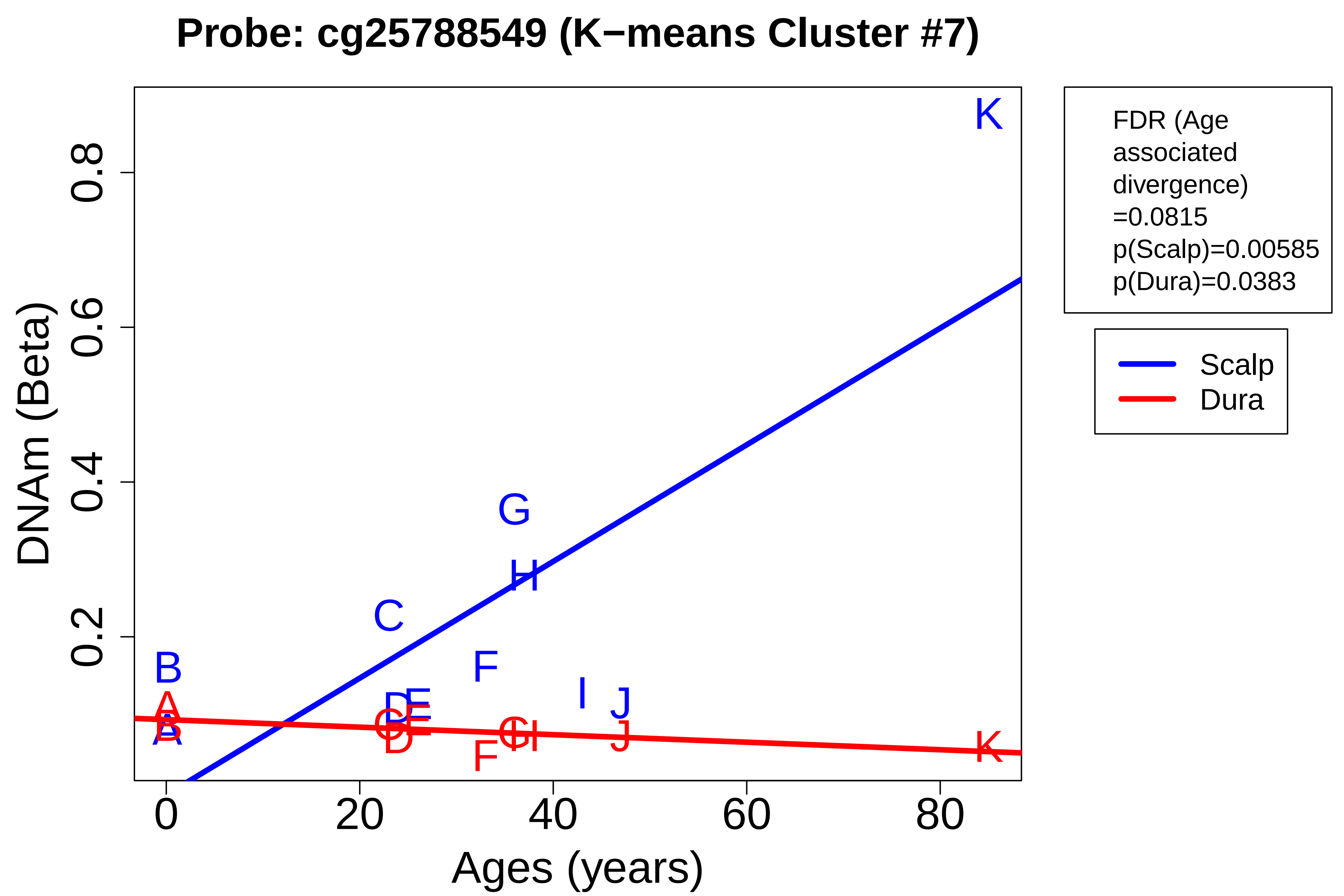

Probe: cg25788549 (K−means Cluster #7)
DNAm (Beta)
0.2
0.4
0.6
0.8
0
20
40
60
80
Ages (years)
A
B
C
D
E
F
G
H
I
J
K
FDR (Age associated divergence) =0.0815
p(Scalp)=0.00585
p(Dura)=0.0383
Scalp
Dura

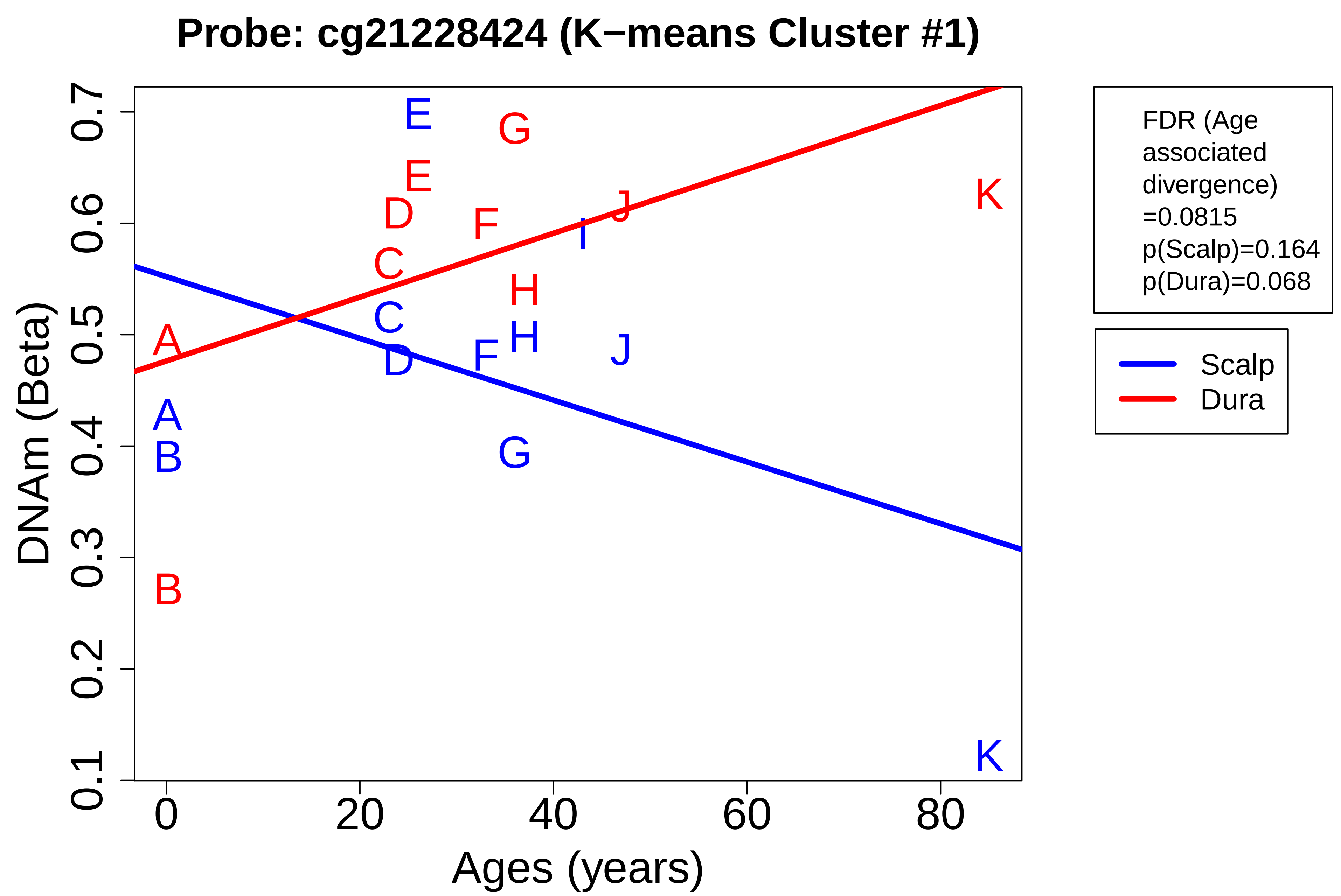

Probe: cg21228424 (K−means Cluster #1)
FDR (Age associated divergence) =0.0815
p(Scalp)=0.164
p(Dura)=0.068
Scalp
Dura
DNAm (Beta)
0.1
0.2
0.3
0.4
0.5
0.6
0.7
Ages (years)
0
20
40
60
80
A
B
C
D
E
F
G
H
I
J
K

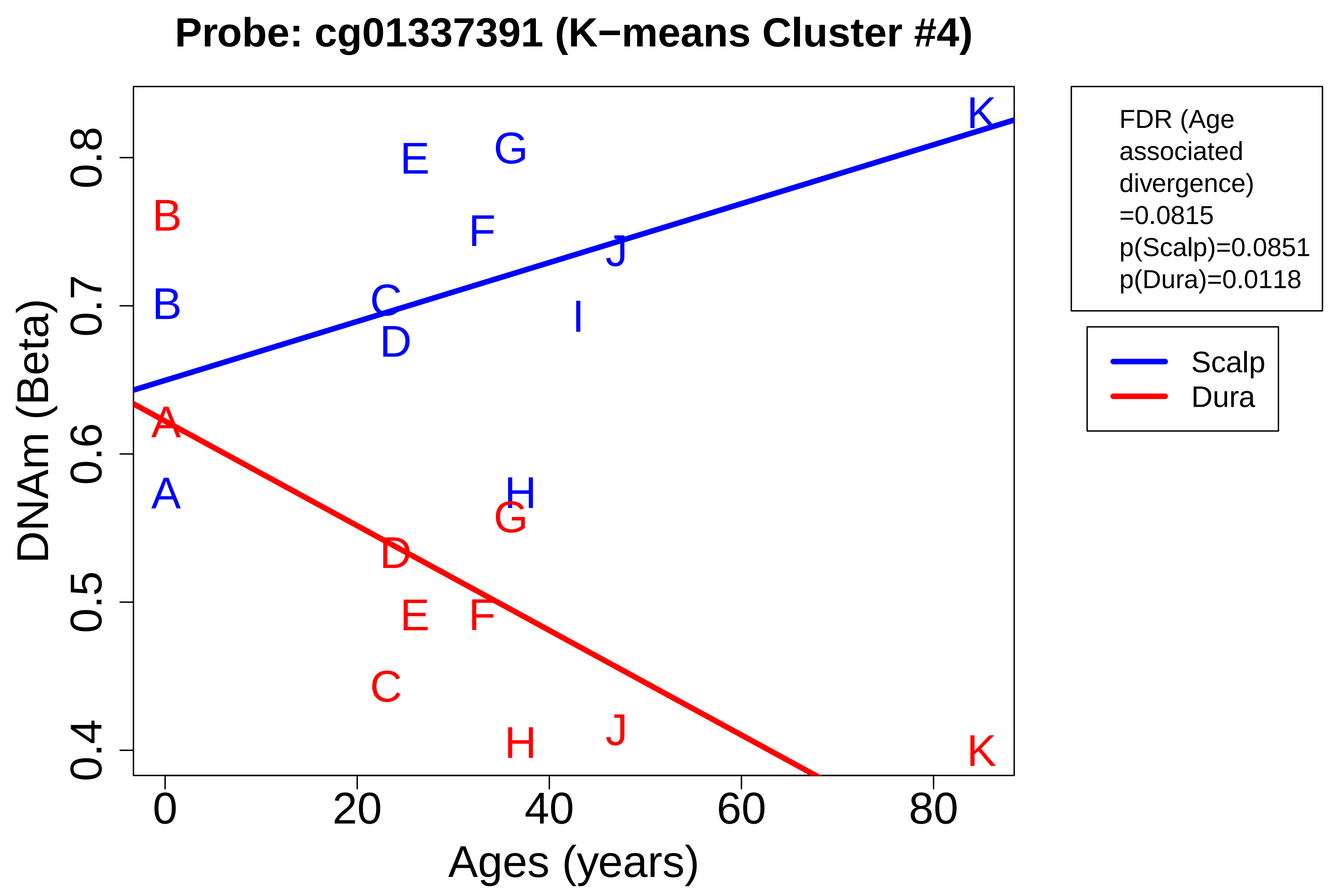
Probe: cg01337391 (K−means Cluster #4)
DNAm (Beta)
0.4
0.5
0.6
0.7
0.8
0
20
40
60
80
Ages (years)
FDR (Age associated divergence) =0.0815
p(Scalp)=0.0851
p(Dura)=0.0118
Scalp
Dura

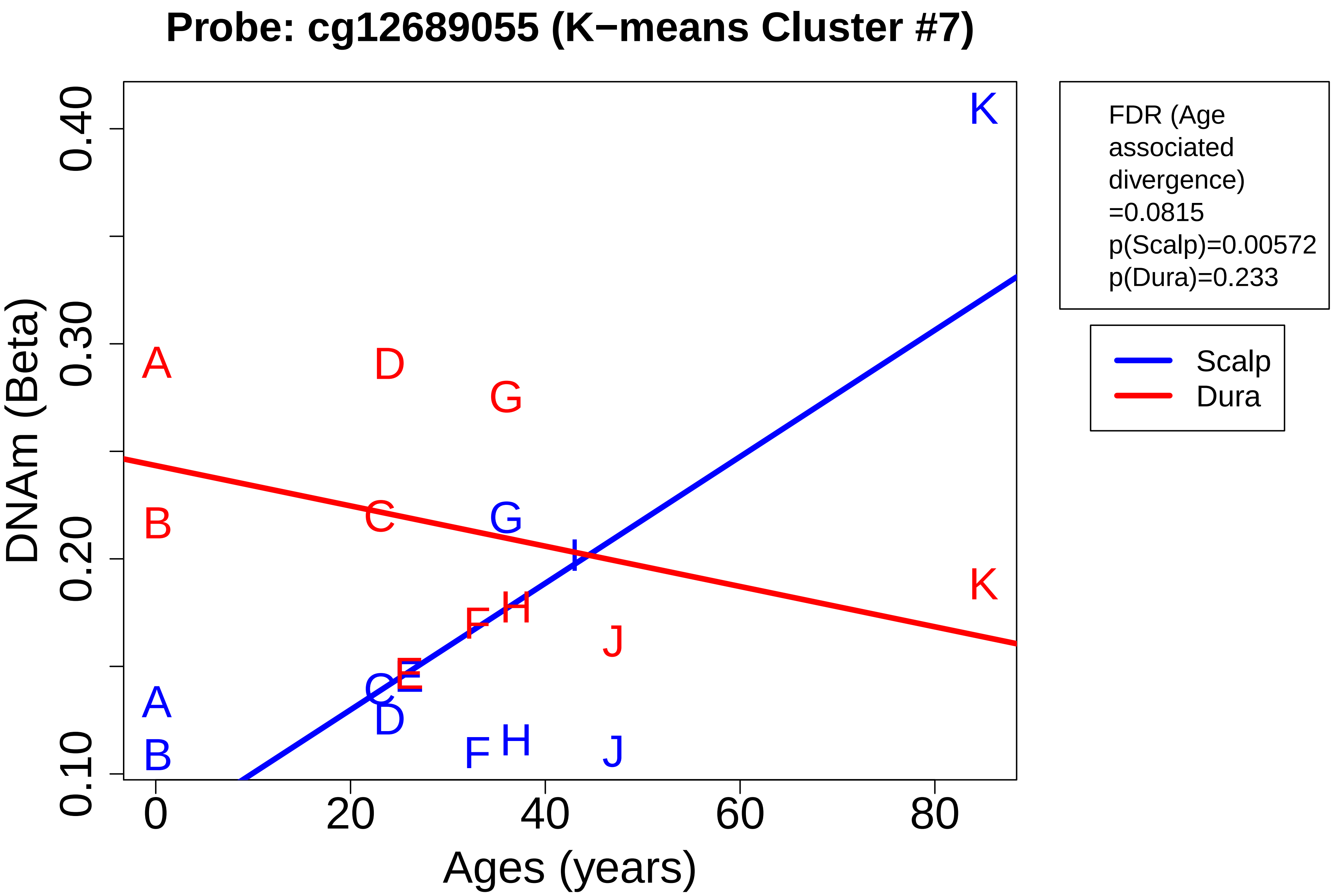
Probe: cg12689055 (K−means Cluster #7)
DNAm (Beta)
0.10
0.20
0.30
0.40
0
20
40
60
80
Ages (years)
FDR (Age associated divergence) =0.0815
p(Scalp)=0.00572
p(Dura)=0.233
Scalp
Dura

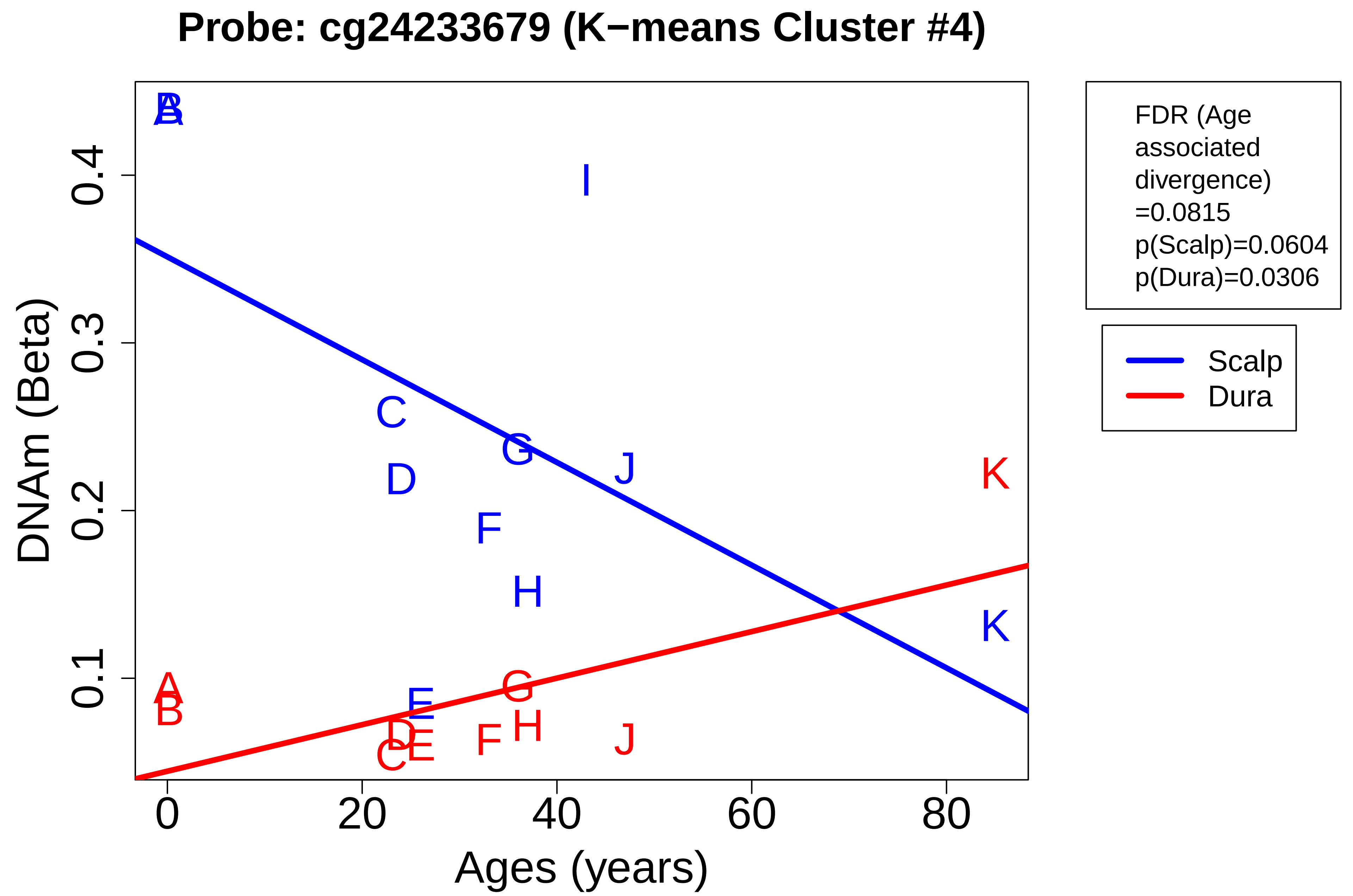

Probe: cg24233679 (K−means Cluster #4)
DNAm (Beta)
0.1
0.2
0.3
0.4
0
20
40
60
80
Ages (years)
FDR (Age associated divergence) =0.0815
p(Scalp)=0.0604
p(Dura)=0.0306
Scalp
Dura

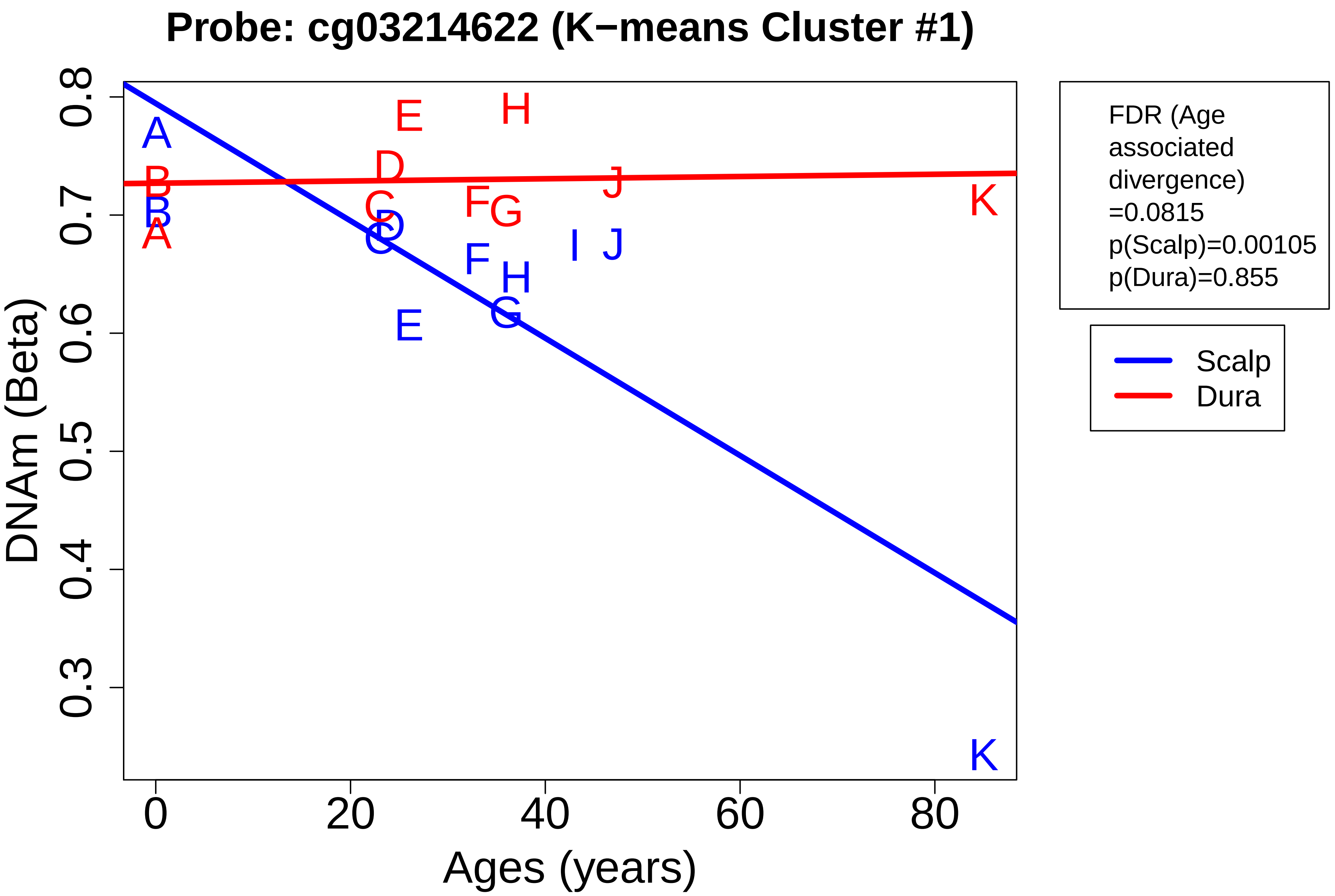

Probe: cg03214622 (K−means Cluster #1)
DNAm (Beta)
0.3
0.4
0.5
0.6
0.7
0.8
Ages (years)
0
20
40
60
80
FDR (Age associated divergence) =0.0815
p(Scalp)=0.00105
p(Dura)=0.855
Scalp
Dura

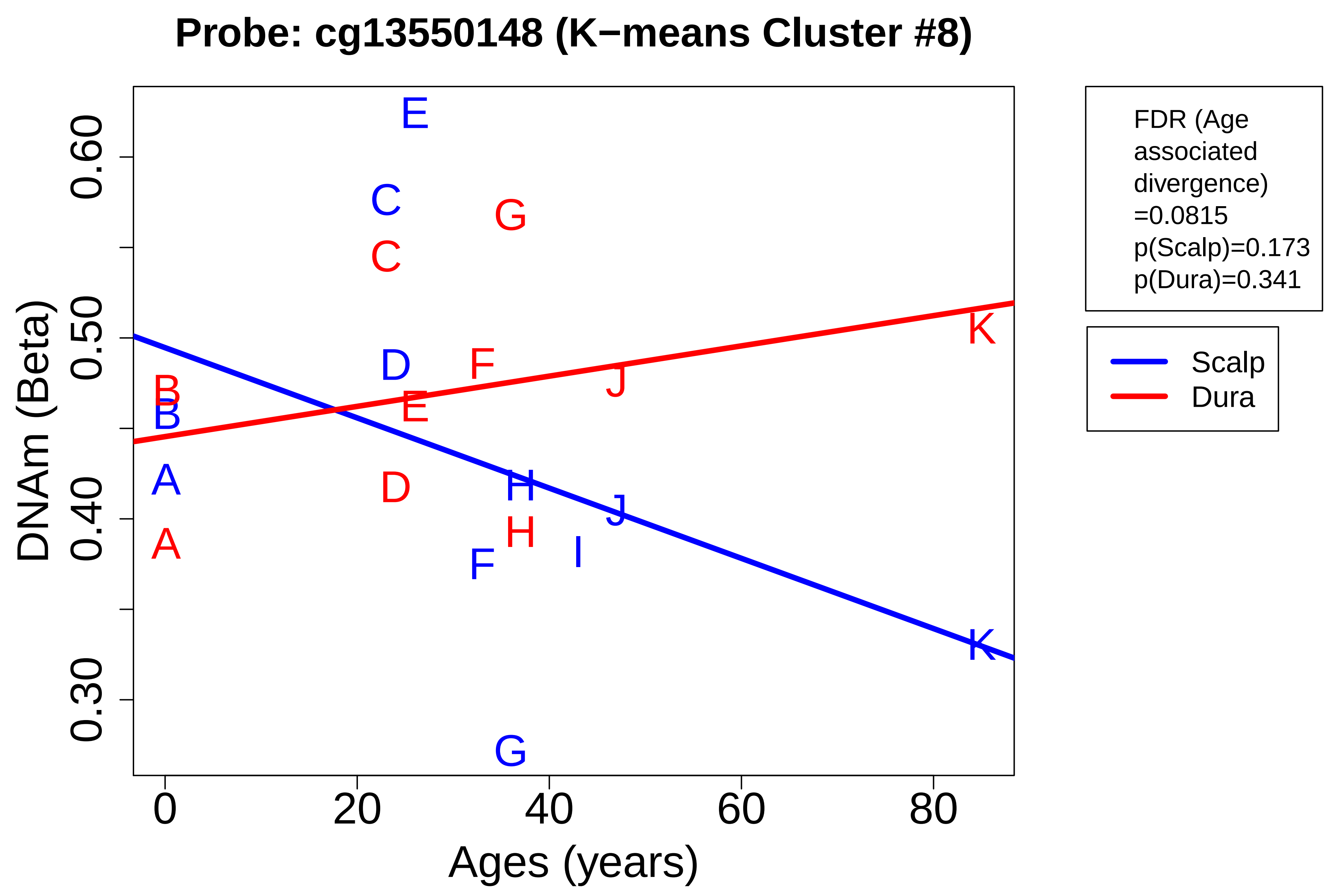

Probe: cg13550148 (K−means Cluster #8)
DNAm (Beta)
0.30
0.40
0.50
0.60
Ages (years)
0
20
40
60
80
FDR (Age associated divergence) =0.0815
p(Scalp)=0.173
p(Dura)=0.341
Scalp
Dura
A
B
C
D
E
F
G
H
I
J
K

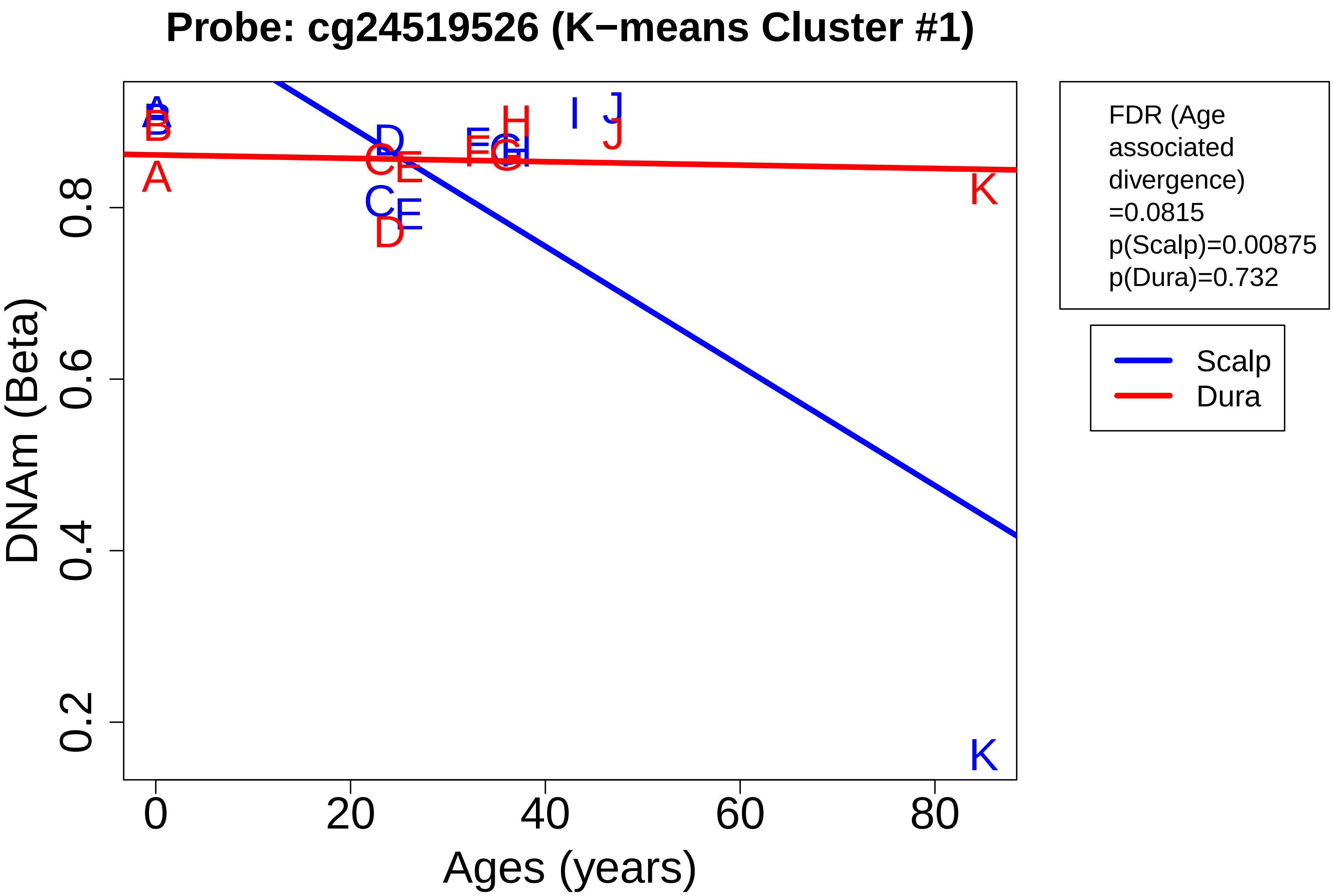

Probe: cg24519526 (K−means Cluster #1)
DNAm (Beta)
0.2
0.4
0.6
0.8
0
20
40
60
80
Ages (years)
A
B
C
D
E
F
G
H
I
J
K
FDR (Age associated divergence) =0.0815
p(Scalp)=0.00875
p(Dura)=0.732
Scalp
Dura

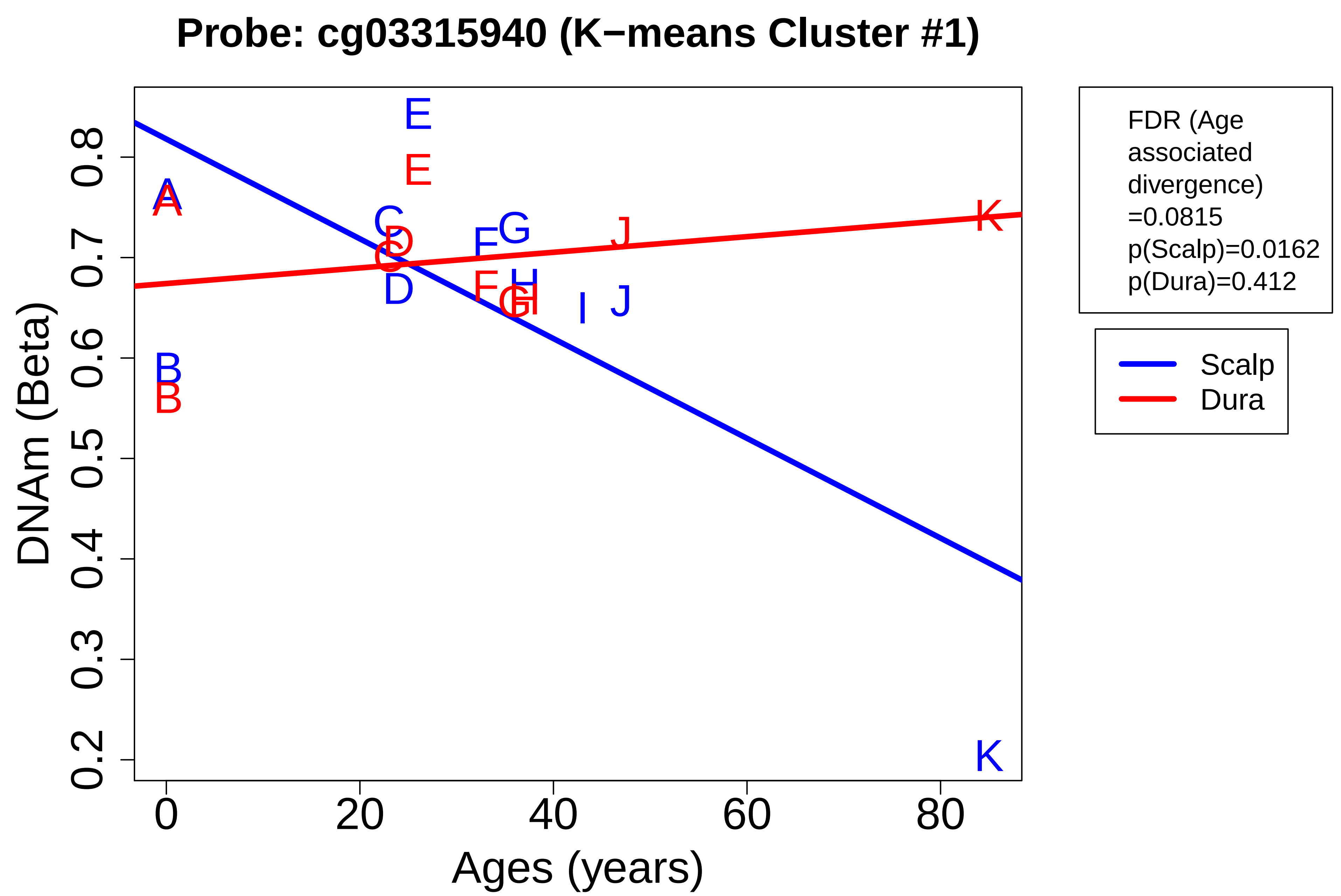
Probe: cg03315940 (K−means Cluster #1)
DNAm (Beta)
0.2
0.3
0.4
0.5
0.6
0.7
0.8
0
20
40
60
80
Ages (years)
A
B
C
D
E
F
G
H
I
J
K
FDR (Age associated divergence) =0.0815
p(Scalp)=0.0162
p(Dura)=0.412
Scalp
Dura

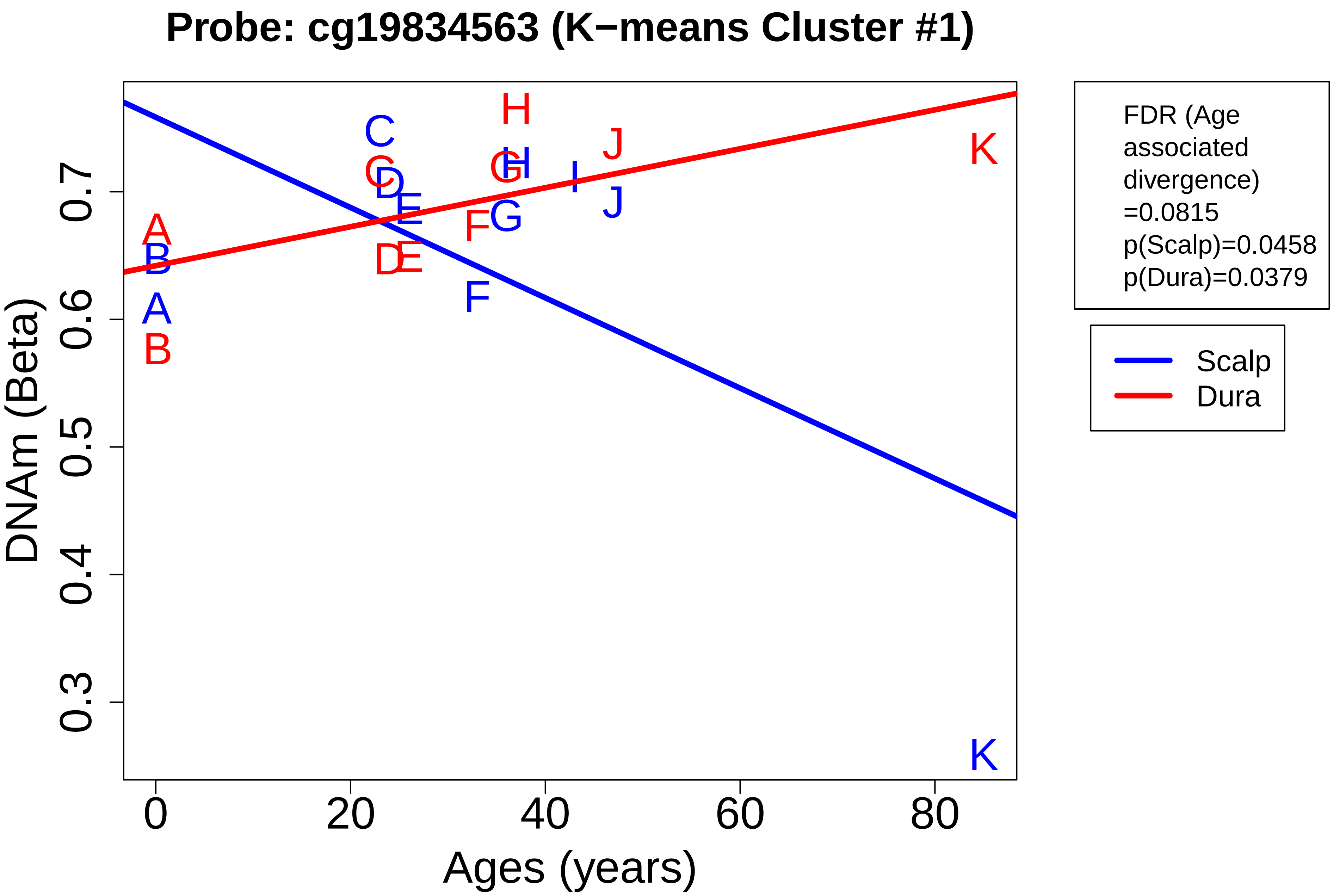

Probe: cg19834563 (K−means Cluster #1)
DNAm (Beta)
0.3
0.4
0.5
0.6
0.7
0
20
40
60
80
Ages (years)
A
B
C
D
E
F
G
H
I
J
K
FDR (Age associated divergence) =0.0815
p(Scalp)=0.0458
p(Dura)=0.0379
Scalp
Dura

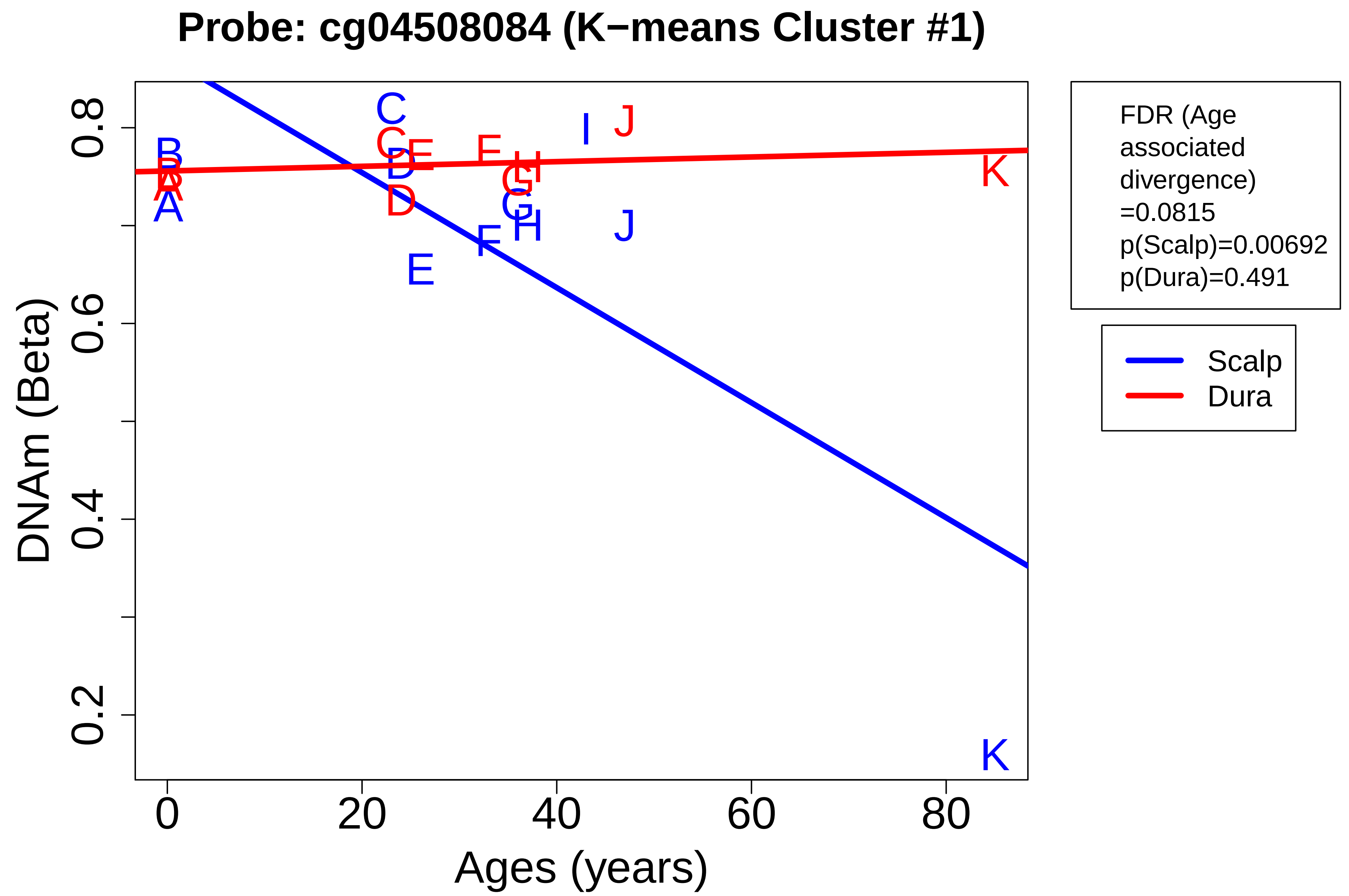

Probe: cg04508084 (K−means Cluster #1)
DNAm (Beta)
0.2
0.4
0.6
0.8
0
20
40
60
80
Ages (years)
FDR (Age associated divergence) =0.0815
p(Scalp)=0.00692
p(Dura)=0.491
Scalp
Dura

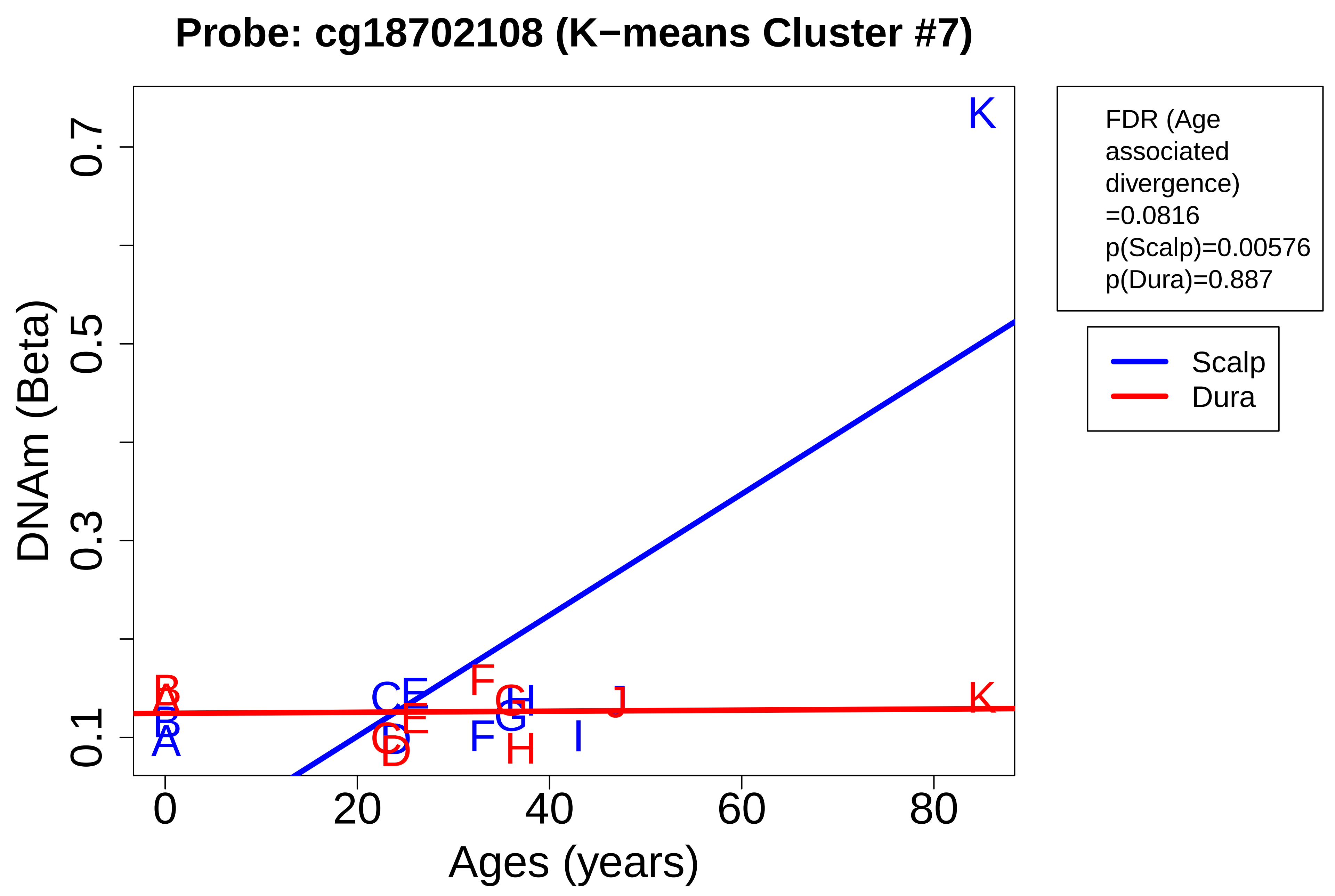

Probe: cg18702108 (K−means Cluster #7)
DNAm (Beta)
0.1
0.3
0.5
0.7
Ages (years)
0
20
40
60
80
FDR (Age associated divergence) =0.0816
p(Scalp)=0.00576
p(Dura)=0.887
Scalp
Dura

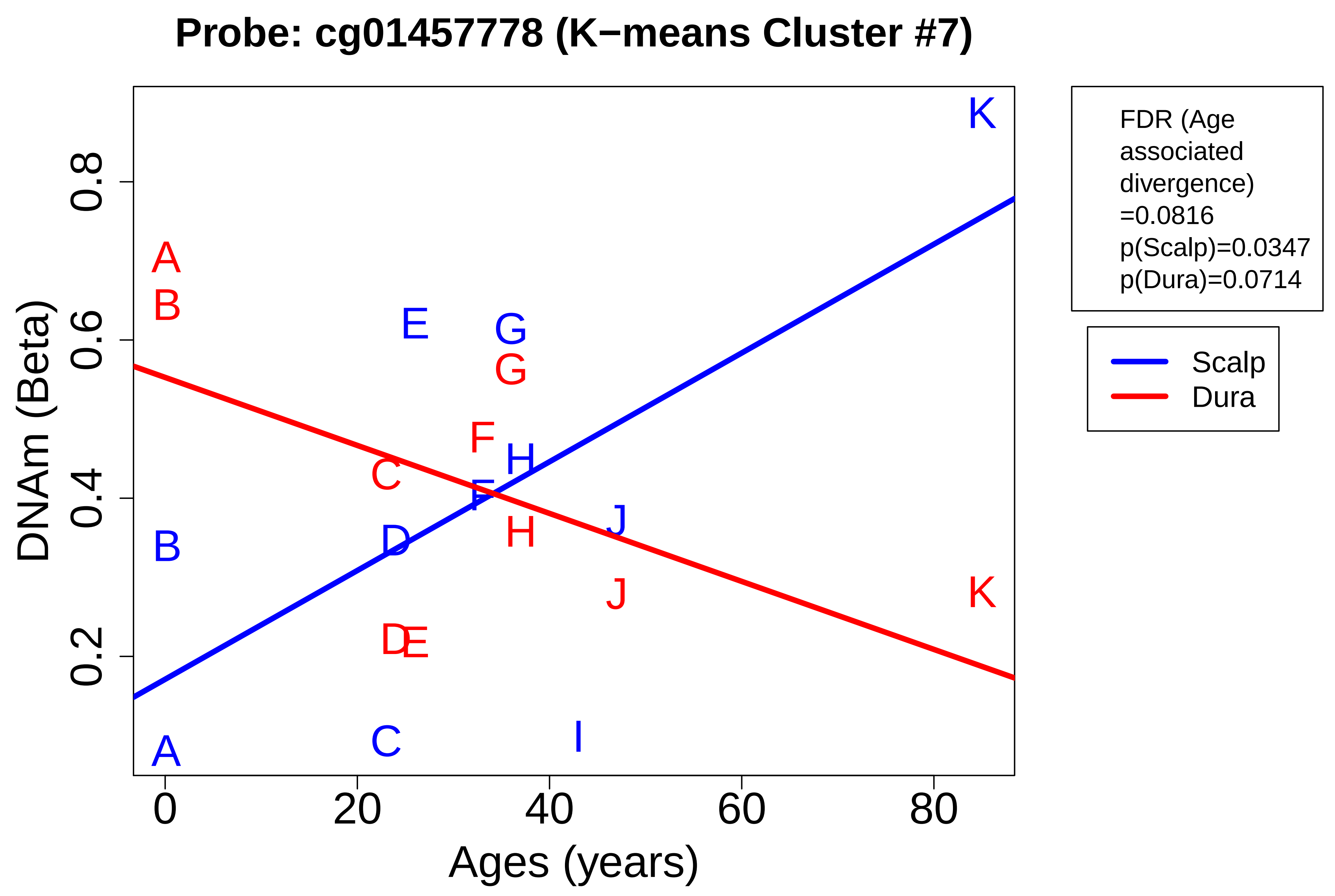

Probe: cg01457778 (K−means Cluster #7)
DNAm (Beta)
0.2
0.4
0.6
0.8
Ages (years)
0
20
40
60
80
FDR (Age associated divergence) =0.0816
p(Scalp)=0.0347
p(Dura)=0.0714
Scalp
Dura
A
B
C
D
E
F
G
H
I
J
K

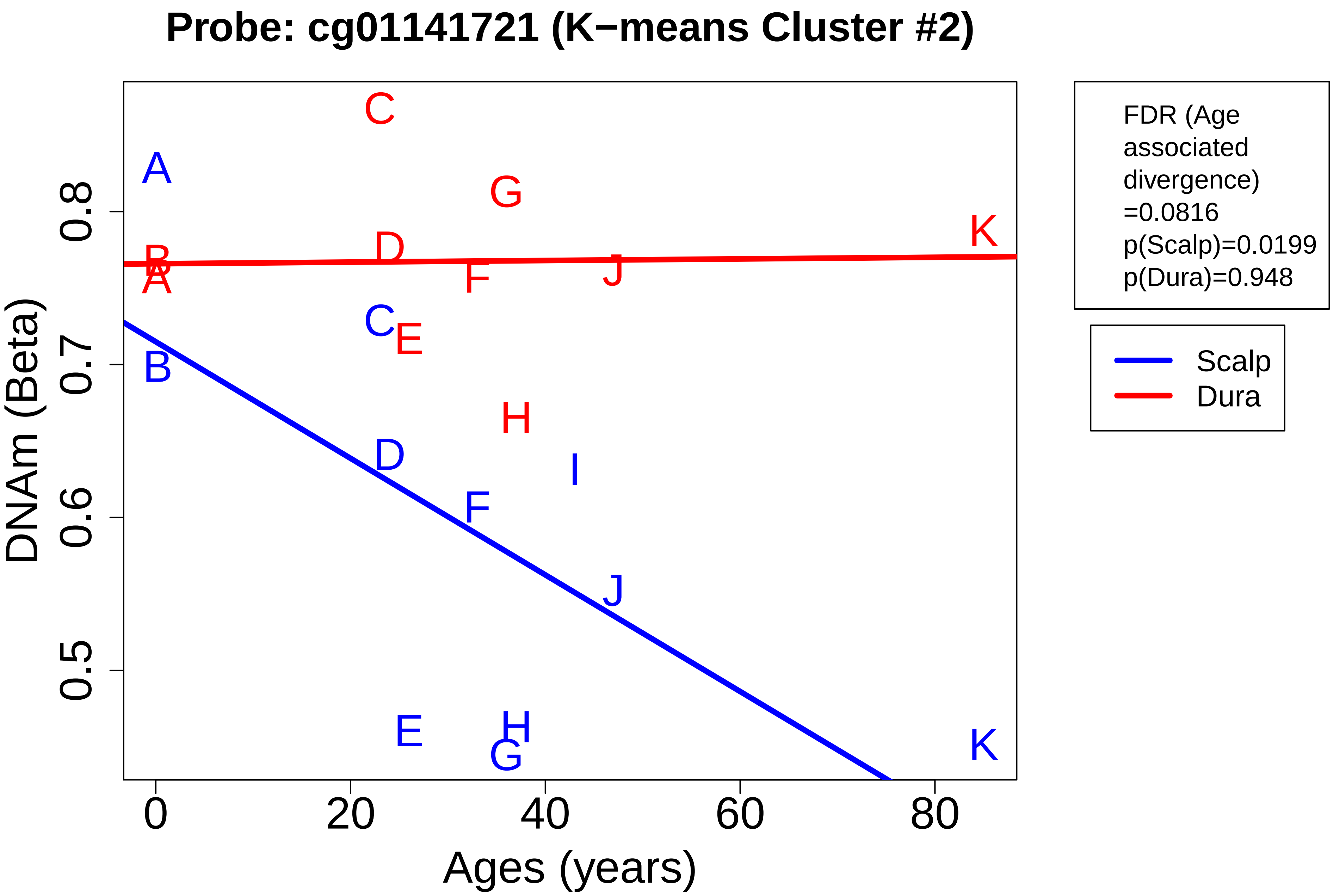

Probe: cg01141721 (K−means Cluster #2)
DNAm (Beta)
0.5
0.6
0.7
0.8
Ages (years)
0
20
40
60
80
A
B
C
D
E
F
G
H
I
J
K
A
B
C
D
E
F
G
H
J
K
FDR (Age associated divergence) =0.0816
p(Scalp)=0.0199
p(Dura)=0.948
Scalp
Dura

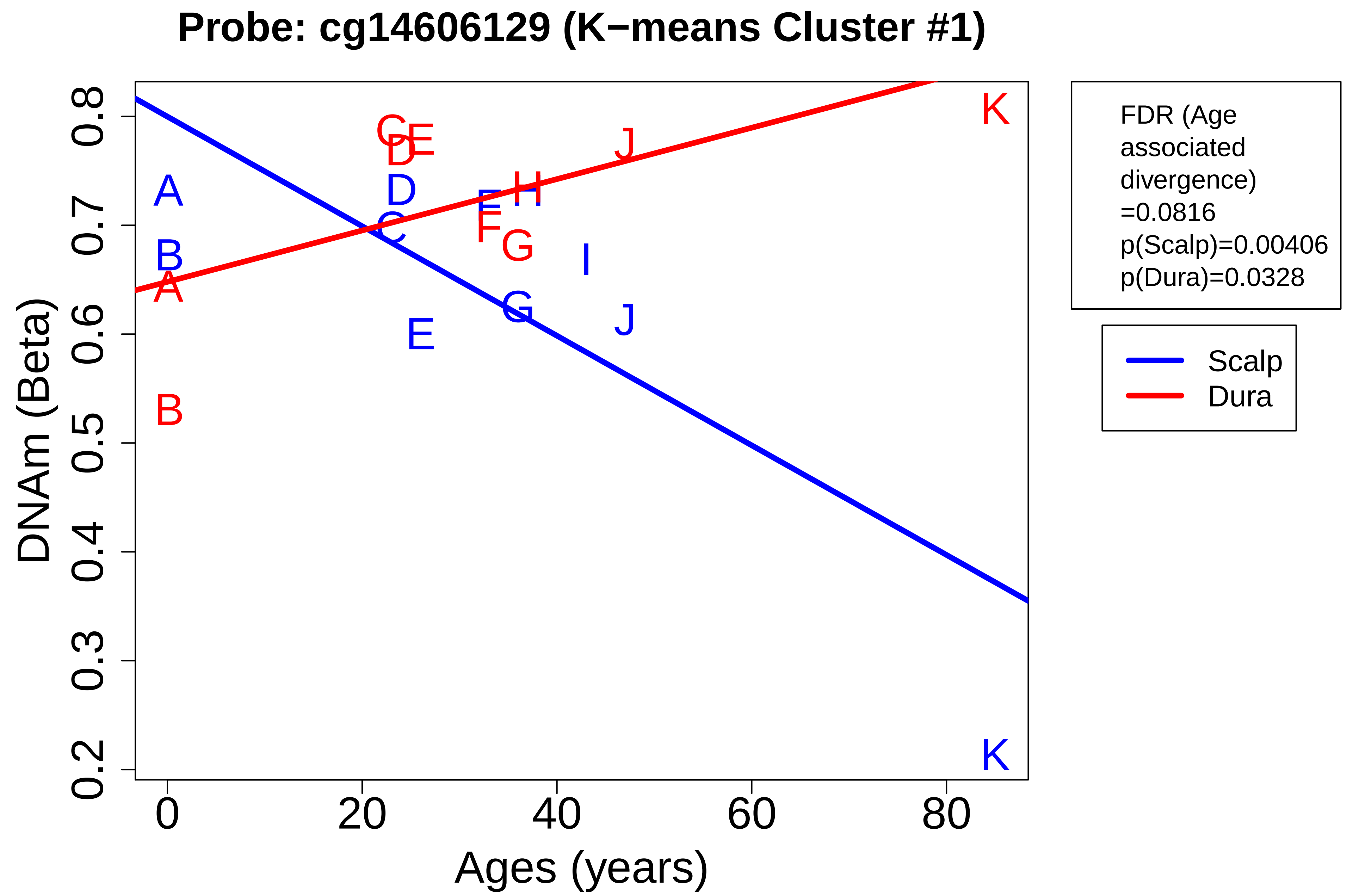
Probe: cg14606129 (K−means Cluster #1)
DNAm (Beta)
0.2
0.3
0.4
0.5
0.6
0.7
0.8
Ages (years)
0
20
40
60
80
FDR (Age associated divergence) =0.0816
p(Scalp)=0.00406
p(Dura)=0.0328
Scalp
Dura

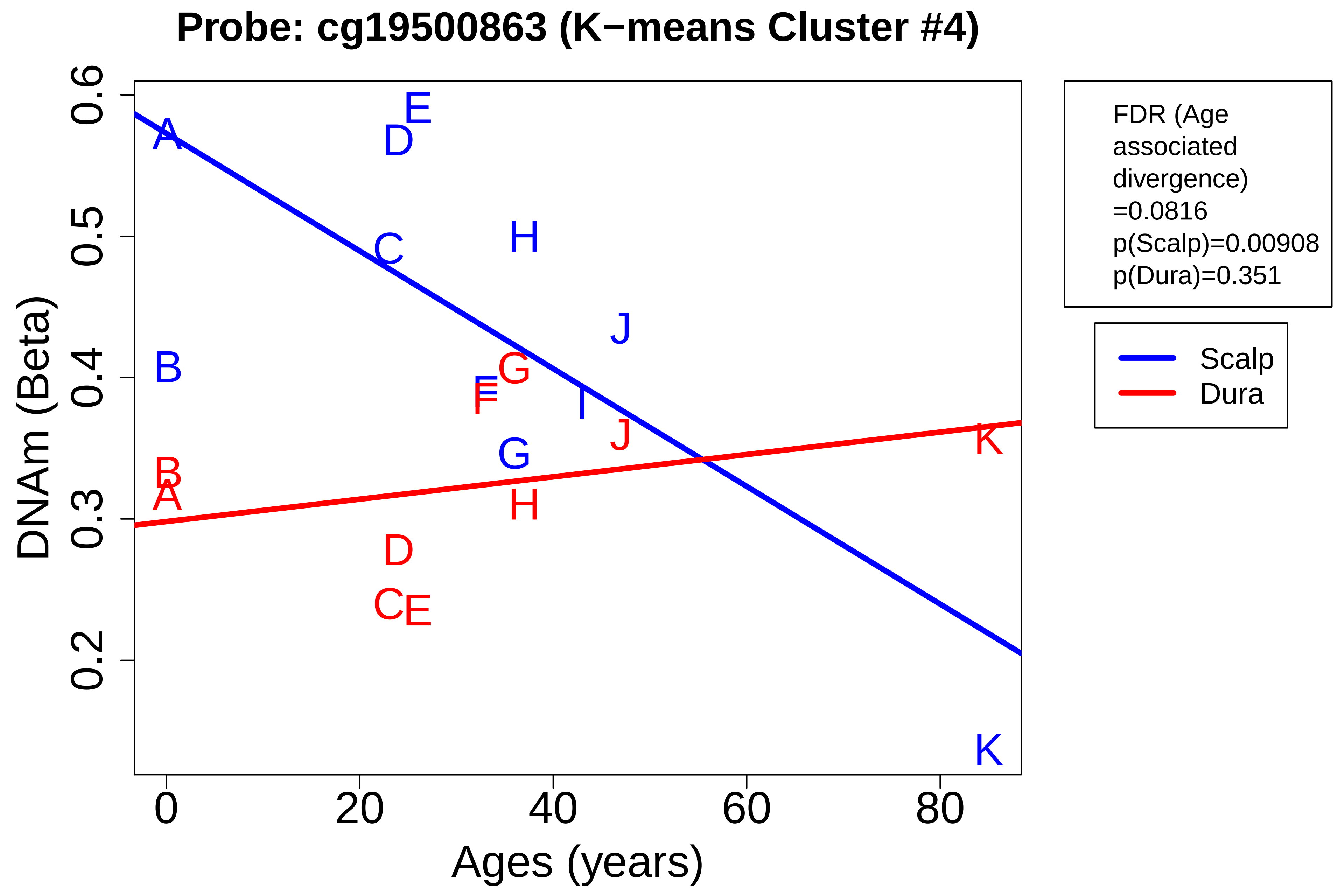

Probe: cg19500863 (K−means Cluster #4)
DNAm (Beta)
0.2
0.3
0.4
0.5
0.6
0
20
40
60
80
Ages (years)
FDR (Age associated divergence) =0.0816
p(Scalp)=0.00908
p(Dura)=0.351
Scalp
Dura

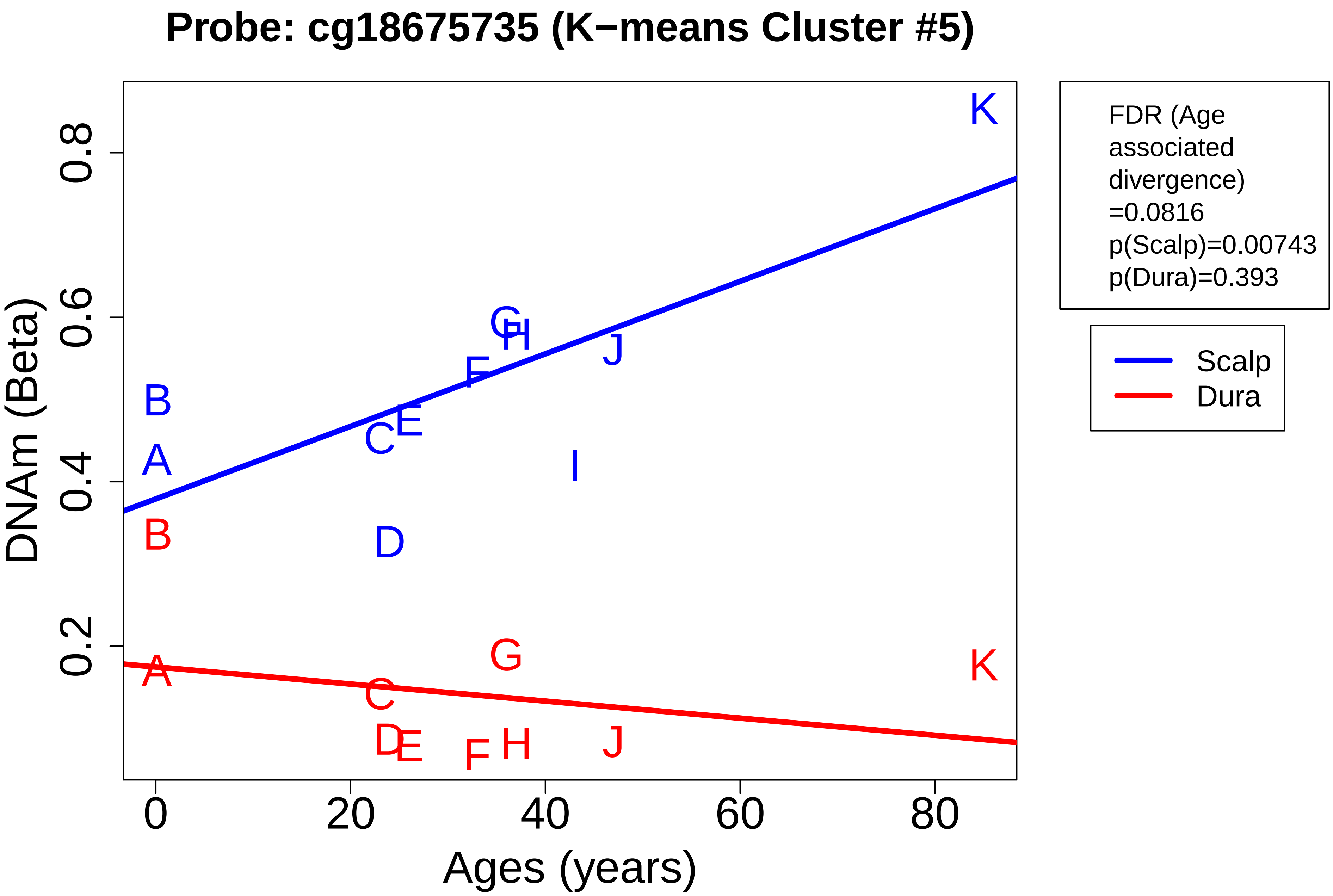
Probe: cg18675735 (K−means Cluster #5)
FDR (Age associated divergence) =0.0816
p(Scalp)=0.00743
p(Dura)=0.393
Scalp
Dura
DNAm (Beta)
0.2
0.4
0.6
0.8
0
20
40
60
80
Ages (years)
A
B
C
D
E
F
G
H
I
J
K

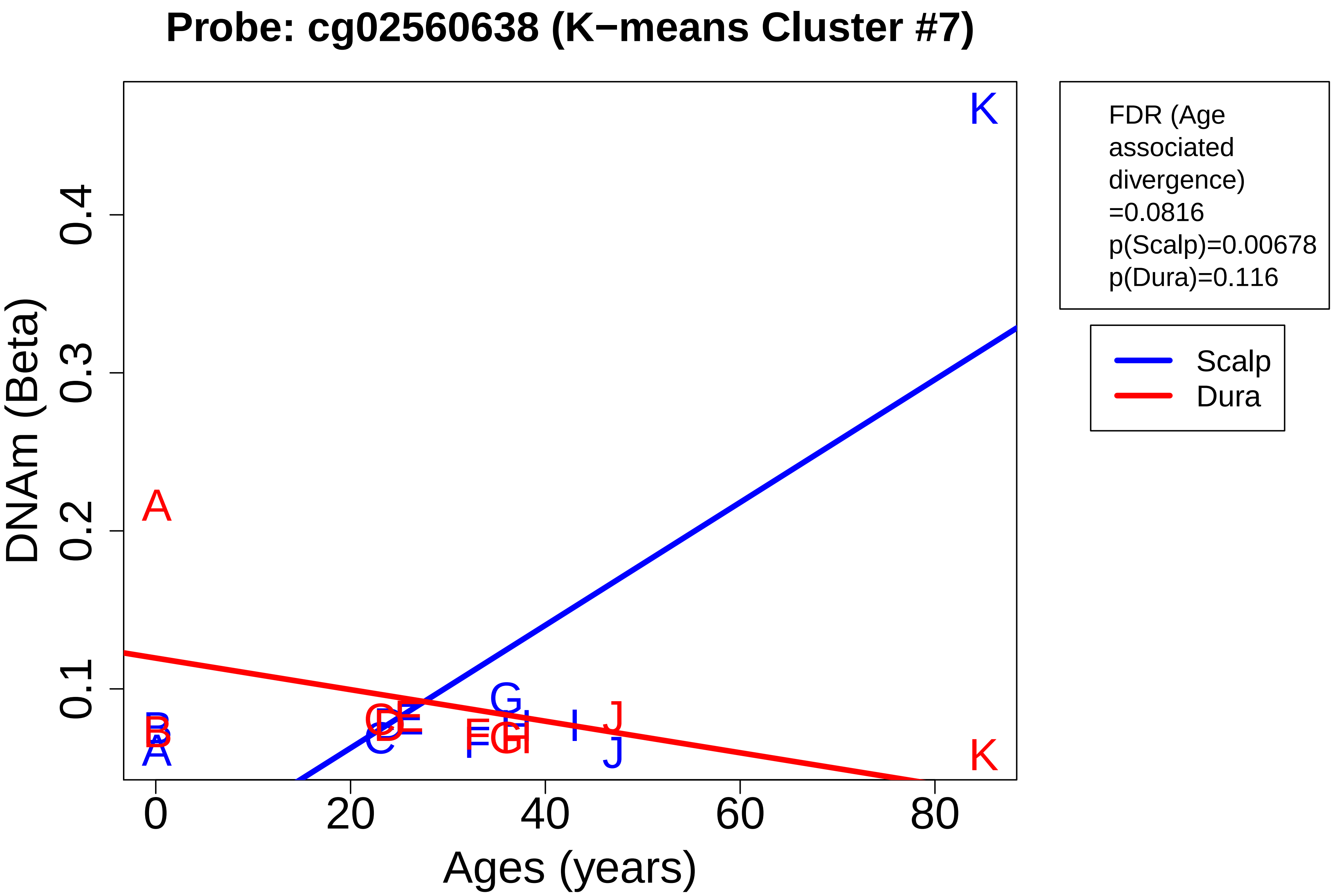
Probe: cg02560638 (K−means Cluster #7)
FDR (Age associated divergence) =0.0816
p(Scalp)=0.00678
p(Dura)=0.116
Scalp
Dura
DNAm (Beta)
Ages (years)
0.1
0.2
0.3
0.4
0
20
40
60
80

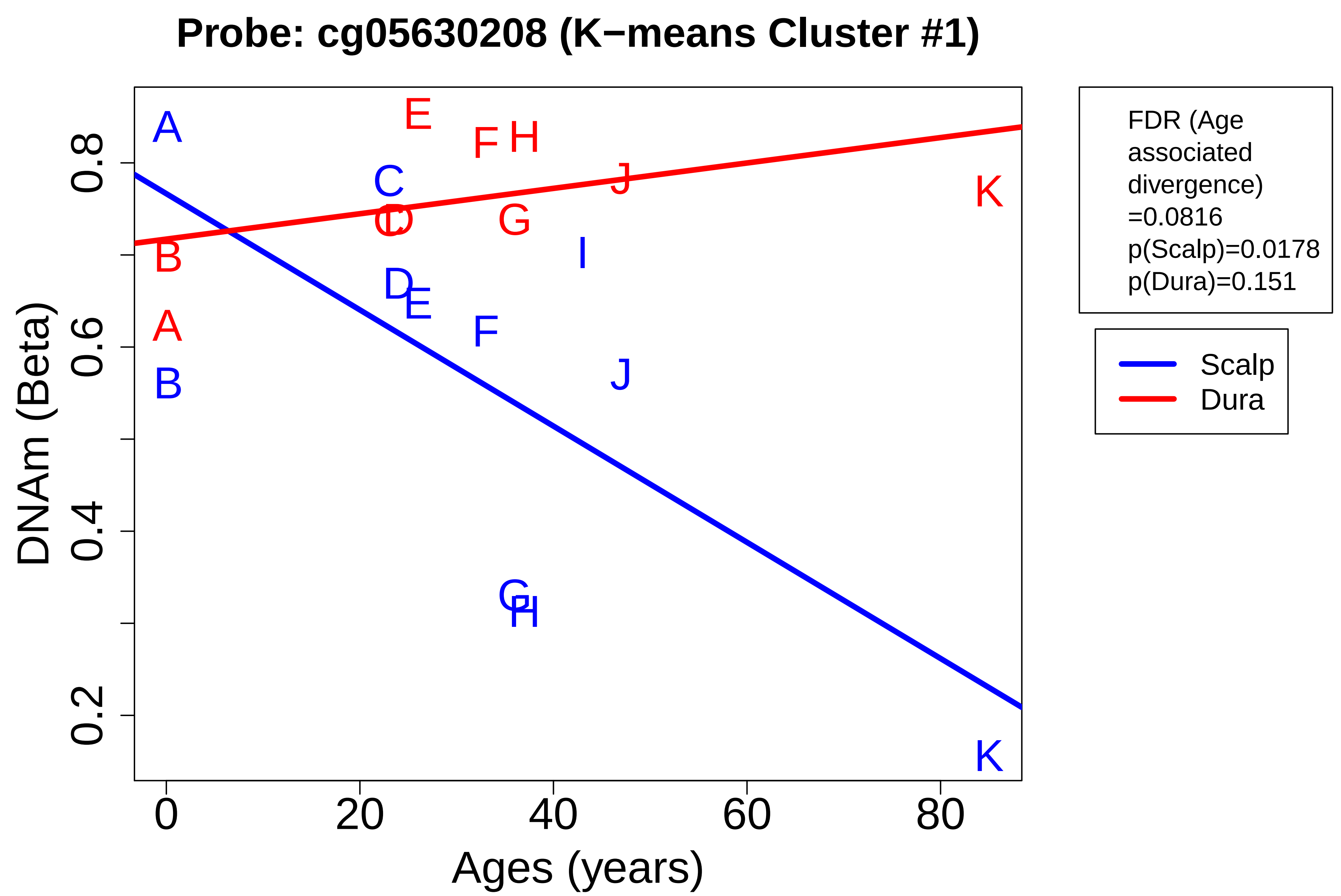

Probe: cg05630208 (K−means Cluster #1)
DNAm (Beta)
0.2
0.4
0.6
0.8
Ages (years)
0
20
40
60
80
FDR (Age associated divergence) =0.0816
p(Scalp)=0.0178
p(Dura)=0.151
Scalp
Dura

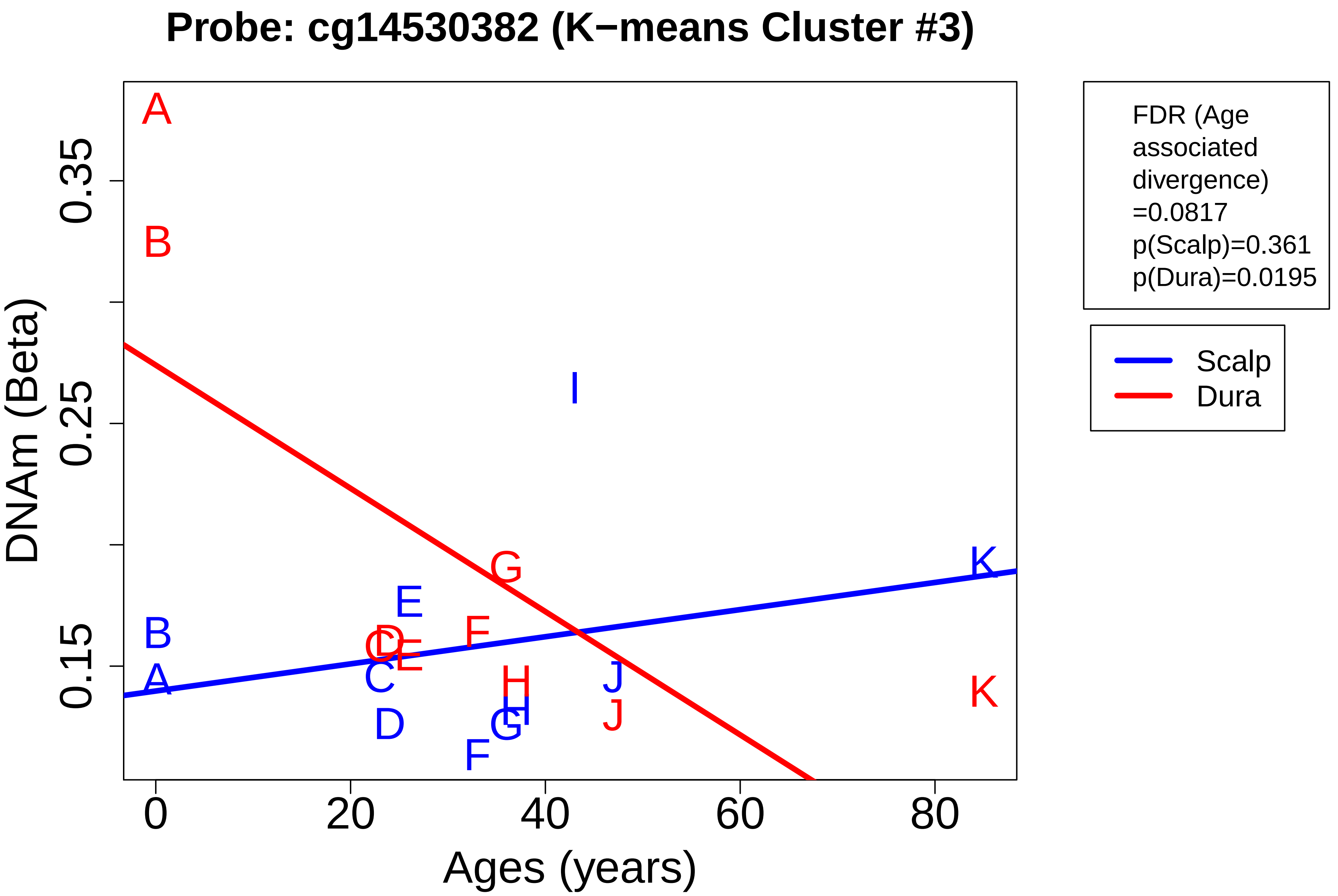

Probe: cg14530382 (K−means Cluster #3)
DNAm (Beta)
0.15
0.25
0.35
Ages (years)
0
20
40
60
80
FDR (Age associated divergence) =0.0817
p(Scalp)=0.361
p(Dura)=0.0195
Scalp
Dura

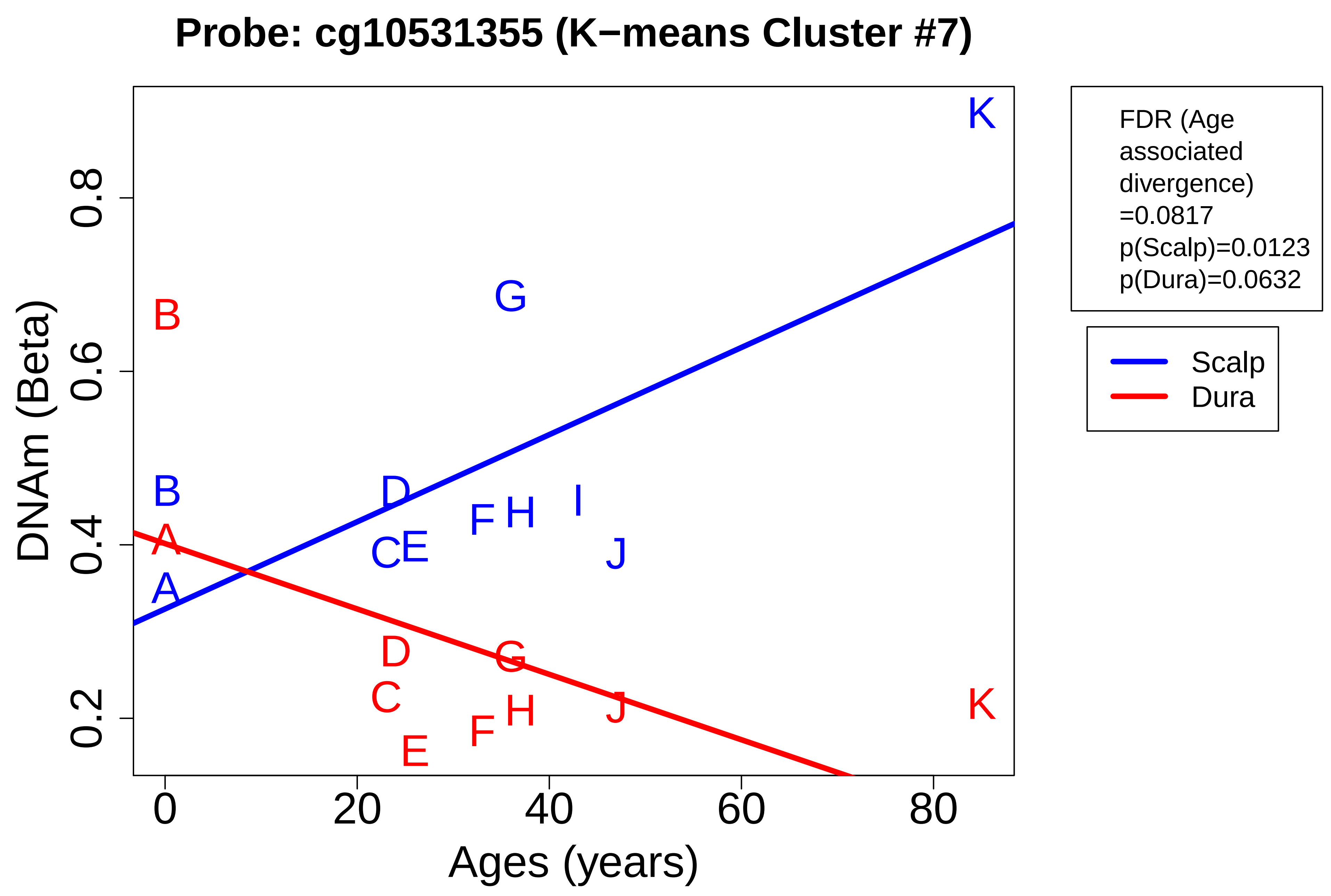

Probe: cg10531355 (K−means Cluster #7)
DNAm (Beta)
0.2
0.4
0.6
0.8
0
20
40
60
80
Ages (years)
K
G
B
B
D
I
F
H
A
CE
J
A
D
G
C
H
J
K
F
E
FDR (Age associated divergence) =0.0817
p(Scalp)=0.0123
p(Dura)=0.0632
Scalp
Dura

Probe: cg13454978 (K−means Cluster #7)

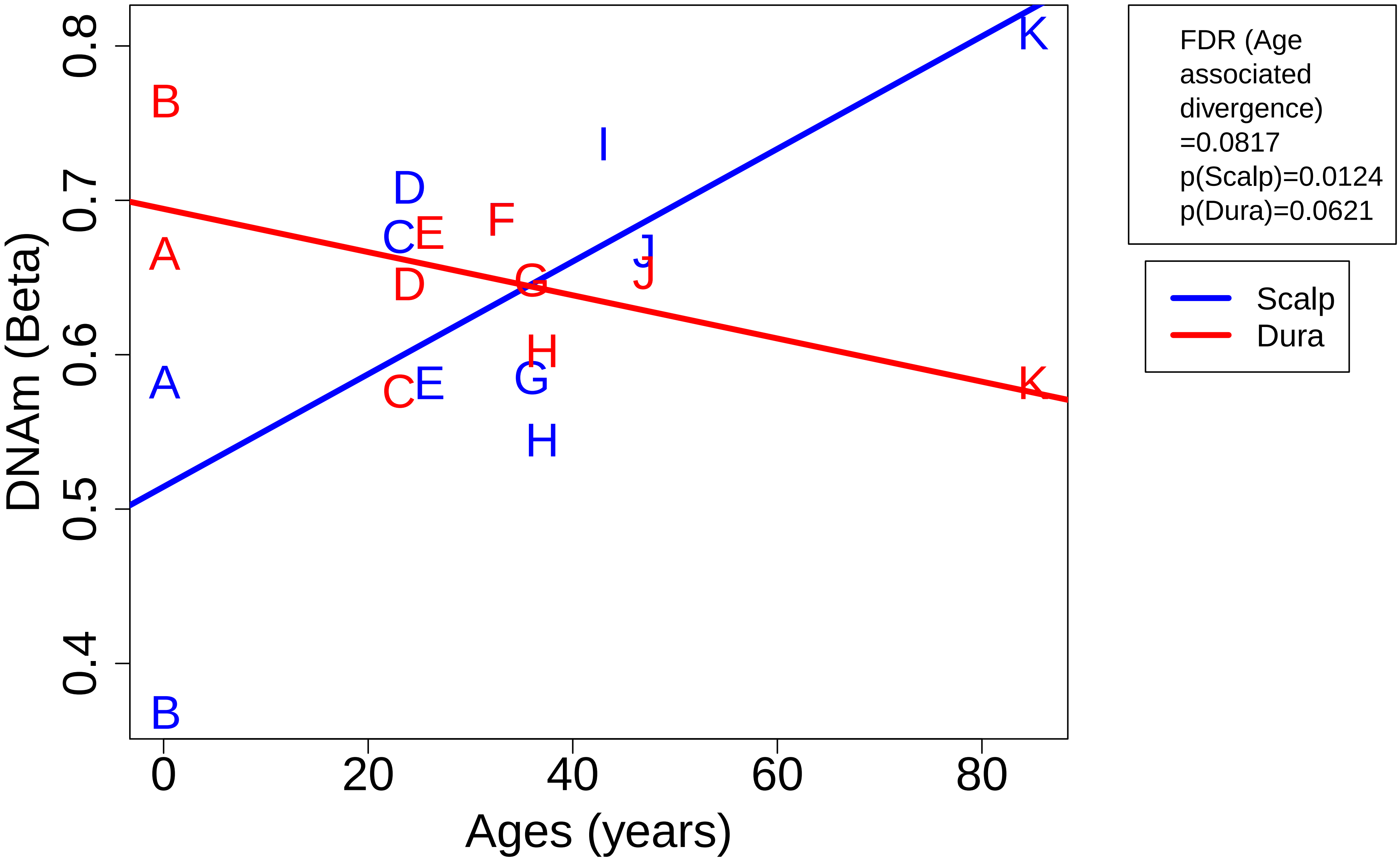

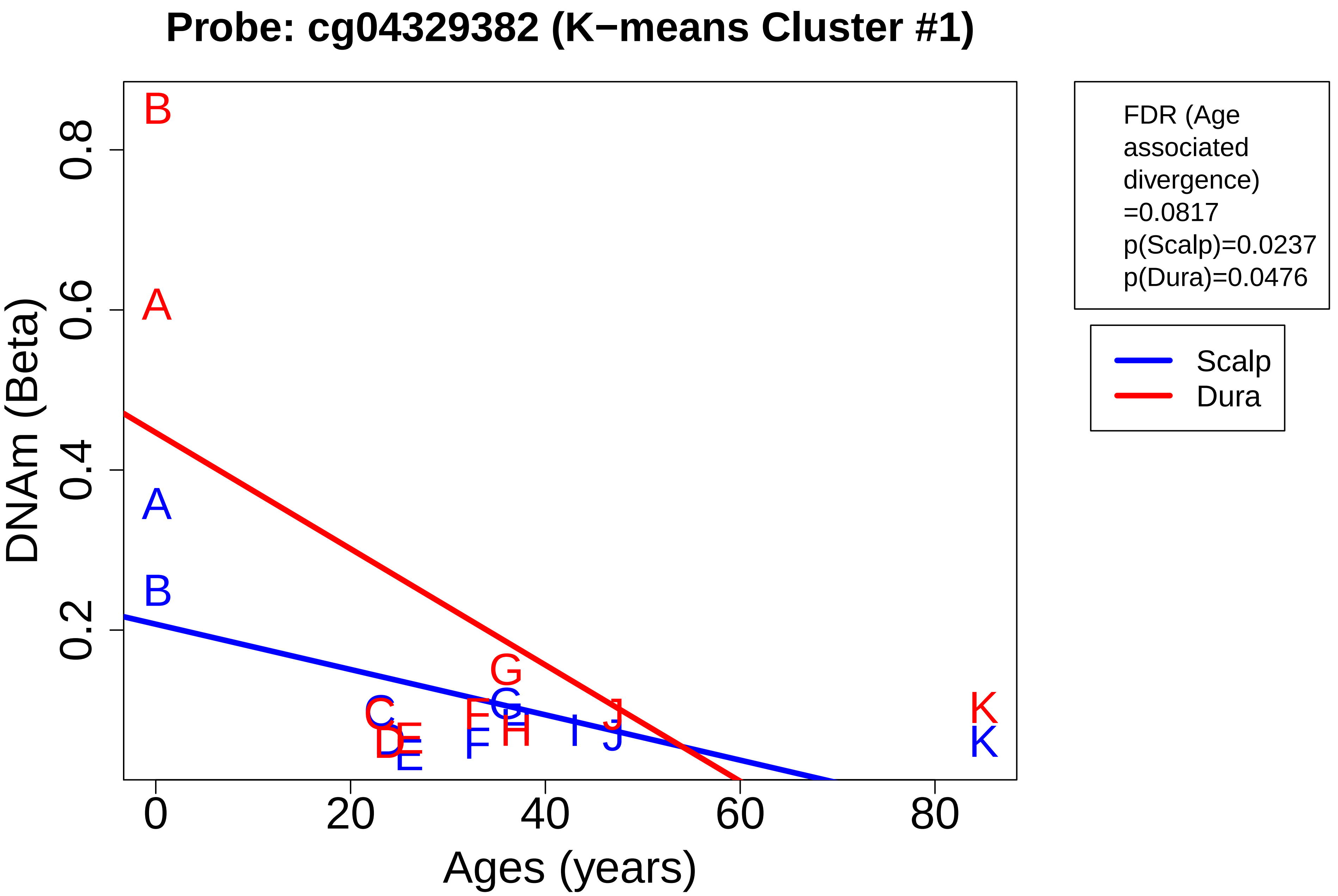

Probe: cg04329382 (K−means Cluster #1)
DNAm (Beta)
0.2
0.4
0.6
0.8
Ages (years)
0
20
40
60
80
FDR (Age associated divergence) =0.0817
p(Scalp)=0.0237
p(Dura)=0.0476
Scalp
Dura

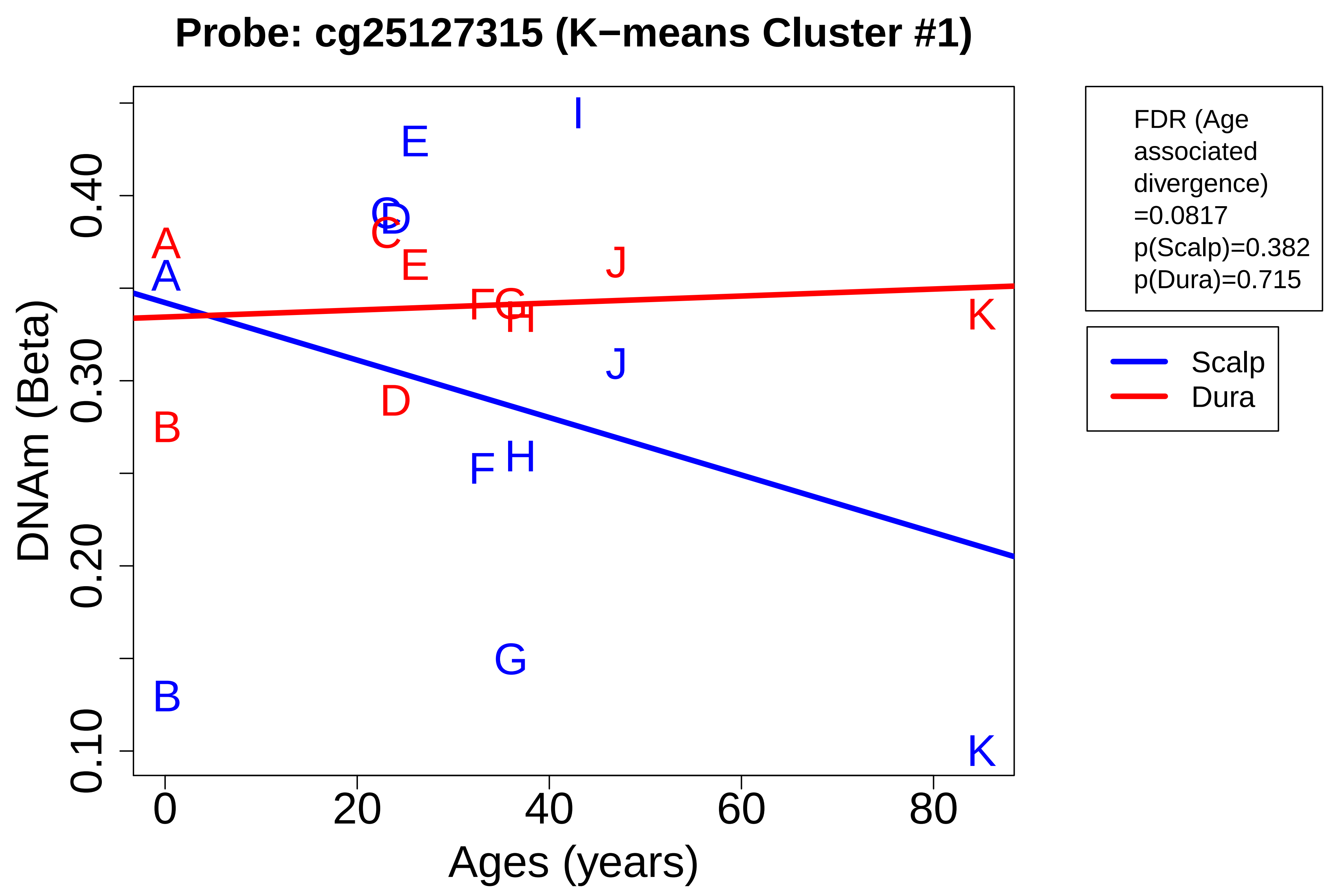

Probe: cg25127315 (K−means Cluster #1)
DNAm (Beta)
0.10
0.20
0.30
0.40
0
20
40
60
80
Ages (years)
FDR (Age associated divergence) =0.0817
p(Scalp)=0.382
p(Dura)=0.715
Scalp
Dura
A
B
C
D
E
F
G
H
I
J
K

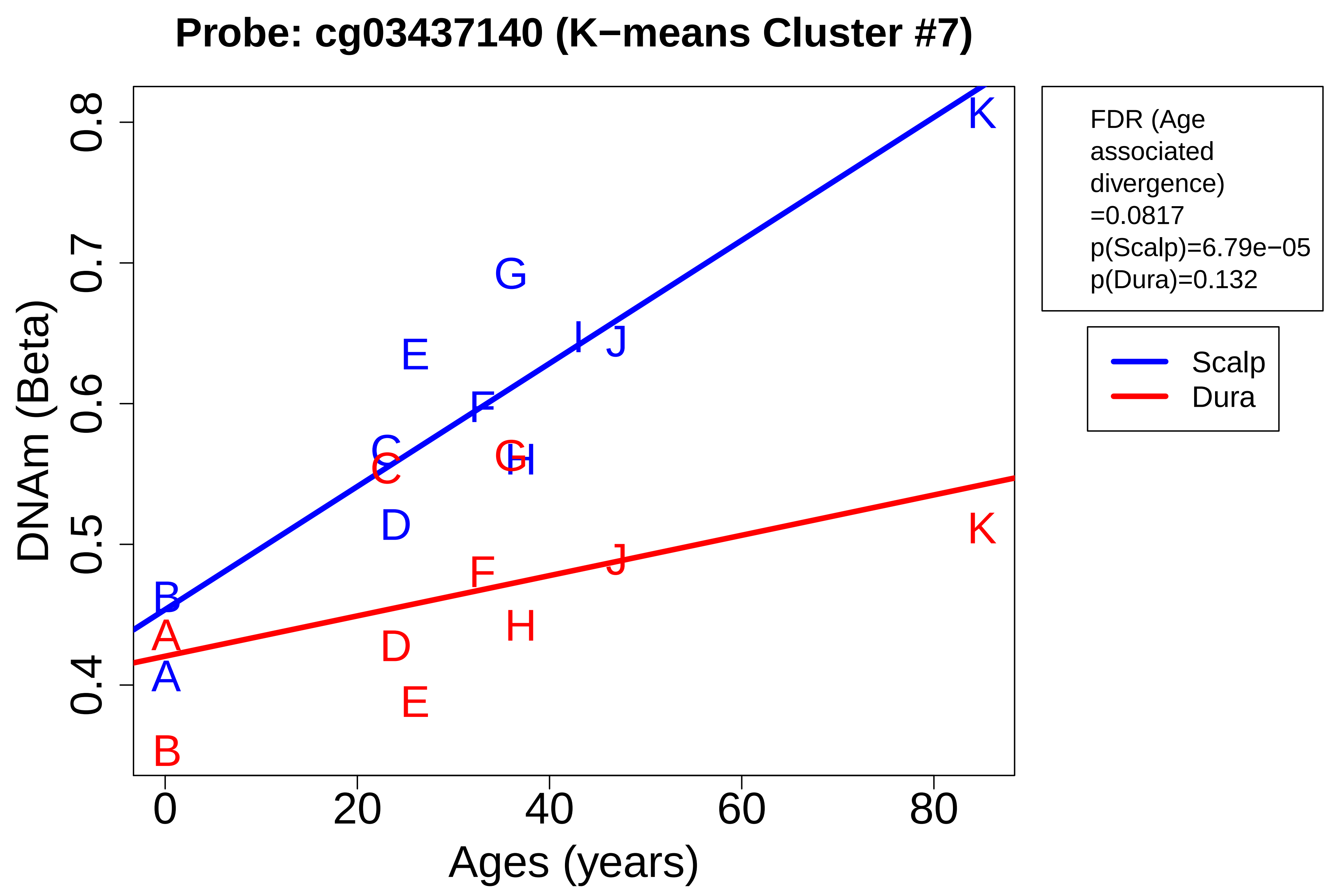

Probe: cg03437140 (K−means Cluster #7)
DNAm (Beta)
0.4
0.5
0.6
0.7
0.8
0
20
40
60
80
Ages (years)
FDR (Age associated divergence) =0.0817
p(Scalp)=6.79e−05
p(Dura)=0.132
Scalp
Dura
A
B
C
D
E
F
G
H
I
J
K

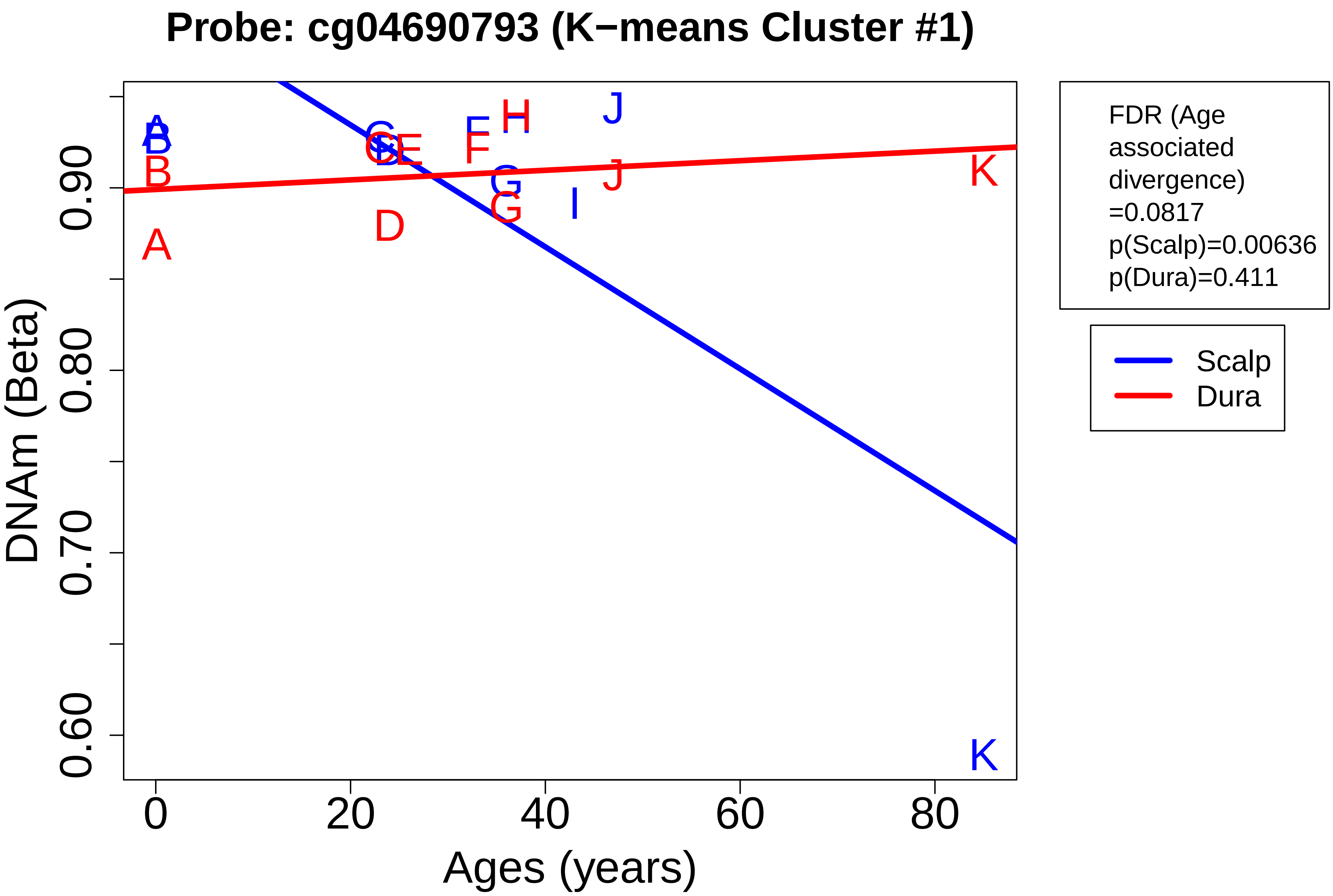
Probe: cg04690793 (K−means Cluster #1)
DNAm (Beta)
0.60
0.70
0.80
0.90
0
20
40
60
80
Ages (years)
A
B
C
D
E
F
G
H
I
J
K
FDR (Age associated divergence) =0.0817
p(Scalp)=0.00636
p(Dura)=0.411
Scalp
Dura

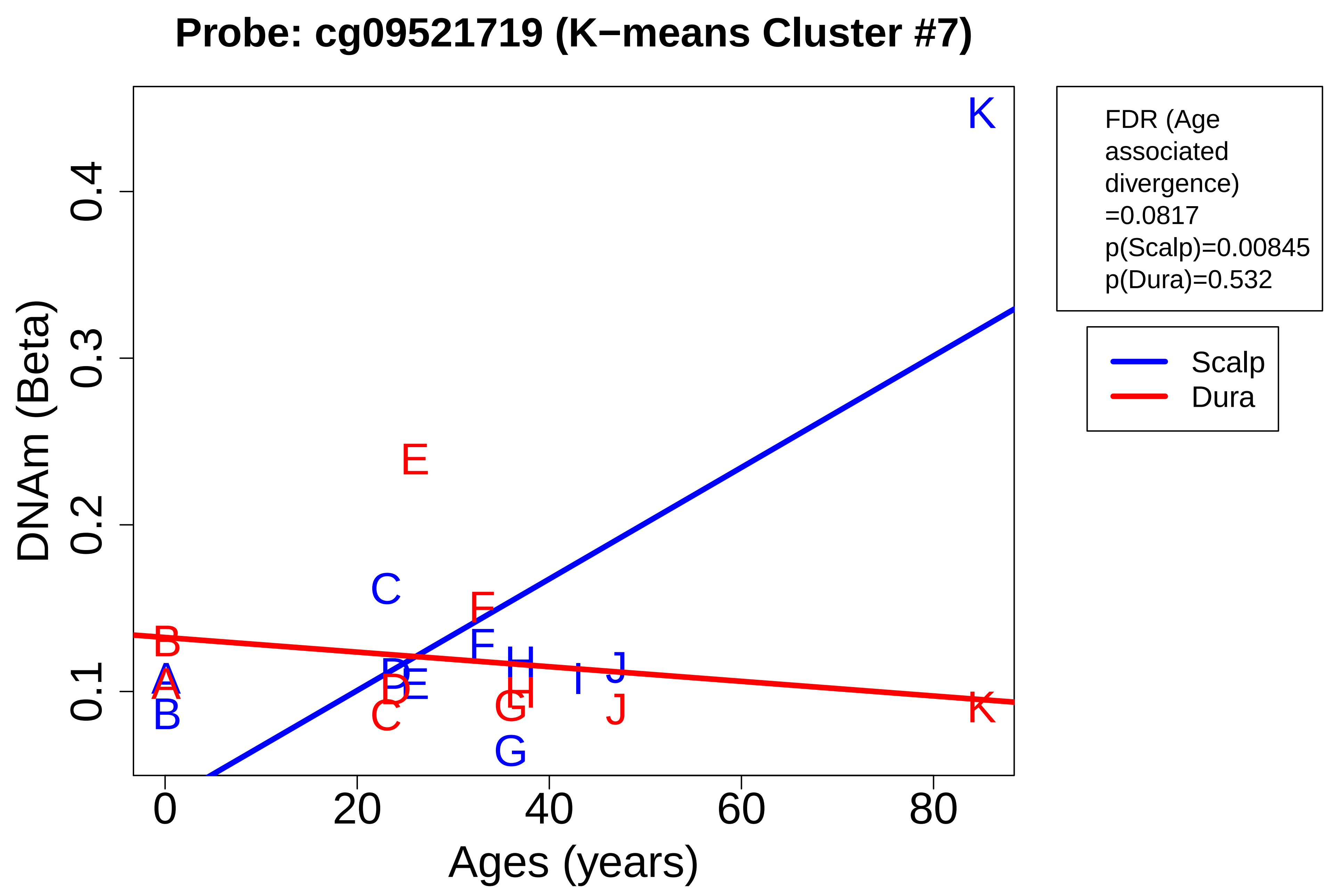

Probe: cg09521719 (K−means Cluster #7)
DNAm (Beta)
0.1
0.2
0.3
0.4
0
20
40
60
80
Ages (years)
FDR (Age associated divergence) =0.0817
p(Scalp)=0.00845
p(Dura)=0.532
Scalp
Dura

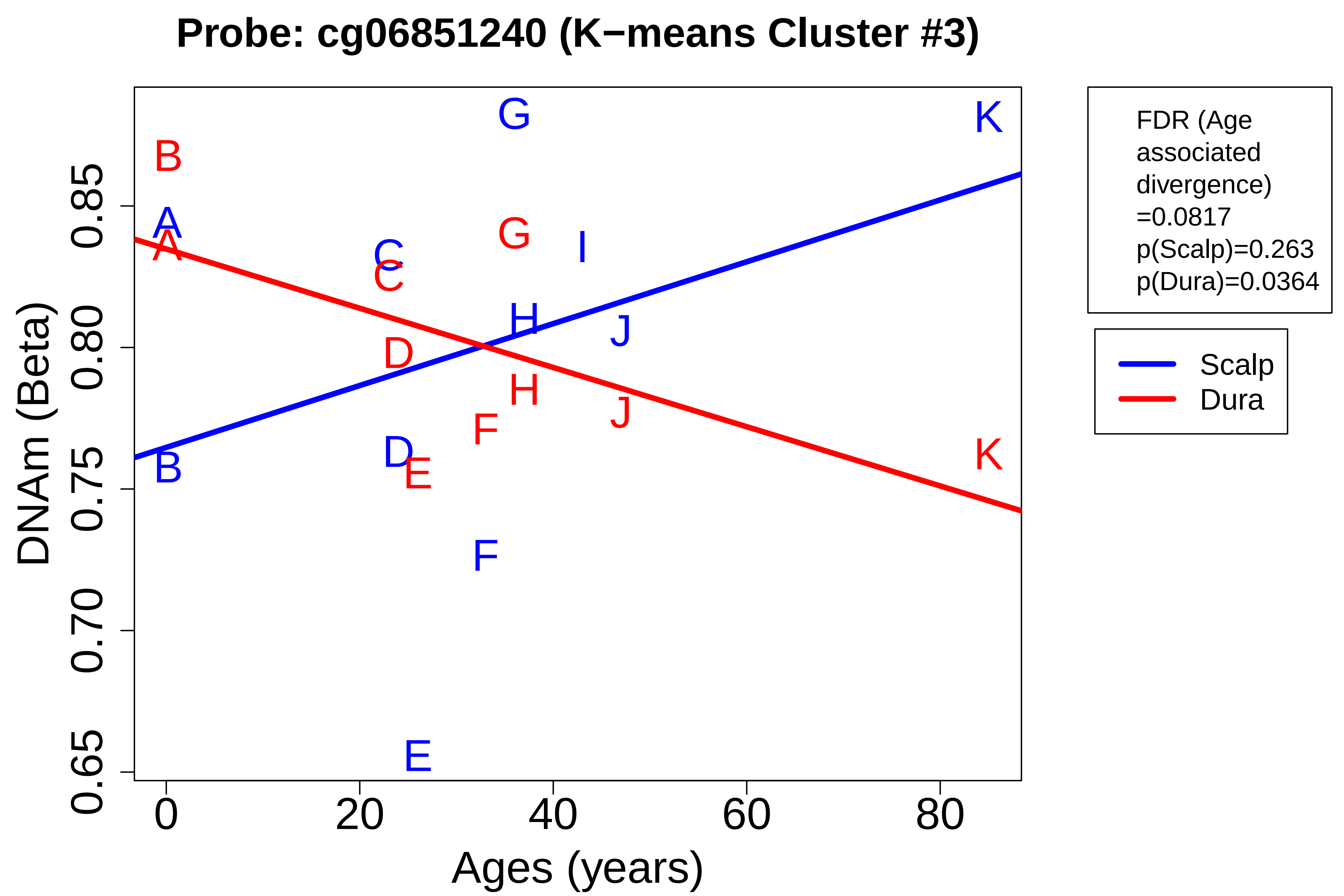

Probe: cg06851240 (K−means Cluster #3)
DNAm (Beta)
0.65
0.70
0.75
0.80
0.85
0
20
40
60
80
Ages (years)
FDR (Age associated divergence) =0.0817
p(Scalp)=0.263
p(Dura)=0.0364
Scalp
Dura
A
B
C
D
E
F
G
H
I
J
K
A
B
C
D
E
F
G
H
J
K

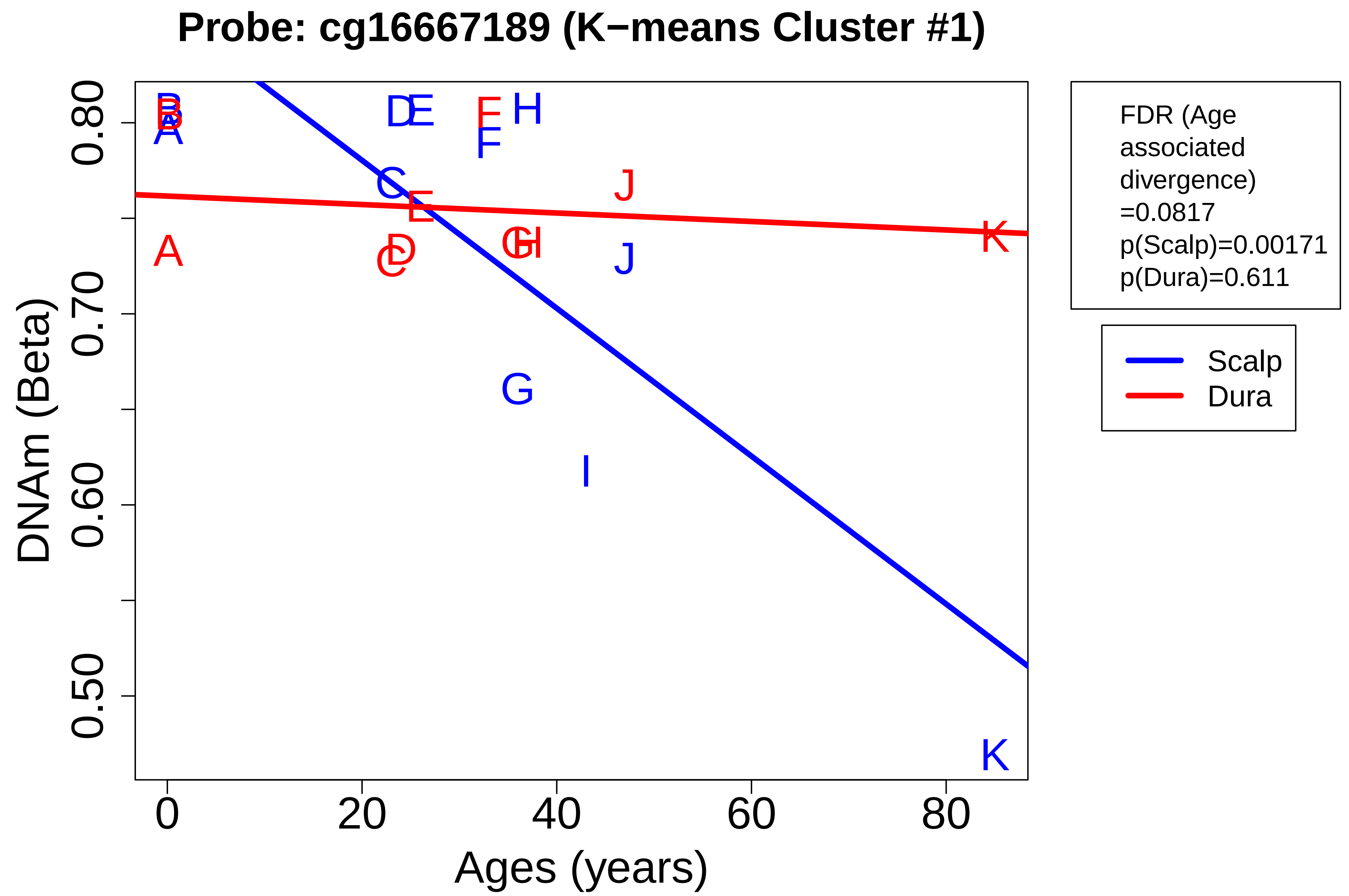

Probe: cg16667189 (K−means Cluster #1)
DNAm (Beta)
0.50
0.60
0.70
0.80
Ages (years)
0
20
40
60
80
FDR (Age associated divergence) =0.0817
p(Scalp)=0.00171
p(Dura)=0.611
Scalp
Dura

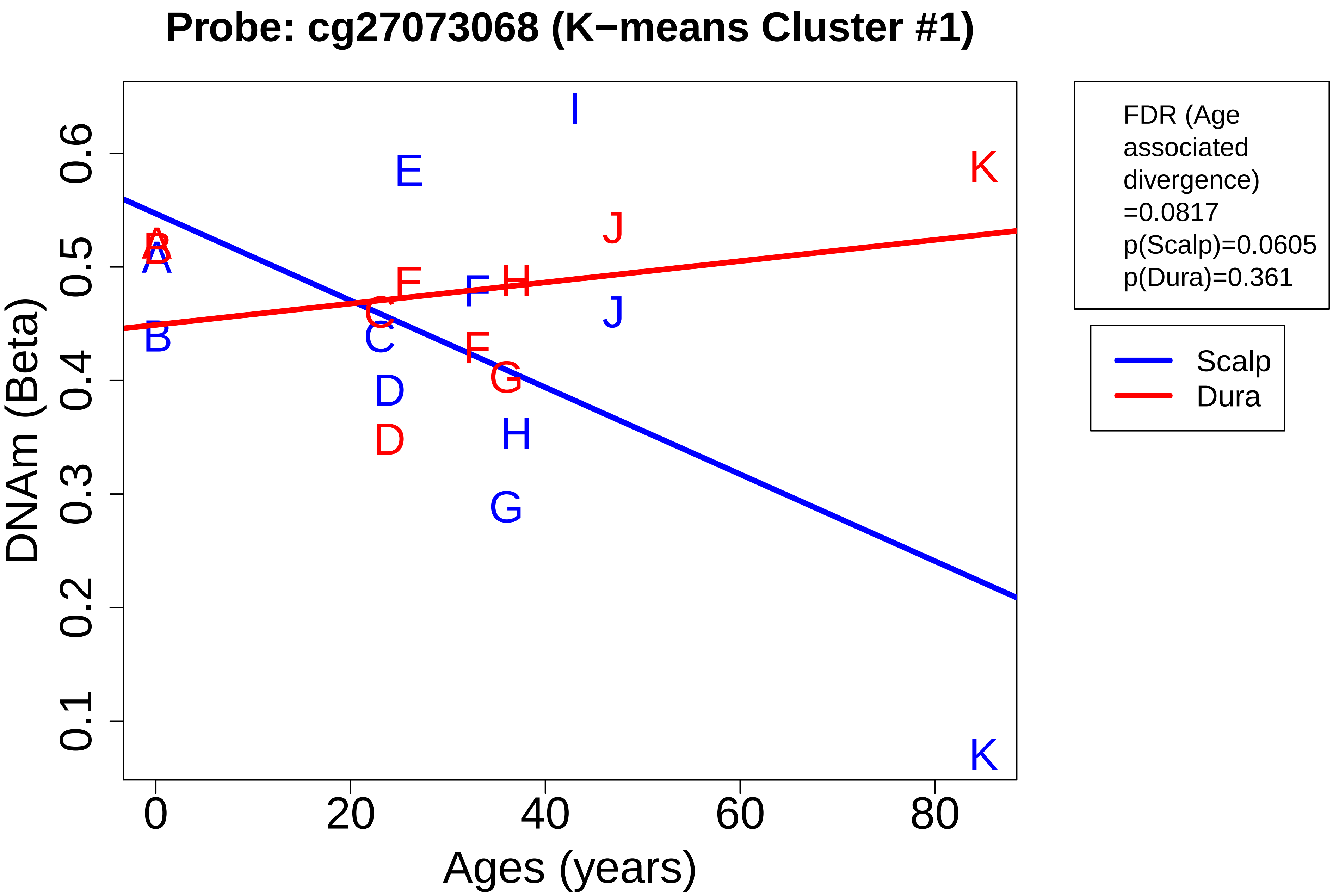

Probe: cg27073068 (K−means Cluster #1)
DNAm (Beta)
0.1
0.2
0.3
0.4
0.5
0.6
Ages (years)
0
20
40
60
80
FDR (Age associated divergence) =0.0817
p(Scalp)=0.0605
p(Dura)=0.361
Scalp
Dura
A
B
C
D
E
F
G
H
I
J
K

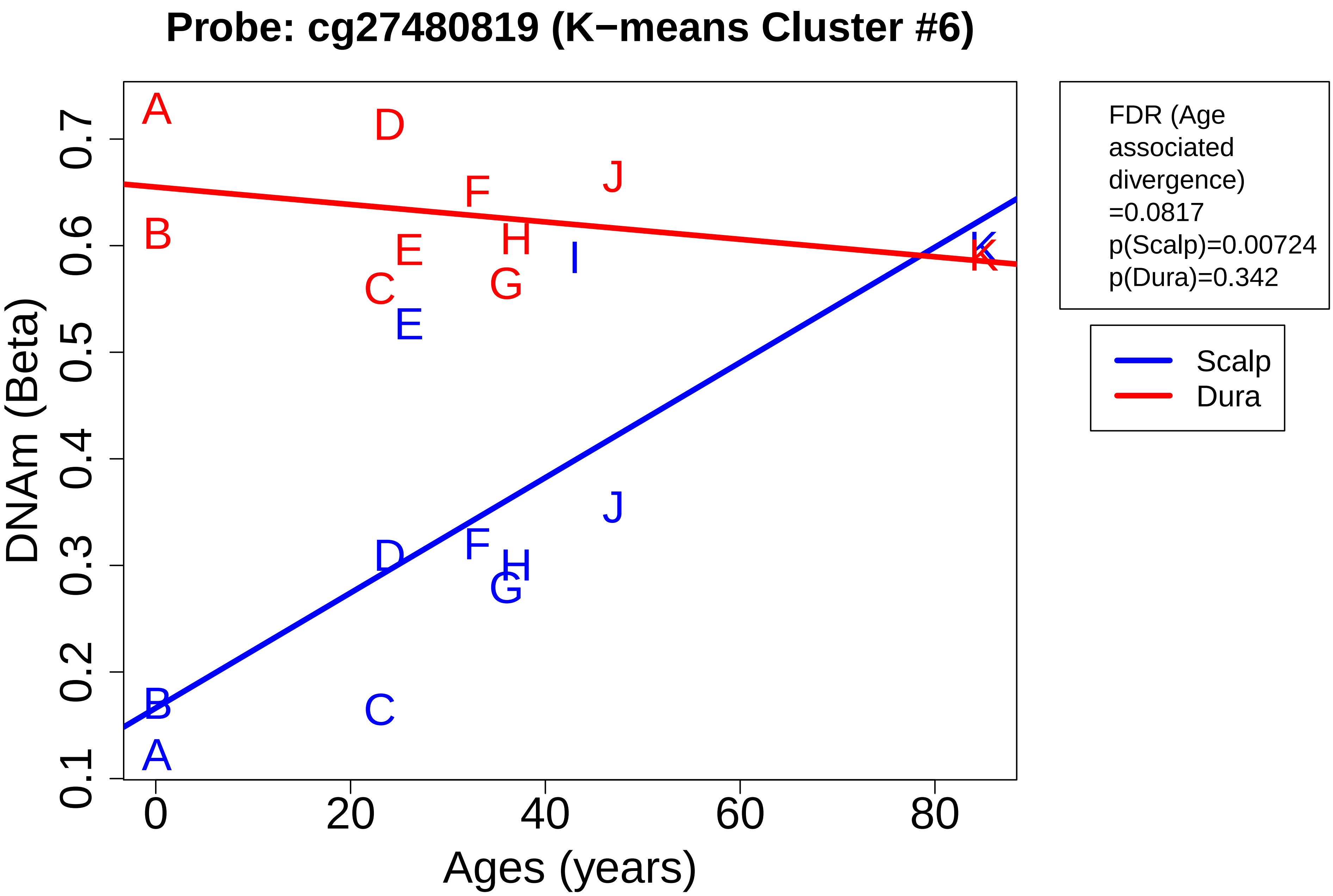

Probe: cg27480819 (K−means Cluster #6)
DNAm (Beta)
0.1
0.2
0.3
0.4
0.5
0.6
0.7
Ages (years)
0
20
40
60
80
A
B
C
D
E
F
G
H
I
J
K
FDR (Age associated divergence) =0.0817
p(Scalp)=0.00724
p(Dura)=0.342
Scalp
Dura

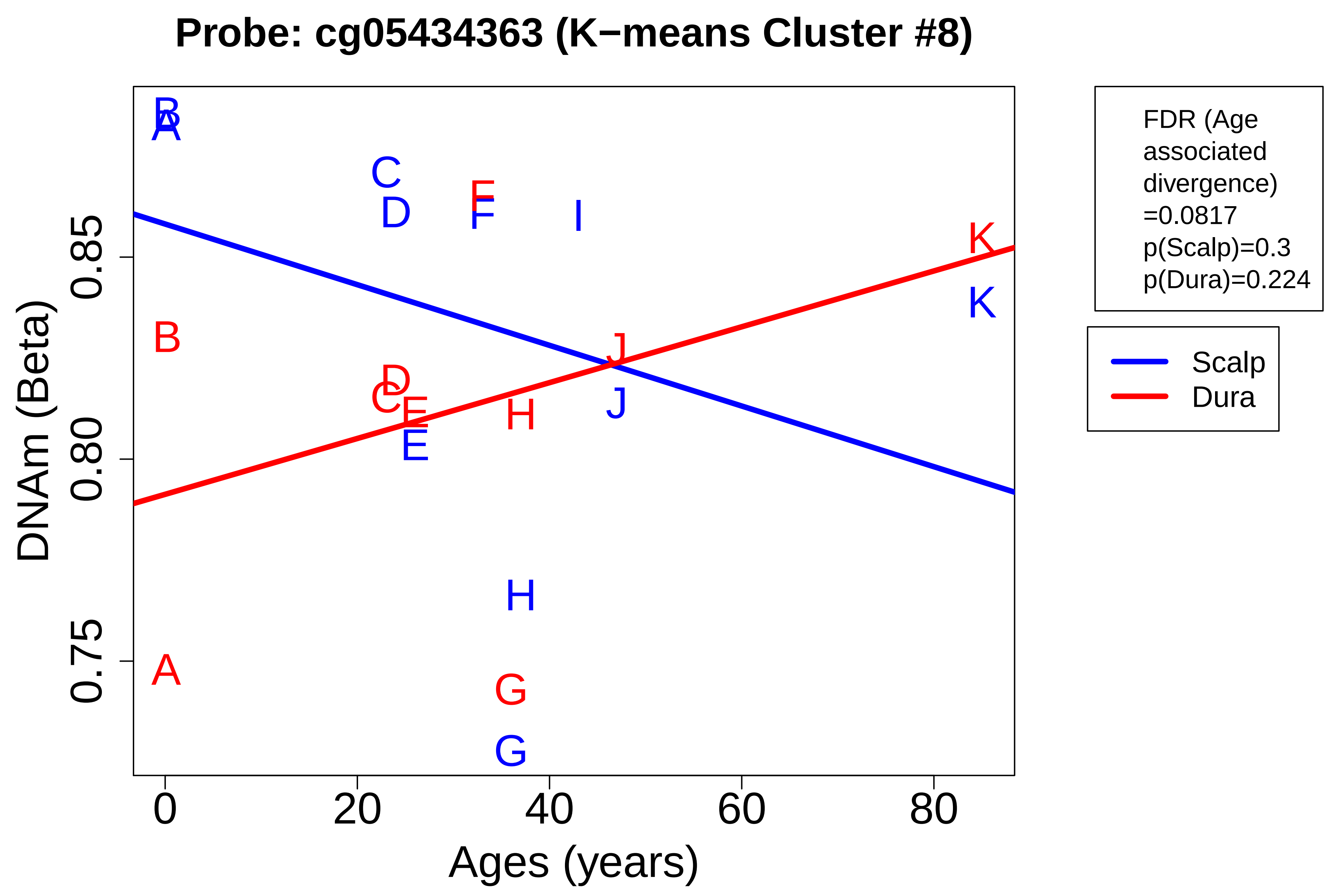

Probe: cg05434363 (K−means Cluster #8)
DNAm (Beta)
0.75
0.80
0.85
Ages (years)
0
20
40
60
80
FDR (Age associated divergence) =0.0817
p(Scalp)=0.3
p(Dura)=0.224
Scalp
Dura

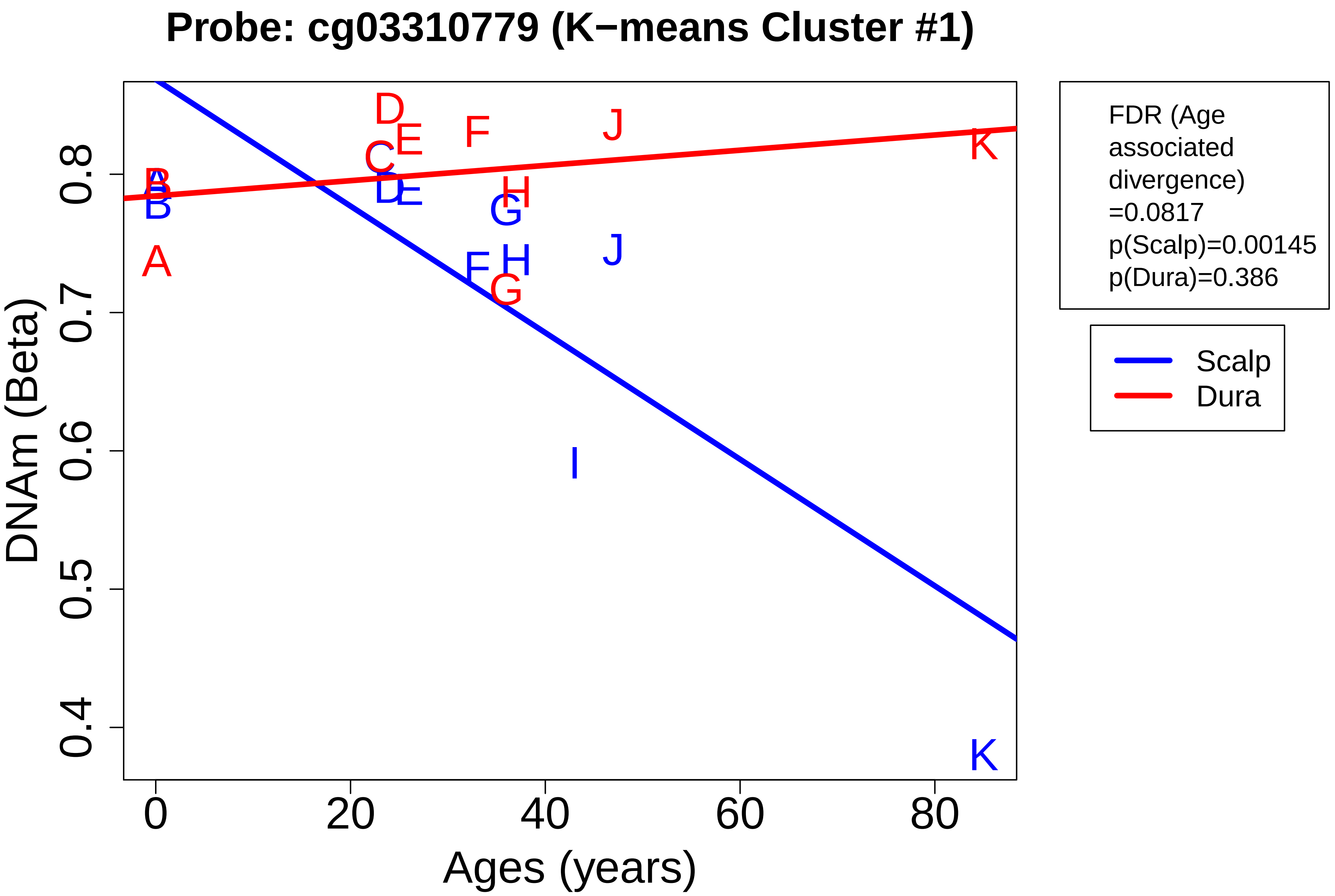
Probe: cg03310779 (K−means Cluster #1)
DNAm (Beta)
0.4
0.5
0.6
0.7
0.8
0
20
40
60
80
Ages (years)
A
B
C
D
E
F
G
H
I
J
K
FDR (Age associated divergence) =0.0817
p(Scalp)=0.00145
p(Dura)=0.386
Scalp
Dura

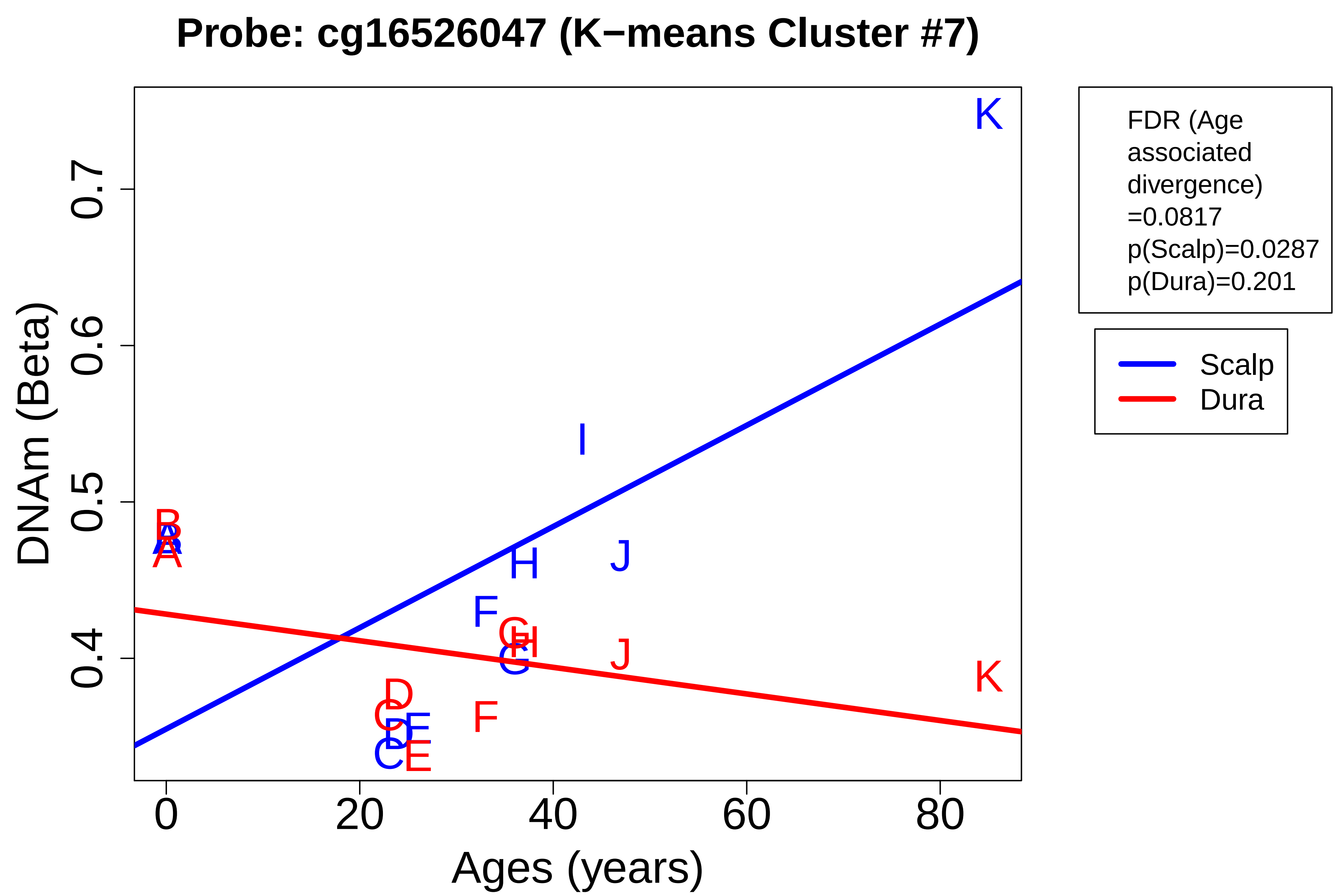
Probe: cg16526047 (K−means Cluster #7)
DNAm (Beta)
Ages (years)
FDR (Age associated divergence) =0.0817
p(Scalp)=0.0287
p(Dura)=0.201
Scalp
Dura

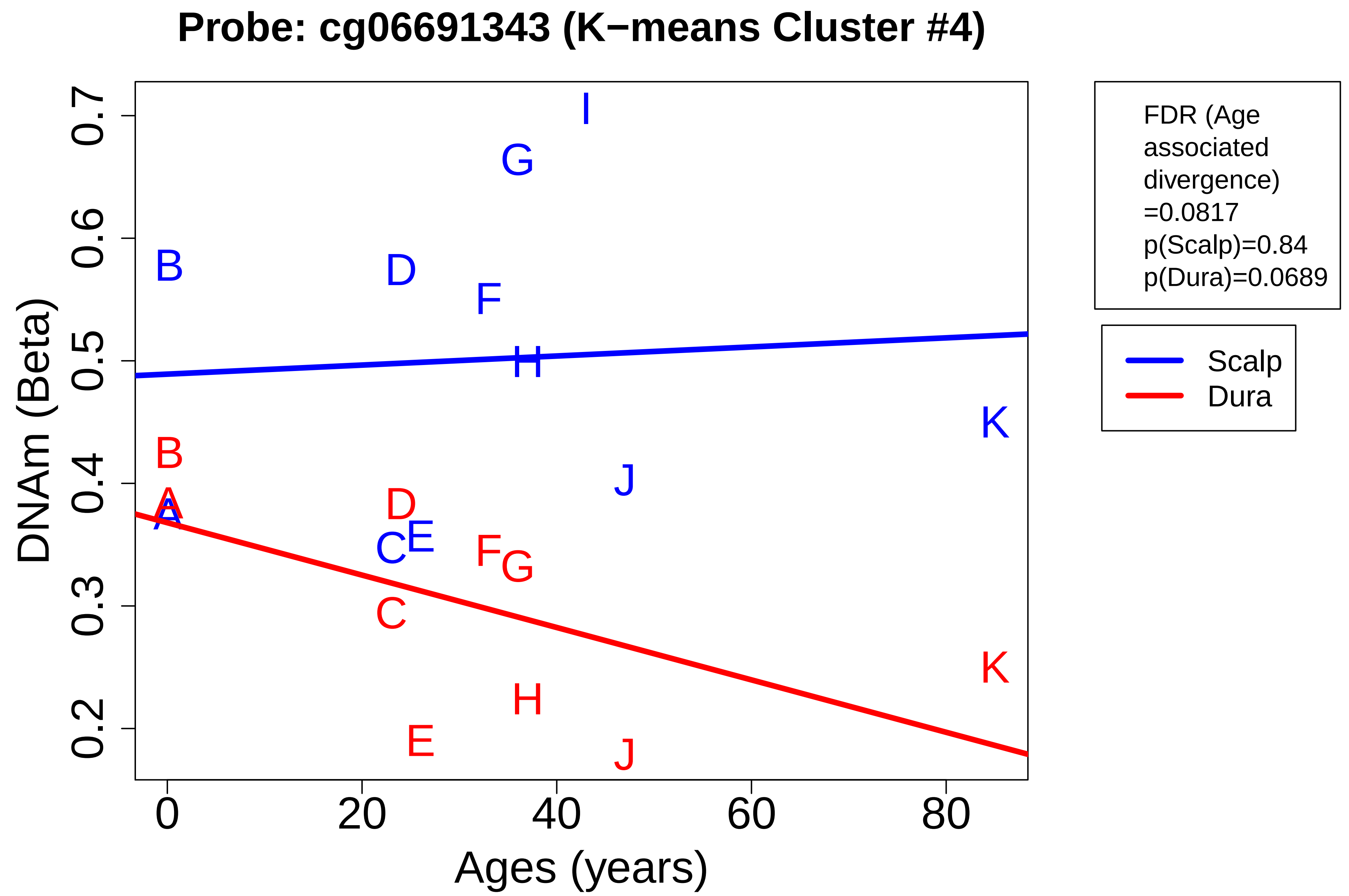

Probe: cg06691343 (K−means Cluster #4)
DNAm (Beta)
Ages (years)
0.2
0.3
0.4
0.5
0.6
0.7
0
20
40
60
80
FDR (Age associated divergence) =0.0817
p(Scalp)=0.84
p(Dura)=0.0689
Scalp
Dura

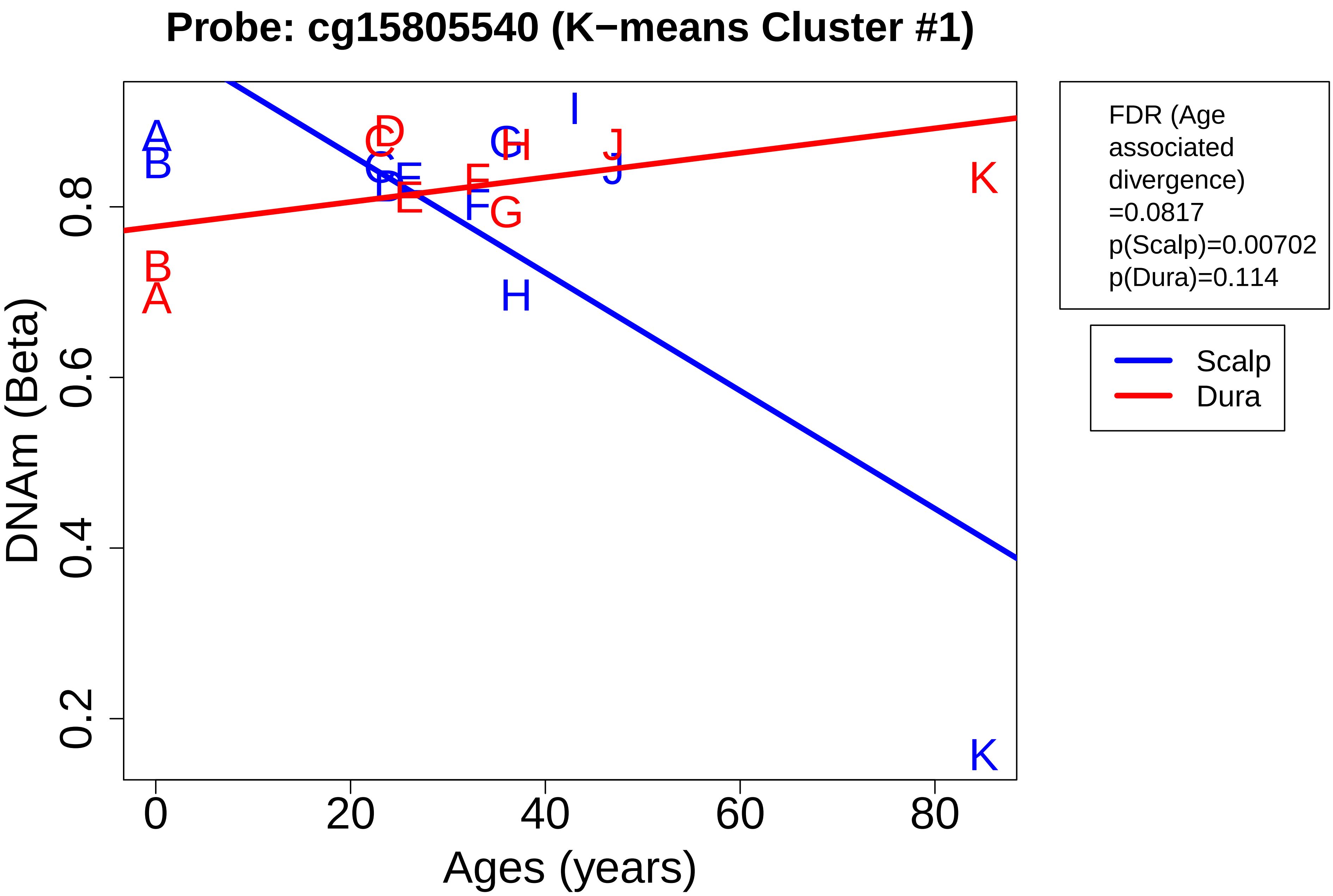
Probe: cg15805540 (K−means Cluster #1)
DNAm (Beta)
0.2
0.4
0.6
0.8
0
20
40
60
80
Ages (years)
FDR (Age associated divergence) =0.0817
p(Scalp)=0.00702
p(Dura)=0.114
Scalp
Dura

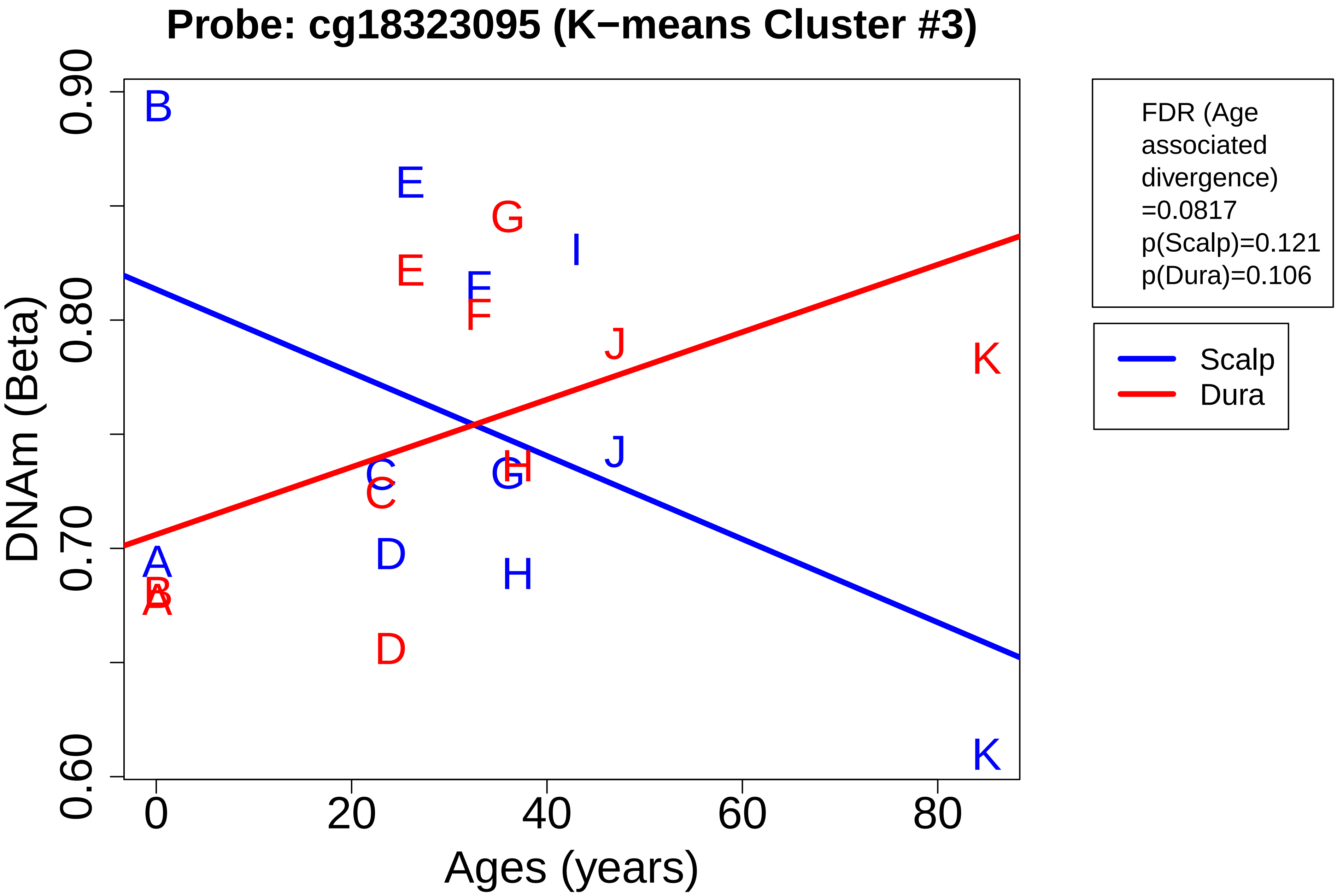

Probe: cg18323095 (K−means Cluster #3)
DNAm (Beta)
0.60
0.70
0.80
0.90
Ages (years)
0
20
40
60
80
FDR (Age associated divergence) =0.0817
p(Scalp)=0.121
p(Dura)=0.106
Scalp
Dura

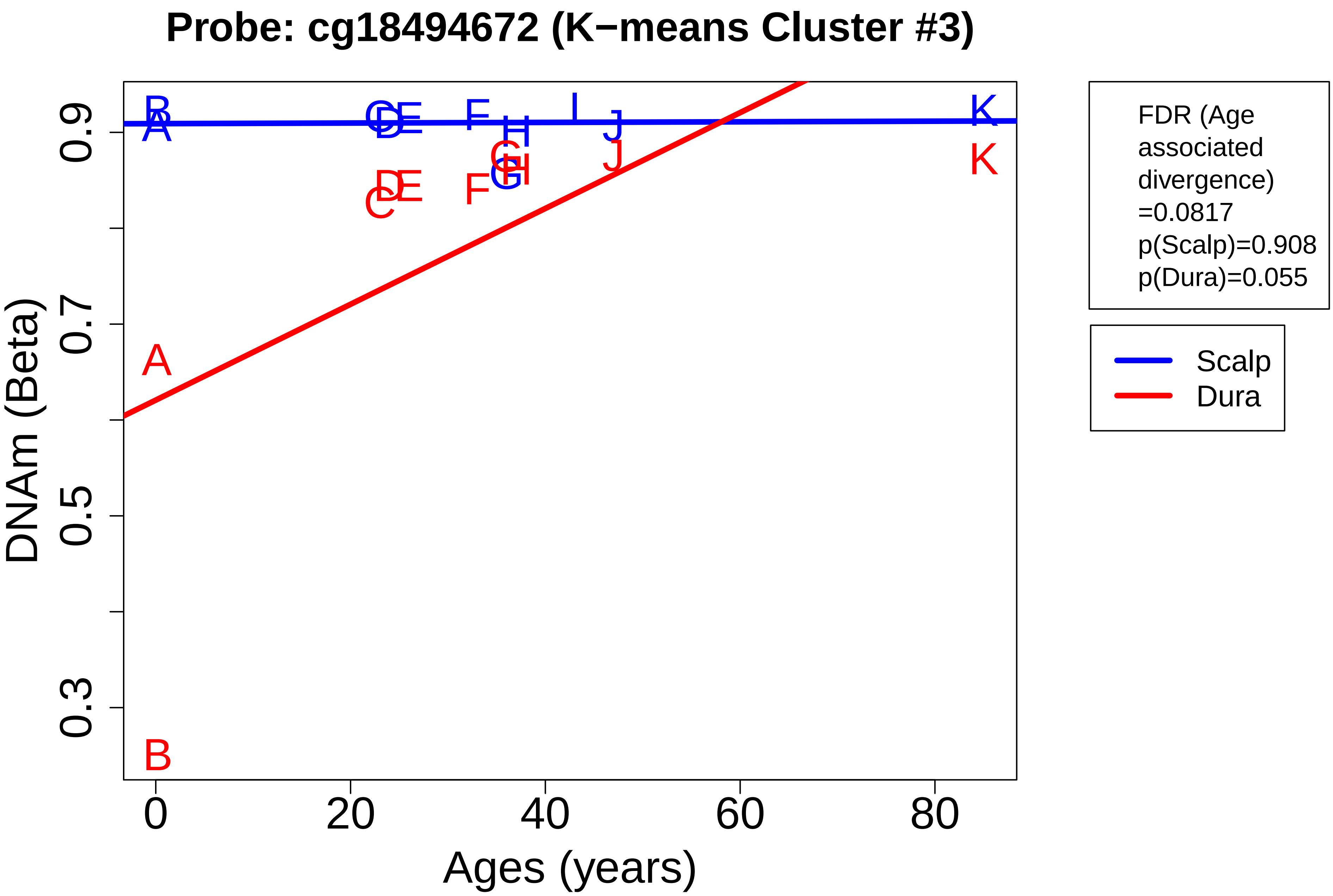

Probe: cg18494672 (K−means Cluster #3)
DNAm (Beta)
0.3
0.5
0.7
0.9
0
20
40
60
80
Ages (years)
FDR (Age associated divergence) =0.0817
p(Scalp)=0.908
p(Dura)=0.055
Scalp
Dura

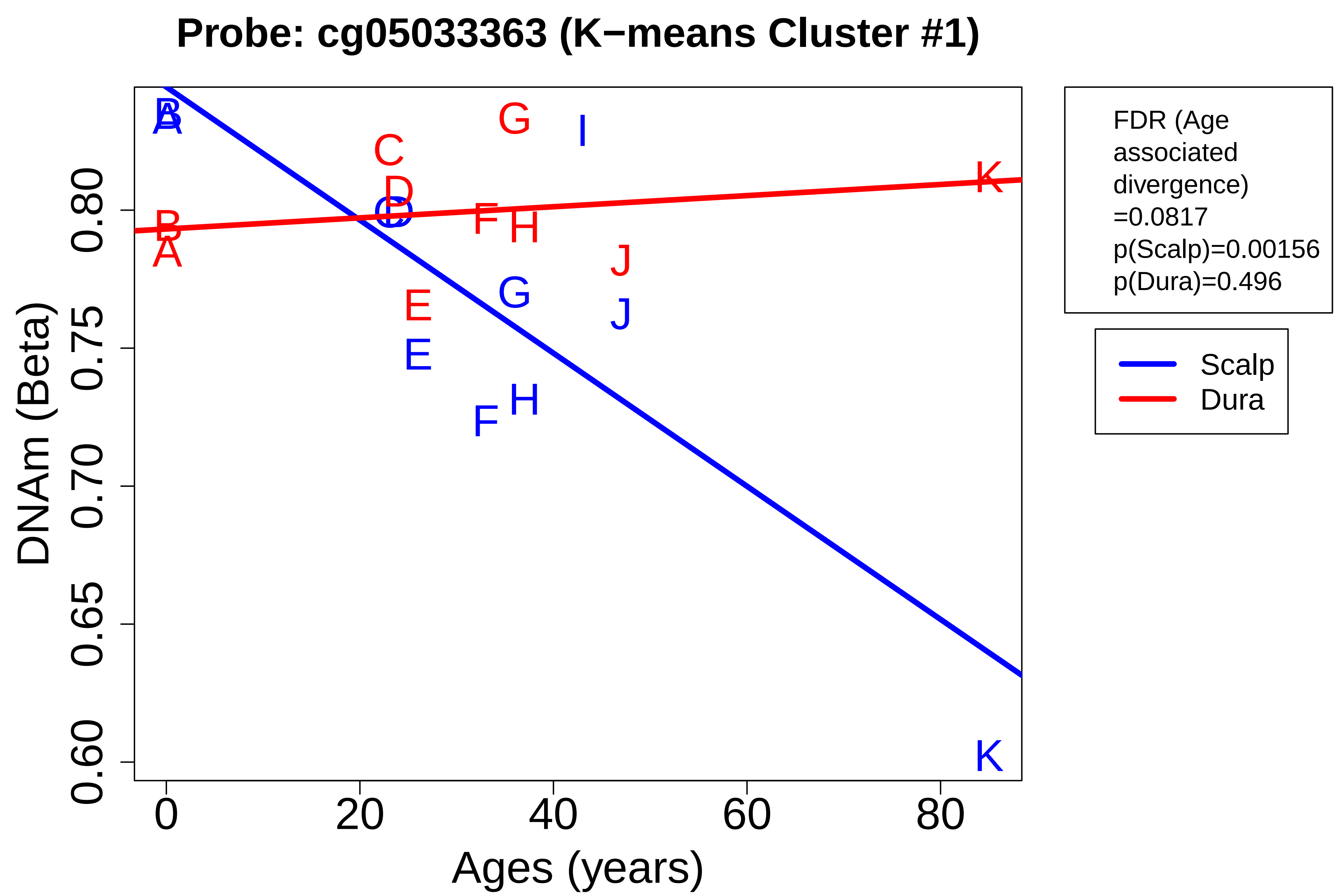

Probe: cg05033363 (K−means Cluster #1)
DNAm (Beta)
Ages (years)
0.60
0.65
0.70
0.75
0.80
0
20
40
60
80
FDR (Age associated divergence) =0.0817
p(Scalp)=0.00156
p(Dura)=0.496
Scalp
Dura

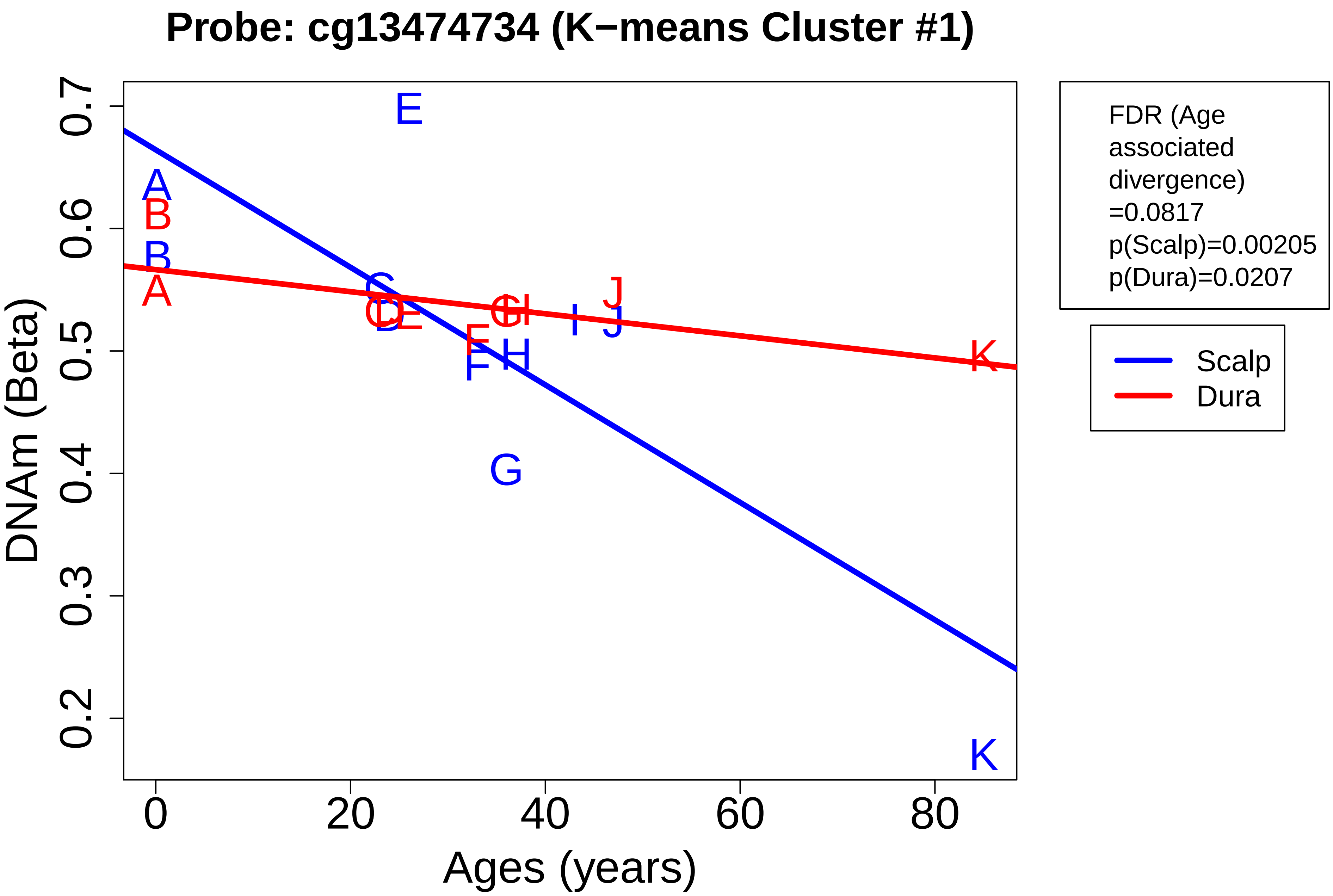

Probe: cg13474734 (K−means Cluster #1)
DNAm (Beta)
0.2
0.3
0.4
0.5
0.6
0.7
Ages (years)
0
20
40
60
80
FDR (Age associated divergence) =0.0817
p(Scalp)=0.00205
p(Dura)=0.0207
Scalp
Dura

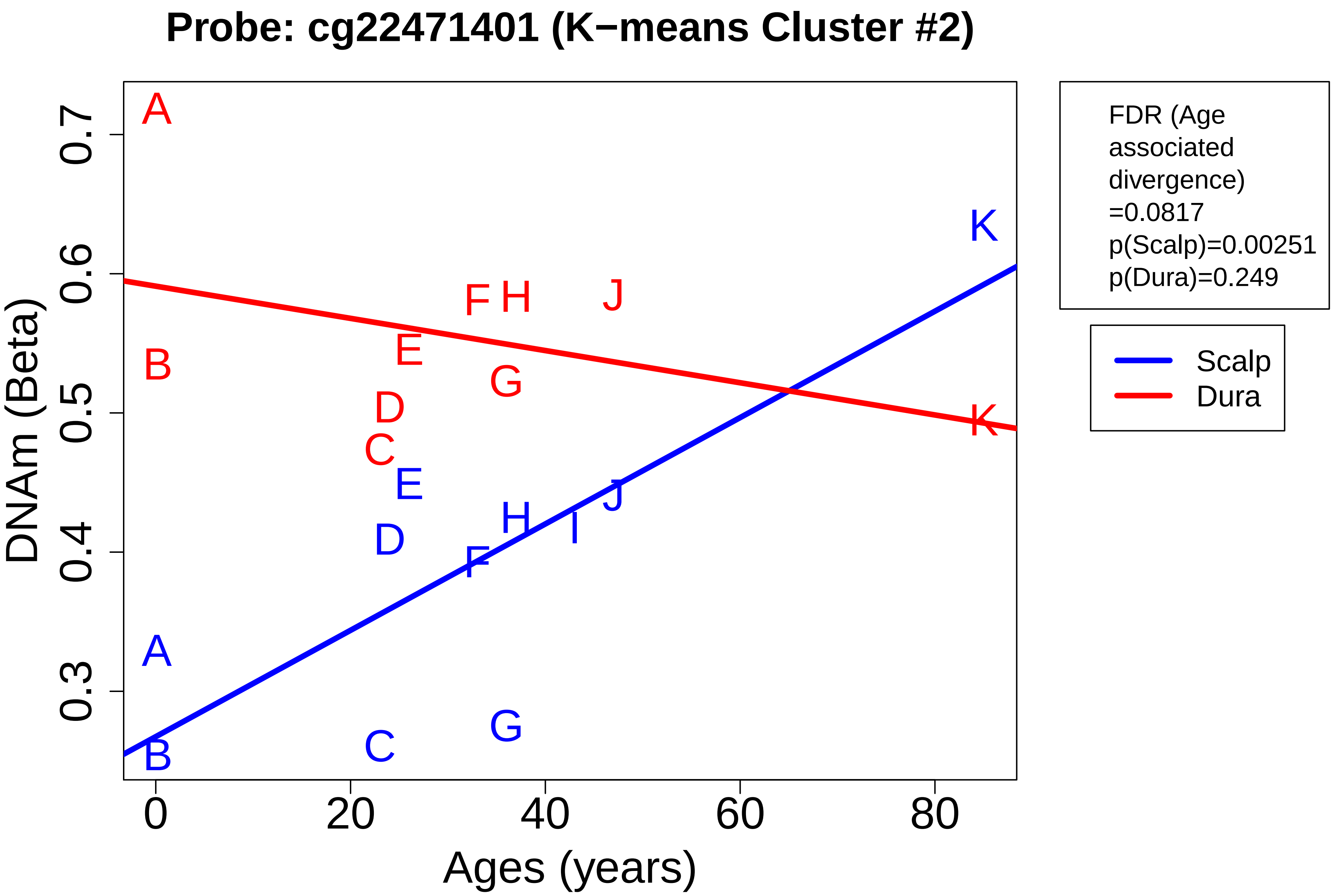

Probe: cg22471401 (K−means Cluster #2)
DNAm (Beta)
0.3
0.4
0.5
0.6
0.7
0
20
40
60
80
Ages (years)
FDR (Age associated divergence) =0.0817
p(Scalp)=0.00251
p(Dura)=0.249
Scalp
Dura

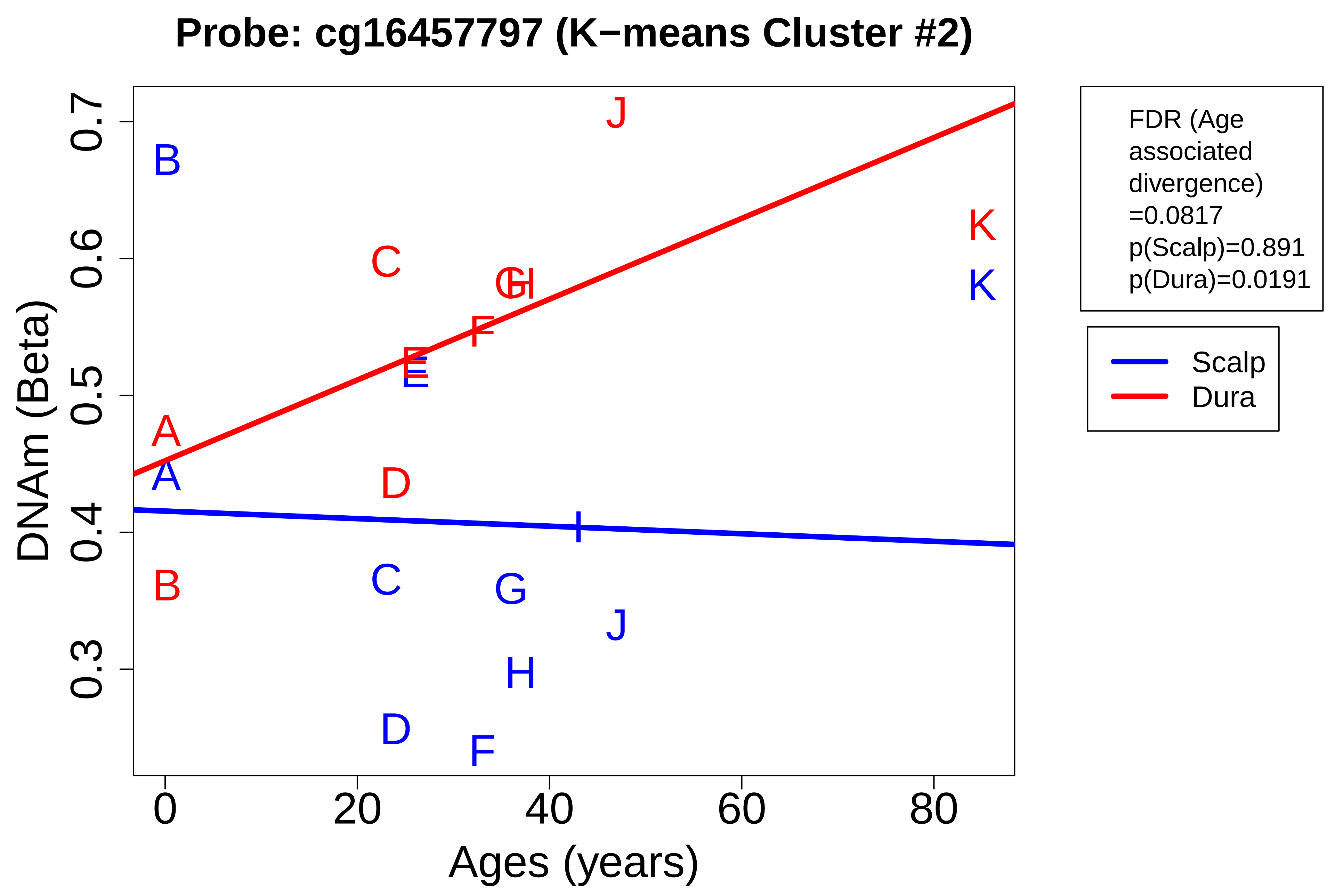

Probe: cg16457797 (K−means Cluster #2)
DNAm (Beta)
0.3
0.4
0.5
0.6
0.7
0
20
40
60
80
Ages (years)
A
B
C
D
E
F
G
H
J
K
FDR (Age associated divergence) =0.0817
p(Scalp)=0.891
p(Dura)=0.0191
Scalp
Dura

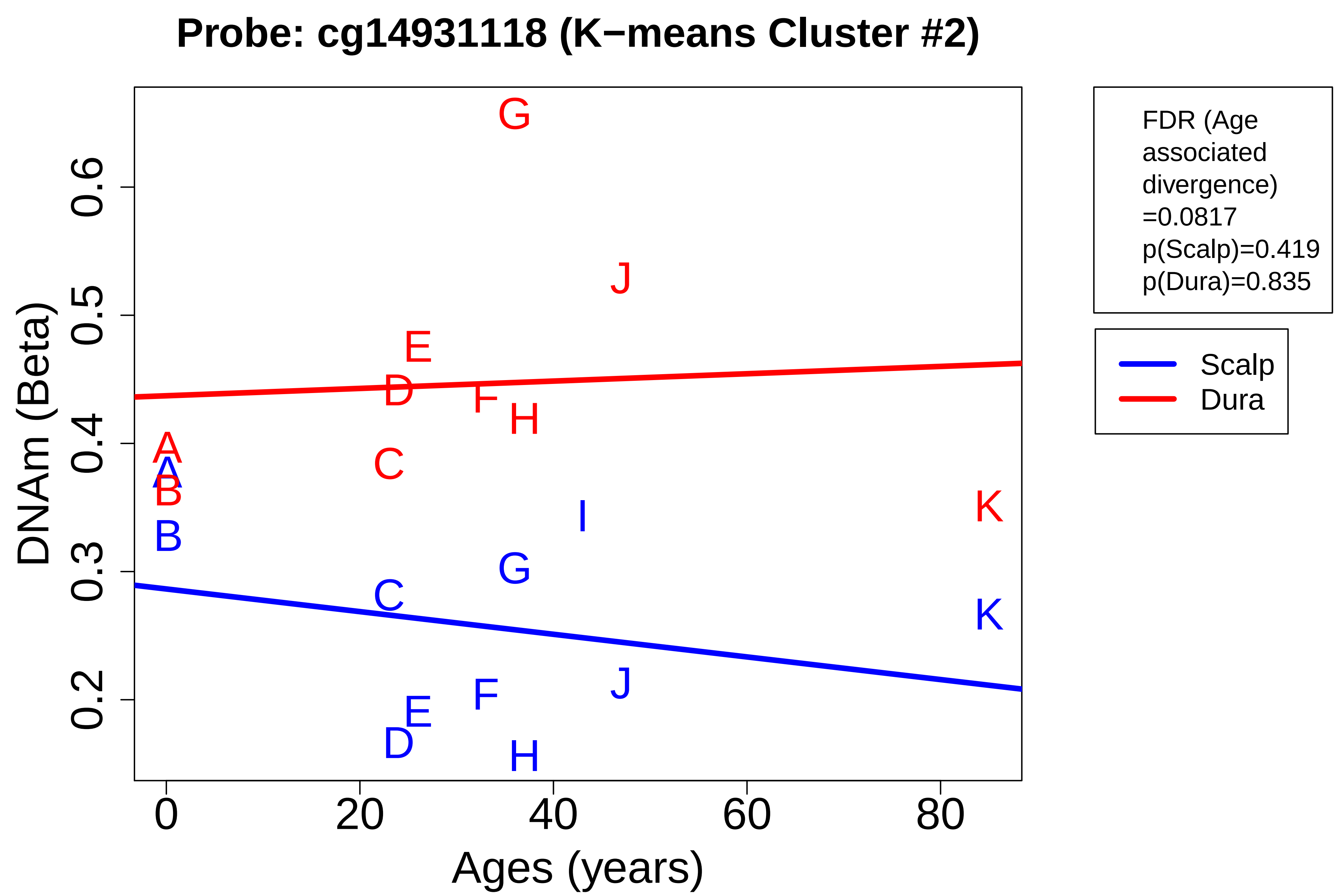

Probe: cg14931118 (K−means Cluster #2)
FDR (Age associated divergence) =0.0817
p(Scalp)=0.419
p(Dura)=0.835
Scalp
Dura
DNAm (Beta)
0.2
0.3
0.4
0.5
0.6
0
20
40
60
80
Ages (years)
A B C D E F G H I J K

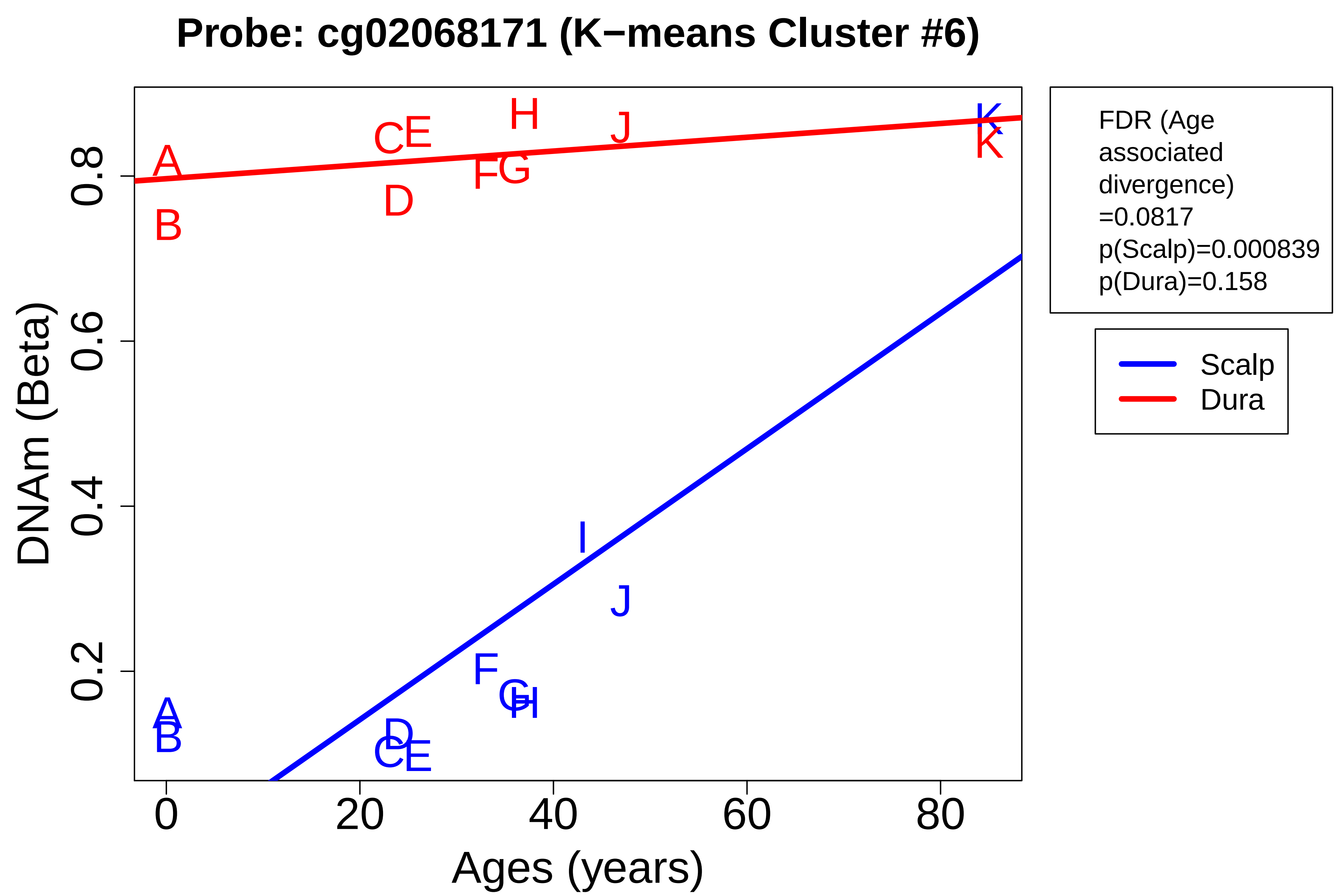

Probe: cg02068171 (K−means Cluster #6)
DNAm (Beta)
0.2
0.4
0.6
0.8
0
20
40
60
80
Ages (years)
FDR (Age associated divergence) =0.0817
p(Scalp)=0.000839
p(Dura)=0.158
Scalp
Dura

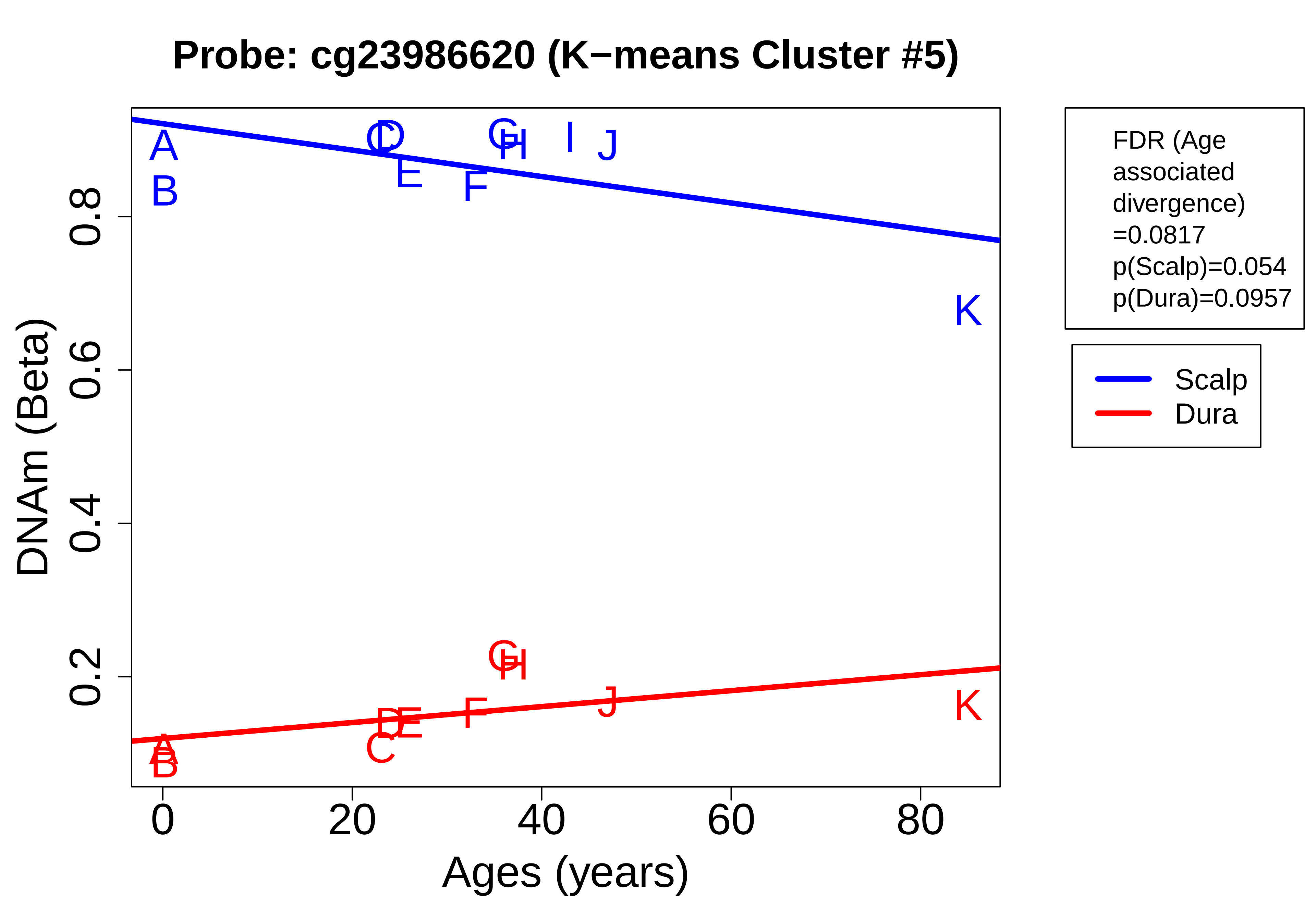

Probe: cg23986620 (K−means Cluster #5)
DNAm (Beta)
Ages (years)
0.2
0.4
0.6
0.8
0
20
40
60
80
A
B
C
D
E
F
G
H
I
J
K
FDR (Age associated divergence) =0.0817
p(Scalp)=0.054
p(Dura)=0.0957
Scalp
Dura

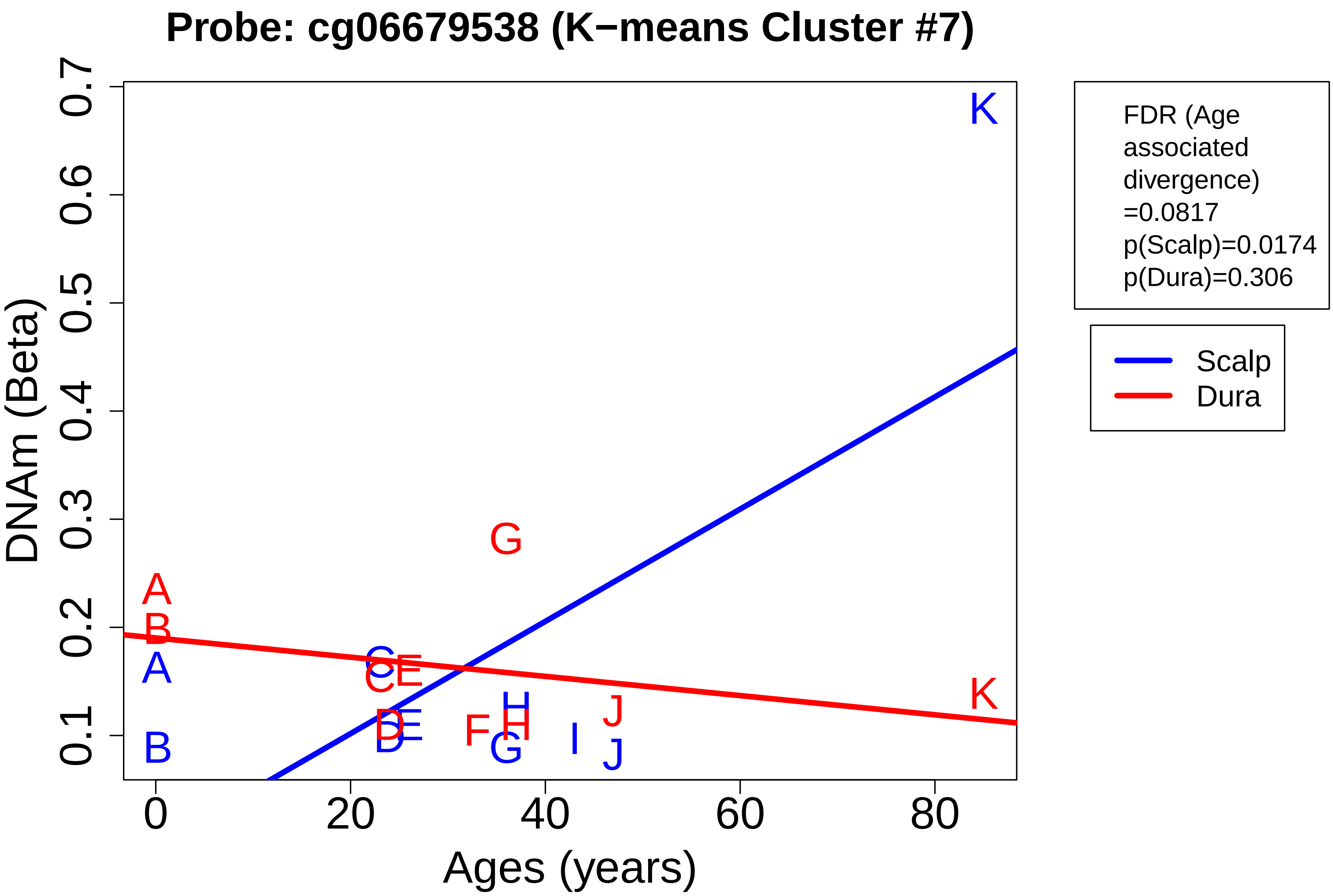

Probe: cg06679538 (K−means Cluster #7)
DNAm (Beta)
0.1
0.2
0.3
0.4
0.5
0.6
0.7
0
20
40
60
80
Ages (years)
FDR (Age associated divergence) =0.0817
p(Scalp)=0.0174
p(Dura)=0.306
Scalp
Dura

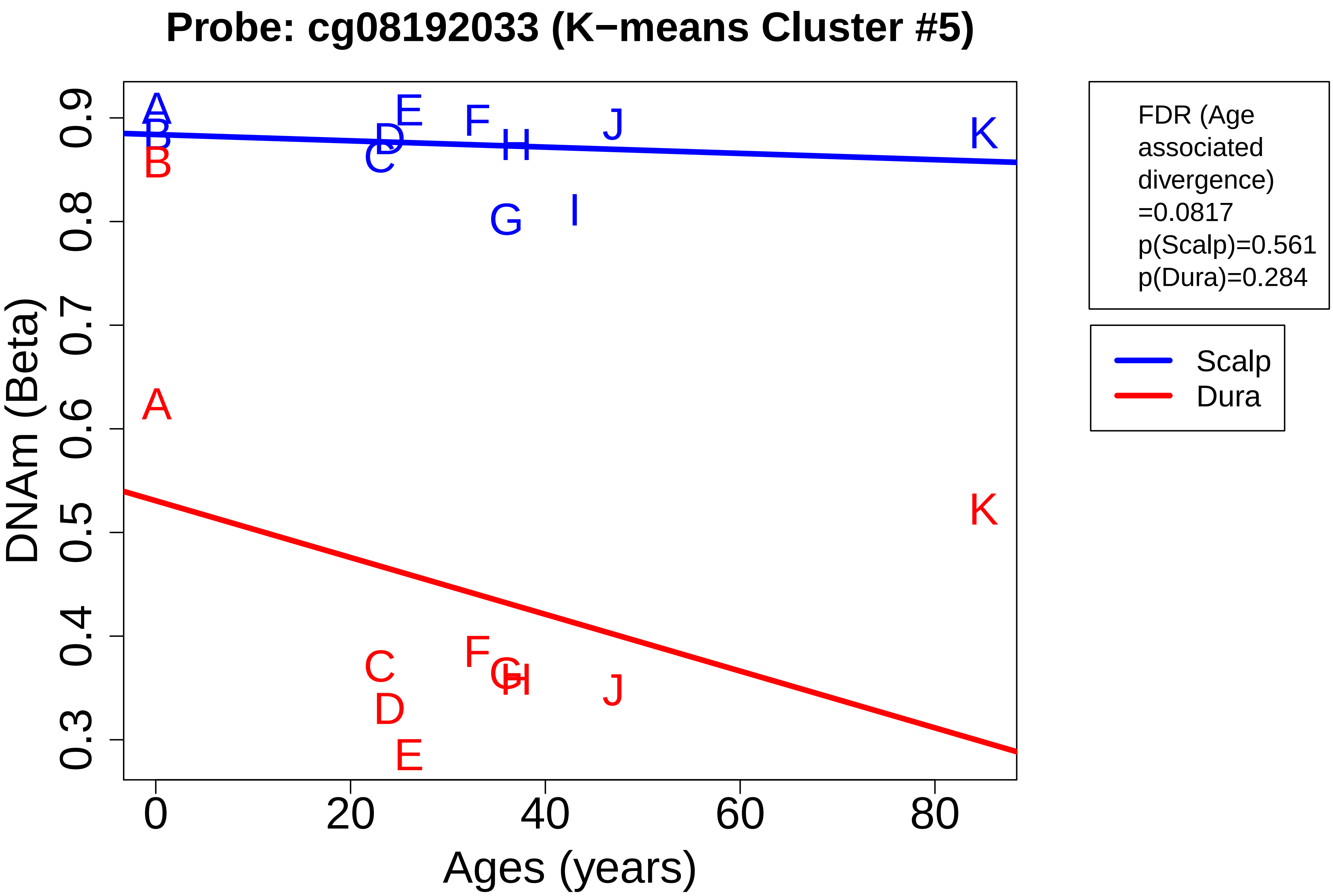

Probe: cg08192033 (K−means Cluster #5)
DNAm (Beta)
0.3
0.4
0.5
0.6
0.7
0.8
0.9
0
20
40
60
80
Ages (years)
A
B
C
D
E
F
G
H
I
J
K
FDR (Age associated divergence) =0.0817
p(Scalp)=0.561
p(Dura)=0.284
Scalp
Dura

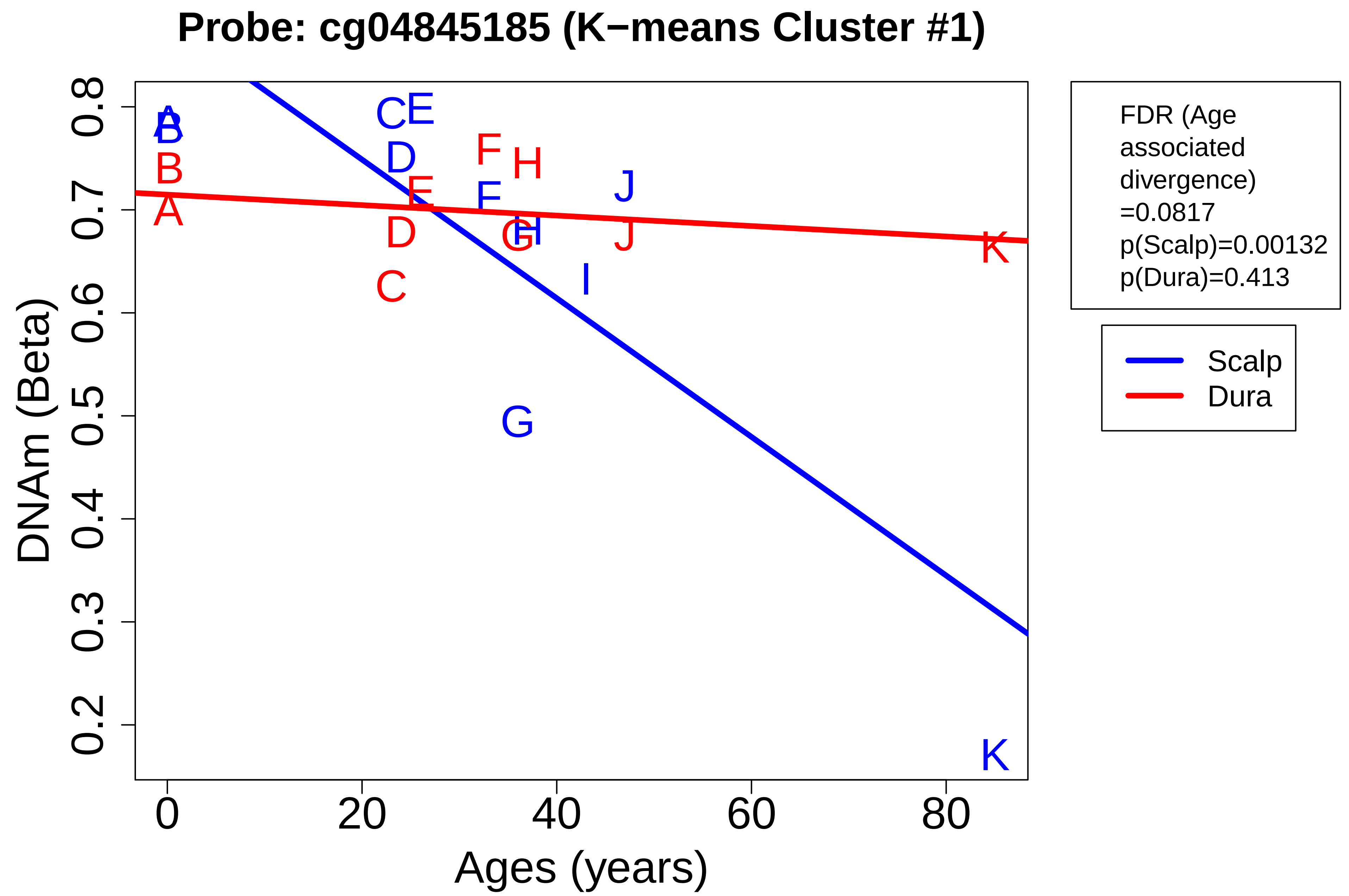
Probe: cg04845185 (K−means Cluster #1)
DNAm (Beta)
Ages (years)
0.2
0.3
0.4
0.5
0.6
0.7
0.8
0
20
40
60
80
FDR (Age associated divergence) =0.0817
p(Scalp)=0.00132
p(Dura)=0.413
Scalp
Dura

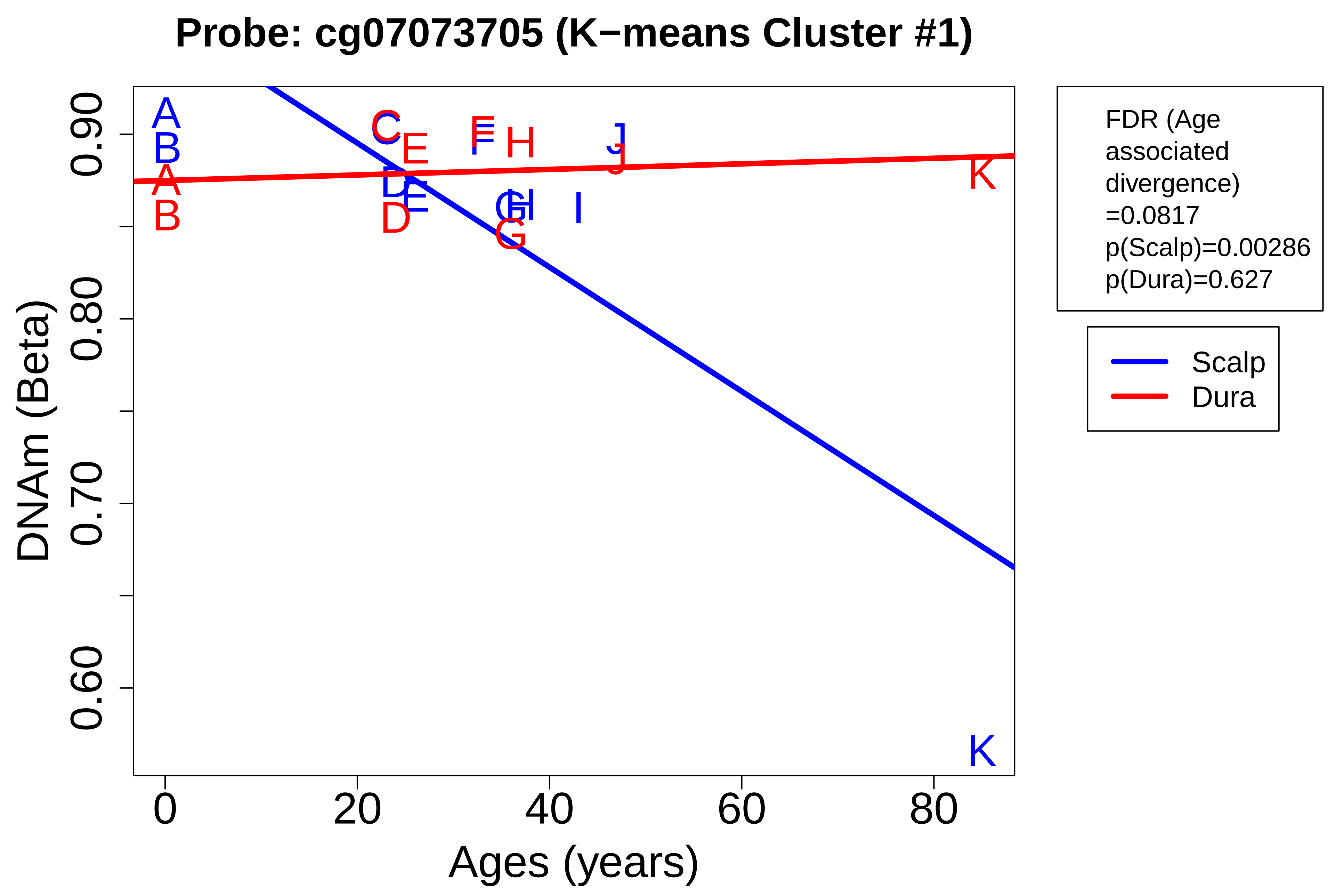

Probe: cg07073705 (K−means Cluster #1)
DNAm (Beta)
0.60
0.70
0.80
0.90
Ages (years)
0
20
40
60
80
A
B
C
D
E
F
G
H
I
J
K
FDR (Age associated divergence) =0.0817
p(Scalp)=0.00286
p(Dura)=0.627
Scalp
Dura

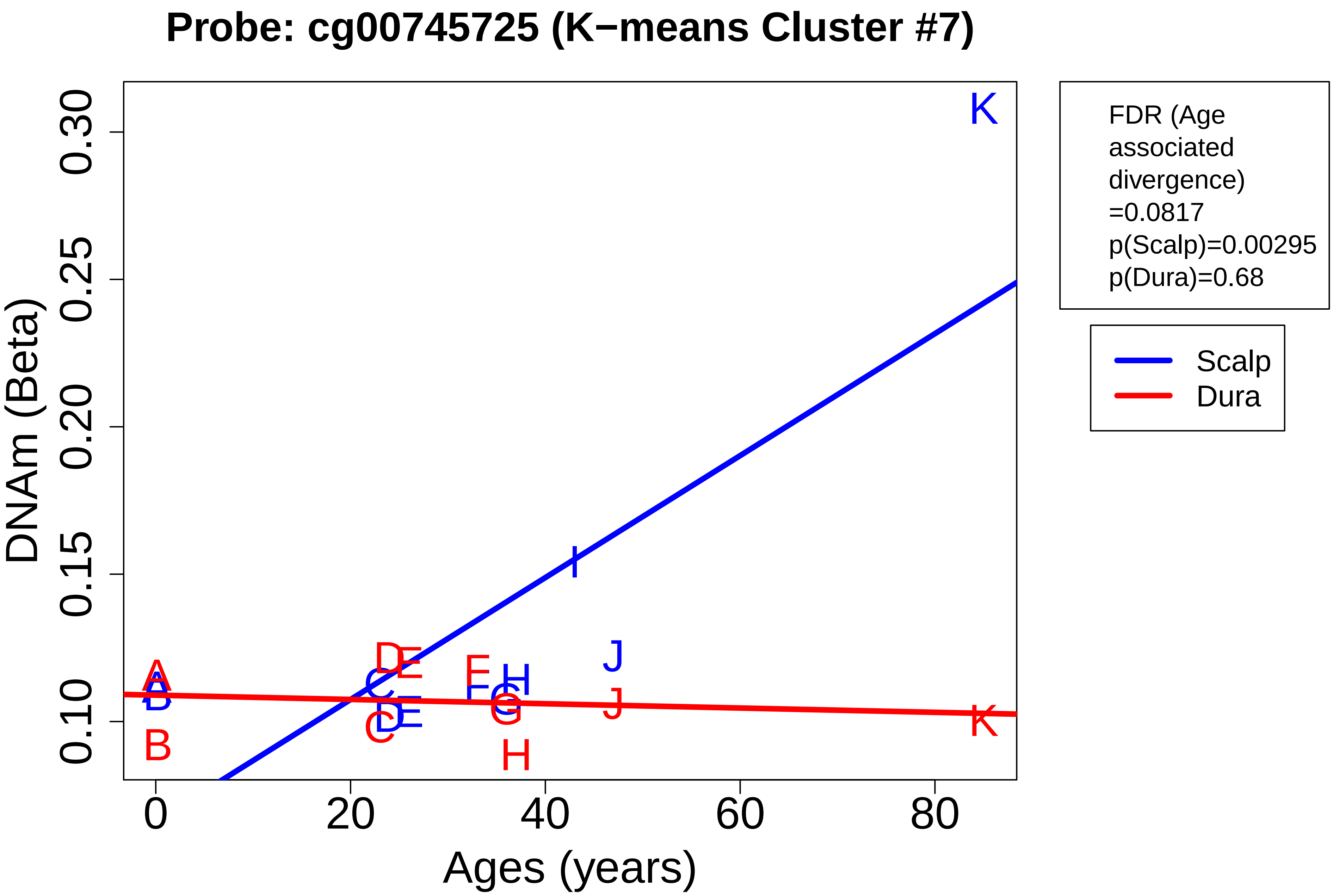

Probe: cg00745725 (K−means Cluster #7)
DNAm (Beta)
0.10
0.15
0.20
0.25
0.30
Ages (years)
0
20
40
60
80
FDR (Age associated divergence) =0.0817
p(Scalp)=0.00295
p(Dura)=0.68
Scalp
Dura

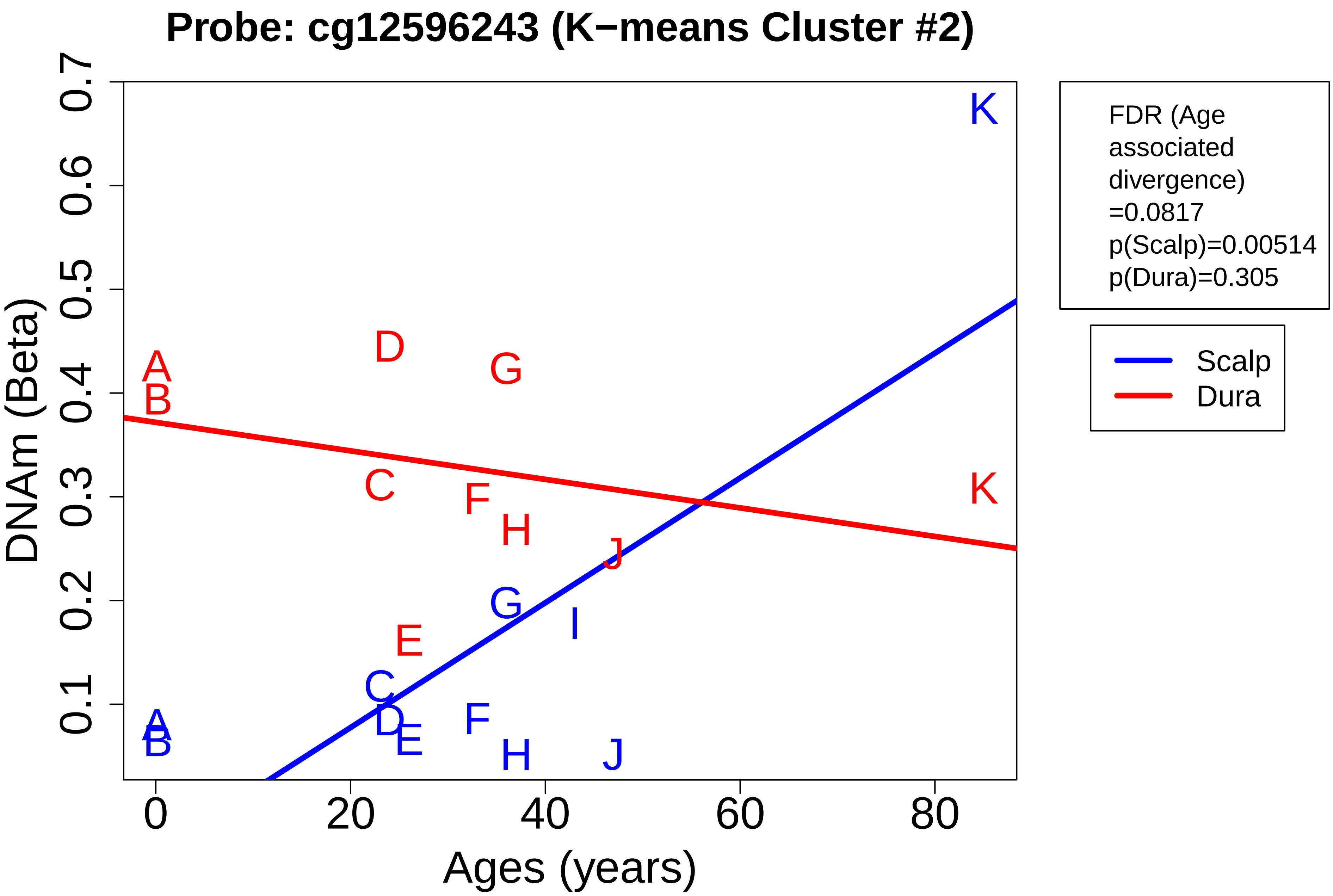
Probe: cg12596243 (K−means Cluster #2)
DNAm (Beta)
0.1
0.2
0.3
0.4
0.5
0.6
0.7
0
20
40
60
80
Ages (years)
FDR (Age associated divergence) =0.0817
p(Scalp)=0.00514
p(Dura)=0.305
Scalp
Dura
A
B
C
D
E
F
G
H
I
J
K

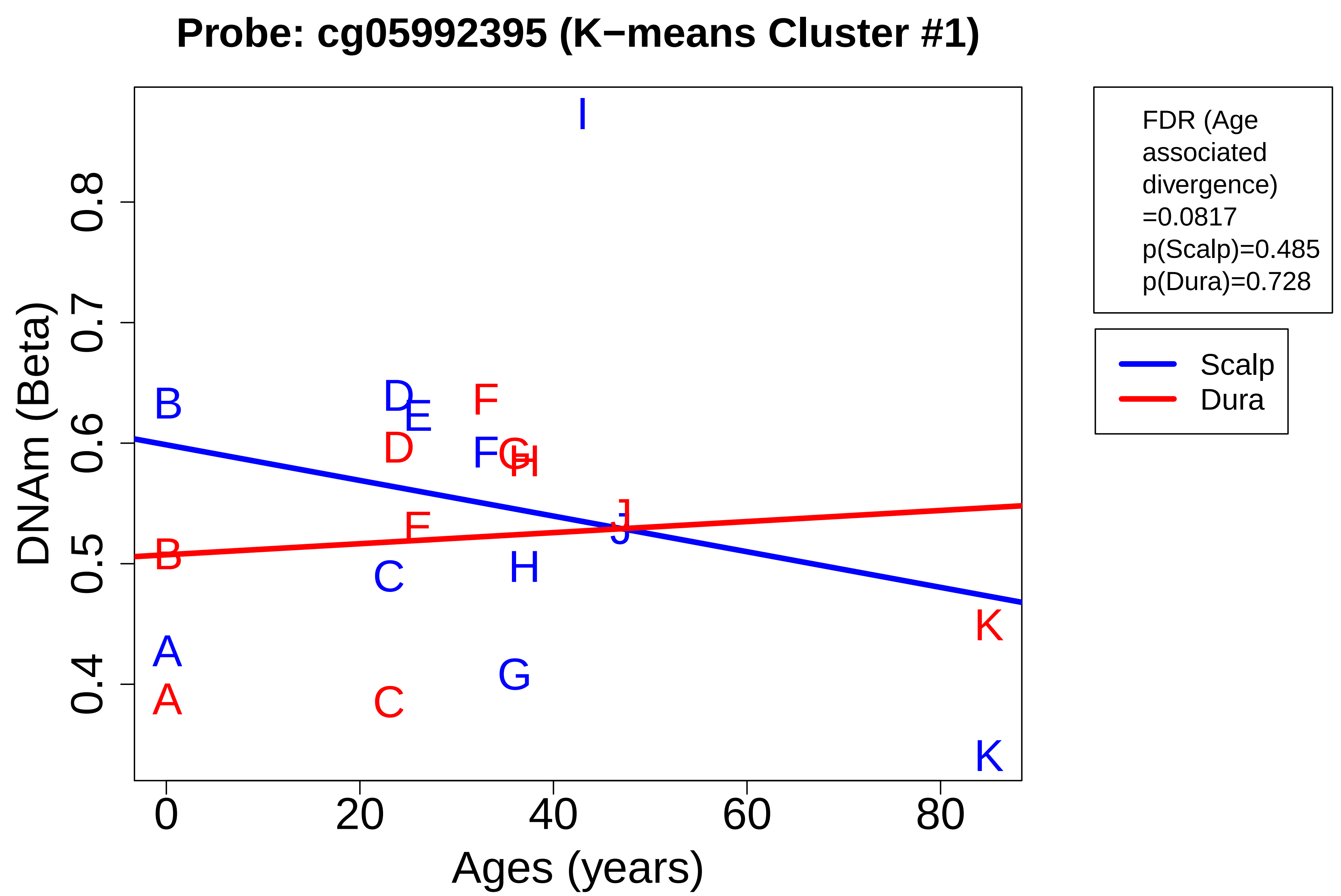

Probe: cg05992395 (K−means Cluster #1)
DNAm (Beta)
0.4
0.5
0.6
0.7
0.8
Ages (years)
0
20
40
60
80
FDR (Age associated divergence) =0.0817
p(Scalp)=0.485
p(Dura)=0.728
Scalp
Dura

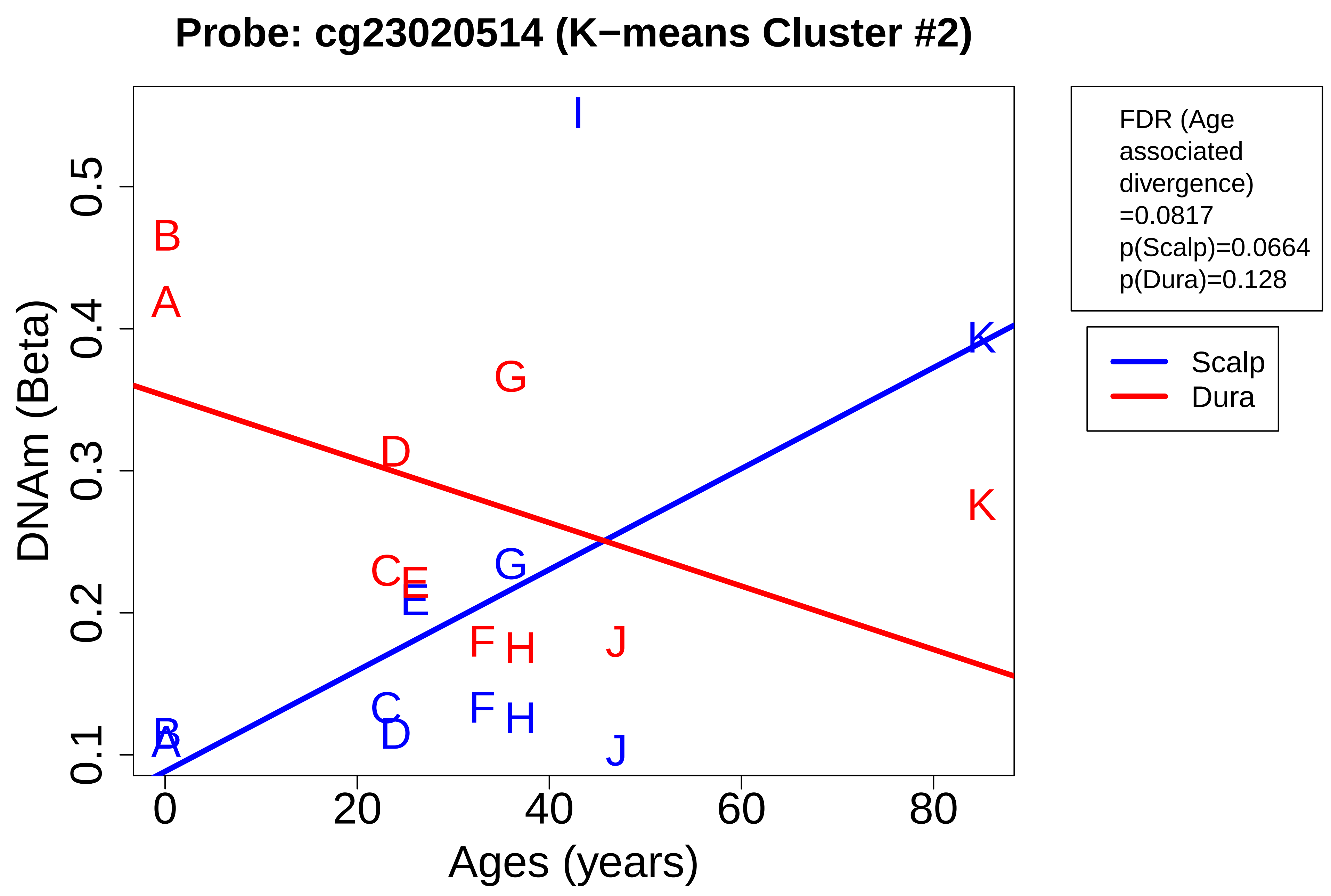

Probe: cg23020514 (K−means Cluster #2)
FDR (Age associated divergence) =0.0817
p(Scalp)=0.0664
p(Dura)=0.128
Scalp
Dura
DNAm (Beta)
Ages (years)
0.1
0.2
0.3
0.4
0.5
0
20
40
60
80

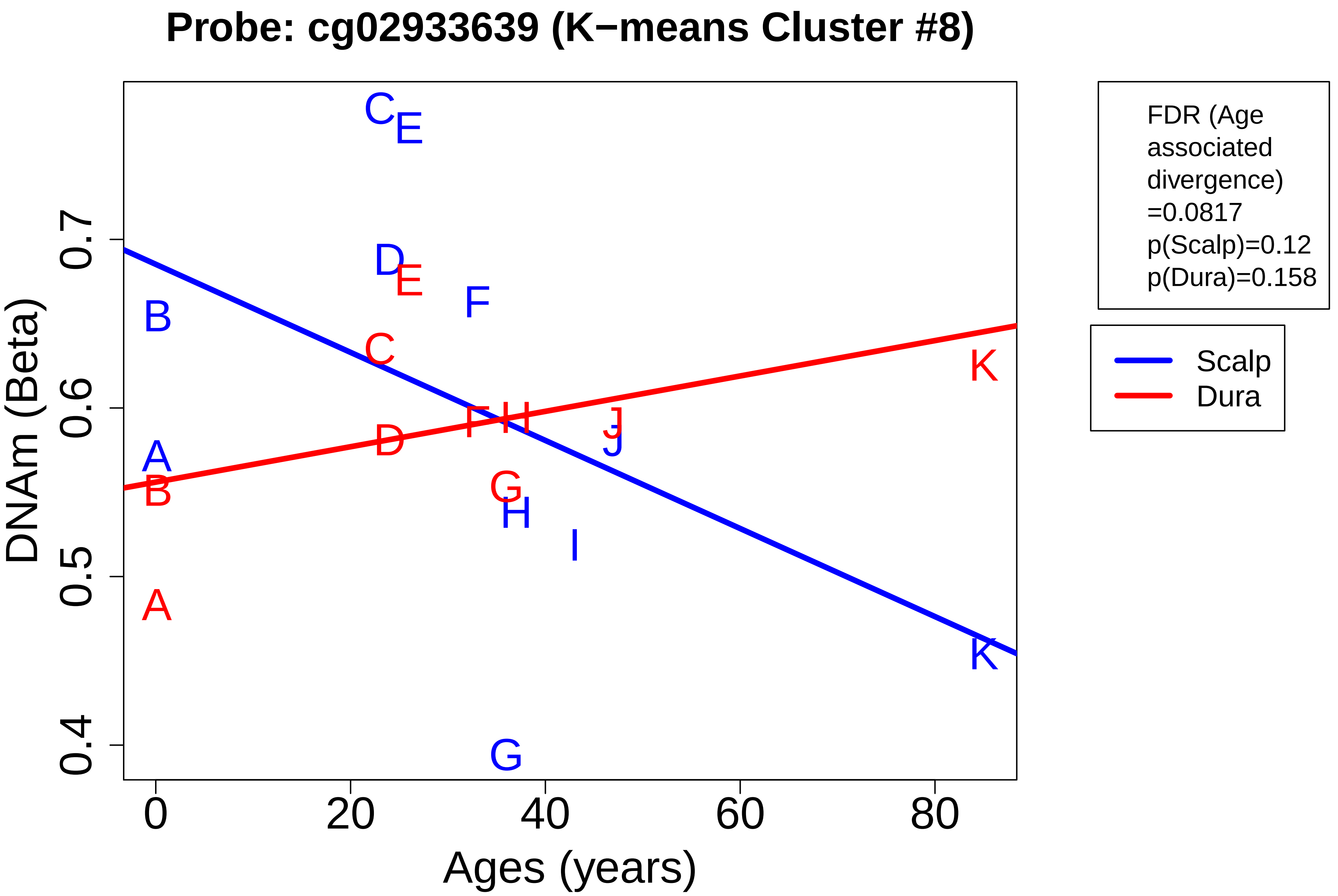

Probe: cg02933639 (K−means Cluster #8)
DNAm (Beta)
0.4
0.5
0.6
0.7
0
20
40
60
80
Ages (years)
FDR (Age associated divergence) =0.0817
p(Scalp)=0.12
p(Dura)=0.158
Scalp
Dura

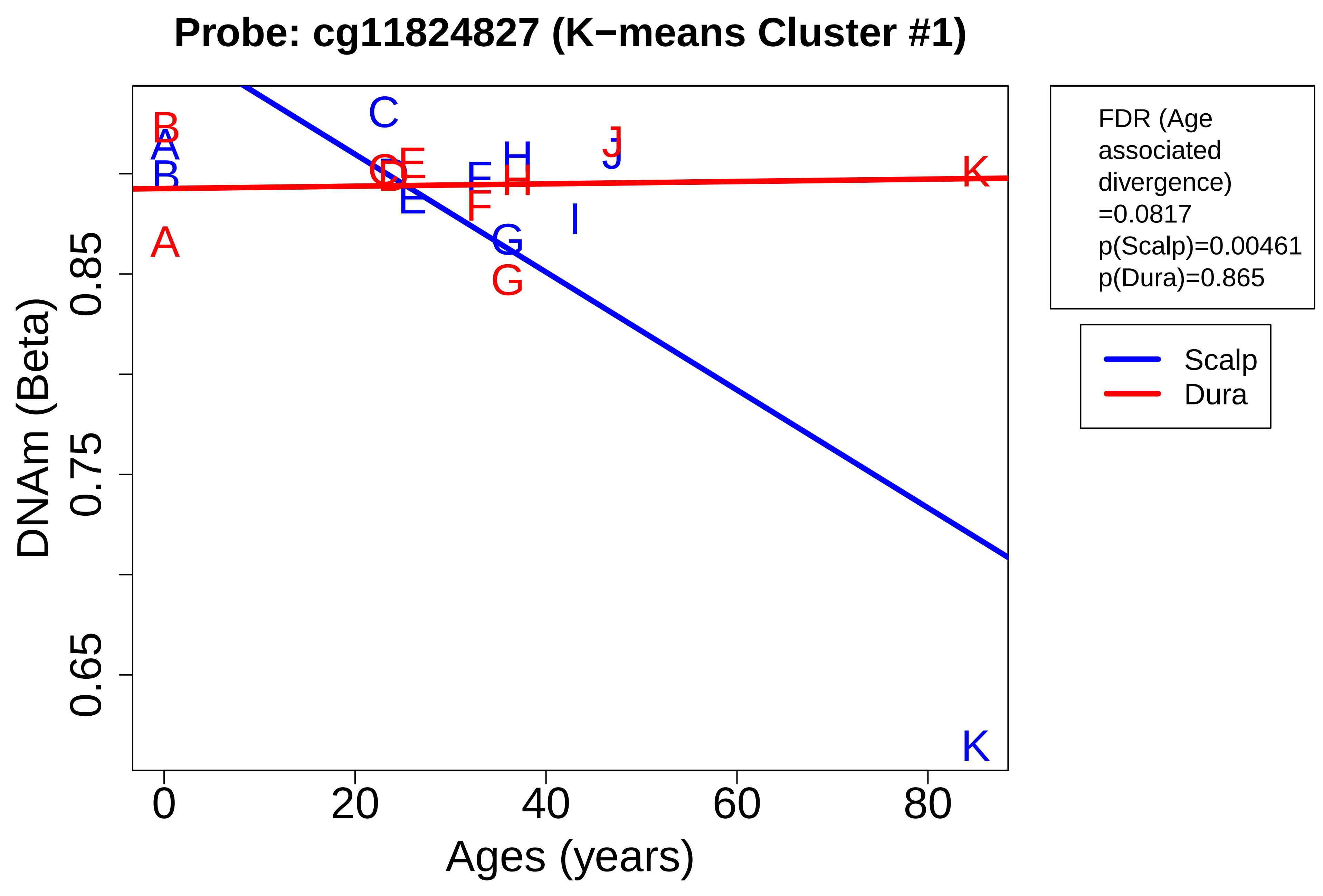

Probe: cg11824827 (K−means Cluster #1)
DNAm (Beta)
0.65
0.75
0.85
Ages (years)
0
20
40
60
80
A B C D E F G H I J K
FDR (Age associated divergence) =0.0817
p(Scalp)=0.00461
p(Dura)=0.865
Scalp
Dura

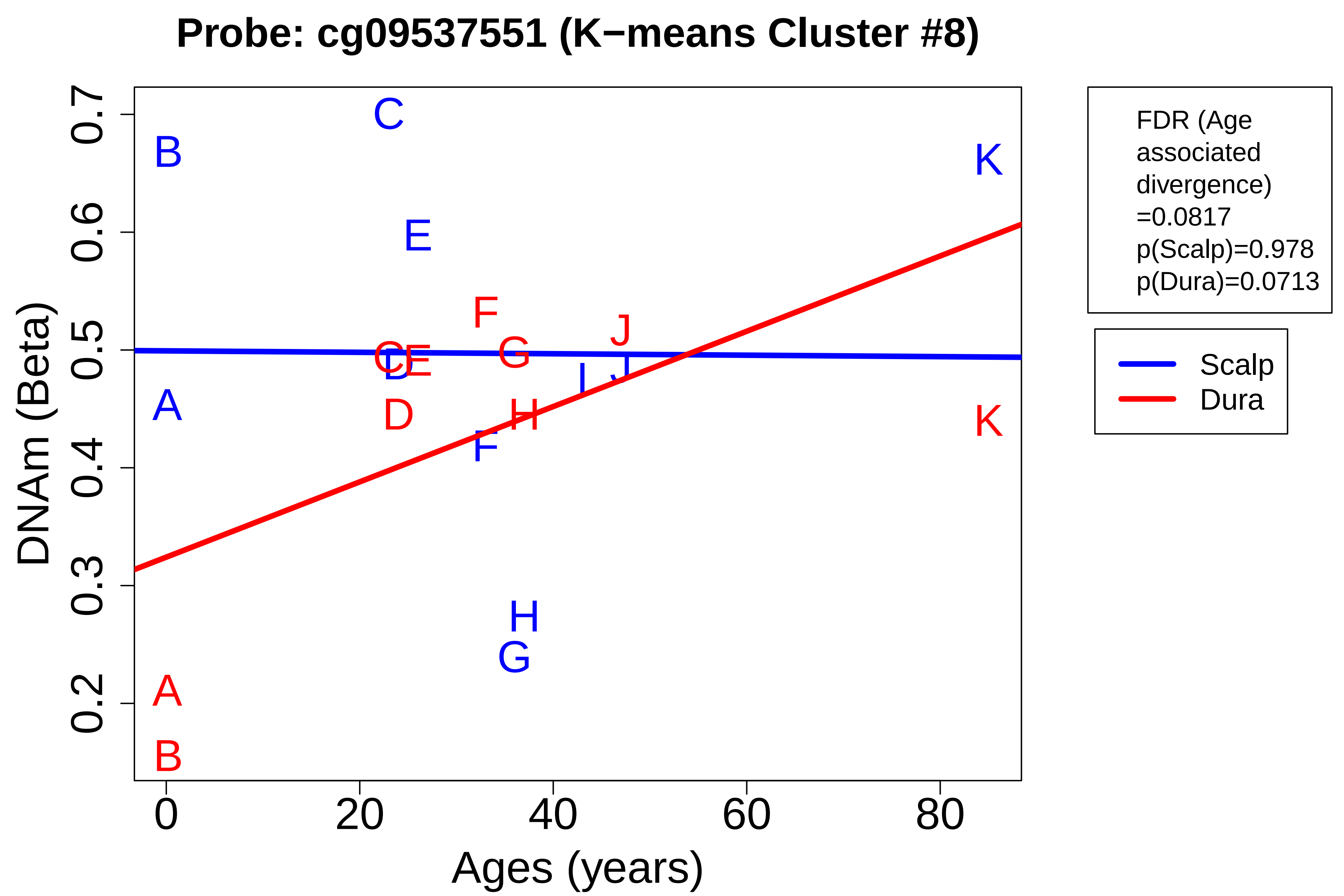

Probe: cg09537551 (K−means Cluster #8)
DNAm (Beta)
0.2
0.3
0.4
0.5
0.6
0.7
0
20
40
60
80
Ages (years)
FDR (Age associated divergence) =0.0817
p(Scalp)=0.978
p(Dura)=0.0713
Scalp
Dura

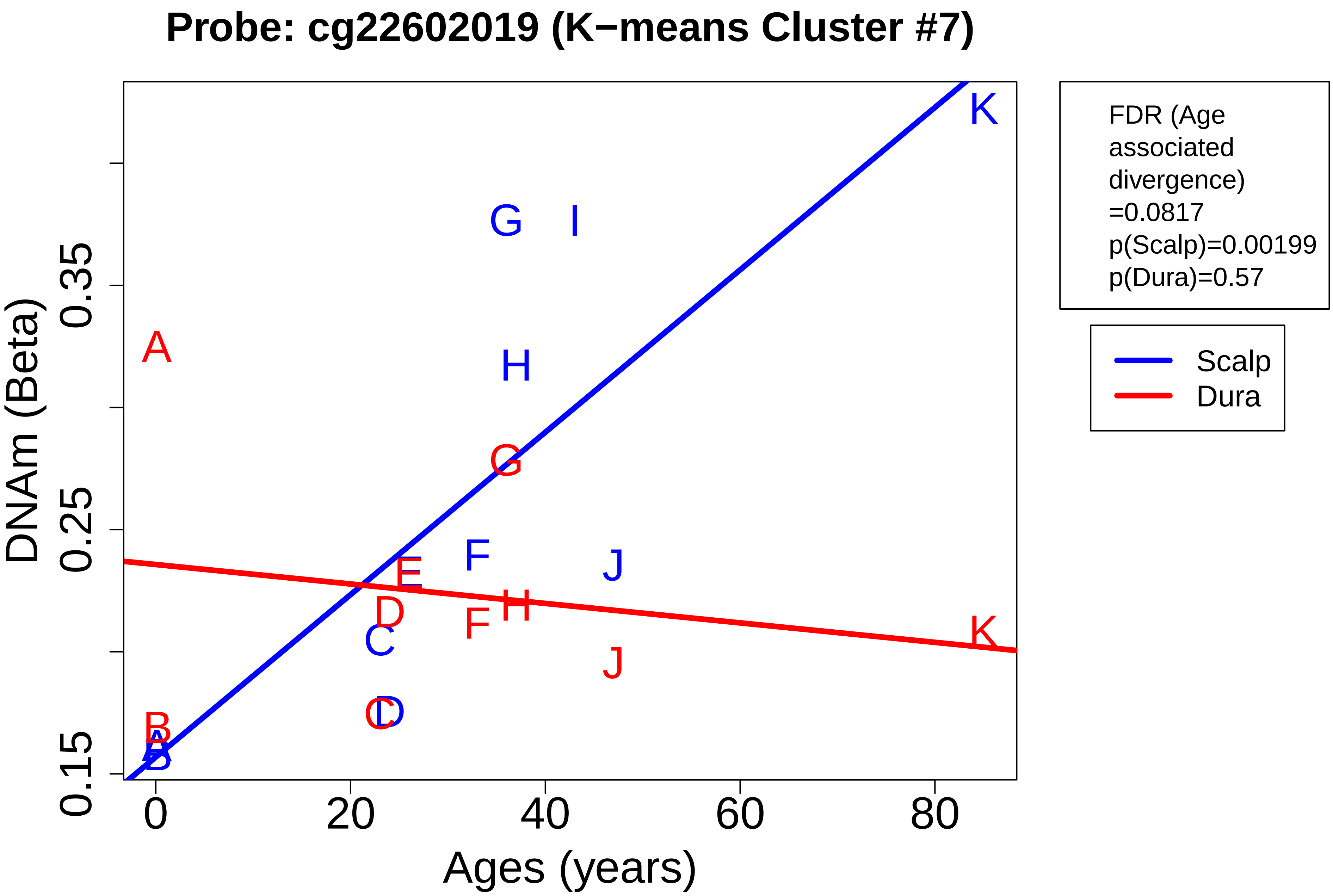

Probe: cg22602019 (K−means Cluster #7)
DNAm (Beta)
0.15
0.25
0.35
0
20
40
60
80
Ages (years)
FDR (Age associated divergence) =0.0817
p(Scalp)=0.00199
p(Dura)=0.57
Scalp
Dura
A
B
C
D
E
F
G
H
I
J
K

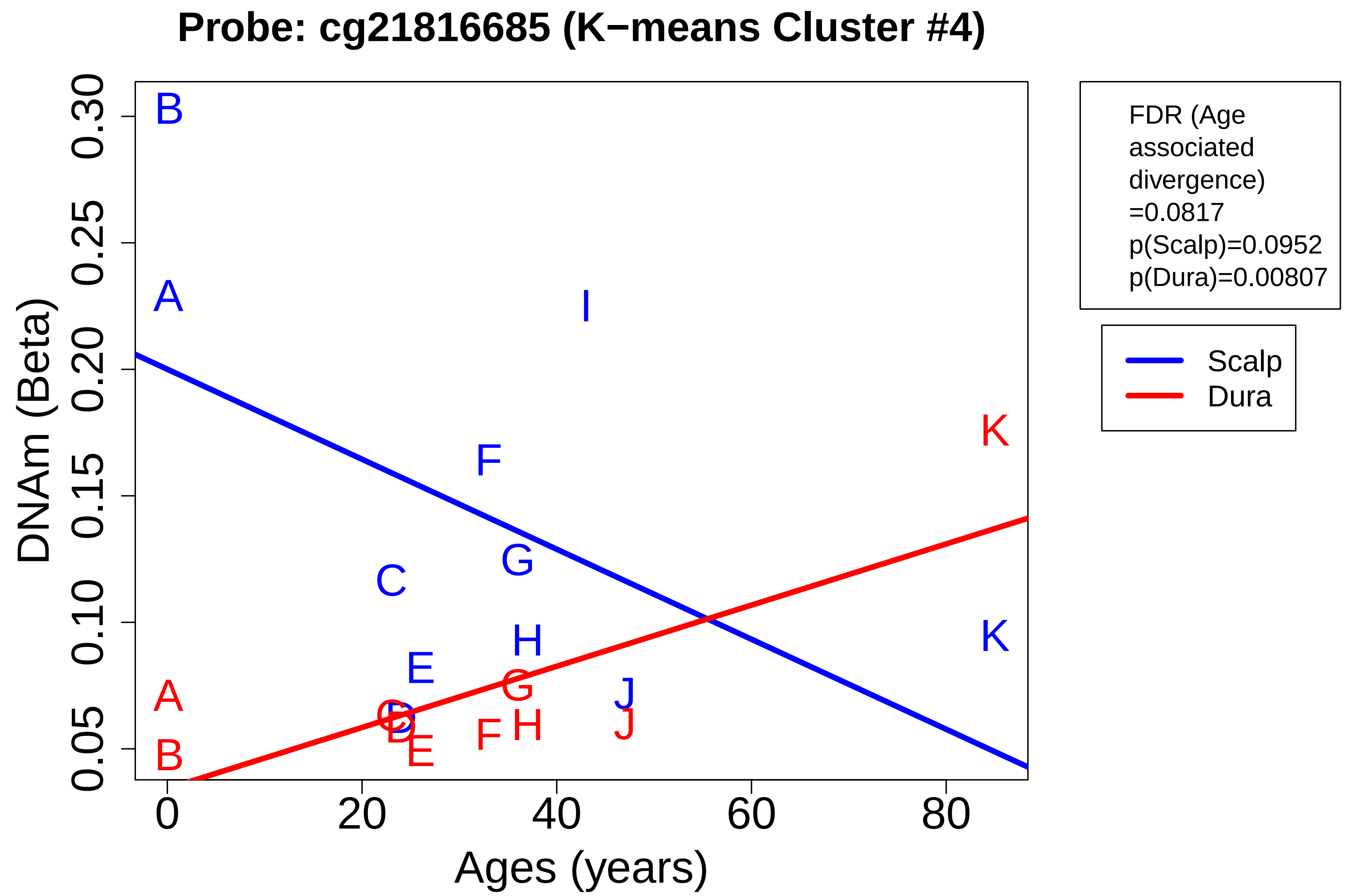

Probe: cg21816685 (K−means Cluster #4)
DNAm (Beta)
0.05
0.10
0.15
0.20
0.25
0.30
0
20
40
60
80
Ages (years)
FDR (Age associated divergence) =0.0817
p(Scalp)=0.0952
p(Dura)=0.00807
Scalp
Dura

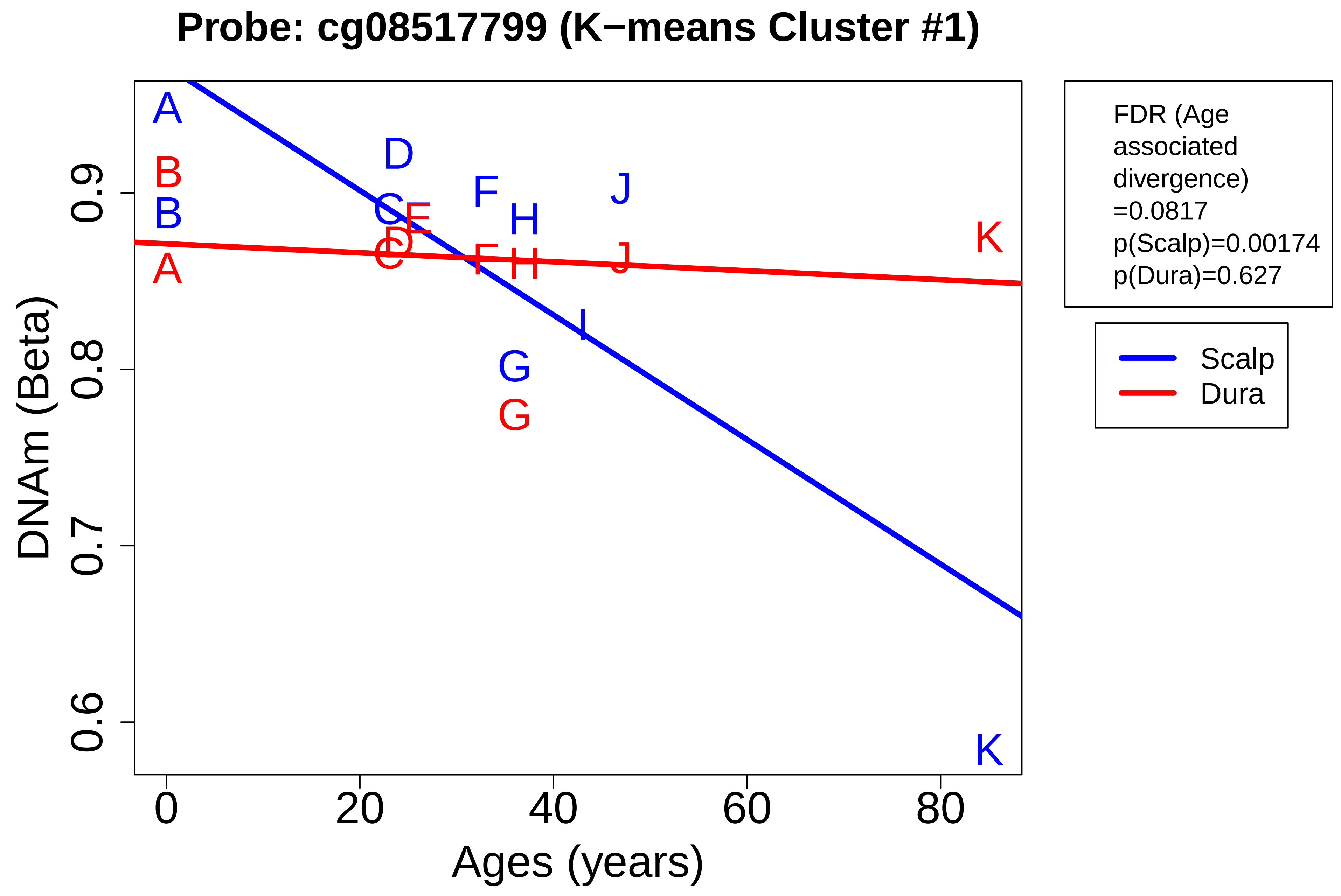

Probe: cg08517799 (K−means Cluster #1)
DNAm (Beta)
0.6
0.7
0.8
0.9
Ages (years)
0
20
40
60
80
A
B
B
A
D
C
E
D
C
F
H
J
F
H
J
K
I
G
G
K
FDR (Age associated divergence) =0.0817
p(Scalp)=0.00174
p(Dura)=0.627
Scalp
Dura

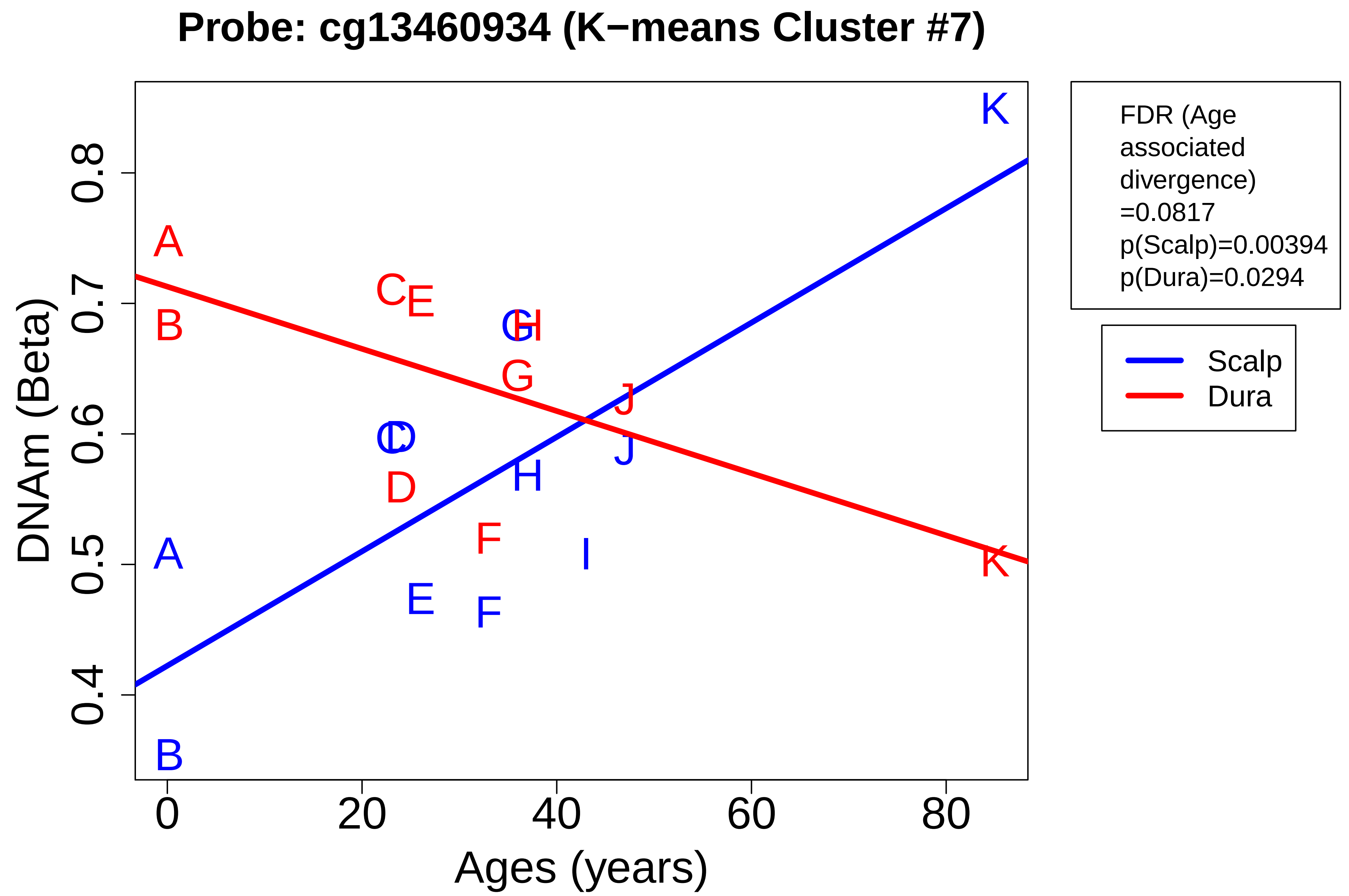

Probe: cg13460934 (K−means Cluster #7)
DNAm (Beta)
0.4
0.5
0.6
0.7
0.8
0
20
40
60
80
Ages (years)
FDR (Age associated divergence) =0.0817
p(Scalp)=0.00394
p(Dura)=0.0294
Scalp
Dura
A
B
C
D
E
F
G
H
I
J
K

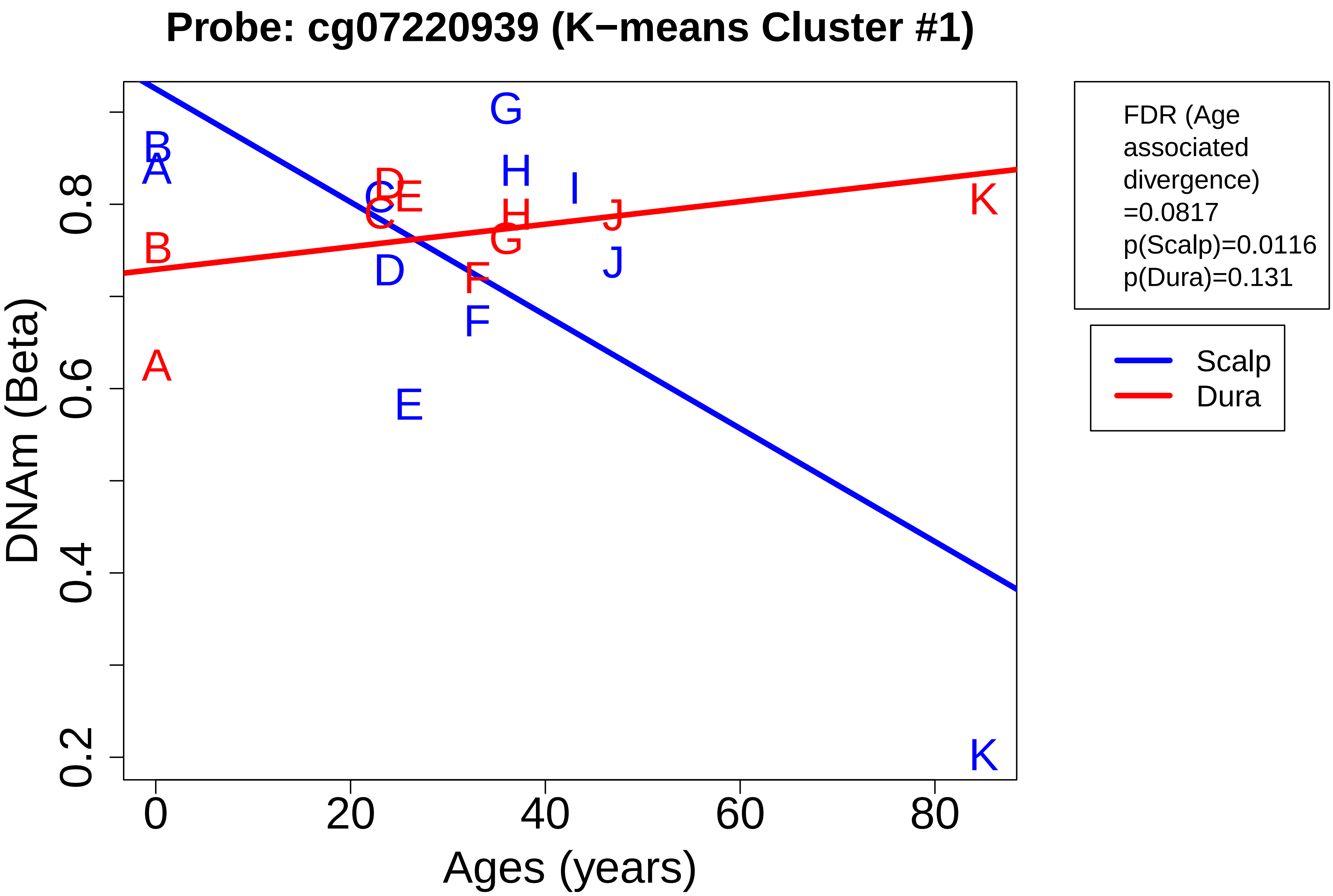

Probe: cg07220939 (K−means Cluster #1)
DNAm (Beta)
0.2
0.4
0.6
0.8
0
20
40
60
80
Ages (years)
FDR (Age associated divergence) =0.0817
p(Scalp)=0.0116
p(Dura)=0.131
Scalp
Dura

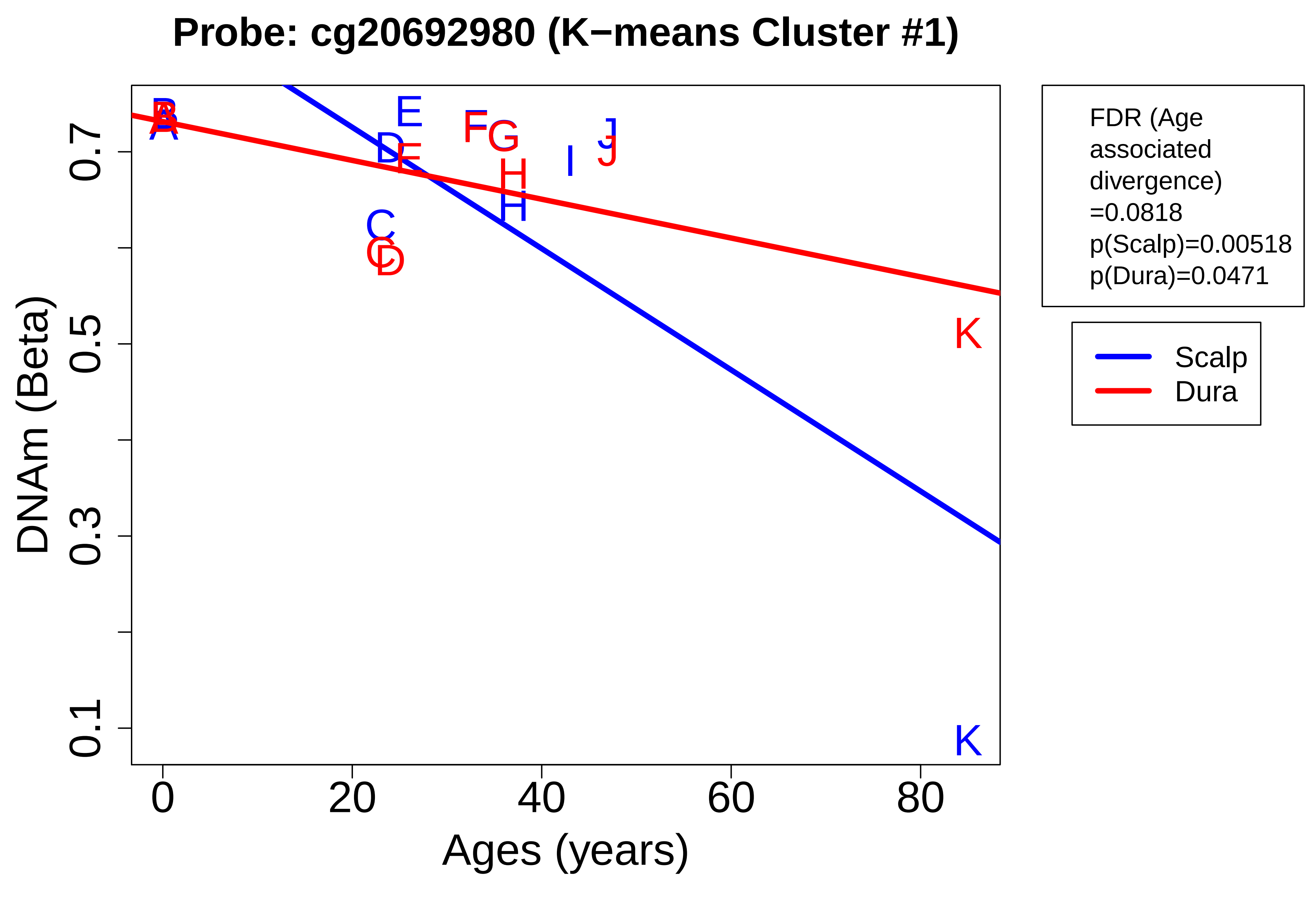

Probe: cg20692980 (K−means Cluster #1)
DNAm (Beta)
0.1
0.3
0.5
0.7
Ages (years)
0
20
40
60
80
FDR (Age associated divergence) =0.0818
p(Scalp)=0.00518
p(Dura)=0.0471
Scalp
Dura

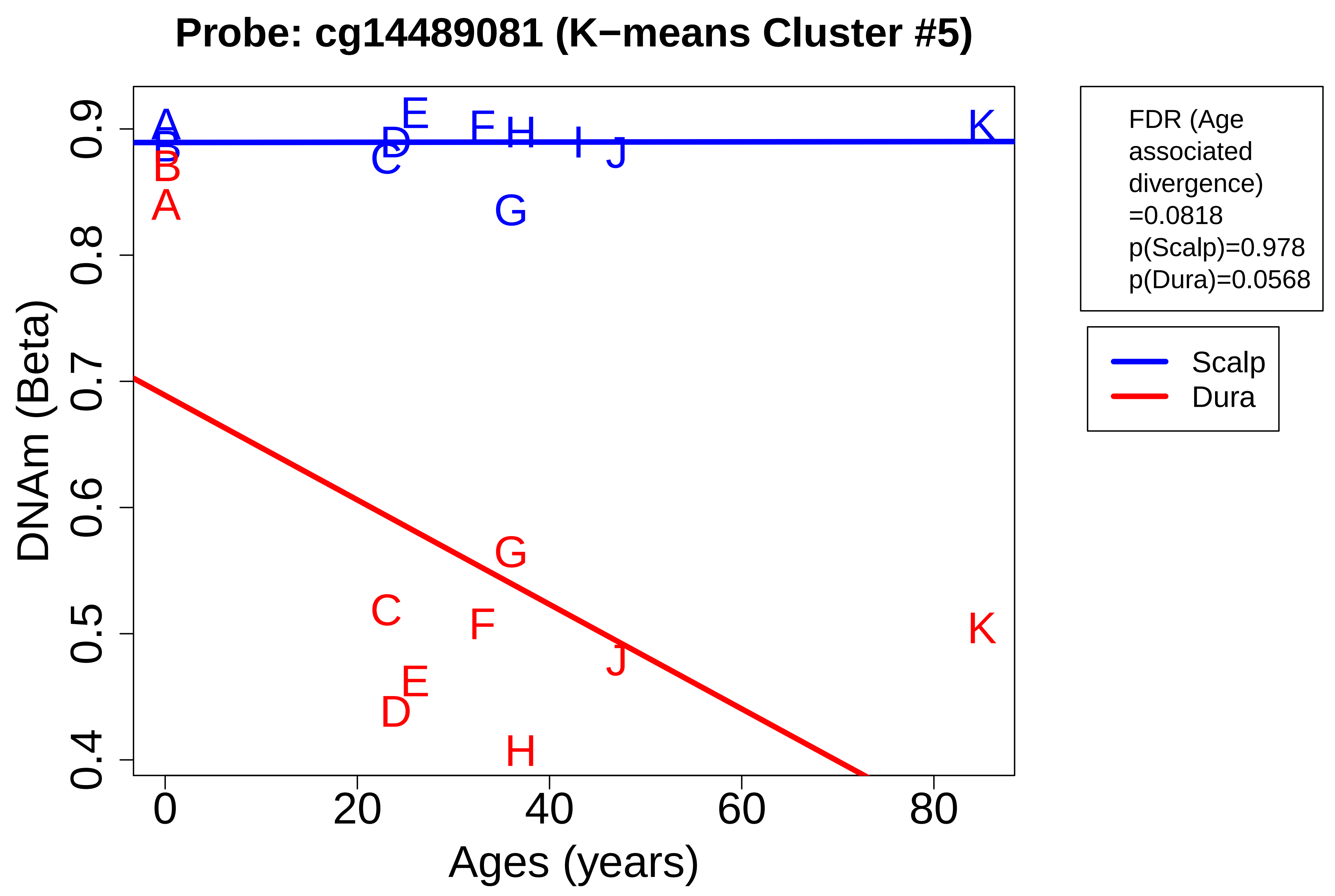
Probe: cg14489081 (K−means Cluster #5)
DNAm (Beta)
0.4
0.5
0.6
0.7
0.8
0.9
Ages (years)
0
20
40
60
80
A B C D E F G H I J K
A B C D E F G H J K
FDR (Age associated divergence) =0.0818
p(Scalp)=0.978
p(Dura)=0.0568
Scalp
Dura

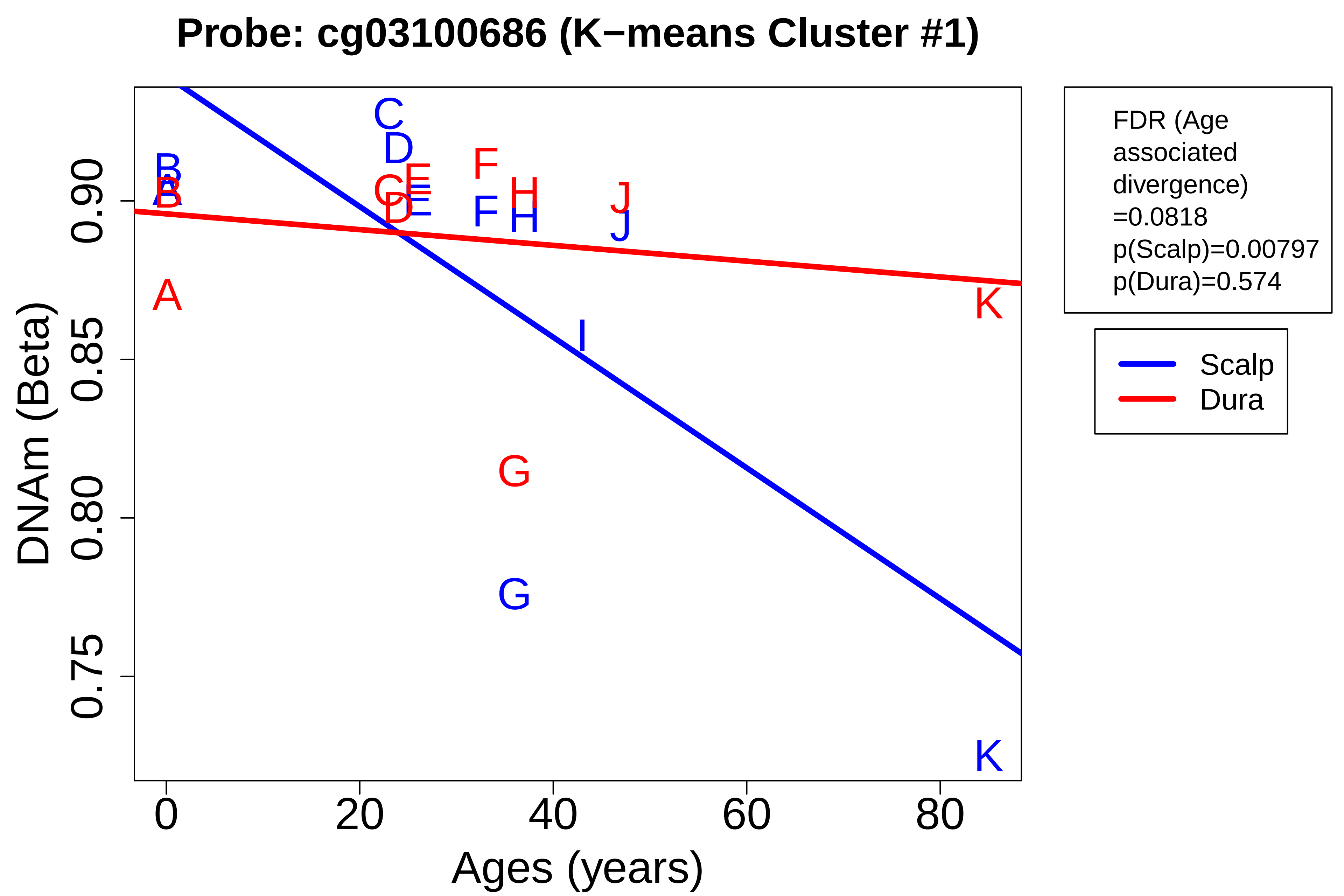
Probe: cg03100686 (K−means Cluster #1)
DNAm (Beta)
0.75
0.80
0.85
0.90
0
20
40
60
80
Ages (years)
A
B
C
D
E
F
G
H
I
J
K
FDR (Age associated divergence) =0.0818
p(Scalp)=0.00797
p(Dura)=0.574
Scalp
Dura

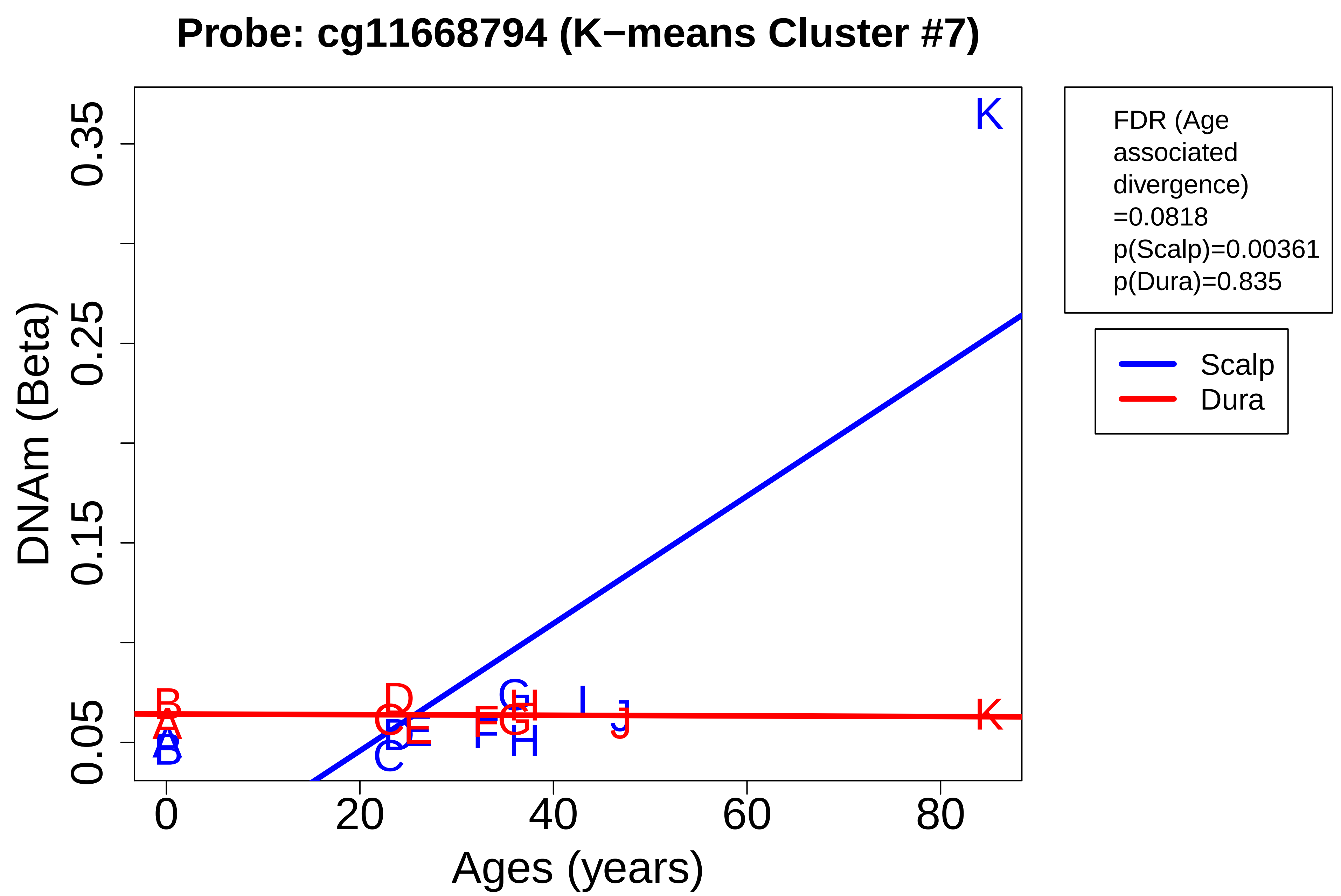

Probe: cg11668794 (K−means Cluster #7)
DNAm (Beta)
0.05
0.15
0.25
0.35
Ages (years)
0
20
40
60
80
FDR (Age associated divergence) =0.0818
p(Scalp)=0.00361
p(Dura)=0.835
Scalp
Dura

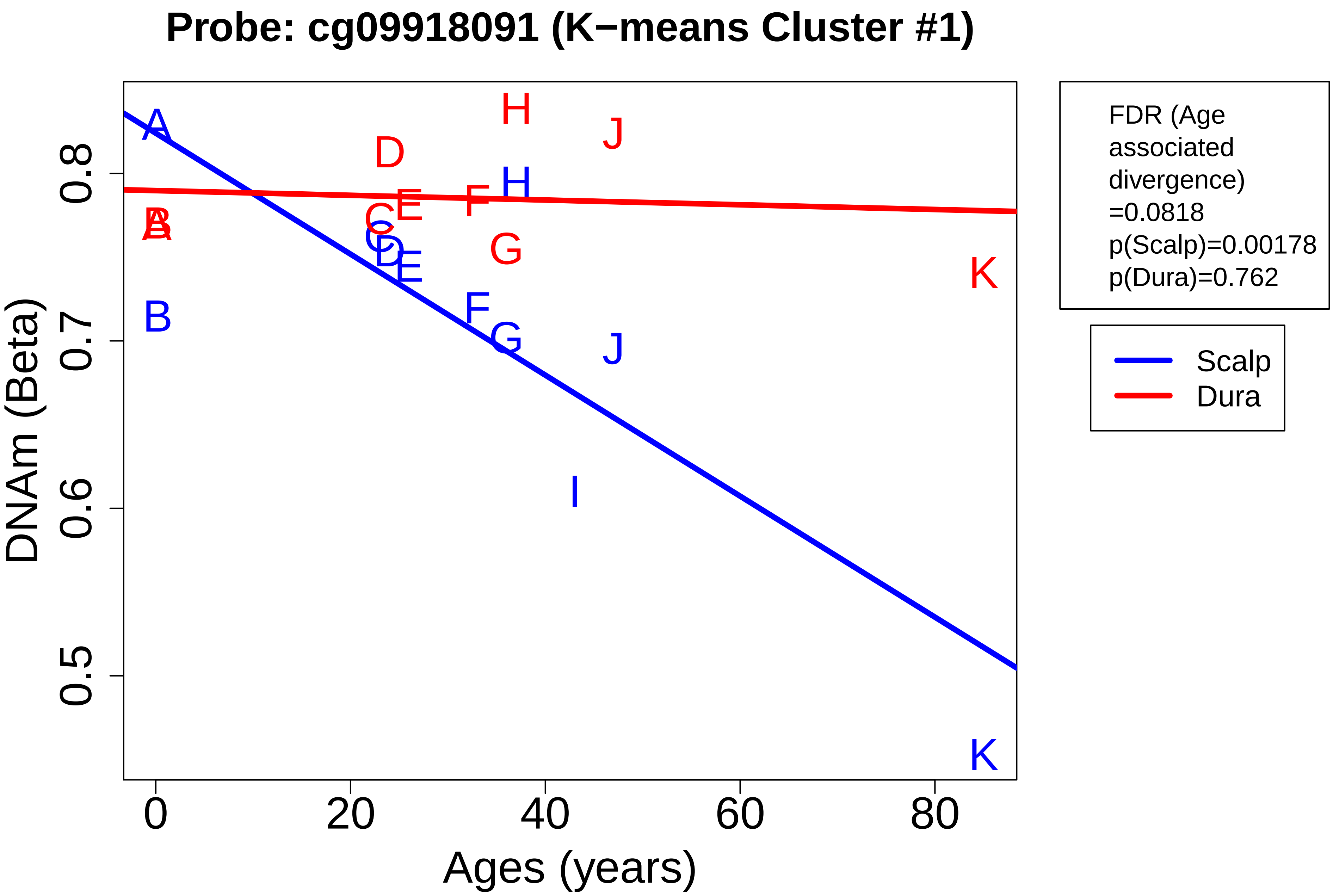

Probe: cg09918091 (K−means Cluster #1)
DNAm (Beta)
0.5
0.6
0.7
0.8
Ages (years)
0
20
40
60
80
A
B
C
D
E
F
G
H
I
J
K
FDR (Age associated divergence) =0.0818
p(Scalp)=0.00178
p(Dura)=0.762
Scalp
Dura

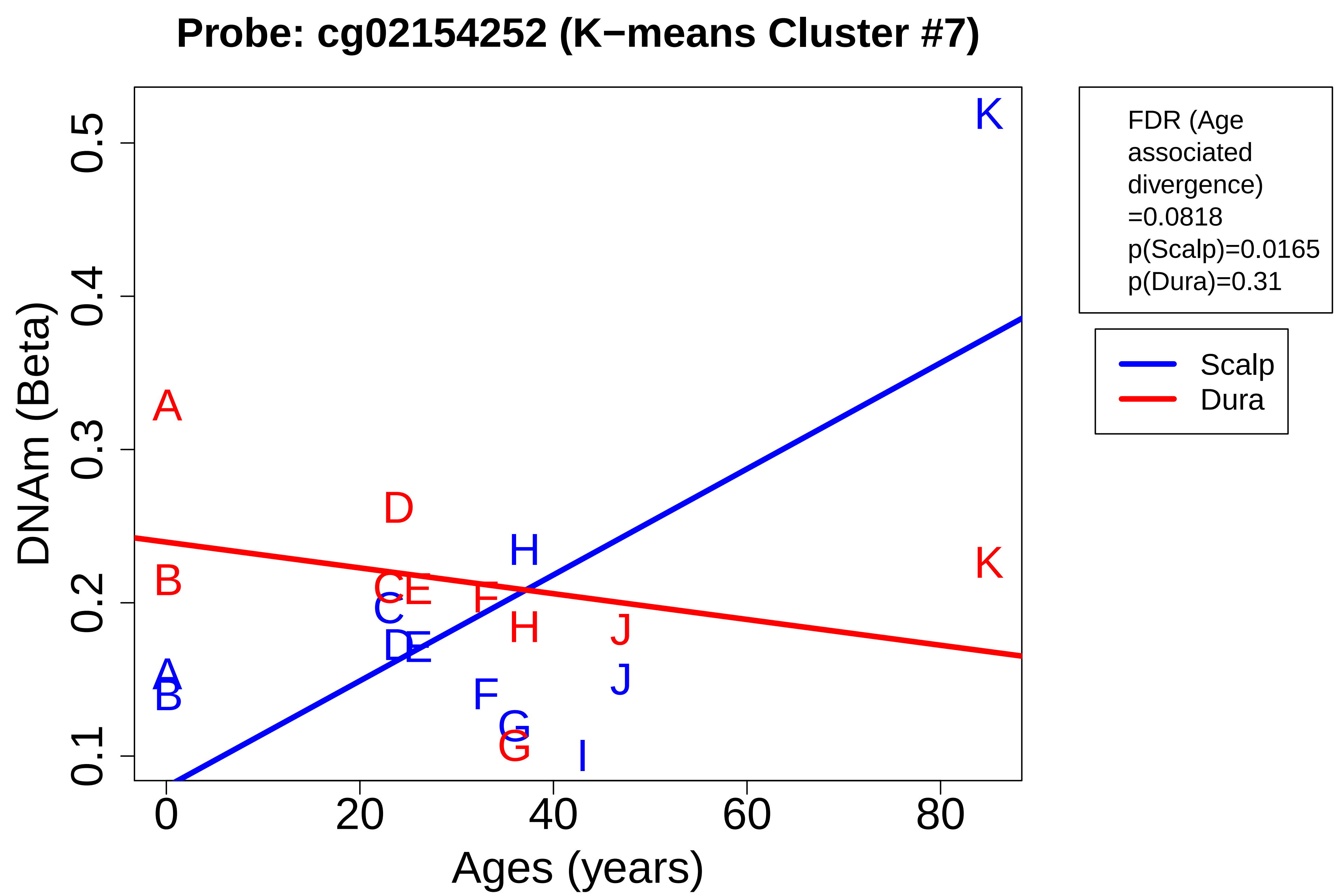

Probe: cg02154252 (K−means Cluster #7)
DNAm (Beta)
0.1
0.2
0.3
0.4
0.5
Ages (years)
0
20
40
60
80
FDR (Age associated divergence) =0.0818
p(Scalp)=0.0165
p(Dura)=0.31
Scalp
Dura
A
B
C
D
E
F
G
H
I
J
K

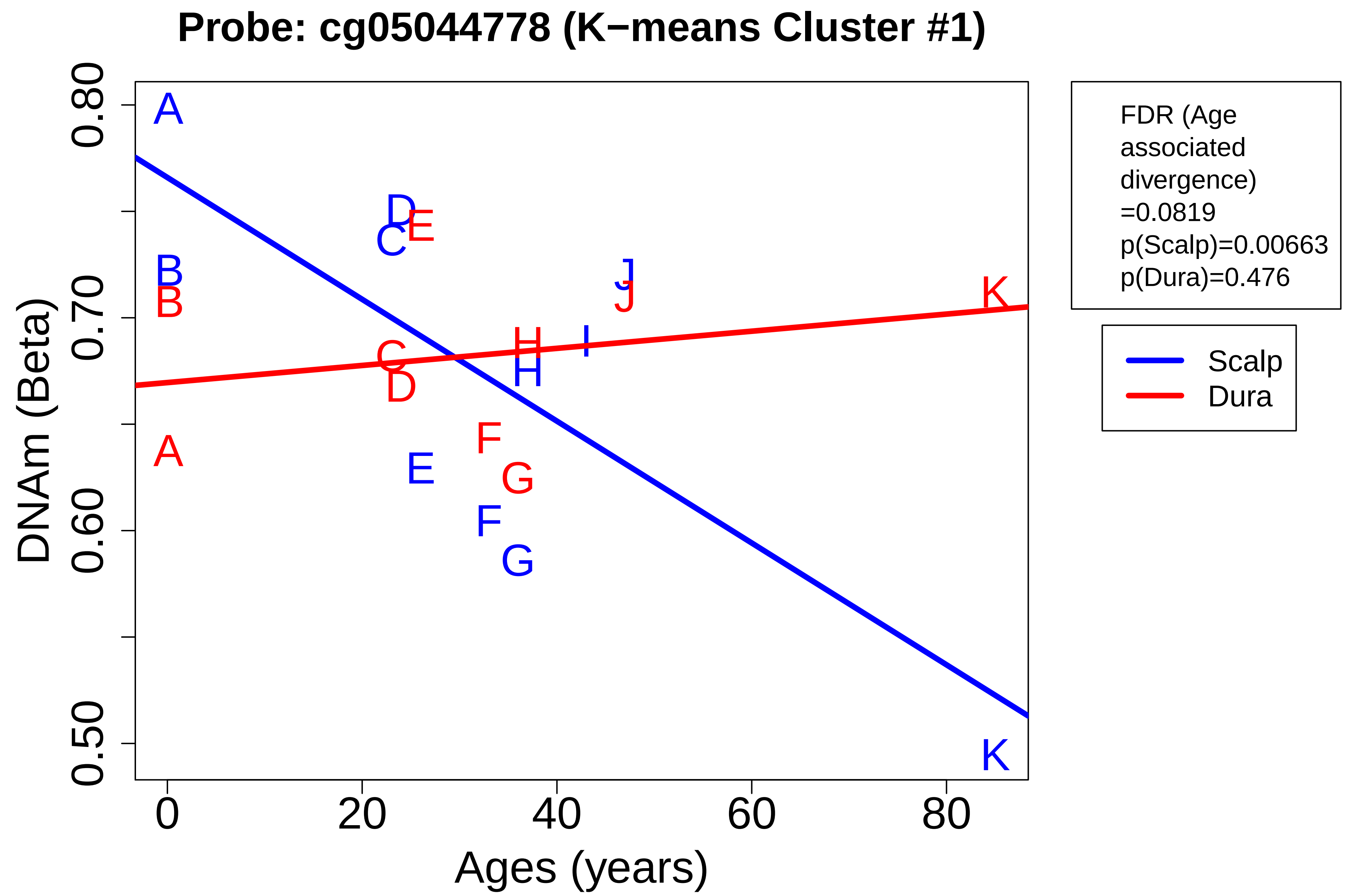

Probe: cg05044778 (K−means Cluster #1)
DNAm (Beta)
0.50
0.60
0.70
0.80
Ages (years)
0
20
40
60
80
A
B
C
D
E
F
G
H
I
J
K
FDR (Age associated divergence) =0.0819
p(Scalp)=0.00663
p(Dura)=0.476
Scalp
Dura

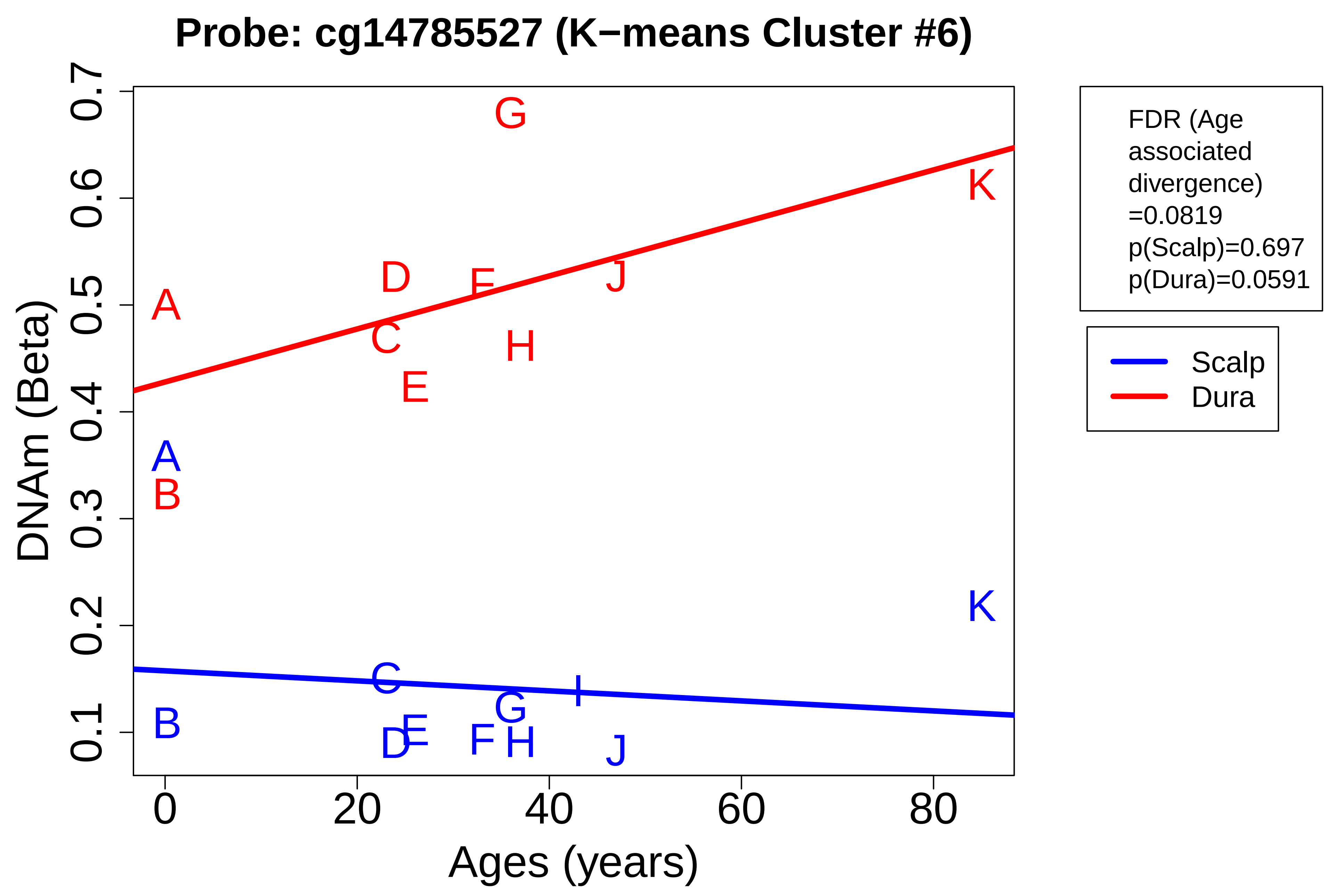

Probe: cg14785527 (K−means Cluster #6)
DNAm (Beta)
Ages (years)
0.1
0.2
0.3
0.4
0.5
0.6
0.7
0
20
40
60
80
FDR (Age associated divergence) =0.0819
p(Scalp)=0.697
p(Dura)=0.0591
Scalp
Dura
A
B
C
D
E
F
G
H
I
J
K

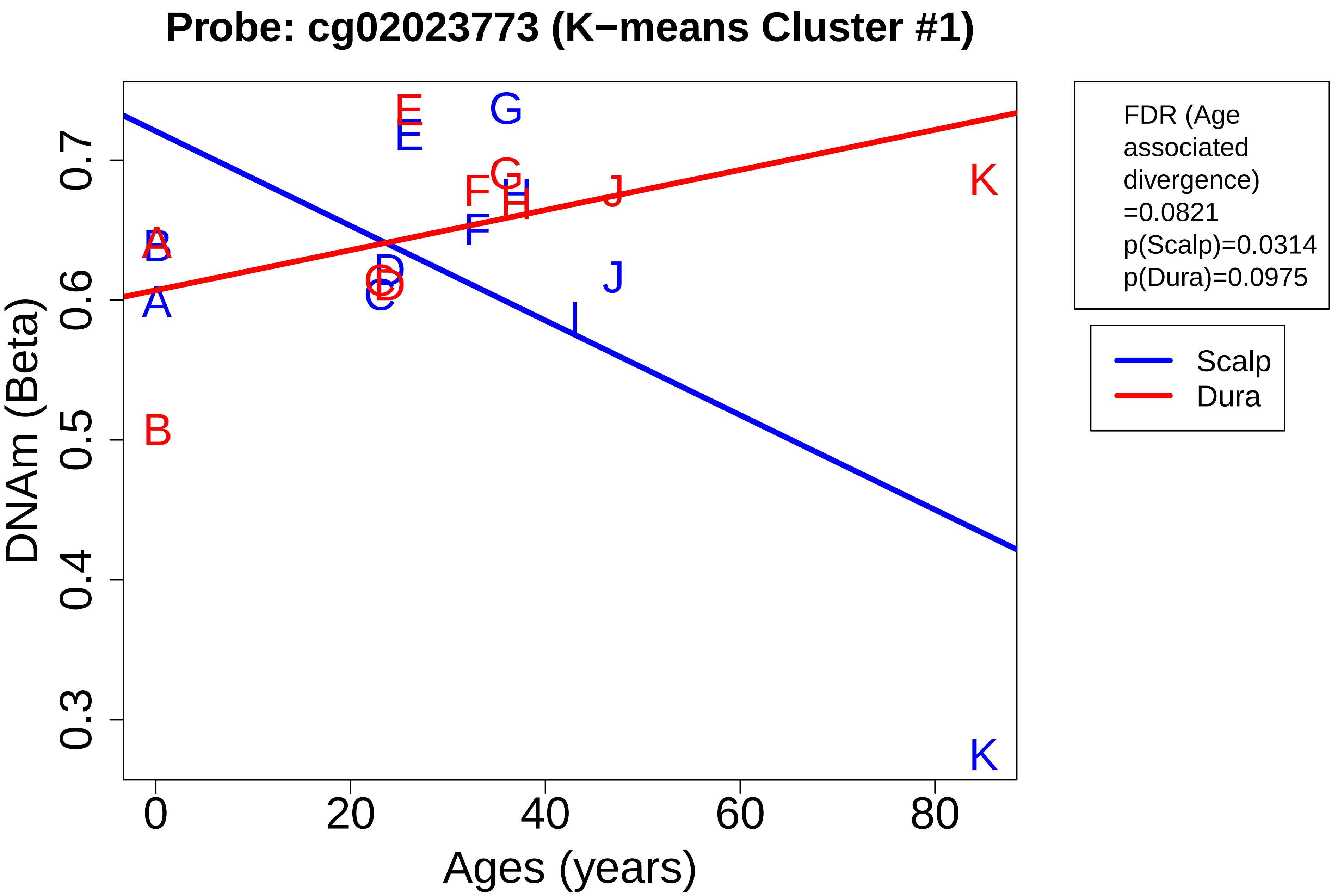

Probe: cg02023773 (K−means Cluster #1)
DNAm (Beta)
0.3
0.4
0.5
0.6
0.7
0
20
40
60
80
Ages (years)
FDR (Age associated divergence) =0.0821
p(Scalp)=0.0314
p(Dura)=0.0975
Scalp
Dura

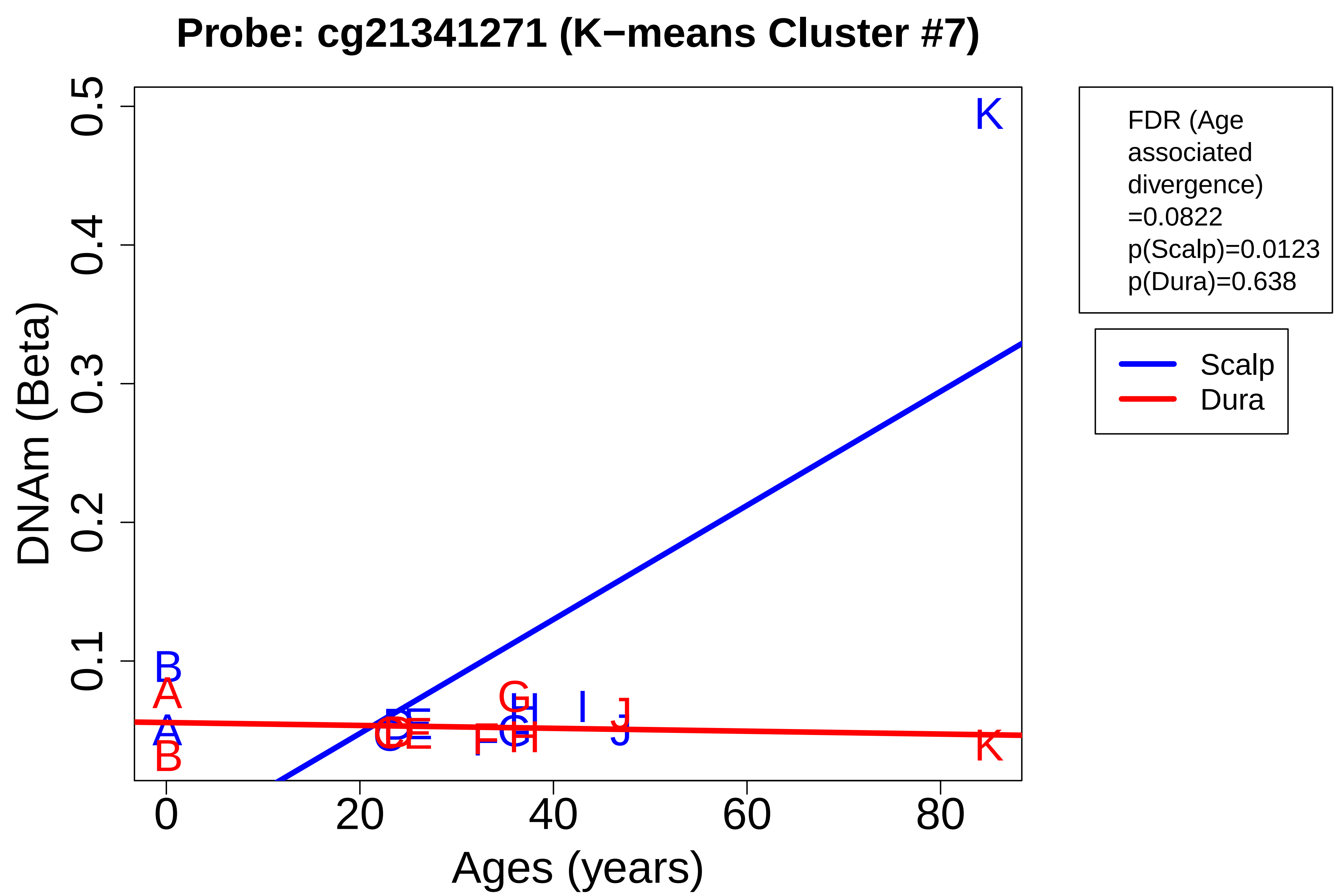
Probe: cg21341271 (K−means Cluster #7)
DNAm (Beta)
0.1
0.2
0.3
0.4
0.5
0
20
40
60
80
Ages (years)
FDR (Age associated divergence) =0.0822
p(Scalp)=0.0123
p(Dura)=0.638
Scalp
Dura

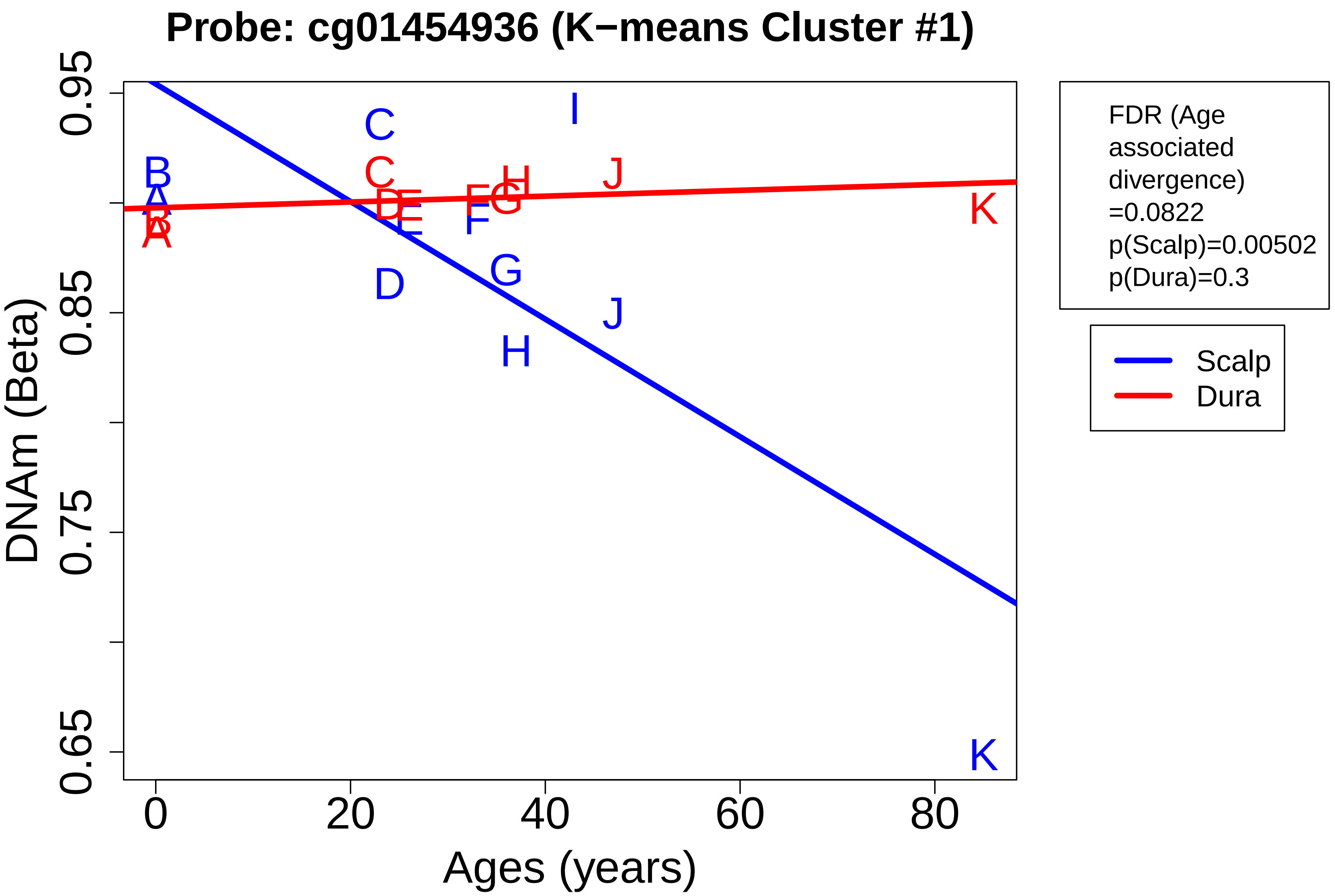

Probe: cg01454936 (K−means Cluster #1)
DNAm (Beta)
0.65
0.75
0.85
0.95
0
20
40
60
80
Ages (years)
A
B
C
D
E
F
G
H
I
J
K
FDR (Age associated divergence) =0.0822
p(Scalp)=0.00502
p(Dura)=0.3
Scalp
Dura

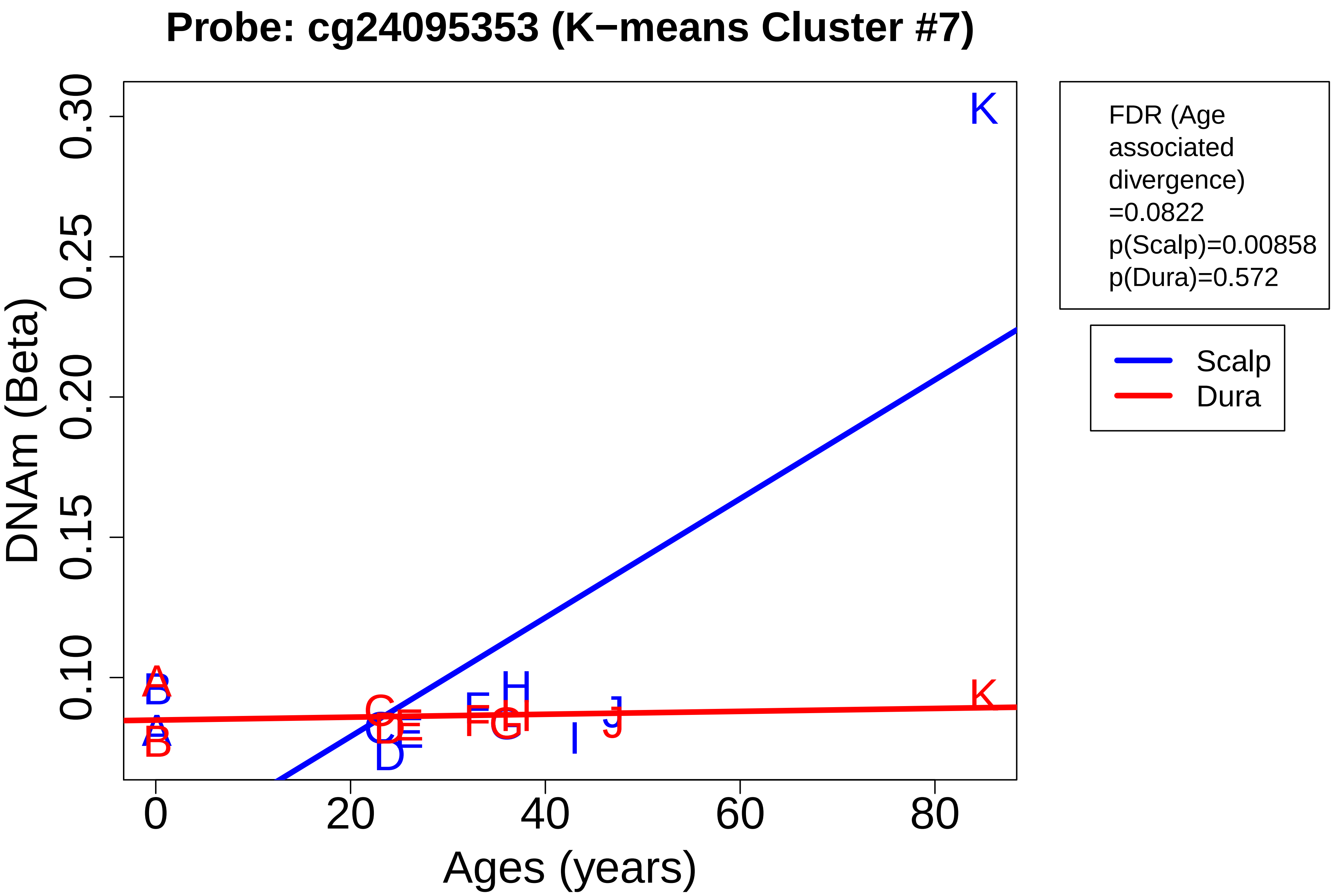
Probe: cg24095353 (K−means Cluster #7)
DNAm (Beta)
0.10
0.15
0.20
0.25
0.30
0
20
40
60
80
Ages (years)
FDR (Age associated divergence) =0.0822
p(Scalp)=0.00858
p(Dura)=0.572
Scalp
Dura

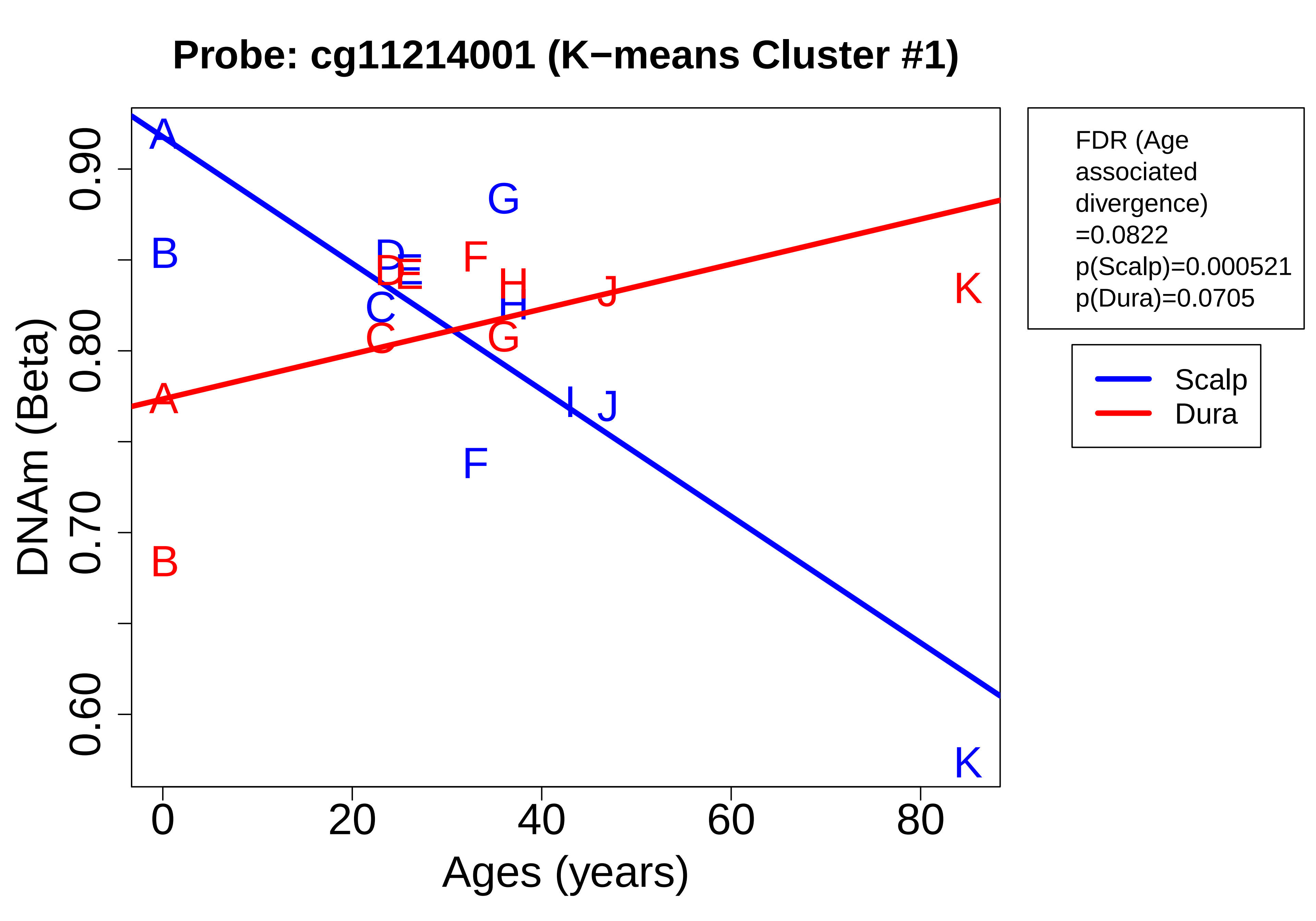

Probe: cg11214001 (K−means Cluster #1)
DNAm (Beta)
0.60
0.70
0.80
0.90
0
20
40
60
80
Ages (years)
FDR (Age associated divergence) =0.0822
p(Scalp)=0.000521
p(Dura)=0.0705
Scalp
Dura

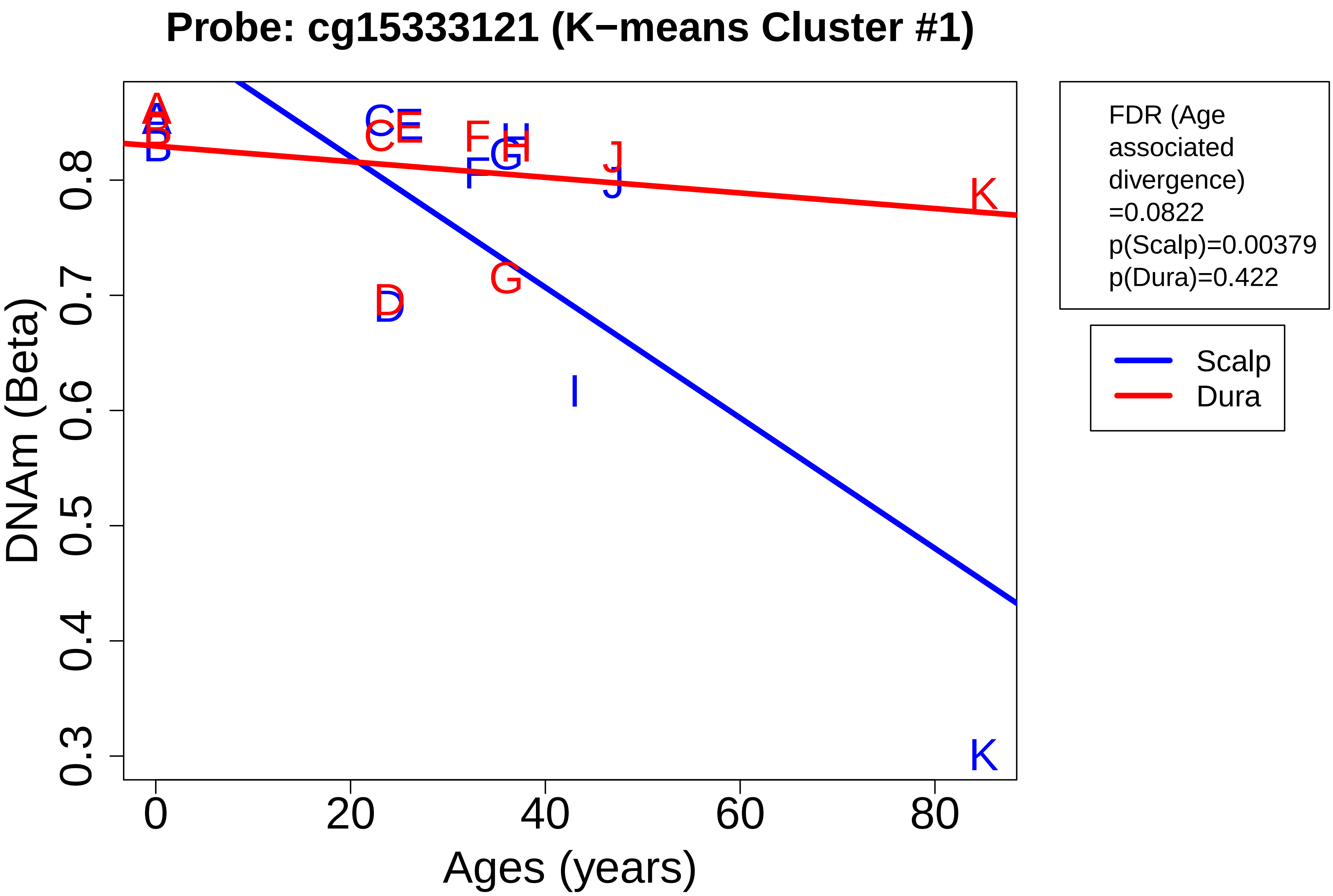
Probe: cg15333121 (K−means Cluster #1)
DNAm (Beta)
0.3
0.4
0.5
0.6
0.7
0.8
0
20
40
60
80
Ages (years)
A
B
C
D
E
F
G
H
I
J
K
FDR (Age associated divergence) =0.0822
p(Scalp)=0.00379
p(Dura)=0.422
Scalp
Dura

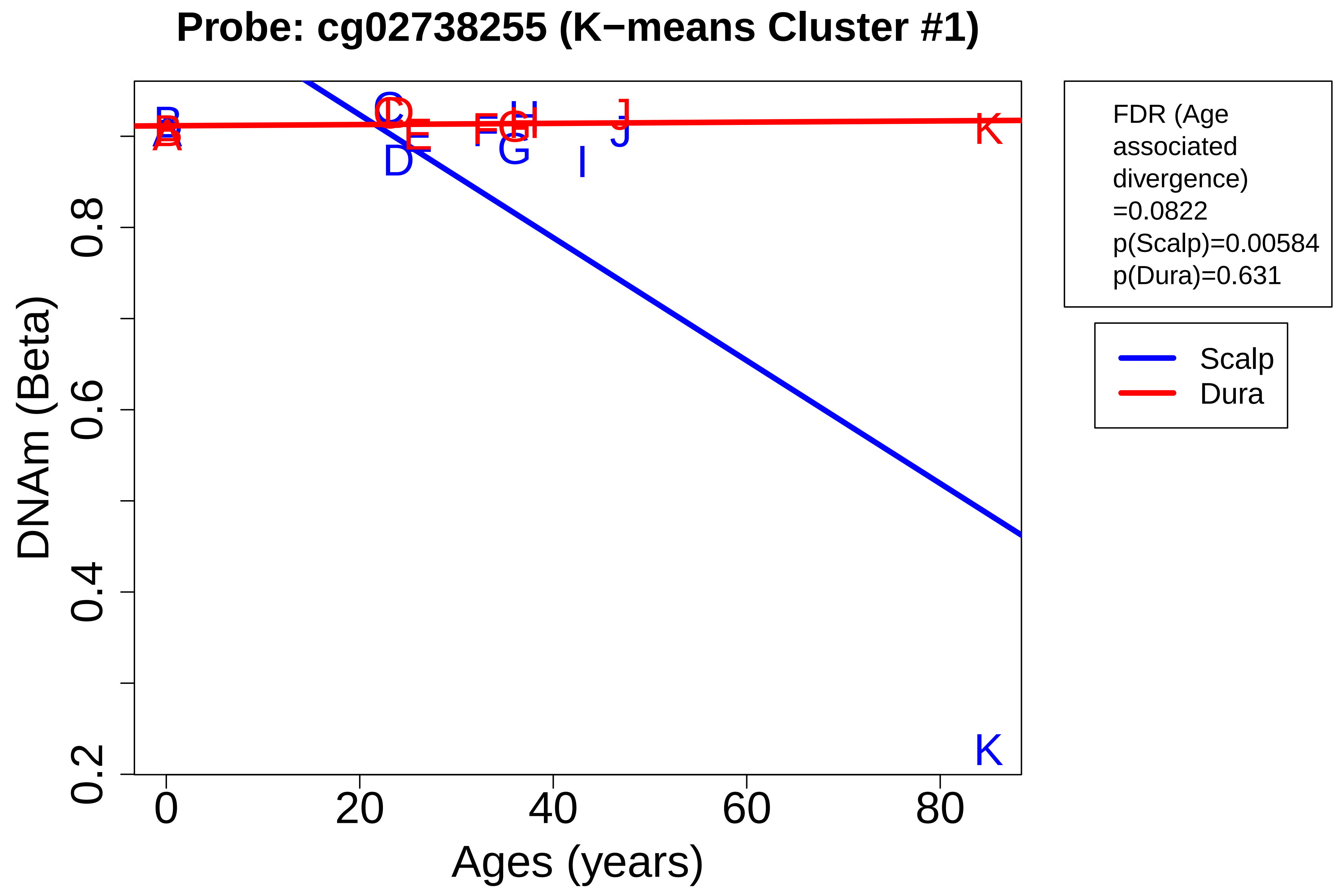

Probe: cg02738255 (K−means Cluster #1)
FDR (Age associated divergence) =0.0822
p(Scalp)=0.00584
p(Dura)=0.631
Scalp
Dura
DNAm (Beta)
Ages (years)
0.2
0.4
0.6
0.8
0
20
40
60
80
A
B
C
D
E
F
G
H
I
J
K

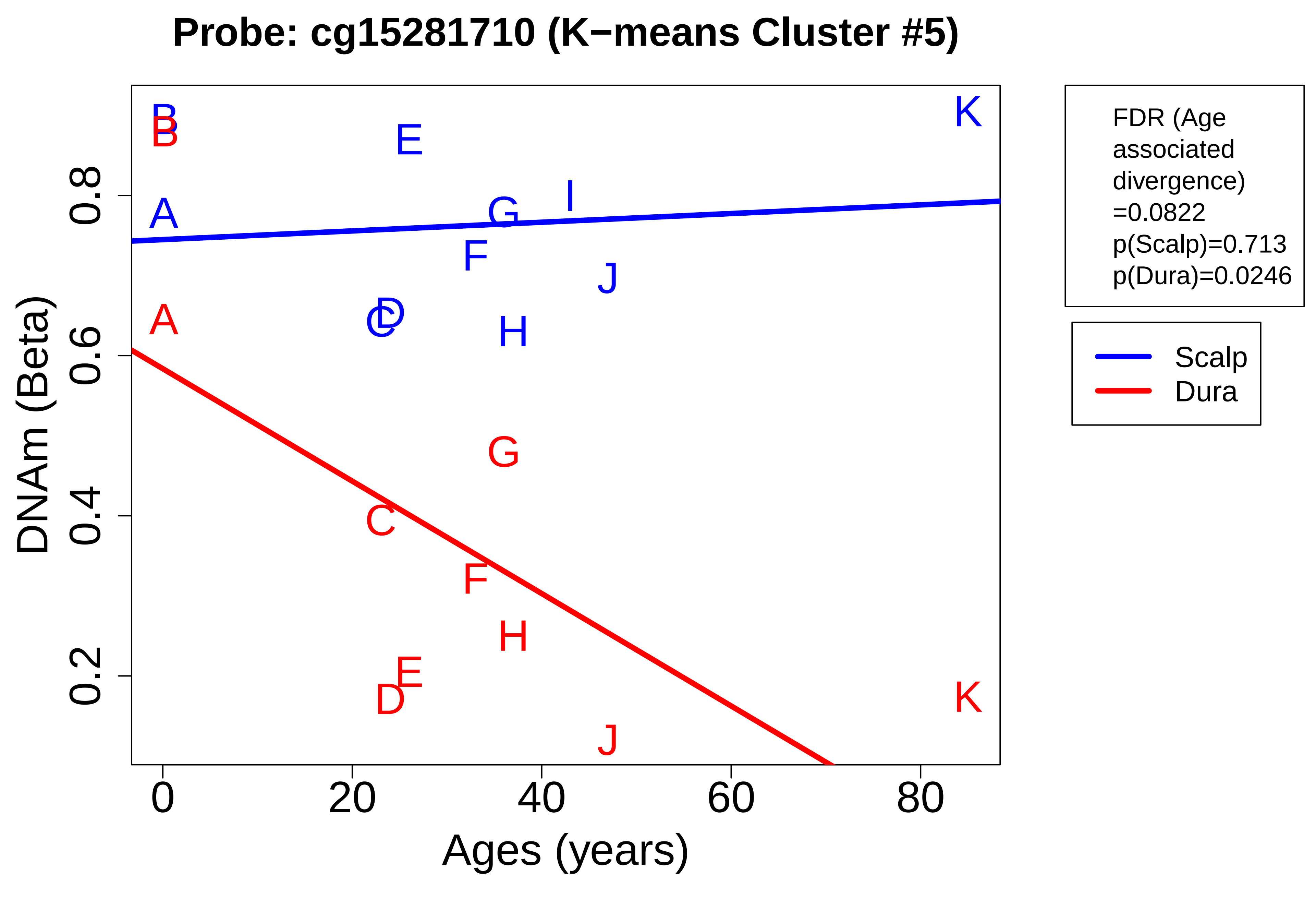
Probe: cg15281710 (K−means Cluster #5)
DNAm (Beta)
0.2
0.4
0.6
0.8
0
20
40
60
80
Ages (years)
A
B
C
D
E
F
G
H
I
J
K
FDR (Age associated divergence) =0.0822
p(Scalp)=0.713
p(Dura)=0.0246
Scalp
Dura

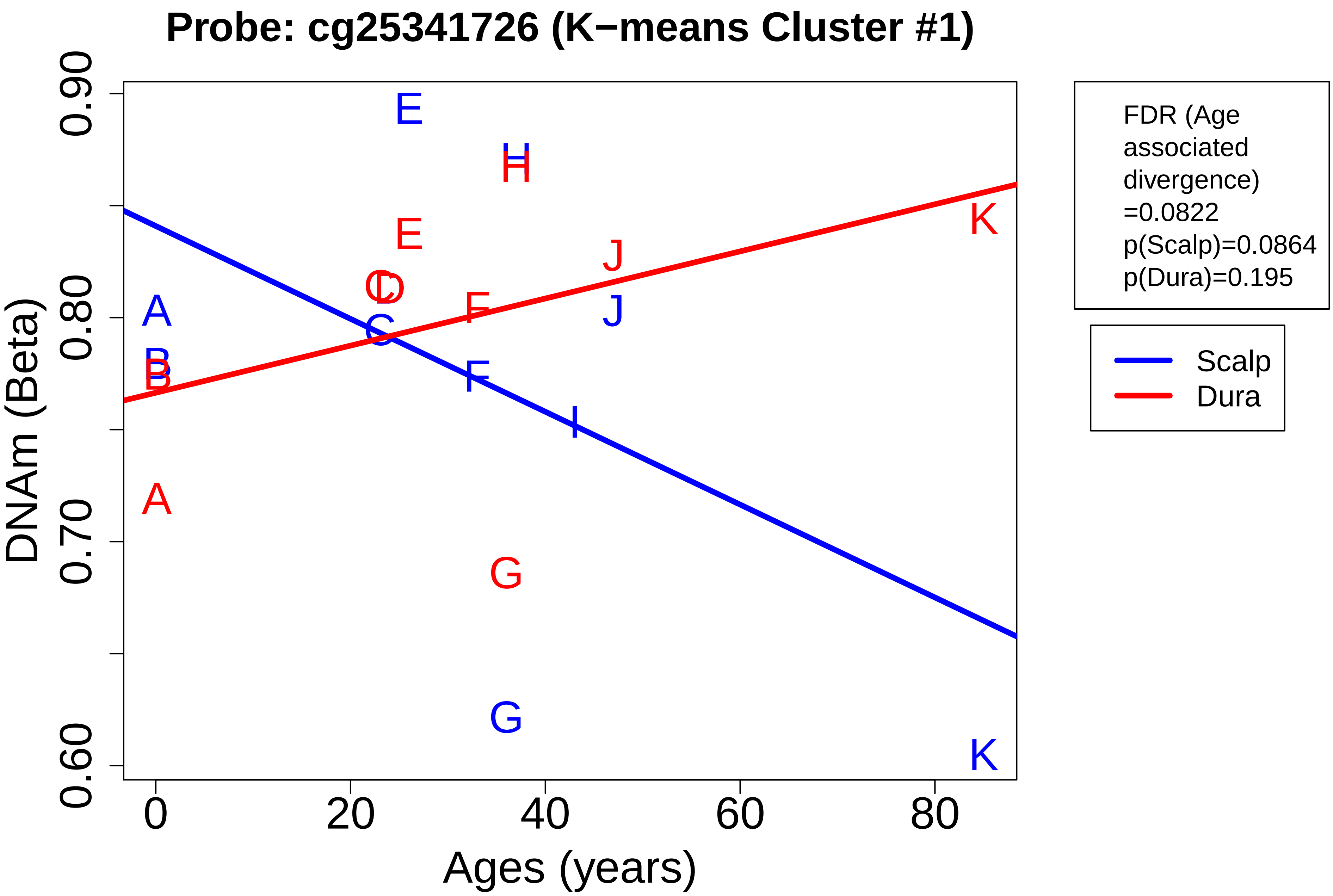

Probe: cg25341726 (K−means Cluster #1)
DNAm (Beta)
0.60
0.70
0.80
0.90
Ages (years)
0
20
40
60
80
FDR (Age associated divergence) =0.0822
p(Scalp)=0.0864
p(Dura)=0.195
Scalp
Dura

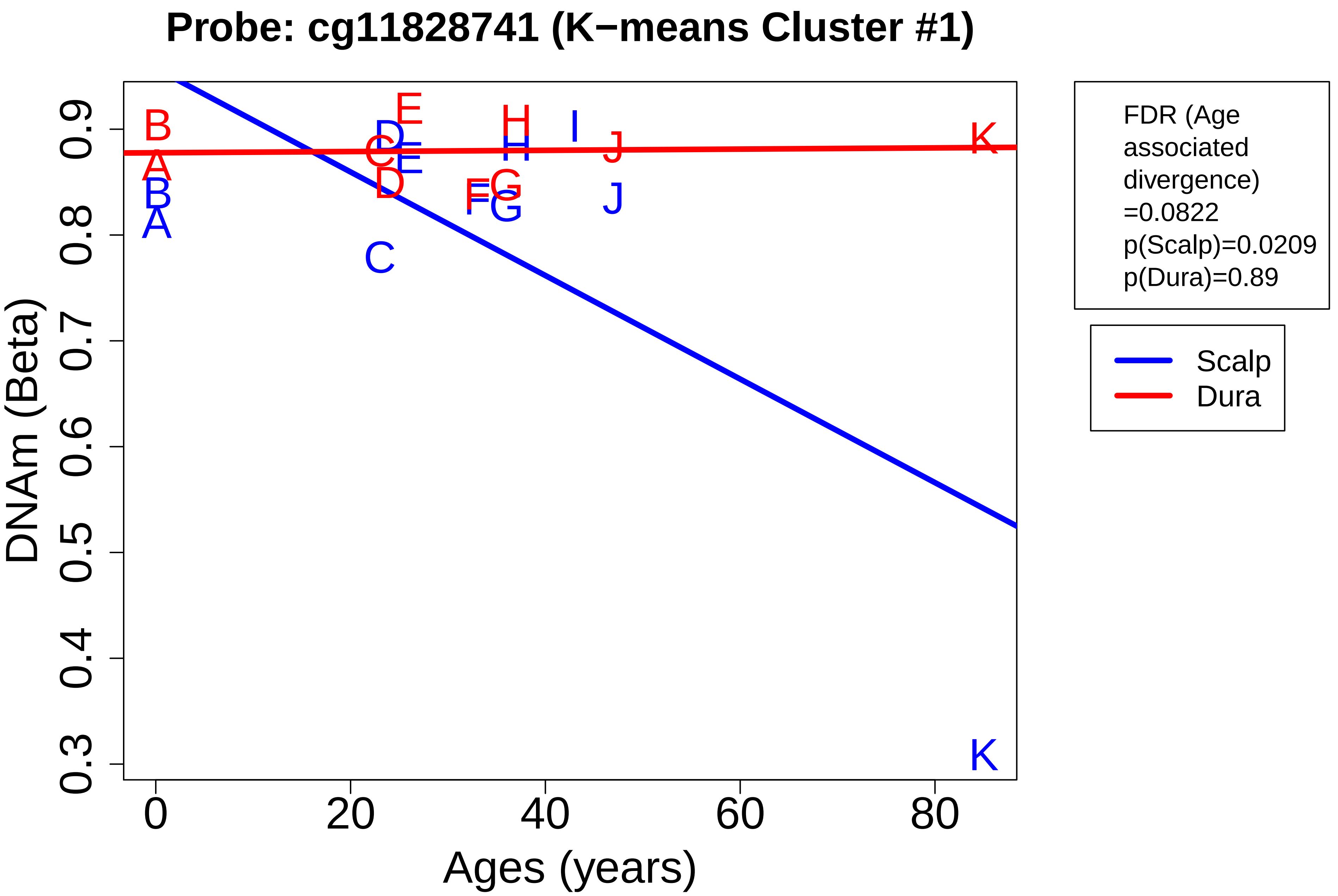

Probe: cg11828741 (K−means Cluster #1)
DNAm (Beta)
Ages (years)
0.3
0.4
0.5
0.6
0.7
0.8
0.9
0
20
40
60
80
FDR (Age associated divergence) =0.0822
p(Scalp)=0.0209
p(Dura)=0.89
Scalp
Dura

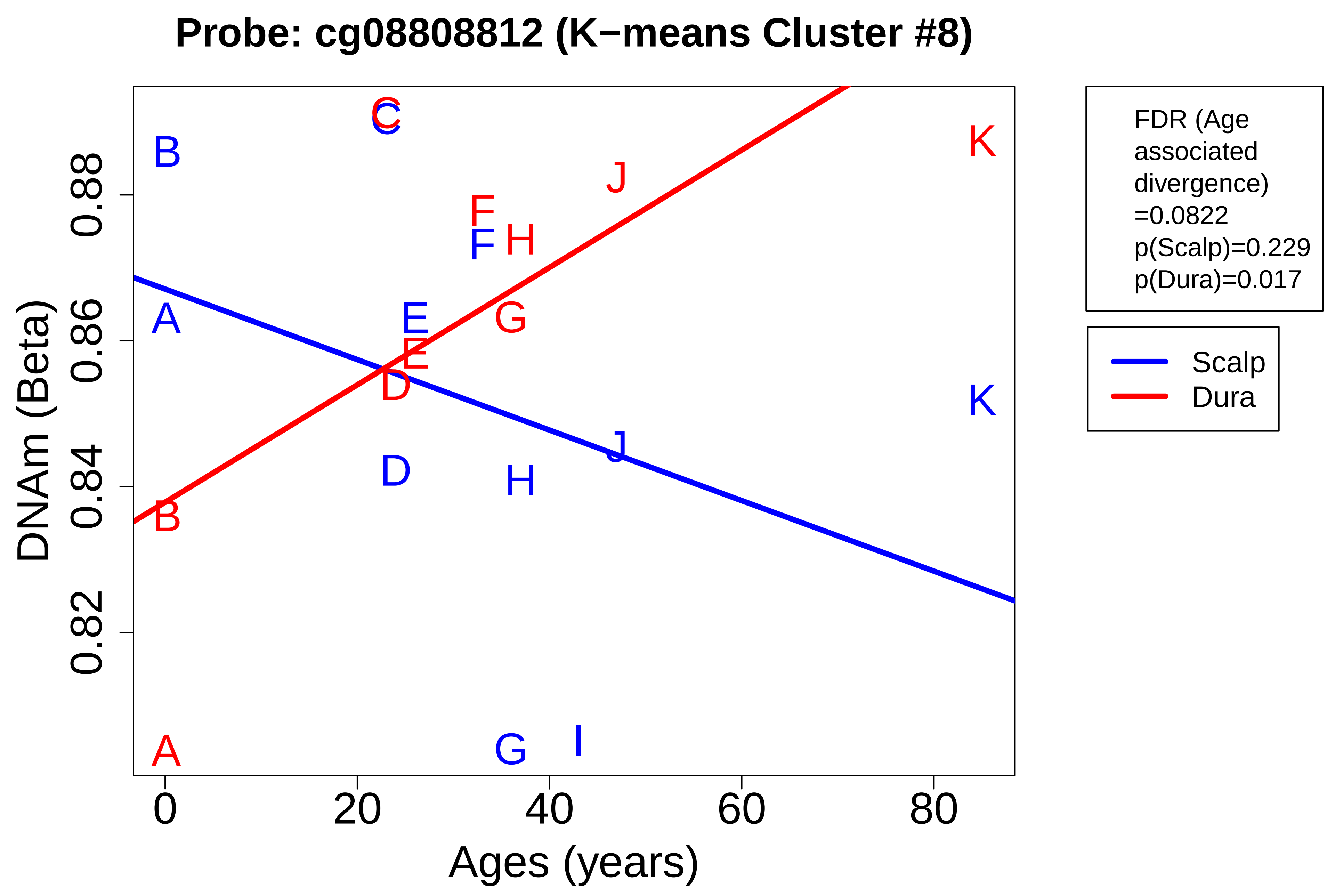

Probe: cg08808812 (K−means Cluster #8)
DNAm (Beta)
0.82
0.84
0.86
0.88
0
20
40
60
80
Ages (years)
FDR (Age associated divergence) =0.0822
p(Scalp)=0.229
p(Dura)=0.017
Scalp
Dura
A
B
C
D
E
F
G
H
I
J
K

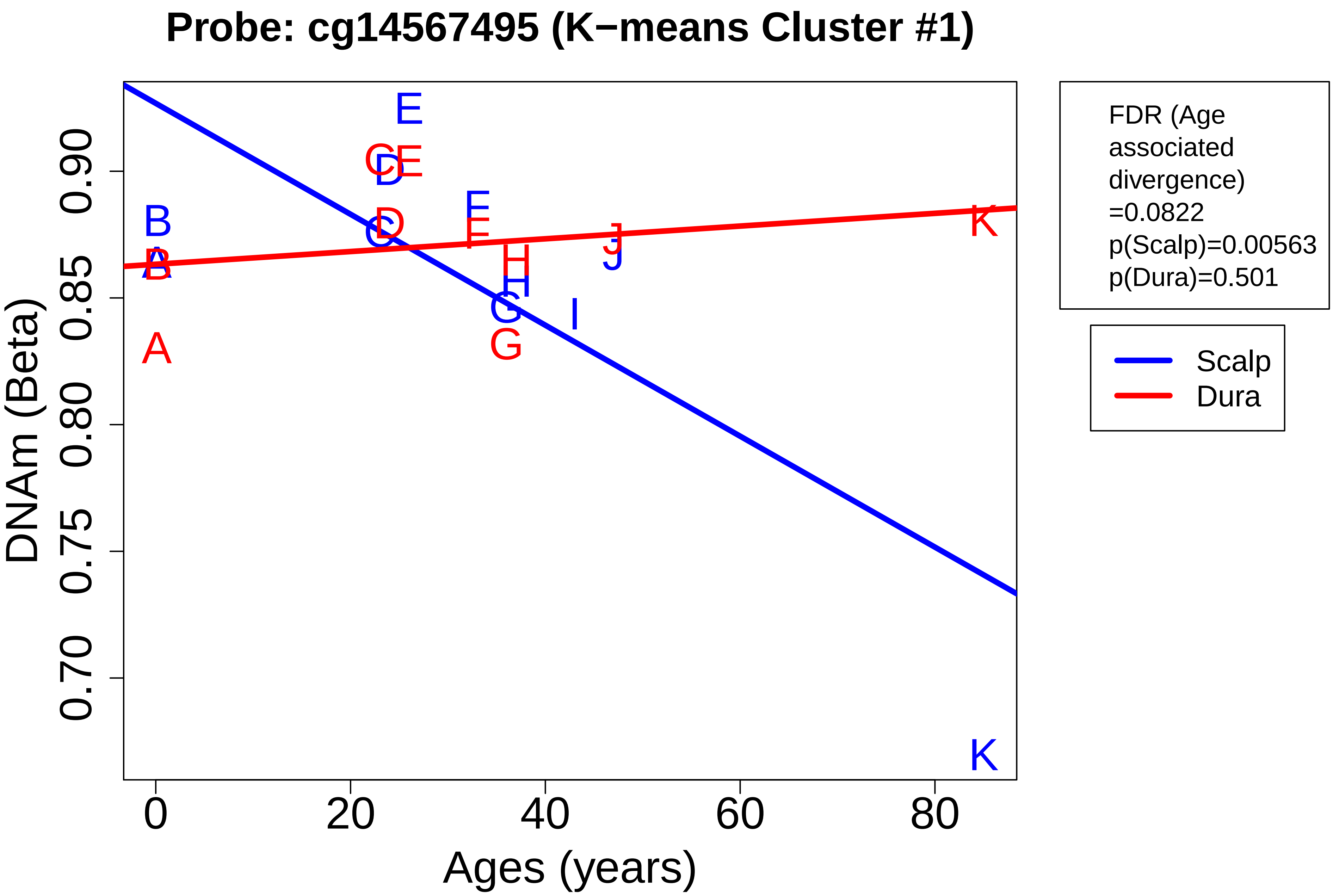

Probe: cg14567495 (K−means Cluster #1)
DNAm (Beta)
0.70
0.75
0.80
0.85
0.90
0
20
40
60
80
Ages (years)
FDR (Age associated divergence) =0.0822
p(Scalp)=0.00563
p(Dura)=0.501
Scalp
Dura

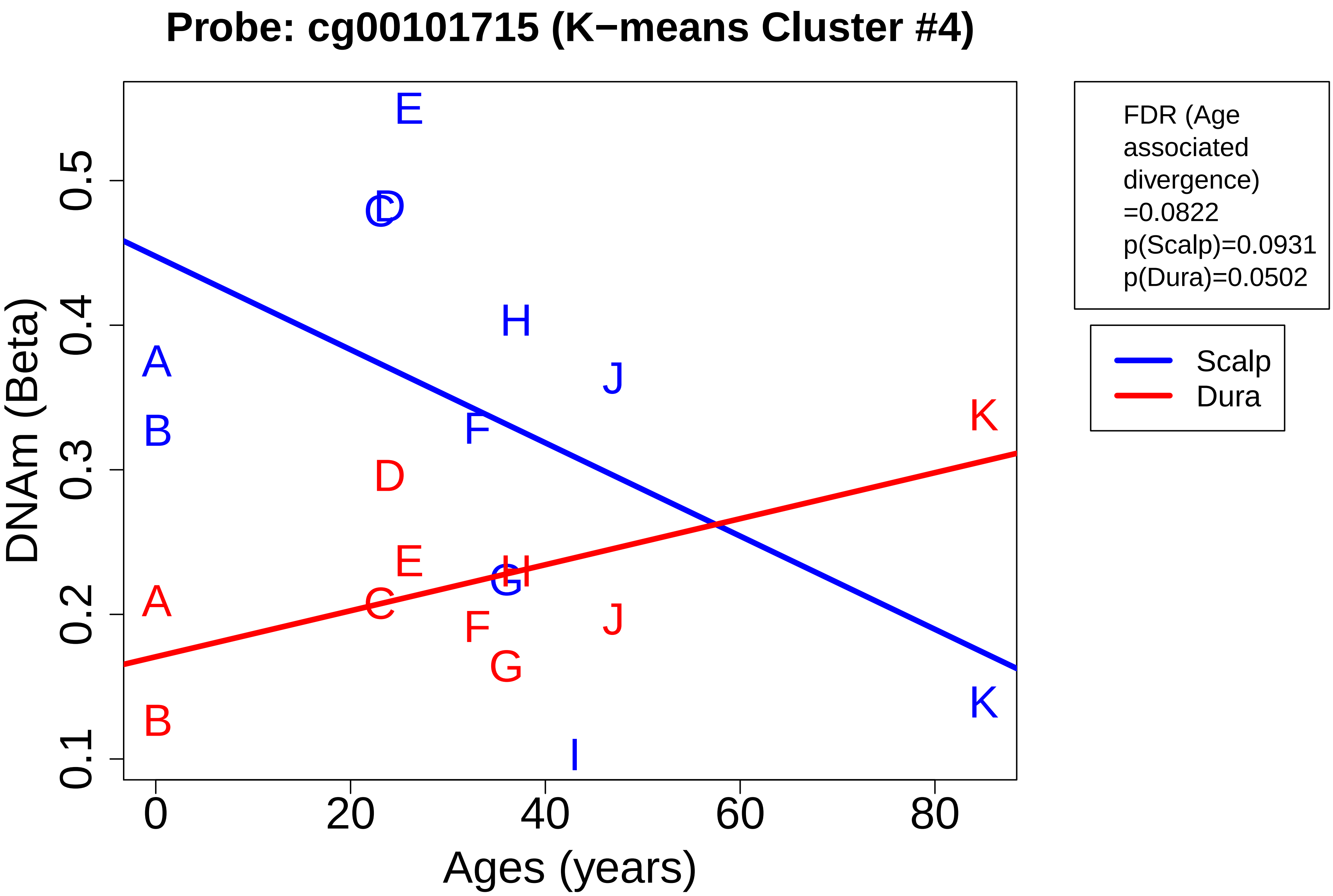

Probe: cg00101715 (K−means Cluster #4)
DNAm (Beta)
0.1
0.2
0.3
0.4
0.5
Ages (years)
0
20
40
60
80
FDR (Age associated divergence) =0.0822
p(Scalp)=0.0931
p(Dura)=0.0502
Scalp
Dura

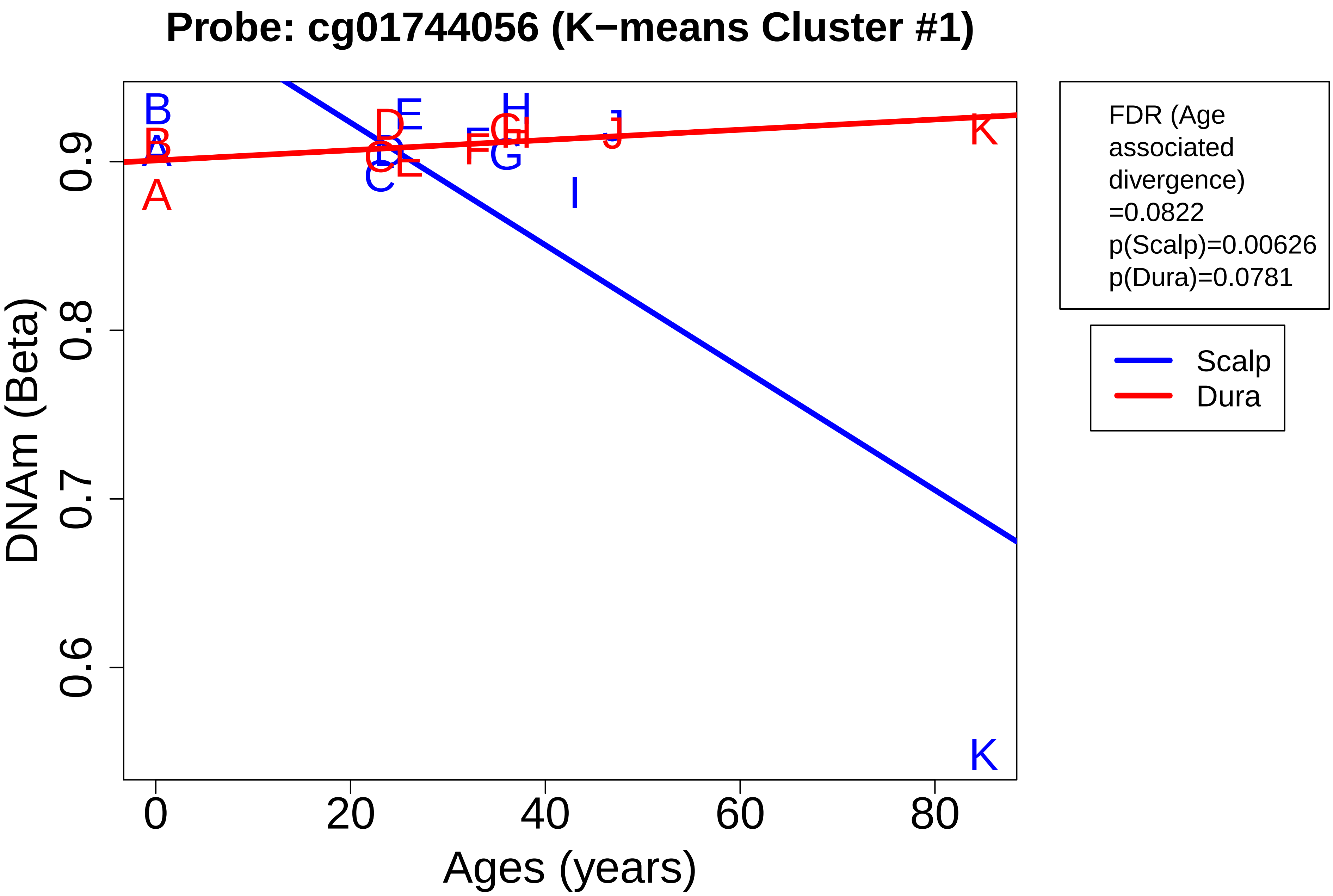

Probe: cg01744056 (K−means Cluster #1)
DNAm (Beta)
0.6
0.7
0.8
0.9
Ages (years)
0
20
40
60
80
FDR (Age associated divergence) =0.0822
p(Scalp)=0.00626
p(Dura)=0.0781
Scalp
Dura

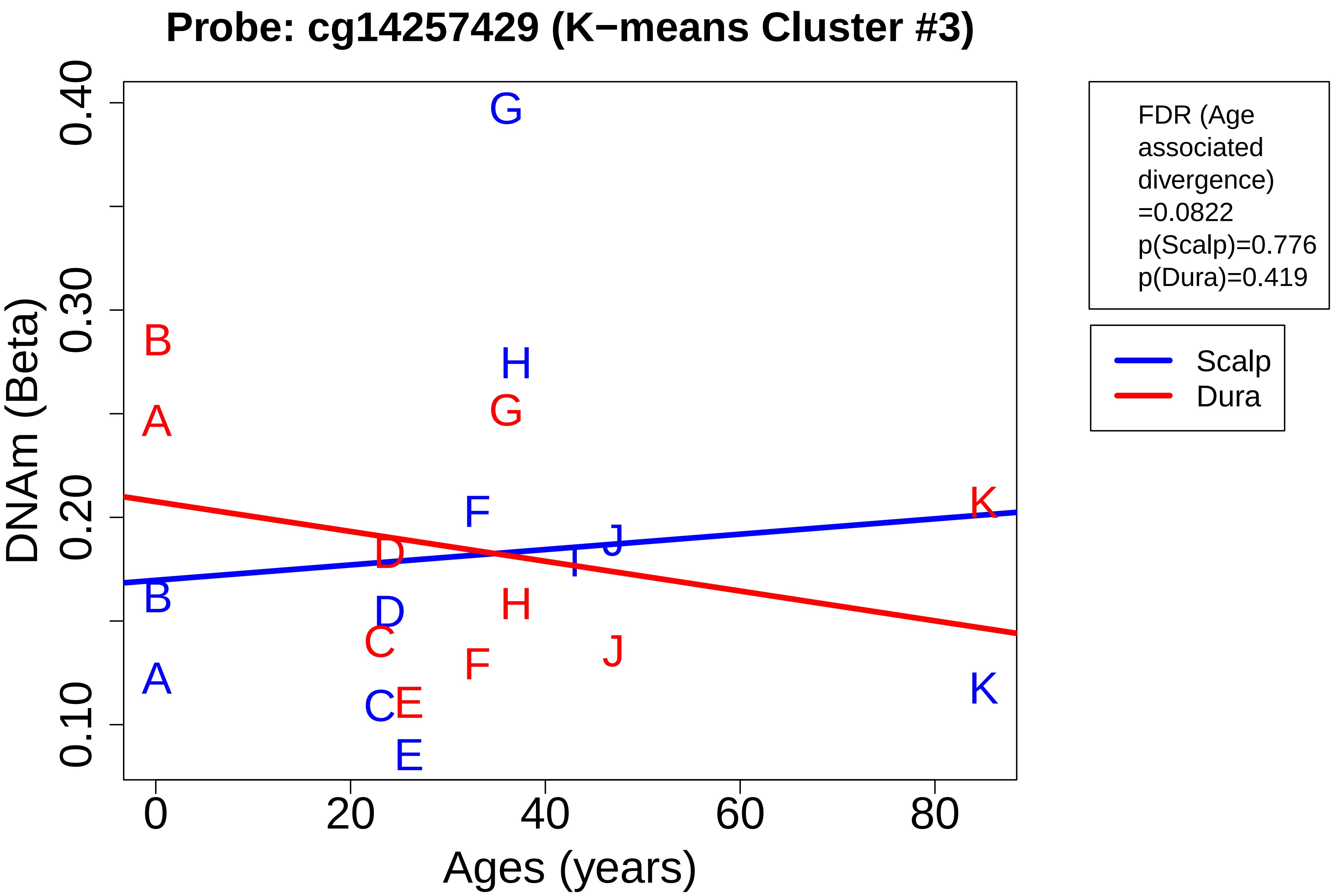

Probe: cg14257429 (K−means Cluster #3)
DNAm (Beta)
0.10
0.20
0.30
0.40
0
20
40
60
80
Ages (years)
FDR (Age associated divergence) =0.0822
p(Scalp)=0.776
p(Dura)=0.419
Scalp
Dura

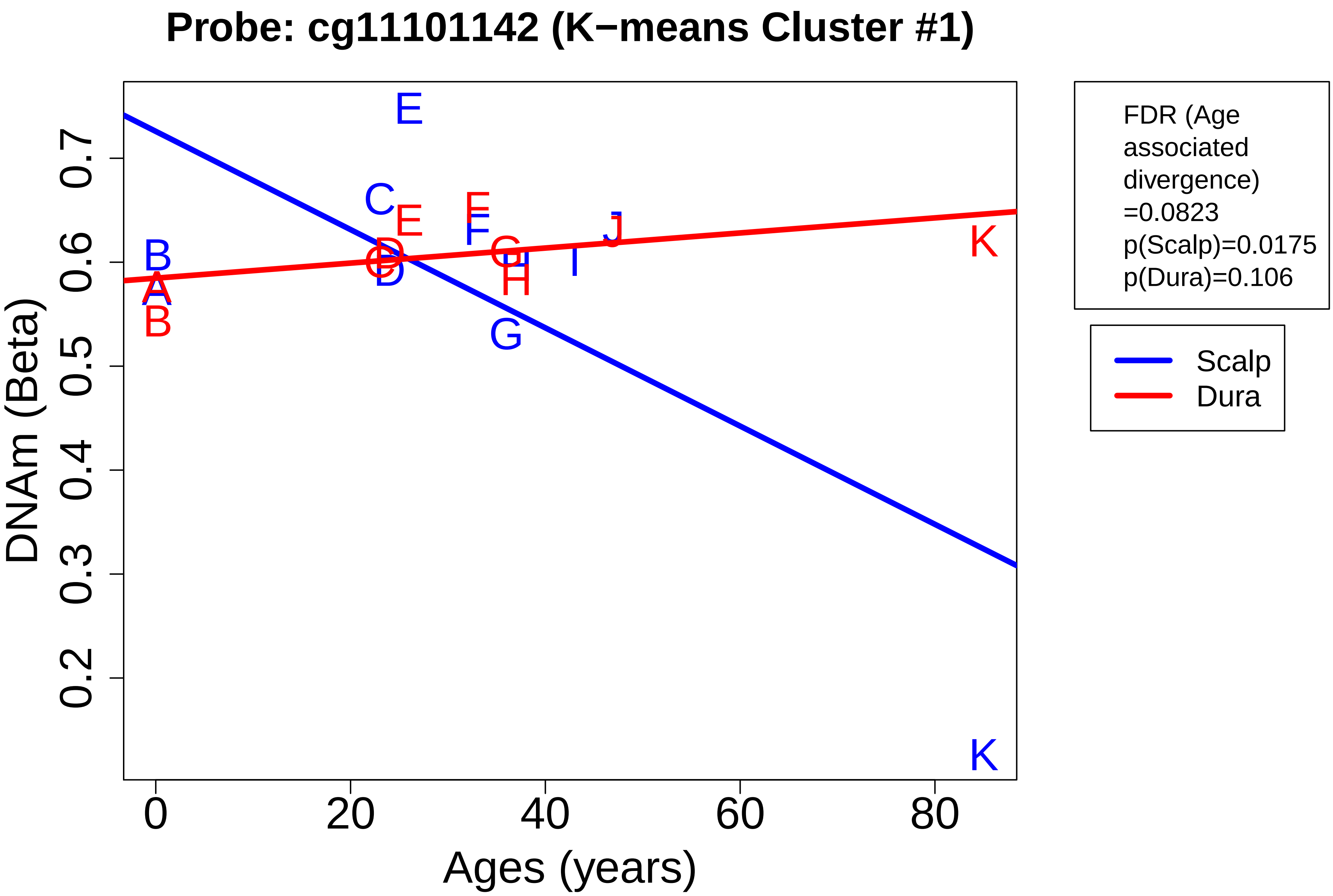
Probe: cg11101142 (K−means Cluster #1)
DNAm (Beta)
Ages (years)
0.2
0.3
0.4
0.5
0.6
0.7
0
20
40
60
80
FDR (Age associated divergence) =0.0823
p(Scalp)=0.0175
p(Dura)=0.106
Scalp
Dura

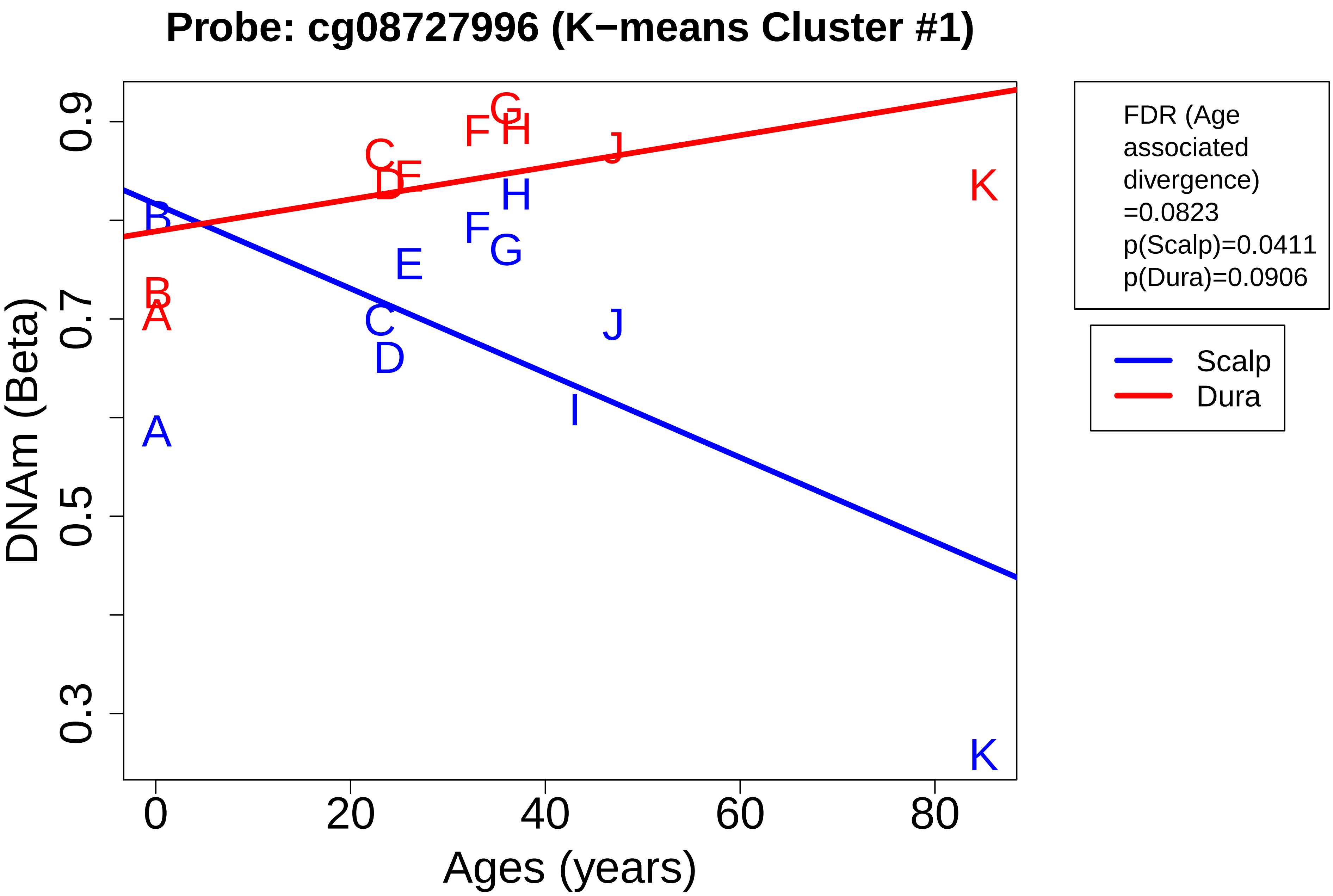
Probe: cg08727996 (K−means Cluster #1)
DNAm (Beta)
0.3
0.5
0.7
0.9
0
20
40
60
80
Ages (years)
FDR (Age associated divergence) =0.0823
p(Scalp)=0.0411
p(Dura)=0.0906
Scalp
Dura

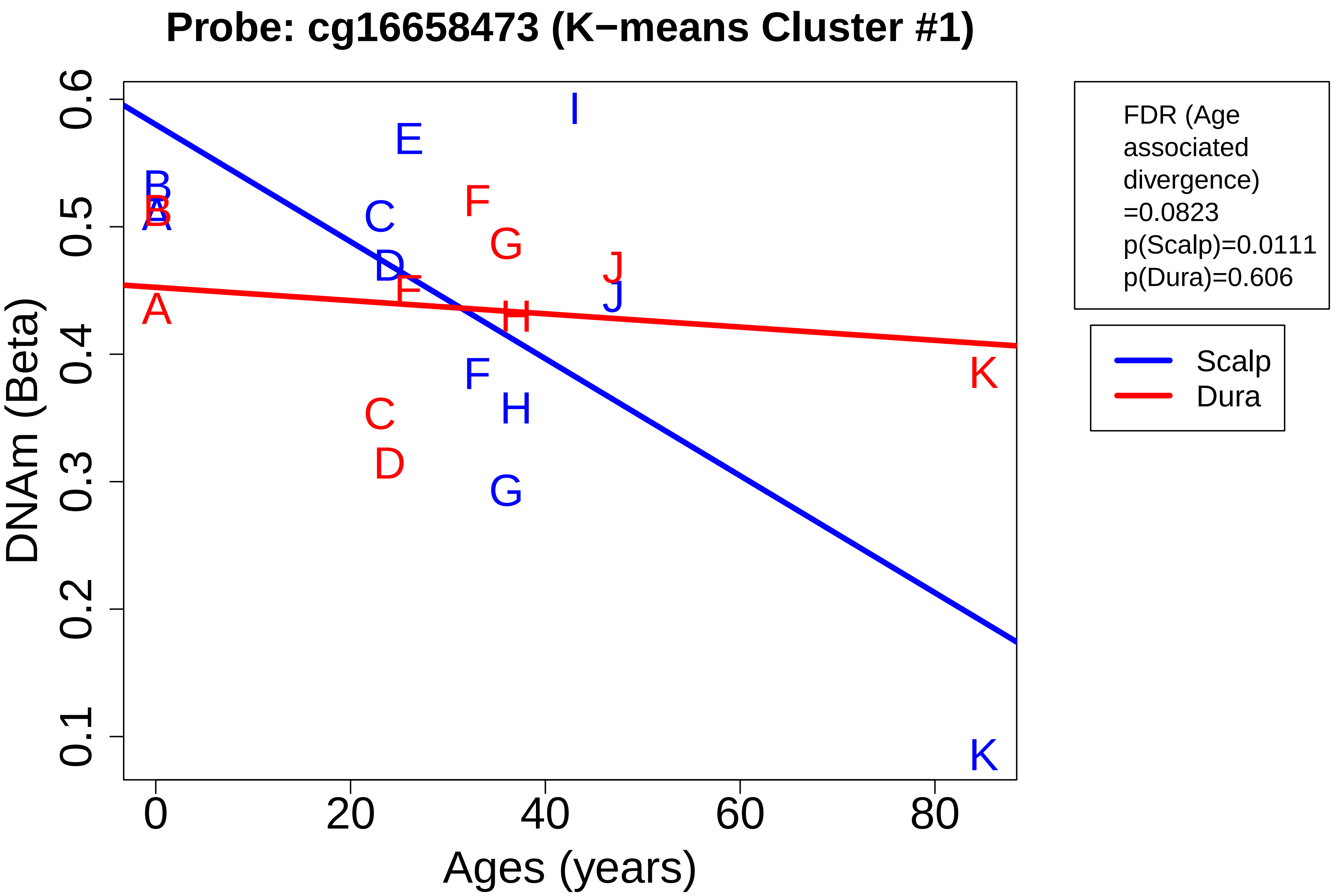

Probe: cg16658473 (K−means Cluster #1)
DNAm (Beta)
0.1
0.2
0.3
0.4
0.5
0.6
0
20
40
60
80
Ages (years)
A
B
C
D
E
F
G
H
I
J
K
FDR (Age associated divergence) =0.0823
p(Scalp)=0.0111
p(Dura)=0.606
Scalp
Dura

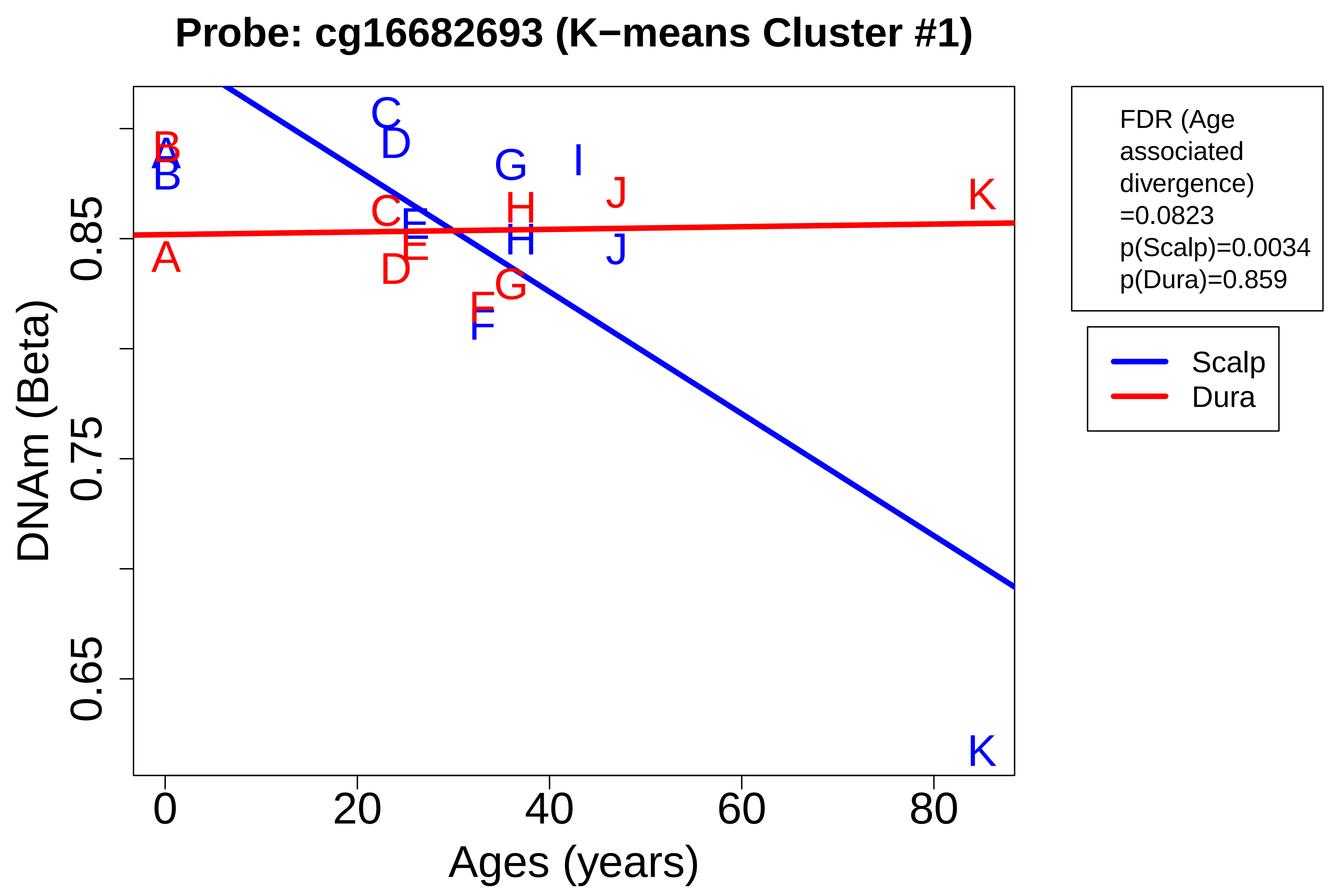

Probe: cg16682693 (K−means Cluster #1)
DNAm (Beta)
0.65
0.75
0.85
0
20
40
60
80
Ages (years)
FDR (Age associated divergence) =0.0823
p(Scalp)=0.0034
p(Dura)=0.859
Scalp
Dura

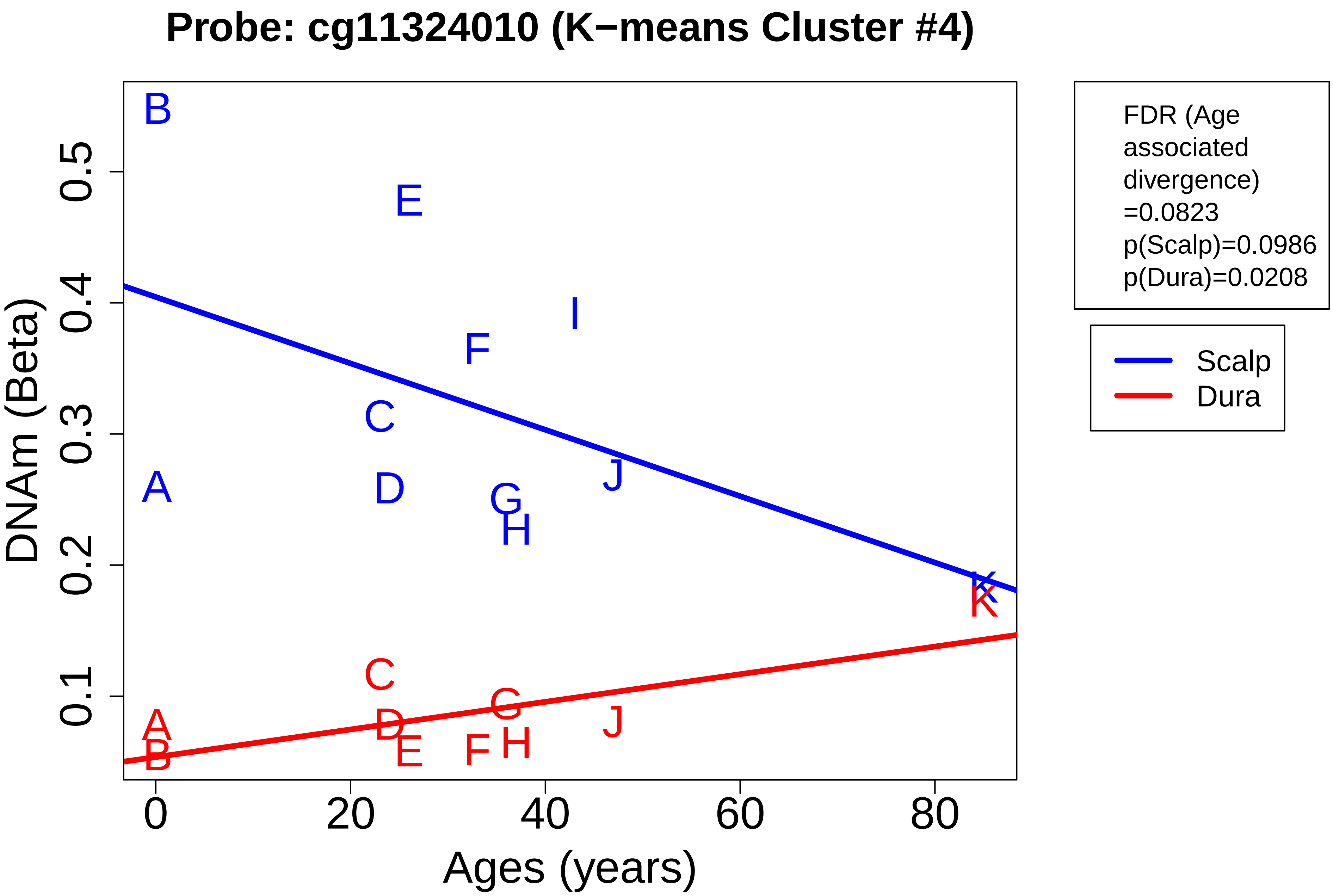

Probe: cg11324010 (K−means Cluster #4)
FDR (Age associated divergence) =0.0823
p(Scalp)=0.0986
p(Dura)=0.0208
Scalp
Dura
DNAm (Beta)
Ages (years)
0.1
0.2
0.3
0.4
0.5
0
20
40
60
80

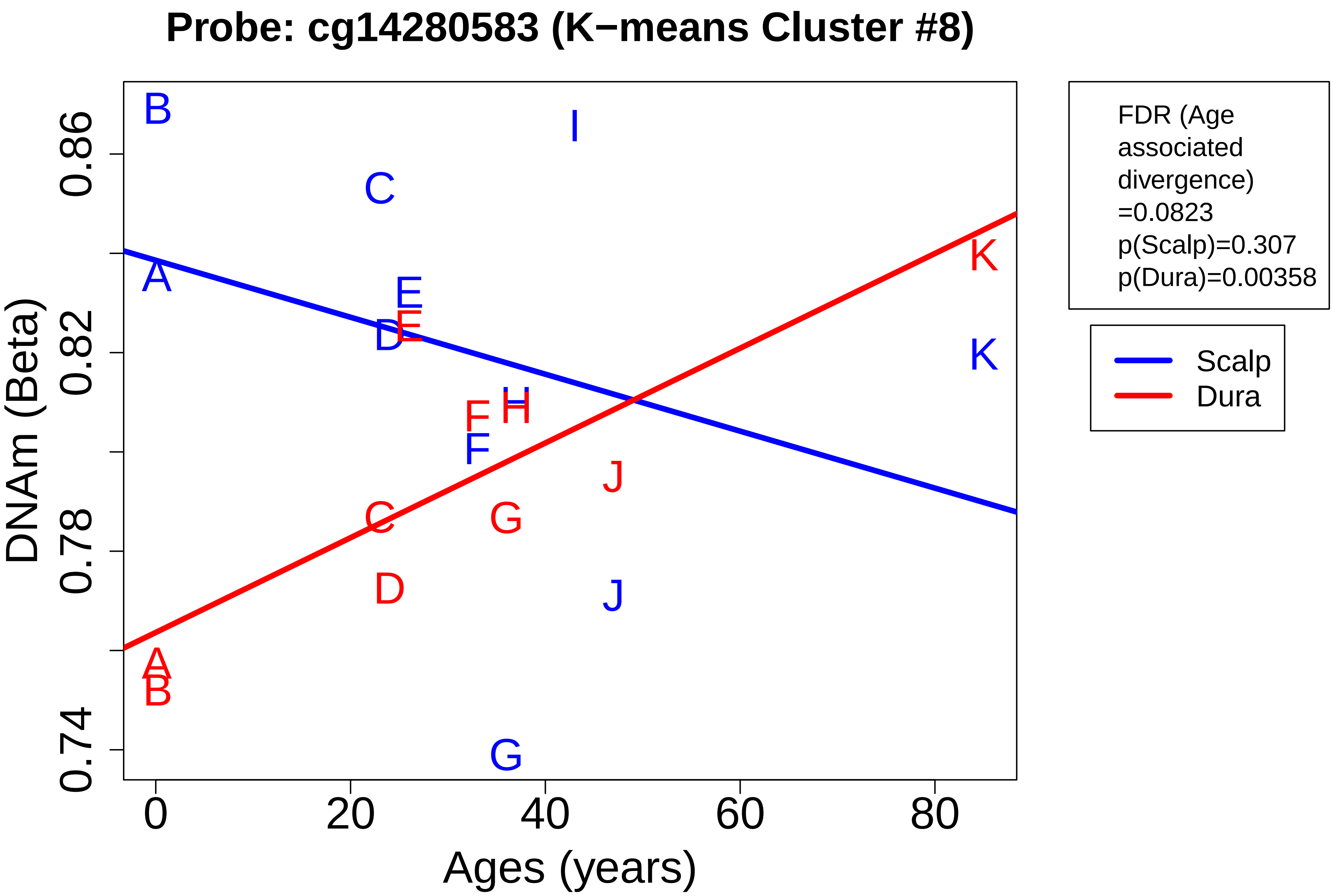

Probe: cg14280583 (K−means Cluster #8)
DNAm (Beta)
0.74
0.78
0.82
0.86
0
20
40
60
80
Ages (years)
FDR (Age associated divergence) =0.0823
p(Scalp)=0.307
p(Dura)=0.00358
Scalp
Dura

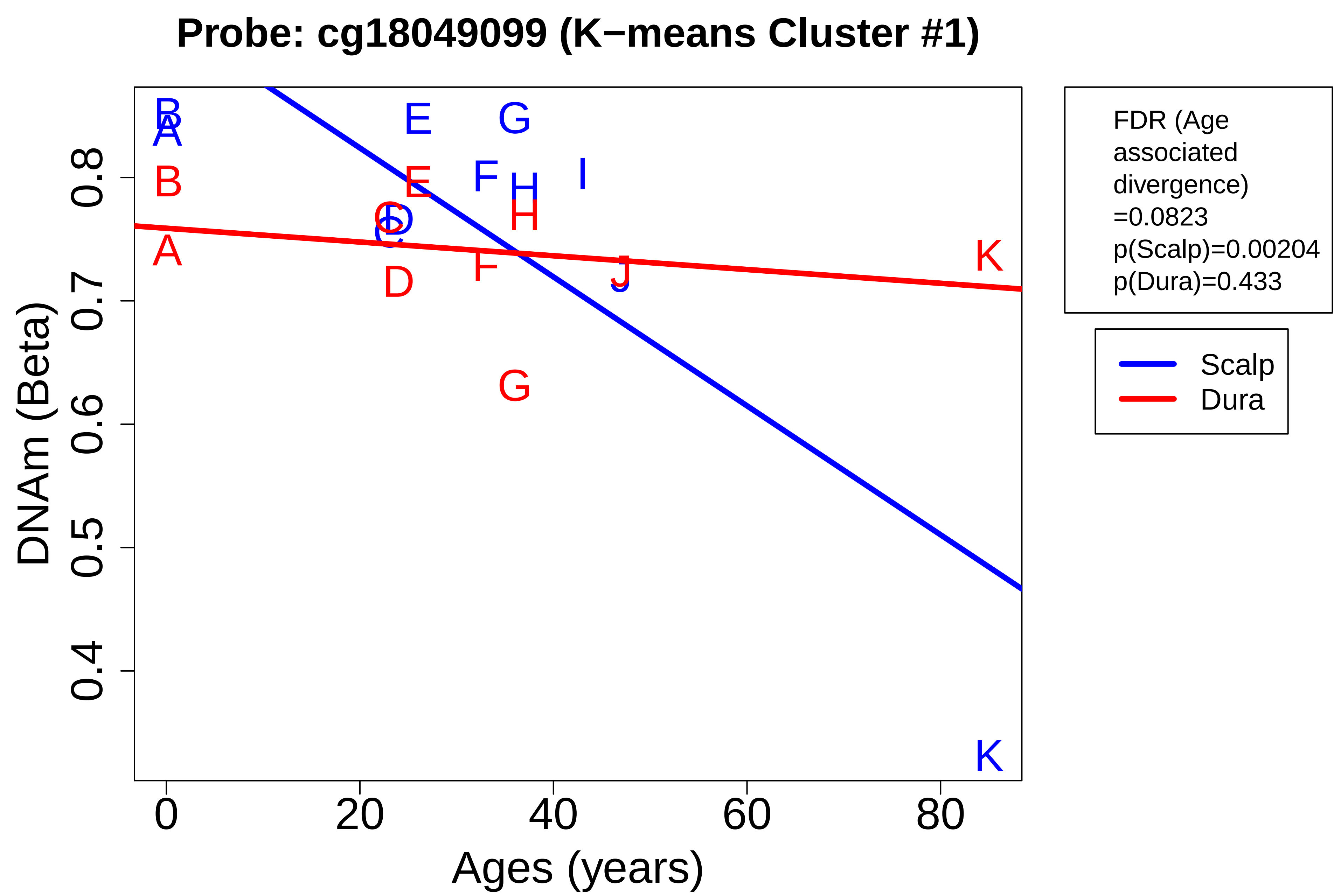

Probe: cg18049099 (K−means Cluster #1)
FDR (Age associated divergence) =0.0823
p(Scalp)=0.00204
p(Dura)=0.433
Scalp
Dura
DNAm (Beta)
Ages (years)
0.4
0.5
0.6
0.7
0.8
0
20
40
60
80
A
B
C
D
E
F
G
H
I
J
K

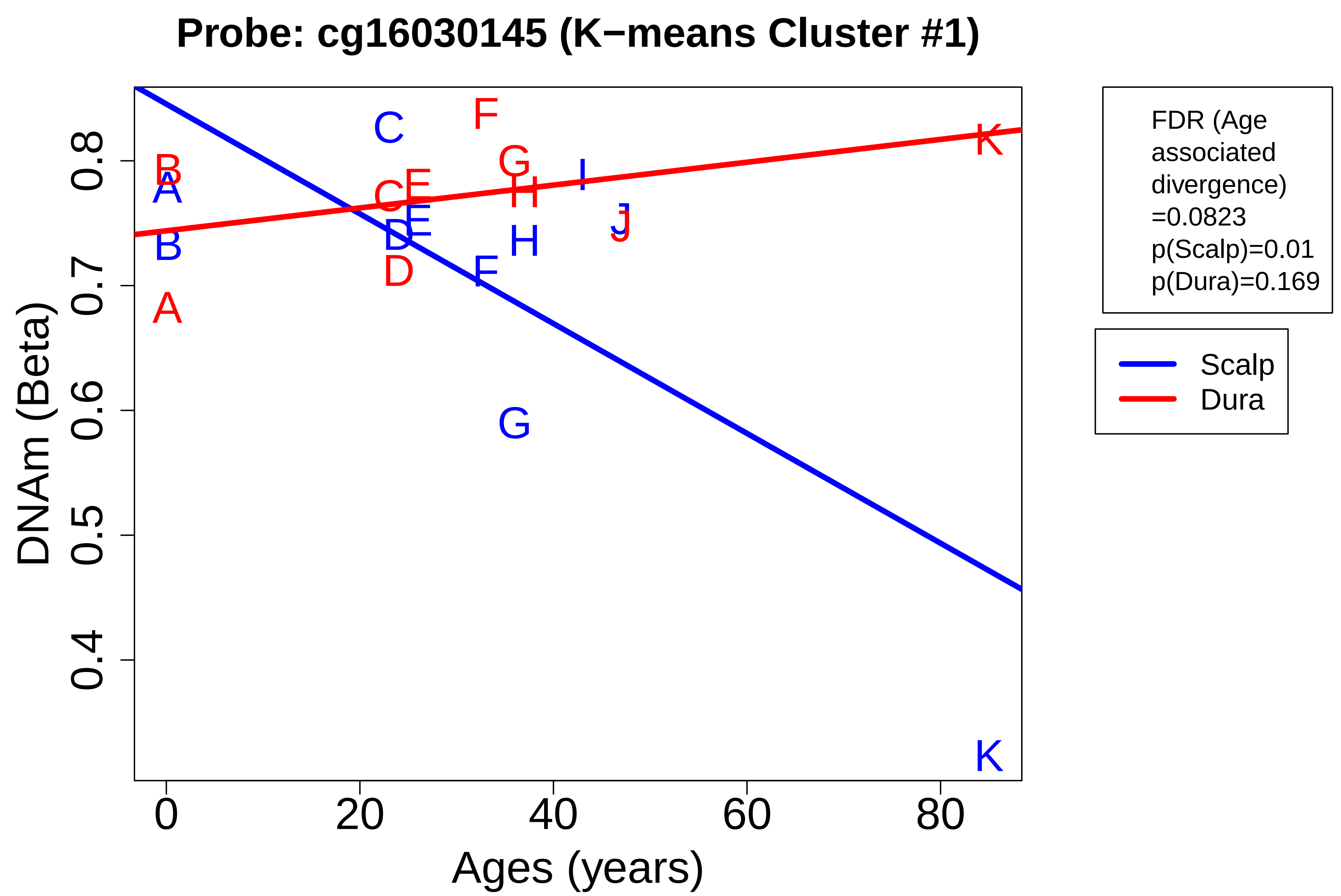

Probe: cg16030145 (K−means Cluster #1)
DNAm (Beta)
0.4
0.5
0.6
0.7
0.8
0
20
40
60
80
Ages (years)
A
B
C
D
E
F
G
H
I
J
K
FDR (Age associated divergence) =0.0823
p(Scalp)=0.01
p(Dura)=0.169
Scalp
Dura

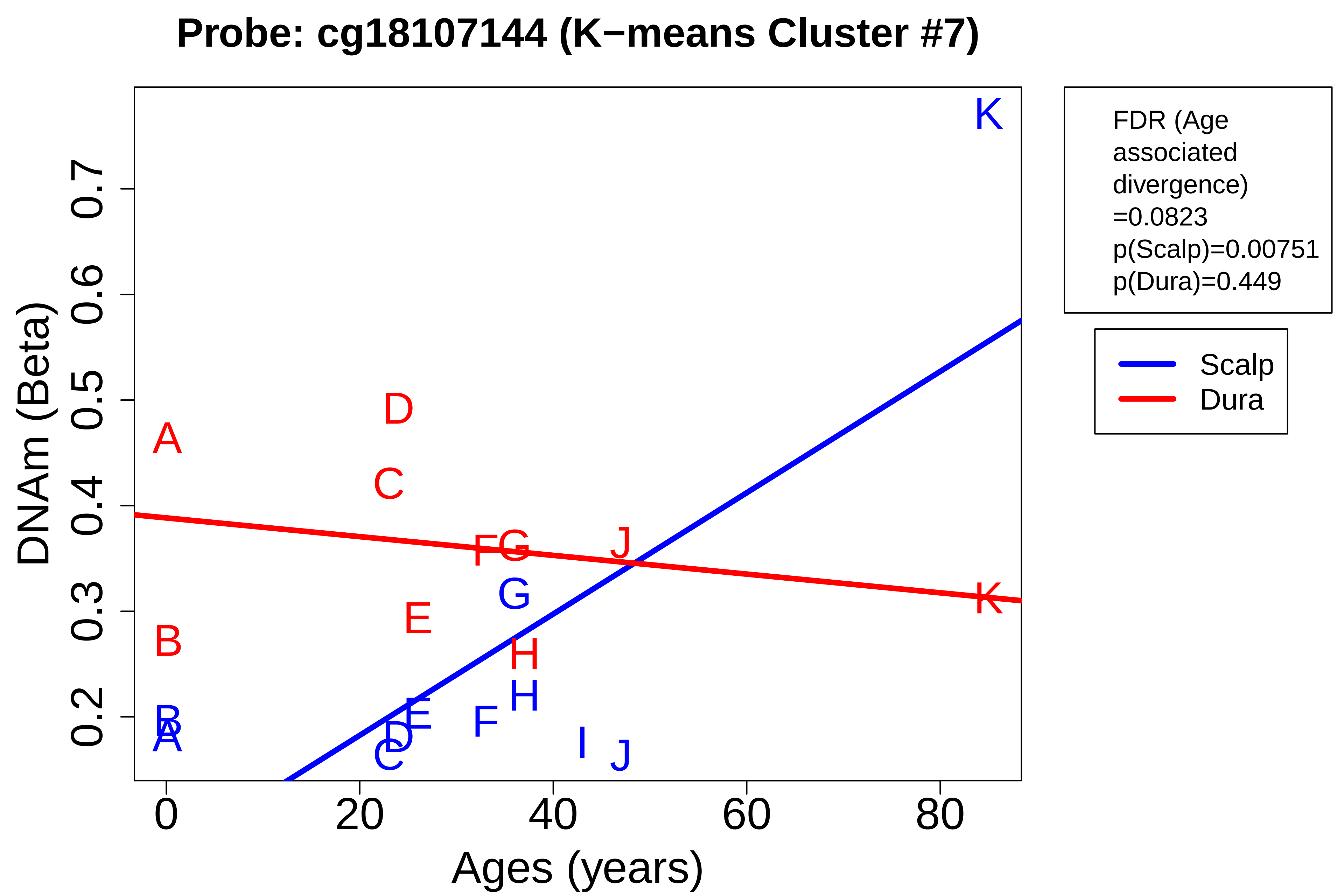

Probe: cg18107144 (K−means Cluster #7)
DNAm (Beta)
0.2
0.3
0.4
0.5
0.6
0.7
0
20
40
60
80
Ages (years)
FDR (Age associated divergence) =0.0823
p(Scalp)=0.00751
p(Dura)=0.449
Scalp
Dura

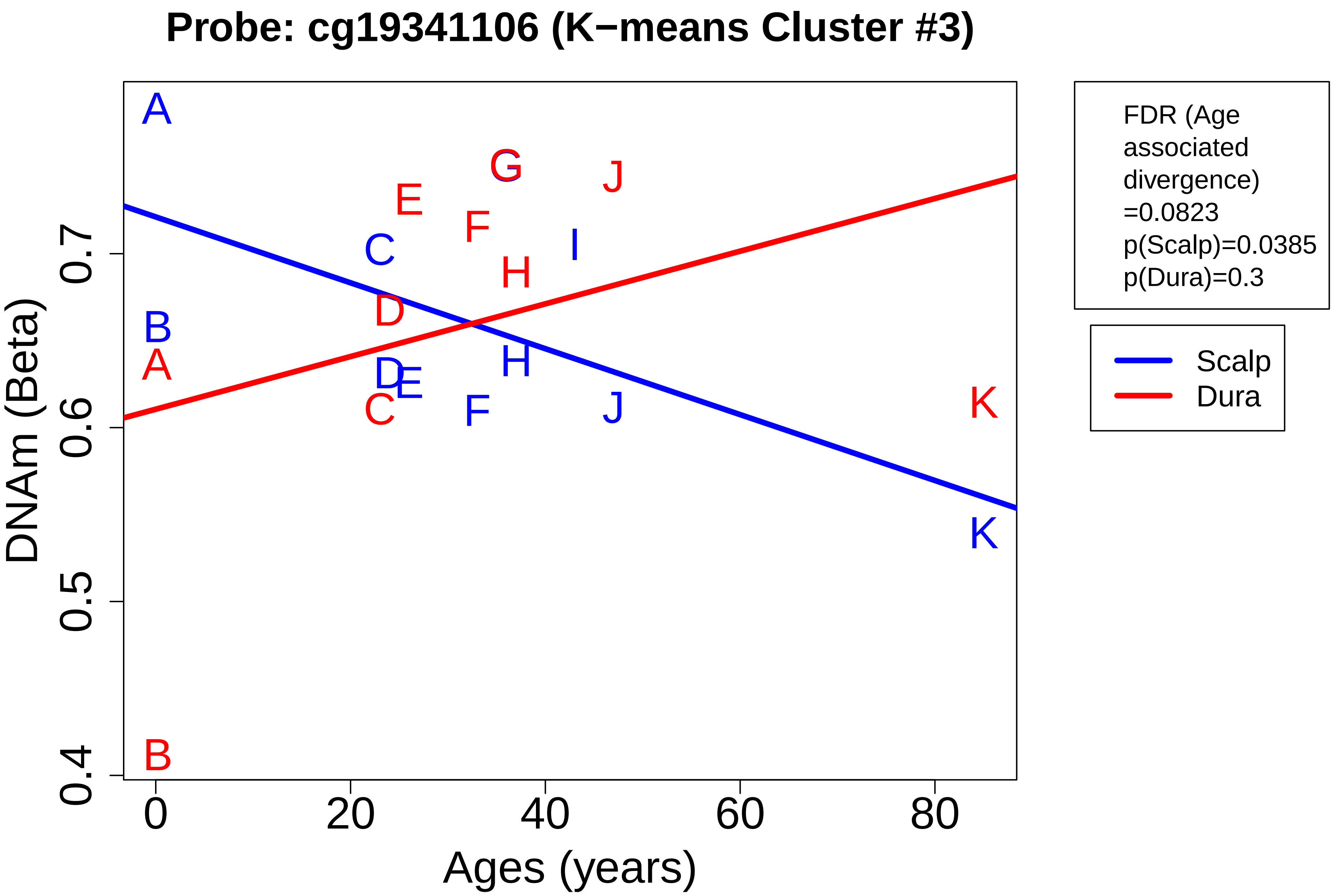

Probe: cg19341106 (K−means Cluster #3)
DNAm (Beta)
0.4
0.5
0.6
0.7
Ages (years)
0
20
40
60
80
FDR (Age associated divergence) =0.0823
p(Scalp)=0.0385
p(Dura)=0.3
Scalp
Dura
A
B
C
D
E
F
G
H
I
J
K

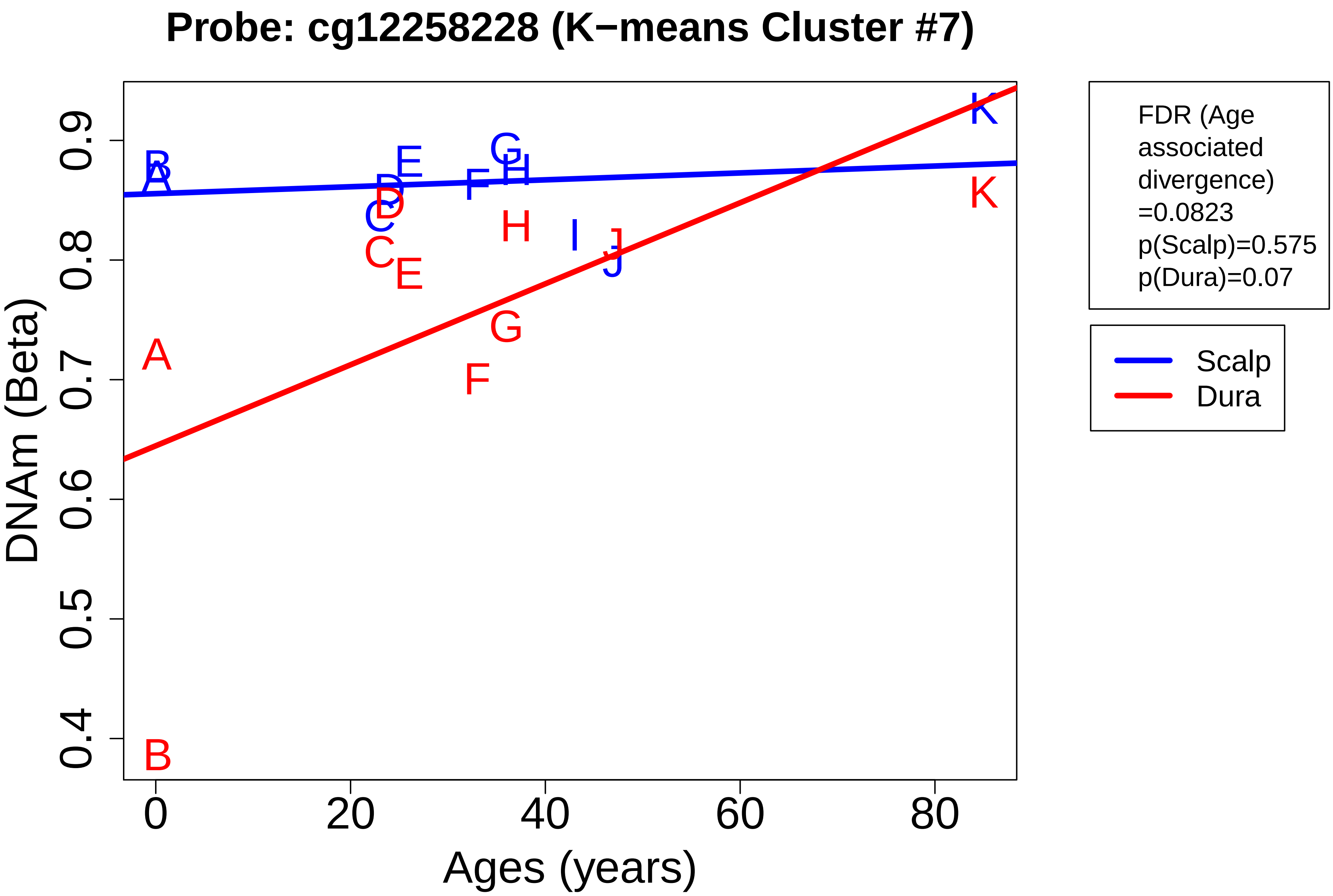

Probe: cg12258228 (K−means Cluster #7)
DNAm (Beta)
0.4
0.5
0.6
0.7
0.8
0.9
0
20
40
60
80
Ages (years)
FDR (Age associated divergence) =0.0823
p(Scalp)=0.575
p(Dura)=0.07
Scalp
Dura

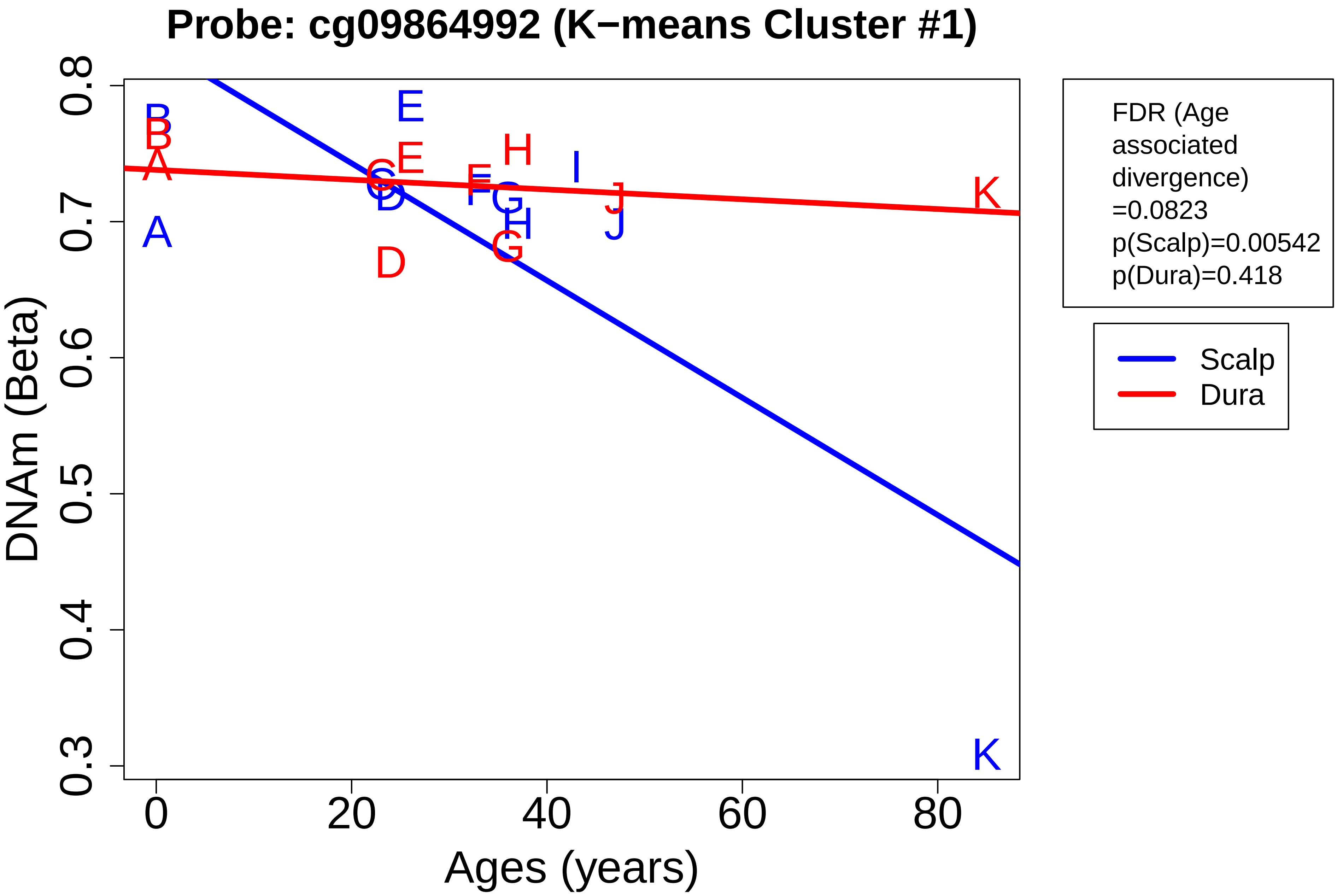

Probe: cg09864992 (K−means Cluster #1)
FDR (Age associated divergence) =0.0823
p(Scalp)=0.00542
p(Dura)=0.418
Scalp
Dura
DNAm (Beta)
0.3
0.4
0.5
0.6
0.7
0.8
0
20
40
60
80
Ages (years)
A
B
C
D
E
F
G
H
I
J
K

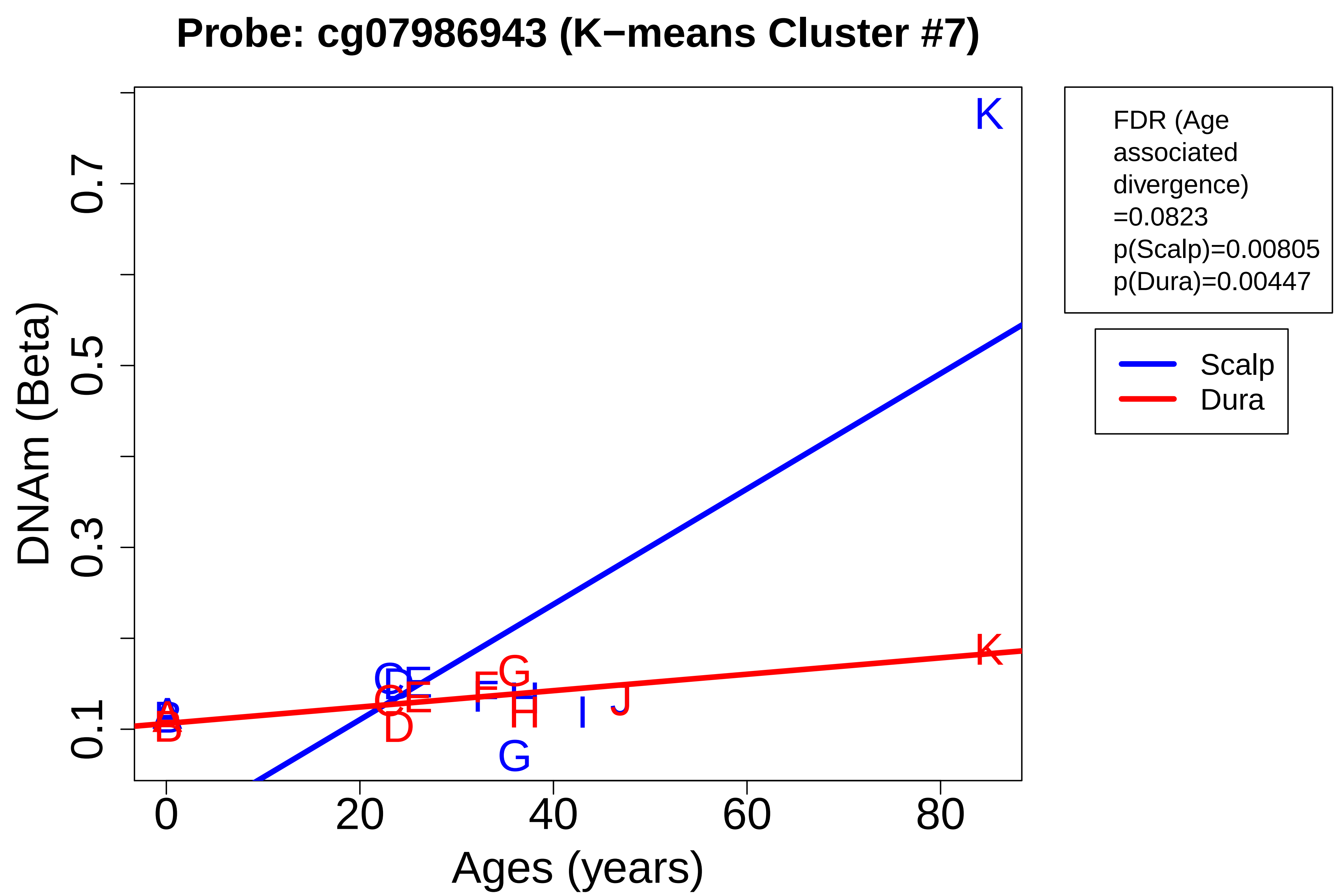

Probe: cg07986943 (K−means Cluster #7)
FDR (Age associated divergence) =0.0823
p(Scalp)=0.00805
p(Dura)=0.00447
Scalp
Dura
DNAm (Beta)
0.1
0.3
0.5
0.7
Ages (years)
0
20
40
60
80
A
B
C
D
E
F
G
H
I
J
K

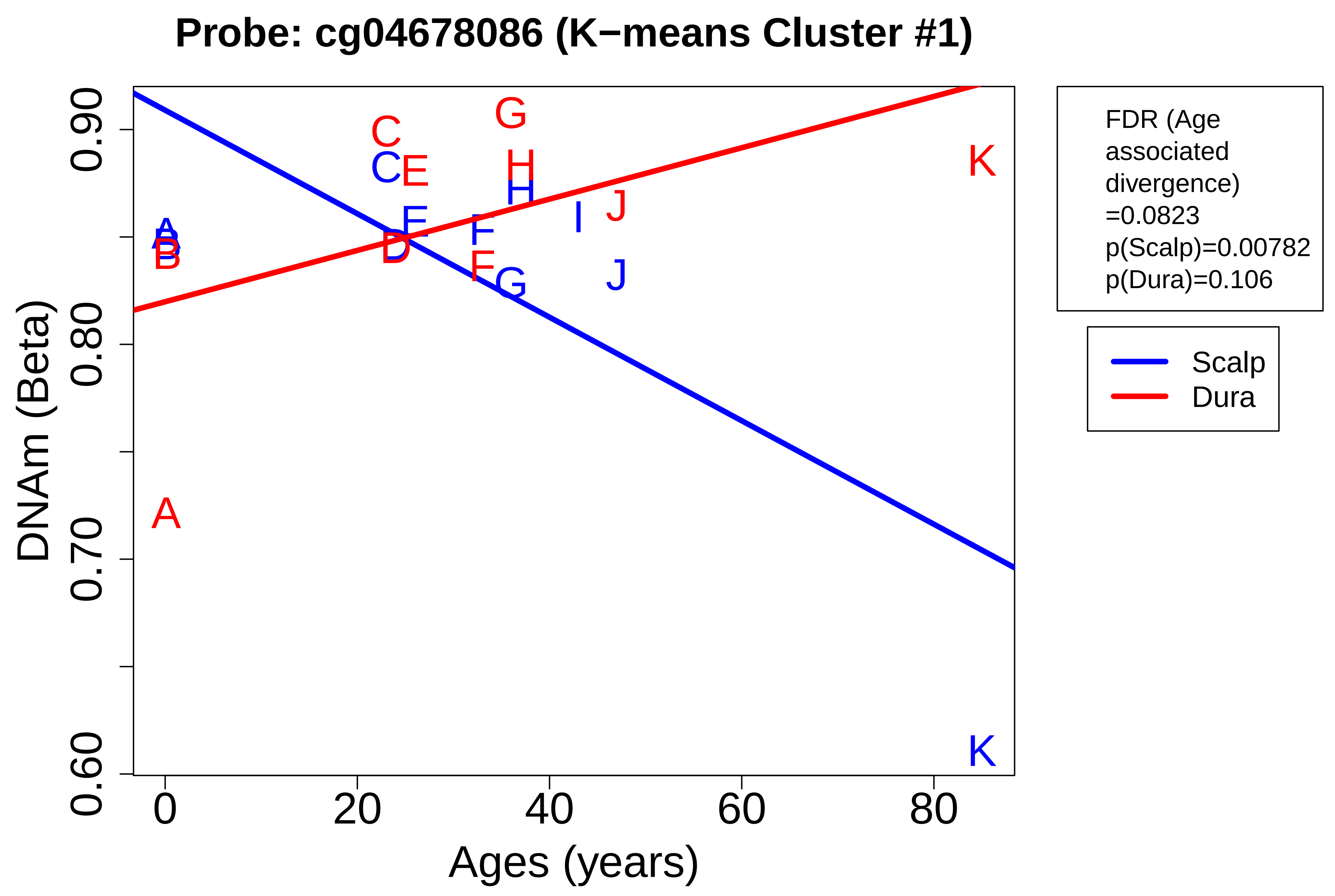

Probe: cg04678086 (K−means Cluster #1)
DNAm (Beta)
0.60
0.70
0.80
0.90
0
20
40
60
80
Ages (years)
A
B
C
D
E
F
G
H
I
J
K
FDR (Age associated divergence) =0.0823
p(Scalp)=0.00782
p(Dura)=0.106
Scalp
Dura

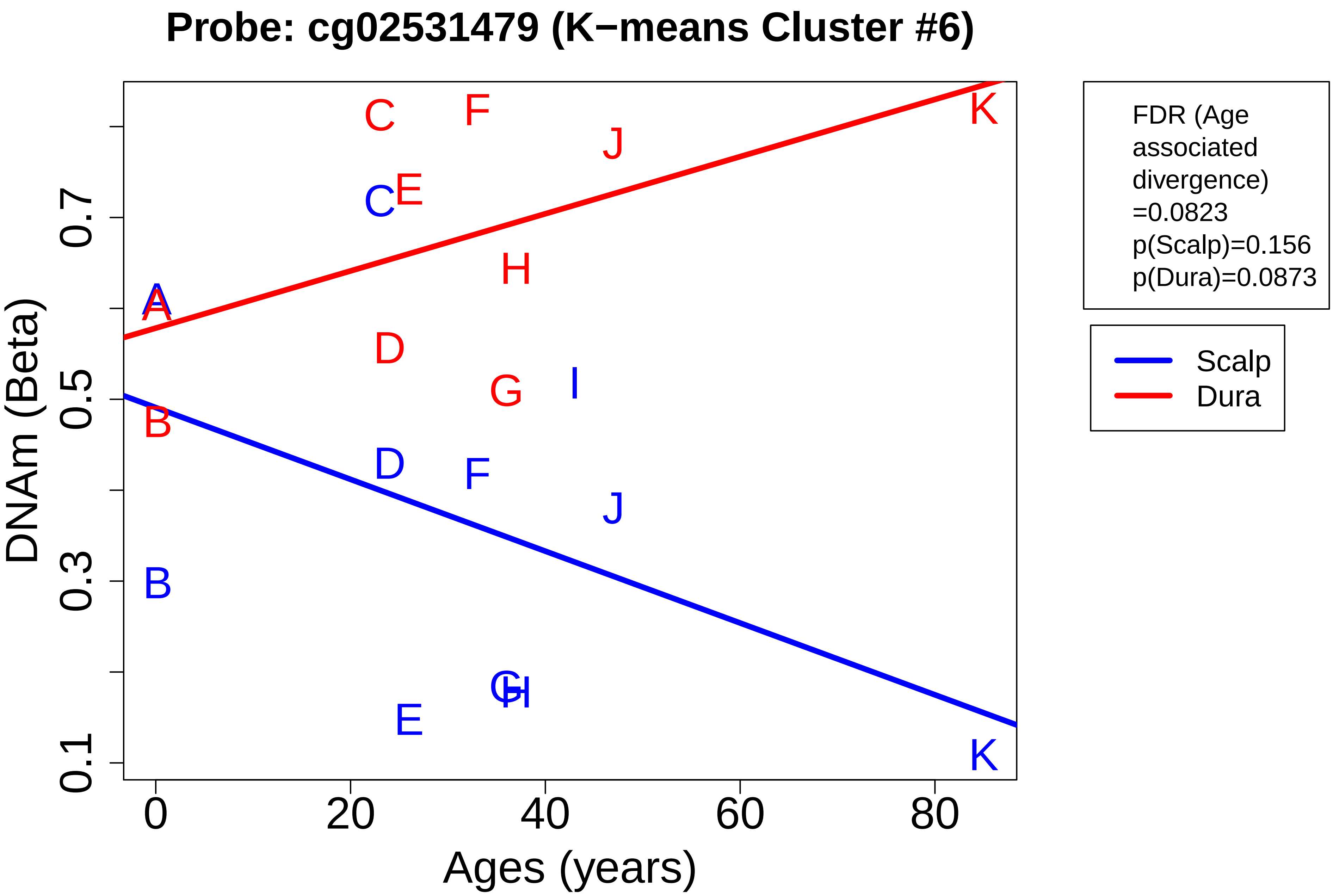

Probe: cg02531479 (K−means Cluster #6)
DNAm (Beta)
Ages (years)
0.1
0.3
0.5
0.7
0
20
40
60
80
FDR (Age associated divergence) =0.0823
p(Scalp)=0.156
p(Dura)=0.0873
Scalp
Dura

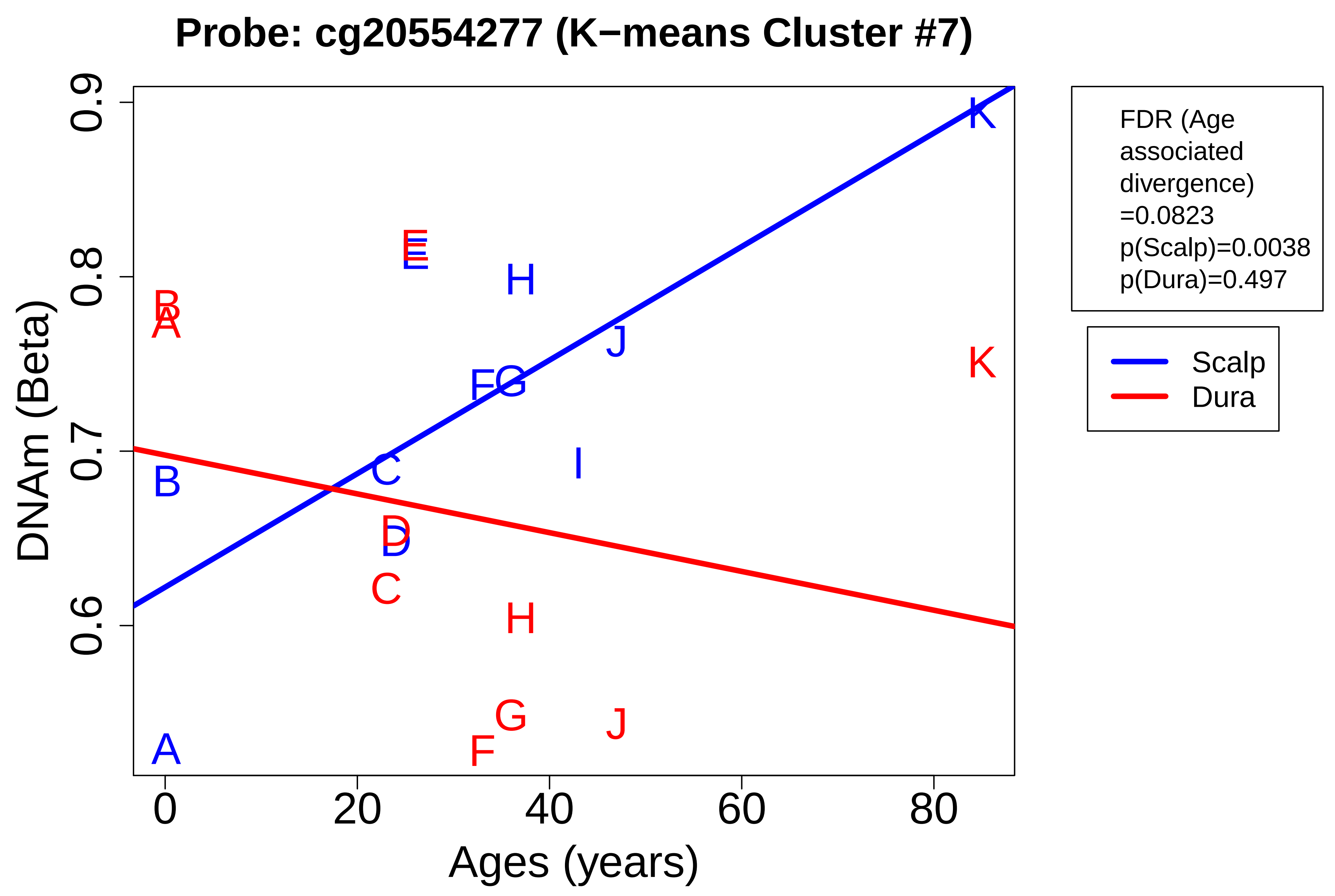

Probe: cg20554277 (K−means Cluster #7)
DNAm (Beta)
0.6
0.7
0.8
0.9
Ages (years)
0
20
40
60
80
FDR (Age associated divergence) =0.0823
p(Scalp)=0.0038
p(Dura)=0.497
Scalp
Dura
A
B
C
D
E
F
G
H
I
J
K

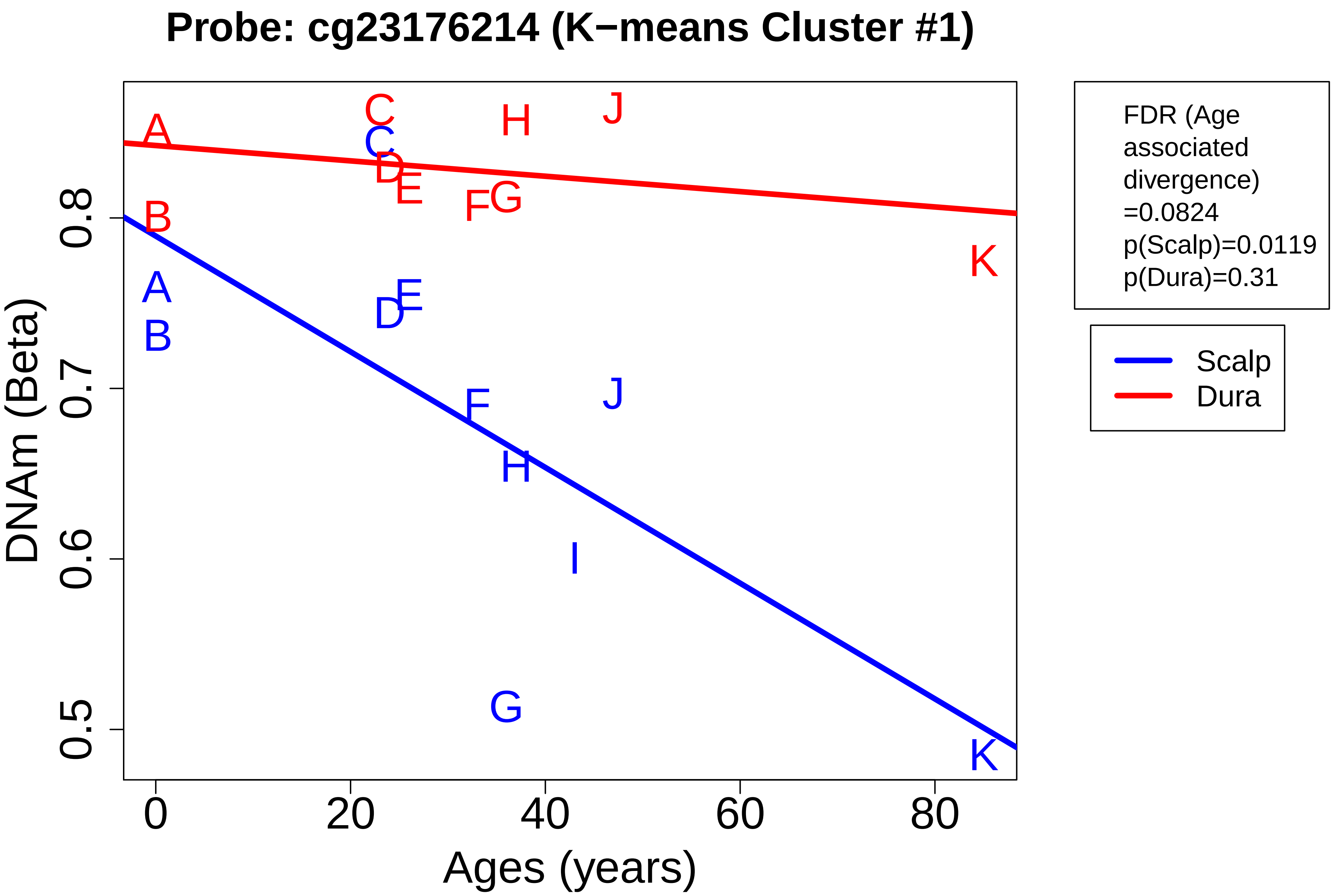

Probe: cg23176214 (K−means Cluster #1)
FDR (Age associated divergence) =0.0824
p(Scalp)=0.0119
p(Dura)=0.31
Scalp
Dura
DNAm (Beta)
0.5
0.6
0.7
0.8
0
20
40
60
80
Ages (years)
A
B
C
D
E
F
G
H
I
J
K

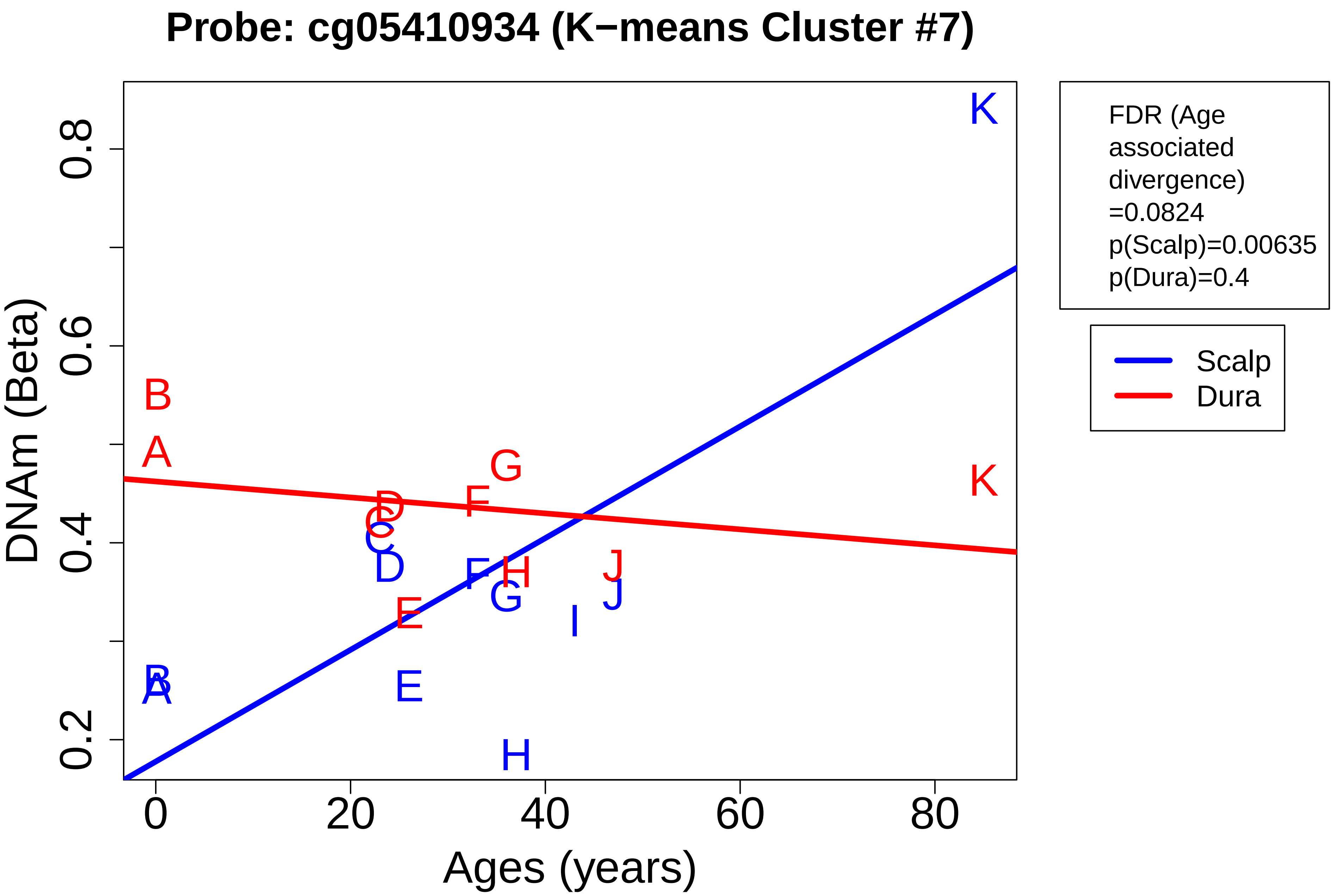

Probe: cg05410934 (K−means Cluster #7)
DNAm (Beta)
0.2
0.4
0.6
0.8
Ages (years)
0
20
40
60
80
FDR (Age associated divergence) =0.0824
p(Scalp)=0.00635
p(Dura)=0.4
Scalp
Dura
A
B
C
D
E
F
G
H
I
J
K

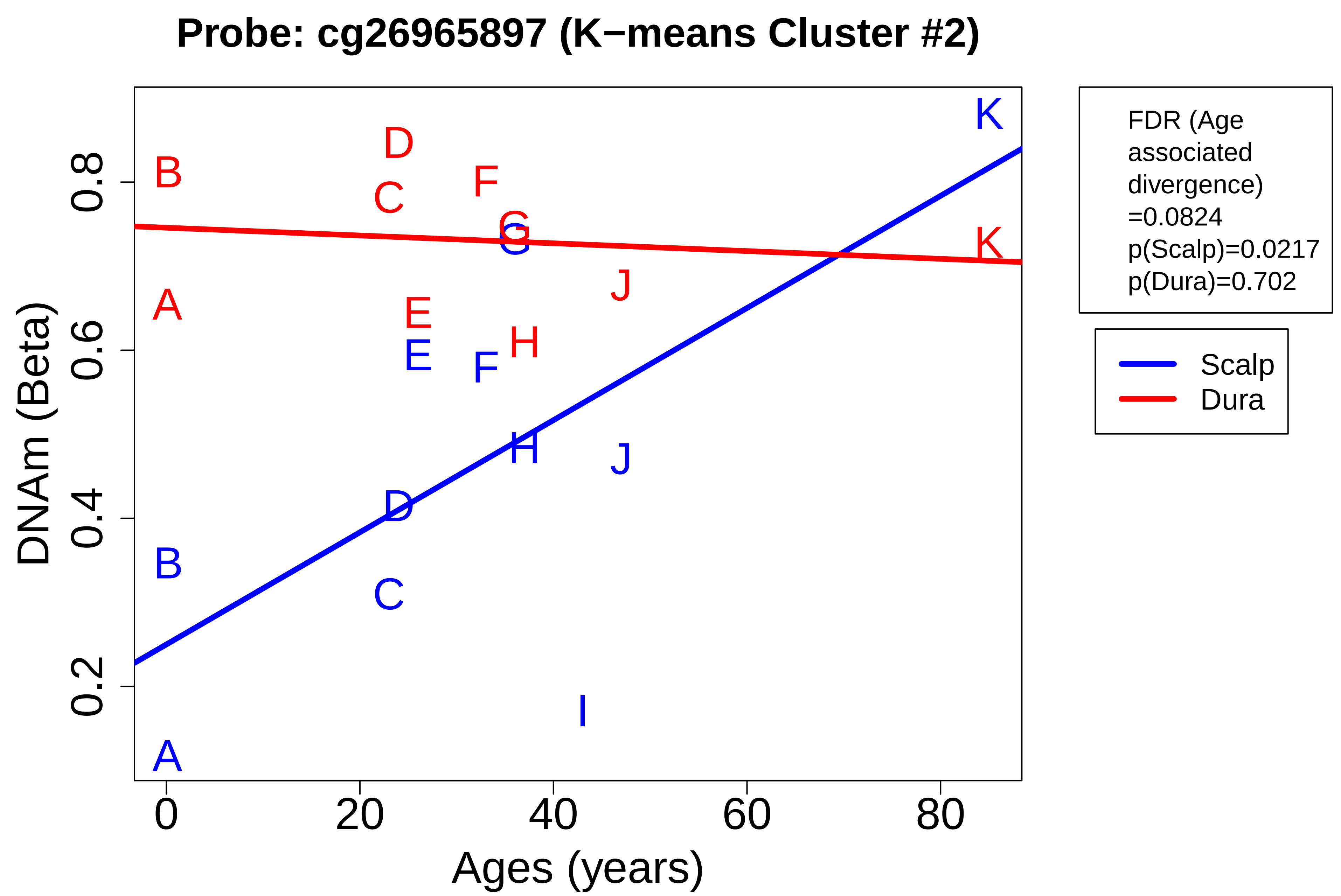

Probe: cg26965897 (K−means Cluster #2)
DNAm (Beta)
0.2
0.4
0.6
0.8
0
20
40
60
80
Ages (years)
A
B
C
D
E
F
G
H
I
J
K
FDR (Age associated divergence) =0.0824
p(Scalp)=0.0217
p(Dura)=0.702
Scalp
Dura

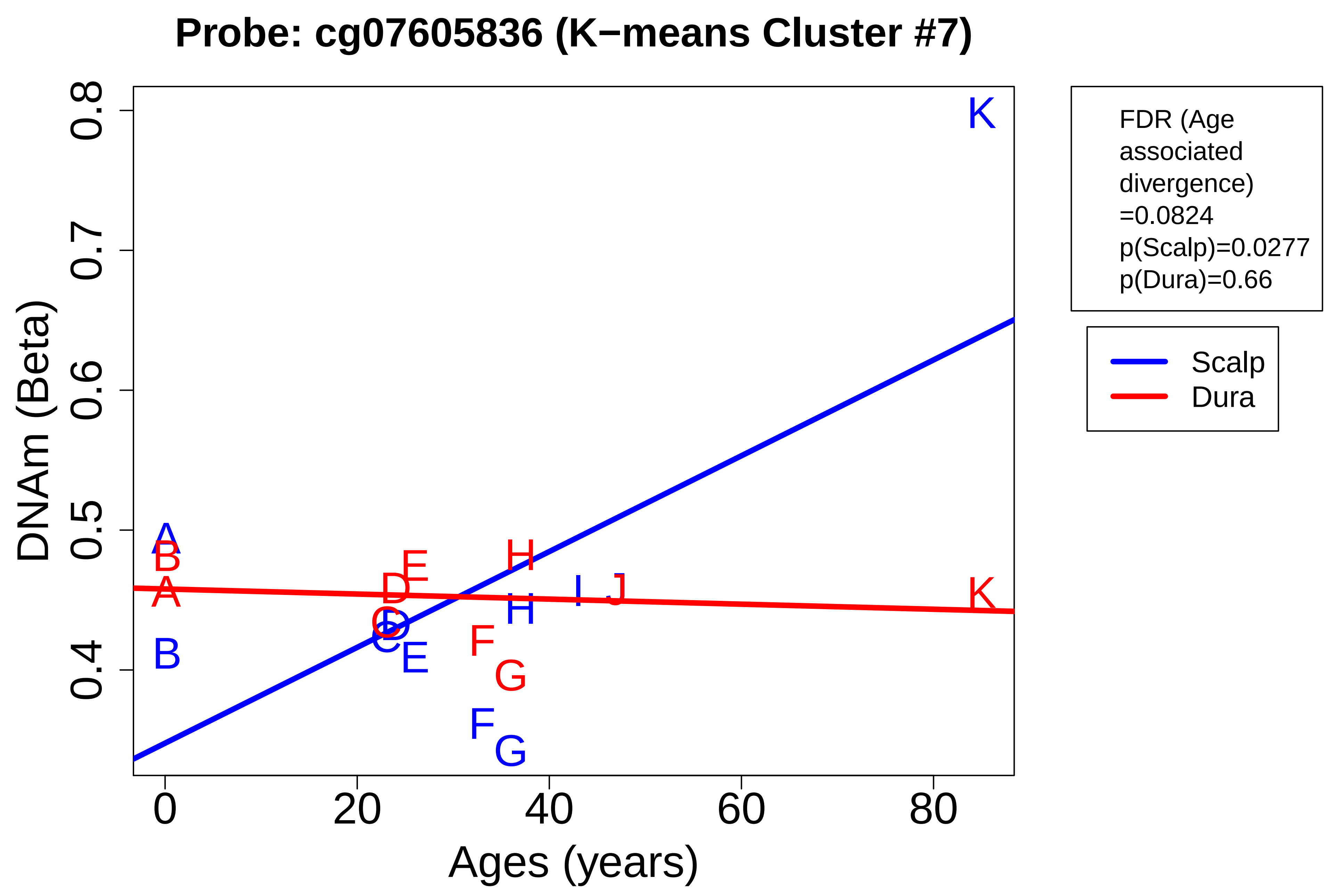

Probe: cg07605836 (K−means Cluster #7)
DNAm (Beta)
0.4
0.5
0.6
0.7
0.8
Ages (years)
0
20
40
60
80
A
B
C
D
E
F
G
H
I
J
K
FDR (Age associated divergence) =0.0824
p(Scalp)=0.0277
p(Dura)=0.66
Scalp
Dura

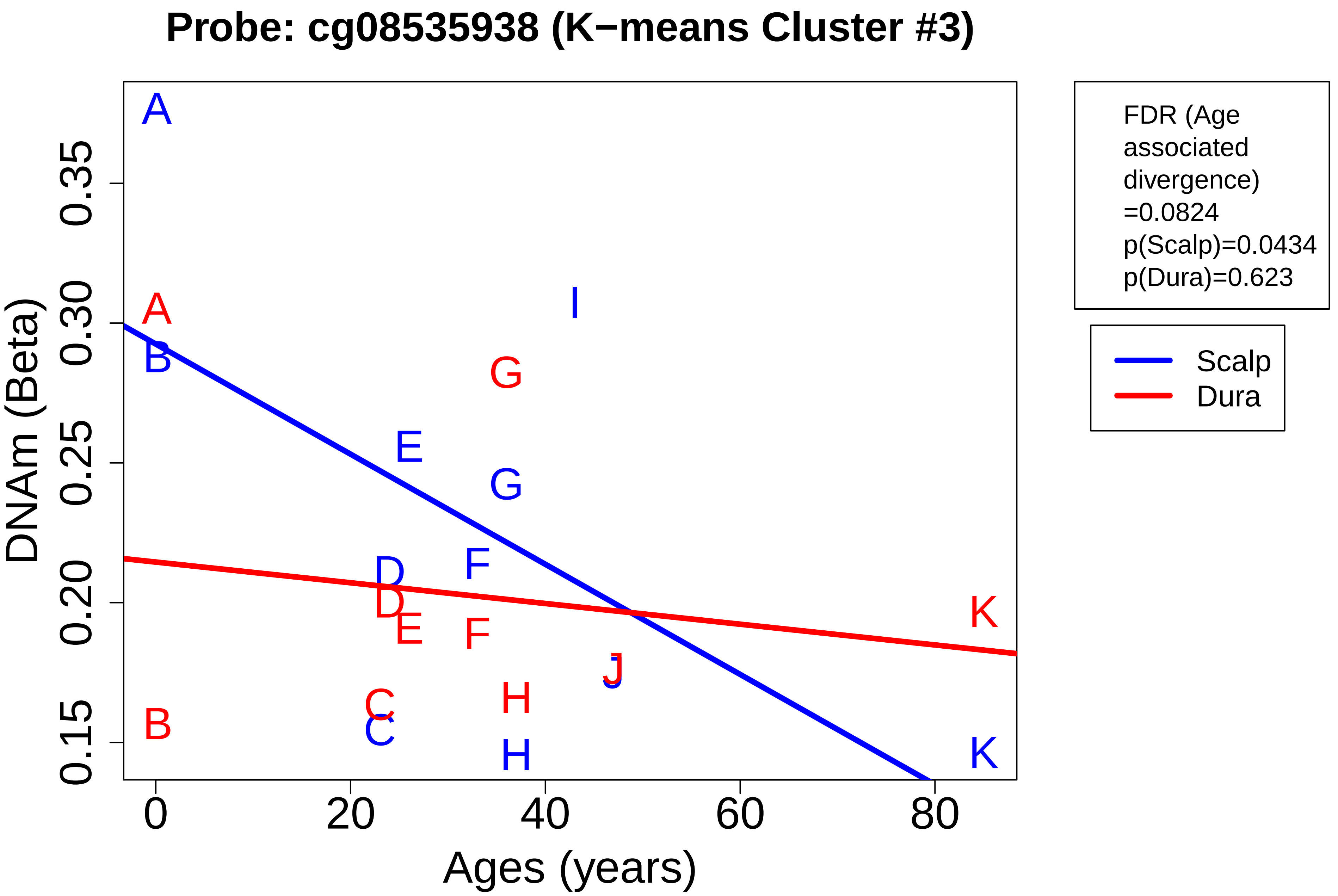

Probe: cg08535938 (K−means Cluster #3)
DNAm (Beta)
0.15
0.20
0.25
0.30
0.35
0
20
40
60
80
Ages (years)
A
B
C
D
E
F
G
H
I
J
K
A
B
C
D
E
F
G
H
J
K
FDR (Age associated divergence) =0.0824
p(Scalp)=0.0434
p(Dura)=0.623
Scalp
Dura

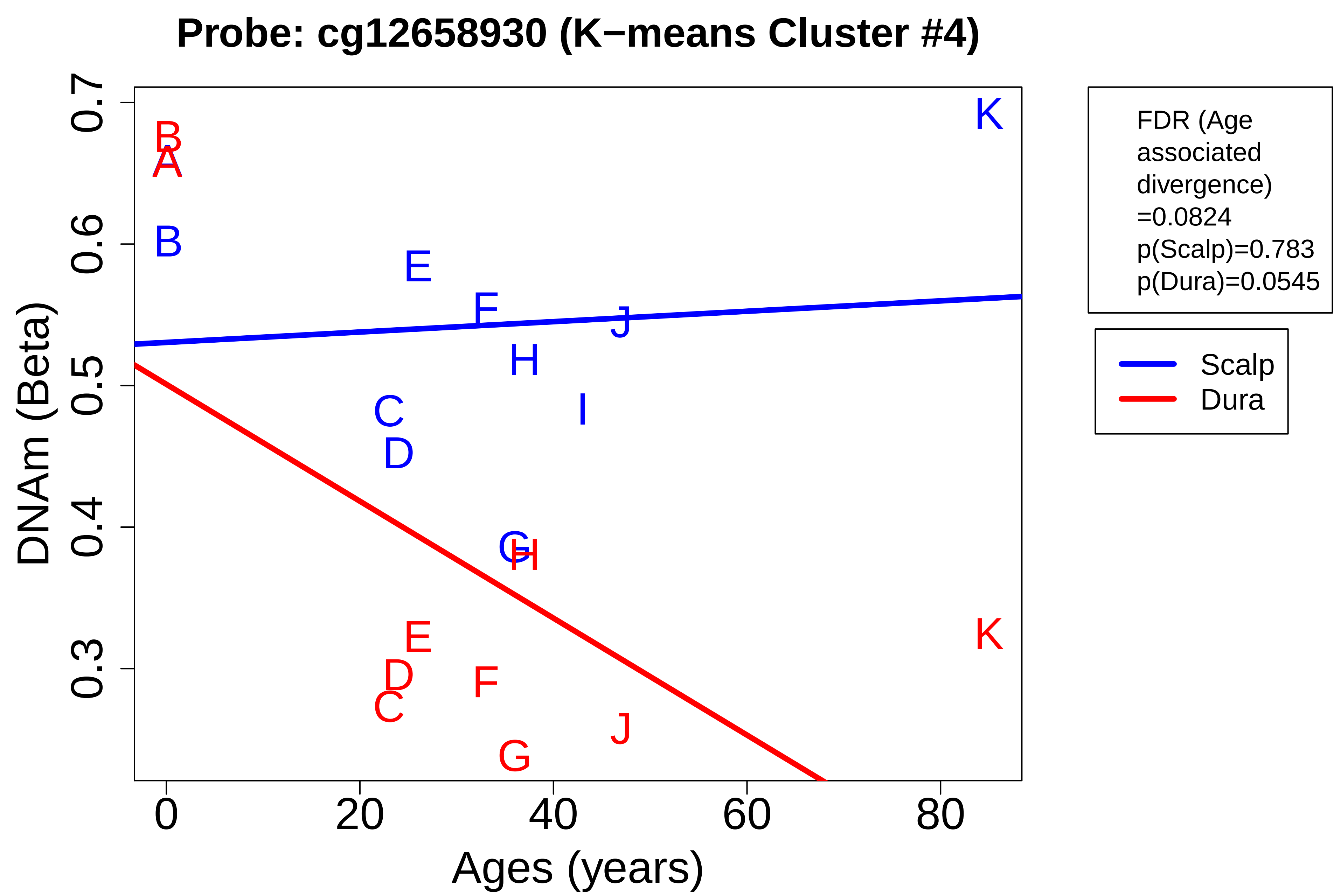

Probe: cg12658930 (K−means Cluster #4)
DNAm (Beta)
0.3
0.4
0.5
0.6
0.7
0
20
40
60
80
Ages (years)
FDR (Age associated divergence) =0.0824
p(Scalp)=0.783
p(Dura)=0.0545
Scalp
Dura

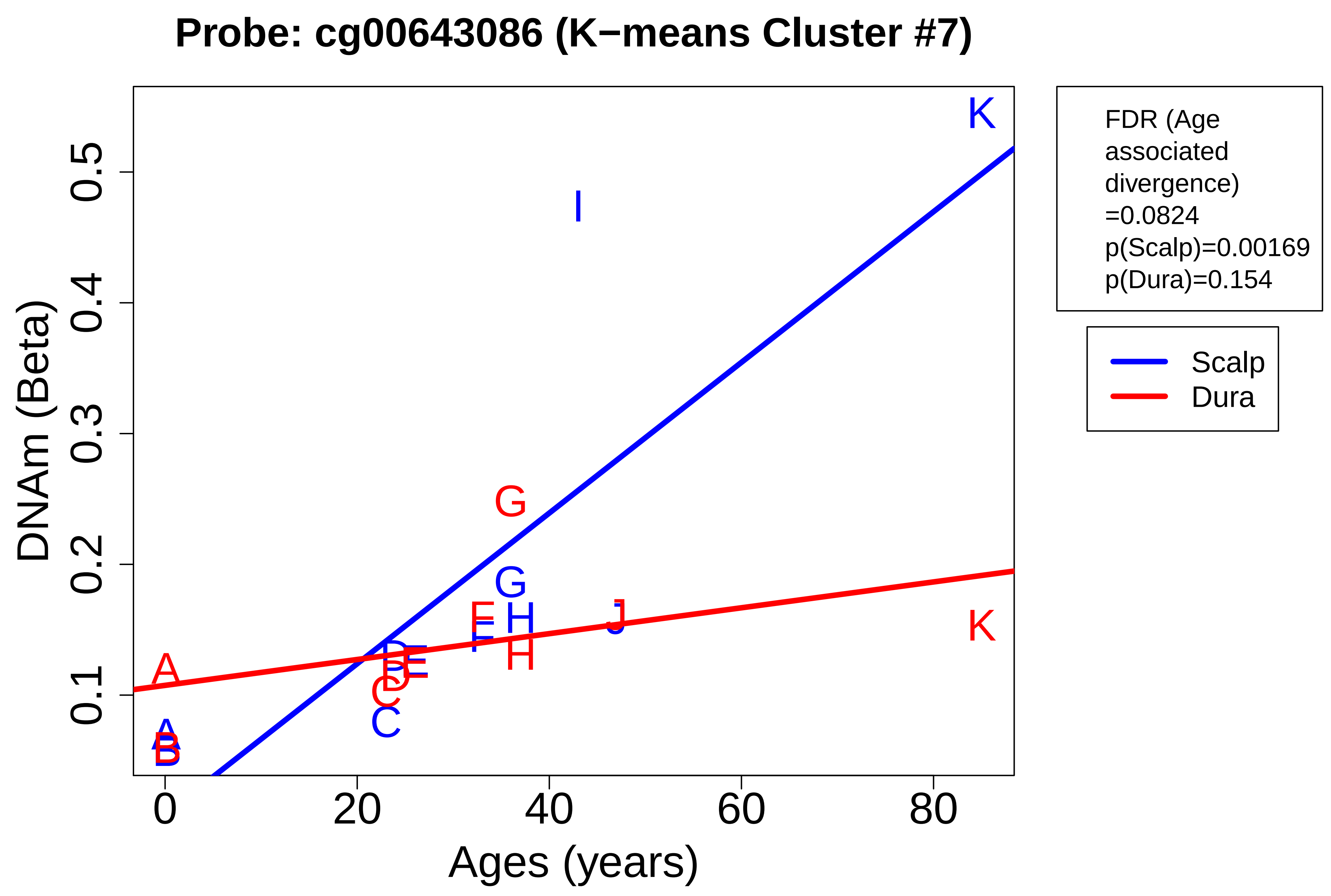

Probe: cg00643086 (K−means Cluster #7)
DNAm (Beta)
0.1
0.2
0.3
0.4
0.5
0
20
40
60
80
Ages (years)
A
B
C
D
E
F
G
H
I
J
K
FDR (Age associated divergence) =0.0824
p(Scalp)=0.00169
p(Dura)=0.154
Scalp
Dura

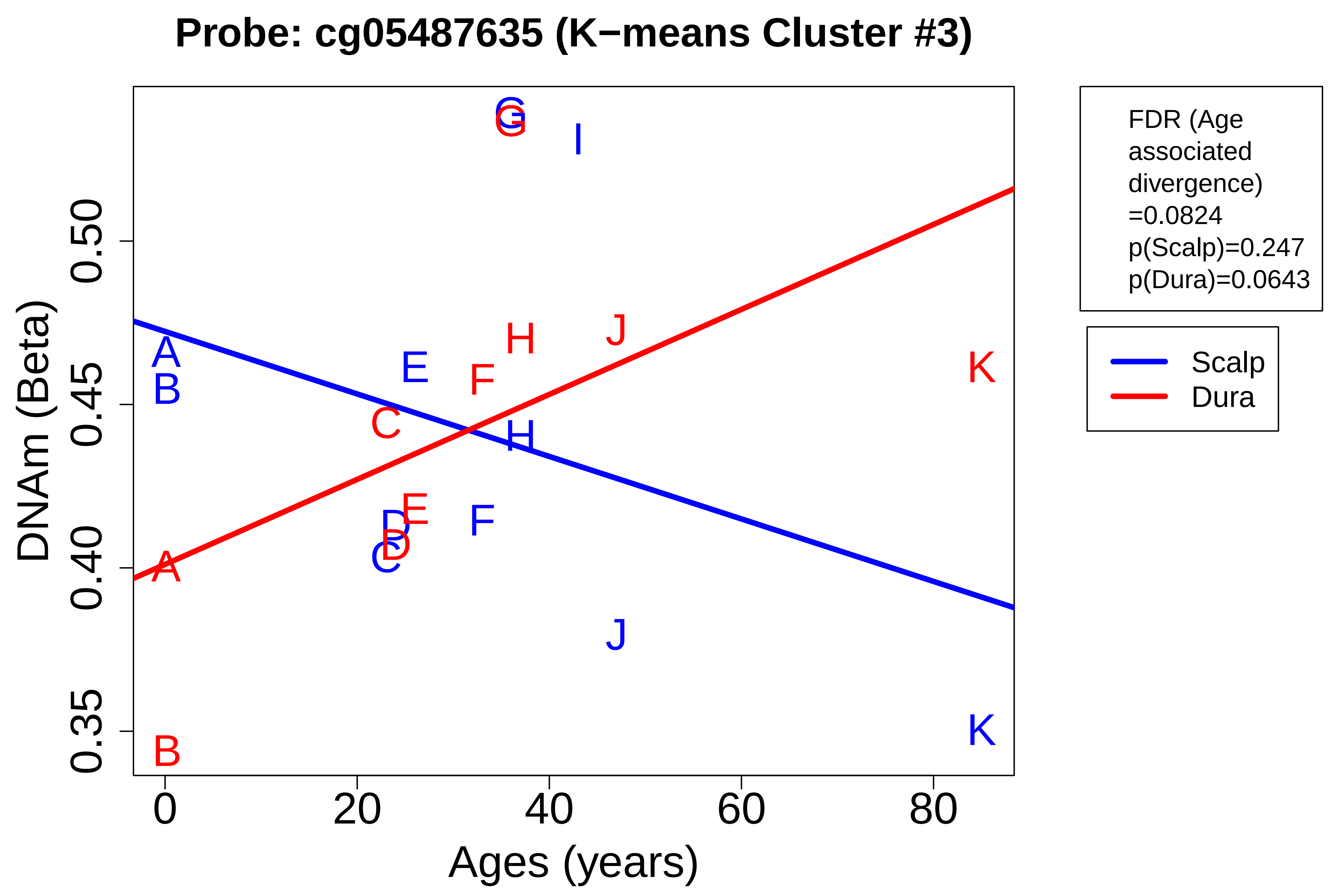

Probe: cg05487635 (K−means Cluster #3)
DNAm (Beta)
0.35
0.40
0.45
0.50
0
20
40
60
80
Ages (years)
FDR (Age associated divergence) =0.0824
p(Scalp)=0.247
p(Dura)=0.0643
Scalp
Dura
A
B
C
D
E
F
G
H
I
J
K

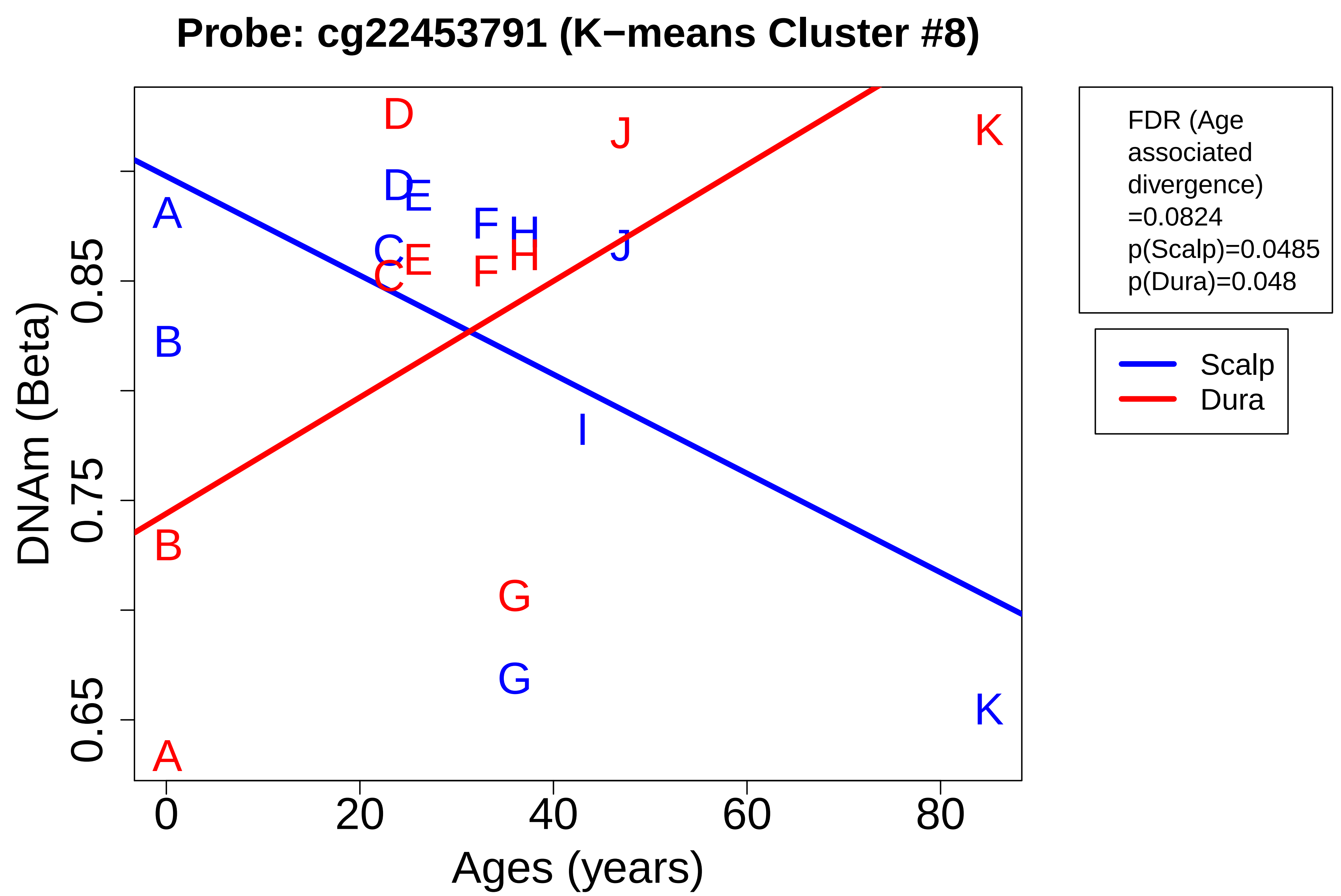

Probe: cg22453791 (K−means Cluster #8)
DNAm (Beta)
0.65
0.75
0.85
Ages (years)
0
20
40
60
80
A
B
C
D
E
F
G
H
I
J
K
FDR (Age associated divergence) =0.0824
p(Scalp)=0.0485
p(Dura)=0.048
Scalp
Dura

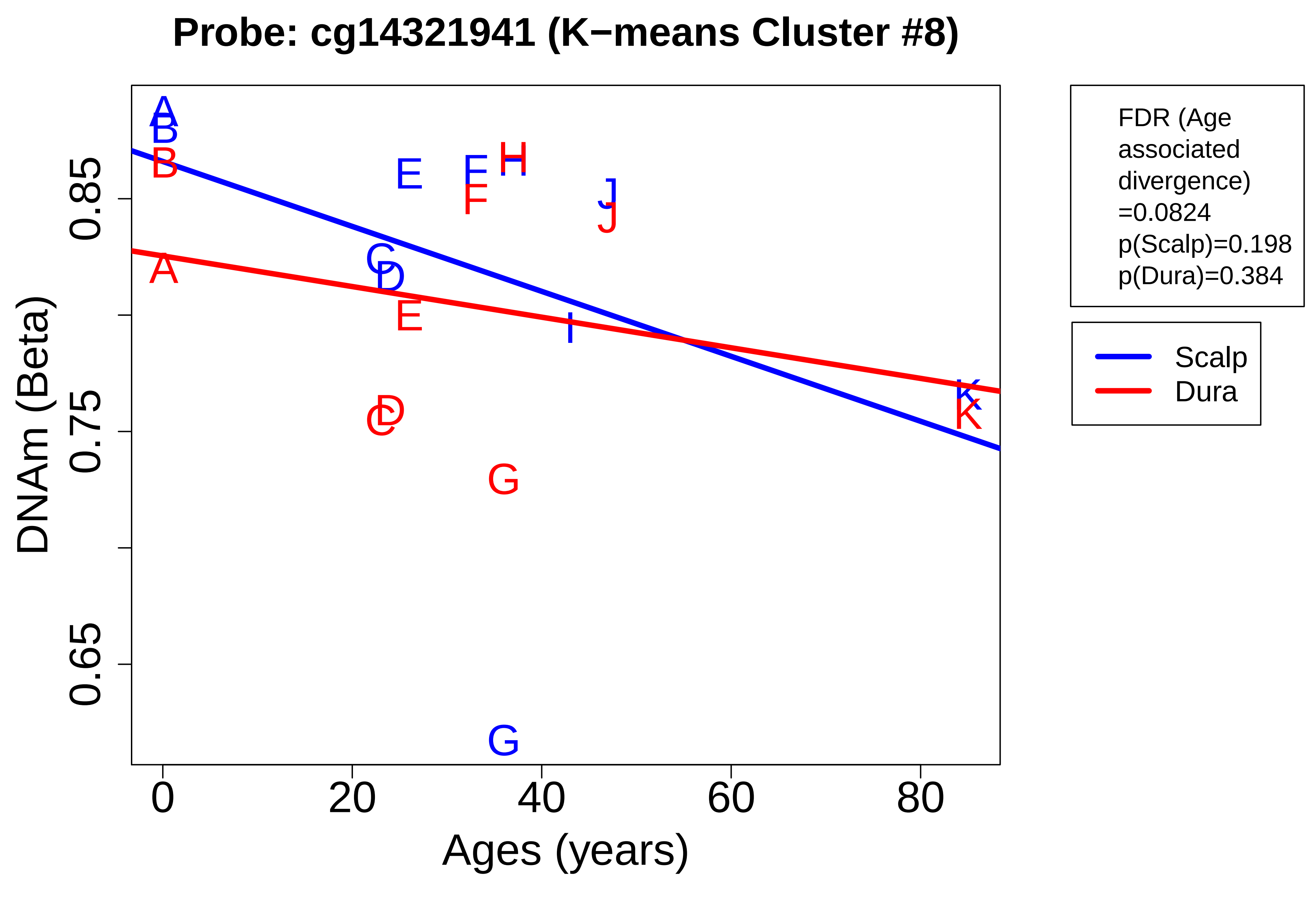

Probe: cg14321941 (K−means Cluster #8)
DNAm (Beta)
0.65
0.75
0.85
Ages (years)
0
20
40
60
80
A
B
B
A
C
D
E
E
F
F
H
H
J
J
I
K
K
D
C
G
G
FDR (Age associated divergence) =0.0824
p(Scalp)=0.198
p(Dura)=0.384
Scalp
Dura

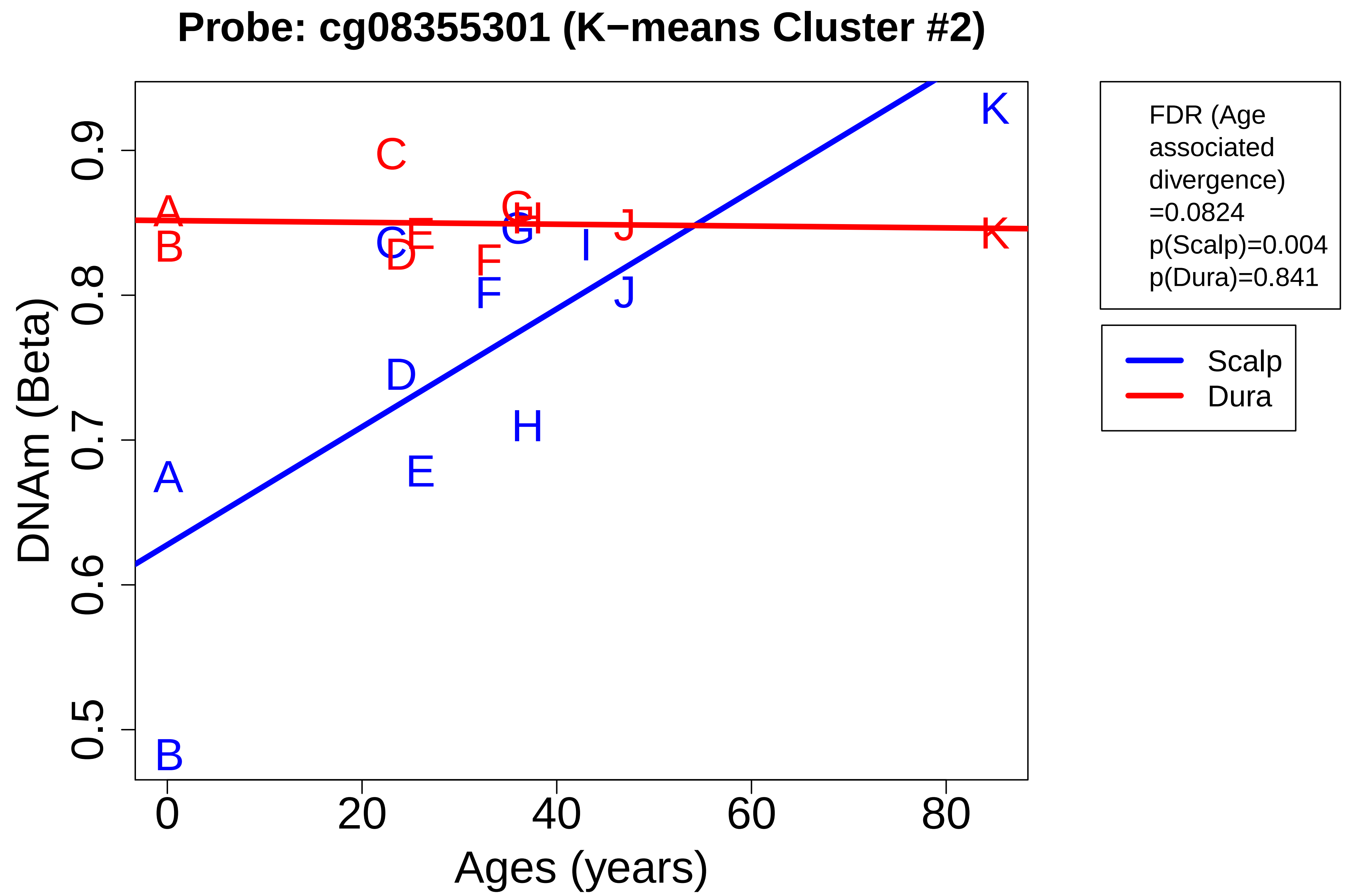
Probe: cg08355301 (K−means Cluster #2)
DNAm (Beta)
0.5
0.6
0.7
0.8
0.9
0
20
40
60
80
Ages (years)
FDR (Age associated divergence) =0.0824
p(Scalp)=0.004
p(Dura)=0.841
Scalp
Dura

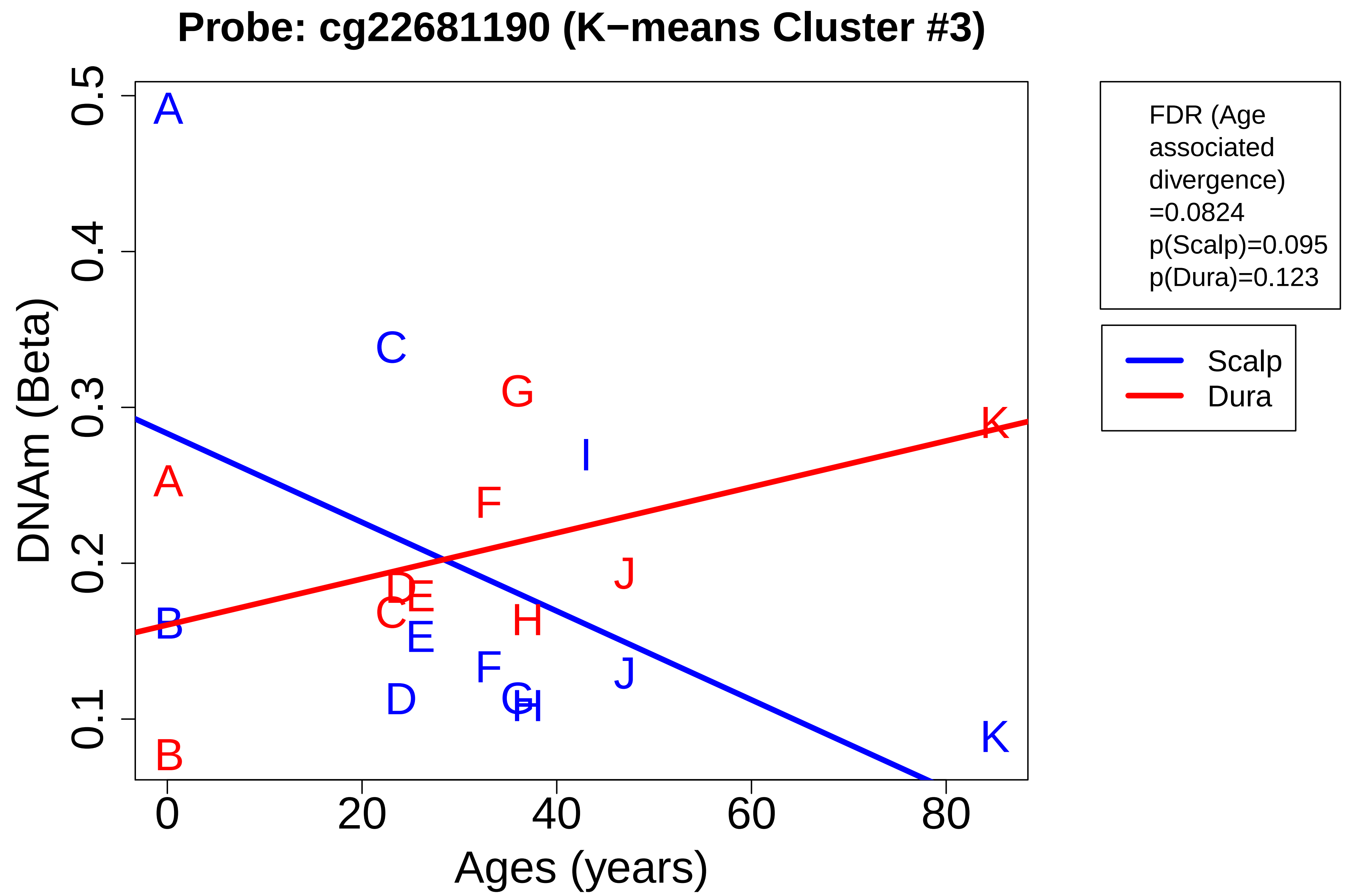

Probe: cg22681190 (K−means Cluster #3)
FDR (Age associated divergence) =0.0824
p(Scalp)=0.095
p(Dura)=0.123
Scalp
Dura
DNAm (Beta)
Ages (years)
0.1
0.2
0.3
0.4
0.5
0
20
40
60
80

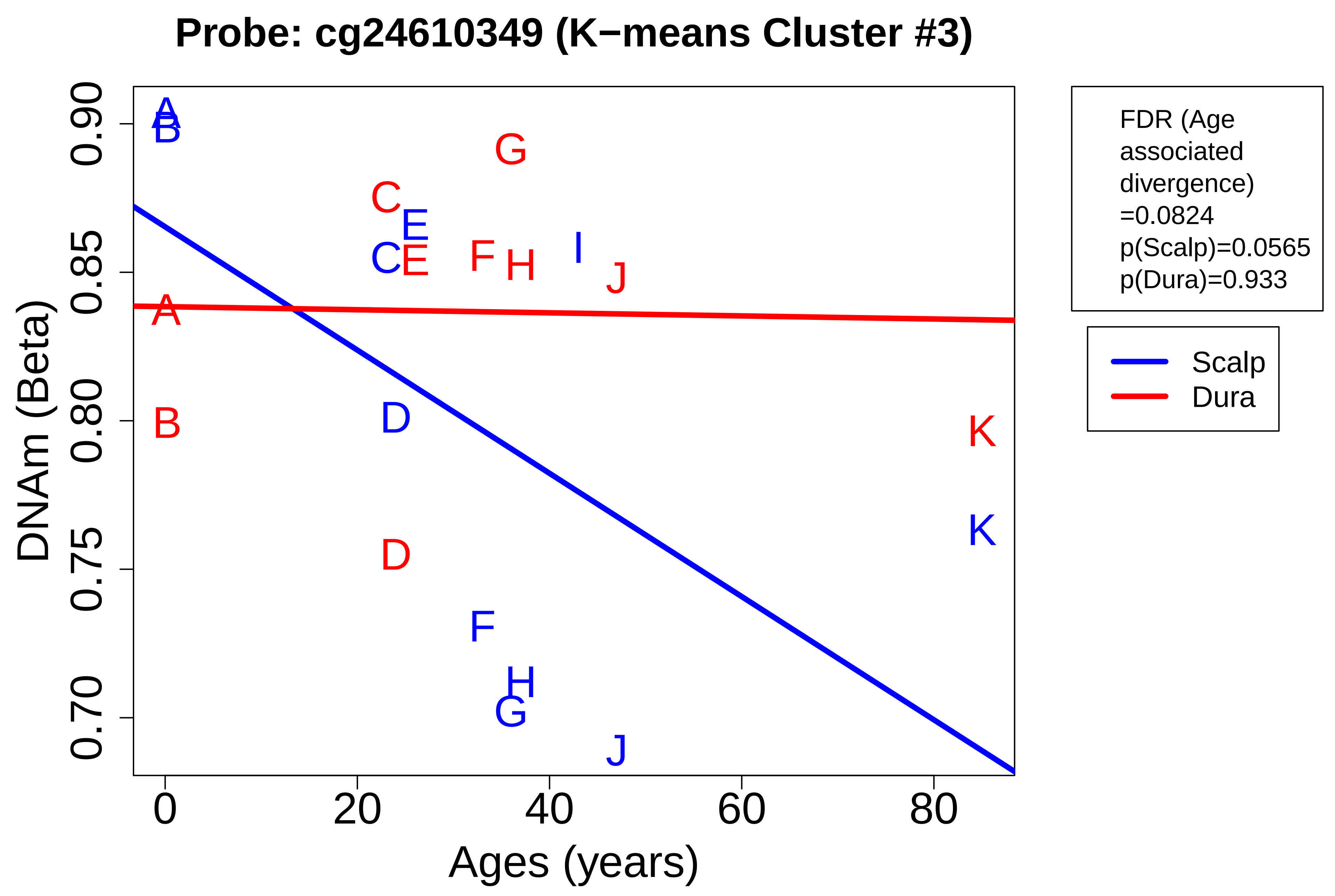

Probe: cg24610349 (K−means Cluster #3)
FDR (Age associated divergence) =0.0824
p(Scalp)=0.0565
p(Dura)=0.933
Scalp
Dura
DNAm (Beta)
0.70
0.75
0.80
0.85
0.90
Ages (years)
0
20
40
60
80
A
B
C
D
E
F
G
H
I
J
K

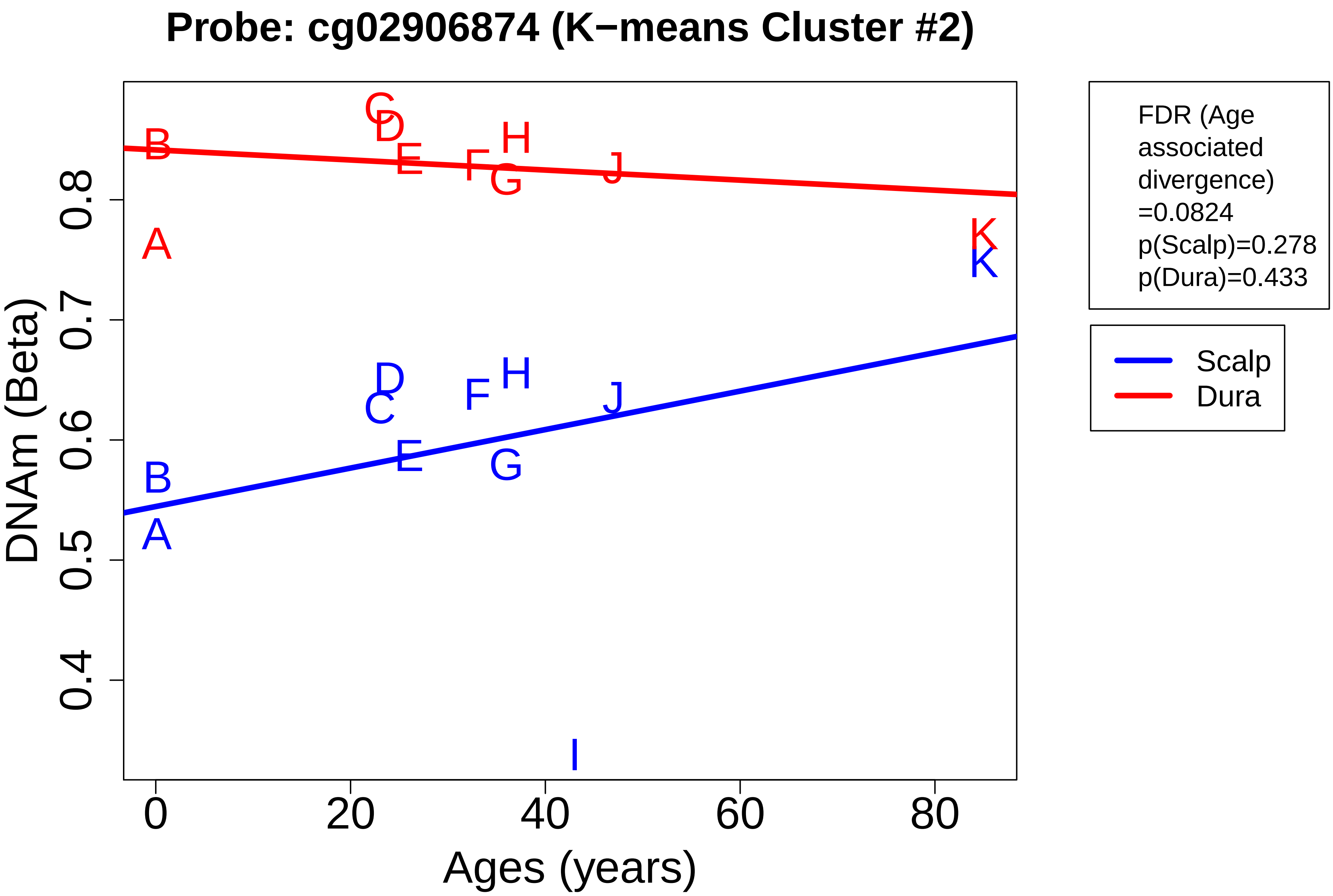

Probe: cg02906874 (K−means Cluster #2)
DNAm (Beta)
0.4
0.5
0.6
0.7
0.8
0
20
40
60
80
Ages (years)
FDR (Age associated divergence) =0.0824
p(Scalp)=0.278
p(Dura)=0.433
Scalp
Dura
A
B
C
D
E
F
G
H
I
J
K

Probe: cg11936410 (K−means Cluster #1)

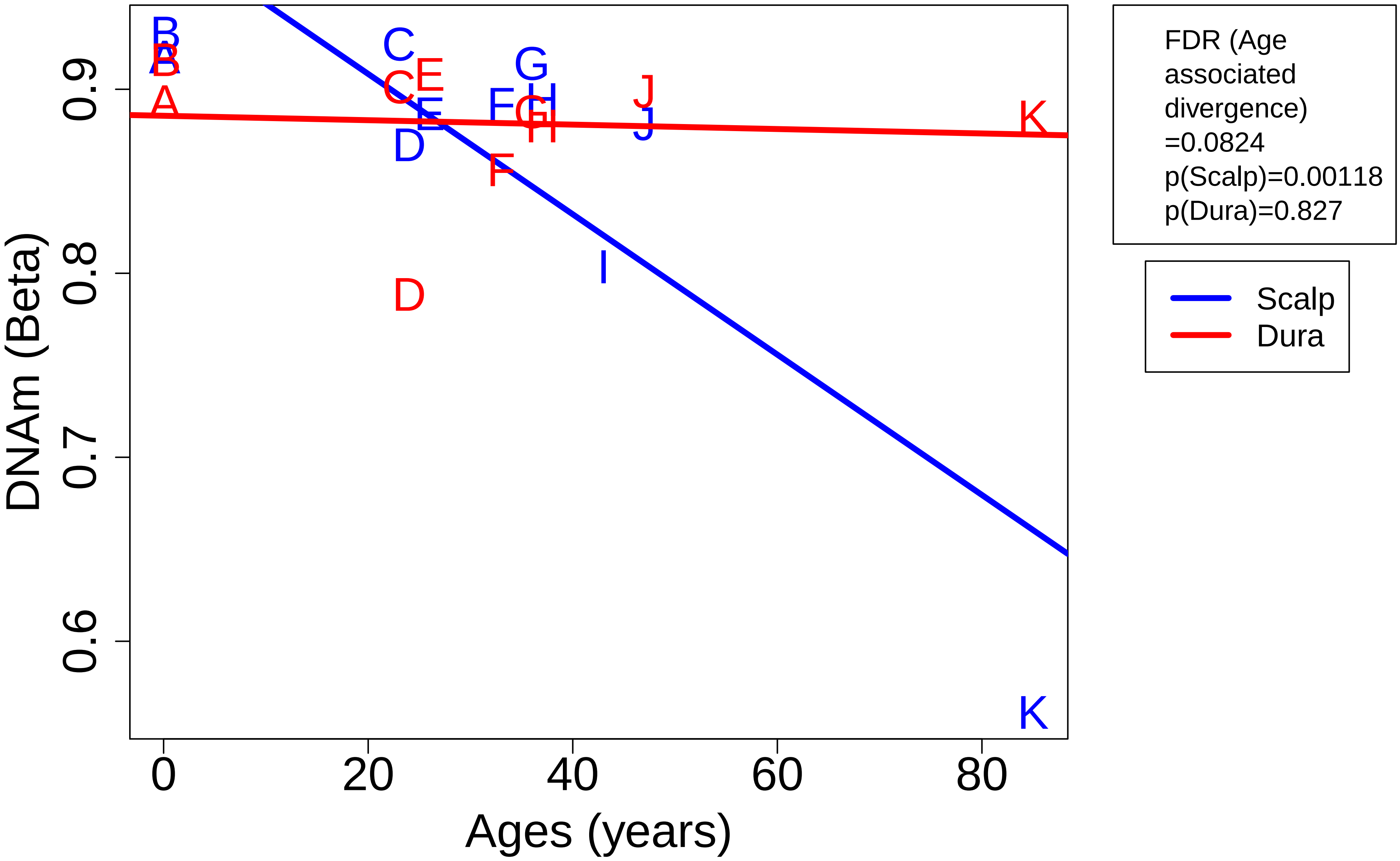

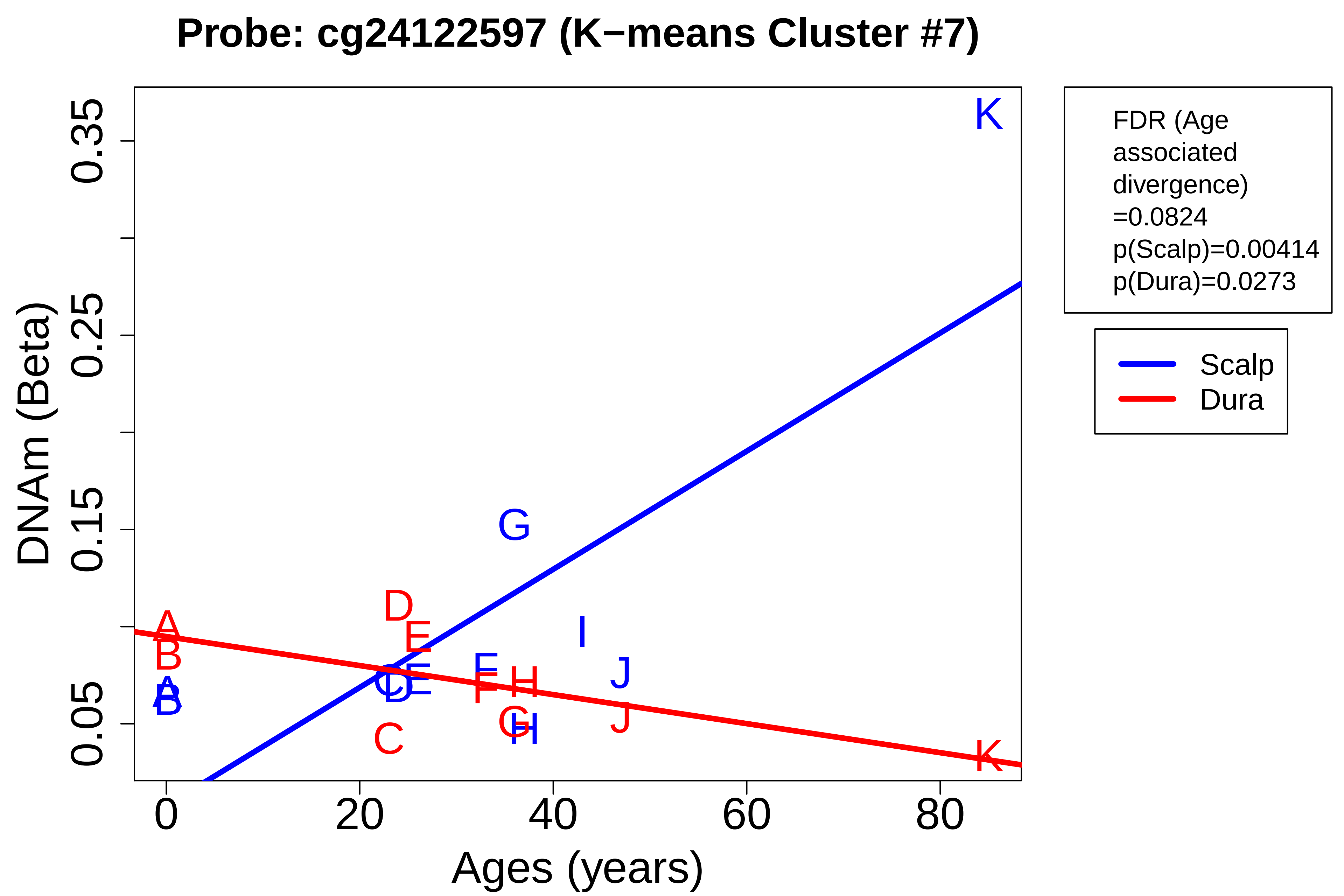
Probe: cg24122597 (K−means Cluster #7)
DNAm (Beta)
0.05
0.15
0.25
0.35
Ages (years)
0
20
40
60
80
FDR (Age associated divergence) =0.0824
p(Scalp)=0.00414
p(Dura)=0.0273
Scalp
Dura
A
B
C
D
E
F
G
H
I
J
K

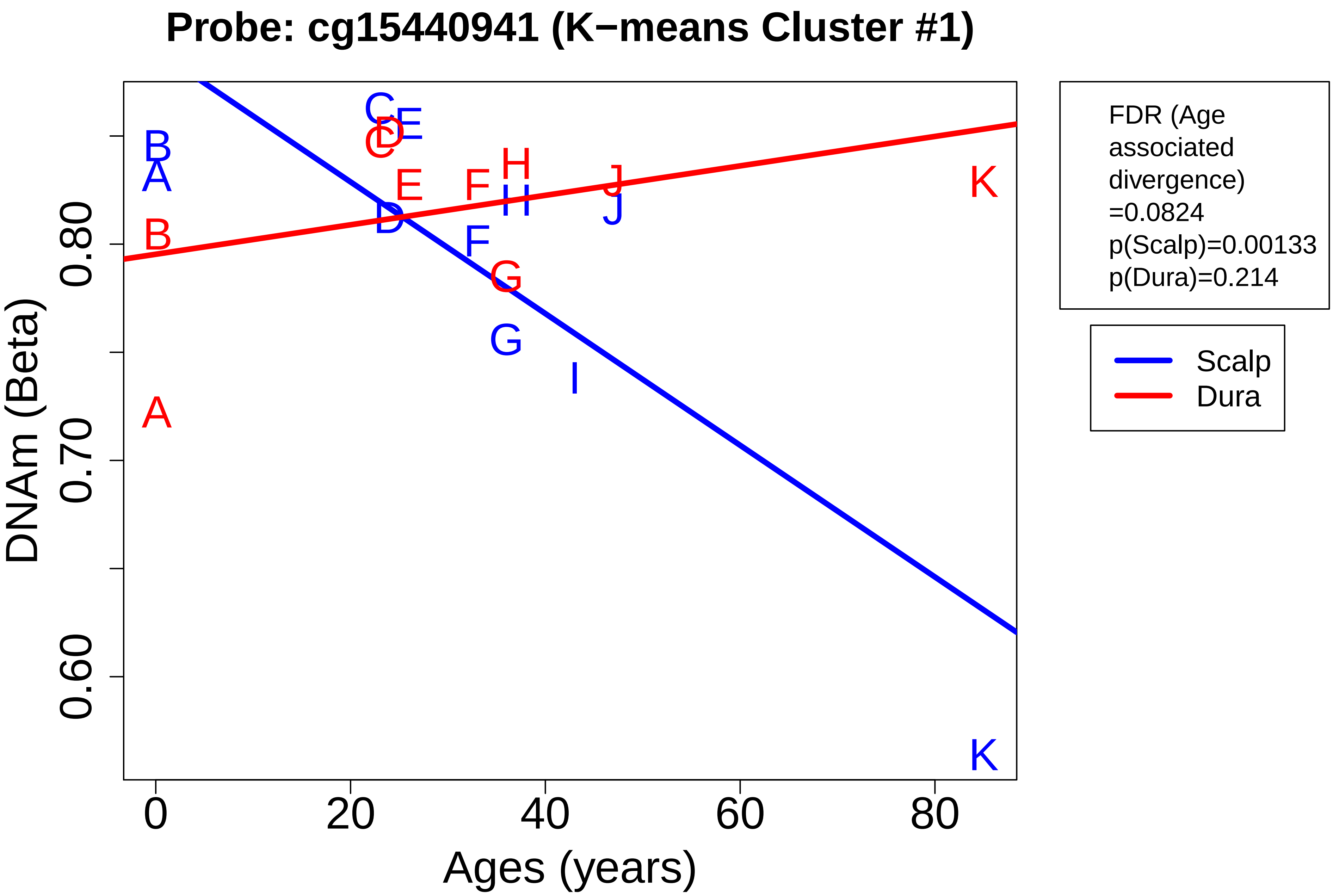
Probe: cg15440941 (K−means Cluster #1)
DNAm (Beta)
0.60
0.70
0.80
0
20
40
60
80
Ages (years)
FDR (Age associated divergence) =0.0824
p(Scalp)=0.00133
p(Dura)=0.214
Scalp
Dura
A
B
C
D
E
F
G
H
I
J
K

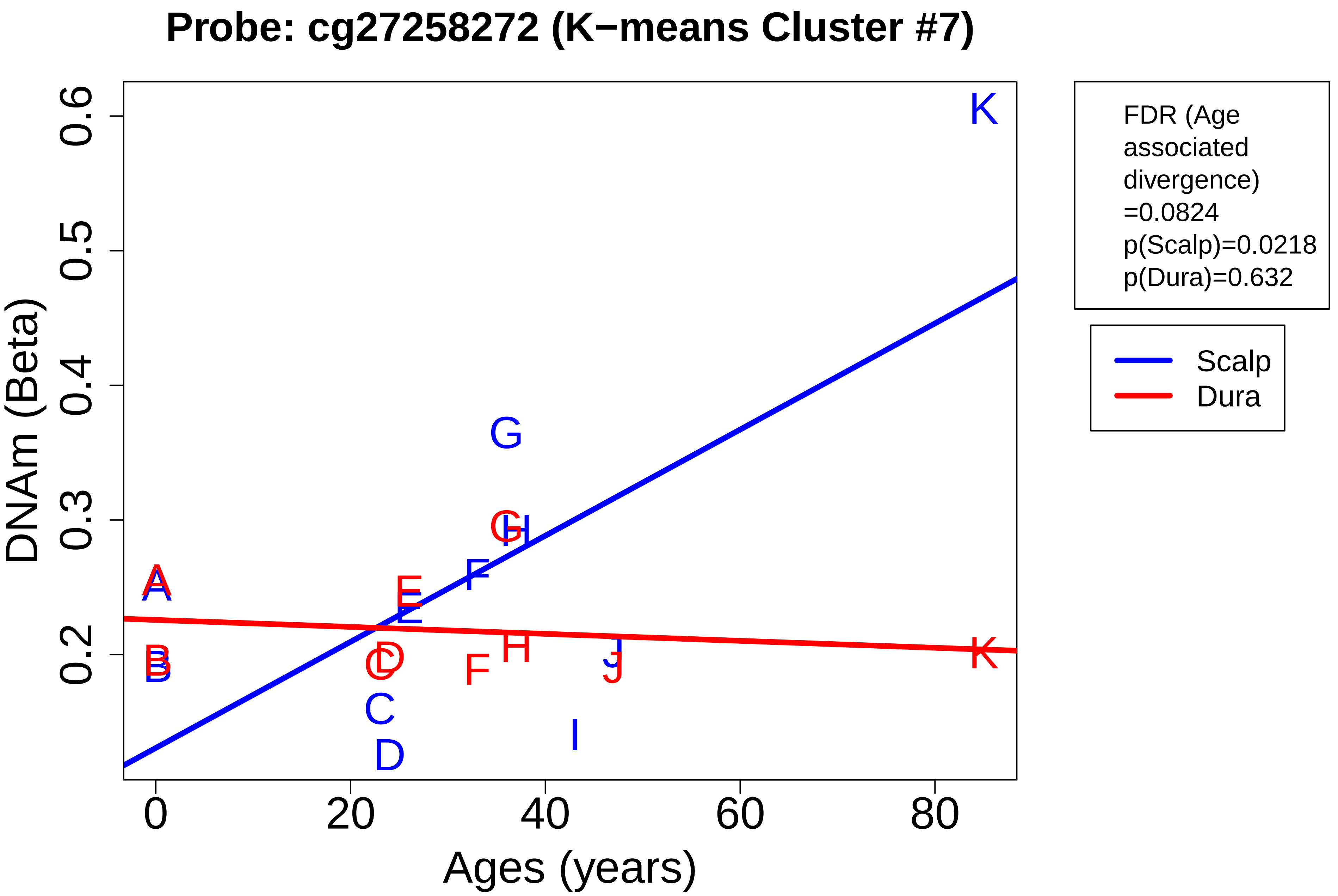

Probe: cg27258272 (K−means Cluster #7)
DNAm (Beta)
0.2
0.3
0.4
0.5
0.6
0
20
40
60
80
Ages (years)
FDR (Age associated divergence) =0.0824
p(Scalp)=0.0218
p(Dura)=0.632
Scalp
Dura

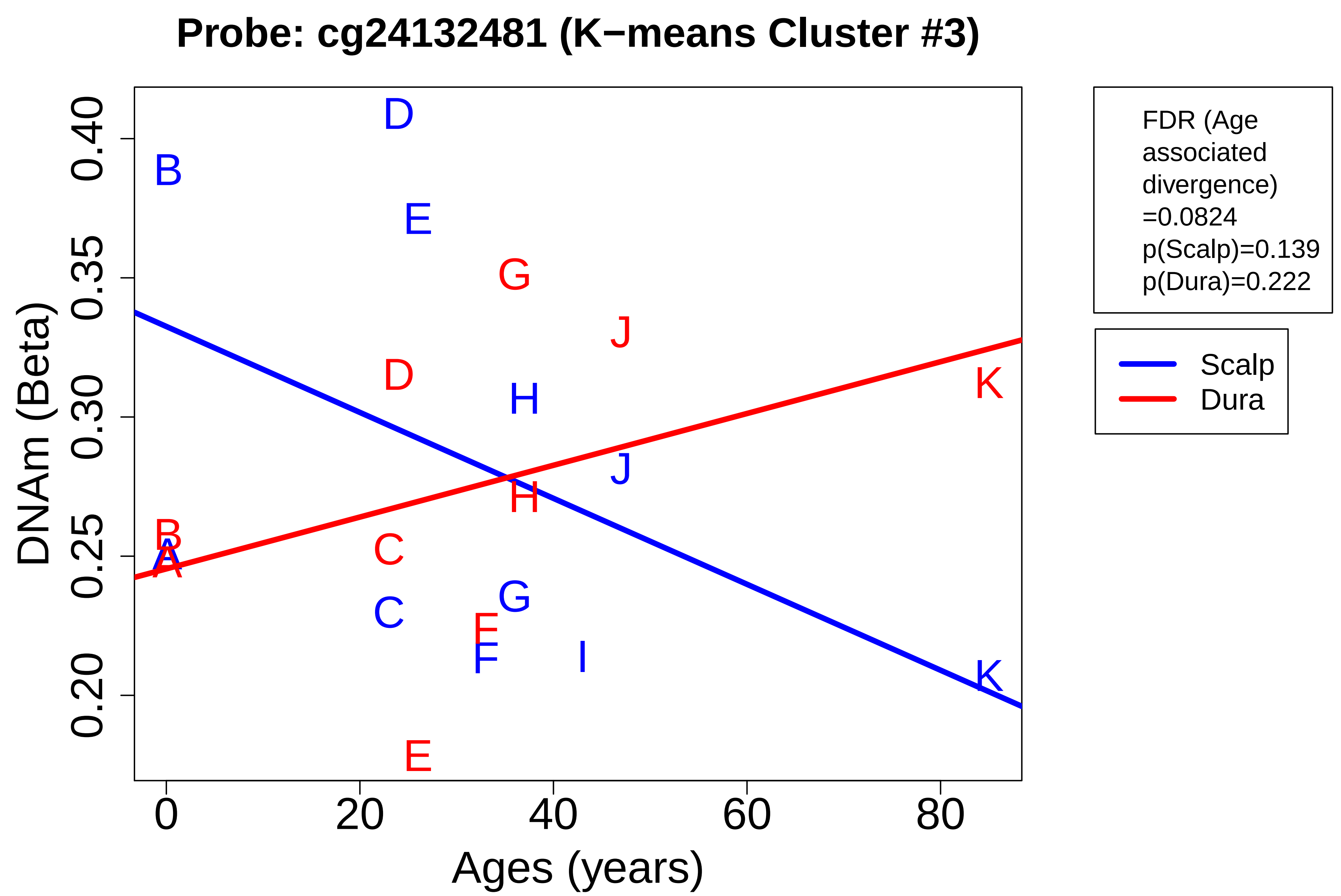

Probe: cg24132481 (K−means Cluster #3)
DNAm (Beta)
0.20
0.25
0.30
0.35
0.40
Ages (years)
0
20
40
60
80
FDR (Age associated divergence) =0.0824
p(Scalp)=0.139
p(Dura)=0.222
Scalp
Dura

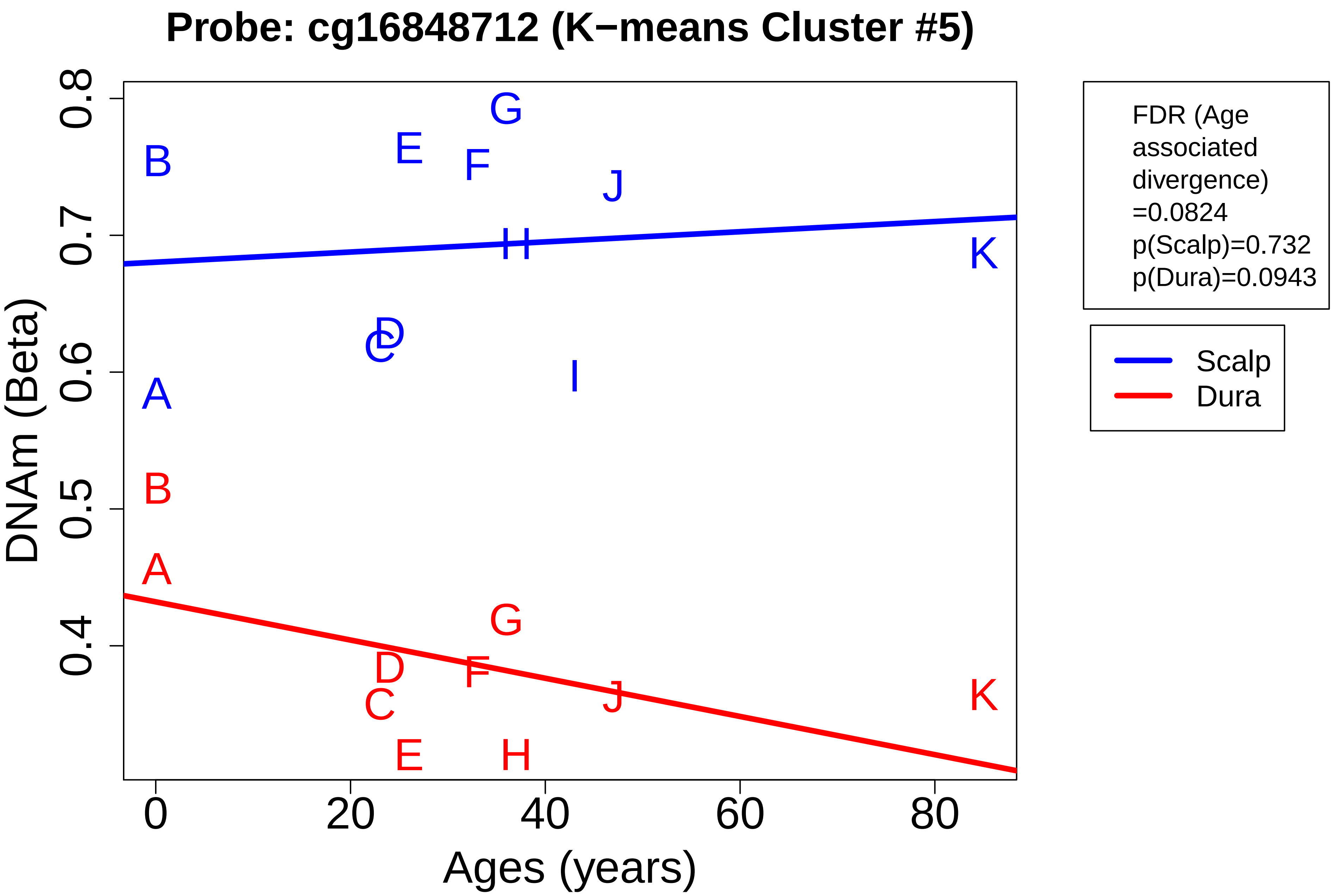

Probe: cg16848712 (K−means Cluster #5)
DNAm (Beta)
0.4
0.5
0.6
0.7
0.8
0
20
40
60
80
Ages (years)
A
B
C
D
E
F
G
H
I
J
K
FDR (Age associated divergence) =0.0824
p(Scalp)=0.732
p(Dura)=0.0943
Scalp
Dura

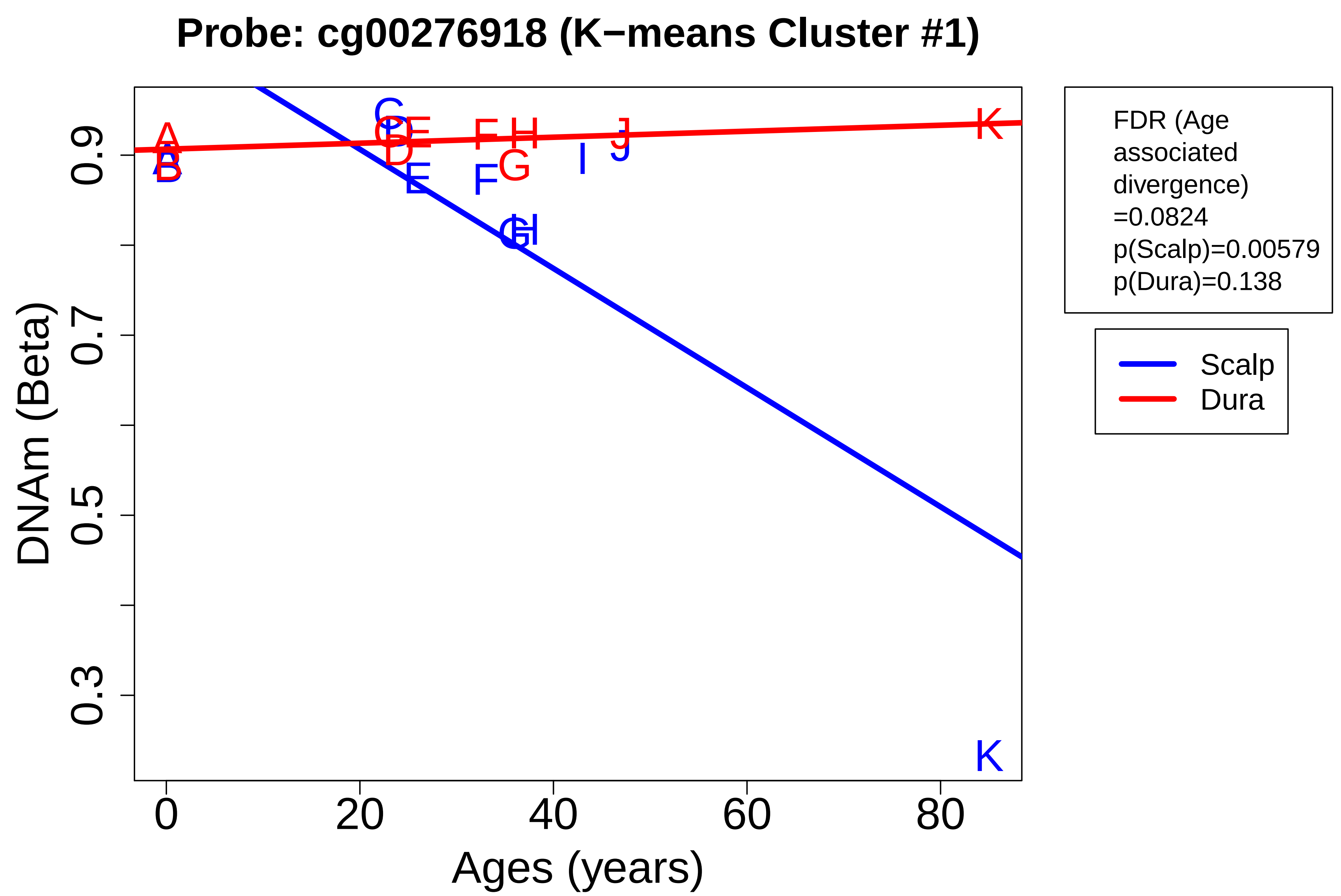
Probe: cg00276918 (K−means Cluster #1)
FDR (Age associated divergence) =0.0824
p(Scalp)=0.00579
p(Dura)=0.138
Scalp
Dura
DNAm (Beta)
0.3
0.5
0.7
0.9
0
20
40
60
80
Ages (years)
A
B
C
D
E
F
G
H
I
J
K

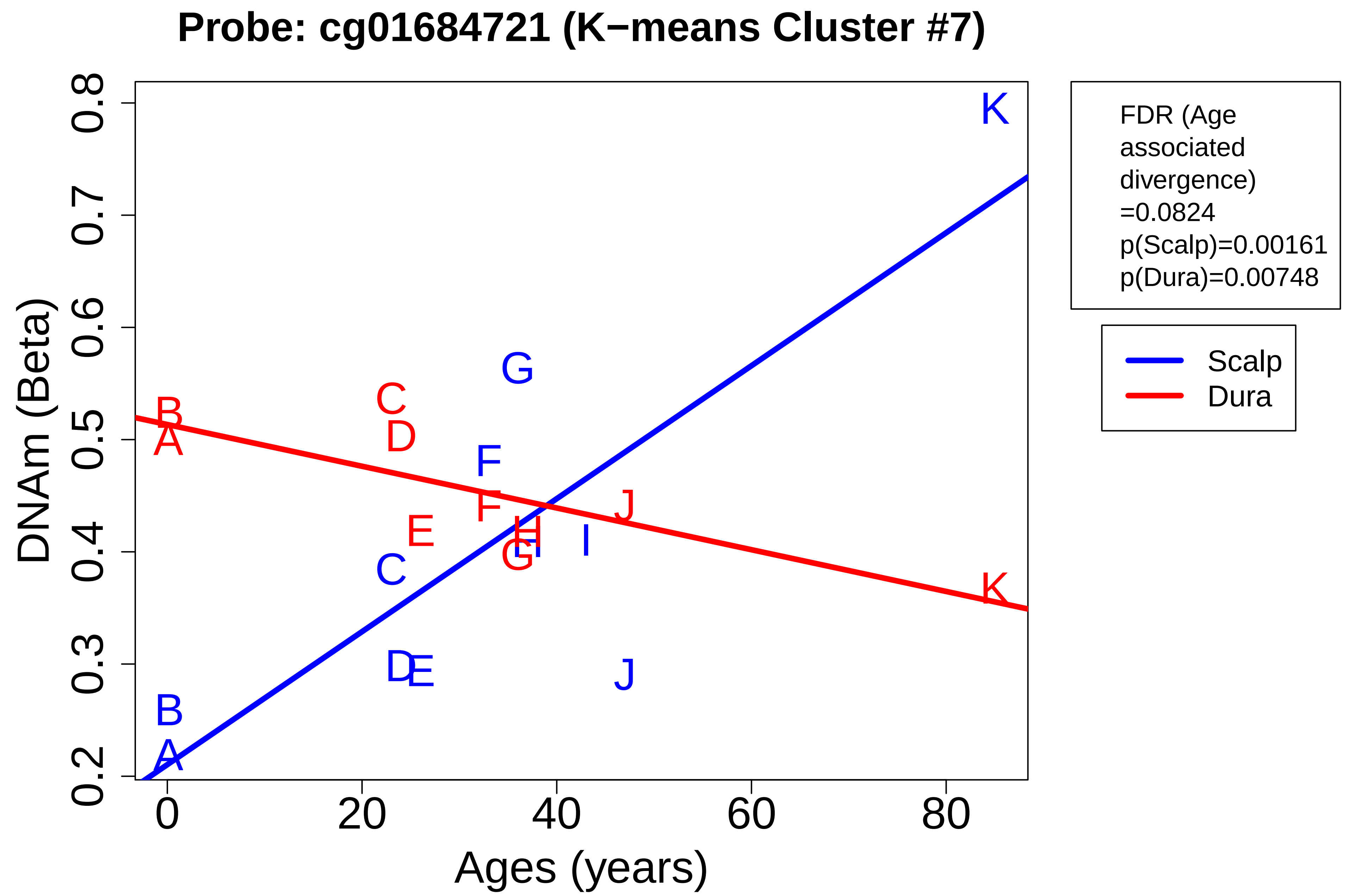

Probe: cg01684721 (K−means Cluster #7)
DNAm (Beta)
Ages (years)
0.2
0.3
0.4
0.5
0.6
0.7
0.8
0
20
40
60
80
FDR (Age associated divergence) =0.0824
p(Scalp)=0.00161
p(Dura)=0.00748
Scalp
Dura

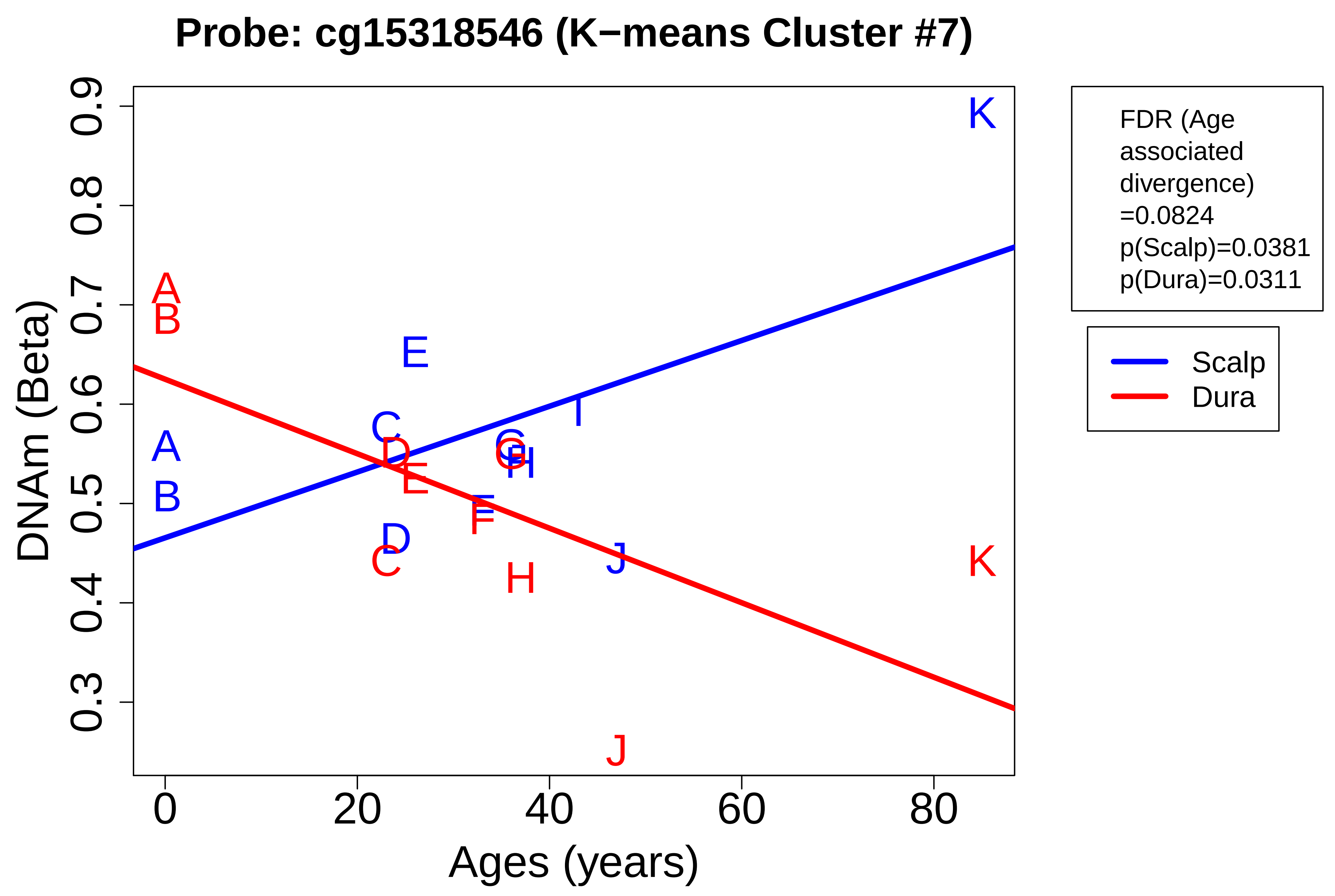

Probe: cg15318546 (K−means Cluster #7)
DNAm (Beta)
0.3
0.4
0.5
0.6
0.7
0.8
0.9
0
20
40
60
80
Ages (years)
FDR (Age associated divergence) =0.0824
p(Scalp)=0.0381
p(Dura)=0.0311
Scalp
Dura

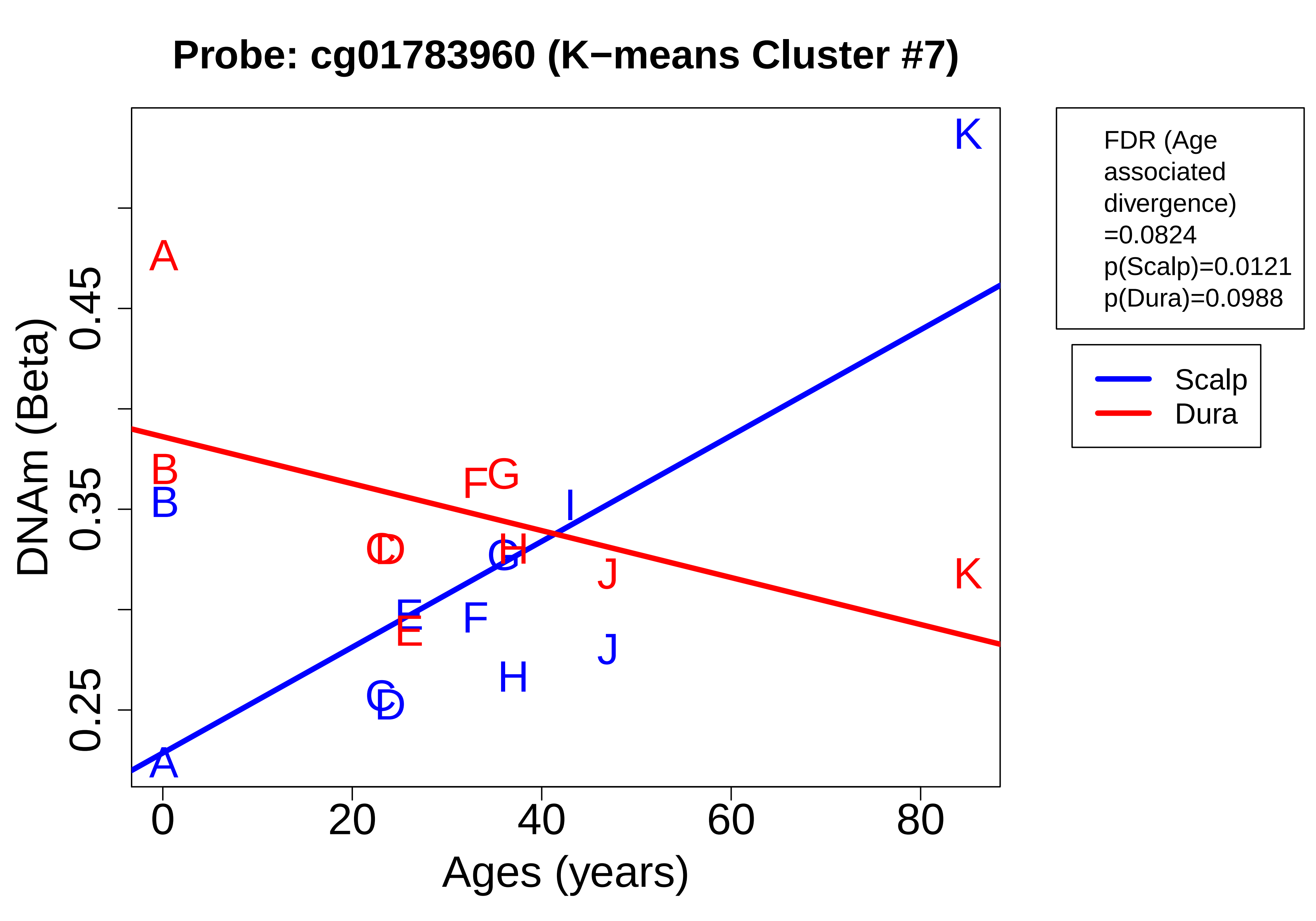

Probe: cg01783960 (K−means Cluster #7)
DNAm (Beta)
0.25
0.35
0.45
Ages (years)
0
20
40
60
80
A
B
C
D
E
F
G
H
I
J
K
FDR (Age associated divergence) =0.0824
p(Scalp)=0.0121
p(Dura)=0.0988
Scalp
Dura

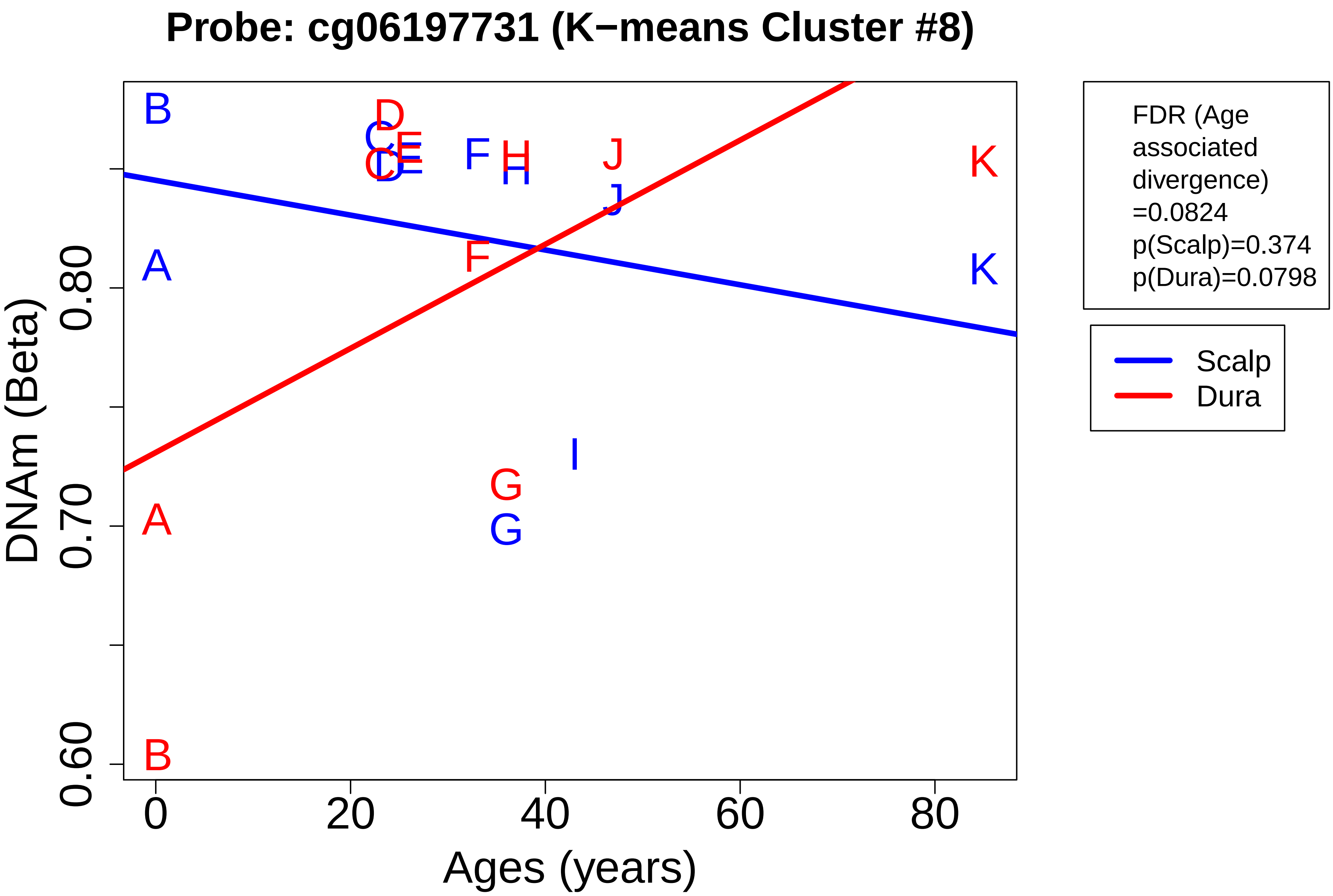

Probe: cg06197731 (K−means Cluster #8)
DNAm (Beta)
0.60
0.70
0.80
Ages (years)
0
20
40
60
80
A
B
C
D
E
F
G
H
I
J
K
FDR (Age associated divergence) =0.0824
p(Scalp)=0.374
p(Dura)=0.0798
Scalp
Dura

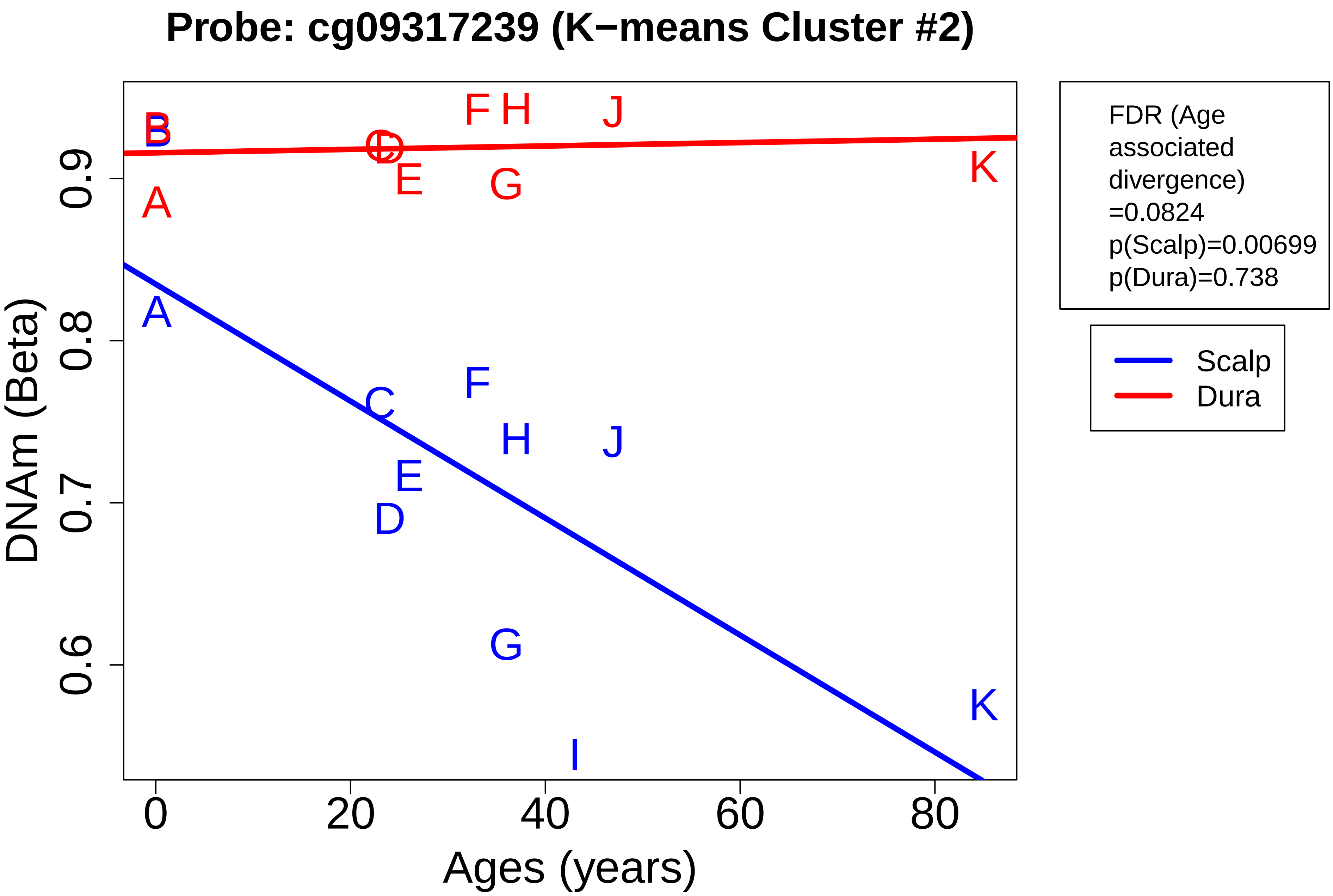

Probe: cg09317239 (K−means Cluster #2)
DNAm (Beta)
0.6
0.7
0.8
0.9
0
20
40
60
80
Ages (years)
A
B
C
D
E
F
G
H
I
J
K
FDR (Age
associated
divergence)
=0.0824
p(Scalp)=0.00699
p(Dura)=0.738
Scalp
Dura

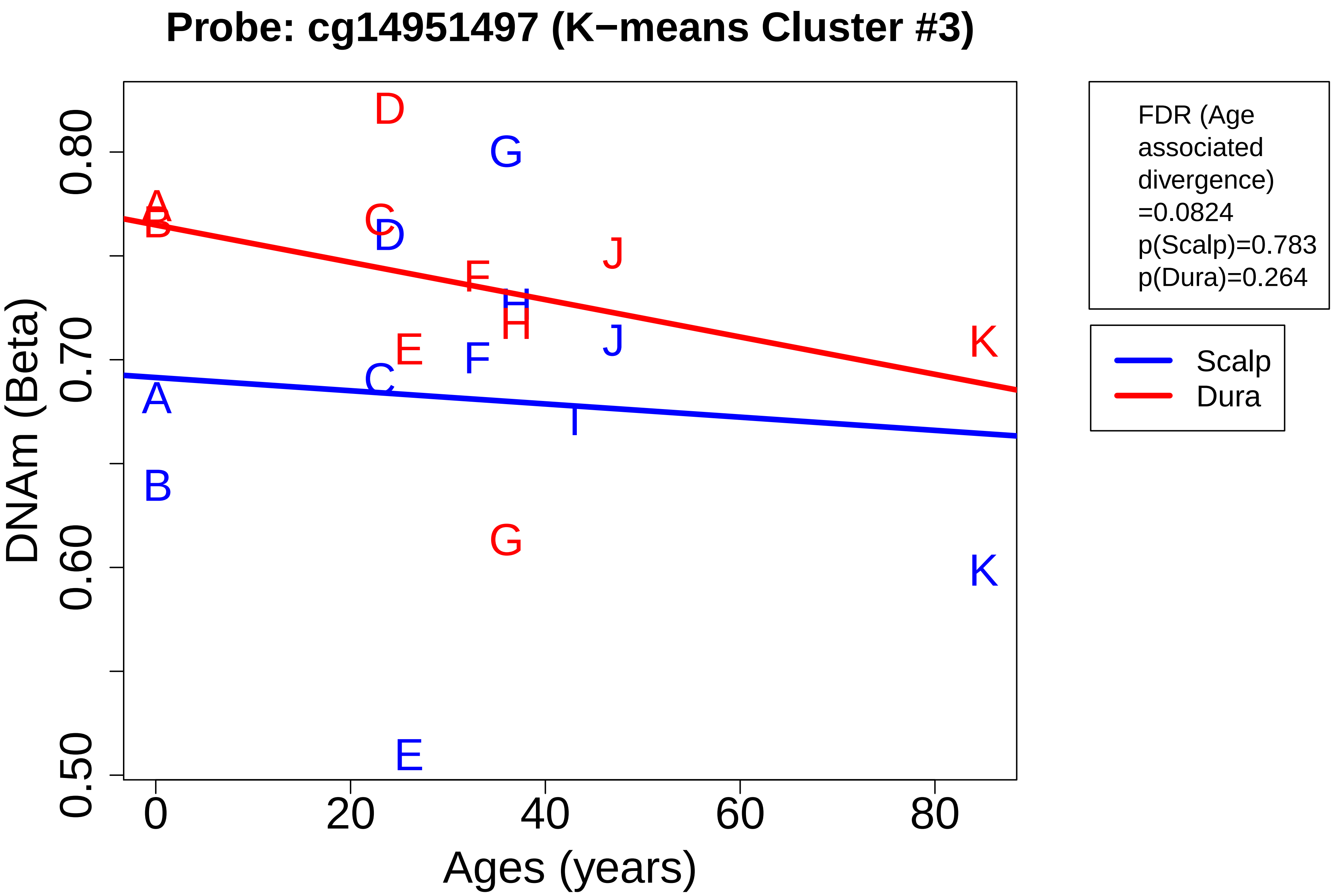

Probe: cg14951497 (K−means Cluster #3)
DNAm (Beta)
0.50
0.60
0.70
0.80
0
20
40
60
80
Ages (years)
FDR (Age associated divergence) =0.0824
p(Scalp)=0.783
p(Dura)=0.264
Scalp
Dura
A
B
C
D
E
F
G
H
I
J
K

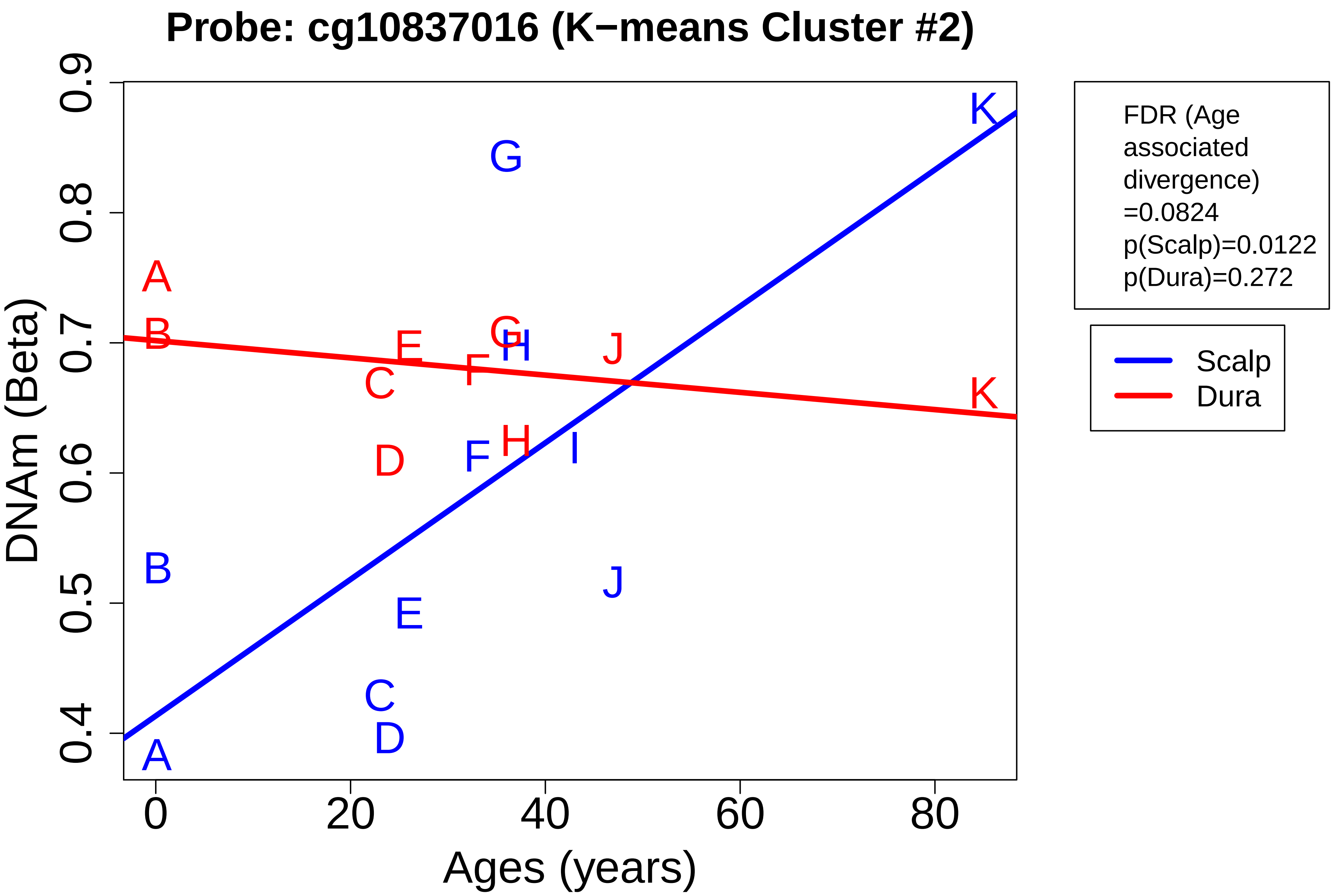

Probe: cg10837016 (K−means Cluster #2)
DNAm (Beta)
0.4
0.5
0.6
0.7
0.8
0.9
Ages (years)
0
20
40
60
80
FDR (Age associated divergence) =0.0824
p(Scalp)=0.0122
p(Dura)=0.272
Scalp
Dura
A B C D E F G H I J K

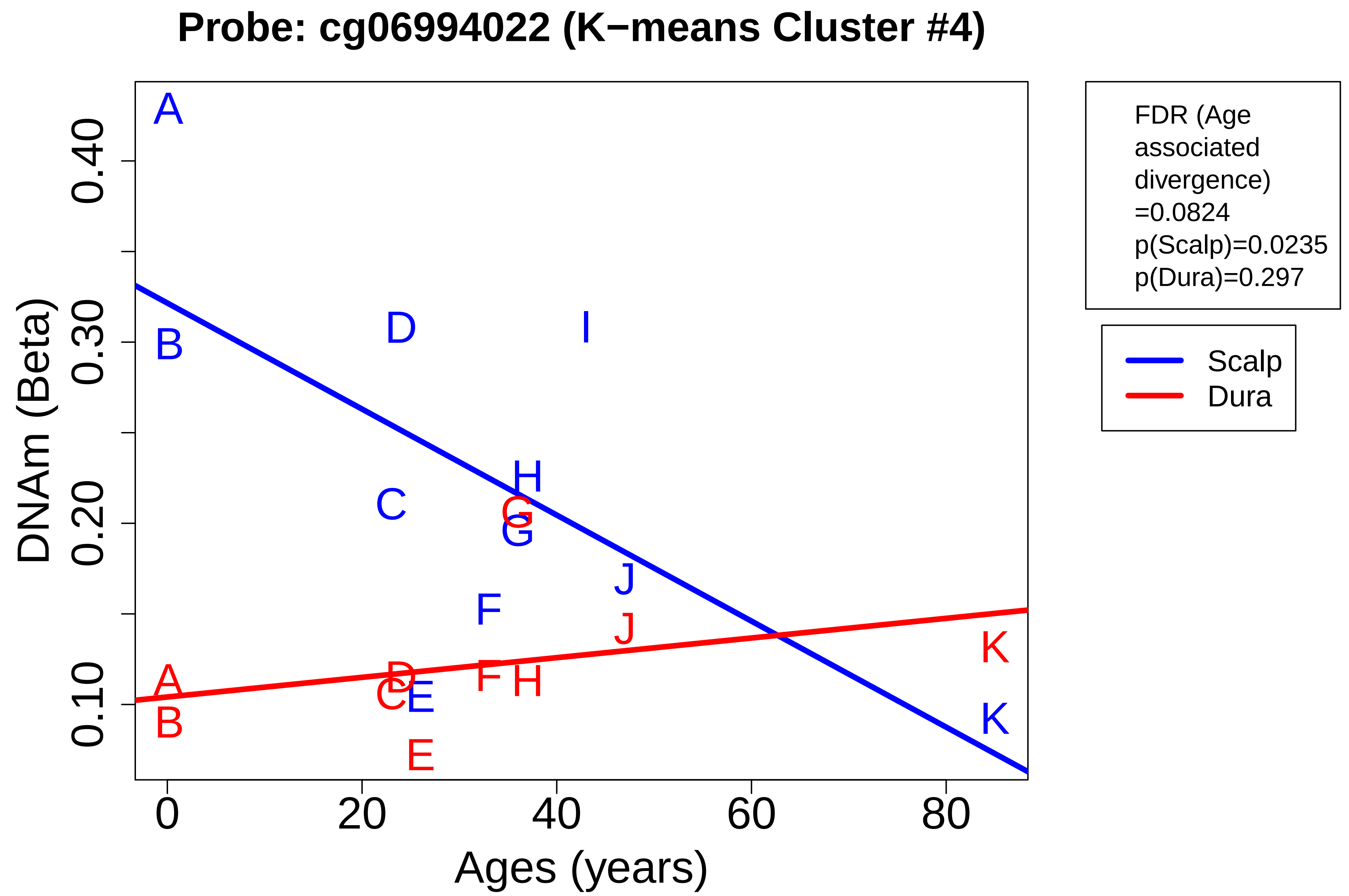

Probe: cg06994022 (K−means Cluster #4)
DNAm (Beta)
0.10
0.20
0.30
0.40
0
20
40
60
80
Ages (years)
A
B
C
D
E
F
G
H
I
J
K
FDR (Age associated divergence) =0.0824
p(Scalp)=0.0235
p(Dura)=0.297
Scalp
Dura

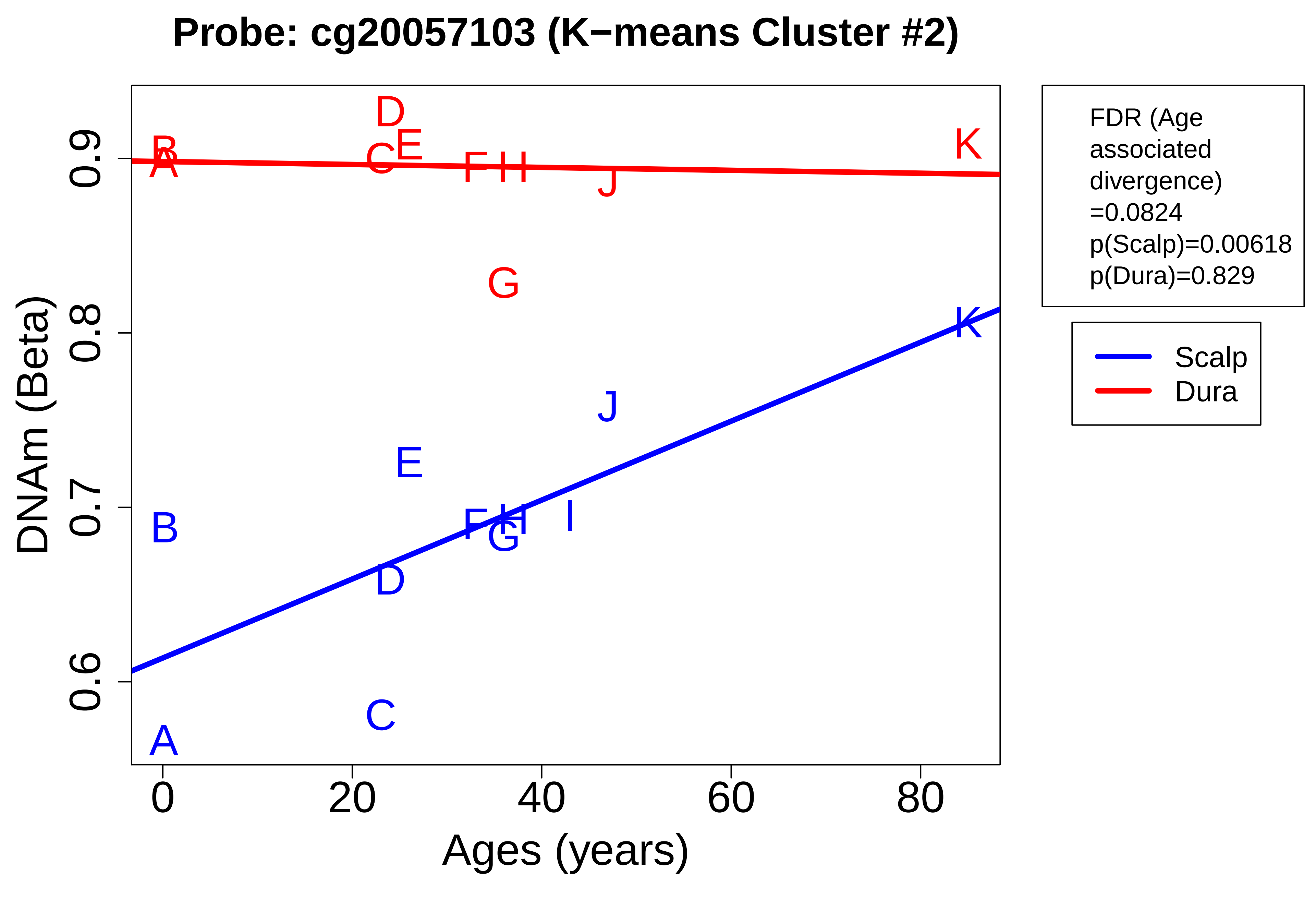

Probe: cg20057103 (K−means Cluster #2)
DNAm (Beta)
0.6
0.7
0.8
0.9
0
20
40
60
80
Ages (years)
FDR (Age associated divergence) =0.0824
p(Scalp)=0.00618
p(Dura)=0.829
Scalp
Dura

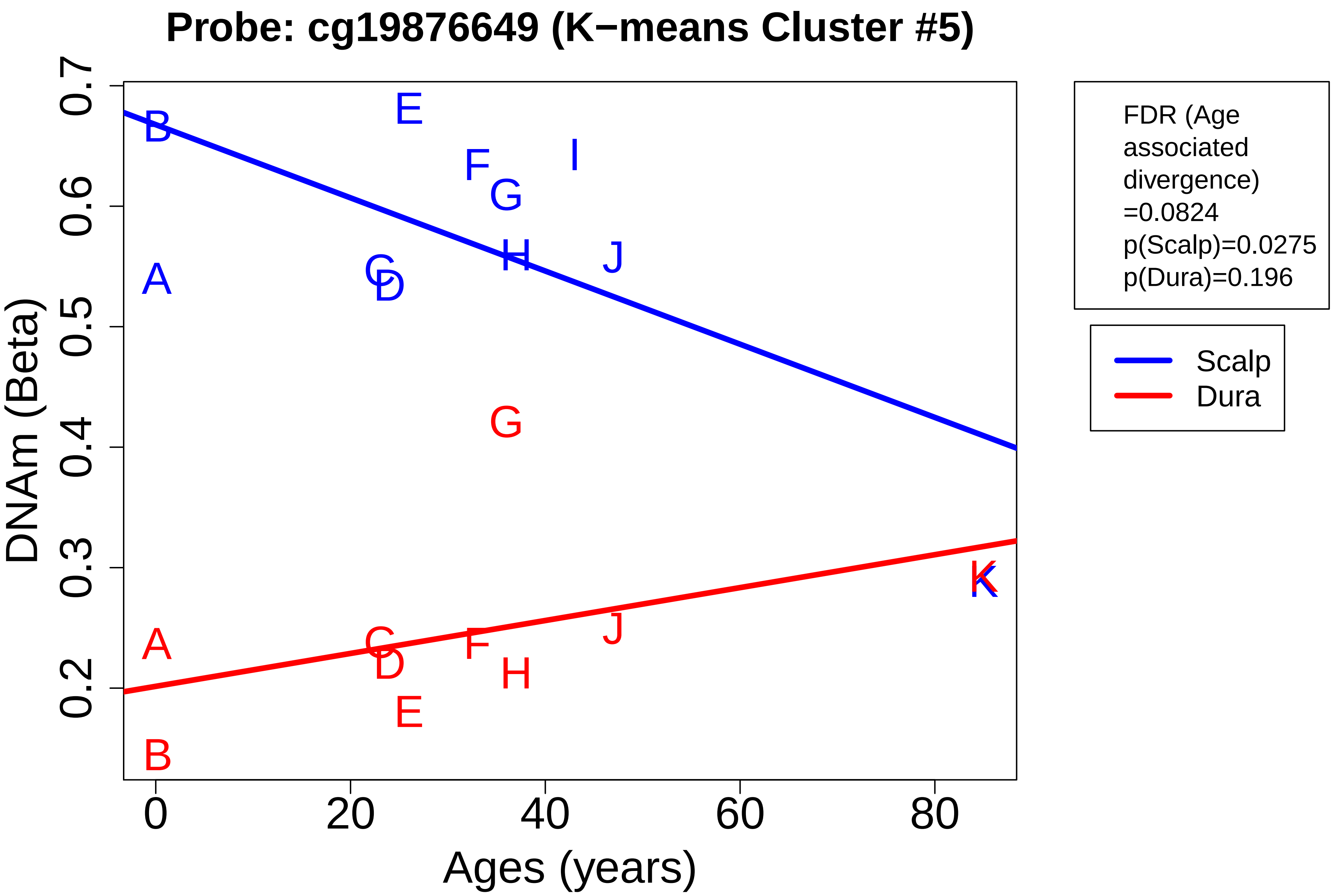

Probe: cg19876649 (K−means Cluster #5)
DNAm (Beta)
0.2
0.3
0.4
0.5
0.6
0.7
Ages (years)
0
20
40
60
80
A
B
C
D
E
F
G
H
I
J
K
FDR (Age associated divergence) =0.0824
p(Scalp)=0.0275
p(Dura)=0.196
Scalp
Dura

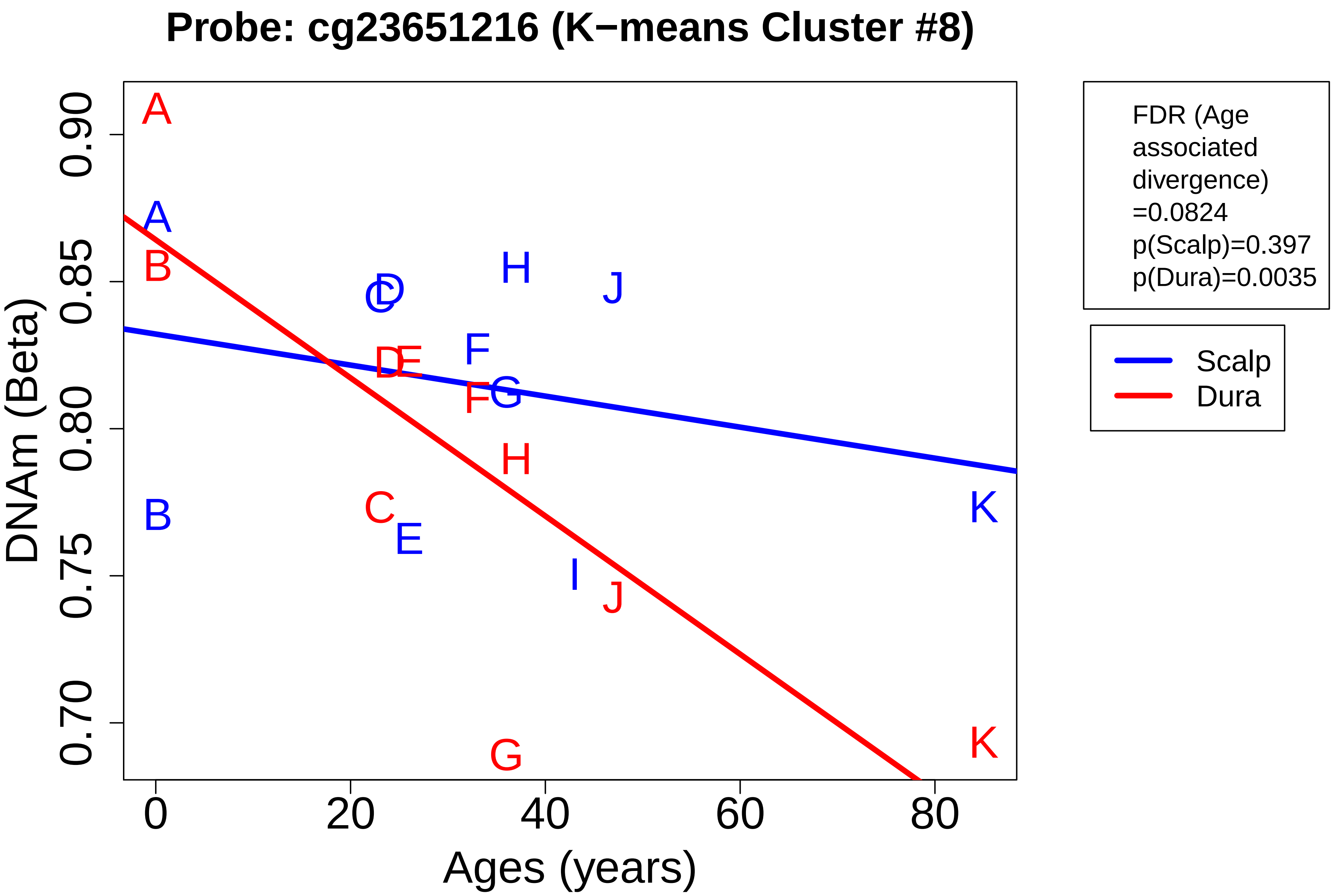

Probe: cg23651216 (K−means Cluster #8)
DNAm (Beta)
0.70
0.75
0.80
0.85
0.90
0
20
40
60
80
Ages (years)
FDR (Age associated divergence) =0.0824
p(Scalp)=0.397
p(Dura)=0.0035
Scalp
Dura

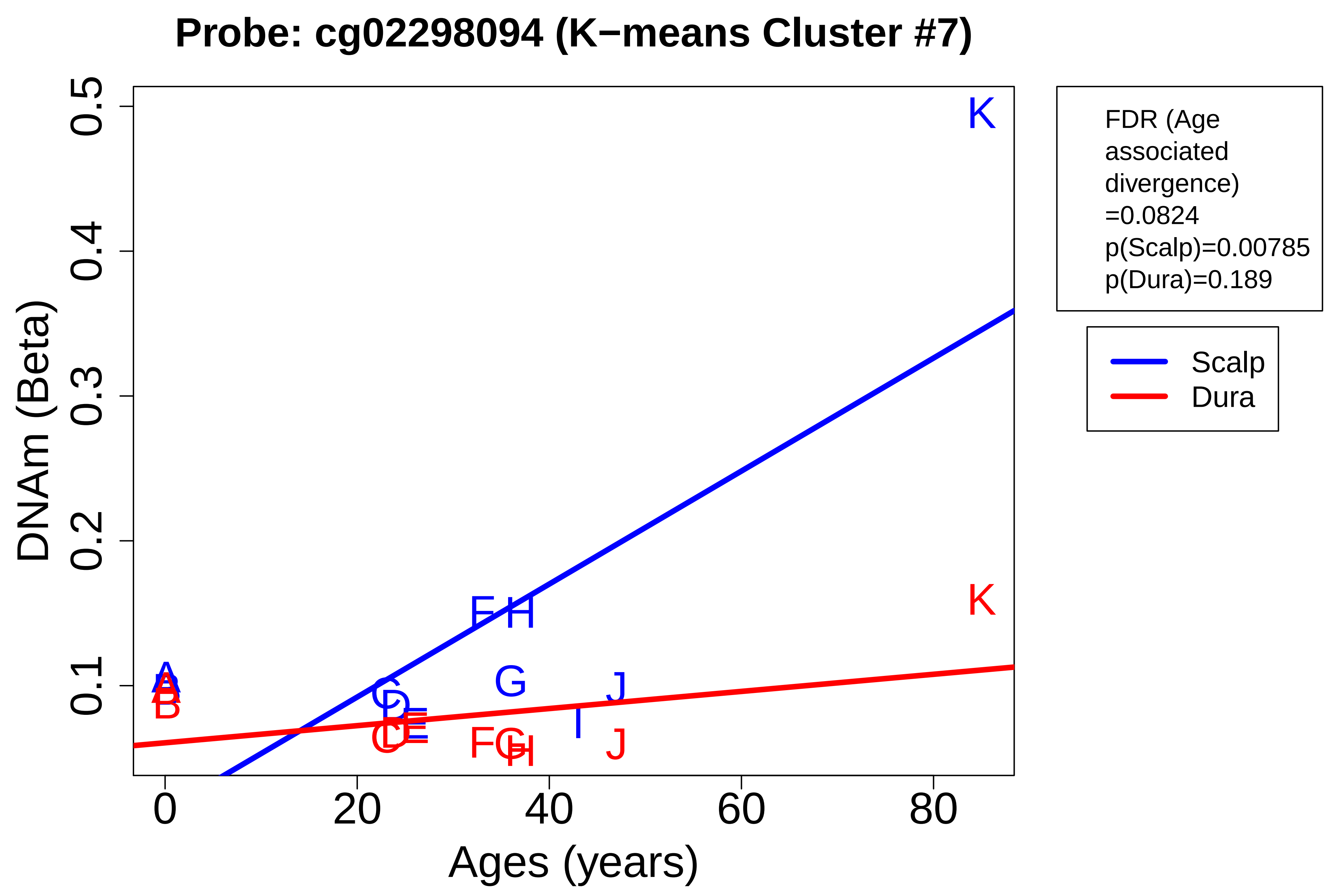

Probe: cg02298094 (K−means Cluster #7)
DNAm (Beta)
0.1
0.2
0.3
0.4
0.5
0
20
40
60
80
Ages (years)
FDR (Age associated divergence) =0.0824
p(Scalp)=0.00785
p(Dura)=0.189
Scalp
Dura

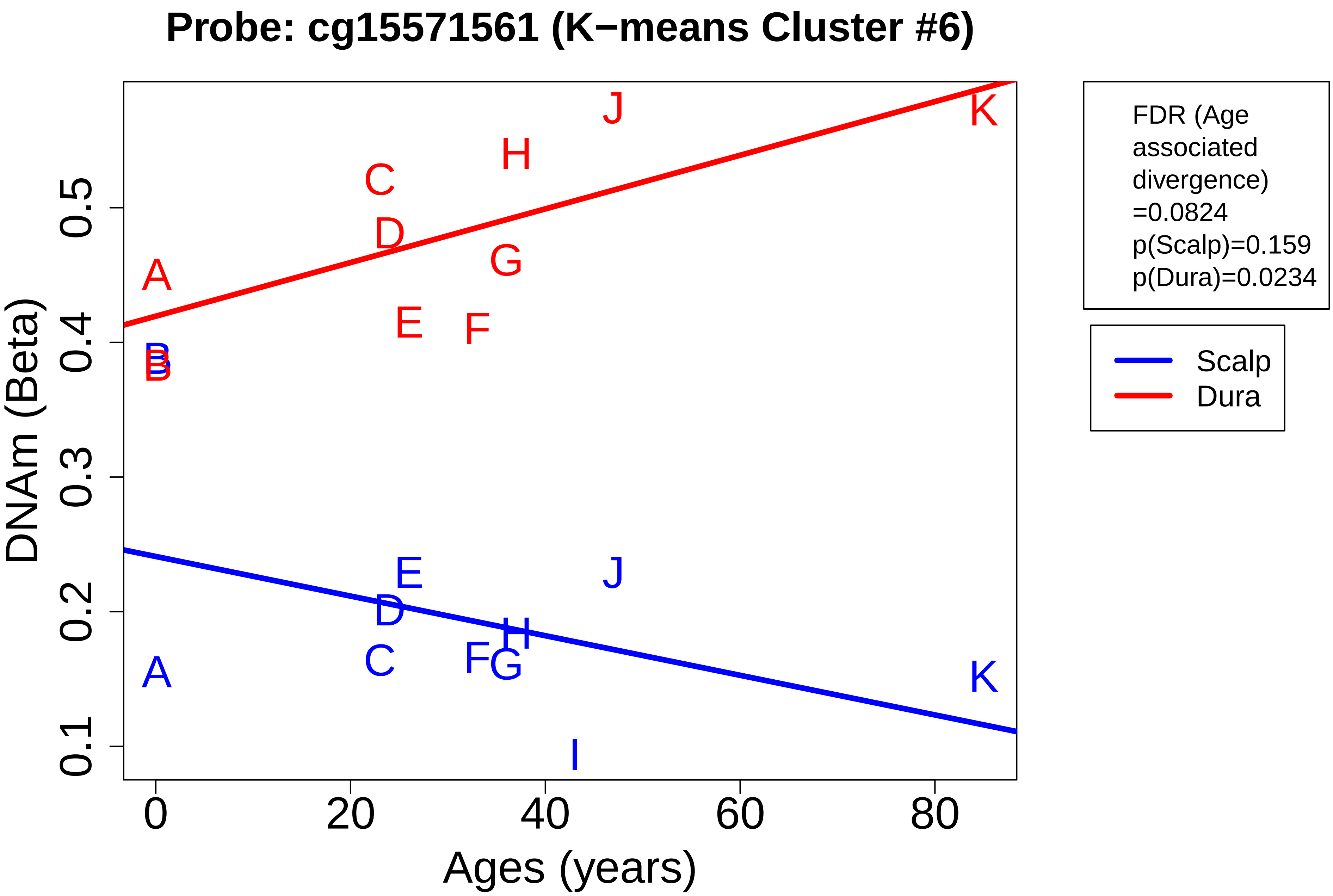

Probe: cg15571561 (K−means Cluster #6)
DNAm (Beta)
0.1
0.2
0.3
0.4
0.5
Ages (years)
0
20
40
60
80
FDR (Age associated divergence) =0.0824
p(Scalp)=0.159
p(Dura)=0.0234
Scalp
Dura

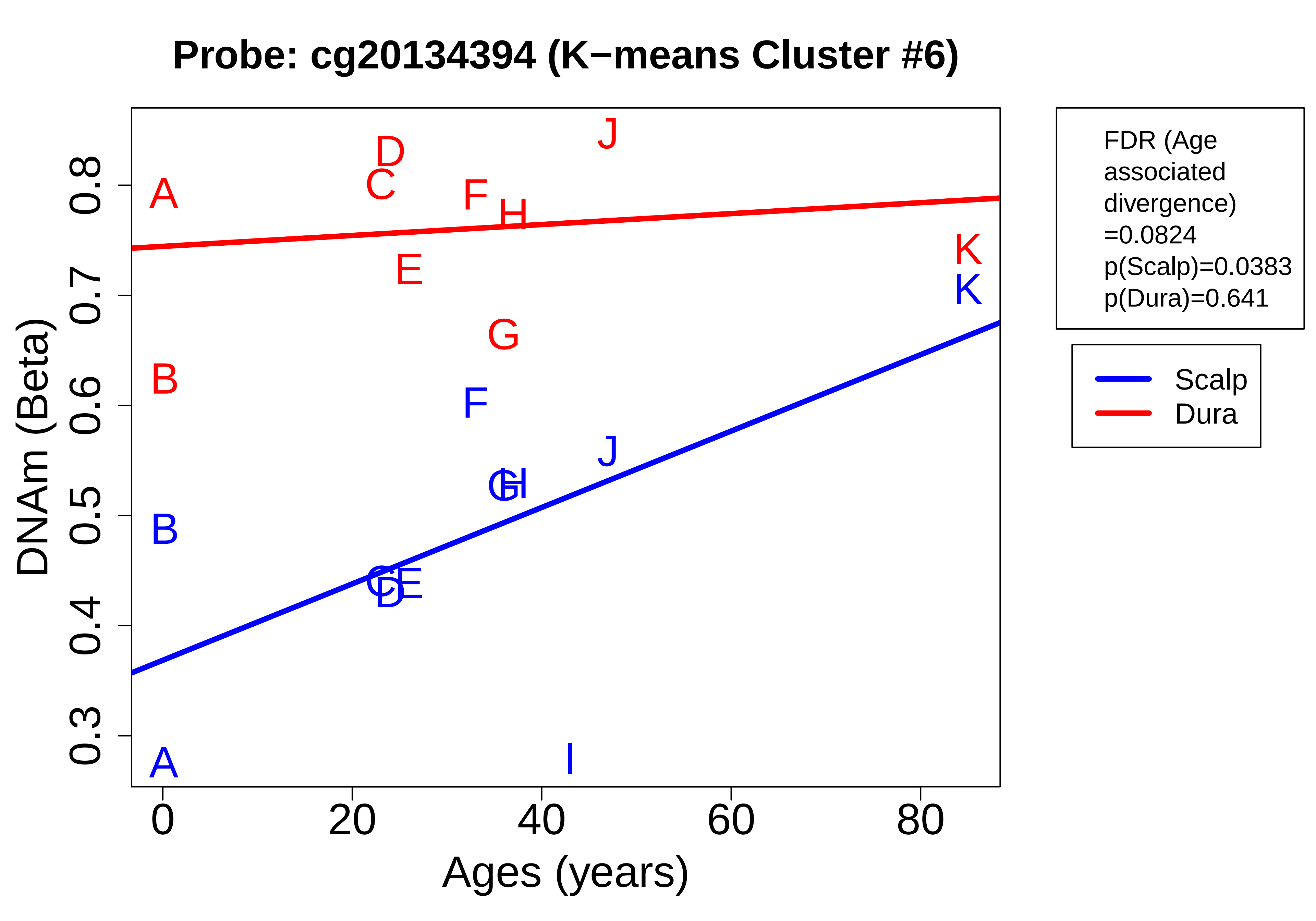

Probe: cg20134394 (K−means Cluster #6)
DNAm (Beta)
0.3
0.4
0.5
0.6
0.7
0.8
0
20
40
60
80
Ages (years)
FDR (Age associated divergence) =0.0824
p(Scalp)=0.0383
p(Dura)=0.641
Scalp
Dura

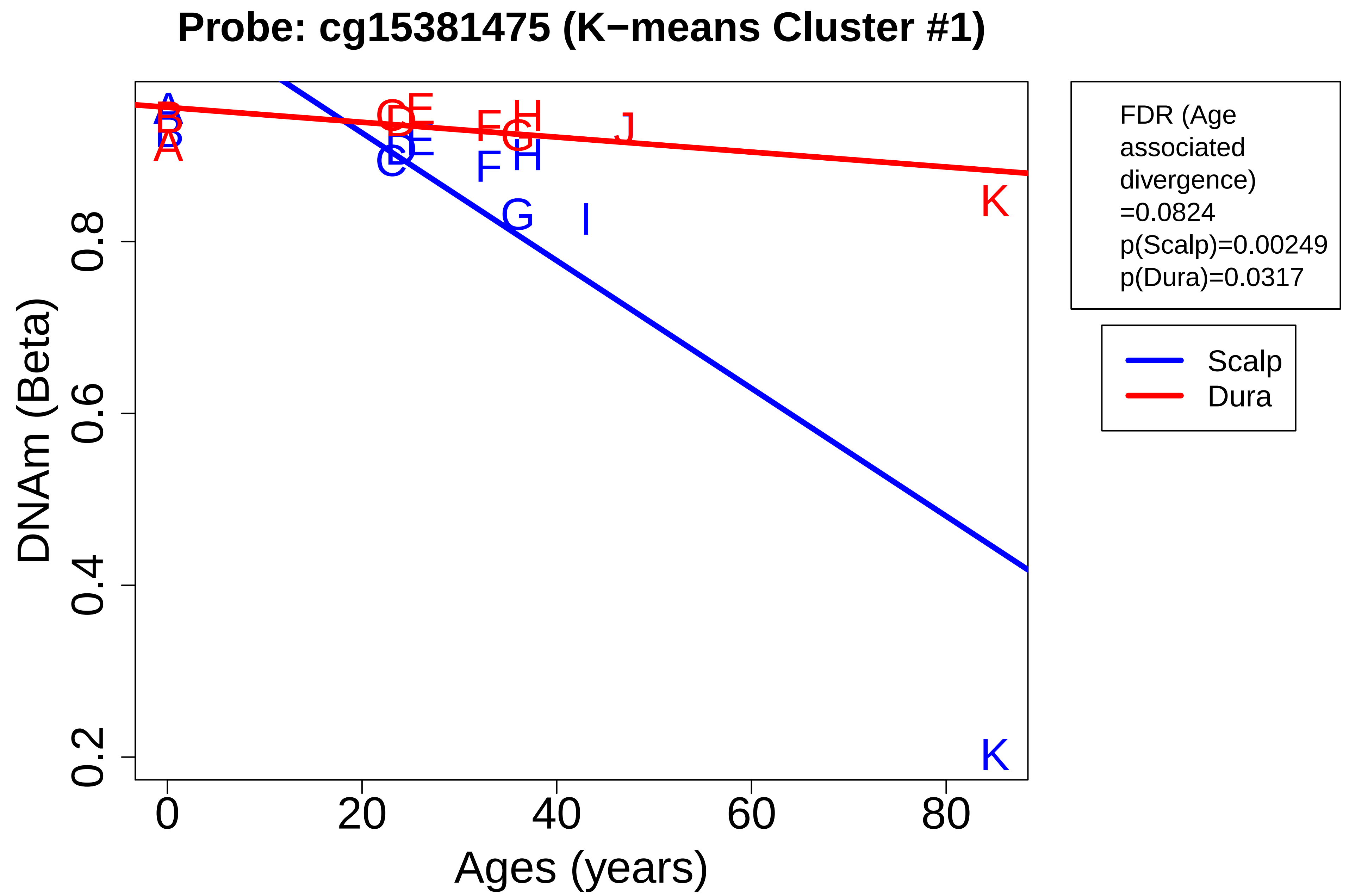

Probe: cg15381475 (K−means Cluster #1)
DNAm (Beta)
0.2
0.4
0.6
0.8
0
20
40
60
80
Ages (years)
A
B
C
D
E
F
G
H
I
J
K
FDR (Age associated divergence) =0.0824
p(Scalp)=0.00249
p(Dura)=0.0317
Scalp
Dura

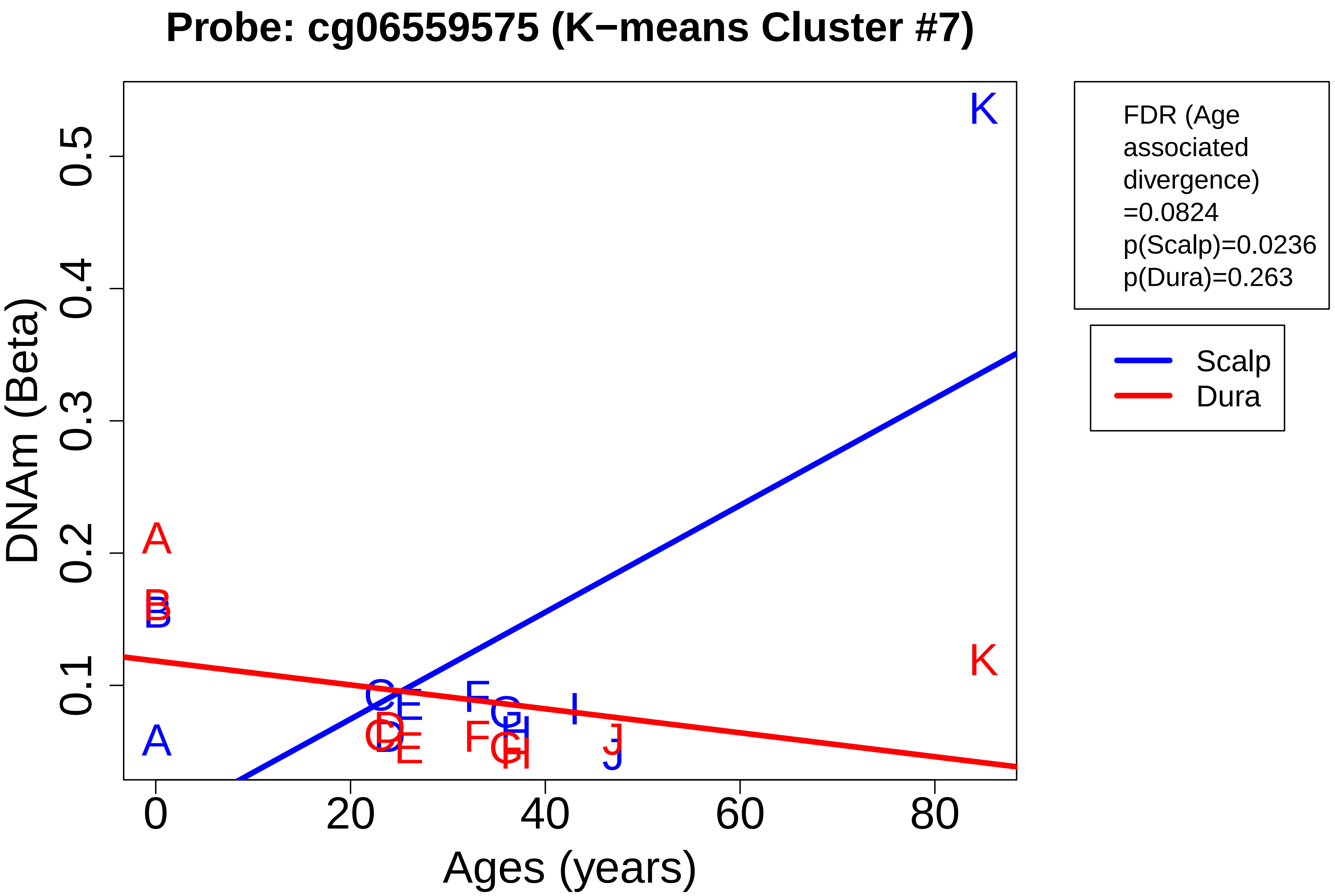
Probe: cg06559575 (K−means Cluster #7)
DNAm (Beta)
Ages (years)
0.1
0.2
0.3
0.4
0.5
0
20
40
60
80
FDR (Age associated divergence) =0.0824
p(Scalp)=0.0236
p(Dura)=0.263
Scalp
Dura
A
B
C
D
E
F
G
H
I
J
K

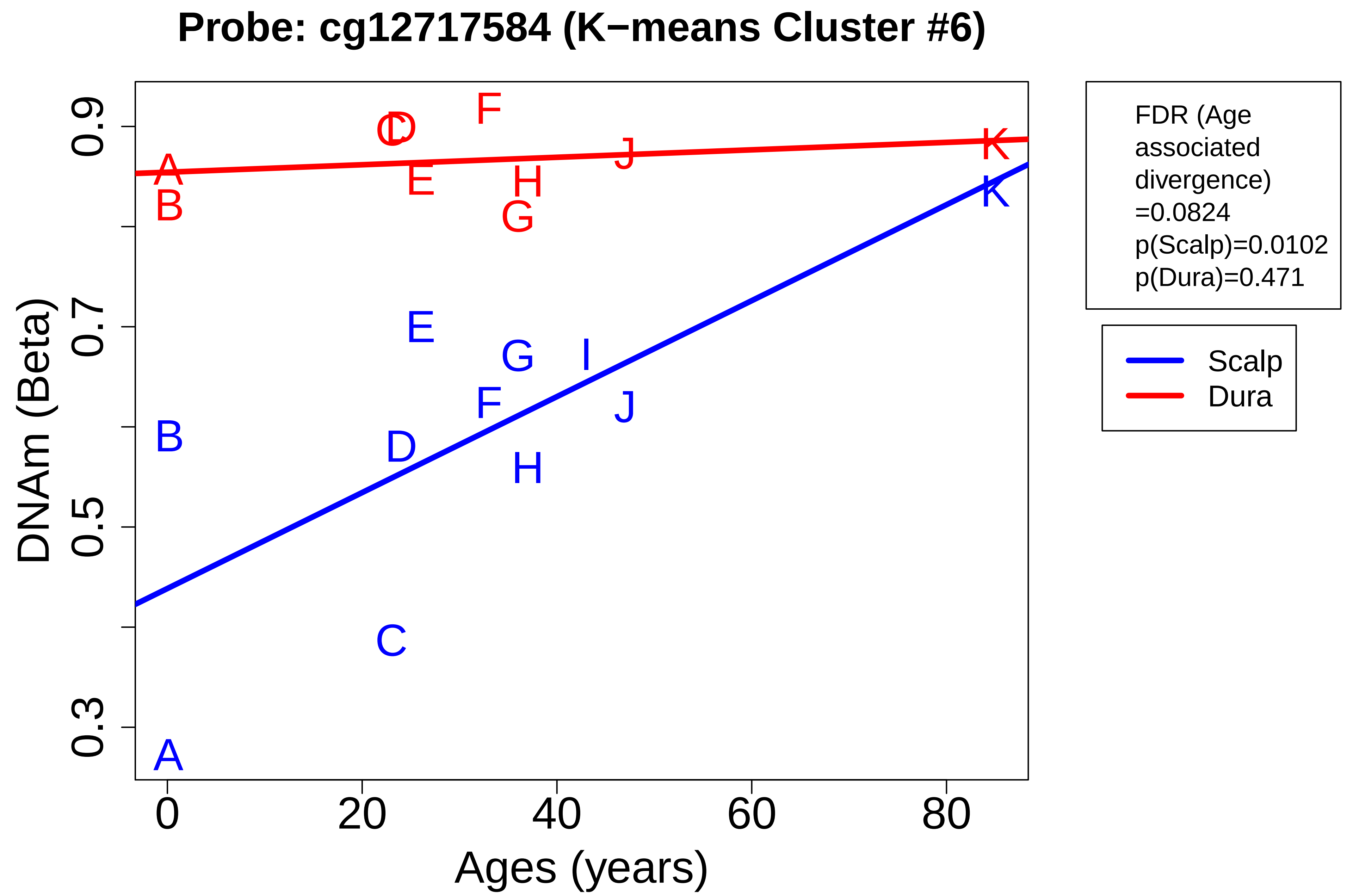

Probe: cg12717584 (K−means Cluster #6)
FDR (Age associated divergence) =0.0824
p(Scalp)=0.0102
p(Dura)=0.471
Scalp
Dura
DNAm (Beta)
0.3
0.5
0.7
0.9
0
20
40
60
80
Ages (years)
A
B
C
D
E
F
G
H
I
J
K

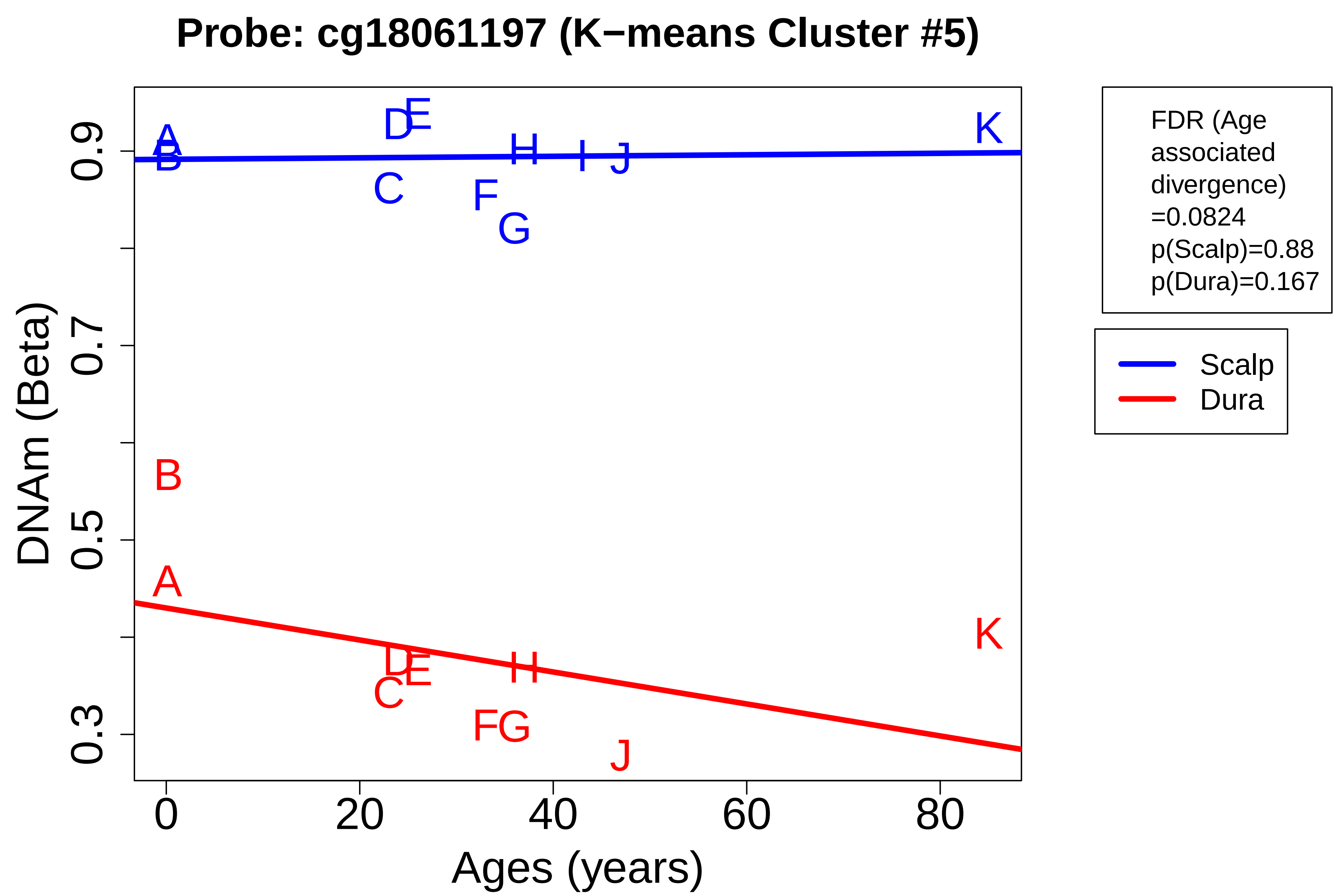
Probe: cg18061197 (K−means Cluster #5)
DNAm (Beta)
0.3
0.5
0.7
0.9
0
20
40
60
80
Ages (years)
FDR (Age associated divergence) =0.0824
p(Scalp)=0.88
p(Dura)=0.167
Scalp
Dura
A
B
C
D
E
F
G
H
I
J
K

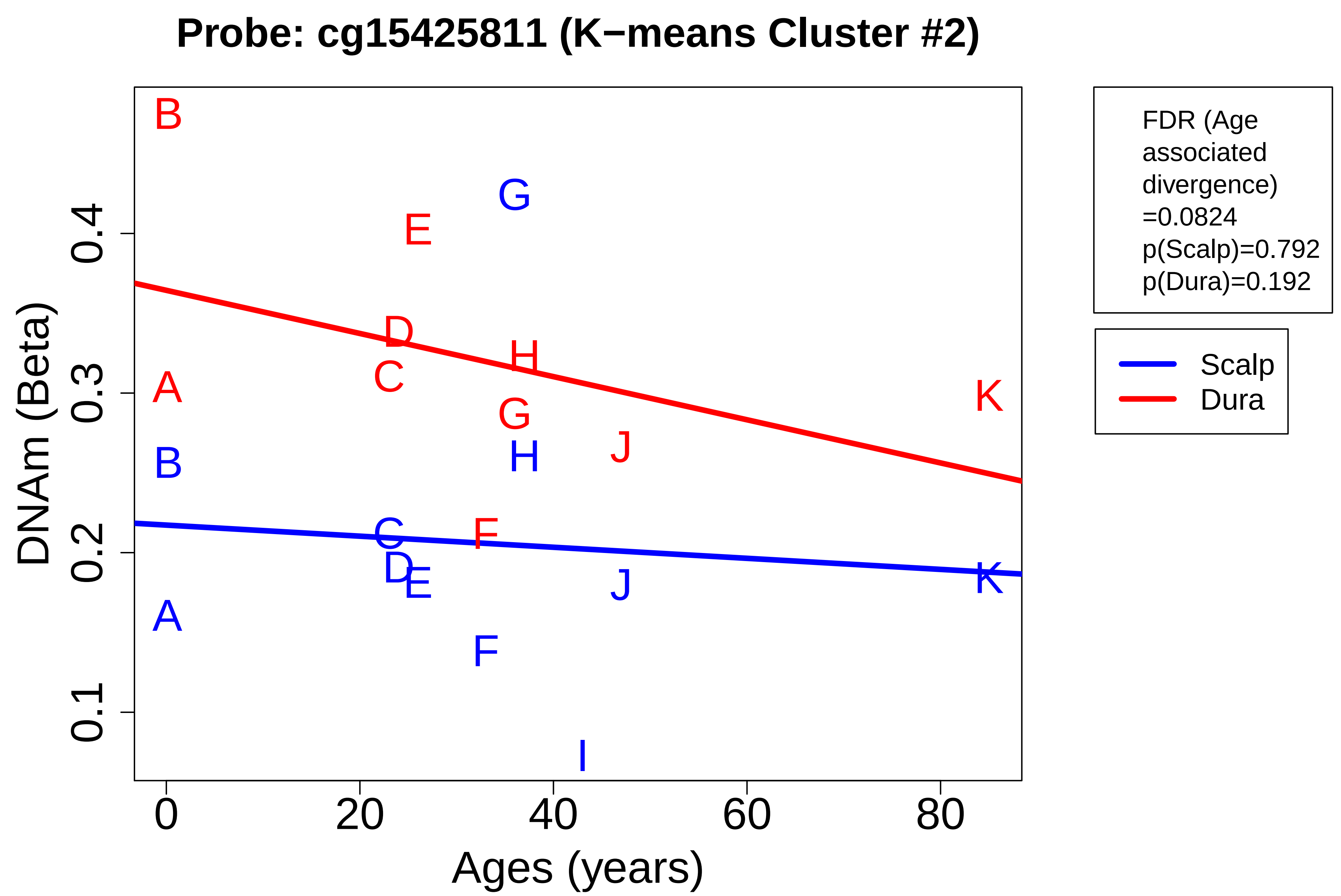

Probe: cg15425811 (K−means Cluster #2)
FDR (Age associated divergence) =0.0824
p(Scalp)=0.792
p(Dura)=0.192
Scalp
Dura
DNAm (Beta)
0.1
0.2
0.3
0.4
0
20
40
60
80
Ages (years)

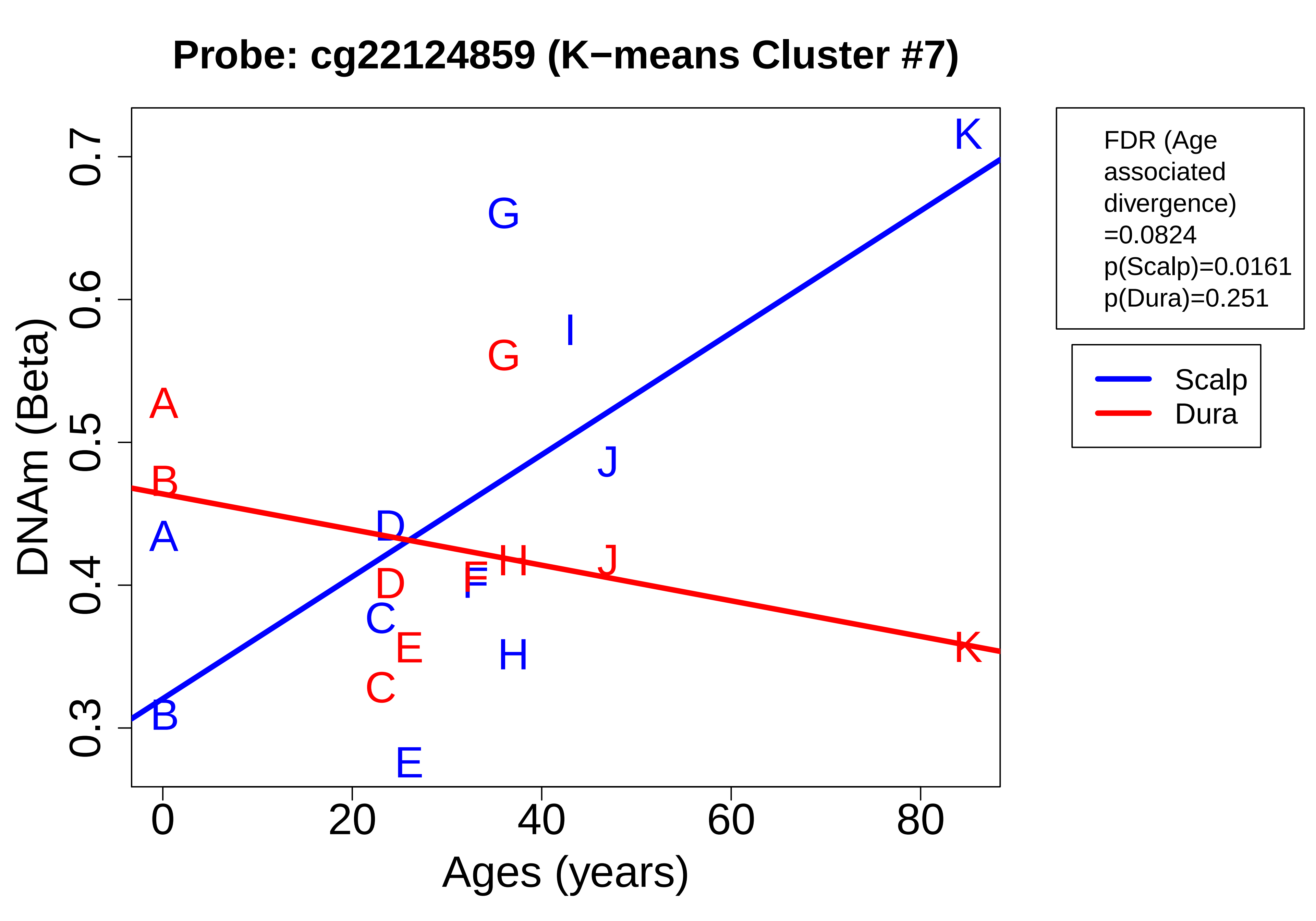
Probe: cg22124859 (K−means Cluster #7)
DNAm (Beta)
Ages (years)
0.3
0.4
0.5
0.6
0.7
0
20
40
60
80
FDR (Age associated divergence) =0.0824
p(Scalp)=0.0161
p(Dura)=0.251
Scalp
Dura
A
B
C
D
E
F
G
H
I
J
K

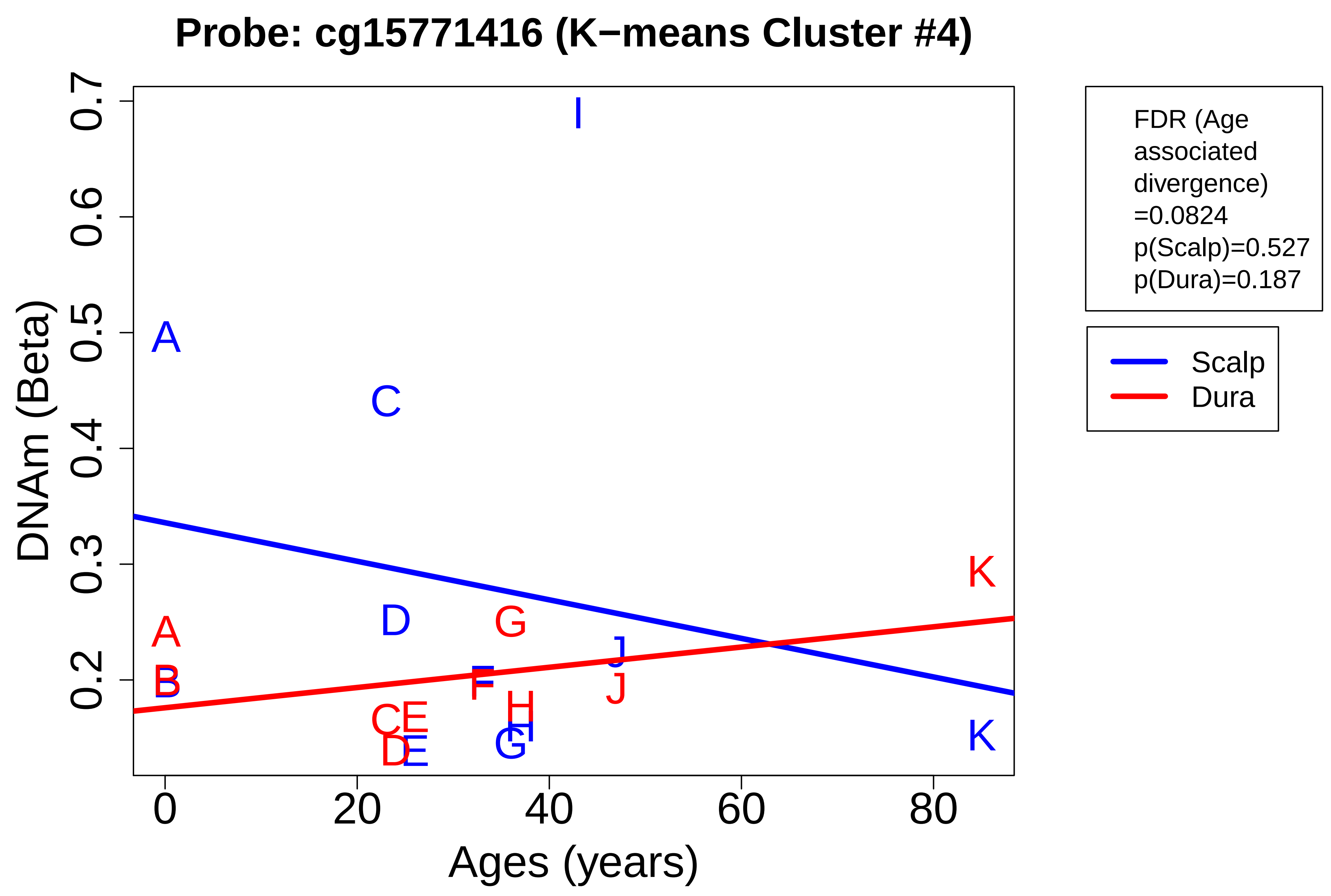

Probe: cg15771416 (K−means Cluster #4)
DNAm (Beta)
0.2
0.3
0.4
0.5
0.6
0.7
Ages (years)
0
20
40
60
80
FDR (Age associated divergence) =0.0824
p(Scalp)=0.527
p(Dura)=0.187
Scalp
Dura

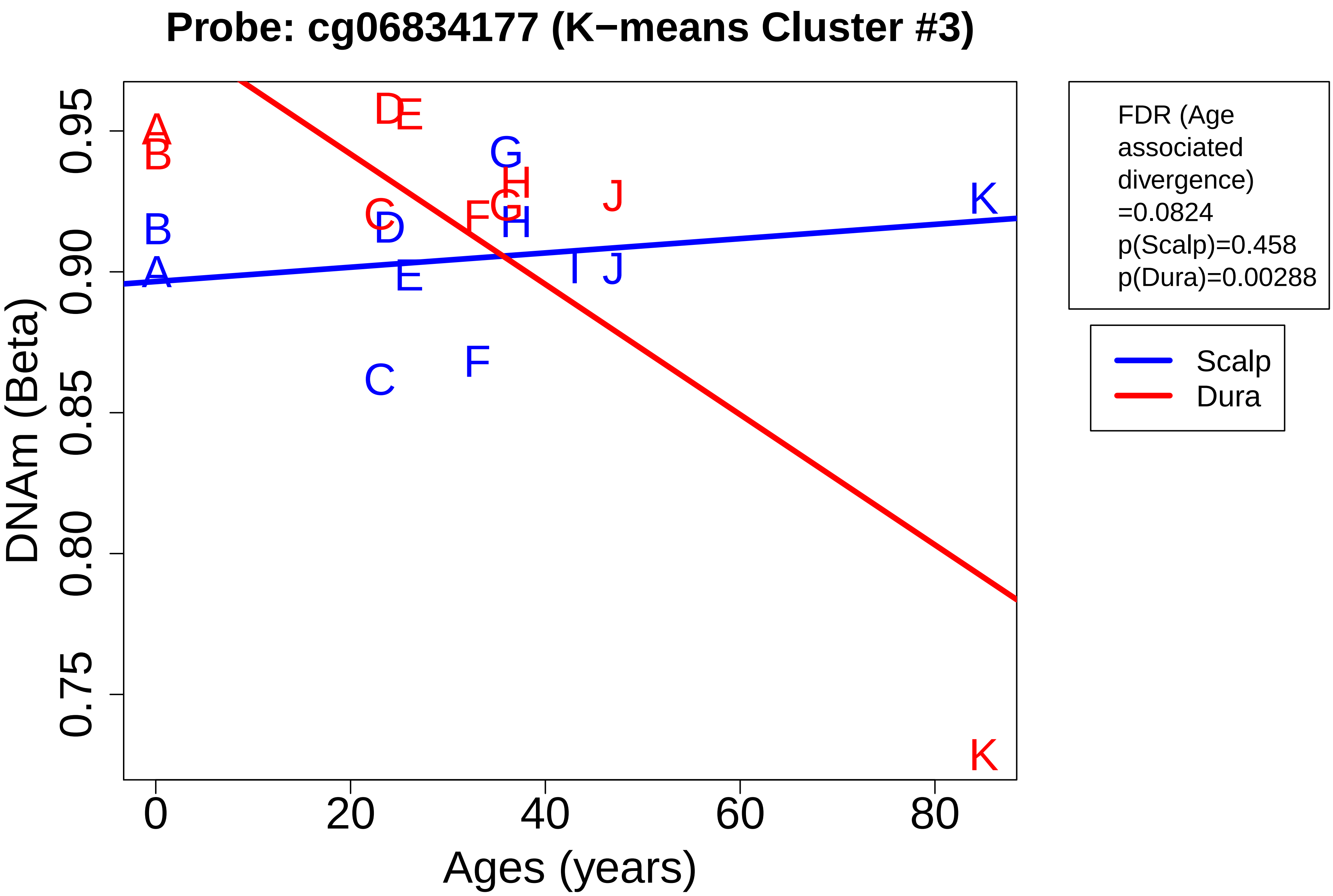

Probe: cg06834177 (K−means Cluster #3)
DNAm (Beta)
0.75
0.80
0.85
0.90
0.95
0
20
40
60
80
Ages (years)
FDR (Age associated divergence) =0.0824
p(Scalp)=0.458
p(Dura)=0.00288
Scalp
Dura

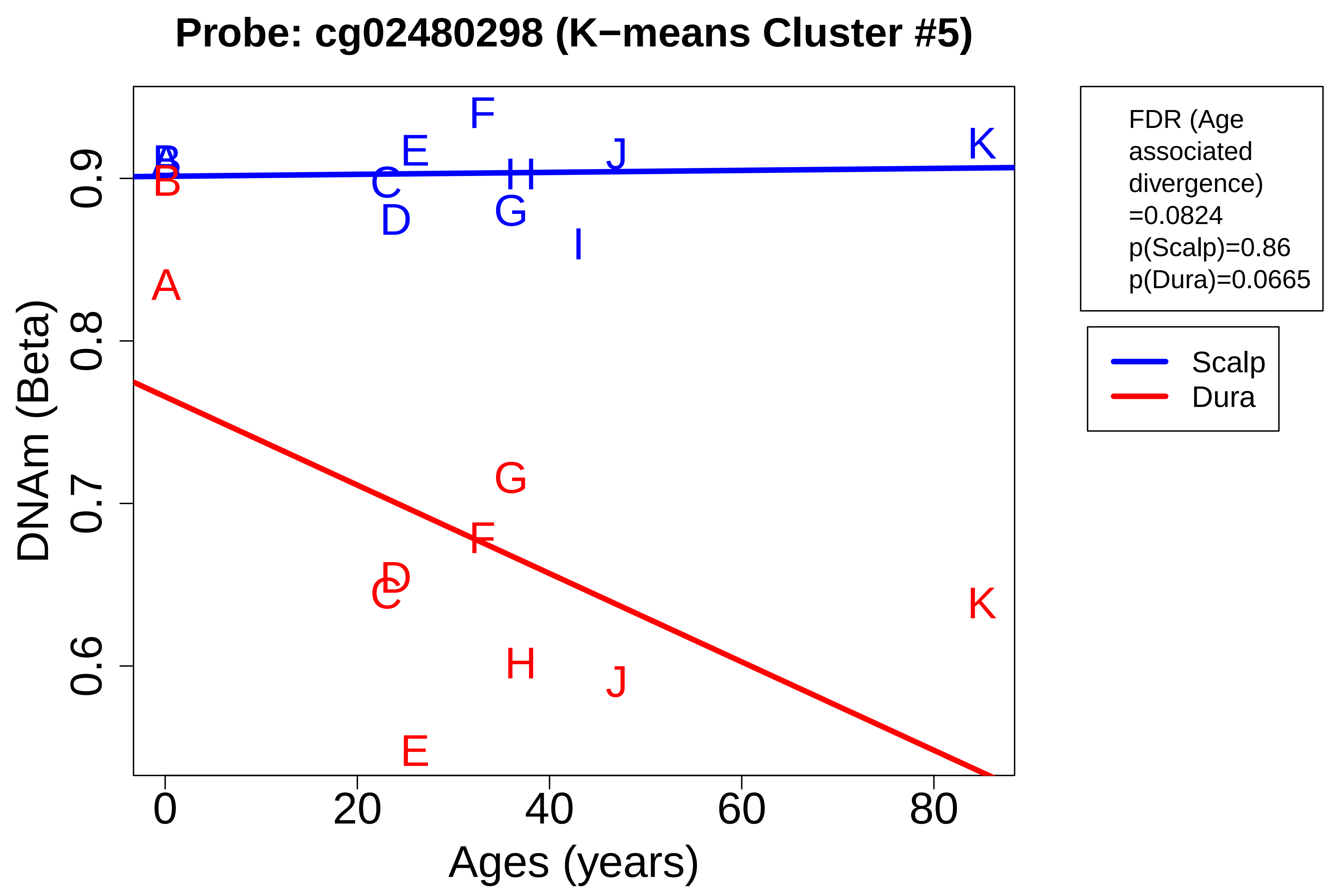

Probe: cg02480298 (K−means Cluster #5)
DNAm (Beta)
0.6
0.7
0.8
0.9
0
20
40
60
80
Ages (years)
A
B
C
D
E
F
G
H
I
J
K
A
B
C
D
E
F
G
H
J
K
FDR (Age associated divergence) =0.0824
p(Scalp)=0.86
p(Dura)=0.0665
Scalp
Dura

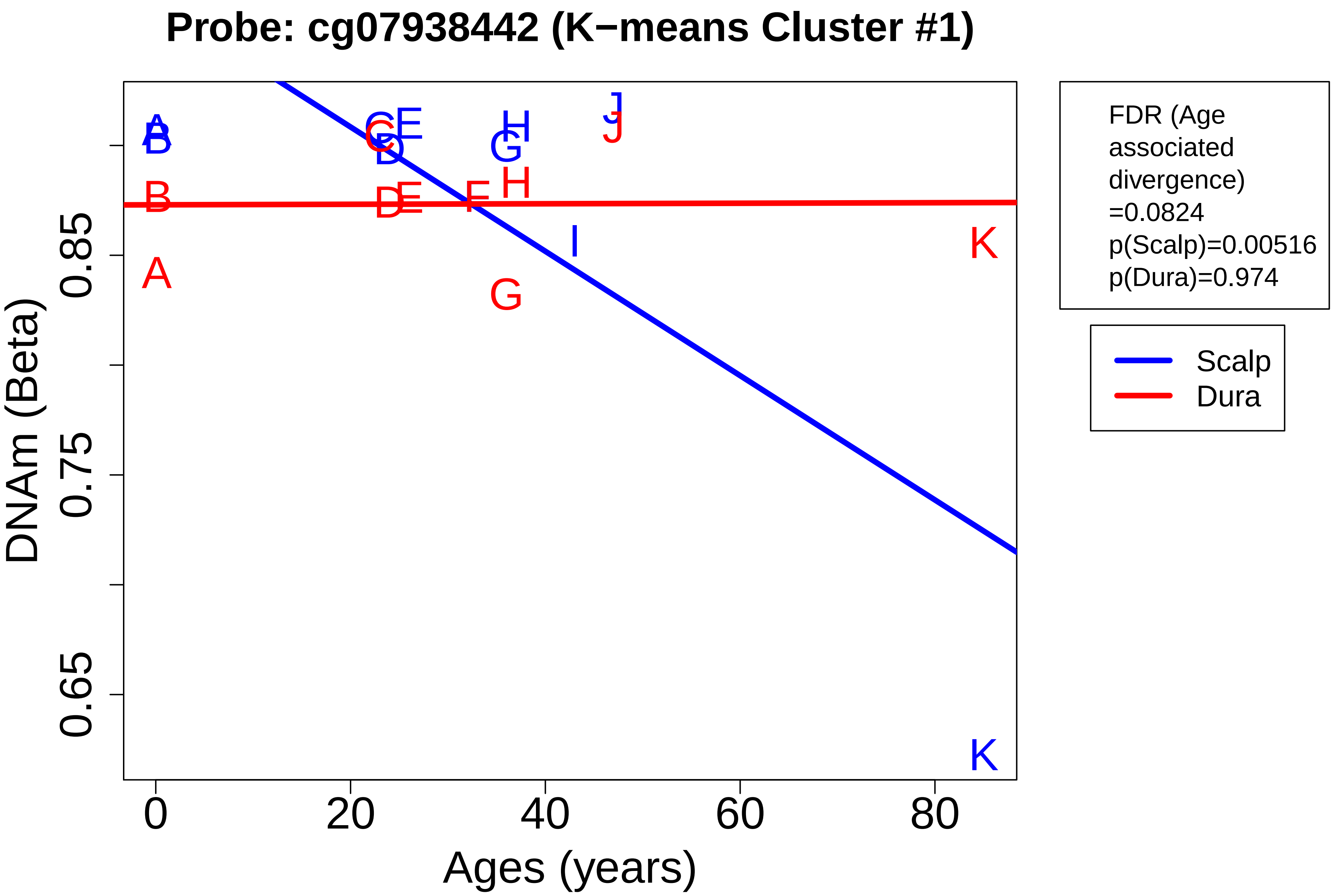

Probe: cg07938442 (K−means Cluster #1)
DNAm (Beta)
0.65
0.75
0.85
Ages (years)
0
20
40
60
80
FDR (Age associated divergence) =0.0824
p(Scalp)=0.00516
p(Dura)=0.974
Scalp
Dura

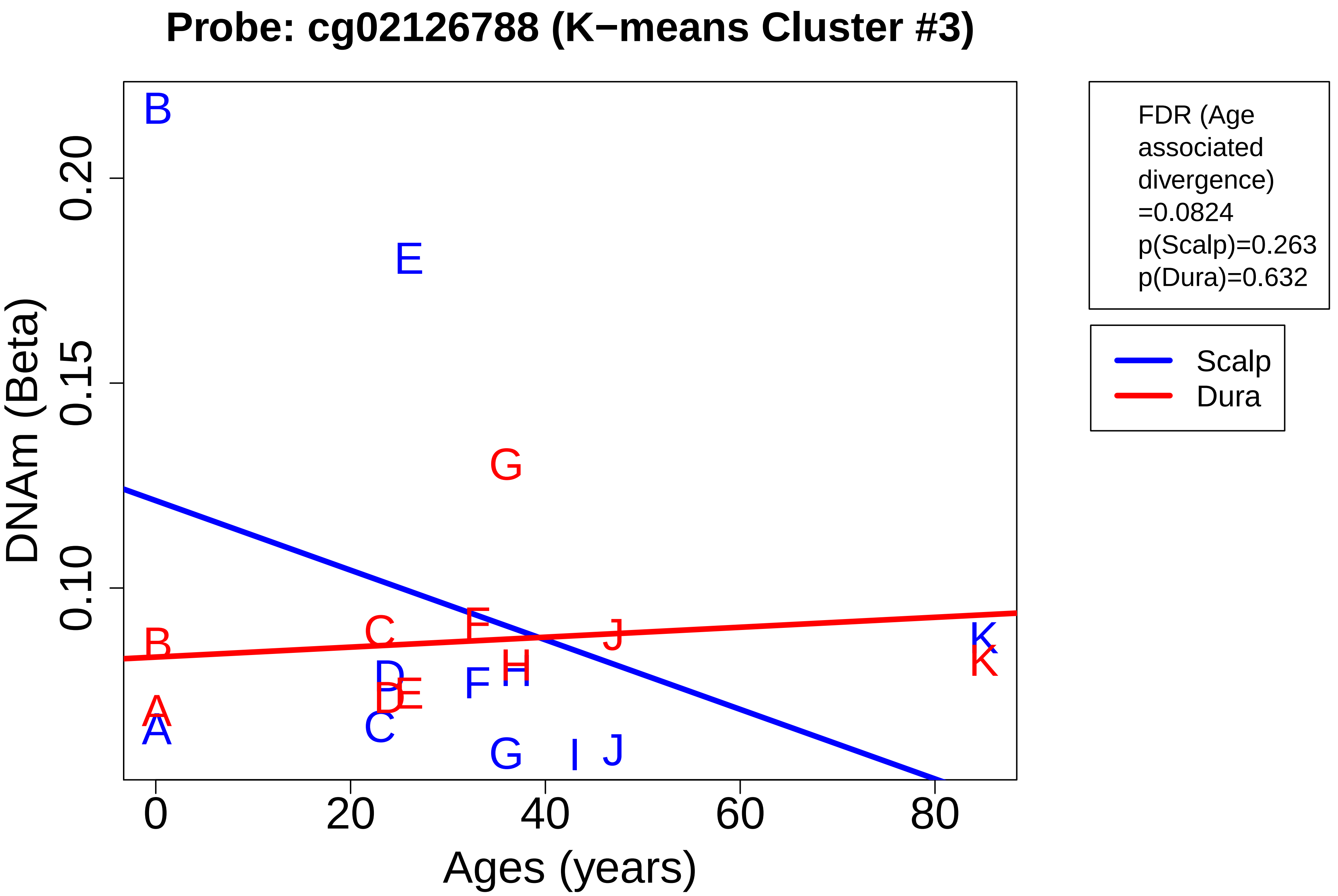

Probe: cg02126788 (K−means Cluster #3)
DNAm (Beta)
0.10
0.15
0.20
Ages (years)
0
20
40
60
80
FDR (Age associated divergence) =0.0824
p(Scalp)=0.263
p(Dura)=0.632
Scalp
Dura

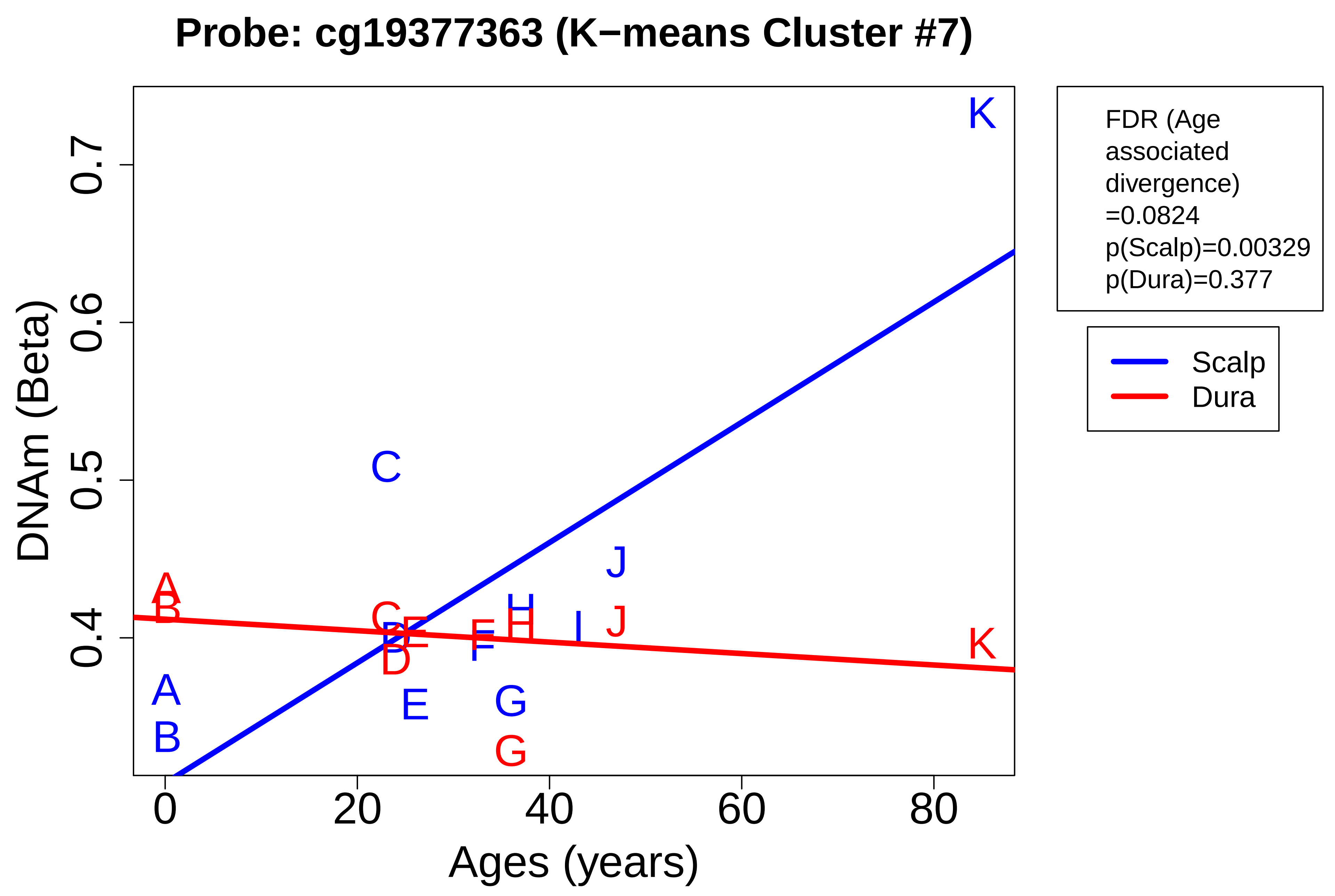
Probe: cg19377363 (K−means Cluster #7)
DNAm (Beta)
0.4
0.5
0.6
0.7
Ages (years)
0
20
40
60
80
FDR (Age associated divergence) =0.0824
p(Scalp)=0.00329
p(Dura)=0.377
Scalp
Dura
A
B
C
D
E
F
G
H
I
J
K

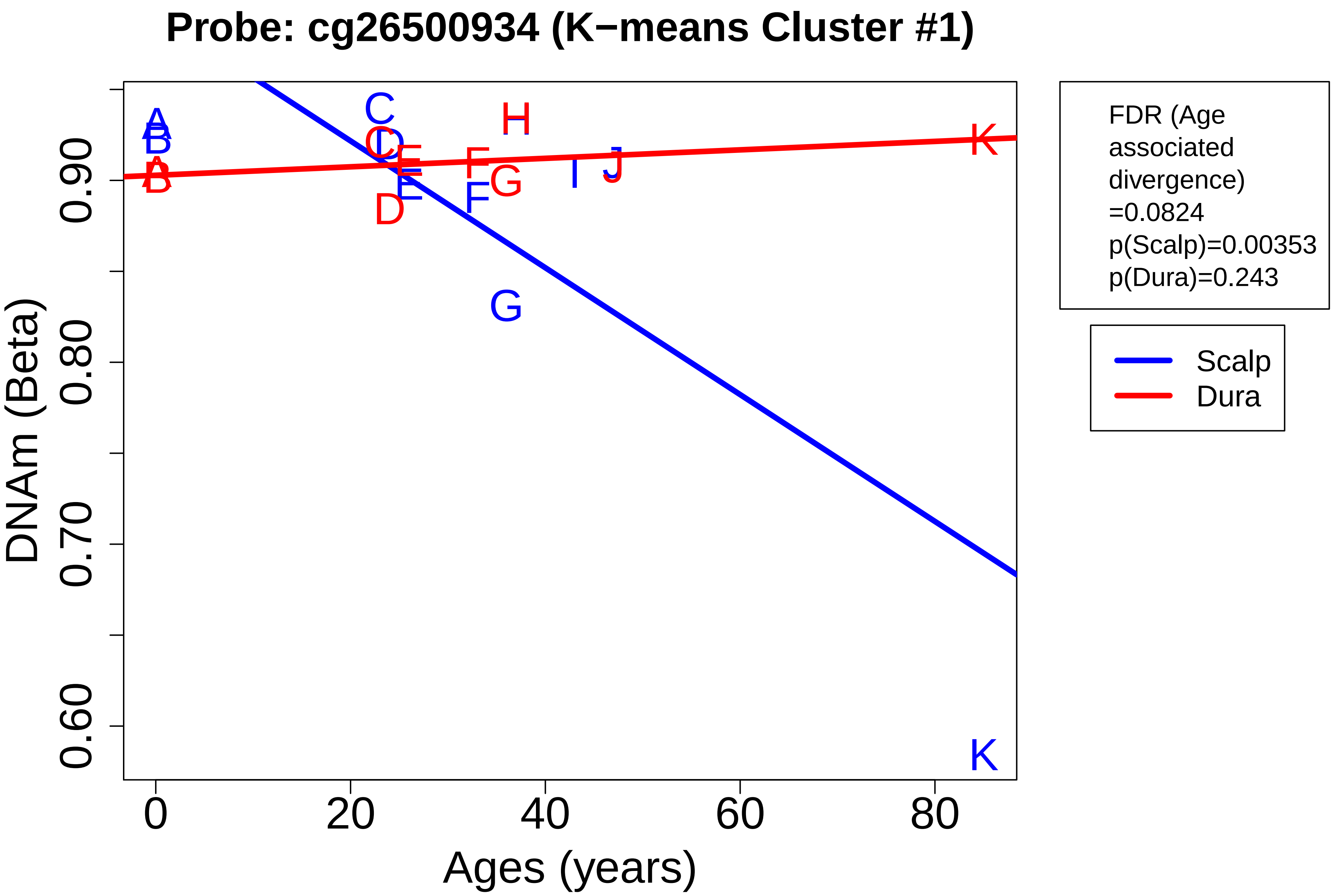

Probe: cg26500934 (K−means Cluster #1)
DNAm (Beta)
0.60
0.70
0.80
0.90
0
20
40
60
80
Ages (years)
FDR (Age associated divergence) =0.0824
p(Scalp)=0.00353
p(Dura)=0.243
Scalp
Dura

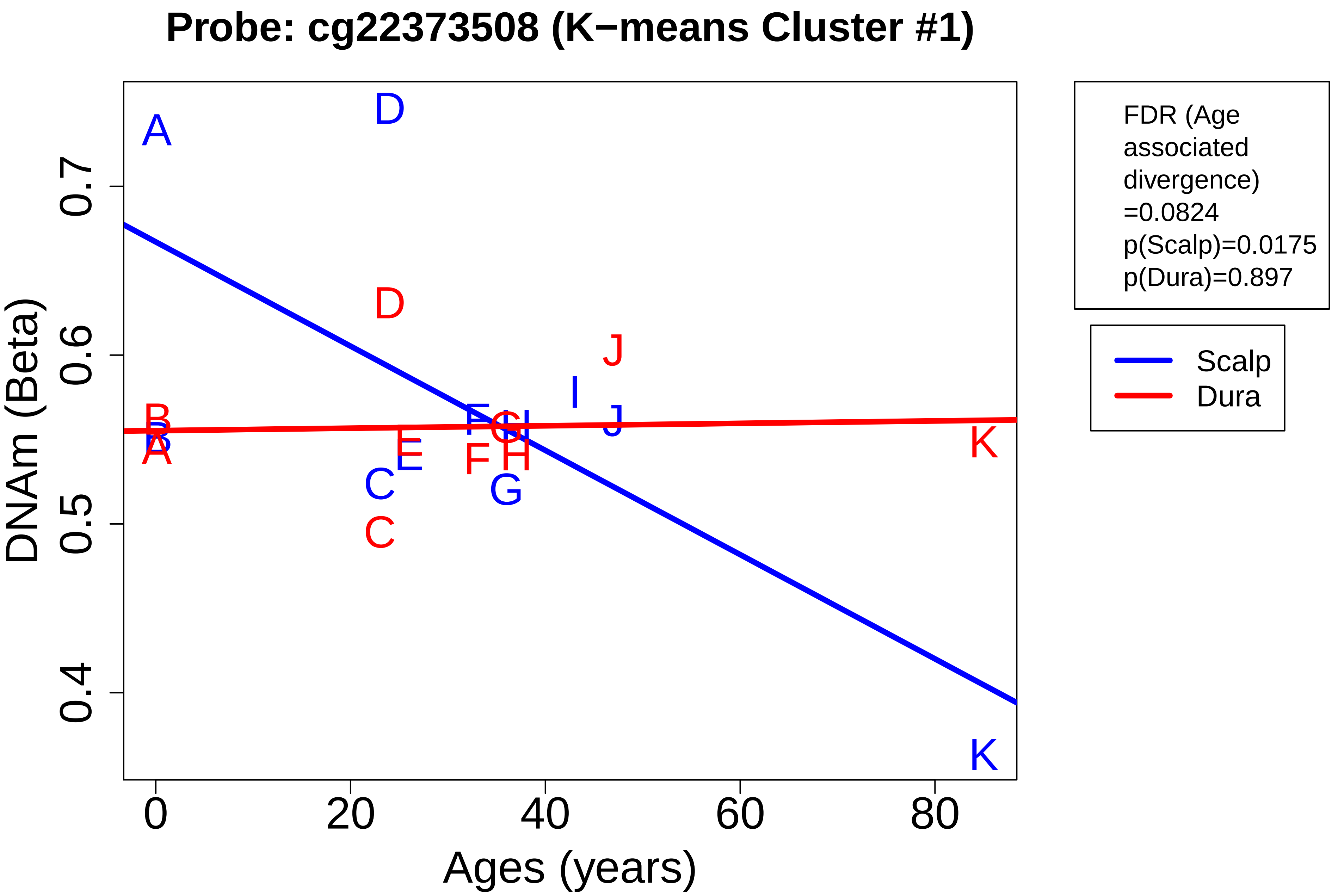

Probe: cg22373508 (K−means Cluster #1)
FDR (Age associated divergence) =0.0824
p(Scalp)=0.0175
p(Dura)=0.897
Scalp
Dura
DNAm (Beta)
0.4
0.5
0.6
0.7
Ages (years)
0
20
40
60
80
A
B
C
D
E
F
G
H
I
J
K

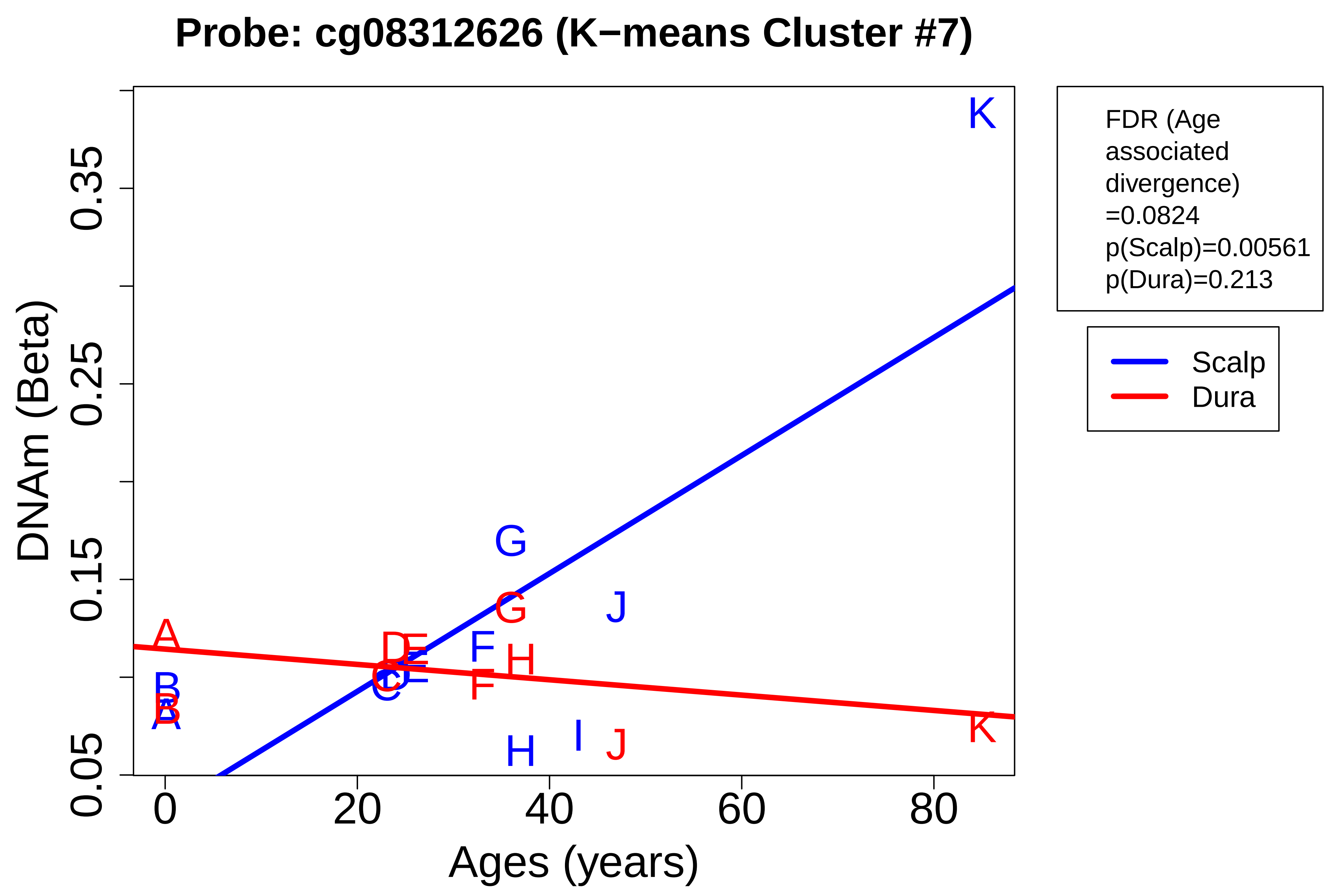

Probe: cg08312626 (K−means Cluster #7)
DNAm (Beta)
0.05
0.15
0.25
0.35
Ages (years)
0
20
40
60
80
FDR (Age associated divergence) =0.0824
p(Scalp)=0.00561
p(Dura)=0.213
Scalp
Dura
A
B
C
D
E
F
G
H
I
J
K

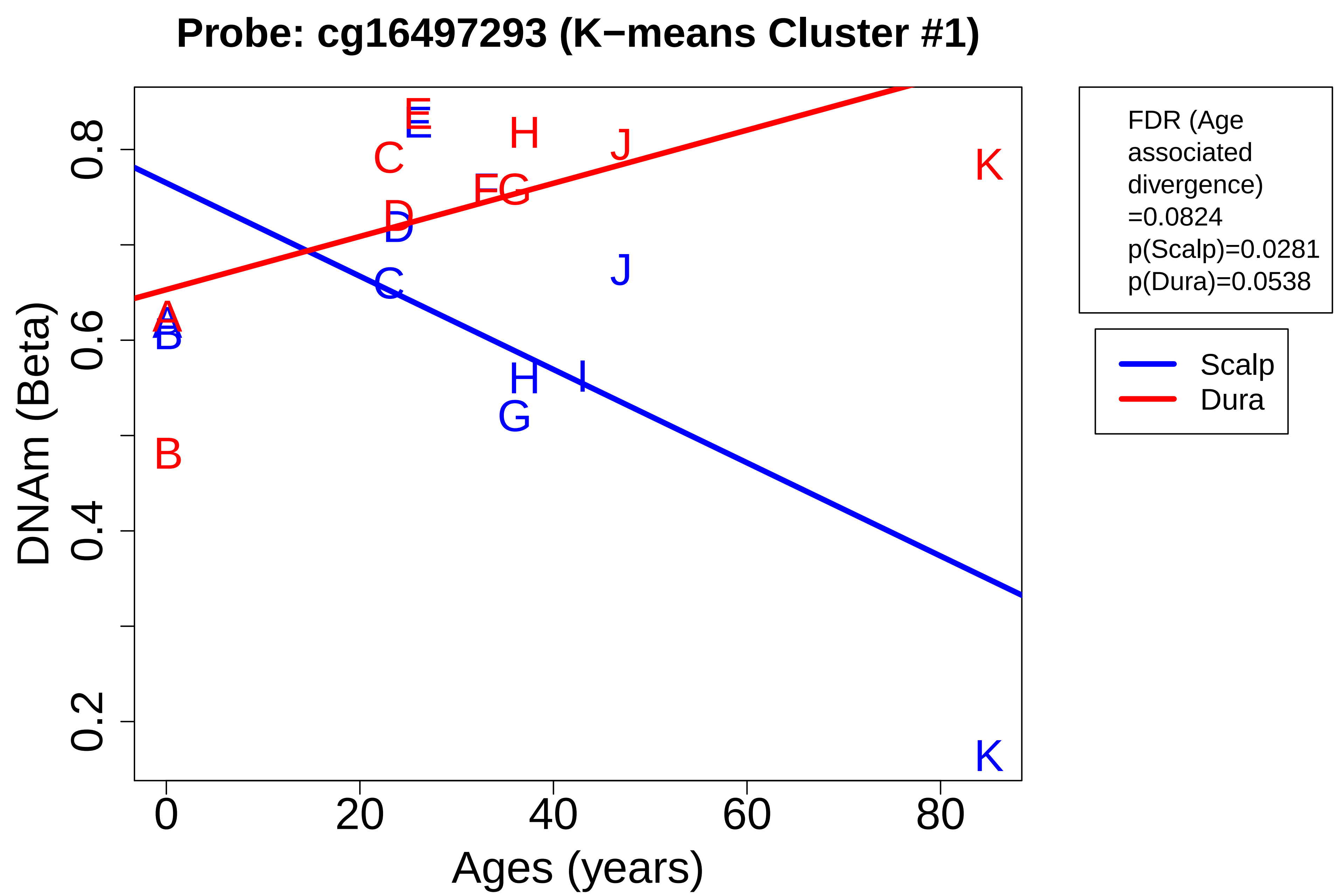

Probe: cg16497293 (K−means Cluster #1)
DNAm (Beta)
0.2
0.4
0.6
0.8
0
20
40
60
80
Ages (years)
A
B
C
D
E
F
G
H
I
J
K
FDR (Age associated divergence) =0.0824
p(Scalp)=0.0281
p(Dura)=0.0538
Scalp
Dura

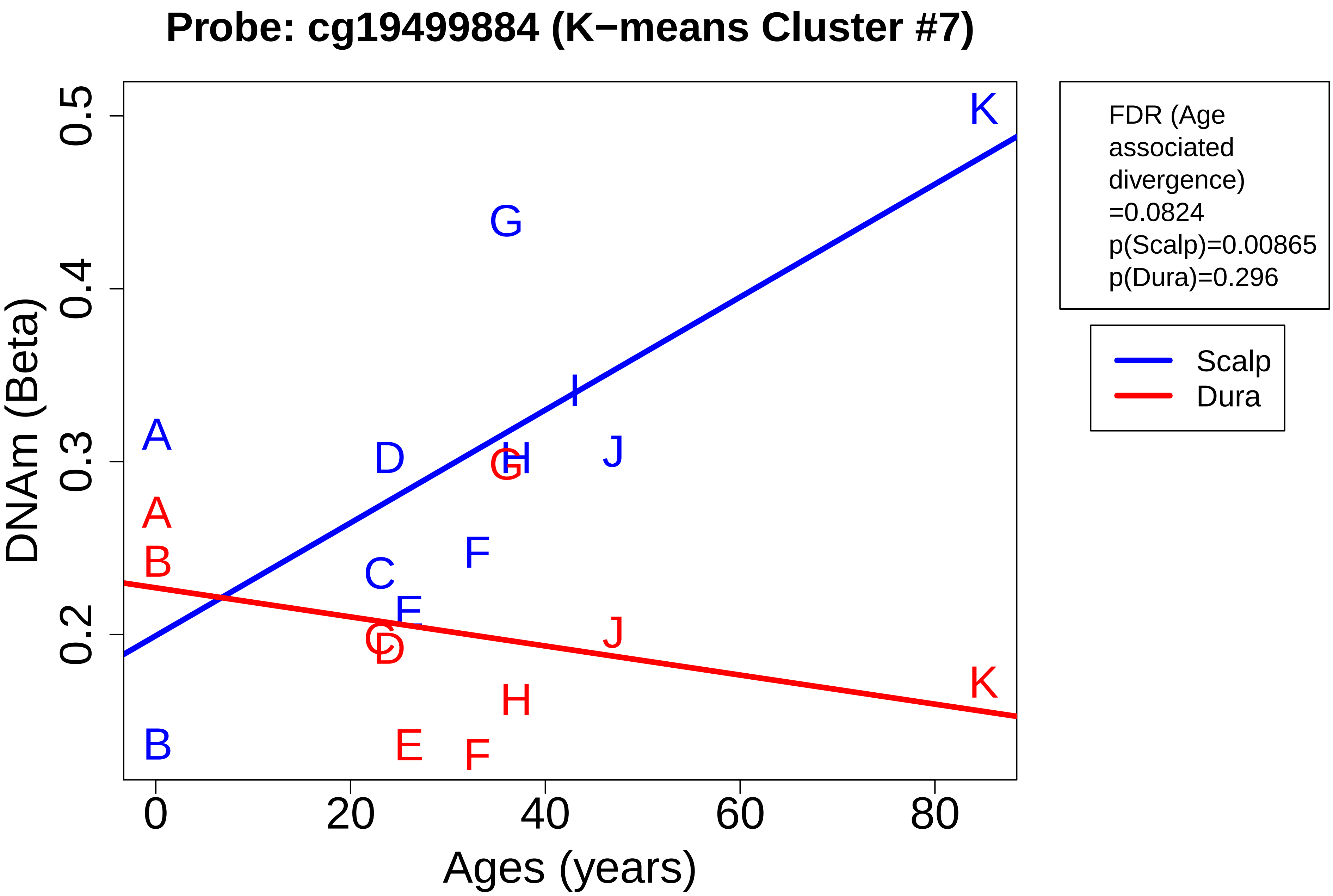

Probe: cg19499884 (K−means Cluster #7)
FDR (Age associated divergence) =0.0824
p(Scalp)=0.00865
p(Dura)=0.296
Scalp
Dura
DNAm (Beta)
Ages (years)
0.2
0.3
0.4
0.5
0
20
40
60
80

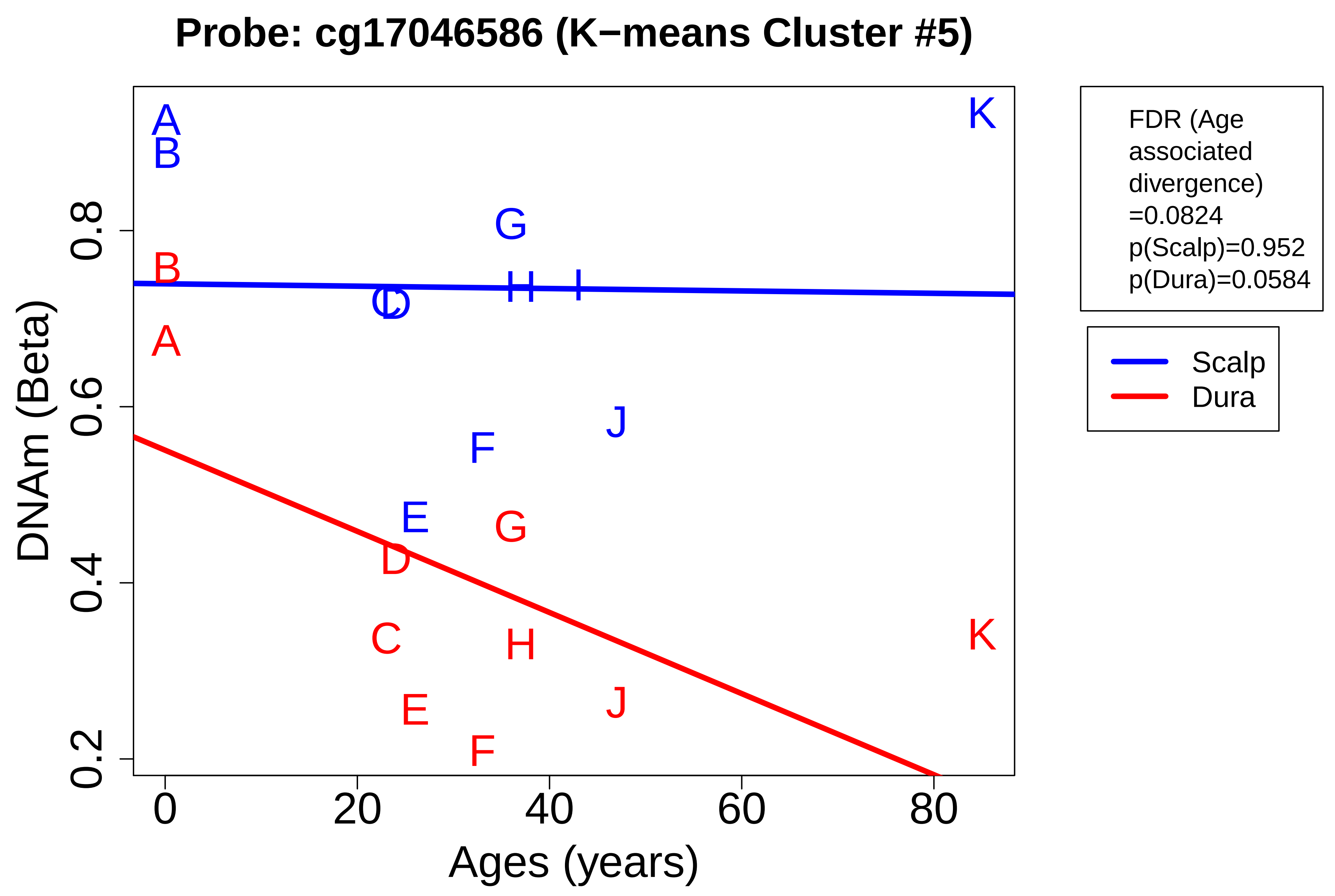

Probe: cg17046586 (K−means Cluster #5)
DNAm (Beta)
0.2
0.4
0.6
0.8
0
20
40
60
80
Ages (years)
FDR (Age associated divergence) =0.0824
p(Scalp)=0.952
p(Dura)=0.0584
Scalp
Dura

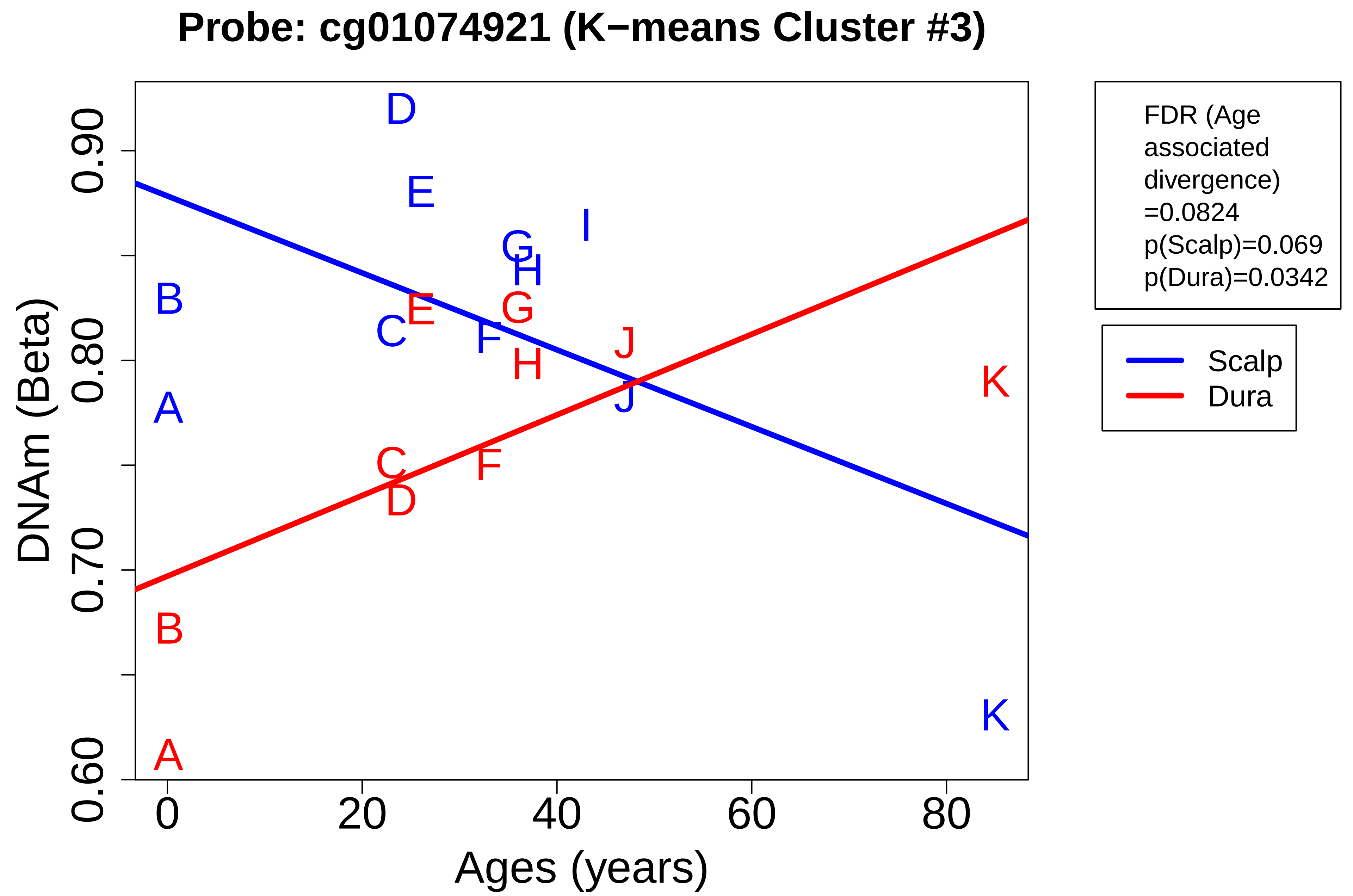

Probe: cg01074921 (K−means Cluster #3)
DNAm (Beta)
0.60
0.70
0.80
0.90
Ages (years)
0
20
40
60
80
FDR (Age associated divergence) =0.0824
p(Scalp)=0.069
p(Dura)=0.0342
Scalp
Dura
A
B
C
D
E
F
G
H
I
J
K

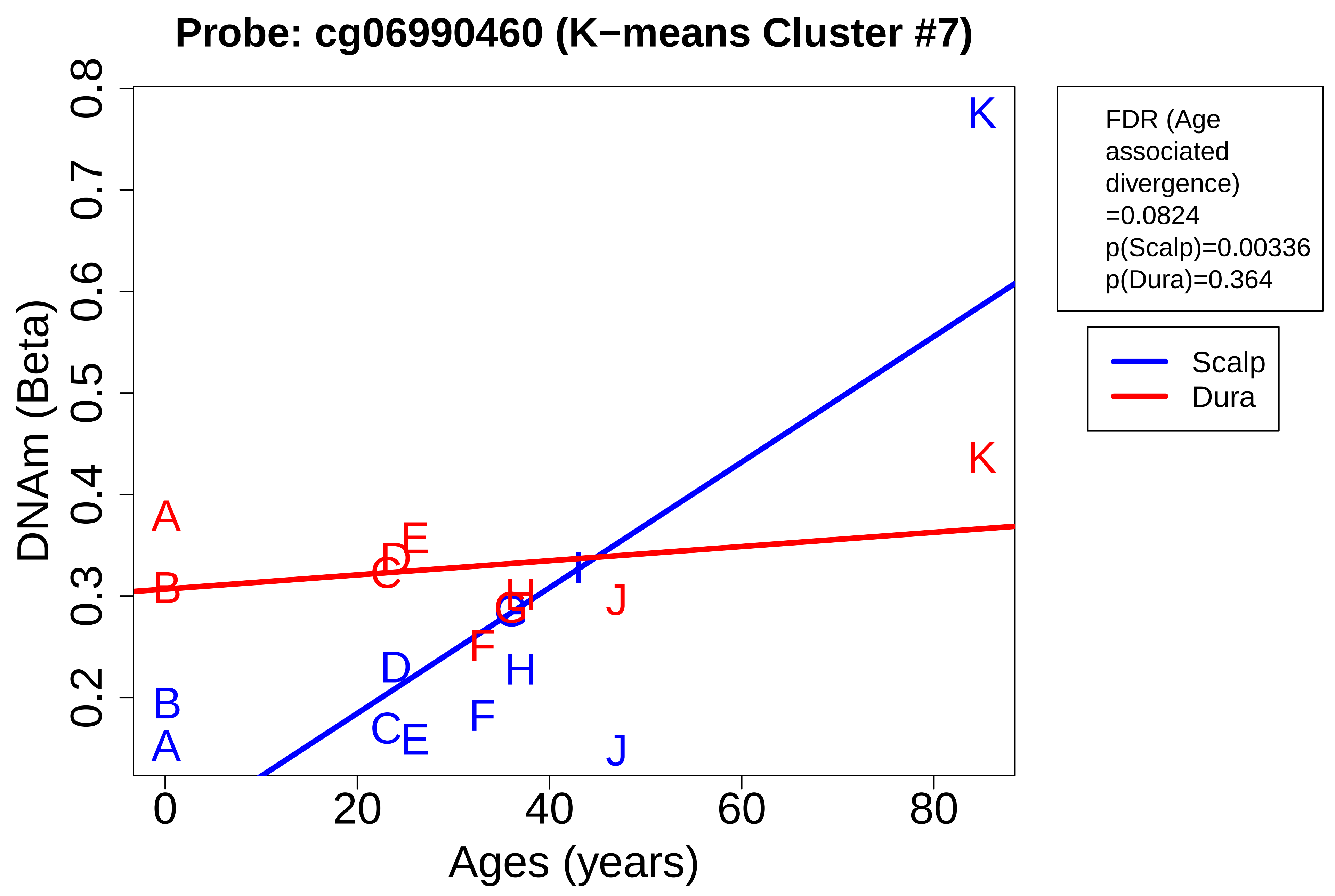

Probe: cg06990460 (K−means Cluster #7)
DNAm (Beta)
0.2
0.3
0.4
0.5
0.6
0.7
0.8
0
20
40
60
80
Ages (years)
FDR (Age associated divergence) =0.0824
p(Scalp)=0.00336
p(Dura)=0.364
Scalp
Dura
A
B
C
D
E
F
G
H
I
J
K

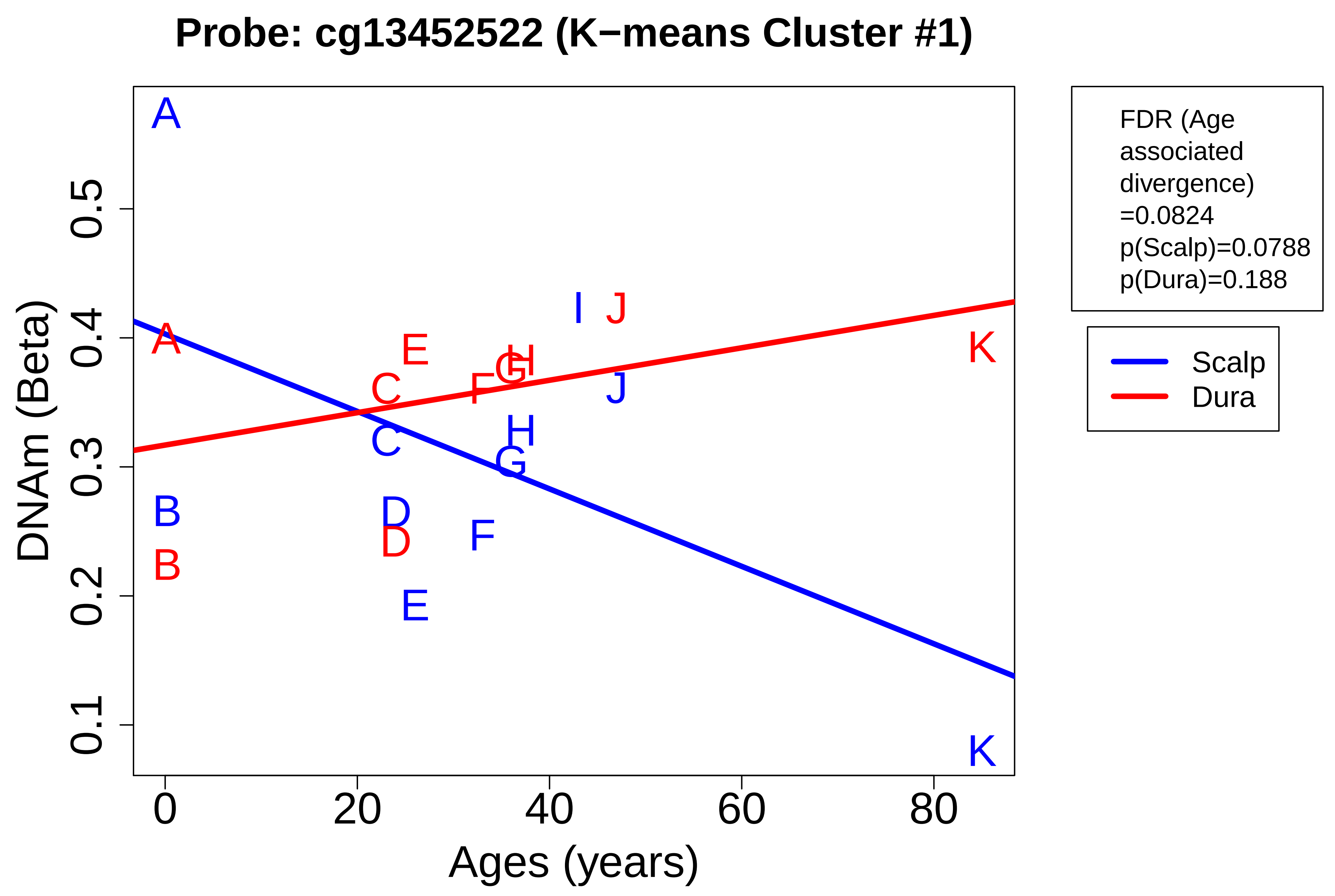
Probe: cg13452522 (K−means Cluster #1)
DNAm (Beta)
0.1
0.2
0.3
0.4
0.5
Ages (years)
0
20
40
60
80
FDR (Age associated divergence) =0.0824
p(Scalp)=0.0788
p(Dura)=0.188
Scalp
Dura

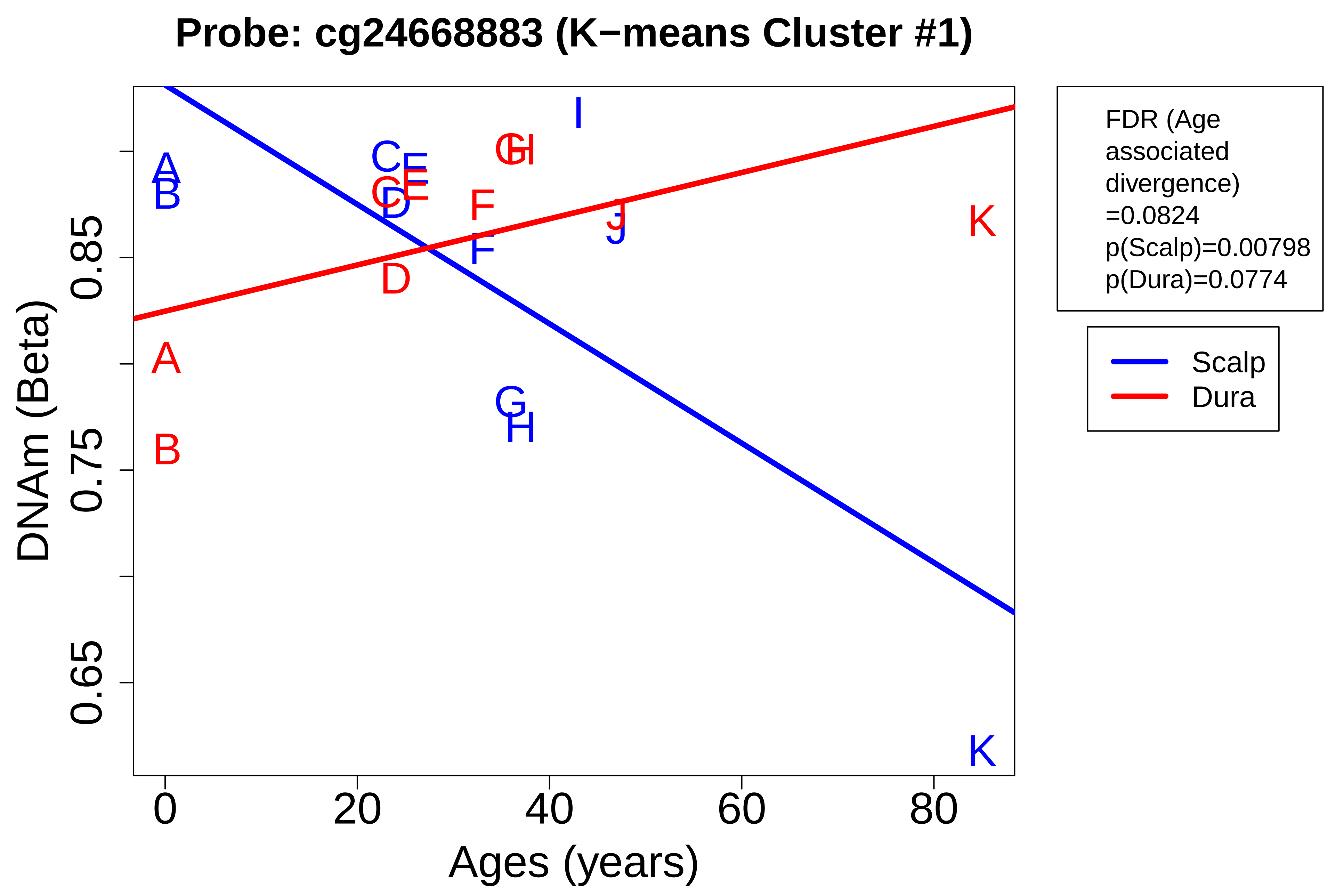

Probe: cg24668883 (K−means Cluster #1)
DNAm (Beta)
0.65
0.75
0.85
0
20
40
60
80
Ages (years)
A
B
C
D
E
F
G
H
I
J
K
FDR (Age associated divergence) =0.0824
p(Scalp)=0.00798
p(Dura)=0.0774
Scalp
Dura

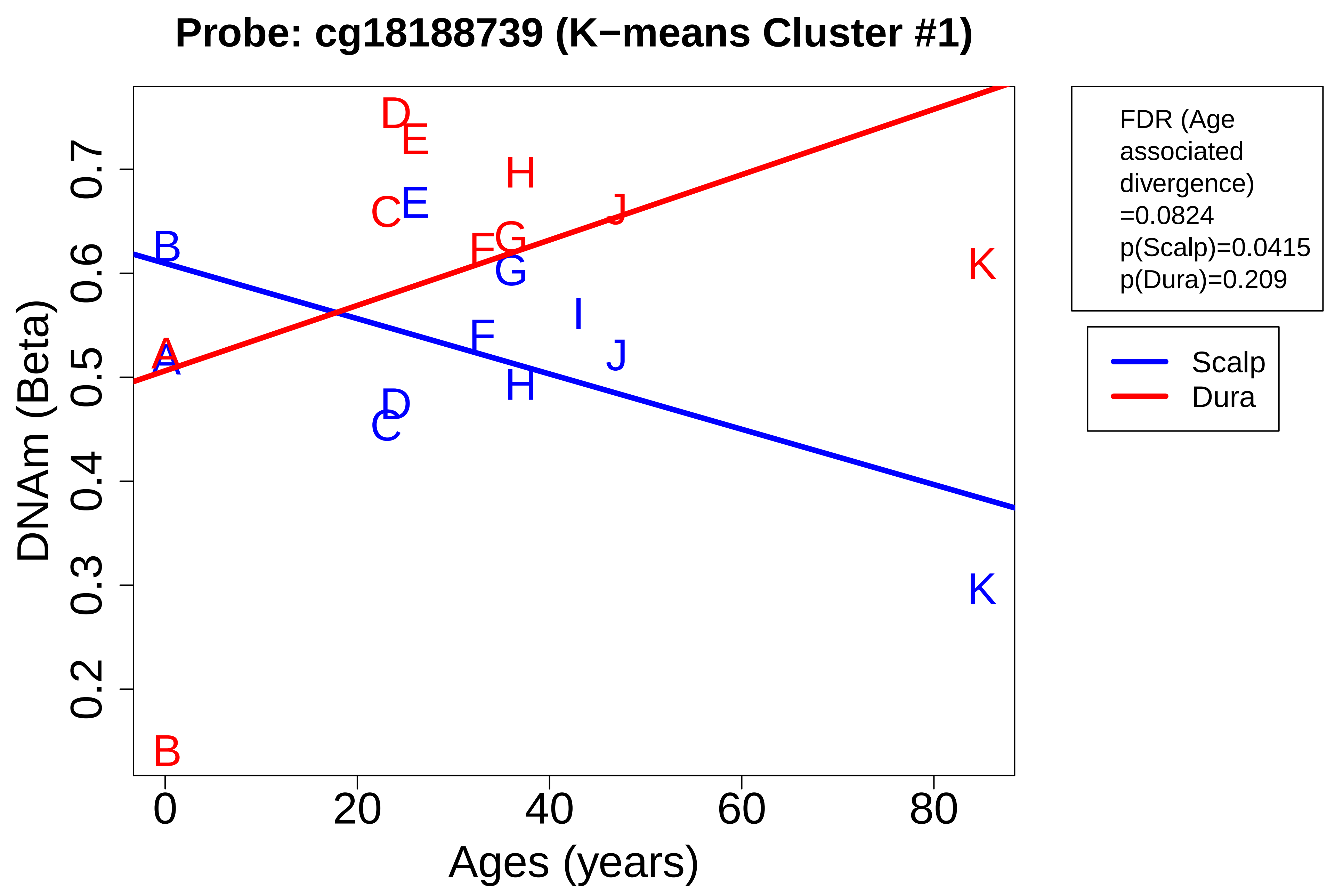

Probe: cg18188739 (K−means Cluster #1)
DNAm (Beta)
0.2
0.3
0.4
0.5
0.6
0.7
0
20
40
60
80
Ages (years)
A
B
C
D
E
F
G
H
I
J
K
FDR (Age associated divergence) =0.0824
p(Scalp)=0.0415
p(Dura)=0.209
Scalp
Dura

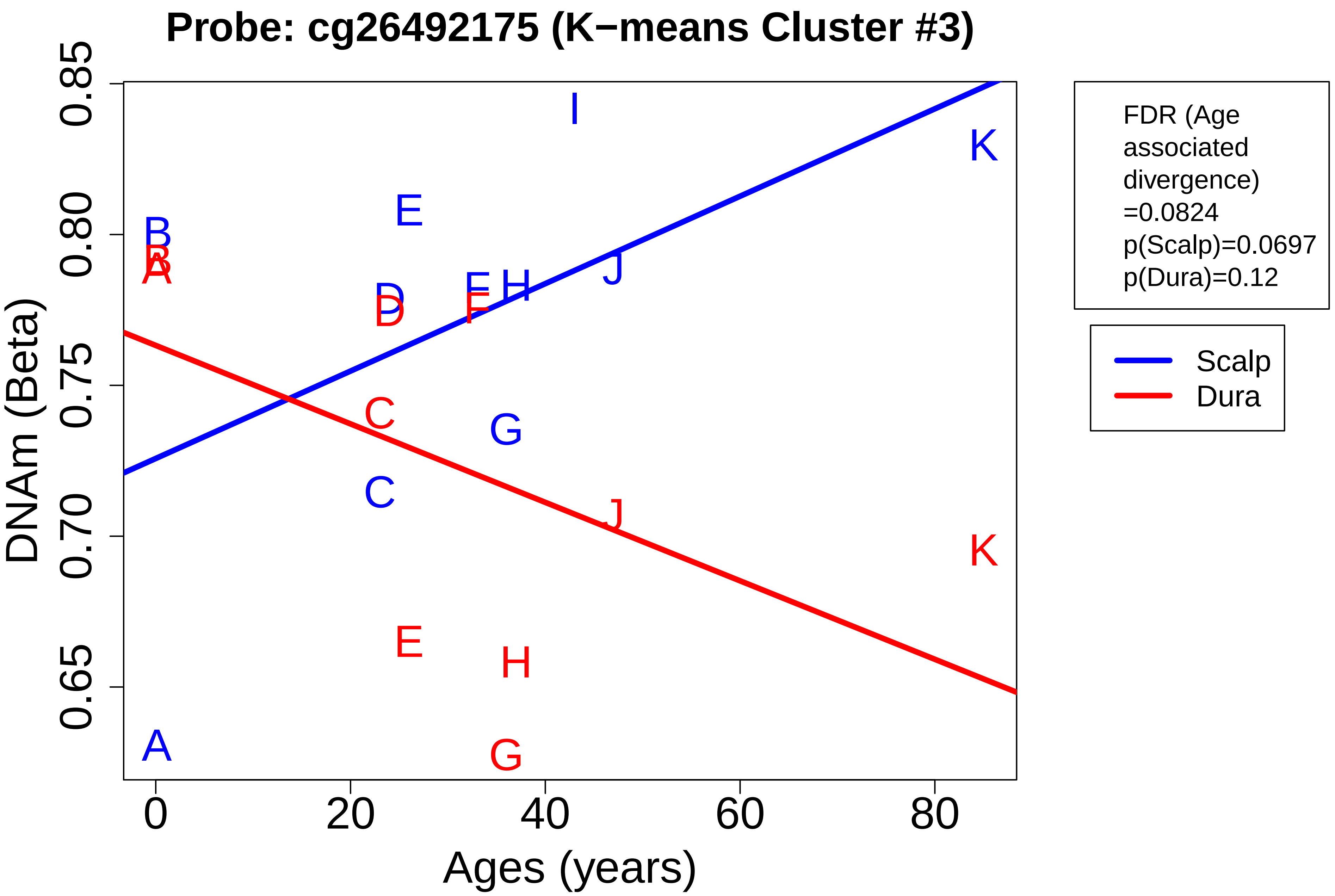

Probe: cg26492175 (K−means Cluster #3)
DNAm (Beta)
0.65
0.70
0.75
0.80
0.85
Ages (years)
0
20
40
60
80
FDR (Age associated divergence) =0.0824
p(Scalp)=0.0697
p(Dura)=0.12
Scalp
Dura

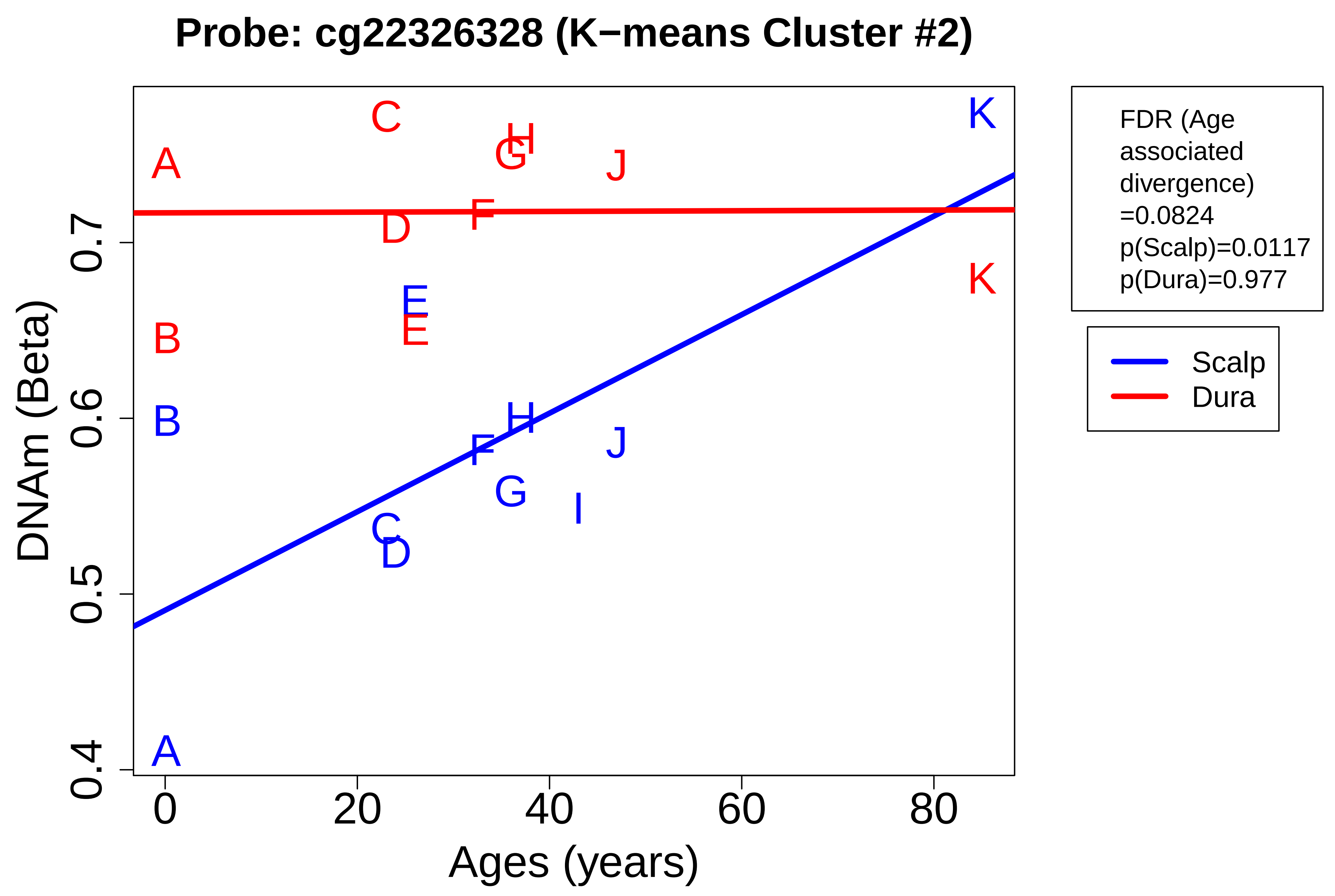

Probe: cg22326328 (K−means Cluster #2)
DNAm (Beta)
0.4
0.5
0.6
0.7
Ages (years)
0
20
40
60
80
A
B
C
D
E
F
G
H
I
J
K
FDR (Age associated divergence) =0.0824
p(Scalp)=0.0117
p(Dura)=0.977
Scalp
Dura

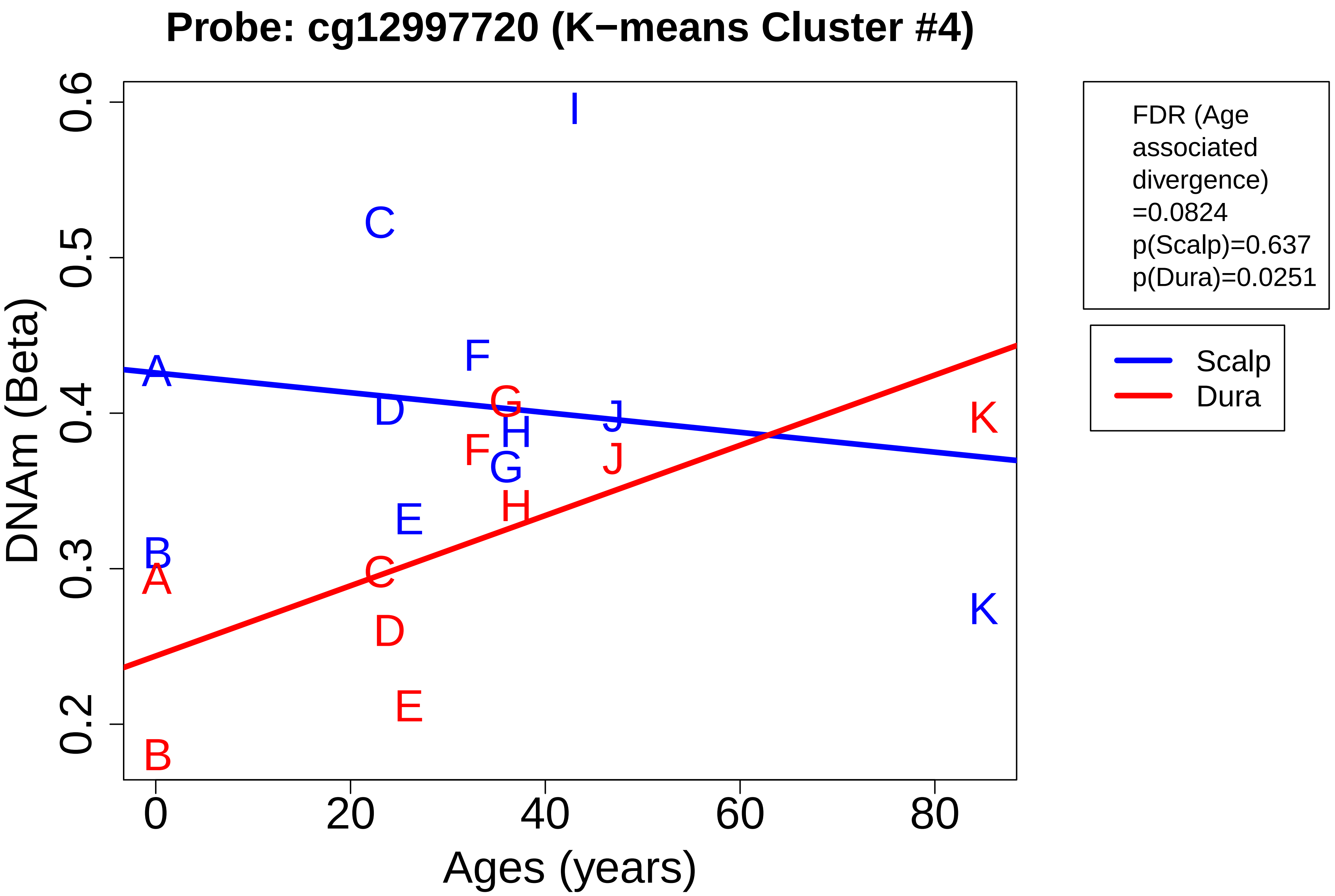

Probe: cg12997720 (K−means Cluster #4)
DNAm (Beta)
0.2
0.3
0.4
0.5
0.6
0
20
40
60
80
Ages (years)
I
C
F
A
G
D
J
H
K
F
G
J
H
E
B
C
A
D
K
E
B
FDR (Age associated divergence) =0.0824
p(Scalp)=0.637
p(Dura)=0.0251
Scalp
Dura

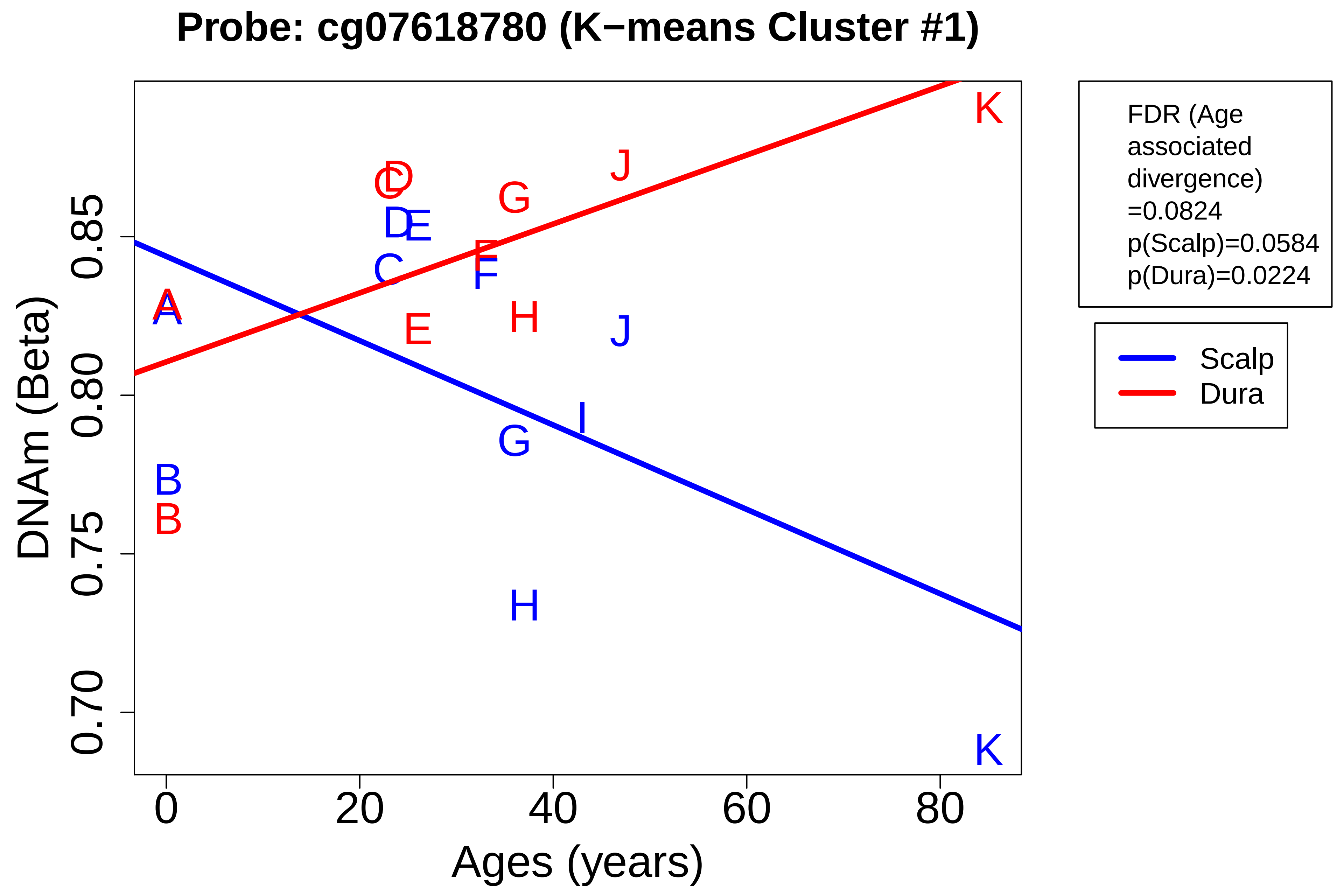

Probe: cg07618780 (K−means Cluster #1)
DNAm (Beta)
0.70
0.75
0.80
0.85
Ages (years)
0
20
40
60
80
A
B
C
D
E
F
G
H
I
J
K
FDR (Age associated divergence) =0.0824
p(Scalp)=0.0584
p(Dura)=0.0224
Scalp
Dura

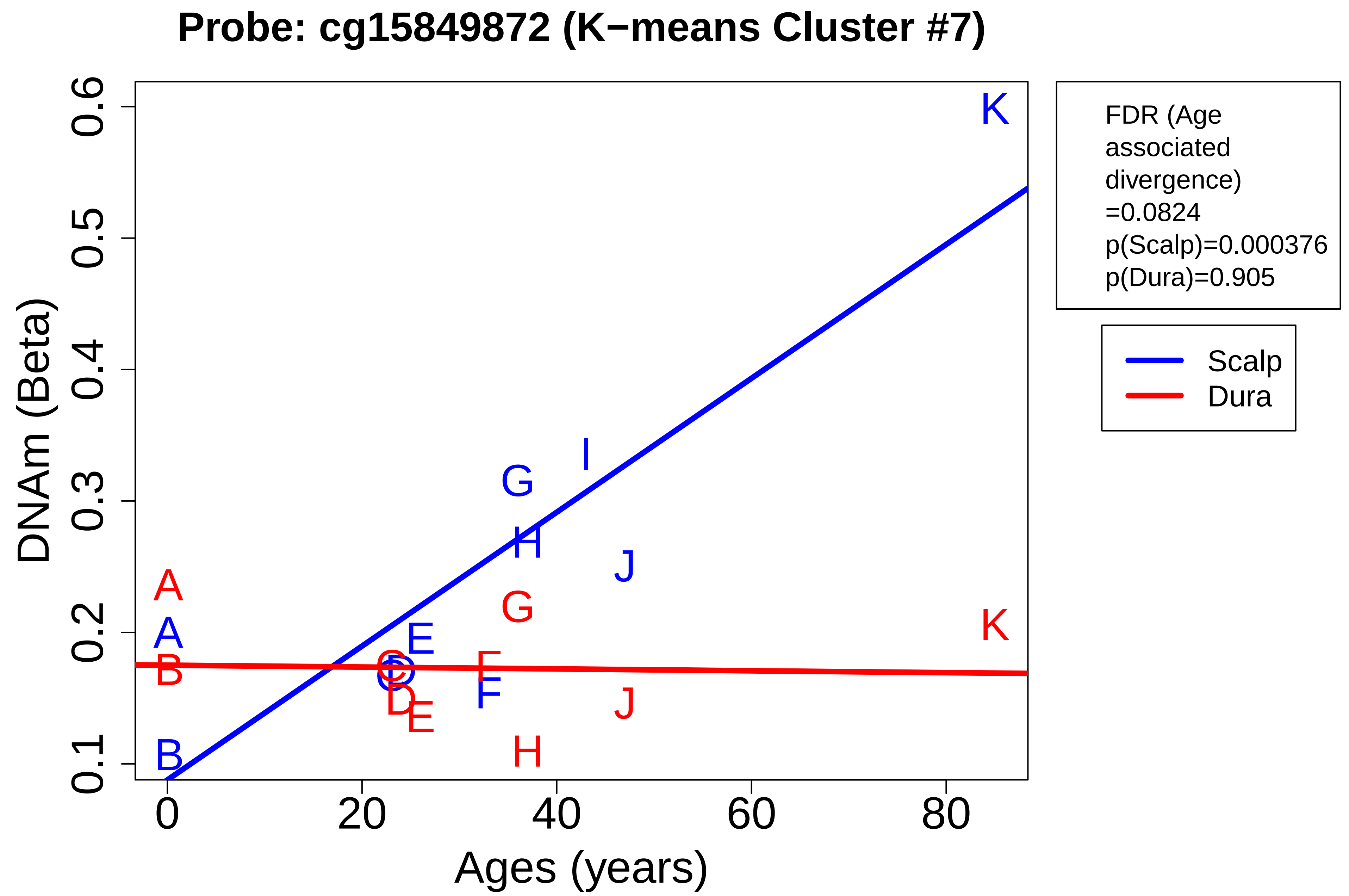

Probe: cg15849872 (K−means Cluster #7)
DNAm (Beta)
Ages (years)
0.1
0.2
0.3
0.4
0.5
0.6
0
20
40
60
80
FDR (Age associated divergence) =0.0824
p(Scalp)=0.000376
p(Dura)=0.905
Scalp
Dura

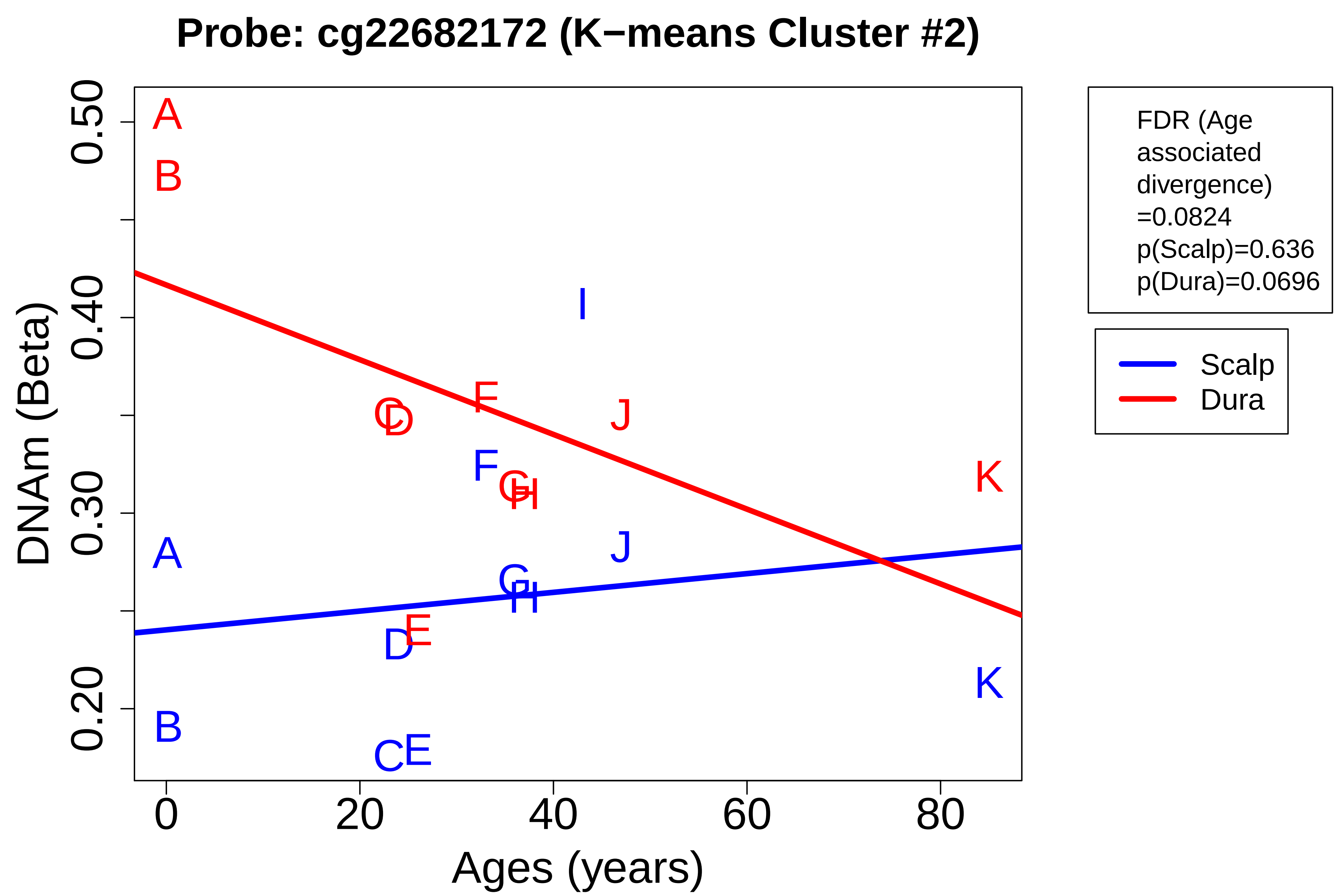

Probe: cg22682172 (K−means Cluster #2)
DNAm (Beta)
0.20
0.30
0.40
0.50
0
20
40
60
80
Ages (years)
A
B
C
D
E
F
G
H
I
J
K
FDR (Age associated divergence) =0.0824
p(Scalp)=0.636
p(Dura)=0.0696
Scalp
Dura

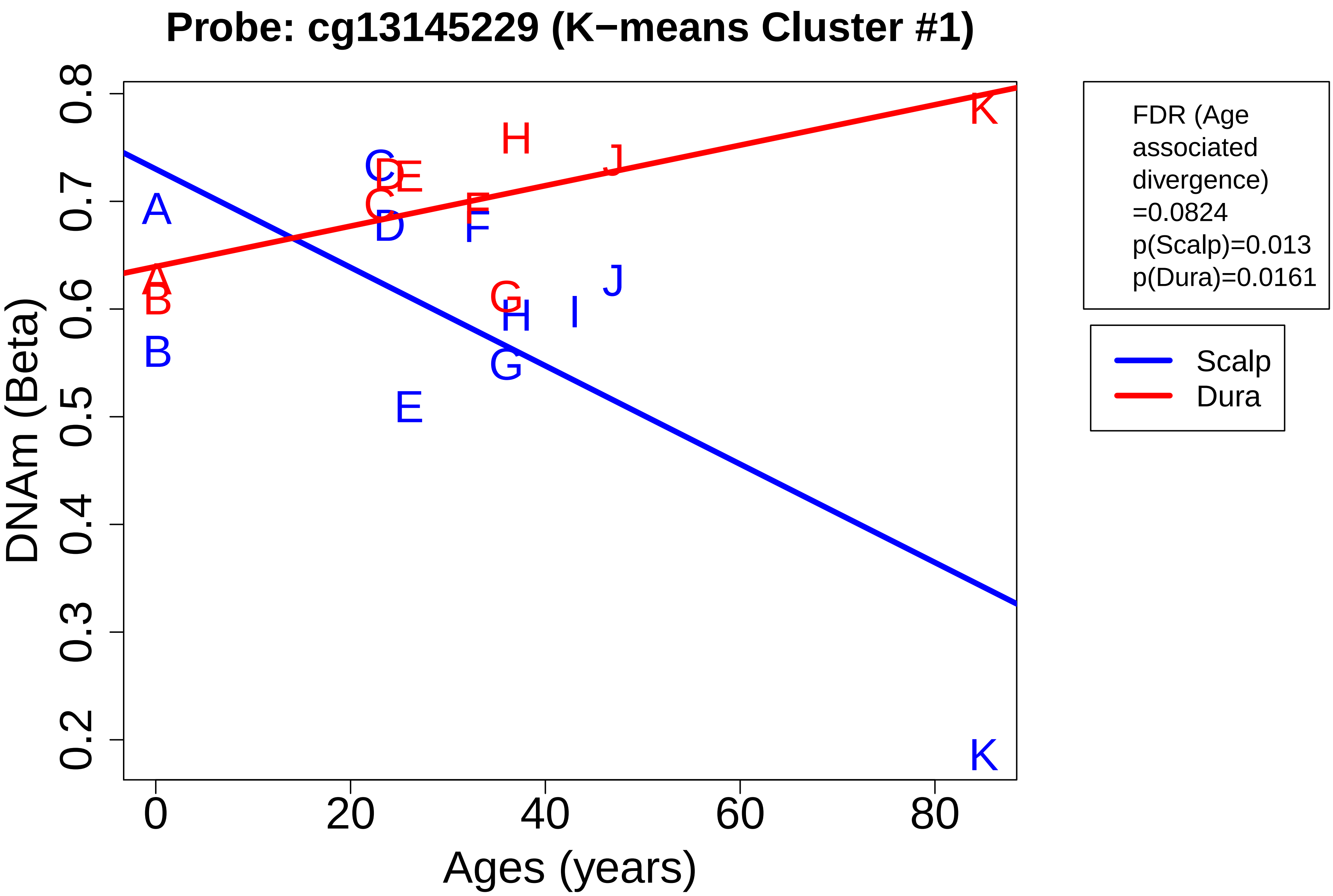

Probe: cg13145229 (K−means Cluster #1)
DNAm (Beta)
0.2
0.3
0.4
0.5
0.6
0.7
0.8
0
20
40
60
80
Ages (years)
A
B
C
D
E
F
G
H
I
J
K
FDR (Age associated divergence) =0.0824
p(Scalp)=0.013
p(Dura)=0.0161
Scalp
Dura

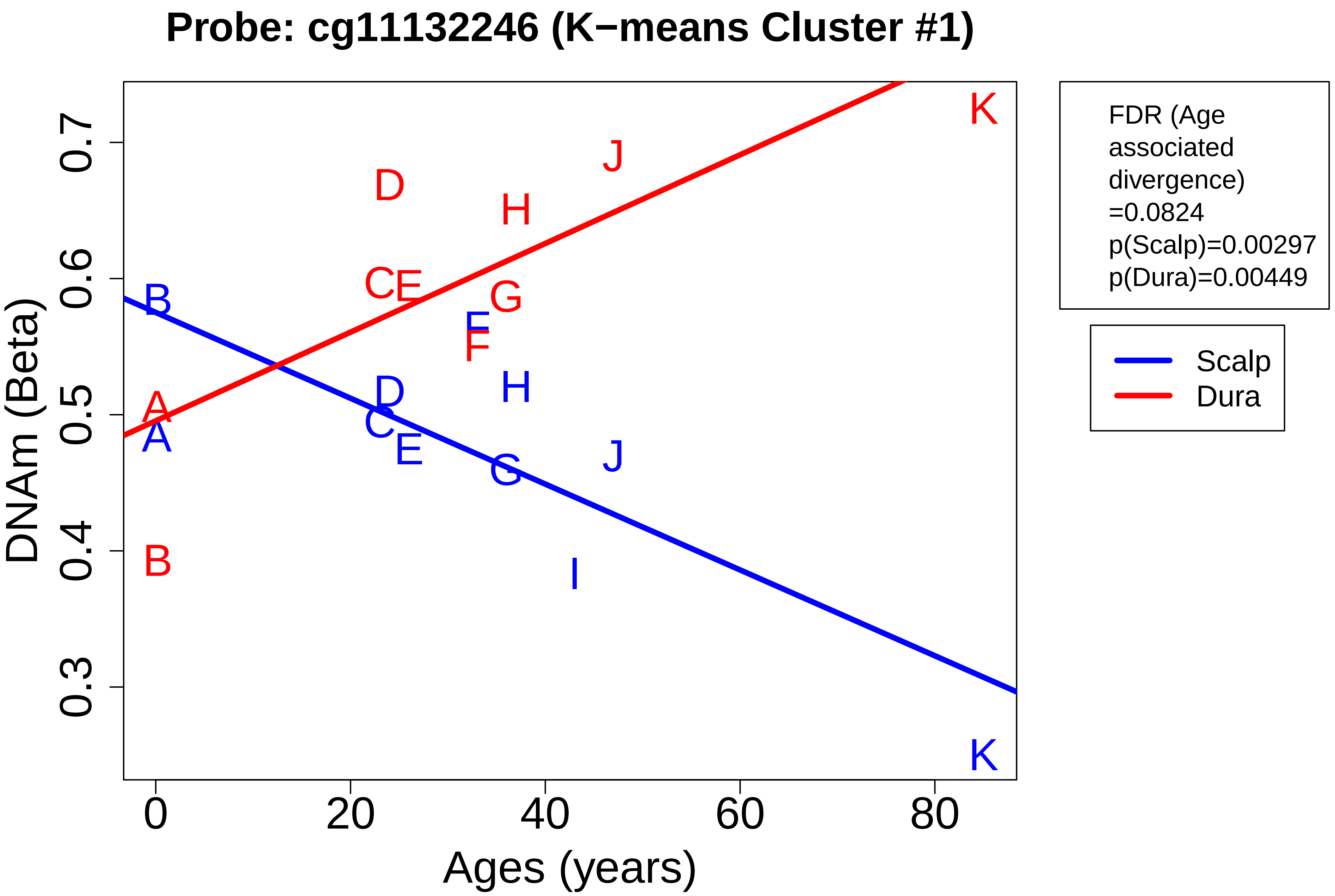

Probe: cg11132246 (K−means Cluster #1)
DNAm (Beta)
Ages (years)
0.3
0.4
0.5
0.6
0.7
0
20
40
60
80
FDR (Age associated divergence) =0.0824
p(Scalp)=0.00297
p(Dura)=0.00449
Scalp
Dura

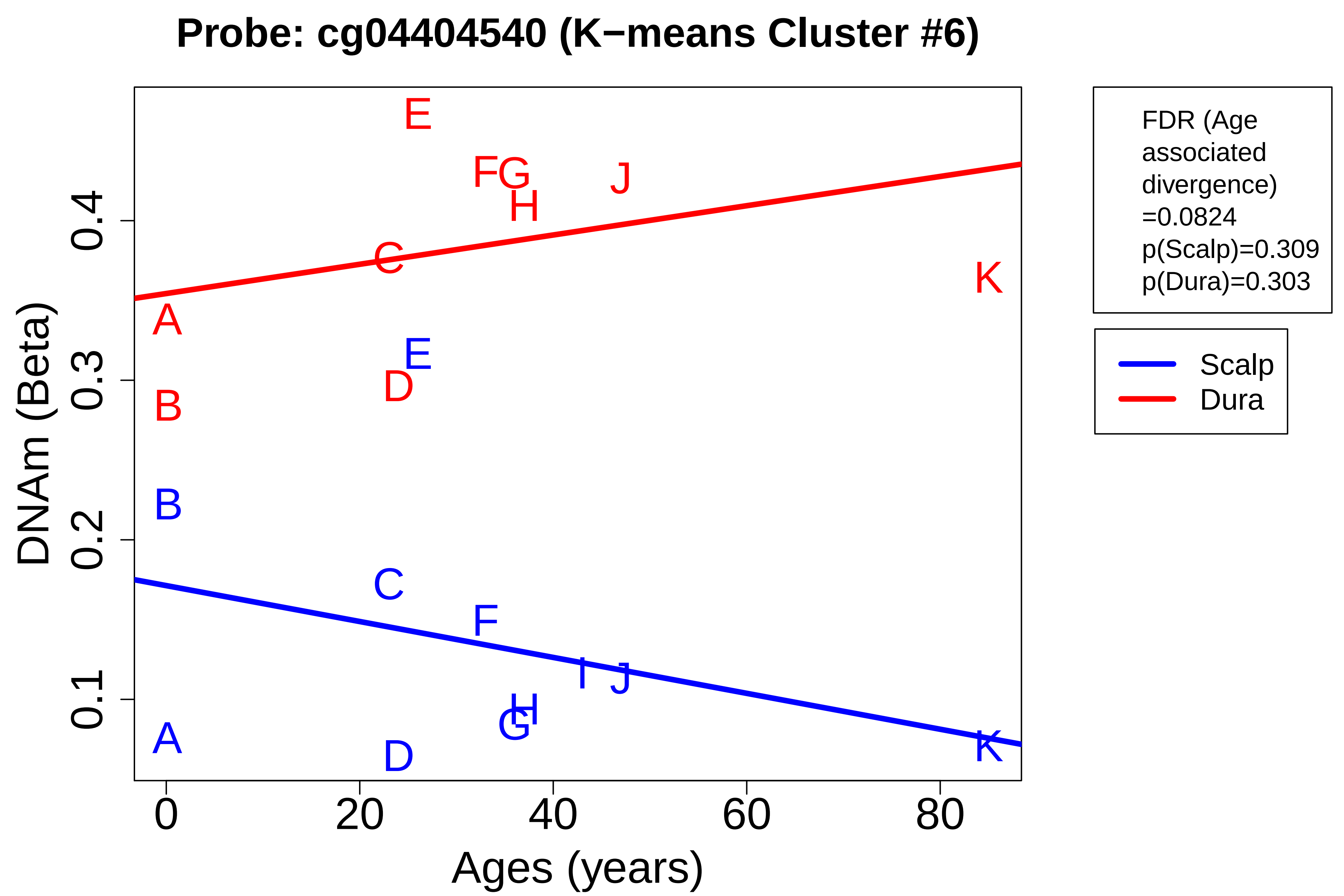
Probe: cg04404540 (K−means Cluster #6)
DNAm (Beta)
0.1
0.2
0.3
0.4
Ages (years)
0
20
40
60
80
FDR (Age associated divergence) =0.0824
p(Scalp)=0.309
p(Dura)=0.303
Scalp
Dura
A
B
C
D
E
F
G
H
I
J
K

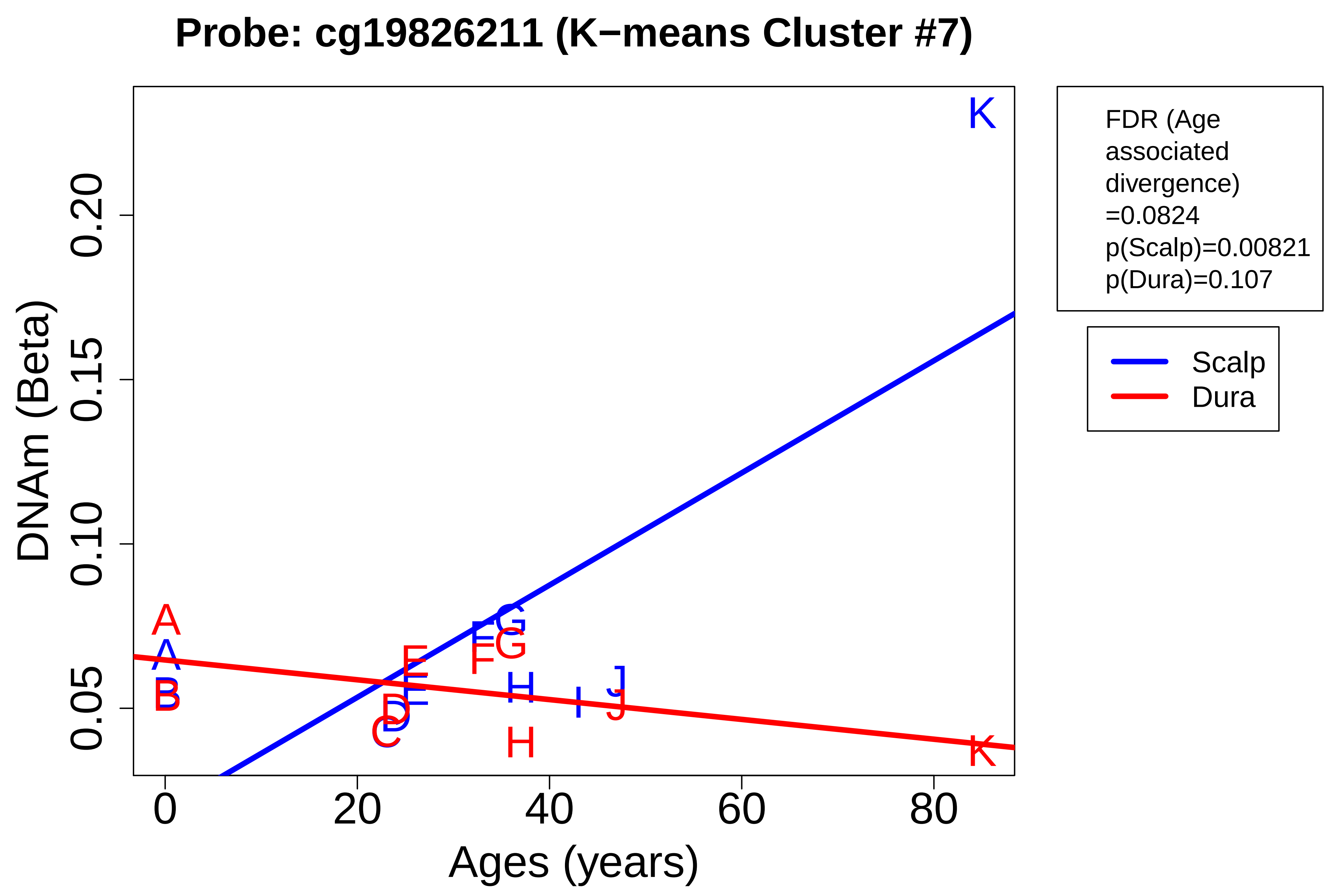

Probe: cg19826211 (K−means Cluster #7)
FDR (Age associated divergence) =0.0824
p(Scalp)=0.00821
p(Dura)=0.107
Scalp
Dura
DNAm (Beta)
0.05
0.10
0.15
0.20
Ages (years)
0
20
40
60
80
A
B
C
D
E
F
G
H
I
J
K

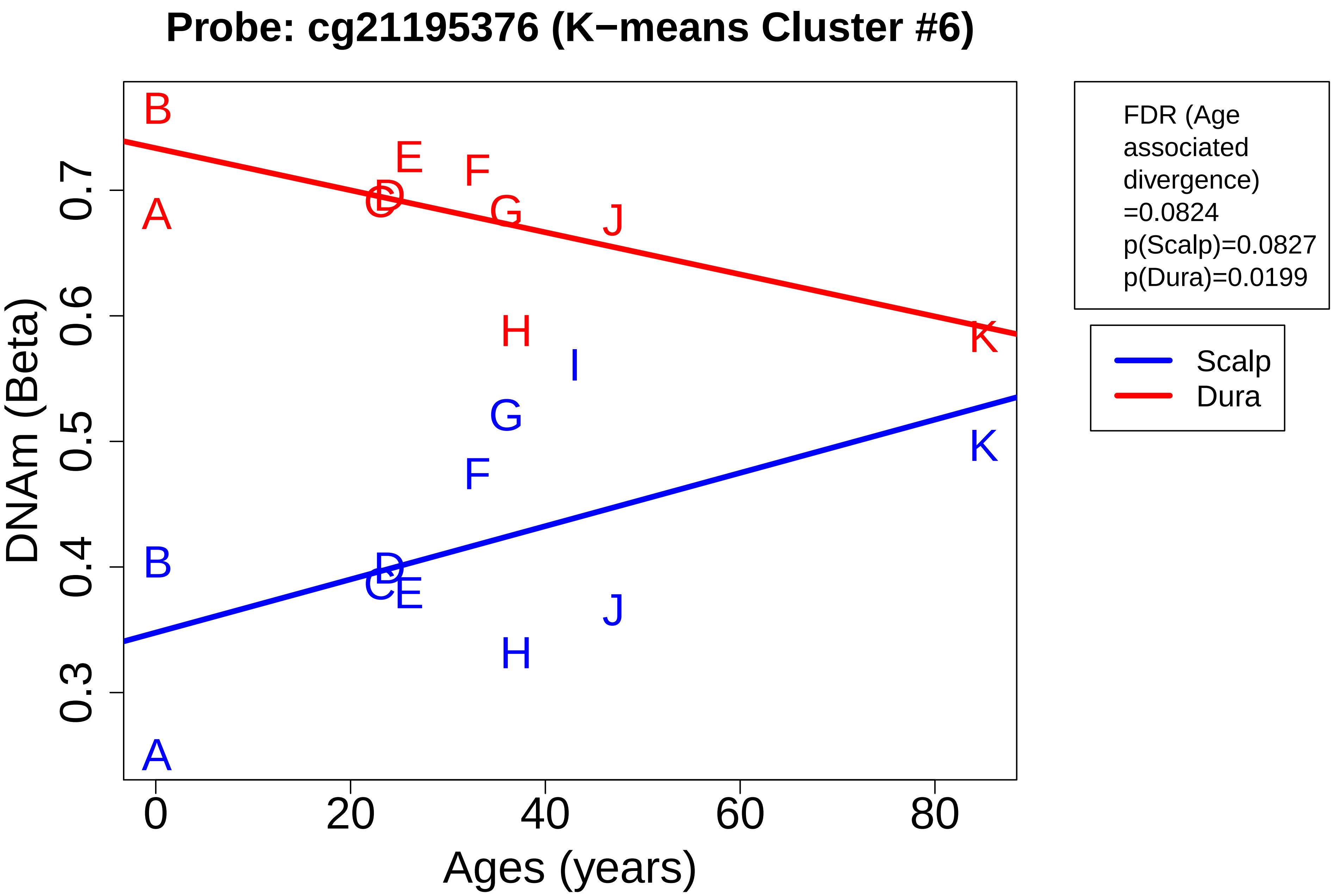
Probe: cg21195376 (K−means Cluster #6)
DNAm (Beta)
Ages (years)
0.3
0.4
0.5
0.6
0.7
0
20
40
60
80
FDR (Age associated divergence) =0.0824
p(Scalp)=0.0827
p(Dura)=0.0199
Scalp
Dura

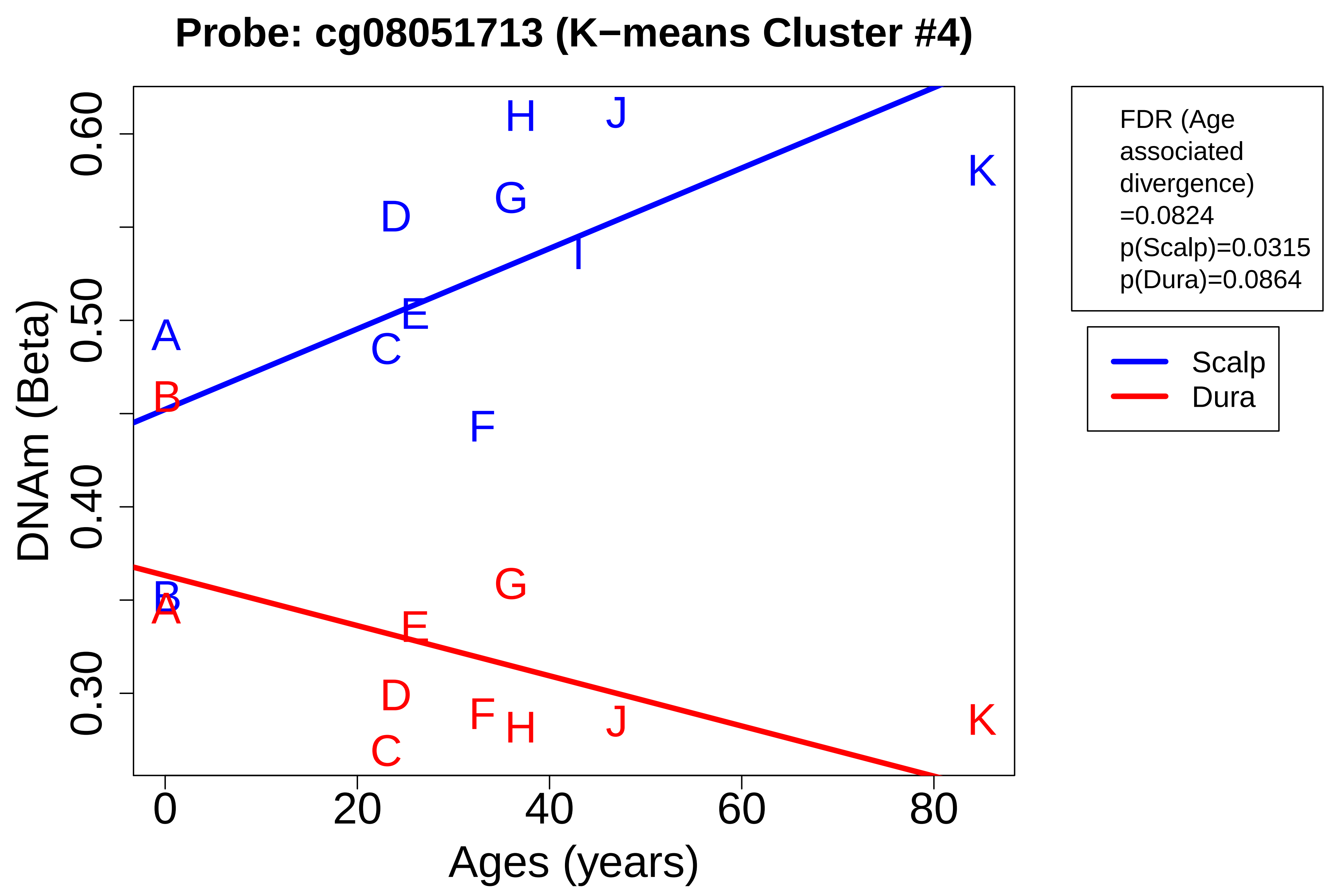

Probe: cg08051713 (K−means Cluster #4)
DNAm (Beta)
0.30
0.40
0.50
0.60
Ages (years)
0
20
40
60
80
A
B
C
D
E
F
G
H
I
J
K
FDR (Age associated divergence) =0.0824
p(Scalp)=0.0315
p(Dura)=0.0864
Scalp
Dura

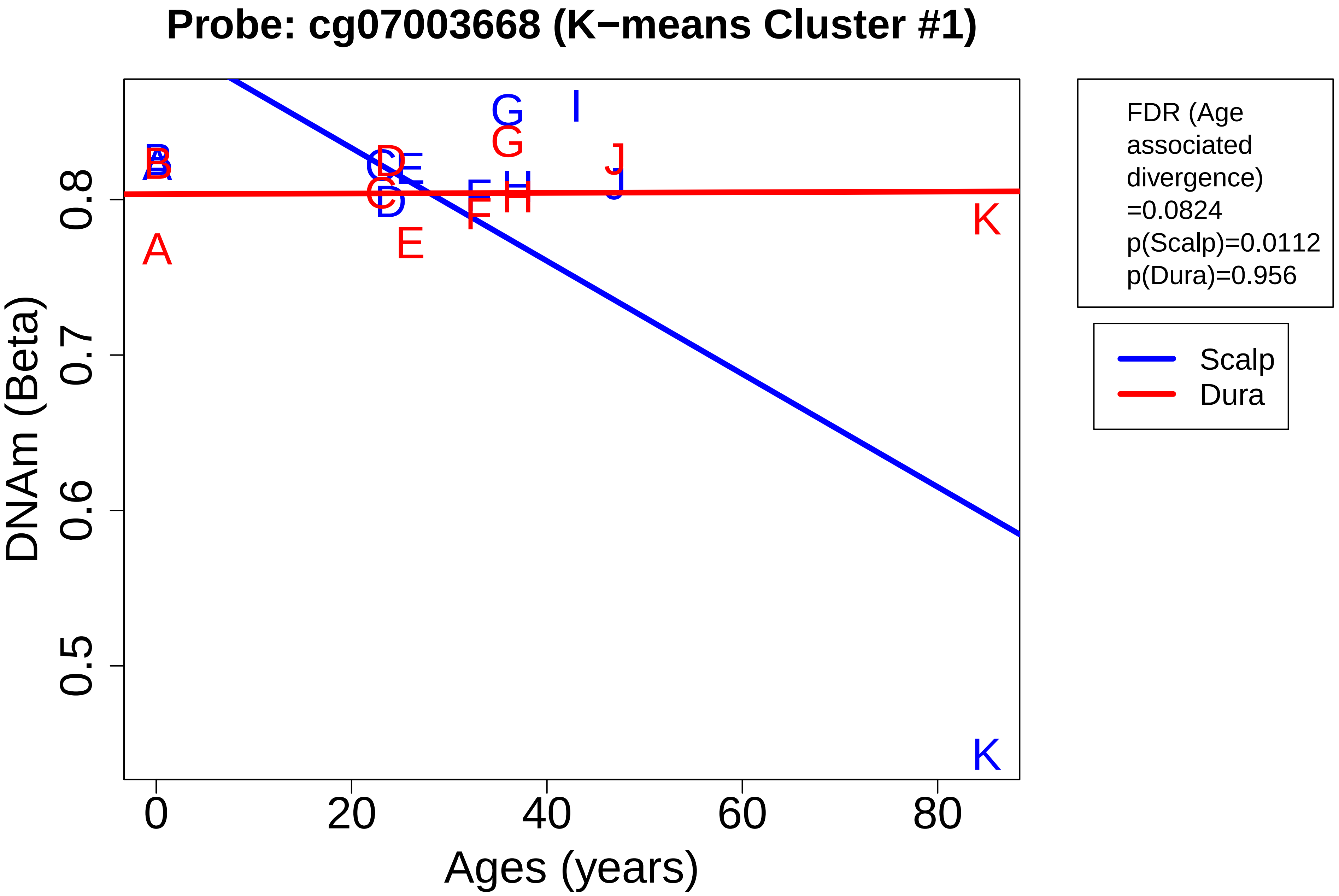

Probe: cg07003668 (K−means Cluster #1)
FDR (Age associated divergence) =0.0824
p(Scalp)=0.0112
p(Dura)=0.956
Scalp
Dura
DNAm (Beta)
0.5
0.6
0.7
0.8
Ages (years)
0
20
40
60
80

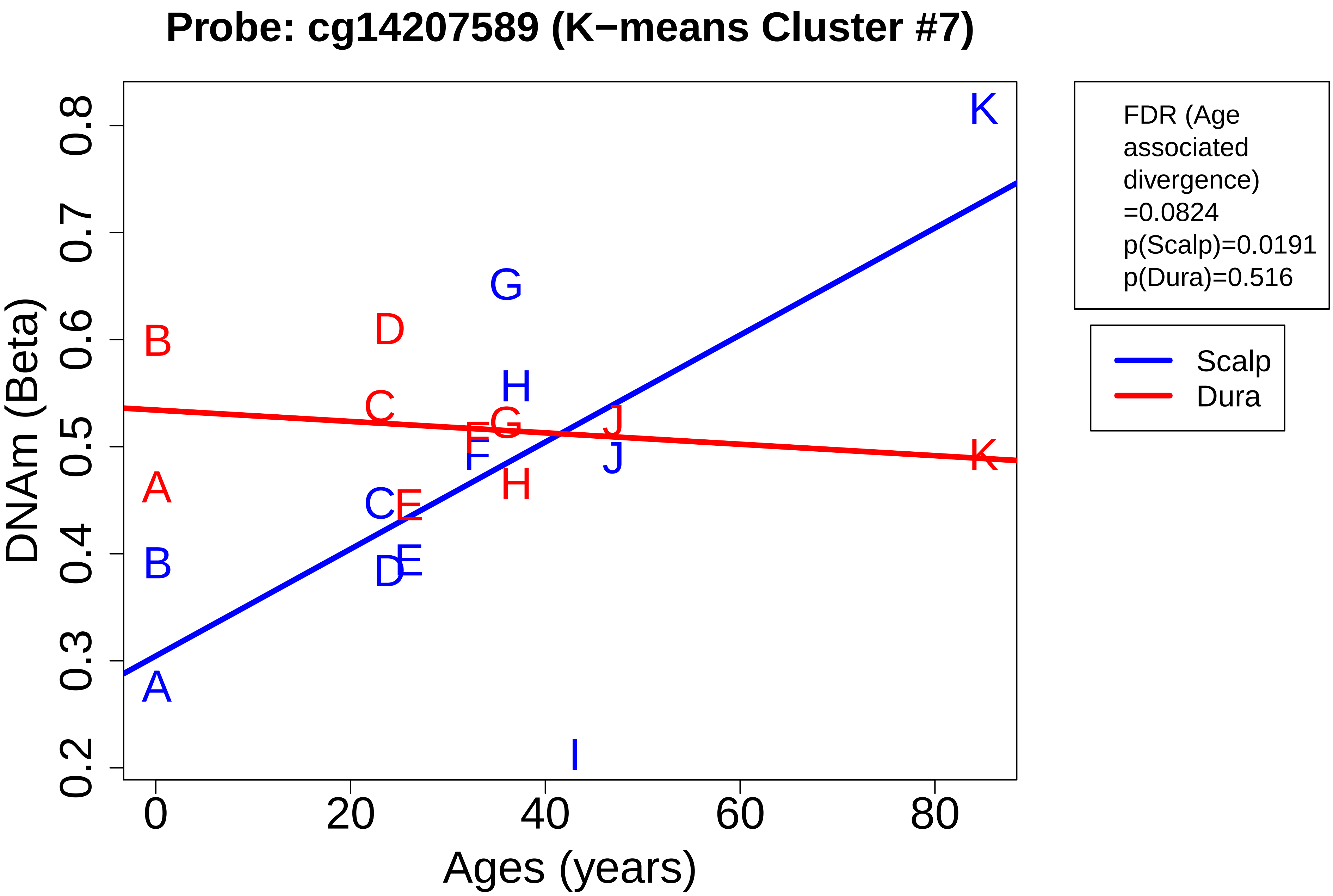

Probe: cg14207589 (K−means Cluster #7)
DNAm (Beta)
0.2
0.3
0.4
0.5
0.6
0.7
0.8
Ages (years)
0
20
40
60
80
A
B
C
D
E
F
G
H
I
J
K
FDR (Age associated divergence) =0.0824
p(Scalp)=0.0191
p(Dura)=0.516
Scalp
Dura

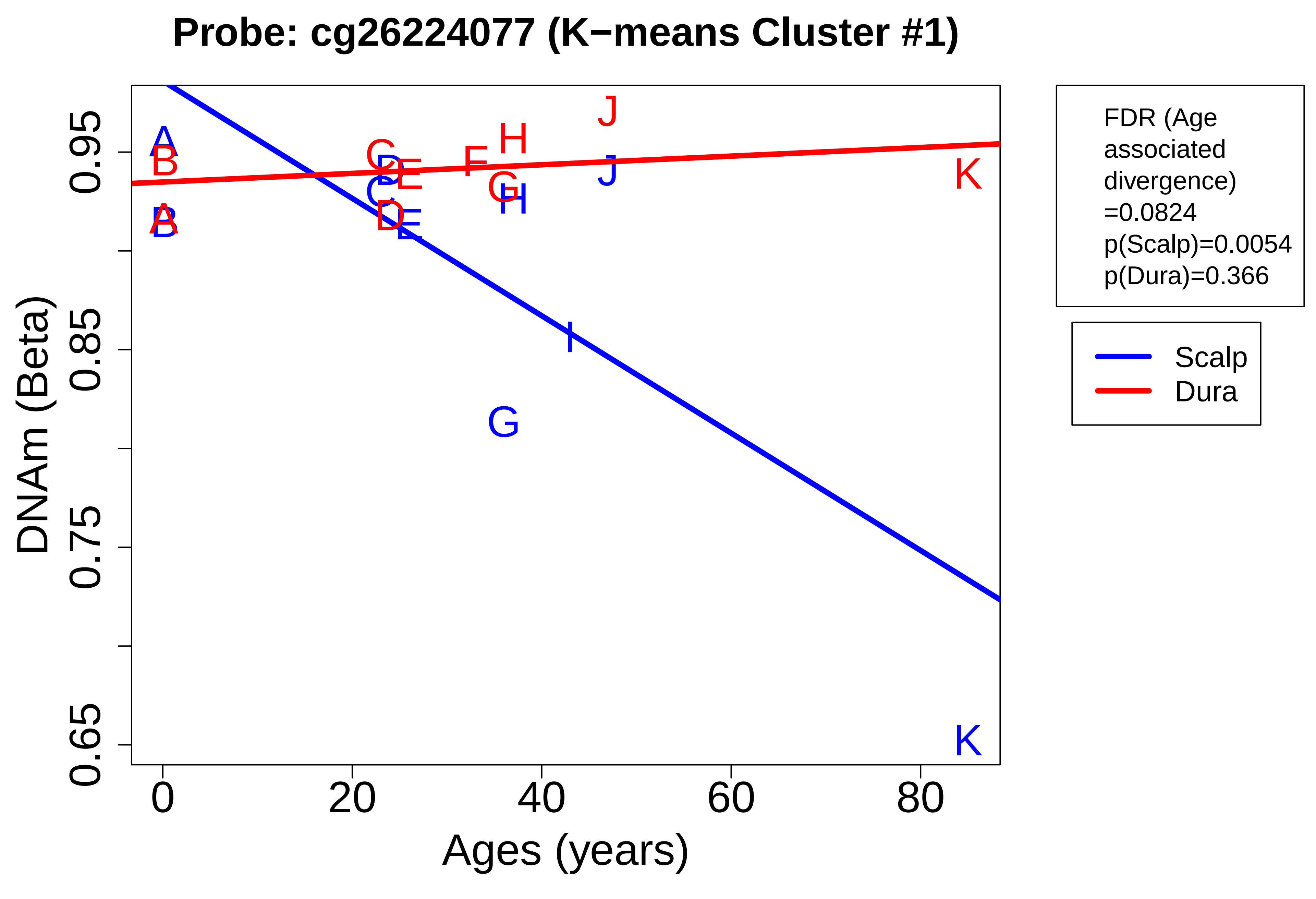

Probe: cg26224077 (K−means Cluster #1)
DNAm (Beta)
0.65
0.75
0.85
0.95
Ages (years)
0
20
40
60
80
FDR (Age associated divergence) =0.0824
p(Scalp)=0.0054
p(Dura)=0.366
Scalp
Dura
A
B
C
D
E
F
G
H
I
J
K

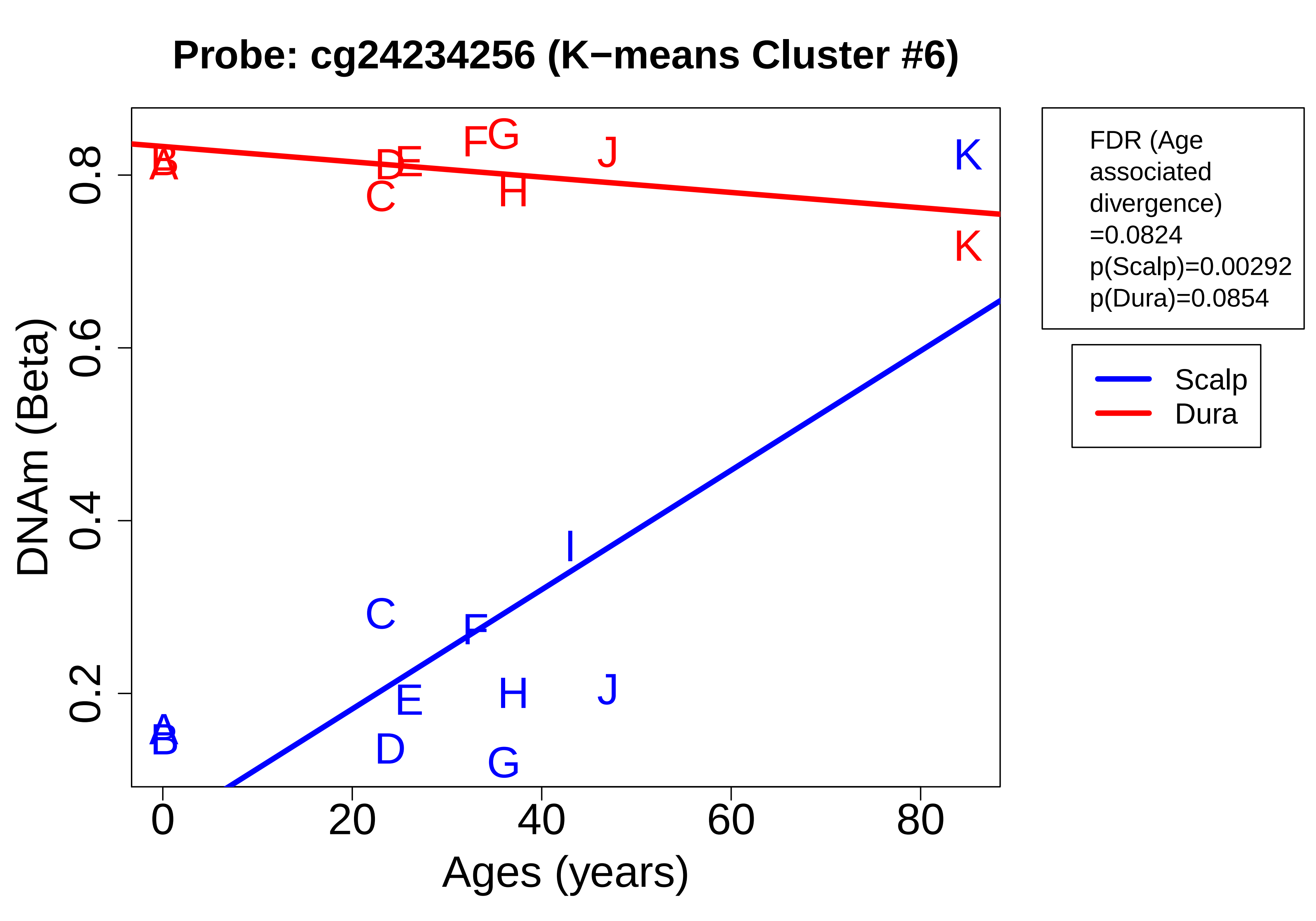

Probe: cg24234256 (K−means Cluster #6)
DNAm (Beta)
0.2
0.4
0.6
0.8
Ages (years)
0
20
40
60
80
A
B
C
D
E
F
G
H
I
J
K
FDR (Age associated divergence) =0.0824
p(Scalp)=0.00292
p(Dura)=0.0854
Scalp
Dura

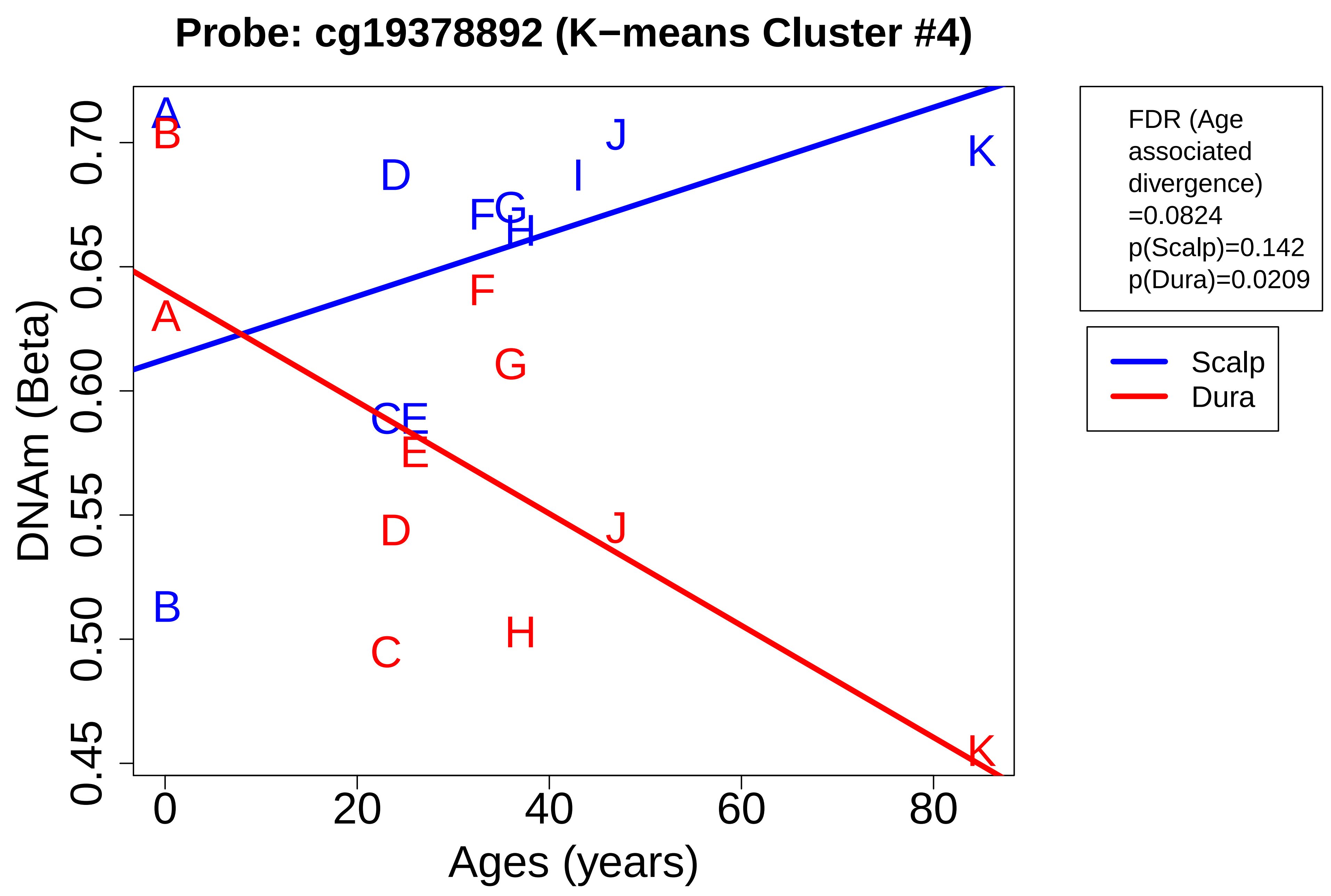

Probe: cg19378892 (K−means Cluster #4)
DNAm (Beta)
0.45
0.50
0.55
0.60
0.65
0.70
0
20
40
60
80
Ages (years)
A
B
A
B
C
D
E
F
G
H
I
J
K
C
D
E
F
G
H
J
K
FDR (Age associated divergence) =0.0824
p(Scalp)=0.142
p(Dura)=0.0209
Scalp
Dura

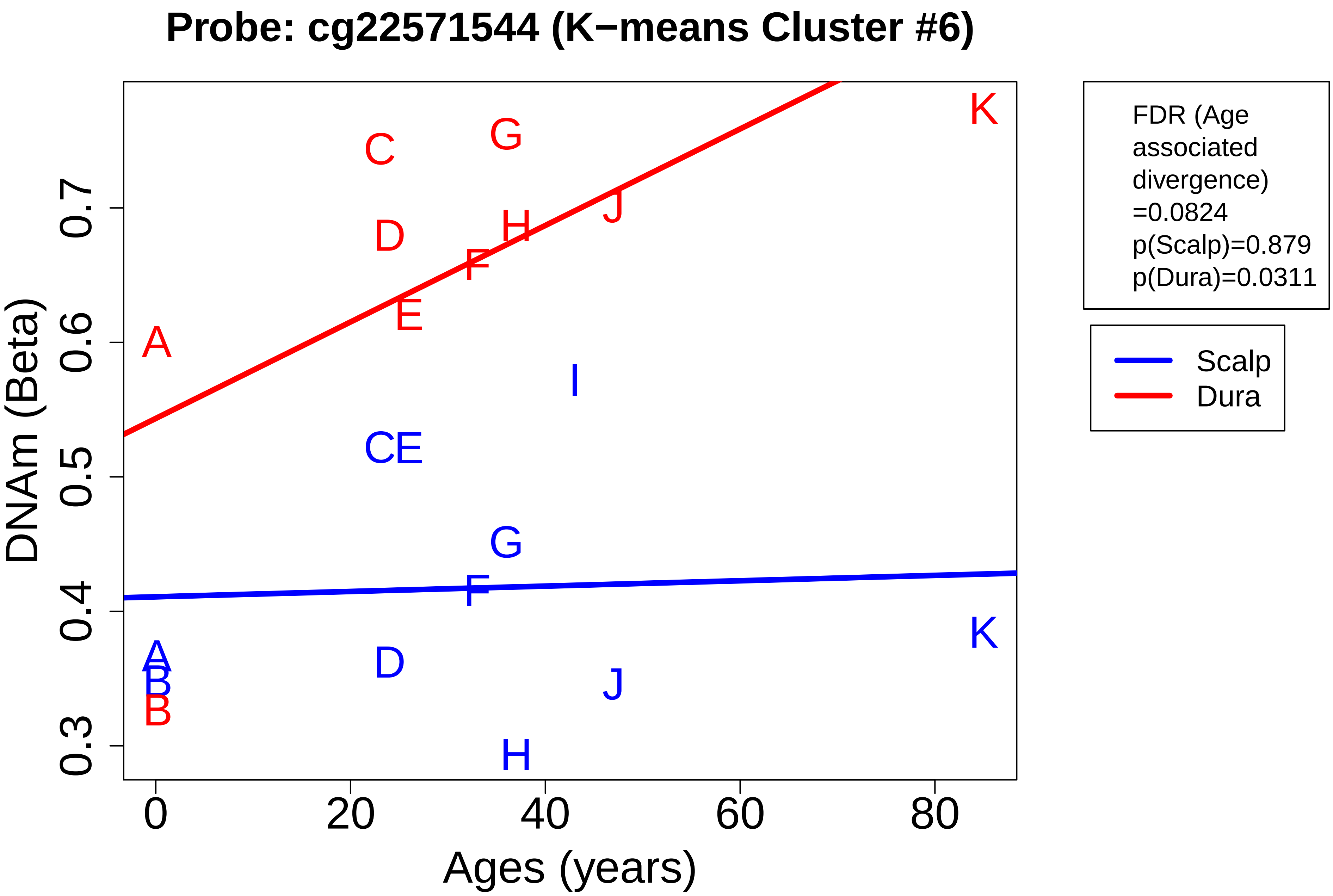
Probe: cg22571544 (K−means Cluster #6)
DNAm (Beta)
0.3
0.4
0.5
0.6
0.7
0
20
40
60
80
Ages (years)
FDR (Age associated divergence) =0.0824
p(Scalp)=0.879
p(Dura)=0.0311
Scalp
Dura
A
B
C
D
E
F
G
H
I
J
K
CE

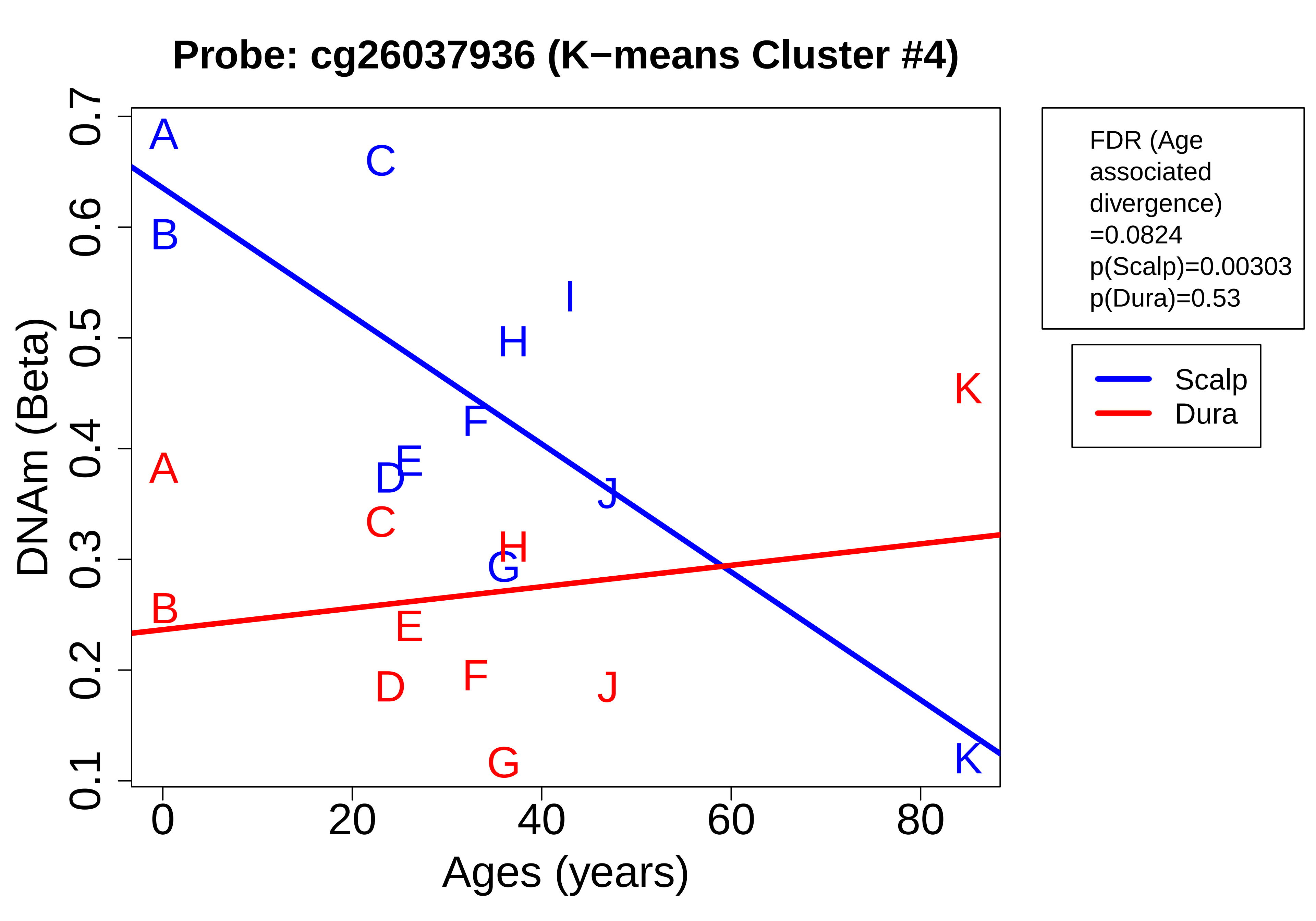

Probe: cg26037936 (K−means Cluster #4)
DNAm (Beta)
0.1
0.2
0.3
0.4
0.5
0.6
0.7
Ages (years)
0
20
40
60
80
A
B
C
D
E
F
G
H
I
J
K
FDR (Age associated divergence) =0.0824
p(Scalp)=0.00303
p(Dura)=0.53
Scalp
Dura

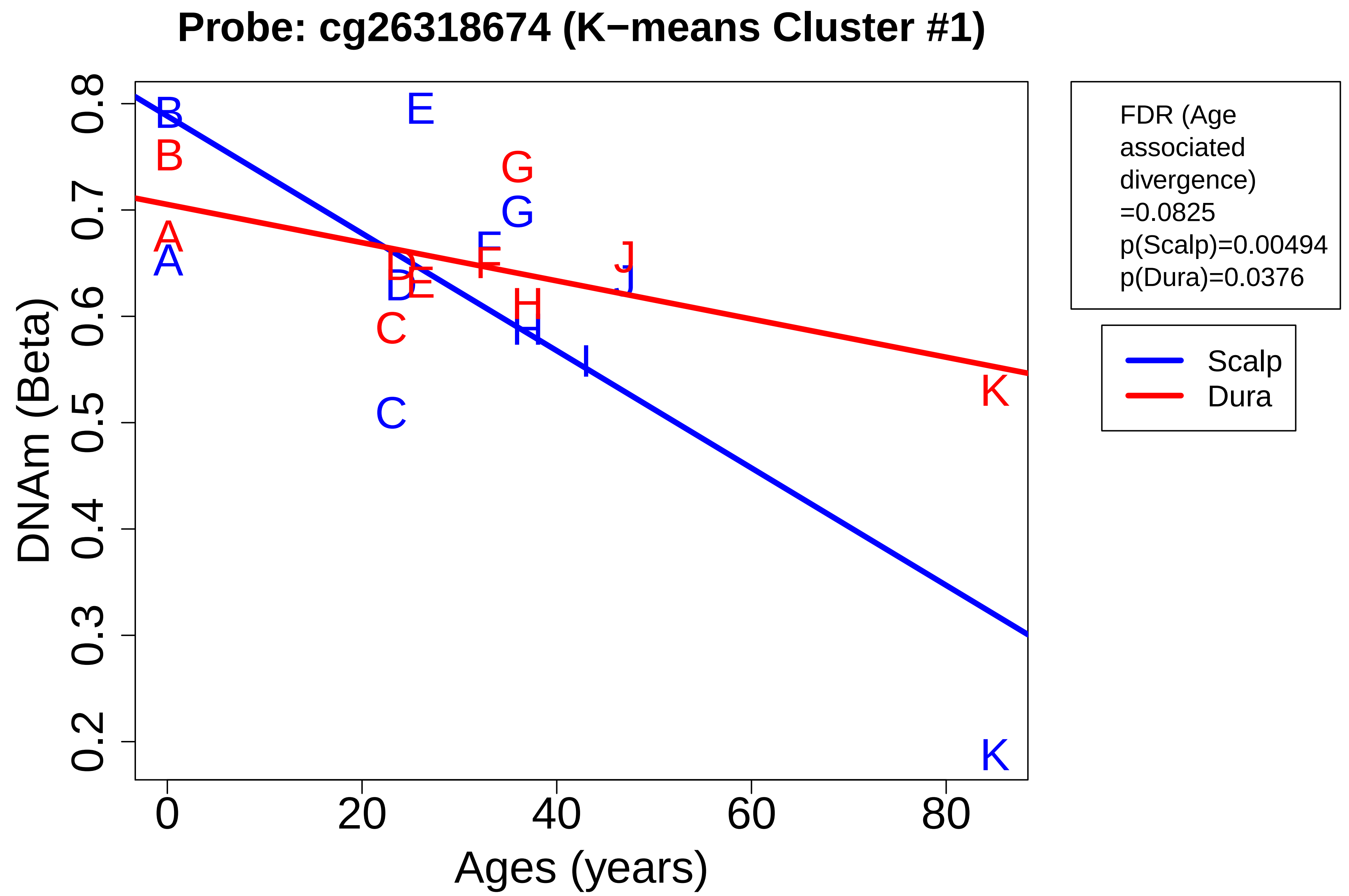

Probe: cg26318674 (K−means Cluster #1)
DNAm (Beta)
0.2
0.3
0.4
0.5
0.6
0.7
0.8
0
20
40
60
80
Ages (years)
B
E
B
G
G
A
A
F
J
D
E
F
D
E
J
H
C
H
I
K
C
K
FDR (Age associated divergence) =0.0825
p(Scalp)=0.00494
p(Dura)=0.0376
Scalp
Dura

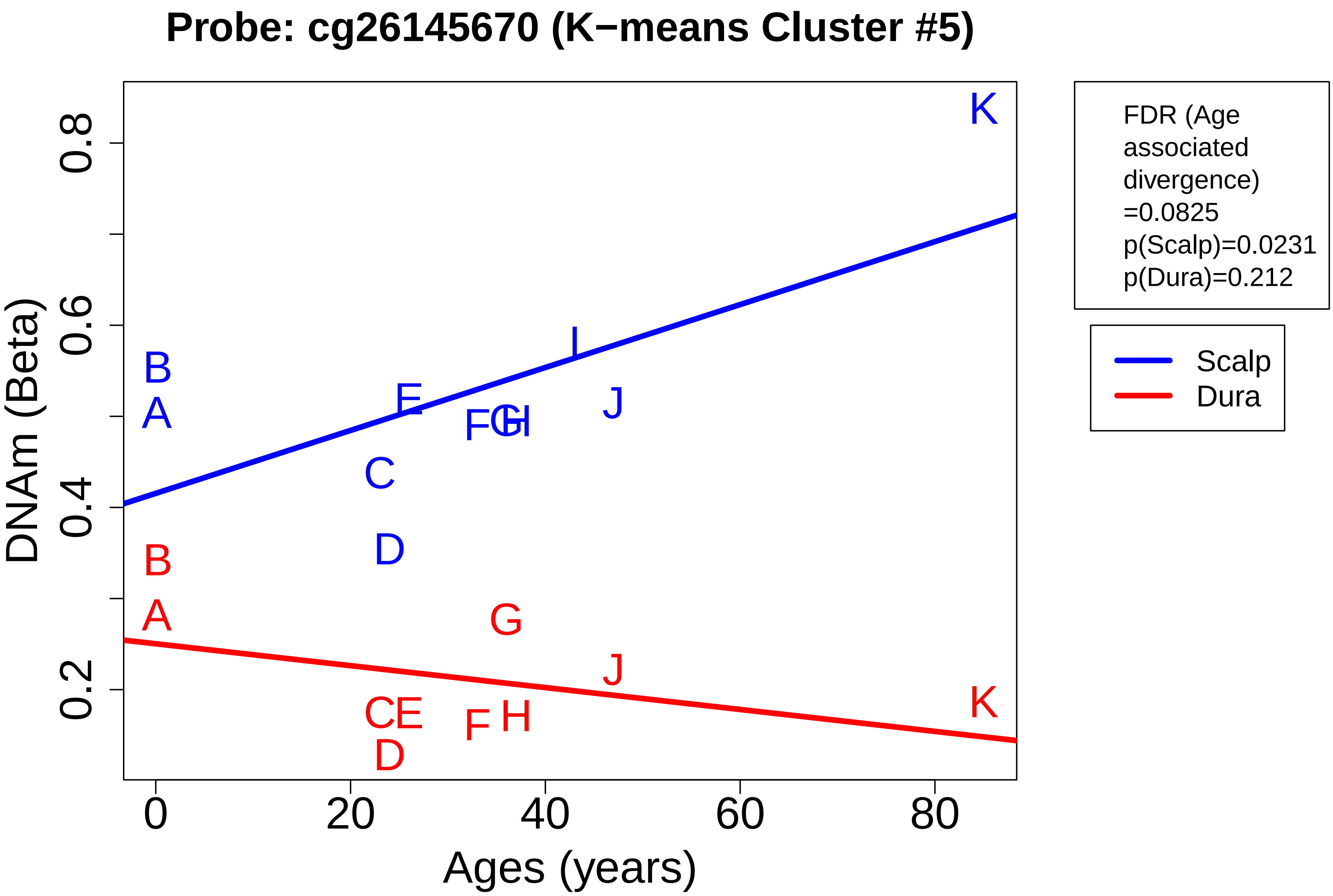

Probe: cg26145670 (K−means Cluster #5)
DNAm (Beta)
0.2
0.4
0.6
0.8
Ages (years)
0
20
40
60
80
FDR (Age associated divergence) =0.0825
p(Scalp)=0.0231
p(Dura)=0.212
Scalp
Dura
A
B
C
D
E
F
G
H
I
J
K

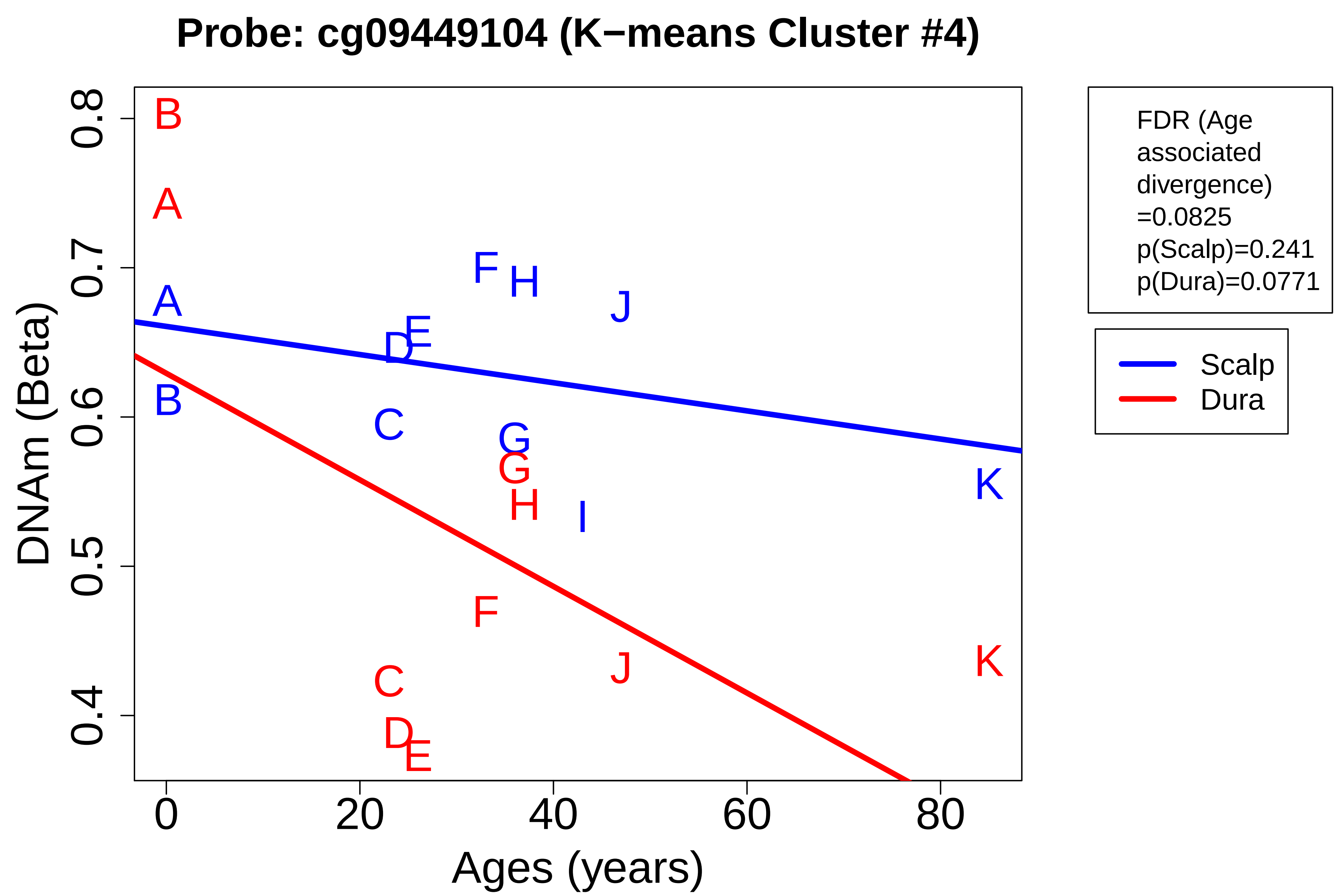

Probe: cg09449104 (K−means Cluster #4)
DNAm (Beta)
0.4
0.5
0.6
0.7
0.8
Ages (years)
0
20
40
60
80
FDR (Age associated divergence) =0.0825
p(Scalp)=0.241
p(Dura)=0.0771
Scalp
Dura

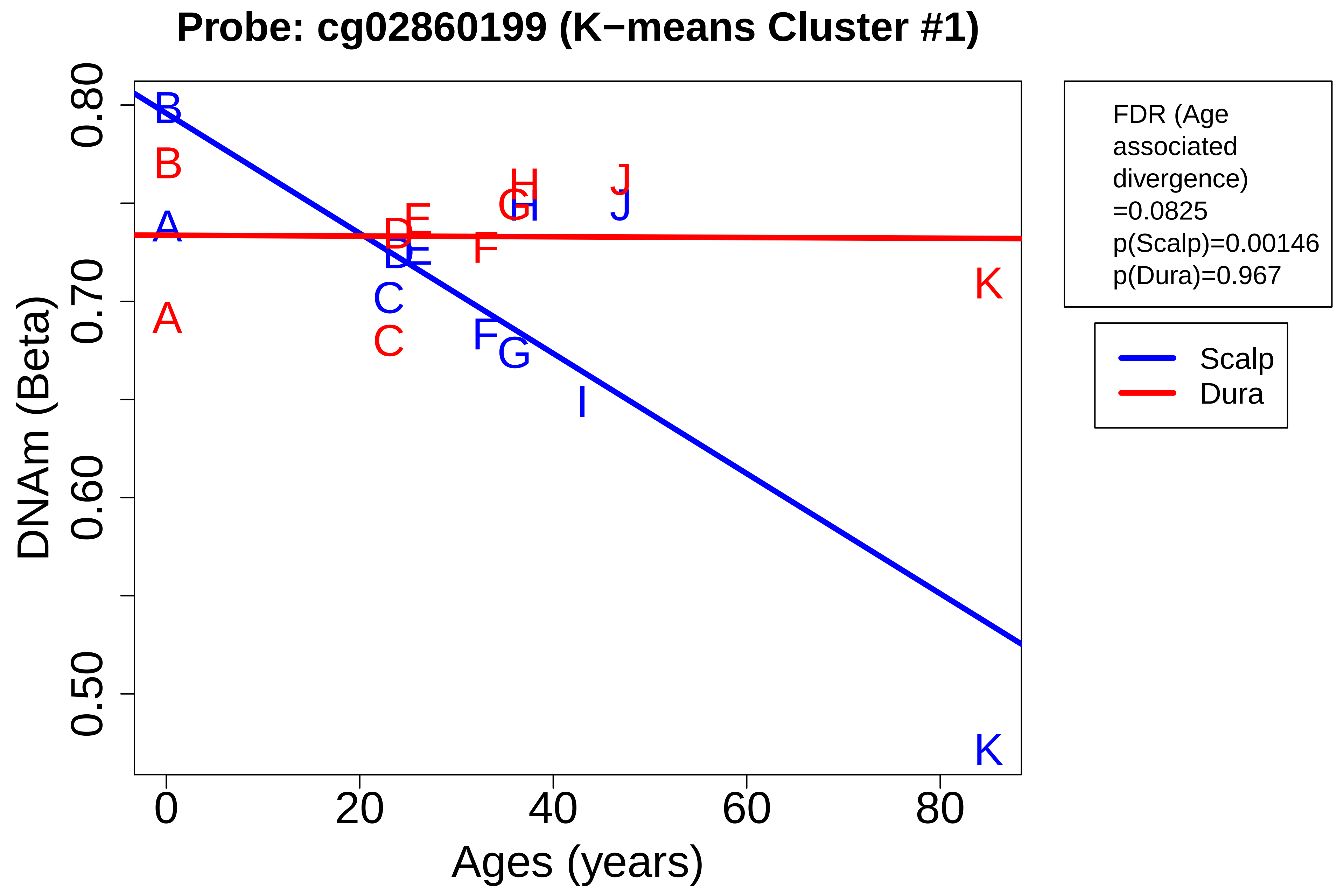

Probe: cg02860199 (K−means Cluster #1)
DNAm (Beta)
0.80
0.70
0.60
0.50
0
20
40
60
80
Ages (years)
A
B
C
D
E
F
G
H
I
J
K
FDR (Age associated divergence) =0.0825
p(Scalp)=0.00146
p(Dura)=0.967
Scalp
Dura

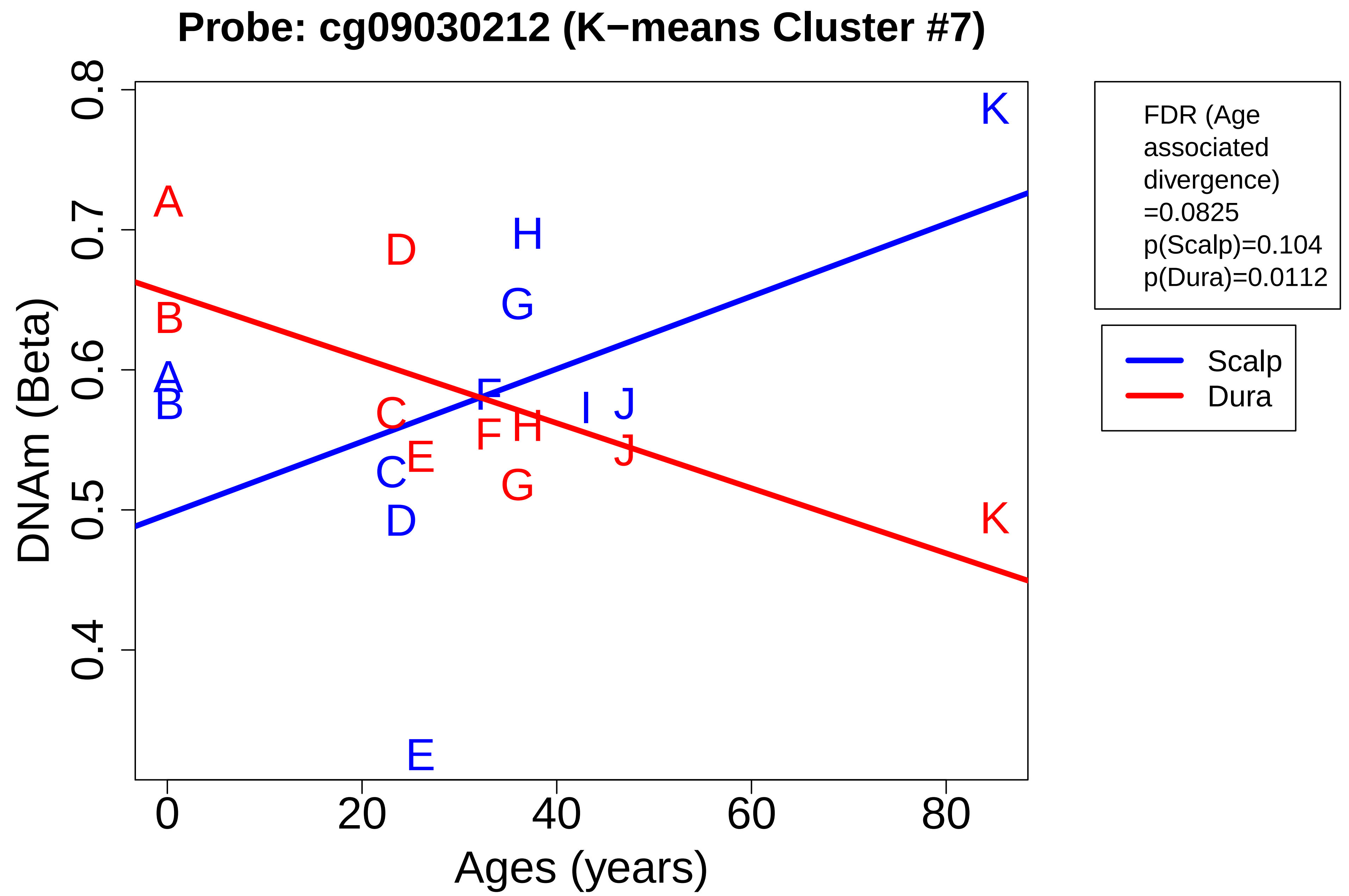
Probe: cg09030212 (K−means Cluster #7)
DNAm (Beta)
0.4
0.5
0.6
0.7
0.8
0
20
40
60
80
Ages (years)
A B C D E F G H I J K
FDR (Age associated divergence) =0.0825
p(Scalp)=0.104
p(Dura)=0.0112
Scalp
Dura

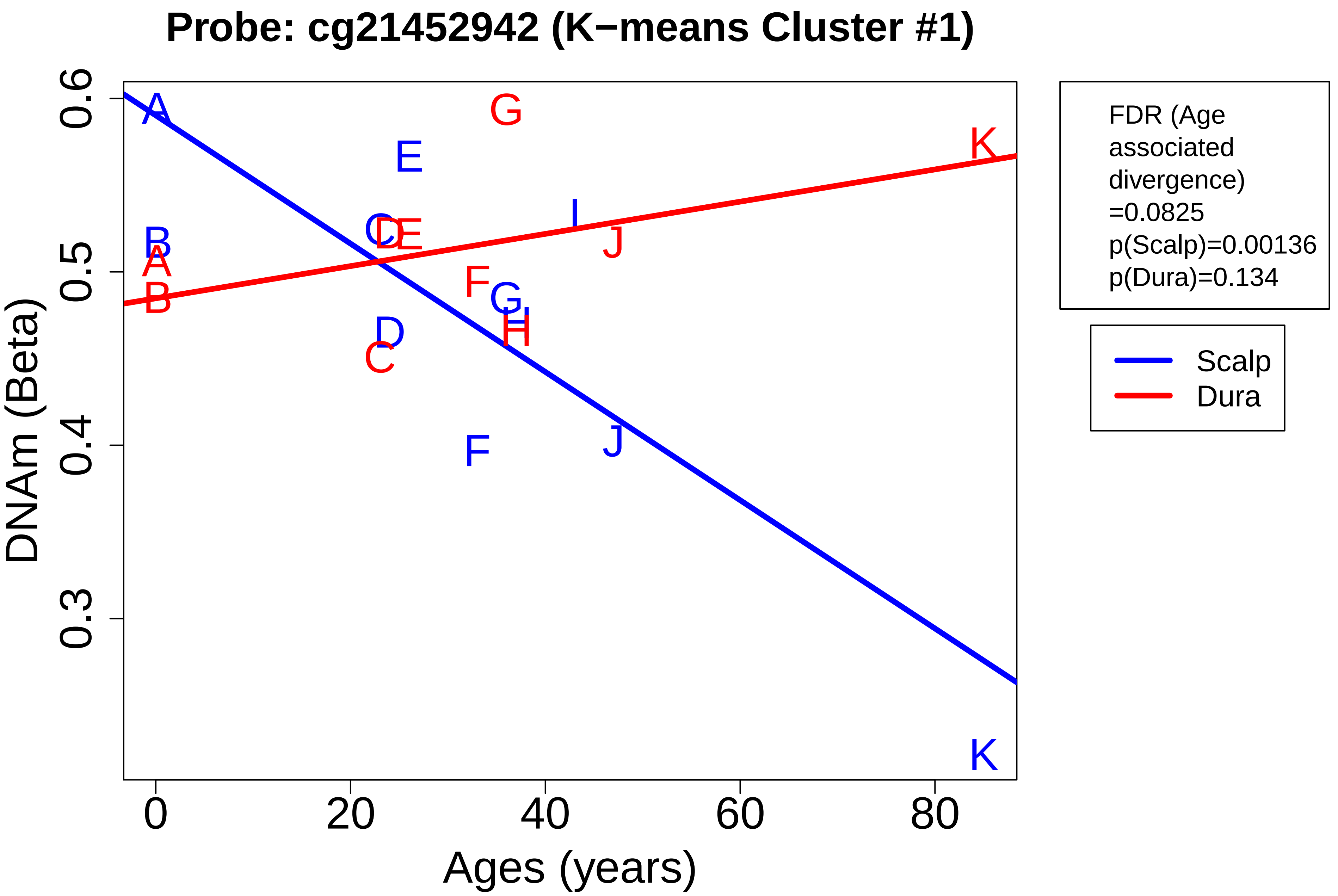
Probe: cg21452942 (K−means Cluster #1)
DNAm (Beta)
0.3
0.4
0.5
0.6
0
20
40
60
80
Ages (years)
A
B
C
D
E
F
G
H
I
J
K
FDR (Age associated divergence) =0.0825
p(Scalp)=0.00136
p(Dura)=0.134
Scalp
Dura

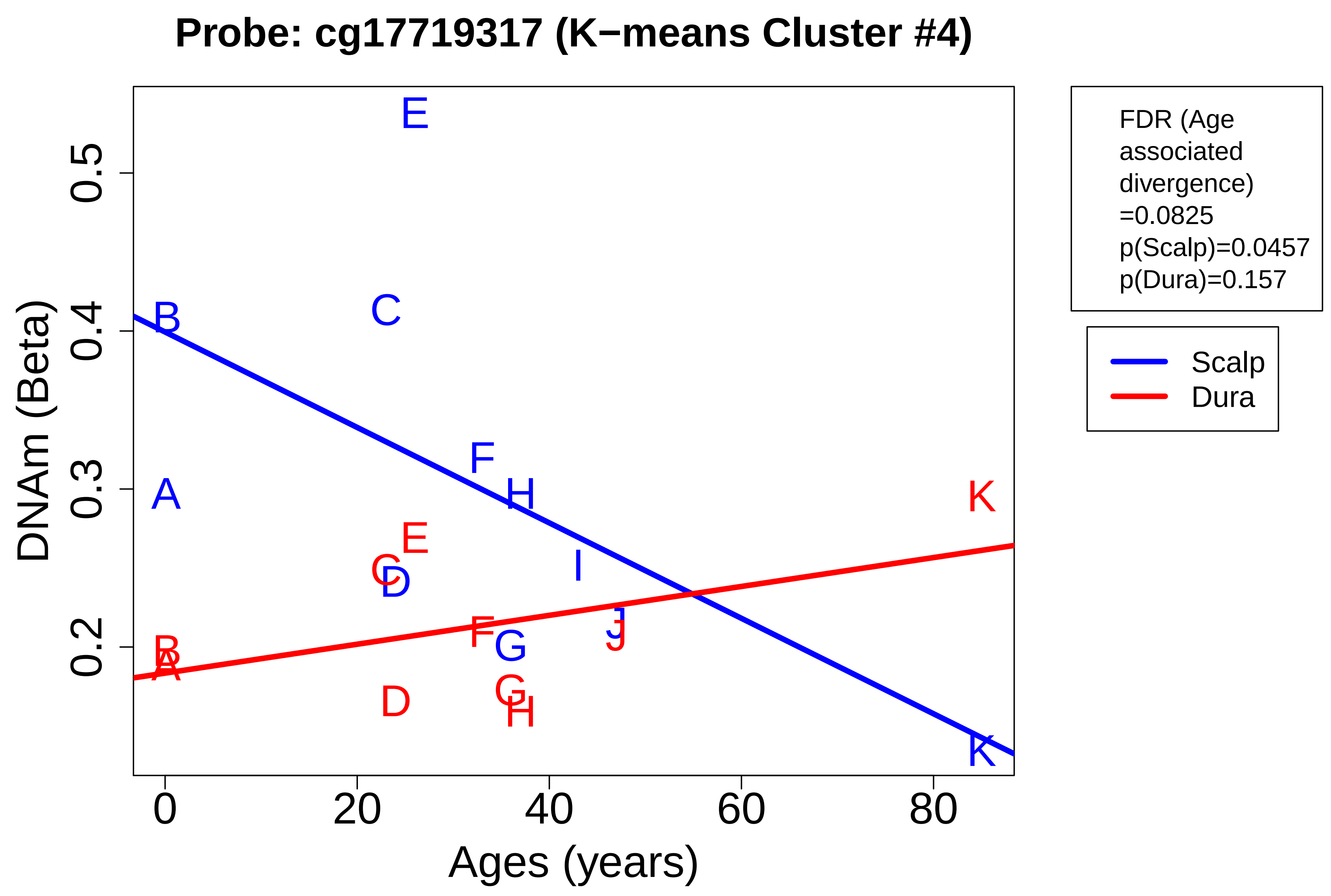

Probe: cg17719317 (K−means Cluster #4)
DNAm (Beta)
0.2
0.3
0.4
0.5
Ages (years)
0
20
40
60
80
FDR (Age associated divergence) =0.0825
p(Scalp)=0.0457
p(Dura)=0.157
Scalp
Dura

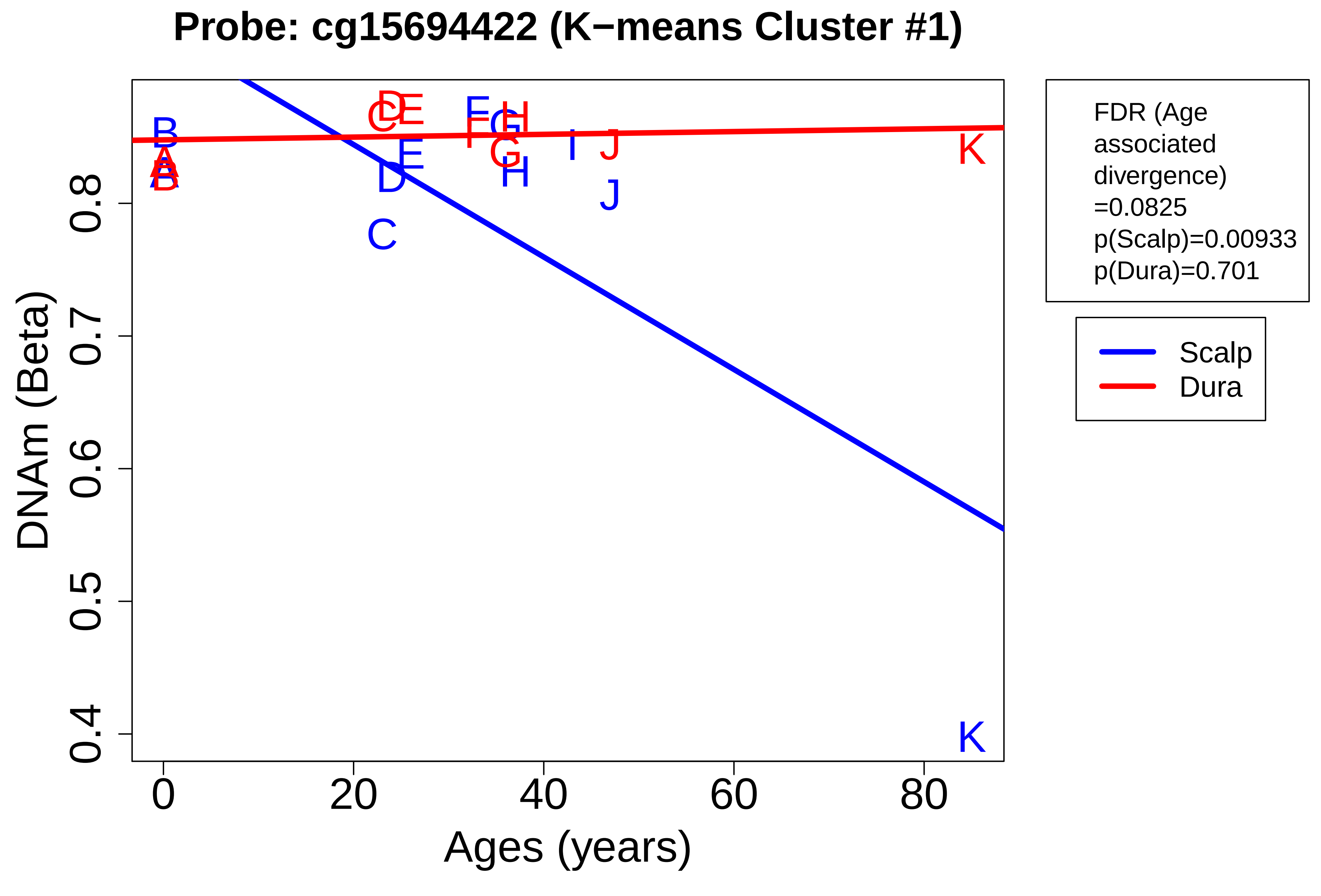

Probe: cg15694422 (K−means Cluster #1)
DNAm (Beta)
0.4
0.5
0.6
0.7
0.8
0
20
40
60
80
Ages (years)
A B C D E F G H I J K
FDR (Age associated divergence) =0.0825
p(Scalp)=0.00933
p(Dura)=0.701
Scalp
Dura

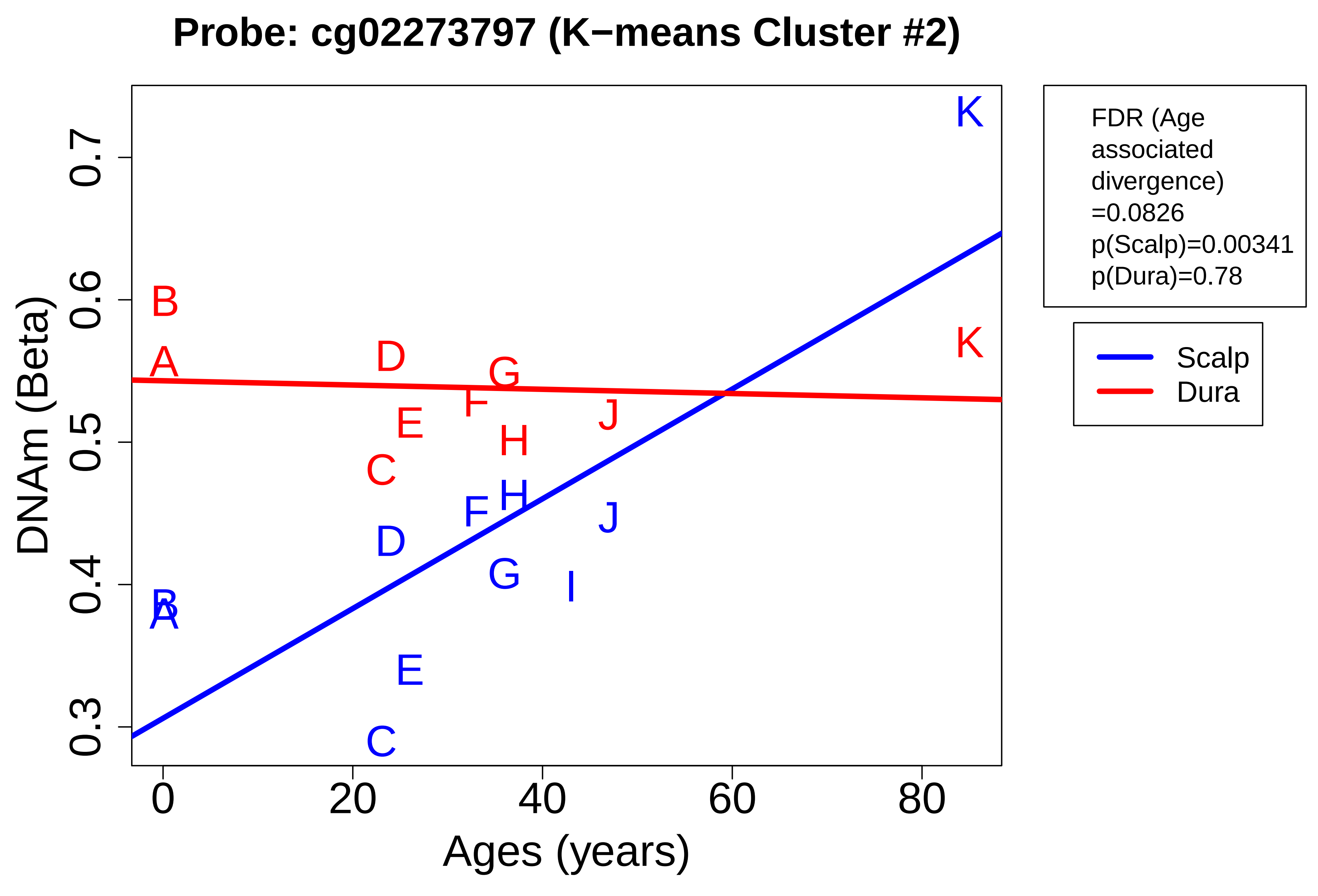

Probe: cg02273797 (K−means Cluster #2)
FDR (Age associated divergence) =0.0826
p(Scalp)=0.00341
p(Dura)=0.78
Scalp
Dura
DNAm (Beta)
0.3
0.4
0.5
0.6
0.7
Ages (years)
0
20
40
60
80
A
B
C
D
E
F
G
H
I
J
K

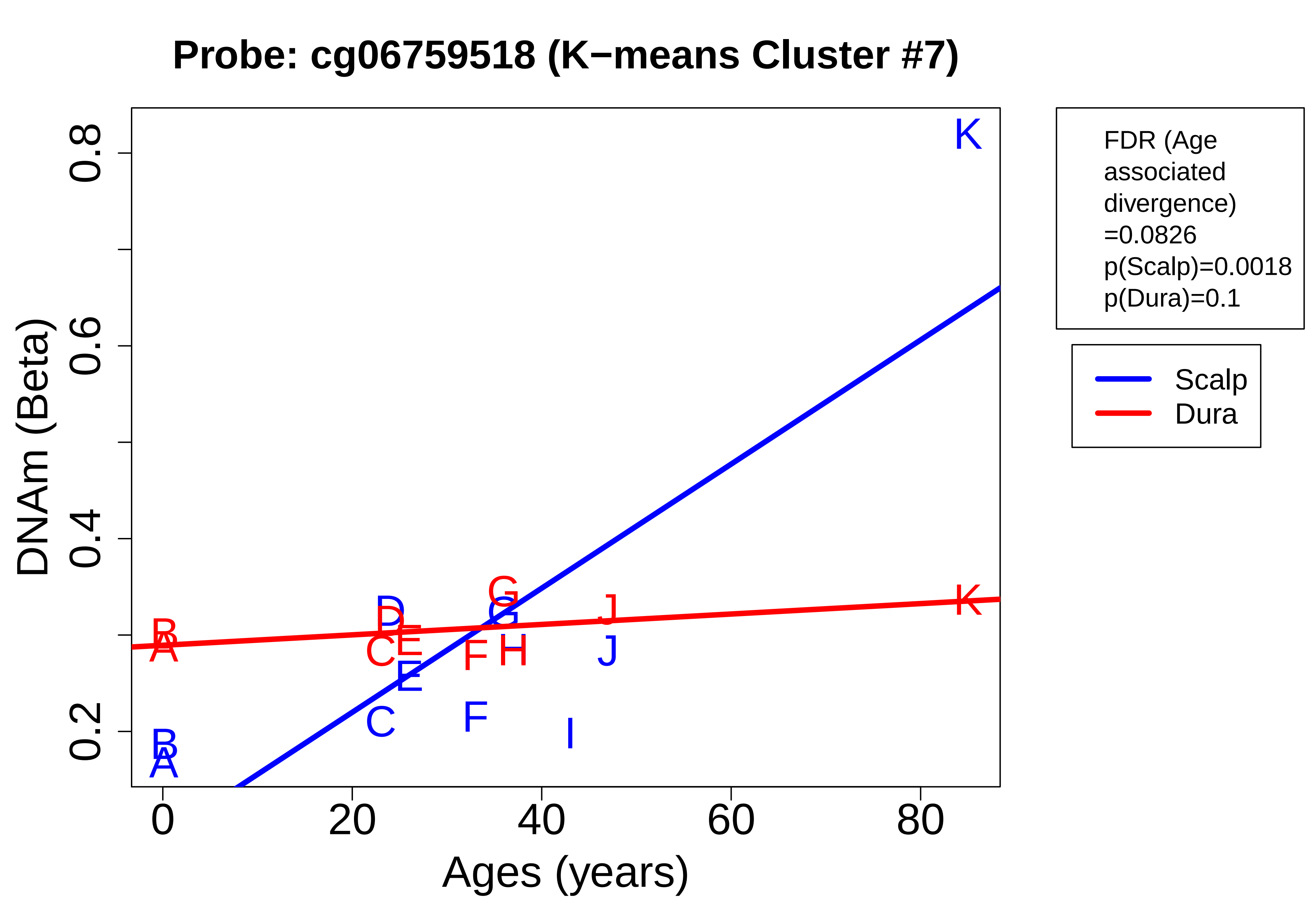

Probe: cg06759518 (K−means Cluster #7)
DNAm (Beta)
0.2
0.4
0.6
0.8
Ages (years)
0
20
40
60
80
FDR (Age associated divergence) =0.0826
p(Scalp)=0.0018
p(Dura)=0.1
Scalp
Dura
A
B
C
D
E
F
G
H
I
J
K

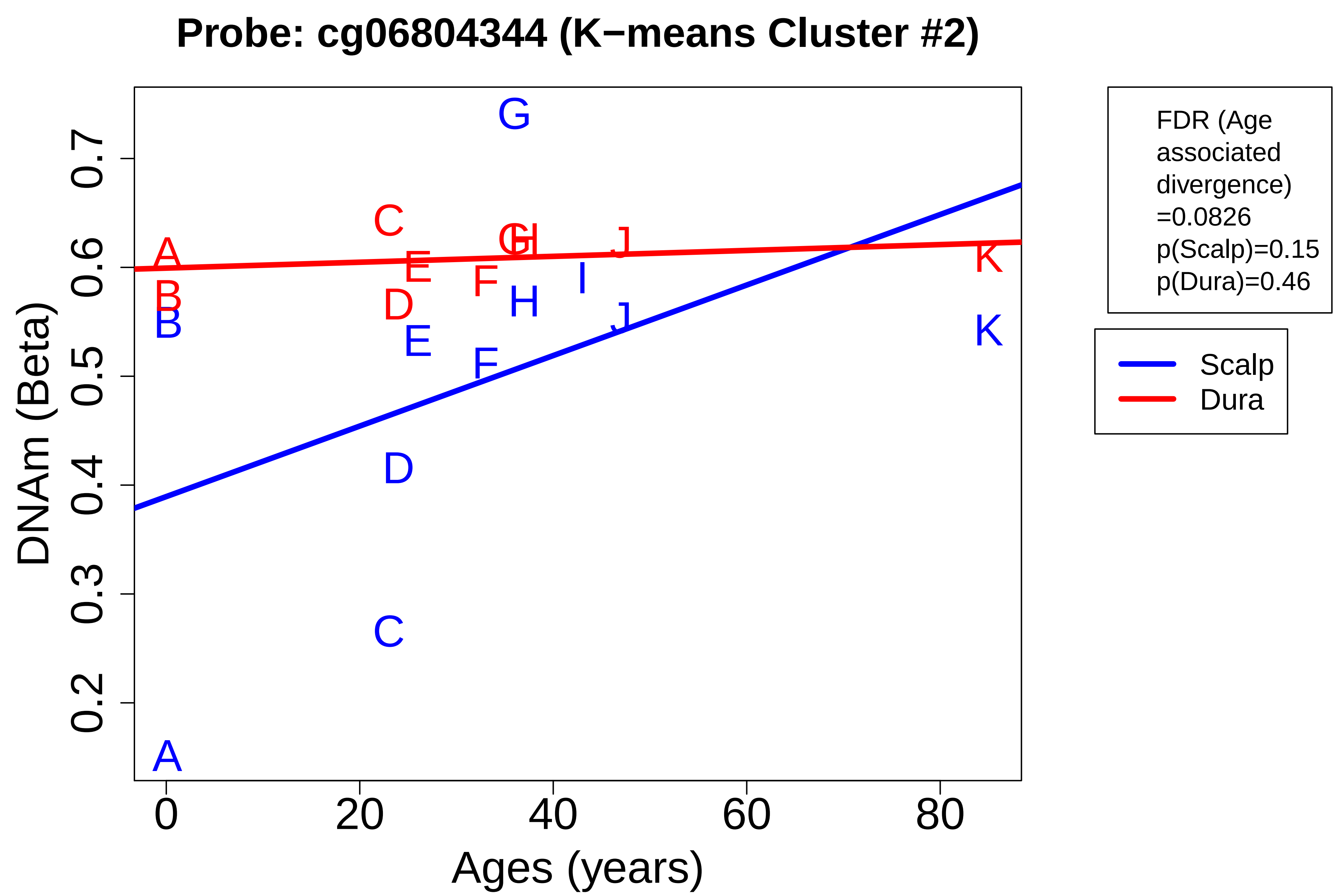

Probe: cg06804344 (K−means Cluster #2)
DNAm (Beta)
Ages (years)
0.2
0.3
0.4
0.5
0.6
0.7
0
20
40
60
80
FDR (Age associated divergence) =0.0826
p(Scalp)=0.15
p(Dura)=0.46
Scalp
Dura

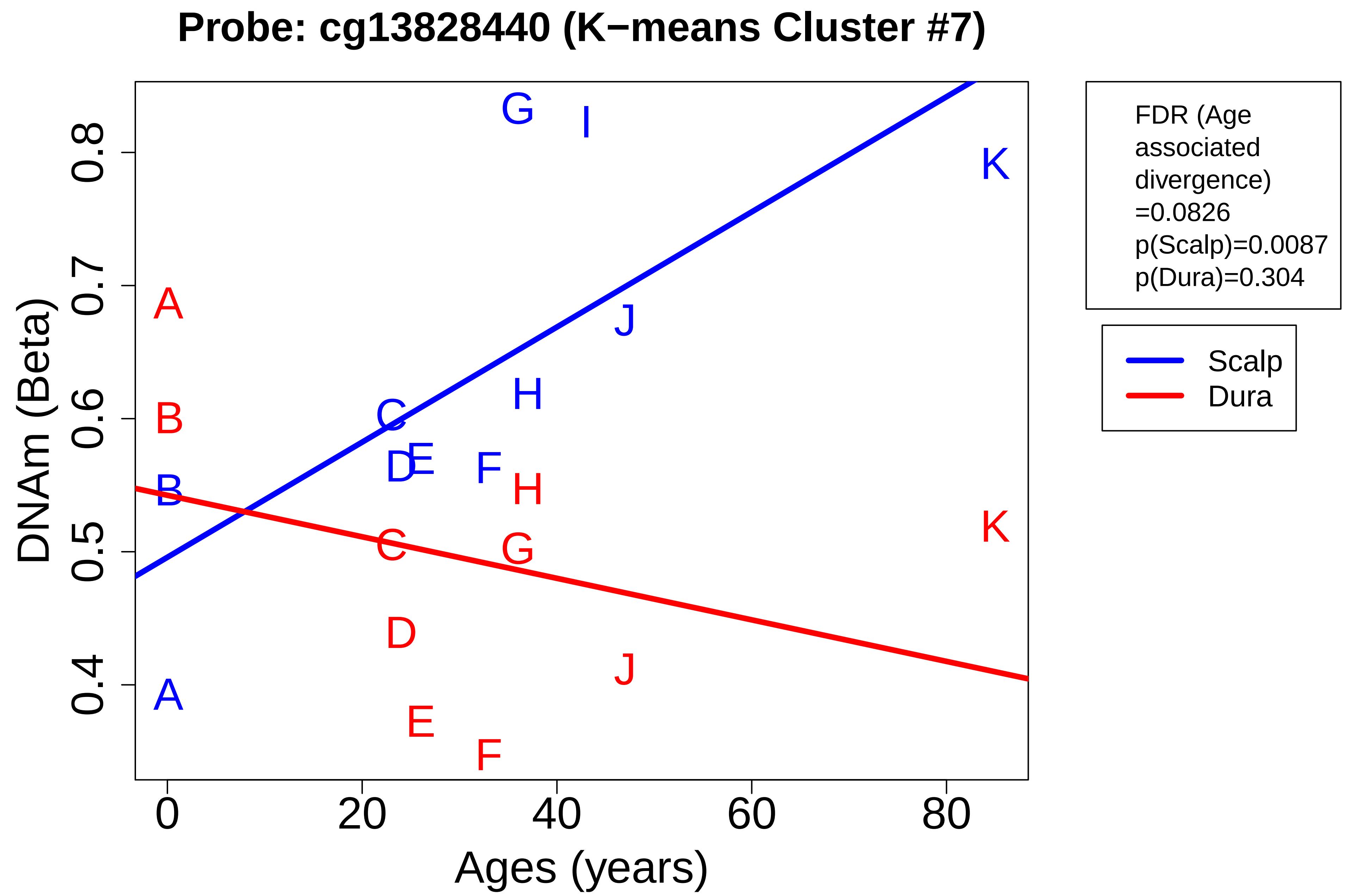

Probe: cg13828440 (K−means Cluster #7)
FDR (Age associated divergence) =0.0826
p(Scalp)=0.0087
p(Dura)=0.304
Scalp
Dura
DNAm (Beta)
0.4
0.5
0.6
0.7
0.8
0
20
40
60
80
Ages (years)

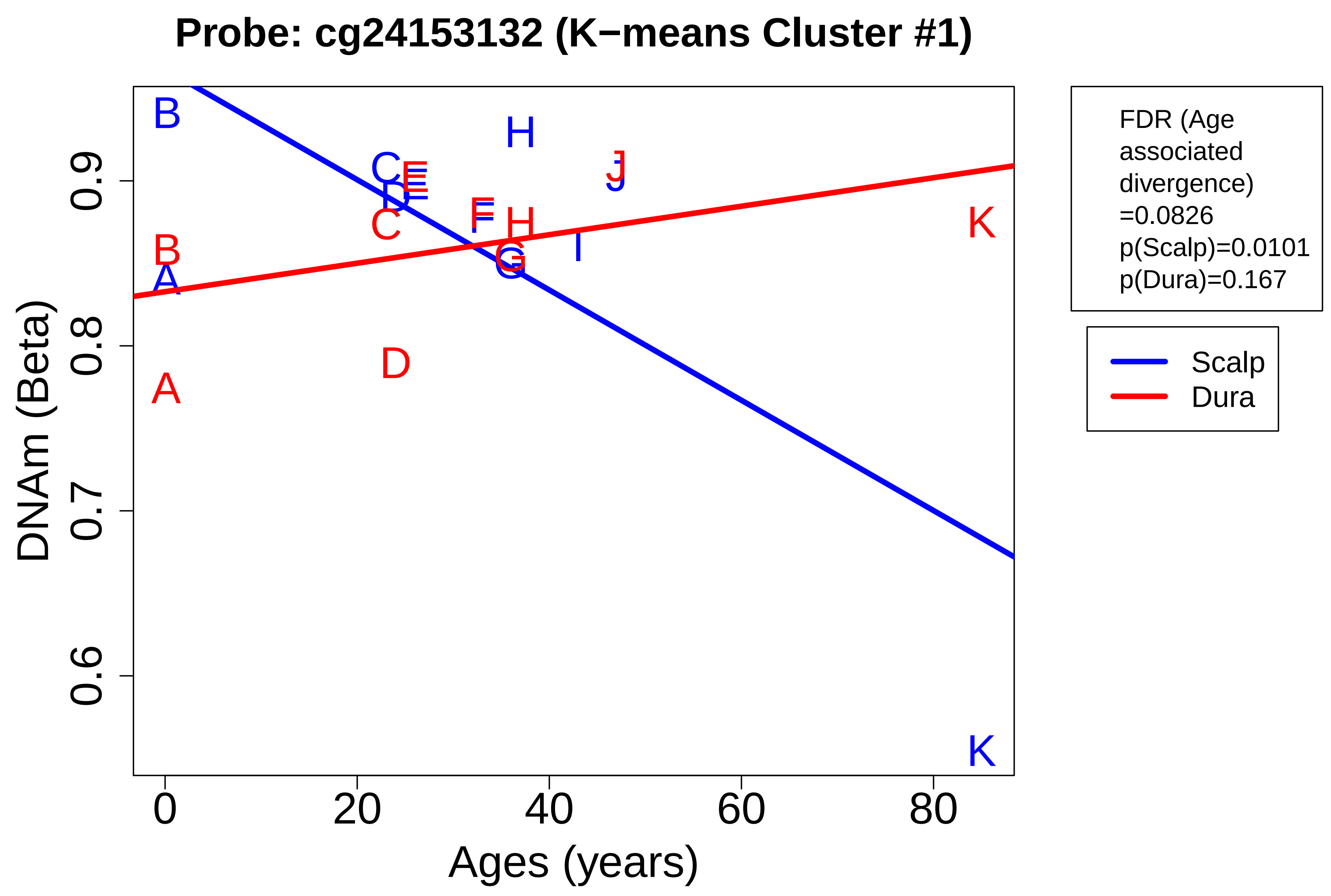

Probe: cg24153132 (K−means Cluster #1)
DNAm (Beta)
0.6
0.7
0.8
0.9
0
20
40
60
80
Ages (years)
FDR (Age associated divergence) =0.0826
p(Scalp)=0.0101
p(Dura)=0.167
Scalp
Dura

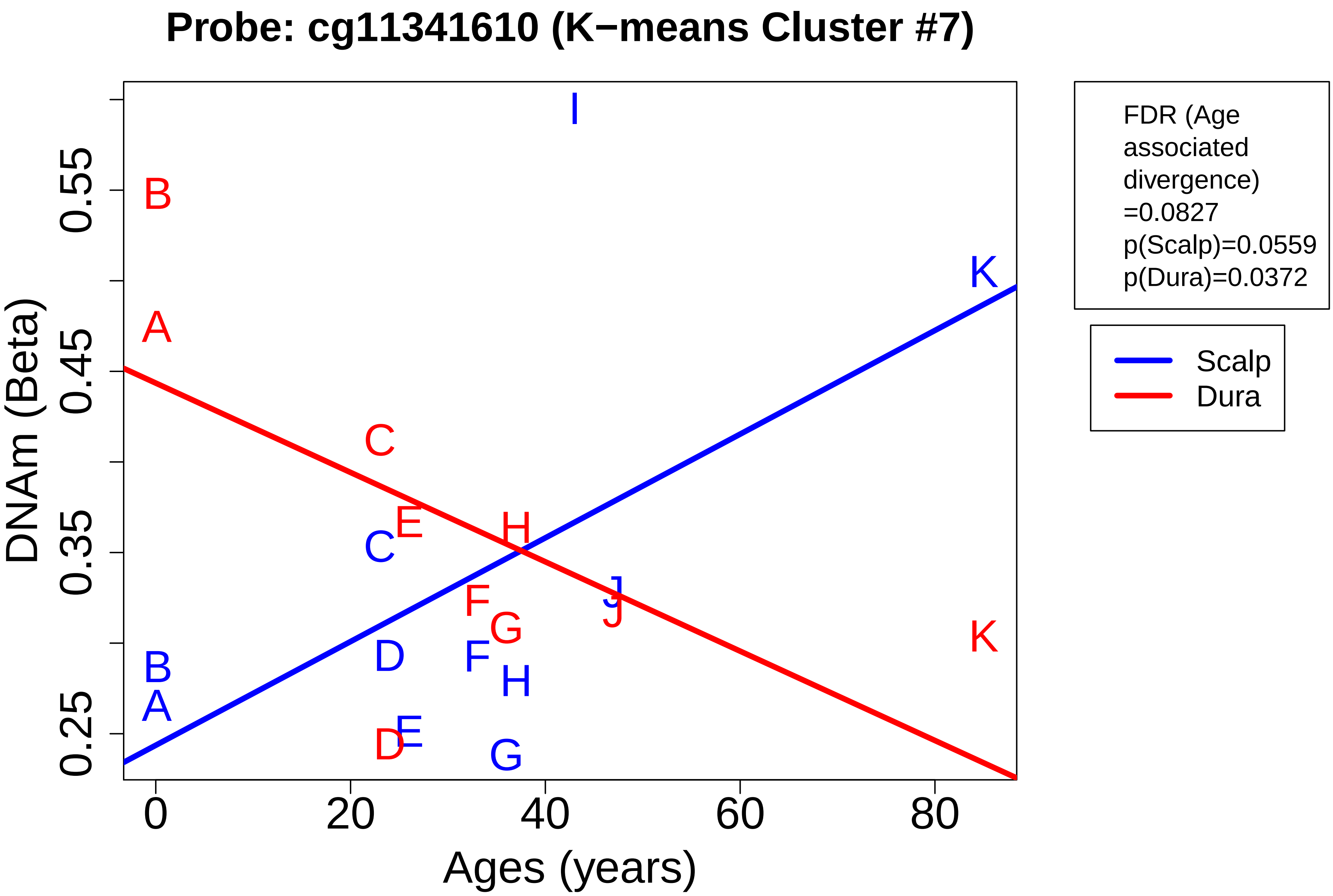

Probe: cg11341610 (K−means Cluster #7)
DNAm (Beta)
0.25
0.35
0.45
0.55
0
20
40
60
80
Ages (years)
FDR (Age associated divergence) =0.0827
p(Scalp)=0.0559
p(Dura)=0.0372
Scalp
Dura
A
B
C
D
E
F
G
H
I
J
K

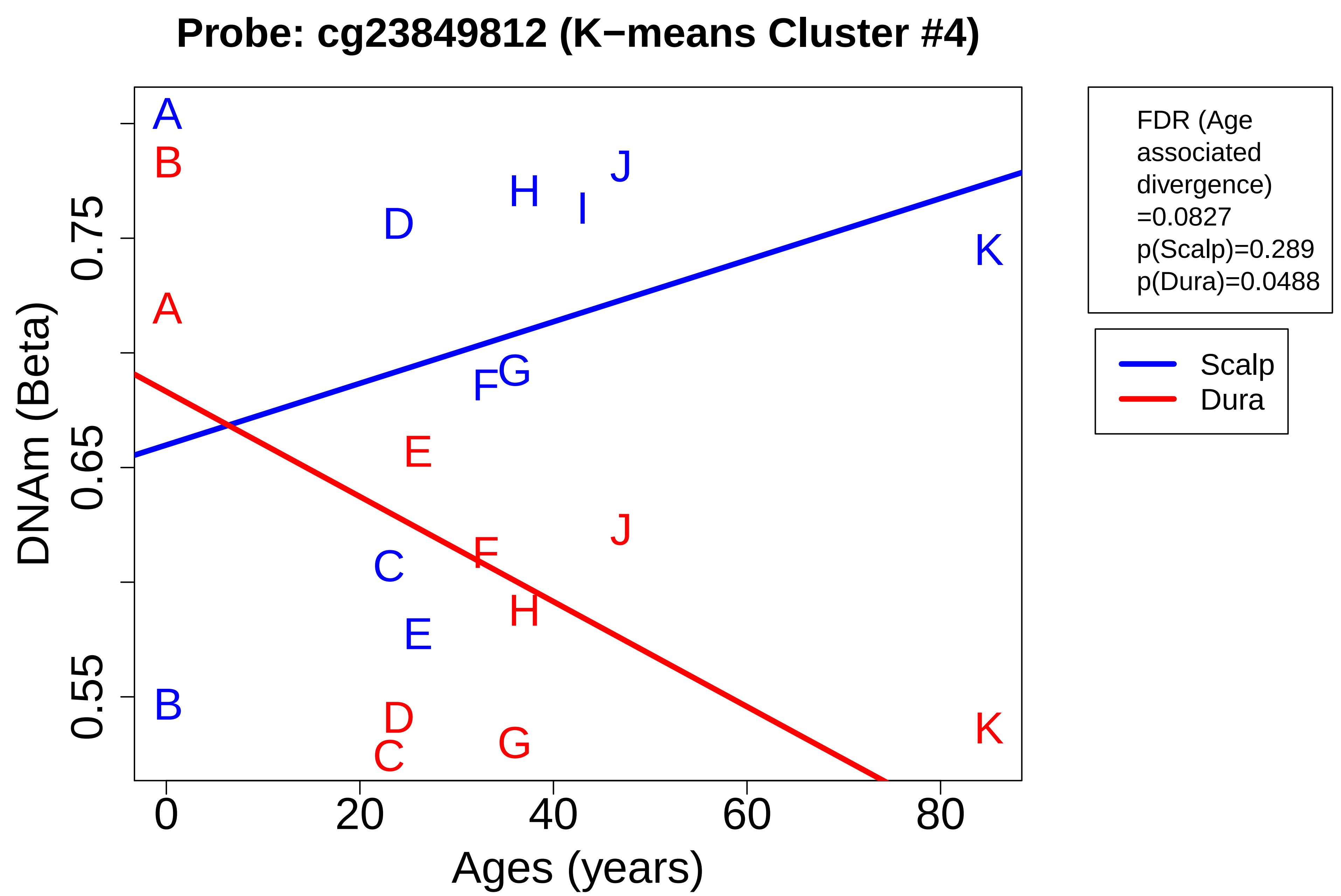
Probe: cg23849812 (K−means Cluster #4)
DNAm (Beta)
0.55
0.65
0.75
0
20
40
60
80
Ages (years)
A
B
A
D
H
I
J
K
G
F
E
J
F
C
H
E
B
D
C
G
K
FDR (Age associated divergence) =0.0827
p(Scalp)=0.289
p(Dura)=0.0488
Scalp
Dura

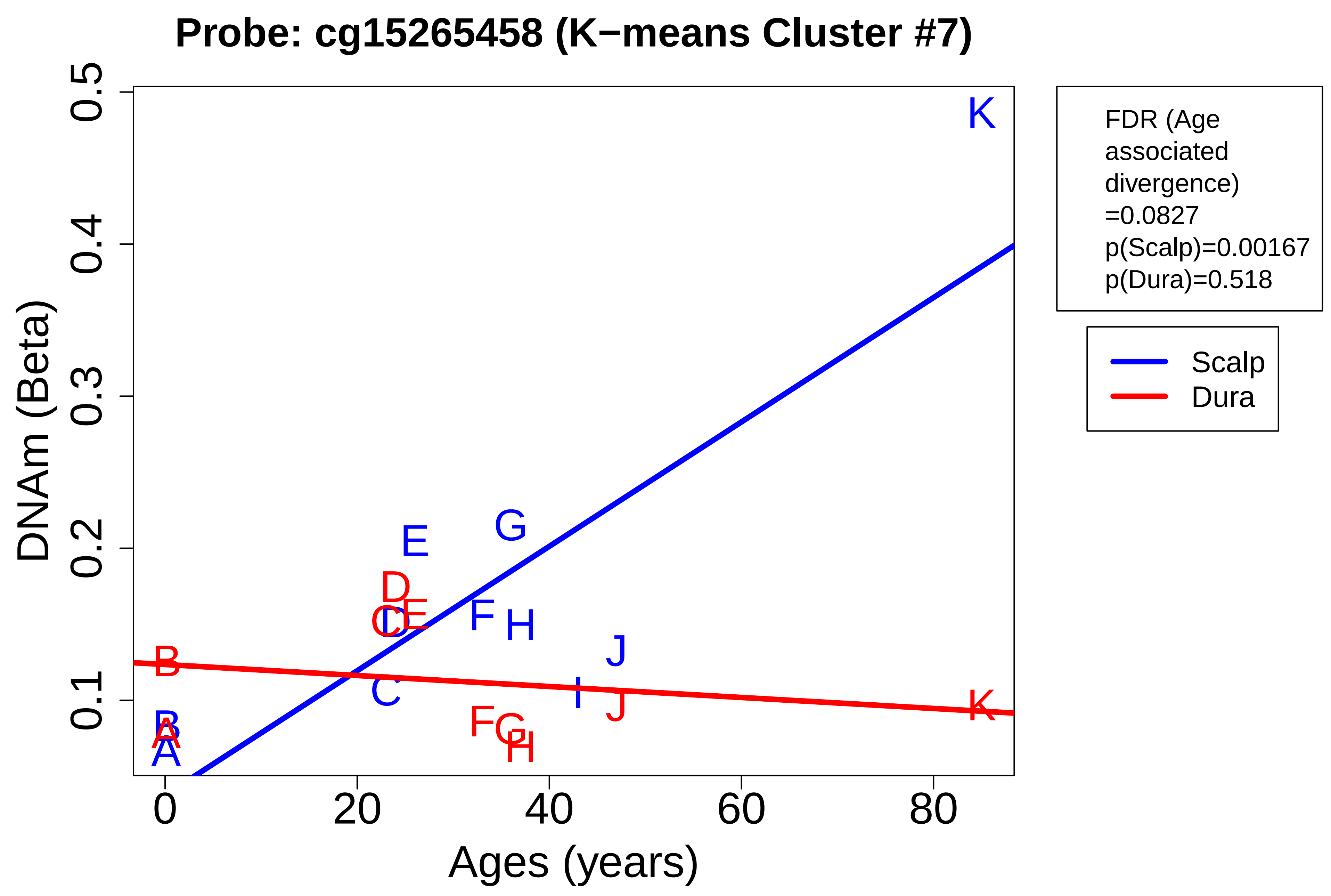
Probe: cg15265458 (K−means Cluster #7)
DNAm (Beta)
0.1
0.2
0.3
0.4
0.5
0
20
40
60
80
Ages (years)
FDR (Age associated divergence) =0.0827
p(Scalp)=0.00167
p(Dura)=0.518
Scalp
Dura

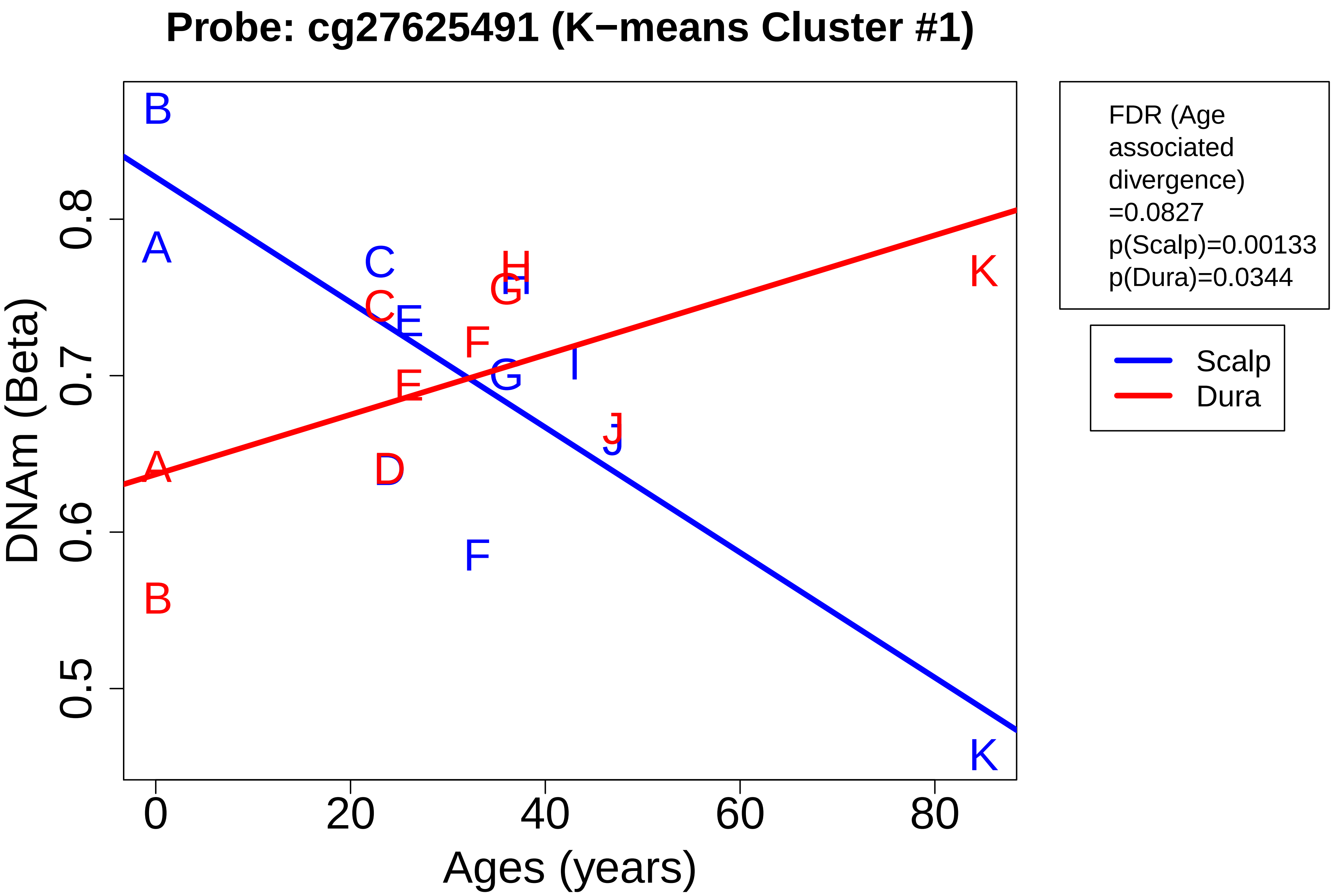
Probe: cg27625491 (K−means Cluster #1)
FDR (Age associated divergence) =0.0827
p(Scalp)=0.00133
p(Dura)=0.0344
Scalp
Dura
DNAm (Beta)
Ages (years)
0.5
0.6
0.7
0.8
0
20
40
60
80
A
B
C
D
E
F
G
H
I
J
K

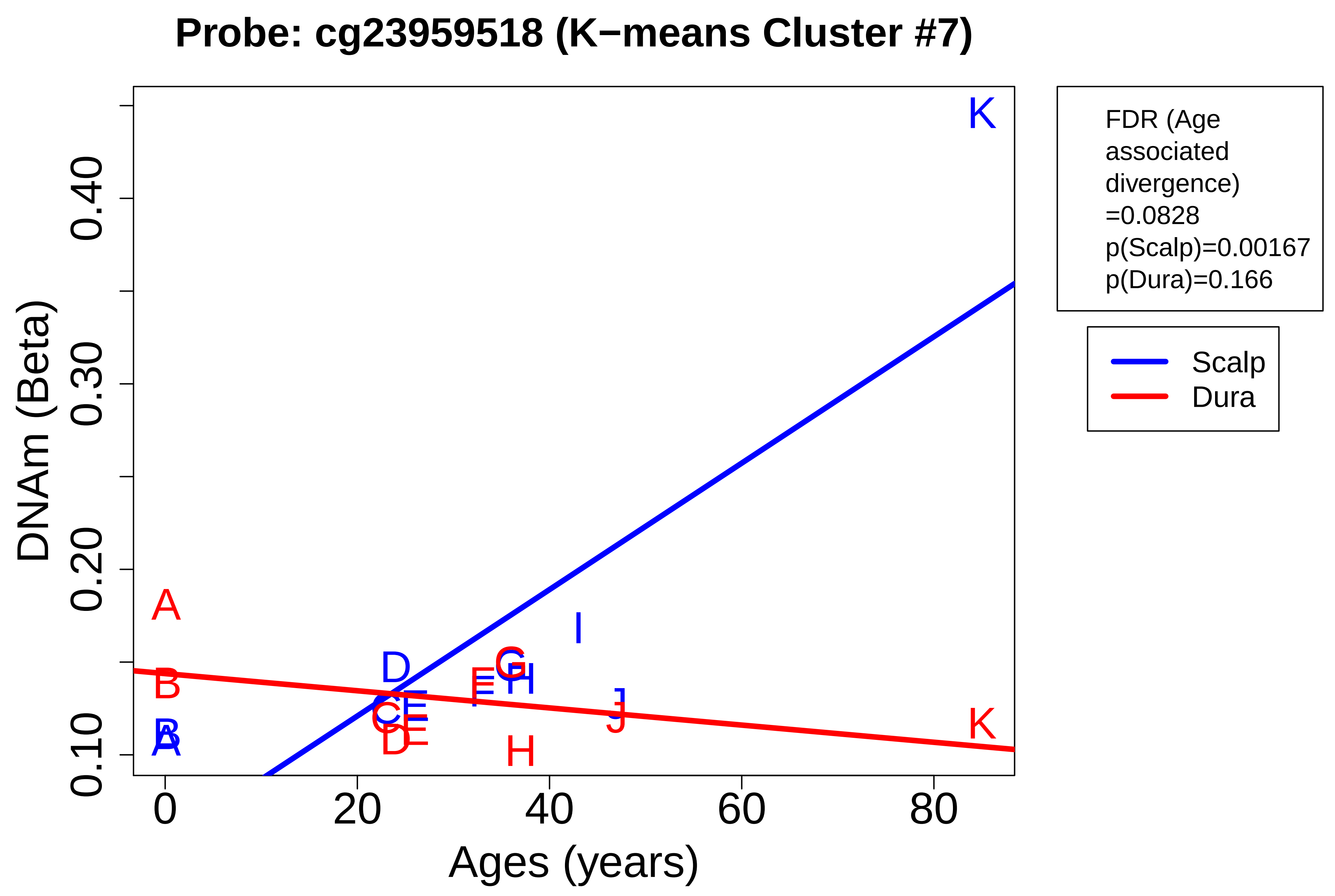

Probe: cg23959518 (K−means Cluster #7)
DNAm (Beta)
0.10
0.20
0.30
0.40
0
20
40
60
80
Ages (years)
FDR (Age associated divergence) =0.0828
p(Scalp)=0.00167
p(Dura)=0.166
Scalp
Dura

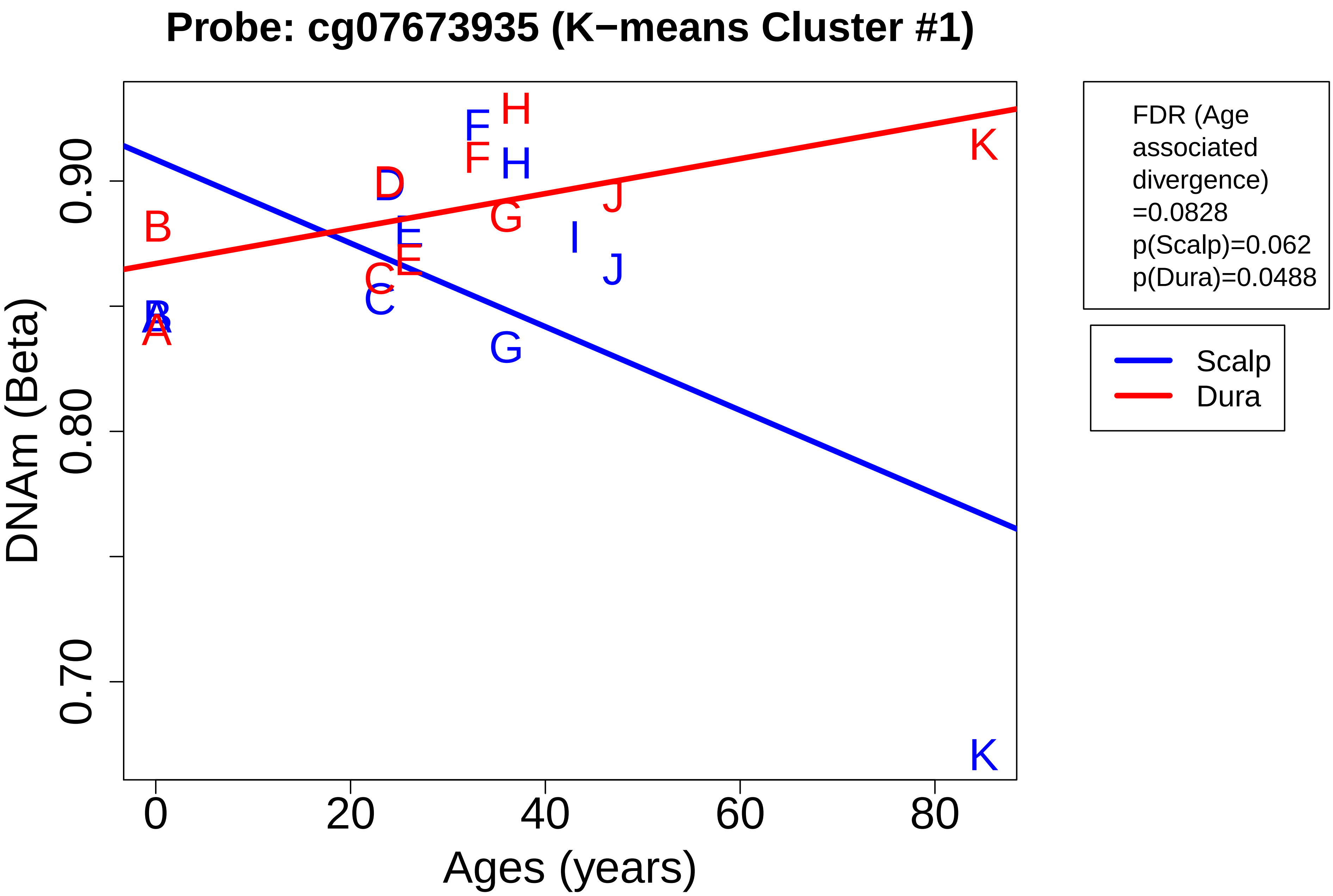

Probe: cg07673935 (K−means Cluster #1)
DNAm (Beta)
0.70
0.80
0.90
0
20
40
60
80
Ages (years)
FDR (Age associated divergence) =0.0828
p(Scalp)=0.062
p(Dura)=0.0488
Scalp
Dura

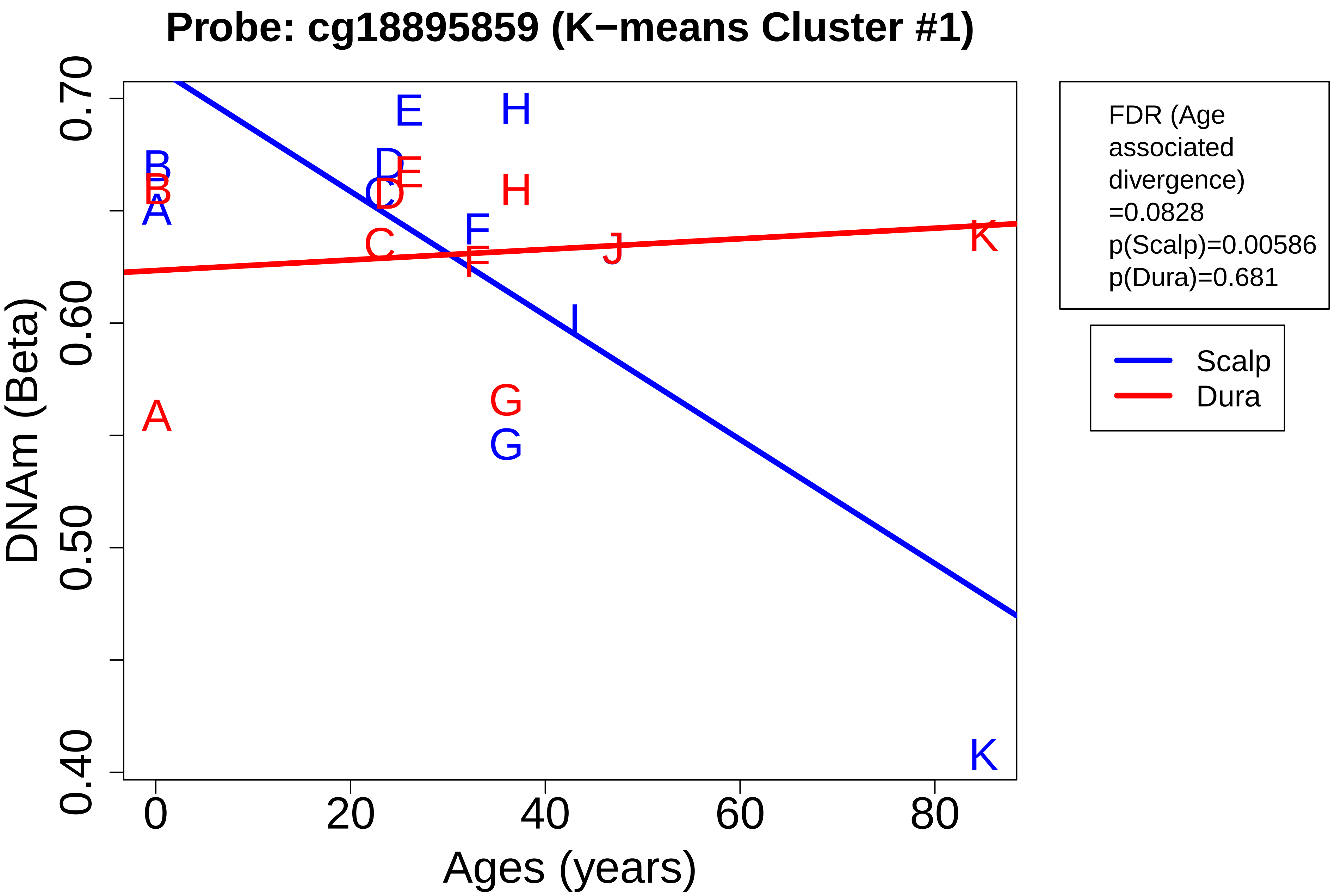

Probe: cg18895859 (K−means Cluster #1)
DNAm (Beta)
0.40
0.50
0.60
0.70
0
20
40
60
80
Ages (years)
FDR (Age associated divergence) =0.0828
p(Scalp)=0.00586
p(Dura)=0.681
Scalp
Dura
A
B
C
D
E
F
G
H
I
J
K

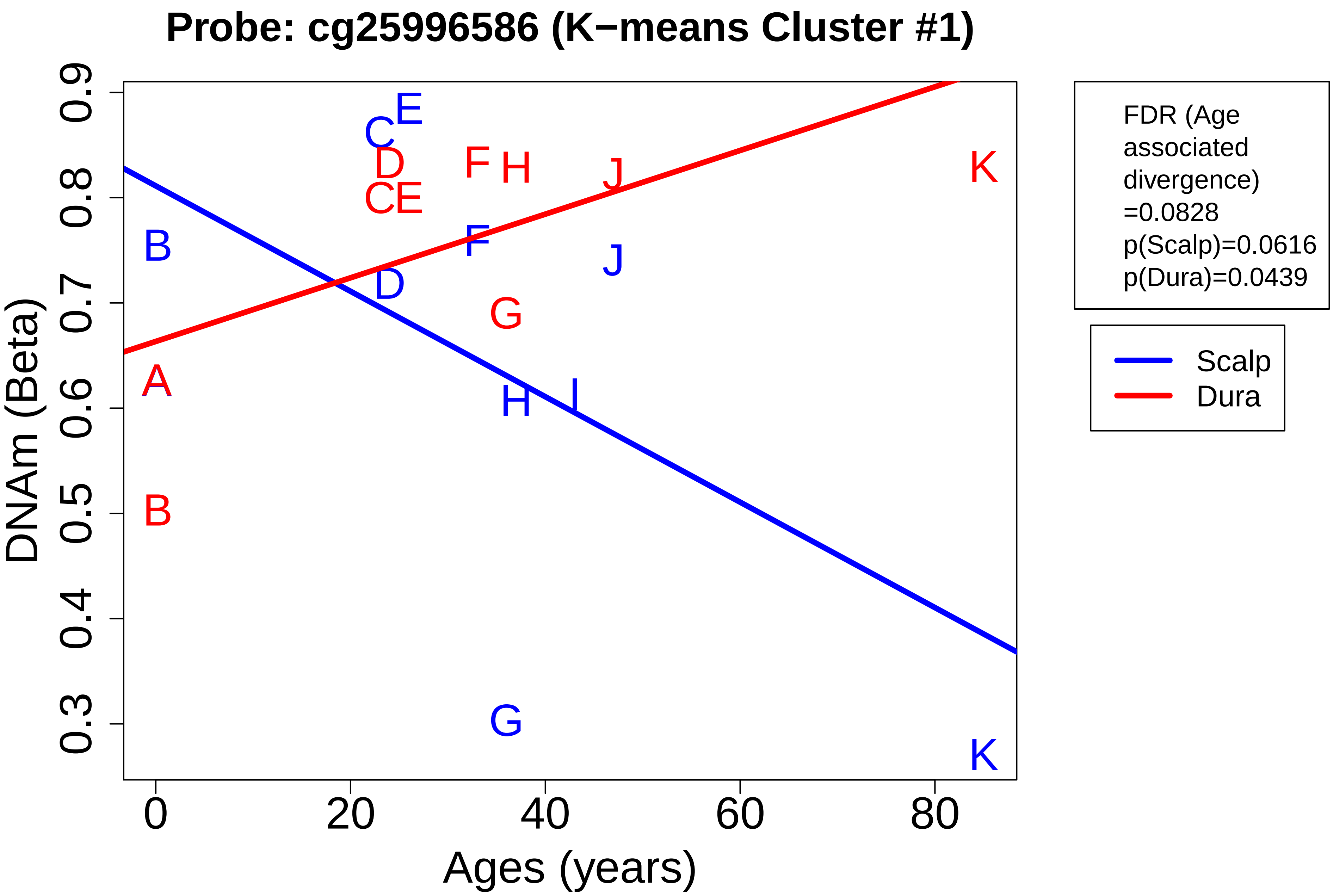

Probe: cg25996586 (K−means Cluster #1)
DNAm (Beta)
0.3
0.4
0.5
0.6
0.7
0.8
0.9
0
20
40
60
80
Ages (years)
FDR (Age associated divergence) =0.0828
p(Scalp)=0.0616
p(Dura)=0.0439
Scalp
Dura

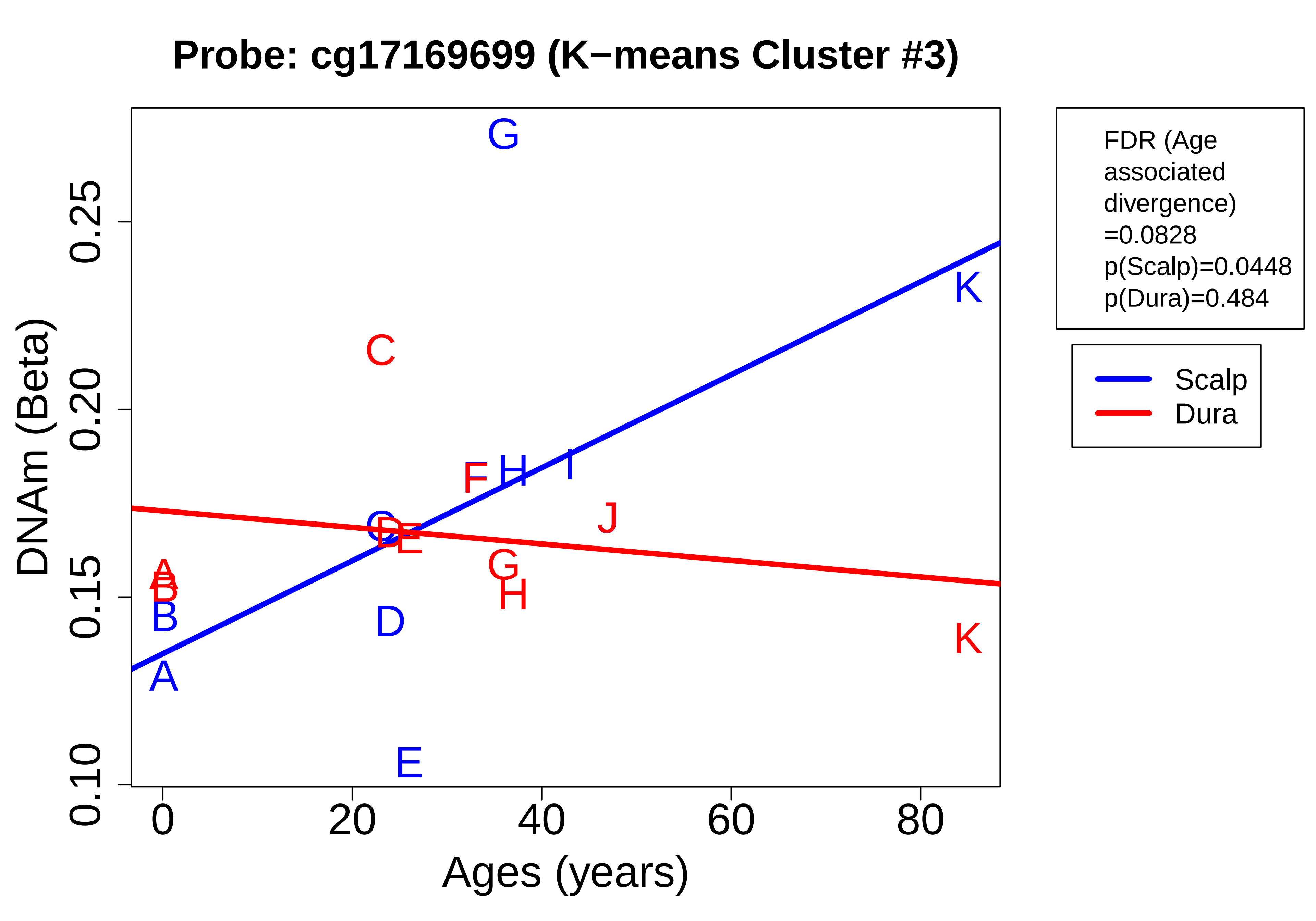

Probe: cg17169699 (K−means Cluster #3)
DNAm (Beta)
0.10
0.15
0.20
0.25
0
20
40
60
80
Ages (years)
FDR (Age associated divergence) =0.0828
p(Scalp)=0.0448
p(Dura)=0.484
Scalp
Dura

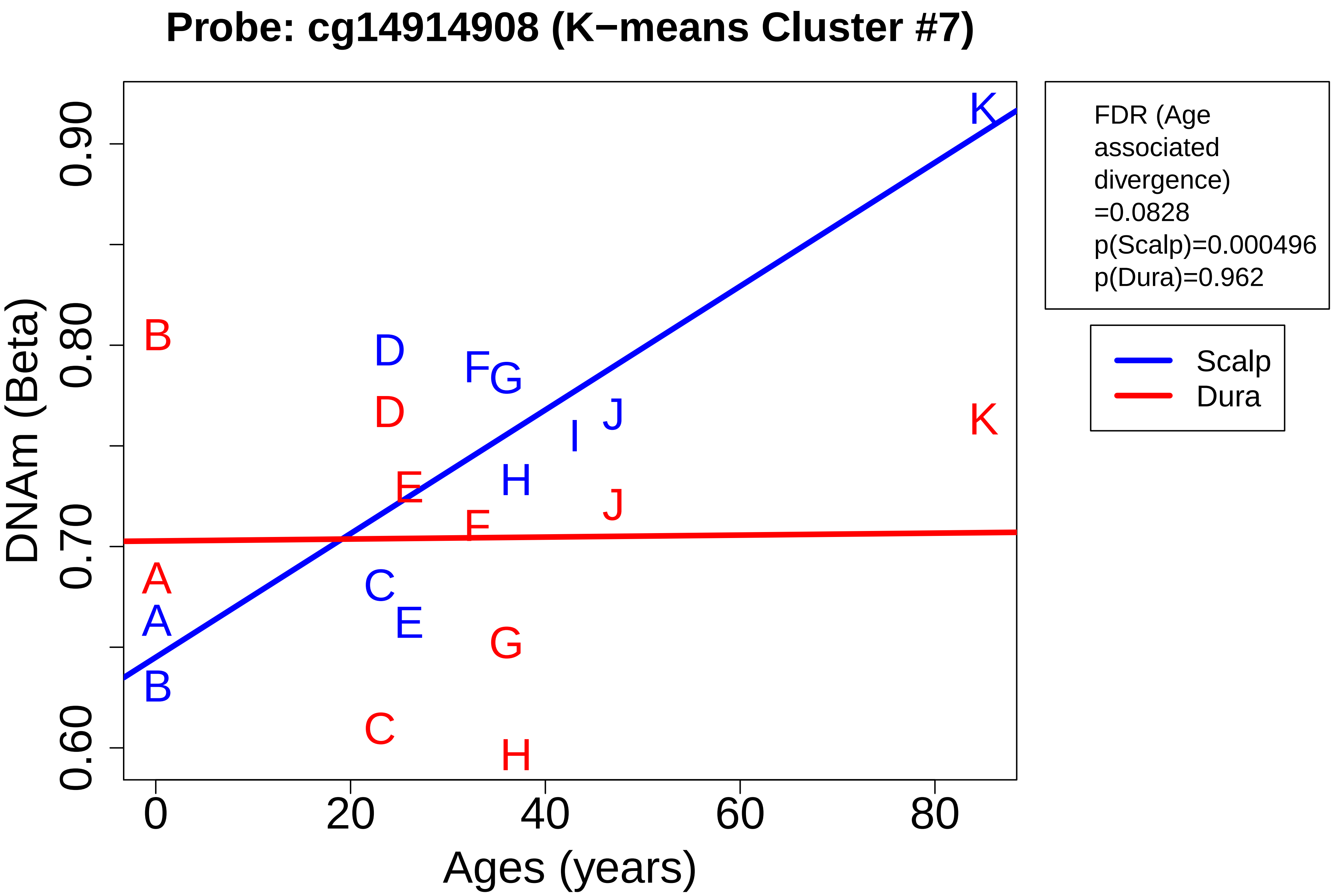

Probe: cg14914908 (K−means Cluster #7)
DNAm (Beta)
0.60
0.70
0.80
0.90
0
20
40
60
80
Ages (years)
FDR (Age associated divergence) =0.0828
p(Scalp)=0.000496
p(Dura)=0.962
Scalp
Dura

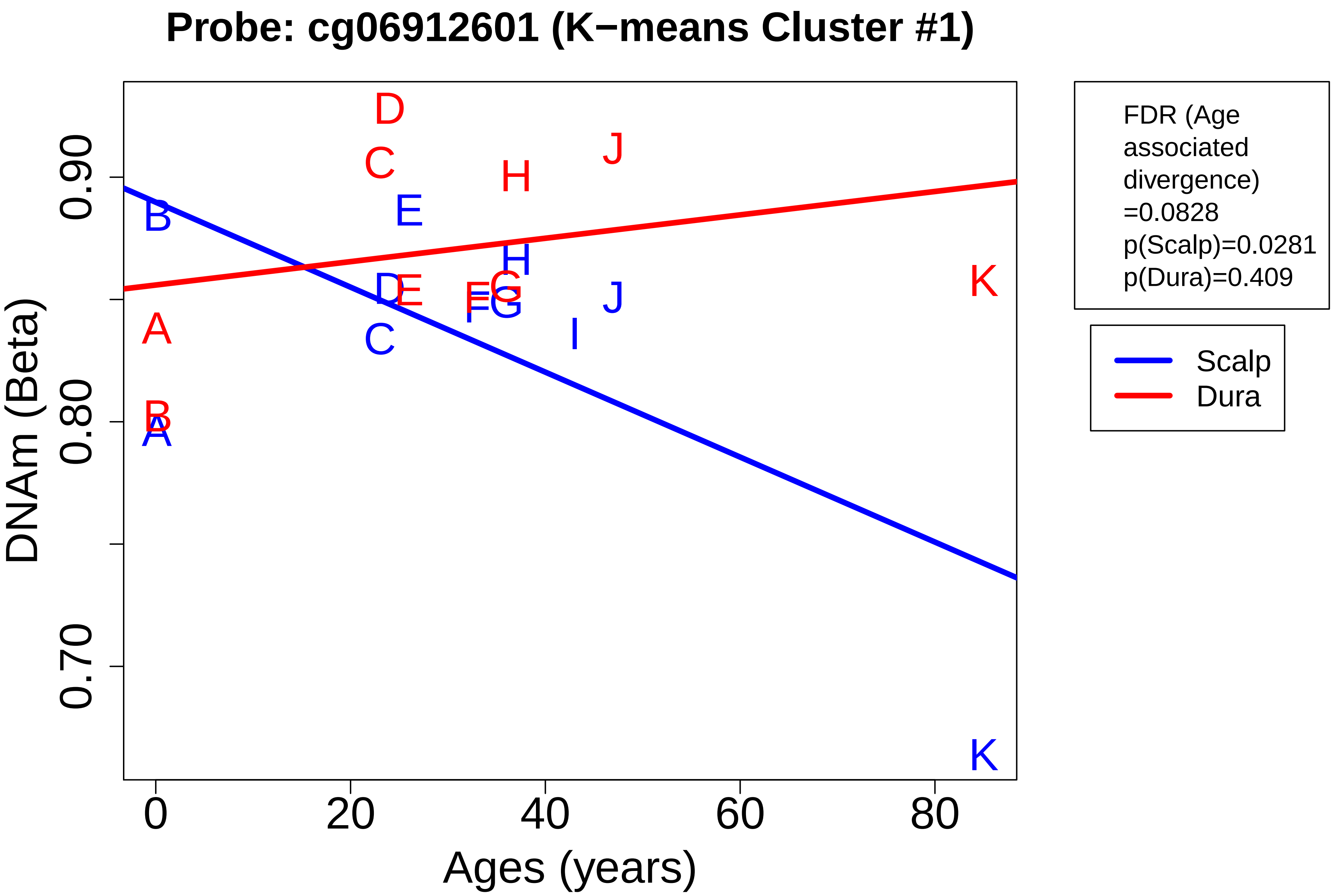

Probe: cg06912601 (K−means Cluster #1)
DNAm (Beta)
0.70
0.80
0.90
0
20
40
60
80
Ages (years)
FDR (Age associated divergence) =0.0828
p(Scalp)=0.0281
p(Dura)=0.409
Scalp
Dura

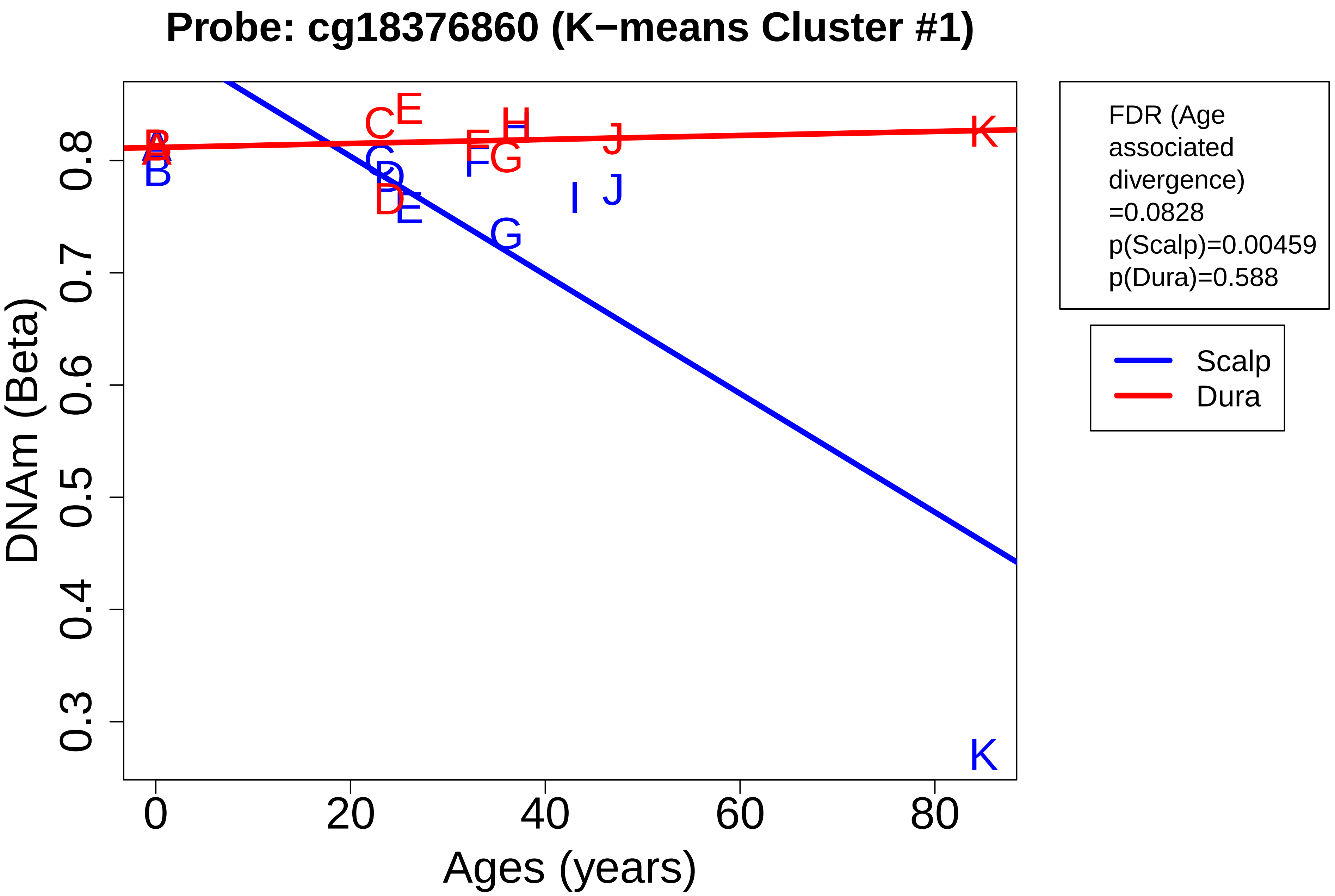

Probe: cg18376860 (K−means Cluster #1)
DNAm (Beta)
0.3
0.4
0.5
0.6
0.7
0.8
Ages (years)
0
20
40
60
80
FDR (Age associated divergence) =0.0828
p(Scalp)=0.00459
p(Dura)=0.588
Scalp
Dura
A
B
C
D
E
F
G
H
I
J
K

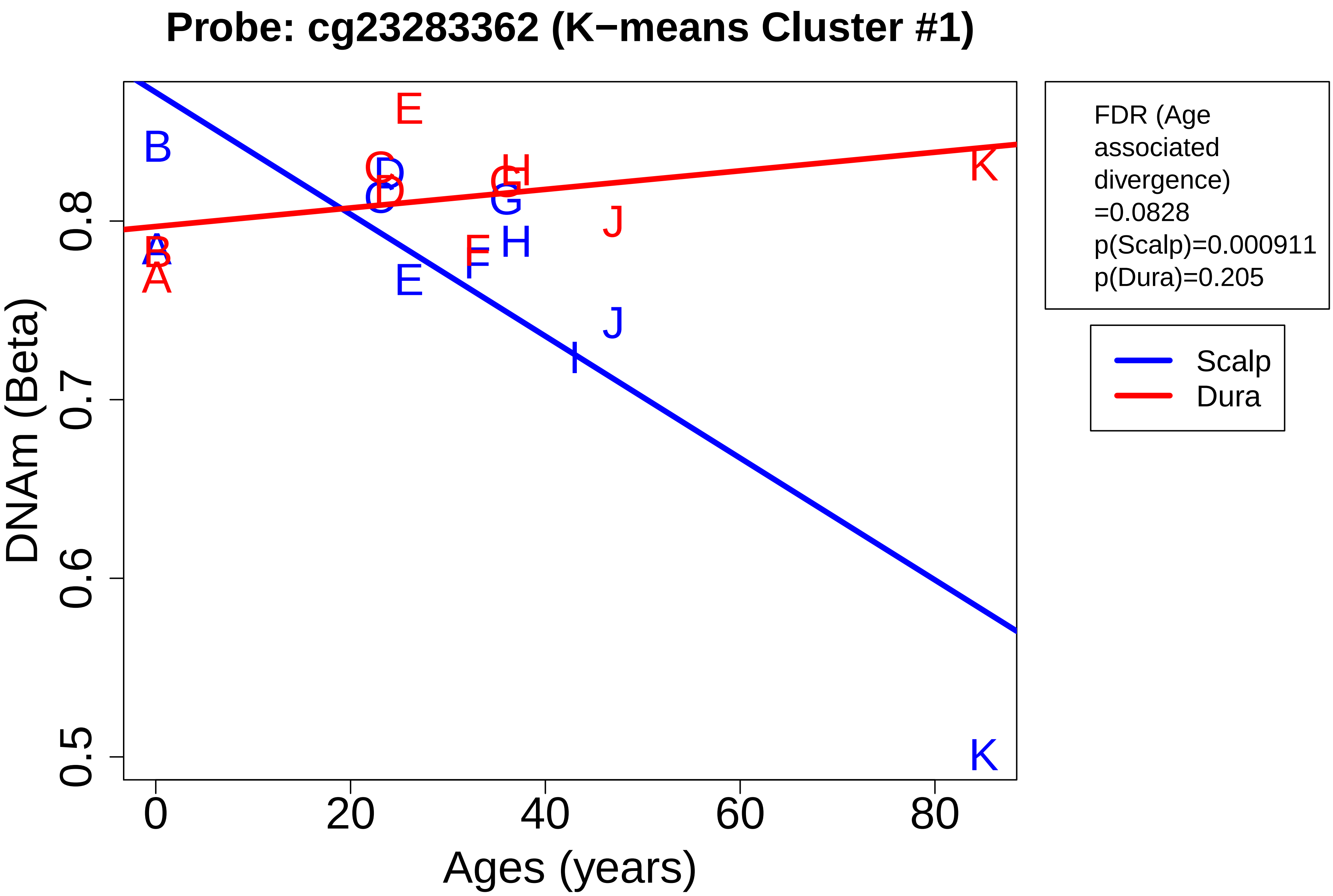

Probe: cg23283362 (K−means Cluster #1)
DNAm (Beta)
0.5
0.6
0.7
0.8
0
20
40
60
80
Ages (years)
FDR (Age associated divergence) =0.0828
p(Scalp)=0.000911
p(Dura)=0.205
Scalp
Dura

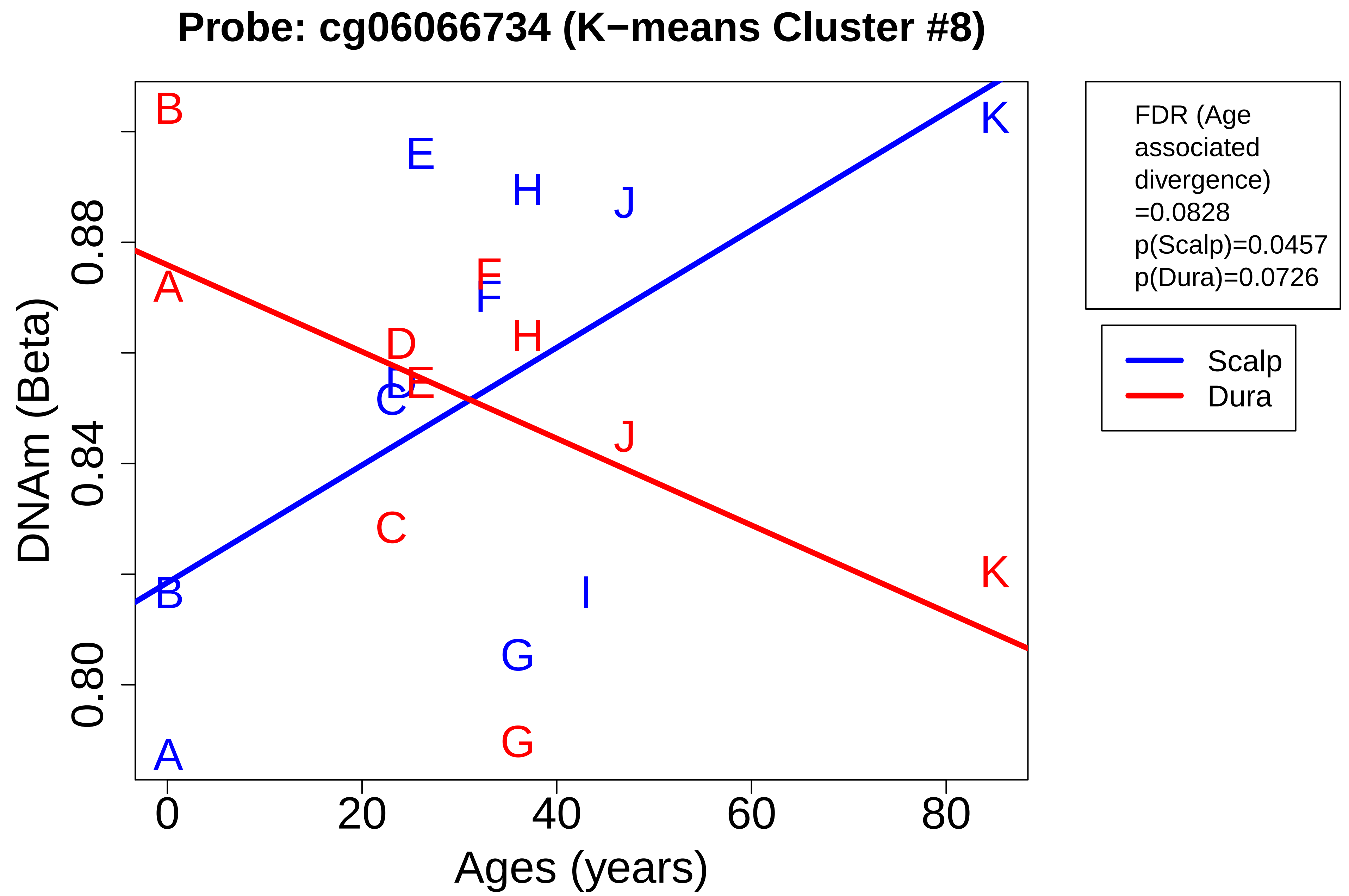

Probe: cg06066734 (K−means Cluster #8)
DNAm (Beta)
0.80
0.84
0.88
Ages (years)
0
20
40
60
80
FDR (Age associated divergence) =0.0828
p(Scalp)=0.0457
p(Dura)=0.0726
Scalp
Dura
A
B
C
D
E
F
G
H
I
J
K

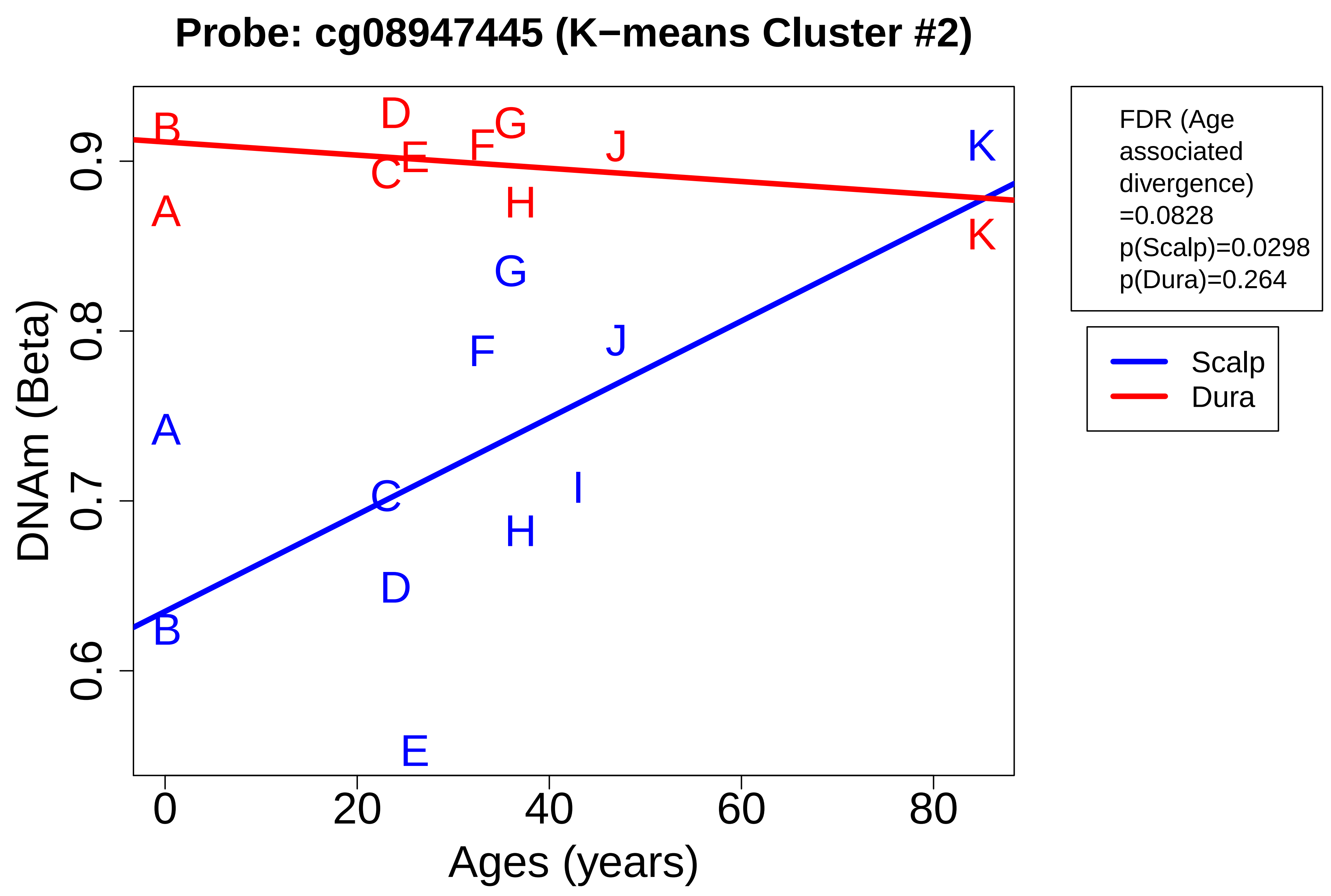

Probe: cg08947445 (K−means Cluster #2)
DNAm (Beta)
0.6
0.7
0.8
0.9
0
20
40
60
80
Ages (years)
FDR (Age associated divergence) =0.0828
p(Scalp)=0.0298
p(Dura)=0.264
Scalp
Dura
A
B
C
D
E
F
G
H
I
J
K

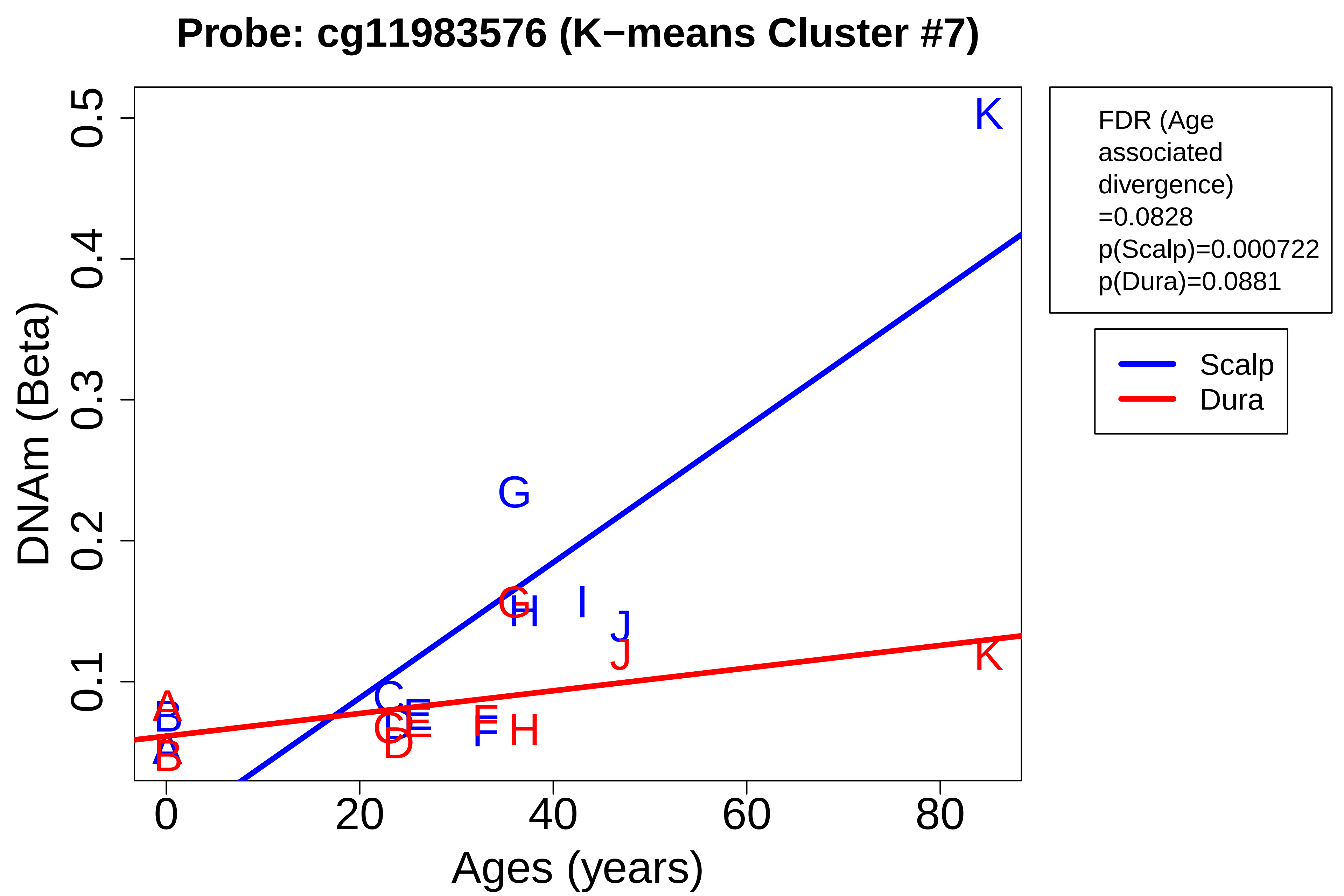

Probe: cg11983576 (K−means Cluster #7)
DNAm (Beta)
0.1
0.2
0.3
0.4
0.5
Ages (years)
0
20
40
60
80
FDR (Age associated divergence) =0.0828
p(Scalp)=0.000722
p(Dura)=0.0881
Scalp
Dura

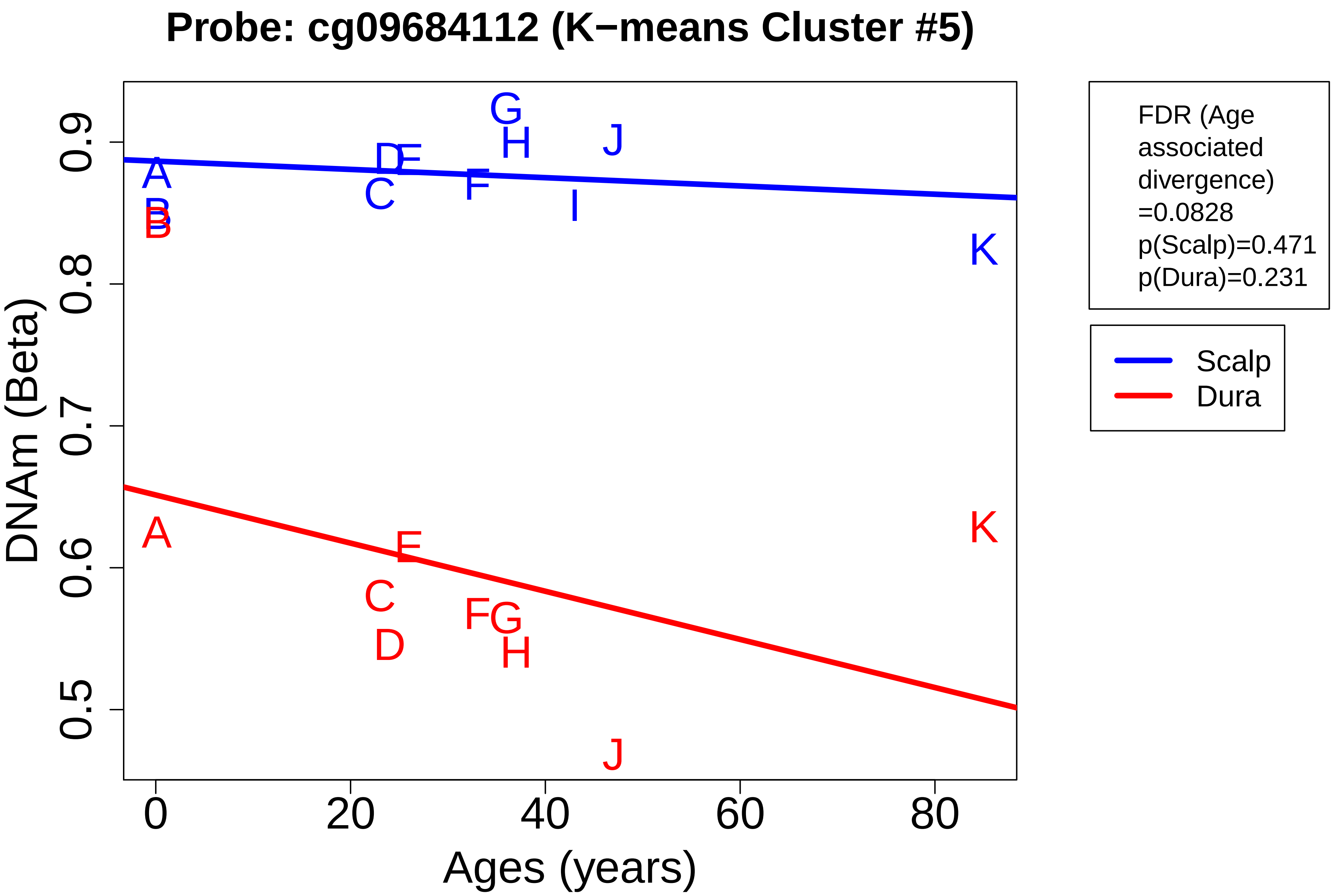

Probe: cg09684112 (K−means Cluster #5)
DNAm (Beta)
0.5
0.6
0.7
0.8
0.9
0
20
40
60
80
Ages (years)
A
B
C
D
E
F
G
H
I
J
K
FDR (Age associated divergence) =0.0828
p(Scalp)=0.471
p(Dura)=0.231
Scalp
Dura

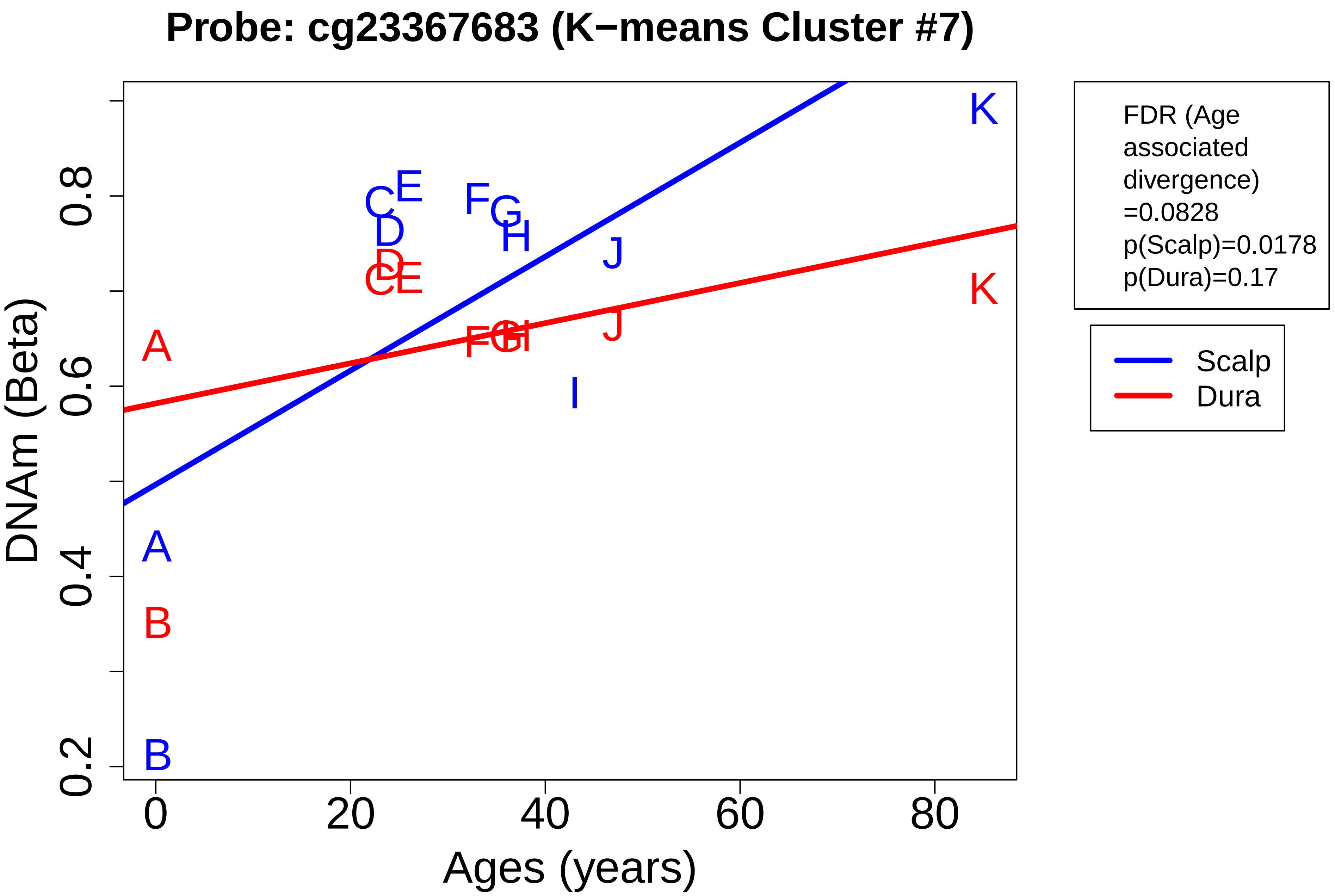
Probe: cg23367683 (K−means Cluster #7)
DNAm (Beta)
0.2
0.4
0.6
0.8
0
20
40
60
80
Ages (years)
A
B
C
D
E
F
G
H
I
J
K
FDR (Age associated divergence) =0.0828
p(Scalp)=0.0178
p(Dura)=0.17
Scalp
Dura

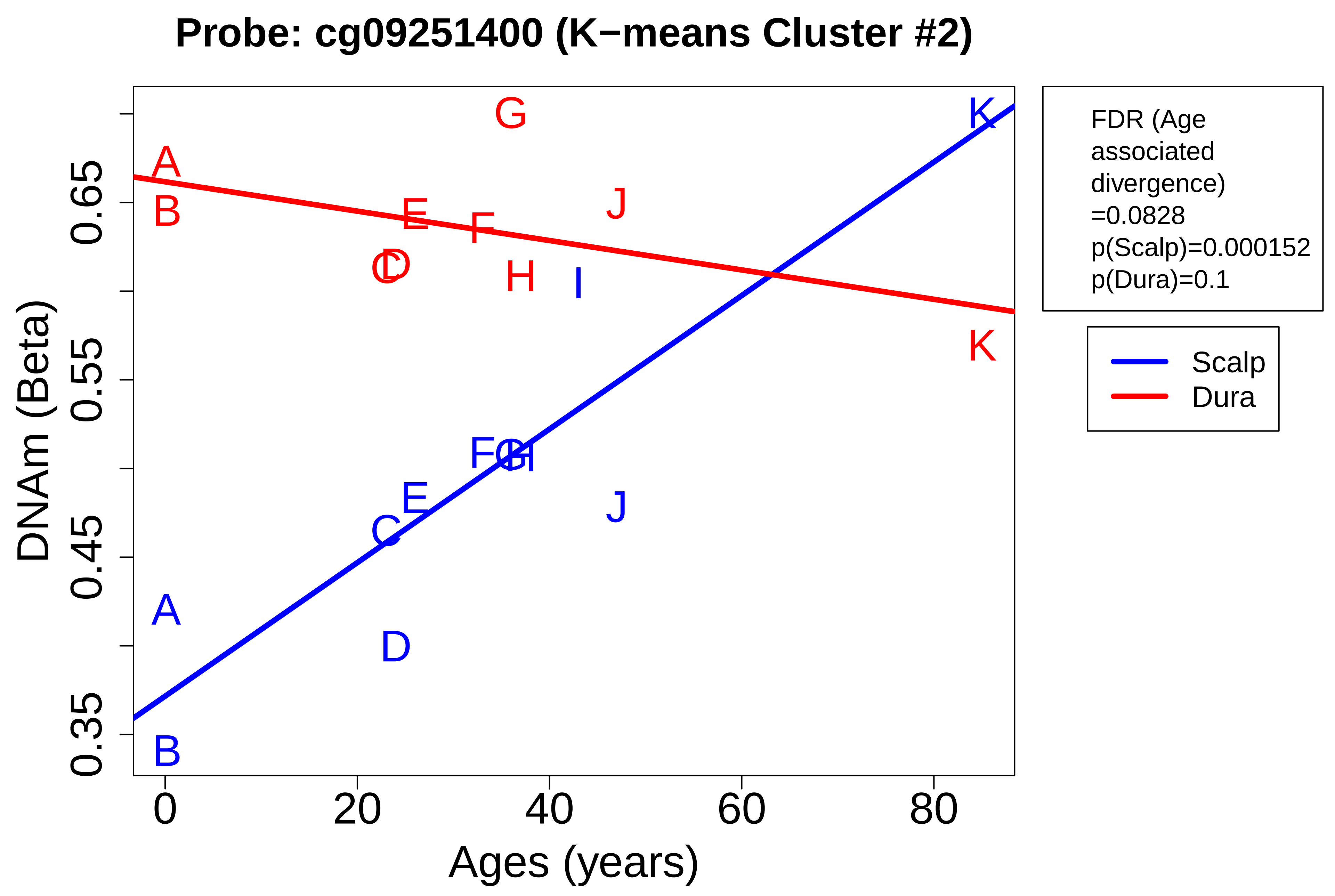
Probe: cg09251400 (K−means Cluster #2)
DNAm (Beta)
0.35
0.45
0.55
0.65
0
20
40
60
80
Ages (years)
FDR (Age associated divergence) =0.0828
p(Scalp)=0.000152
p(Dura)=0.1
Scalp
Dura

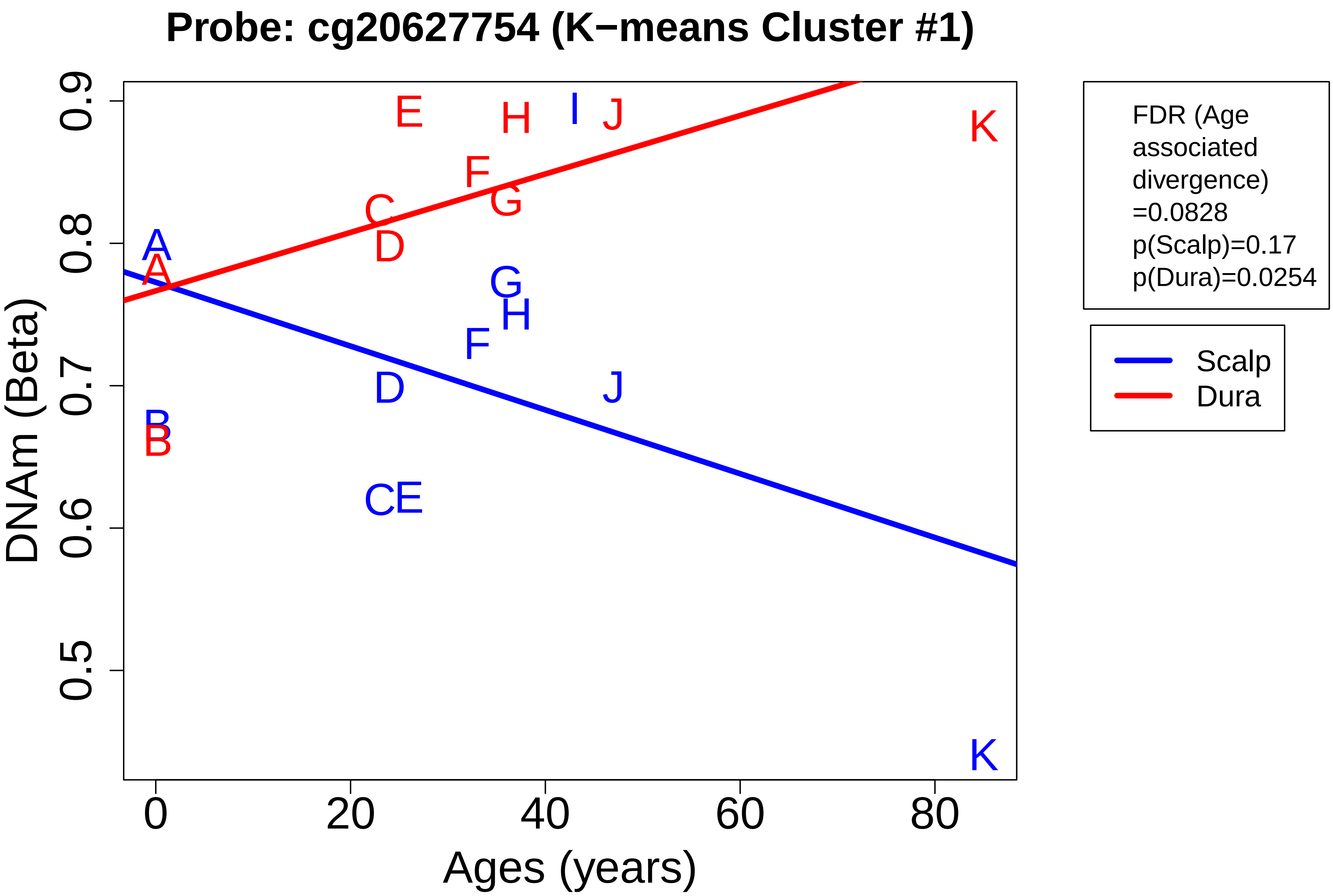
Probe: cg20627754 (K−means Cluster #1)
DNAm (Beta)
0.5
0.6
0.7
0.8
0.9
0
20
40
60
80
Ages (years)
A
B
C
D
E
F
G
H
I
J
K
A
B
CE
D
F
G
H
J
K
FDR (Age associated divergence) =0.0828
p(Scalp)=0.17
p(Dura)=0.0254
Scalp
Dura

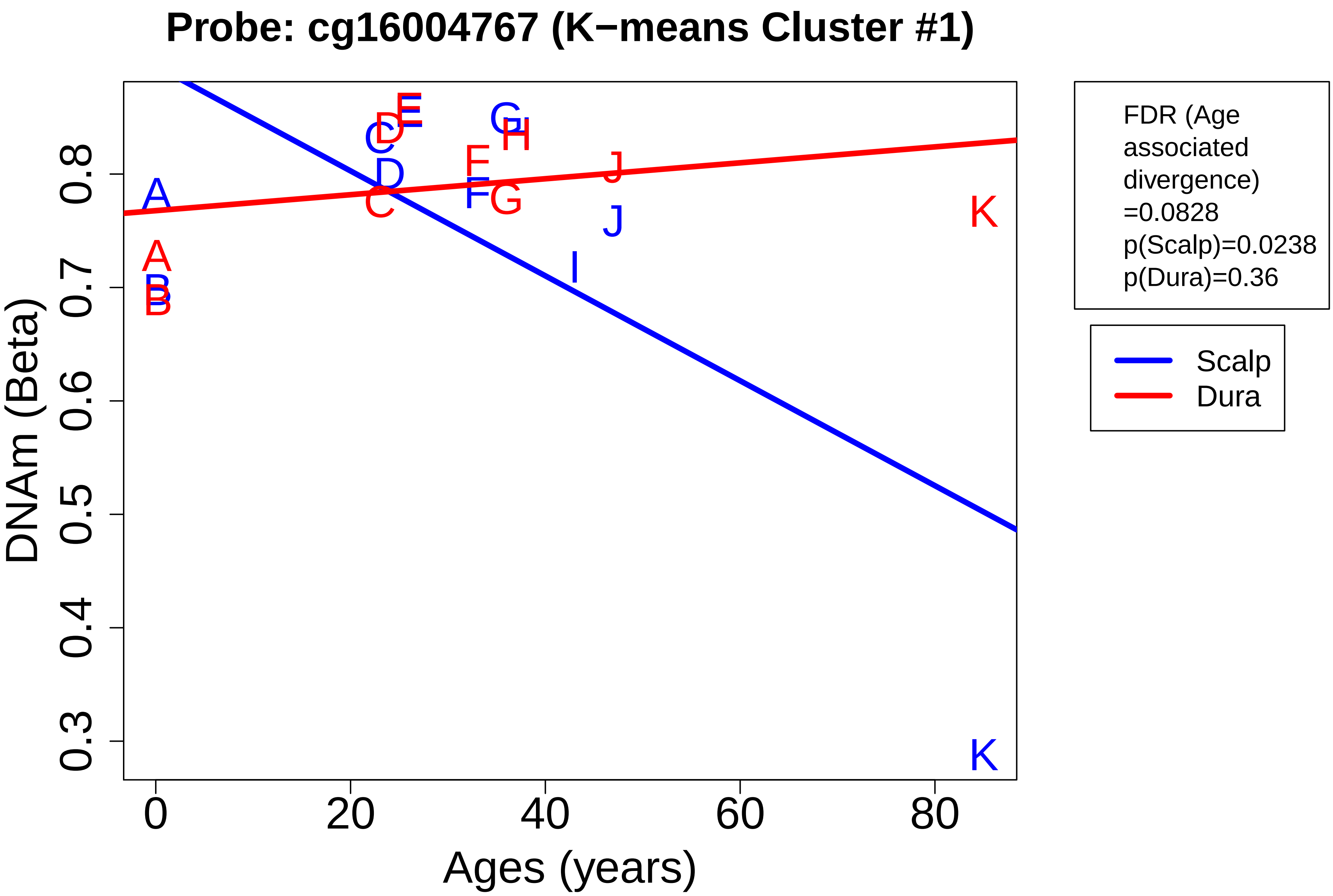
Probe: cg16004767 (K−means Cluster #1)
DNAm (Beta)
0.3
0.4
0.5
0.6
0.7
0.8
0
20
40
60
80
Ages (years)
A
B
C
D
E
F
G
H
I
J
K
FDR (Age associated divergence) =0.0828
p(Scalp)=0.0238
p(Dura)=0.36
Scalp
Dura

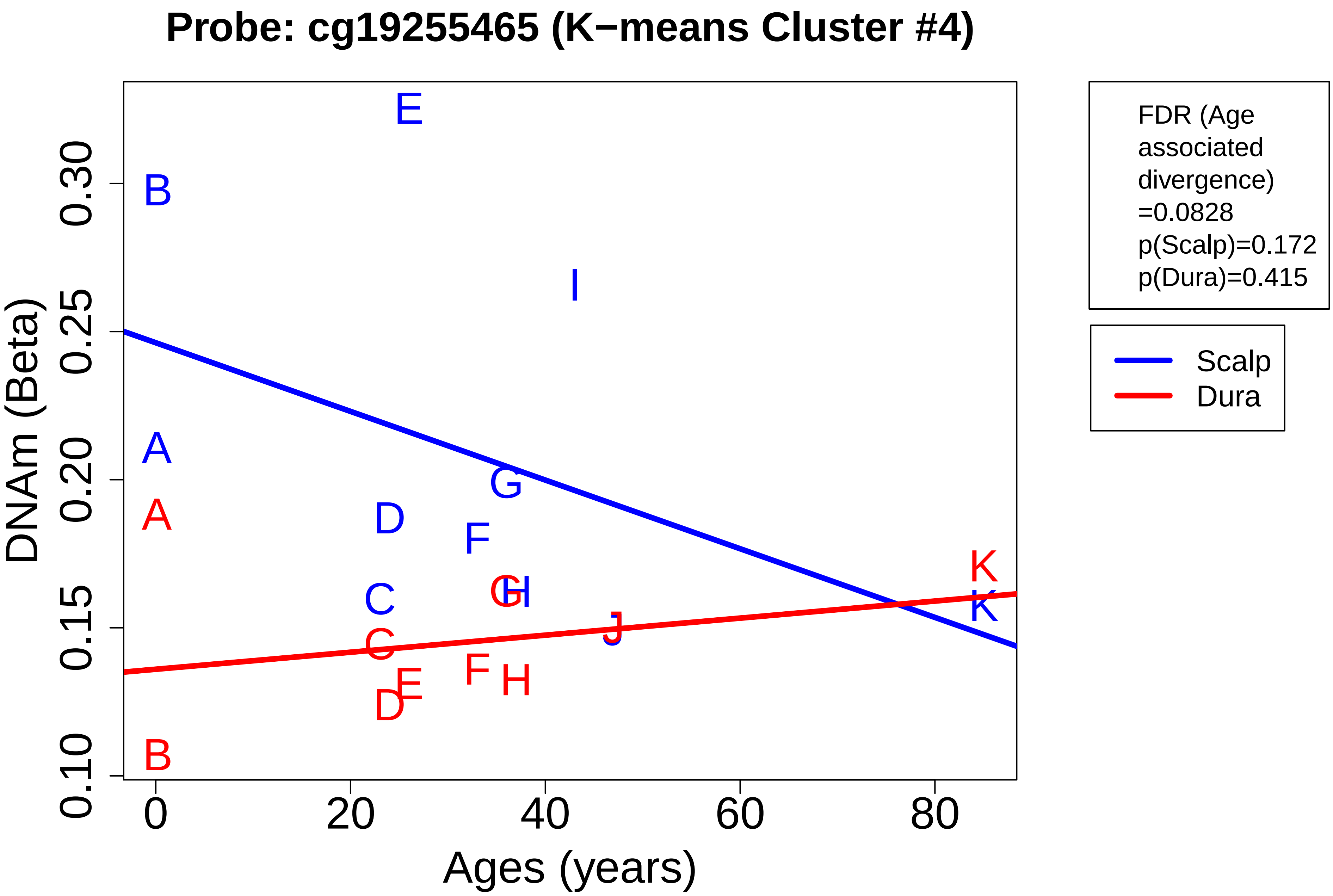

Probe: cg19255465 (K−means Cluster #4)
DNAm (Beta)
0.10
0.15
0.20
0.25
0.30
0
20
40
60
80
Ages (years)
FDR (Age associated divergence) =0.0828
p(Scalp)=0.172
p(Dura)=0.415
Scalp
Dura
A
B
C
D
E
F
G
H
I
J
K

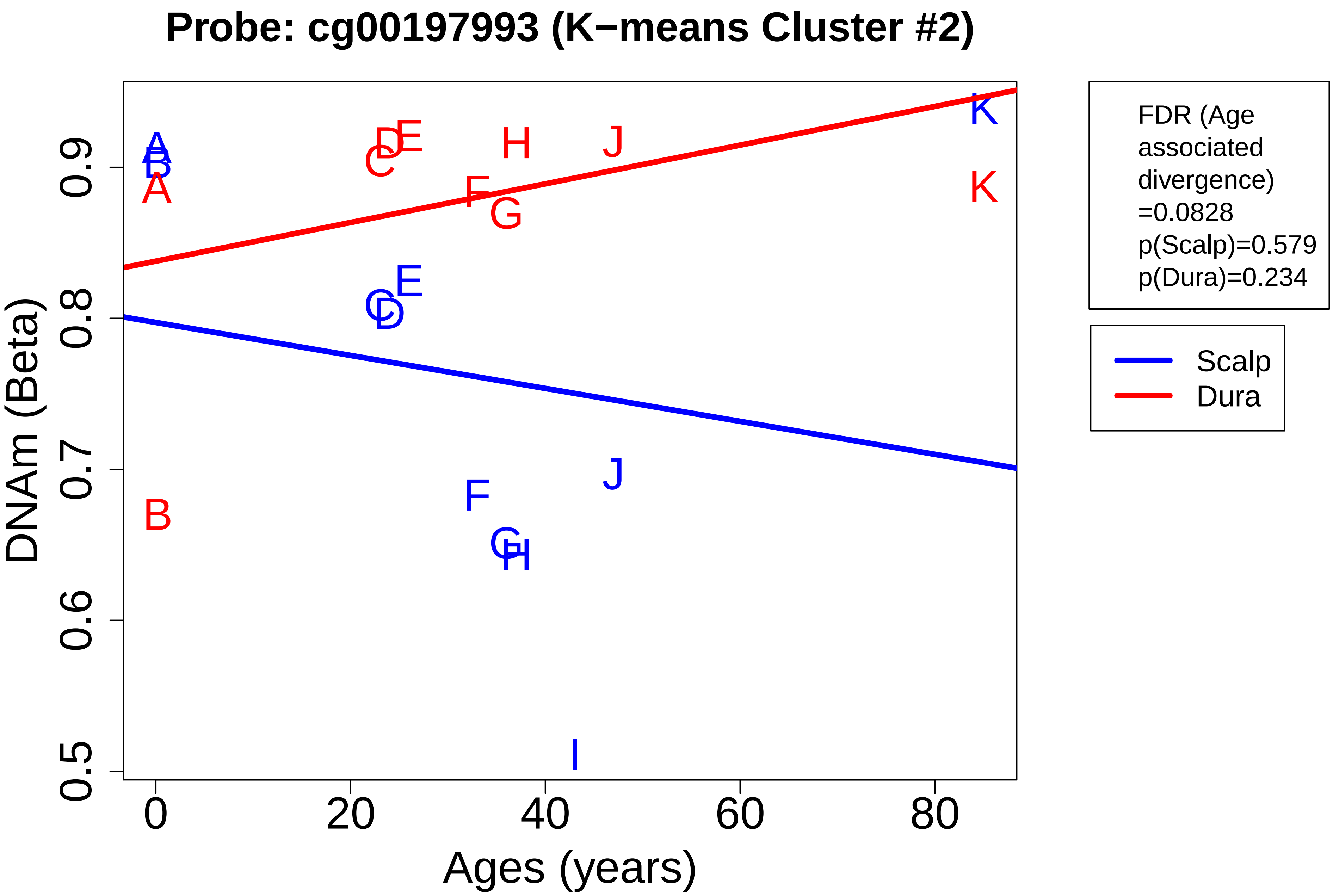

Probe: cg00197993 (K−means Cluster #2)
DNAm (Beta)
0.5
0.6
0.7
0.8
0.9
0
20
40
60
80
Ages (years)
FDR (Age associated divergence) =0.0828
p(Scalp)=0.579
p(Dura)=0.234
Scalp
Dura

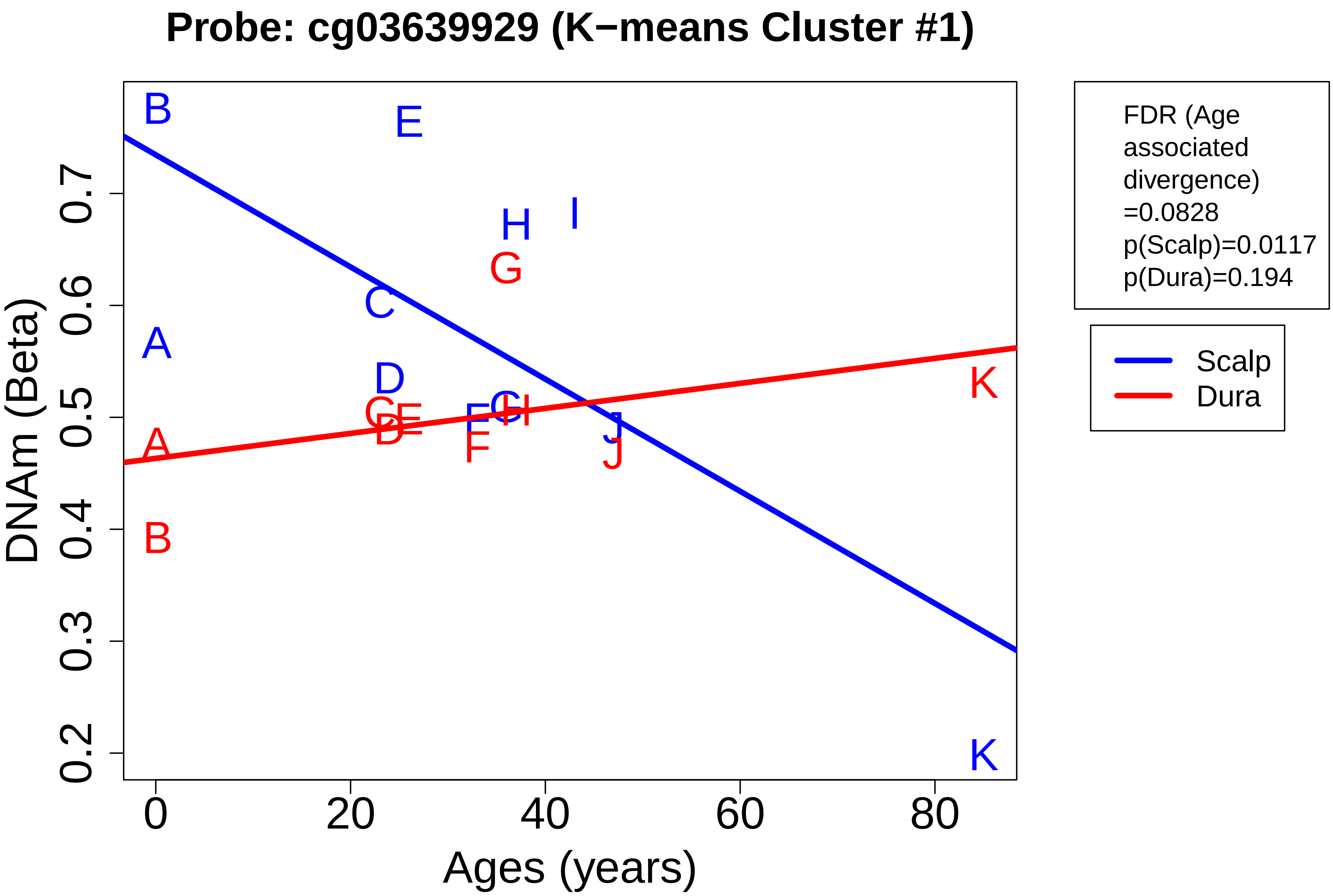

Probe: cg03639929 (K−means Cluster #1)
FDR (Age associated divergence) =0.0828
p(Scalp)=0.0117
p(Dura)=0.194
Scalp
Dura
DNAm (Beta)
0.2
0.3
0.4
0.5
0.6
0.7
Ages (years)
0
20
40
60
80

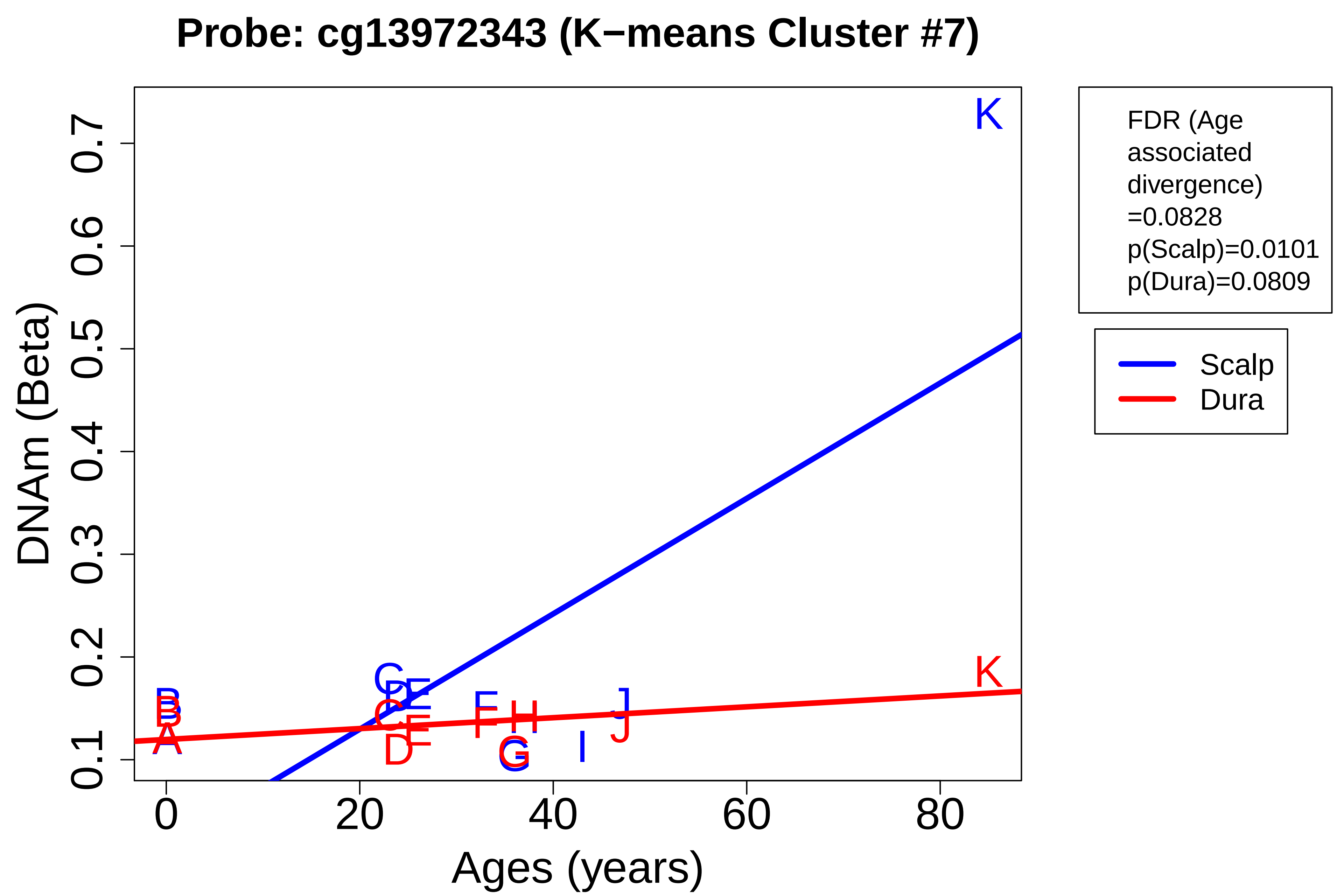

Probe: cg13972343 (K−means Cluster #7)
DNAm (Beta)
0.1
0.2
0.3
0.4
0.5
0.6
0.7
Ages (years)
0
20
40
60
80
FDR (Age associated divergence) =0.0828
p(Scalp)=0.0101
p(Dura)=0.0809
Scalp
Dura

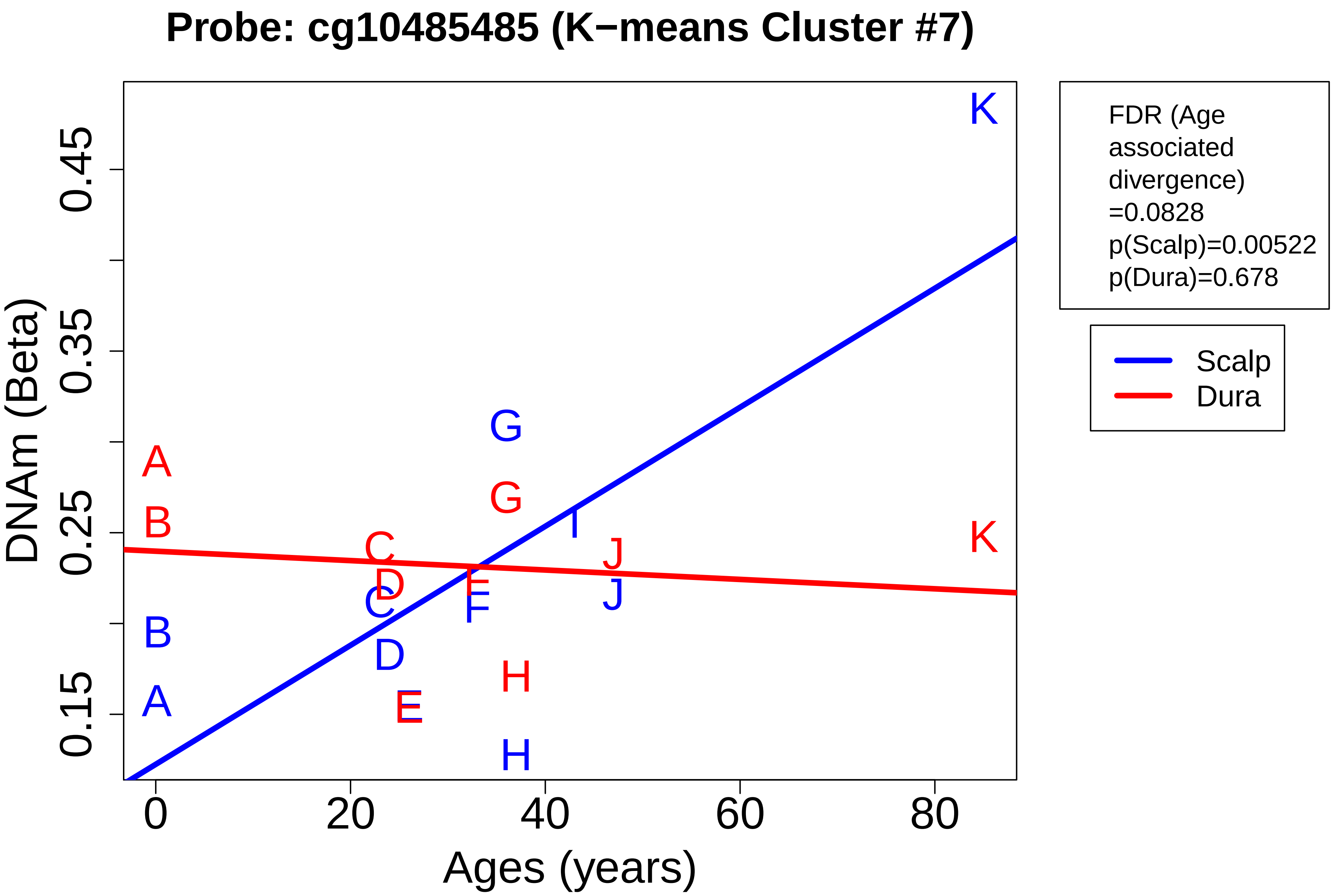
Probe: cg10485485 (K−means Cluster #7)
DNAm (Beta)
0.15
0.25
0.35
0.45
0
20
40
60
80
Ages (years)
FDR (Age associated divergence) =0.0828
p(Scalp)=0.00522
p(Dura)=0.678
Scalp
Dura

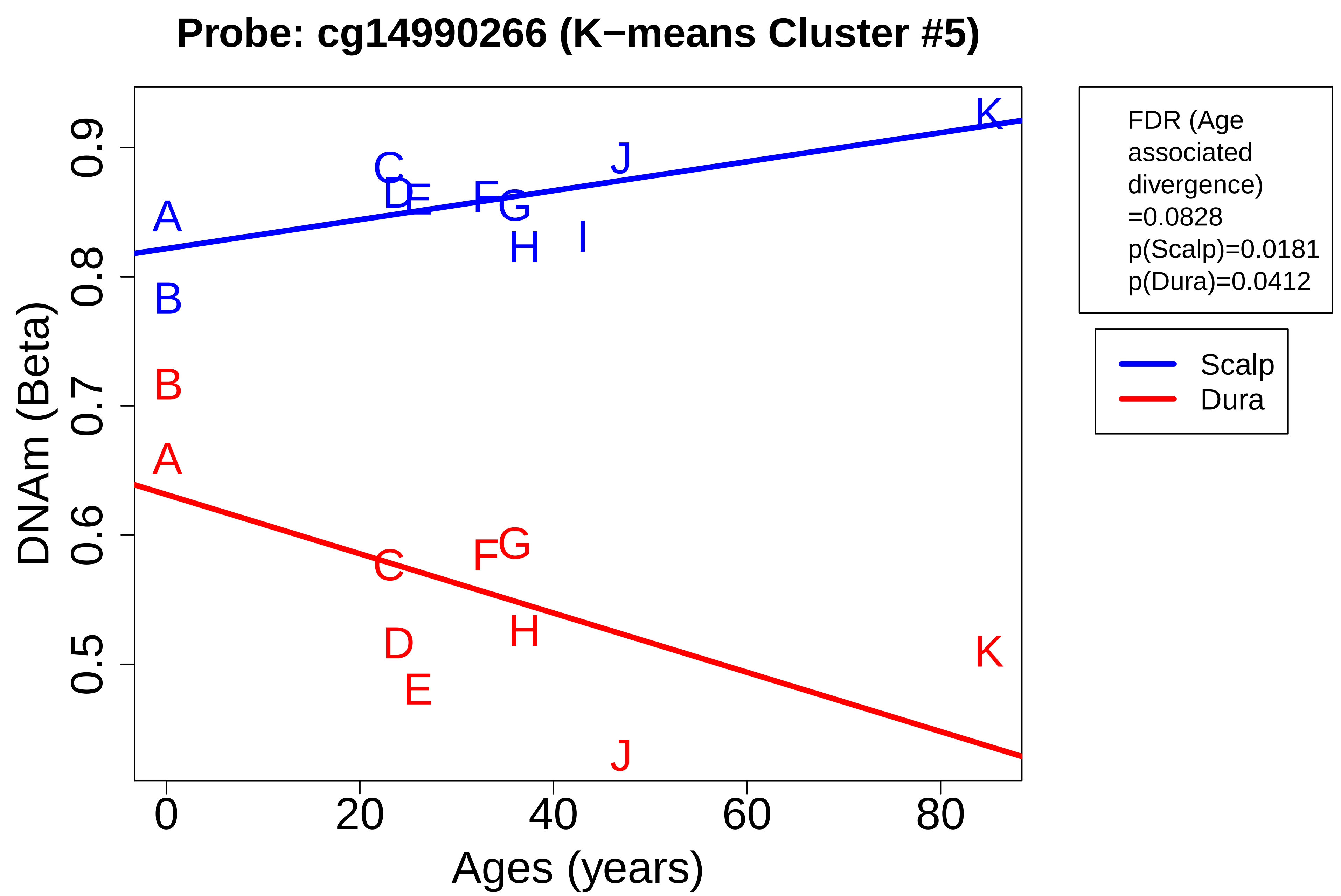

Probe: cg14990266 (K−means Cluster #5)
DNAm (Beta)
0.5
0.6
0.7
0.8
0.9
0
20
40
60
80
Ages (years)
FDR (Age associated divergence) =0.0828
p(Scalp)=0.0181
p(Dura)=0.0412
Scalp
Dura

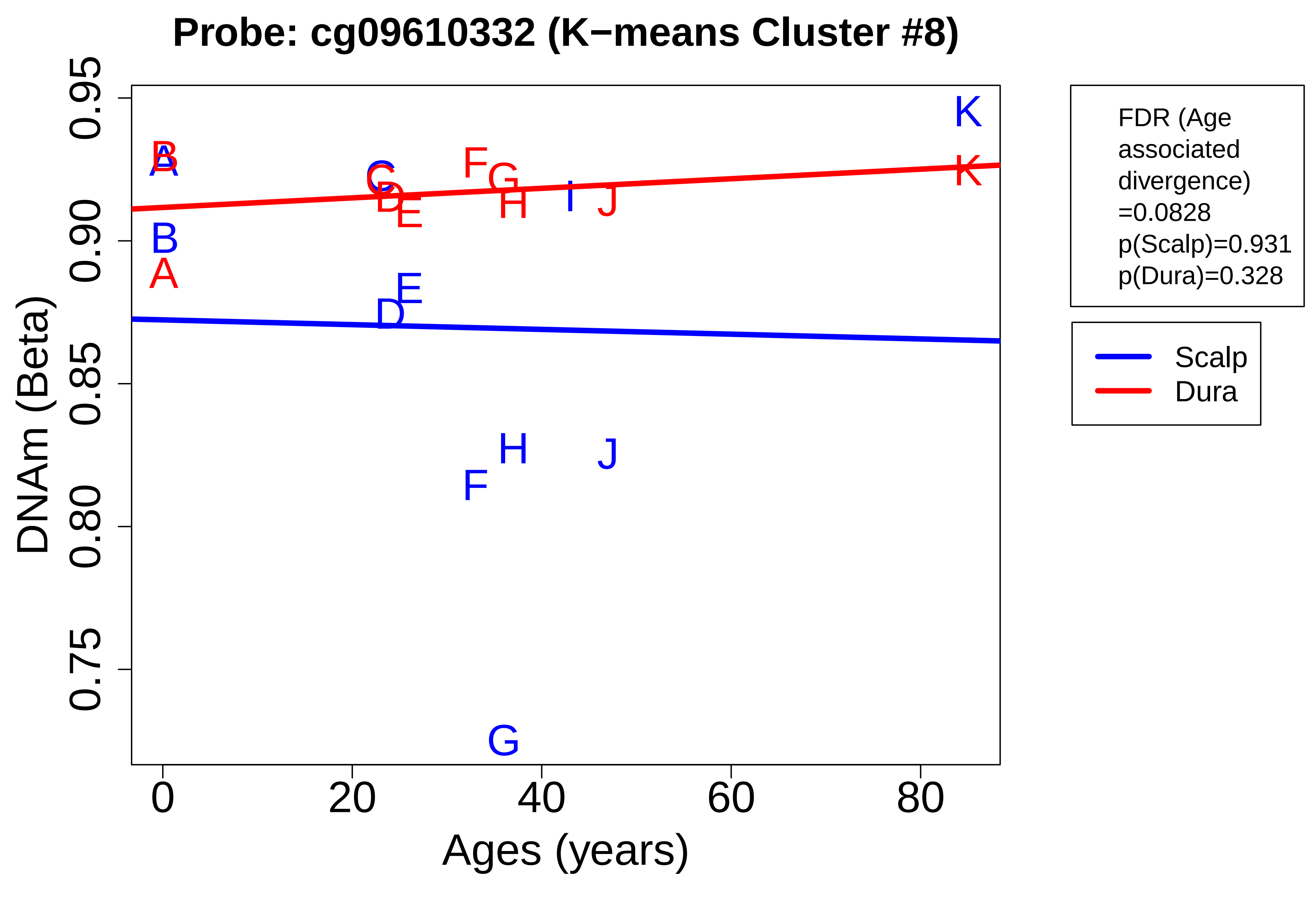

Probe: cg09610332 (K−means Cluster #8)
DNAm (Beta)
0.75
0.80
0.85
0.90
0.95
0
20
40
60
80
Ages (years)
FDR (Age associated divergence) =0.0828
p(Scalp)=0.931
p(Dura)=0.328
Scalp
Dura

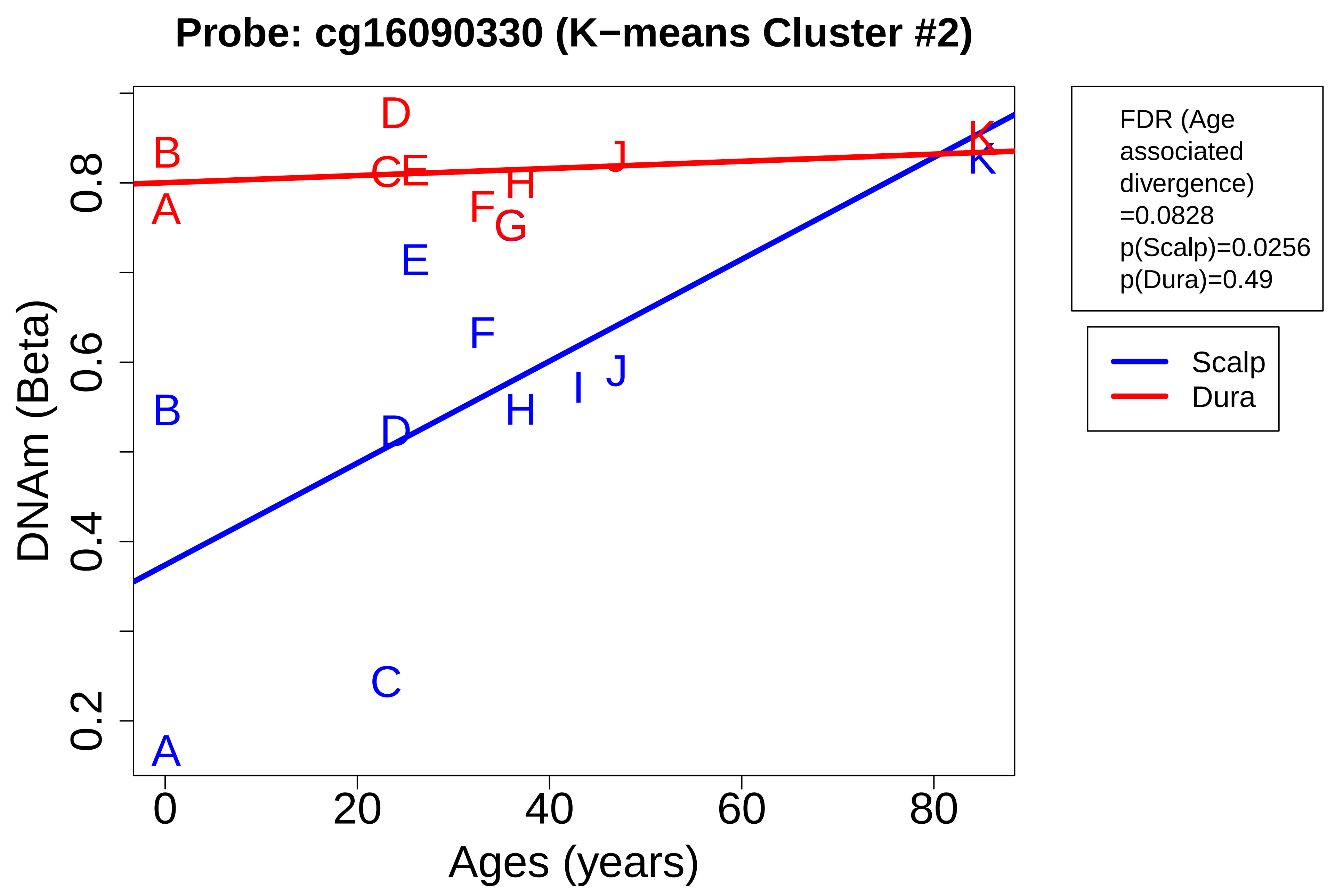

Probe: cg16090330 (K−means Cluster #2)
FDR (Age associated divergence) =0.0828
p(Scalp)=0.0256
p(Dura)=0.49
Scalp
Dura
DNAm (Beta)
0.2
0.4
0.6
0.8
0
20
40
60
80
Ages (years)
A
B
C
D
E
F
G
H
I
J
K

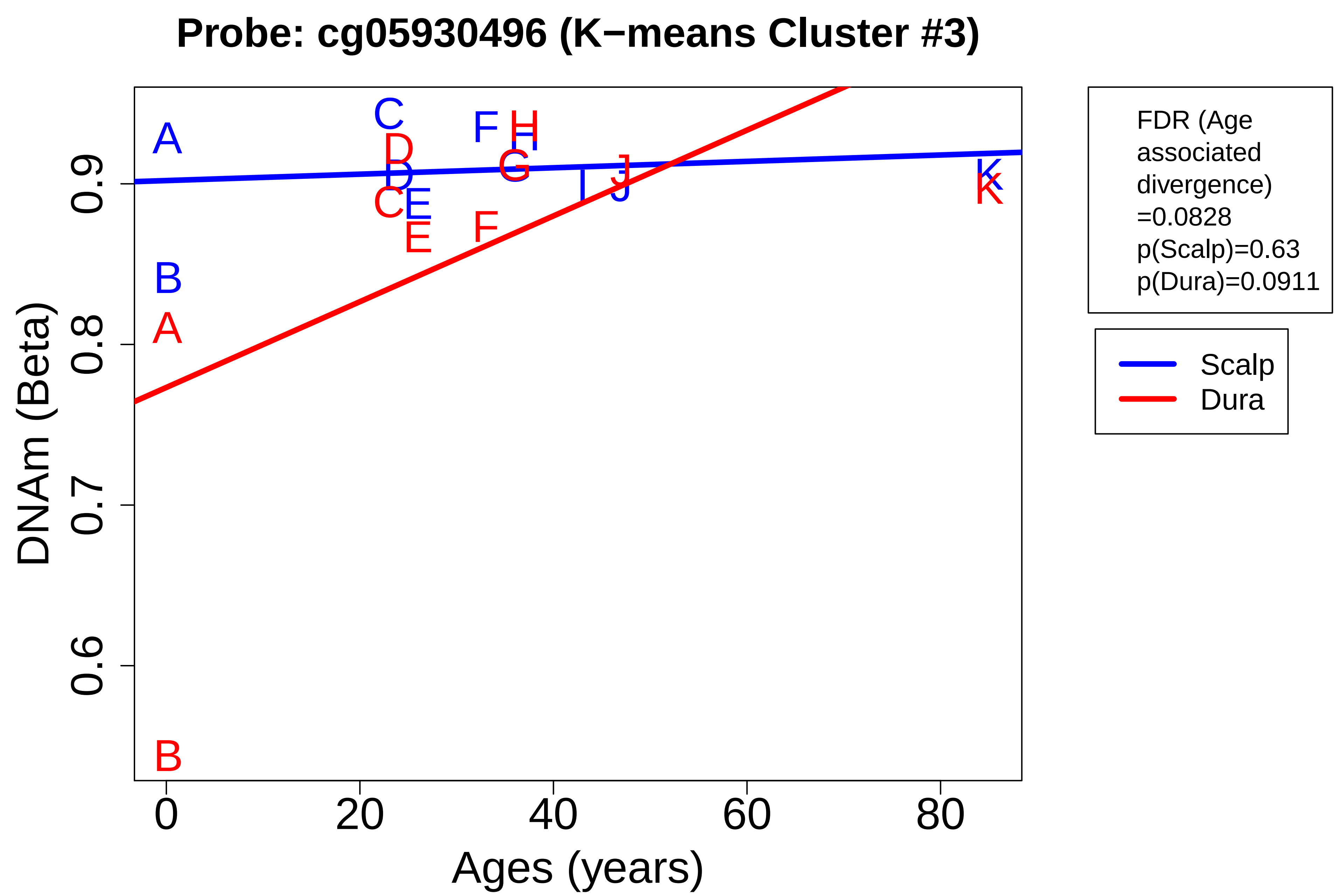

Probe: cg05930496 (K−means Cluster #3)
DNAm (Beta)
0.6
0.7
0.8
0.9
Ages (years)
0
20
40
60
80
FDR (Age associated divergence) =0.0828
p(Scalp)=0.63
p(Dura)=0.0911
Scalp
Dura

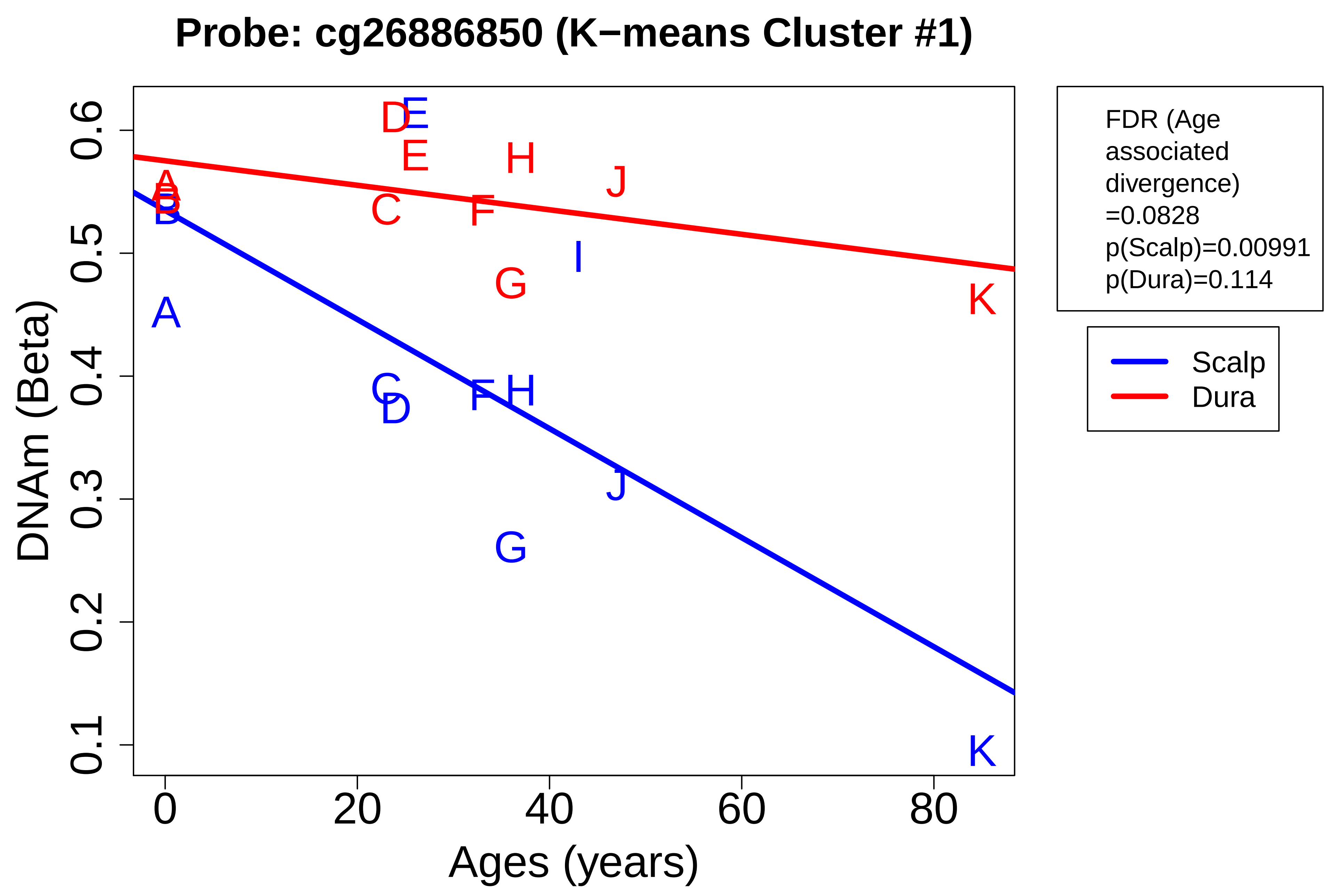
Probe: cg26886850 (K−means Cluster #1)
DNAm (Beta)
Ages (years)
0.1
0.2
0.3
0.4
0.5
0.6
0
20
40
60
80
A
B
C
D
E
F
G
H
I
J
K
FDR (Age associated divergence) =0.0828
p(Scalp)=0.00991
p(Dura)=0.114
Scalp
Dura

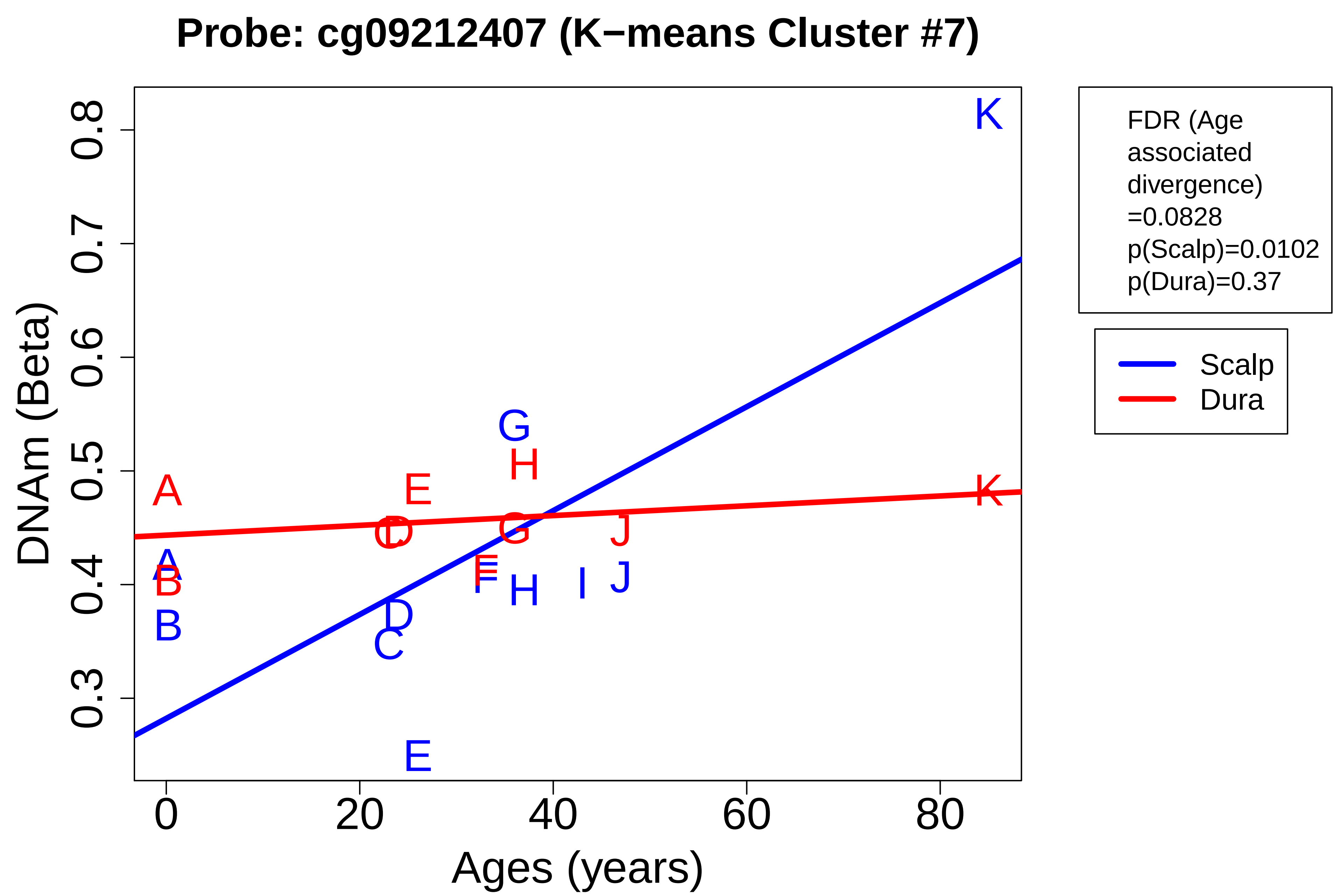

Probe: cg09212407 (K−means Cluster #7)
FDR (Age associated divergence) =0.0828
p(Scalp)=0.0102
p(Dura)=0.37
Scalp
Dura
DNAm (Beta)
Ages (years)
0.3
0.4
0.5
0.6
0.7
0.8
0
20
40
60
80

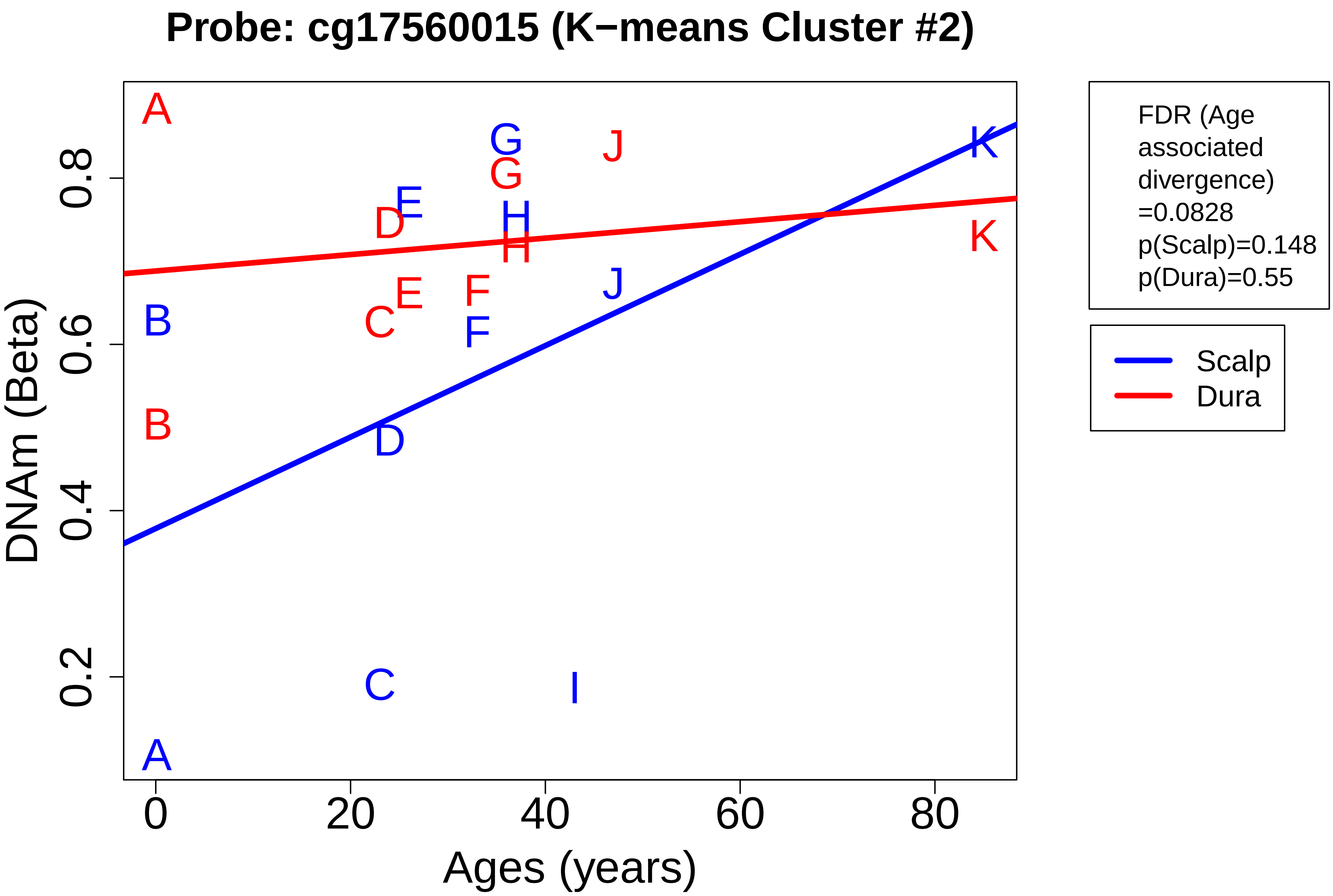
Probe: cg17560015 (K−means Cluster #2)
DNAm (Beta)
0.2
0.4
0.6
0.8
0
20
40
60
80
Ages (years)
A
B
C
D
E
F
G
H
I
J
K
FDR (Age associated divergence) =0.0828
p(Scalp)=0.148
p(Dura)=0.55
Scalp
Dura

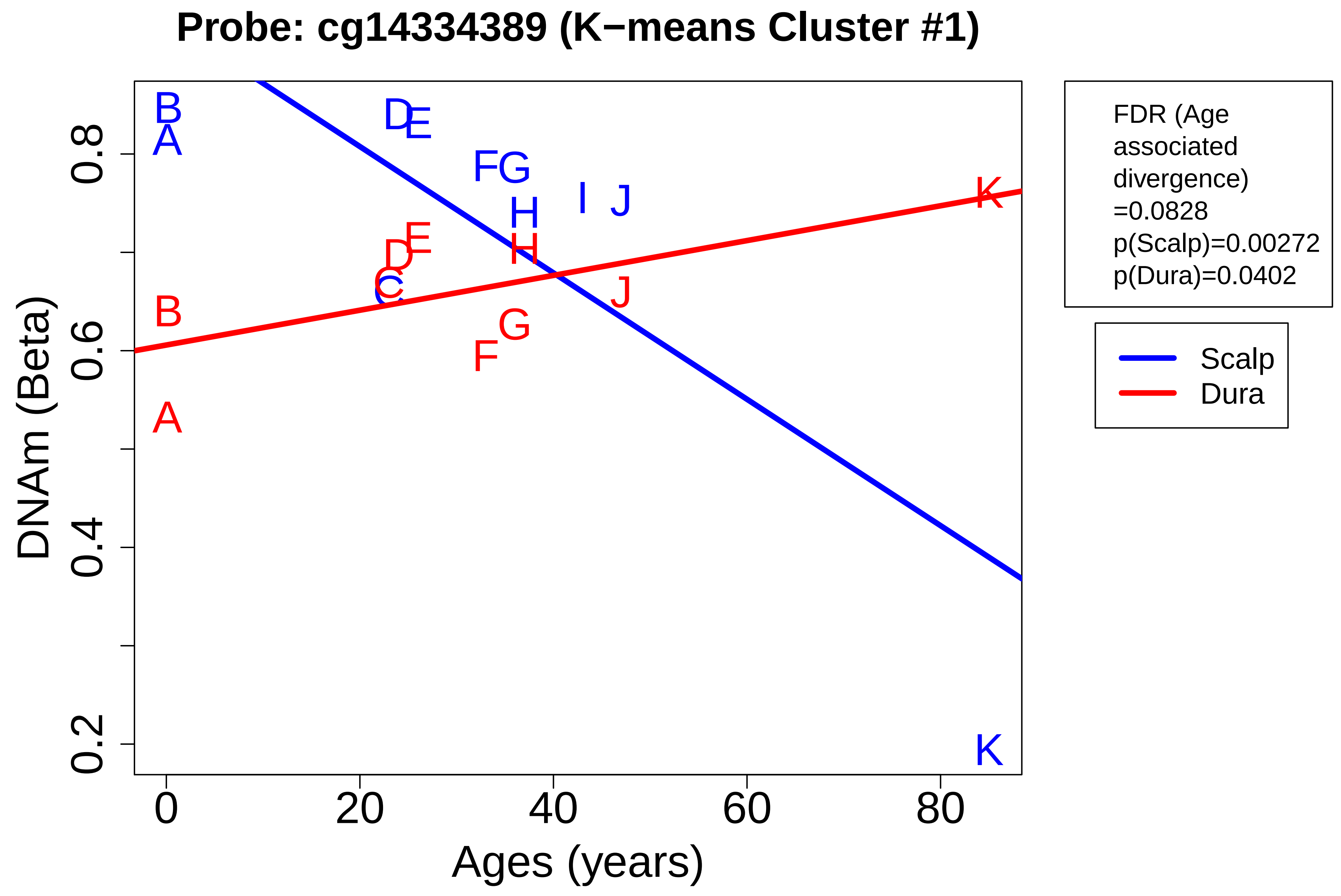
Probe: cg14334389 (K−means Cluster #1)
DNAm (Beta)
0.2
0.4
0.6
0.8
Ages (years)
0
20
40
60
80
A
B
C
D
E
F
G
H
I
J
K
FDR (Age associated divergence) =0.0828
p(Scalp)=0.00272
p(Dura)=0.0402
Scalp
Dura

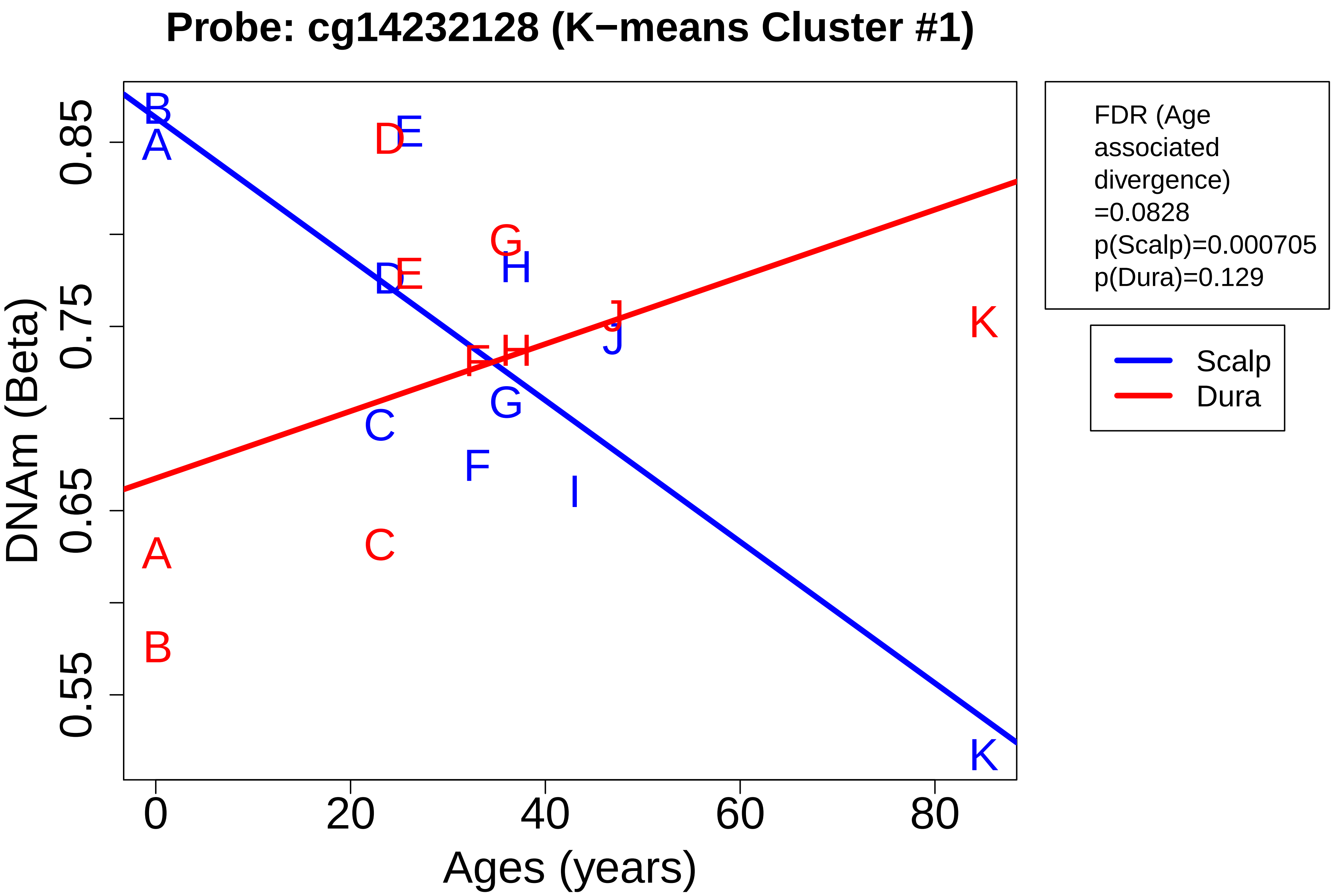

Probe: cg14232128 (K−means Cluster #1)
DNAm (Beta)
0.55
0.65
0.75
0.85
Ages (years)
0
20
40
60
80
FDR (Age associated divergence) =0.0828
p(Scalp)=0.000705
p(Dura)=0.129
Scalp
Dura

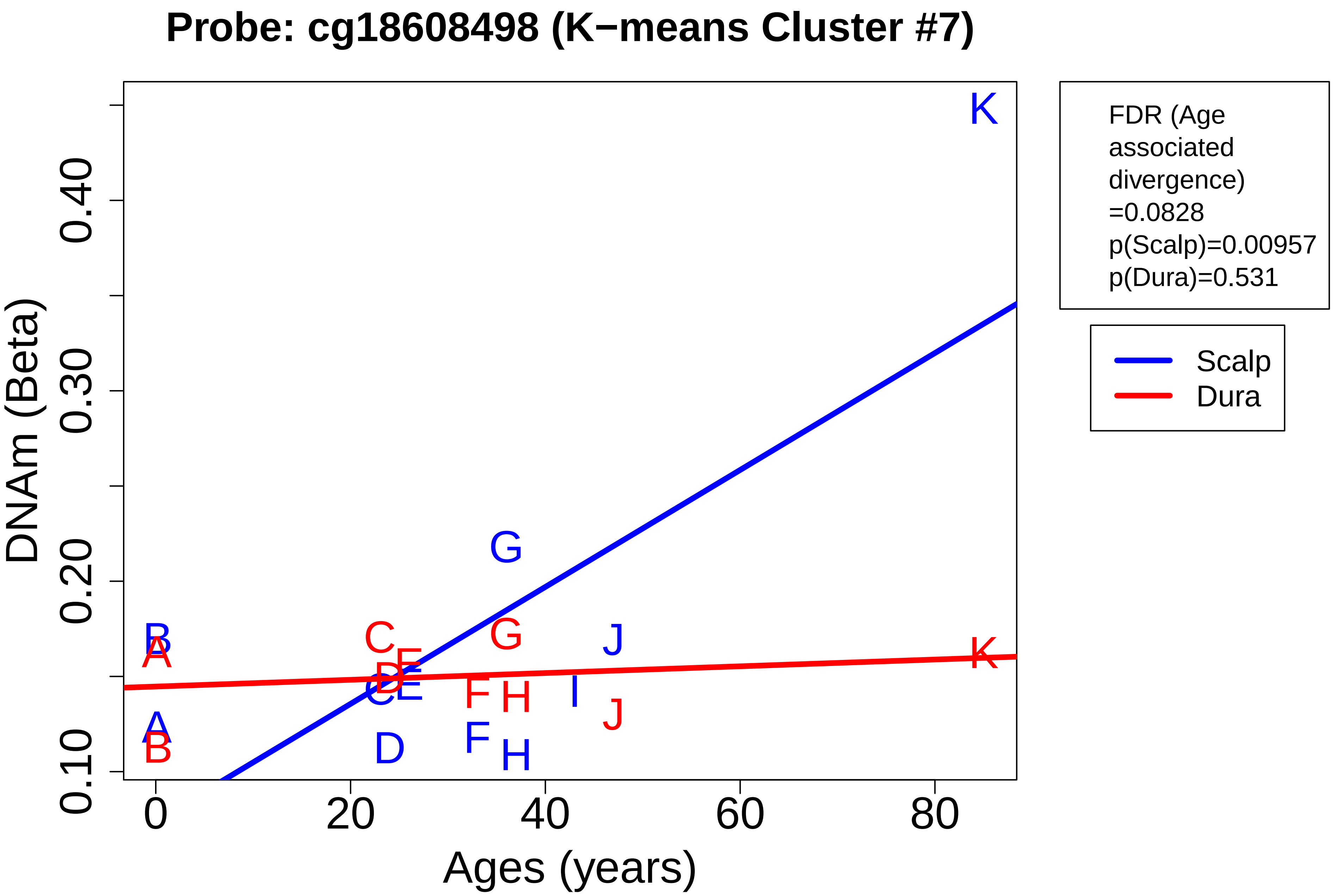

Probe: cg18608498 (K−means Cluster #7)
FDR (Age associated divergence) =0.0828
p(Scalp)=0.00957
p(Dura)=0.531
Scalp
Dura
DNAm (Beta)
0.10
0.20
0.30
0.40
Ages (years)
0
20
40
60
80

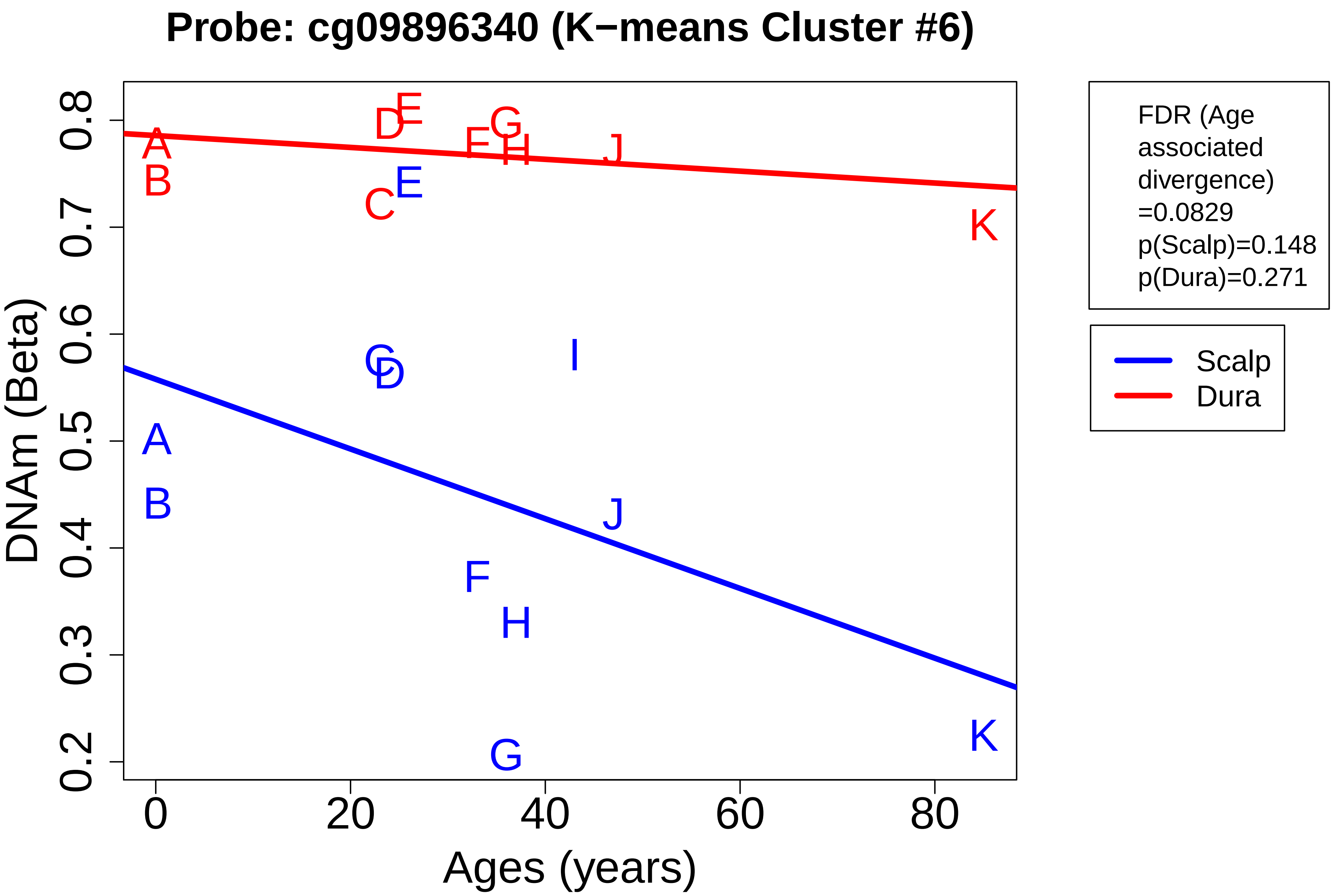

Probe: cg09896340 (K−means Cluster #6)
DNAm (Beta)
0.2
0.3
0.4
0.5
0.6
0.7
0.8
0
20
40
60
80
Ages (years)
FDR (Age associated divergence) =0.0829
p(Scalp)=0.148
p(Dura)=0.271
Scalp
Dura
A
B
C
D
E
F
G
H
I
J
K

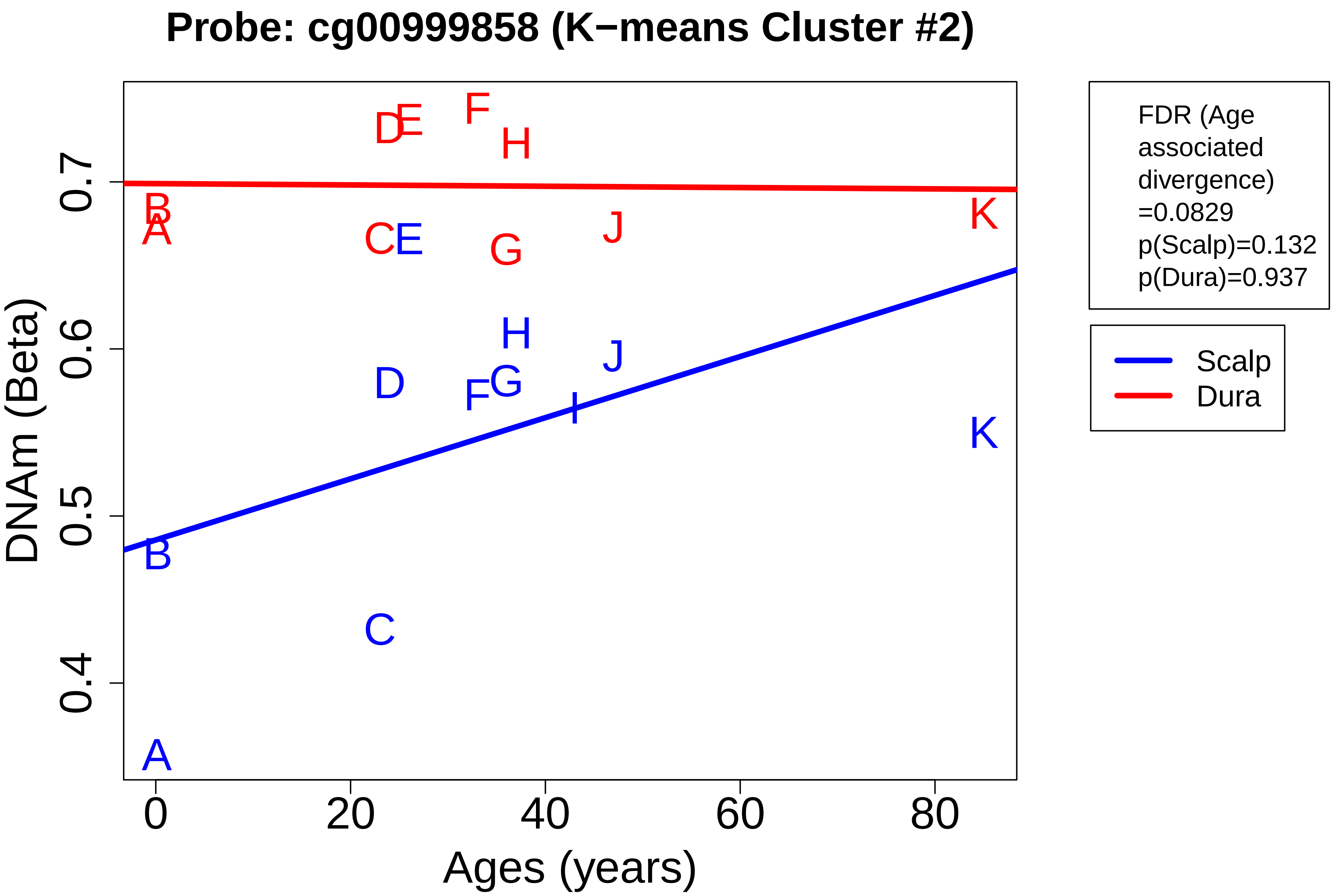

Probe: cg00999858 (K−means Cluster #2)
FDR (Age associated divergence) =0.0829
p(Scalp)=0.132
p(Dura)=0.937
Scalp
Dura
DNAm (Beta)
Ages (years)
0.4
0.5
0.6
0.7
0
20
40
60
80

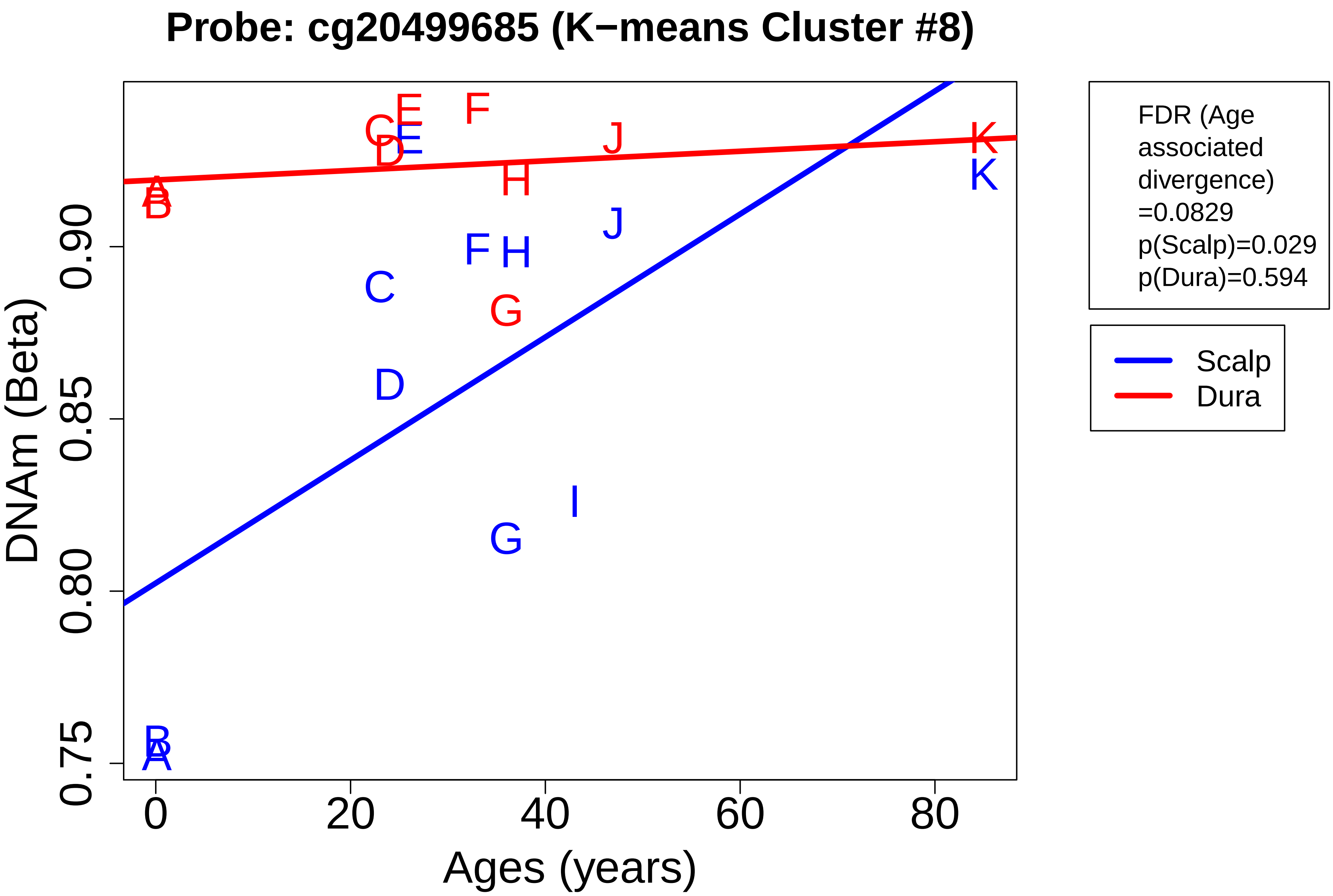

Probe: cg20499685 (K−means Cluster #8)
DNAm (Beta)
0.75
0.80
0.85
0.90
0
20
40
60
80
Ages (years)
FDR (Age associated divergence) =0.0829
p(Scalp)=0.029
p(Dura)=0.594
Scalp
Dura

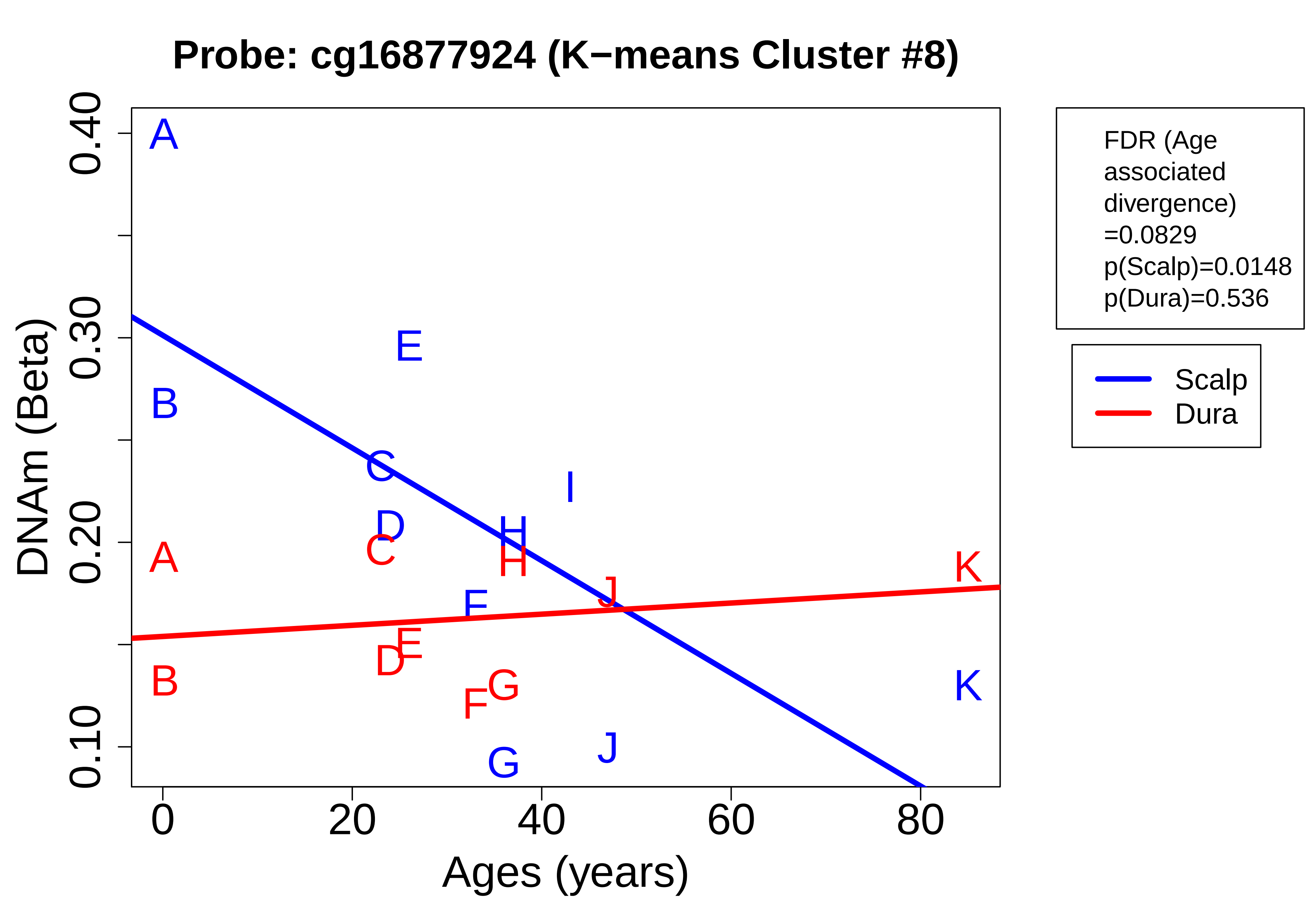

Probe: cg16877924 (K−means Cluster #8)
DNAm (Beta)
0.10
0.20
0.30
0.40
Ages (years)
0
20
40
60
80
A
B
C
D
E
F
G
H
I
J
K
FDR (Age associated divergence) =0.0829
p(Scalp)=0.0148
p(Dura)=0.536
Scalp
Dura

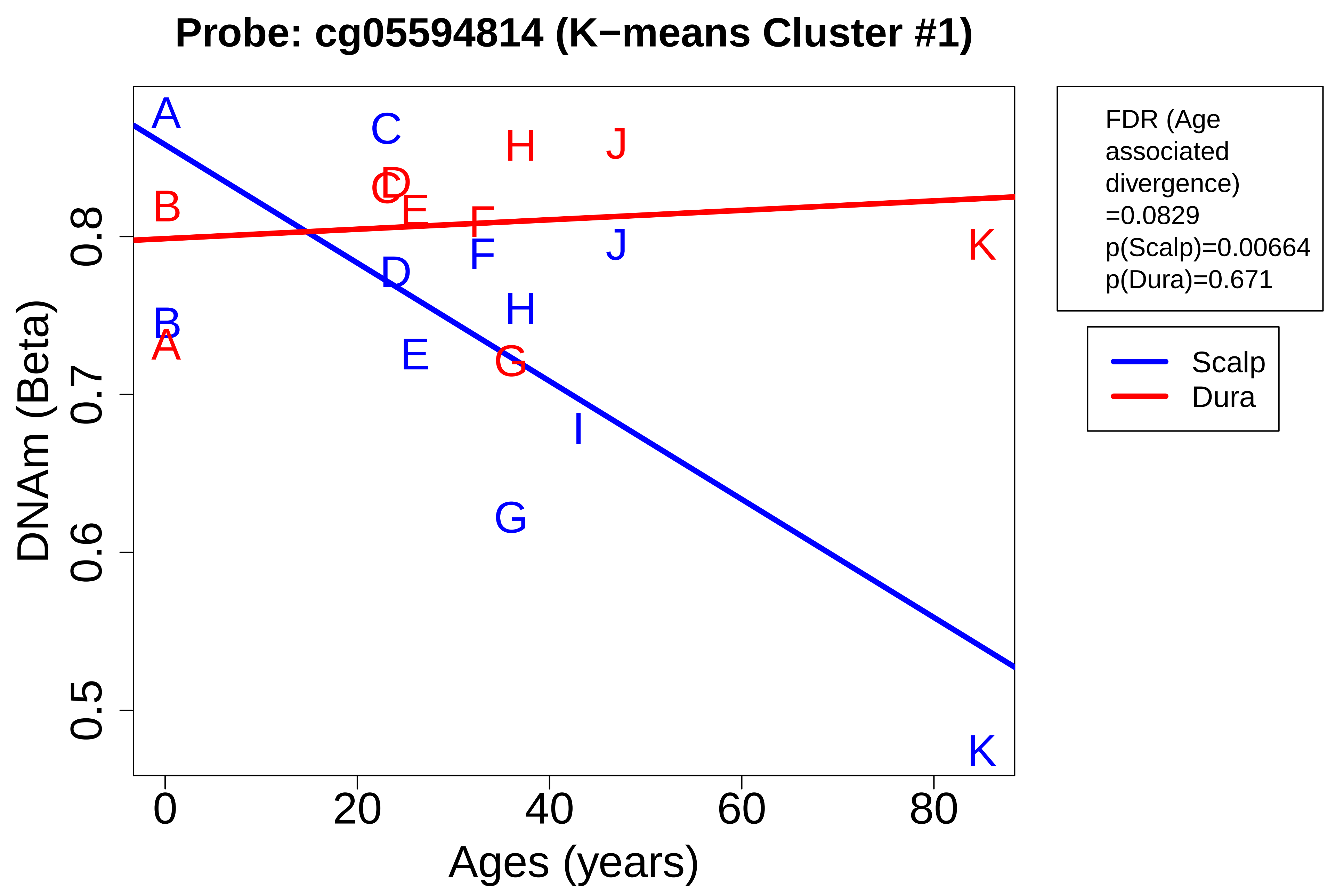

Probe: cg05594814 (K−means Cluster #1)
DNAm (Beta)
Ages (years)
0.5
0.6
0.7
0.8
0
20
40
60
80
A
B
C
D
E
F
G
H
I
J
K
FDR (Age associated divergence) =0.0829
p(Scalp)=0.00664
p(Dura)=0.671
Scalp
Dura

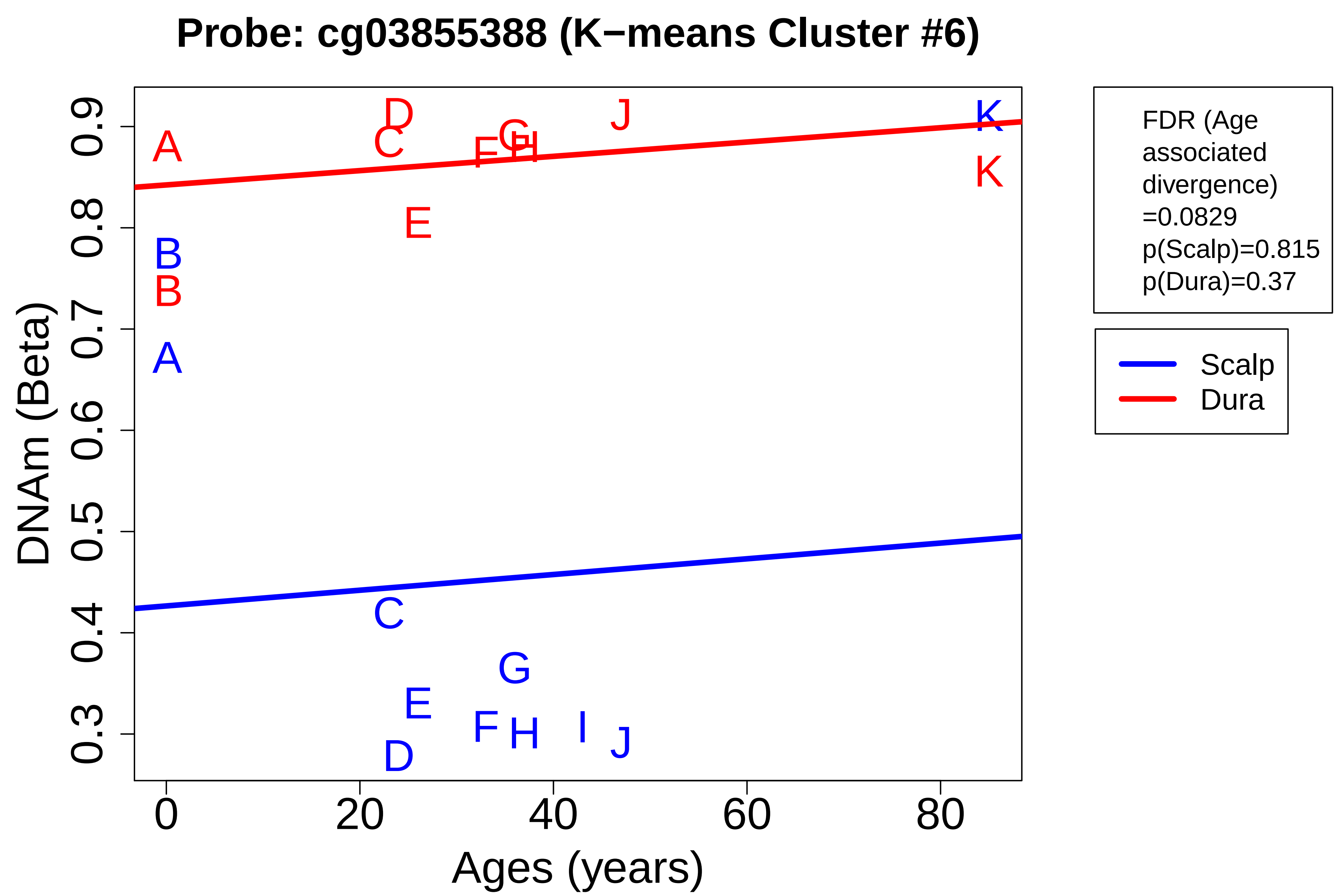
Probe: cg03855388 (K−means Cluster #6)
DNAm (Beta)
0.3
0.4
0.5
0.6
0.7
0.8
0.9
0
20
40
60
80
Ages (years)
A
B
C
D
E
F
G
H
I
J
K
FDR (Age associated divergence) =0.0829
p(Scalp)=0.815
p(Dura)=0.37
Scalp
Dura

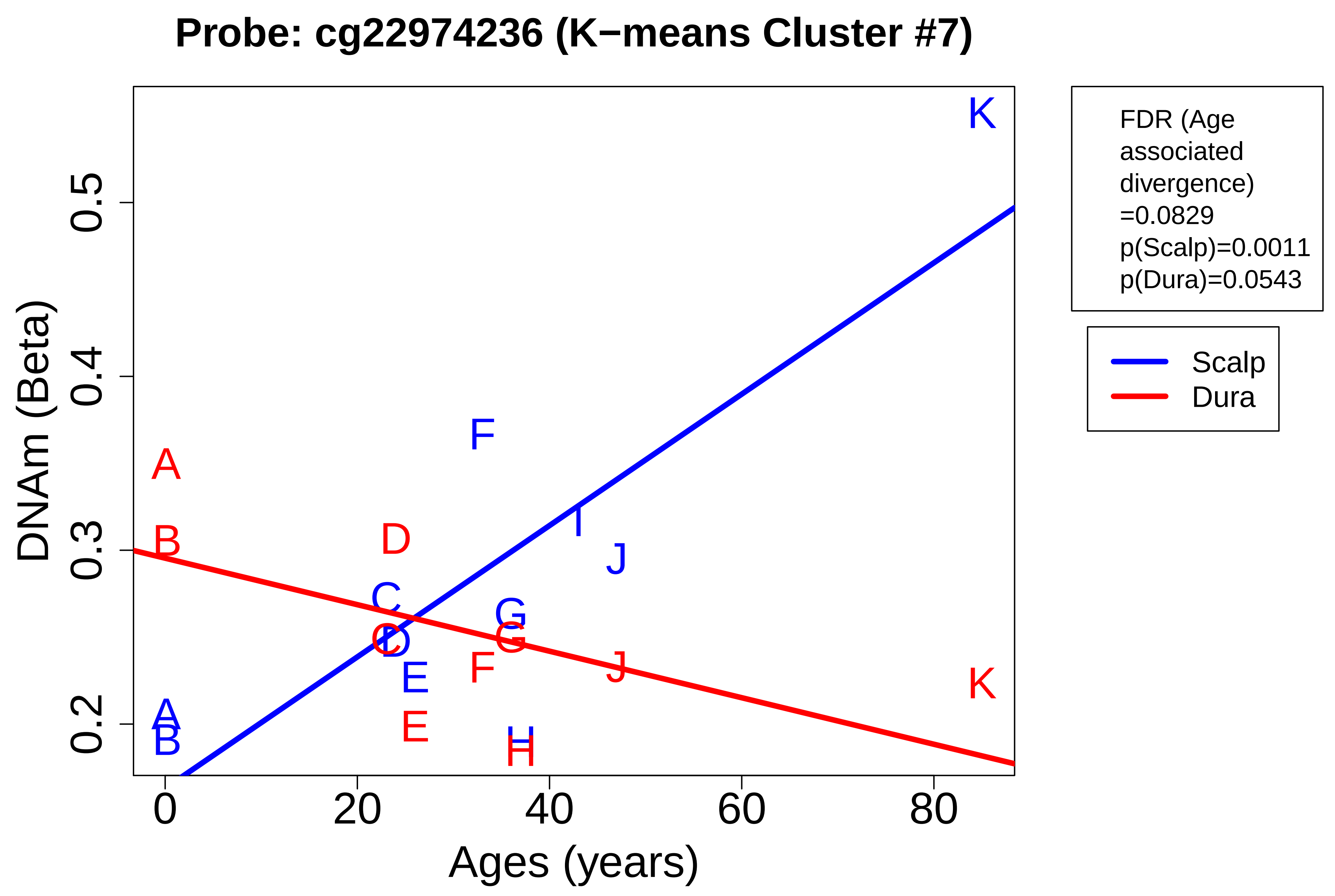

Probe: cg22974236 (K−means Cluster #7)
DNAm (Beta)
0.2
0.3
0.4
0.5
0
20
40
60
80
Ages (years)
FDR (Age associated divergence) =0.0829
p(Scalp)=0.0011
p(Dura)=0.0543
Scalp
Dura
A
B
C
D
E
F
G
H
I
J
K

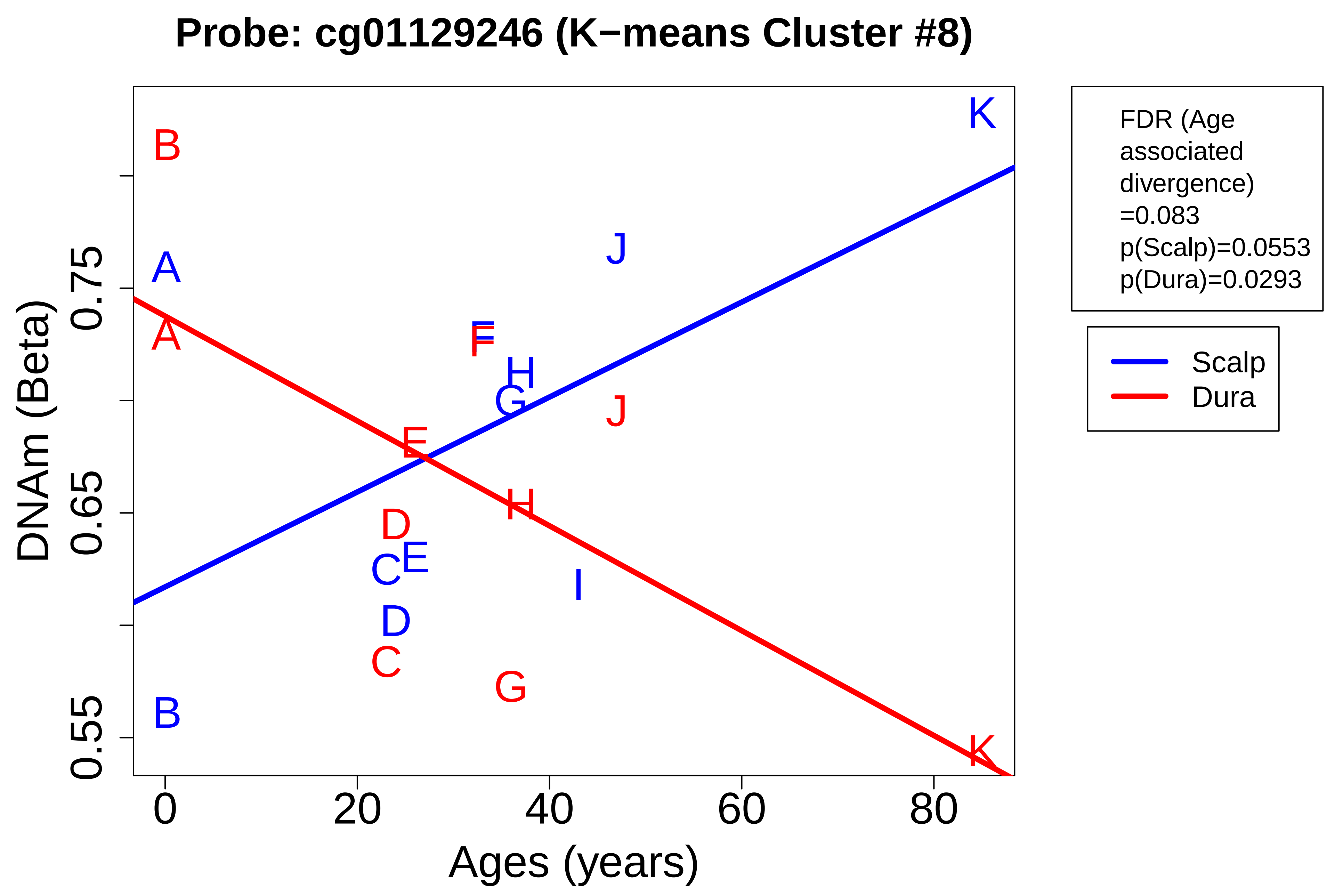
Probe: cg01129246 (K−means Cluster #8)
FDR (Age associated divergence) =0.083
p(Scalp)=0.0553
p(Dura)=0.0293
Scalp
Dura
DNAm (Beta)
0.55
0.65
0.75
Ages (years)
0
20
40
60
80
A
B
C
D
E
F
G
H
I
J
K

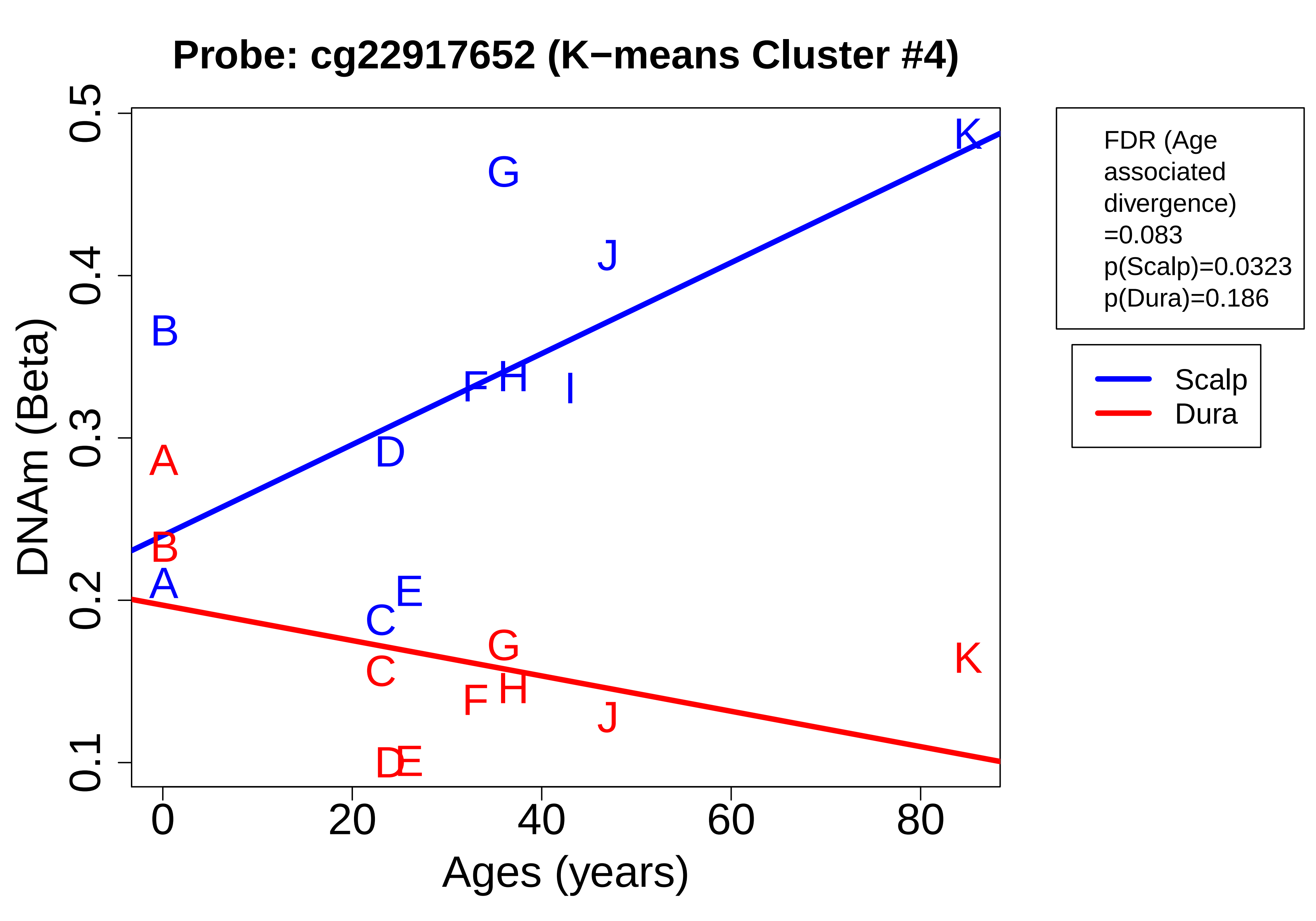

Probe: cg22917652 (K−means Cluster #4)
DNAm (Beta)
0.1
0.2
0.3
0.4
0.5
0
20
40
60
80
Ages (years)
FDR (Age associated divergence) =0.083
p(Scalp)=0.0323
p(Dura)=0.186
Scalp
Dura

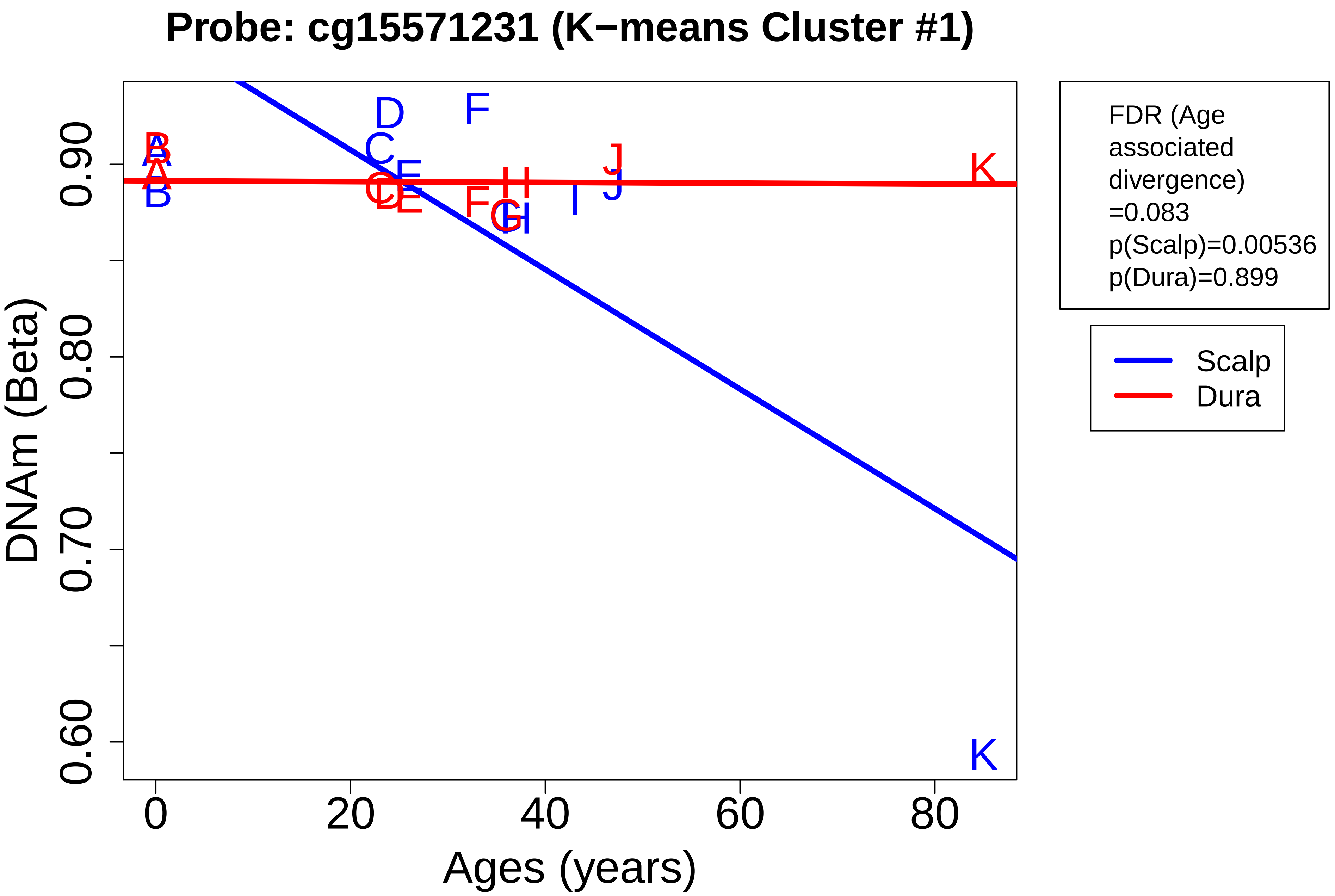
Probe: cg15571231 (K−means Cluster #1)
DNAm (Beta)
0.60
0.70
0.80
0.90
0
20
40
60
80
Ages (years)
A
B
C
D
E
F
G
H
I
J
K
FDR (Age associated divergence) =0.083
p(Scalp)=0.00536
p(Dura)=0.899
Scalp
Dura

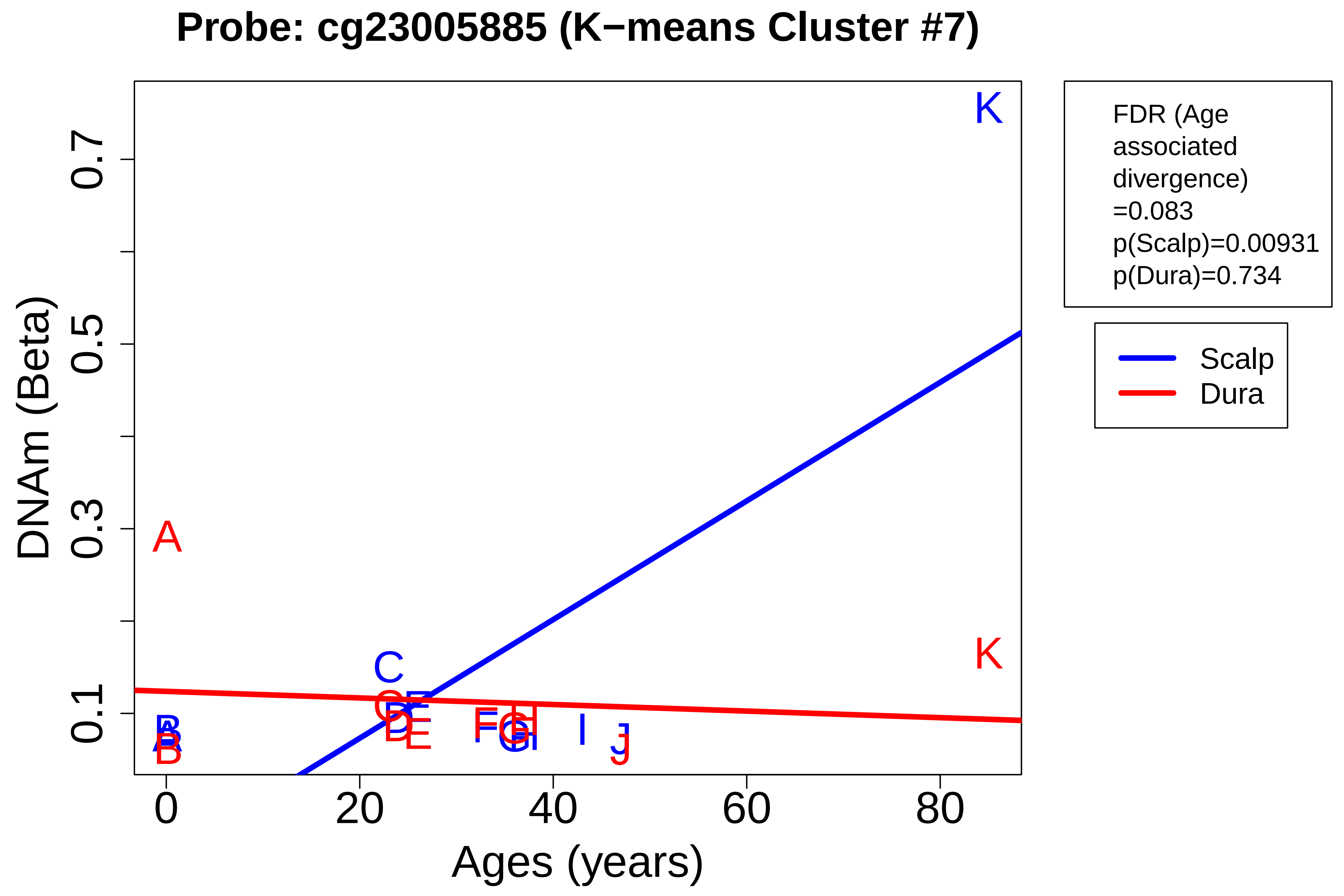

Probe: cg23005885 (K−means Cluster #7)
DNAm (Beta)
0.1
0.3
0.5
0.7
Ages (years)
0
20
40
60
80
FDR (Age associated divergence) =0.083
p(Scalp)=0.00931
p(Dura)=0.734
Scalp
Dura

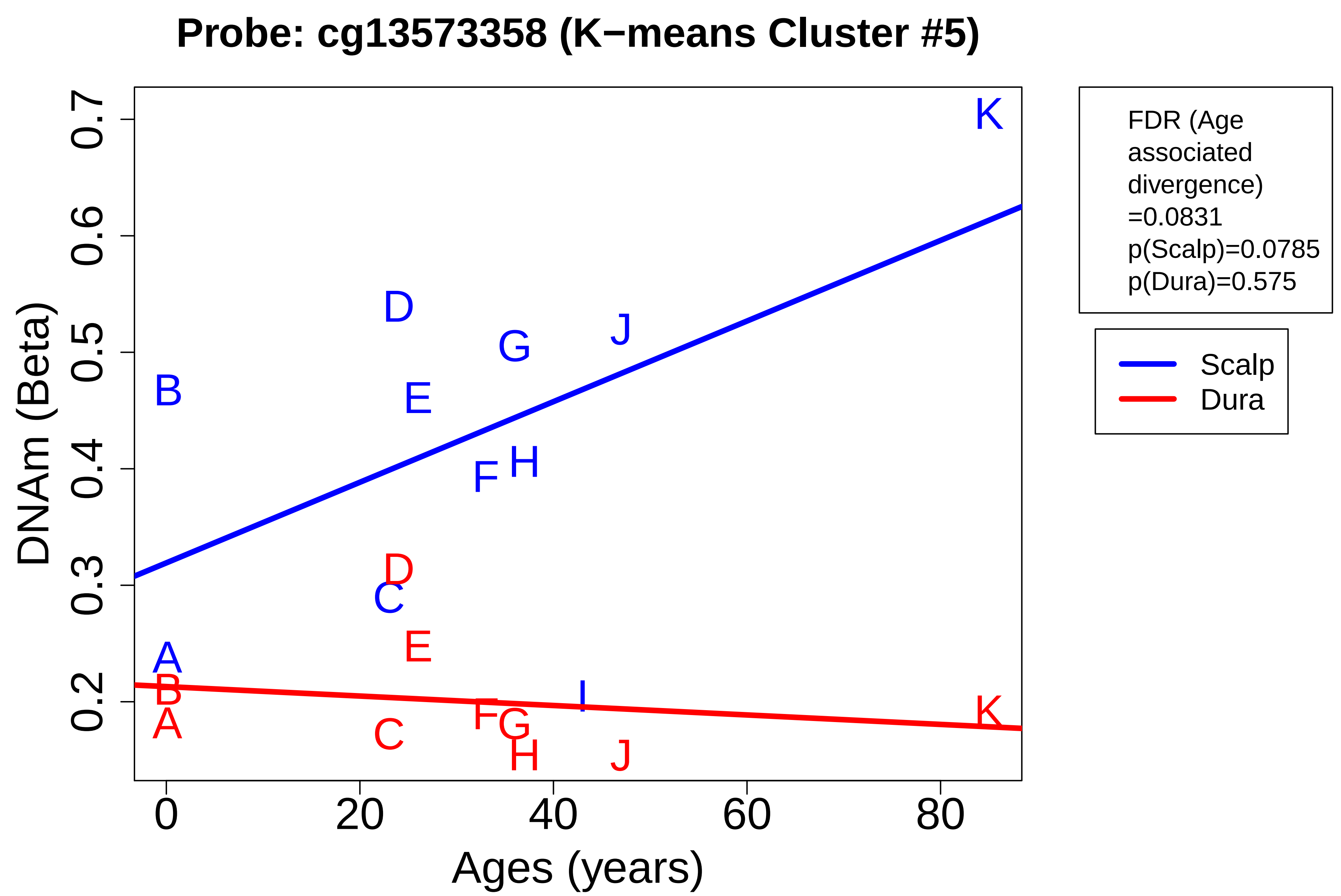

Probe: cg13573358 (K−means Cluster #5)
DNAm (Beta)
0.2
0.3
0.4
0.5
0.6
0.7
Ages (years)
0
20
40
60
80
FDR (Age associated divergence) =0.0831
p(Scalp)=0.0785
p(Dura)=0.575
Scalp
Dura
A
B
C
D
E
F
G
H
I
J
K

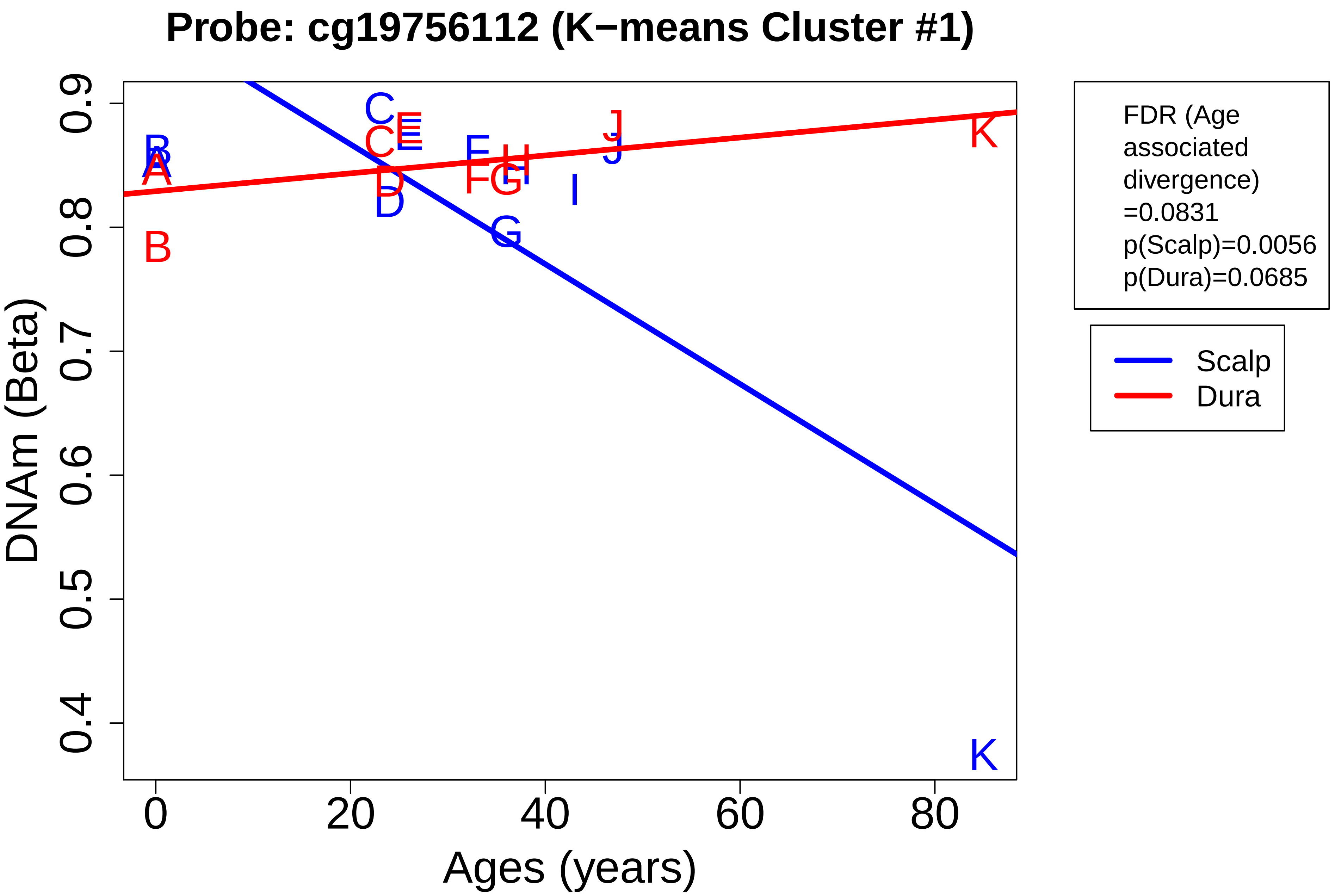
Probe: cg19756112 (K−means Cluster #1)
DNAm (Beta)
0.4
0.5
0.6
0.7
0.8
0.9
0
20
40
60
80
Ages (years)
A
B
C
D
E
F
G
H
I
J
K
FDR (Age associated divergence) =0.0831
p(Scalp)=0.0056
p(Dura)=0.0685
Scalp
Dura

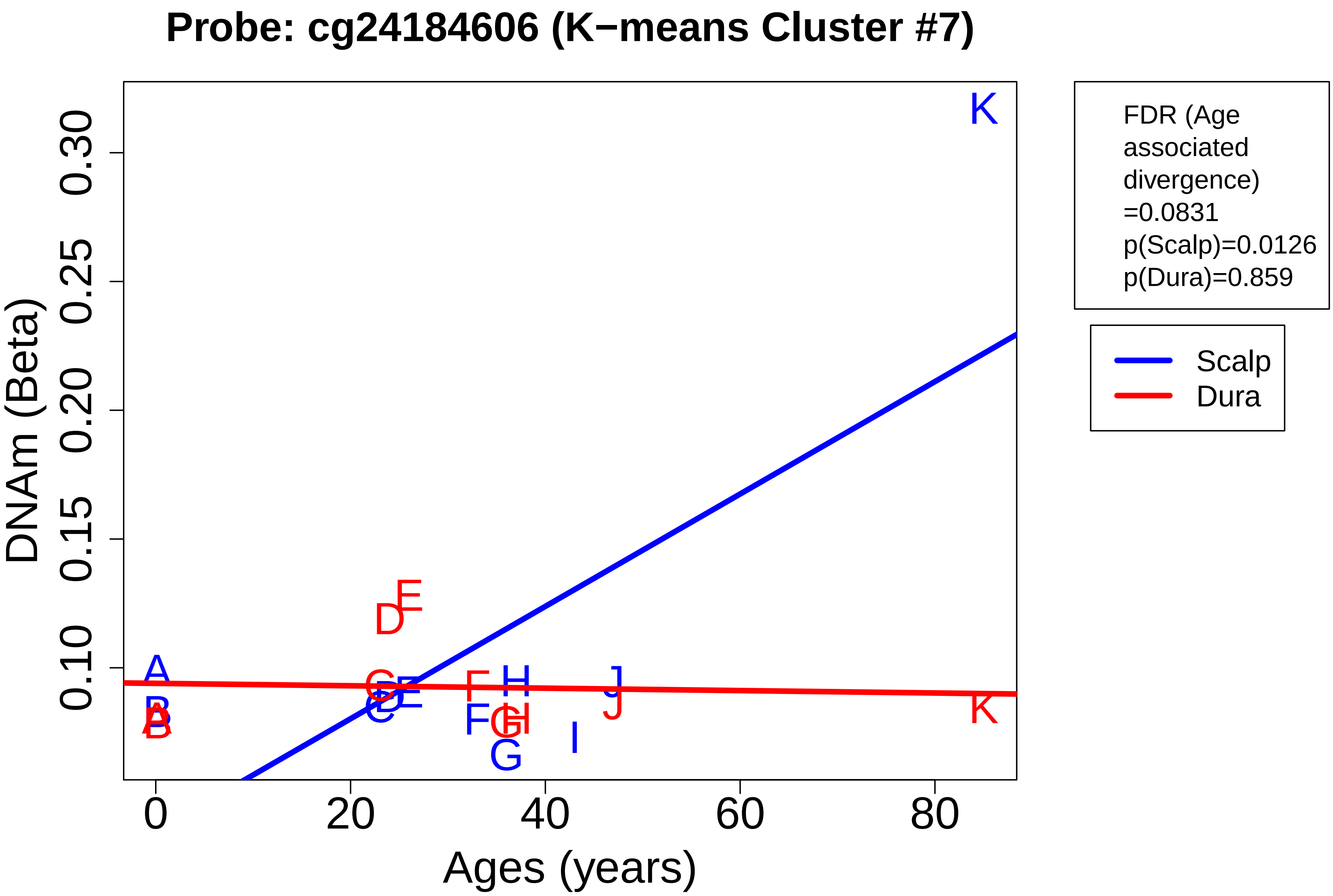
Probe: cg24184606 (K−means Cluster #7)
DNAm (Beta)
0.10
0.15
0.20
0.25
0.30
0
20
40
60
80
Ages (years)
FDR (Age associated divergence) =0.0831
p(Scalp)=0.0126
p(Dura)=0.859
Scalp
Dura

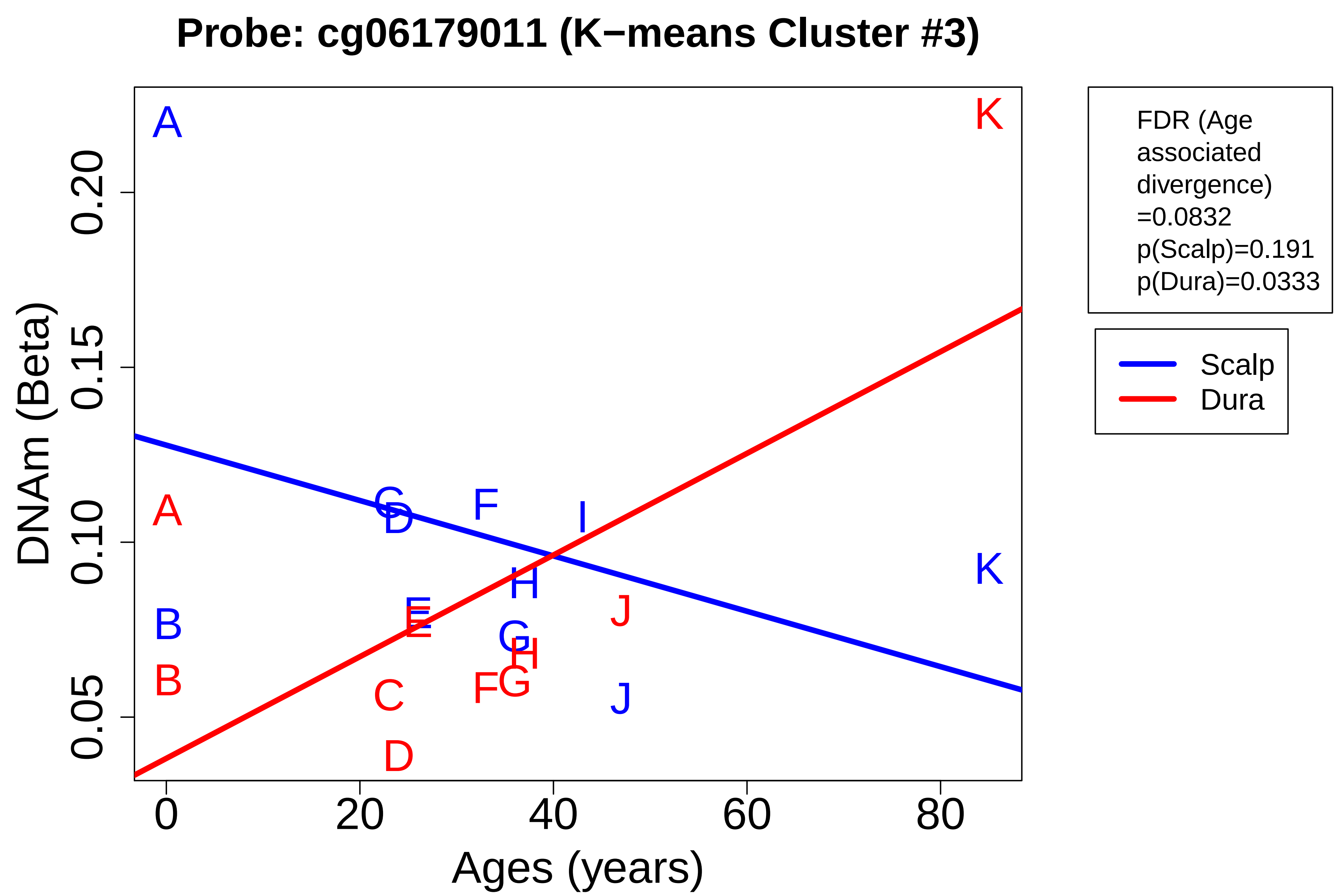

Probe: cg06179011 (K−means Cluster #3)
DNAm (Beta)
0.05
0.10
0.15
0.20
Ages (years)
0
20
40
60
80
A
B
C
D
E
F
G
H
I
J
K
FDR (Age associated divergence) =0.0832
p(Scalp)=0.191
p(Dura)=0.0333
Scalp
Dura

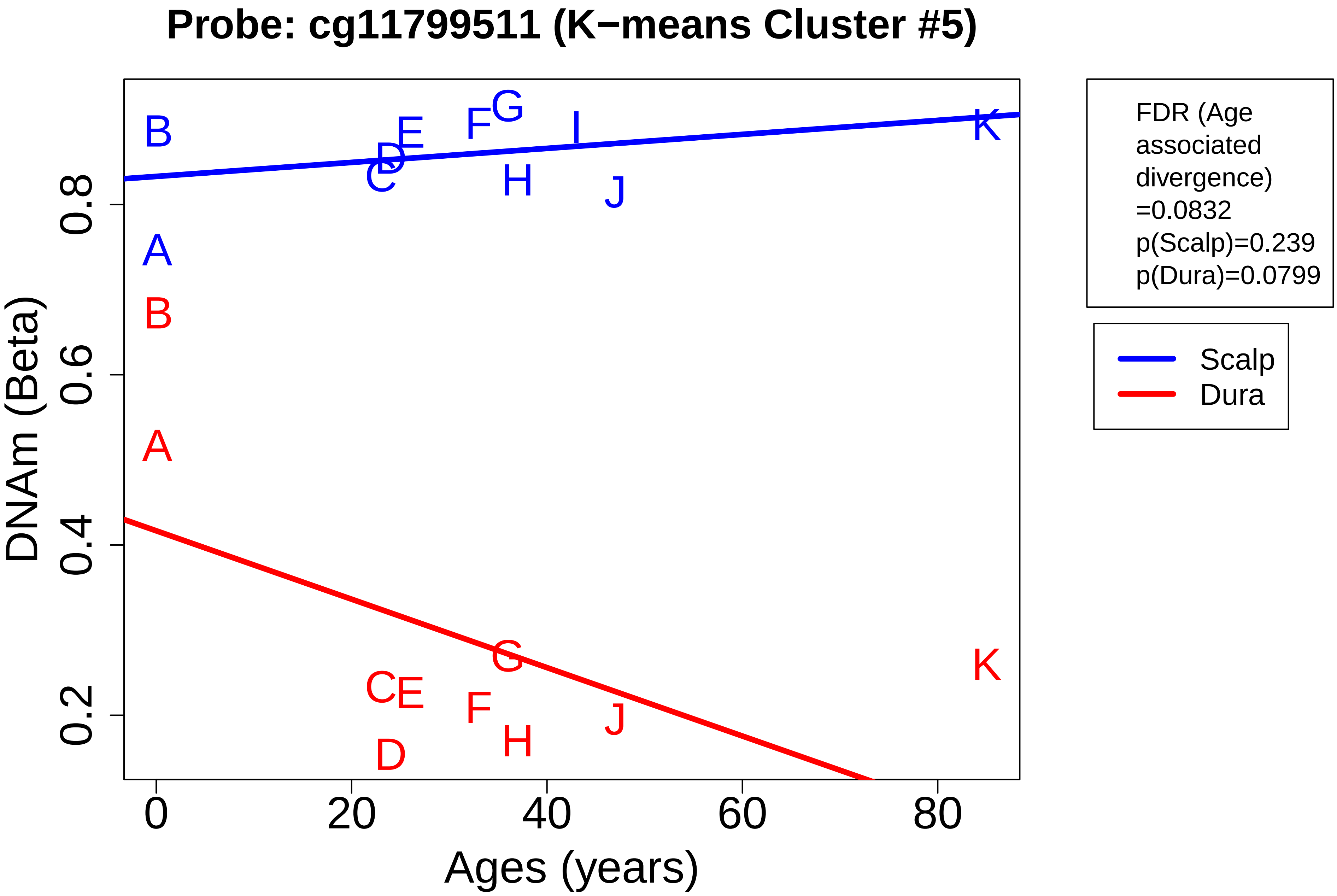
Probe: cg11799511 (K−means Cluster #5)
DNAm (Beta)
0.2
0.4
0.6
0.8
0
20
40
60
80
Ages (years)
A
B
C
D
E
F
G
H
I
J
K
FDR (Age associated divergence) =0.0832
p(Scalp)=0.239
p(Dura)=0.0799
Scalp
Dura

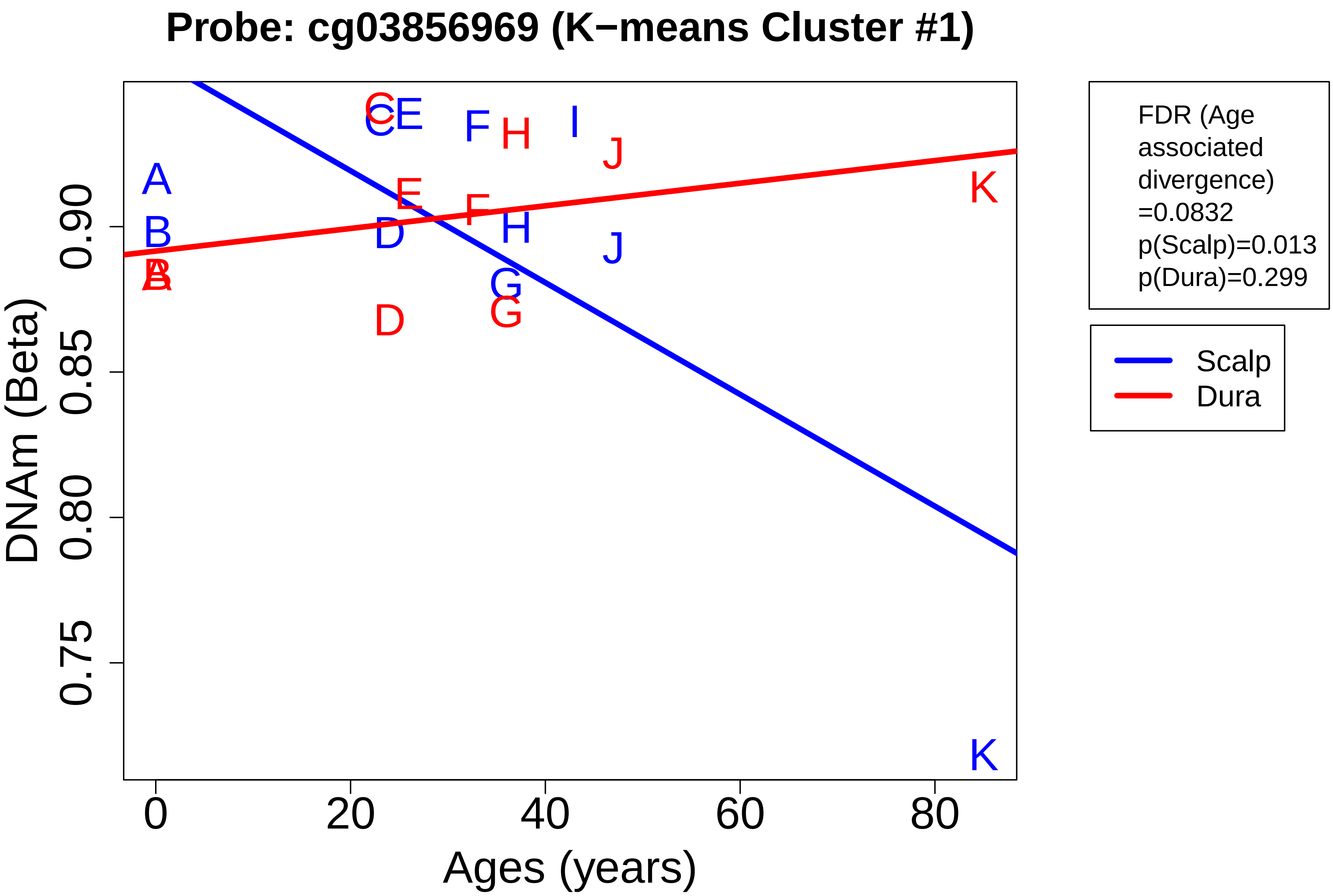

Probe: cg03856969 (K−means Cluster #1)
DNAm (Beta)
0.75
0.80
0.85
0.90
0
20
40
60
80
Ages (years)
FDR (Age associated divergence) =0.0832
p(Scalp)=0.013
p(Dura)=0.299
Scalp
Dura

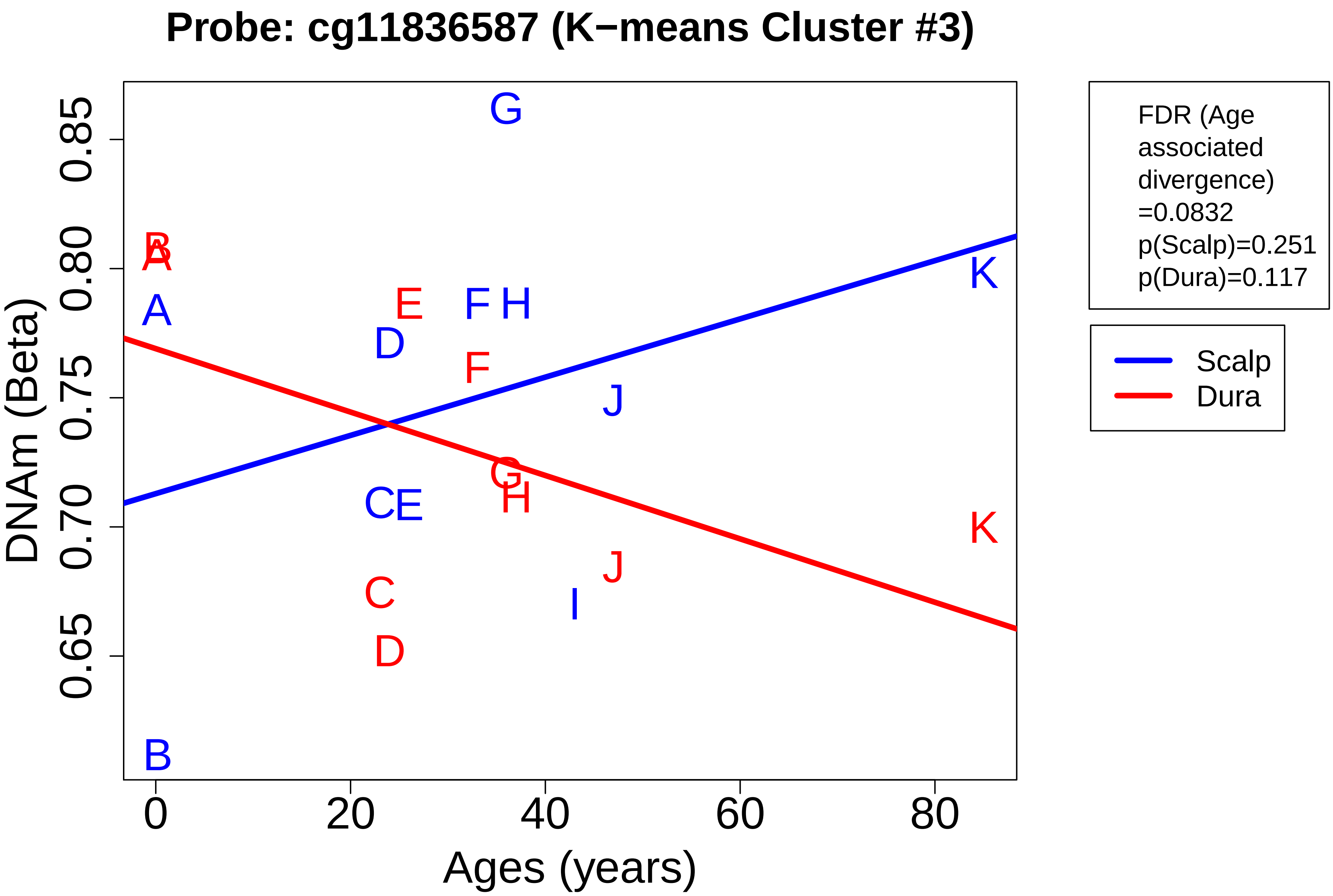

Probe: cg11836587 (K−means Cluster #3)
DNAm (Beta)
0.65
0.70
0.75
0.80
0.85
0
20
40
60
80
Ages (years)
FDR (Age associated divergence) =0.0832
p(Scalp)=0.251
p(Dura)=0.117
Scalp
Dura

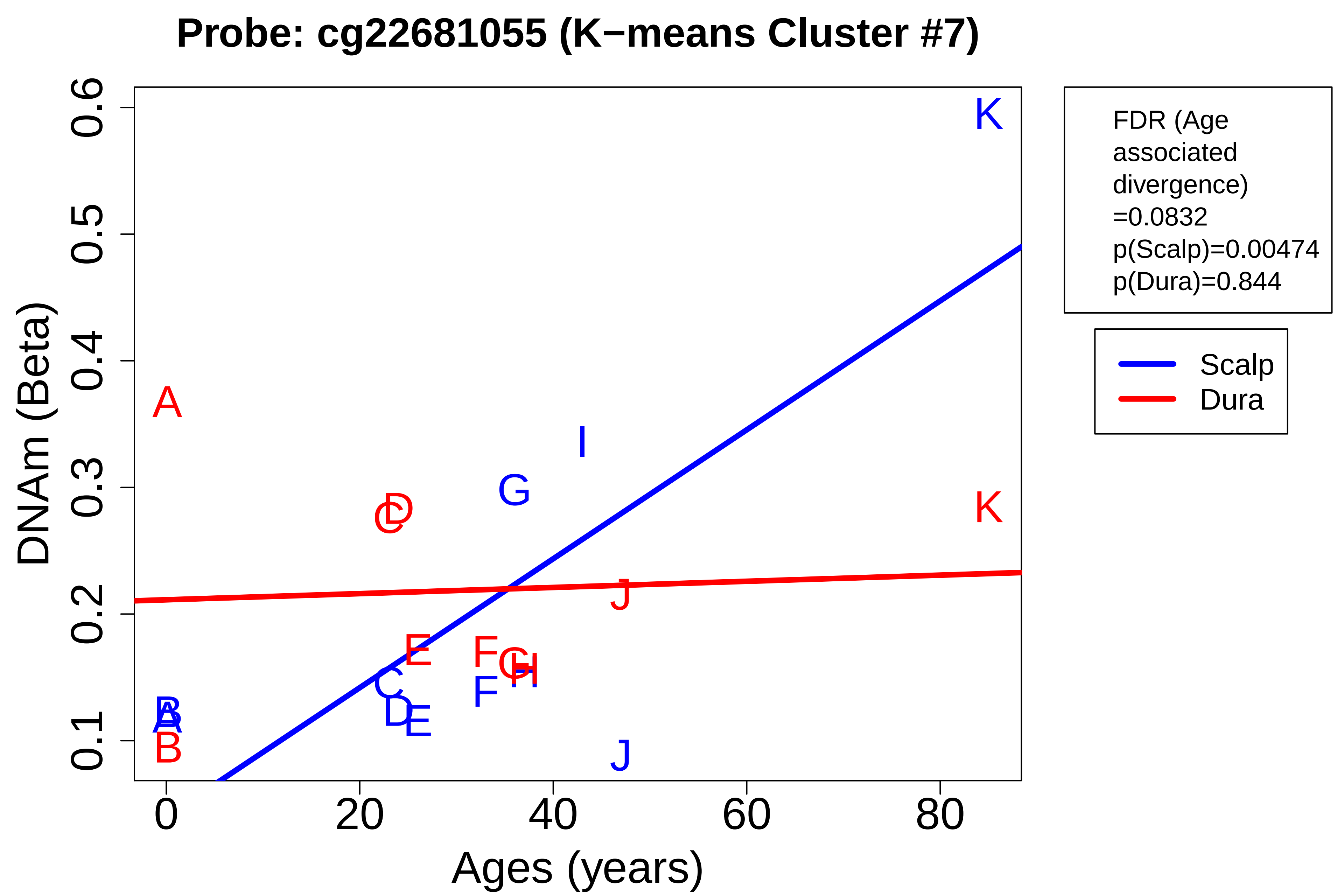
Probe: cg22681055 (K−means Cluster #7)
DNAm (Beta)
Ages (years)
0.1
0.2
0.3
0.4
0.5
0.6
0
20
40
60
80
FDR (Age associated divergence) =0.0832
p(Scalp)=0.00474
p(Dura)=0.844
Scalp
Dura

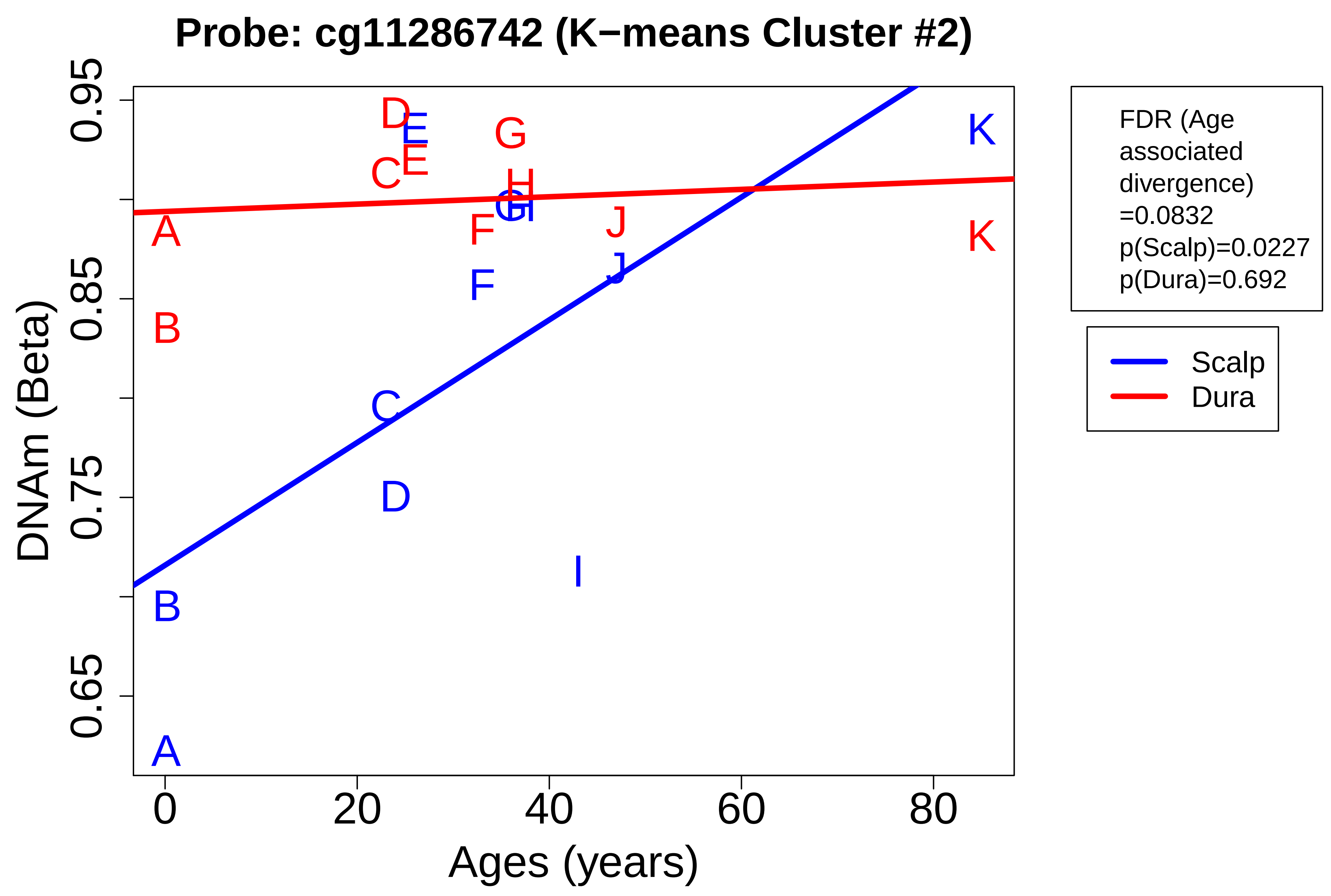

Probe: cg11286742 (K−means Cluster #2)
DNAm (Beta)
0.65
0.75
0.85
0.95
Ages (years)
0
20
40
60
80
FDR (Age associated divergence) =0.0832
p(Scalp)=0.0227
p(Dura)=0.692
Scalp
Dura

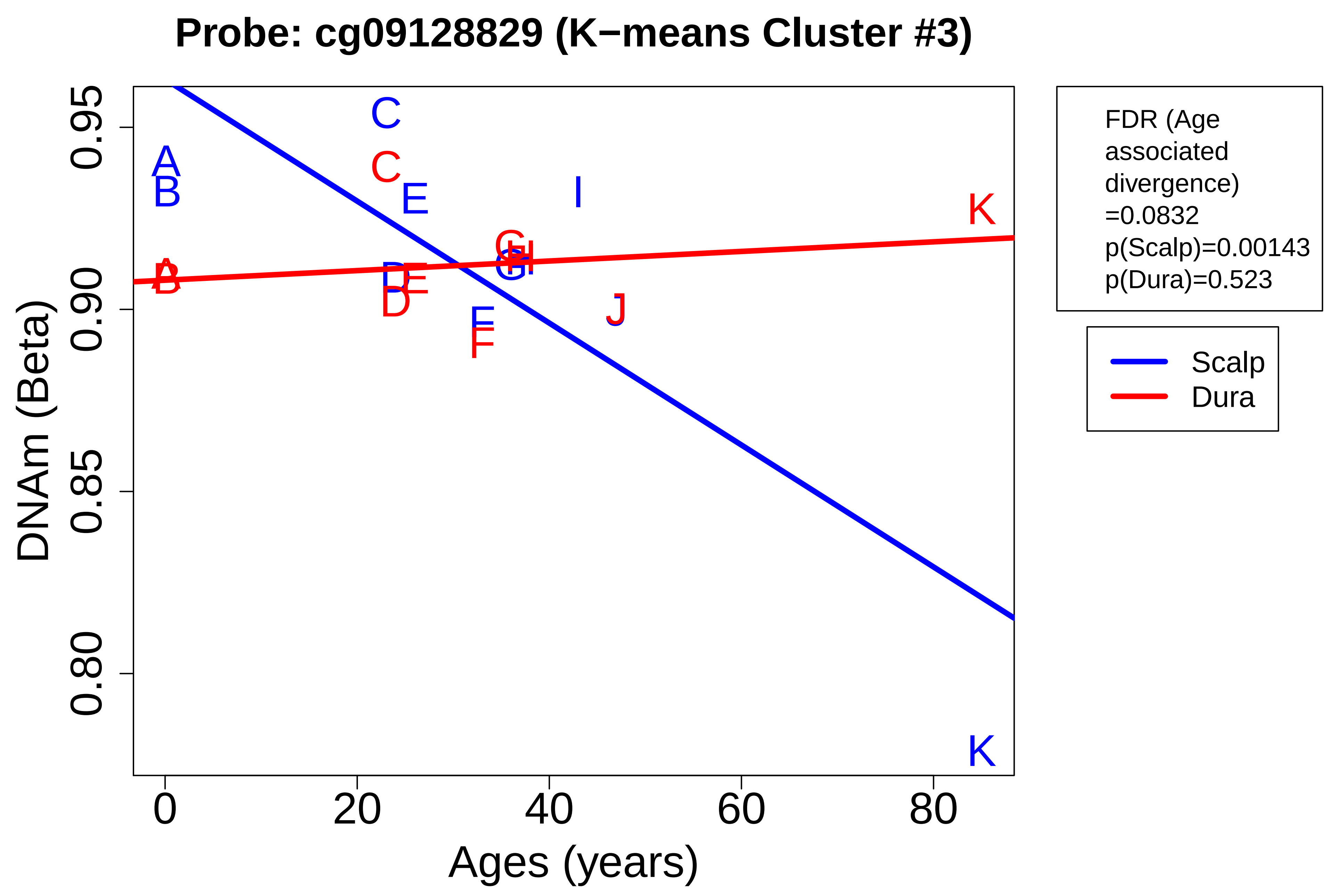

Probe: cg09128829 (K−means Cluster #3)
DNAm (Beta)
0.80
0.85
0.90
0.95
0
20
40
60
80
Ages (years)
FDR (Age associated divergence) =0.0832
p(Scalp)=0.00143
p(Dura)=0.523
Scalp
Dura

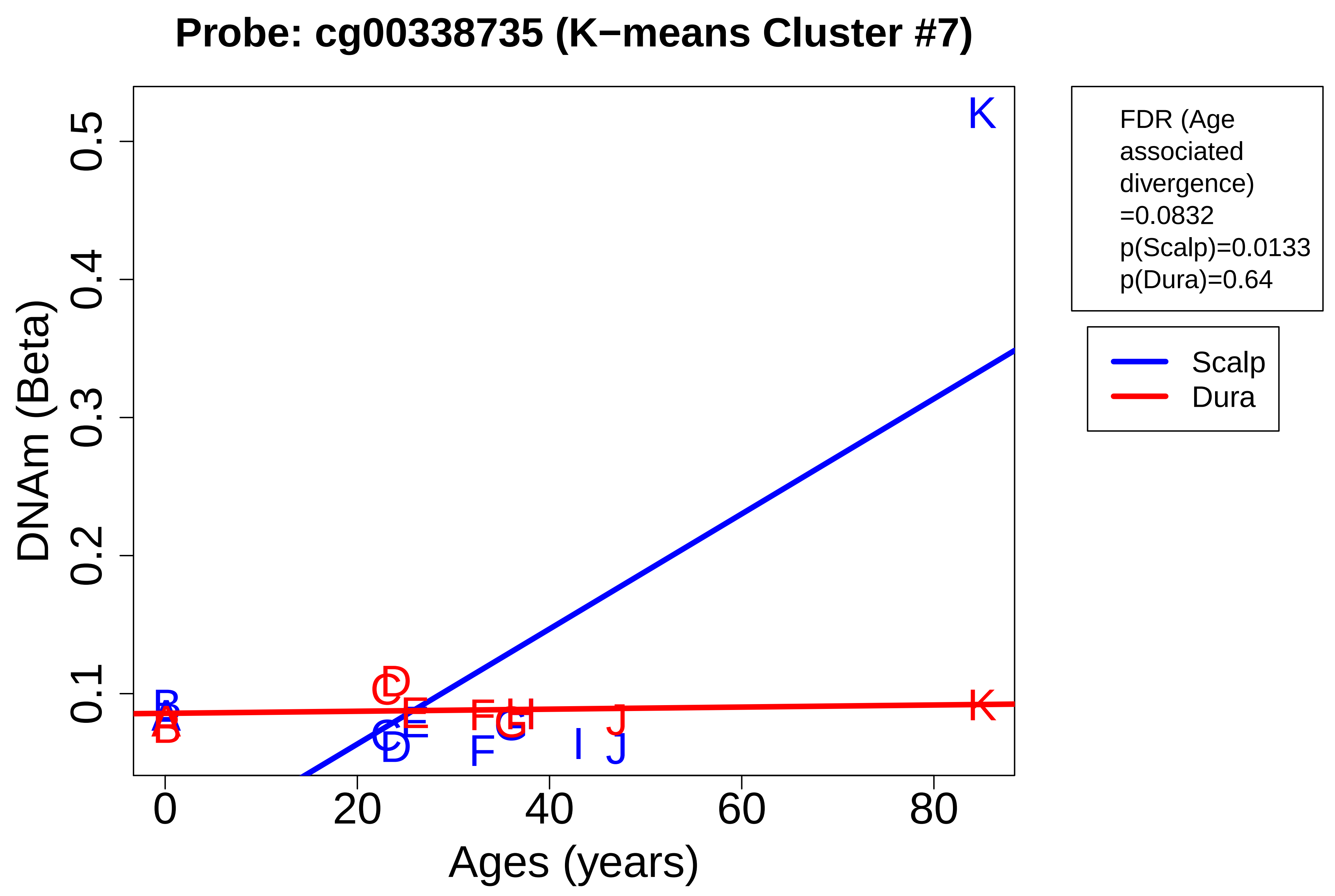

Probe: cg00338735 (K−means Cluster #7)
DNAm (Beta)
0.1
0.2
0.3
0.4
0.5
Ages (years)
0
20
40
60
80
FDR (Age associated divergence) =0.0832
p(Scalp)=0.0133
p(Dura)=0.64
Scalp
Dura

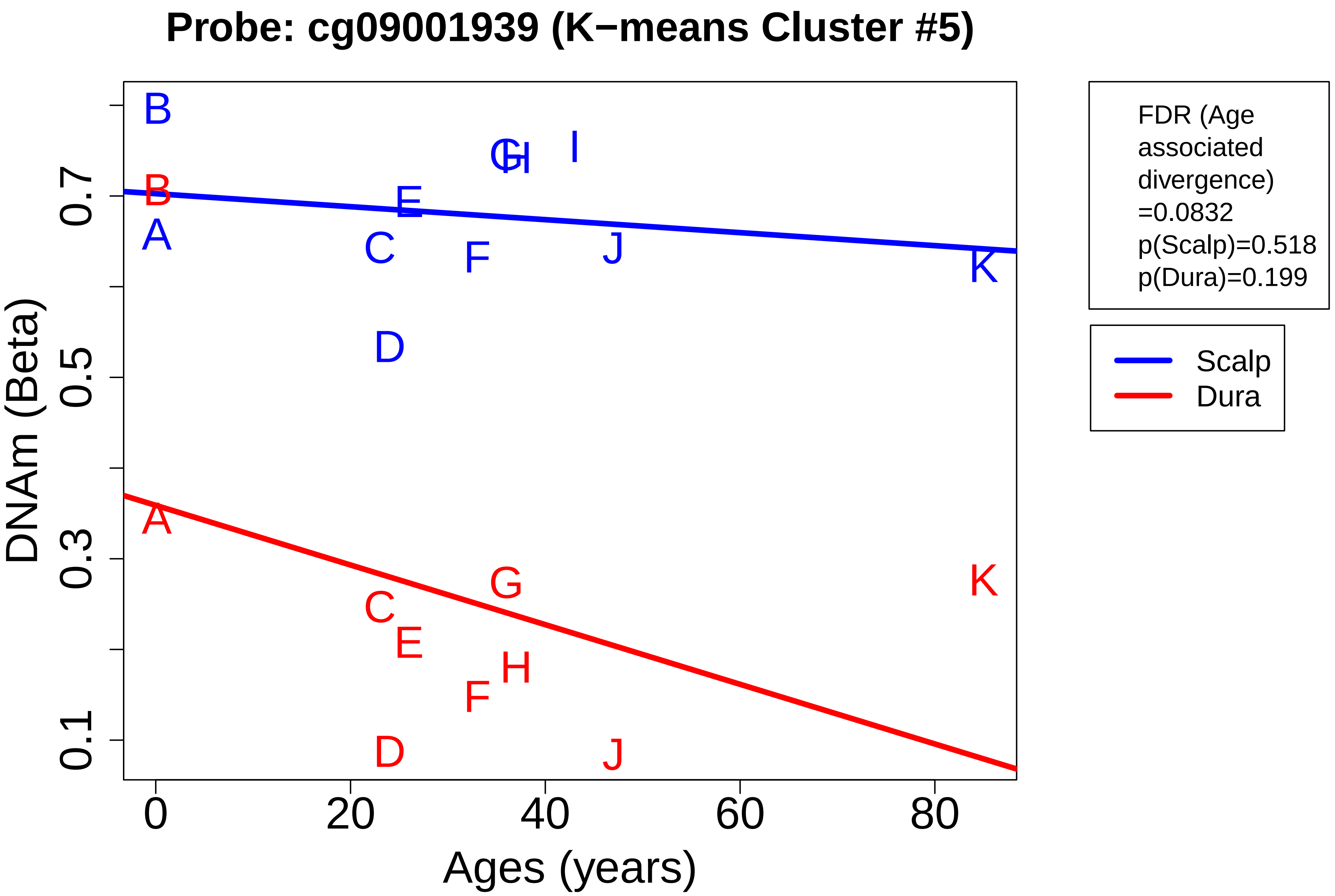
Probe: cg09001939 (K−means Cluster #5)
DNAm (Beta)
0.1
0.3
0.5
0.7
Ages (years)
0
20
40
60
80
FDR (Age associated divergence) =0.0832
p(Scalp)=0.518
p(Dura)=0.199
Scalp
Dura

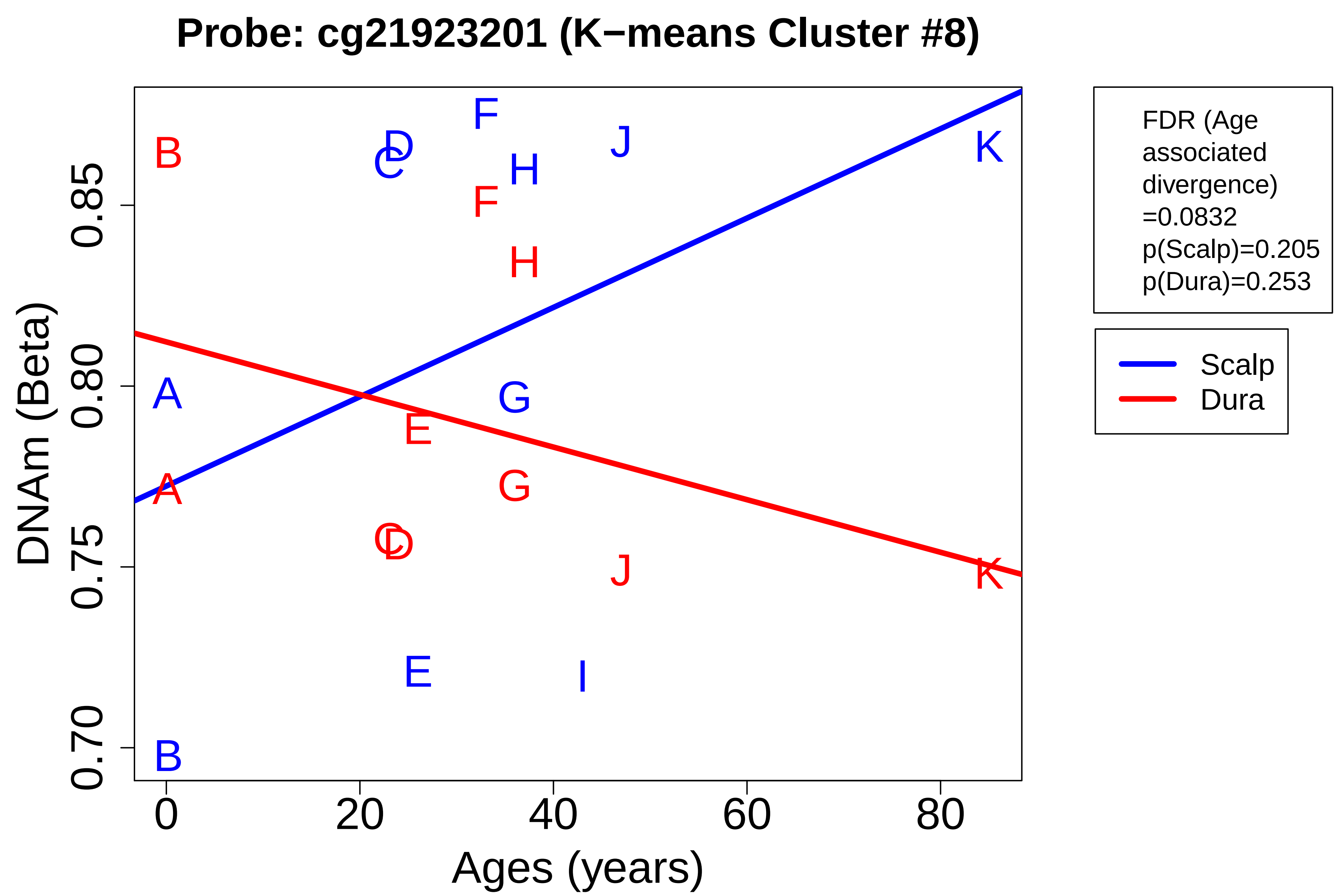

Probe: cg21923201 (K−means Cluster #8)
DNAm (Beta)
0.70
0.75
0.80
0.85
Ages (years)
0
20
40
60
80
FDR (Age associated divergence) =0.0832
p(Scalp)=0.205
p(Dura)=0.253
Scalp
Dura

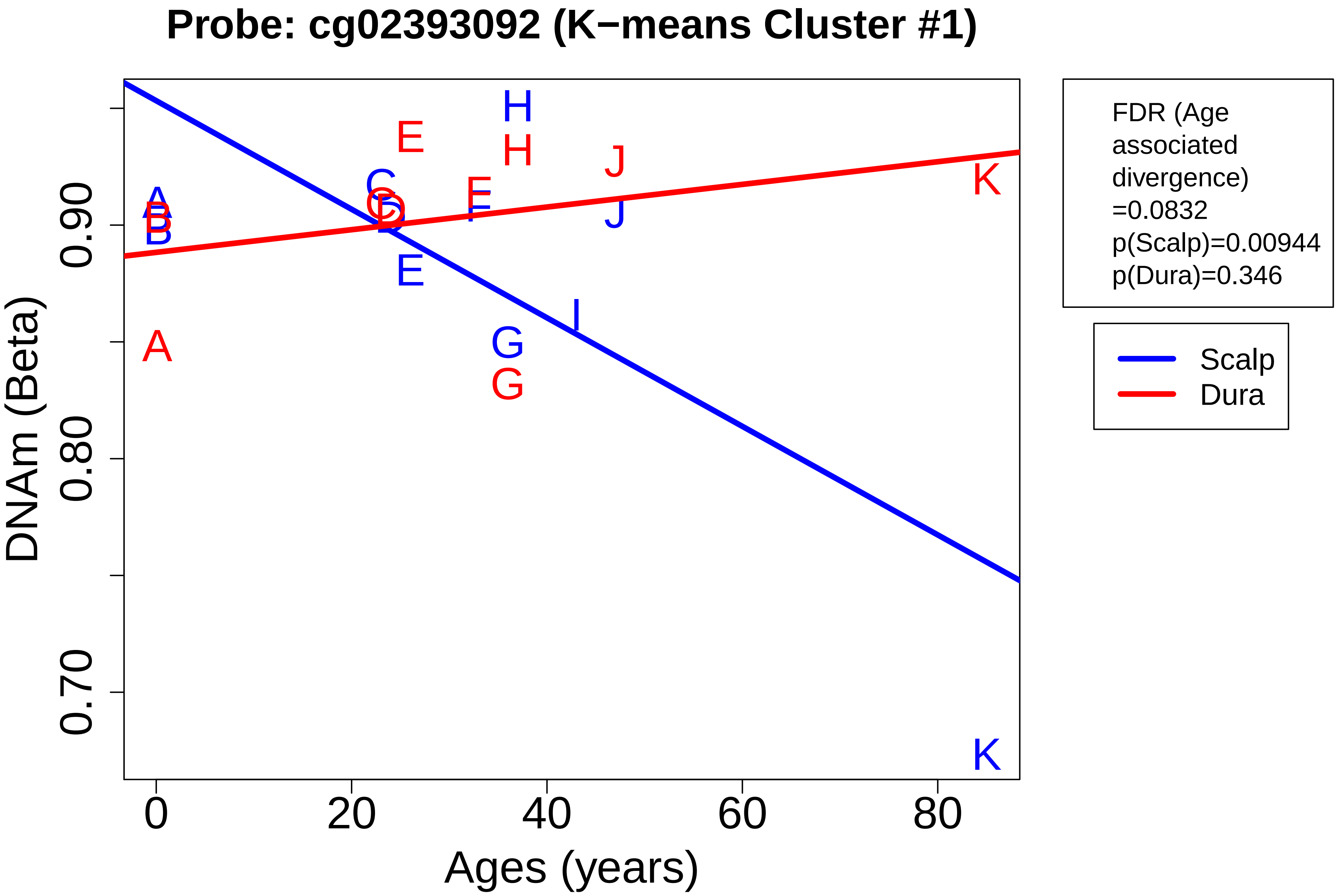
Probe: cg02393092 (K−means Cluster #1)
DNAm (Beta)
0.70
0.80
0.90
0
20
40
60
80
Ages (years)
FDR (Age associated divergence) =0.0832
p(Scalp)=0.00944
p(Dura)=0.346
Scalp
Dura

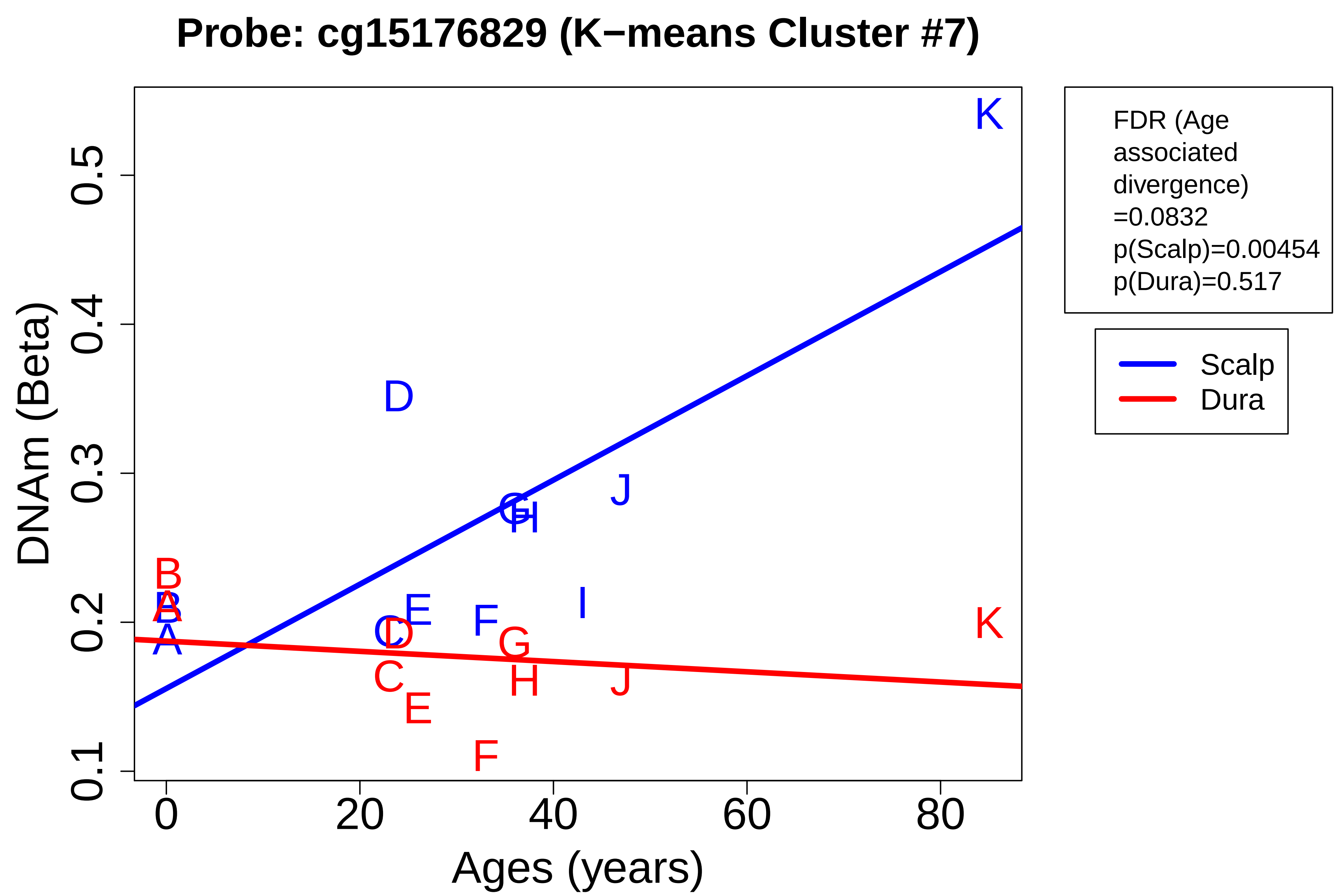

Probe: cg15176829 (K−means Cluster #7)
DNAm (Beta)
0.1
0.2
0.3
0.4
0.5
0
20
40
60
80
Ages (years)
FDR (Age associated divergence) =0.0832
p(Scalp)=0.00454
p(Dura)=0.517
Scalp
Dura

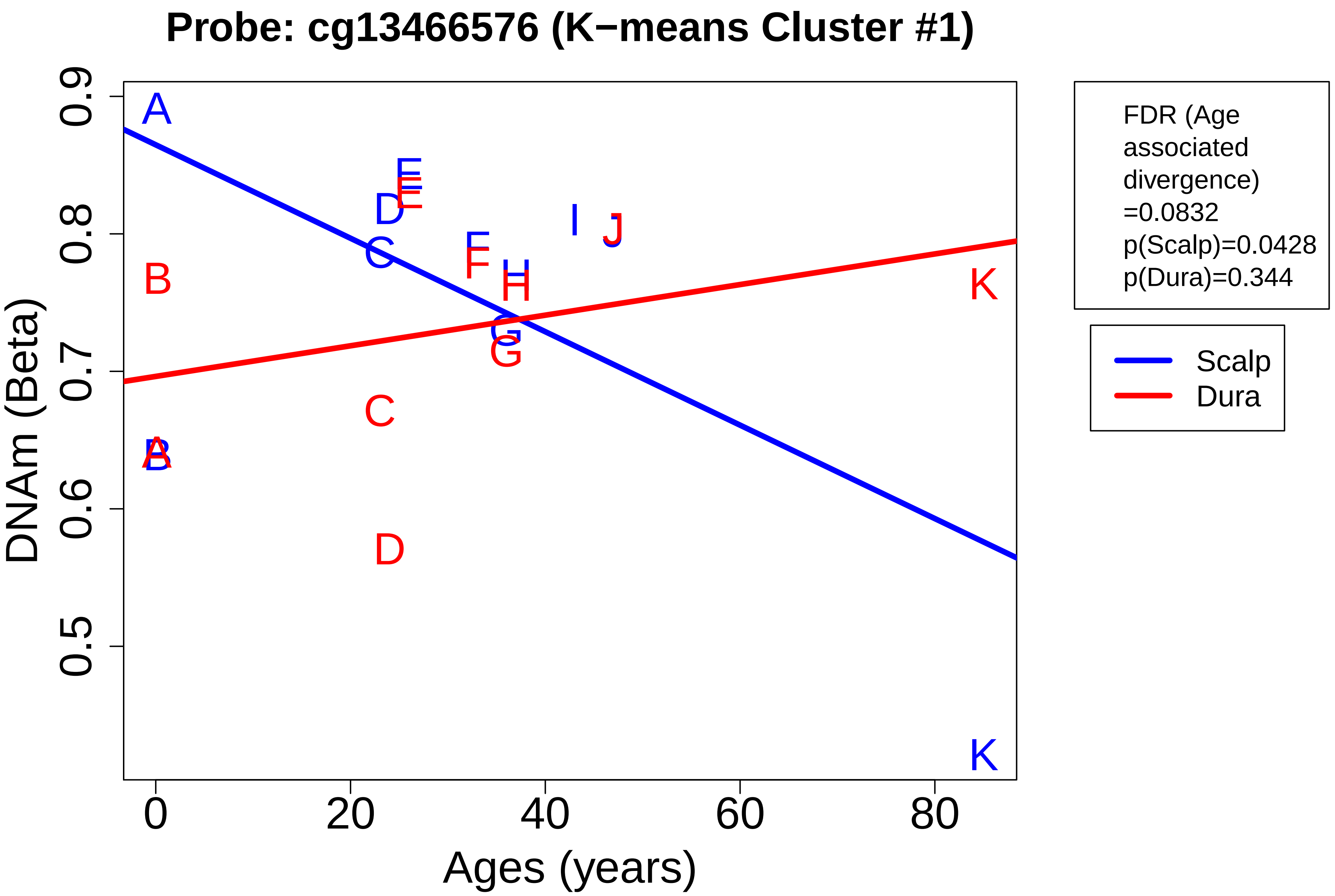

Probe: cg13466576 (K−means Cluster #1)
FDR (Age associated divergence) =0.0832
p(Scalp)=0.0428
p(Dura)=0.344
Scalp
Dura
DNAm (Beta)
0.5
0.6
0.7
0.8
0.9
0
20
40
60
80
Ages (years)
A B C D E F G H I J K

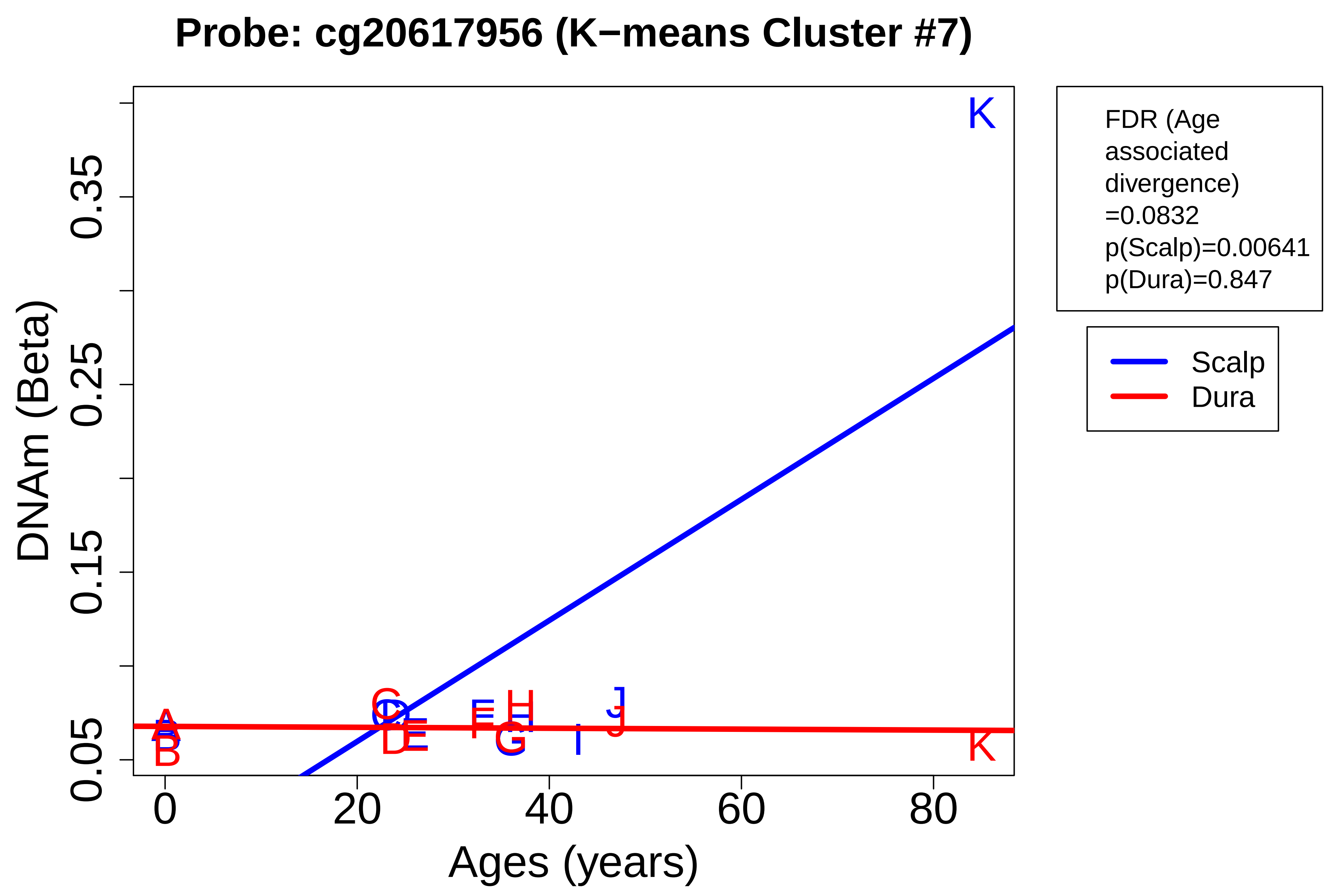

Probe: cg20617956 (K−means Cluster #7)
DNAm (Beta)
0.05
0.15
0.25
0.35
0
20
40
60
80
Ages (years)
A
B
C
D
E
F
G
H
I
J
K
FDR (Age associated divergence) =0.0832
p(Scalp)=0.00641
p(Dura)=0.847
Scalp
Dura

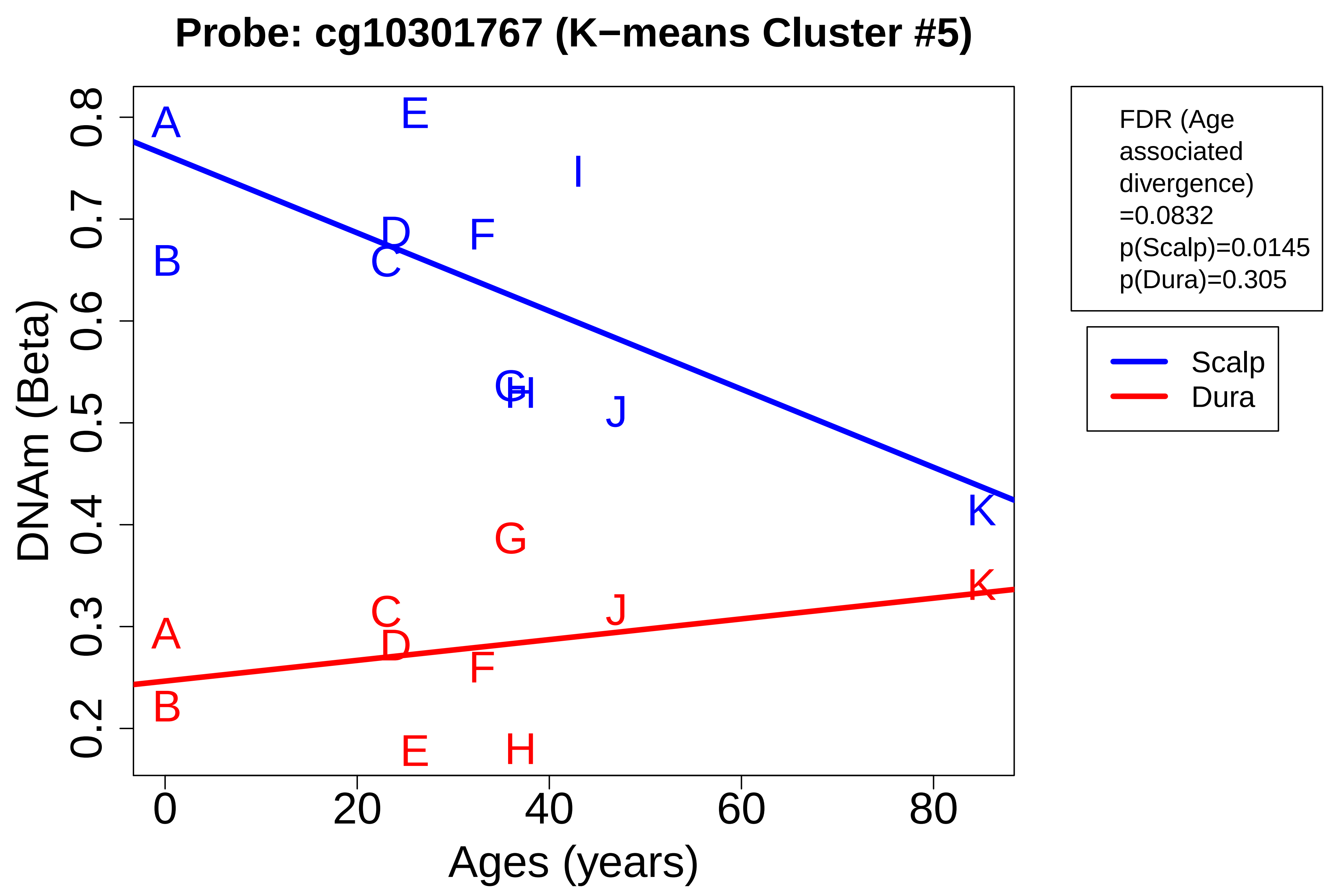

Probe: cg10301767 (K−means Cluster #5)
DNAm (Beta)
0.2
0.3
0.4
0.5
0.6
0.7
0.8
Ages (years)
0
20
40
60
80
A
B
C
D
E
F
G
H
I
J
K
FDR (Age associated divergence) =0.0832
p(Scalp)=0.0145
p(Dura)=0.305
Scalp
Dura

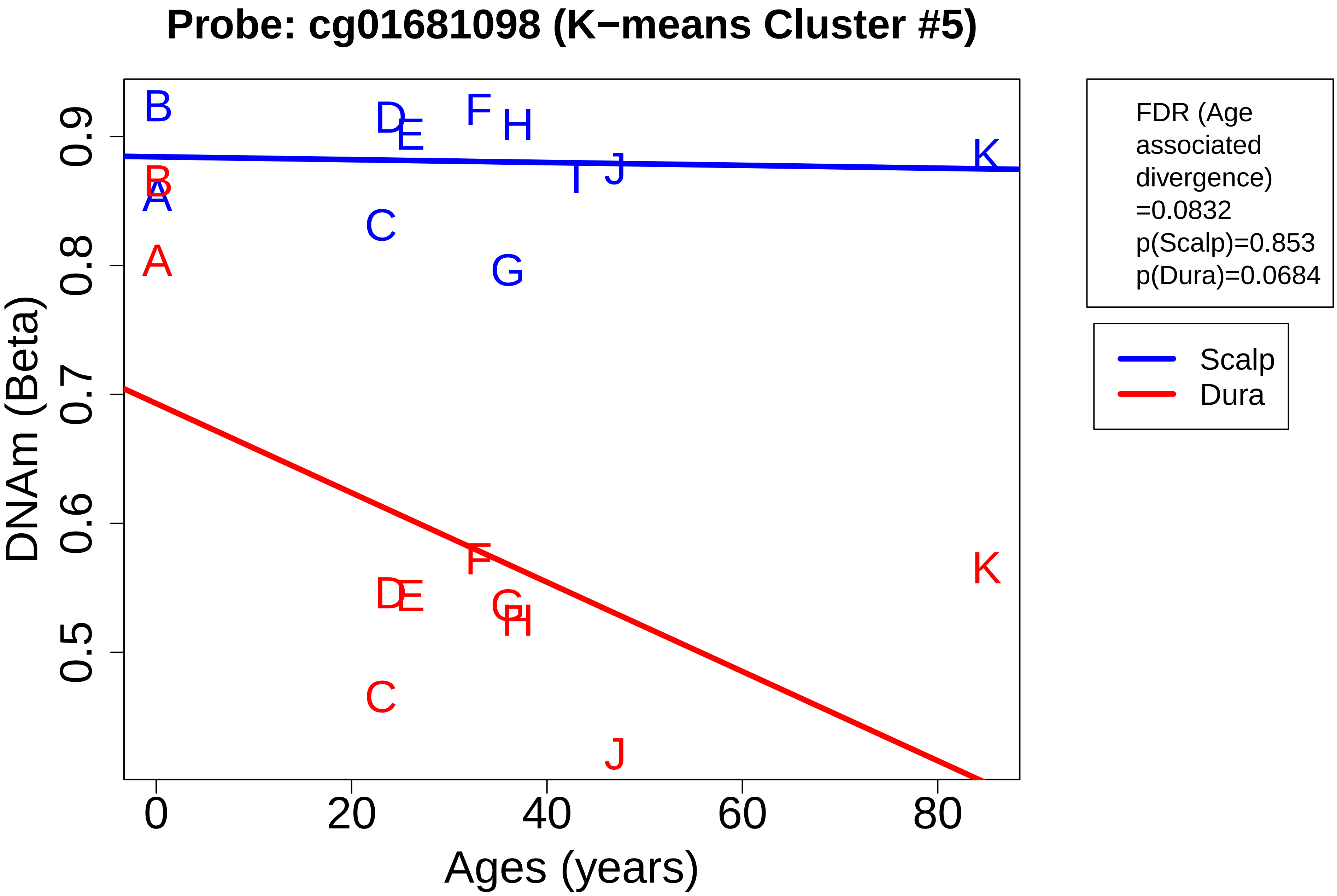

Probe: cg01681098 (K−means Cluster #5)
DNAm (Beta)
0.5
0.6
0.7
0.8
0.9
Ages (years)
0
20
40
60
80
FDR (Age associated divergence) =0.0832
p(Scalp)=0.853
p(Dura)=0.0684
Scalp
Dura

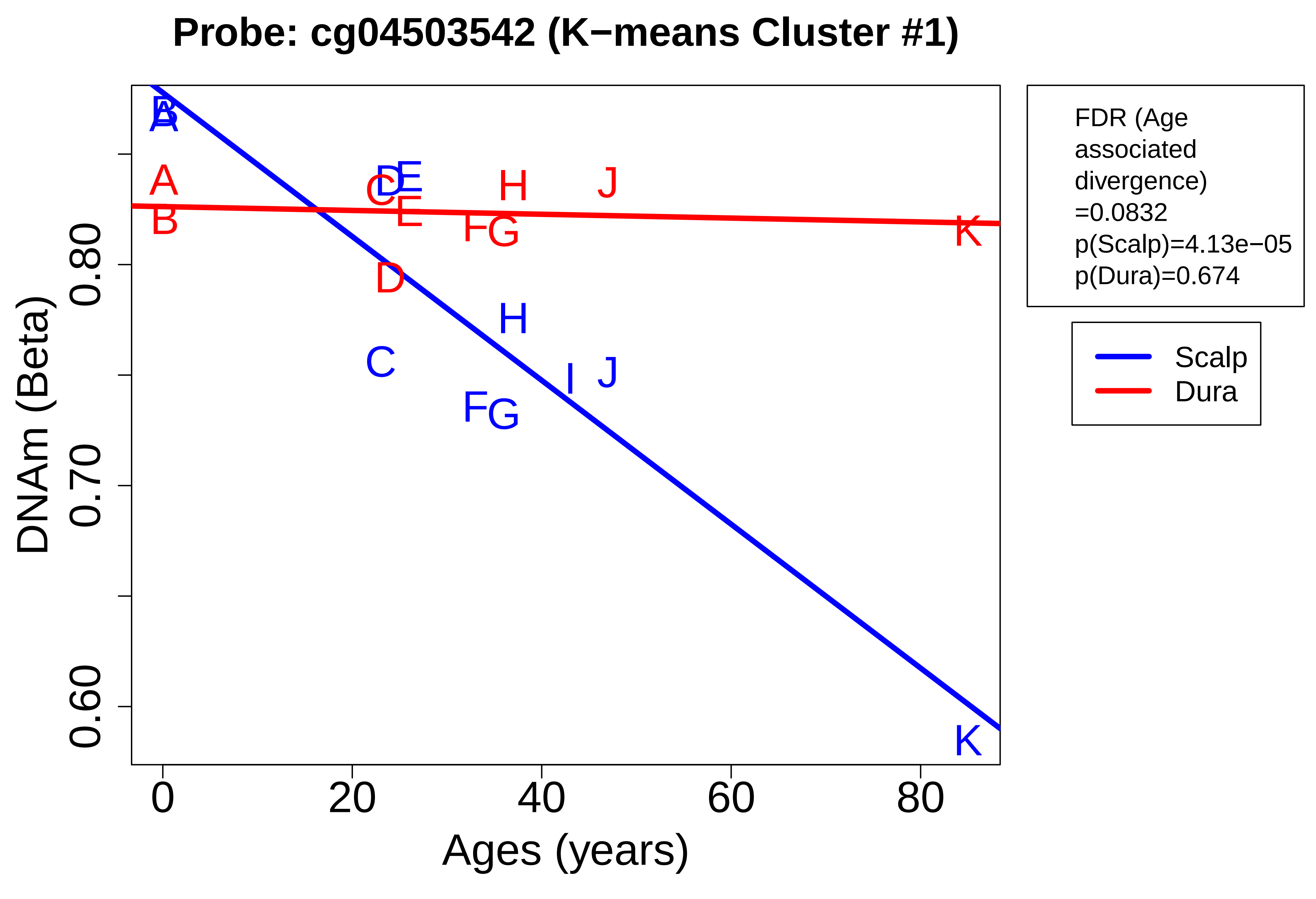

Probe: cg04503542 (K−means Cluster #1)
DNAm (Beta)
0.60
0.70
0.80
0
20
40
60
80
Ages (years)
FDR (Age associated divergence) =0.0832
p(Scalp)=4.13e−05
p(Dura)=0.674
Scalp
Dura

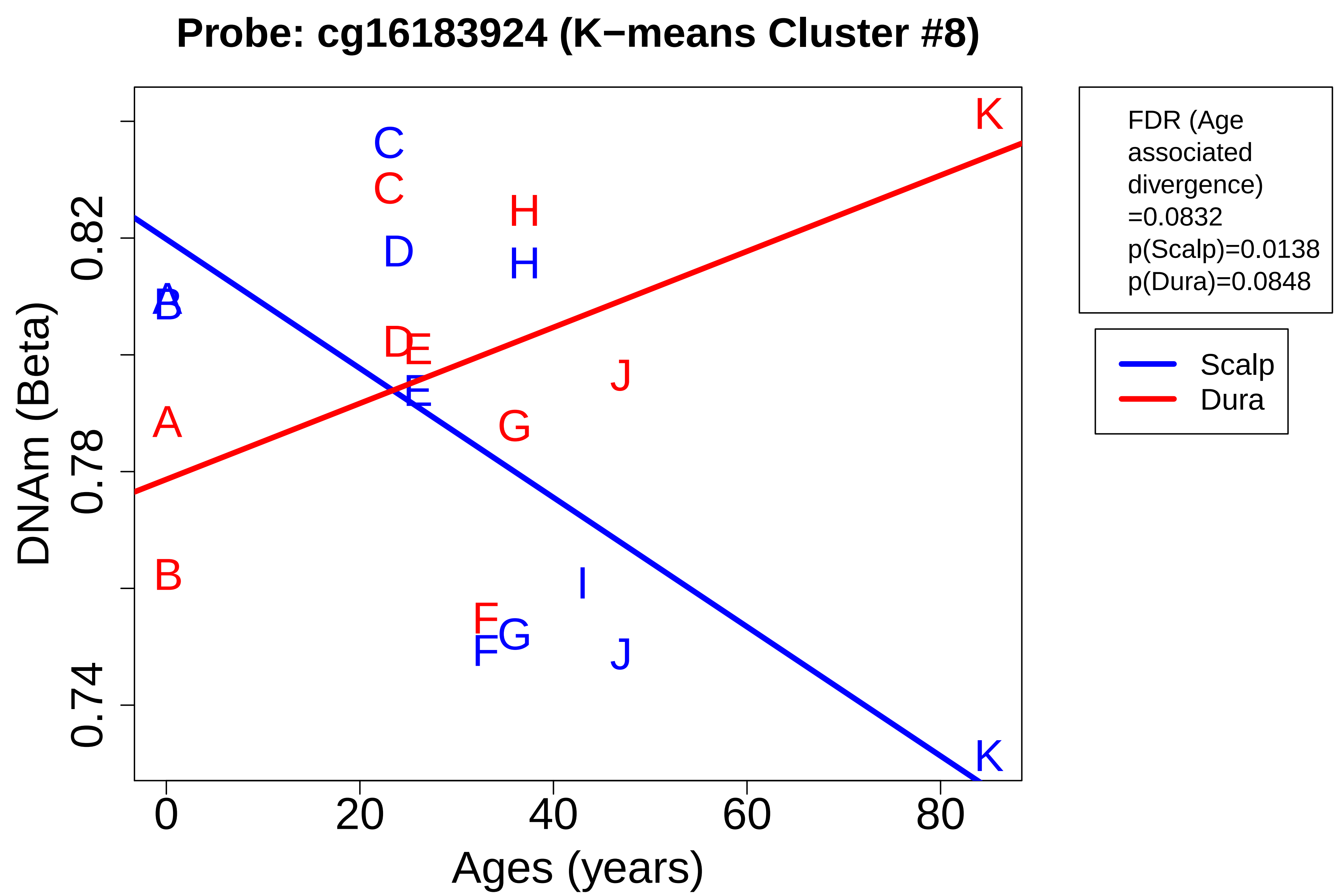

Probe: cg16183924 (K−means Cluster #8)
DNAm (Beta)
0.74
0.78
0.82
Ages (years)
0
20
40
60
80
FDR (Age associated divergence) =0.0832
p(Scalp)=0.0138
p(Dura)=0.0848
Scalp
Dura
A
B
C
D
E
F
G
H
I
J
K

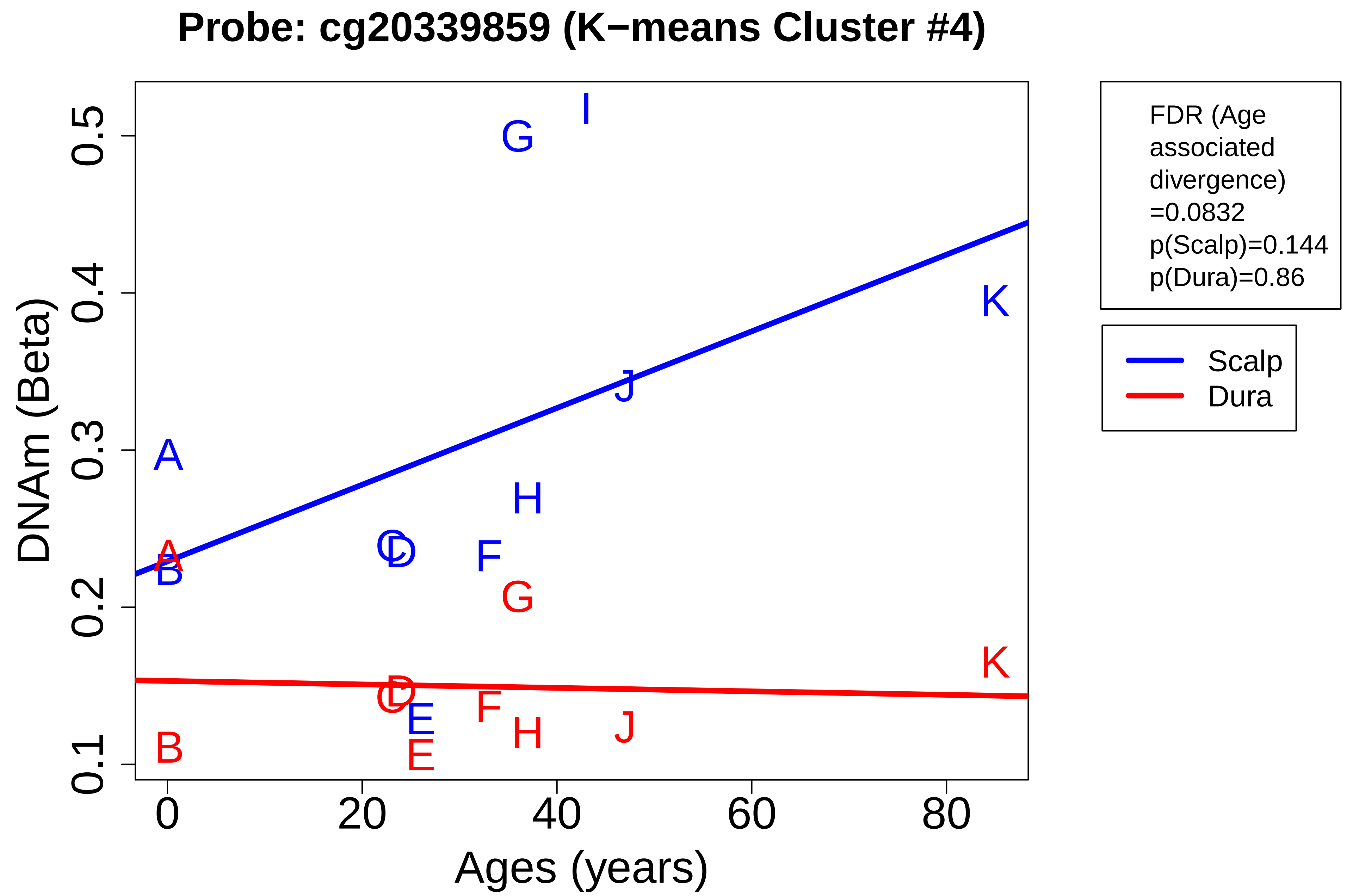

Probe: cg20339859 (K−means Cluster #4)
DNAm (Beta)
0.1
0.2
0.3
0.4
0.5
0
20
40
60
80
Ages (years)
FDR (Age associated divergence) =0.0832
p(Scalp)=0.144
p(Dura)=0.86
Scalp
Dura
A
B
C
D
E
F
G
H
I
J
K

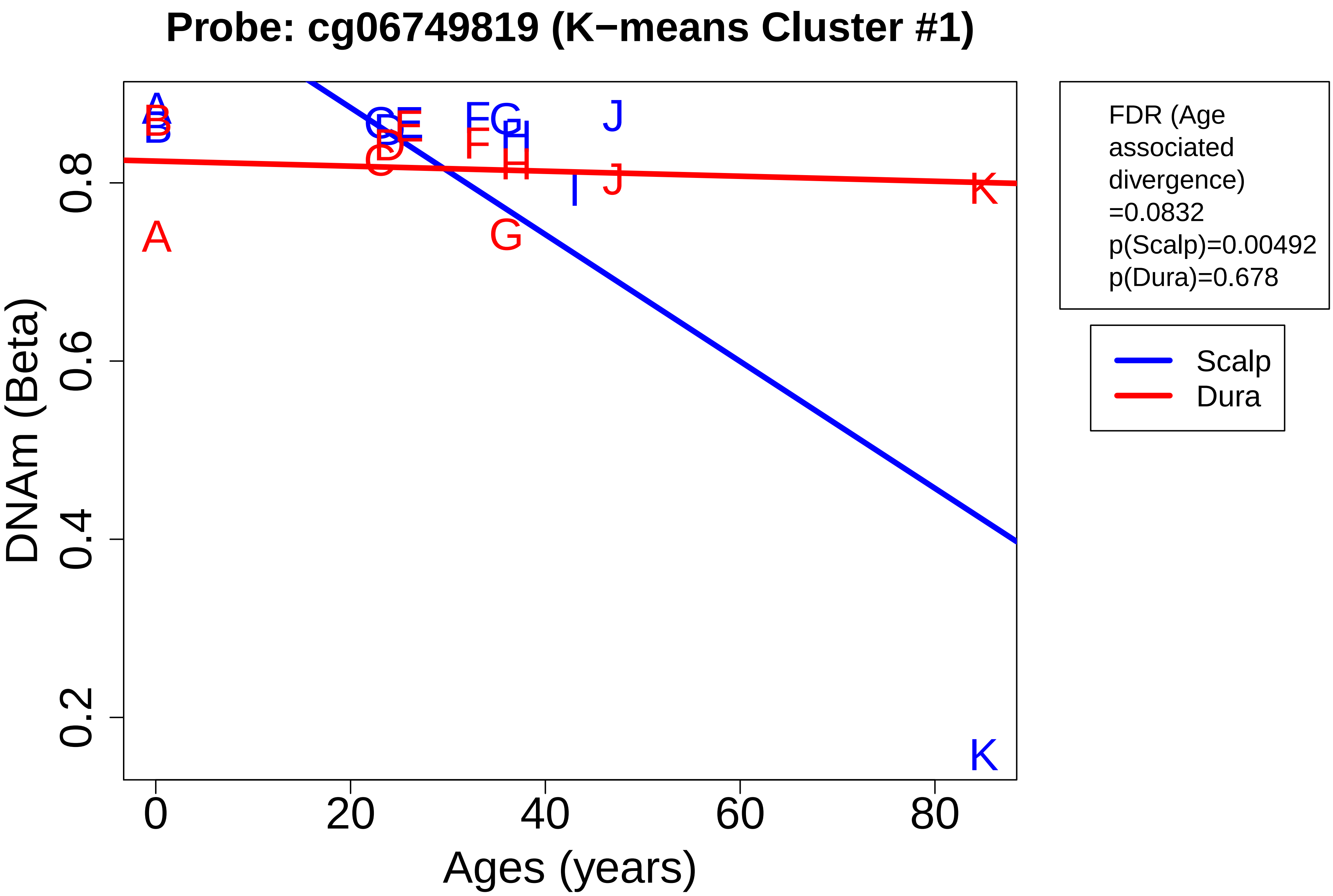

Probe: cg06749819 (K−means Cluster #1)
DNAm (Beta)
0.2
0.4
0.6
0.8
0
20
40
60
80
Ages (years)
FDR (Age associated divergence) =0.0832
p(Scalp)=0.00492
p(Dura)=0.678
Scalp
Dura

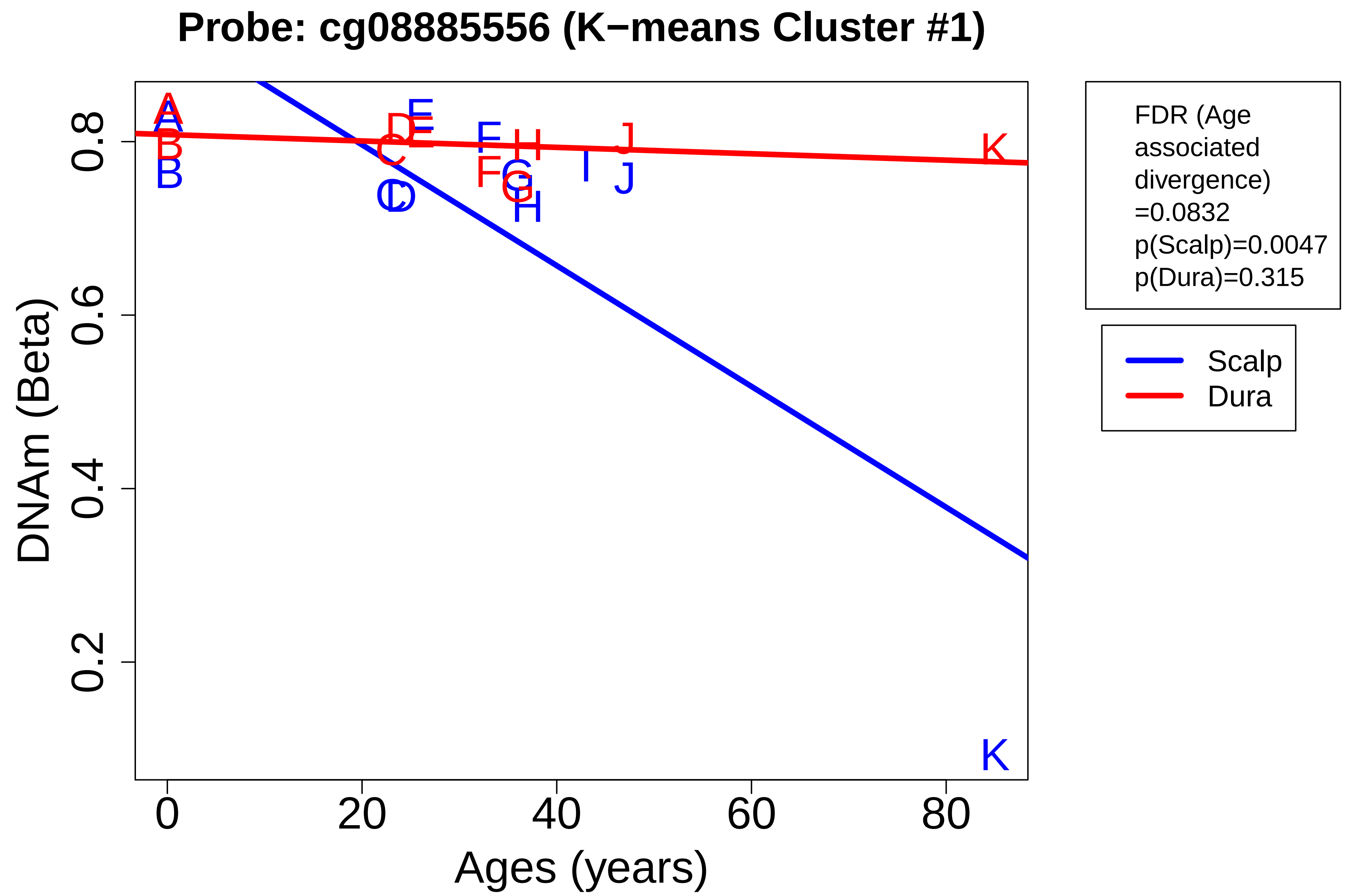

Probe: cg08885556 (K−means Cluster #1)
DNAm (Beta)
0.2
0.4
0.6
0.8
0
20
40
60
80
Ages (years)
FDR (Age associated divergence) =0.0832
p(Scalp)=0.0047
p(Dura)=0.315
Scalp
Dura

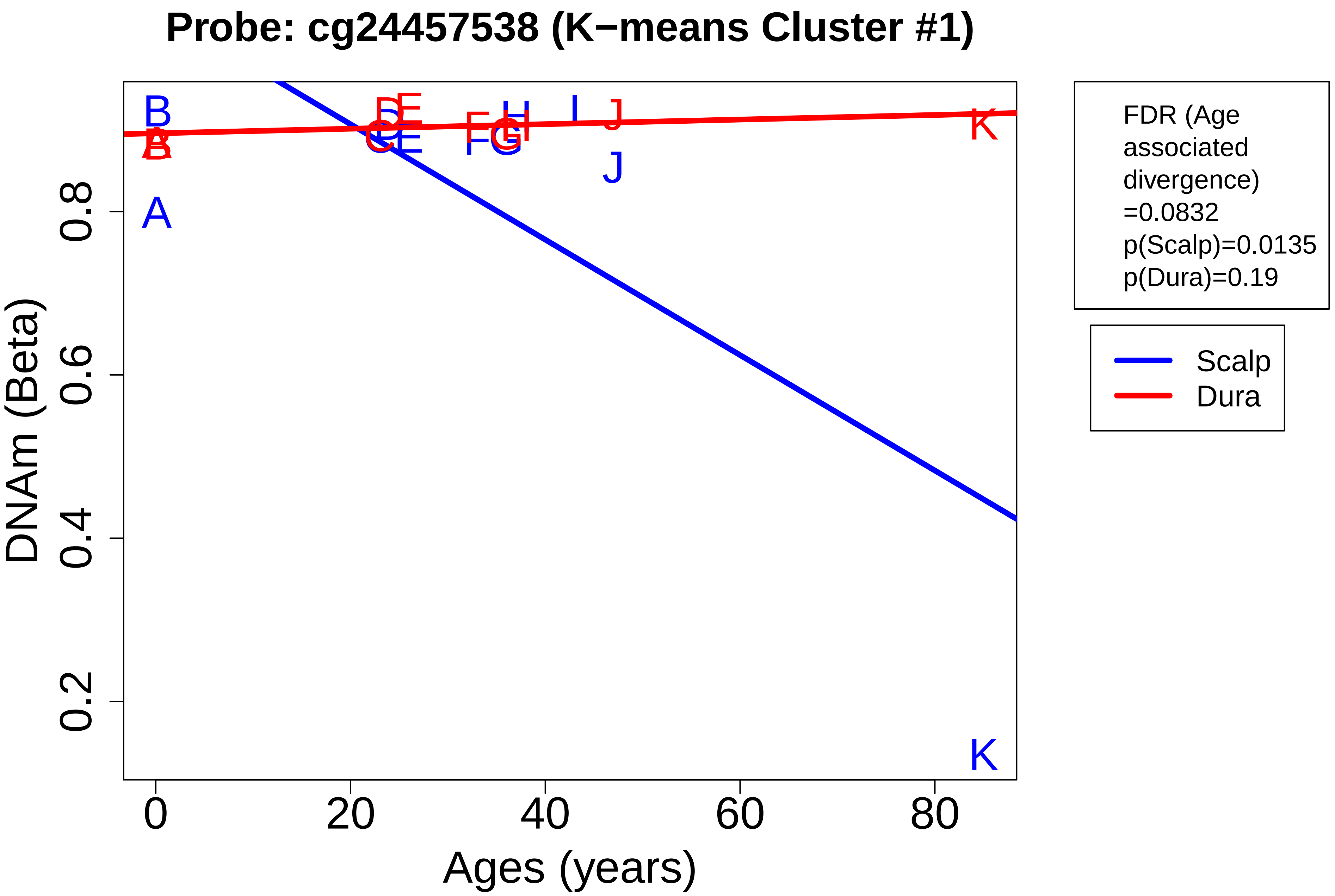

Probe: cg24457538 (K−means Cluster #1)
DNAm (Beta)
Ages (years)
0.2
0.4
0.6
0.8
0
20
40
60
80
FDR (Age associated divergence) =0.0832
p(Scalp)=0.0135
p(Dura)=0.19
Scalp
Dura

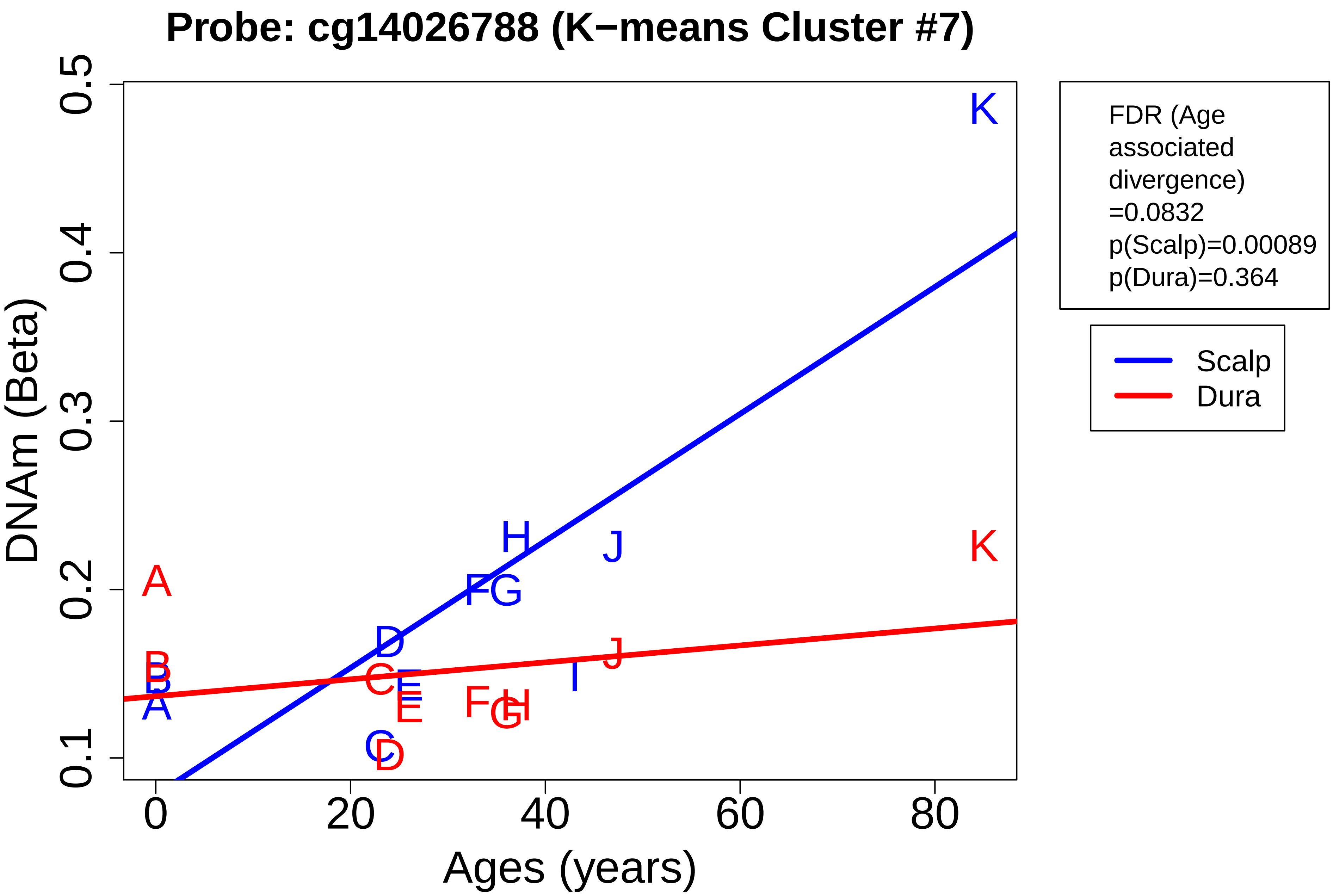

Probe: cg14026788 (K−means Cluster #7)
DNAm (Beta)
0.1
0.2
0.3
0.4
0.5
0
20
40
60
80
Ages (years)
FDR (Age associated divergence) =0.0832
p(Scalp)=0.00089
p(Dura)=0.364
Scalp
Dura

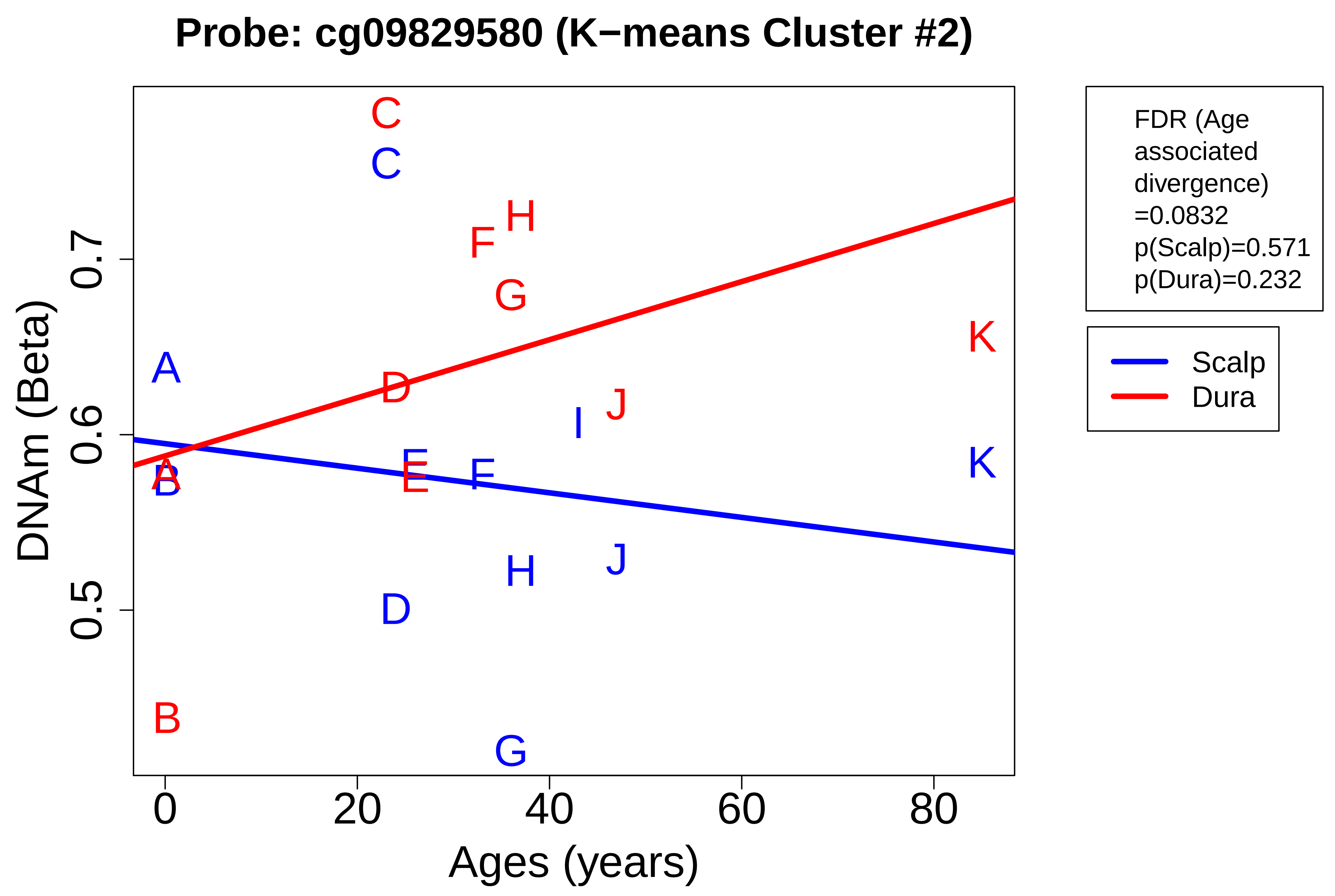

Probe: cg09829580 (K−means Cluster #2)
DNAm (Beta)
0.5
0.6
0.7
Ages (years)
0
20
40
60
80
FDR (Age associated divergence) =0.0832
p(Scalp)=0.571
p(Dura)=0.232
Scalp
Dura

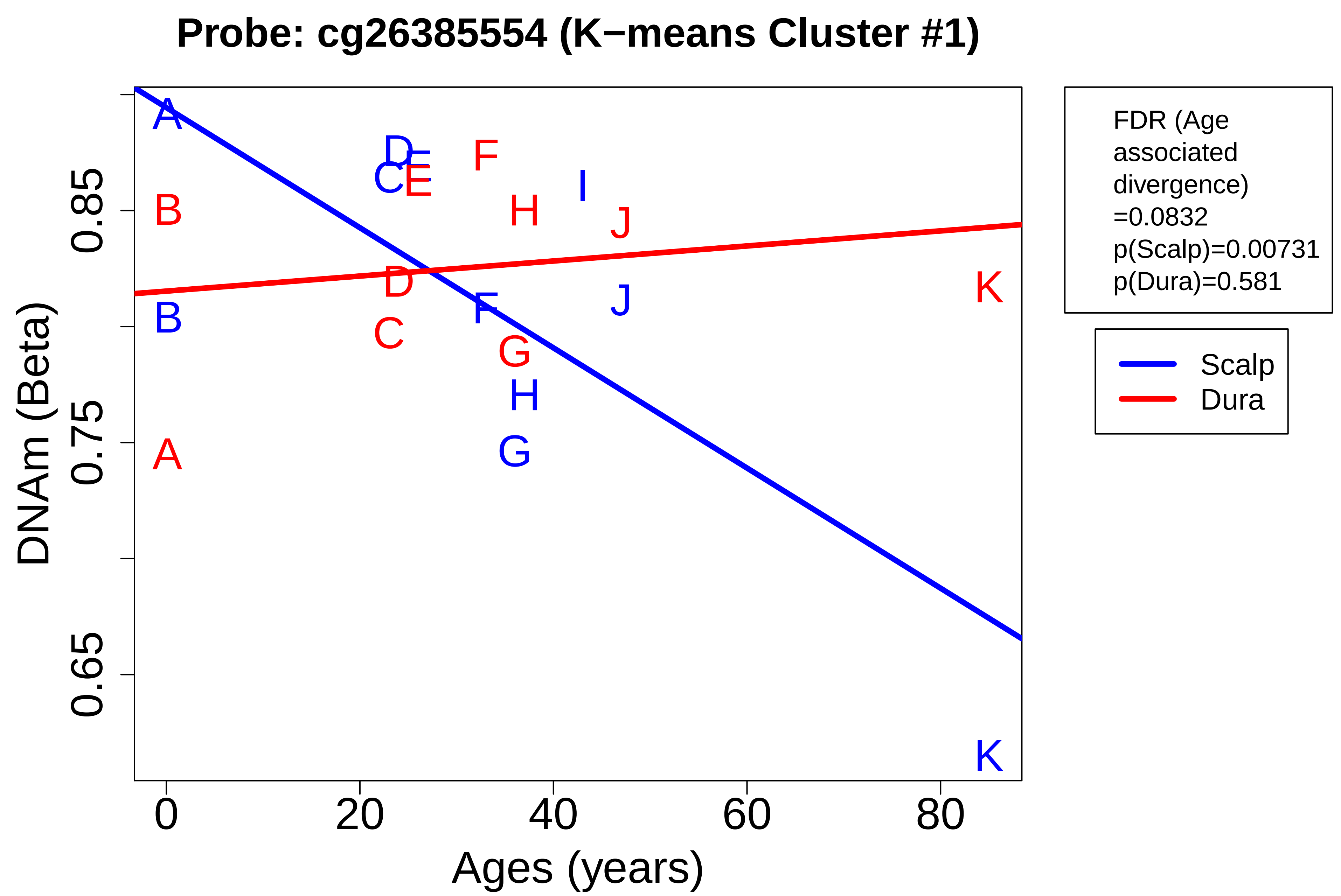

Probe: cg26385554 (K−means Cluster #1)
DNAm (Beta)
0.65
0.75
0.85
Ages (years)
0
20
40
60
80
A
B
C
D
E
F
G
H
I
J
K
FDR (Age associated divergence) =0.0832
p(Scalp)=0.00731
p(Dura)=0.581
Scalp
Dura

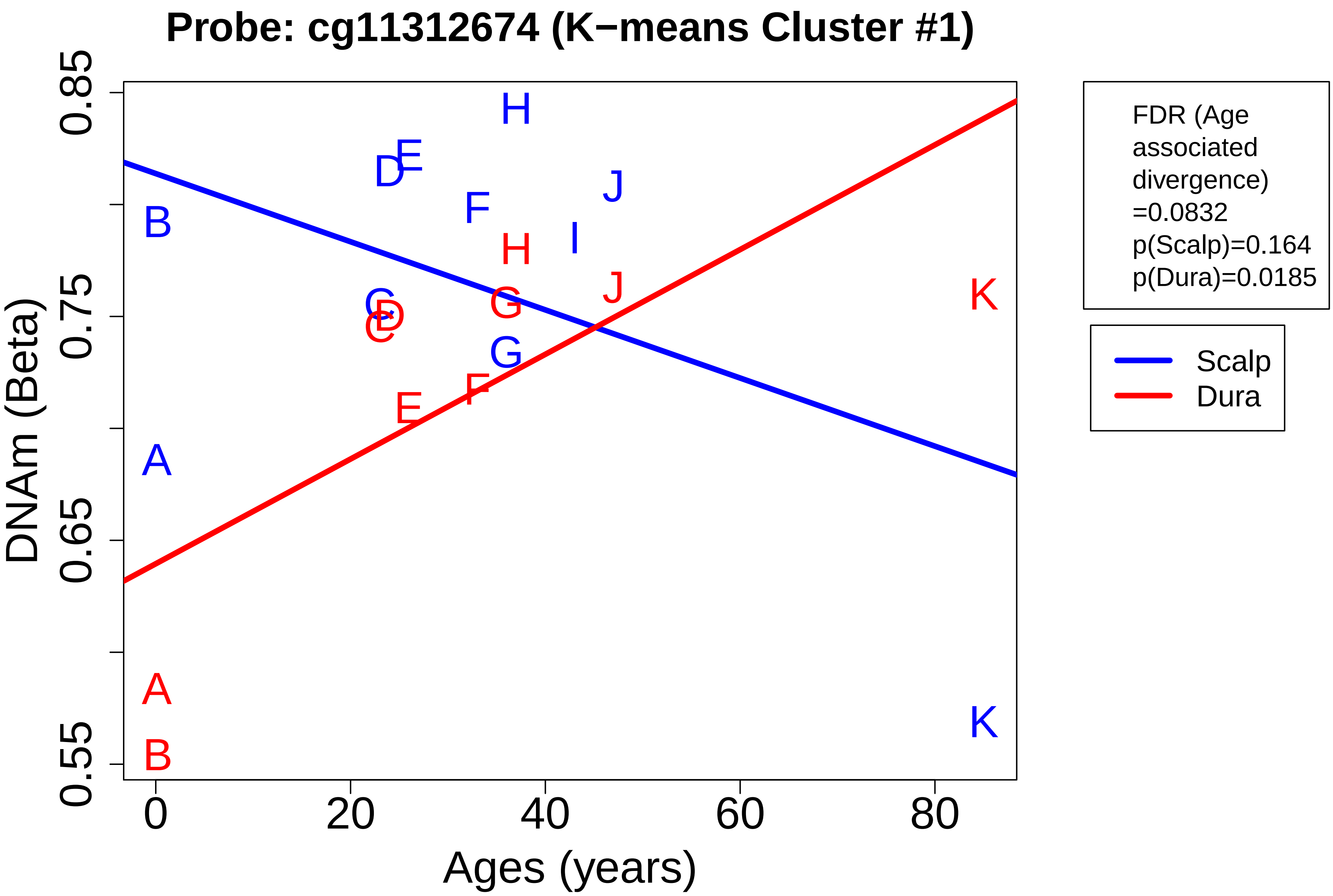

Probe: cg11312674 (K−means Cluster #1)
DNAm (Beta)
0.55
0.65
0.75
0.85
0
20
40
60
80
Ages (years)
FDR (Age associated divergence) =0.0832
p(Scalp)=0.164
p(Dura)=0.0185
Scalp
Dura

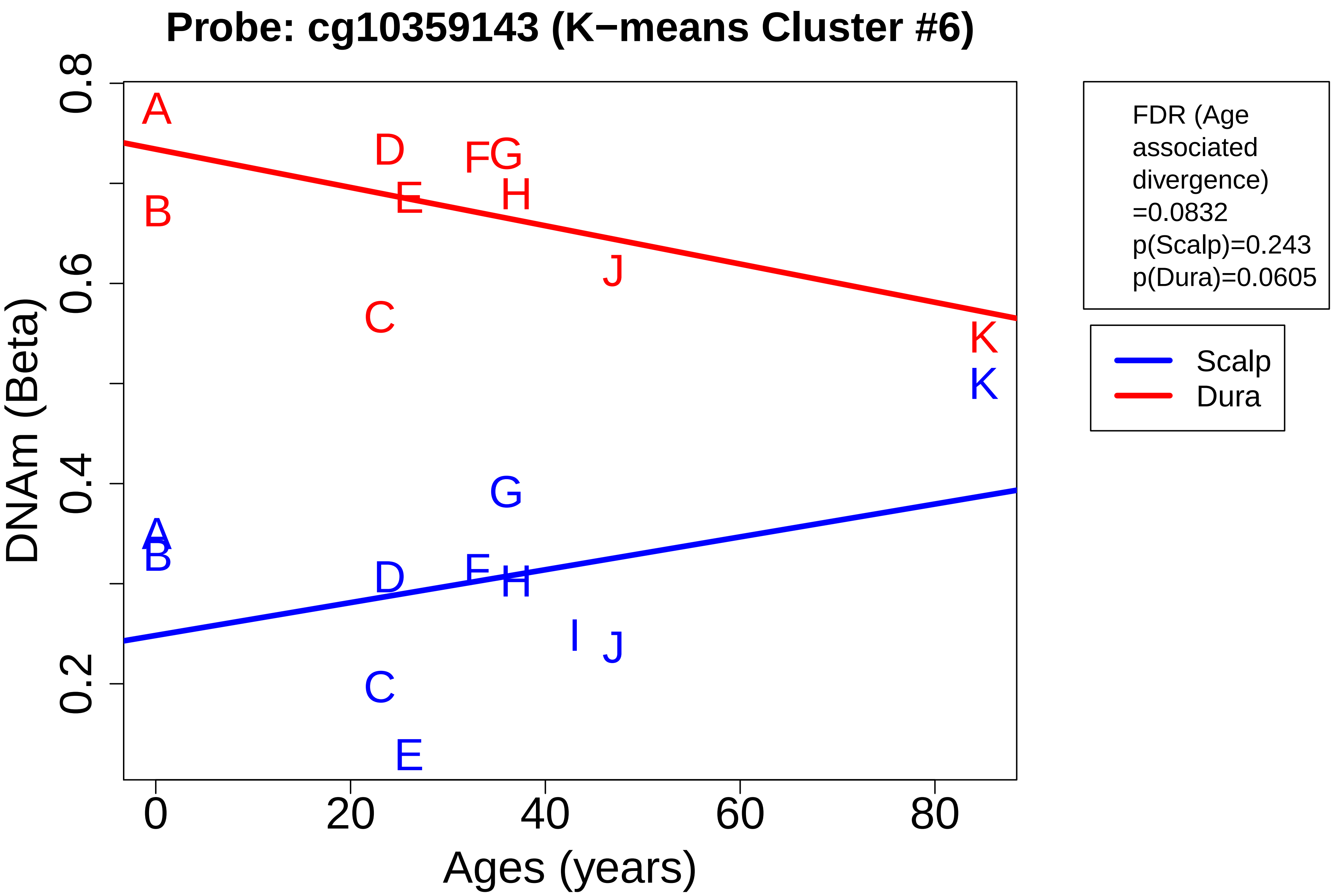
Probe: cg10359143 (K−means Cluster #6)
DNAm (Beta)
0.2
0.4
0.6
0.8
Ages (years)
0
20
40
60
80
A
B
C
D
E
F
G
H
I
J
K
FDR (Age associated divergence) =0.0832
p(Scalp)=0.243
p(Dura)=0.0605
Scalp
Dura

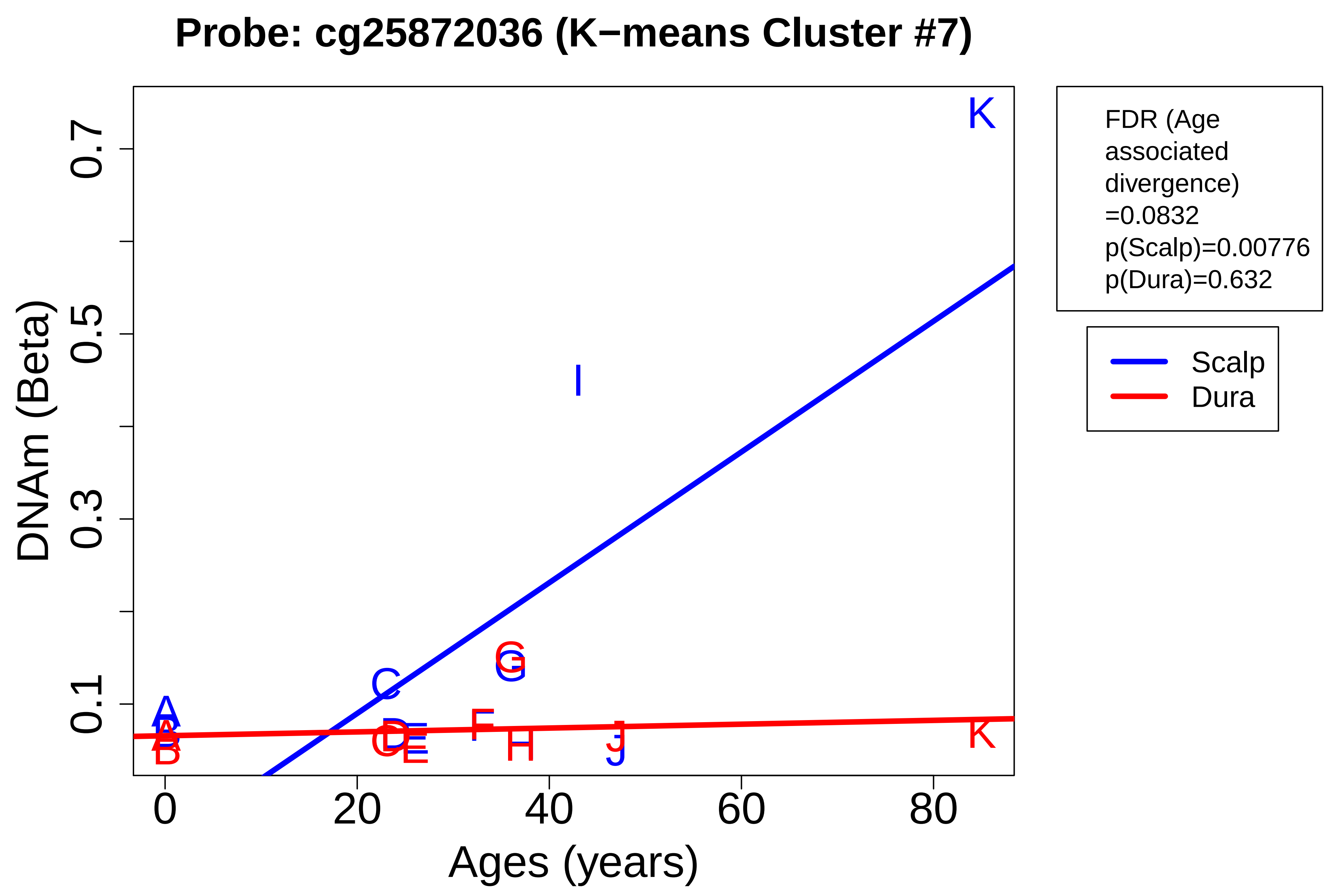

Probe: cg25872036 (K−means Cluster #7)
DNAm (Beta)
0.1
0.3
0.5
0.7
Ages (years)
0
20
40
60
80
A
B
C
D
E
F
G
H
I
J
K
FDR (Age associated divergence) =0.0832
p(Scalp)=0.00776
p(Dura)=0.632
Scalp
Dura

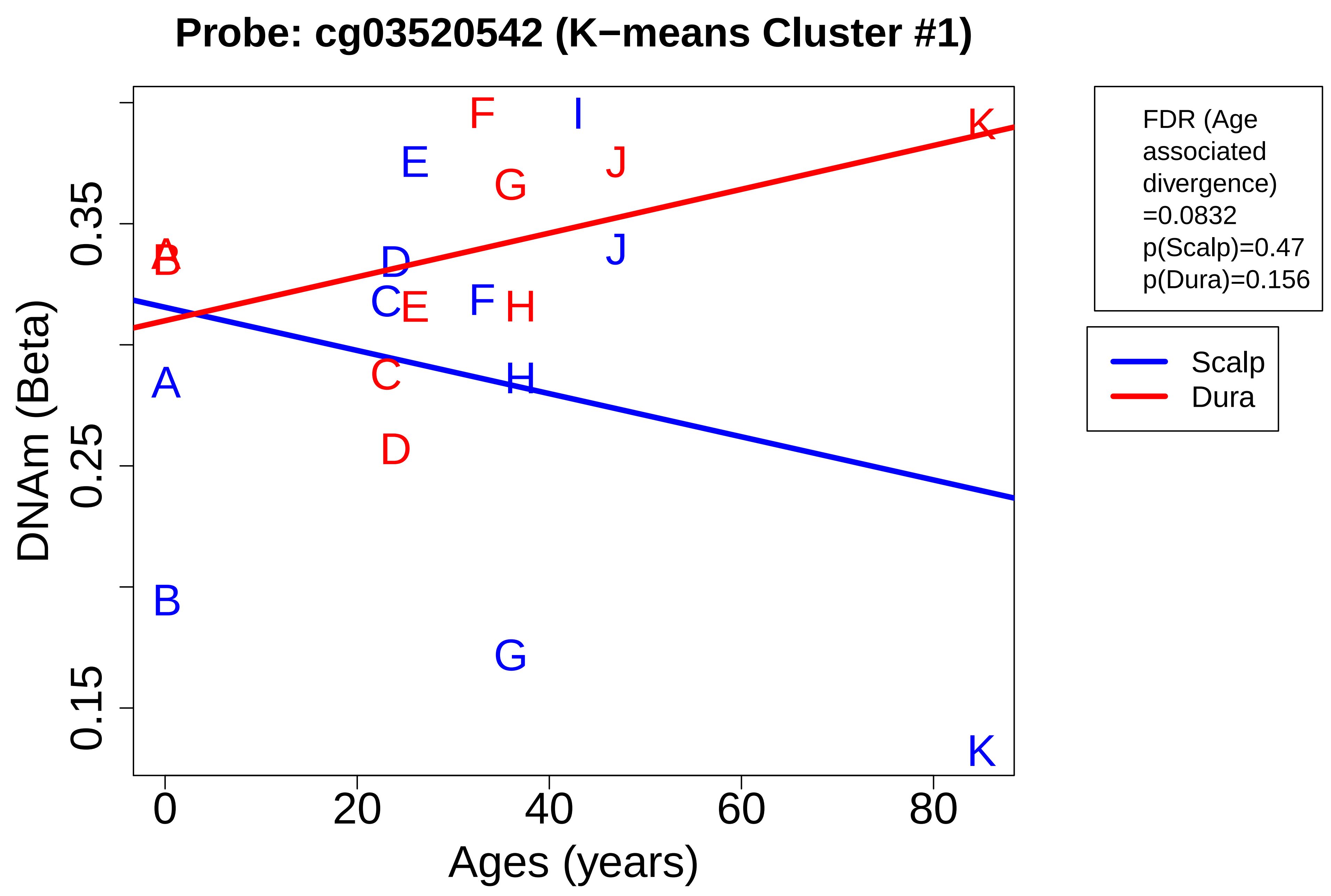
Probe: cg03520542 (K−means Cluster #1)
DNAm (Beta)
0.15
0.25
0.35
0
20
40
60
80
Ages (years)
A
B
C
D
E
F
G
H
I
J
K
FDR (Age associated divergence) =0.0832
p(Scalp)=0.47
p(Dura)=0.156
Scalp
Dura

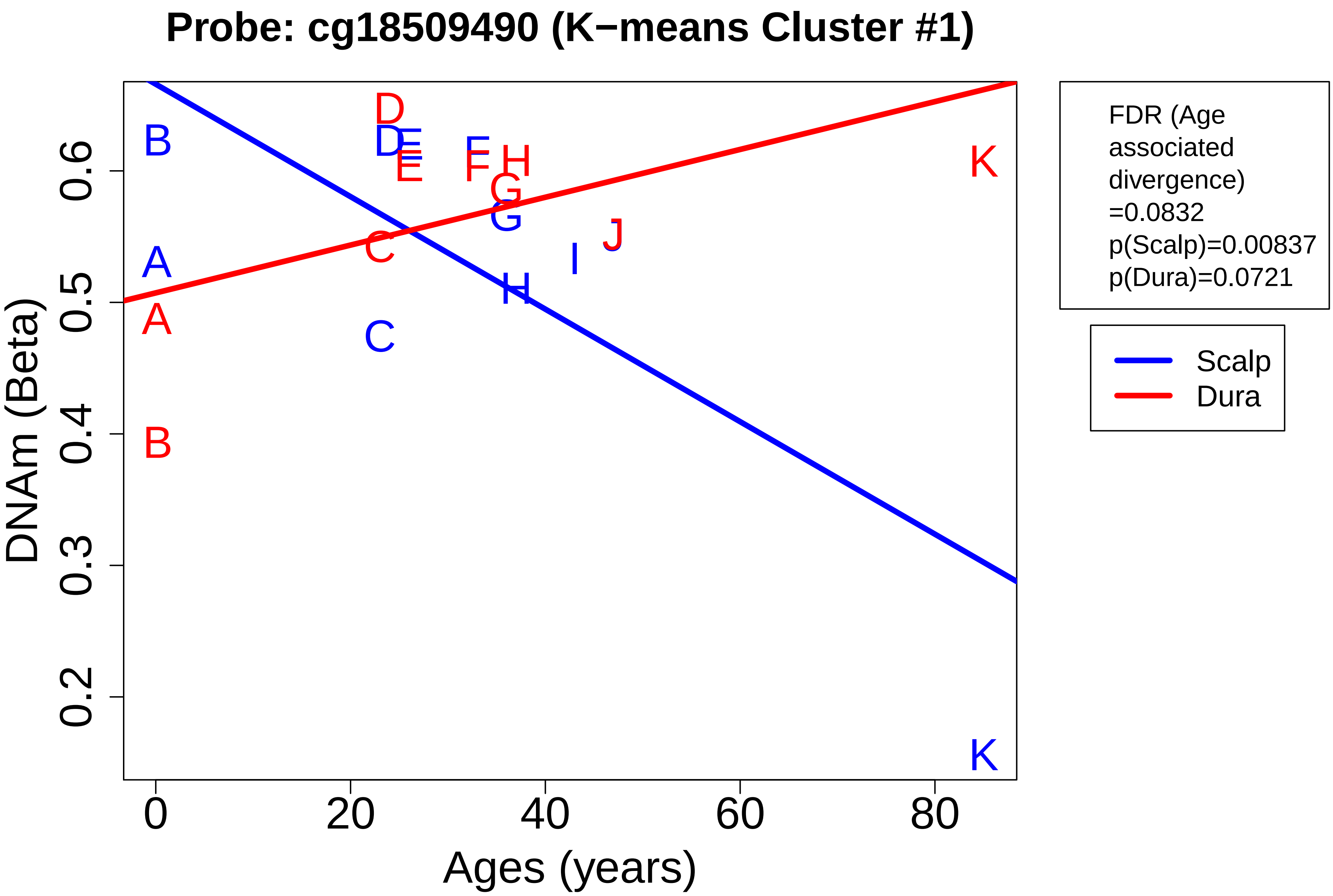

Probe: cg18509490 (K−means Cluster #1)
DNAm (Beta)
0.2
0.3
0.4
0.5
0.6
Ages (years)
0
20
40
60
80
A
B
C
D
E
F
G
H
I
J
K
FDR (Age associated divergence) =0.0832
p(Scalp)=0.00837
p(Dura)=0.0721
Scalp
Dura

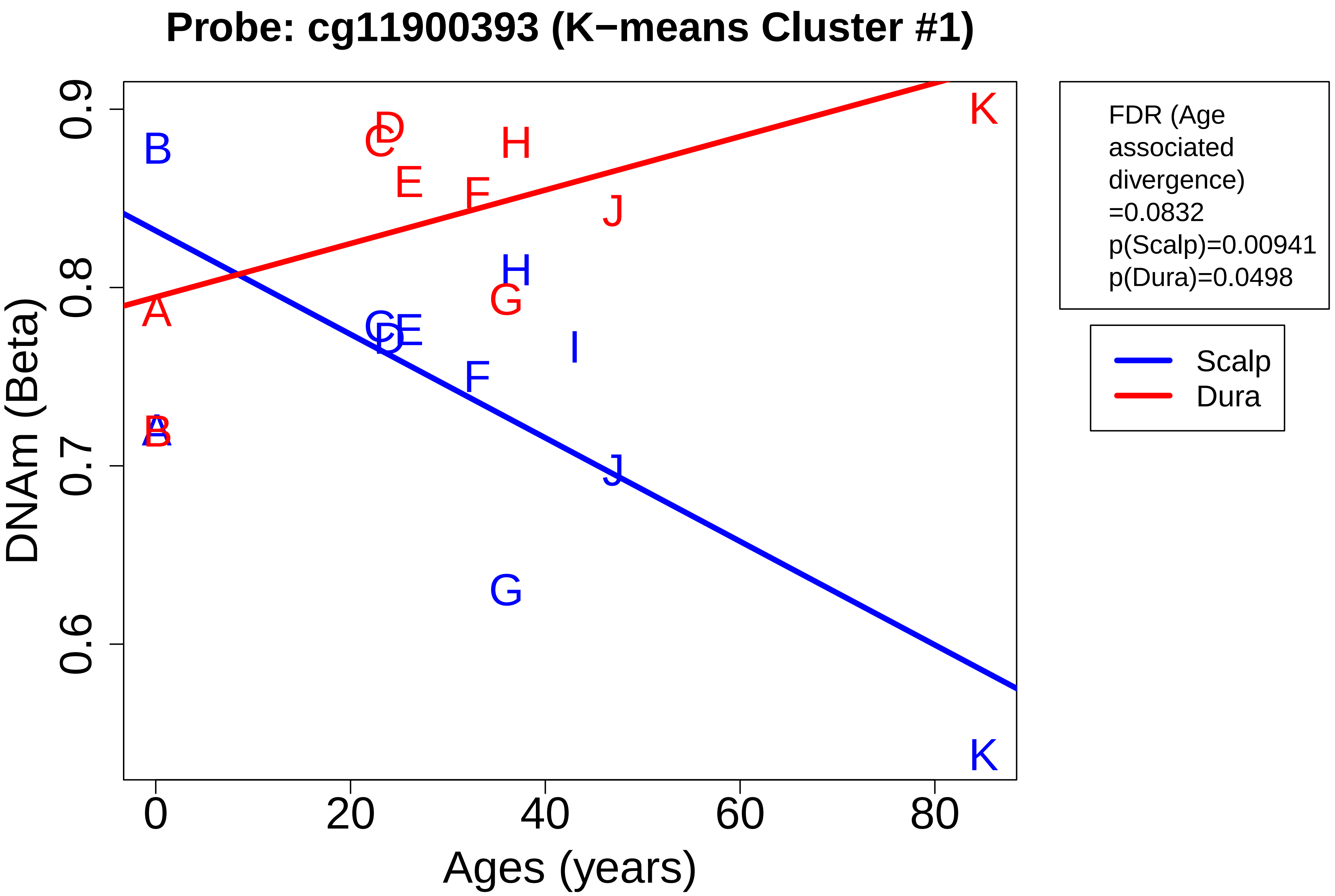
Probe: cg11900393 (K−means Cluster #1)
DNAm (Beta)
0.6
0.7
0.8
0.9
0
20
40
60
80
Ages (years)
FDR (Age associated divergence) =0.0832
p(Scalp)=0.00941
p(Dura)=0.0498
Scalp
Dura

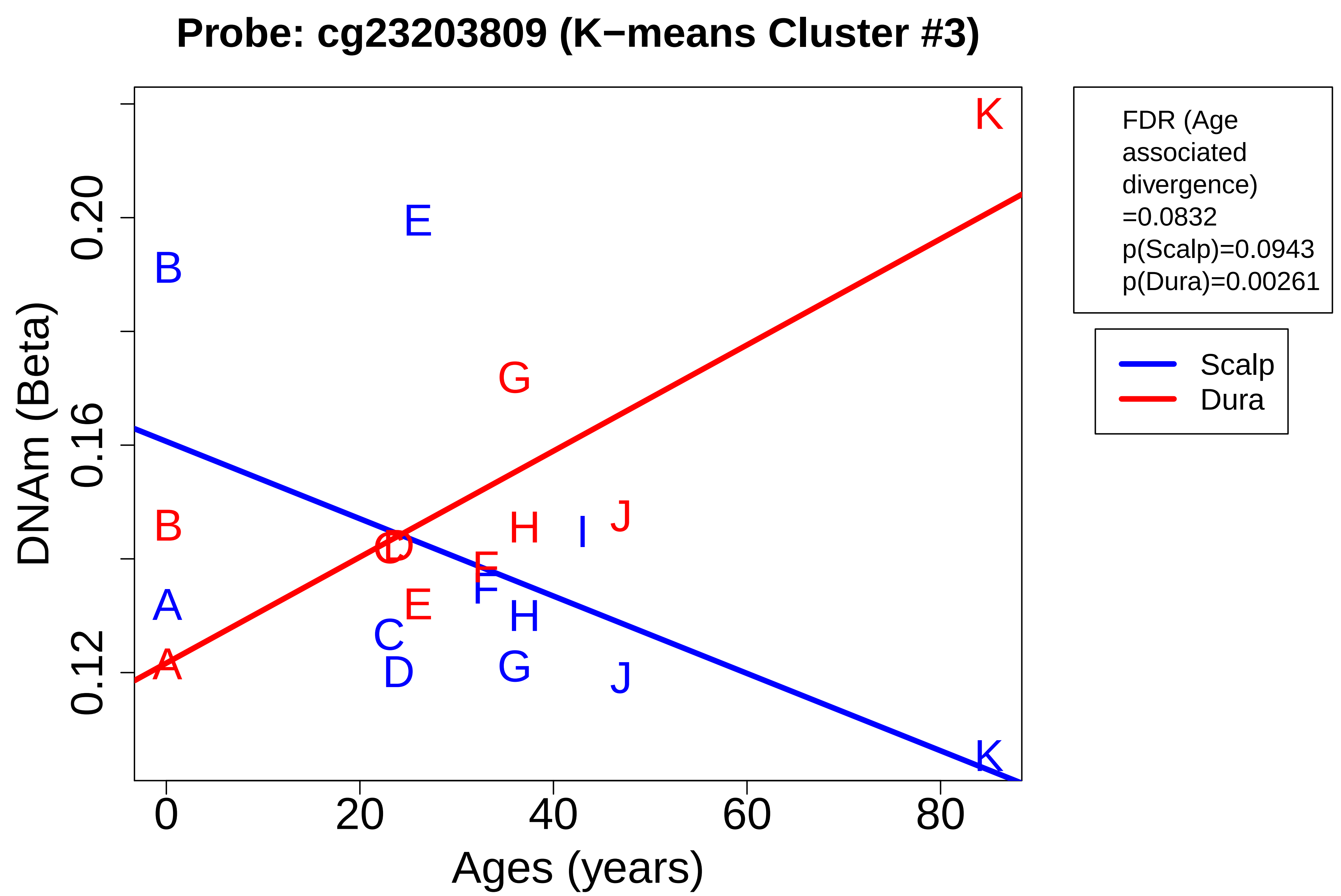
Probe: cg23203809 (K−means Cluster #3)
DNAm (Beta)
0.12
0.16
0.20
0
20
40
60
80
Ages (years)
A
B
C
D
E
F
G
H
I
J
K
FDR (Age associated divergence) =0.0832
p(Scalp)=0.0943
p(Dura)=0.00261
Scalp
Dura

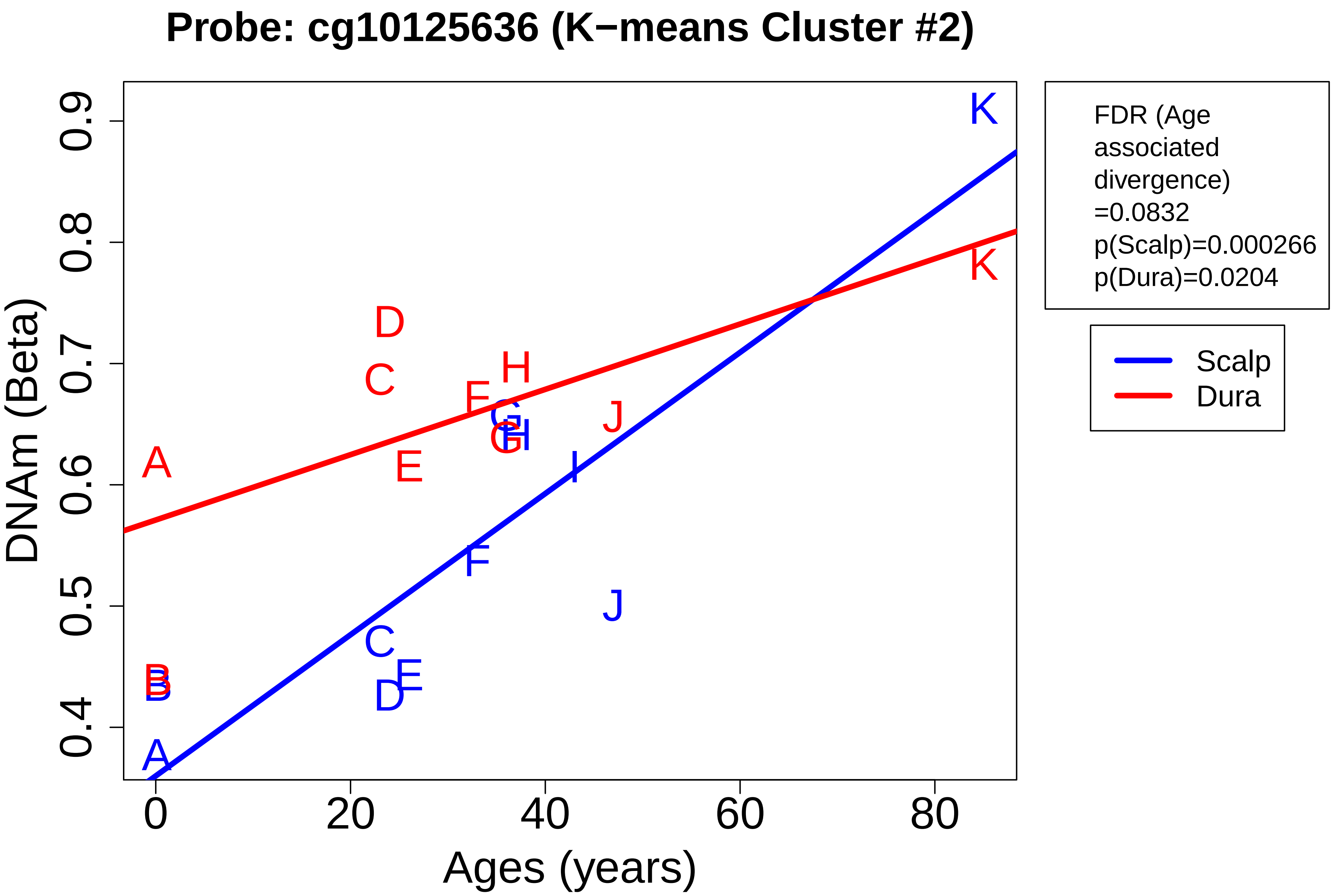

Probe: cg10125636 (K−means Cluster #2)
DNAm (Beta)
0.4
0.5
0.6
0.7
0.8
0.9
Ages (years)
0
20
40
60
80
FDR (Age associated divergence) =0.0832
p(Scalp)=0.000266
p(Dura)=0.0204
Scalp
Dura

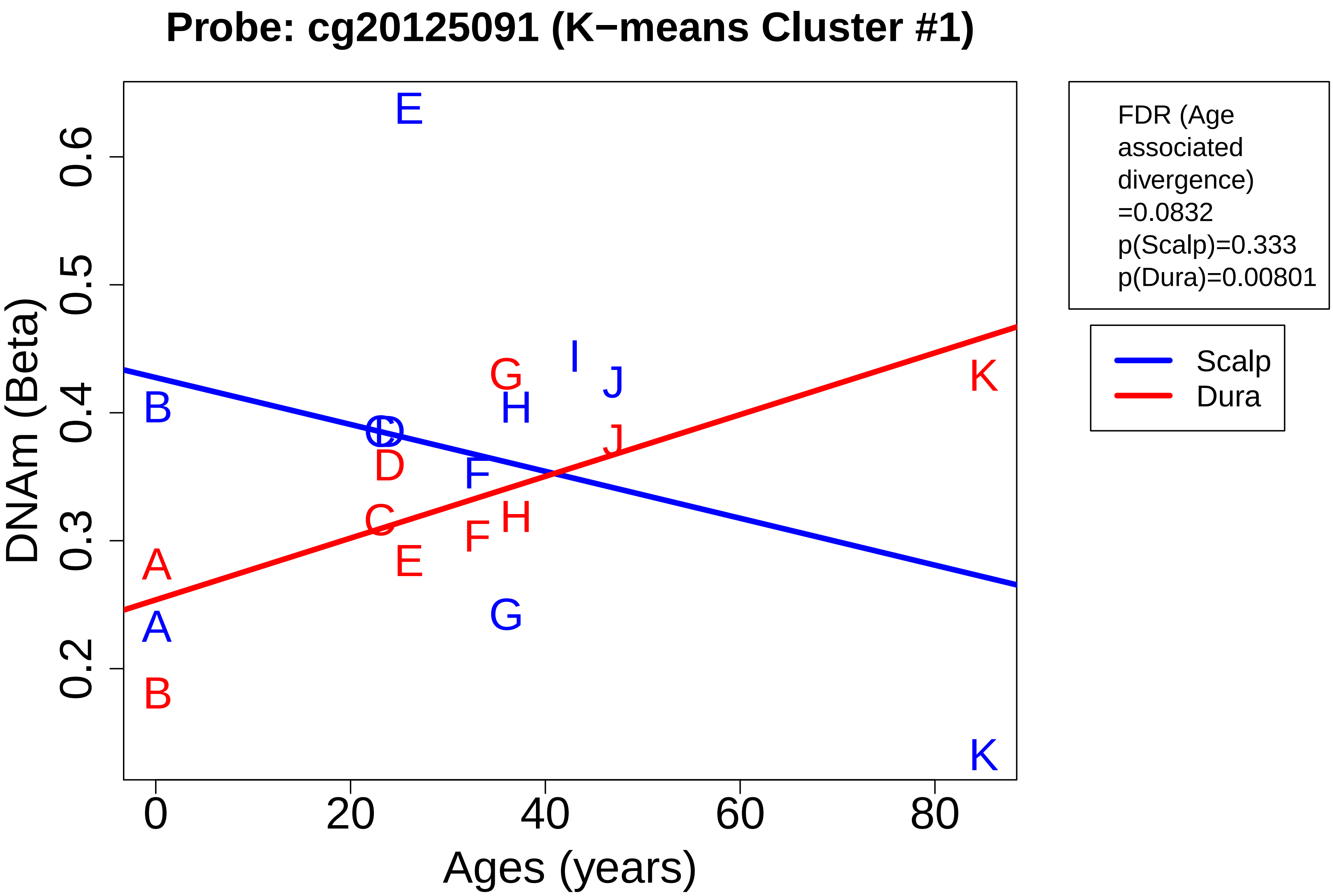

Probe: cg20125091 (K−means Cluster #1)
DNAm (Beta)
0.2
0.3
0.4
0.5
0.6
0
20
40
60
80
Ages (years)
FDR (Age associated divergence) =0.0832
p(Scalp)=0.333
p(Dura)=0.00801
Scalp
Dura

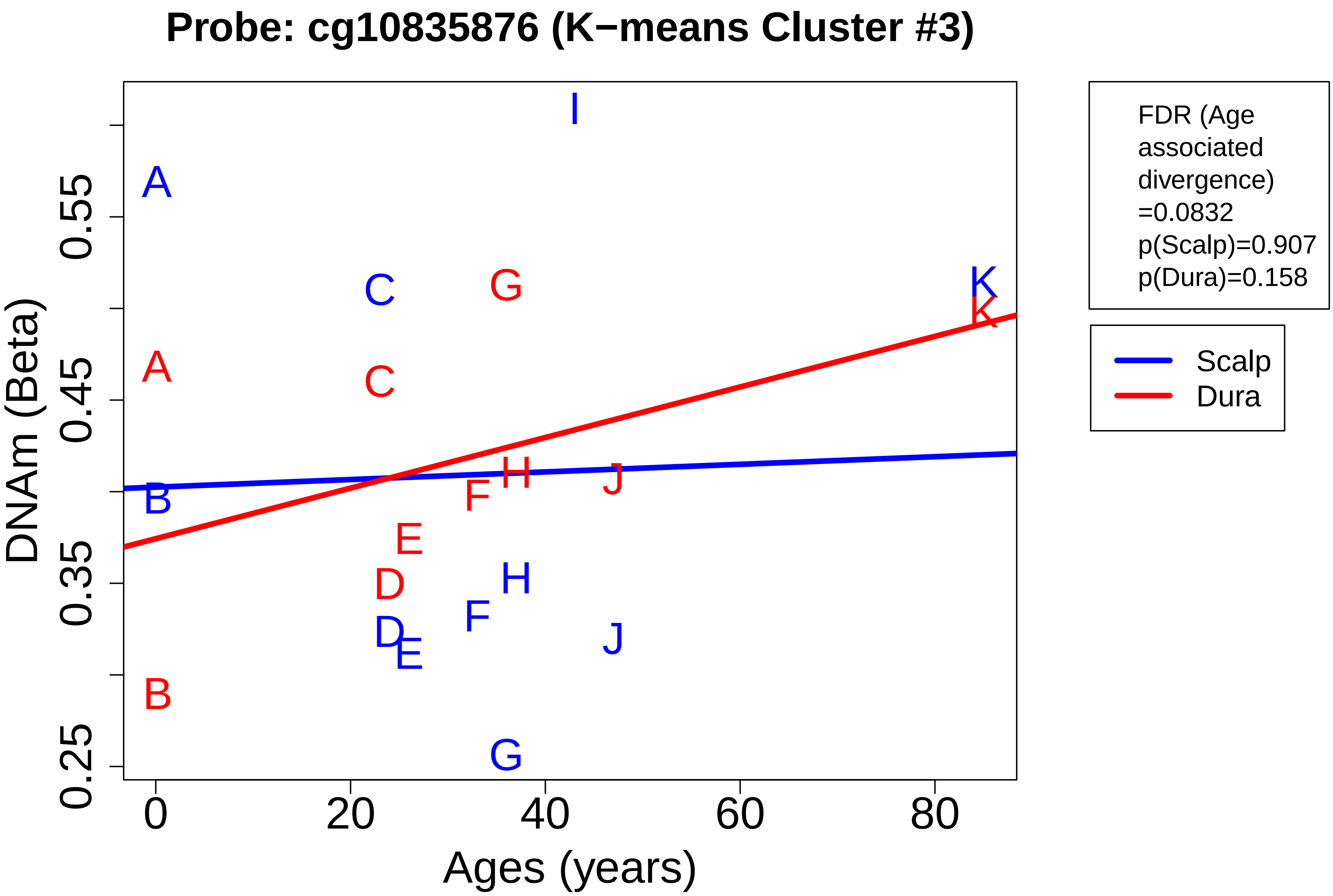

Probe: cg10835876 (K−means Cluster #3)
DNAm (Beta)
0.25
0.35
0.45
0.55
0
20
40
60
80
Ages (years)
FDR (Age associated divergence) =0.0832
p(Scalp)=0.907
p(Dura)=0.158
Scalp
Dura
A
B
C
D
E
F
G
H
I
J
K

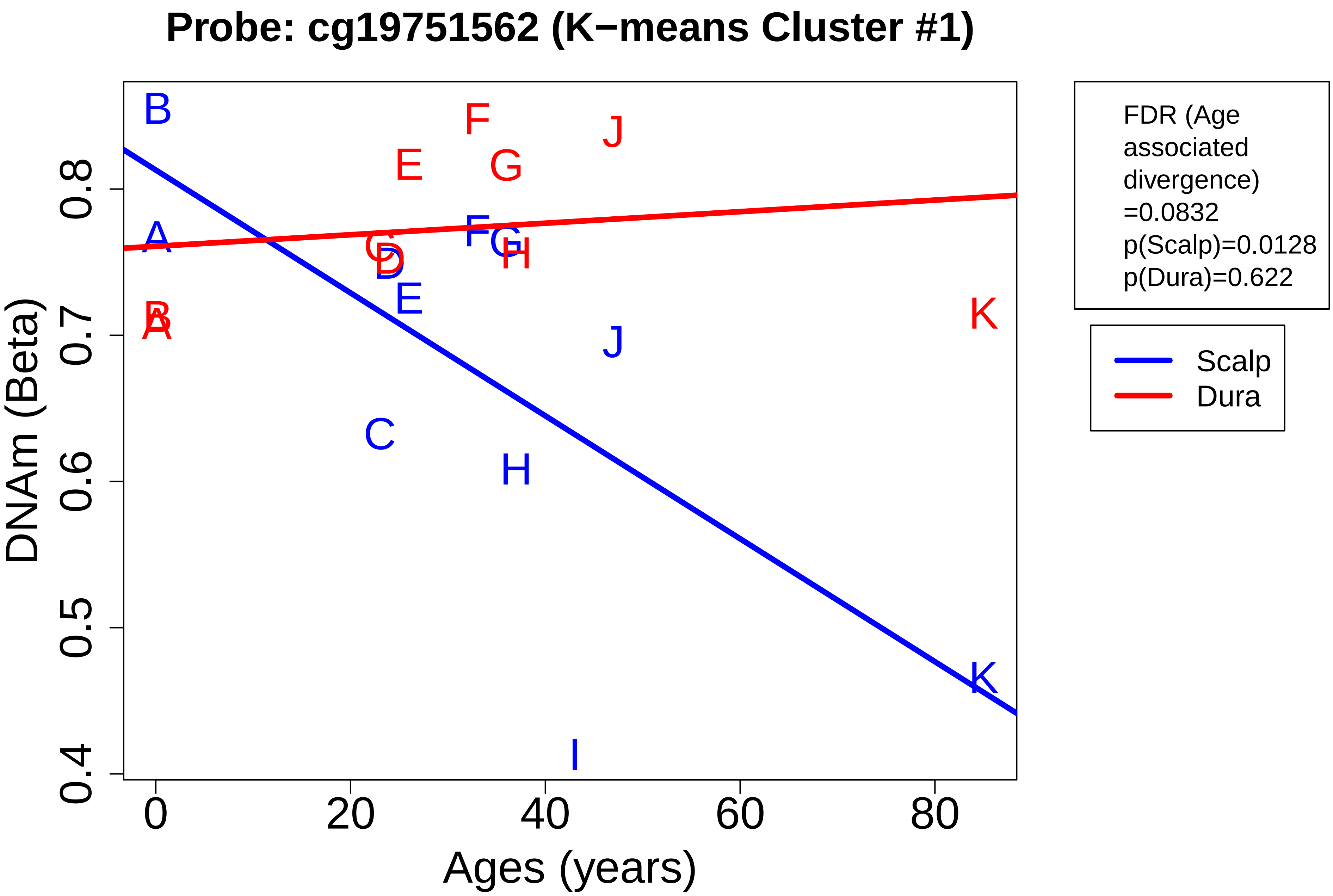

Probe: cg19751562 (K−means Cluster #1)
DNAm (Beta)
0.4
0.5
0.6
0.7
0.8
0
20
40
60
80
Ages (years)
FDR (Age associated divergence) =0.0832
p(Scalp)=0.0128
p(Dura)=0.622
Scalp
Dura

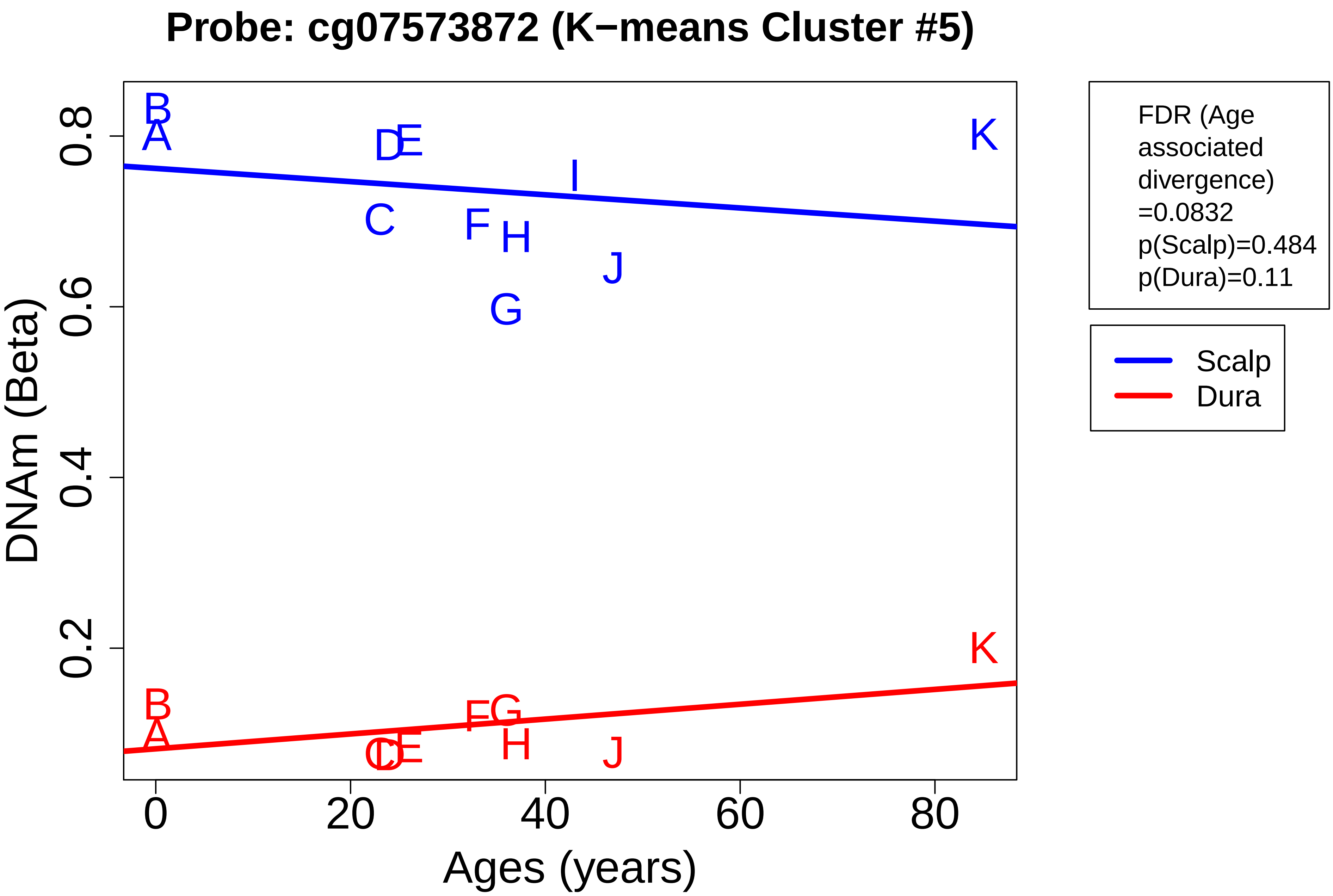

Probe: cg07573872 (K−means Cluster #5)
DNAm (Beta)
0.2
0.4
0.6
0.8
Ages (years)
0
20
40
60
80
FDR (Age associated divergence) =0.0832
p(Scalp)=0.484
p(Dura)=0.11
Scalp
Dura
A
B
C
D
E
F
G
H
I
J
K

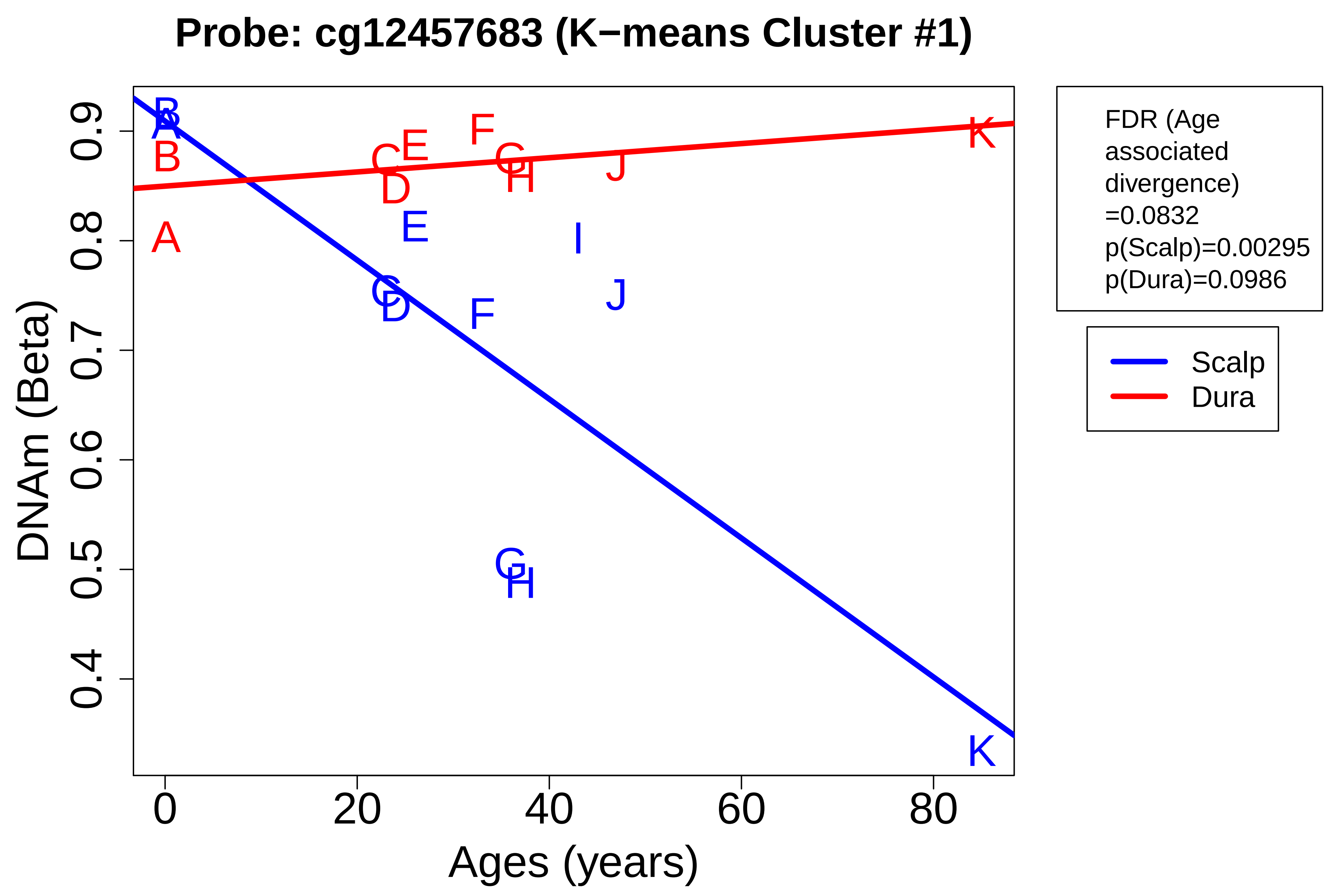

Probe: cg12457683 (K−means Cluster #1)
FDR (Age associated divergence) =0.0832
p(Scalp)=0.00295
p(Dura)=0.0986
Scalp
Dura
DNAm (Beta)
0.4
0.5
0.6
0.7
0.8
0.9
Ages (years)
0
20
40
60
80
A
B
C
D
E
F
G
H
I
J
K

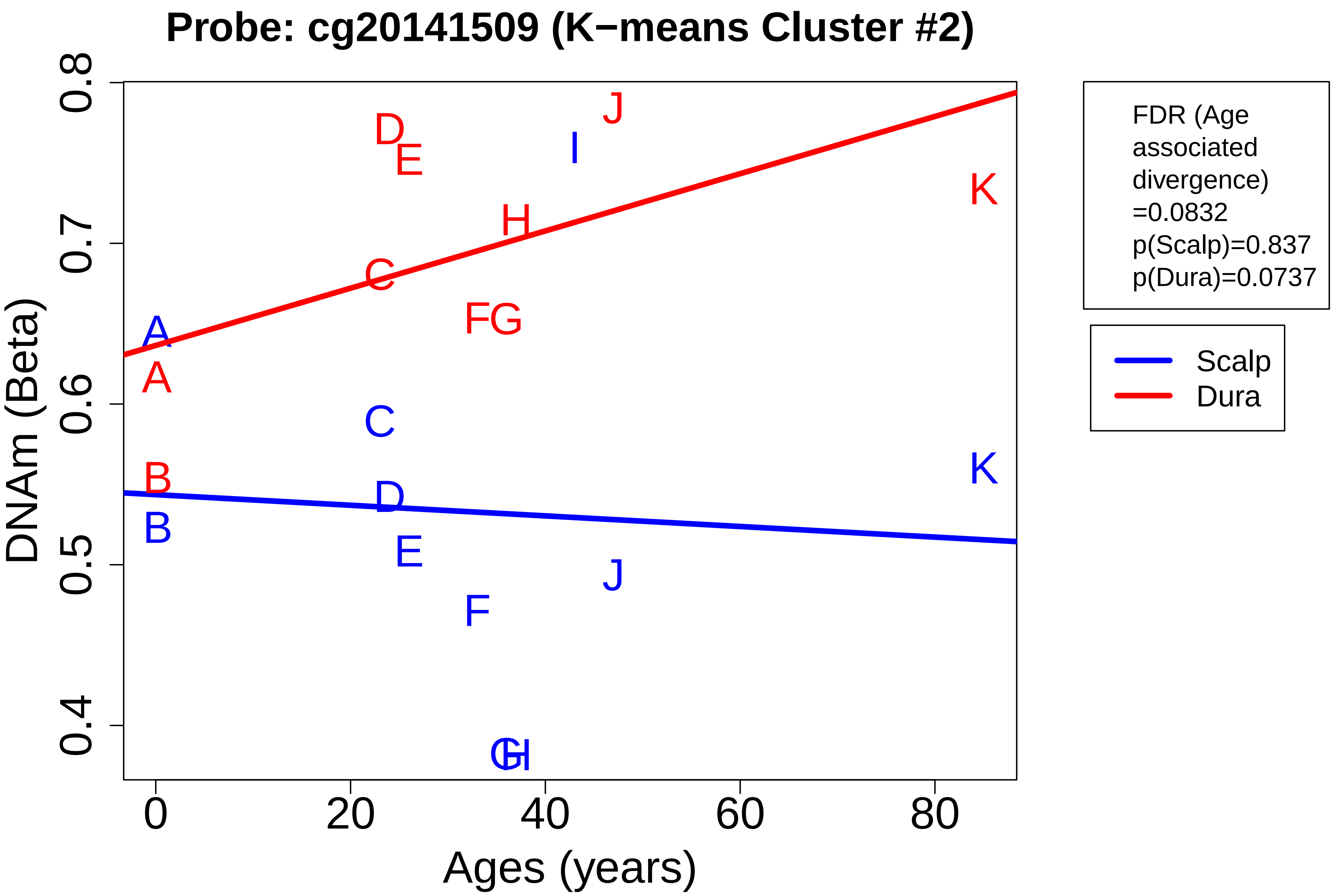

Probe: cg20141509 (K−means Cluster #2)
DNAm (Beta)
Ages (years)
0.4
0.5
0.6
0.7
0.8
0
20
40
60
80
FDR (Age associated divergence) =0.0832
p(Scalp)=0.837
p(Dura)=0.0737
Scalp
Dura

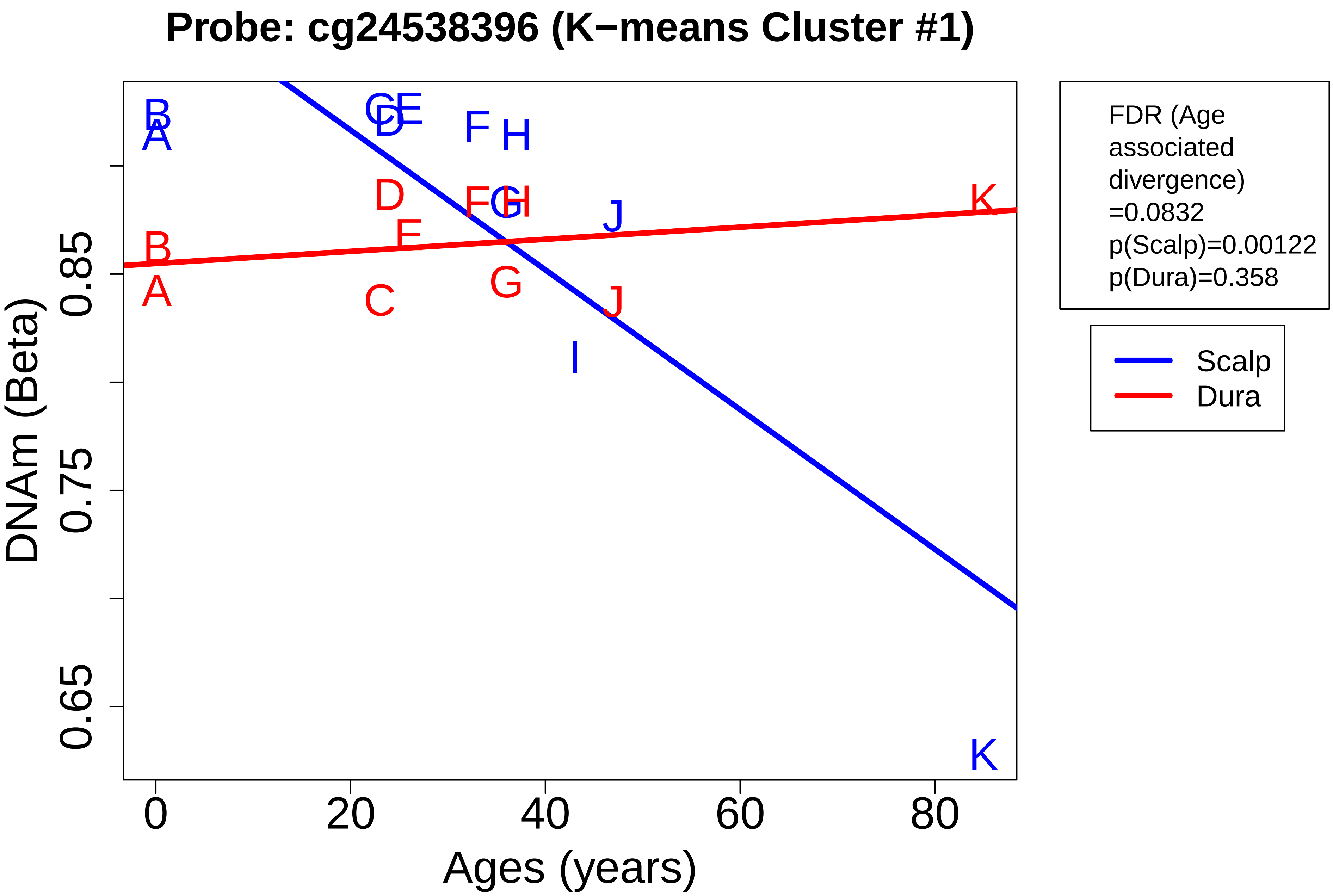

Probe: cg24538396 (K−means Cluster #1)
FDR (Age associated divergence) =0.0832
p(Scalp)=0.00122
p(Dura)=0.358
Scalp
Dura
DNAm (Beta)
0.65
0.75
0.85
Ages (years)
0
20
40
60
80
A
B
C
D
E
F
G
H
I
J
K

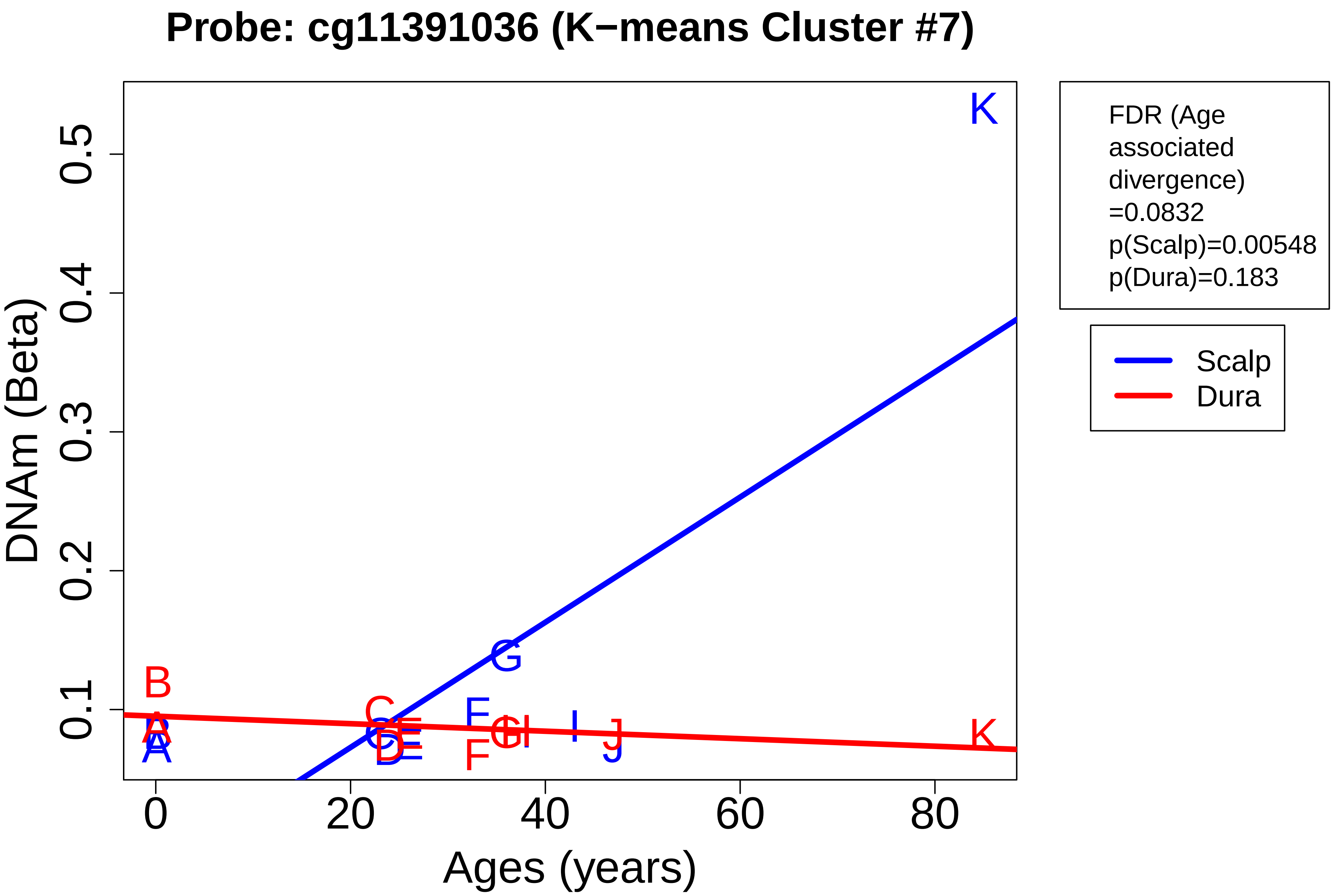

Probe: cg11391036 (K−means Cluster #7)
DNAm (Beta)
0.1
0.2
0.3
0.4
0.5
0
20
40
60
80
Ages (years)
FDR (Age associated divergence) =0.0832
p(Scalp)=0.00548
p(Dura)=0.183
Scalp
Dura

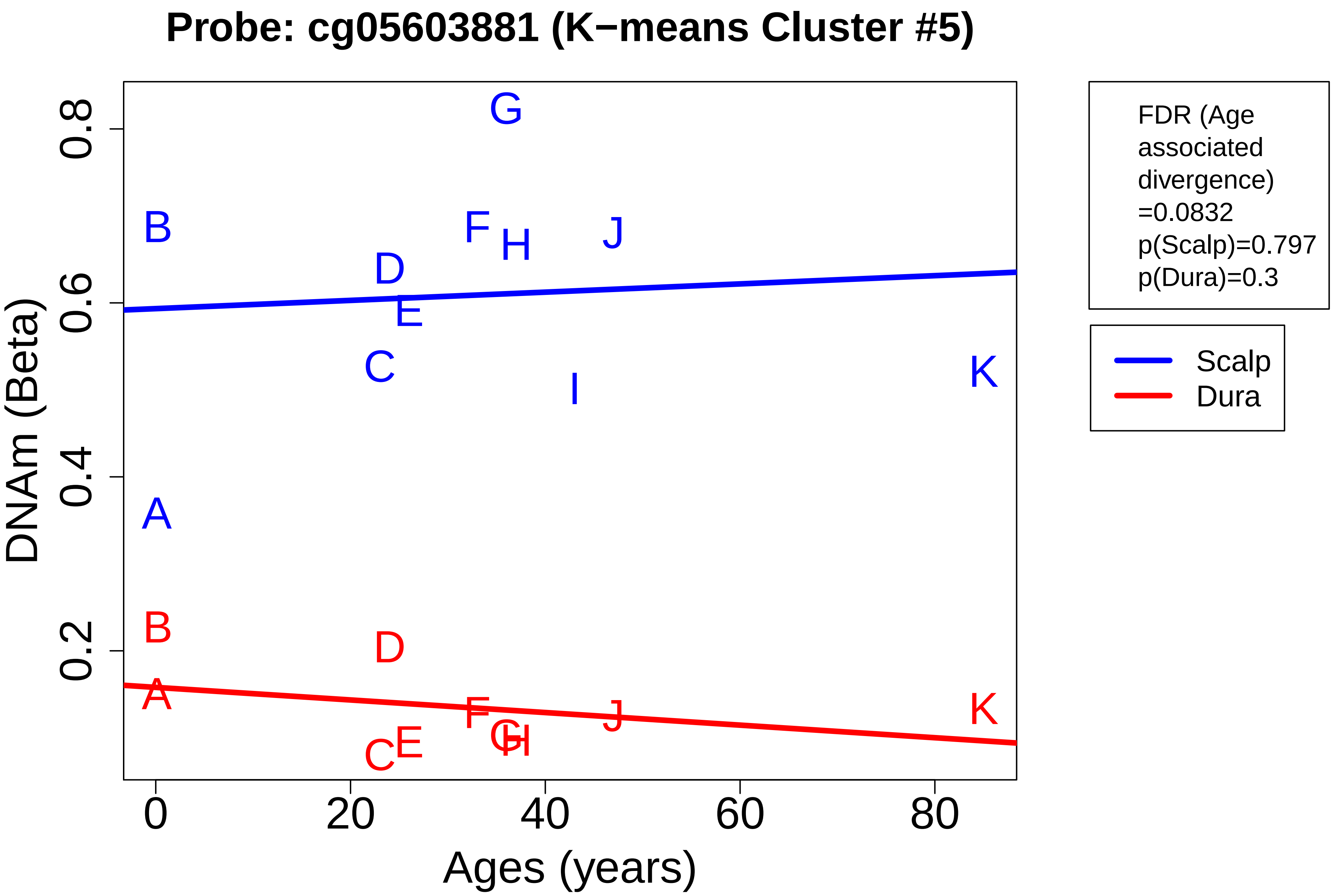

Probe: cg05603881 (K−means Cluster #5)
FDR (Age associated divergence) =0.0832
p(Scalp)=0.797
p(Dura)=0.3
Scalp
Dura
DNAm (Beta)
0.2
0.4
0.6
0.8
0
20
40
60
80
Ages (years)
A
B
C
D
E
F
G
H
I
J
K

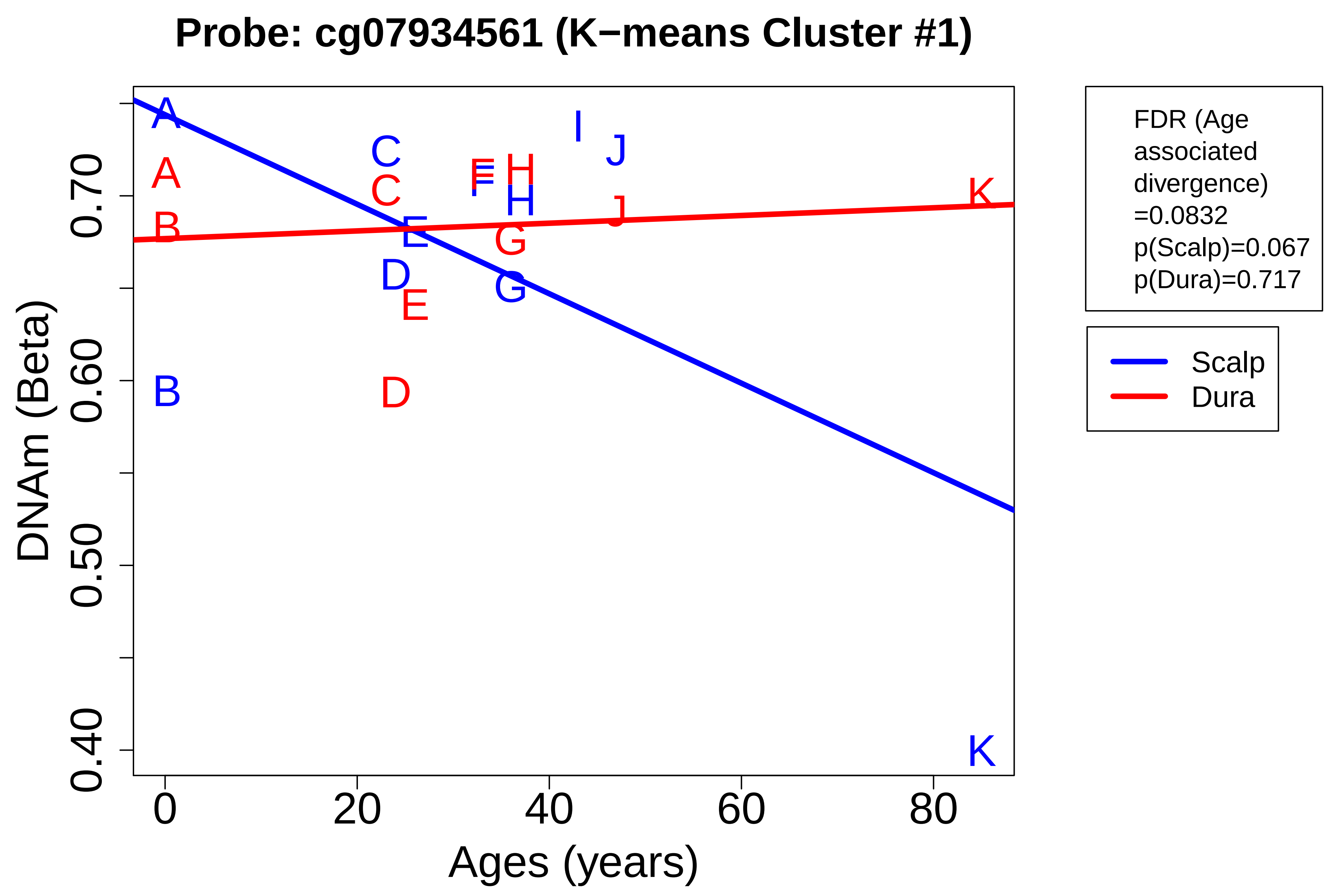

Probe: cg07934561 (K−means Cluster #1)
DNAm (Beta)
0.40
0.50
0.60
0.70
Ages (years)
0
20
40
60
80
A
B
C
D
E
F
G
H
I
J
K
FDR (Age associated divergence) =0.0832
p(Scalp)=0.067
p(Dura)=0.717
Scalp
Dura

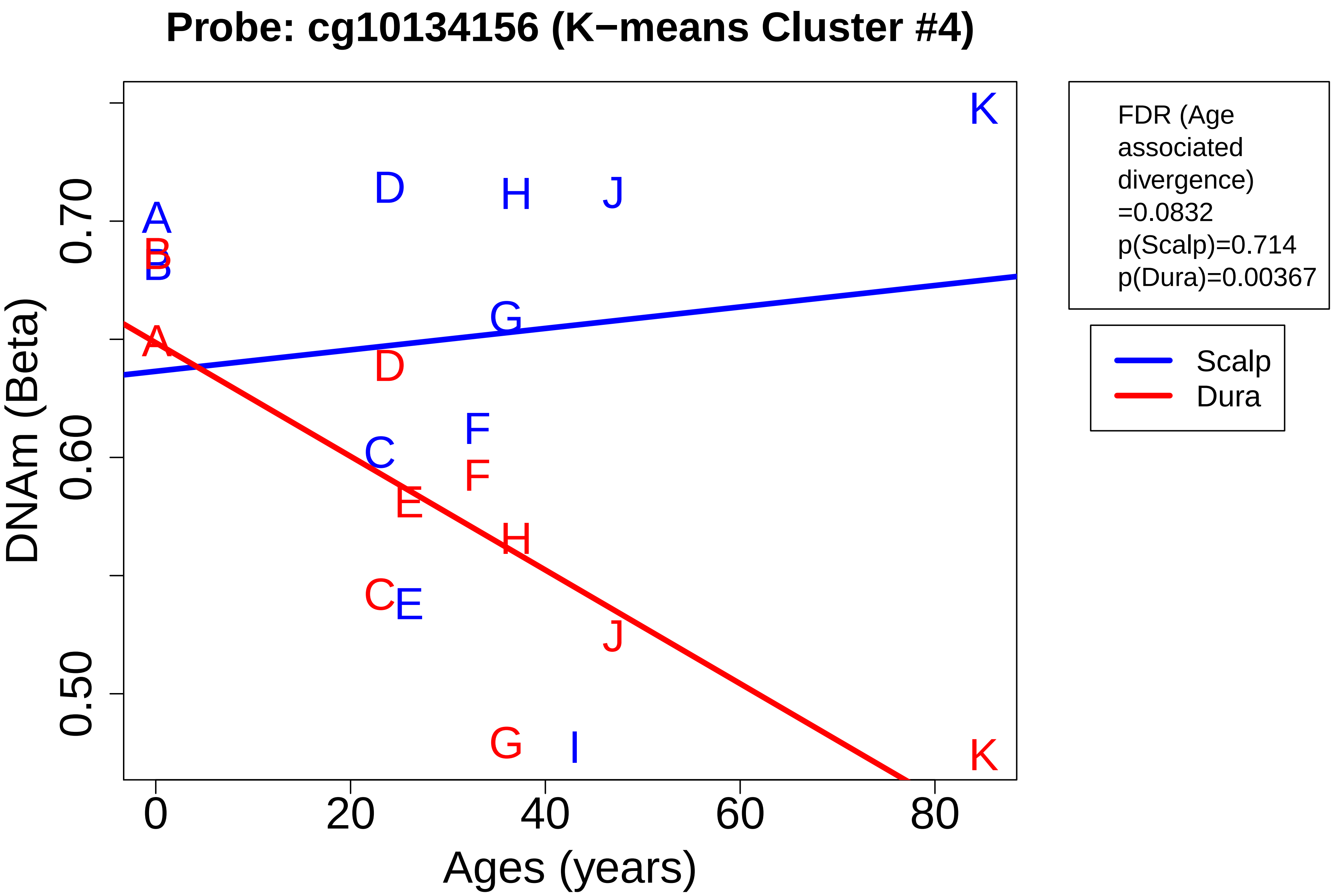

Probe: cg10134156 (K−means Cluster #4)
DNAm (Beta)
0.50
0.60
0.70
Ages (years)
0
20
40
60
80
A
B
C
D
E
F
G
H
I
J
K
FDR (Age associated divergence) =0.0832
p(Scalp)=0.714
p(Dura)=0.00367
Scalp
Dura

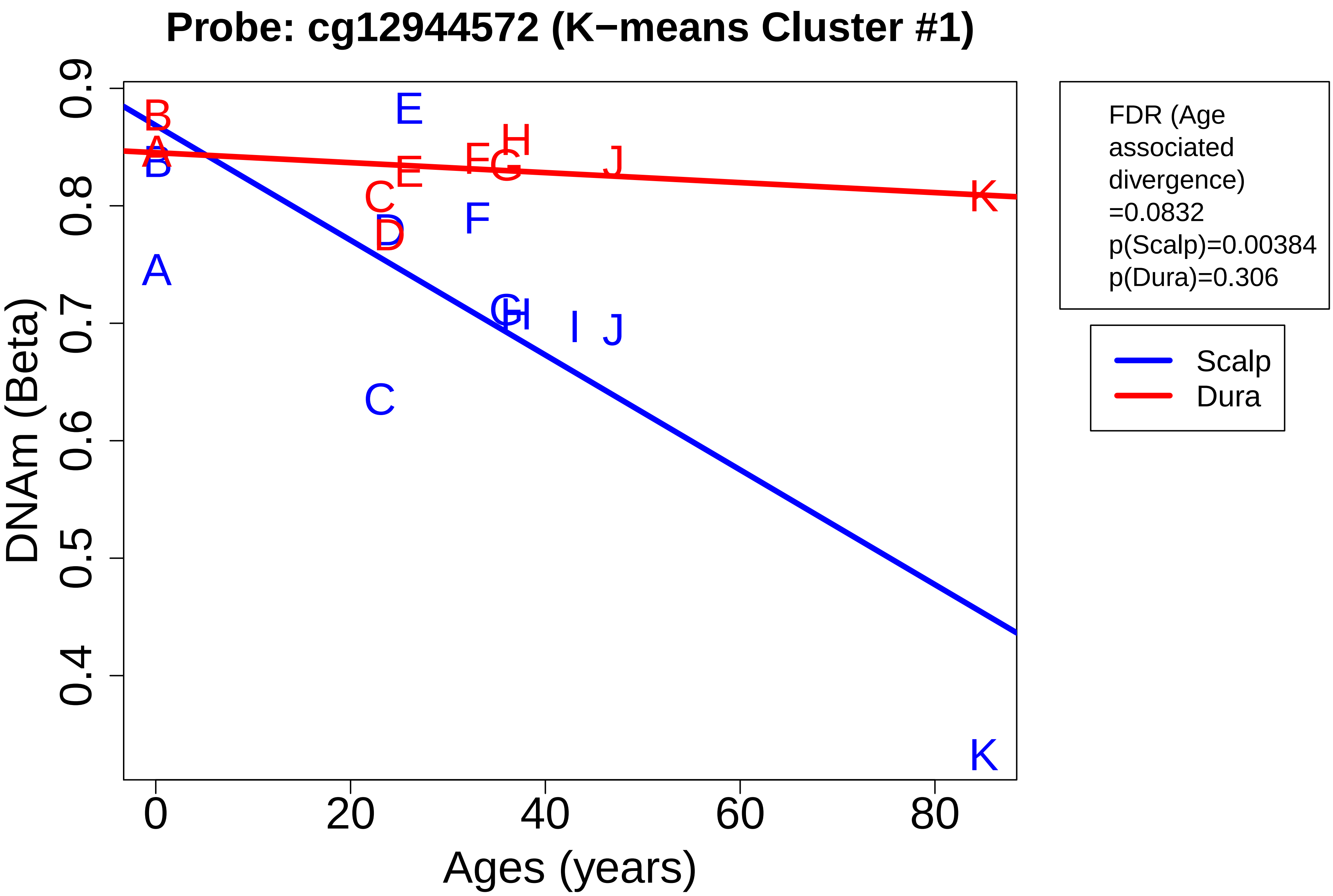
Probe: cg12944572 (K−means Cluster #1)
DNAm (Beta)
0.4
0.5
0.6
0.7
0.8
0.9
0
20
40
60
80
Ages (years)
FDR (Age associated divergence) =0.0832
p(Scalp)=0.00384
p(Dura)=0.306
Scalp
Dura

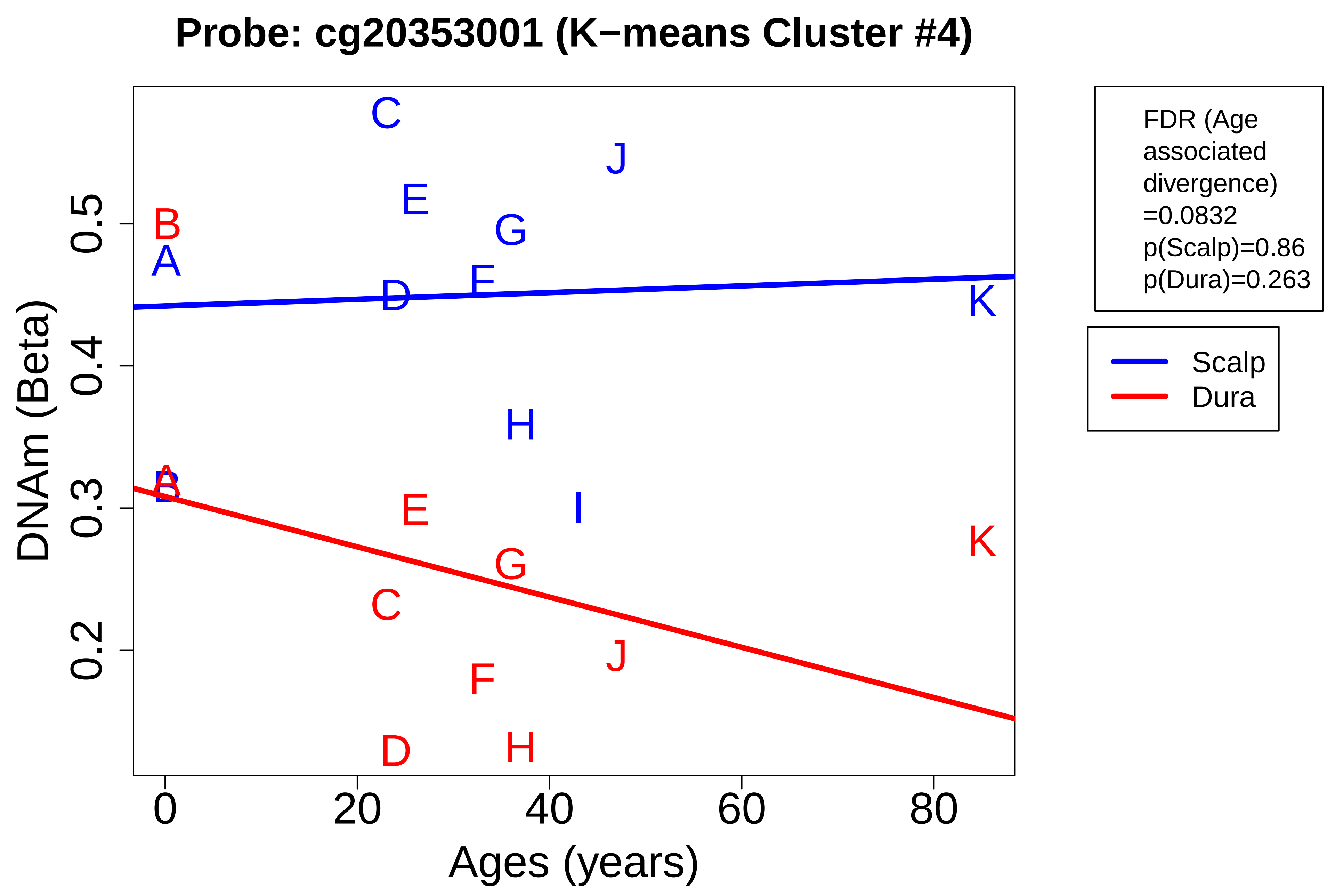

Probe: cg20353001 (K−means Cluster #4)
DNAm (Beta)
0.2
0.3
0.4
0.5
0
20
40
60
80
Ages (years)
A
B
C
D
E
F
G
H
I
J
K
FDR (Age associated divergence) =0.0832
p(Scalp)=0.86
p(Dura)=0.263
Scalp
Dura

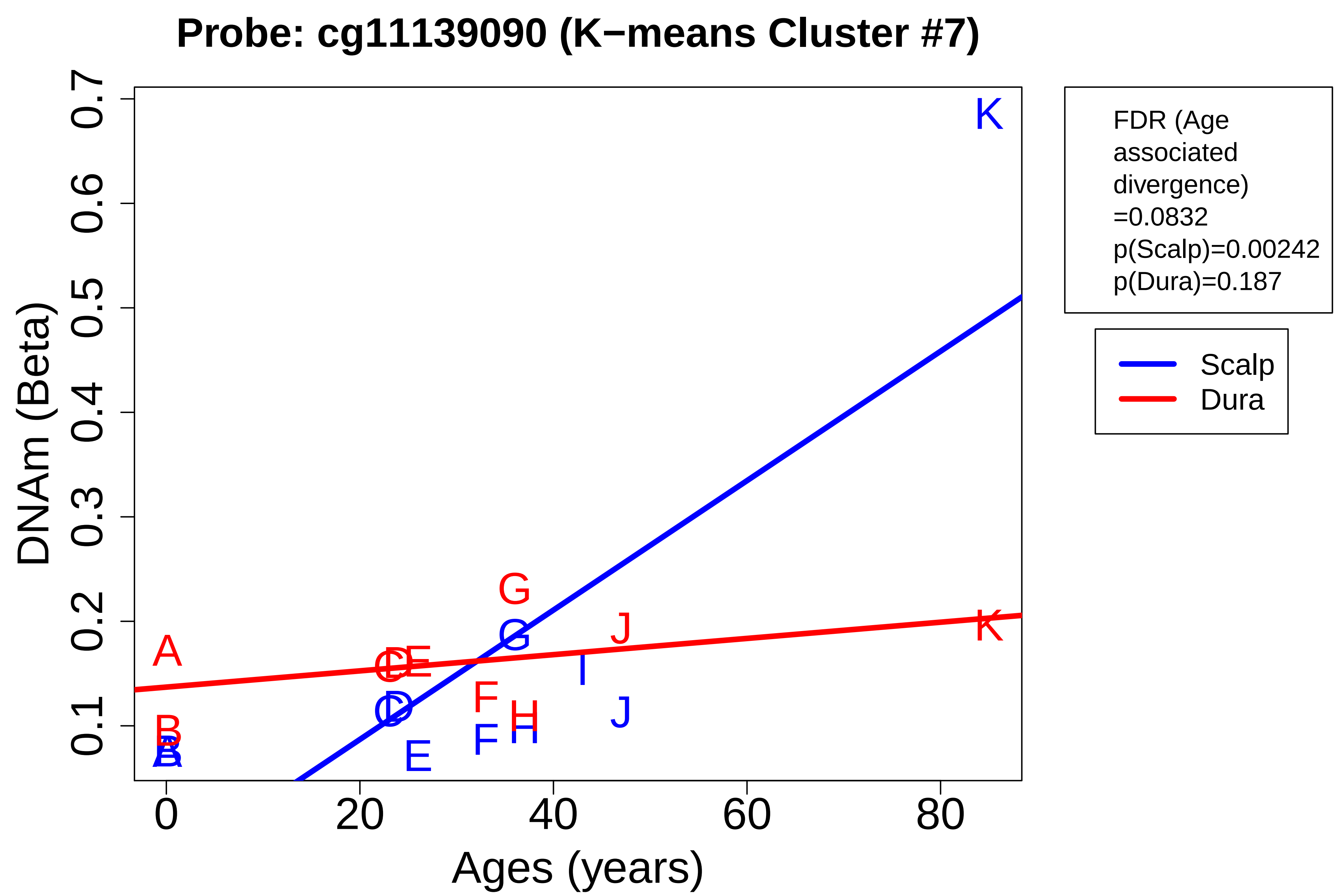

Probe: cg11139090 (K−means Cluster #7)
DNAm (Beta)
0.1
0.2
0.3
0.4
0.5
0.6
0.7
0
20
40
60
80
Ages (years)
A
B
C
D
E
F
G
H
I
J
K
FDR (Age associated divergence) =0.0832
p(Scalp)=0.00242
p(Dura)=0.187
Scalp
Dura

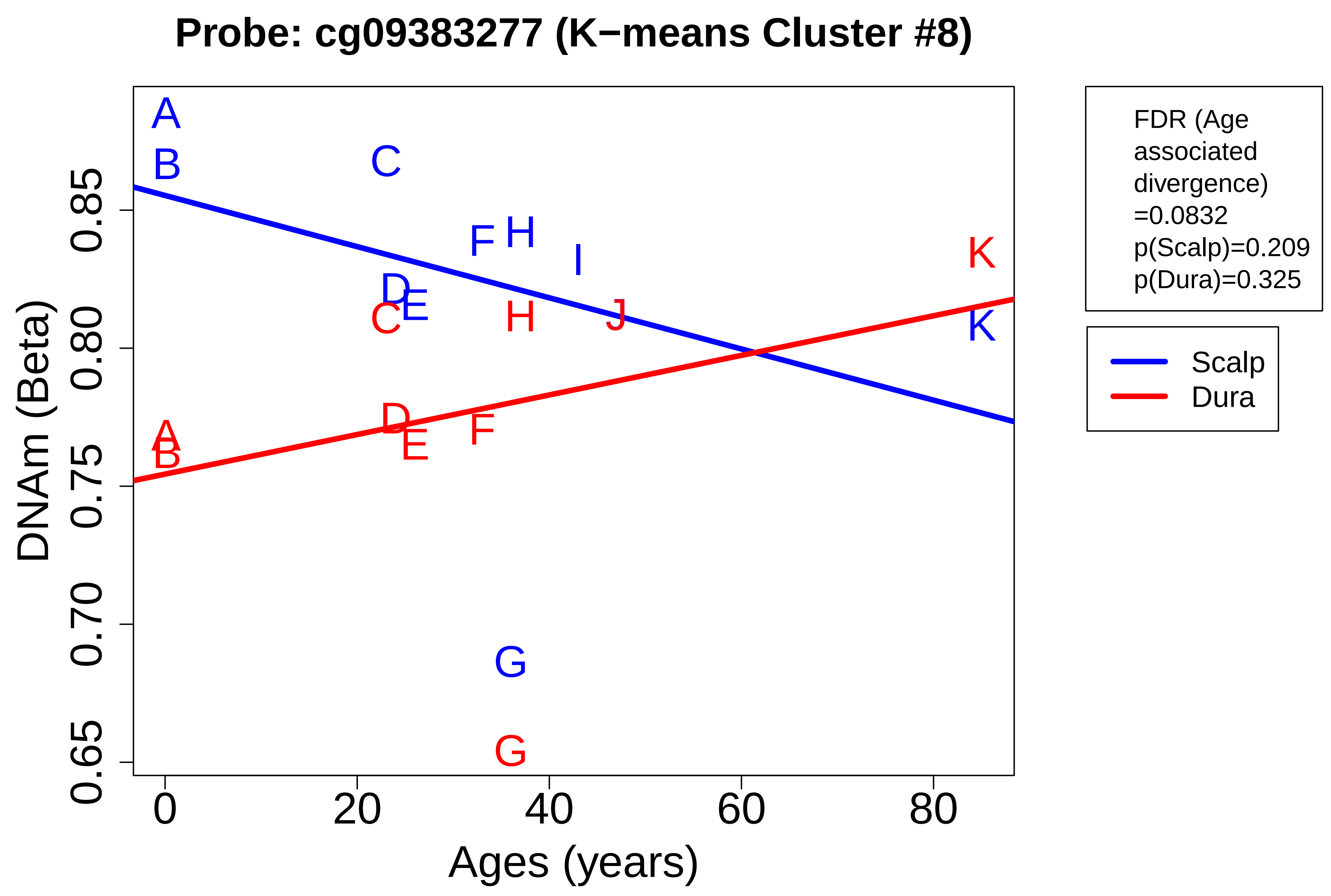

Probe: cg09383277 (K−means Cluster #8)
FDR (Age associated divergence) =0.0832
p(Scalp)=0.209
p(Dura)=0.325
Scalp
Dura
DNAm (Beta)
Ages (years)
0.65
0.70
0.75
0.80
0.85
0
20
40
60
80

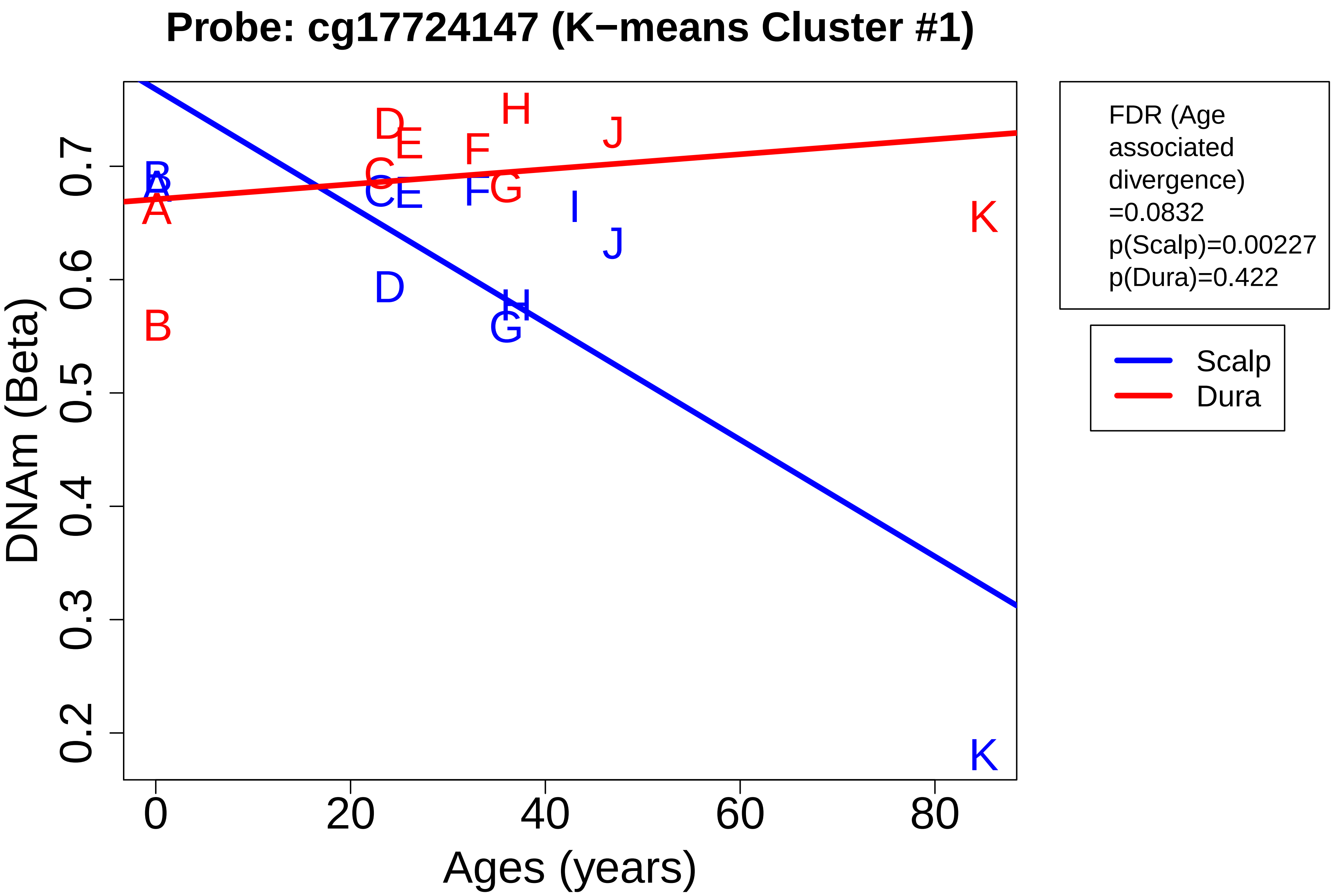

Probe: cg17724147 (K−means Cluster #1)
DNAm (Beta)
0.2
0.3
0.4
0.5
0.6
0.7
Ages (years)
0
20
40
60
80
FDR (Age associated divergence) =0.0832
p(Scalp)=0.00227
p(Dura)=0.422
Scalp
Dura

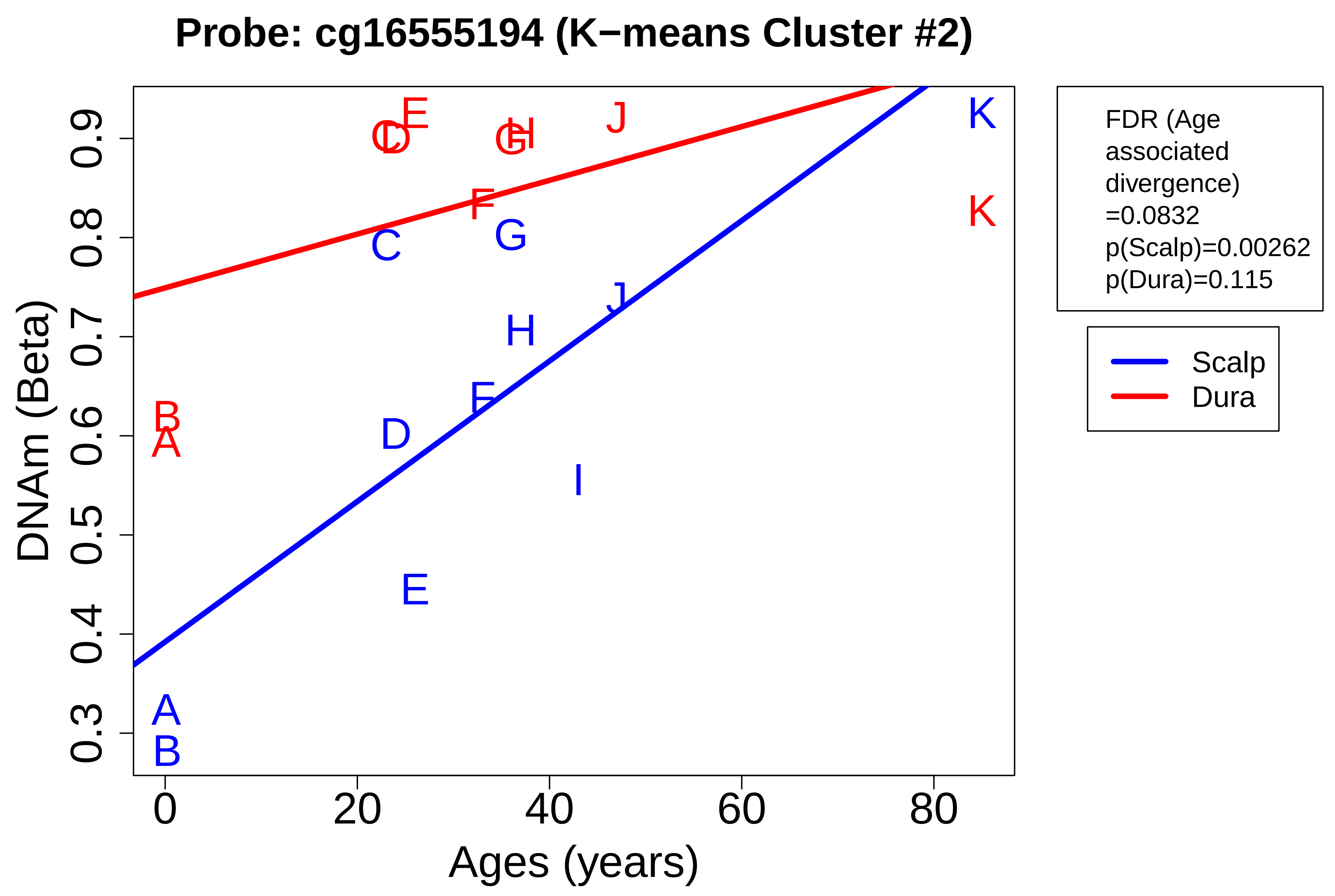
Probe: cg16555194 (K−means Cluster #2)
DNAm (Beta)
0.3
0.4
0.5
0.6
0.7
0.8
0.9
0
20
40
60
80
Ages (years)
A
B
C
D
E
F
G
H
I
J
K
FDR (Age associated divergence) =0.0832
p(Scalp)=0.00262
p(Dura)=0.115
Scalp
Dura

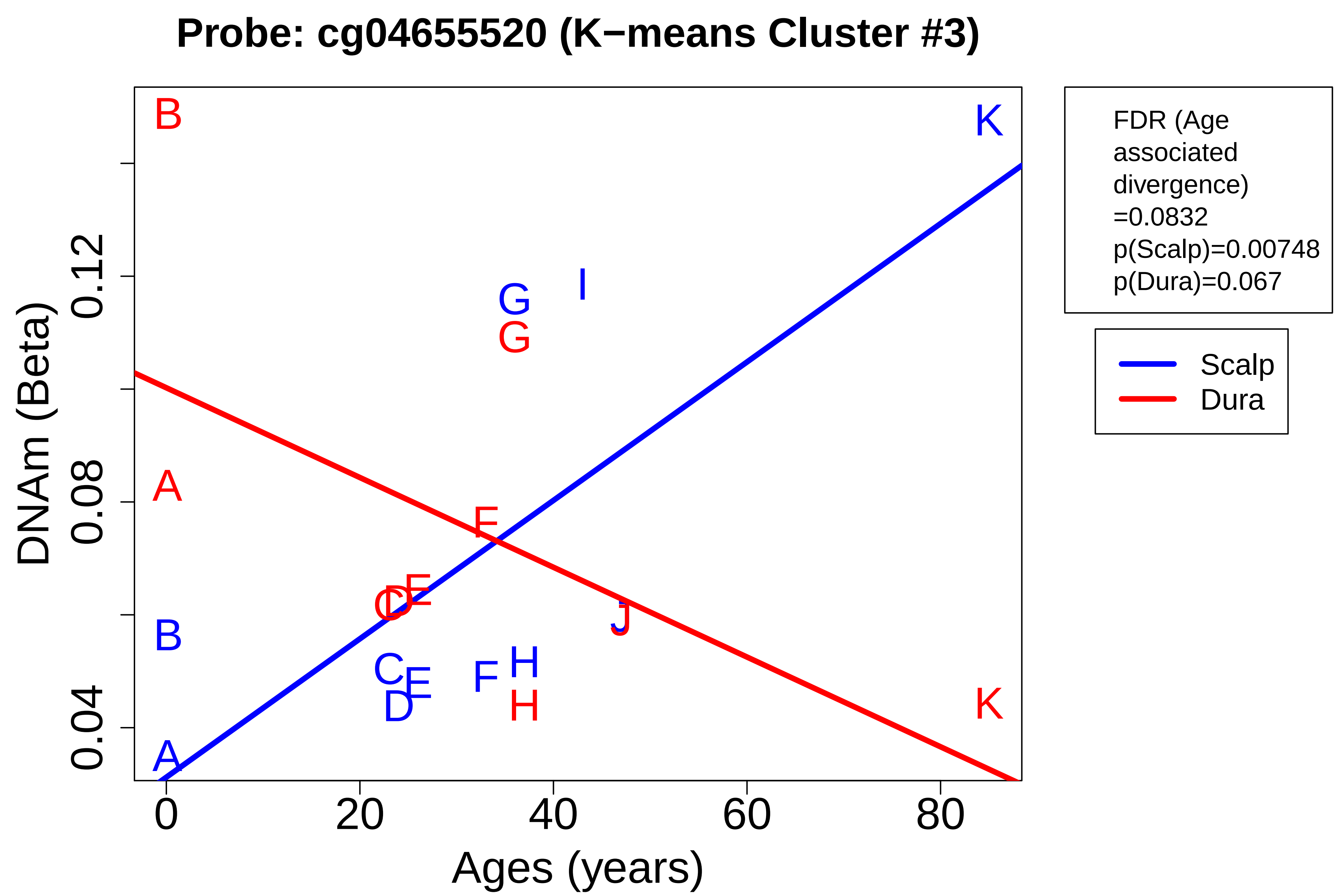

Probe: cg04655520 (K−means Cluster #3)
DNAm (Beta)
0.04
0.08
0.12
0
20
40
60
80
Ages (years)
FDR (Age associated divergence) =0.0832
p(Scalp)=0.00748
p(Dura)=0.067
Scalp
Dura

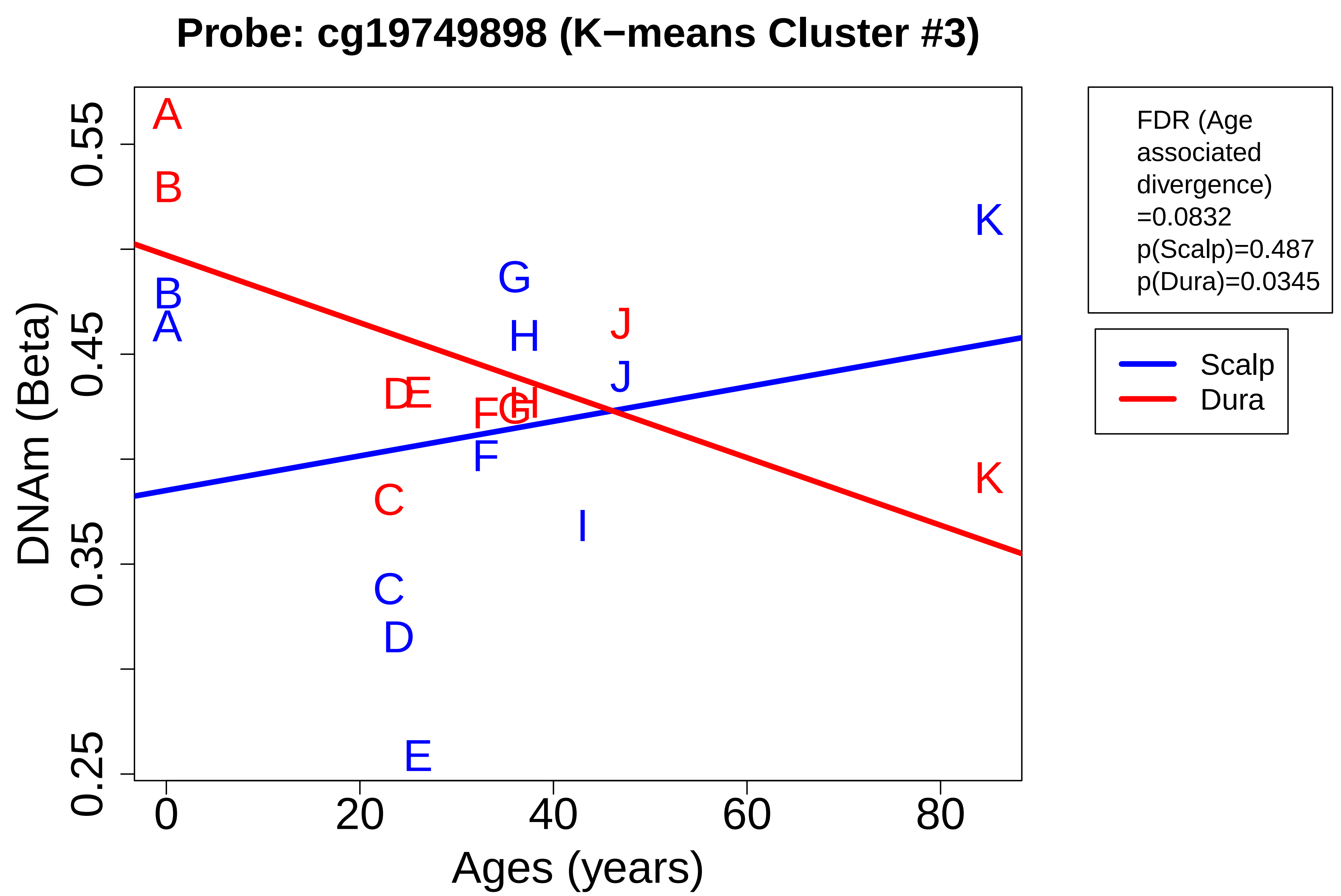

Probe: cg19749898 (K−means Cluster #3)
DNAm (Beta)
0.25
0.35
0.45
0.55
0
20
40
60
80
Ages (years)
A
B
C
D
E
F
G
H
I
J
K
FDR (Age associated divergence) =0.0832
p(Scalp)=0.487
p(Dura)=0.0345
Scalp
Dura

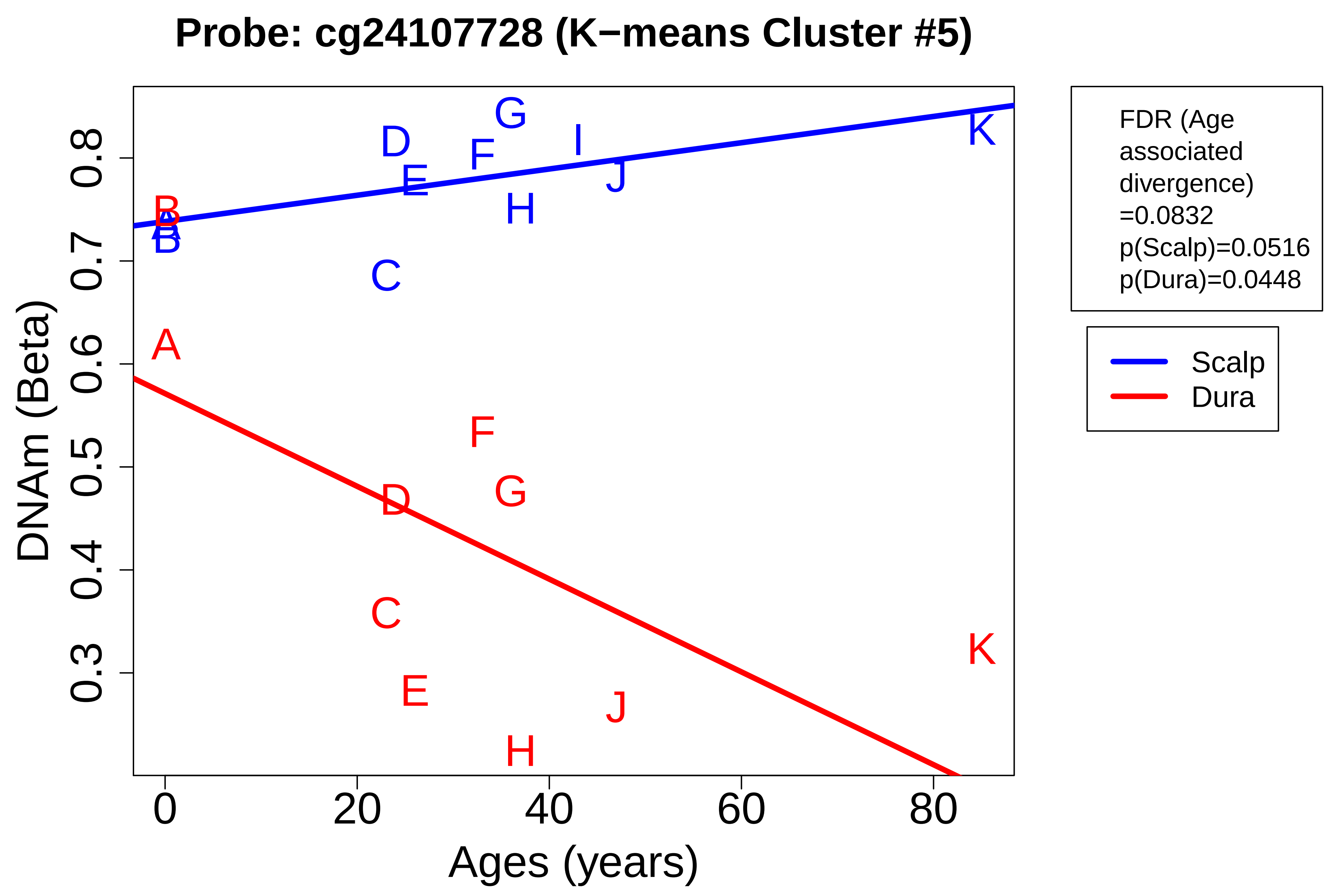

Probe: cg24107728 (K−means Cluster #5)
DNAm (Beta)
0.3
0.4
0.5
0.6
0.7
0.8
0
20
40
60
80
Ages (years)
FDR (Age associated divergence) =0.0832
p(Scalp)=0.0516
p(Dura)=0.0448
Scalp
Dura

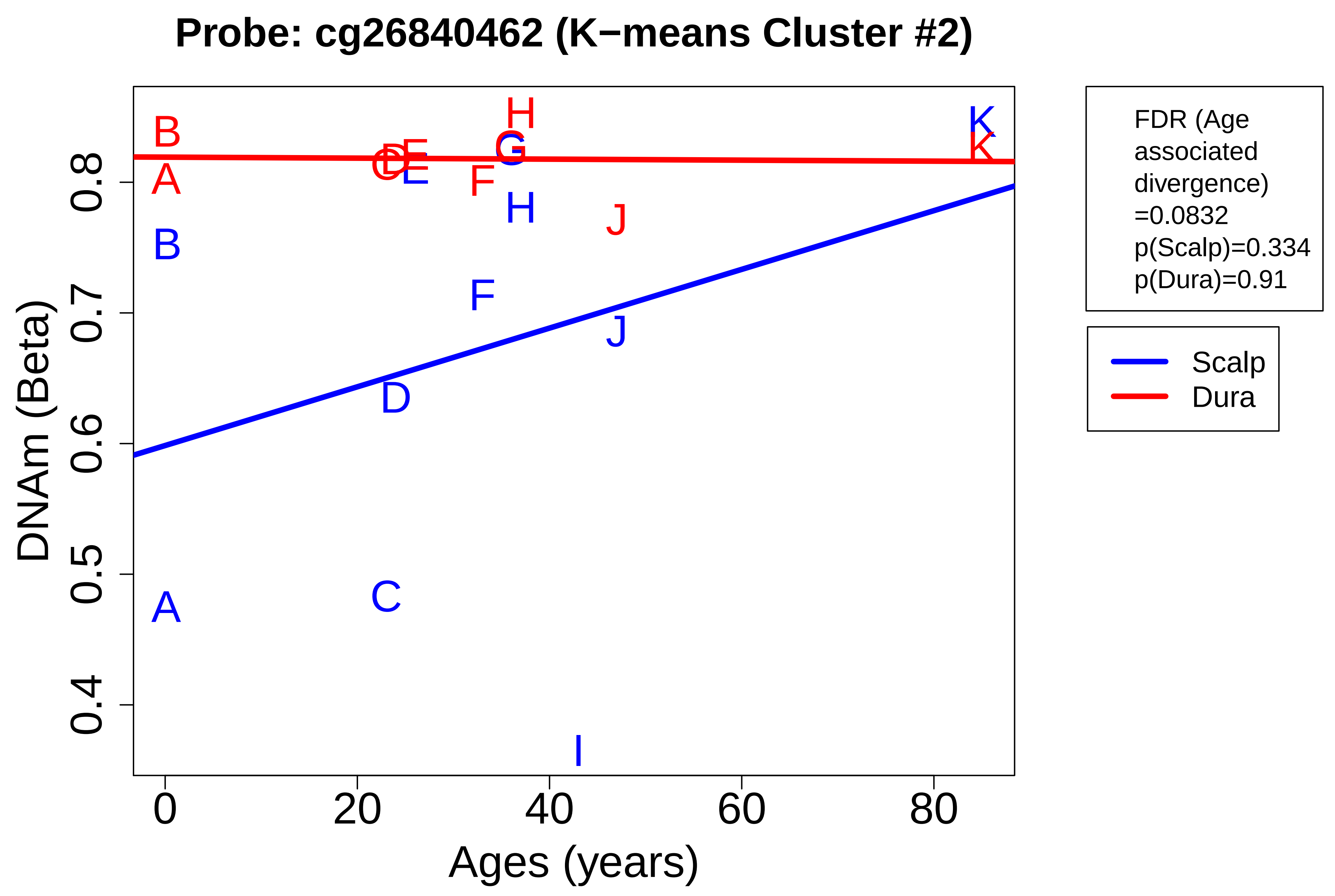

Probe: cg26840462 (K−means Cluster #2)
DNAm (Beta)
0.4
0.5
0.6
0.7
0.8
0
20
40
60
80
Ages (years)
FDR (Age associated divergence) =0.0832
p(Scalp)=0.334
p(Dura)=0.91
Scalp
Dura

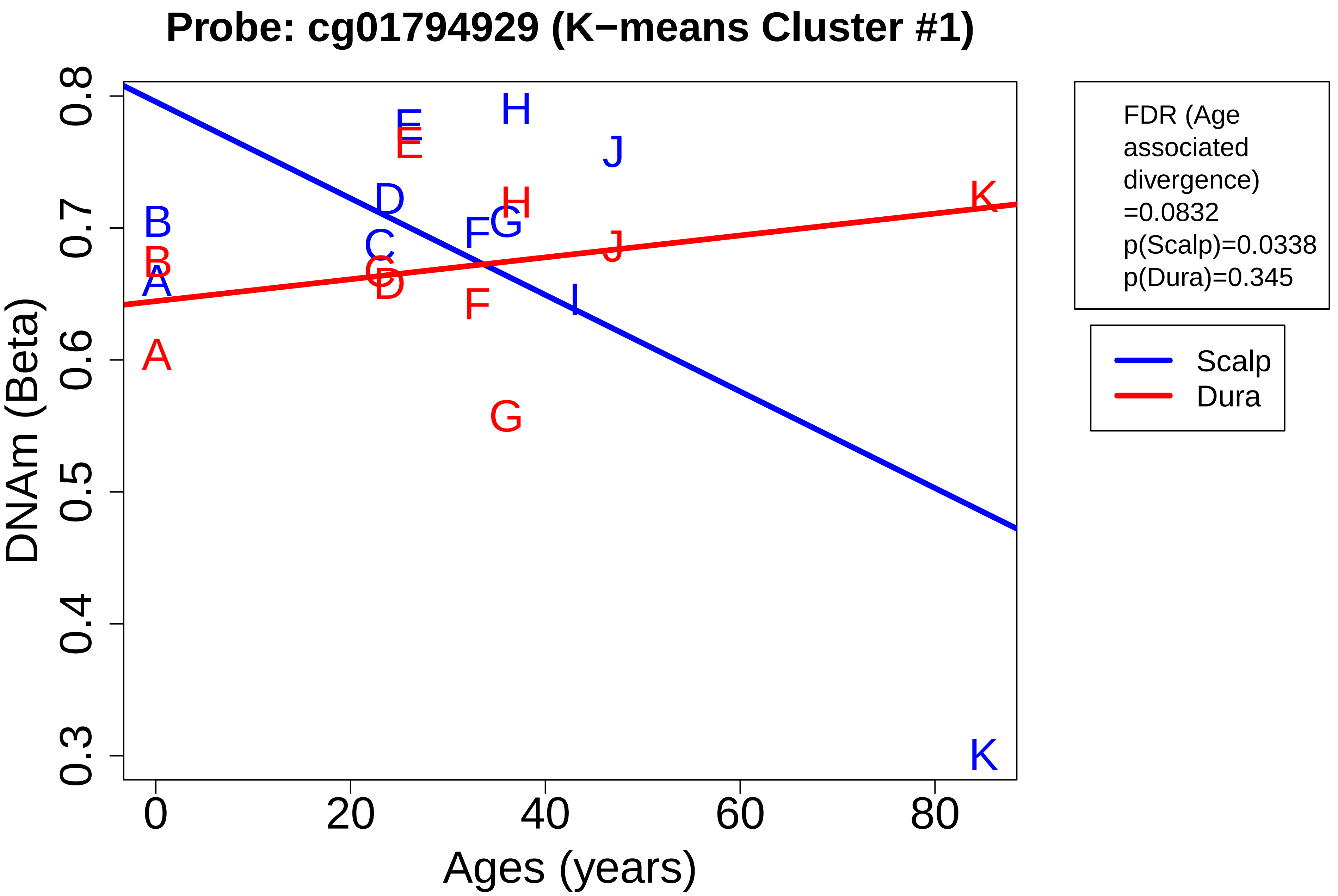
Probe: cg01794929 (K−means Cluster #1)
DNAm (Beta)
Ages (years)
0.3
0.4
0.5
0.6
0.7
0.8
0
20
40
60
80
A B C D E F G H I J K
FDR (Age associated divergence) =0.0832
p(Scalp)=0.0338
p(Dura)=0.345
Scalp
Dura

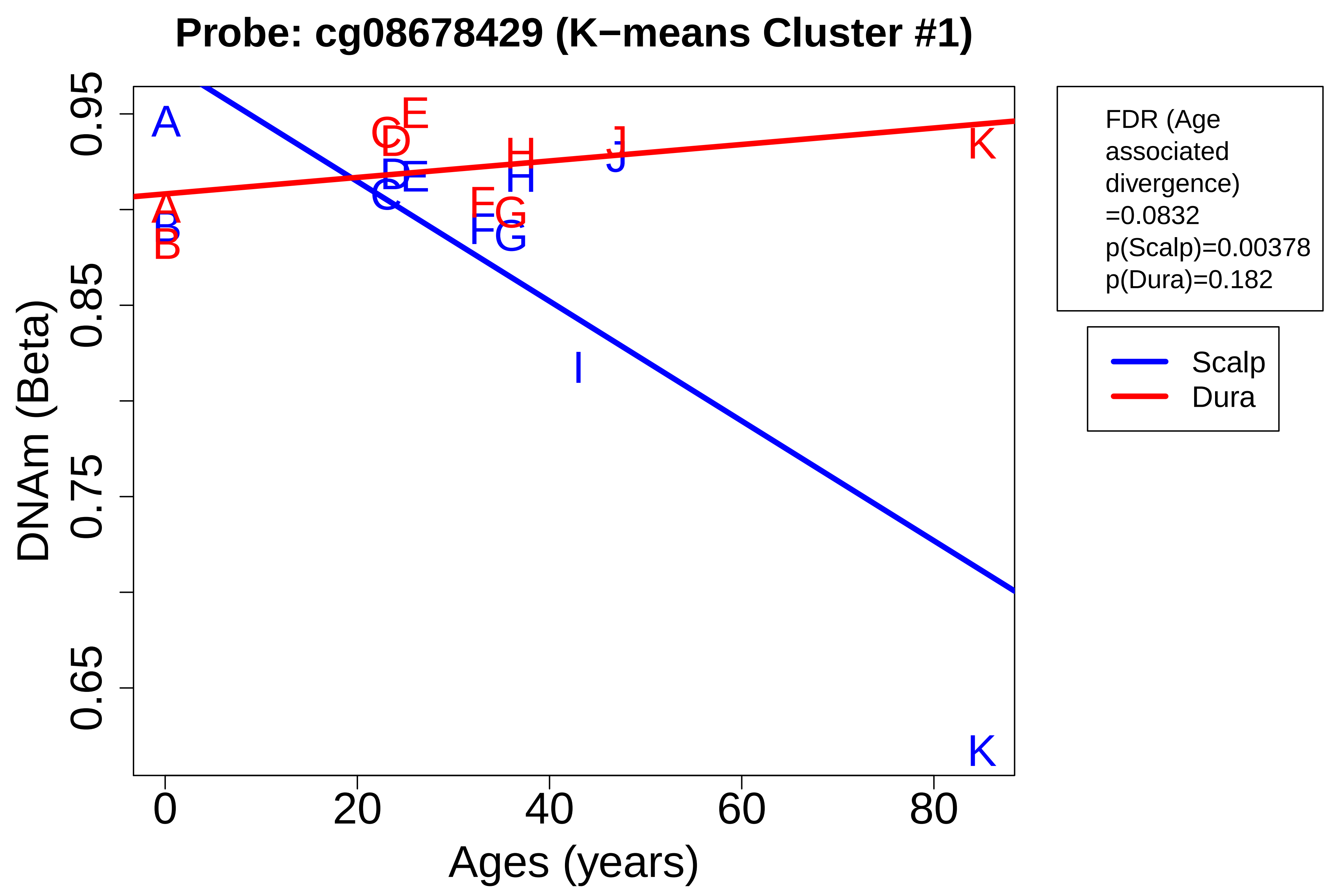
Probe: cg08678429 (K−means Cluster #1)
DNAm (Beta)
0.65
0.75
0.85
0.95
0
20
40
60
80
Ages (years)
A
B
C
D
E
F
G
H
I
J
K
FDR (Age associated divergence) =0.0832
p(Scalp)=0.00378
p(Dura)=0.182
Scalp
Dura

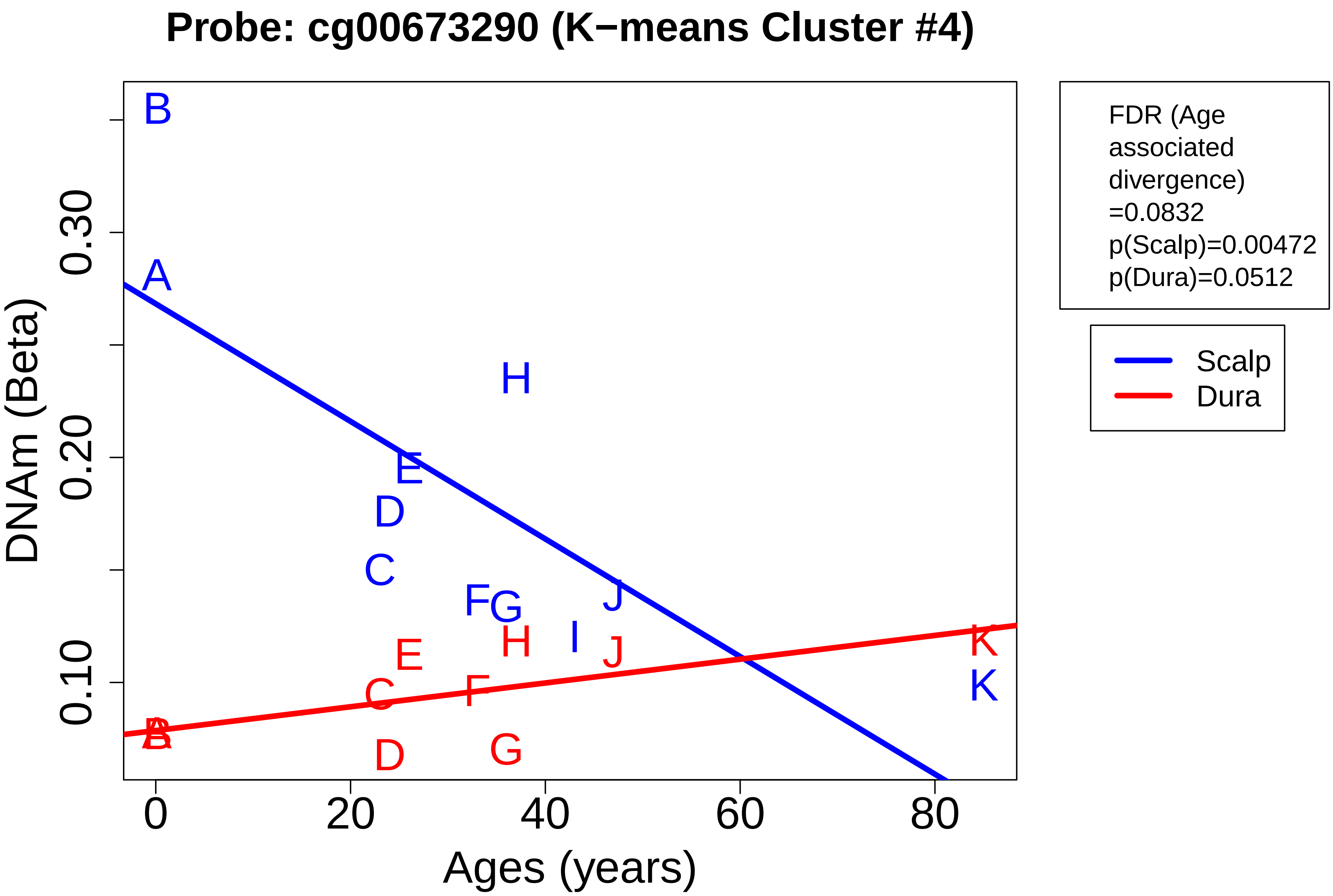
Probe: cg00673290 (K−means Cluster #4)
DNAm (Beta)
0.10
0.20
0.30
0
20
40
60
80
Ages (years)
A
B
C
D
E
F
G
H
I
J
K
FDR (Age associated divergence) =0.0832
p(Scalp)=0.00472
p(Dura)=0.0512
Scalp
Dura

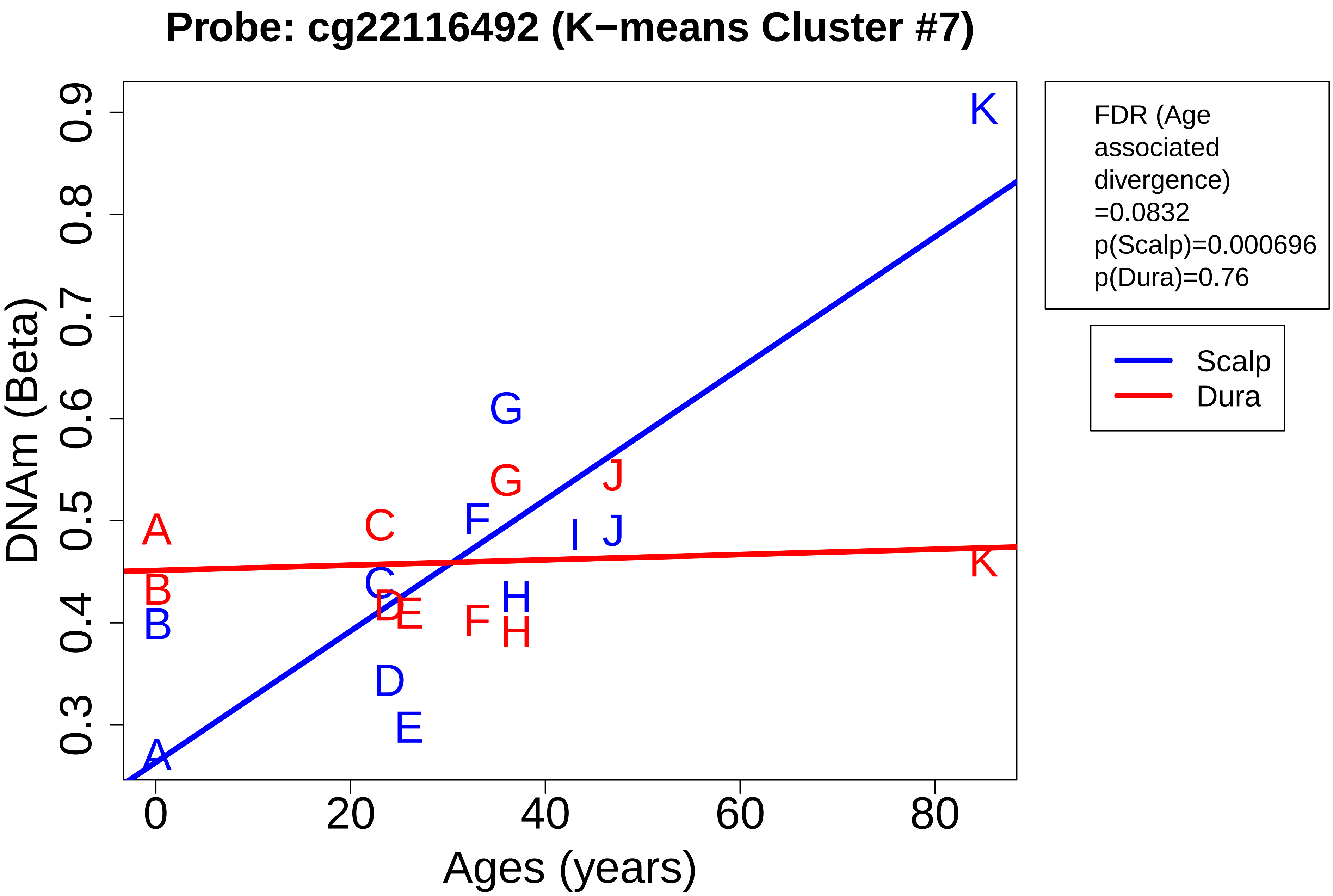
Probe: cg22116492 (K−means Cluster #7)
DNAm (Beta)
0.3
0.4
0.5
0.6
0.7
0.8
0.9
Ages (years)
0
20
40
60
80
FDR (Age associated divergence) =0.0832
p(Scalp)=0.000696
p(Dura)=0.76
Scalp
Dura
A
B
C
D
E
F
G
H
I
J
K

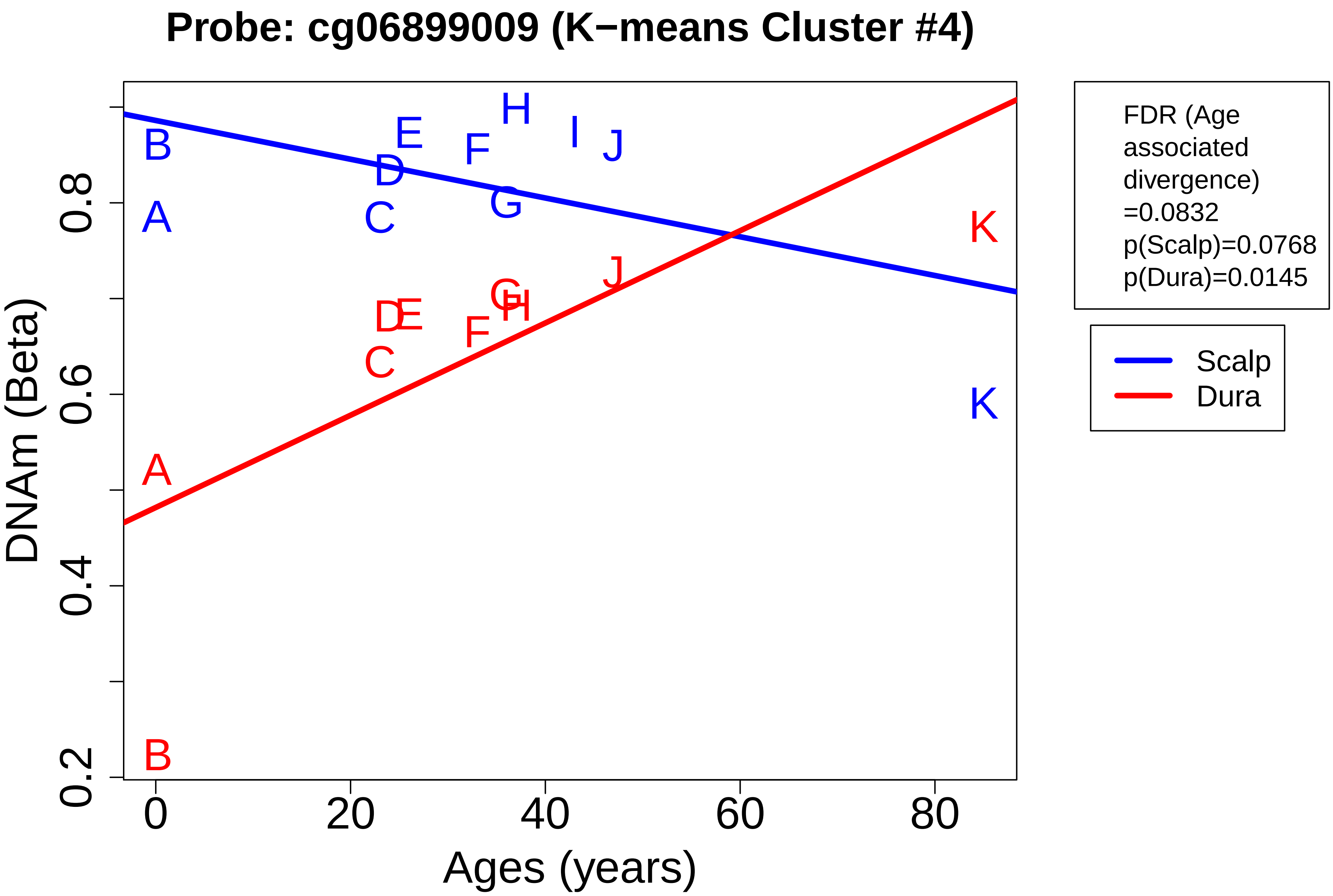

Probe: cg06899009 (K−means Cluster #4)
DNAm (Beta)
0.2
0.4
0.6
0.8
Ages (years)
0
20
40
60
80
A
B
C
D
E
F
G
H
I
J
K
FDR (Age associated divergence) =0.0832
p(Scalp)=0.0768
p(Dura)=0.0145
Scalp
Dura

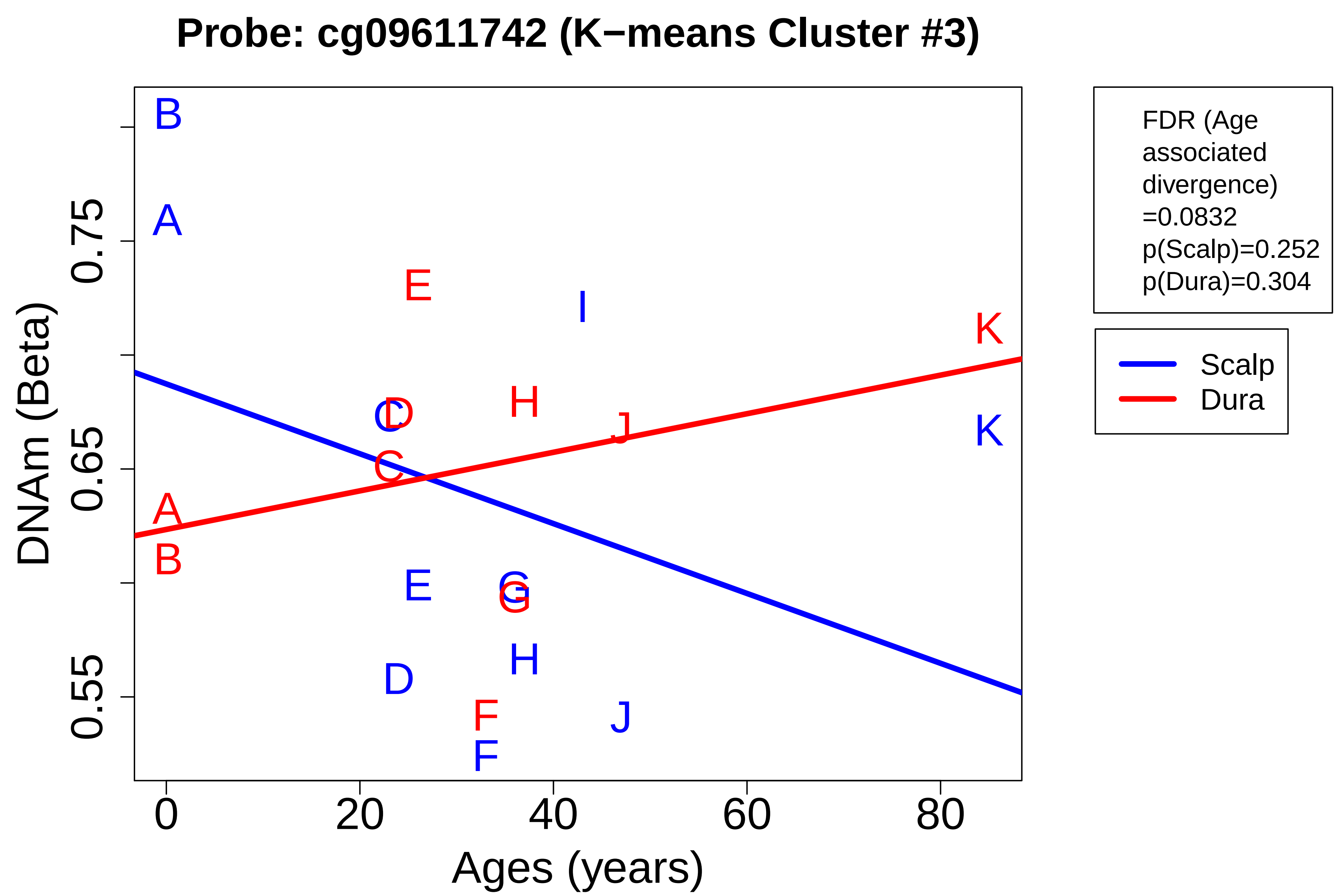

Probe: cg09611742 (K−means Cluster #3)
DNAm (Beta)
0.55
0.65
0.75
Ages (years)
0
20
40
60
80
A
B
C
D
E
F
G
H
I
J
K
FDR (Age associated divergence) =0.0832
p(Scalp)=0.252
p(Dura)=0.304
Scalp
Dura

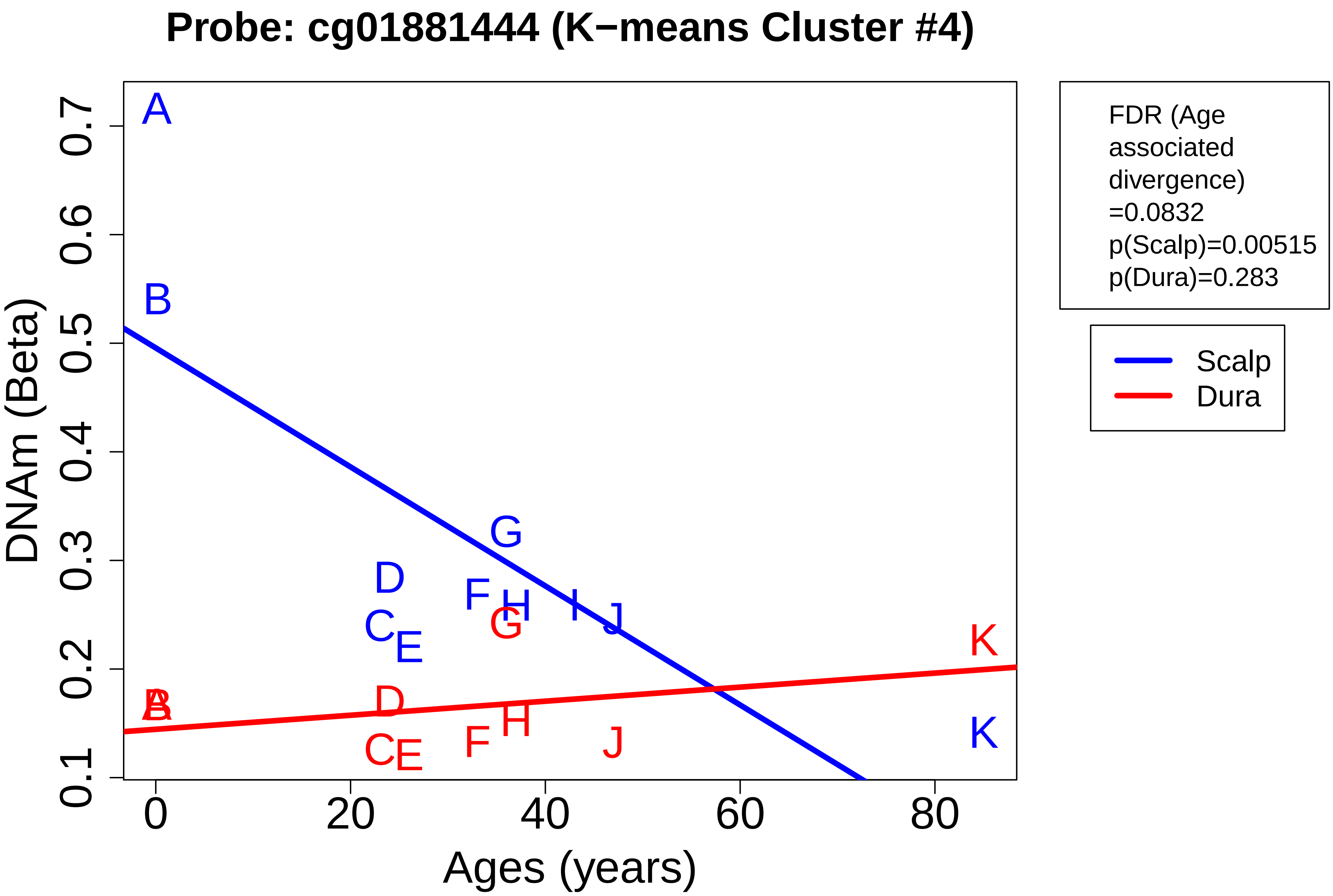
Probe: cg01881444 (K−means Cluster #4)
FDR (Age associated divergence) =0.0832
p(Scalp)=0.00515
p(Dura)=0.283
Scalp
Dura
DNAm (Beta)
Ages (years)
0.1
0.2
0.3
0.4
0.5
0.6
0.7
0
20
40
60
80
A
B
C
D
E
F
G
H
I
J
K

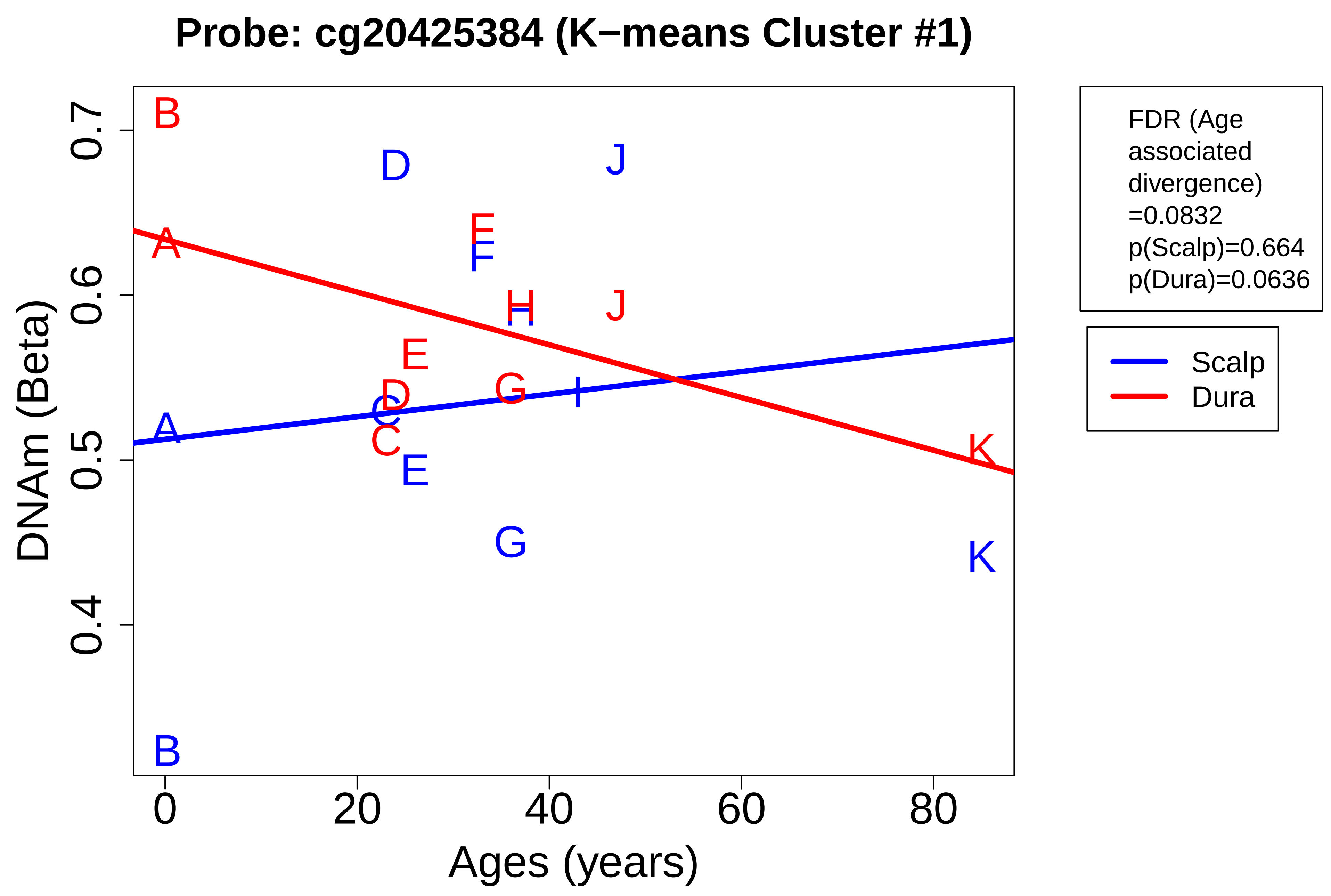

Probe: cg20425384 (K−means Cluster #1)
DNAm (Beta)
0.4
0.5
0.6
0.7
0
20
40
60
80
Ages (years)
FDR (Age associated divergence) =0.0832
p(Scalp)=0.664
p(Dura)=0.0636
Scalp
Dura

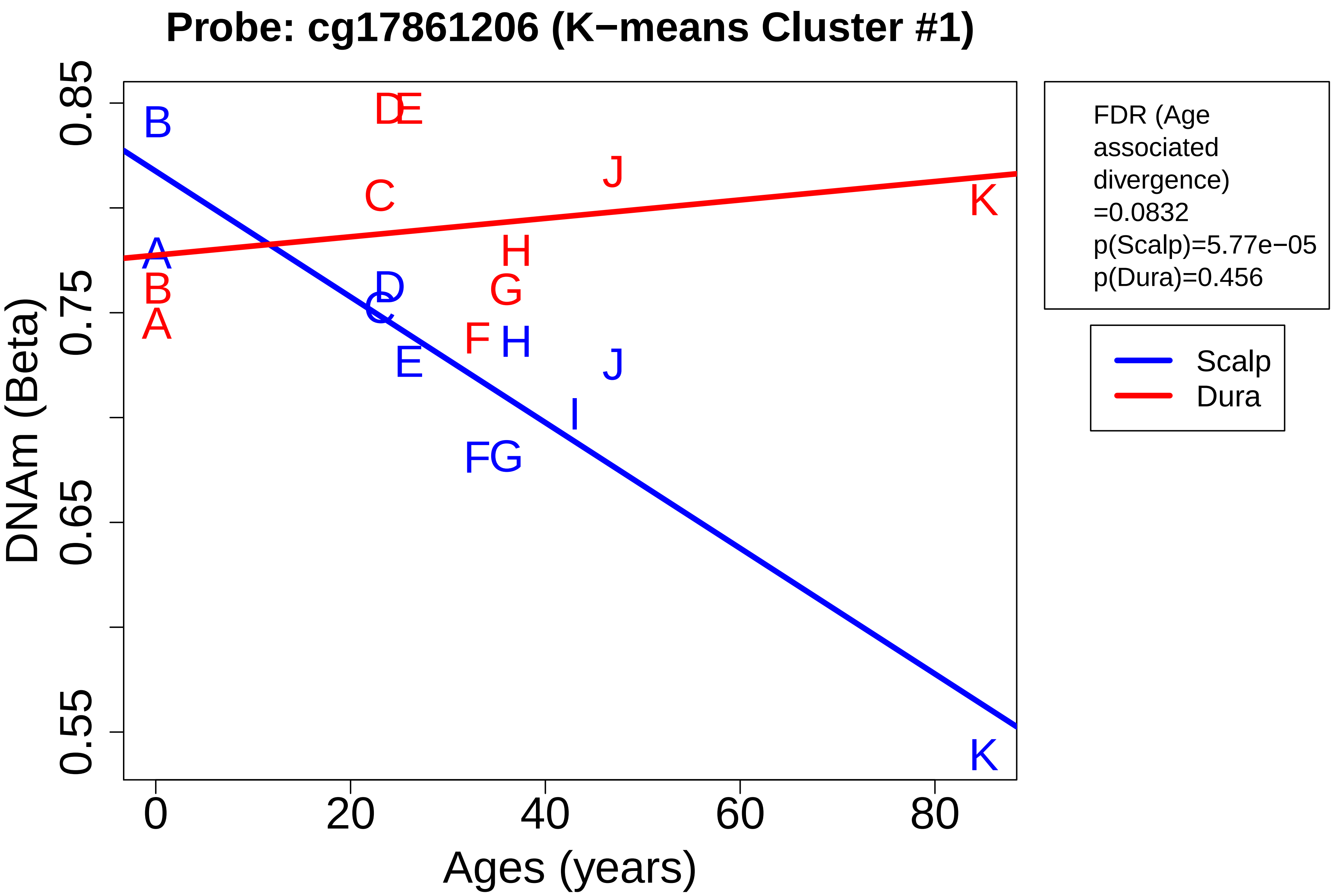

Probe: cg17861206 (K−means Cluster #1)
DNAm (Beta)
0.85
0.75
0.65
0.55
0
20
40
60
80
Ages (years)
FDR (Age
associated
divergence)
=0.0832
p(Scalp)=5.77e−05
p(Dura)=0.456
Scalp
Dura

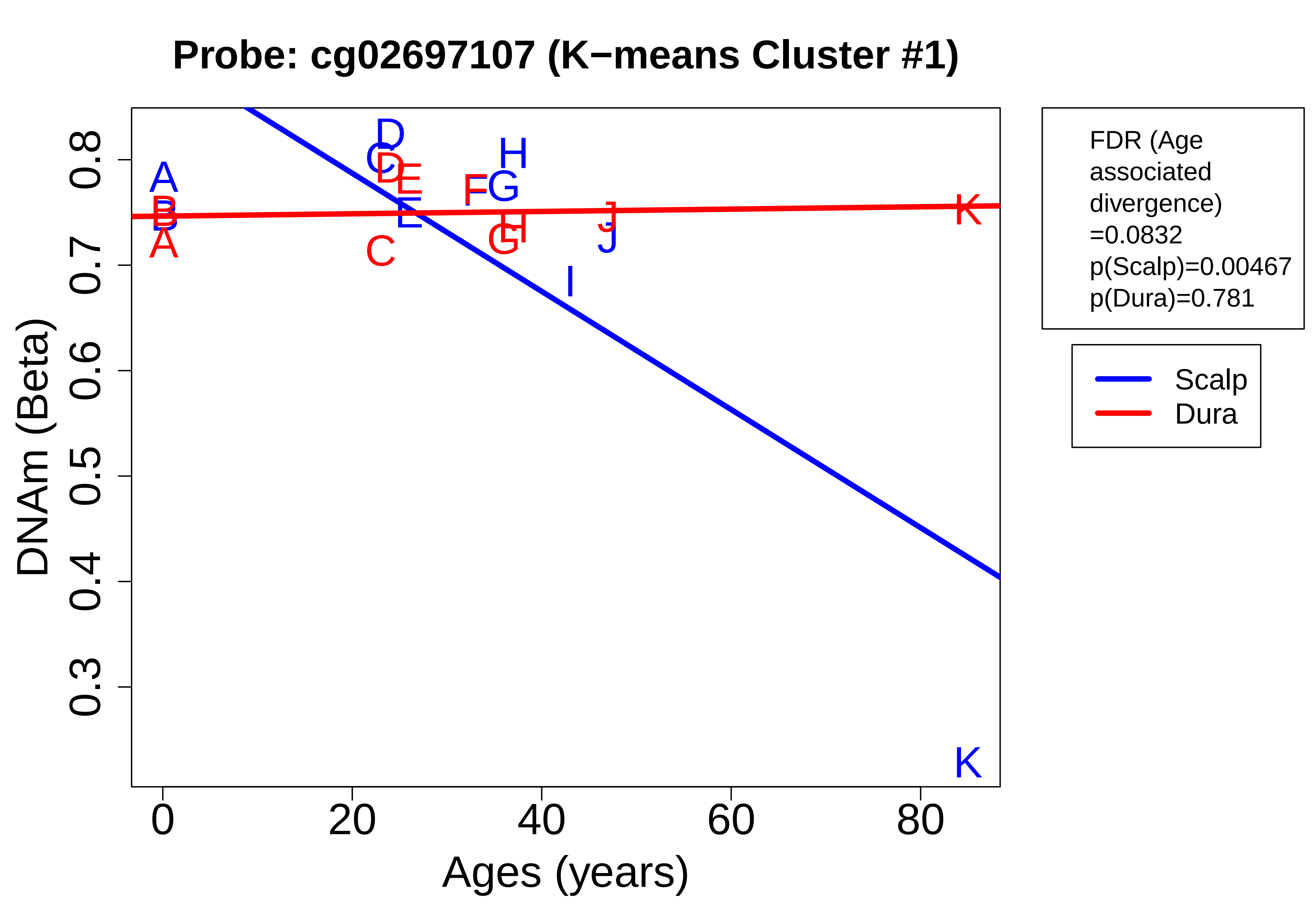
Probe: cg02697107 (K−means Cluster #1)
DNAm (Beta)
0.3
0.4
0.5
0.6
0.7
0.8
0
20
40
60
80
Ages (years)
A
B
C
D
E
F
G
H
I
J
K
FDR (Age associated divergence) =0.0832
p(Scalp)=0.00467
p(Dura)=0.781
Scalp
Dura

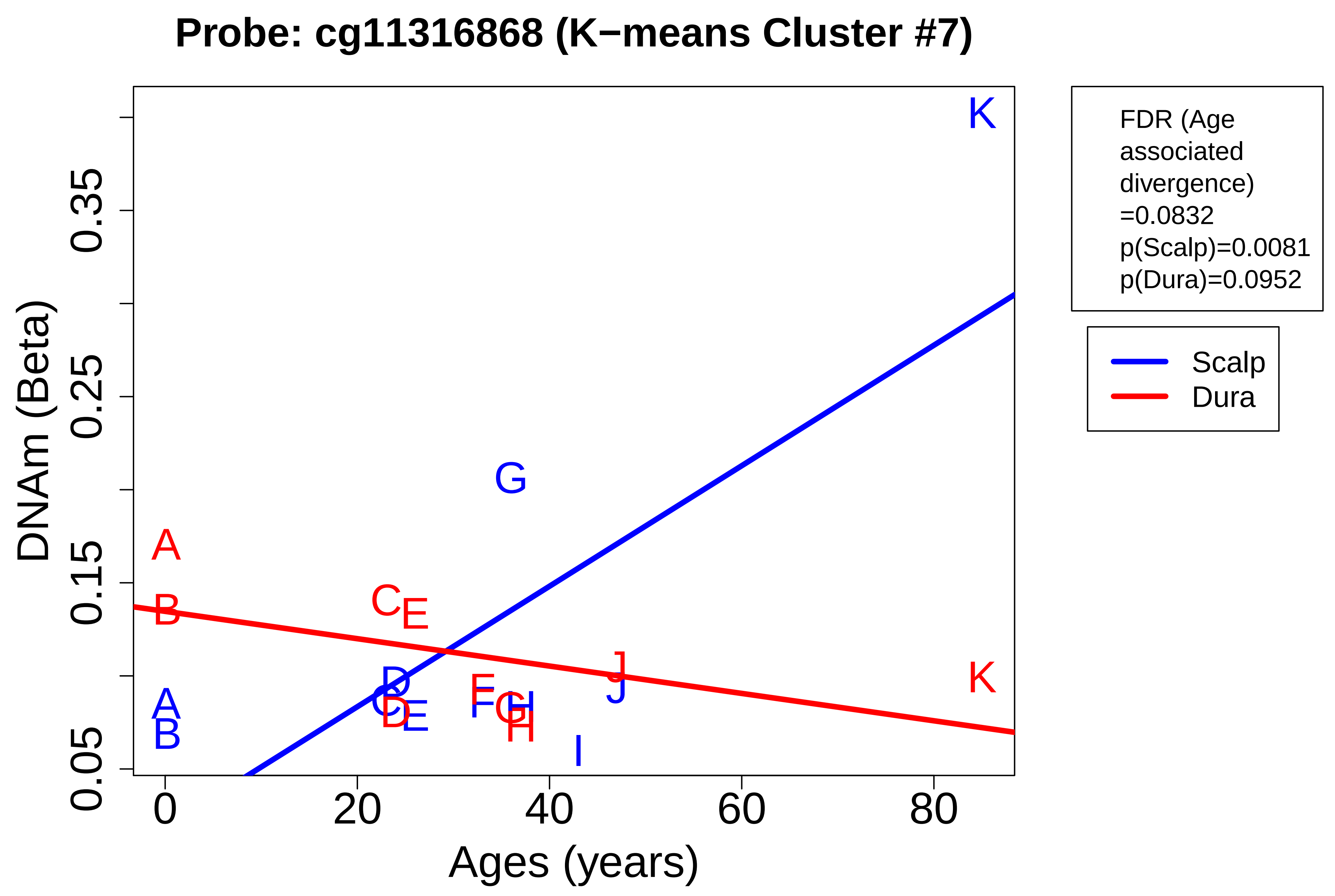

Probe: cg11316868 (K−means Cluster #7)
DNAm (Beta)
0.05
0.15
0.25
0.35
0
20
40
60
80
Ages (years)
A
B
C
D
E
F
G
H
I
J
K
FDR (Age associated divergence) =0.0832
p(Scalp)=0.0081
p(Dura)=0.0952
Scalp
Dura

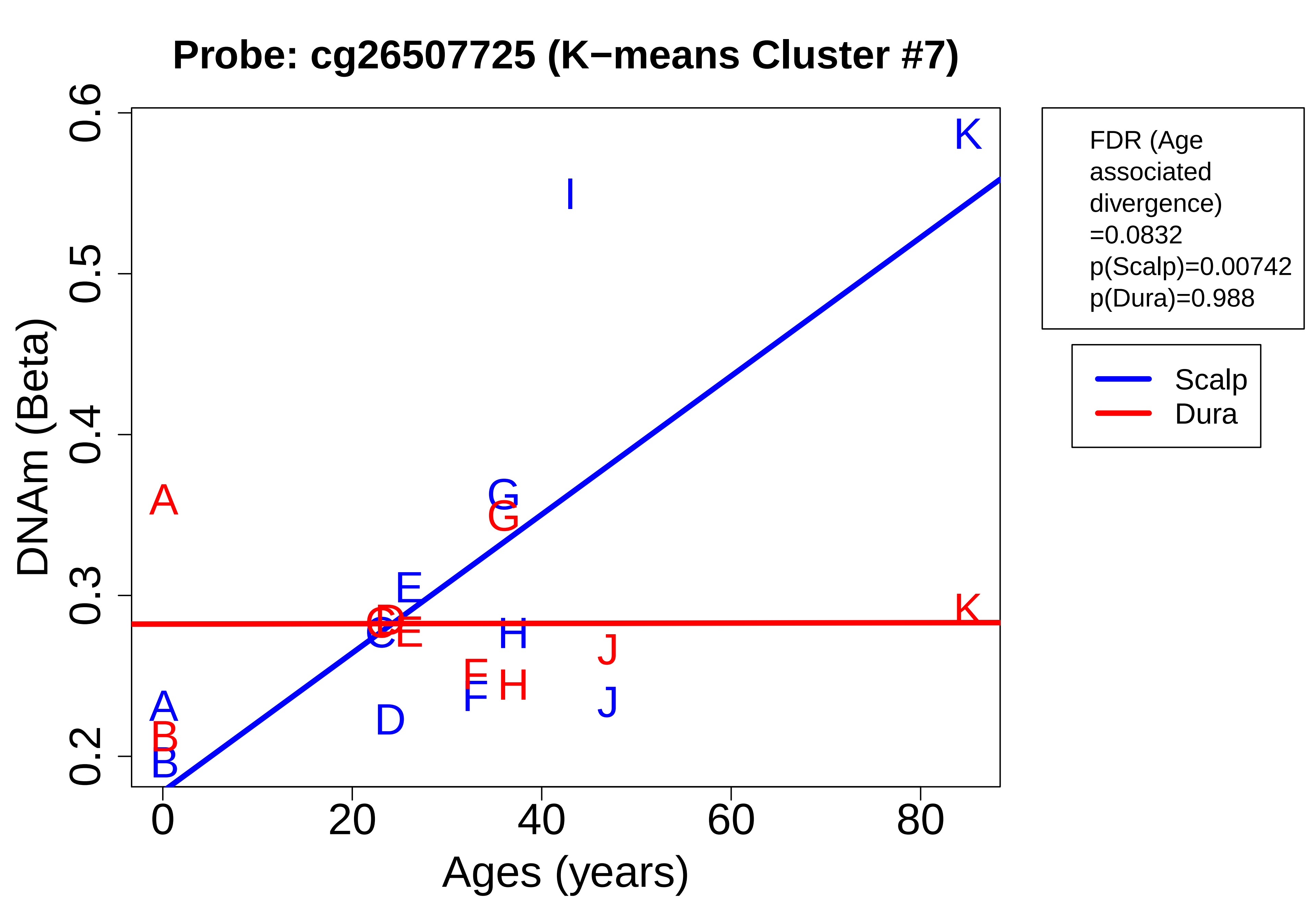

Probe: cg26507725 (K−means Cluster #7)
DNAm (Beta)
0.2
0.3
0.4
0.5
0.6
0
20
40
60
80
Ages (years)
FDR (Age associated divergence) =0.0832
p(Scalp)=0.00742
p(Dura)=0.988
Scalp
Dura
A
B
C
D
E
F
G
H
I
J
K

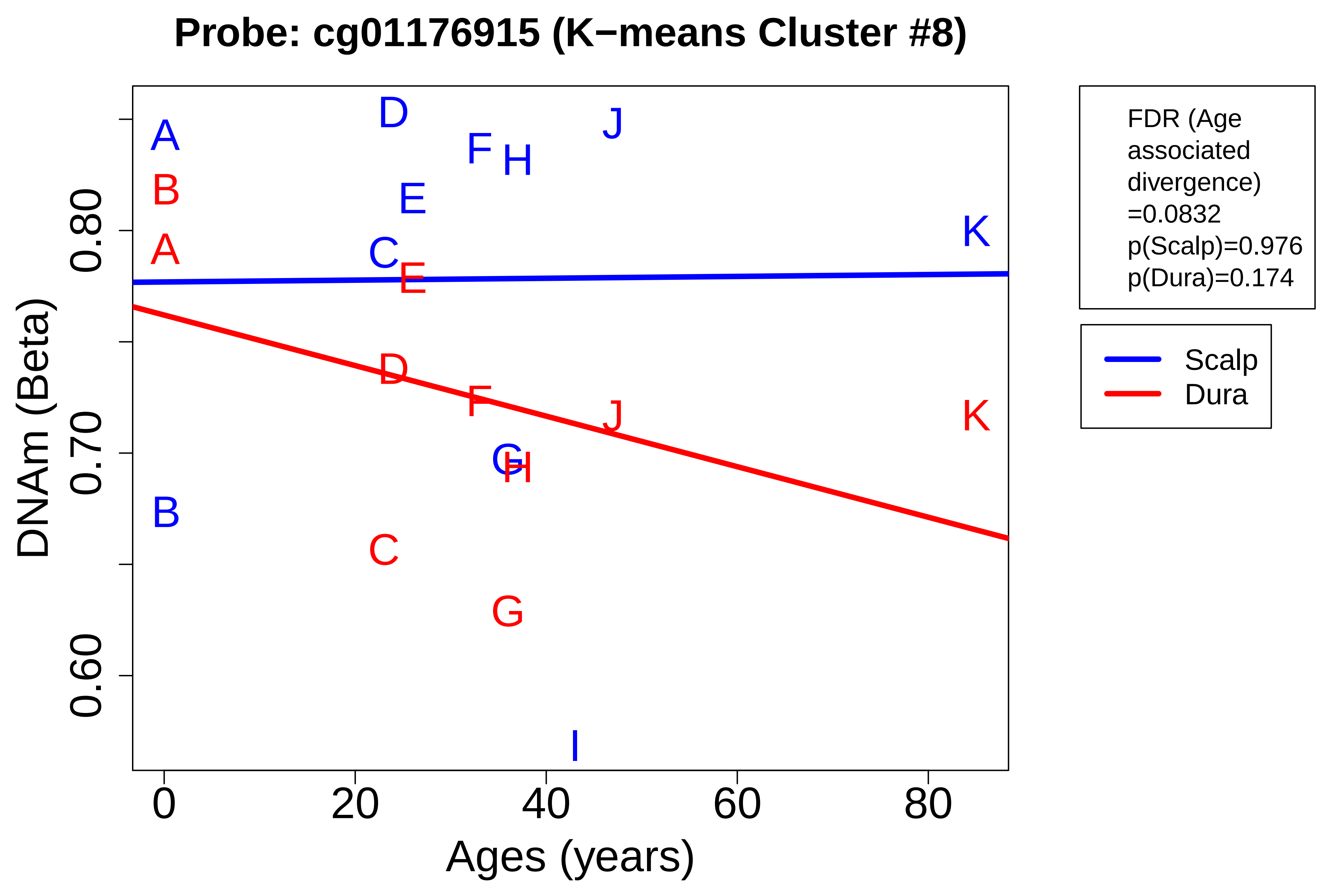

Probe: cg01176915 (K−means Cluster #8)
DNAm (Beta)
0.60
0.70
0.80
0
20
40
60
80
Ages (years)
A
B
A
B
C
C
D
D
E
E
F
F
G
G
H
H
I
J
J
K
K
FDR (Age associated divergence) =0.0832
p(Scalp)=0.976
p(Dura)=0.174
Scalp
Dura

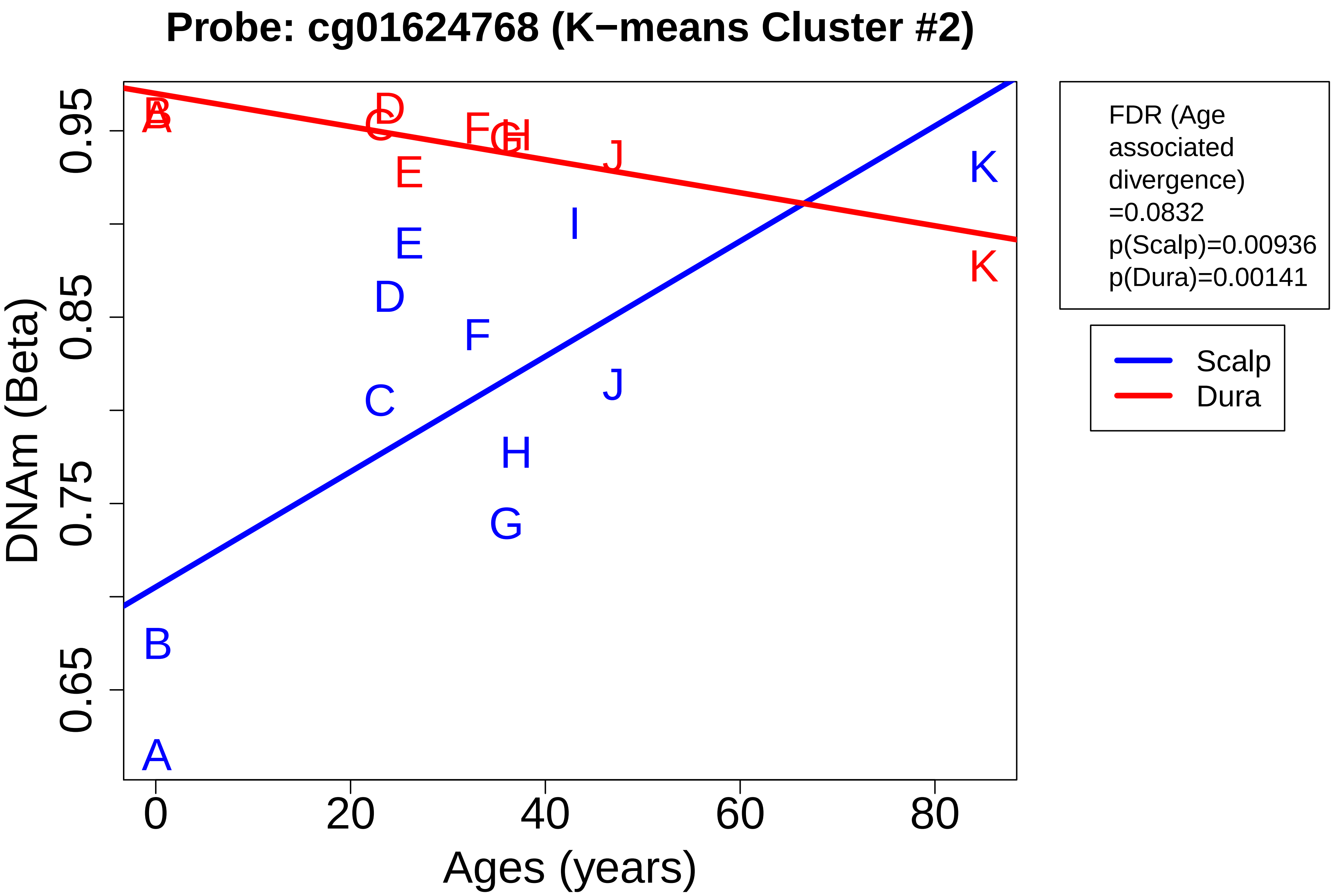

Probe: cg01624768 (K−means Cluster #2)
DNAm (Beta)
0.65
0.75
0.85
0.95
Ages (years)
0
20
40
60
80
FDR (Age associated divergence) =0.0832
p(Scalp)=0.00936
p(Dura)=0.00141
Scalp
Dura

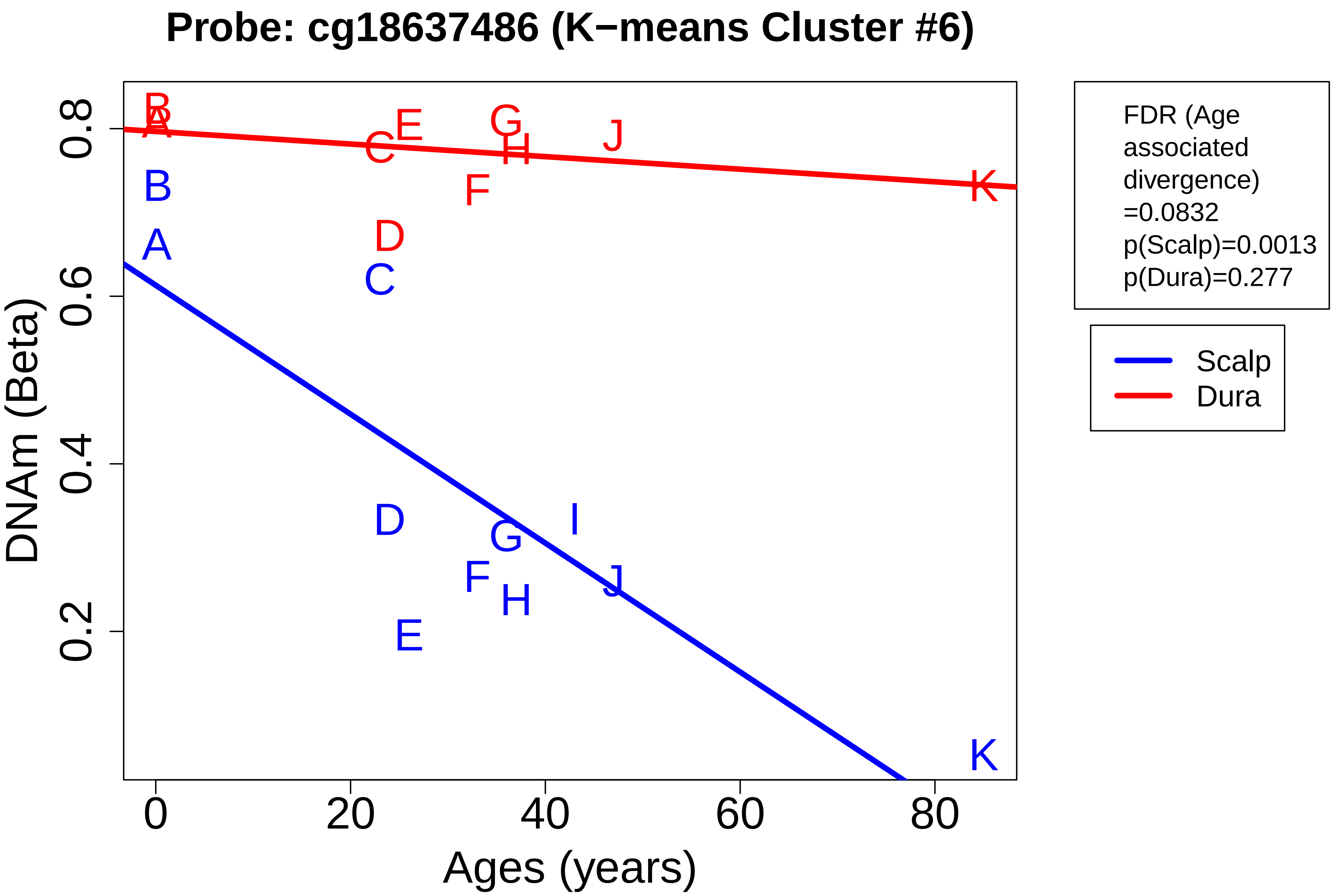

Probe: cg18637486 (K−means Cluster #6)
DNAm (Beta)
0.2
0.4
0.6
0.8
0
20
40
60
80
Ages (years)
FDR (Age associated divergence) =0.0832
p(Scalp)=0.0013
p(Dura)=0.277
Scalp
Dura

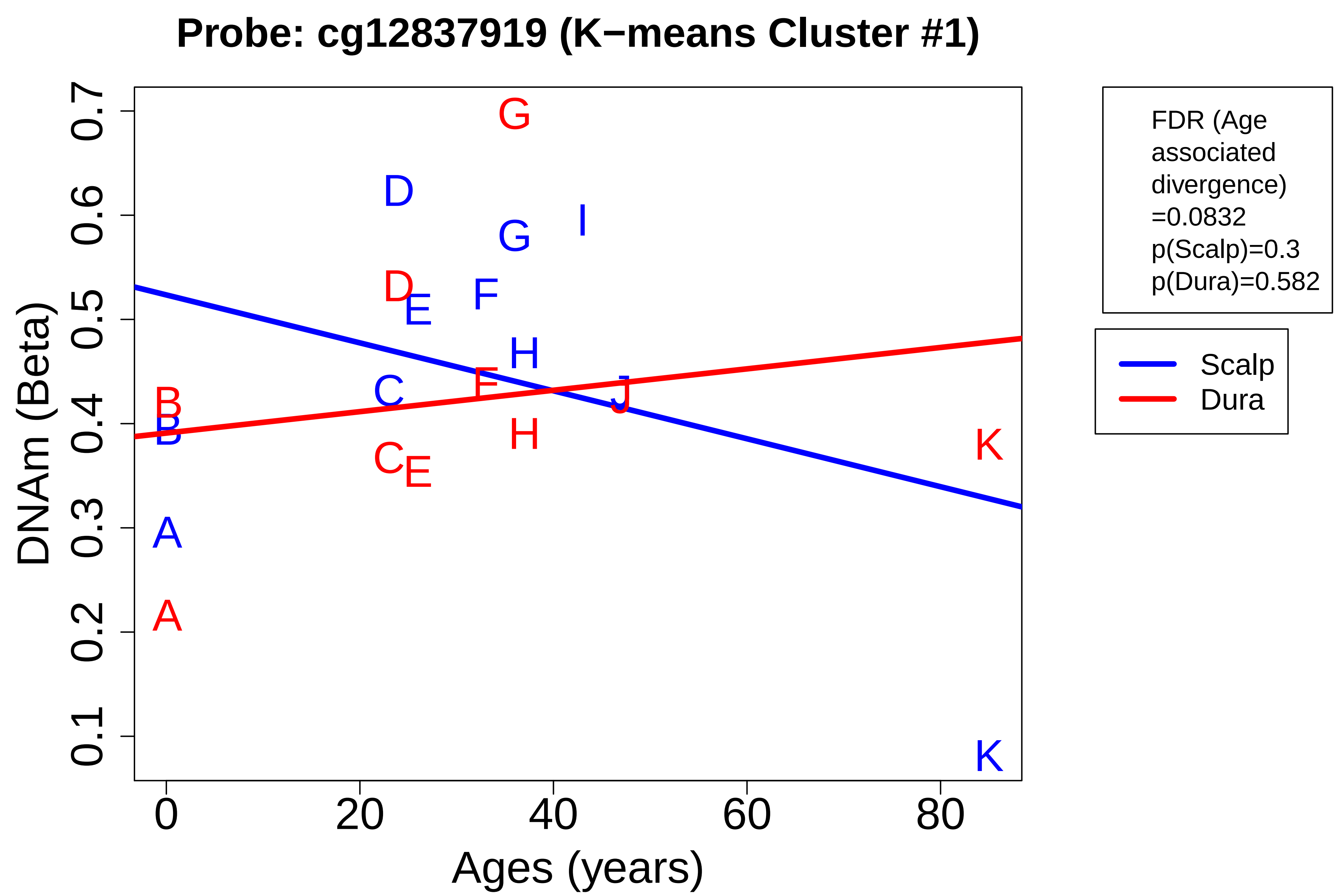

Probe: cg12837919 (K−means Cluster #1)
DNAm (Beta)
0.1
0.2
0.3
0.4
0.5
0.6
0.7
Ages (years)
0
20
40
60
80
FDR (Age associated divergence) =0.0832
p(Scalp)=0.3
p(Dura)=0.582
Scalp
Dura

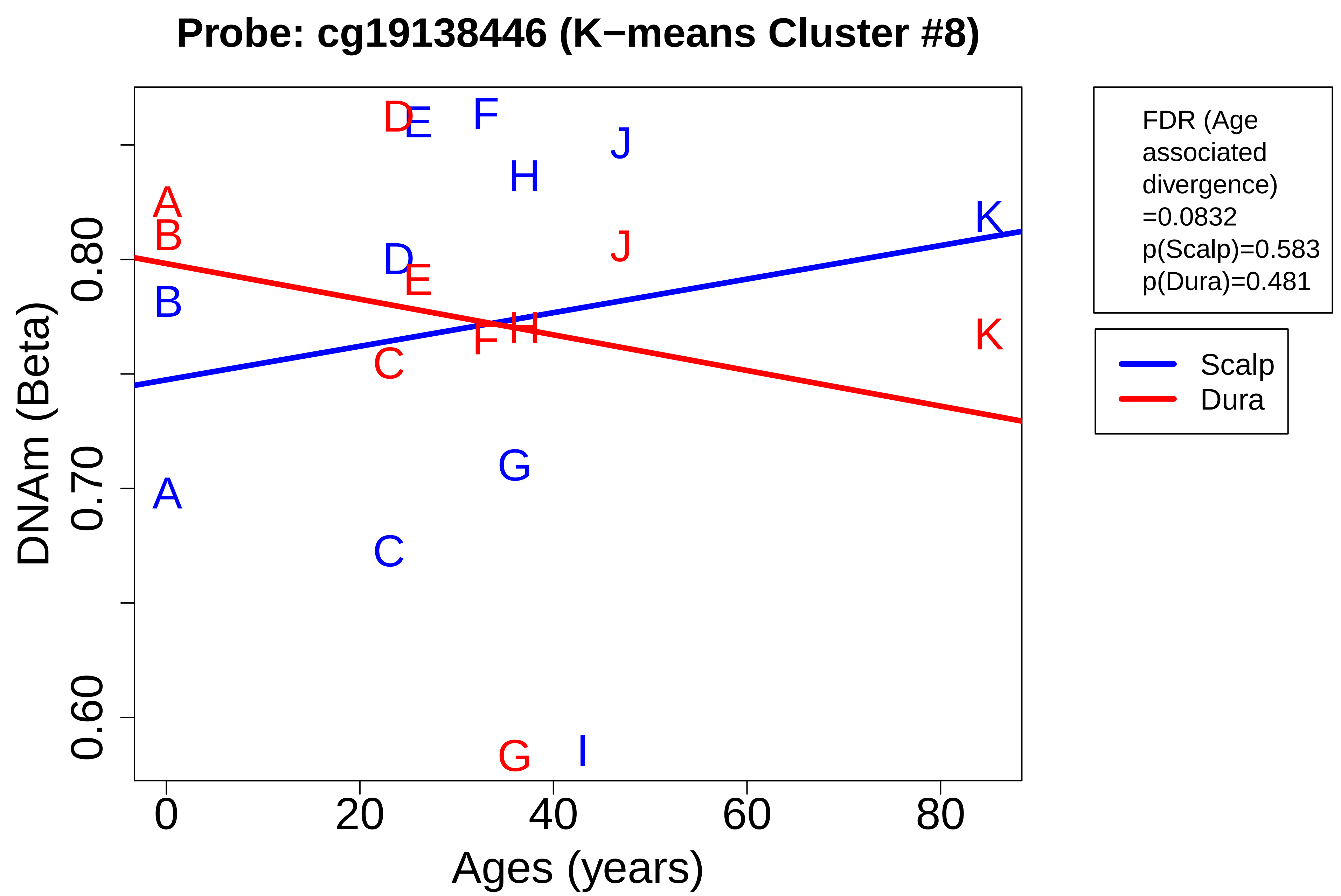

Probe: cg19138446 (K−means Cluster #8)
DNAm (Beta)
0.60
0.70
0.80
Ages (years)
0
20
40
60
80
FDR (Age associated divergence) =0.0832
p(Scalp)=0.583
p(Dura)=0.481
Scalp
Dura
A
B
C
D
E
F
G
H
I
J
K

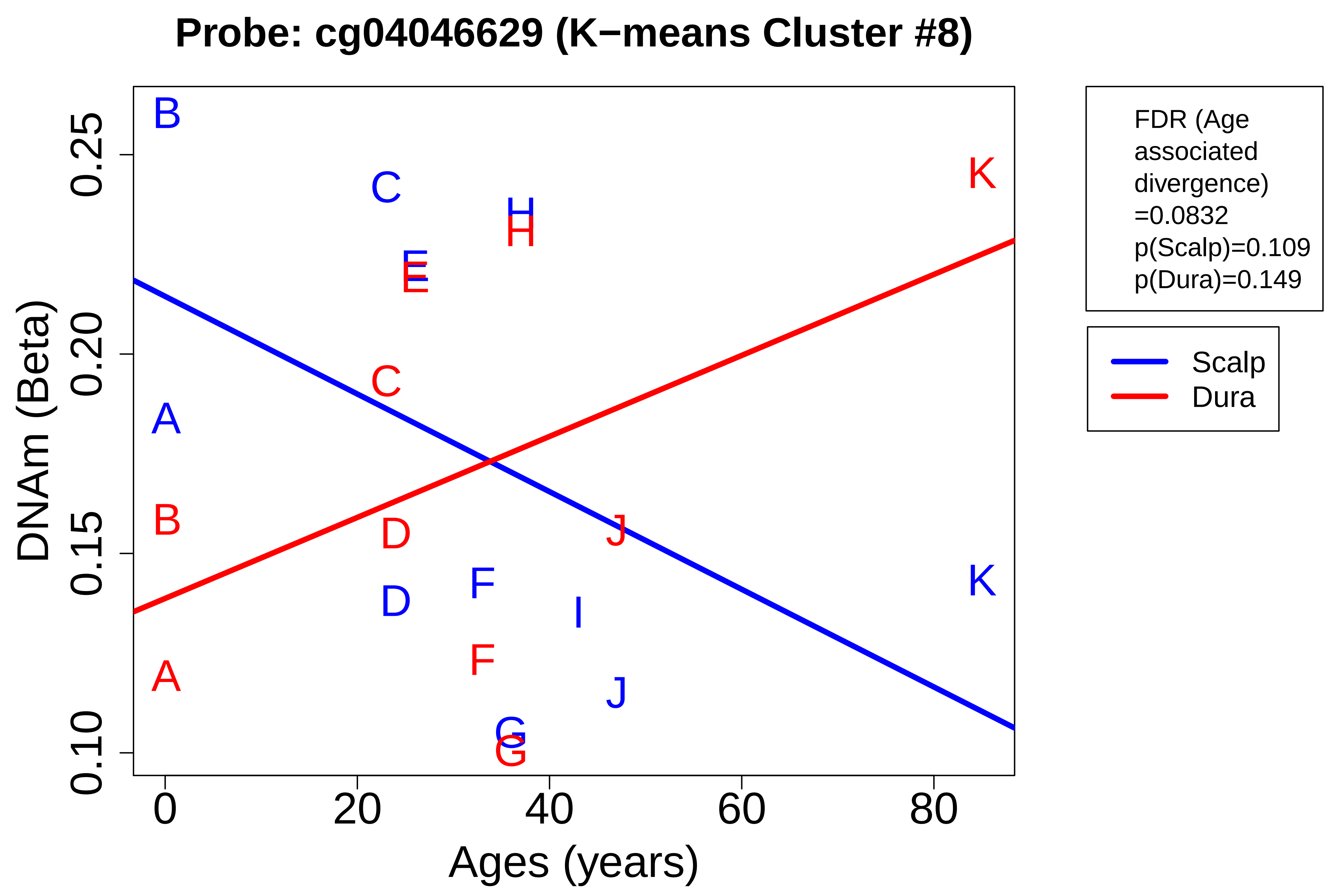
Probe: cg04046629 (K−means Cluster #8)
FDR (Age associated divergence) =0.0832
p(Scalp)=0.109
p(Dura)=0.149
Scalp
Dura
DNAm (Beta)
0.10
0.15
0.20
0.25
Ages (years)
0
20
40
60
80
A
B
C
D
E
F
G
H
I
J
K

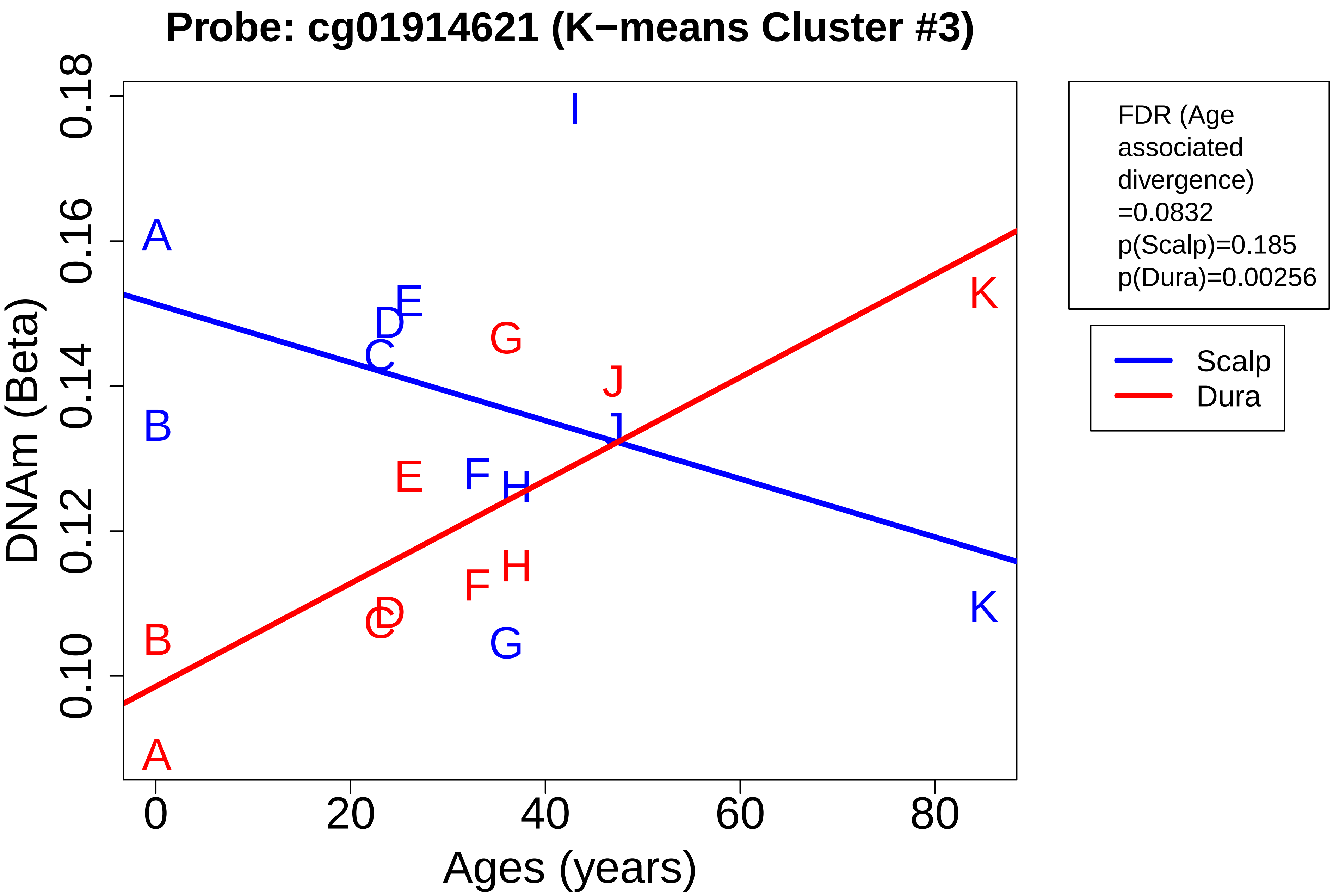
Probe: cg01914621 (K−means Cluster #3)
DNAm (Beta)
0.10
0.12
0.14
0.16
0.18
Ages (years)
0
20
40
60
80
A
B
C
D
E
F
G
H
I
J
K
FDR (Age associated divergence) =0.0832
p(Scalp)=0.185
p(Dura)=0.00256
Scalp
Dura

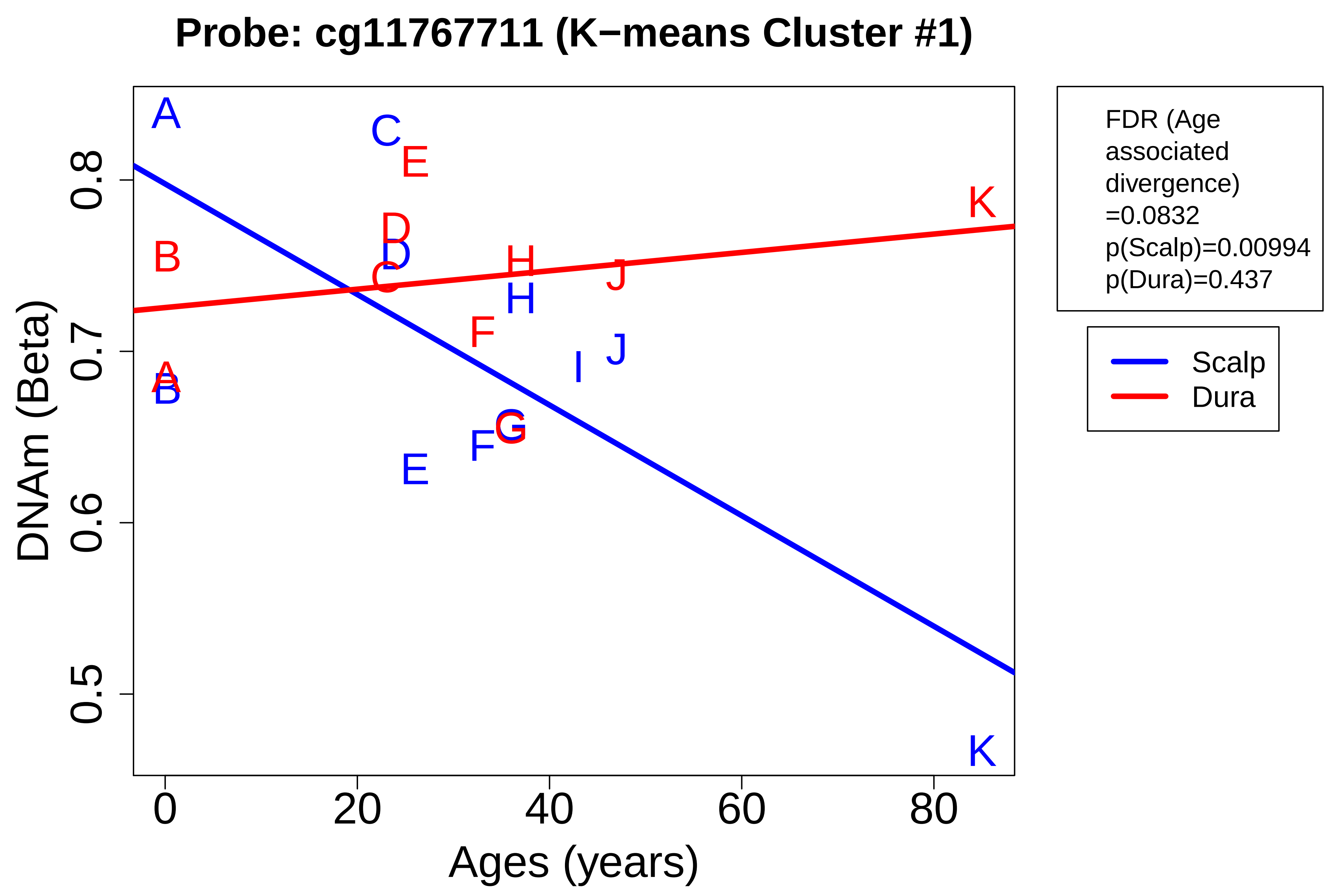

Probe: cg11767711 (K−means Cluster #1)
DNAm (Beta)
0.5
0.6
0.7
0.8
0
20
40
60
80
Ages (years)
A
B
C
D
E
F
G
H
I
J
K
FDR (Age associated divergence) =0.0832
p(Scalp)=0.00994
p(Dura)=0.437
Scalp
Dura

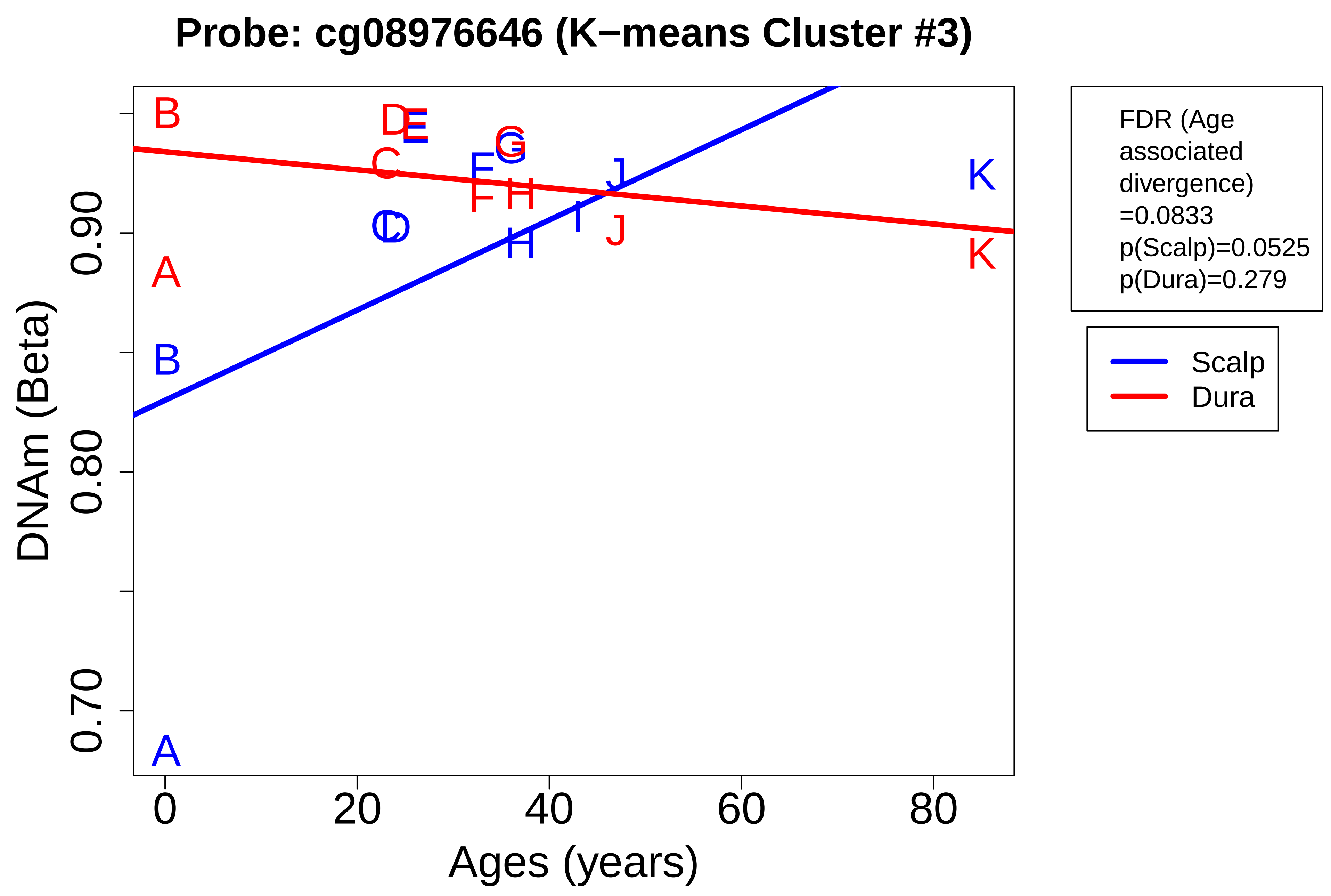

Probe: cg08976646 (K−means Cluster #3)
DNAm (Beta)
0.70
0.80
0.90
0
20
40
60
80
Ages (years)
FDR (Age associated divergence) =0.0833
p(Scalp)=0.0525
p(Dura)=0.279
Scalp
Dura
A
B
C
D
E
F
G
H
I
J
K

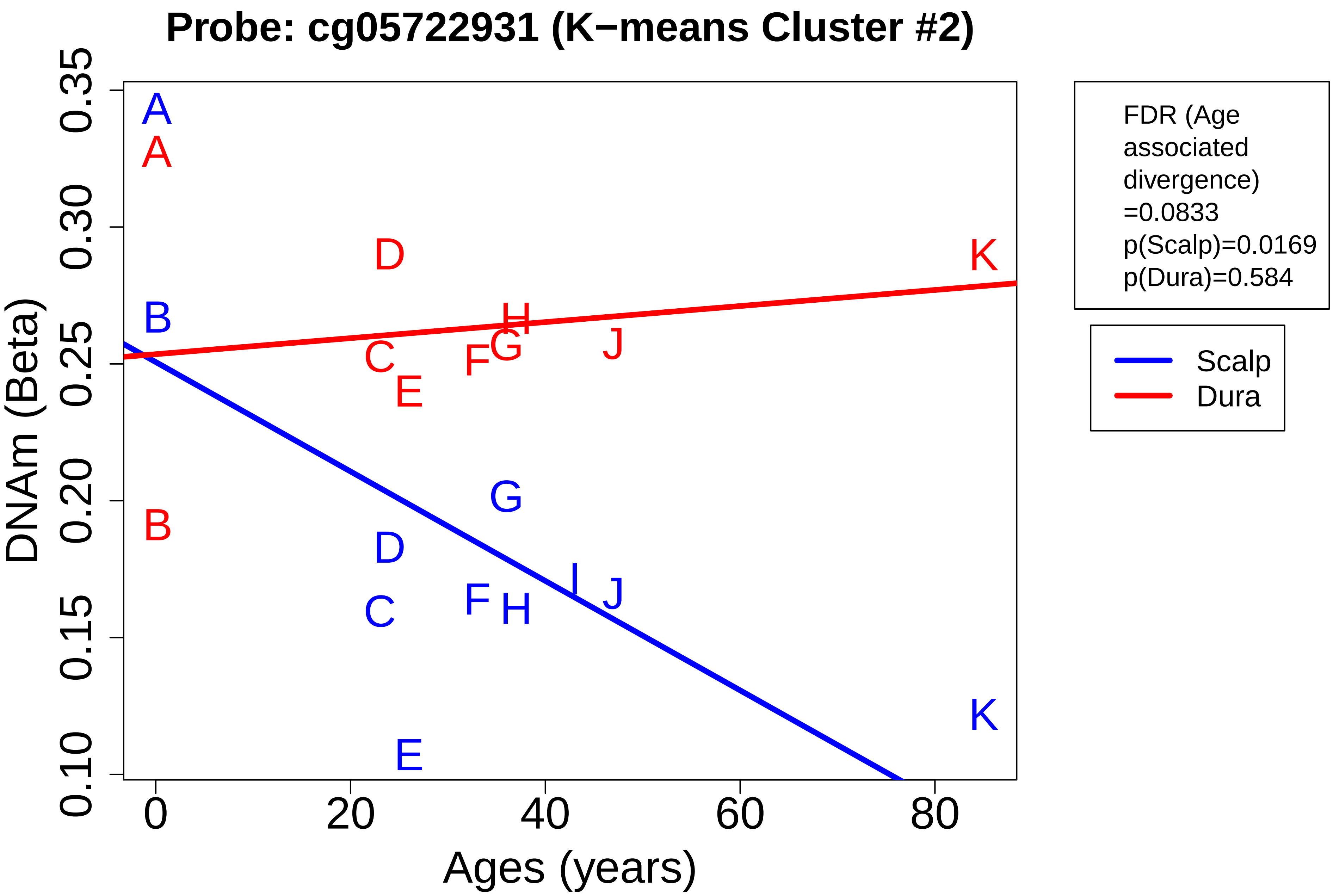

Probe: cg05722931 (K−means Cluster #2)
DNAm (Beta)
0.10
0.15
0.20
0.25
0.30
0.35
Ages (years)
0
20
40
60
80
A
A
B
B
C
C
D
D
E
E
F
F
G
G
H
H
I
J
J
K
K
FDR (Age associated divergence) =0.0833
p(Scalp)=0.0169
p(Dura)=0.584
Scalp
Dura

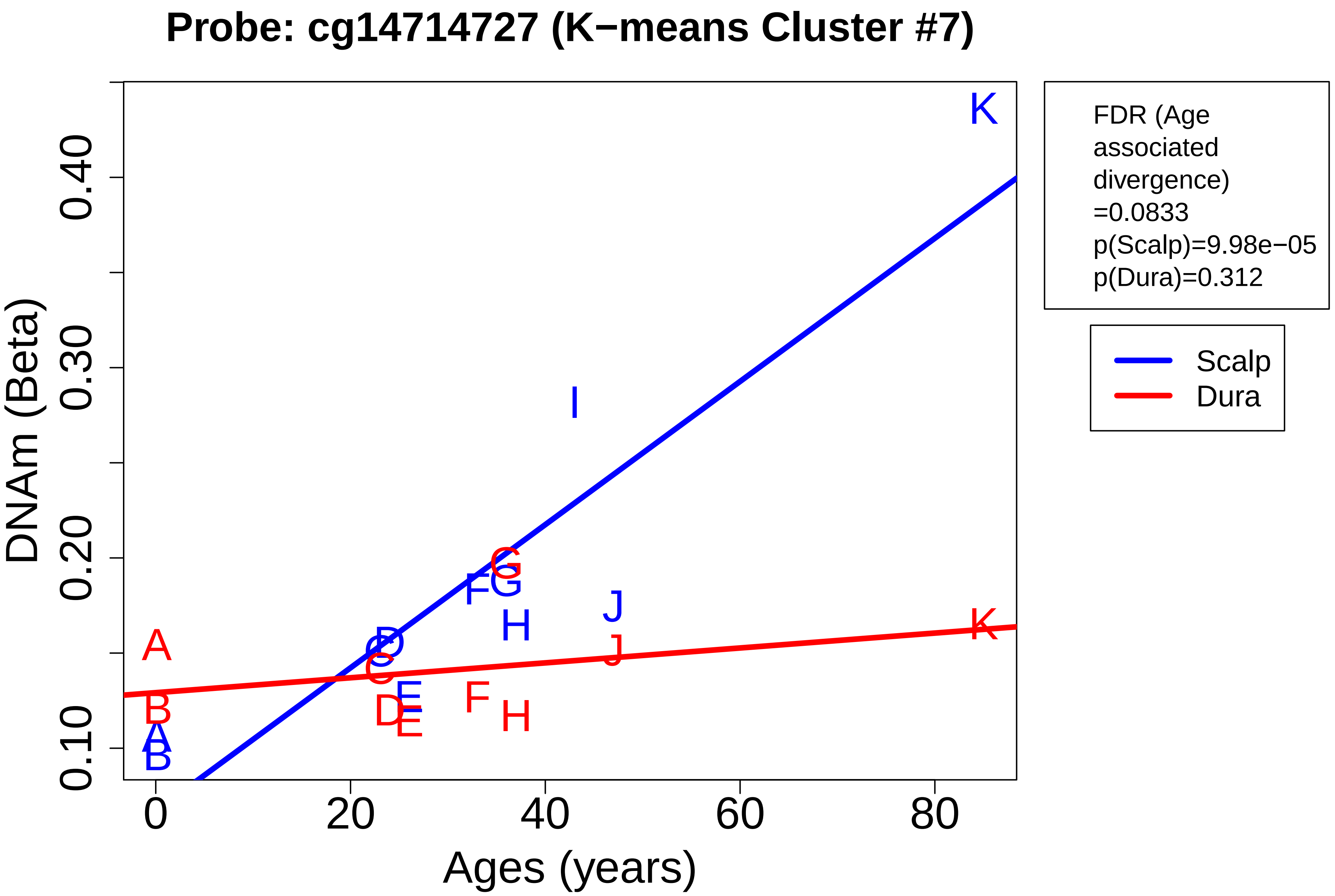
Probe: cg14714727 (K−means Cluster #7)
FDR (Age associated divergence) =0.0833
p(Scalp)=9.98e−05
p(Dura)=0.312
Scalp
Dura
DNAm (Beta)
Ages (years)
0.10
0.20
0.30
0.40
0
20
40
60
80
A
B
C
D
E
F
G
H
I
J
K

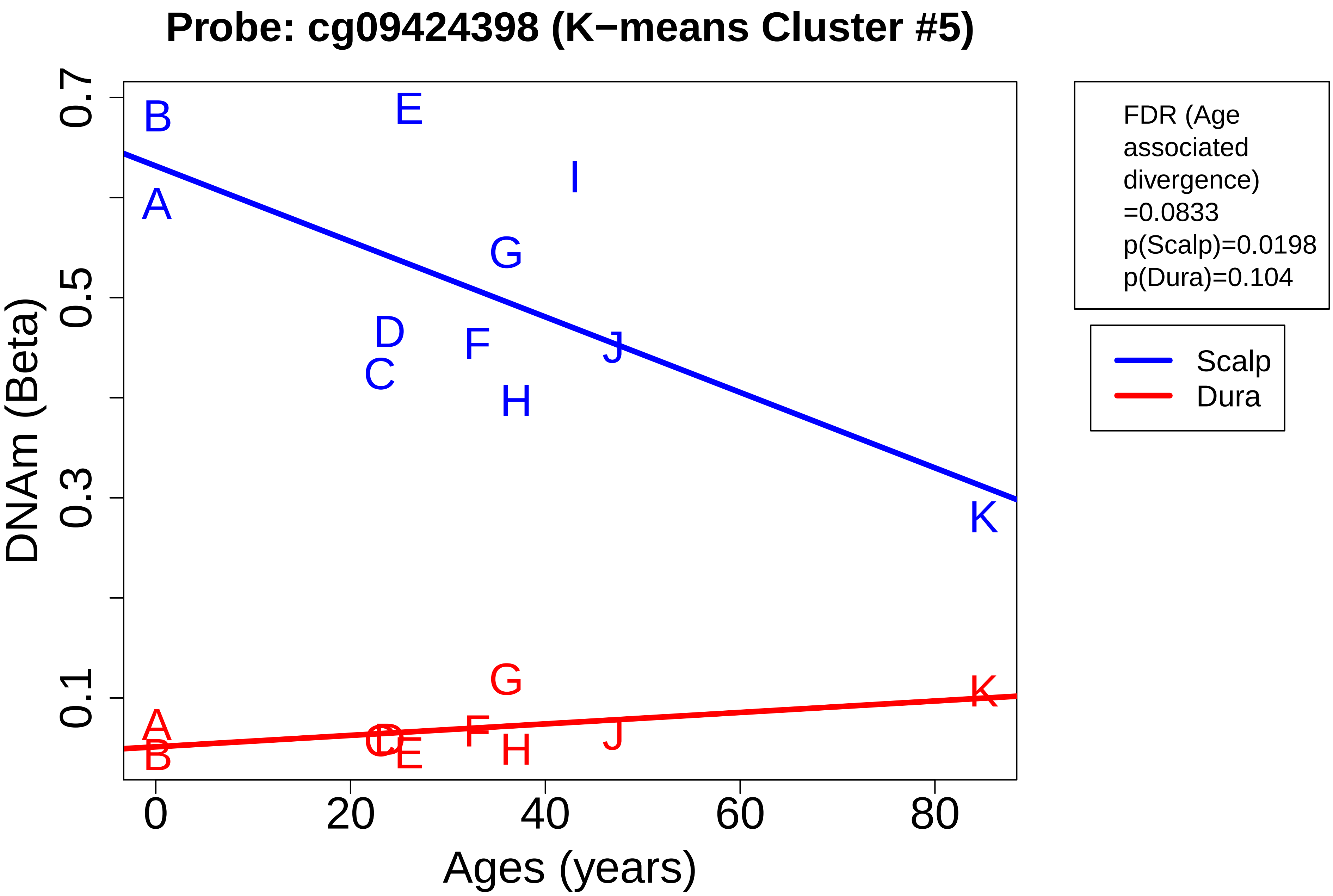
Probe: cg09424398 (K−means Cluster #5)
DNAm (Beta)
0.7
0.5
0.3
0.1
B
E
I
A
G
D
F
J
C
H
K
G
K
A
D
C
F
J
B
E
H
0
20
40
60
80
Ages (years)
FDR (Age associated divergence) =0.0833
p(Scalp)=0.0198
p(Dura)=0.104
Scalp
Dura

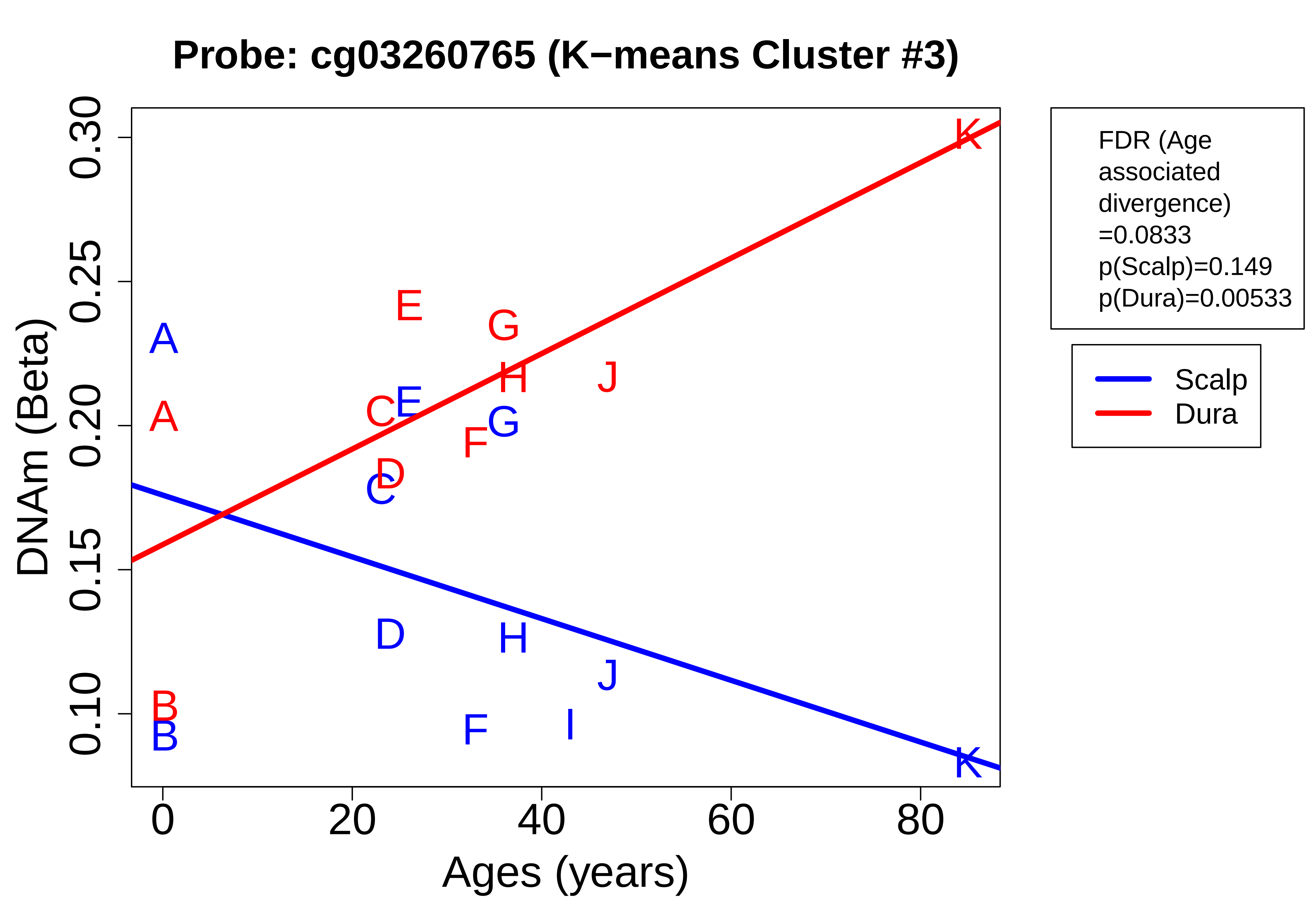

Probe: cg03260765 (K−means Cluster #3)
DNAm (Beta)
0.10
0.15
0.20
0.25
0.30
Ages (years)
0
20
40
60
80
A
B
C
D
E
F
G
H
I
J
K
FDR (Age associated divergence) =0.0833
p(Scalp)=0.149
p(Dura)=0.00533
Scalp
Dura

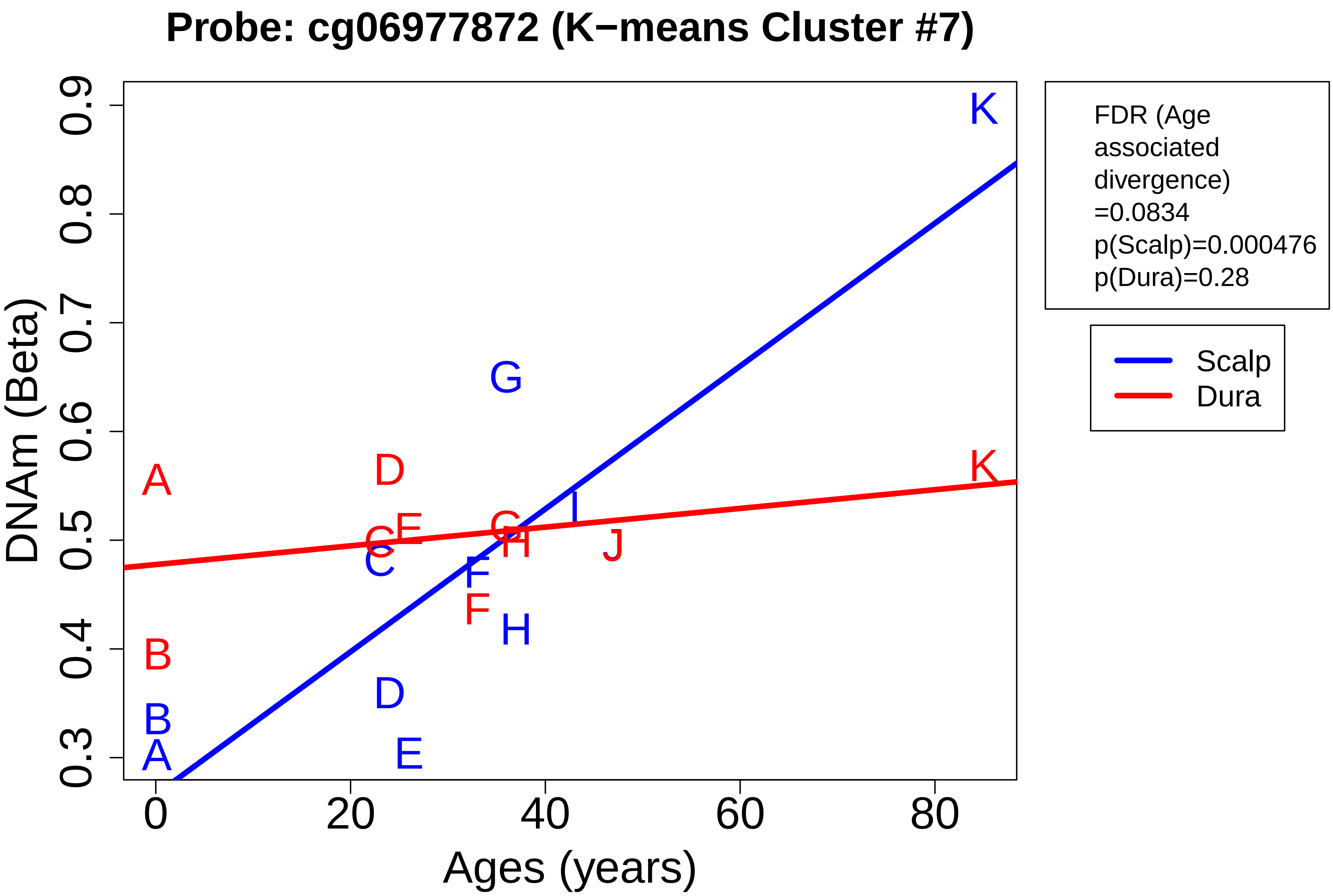

Probe: cg06977872 (K−means Cluster #7)
DNAm (Beta)
0.3
0.4
0.5
0.6
0.7
0.8
0.9
0
20
40
60
80
Ages (years)
A
B
C
D
E
F
G
H
I
J
K
FDR (Age associated divergence) =0.0834
p(Scalp)=0.000476
p(Dura)=0.28
Scalp
Dura

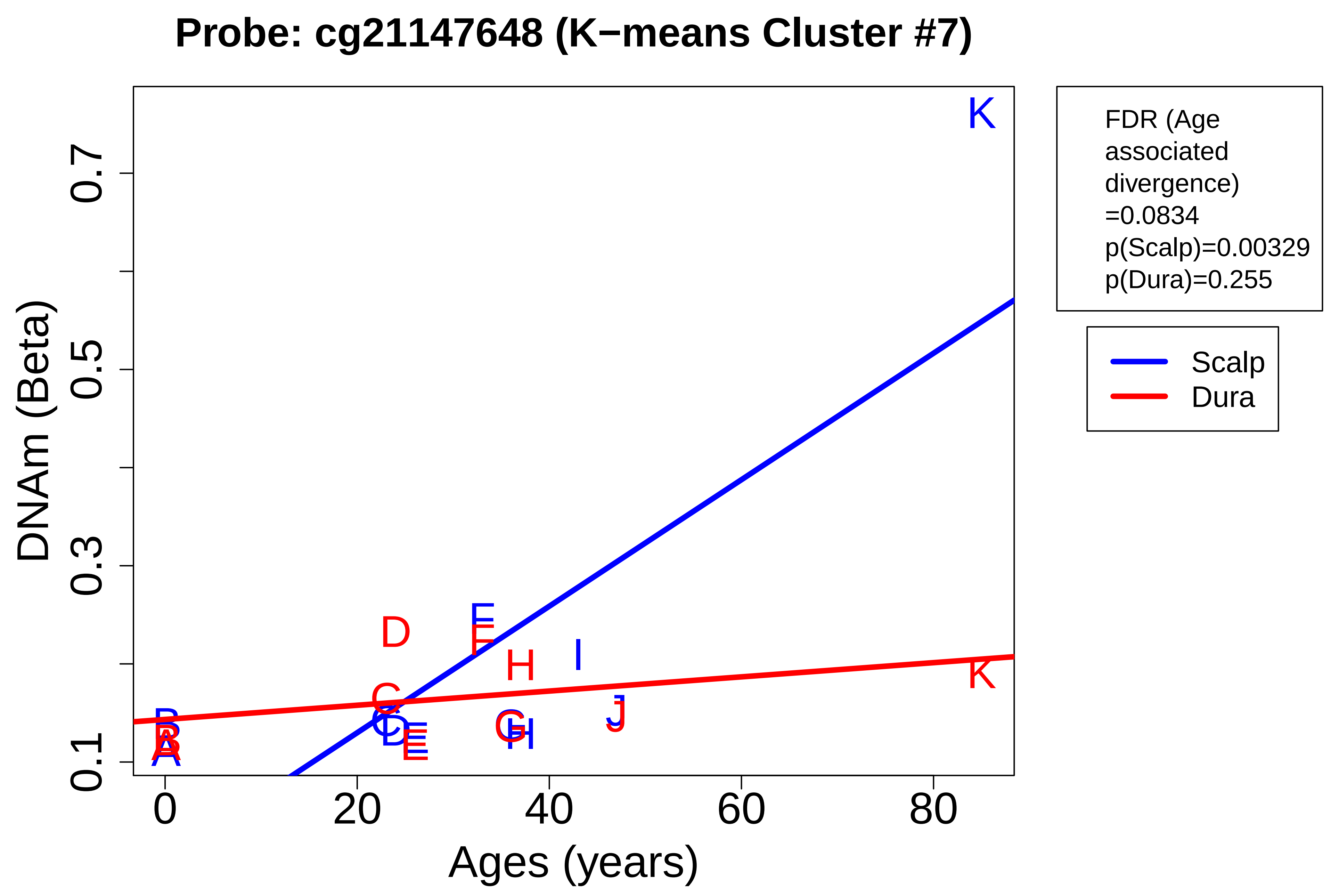

Probe: cg21147648 (K−means Cluster #7)
DNAm (Beta)
0.1
0.3
0.5
0.7
0
20
40
60
80
Ages (years)
FDR (Age associated divergence) =0.0834
p(Scalp)=0.00329
p(Dura)=0.255
Scalp
Dura
A
B
C
D
E
F
G
H
I
J
K

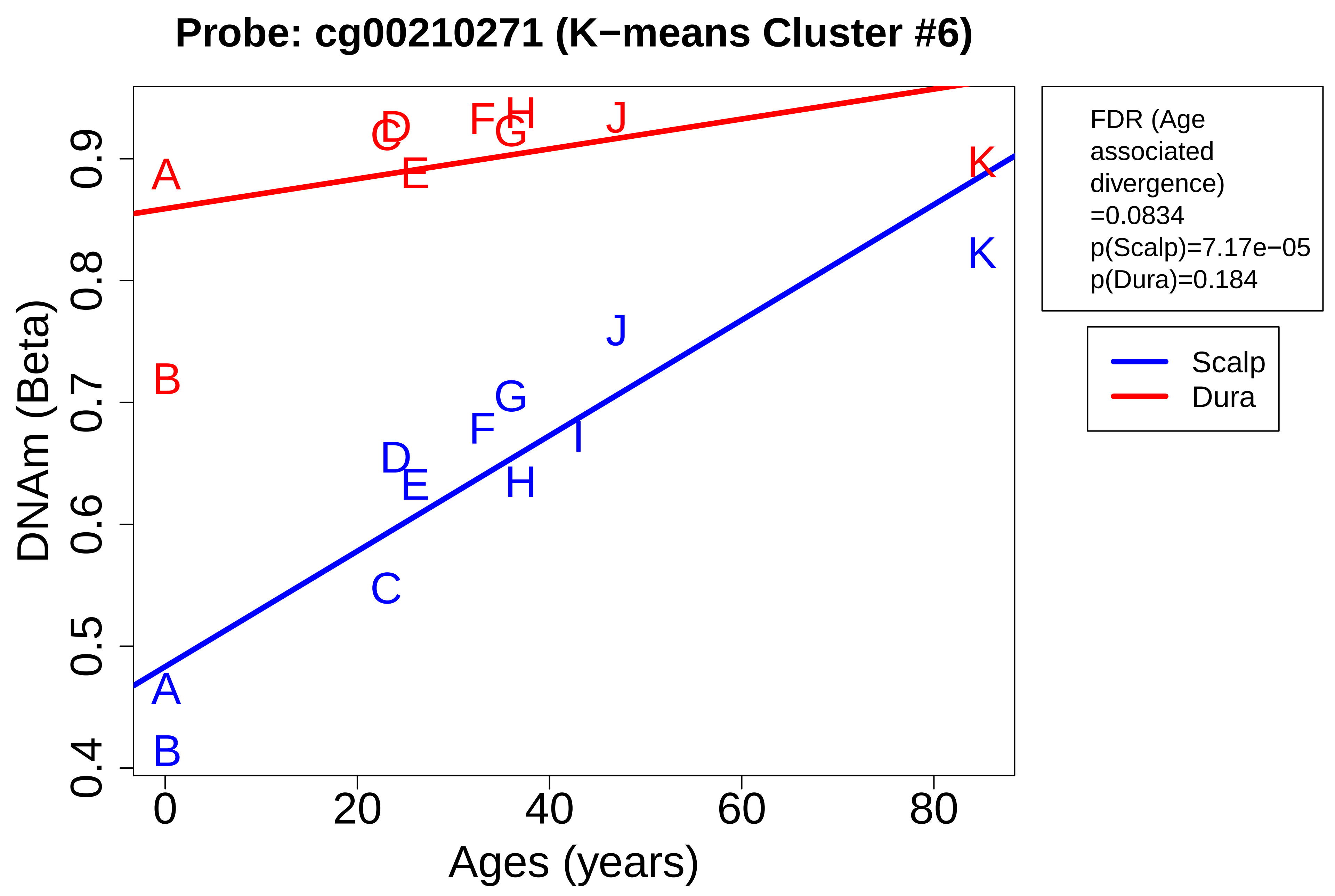

Probe: cg00210271 (K−means Cluster #6)
FDR (Age associated divergence) =0.0834
p(Scalp)=7.17e−05
p(Dura)=0.184
Scalp
Dura
DNAm (Beta)
Ages (years)
0.4
0.5
0.6
0.7
0.8
0.9
0
20
40
60
80
A
B
C
D
E
F
G
H
I
J
K

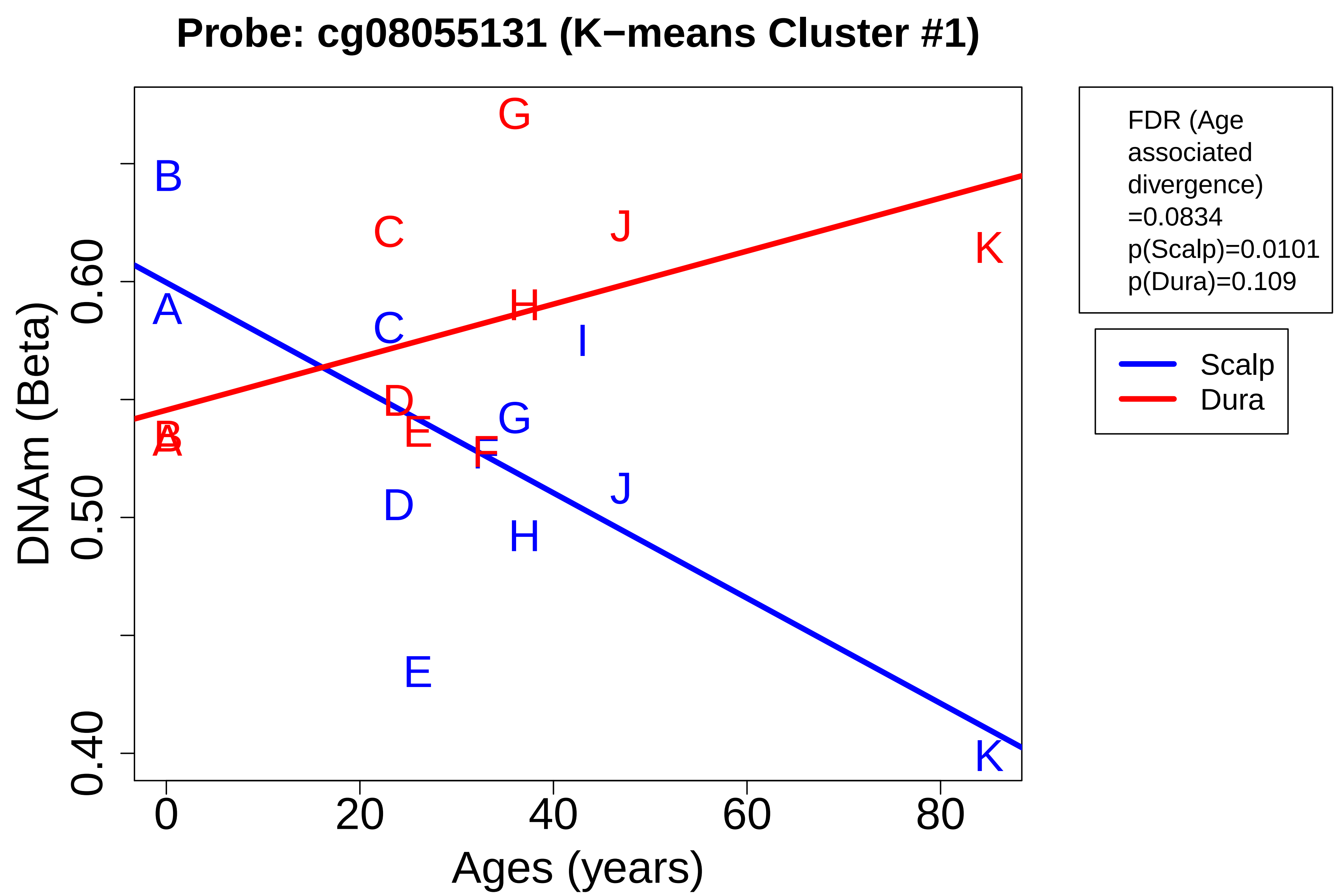

Probe: cg08055131 (K−means Cluster #1)
DNAm (Beta)
0.40
0.50
0.60
0
20
40
60
80
Ages (years)
FDR (Age associated divergence) =0.0834
p(Scalp)=0.0101
p(Dura)=0.109
Scalp
Dura
A
B
C
D
E
F
G
H
I
J
K

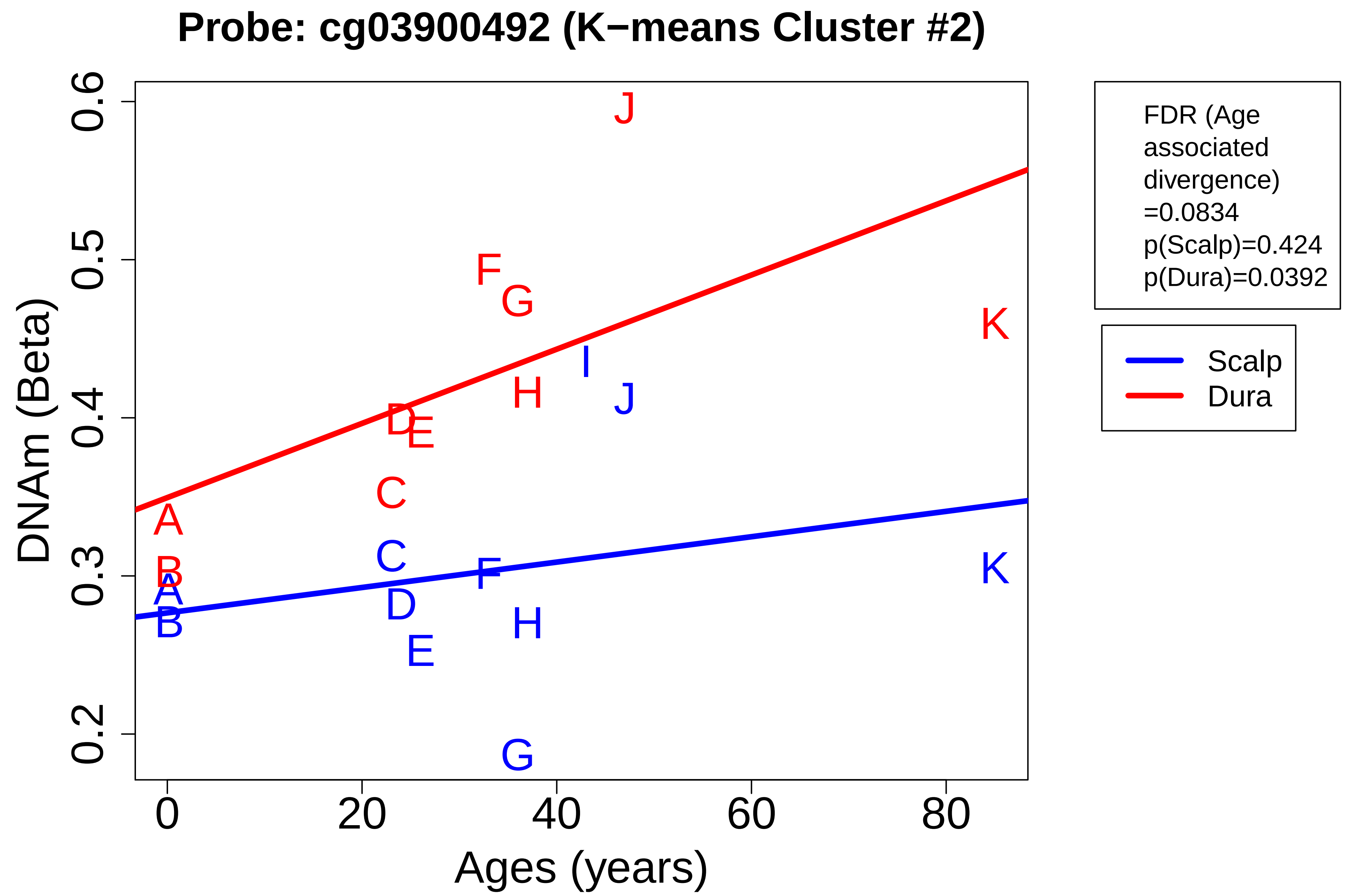

Probe: cg03900492 (K−means Cluster #2)
DNAm (Beta)
0.2
0.3
0.4
0.5
0.6
Ages (years)
0
20
40
60
80
FDR (Age associated divergence) =0.0834
p(Scalp)=0.424
p(Dura)=0.0392
Scalp
Dura
A
B
C
D
E
F
G
H
I
J
K

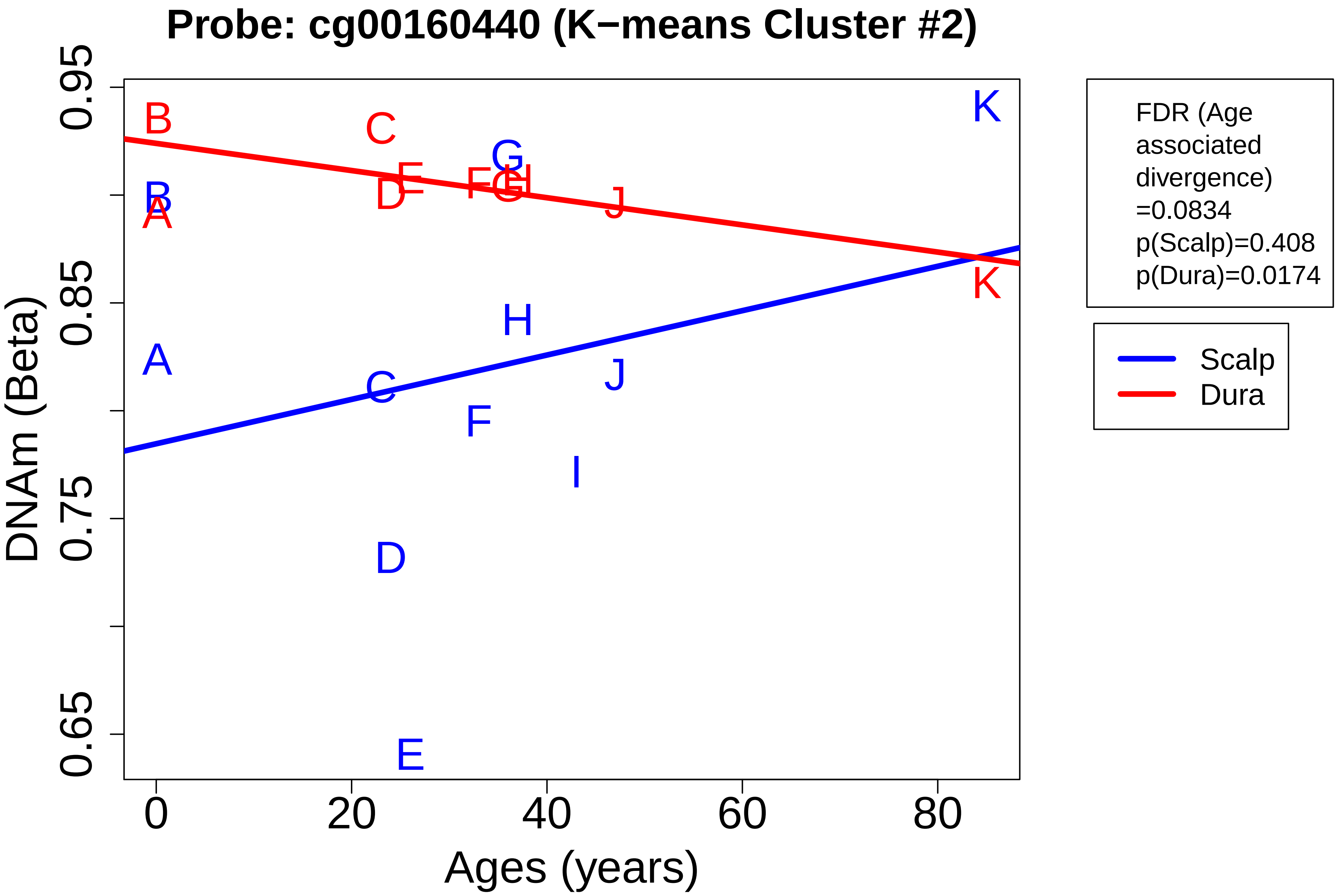

Probe: cg00160440 (K−means Cluster #2)
DNAm (Beta)
0.65
0.75
0.85
0.95
0
20
40
60
80
Ages (years)
FDR (Age associated divergence) =0.0834
p(Scalp)=0.408
p(Dura)=0.0174
Scalp
Dura

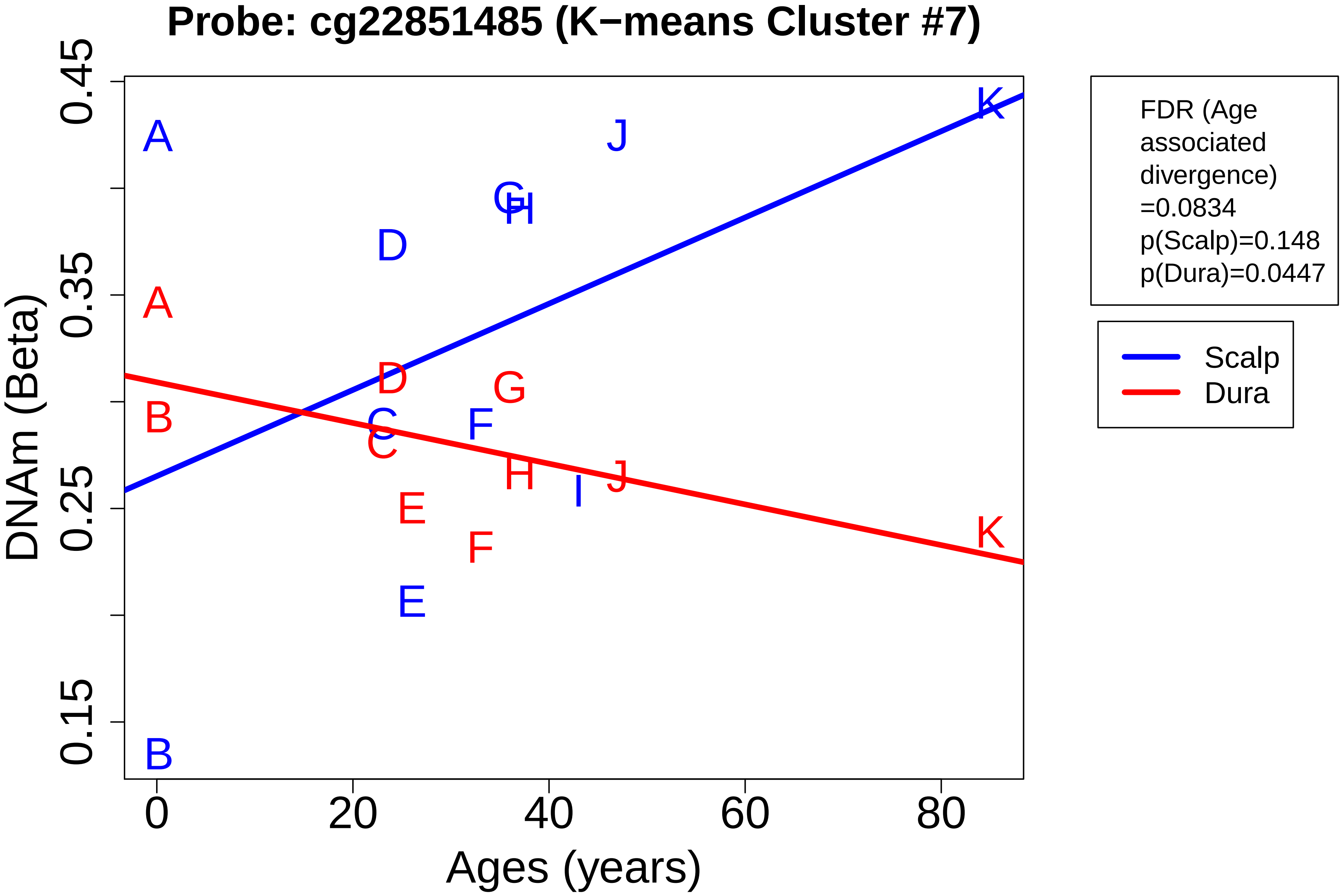

Probe: cg22851485 (K−means Cluster #7)
DNAm (Beta)
0.45
0.35
0.25
0.15
0
20
40
60
80
Ages (years)
A
B
C
D
E
F
G
H
I
J
K
FDR (Age associated divergence) =0.0834
p(Scalp)=0.148
p(Dura)=0.0447
Scalp
Dura

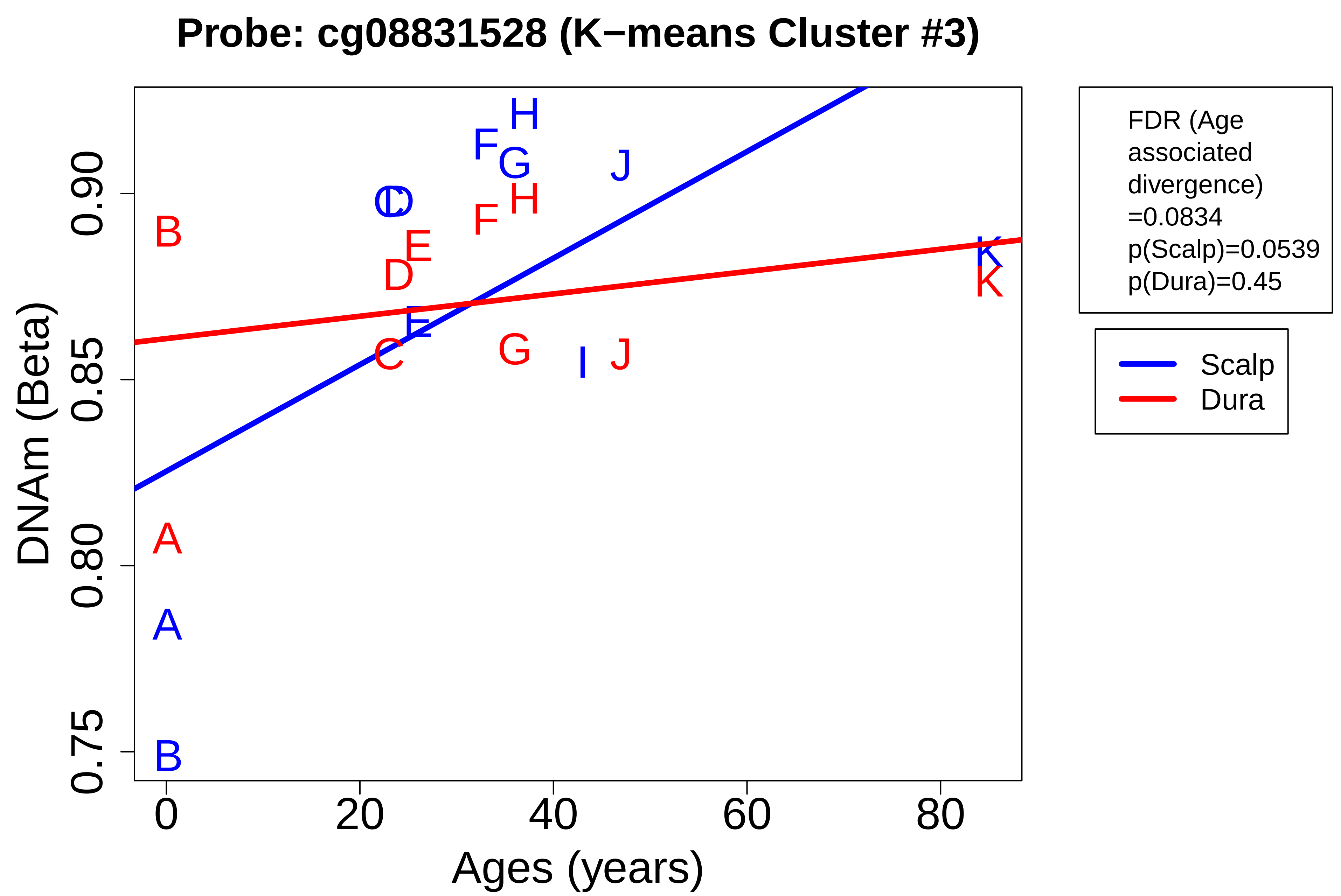

Probe: cg08831528 (K−means Cluster #3)
DNAm (Beta)
0.75
0.80
0.85
0.90
Ages (years)
0
20
40
60
80
FDR (Age associated divergence) =0.0834
p(Scalp)=0.0539
p(Dura)=0.45
Scalp
Dura

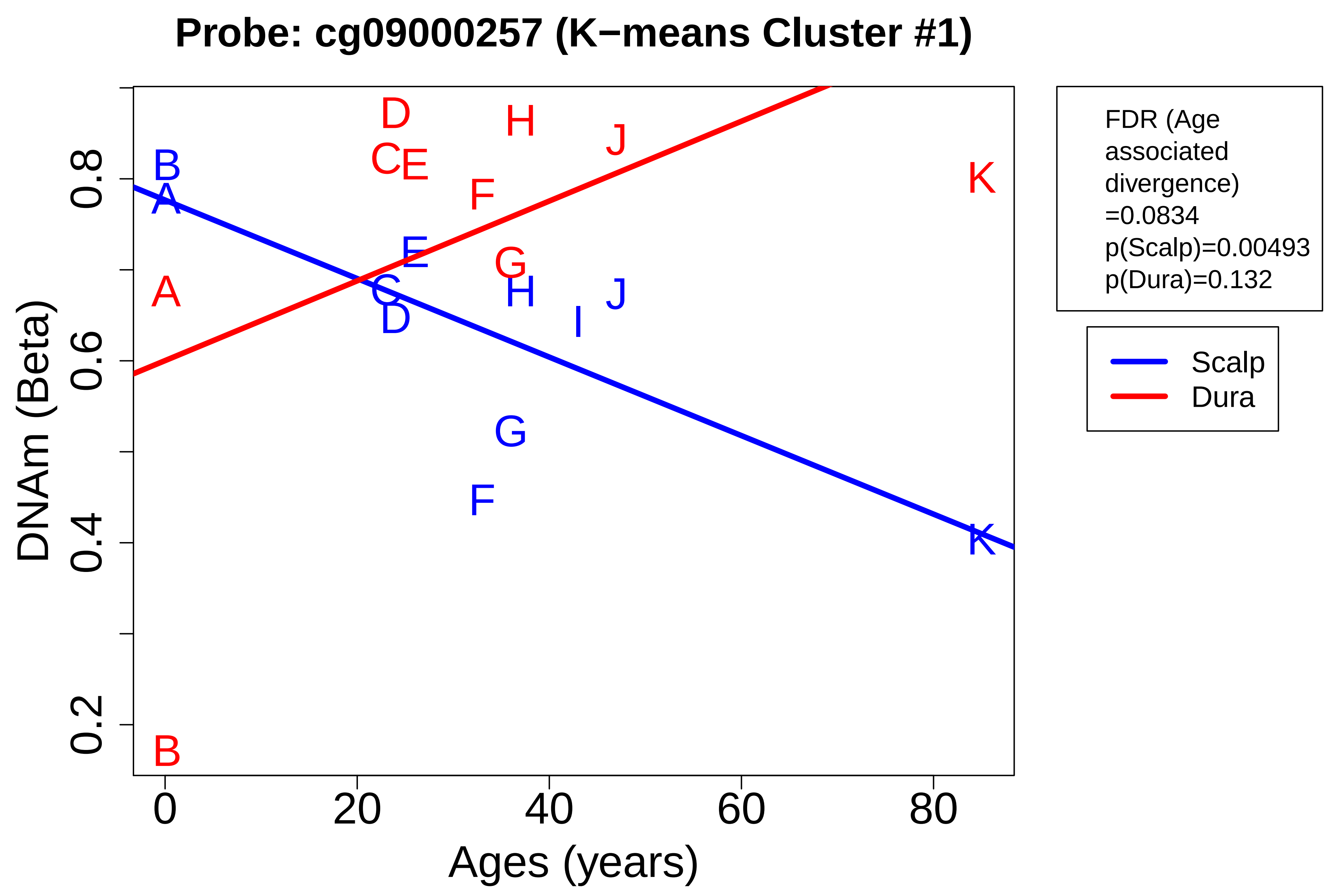

Probe: cg09000257 (K−means Cluster #1)
DNAm (Beta)
0.2
0.4
0.6
0.8
0
20
40
60
80
Ages (years)
FDR (Age associated divergence) =0.0834
p(Scalp)=0.00493
p(Dura)=0.132
Scalp
Dura

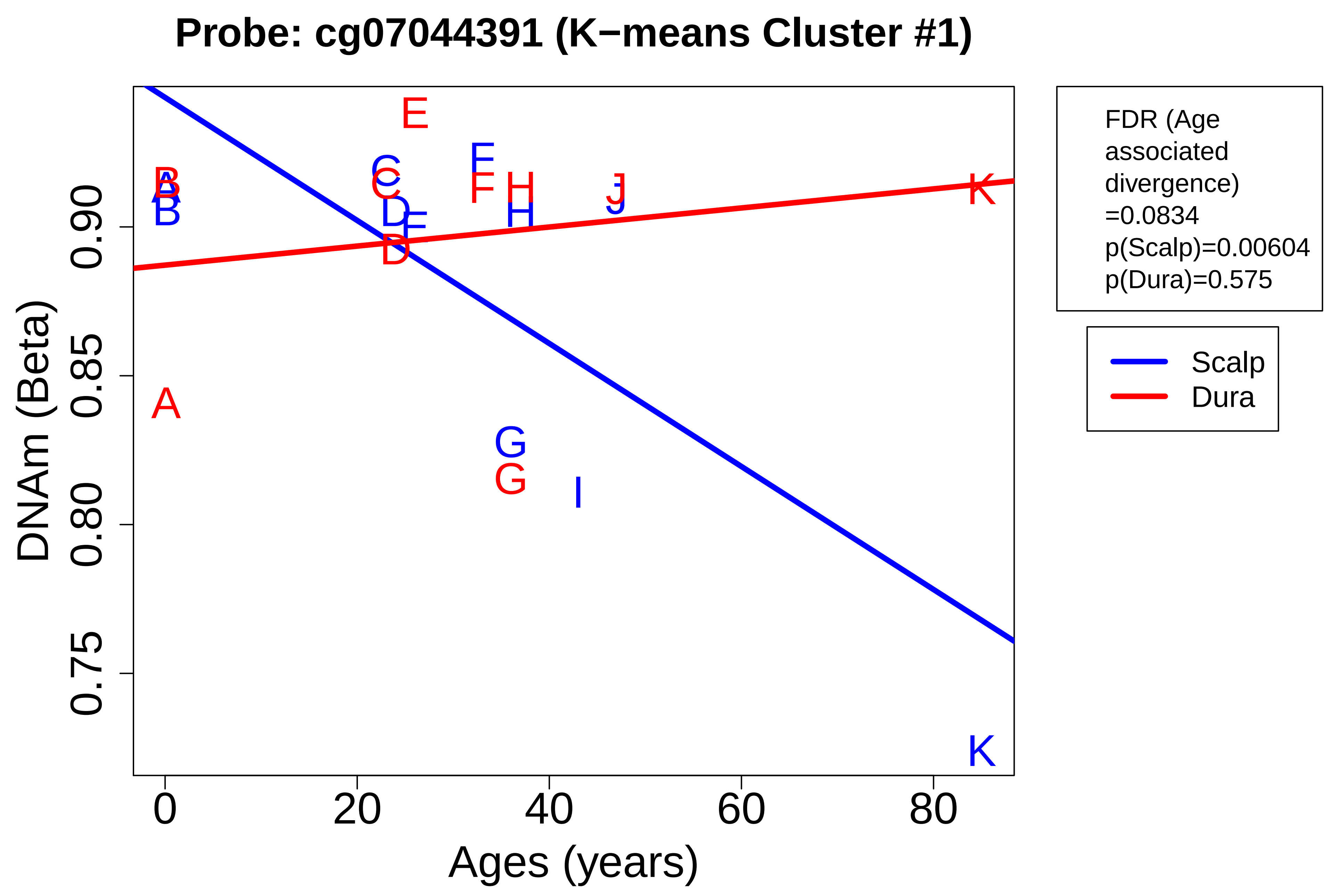

Probe: cg07044391 (K−means Cluster #1)
FDR (Age associated divergence) =0.0834
p(Scalp)=0.00604
p(Dura)=0.575
Scalp
Dura
DNAm (Beta)
0.75
0.80
0.85
0.90
Ages (years)
0
20
40
60
80
A
B
C
D
E
F
G
H
I
J
K

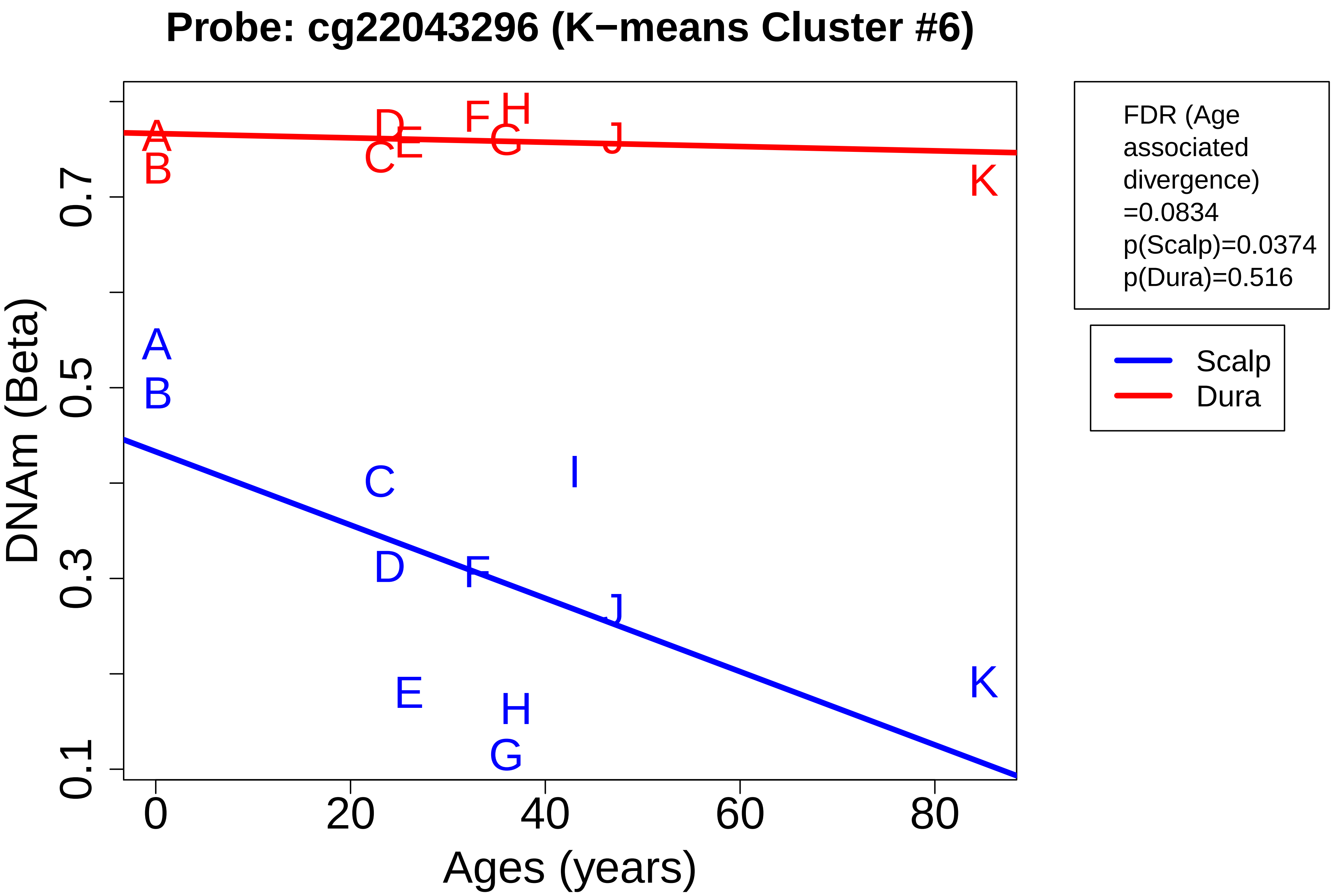

Probe: cg22043296 (K−means Cluster #6)
DNAm (Beta)
0.1
0.3
0.5
0.7
0
20
40
60
80
Ages (years)
A
B
C
D
E
F
G
H
J
K
A
B
C
I
D
F
J
E
H
G
K
FDR (Age associated divergence) =0.0834
p(Scalp)=0.0374
p(Dura)=0.516
Scalp
Dura

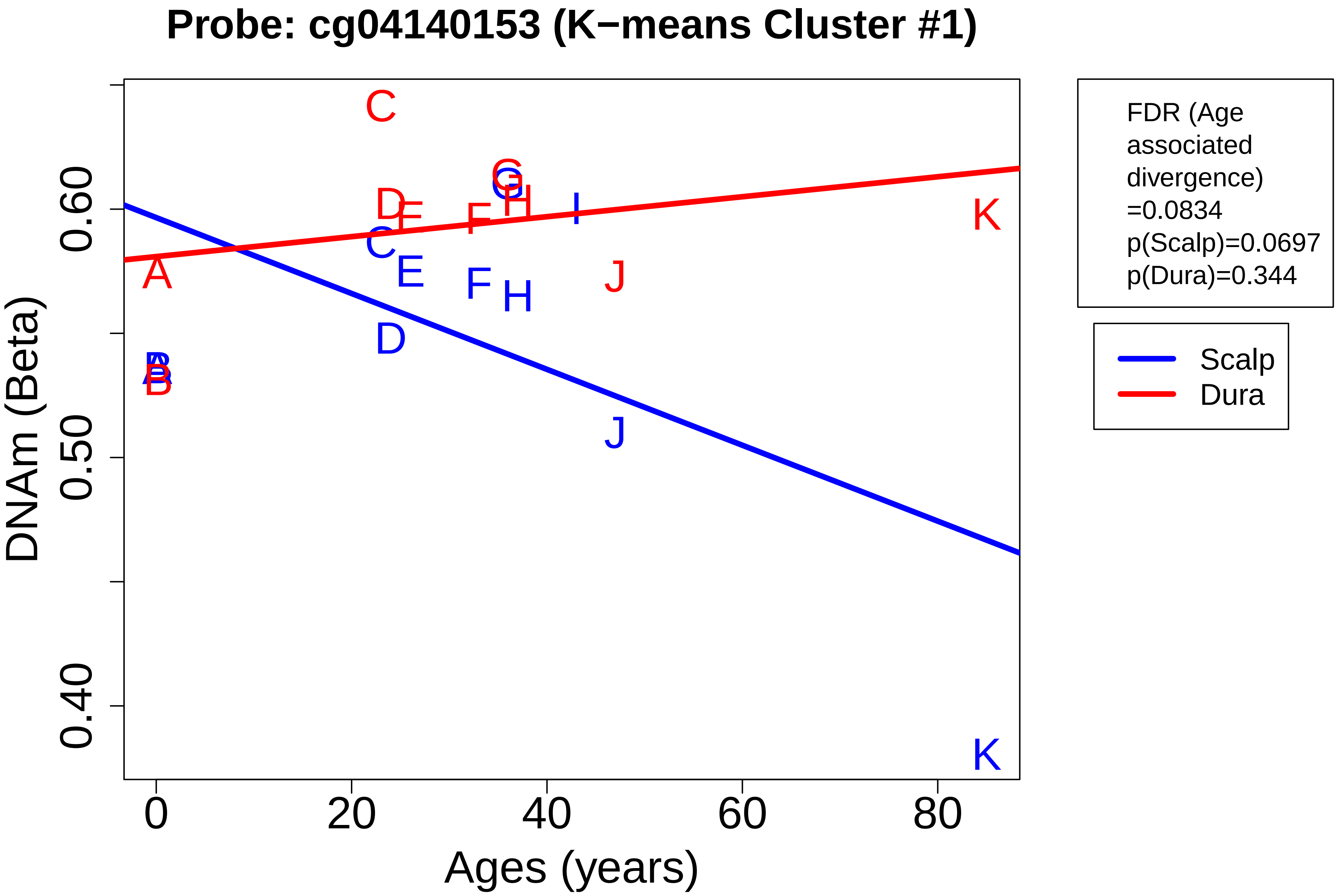

Probe: cg04140153 (K−means Cluster #1)
DNAm (Beta)
0.40
0.50
0.60
Ages (years)
0
20
40
60
80
FDR (Age associated divergence) =0.0834
p(Scalp)=0.0697
p(Dura)=0.344
Scalp
Dura
A B C D E F G H I J K

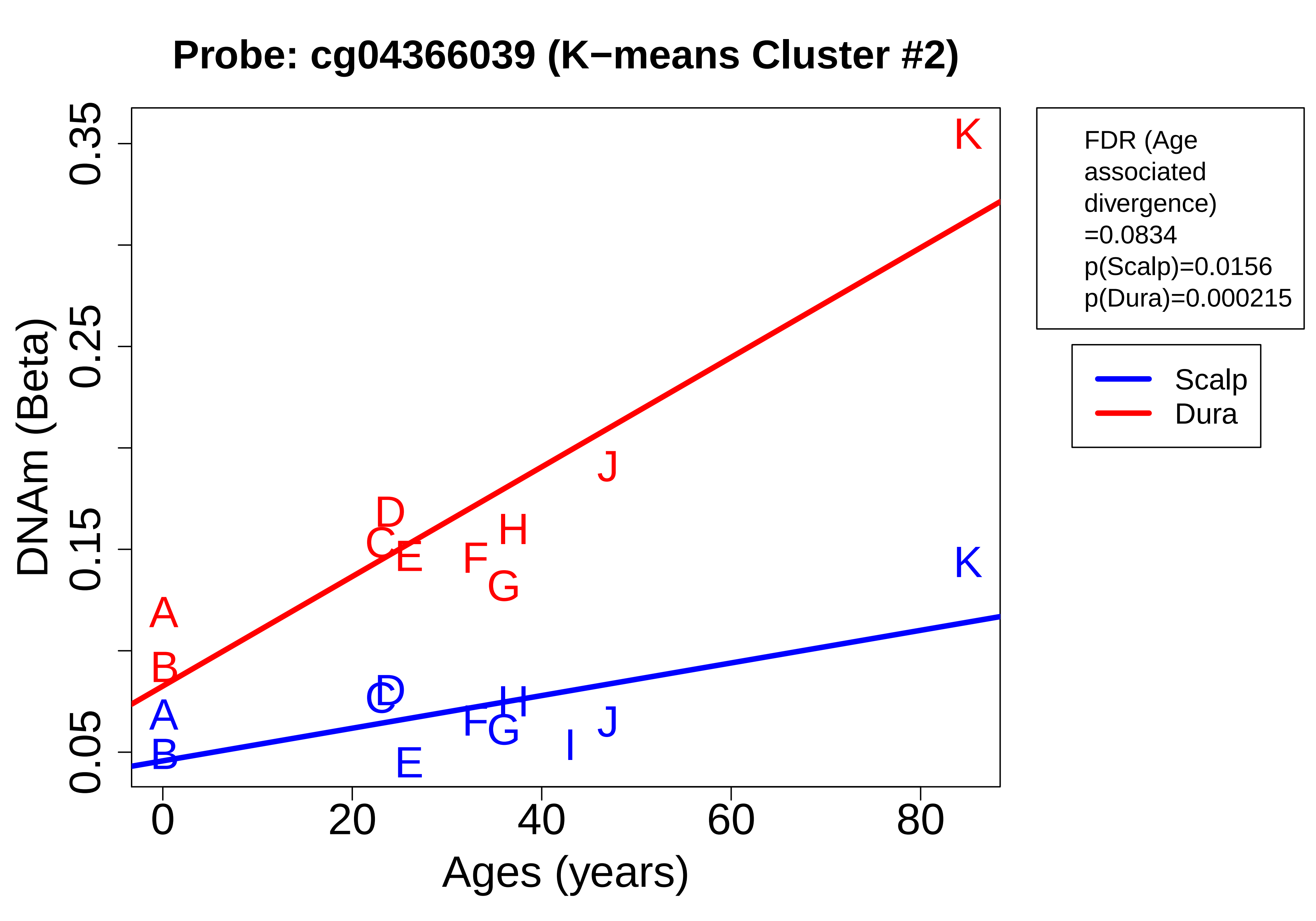

Probe: cg04366039 (K−means Cluster #2)
DNAm (Beta)
0.05
0.15
0.25
0.35
Ages (years)
0
20
40
60
80
FDR (Age associated divergence) =0.0834
p(Scalp)=0.0156
p(Dura)=0.000215
Scalp
Dura
A
B
C
D
E
F
G
H
I
J
K

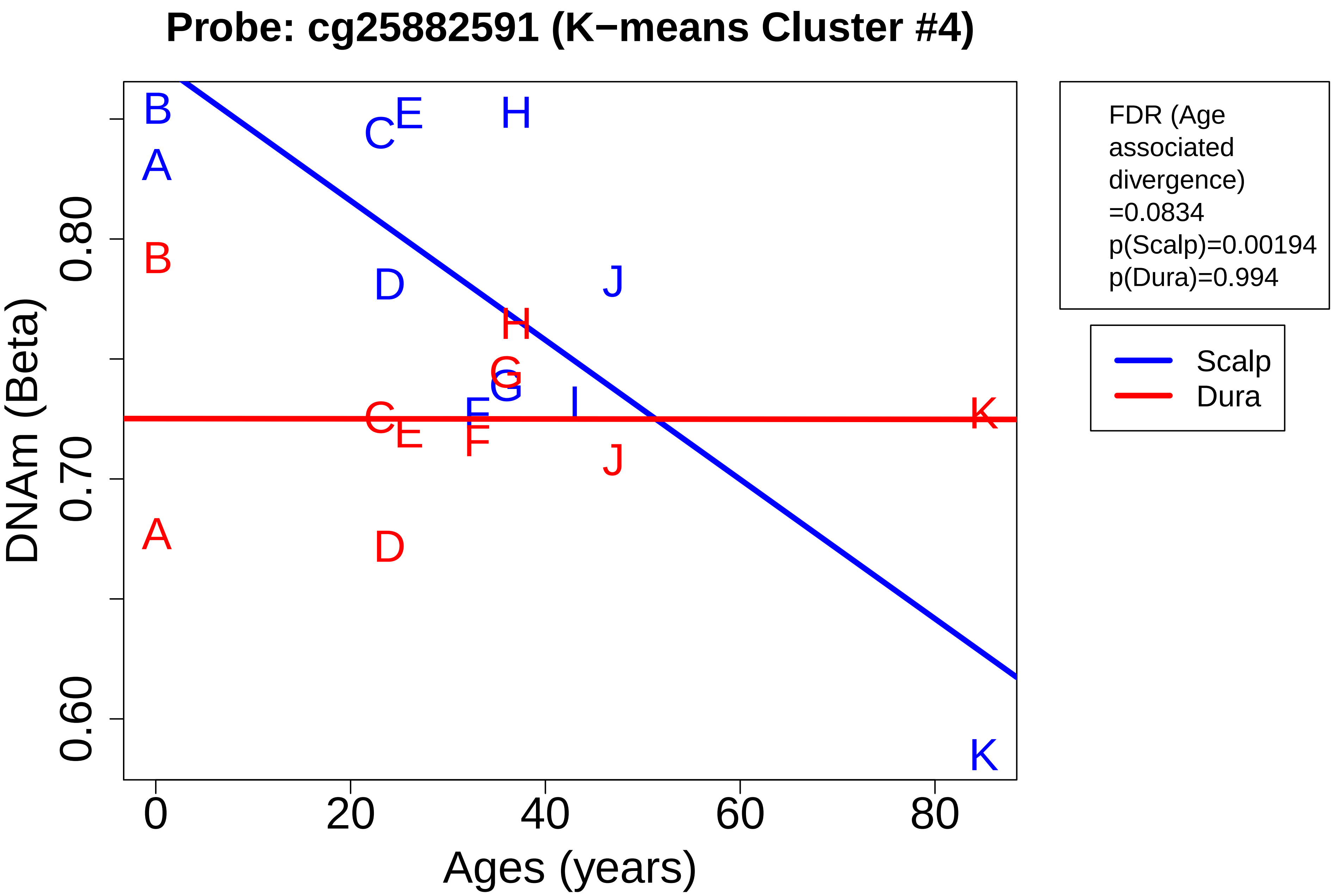

Probe: cg25882591 (K−means Cluster #4)
DNAm (Beta)
0.60
0.70
0.80
Ages (years)
0
20
40
60
80
FDR (Age associated divergence) =0.0834
p(Scalp)=0.00194
p(Dura)=0.994
Scalp
Dura

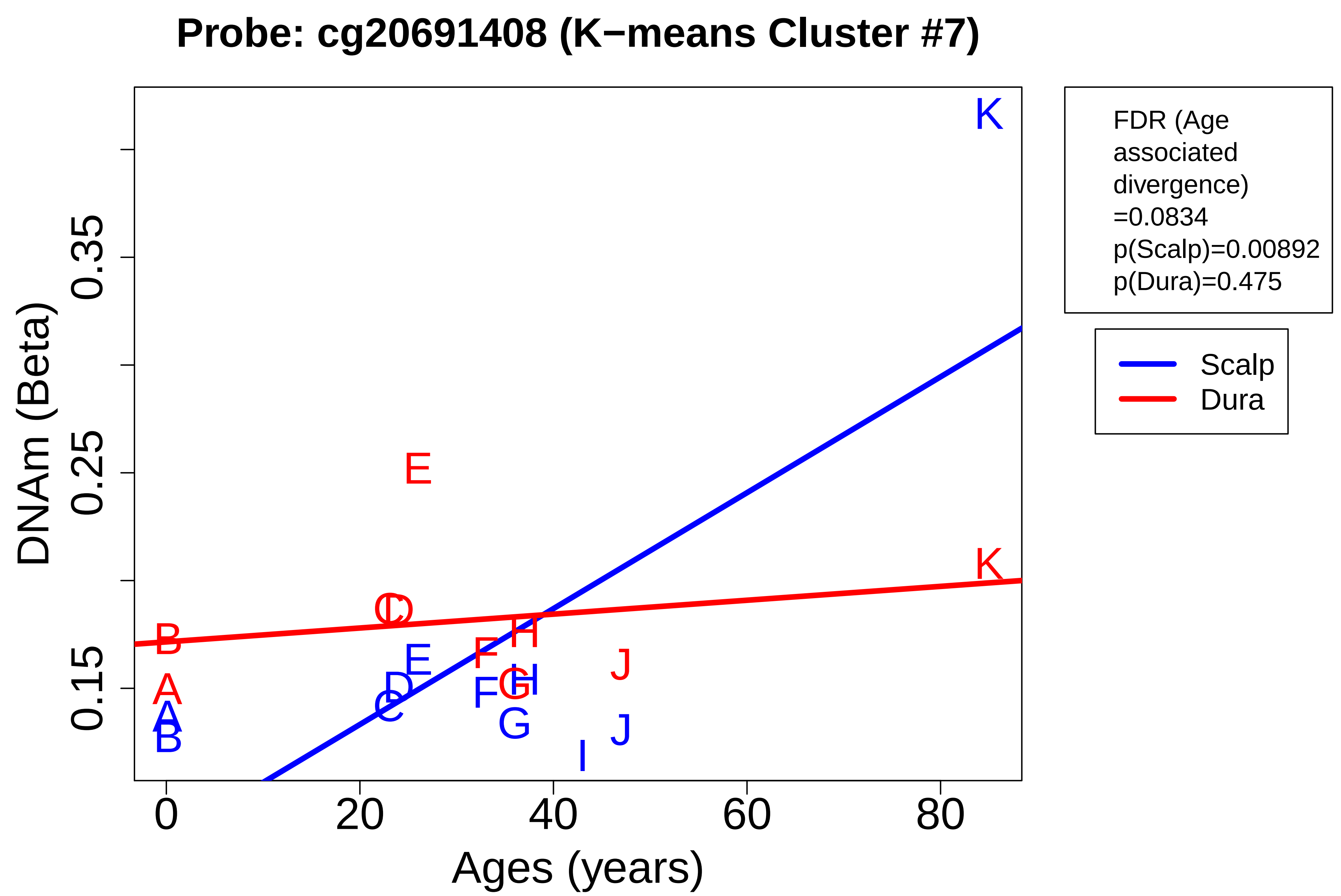

Probe: cg20691408 (K−means Cluster #7)
DNAm (Beta)
0.15
0.25
0.35
0
20
40
60
80
Ages (years)
FDR (Age associated divergence) =0.0834
p(Scalp)=0.00892
p(Dura)=0.475
Scalp
Dura

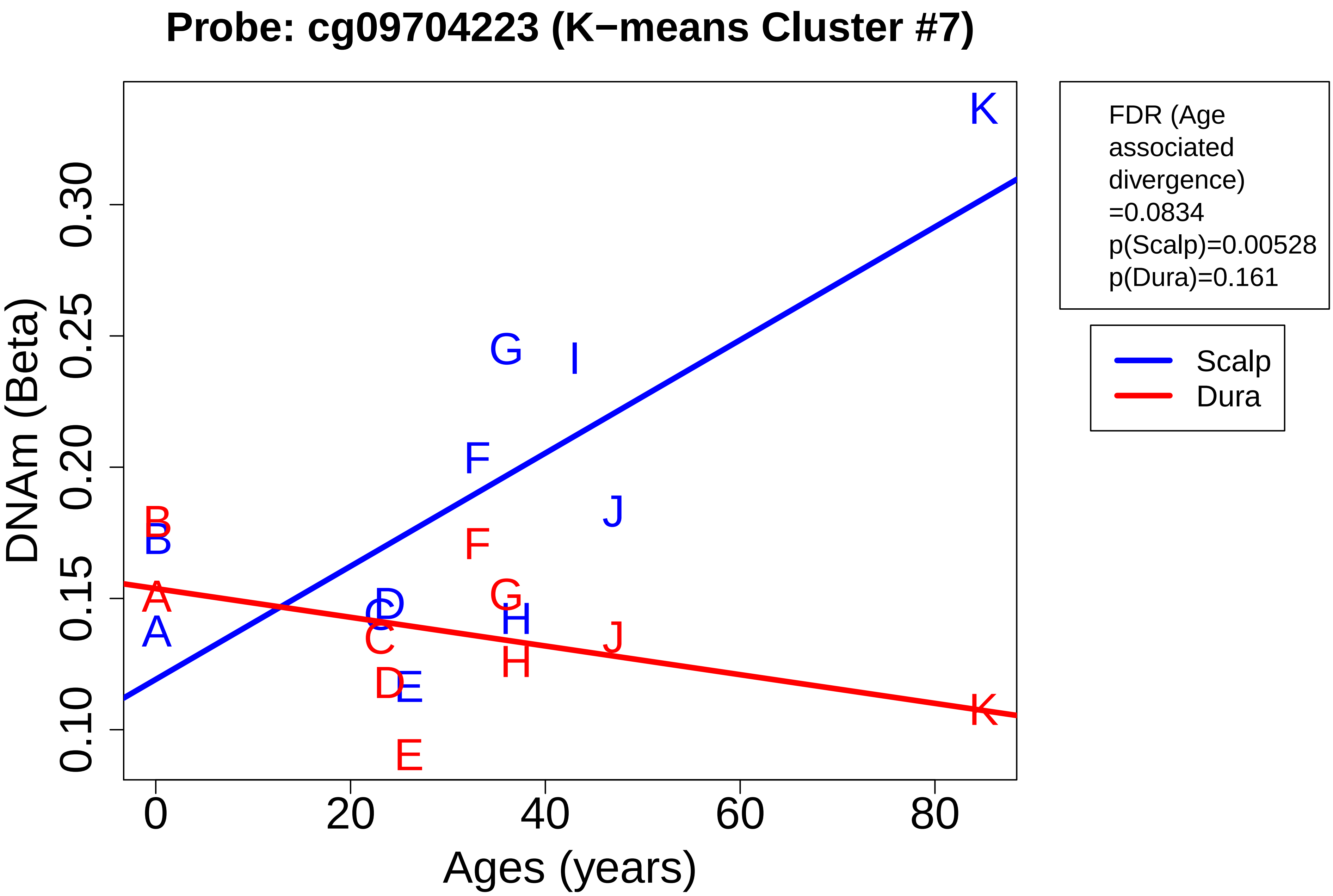
Probe: cg09704223 (K−means Cluster #7)
FDR (Age associated divergence) =0.0834
p(Scalp)=0.00528
p(Dura)=0.161
Scalp
Dura
DNAm (Beta)
0.10
0.15
0.20
0.25
0.30
0
20
40
60
80
Ages (years)
A
B
C
D
E
F
G
H
I
J
K

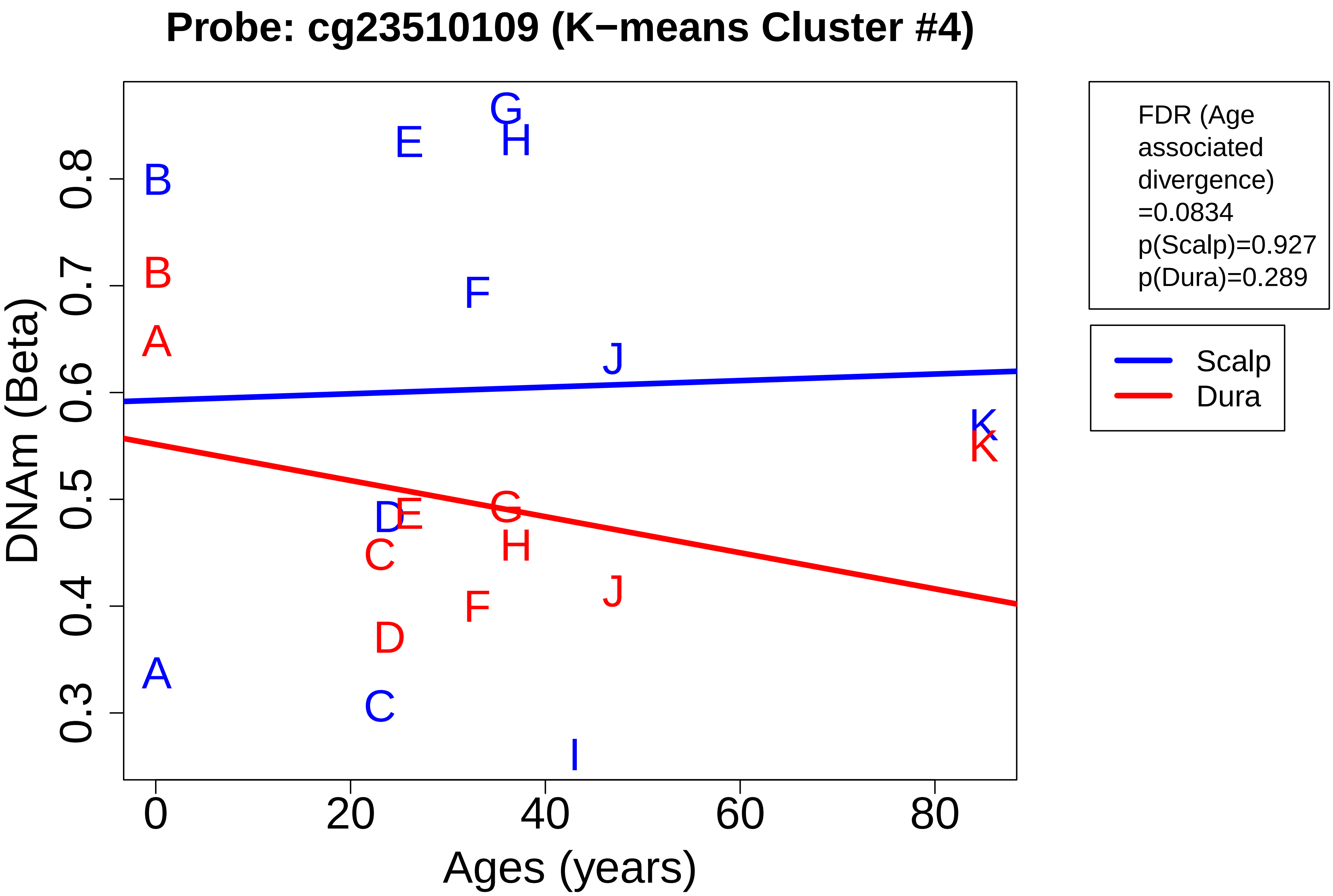
Probe: cg23510109 (K−means Cluster #4)
DNAm (Beta)
0.3
0.4
0.5
0.6
0.7
0.8
0
20
40
60
80
Ages (years)
FDR (Age associated divergence) =0.0834
p(Scalp)=0.927
p(Dura)=0.289
Scalp
Dura

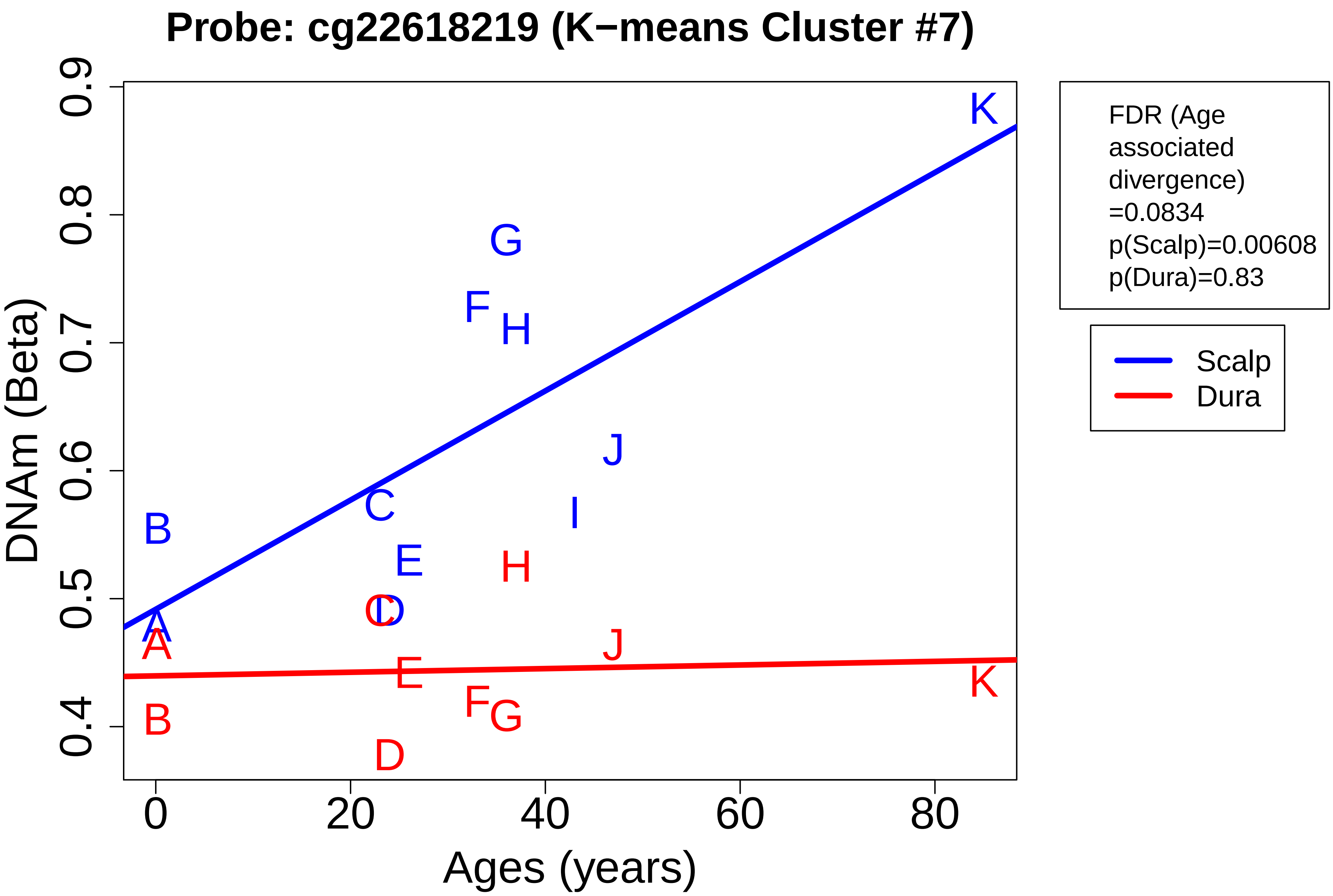

Probe: cg22618219 (K−means Cluster #7)
DNAm (Beta)
Ages (years)
0.4
0.5
0.6
0.7
0.8
0.9
0
20
40
60
80
FDR (Age associated divergence) =0.0834
p(Scalp)=0.00608
p(Dura)=0.83
Scalp
Dura

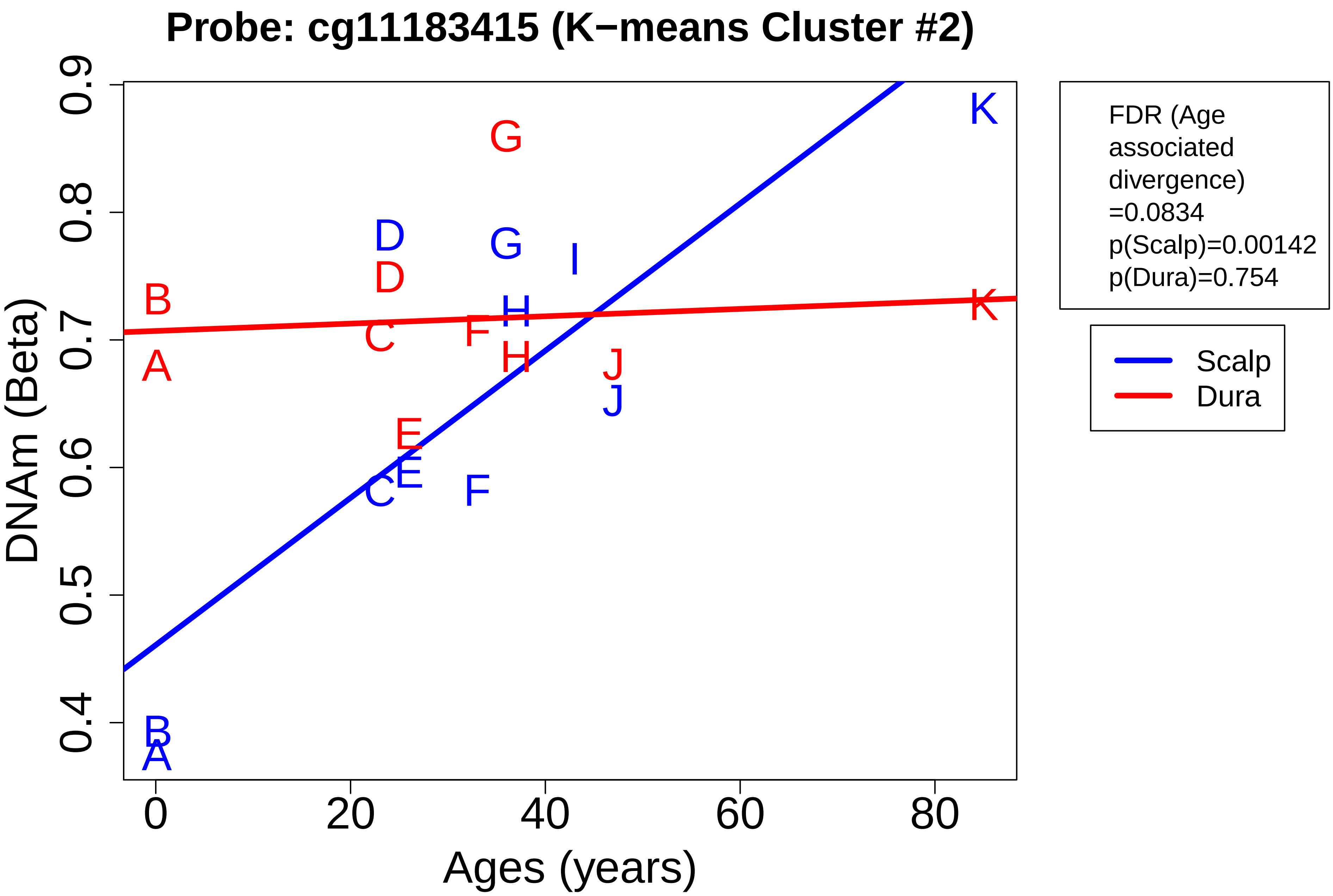
Probe: cg11183415 (K−means Cluster #2)
DNAm (Beta)
0.4
0.5
0.6
0.7
0.8
0.9
Ages (years)
0
20
40
60
80
FDR (Age associated divergence) =0.0834
p(Scalp)=0.00142
p(Dura)=0.754
Scalp
Dura

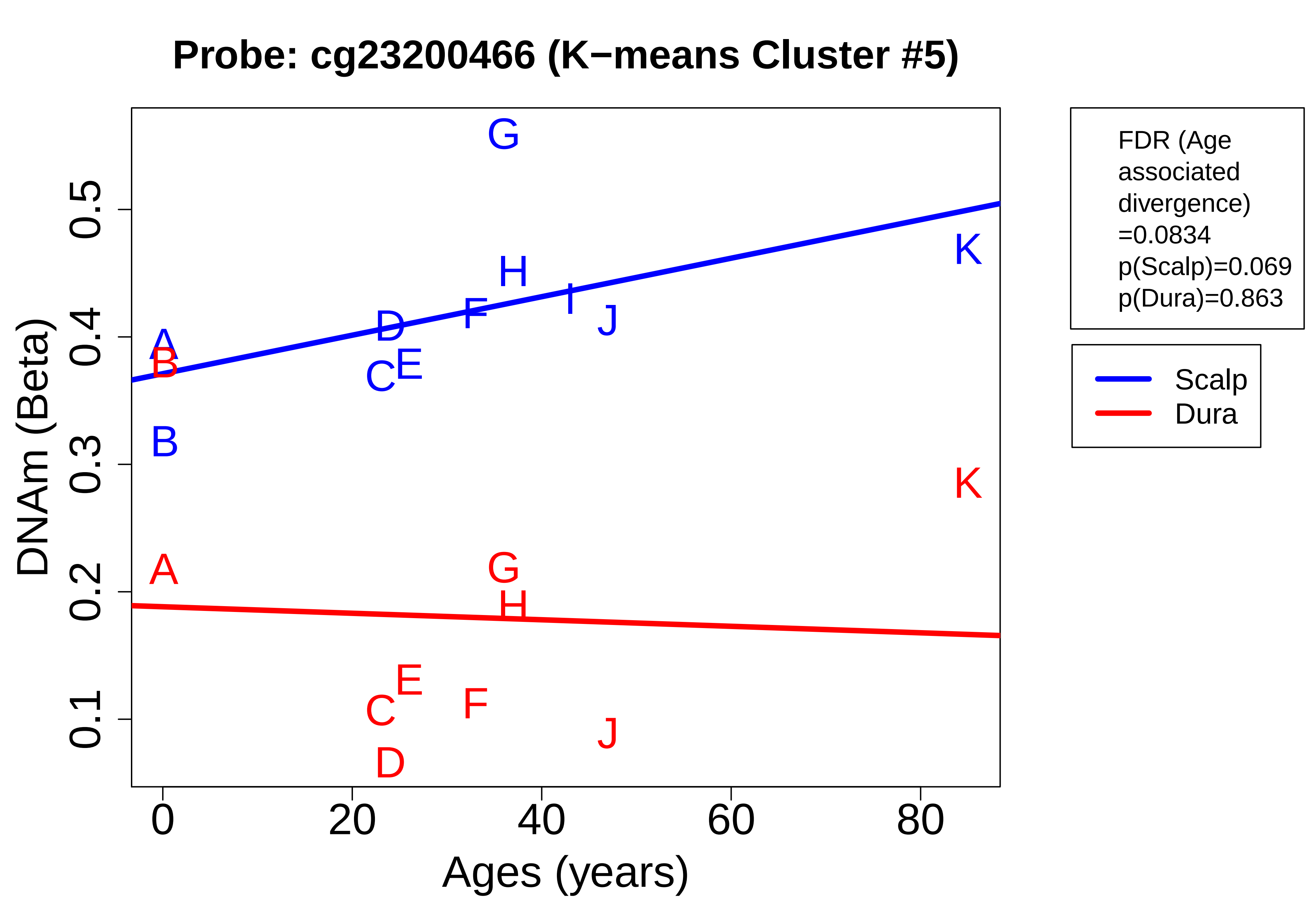

Probe: cg23200466 (K−means Cluster #5)
FDR (Age associated divergence) =0.0834
p(Scalp)=0.069
p(Dura)=0.863
Scalp
Dura
DNAm (Beta)
Ages (years)
0.1
0.2
0.3
0.4
0.5
0
20
40
60
80

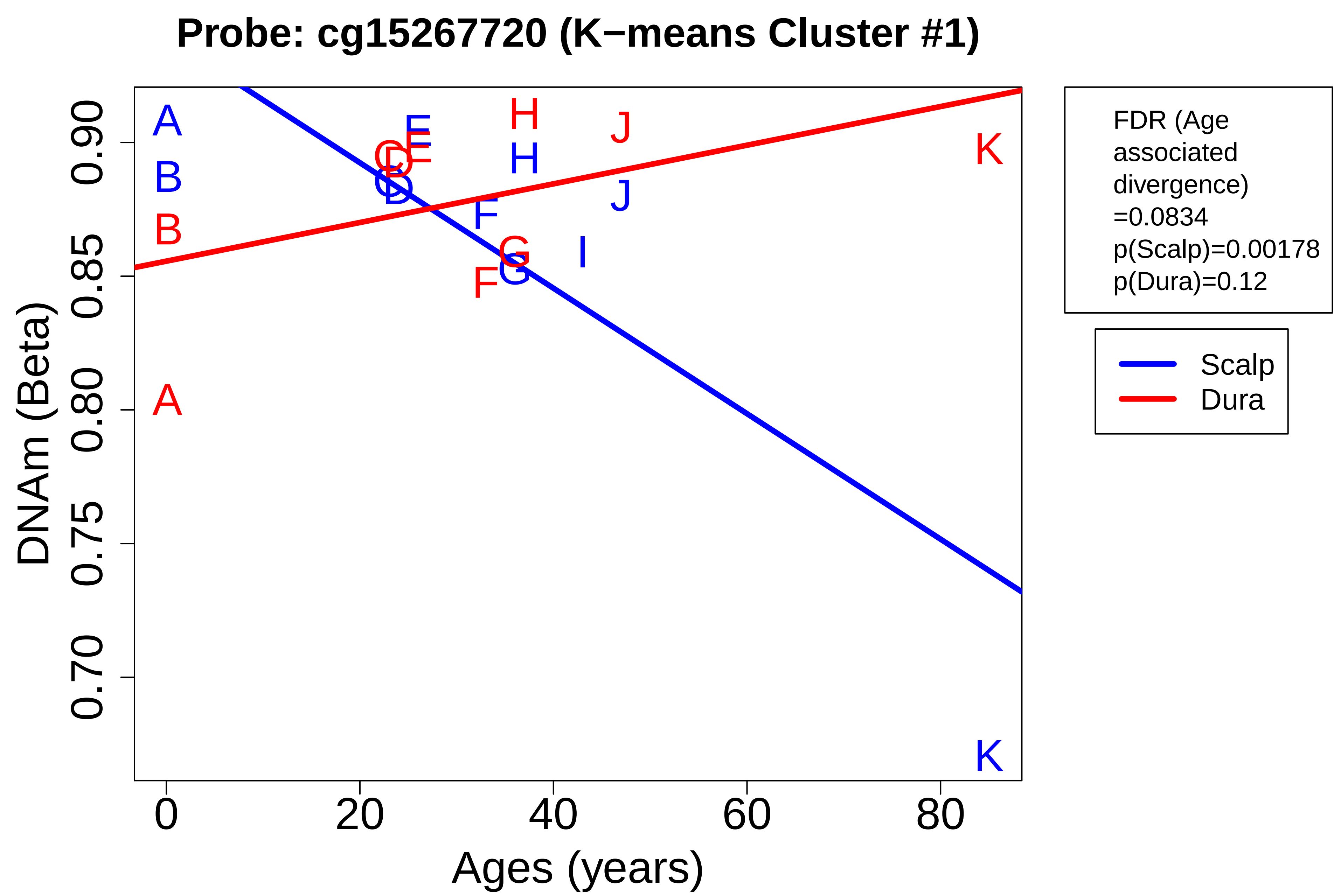

Probe: cg15267720 (K−means Cluster #1)
DNAm (Beta)
0.70
0.75
0.80
0.85
0.90
Ages (years)
0
20
40
60
80
A
B
C
D
E
F
G
H
I
J
K
FDR (Age associated divergence) =0.0834
p(Scalp)=0.00178
p(Dura)=0.12
Scalp
Dura

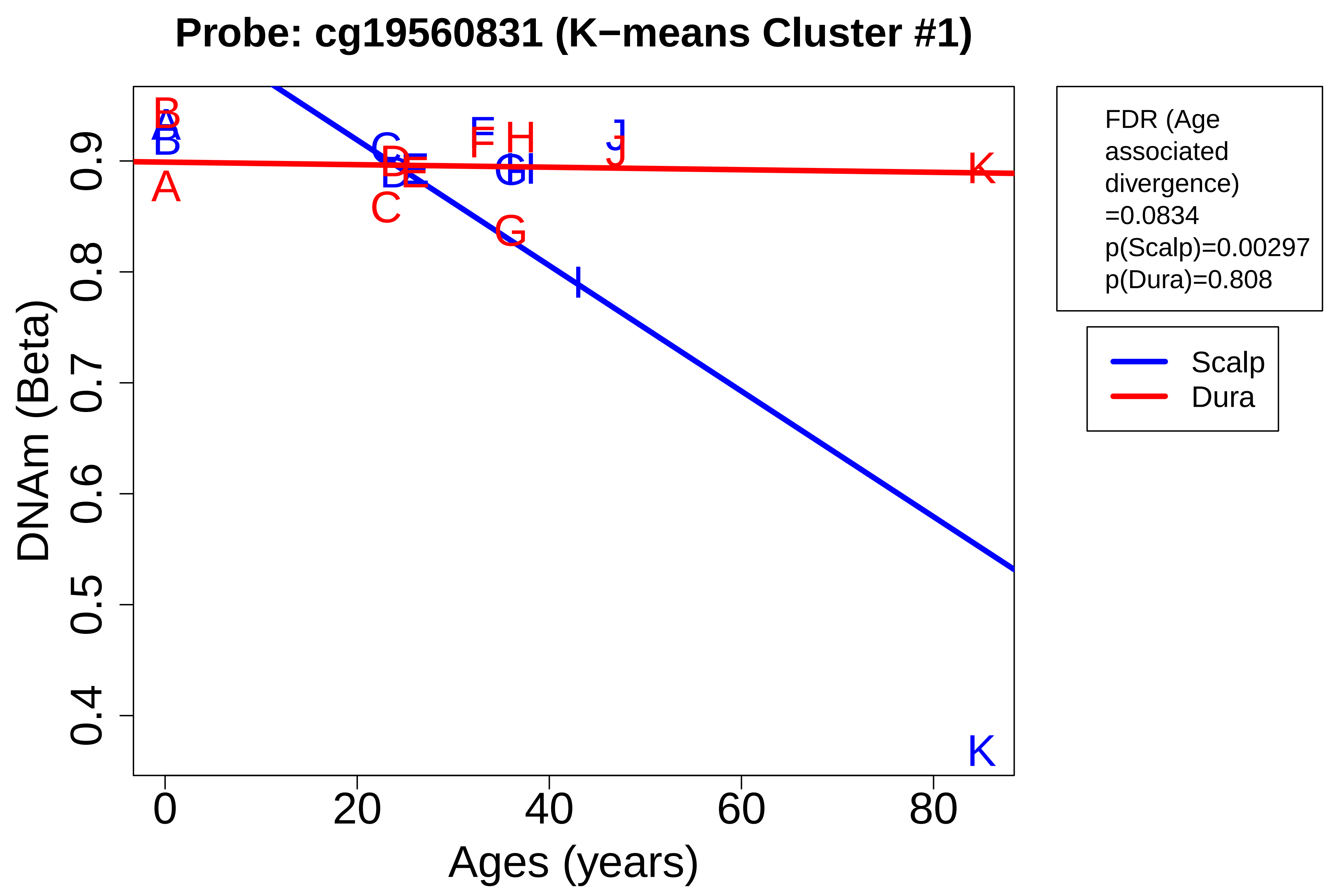

Probe: cg19560831 (K−means Cluster #1)
DNAm (Beta)
Ages (years)
0.4
0.5
0.6
0.7
0.8
0.9
0
20
40
60
80
FDR (Age associated divergence) =0.0834
p(Scalp)=0.00297
p(Dura)=0.808
Scalp
Dura

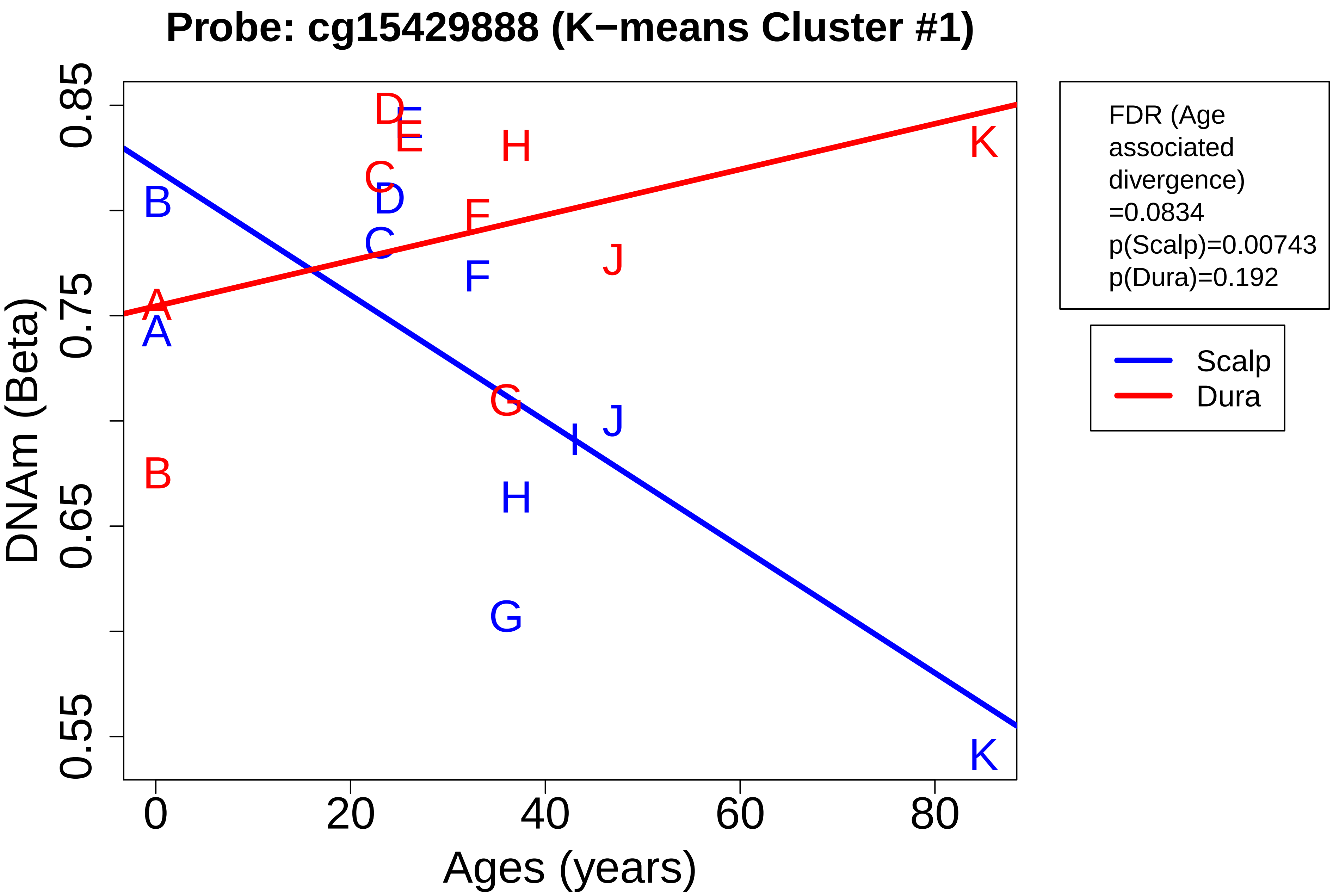

Probe: cg15429888 (K−means Cluster #1)
DNAm (Beta)
0.55
0.65
0.75
0.85
0
20
40
60
80
Ages (years)
FDR (Age associated divergence) =0.0834
p(Scalp)=0.00743
p(Dura)=0.192
Scalp
Dura

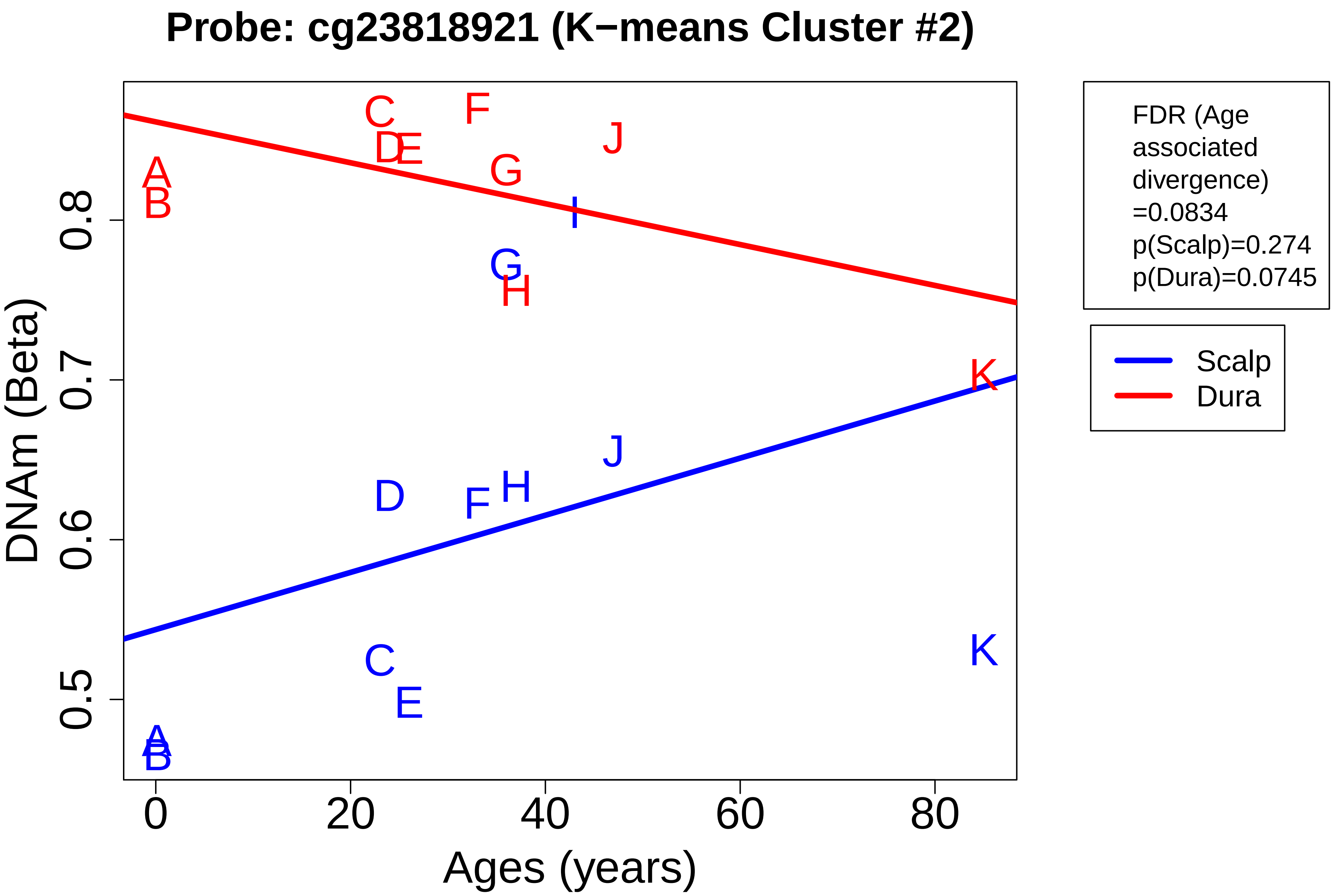

Probe: cg23818921 (K−means Cluster #2)
DNAm (Beta)
0.5
0.6
0.7
0.8
0
20
40
60
80
Ages (years)
FDR (Age associated divergence) =0.0834
p(Scalp)=0.274
p(Dura)=0.0745
Scalp
Dura

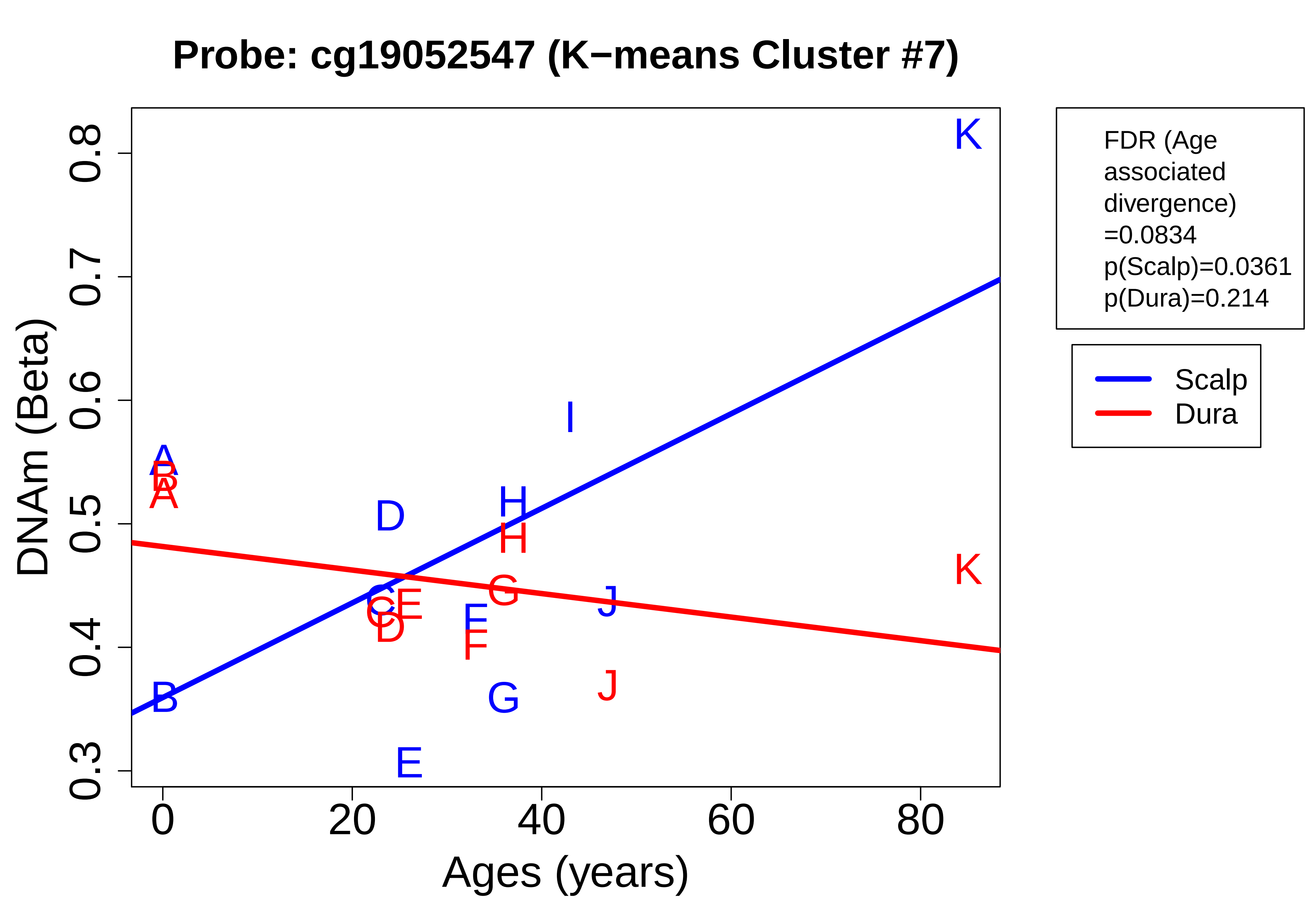
Probe: cg19052547 (K−means Cluster #7)
DNAm (Beta)
0.3
0.4
0.5
0.6
0.7
0.8
Ages (years)
0
20
40
60
80
FDR (Age associated divergence) =0.0834
p(Scalp)=0.0361
p(Dura)=0.214
Scalp
Dura

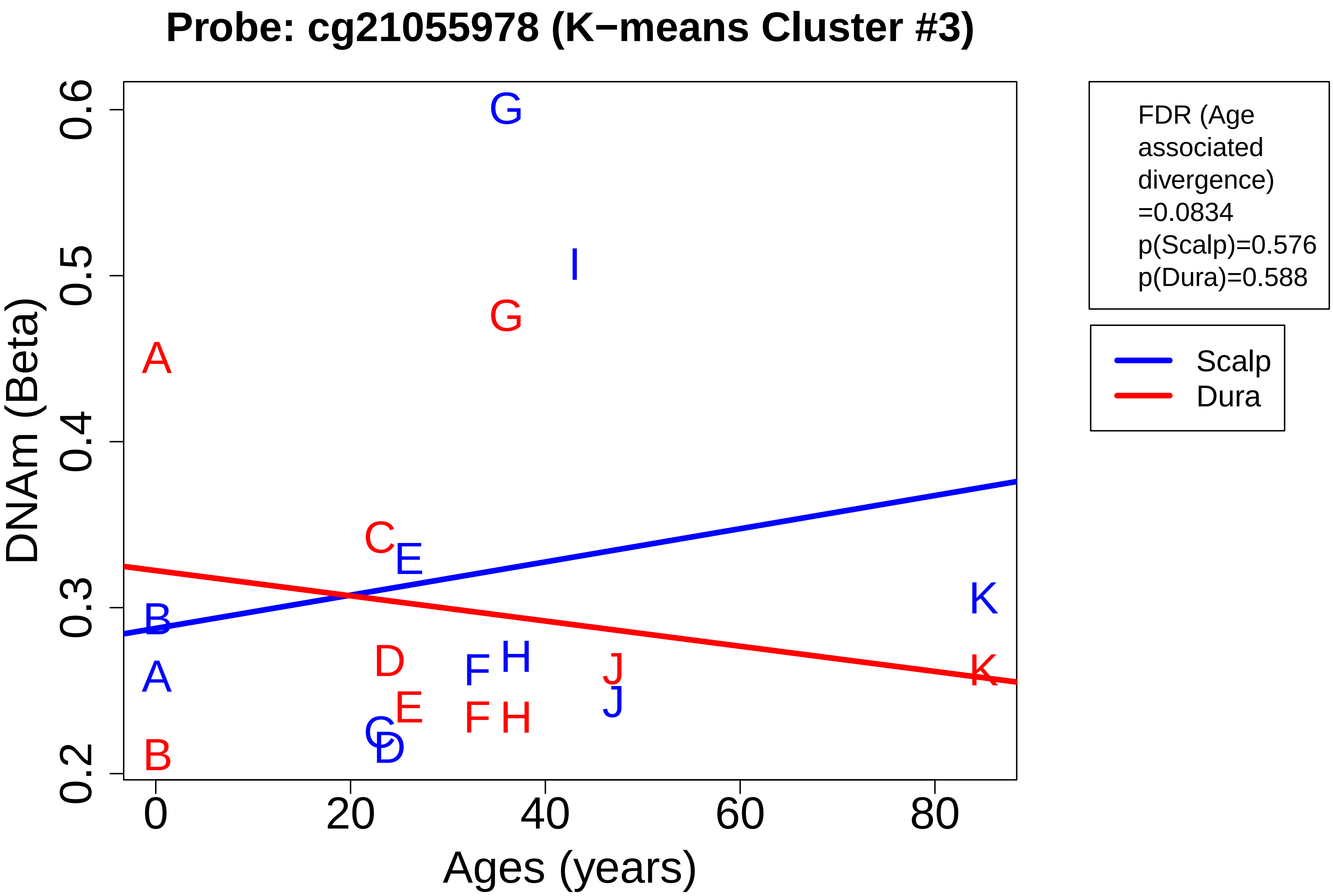
Probe: cg21055978 (K−means Cluster #3)
DNAm (Beta)
Ages (years)
0.2
0.3
0.4
0.5
0.6
0
20
40
60
80
FDR (Age associated divergence) =0.0834
p(Scalp)=0.576
p(Dura)=0.588
Scalp
Dura

Probe: cg15161251 (K−means Cluster #1)

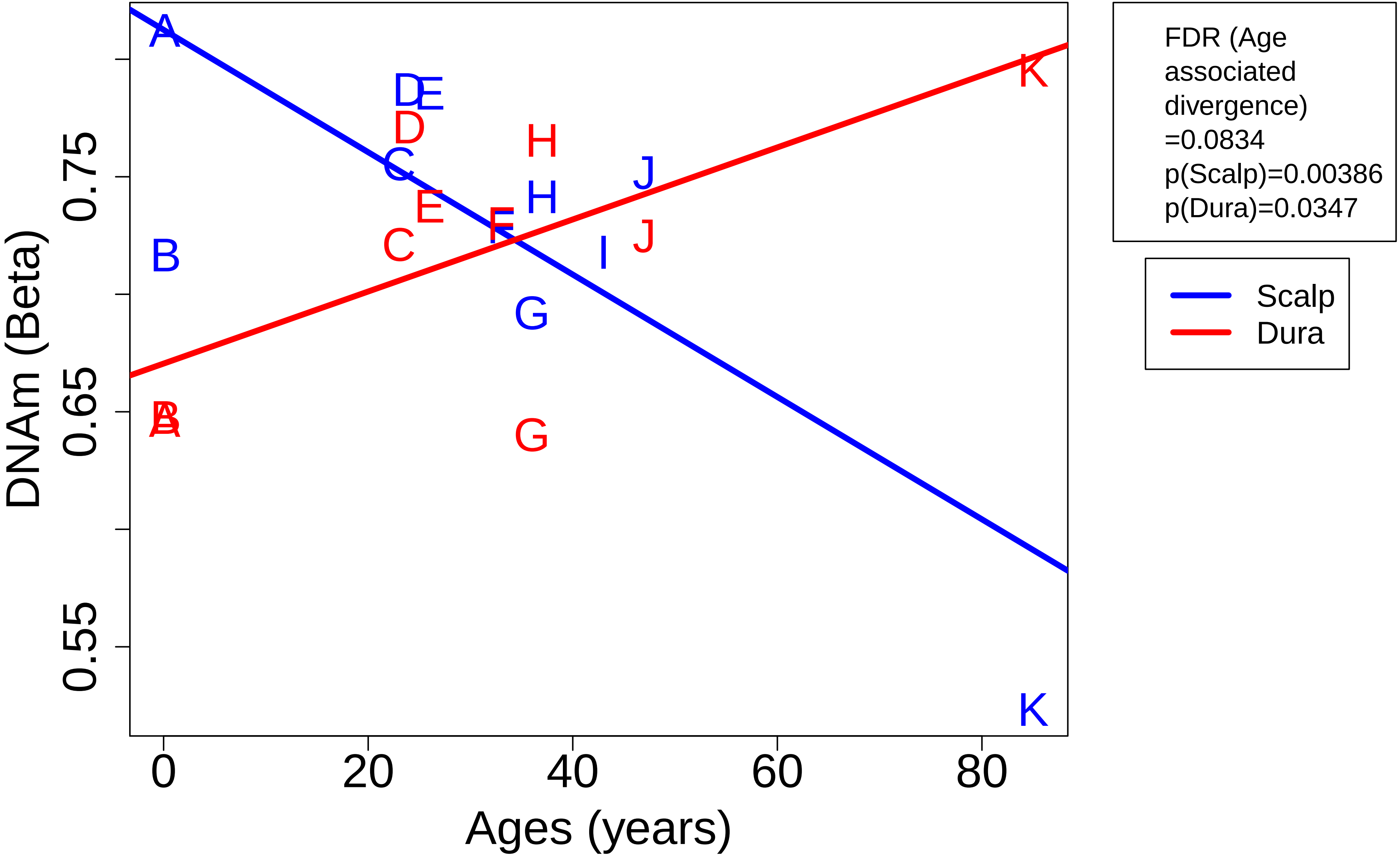

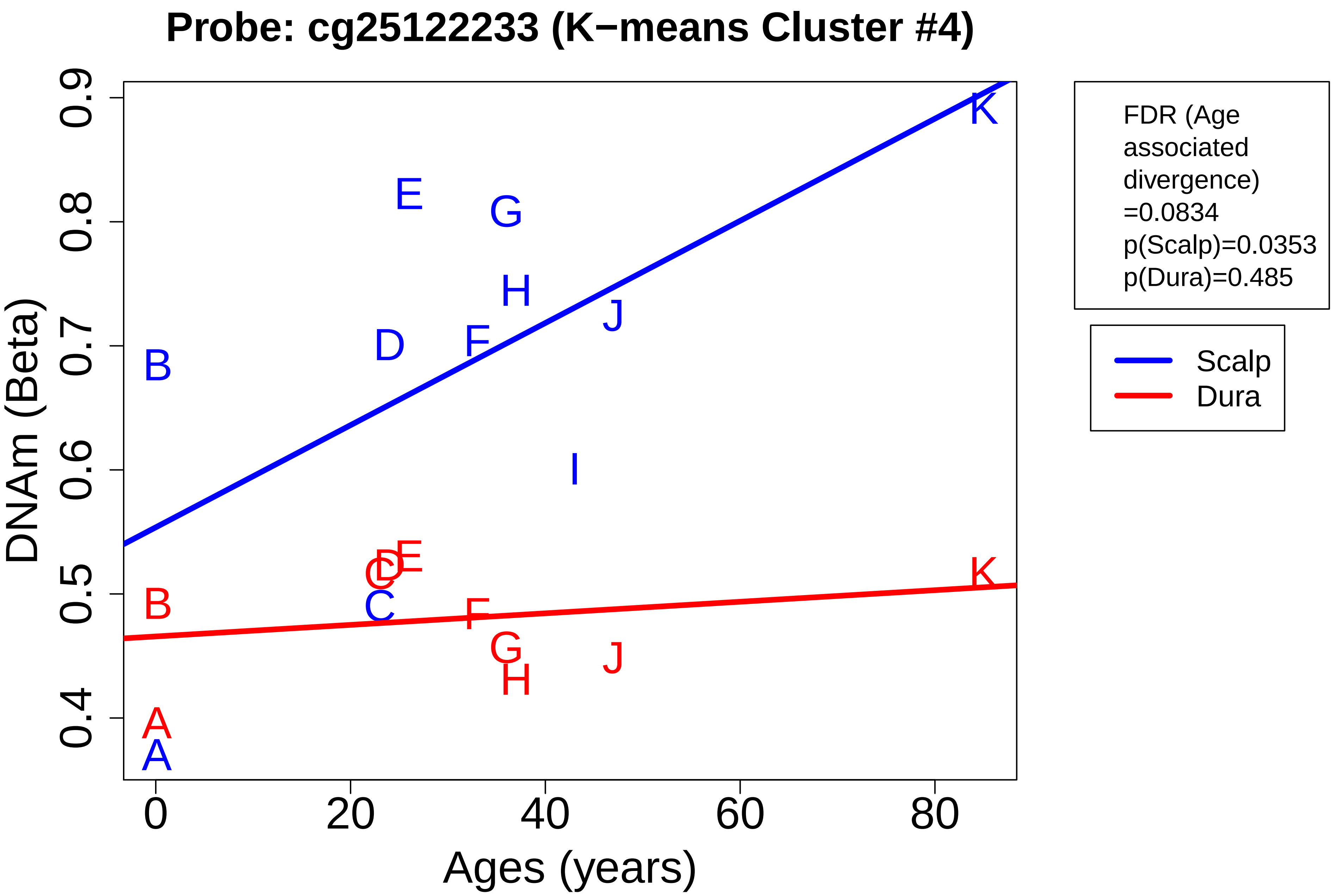

Probe: cg25122233 (K−means Cluster #4)
DNAm (Beta)
0.4
0.5
0.6
0.7
0.8
0.9
Ages (years)
0
20
40
60
80
A B C D E F G H I J K
FDR (Age associated divergence) =0.0834
p(Scalp)=0.0353
p(Dura)=0.485
Scalp
Dura

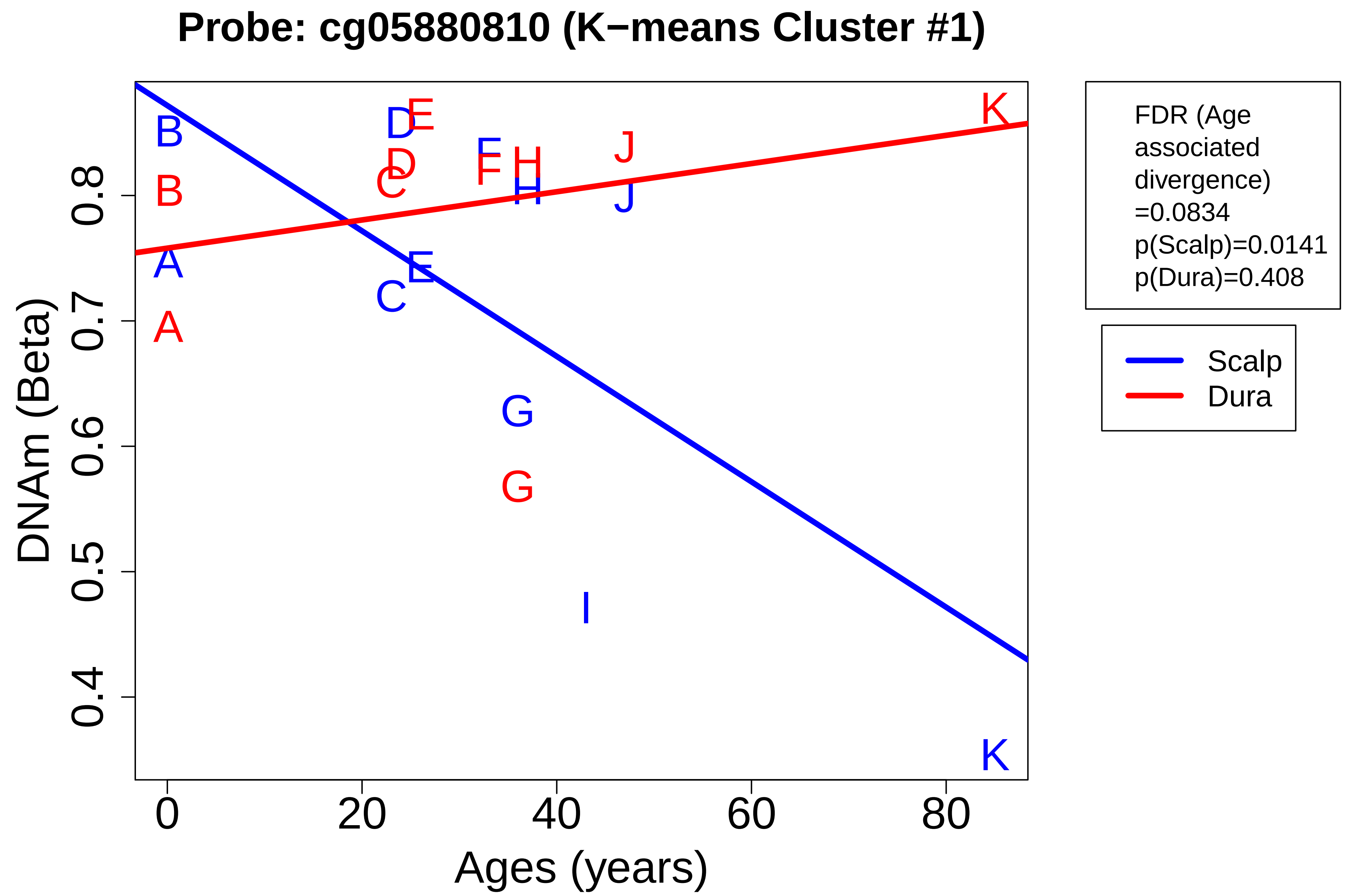

Probe: cg05880810 (K−means Cluster #1)
FDR (Age associated divergence) =0.0834
p(Scalp)=0.0141
p(Dura)=0.408
Scalp
Dura
DNAm (Beta)
0.4
0.5
0.6
0.7
0.8
Ages (years)
0
20
40
60
80
A
B
C
D
E
F
G
H
I
J
K

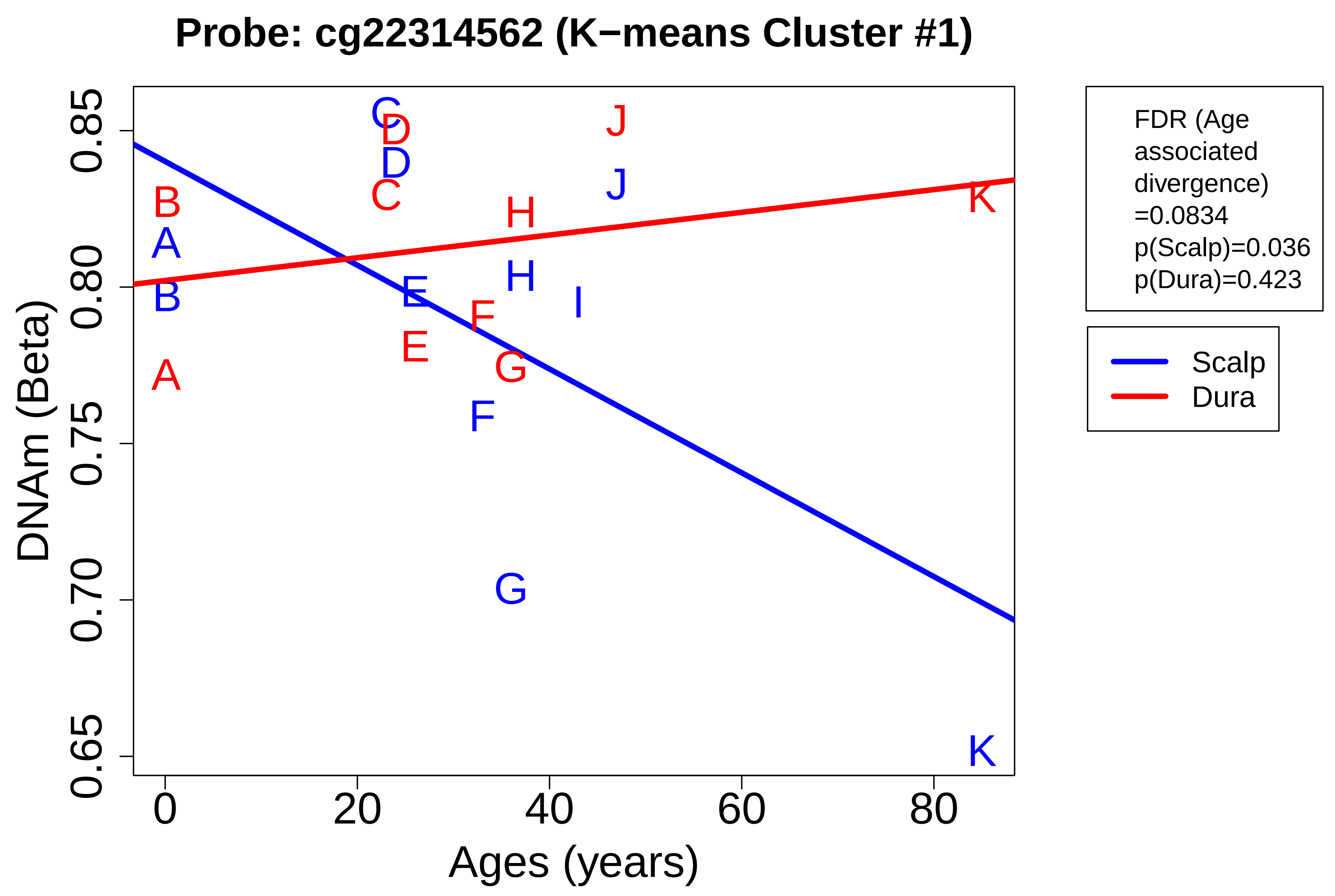

Probe: cg22314562 (K−means Cluster #1)
DNAm (Beta)
0.65
0.70
0.75
0.80
0.85
Ages (years)
0
20
40
60
80
C
D
D
C
J
J
B
A
B
A
H
H
E
E
F
F
G
G
I
K
K
FDR (Age associated divergence) =0.0834
p(Scalp)=0.036
p(Dura)=0.423
Scalp
Dura

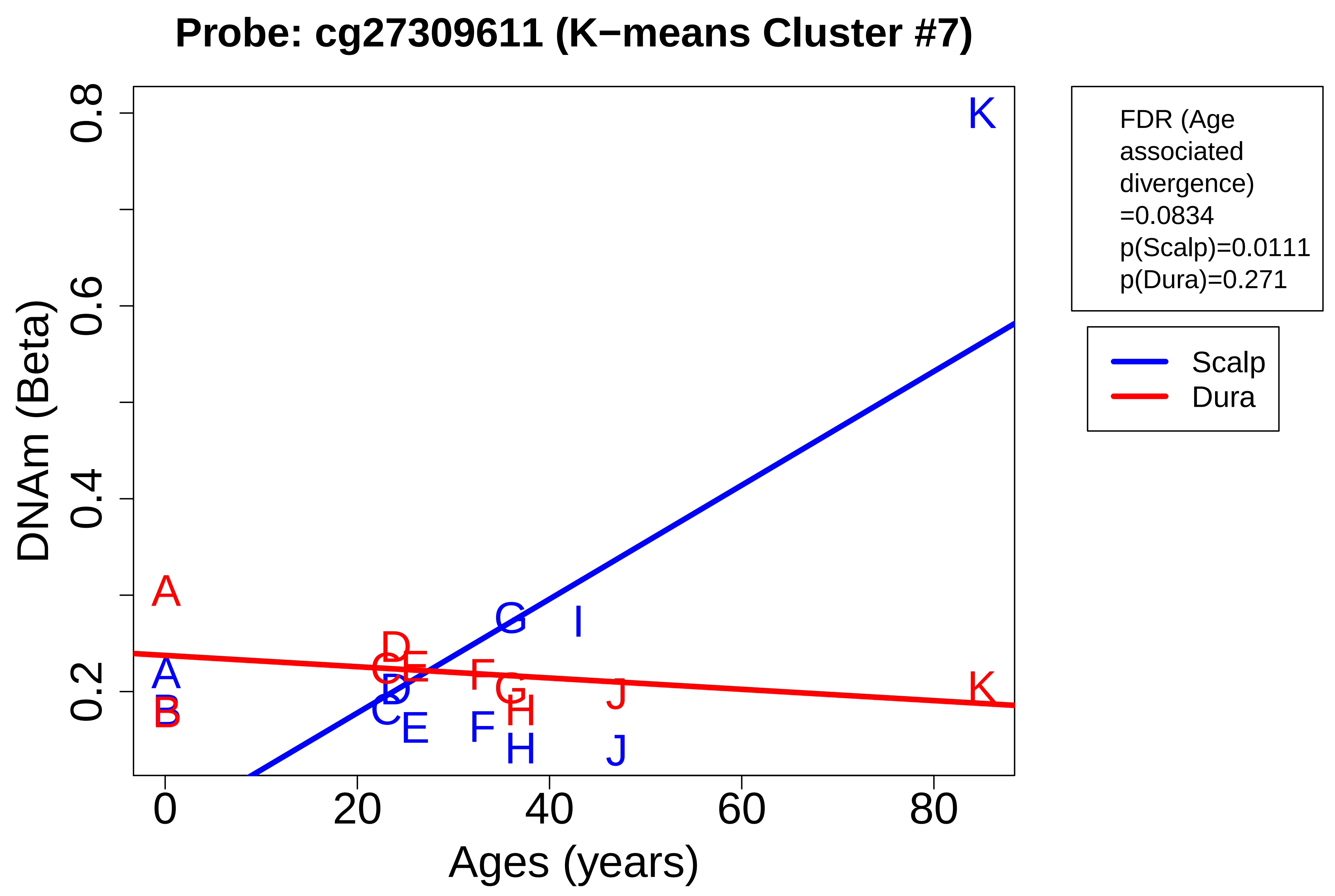

Probe: cg27309611 (K−means Cluster #7)
DNAm (Beta)
0.2
0.4
0.6
0.8
Ages (years)
0
20
40
60
80
FDR (Age associated divergence) =0.0834
p(Scalp)=0.0111
p(Dura)=0.271
Scalp
Dura

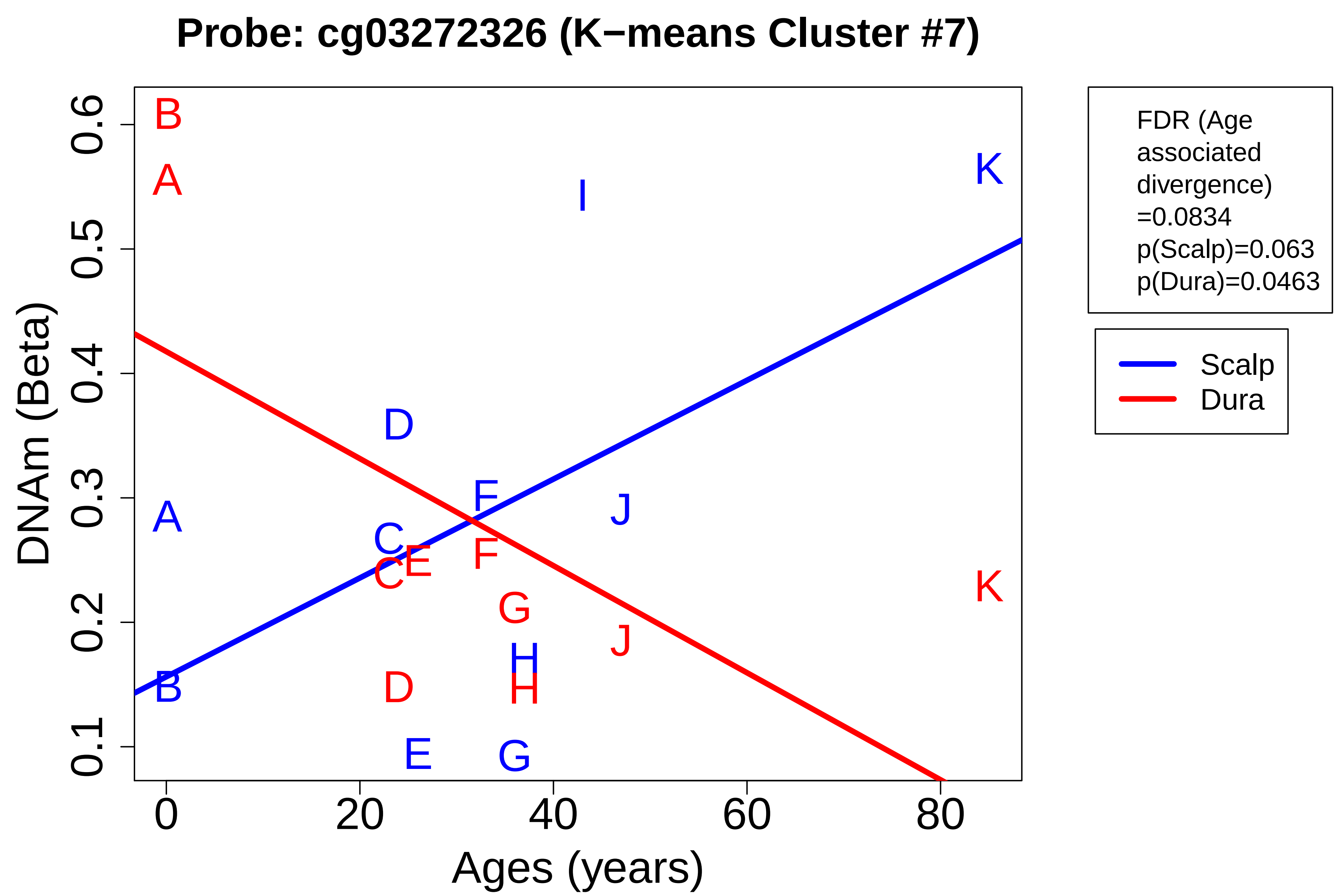

Probe: cg03272326 (K−means Cluster #7)
DNAm (Beta)
0.1
0.2
0.3
0.4
0.5
0.6
0
20
40
60
80
Ages (years)
B
A
K
I
D
F
J
A
C
E
C
F
K
G
J
H
B
D
H
E
G
FDR (Age associated divergence) =0.0834
p(Scalp)=0.063
p(Dura)=0.0463
Scalp
Dura

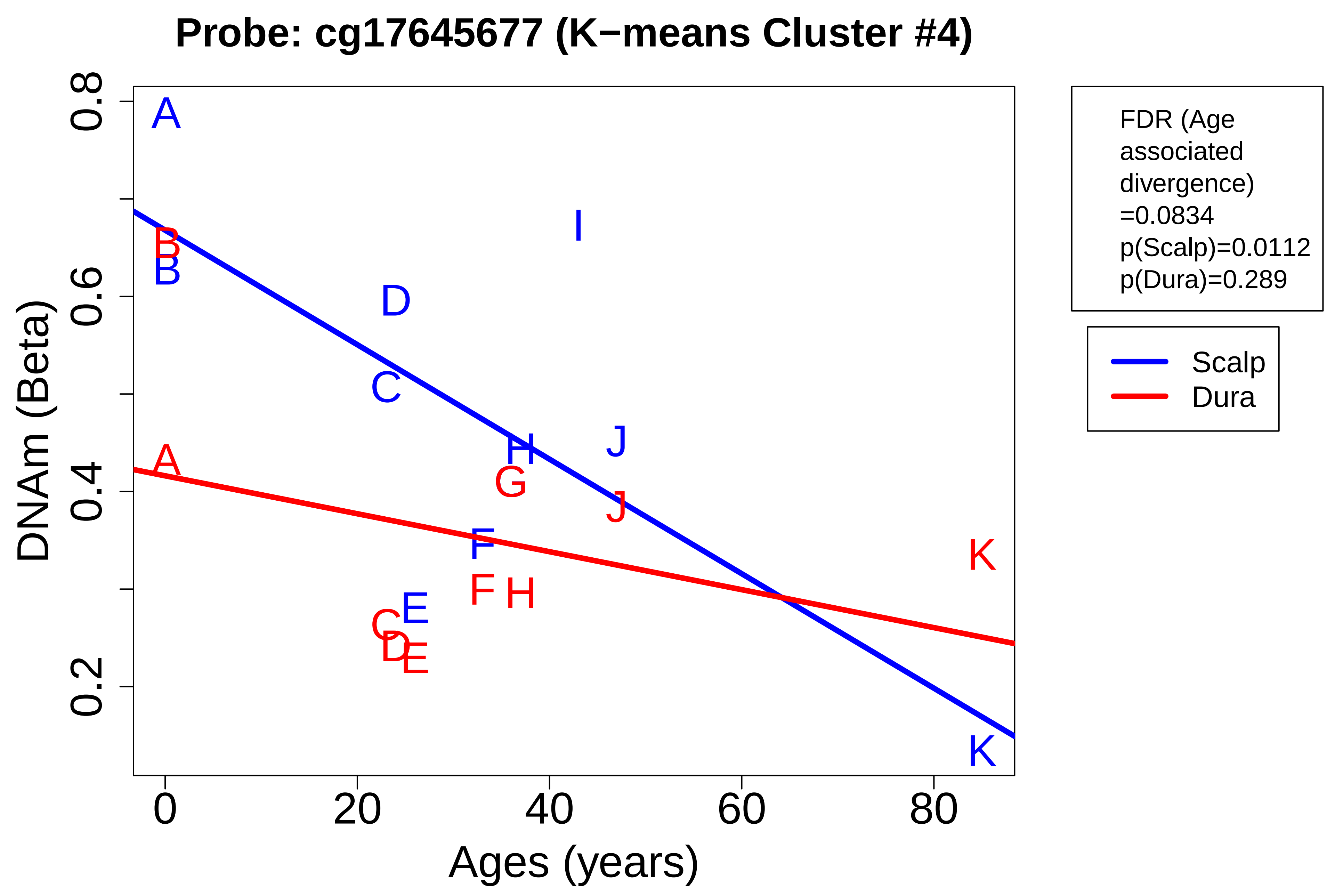

Probe: cg17645677 (K−means Cluster #4)
DNAm (Beta)
0.2
0.4
0.6
0.8
0
20
40
60
80
Ages (years)
FDR (Age associated divergence) =0.0834
p(Scalp)=0.0112
p(Dura)=0.289
Scalp
Dura

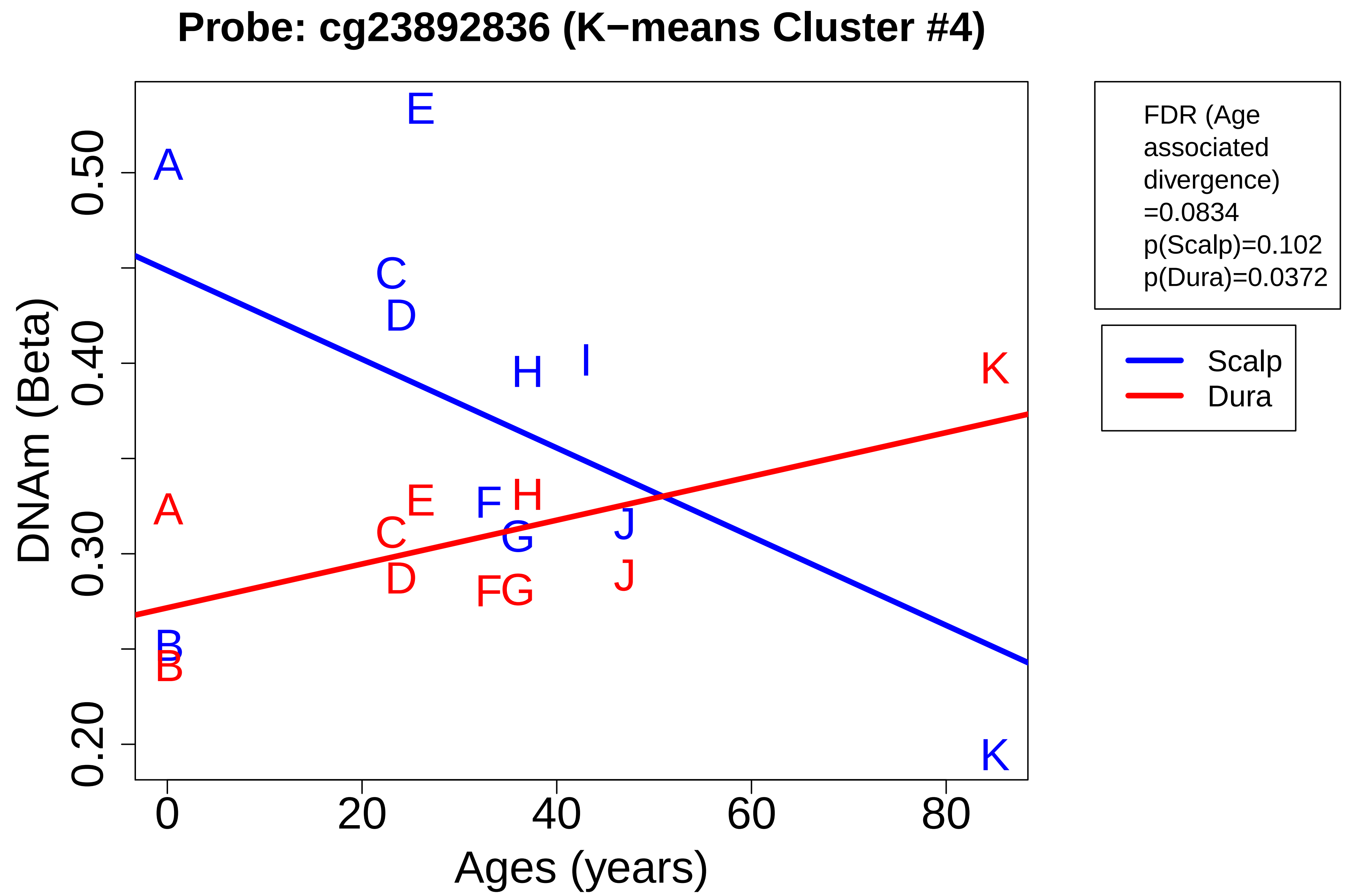

Probe: cg23892836 (K−means Cluster #4)
DNAm (Beta)
0.20
0.30
0.40
0.50
0
20
40
60
80
Ages (years)
A
B
C
D
E
F
G
H
I
J
K
FDR (Age associated divergence) =0.0834
p(Scalp)=0.102
p(Dura)=0.0372
Scalp
Dura

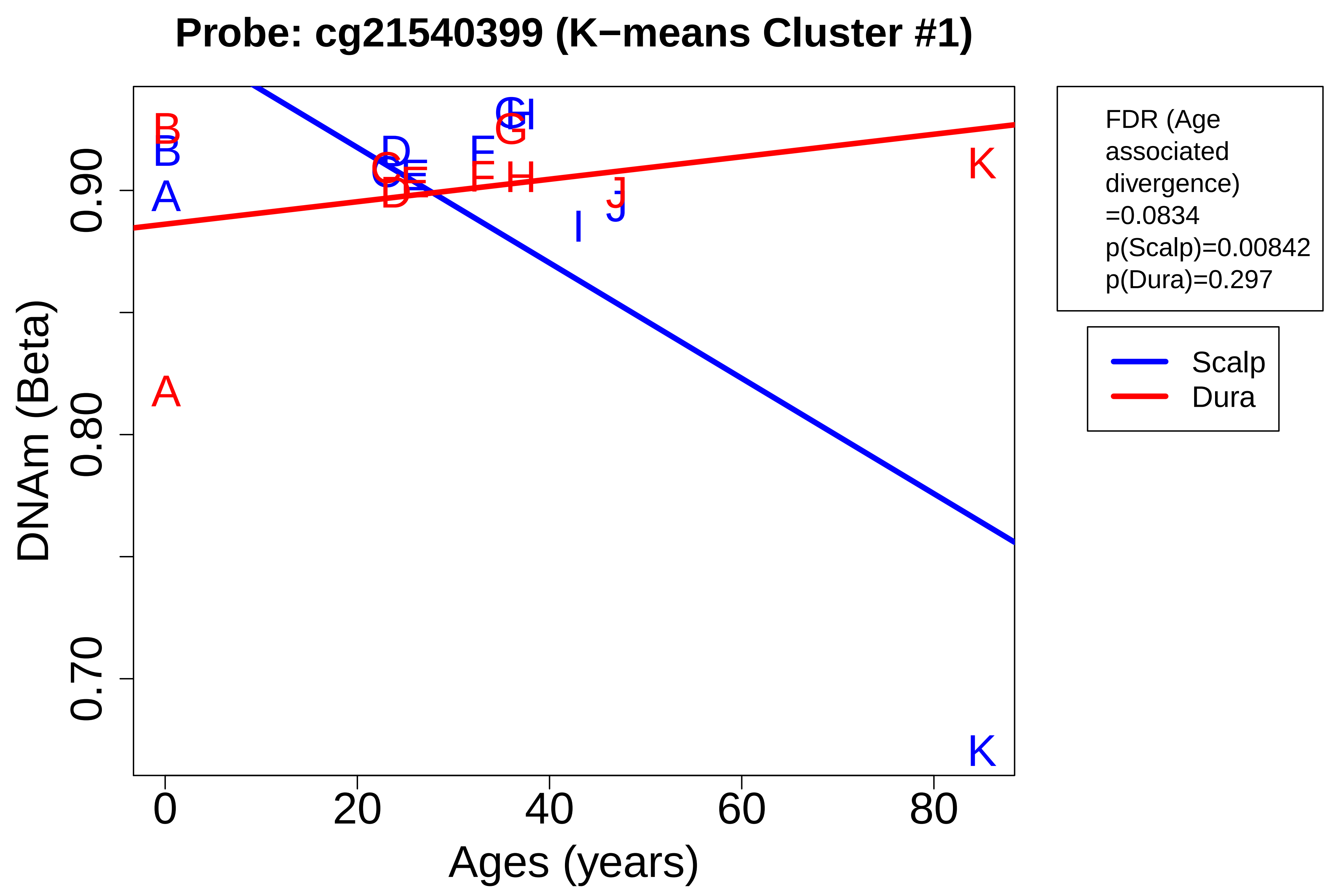

Probe: cg21540399 (K−means Cluster #1)
DNAm (Beta)
0.70
0.80
0.90
Ages (years)
0
20
40
60
80
A
B
C
D
E
F
G
H
I
J
K
FDR (Age associated divergence) =0.0834
p(Scalp)=0.00842
p(Dura)=0.297
Scalp
Dura

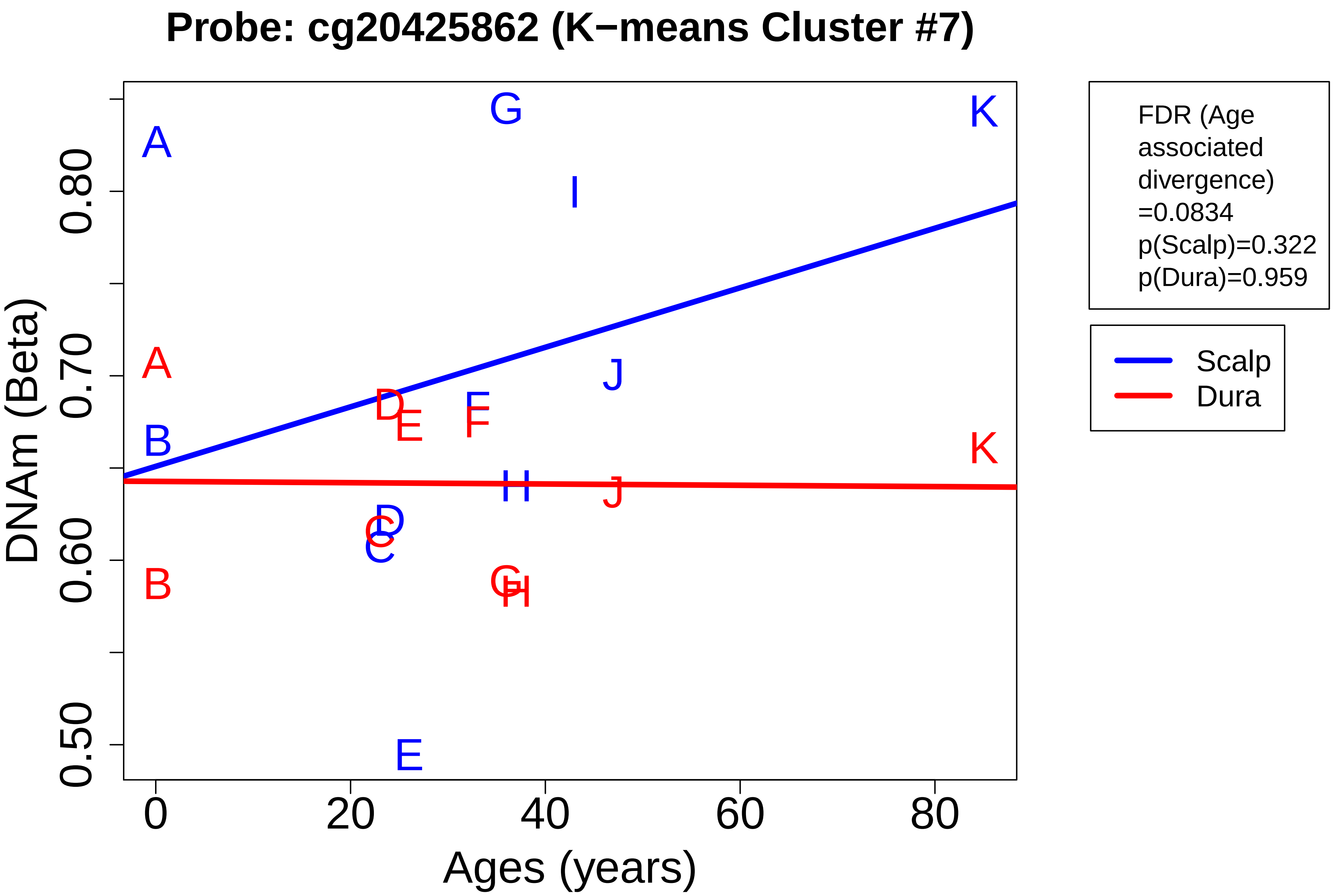

Probe: cg20425862 (K−means Cluster #7)
DNAm (Beta)
0.50
0.60
0.70
0.80
Ages (years)
0
20
40
60
80
A
B
C
D
E
F
G
H
I
J
K
A
B
C
D
E
F
G
H
J
K
FDR (Age associated divergence) =0.0834
p(Scalp)=0.322
p(Dura)=0.959
Scalp
Dura

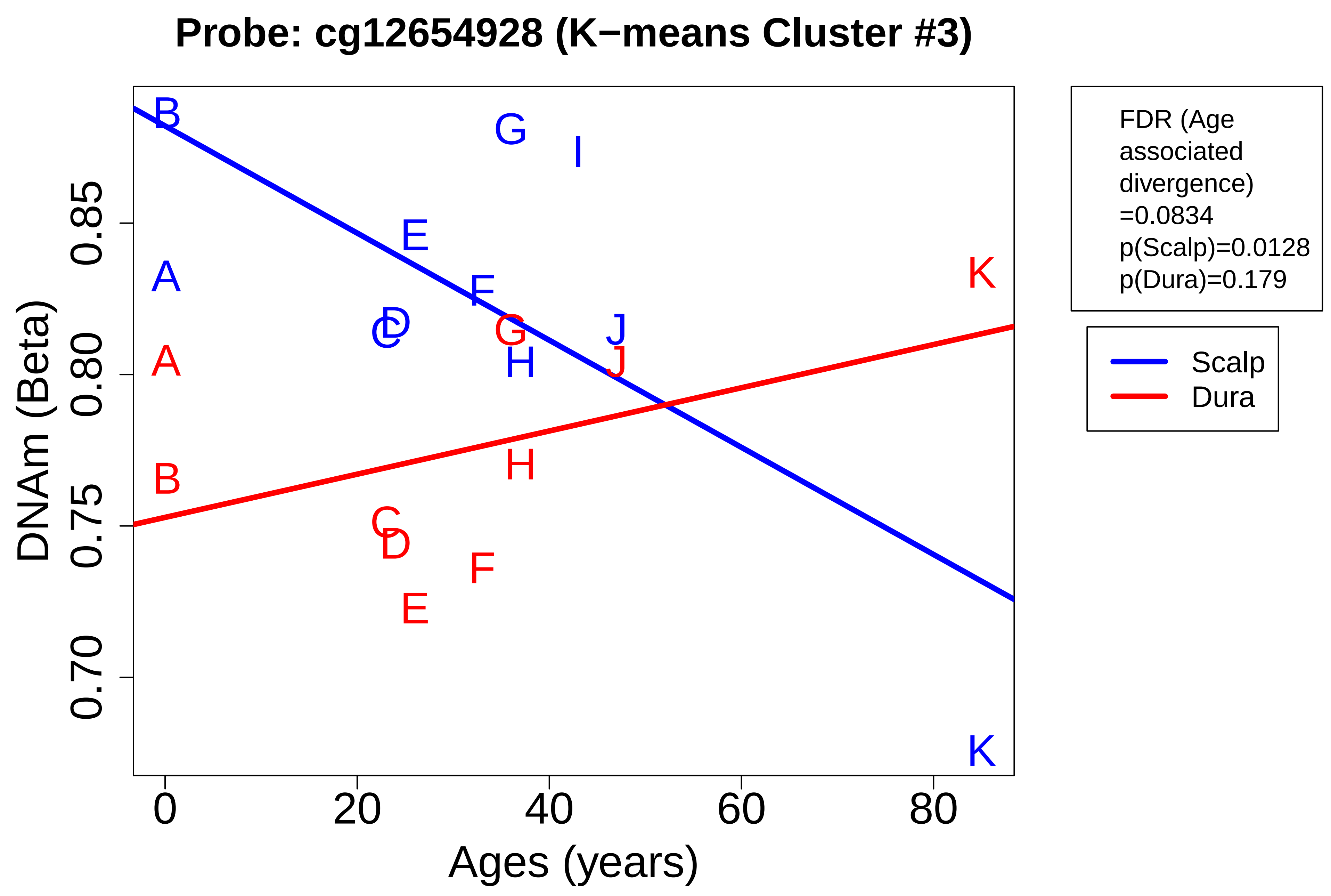

Probe: cg12654928 (K−means Cluster #3)
DNAm (Beta)
0.70
0.75
0.80
0.85
Ages (years)
0
20
40
60
80
FDR (Age associated divergence) =0.0834
p(Scalp)=0.0128
p(Dura)=0.179
Scalp
Dura
A
B
C
D
E
F
G
H
I
J
K

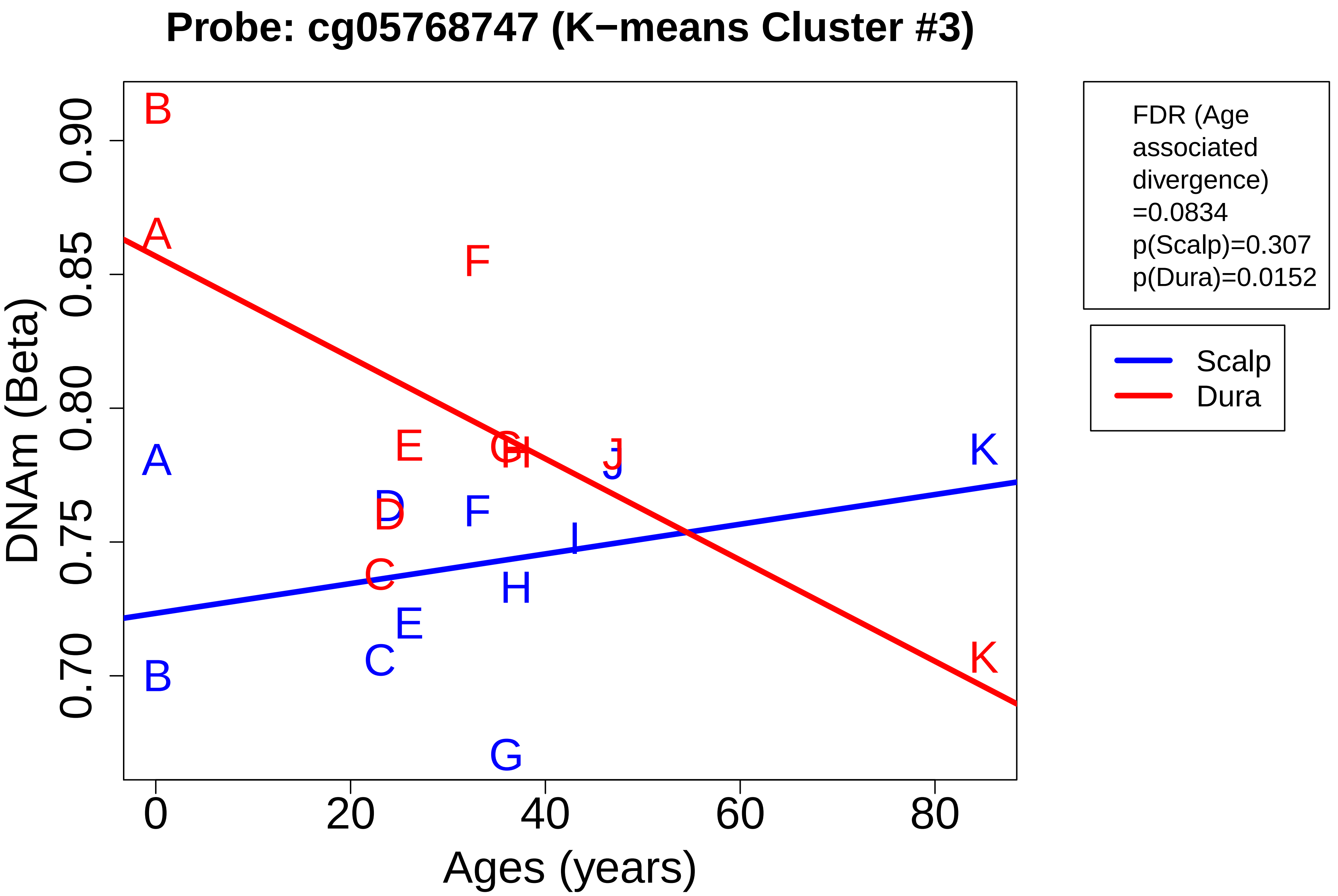

Probe: cg05768747 (K−means Cluster #3)
DNAm (Beta)
0.70
0.75
0.80
0.85
0.90
Ages (years)
0
20
40
60
80
FDR (Age associated divergence) =0.0834
p(Scalp)=0.307
p(Dura)=0.0152
Scalp
Dura
A
B
C
D
E
F
G
H
I
J
K

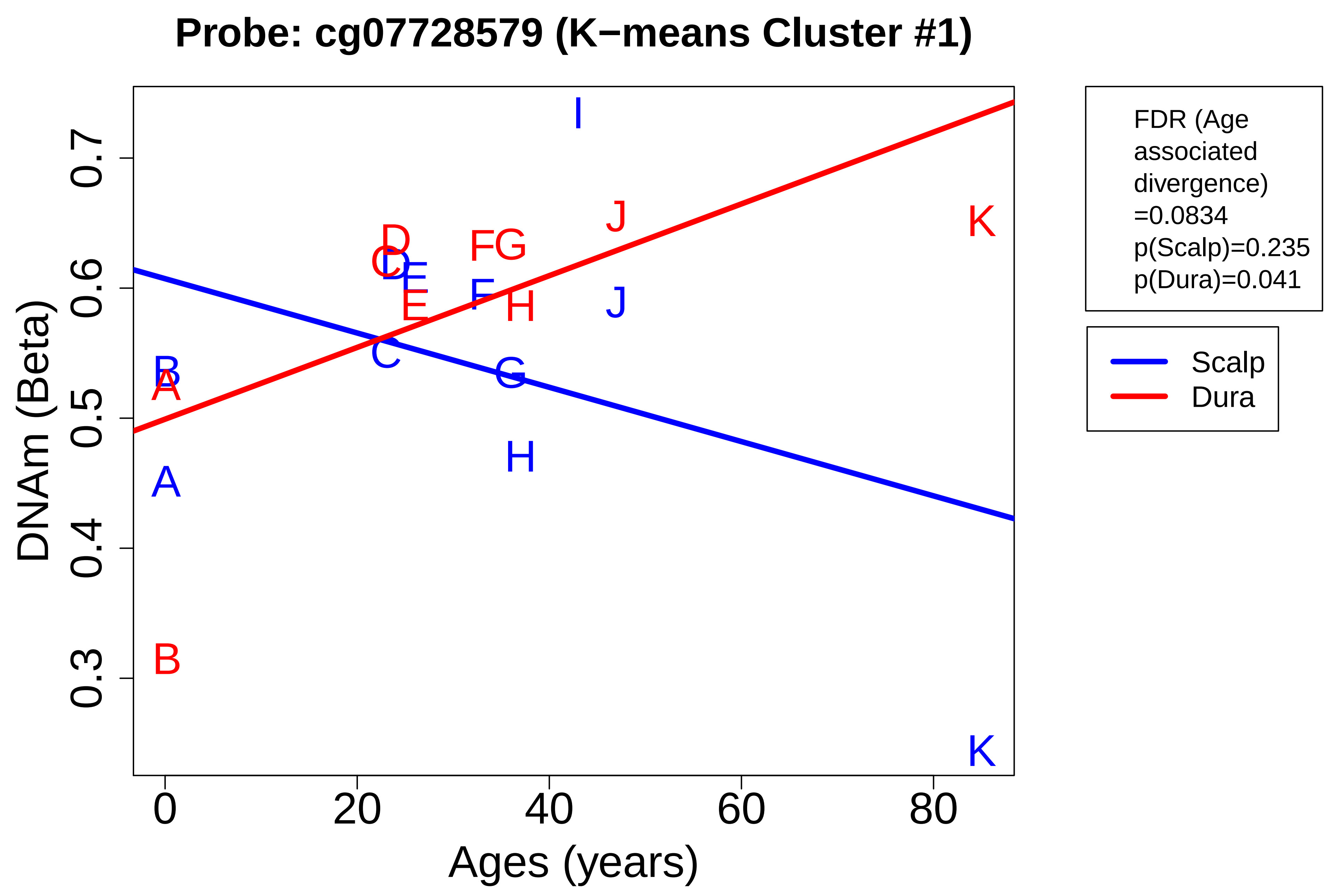
Probe: cg07728579 (K−means Cluster #1)
DNAm (Beta)
0.3
0.4
0.5
0.6
0.7
Ages (years)
0
20
40
60
80
FDR (Age associated divergence) =0.0834
p(Scalp)=0.235
p(Dura)=0.041
Scalp
Dura

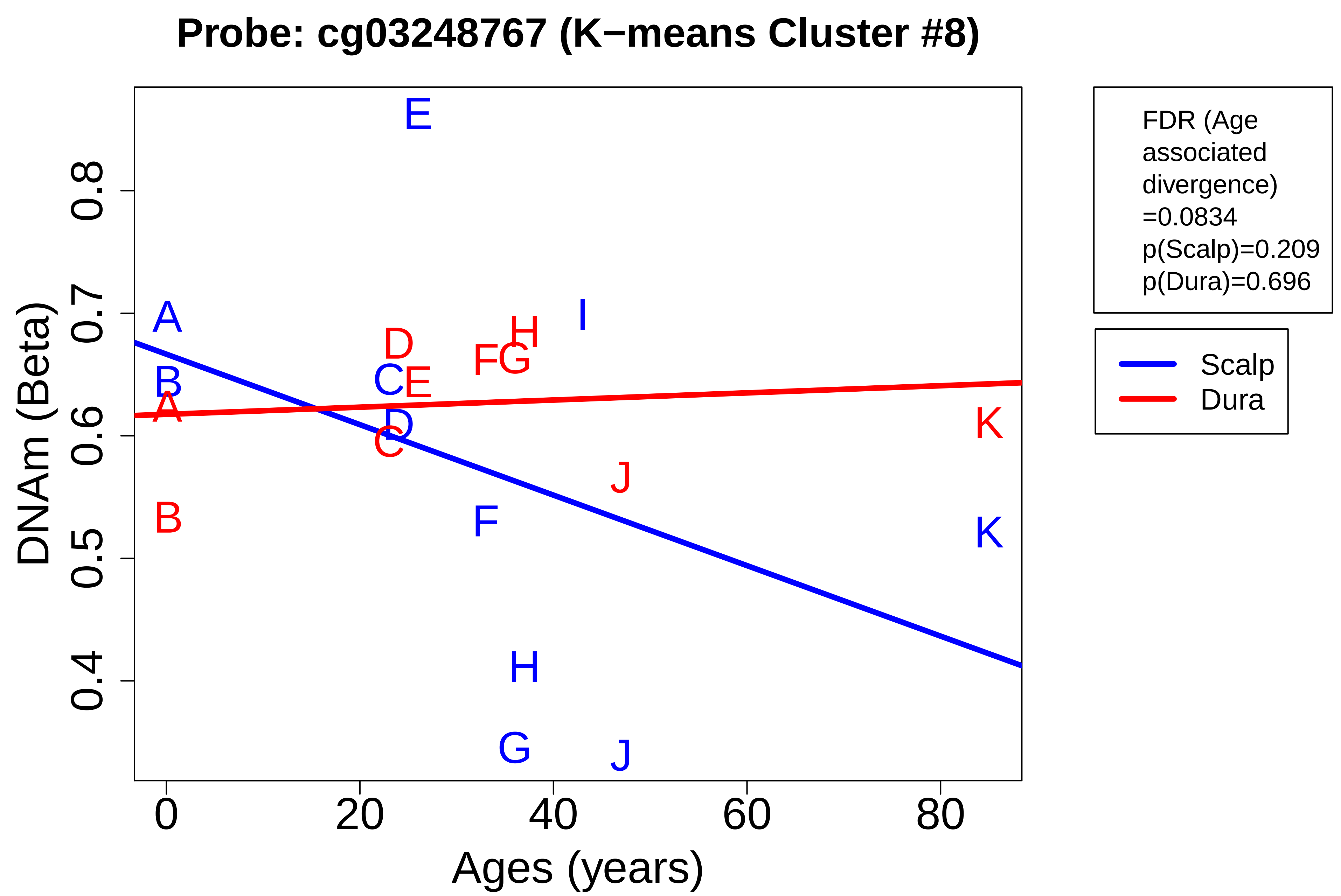

Probe: cg03248767 (K−means Cluster #8)
DNAm (Beta)
0.4
0.5
0.6
0.7
0.8
0
20
40
60
80
Ages (years)
E
A
B
A
D
C
E
D
C
F
G
H
I
K
J
B
F
K
H
G
J
FDR (Age associated divergence) =0.0834
p(Scalp)=0.209
p(Dura)=0.696
Scalp
Dura

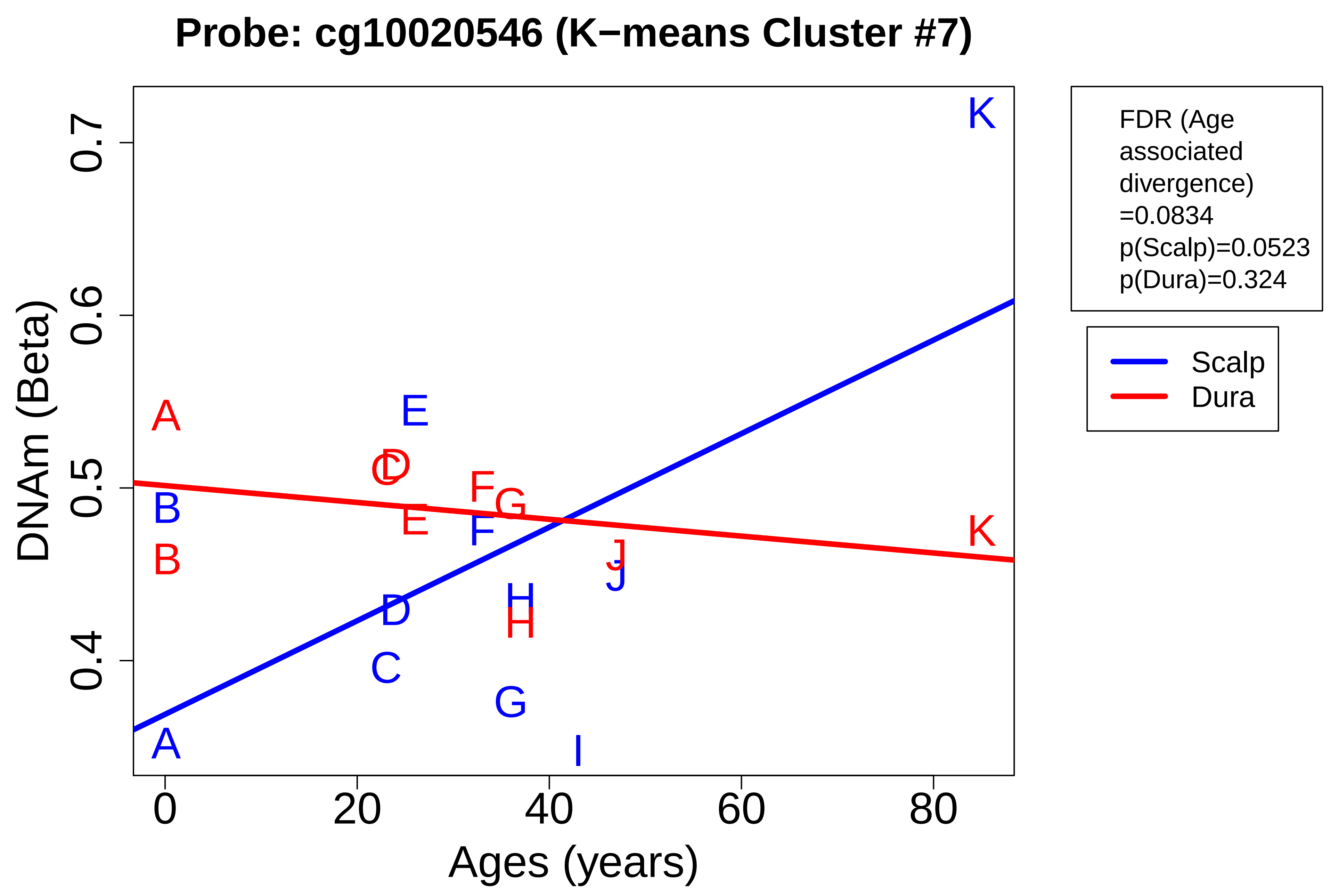

Probe: cg10020546 (K−means Cluster #7)
DNAm (Beta)
0.4
0.5
0.6
0.7
Ages (years)
0
20
40
60
80
A
B
C
D
E
F
G
H
I
J
K
FDR (Age associated divergence) =0.0834
p(Scalp)=0.0523
p(Dura)=0.324
Scalp
Dura

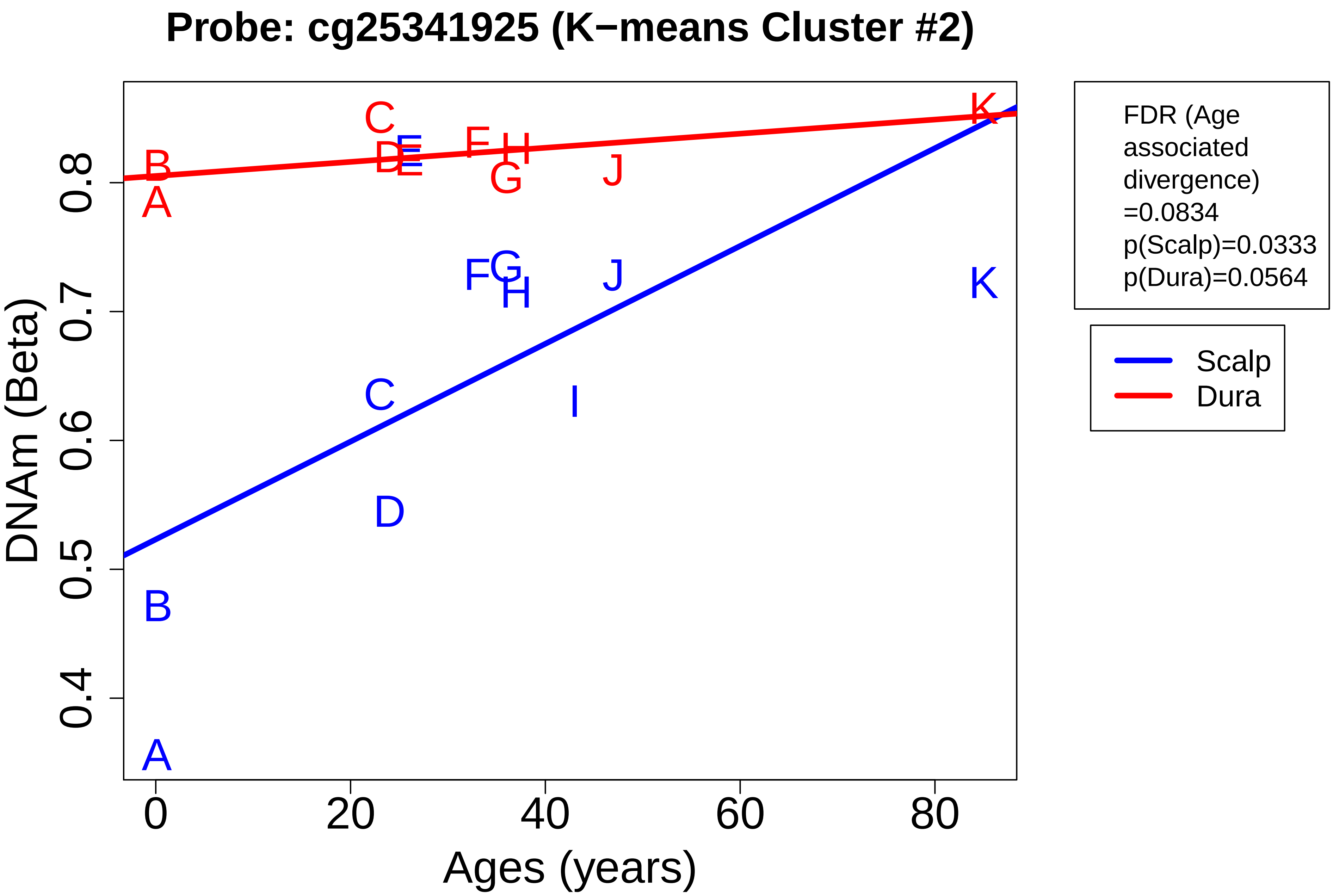

Probe: cg25341925 (K−means Cluster #2)
DNAm (Beta)
0.4
0.5
0.6
0.7
0.8
Ages (years)
0
20
40
60
80
FDR (Age associated divergence) =0.0834
p(Scalp)=0.0333
p(Dura)=0.0564
Scalp
Dura

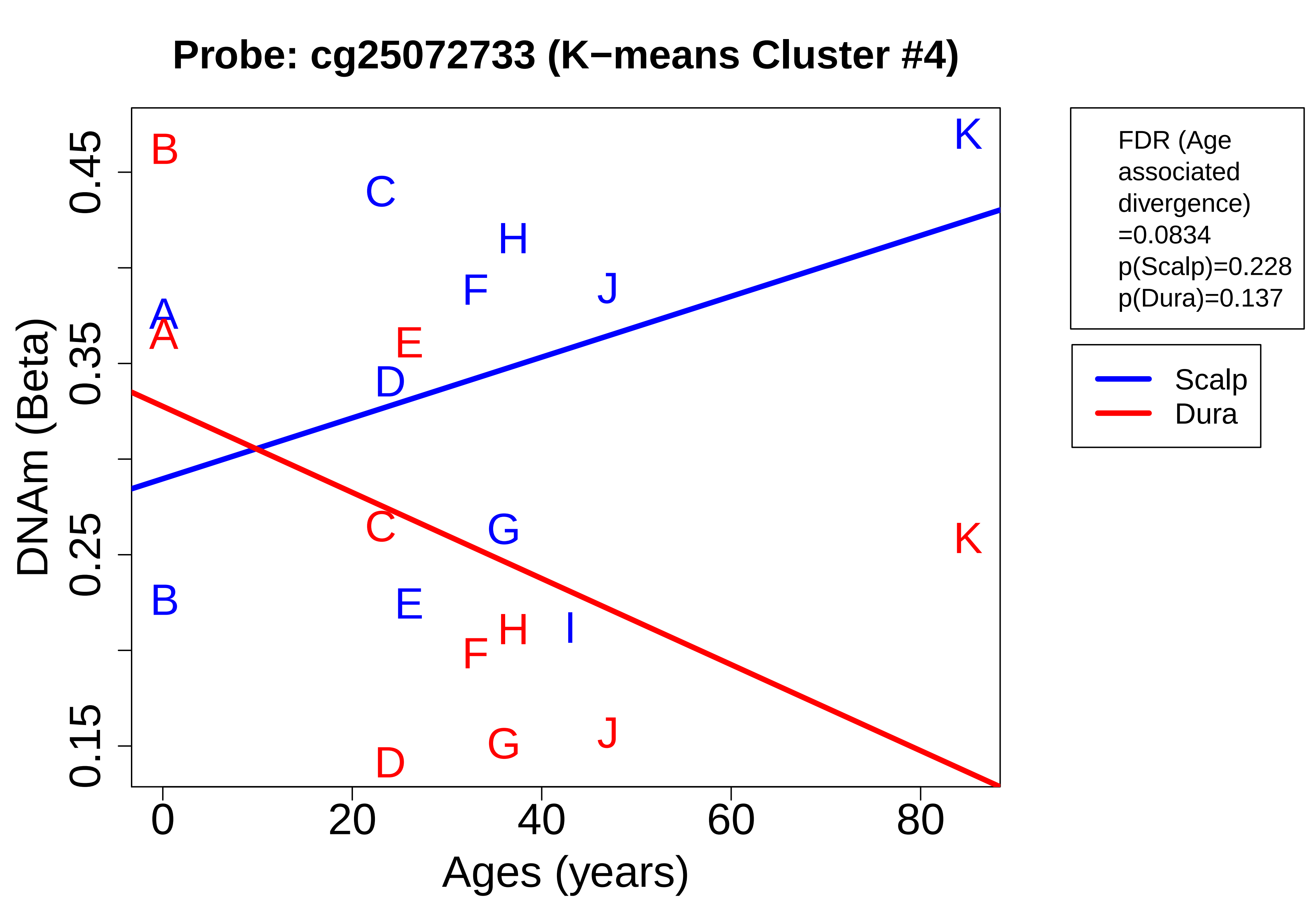

Probe: cg25072733 (K−means Cluster #4)
DNAm (Beta)
0.15
0.25
0.35
0.45
Ages (years)
0
20
40
60
80
B
C
H
F
J
A
A
E
D
K
C
G
K
B
E
H
I
F
J
G
D
FDR (Age associated divergence) =0.0834
p(Scalp)=0.228
p(Dura)=0.137
Scalp
Dura

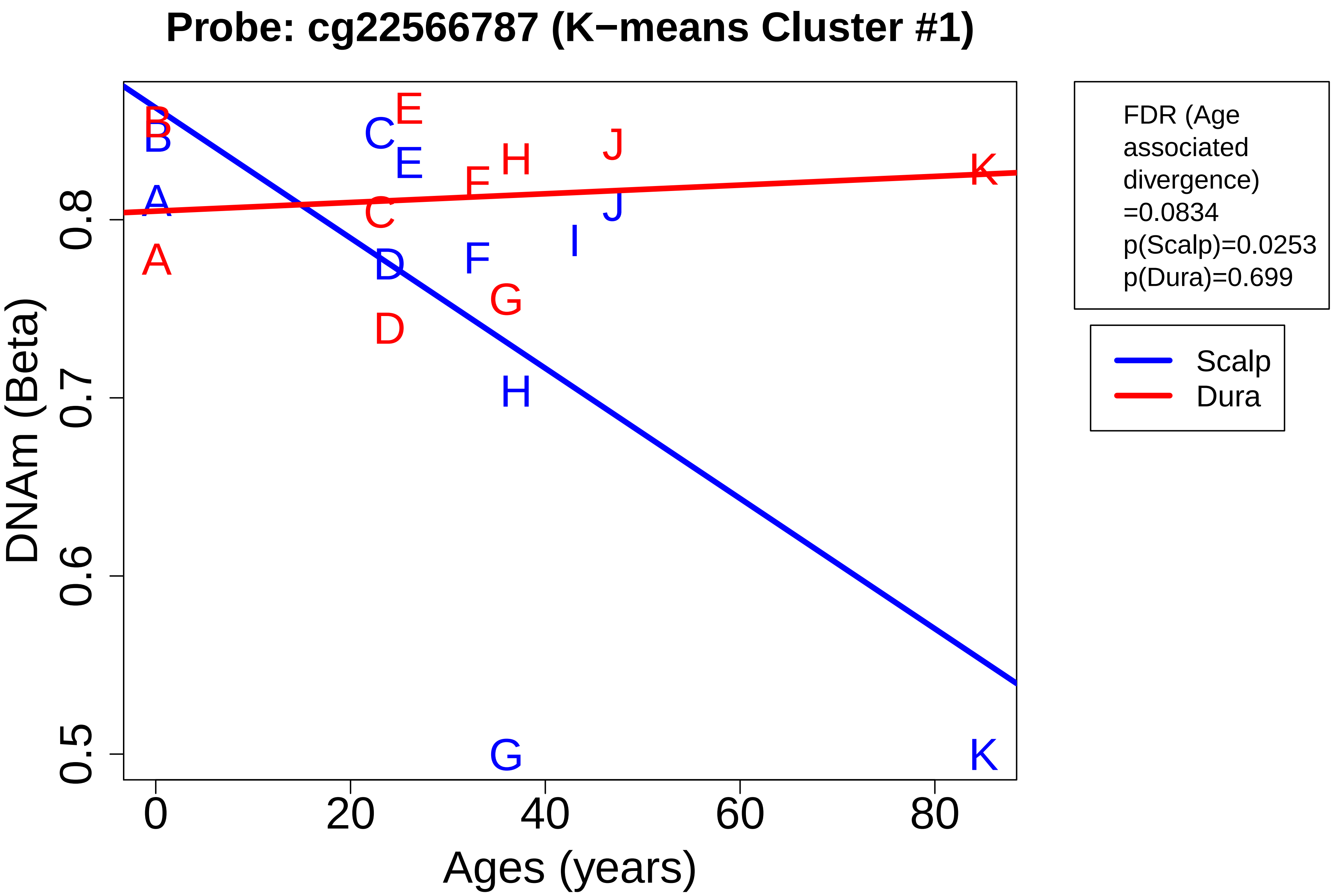

Probe: cg22566787 (K−means Cluster #1)
FDR (Age associated divergence) =0.0834
p(Scalp)=0.0253
p(Dura)=0.699
Scalp
Dura
DNAm (Beta)
0.5
0.6
0.7
0.8
0
20
40
60
80
Ages (years)
A
B
C
D
E
F
G
H
I
J
K

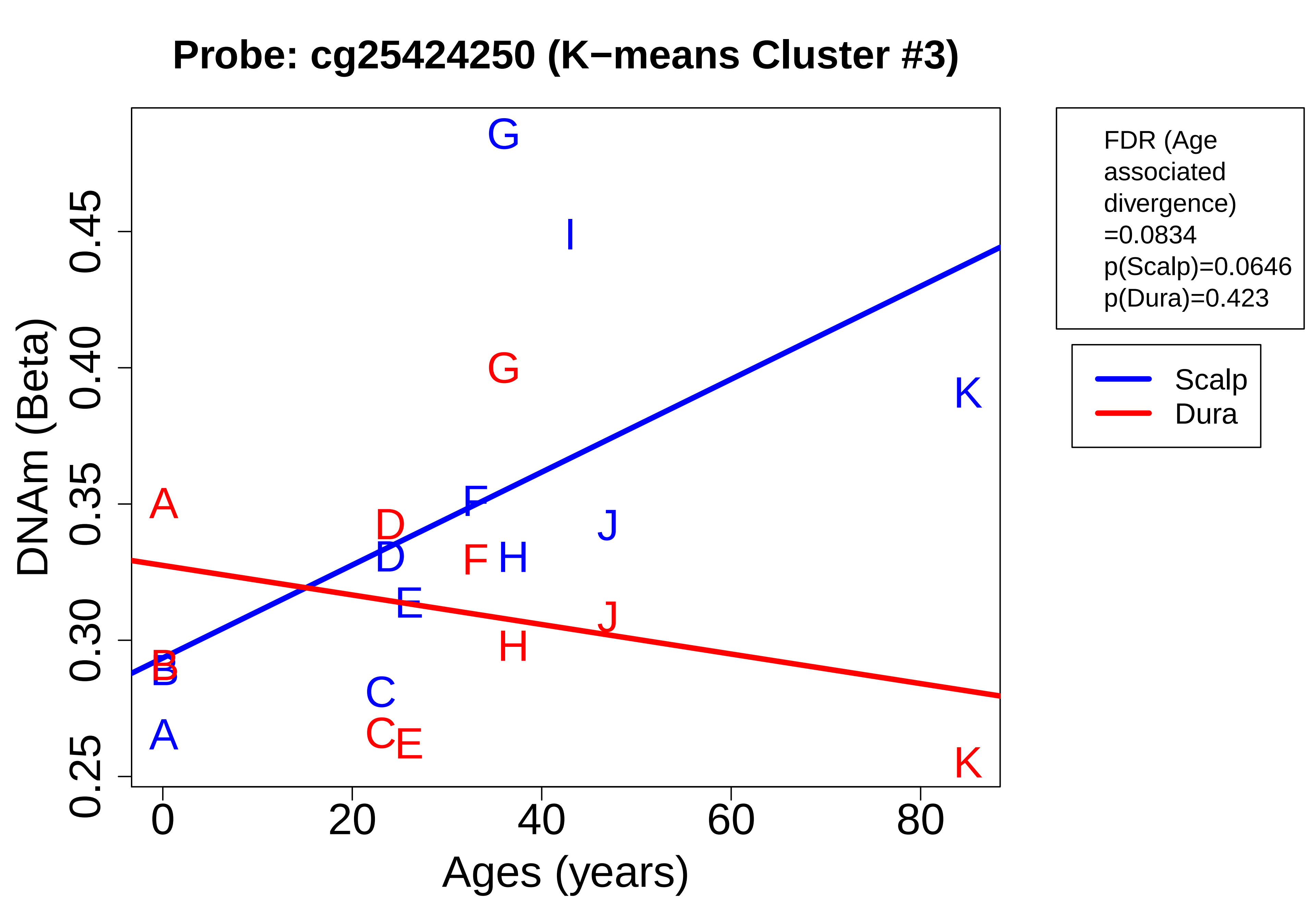
Probe: cg25424250 (K−means Cluster #3)
DNAm (Beta)
0.25
0.30
0.35
0.40
0.45
Ages (years)
0
20
40
60
80
FDR (Age associated divergence) =0.0834
p(Scalp)=0.0646
p(Dura)=0.423
Scalp
Dura
A
B
C
D
E
F
G
H
I
J
K

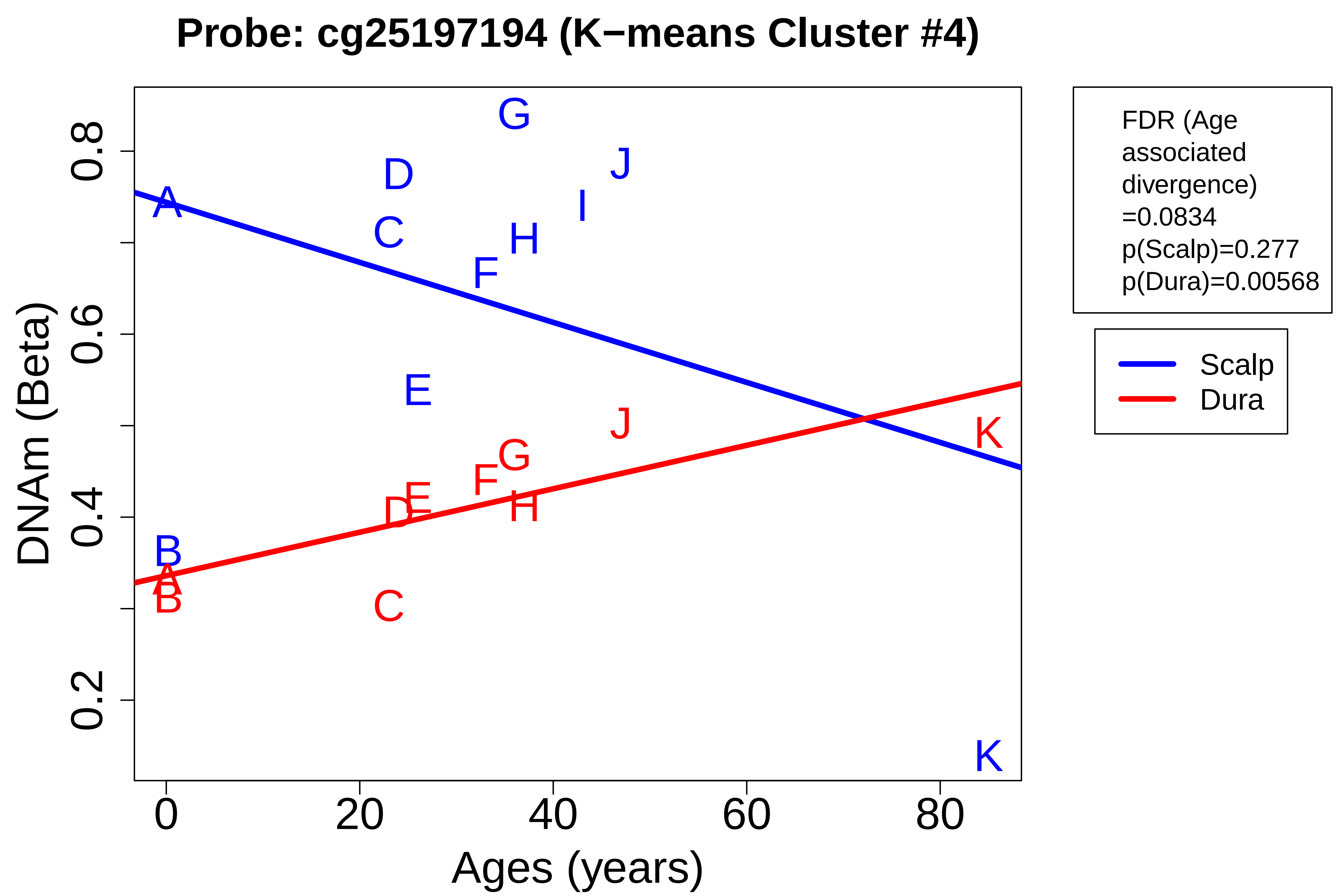

Probe: cg25197194 (K−means Cluster #4)
DNAm (Beta)
0.2
0.4
0.6
0.8
Ages (years)
0
20
40
60
80
A
B
C
D
E
F
G
H
I
J
K
FDR (Age associated divergence) =0.0834
p(Scalp)=0.277
p(Dura)=0.00568
Scalp
Dura

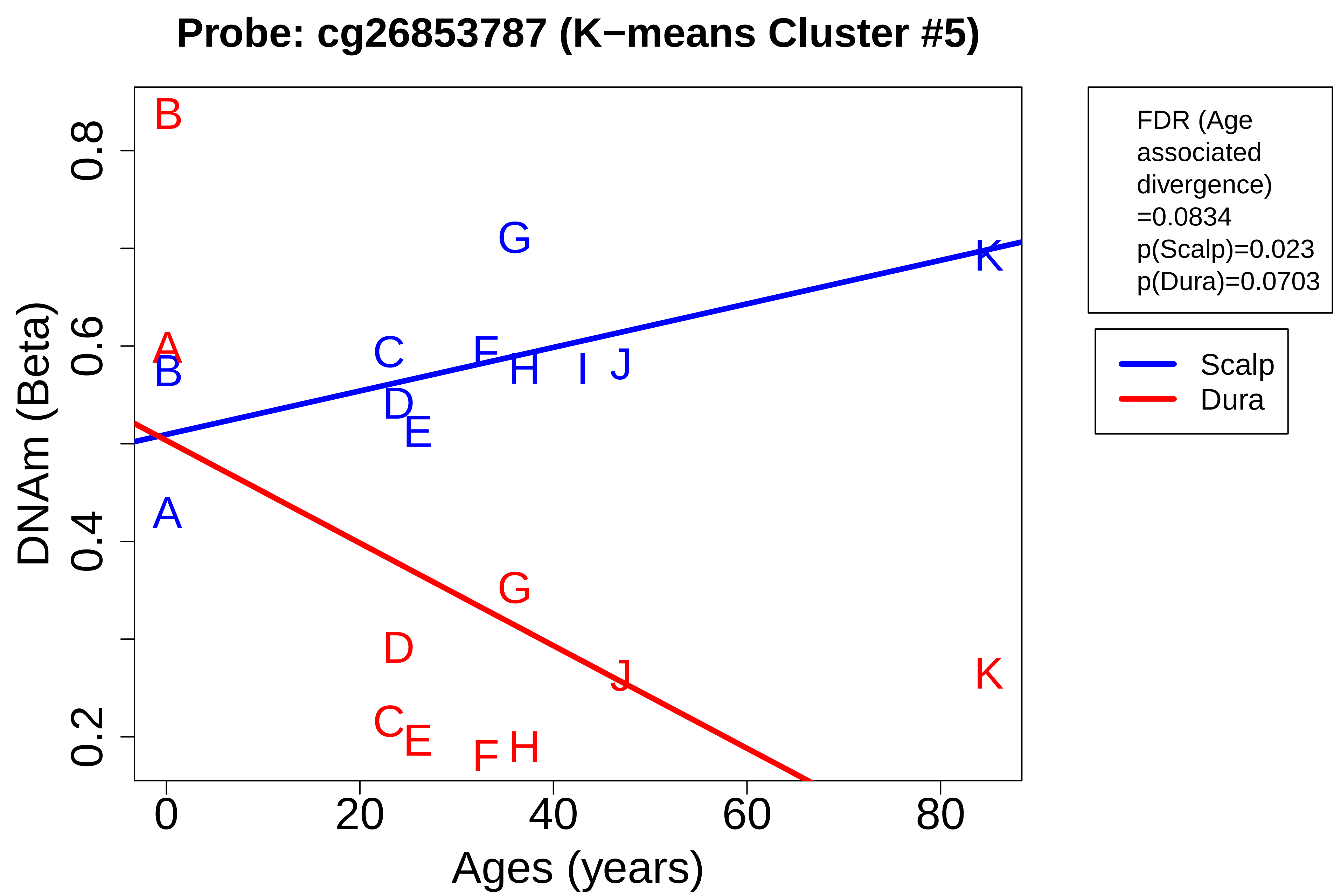

Probe: cg26853787 (K−means Cluster #5)
DNAm (Beta)
0.2
0.4
0.6
0.8
Ages (years)
0
20
40
60
80
FDR (Age associated divergence) =0.0834
p(Scalp)=0.023
p(Dura)=0.0703
Scalp
Dura
A
B
C
D
E
F
G
H
I
J
K

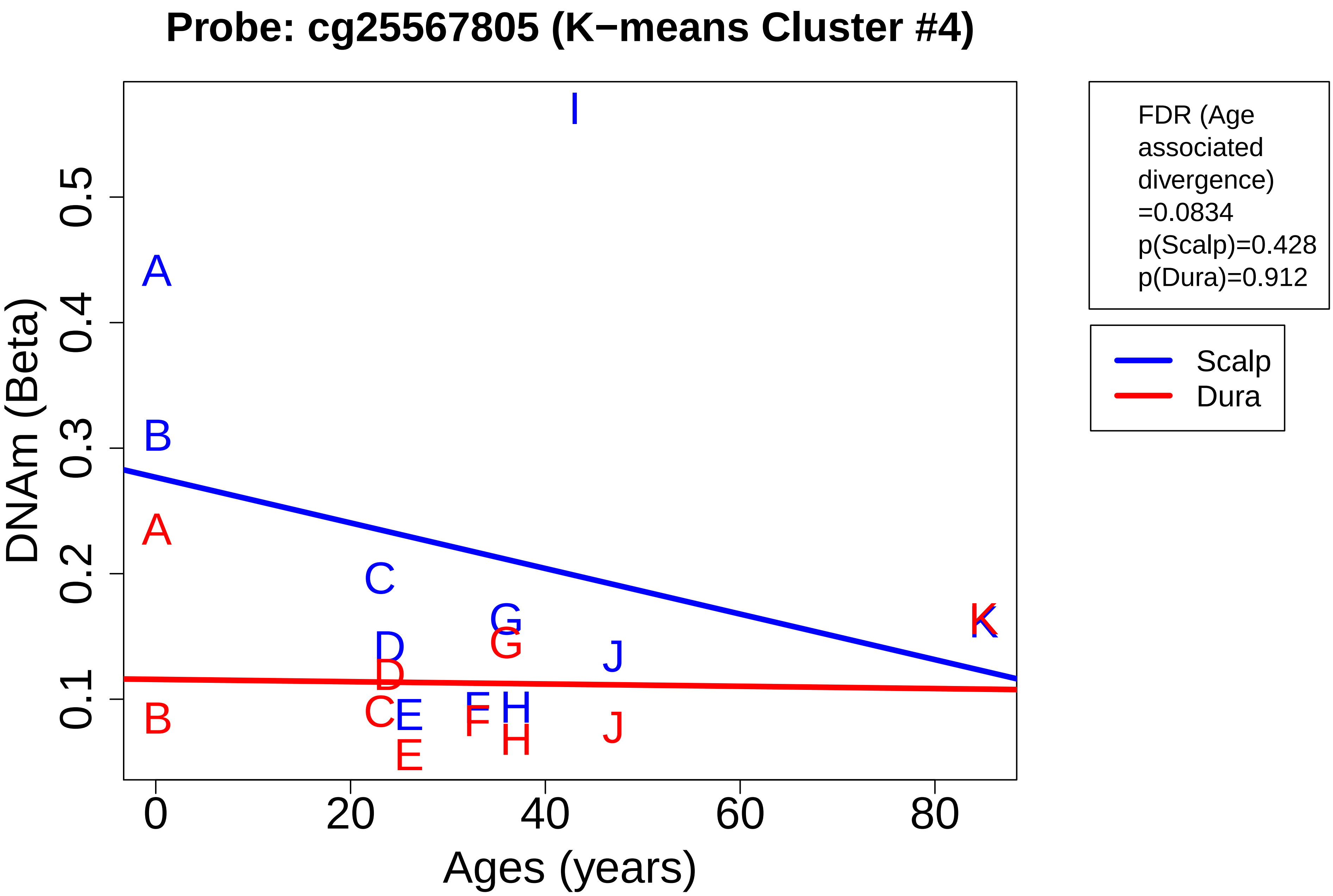
Probe: cg25567805 (K−means Cluster #4)
DNAm (Beta)
0.1
0.2
0.3
0.4
0.5
0
20
40
60
80
Ages (years)
A
B
C
D
E
F
G
H
I
J
K
FDR (Age associated divergence) =0.0834
p(Scalp)=0.428
p(Dura)=0.912
Scalp
Dura

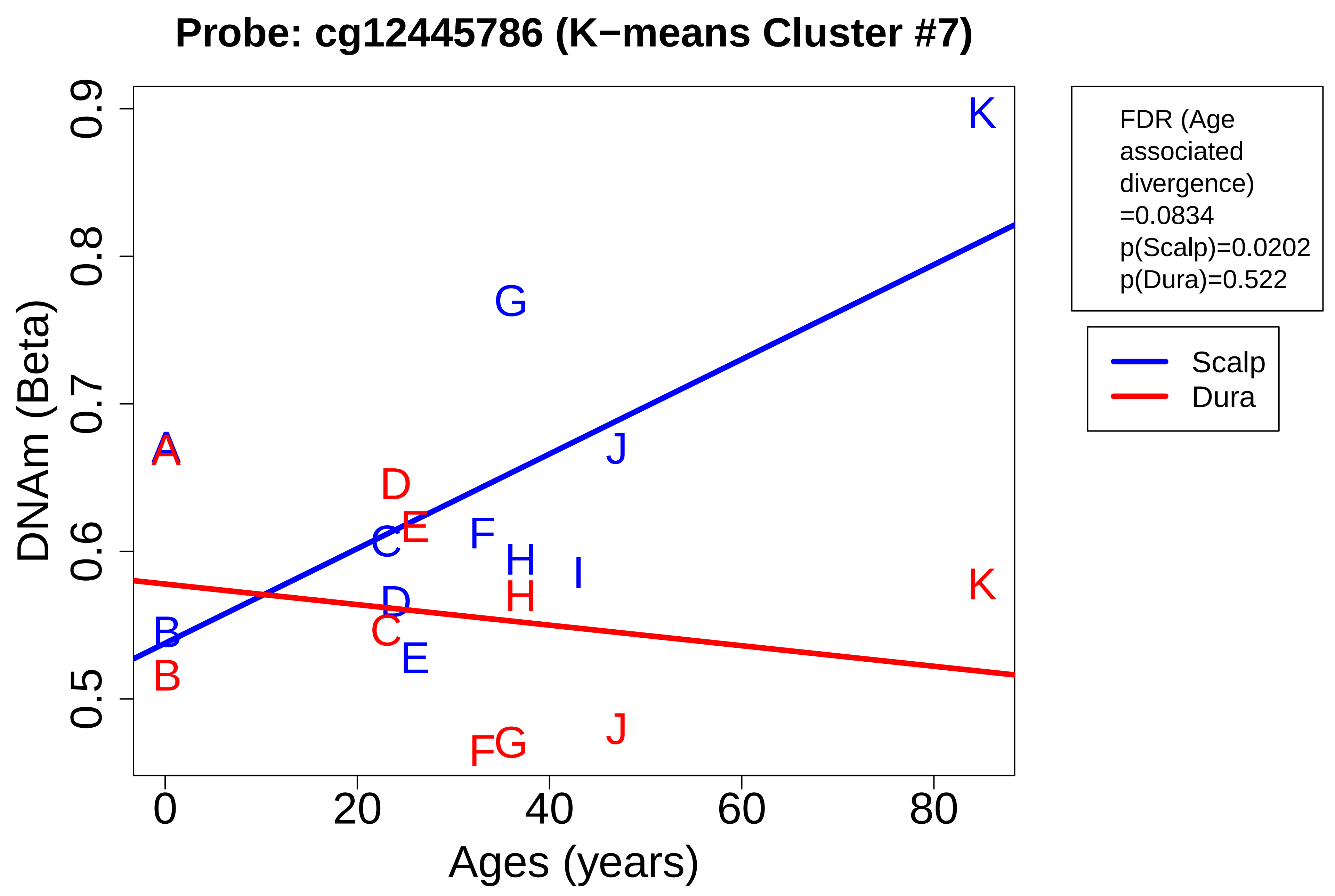

Probe: cg12445786 (K−means Cluster #7)
DNAm (Beta)
0.5
0.6
0.7
0.8
0.9
0
20
40
60
80
Ages (years)
FDR (Age associated divergence) =0.0834
p(Scalp)=0.0202
p(Dura)=0.522
Scalp
Dura

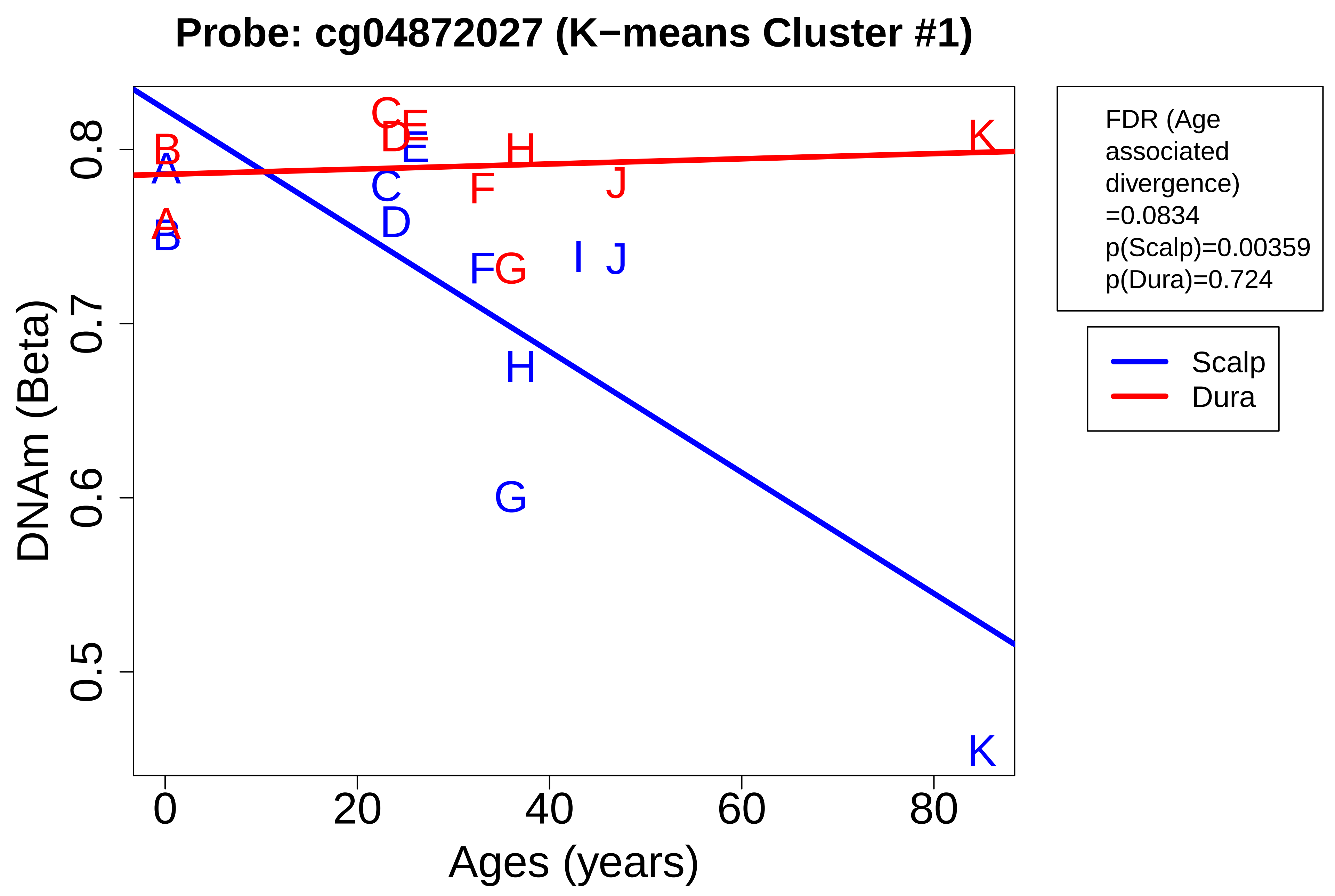

Probe: cg04872027 (K−means Cluster #1)
DNAm (Beta)
0.5
0.6
0.7
0.8
Ages (years)
0
20
40
60
80
FDR (Age associated divergence) =0.0834
p(Scalp)=0.00359
p(Dura)=0.724
Scalp
Dura

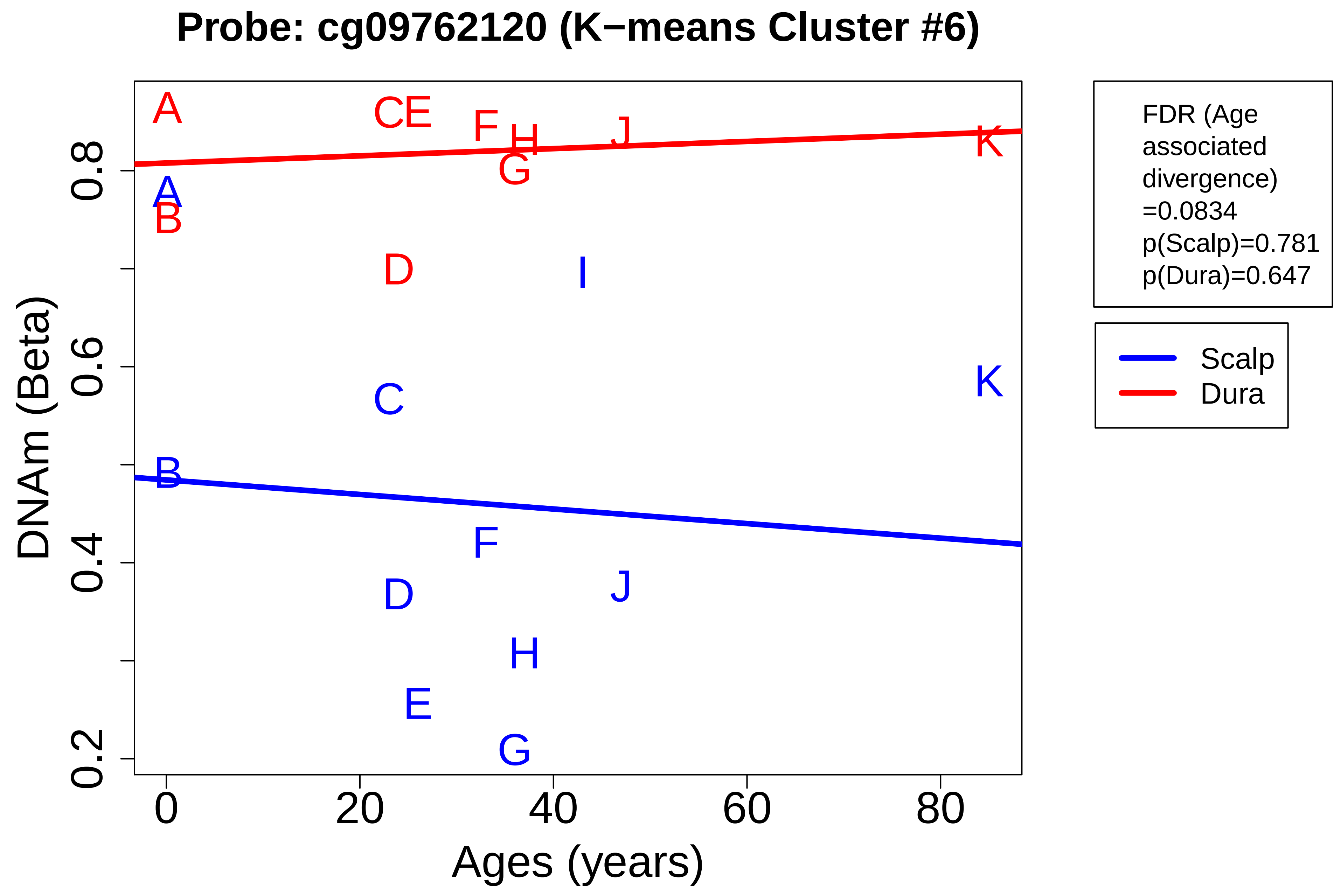

Probe: cg09762120 (K−means Cluster #6)
DNAm (Beta)
0.2
0.4
0.6
0.8
Ages (years)
0
20
40
60
80
FDR (Age associated divergence) =0.0834
p(Scalp)=0.781
p(Dura)=0.647
Scalp
Dura
A B C D E F G H I J K

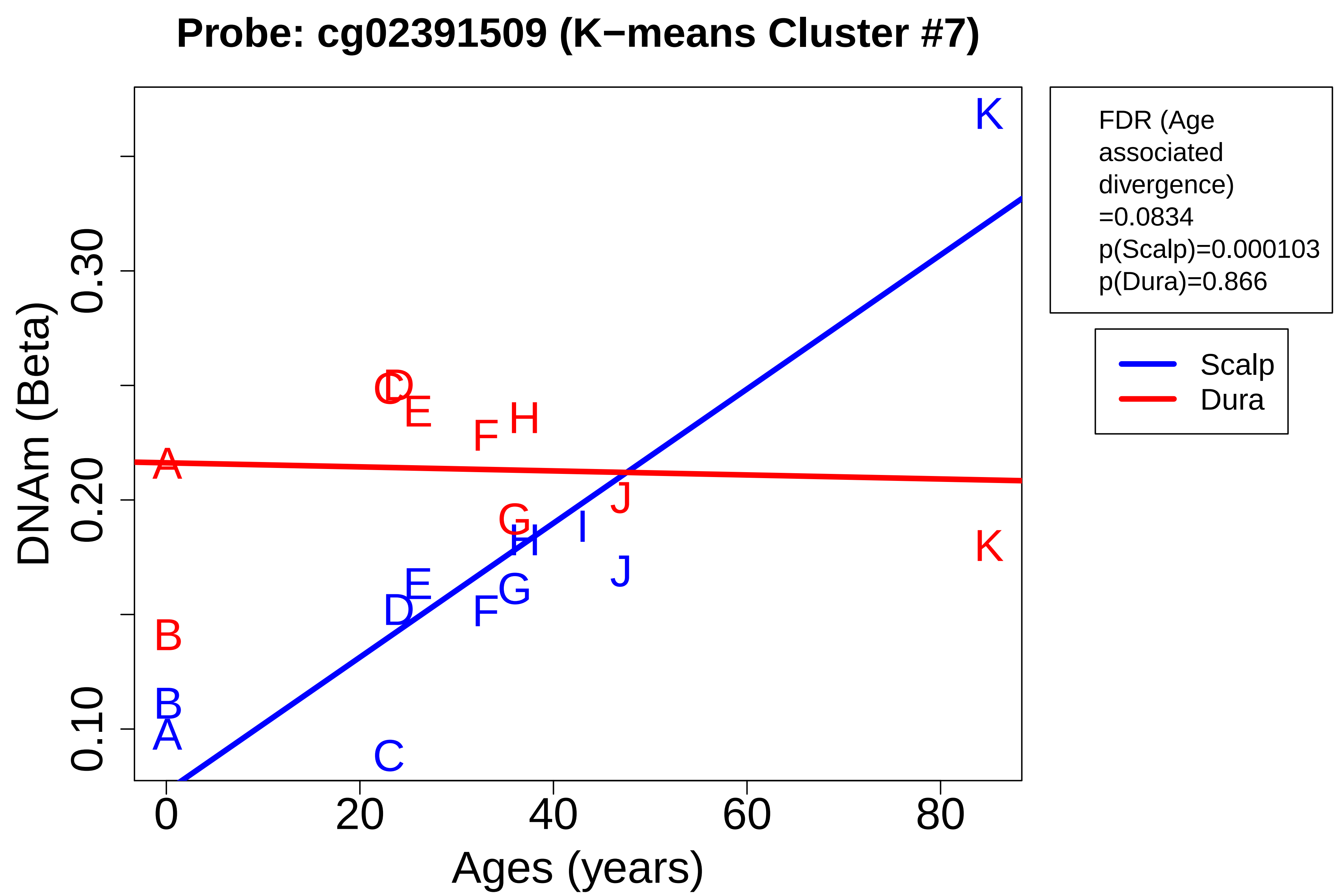

Probe: cg02391509 (K−means Cluster #7)
DNAm (Beta)
0.10
0.20
0.30
0
20
40
60
80
Ages (years)
FDR (Age associated divergence) =0.0834
p(Scalp)=0.000103
p(Dura)=0.866
Scalp
Dura

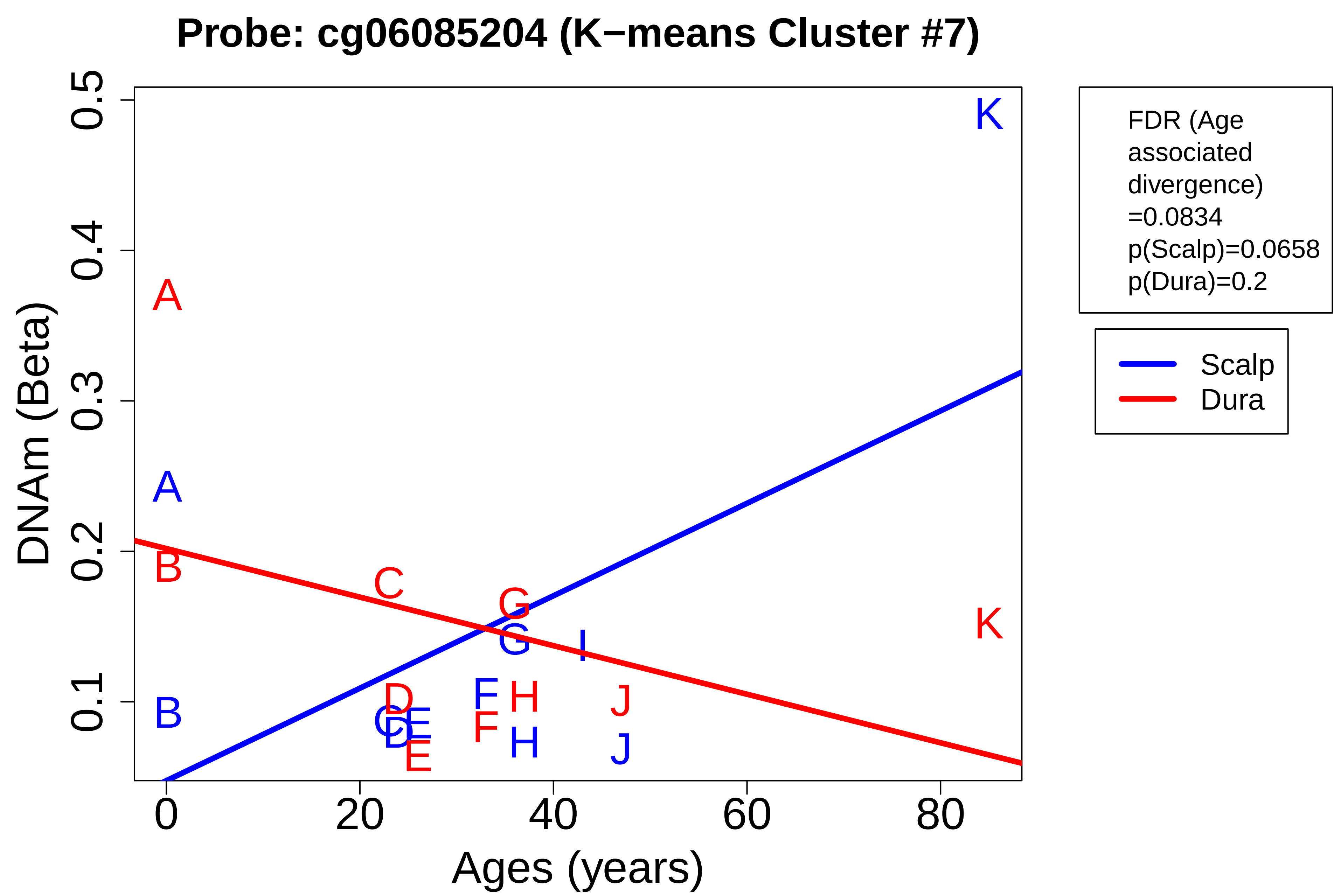

Probe: cg06085204 (K−means Cluster #7)
DNAm (Beta)
0.1
0.2
0.3
0.4
0.5
0
20
40
60
80
Ages (years)
FDR (Age associated divergence) =0.0834
p(Scalp)=0.0658
p(Dura)=0.2
Scalp
Dura
A
B
C
D
E
F
G
H
I
J
K

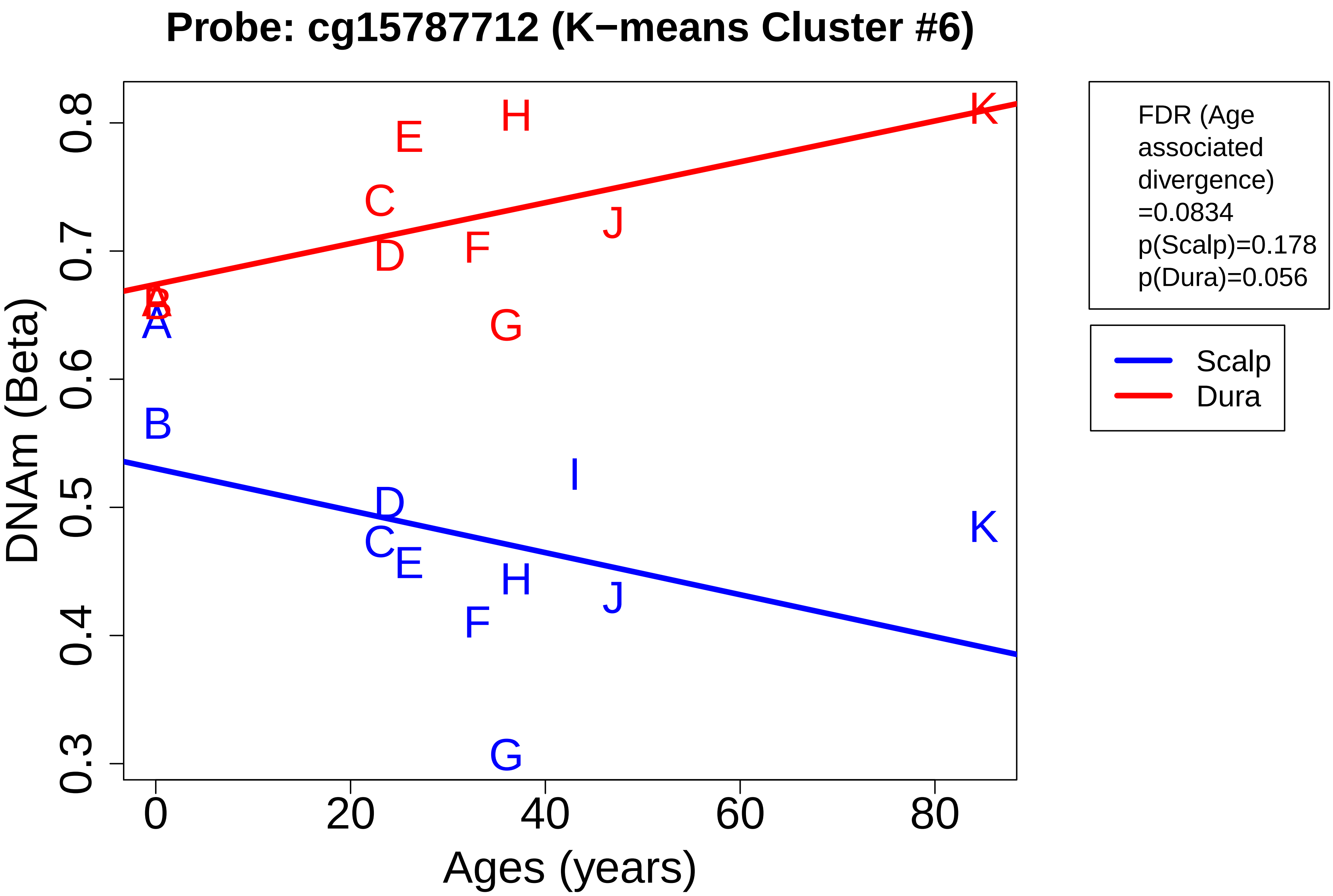

Probe: cg15787712 (K−means Cluster #6)
DNAm (Beta)
Ages (years)
0.3
0.4
0.5
0.6
0.7
0.8
0
20
40
60
80
FDR (Age associated divergence) =0.0834
p(Scalp)=0.178
p(Dura)=0.056
Scalp
Dura

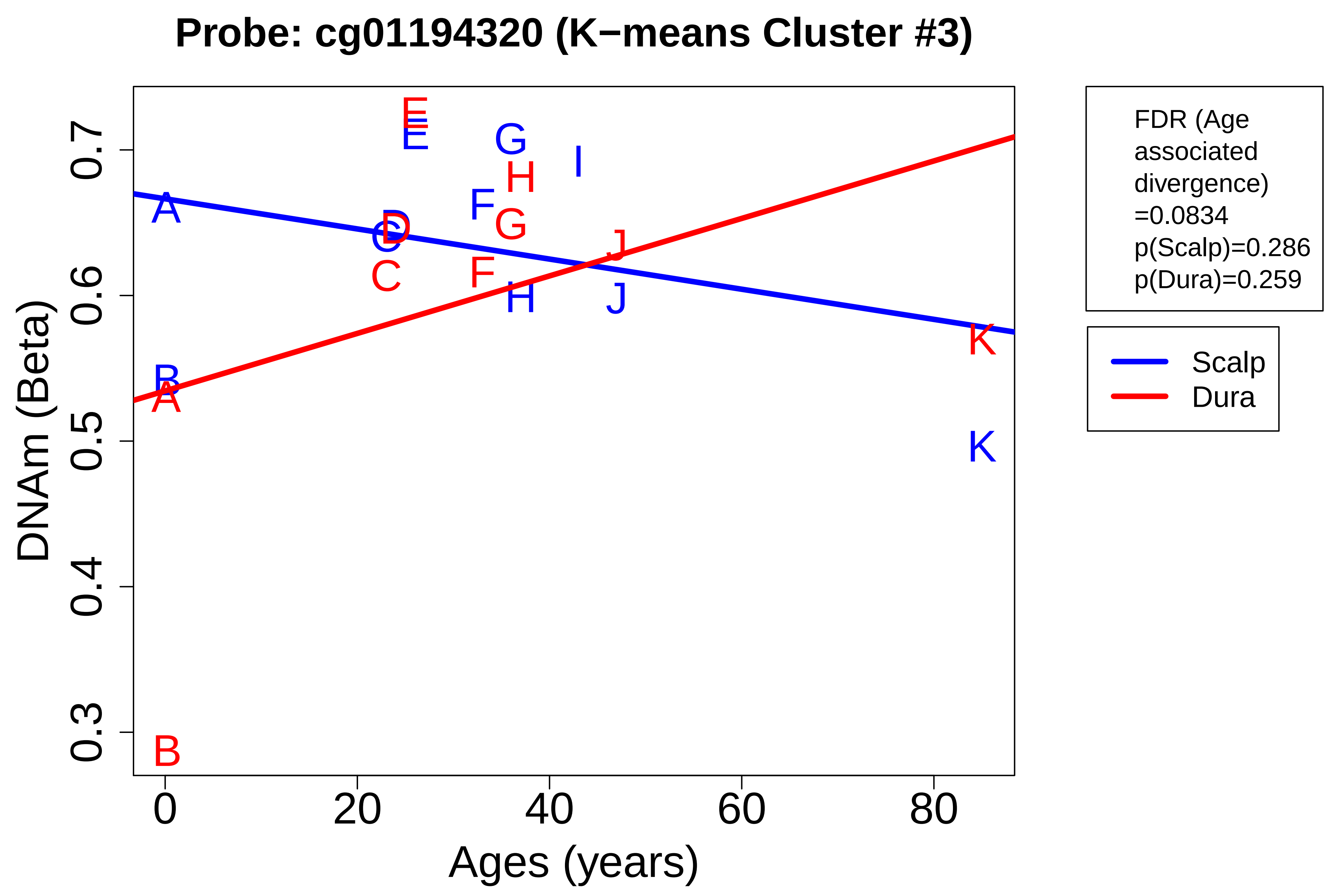

Probe: cg01194320 (K−means Cluster #3)
DNAm (Beta)
0.3
0.4
0.5
0.6
0.7
0
20
40
60
80
Ages (years)
A
B
C
D
E
F
G
H
I
J
K
FDR (Age associated divergence) =0.0834
p(Scalp)=0.286
p(Dura)=0.259
Scalp
Dura

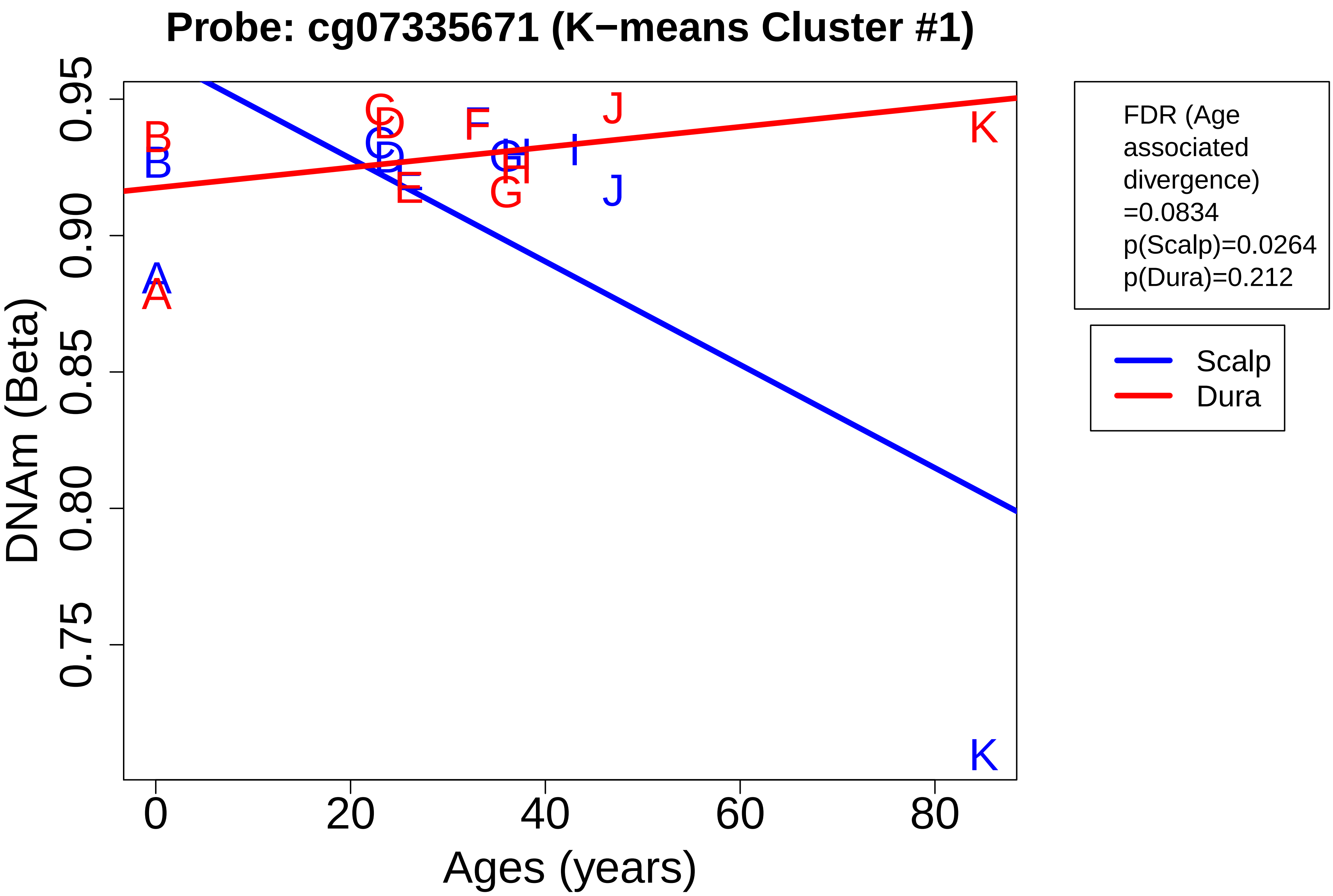

Probe: cg07335671 (K−means Cluster #1)
DNAm (Beta)
0.75
0.80
0.85
0.90
0.95
0
20
40
60
80
Ages (years)
FDR (Age associated divergence) =0.0834
p(Scalp)=0.0264
p(Dura)=0.212
Scalp
Dura

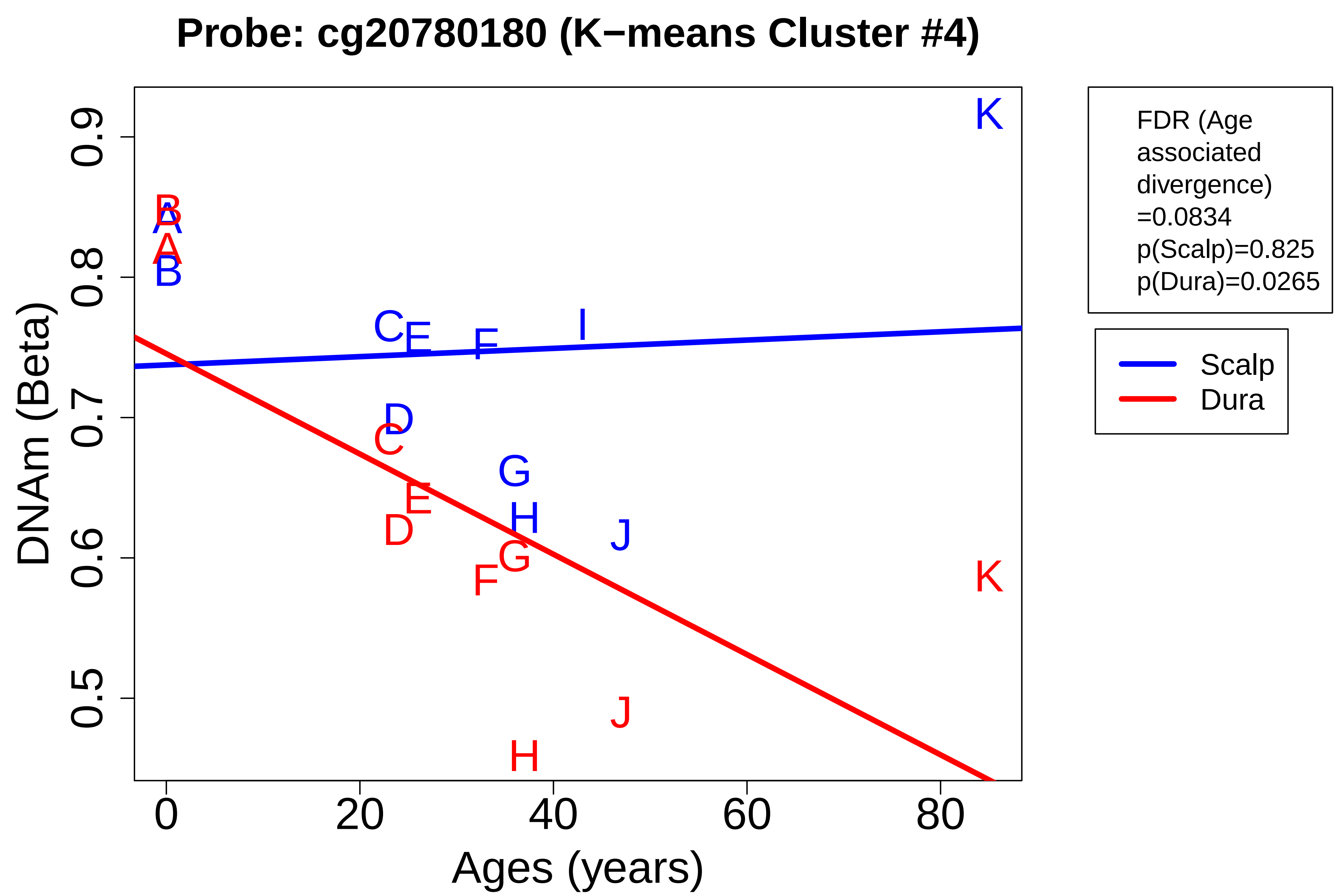

Probe: cg20780180 (K−means Cluster #4)
DNAm (Beta)
0.5
0.6
0.7
0.8
0.9
Ages (years)
0
20
40
60
80
A
B
C
D
E
F
G
H
I
J
K
FDR (Age associated divergence) =0.0834
p(Scalp)=0.825
p(Dura)=0.0265
Scalp
Dura

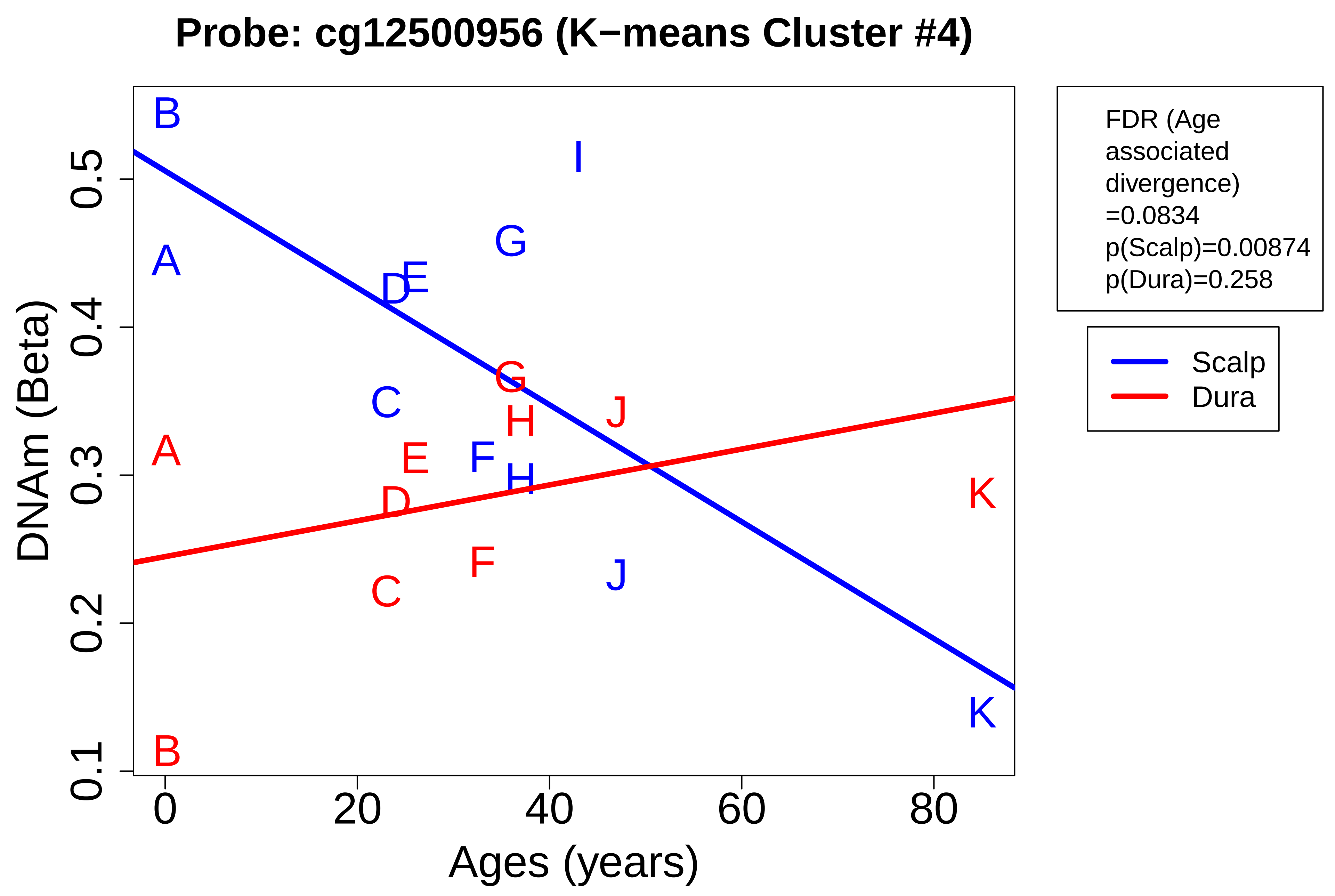

Probe: cg12500956 (K−means Cluster #4)
DNAm (Beta)
Ages (years)
0.1
0.2
0.3
0.4
0.5
0
20
40
60
80
FDR (Age associated divergence) =0.0834
p(Scalp)=0.00874
p(Dura)=0.258
Scalp
Dura
A
B
C
D
E
F
G
H
I
J
K

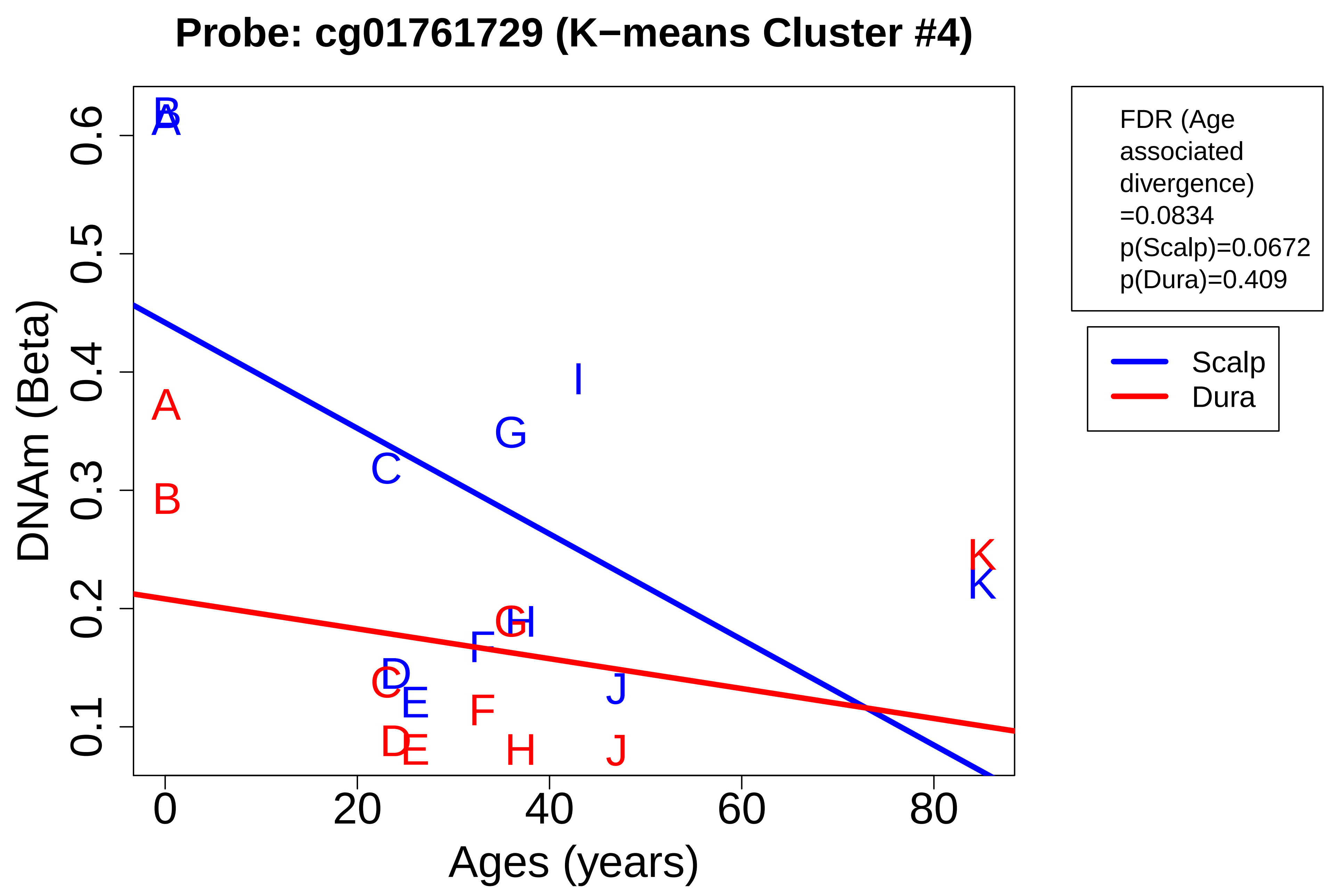
Probe: cg01761729 (K−means Cluster #4)
DNAm (Beta)
0.1
0.2
0.3
0.4
0.5
0.6
0
20
40
60
80
Ages (years)
FDR (Age associated divergence) =0.0834
p(Scalp)=0.0672
p(Dura)=0.409
Scalp
Dura

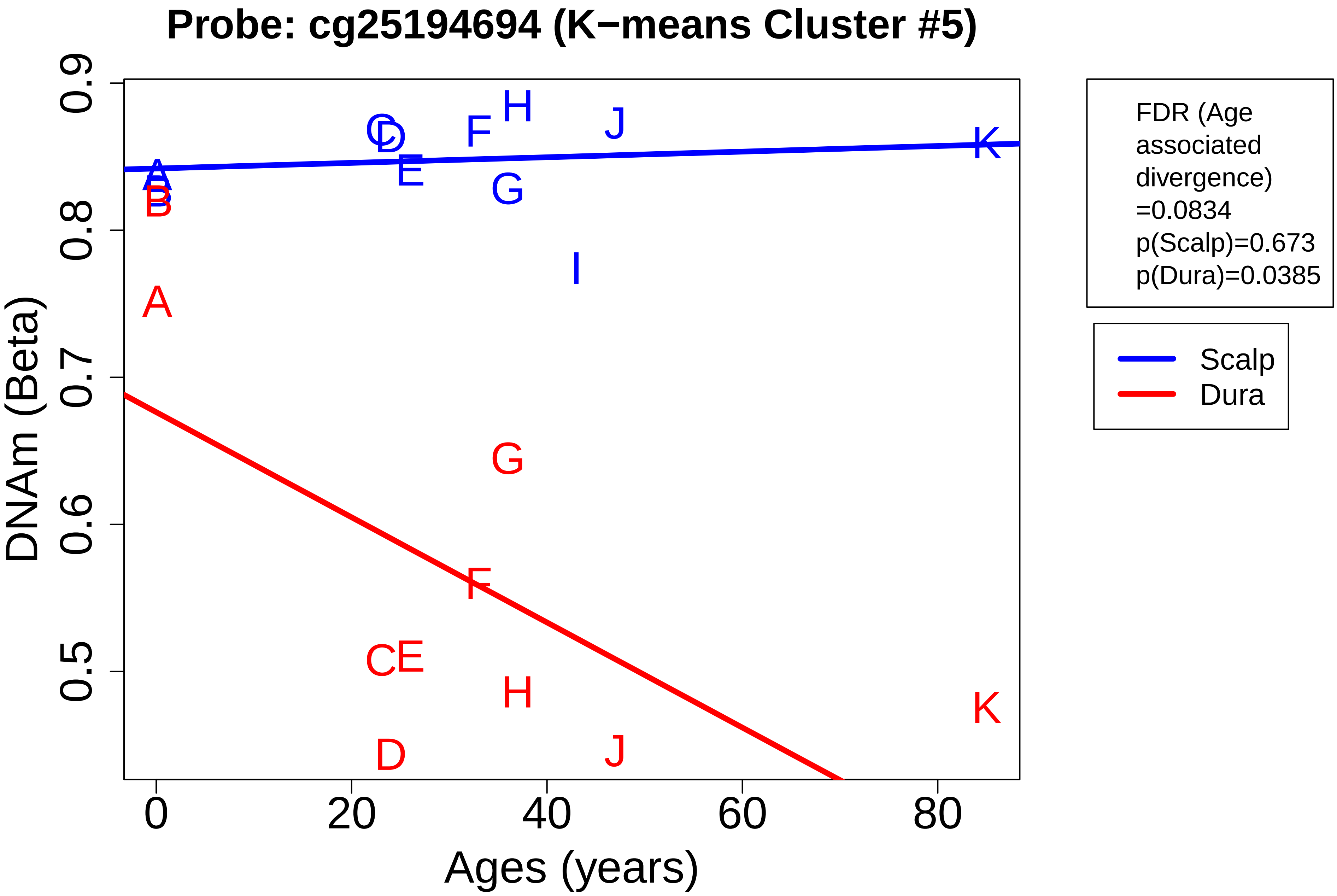
Probe: cg25194694 (K−means Cluster #5)
DNAm (Beta)
0.5
0.6
0.7
0.8
0.9
0
20
40
60
80
Ages (years)
A
B
C
D
E
F
G
H
I
J
K
FDR (Age associated divergence) =0.0834
p(Scalp)=0.673
p(Dura)=0.0385
Scalp
Dura

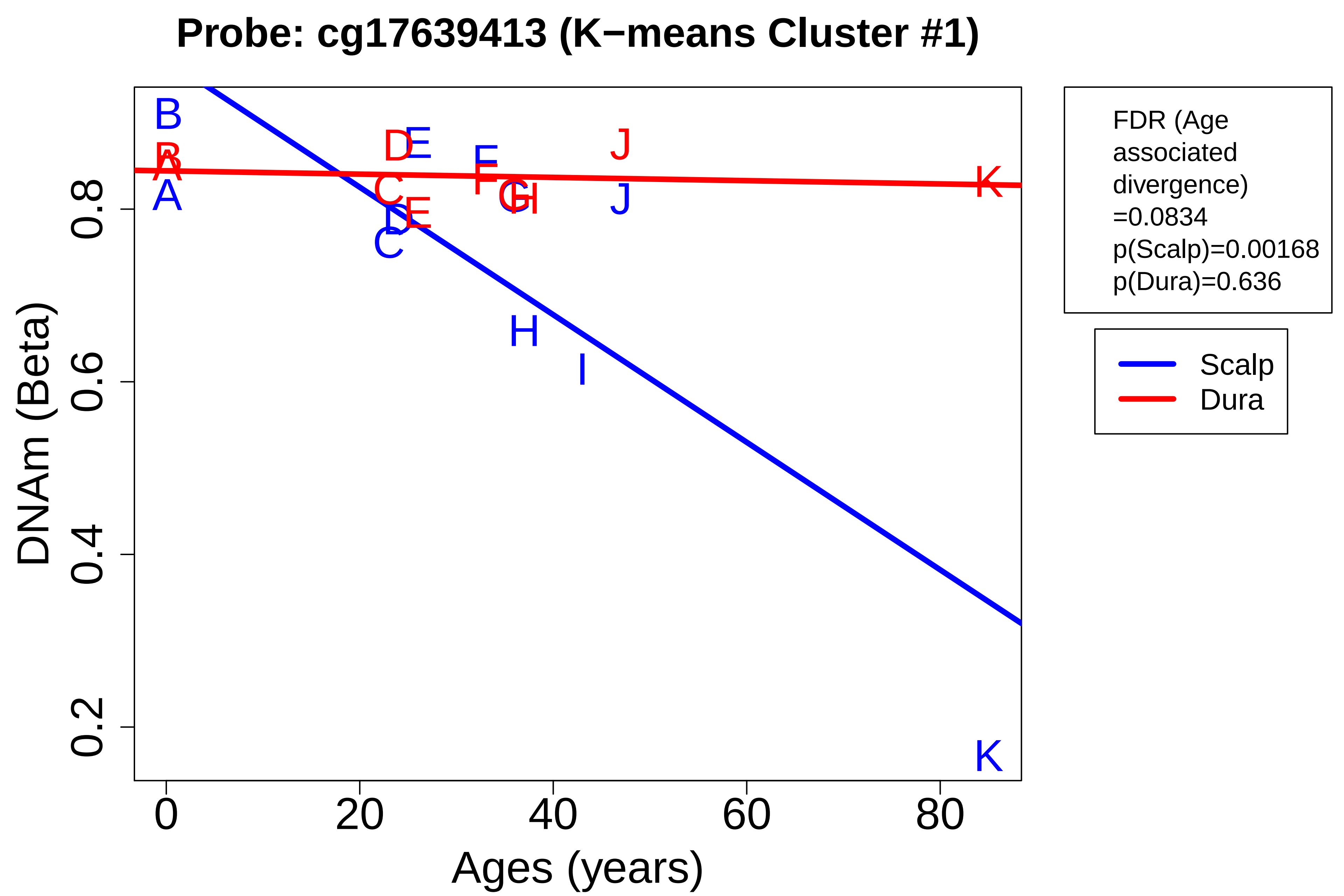

Probe: cg17639413 (K−means Cluster #1)
DNAm (Beta)
0.2
0.4
0.6
0.8
Ages (years)
0
20
40
60
80
FDR (Age associated divergence) =0.0834
p(Scalp)=0.00168
p(Dura)=0.636
Scalp
Dura

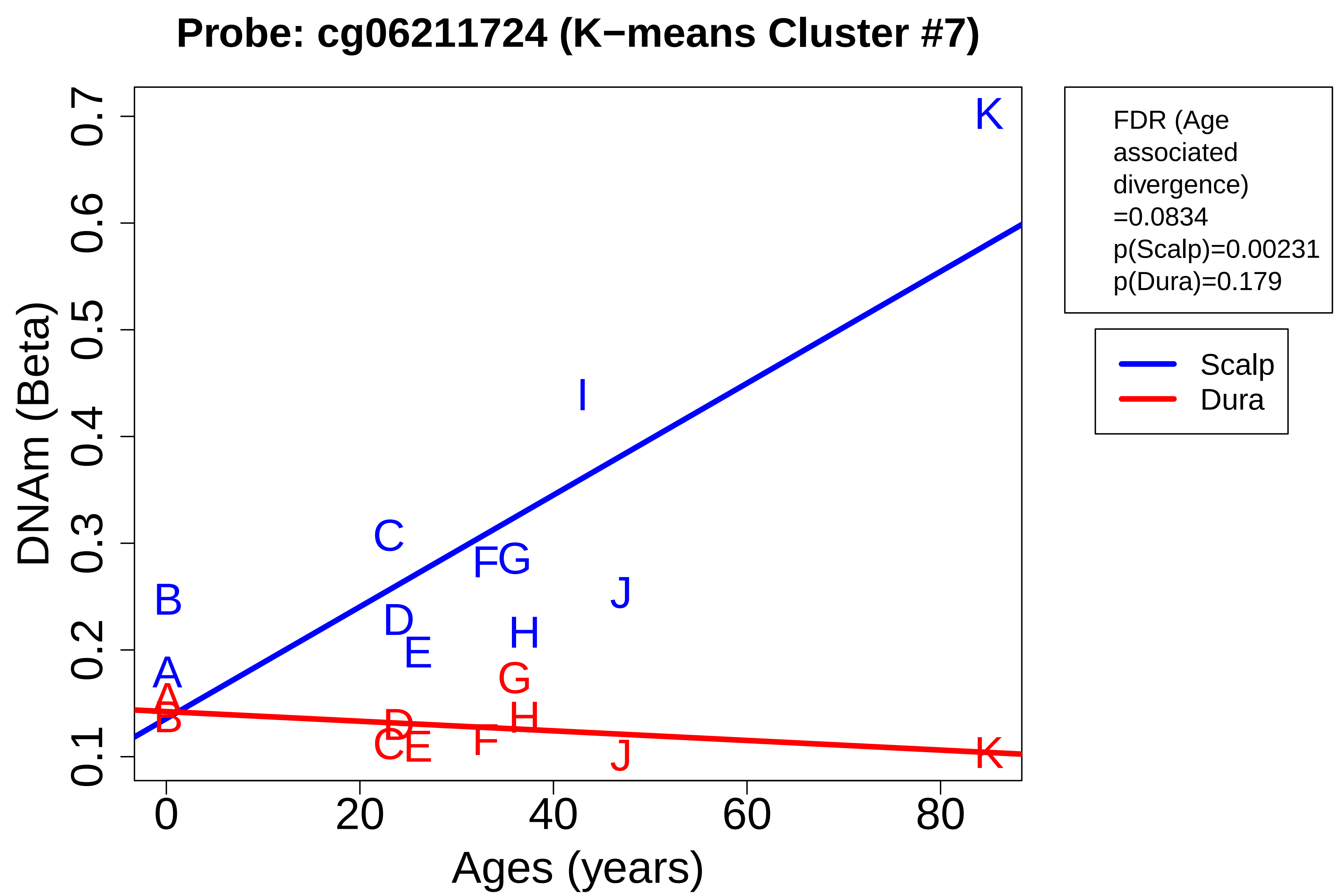

Probe: cg06211724 (K−means Cluster #7)
DNAm (Beta)
0.1
0.2
0.3
0.4
0.5
0.6
0.7
Ages (years)
0
20
40
60
80
FDR (Age associated divergence) =0.0834
p(Scalp)=0.00231
p(Dura)=0.179
Scalp
Dura

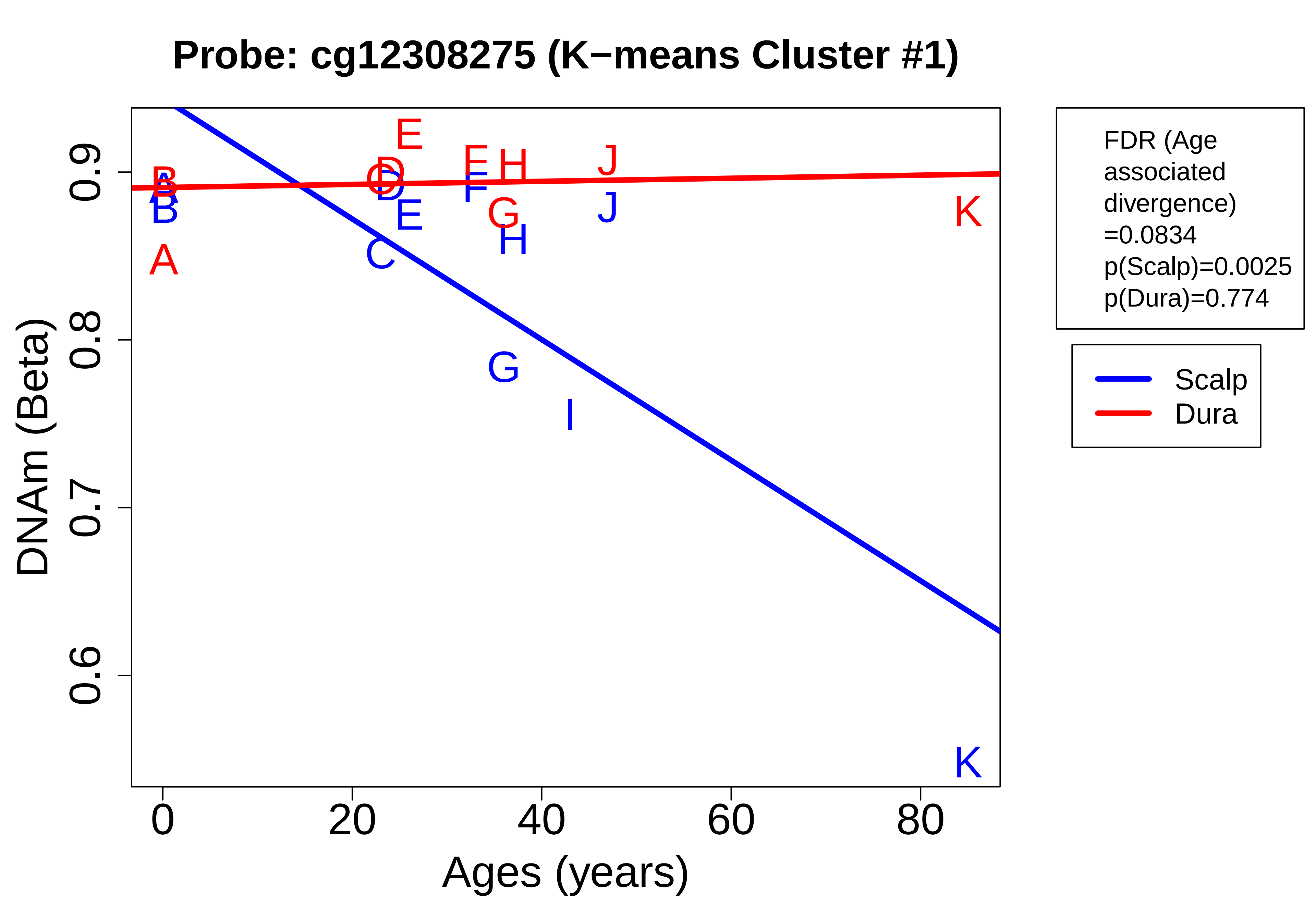
Probe: cg12308275 (K−means Cluster #1)
DNAm (Beta)
0.6
0.7
0.8
0.9
Ages (years)
0
20
40
60
80
FDR (Age associated divergence) =0.0834
p(Scalp)=0.0025
p(Dura)=0.774
Scalp
Dura
A
B
C
D
E
F
G
H
I
J
K

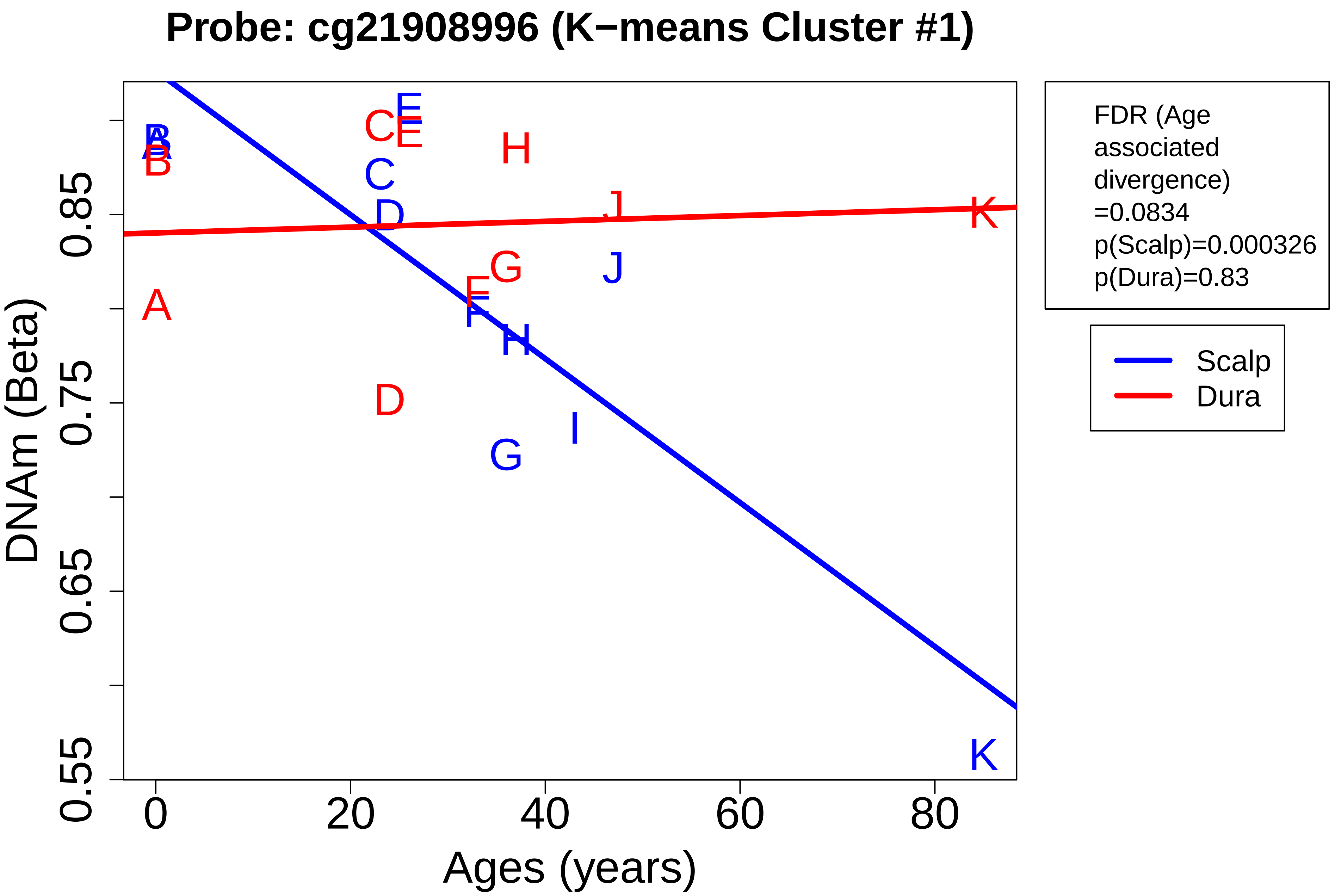

Probe: cg21908996 (K−means Cluster #1)
DNAm (Beta)
0.55
0.65
0.75
0.85
0
20
40
60
80
Ages (years)
FDR (Age associated divergence) =0.0834
p(Scalp)=0.000326
p(Dura)=0.83
Scalp
Dura
A
B
C
D
E
F
G
H
I
J
K

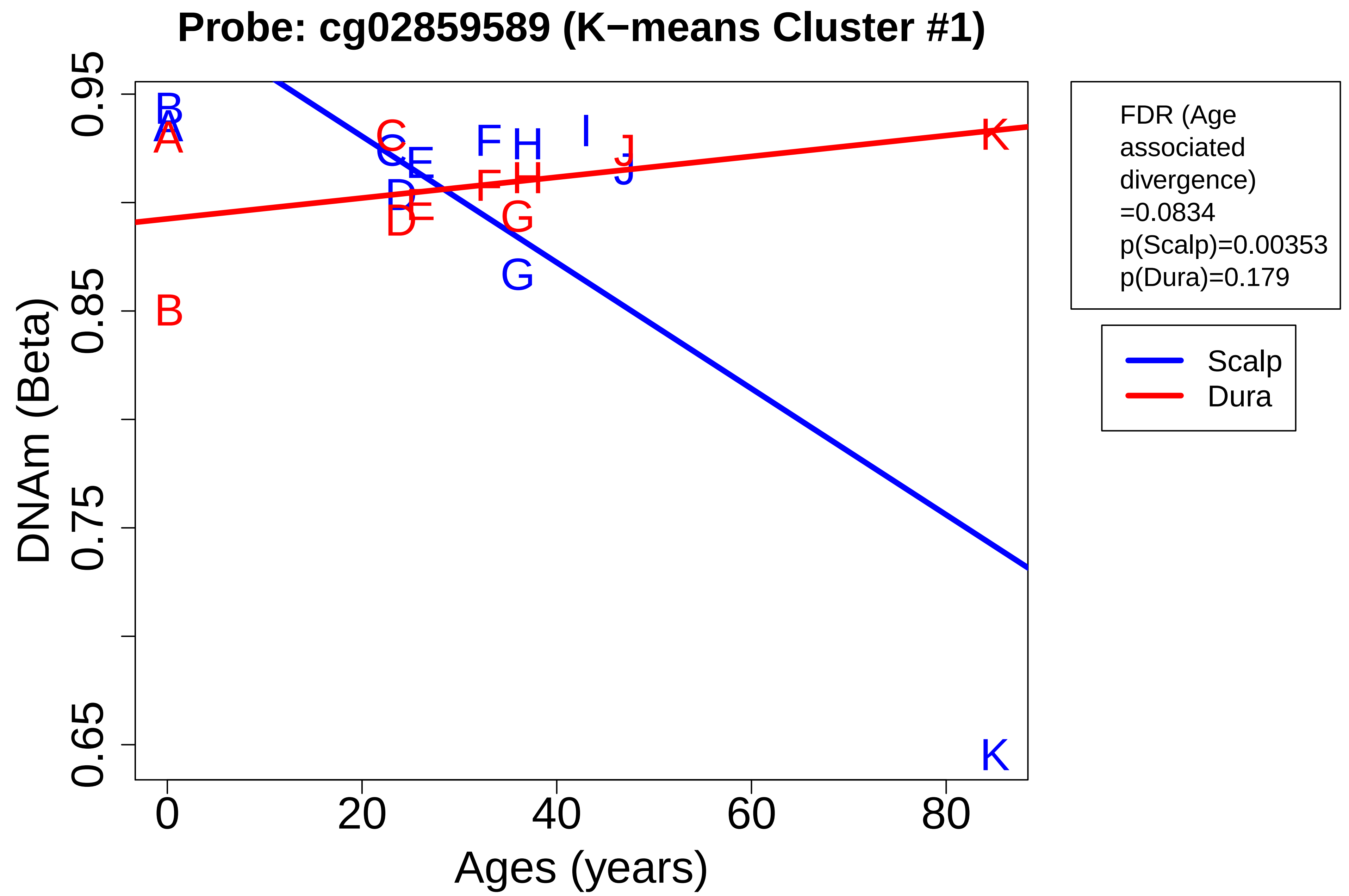

Probe: cg02859589 (K−means Cluster #1)
DNAm (Beta)
0.95
0.85
0.75
0.65
0
20
40
60
80
Ages (years)
A
B
C
D
E
F
G
H
I
J
K
FDR (Age associated divergence) =0.0834
p(Scalp)=0.00353
p(Dura)=0.179
Scalp
Dura

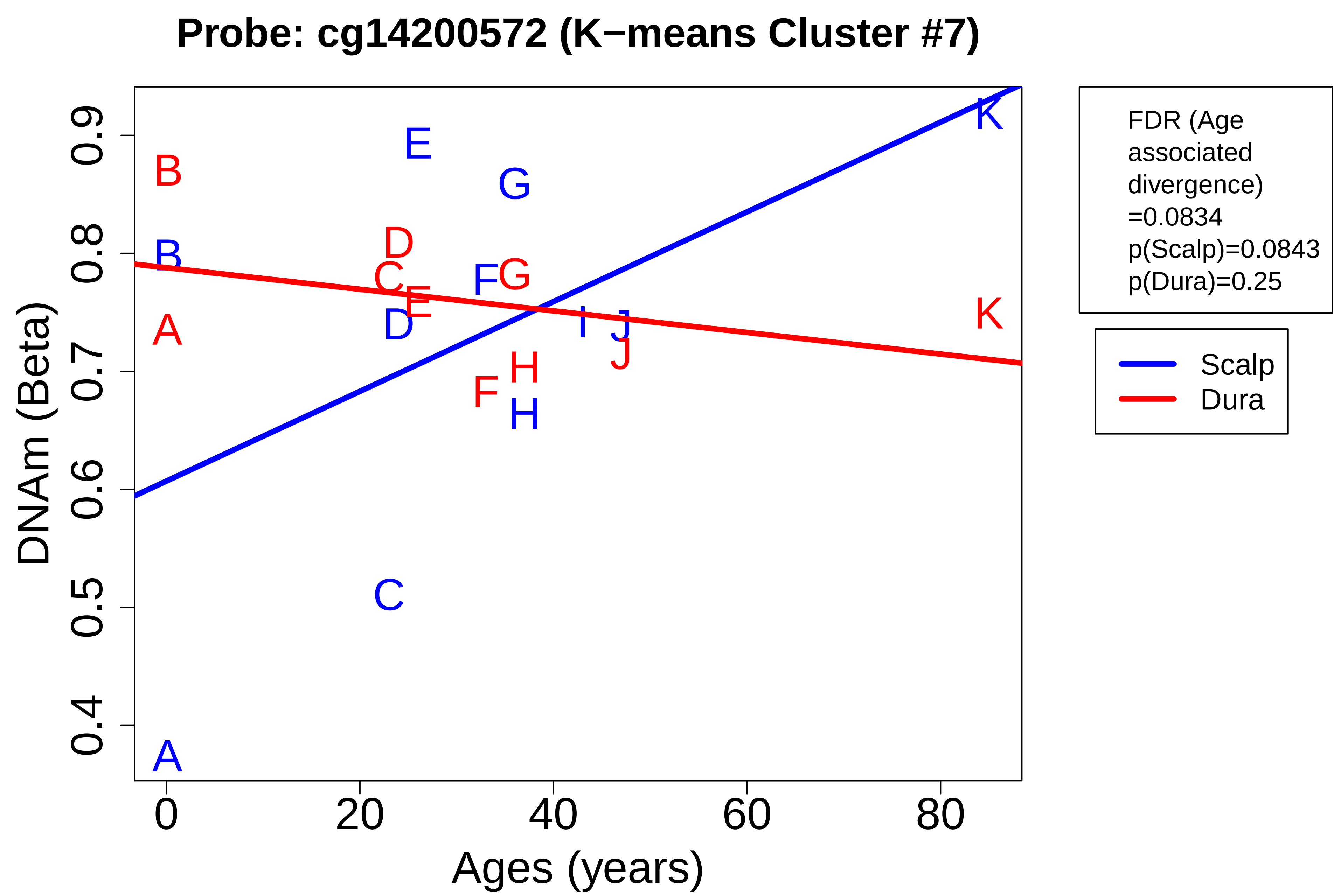
Probe: cg14200572 (K−means Cluster #7)
DNAm (Beta)
0.4
0.5
0.6
0.7
0.8
0.9
0
20
40
60
80
Ages (years)
FDR (Age associated divergence) =0.0834
p(Scalp)=0.0843
p(Dura)=0.25
Scalp
Dura
A
B
C
D
E
F
G
H
I
J
K

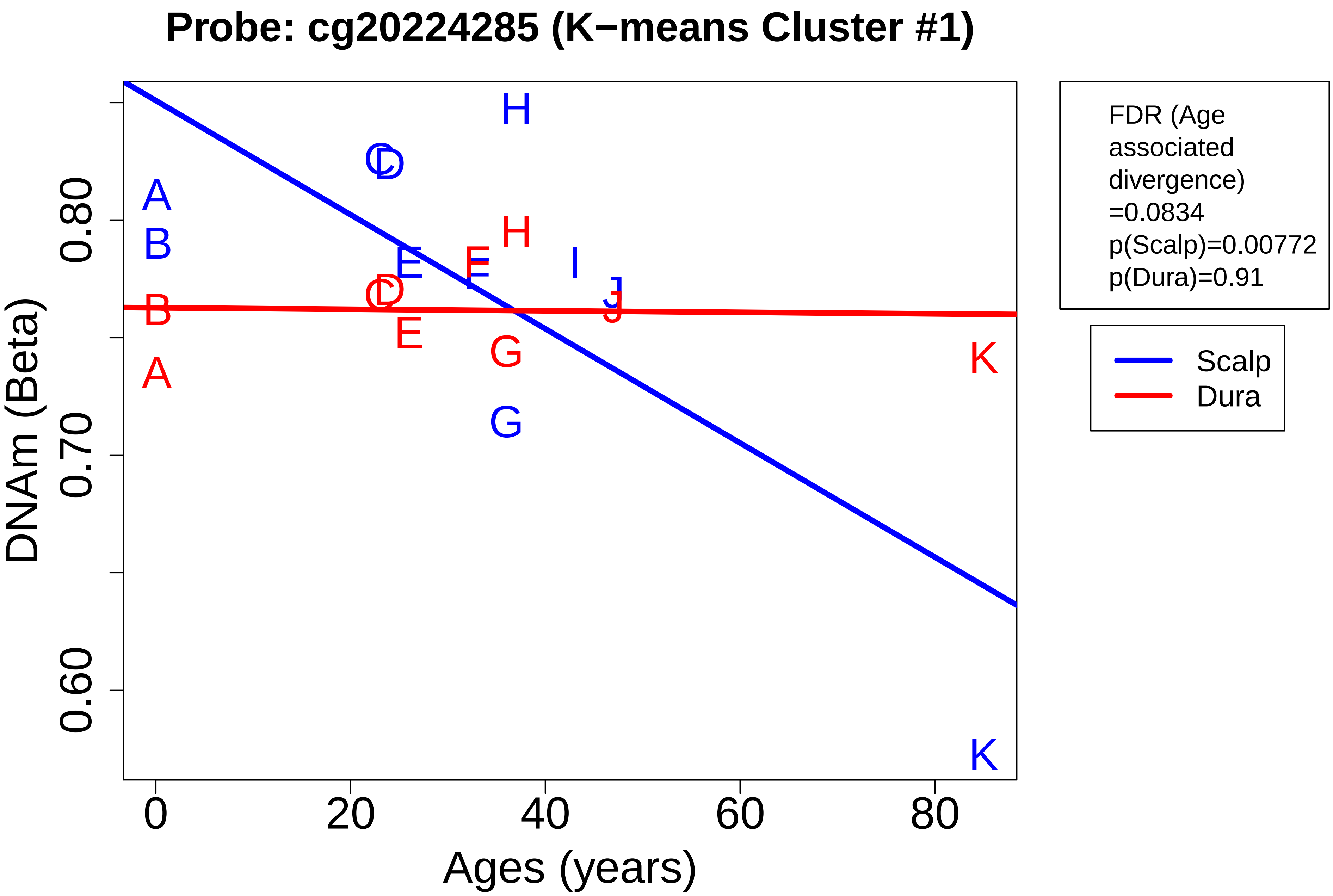

Probe: cg20224285 (K−means Cluster #1)
DNAm (Beta)
0.60
0.70
0.80
0
20
40
60
80
Ages (years)
FDR (Age associated divergence) =0.0834
p(Scalp)=0.00772
p(Dura)=0.91
Scalp
Dura
A
B
C
D
E
F
G
H
I
J
K

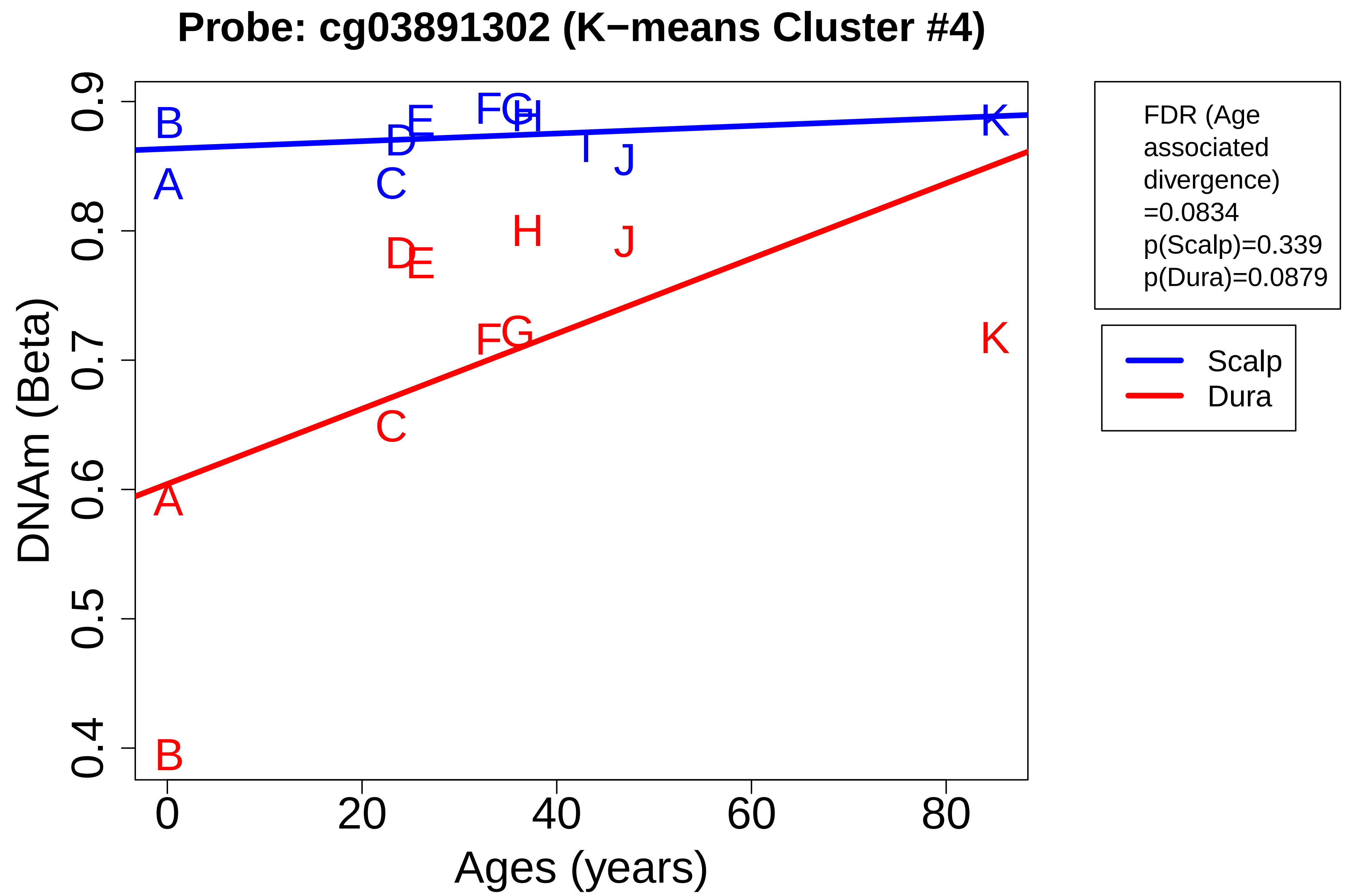

Probe: cg03891302 (K−means Cluster #4)
DNAm (Beta)
Ages (years)
0.4
0.5
0.6
0.7
0.8
0.9
0
20
40
60
80
FDR (Age associated divergence) =0.0834
p(Scalp)=0.339
p(Dura)=0.0879
Scalp
Dura

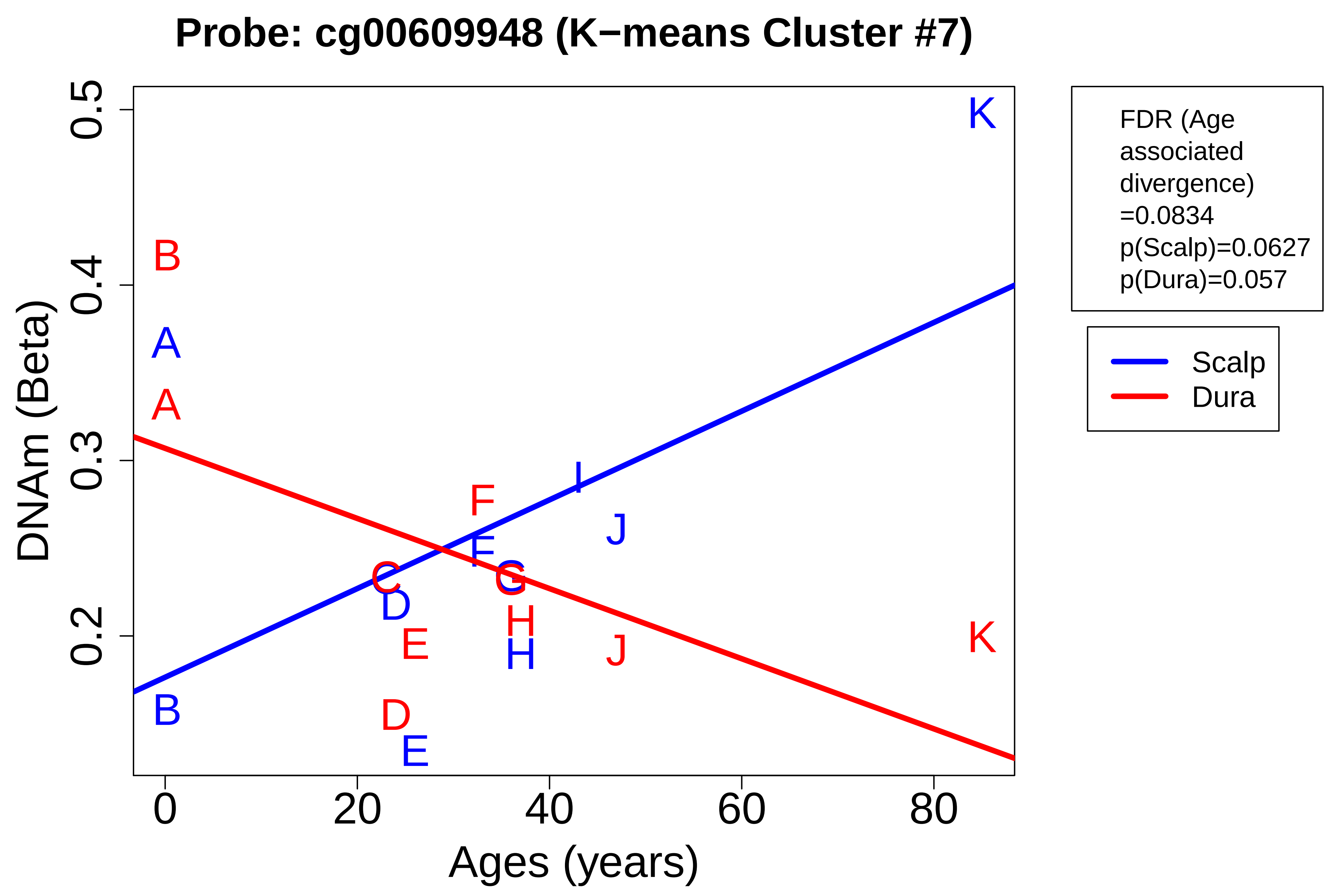
Probe: cg00609948 (K−means Cluster #7)
DNAm (Beta)
0.2
0.3
0.4
0.5
Ages (years)
0
20
40
60
80
FDR (Age associated divergence) =0.0834
p(Scalp)=0.0627
p(Dura)=0.057
Scalp
Dura
A
B
C
D
E
F
G
H
I
J
K

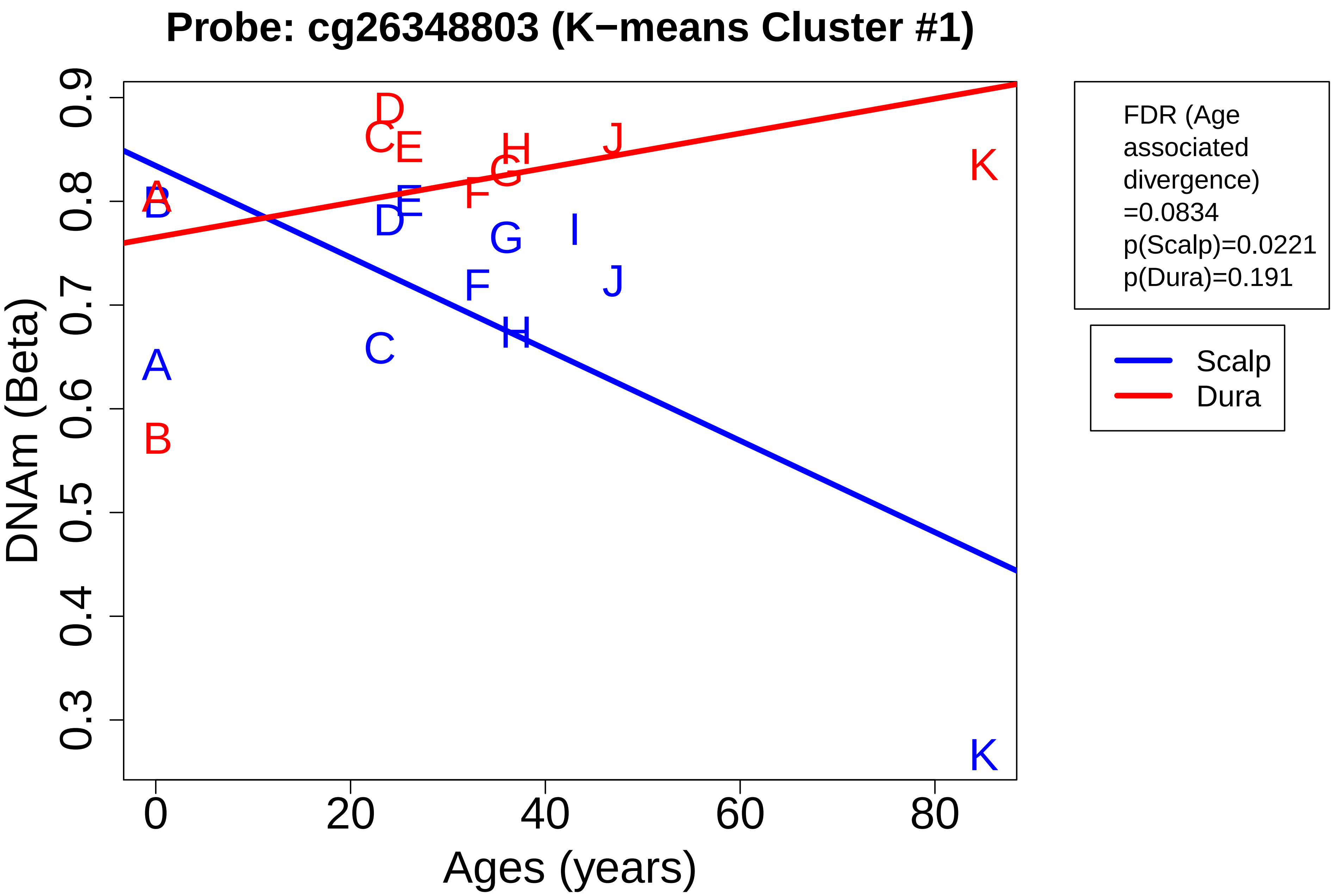

Probe: cg26348803 (K−means Cluster #1)
DNAm (Beta)
0.3
0.4
0.5
0.6
0.7
0.8
0.9
0
20
40
60
80
Ages (years)
A
B
C
D
E
F
G
H
I
J
K
FDR (Age associated divergence) =0.0834
p(Scalp)=0.0221
p(Dura)=0.191
Scalp
Dura

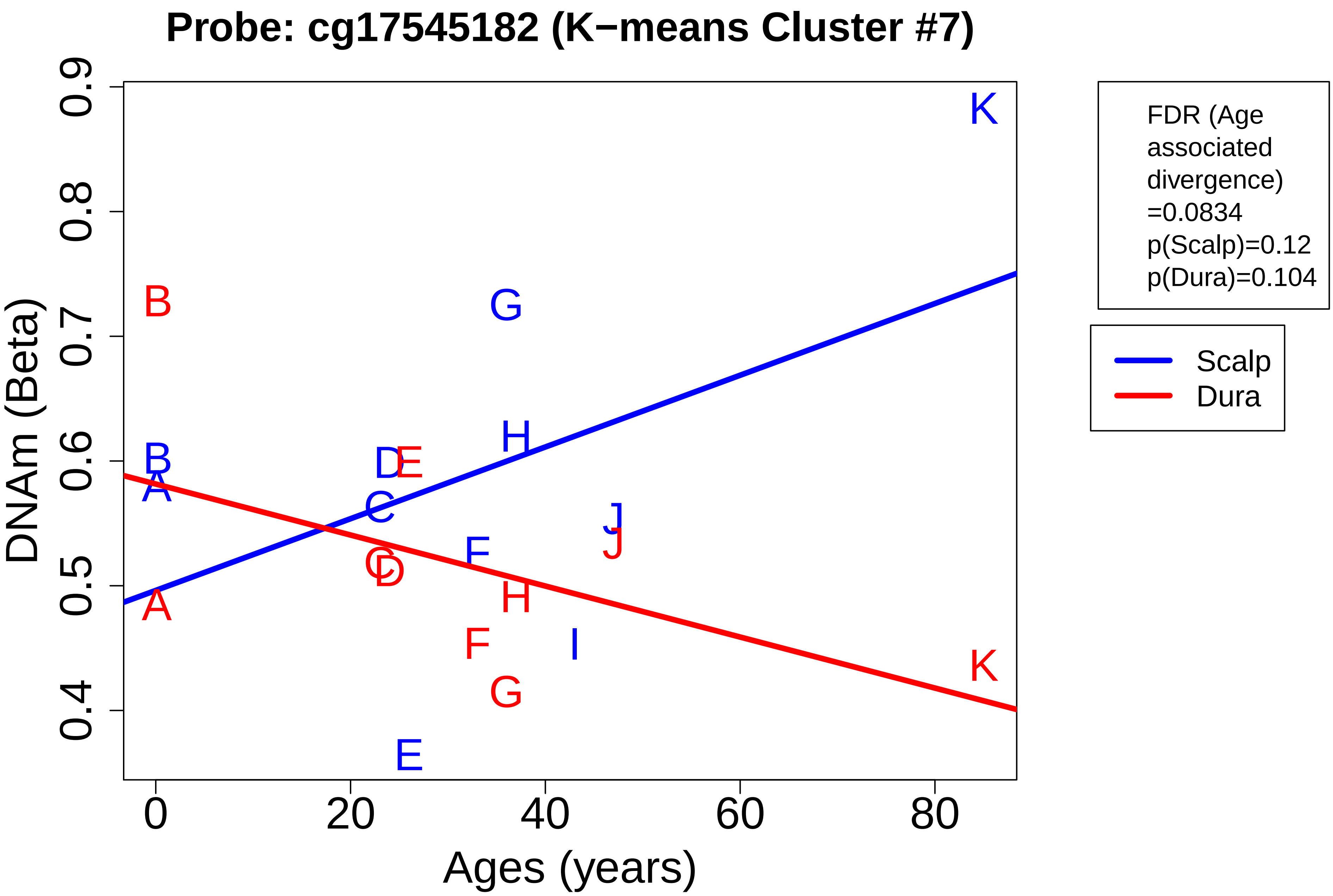
Probe: cg17545182 (K−means Cluster #7)
DNAm (Beta)
0.4
0.5
0.6
0.7
0.8
0.9
0
20
40
60
80
Ages (years)
FDR (Age associated divergence) =0.0834
p(Scalp)=0.12
p(Dura)=0.104
Scalp
Dura

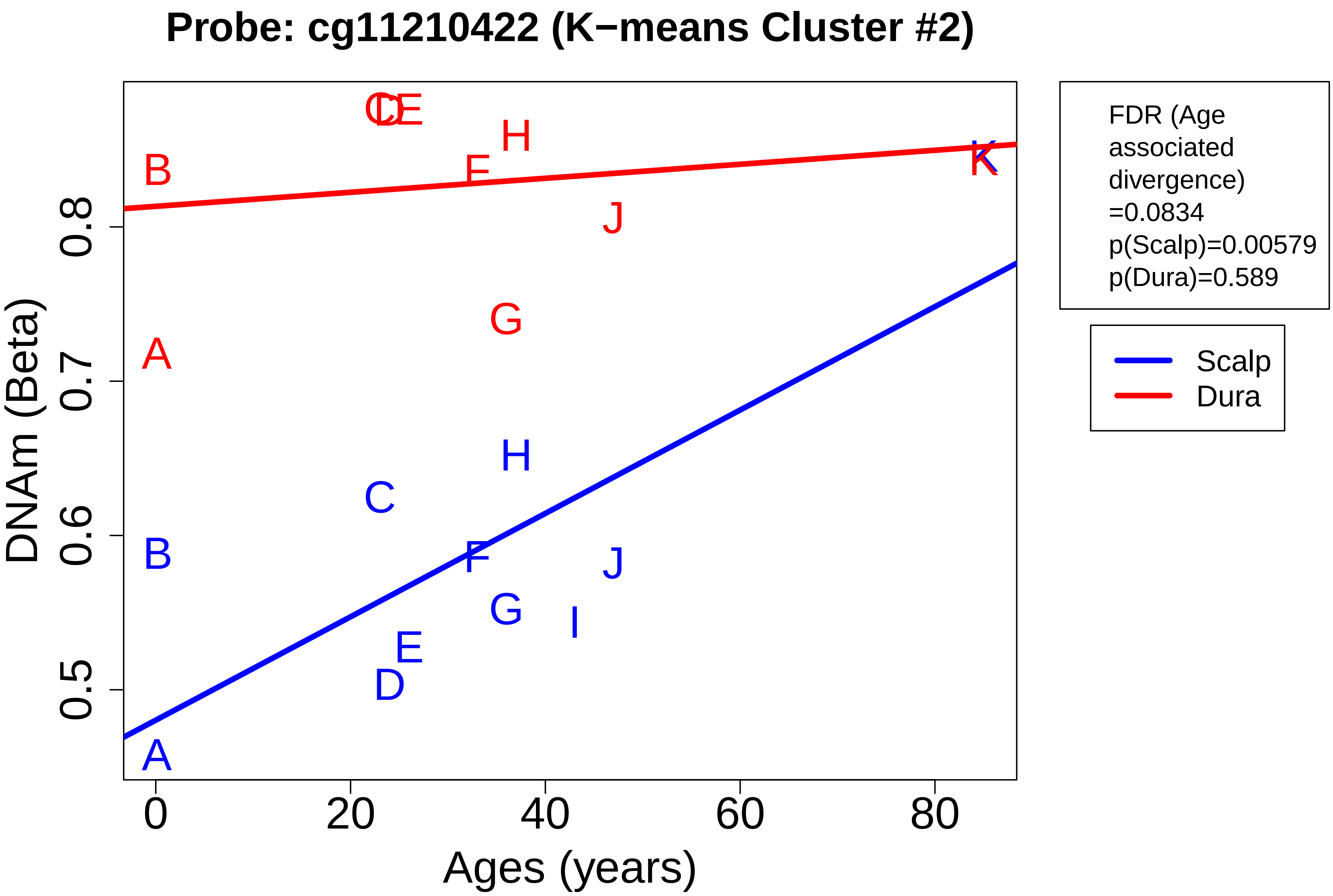

Probe: cg11210422 (K−means Cluster #2)
DNAm (Beta)
0.5
0.6
0.7
0.8
0
20
40
60
80
Ages (years)
FDR (Age associated divergence) =0.0834
p(Scalp)=0.00579
p(Dura)=0.589
Scalp
Dura

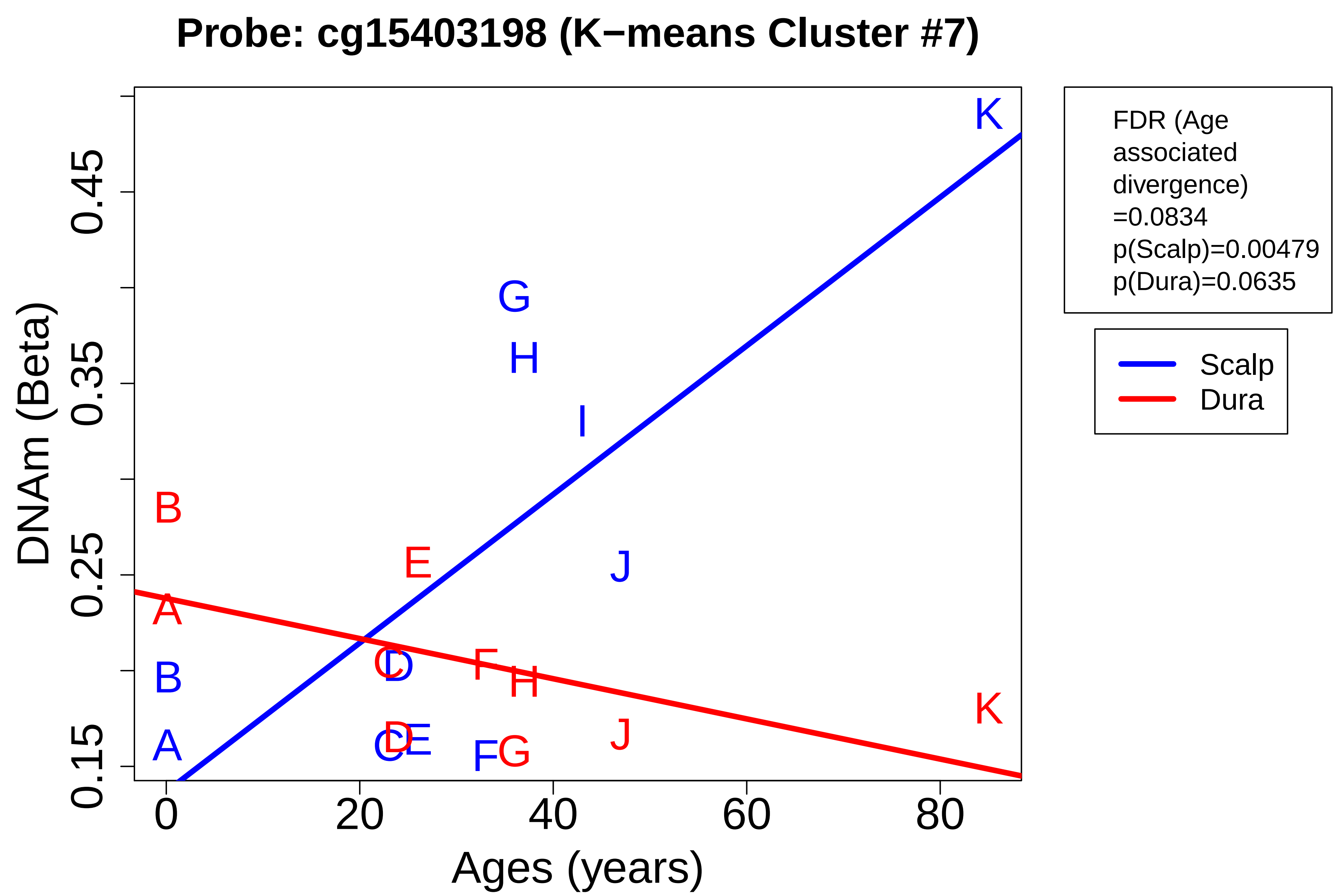

Probe: cg15403198 (K−means Cluster #7)
DNAm (Beta)
0.15
0.25
0.35
0.45
0
20
40
60
80
Ages (years)
FDR (Age associated divergence) =0.0834
p(Scalp)=0.00479
p(Dura)=0.0635
Scalp
Dura

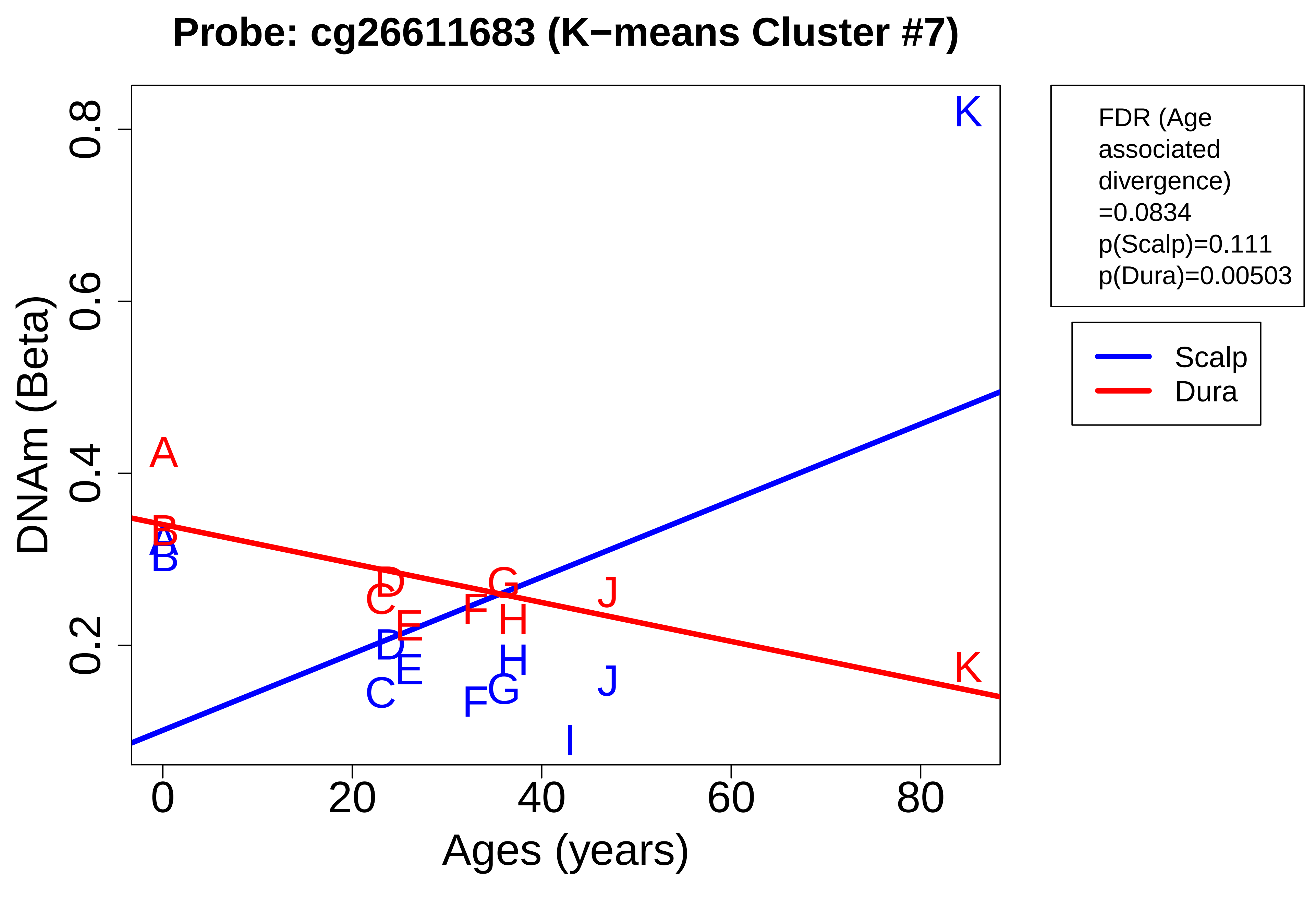

Probe: cg26611683 (K−means Cluster #7)
DNAm (Beta)
0.2
0.4
0.6
0.8
0
20
40
60
80
Ages (years)
A
B
C
D
E
F
G
H
I
J
K
FDR (Age associated divergence) =0.0834
p(Scalp)=0.111
p(Dura)=0.00503
Scalp
Dura

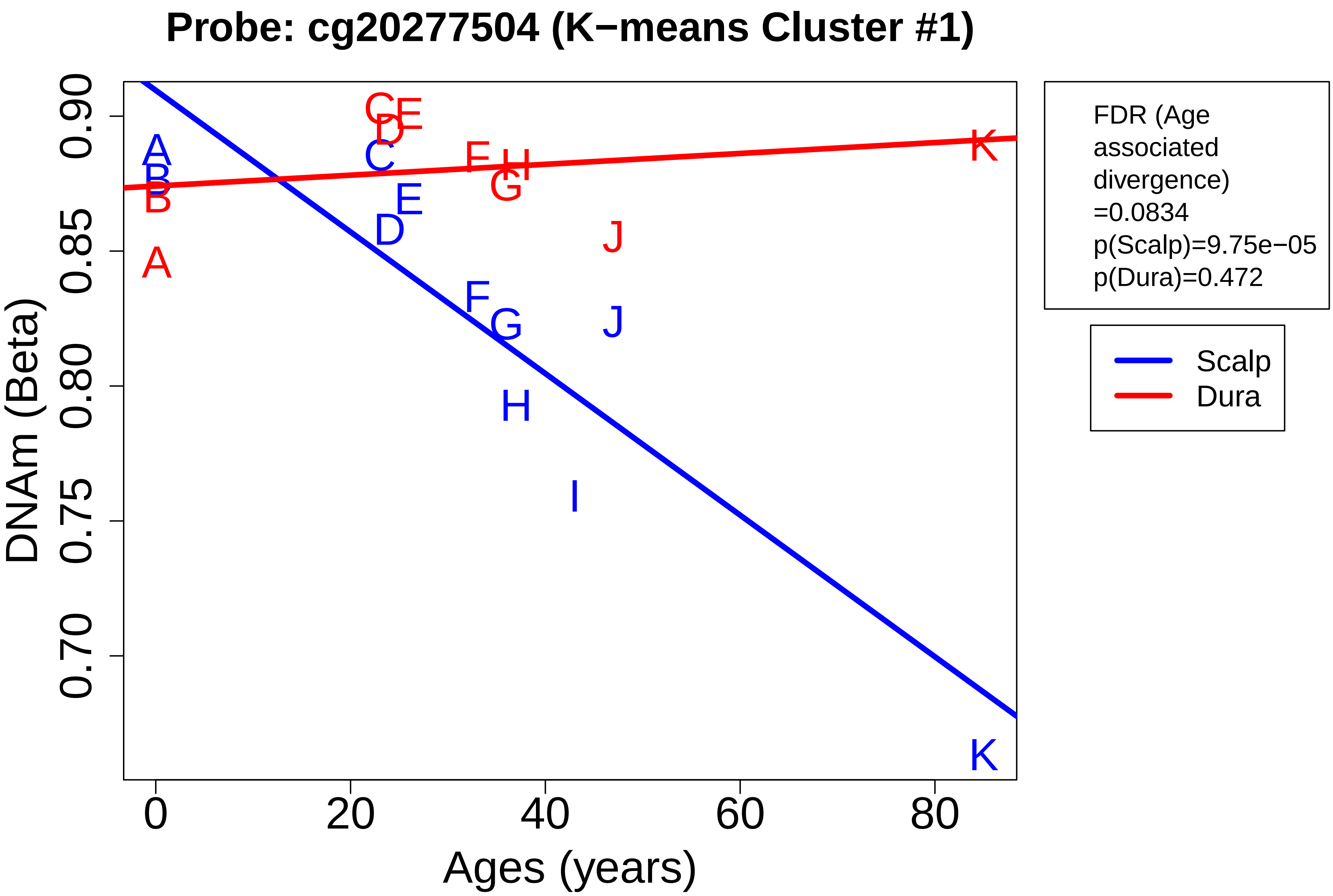

Probe: cg20277504 (K−means Cluster #1)
DNAm (Beta)
0.70
0.75
0.80
0.85
0.90
0
20
40
60
80
Ages (years)
FDR (Age associated divergence) =0.0834
p(Scalp)=9.75e−05
p(Dura)=0.472
Scalp
Dura

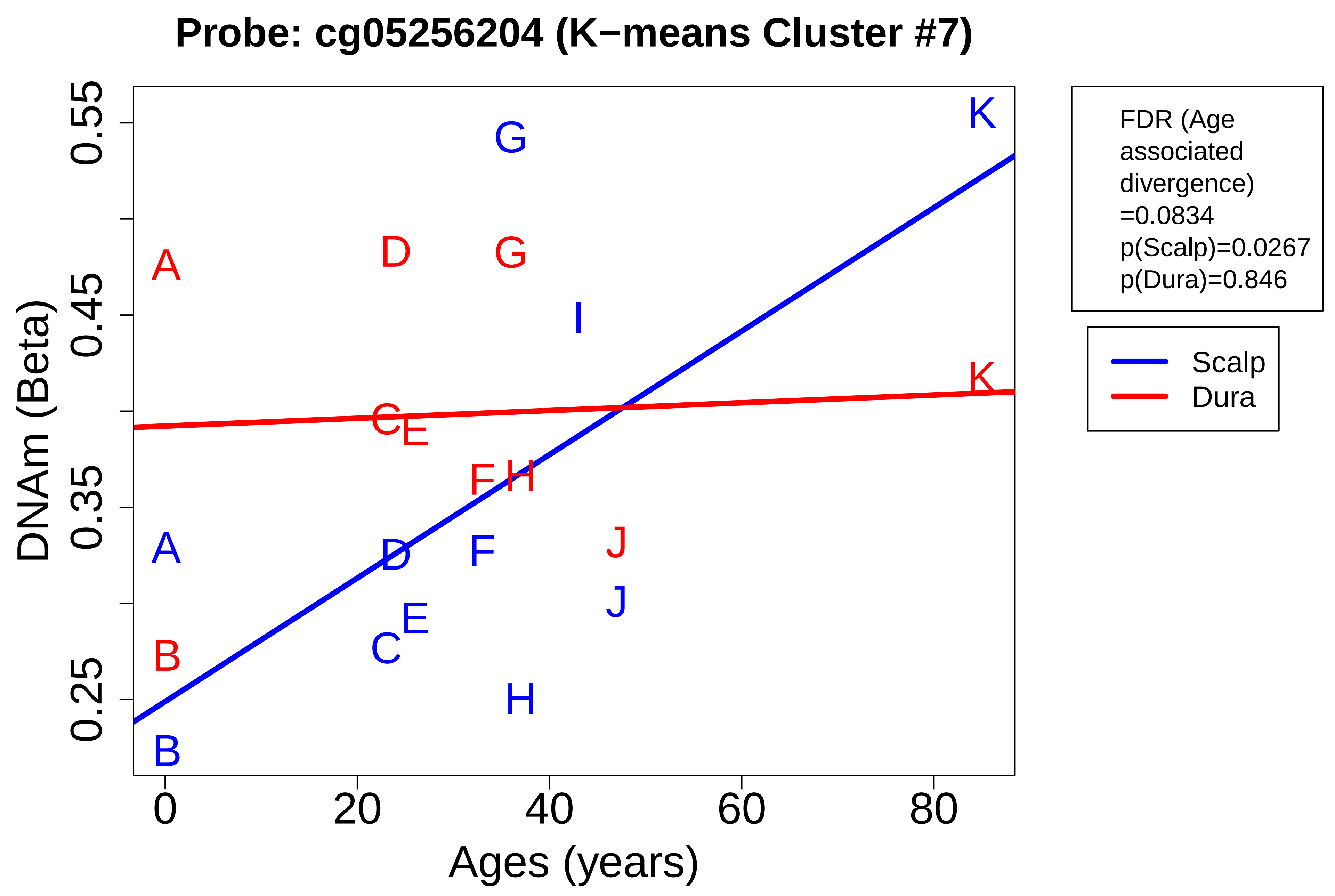

Probe: cg05256204 (K−means Cluster #7)
DNAm (Beta)
0.25
0.35
0.45
0.55
Ages (years)
0
20
40
60
80
FDR (Age associated divergence) =0.0834
p(Scalp)=0.0267
p(Dura)=0.846
Scalp
Dura

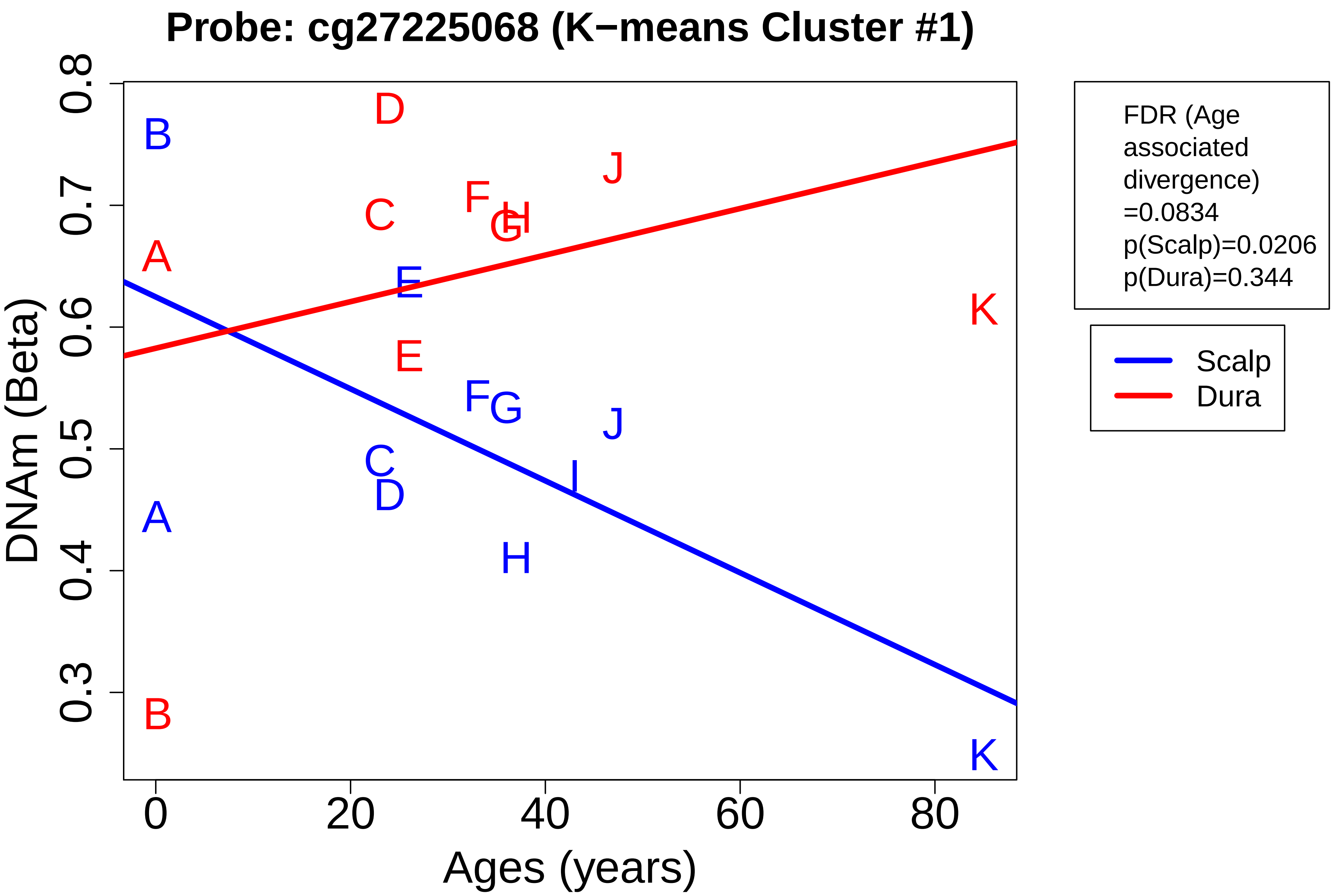
Probe: cg27225068 (K−means Cluster #1)
DNAm (Beta)
0.3
0.4
0.5
0.6
0.7
0.8
0
20
40
60
80
Ages (years)
FDR (Age associated divergence) =0.0834
p(Scalp)=0.0206
p(Dura)=0.344
Scalp
Dura

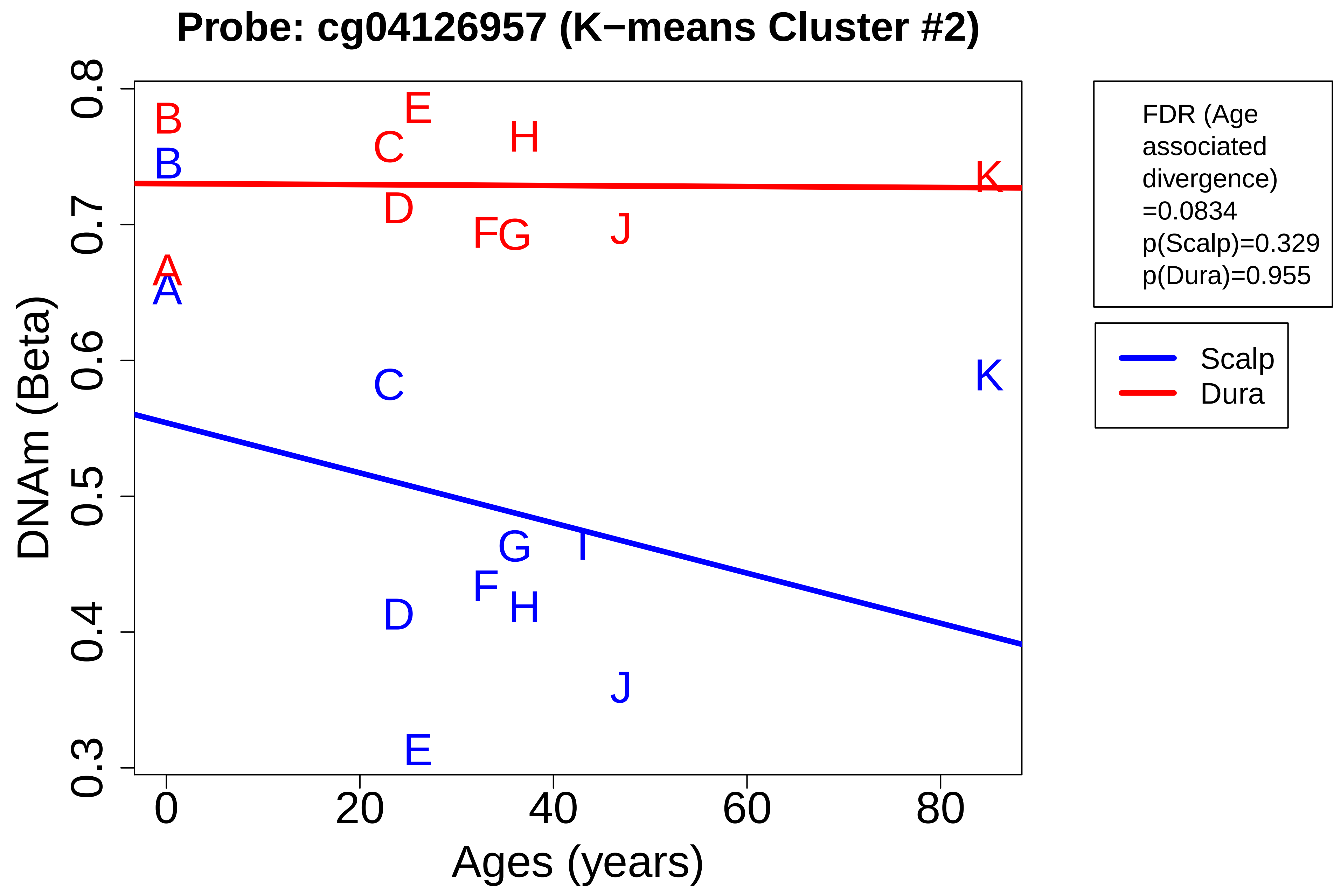
Probe: cg04126957 (K−means Cluster #2)
DNAm (Beta)
0.3
0.4
0.5
0.6
0.7
0.8
0
20
40
60
80
Ages (years)
B
E
C
H
B
K
D
FG
J
A
A
C
K
G
I
F
H
D
J
E
FDR (Age associated divergence) =0.0834
p(Scalp)=0.329
p(Dura)=0.955
Scalp
Dura

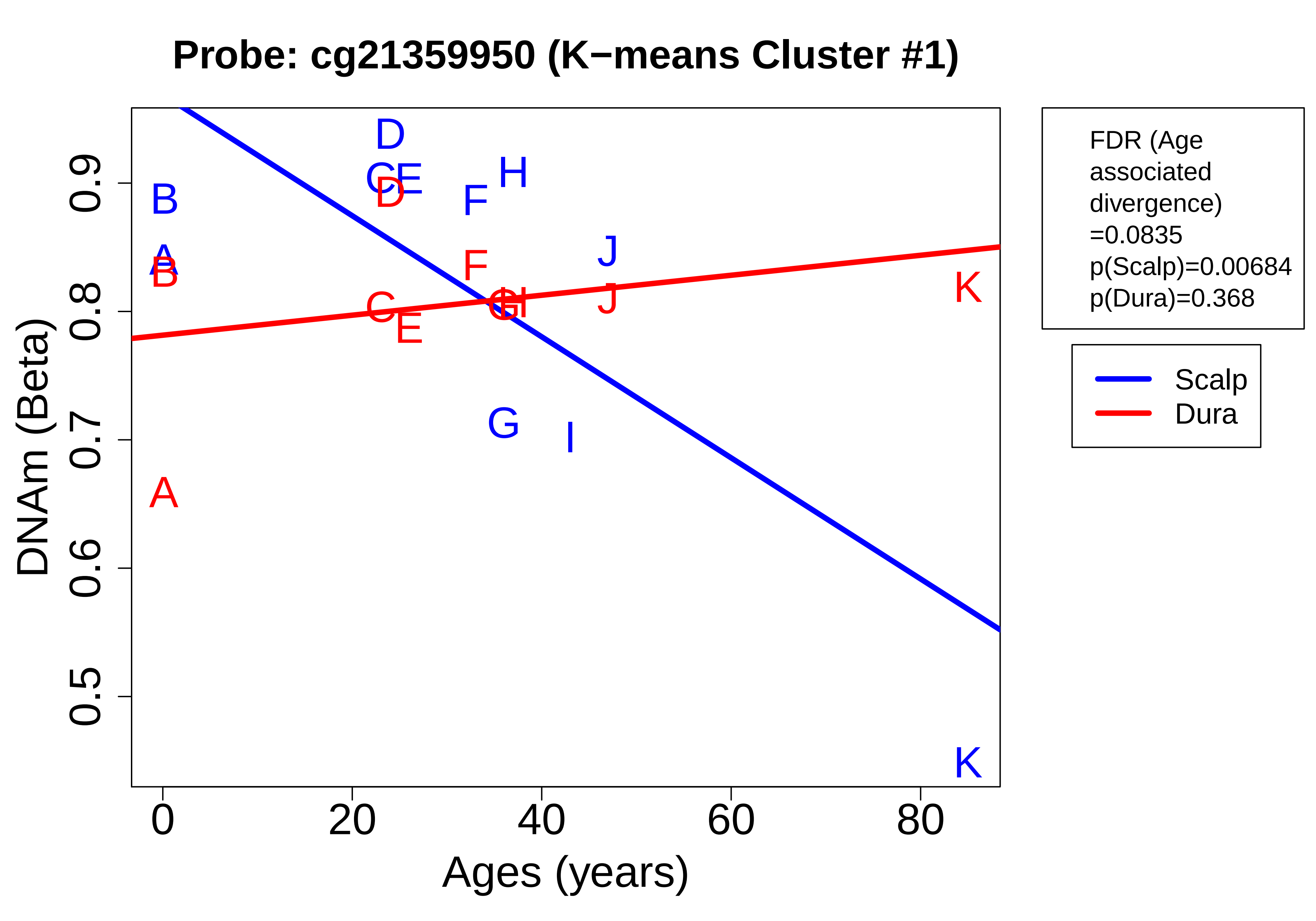
Probe: cg21359950 (K−means Cluster #1)
DNAm (Beta)
Ages (years)
0.5
0.6
0.7
0.8
0.9
0
20
40
60
80
FDR (Age associated divergence) =0.0835
p(Scalp)=0.00684
p(Dura)=0.368
Scalp
Dura
A
B
C
D
E
F
G
H
I
J
K

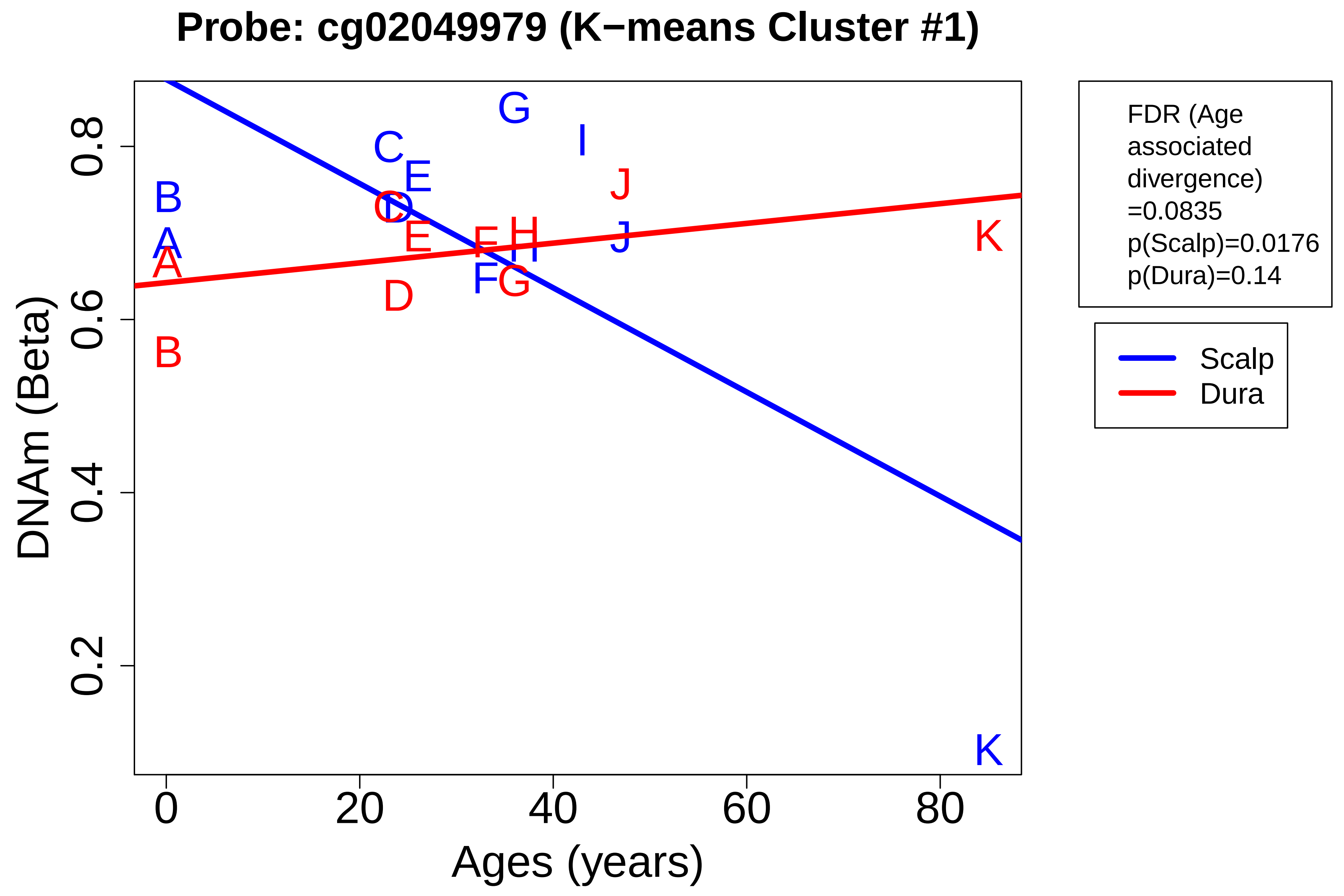

Probe: cg02049979 (K−means Cluster #1)
DNAm (Beta)
0.2
0.4
0.6
0.8
0
20
40
60
80
Ages (years)
A B C D E F G H I J K
A B C D E F G H J K
FDR (Age associated divergence) =0.0835
p(Scalp)=0.0176
p(Dura)=0.14
Scalp
Dura

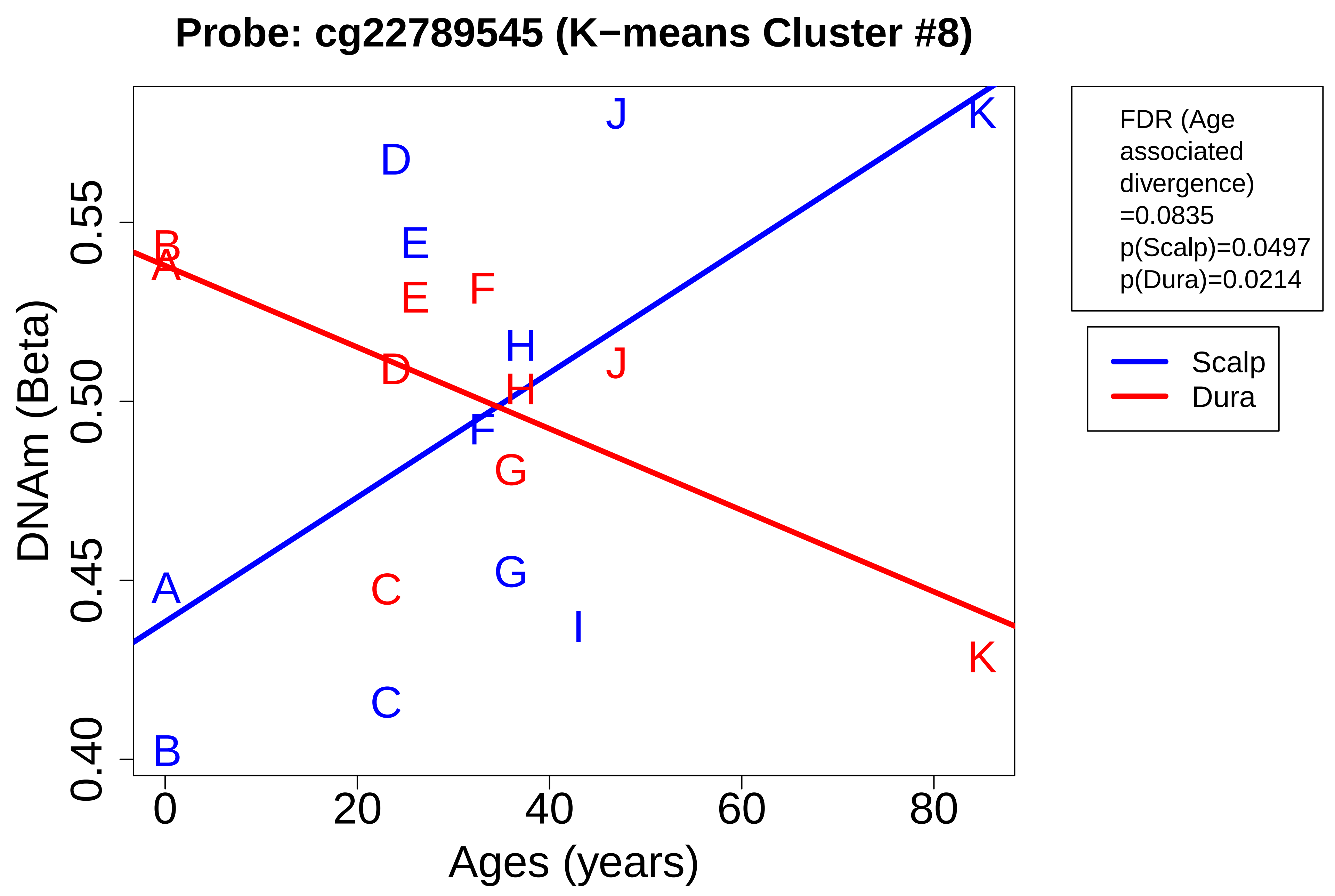

Probe: cg22789545 (K−means Cluster #8)
DNAm (Beta)
0.40
0.45
0.50
0.55
0
20
40
60
80
Ages (years)
A
B
C
D
E
F
G
H
I
J
K
FDR (Age associated divergence) =0.0835
p(Scalp)=0.0497
p(Dura)=0.0214
Scalp
Dura

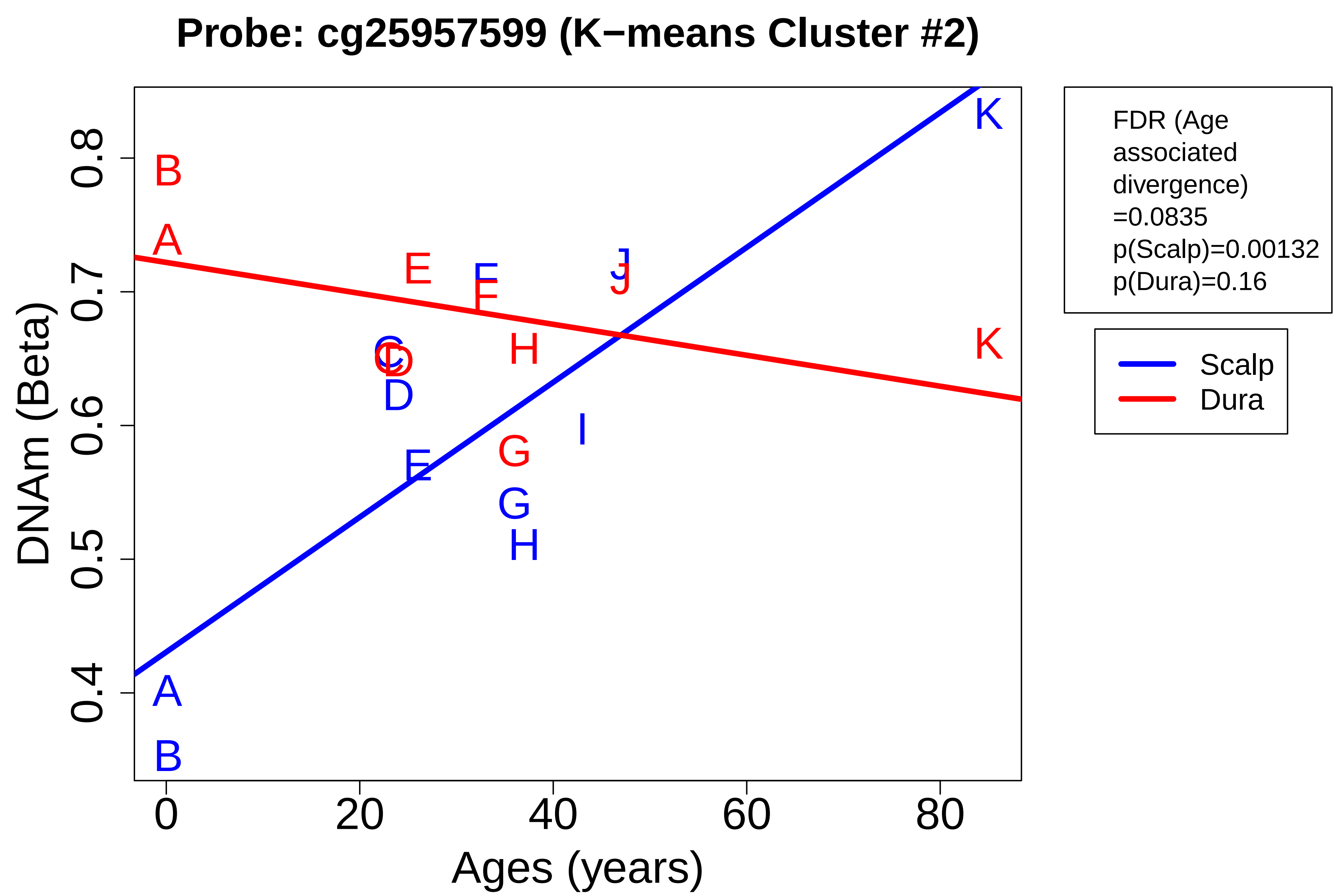

Probe: cg25957599 (K−means Cluster #2)
DNAm (Beta)
0.4
0.5
0.6
0.7
0.8
Ages (years)
0
20
40
60
80
FDR (Age associated divergence) =0.0835
p(Scalp)=0.00132
p(Dura)=0.16
Scalp
Dura

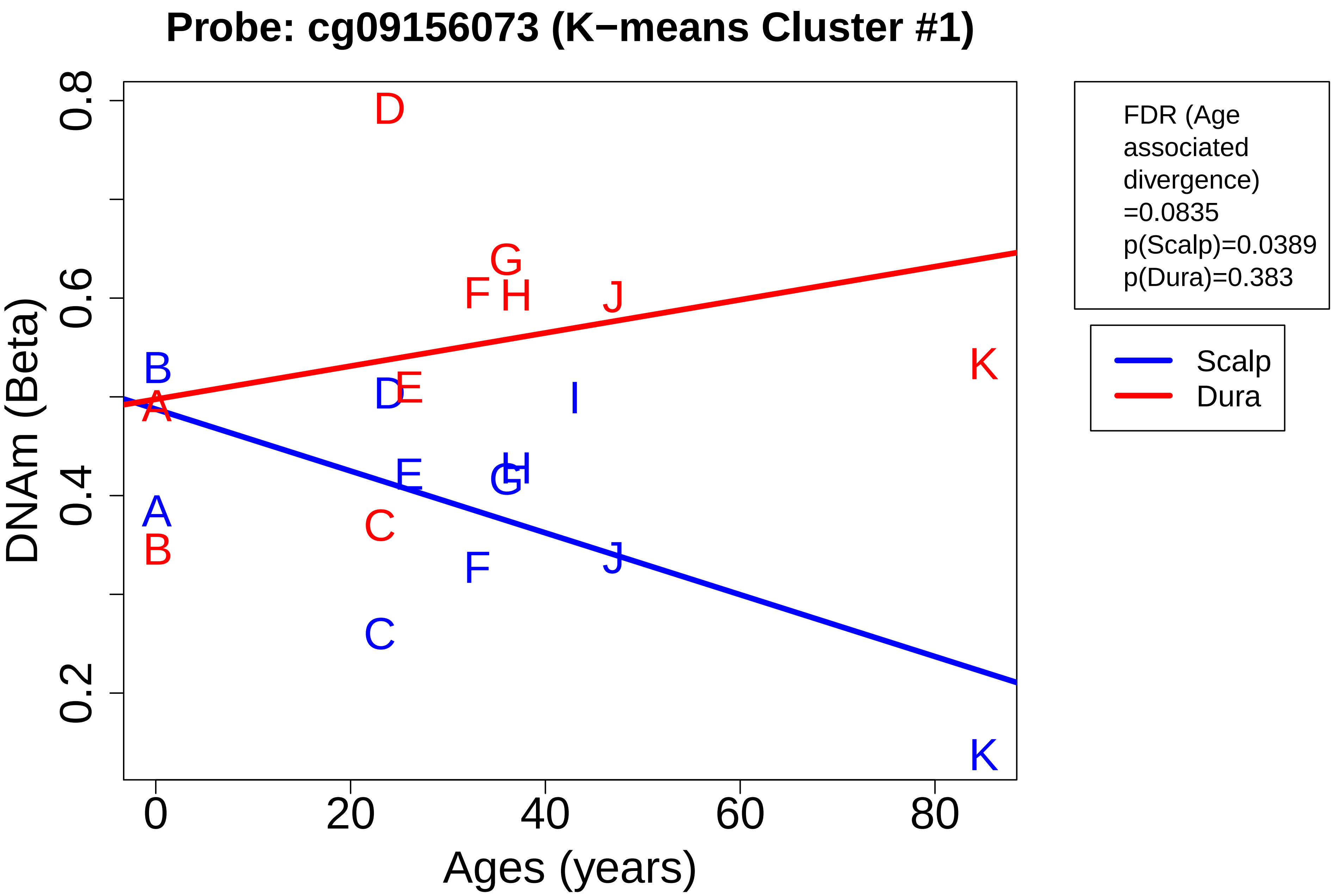

Probe: cg09156073 (K−means Cluster #1)
DNAm (Beta)
0.2
0.4
0.6
0.8
Ages (years)
0
20
40
60
80
FDR (Age associated divergence) =0.0835
p(Scalp)=0.0389
p(Dura)=0.383
Scalp
Dura
A
B
C
D
E
F
G
H
I
J
K

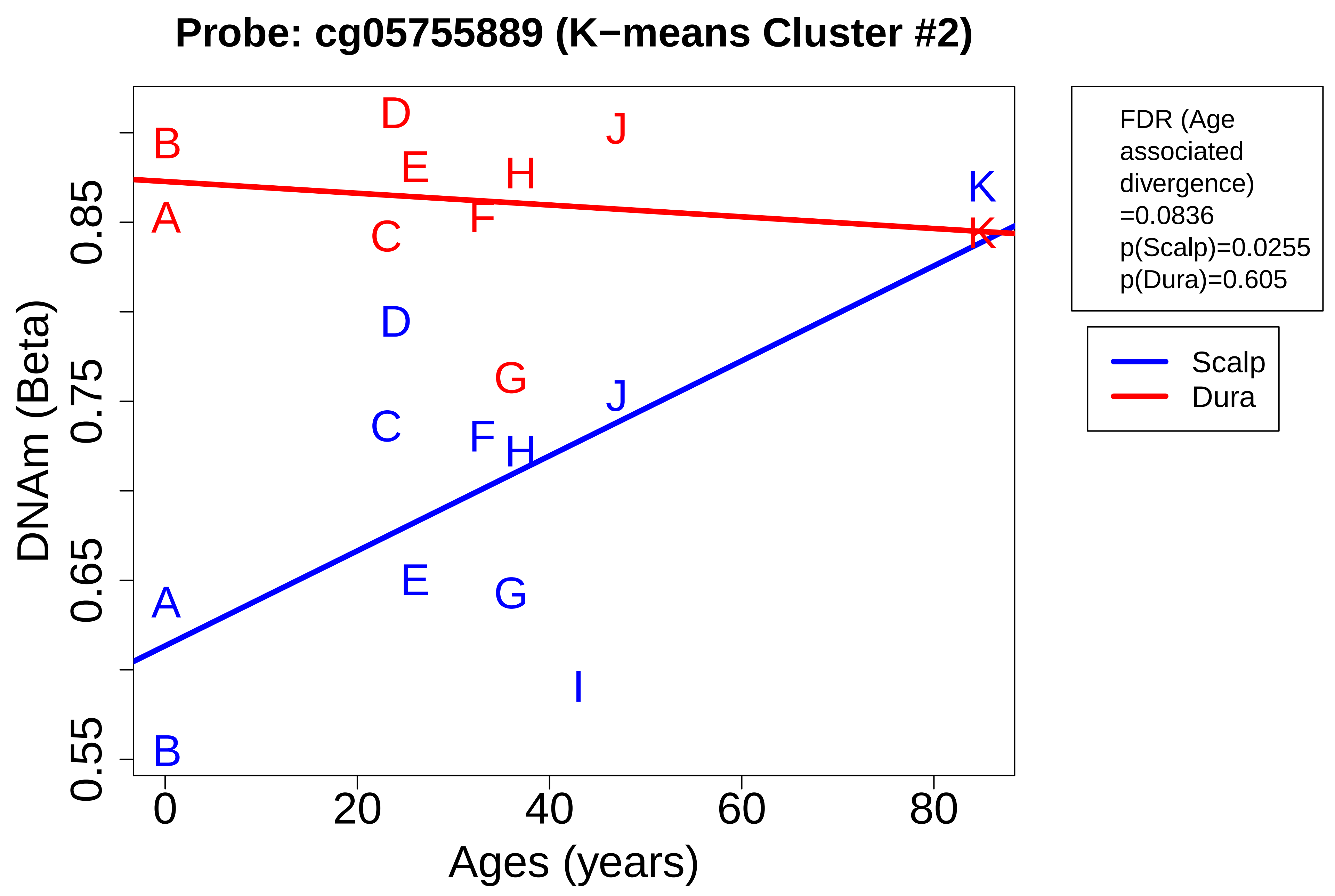

Probe: cg05755889 (K−means Cluster #2)
DNAm (Beta)
0.55
0.65
0.75
0.85
0
20
40
60
80
Ages (years)
FDR (Age associated divergence) =0.0836
p(Scalp)=0.0255
p(Dura)=0.605
Scalp
Dura

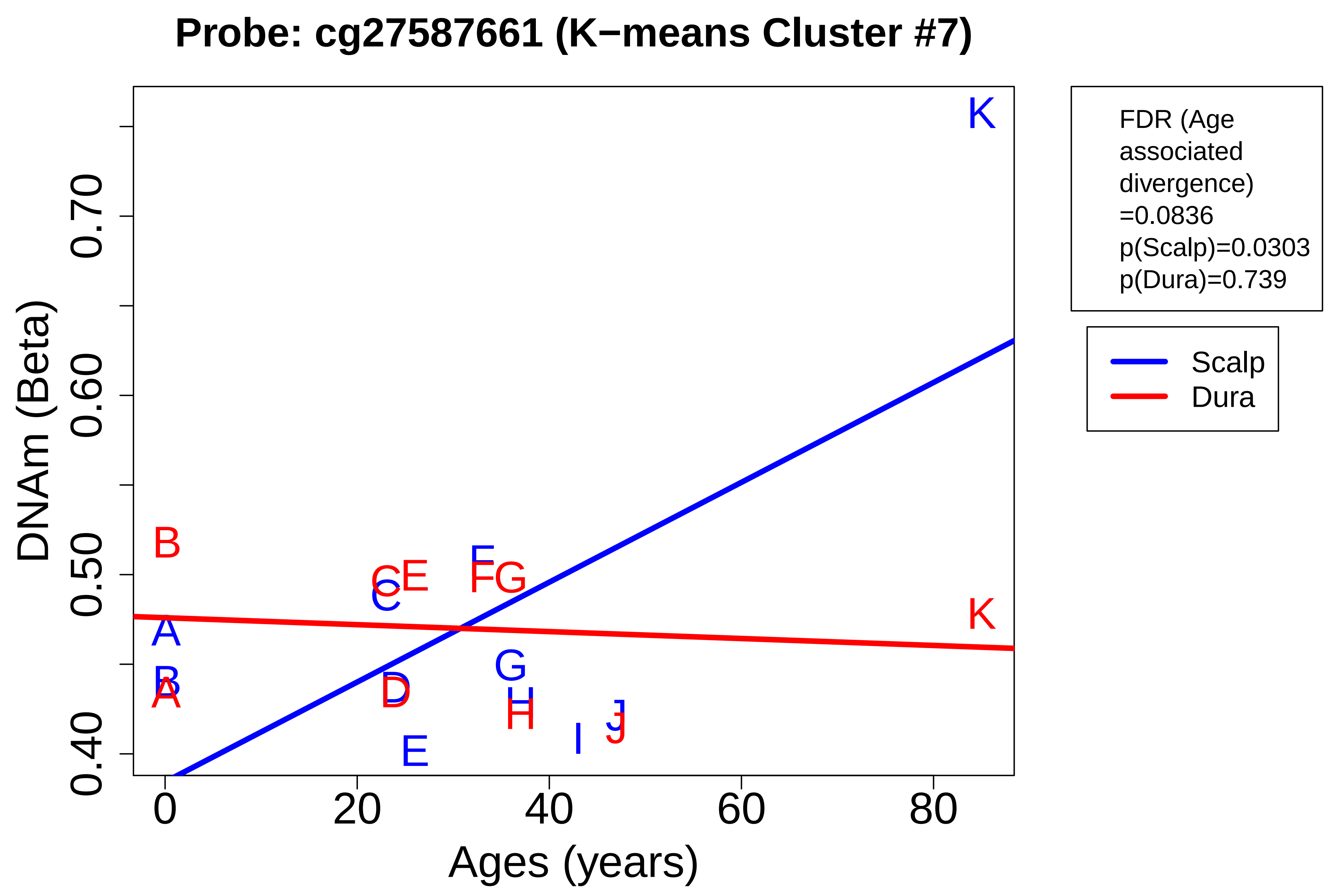

Probe: cg27587661 (K−means Cluster #7)
DNAm (Beta)
0.40
0.50
0.60
0.70
Ages (years)
0
20
40
60
80
FDR (Age associated divergence) =0.0836
p(Scalp)=0.0303
p(Dura)=0.739
Scalp
Dura
A
B
C
D
E
F
G
H
I
J
K

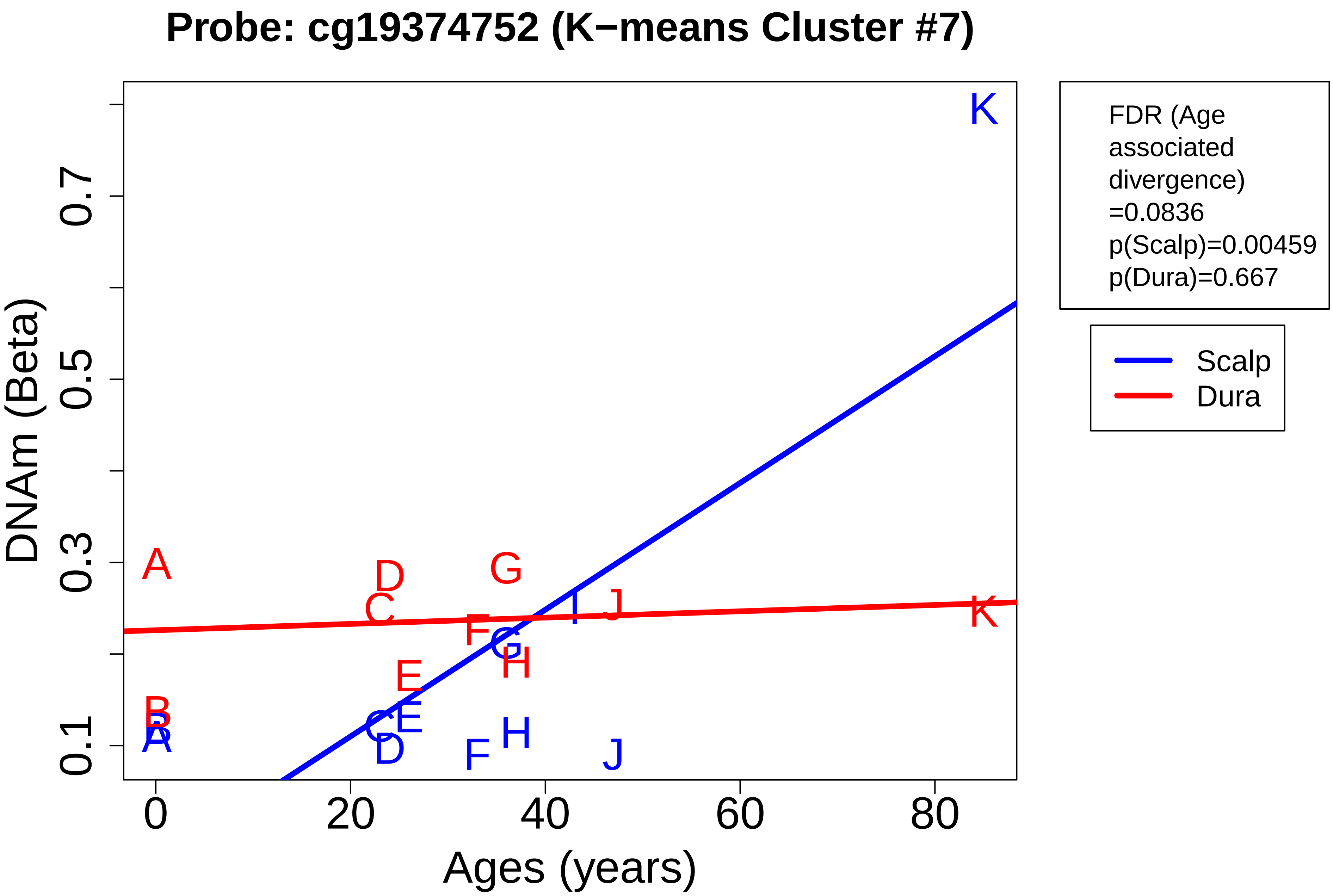

Probe: cg19374752 (K−means Cluster #7)
DNAm (Beta)
0.1
0.3
0.5
0.7
0
20
40
60
80
Ages (years)
FDR (Age associated divergence) =0.0836
p(Scalp)=0.00459
p(Dura)=0.667
Scalp
Dura

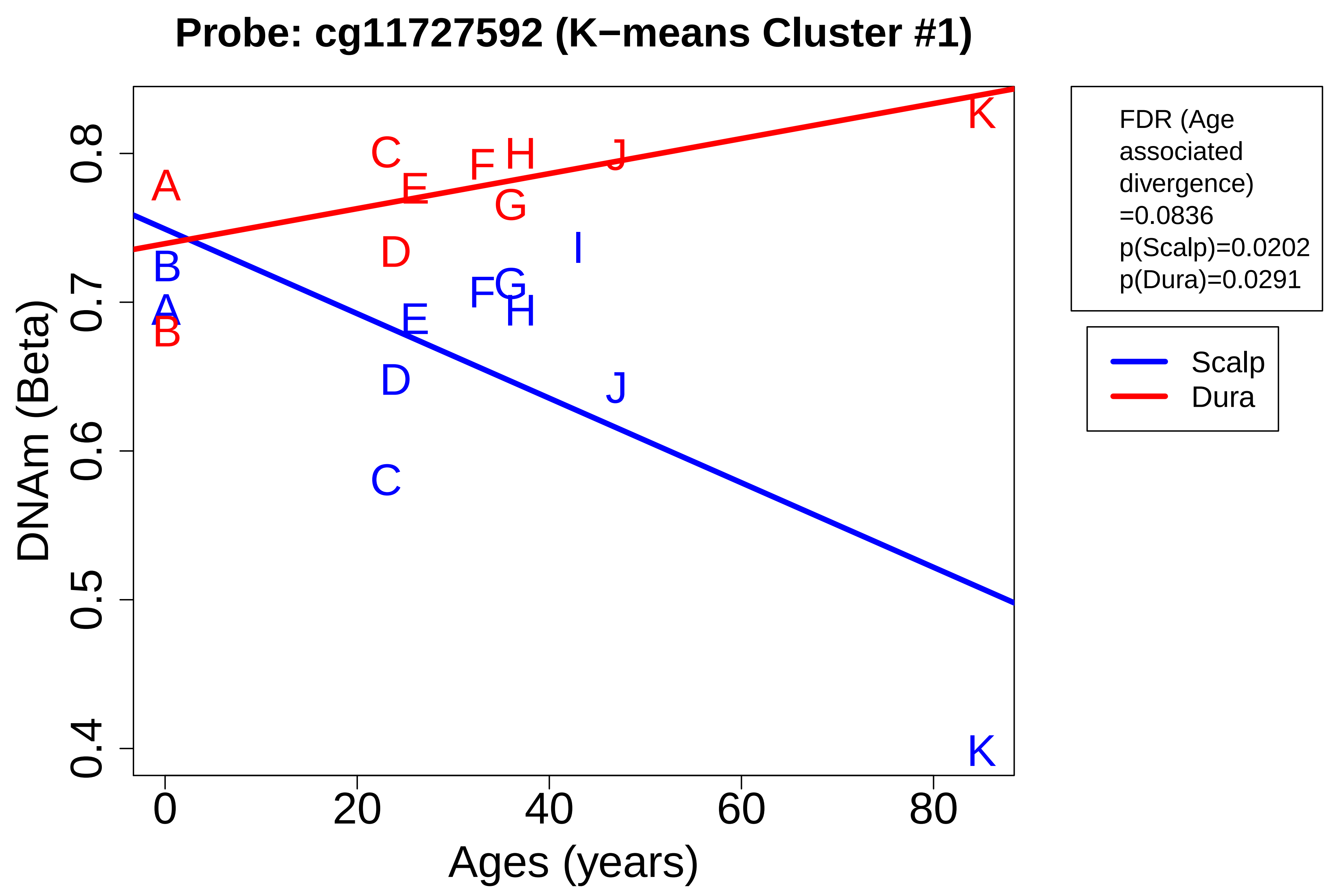
Probe: cg11727592 (K−means Cluster #1)
FDR (Age associated divergence) =0.0836
p(Scalp)=0.0202
p(Dura)=0.0291
Scalp
Dura
DNAm (Beta)
Ages (years)
0.4
0.5
0.6
0.7
0.8
0
20
40
60
80
A
B
C
D
E
F
G
H
I
J
K

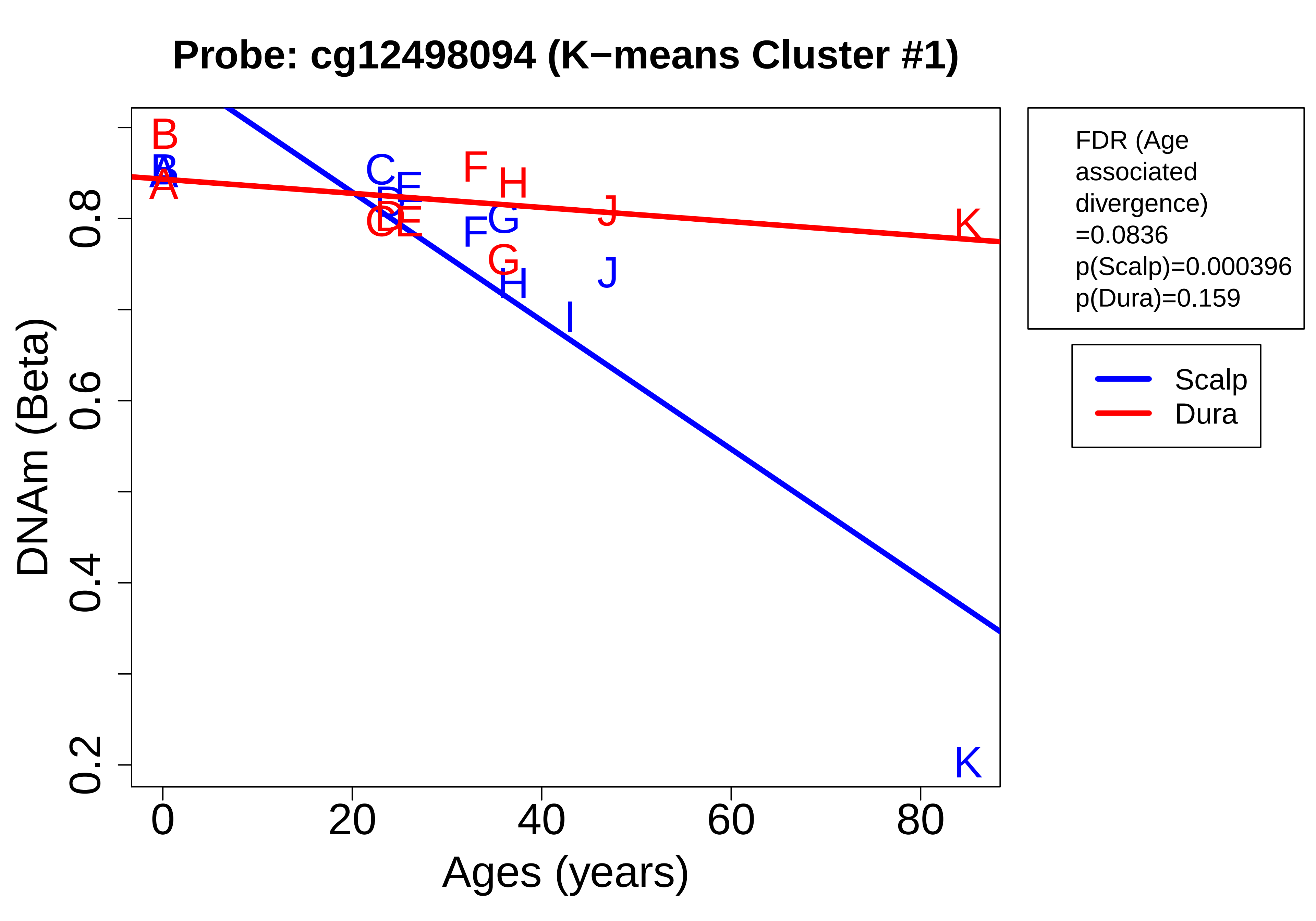

Probe: cg12498094 (K−means Cluster #1)
DNAm (Beta)
0.2
0.4
0.6
0.8
0
20
40
60
80
Ages (years)
FDR (Age associated divergence) =0.0836
p(Scalp)=0.000396
p(Dura)=0.159
Scalp
Dura

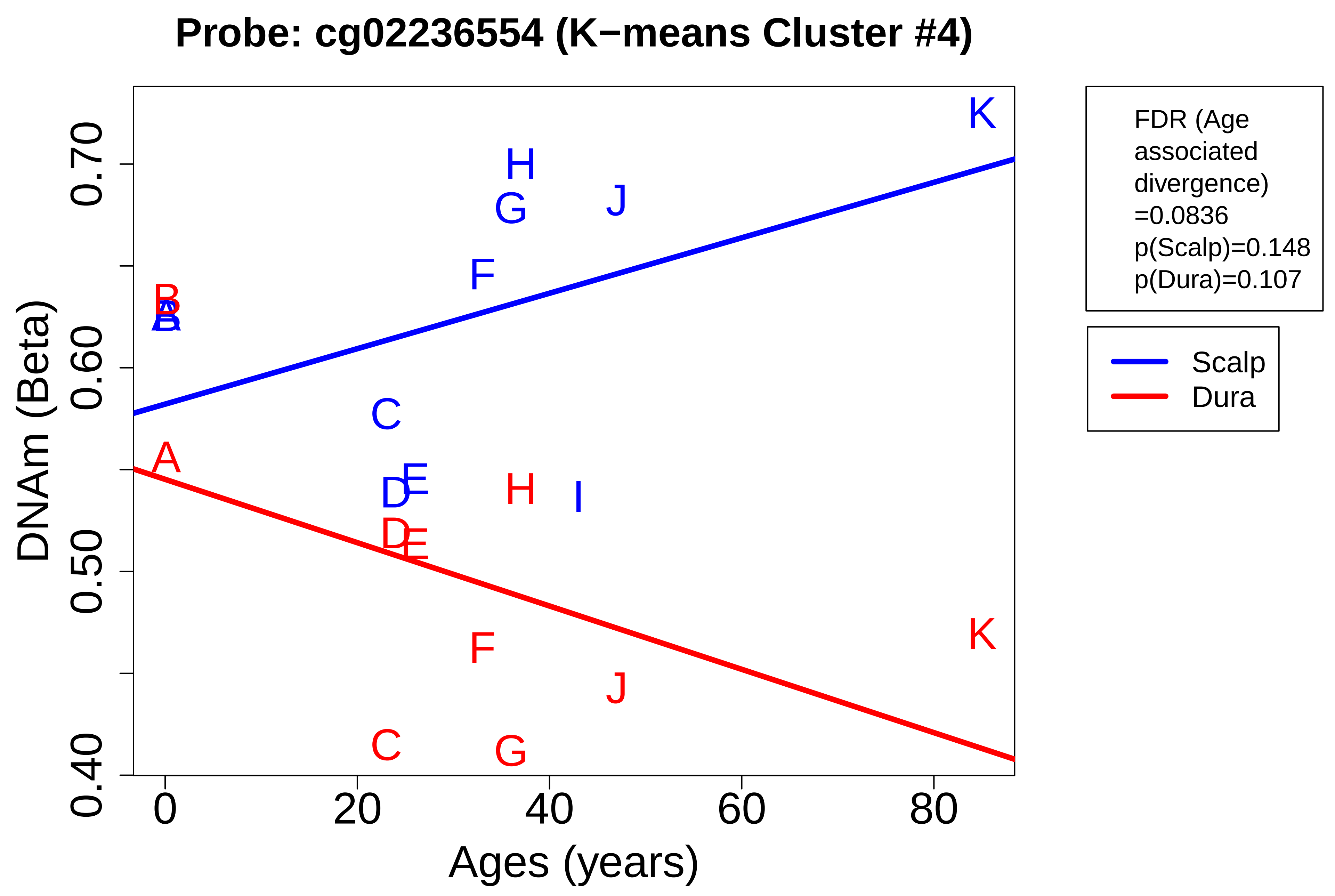
Probe: cg02236554 (K−means Cluster #4)
DNAm (Beta)
0.40
0.50
0.60
0.70
0
20
40
60
80
Ages (years)
FDR (Age associated divergence) =0.0836
p(Scalp)=0.148
p(Dura)=0.107
Scalp
Dura
A
B
C
D
E
F
G
H
I
J
K

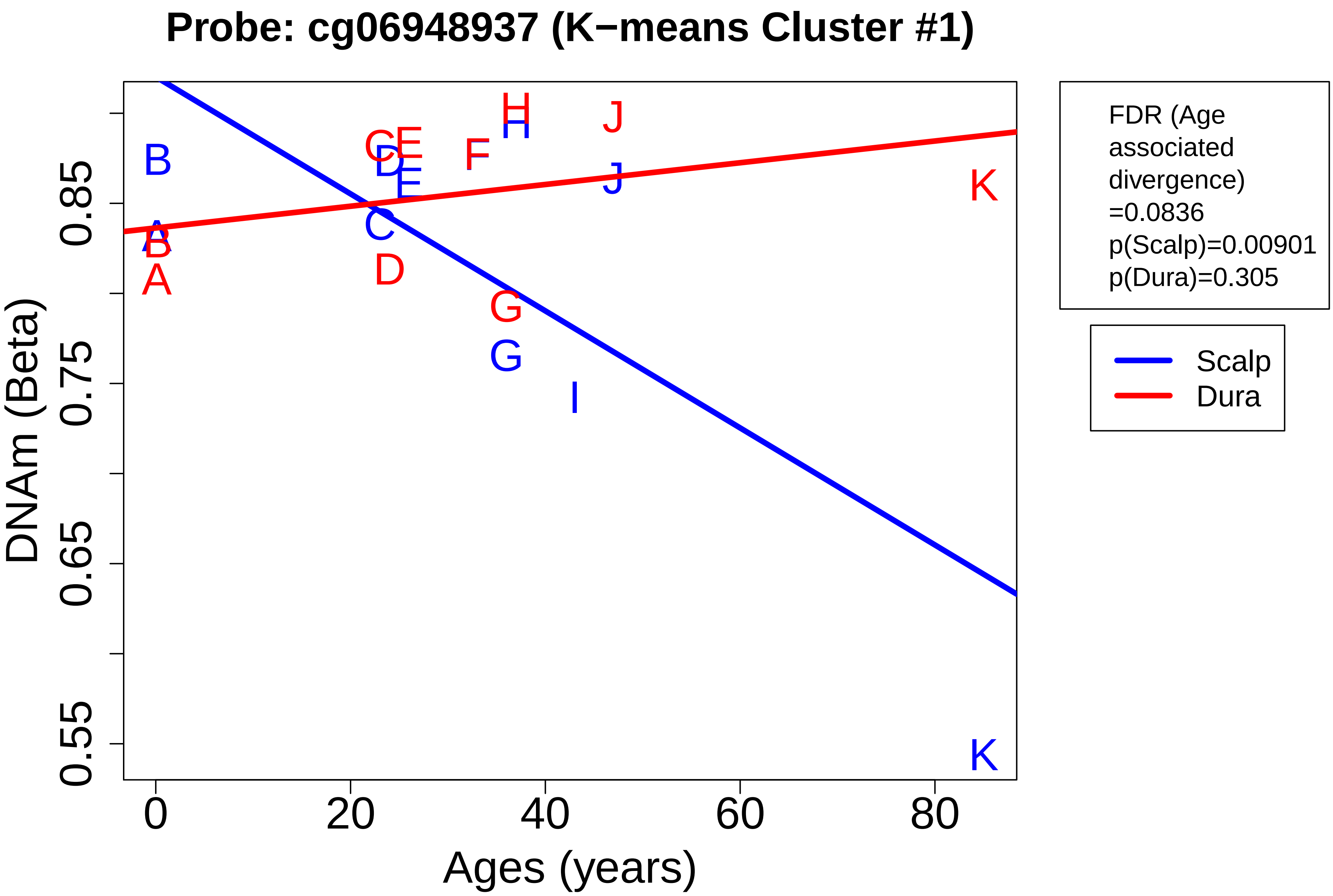

Probe: cg06948937 (K−means Cluster #1)
DNAm (Beta)
0.55
0.65
0.75
0.85
0
20
40
60
80
Ages (years)
FDR (Age associated divergence) =0.0836
p(Scalp)=0.00901
p(Dura)=0.305
Scalp
Dura

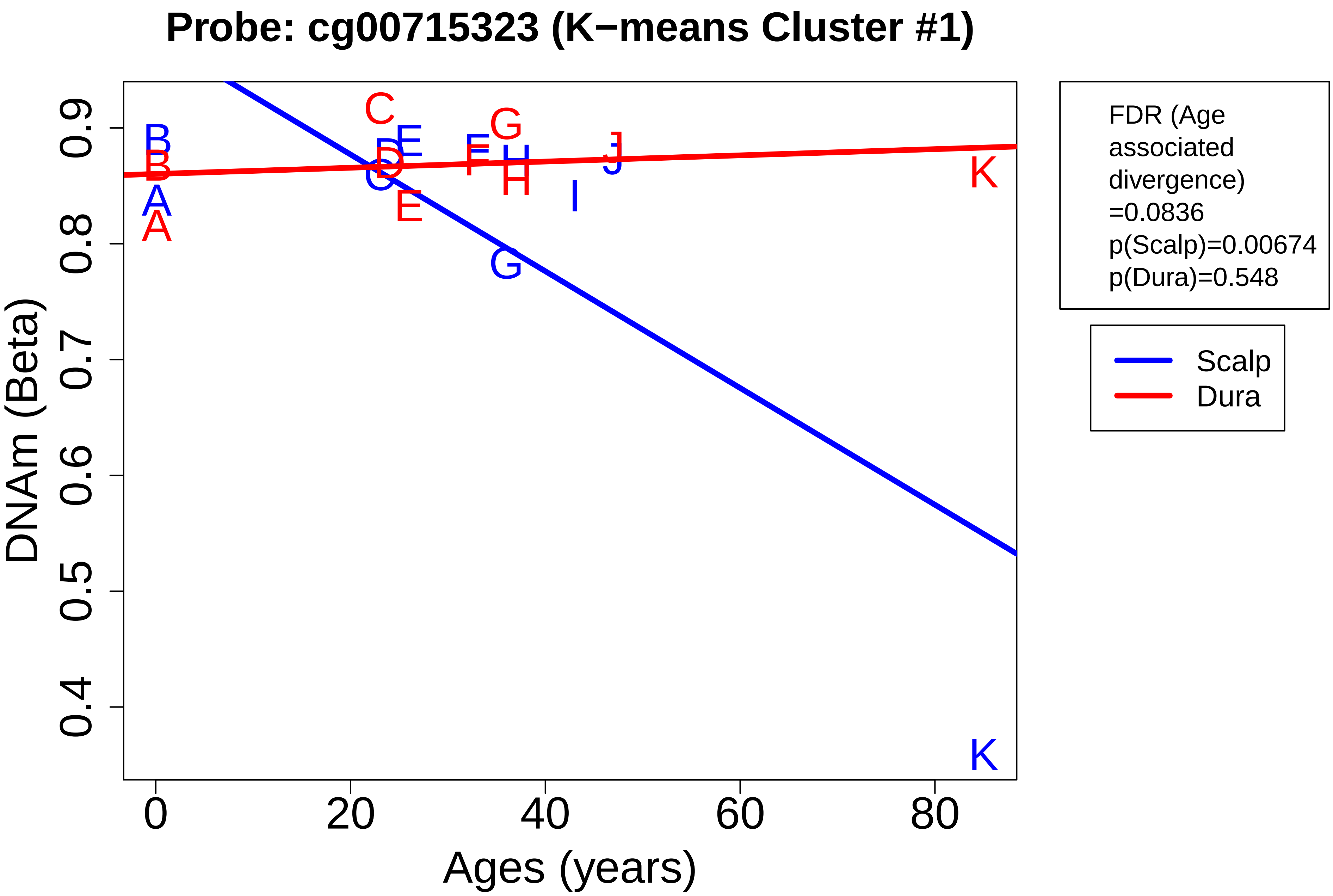
Probe: cg00715323 (K−means Cluster #1)
DNAm (Beta)
0.4
0.5
0.6
0.7
0.8
0.9
0
20
40
60
80
Ages (years)
FDR (Age associated divergence) =0.0836
p(Scalp)=0.00674
p(Dura)=0.548
Scalp
Dura

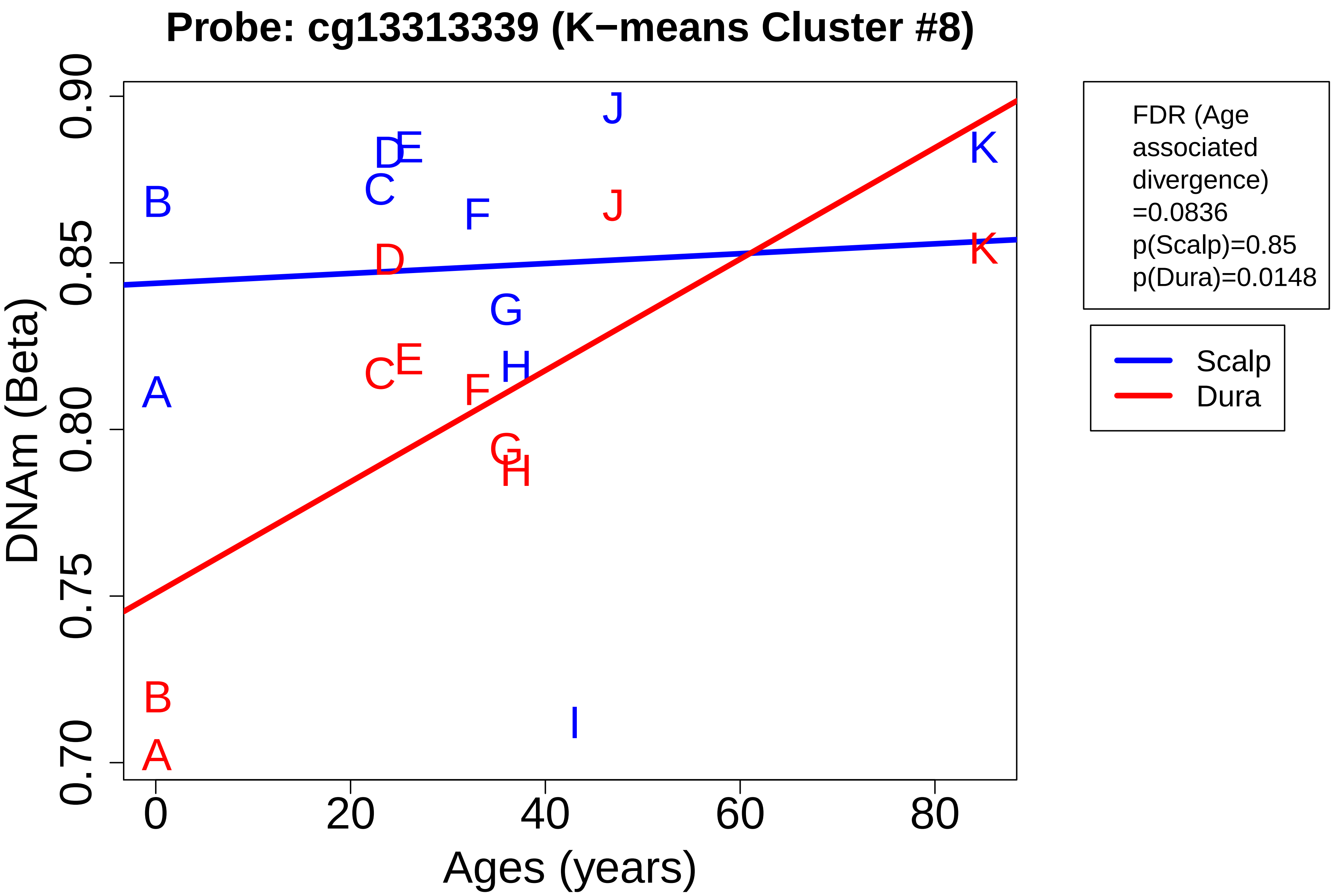
Probe: cg13313339 (K−means Cluster #8)
DNAm (Beta)
0.70
0.75
0.80
0.85
0.90
Ages (years)
0
20
40
60
80
FDR (Age associated divergence) =0.0836
p(Scalp)=0.85
p(Dura)=0.0148
Scalp
Dura

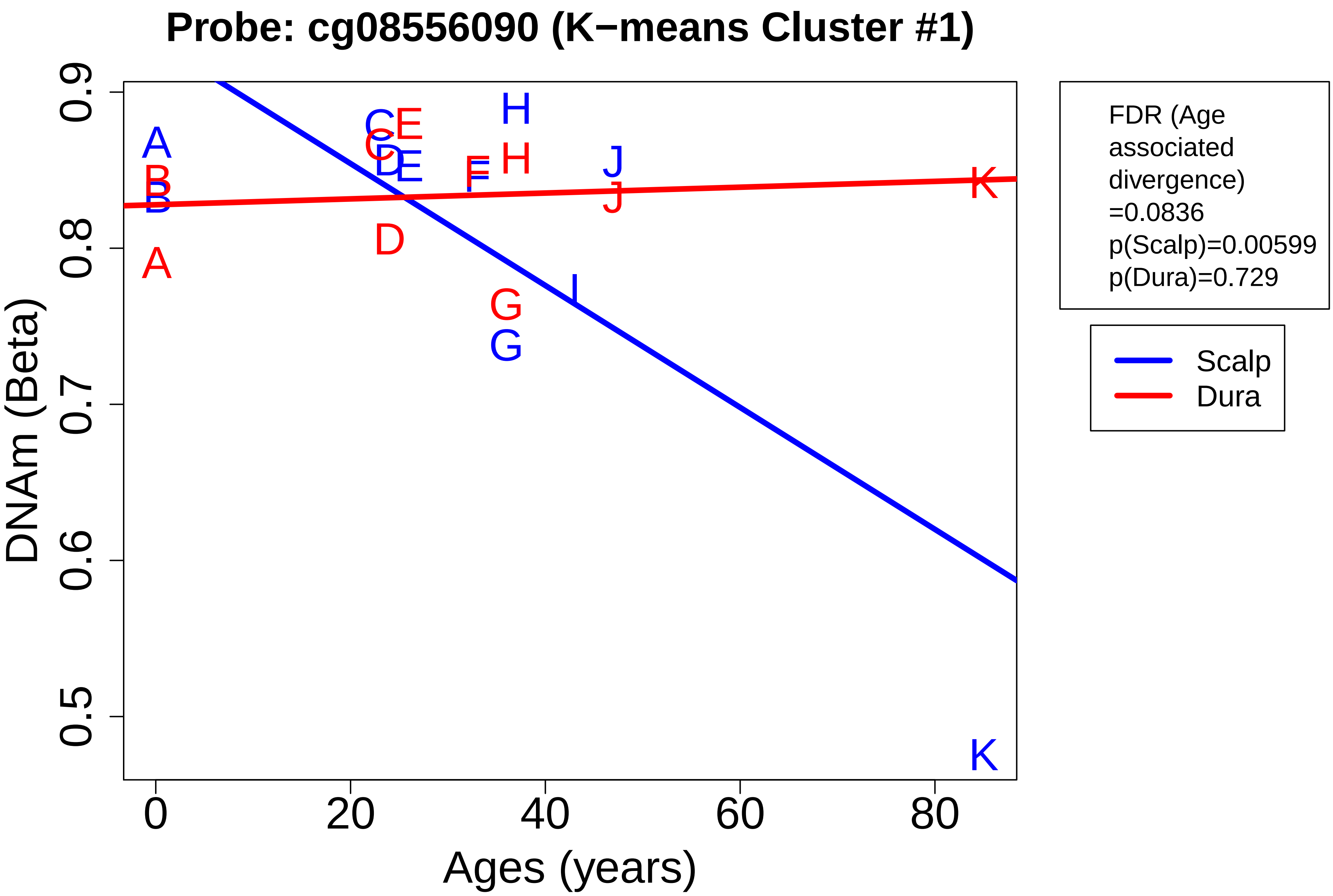

Probe: cg08556090 (K−means Cluster #1)
DNAm (Beta)
0.5
0.6
0.7
0.8
0.9
Ages (years)
0
20
40
60
80
FDR (Age associated divergence) =0.0836
p(Scalp)=0.00599
p(Dura)=0.729
Scalp
Dura

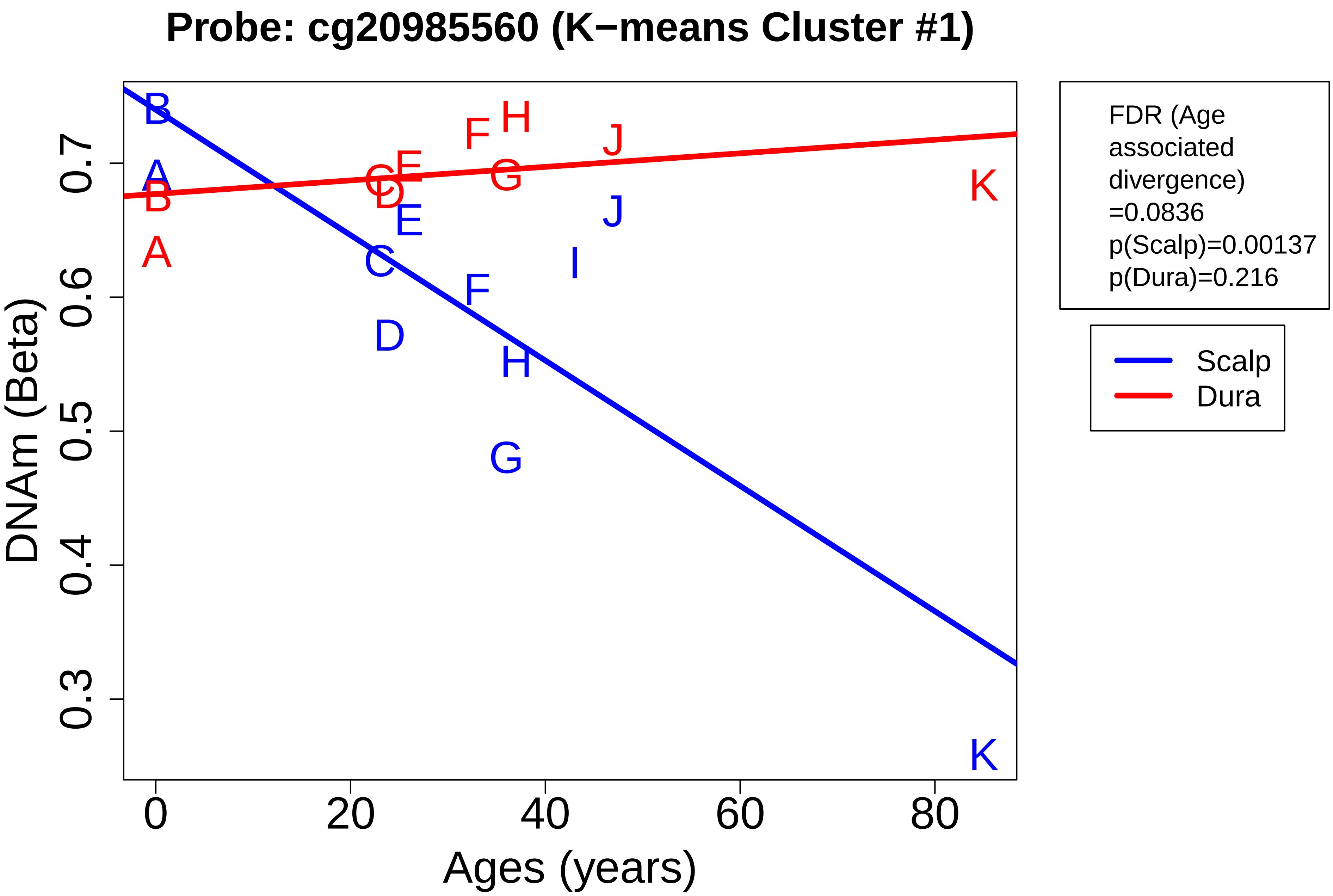

Probe: cg20985560 (K−means Cluster #1)
DNAm (Beta)
0.3
0.4
0.5
0.6
0.7
0
20
40
60
80
Ages (years)
FDR (Age associated divergence) =0.0836
p(Scalp)=0.00137
p(Dura)=0.216
Scalp
Dura

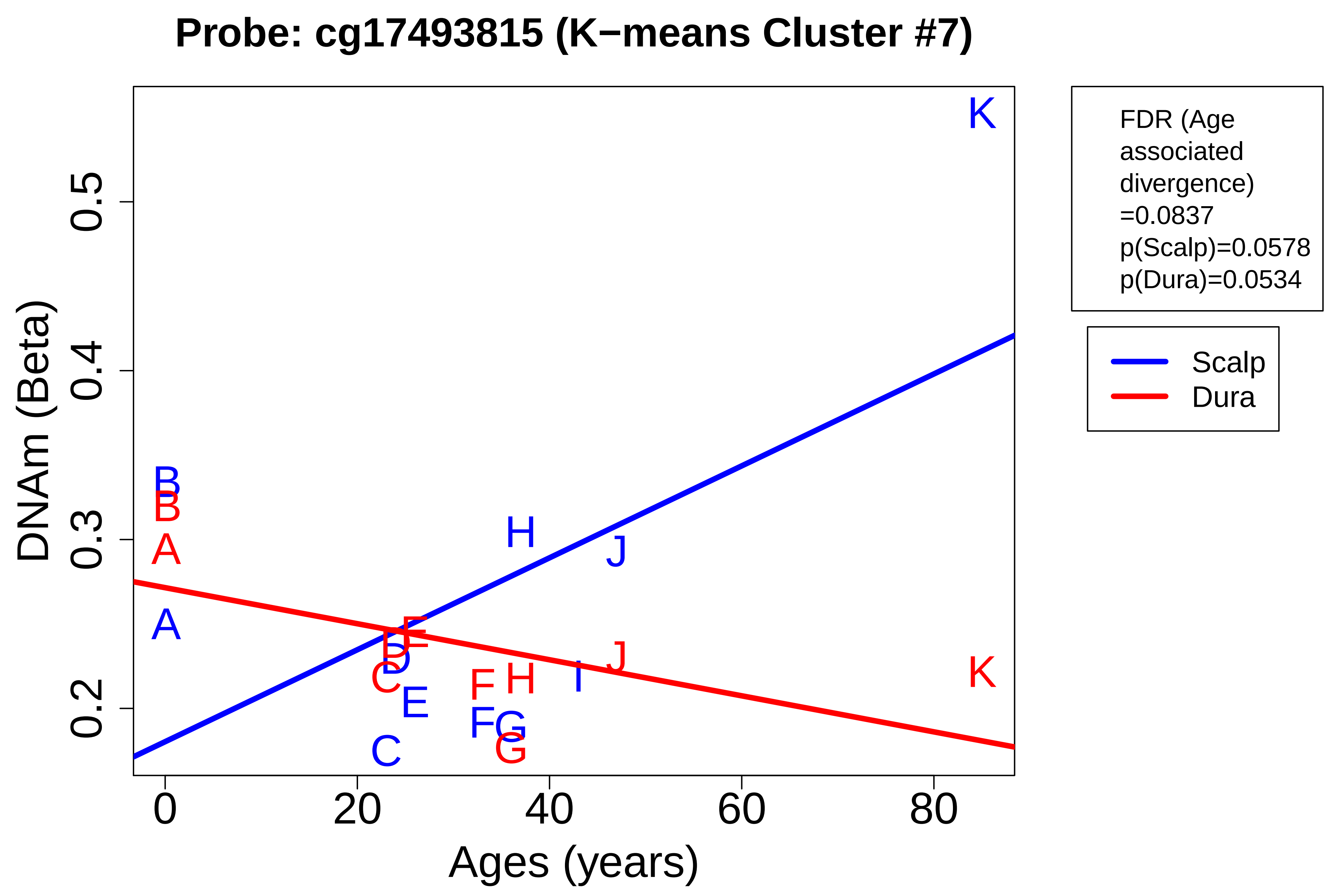

Probe: cg17493815 (K−means Cluster #7)
FDR (Age associated divergence) =0.0837
p(Scalp)=0.0578
p(Dura)=0.0534
Scalp
Dura
DNAm (Beta)
0.2
0.3
0.4
0.5
Ages (years)
0
20
40
60
80

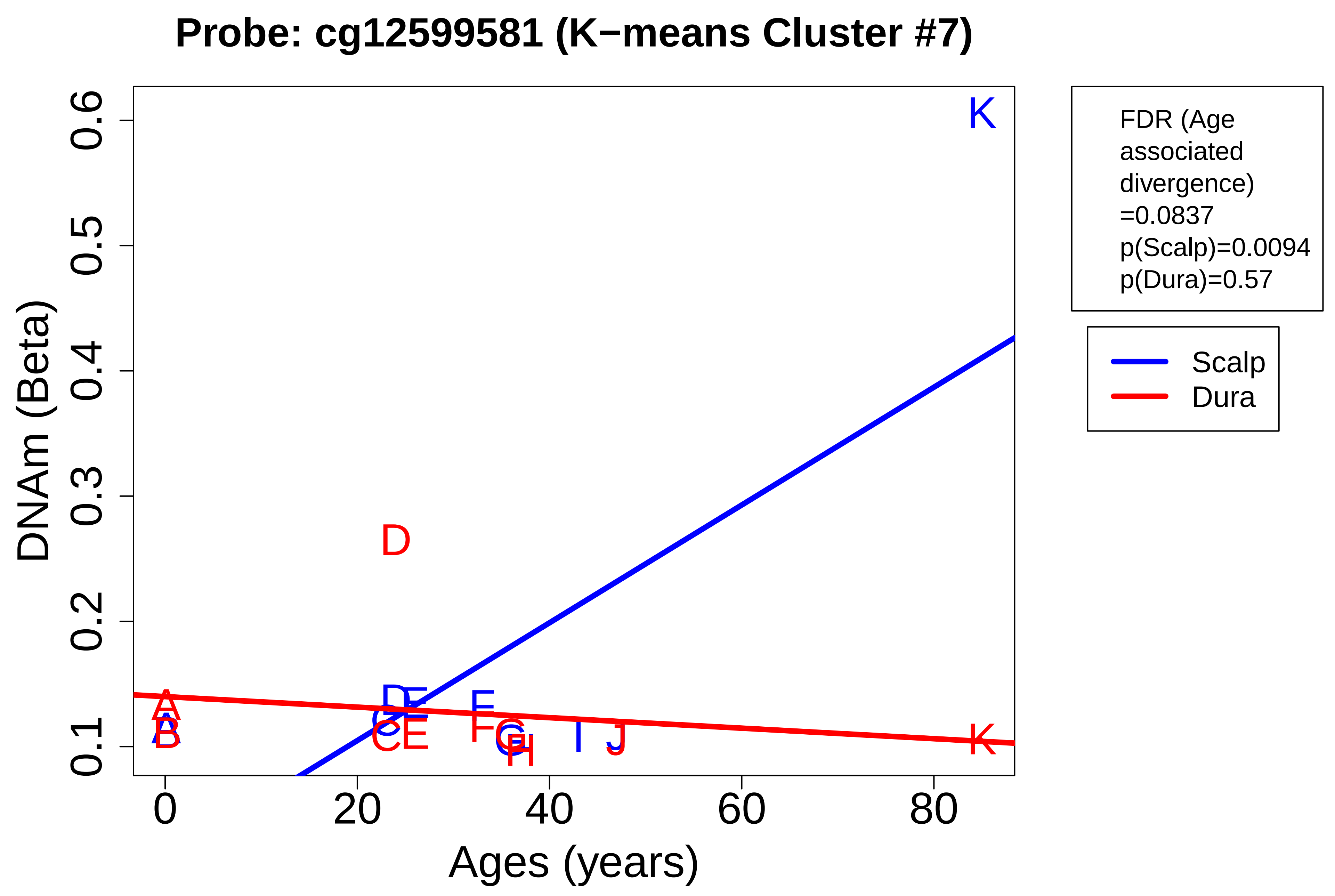
Probe: cg12599581 (K−means Cluster #7)
DNAm (Beta)
0.1
0.2
0.3
0.4
0.5
0.6
0
20
40
60
80
Ages (years)
FDR (Age associated divergence) =0.0837
p(Scalp)=0.0094
p(Dura)=0.57
Scalp
Dura

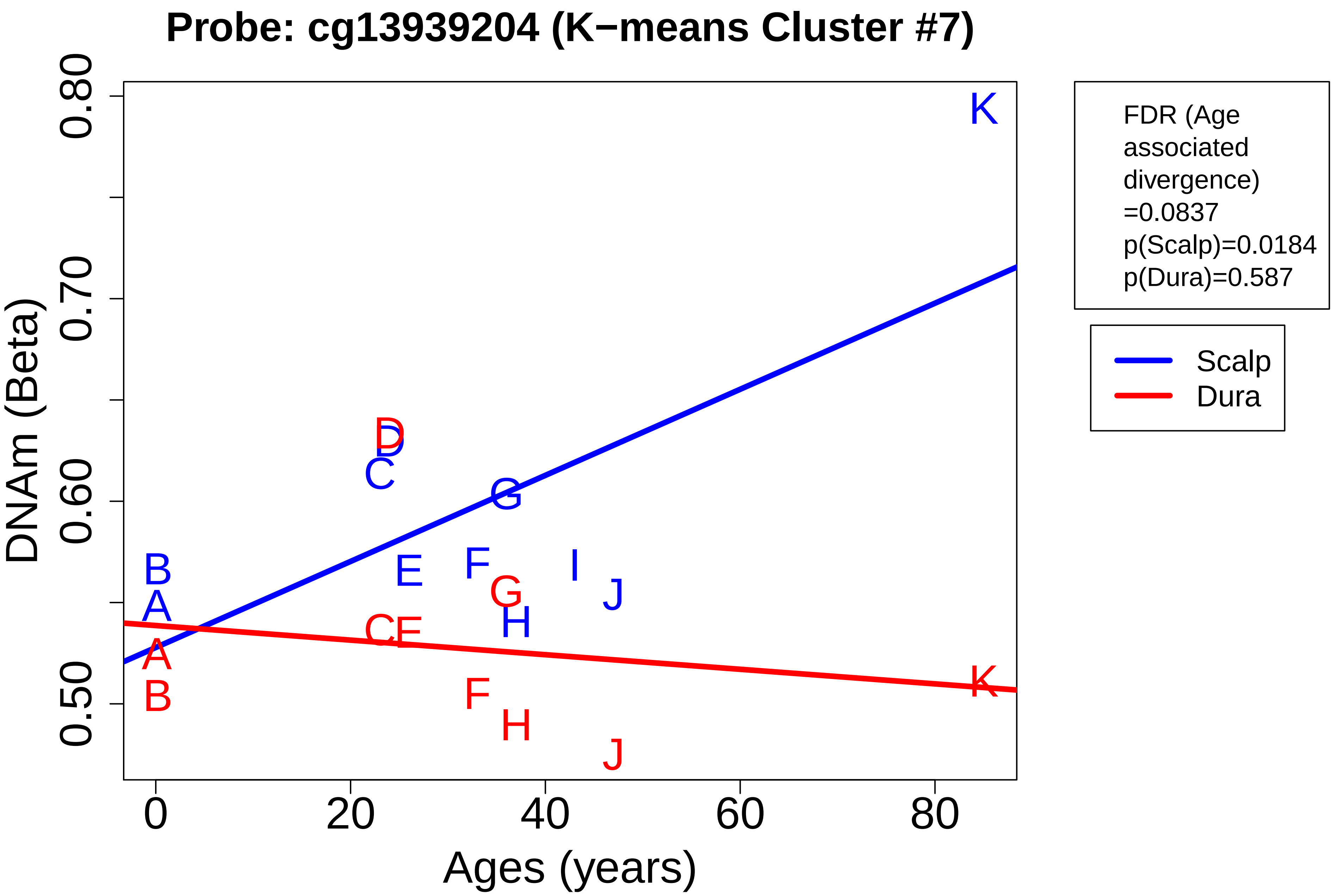

Probe: cg13939204 (K−means Cluster #7)
DNAm (Beta)
0.50
0.60
0.70
0.80
Ages (years)
0
20
40
60
80
A
B
C
D
E
F
G
H
I
J
K
FDR (Age associated divergence) =0.0837
p(Scalp)=0.0184
p(Dura)=0.587
Scalp
Dura

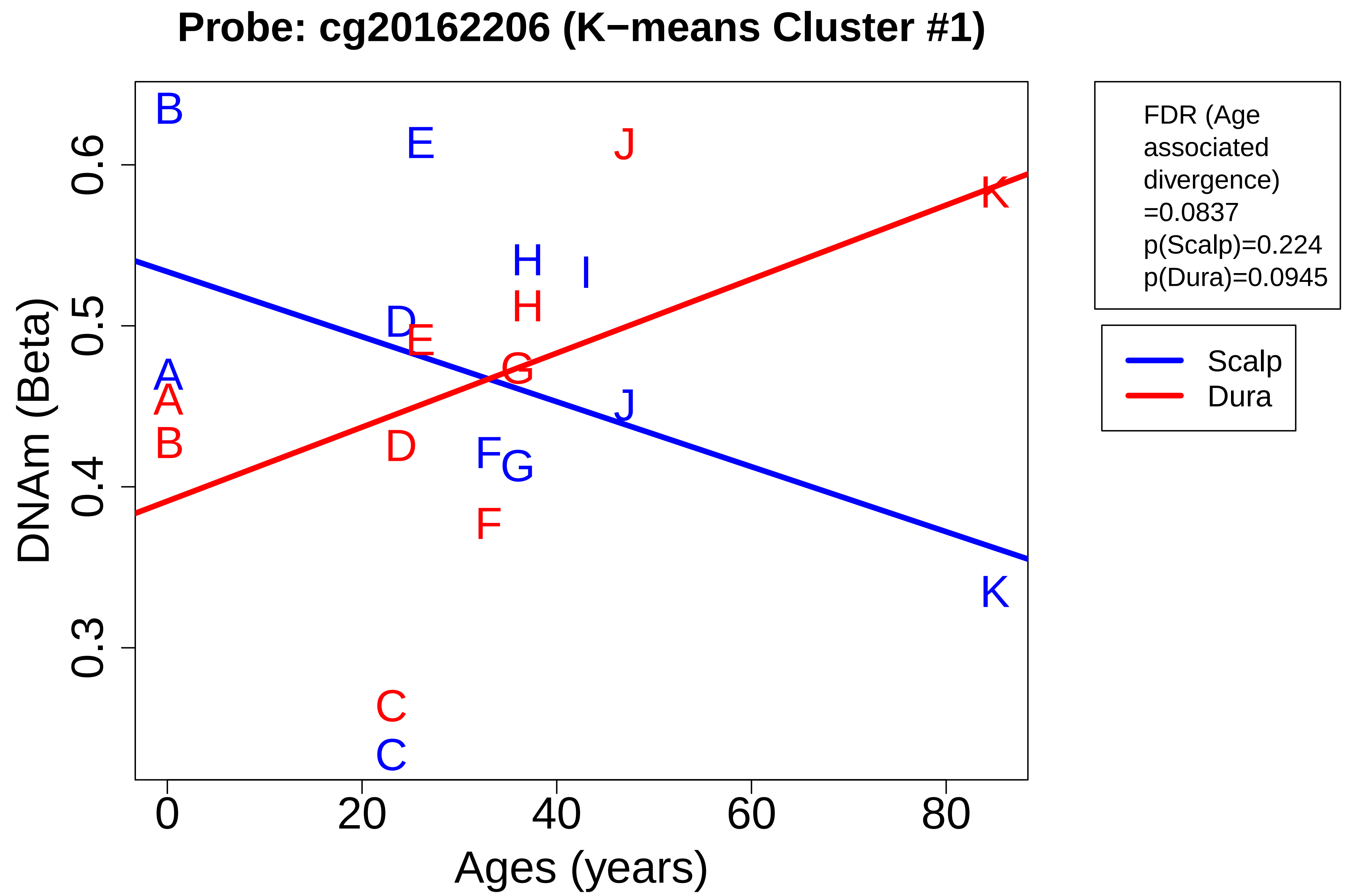

Probe: cg20162206 (K−means Cluster #1)
FDR (Age associated divergence) =0.0837
p(Scalp)=0.224
p(Dura)=0.0945
Scalp
Dura
DNAm (Beta)
0.3
0.4
0.5
0.6
0
20
40
60
80
Ages (years)

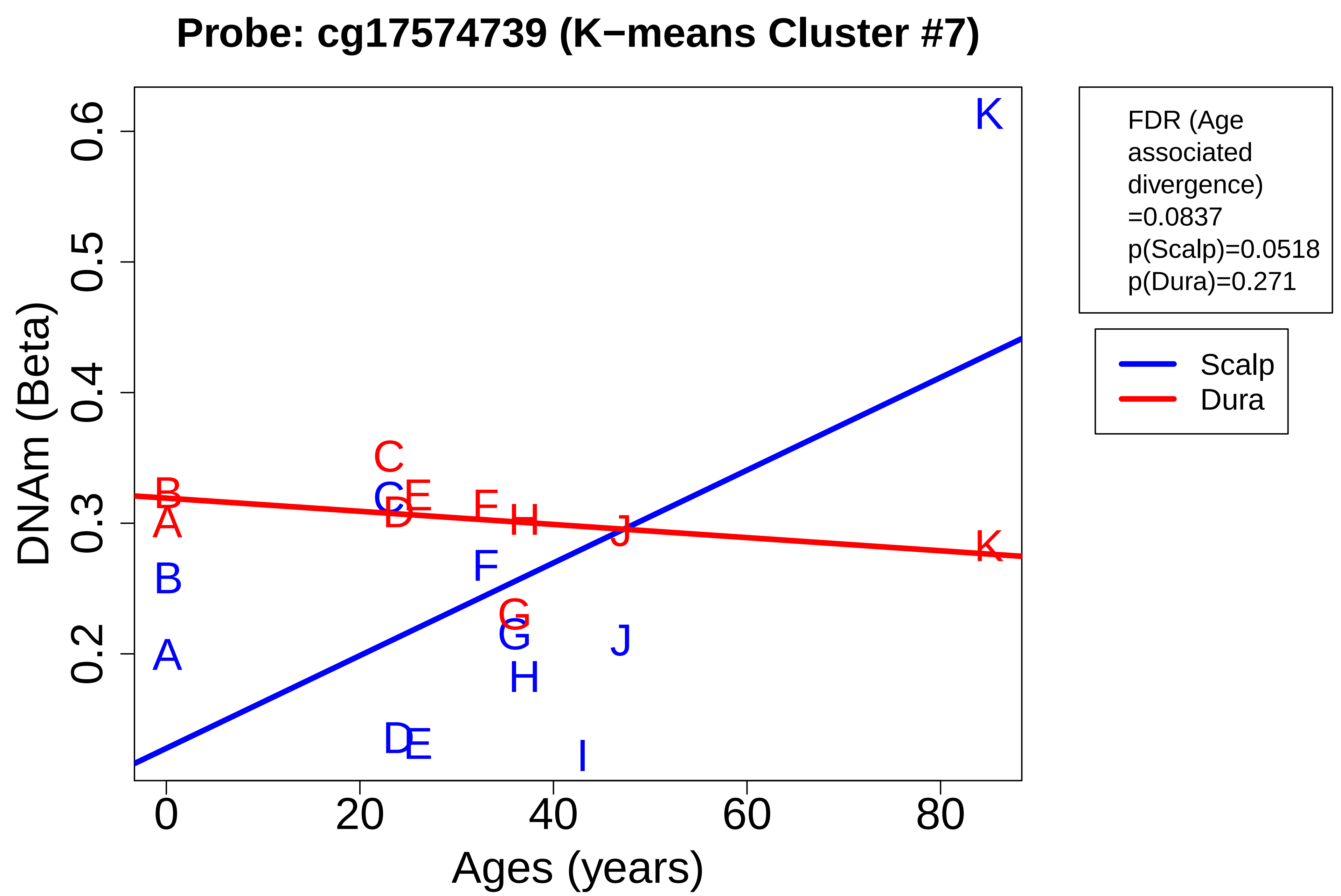

Probe: cg17574739 (K−means Cluster #7)
DNAm (Beta)
0.2
0.3
0.4
0.5
0.6
Ages (years)
0
20
40
60
80
A
B
C
D
E
F
G
H
I
J
K
FDR (Age associated divergence) =0.0837
p(Scalp)=0.0518
p(Dura)=0.271
Scalp
Dura

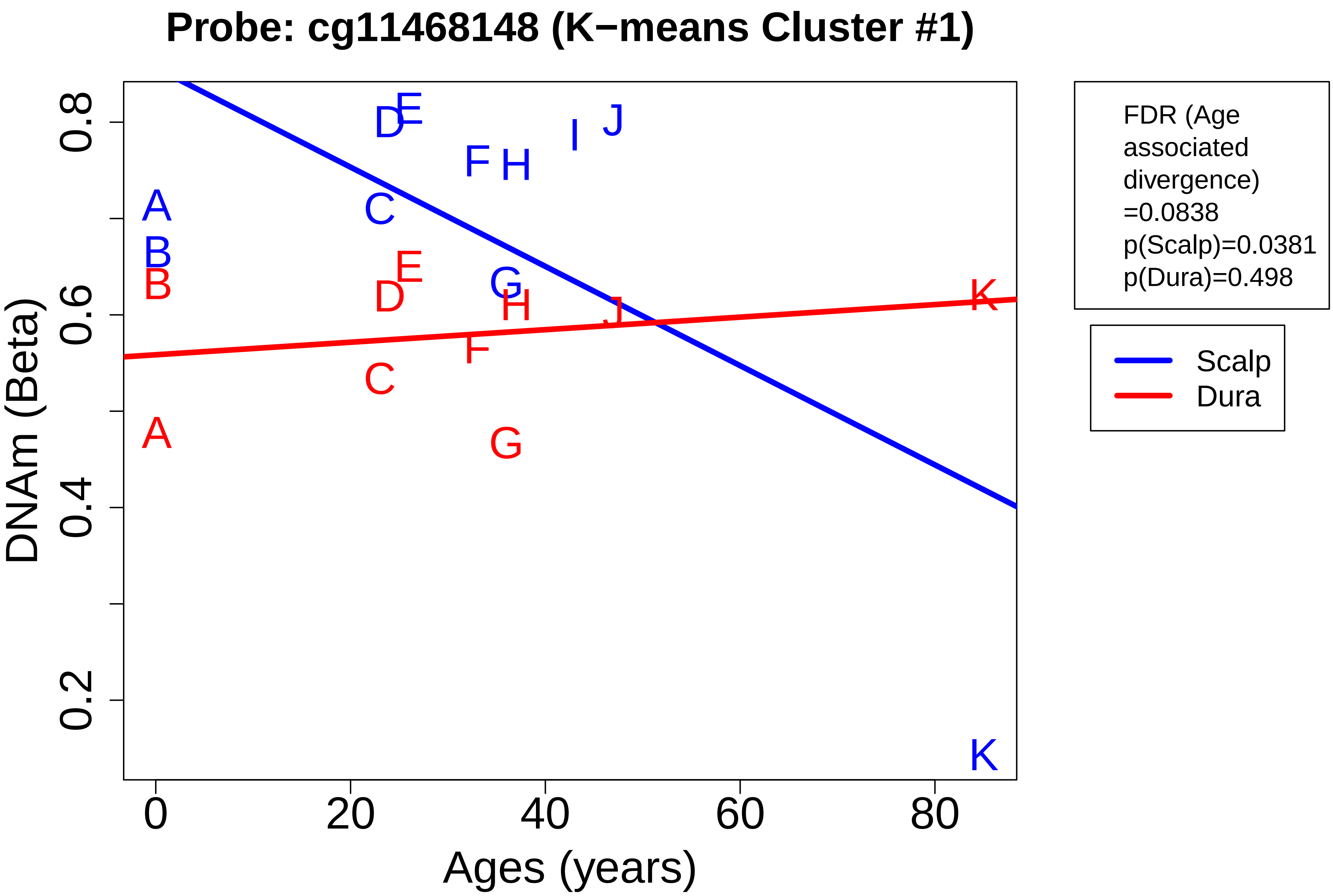

Probe: cg11468148 (K−means Cluster #1)
DNAm (Beta)
0.2
0.4
0.6
0.8
0
20
40
60
80
Ages (years)
A
B
C
D
E
F
G
H
I
J
K
FDR (Age associated divergence) =0.0838
p(Scalp)=0.0381
p(Dura)=0.498
Scalp
Dura

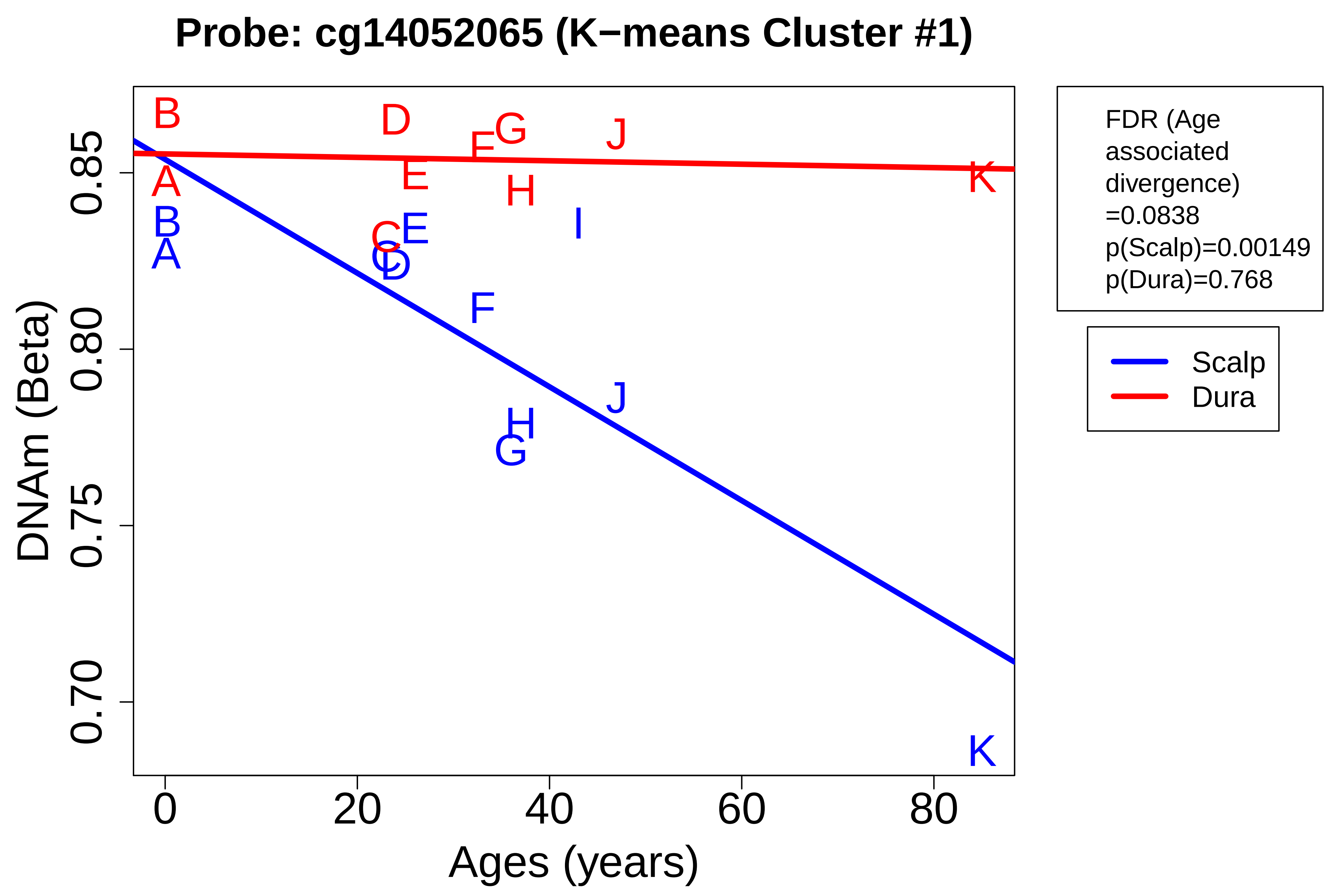

Probe: cg14052065 (K−means Cluster #1)
DNAm (Beta)
0.70
0.75
0.80
0.85
Ages (years)
0
20
40
60
80
B
D
G
F
J
A
E
H
K
B
A
C
E
I
C
D
F
J
H
G
K
FDR (Age associated divergence) =0.0838
p(Scalp)=0.00149
p(Dura)=0.768
Scalp
Dura

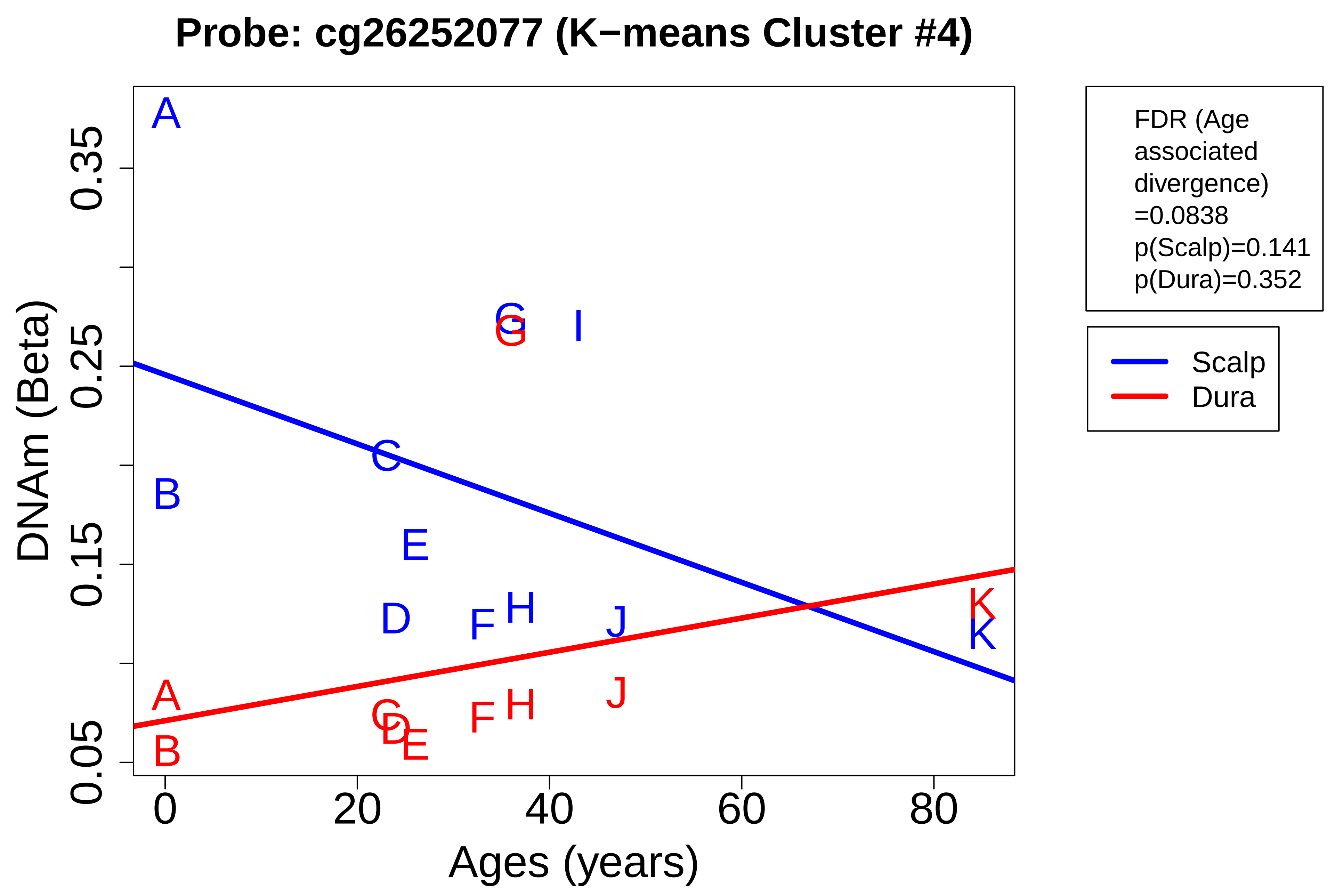

Probe: cg26252077 (K−means Cluster #4)
DNAm (Beta)
0.05
0.15
0.25
0.35
Ages (years)
0
20
40
60
80
A
B
C
D
E
F
G
H
I
J
K
FDR (Age associated divergence) =0.0838
p(Scalp)=0.141
p(Dura)=0.352
Scalp
Dura

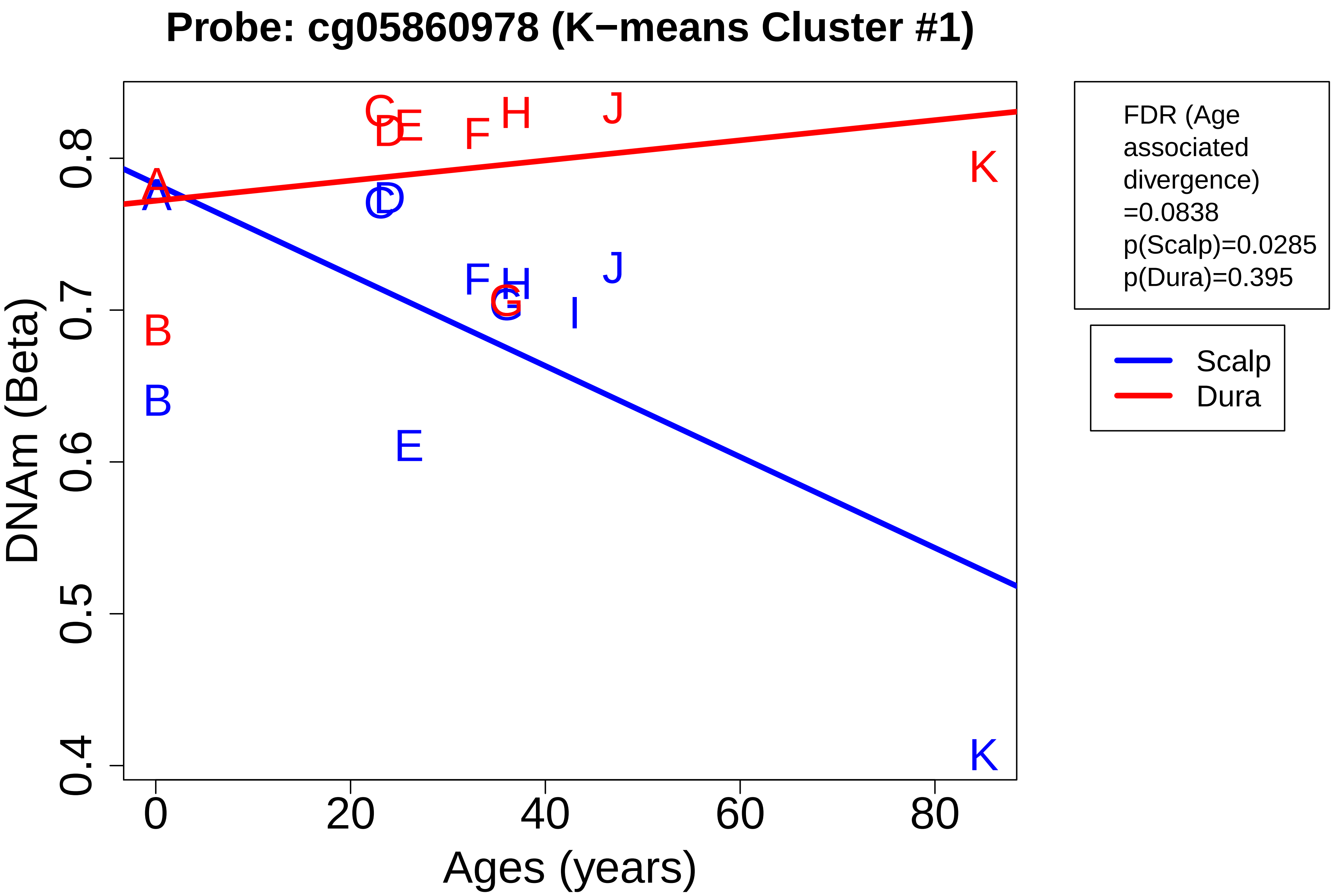

Probe: cg05860978 (K−means Cluster #1)
DNAm (Beta)
0.4
0.5
0.6
0.7
0.8
0
20
40
60
80
Ages (years)
FDR (Age associated divergence) =0.0838
p(Scalp)=0.0285
p(Dura)=0.395
Scalp
Dura

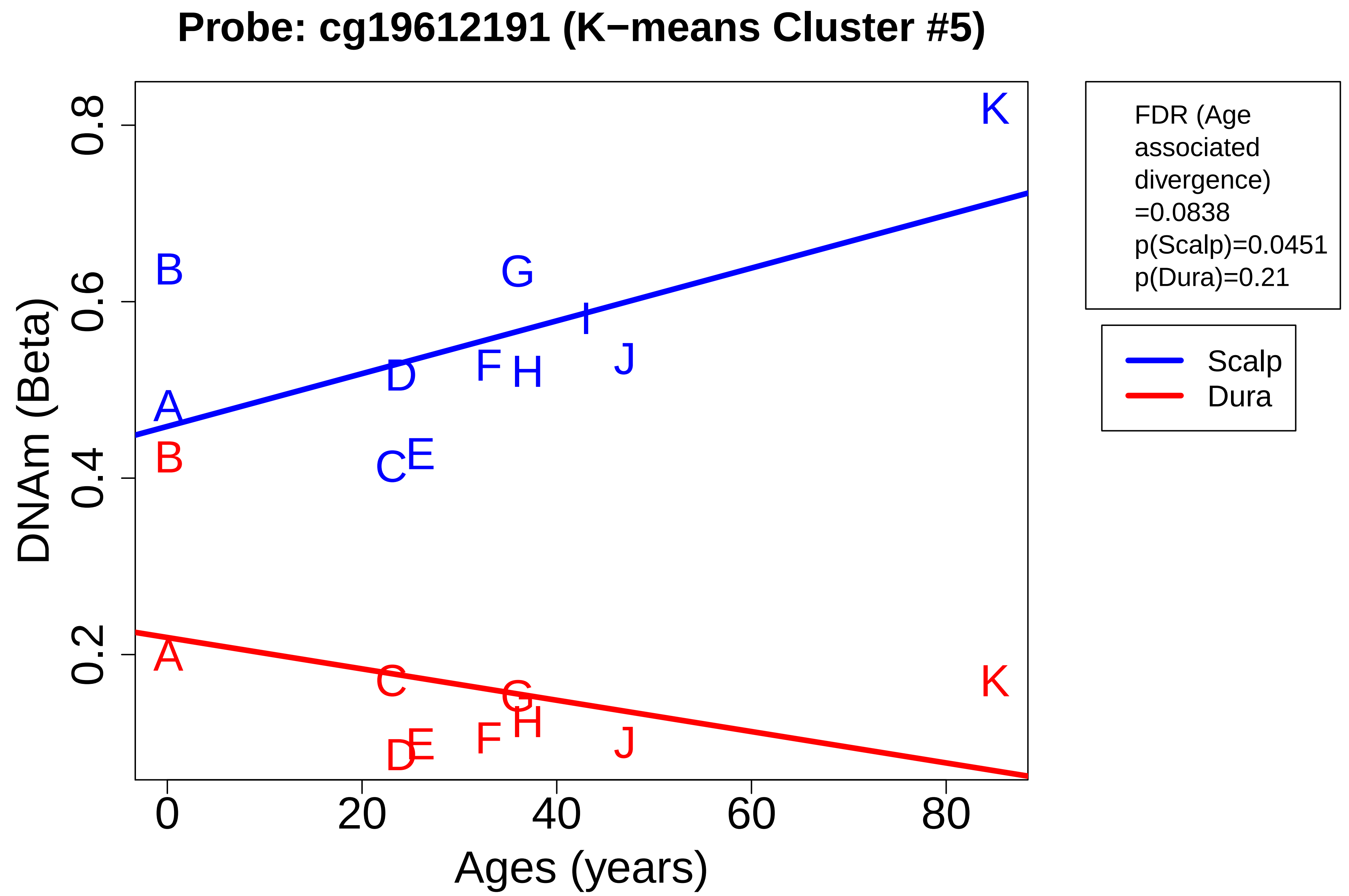

Probe: cg19612191 (K−means Cluster #5)
DNAm (Beta)
0.2
0.4
0.6
0.8
0
20
40
60
80
Ages (years)
A
B
C
D
E
F
G
H
I
J
K
FDR (Age associated divergence) =0.0838
p(Scalp)=0.0451
p(Dura)=0.21
Scalp
Dura

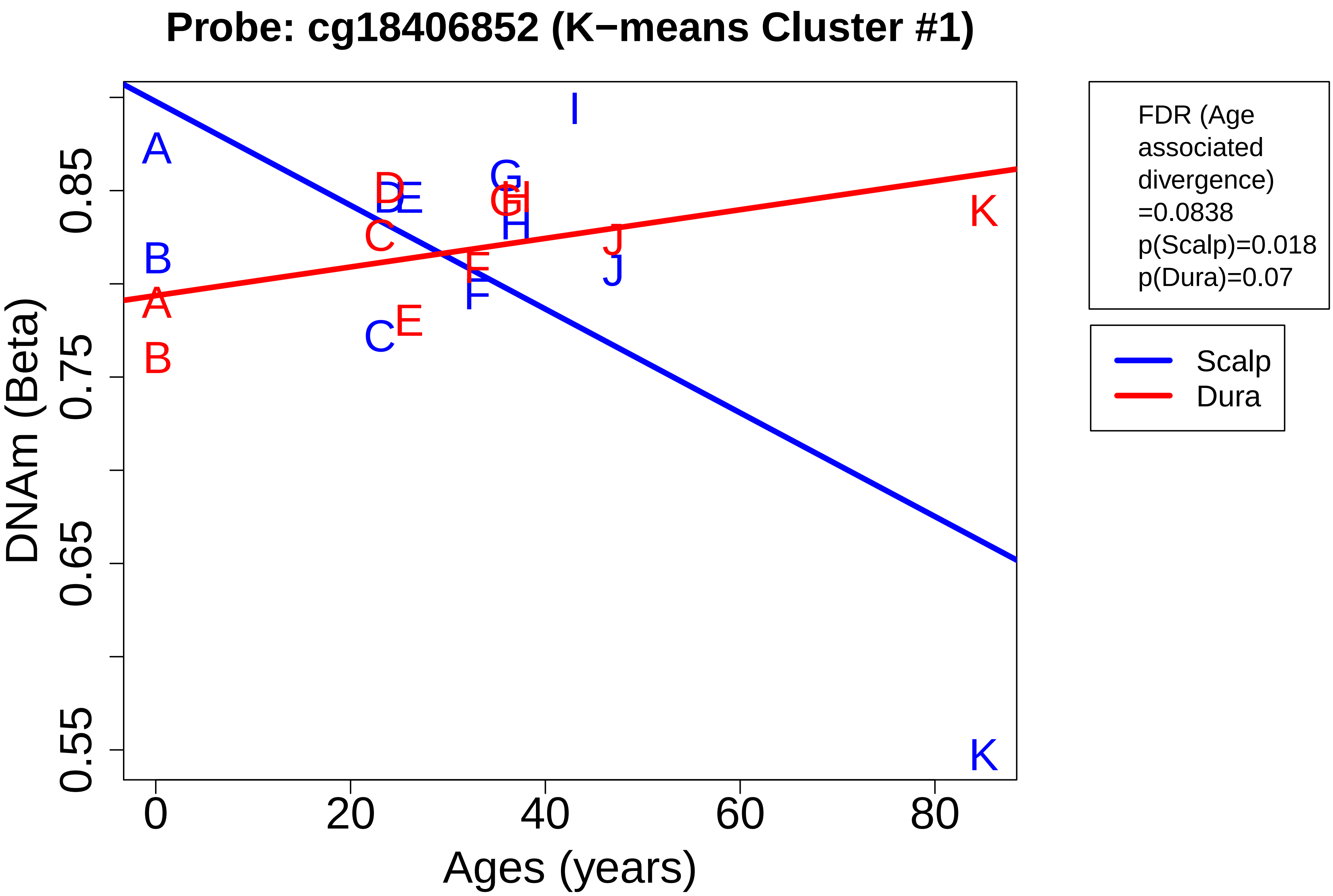

Probe: cg18406852 (K−means Cluster #1)
DNAm (Beta)
0.55
0.65
0.75
0.85
Ages (years)
0
20
40
60
80
A
B
C
D
E
F
G
H
I
J
K
FDR (Age associated divergence) =0.0838
p(Scalp)=0.018
p(Dura)=0.07
Scalp
Dura

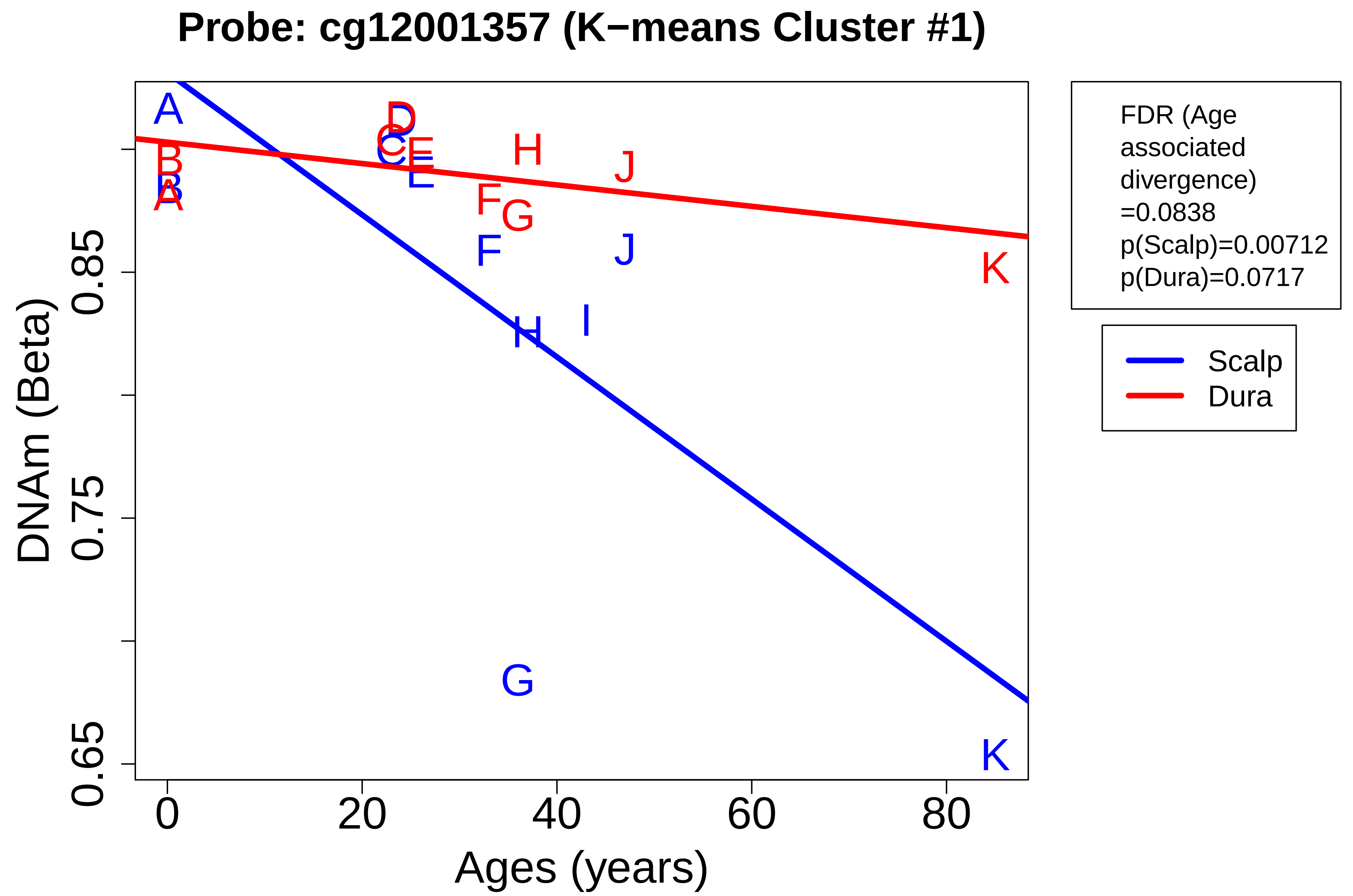

Probe: cg12001357 (K−means Cluster #1)
DNAm (Beta)
0.65
0.75
0.85
Ages (years)
0
20
40
60
80
FDR (Age associated divergence) =0.0838
p(Scalp)=0.00712
p(Dura)=0.0717
Scalp
Dura
A
B
C
D
E
F
G
H
I
J
K

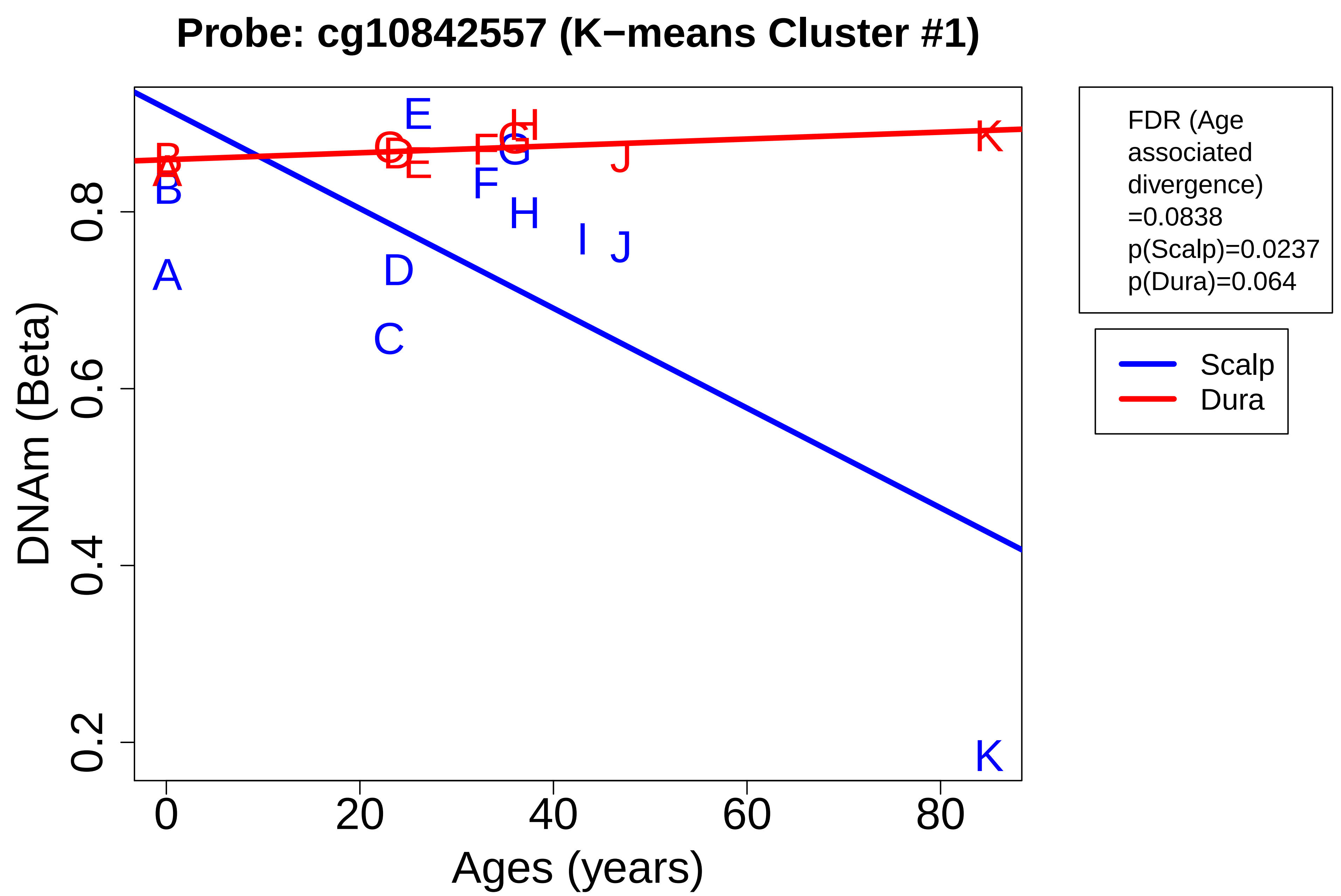

Probe: cg10842557 (K−means Cluster #1)
DNAm (Beta)
0.2
0.4
0.6
0.8
Ages (years)
0
20
40
60
80
FDR (Age associated divergence) =0.0838
p(Scalp)=0.0237
p(Dura)=0.064
Scalp
Dura
A B C D E F G H I J K

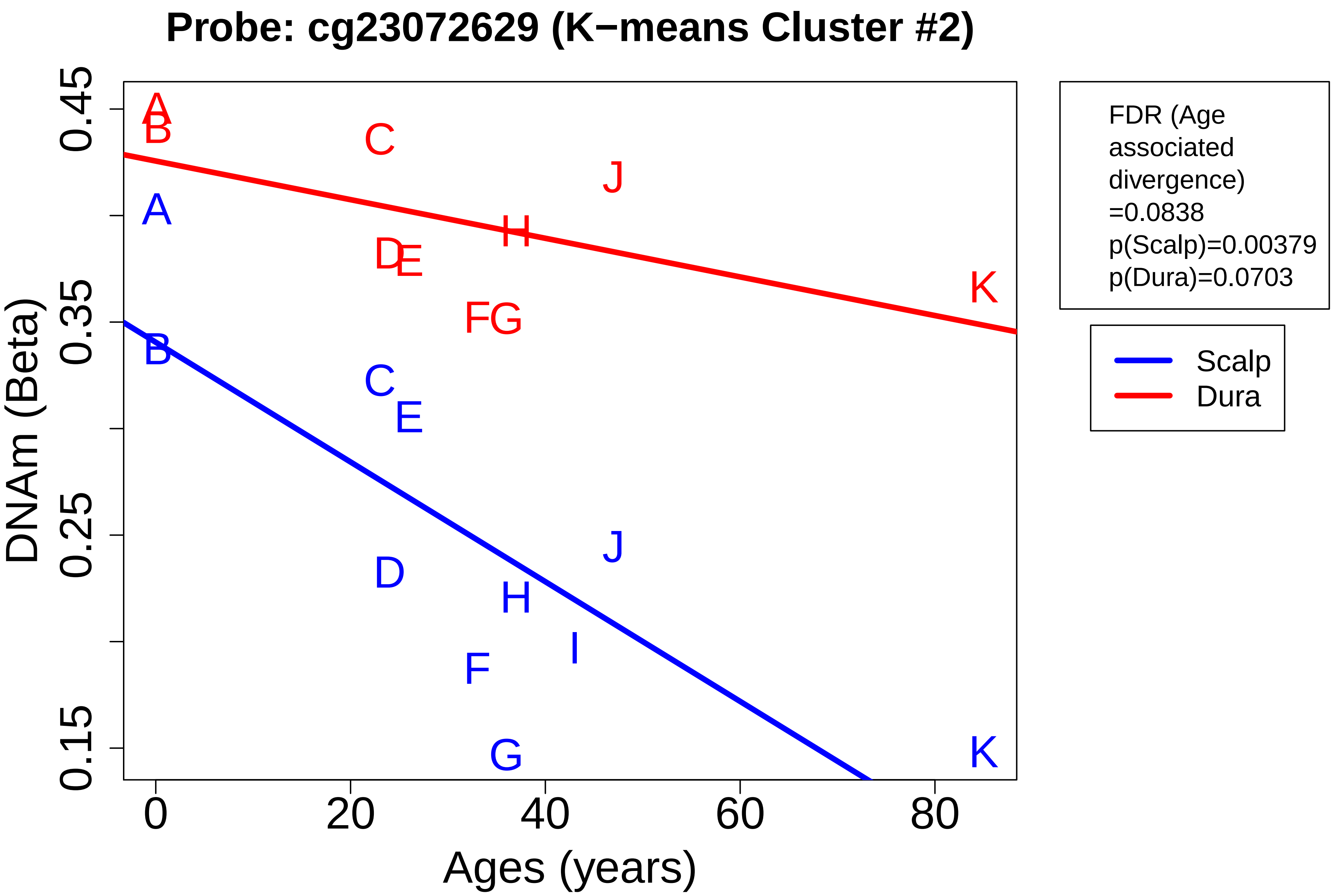

Probe: cg23072629 (K−means Cluster #2)
DNAm (Beta)
0.15
0.25
0.35
0.45
Ages (years)
0
20
40
60
80
A
B
C
D
E
F
G
H
I
J
K
FDR (Age associated divergence) =0.0838
p(Scalp)=0.00379
p(Dura)=0.0703
Scalp
Dura

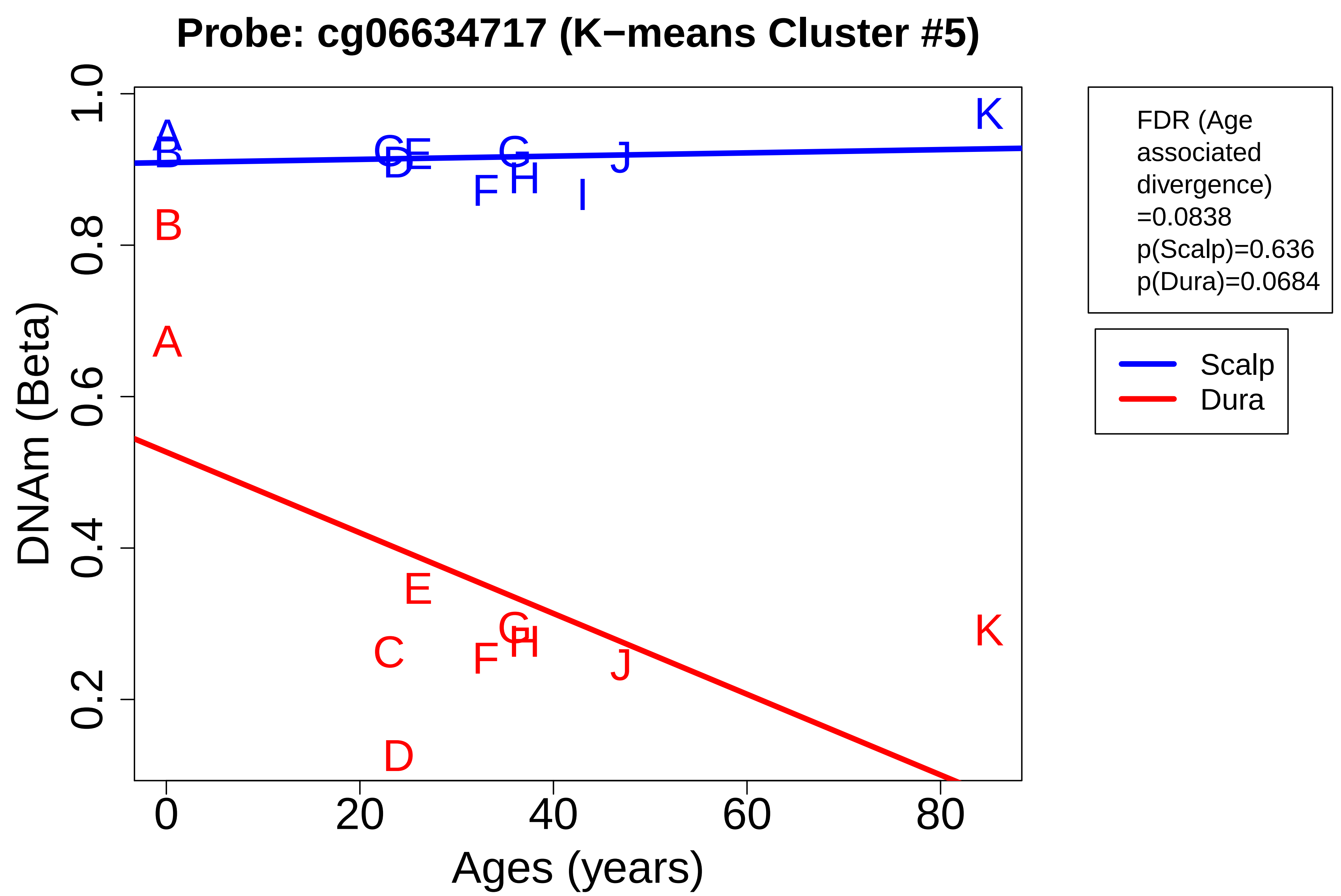

Probe: cg06634717 (K−means Cluster #5)
DNAm (Beta)
1.0
0.8
0.6
0.4
0.2
0
20
40
60
80
Ages (years)
A
B
C
D
E
F
G
H
I
J
K
FDR (Age associated divergence) =0.0838
p(Scalp)=0.636
p(Dura)=0.0684
Scalp
Dura

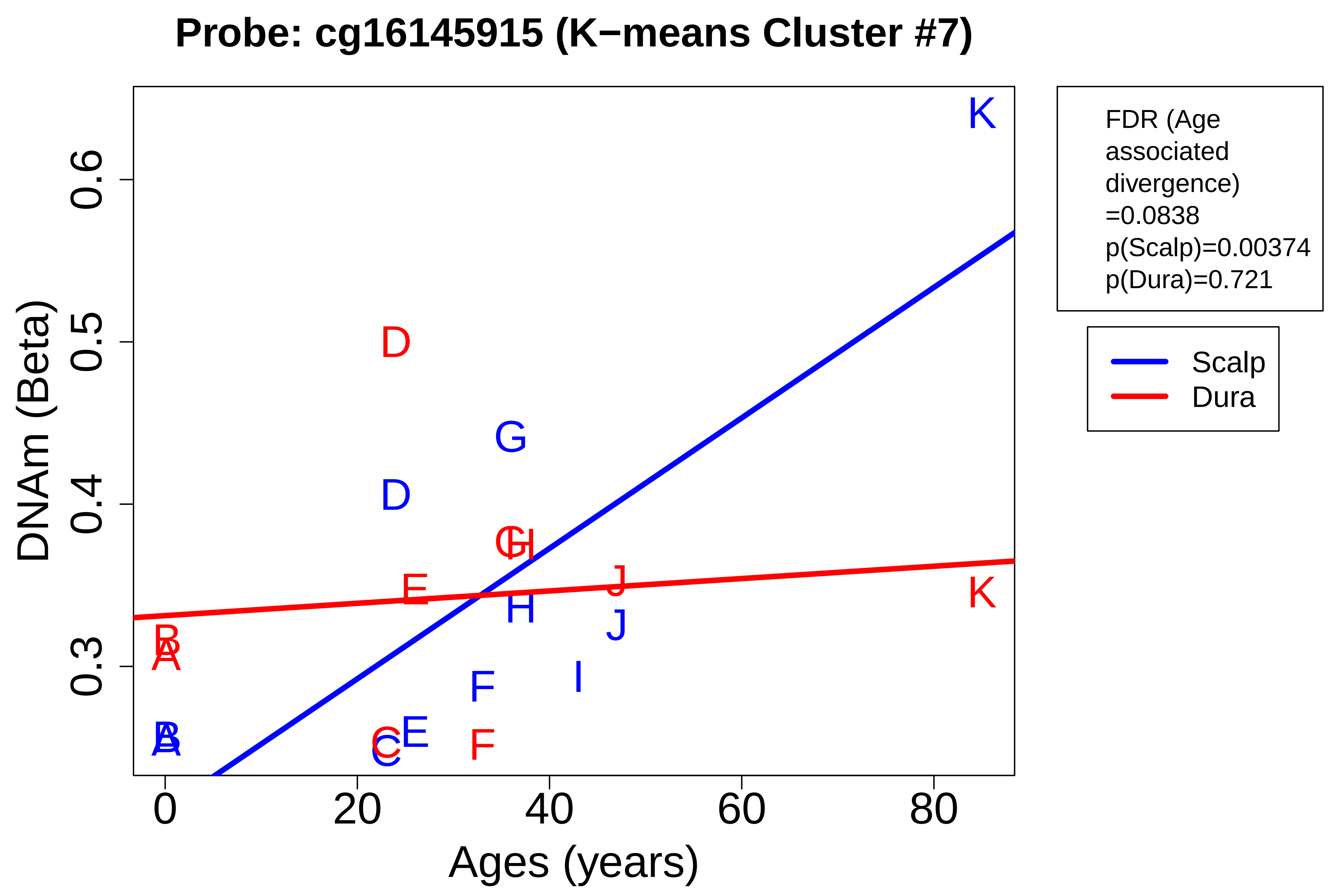

Probe: cg16145915 (K−means Cluster #7)
DNAm (Beta)
0.3
0.4
0.5
0.6
0
20
40
60
80
Ages (years)
FDR (Age associated divergence) =0.0838
p(Scalp)=0.00374
p(Dura)=0.721
Scalp
Dura
A
B
C
D
E
F
G
H
I
J
K

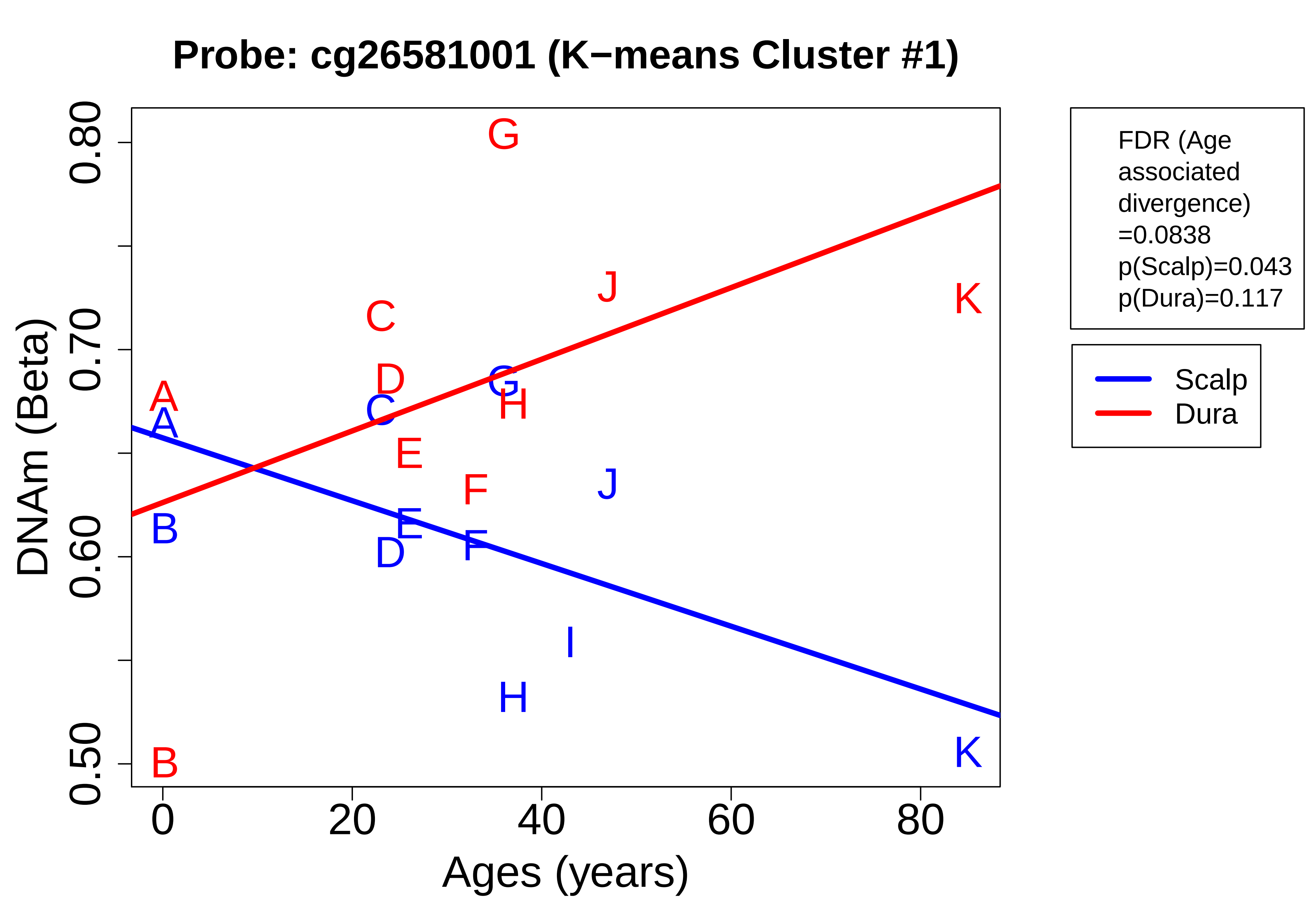

Probe: cg26581001 (K−means Cluster #1)
DNAm (Beta)
0.50
0.60
0.70
0.80
Ages (years)
0
20
40
60
80
FDR (Age associated divergence) =0.0838
p(Scalp)=0.043
p(Dura)=0.117
Scalp
Dura
A
B
C
D
E
F
G
H
I
J
K

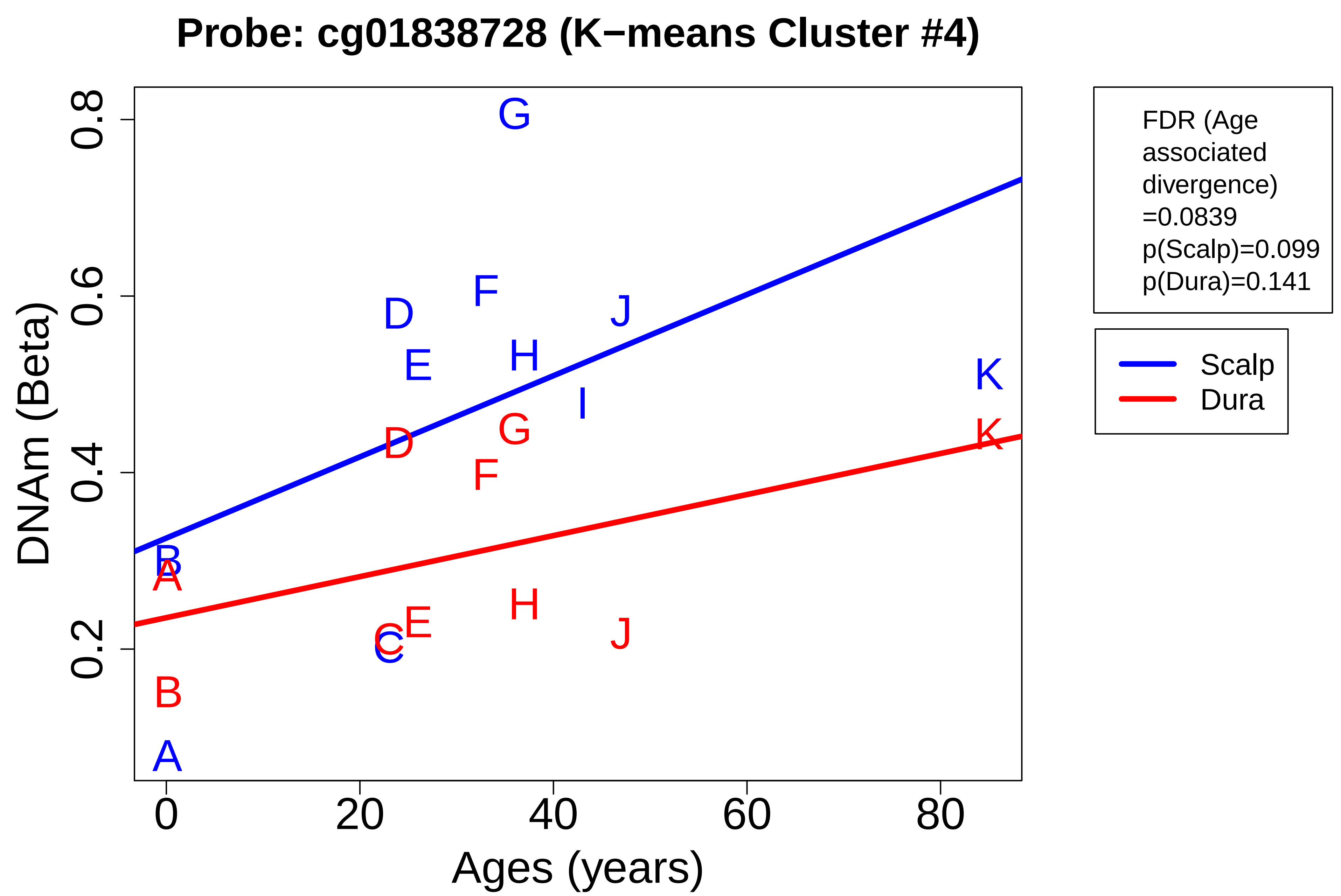
Probe: cg01838728 (K−means Cluster #4)
DNAm (Beta)
0.2
0.4
0.6
0.8
Ages (years)
0
20
40
60
80
A
B
C
D
E
F
G
H
I
J
K
FDR (Age associated divergence) =0.0839
p(Scalp)=0.099
p(Dura)=0.141
Scalp
Dura

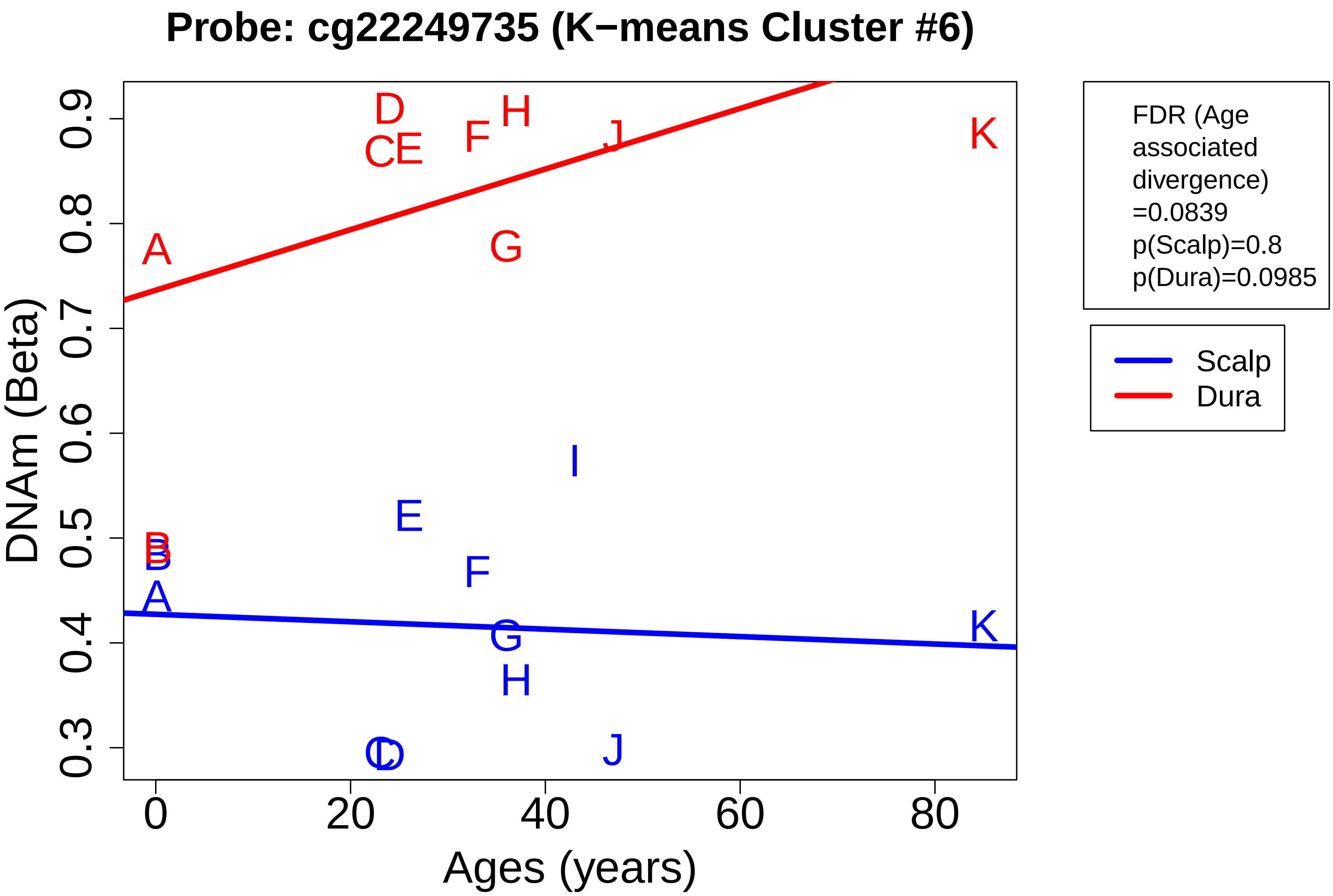

Probe: cg22249735 (K−means Cluster #6)
DNAm (Beta)
0.3
0.4
0.5
0.6
0.7
0.8
0.9
Ages (years)
0
20
40
60
80
A
B
C
D
E
F
G
H
I
J
K
FDR (Age associated divergence) =0.0839
p(Scalp)=0.8
p(Dura)=0.0985
Scalp
Dura

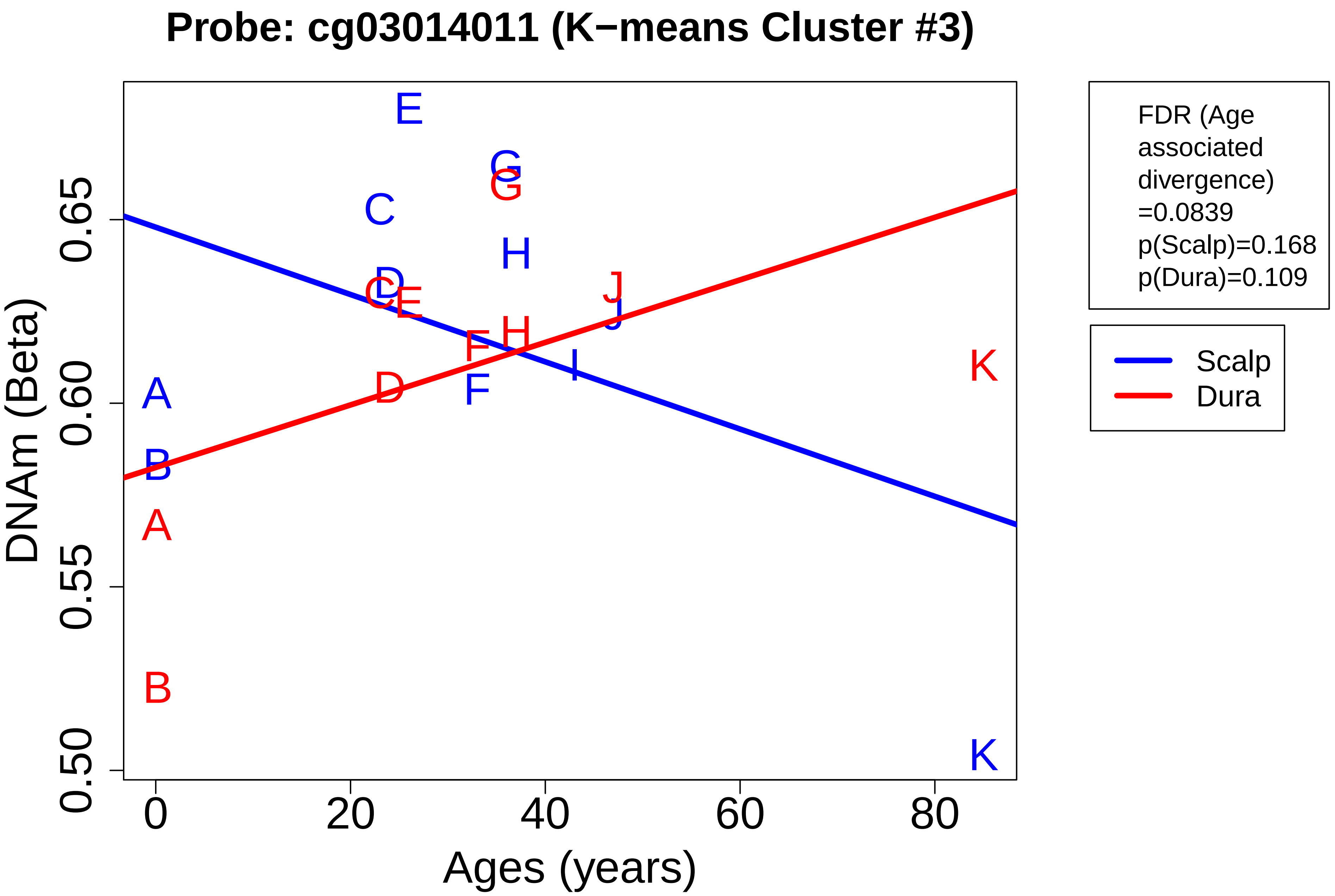

Probe: cg03014011 (K−means Cluster #3)
FDR (Age associated divergence) =0.0839
p(Scalp)=0.168
p(Dura)=0.109
Scalp
Dura
DNAm (Beta)
0.50
0.55
0.60
0.65
0
20
40
60
80
Ages (years)
A
B
C
D
E
F
G
H
I
J
K

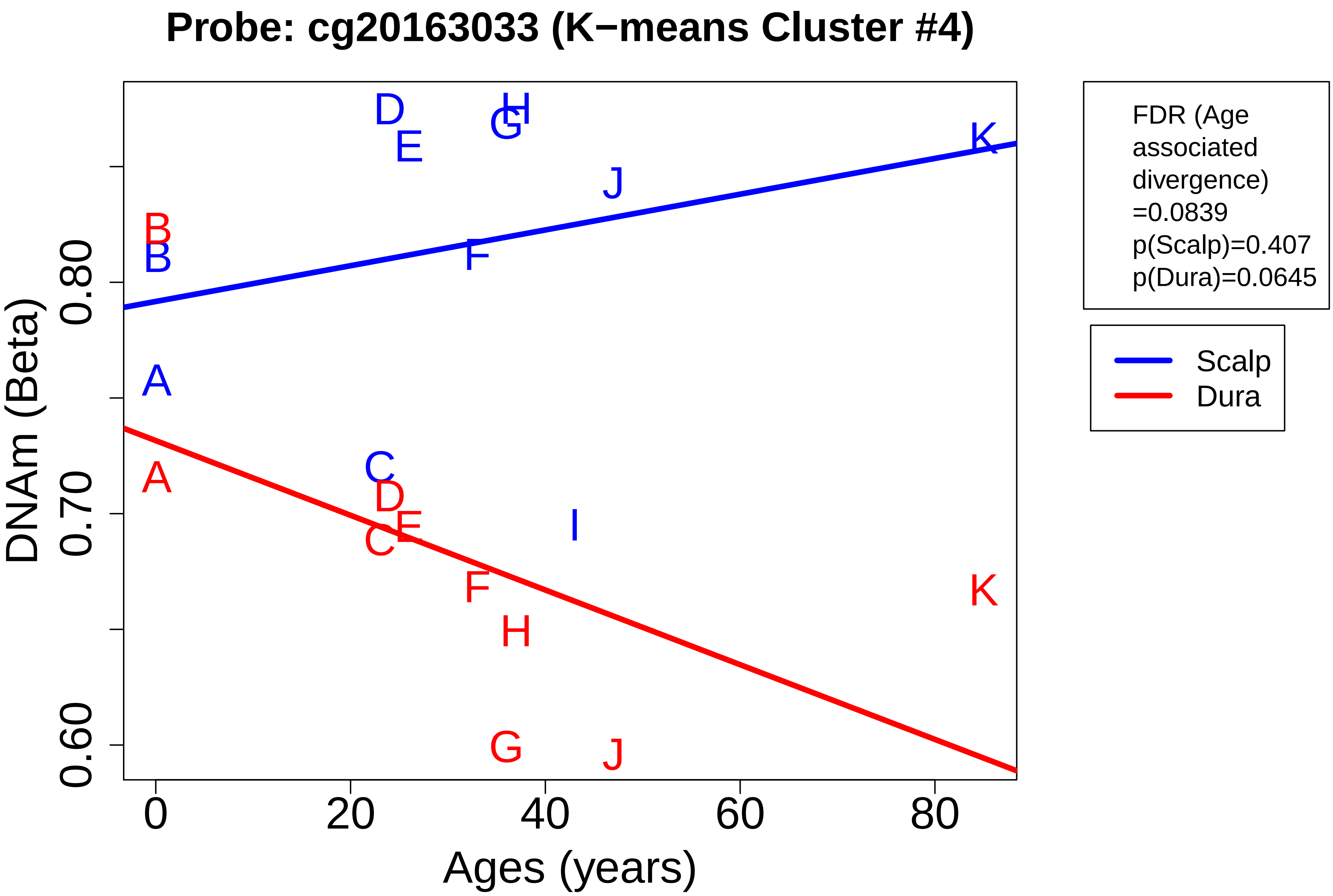

Probe: cg20163033 (K−means Cluster #4)
FDR (Age associated divergence) =0.0839
p(Scalp)=0.407
p(Dura)=0.0645
Scalp
Dura
DNAm (Beta)
0.60
0.70
0.80
Ages (years)
0
20
40
60
80
A
B
C
D
E
F
G
H
I
J
K

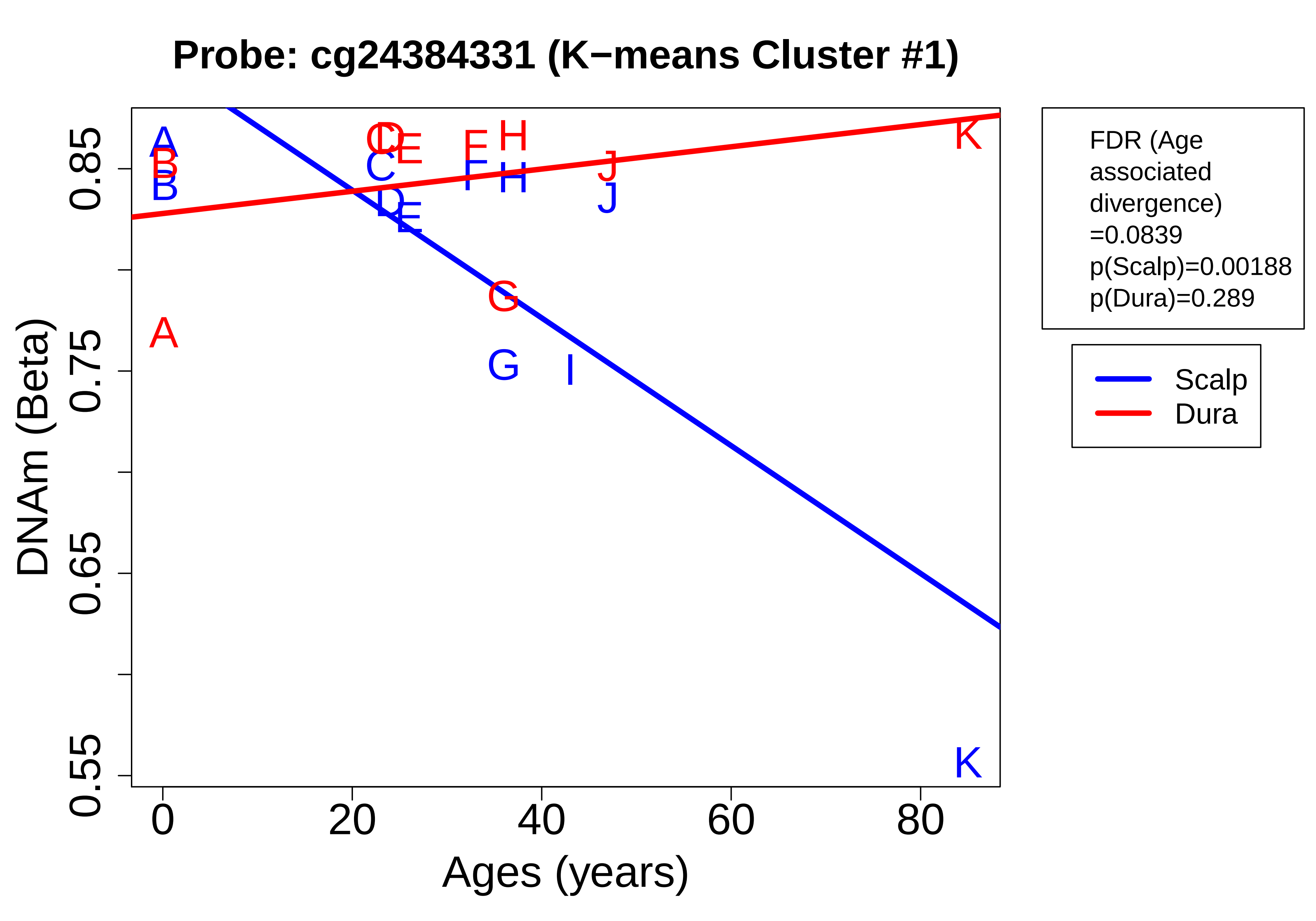

Probe: cg24384331 (K−means Cluster #1)
DNAm (Beta)
0.55
0.65
0.75
0.85
0
20
40
60
80
Ages (years)
A
B
C
D
E
F
G
H
I
J
K
FDR (Age associated divergence) =0.0839
p(Scalp)=0.00188
p(Dura)=0.289
Scalp
Dura

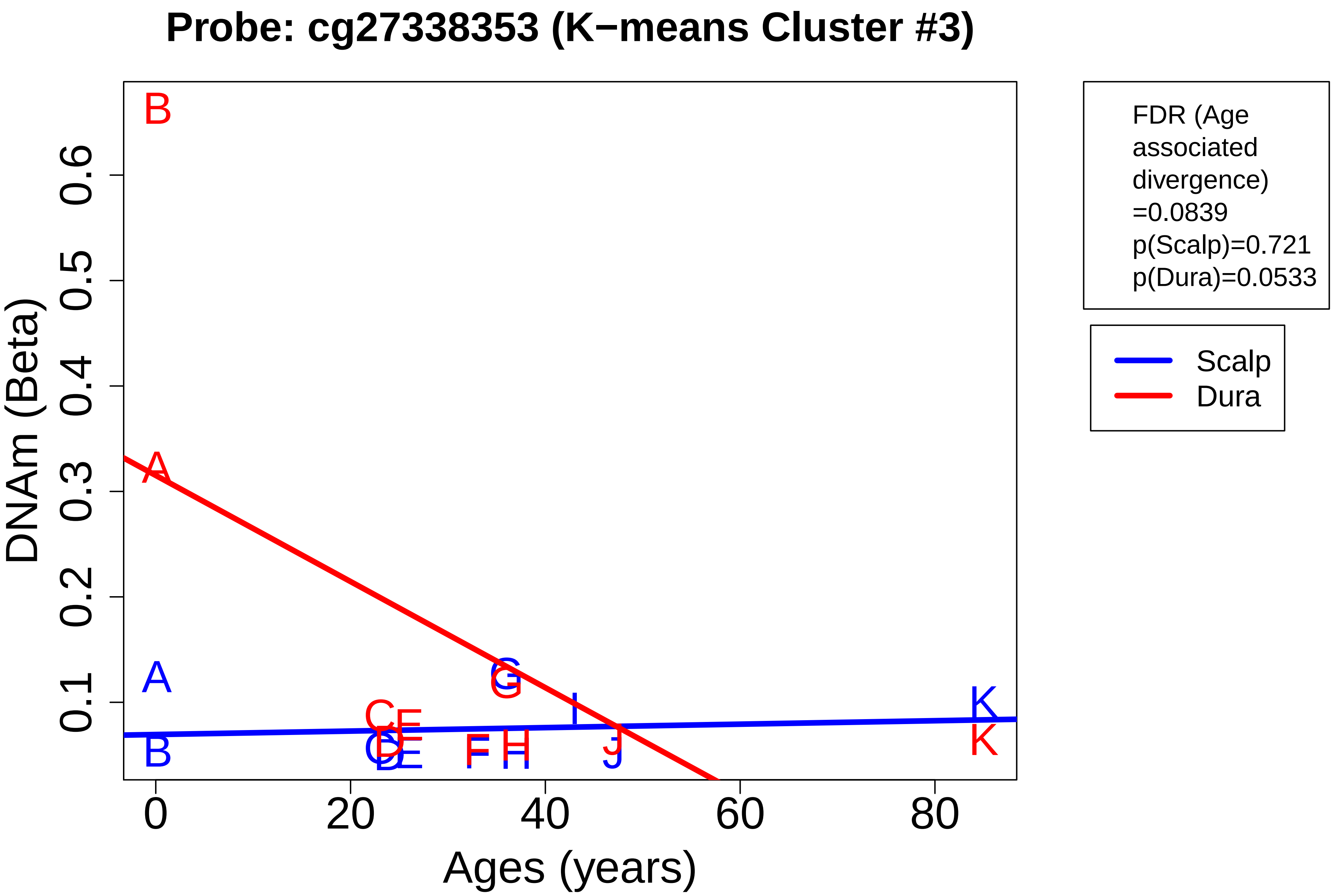

Probe: cg27338353 (K−means Cluster #3)
DNAm (Beta)
0.1
0.2
0.3
0.4
0.5
0.6
Ages (years)
0
20
40
60
80
FDR (Age associated divergence) =0.0839
p(Scalp)=0.721
p(Dura)=0.0533
Scalp
Dura

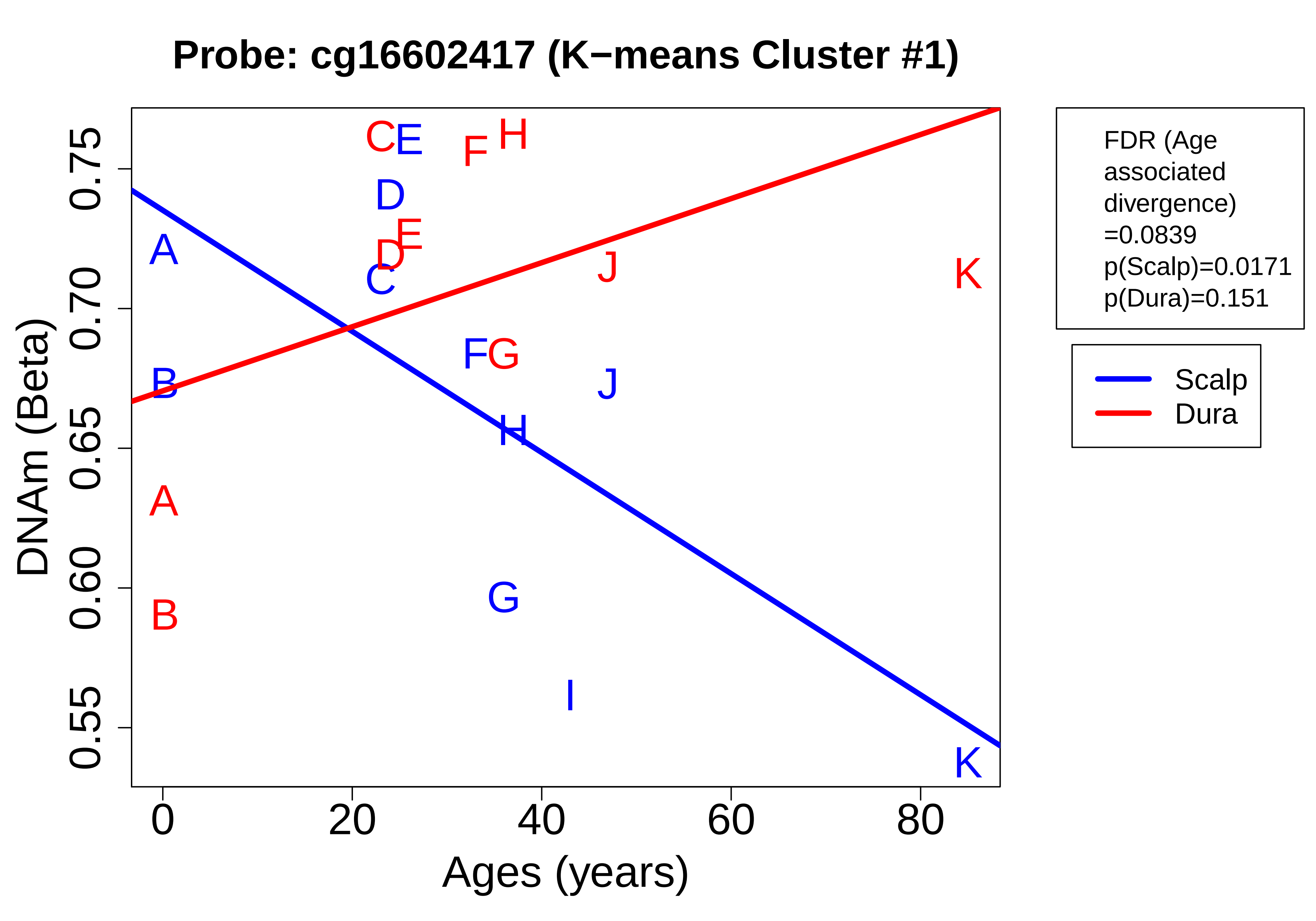

Probe: cg16602417 (K−means Cluster #1)
DNAm (Beta)
0.55
0.60
0.65
0.70
0.75
0
20
40
60
80
Ages (years)
A
B
C
D
E
F
G
H
I
J
K
FDR (Age associated divergence) =0.0839
p(Scalp)=0.0171
p(Dura)=0.151
Scalp
Dura

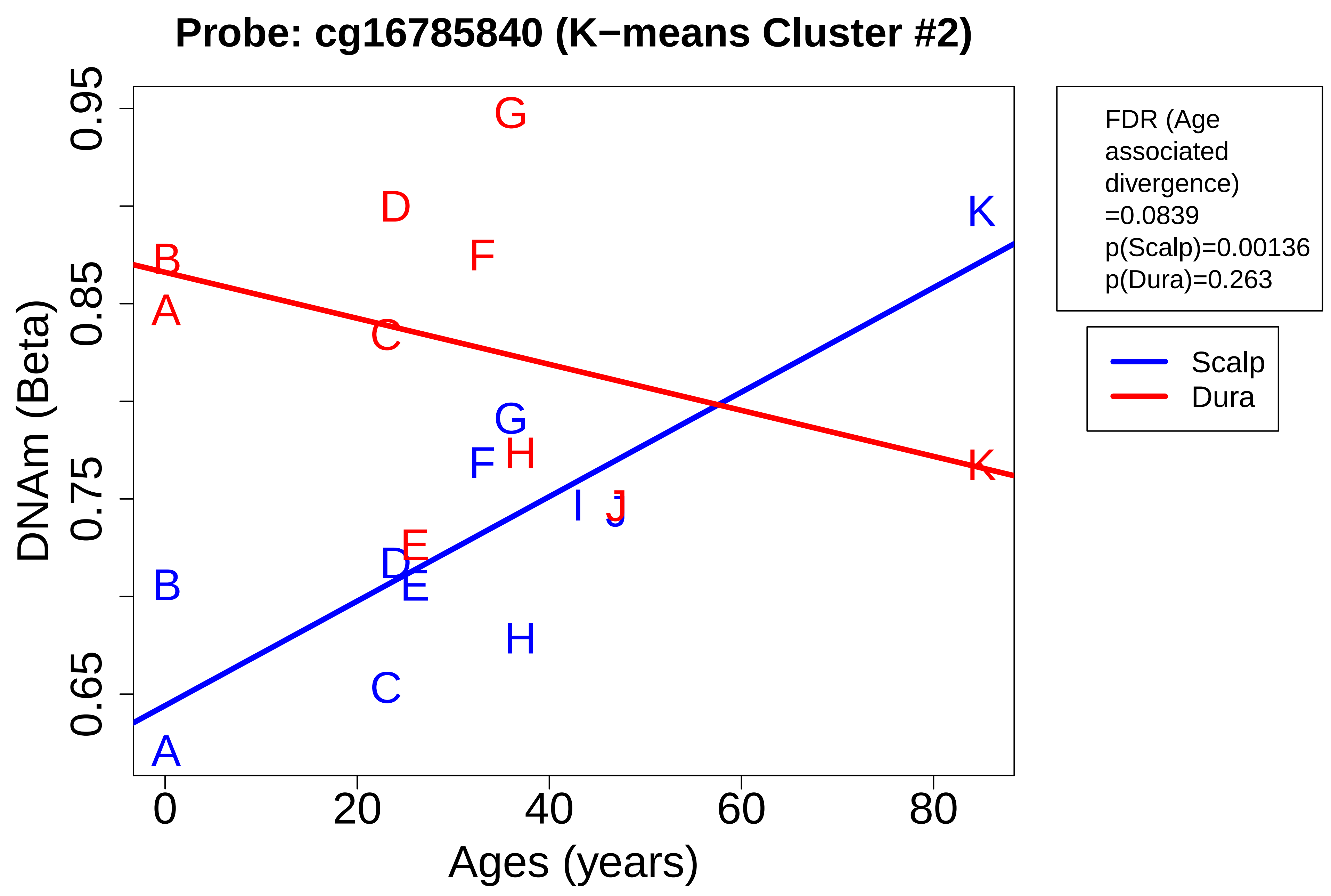

Probe: cg16785840 (K−means Cluster #2)
DNAm (Beta)
0.65
0.75
0.85
0.95
0
20
40
60
80
Ages (years)
FDR (Age
associated
divergence)
=0.0839
p(Scalp)=0.00136
p(Dura)=0.263
Scalp
Dura
A
B
C
D
E
F
G
H
I
J
K

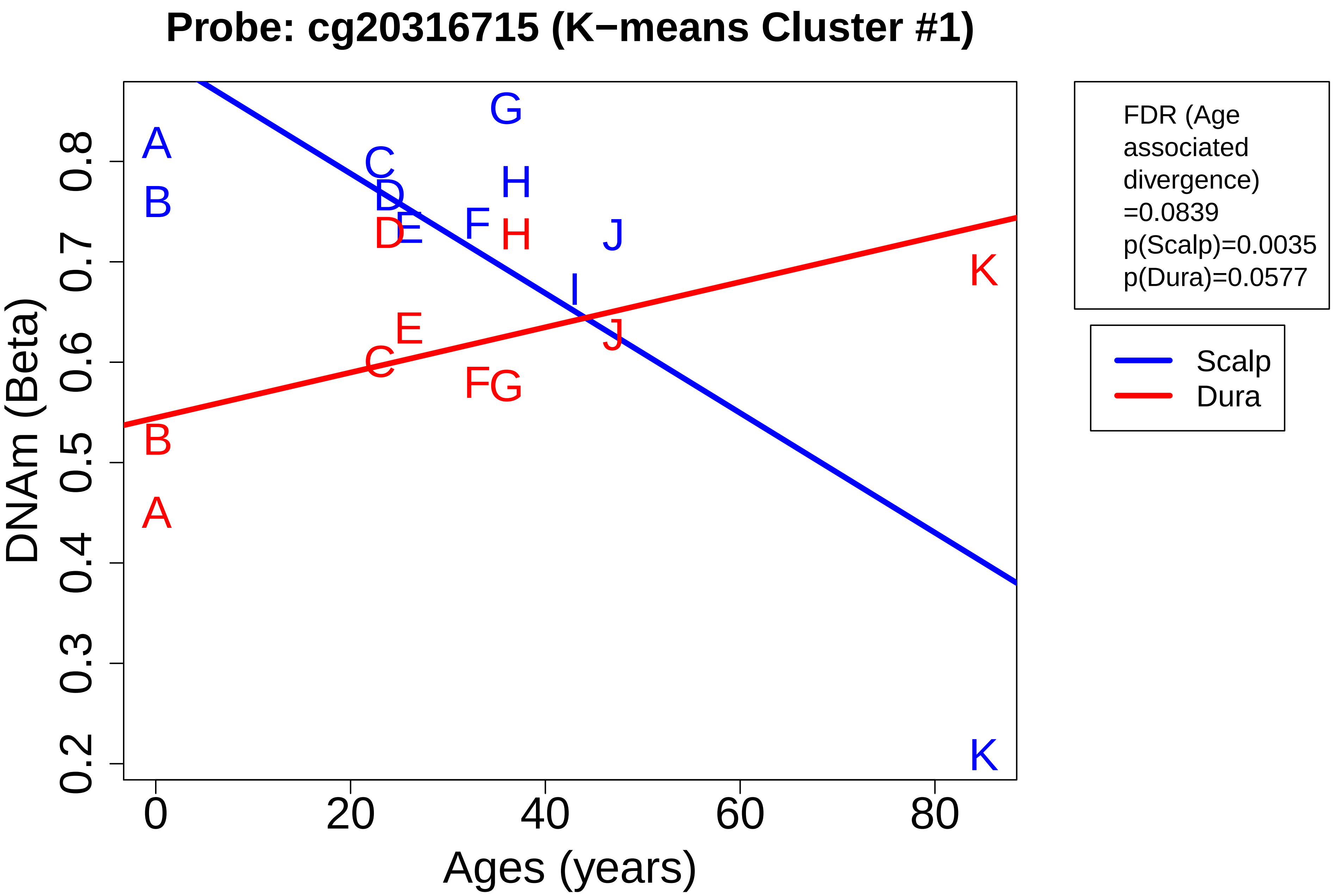

Probe: cg20316715 (K−means Cluster #1)
DNAm (Beta)
0.2
0.3
0.4
0.5
0.6
0.7
0.8
0
20
40
60
80
Ages (years)
A
B
C
D
E
F
G
H
I
J
K
FDR (Age associated divergence) =0.0839
p(Scalp)=0.0035
p(Dura)=0.0577
Scalp
Dura

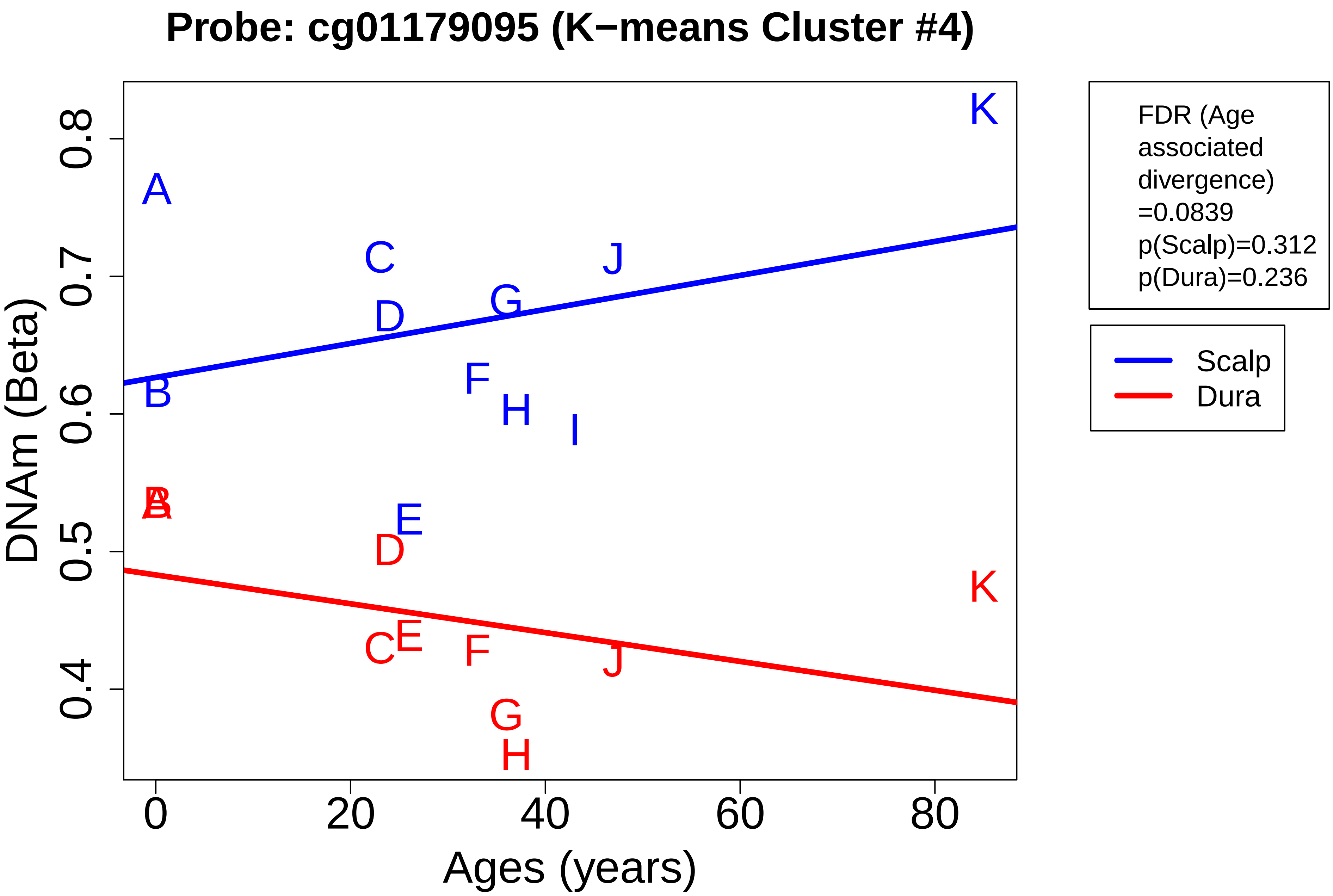

Probe: cg01179095 (K−means Cluster #4)
DNAm (Beta)
0.4
0.5
0.6
0.7
0.8
0
20
40
60
80
Ages (years)
A
B
C
D
E
F
G
H
I
J
K
FDR (Age associated divergence) =0.0839
p(Scalp)=0.312
p(Dura)=0.236
Scalp
Dura

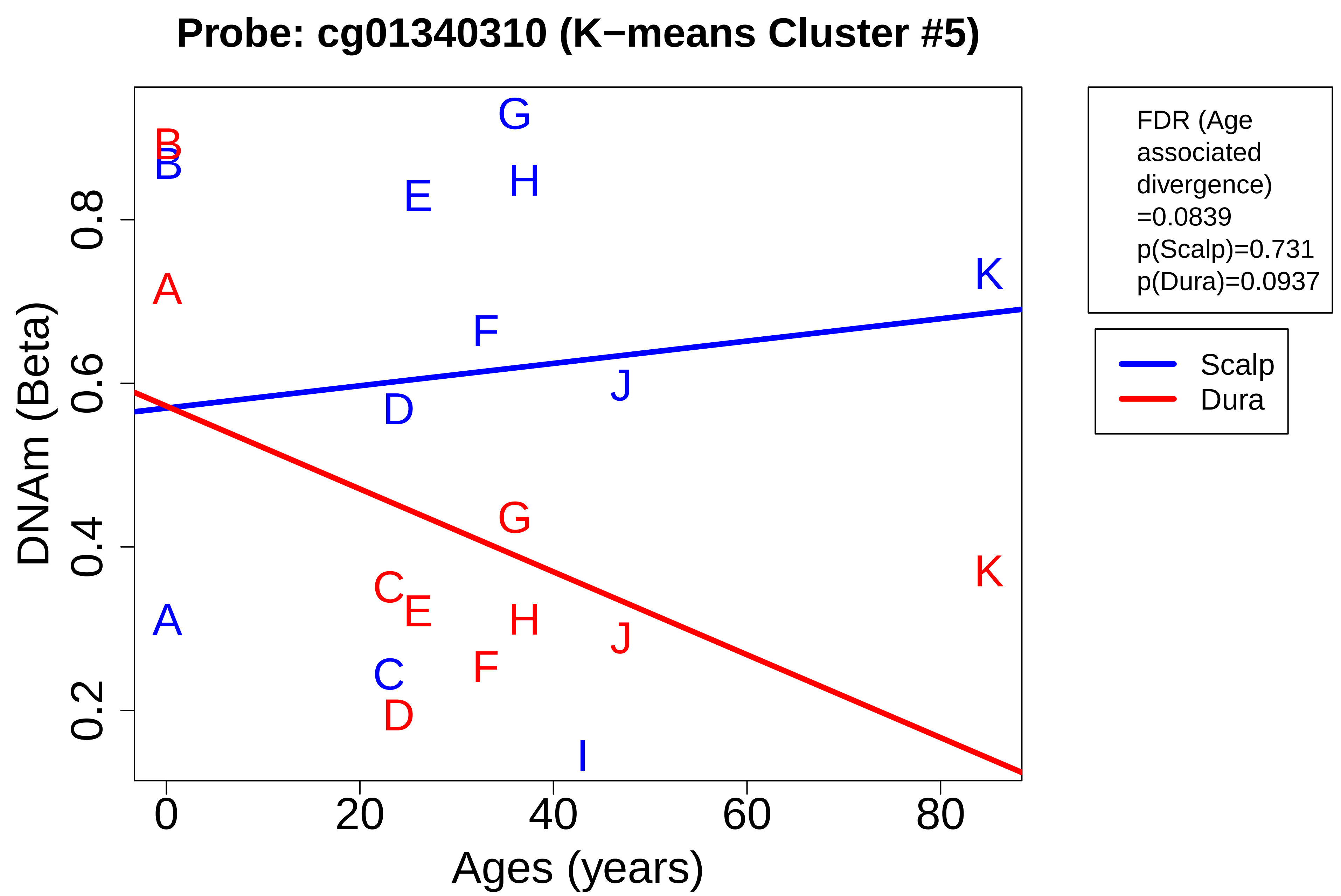

Probe: cg01340310 (K−means Cluster #5)
DNAm (Beta)
0.2
0.4
0.6
0.8
Ages (years)
0
20
40
60
80
B
B
G
E
H
A
K
F
J
D
G
C
E
K
A
H
J
F
C
D
I
FDR (Age associated divergence) =0.0839
p(Scalp)=0.731
p(Dura)=0.0937
Scalp
Dura

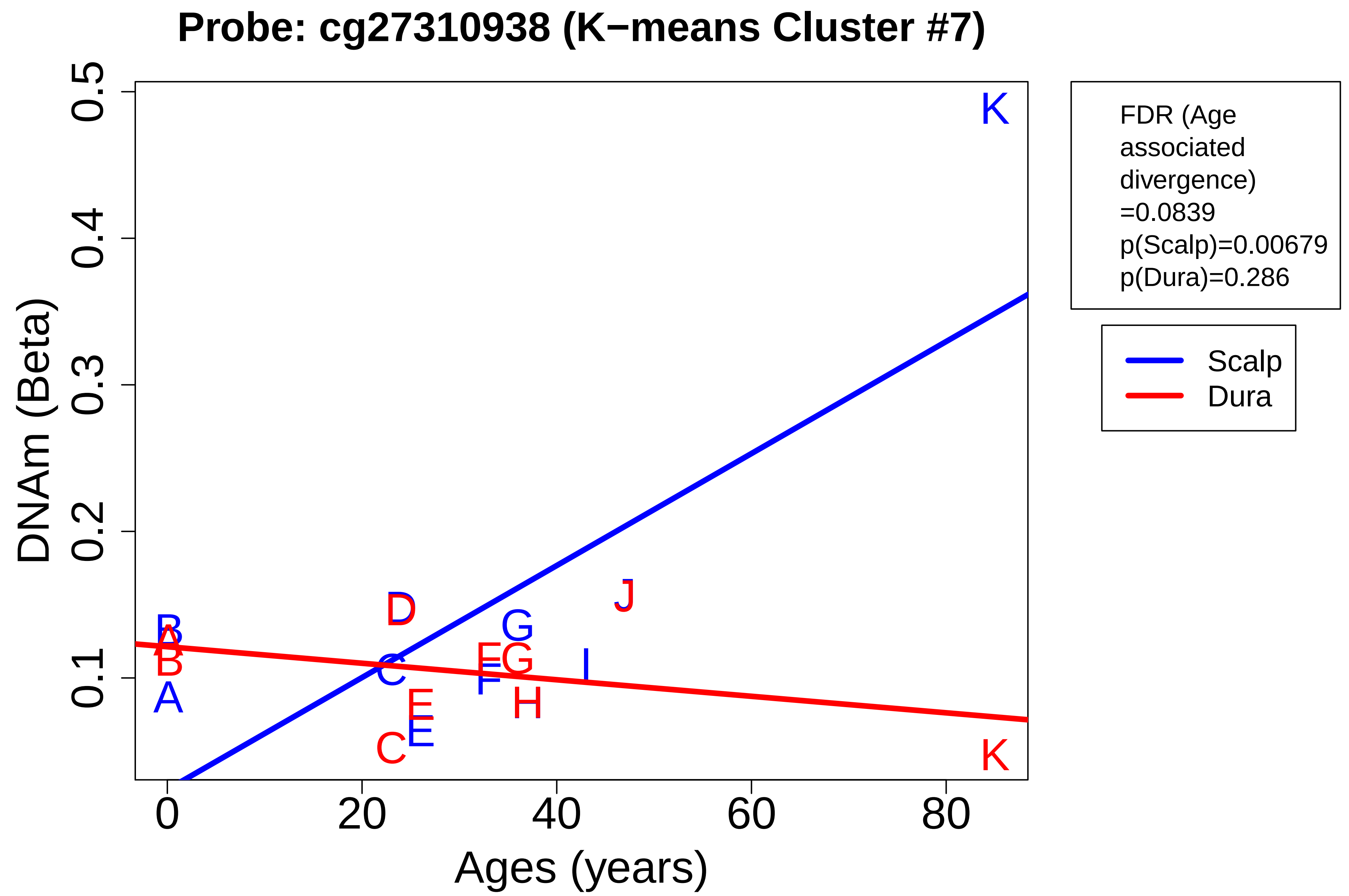
Probe: cg27310938 (K−means Cluster #7)
DNAm (Beta)
0.1
0.2
0.3
0.4
0.5
0
20
40
60
80
Ages (years)
A
B
C
D
E
F
G
H
I
J
K
FDR (Age associated divergence) =0.0839
p(Scalp)=0.00679
p(Dura)=0.286
Scalp
Dura

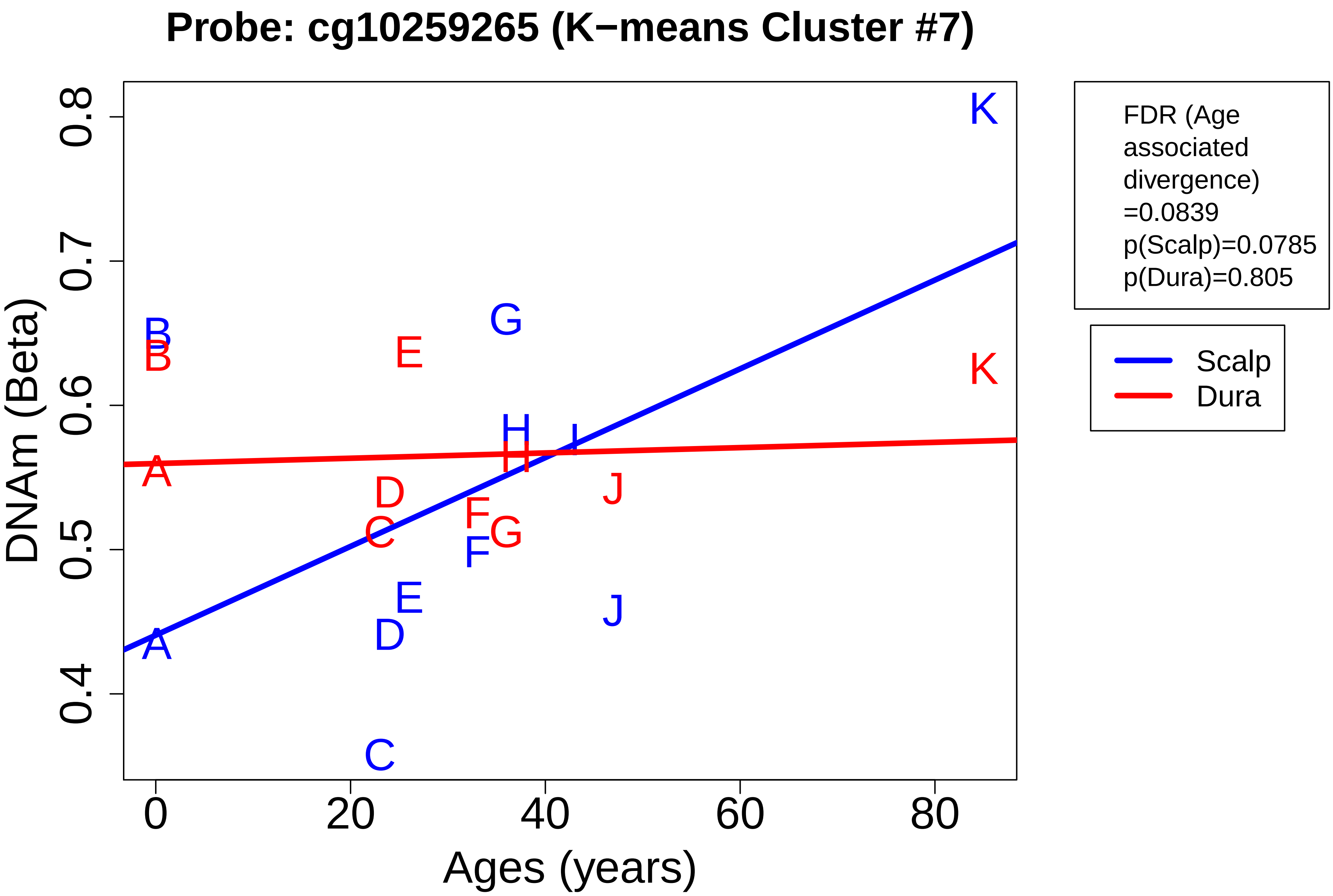

Probe: cg10259265 (K−means Cluster #7)
DNAm (Beta)
0.4
0.5
0.6
0.7
0.8
0
20
40
60
80
Ages (years)
FDR (Age associated divergence) =0.0839
p(Scalp)=0.0785
p(Dura)=0.805
Scalp
Dura

Probe: cg20510272 (K−means Cluster #7)

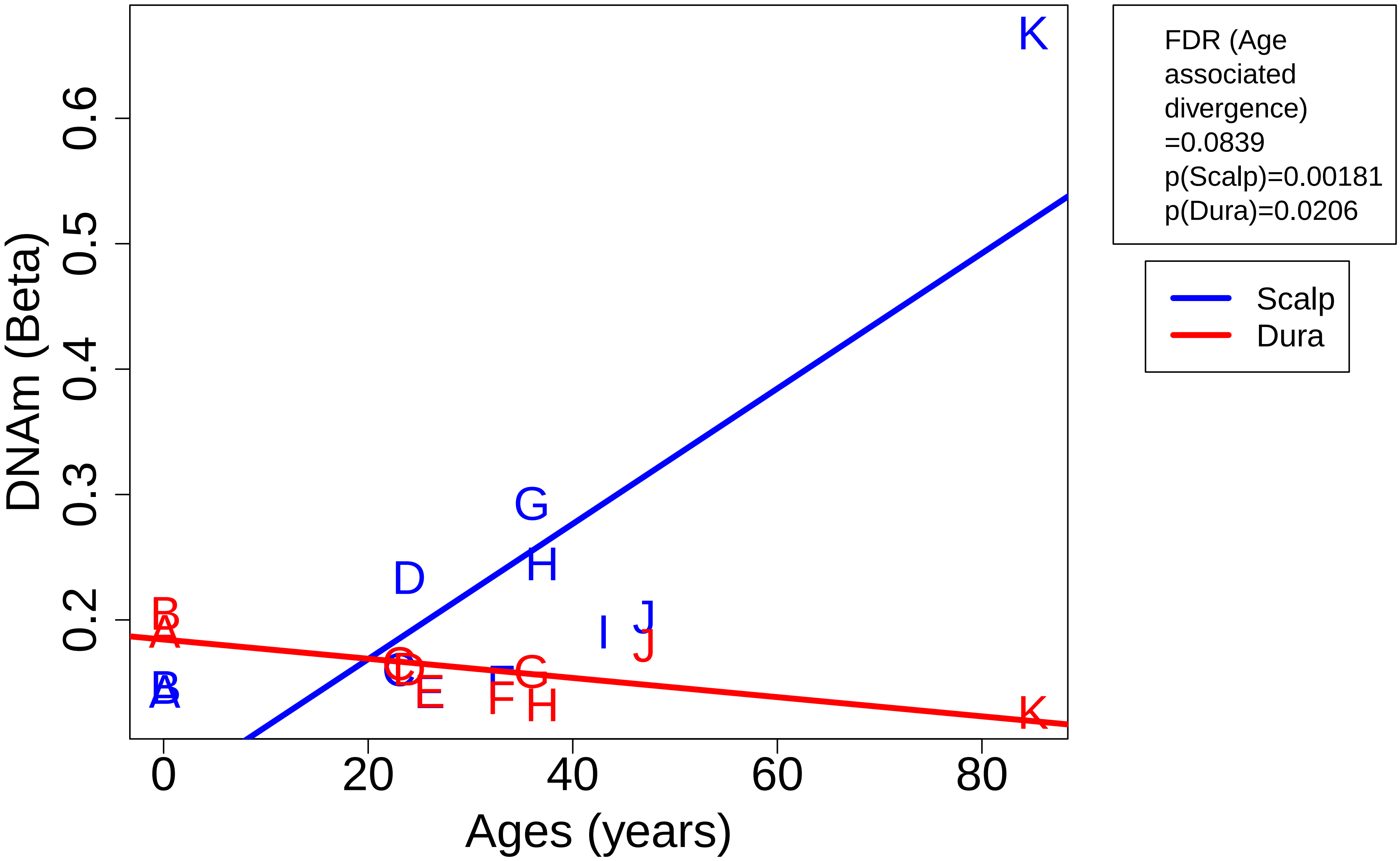

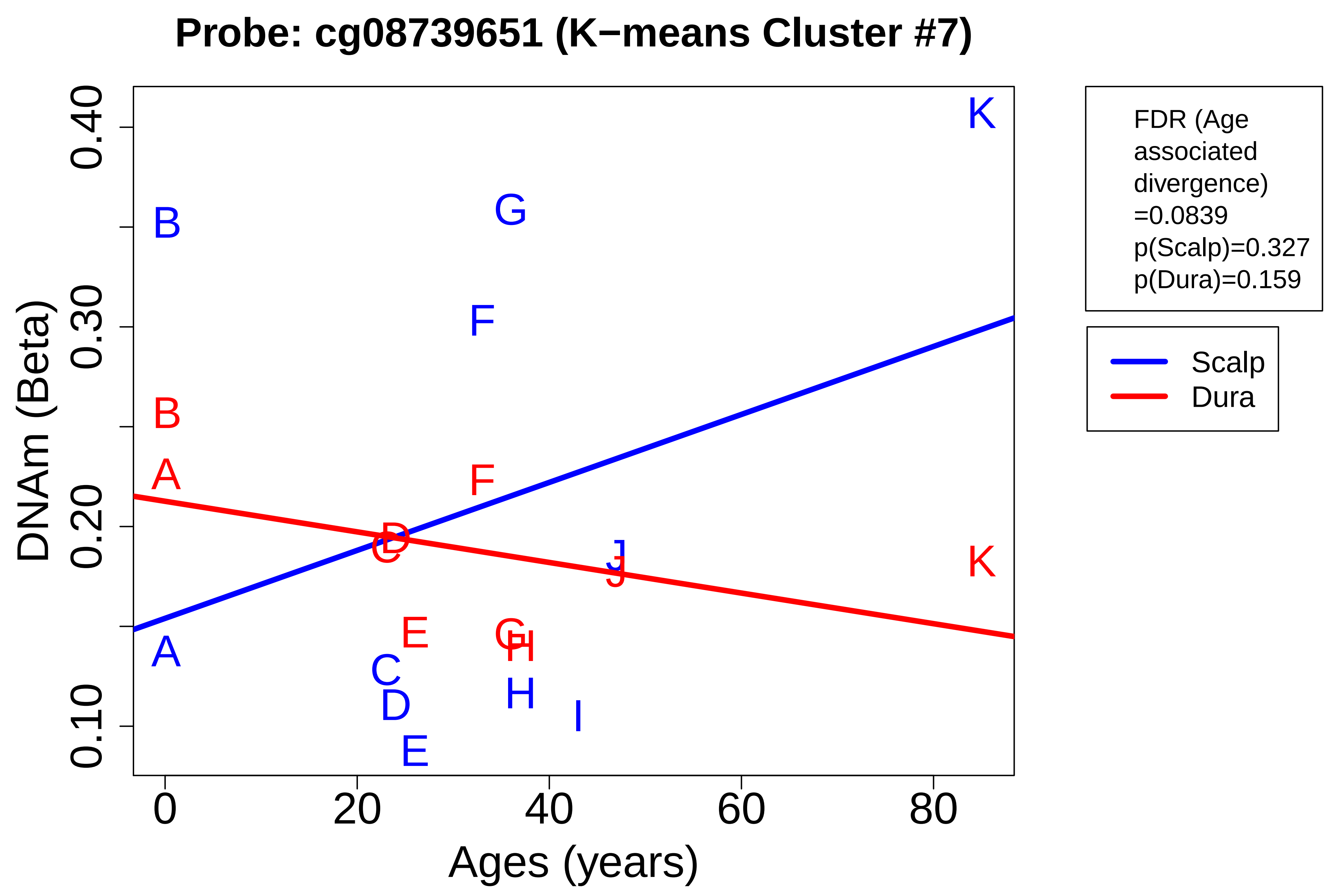

Probe: cg08739651 (K−means Cluster #7)
DNAm (Beta)
0.10
0.20
0.30
0.40
Ages (years)
0
20
40
60
80
FDR (Age associated divergence) =0.0839
p(Scalp)=0.327
p(Dura)=0.159
Scalp
Dura

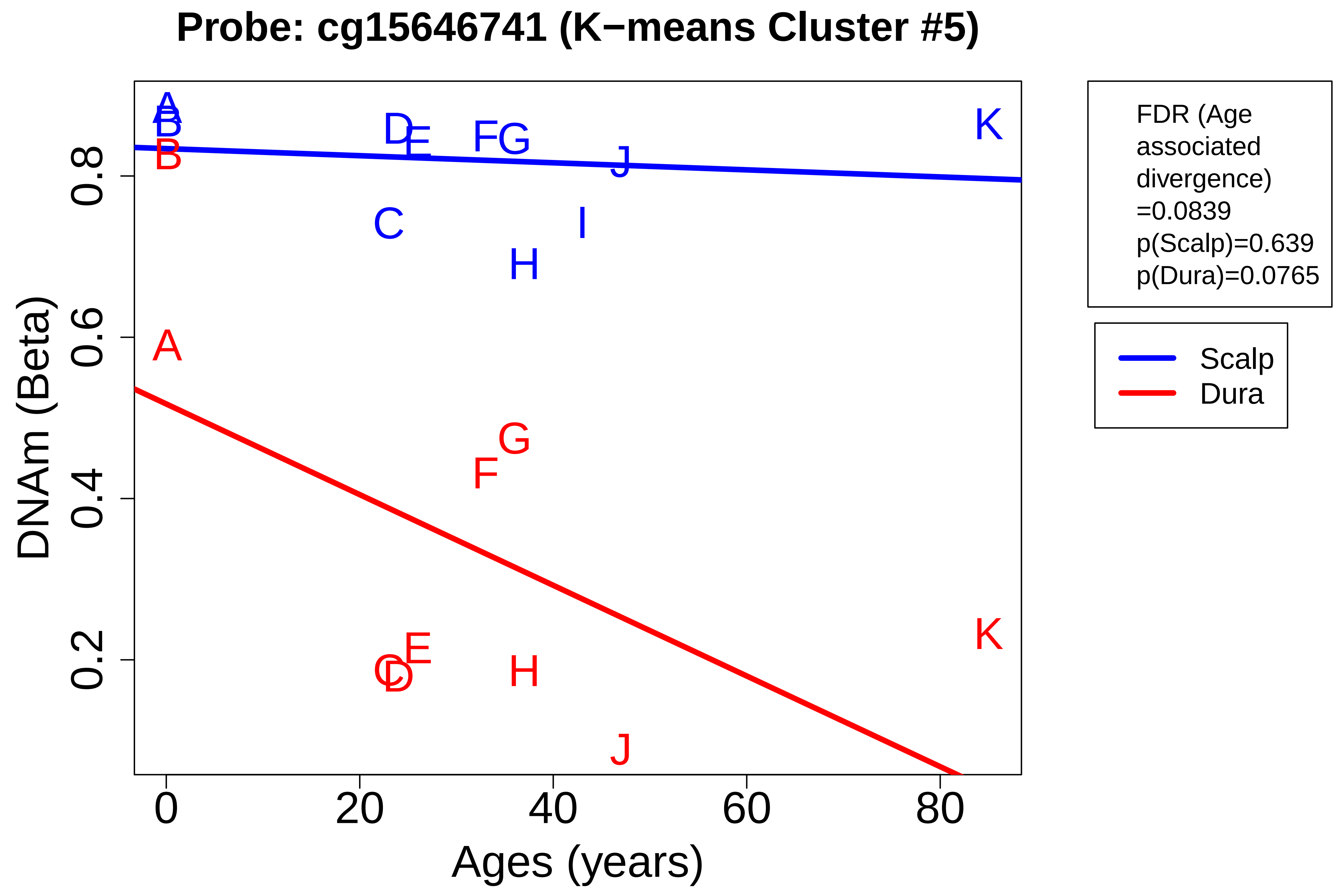

Probe: cg15646741 (K−means Cluster #5)
DNAm (Beta)
0.2
0.4
0.6
0.8
0
20
40
60
80
Ages (years)
A
B
C
D
E
F
G
H
I
J
K
FDR (Age associated divergence) =0.0839
p(Scalp)=0.639
p(Dura)=0.0765
Scalp
Dura

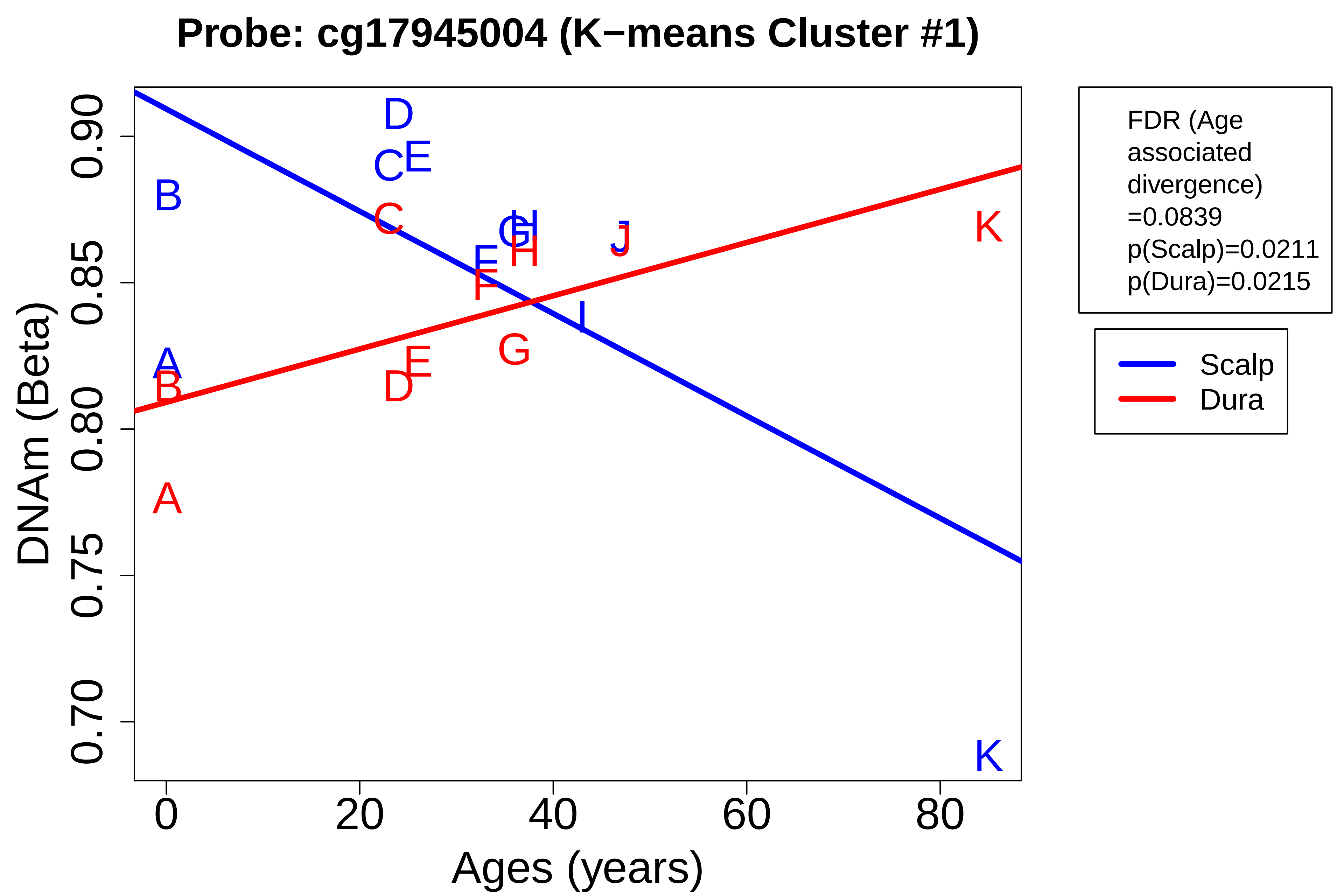

Probe: cg17945004 (K−means Cluster #1)
DNAm (Beta)
0.70
0.75
0.80
0.85
0.90
Ages (years)
0
20
40
60
80
FDR (Age associated divergence) =0.0839
p(Scalp)=0.0211
p(Dura)=0.0215
Scalp
Dura

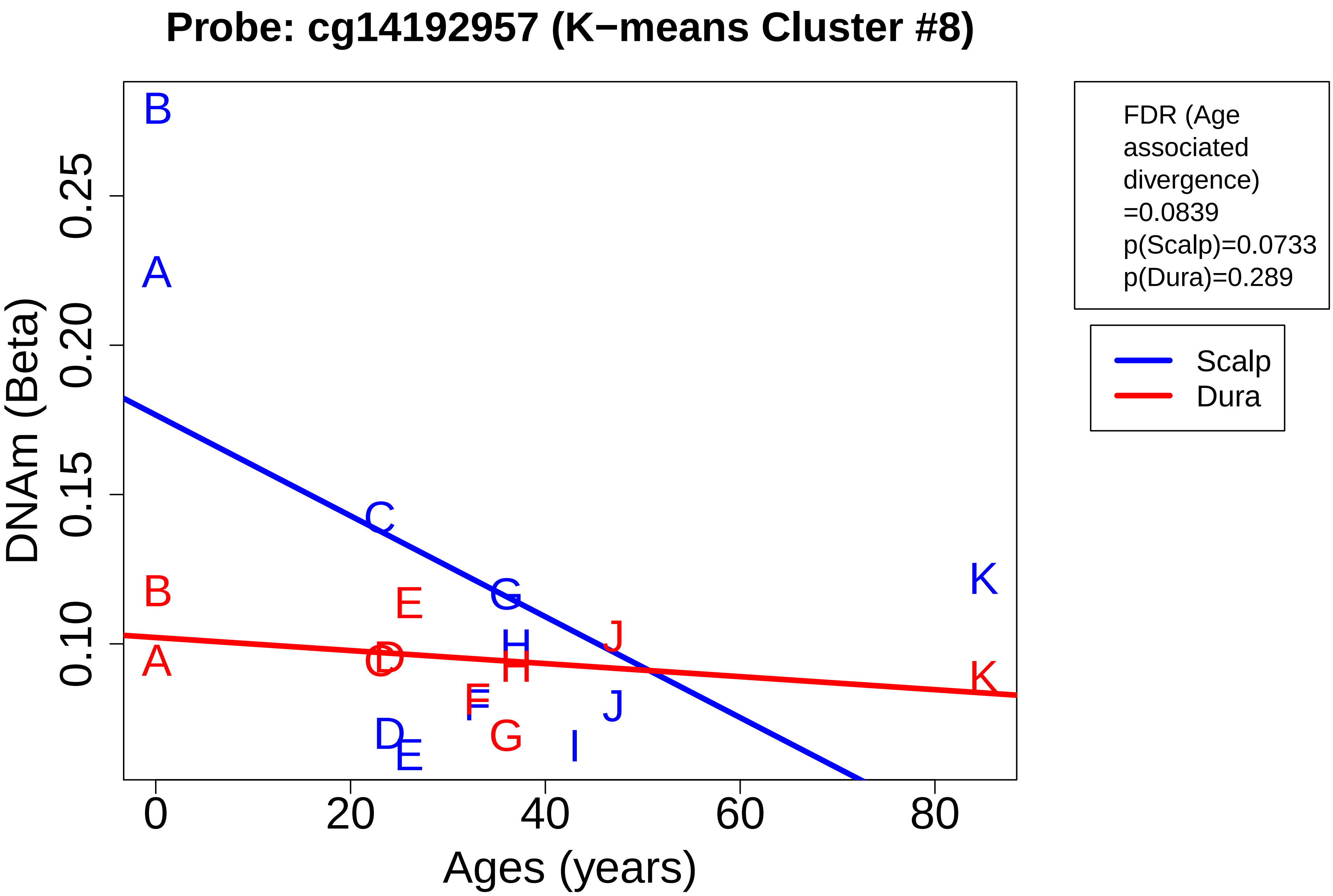
Probe: cg14192957 (K−means Cluster #8)
DNAm (Beta)
0.10
0.15
0.20
0.25
0
20
40
60
80
Ages (years)
FDR (Age associated divergence) =0.0839
p(Scalp)=0.0733
p(Dura)=0.289
Scalp
Dura

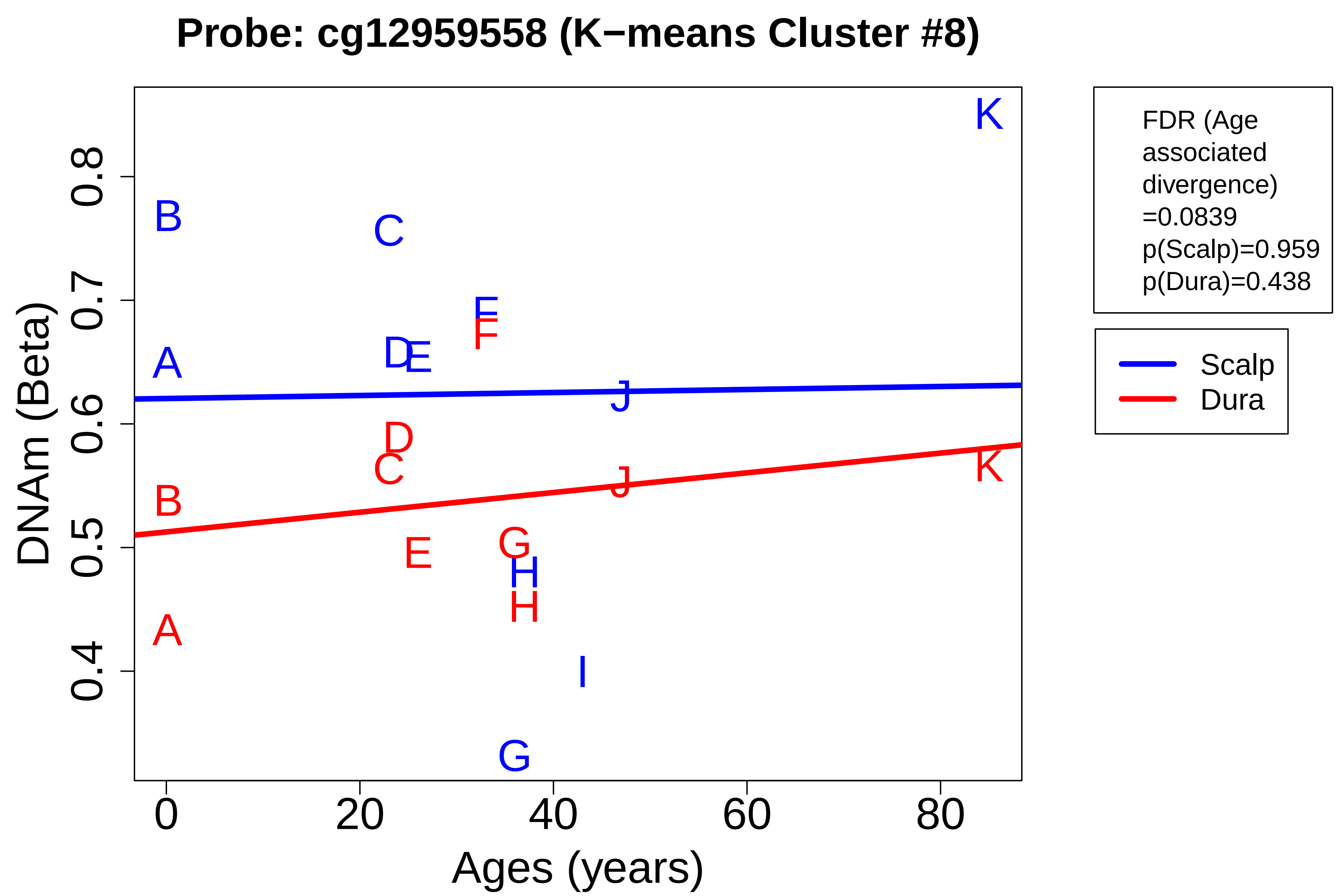

Probe: cg12959558 (K−means Cluster #8)
DNAm (Beta)
0.4
0.5
0.6
0.7
0.8
0
20
40
60
80
Ages (years)
A
B
C
D
E
F
G
H
I
J
K
FDR (Age associated divergence) =0.0839
p(Scalp)=0.959
p(Dura)=0.438
Scalp
Dura

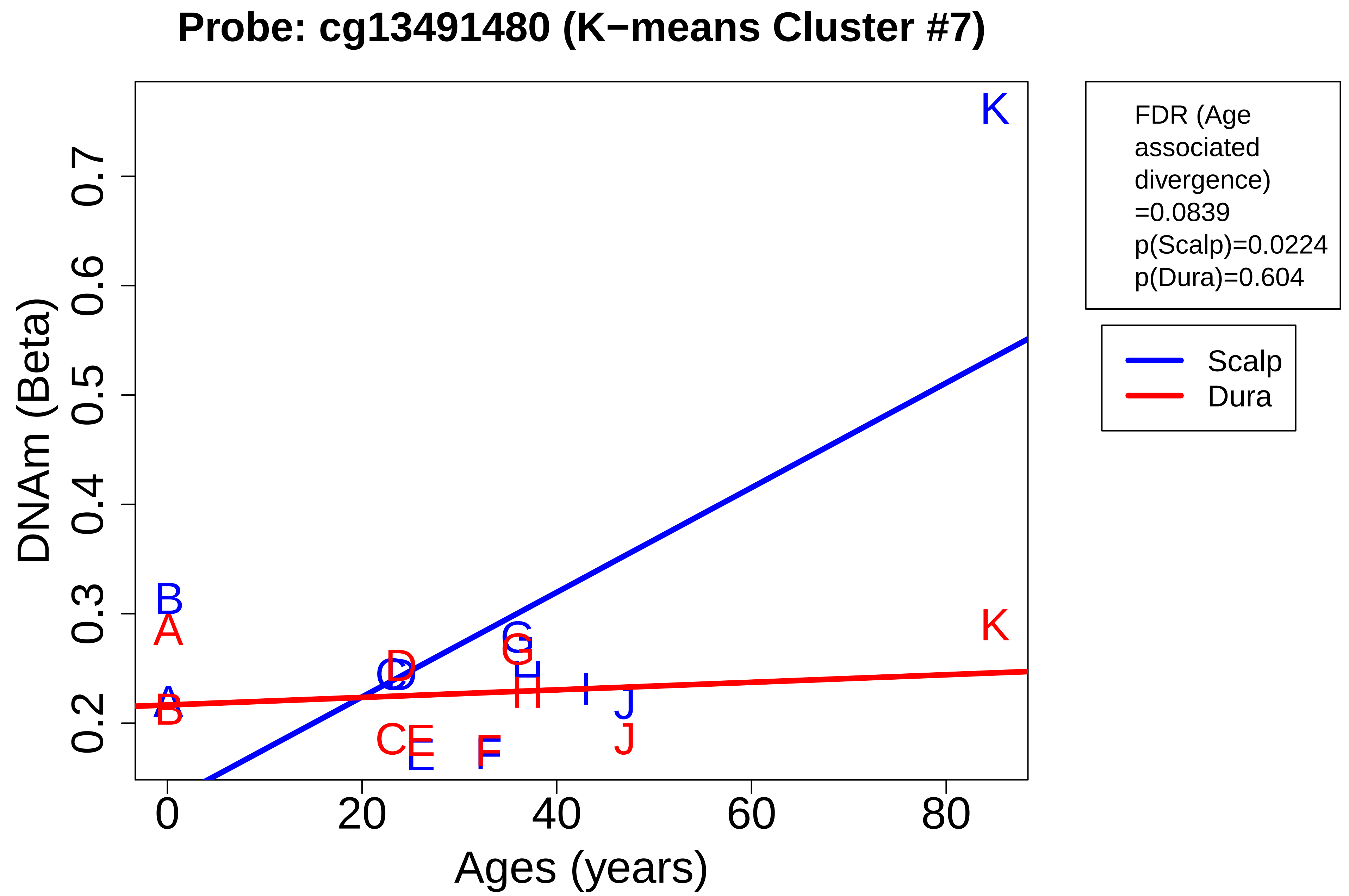
Probe: cg13491480 (K−means Cluster #7)
DNAm (Beta)
0.2
0.3
0.4
0.5
0.6
0.7
0
20
40
60
80
Ages (years)
FDR (Age associated divergence) =0.0839
p(Scalp)=0.0224
p(Dura)=0.604
Scalp
Dura

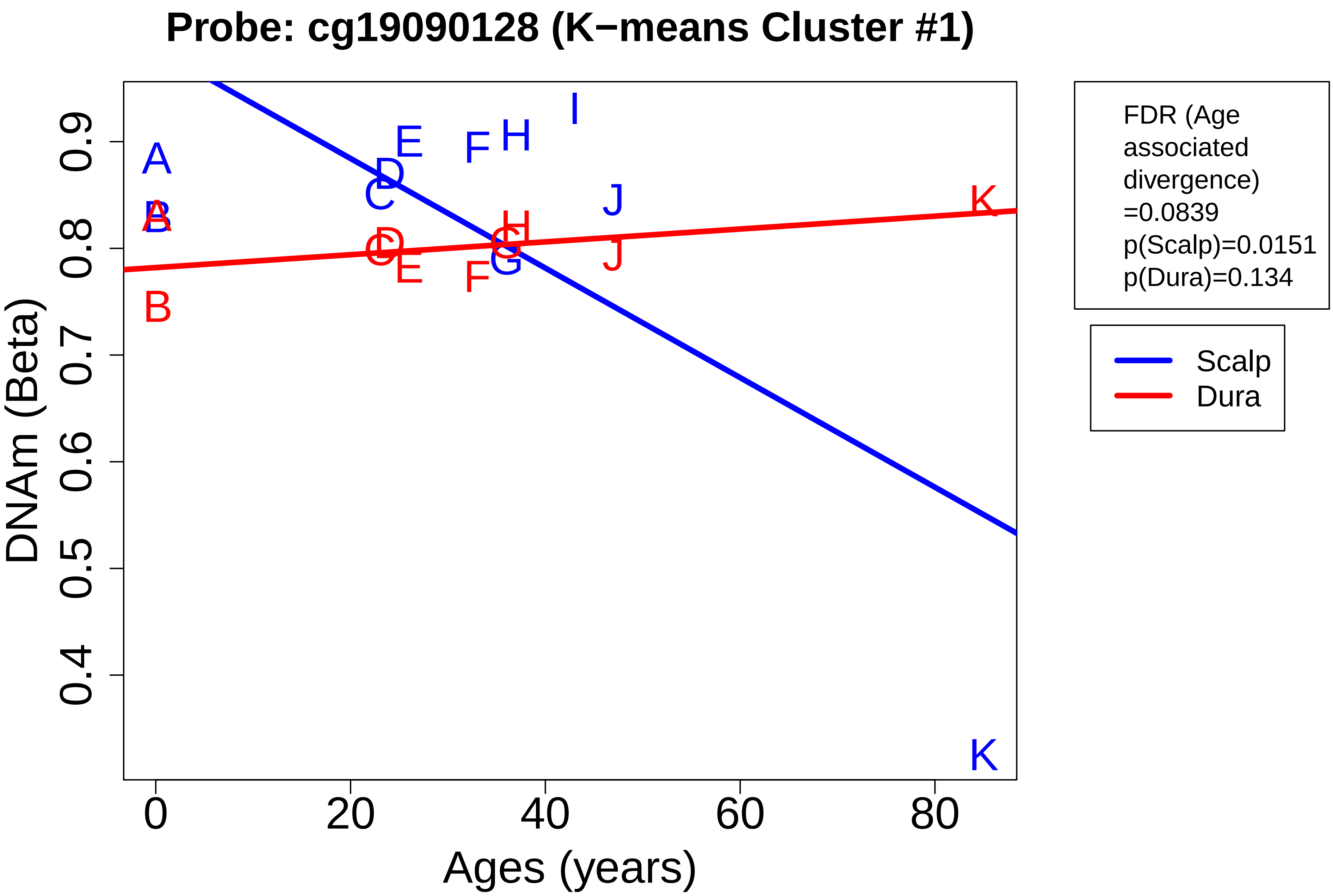

Probe: cg19090128 (K−means Cluster #1)
DNAm (Beta)
0.4
0.5
0.6
0.7
0.8
0.9
0
20
40
60
80
Ages (years)
A
B
C
D
E
F
G
H
I
J
K
FDR (Age associated divergence) =0.0839
p(Scalp)=0.0151
p(Dura)=0.134
Scalp
Dura

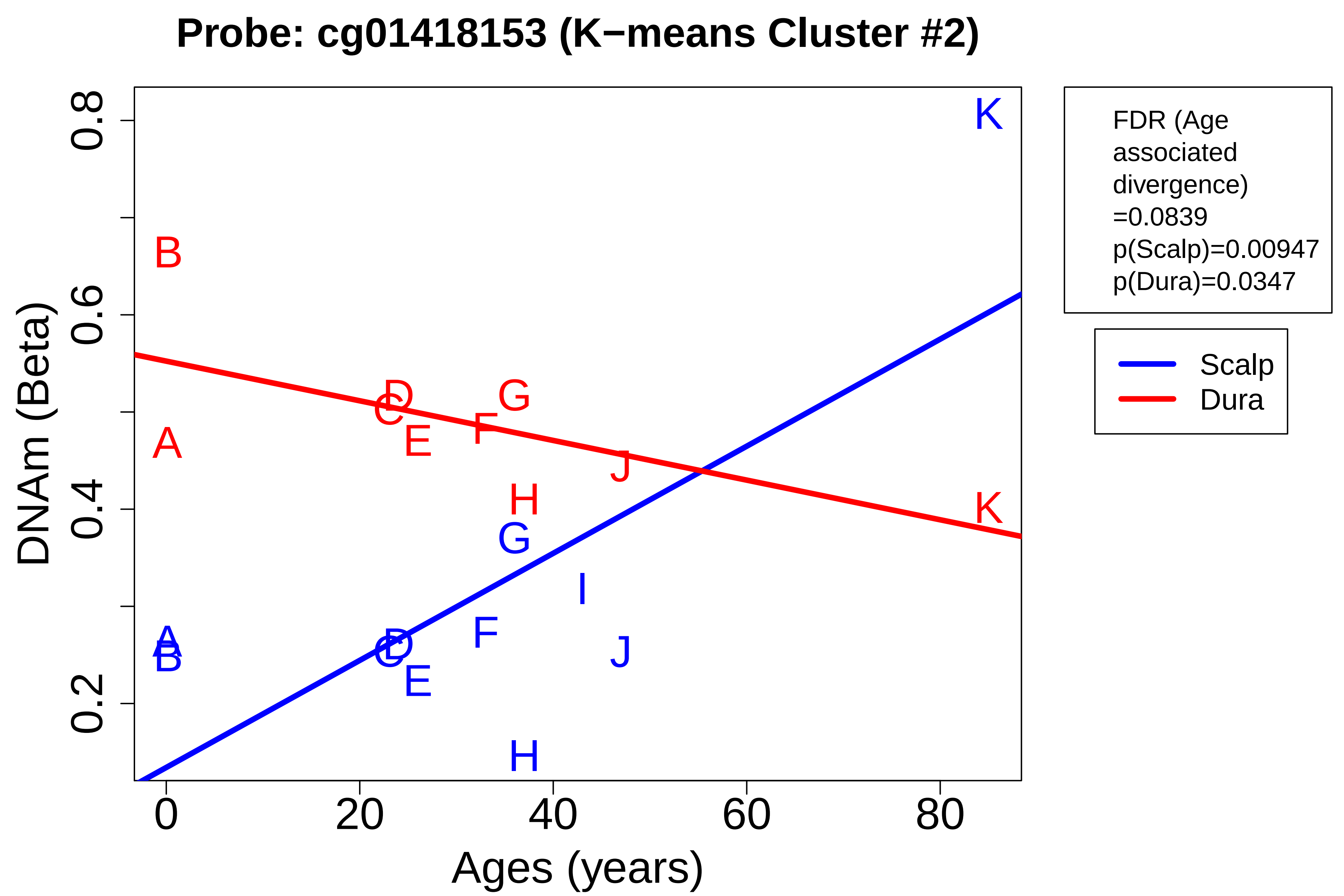

Probe: cg01418153 (K−means Cluster #2)
DNAm (Beta)
0.2
0.4
0.6
0.8
0
20
40
60
80
Ages (years)
FDR (Age associated divergence) =0.0839
p(Scalp)=0.00947
p(Dura)=0.0347
Scalp
Dura
A
B
C
D
E
F
G
H
I
J
K

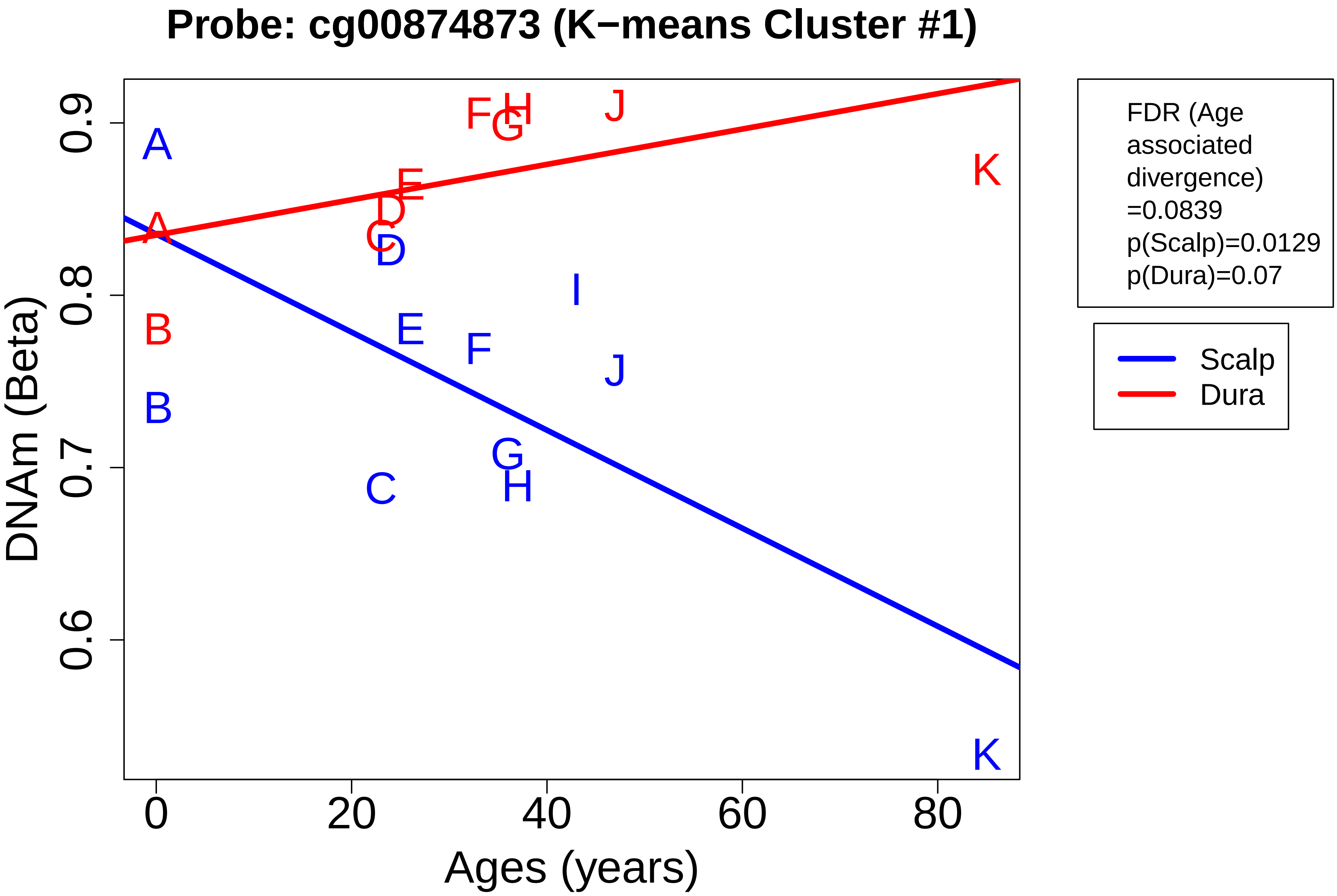

Probe: cg00874873 (K−means Cluster #1)
DNAm (Beta)
0.6
0.7
0.8
0.9
0
20
40
60
80
Ages (years)
A
B
C
D
E
F
G
H
I
J
K
FDR (Age associated divergence) =0.0839
p(Scalp)=0.0129
p(Dura)=0.07
Scalp
Dura

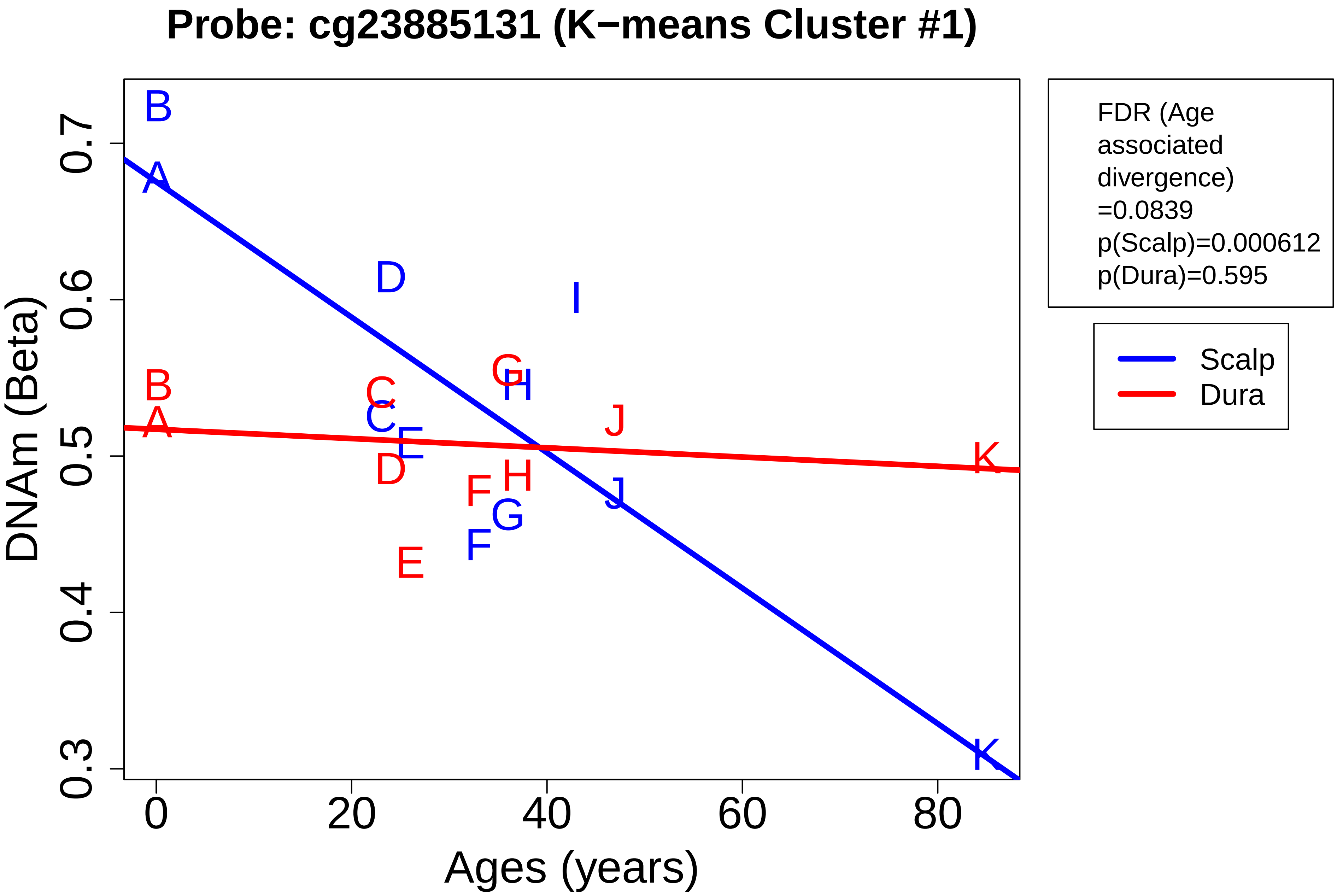

Probe: cg23885131 (K−means Cluster #1)
DNAm (Beta)
0.3
0.4
0.5
0.6
0.7
0
20
40
60
80
Ages (years)
FDR (Age associated divergence) =0.0839
p(Scalp)=0.000612
p(Dura)=0.595
Scalp
Dura

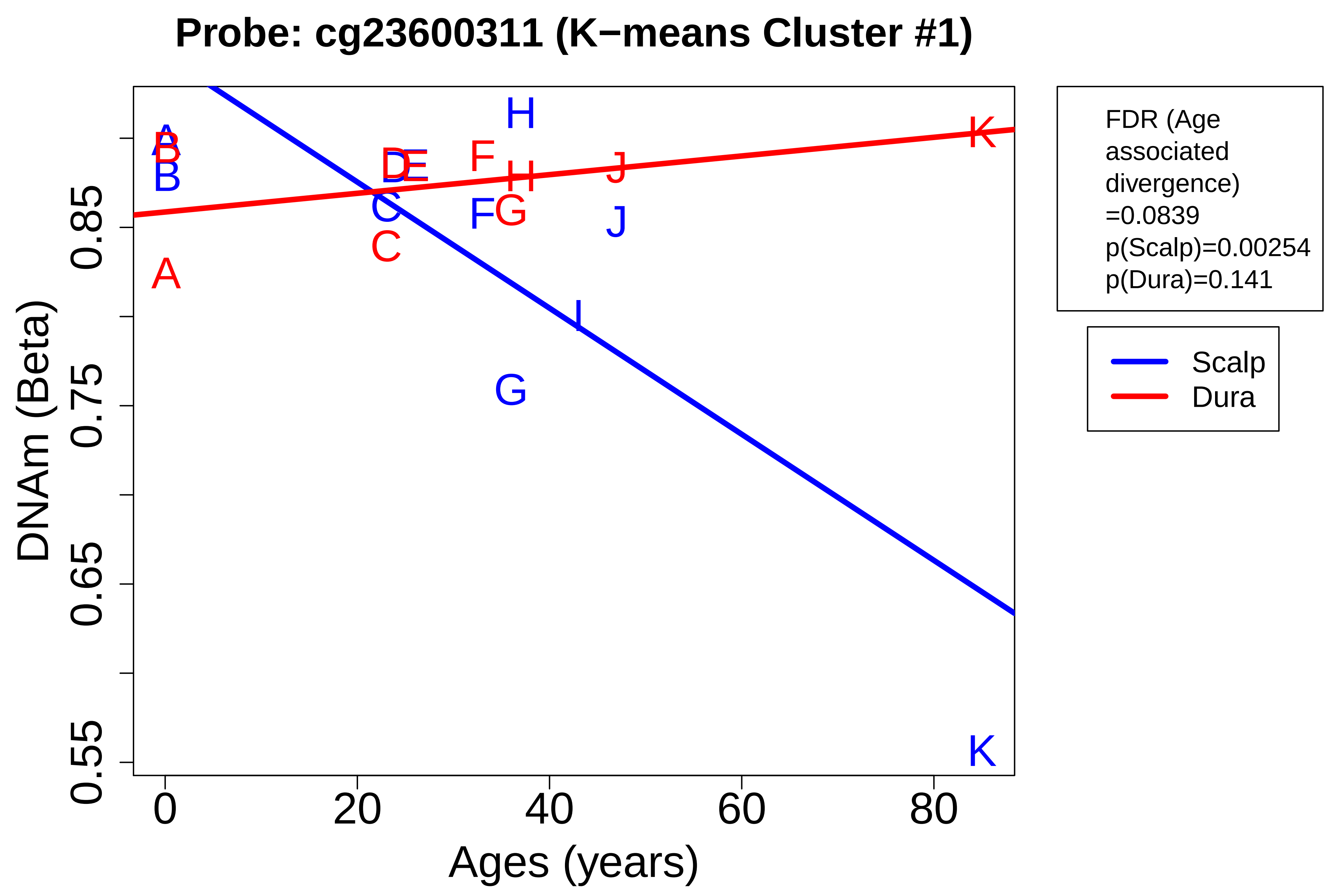

Probe: cg23600311 (K−means Cluster #1)
DNAm (Beta)
0.55
0.65
0.75
0.85
0
20
40
60
80
Ages (years)
A
B
C
D
E
F
G
H
I
J
K
FDR (Age associated divergence) =0.0839
p(Scalp)=0.00254
p(Dura)=0.141
Scalp
Dura

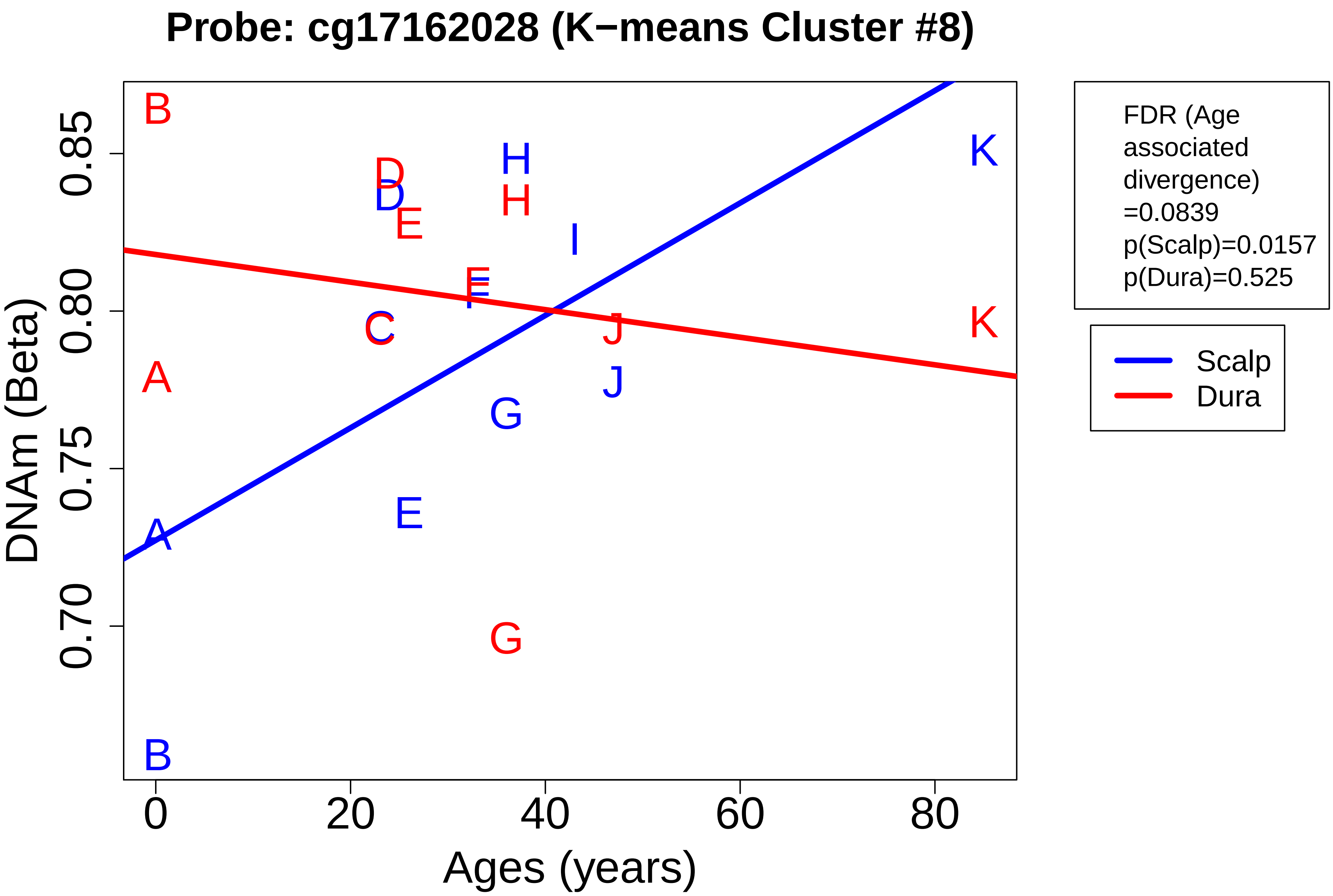

Probe: cg17162028 (K−means Cluster #8)
DNAm (Beta)
0.70
0.75
0.80
0.85
Ages (years)
0
20
40
60
80
FDR (Age associated divergence) =0.0839
p(Scalp)=0.0157
p(Dura)=0.525
Scalp
Dura

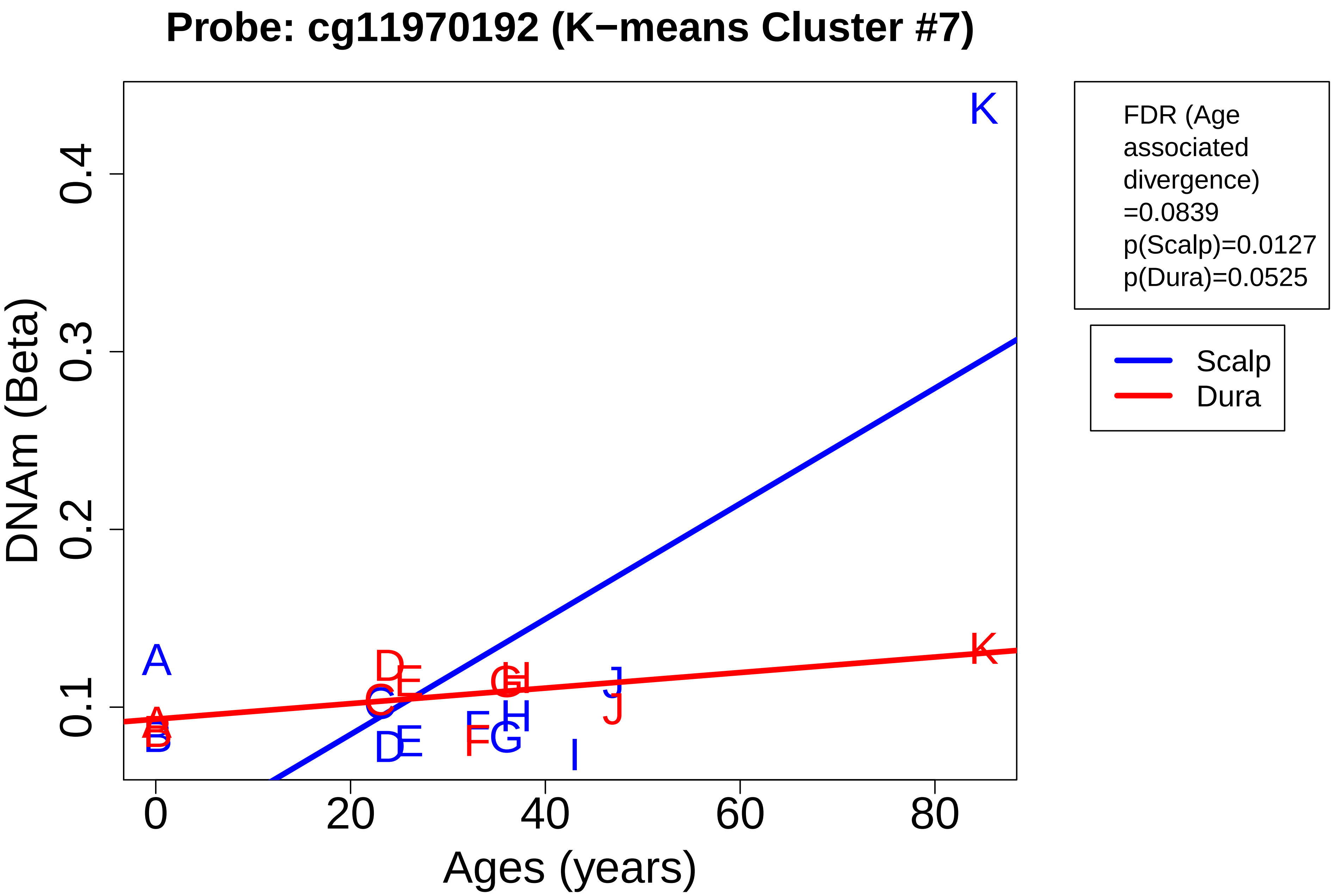

Probe: cg11970192 (K−means Cluster #7)
DNAm (Beta)
0.1
0.2
0.3
0.4
0
20
40
60
80
Ages (years)
A
B
C
D
E
F
G
H
I
J
K
FDR (Age associated divergence) =0.0839
p(Scalp)=0.0127
p(Dura)=0.0525
Scalp
Dura

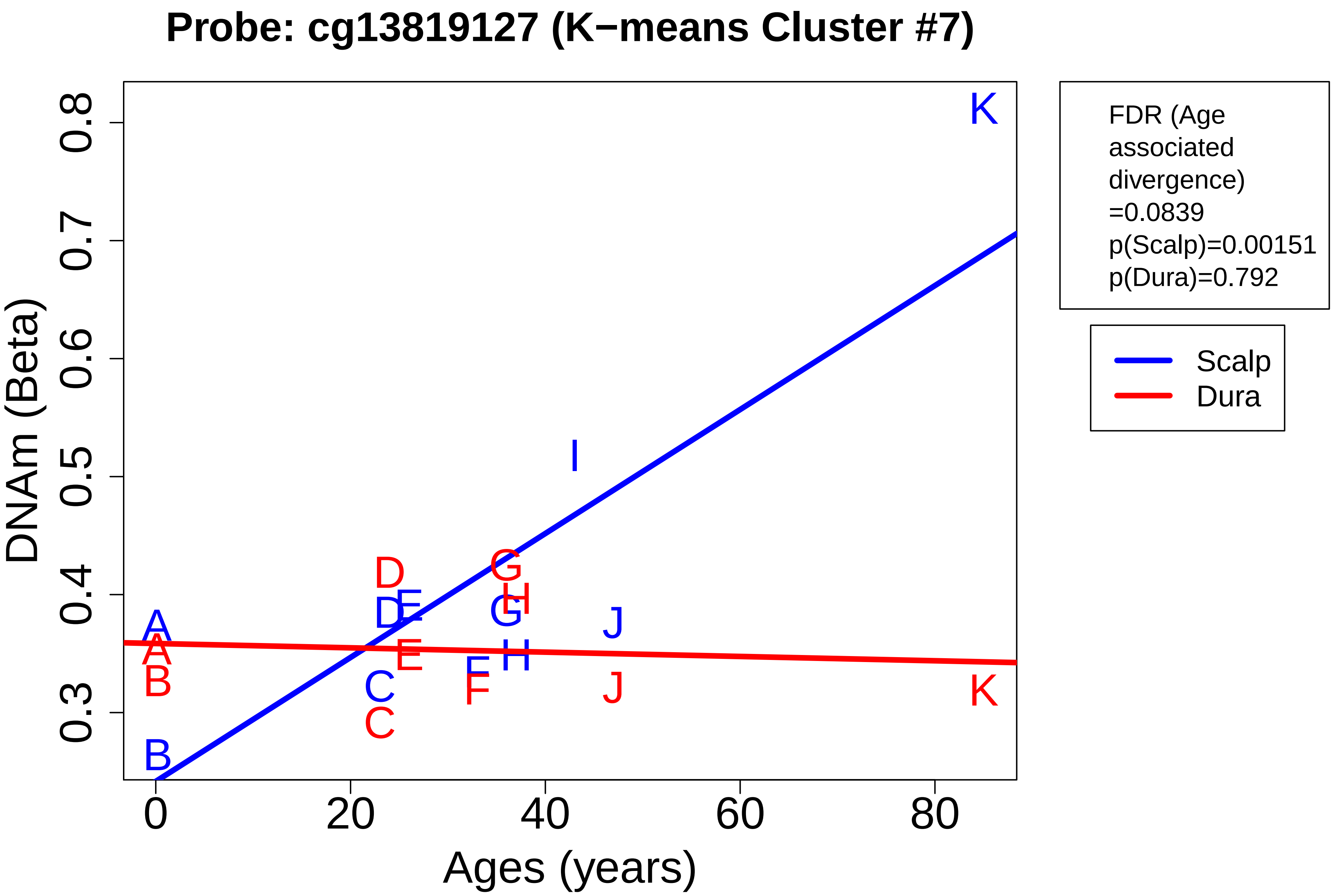

Probe: cg13819127 (K−means Cluster #7)
FDR (Age associated divergence) =0.0839
p(Scalp)=0.00151
p(Dura)=0.792
Scalp
Dura
DNAm (Beta)
Ages (years)
0.3
0.4
0.5
0.6
0.7
0.8
0
20
40
60
80
A
B
C
D
E
F
G
H
I
J
K

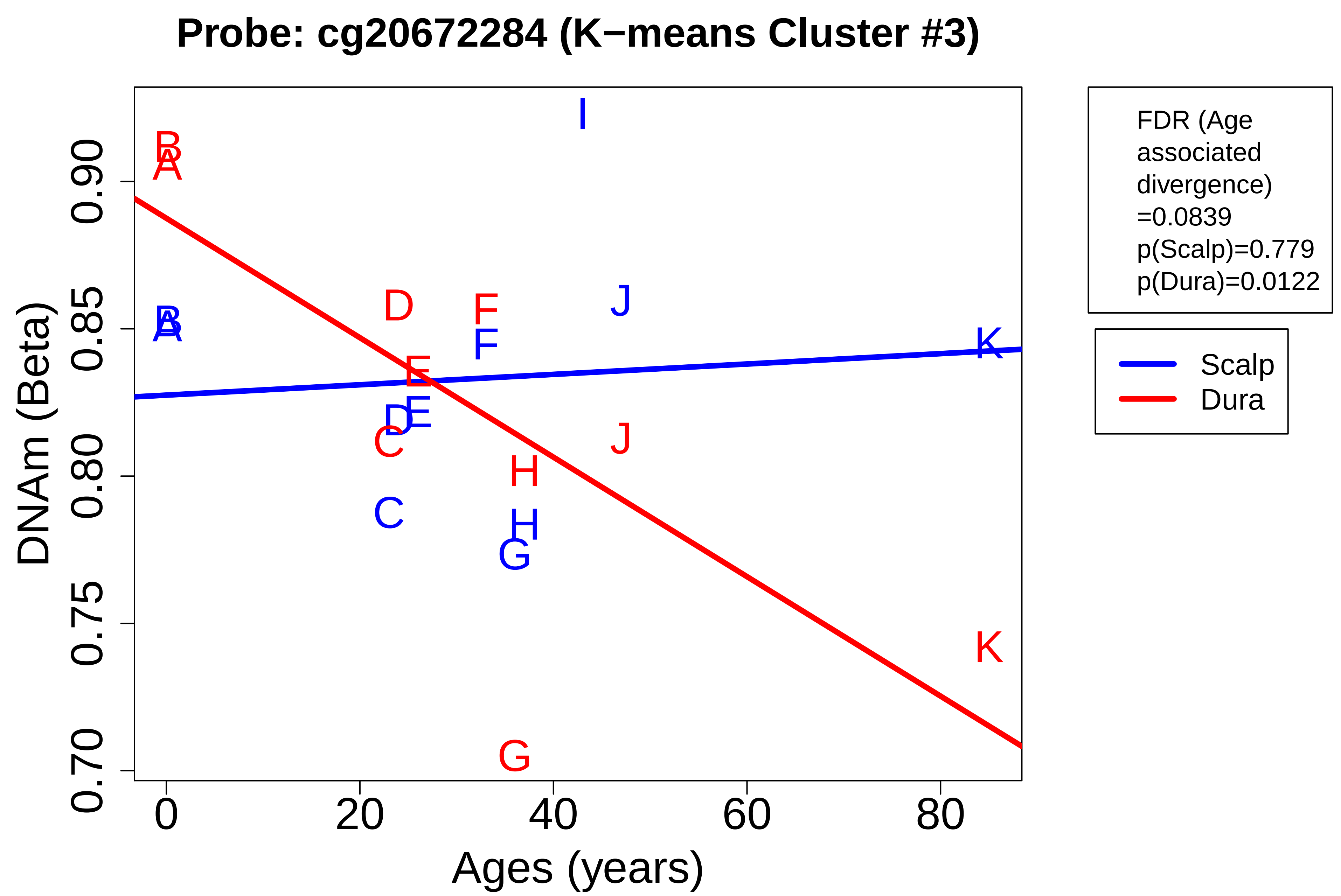

Probe: cg20672284 (K−means Cluster #3)
DNAm (Beta)
0.70
0.75
0.80
0.85
0.90
0
20
40
60
80
Ages (years)
FDR (Age associated divergence) =0.0839
p(Scalp)=0.779
p(Dura)=0.0122
Scalp
Dura

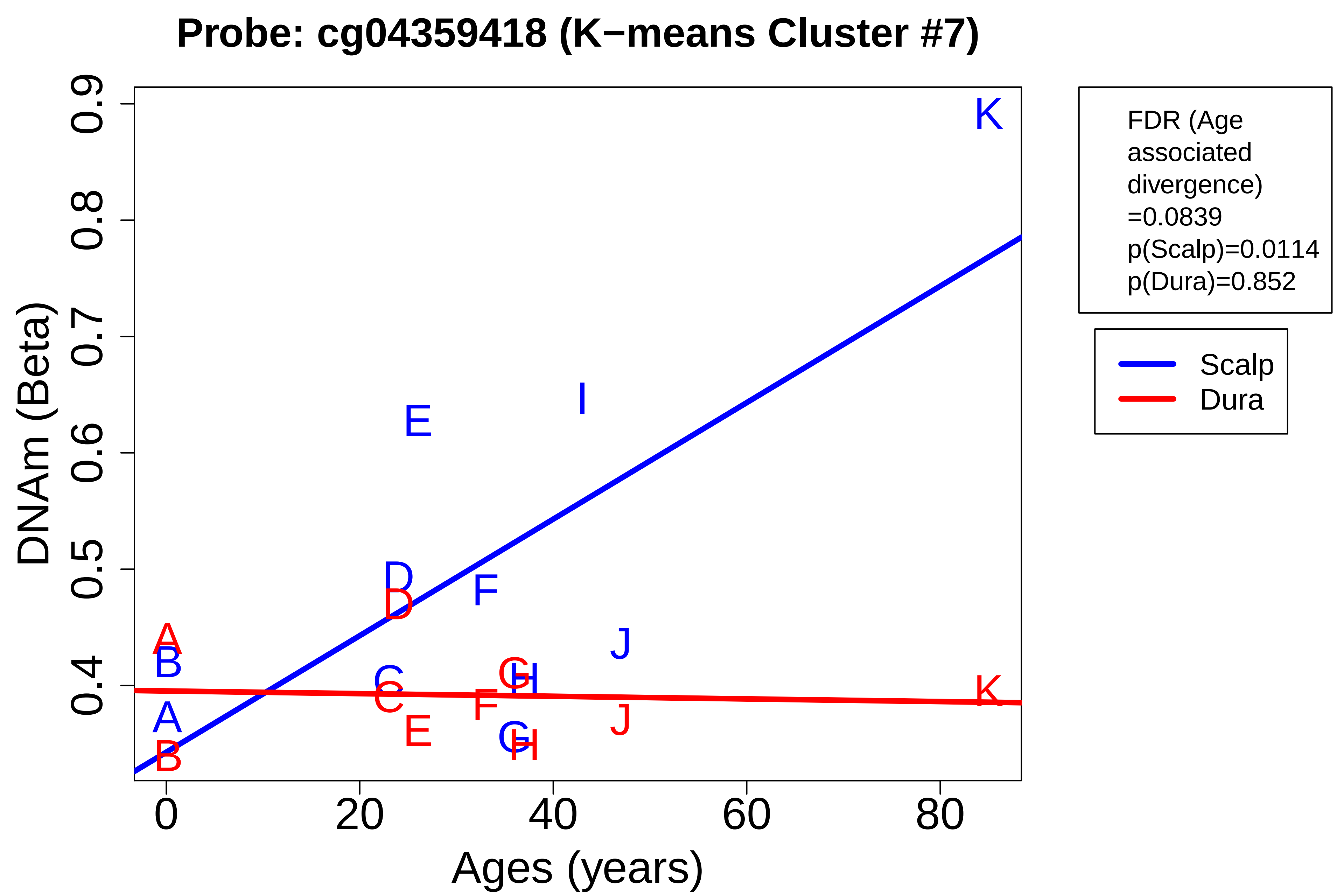

Probe: cg04359418 (K−means Cluster #7)
DNAm (Beta)
0.4
0.5
0.6
0.7
0.8
0.9
Ages (years)
0
20
40
60
80
FDR (Age associated divergence) =0.0839
p(Scalp)=0.0114
p(Dura)=0.852
Scalp
Dura

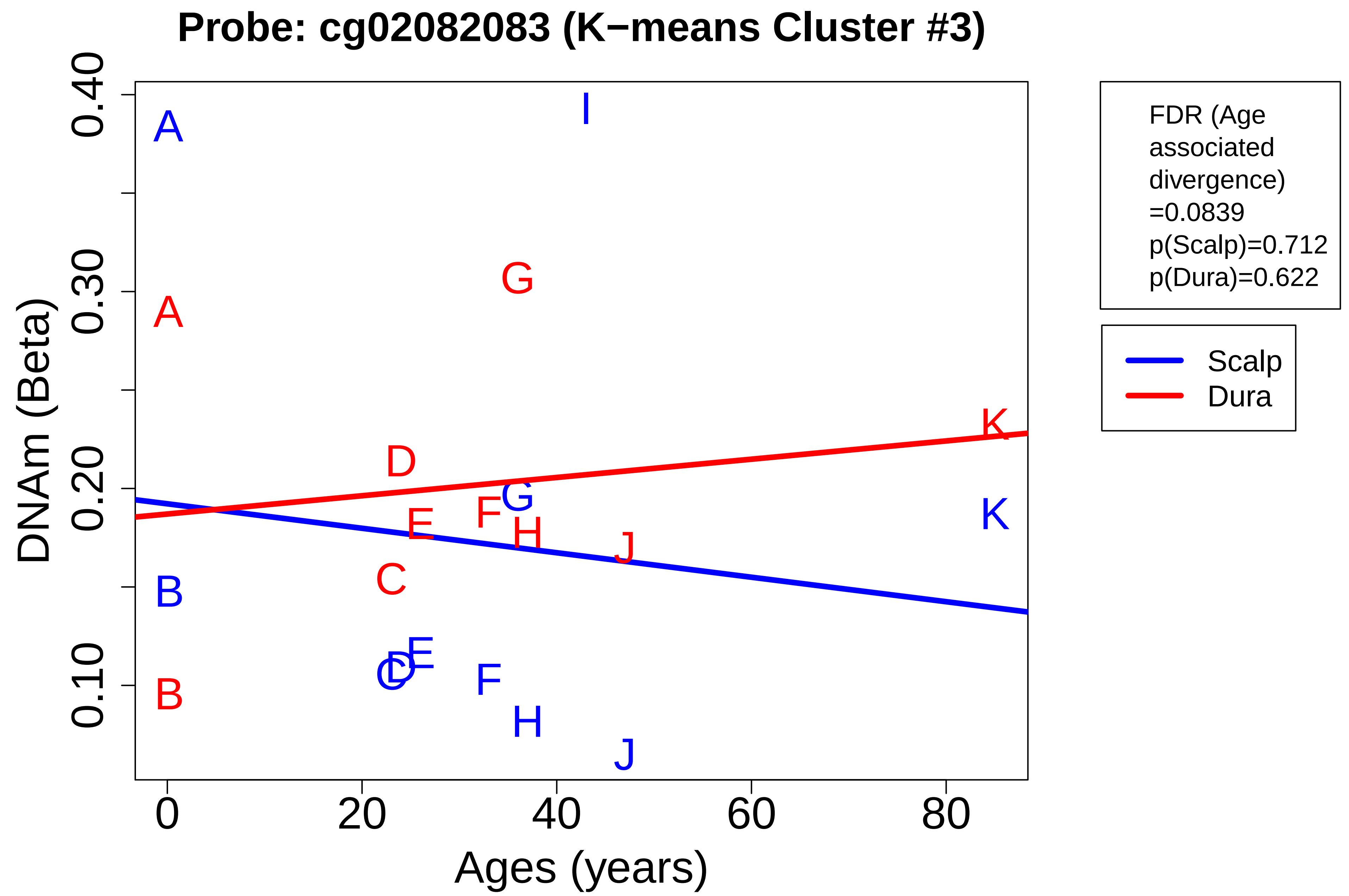

Probe: cg02082083 (K−means Cluster #3)
DNAm (Beta)
0.10
0.20
0.30
0.40
Ages (years)
0
20
40
60
80
A
B
C
D
E
F
G
H
I
J
K
FDR (Age associated divergence) =0.0839
p(Scalp)=0.712
p(Dura)=0.622
Scalp
Dura

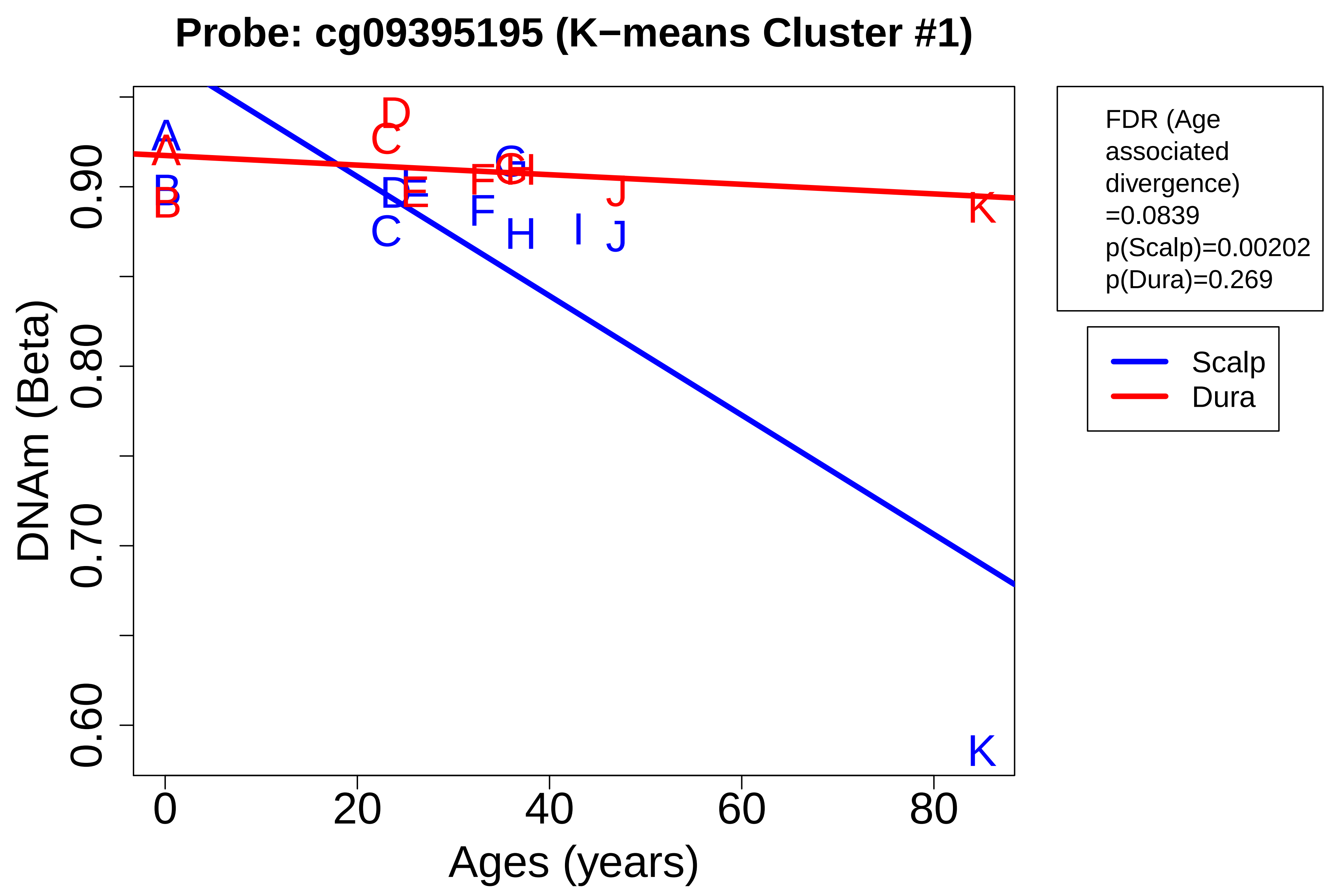

Probe: cg09395195 (K−means Cluster #1)
FDR (Age associated divergence) =0.0839
p(Scalp)=0.00202
p(Dura)=0.269
Scalp
Dura
DNAm (Beta)
0.60
0.70
0.80
0.90
Ages (years)
0
20
40
60
80

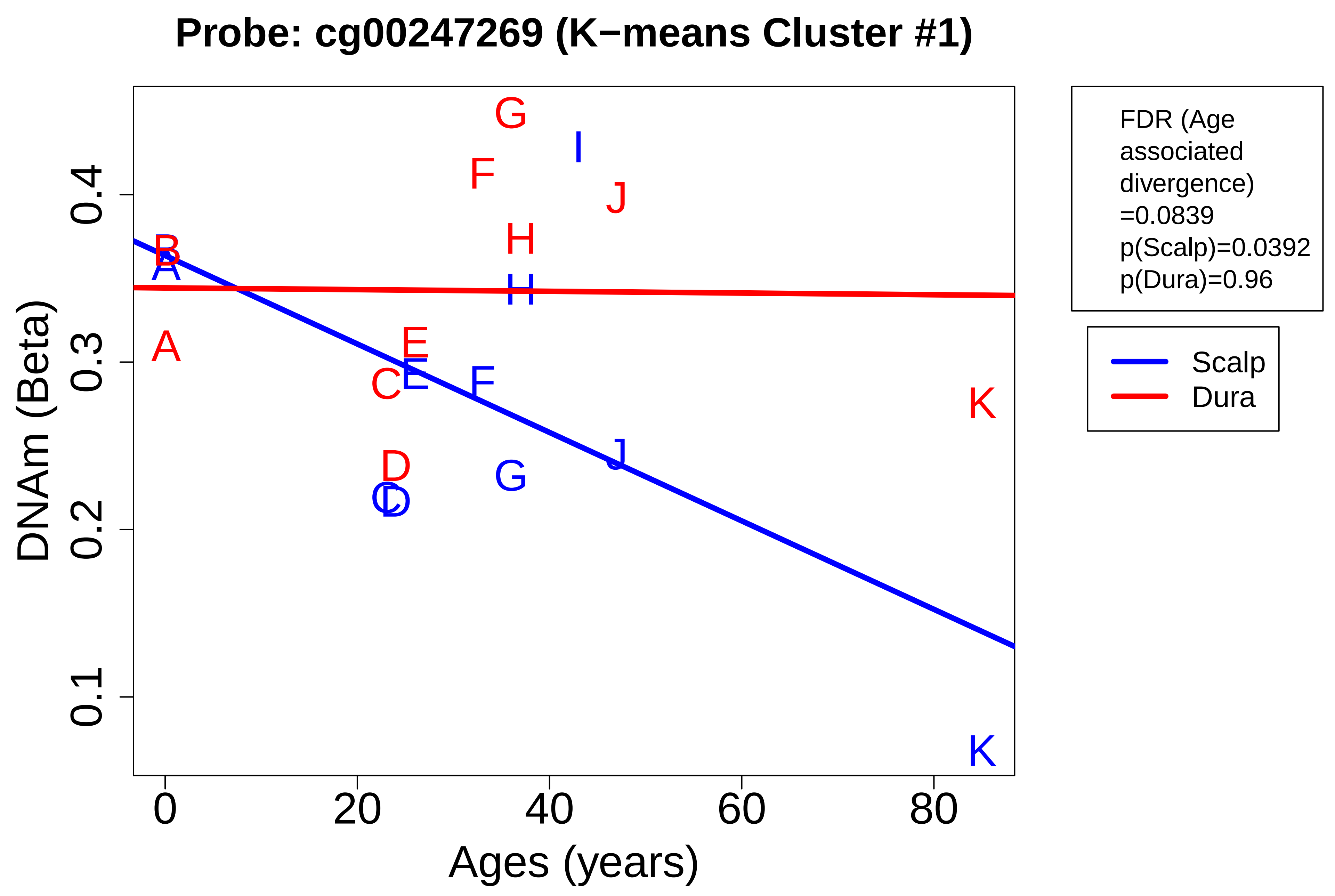

Probe: cg00247269 (K−means Cluster #1)
DNAm (Beta)
0.1
0.2
0.3
0.4
0
20
40
60
80
Ages (years)
G
I
F
J
H
B
A
A
E
C
E
F
K
D
J
G
C
D
K
H
FDR (Age associated divergence) =0.0839
p(Scalp)=0.0392
p(Dura)=0.96
Scalp
Dura

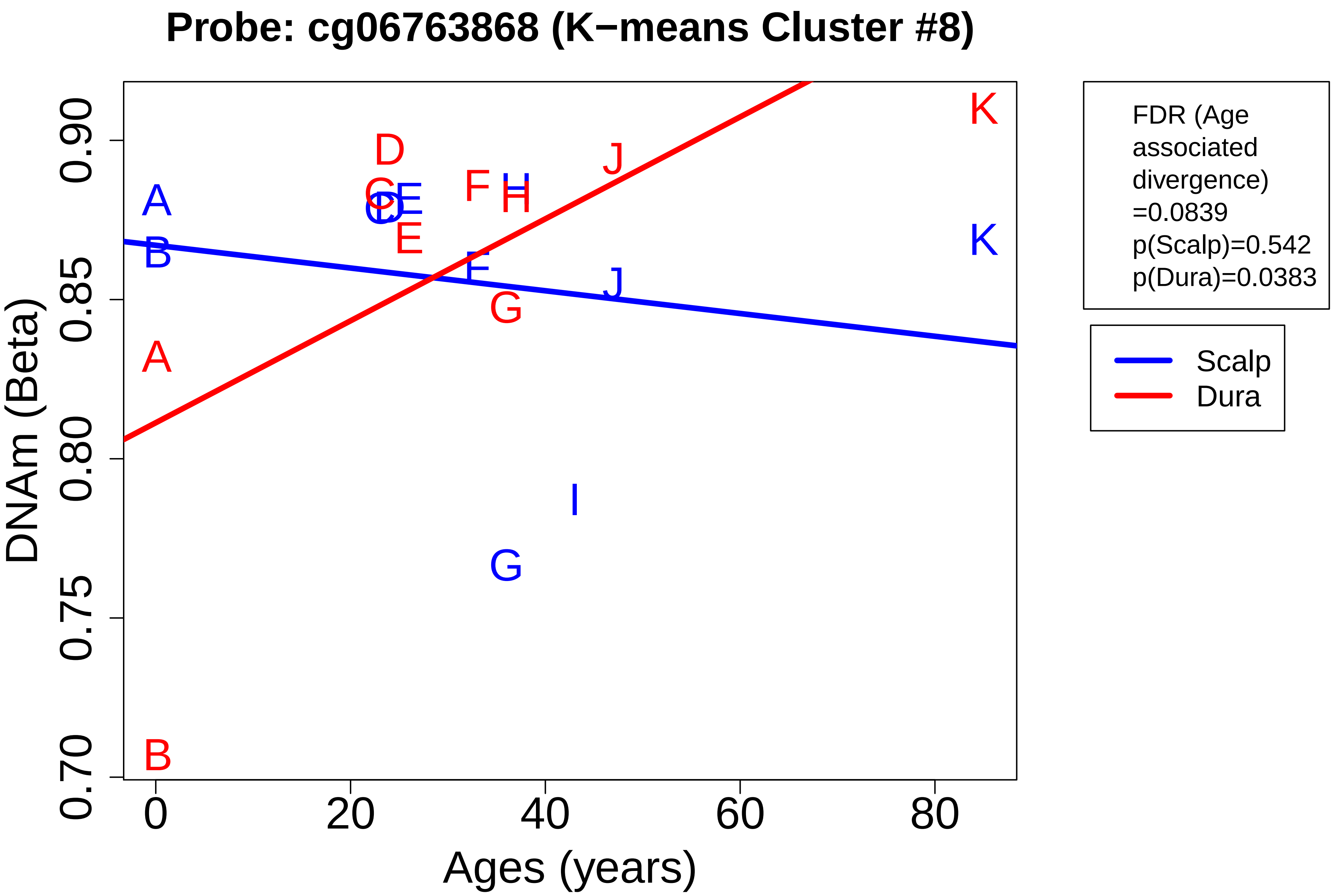

Probe: cg06763868 (K−means Cluster #8)
DNAm (Beta)
0.70
0.75
0.80
0.85
0.90
0
20
40
60
80
Ages (years)
FDR (Age associated divergence) =0.0839
p(Scalp)=0.542
p(Dura)=0.0383
Scalp
Dura

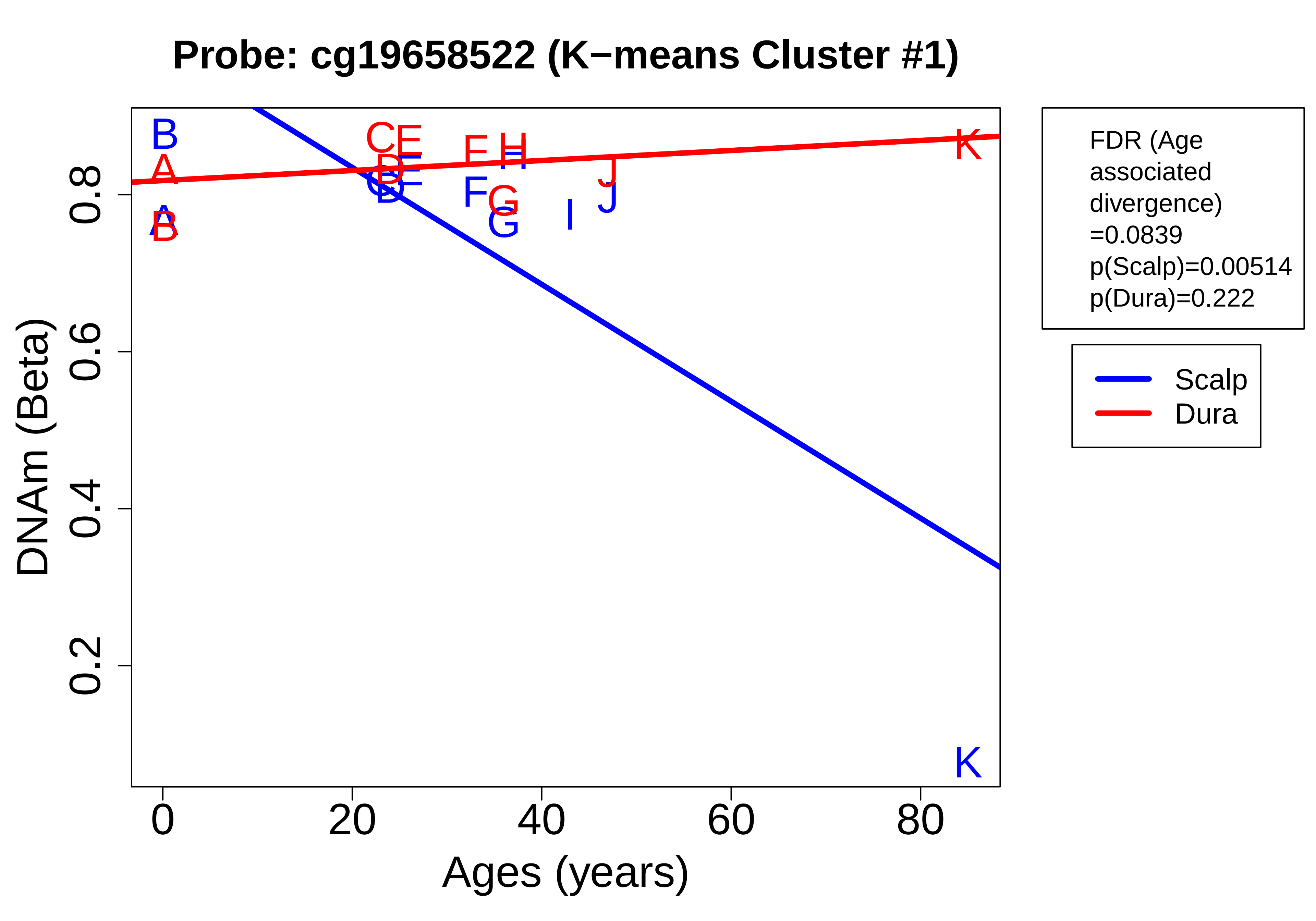
Probe: cg19658522 (K−means Cluster #1)
DNAm (Beta)
0.2
0.4
0.6
0.8
Ages (years)
0
20
40
60
80
FDR (Age associated divergence) =0.0839
p(Scalp)=0.00514
p(Dura)=0.222
Scalp
Dura

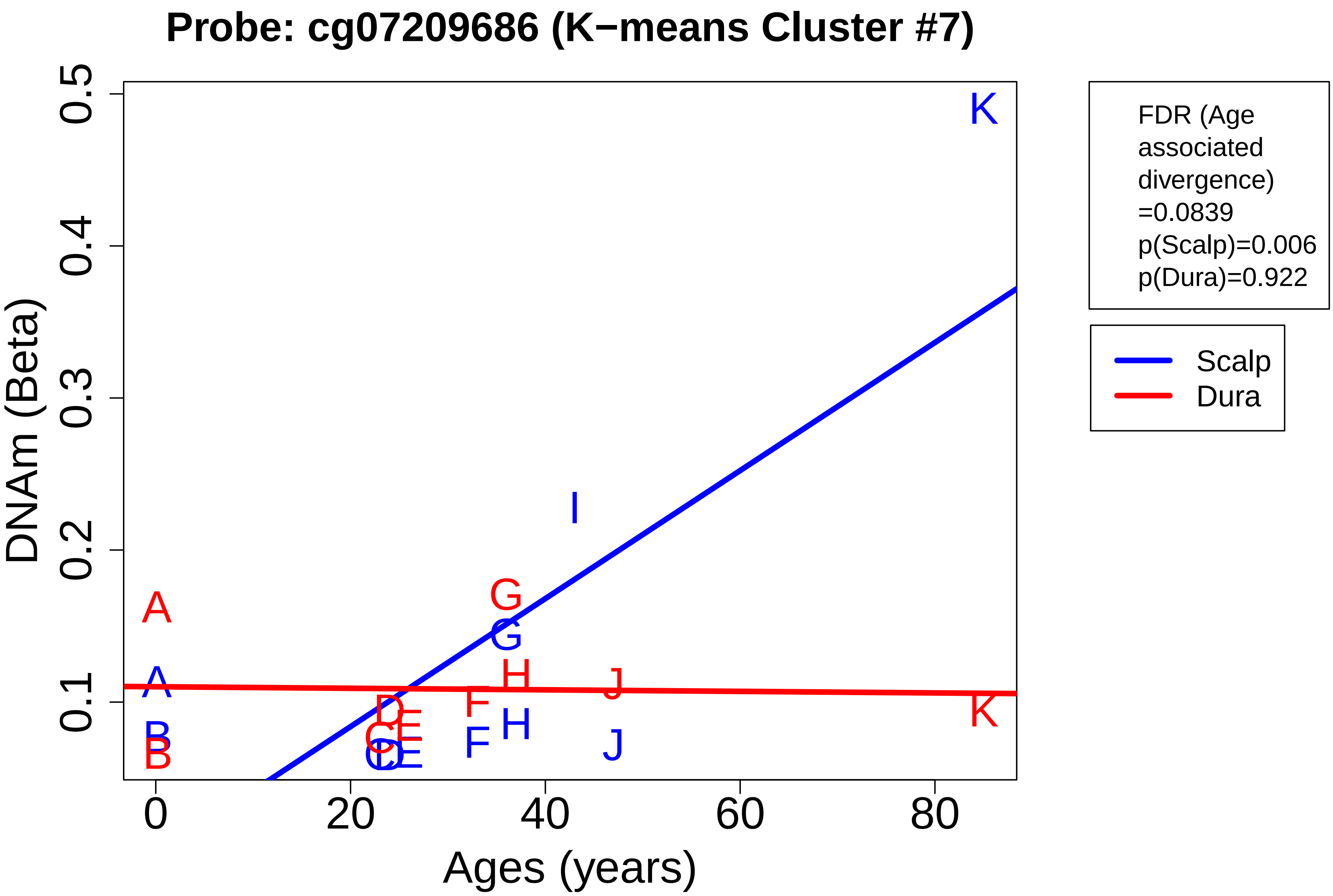

Probe: cg07209686 (K−means Cluster #7)
DNAm (Beta)
0.1
0.2
0.3
0.4
0.5
0
20
40
60
80
Ages (years)
FDR (Age associated divergence) =0.0839
p(Scalp)=0.006
p(Dura)=0.922
Scalp
Dura

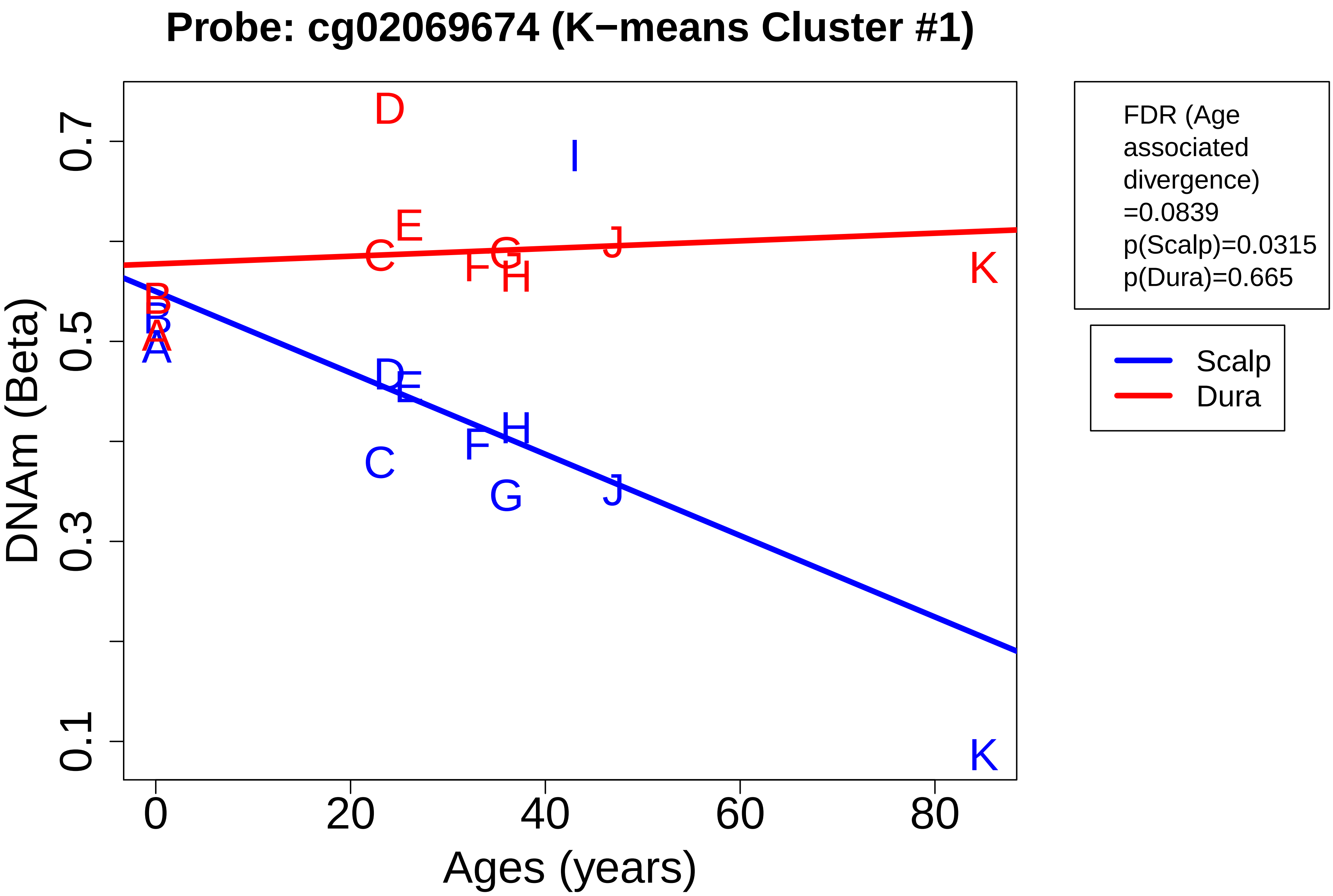

Probe: cg02069674 (K−means Cluster #1)
DNAm (Beta)
0.1
0.3
0.5
0.7
0
20
40
60
80
Ages (years)
FDR (Age associated divergence) =0.0839
p(Scalp)=0.0315
p(Dura)=0.665
Scalp
Dura

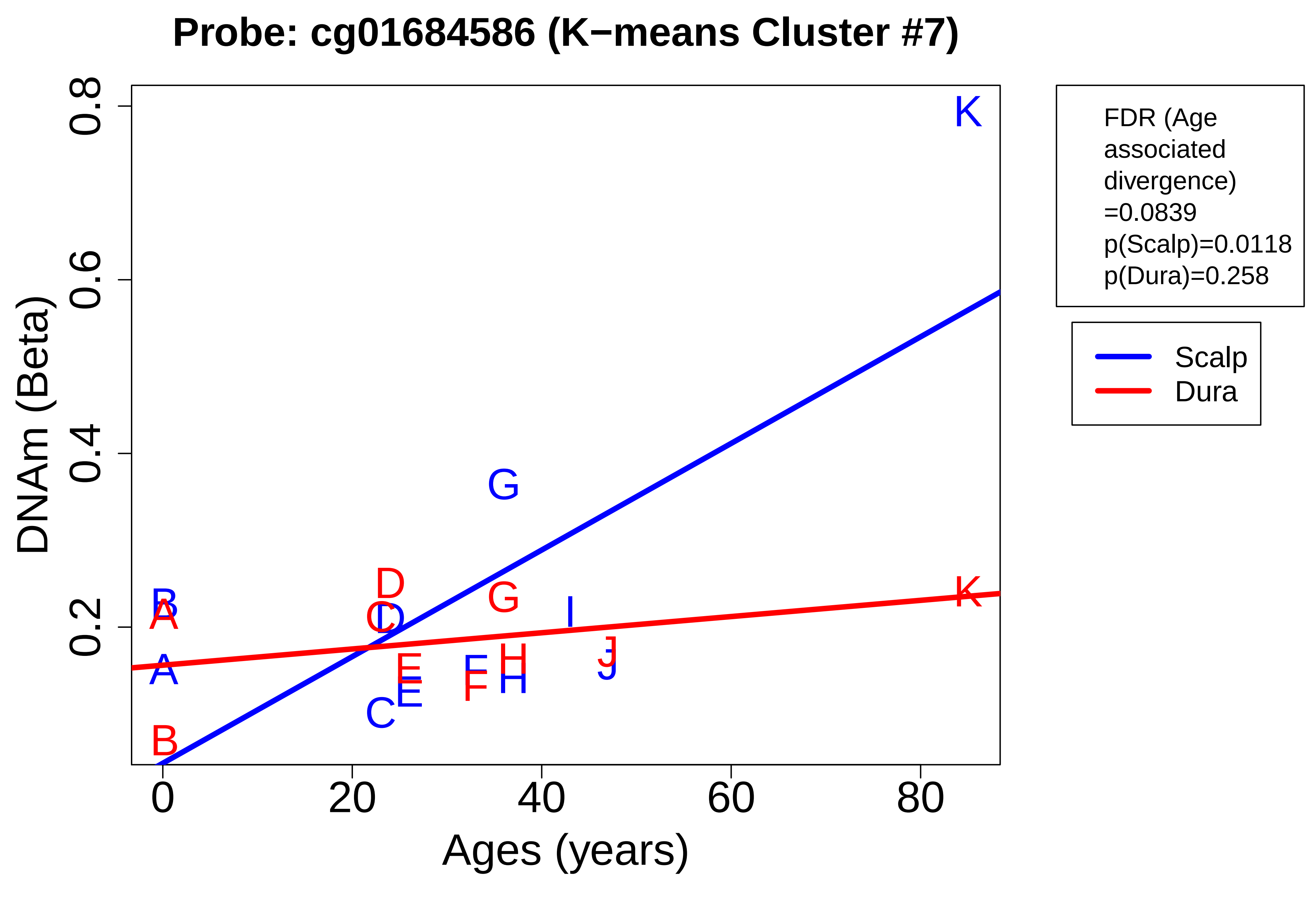
Probe: cg01684586 (K−means Cluster #7)
DNAm (Beta)
0.2
0.4
0.6
0.8
0
20
40
60
80
Ages (years)
FDR (Age associated divergence) =0.0839
p(Scalp)=0.0118
p(Dura)=0.258
Scalp
Dura
A
B
C
D
E
F
G
H
I
J
K

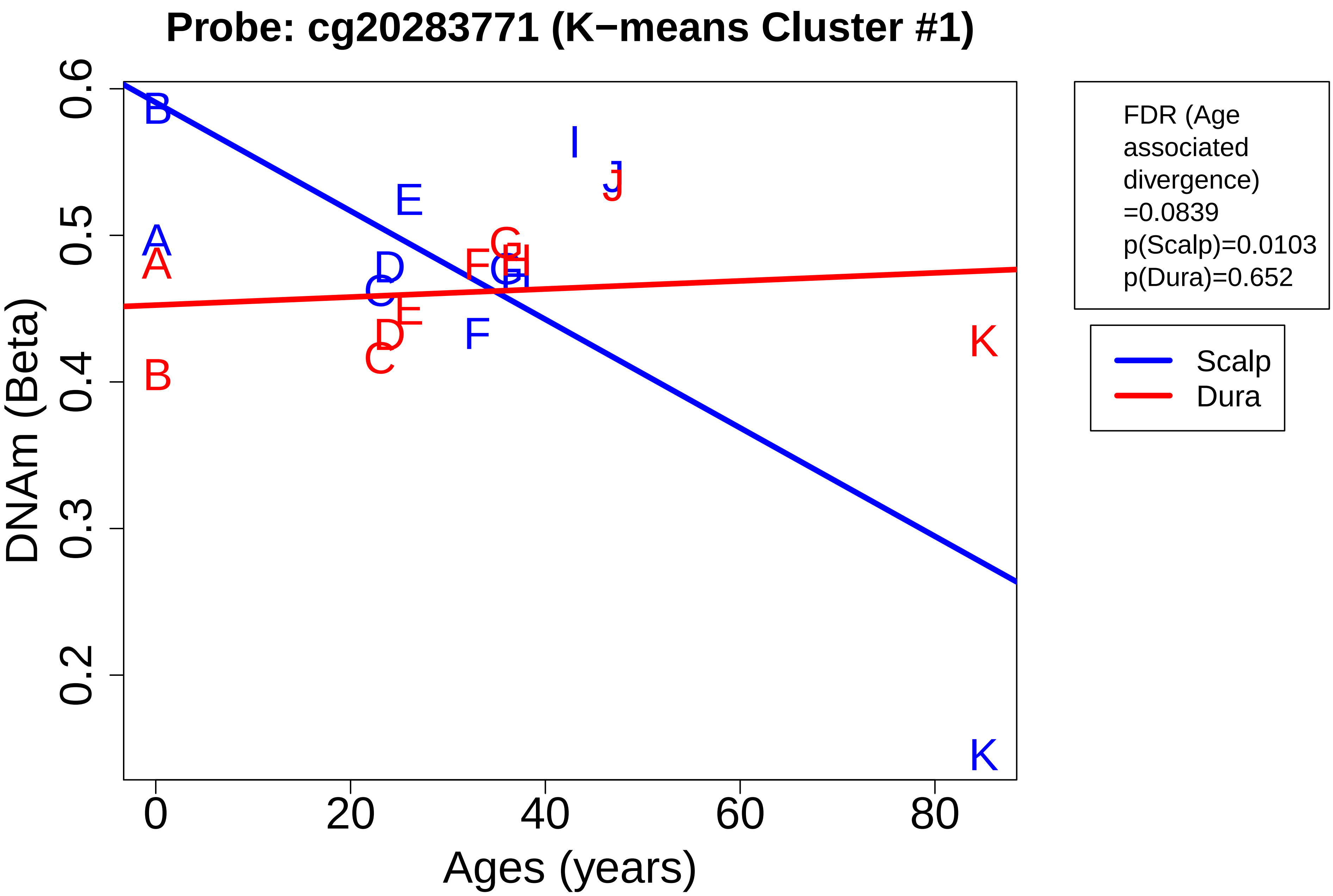

Probe: cg20283771 (K−means Cluster #1)
DNAm (Beta)
Ages (years)
0.2
0.3
0.4
0.5
0.6
0
20
40
60
80
FDR (Age associated divergence) =0.0839
p(Scalp)=0.0103
p(Dura)=0.652
Scalp
Dura
A
B
C
D
E
F
G
H
I
J
K

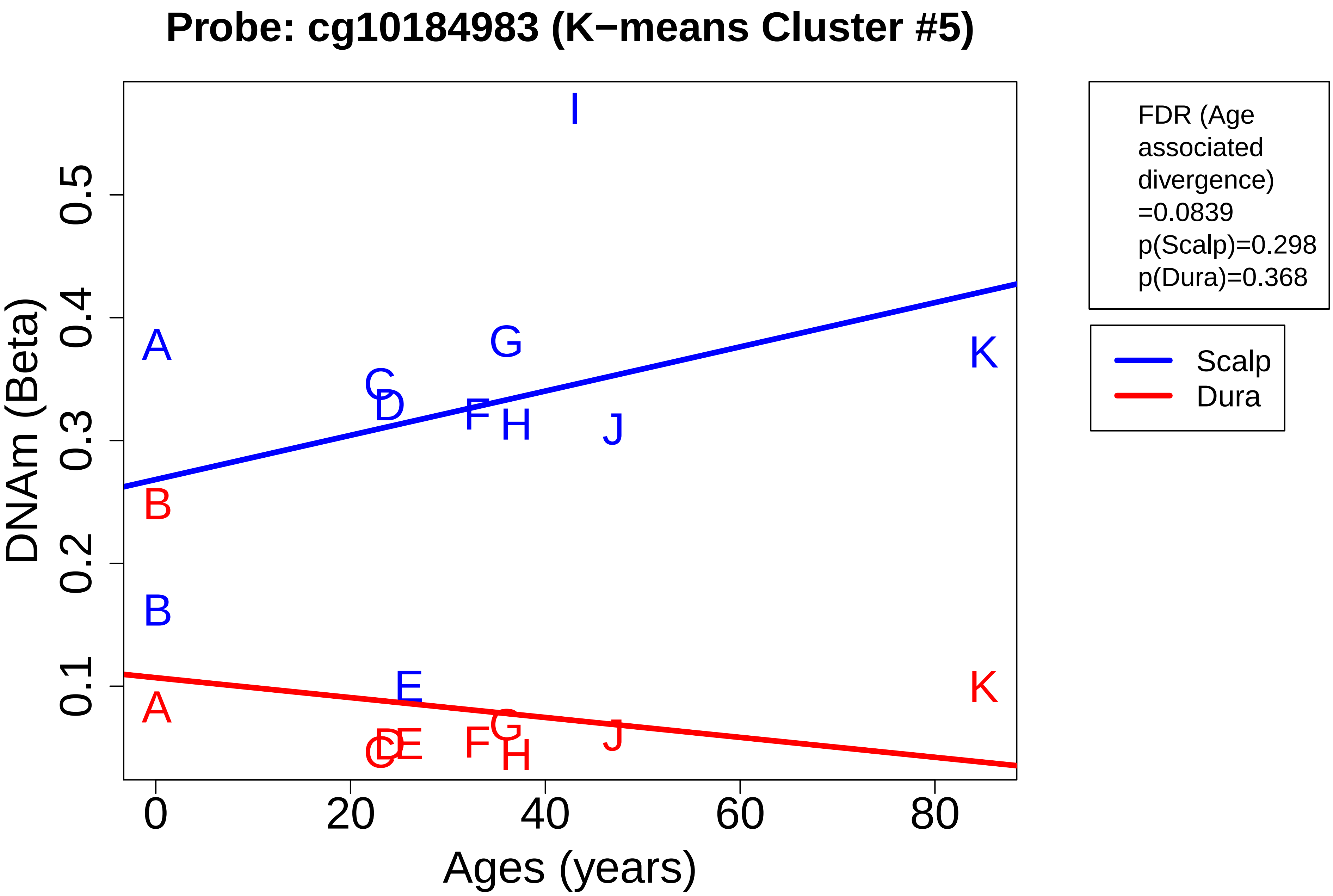
Probe: cg10184983 (K−means Cluster #5)
DNAm (Beta)
0.1
0.2
0.3
0.4
0.5
0
20
40
60
80
Ages (years)
FDR (Age associated divergence) =0.0839
p(Scalp)=0.298
p(Dura)=0.368
Scalp
Dura
A
B
C
D
E
F
G
H
I
J
K

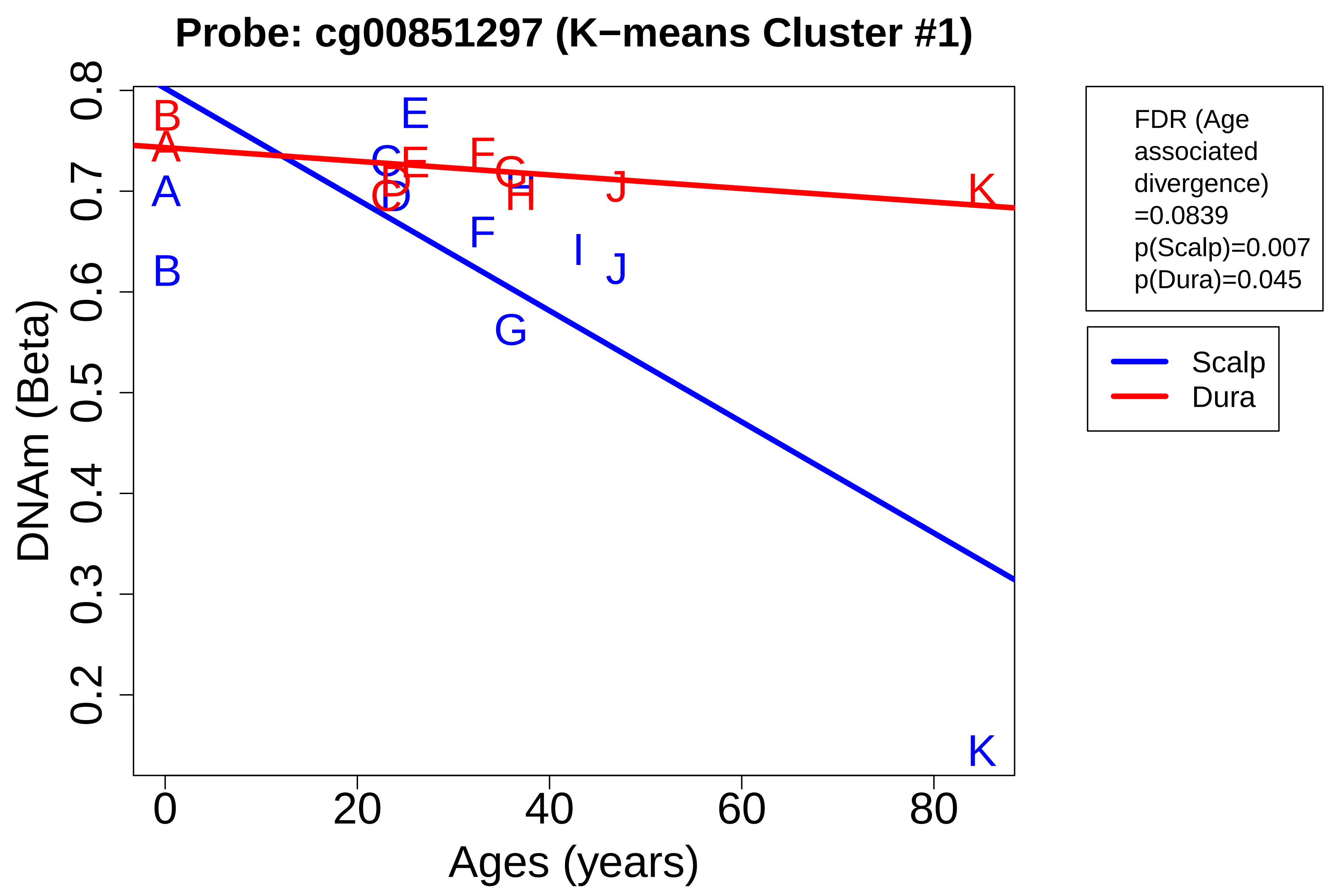
Probe: cg00851297 (K−means Cluster #1)
DNAm (Beta)
0.2
0.3
0.4
0.5
0.6
0.7
0.8
Ages (years)
0
20
40
60
80
FDR (Age associated divergence) =0.0839
p(Scalp)=0.007
p(Dura)=0.045
Scalp
Dura

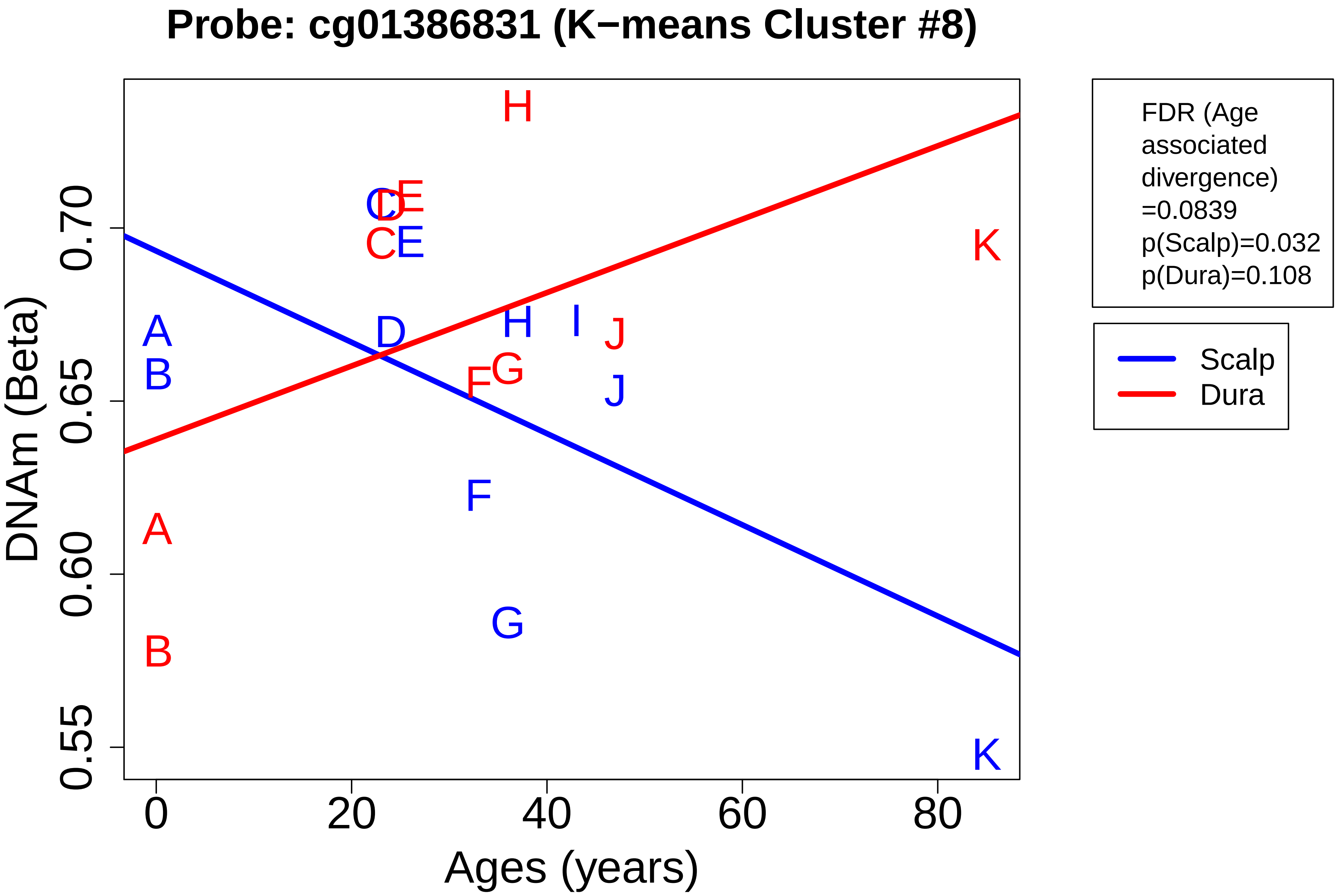
Probe: cg01386831 (K−means Cluster #8)
DNAm (Beta)
0.55
0.60
0.65
0.70
0
20
40
60
80
Ages (years)
A
B
C
D
E
F
G
H
I
J
K
FDR (Age associated divergence) =0.0839
p(Scalp)=0.032
p(Dura)=0.108
Scalp
Dura

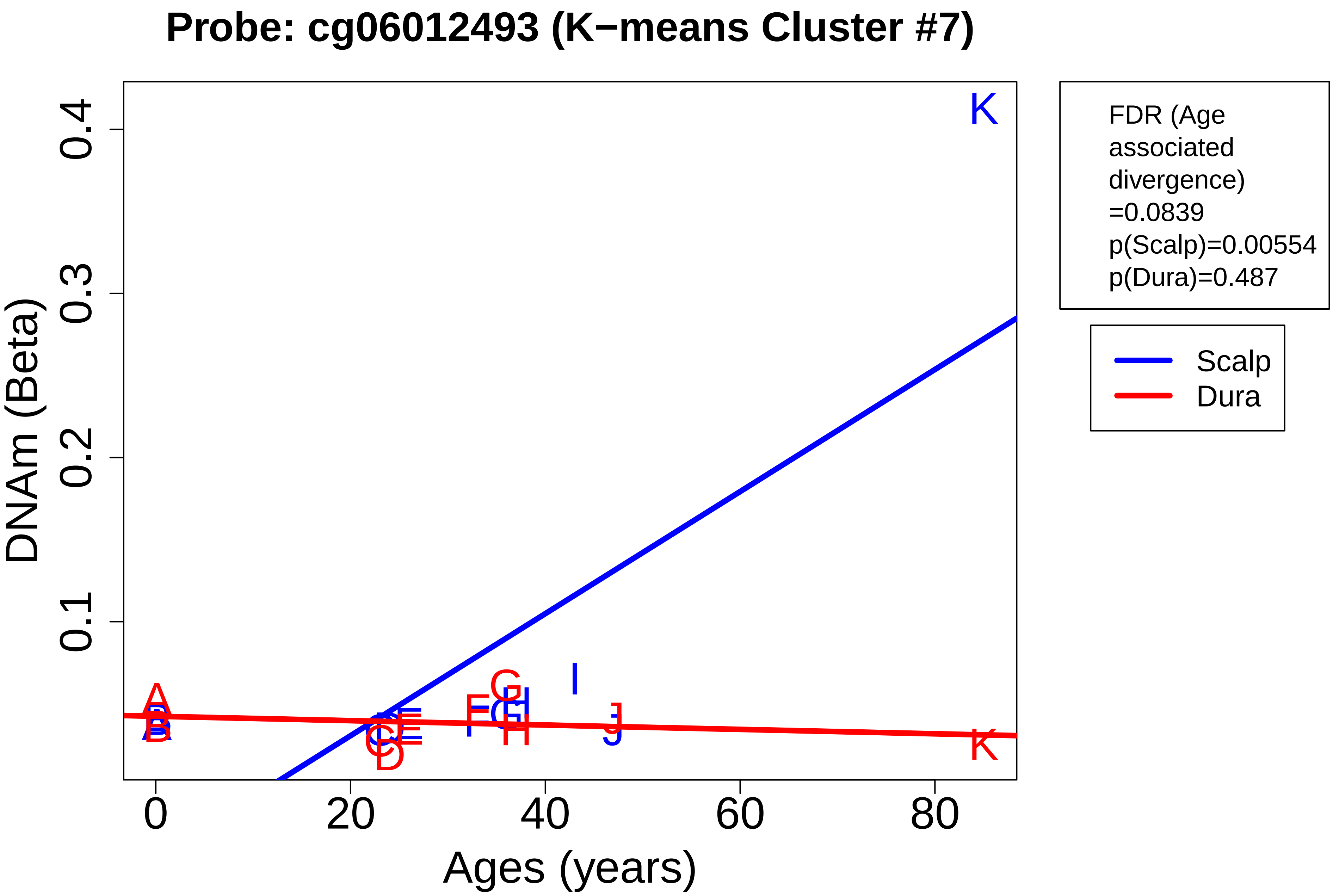
Probe: cg06012493 (K−means Cluster #7)
DNAm (Beta)
0.1
0.2
0.3
0.4
Ages (years)
0
20
40
60
80
FDR (Age associated divergence) =0.0839
p(Scalp)=0.00554
p(Dura)=0.487
Scalp
Dura

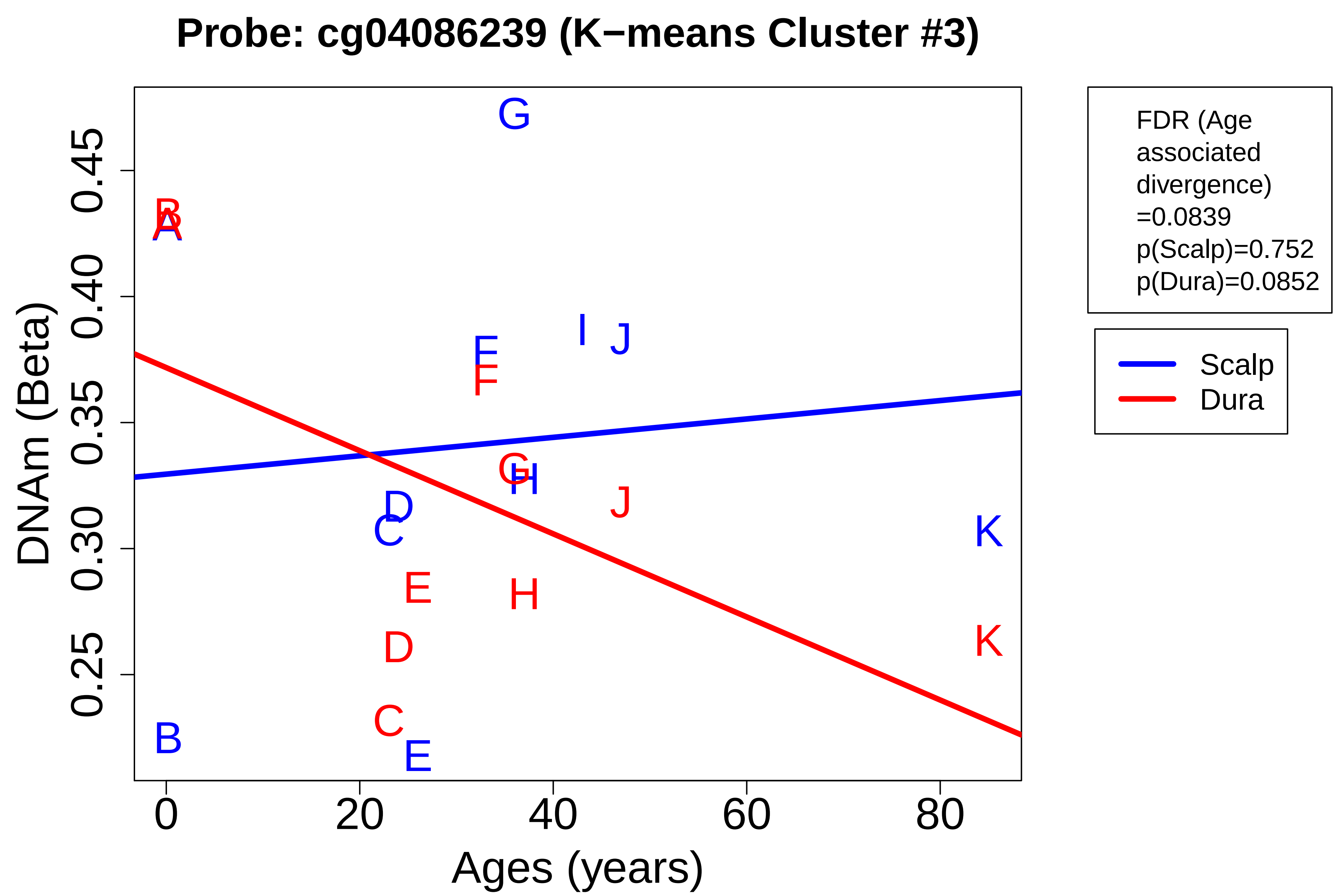

Probe: cg04086239 (K−means Cluster #3)
DNAm (Beta)
0.25
0.30
0.35
0.40
0.45
Ages (years)
0
20
40
60
80
A
B
C
D
E
F
G
H
I
J
K
FDR (Age associated divergence) =0.0839
p(Scalp)=0.752
p(Dura)=0.0852
Scalp
Dura

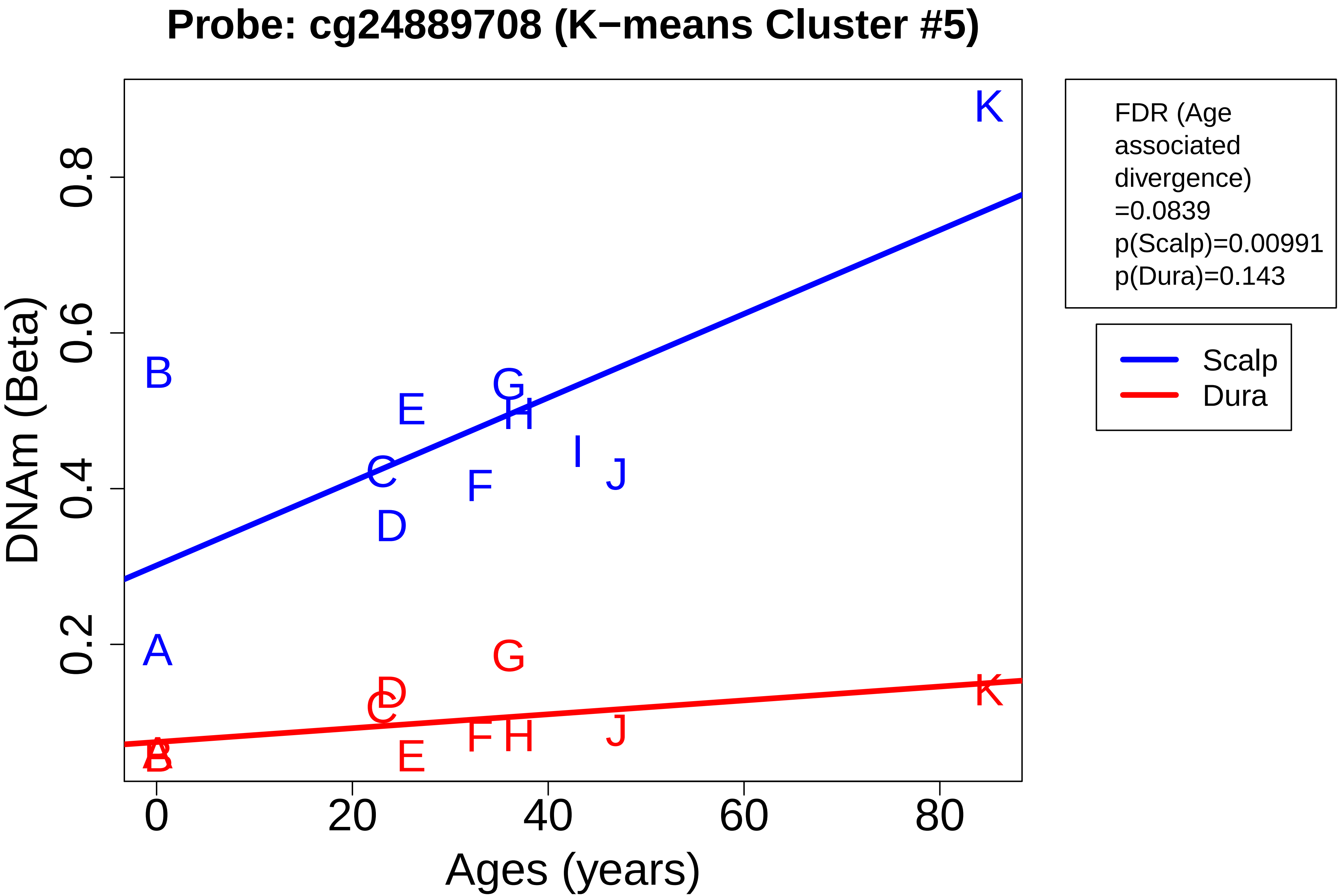

Probe: cg24889708 (K−means Cluster #5)
DNAm (Beta)
0.2
0.4
0.6
0.8
0
20
40
60
80
Ages (years)
FDR (Age associated divergence) =0.0839
p(Scalp)=0.00991
p(Dura)=0.143
Scalp
Dura

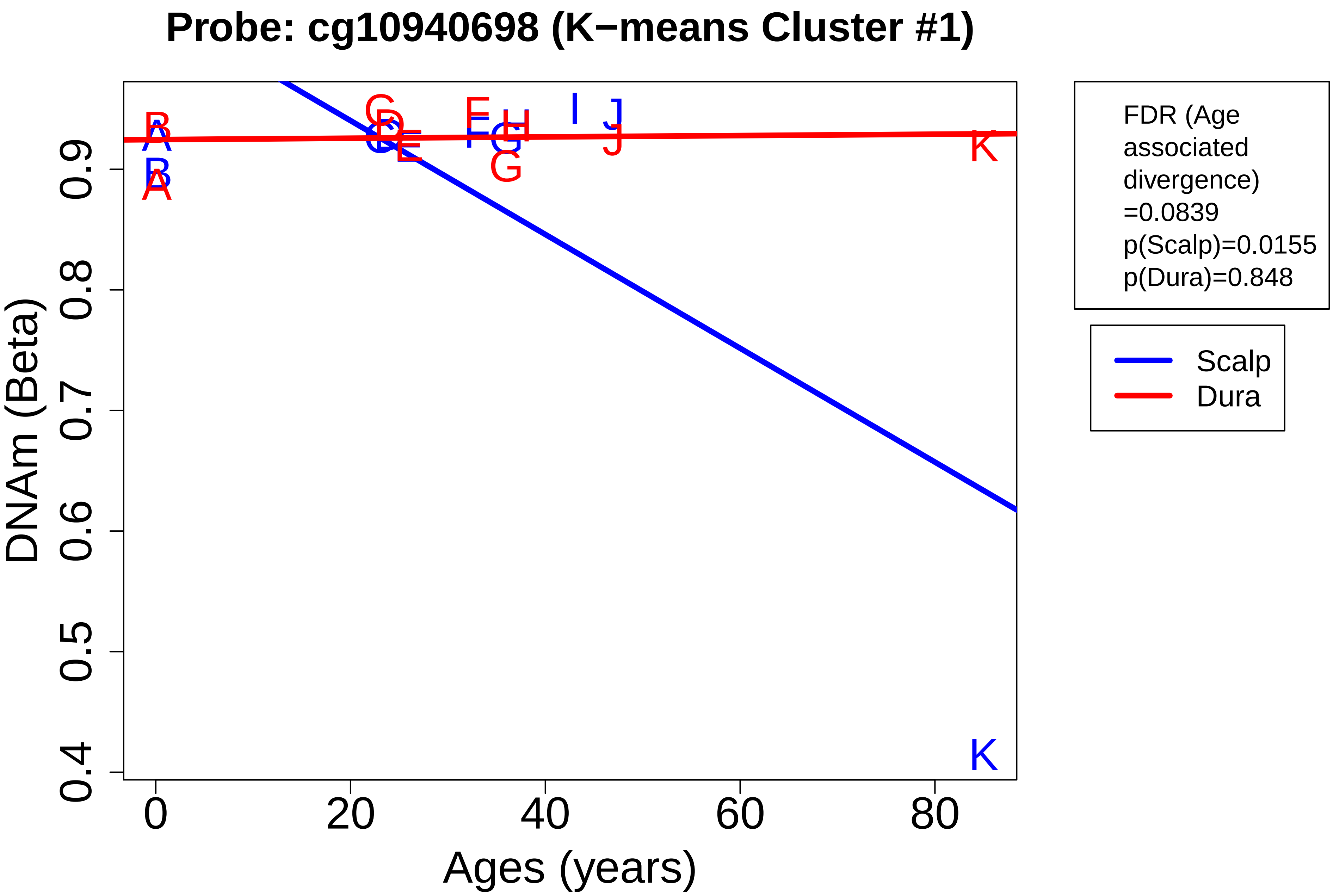

Probe: cg10940698 (K−means Cluster #1)
DNAm (Beta)
0.4
0.5
0.6
0.7
0.8
0.9
0
20
40
60
80
Ages (years)
FDR (Age associated divergence) =0.0839
p(Scalp)=0.0155
p(Dura)=0.848
Scalp
Dura

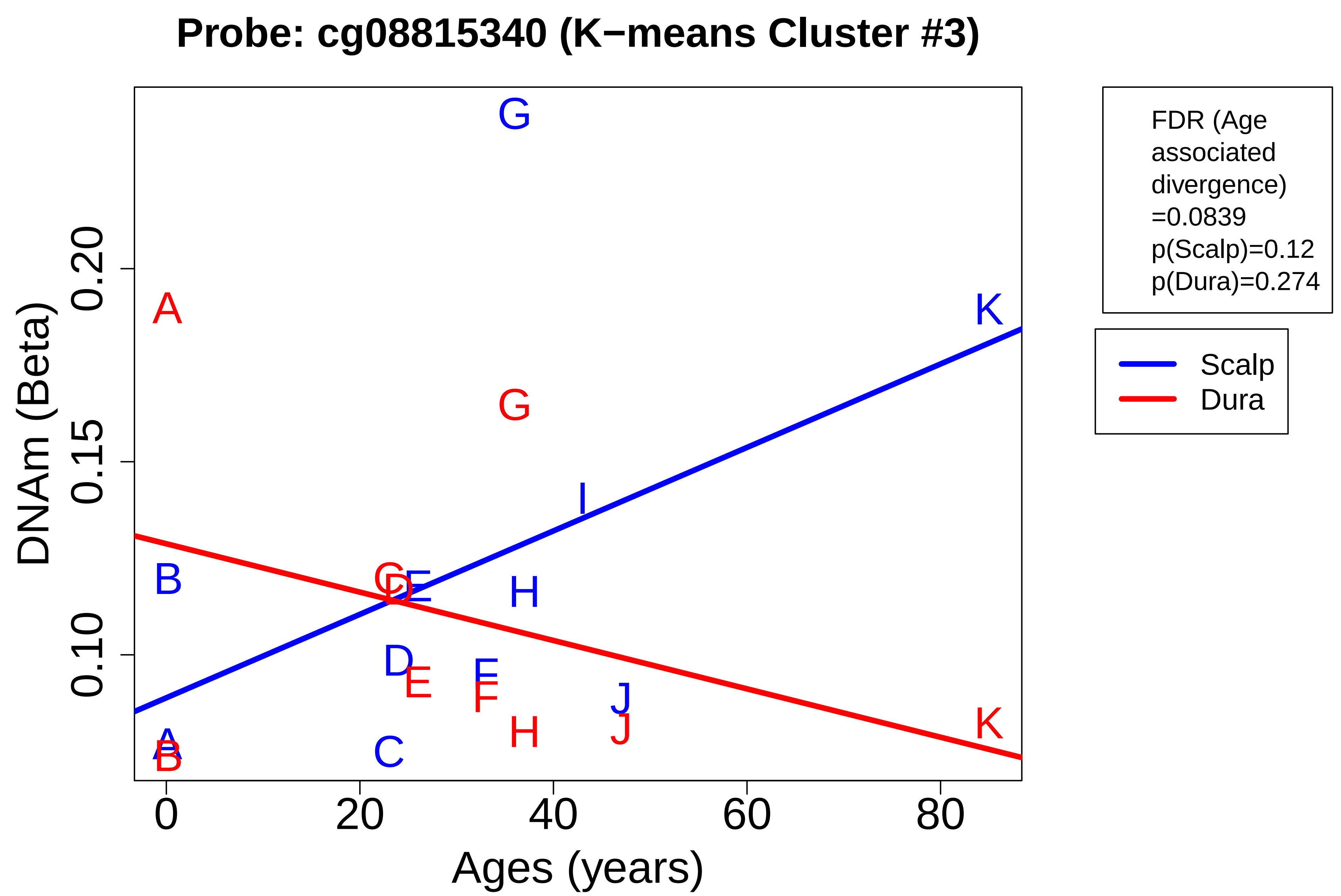
Probe: cg08815340 (K−means Cluster #3)
FDR (Age associated divergence) =0.0839
p(Scalp)=0.12
p(Dura)=0.274
Scalp
Dura
DNAm (Beta)
0.10
0.15
0.20
0
20
40
60
80
Ages (years)
A
B
C
D
E
F
G
H
I
J
K

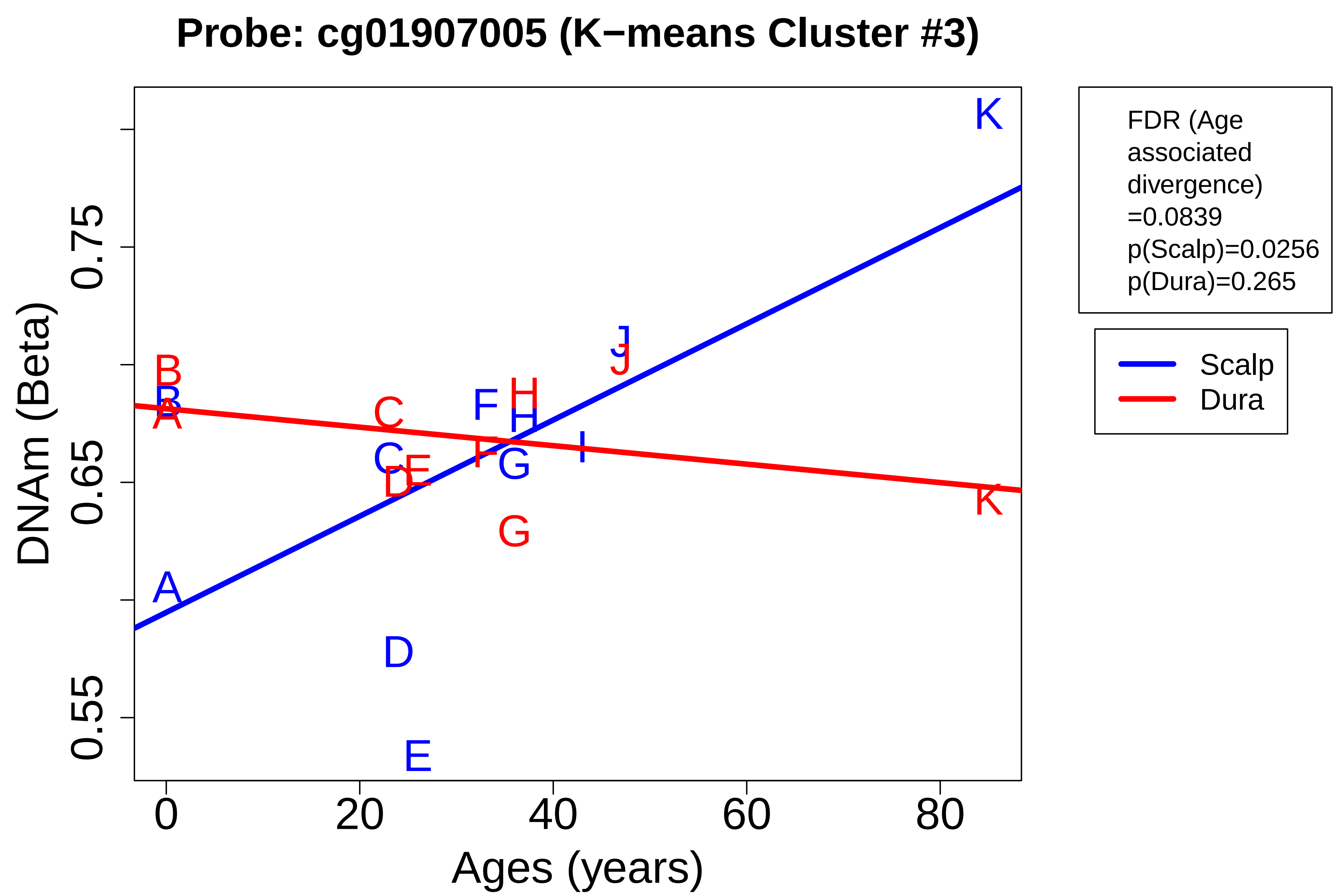

Probe: cg01907005 (K−means Cluster #3)
DNAm (Beta)
0.55
0.65
0.75
0
20
40
60
80
Ages (years)
FDR (Age associated divergence) =0.0839
p(Scalp)=0.0256
p(Dura)=0.265
Scalp
Dura
A
B
C
D
E
F
G
H
I
J
K

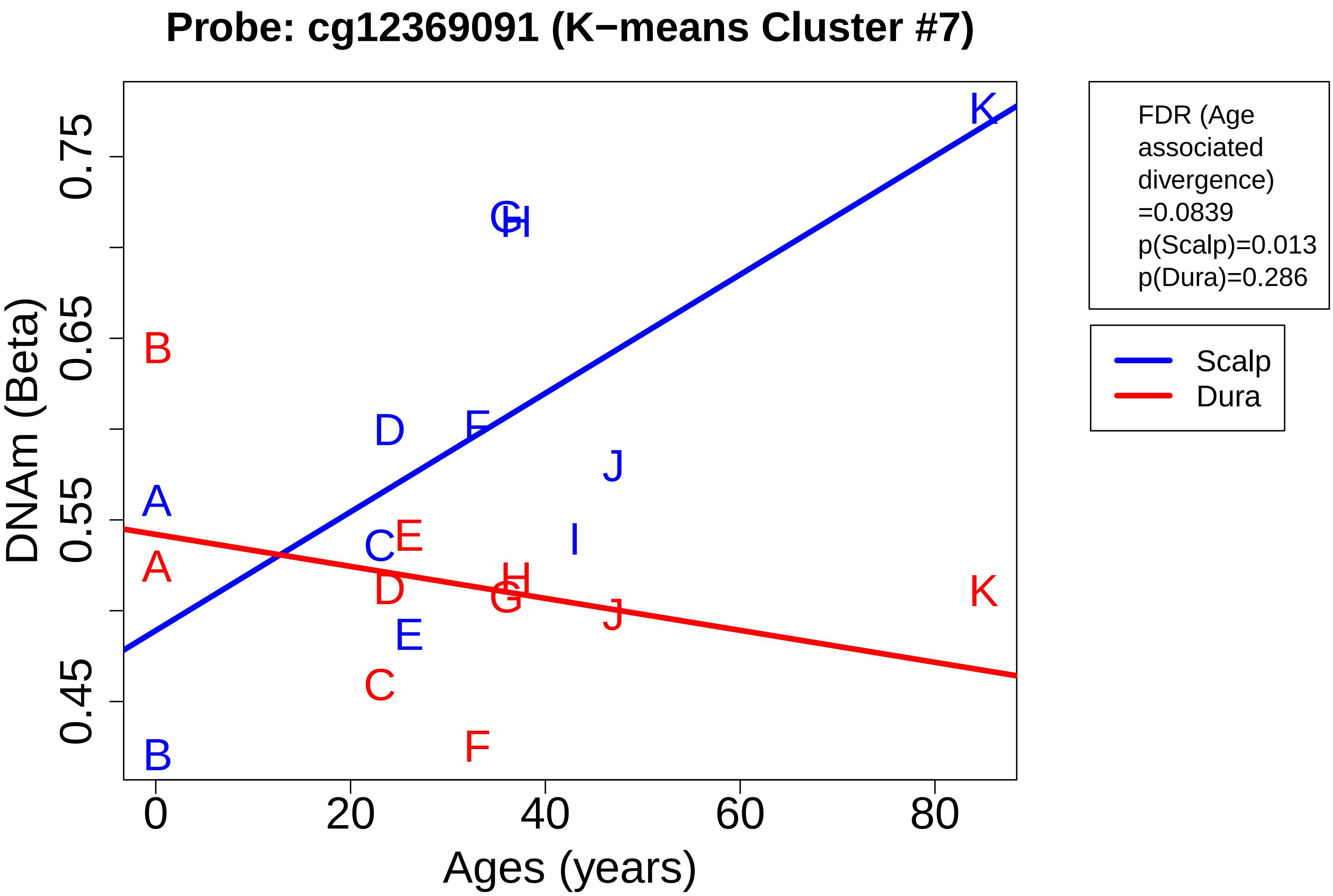

Probe: cg12369091 (K−means Cluster #7)
DNAm (Beta)
0.45
0.55
0.65
0.75
Ages (years)
0
20
40
60
80
FDR (Age associated divergence) =0.0839
p(Scalp)=0.013
p(Dura)=0.286
Scalp
Dura

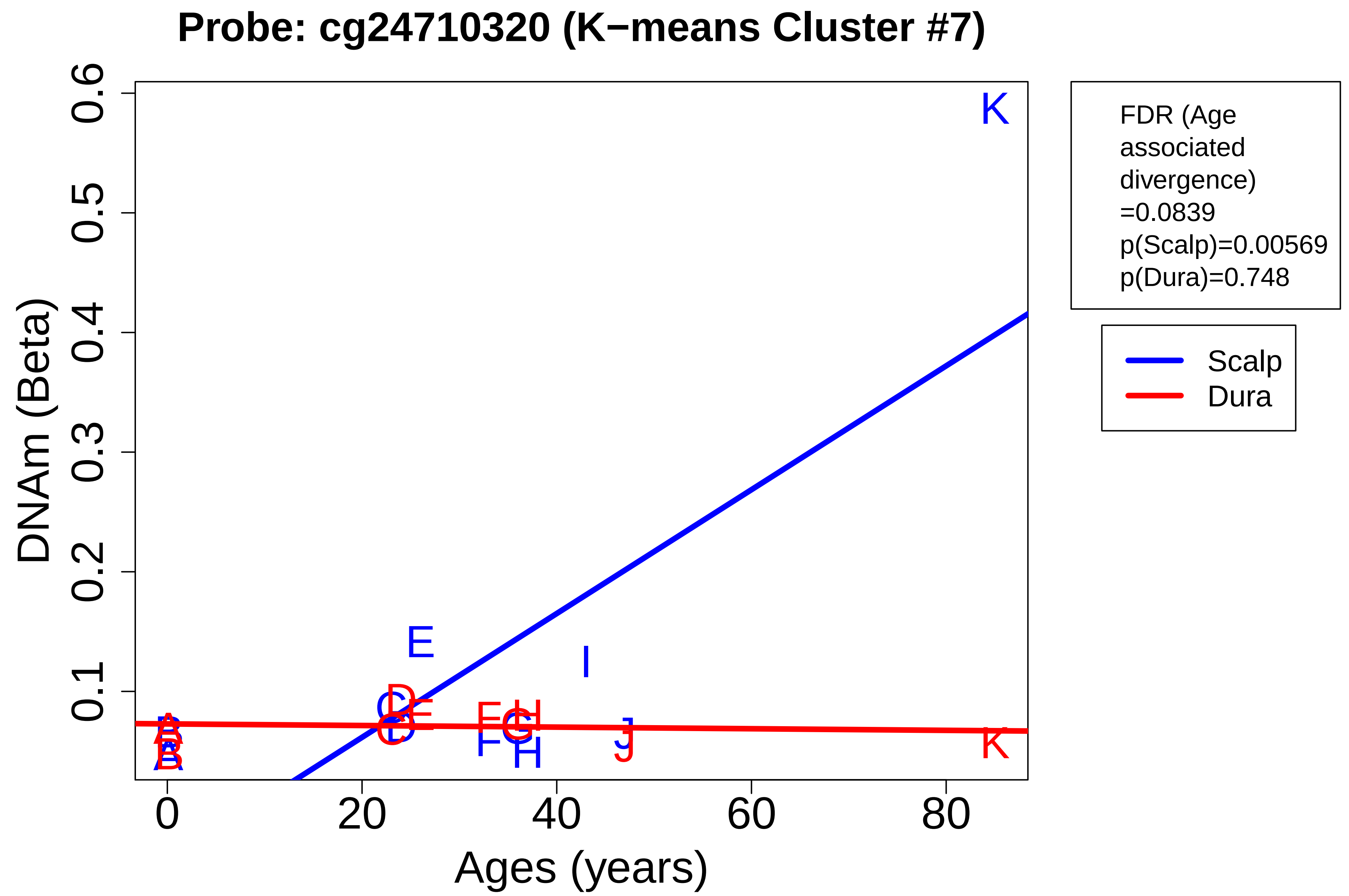

Probe: cg24710320 (K−means Cluster #7)
DNAm (Beta)
0.1
0.2
0.3
0.4
0.5
0.6
0
20
40
60
80
Ages (years)
FDR (Age associated divergence) =0.0839
p(Scalp)=0.00569
p(Dura)=0.748
Scalp
Dura

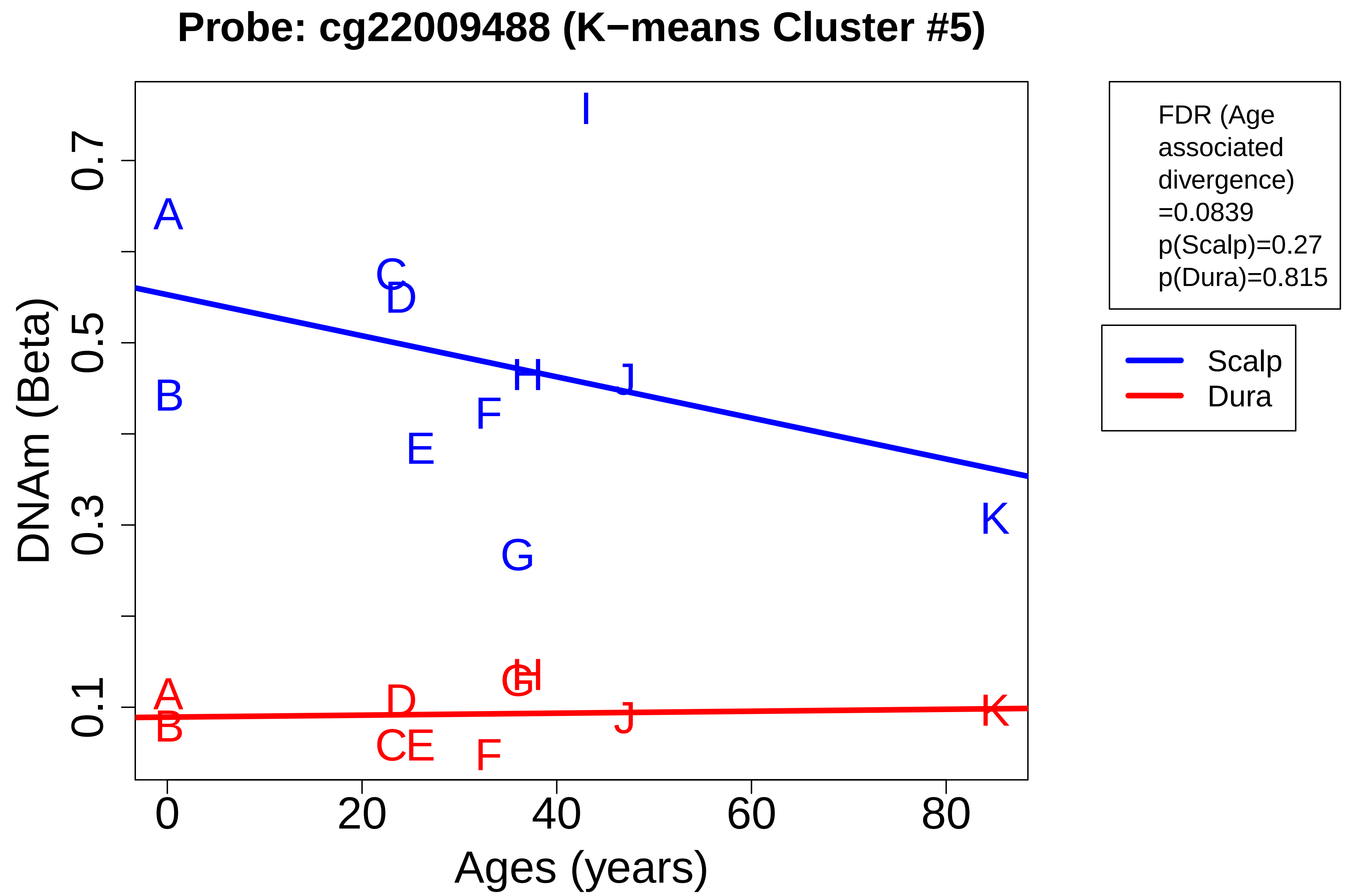

Probe: cg22009488 (K−means Cluster #5)
DNAm (Beta)
0.1
0.3
0.5
0.7
0
20
40
60
80
Ages (years)
A
B
C
D
E
F
G
H
I
J
K
A
B
CE
D
F
GH
J
K
FDR (Age associated divergence) =0.0839
p(Scalp)=0.27
p(Dura)=0.815
Scalp
Dura

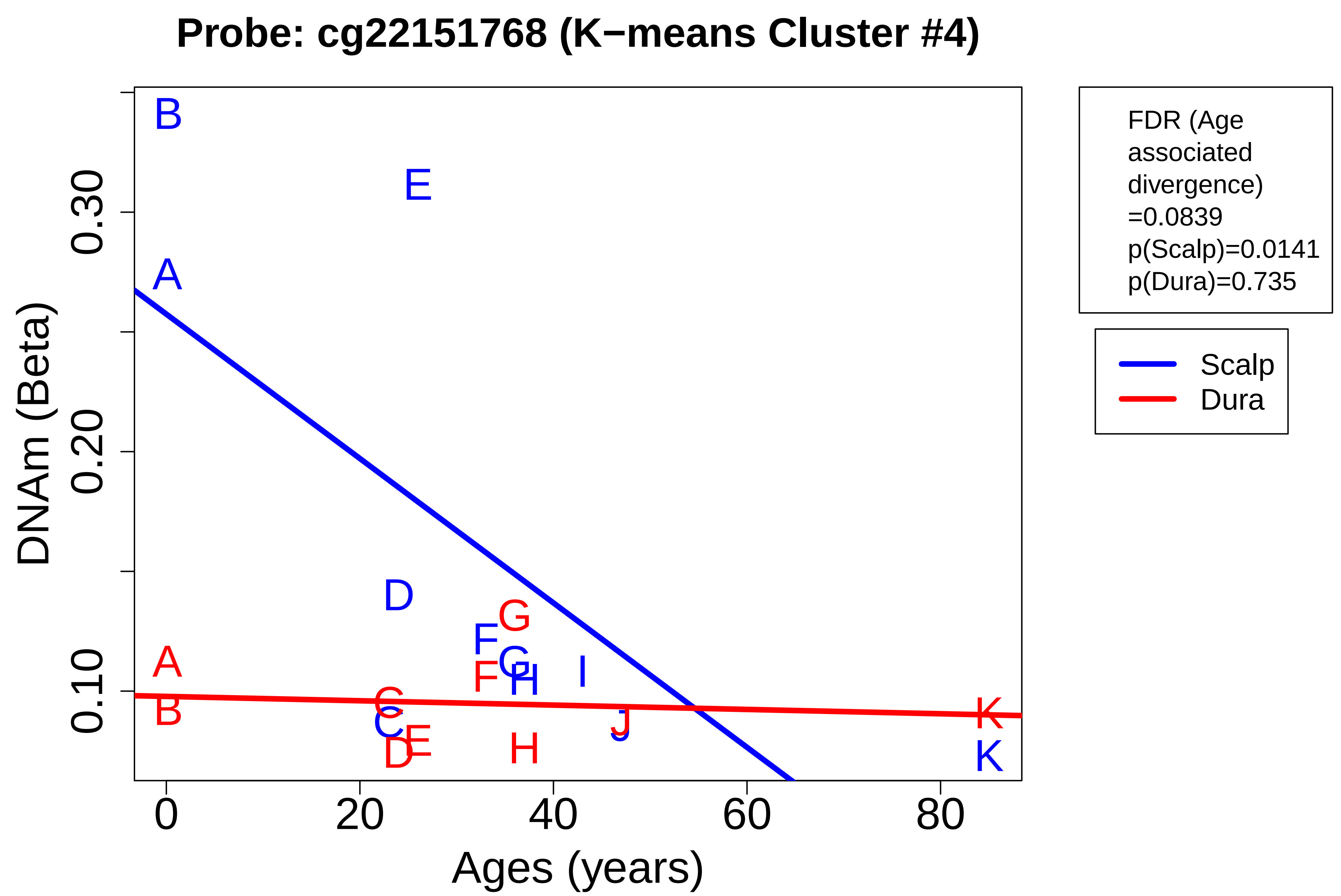

Probe: cg22151768 (K−means Cluster #4)
DNAm (Beta)
0.10
0.20
0.30
0
20
40
60
80
Ages (years)
A
B
C
D
E
F
G
H
I
J
K
FDR (Age associated divergence) =0.0839
p(Scalp)=0.0141
p(Dura)=0.735
Scalp
Dura

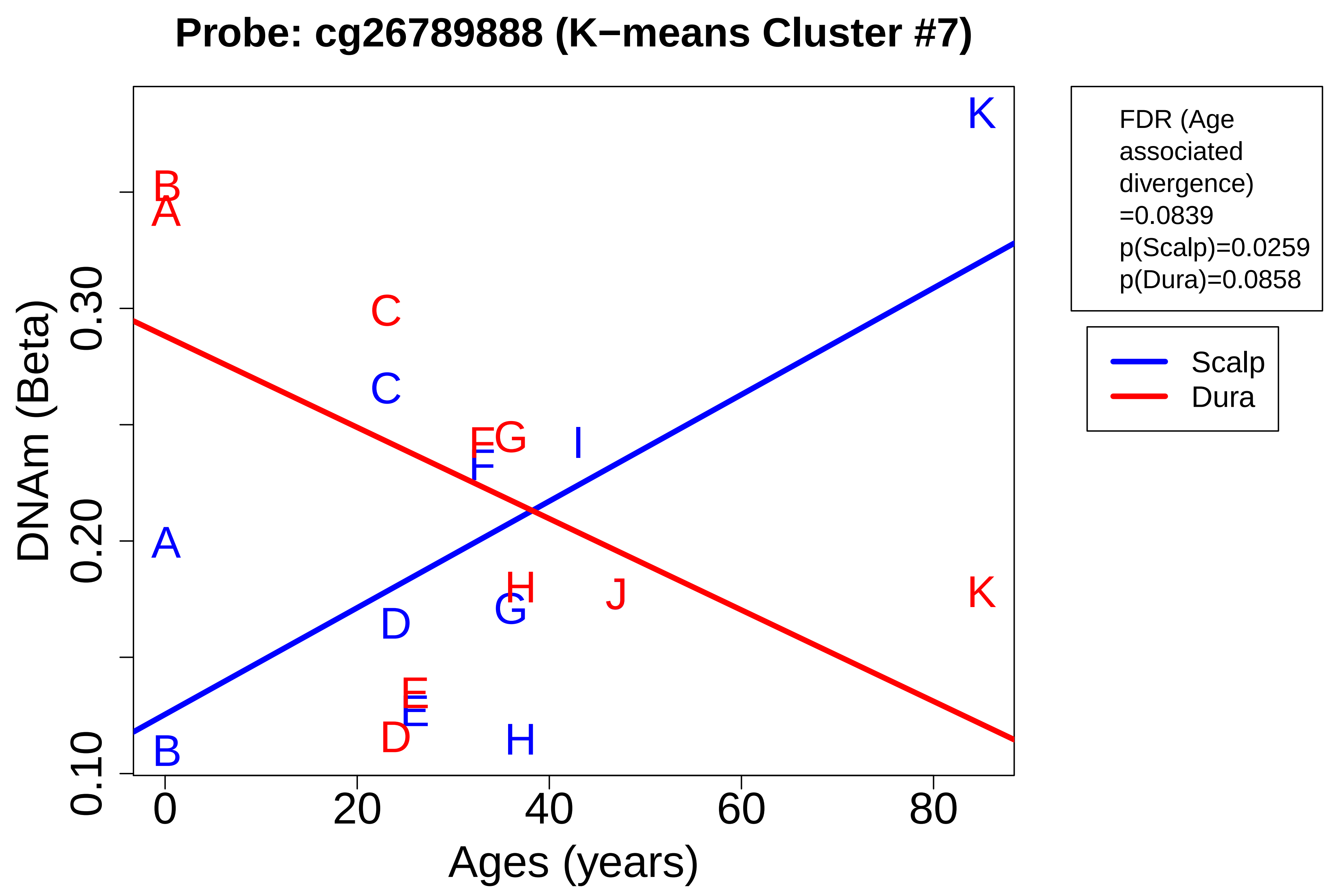

Probe: cg26789888 (K−means Cluster #7)
DNAm (Beta)
Ages (years)
0.10
0.20
0.30
0
20
40
60
80
FDR (Age associated divergence) =0.0839
p(Scalp)=0.0259
p(Dura)=0.0858
Scalp
Dura
A
B
C
D
E
F
G
H
I
J
K

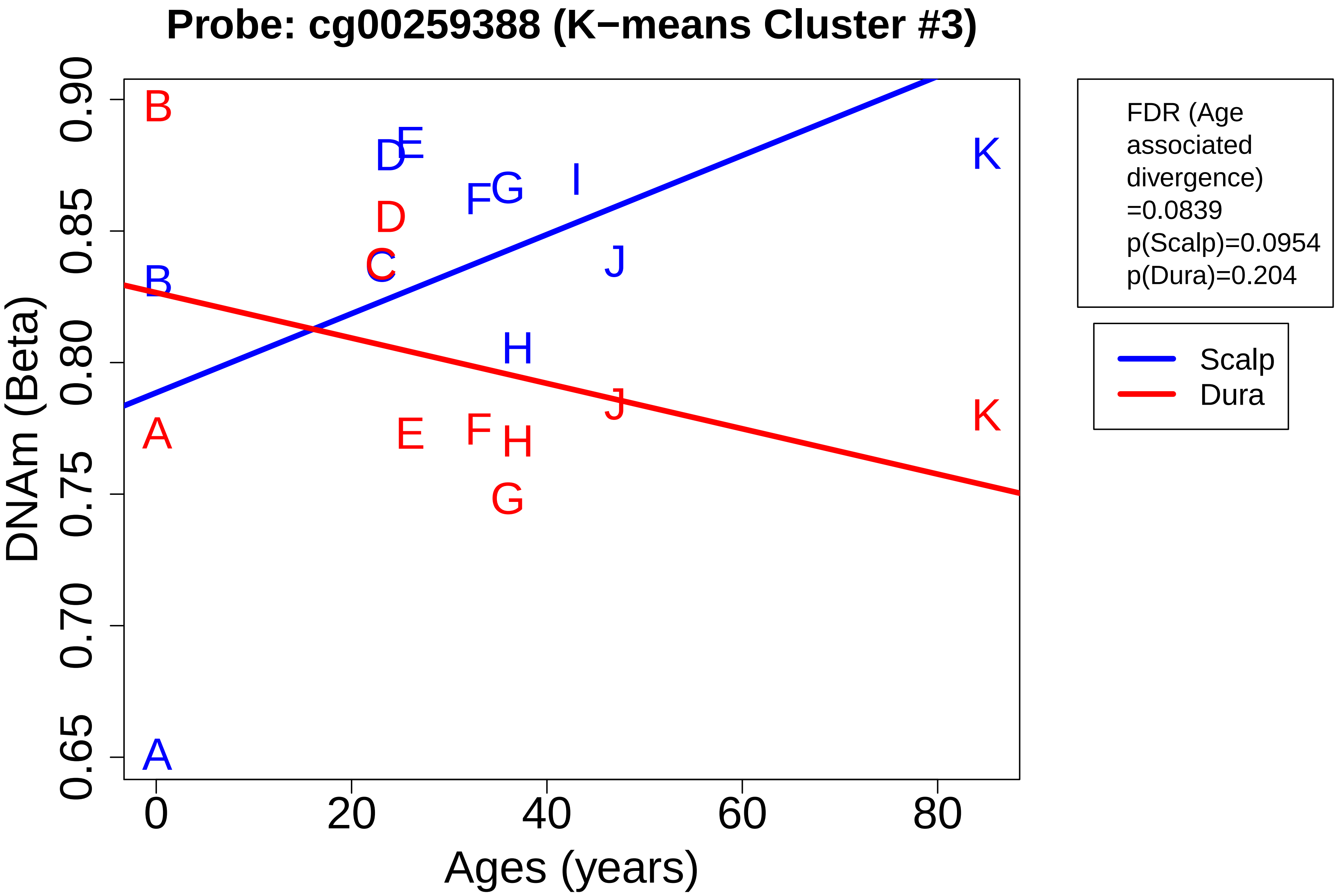

Probe: cg00259388 (K−means Cluster #3)
DNAm (Beta)
0.65
0.70
0.75
0.80
0.85
0.90
Ages (years)
0
20
40
60
80
FDR (Age associated divergence) =0.0839
p(Scalp)=0.0954
p(Dura)=0.204
Scalp
Dura

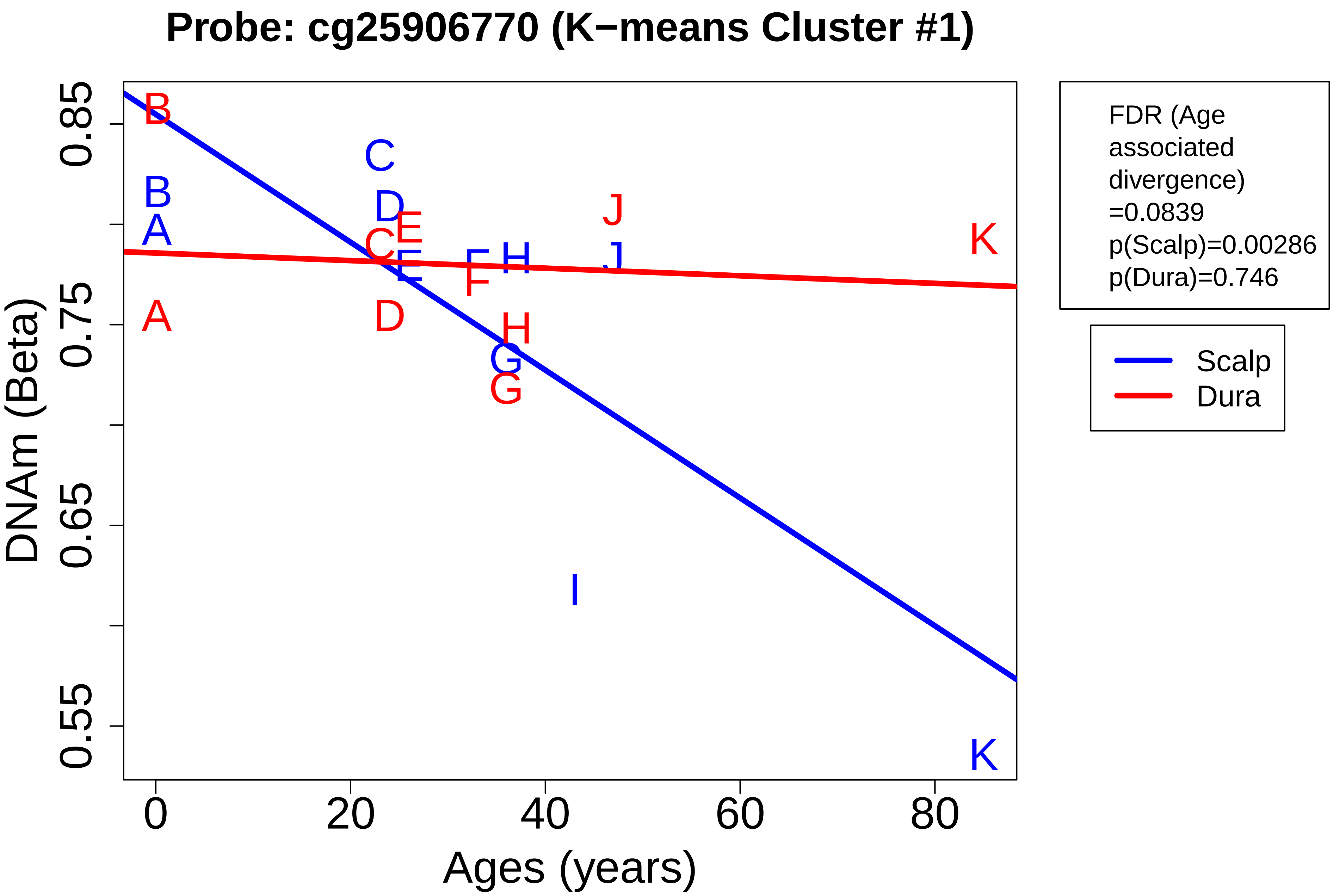

Probe: cg25906770 (K−means Cluster #1)
DNAm (Beta)
0.55
0.65
0.75
0.85
Ages (years)
0
20
40
60
80
FDR (Age associated divergence) =0.0839
p(Scalp)=0.00286
p(Dura)=0.746
Scalp
Dura

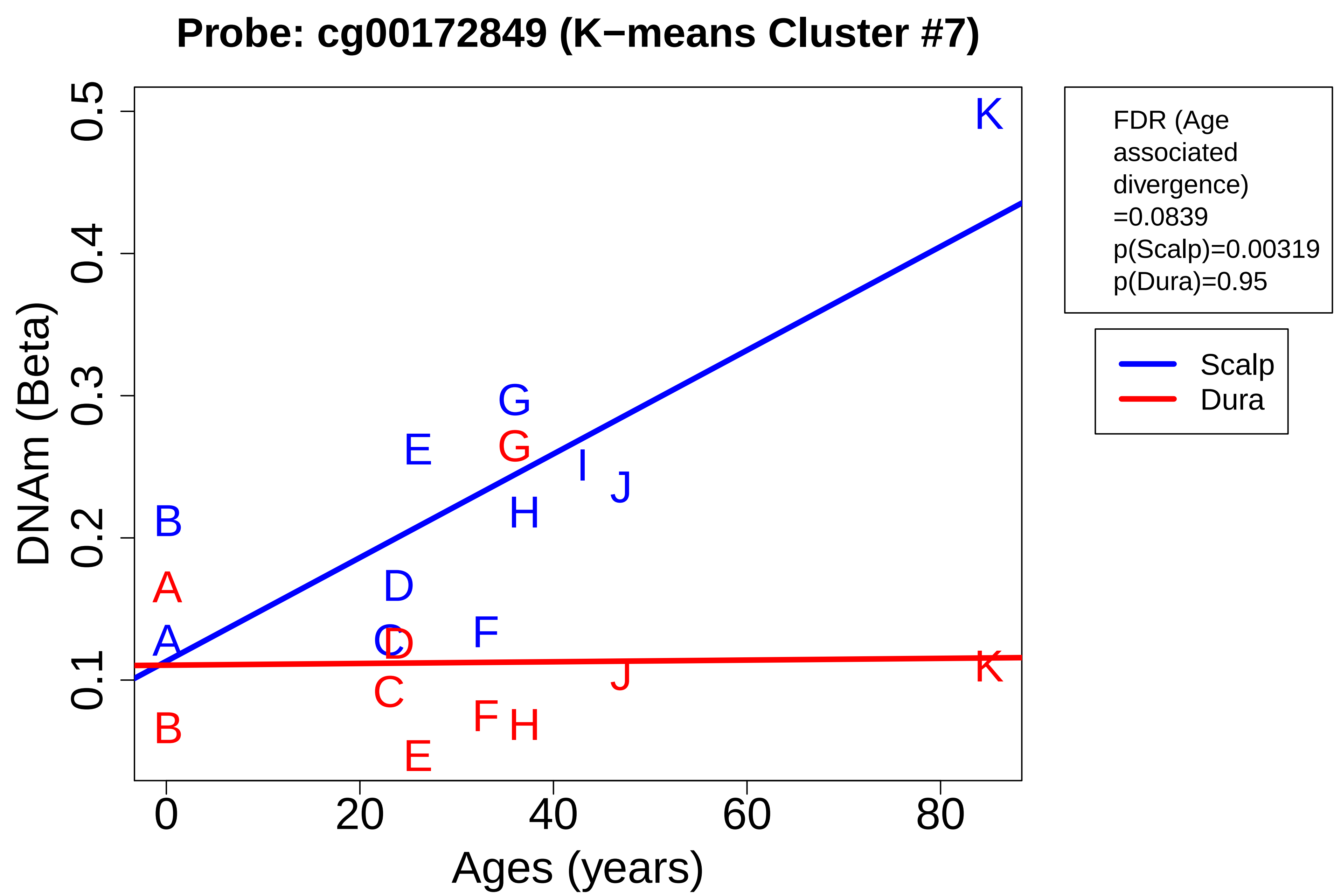

Probe: cg00172849 (K−means Cluster #7)
DNAm (Beta)
0.1
0.2
0.3
0.4
0.5
Ages (years)
0
20
40
60
80
FDR (Age associated divergence) =0.0839
p(Scalp)=0.00319
p(Dura)=0.95
Scalp
Dura
A
B
C
D
E
F
G
H
I
J
K

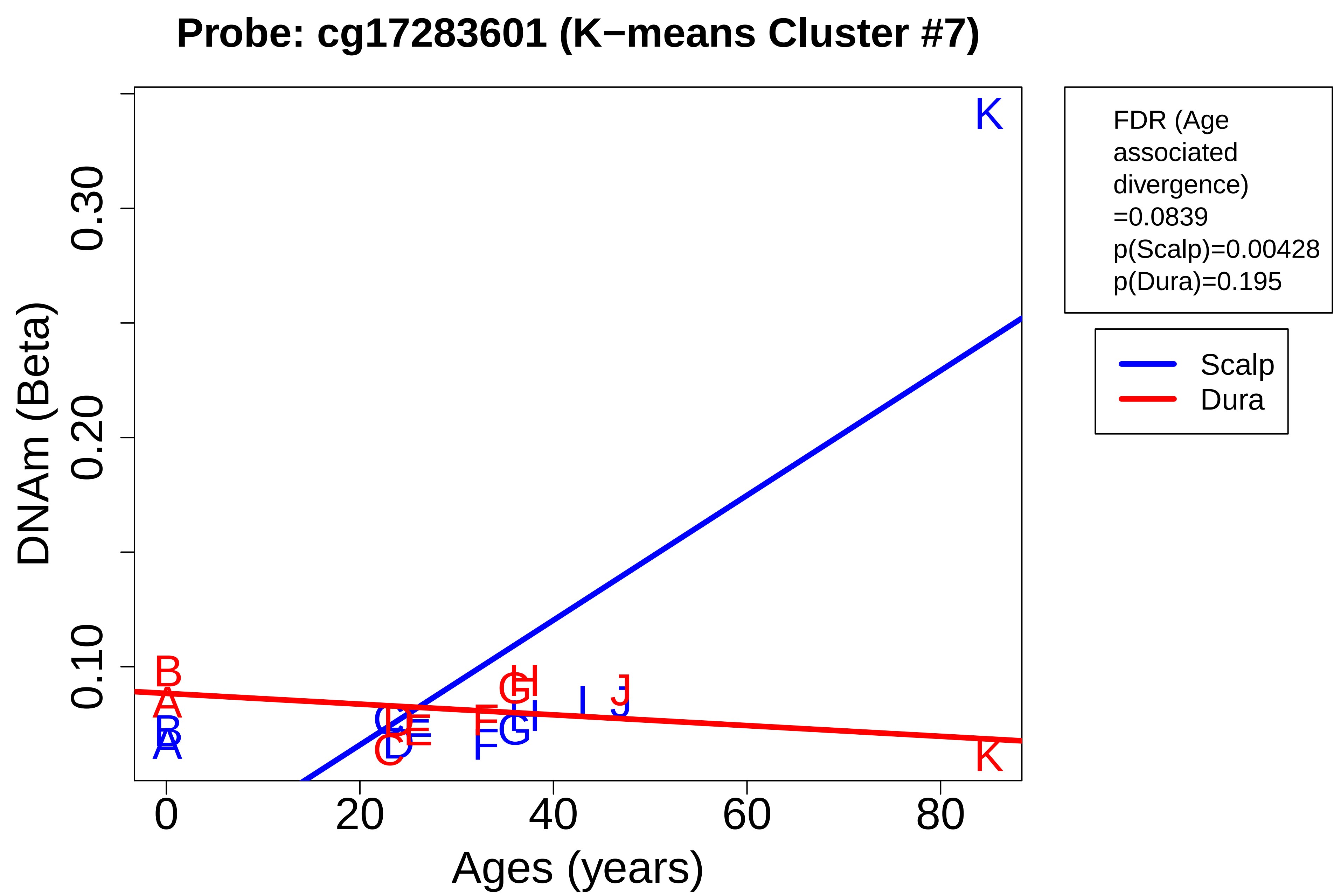

Probe: cg17283601 (K−means Cluster #7)
DNAm (Beta)
0.10
0.20
0.30
Ages (years)
0
20
40
60
80
FDR (Age associated divergence) =0.0839
p(Scalp)=0.00428
p(Dura)=0.195
Scalp
Dura

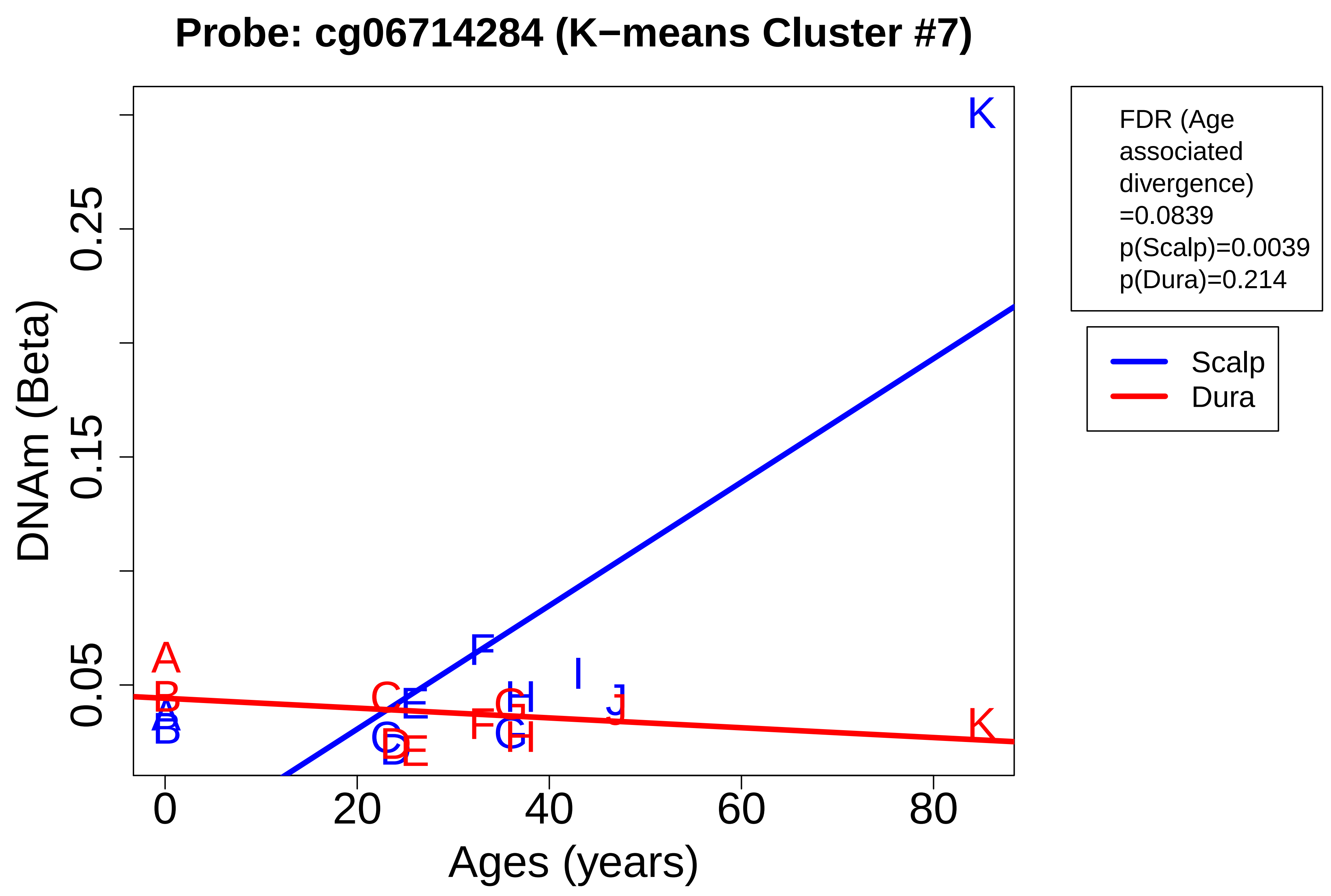

Probe: cg06714284 (K−means Cluster #7)
DNAm (Beta)
0.05
0.15
0.25
Ages (years)
0
20
40
60
80
FDR (Age associated divergence) =0.0839
p(Scalp)=0.0039
p(Dura)=0.214
Scalp
Dura
A
B
C
D
E
F
G
H
I
J
K

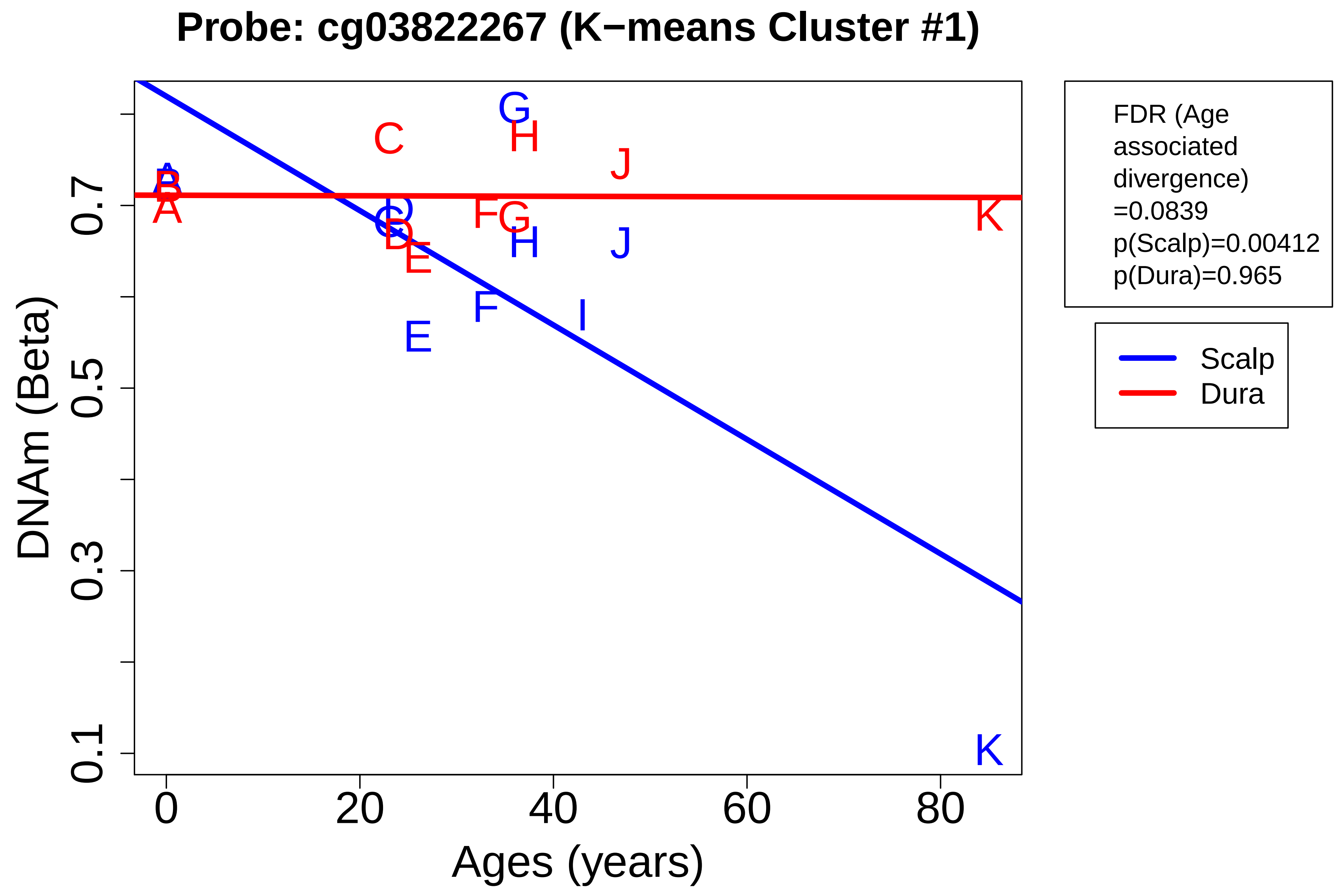
Probe: cg03822267 (K−means Cluster #1)
FDR (Age associated divergence) =0.0839
p(Scalp)=0.00412
p(Dura)=0.965
Scalp
Dura
DNAm (Beta)
0.1
0.3
0.5
0.7
0
20
40
60
80
Ages (years)
A
B
C
D
E
F
G
H
I
J
K

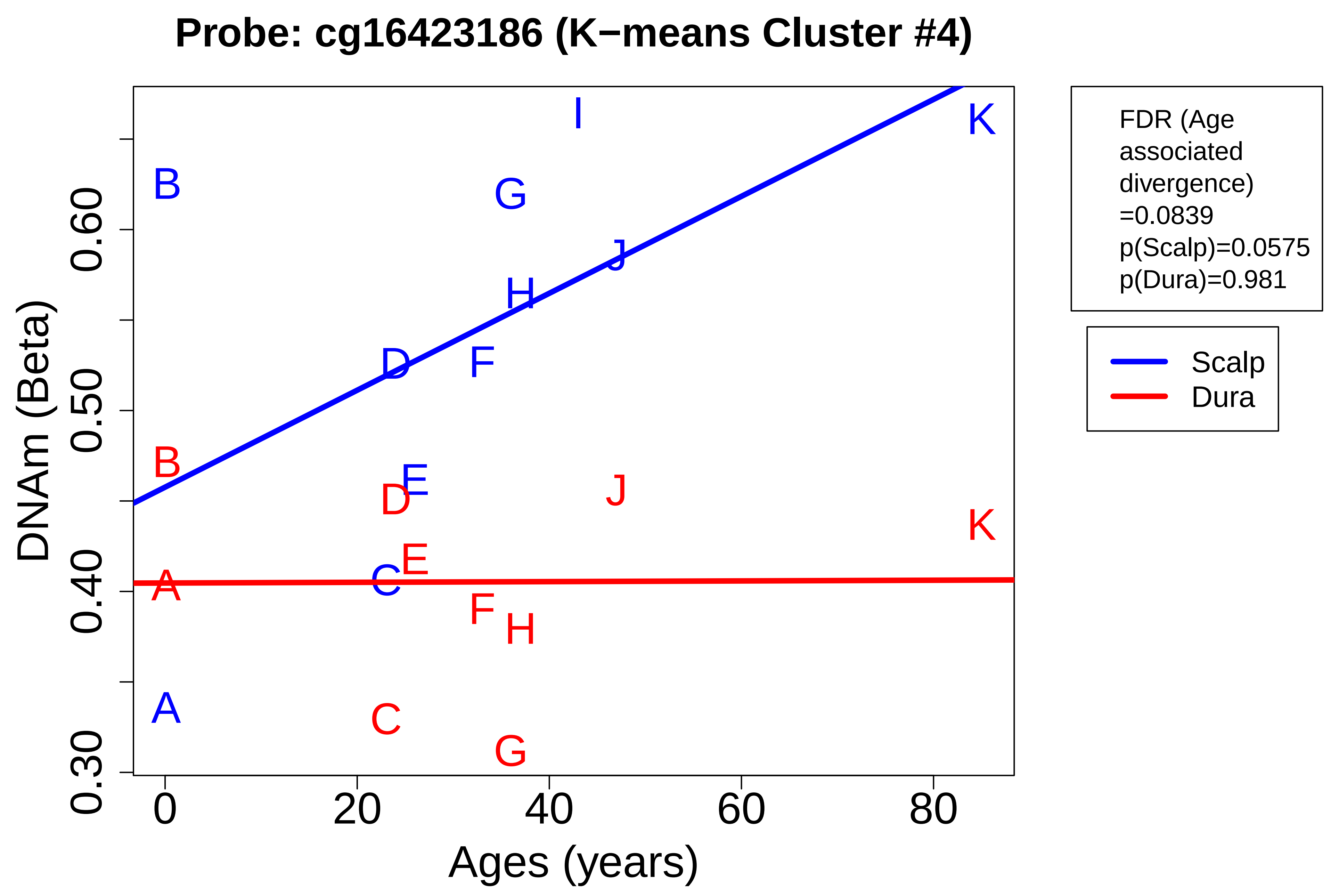

Probe: cg16423186 (K−means Cluster #4)
DNAm (Beta)
0.30
0.40
0.50
0.60
0
20
40
60
80
Ages (years)
A
B
C
D
E
F
G
H
I
J
K
FDR (Age associated divergence) =0.0839
p(Scalp)=0.0575
p(Dura)=0.981
Scalp
Dura

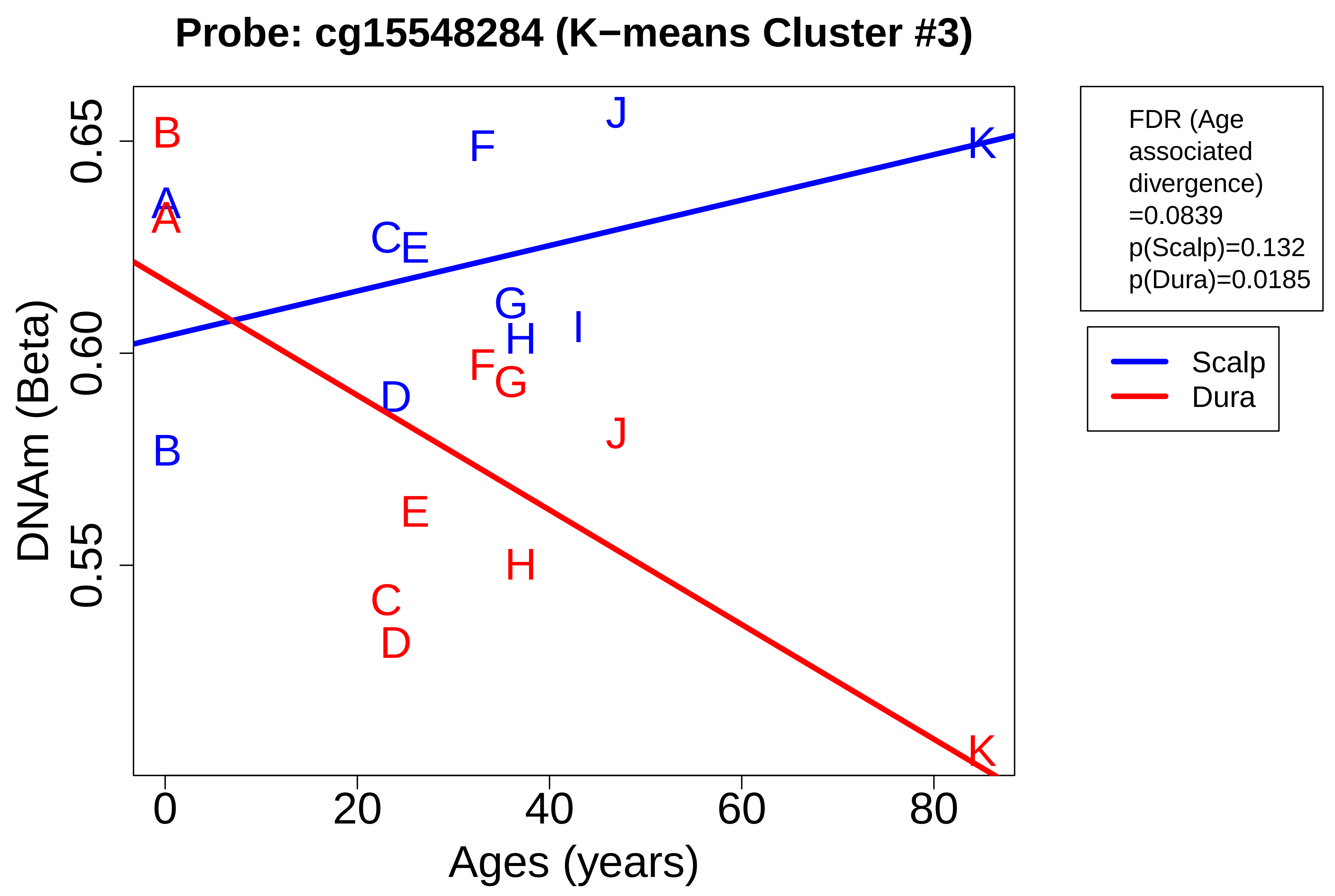
Probe: cg15548284 (K−means Cluster #3)
DNAm (Beta)
0.55
0.60
0.65
Ages (years)
0
20
40
60
80
FDR (Age associated divergence) =0.0839
p(Scalp)=0.132
p(Dura)=0.0185
Scalp
Dura

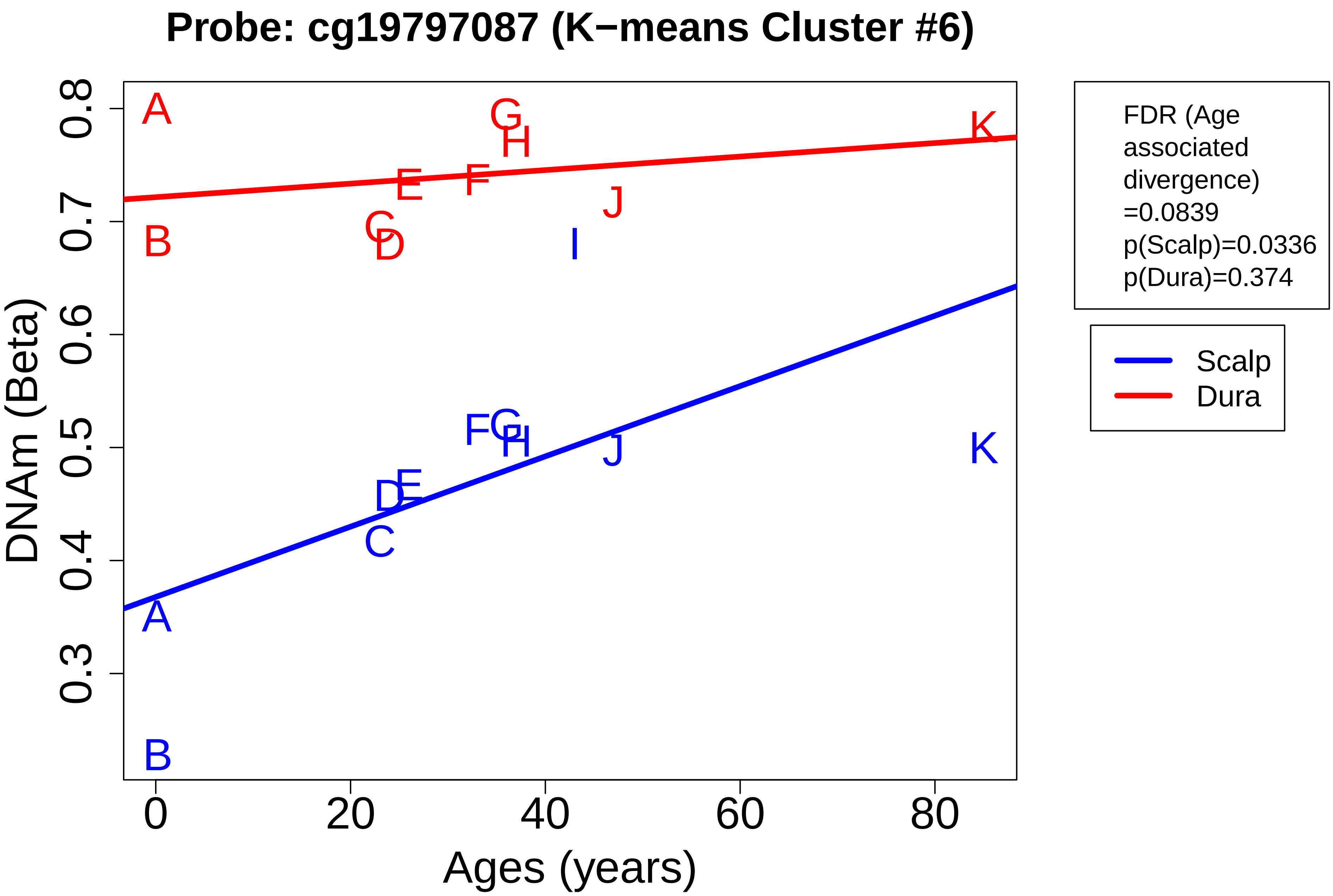

Probe: cg19797087 (K−means Cluster #6)
DNAm (Beta)
0.3
0.4
0.5
0.6
0.7
0.8
0
20
40
60
80
Ages (years)
A
B
C
D
E
F
G
H
I
J
K
FDR (Age associated divergence) =0.0839
p(Scalp)=0.0336
p(Dura)=0.374
Scalp
Dura

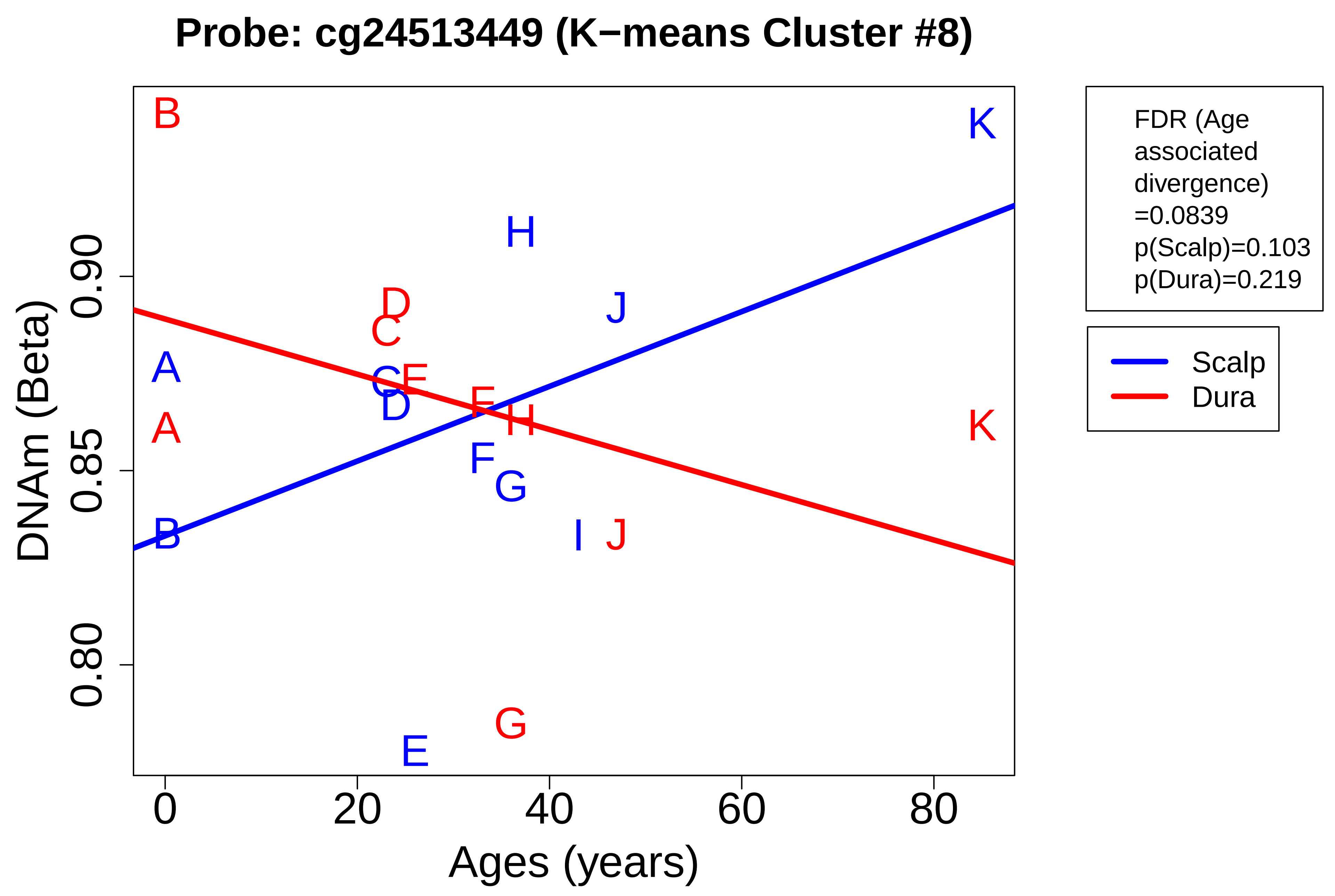

Probe: cg24513449 (K−means Cluster #8)
DNAm (Beta)
0.80
0.85
0.90
Ages (years)
0
20
40
60
80
FDR (Age associated divergence) =0.0839
p(Scalp)=0.103
p(Dura)=0.219
Scalp
Dura

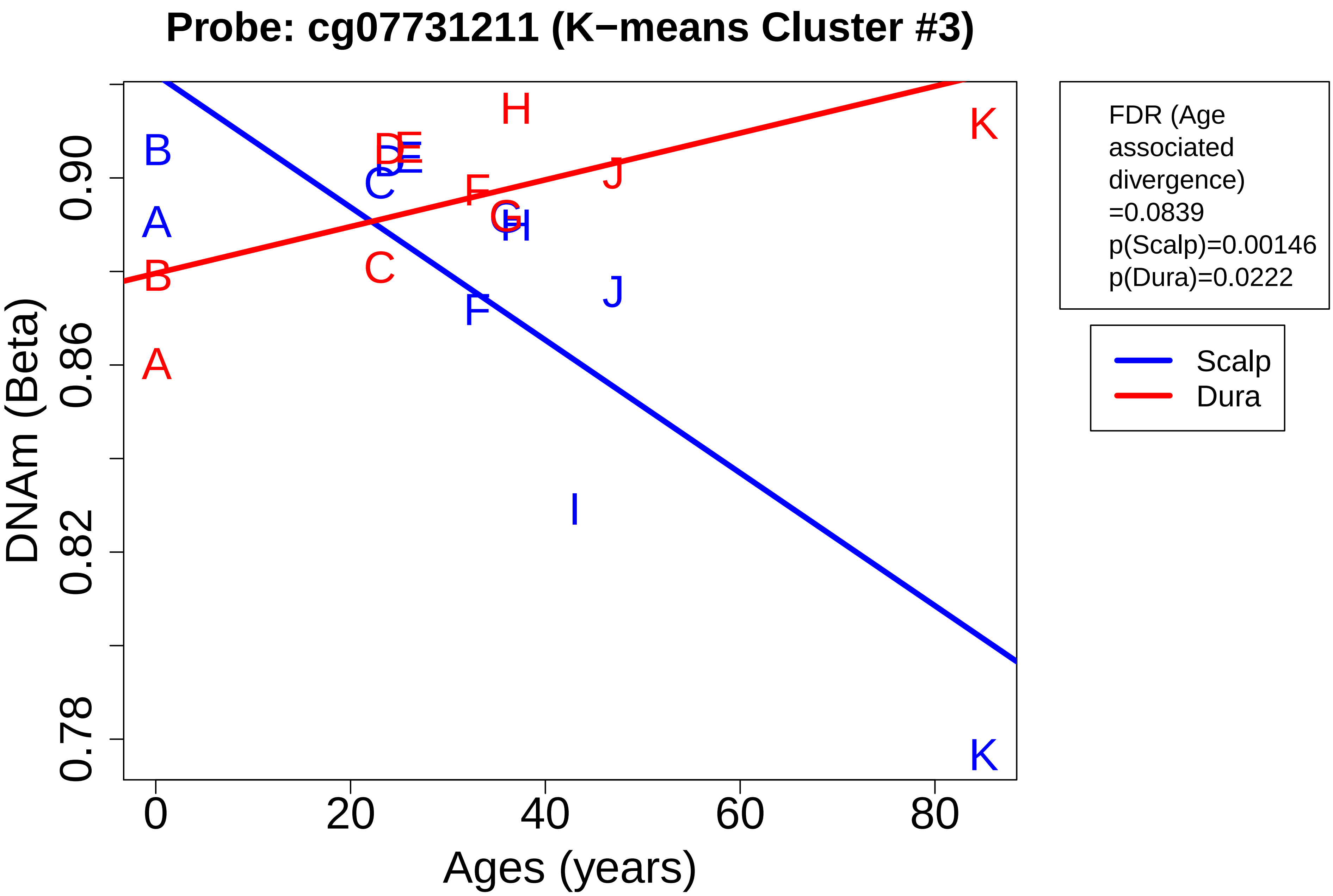

Probe: cg07731211 (K−means Cluster #3)
DNAm (Beta)
0.78
0.82
0.86
0.90
0
20
40
60
80
Ages (years)
FDR (Age associated divergence) =0.0839
p(Scalp)=0.00146
p(Dura)=0.0222
Scalp
Dura

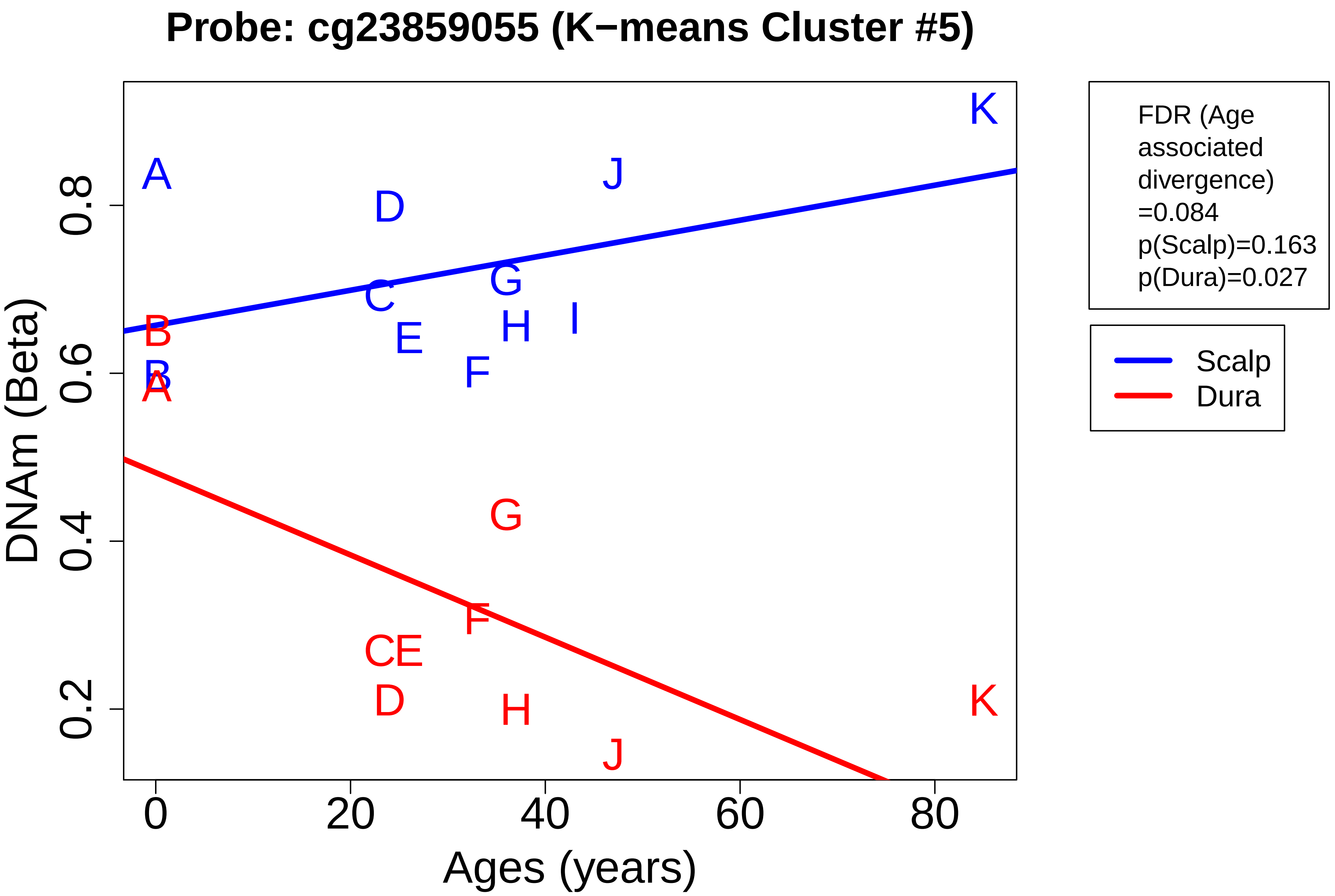
Probe: cg23859055 (K−means Cluster #5)
DNAm (Beta)
Ages (years)
0.2
0.4
0.6
0.8
0
20
40
60
80
A
B
C
D
E
F
G
H
I
J
K
FDR (Age associated divergence) =0.084
p(Scalp)=0.163
p(Dura)=0.027
Scalp
Dura

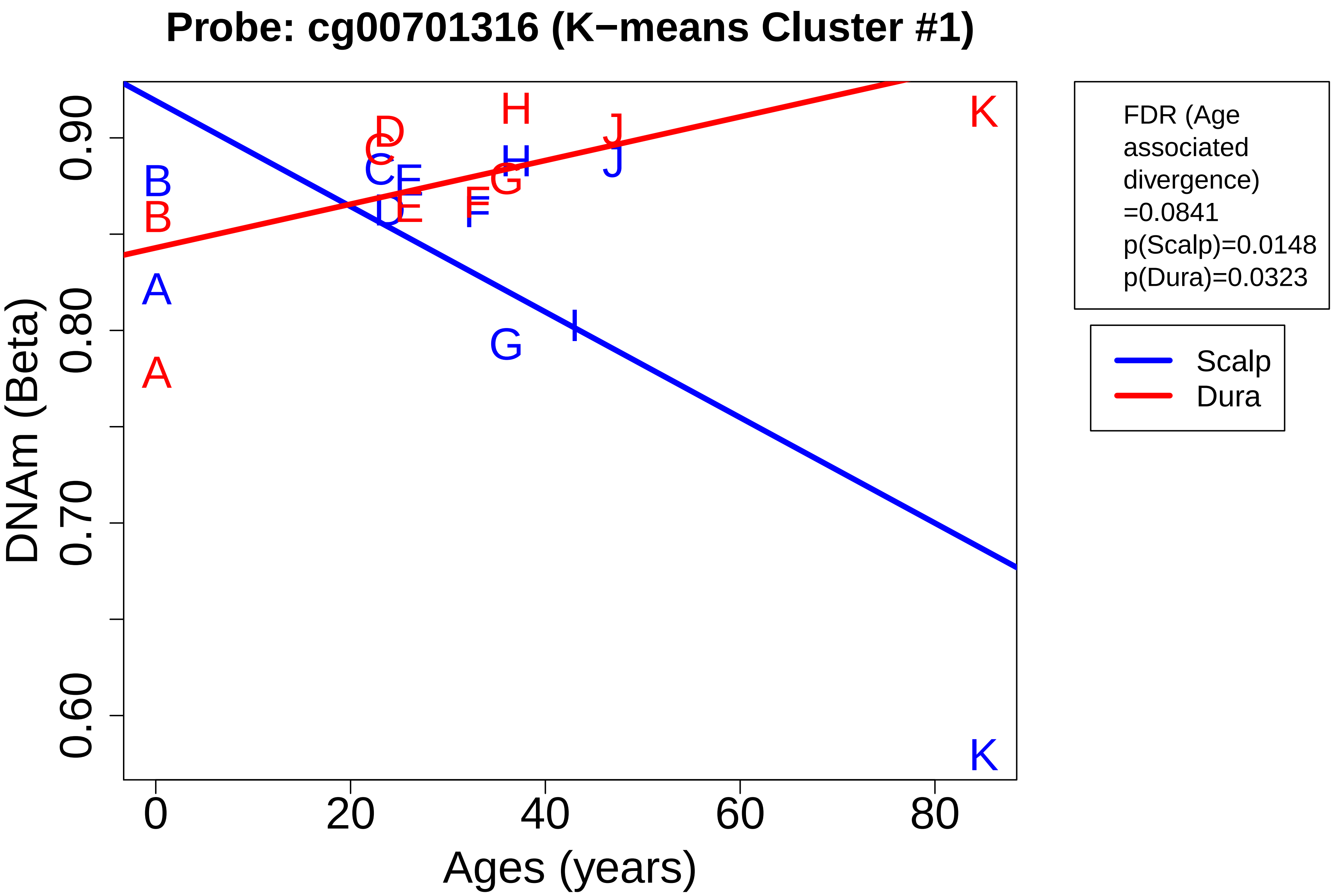

Probe: cg00701316 (K−means Cluster #1)
DNAm (Beta)
0.60
0.70
0.80
0.90
0
20
40
60
80
Ages (years)
FDR (Age associated divergence) =0.0841
p(Scalp)=0.0148
p(Dura)=0.0323
Scalp
Dura

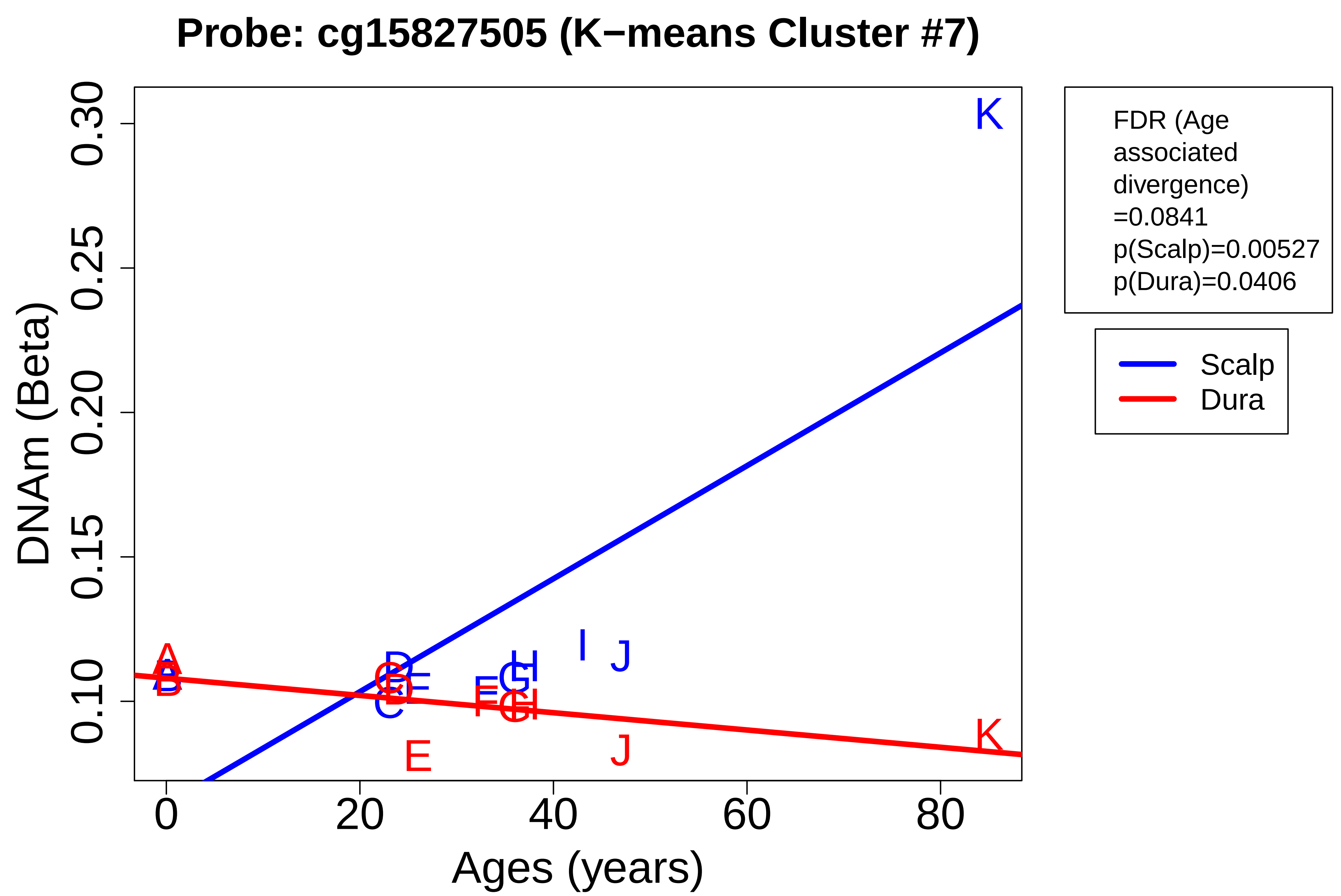

Probe: cg15827505 (K−means Cluster #7)
DNAm (Beta)
0.10
0.15
0.20
0.25
0.30
0
20
40
60
80
Ages (years)
FDR (Age associated divergence) =0.0841
p(Scalp)=0.00527
p(Dura)=0.0406
Scalp
Dura

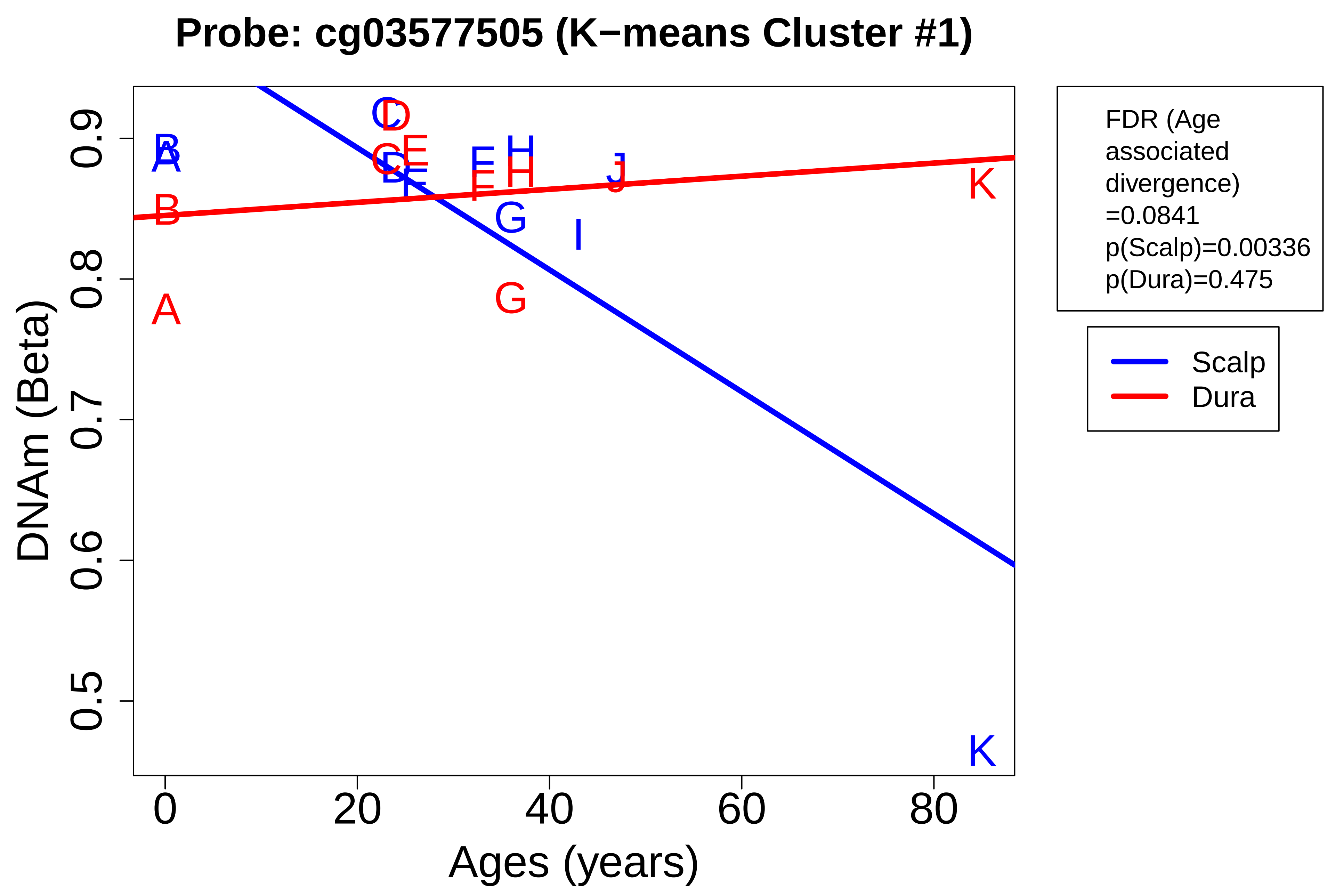

Probe: cg03577505 (K−means Cluster #1)
DNAm (Beta)
0.5
0.6
0.7
0.8
0.9
0
20
40
60
80
Ages (years)
FDR (Age associated divergence) =0.0841
p(Scalp)=0.00336
p(Dura)=0.475
Scalp
Dura

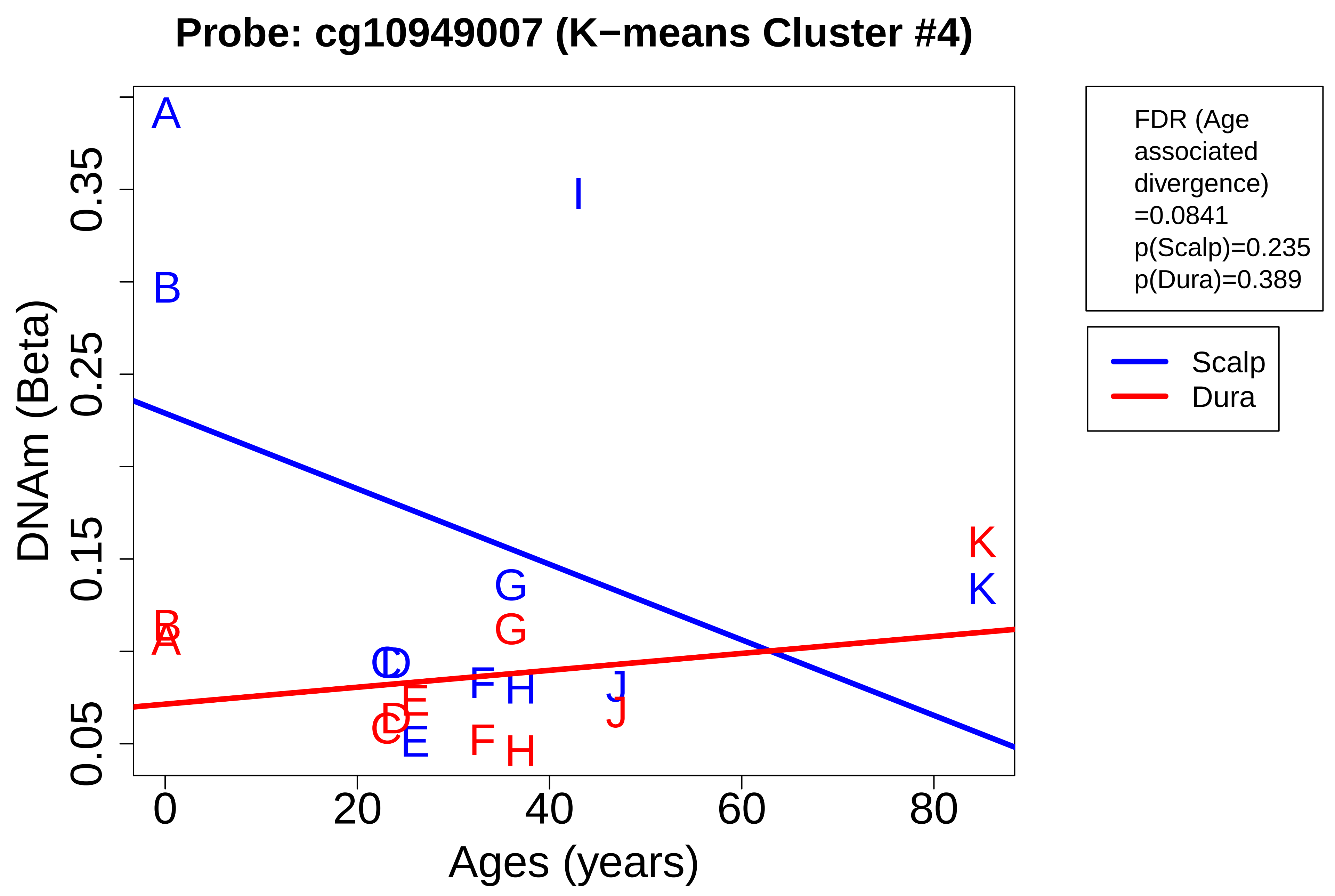

Probe: cg10949007 (K−means Cluster #4)
FDR (Age associated divergence) =0.0841
p(Scalp)=0.235
p(Dura)=0.389
Scalp
Dura
DNAm (Beta)
0.05
0.15
0.25
0.35
0
20
40
60
80
Ages (years)
A
B
C
D
E
F
G
H
I
J
K

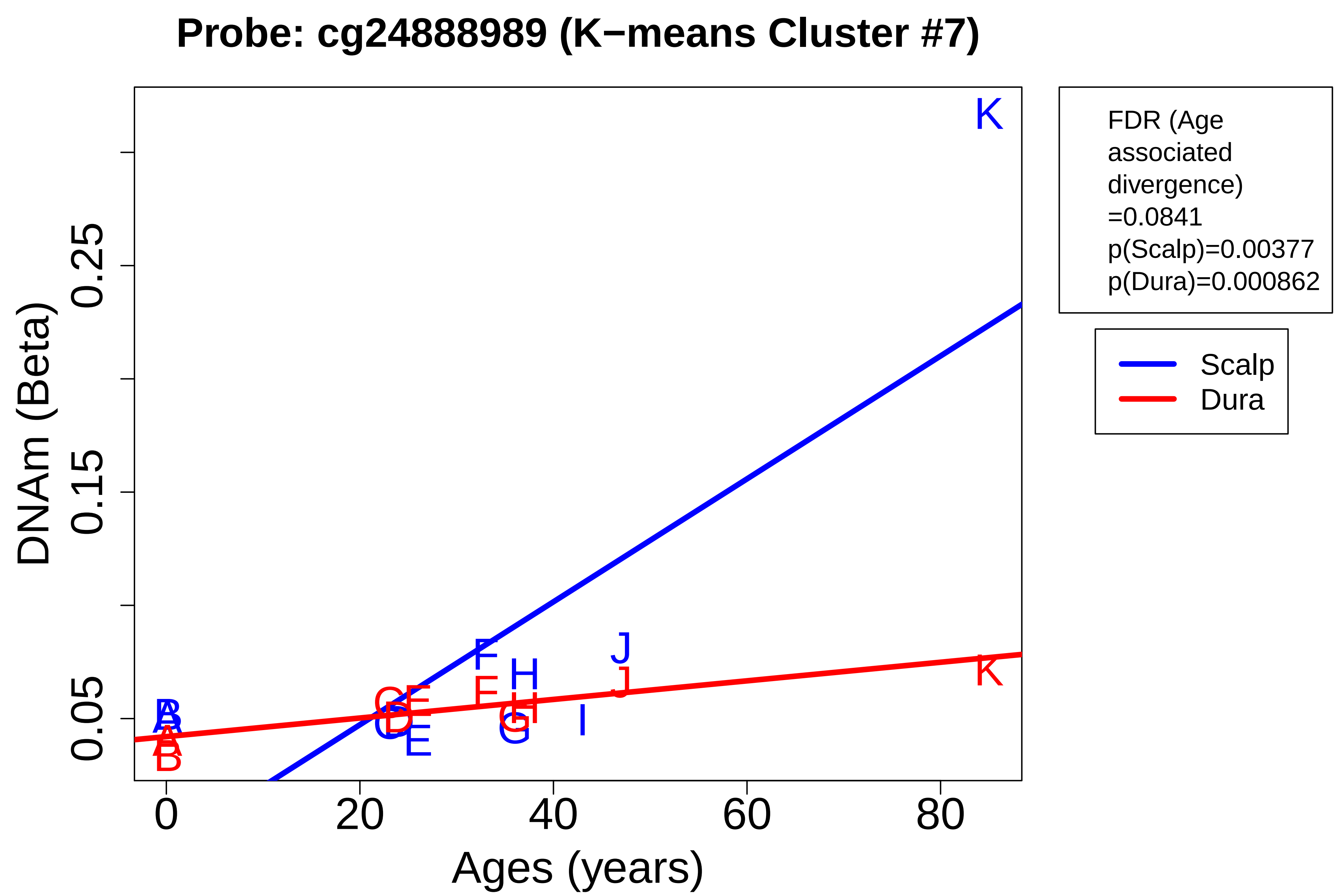

Probe: cg24888989 (K−means Cluster #7)
DNAm (Beta)
0.05
0.15
0.25
0
20
40
60
80
Ages (years)
FDR (Age associated divergence) =0.0841
p(Scalp)=0.00377
p(Dura)=0.000862
Scalp
Dura

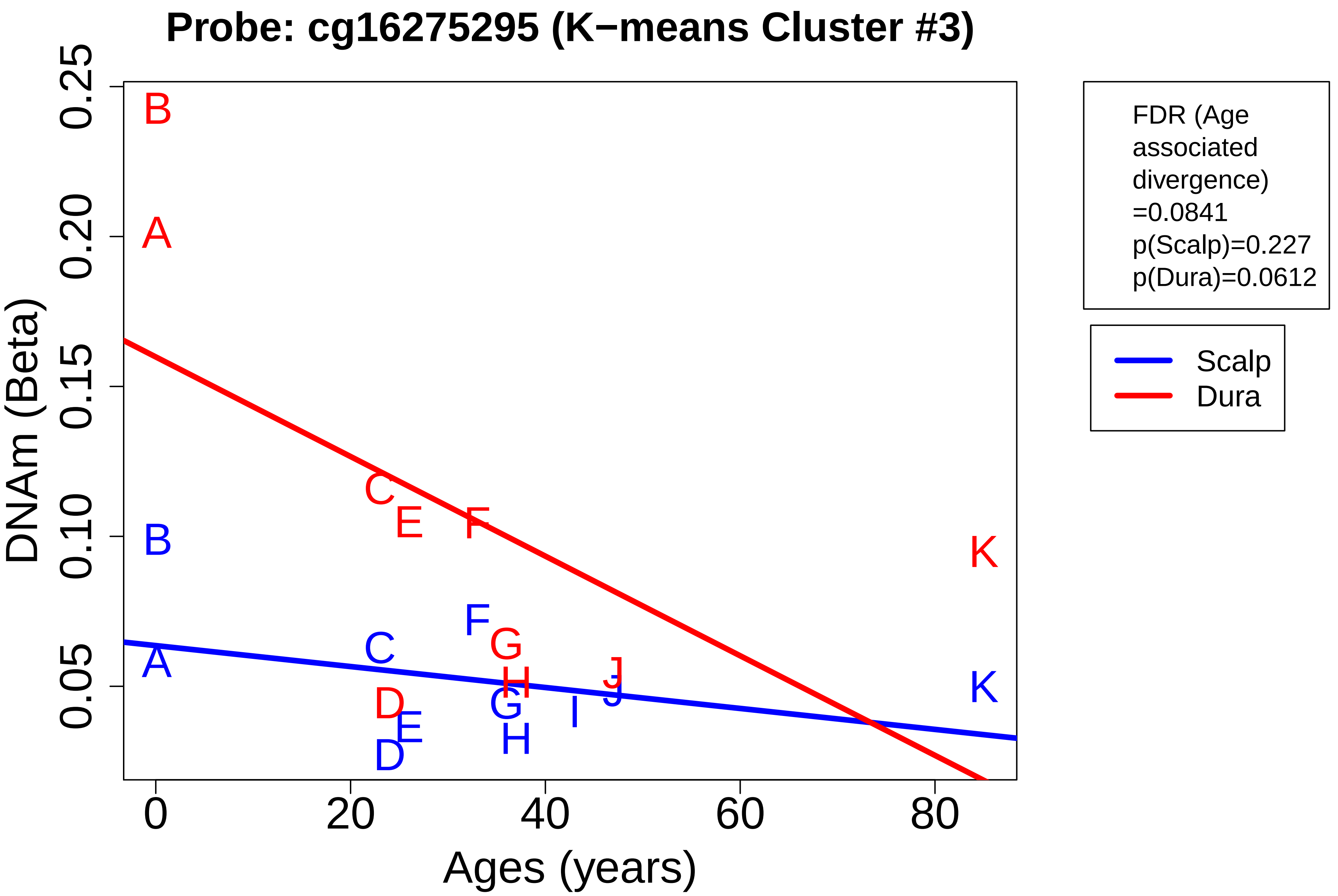

Probe: cg16275295 (K−means Cluster #3)
DNAm (Beta)
0.05
0.10
0.15
0.20
0.25
0
20
40
60
80
Ages (years)
A B C D E F G H I J K
FDR (Age associated divergence) =0.0841
p(Scalp)=0.227
p(Dura)=0.0612
Scalp
Dura

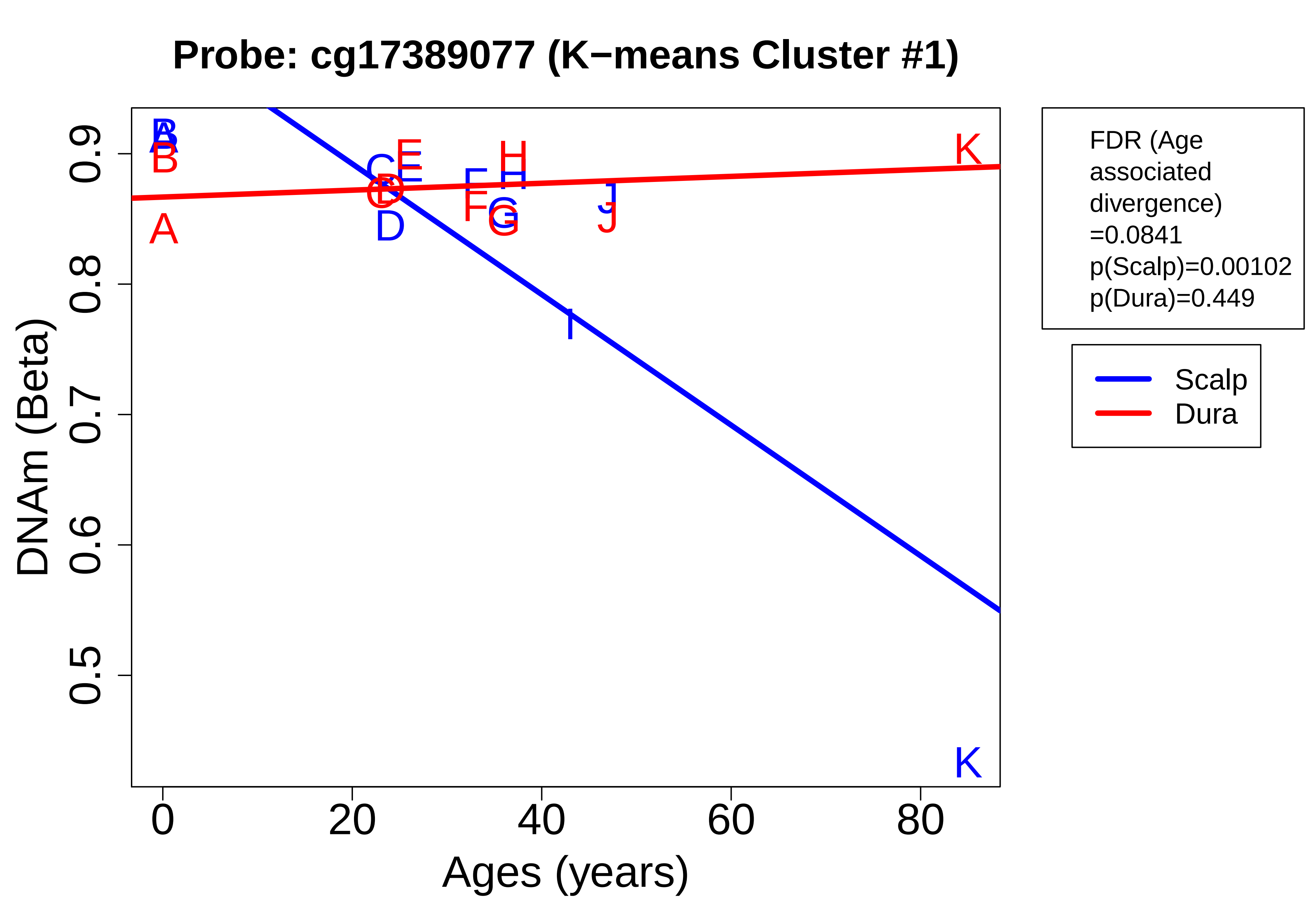

Probe: cg17389077 (K−means Cluster #1)
DNAm (Beta)
0.5
0.6
0.7
0.8
0.9
Ages (years)
0
20
40
60
80
FDR (Age associated divergence) =0.0841
p(Scalp)=0.00102
p(Dura)=0.449
Scalp
Dura

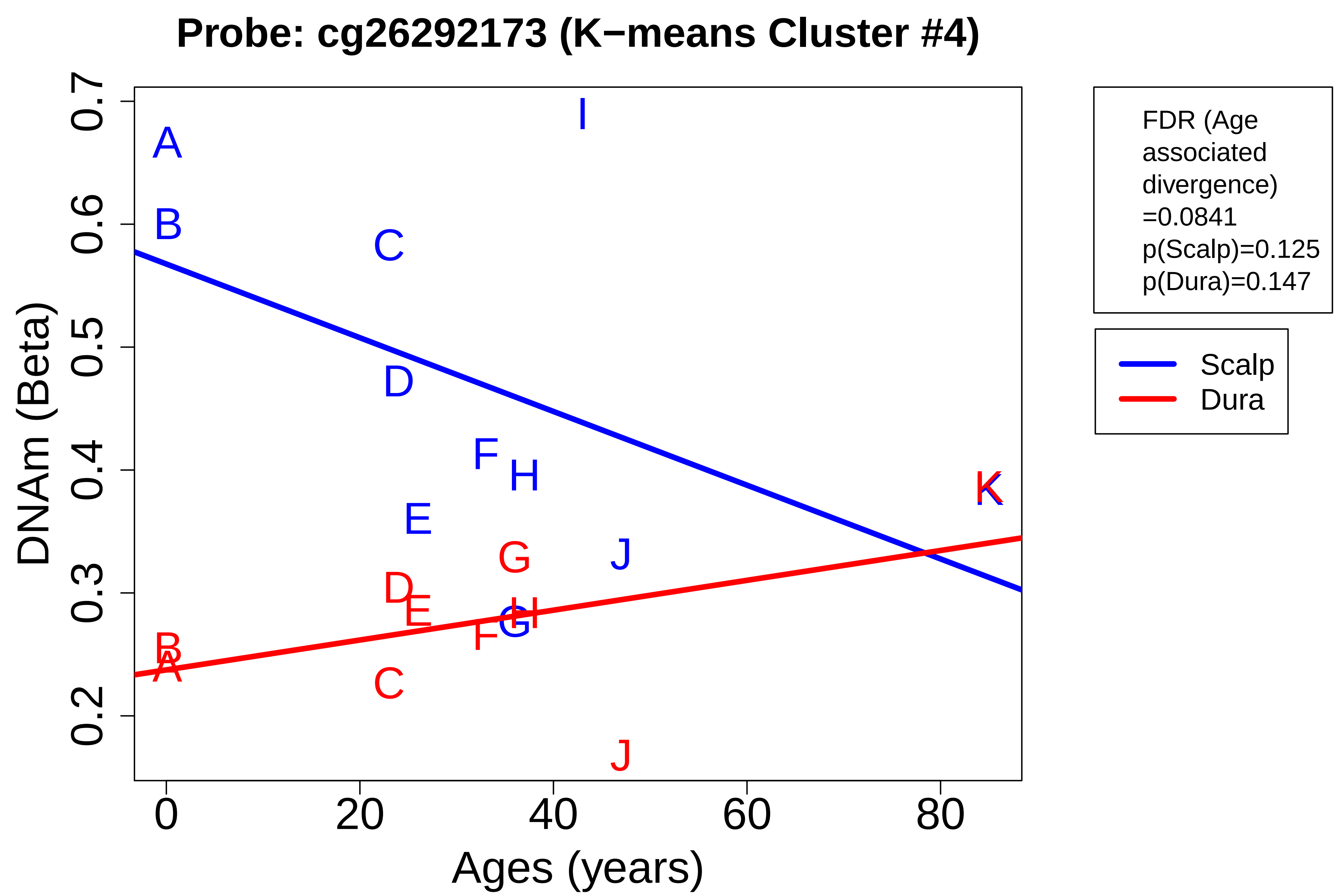
Probe: cg26292173 (K−means Cluster #4)
DNAm (Beta)
0.2
0.3
0.4
0.5
0.6
0.7
Ages (years)
0
20
40
60
80
A
B
C
D
E
F
G
H
I
J
K
FDR (Age associated divergence) =0.0841
p(Scalp)=0.125
p(Dura)=0.147
Scalp
Dura

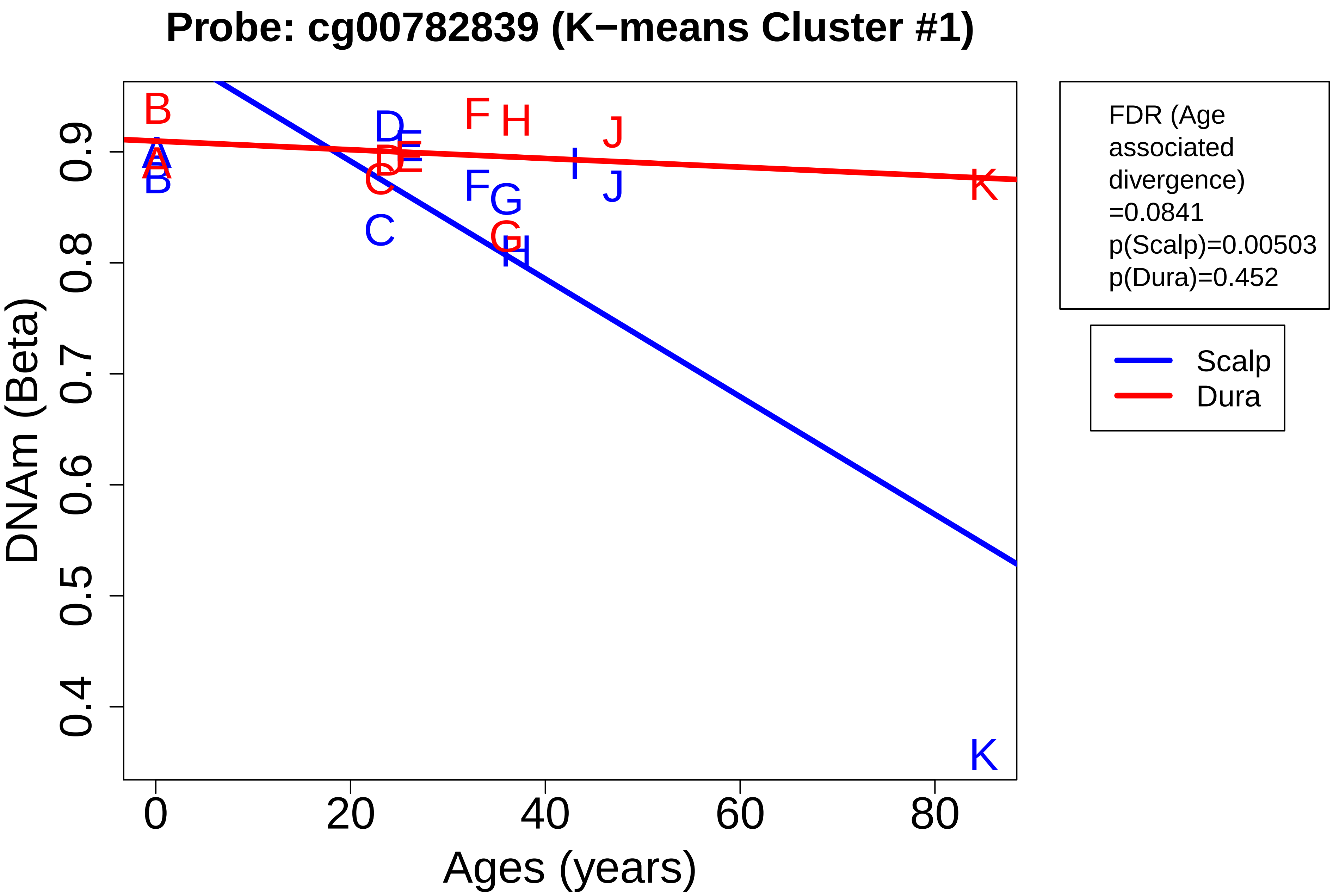

Probe: cg00782839 (K−means Cluster #1)
DNAm (Beta)
Ages (years)
0.4
0.5
0.6
0.7
0.8
0.9
0
20
40
60
80
FDR (Age associated divergence) =0.0841
p(Scalp)=0.00503
p(Dura)=0.452
Scalp
Dura

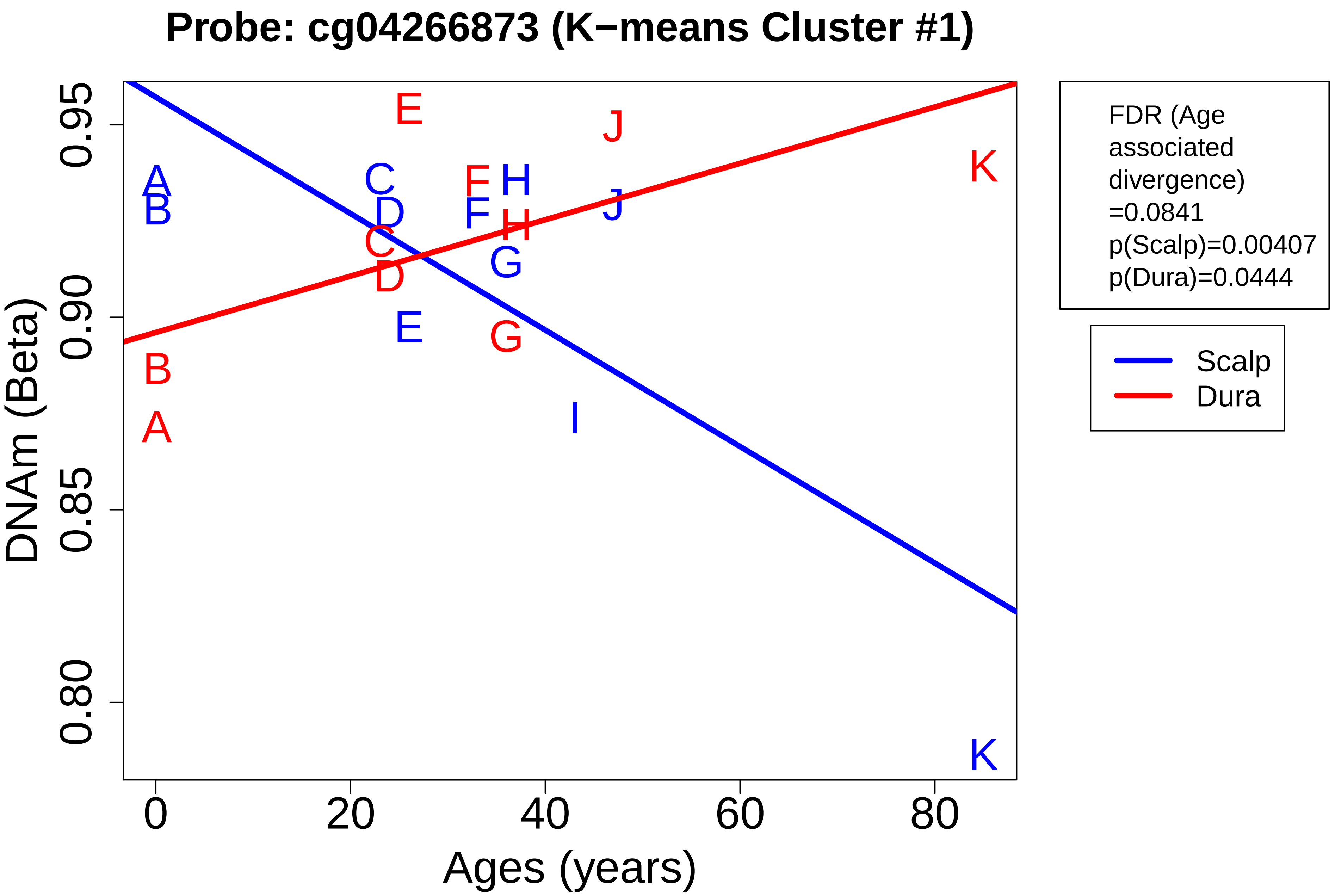
Probe: cg04266873 (K−means Cluster #1)
DNAm (Beta)
0.80
0.85
0.90
0.95
Ages (years)
0
20
40
60
80
A
B
C
D
E
F
G
H
I
J
K
FDR (Age associated divergence) =0.0841
p(Scalp)=0.00407
p(Dura)=0.0444
Scalp
Dura

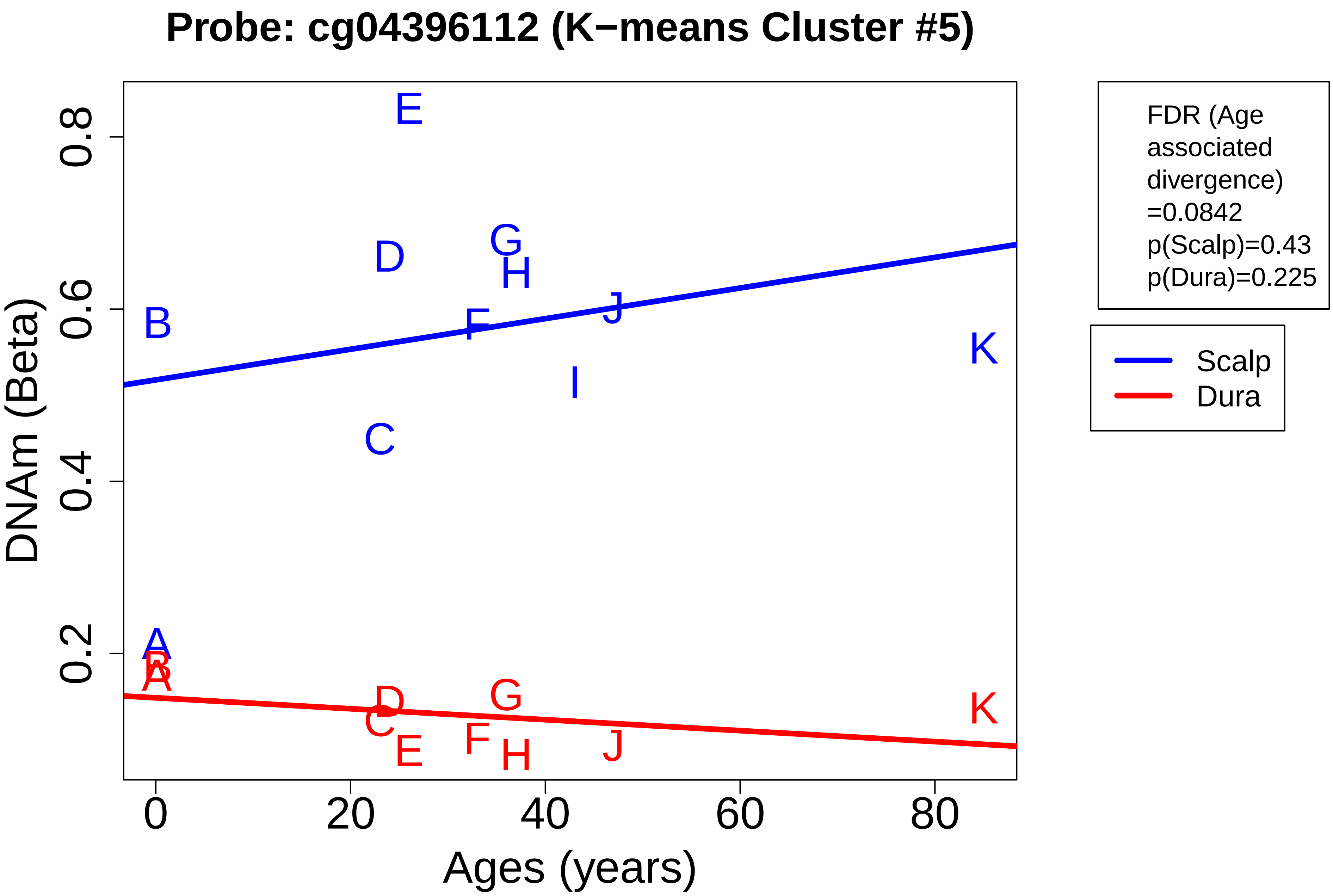

Probe: cg04396112 (K−means Cluster #5)
DNAm (Beta)
0.2
0.4
0.6
0.8
0
20
40
60
80
Ages (years)
E
D
G
H
B
F
J
K
I
C
A
B
A
D
G
C
K
F
E
H
J
FDR (Age associated divergence) =0.0842
p(Scalp)=0.43
p(Dura)=0.225
Scalp
Dura

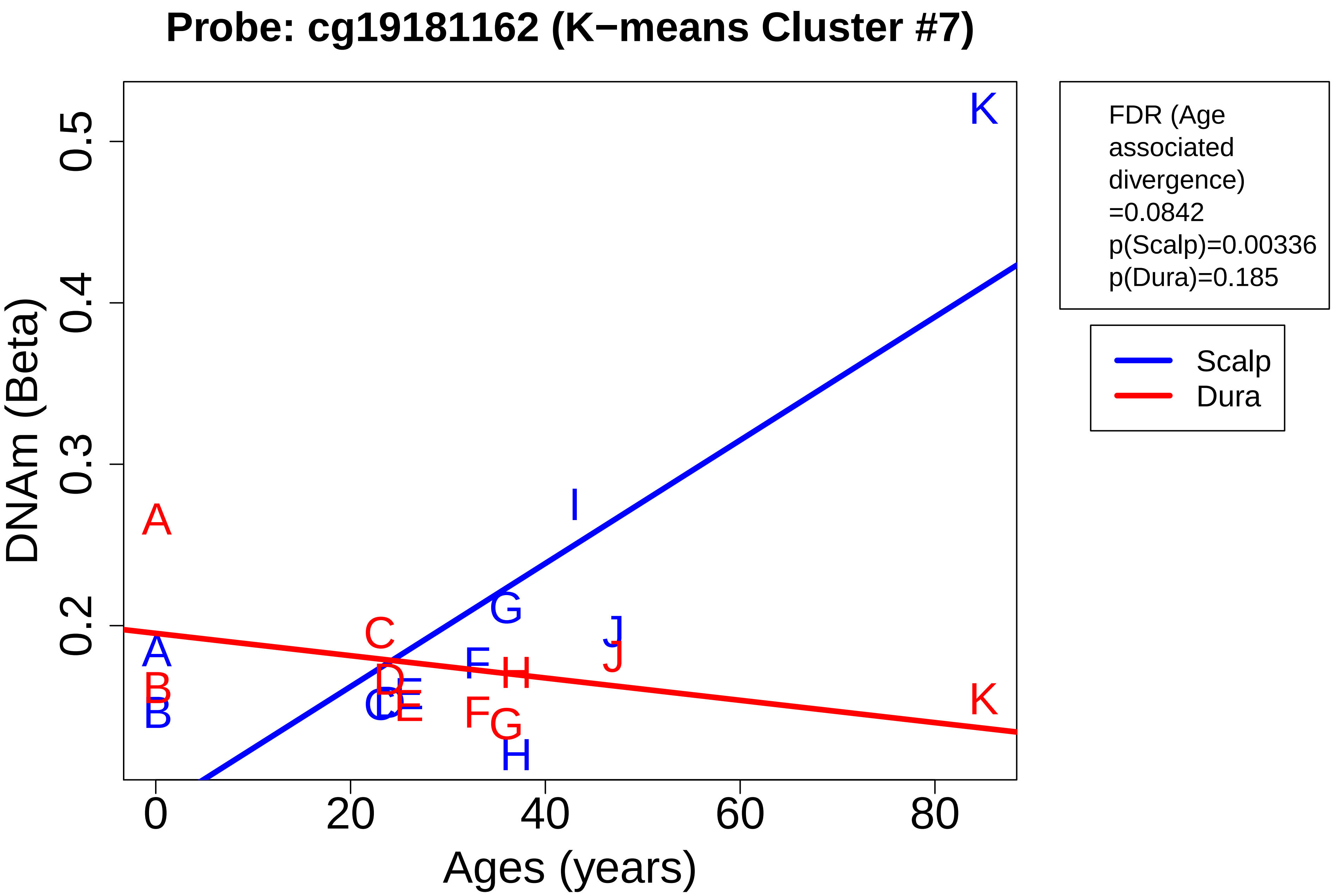

Probe: cg19181162 (K−means Cluster #7)
FDR (Age associated divergence) =0.0842
p(Scalp)=0.00336
p(Dura)=0.185
Scalp
Dura
DNAm (Beta)
0.2
0.3
0.4
0.5
0
20
40
60
80
Ages (years)

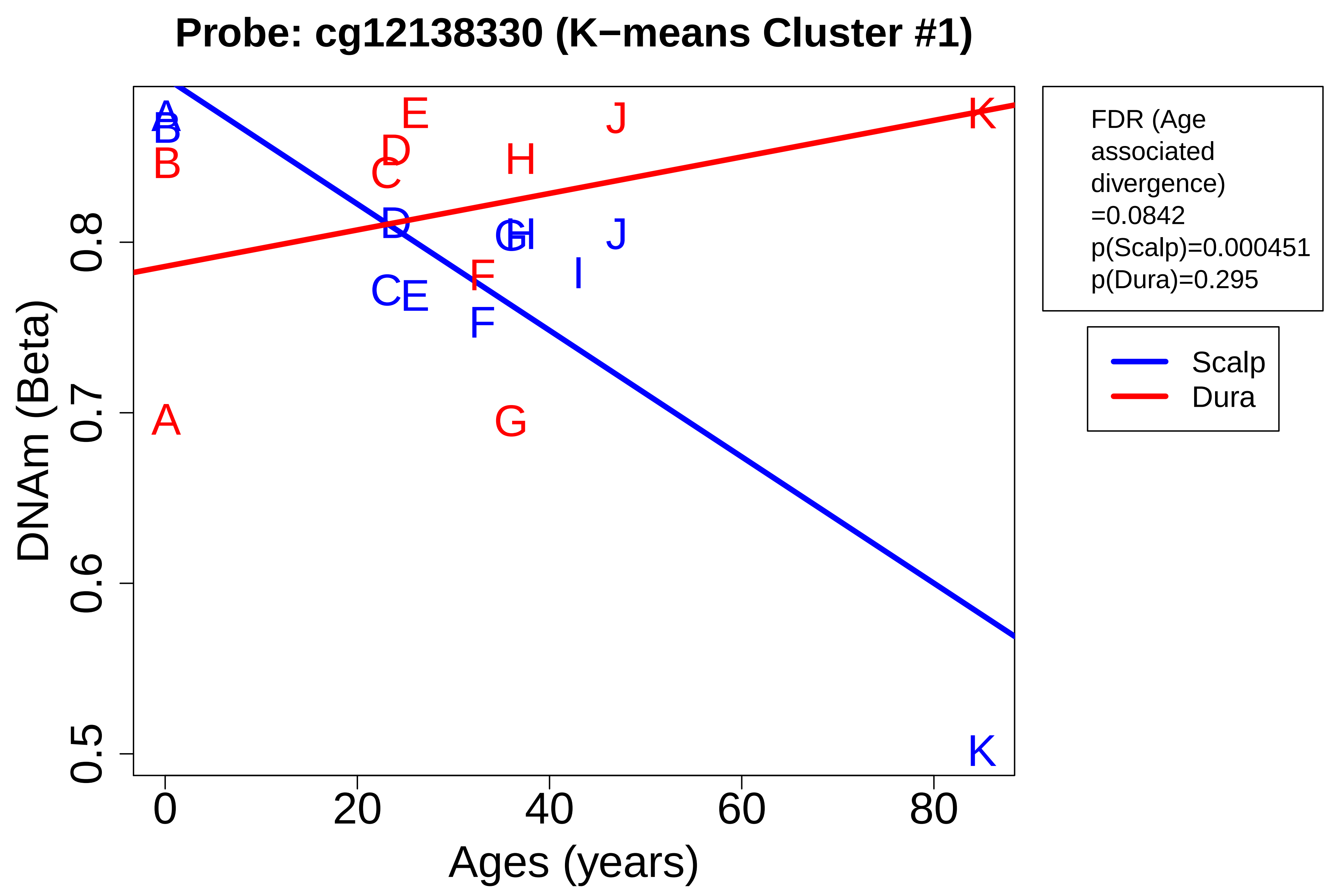

Probe: cg12138330 (K−means Cluster #1)
DNAm (Beta)
0.5
0.6
0.7
0.8
Ages (years)
0
20
40
60
80
FDR (Age associated divergence) =0.0842
p(Scalp)=0.000451
p(Dura)=0.295
Scalp
Dura

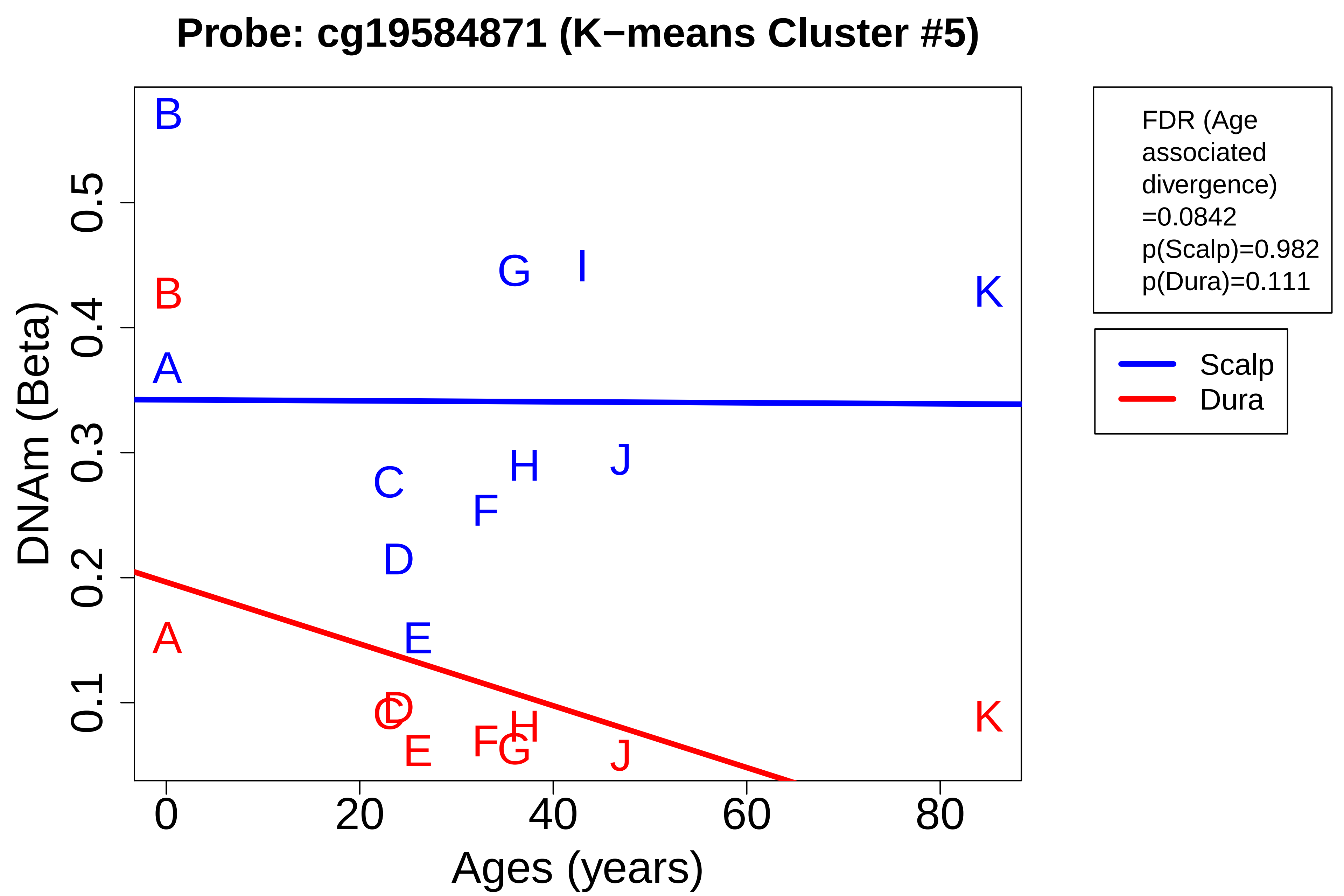

Probe: cg19584871 (K−means Cluster #5)
FDR (Age associated divergence) =0.0842
p(Scalp)=0.982
p(Dura)=0.111
Scalp
Dura
DNAm (Beta)
0.1
0.2
0.3
0.4
0.5
0
20
40
60
80
Ages (years)
A
B
C
D
E
F
G
H
I
J
K

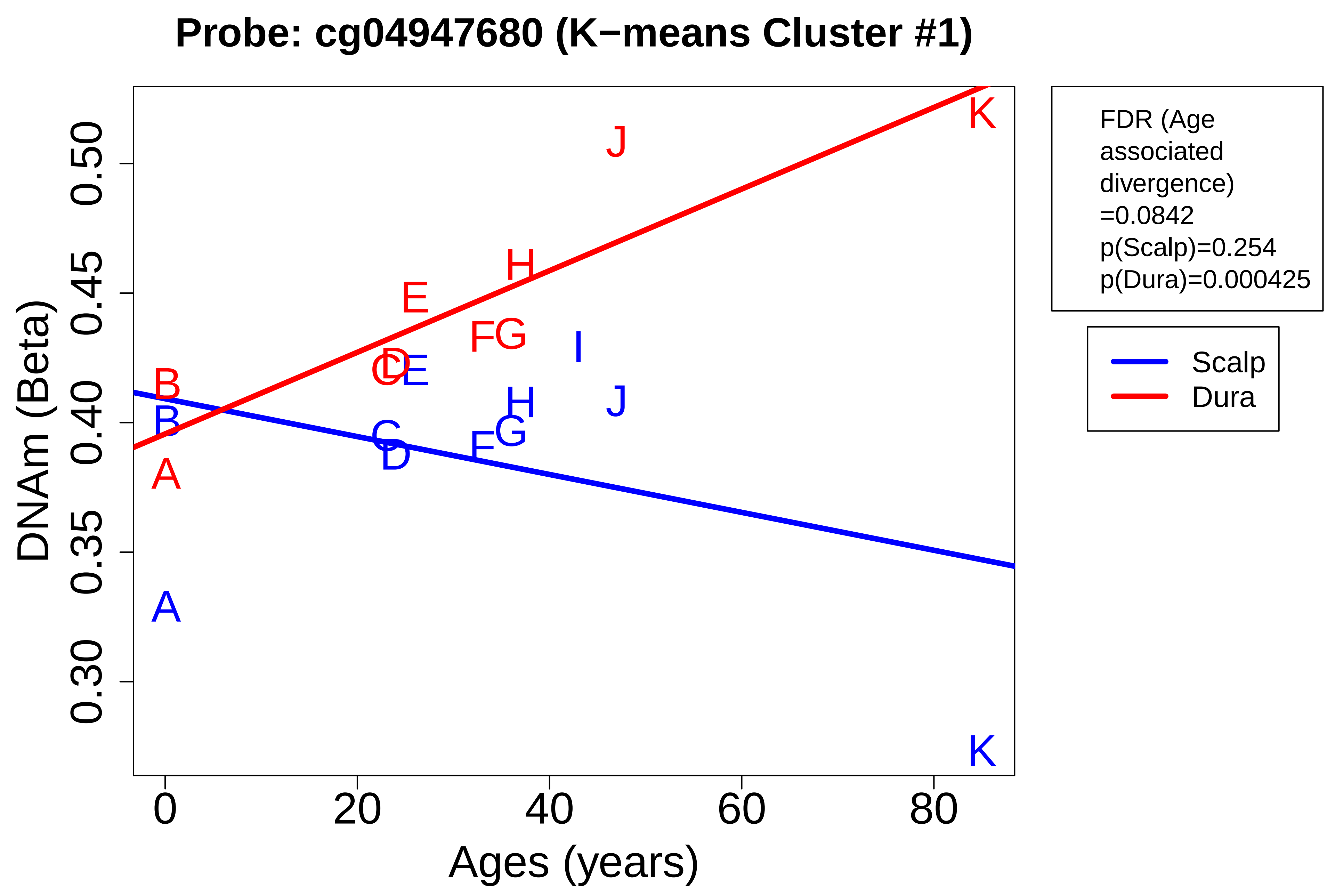

Probe: cg04947680 (K−means Cluster #1)
FDR (Age associated divergence) =0.0842
p(Scalp)=0.254
p(Dura)=0.000425
Scalp
Dura
DNAm (Beta)
0.30
0.35
0.40
0.45
0.50
0
20
40
60
80
Ages (years)

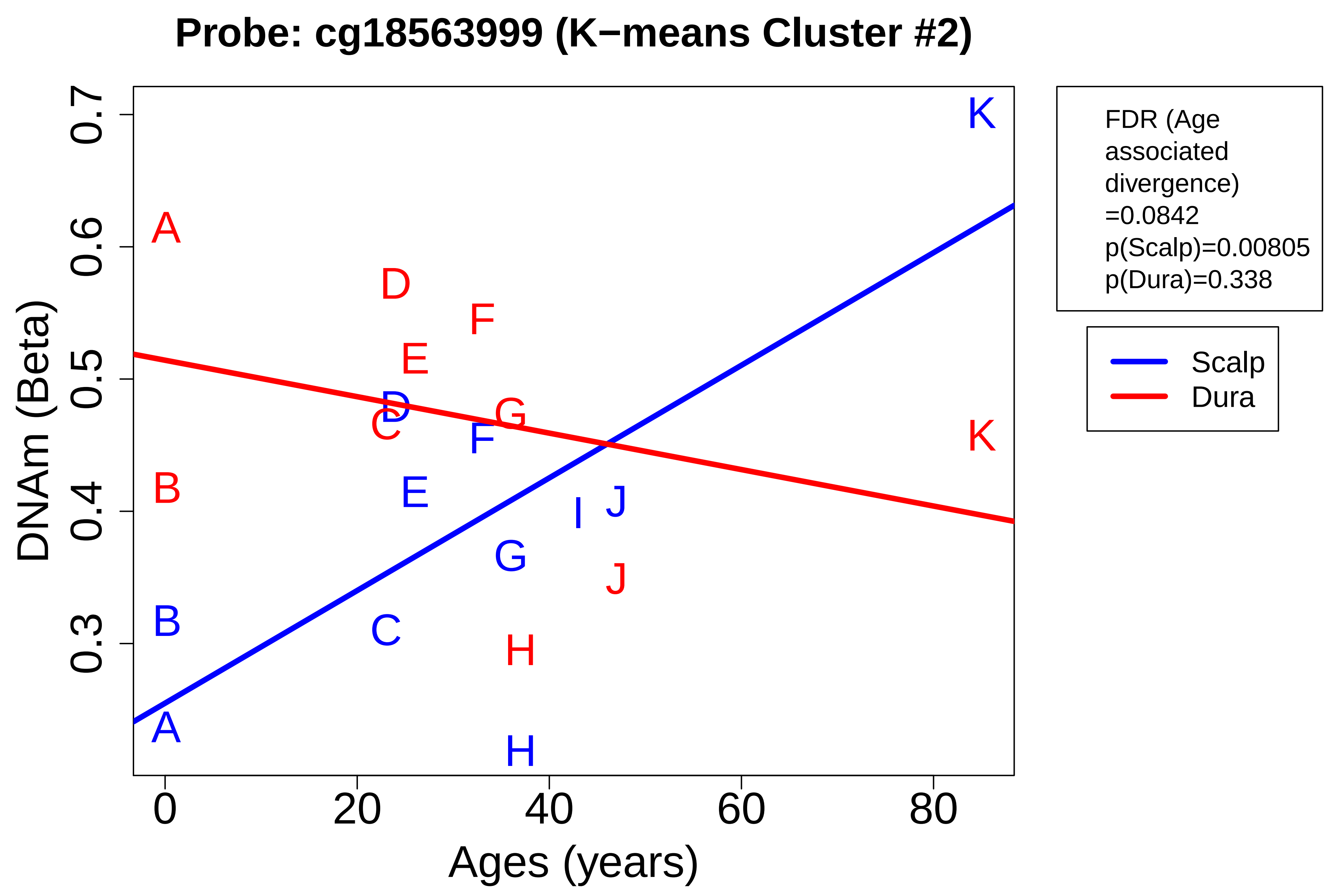

Probe: cg18563999 (K−means Cluster #2)
FDR (Age associated divergence) =0.0842
p(Scalp)=0.00805
p(Dura)=0.338
Scalp
Dura
DNAm (Beta)
0.3
0.4
0.5
0.6
0.7
0
20
40
60
80
Ages (years)
A
B
C
D
E
F
G
H
I
J
K

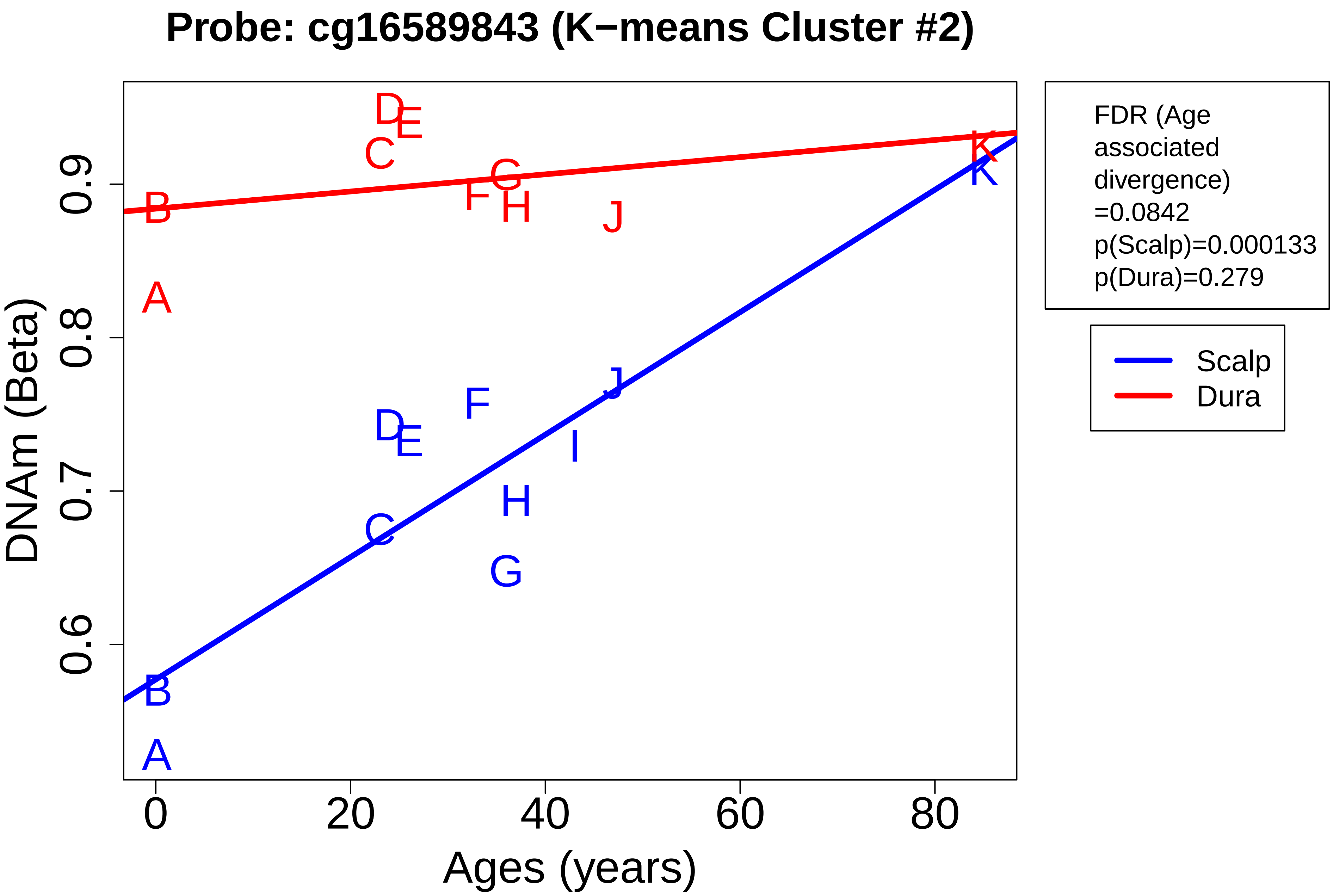

Probe: cg16589843 (K−means Cluster #2)
DNAm (Beta)
0.6
0.7
0.8
0.9
0
20
40
60
80
Ages (years)
FDR (Age associated divergence) =0.0842
p(Scalp)=0.000133
p(Dura)=0.279
Scalp
Dura
A
B
C
D
E
F
G
H
I
J
K

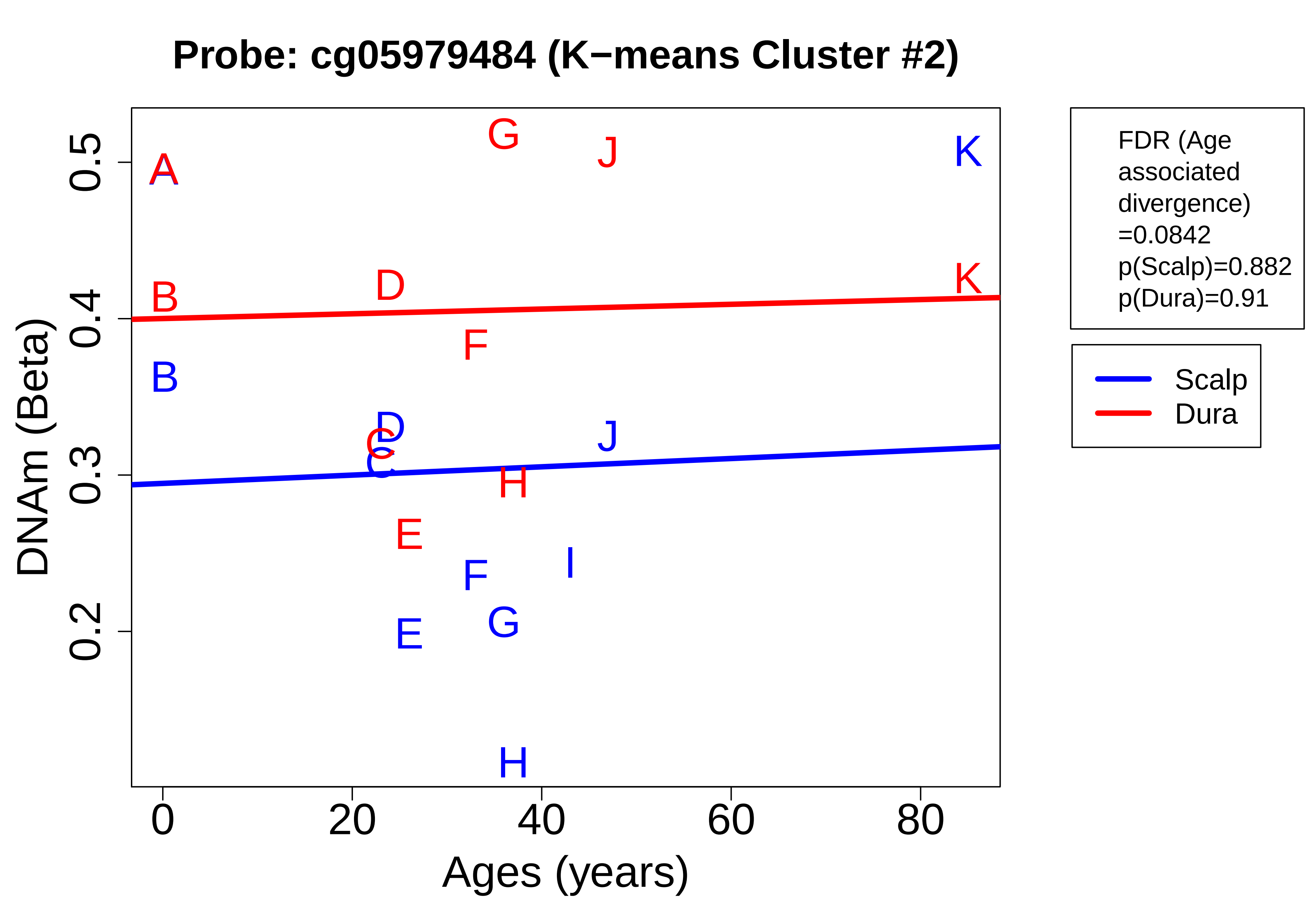

Probe: cg05979484 (K−means Cluster #2)
DNAm (Beta)
0.2
0.3
0.4
0.5
0
20
40
60
80
Ages (years)
A
B
C
D
E
F
G
H
I
J
K
FDR (Age associated divergence) =0.0842
p(Scalp)=0.882
p(Dura)=0.91
Scalp
Dura

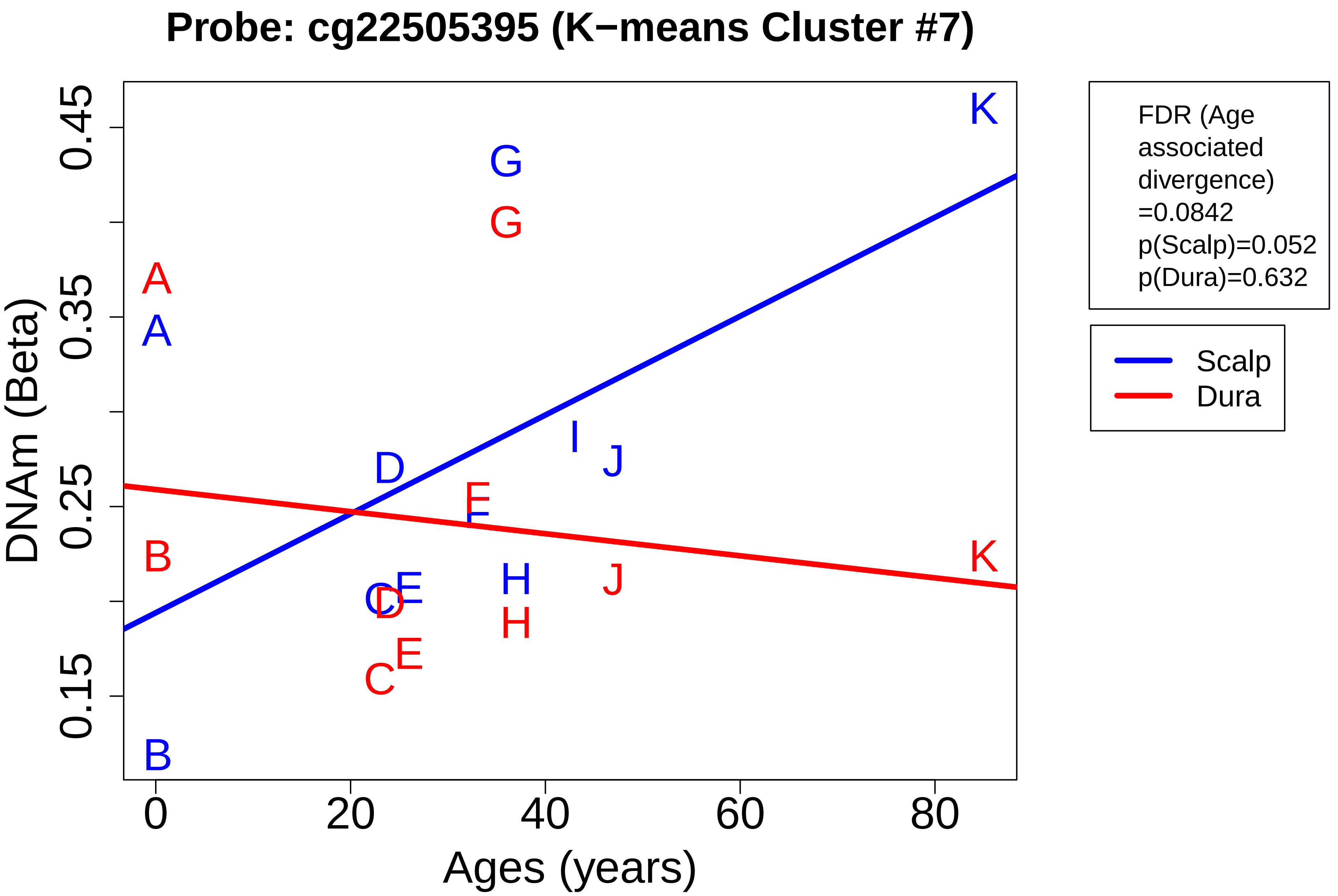

Probe: cg22505395 (K−means Cluster #7)
DNAm (Beta)
0.15
0.25
0.35
0.45
Ages (years)
0
20
40
60
80
FDR (Age associated divergence) =0.0842
p(Scalp)=0.052
p(Dura)=0.632
Scalp
Dura

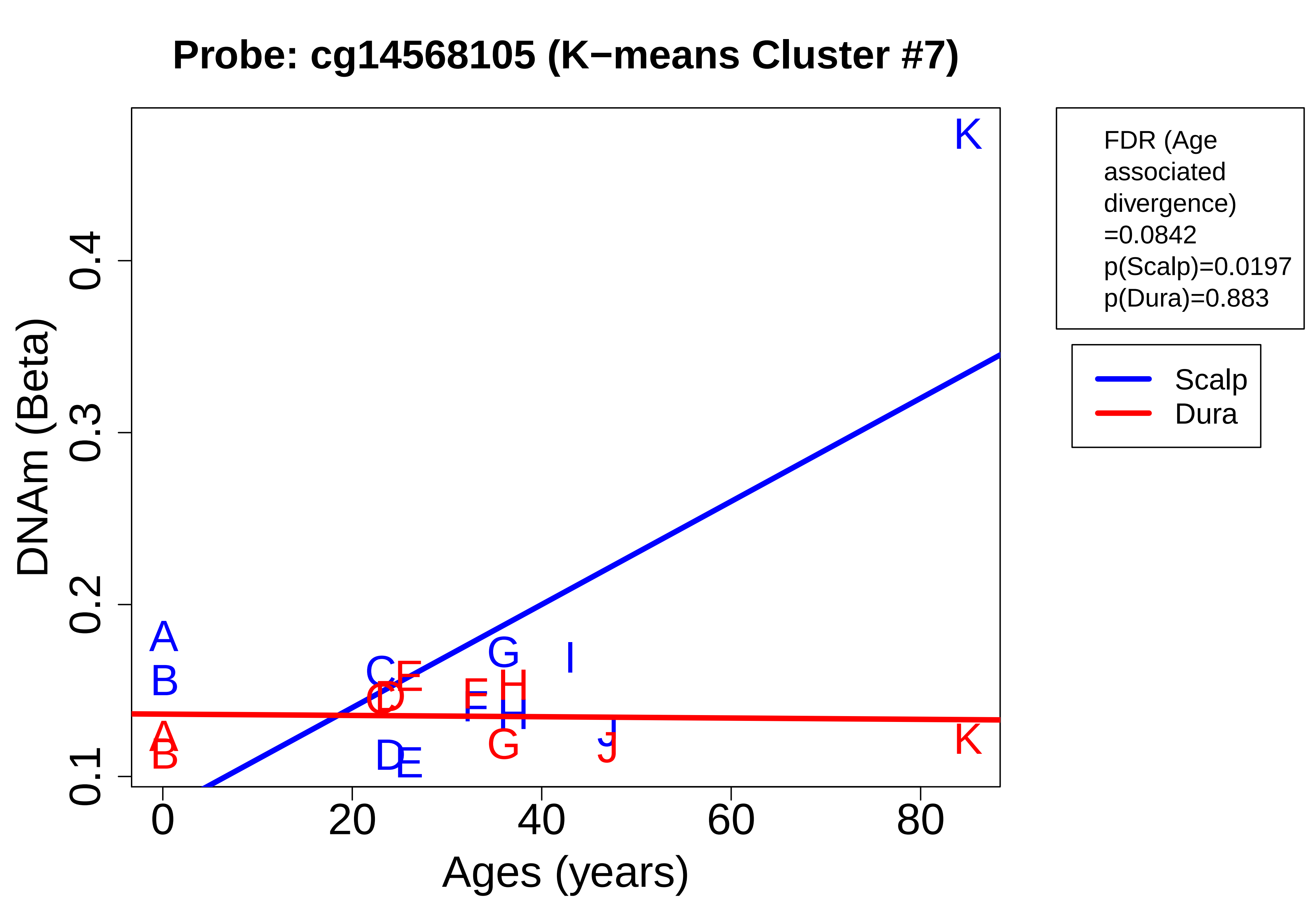

Probe: cg14568105 (K−means Cluster #7)
DNAm (Beta)
Ages (years)
0.1
0.2
0.3
0.4
0
20
40
60
80
FDR (Age associated divergence) =0.0842
p(Scalp)=0.0197
p(Dura)=0.883
Scalp
Dura

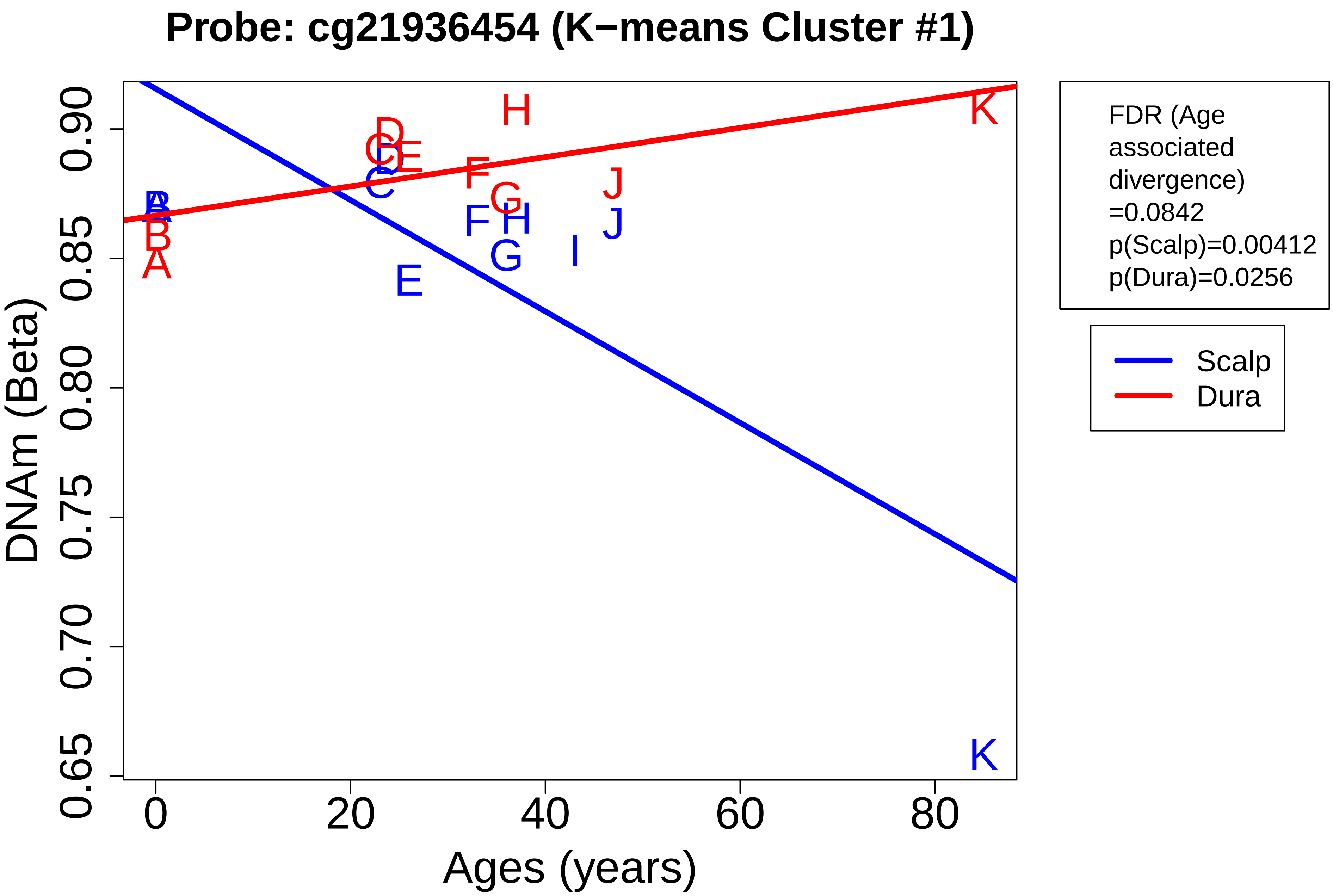
Probe: cg21936454 (K−means Cluster #1)
DNAm (Beta)
0.65
0.70
0.75
0.80
0.85
0.90
0
20
40
60
80
Ages (years)
FDR (Age associated divergence) =0.0842
p(Scalp)=0.00412
p(Dura)=0.0256
Scalp
Dura

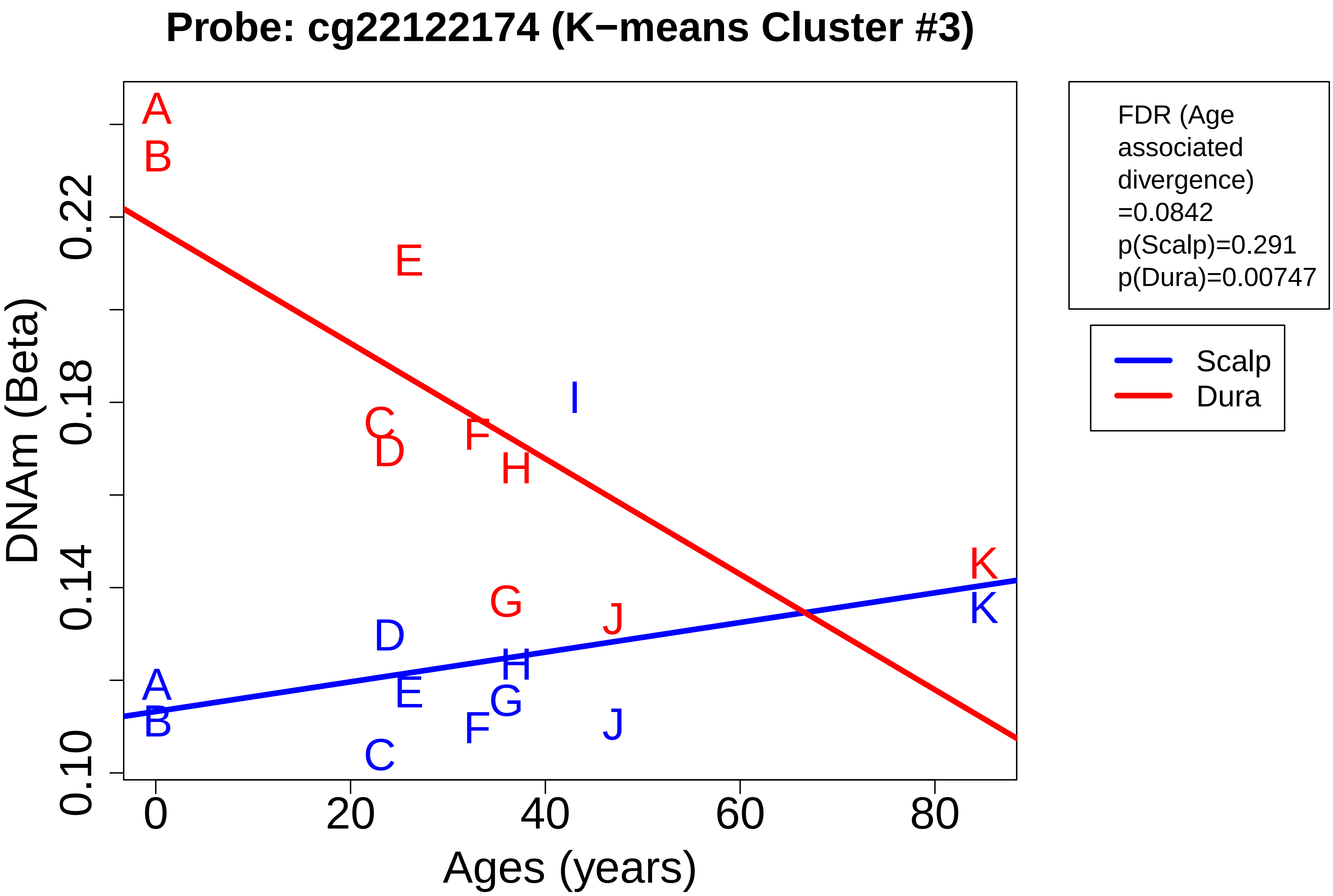

Probe: cg22122174 (K−means Cluster #3)
FDR (Age associated divergence) =0.0842
p(Scalp)=0.291
p(Dura)=0.00747
Scalp
Dura
DNAm (Beta)
0.10
0.14
0.18
0.22
0
20
40
60
80
Ages (years)
A
B
C
D
E
F
G
H
I
J
K

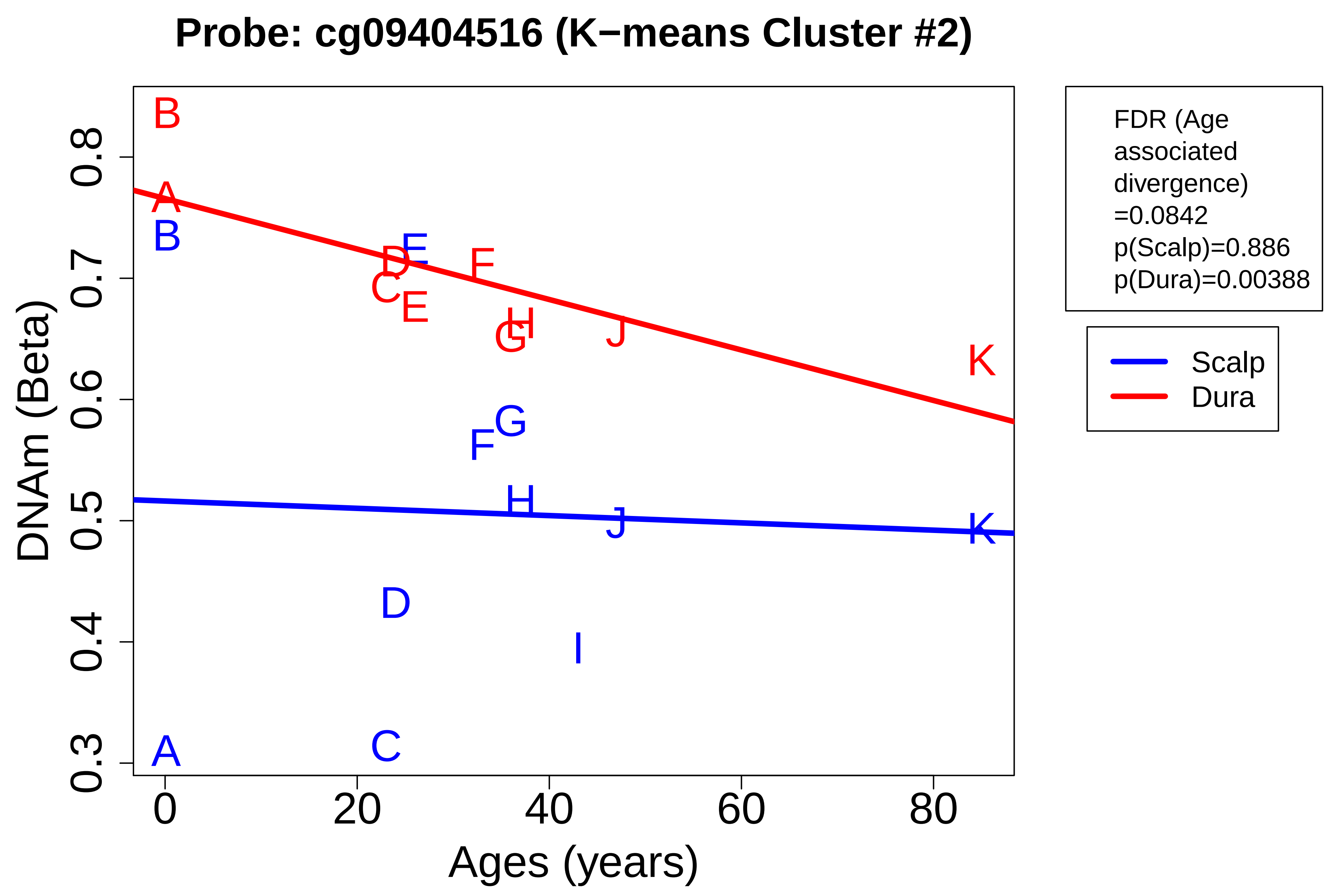

Probe: cg09404516 (K−means Cluster #2)
DNAm (Beta)
0.3
0.4
0.5
0.6
0.7
0.8
Ages (years)
0
20
40
60
80
FDR (Age associated divergence) =0.0842
p(Scalp)=0.886
p(Dura)=0.00388
Scalp
Dura

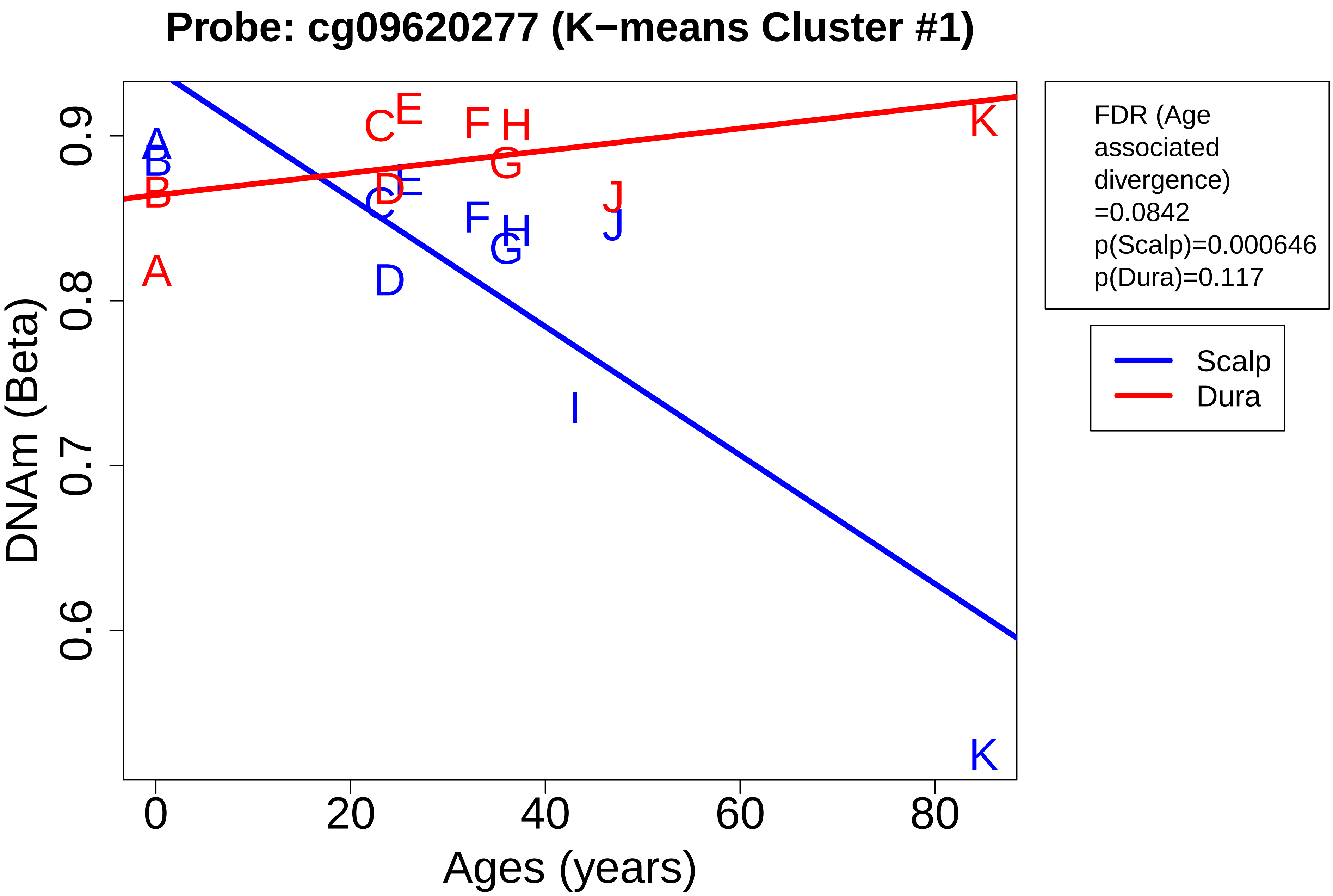

Probe: cg09620277 (K−means Cluster #1)
DNAm (Beta)
Ages (years)
0.6
0.7
0.8
0.9
0
20
40
60
80
FDR (Age associated divergence) =0.0842
p(Scalp)=0.000646
p(Dura)=0.117
Scalp
Dura

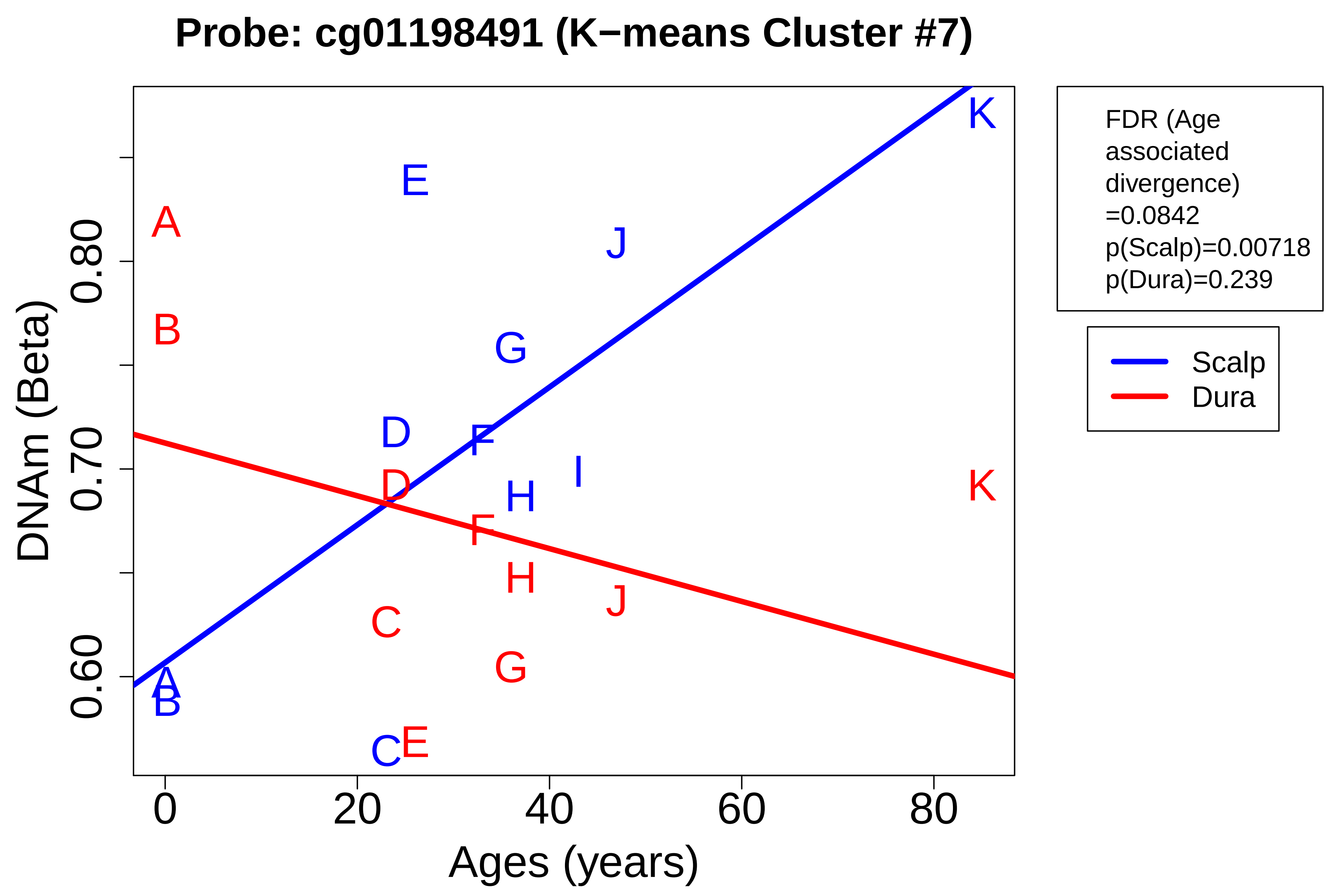
Probe: cg01198491 (K−means Cluster #7)
DNAm (Beta)
0.60
0.70
0.80
0
20
40
60
80
Ages (years)
A
B
C
D
E
F
G
H
I
J
K
FDR (Age associated divergence) =0.0842
p(Scalp)=0.00718
p(Dura)=0.239
Scalp
Dura

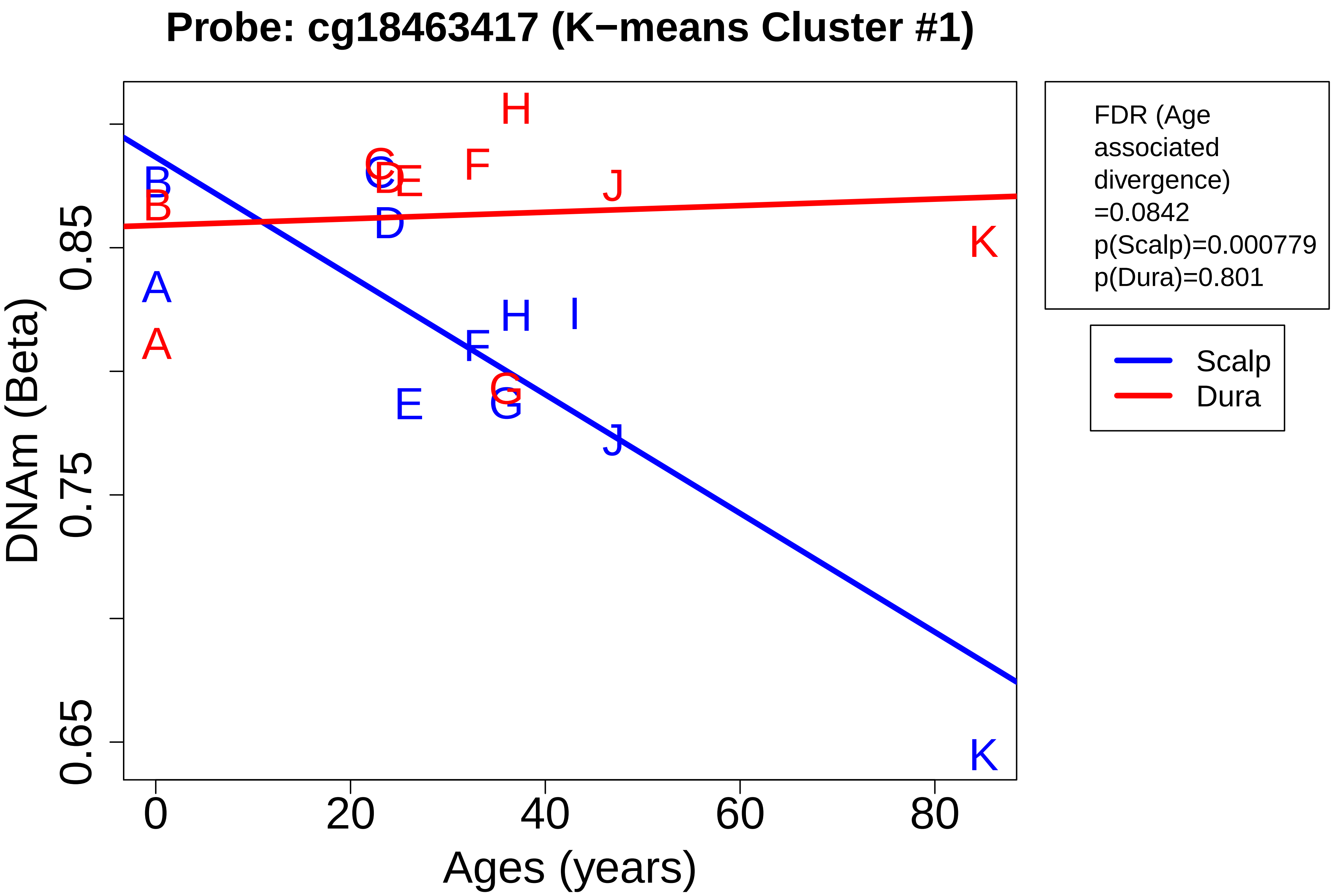
Probe: cg18463417 (K−means Cluster #1)
FDR (Age associated divergence) =0.0842
p(Scalp)=0.000779
p(Dura)=0.801
Scalp
Dura
DNAm (Beta)
0.65
0.75
0.85
0
20
40
60
80
Ages (years)
A
B
C
D
E
F
G
H
I
J
K

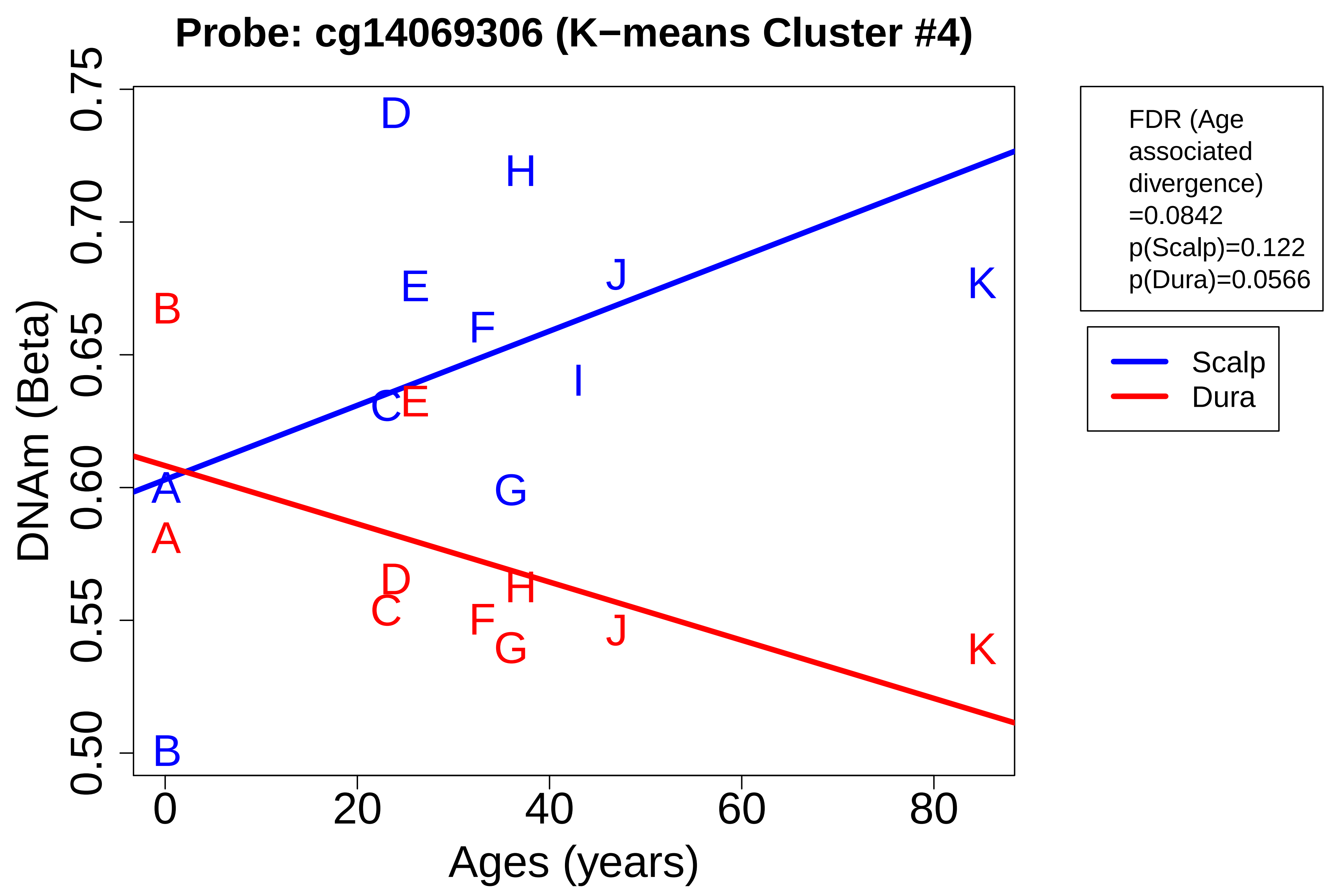

Probe: cg14069306 (K−means Cluster #4)
DNAm (Beta)
0.50
0.55
0.60
0.65
0.70
0.75
0
20
40
60
80
Ages (years)
A
B
C
D
E
F
G
H
I
J
K
FDR (Age associated divergence) =0.0842
p(Scalp)=0.122
p(Dura)=0.0566
Scalp
Dura

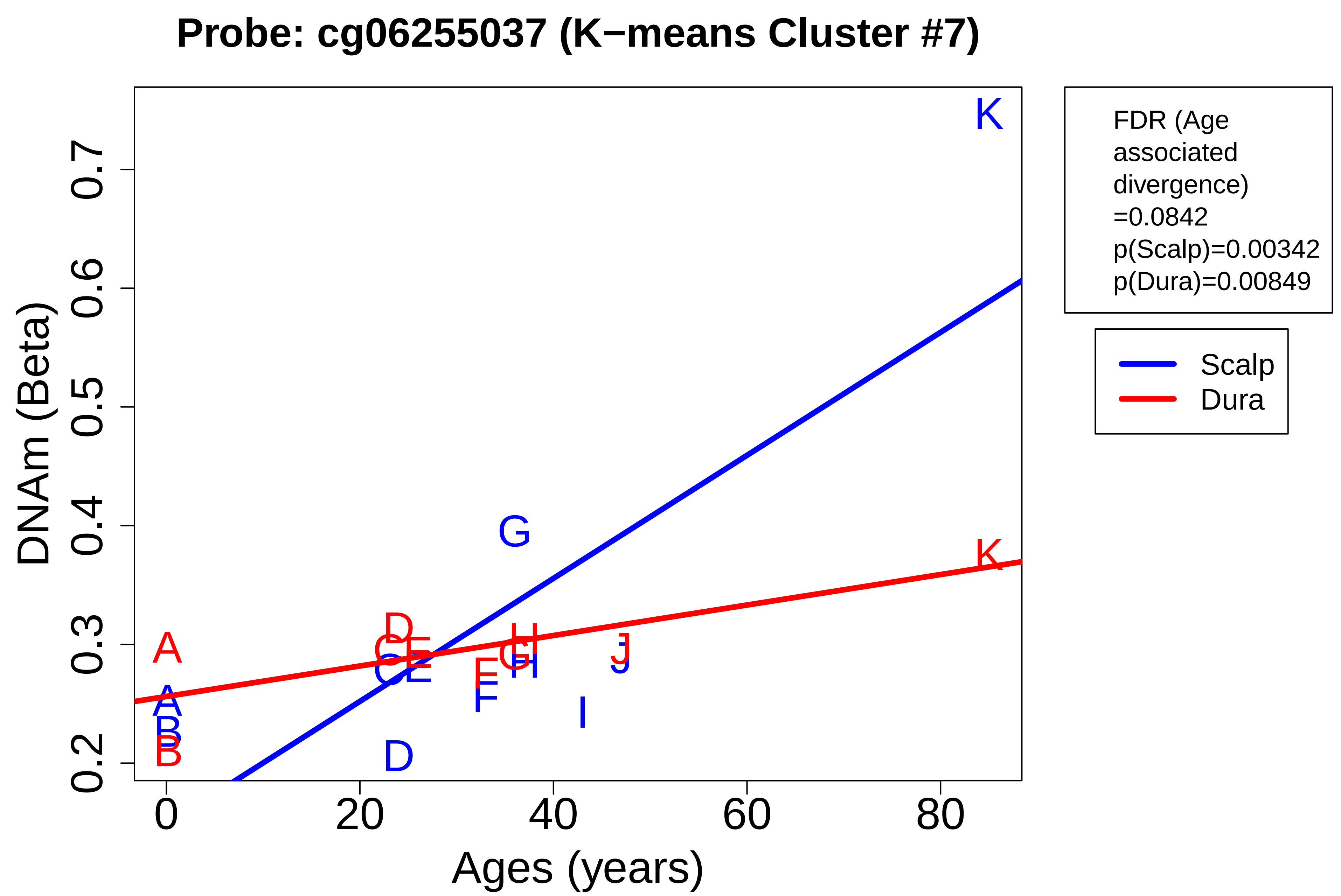

Probe: cg06255037 (K−means Cluster #7)
DNAm (Beta)
0.2
0.3
0.4
0.5
0.6
0.7
Ages (years)
0
20
40
60
80
FDR (Age associated divergence) =0.0842
p(Scalp)=0.00342
p(Dura)=0.00849
Scalp
Dura
A
B
C
D
E
F
G
H
I
J
K

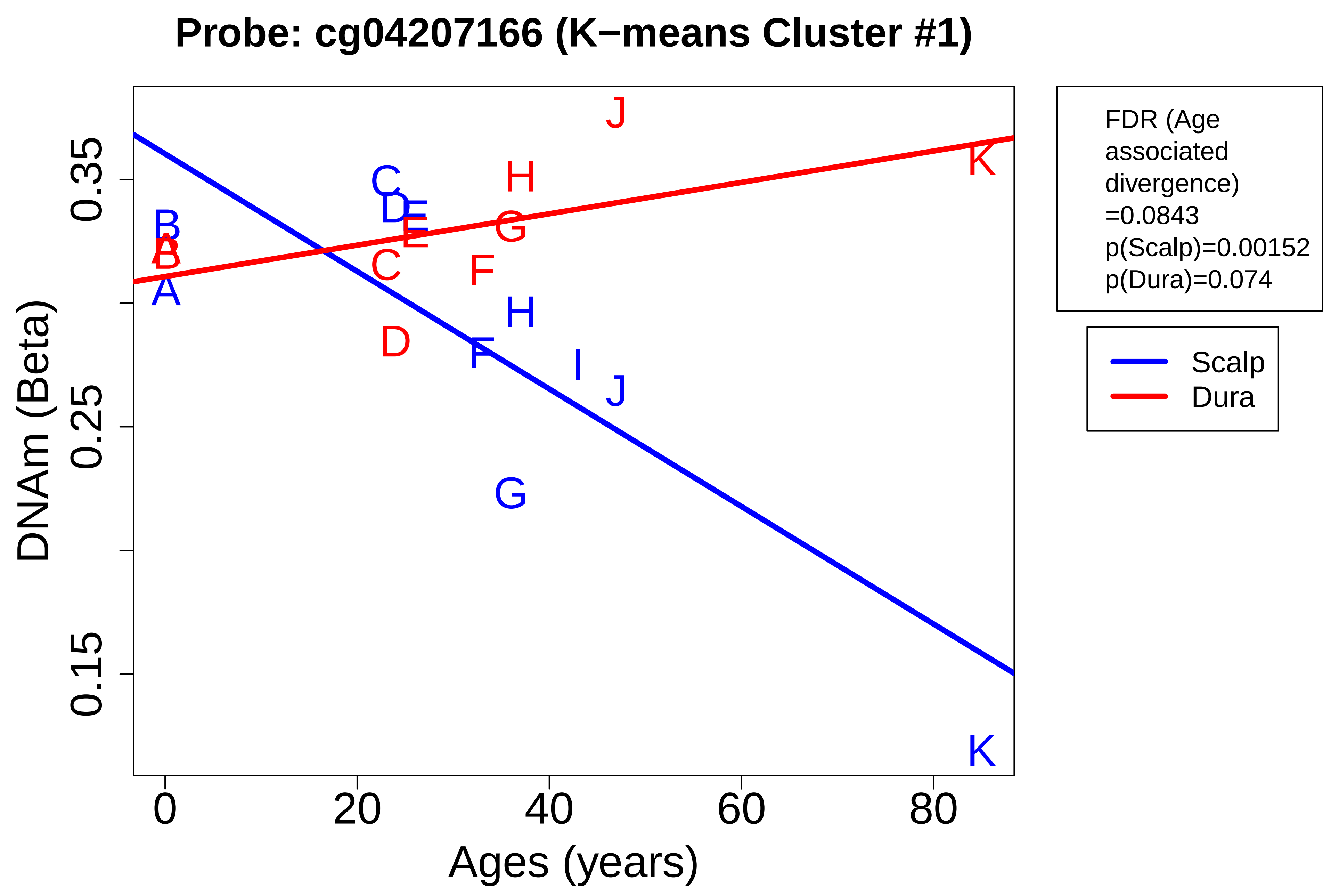

Probe: cg04207166 (K−means Cluster #1)
FDR (Age associated divergence) =0.0843
p(Scalp)=0.00152
p(Dura)=0.074
Scalp
Dura
DNAm (Beta)
0.15
0.25
0.35
0
20
40
60
80
Ages (years)
A
B
C
D
E
F
G
H
I
J
K

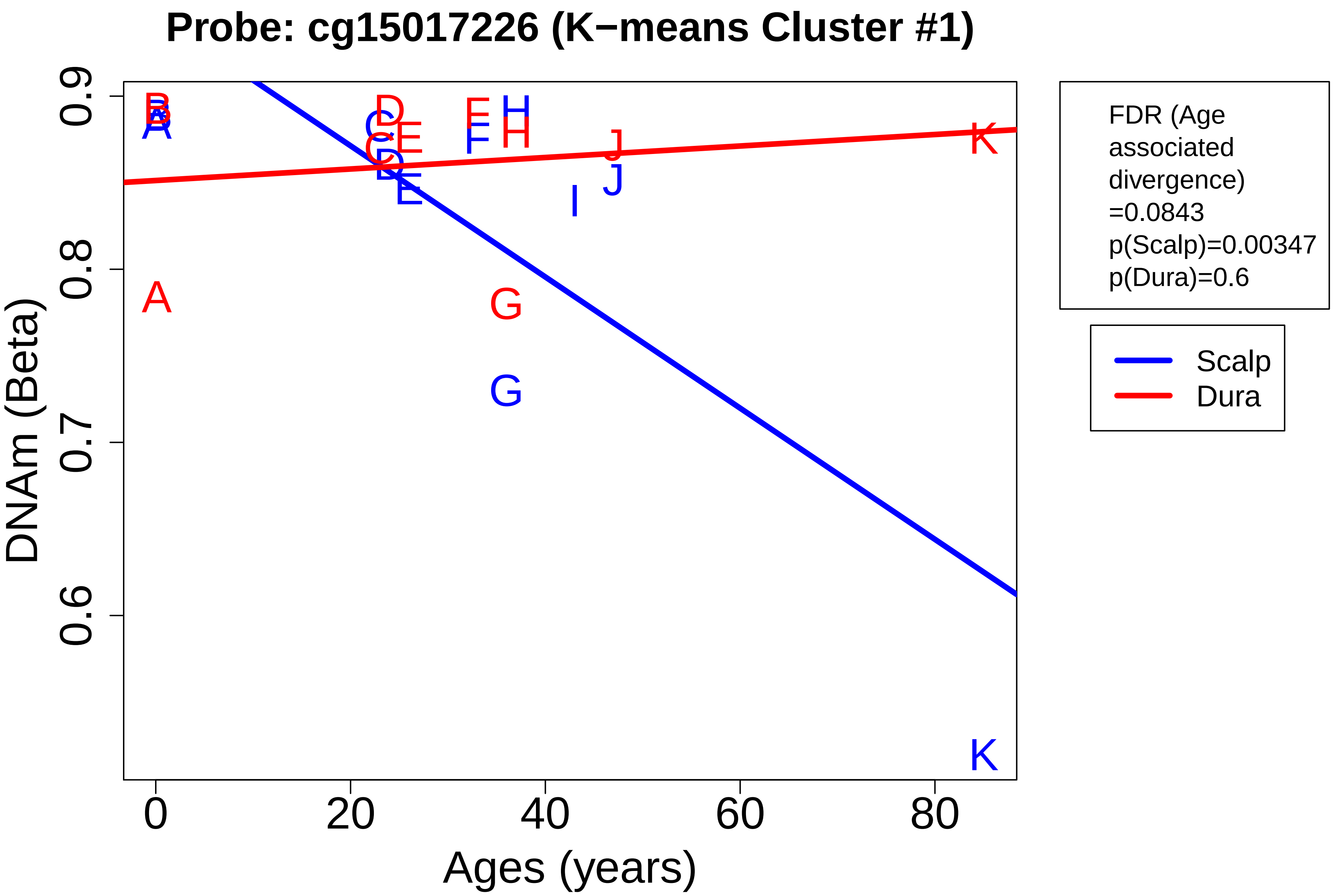

Probe: cg15017226 (K−means Cluster #1)
DNAm (Beta)
0.6
0.7
0.8
0.9
0
20
40
60
80
Ages (years)
FDR (Age associated divergence) =0.0843
p(Scalp)=0.00347
p(Dura)=0.6
Scalp
Dura

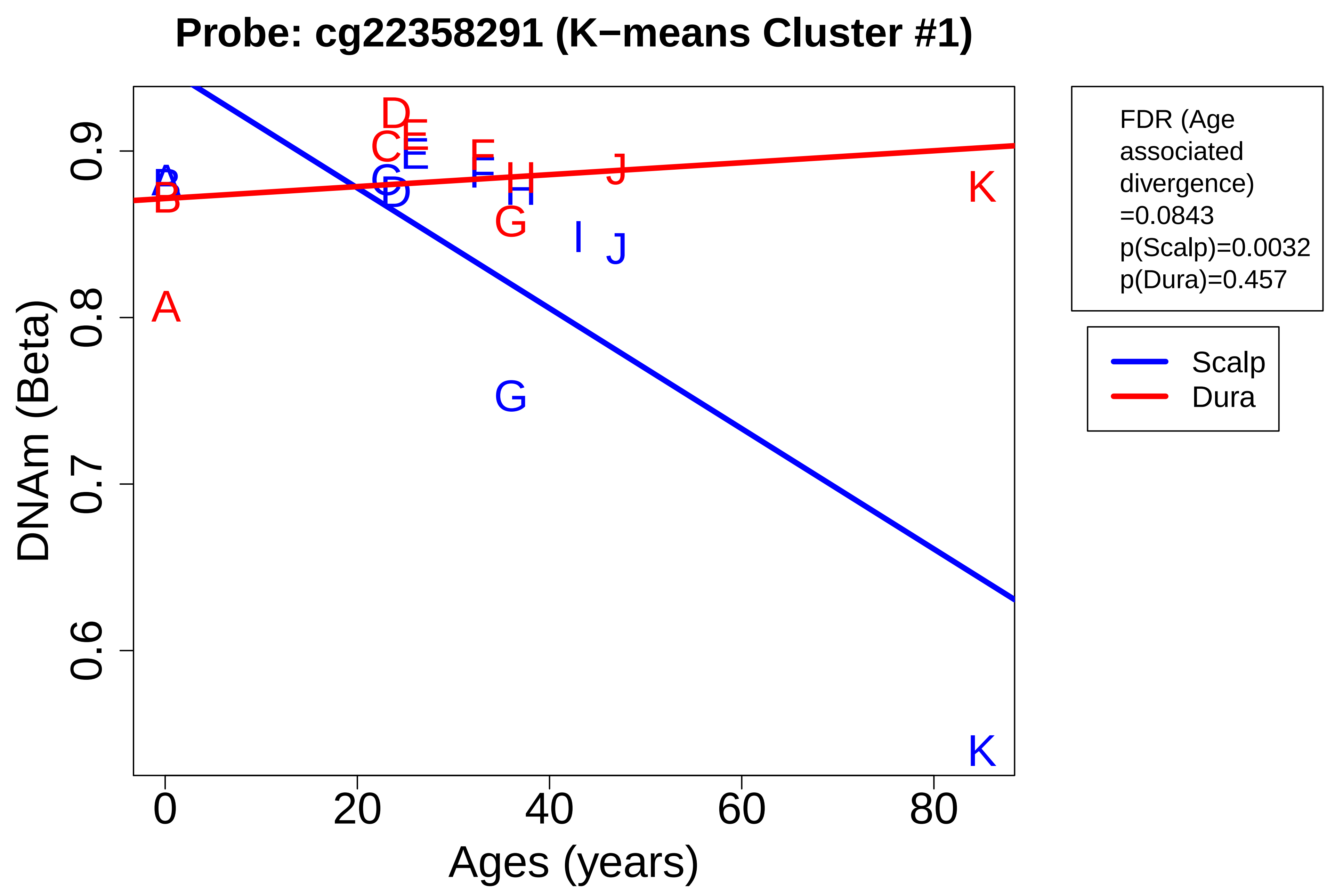

Probe: cg22358291 (K−means Cluster #1)
DNAm (Beta)
0.6
0.7
0.8
0.9
0
20
40
60
80
Ages (years)
A
B
C
D
E
F
G
H
I
J
K
FDR (Age associated divergence) =0.0843
p(Scalp)=0.0032
p(Dura)=0.457
Scalp
Dura

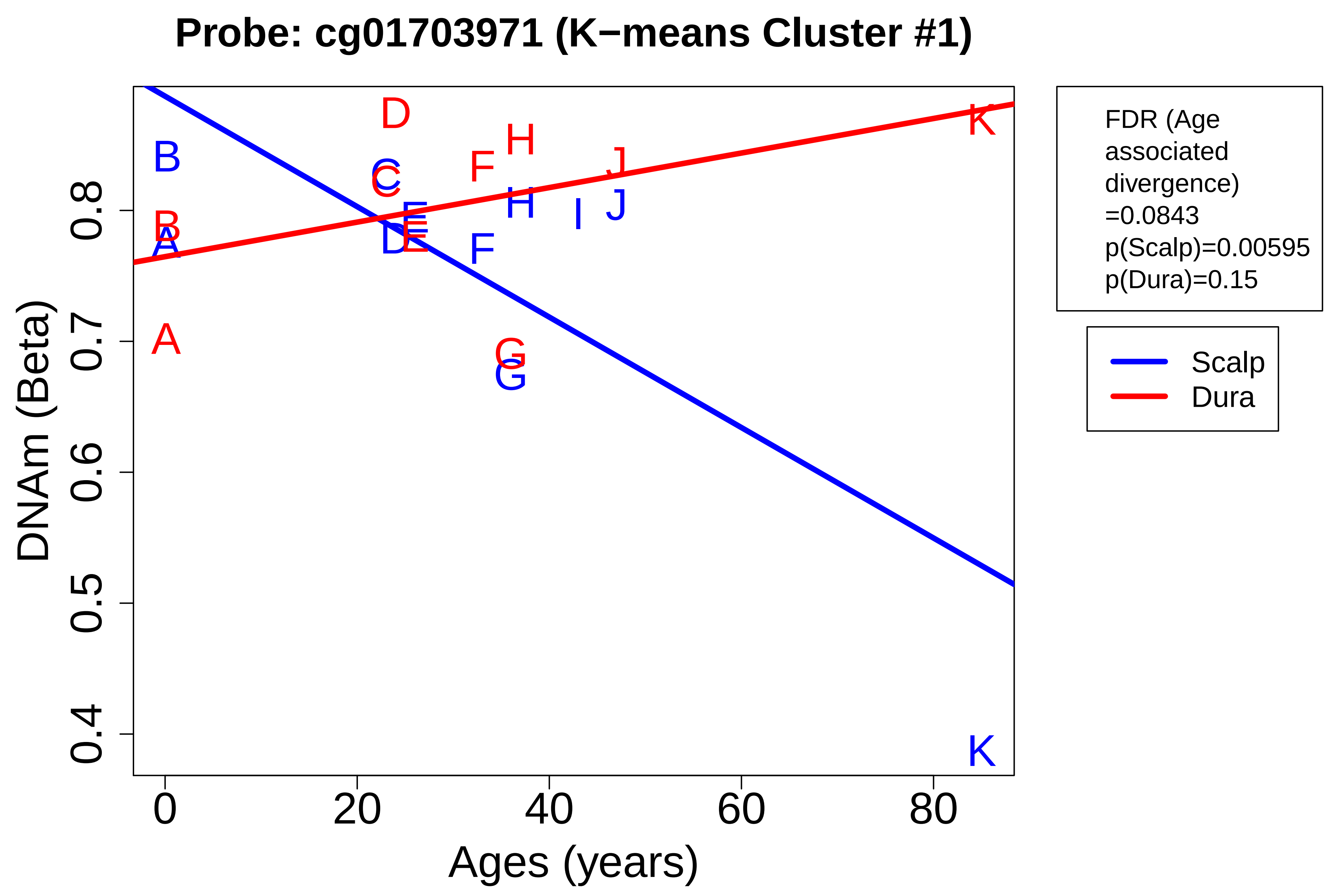

Probe: cg01703971 (K−means Cluster #1)
DNAm (Beta)
Ages (years)
0.4
0.5
0.6
0.7
0.8
0
20
40
60
80
FDR (Age associated divergence) =0.0843
p(Scalp)=0.00595
p(Dura)=0.15
Scalp
Dura

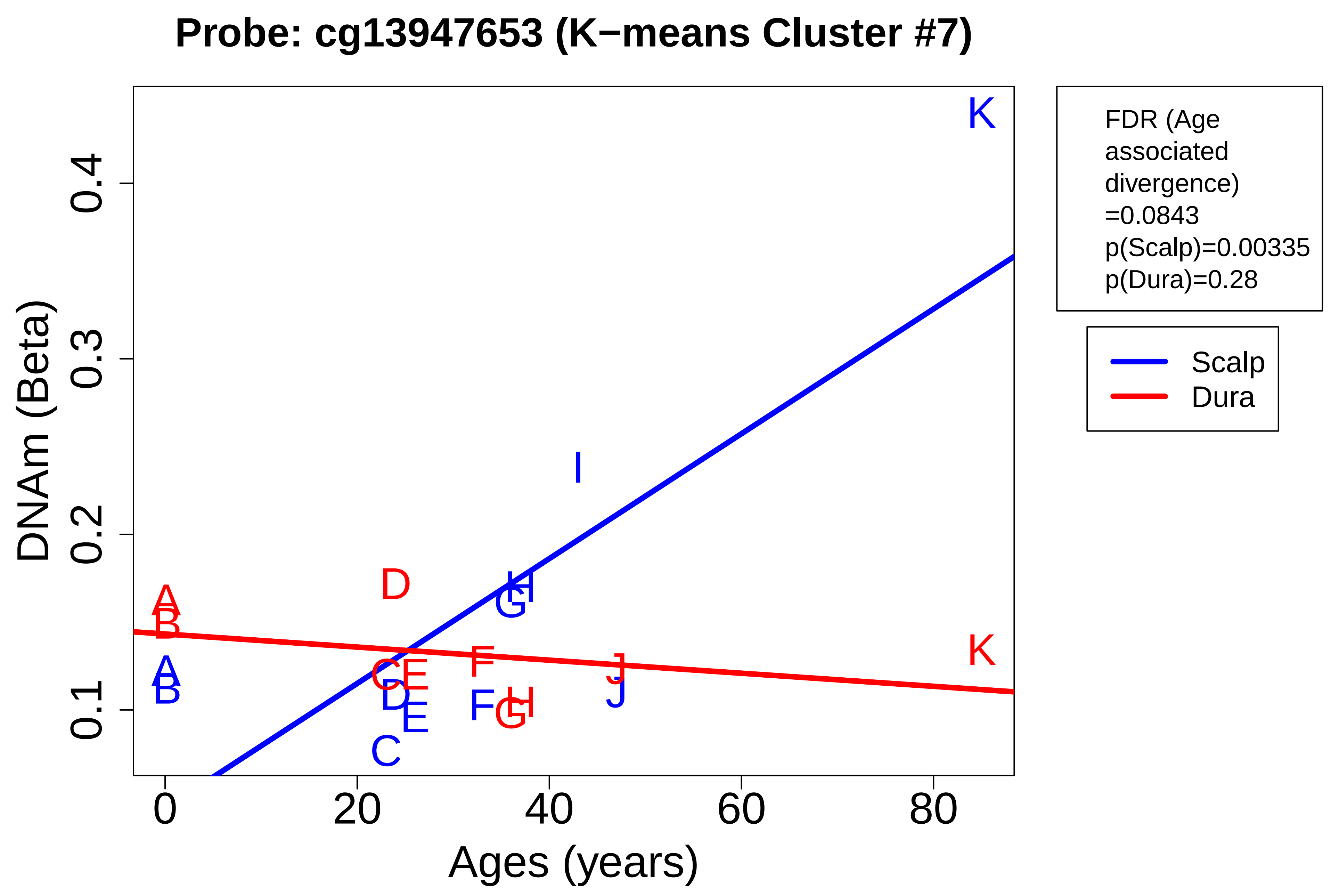

Probe: cg13947653 (K−means Cluster #7)
DNAm (Beta)
Ages (years)
0.1
0.2
0.3
0.4
0
20
40
60
80
FDR (Age associated divergence) =0.0843
p(Scalp)=0.00335
p(Dura)=0.28
Scalp
Dura
A
B
C
D
E
F
G
H
I
J
K

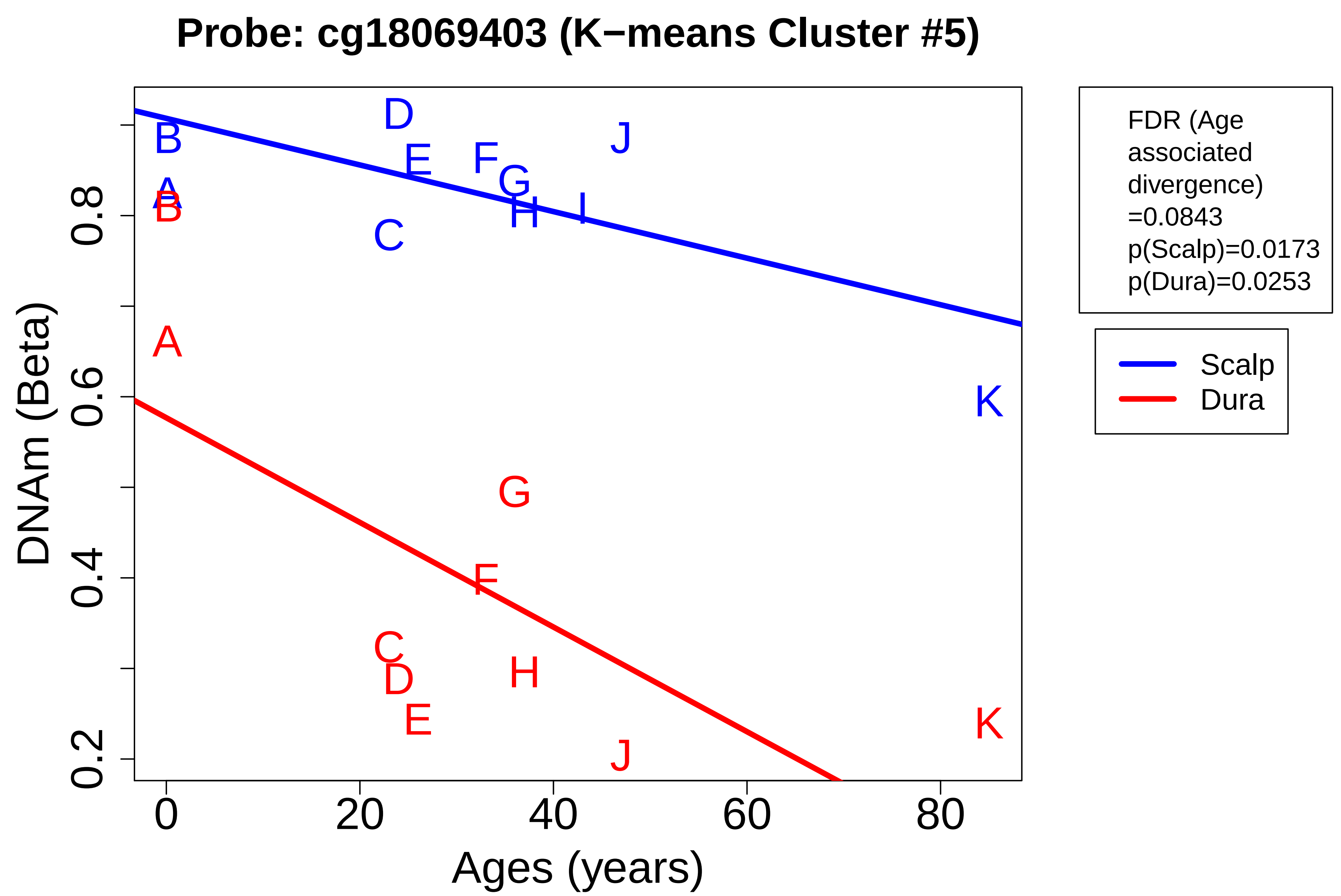

Probe: cg18069403 (K−means Cluster #5)
DNAm (Beta)
0.2
0.4
0.6
0.8
Ages (years)
0
20
40
60
80
FDR (Age associated divergence) =0.0843
p(Scalp)=0.0173
p(Dura)=0.0253
Scalp
Dura

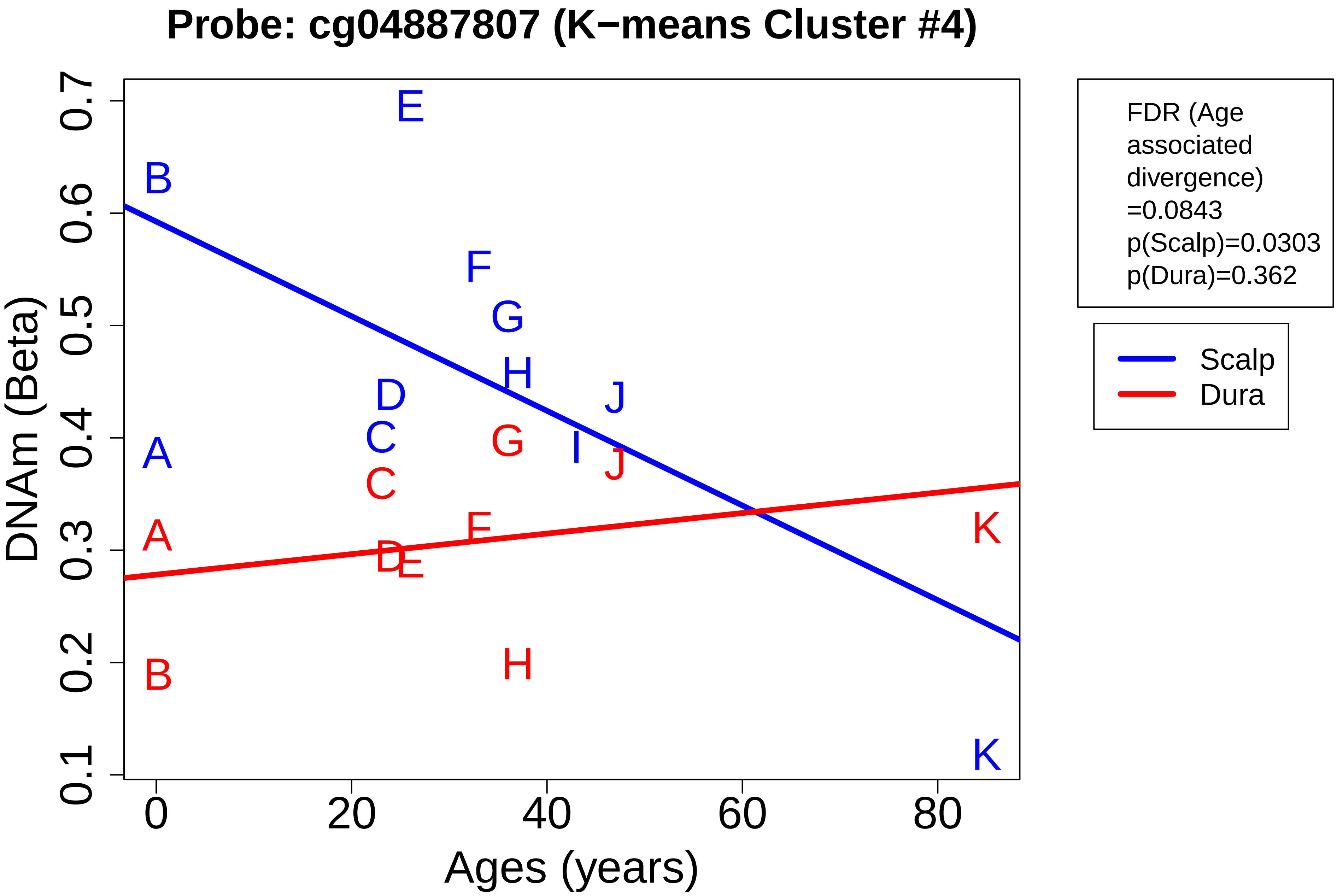

Probe: cg04887807 (K−means Cluster #4)
DNAm (Beta)
0.1
0.2
0.3
0.4
0.5
0.6
0.7
Ages (years)
0
20
40
60
80
A
B
C
D
E
F
G
H
I
J
K
FDR (Age associated divergence) =0.0843
p(Scalp)=0.0303
p(Dura)=0.362
Scalp
Dura

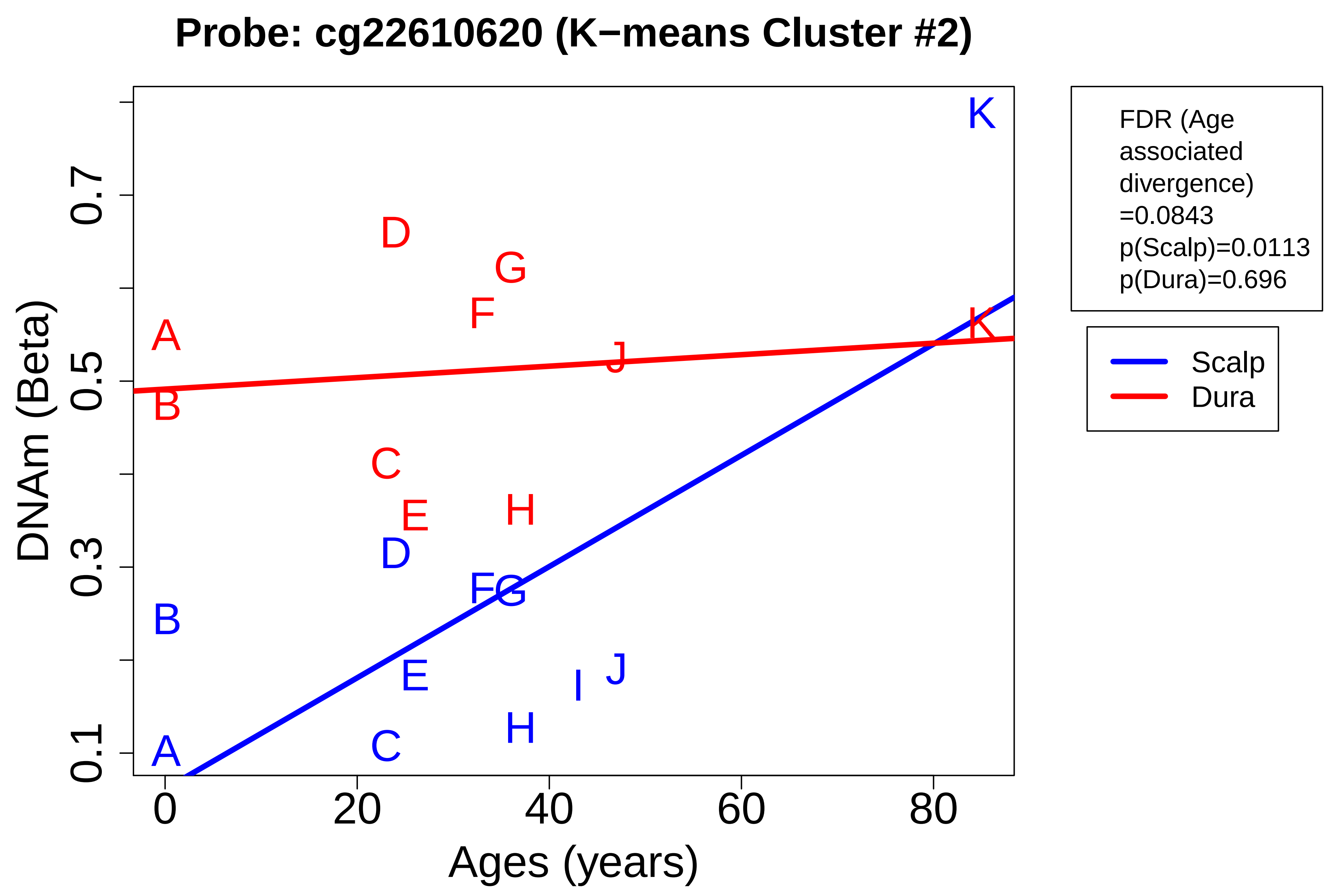

Probe: cg22610620 (K−means Cluster #2)
DNAm (Beta)
0.1
0.3
0.5
0.7
0
20
40
60
80
Ages (years)
A
B
C
D
E
F
G
H
I
J
K
FDR (Age associated divergence) =0.0843
p(Scalp)=0.0113
p(Dura)=0.696
Scalp
Dura

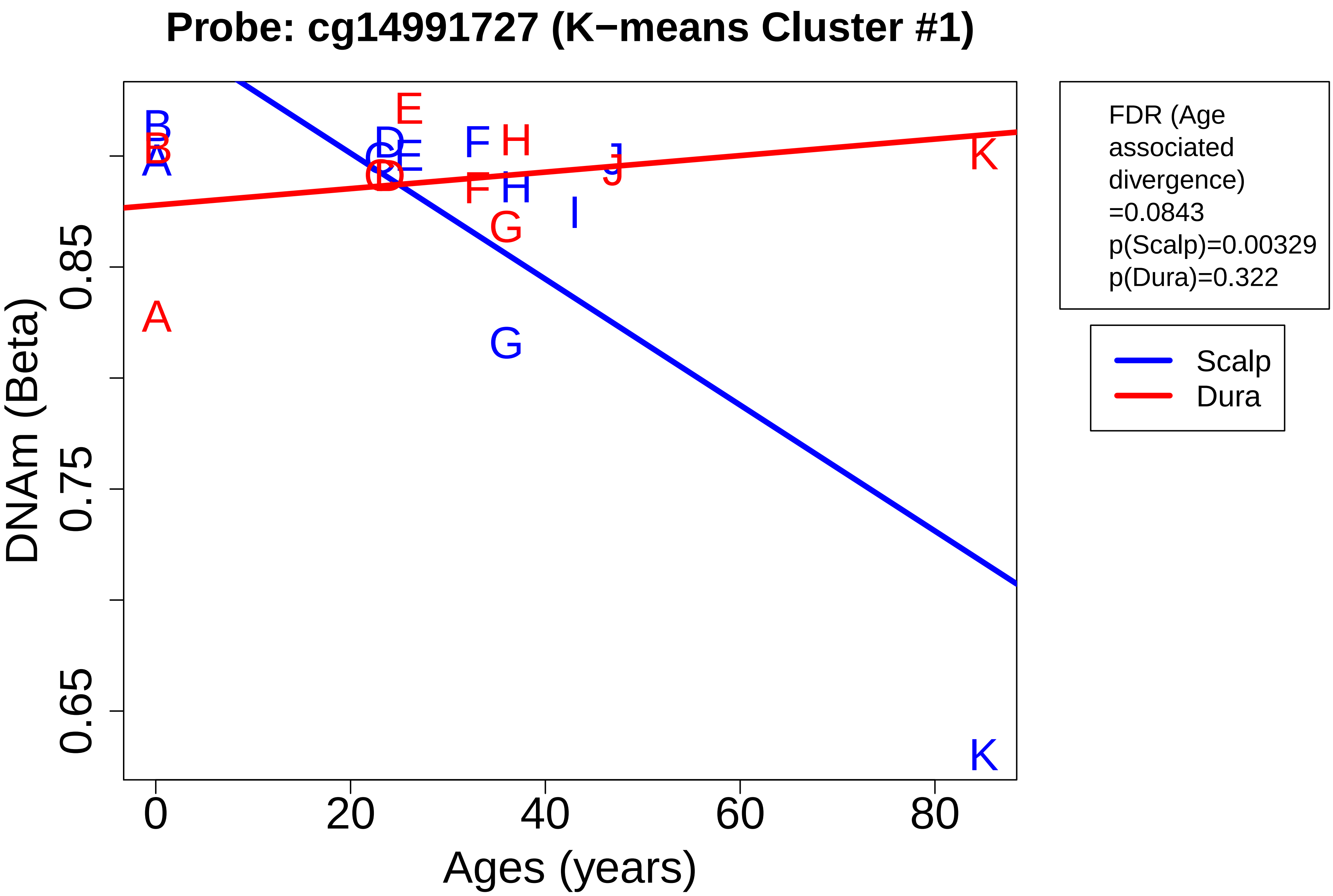

Probe: cg14991727 (K−means Cluster #1)
DNAm (Beta)
0.65
0.75
0.85
Ages (years)
0
20
40
60
80
FDR (Age associated divergence) =0.0843
p(Scalp)=0.00329
p(Dura)=0.322
Scalp
Dura

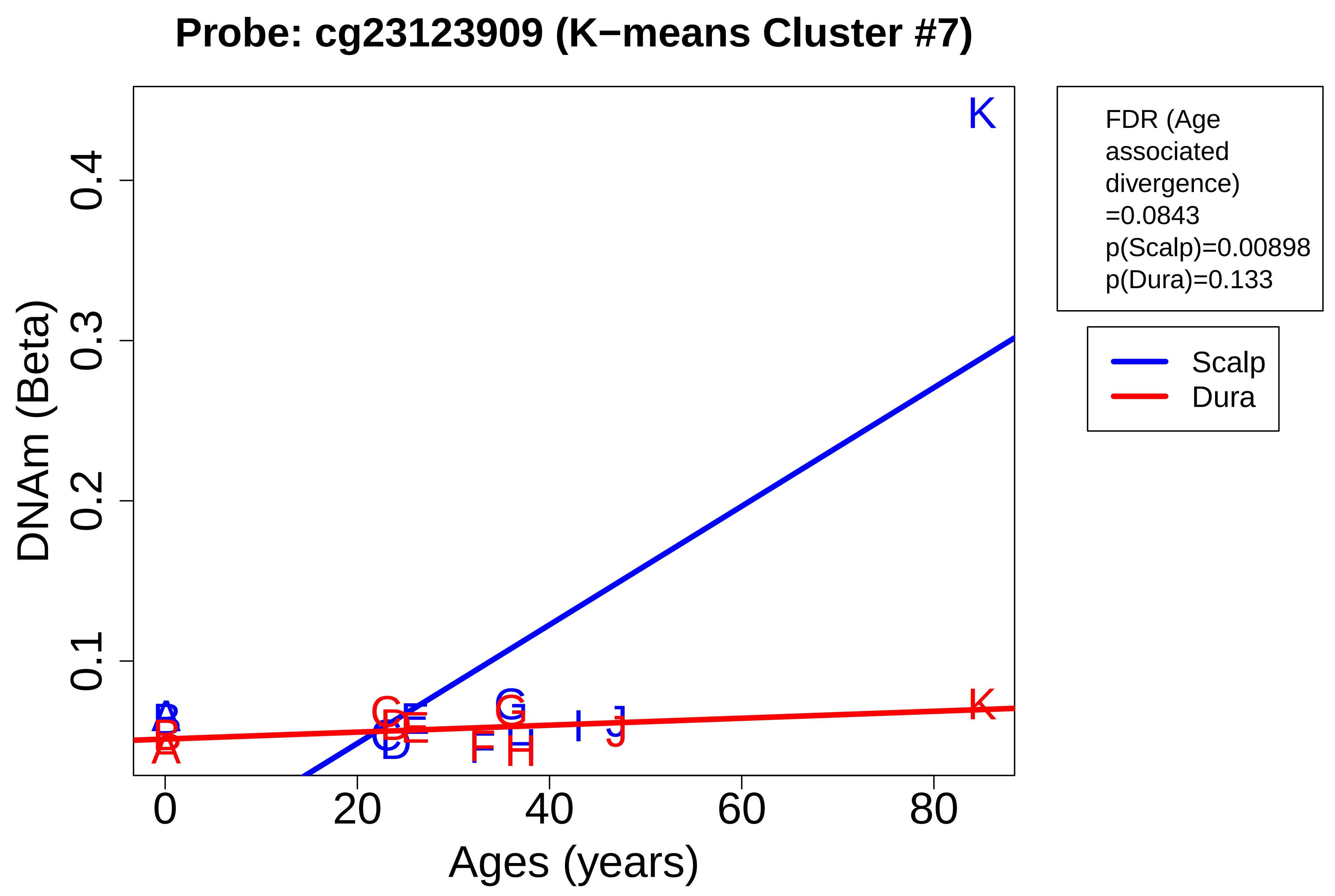

Probe: cg23123909 (K−means Cluster #7)
DNAm (Beta)
0.1
0.2
0.3
0.4
0
20
40
60
80
Ages (years)
FDR (Age associated divergence) =0.0843
p(Scalp)=0.00898
p(Dura)=0.133
Scalp
Dura

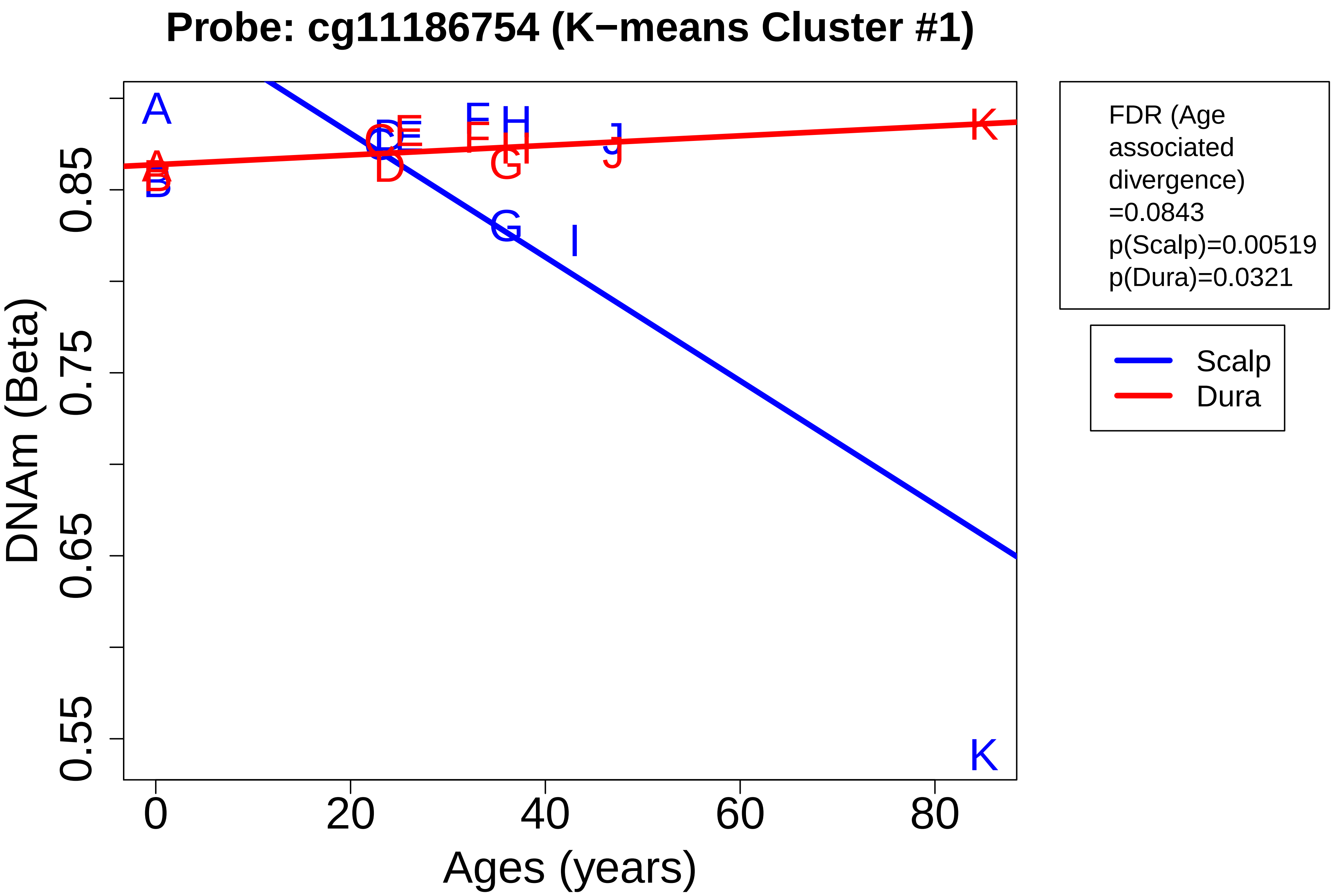

Probe: cg11186754 (K−means Cluster #1)
DNAm (Beta)
0.55
0.65
0.75
0.85
Ages (years)
0
20
40
60
80
FDR (Age associated divergence) =0.0843
p(Scalp)=0.00519
p(Dura)=0.0321
Scalp
Dura

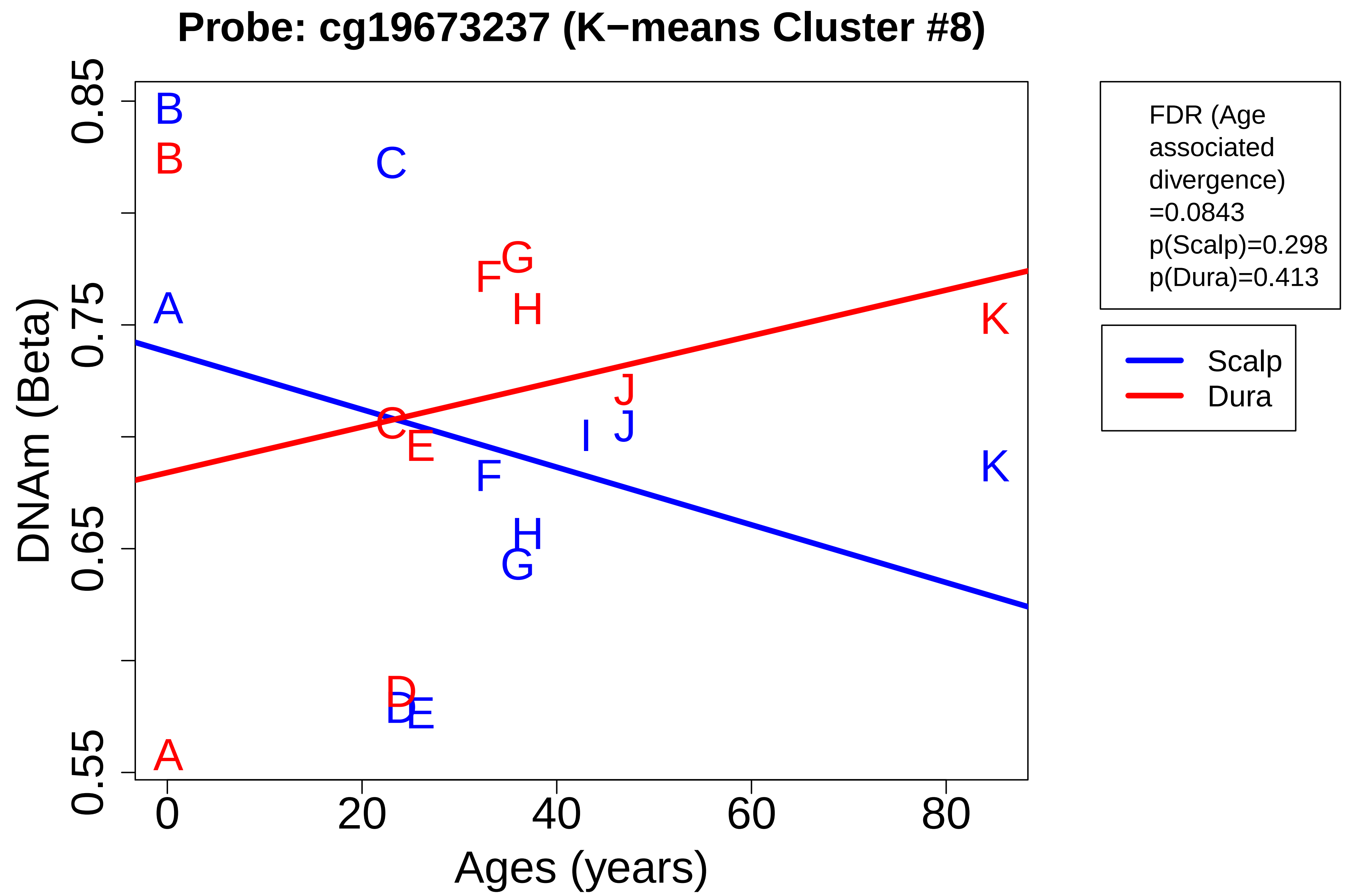

Probe: cg19673237 (K−means Cluster #8)
DNAm (Beta)
0.55
0.65
0.75
0.85
0
20
40
60
80
Ages (years)
A
B
C
D
E
F
G
H
I
J
K
FDR (Age associated divergence) =0.0843
p(Scalp)=0.298
p(Dura)=0.413
Scalp
Dura

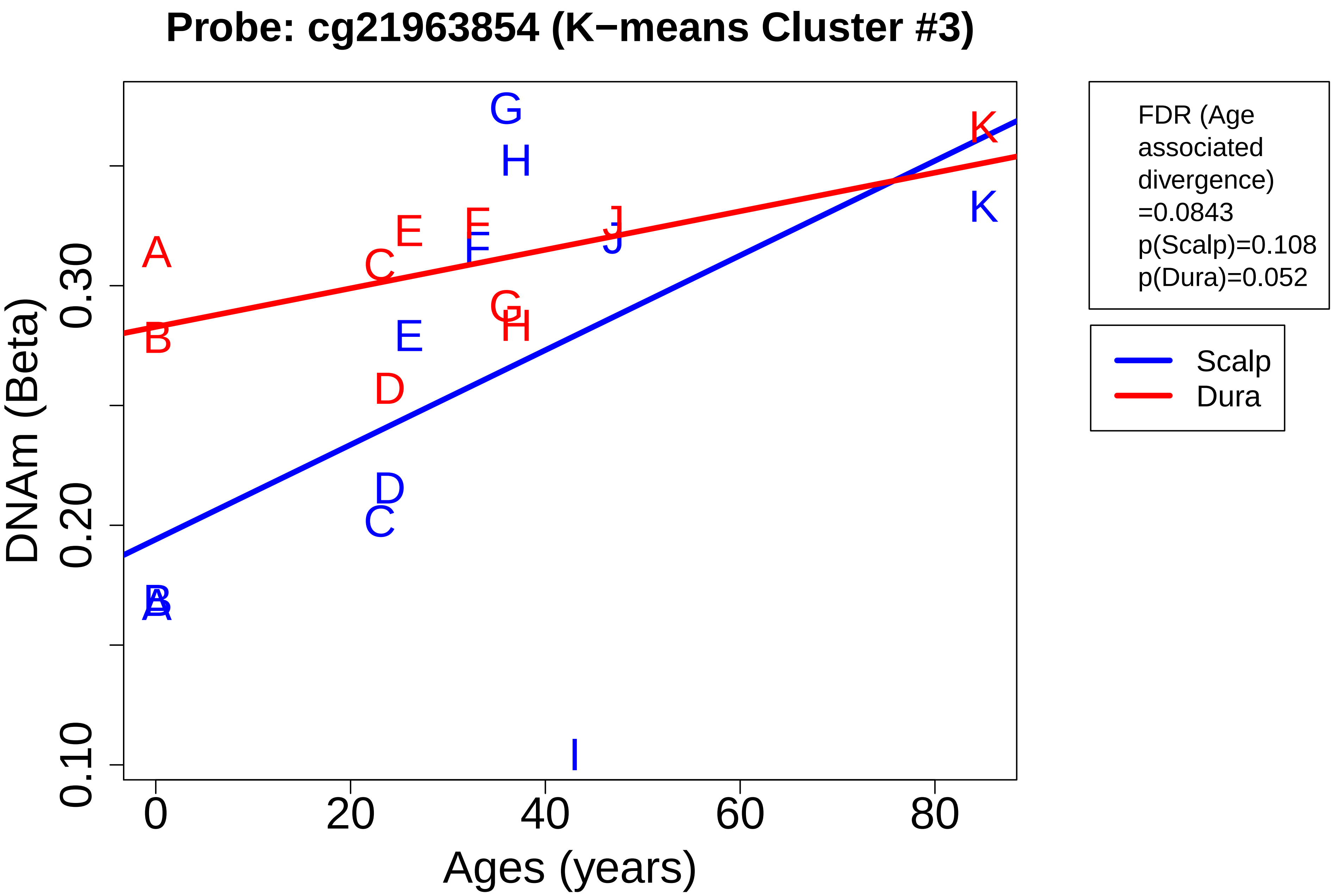
Probe: cg21963854 (K−means Cluster #3)
DNAm (Beta)
0.10
0.20
0.30
0
20
40
60
80
Ages (years)
A
B
C
D
E
F
G
H
I
J
K
FDR (Age associated divergence) =0.0843
p(Scalp)=0.108
p(Dura)=0.052
Scalp
Dura

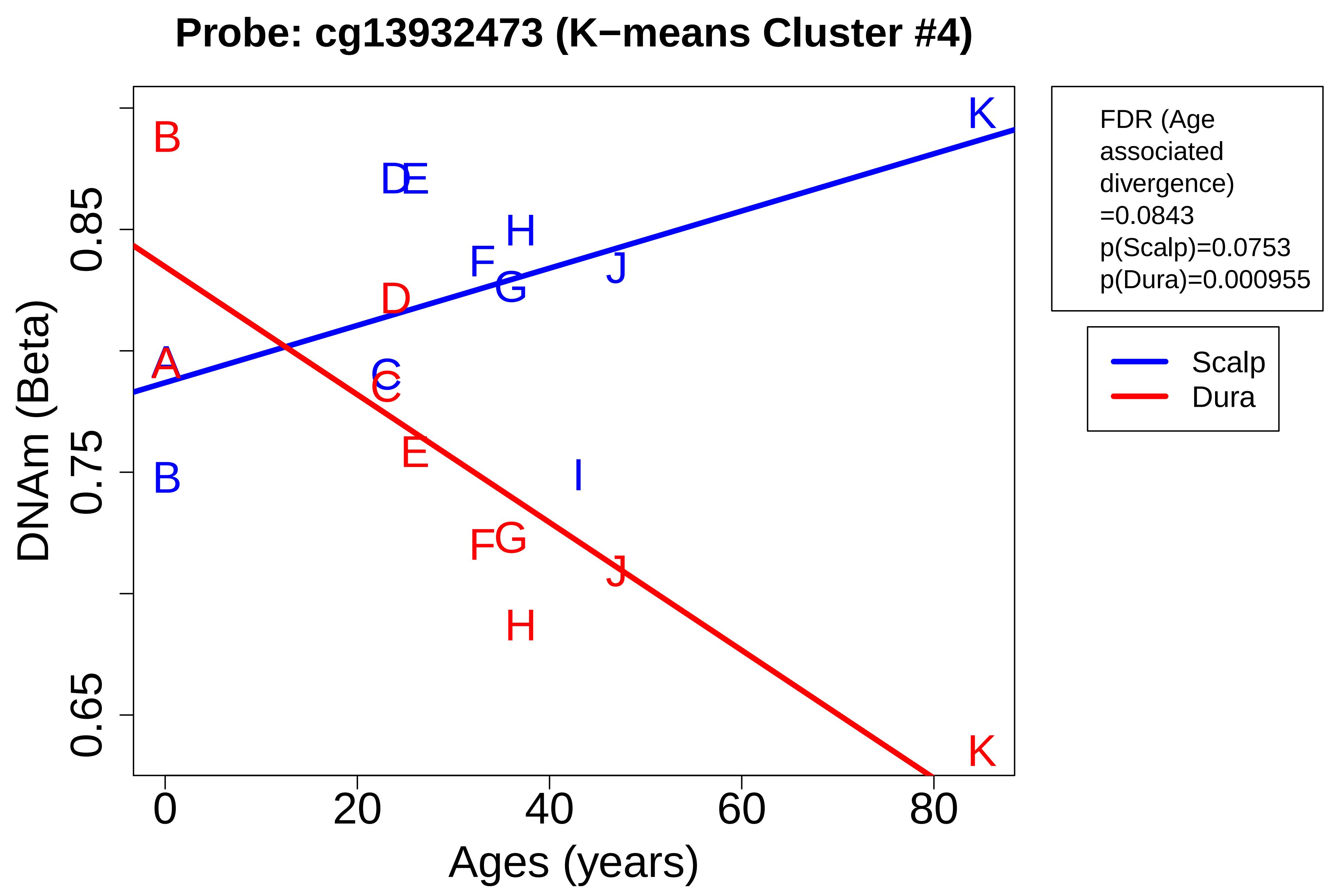
Probe: cg13932473 (K−means Cluster #4)
DNAm (Beta)
0.65
0.75
0.85
0
20
40
60
80
Ages (years)
FDR (Age associated divergence) =0.0843
p(Scalp)=0.0753
p(Dura)=0.000955
Scalp
Dura
A B C D E F G H I J K

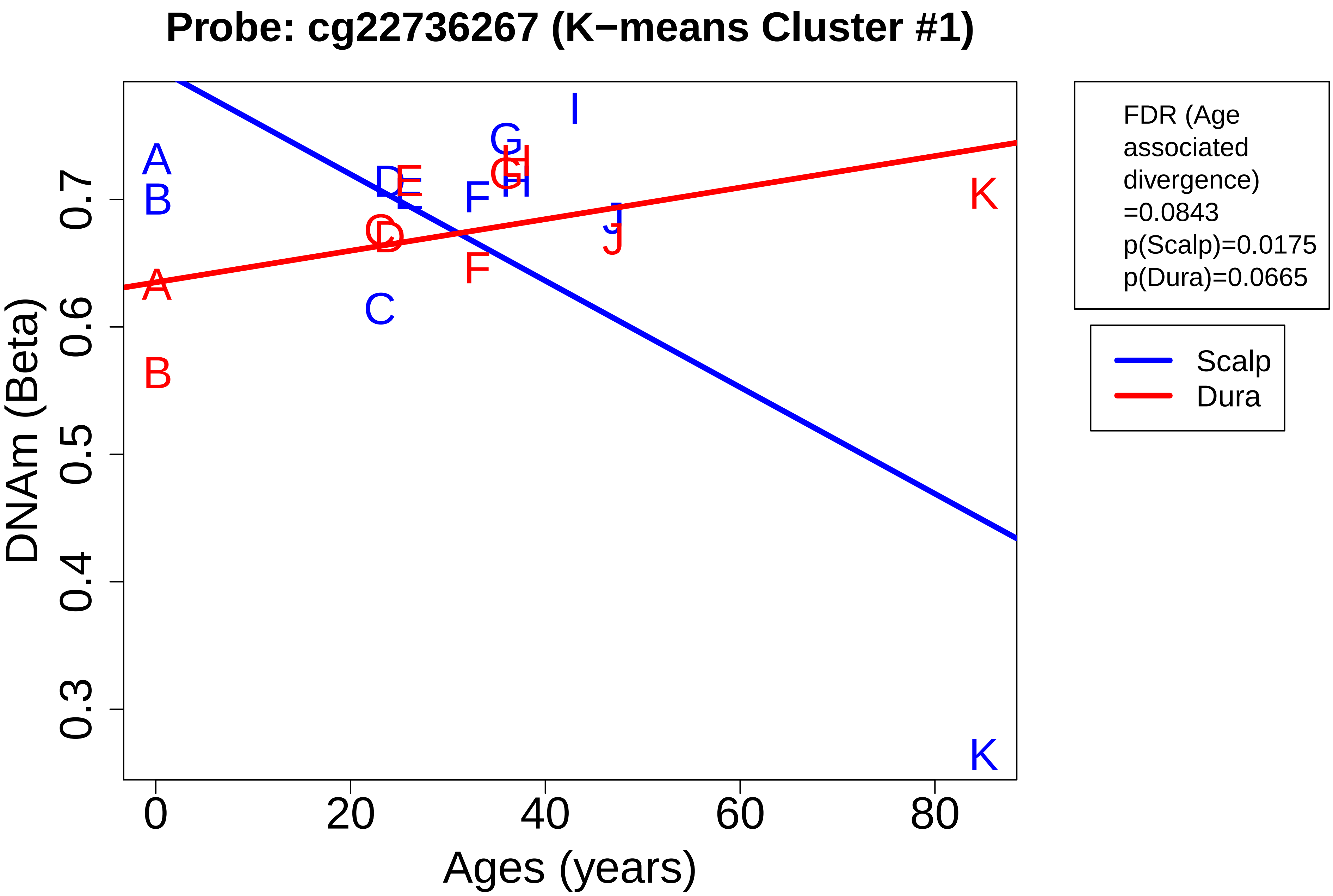

Probe: cg22736267 (K−means Cluster #1)
DNAm (Beta)
0.3
0.4
0.5
0.6
0.7
0
20
40
60
80
Ages (years)
FDR (Age associated divergence) =0.0843
p(Scalp)=0.0175
p(Dura)=0.0665
Scalp
Dura

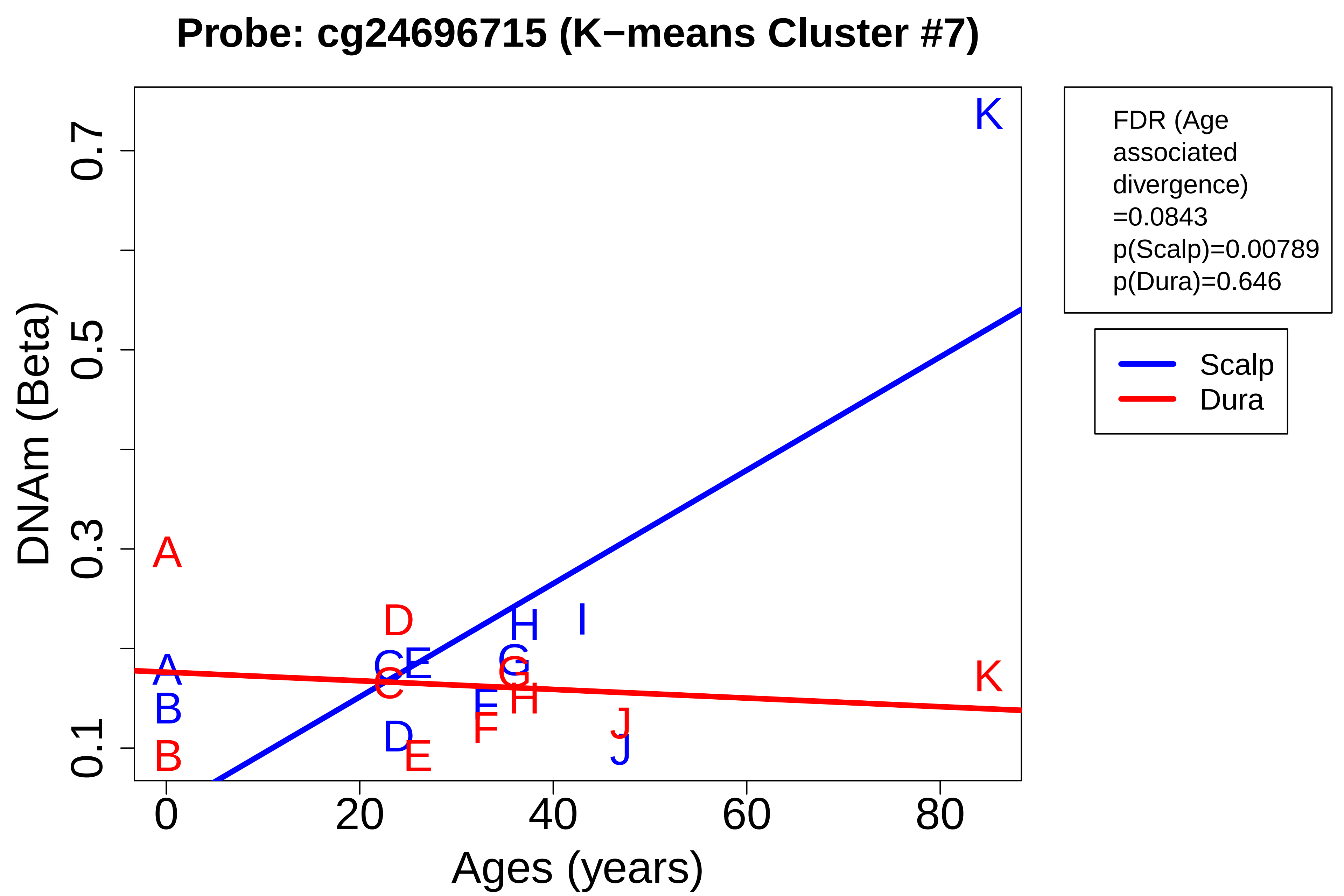
Probe: cg24696715 (K−means Cluster #7)
DNAm (Beta)
0.1
0.3
0.5
0.7
0
20
40
60
80
Ages (years)
A
B
C
D
E
F
G
H
I
J
K
FDR (Age associated divergence) =0.0843
p(Scalp)=0.00789
p(Dura)=0.646
Scalp
Dura

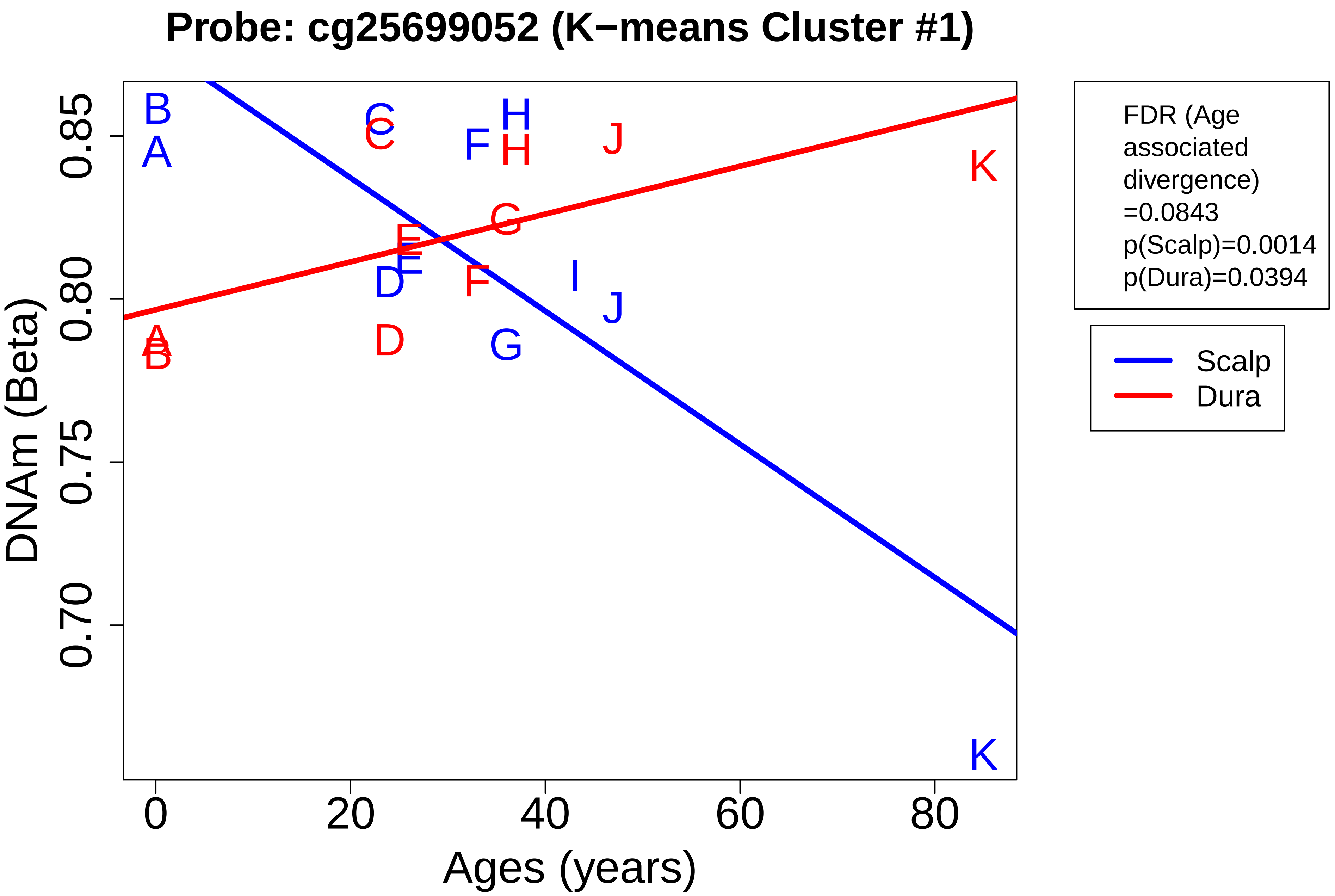

Probe: cg25699052 (K−means Cluster #1)
DNAm (Beta)
0.70
0.75
0.80
0.85
Ages (years)
0
20
40
60
80
B
A
C
C
F
H
H
J
K
G
E
E
D
F
I
J
A
B
D
G
K
FDR (Age associated divergence) =0.0843
p(Scalp)=0.0014
p(Dura)=0.0394
Scalp
Dura

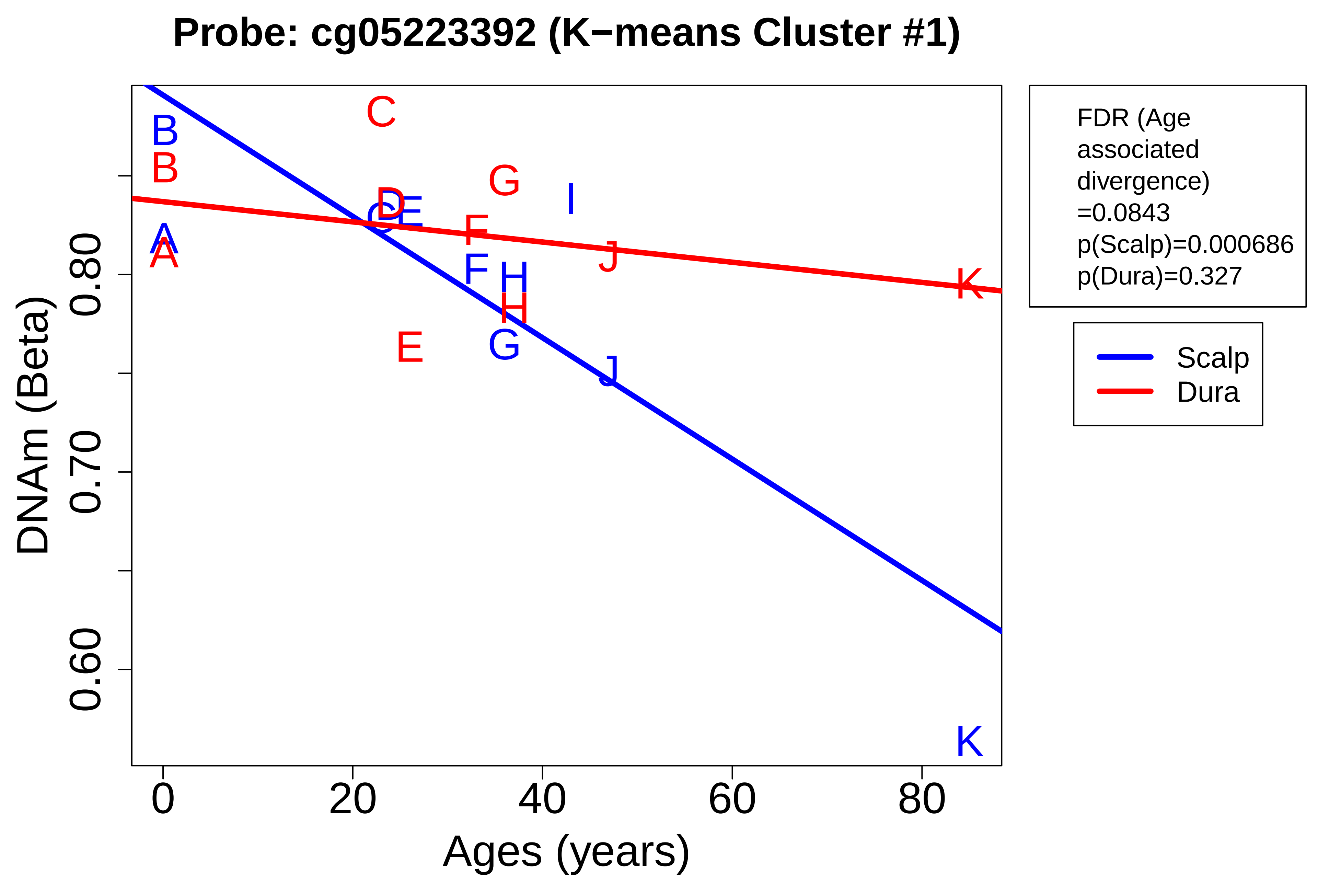
Probe: cg05223392 (K−means Cluster #1)
DNAm (Beta)
0.60
0.70
0.80
0
20
40
60
80
Ages (years)
FDR (Age associated divergence) =0.0843
p(Scalp)=0.000686
p(Dura)=0.327
Scalp
Dura

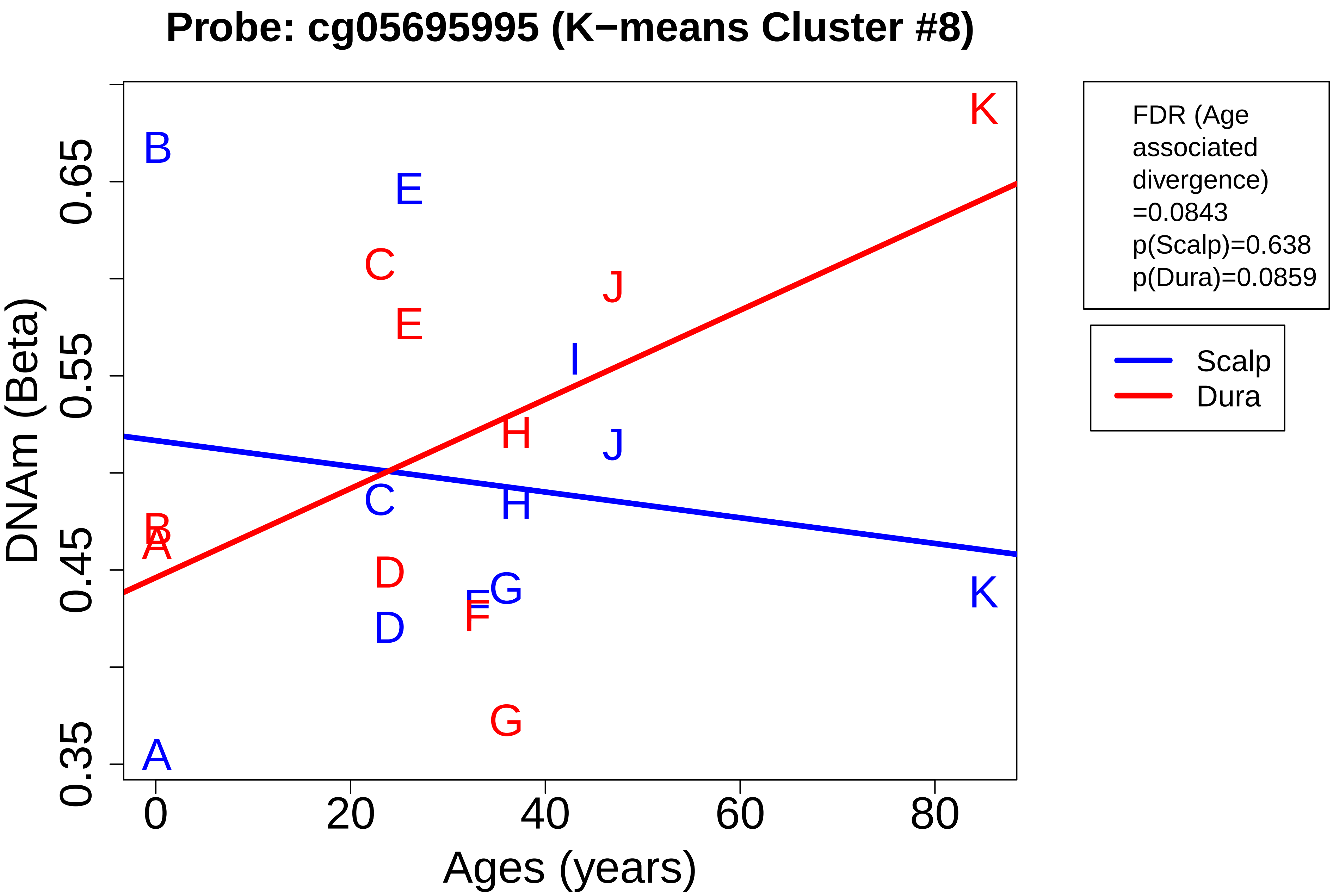

Probe: cg05695995 (K−means Cluster #8)
DNAm (Beta)
0.35
0.45
0.55
0.65
0
20
40
60
80
Ages (years)
A
B
C
D
E
F
G
H
I
J
K
FDR (Age associated divergence) =0.0843
p(Scalp)=0.638
p(Dura)=0.0859
Scalp
Dura

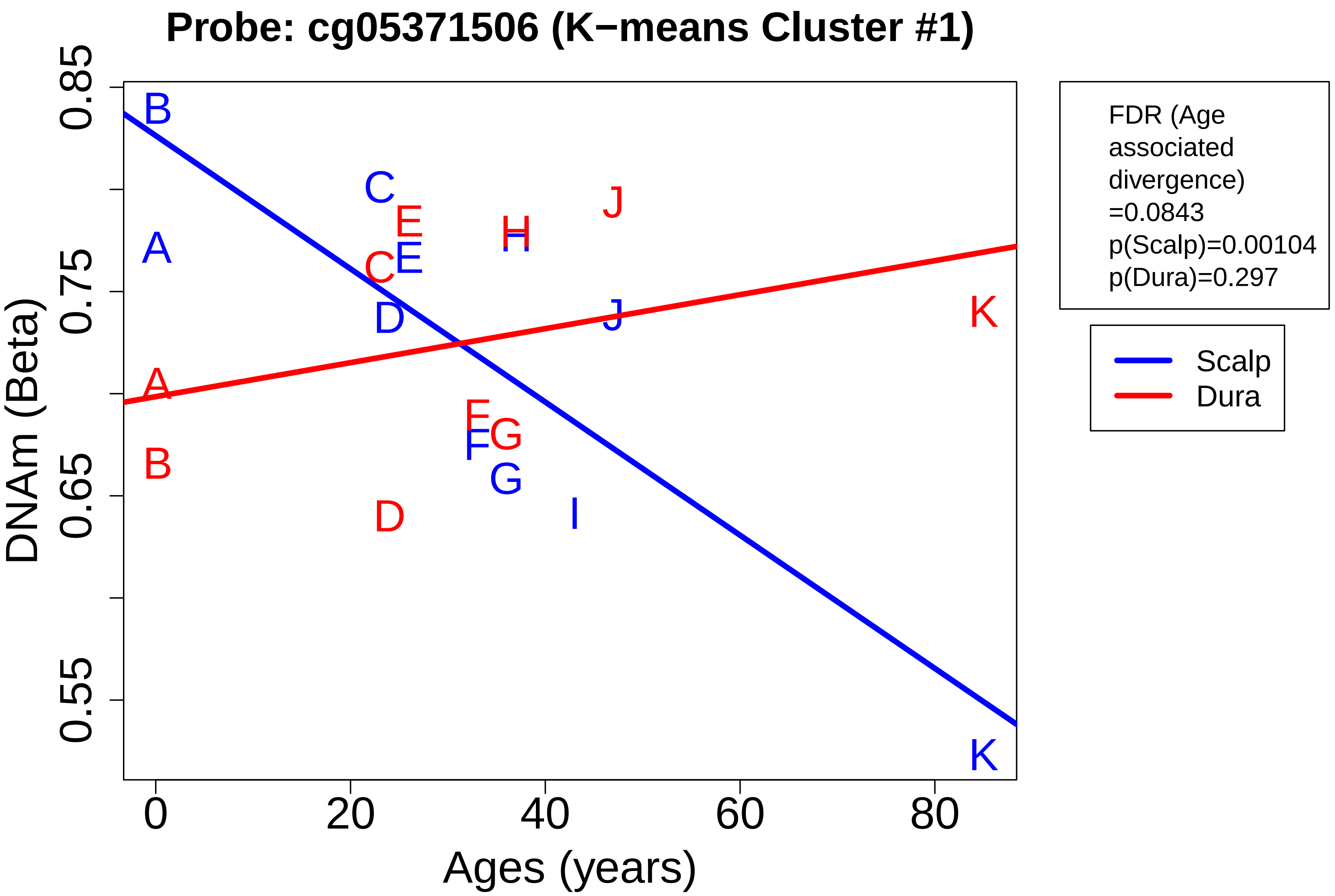

Probe: cg05371506 (K−means Cluster #1)
DNAm (Beta)
0.55
0.65
0.75
0.85
Ages (years)
0
20
40
60
80
B
A
C
E
E
C
D
H
J
J
K
A
B
D
F
F
G
G
I
K
FDR (Age associated divergence) =0.0843
p(Scalp)=0.00104
p(Dura)=0.297
Scalp
Dura

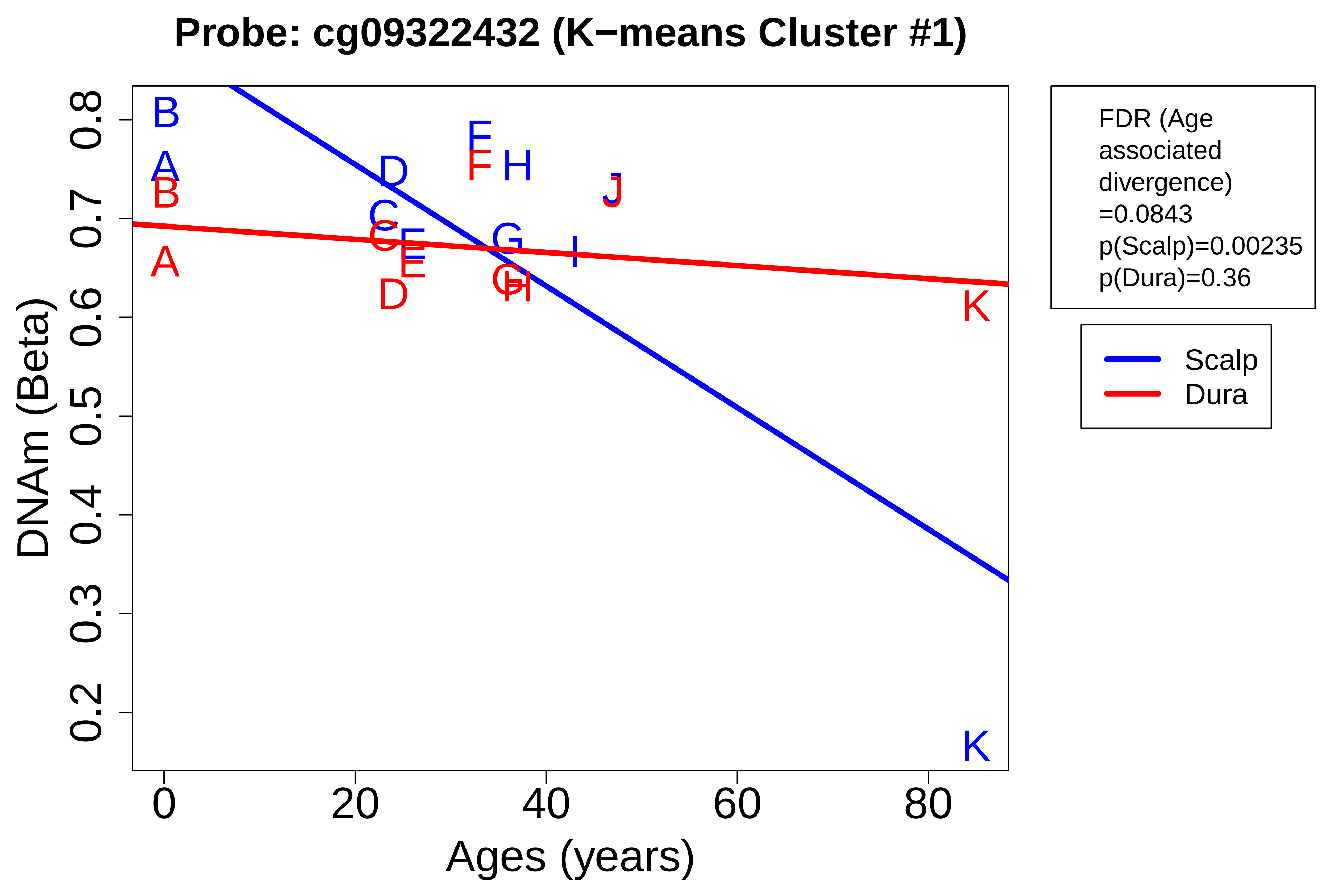
Probe: cg09322432 (K−means Cluster #1)
DNAm (Beta)
Ages (years)
0.2
0.3
0.4
0.5
0.6
0.7
0.8
0
20
40
60
80
A
B
C
D
E
F
G
H
I
J
K
FDR (Age associated divergence) =0.0843
p(Scalp)=0.00235
p(Dura)=0.36
Scalp
Dura

Probe: cg05784856 (K−means Cluster #4)

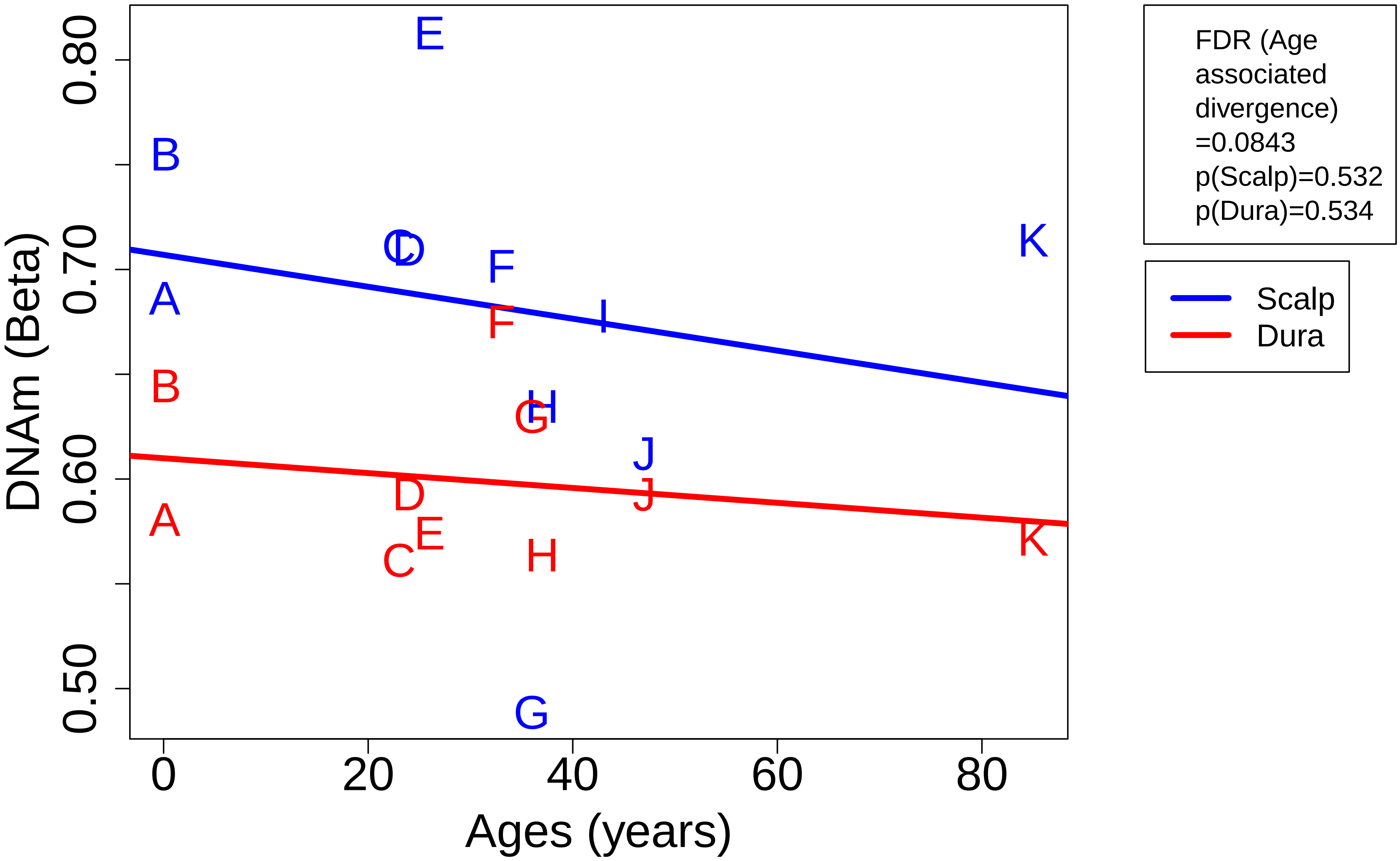

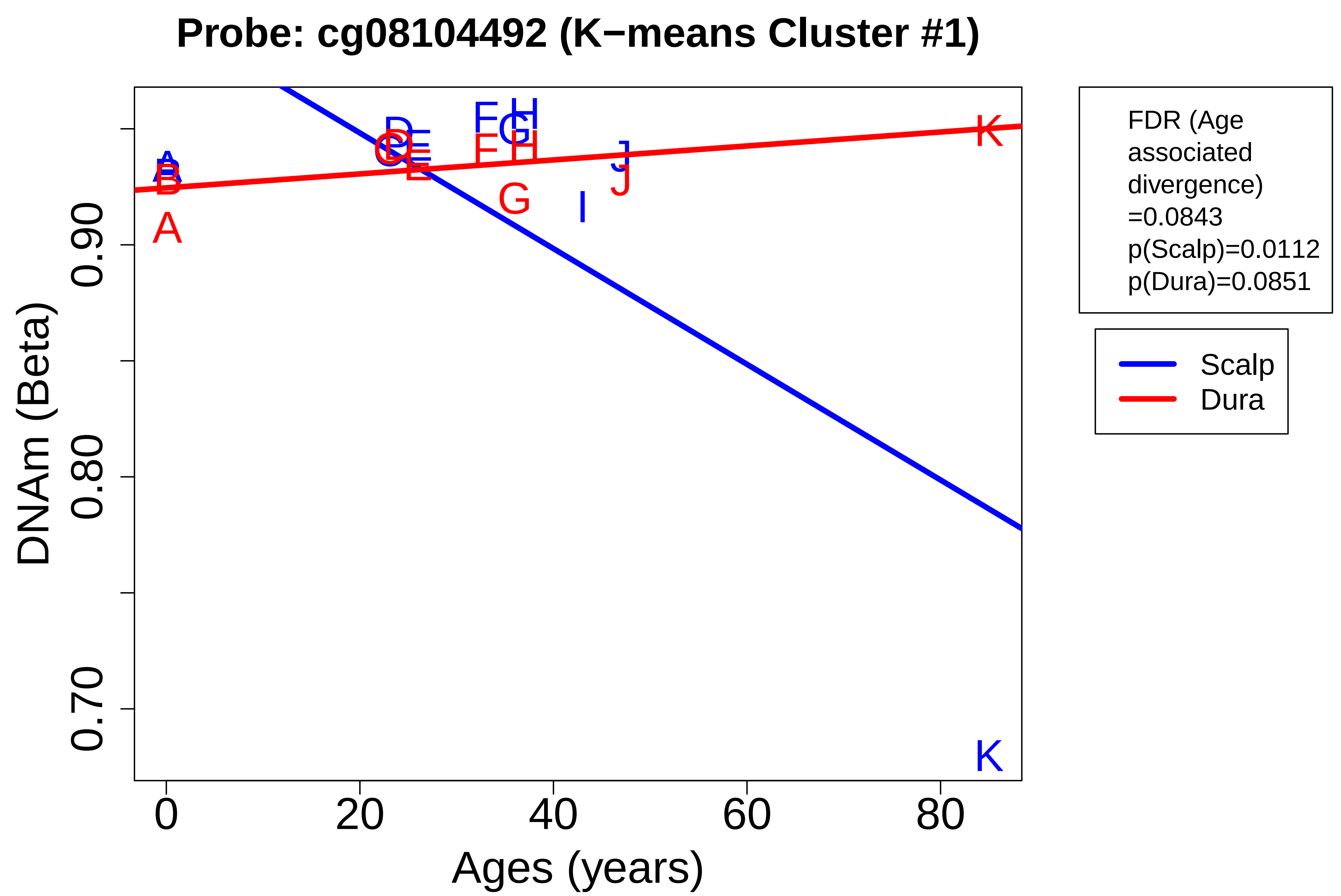
Probe: cg08104492 (K−means Cluster #1)
DNAm (Beta)
0.70
0.80
0.90
Ages (years)
0
20
40
60
80
A
B
C
D
E
F
G
H
I
J
K
FDR (Age associated divergence) =0.0843
p(Scalp)=0.0112
p(Dura)=0.0851
Scalp
Dura

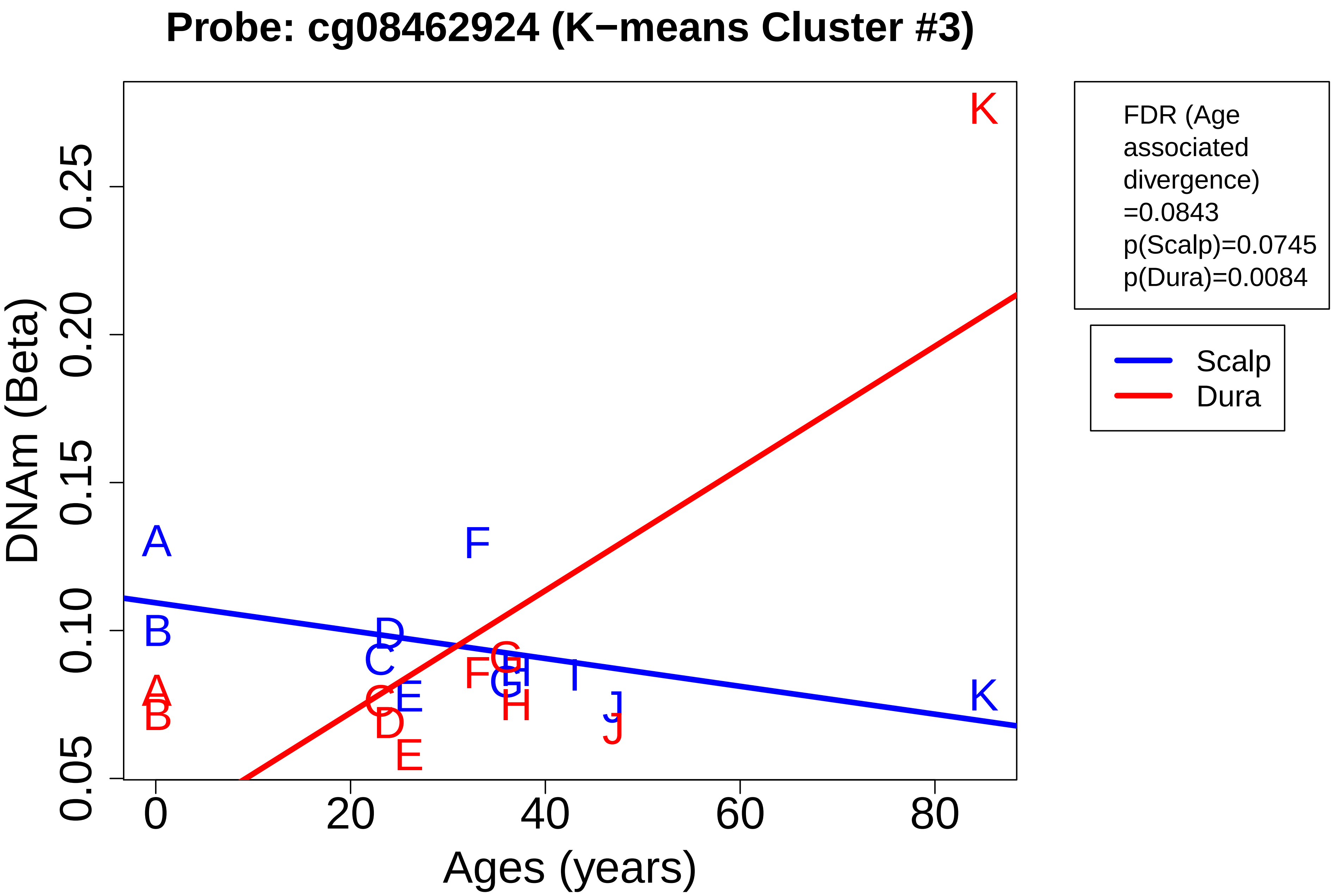

Probe: cg08462924 (K−means Cluster #3)
DNAm (Beta)
0.05
0.10
0.15
0.20
0.25
0
20
40
60
80
Ages (years)
FDR (Age associated divergence) =0.0843
p(Scalp)=0.0745
p(Dura)=0.0084
Scalp
Dura
A
B
C
D
E
F
G
H
I
J
K

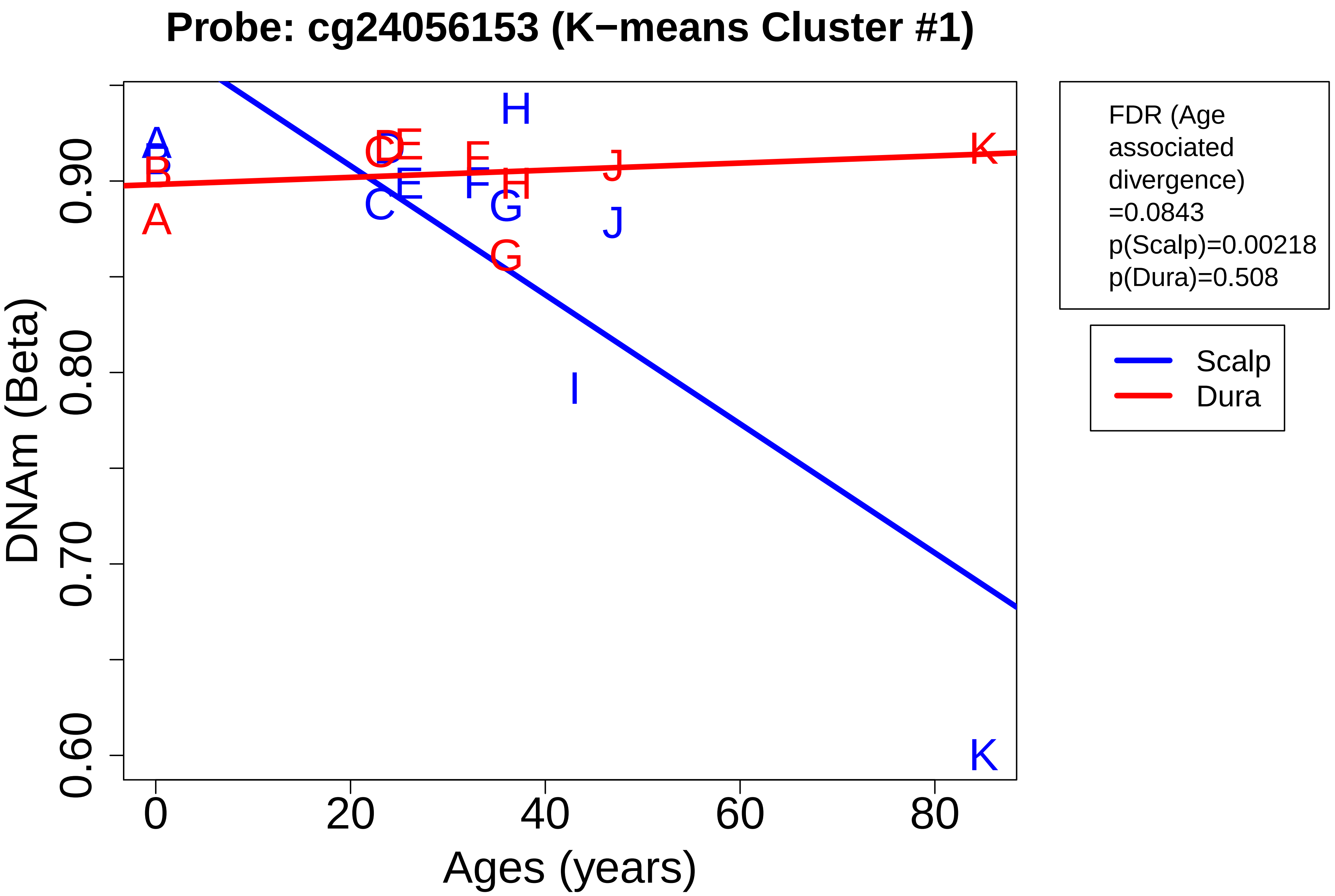
Probe: cg24056153 (K−means Cluster #1)
DNAm (Beta)
0.60
0.70
0.80
0.90
0
20
40
60
80
Ages (years)
FDR (Age associated divergence) =0.0843
p(Scalp)=0.00218
p(Dura)=0.508
Scalp
Dura

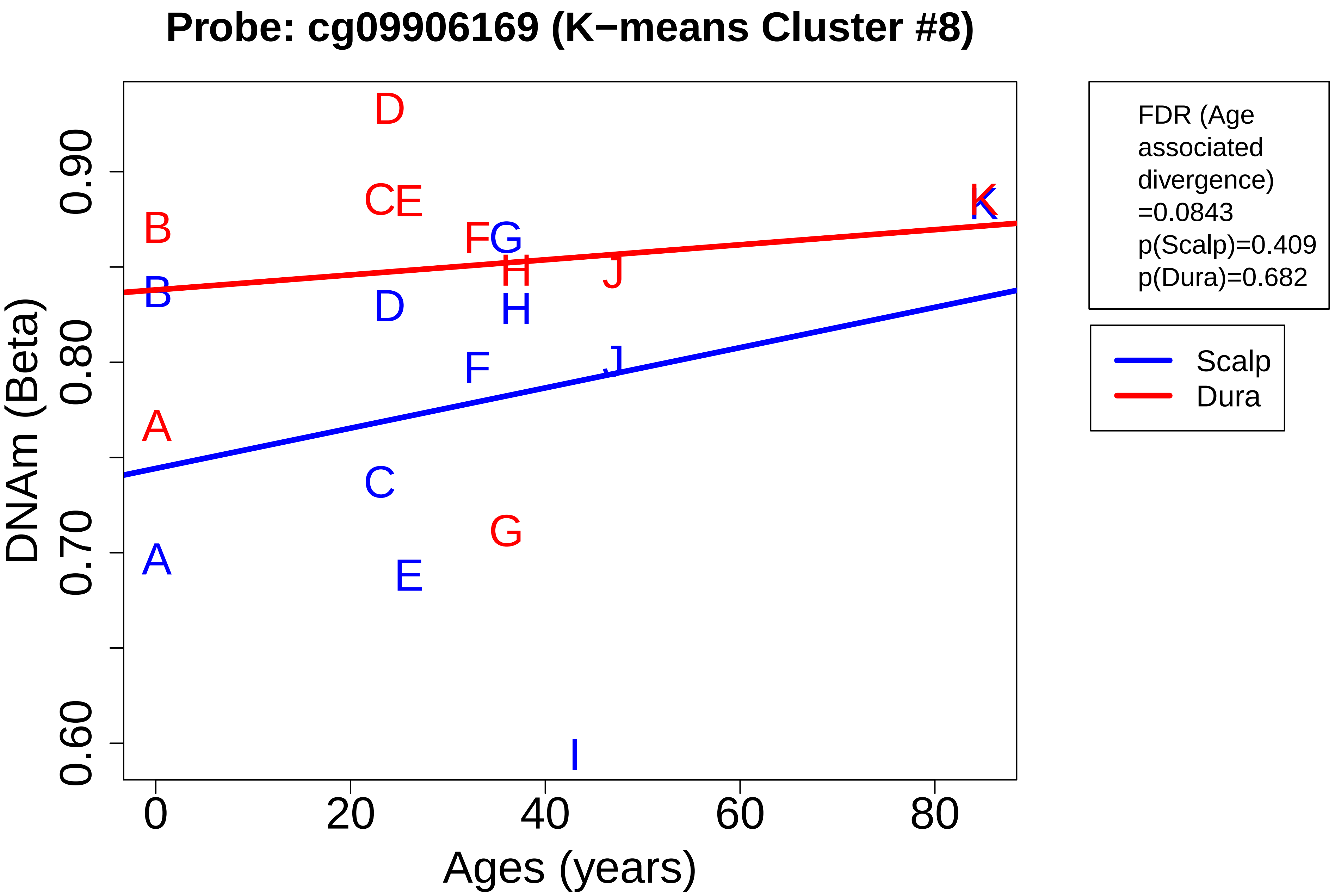
Probe: cg09906169 (K−means Cluster #8)
DNAm (Beta)
0.60
0.70
0.80
0.90
0
20
40
60
80
Ages (years)
FDR (Age associated divergence) =0.0843
p(Scalp)=0.409
p(Dura)=0.682
Scalp
Dura
A
B
C
D
E
F
G
H
I
J
K

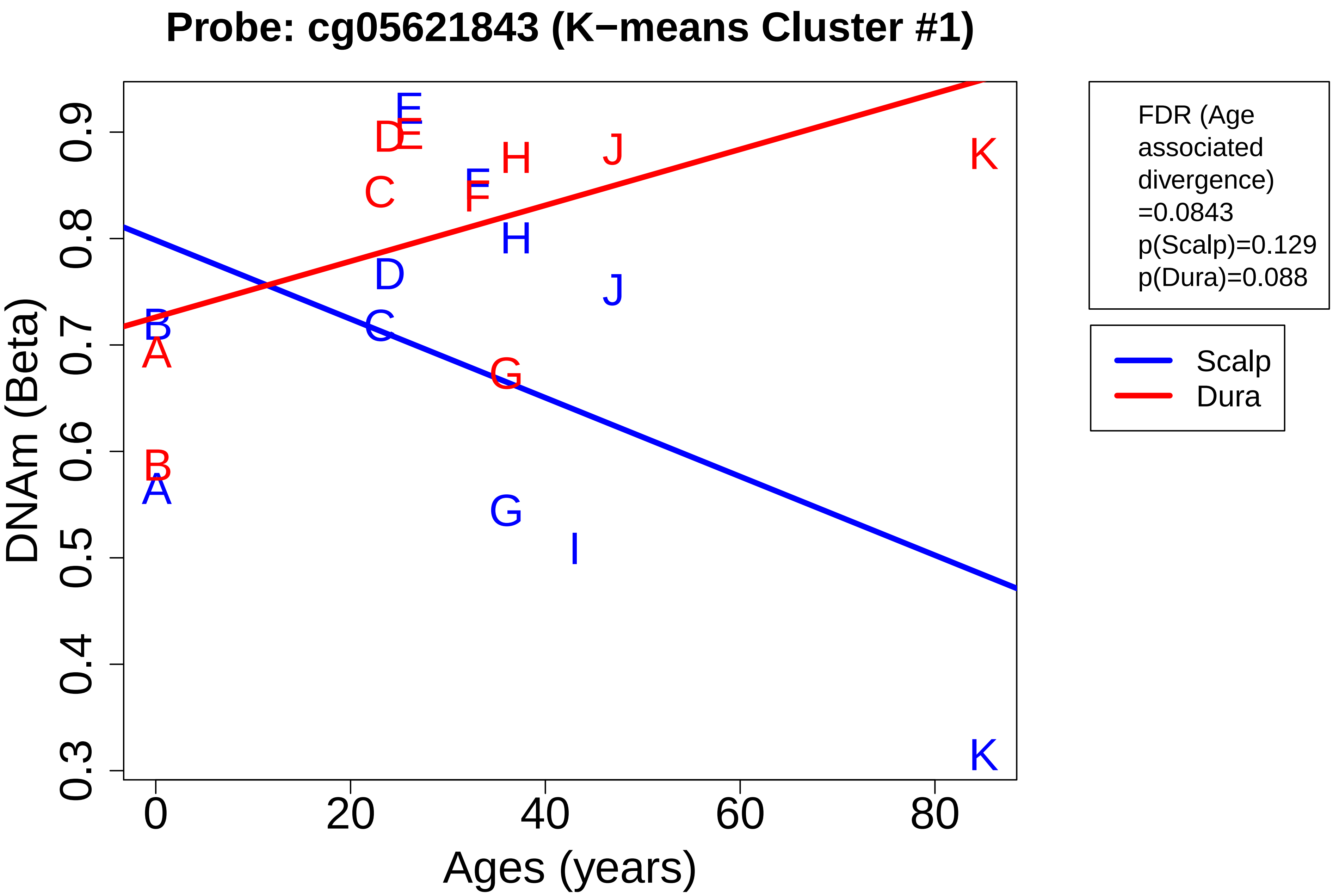

Probe: cg05621843 (K−means Cluster #1)
DNAm (Beta)
0.3
0.4
0.5
0.6
0.7
0.8
0.9
0
20
40
60
80
Ages (years)
FDR (Age associated divergence) =0.0843
p(Scalp)=0.129
p(Dura)=0.088
Scalp
Dura

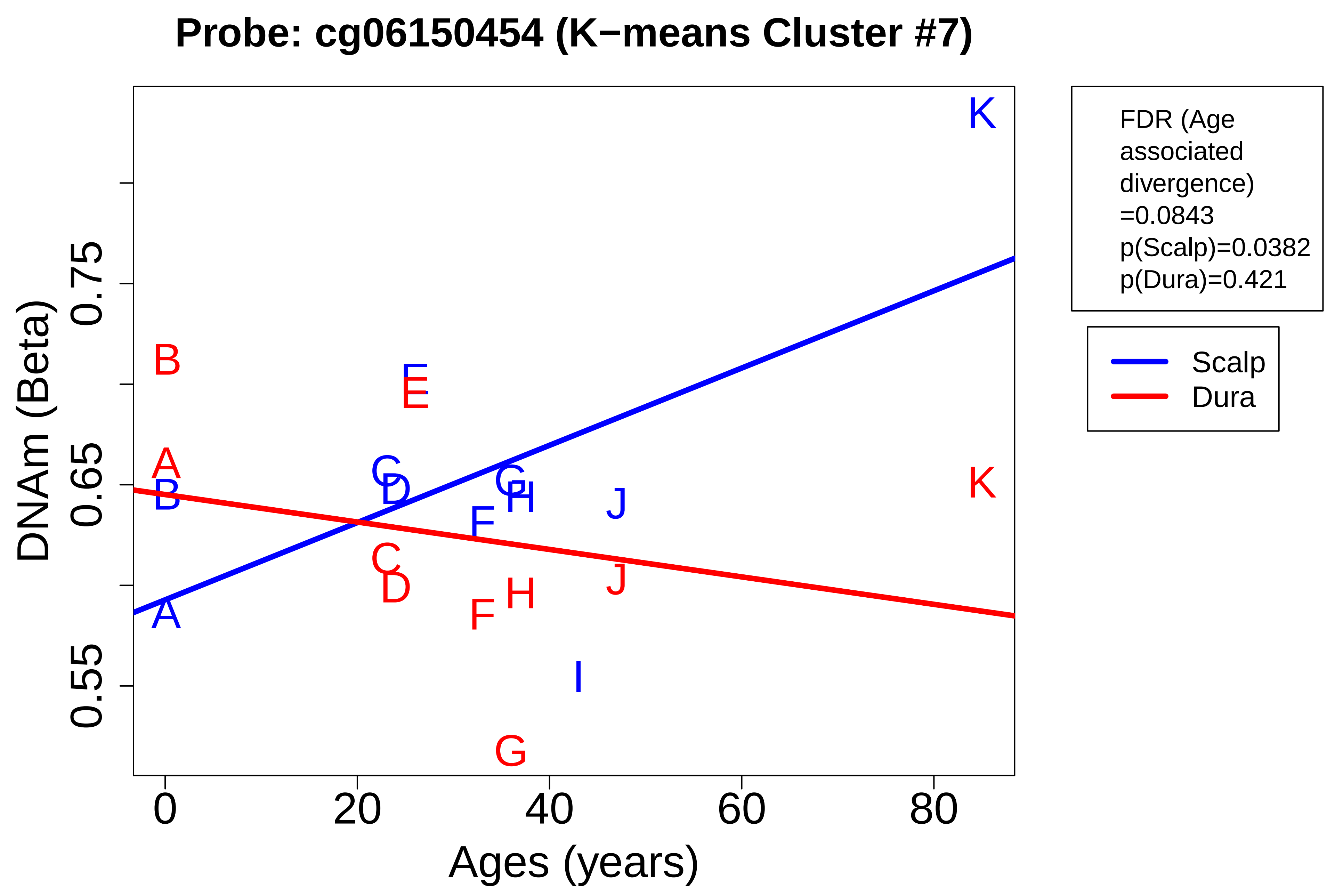

Probe: cg06150454 (K−means Cluster #7)
FDR (Age associated divergence) =0.0843
p(Scalp)=0.0382
p(Dura)=0.421
Scalp
Dura
DNAm (Beta)
0.55
0.65
0.75
0
20
40
60
80
Ages (years)
A
B
C
D
E
F
G
H
I
J
K

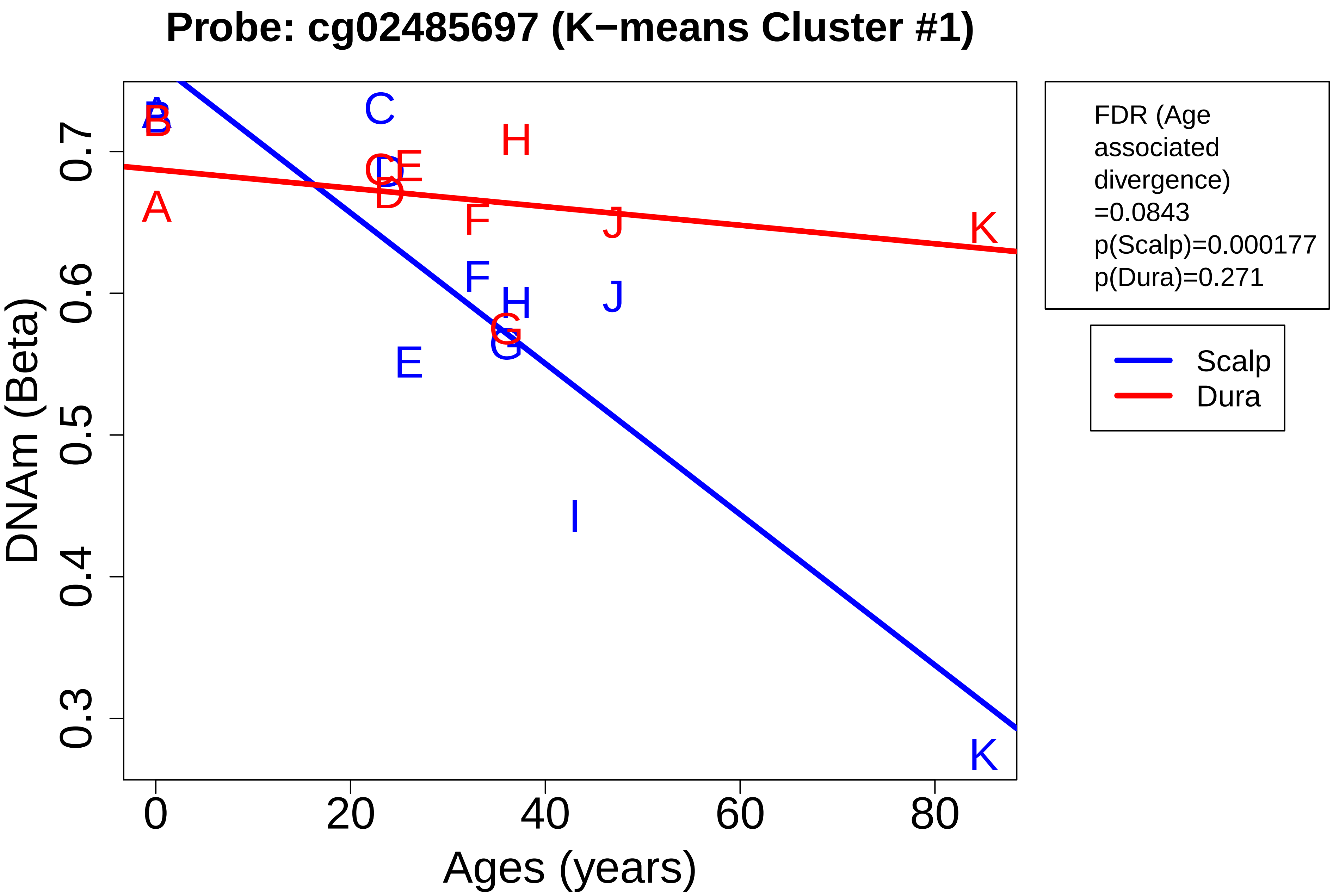

Probe: cg02485697 (K−means Cluster #1)
DNAm (Beta)
0.3
0.4
0.5
0.6
0.7
0
20
40
60
80
Ages (years)
FDR (Age associated divergence) =0.0843
p(Scalp)=0.000177
p(Dura)=0.271
Scalp
Dura

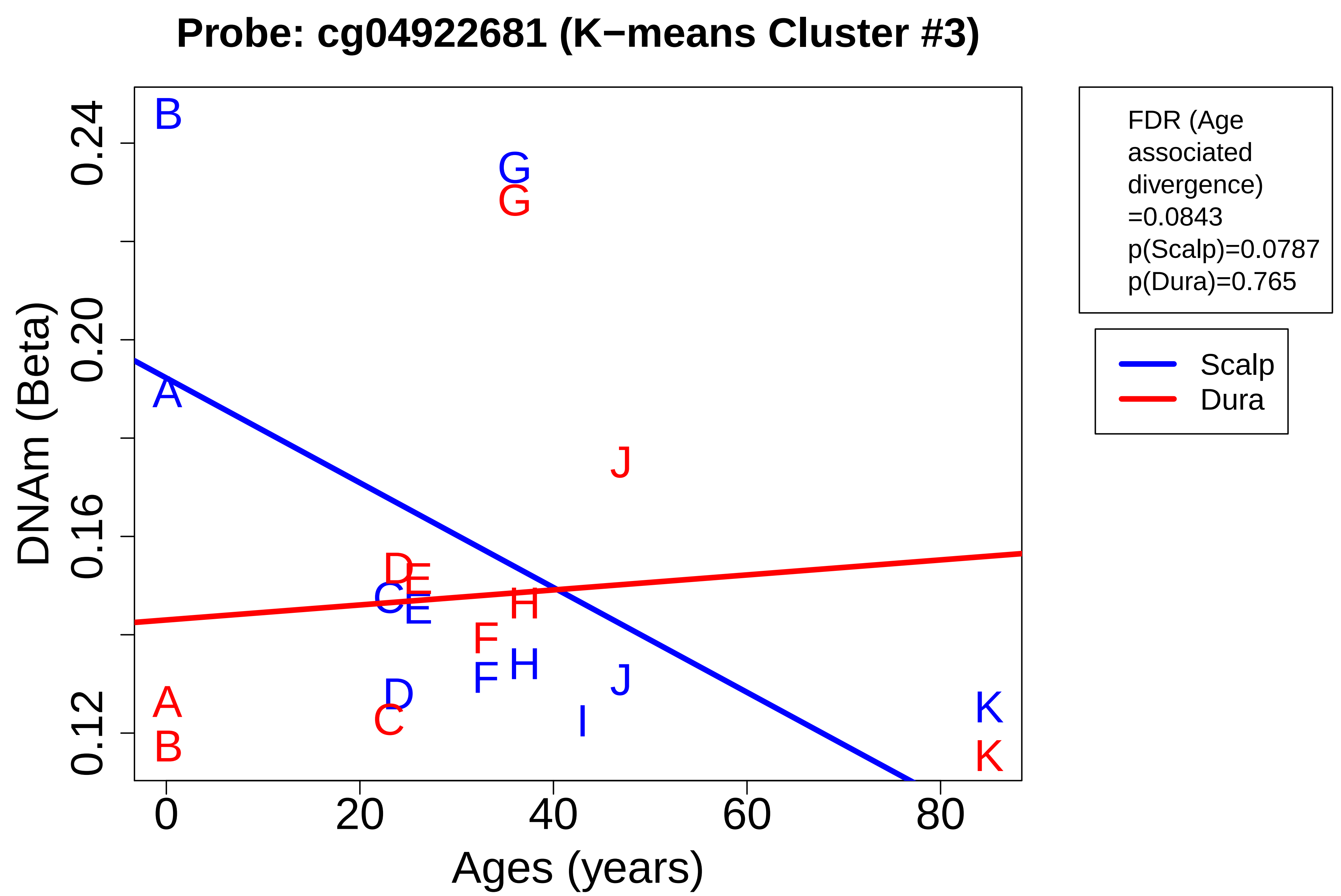

Probe: cg04922681 (K−means Cluster #3)
DNAm (Beta)
0.12
0.16
0.20
0.24
Ages (years)
0
20
40
60
80
FDR (Age associated divergence) =0.0843
p(Scalp)=0.0787
p(Dura)=0.765
Scalp
Dura
A
B
C
D
E
F
G
H
I
J
K

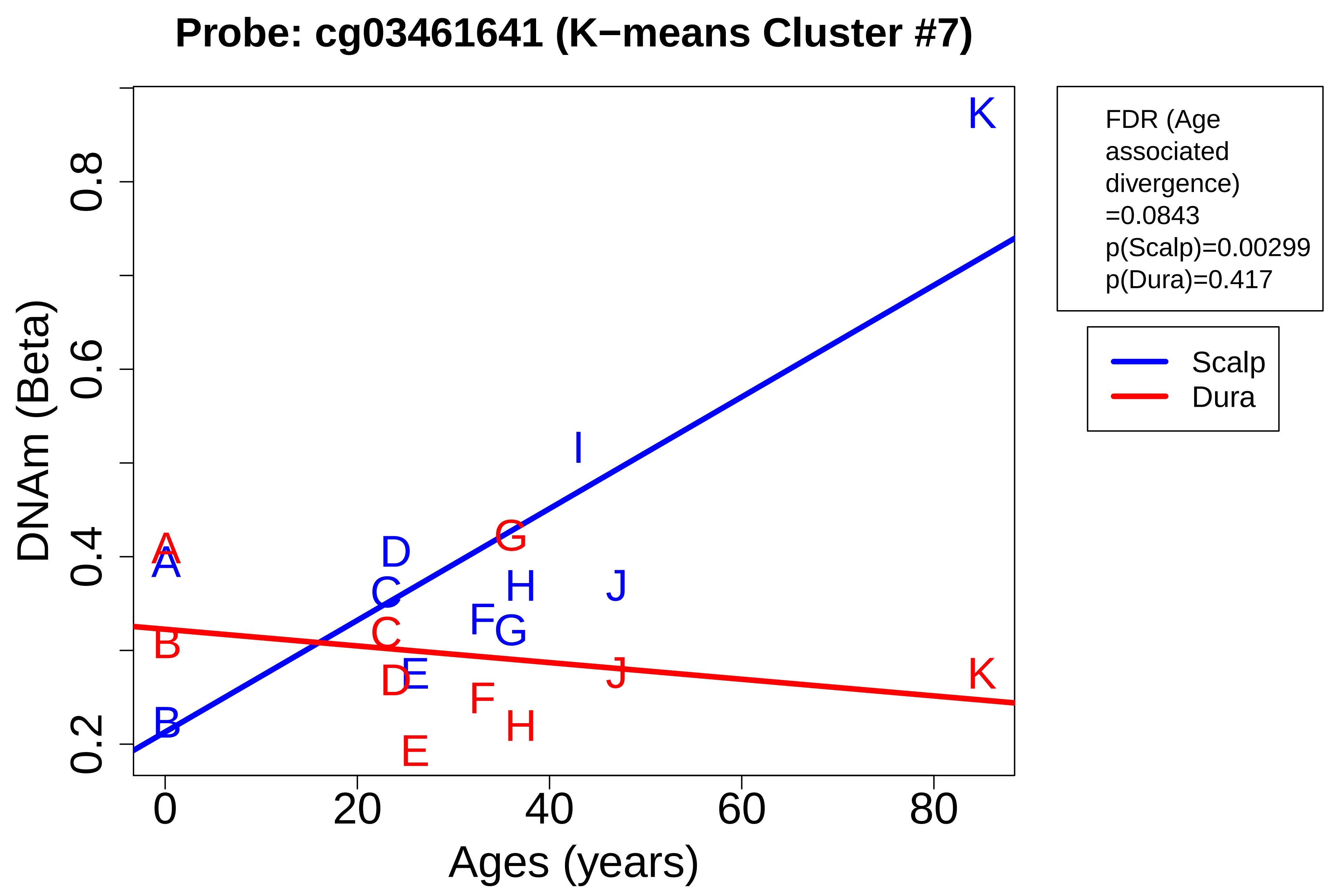

Probe: cg03461641 (K−means Cluster #7)
FDR (Age associated divergence) =0.0843
p(Scalp)=0.00299
p(Dura)=0.417
Scalp
Dura
DNAm (Beta)
0.2
0.4
0.6
0.8
0
20
40
60
80
Ages (years)
A
B
C
D
E
F
G
H
I
J
K

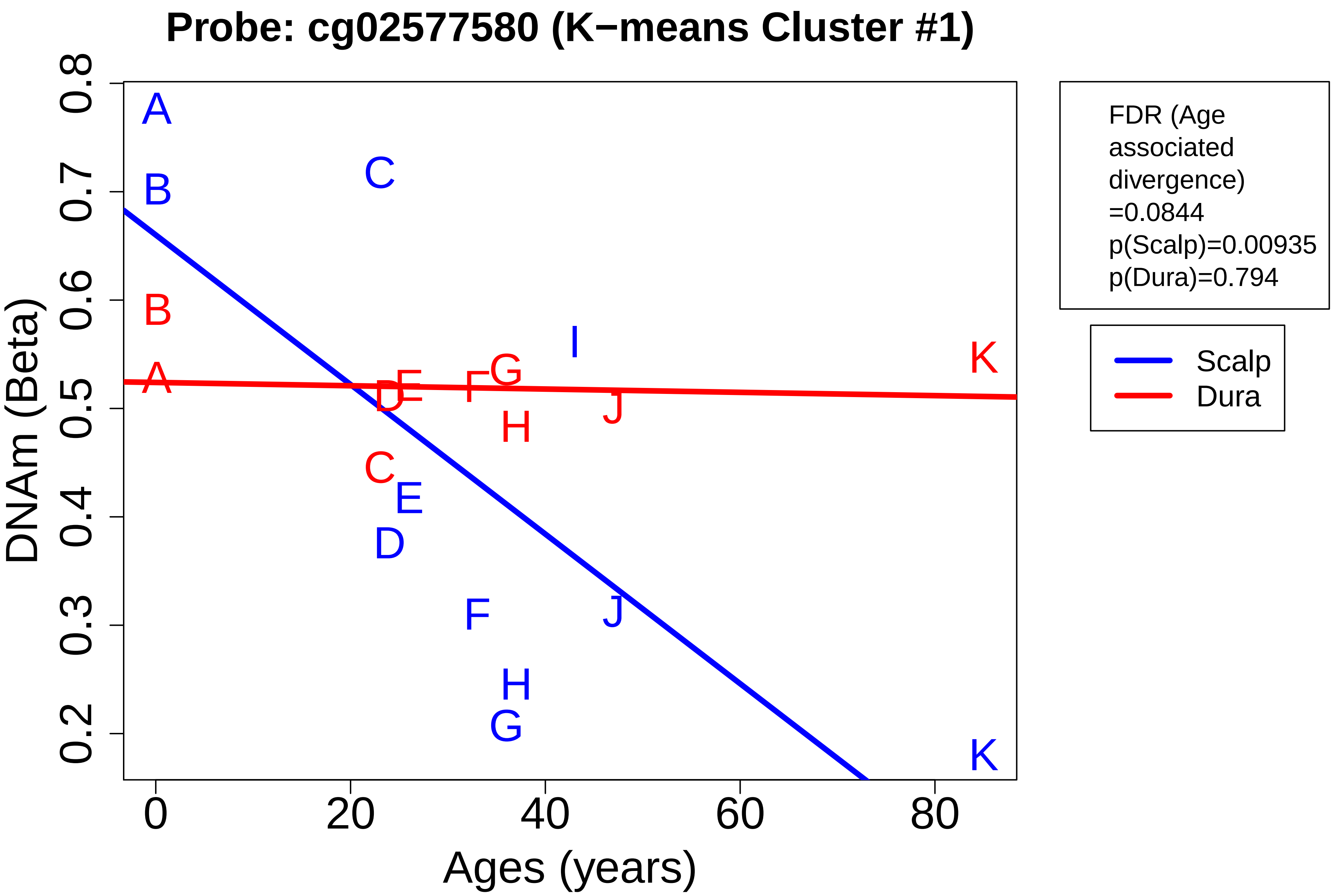

Probe: cg02577580 (K−means Cluster #1)
FDR (Age associated divergence) =0.0844
p(Scalp)=0.00935
p(Dura)=0.794
Scalp
Dura
DNAm (Beta)
Ages (years)
0.2
0.3
0.4
0.5
0.6
0.7
0.8
0
20
40
60
80
A
B
C
D
E
F
G
H
I
J
K

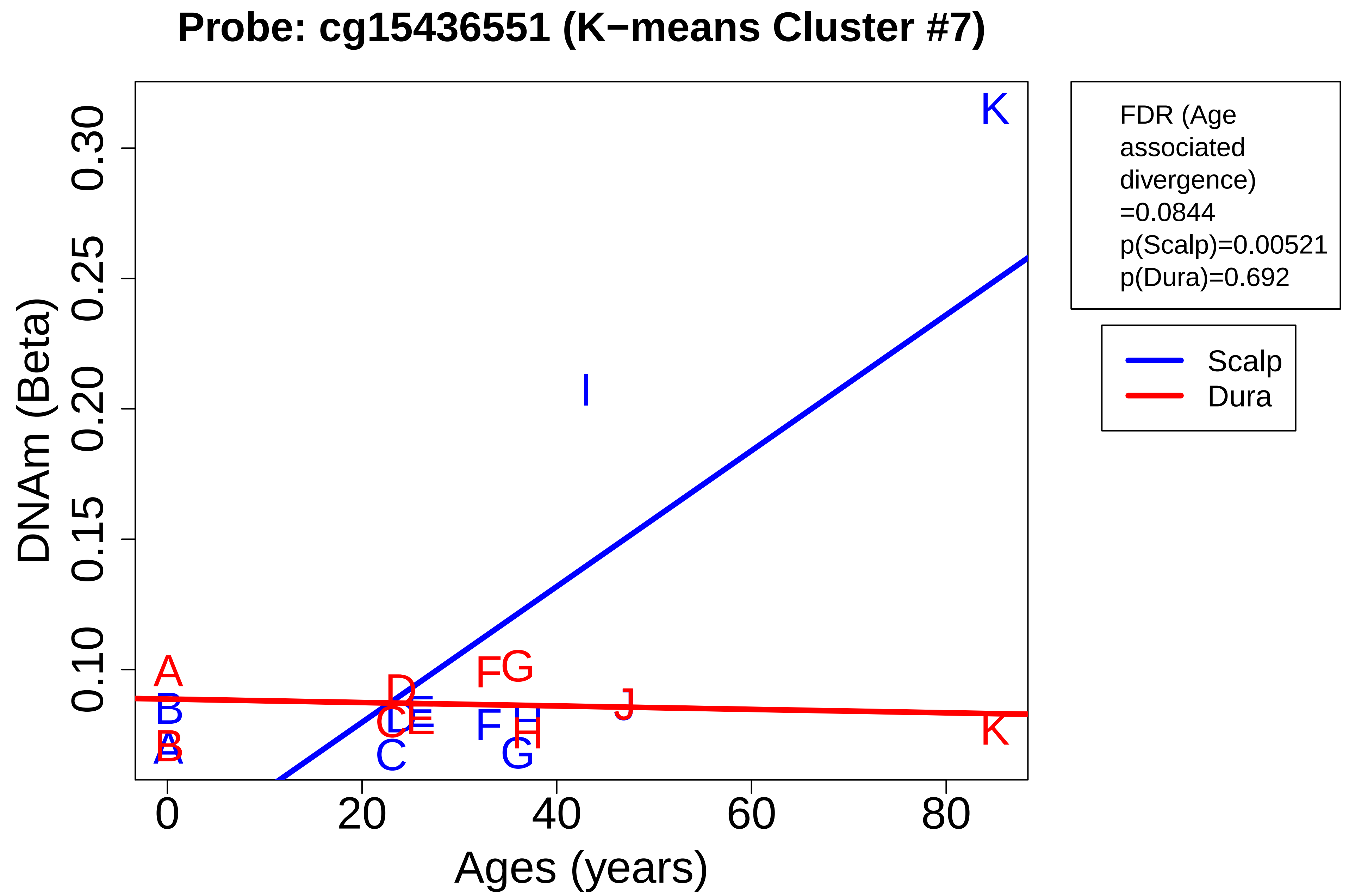
Probe: cg15436551 (K−means Cluster #7)
DNAm (Beta)
0.10
0.15
0.20
0.25
0.30
0
20
40
60
80
Ages (years)
FDR (Age associated divergence) =0.0844
p(Scalp)=0.00521
p(Dura)=0.692
Scalp
Dura

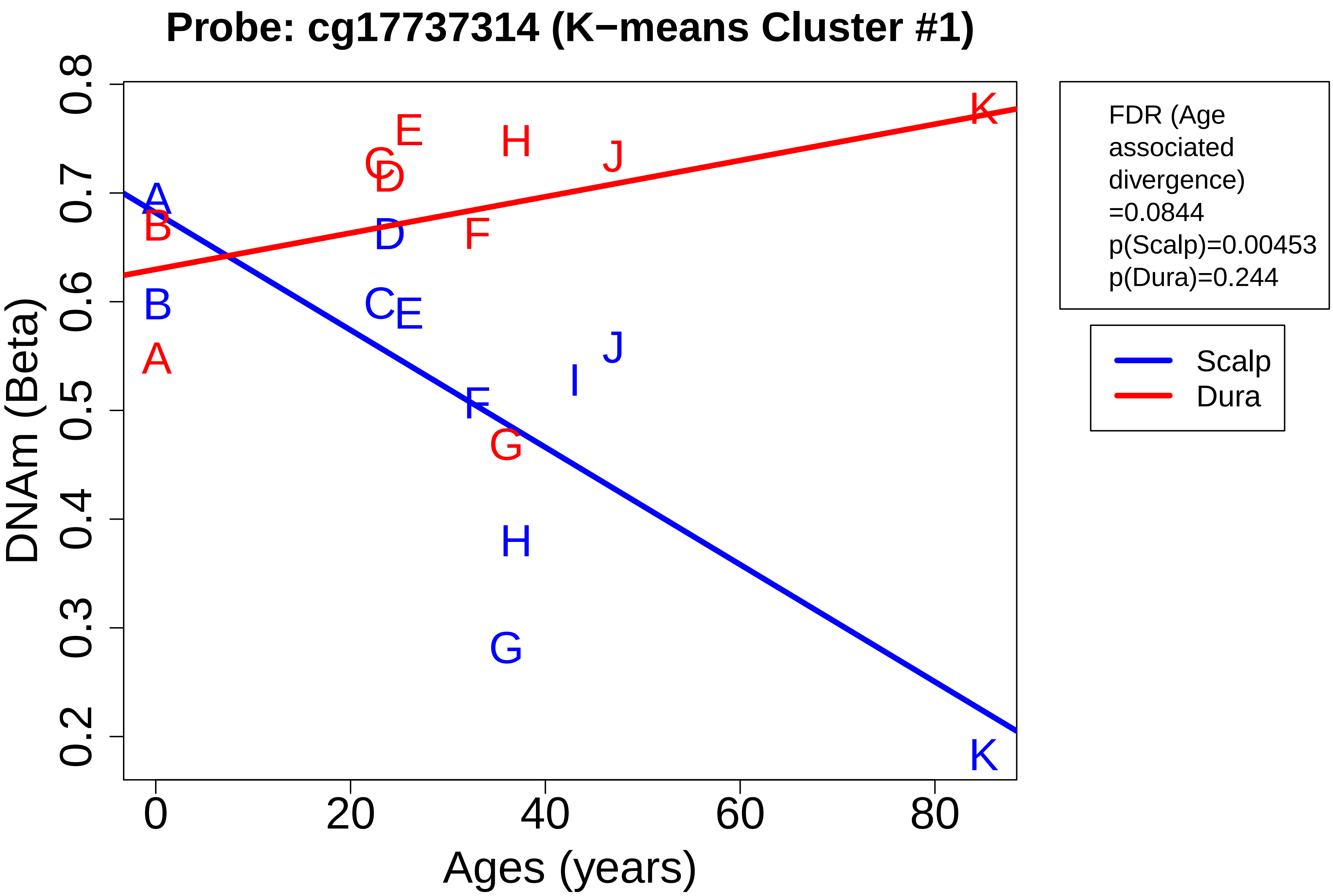

Probe: cg17737314 (K−means Cluster #1)
DNAm (Beta)
0.2
0.3
0.4
0.5
0.6
0.7
0.8
0
20
40
60
80
Ages (years)
A
B
C
D
E
F
G
H
I
J
K
FDR (Age associated divergence) =0.0844
p(Scalp)=0.00453
p(Dura)=0.244
Scalp
Dura

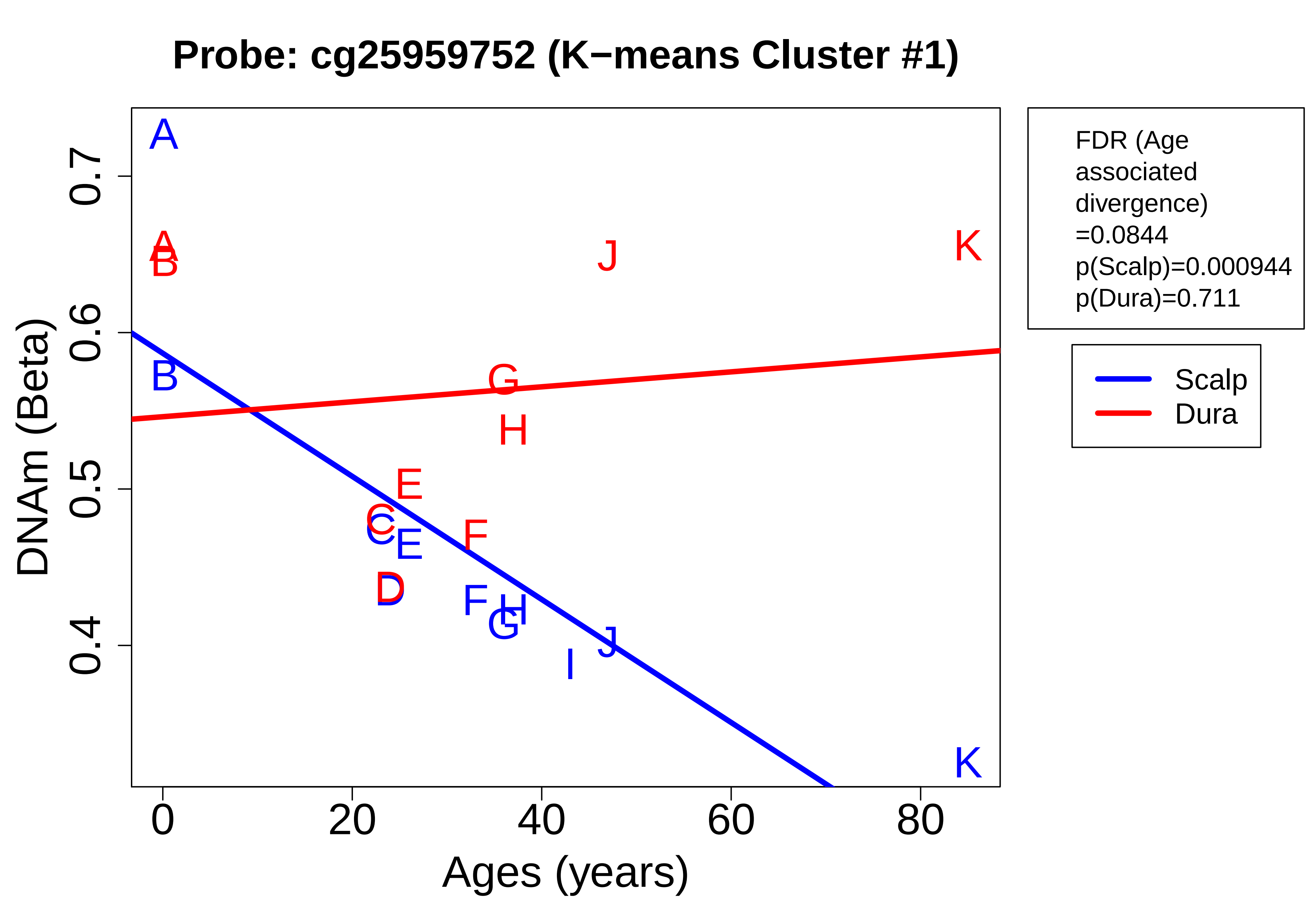

Probe: cg25959752 (K−means Cluster #1)
DNAm (Beta)
0.4
0.5
0.6
0.7
Ages (years)
0
20
40
60
80
FDR (Age associated divergence) =0.0844
p(Scalp)=0.000944
p(Dura)=0.711
Scalp
Dura
A
B
C
D
E
F
G
H
I
J
K

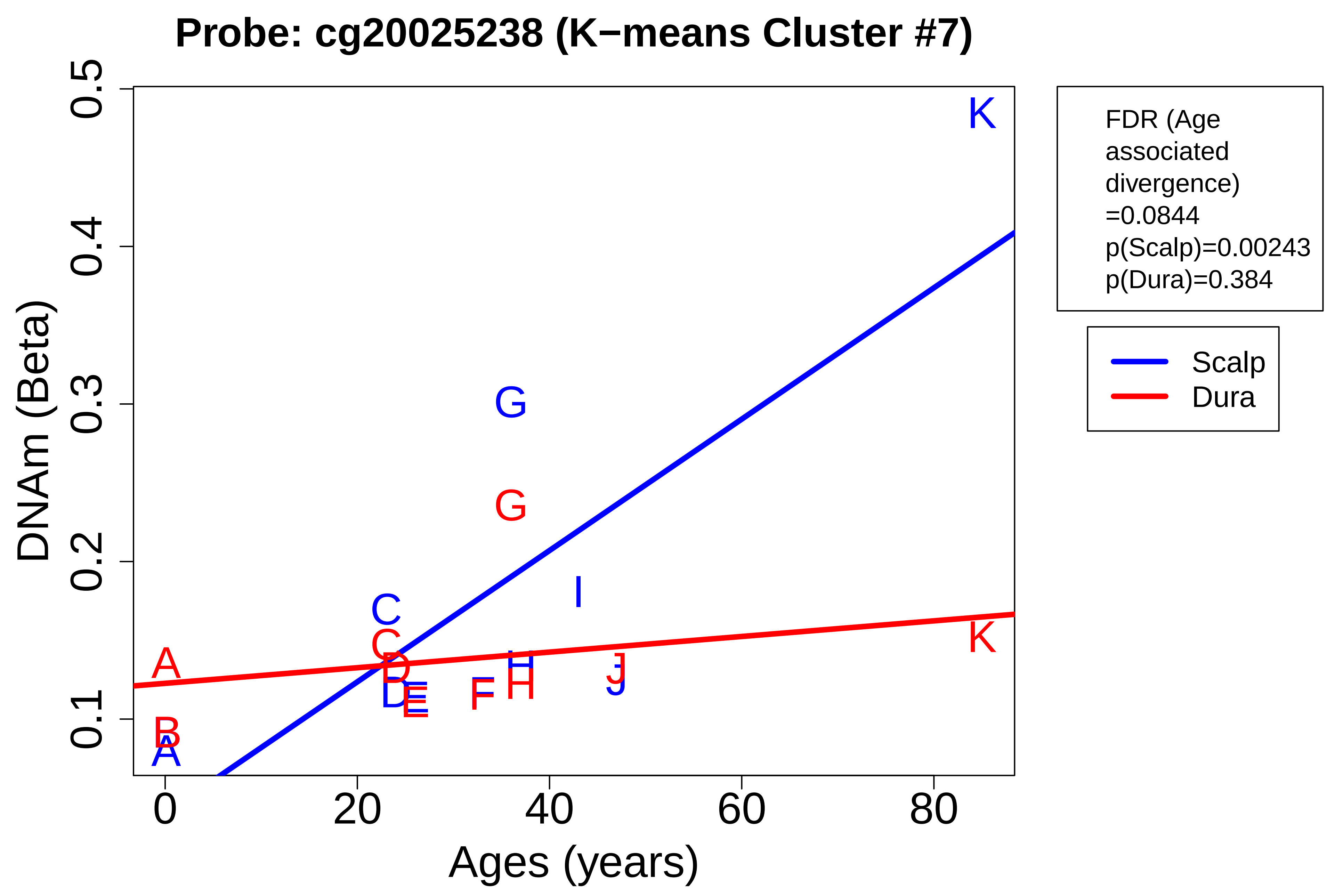

Probe: cg20025238 (K−means Cluster #7)
DNAm (Beta)
0.1
0.2
0.3
0.4
0.5
Ages (years)
0
20
40
60
80
A
B
C
D
E
F
G
H
I
J
K
FDR (Age associated divergence) =0.0844
p(Scalp)=0.00243
p(Dura)=0.384
Scalp
Dura

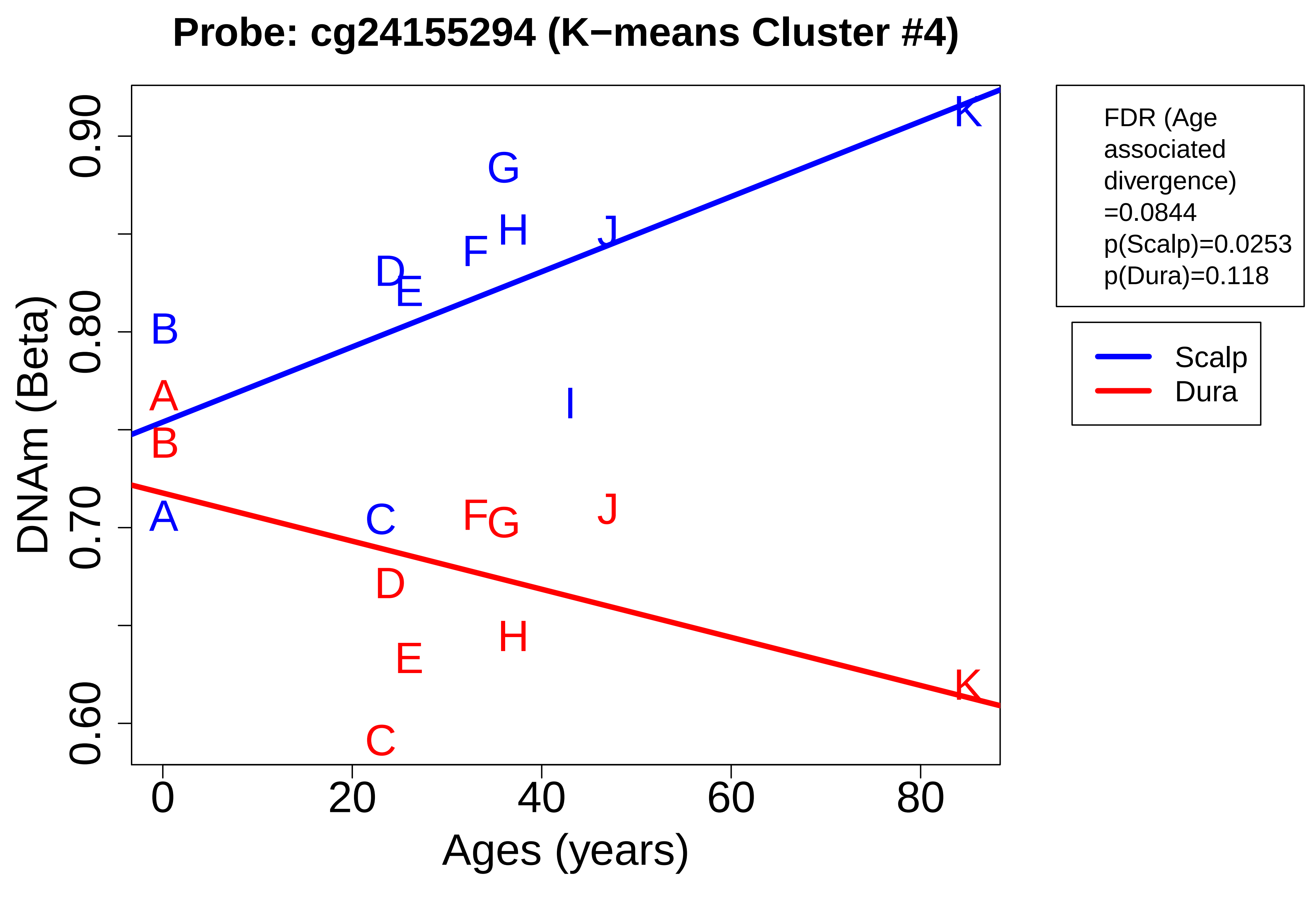

Probe: cg24155294 (K−means Cluster #4)
DNAm (Beta)
0.60
0.70
0.80
0.90
Ages (years)
0
20
40
60
80
FDR (Age associated divergence) =0.0844
p(Scalp)=0.0253
p(Dura)=0.118
Scalp
Dura

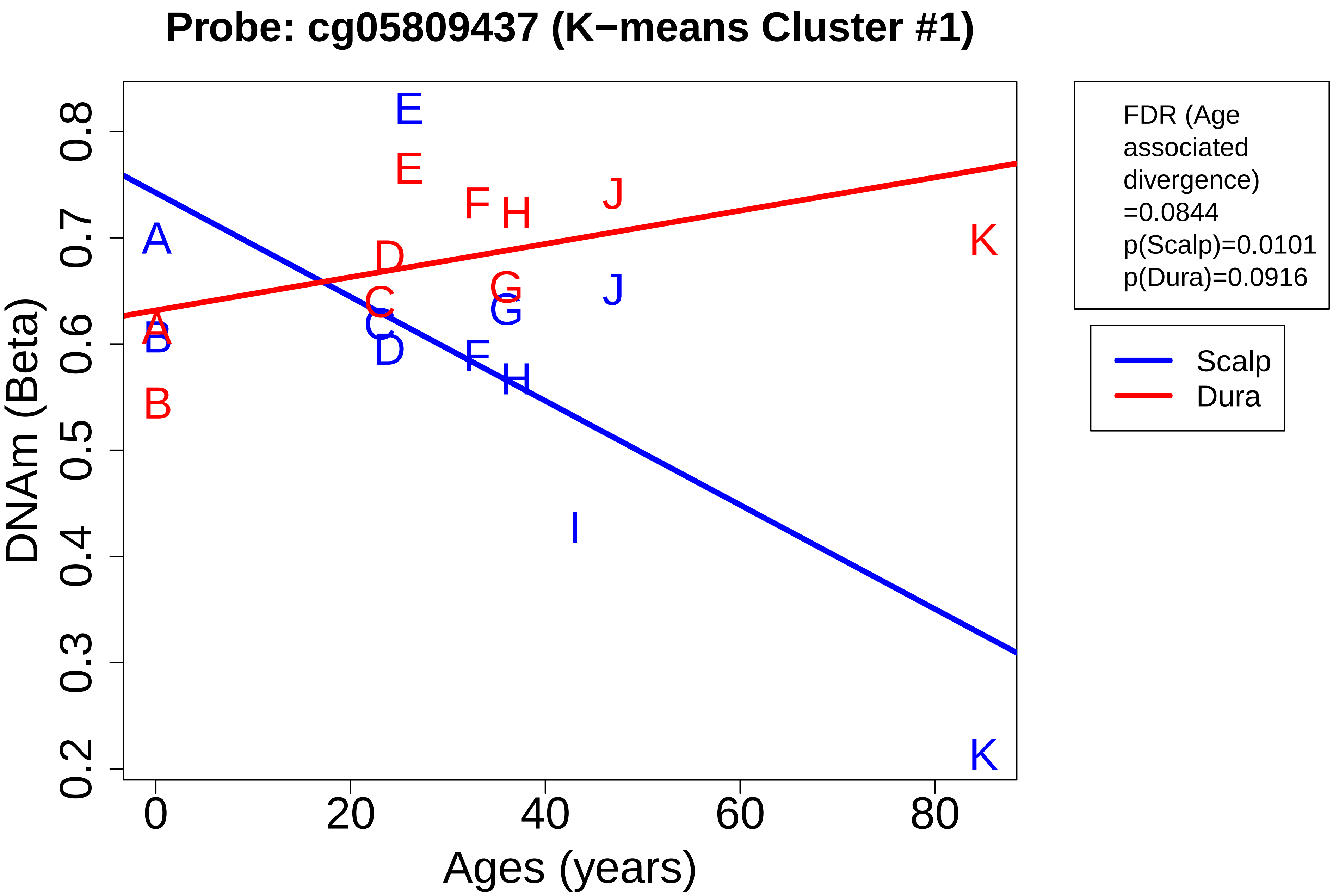

Probe: cg05809437 (K−means Cluster #1)
FDR (Age associated divergence) =0.0844
p(Scalp)=0.0101
p(Dura)=0.0916
Scalp
Dura
DNAm (Beta)
0.2
0.3
0.4
0.5
0.6
0.7
0.8
0
20
40
60
80
Ages (years)
A
B
C
D
E
F
G
H
I
J
K

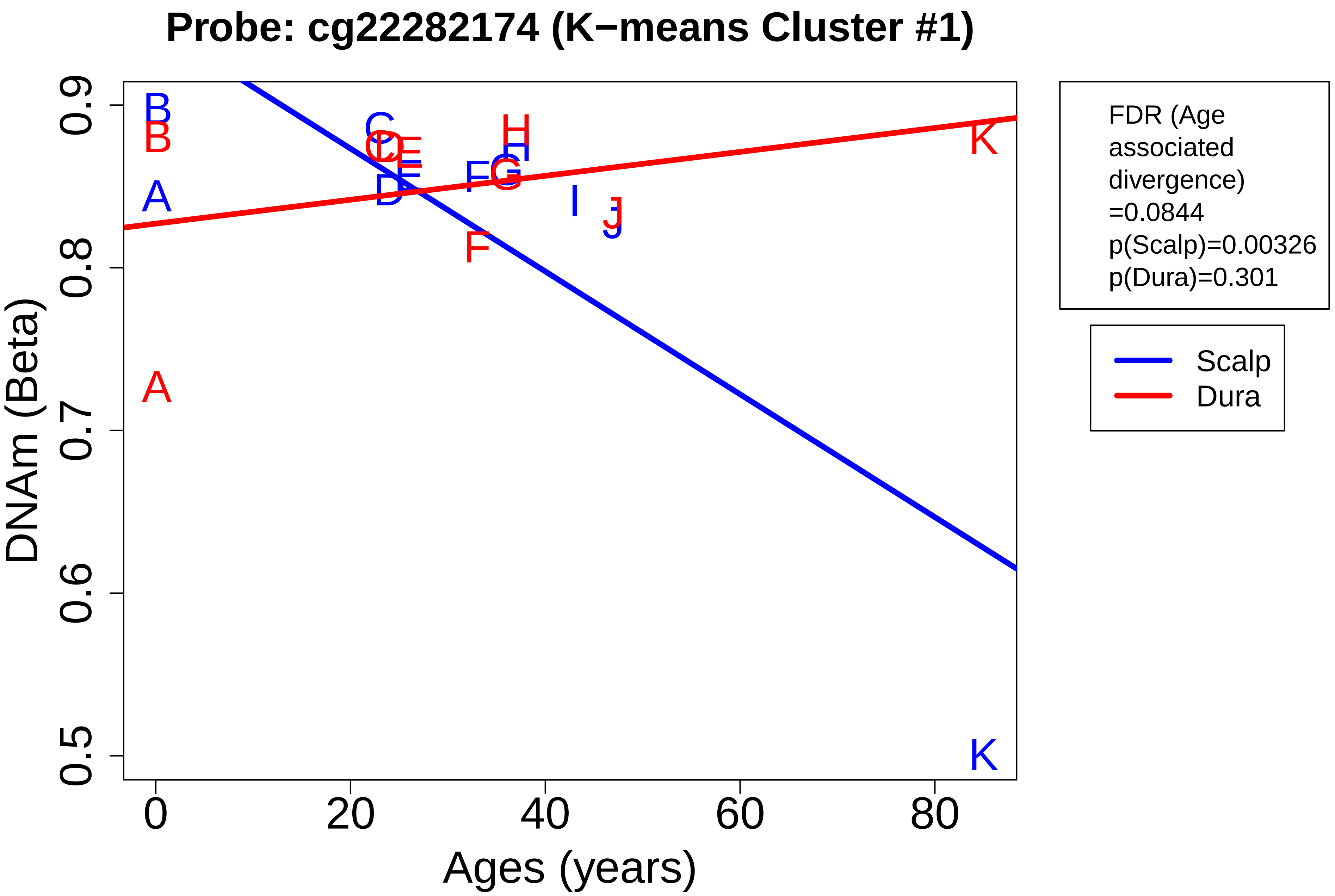

Probe: cg22282174 (K−means Cluster #1)
DNAm (Beta)
0.5
0.6
0.7
0.8
0.9
0
20
40
60
80
Ages (years)
FDR (Age associated divergence) =0.0844
p(Scalp)=0.00326
p(Dura)=0.301
Scalp
Dura

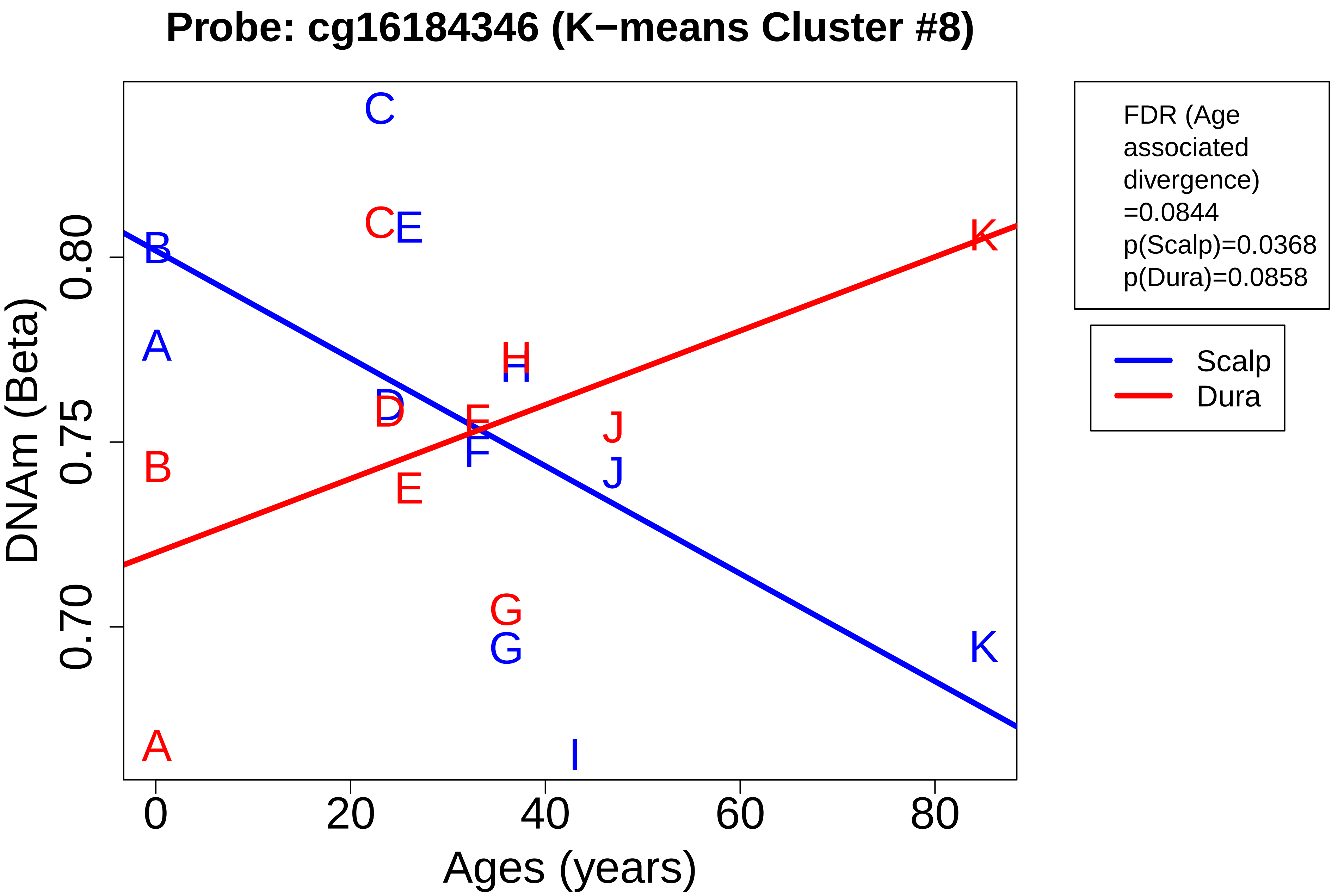

Probe: cg16184346 (K−means Cluster #8)
DNAm (Beta)
0.70
0.75
0.80
Ages (years)
0
20
40
60
80
FDR (Age associated divergence) =0.0844
p(Scalp)=0.0368
p(Dura)=0.0858
Scalp
Dura
A
B
C
D
E
F
G
H
I
J
K

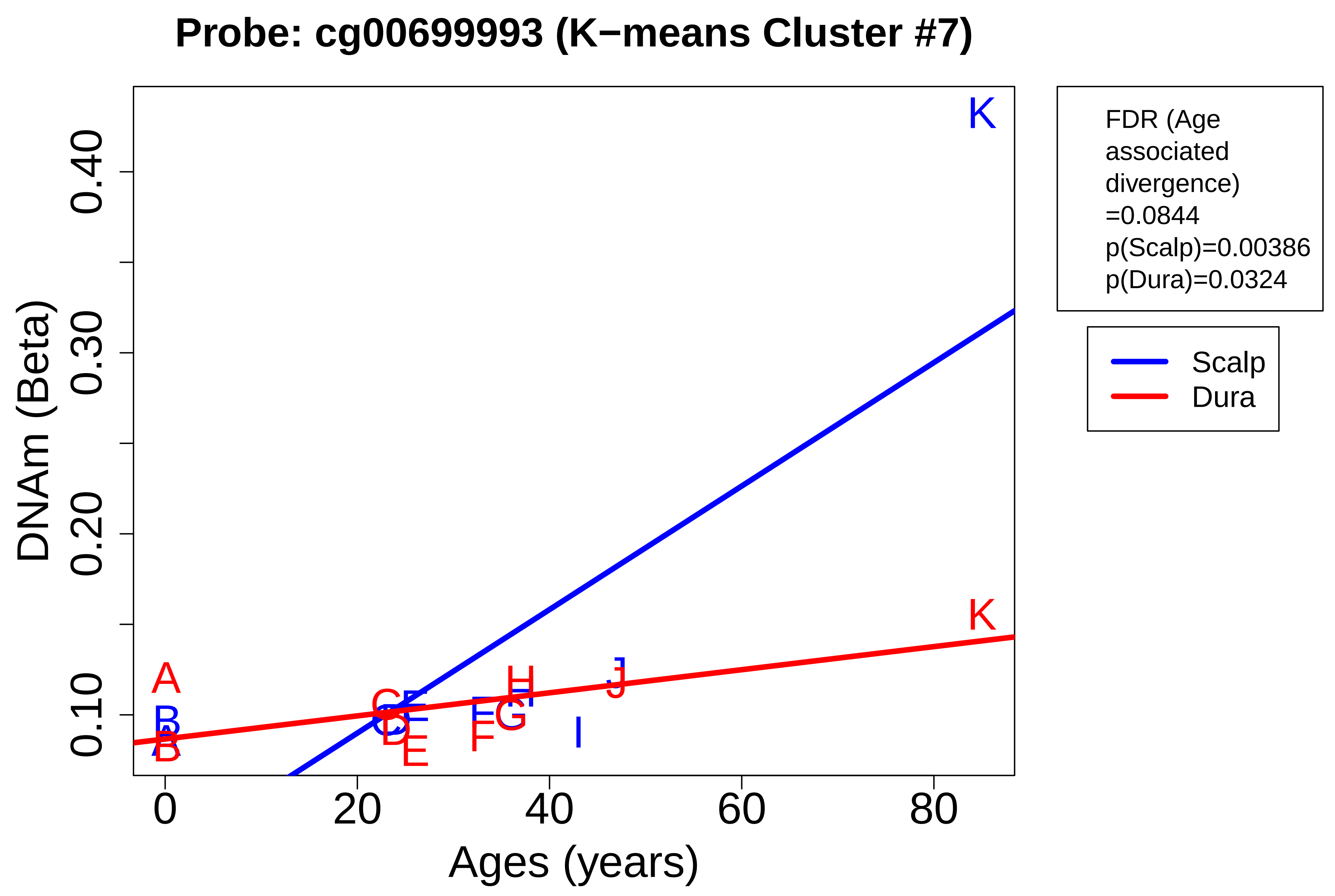

Probe: cg00699993 (K−means Cluster #7)
DNAm (Beta)
0.10
0.20
0.30
0.40
0
20
40
60
80
Ages (years)
FDR (Age associated divergence) =0.0844
p(Scalp)=0.00386
p(Dura)=0.0324
Scalp
Dura

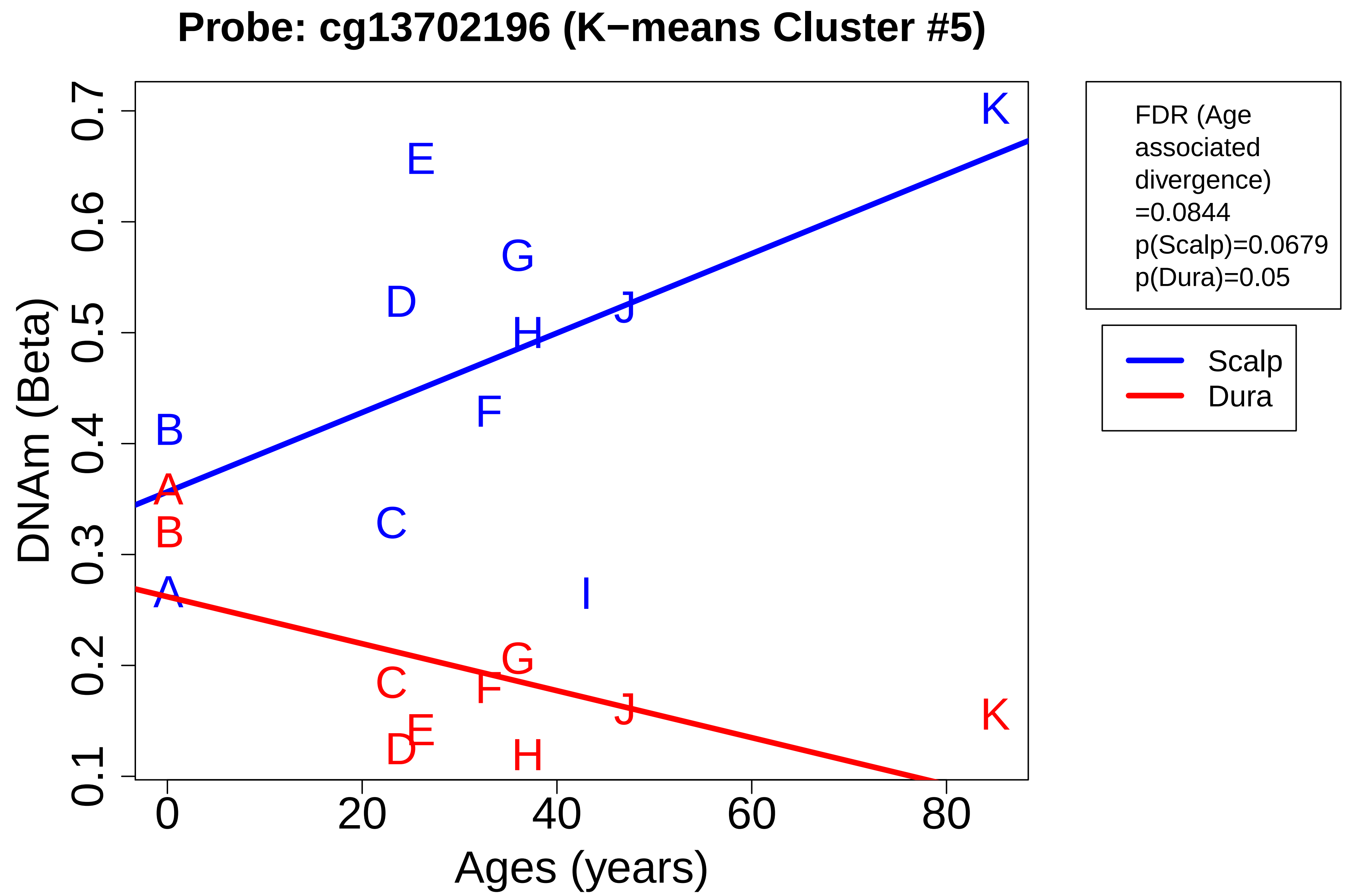

Probe: cg13702196 (K−means Cluster #5)
DNAm (Beta)
0.1
0.2
0.3
0.4
0.5
0.6
0.7
0
20
40
60
80
Ages (years)
FDR (Age associated divergence) =0.0844
p(Scalp)=0.0679
p(Dura)=0.05
Scalp
Dura
A
B
C
D
E
F
G
H
I
J
K

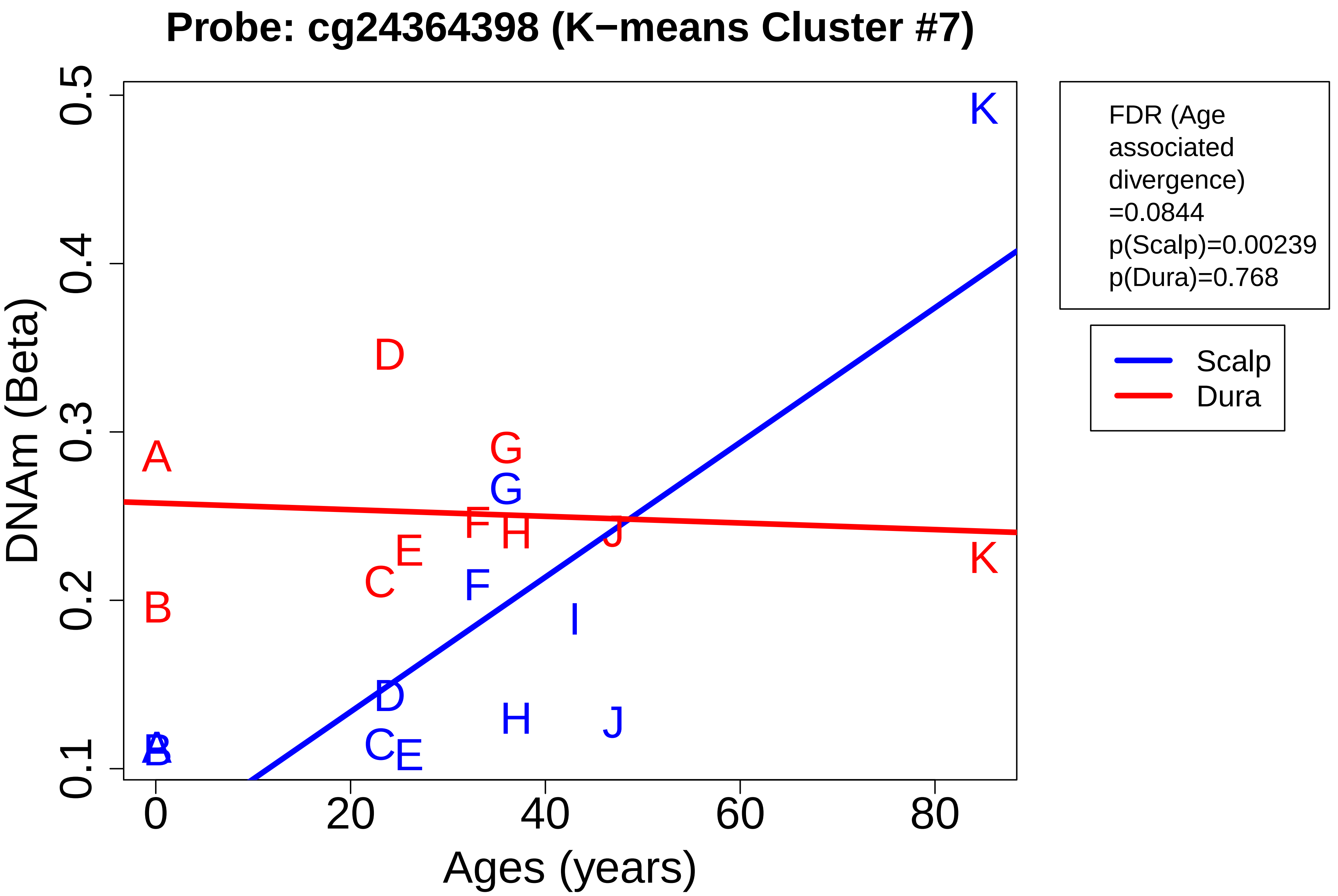

Probe: cg24364398 (K−means Cluster #7)
DNAm (Beta)
Ages (years)
0.1
0.2
0.3
0.4
0.5
0
20
40
60
80
FDR (Age associated divergence) =0.0844
p(Scalp)=0.00239
p(Dura)=0.768
Scalp
Dura

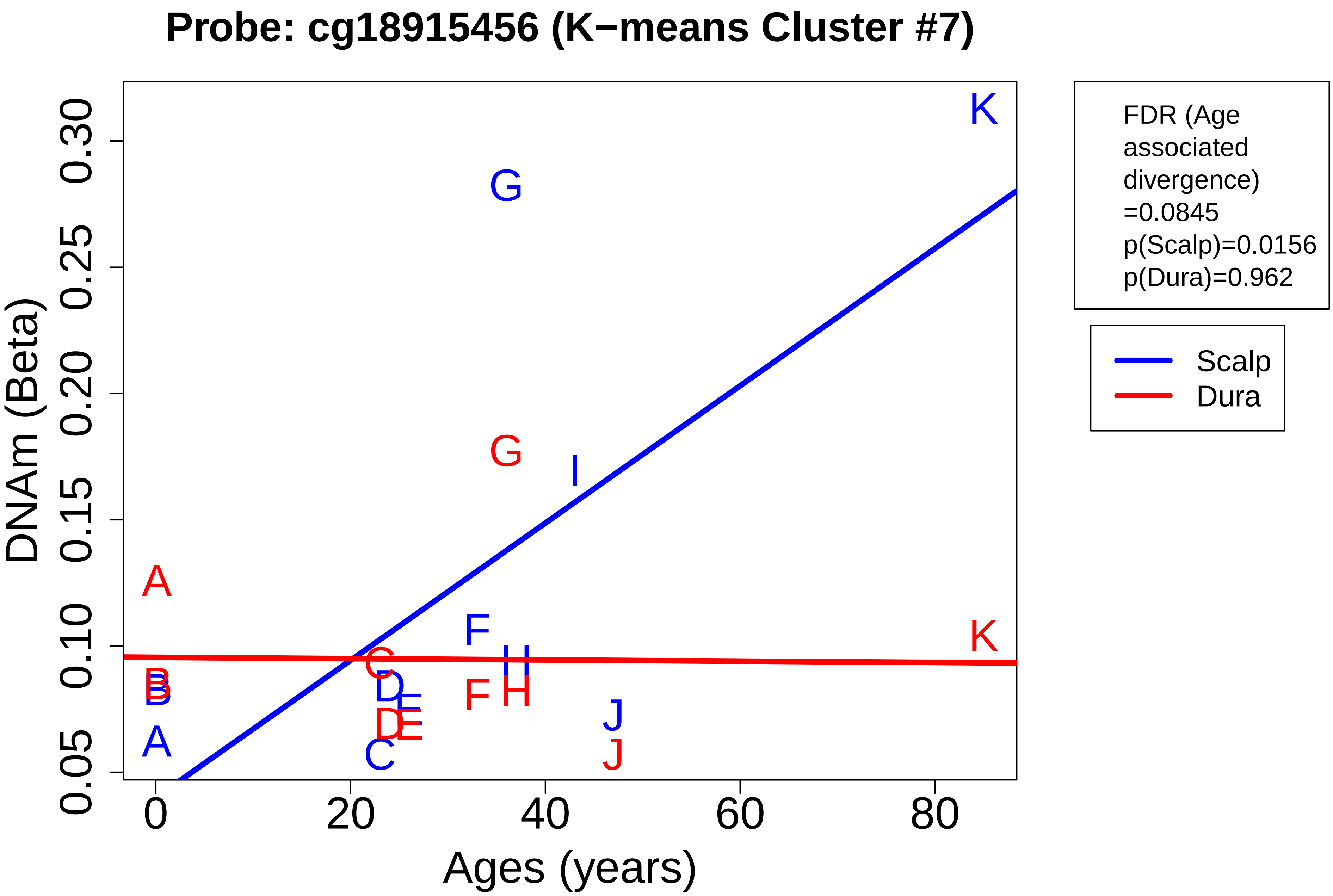
Probe: cg18915456 (K−means Cluster #7)
DNAm (Beta)
0.05
0.10
0.15
0.20
0.25
0.30
0
20
40
60
80
Ages (years)
FDR (Age associated divergence) =0.0845
p(Scalp)=0.0156
p(Dura)=0.962
Scalp
Dura

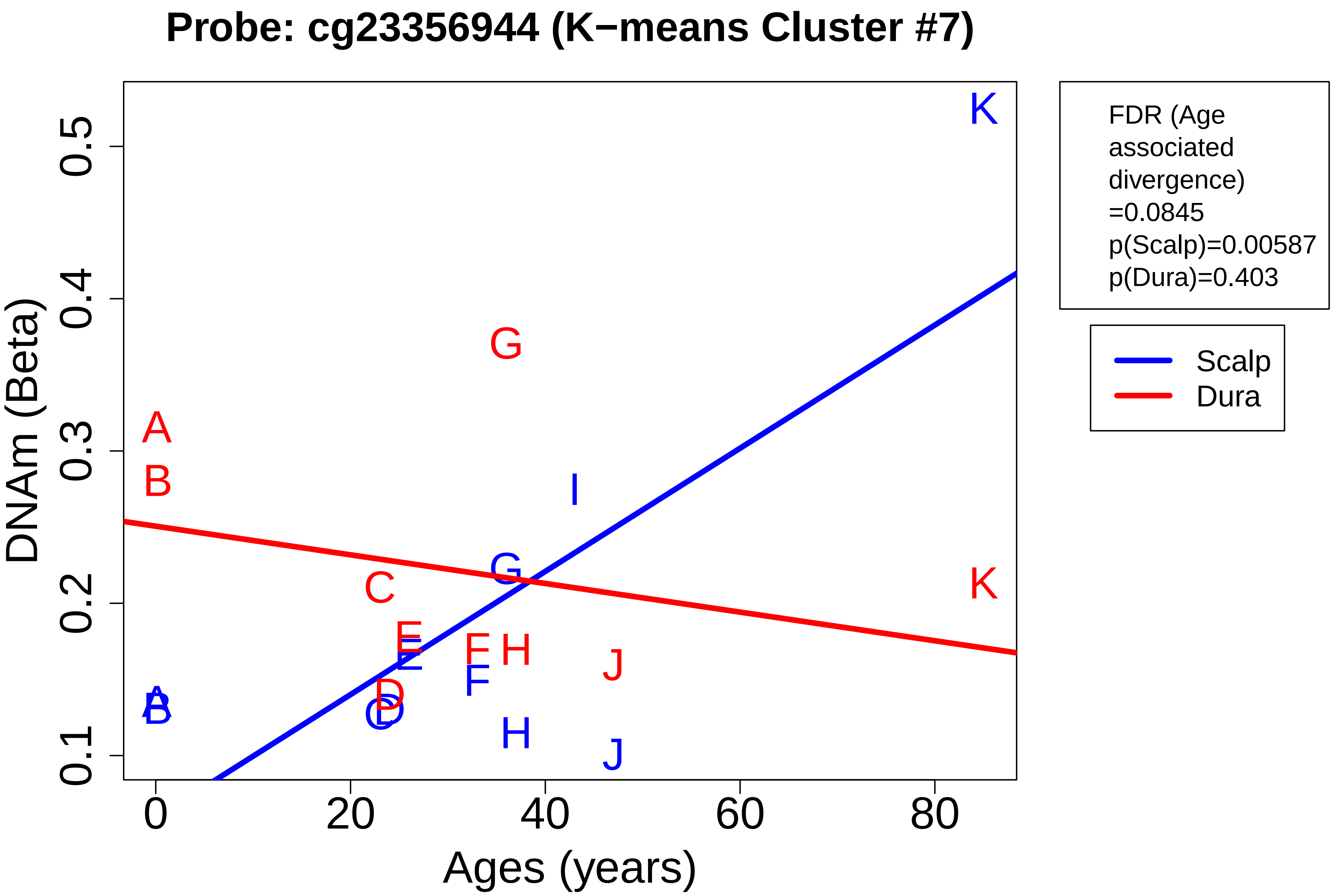
Probe: cg23356944 (K−means Cluster #7)
DNAm (Beta)
0.1
0.2
0.3
0.4
0.5
0
20
40
60
80
Ages (years)
FDR (Age associated divergence) =0.0845
p(Scalp)=0.00587
p(Dura)=0.403
Scalp
Dura

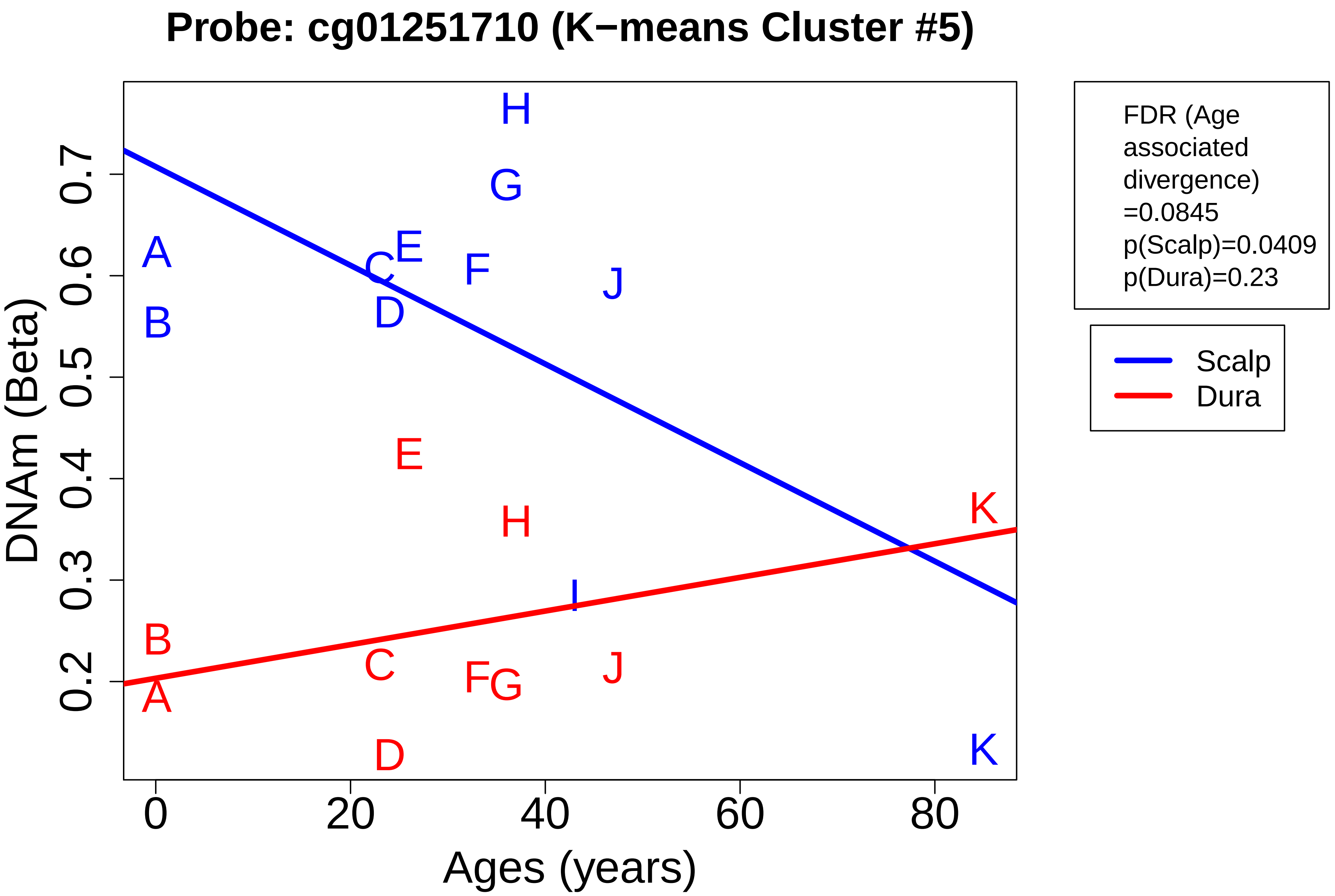

Probe: cg01251710 (K−means Cluster #5)
DNAm (Beta)
0.2
0.3
0.4
0.5
0.6
0.7
0
20
40
60
80
Ages (years)
A
B
C
D
E
F
G
H
I
J
K
FDR (Age associated divergence) =0.0845
p(Scalp)=0.0409
p(Dura)=0.23
Scalp
Dura

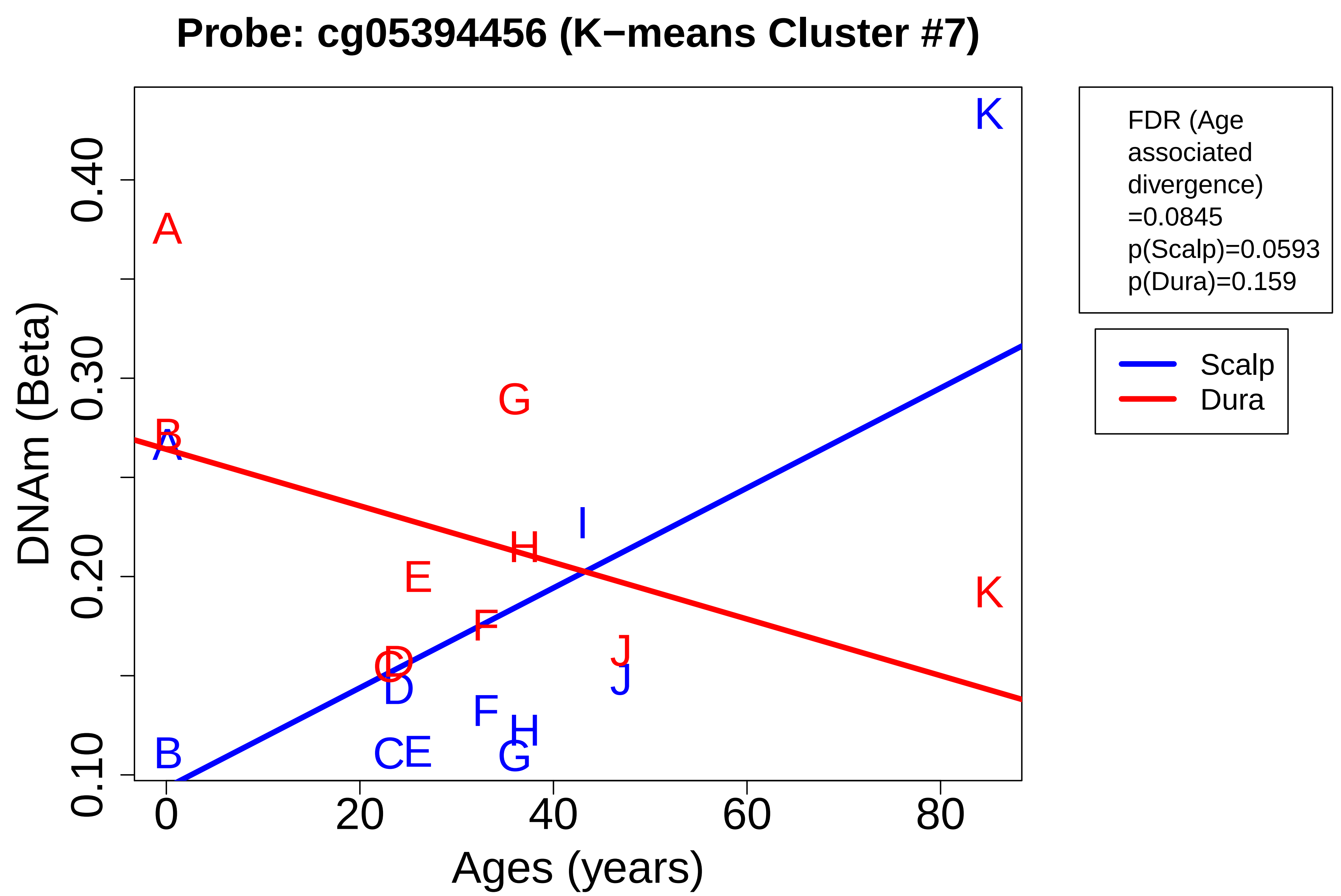

Probe: cg05394456 (K−means Cluster #7)
DNAm (Beta)
0.10
0.20
0.30
0.40
0
20
40
60
80
Ages (years)
FDR (Age associated divergence) =0.0845
p(Scalp)=0.0593
p(Dura)=0.159
Scalp
Dura

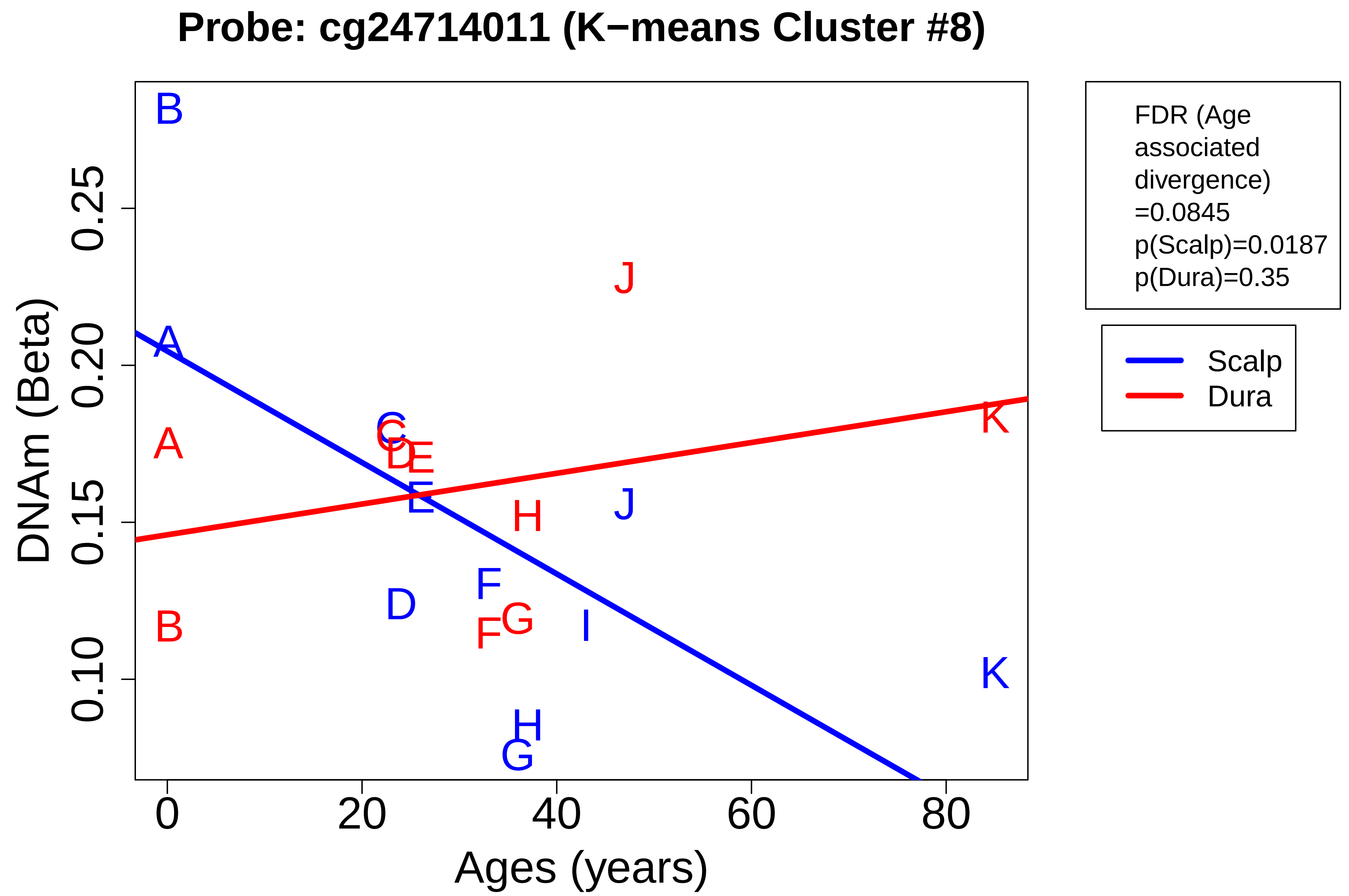

Probe: cg24714011 (K−means Cluster #8)
DNAm (Beta)
0.10
0.15
0.20
0.25
0
20
40
60
80
Ages (years)
FDR (Age associated divergence) =0.0845
p(Scalp)=0.0187
p(Dura)=0.35
Scalp
Dura

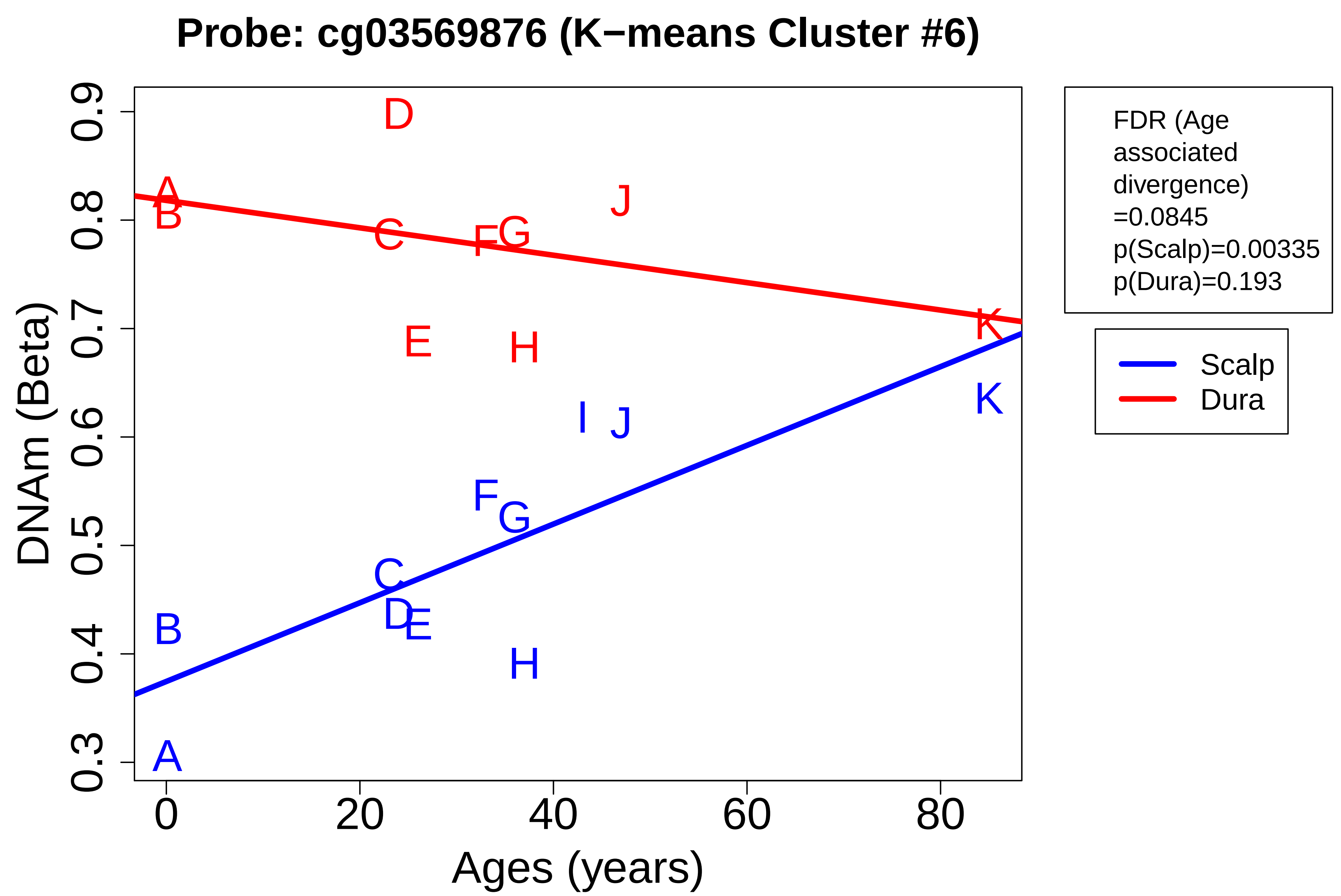

Probe: cg03569876 (K−means Cluster #6)
DNAm (Beta)
0.3
0.4
0.5
0.6
0.7
0.8
0.9
Ages (years)
0
20
40
60
80
A
B
C
D
E
F
G
H
I
J
K
FDR (Age associated divergence) =0.0845
p(Scalp)=0.00335
p(Dura)=0.193
Scalp
Dura

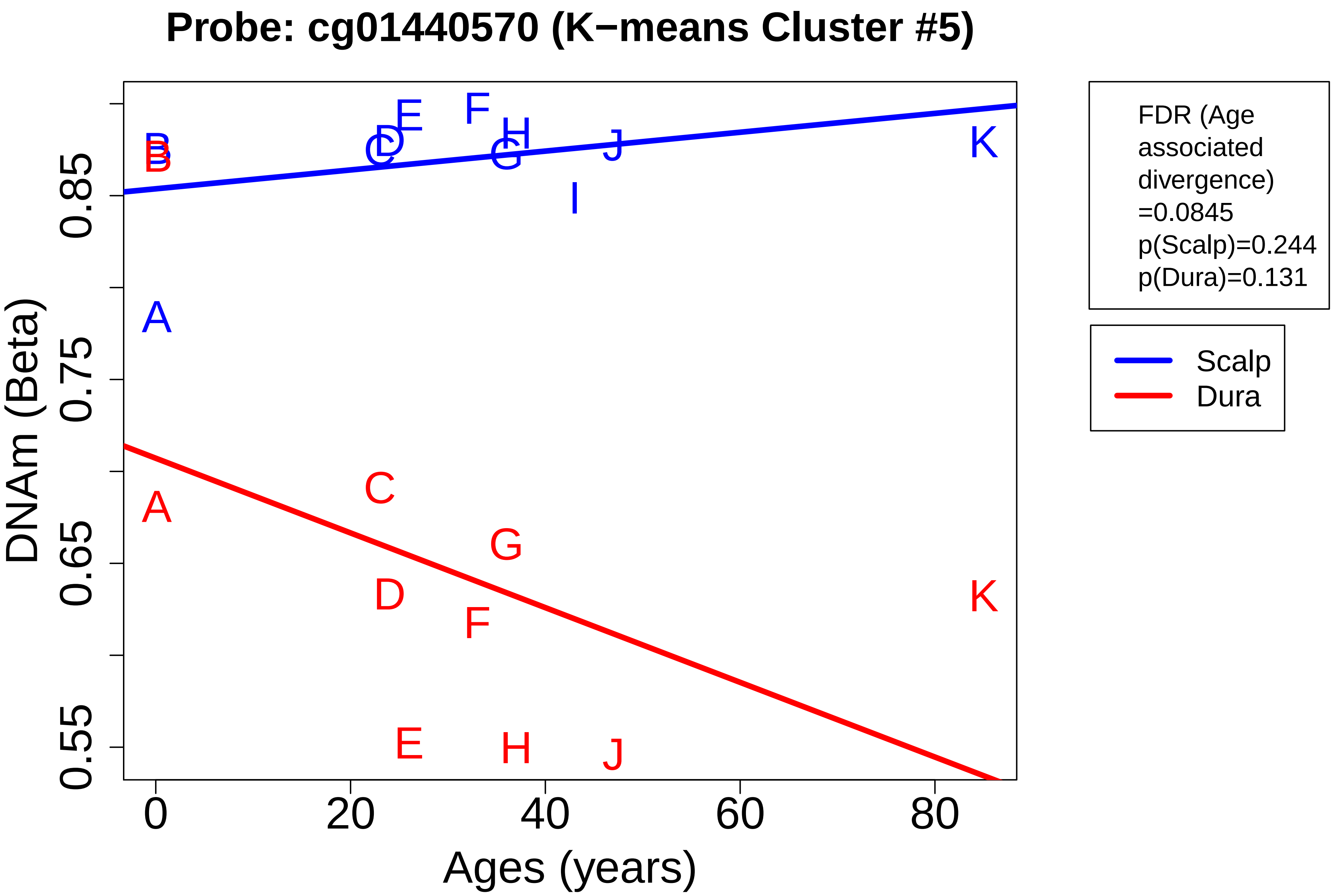

Probe: cg01440570 (K−means Cluster #5)
DNAm (Beta)
0.55
0.65
0.75
0.85
Ages (years)
0
20
40
60
80
FDR (Age associated divergence) =0.0845
p(Scalp)=0.244
p(Dura)=0.131
Scalp
Dura

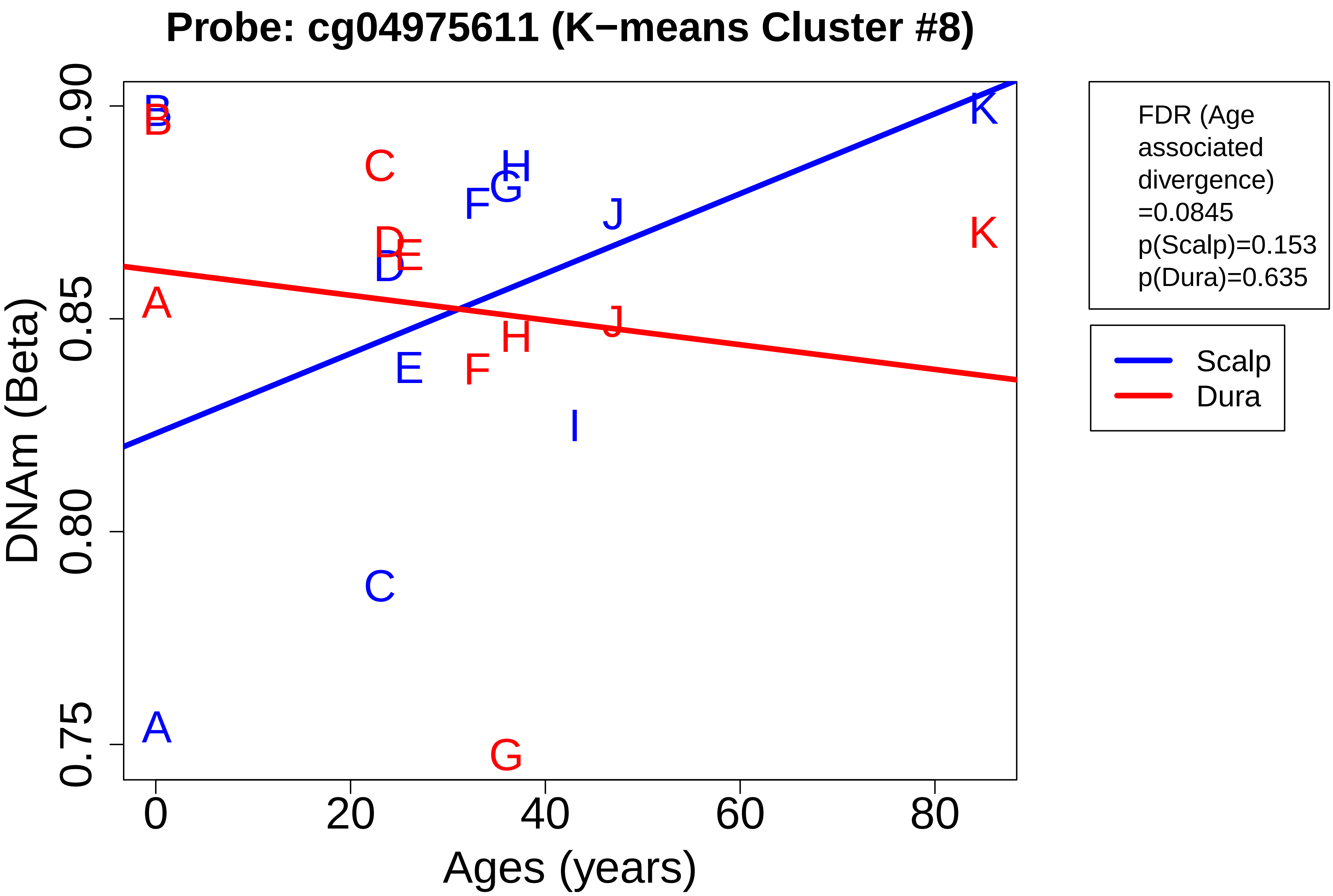
Probe: cg04975611 (K−means Cluster #8)
DNAm (Beta)
0.75
0.80
0.85
0.90
0
20
40
60
80
Ages (years)
A
B
C
D
E
F
G
H
I
J
K
FDR (Age associated divergence) =0.0845
p(Scalp)=0.153
p(Dura)=0.635
Scalp
Dura

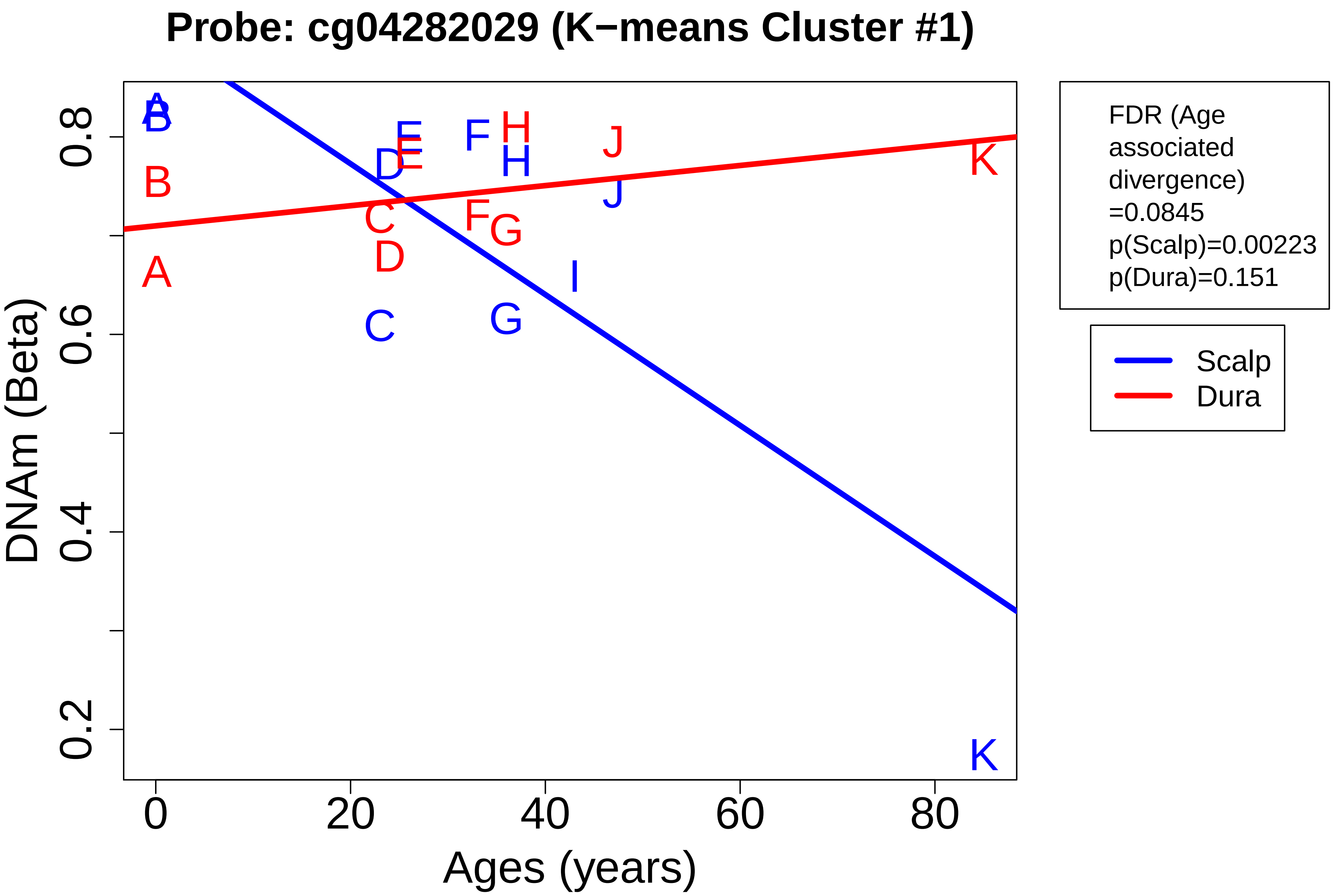

Probe: cg04282029 (K−means Cluster #1)
DNAm (Beta)
Ages (years)
0.2
0.4
0.6
0.8
0
20
40
60
80
FDR (Age associated divergence) =0.0845
p(Scalp)=0.00223
p(Dura)=0.151
Scalp
Dura

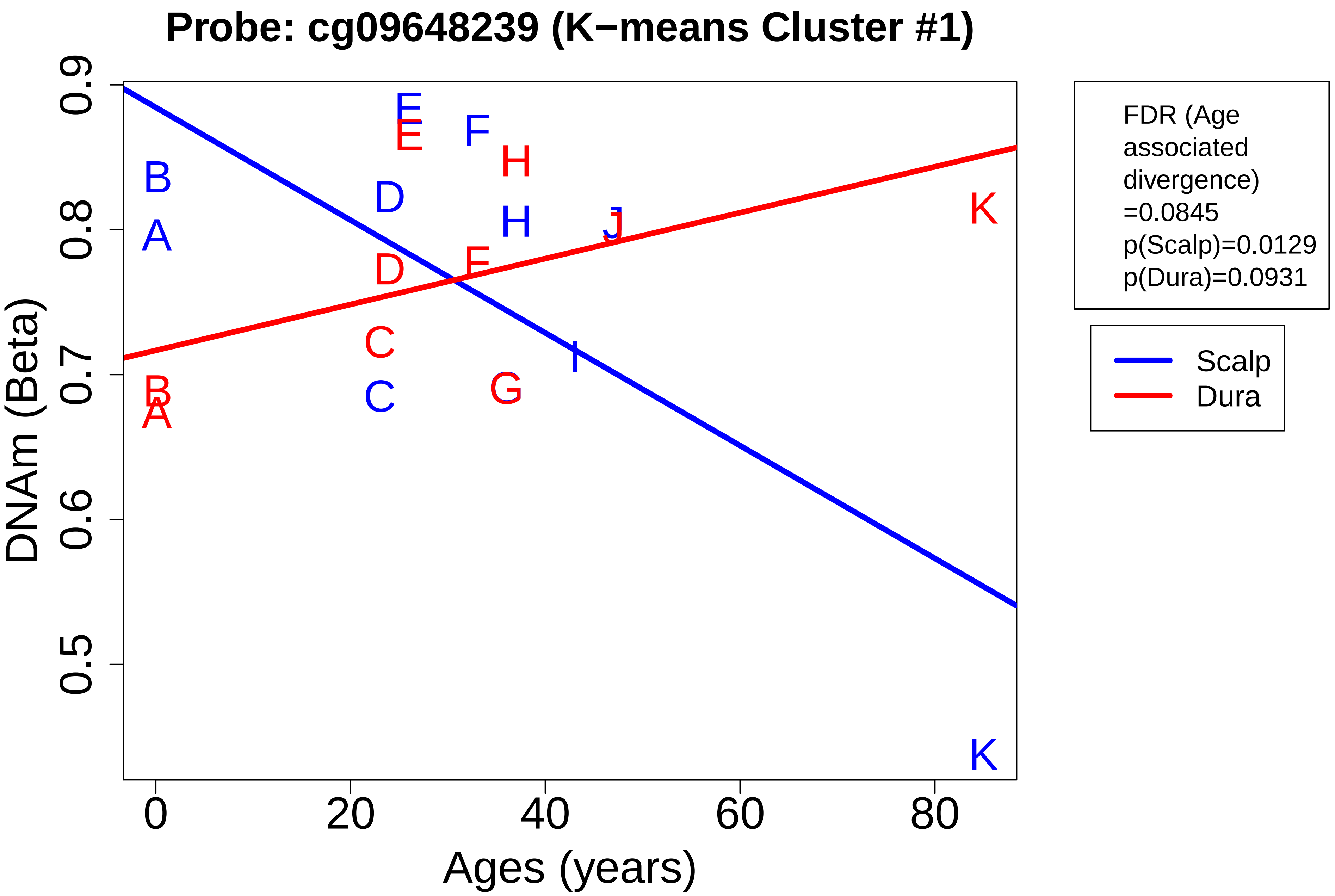

Probe: cg09648239 (K−means Cluster #1)
DNAm (Beta)
0.5
0.6
0.7
0.8
0.9
0
20
40
60
80
Ages (years)
FDR (Age associated divergence) =0.0845
p(Scalp)=0.0129
p(Dura)=0.0931
Scalp
Dura
A
B
C
D
E
F
G
H
I
J
K

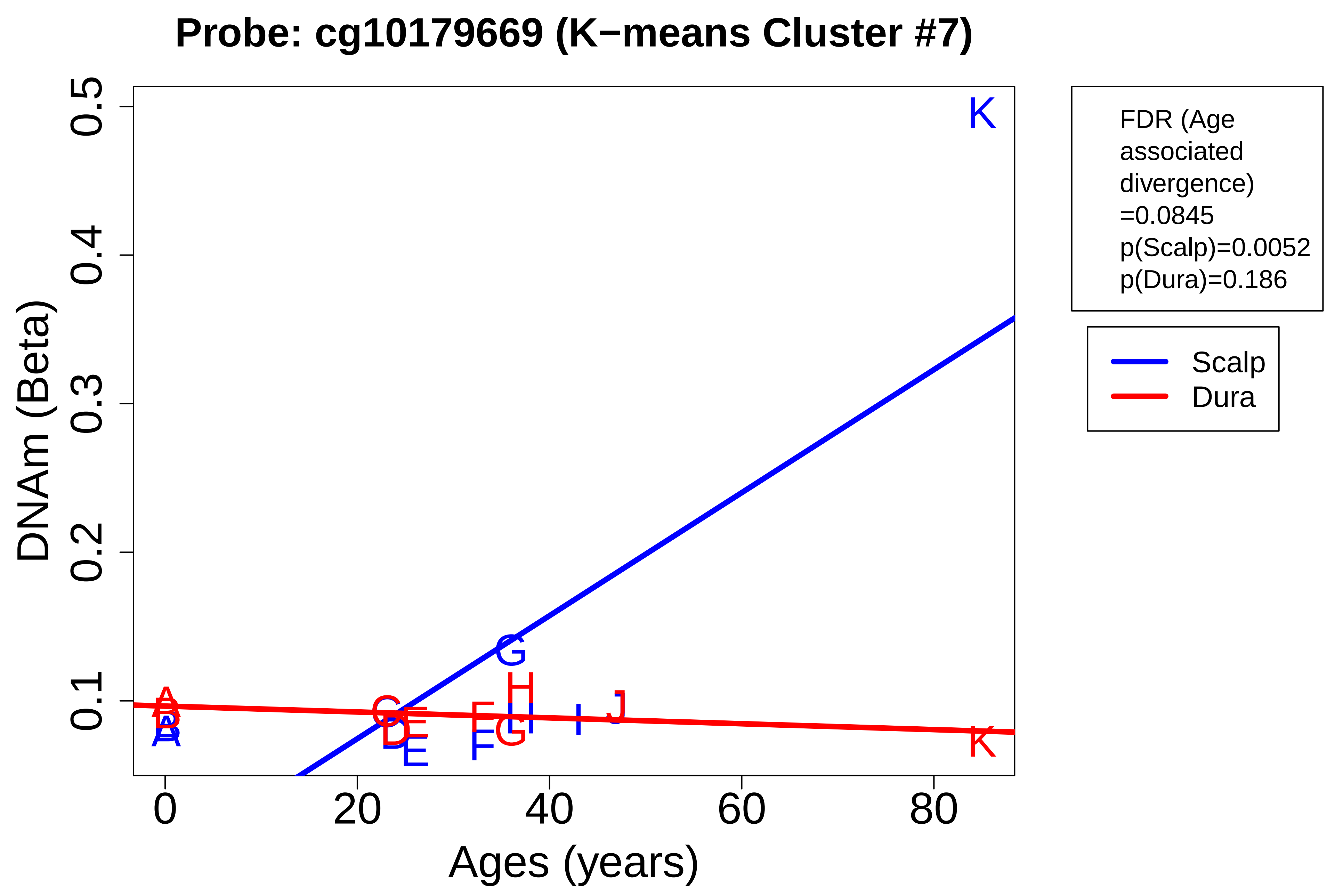

Probe: cg10179669 (K−means Cluster #7)
DNAm (Beta)
0.1
0.2
0.3
0.4
0.5
0
20
40
60
80
Ages (years)
FDR (Age associated divergence) =0.0845
p(Scalp)=0.0052
p(Dura)=0.186
Scalp
Dura

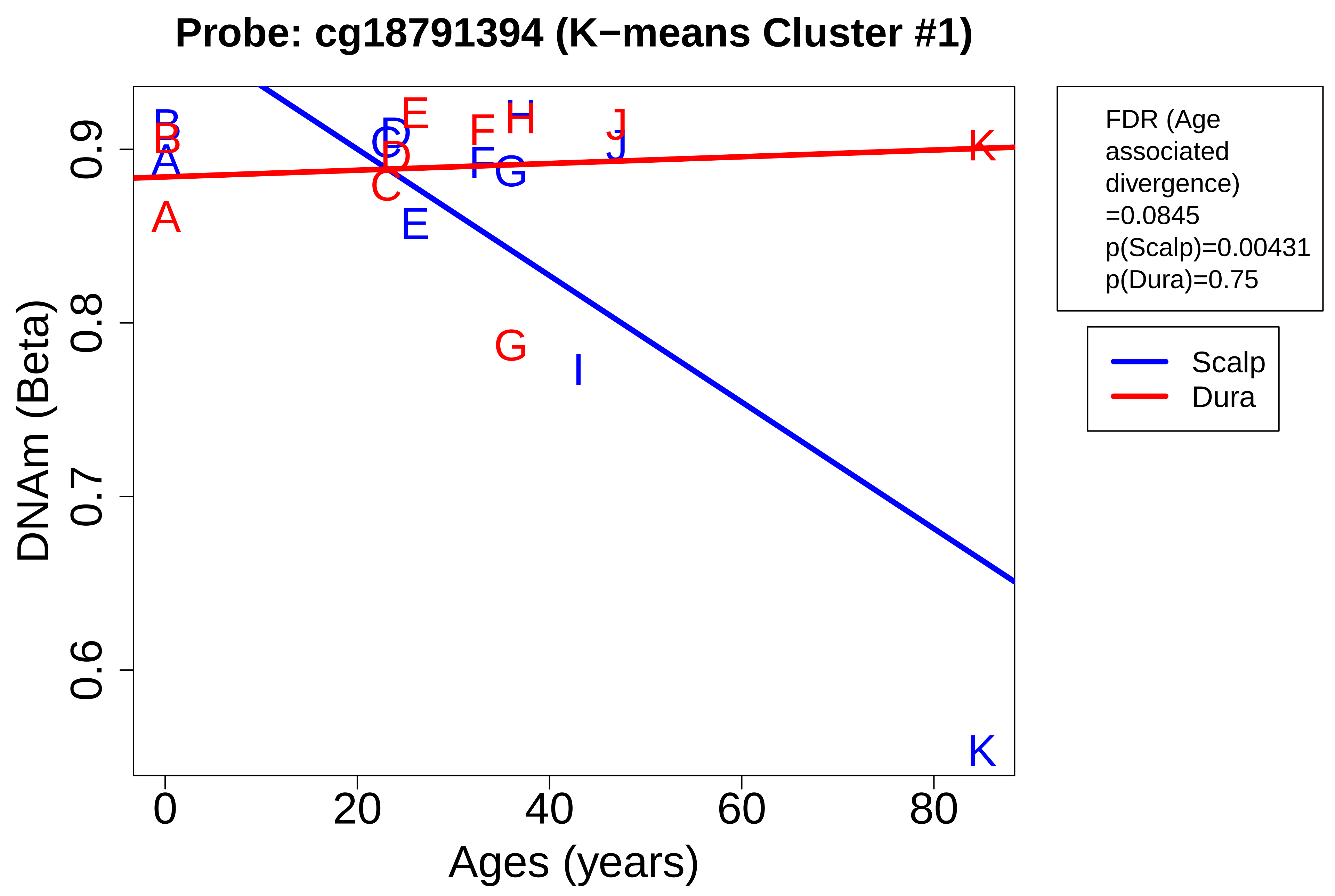

Probe: cg18791394 (K−means Cluster #1)
FDR (Age associated divergence) =0.0845
p(Scalp)=0.00431
p(Dura)=0.75
Scalp
Dura
DNAm (Beta)
0.6
0.7
0.8
0.9
0
20
40
60
80
Ages (years)

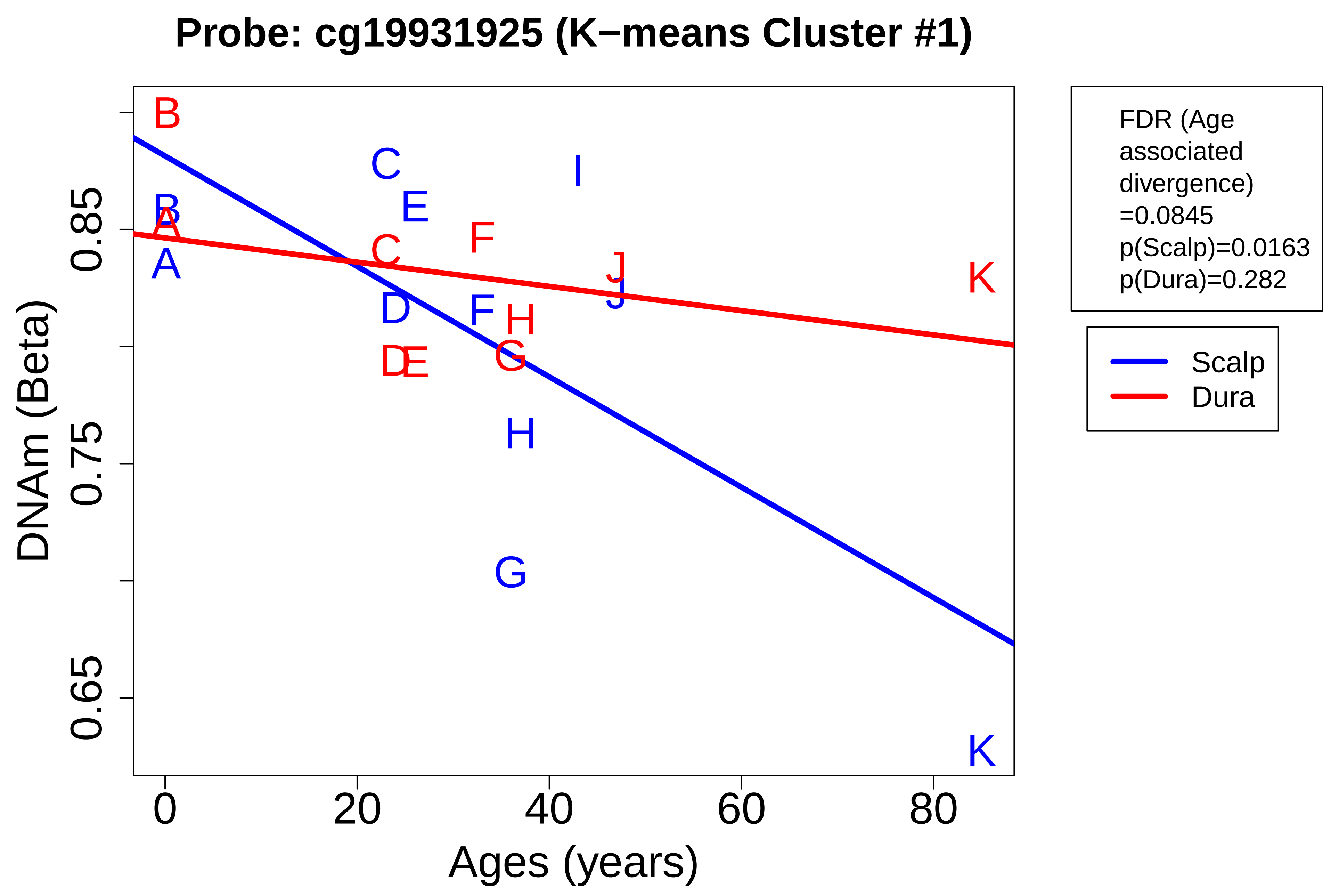

Probe: cg19931925 (K−means Cluster #1)
DNAm (Beta)
0.65
0.75
0.85
0
20
40
60
80
Ages (years)
A
B
C
D
E
F
G
H
I
J
K
FDR (Age associated divergence) =0.0845
p(Scalp)=0.0163
p(Dura)=0.282
Scalp
Dura

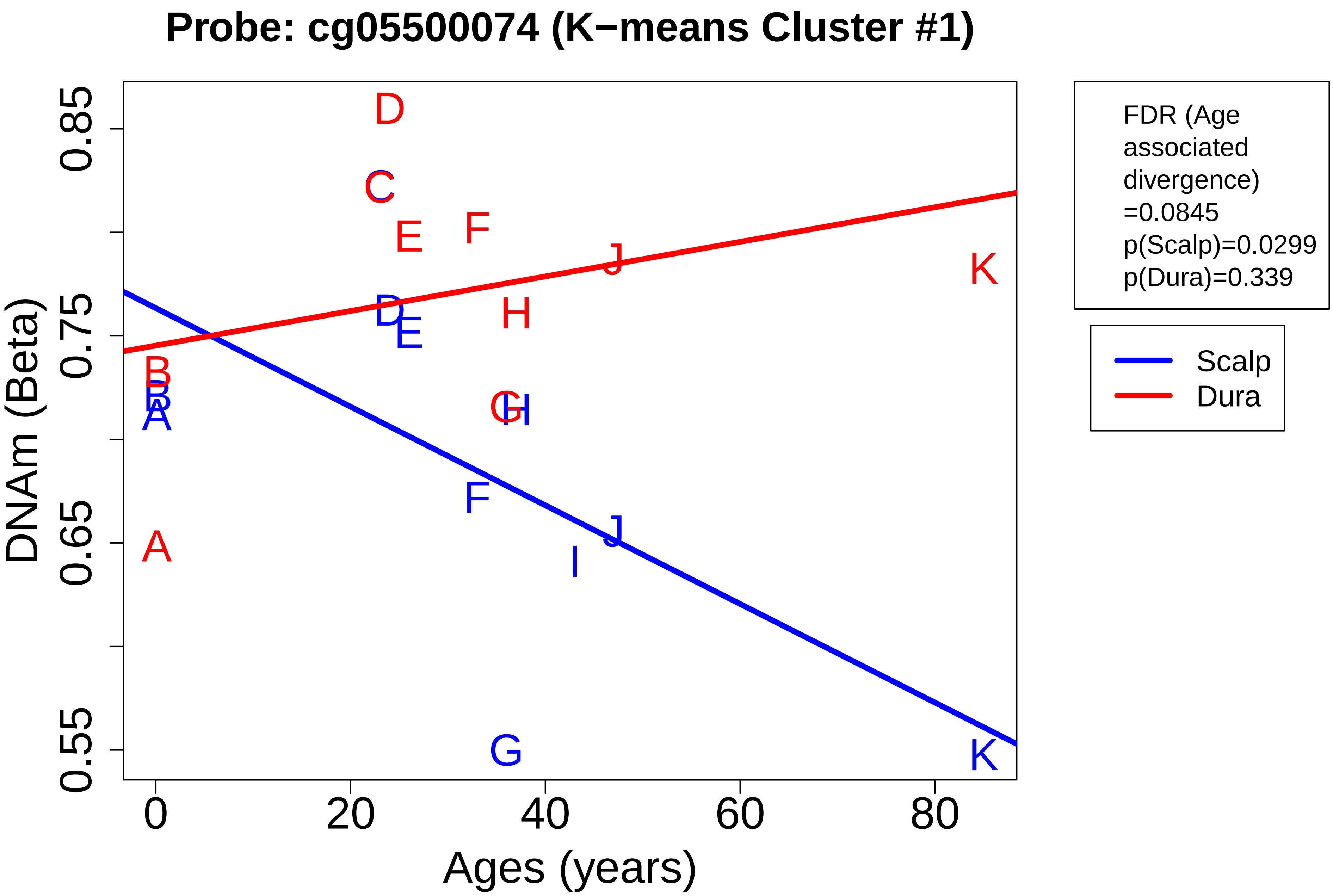

Probe: cg05500074 (K−means Cluster #1)
DNAm (Beta)
0.55
0.65
0.75
0.85
Ages (years)
0
20
40
60
80
FDR (Age associated divergence) =0.0845
p(Scalp)=0.0299
p(Dura)=0.339
Scalp
Dura

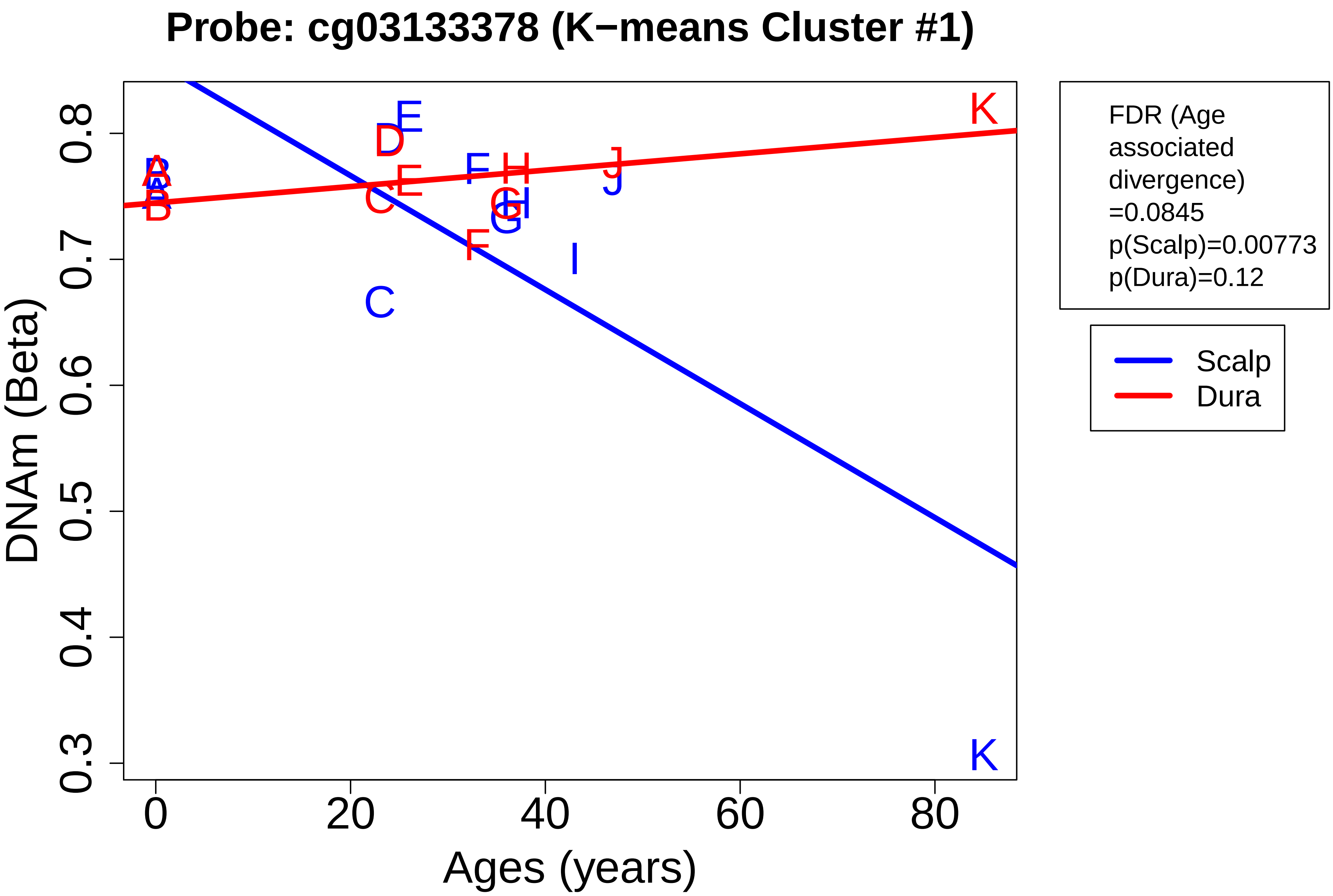

Probe: cg03133378 (K−means Cluster #1)
DNAm (Beta)
0.3
0.4
0.5
0.6
0.7
0.8
0
20
40
60
80
Ages (years)
FDR (Age associated divergence) =0.0845
p(Scalp)=0.00773
p(Dura)=0.12
Scalp
Dura

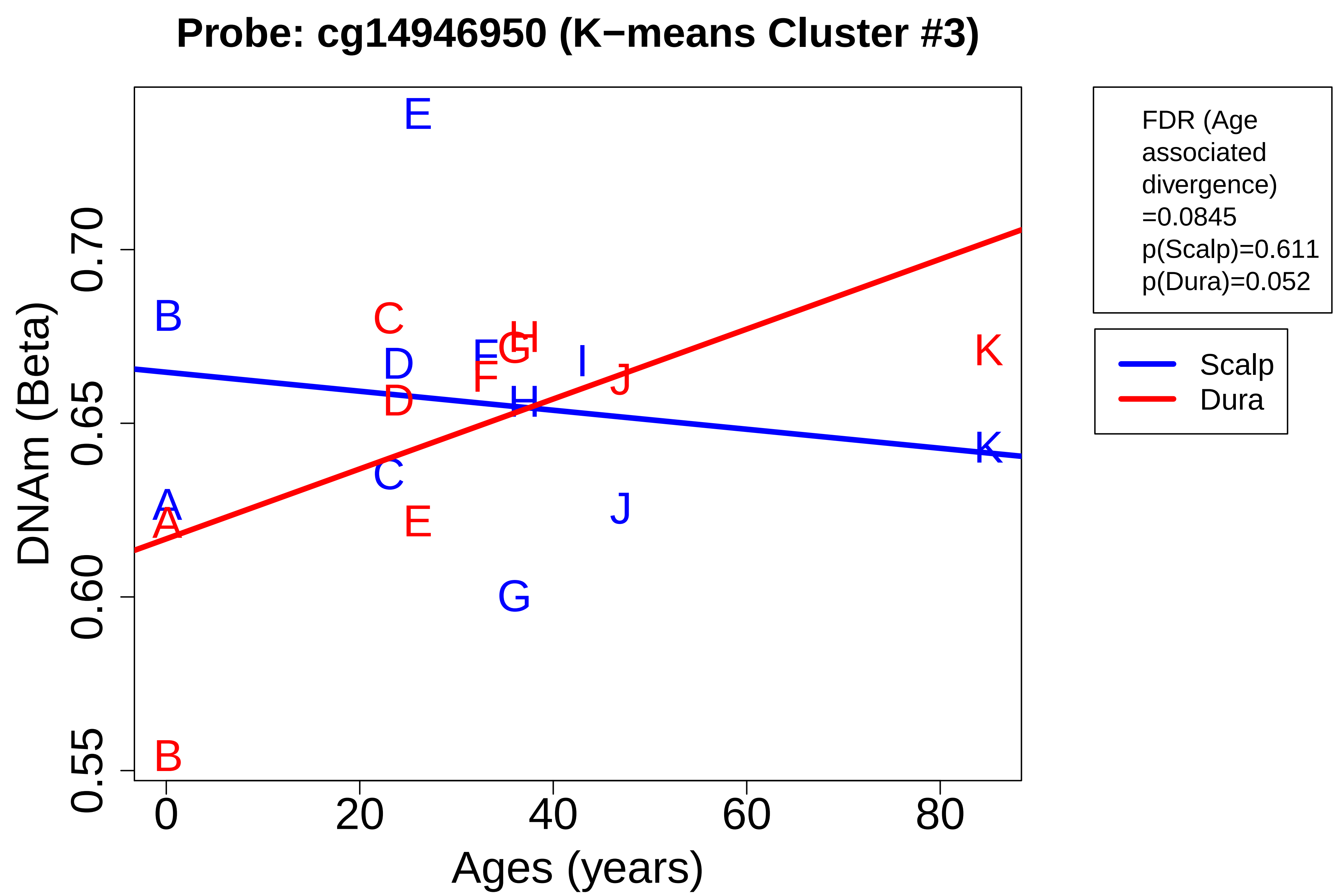

Probe: cg14946950 (K−means Cluster #3)
DNAm (Beta)
0.55
0.60
0.65
0.70
0
20
40
60
80
Ages (years)
FDR (Age
associated
divergence)
=0.0845
p(Scalp)=0.611
p(Dura)=0.052
Scalp
Dura

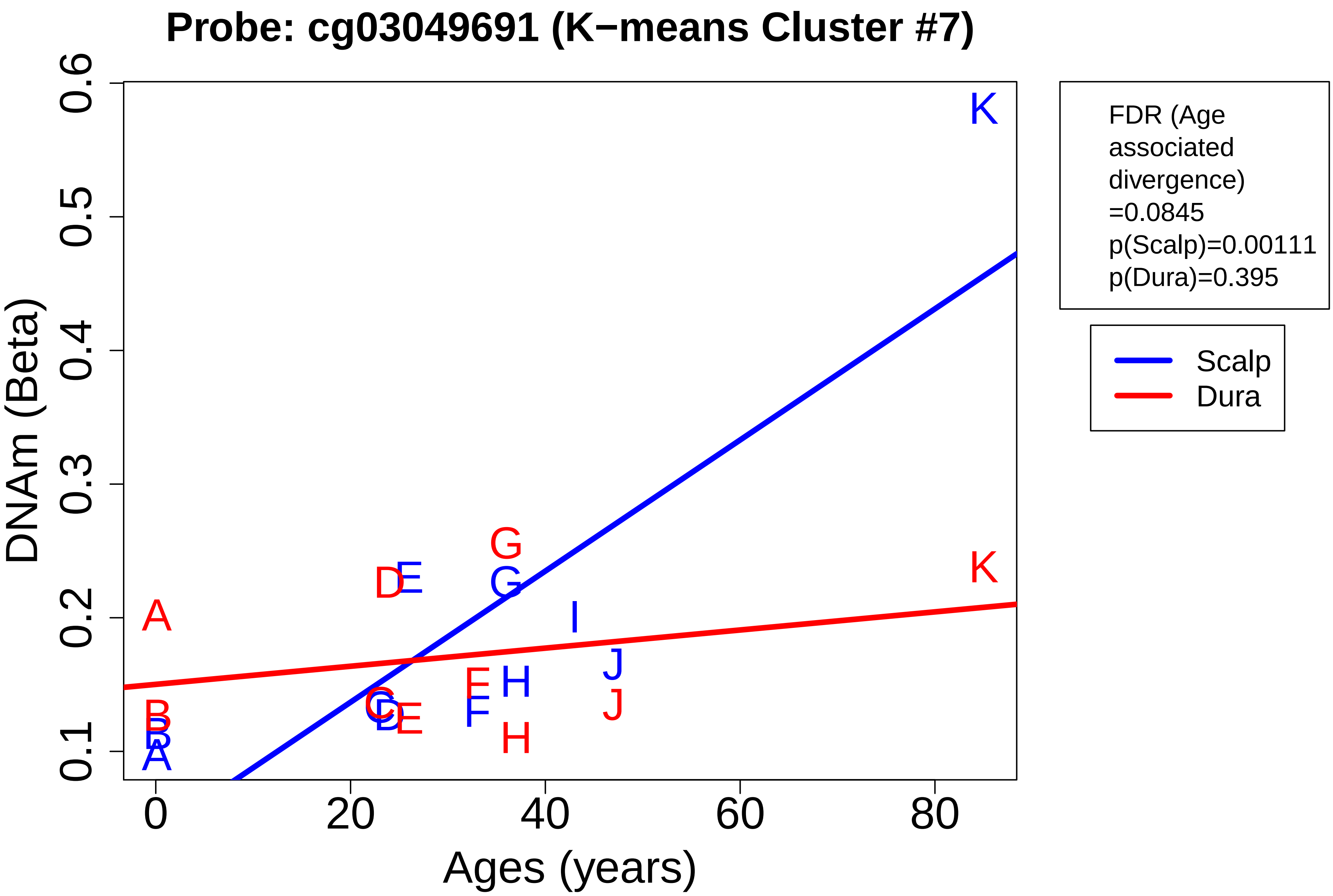

Probe: cg03049691 (K−means Cluster #7)
DNAm (Beta)
0.1
0.2
0.3
0.4
0.5
0.6
Ages (years)
0
20
40
60
80
FDR (Age associated divergence) =0.0845
p(Scalp)=0.00111
p(Dura)=0.395
Scalp
Dura
A
B
C
D
E
F
G
H
I
J
K

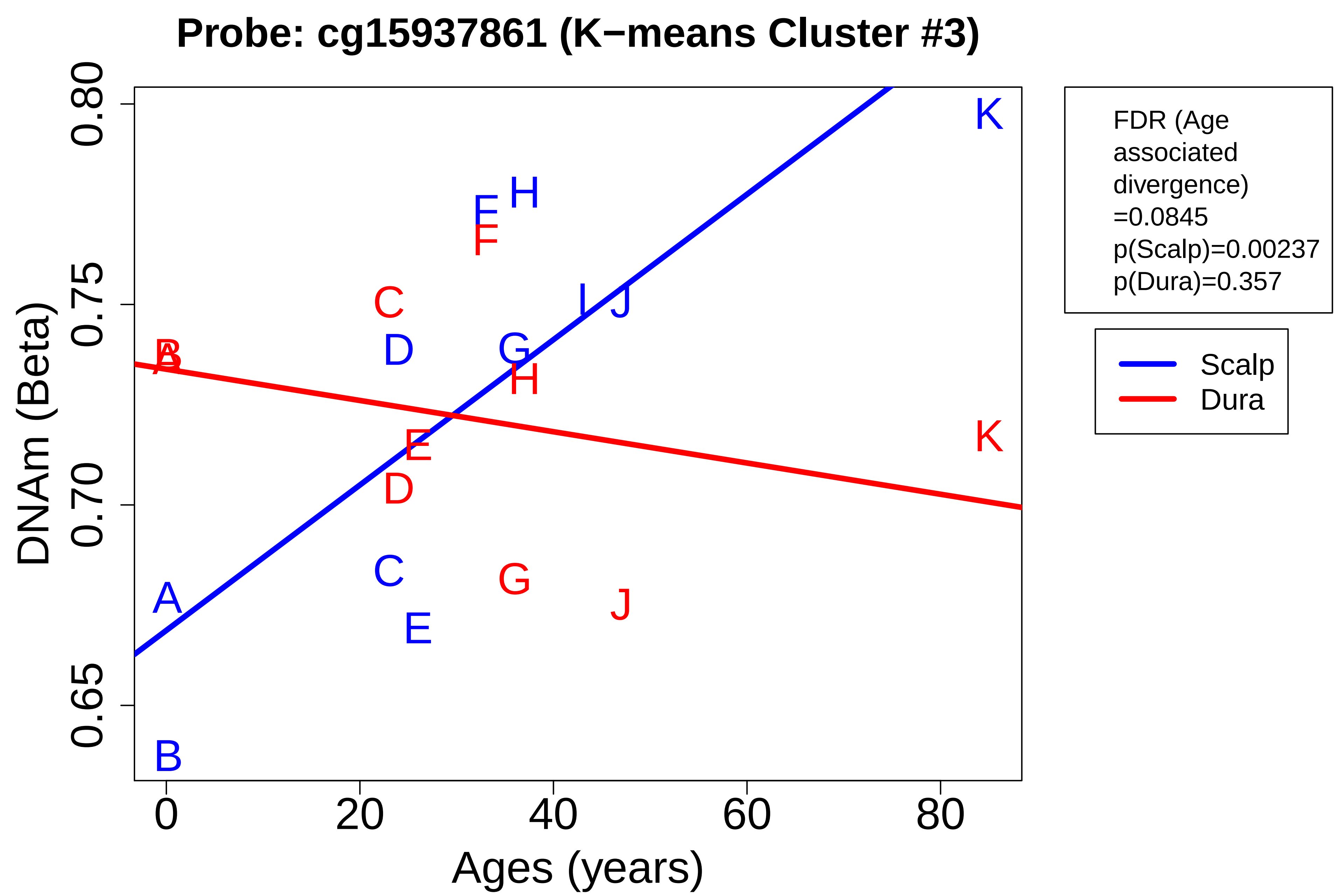

Probe: cg15937861 (K−means Cluster #3)
DNAm (Beta)
0.65
0.70
0.75
0.80
0
20
40
60
80
Ages (years)
FDR (Age associated divergence) =0.0845
p(Scalp)=0.00237
p(Dura)=0.357
Scalp
Dura

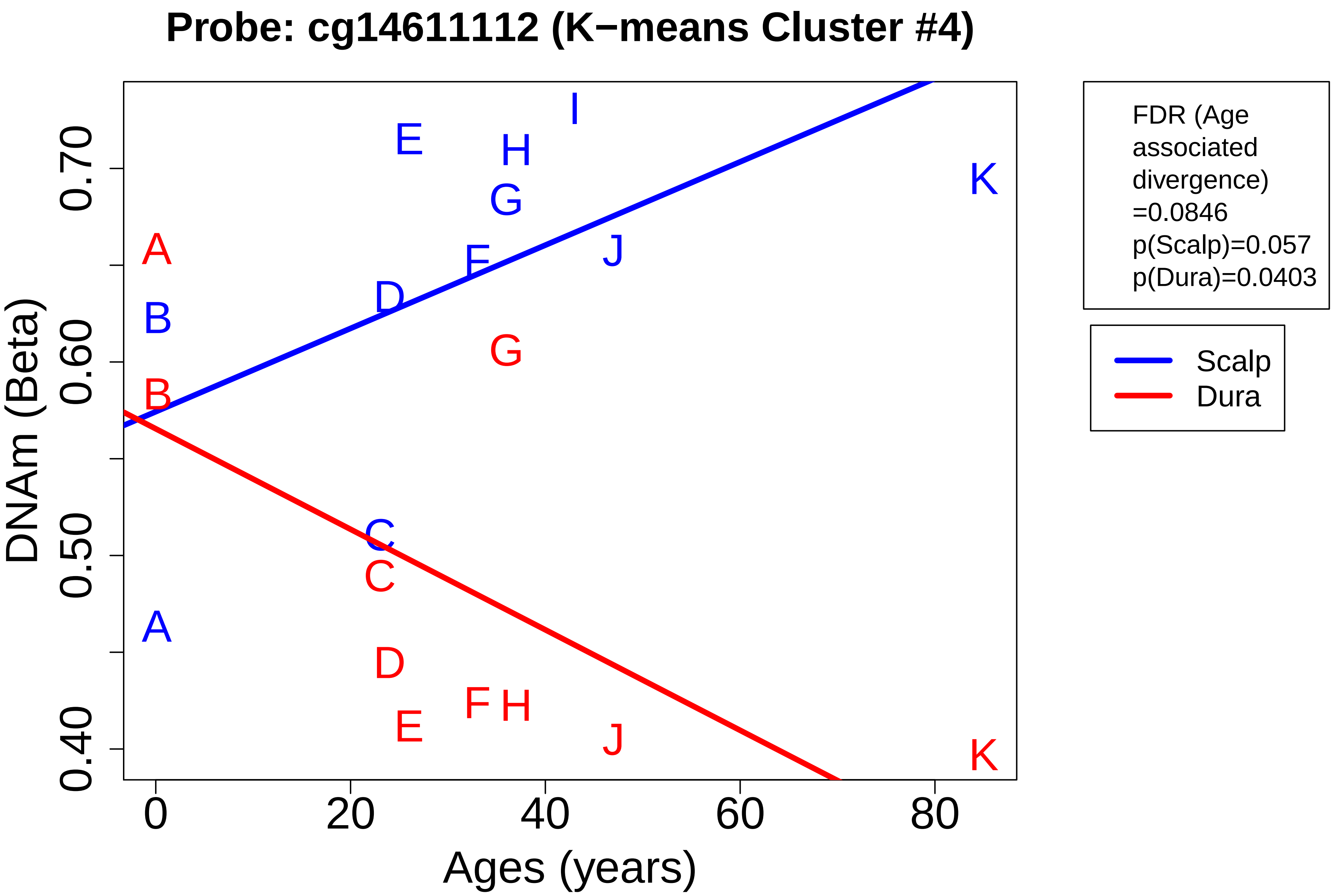
Probe: cg14611112 (K−means Cluster #4)
DNAm (Beta)
0.40
0.50
0.60
0.70
Ages (years)
0
20
40
60
80
FDR (Age associated divergence) =0.0846
p(Scalp)=0.057
p(Dura)=0.0403
Scalp
Dura
A
B
C
D
E
F
G
H
I
J
K

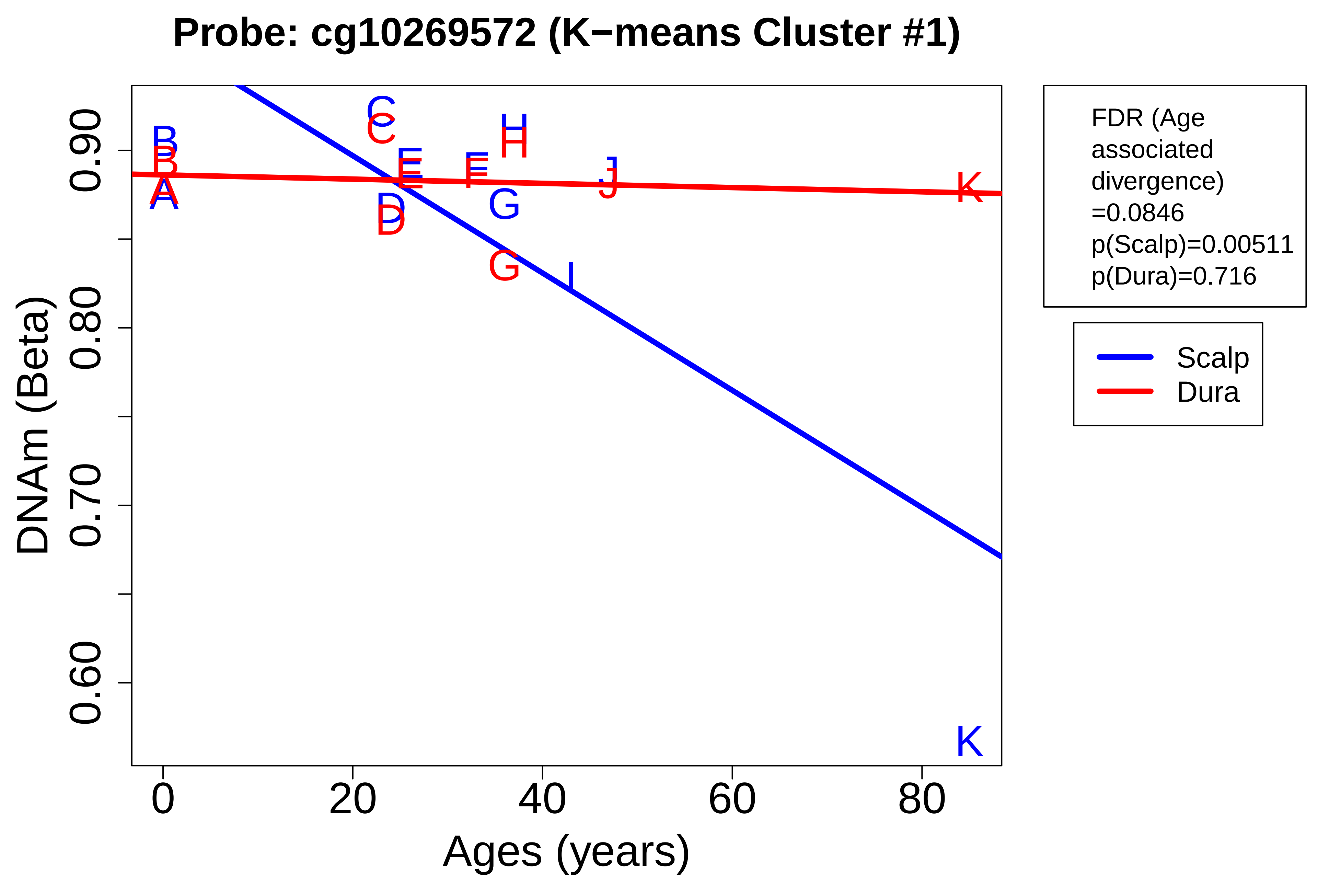

Probe: cg10269572 (K−means Cluster #1)
DNAm (Beta)
0.60
0.70
0.80
0.90
0
20
40
60
80
Ages (years)
FDR (Age associated divergence) =0.0846
p(Scalp)=0.00511
p(Dura)=0.716
Scalp
Dura

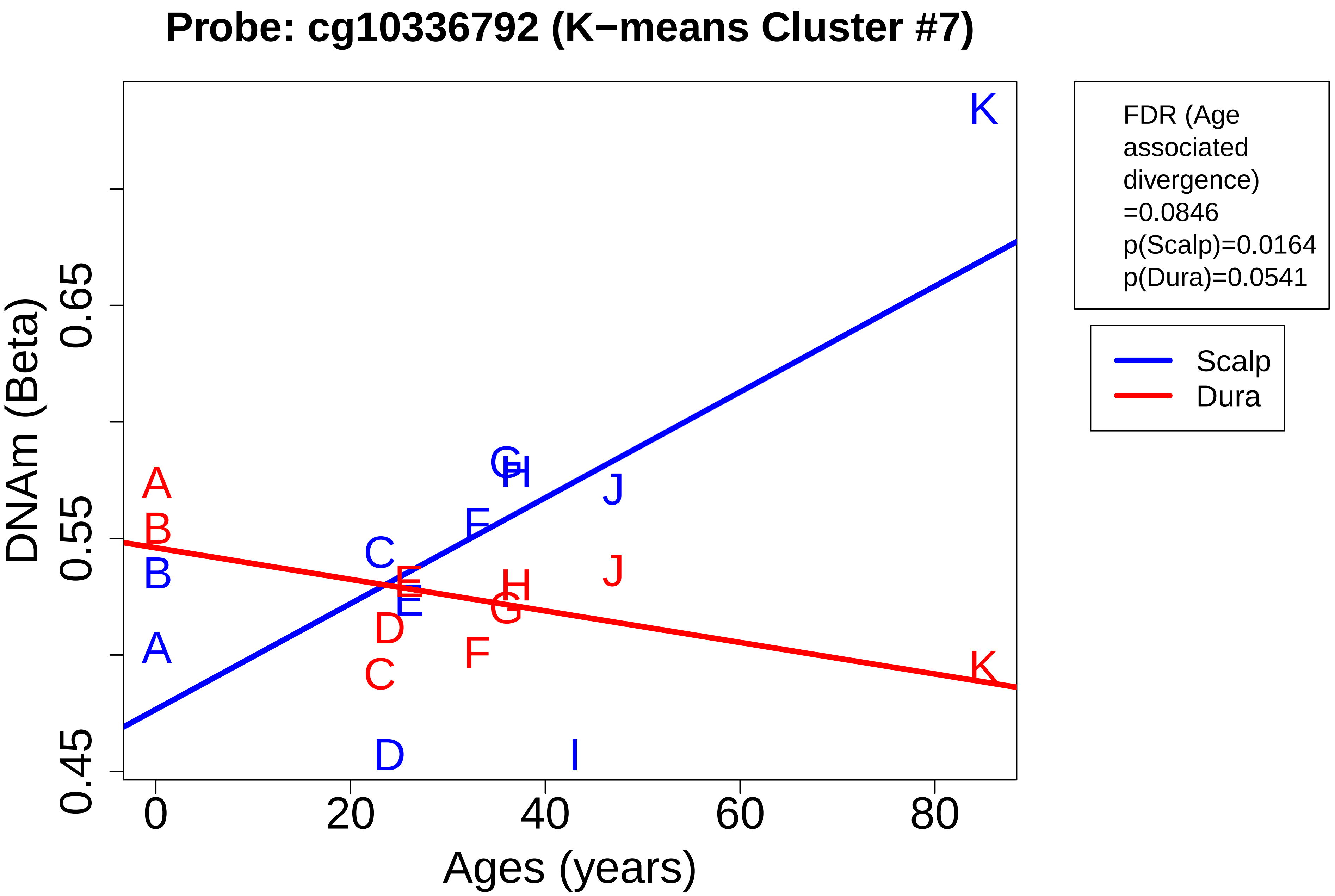

Probe: cg10336792 (K−means Cluster #7)
DNAm (Beta)
0.45
0.55
0.65
0
20
40
60
80
Ages (years)
FDR (Age associated divergence) =0.0846
p(Scalp)=0.0164
p(Dura)=0.0541
Scalp
Dura
A
B
C
D
E
F
G
H
I
J
K

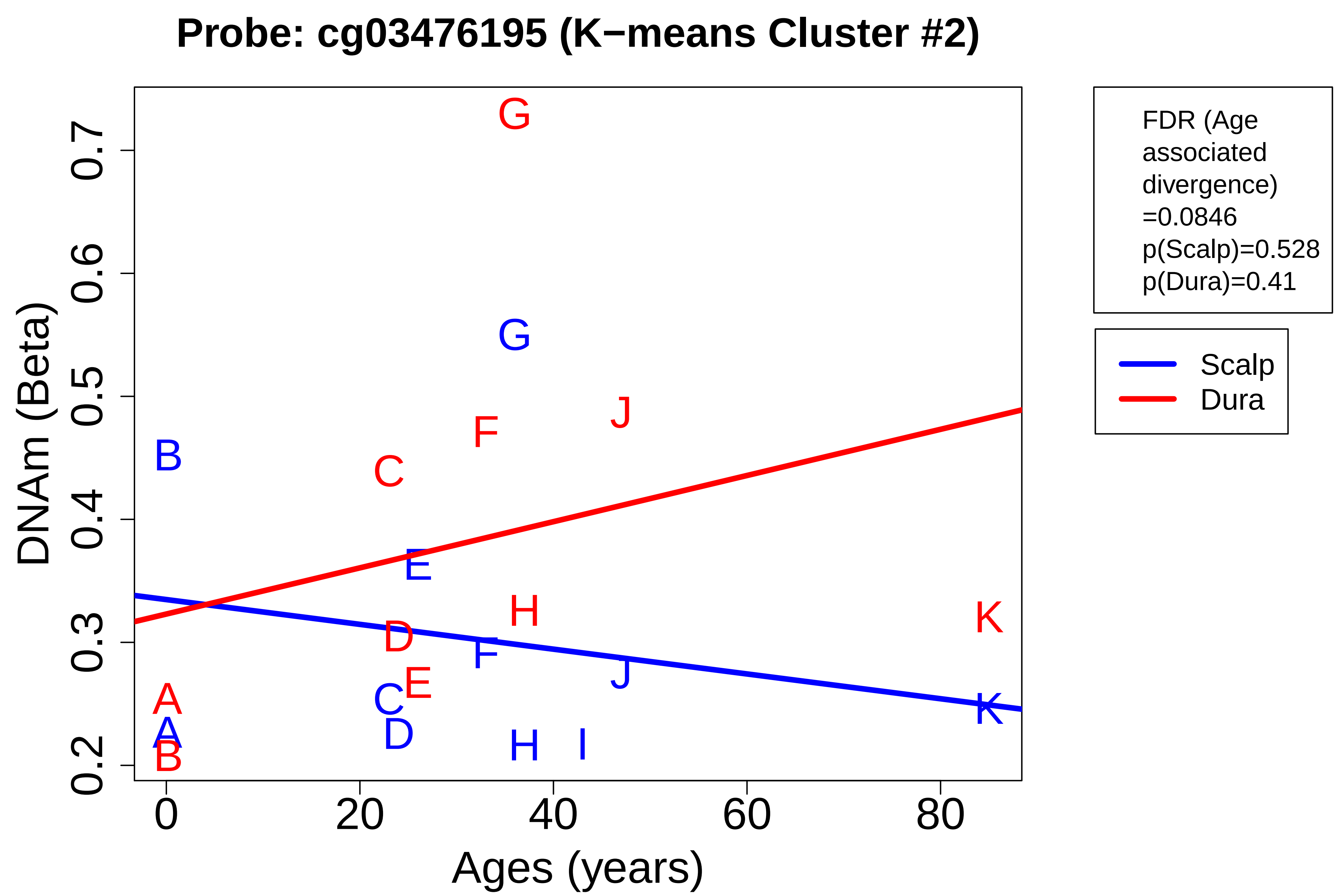

Probe: cg03476195 (K−means Cluster #2)
DNAm (Beta)
0.2
0.3
0.4
0.5
0.6
0.7
Ages (years)
0
20
40
60
80
FDR (Age associated divergence) =0.0846
p(Scalp)=0.528
p(Dura)=0.41
Scalp
Dura

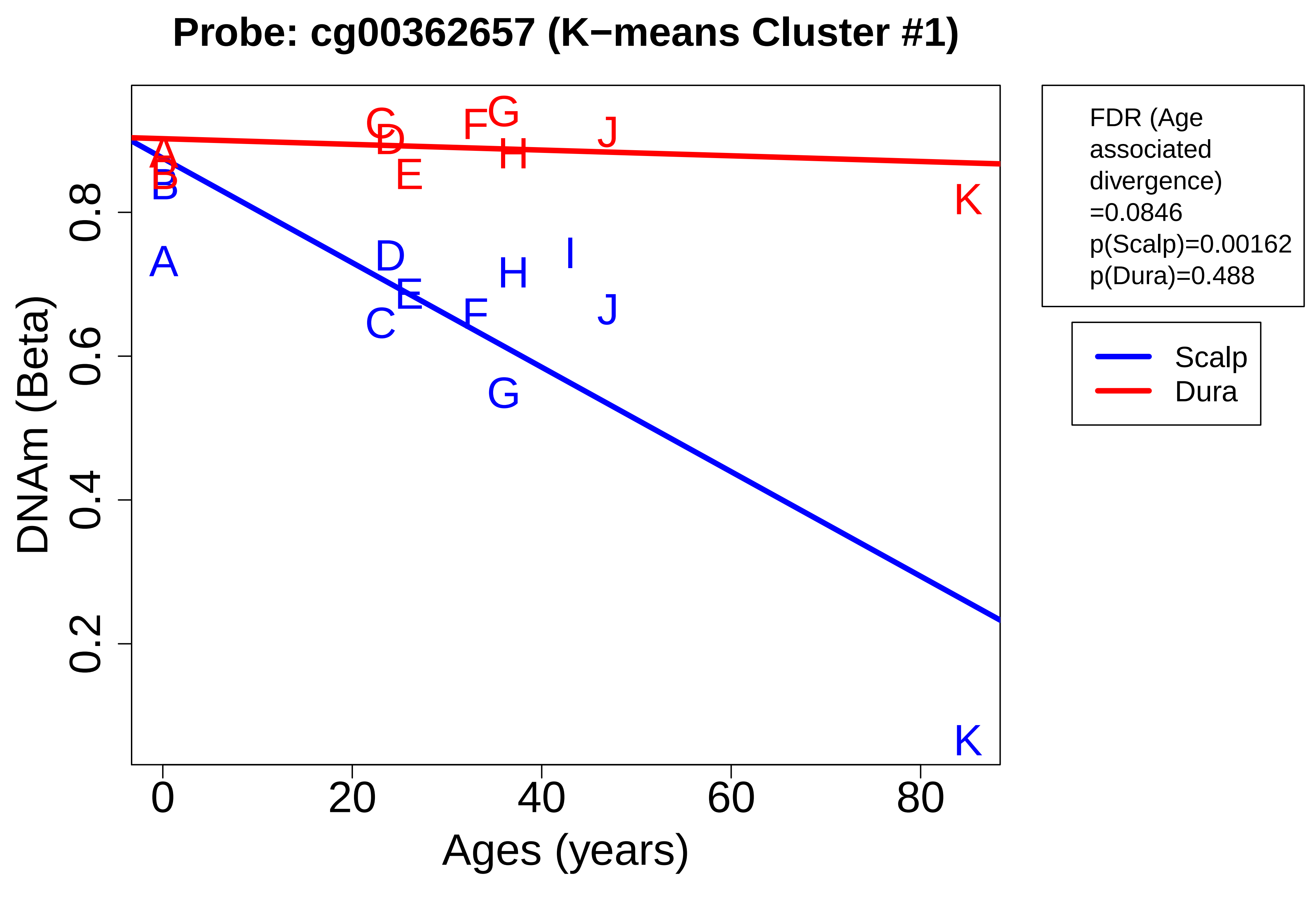

Probe: cg00362657 (K−means Cluster #1)
DNAm (Beta)
0.2
0.4
0.6
0.8
0
20
40
60
80
Ages (years)
FDR (Age associated divergence) =0.0846
p(Scalp)=0.00162
p(Dura)=0.488
Scalp
Dura

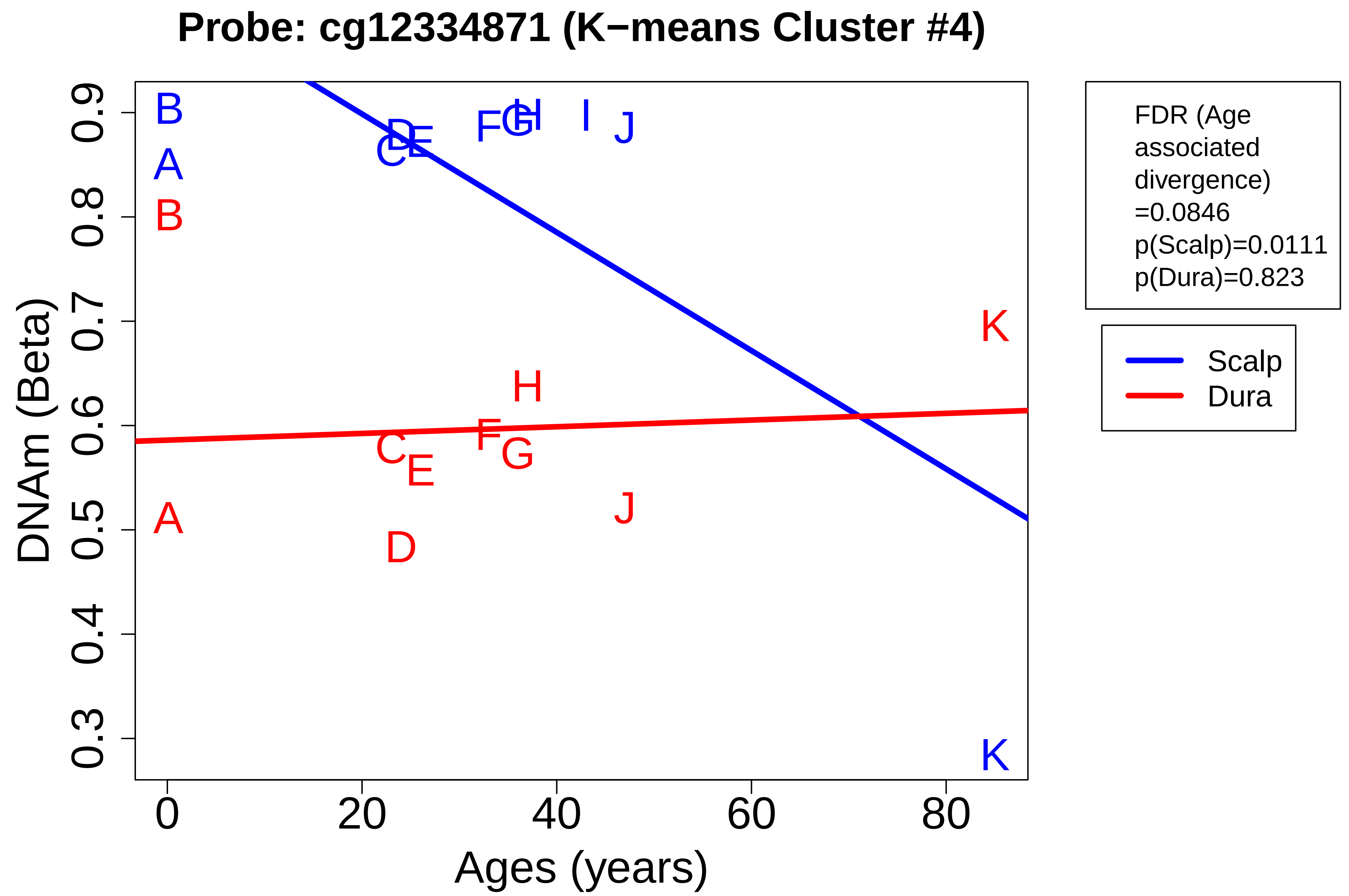

Probe: cg12334871 (K−means Cluster #4)
DNAm (Beta)
Ages (years)
0.3
0.4
0.5
0.6
0.7
0.8
0.9
0
20
40
60
80
FDR (Age associated divergence) =0.0846
p(Scalp)=0.0111
p(Dura)=0.823
Scalp
Dura

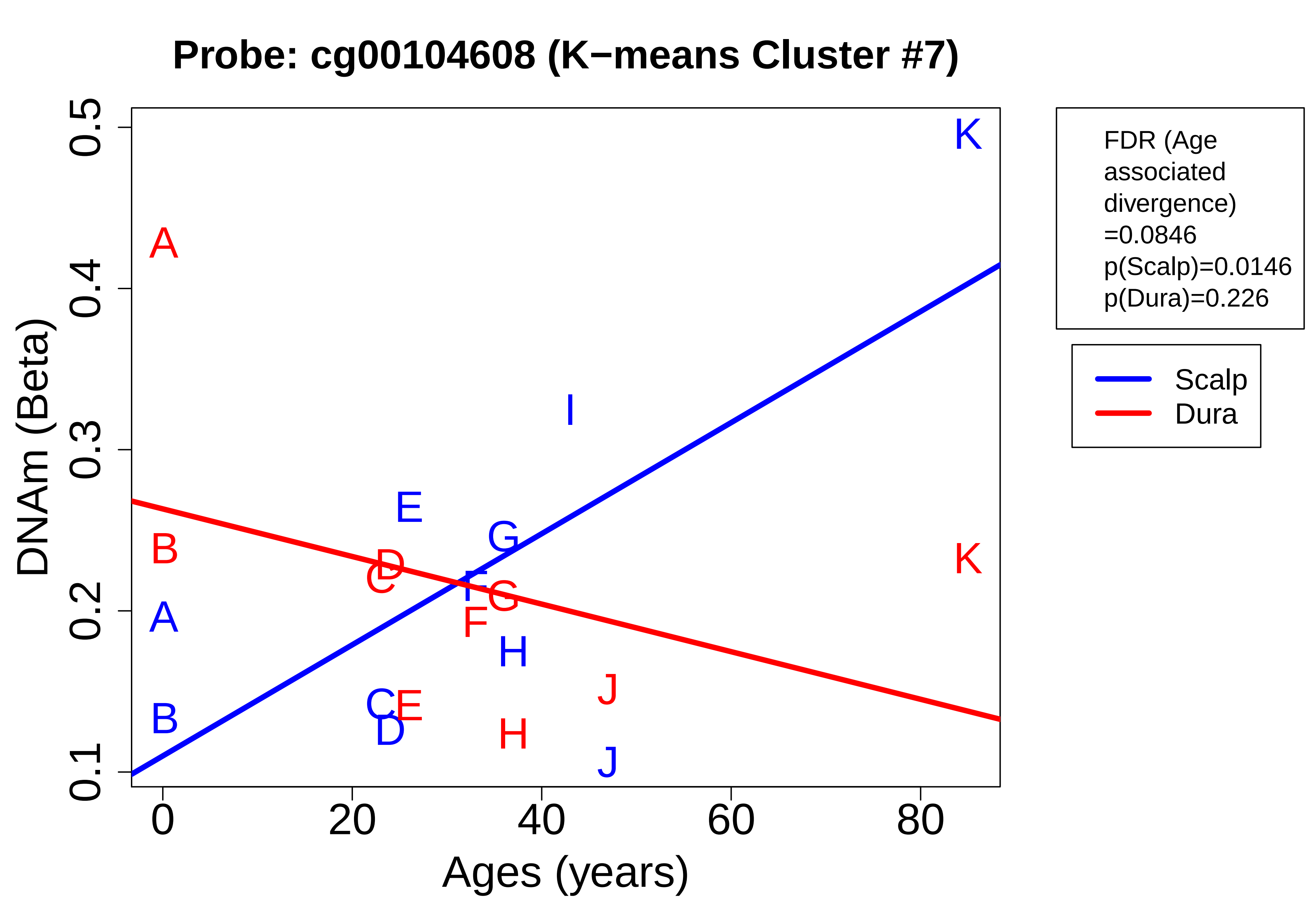
Probe: cg00104608 (K−means Cluster #7)
DNAm (Beta)
Ages (years)
0.1
0.2
0.3
0.4
0.5
0
20
40
60
80
FDR (Age associated divergence) =0.0846
p(Scalp)=0.0146
p(Dura)=0.226
Scalp
Dura
A
B
C
D
E
F
G
H
I
J
K

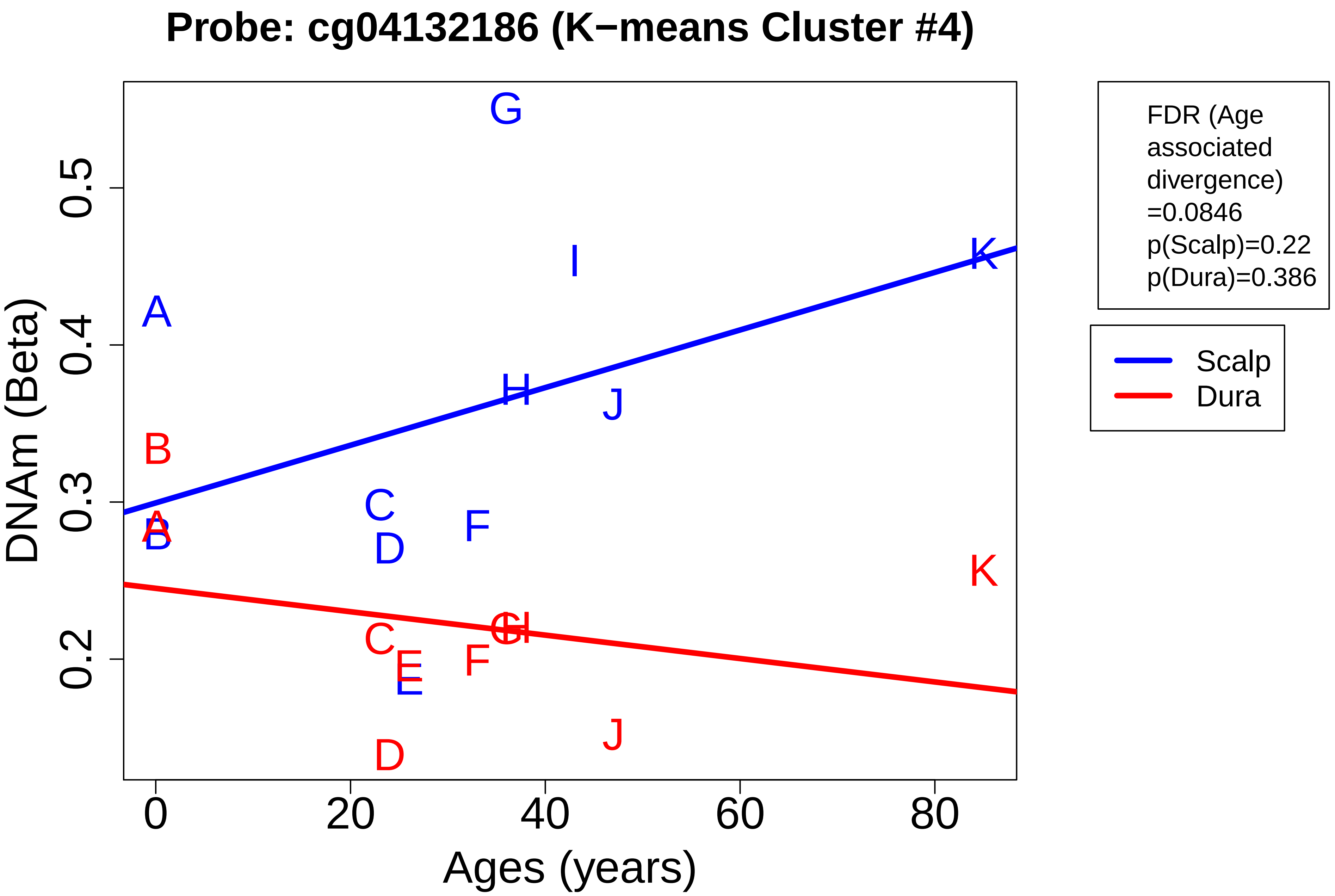

Probe: cg04132186 (K−means Cluster #4)
DNAm (Beta)
0.2
0.3
0.4
0.5
0
20
40
60
80
Ages (years)
A
B
C
D
E
F
G
H
I
J
K
FDR (Age associated divergence) =0.0846
p(Scalp)=0.22
p(Dura)=0.386
Scalp
Dura

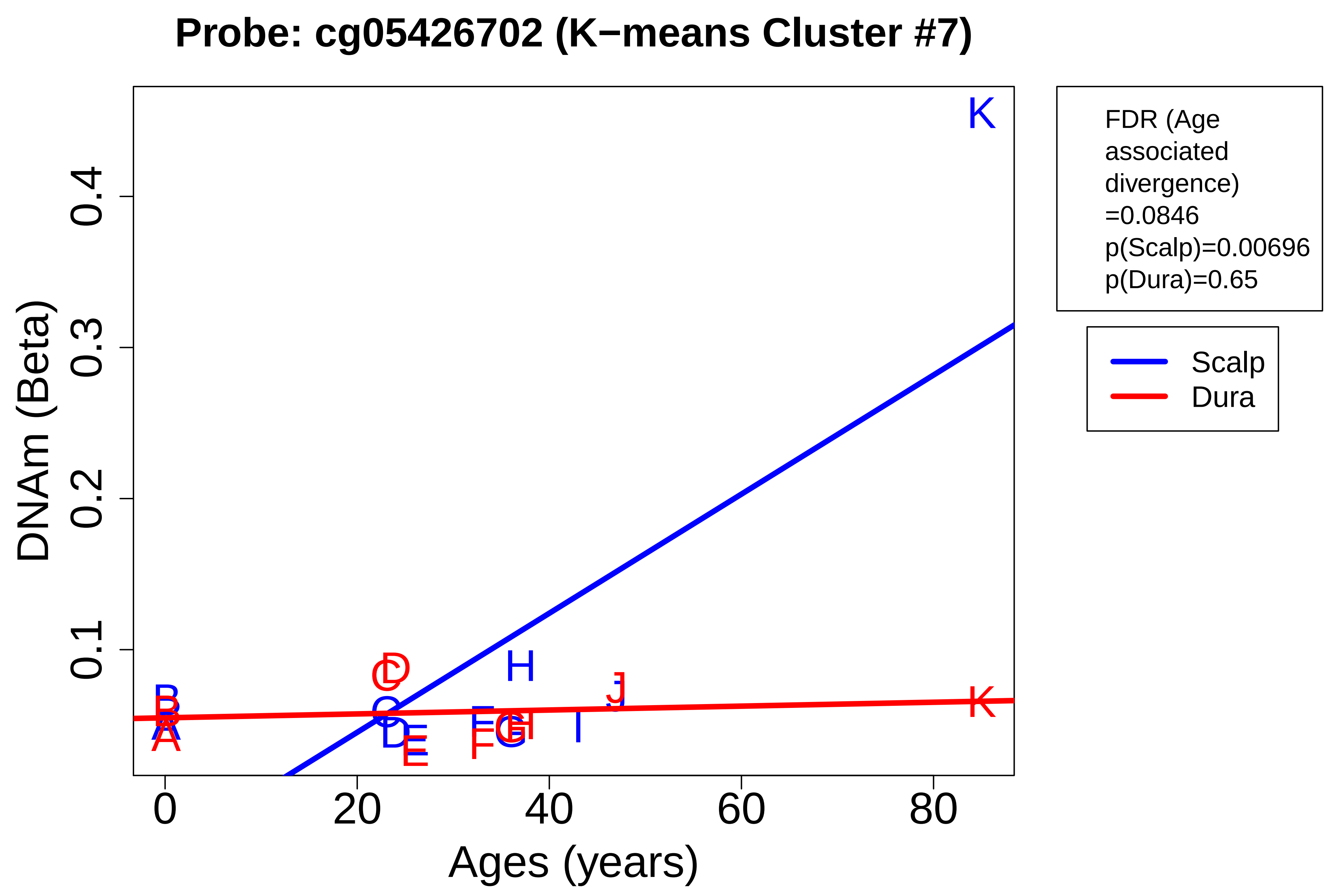

Probe: cg05426702 (K−means Cluster #7)
DNAm (Beta)
0.1
0.2
0.3
0.4
0
20
40
60
80
Ages (years)
FDR (Age associated divergence) =0.0846
p(Scalp)=0.00696
p(Dura)=0.65
Scalp
Dura

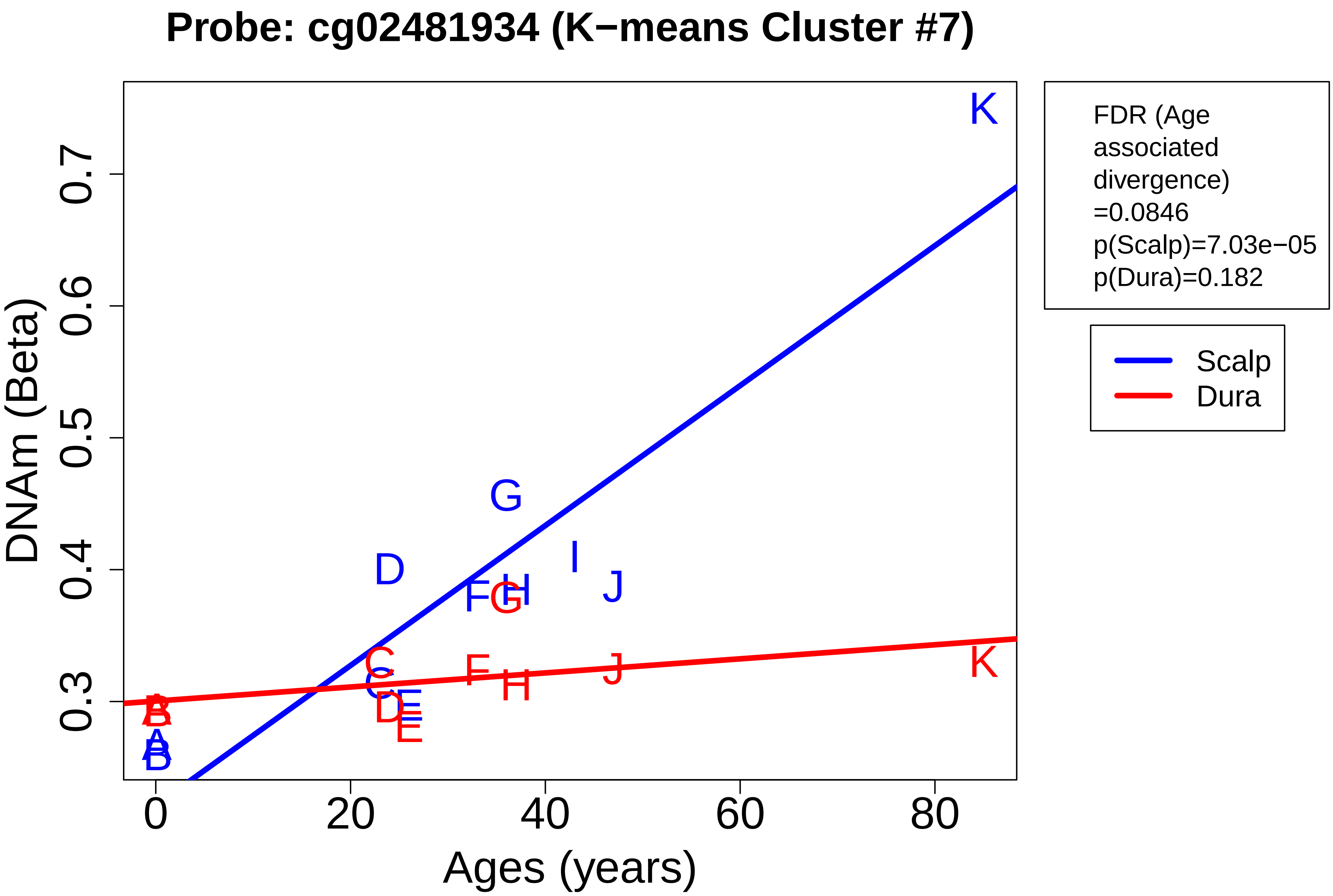

Probe: cg02481934 (K−means Cluster #7)
DNAm (Beta)
0.3
0.4
0.5
0.6
0.7
0
20
40
60
80
Ages (years)
FDR (Age associated divergence) =0.0846
p(Scalp)=7.03e−05
p(Dura)=0.182
Scalp
Dura

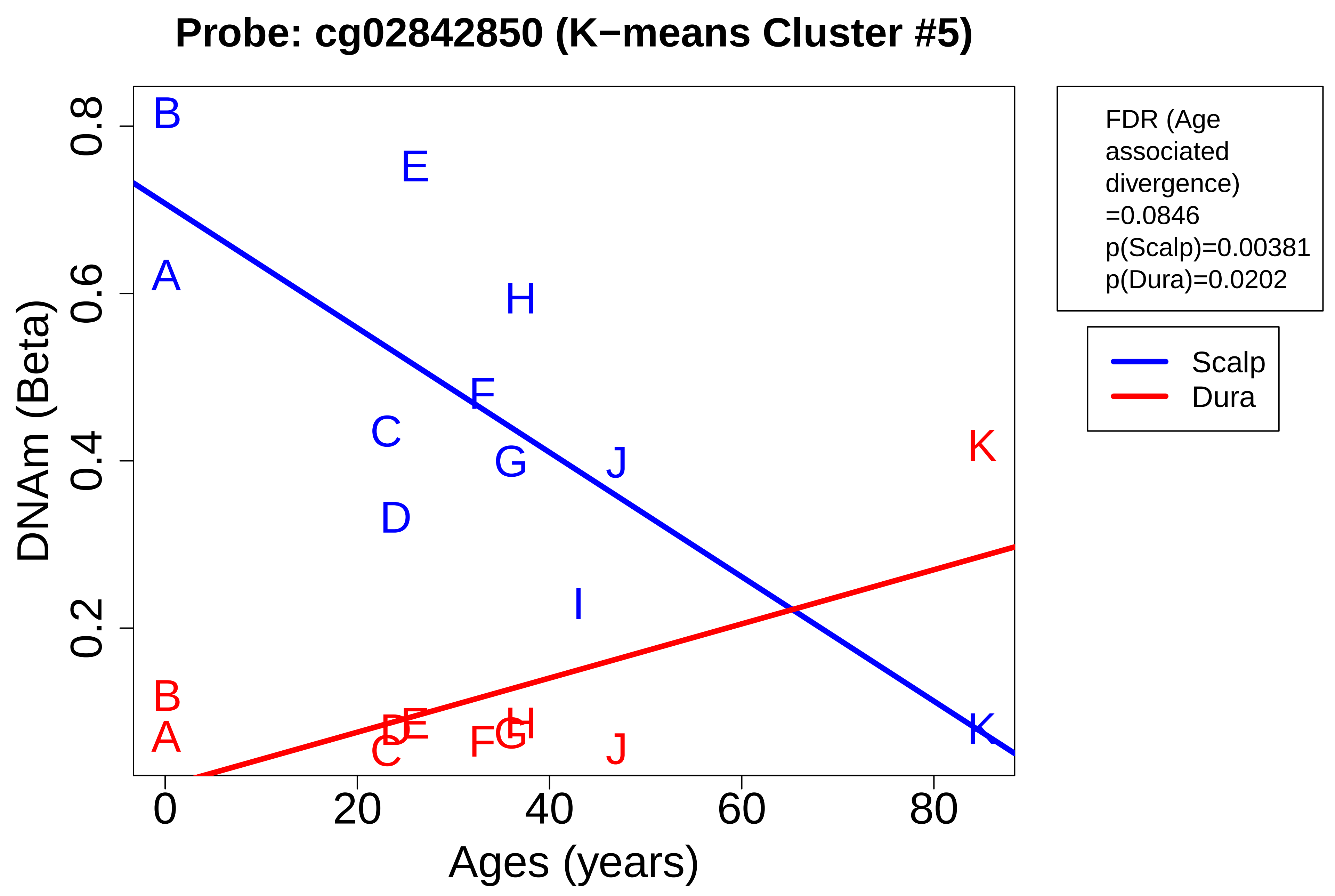

Probe: cg02842850 (K−means Cluster #5)
DNAm (Beta)
0.2
0.4
0.6
0.8
Ages (years)
0
20
40
60
80
FDR (Age associated divergence) =0.0846
p(Scalp)=0.00381
p(Dura)=0.0202
Scalp
Dura
A
B
C
D
E
F
G
H
I
J
K

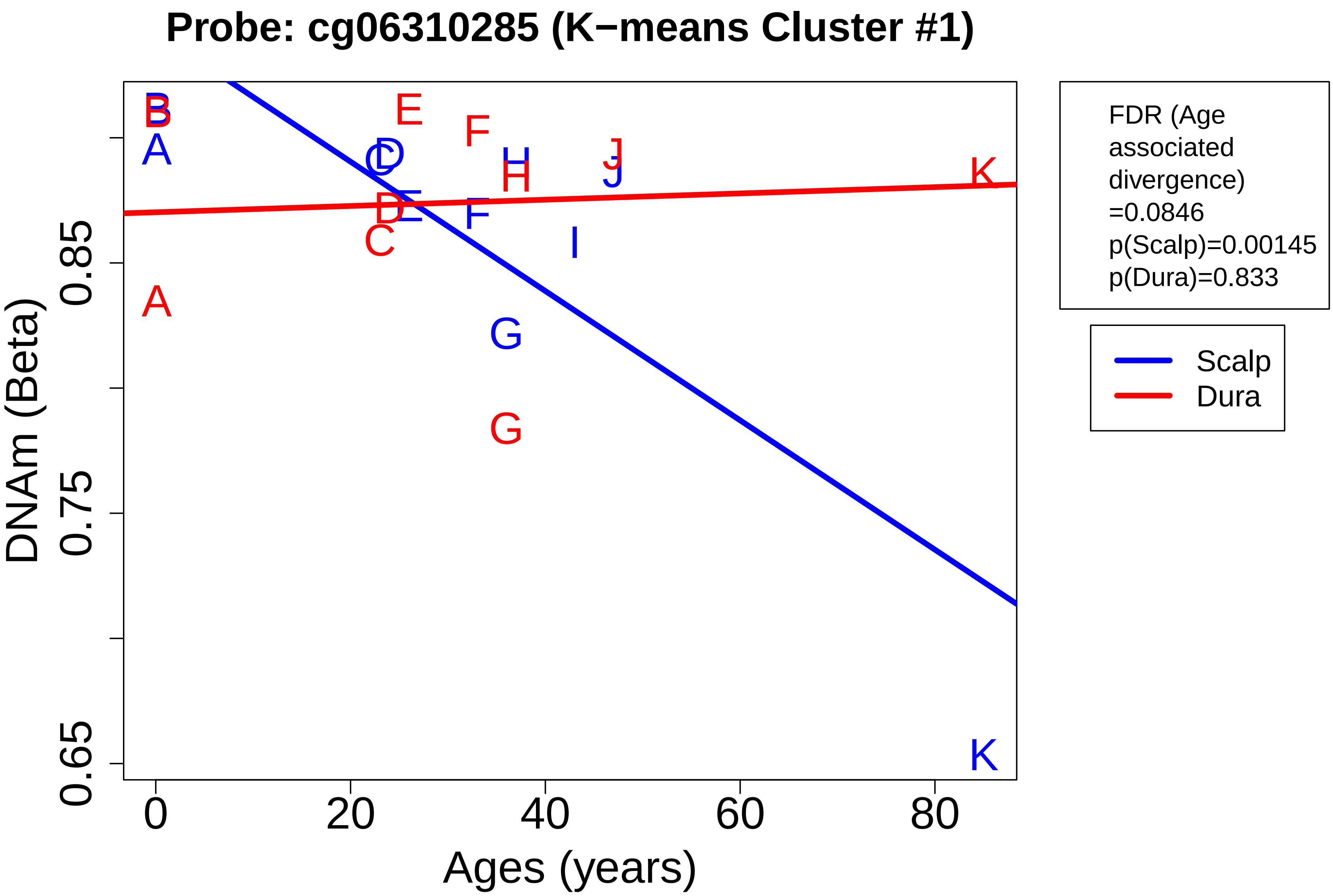
Probe: cg06310285 (K−means Cluster #1)
DNAm (Beta)
0.65
0.75
0.85
Ages (years)
0
20
40
60
80
FDR (Age associated divergence) =0.0846
p(Scalp)=0.00145
p(Dura)=0.833
Scalp
Dura

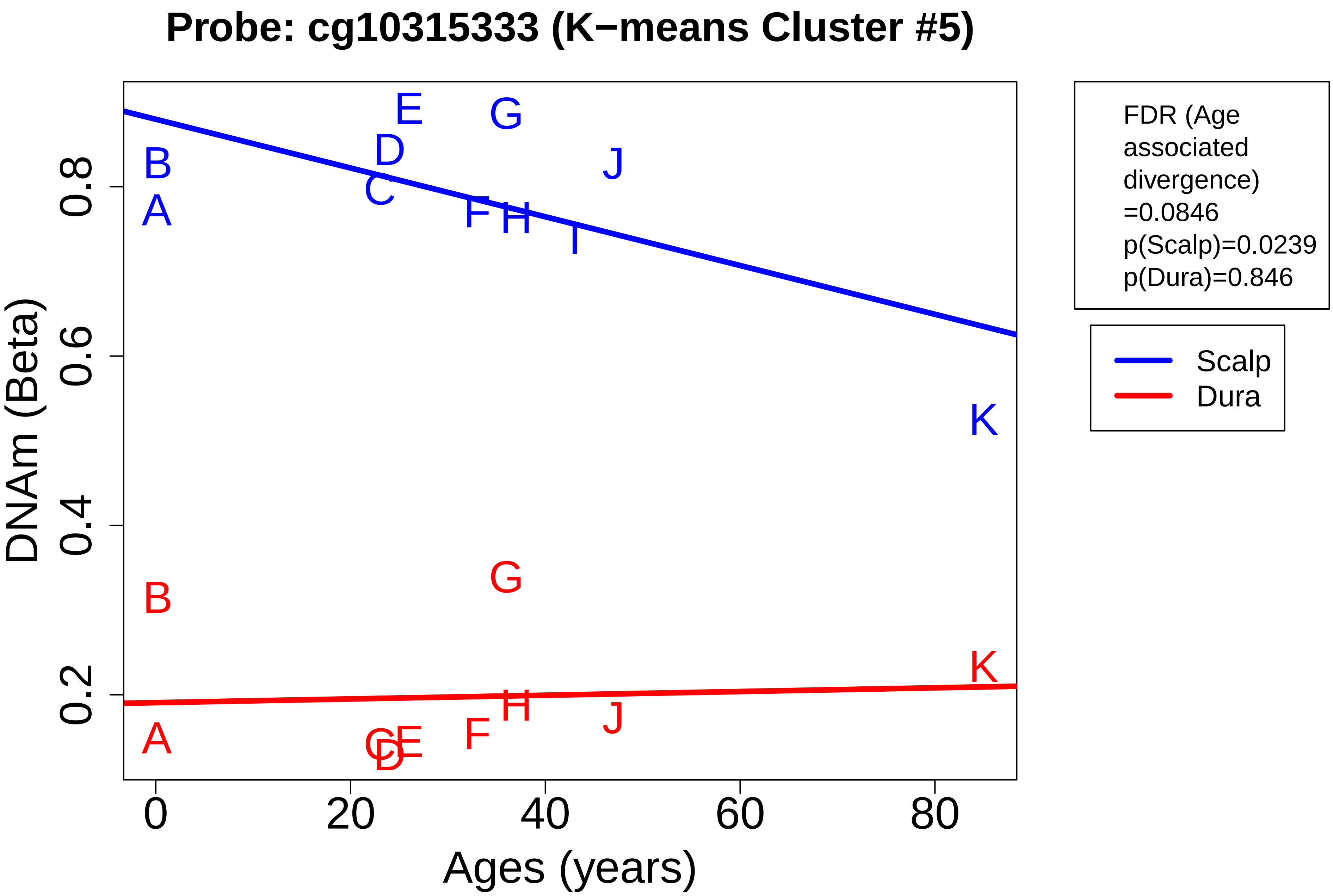

Probe: cg10315333 (K−means Cluster #5)
DNAm (Beta)
0.2
0.4
0.6
0.8
0
20
40
60
80
Ages (years)
E
G
D
B
J
C
A
F
H
I
K
G
B
K
H
J
A
C
E
F
D
FDR (Age associated divergence) =0.0846
p(Scalp)=0.0239
p(Dura)=0.846
Scalp
Dura

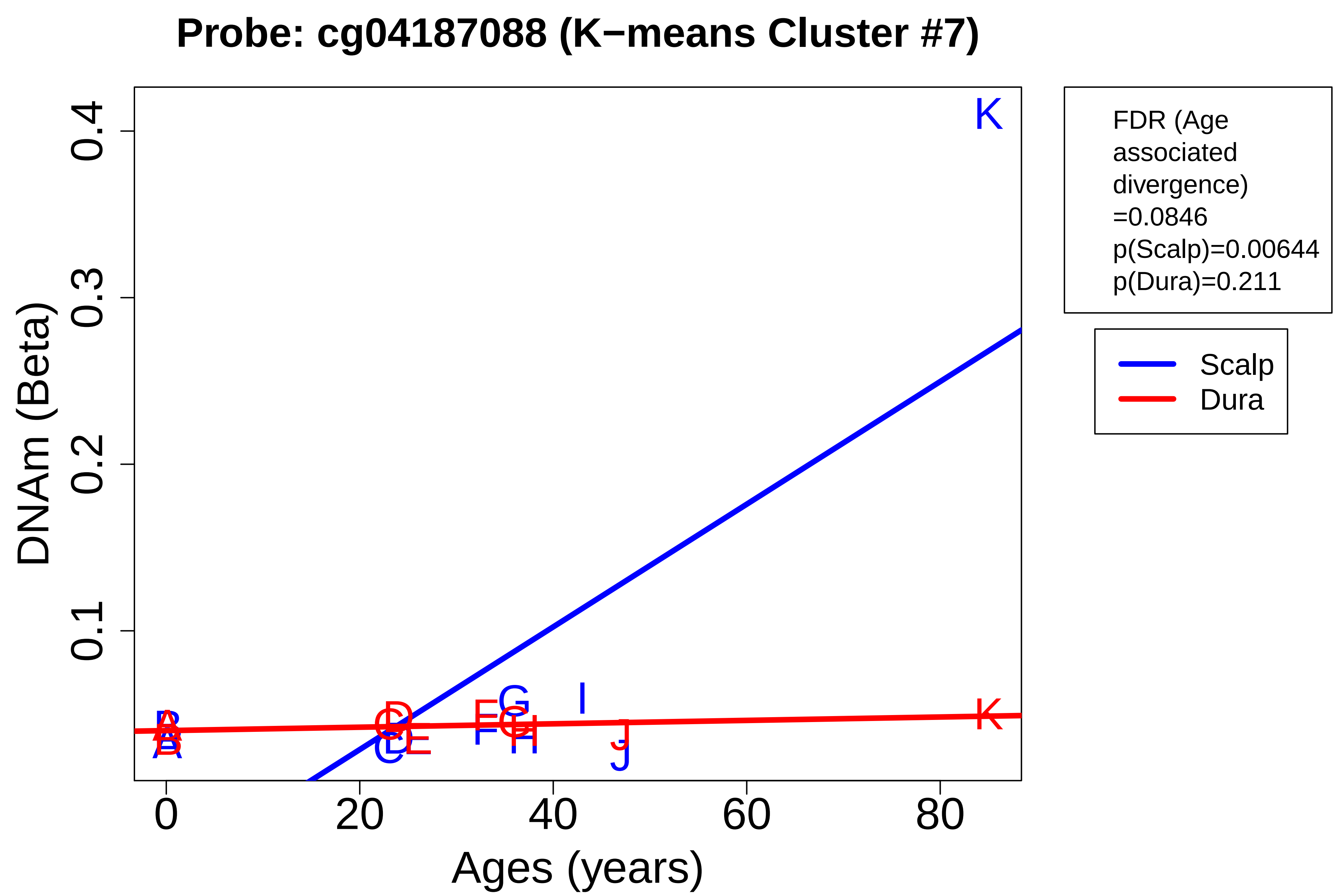
Probe: cg04187088 (K−means Cluster #7)
DNAm (Beta)
0.1
0.2
0.3
0.4
Ages (years)
0
20
40
60
80
FDR (Age associated divergence) =0.0846
p(Scalp)=0.00644
p(Dura)=0.211
Scalp
Dura

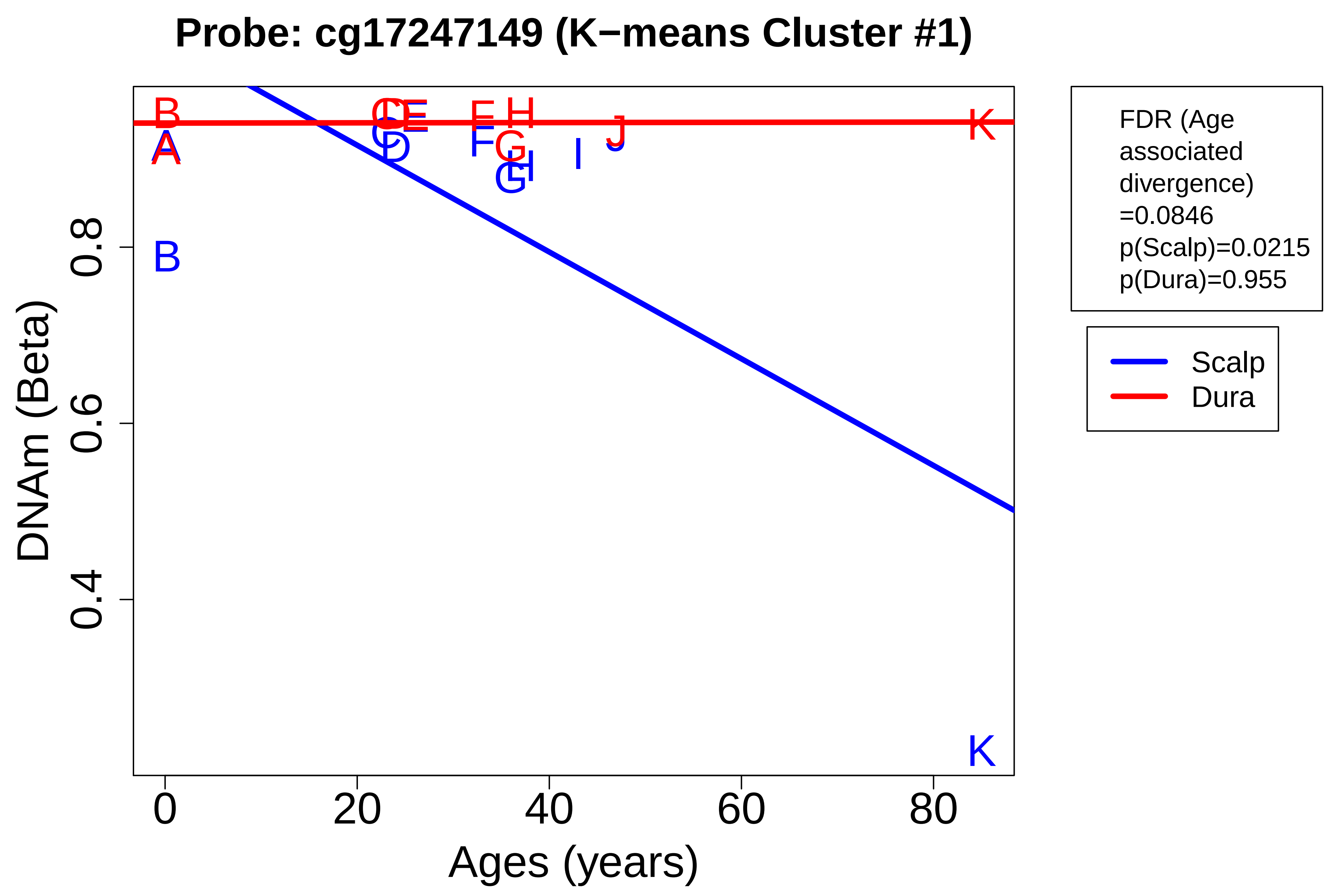
Probe: cg17247149 (K−means Cluster #1)
DNAm (Beta)
0.4
0.6
0.8
0
20
40
60
80
Ages (years)
A
B
C
D
E
F
G
H
I
J
K
FDR (Age associated divergence) =0.0846
p(Scalp)=0.0215
p(Dura)=0.955
Scalp
Dura

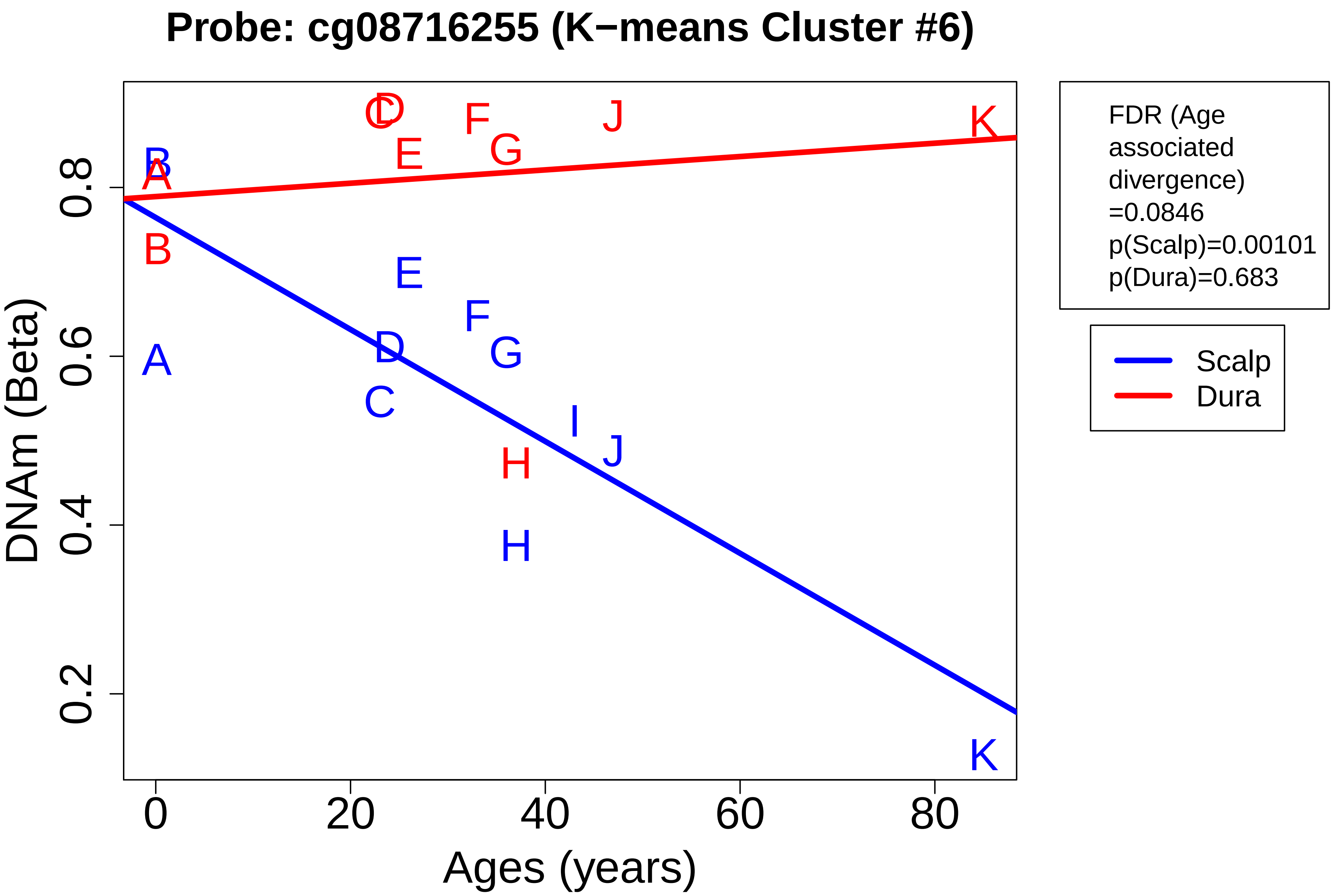
Probe: cg08716255 (K−means Cluster #6)
DNAm (Beta)
0.2
0.4
0.6
0.8
0
20
40
60
80
Ages (years)
FDR (Age associated divergence) =0.0846
p(Scalp)=0.00101
p(Dura)=0.683
Scalp
Dura
A
B
C
D
E
F
G
H
I
J
K

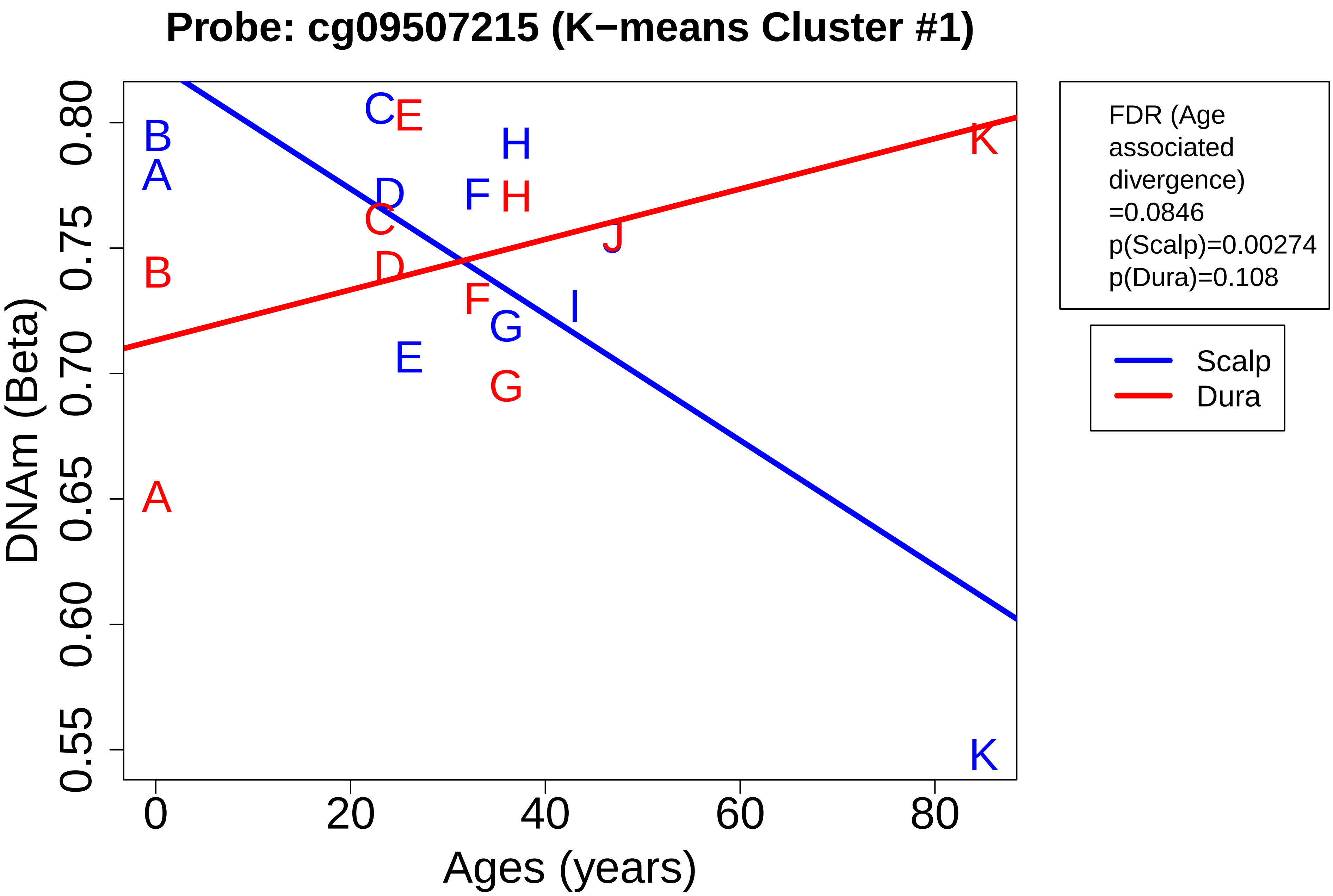

Probe: cg09507215 (K−means Cluster #1)
DNAm (Beta)
0.55
0.60
0.65
0.70
0.75
0.80
0
20
40
60
80
Ages (years)
A
B
C
D
E
F
G
H
I
J
K
FDR (Age associated divergence) =0.0846
p(Scalp)=0.00274
p(Dura)=0.108
Scalp
Dura

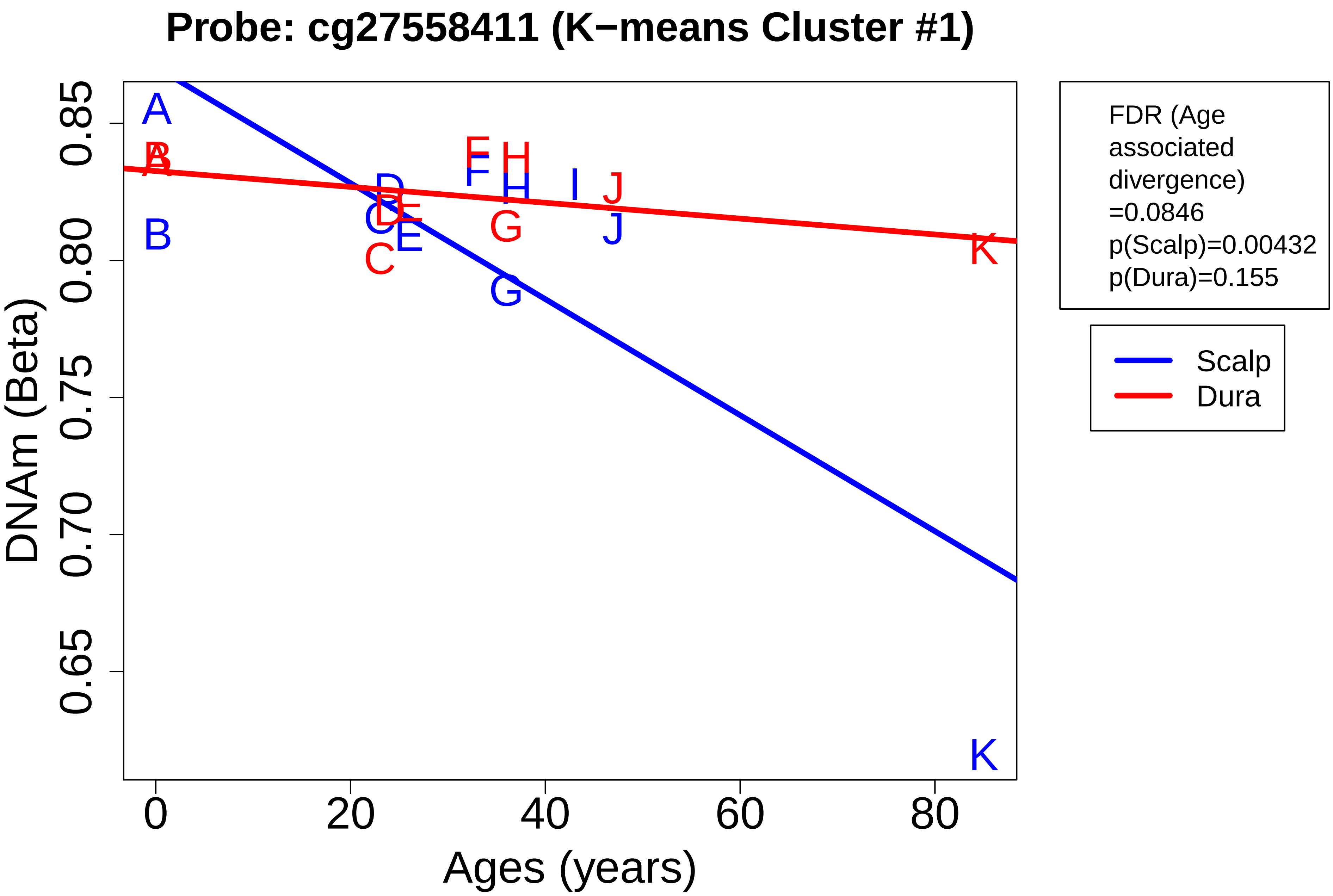

Probe: cg27558411 (K−means Cluster #1)
DNAm (Beta)
0.65
0.70
0.75
0.80
0.85
Ages (years)
0
20
40
60
80
FDR (Age associated divergence) =0.0846
p(Scalp)=0.00432
p(Dura)=0.155
Scalp
Dura

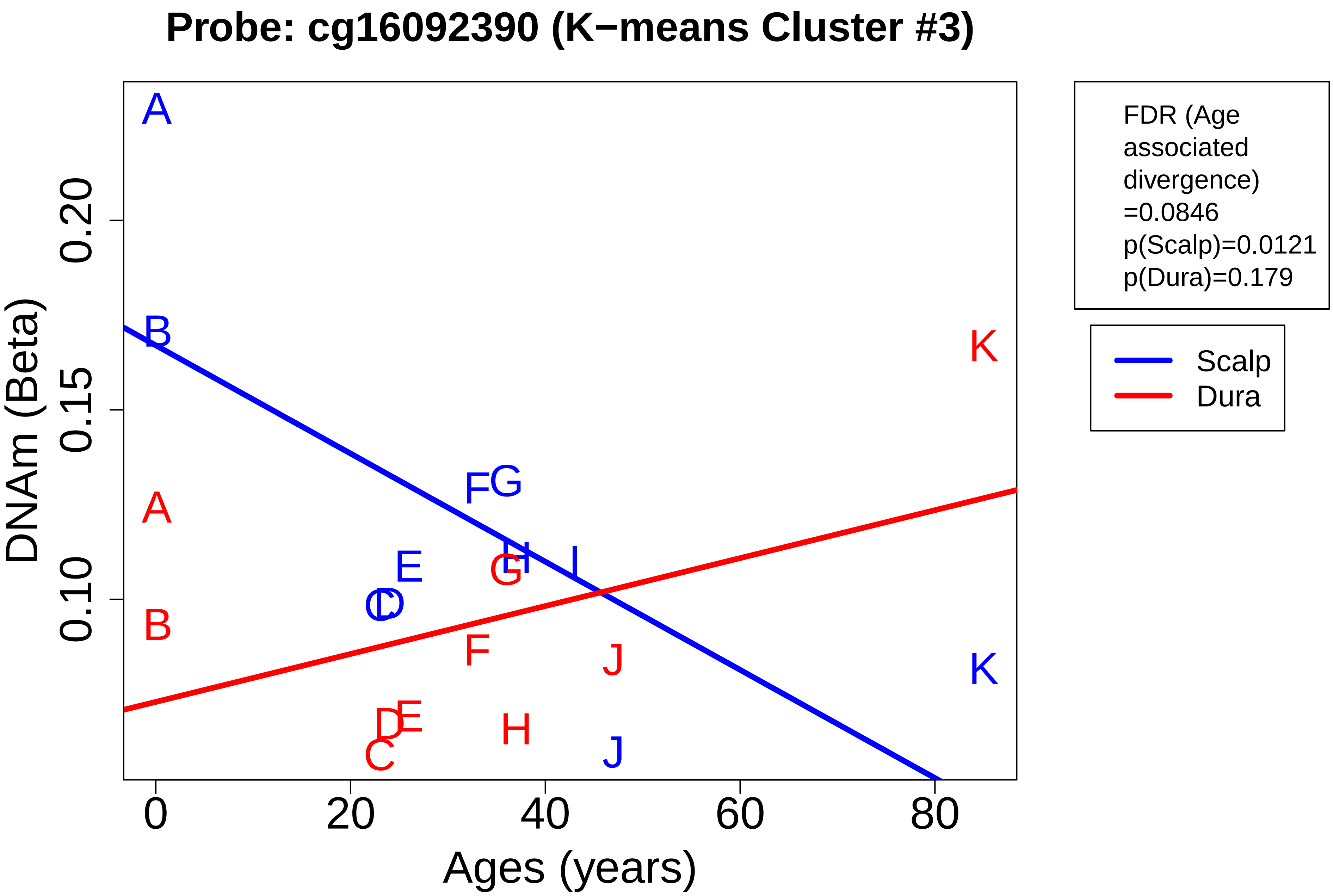

Probe: cg16092390 (K−means Cluster #3)
DNAm (Beta)
0.10
0.15
0.20
Ages (years)
0
20
40
60
80
A
B
C
D
E
F
G
H
I
J
K
FDR (Age associated divergence) =0.0846
p(Scalp)=0.0121
p(Dura)=0.179
Scalp
Dura

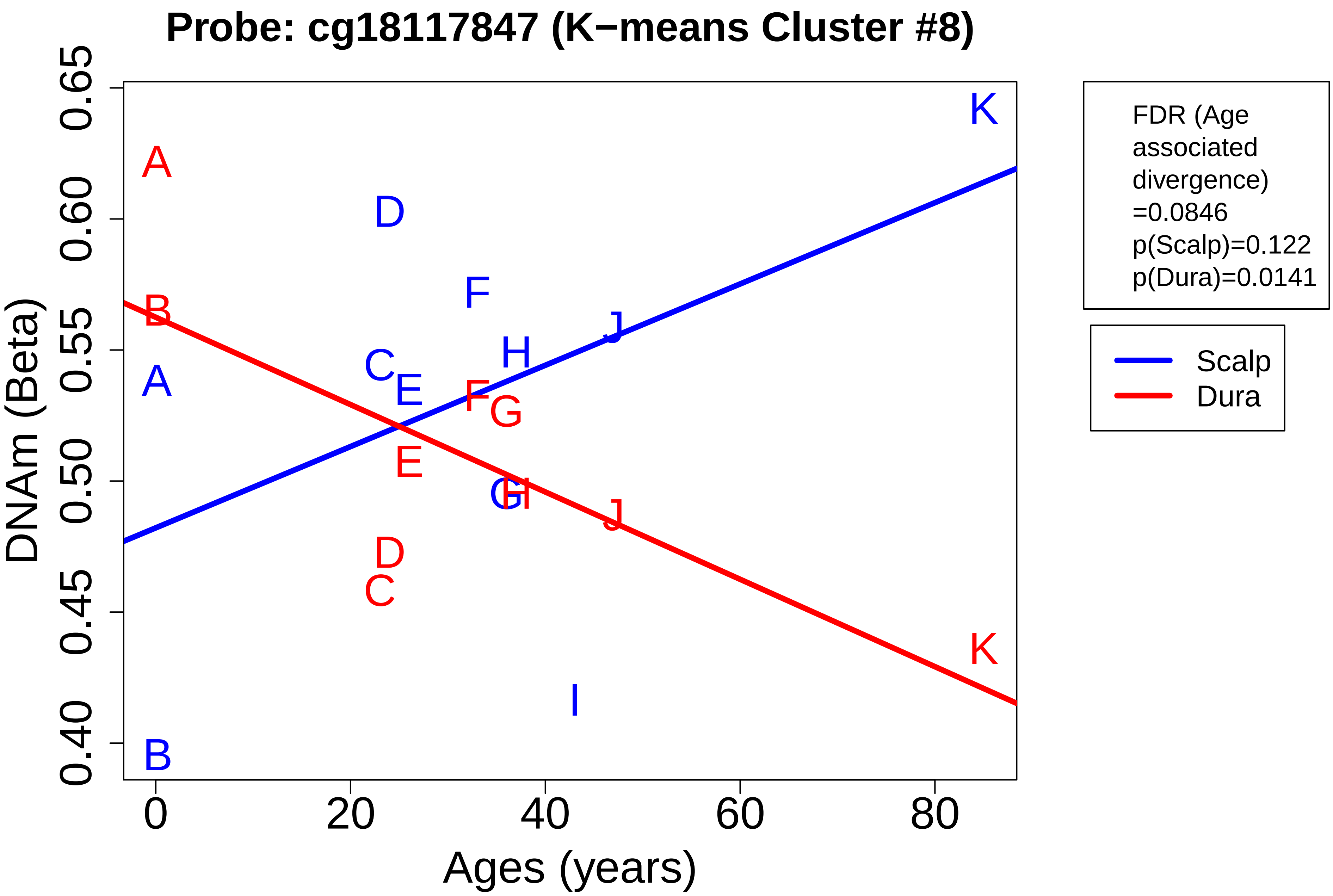

Probe: cg18117847 (K−means Cluster #8)
DNAm (Beta)
0.40
0.45
0.50
0.55
0.60
0.65
0
20
40
60
80
Ages (years)
FDR (Age associated divergence) =0.0846
p(Scalp)=0.122
p(Dura)=0.0141
Scalp
Dura
A
B
C
D
E
F
G
H
I
J
K

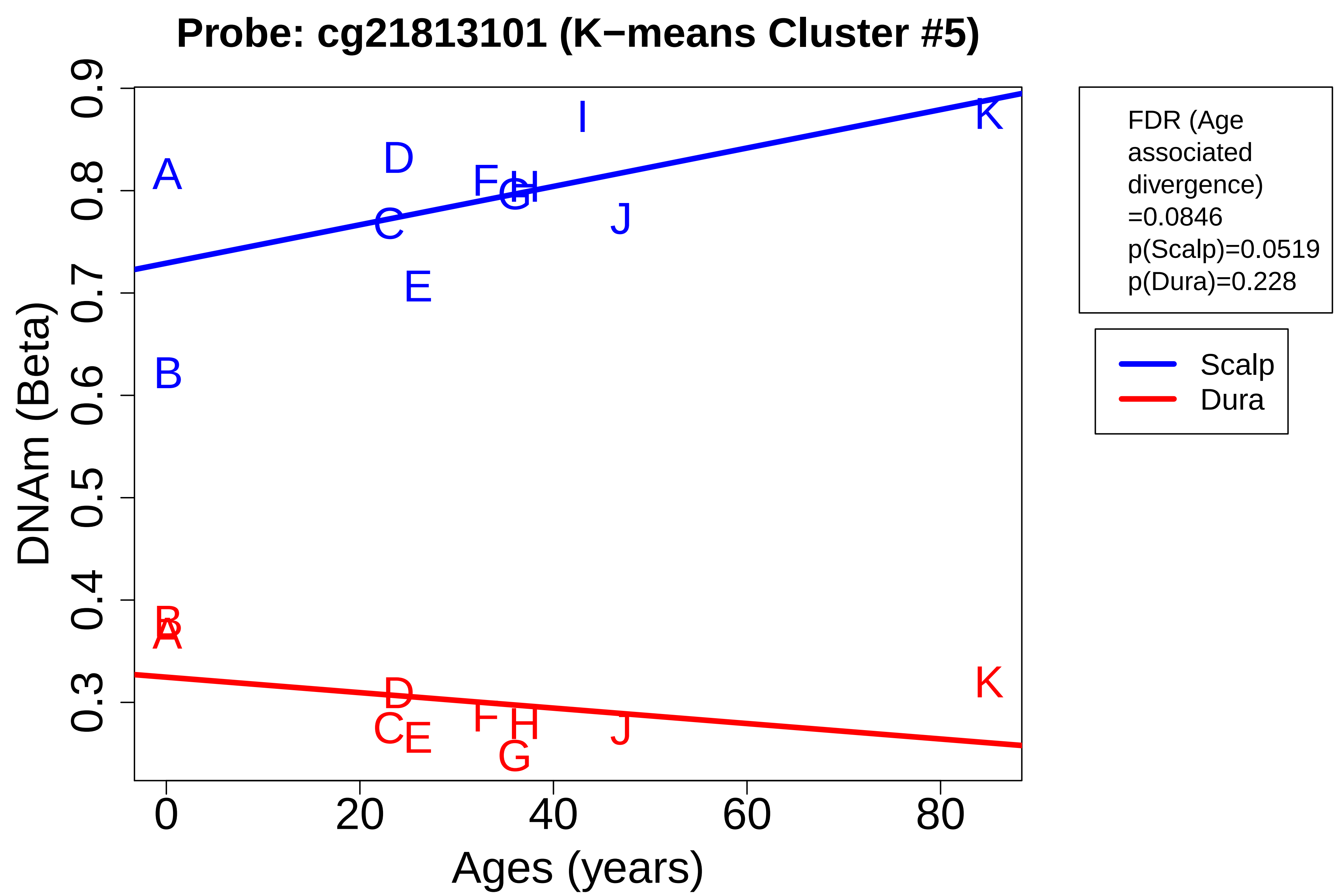
Probe: cg21813101 (K−means Cluster #5)
DNAm (Beta)
0.3
0.4
0.5
0.6
0.7
0.8
0.9
0
20
40
60
80
Ages (years)
A
B
C
D
E
F
G
H
I
J
K
FDR (Age associated divergence) =0.0846
p(Scalp)=0.0519
p(Dura)=0.228
Scalp
Dura

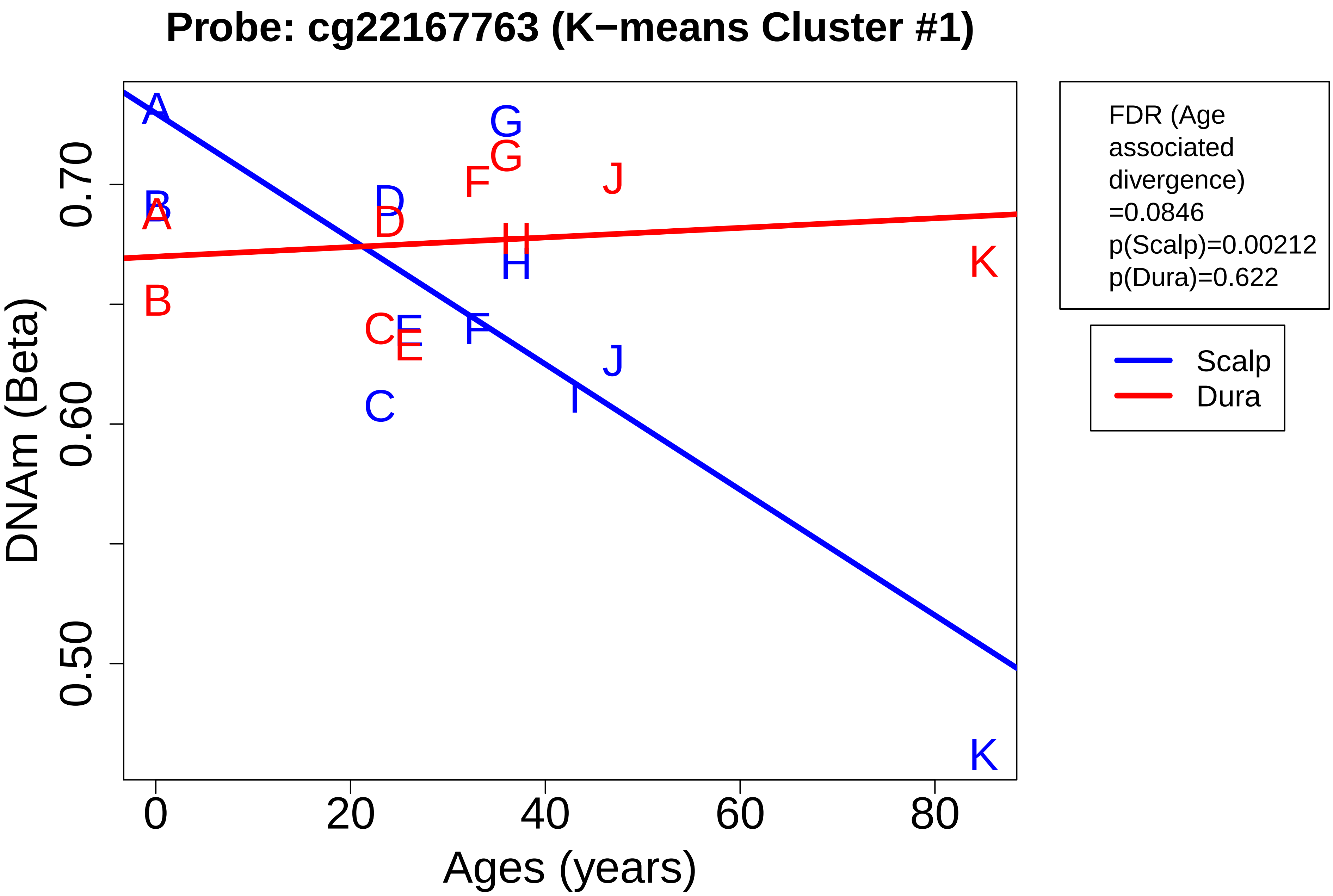
Probe: cg22167763 (K−means Cluster #1)
FDR (Age associated divergence) =0.0846
p(Scalp)=0.00212
p(Dura)=0.622
Scalp
Dura
DNAm (Beta)
0.50
0.60
0.70
Ages (years)
0
20
40
60
80
A
B
C
D
E
F
G
H
I
J
K
A
B
C
D
E
F
G
H
J
K

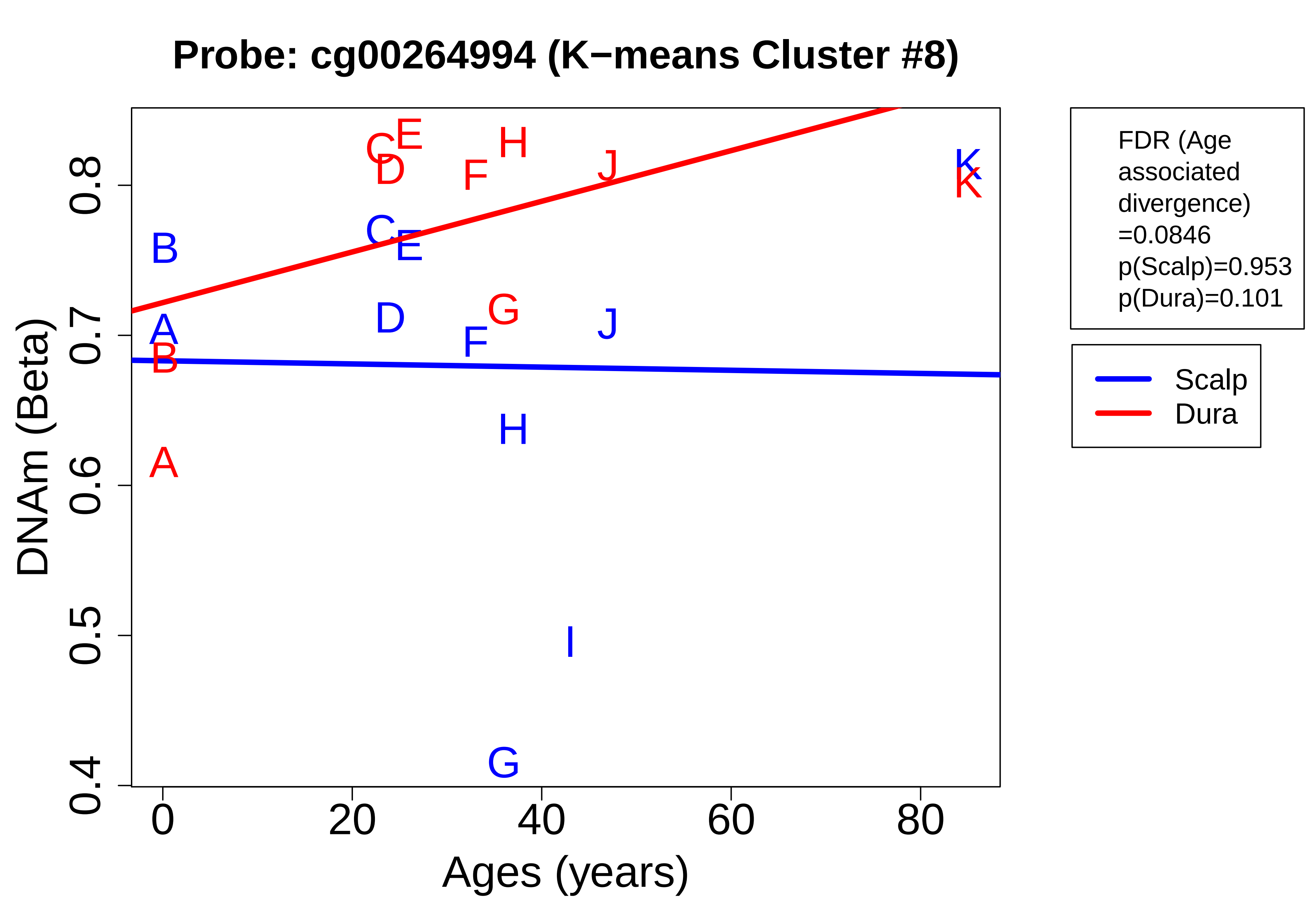

Probe: cg00264994 (K−means Cluster #8)
DNAm (Beta)
0.4
0.5
0.6
0.7
0.8
0
20
40
60
80
Ages (years)
A
B
C
D
E
F
G
H
I
J
K
FDR (Age associated divergence) =0.0846
p(Scalp)=0.953
p(Dura)=0.101
Scalp
Dura

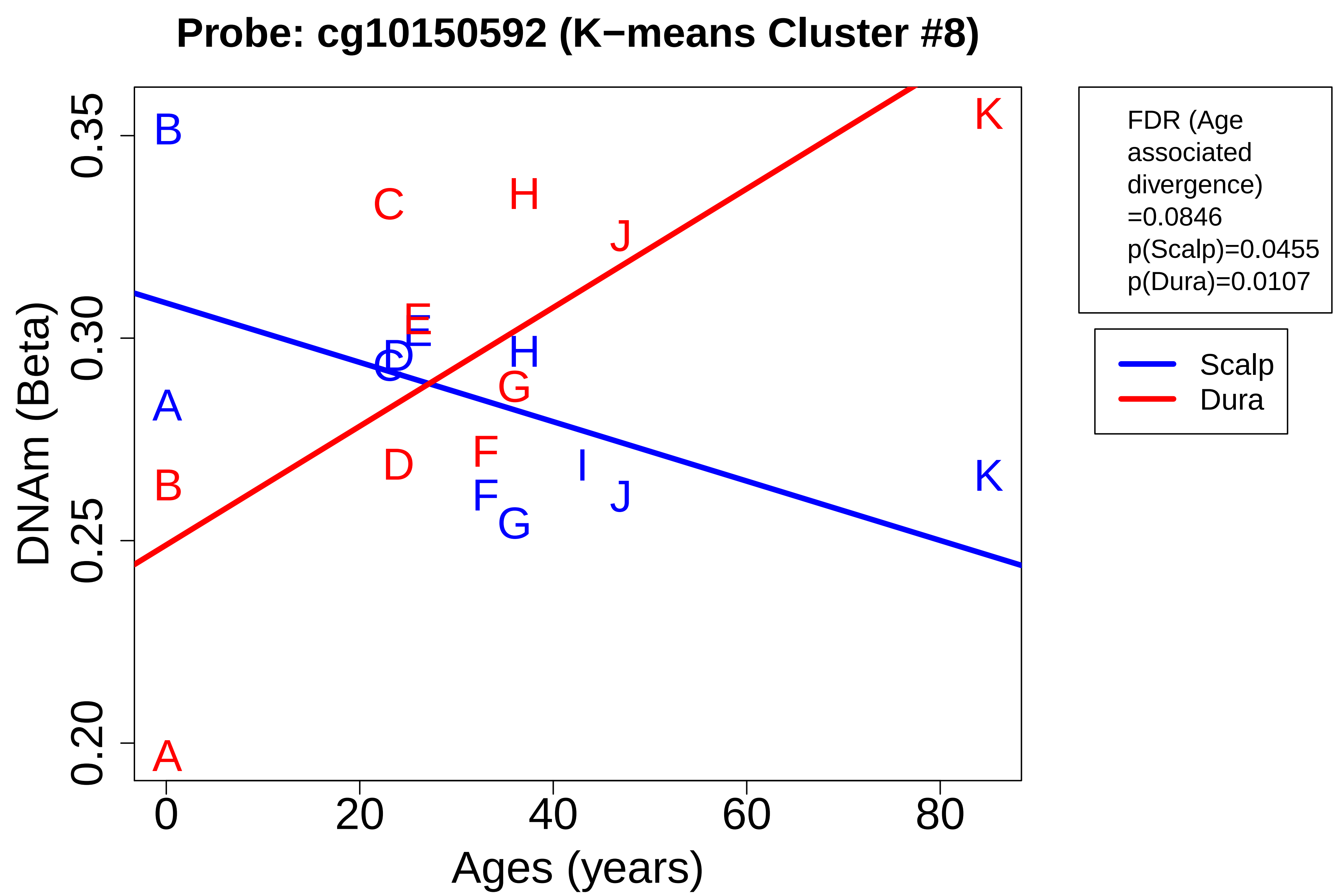
Probe: cg10150592 (K−means Cluster #8)
DNAm (Beta)
0.20
0.25
0.30
0.35
Ages (years)
0
20
40
60
80
FDR (Age associated divergence) =0.0846
p(Scalp)=0.0455
p(Dura)=0.0107
Scalp
Dura

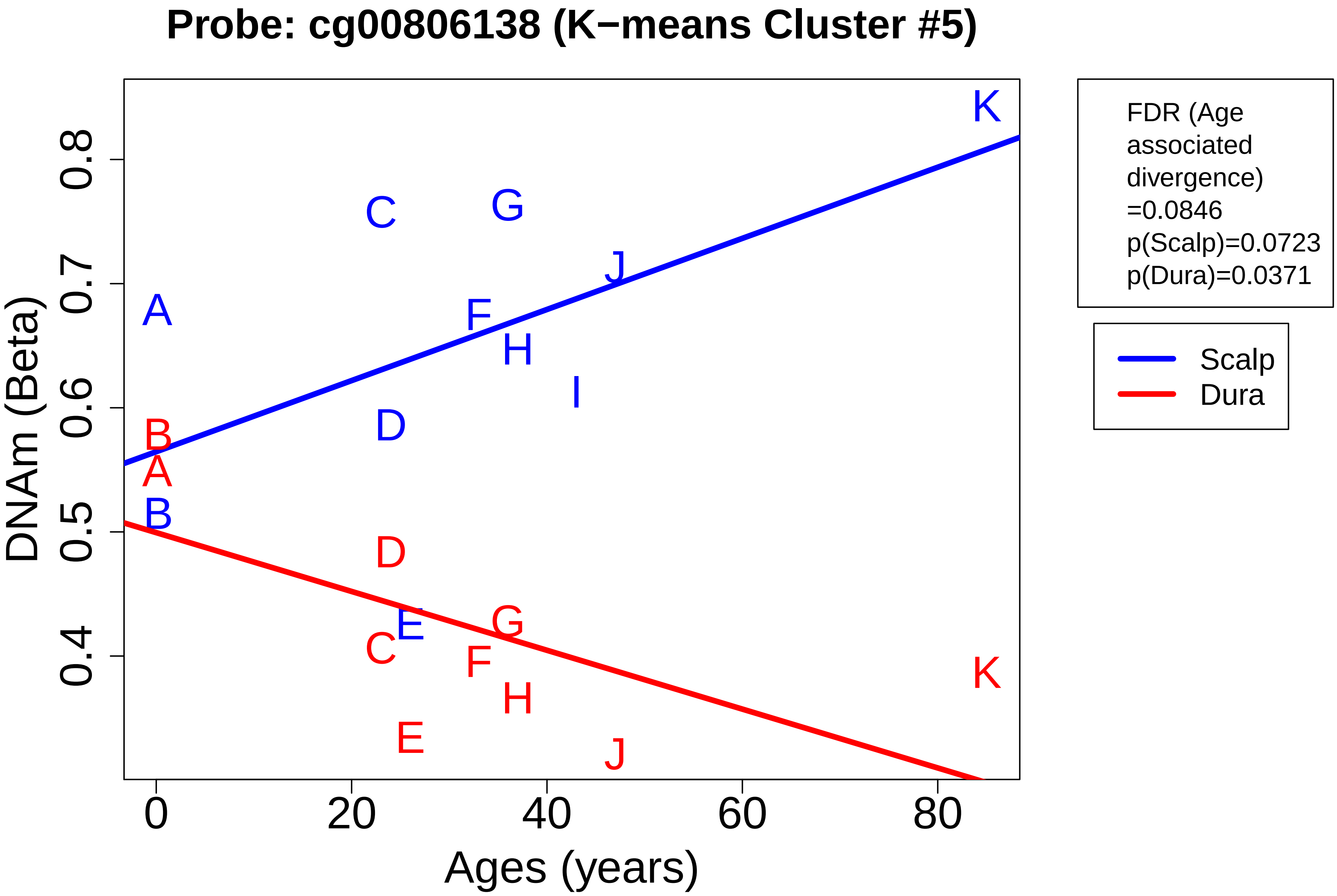
Probe: cg00806138 (K−means Cluster #5)
DNAm (Beta)
0.4
0.5
0.6
0.7
0.8
Ages (years)
0
20
40
60
80
FDR (Age associated divergence) =0.0846
p(Scalp)=0.0723
p(Dura)=0.0371
Scalp
Dura

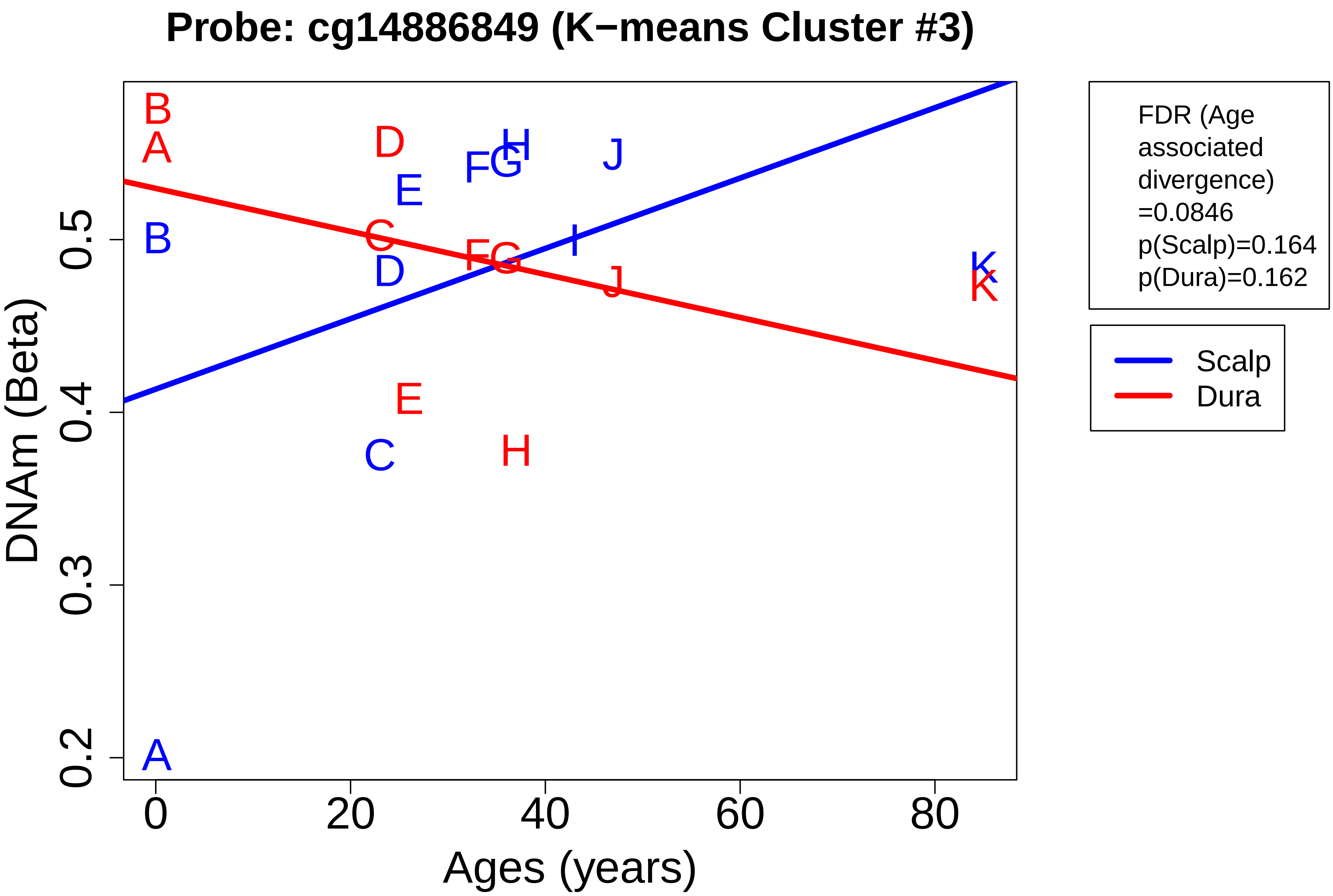

Probe: cg14886849 (K−means Cluster #3)
DNAm (Beta)
Ages (years)
0.2
0.3
0.4
0.5
0
20
40
60
80
FDR (Age associated divergence) =0.0846
p(Scalp)=0.164
p(Dura)=0.162
Scalp
Dura

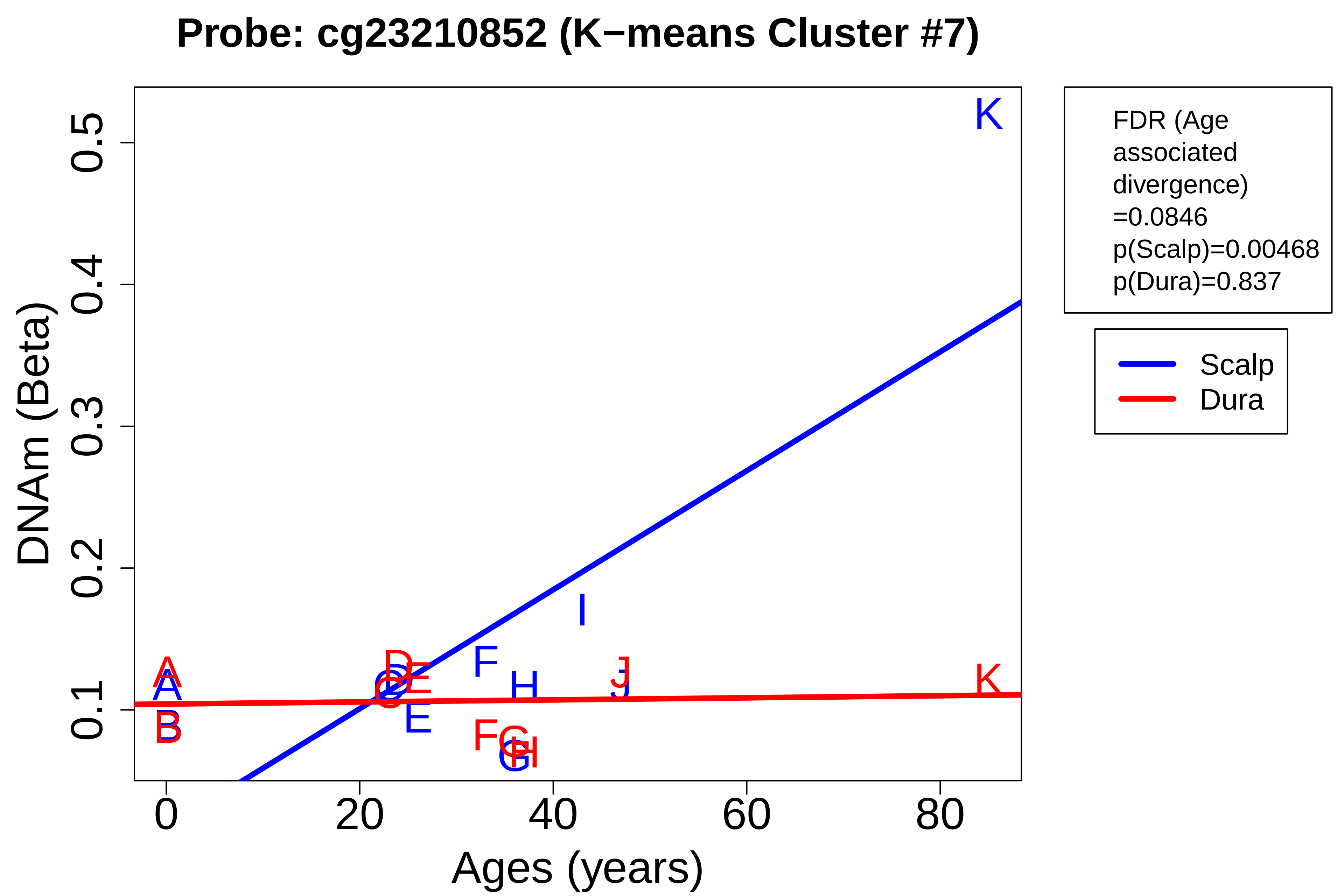

Probe: cg23210852 (K−means Cluster #7)
DNAm (Beta)
0.1
0.2
0.3
0.4
0.5
Ages (years)
0
20
40
60
80
FDR (Age associated divergence) =0.0846
p(Scalp)=0.00468
p(Dura)=0.837
Scalp
Dura

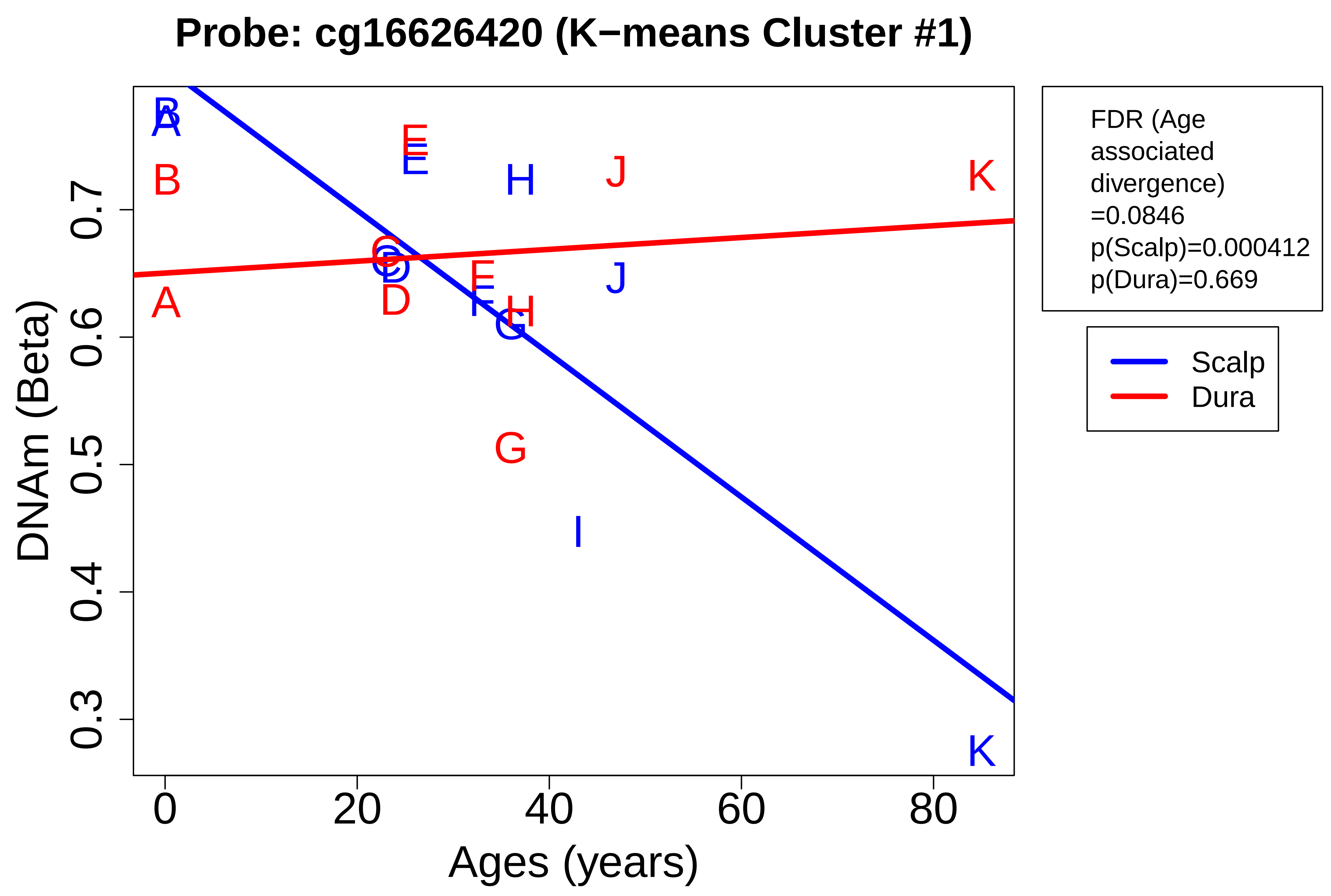

Probe: cg16626420 (K−means Cluster #1)
DNAm (Beta)
0.3
0.4
0.5
0.6
0.7
0
20
40
60
80
Ages (years)
A
B
C
D
E
F
G
H
I
J
K
FDR (Age associated divergence) =0.0846
p(Scalp)=0.000412
p(Dura)=0.669
Scalp
Dura

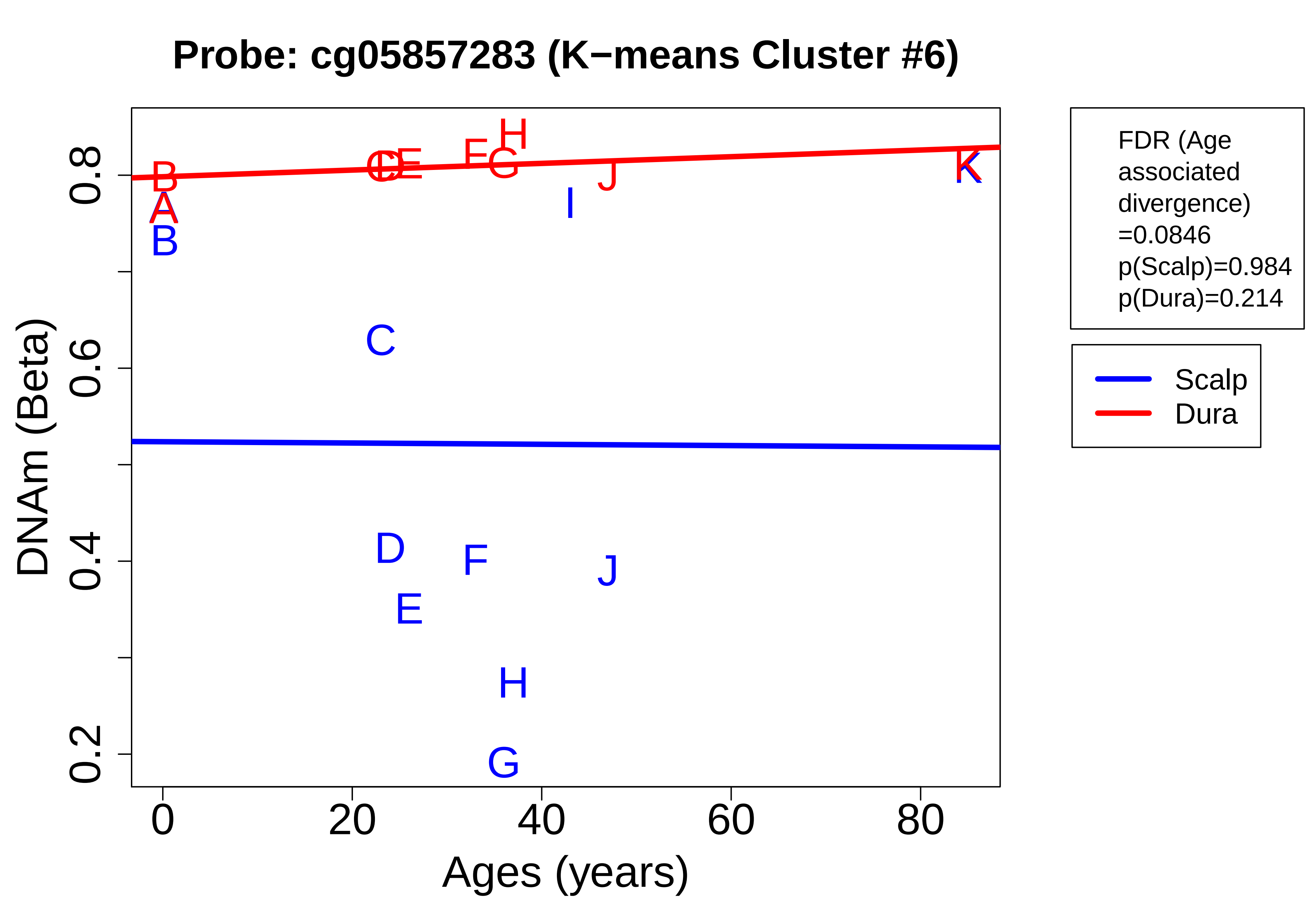

Probe: cg05857283 (K−means Cluster #6)
DNAm (Beta)
0.2
0.4
0.6
0.8
0
20
40
60
80
Ages (years)
FDR (Age associated divergence) =0.0846
p(Scalp)=0.984
p(Dura)=0.214
Scalp
Dura

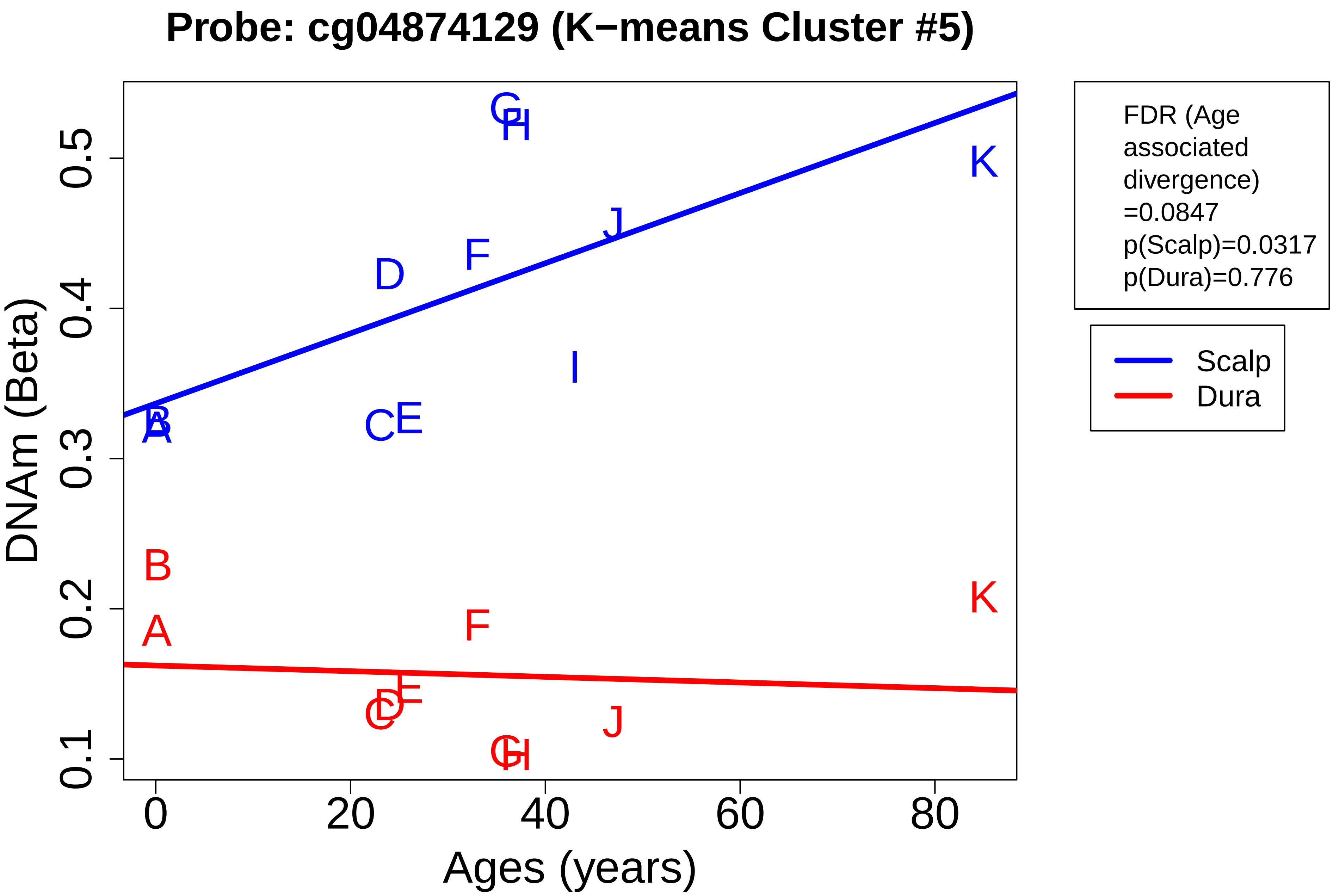
Probe: cg04874129 (K−means Cluster #5)
DNAm (Beta)
0.1
0.2
0.3
0.4
0.5
0
20
40
60
80
Ages (years)
FDR (Age associated divergence) =0.0847
p(Scalp)=0.0317
p(Dura)=0.776
Scalp
Dura
A
B
C
D
E
F
G
H
I
J
K

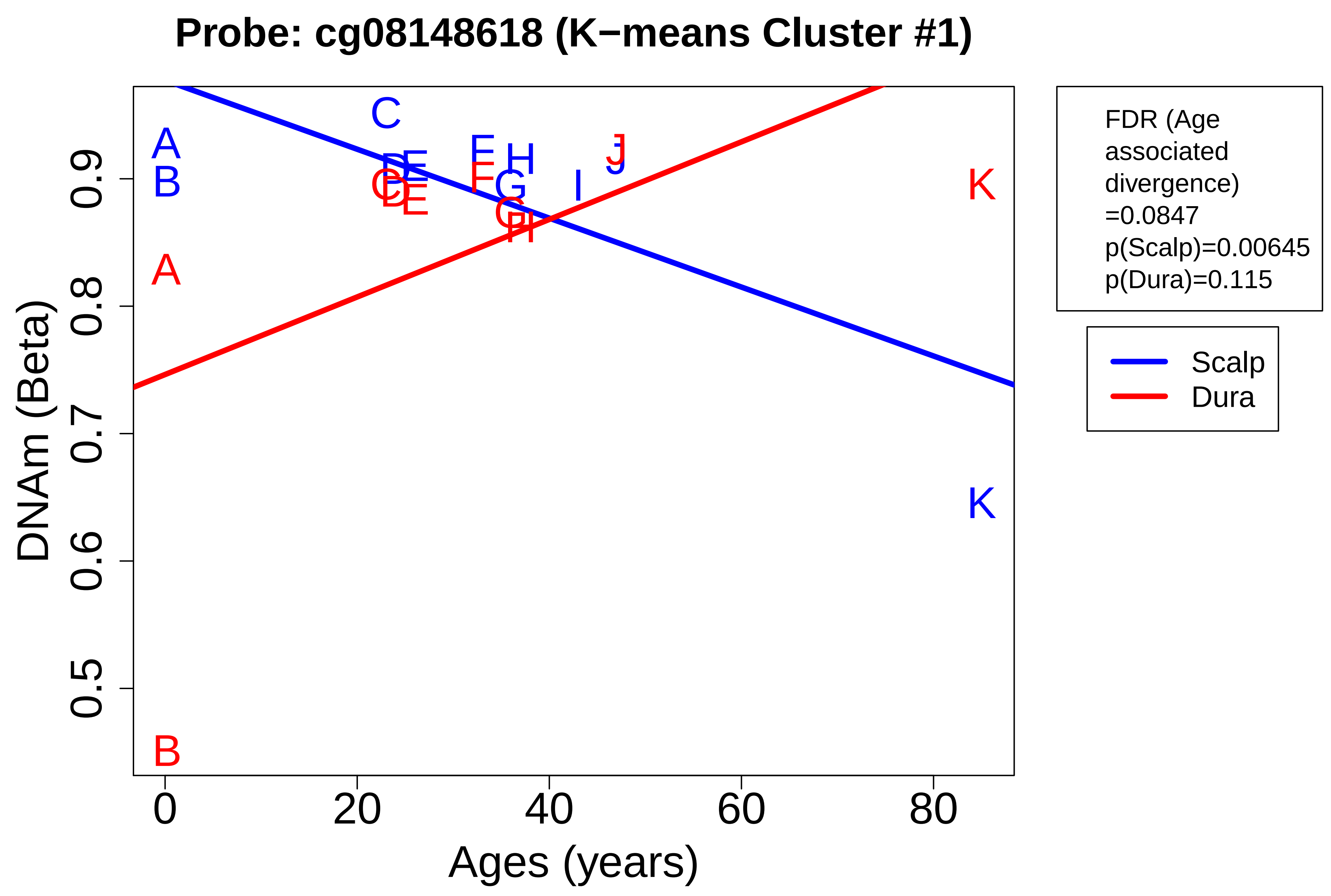

Probe: cg08148618 (K−means Cluster #1)
DNAm (Beta)
0.5
0.6
0.7
0.8
0.9
0
20
40
60
80
Ages (years)
A
B
C
D
E
F
G
H
I
J
K
FDR (Age associated divergence) =0.0847
p(Scalp)=0.00645
p(Dura)=0.115
Scalp
Dura

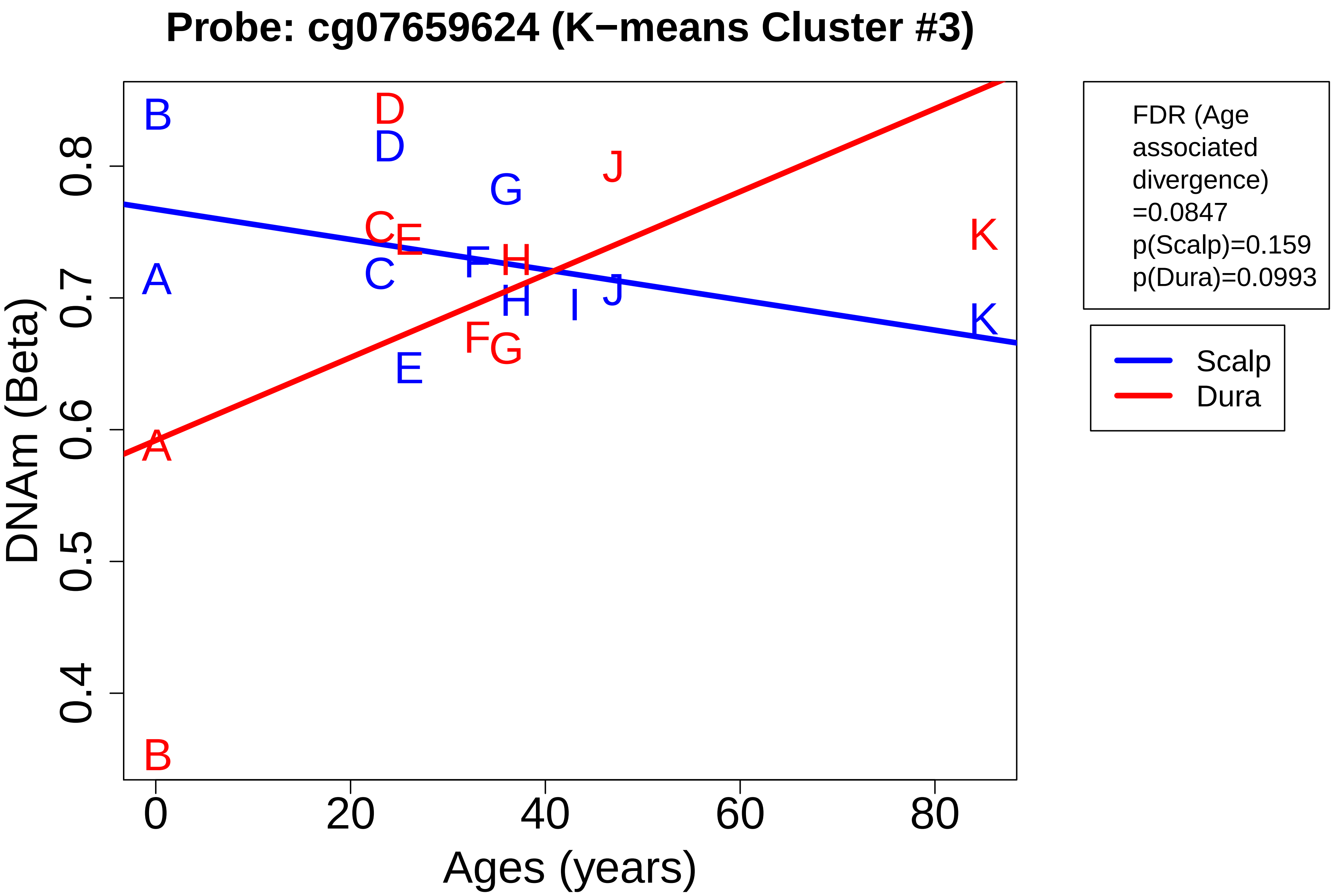

Probe: cg07659624 (K−means Cluster #3)
DNAm (Beta)
0.4
0.5
0.6
0.7
0.8
Ages (years)
0
20
40
60
80
FDR (Age associated divergence) =0.0847
p(Scalp)=0.159
p(Dura)=0.0993
Scalp
Dura

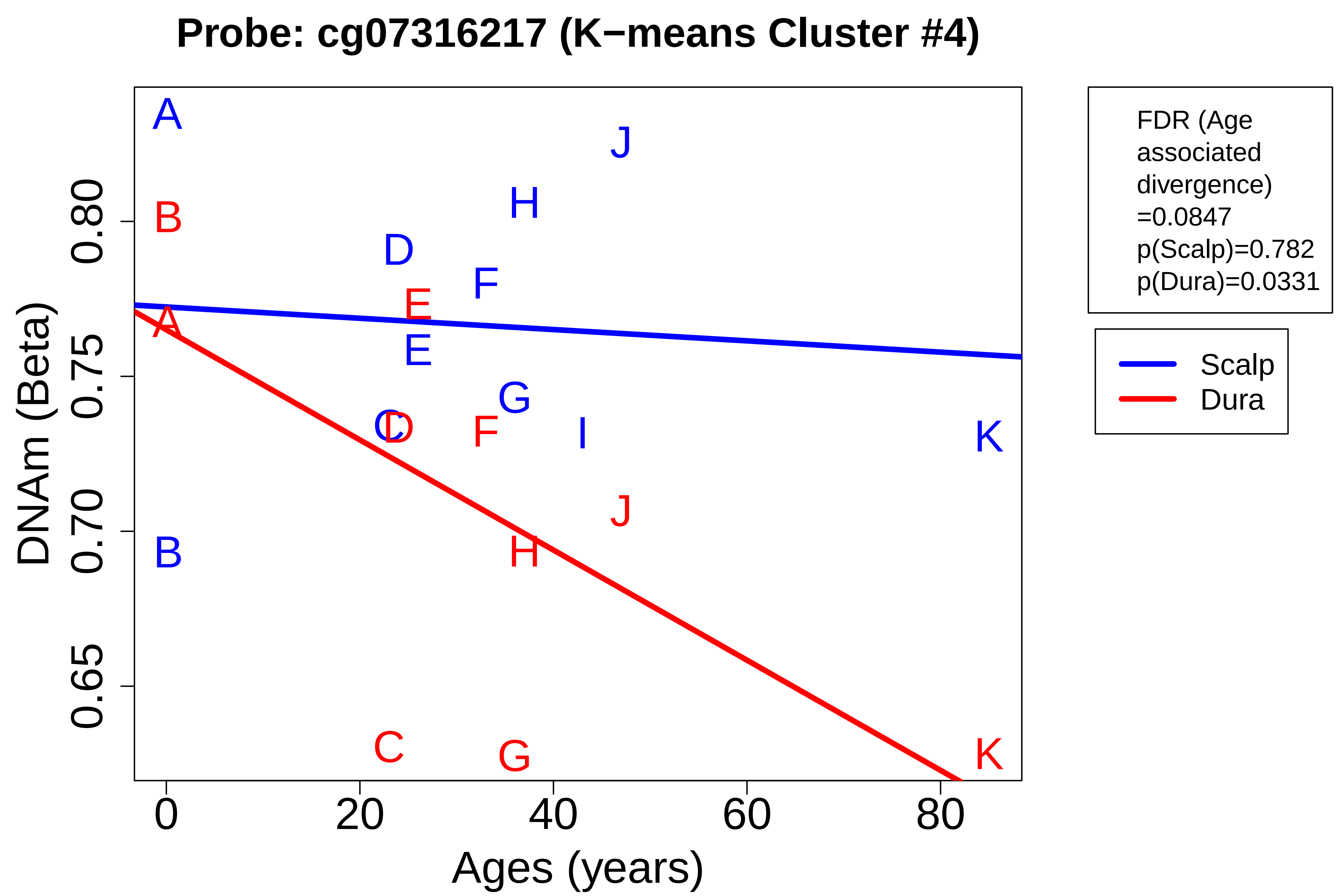

Probe: cg07316217 (K−means Cluster #4)
FDR (Age associated divergence) =0.0847
p(Scalp)=0.782
p(Dura)=0.0331
Scalp
Dura
DNAm (Beta)
0.65
0.70
0.75
0.80
Ages (years)
0
20
40
60
80
A
B
C
D
E
F
G
H
I
J
K

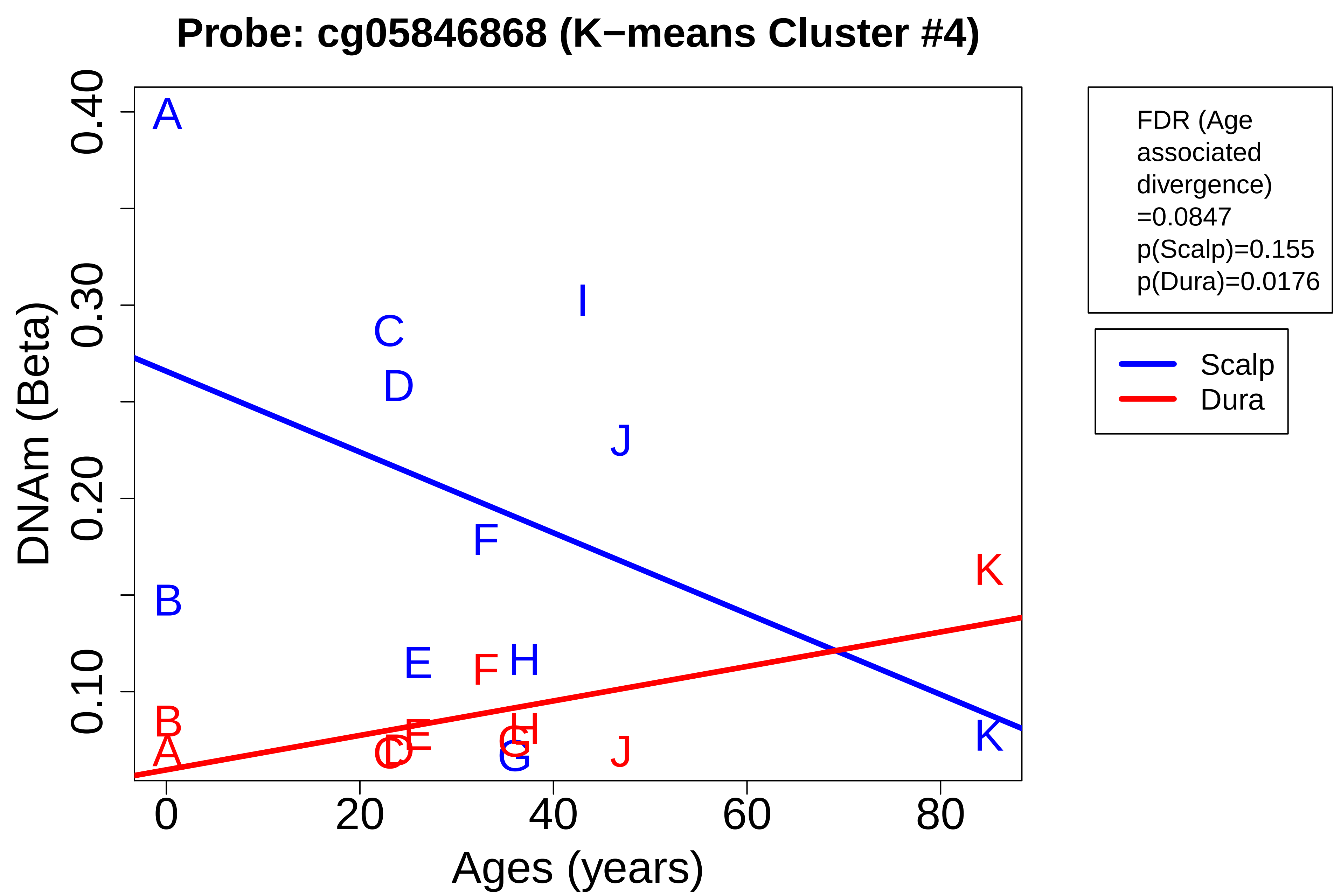
Probe: cg05846868 (K−means Cluster #4)
DNAm (Beta)
0.10
0.20
0.30
0.40
0
20
40
60
80
Ages (years)
A
B
C
D
E
F
G
H
I
J
K
FDR (Age associated divergence) =0.0847
p(Scalp)=0.155
p(Dura)=0.0176
Scalp
Dura

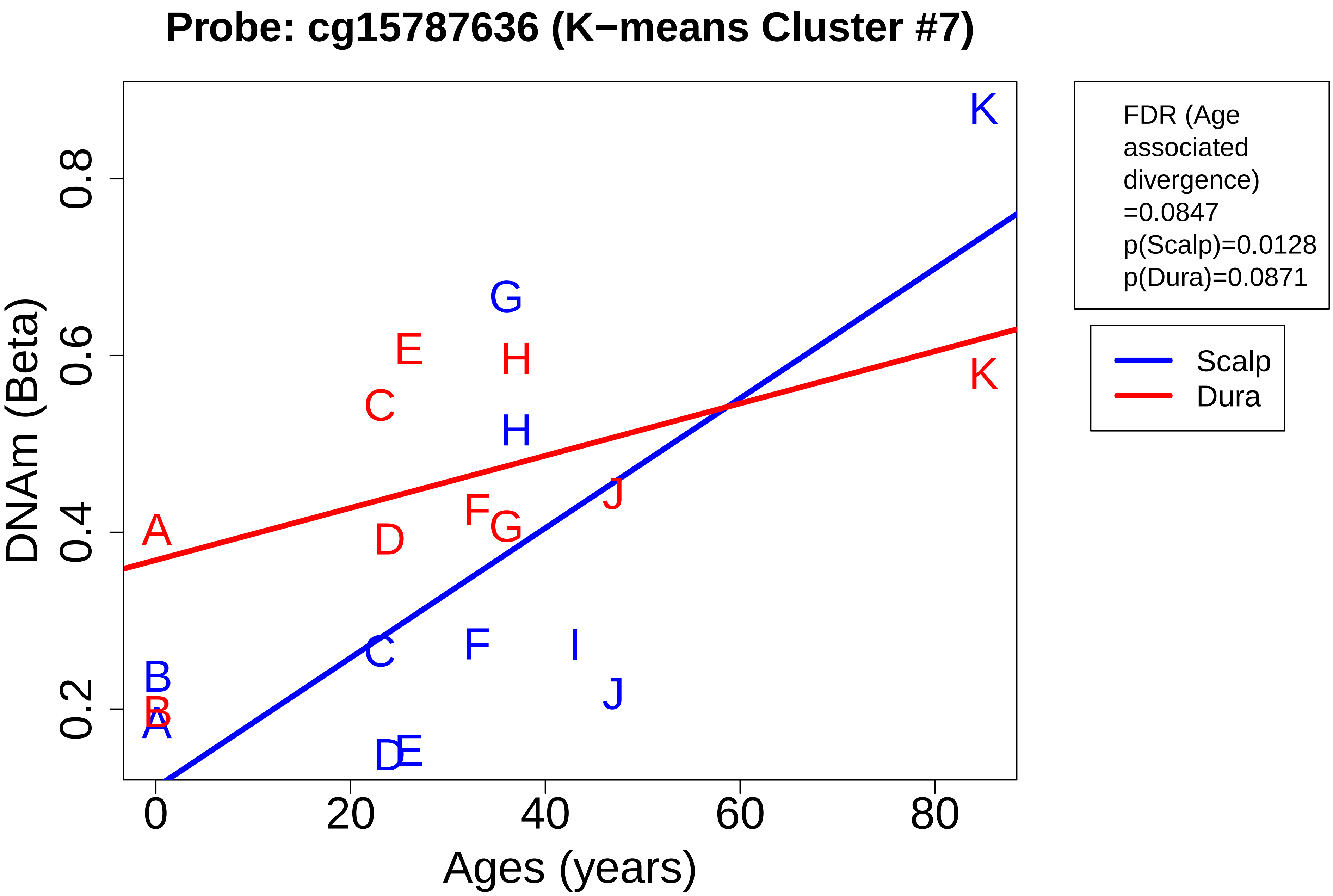
Probe: cg15787636 (K−means Cluster #7)
DNAm (Beta)
0.2
0.4
0.6
0.8
0
20
40
60
80
Ages (years)
FDR (Age associated divergence) =0.0847
p(Scalp)=0.0128
p(Dura)=0.0871
Scalp
Dura
A
B
C
D
E
F
G
H
I
J
K

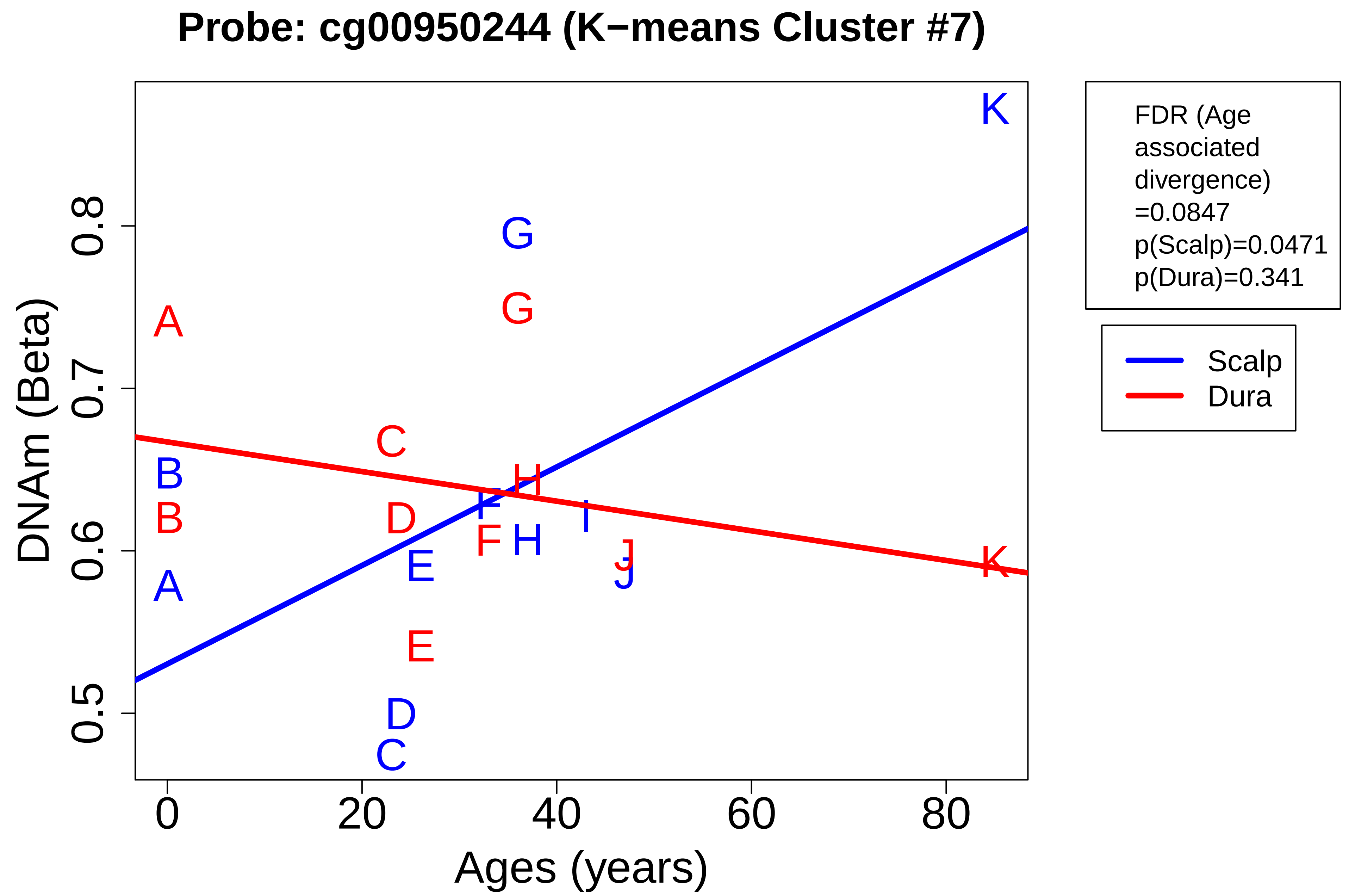

Probe: cg00950244 (K−means Cluster #7)
DNAm (Beta)
0.5
0.6
0.7
0.8
0
20
40
60
80
Ages (years)
A
B
C
D
E
F
G
H
I
J
K
FDR (Age associated divergence) =0.0847
p(Scalp)=0.0471
p(Dura)=0.341
Scalp
Dura

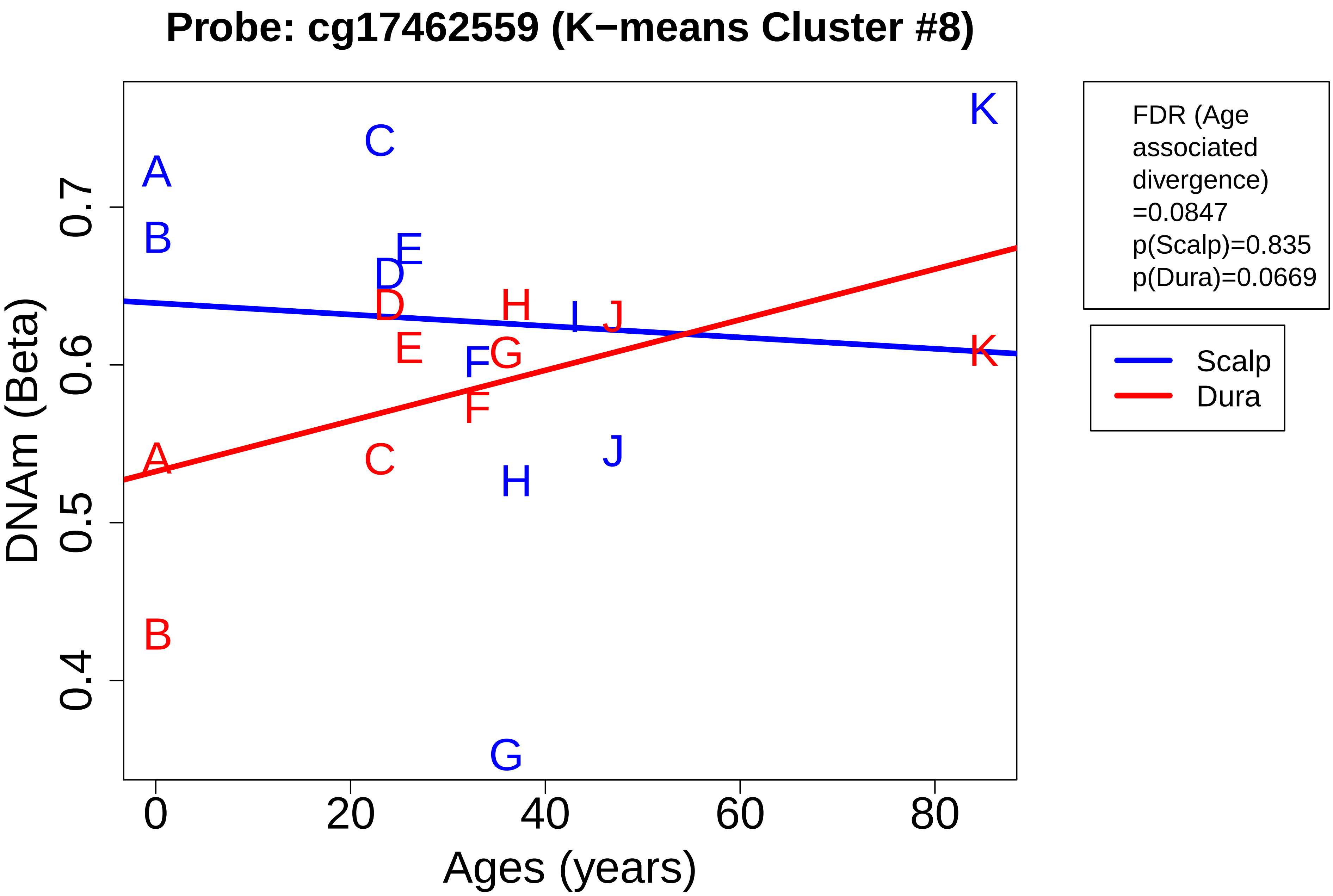
Probe: cg17462559 (K−means Cluster #8)
DNAm (Beta)
0.4
0.5
0.6
0.7
0
20
40
60
80
Ages (years)
FDR (Age associated divergence) =0.0847
p(Scalp)=0.835
p(Dura)=0.0669
Scalp
Dura

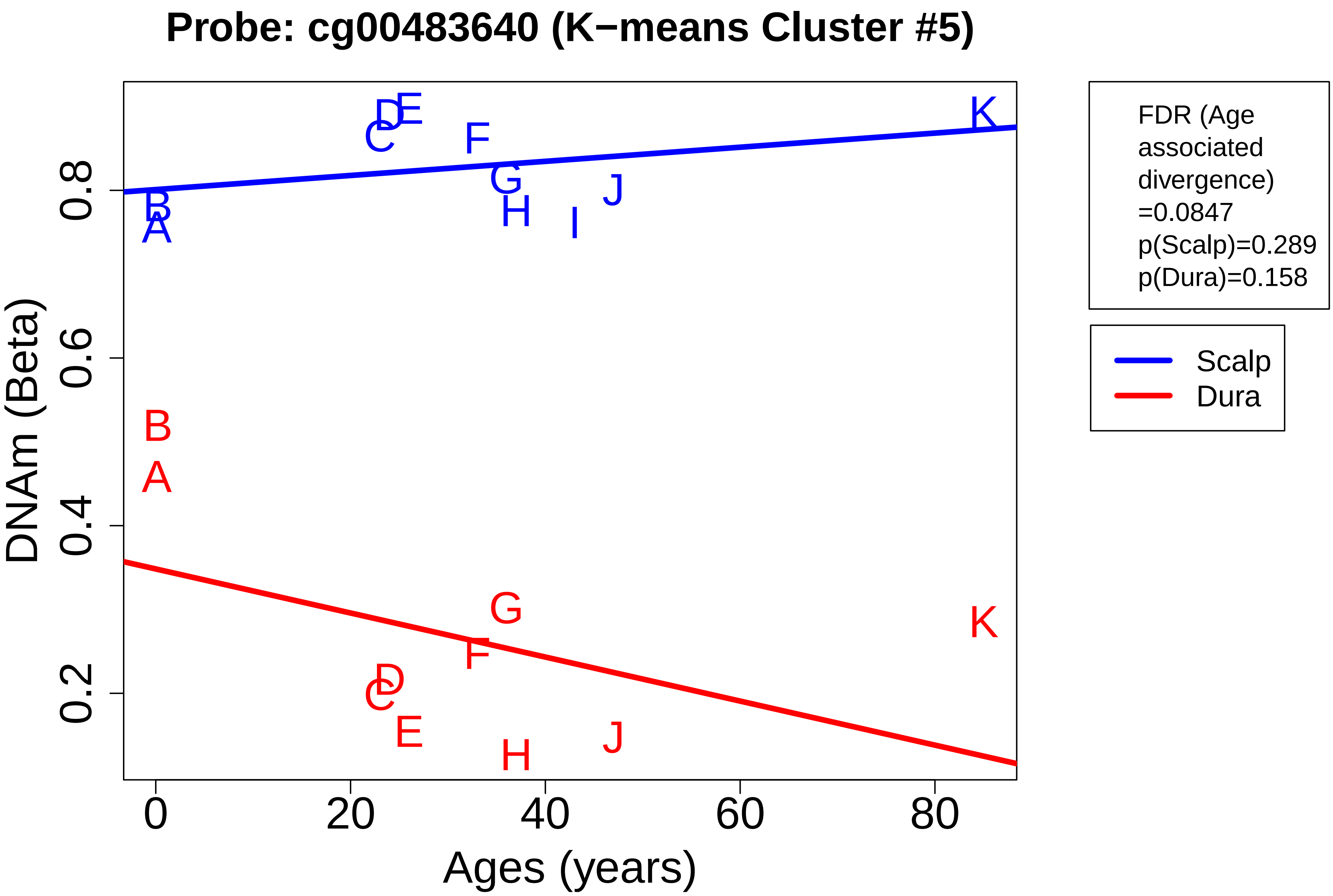

Probe: cg00483640 (K−means Cluster #5)
DNAm (Beta)
0.2
0.4
0.6
0.8
Ages (years)
0
20
40
60
80
A
B
C
D
E
F
G
H
I
J
K
A
B
C
D
E
F
G
H
J
K
FDR (Age associated divergence) =0.0847
p(Scalp)=0.289
p(Dura)=0.158
Scalp
Dura

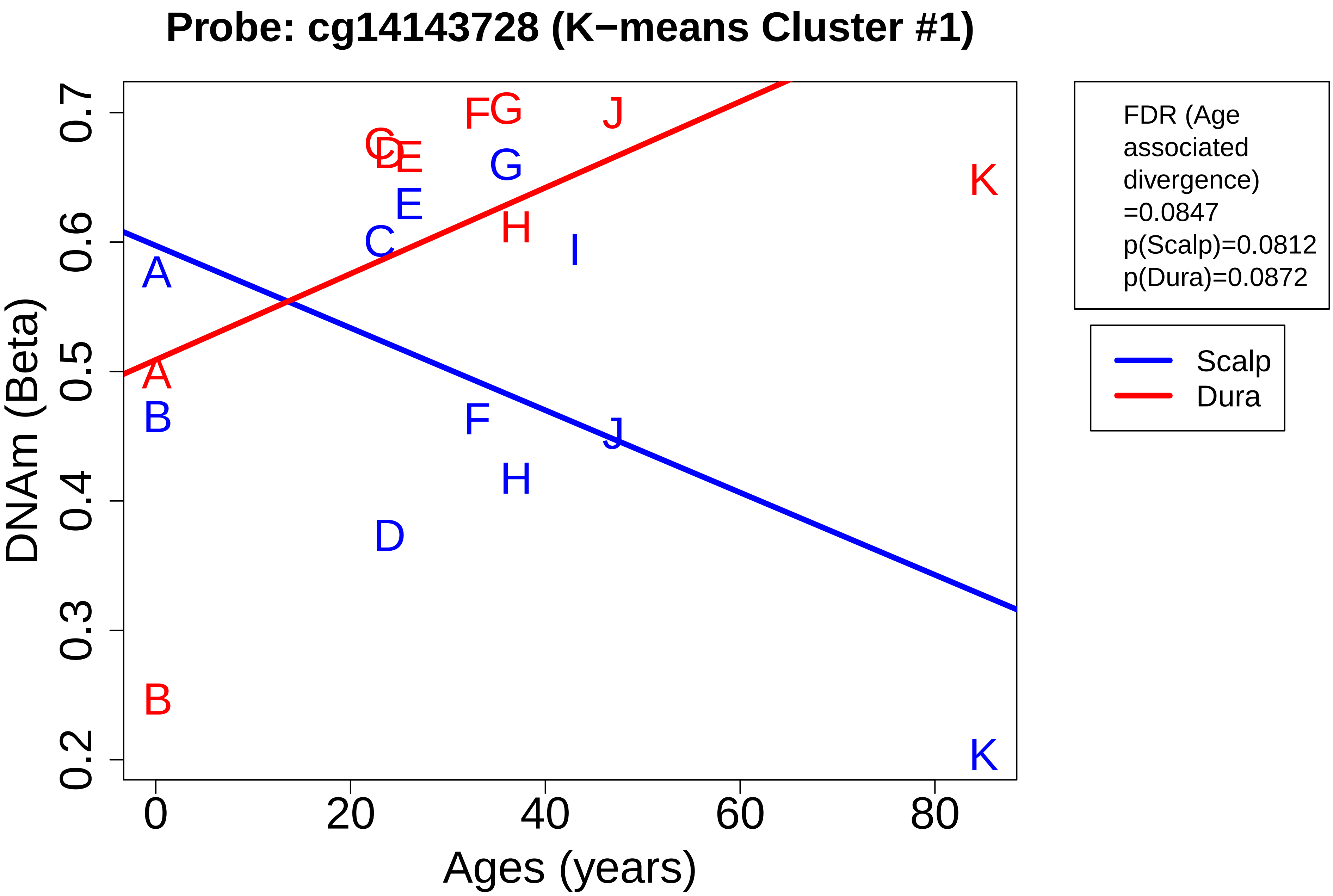
Probe: cg14143728 (K−means Cluster #1)
DNAm (Beta)
Ages (years)
0.2
0.3
0.4
0.5
0.6
0.7
0
20
40
60
80
FDR (Age associated divergence) =0.0847
p(Scalp)=0.0812
p(Dura)=0.0872
Scalp
Dura

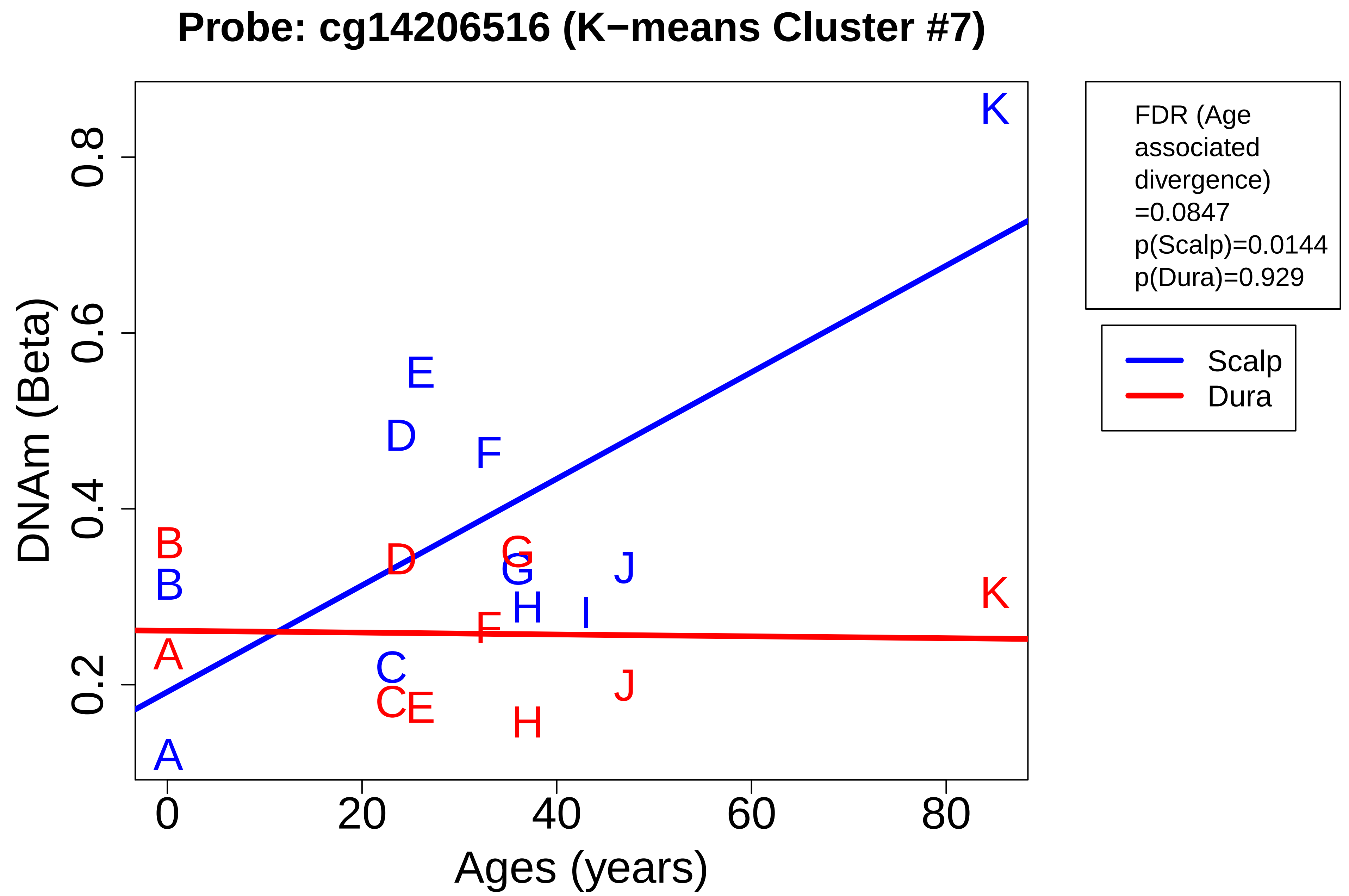

Probe: cg14206516 (K−means Cluster #7)
DNAm (Beta)
0.2
0.4
0.6
0.8
Ages (years)
0
20
40
60
80
FDR (Age associated divergence) =0.0847
p(Scalp)=0.0144
p(Dura)=0.929
Scalp
Dura
A
B
C
D
E
F
G
H
I
J
K

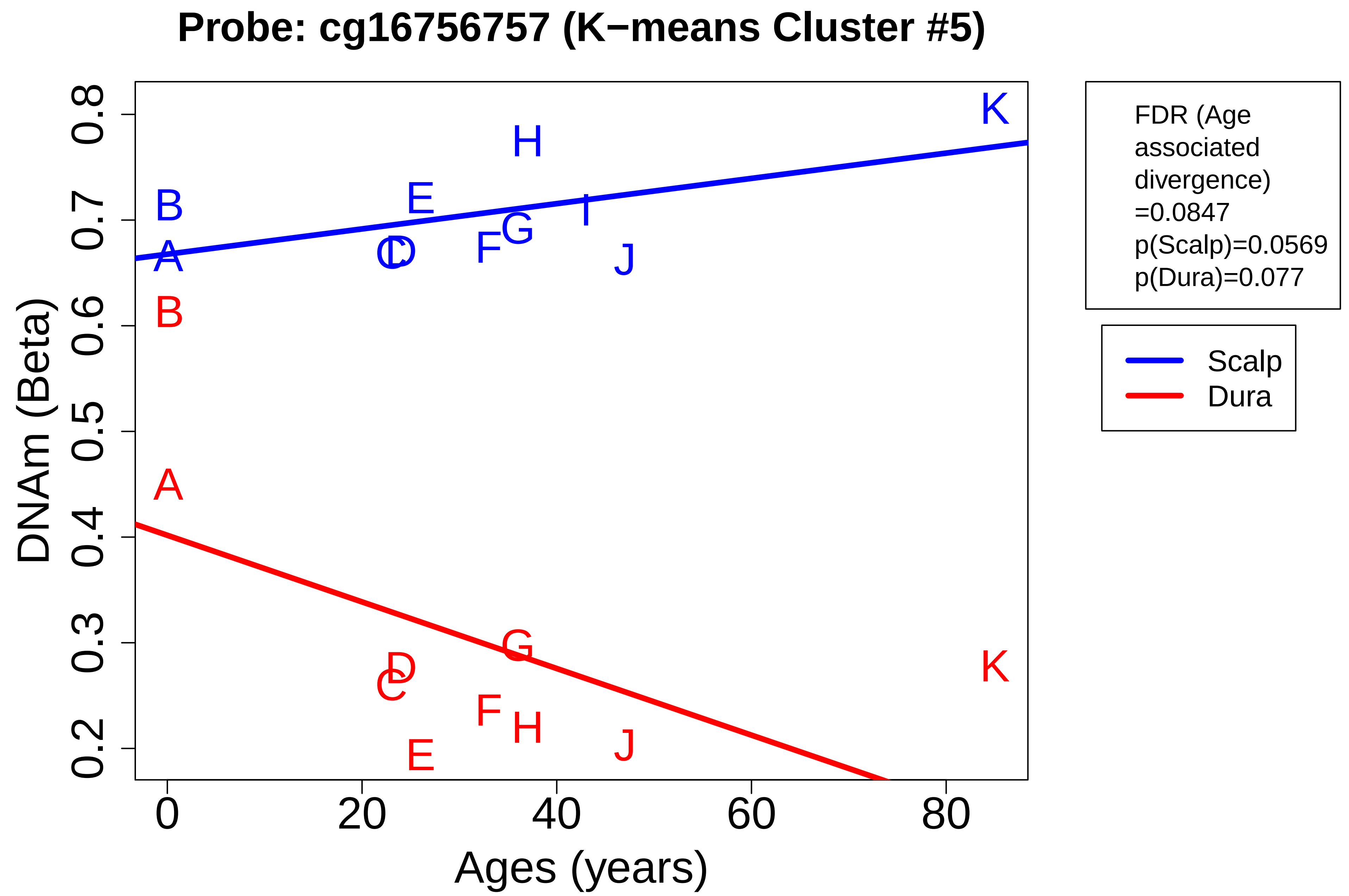

Probe: cg16756757 (K−means Cluster #5)
DNAm (Beta)
0.2
0.3
0.4
0.5
0.6
0.7
0.8
Ages (years)
0
20
40
60
80
FDR (Age associated divergence) =0.0847
p(Scalp)=0.0569
p(Dura)=0.077
Scalp
Dura

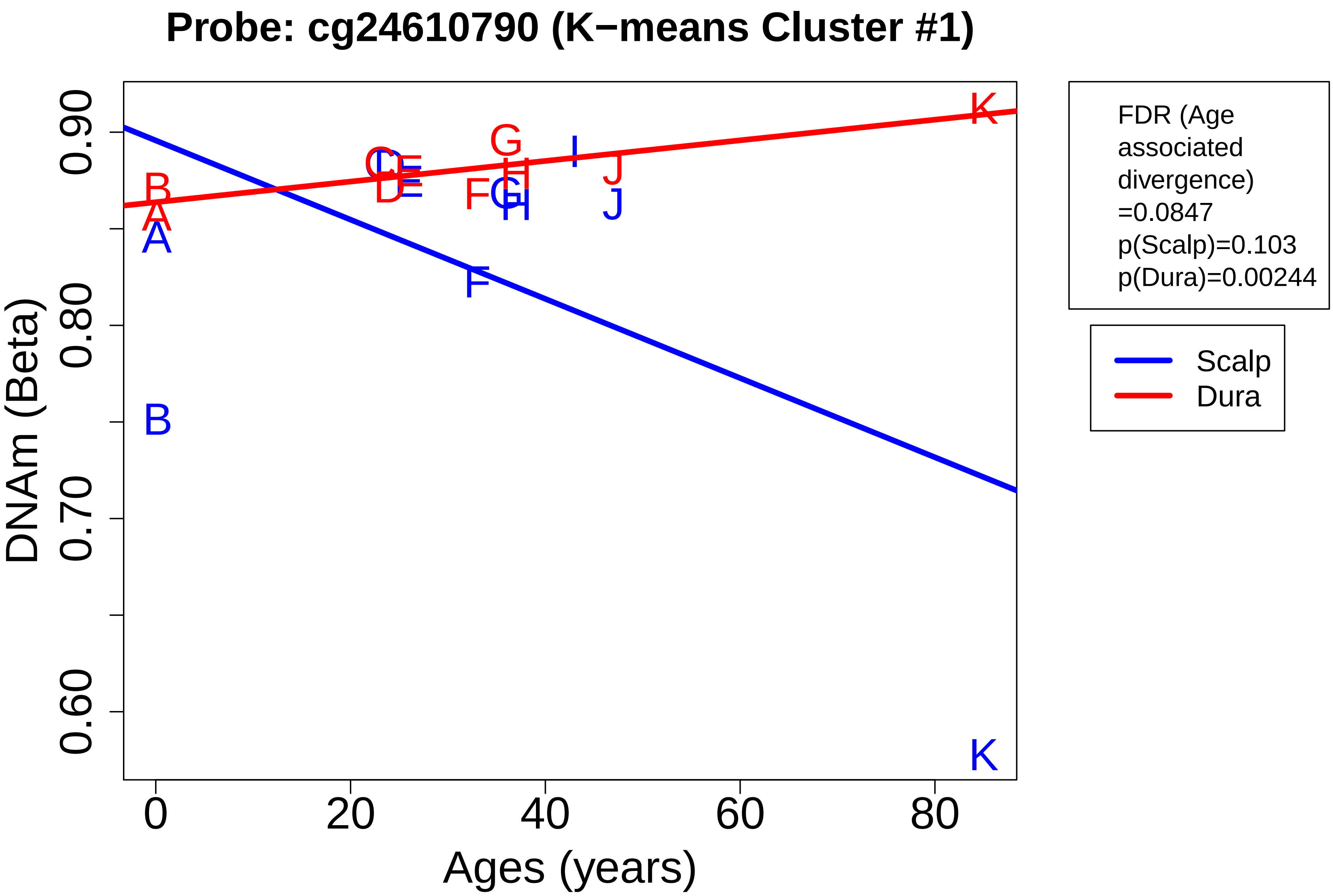

Probe: cg24610790 (K−means Cluster #1)
DNAm (Beta)
0.60
0.70
0.80
0.90
0
20
40
60
80
Ages (years)
FDR (Age associated divergence) =0.0847
p(Scalp)=0.103
p(Dura)=0.00244
Scalp
Dura

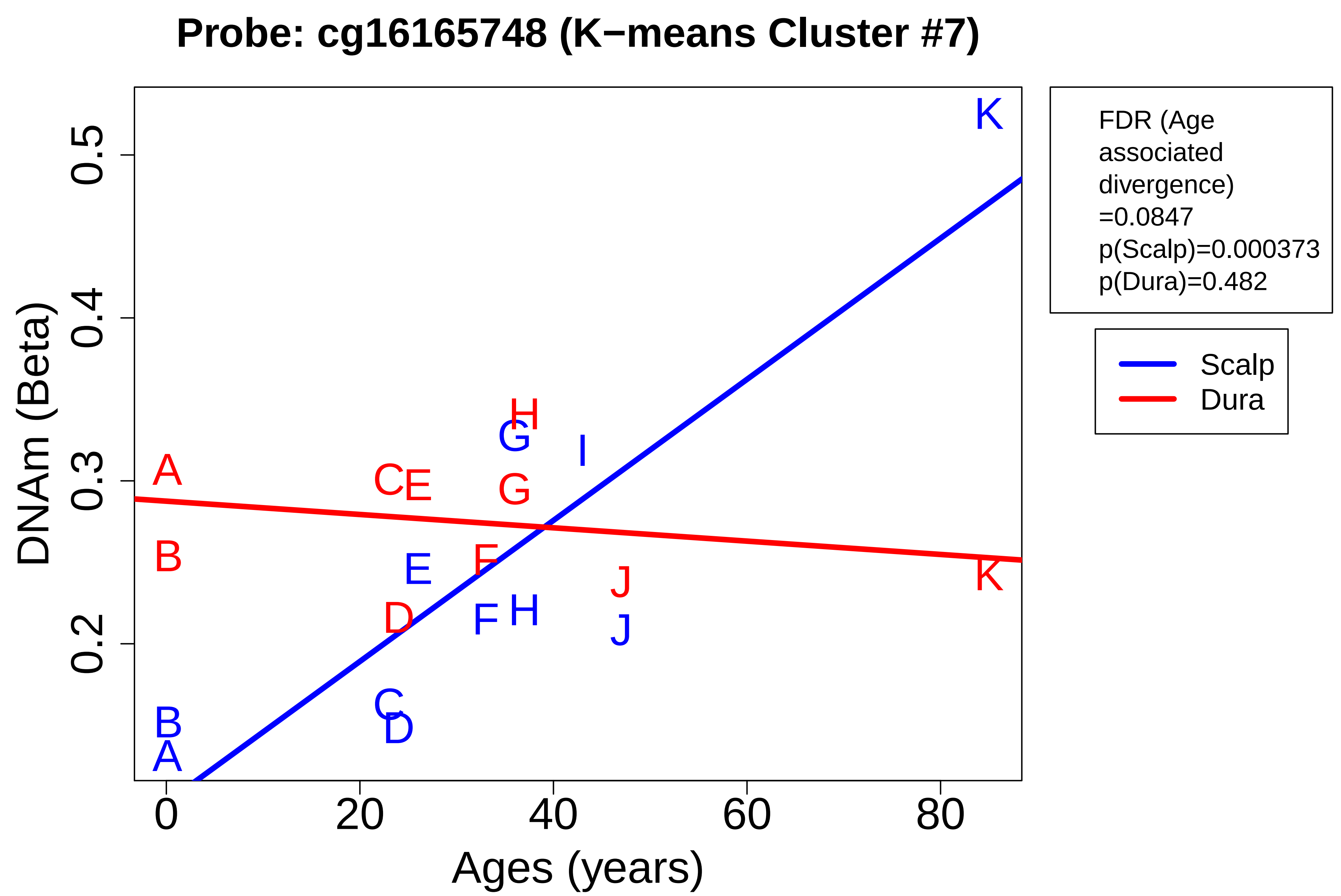

Probe: cg16165748 (K−means Cluster #7)
DNAm (Beta)
0.2
0.3
0.4
0.5
Ages (years)
0
20
40
60
80
A
B
C
D
E
F
G
H
I
J
K
FDR (Age associated divergence) =0.0847
p(Scalp)=0.000373
p(Dura)=0.482
Scalp
Dura

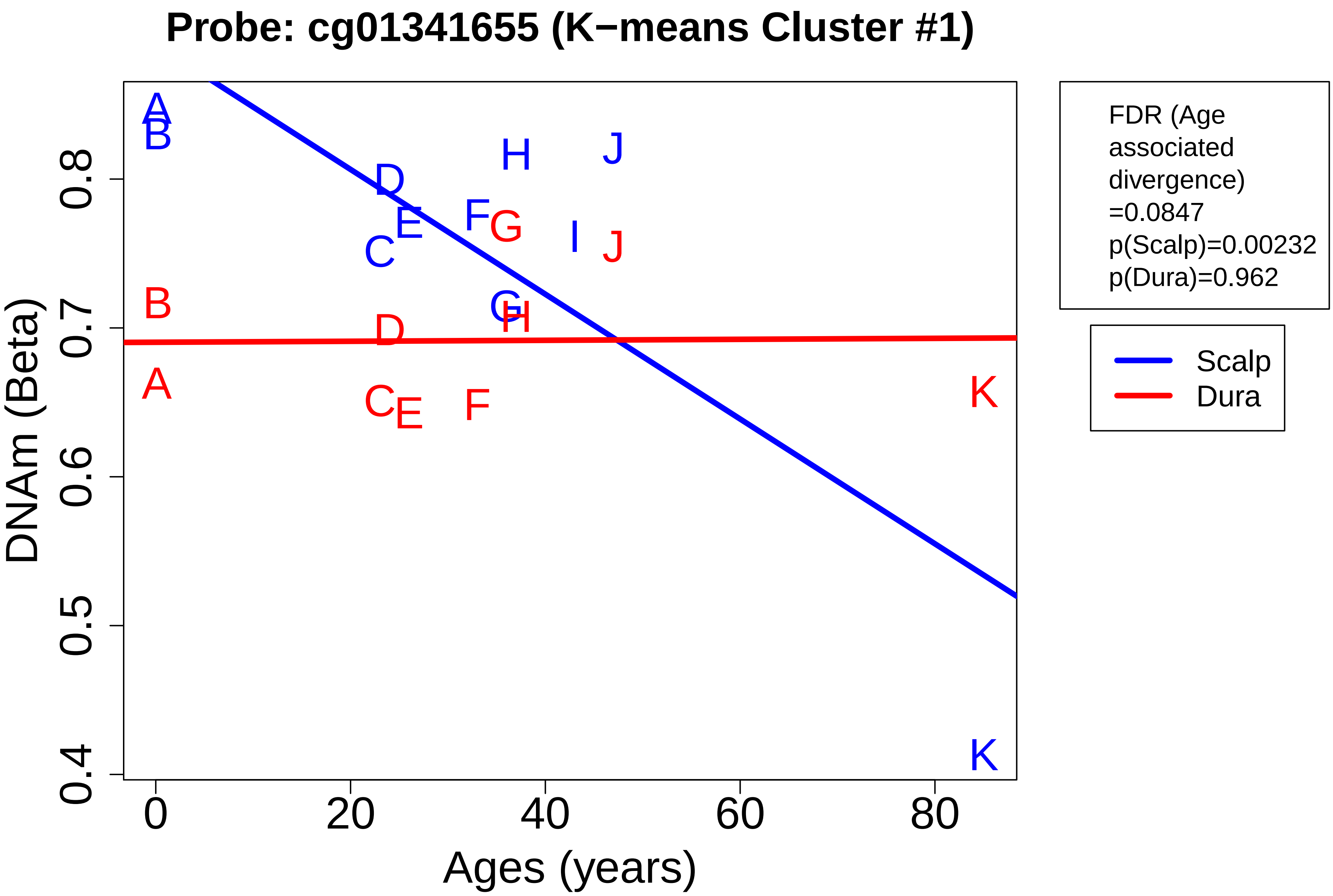

Probe: cg01341655 (K−means Cluster #1)
FDR (Age associated divergence) =0.0847
p(Scalp)=0.00232
p(Dura)=0.962
Scalp
Dura
DNAm (Beta)
0.4
0.5
0.6
0.7
0.8
0
20
40
60
80
Ages (years)
A
B
C
D
E
F
G
H
I
J
K

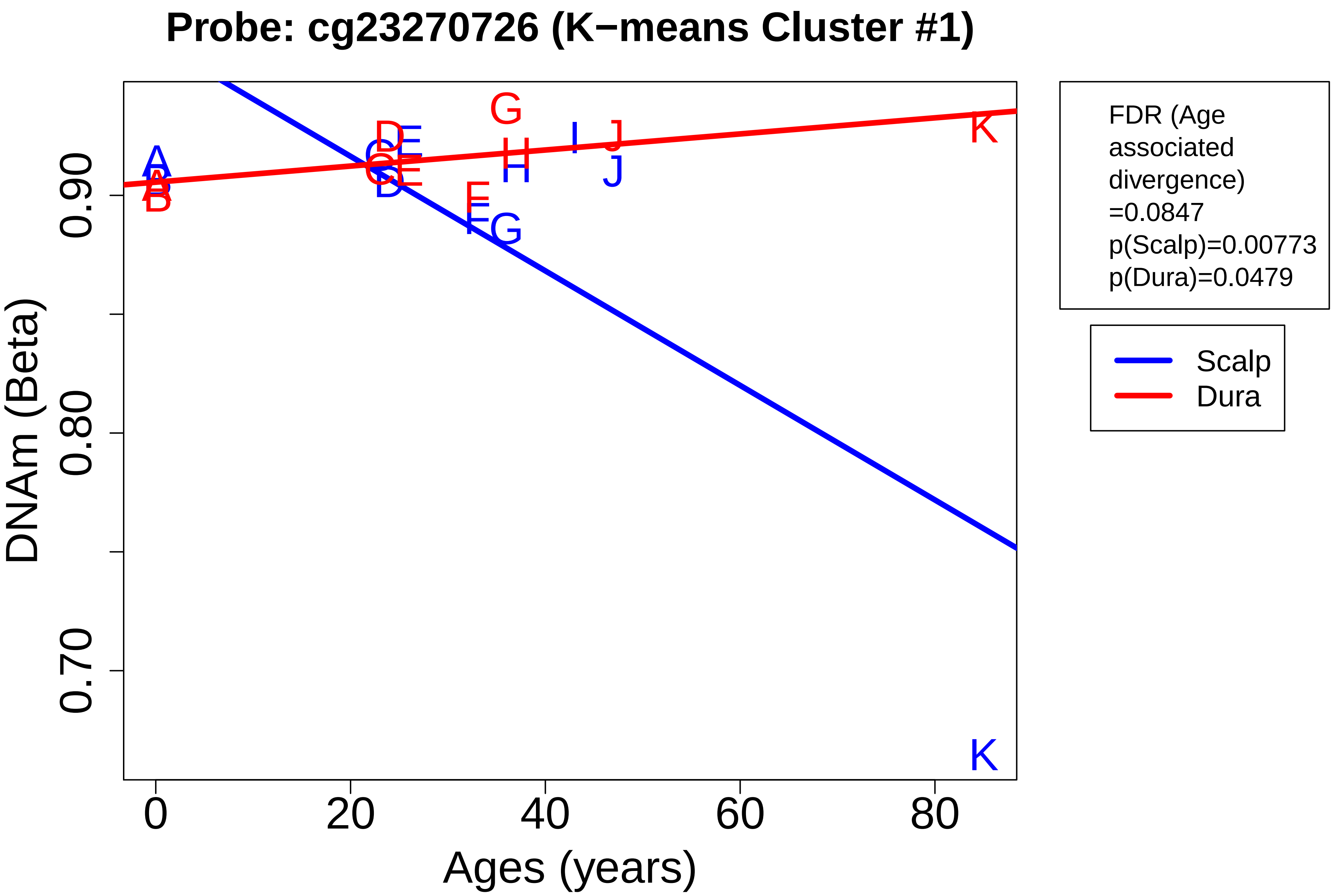

Probe: cg23270726 (K−means Cluster #1)
DNAm (Beta)
0.70
0.80
0.90
Ages (years)
0
20
40
60
80
A
B
C
D
E
F
G
H
I
J
K
FDR (Age associated divergence) =0.0847
p(Scalp)=0.00773
p(Dura)=0.0479
Scalp
Dura

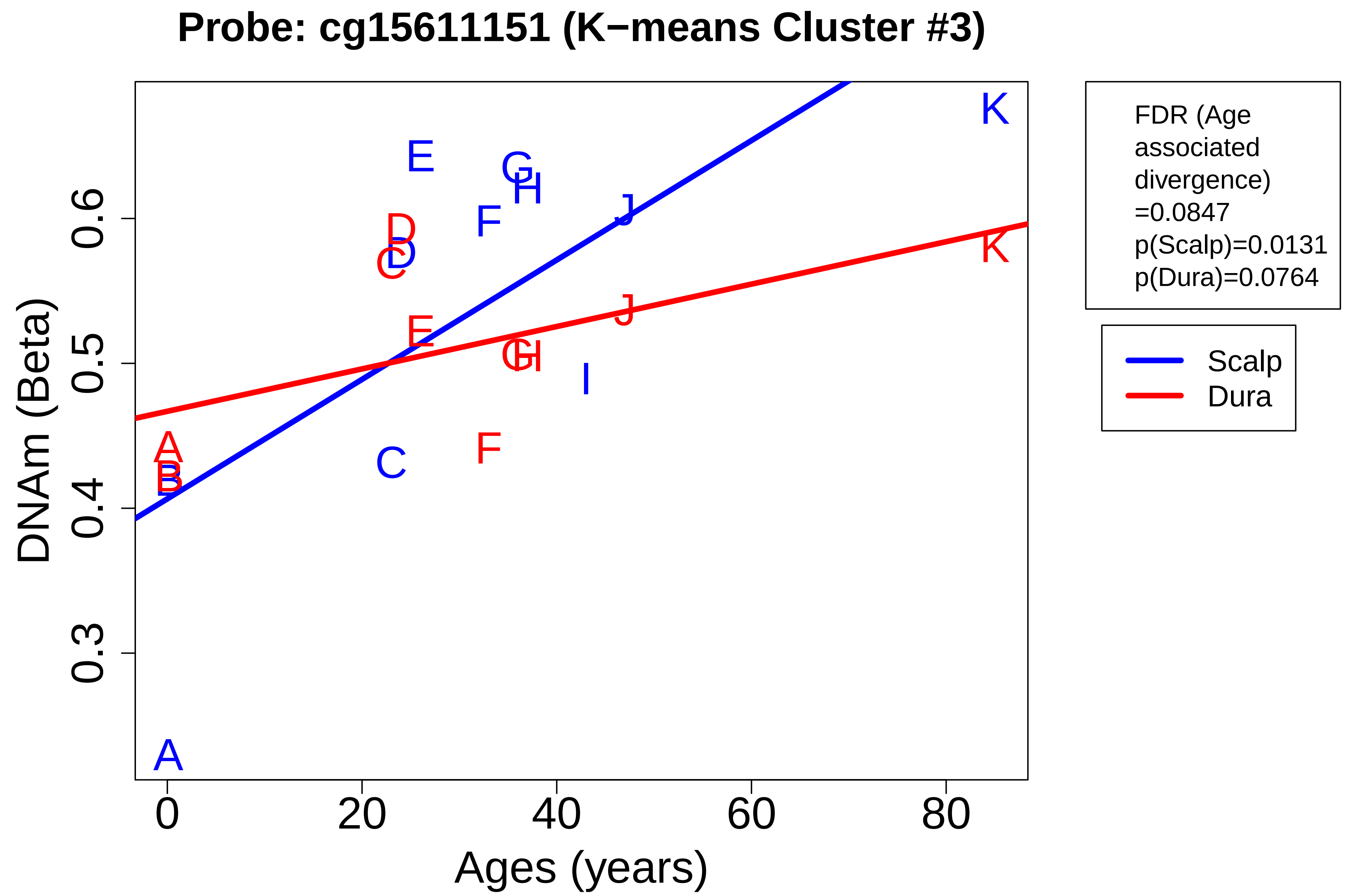

Probe: cg15611151 (K−means Cluster #3)
FDR (Age associated divergence) =0.0847
p(Scalp)=0.0131
p(Dura)=0.0764
Scalp
Dura
DNAm (Beta)
0.3
0.4
0.5
0.6
Ages (years)
0
20
40
60
80
A
B
C
D
E
F
G
H
I
J
K

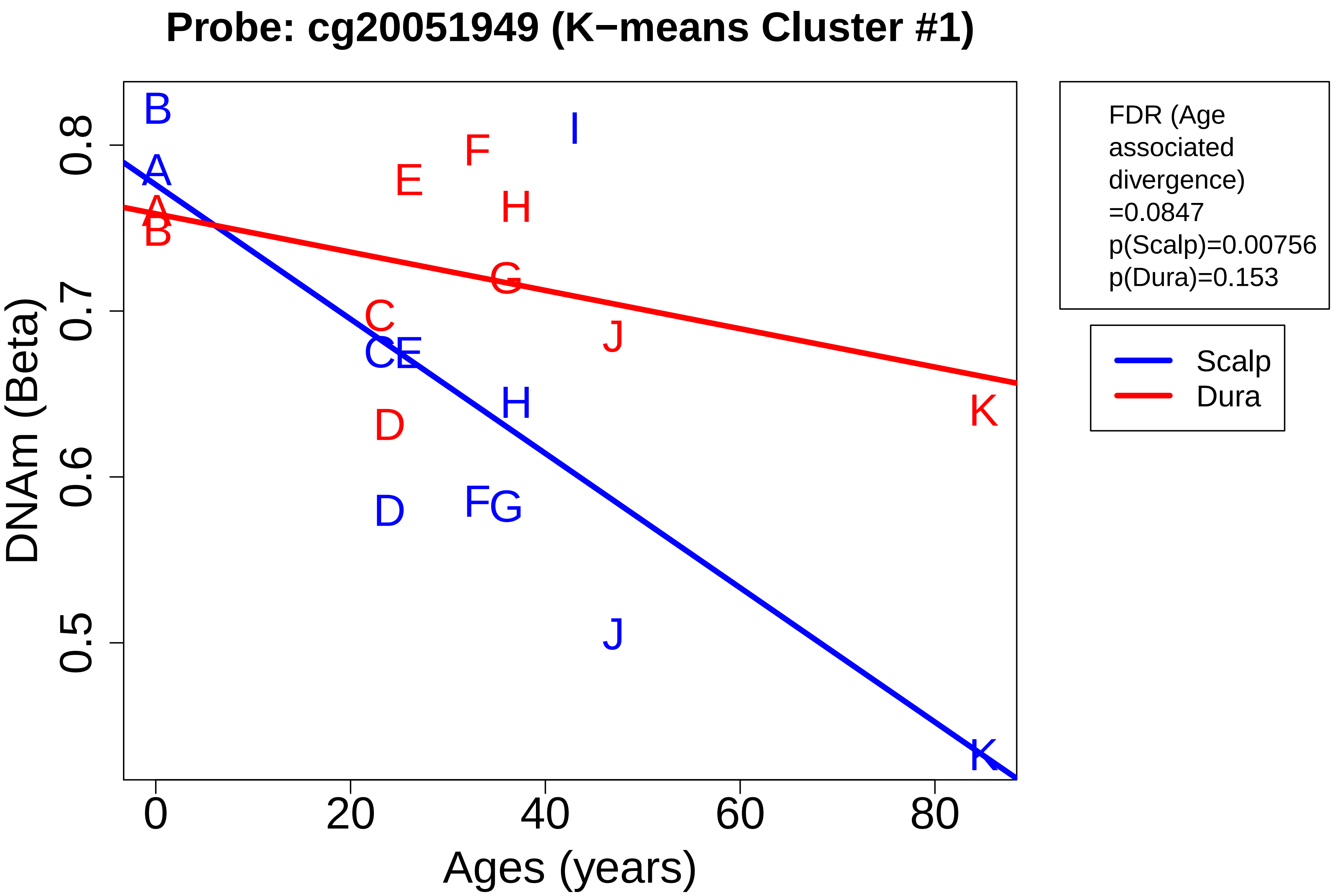

Probe: cg20051949 (K−means Cluster #1)
DNAm (Beta)
Ages (years)
0.5
0.6
0.7
0.8
0
20
40
60
80
FDR (Age associated divergence) =0.0847
p(Scalp)=0.00756
p(Dura)=0.153
Scalp
Dura

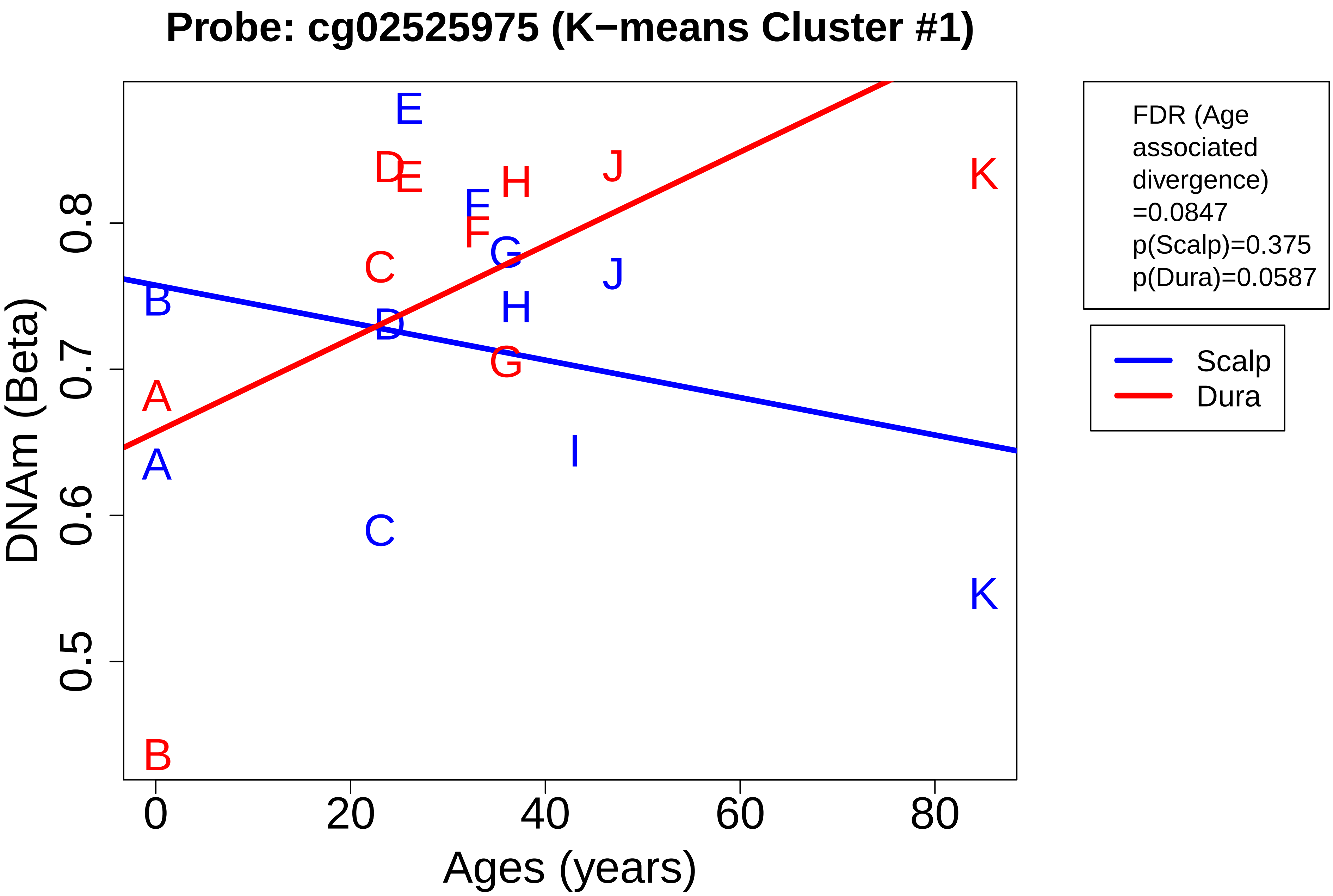

Probe: cg02525975 (K−means Cluster #1)
FDR (Age associated divergence) =0.0847
p(Scalp)=0.375
p(Dura)=0.0587
Scalp
Dura
DNAm (Beta)
0.5
0.6
0.7
0.8
0
20
40
60
80
Ages (years)

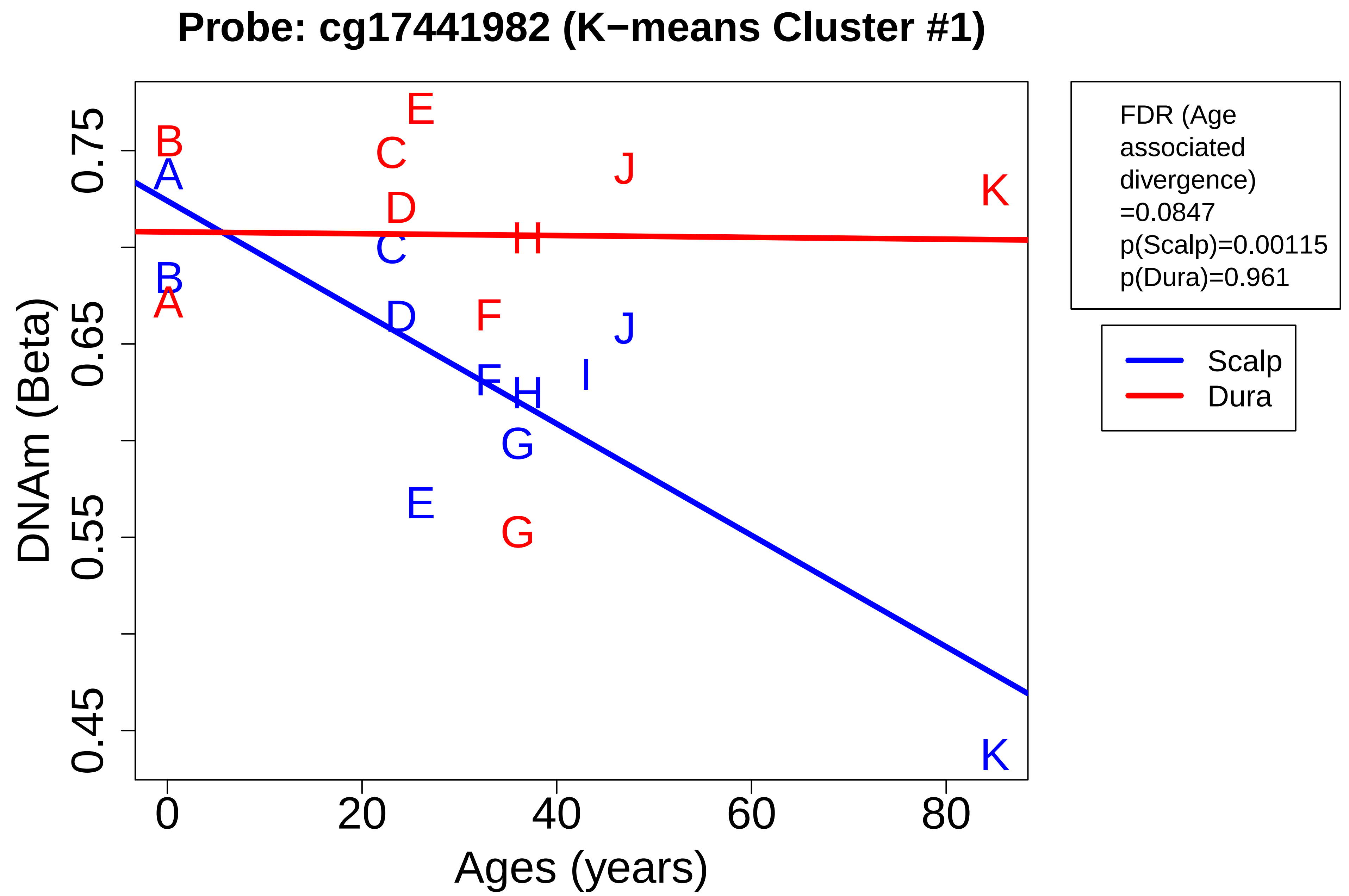

Probe: cg17441982 (K−means Cluster #1)
DNAm (Beta)
0.45
0.55
0.65
0.75
Ages (years)
0
20
40
60
80
A
B
C
D
E
F
G
H
I
J
K
FDR (Age associated divergence) =0.0847
p(Scalp)=0.00115
p(Dura)=0.961
Scalp
Dura

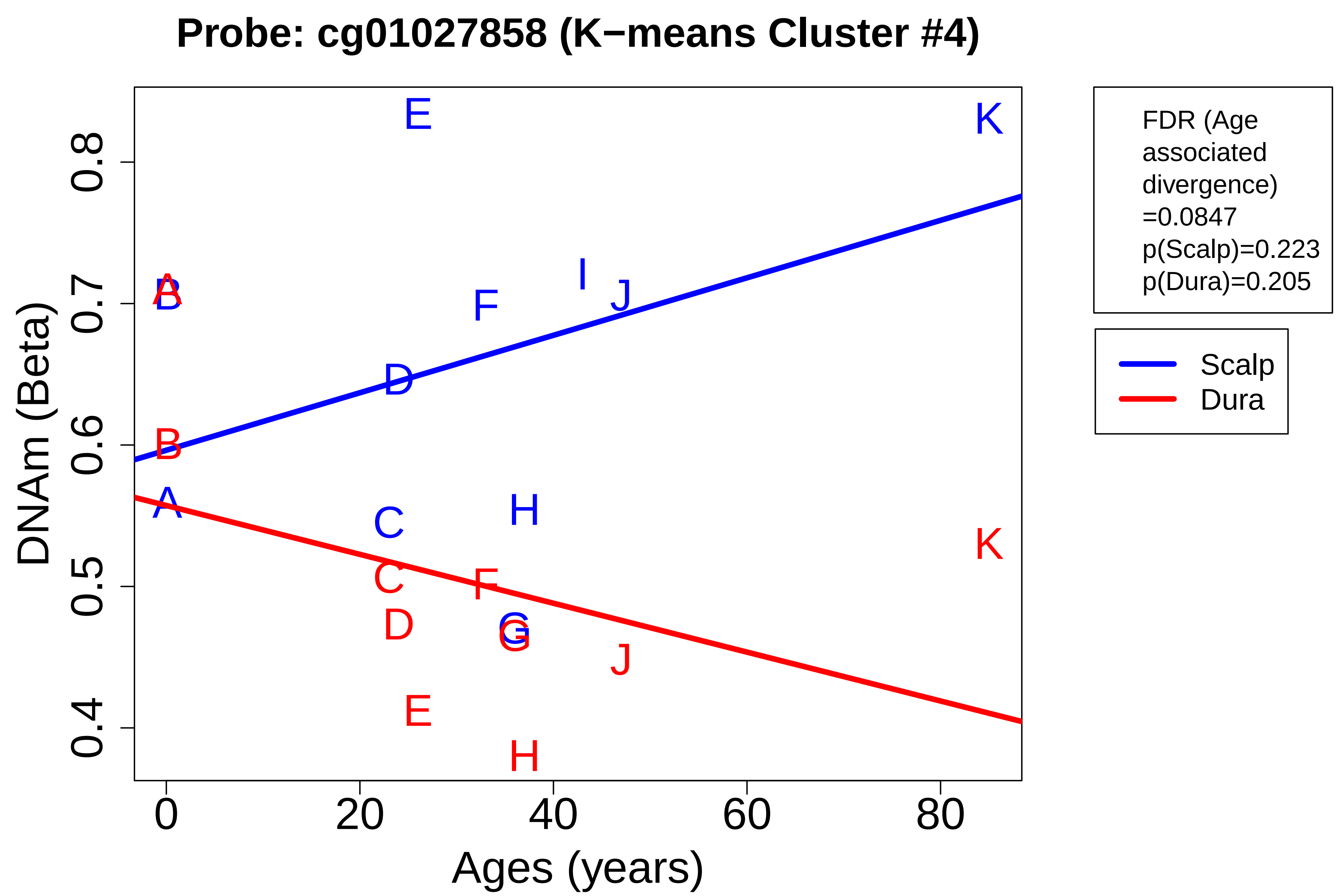
Probe: cg01027858 (K−means Cluster #4)
DNAm (Beta)
Ages (years)
0.4
0.5
0.6
0.7
0.8
0
20
40
60
80
FDR (Age associated divergence) =0.0847
p(Scalp)=0.223
p(Dura)=0.205
Scalp
Dura
A
B
C
D
E
F
G
H
I
J
K

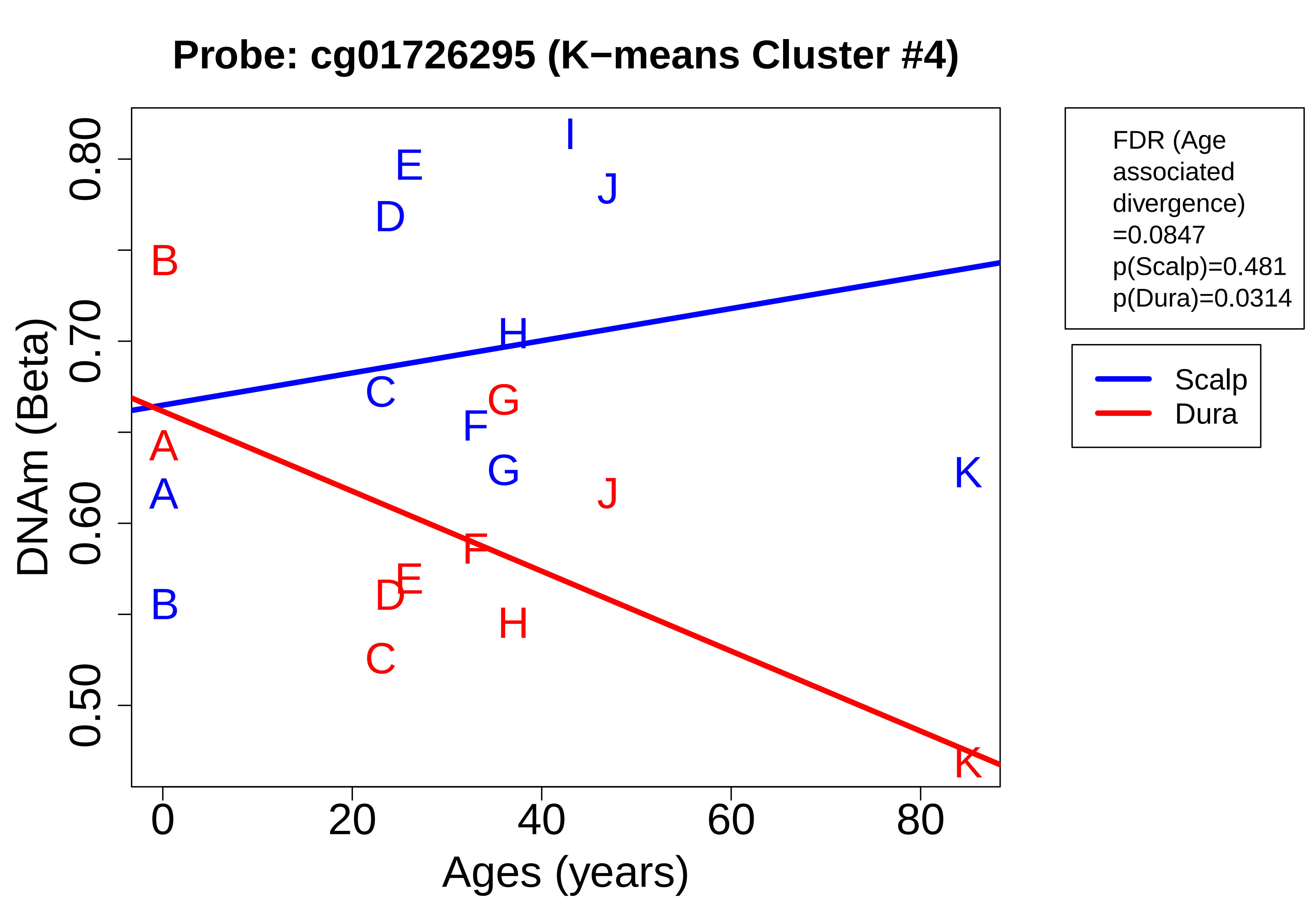
Probe: cg01726295 (K−means Cluster #4)
FDR (Age associated divergence) =0.0847
p(Scalp)=0.481
p(Dura)=0.0314
Scalp
Dura
DNAm (Beta)
0.50
0.60
0.70
0.80
0
20
40
60
80
Ages (years)
A
B
C
D
E
F
G
H
I
J
K

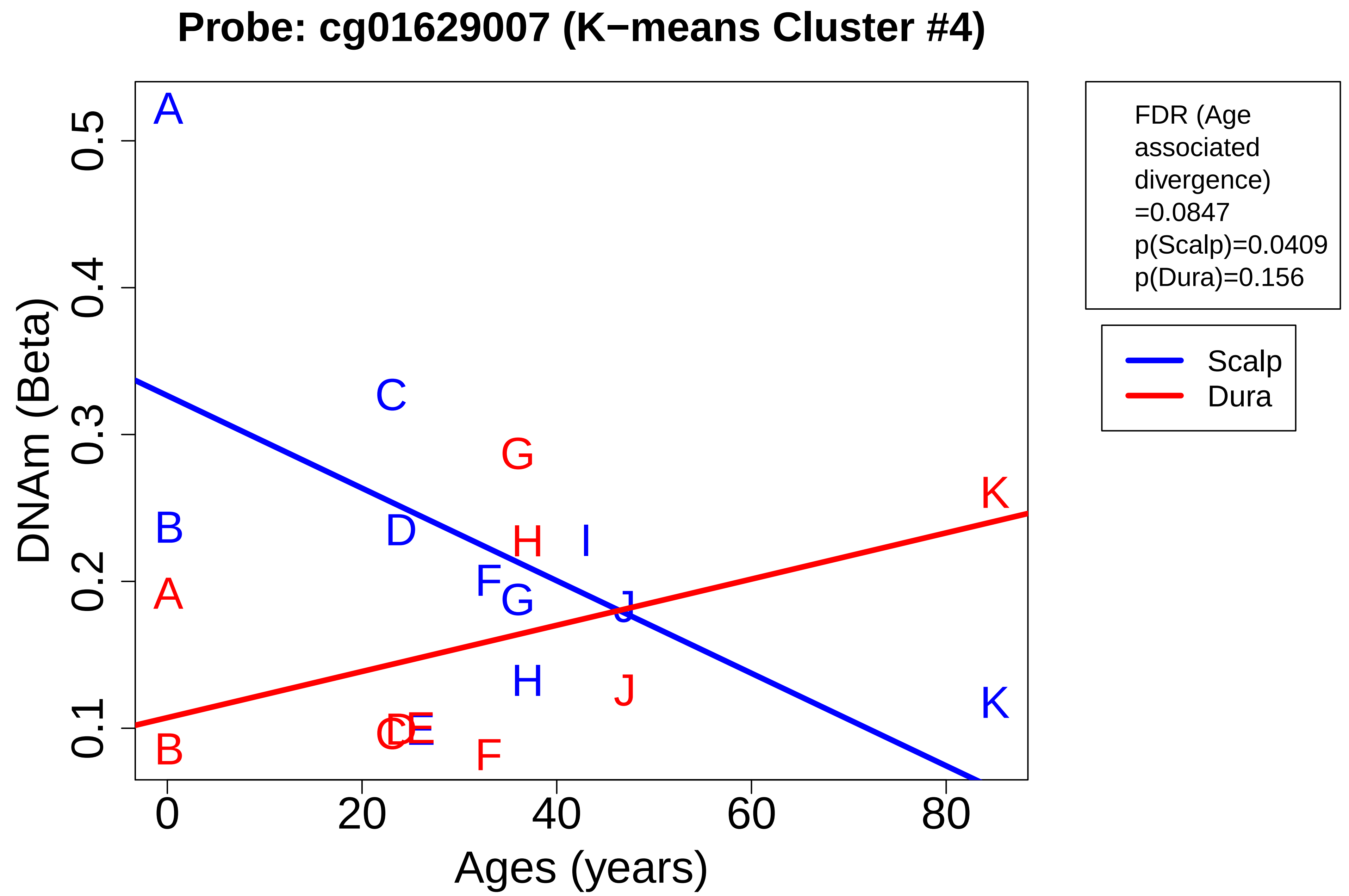

Probe: cg01629007 (K−means Cluster #4)
DNAm (Beta)
0.1
0.2
0.3
0.4
0.5
Ages (years)
0
20
40
60
80
A
B
C
D
E
F
G
H
I
J
K
FDR (Age associated divergence) =0.0847
p(Scalp)=0.0409
p(Dura)=0.156
Scalp
Dura

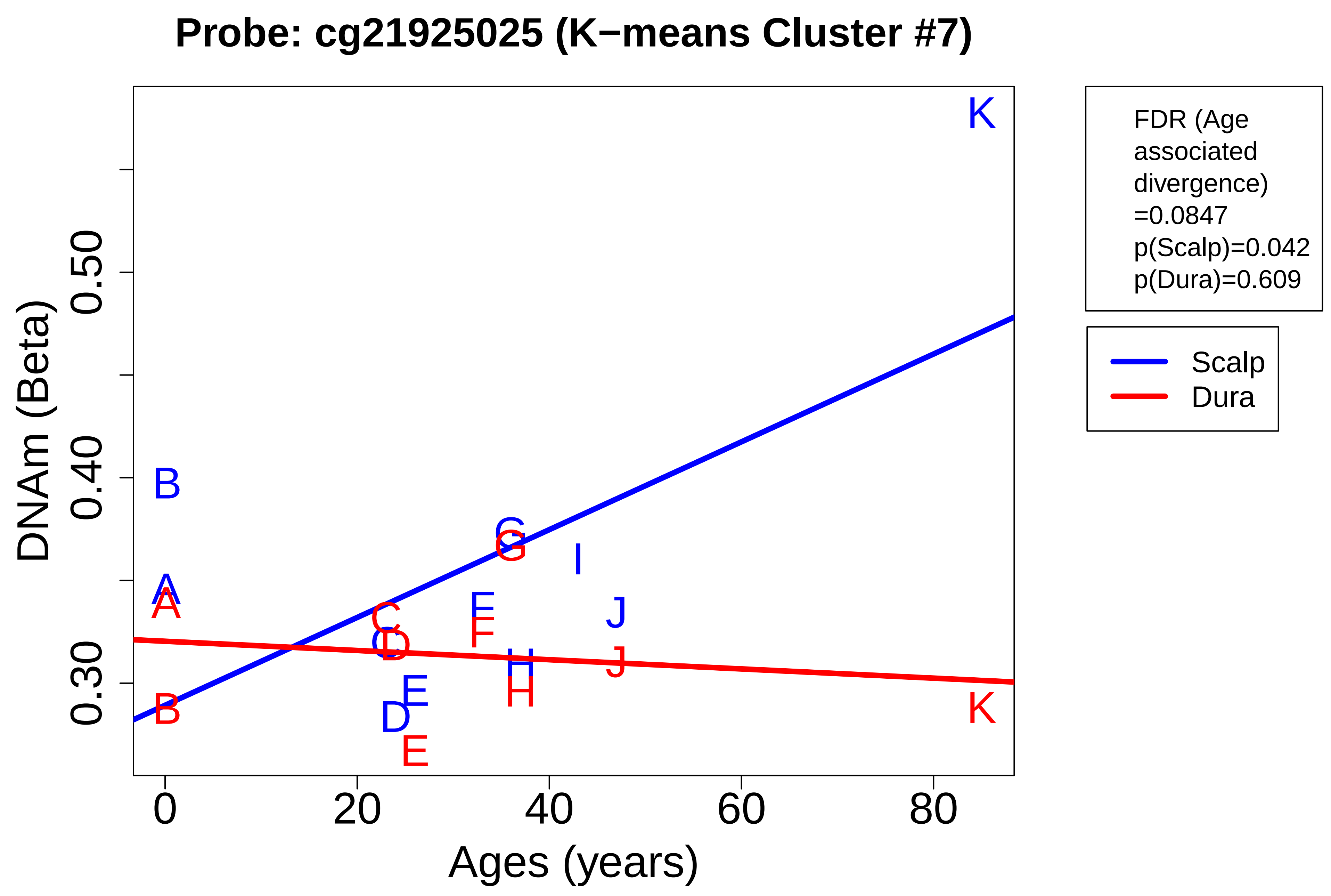

Probe: cg21925025 (K−means Cluster #7)
DNAm (Beta)
0.30
0.40
0.50
0
20
40
60
80
Ages (years)
A
B
C
D
E
F
G
H
I
J
K
FDR (Age associated divergence) =0.0847
p(Scalp)=0.042
p(Dura)=0.609
Scalp
Dura

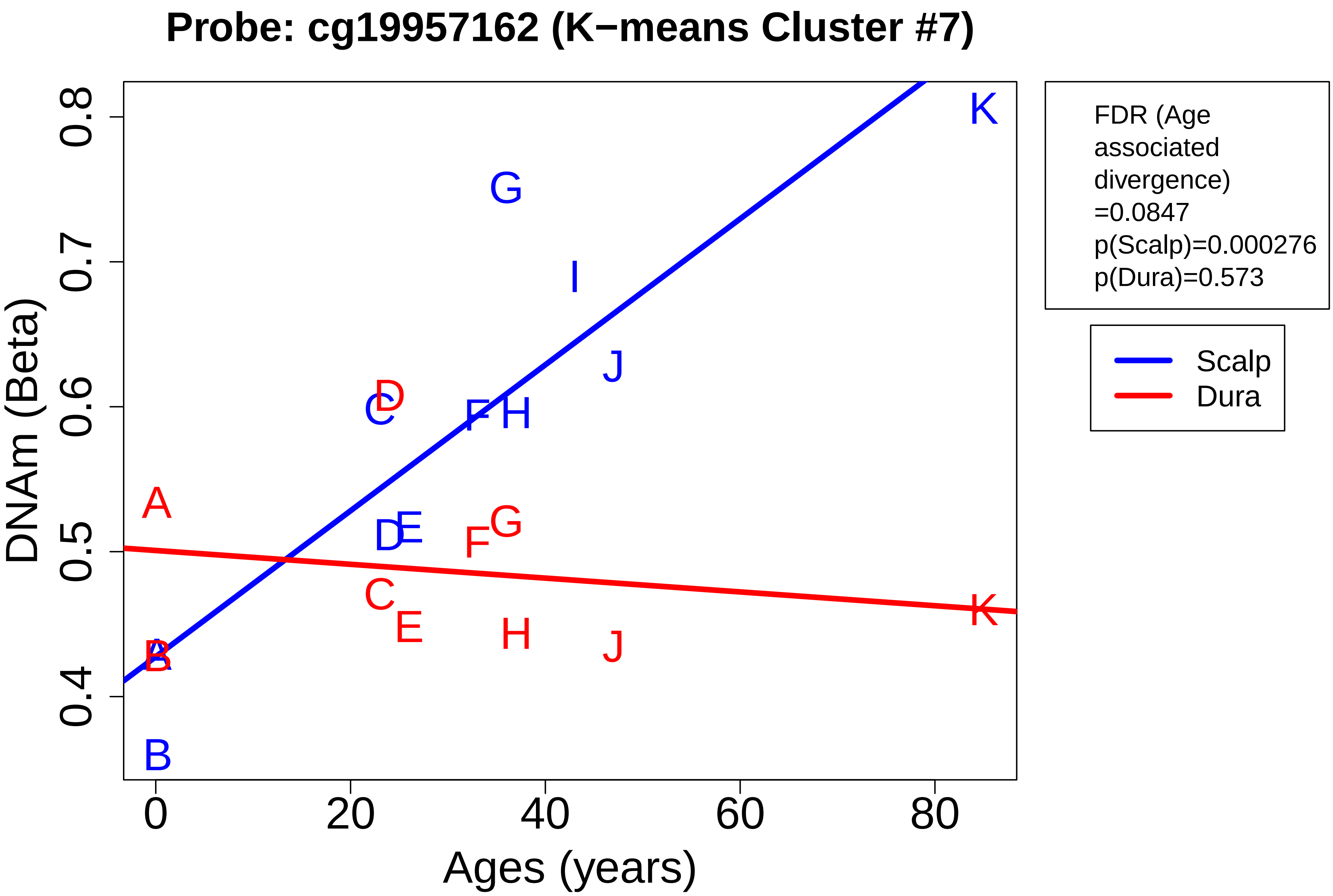

Probe: cg19957162 (K−means Cluster #7)
DNAm (Beta)
0.4
0.5
0.6
0.7
0.8
Ages (years)
0
20
40
60
80
FDR (Age associated divergence) =0.0847
p(Scalp)=0.000276
p(Dura)=0.573
Scalp
Dura

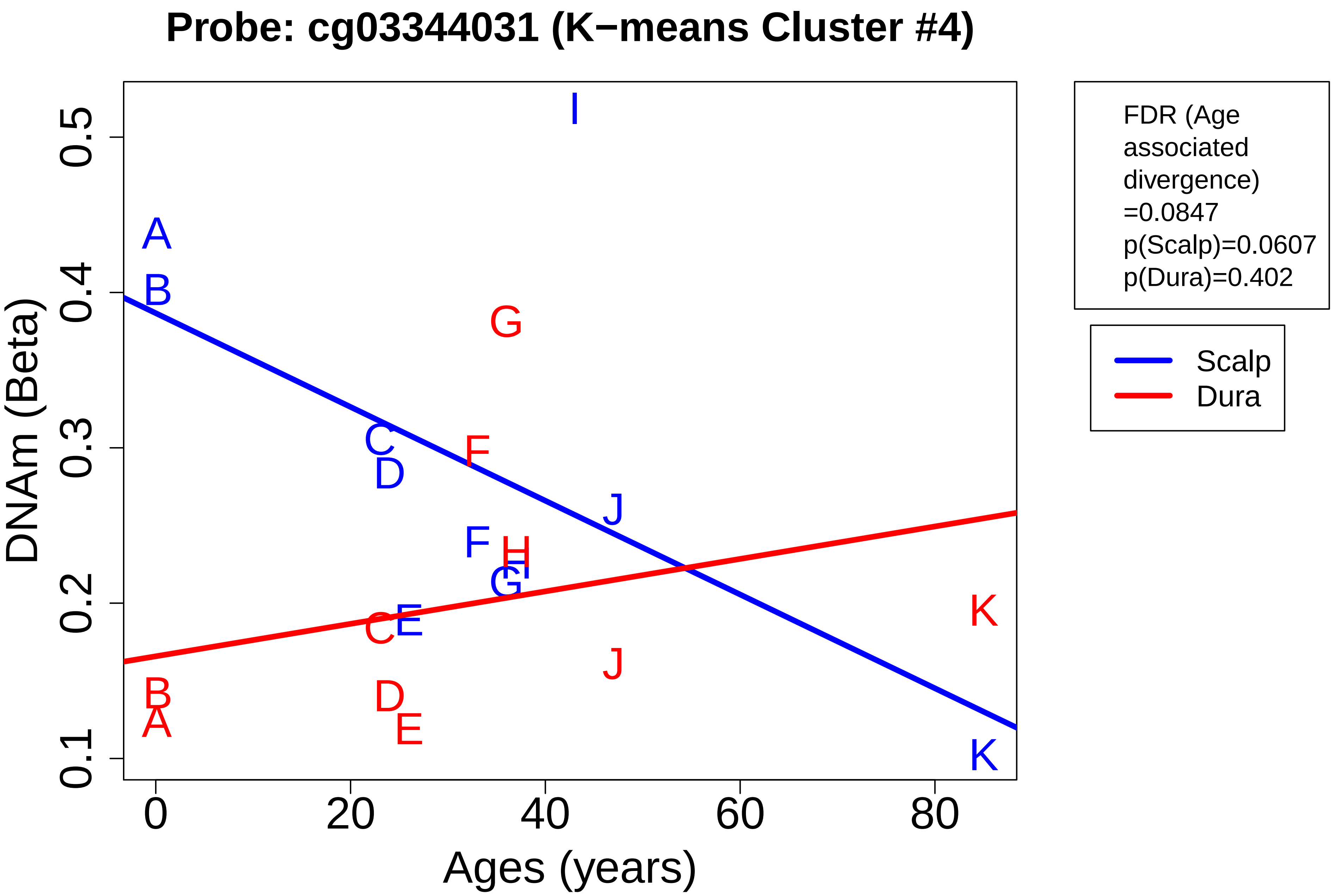

Probe: cg03344031 (K−means Cluster #4)
DNAm (Beta)
0.1
0.2
0.3
0.4
0.5
Ages (years)
0
20
40
60
80
A
B
C
D
E
F
G
H
I
J
K
FDR (Age associated divergence) =0.0847
p(Scalp)=0.0607
p(Dura)=0.402
Scalp
Dura

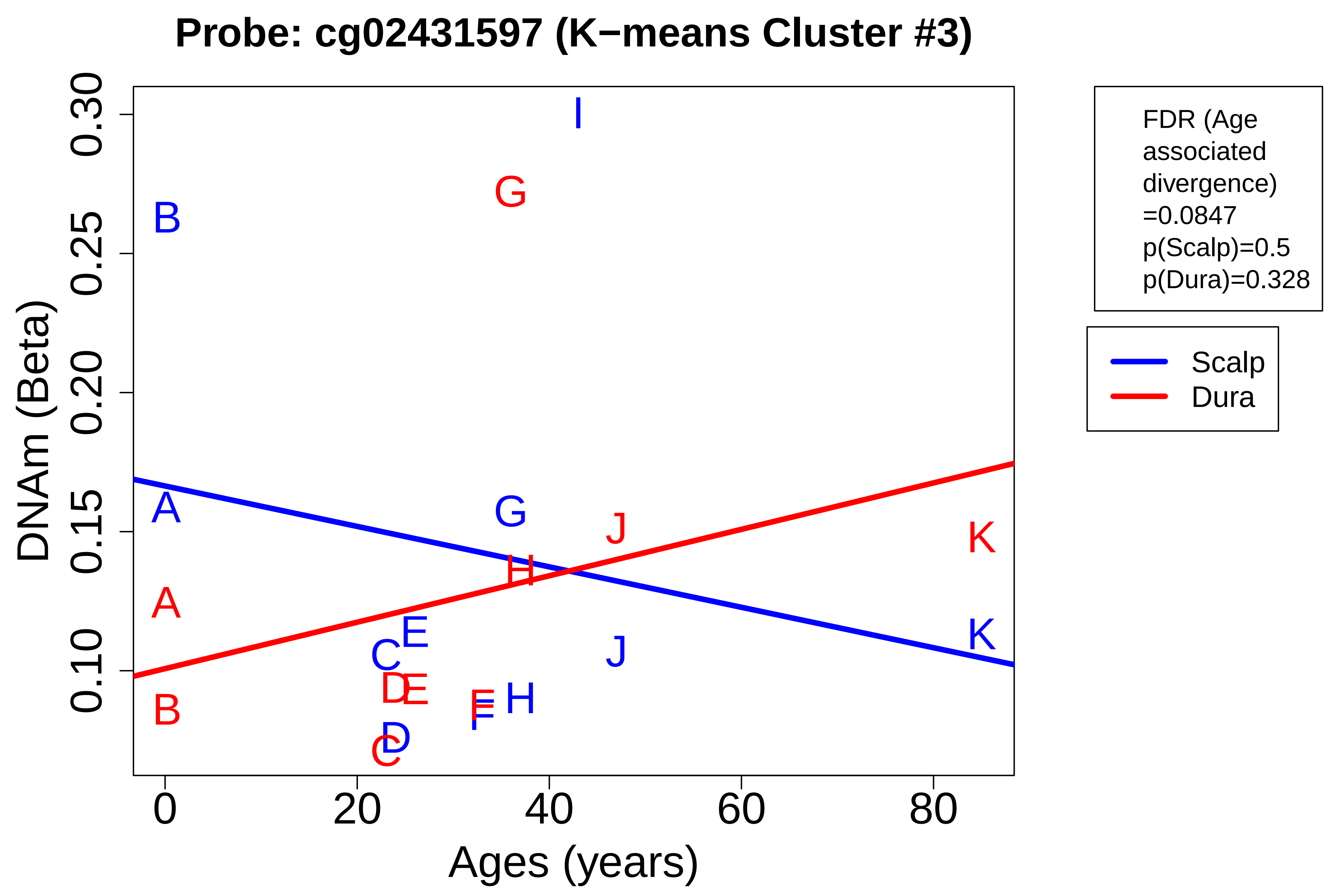

Probe: cg02431597 (K−means Cluster #3)
DNAm (Beta)
0.10
0.15
0.20
0.25
0.30
Ages (years)
0
20
40
60
80
A
B
C
D
E
F
G
H
I
J
K
FDR (Age associated divergence) =0.0847
p(Scalp)=0.5
p(Dura)=0.328
Scalp
Dura

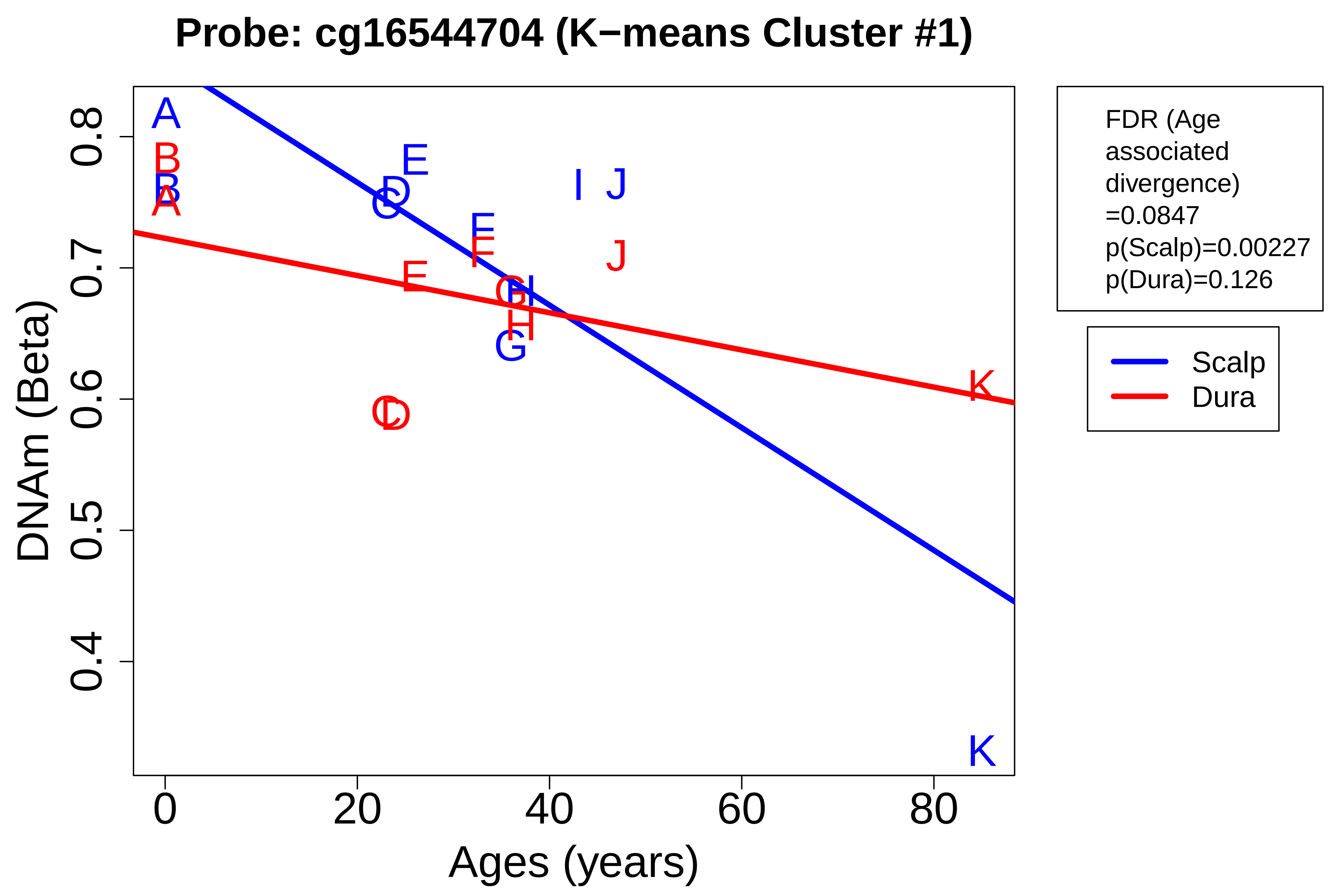

Probe: cg16544704 (K−means Cluster #1)
FDR (Age associated divergence) =0.0847
p(Scalp)=0.00227
p(Dura)=0.126
Scalp
Dura
DNAm (Beta)
0.4
0.5
0.6
0.7
0.8
0
20
40
60
80
Ages (years)

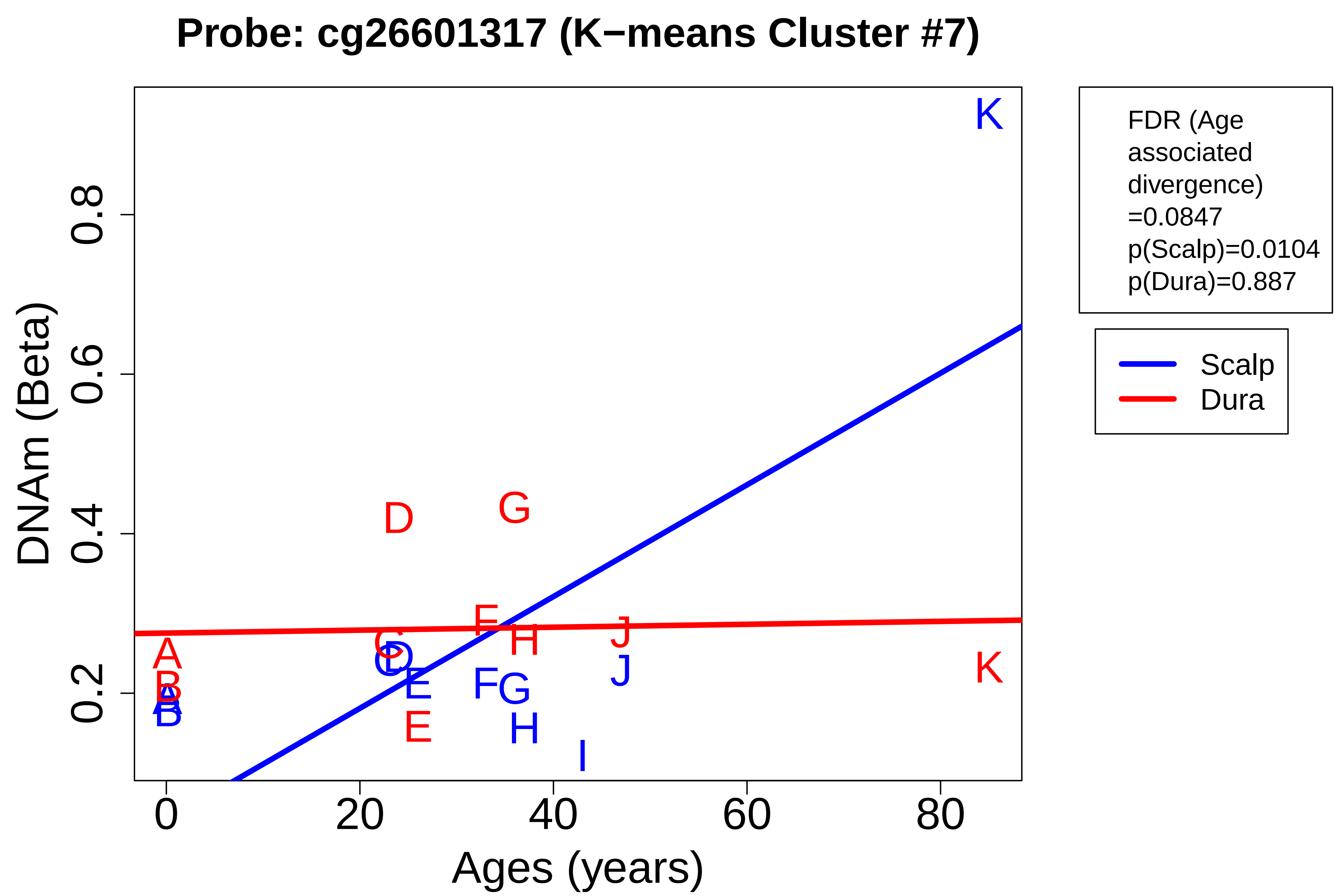

Probe: cg26601317 (K−means Cluster #7)
DNAm (Beta)
0.2
0.4
0.6
0.8
0
20
40
60
80
Ages (years)
FDR (Age associated divergence) =0.0847
p(Scalp)=0.0104
p(Dura)=0.887
Scalp
Dura
A
B
C
D
E
F
G
H
I
J
K

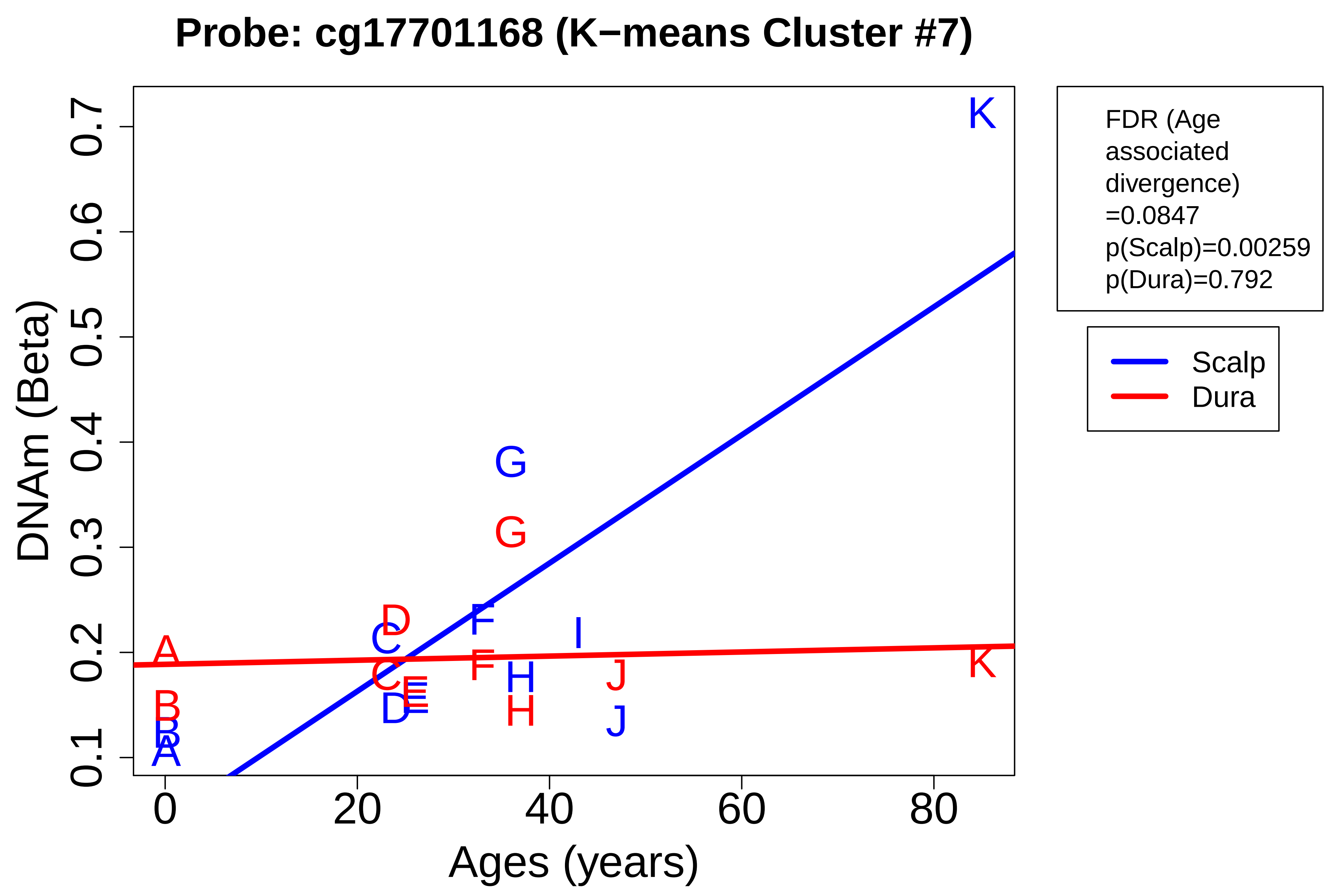

Probe: cg17701168 (K−means Cluster #7)
FDR (Age associated divergence) =0.0847
p(Scalp)=0.00259
p(Dura)=0.792
Scalp
Dura
DNAm (Beta)
0.1
0.2
0.3
0.4
0.5
0.6
0.7
0
20
40
60
80
Ages (years)

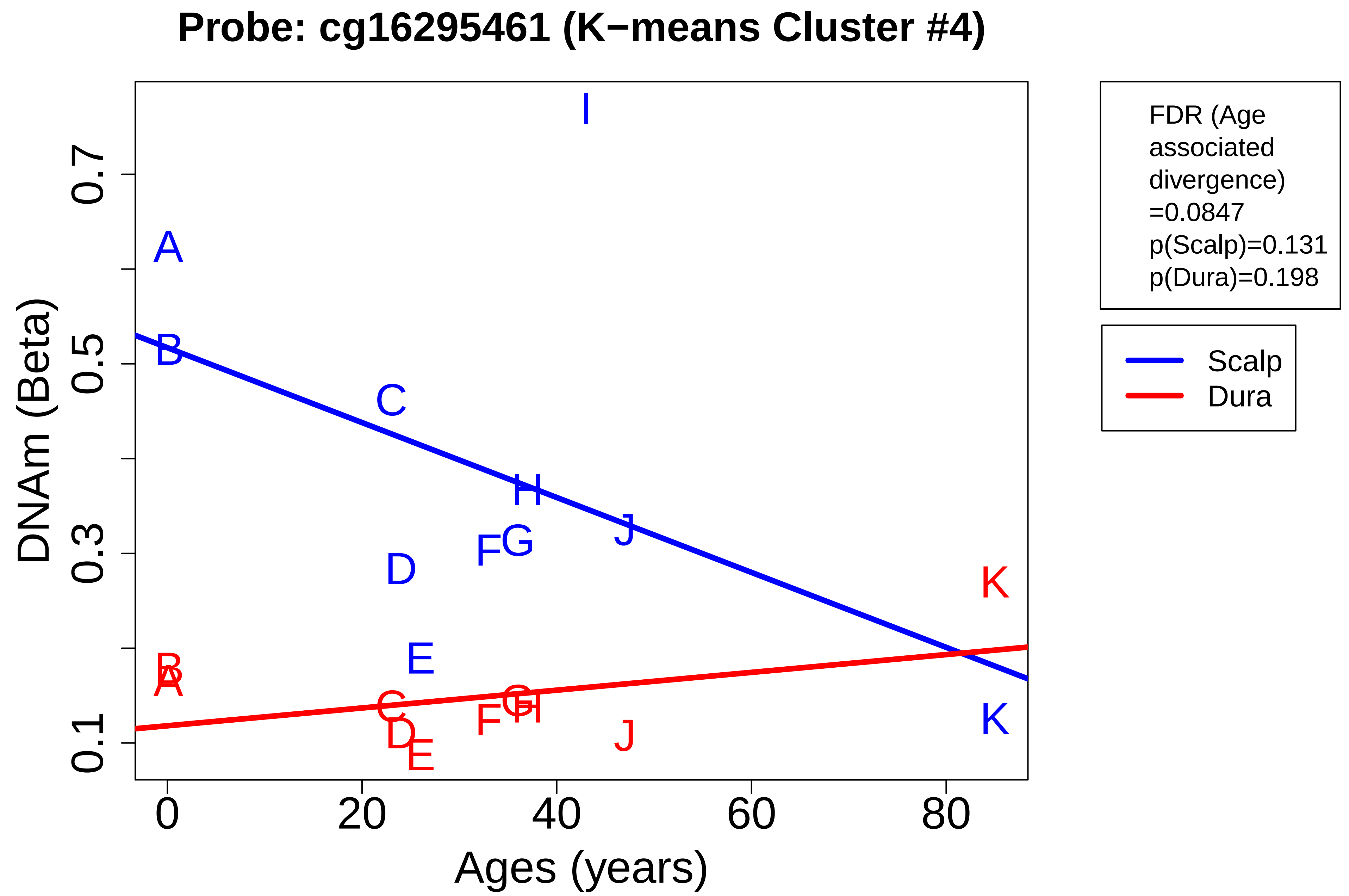
Probe: cg16295461 (K−means Cluster #4)
DNAm (Beta)
0.1
0.3
0.5
0.7
Ages (years)
0
20
40
60
80
A
B
C
D
E
F
G
H
I
J
K
FDR (Age associated divergence) =0.0847
p(Scalp)=0.131
p(Dura)=0.198
Scalp
Dura

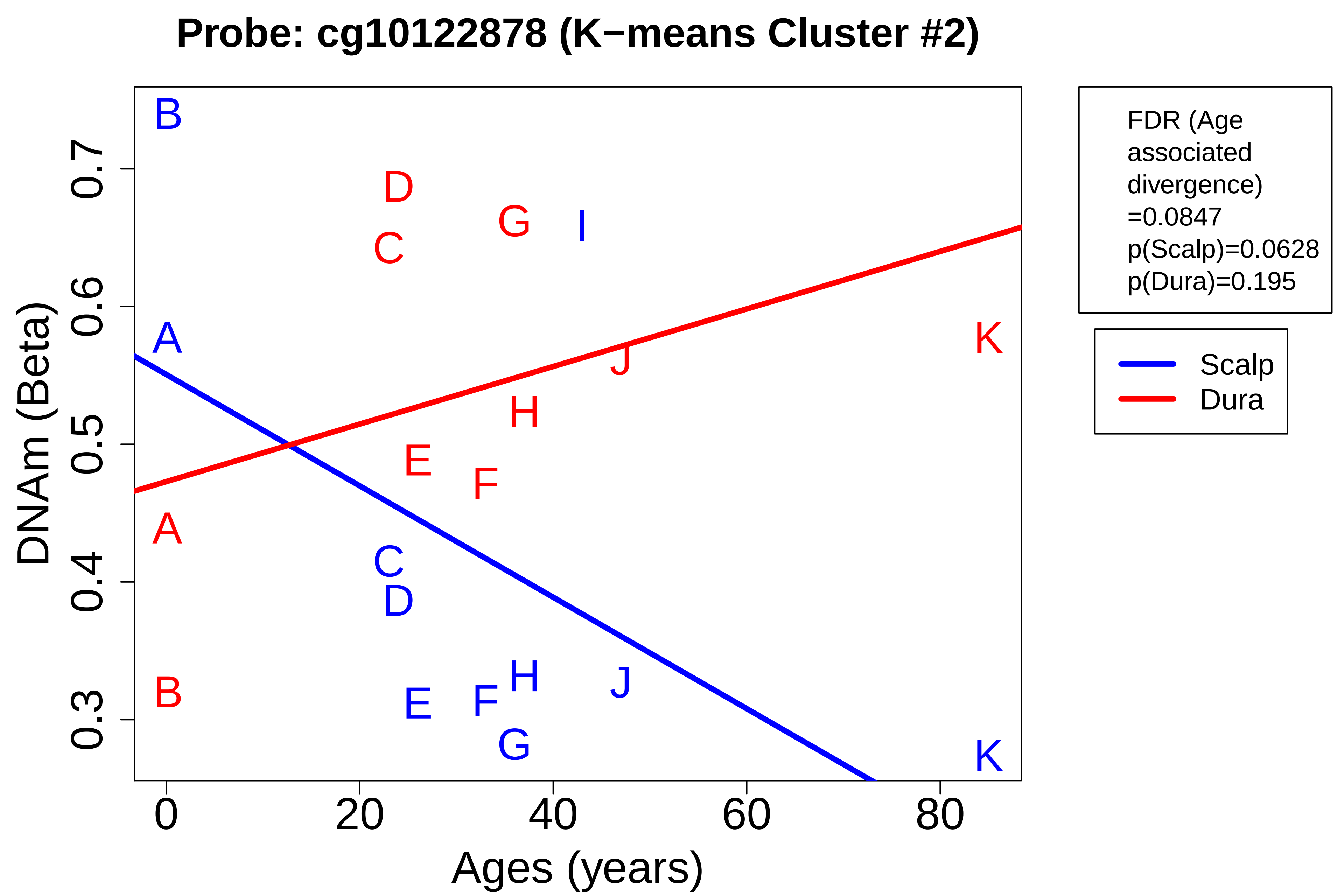
Probe: cg10122878 (K−means Cluster #2)
DNAm (Beta)
0.3
0.4
0.5
0.6
0.7
Ages (years)
0
20
40
60
80
FDR (Age associated divergence) =0.0847
p(Scalp)=0.0628
p(Dura)=0.195
Scalp
Dura
A
B
C
D
E
F
G
H
I
J
K

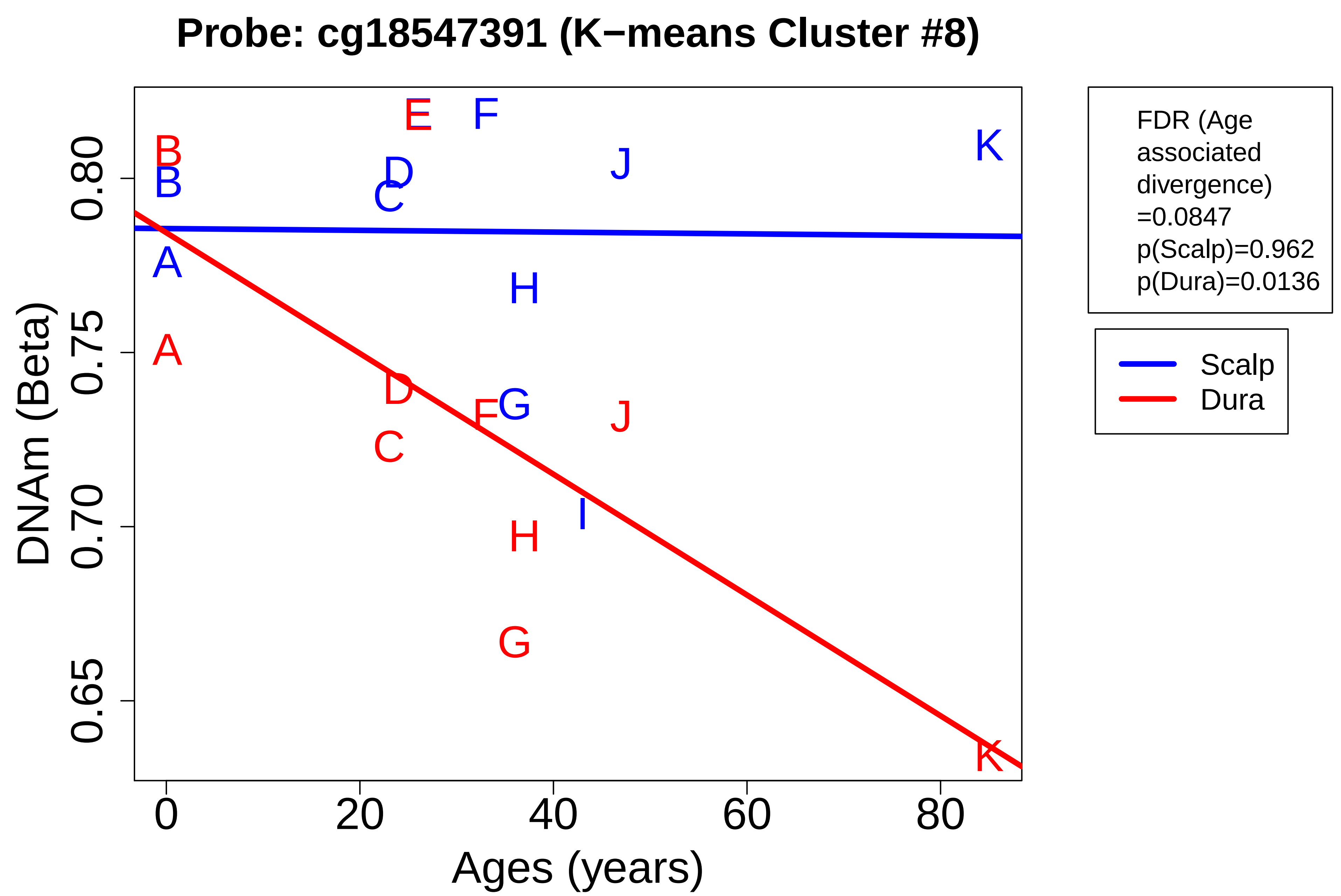
Probe: cg18547391 (K−means Cluster #8)
DNAm (Beta)
0.65
0.70
0.75
0.80
0
20
40
60
80
Ages (years)
A
B
C
D
E
F
G
H
I
J
K
FDR (Age associated divergence) =0.0847
p(Scalp)=0.962
p(Dura)=0.0136
Scalp
Dura

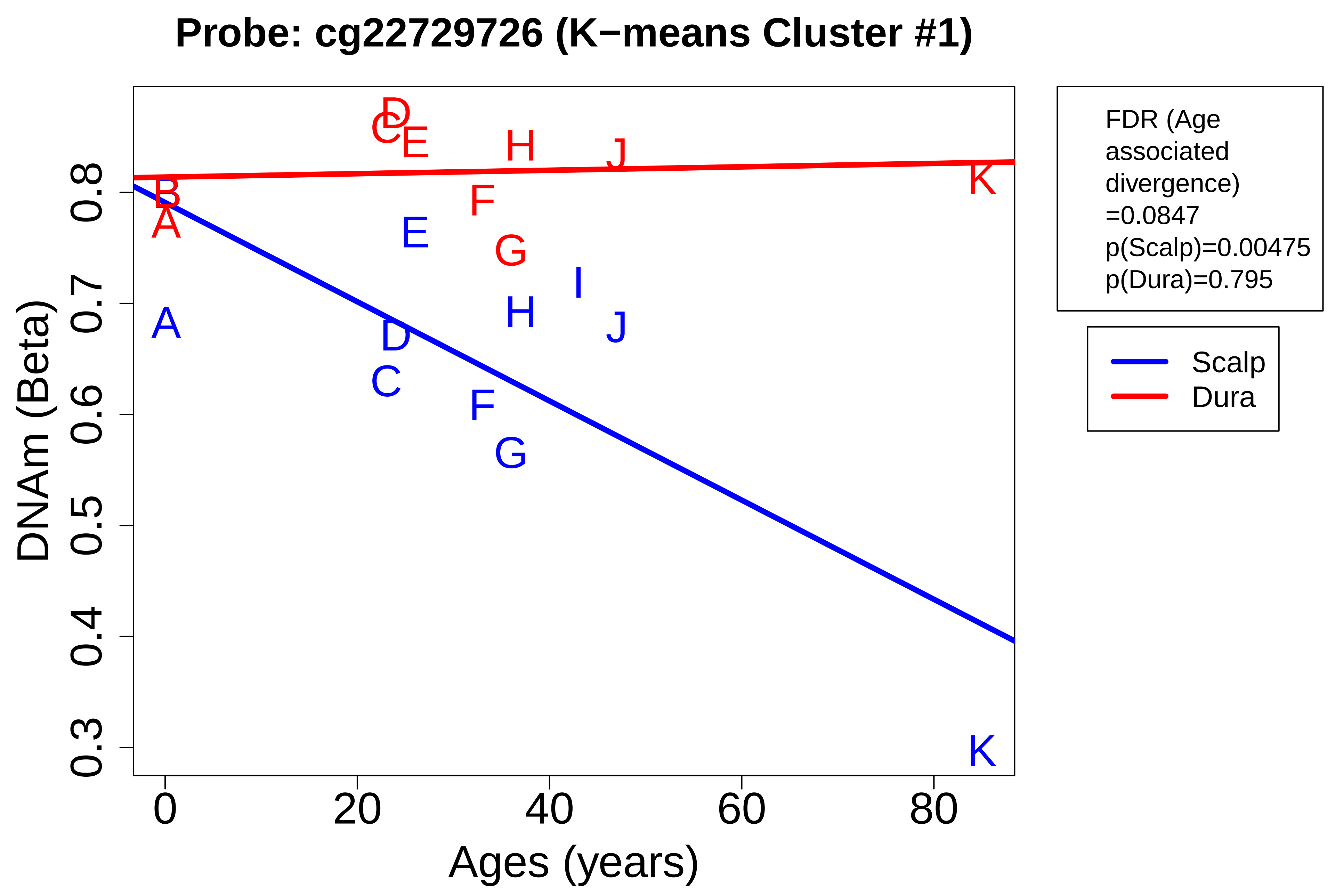

Probe: cg22729726 (K−means Cluster #1)
DNAm (Beta)
0.3
0.4
0.5
0.6
0.7
0.8
0
20
40
60
80
Ages (years)
A
B
C
D
E
F
G
H
I
J
K
FDR (Age associated divergence) =0.0847
p(Scalp)=0.00475
p(Dura)=0.795
Scalp
Dura

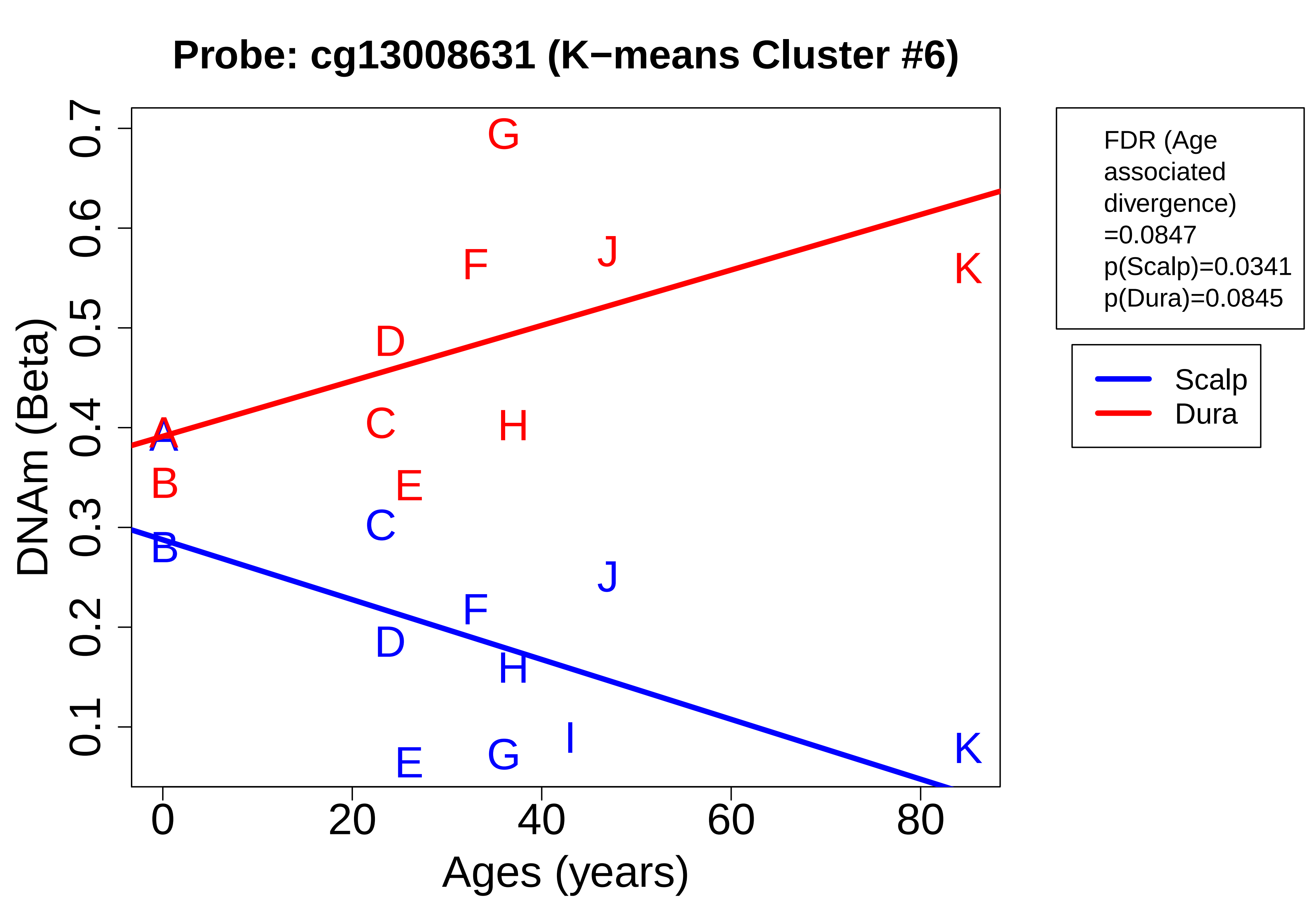

Probe: cg13008631 (K−means Cluster #6)
DNAm (Beta)
0.1
0.2
0.3
0.4
0.5
0.6
0.7
Ages (years)
0
20
40
60
80
FDR (Age associated divergence) =0.0847
p(Scalp)=0.0341
p(Dura)=0.0845
Scalp
Dura

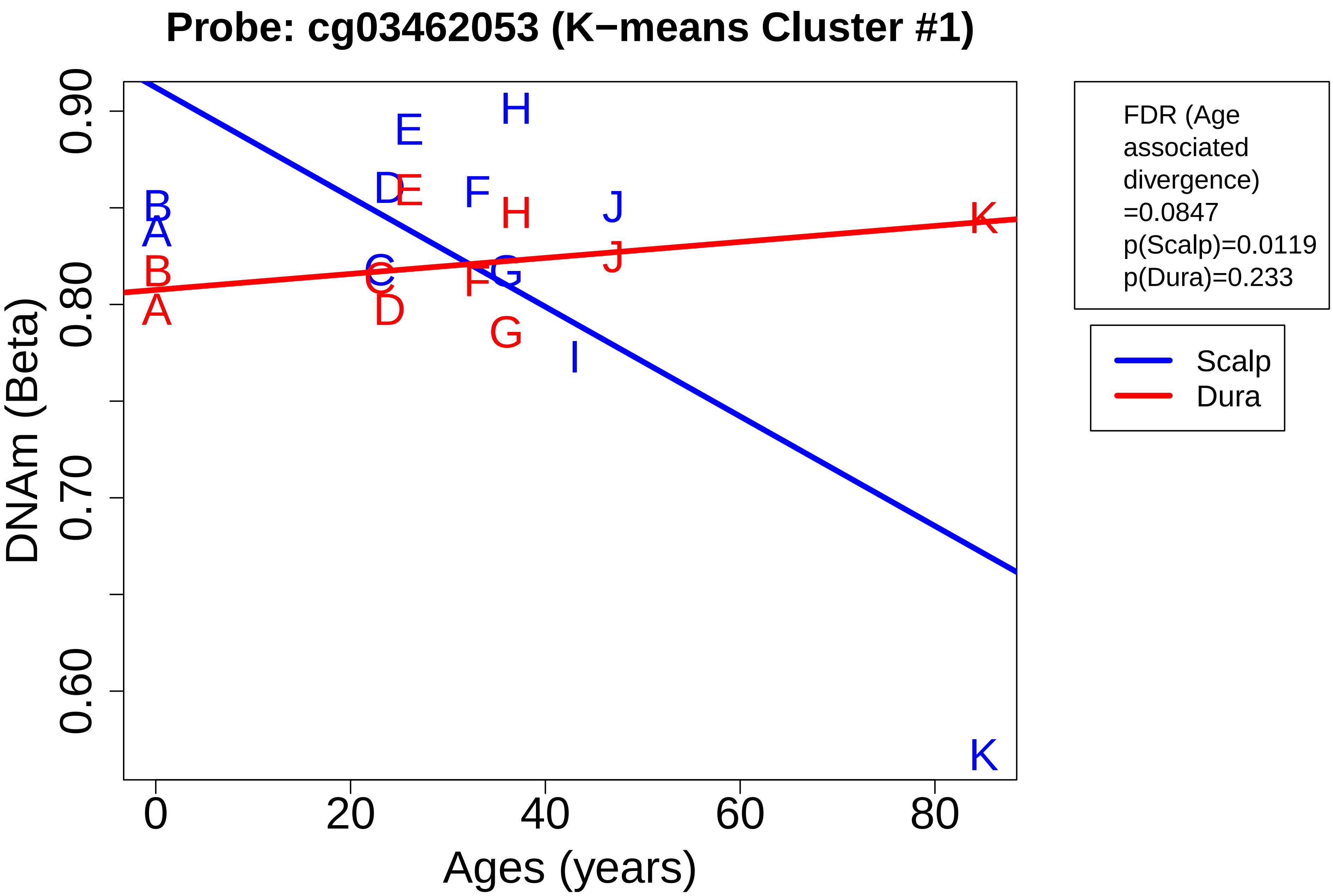

Probe: cg03462053 (K−means Cluster #1)
DNAm (Beta)
0.60
0.70
0.80
0.90
Ages (years)
0
20
40
60
80
FDR (Age associated divergence) =0.0847
p(Scalp)=0.0119
p(Dura)=0.233
Scalp
Dura

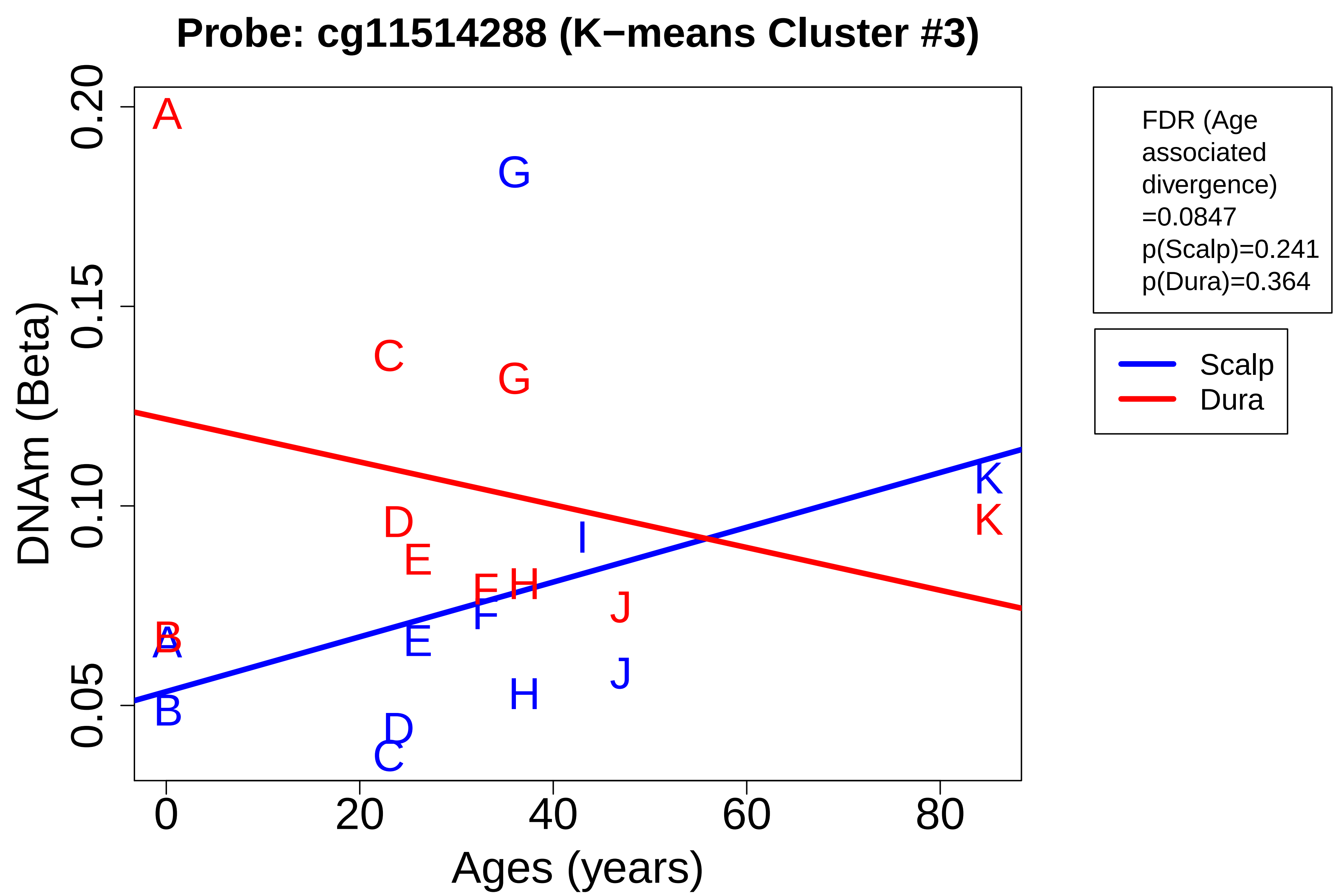

Probe: cg11514288 (K−means Cluster #3)
DNAm (Beta)
0.05
0.10
0.15
0.20
Ages (years)
0
20
40
60
80
A
B
C
D
E
F
G
H
I
J
K
FDR (Age associated divergence) =0.0847
p(Scalp)=0.241
p(Dura)=0.364
Scalp
Dura

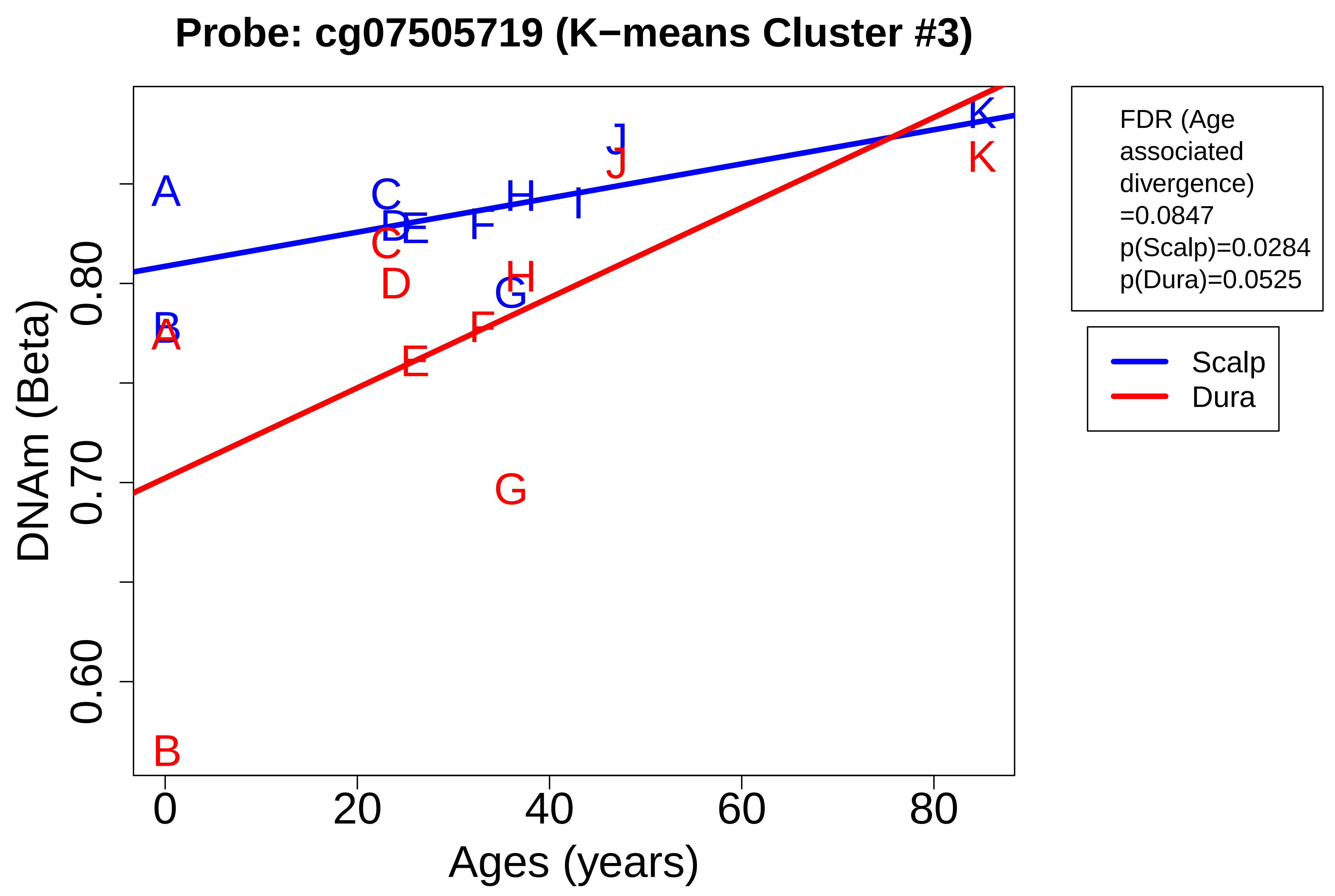

Probe: cg07505719 (K−means Cluster #3)
DNAm (Beta)
0.60
0.70
0.80
0
20
40
60
80
Ages (years)
A
B
C
D
E
F
G
H
I
J
K
FDR (Age associated divergence) =0.0847
p(Scalp)=0.0284
p(Dura)=0.0525
Scalp
Dura

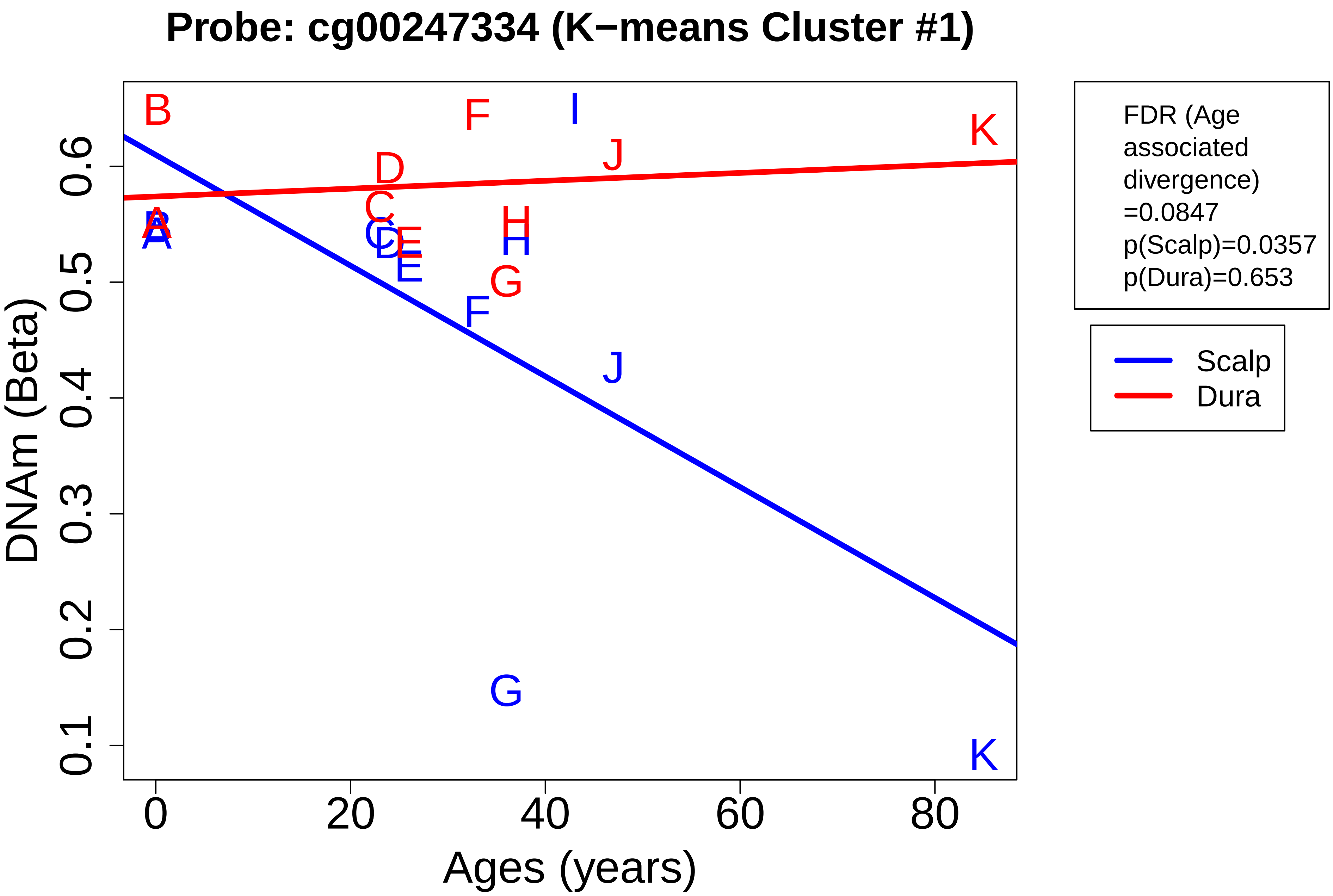

Probe: cg00247334 (K−means Cluster #1)
DNAm (Beta)
0.1
0.2
0.3
0.4
0.5
0.6
0
20
40
60
80
Ages (years)
FDR (Age associated divergence) =0.0847
p(Scalp)=0.0357
p(Dura)=0.653
Scalp
Dura

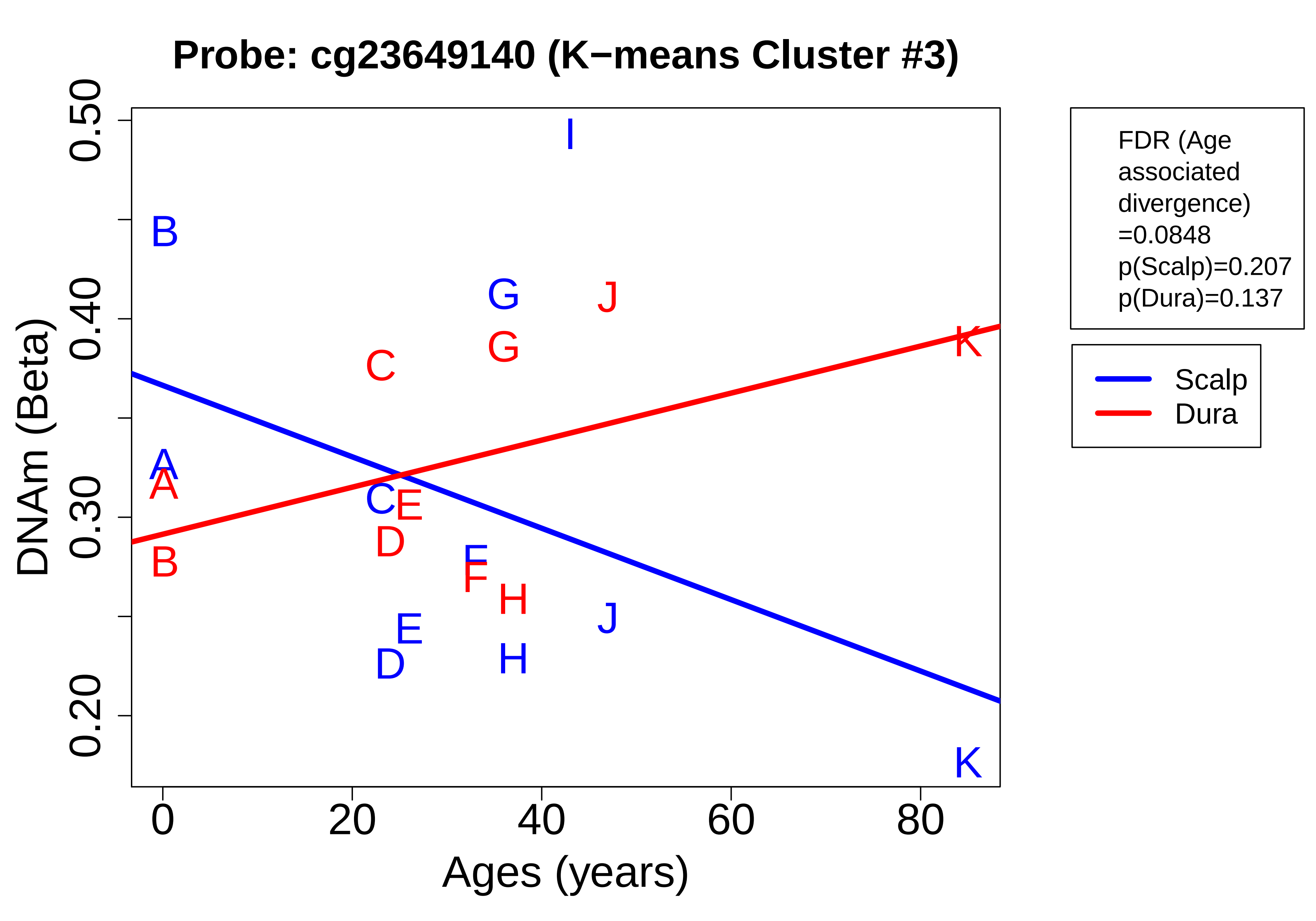

Probe: cg23649140 (K−means Cluster #3)
DNAm (Beta)
0.20
0.30
0.40
0.50
Ages (years)
0
20
40
60
80
FDR (Age associated divergence) =0.0848
p(Scalp)=0.207
p(Dura)=0.137
Scalp
Dura

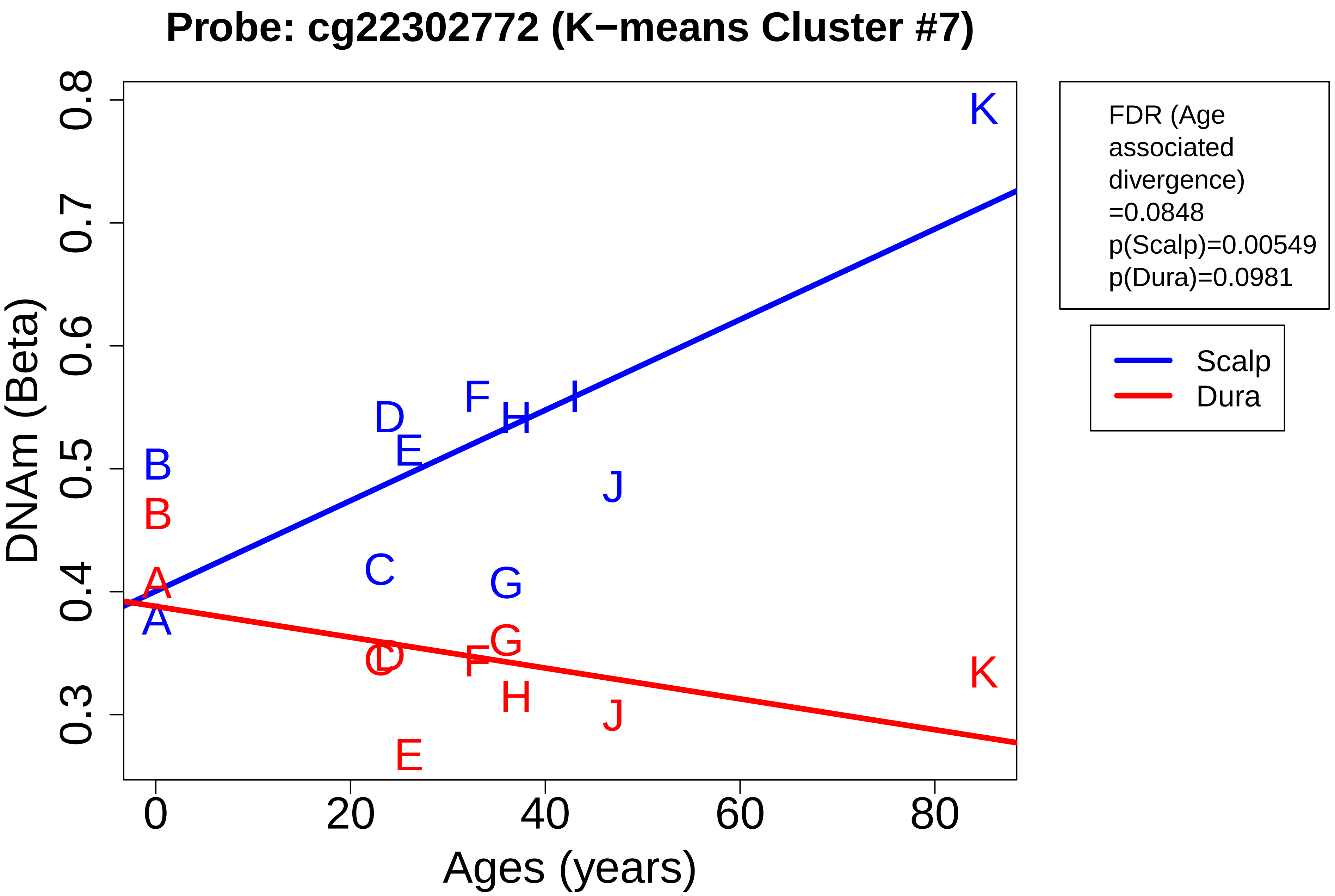
Probe: cg22302772 (K−means Cluster #7)
DNAm (Beta)
0.3
0.4
0.5
0.6
0.7
0.8
0
20
40
60
80
Ages (years)
A
B
C
D
E
F
G
H
I
J
K
FDR (Age associated divergence) =0.0848
p(Scalp)=0.00549
p(Dura)=0.0981
Scalp
Dura

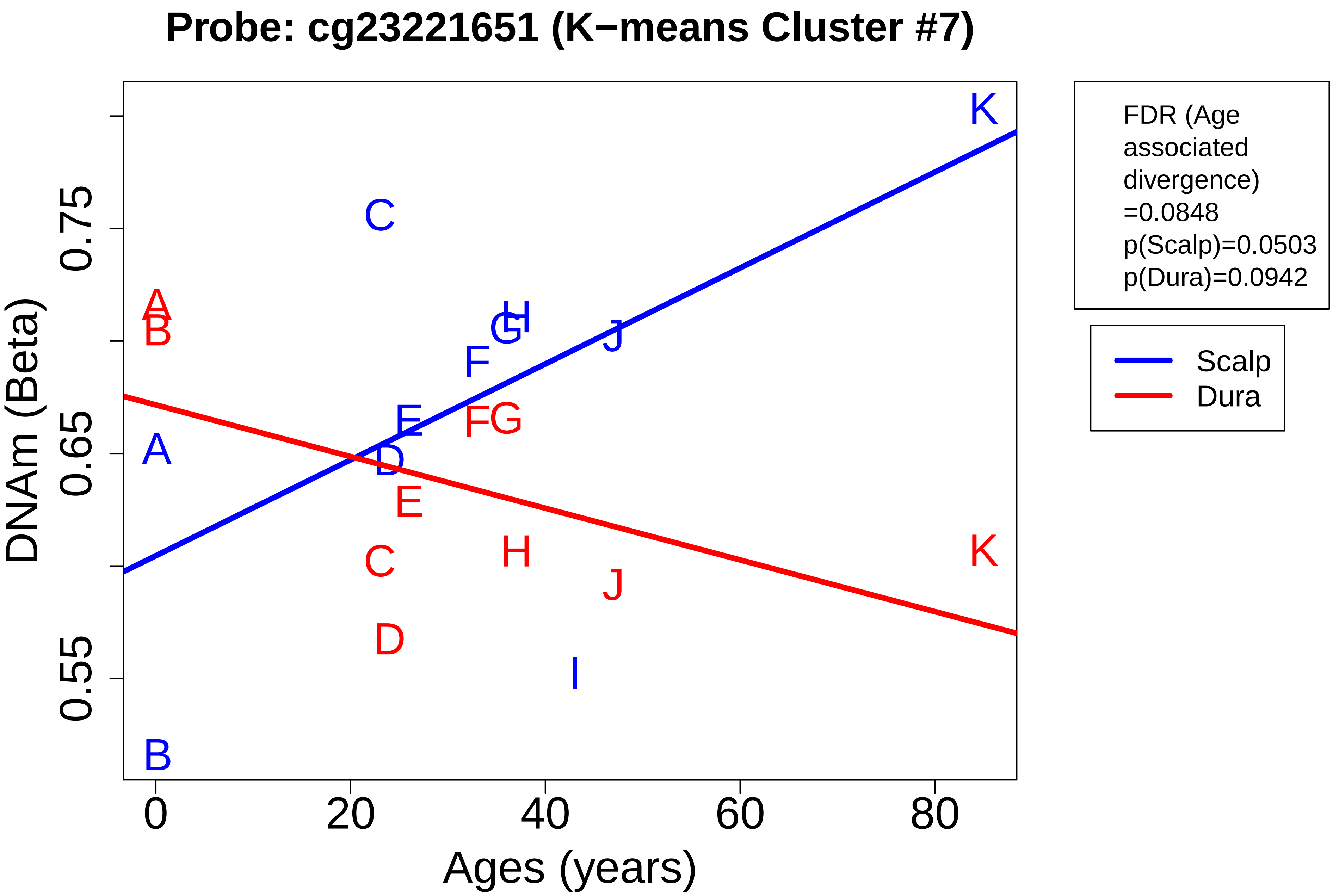
Probe: cg23221651 (K−means Cluster #7)
DNAm (Beta)
0.55
0.65
0.75
0
20
40
60
80
Ages (years)
FDR (Age associated divergence) =0.0848
p(Scalp)=0.0503
p(Dura)=0.0942
Scalp
Dura

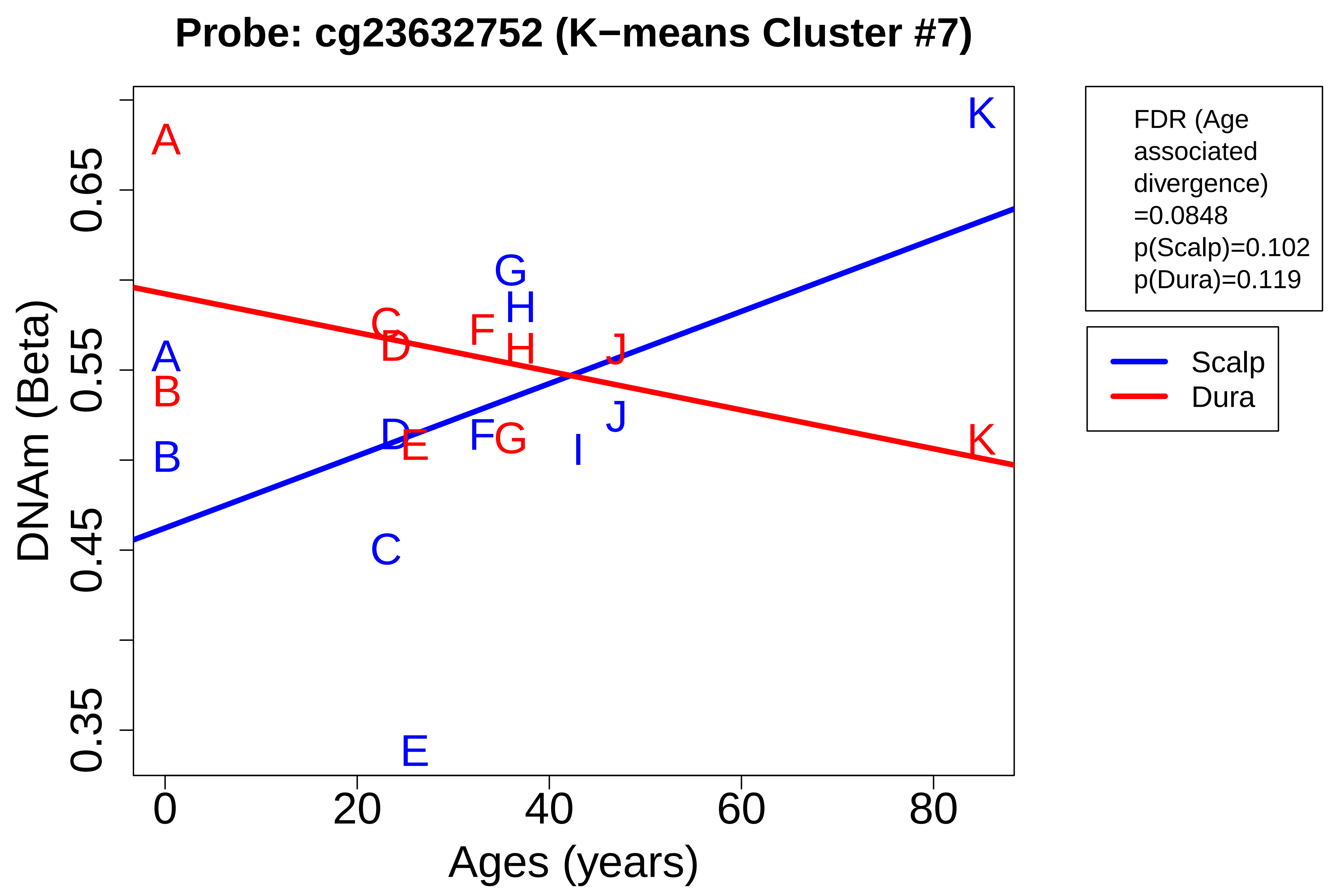

Probe: cg23632752 (K−means Cluster #7)
DNAm (Beta)
0.35
0.45
0.55
0.65
0
20
40
60
80
Ages (years)
A
B
C
D
E
F
G
H
I
J
K
FDR (Age associated divergence) =0.0848
p(Scalp)=0.102
p(Dura)=0.119
Scalp
Dura

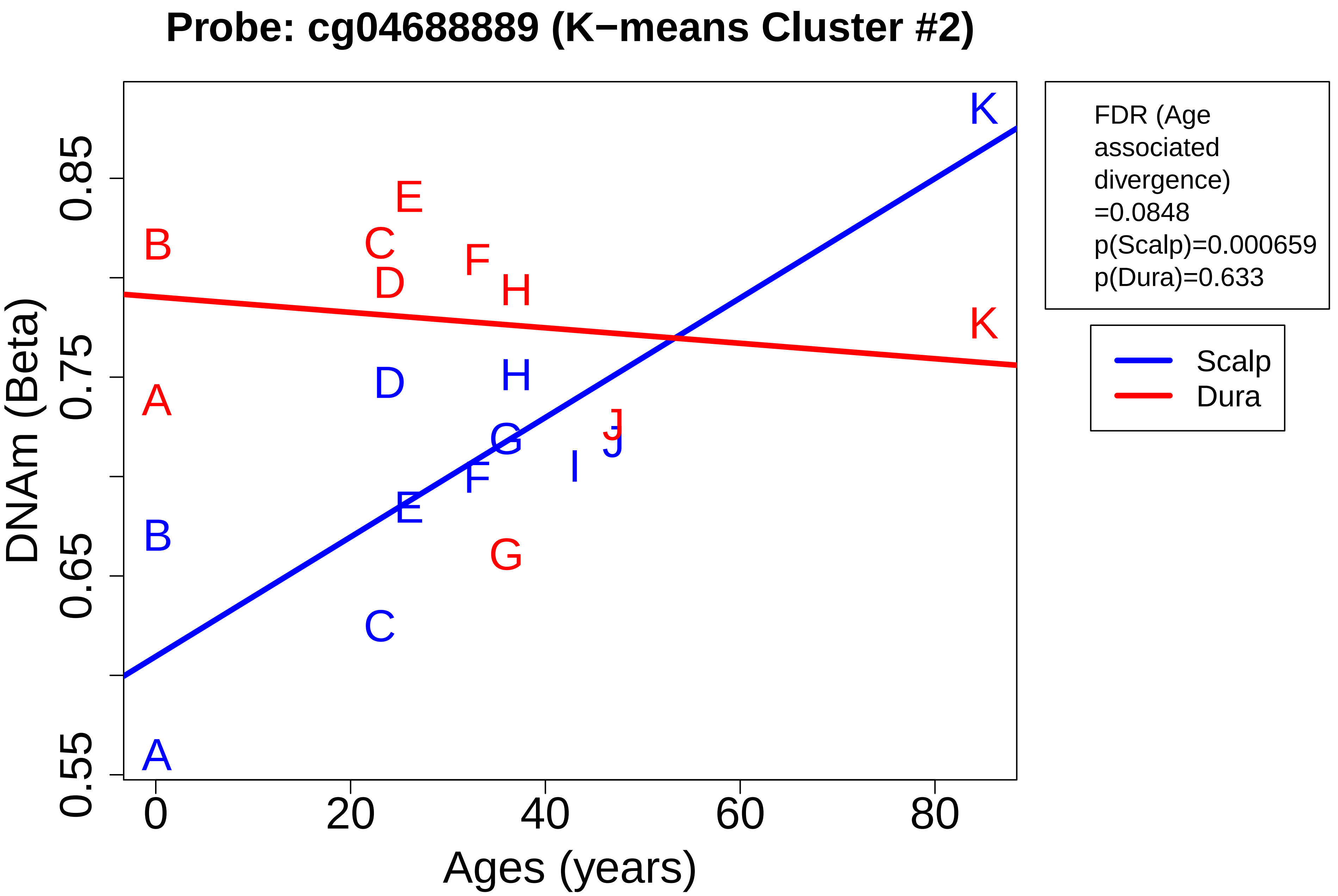
Probe: cg04688889 (K−means Cluster #2)
DNAm (Beta)
0.55
0.65
0.75
0.85
Ages (years)
0
20
40
60
80
FDR (Age associated divergence) =0.0848
p(Scalp)=0.000659
p(Dura)=0.633
Scalp
Dura
A
B
C
D
E
F
G
H
I
J
K

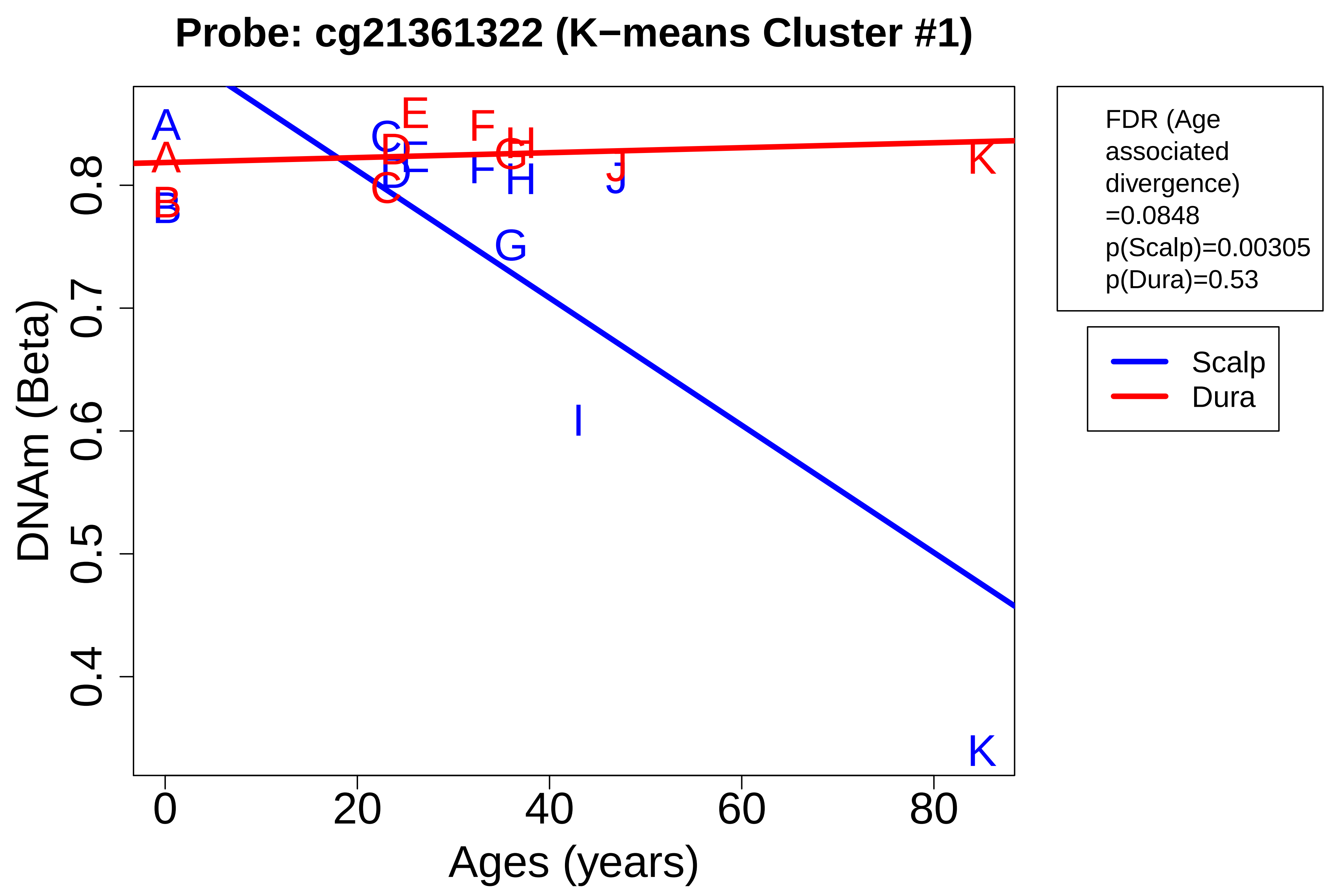

Probe: cg21361322 (K−means Cluster #1)
DNAm (Beta)
0.4
0.5
0.6
0.7
0.8
Ages (years)
0
20
40
60
80
FDR (Age associated divergence) =0.0848
p(Scalp)=0.00305
p(Dura)=0.53
Scalp
Dura
A
B
C
D
E
F
G
H
I
J
K

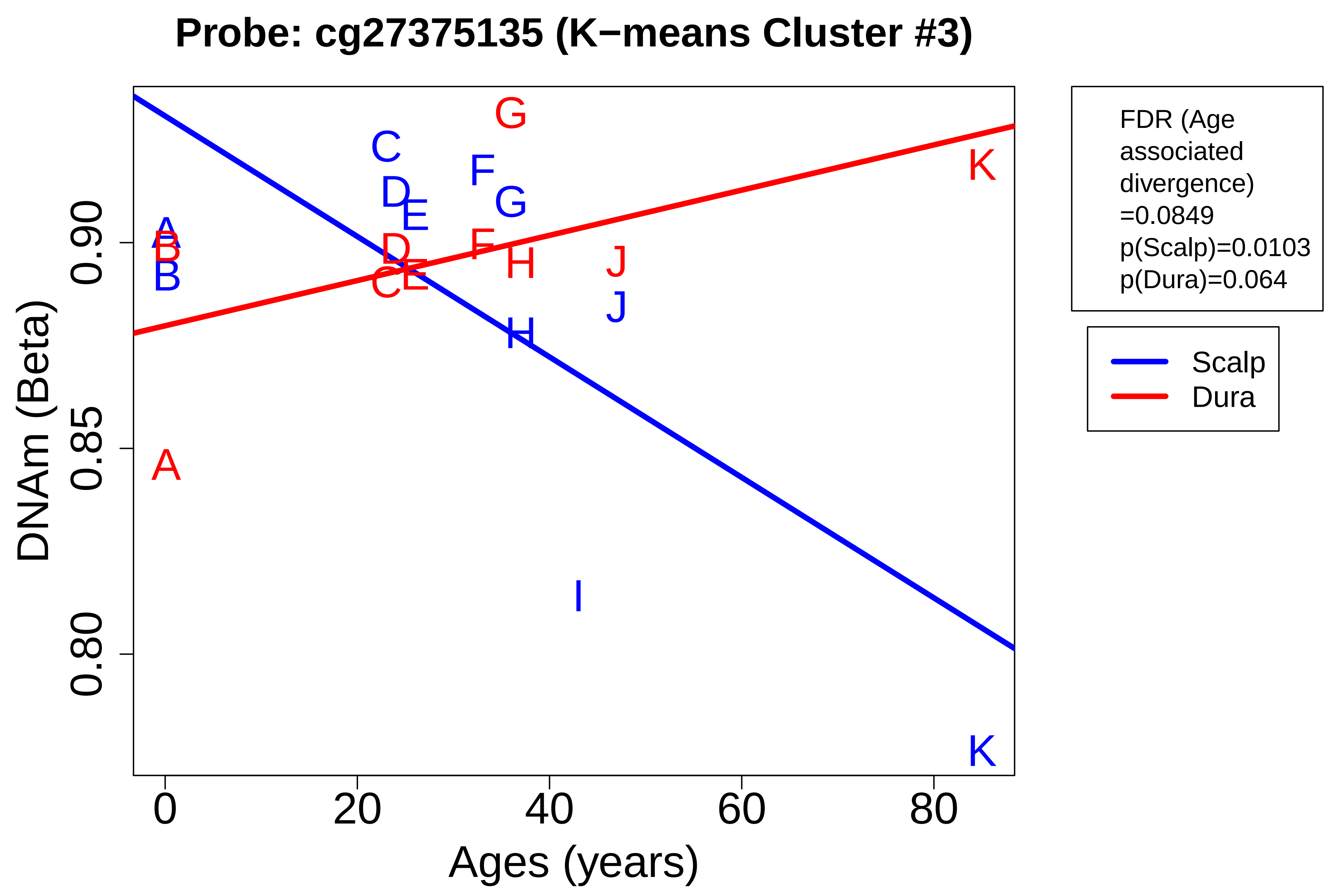
Probe: cg27375135 (K−means Cluster #3)
DNAm (Beta)
0.80
0.85
0.90
Ages (years)
0
20
40
60
80
FDR (Age associated divergence) =0.0849
p(Scalp)=0.0103
p(Dura)=0.064
Scalp
Dura
A
B
C
D
E
F
G
H
I
J
K

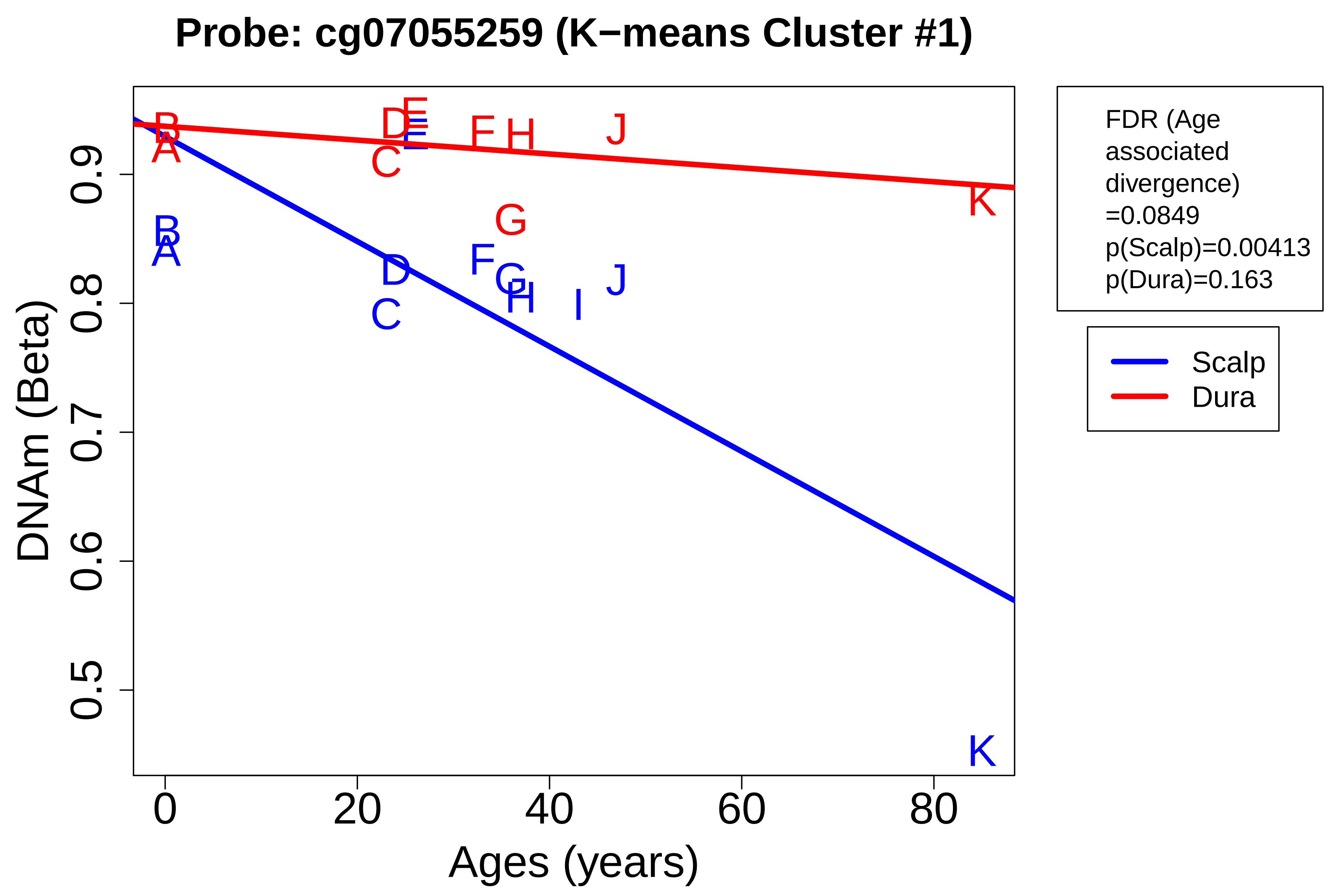

Probe: cg07055259 (K−means Cluster #1)
DNAm (Beta)
0.5
0.6
0.7
0.8
0.9
0
20
40
60
80
Ages (years)
A
B
C
D
E
F
G
H
I
J
K
FDR (Age associated divergence) =0.0849
p(Scalp)=0.00413
p(Dura)=0.163
Scalp
Dura

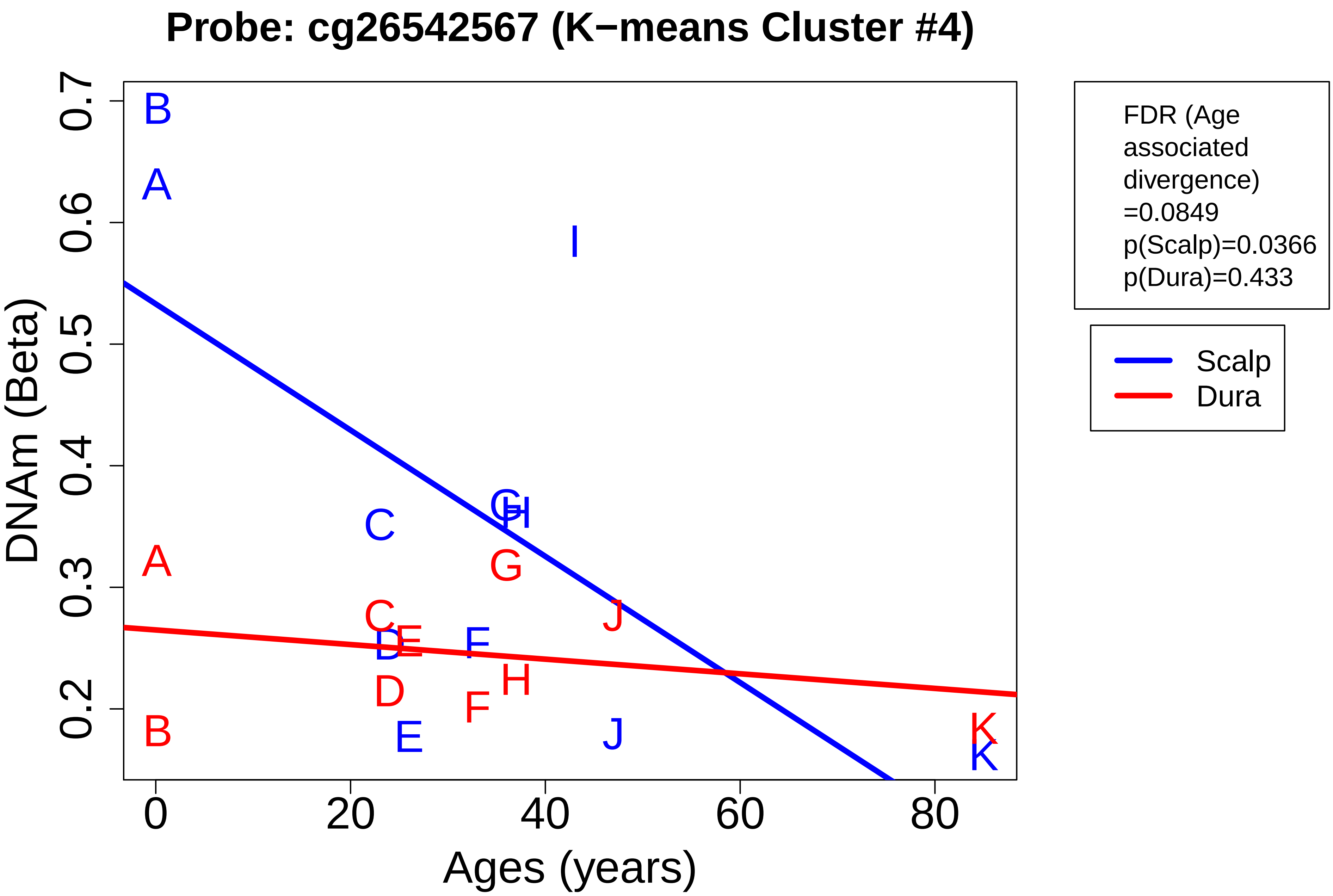

Probe: cg26542567 (K−means Cluster #4)
DNAm (Beta)
0.2
0.3
0.4
0.5
0.6
0.7
Ages (years)
0
20
40
60
80
FDR (Age associated divergence) =0.0849
p(Scalp)=0.0366
p(Dura)=0.433
Scalp
Dura

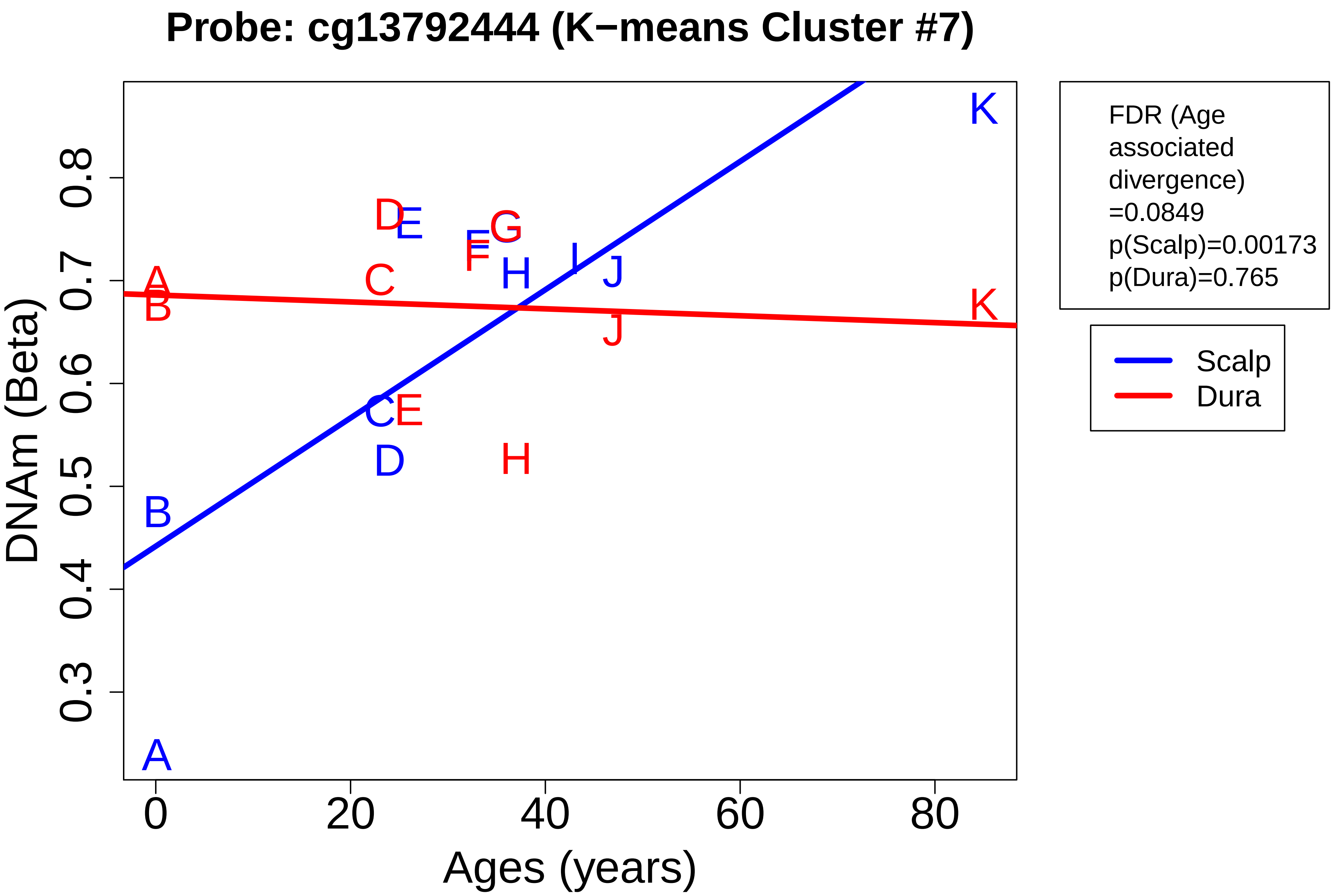

Probe: cg13792444 (K−means Cluster #7)
FDR (Age associated divergence) =0.0849
p(Scalp)=0.00173
p(Dura)=0.765
Scalp
Dura
DNAm (Beta)
0.3
0.4
0.5
0.6
0.7
0.8
0
20
40
60
80
Ages (years)
A
B
C
D
E
F
G
H
I
J
K

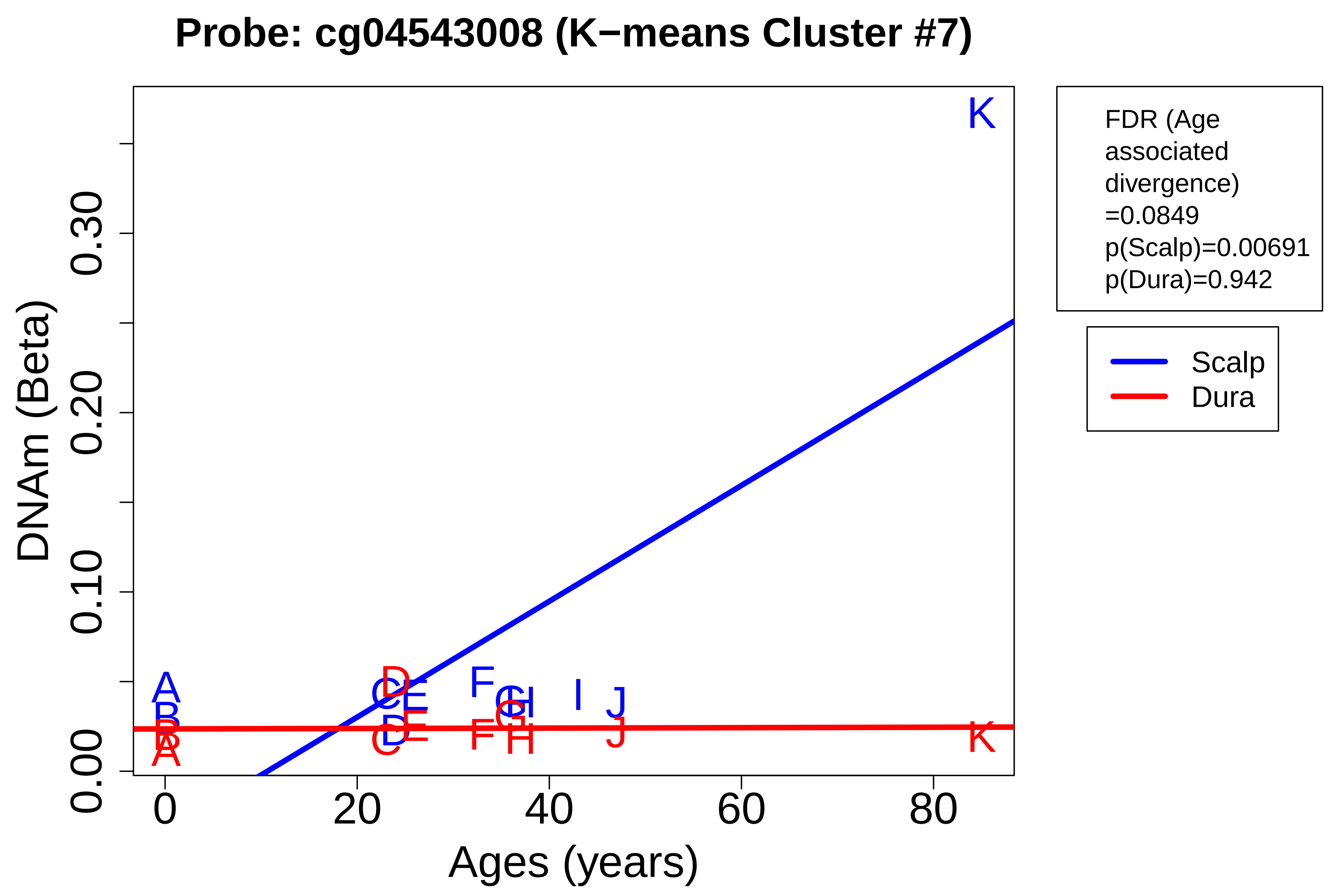

Probe: cg04543008 (K−means Cluster #7)
DNAm (Beta)
0.00
0.10
0.20
0.30
0
20
40
60
80
Ages (years)
FDR (Age associated divergence) =0.0849
p(Scalp)=0.00691
p(Dura)=0.942
Scalp
Dura
A
B
C
D
E
F
G
H
I
J
K

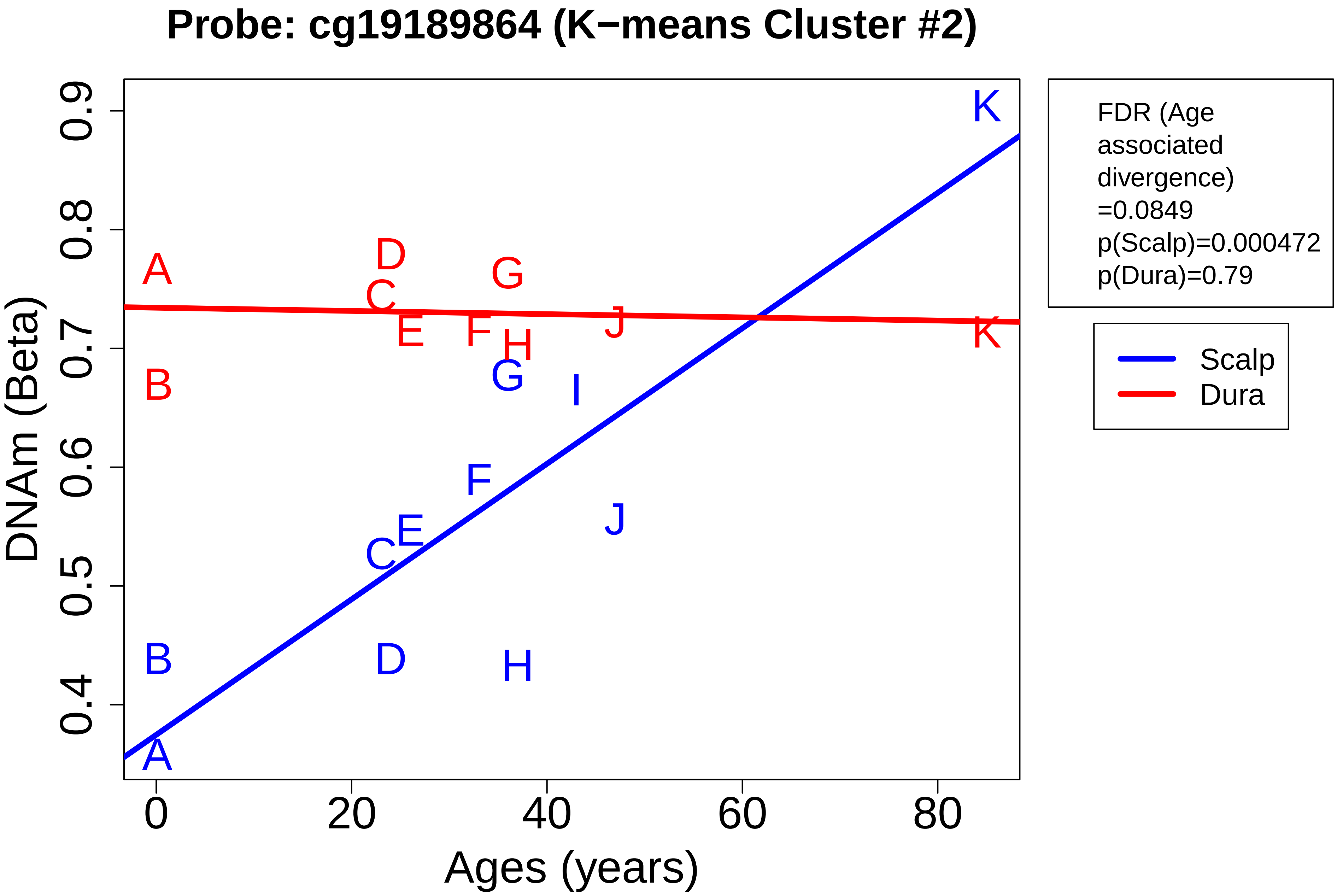
Probe: cg19189864 (K−means Cluster #2)
DNAm (Beta)
0.4
0.5
0.6
0.7
0.8
0.9
0
20
40
60
80
Ages (years)
FDR (Age associated divergence) =0.0849
p(Scalp)=0.000472
p(Dura)=0.79
Scalp
Dura
A
B
C
D
E
F
G
H
I
J
K

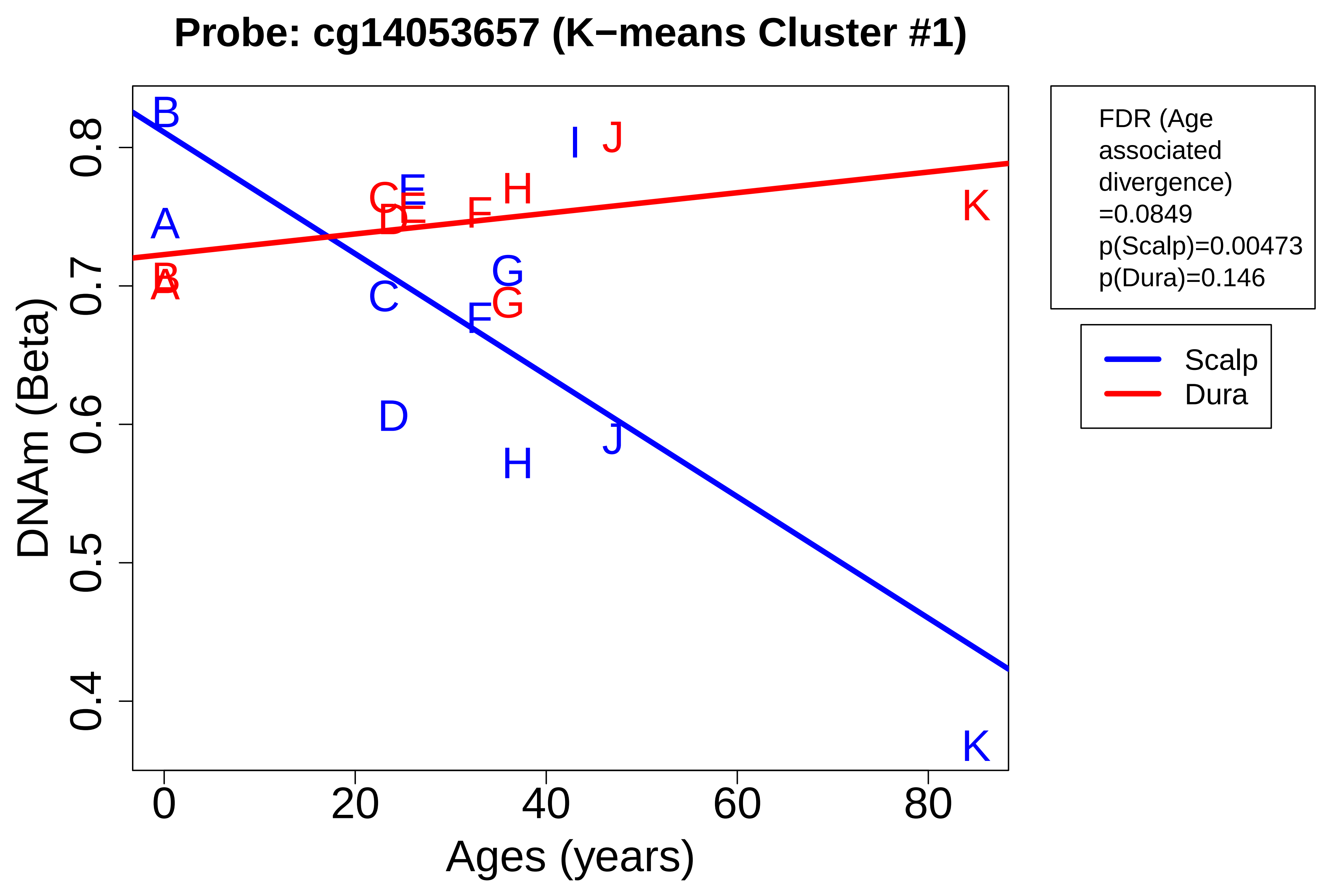
Probe: cg14053657 (K−means Cluster #1)
DNAm (Beta)
0.4
0.5
0.6
0.7
0.8
0
20
40
60
80
Ages (years)
FDR (Age associated divergence) =0.0849
p(Scalp)=0.00473
p(Dura)=0.146
Scalp
Dura

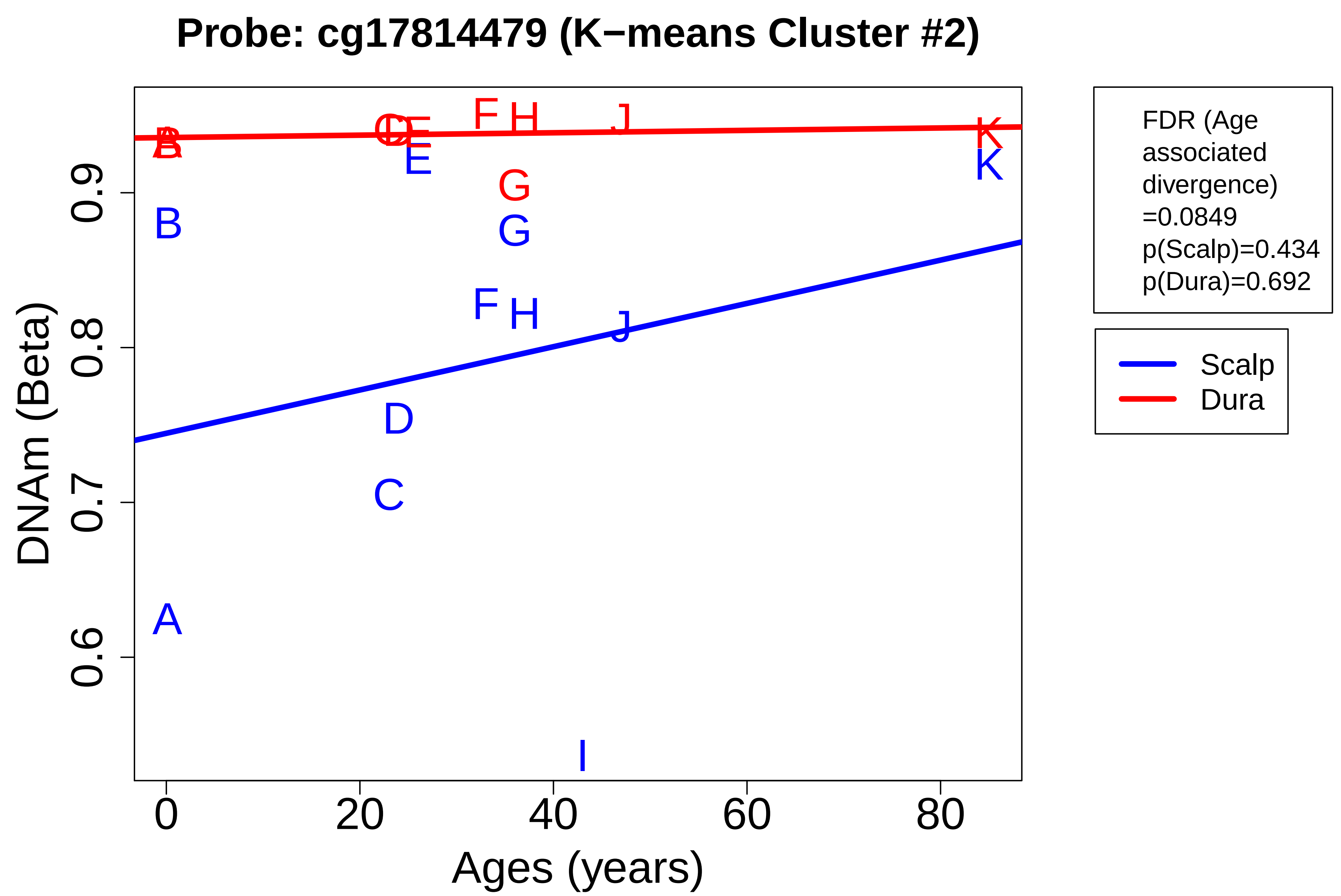

Probe: cg17814479 (K−means Cluster #2)
DNAm (Beta)
0.6
0.7
0.8
0.9
Ages (years)
0
20
40
60
80
A
B
C
D
E
F
G
H
I
J
K
FDR (Age associated divergence) =0.0849
p(Scalp)=0.434
p(Dura)=0.692
Scalp
Dura

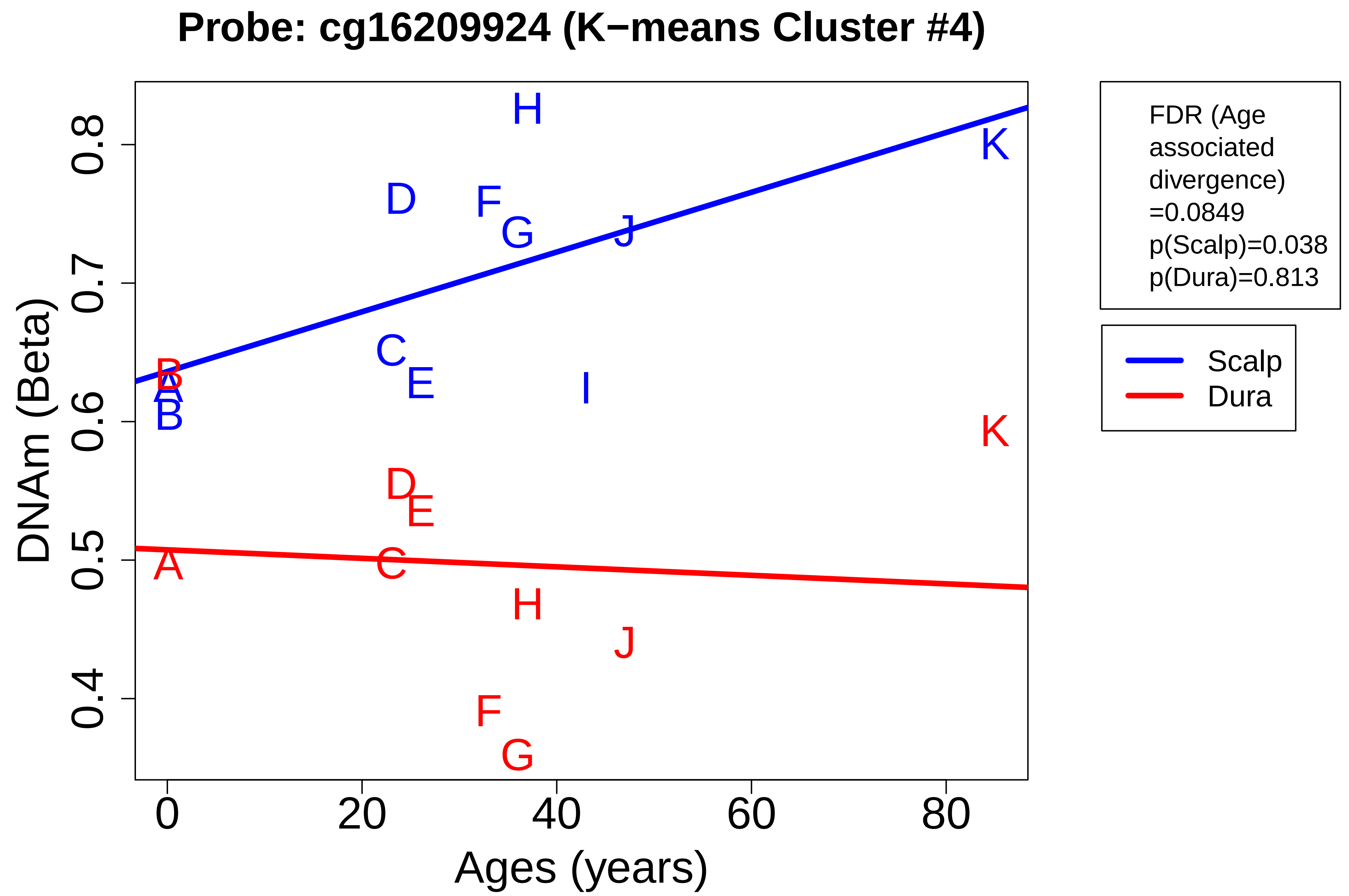

Probe: cg16209924 (K−means Cluster #4)
DNAm (Beta)
0.4
0.5
0.6
0.7
0.8
Ages (years)
0
20
40
60
80
H
K
D
F
G
J
C
E
I
B
A
B
K
D
E
C
A
H
J
F
G
FDR (Age associated divergence) =0.0849
p(Scalp)=0.038
p(Dura)=0.813
Scalp
Dura

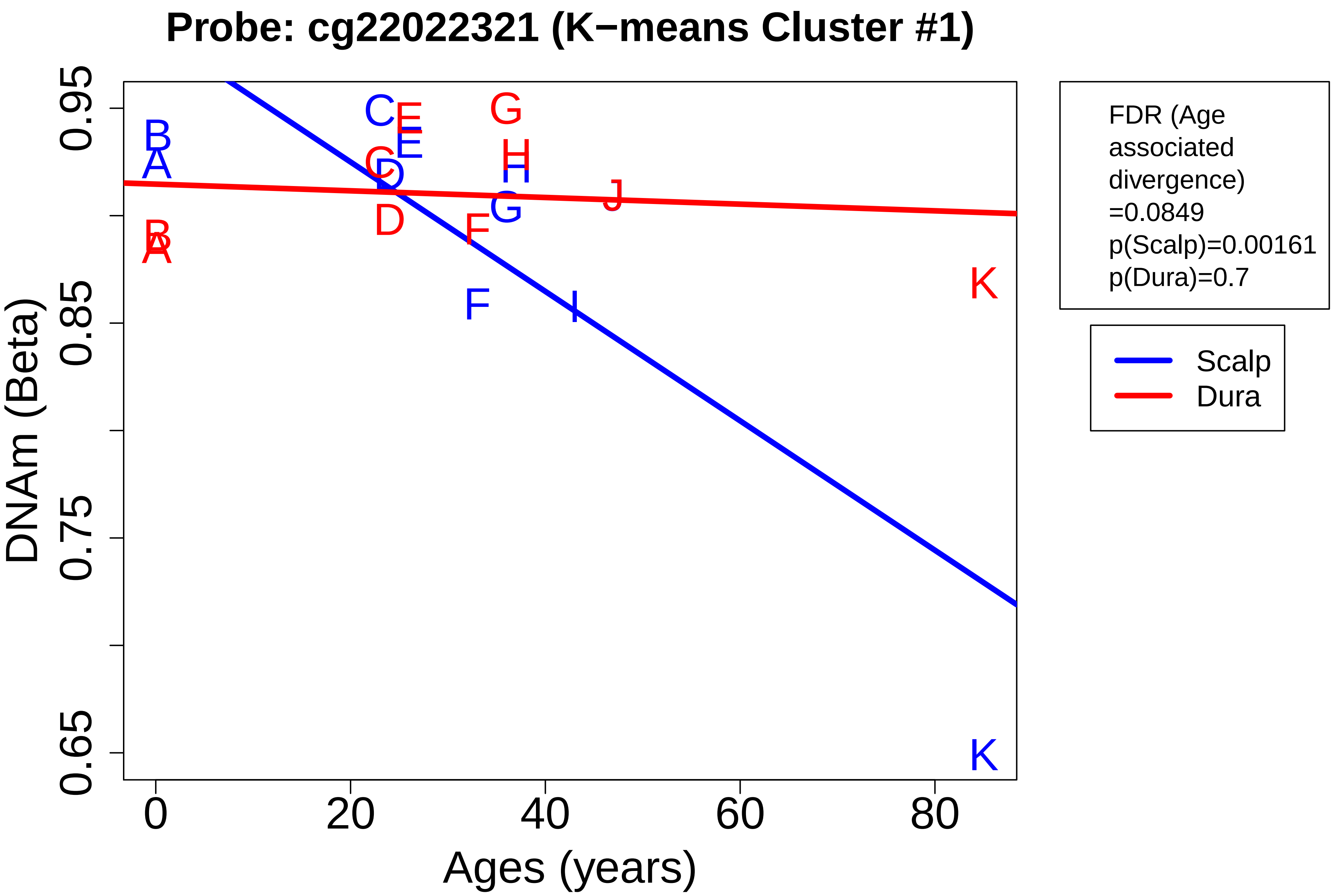
Probe: cg22022321 (K−means Cluster #1)
DNAm (Beta)
0.95
0.85
0.75
0.65
Ages (years)
0
20
40
60
80
FDR (Age associated divergence) =0.0849
p(Scalp)=0.00161
p(Dura)=0.7
Scalp
Dura
A
B
C
D
E
F
G
H
I
J
K

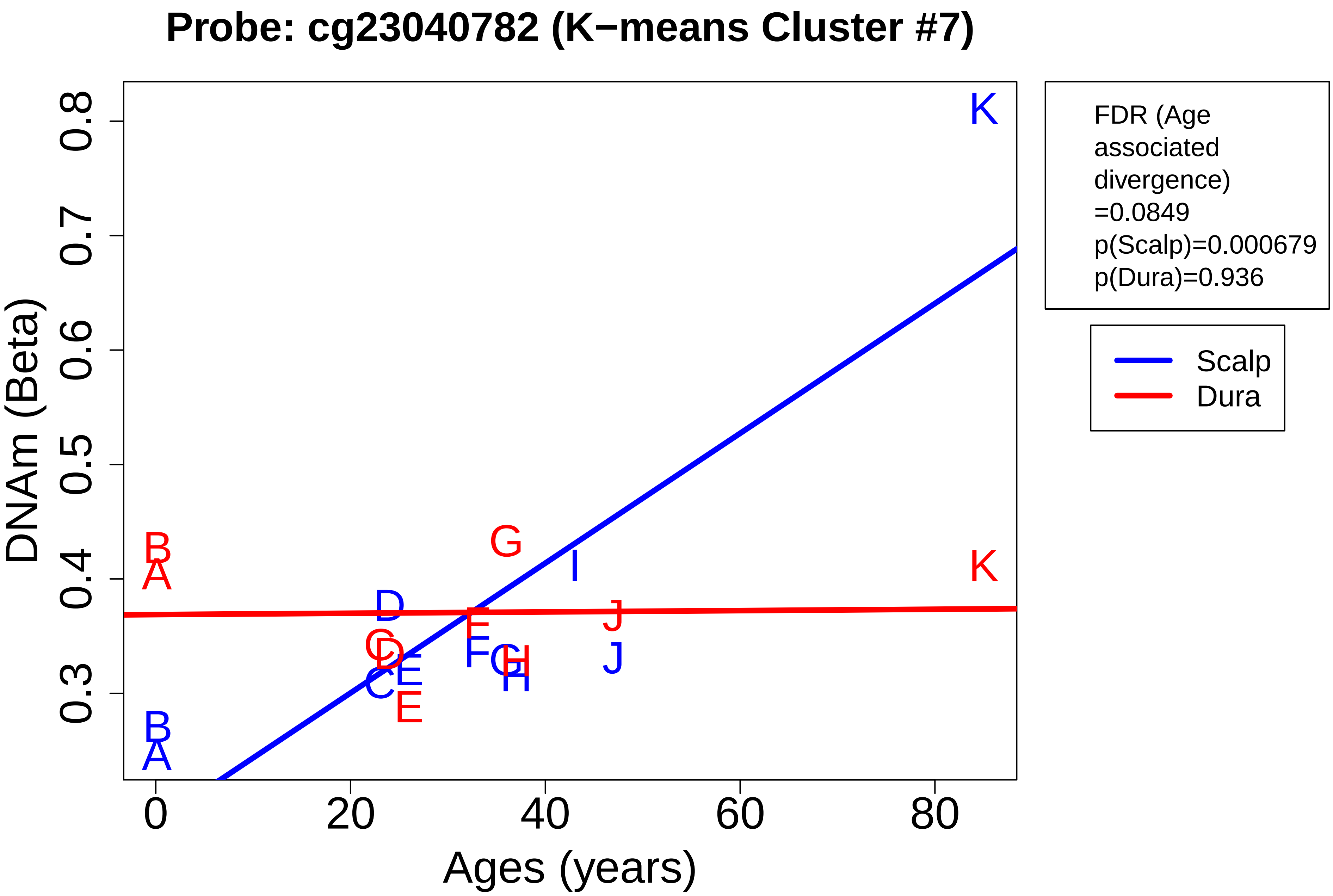

Probe: cg23040782 (K−means Cluster #7)
DNAm (Beta)
0.3
0.4
0.5
0.6
0.7
0.8
0
20
40
60
80
Ages (years)
FDR (Age associated divergence) =0.0849
p(Scalp)=0.000679
p(Dura)=0.936
Scalp
Dura

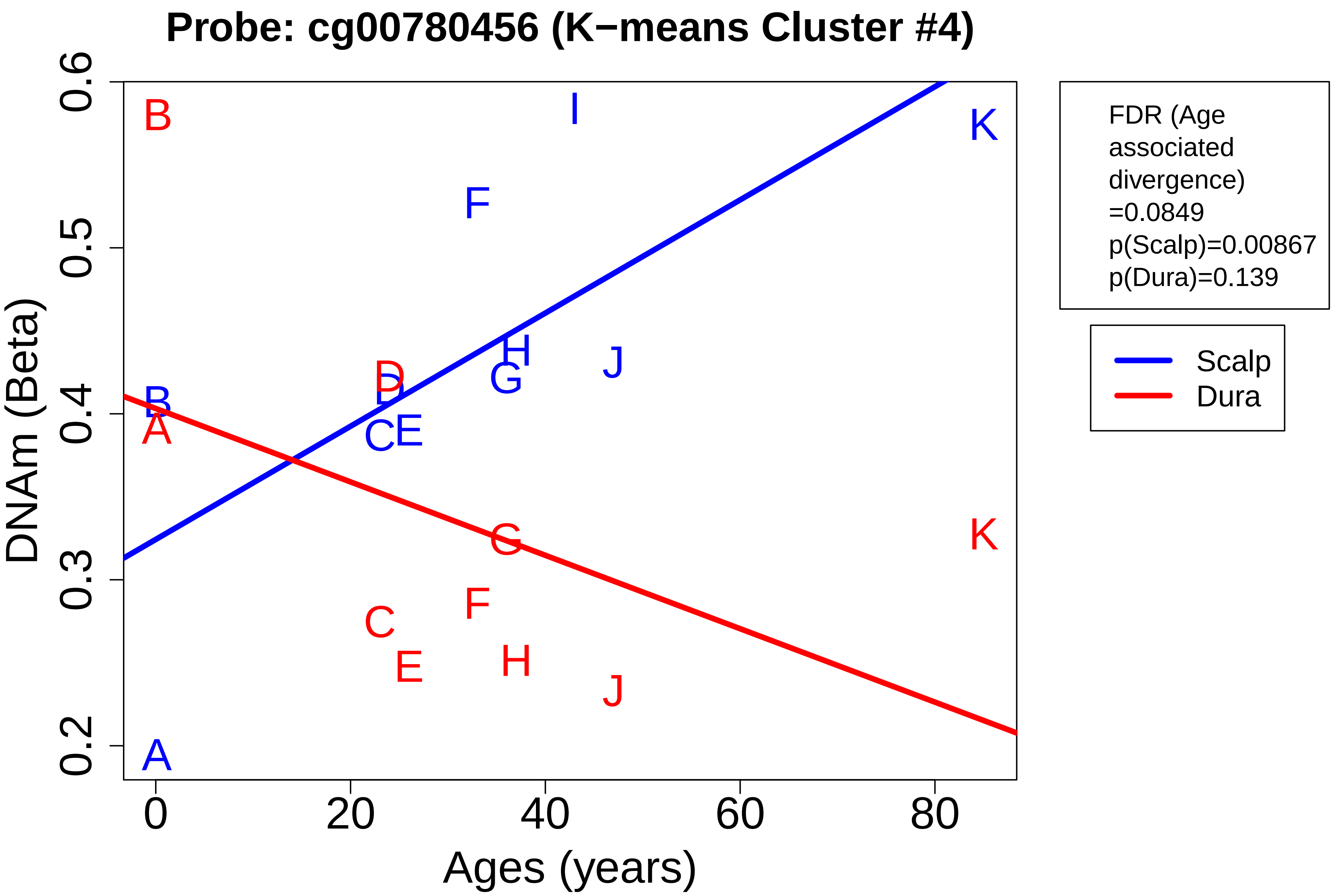

Probe: cg00780456 (K−means Cluster #4)
DNAm (Beta)
0.2
0.3
0.4
0.5
0.6
0
20
40
60
80
Ages (years)
FDR (Age associated divergence) =0.0849
p(Scalp)=0.00867
p(Dura)=0.139
Scalp
Dura
A
B
C
D
E
F
G
H
I
J
K

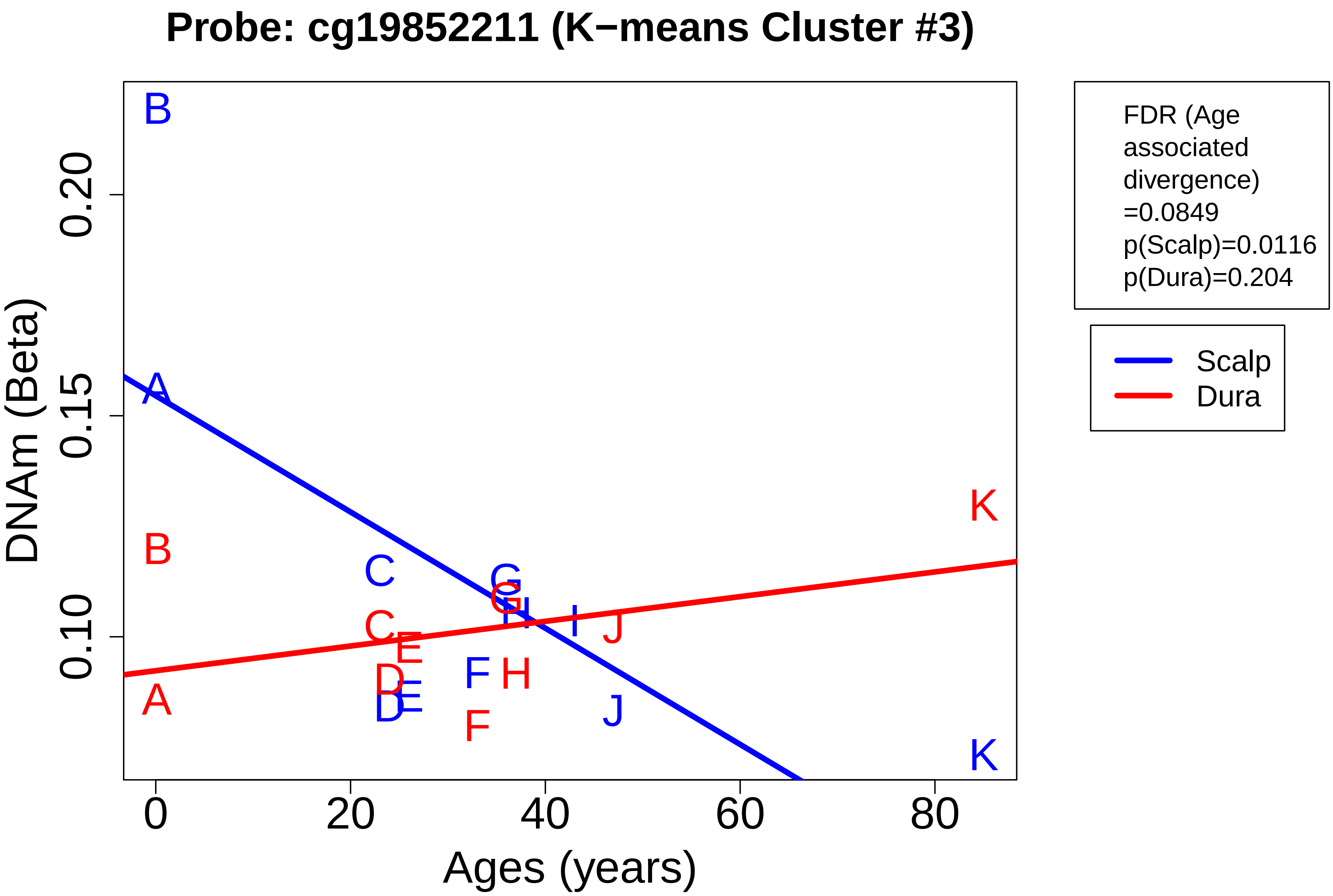

Probe: cg19852211 (K−means Cluster #3)
DNAm (Beta)
0.10
0.15
0.20
0
20
40
60
80
Ages (years)
FDR (Age associated divergence) =0.0849
p(Scalp)=0.0116
p(Dura)=0.204
Scalp
Dura

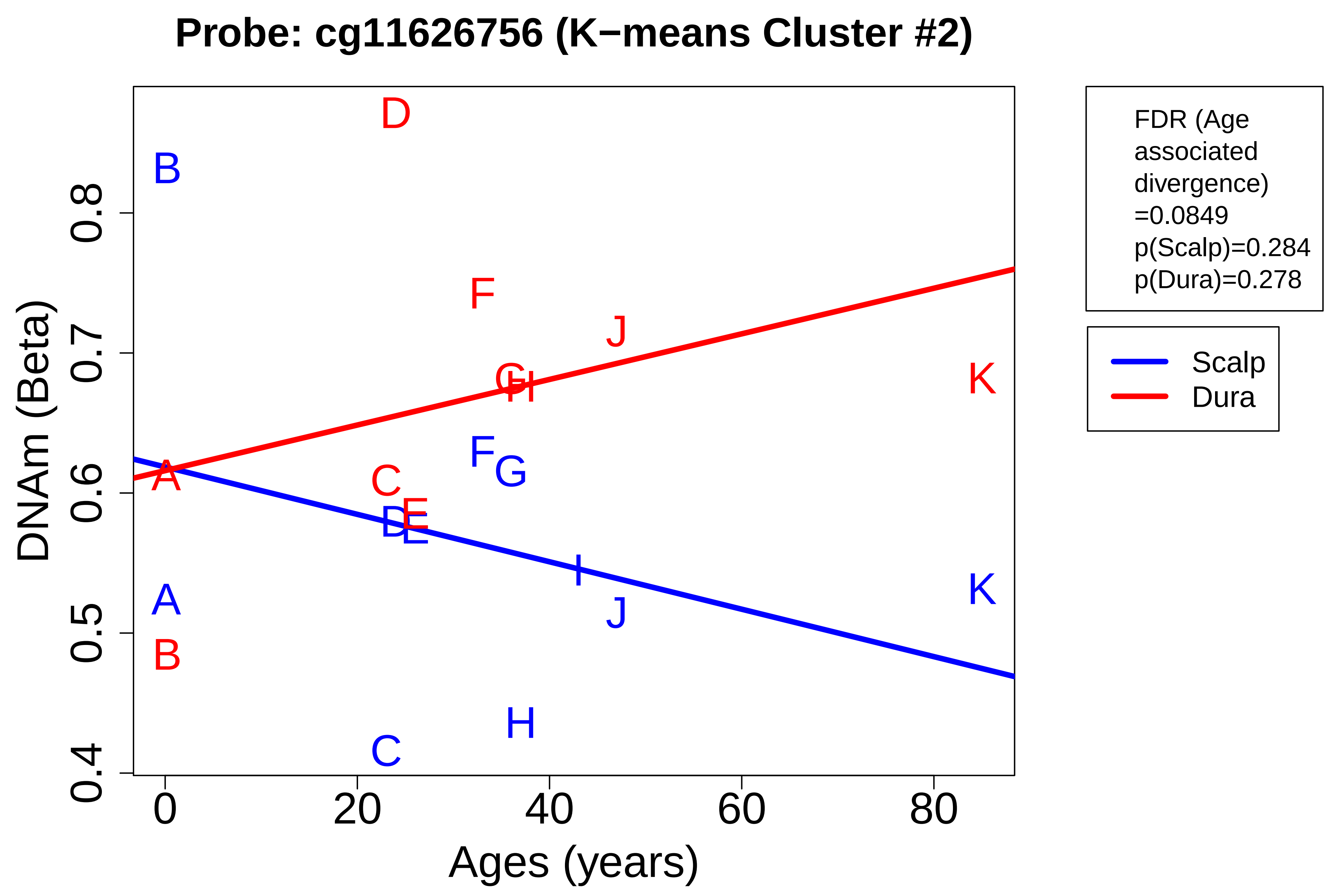

Probe: cg11626756 (K−means Cluster #2)
DNAm (Beta)
0.4
0.5
0.6
0.7
0.8
0
20
40
60
80
Ages (years)
FDR (Age associated divergence) =0.0849
p(Scalp)=0.284
p(Dura)=0.278
Scalp
Dura
A
B
C
D
E
F
G
H
I
J
K

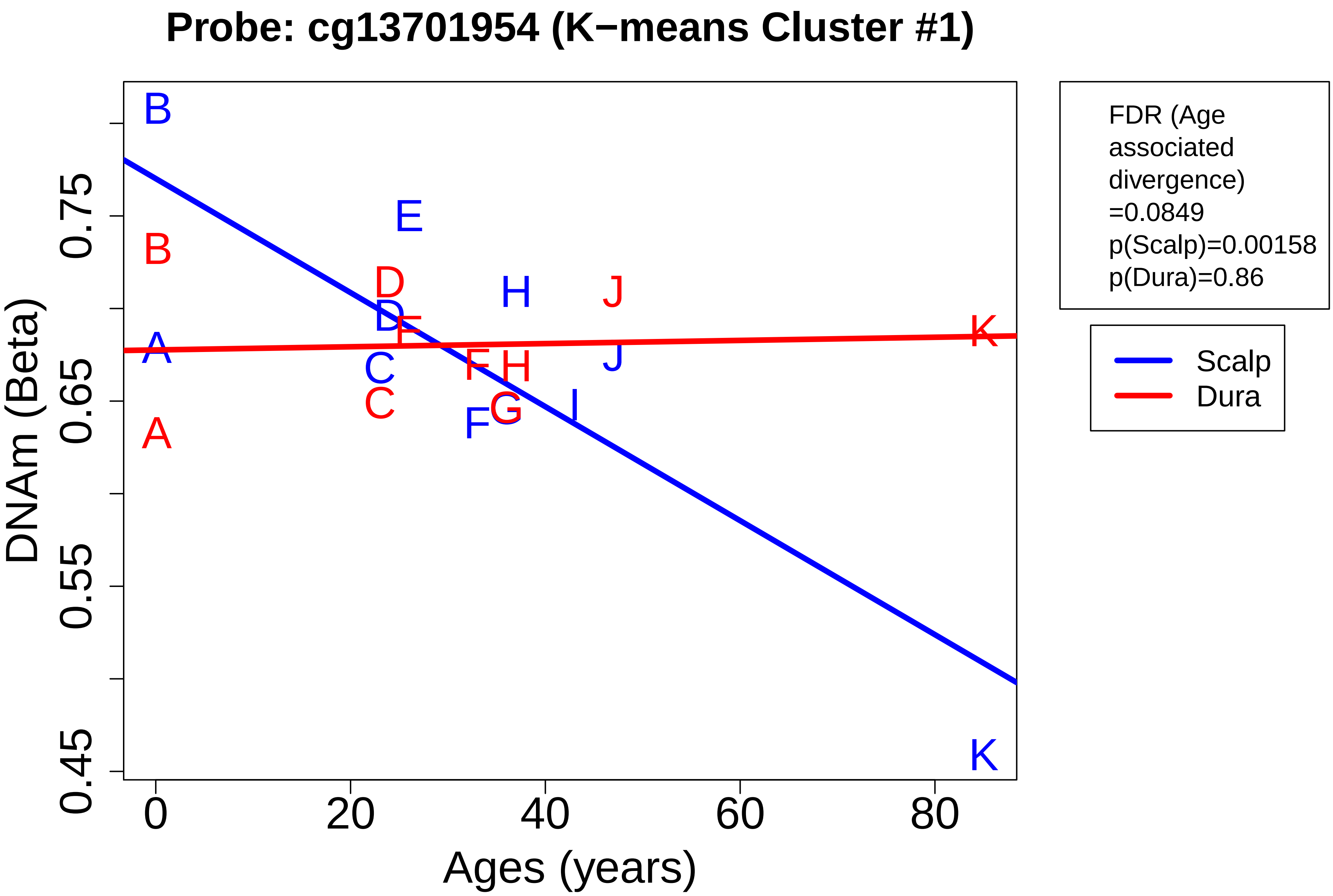

Probe: cg13701954 (K−means Cluster #1)
DNAm (Beta)
0.45
0.55
0.65
0.75
0
20
40
60
80
Ages (years)
A
B
C
D
E
F
G
H
I
J
K
FDR (Age associated divergence) =0.0849
p(Scalp)=0.00158
p(Dura)=0.86
Scalp
Dura

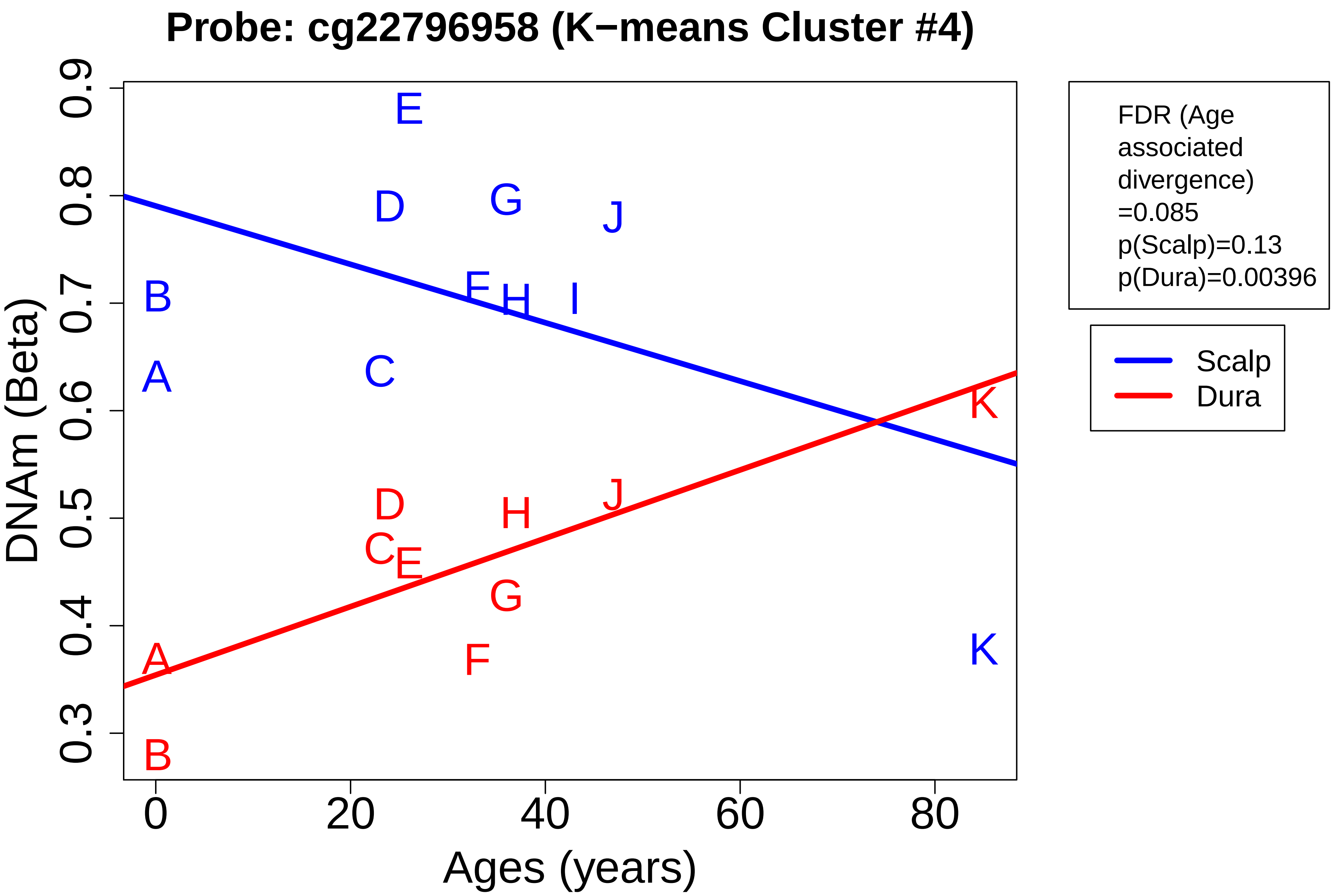

Probe: cg22796958 (K−means Cluster #4)
DNAm (Beta)
0.3
0.4
0.5
0.6
0.7
0.8
0.9
0
20
40
60
80
Ages (years)
FDR (Age associated divergence) =0.085
p(Scalp)=0.13
p(Dura)=0.00396
Scalp
Dura

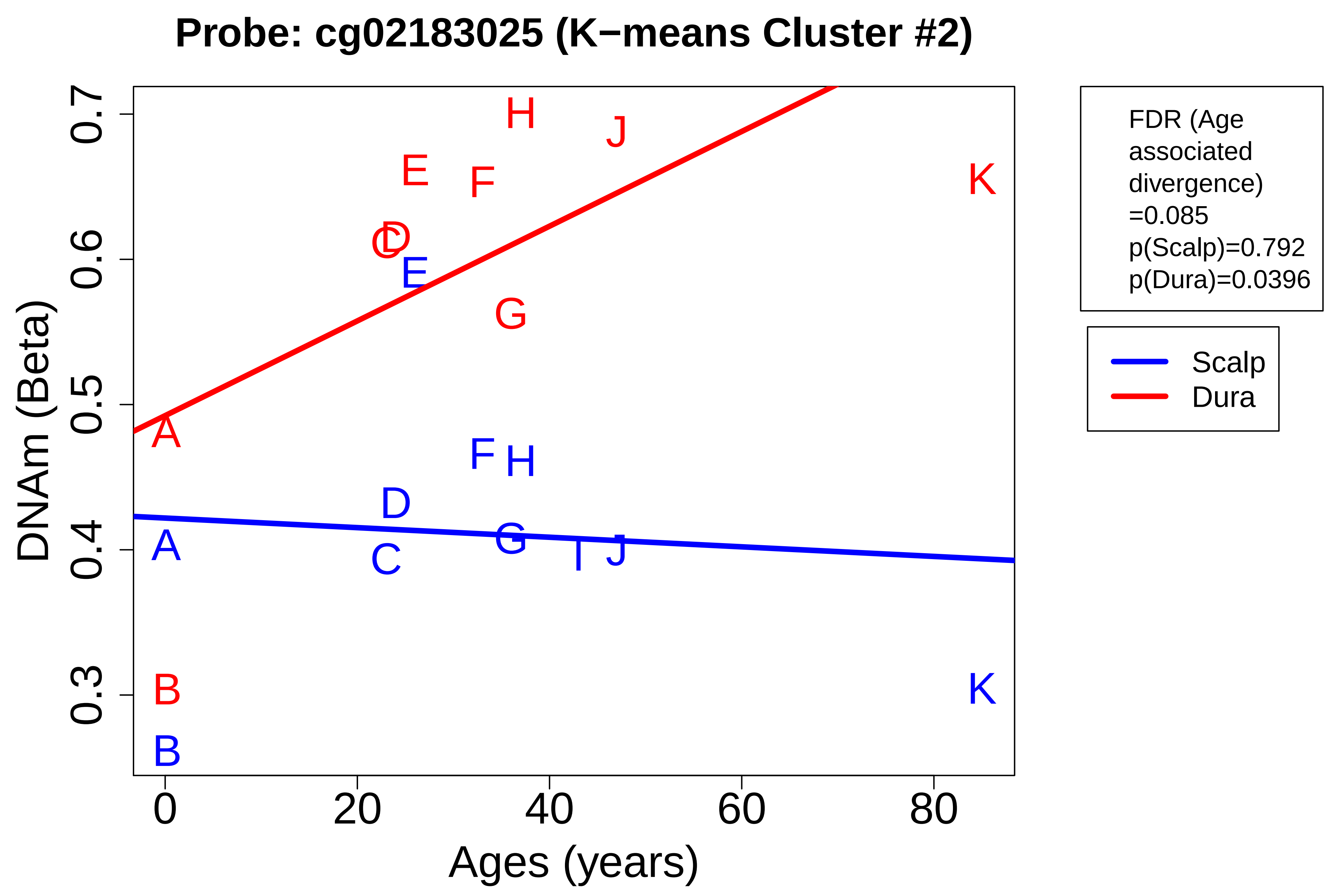
Probe: cg02183025 (K−means Cluster #2)
DNAm (Beta)
0.3
0.4
0.5
0.6
0.7
0
20
40
60
80
Ages (years)
A
B
C
D
E
F
G
H
I
J
K
FDR (Age associated divergence) =0.085
p(Scalp)=0.792
p(Dura)=0.0396
Scalp
Dura

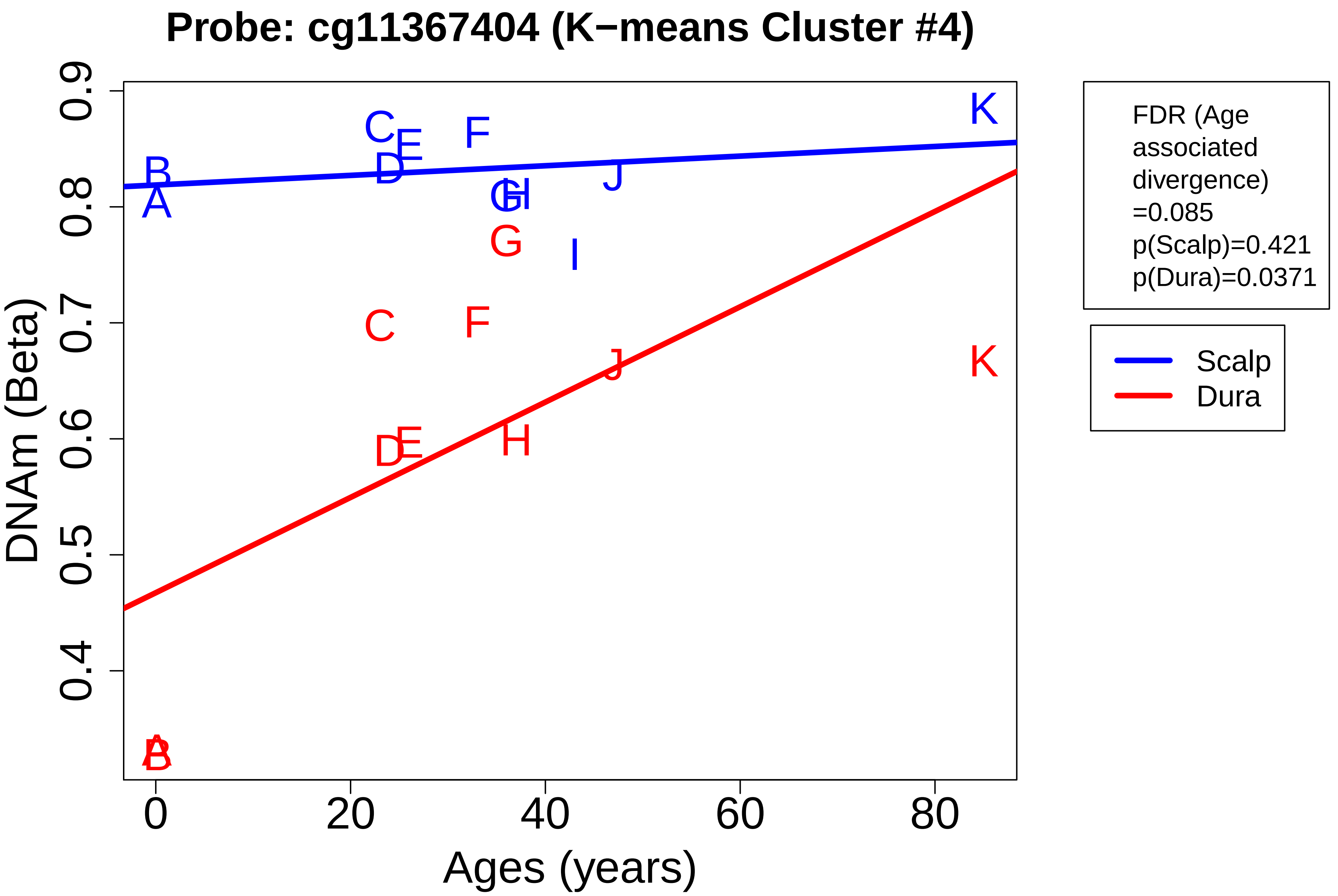
Probe: cg11367404 (K−means Cluster #4)
DNAm (Beta)
Ages (years)
0.4
0.5
0.6
0.7
0.8
0.9
0
20
40
60
80
FDR (Age associated divergence) =0.085
p(Scalp)=0.421
p(Dura)=0.0371
Scalp
Dura

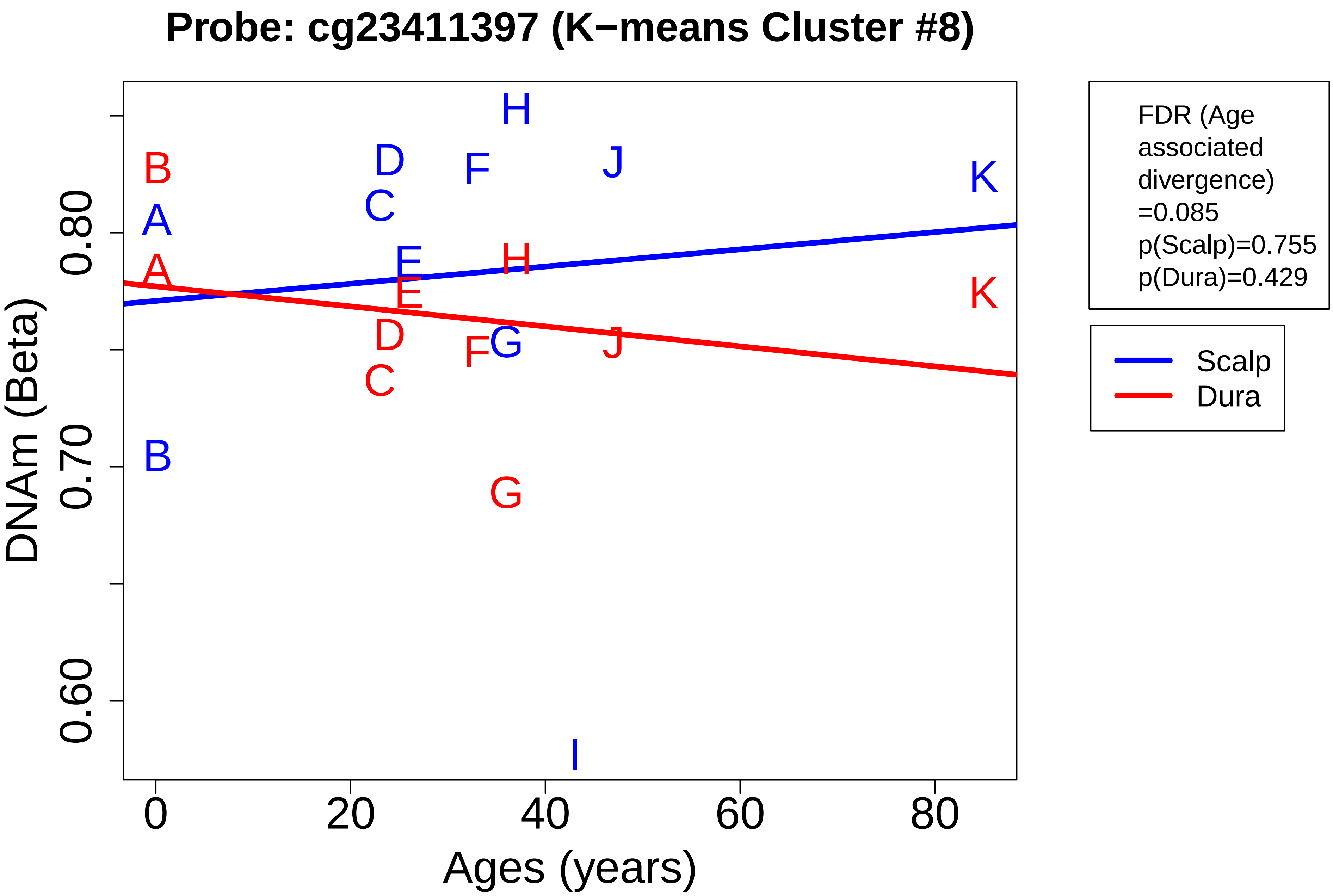

Probe: cg23411397 (K−means Cluster #8)
FDR (Age associated divergence) =0.085
p(Scalp)=0.755
p(Dura)=0.429
Scalp
Dura
DNAm (Beta)
0.60
0.70
0.80
0
20
40
60
80
Ages (years)
A
B
C
D
E
F
G
H
I
J
K

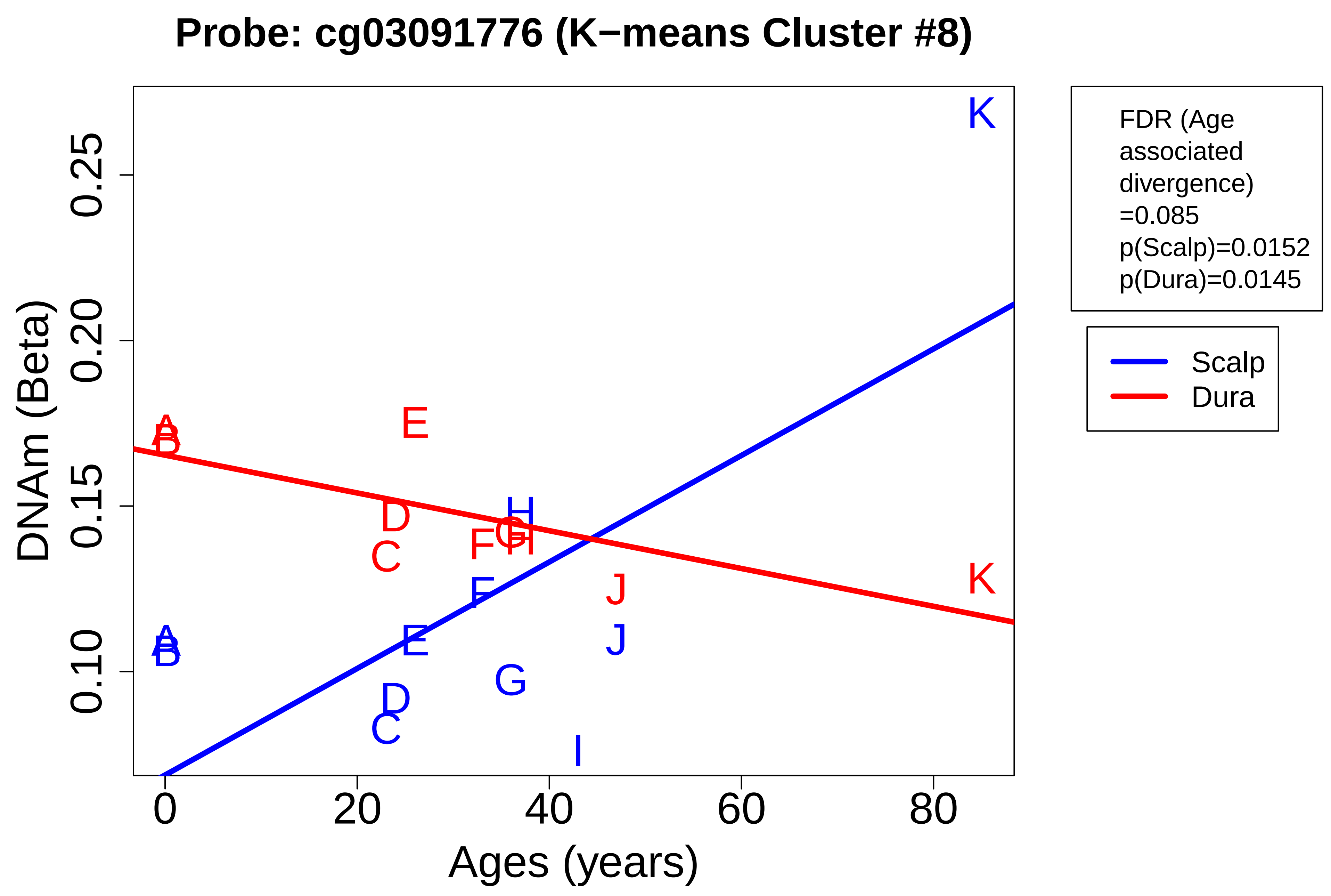

Probe: cg03091776 (K−means Cluster #8)
DNAm (Beta)
0.10
0.15
0.20
0.25
Ages (years)
0
20
40
60
80
A
B
C
D
E
F
G
H
I
J
K
FDR (Age associated divergence) =0.085
p(Scalp)=0.0152
p(Dura)=0.0145
Scalp
Dura

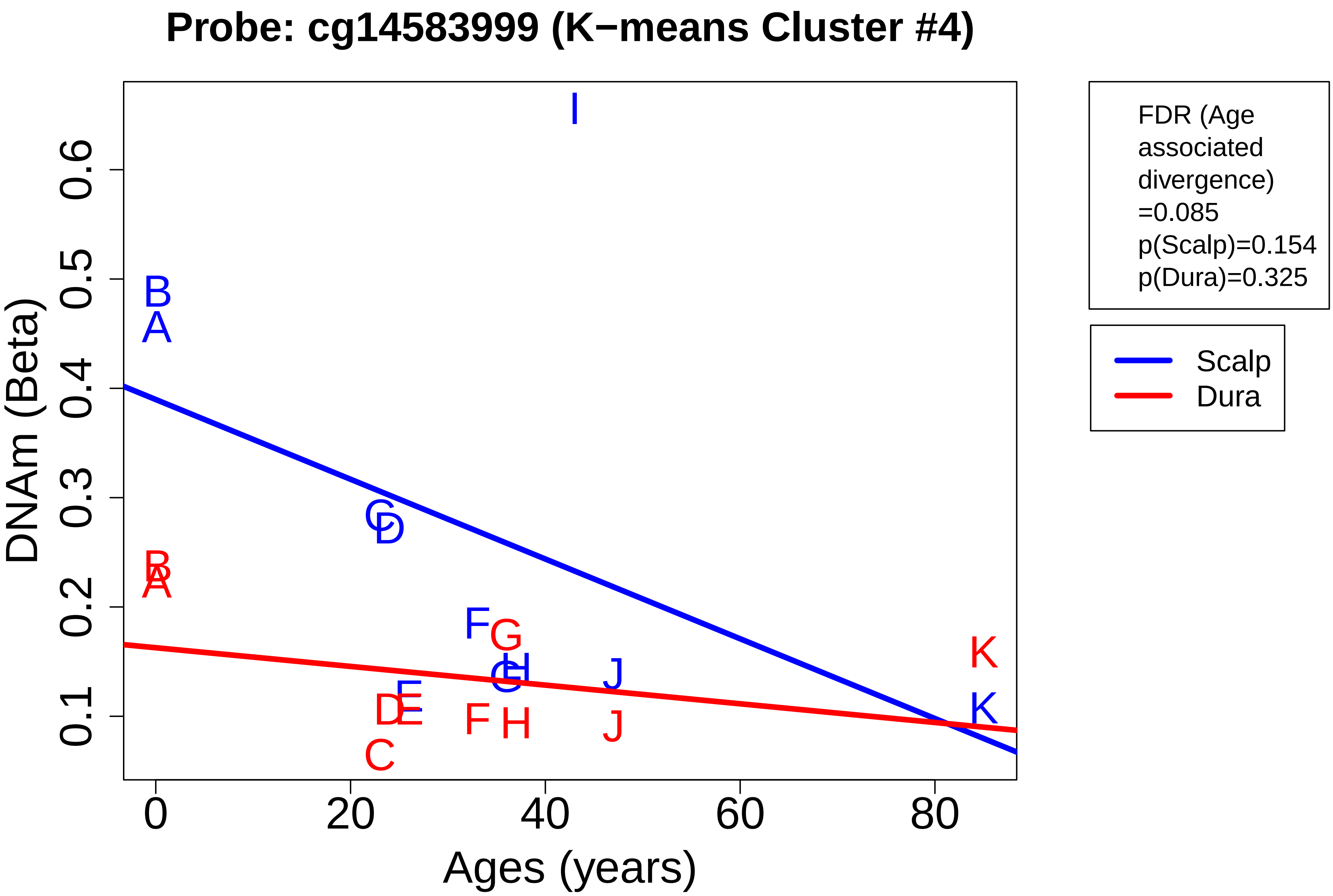
Probe: cg14583999 (K−means Cluster #4)
DNAm (Beta)
Ages (years)
FDR (Age associated divergence) =0.085
p(Scalp)=0.154
p(Dura)=0.325
Scalp
Dura

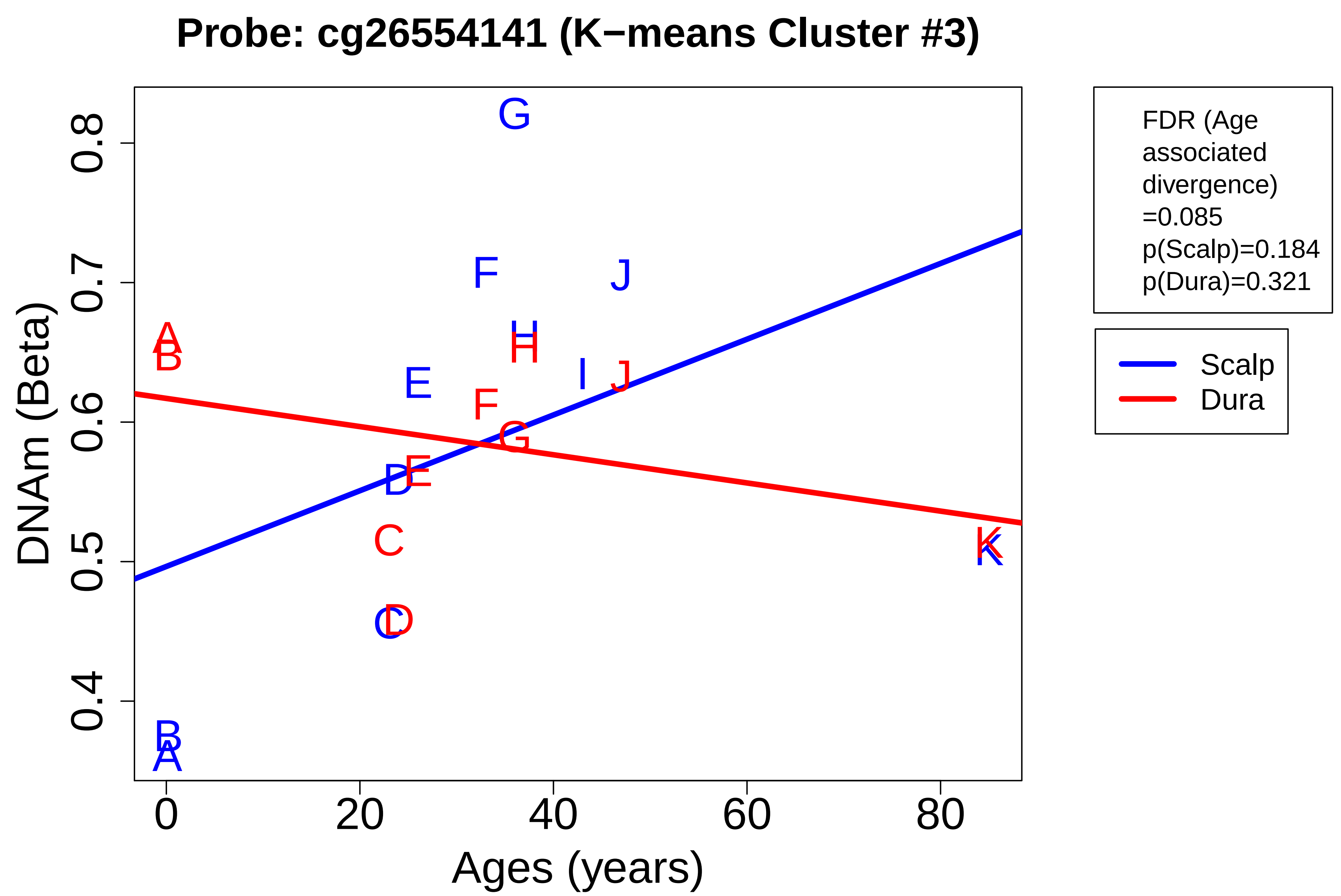

Probe: cg26554141 (K−means Cluster #3)
DNAm (Beta)
Ages (years)
0.4
0.5
0.6
0.7
0.8
0
20
40
60
80
FDR (Age associated divergence) =0.085
p(Scalp)=0.184
p(Dura)=0.321
Scalp
Dura

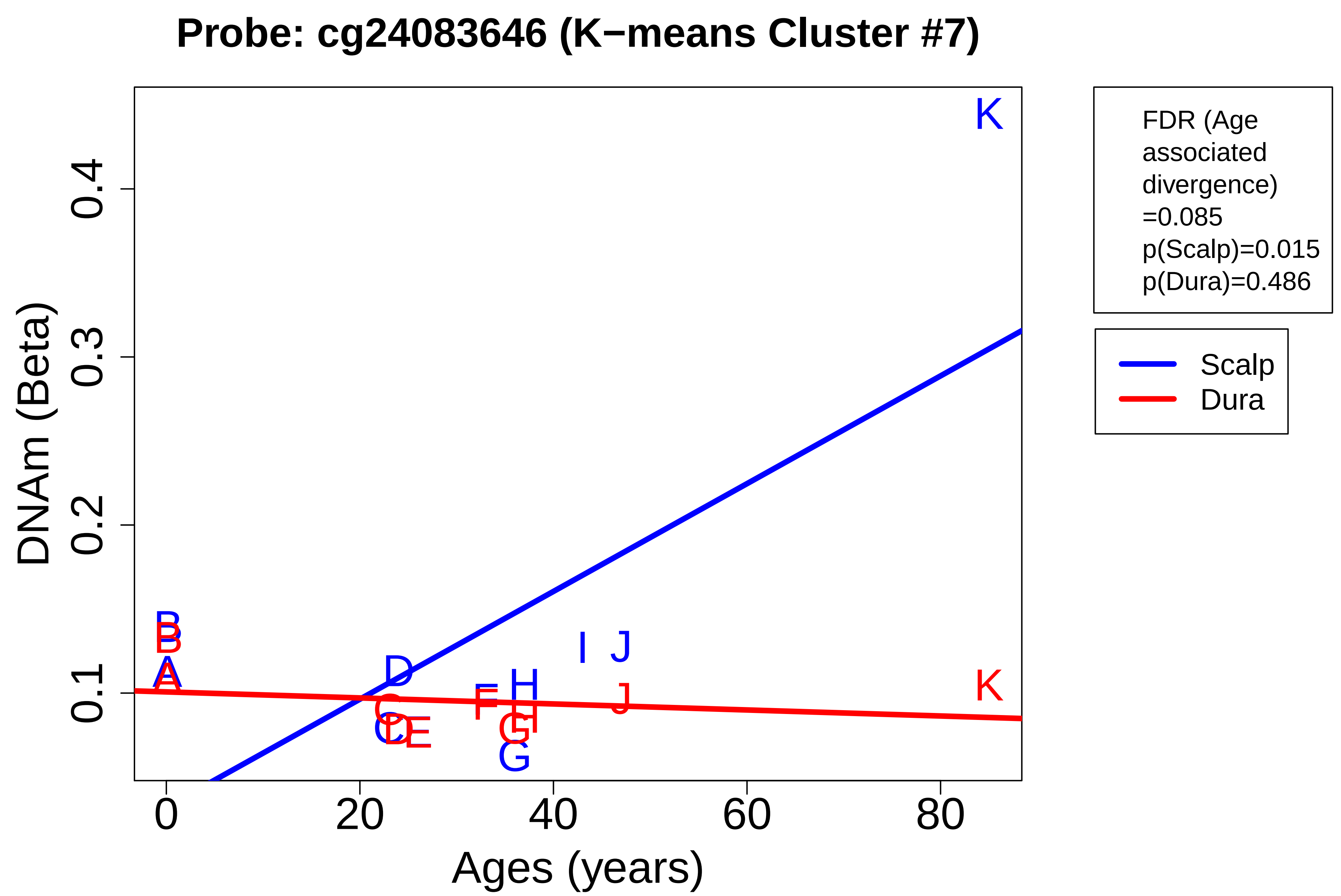

Probe: cg24083646 (K−means Cluster #7)
DNAm (Beta)
0.1
0.2
0.3
0.4
Ages (years)
0
20
40
60
80
FDR (Age associated divergence) =0.085
p(Scalp)=0.015
p(Dura)=0.486
Scalp
Dura

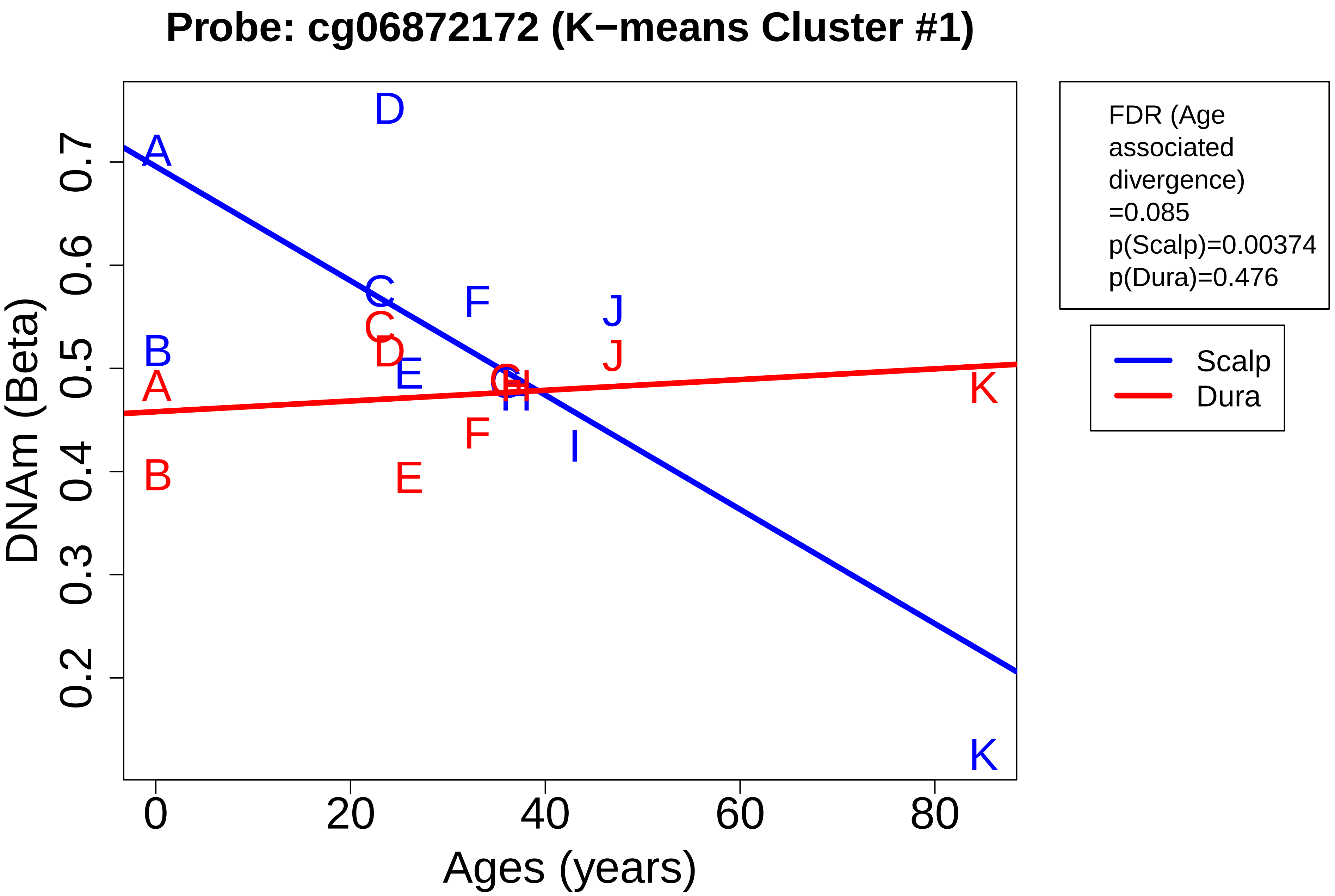

Probe: cg06872172 (K−means Cluster #1)
DNAm (Beta)
Ages (years)
0.2
0.3
0.4
0.5
0.6
0.7
0
20
40
60
80
FDR (Age associated divergence) =0.085
p(Scalp)=0.00374
p(Dura)=0.476
Scalp
Dura

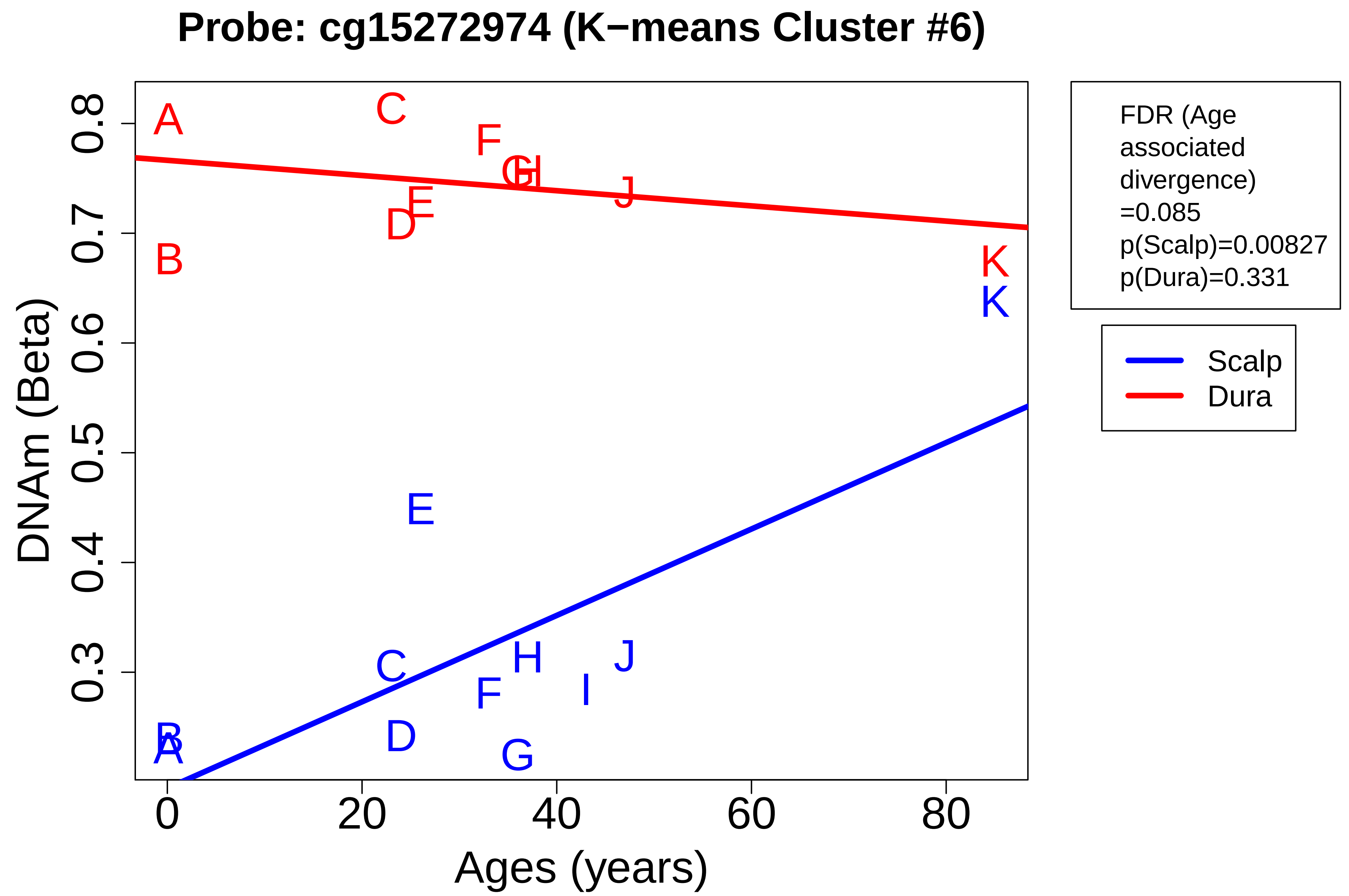

Probe: cg15272974 (K−means Cluster #6)
DNAm (Beta)
0.3
0.4
0.5
0.6
0.7
0.8
0
20
40
60
80
Ages (years)
A
B
C
D
E
F
G
H
J
K
A
B
C
D
E
F
G
H
I
J
K
FDR (Age associated divergence) =0.085
p(Scalp)=0.00827
p(Dura)=0.331
Scalp
Dura

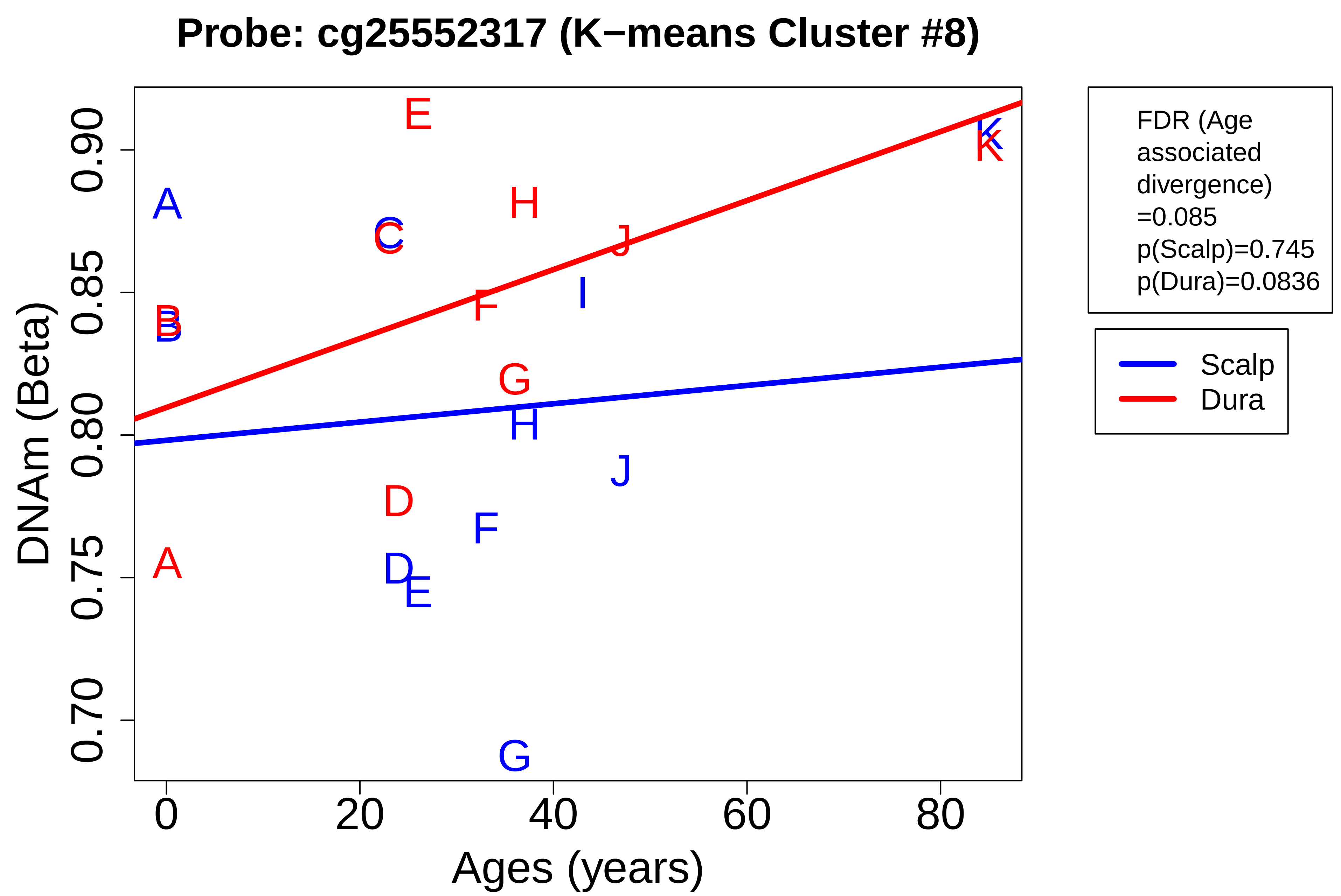

Probe: cg25552317 (K−means Cluster #8)
DNAm (Beta)
0.70
0.75
0.80
0.85
0.90
Ages (years)
0
20
40
60
80
A
B
C
D
E
F
G
H
I
J
K
FDR (Age associated divergence) =0.085
p(Scalp)=0.745
p(Dura)=0.0836
Scalp
Dura

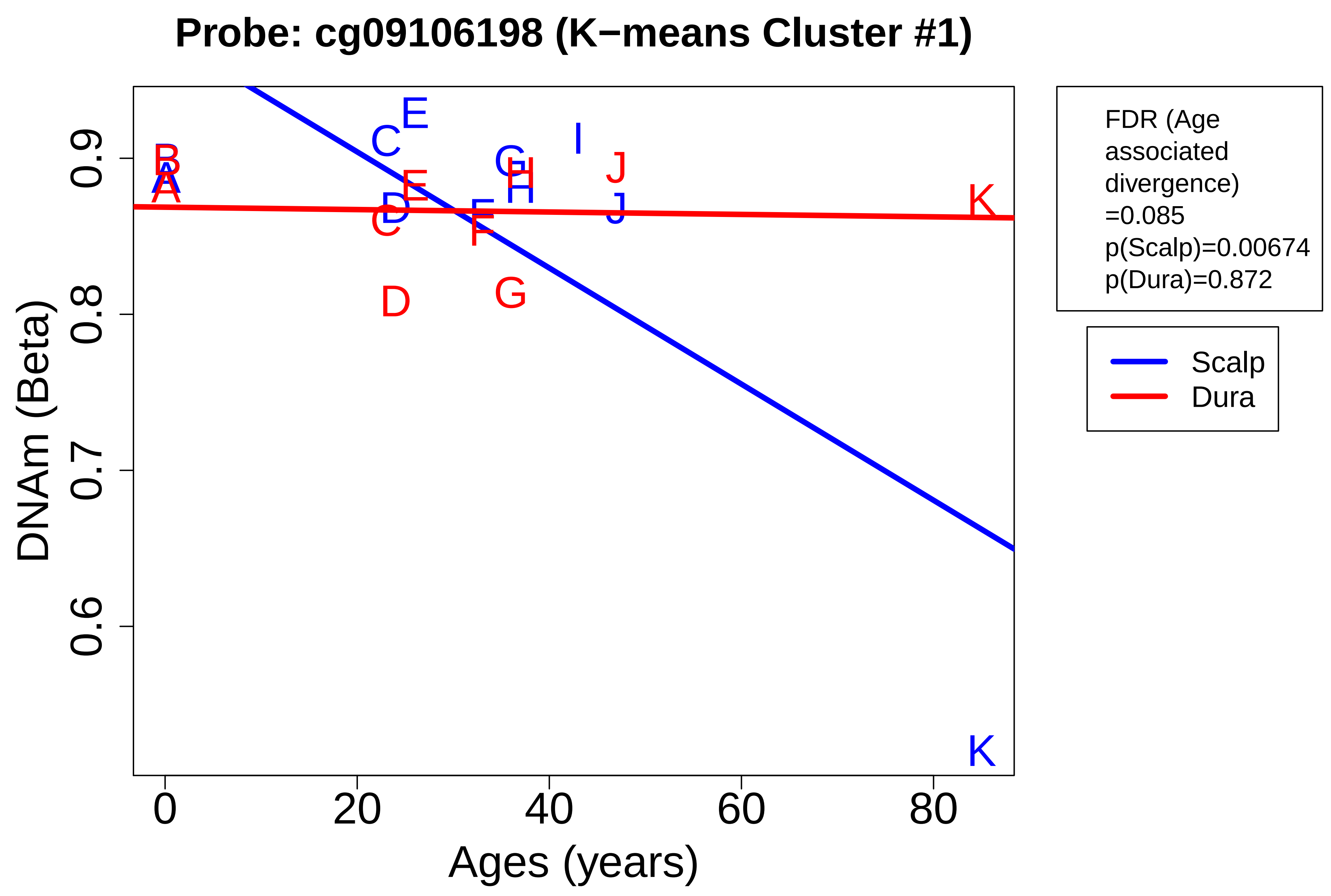

Probe: cg09106198 (K−means Cluster #1)
DNAm (Beta)
0.6
0.7
0.8
0.9
0
20
40
60
80
Ages (years)
FDR (Age associated divergence) =0.085
p(Scalp)=0.00674
p(Dura)=0.872
Scalp
Dura

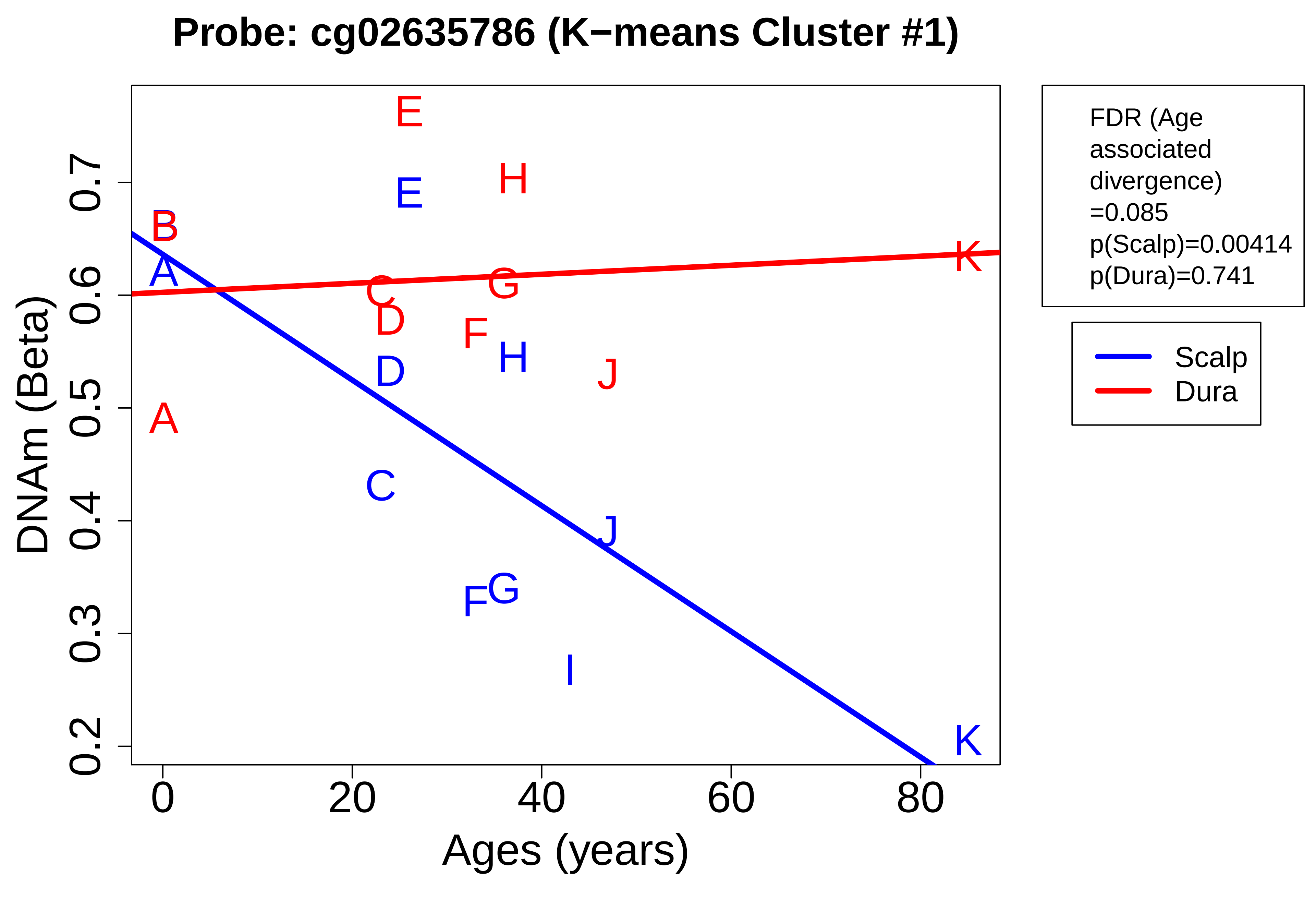
Probe: cg02635786 (K−means Cluster #1)
DNAm (Beta)
0.2
0.3
0.4
0.5
0.6
0.7
Ages (years)
0
20
40
60
80
FDR (Age associated divergence) =0.085
p(Scalp)=0.00414
p(Dura)=0.741
Scalp
Dura

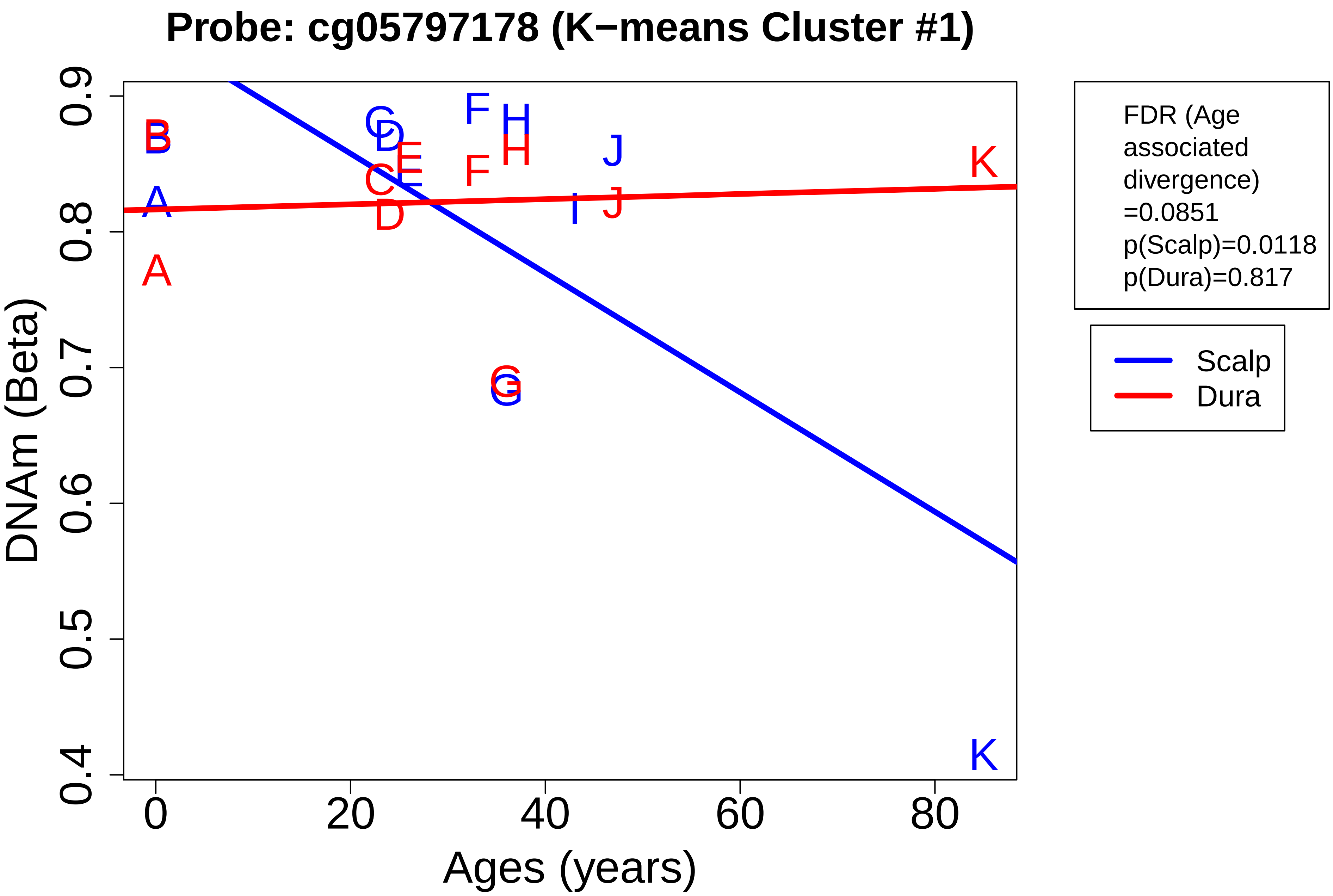
Probe: cg05797178 (K−means Cluster #1)
DNAm (Beta)
Ages (years)
0.4
0.5
0.6
0.7
0.8
0.9
0
20
40
60
80
A
B
C
D
E
F
G
H
I
J
K
A
B
C
D
E
F
G
H
J
K
FDR (Age associated divergence) =0.0851
p(Scalp)=0.0118
p(Dura)=0.817
Scalp
Dura

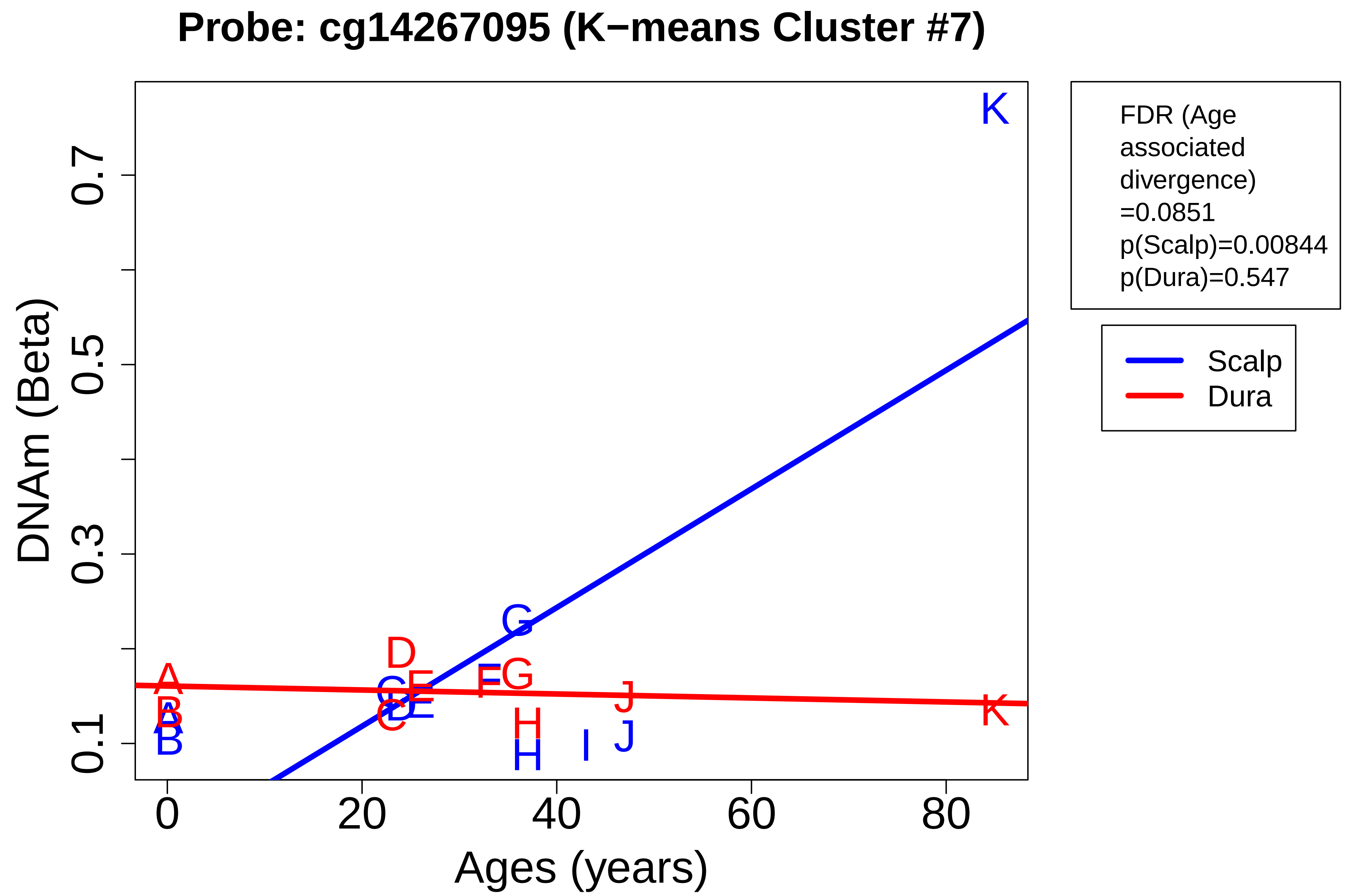

Probe: cg14267095 (K−means Cluster #7)
DNAm (Beta)
0.1
0.3
0.5
0.7
0
20
40
60
80
Ages (years)
FDR (Age associated divergence) =0.0851
p(Scalp)=0.00844
p(Dura)=0.547
Scalp
Dura

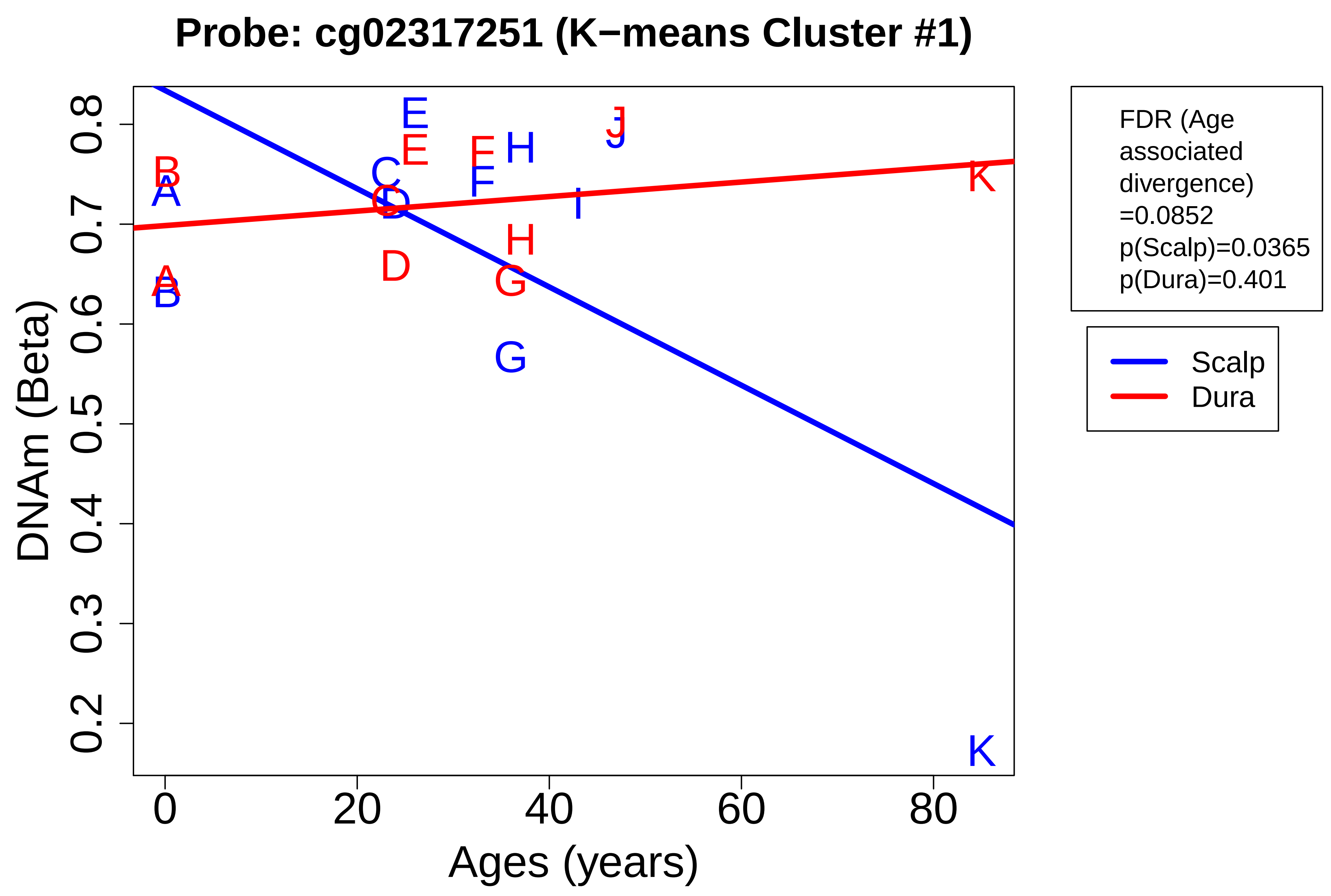

Probe: cg02317251 (K−means Cluster #1)
DNAm (Beta)
0.2
0.3
0.4
0.5
0.6
0.7
0.8
Ages (years)
0
20
40
60
80
FDR (Age associated divergence) =0.0852
p(Scalp)=0.0365
p(Dura)=0.401
Scalp
Dura

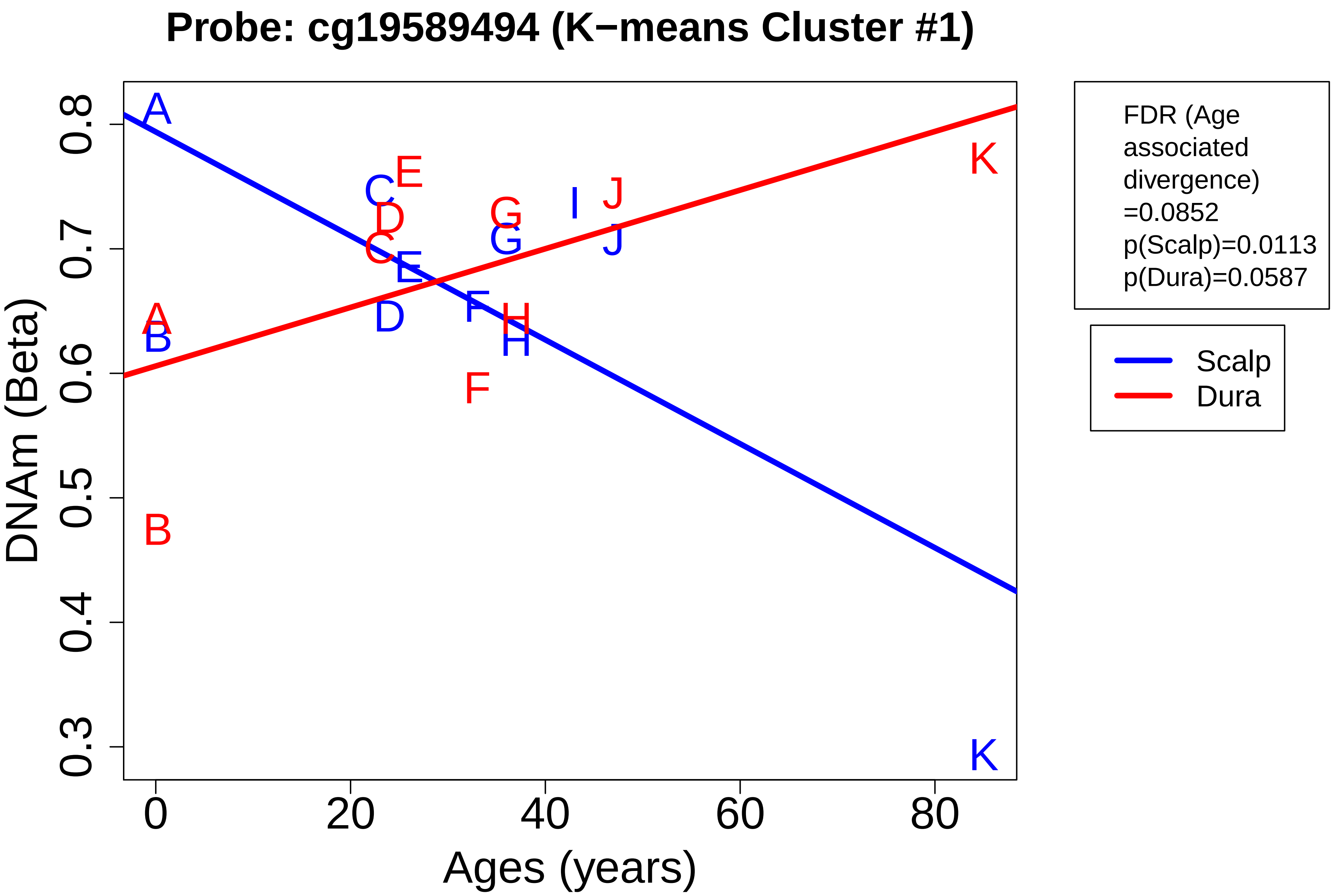

Probe: cg19589494 (K−means Cluster #1)
DNAm (Beta)
0.3
0.4
0.5
0.6
0.7
0.8
0
20
40
60
80
Ages (years)
FDR (Age associated divergence) =0.0852
p(Scalp)=0.0113
p(Dura)=0.0587
Scalp
Dura

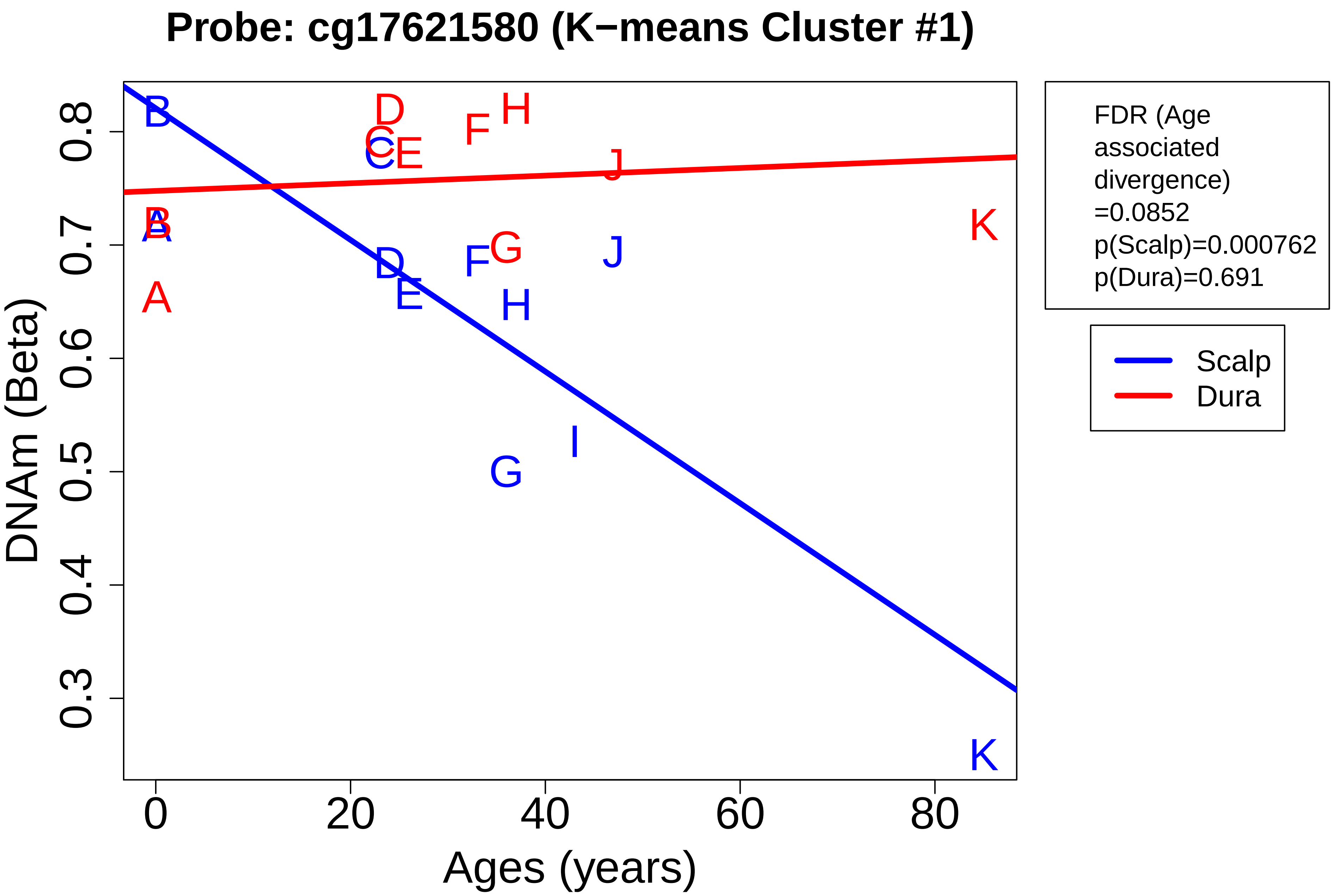

Probe: cg17621580 (K−means Cluster #1)
DNAm (Beta)
0.3
0.4
0.5
0.6
0.7
0.8
0
20
40
60
80
Ages (years)
FDR (Age associated divergence) =0.0852
p(Scalp)=0.000762
p(Dura)=0.691
Scalp
Dura

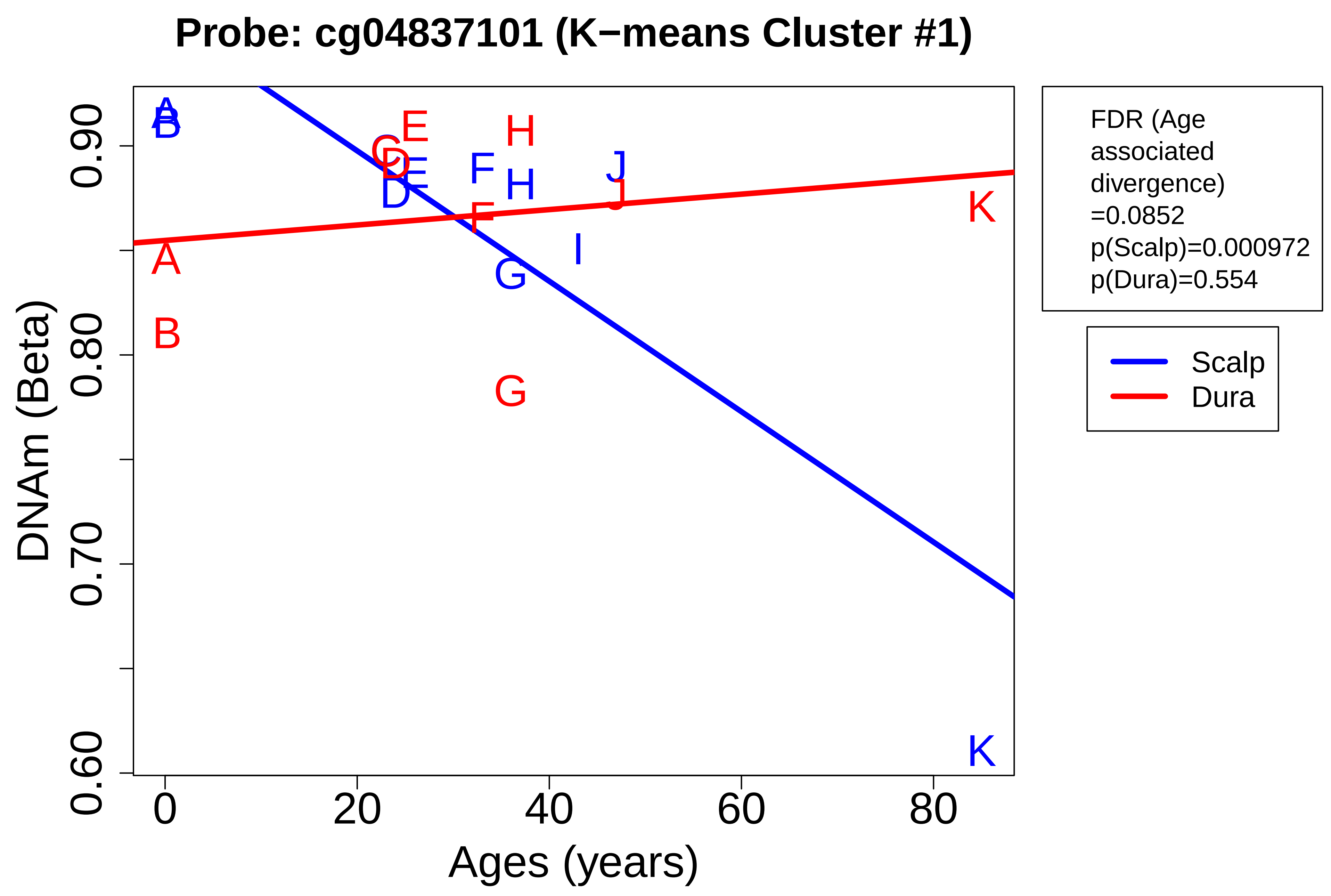

Probe: cg04837101 (K−means Cluster #1)
DNAm (Beta)
0.60
0.70
0.80
0.90
0
20
40
60
80
Ages (years)
A
B
C
D
E
F
G
H
I
J
K
FDR (Age associated divergence) =0.0852
p(Scalp)=0.000972
p(Dura)=0.554
Scalp
Dura

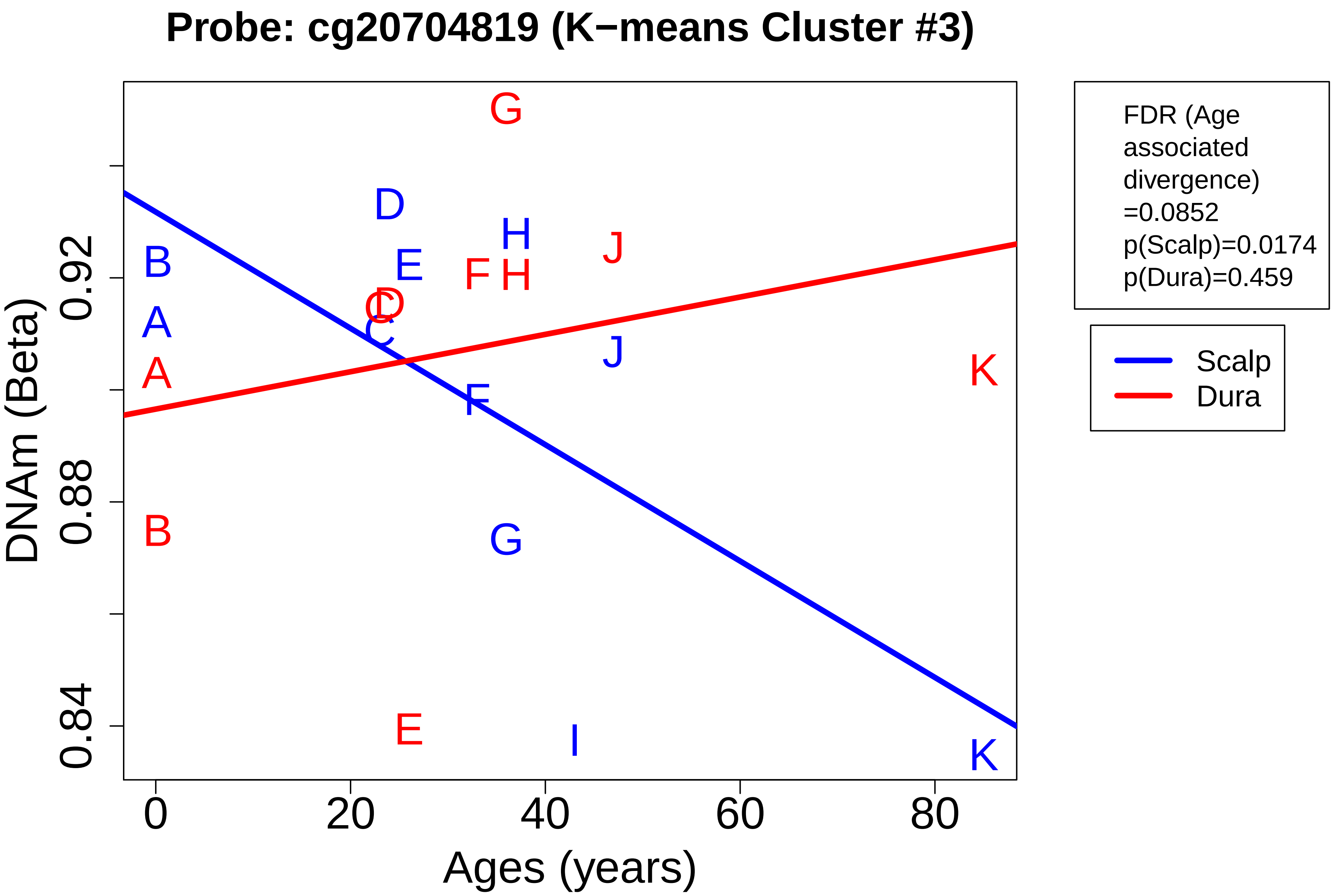

Probe: cg20704819 (K−means Cluster #3)
DNAm (Beta)
0.84
0.88
0.92
Ages (years)
0
20
40
60
80
FDR (Age associated divergence) =0.0852
p(Scalp)=0.0174
p(Dura)=0.459
Scalp
Dura

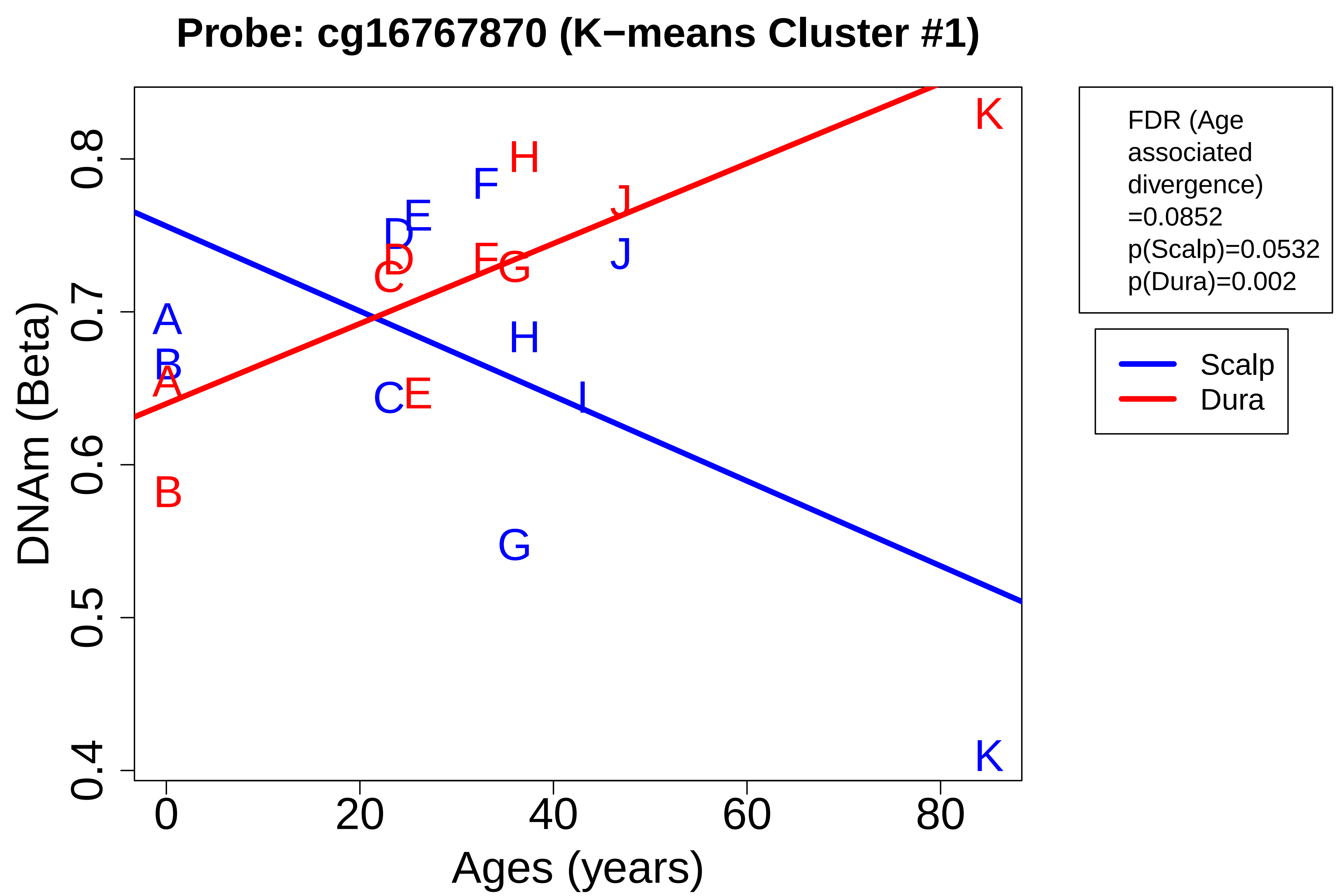

Probe: cg16767870 (K−means Cluster #1)
DNAm (Beta)
0.4
0.5
0.6
0.7
0.8
Ages (years)
0
20
40
60
80
FDR (Age associated divergence) =0.0852
p(Scalp)=0.0532
p(Dura)=0.002
Scalp
Dura

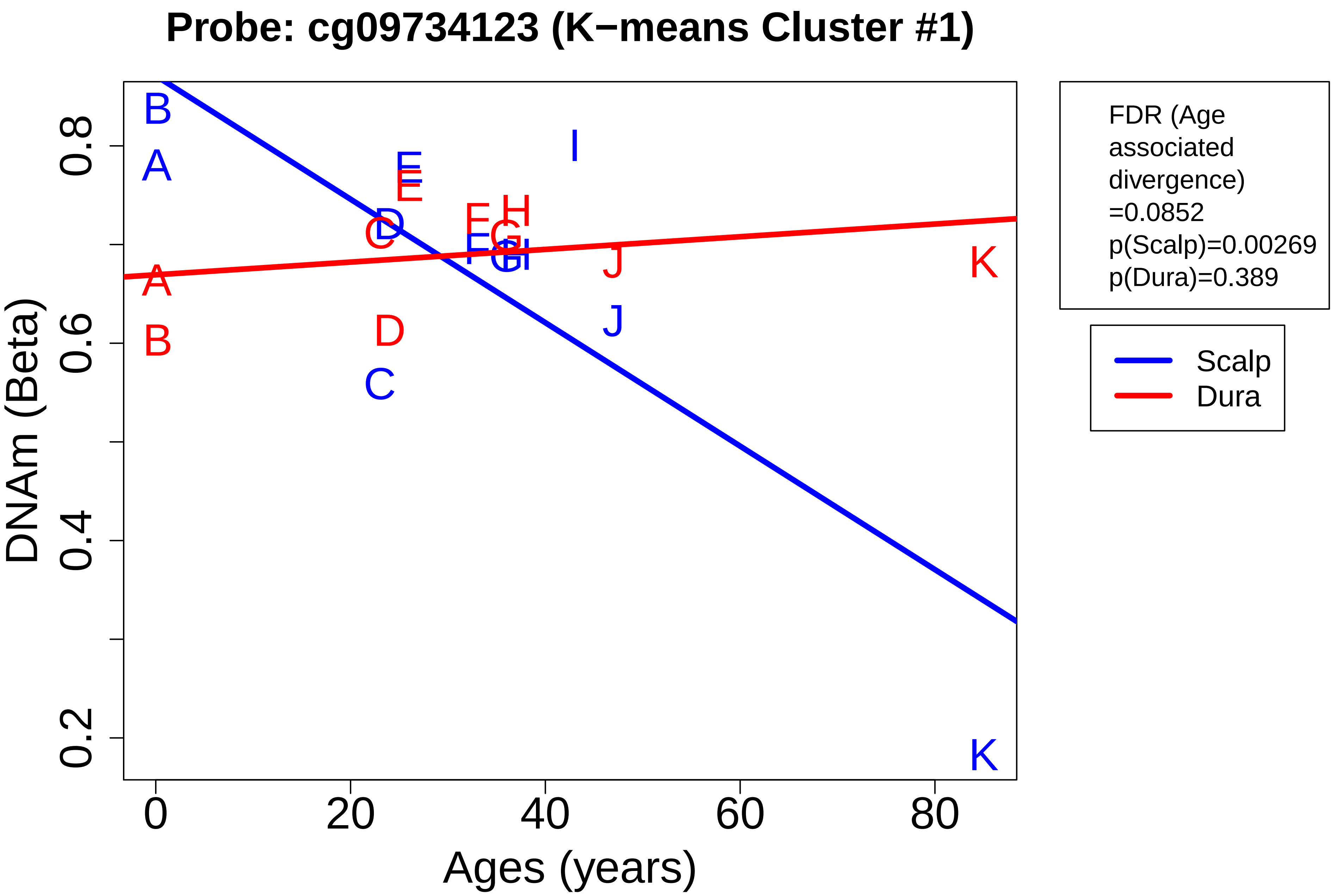

Probe: cg09734123 (K−means Cluster #1)
DNAm (Beta)
0.2
0.4
0.6
0.8
0
20
40
60
80
Ages (years)
FDR (Age associated divergence) =0.0852
p(Scalp)=0.00269
p(Dura)=0.389
Scalp
Dura

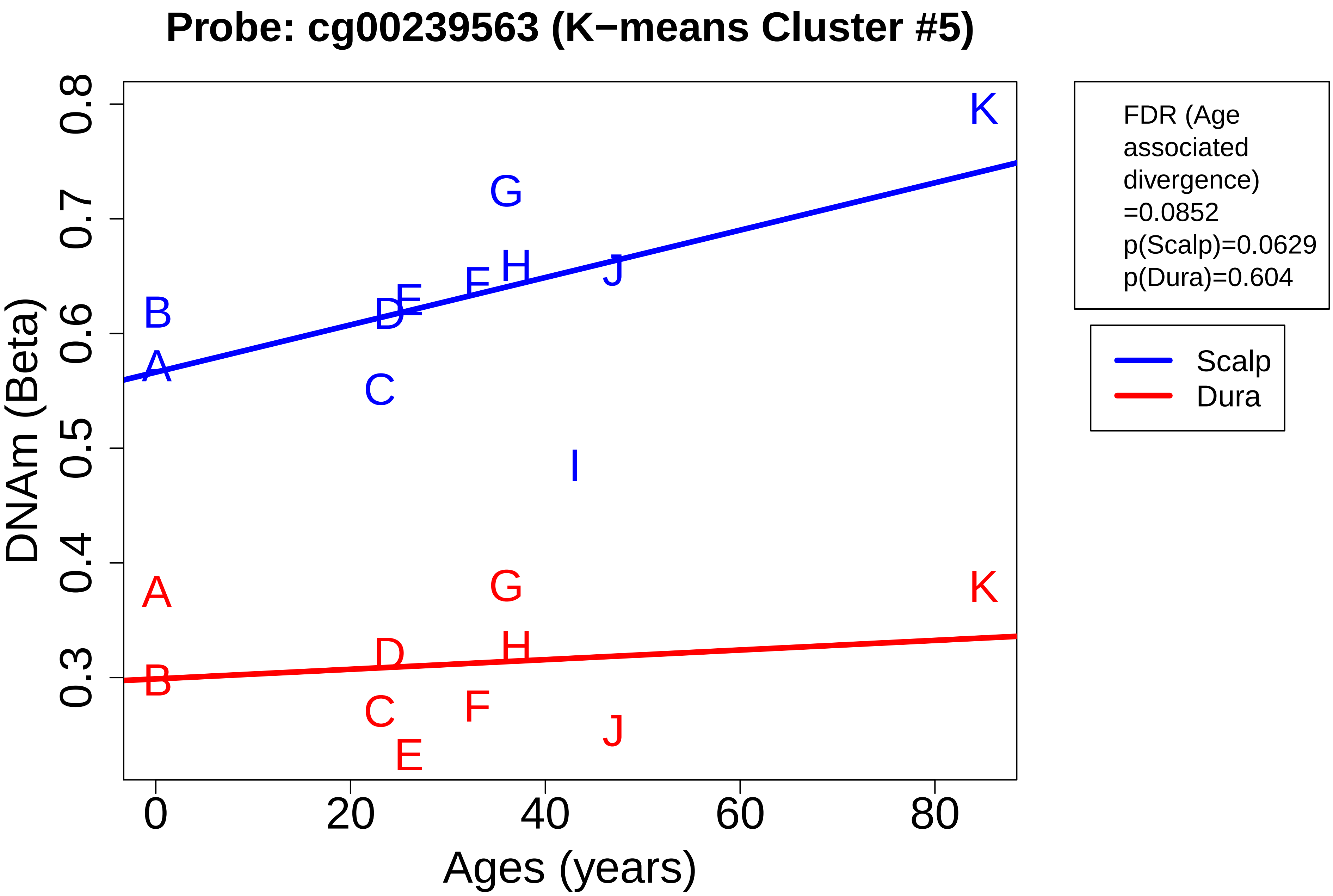
Probe: cg00239563 (K−means Cluster #5)
DNAm (Beta)
0.3
0.4
0.5
0.6
0.7
0.8
0
20
40
60
80
Ages (years)
A
B
C
D
E
F
G
H
I
J
K
FDR (Age associated divergence) =0.0852
p(Scalp)=0.0629
p(Dura)=0.604
Scalp
Dura

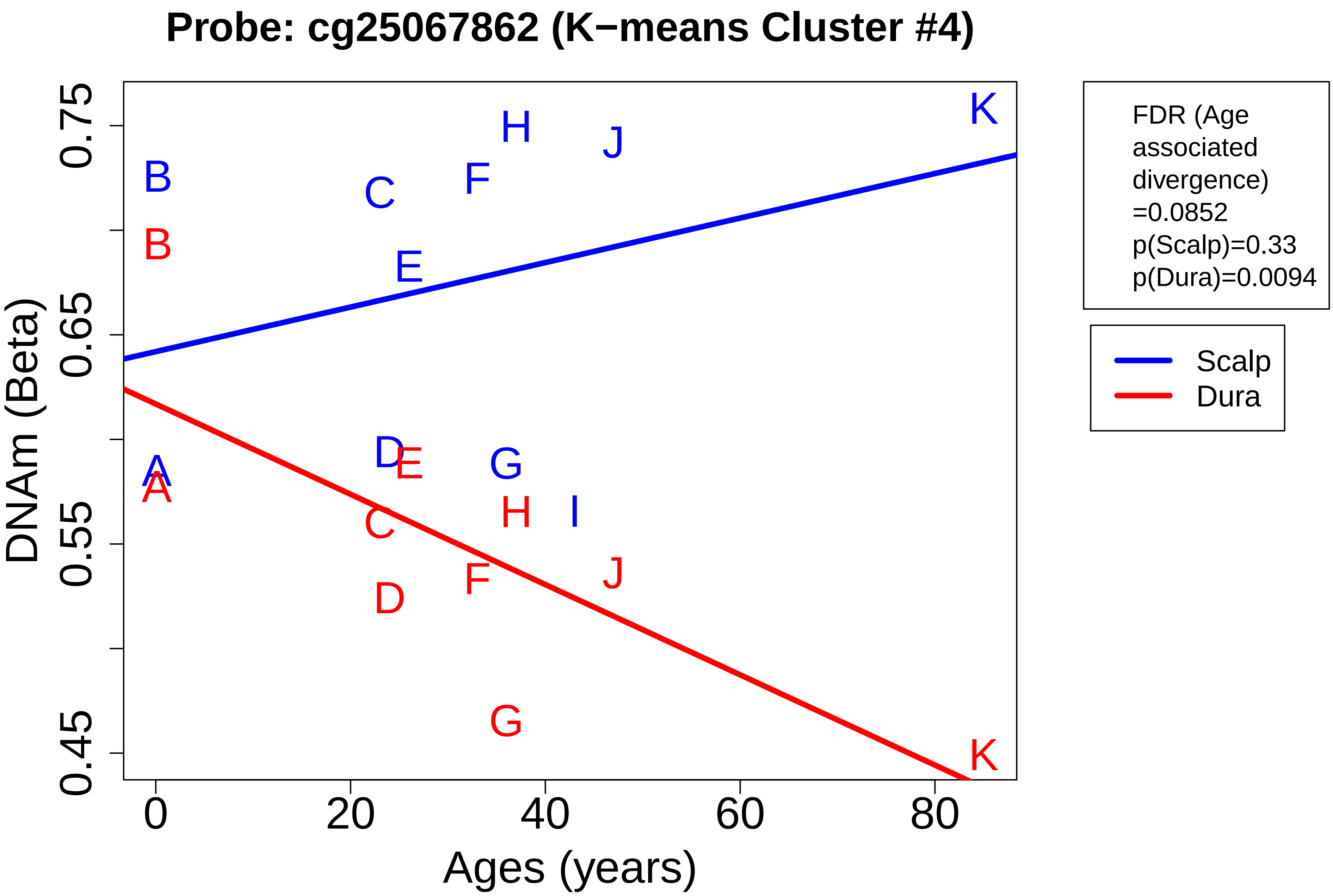
Probe: cg25067862 (K−means Cluster #4)
FDR (Age associated divergence) =0.0852
p(Scalp)=0.33
p(Dura)=0.0094
Scalp
Dura
DNAm (Beta)
0.45
0.55
0.65
0.75
Ages (years)
0
20
40
60
80
A
B
C
D
E
F
G
H
I
J
K

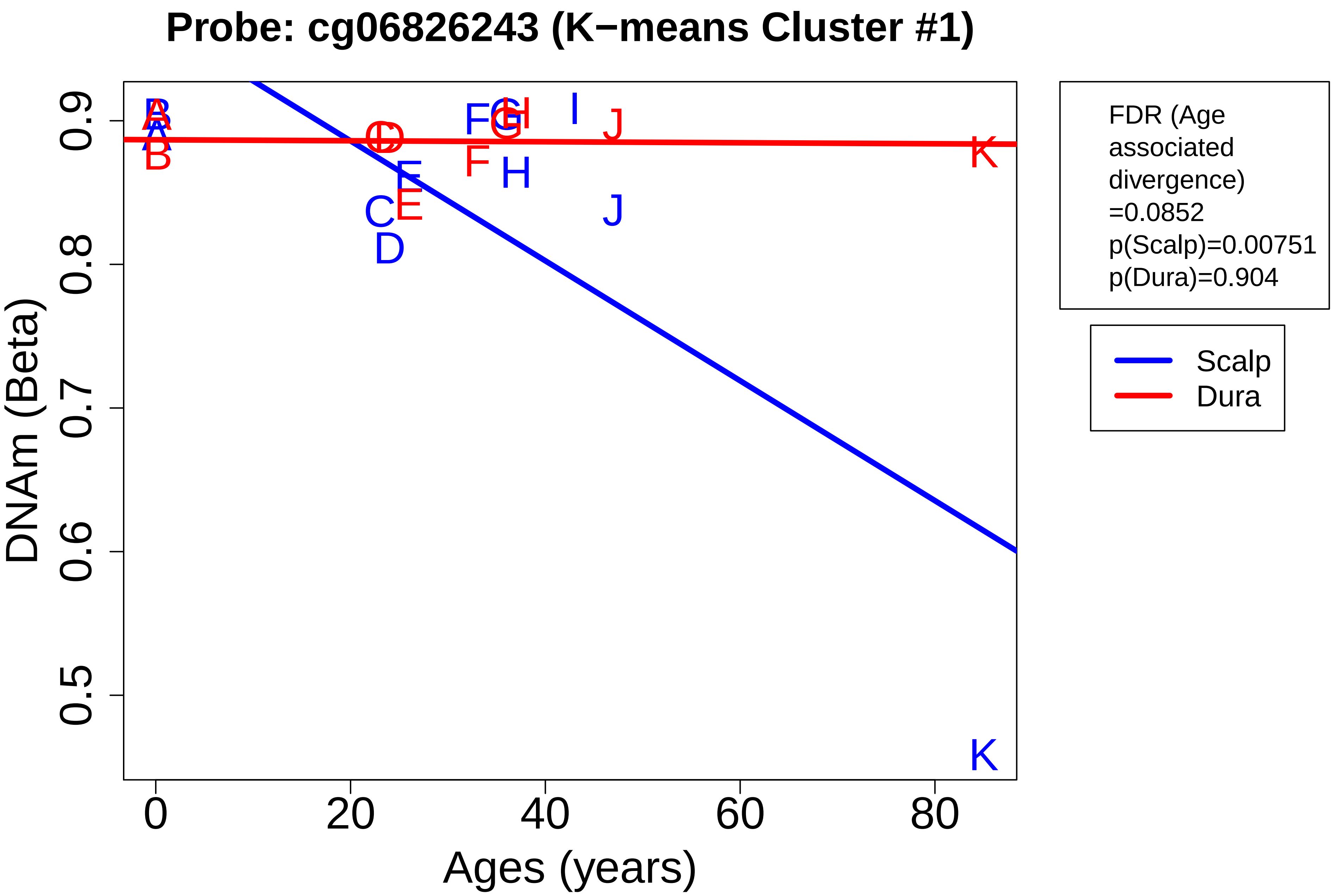
Probe: cg06826243 (K−means Cluster #1)
DNAm (Beta)
Ages (years)
0.5
0.6
0.7
0.8
0.9
0
20
40
60
80
FDR (Age associated divergence) =0.0852
p(Scalp)=0.00751
p(Dura)=0.904
Scalp
Dura

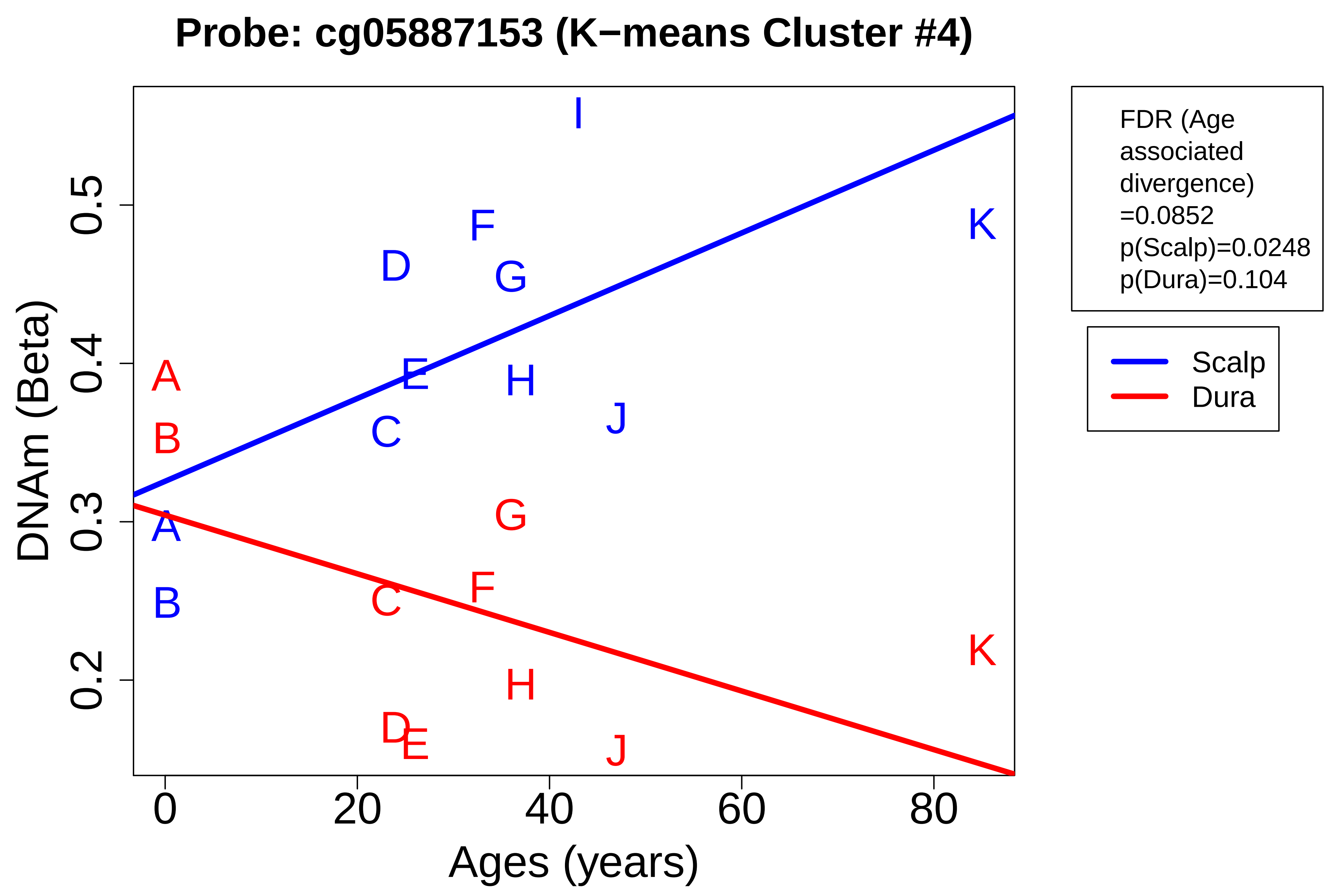

Probe: cg05887153 (K−means Cluster #4)
FDR (Age associated divergence) =0.0852
p(Scalp)=0.0248
p(Dura)=0.104
Scalp
Dura
DNAm (Beta)
0.2
0.3
0.4
0.5
0
20
40
60
80
Ages (years)
A
B
C
D
E
F
G
H
I
J
K

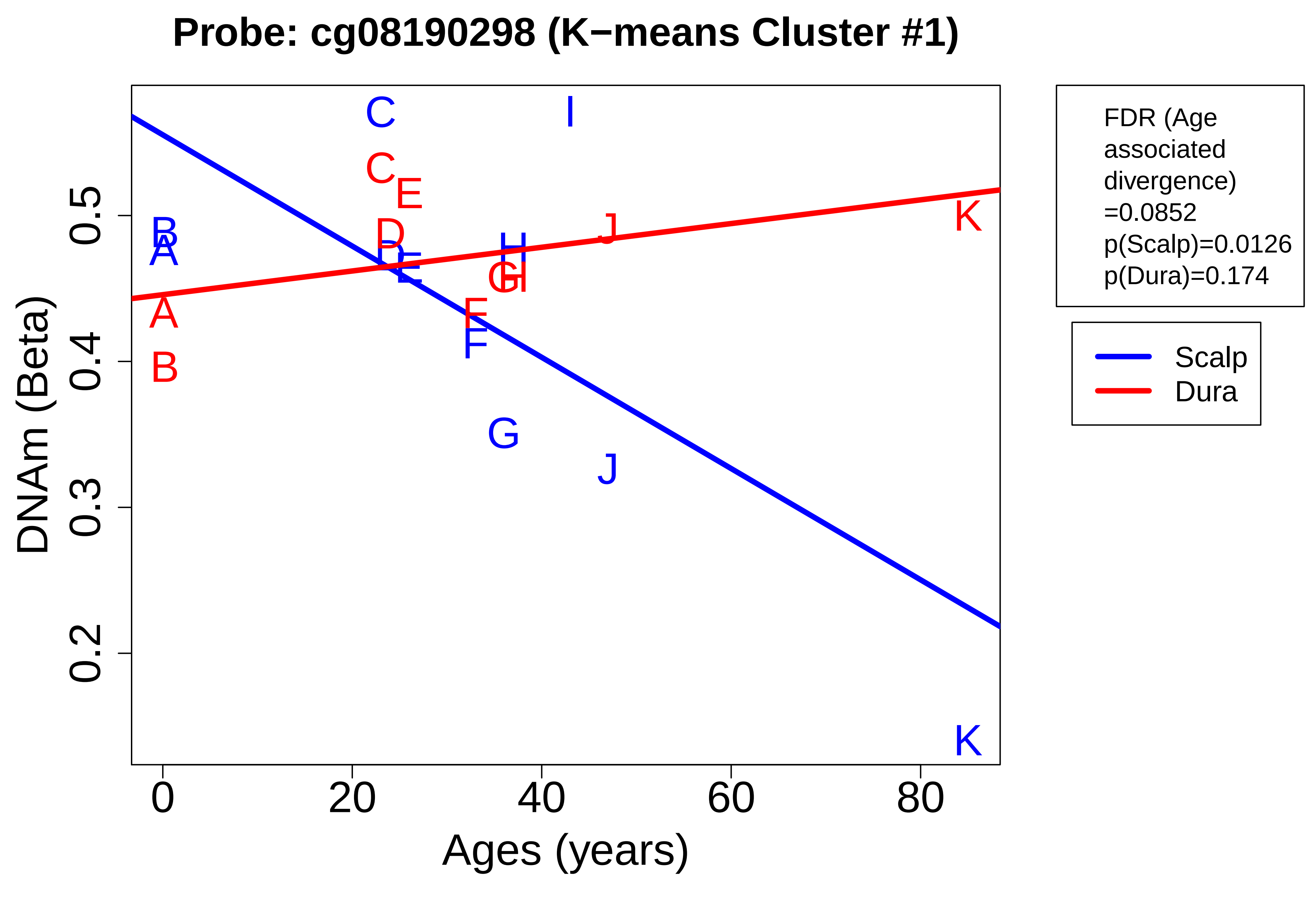

Probe: cg08190298 (K−means Cluster #1)
DNAm (Beta)
0.2
0.3
0.4
0.5
Ages (years)
0
20
40
60
80
A
B
C
D
E
F
G
H
I
J
K
FDR (Age associated divergence) =0.0852
p(Scalp)=0.0126
p(Dura)=0.174
Scalp
Dura

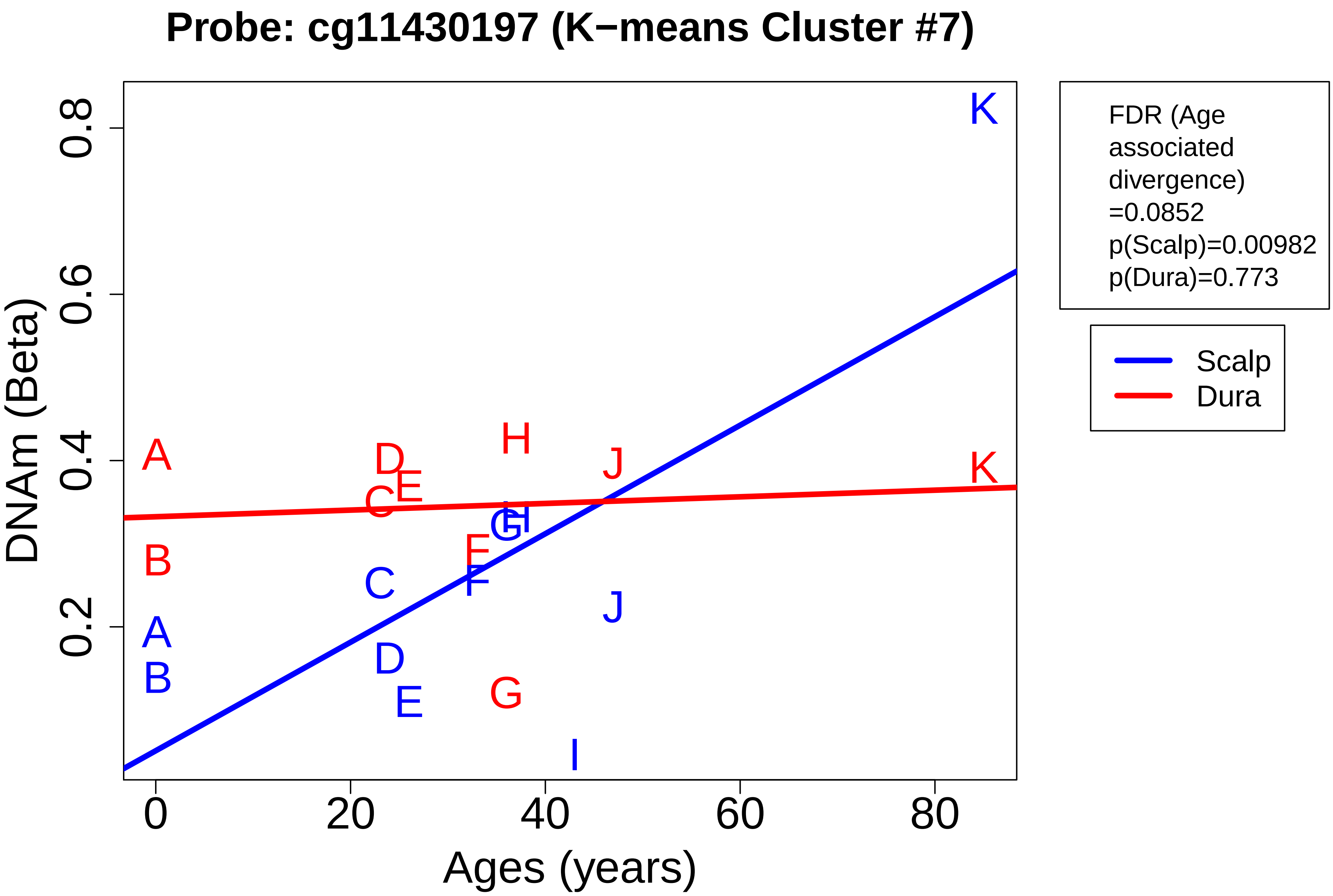
Probe: cg11430197 (K−means Cluster #7)
DNAm (Beta)
0.2
0.4
0.6
0.8
0
20
40
60
80
Ages (years)
FDR (Age associated divergence) =0.0852
p(Scalp)=0.00982
p(Dura)=0.773
Scalp
Dura

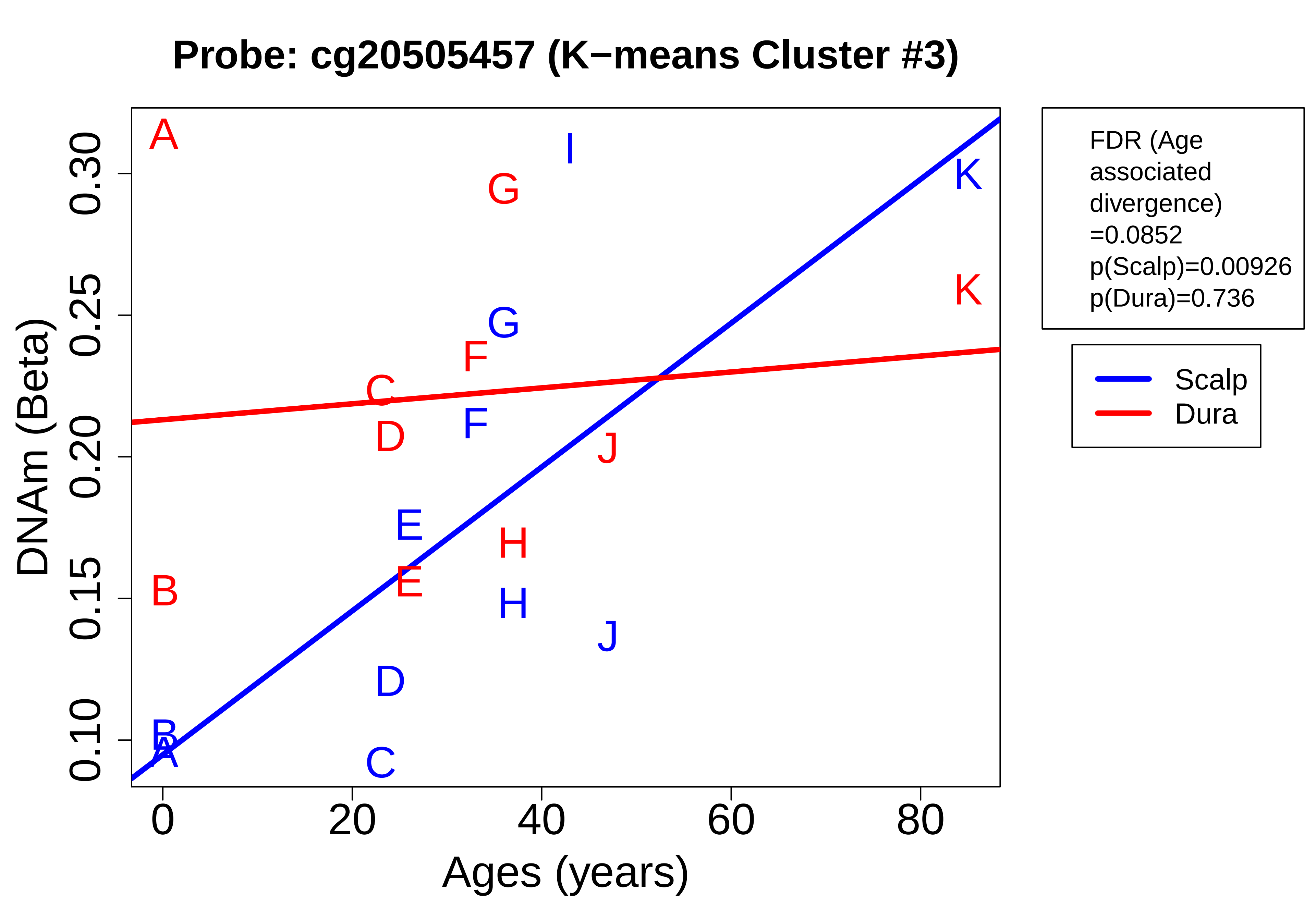
Probe: cg20505457 (K−means Cluster #3)
DNAm (Beta)
0.10
0.15
0.20
0.25
0.30
Ages (years)
0
20
40
60
80
FDR (Age associated divergence) =0.0852
p(Scalp)=0.00926
p(Dura)=0.736
Scalp
Dura
A
B
C
D
E
F
G
H
I
J
K

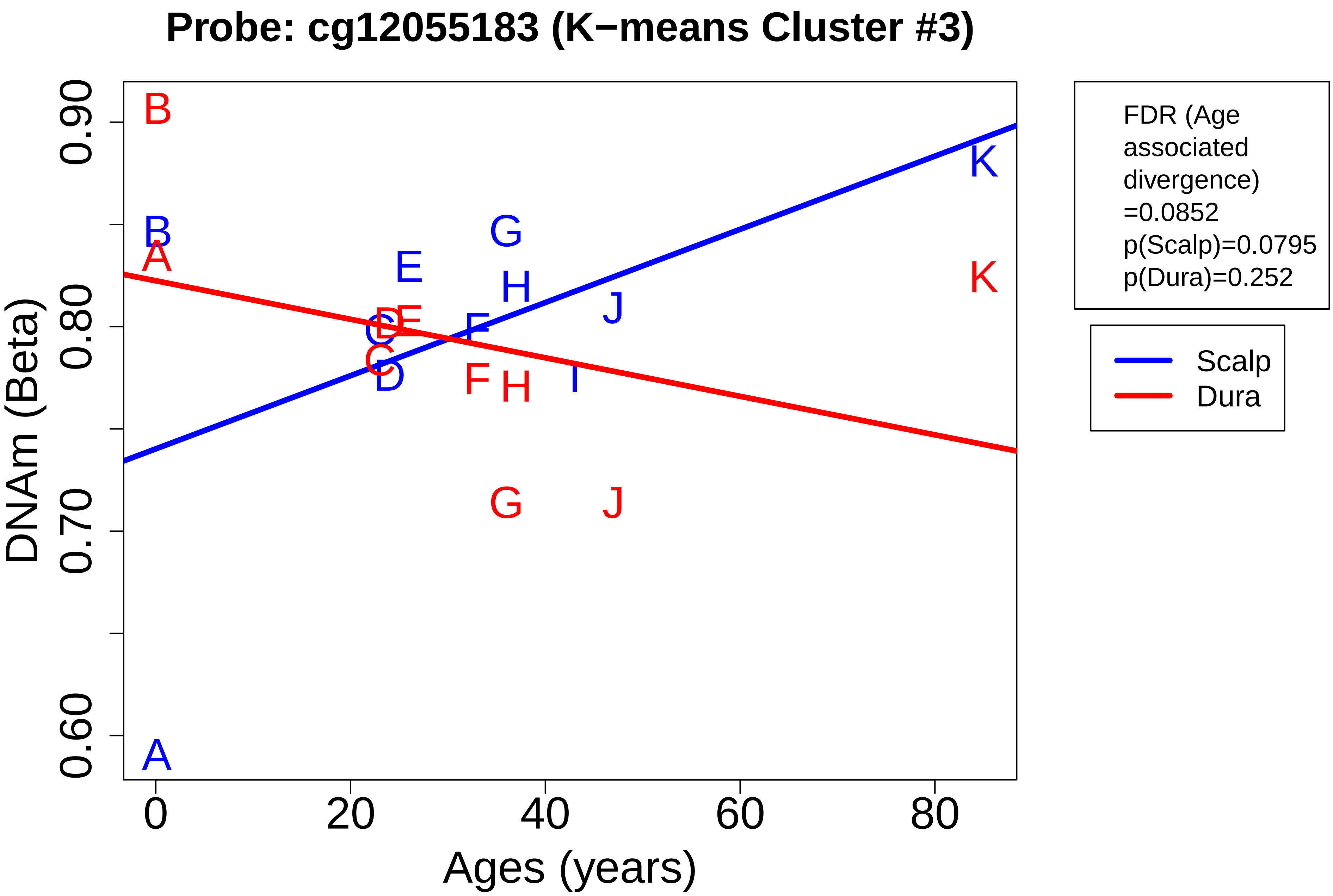

Probe: cg12055183 (K−means Cluster #3)
DNAm (Beta)
0.60
0.70
0.80
0.90
Ages (years)
0
20
40
60
80
FDR (Age associated divergence) =0.0852
p(Scalp)=0.0795
p(Dura)=0.252
Scalp
Dura

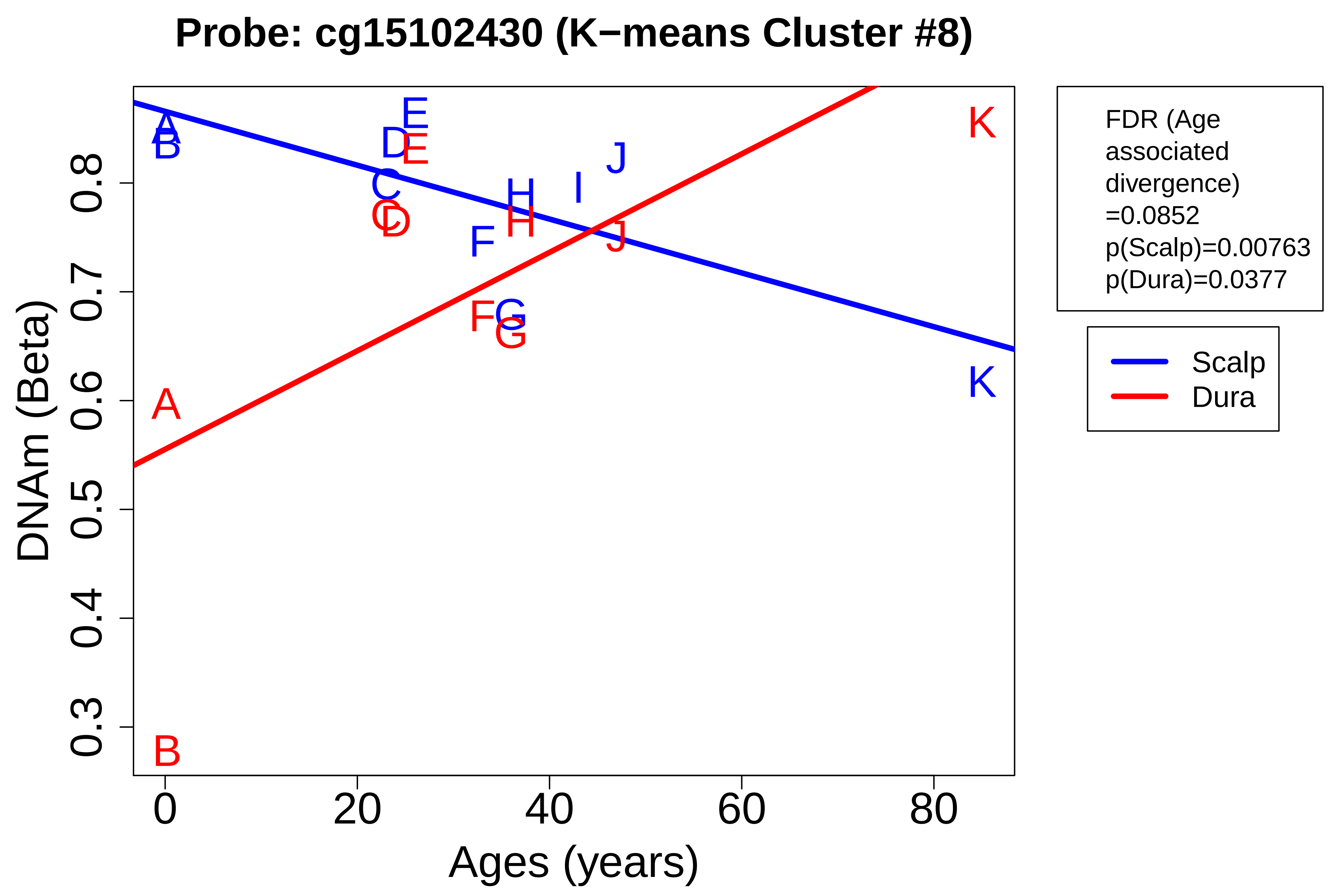

Probe: cg15102430 (K−means Cluster #8)
DNAm (Beta)
0.3
0.4
0.5
0.6
0.7
0.8
0
20
40
60
80
Ages (years)
FDR (Age associated divergence) =0.0852
p(Scalp)=0.00763
p(Dura)=0.0377
Scalp
Dura

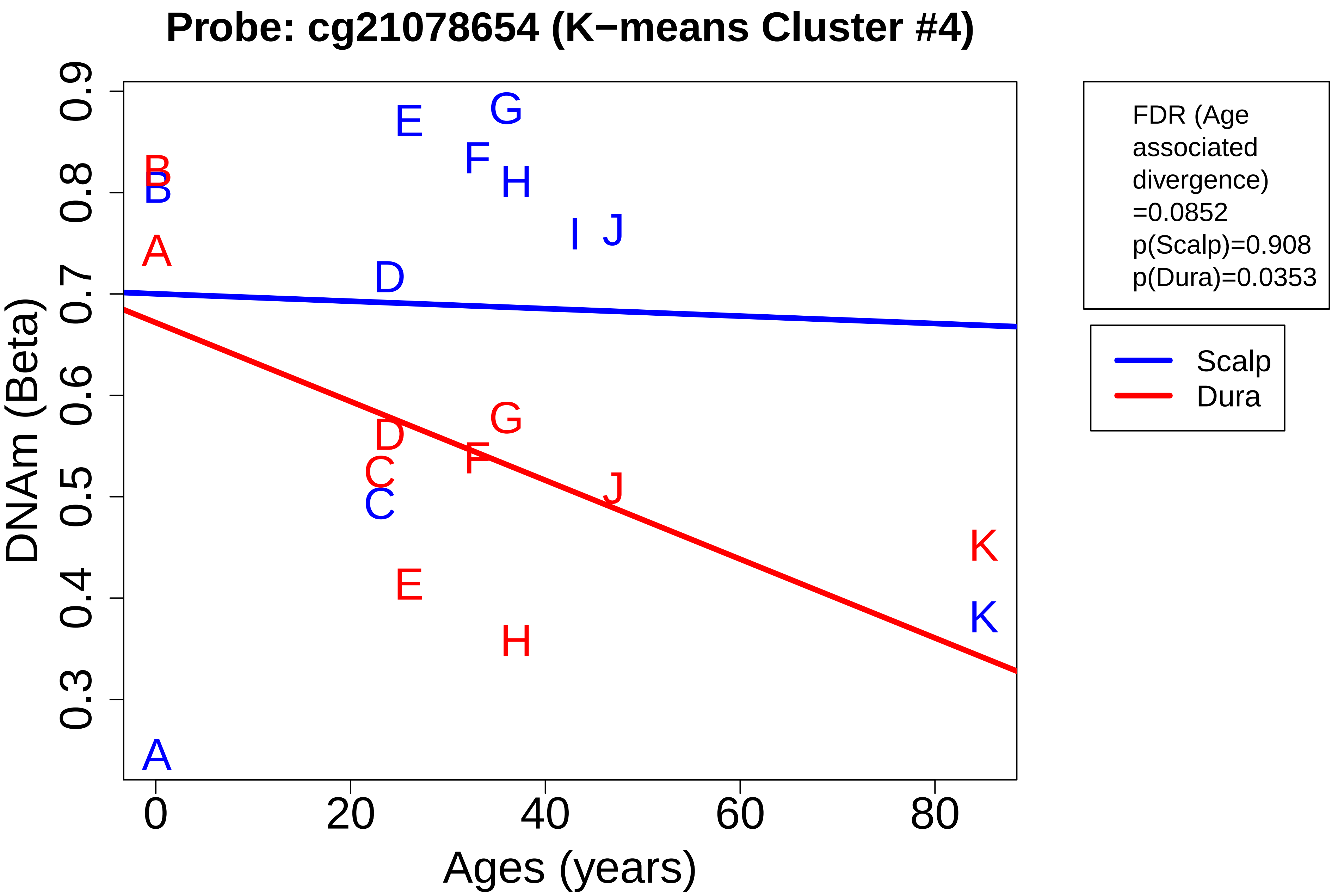

Probe: cg21078654 (K−means Cluster #4)
DNAm (Beta)
0.3
0.4
0.5
0.6
0.7
0.8
0.9
0
20
40
60
80
Ages (years)
FDR (Age associated divergence) =0.0852
p(Scalp)=0.908
p(Dura)=0.0353
Scalp
Dura

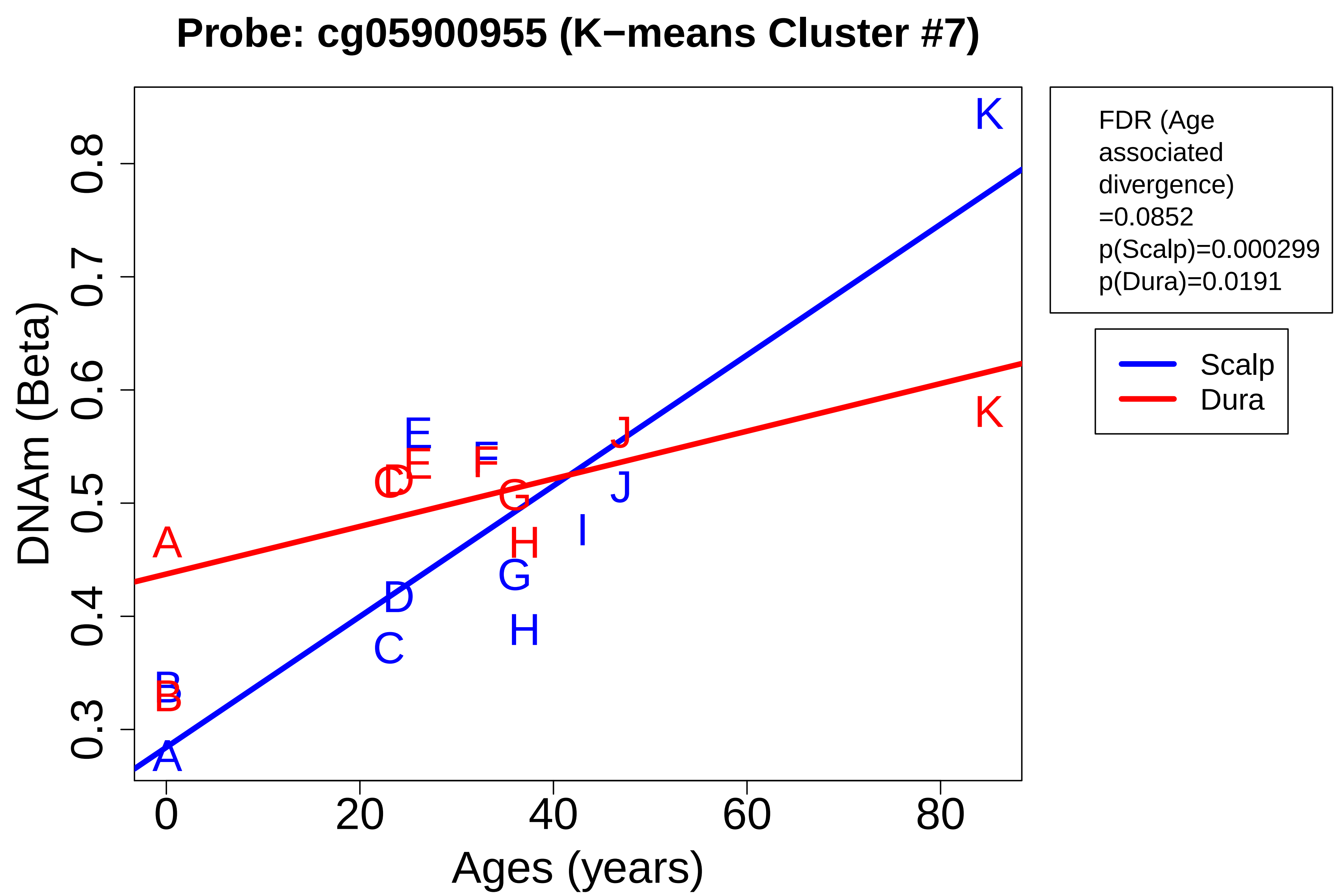

Probe: cg05900955 (K−means Cluster #7)
FDR (Age associated divergence) =0.0852
p(Scalp)=0.000299
p(Dura)=0.0191
Scalp
Dura
DNAm (Beta)
0.3
0.4
0.5
0.6
0.7
0.8
0
20
40
60
80
Ages (years)
A
B
C
D
E
F
G
H
I
J
K

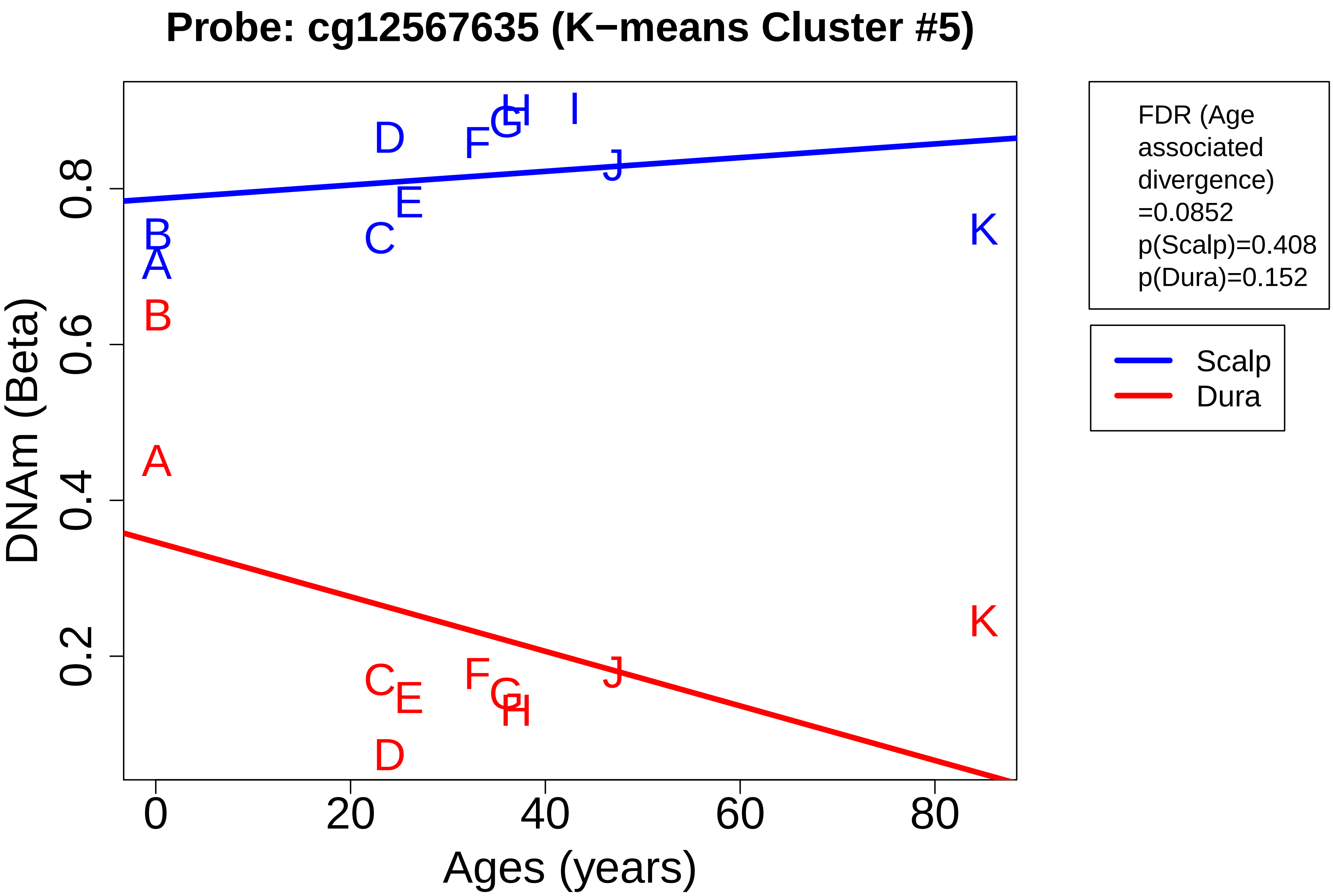

Probe: cg12567635 (K−means Cluster #5)
DNAm (Beta)
0.2
0.4
0.6
0.8
0
20
40
60
80
Ages (years)
FDR (Age associated divergence) =0.0852
p(Scalp)=0.408
p(Dura)=0.152
Scalp
Dura

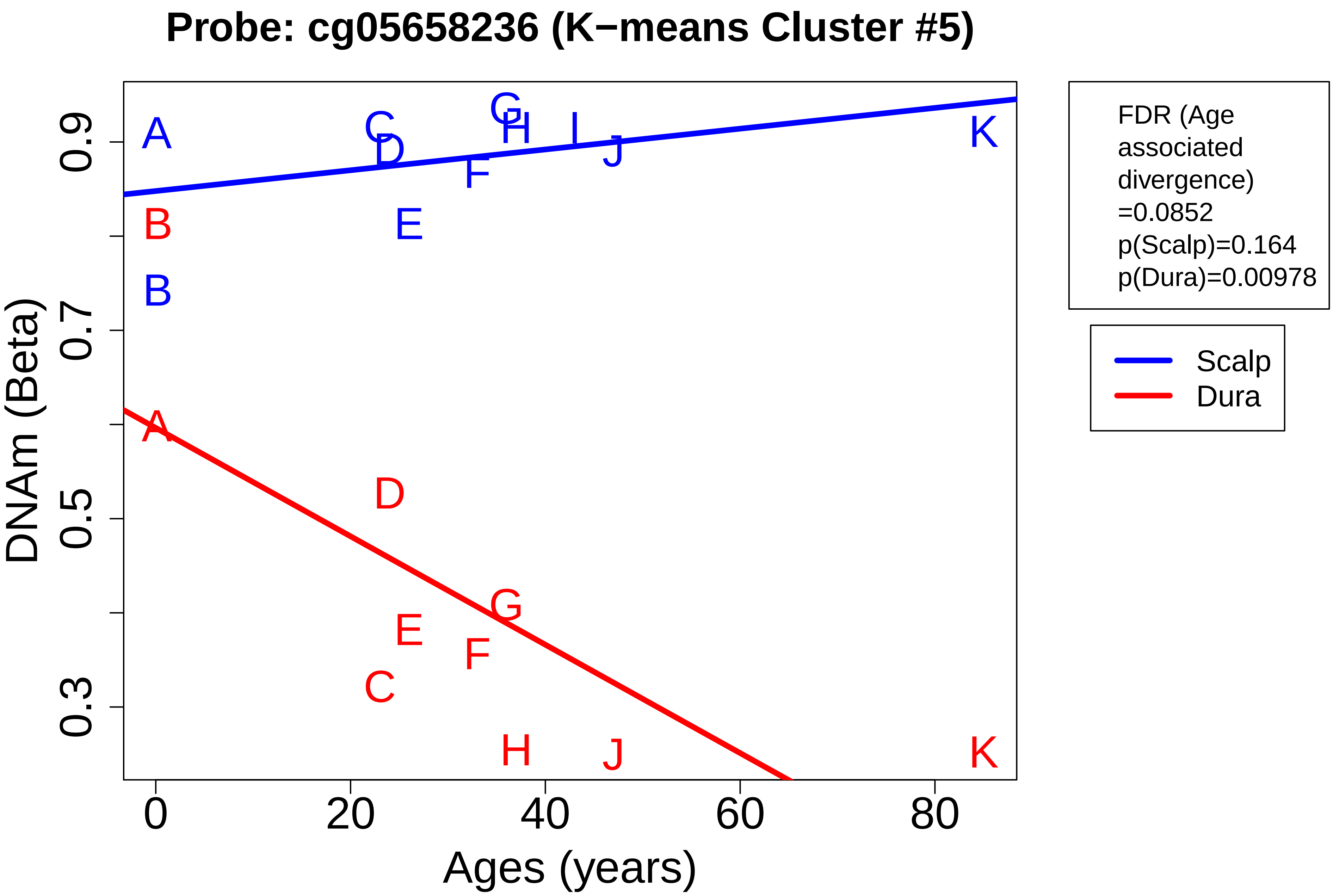

Probe: cg05658236 (K−means Cluster #5)
DNAm (Beta)
0.3
0.5
0.7
0.9
0
20
40
60
80
Ages (years)
FDR (Age associated divergence) =0.0852
p(Scalp)=0.164
p(Dura)=0.00978
Scalp
Dura

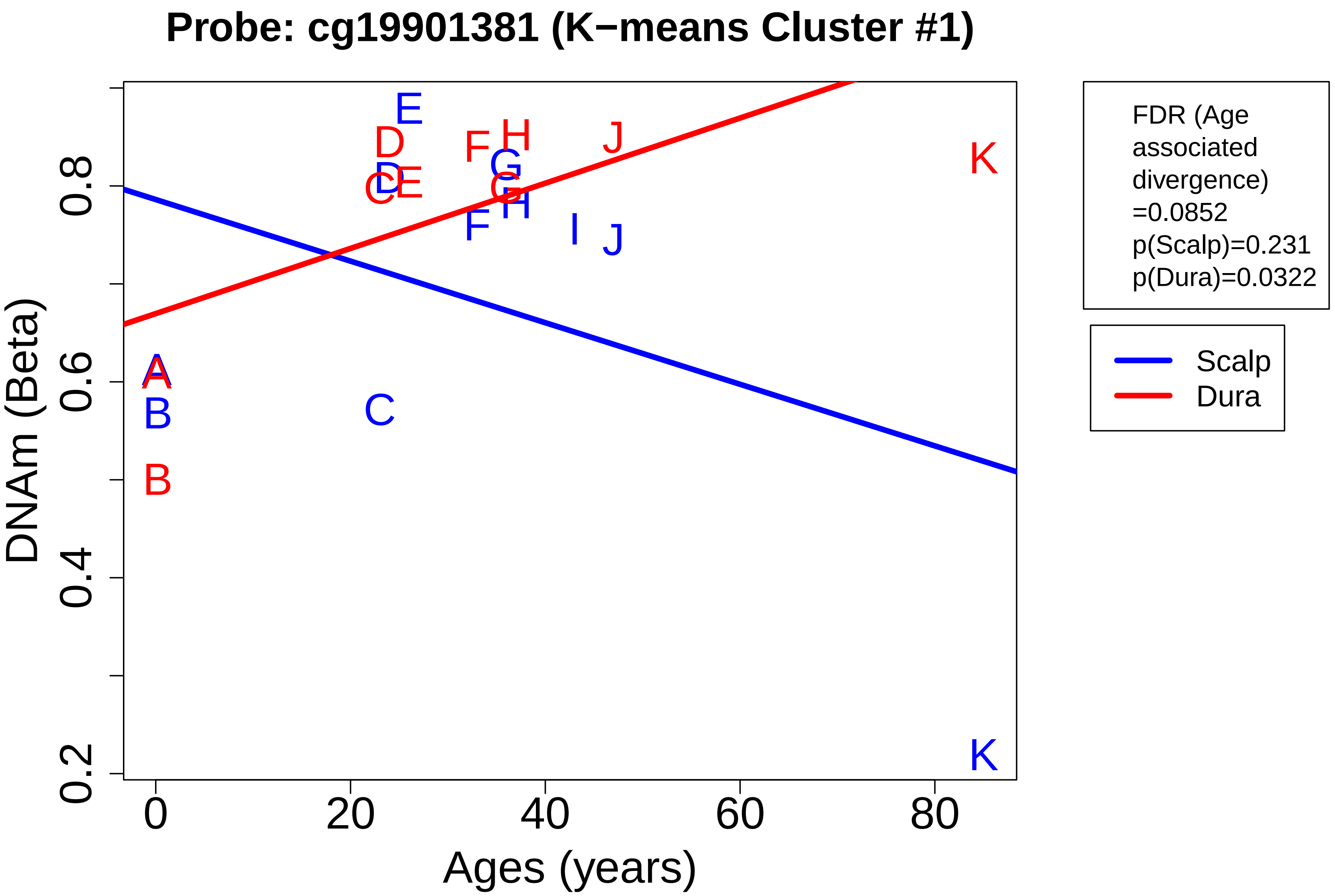

Probe: cg19901381 (K−means Cluster #1)
DNAm (Beta)
0.2
0.4
0.6
0.8
0
20
40
60
80
Ages (years)
FDR (Age associated divergence) =0.0852
p(Scalp)=0.231
p(Dura)=0.0322
Scalp
Dura
A
B
C
D
E
F
G
H
I
J
K

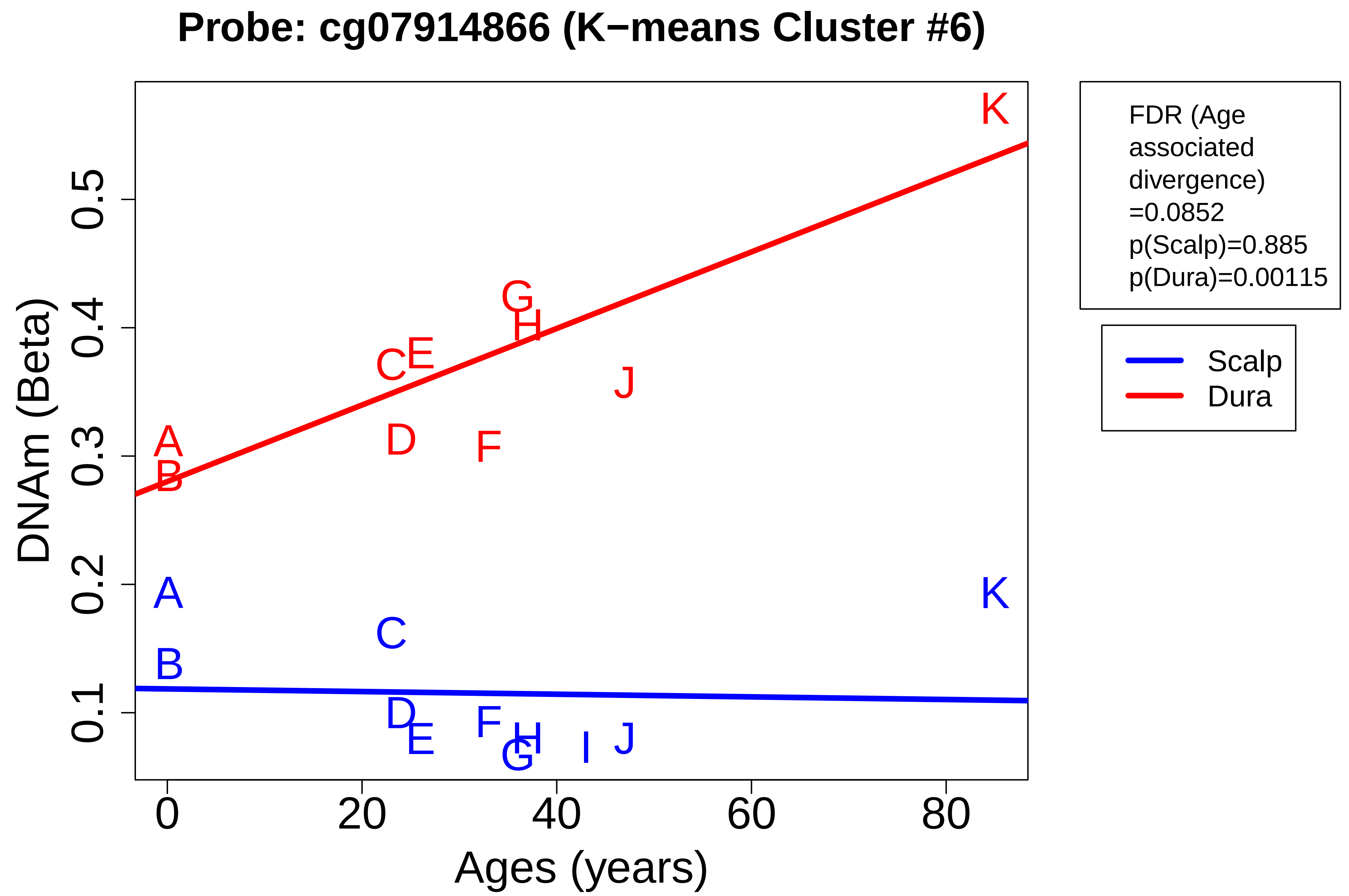

Probe: cg07914866 (K−means Cluster #6)
DNAm (Beta)
0.1
0.2
0.3
0.4
0.5
0
20
40
60
80
Ages (years)
A
B
C
D
E
F
G
H
J
K
A
B
C
D
E
F
G
H
I
J
K
FDR (Age associated divergence) =0.0852
p(Scalp)=0.885
p(Dura)=0.00115
Scalp
Dura

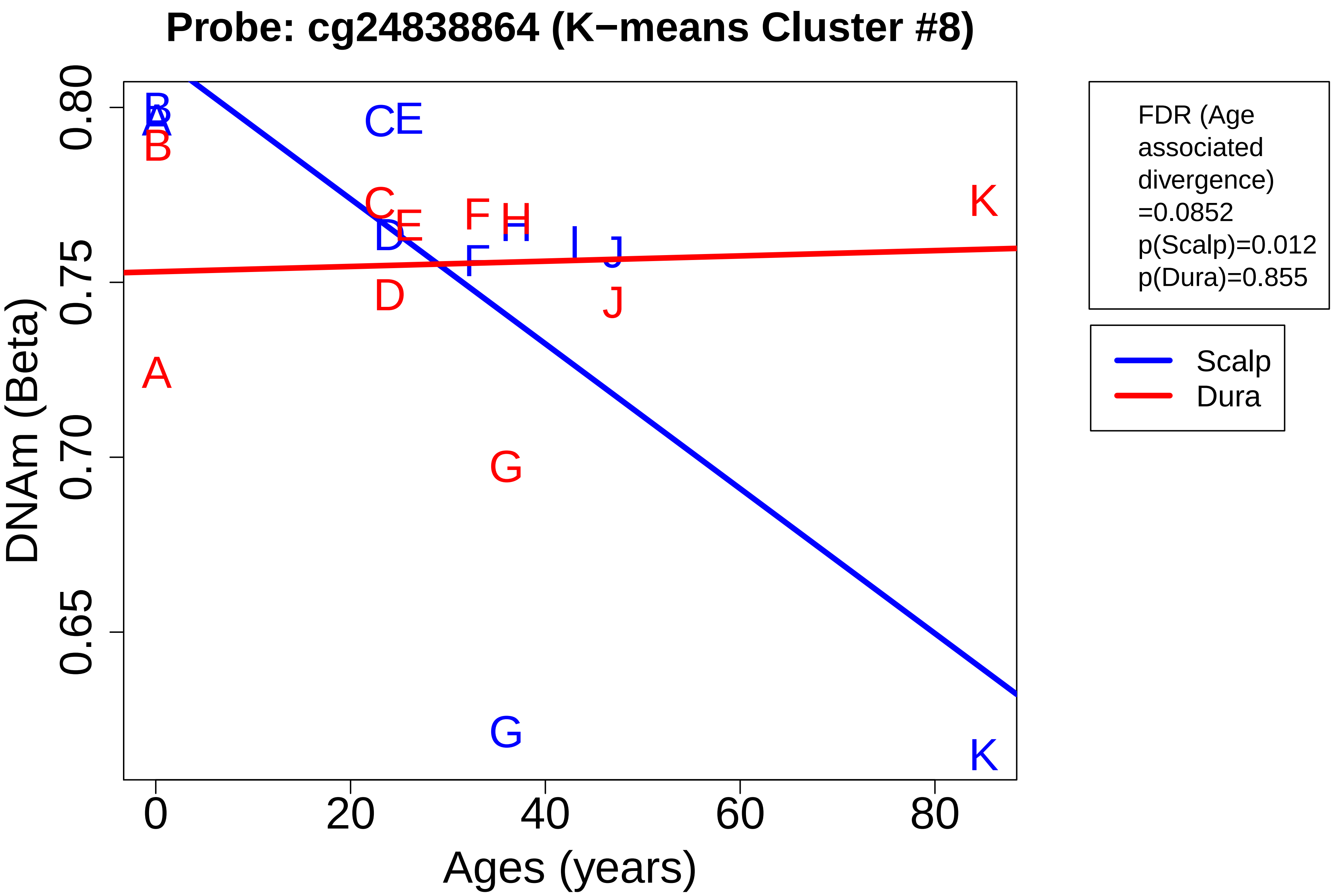
Probe: cg24838864 (K−means Cluster #8)
DNAm (Beta)
0.65
0.70
0.75
0.80
0
20
40
60
80
Ages (years)
FDR (Age associated divergence) =0.0852
p(Scalp)=0.012
p(Dura)=0.855
Scalp
Dura
A
B
C
D
E
F
G
H
I
J
K

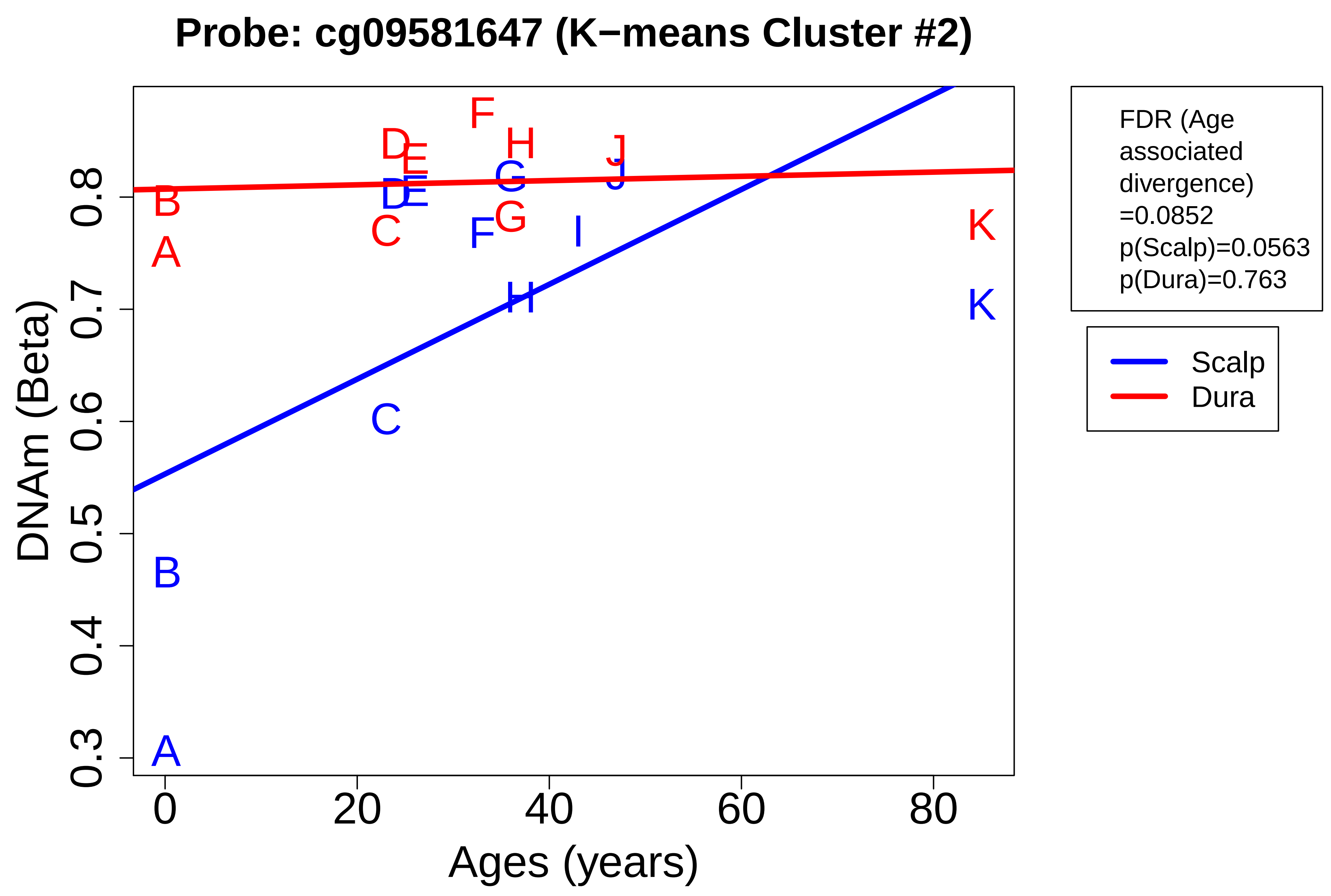

Probe: cg09581647 (K−means Cluster #2)
DNAm (Beta)
0.3
0.4
0.5
0.6
0.7
0.8
0
20
40
60
80
Ages (years)
FDR (Age associated divergence) =0.0852
p(Scalp)=0.0563
p(Dura)=0.763
Scalp
Dura

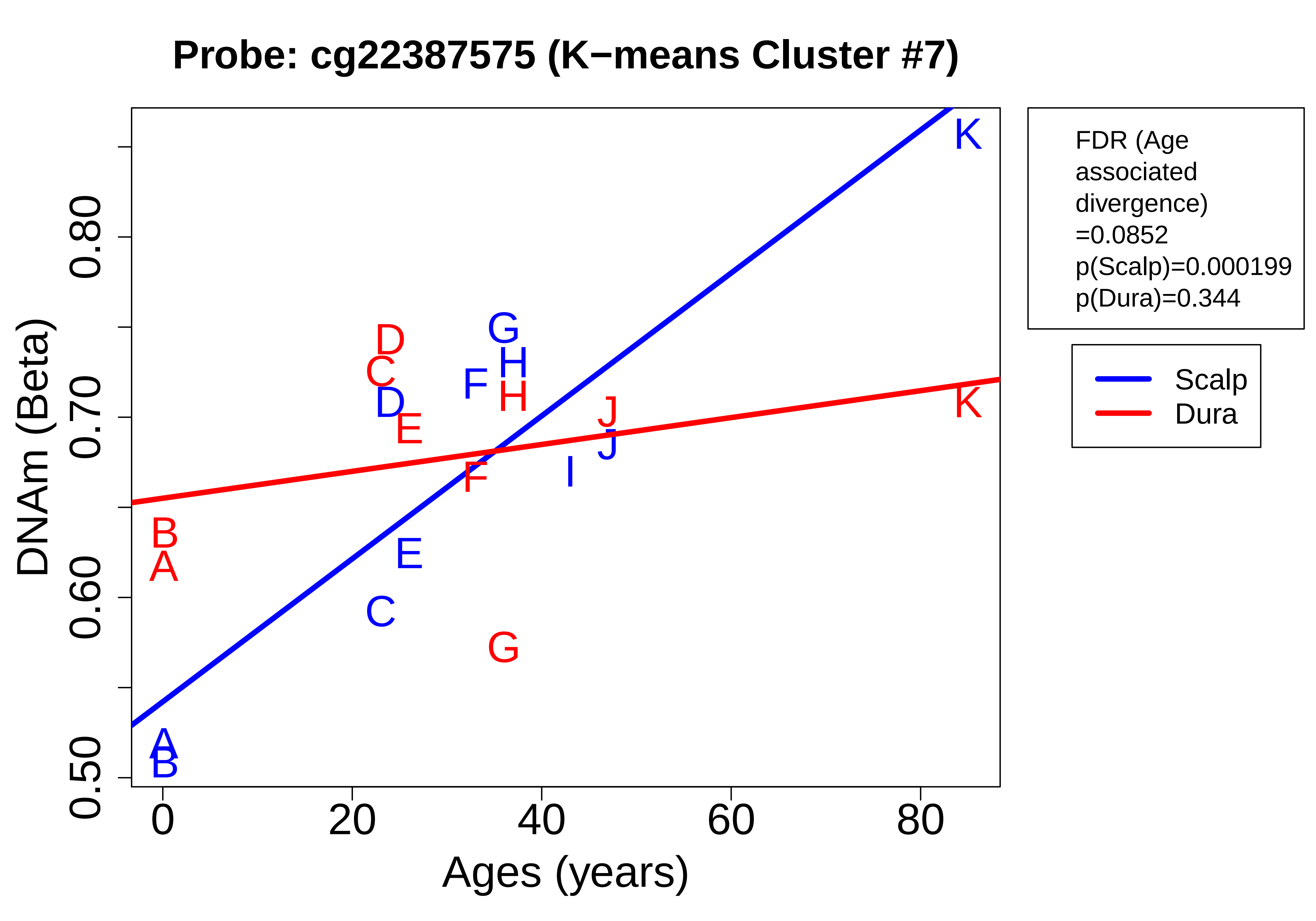
Probe: cg22387575 (K−means Cluster #7)
DNAm (Beta)
0.50
0.60
0.70
0.80
0
20
40
60
80
Ages (years)
FDR (Age associated divergence) =0.0852
p(Scalp)=0.000199
p(Dura)=0.344
Scalp
Dura
A
B
C
D
E
F
G
H
I
J
K

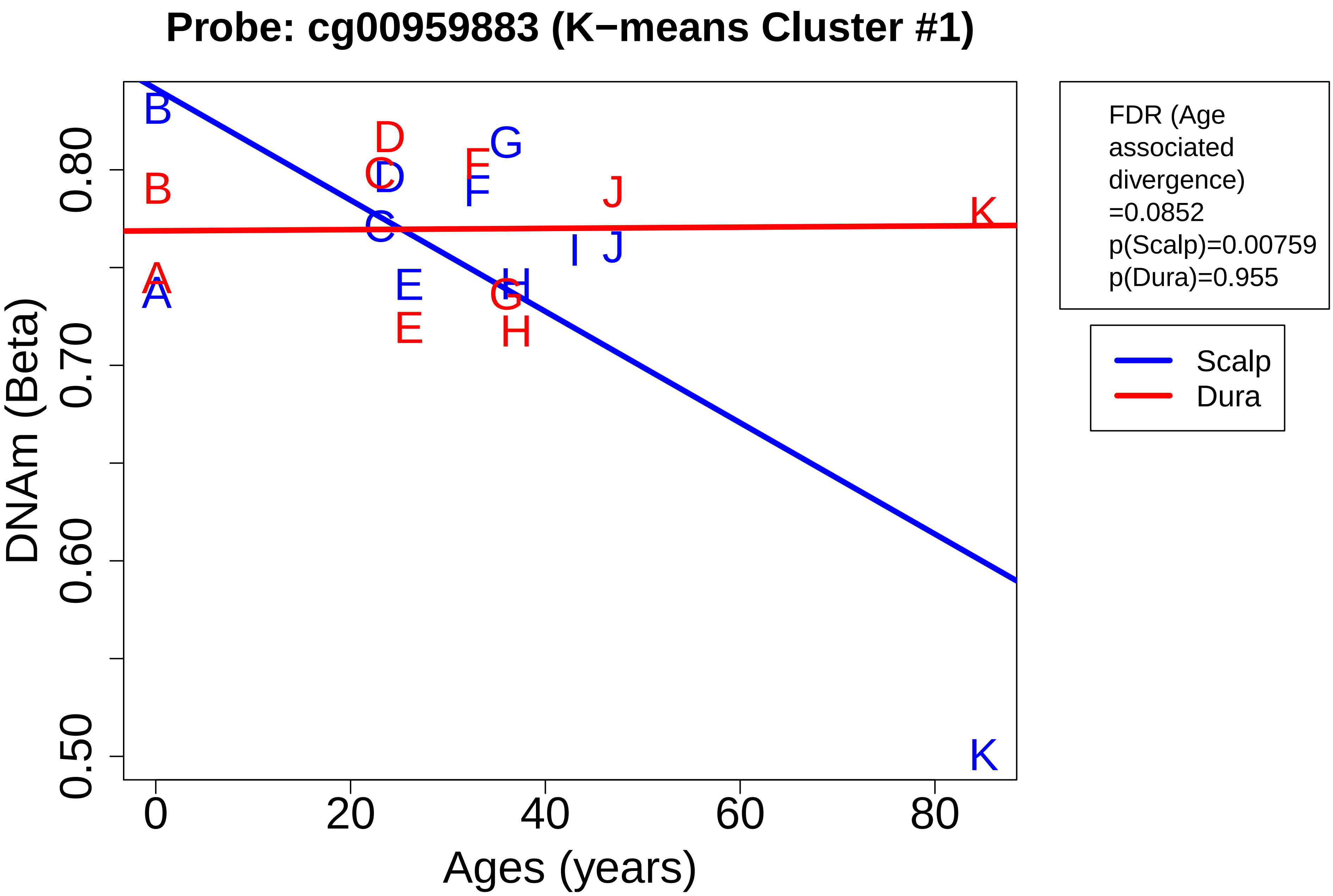
Probe: cg00959883 (K−means Cluster #1)
DNAm (Beta)
0.50
0.60
0.70
0.80
Ages (years)
0
20
40
60
80
FDR (Age associated divergence) =0.0852
p(Scalp)=0.00759
p(Dura)=0.955
Scalp
Dura

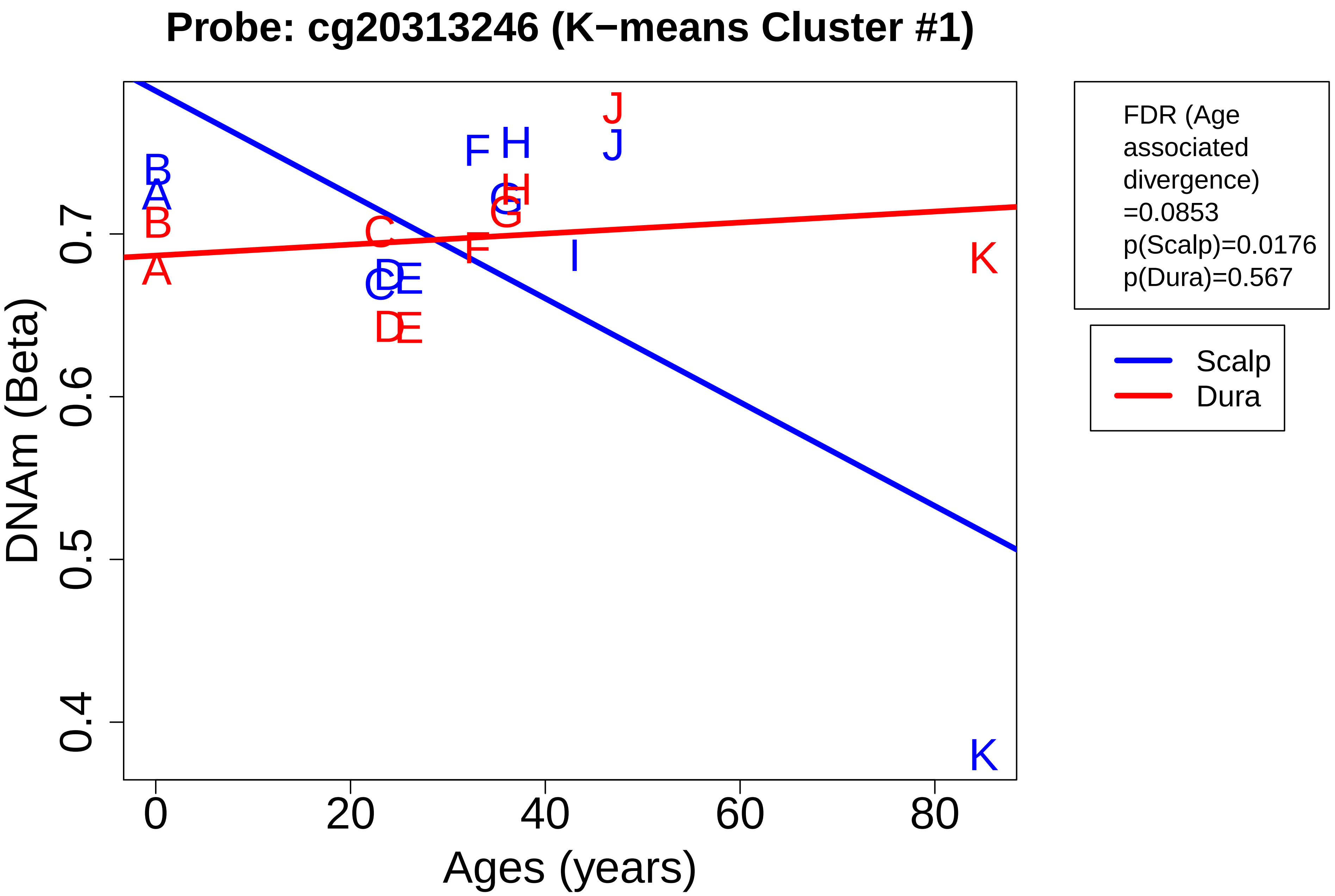

Probe: cg20313246 (K−means Cluster #1)
DNAm (Beta)
0.4
0.5
0.6
0.7
0
20
40
60
80
Ages (years)
A
B
C
D
E
F
G
H
I
J
K
FDR (Age associated divergence) =0.0853
p(Scalp)=0.0176
p(Dura)=0.567
Scalp
Dura

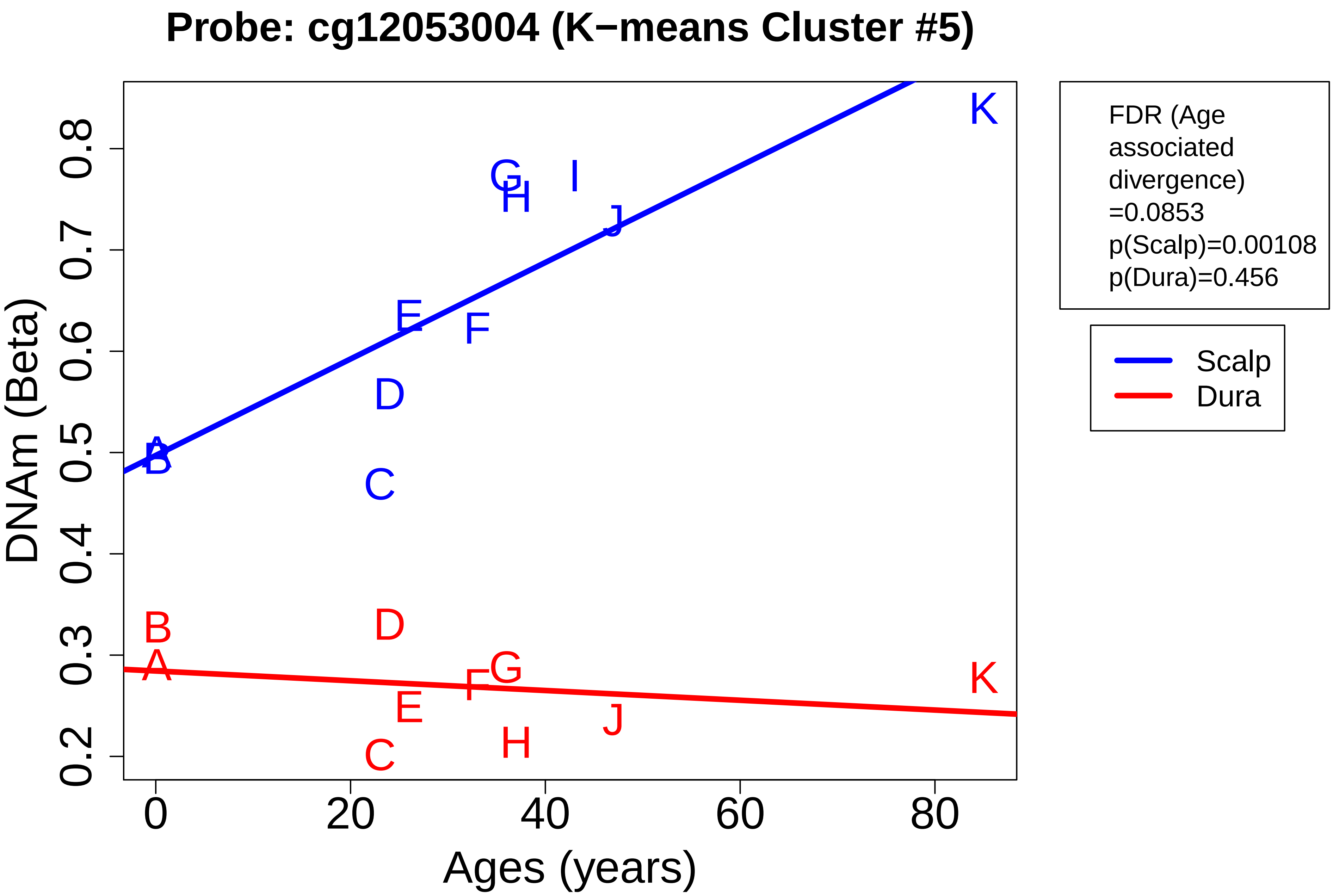
Probe: cg12053004 (K−means Cluster #5)
DNAm (Beta)
Ages (years)
0.2
0.3
0.4
0.5
0.6
0.7
0.8
0
20
40
60
80
FDR (Age associated divergence) =0.0853
p(Scalp)=0.00108
p(Dura)=0.456
Scalp
Dura
A
B
C
D
E
F
G
H
I
J
K

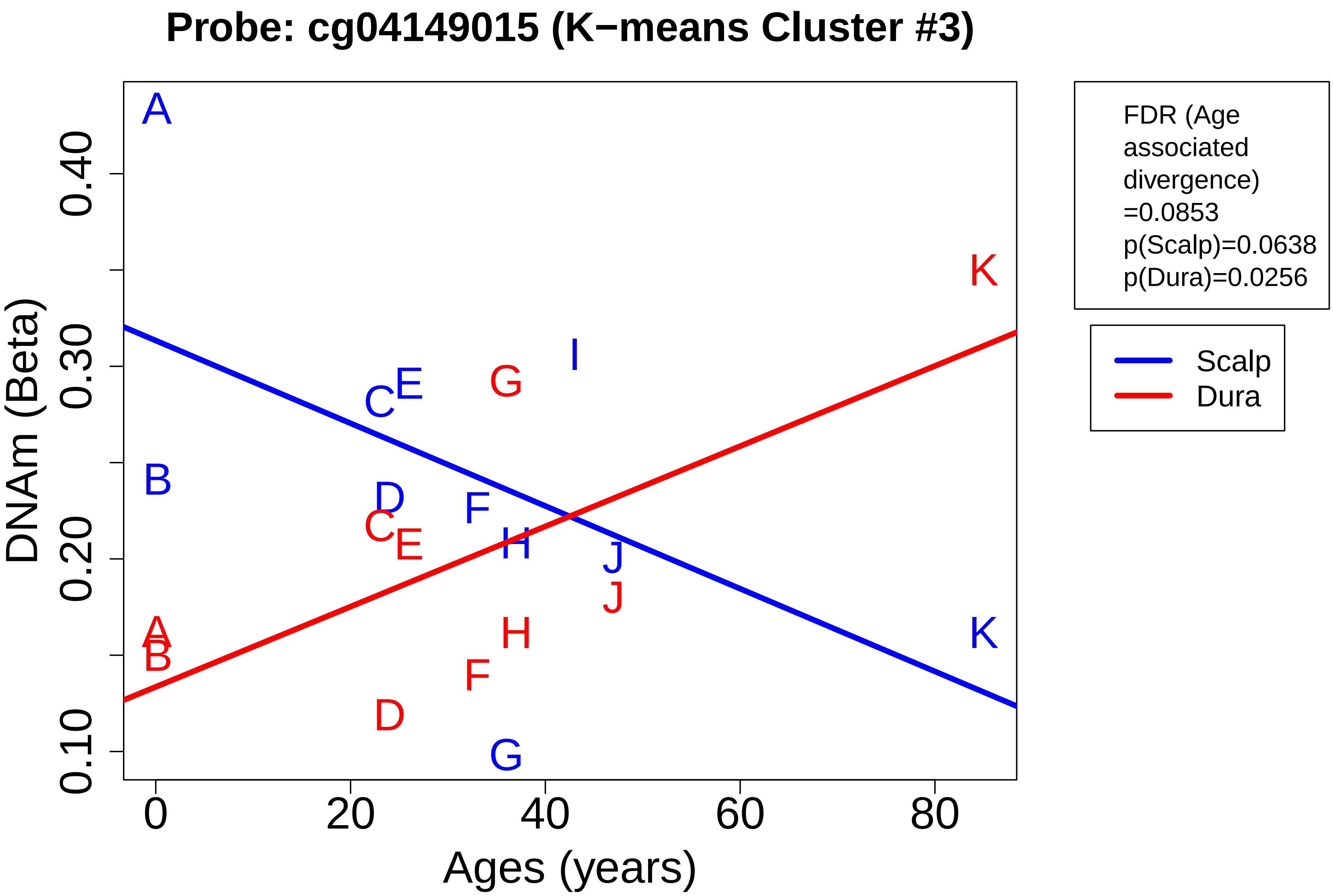
Probe: cg04149015 (K−means Cluster #3)
FDR (Age associated divergence) =0.0853
p(Scalp)=0.0638
p(Dura)=0.0256
Scalp
Dura
DNAm (Beta)
0.10
0.20
0.30
0.40
0
20
40
60
80
Ages (years)
A
B
C
D
E
F
G
H
I
J
K

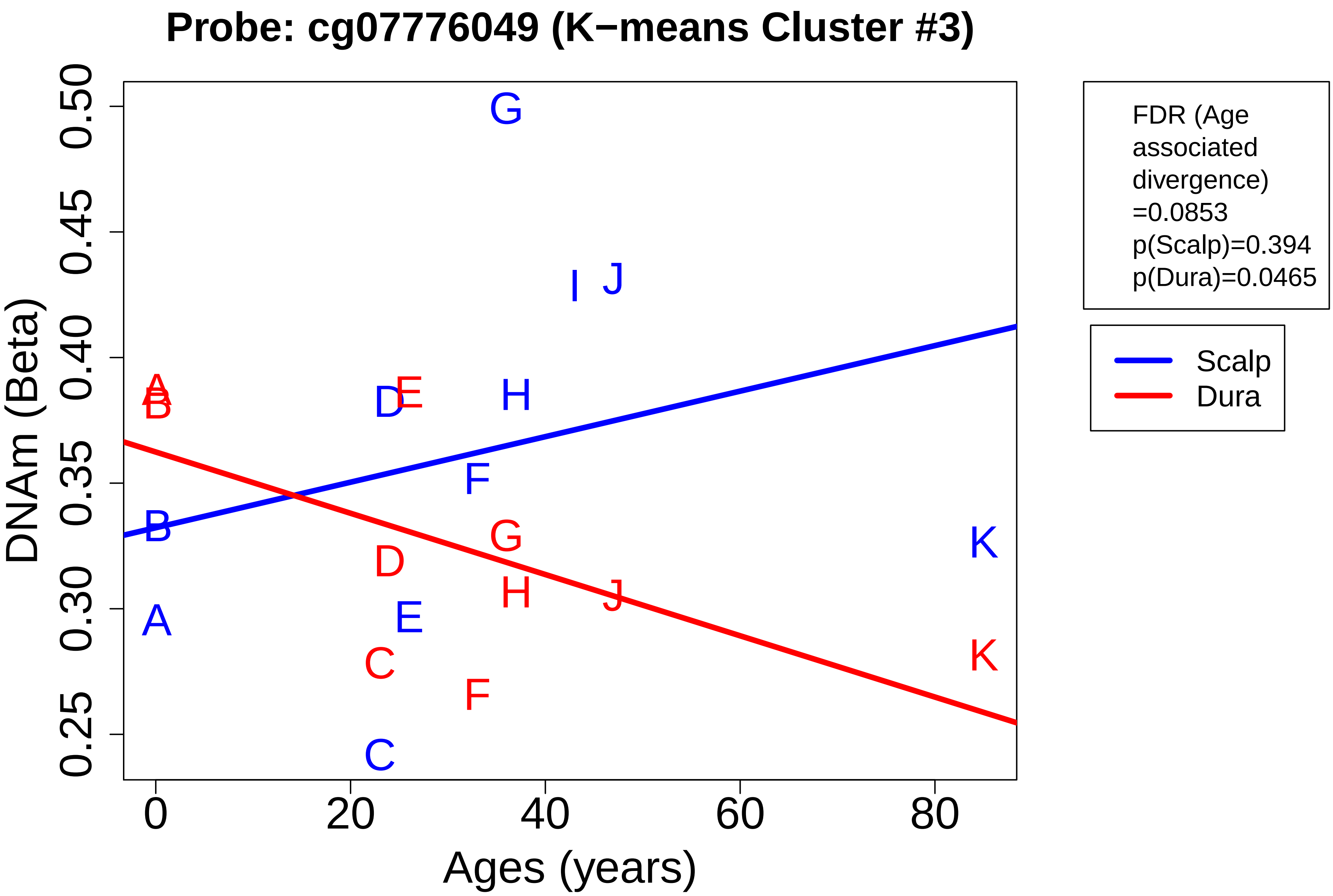
Probe: cg07776049 (K−means Cluster #3)
DNAm (Beta)
0.25
0.30
0.35
0.40
0.45
0.50
0
20
40
60
80
Ages (years)
FDR (Age associated divergence) =0.0853
p(Scalp)=0.394
p(Dura)=0.0465
Scalp
Dura
A
B
C
D
E
F
G
H
I
J
K

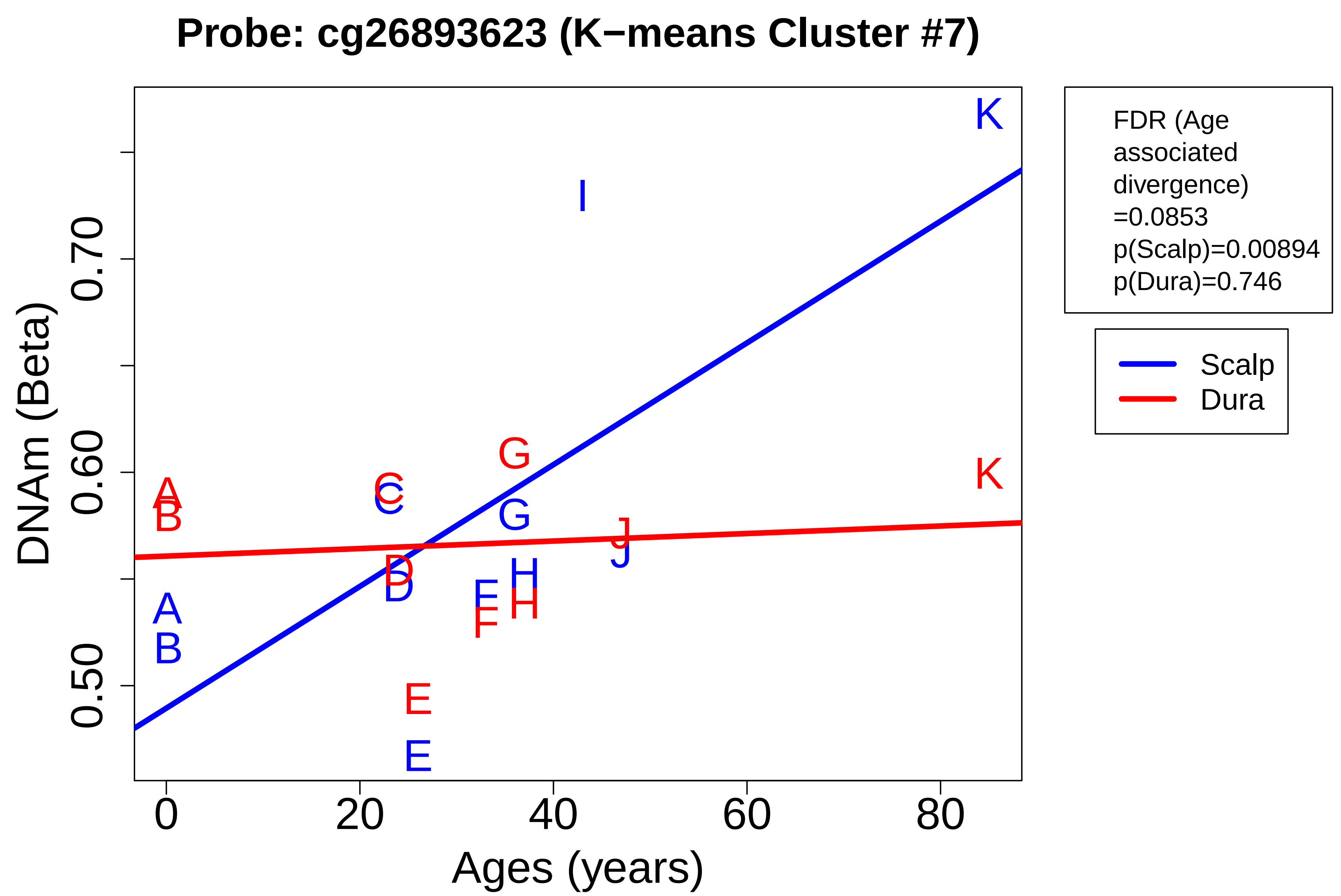

Probe: cg26893623 (K−means Cluster #7)
FDR (Age associated divergence) =0.0853
p(Scalp)=0.00894
p(Dura)=0.746
Scalp
Dura
DNAm (Beta)
0.50
0.60
0.70
Ages (years)
0
20
40
60
80
A
B
C
D
E
F
G
H
I
J
K

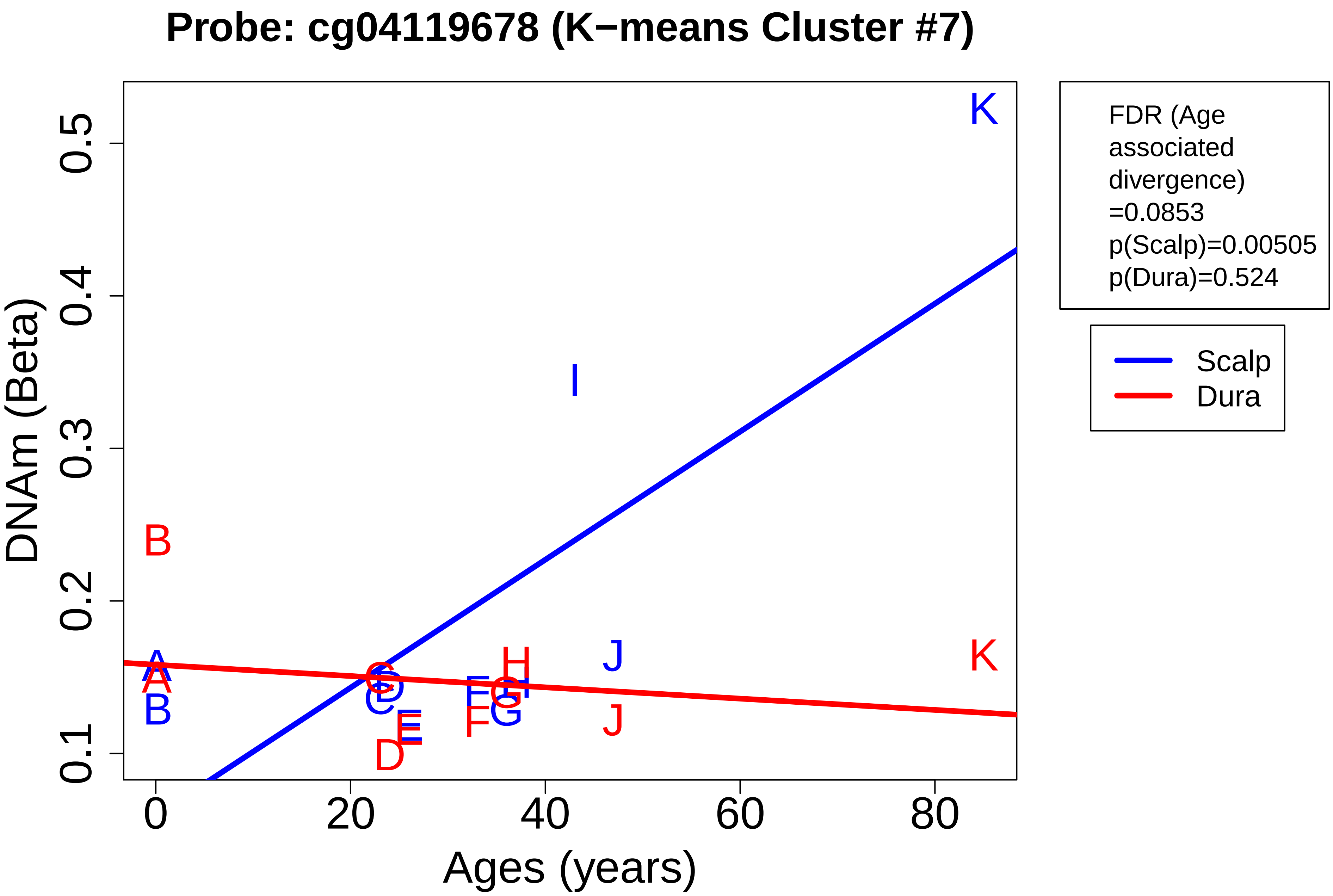

Probe: cg04119678 (K−means Cluster #7)
DNAm (Beta)
0.1
0.2
0.3
0.4
0.5
0
20
40
60
80
Ages (years)
FDR (Age associated divergence) =0.0853
p(Scalp)=0.00505
p(Dura)=0.524
Scalp
Dura

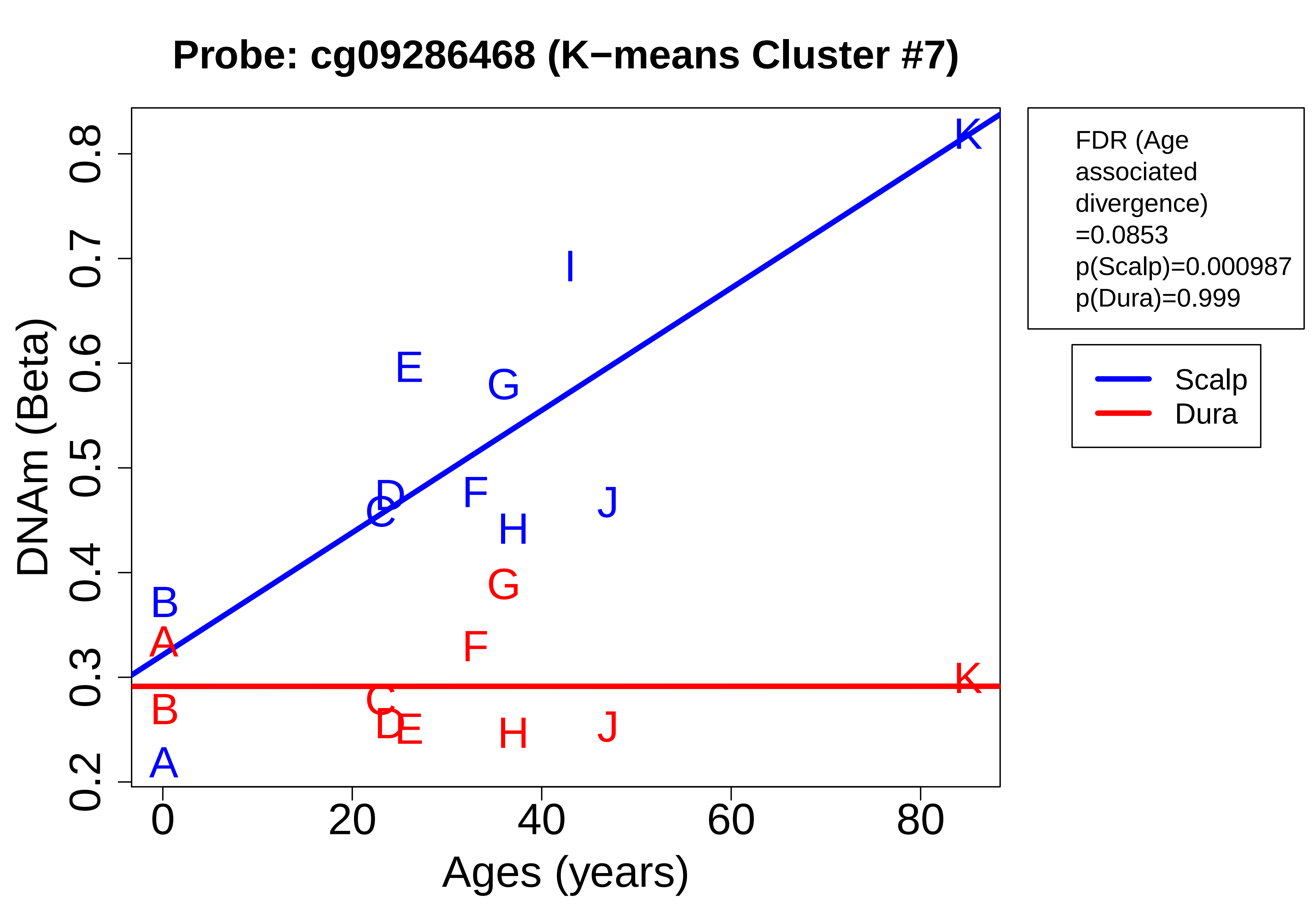

Probe: cg09286468 (K−means Cluster #7)
DNAm (Beta)
Ages (years)
0.2
0.3
0.4
0.5
0.6
0.7
0.8
0
20
40
60
80
FDR (Age associated divergence) =0.0853
p(Scalp)=0.000987
p(Dura)=0.999
Scalp
Dura

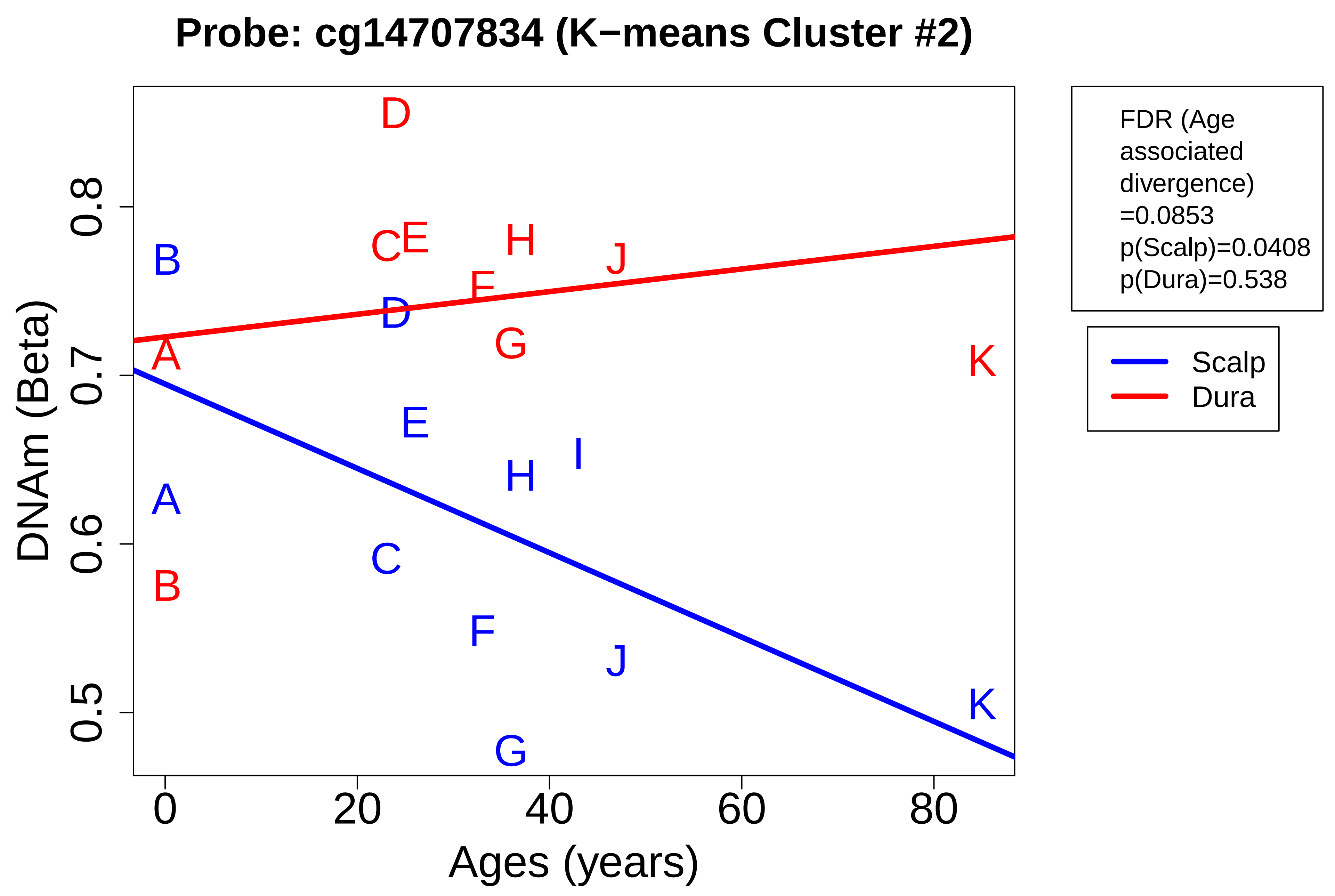

Probe: cg14707834 (K−means Cluster #2)
FDR (Age associated divergence) =0.0853
p(Scalp)=0.0408
p(Dura)=0.538
Scalp
Dura
DNAm (Beta)
Ages (years)
0.5
0.6
0.7
0.8
0
20
40
60
80

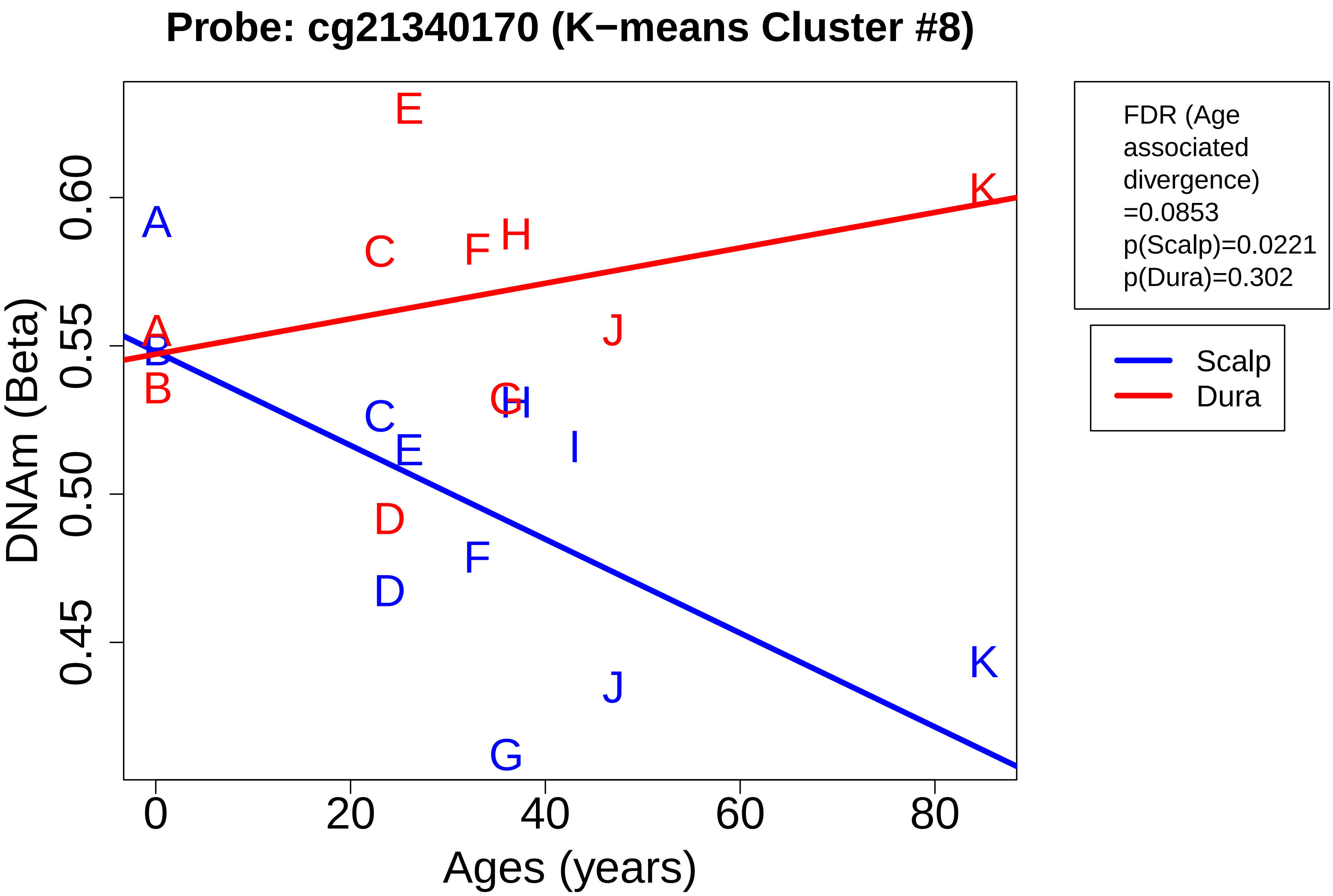

Probe: cg21340170 (K−means Cluster #8)
DNAm (Beta)
0.45
0.50
0.55
0.60
Ages (years)
0
20
40
60
80
FDR (Age associated divergence) =0.0853
p(Scalp)=0.0221
p(Dura)=0.302
Scalp
Dura

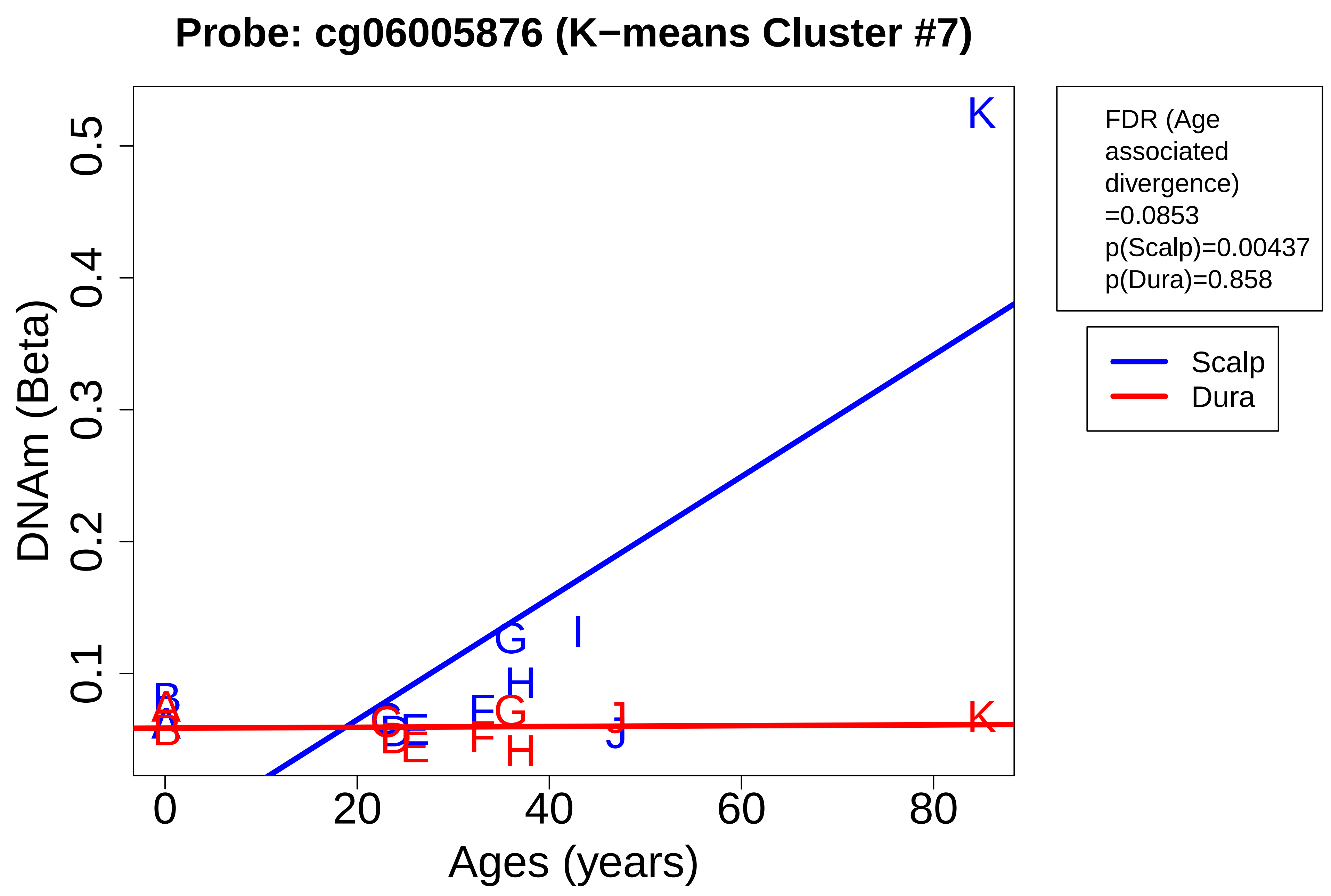
Probe: cg06005876 (K−means Cluster #7)
DNAm (Beta)
0.1
0.2
0.3
0.4
0.5
0
20
40
60
80
Ages (years)
FDR (Age associated divergence) =0.0853
p(Scalp)=0.00437
p(Dura)=0.858
Scalp
Dura
A
B
C
D
E
F
G
H
I
J
K

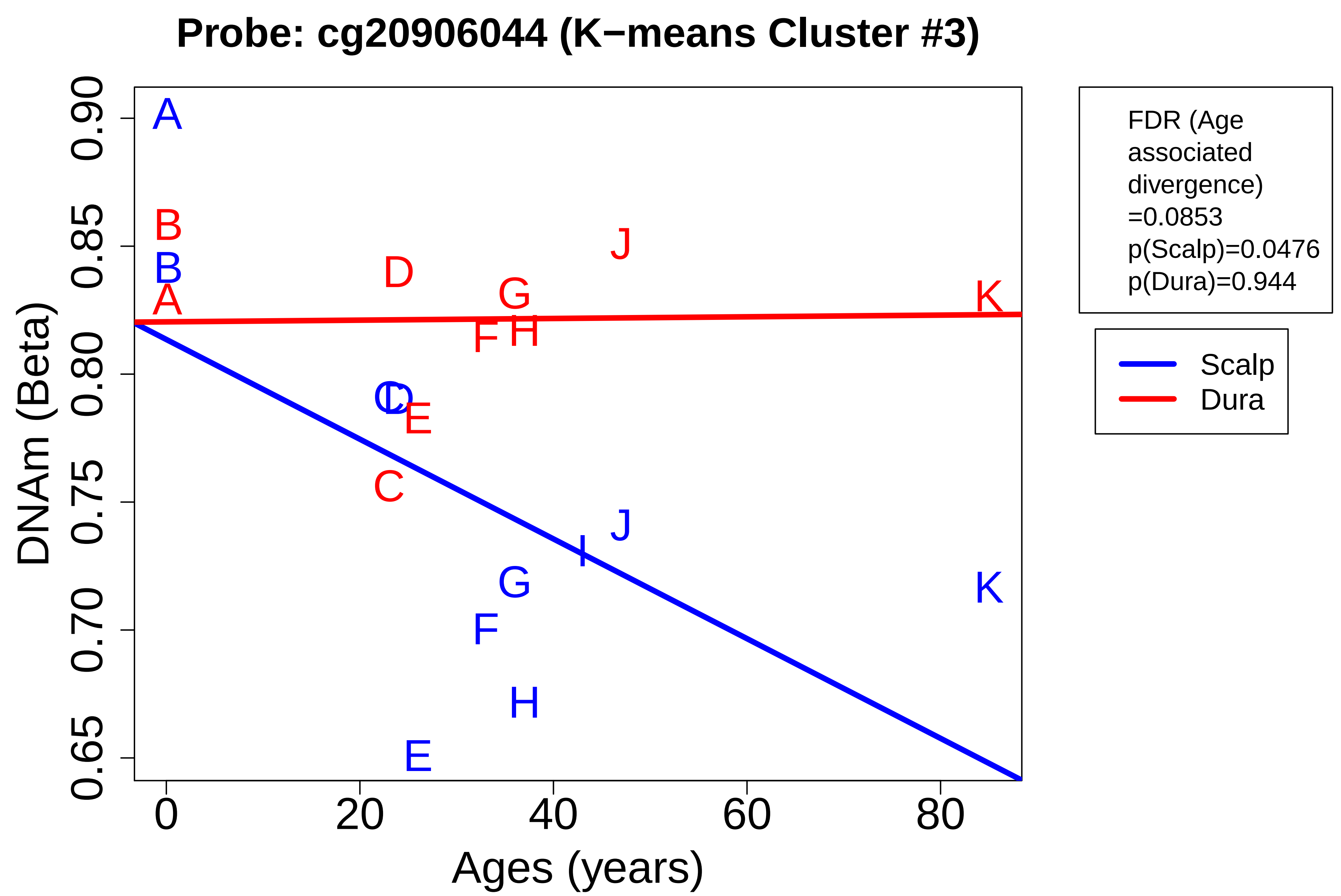

Probe: cg20906044 (K−means Cluster #3)
DNAm (Beta)
0.65
0.70
0.75
0.80
0.85
0.90
0
20
40
60
80
Ages (years)
A
B
C
D
E
F
G
H
I
J
K
FDR (Age associated divergence) =0.0853
p(Scalp)=0.0476
p(Dura)=0.944
Scalp
Dura

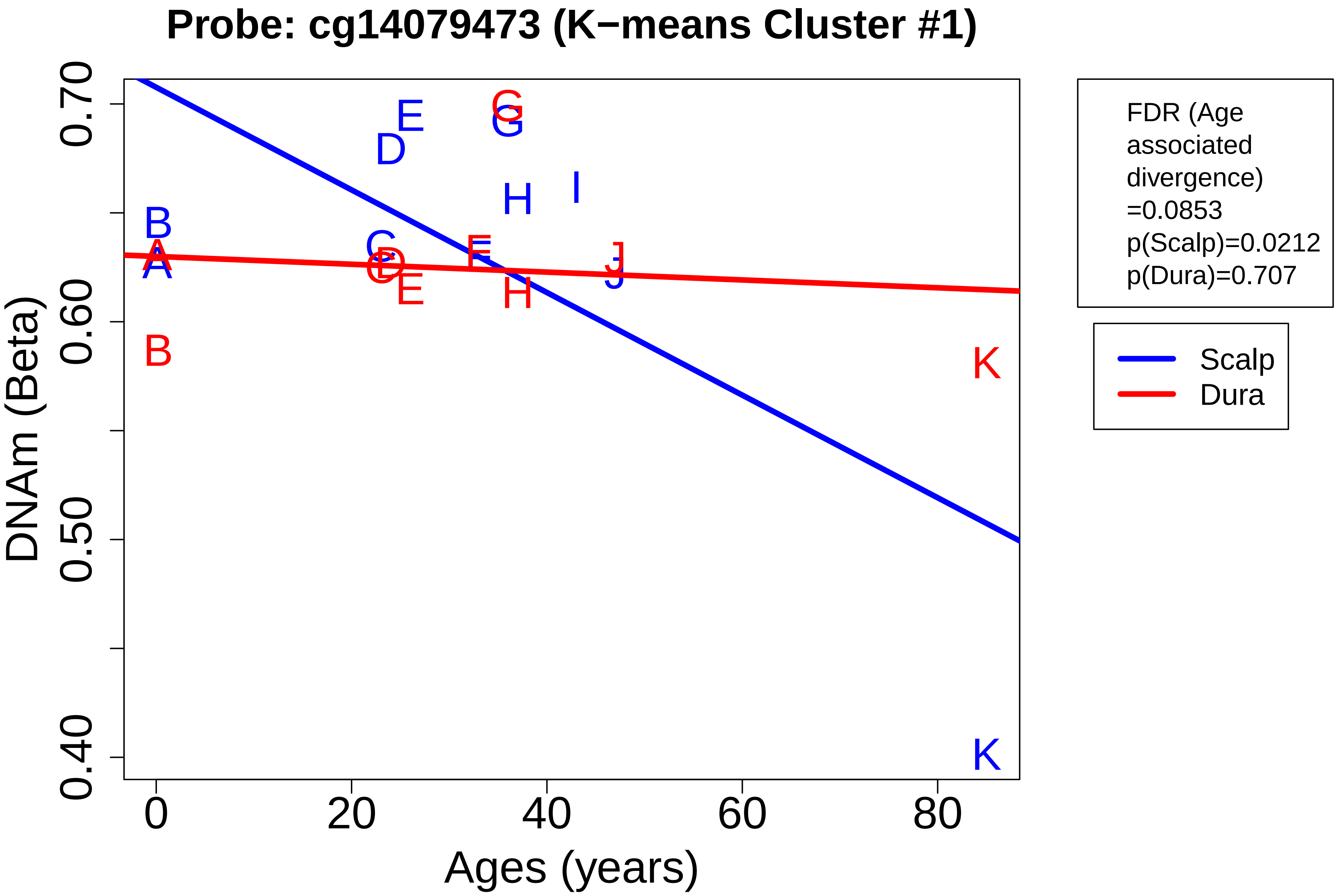

Probe: cg14079473 (K−means Cluster #1)
DNAm (Beta)
0.40
0.50
0.60
0.70
Ages (years)
0
20
40
60
80
FDR (Age associated divergence) =0.0853
p(Scalp)=0.0212
p(Dura)=0.707
Scalp
Dura
A
B
C
D
E
F
G
H
I
J
K

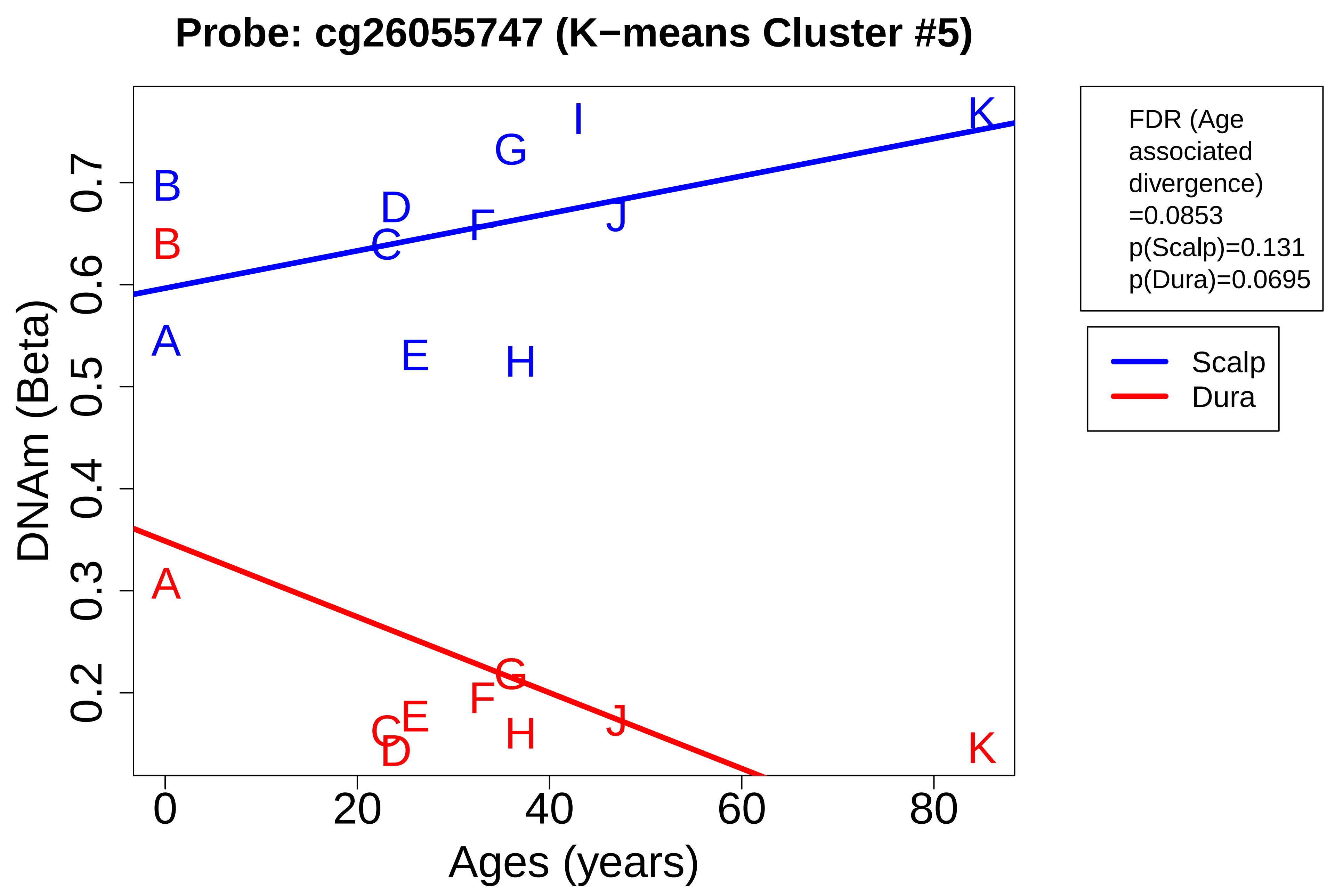

Probe: cg26055747 (K−means Cluster #5)
FDR (Age associated divergence) =0.0853
p(Scalp)=0.131
p(Dura)=0.0695
Scalp
Dura
DNAm (Beta)
Ages (years)
0.2
0.3
0.4
0.5
0.6
0.7
0
20
40
60
80
A
B
C
D
E
F
G
H
I
J
K

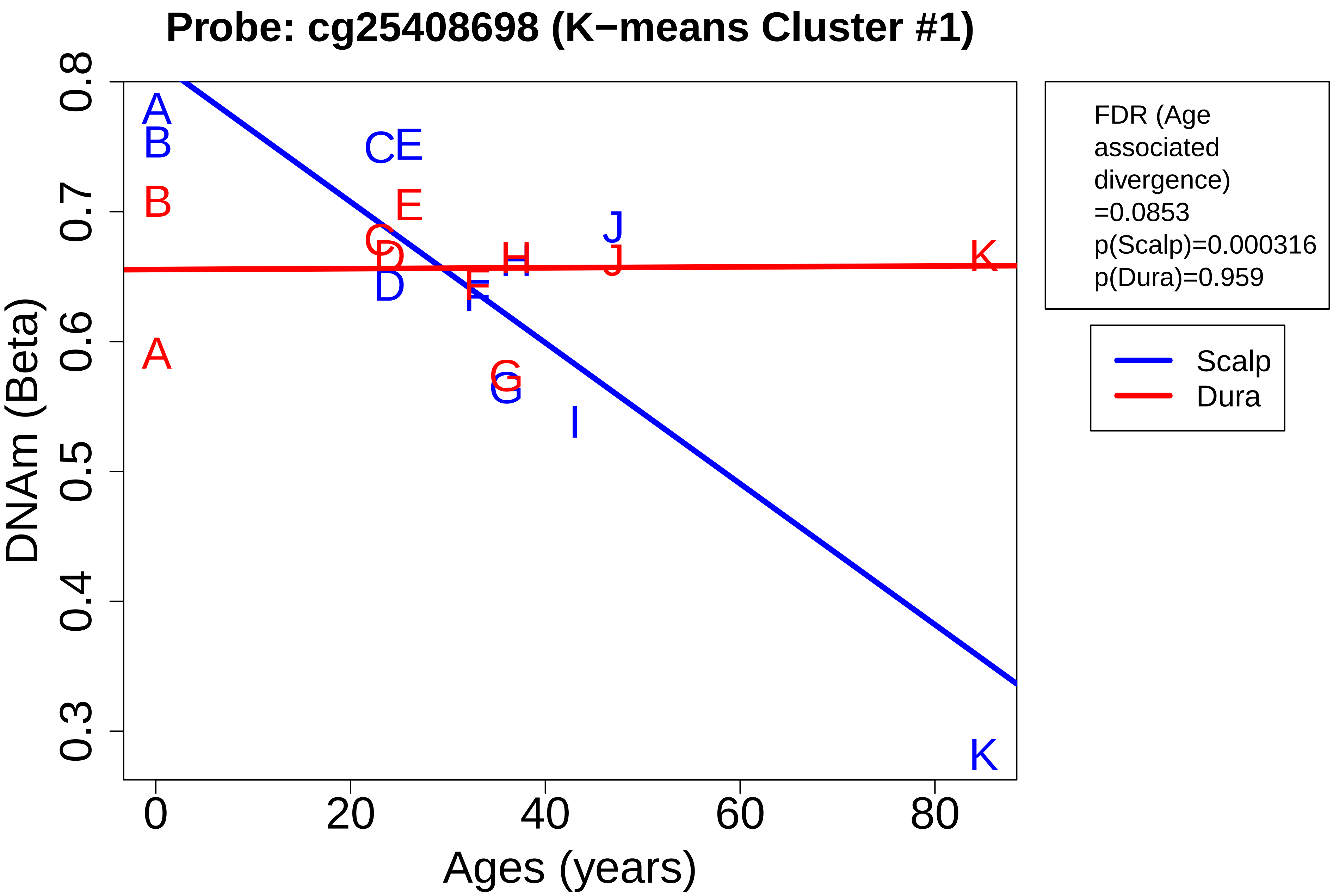

Probe: cg25408698 (K−means Cluster #1)
DNAm (Beta)
0.3
0.4
0.5
0.6
0.7
0.8
0
20
40
60
80
Ages (years)
FDR (Age associated divergence) =0.0853
p(Scalp)=0.000316
p(Dura)=0.959
Scalp
Dura

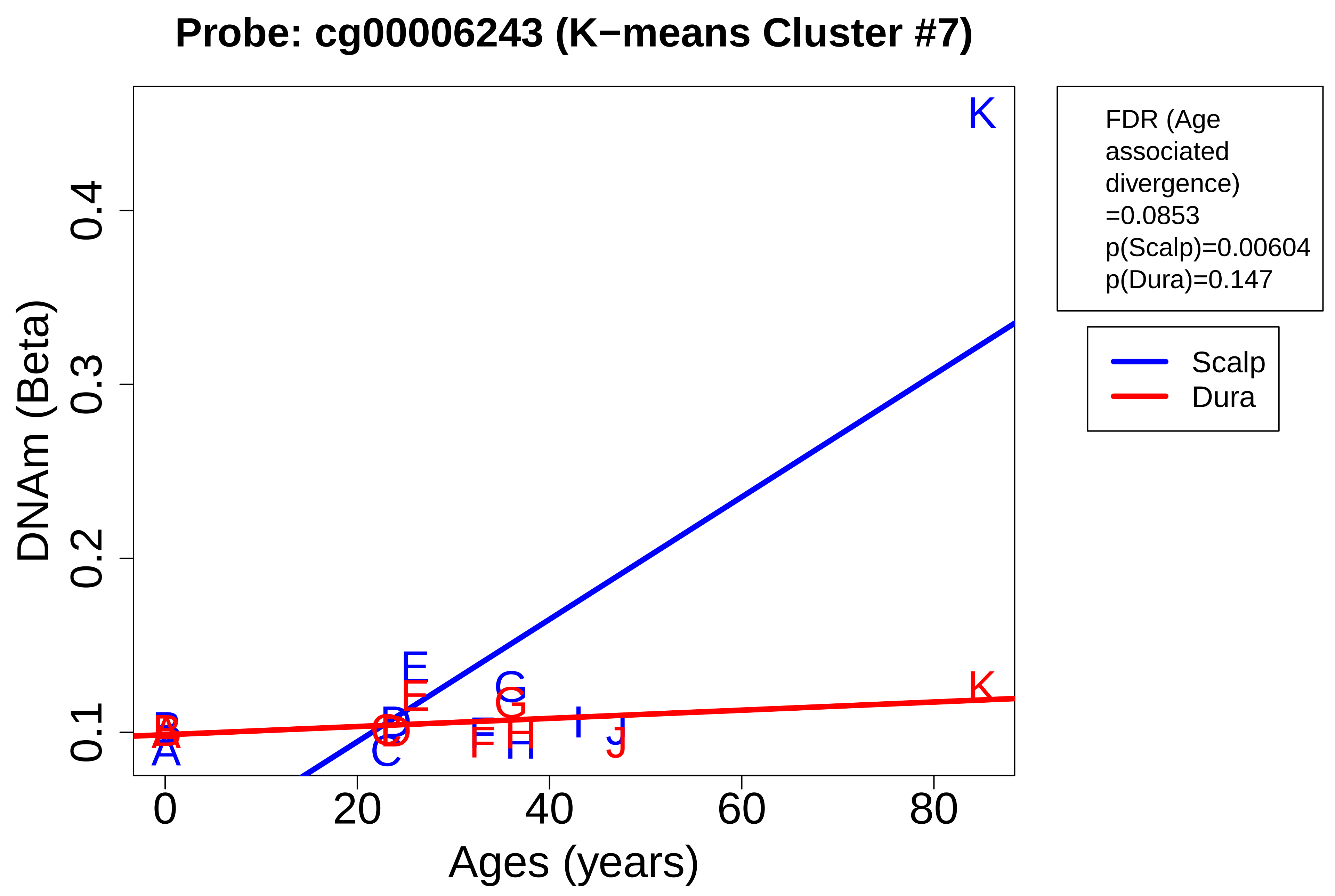

Probe: cg00006243 (K−means Cluster #7)
FDR (Age associated divergence) =0.0853
p(Scalp)=0.00604
p(Dura)=0.147
Scalp
Dura
DNAm (Beta)
0.1
0.2
0.3
0.4
0
20
40
60
80
Ages (years)

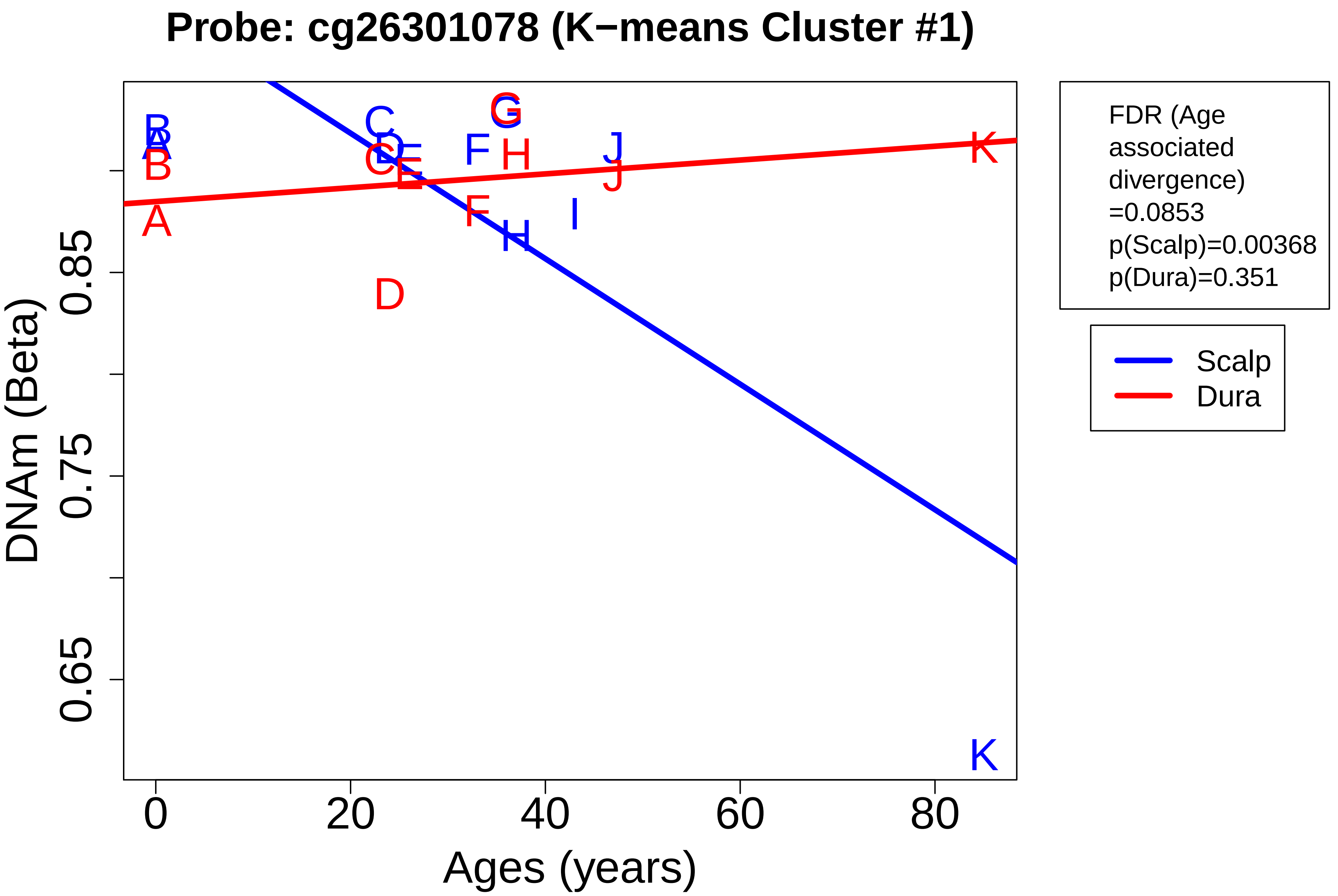

Probe: cg26301078 (K−means Cluster #1)
DNAm (Beta)
0.65
0.75
0.85
0
20
40
60
80
Ages (years)
FDR (Age associated divergence) =0.0853
p(Scalp)=0.00368
p(Dura)=0.351
Scalp
Dura

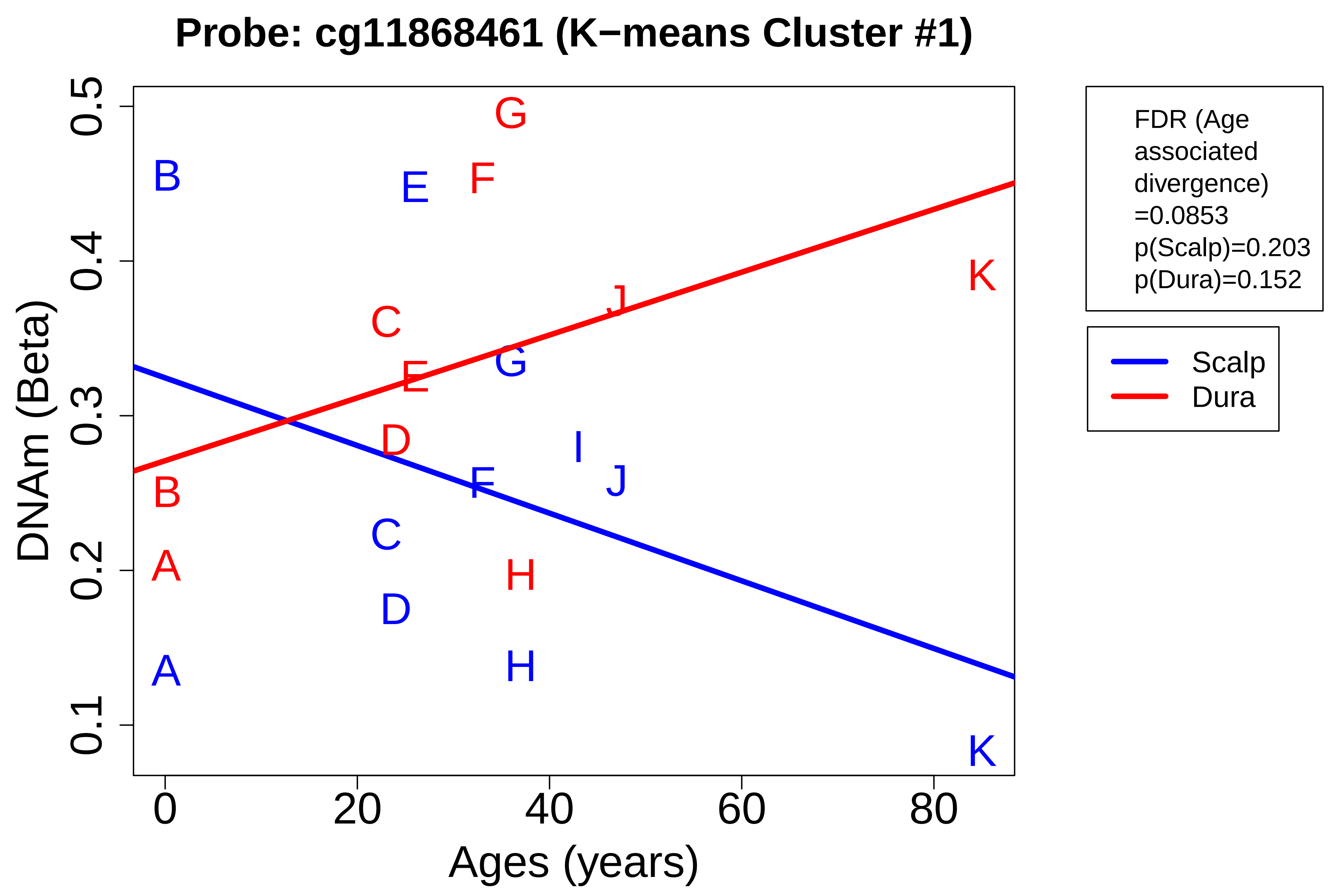

Probe: cg11868461 (K−means Cluster #1)
DNAm (Beta)
0.1
0.2
0.3
0.4
0.5
Ages (years)
0
20
40
60
80
FDR (Age associated divergence) =0.0853
p(Scalp)=0.203
p(Dura)=0.152
Scalp
Dura
A B C D E F G H I J K

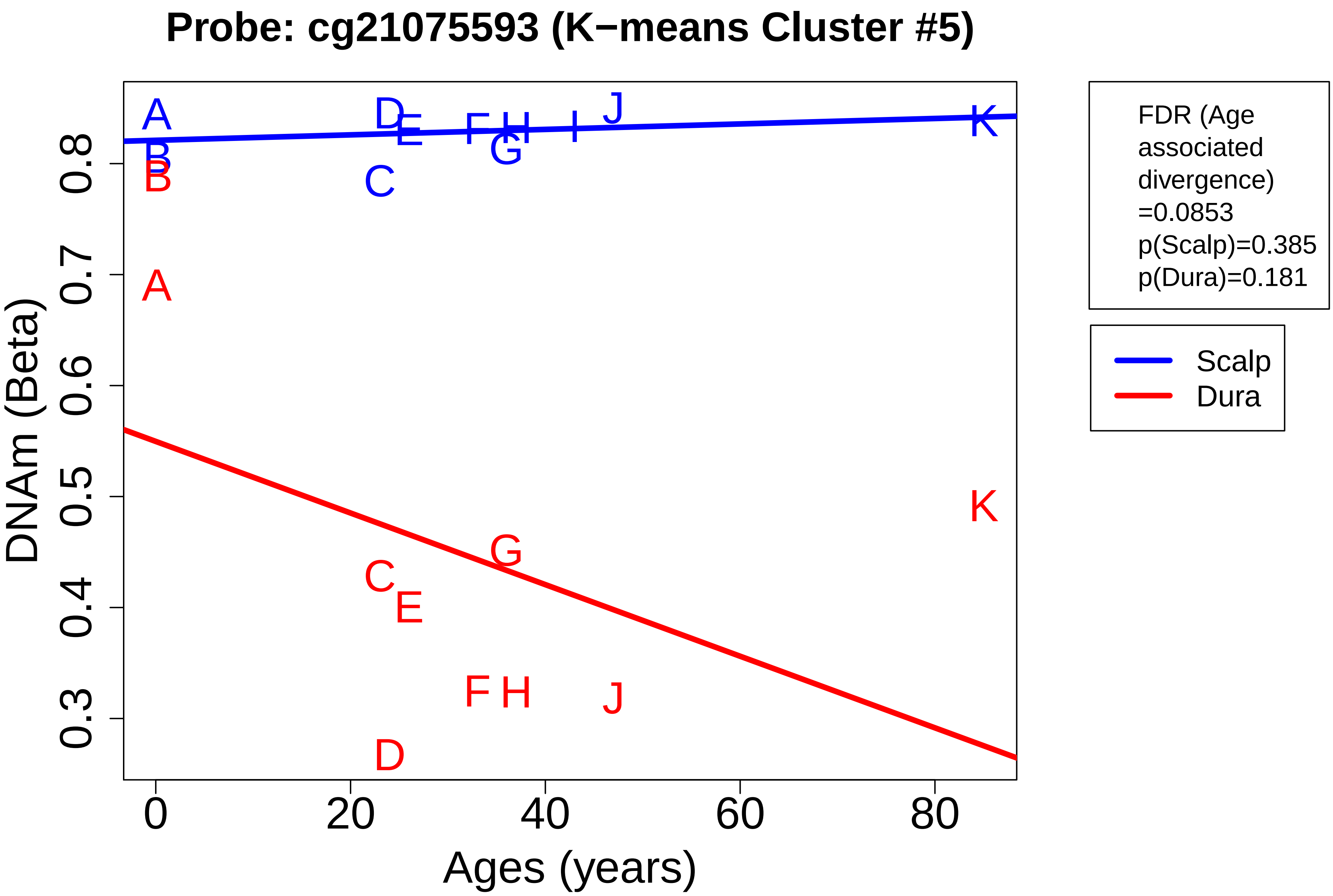

Probe: cg21075593 (K−means Cluster #5)
DNAm (Beta)
Ages (years)
0.3
0.4
0.5
0.6
0.7
0.8
0
20
40
60
80
FDR (Age associated divergence) =0.0853
p(Scalp)=0.385
p(Dura)=0.181
Scalp
Dura

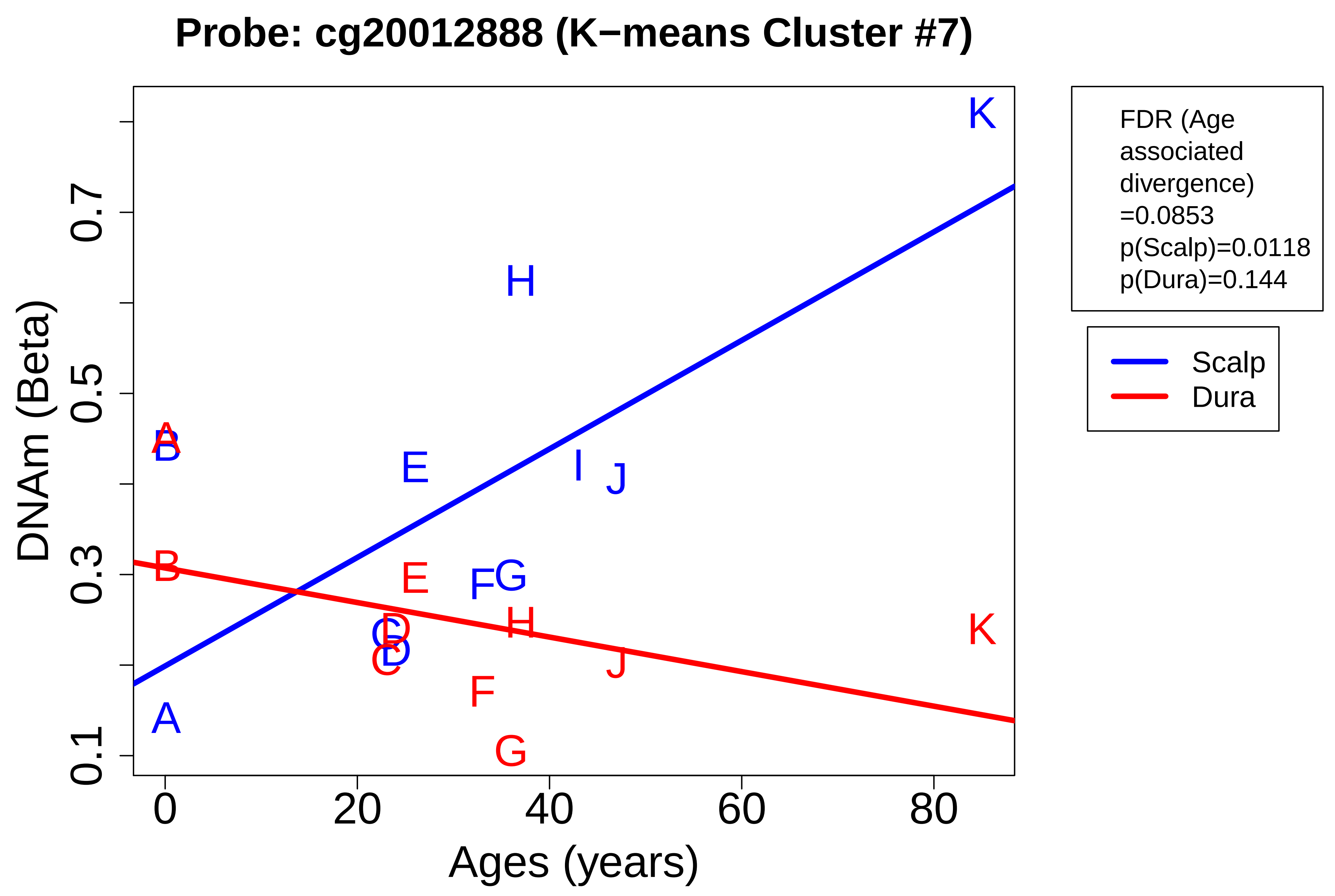

Probe: cg20012888 (K−means Cluster #7)
DNAm (Beta)
0.1
0.3
0.5
0.7
0
20
40
60
80
Ages (years)
FDR (Age associated divergence) =0.0853
p(Scalp)=0.0118
p(Dura)=0.144
Scalp
Dura

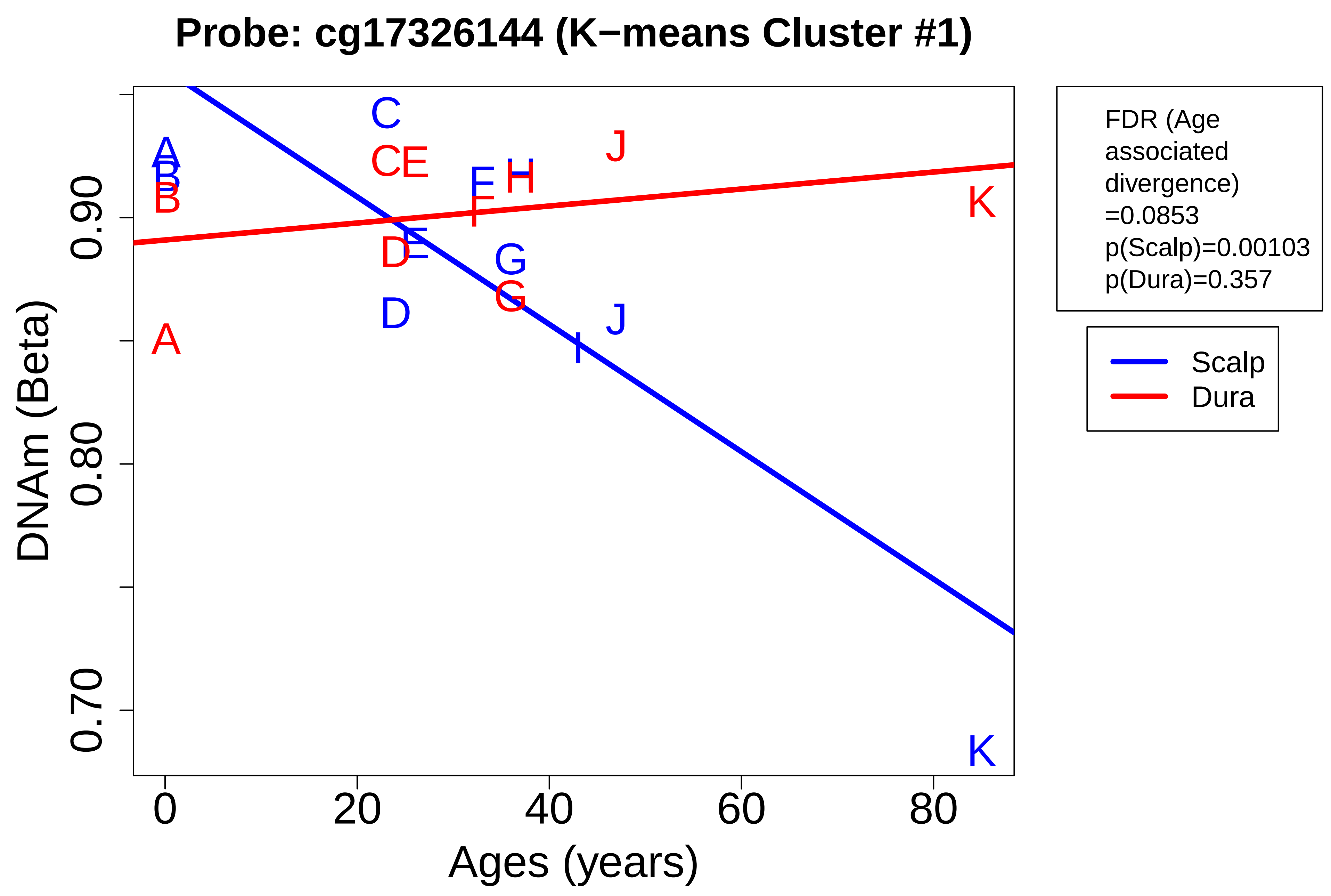

Probe: cg17326144 (K−means Cluster #1)
DNAm (Beta)
0.70
0.80
0.90
0
20
40
60
80
Ages (years)
FDR (Age associated divergence) =0.0853
p(Scalp)=0.00103
p(Dura)=0.357
Scalp
Dura

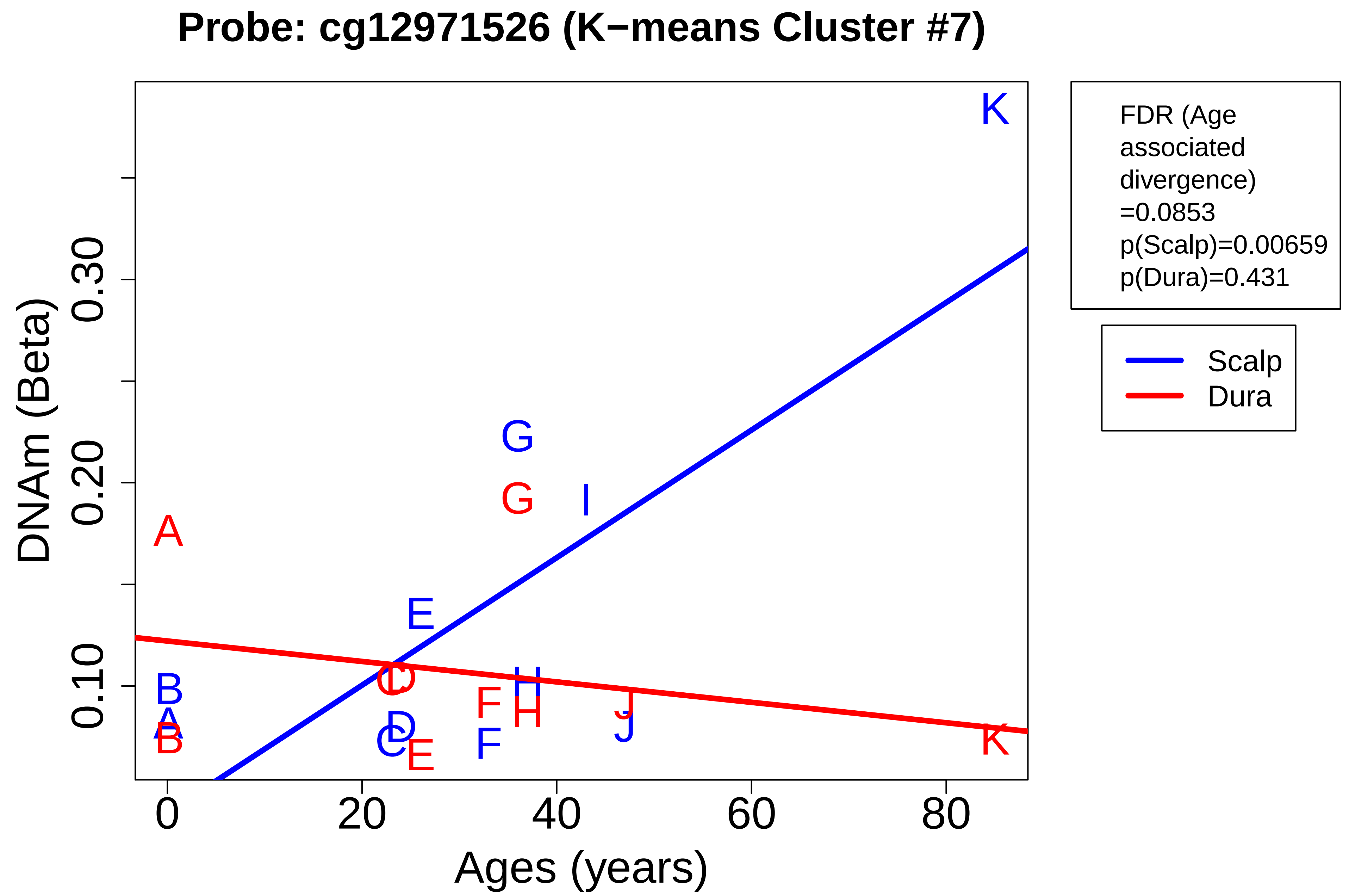

Probe: cg12971526 (K−means Cluster #7)
DNAm (Beta)
0.10
0.20
0.30
Ages (years)
0
20
40
60
80
A
B
C
D
E
F
G
H
I
J
K
FDR (Age associated divergence) =0.0853
p(Scalp)=0.00659
p(Dura)=0.431
Scalp
Dura

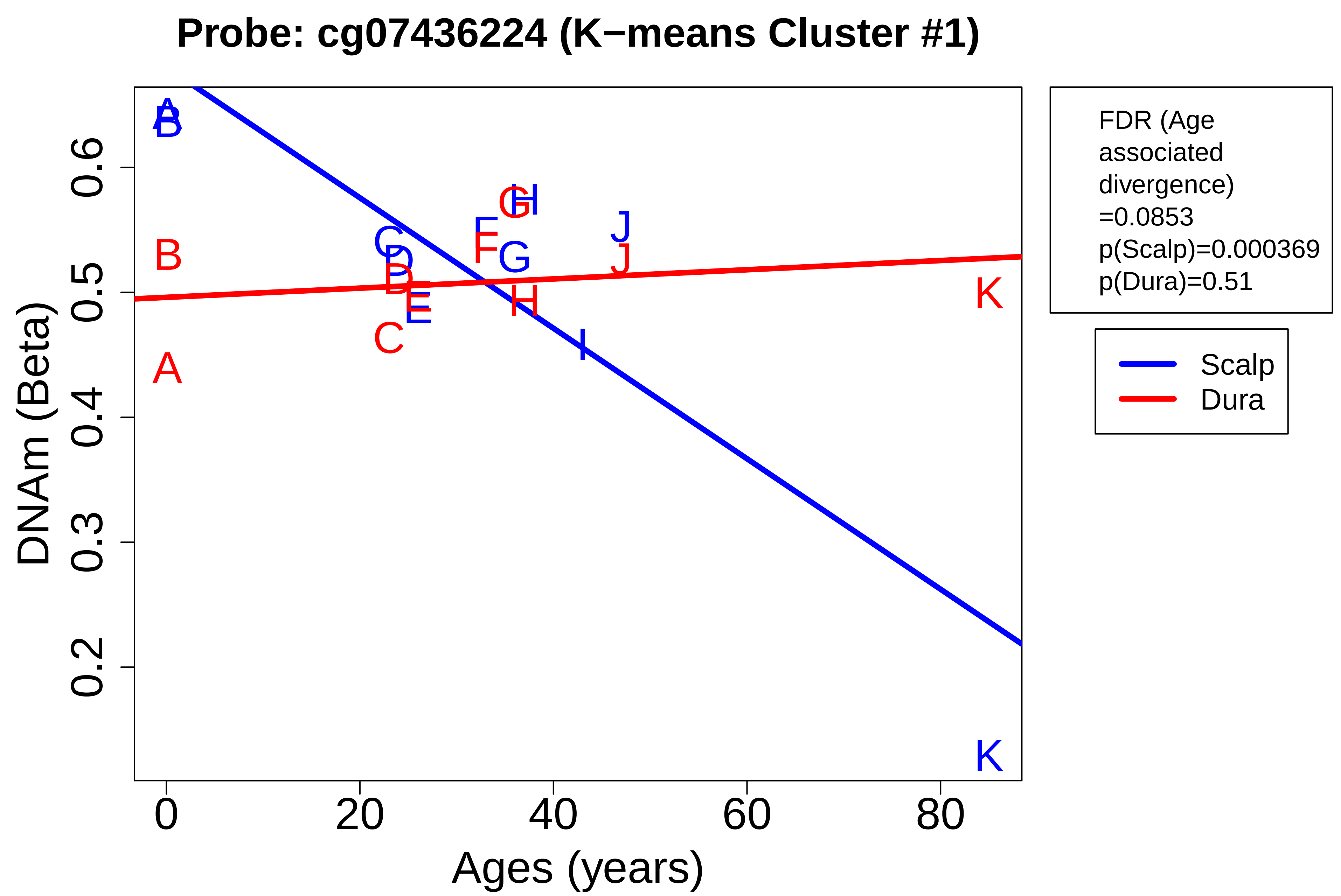

Probe: cg07436224 (K−means Cluster #1)
DNAm (Beta)
0.2
0.3
0.4
0.5
0.6
Ages (years)
0
20
40
60
80
FDR (Age associated divergence) =0.0853
p(Scalp)=0.000369
p(Dura)=0.51
Scalp
Dura

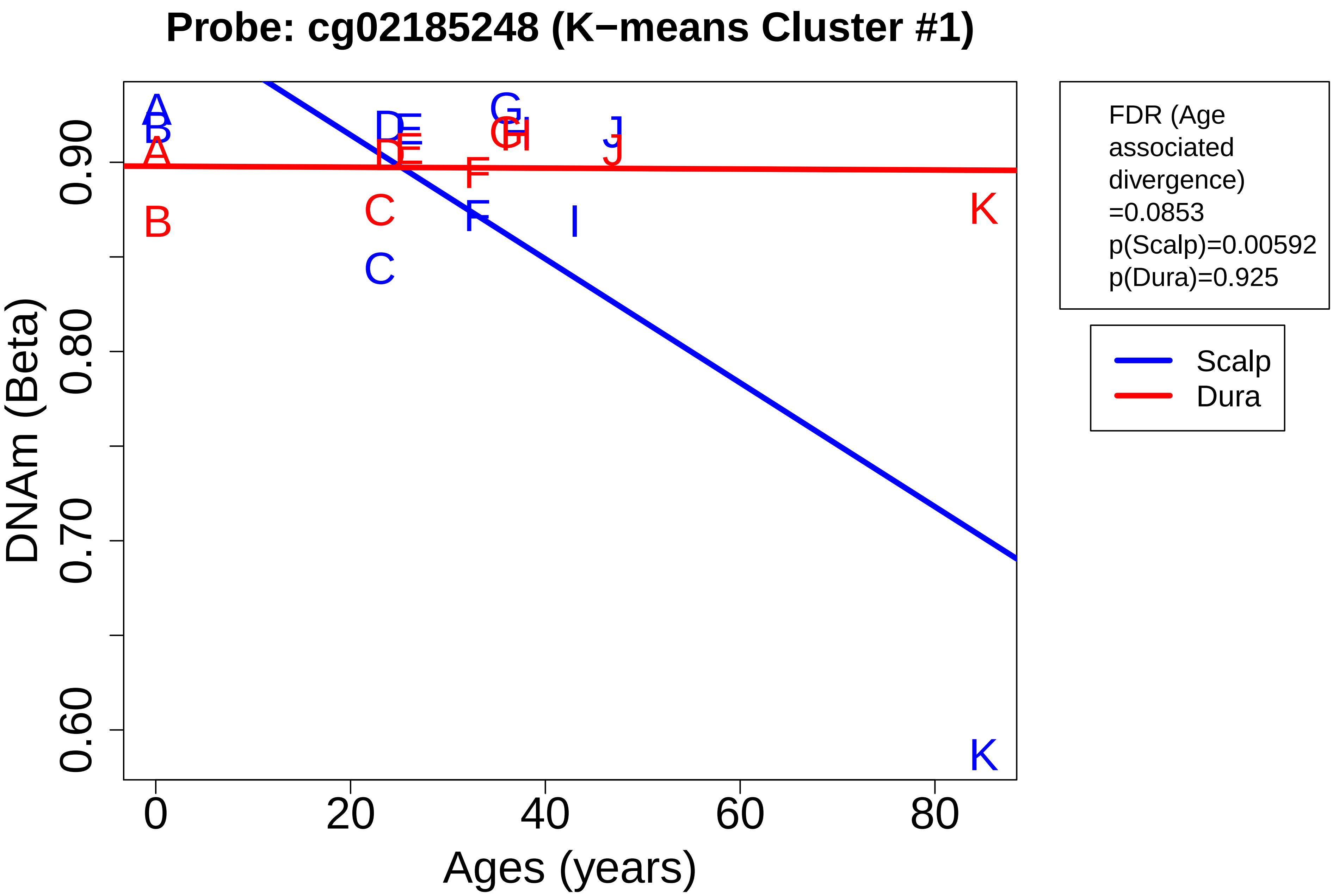
Probe: cg02185248 (K−means Cluster #1)
DNAm (Beta)
0.60
0.70
0.80
0.90
Ages (years)
0
20
40
60
80
A
B
C
D
E
F
G
H
I
J
K
FDR (Age associated divergence) =0.0853
p(Scalp)=0.00592
p(Dura)=0.925
Scalp
Dura

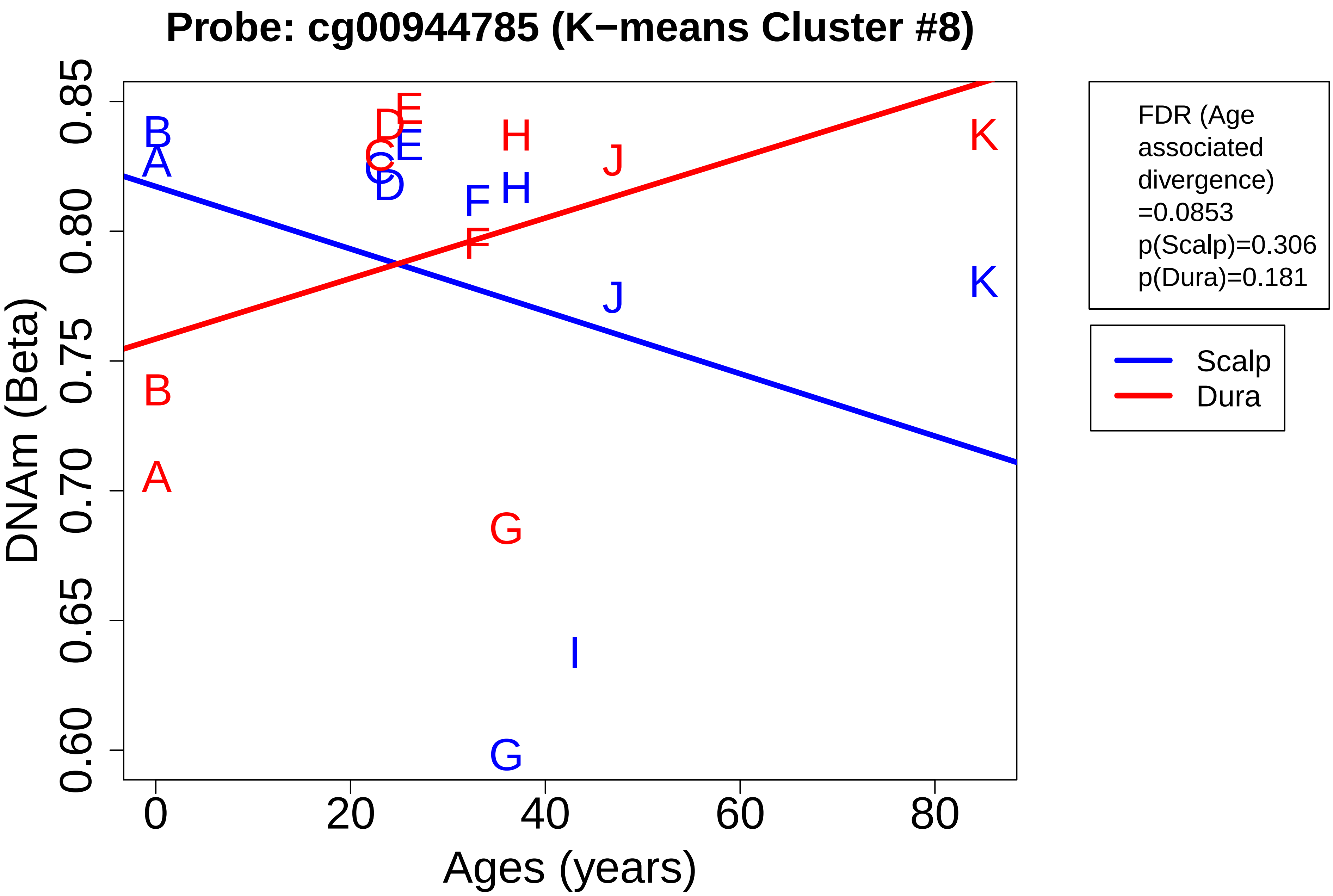

Probe: cg00944785 (K−means Cluster #8)
DNAm (Beta)
0.60
0.65
0.70
0.75
0.80
0.85
0
20
40
60
80
Ages (years)
FDR (Age associated divergence) =0.0853
p(Scalp)=0.306
p(Dura)=0.181
Scalp
Dura

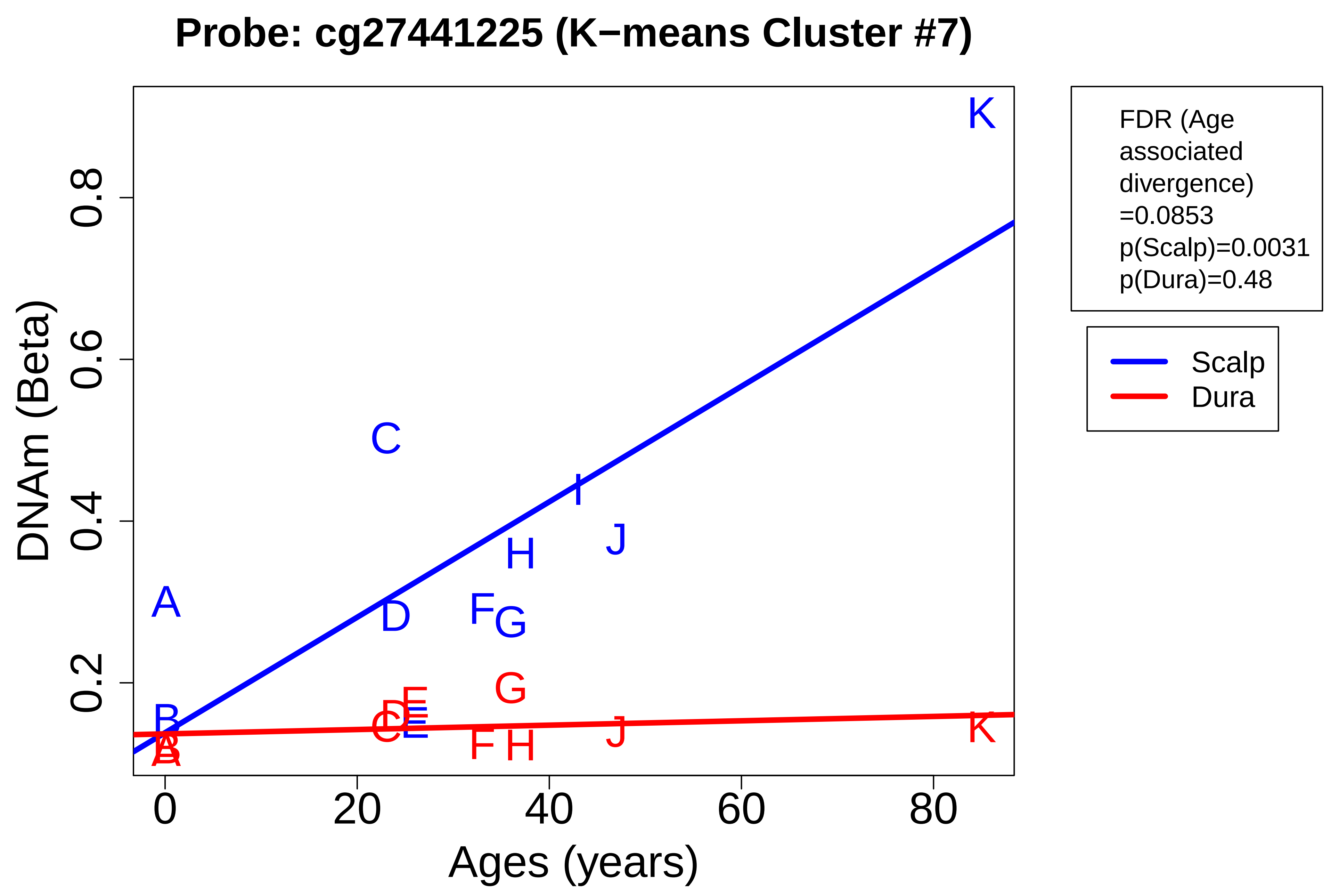

Probe: cg27441225 (K−means Cluster #7)
DNAm (Beta)
0.2
0.4
0.6
0.8
0
20
40
60
80
Ages (years)
FDR (Age associated divergence) =0.0853
p(Scalp)=0.0031
p(Dura)=0.48
Scalp
Dura

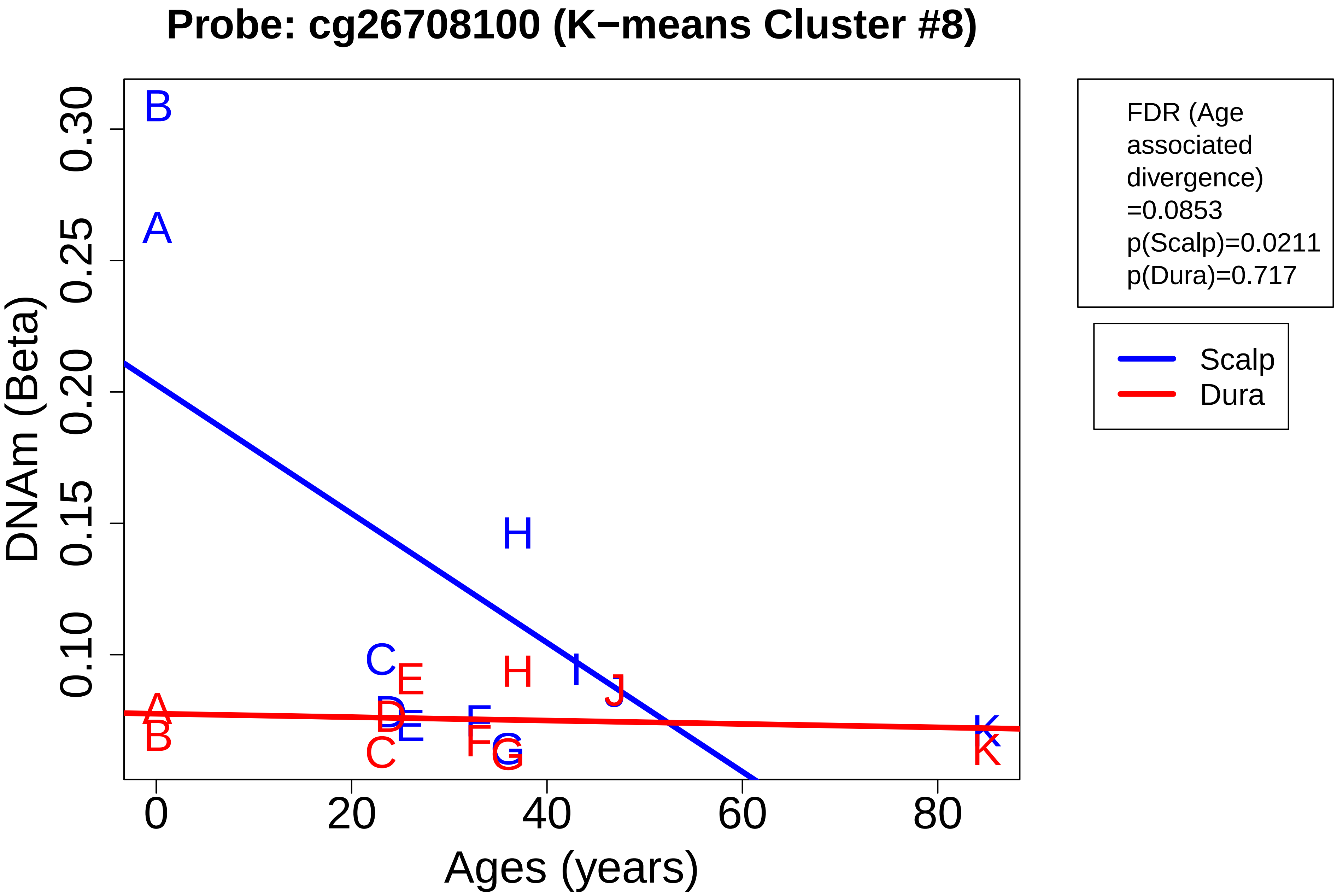

Probe: cg26708100 (K−means Cluster #8)
DNAm (Beta)
0.10
0.15
0.20
0.25
0.30
0
20
40
60
80
Ages (years)
FDR (Age associated divergence) =0.0853
p(Scalp)=0.0211
p(Dura)=0.717
Scalp
Dura

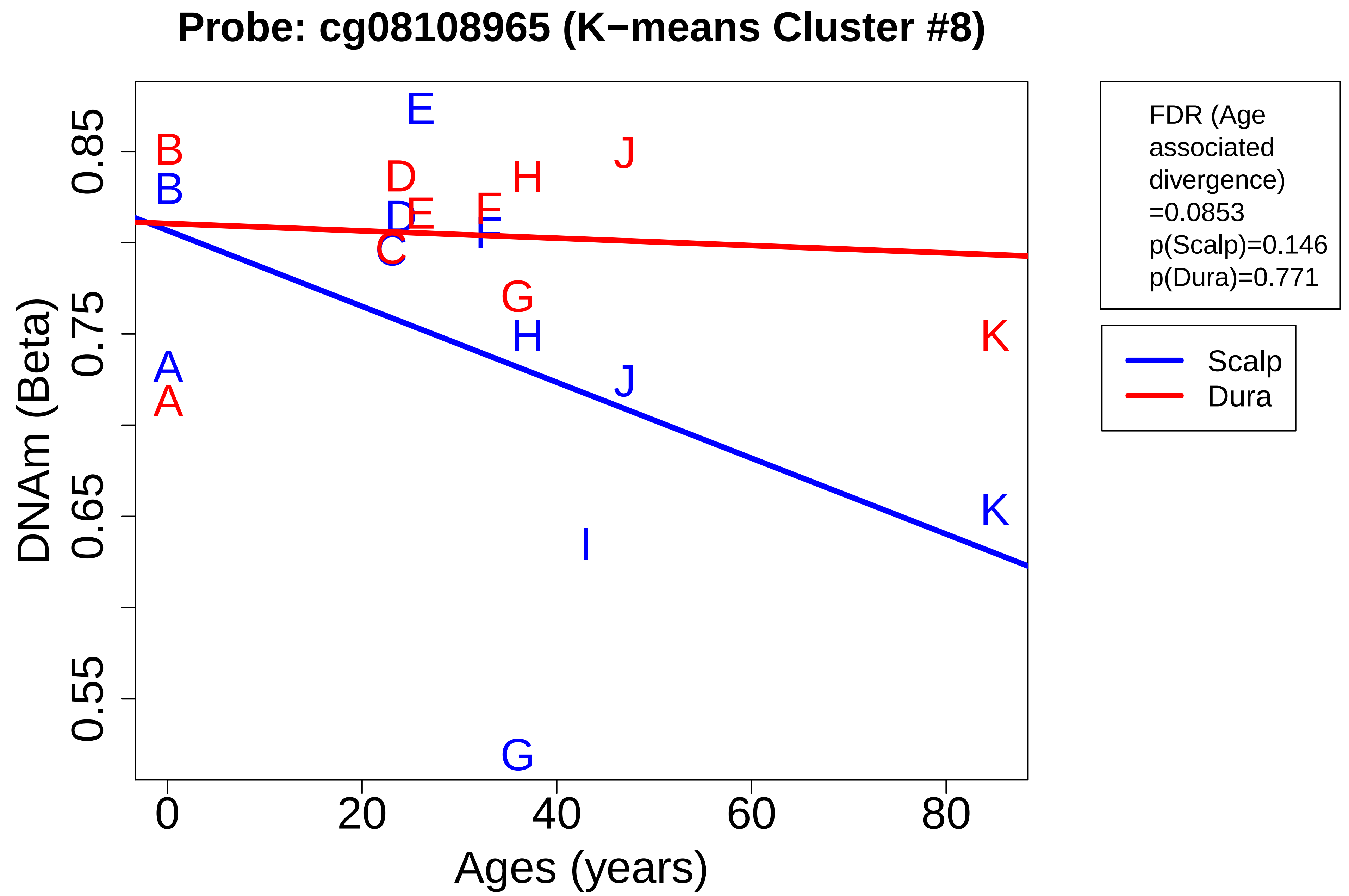

Probe: cg08108965 (K−means Cluster #8)
DNAm (Beta)
0.55
0.65
0.75
0.85
Ages (years)
0
20
40
60
80
FDR (Age associated divergence) =0.0853
p(Scalp)=0.146
p(Dura)=0.771
Scalp
Dura
A
B
C
D
E
F
G
H
I
J
K

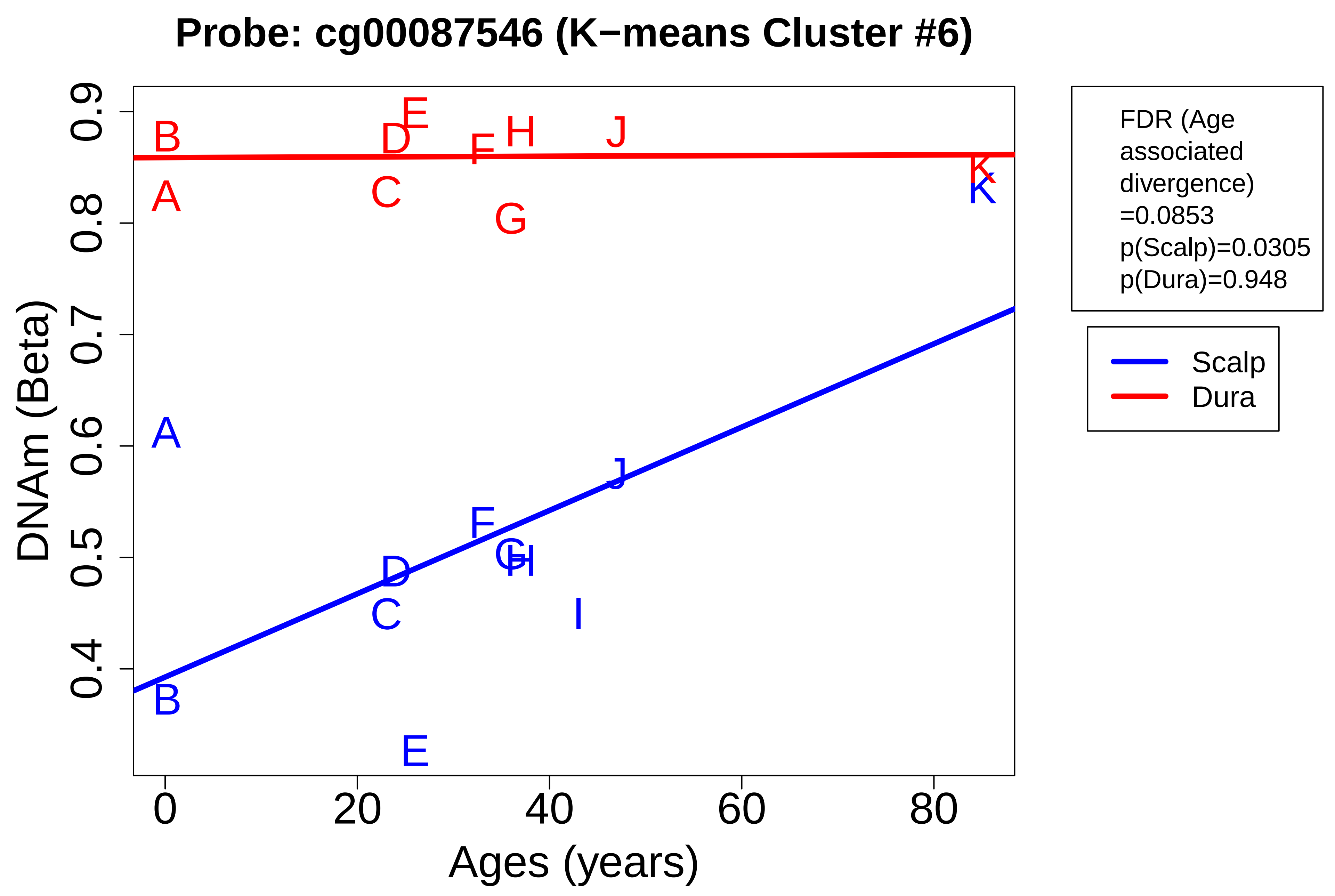

Probe: cg00087546 (K−means Cluster #6)
DNAm (Beta)
0.4
0.5
0.6
0.7
0.8
0.9
0
20
40
60
80
Ages (years)
FDR (Age associated divergence) =0.0853
p(Scalp)=0.0305
p(Dura)=0.948
Scalp
Dura

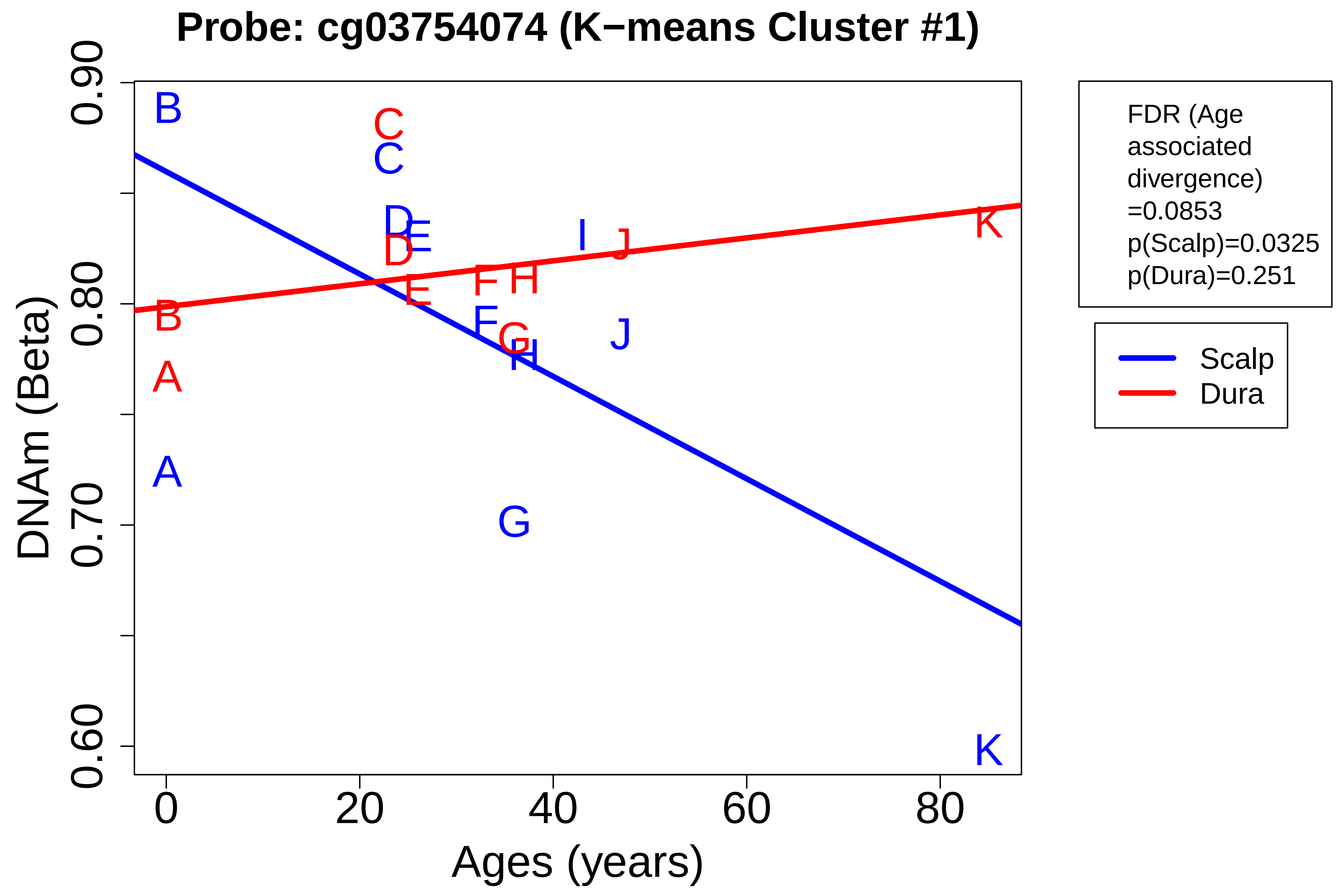

Probe: cg03754074 (K−means Cluster #1)
DNAm (Beta)
0.60
0.70
0.80
0.90
Ages (years)
0
20
40
60
80
A
B
C
D
E
F
G
H
I
J
K
FDR (Age
associated
divergence)
=0.0853
p(Scalp)=0.0325
p(Dura)=0.251
Scalp
Dura

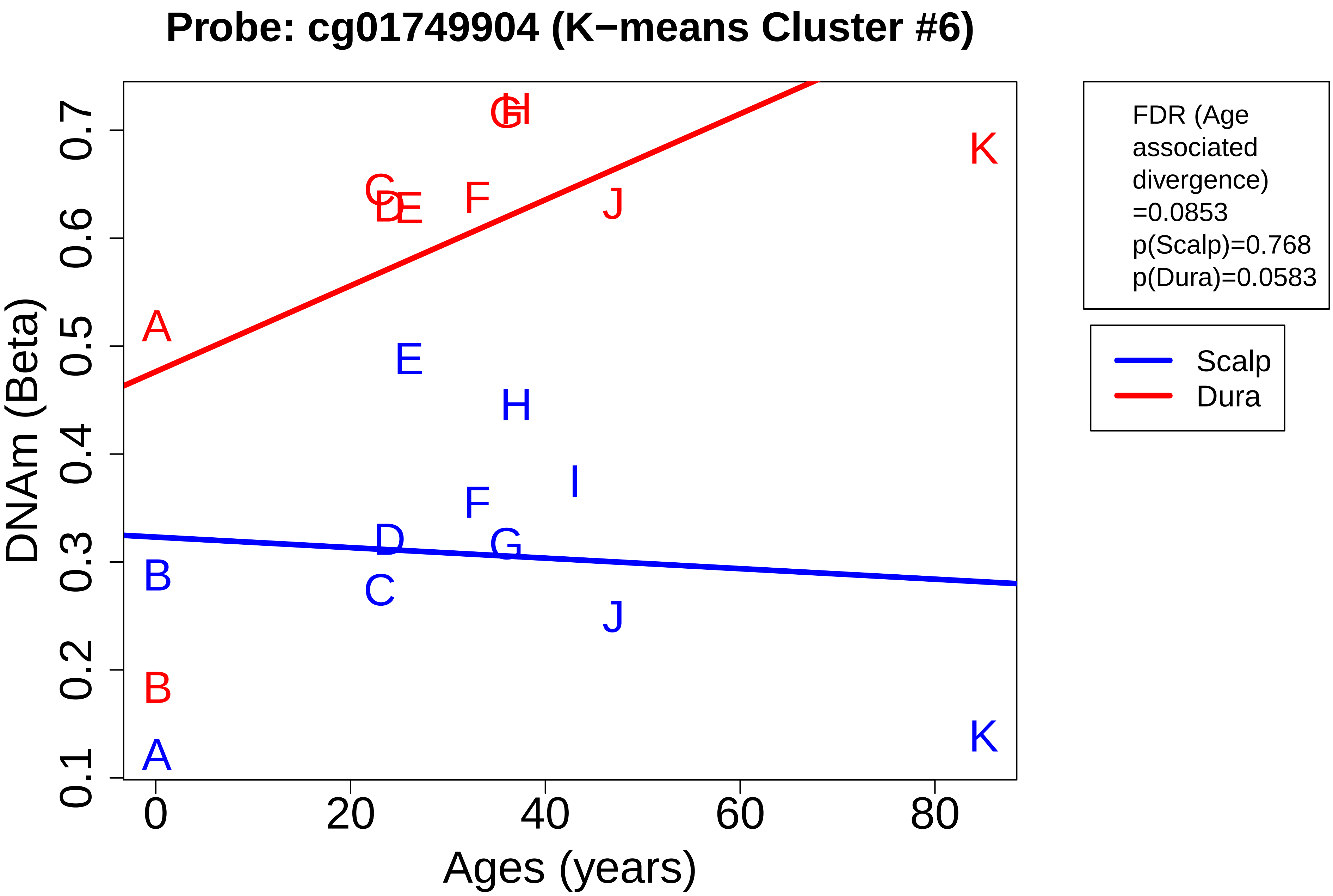

Probe: cg01749904 (K−means Cluster #6)
DNAm (Beta)
Ages (years)
0.1
0.2
0.3
0.4
0.5
0.6
0.7
0
20
40
60
80
FDR (Age associated divergence) =0.0853
p(Scalp)=0.768
p(Dura)=0.0583
Scalp
Dura

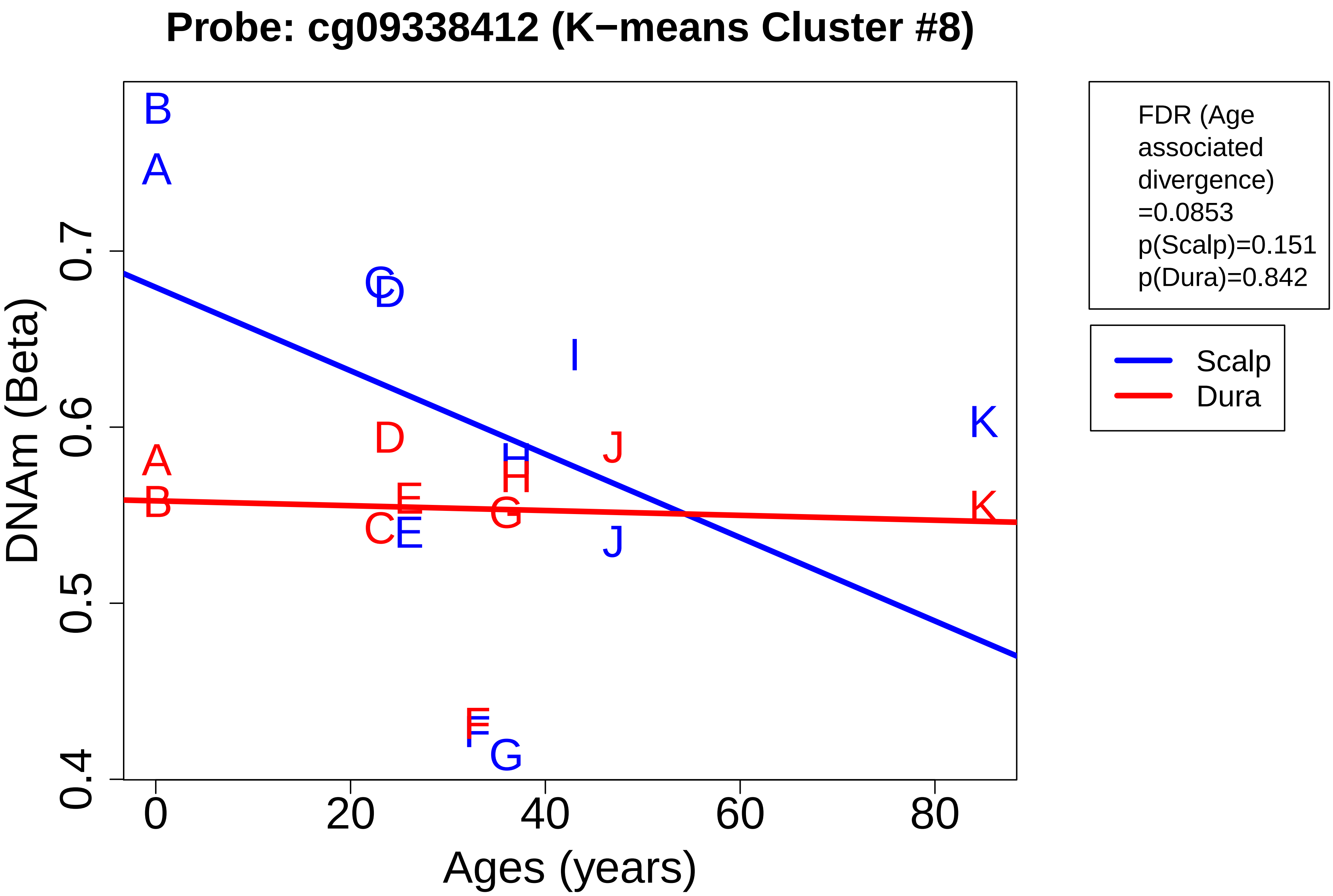

Probe: cg09338412 (K−means Cluster #8)
FDR (Age associated divergence) =0.0853
p(Scalp)=0.151
p(Dura)=0.842
Scalp
Dura
DNAm (Beta)
Ages (years)
0.4
0.5
0.6
0.7
0
20
40
60
80

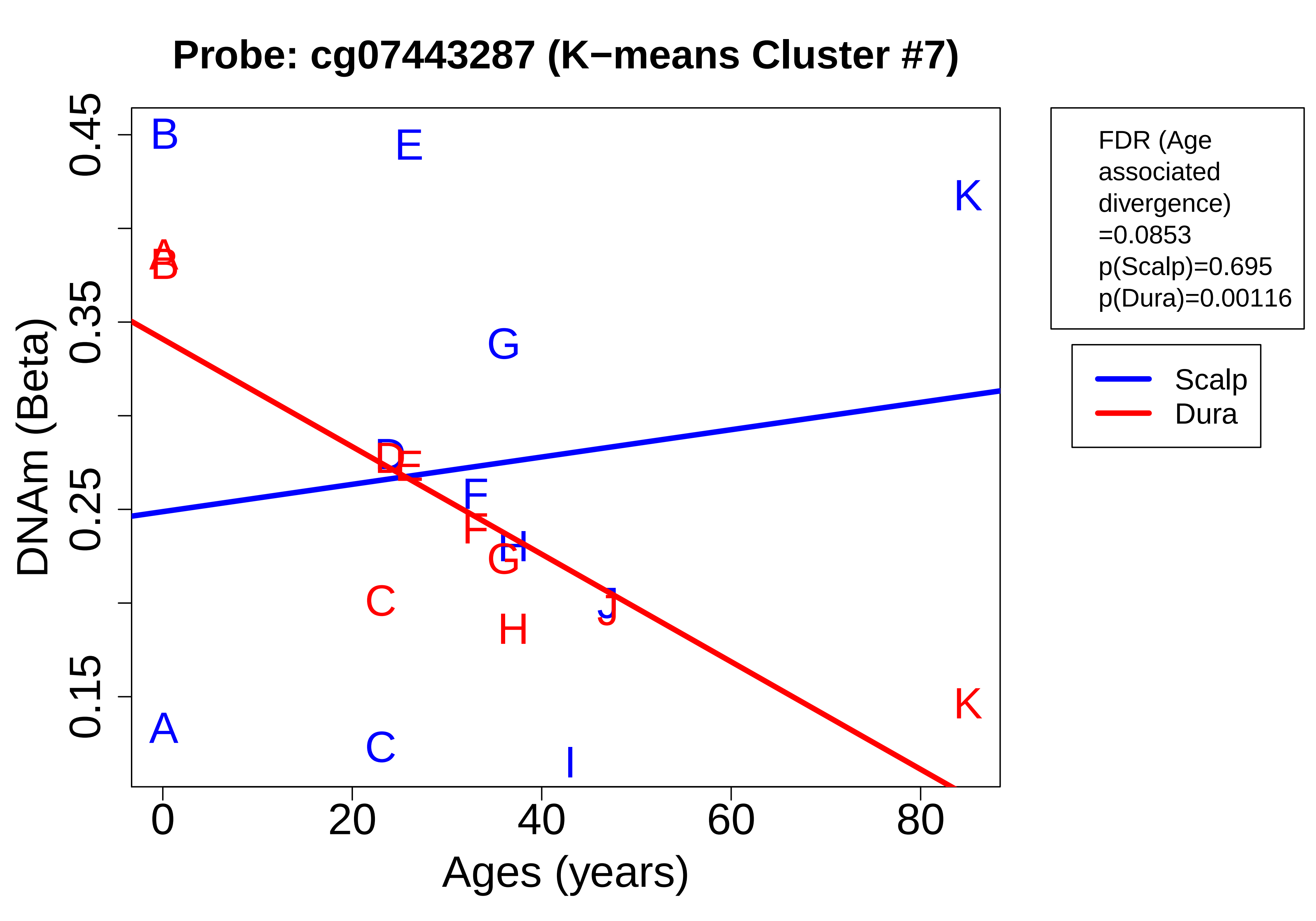
Probe: cg07443287 (K−means Cluster #7)
DNAm (Beta)
Ages (years)
0.15
0.25
0.35
0.45
0
20
40
60
80
FDR (Age associated divergence) =0.0853
p(Scalp)=0.695
p(Dura)=0.00116
Scalp
Dura

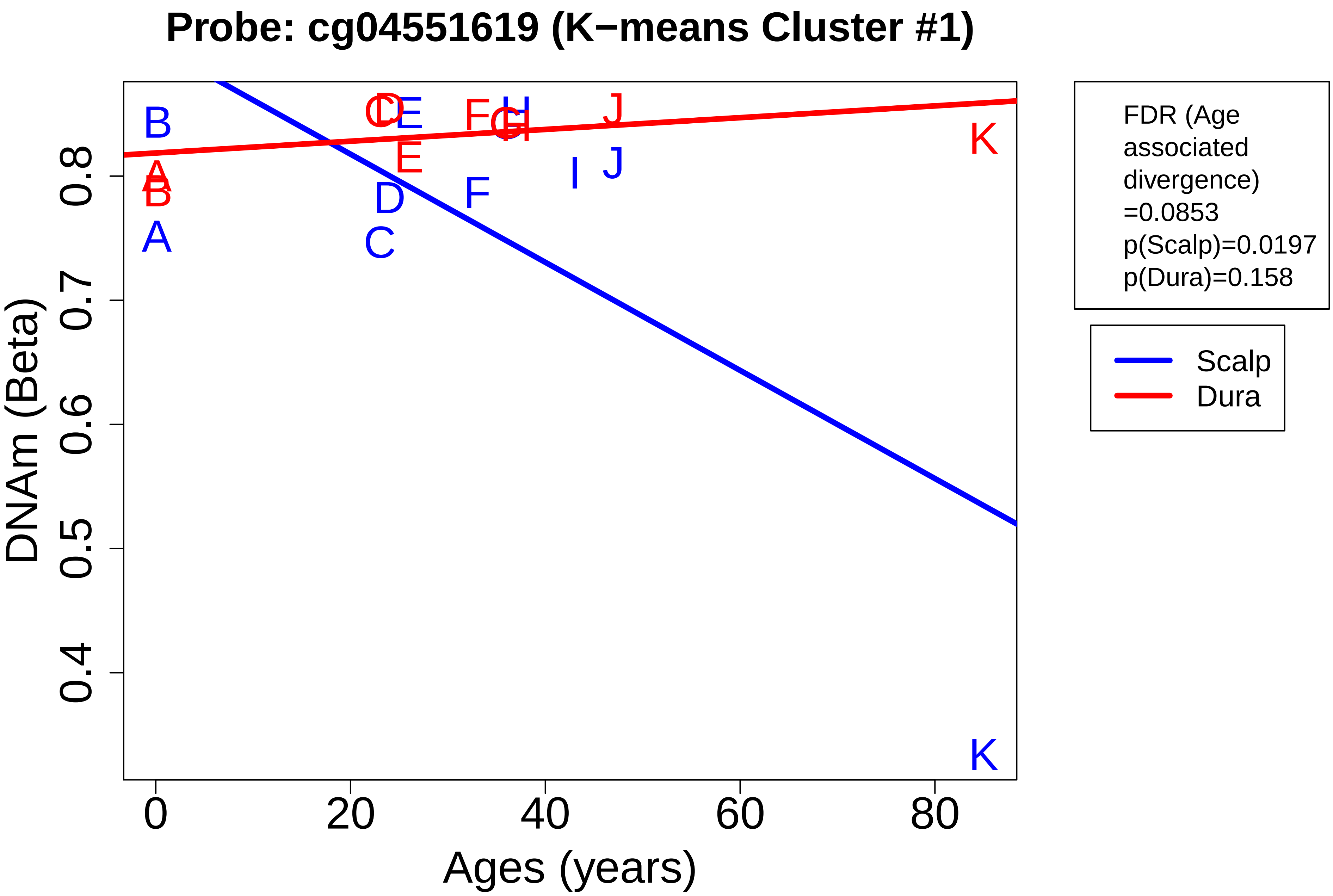

Probe: cg04551619 (K−means Cluster #1)
DNAm (Beta)
0.4
0.5
0.6
0.7
0.8
Ages (years)
0
20
40
60
80
FDR (Age associated divergence) =0.0853
p(Scalp)=0.0197
p(Dura)=0.158
Scalp
Dura

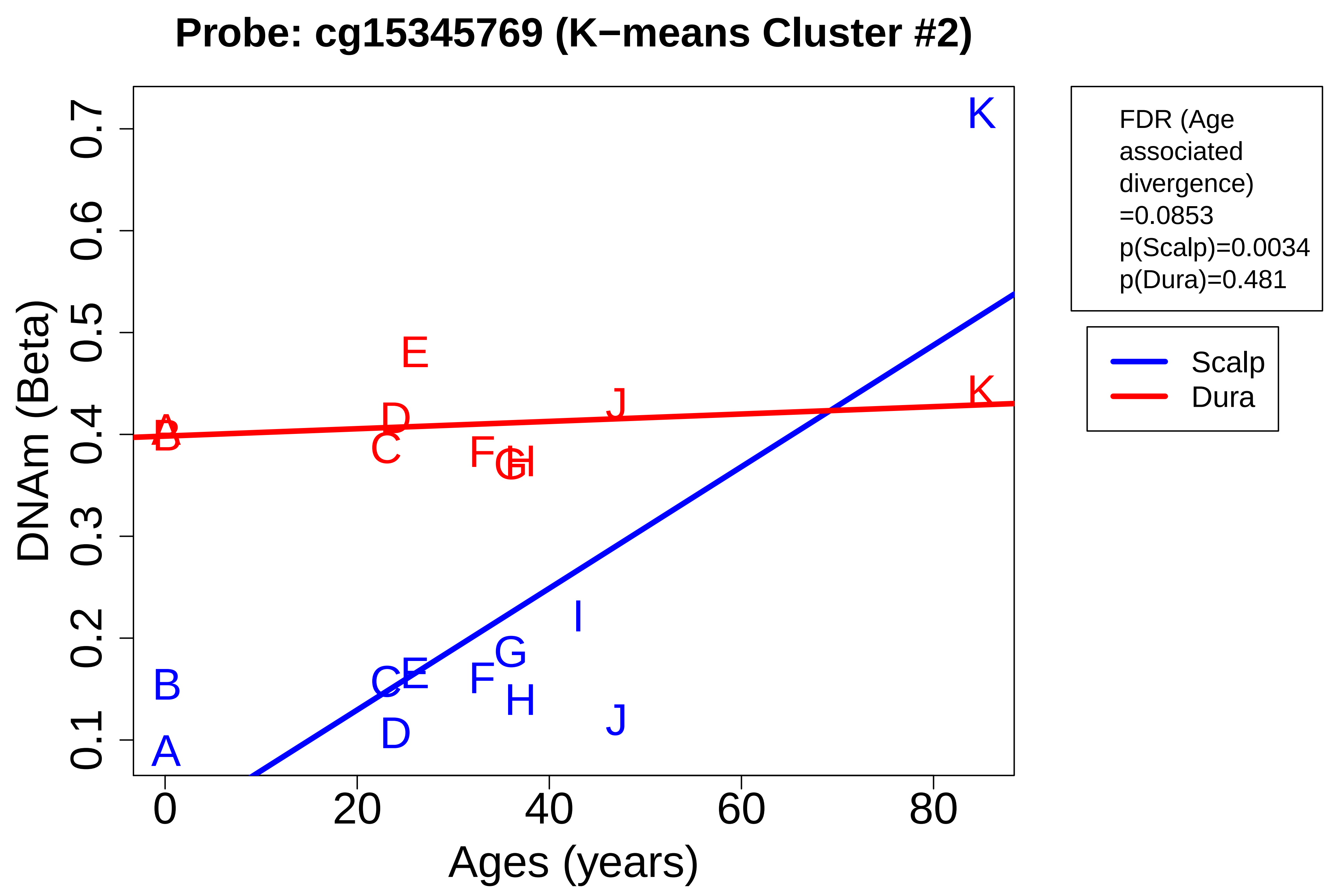

Probe: cg15345769 (K−means Cluster #2)
DNAm (Beta)
0.1
0.2
0.3
0.4
0.5
0.6
0.7
Ages (years)
0
20
40
60
80
FDR (Age associated divergence) =0.0853
p(Scalp)=0.0034
p(Dura)=0.481
Scalp
Dura
A
B
C
D
E
F
G
H
I
J
K

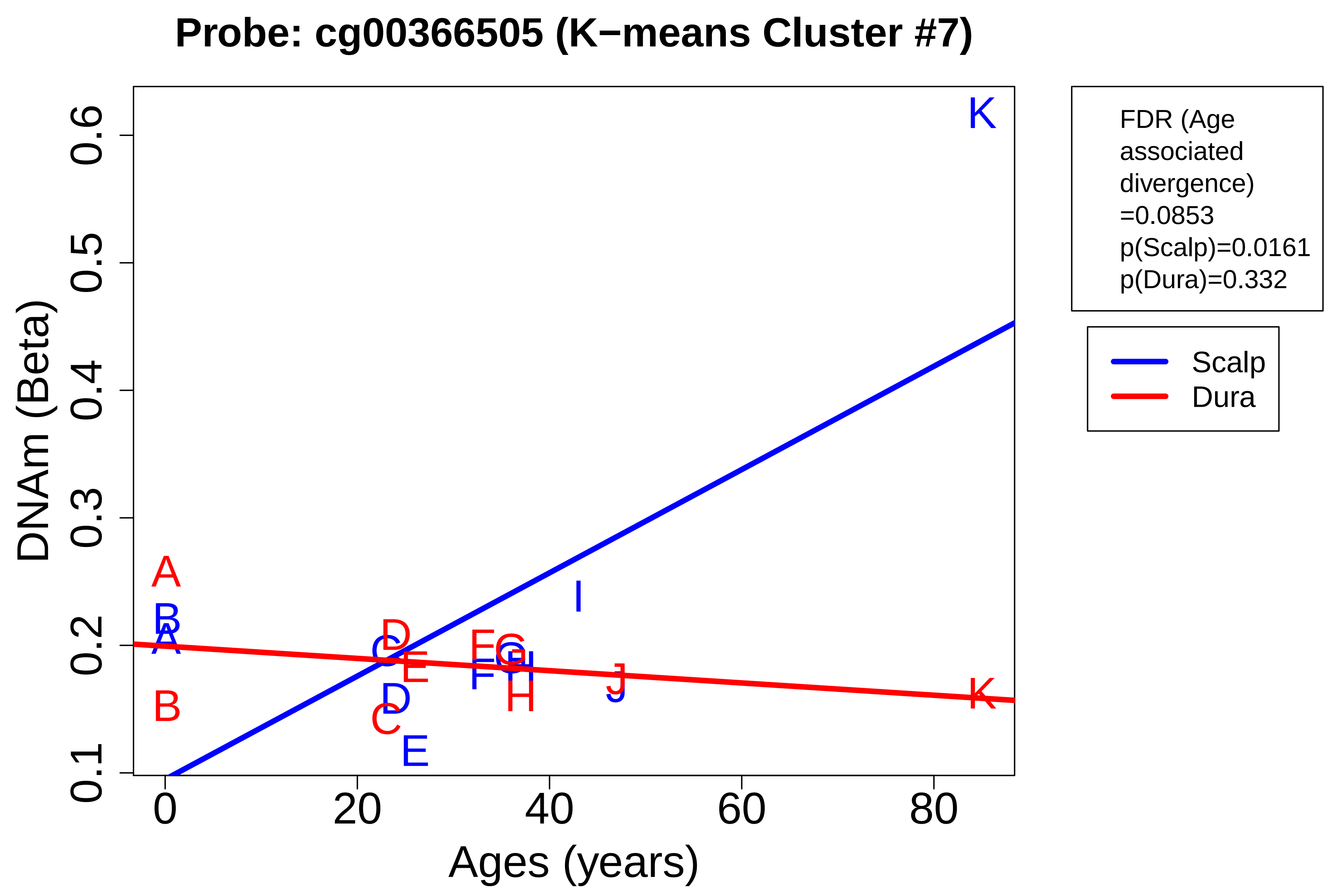
Probe: cg00366505 (K−means Cluster #7)
DNAm (Beta)
0.1
0.2
0.3
0.4
0.5
0.6
Ages (years)
0
20
40
60
80
FDR (Age associated divergence) =0.0853
p(Scalp)=0.0161
p(Dura)=0.332
Scalp
Dura

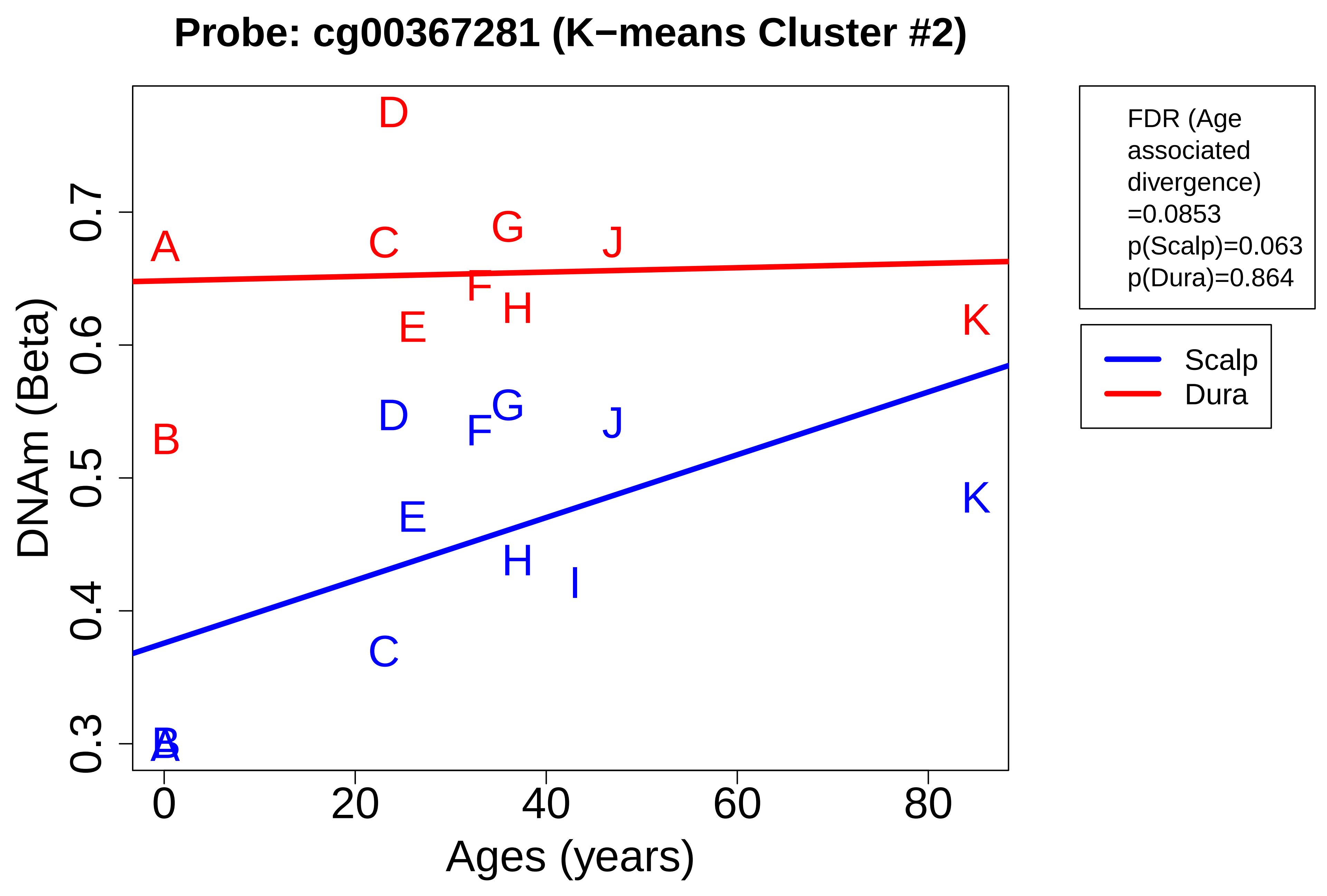

Probe: cg00367281 (K−means Cluster #2)
DNAm (Beta)
0.3
0.4
0.5
0.6
0.7
0
20
40
60
80
Ages (years)
FDR (Age associated divergence) =0.0853
p(Scalp)=0.063
p(Dura)=0.864
Scalp
Dura
A
B
C
D
E
F
G
H
I
J
K

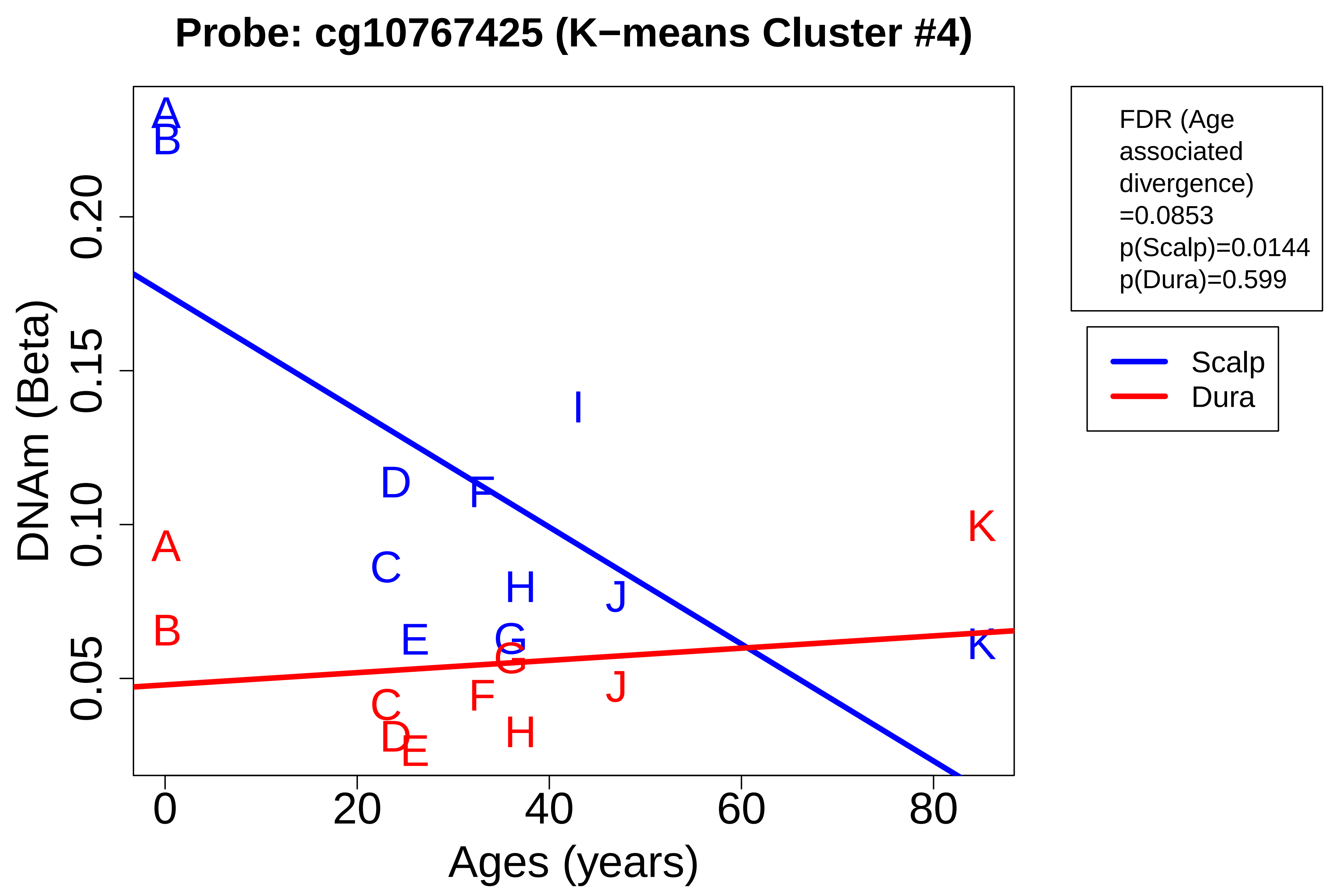

Probe: cg10767425 (K−means Cluster #4)
DNAm (Beta)
0.05
0.10
0.15
0.20
0
20
40
60
80
Ages (years)
FDR (Age associated divergence) =0.0853
p(Scalp)=0.0144
p(Dura)=0.599
Scalp
Dura
A
B
C
D
E
F
G
H
I
J
K

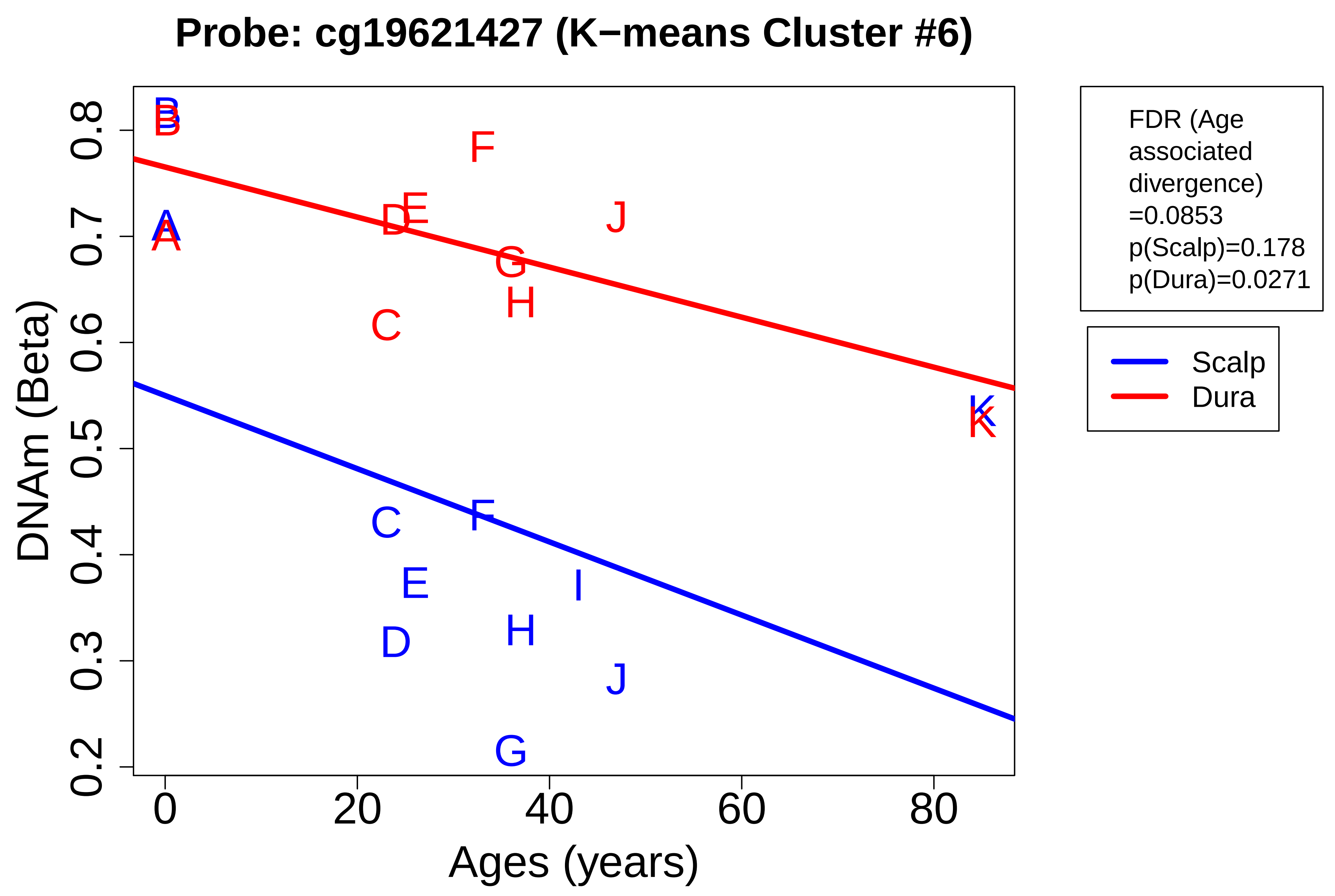

Probe: cg19621427 (K−means Cluster #6)
DNAm (Beta)
0.2
0.3
0.4
0.5
0.6
0.7
0.8
Ages (years)
0
20
40
60
80
FDR (Age associated divergence) =0.0853
p(Scalp)=0.178
p(Dura)=0.0271
Scalp
Dura
A
B
C
D
E
F
G
H
I
J
K

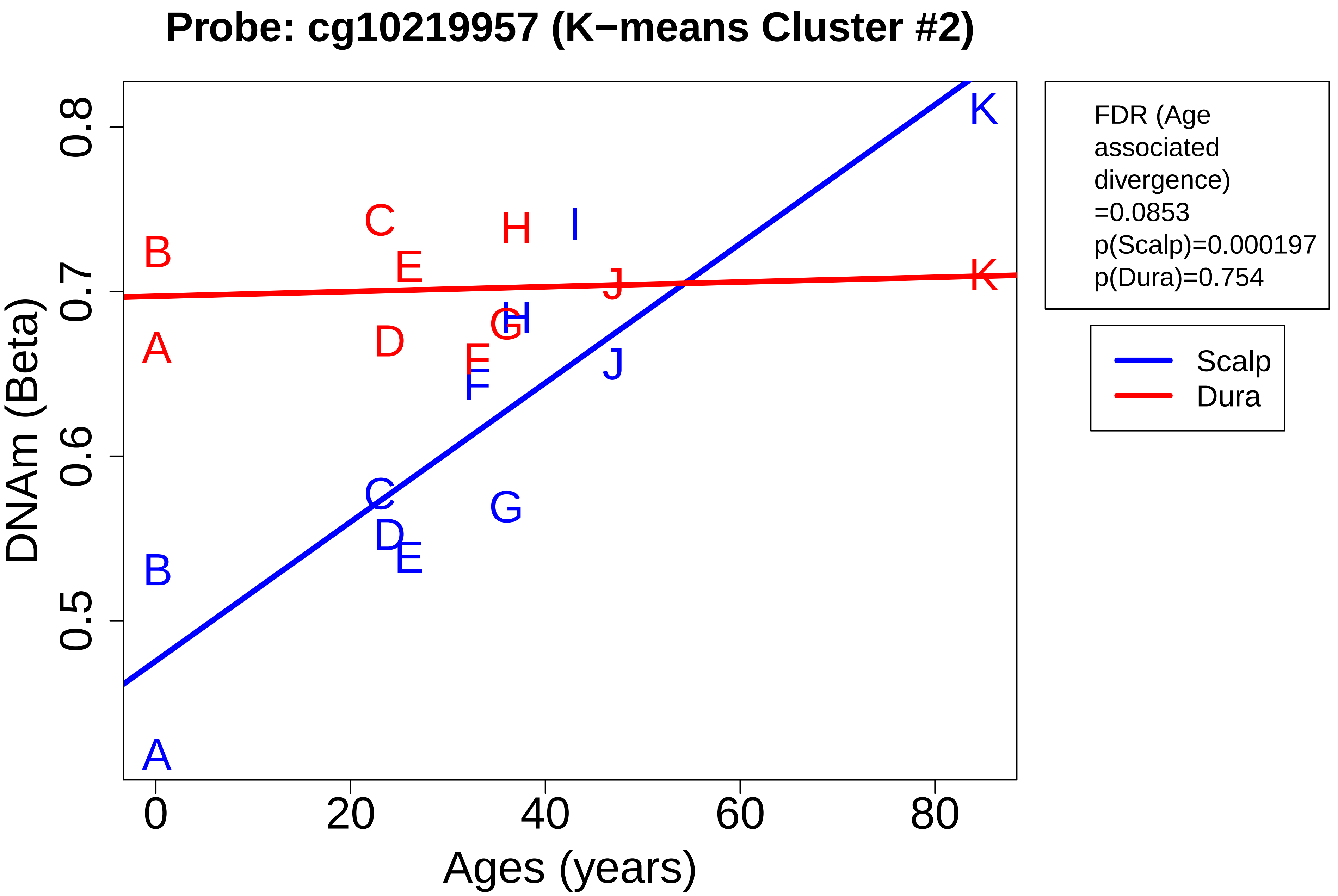

Probe: cg10219957 (K−means Cluster #2)
FDR (Age associated divergence) =0.0853
p(Scalp)=0.000197
p(Dura)=0.754
Scalp
Dura
DNAm (Beta)
0.5
0.6
0.7
0.8
Ages (years)
0
20
40
60
80
A B C D E F G H I J K

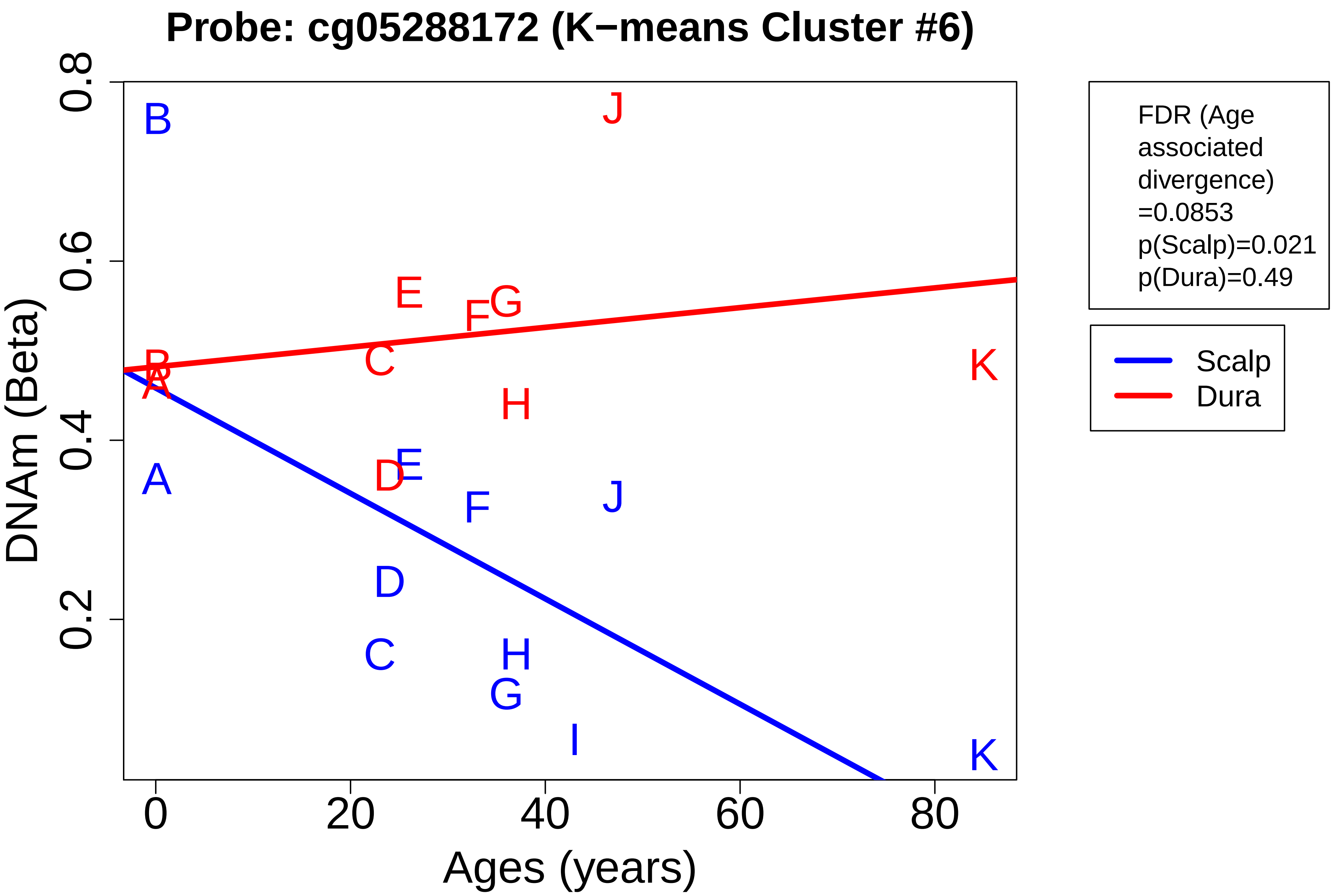

Probe: cg05288172 (K−means Cluster #6)
DNAm (Beta)
0.8
0.6
0.4
0.2
0
20
40
60
80
Ages (years)
FDR (Age associated divergence) =0.0853
p(Scalp)=0.021
p(Dura)=0.49
Scalp
Dura

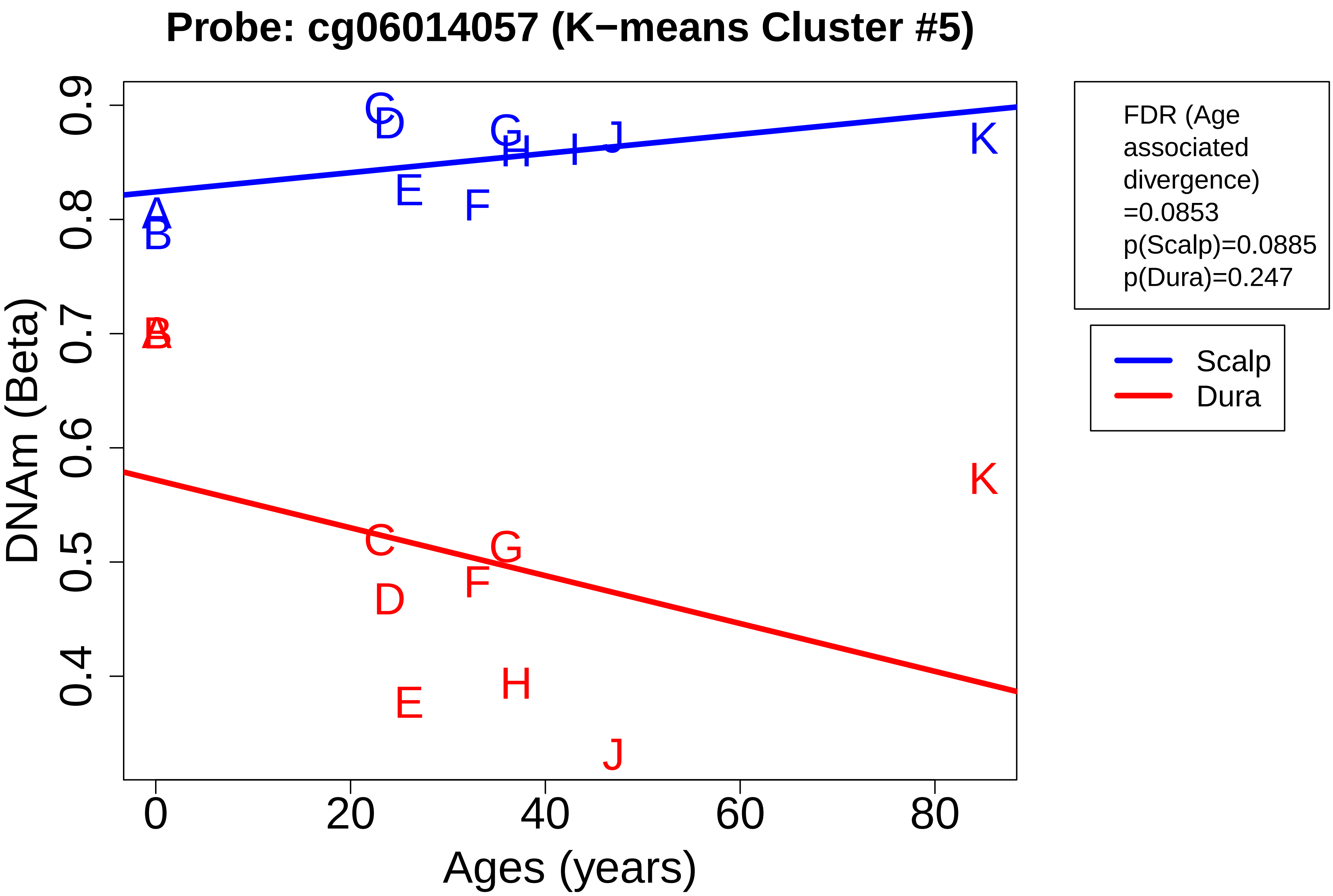
Probe: cg06014057 (K−means Cluster #5)
DNAm (Beta)
Ages (years)
0.4
0.5
0.6
0.7
0.8
0.9
0
20
40
60
80
FDR (Age associated divergence) =0.0853
p(Scalp)=0.0885
p(Dura)=0.247
Scalp
Dura

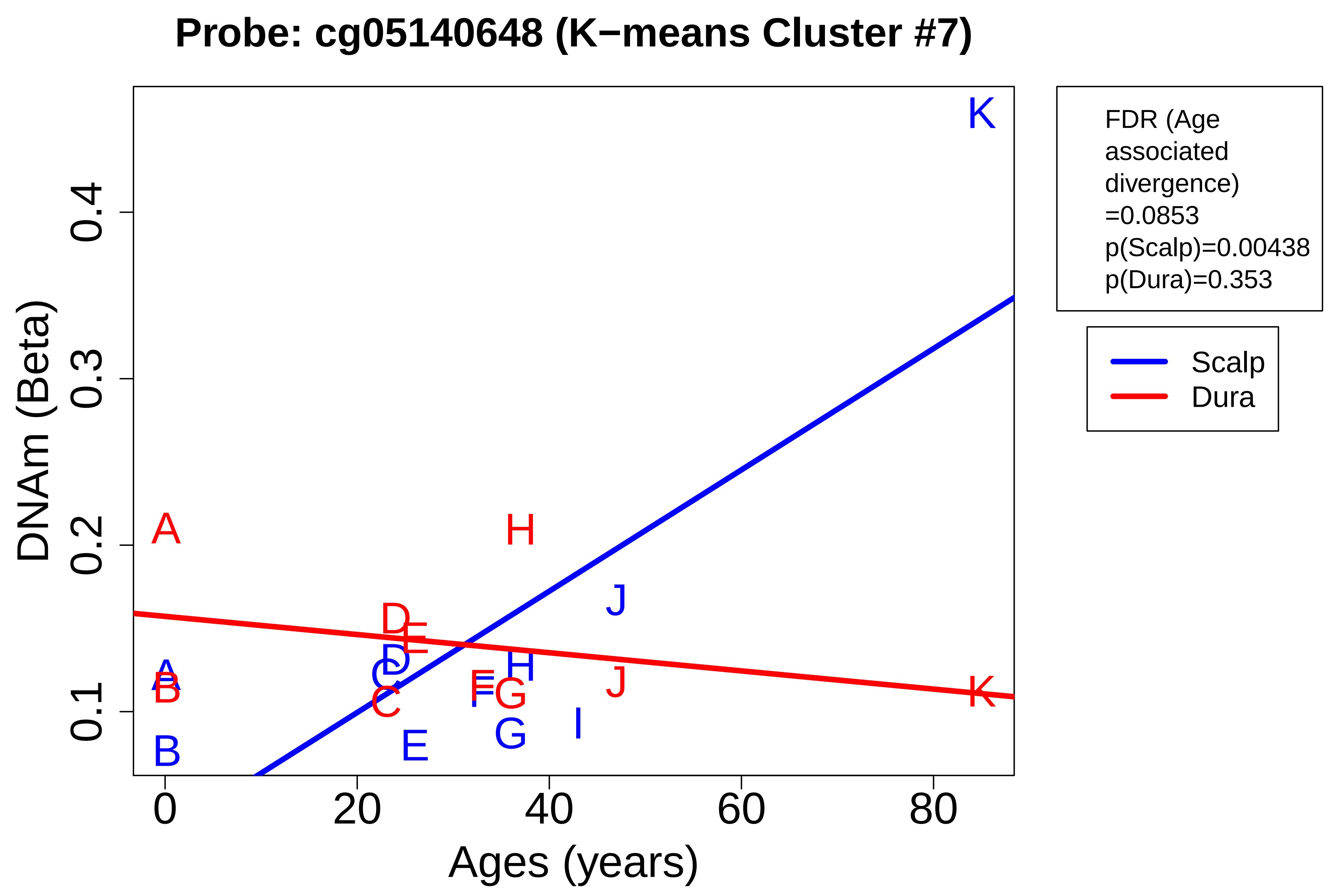

Probe: cg05140648 (K−means Cluster #7)
DNAm (Beta)
0.1
0.2
0.3
0.4
Ages (years)
0
20
40
60
80
FDR (Age associated divergence) =0.0853
p(Scalp)=0.00438
p(Dura)=0.353
Scalp
Dura
A
B
C
D
E
F
G
H
I
J
K

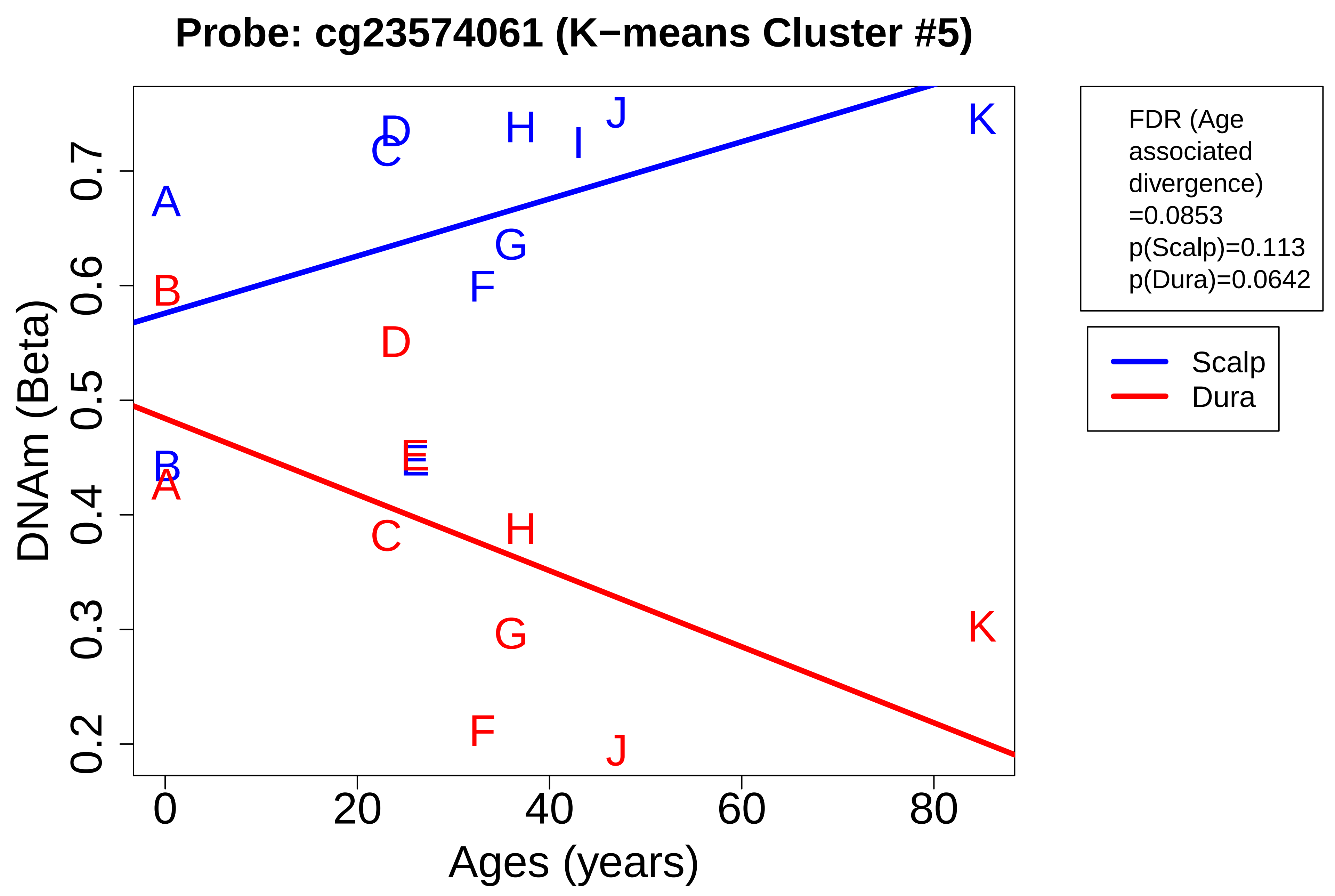

Probe: cg23574061 (K−means Cluster #5)
DNAm (Beta)
0.2
0.3
0.4
0.5
0.6
0.7
0
20
40
60
80
Ages (years)
A
B
C
D
E
F
G
H
I
J
K
A
B
C
D
E
F
G
H
J
K
FDR (Age associated divergence) =0.0853
p(Scalp)=0.113
p(Dura)=0.0642
Scalp
Dura

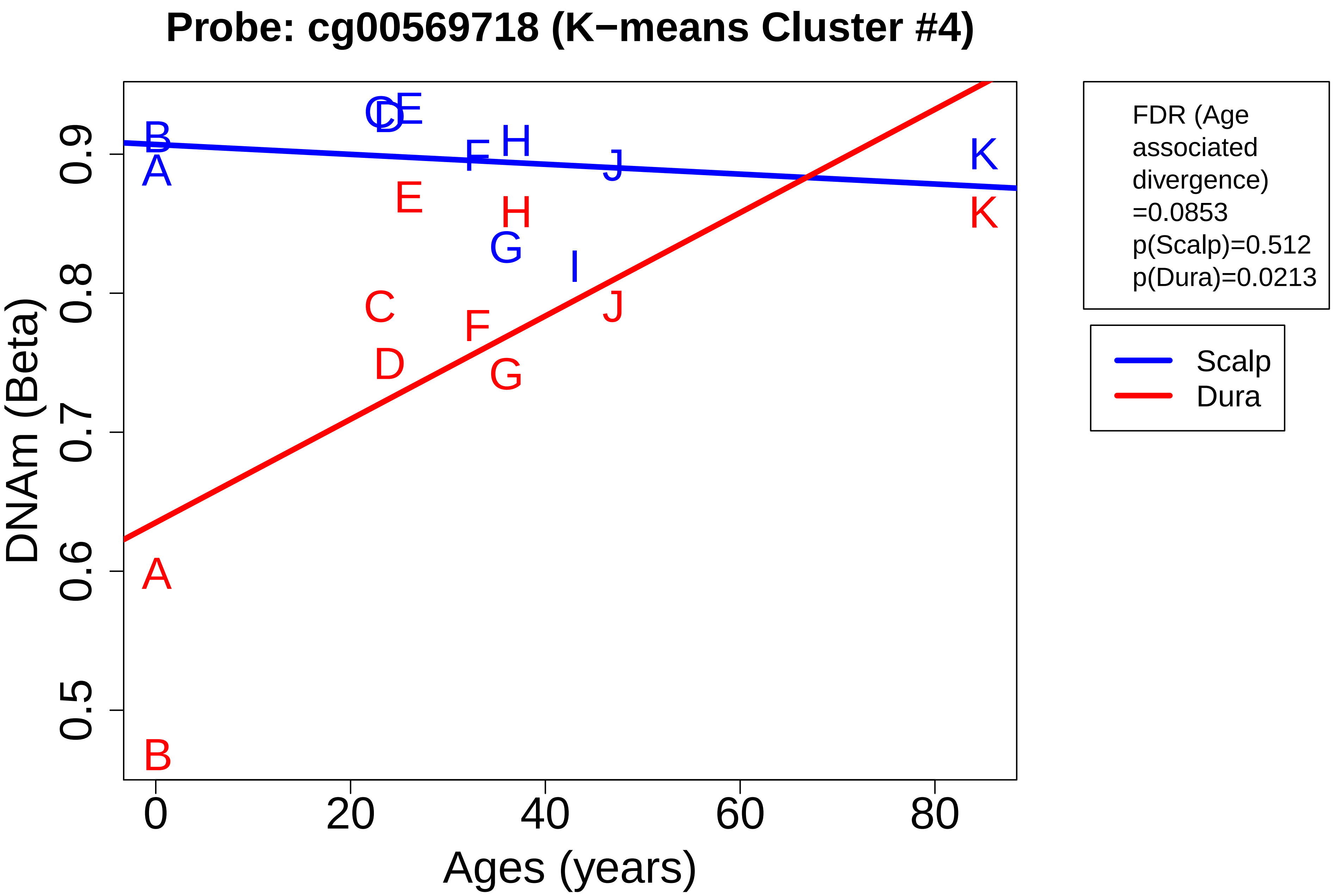

Probe: cg00569718 (K−means Cluster #4)
DNAm (Beta)
0.5
0.6
0.7
0.8
0.9
0
20
40
60
80
Ages (years)
FDR (Age associated divergence) =0.0853
p(Scalp)=0.512
p(Dura)=0.0213
Scalp
Dura

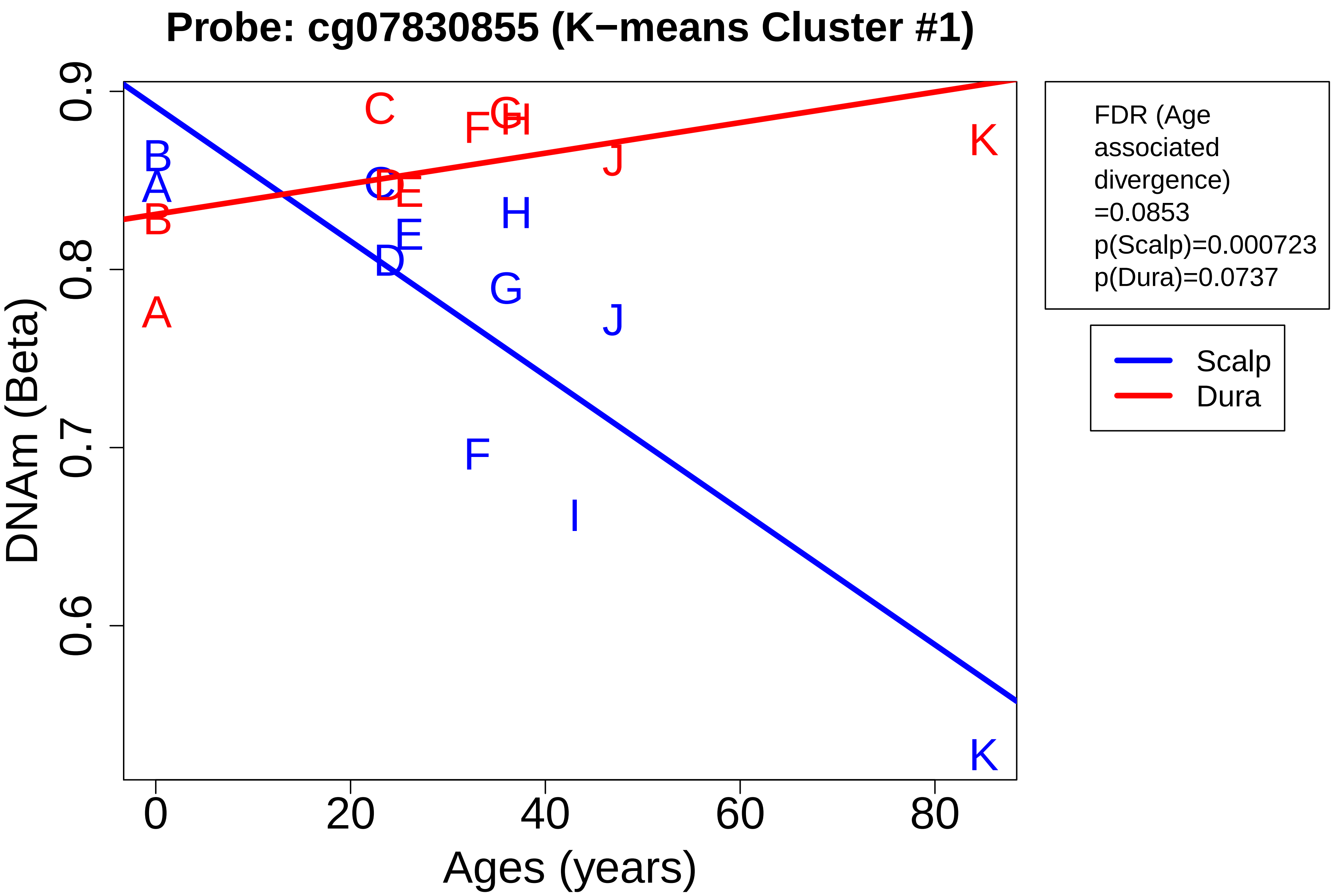
Probe: cg07830855 (K−means Cluster #1)
DNAm (Beta)
0.6
0.7
0.8
0.9
Ages (years)
0
20
40
60
80
FDR (Age associated divergence) =0.0853
p(Scalp)=0.000723
p(Dura)=0.0737
Scalp
Dura
A
B
C
D
E
F
G
H
I
J
K

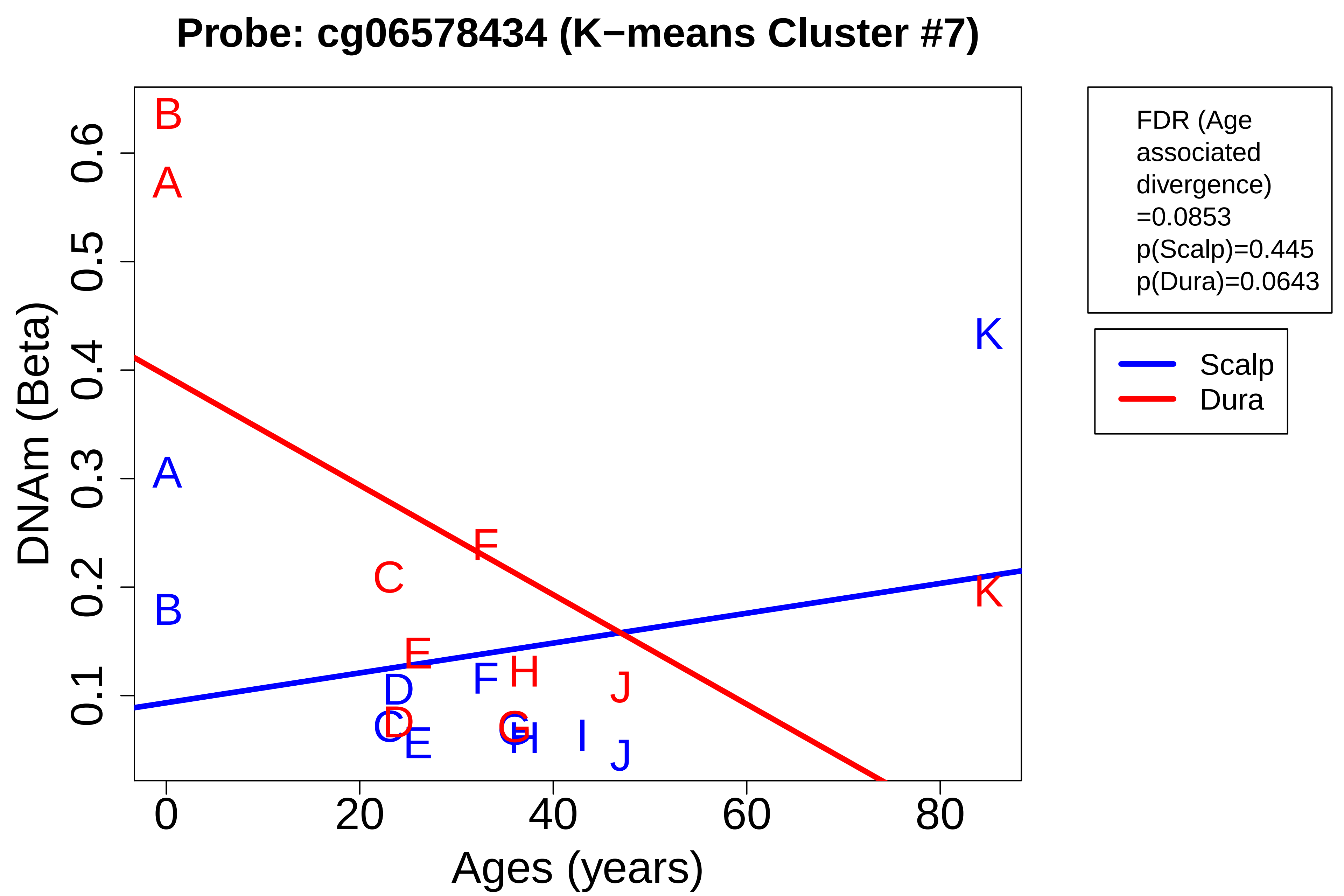
Probe: cg06578434 (K−means Cluster #7)
DNAm (Beta)
Ages (years)
0.1
0.2
0.3
0.4
0.5
0.6
0
20
40
60
80
FDR (Age associated divergence) =0.0853
p(Scalp)=0.445
p(Dura)=0.0643
Scalp
Dura
A
B
C
D
E
F
G
H
I
J
K

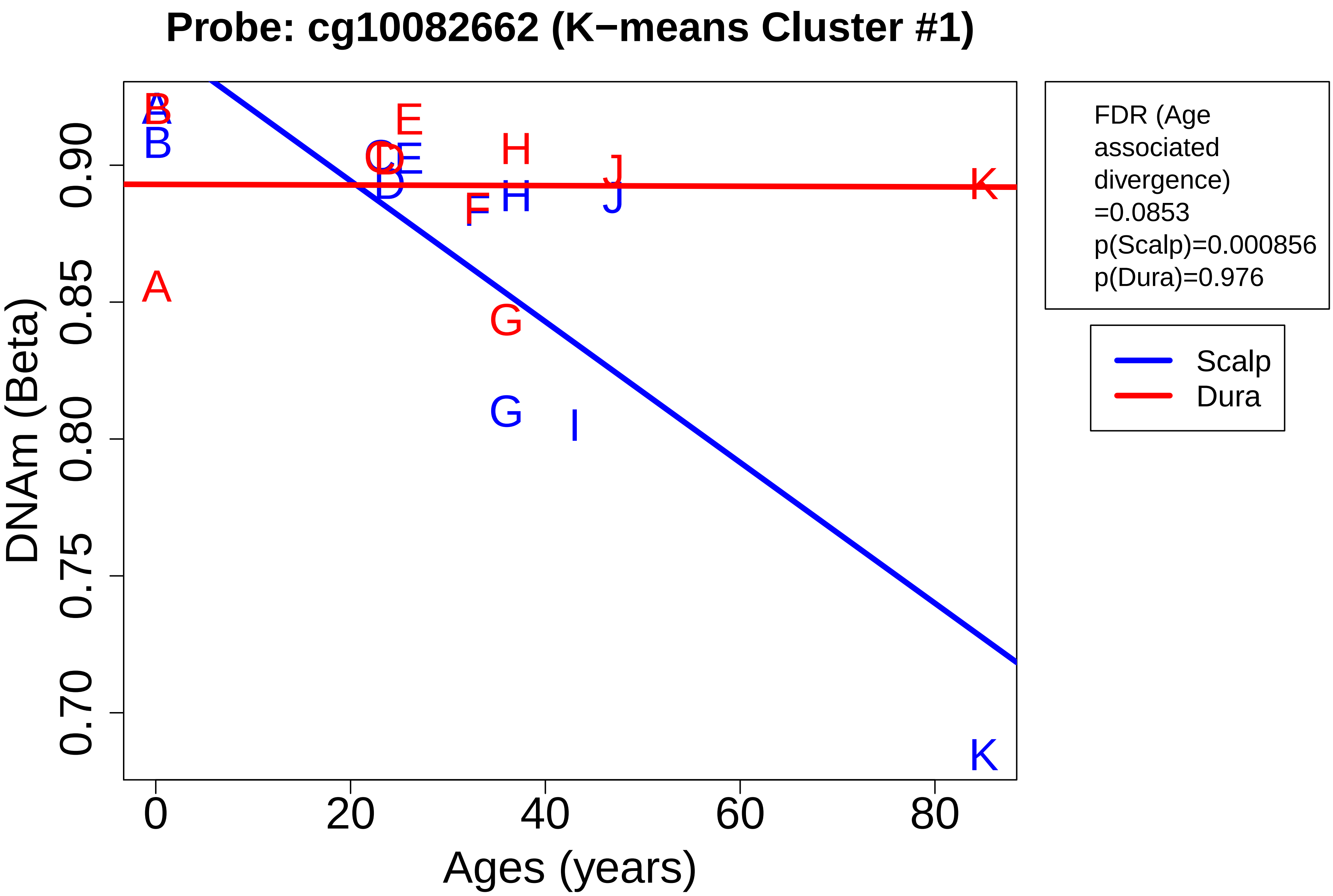

Probe: cg10082662 (K−means Cluster #1)
FDR (Age associated divergence) =0.0853
p(Scalp)=0.000856
p(Dura)=0.976
Scalp
Dura
DNAm (Beta)
0.70
0.75
0.80
0.85
0.90
0
20
40
60
80
Ages (years)
A
B
C
D
E
F
G
H
I
J
K

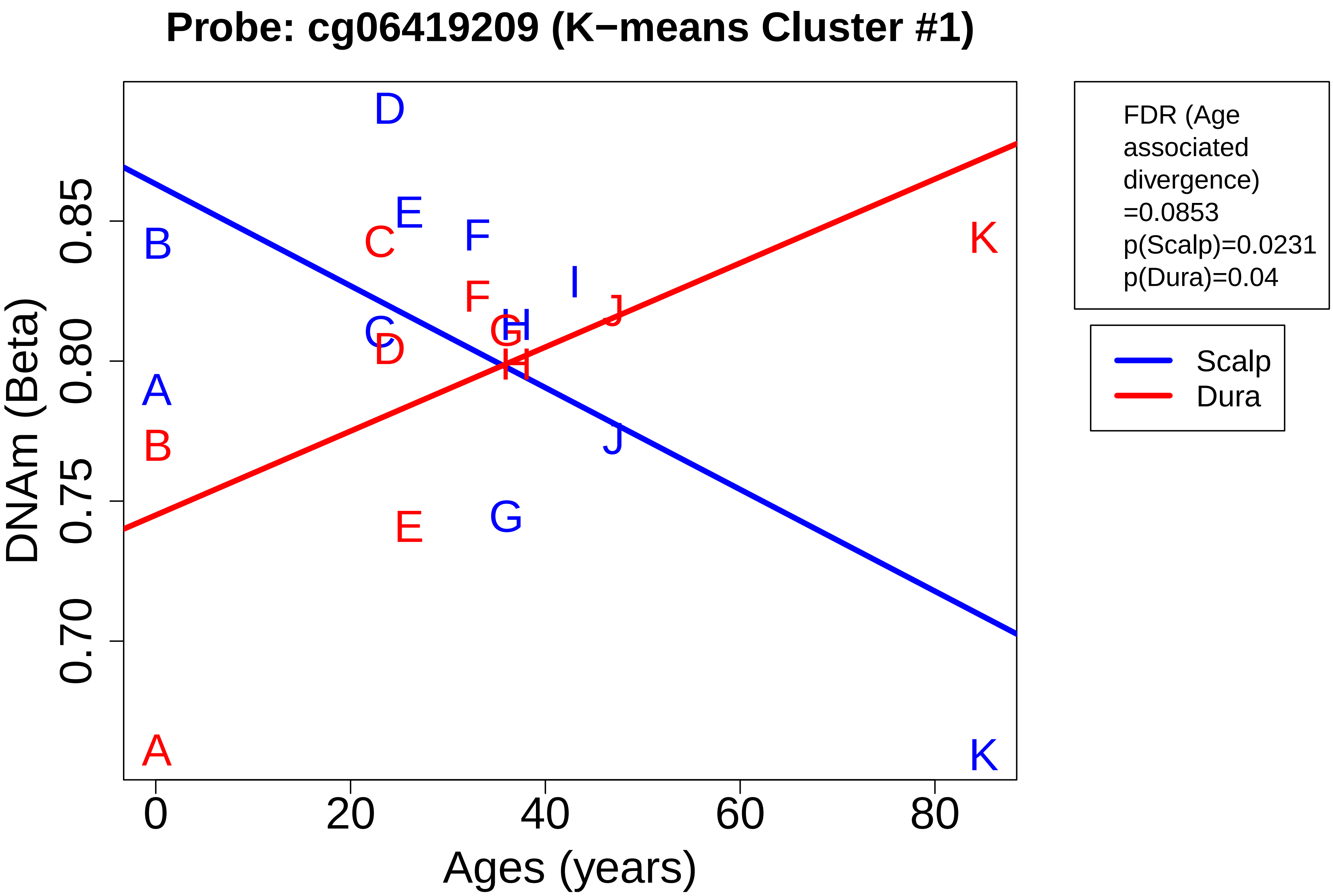

Probe: cg06419209 (K−means Cluster #1)
DNAm (Beta)
0.70
0.75
0.80
0.85
0
20
40
60
80
Ages (years)
FDR (Age associated divergence) =0.0853
p(Scalp)=0.0231
p(Dura)=0.04
Scalp
Dura

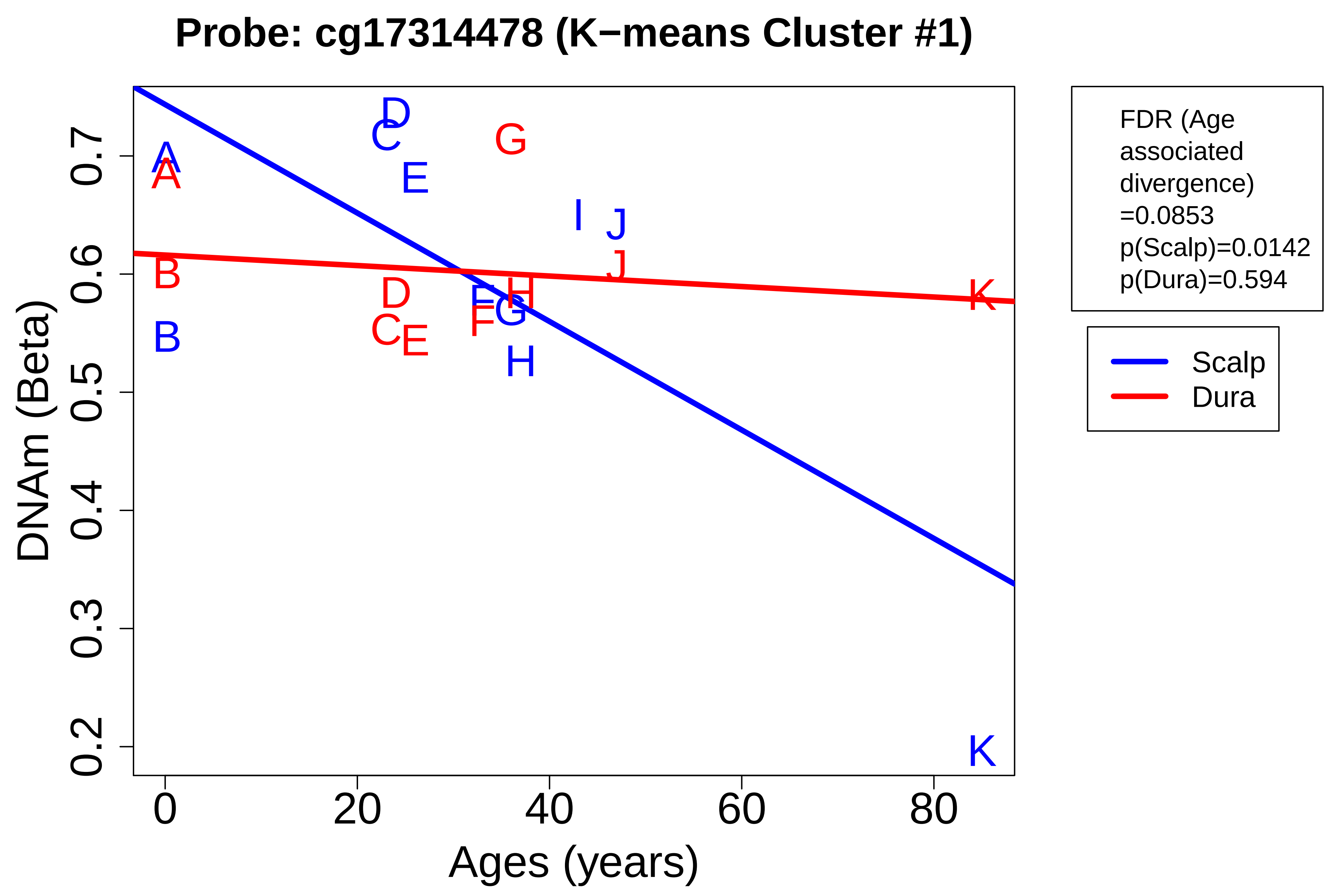

Probe: cg17314478 (K−means Cluster #1)
DNAm (Beta)
0.2
0.3
0.4
0.5
0.6
0.7
0
20
40
60
80
Ages (years)
A
A
B
B
C
C
D
D
E
E
F
F
G
G
H
H
I
J
J
K
K
FDR (Age associated divergence) =0.0853
p(Scalp)=0.0142
p(Dura)=0.594
Scalp
Dura

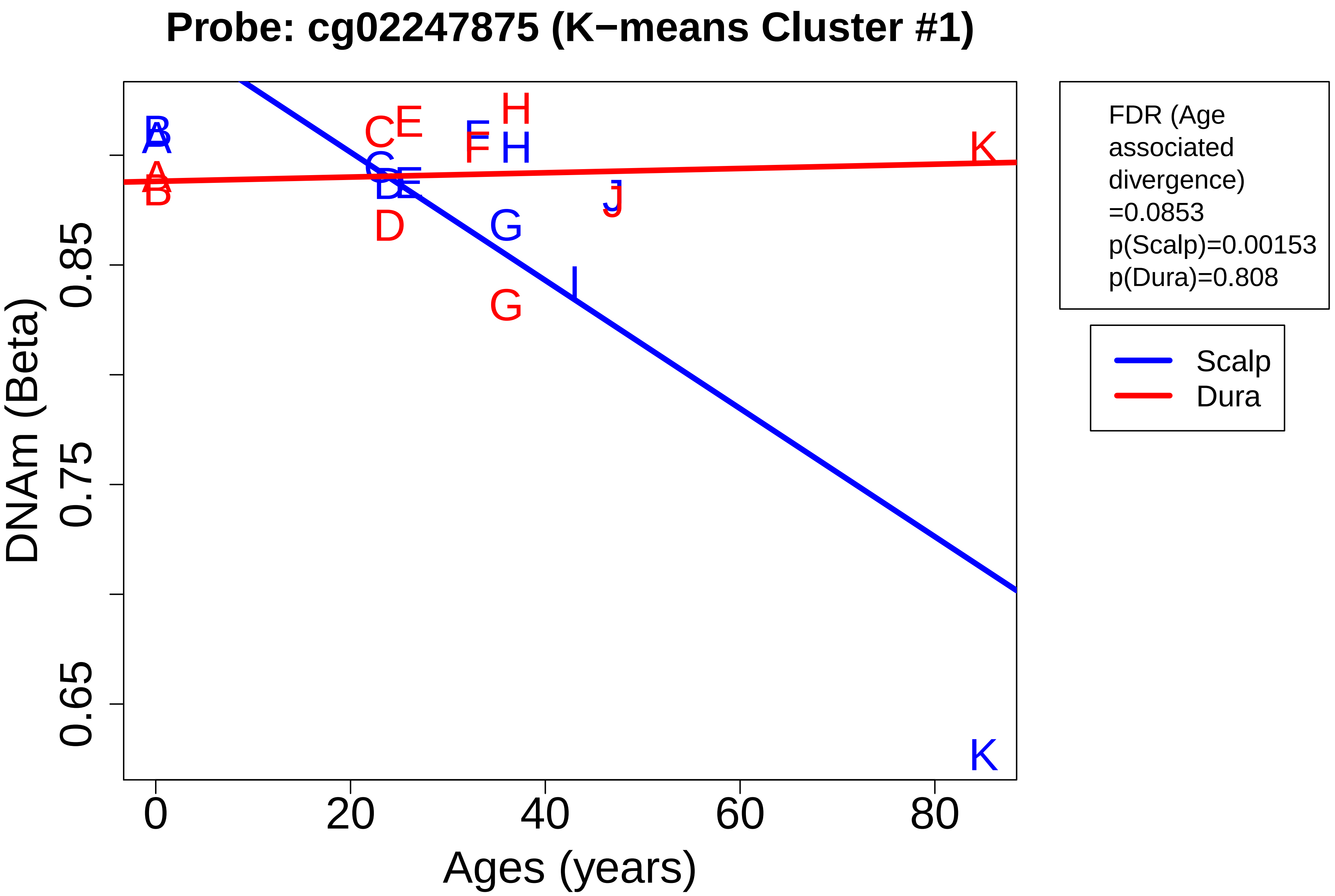

Probe: cg02247875 (K−means Cluster #1)
DNAm (Beta)
0.65
0.75
0.85
Ages (years)
0
20
40
60
80
FDR (Age associated divergence) =0.0853
p(Scalp)=0.00153
p(Dura)=0.808
Scalp
Dura

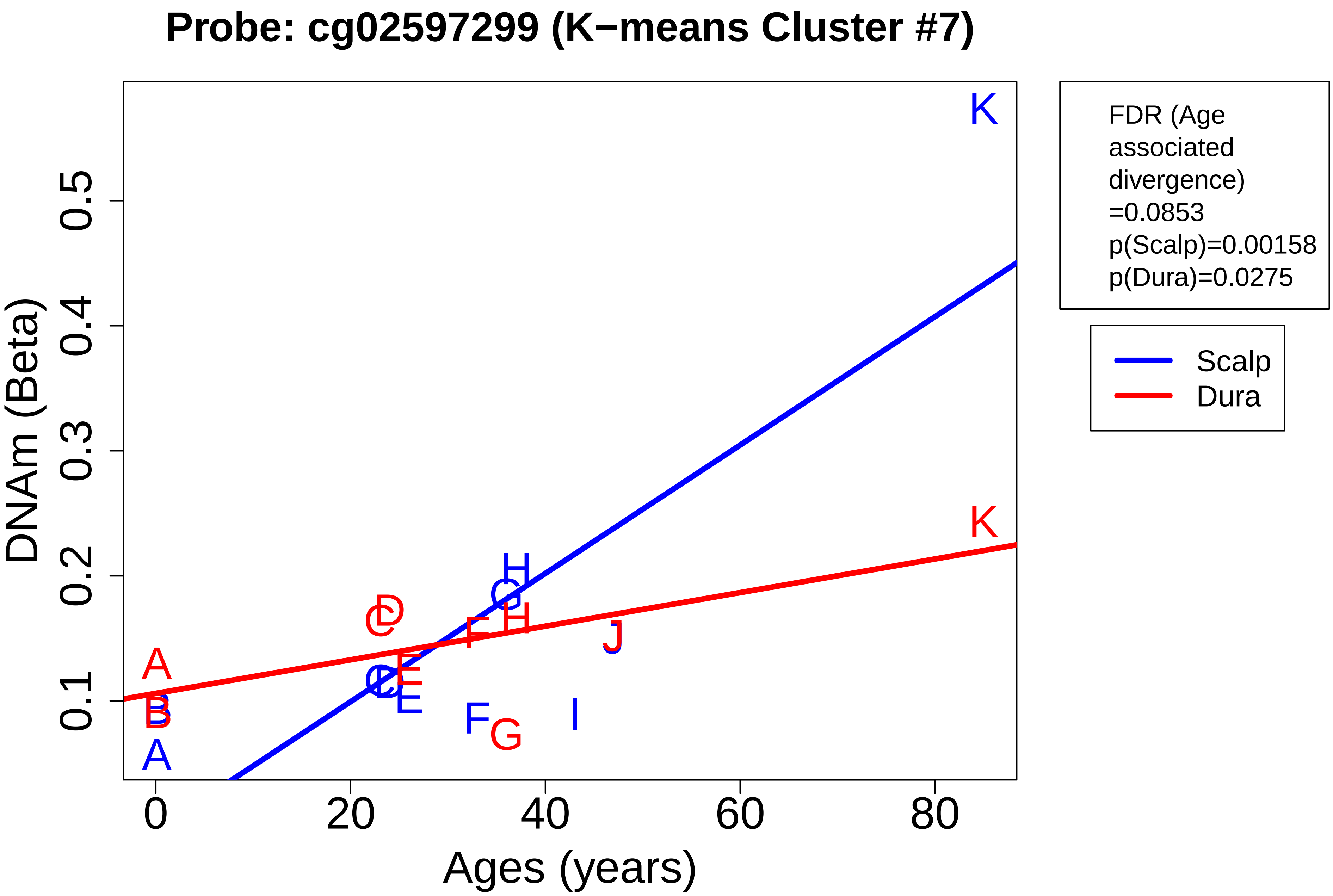

Probe: cg02597299 (K−means Cluster #7)
DNAm (Beta)
0.1
0.2
0.3
0.4
0.5
0
20
40
60
80
Ages (years)
FDR (Age associated divergence) =0.0853
p(Scalp)=0.00158
p(Dura)=0.0275
Scalp
Dura

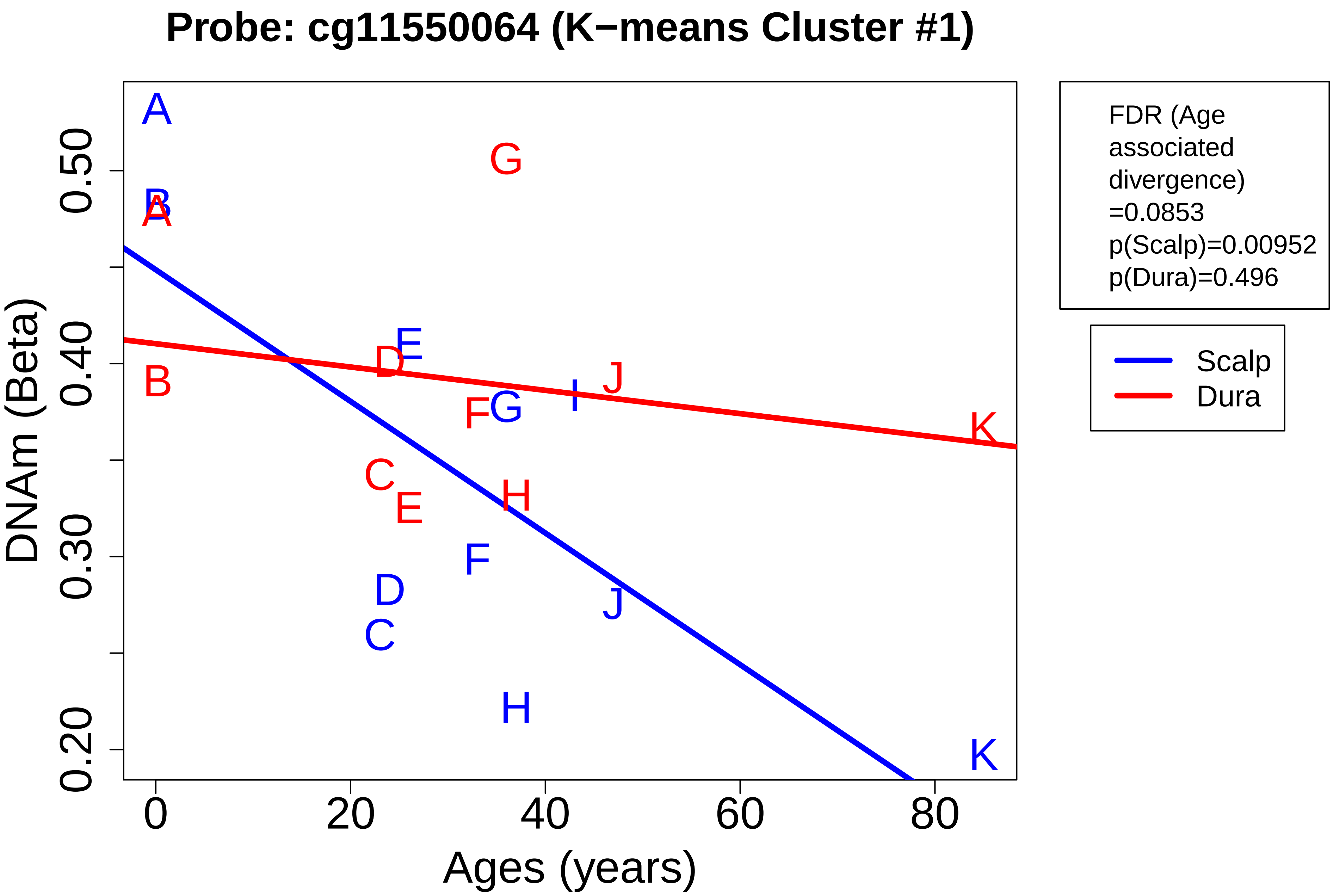
Probe: cg11550064 (K−means Cluster #1)
DNAm (Beta)
0.20
0.30
0.40
0.50
Ages (years)
0
20
40
60
80
FDR (Age associated divergence) =0.0853
p(Scalp)=0.00952
p(Dura)=0.496
Scalp
Dura
A
B
C
D
E
F
G
H
I
J
K

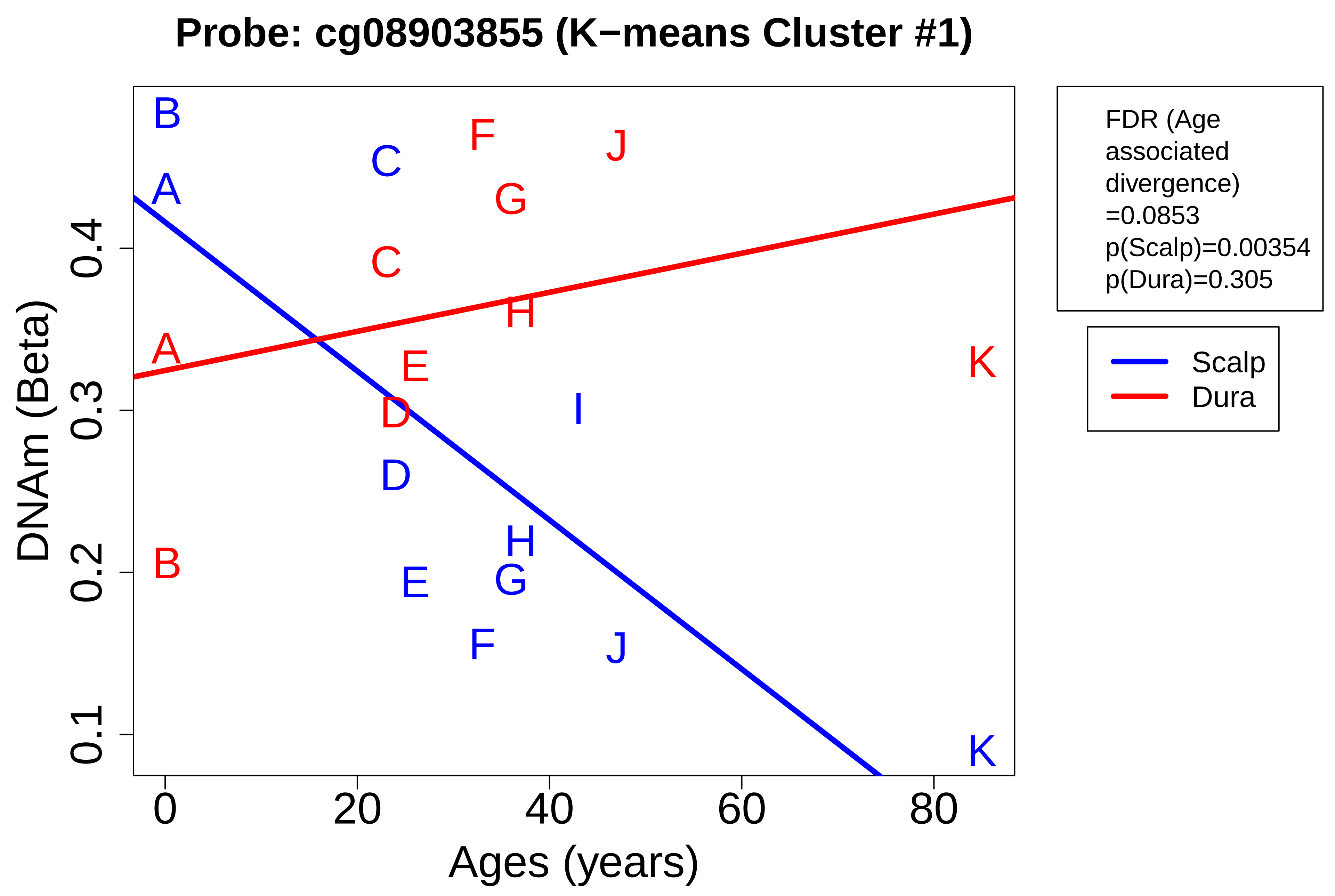
Probe: cg08903855 (K−means Cluster #1)
FDR (Age associated divergence) =0.0853
p(Scalp)=0.00354
p(Dura)=0.305
Scalp
Dura
DNAm (Beta)
0.1
0.2
0.3
0.4
Ages (years)
0
20
40
60
80
A
B
C
D
E
F
G
H
I
J
K

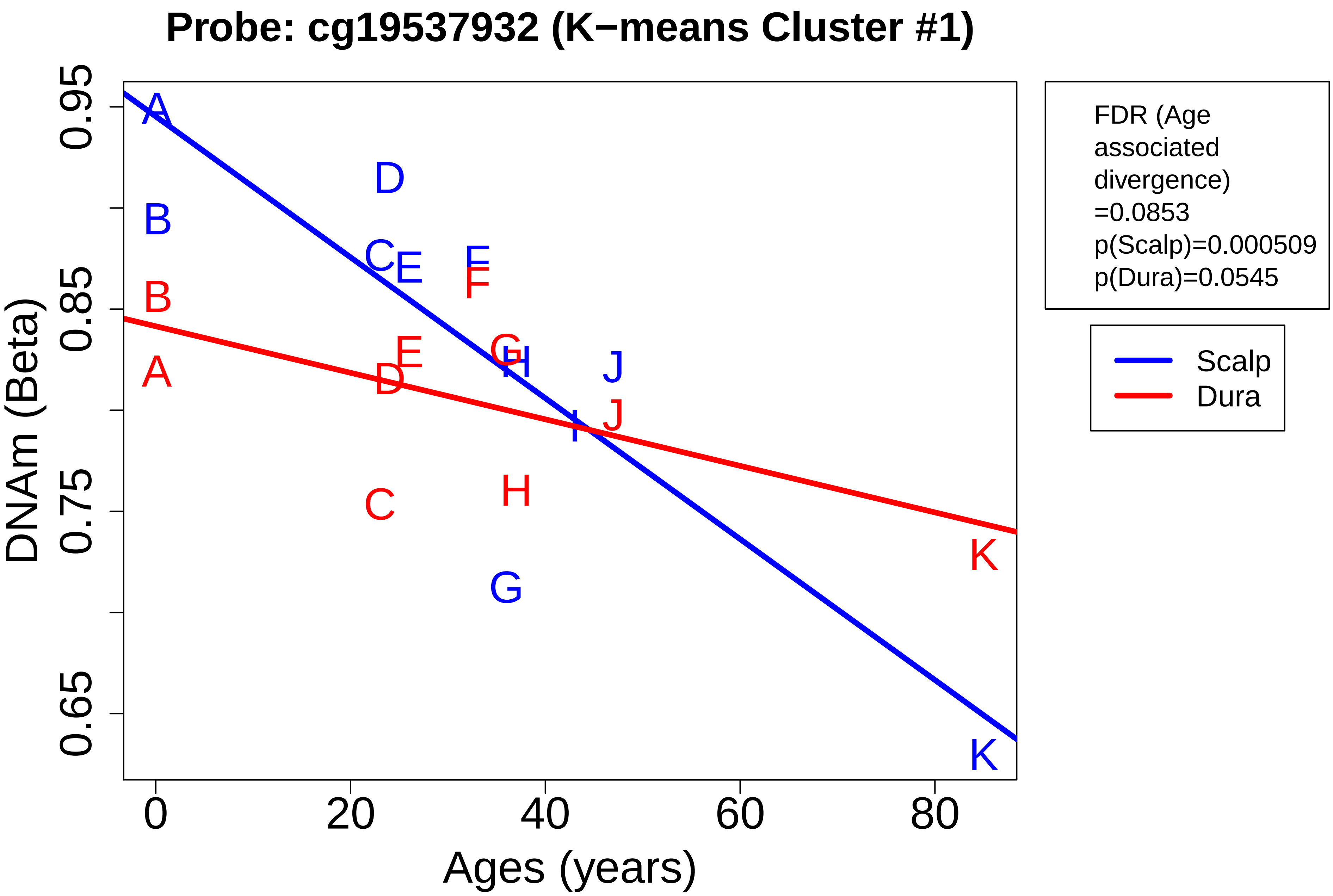

Probe: cg19537932 (K−means Cluster #1)
DNAm (Beta)
0.95
0.85
0.75
0.65
Ages (years)
0
20
40
60
80
FDR (Age associated divergence) =0.0853
p(Scalp)=0.000509
p(Dura)=0.0545
Scalp
Dura
A
B
C
D
E
F
G
H
I
J
K

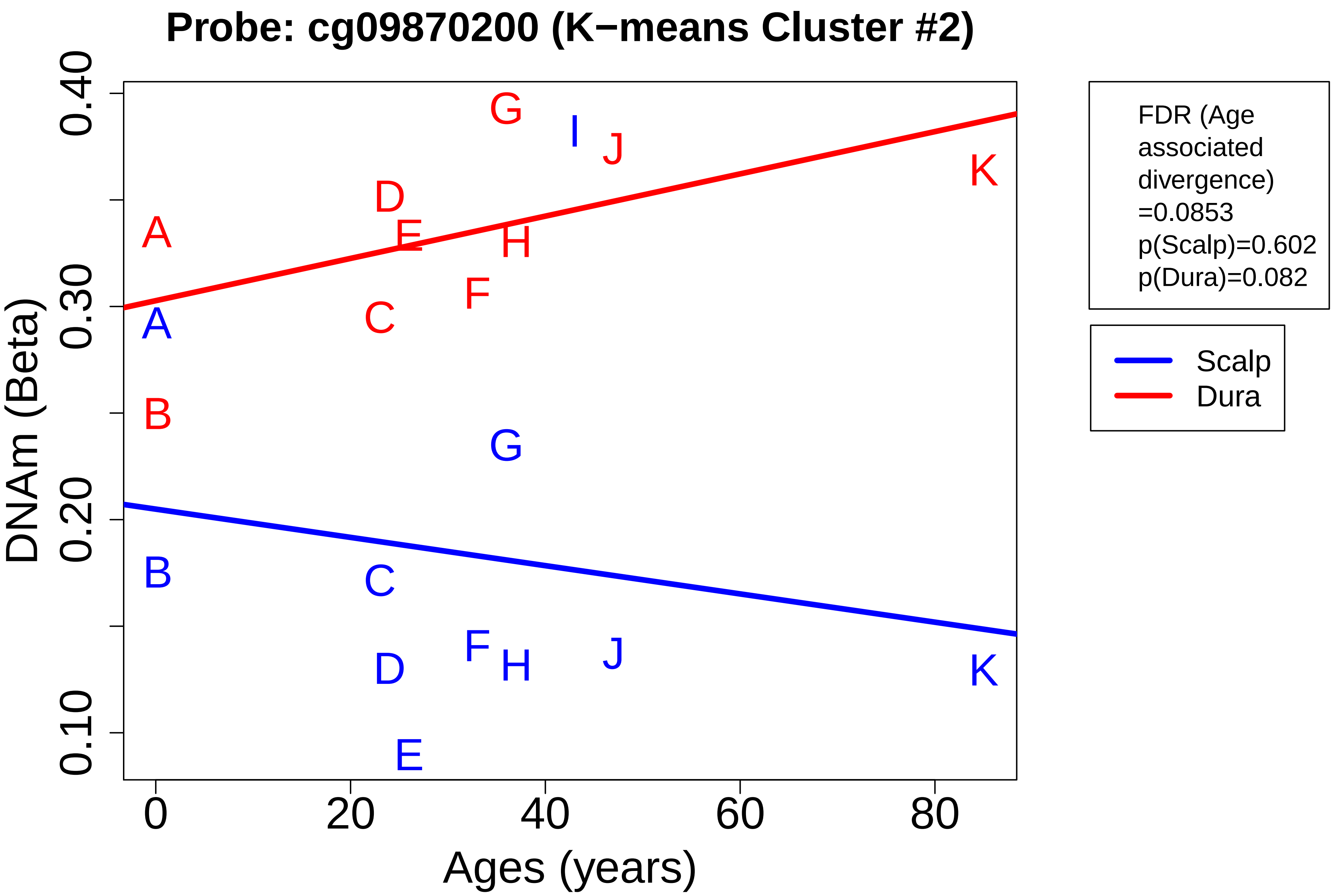
Probe: cg09870200 (K−means Cluster #2)
DNAm (Beta)
0.10
0.20
0.30
0.40
0
20
40
60
80
Ages (years)
A
B
C
D
E
F
G
H
I
J
K
A
B
C
D
E
F
G
H
J
K
FDR (Age associated divergence) =0.0853
p(Scalp)=0.602
p(Dura)=0.082
Scalp
Dura

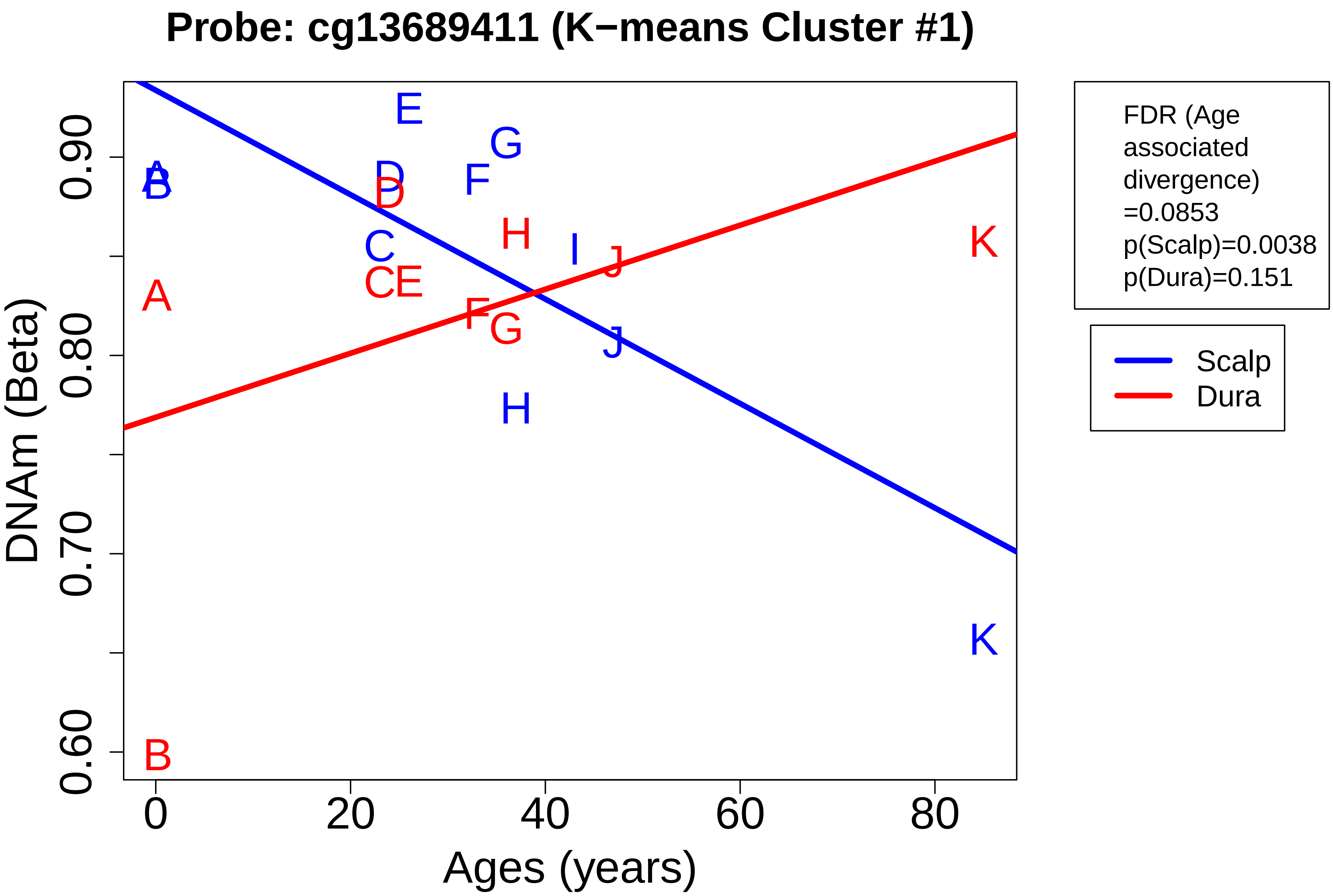

Probe: cg13689411 (K−means Cluster #1)
DNAm (Beta)
0.60
0.70
0.80
0.90
Ages (years)
0
20
40
60
80
A
B
C
D
E
F
G
H
I
J
K
CE
FDR (Age associated divergence) =0.0853
p(Scalp)=0.0038
p(Dura)=0.151
Scalp
Dura

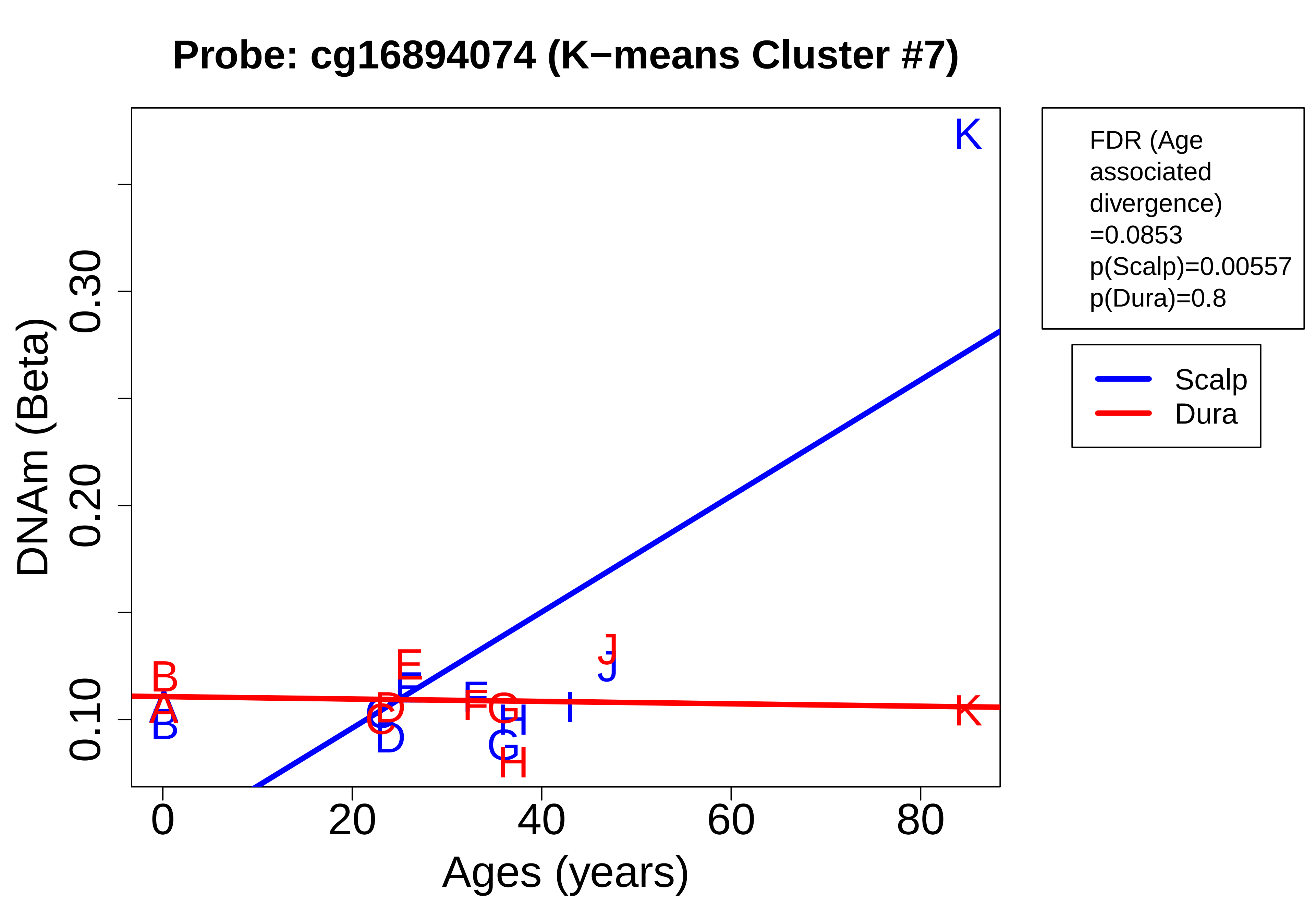

Probe: cg16894074 (K−means Cluster #7)
DNAm (Beta)
0.10
0.20
0.30
Ages (years)
0
20
40
60
80
FDR (Age associated divergence) =0.0853
p(Scalp)=0.00557
p(Dura)=0.8
Scalp
Dura
A
B
C
D
E
F
G
H
I
J
K

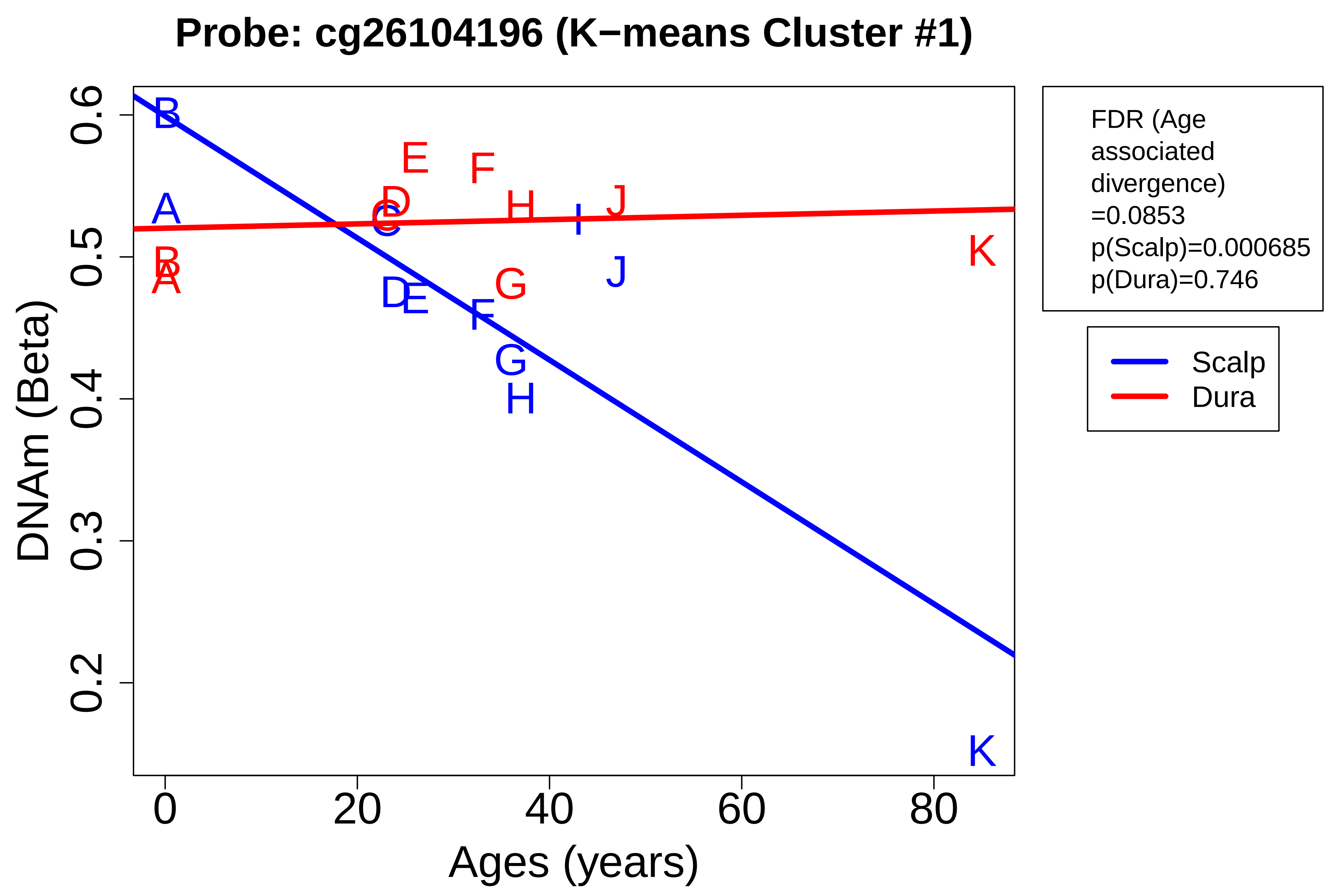

Probe: cg26104196 (K−means Cluster #1)
DNAm (Beta)
0.2
0.3
0.4
0.5
0.6
Ages (years)
0
20
40
60
80
A
B
C
D
E
F
G
H
I
J
K
FDR (Age associated divergence) =0.0853
p(Scalp)=0.000685
p(Dura)=0.746
Scalp
Dura

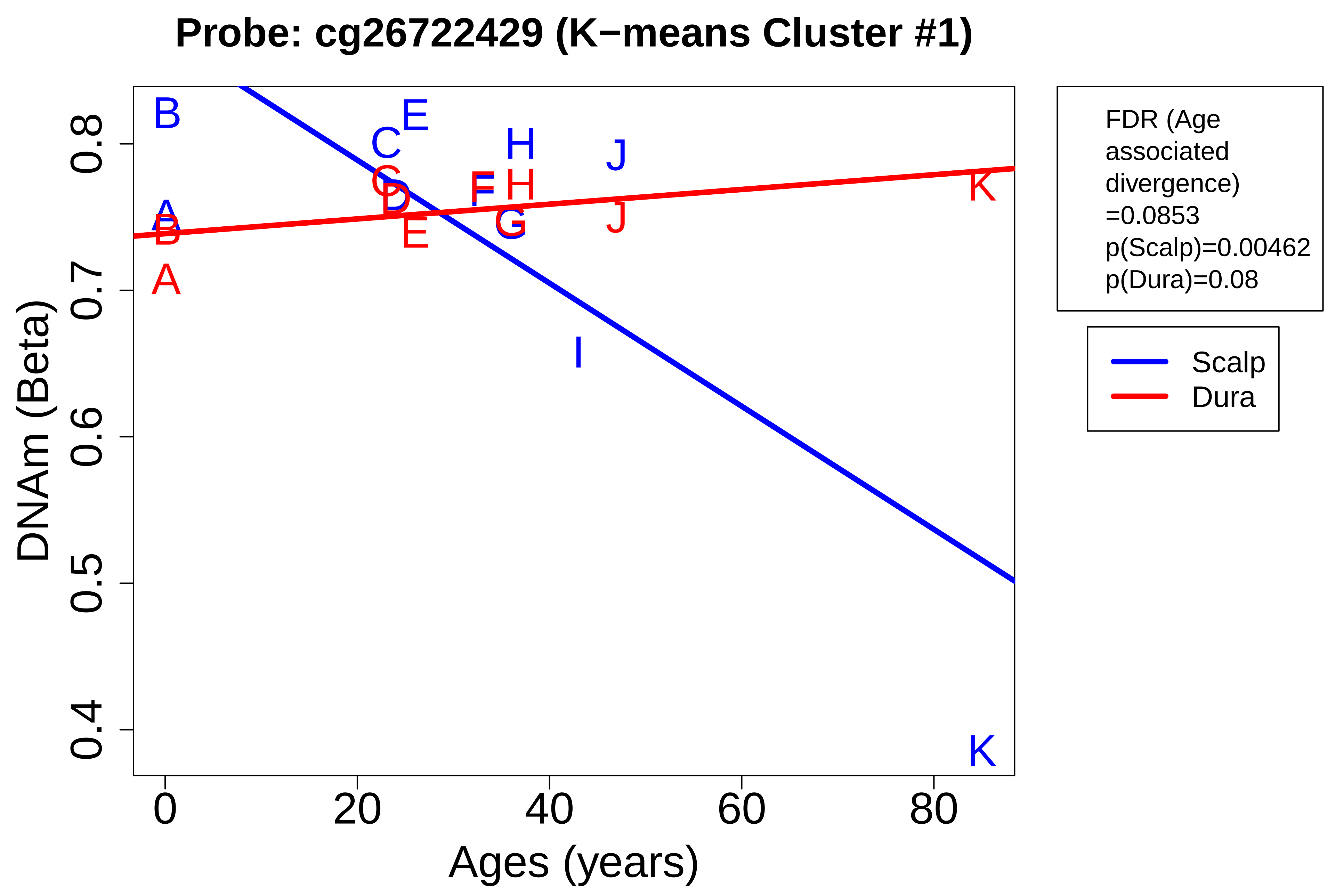
Probe: cg26722429 (K−means Cluster #1)
DNAm (Beta)
0.4
0.5
0.6
0.7
0.8
Ages (years)
0
20
40
60
80
A
B
C
D
E
F
G
H
I
J
K
FDR (Age associated divergence) =0.0853
p(Scalp)=0.00462
p(Dura)=0.08
Scalp
Dura

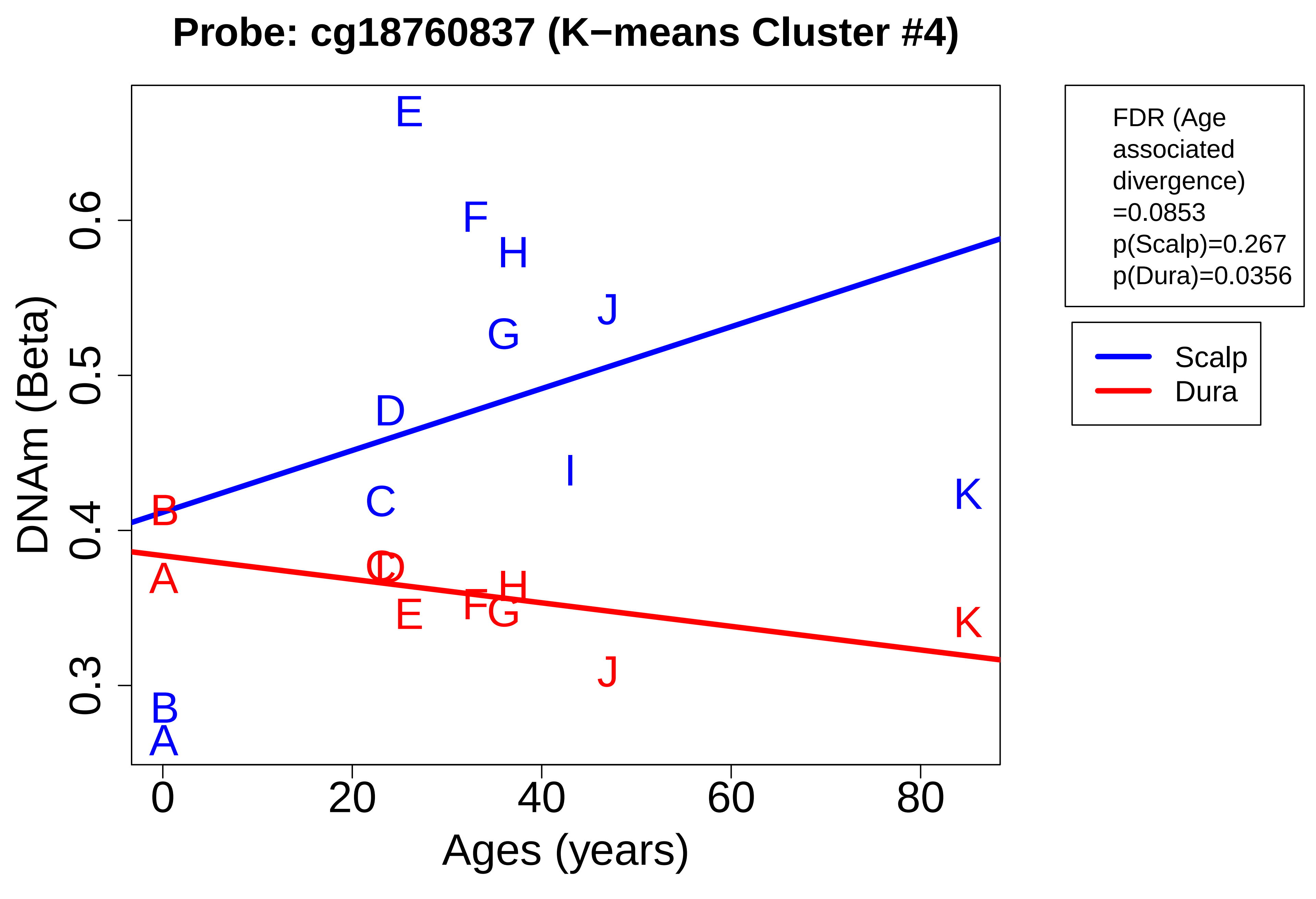

Probe: cg18760837 (K−means Cluster #4)
DNAm (Beta)
0.3
0.4
0.5
0.6
0
20
40
60
80
Ages (years)
FDR (Age associated divergence) =0.0853
p(Scalp)=0.267
p(Dura)=0.0356
Scalp
Dura
A
B
C
D
E
F
G
H
I
J
K

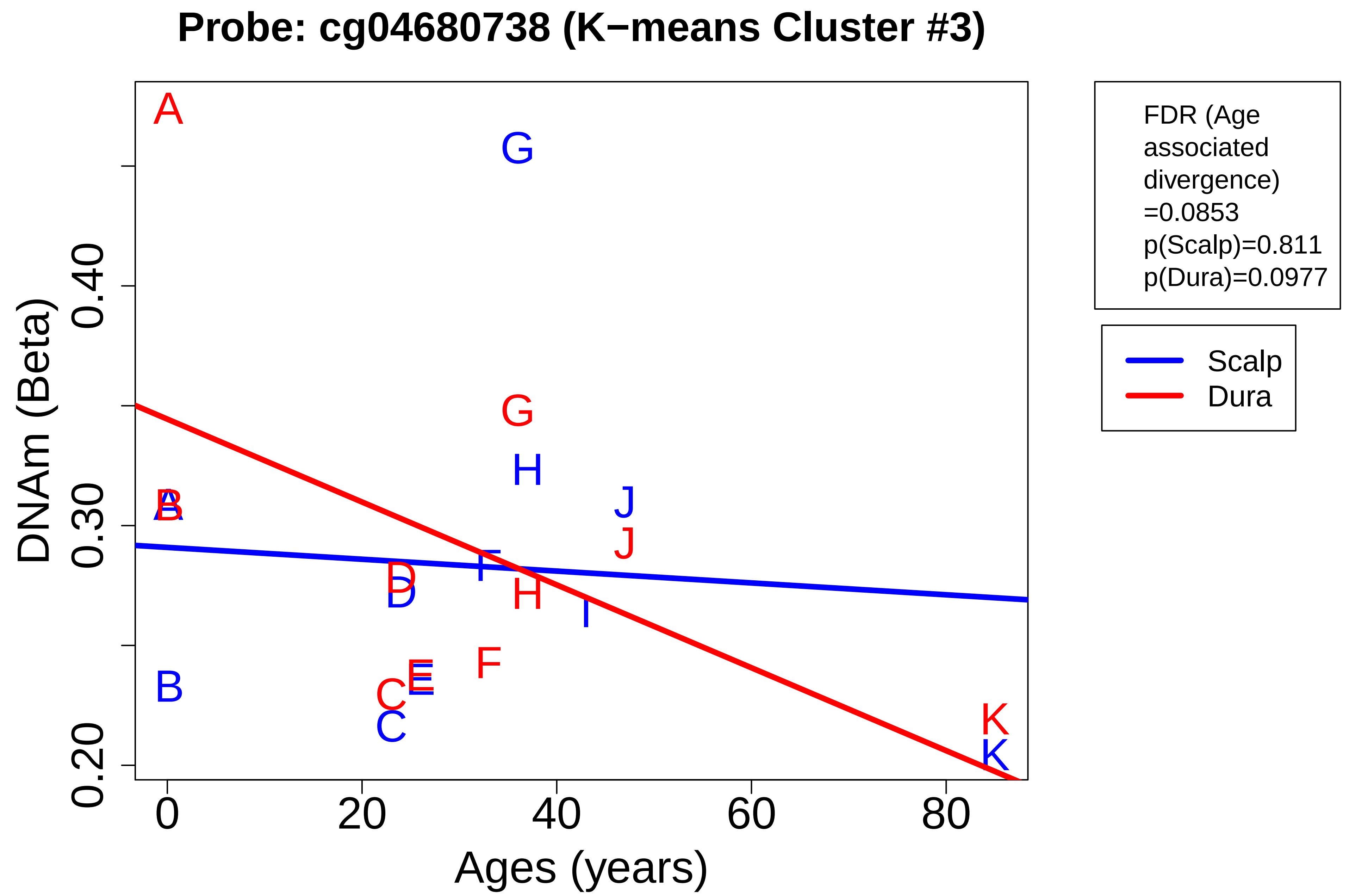
Probe: cg04680738 (K−means Cluster #3)
DNAm (Beta)
0.20
0.30
0.40
0
20
40
60
80
Ages (years)
A
G
G
H
B
A
J
J
F
D
D
H
I
B
F
E
C
E
C
K
K
FDR (Age associated divergence) =0.0853
p(Scalp)=0.811
p(Dura)=0.0977
Scalp
Dura

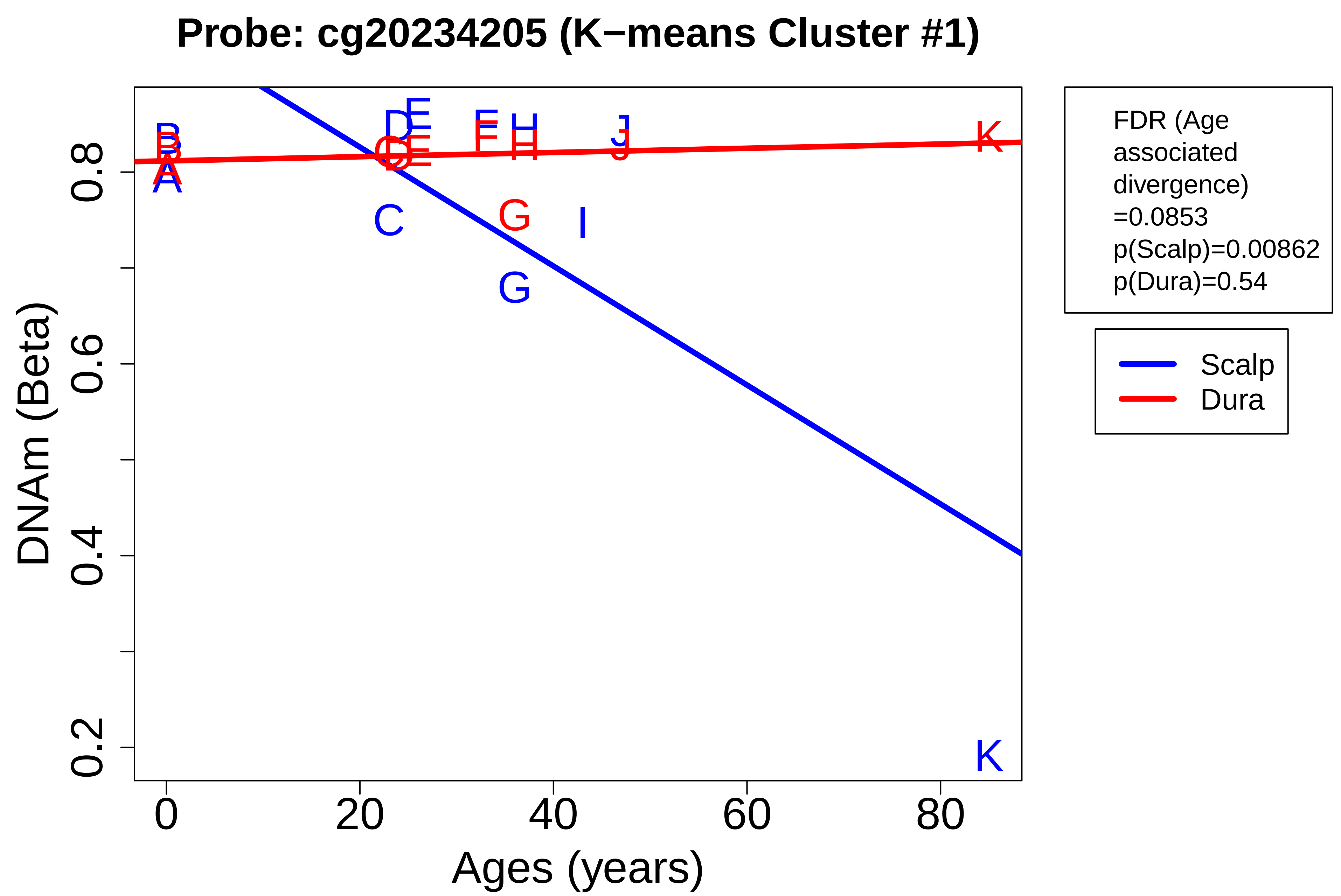

Probe: cg20234205 (K−means Cluster #1)
DNAm (Beta)
0.2
0.4
0.6
0.8
0
20
40
60
80
Ages (years)
FDR (Age associated divergence) =0.0853
p(Scalp)=0.00862
p(Dura)=0.54
Scalp
Dura

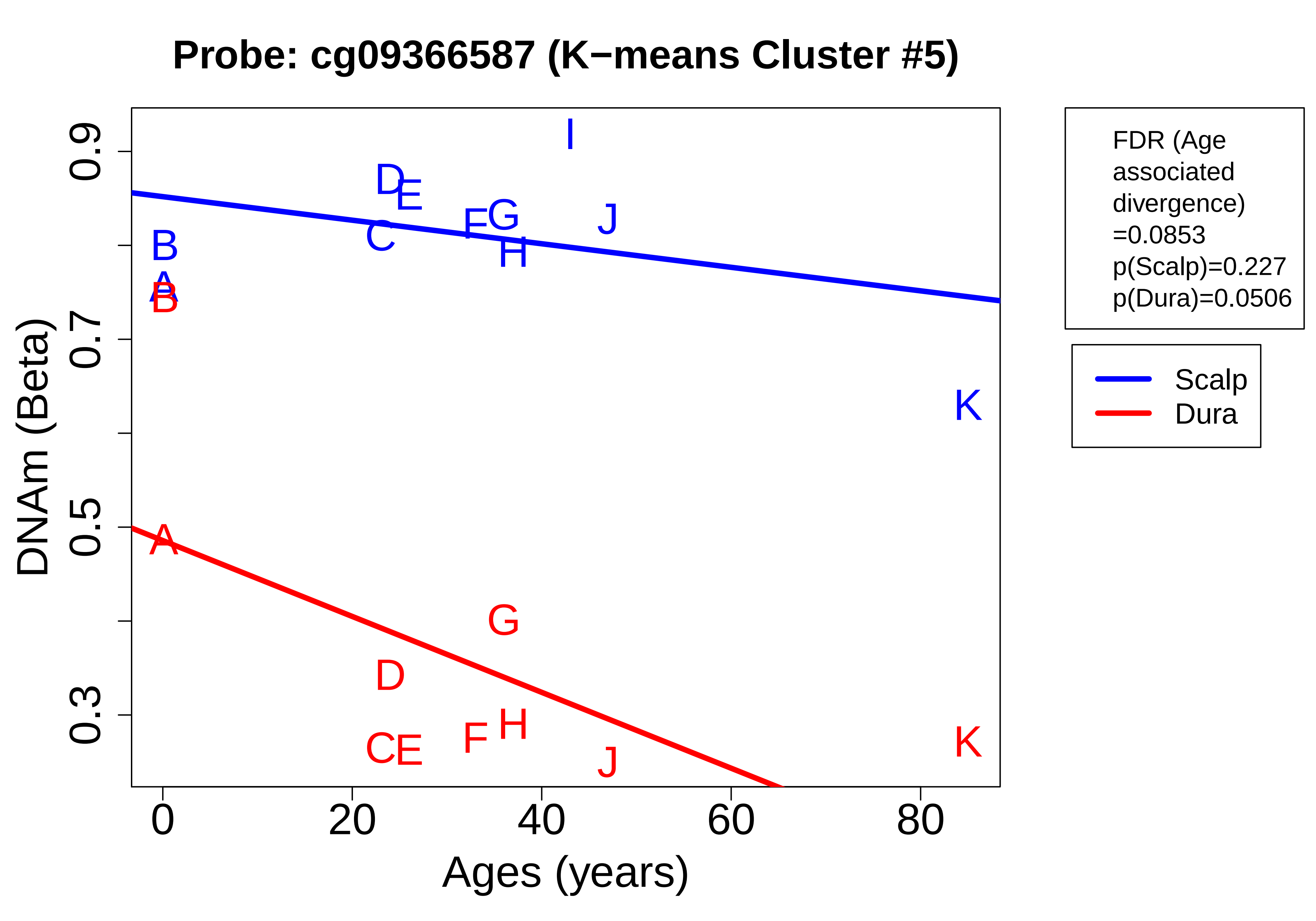

Probe: cg09366587 (K−means Cluster #5)
DNAm (Beta)
0.3
0.5
0.7
0.9
Ages (years)
0
20
40
60
80
FDR (Age associated divergence) =0.0853
p(Scalp)=0.227
p(Dura)=0.0506
Scalp
Dura

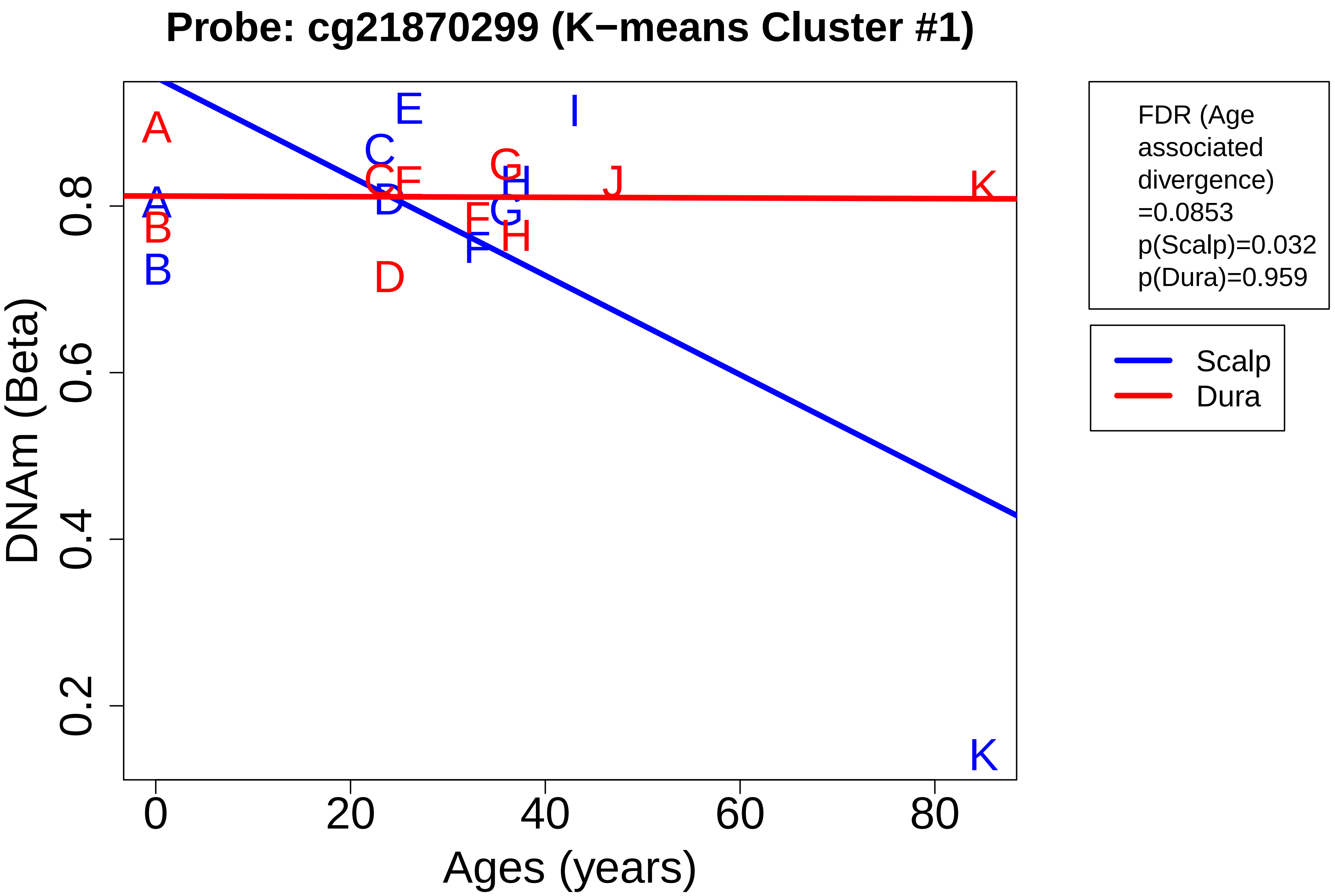
Probe: cg21870299 (K−means Cluster #1)
DNAm (Beta)
0.2
0.4
0.6
0.8
0
20
40
60
80
Ages (years)
FDR (Age associated divergence) =0.0853
p(Scalp)=0.032
p(Dura)=0.959
Scalp
Dura
A
B
C
D
E
F
G
H
I
J
K

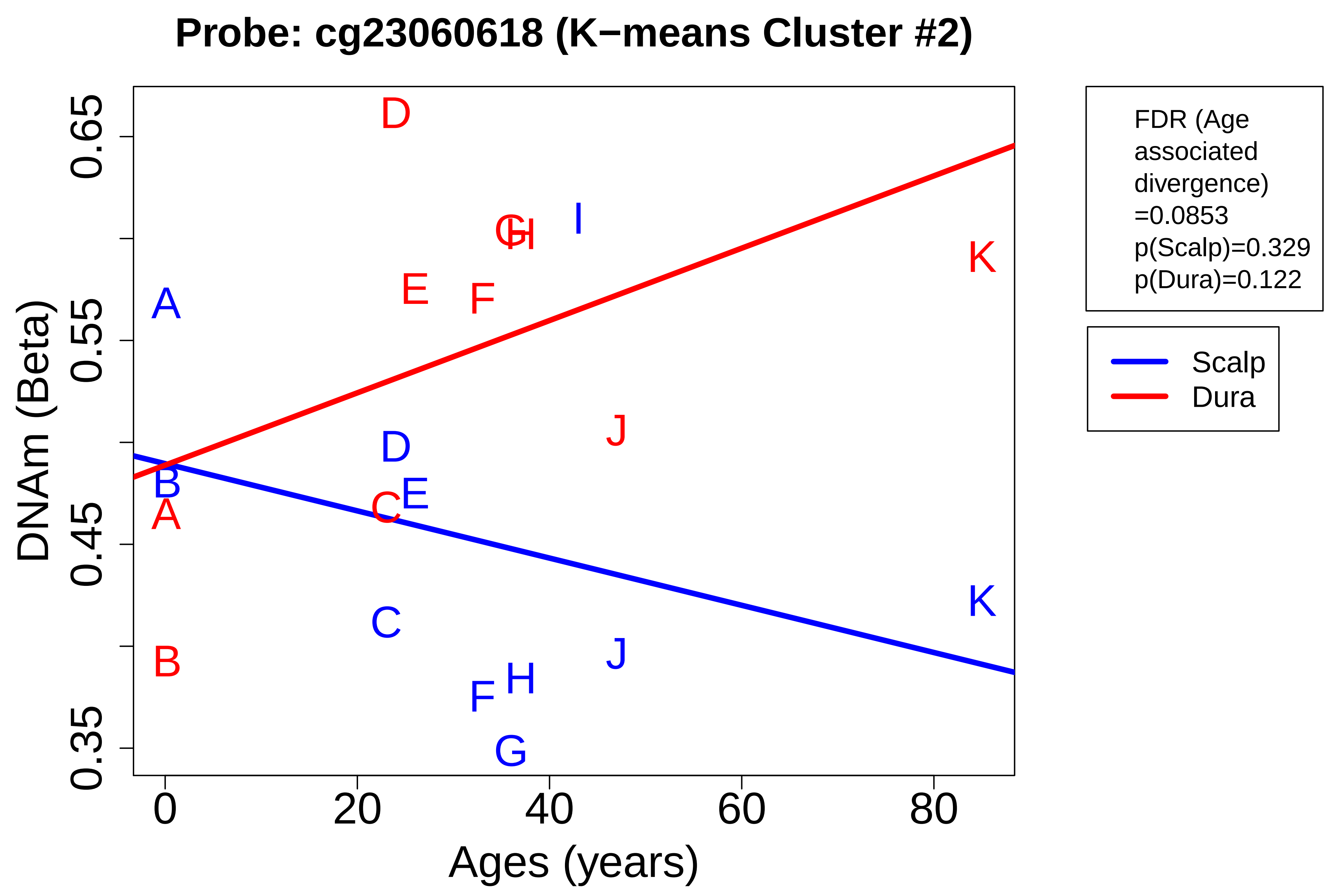

Probe: cg23060618 (K−means Cluster #2)
DNAm (Beta)
0.35
0.45
0.55
0.65
Ages (years)
0
20
40
60
80
A
B
C
D
E
F
G
H
I
J
K
FDR (Age associated divergence) =0.0853
p(Scalp)=0.329
p(Dura)=0.122
Scalp
Dura

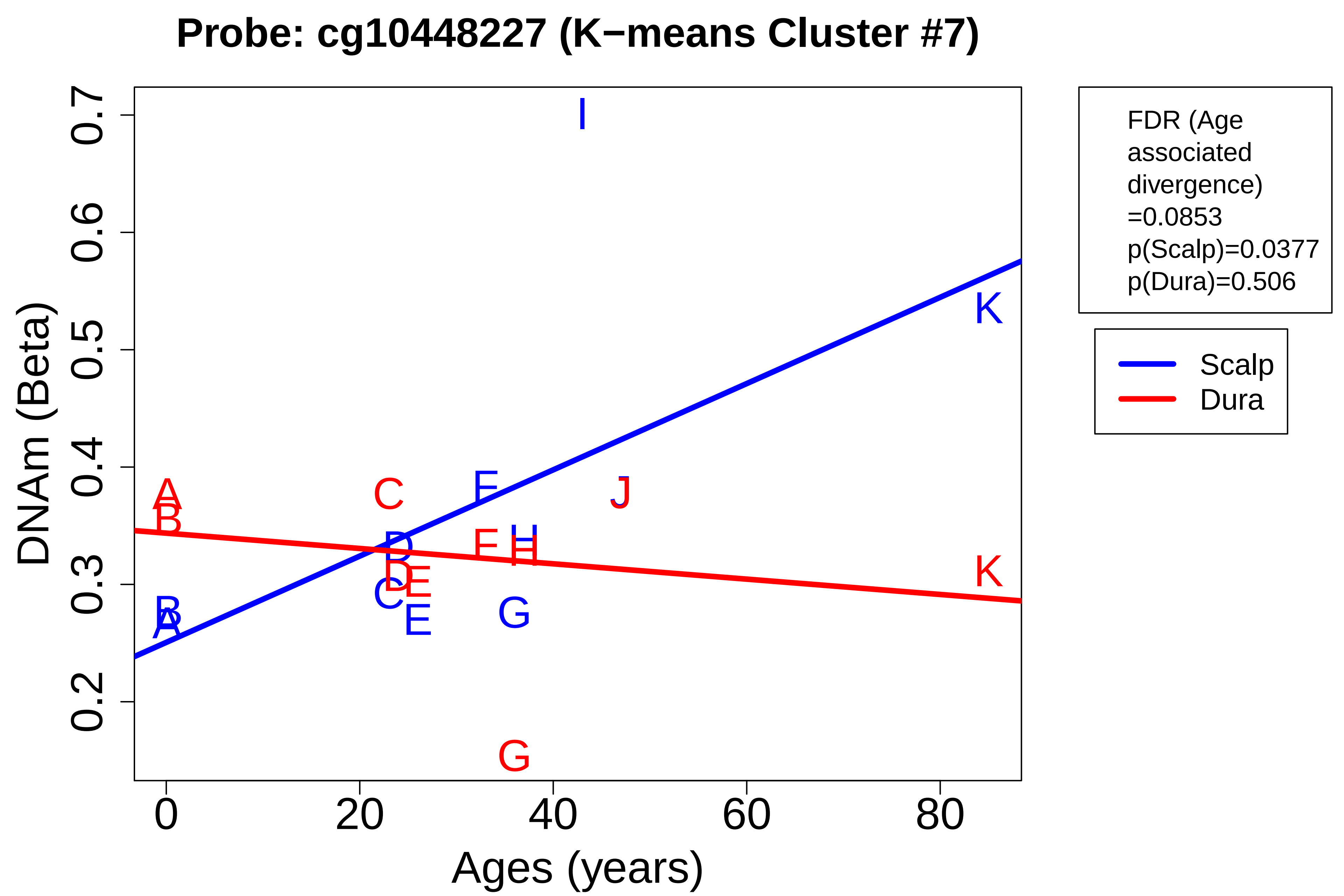
Probe: cg10448227 (K−means Cluster #7)
DNAm (Beta)
0.2
0.3
0.4
0.5
0.6
0.7
0
20
40
60
80
Ages (years)
FDR (Age associated divergence) =0.0853
p(Scalp)=0.0377
p(Dura)=0.506
Scalp
Dura
A
B
C
D
E
F
G
H
I
J
K

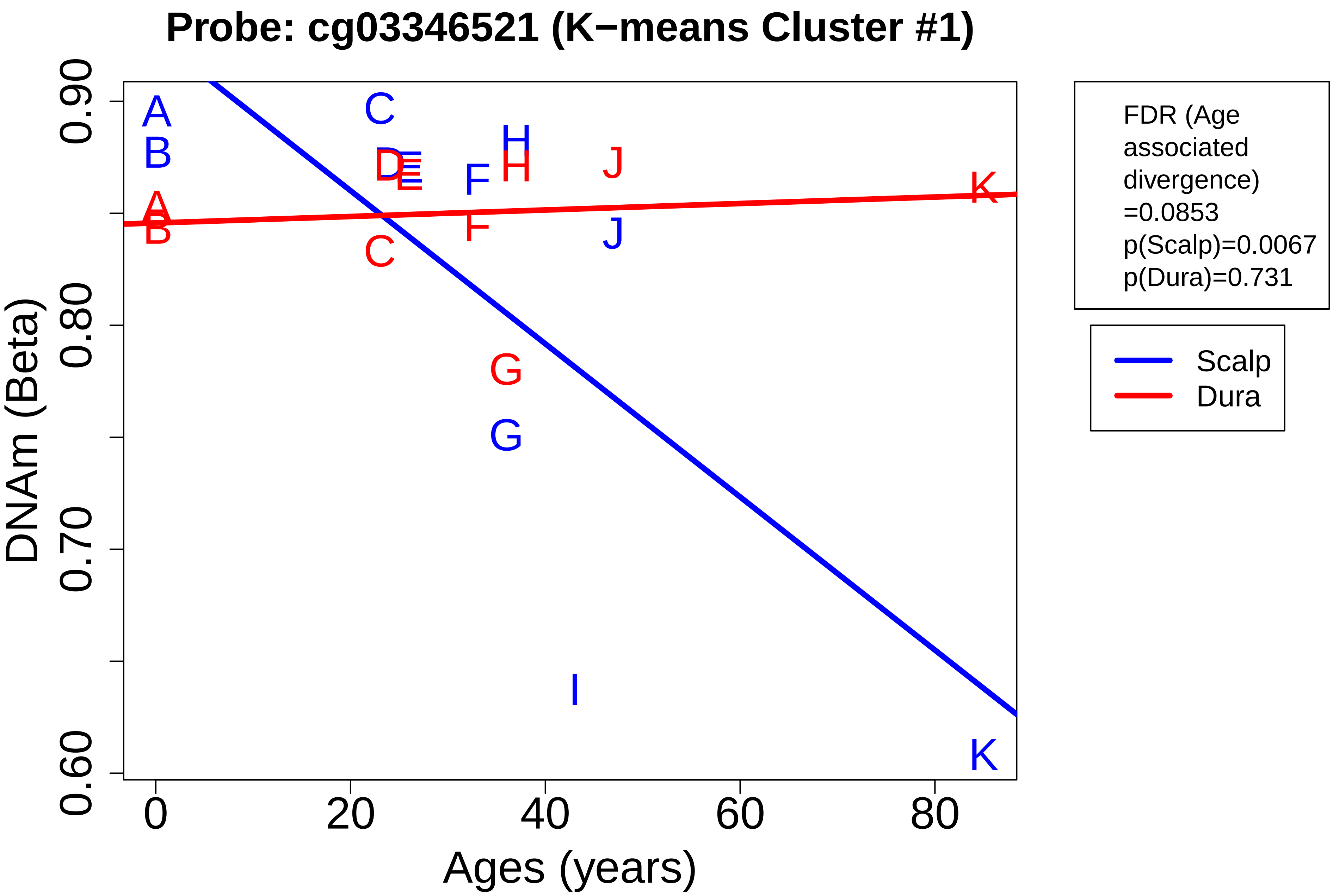

Probe: cg03346521 (K−means Cluster #1)
DNAm (Beta)
0.60
0.70
0.80
0.90
0
20
40
60
80
Ages (years)
FDR (Age associated divergence) =0.0853
p(Scalp)=0.0067
p(Dura)=0.731
Scalp
Dura
A
B
C
D
E
F
G
H
I
J
K

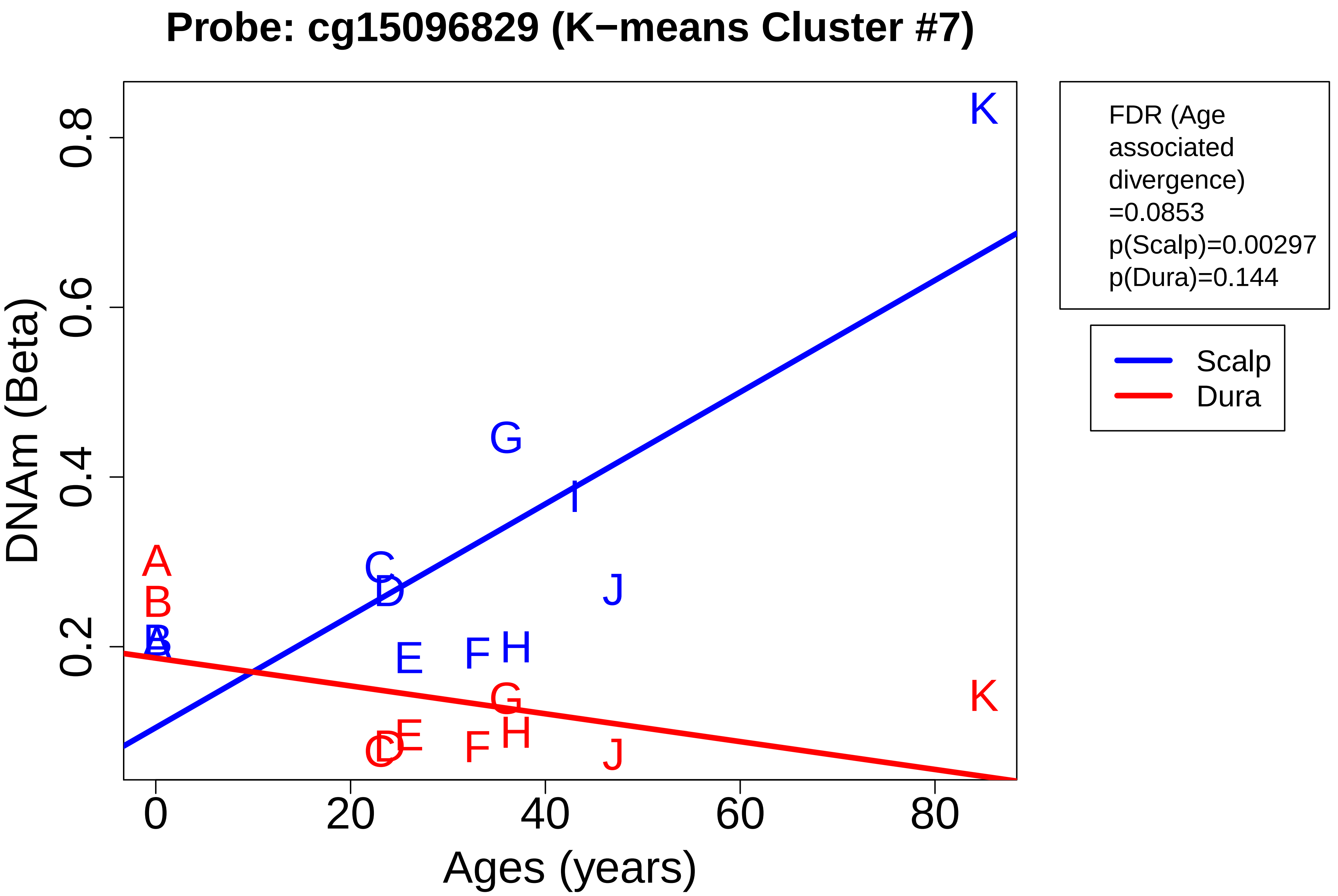

Probe: cg15096829 (K−means Cluster #7)
DNAm (Beta)
0.2
0.4
0.6
0.8
0
20
40
60
80
Ages (years)
A
B
C
D
E
F
G
H
I
J
K
FDR (Age associated divergence) =0.0853
p(Scalp)=0.00297
p(Dura)=0.144
Scalp
Dura

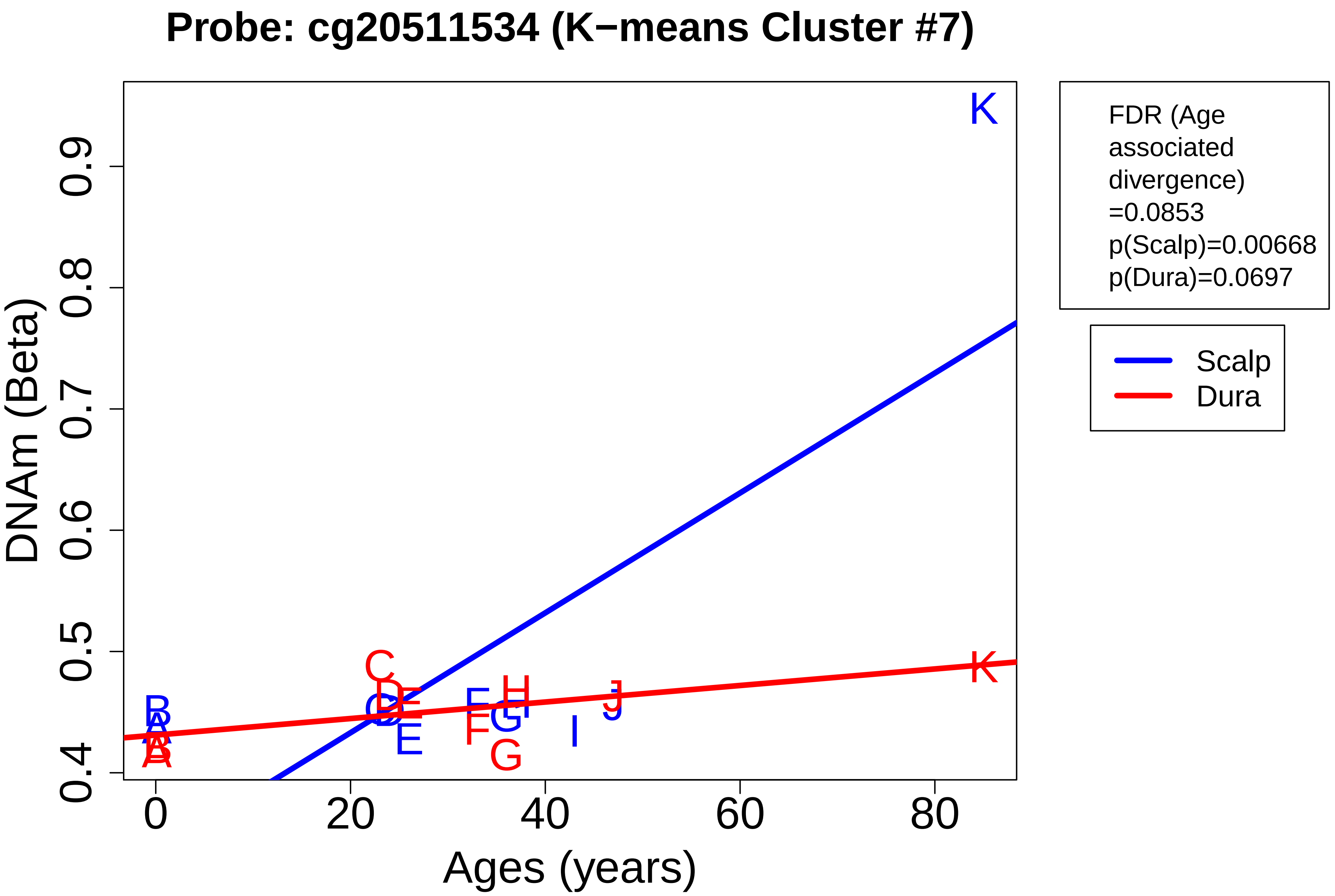
Probe: cg20511534 (K−means Cluster #7)
DNAm (Beta)
0.4
0.5
0.6
0.7
0.8
0.9
0
20
40
60
80
Ages (years)
FDR (Age associated divergence) =0.0853
p(Scalp)=0.00668
p(Dura)=0.0697
Scalp
Dura

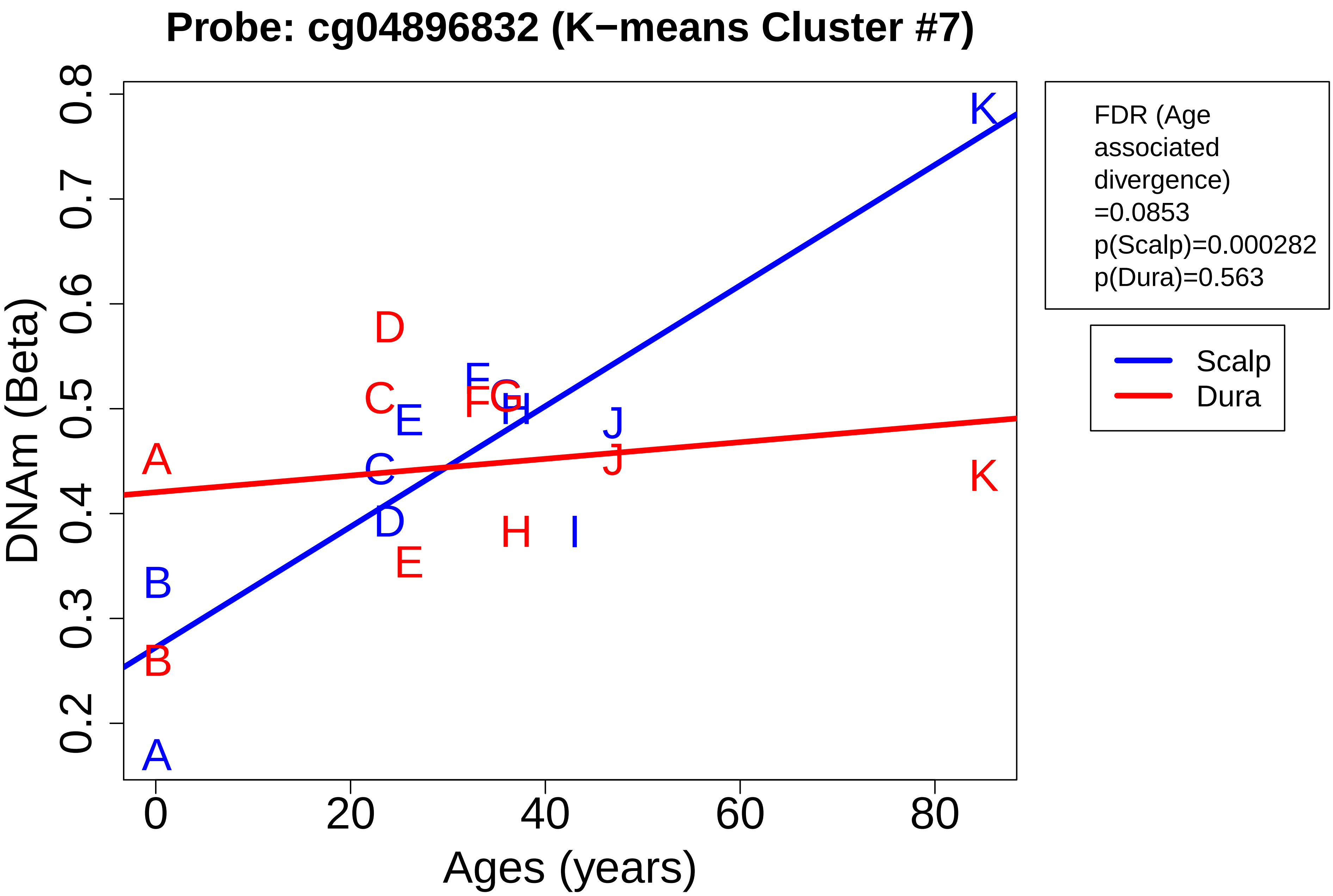

Probe: cg04896832 (K−means Cluster #7)
DNAm (Beta)
Ages (years)
0.2
0.3
0.4
0.5
0.6
0.7
0.8
0
20
40
60
80
FDR (Age associated divergence) =0.0853
p(Scalp)=0.000282
p(Dura)=0.563
Scalp
Dura

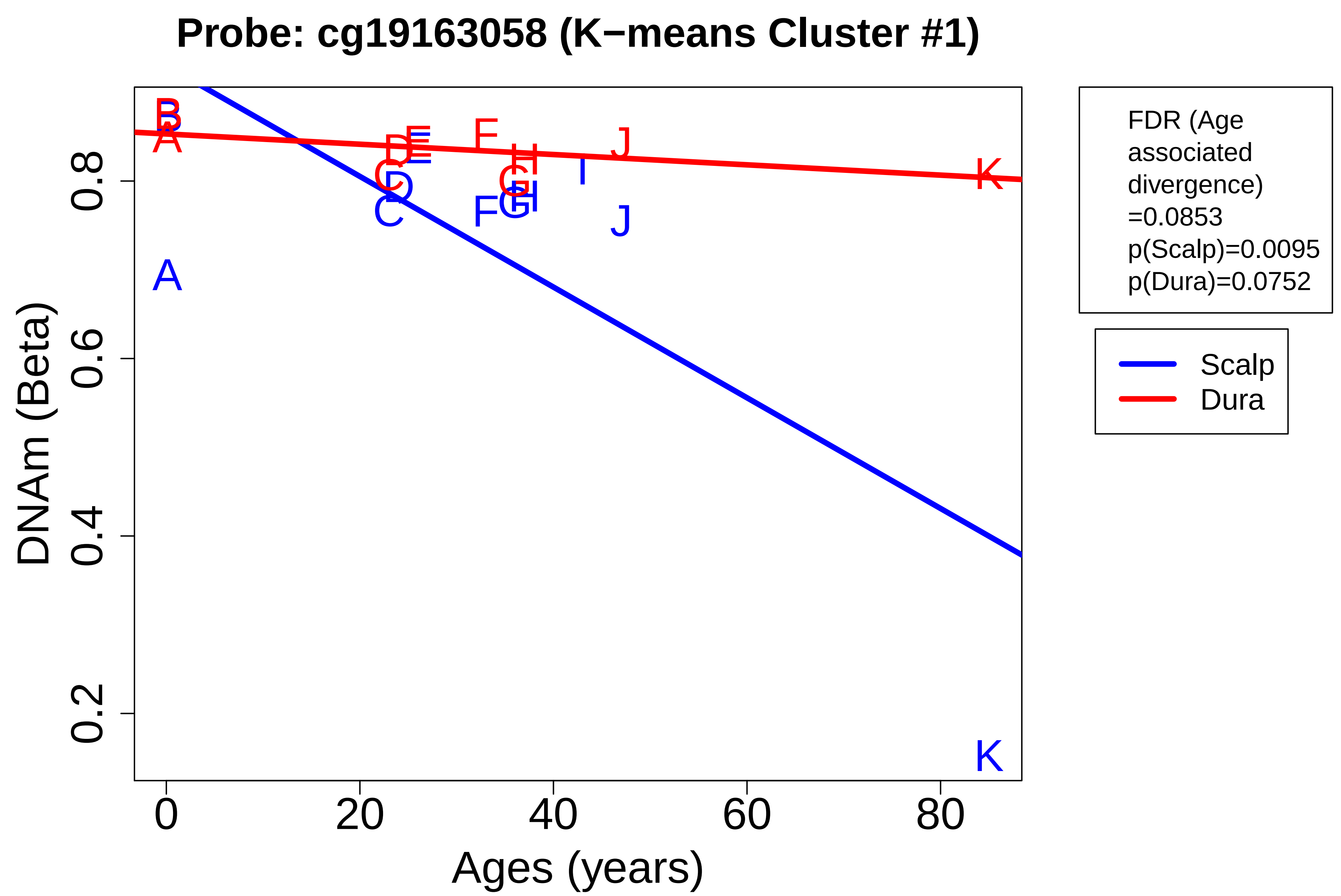

Probe: cg19163058 (K−means Cluster #1)
DNAm (Beta)
0.2
0.4
0.6
0.8
0
20
40
60
80
Ages (years)
A
B
C
D
E
F
G
H
I
J
K
FDR (Age associated divergence) =0.0853
p(Scalp)=0.0095
p(Dura)=0.0752
Scalp
Dura

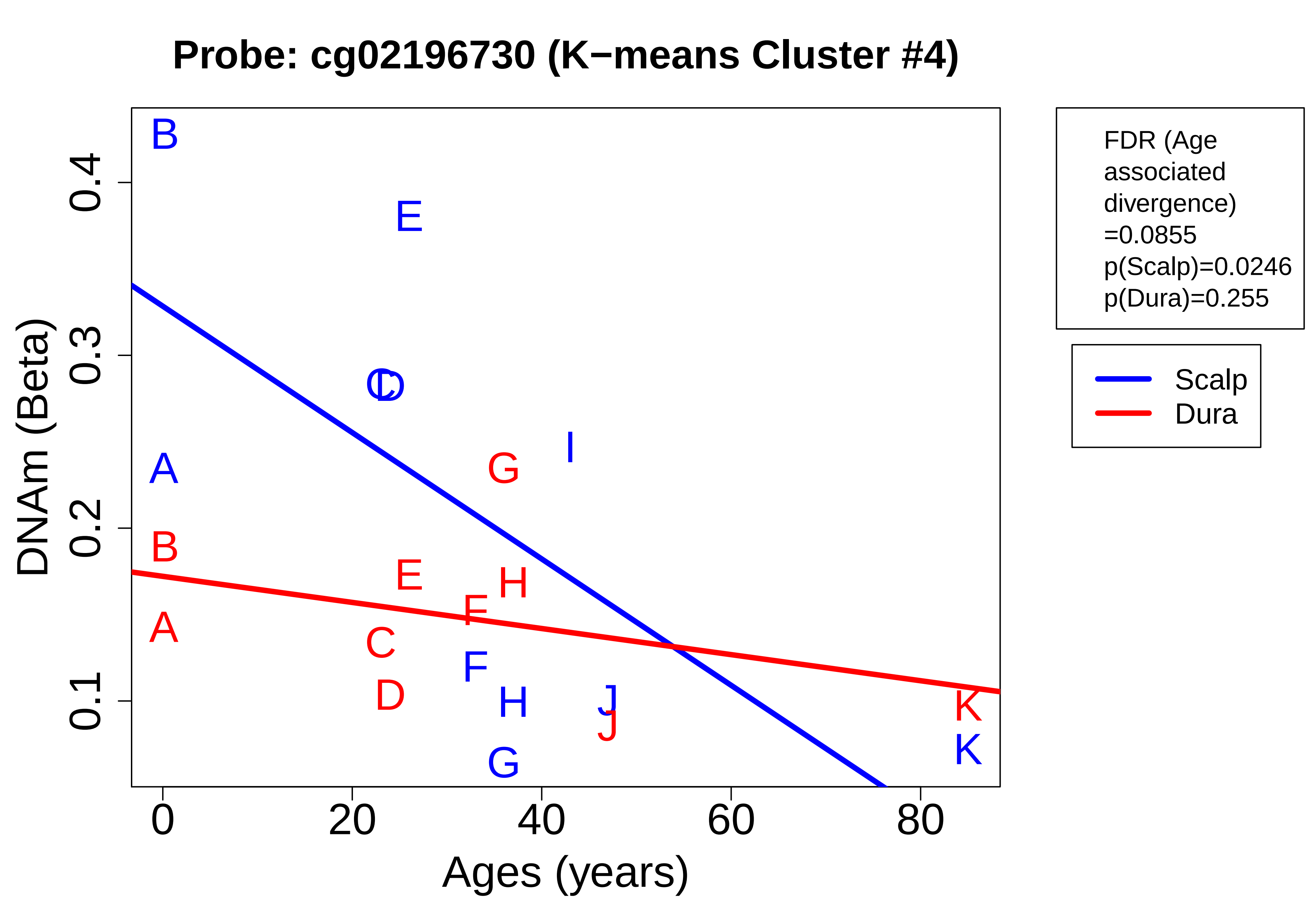

Probe: cg02196730 (K−means Cluster #4)
DNAm (Beta)
0.1
0.2
0.3
0.4
Ages (years)
0
20
40
60
80
FDR (Age associated divergence) =0.0855
p(Scalp)=0.0246
p(Dura)=0.255
Scalp
Dura
A
B
C
D
E
F
G
H
I
J
K

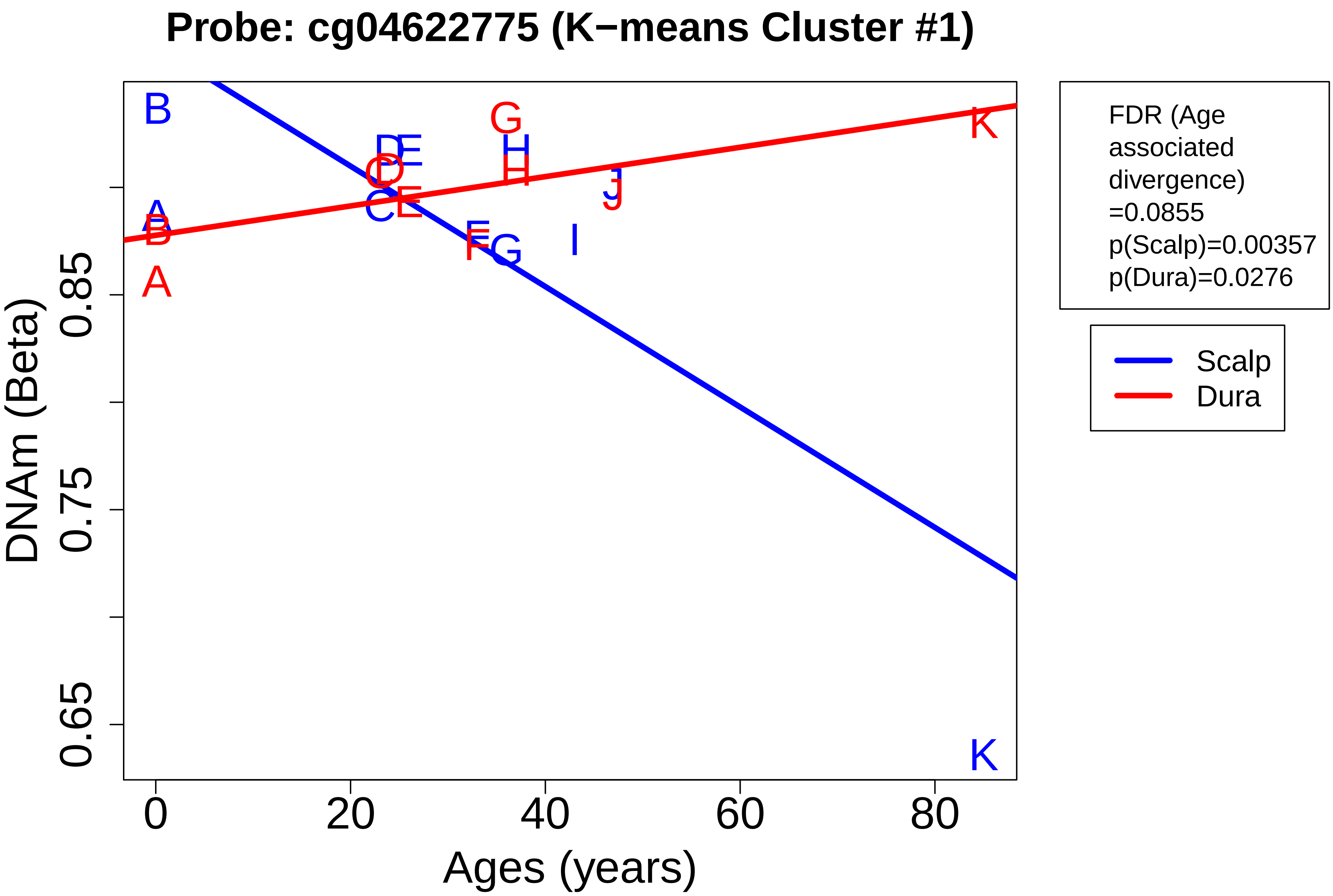
Probe: cg04622775 (K−means Cluster #1)
DNAm (Beta)
0.65
0.75
0.85
Ages (years)
0
20
40
60
80
FDR (Age associated divergence) =0.0855
p(Scalp)=0.00357
p(Dura)=0.0276
Scalp
Dura

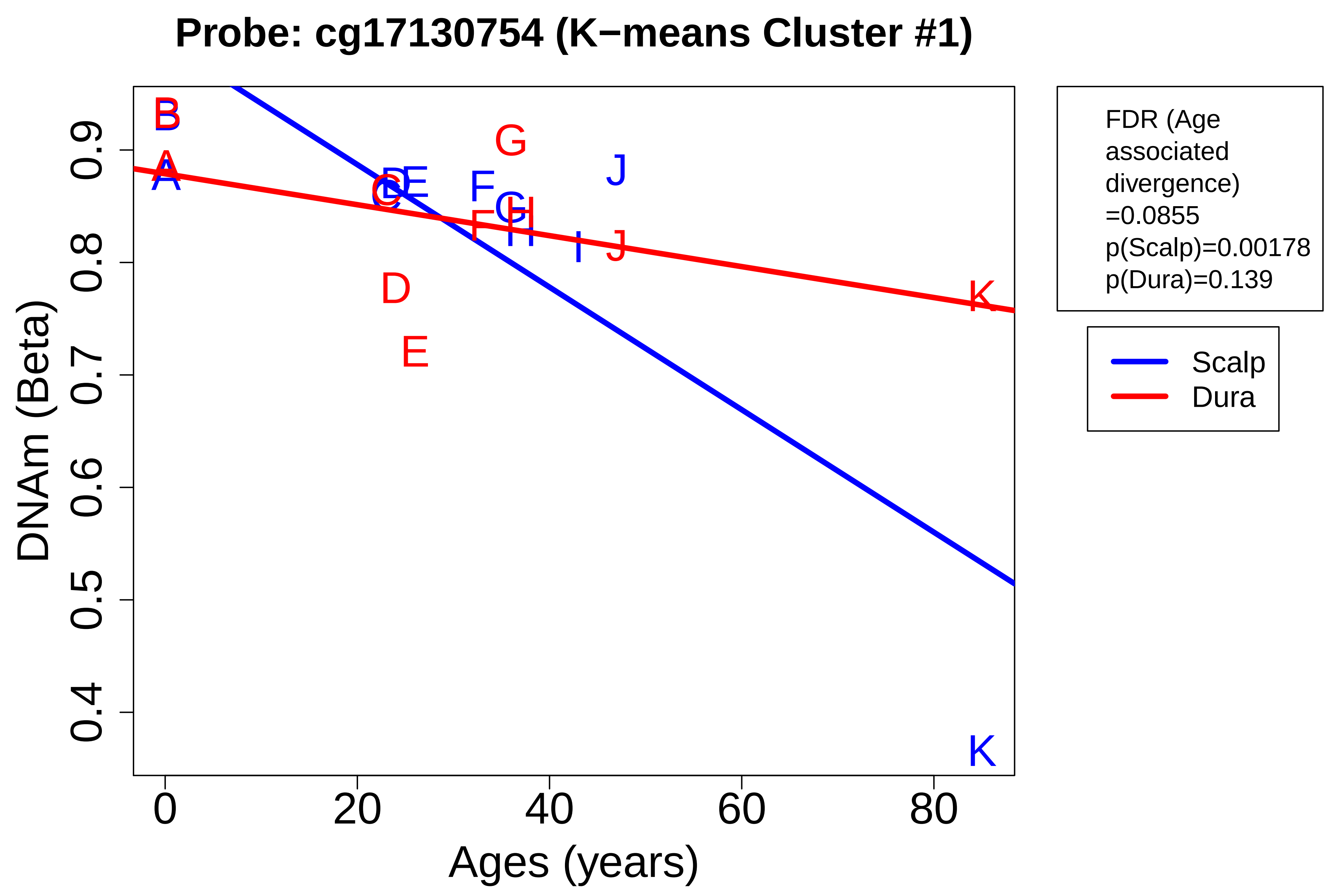

Probe: cg17130754 (K−means Cluster #1)
DNAm (Beta)
0.4
0.5
0.6
0.7
0.8
0.9
Ages (years)
0
20
40
60
80
A
B
C
D
E
F
G
H
I
J
K
FDR (Age associated divergence) =0.0855
p(Scalp)=0.00178
p(Dura)=0.139
Scalp
Dura

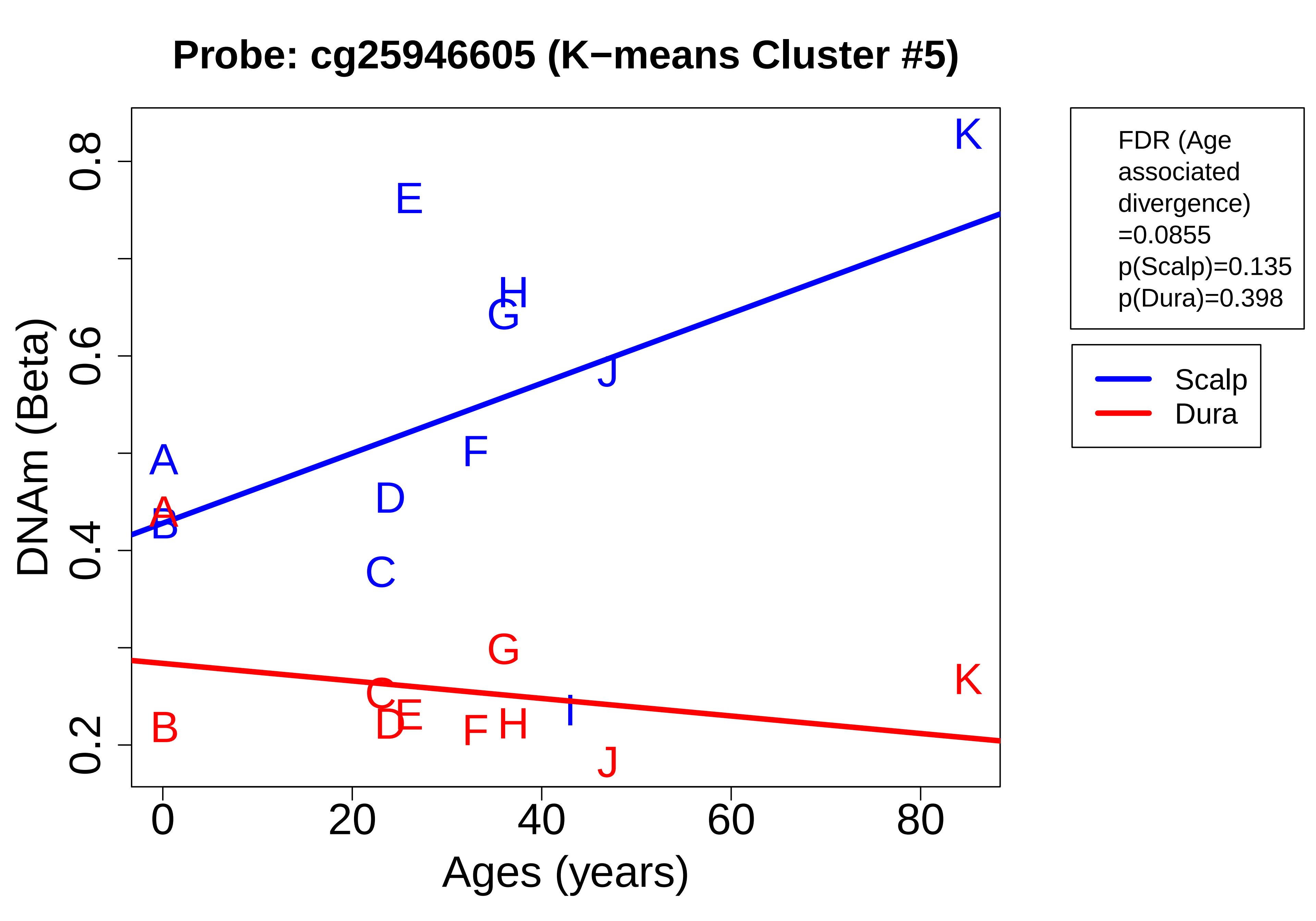

Probe: cg25946605 (K−means Cluster #5)
DNAm (Beta)
0.2
0.4
0.6
0.8
Ages (years)
0
20
40
60
80
A
B
C
D
E
F
G
H
I
J
K
FDR (Age associated divergence) =0.0855
p(Scalp)=0.135
p(Dura)=0.398
Scalp
Dura

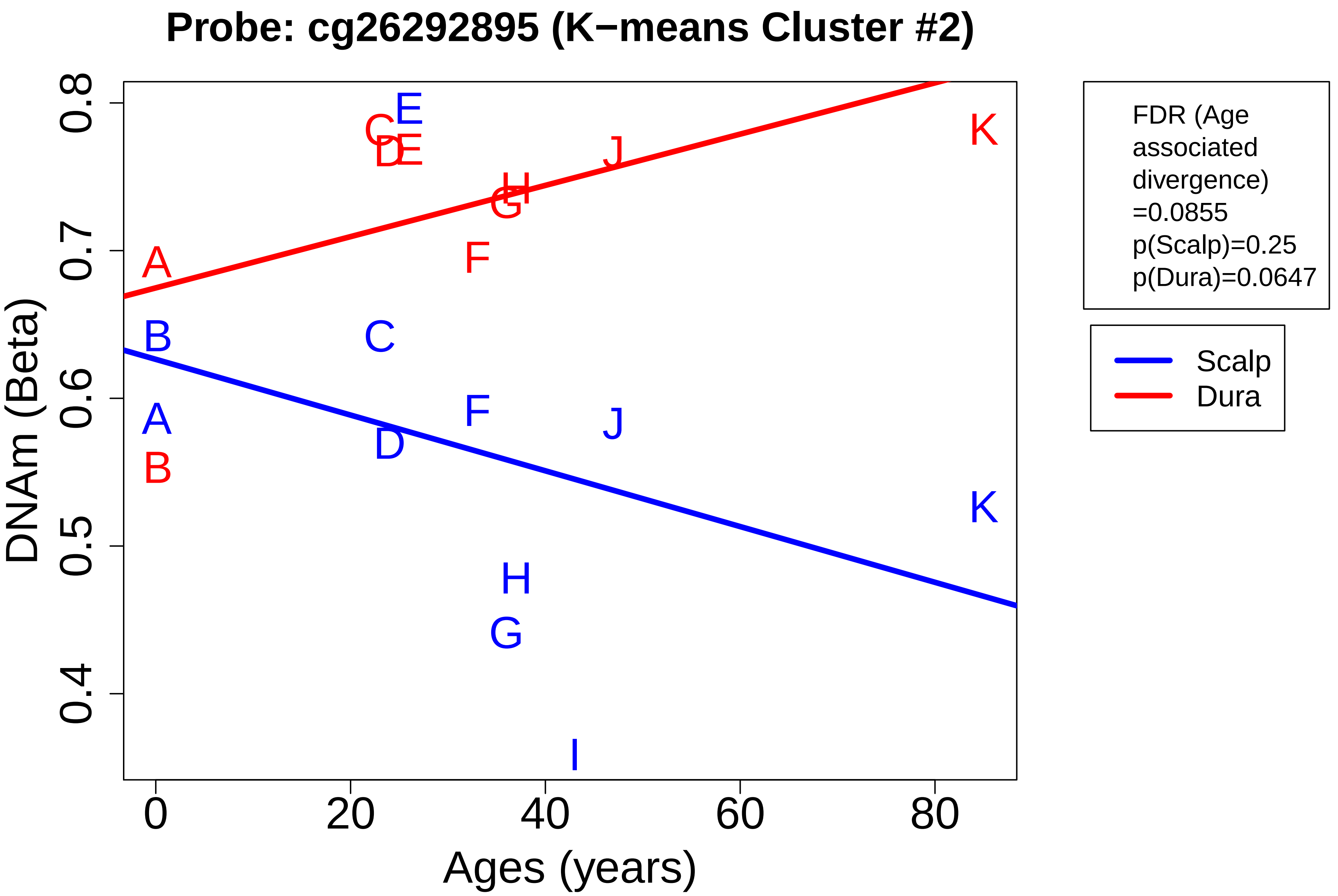

Probe: cg26292895 (K−means Cluster #2)
DNAm (Beta)
0.4
0.5
0.6
0.7
0.8
0
20
40
60
80
Ages (years)
A
B
C
D
E
F
G
H
I
J
K
FDR (Age associated divergence) =0.0855
p(Scalp)=0.25
p(Dura)=0.0647
Scalp
Dura

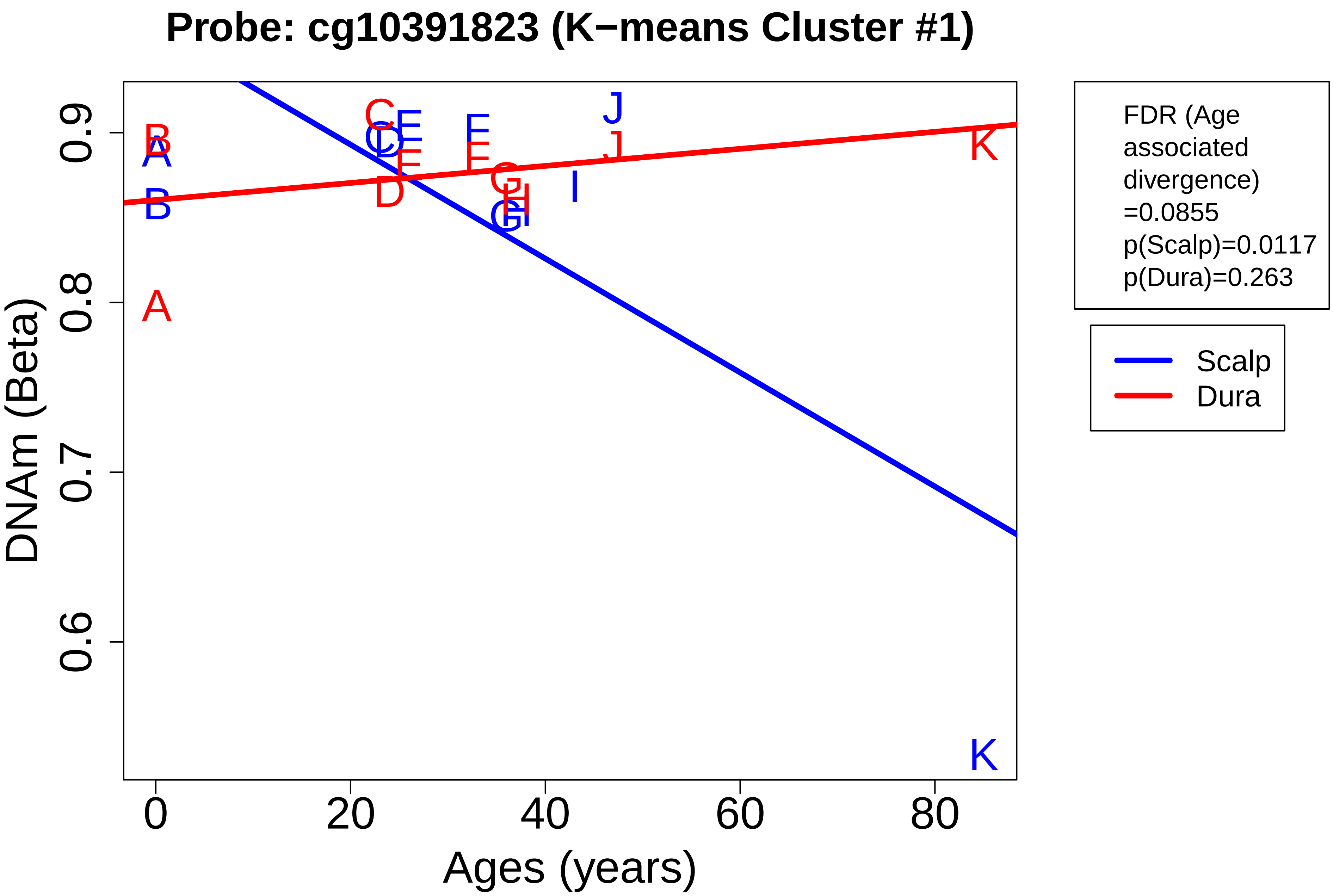
Probe: cg10391823 (K−means Cluster #1)
DNAm (Beta)
0.6
0.7
0.8
0.9
0
20
40
60
80
Ages (years)
FDR (Age associated divergence) =0.0855
p(Scalp)=0.0117
p(Dura)=0.263
Scalp
Dura

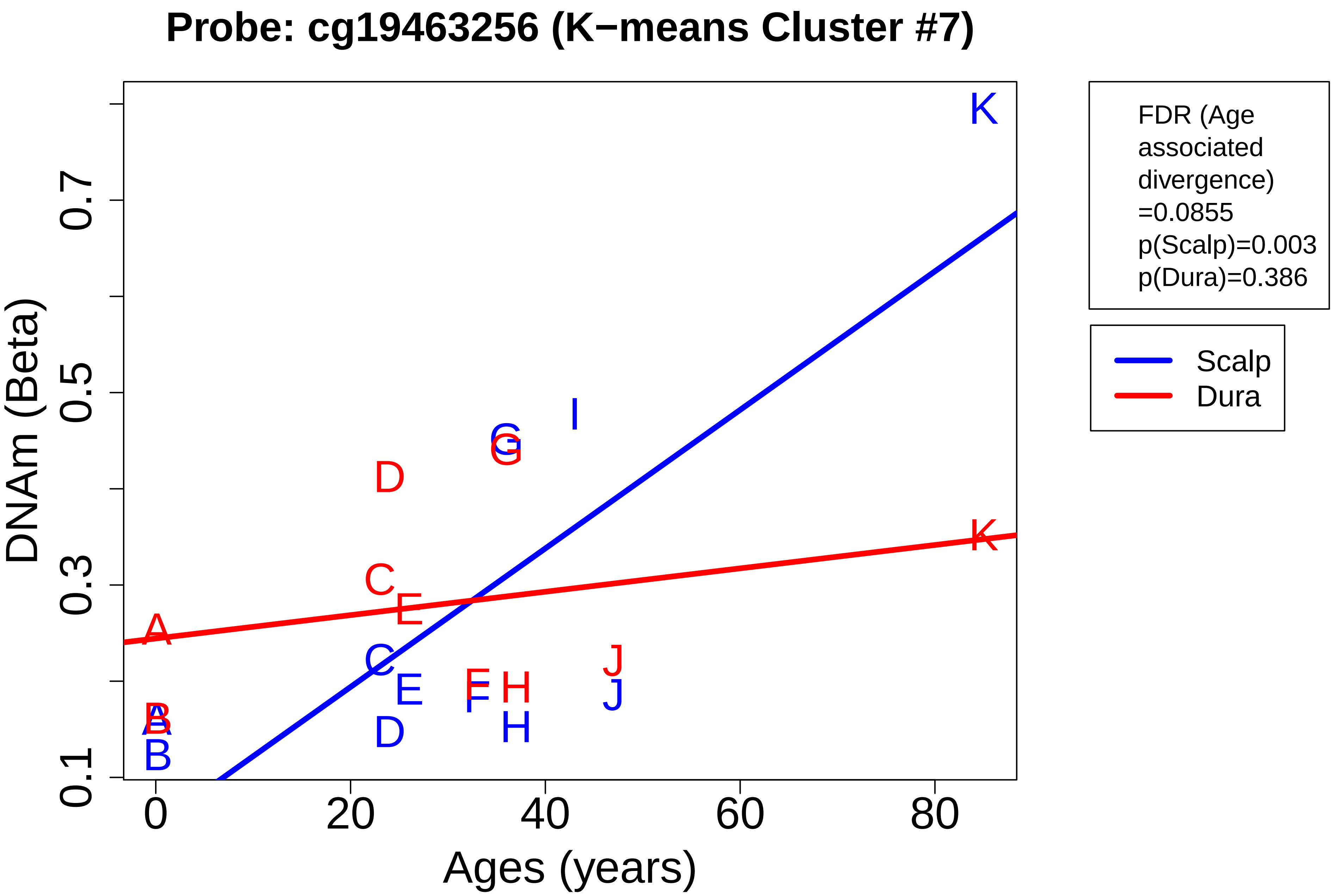

Probe: cg19463256 (K−means Cluster #7)
DNAm (Beta)
0.1
0.3
0.5
0.7
0
20
40
60
80
Ages (years)
K
I
G
G
D
C
E
A
C
E
F
H
J
F
J
A
B
B
D
H
K
FDR (Age associated divergence) =0.0855
p(Scalp)=0.003
p(Dura)=0.386
Scalp
Dura

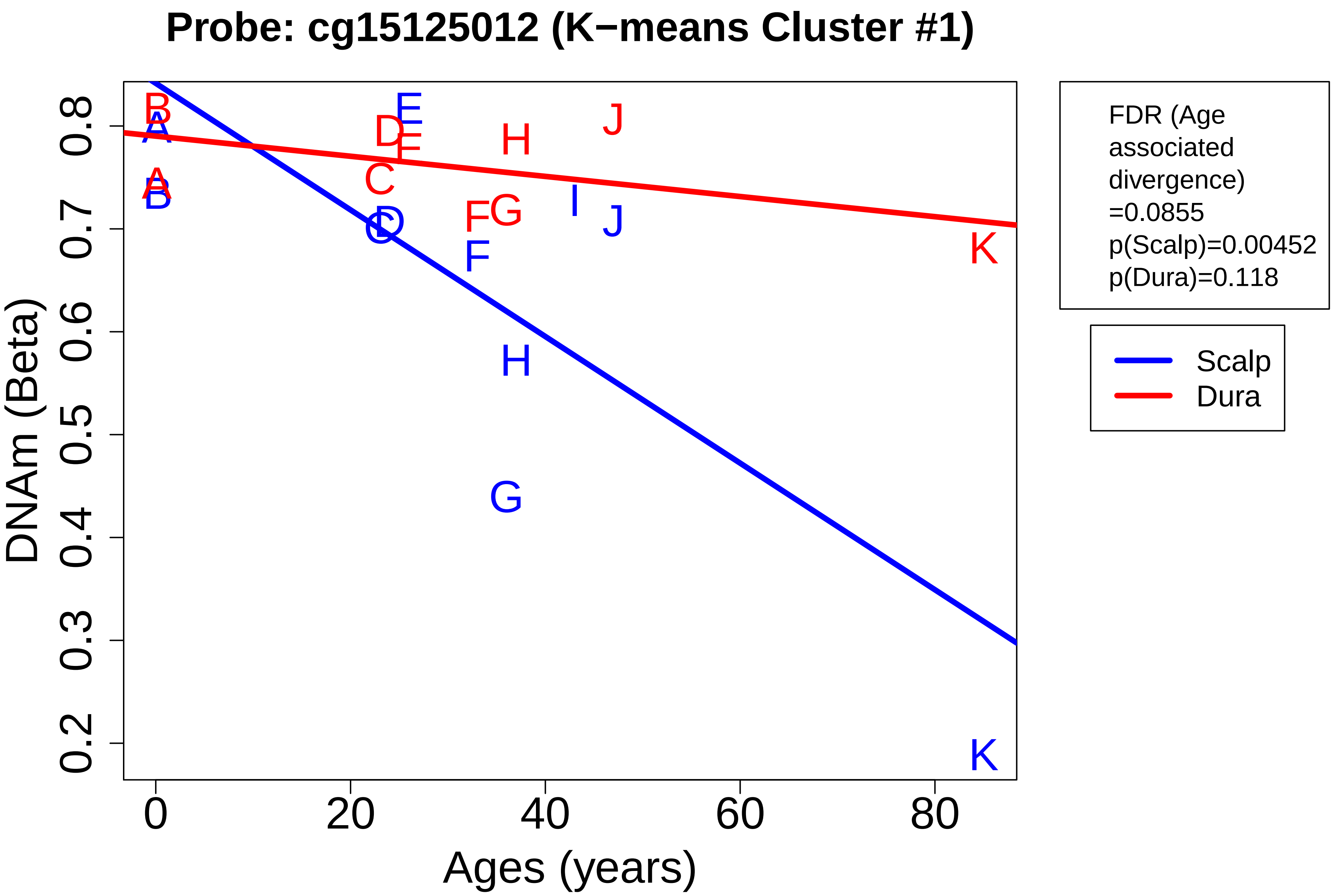

Probe: cg15125012 (K−means Cluster #1)
DNAm (Beta)
0.2
0.3
0.4
0.5
0.6
0.7
0.8
0
20
40
60
80
Ages (years)
A
B
C
D
E
F
G
H
I
J
K
FDR (Age associated divergence) =0.0855
p(Scalp)=0.00452
p(Dura)=0.118
Scalp
Dura

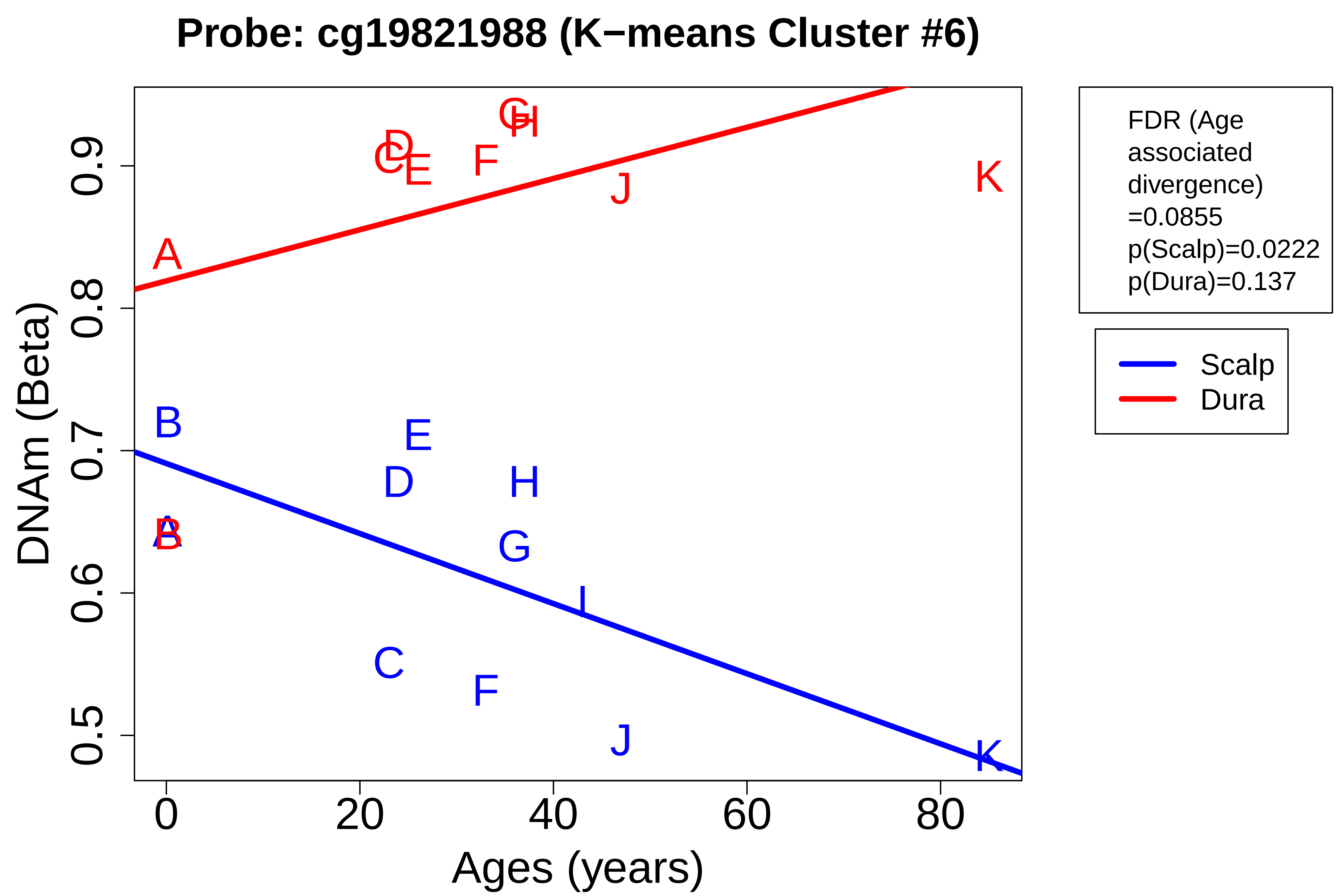

Probe: cg19821988 (K−means Cluster #6)
DNAm (Beta)
0.5
0.6
0.7
0.8
0.9
Ages (years)
0
20
40
60
80
FDR (Age associated divergence) =0.0855
p(Scalp)=0.0222
p(Dura)=0.137
Scalp
Dura

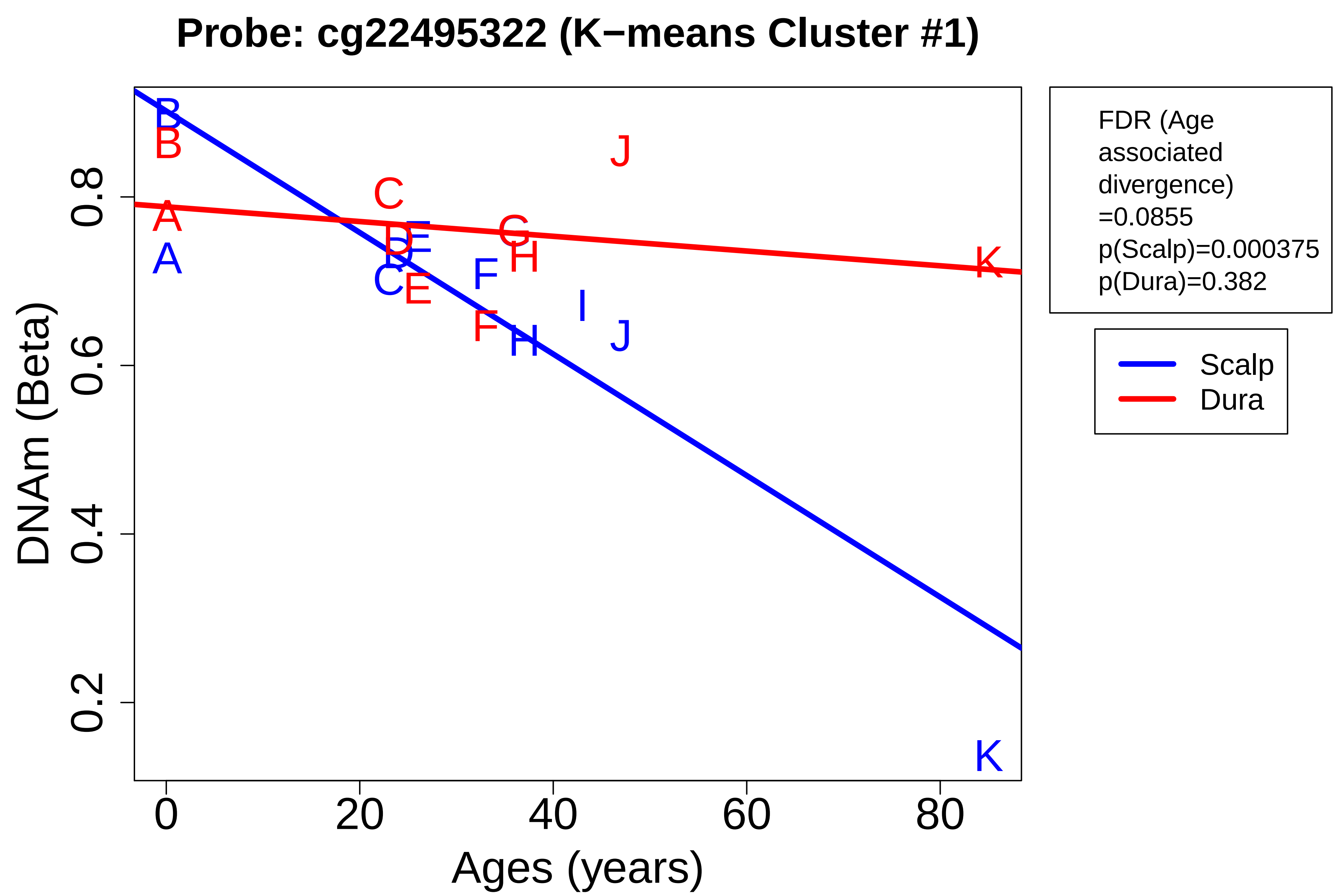
Probe: cg22495322 (K−means Cluster #1)
DNAm (Beta)
0.2
0.4
0.6
0.8
0
20
40
60
80
Ages (years)
FDR (Age associated divergence) =0.0855
p(Scalp)=0.000375
p(Dura)=0.382
Scalp
Dura
A
B
C
D
E
F
G
H
I
J
K

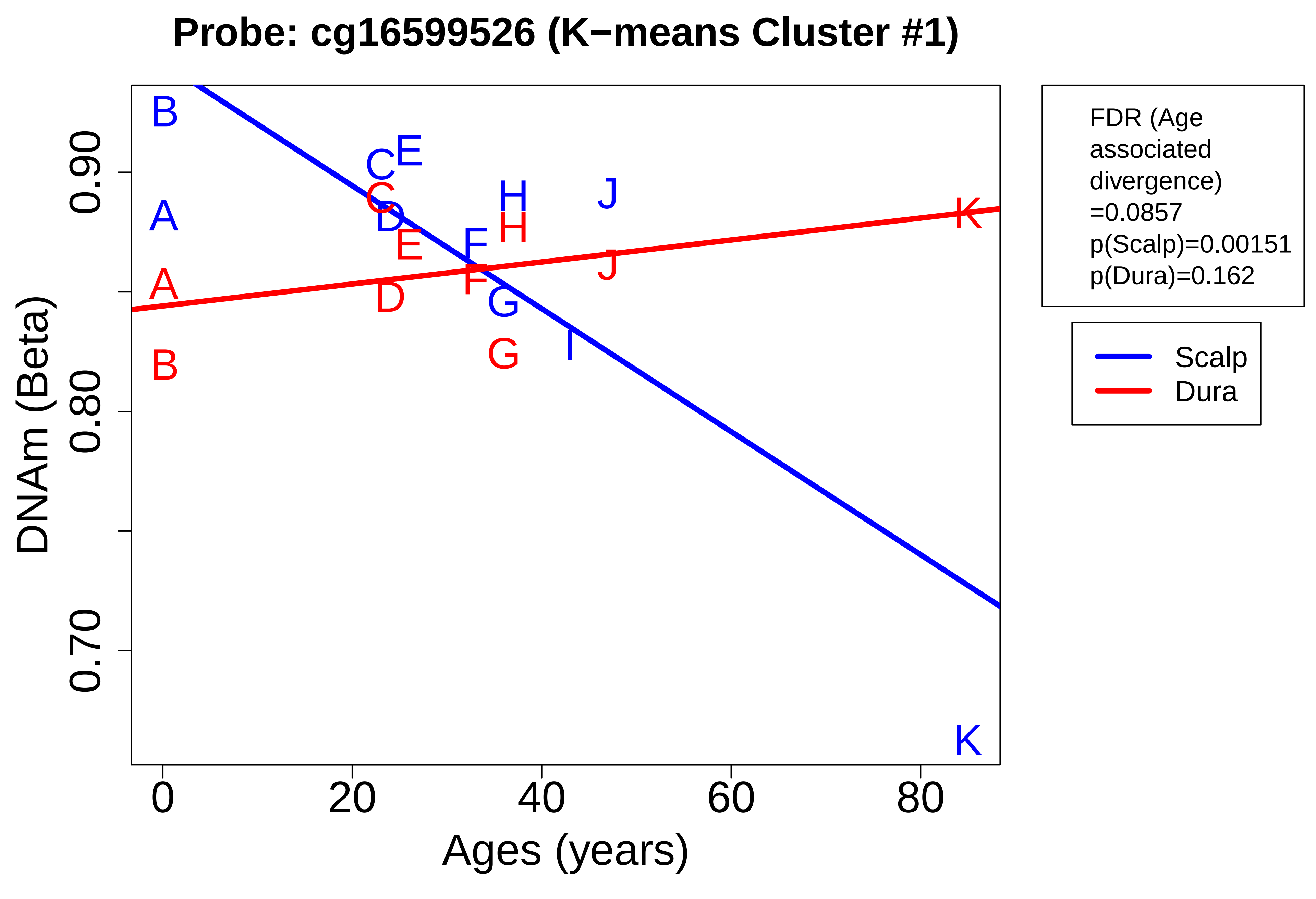

Probe: cg16599526 (K−means Cluster #1)
DNAm (Beta)
0.70
0.80
0.90
Ages (years)
0
20
40
60
80
FDR (Age associated divergence) =0.0857
p(Scalp)=0.00151
p(Dura)=0.162
Scalp
Dura

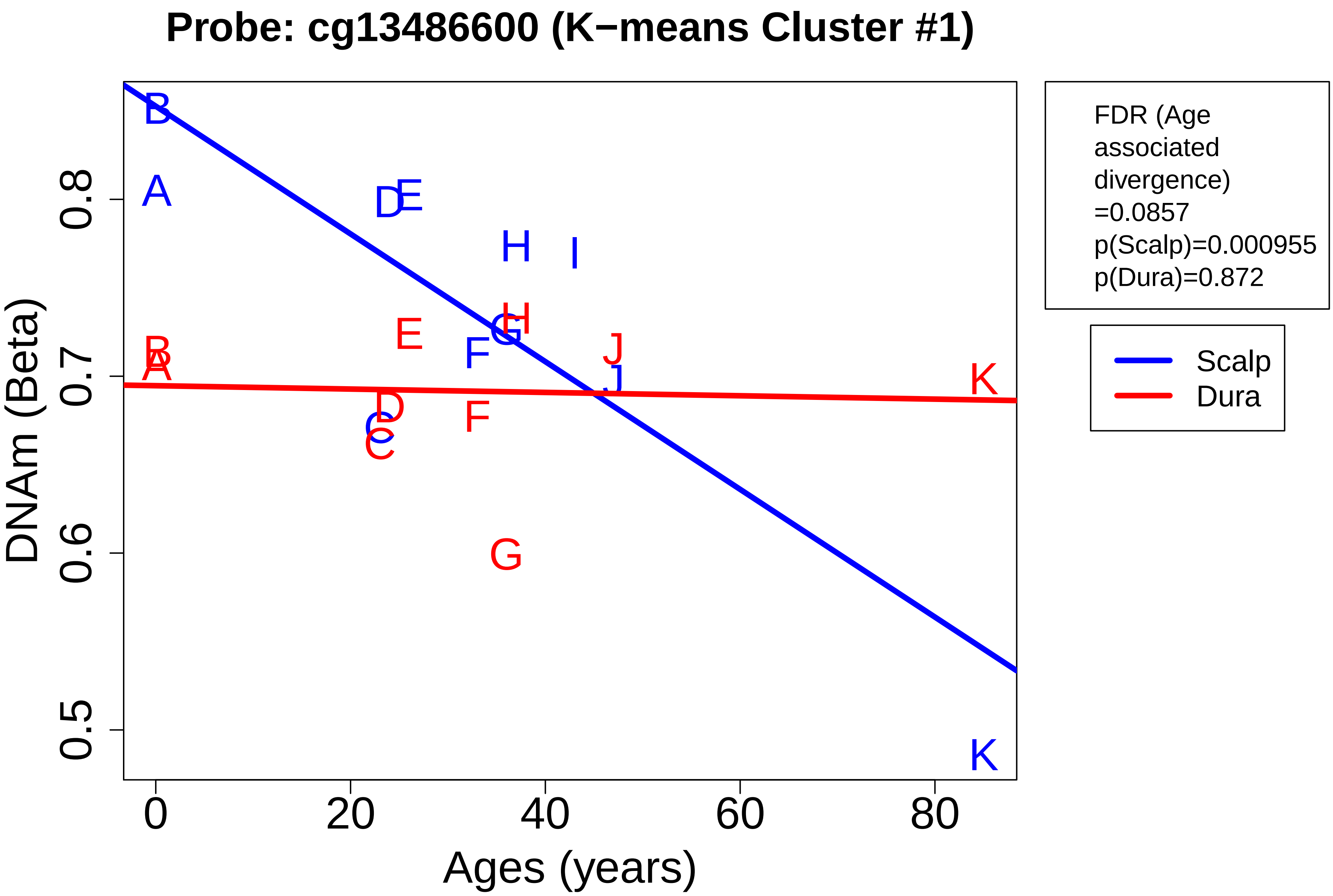

Probe: cg13486600 (K−means Cluster #1)
DNAm (Beta)
0.5
0.6
0.7
0.8
0
20
40
60
80
Ages (years)
FDR (Age associated divergence) =0.0857
p(Scalp)=0.000955
p(Dura)=0.872
Scalp
Dura

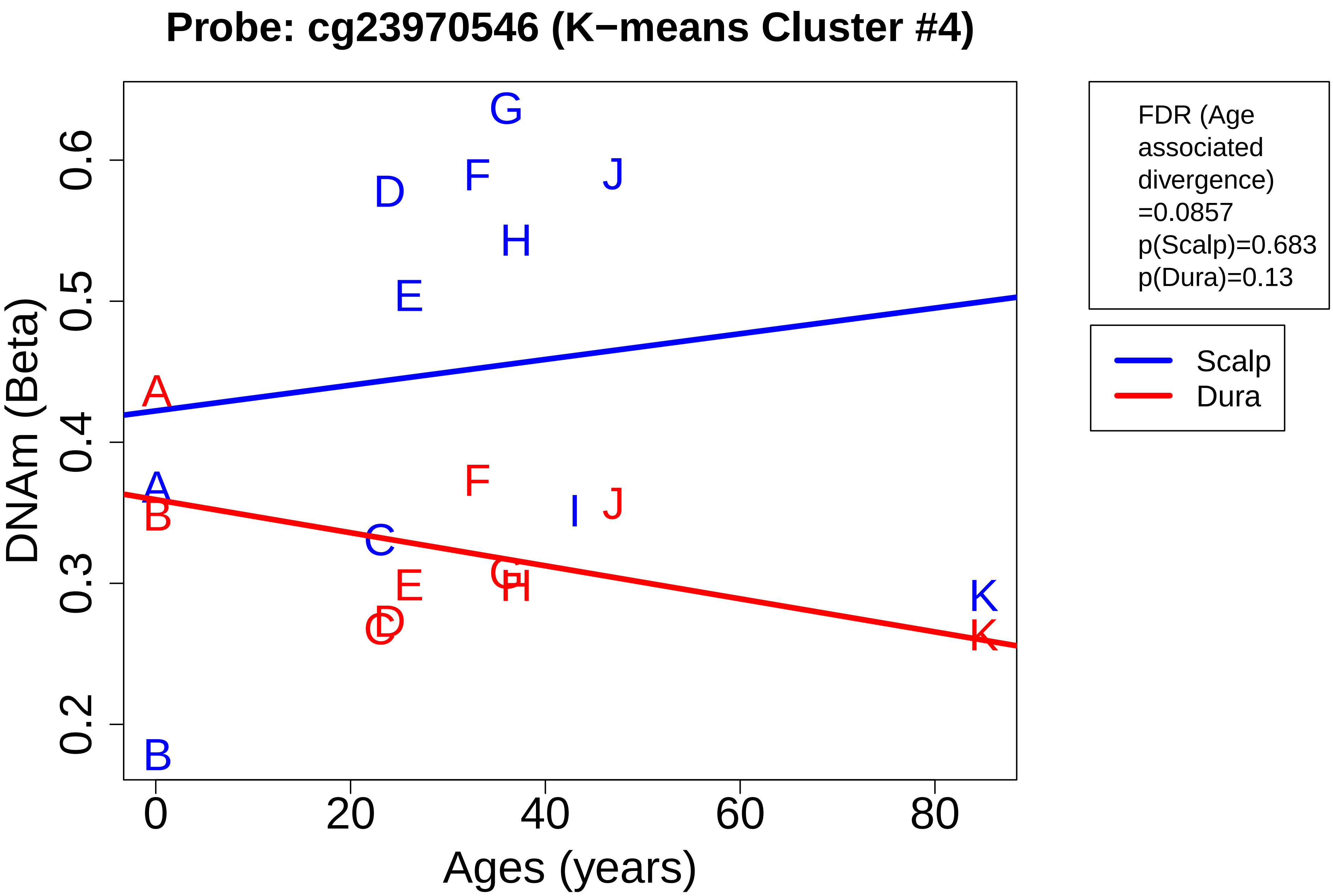

Probe: cg23970546 (K−means Cluster #4)
FDR (Age associated divergence) =0.0857
p(Scalp)=0.683
p(Dura)=0.13
Scalp
Dura
DNAm (Beta)
0.2
0.3
0.4
0.5
0.6
Ages (years)
0
20
40
60
80

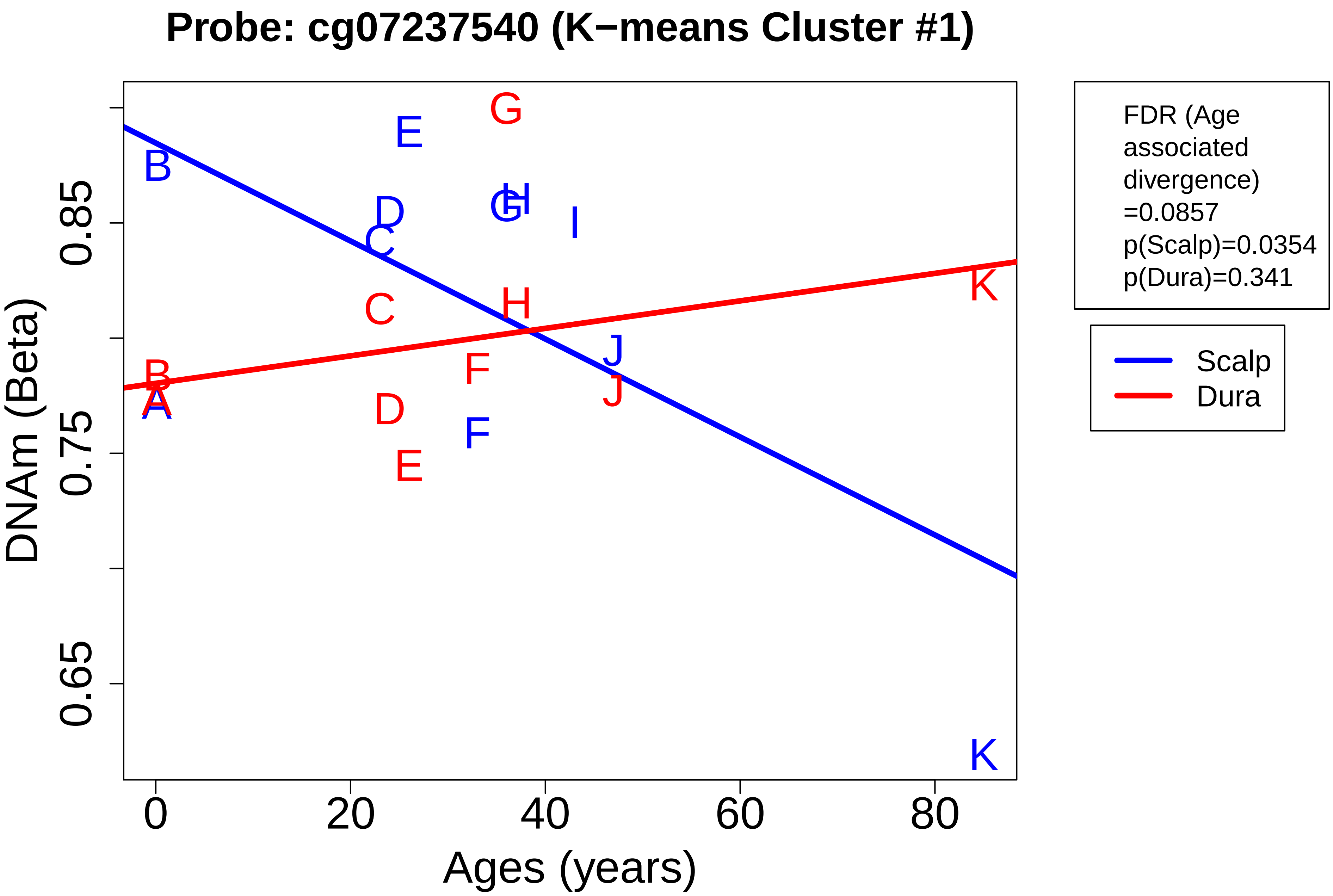

Probe: cg07237540 (K−means Cluster #1)
DNAm (Beta)
0.65
0.75
0.85
Ages (years)
0
20
40
60
80
FDR (Age associated divergence) =0.0857
p(Scalp)=0.0354
p(Dura)=0.341
Scalp
Dura

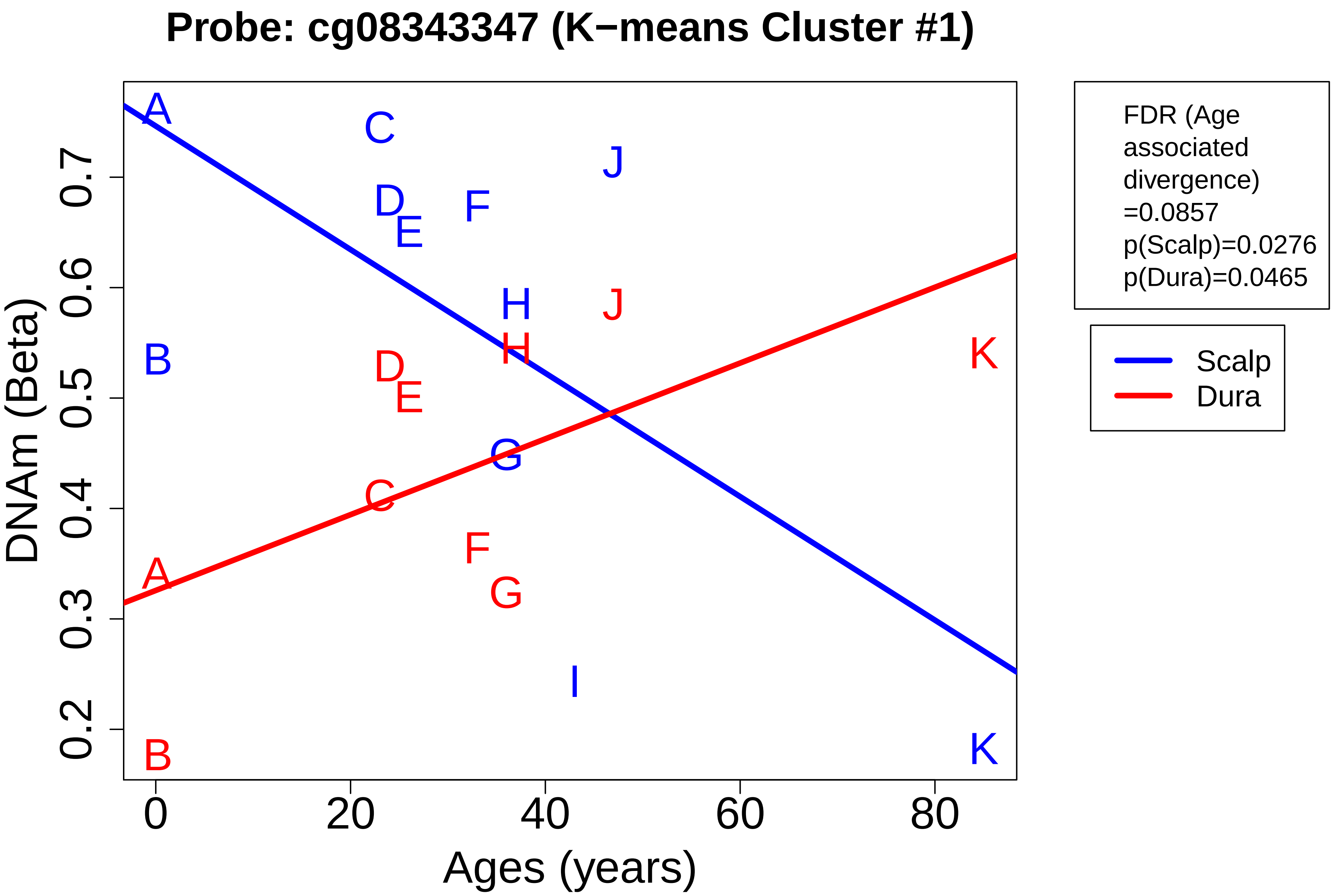

Probe: cg08343347 (K−means Cluster #1)
DNAm (Beta)
0.2
0.3
0.4
0.5
0.6
0.7
Ages (years)
0
20
40
60
80
A
B
C
D
E
F
G
H
I
J
K
FDR (Age associated divergence) =0.0857
p(Scalp)=0.0276
p(Dura)=0.0465
Scalp
Dura

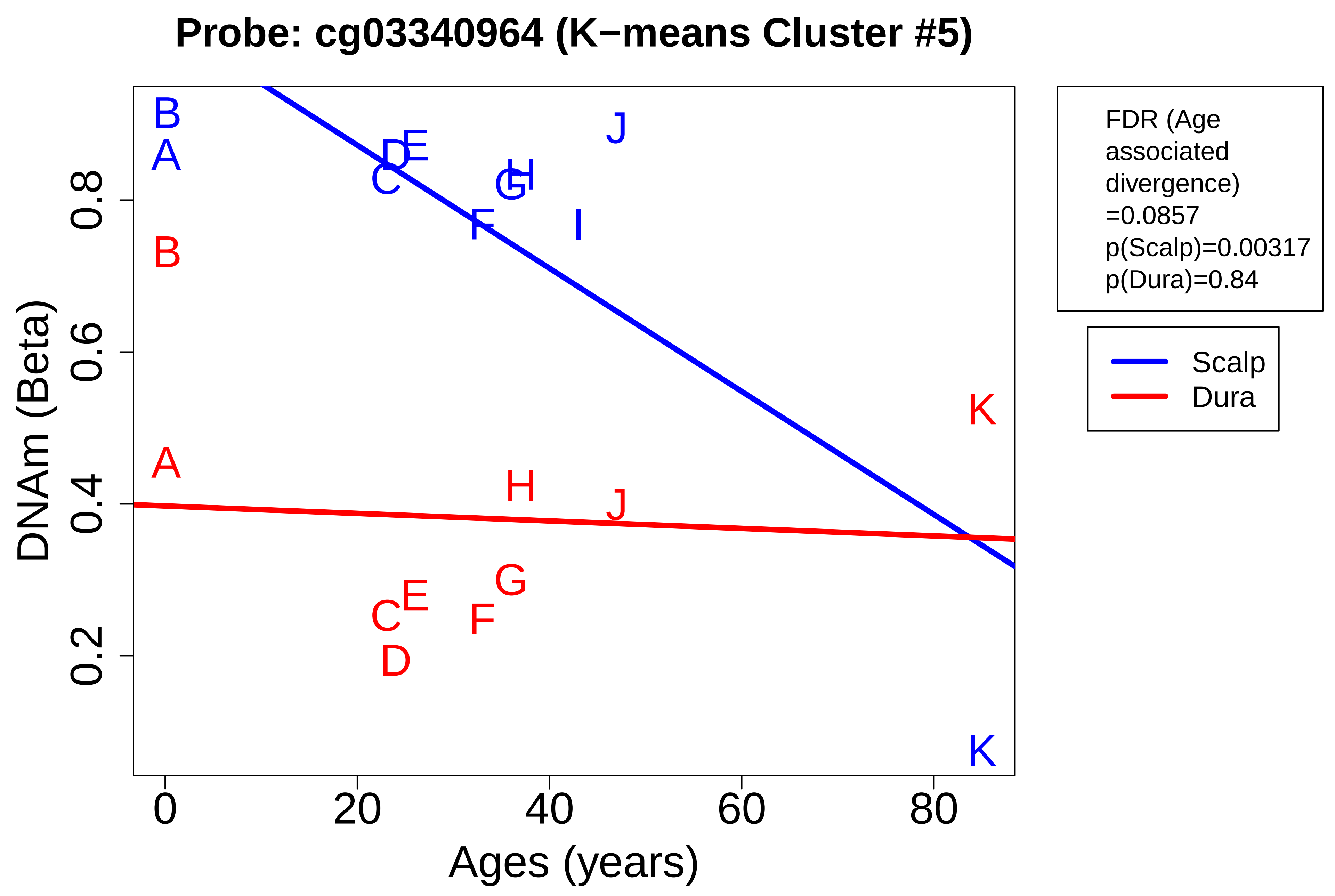

Probe: cg03340964 (K−means Cluster #5)
DNAm (Beta)
0.2
0.4
0.6
0.8
0
20
40
60
80
Ages (years)
FDR (Age associated divergence) =0.0857
p(Scalp)=0.00317
p(Dura)=0.84
Scalp
Dura

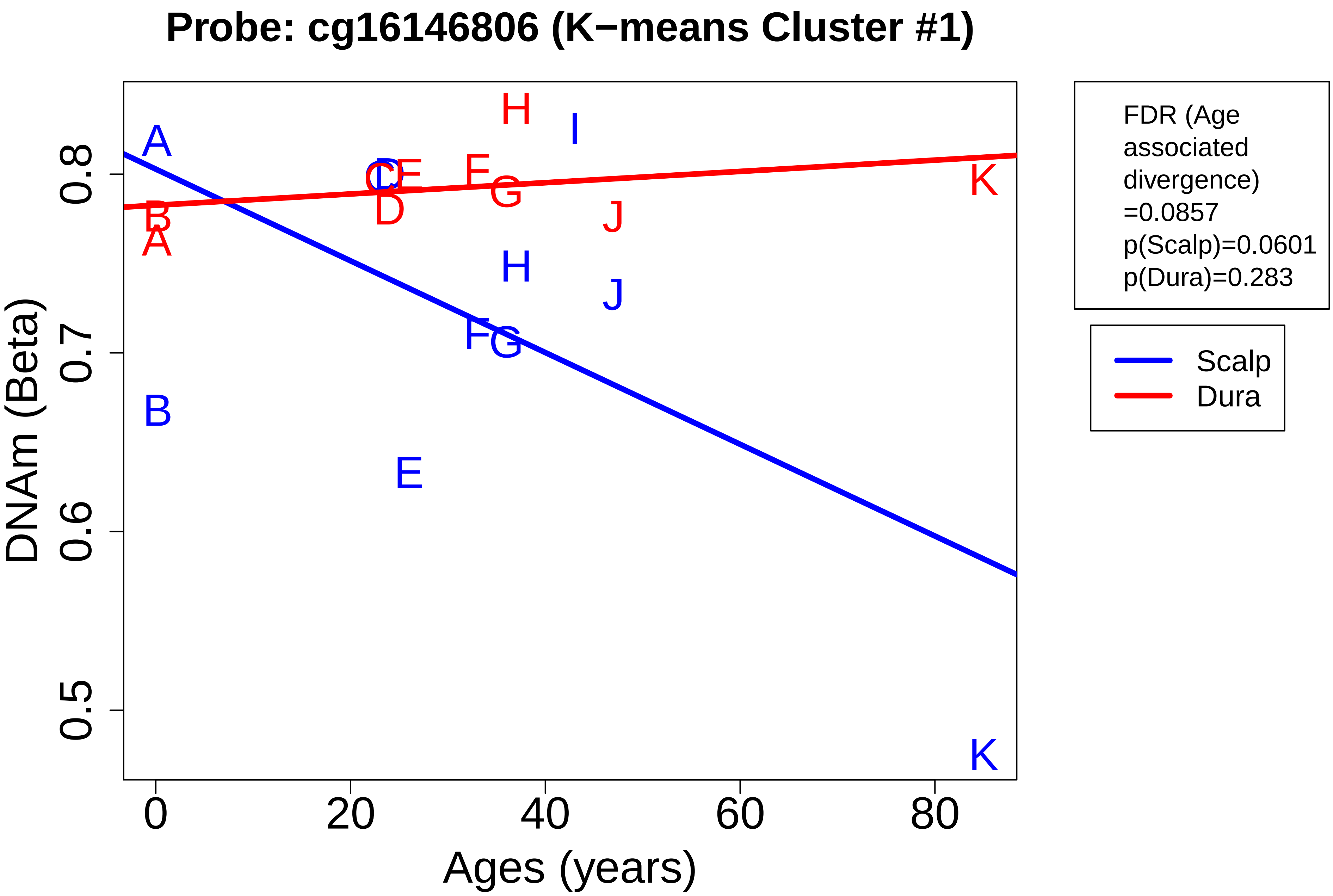

Probe: cg16146806 (K−means Cluster #1)
DNAm (Beta)
0.5
0.6
0.7
0.8
Ages (years)
0
20
40
60
80
FDR (Age associated divergence) =0.0857
p(Scalp)=0.0601
p(Dura)=0.283
Scalp
Dura
A
B
C
D
E
F
G
H
I
J
K

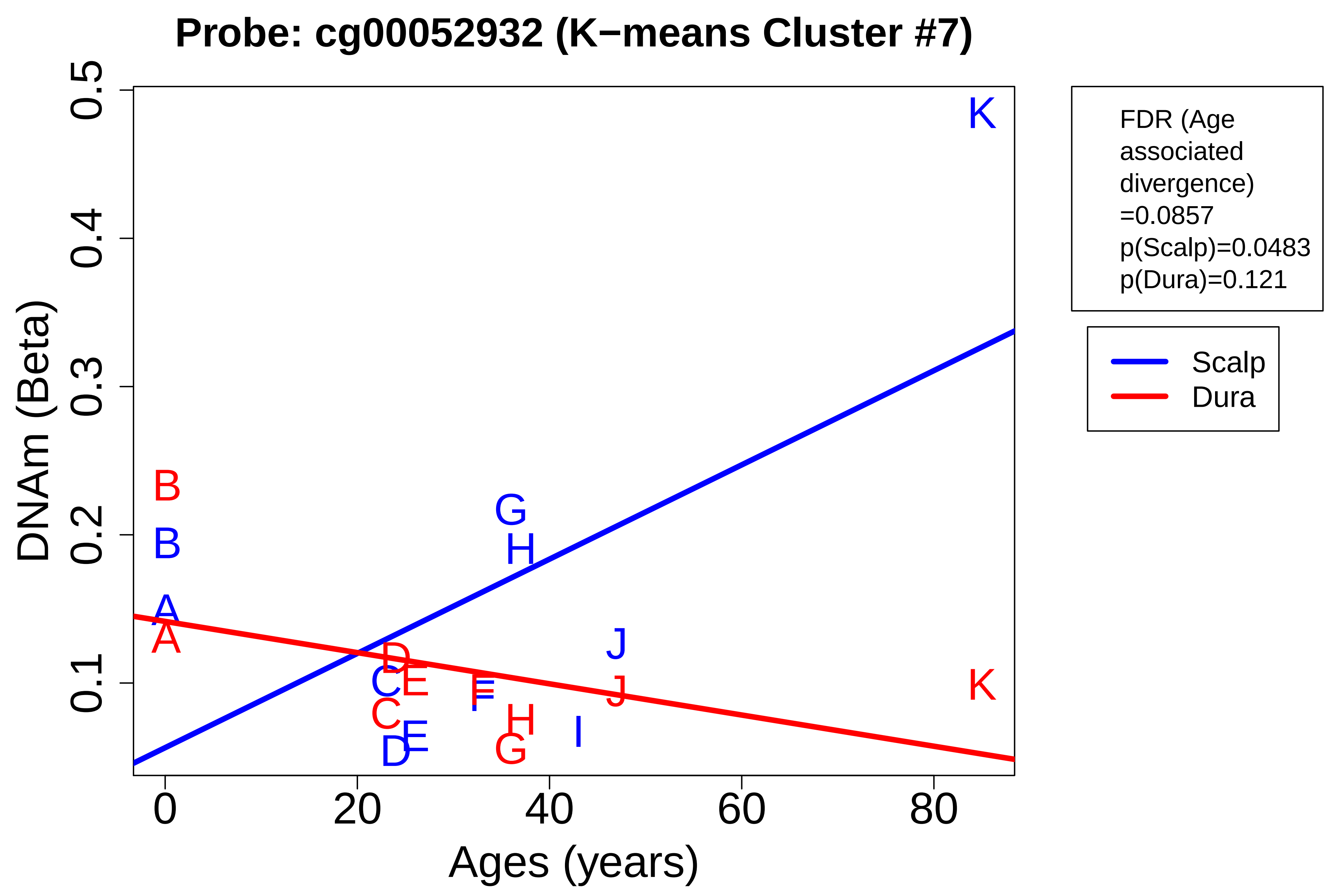
Probe: cg00052932 (K−means Cluster #7)
DNAm (Beta)
0.1
0.2
0.3
0.4
0.5
Ages (years)
0
20
40
60
80
FDR (Age associated divergence) =0.0857
p(Scalp)=0.0483
p(Dura)=0.121
Scalp
Dura
A B C D E F G H I J K

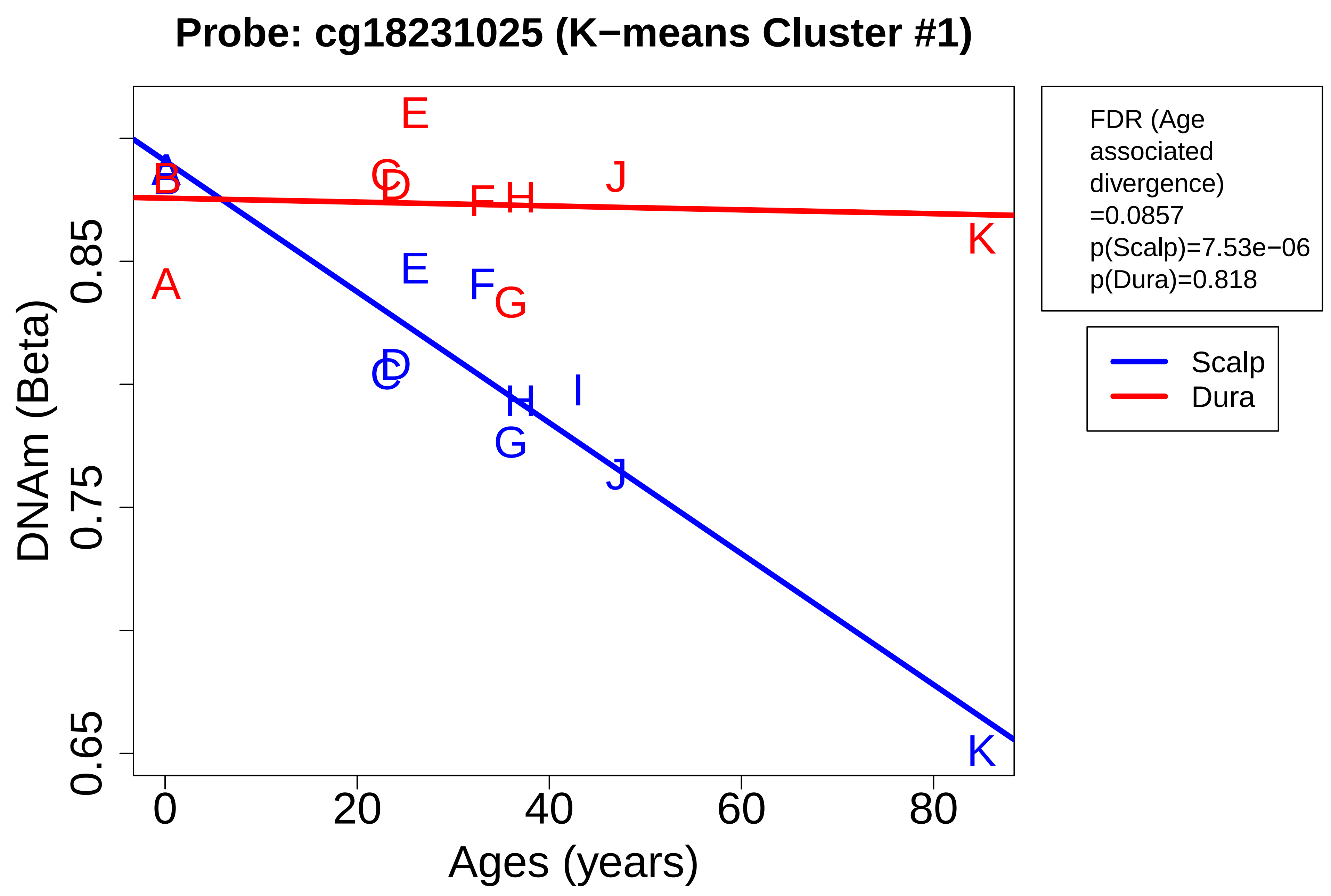

Probe: cg18231025 (K−means Cluster #1)
DNAm (Beta)
0.65
0.75
0.85
0
20
40
60
80
Ages (years)
FDR (Age associated divergence) =0.0857
p(Scalp)=7.53e−06
p(Dura)=0.818
Scalp
Dura
A
B
C
D
E
F
G
H
I
J
K

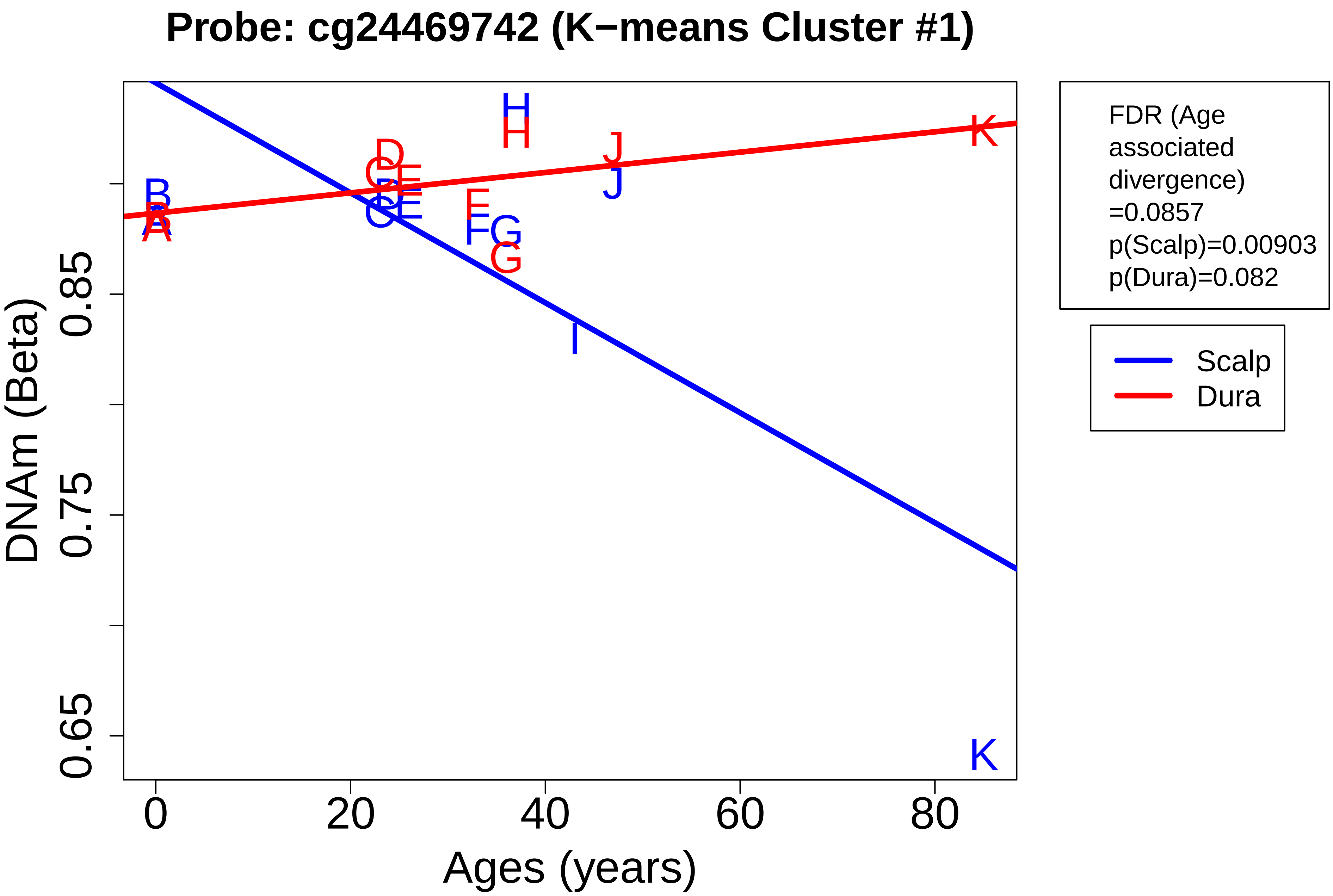
Probe: cg24469742 (K−means Cluster #1)
DNAm (Beta)
0.65
0.75
0.85
Ages (years)
0
20
40
60
80
FDR (Age associated divergence) =0.0857
p(Scalp)=0.00903
p(Dura)=0.082
Scalp
Dura

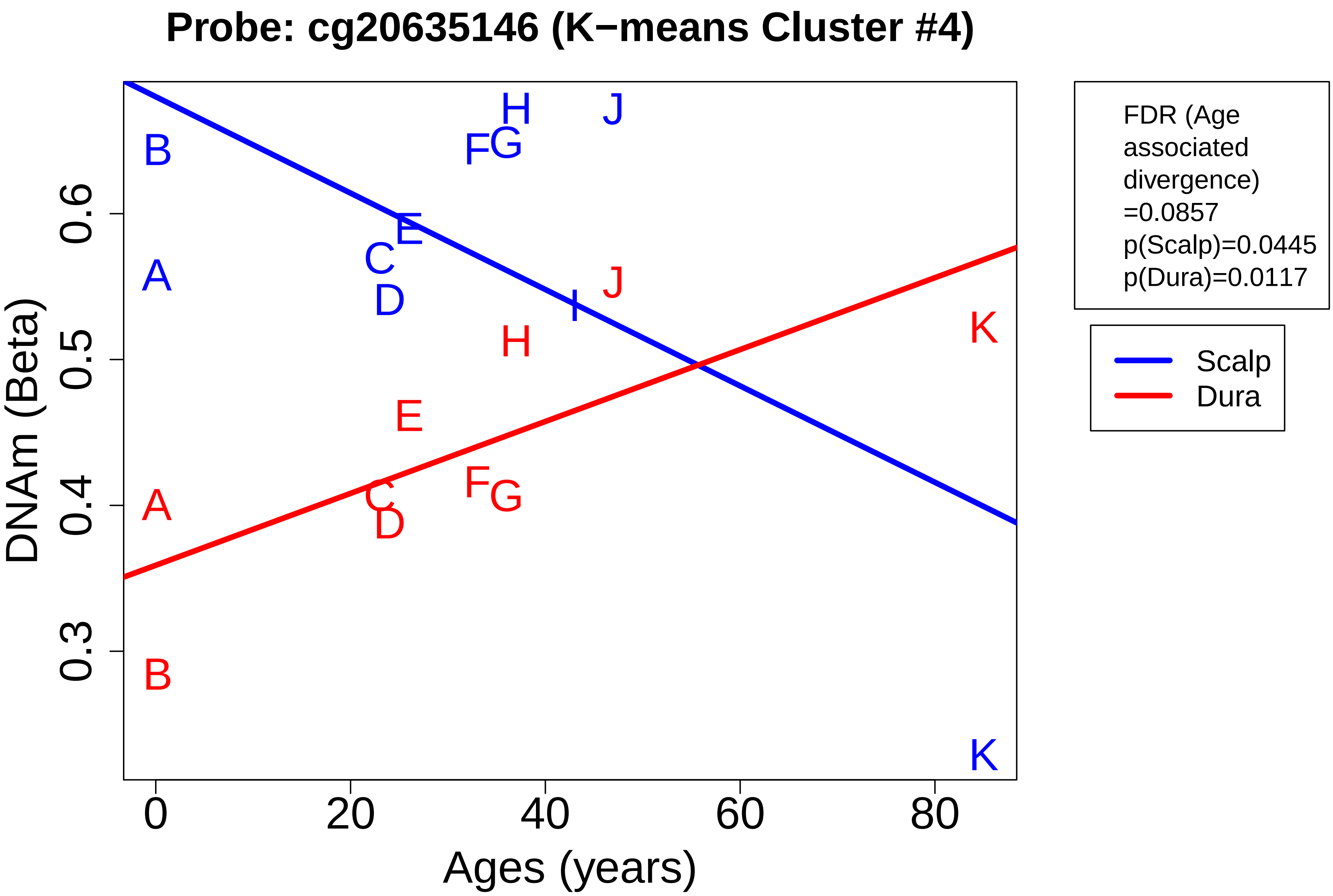

Probe: cg20635146 (K−means Cluster #4)
FDR (Age associated divergence) =0.0857
p(Scalp)=0.0445
p(Dura)=0.0117
Scalp
Dura
DNAm (Beta)
0.3
0.4
0.5
0.6
0
20
40
60
80
Ages (years)
A
B
C
D
E
F
G
H
I
J
K

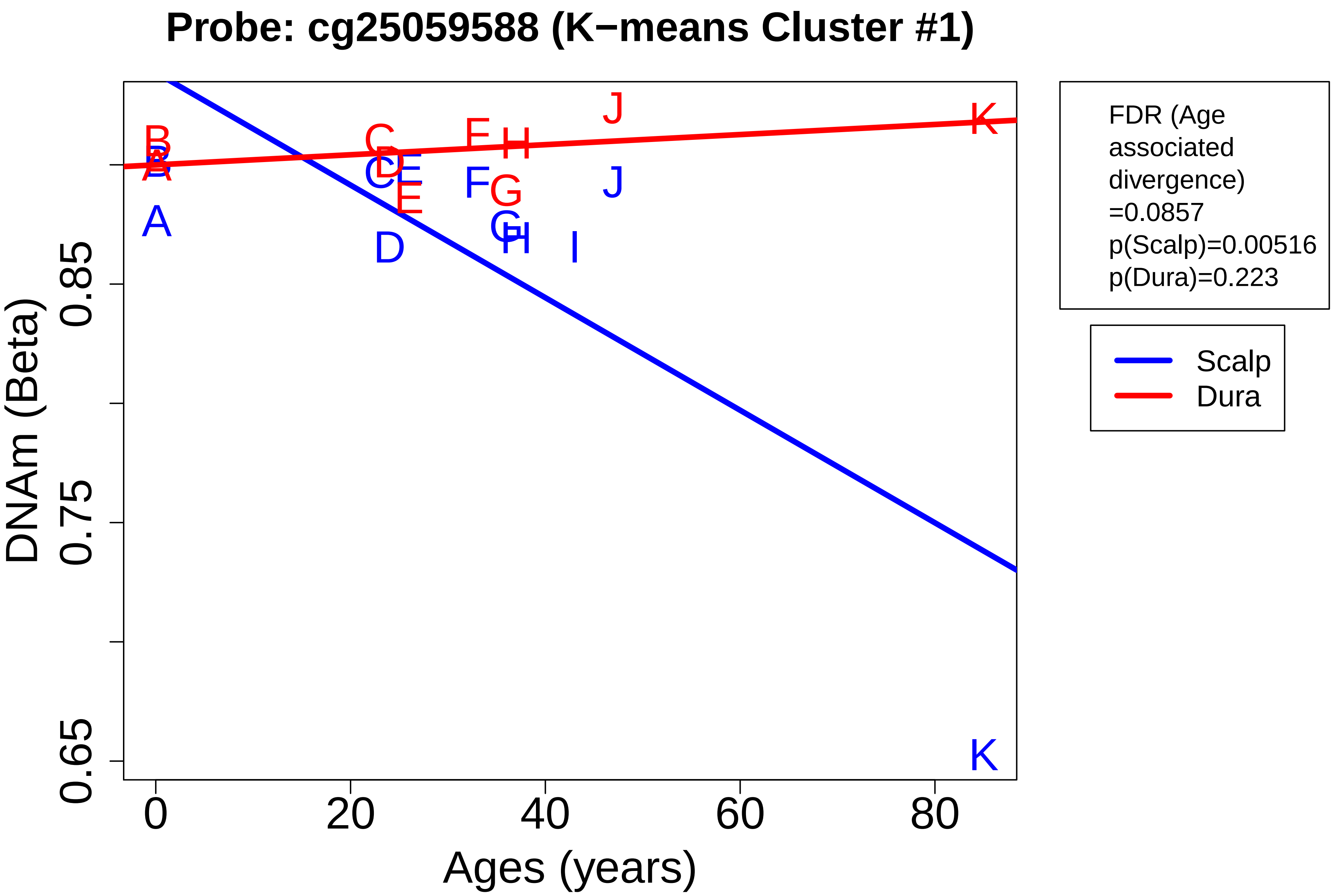
Probe: cg25059588 (K−means Cluster #1)
DNAm (Beta)
0.65
0.75
0.85
Ages (years)
0
20
40
60
80
A
B
C
D
E
F
G
H
I
J
K
FDR (Age associated divergence) =0.0857
p(Scalp)=0.00516
p(Dura)=0.223
Scalp
Dura

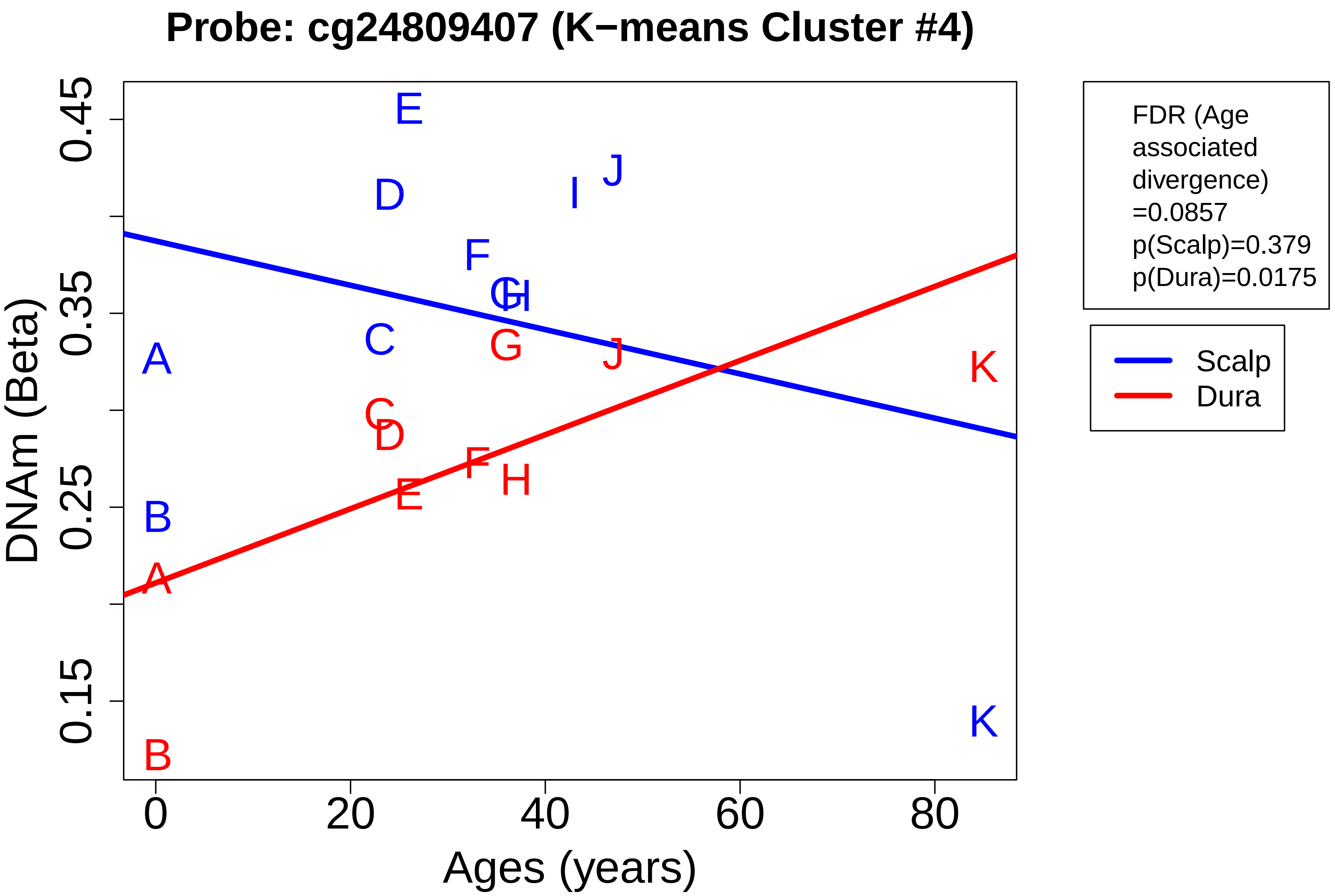
Probe: cg24809407 (K−means Cluster #4)
DNAm (Beta)
0.15
0.25
0.35
0.45
Ages (years)
0
20
40
60
80
FDR (Age associated divergence) =0.0857
p(Scalp)=0.379
p(Dura)=0.0175
Scalp
Dura
A
B
C
D
E
F
G
H
I
J
K

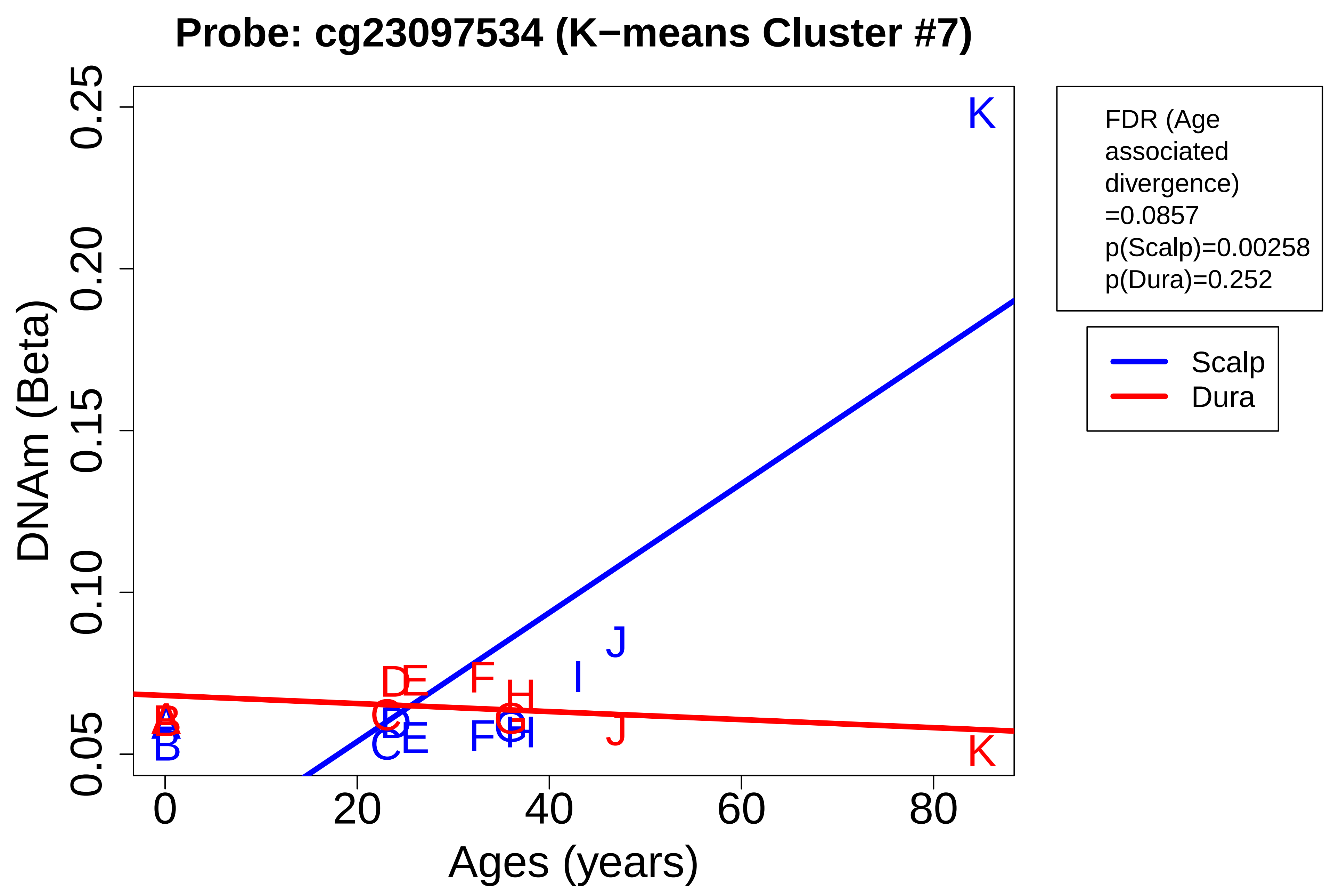

Probe: cg23097534 (K−means Cluster #7)
DNAm (Beta)
0.05
0.10
0.15
0.20
0.25
0
20
40
60
80
Ages (years)
FDR (Age associated divergence) =0.0857
p(Scalp)=0.00258
p(Dura)=0.252
Scalp
Dura

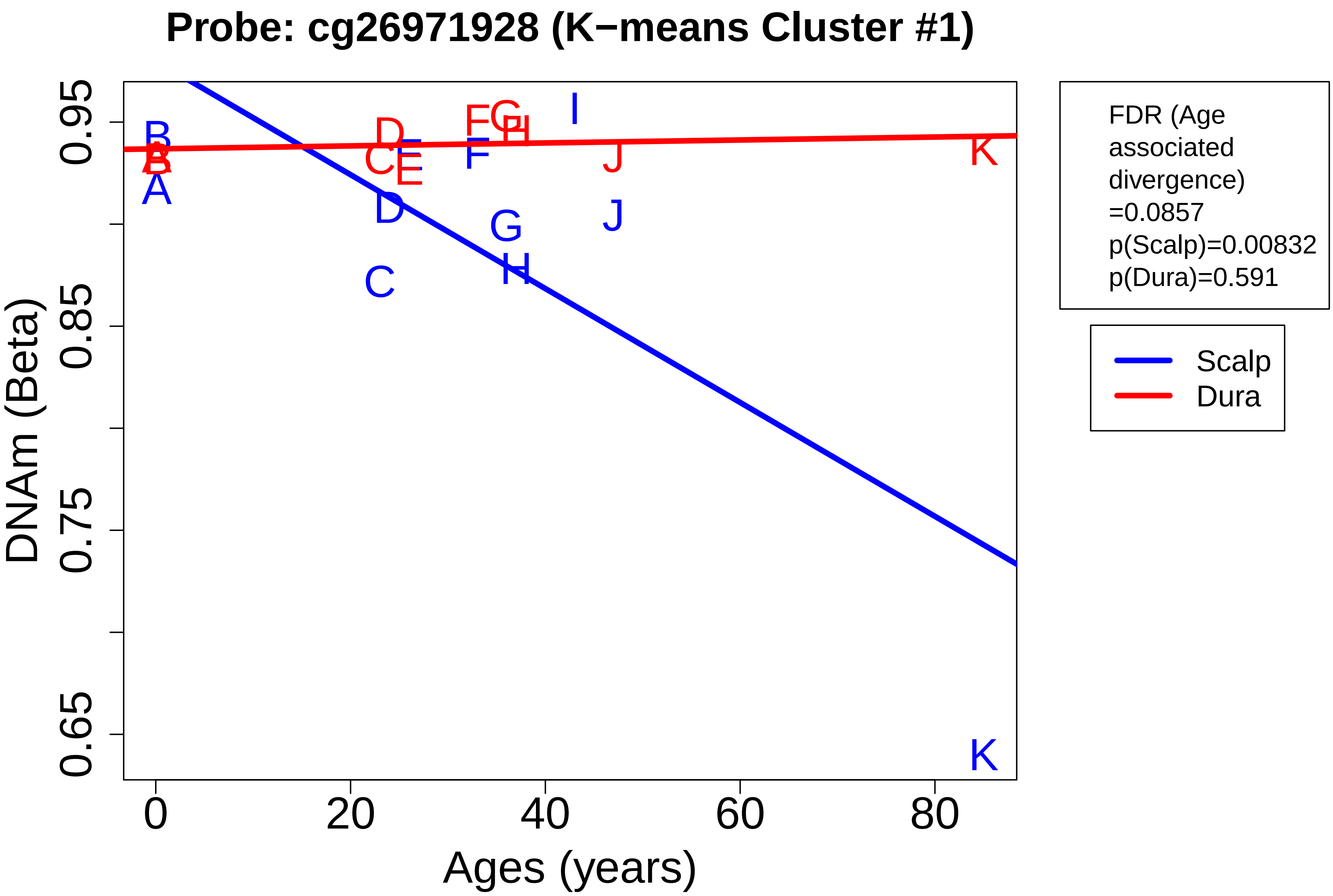

Probe: cg26971928 (K−means Cluster #1)
DNAm (Beta)
0.65
0.75
0.85
0.95
0
20
40
60
80
Ages (years)
FDR (Age associated divergence) =0.0857
p(Scalp)=0.00832
p(Dura)=0.591
Scalp
Dura

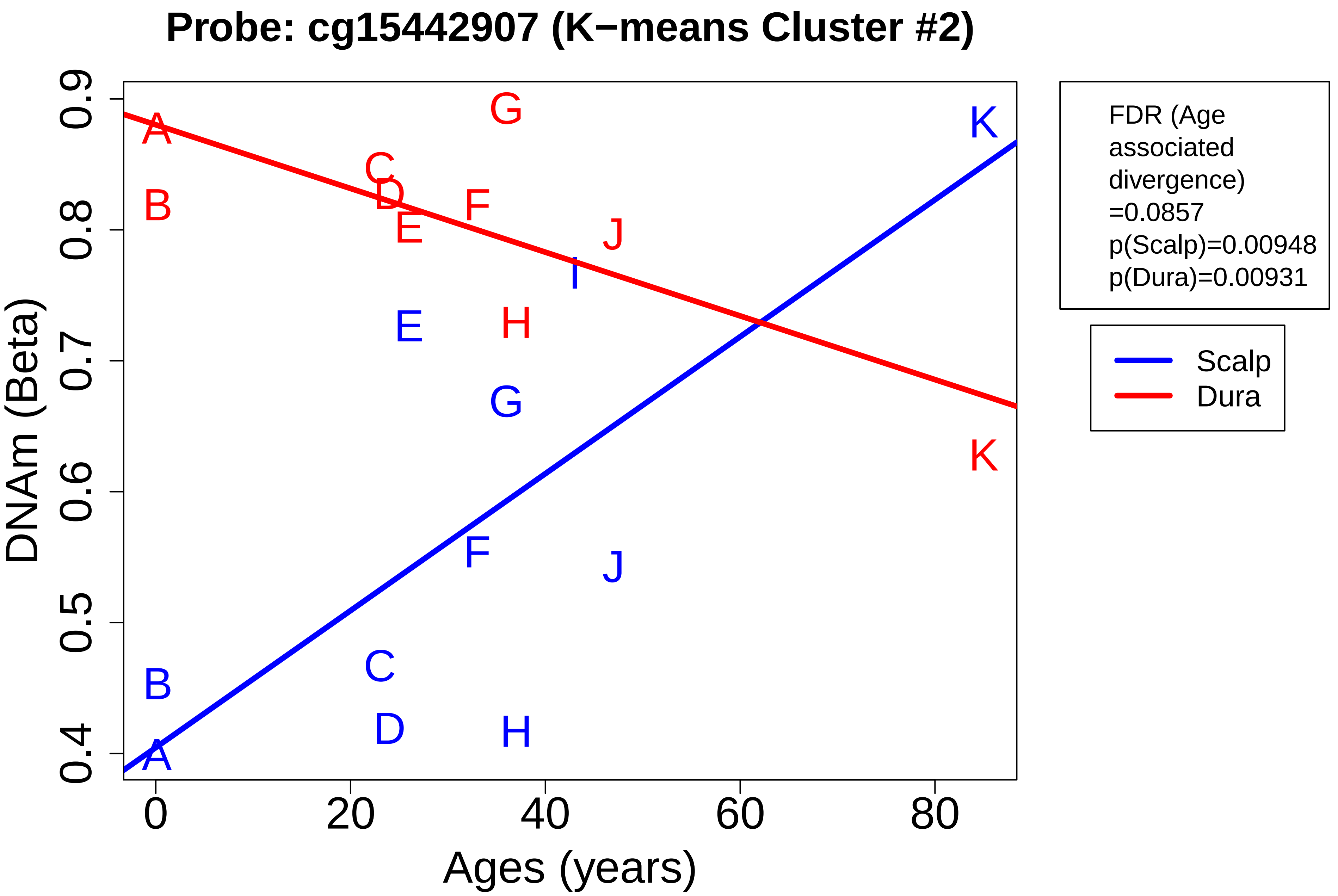

Probe: cg15442907 (K−means Cluster #2)
DNAm (Beta)
0.4
0.5
0.6
0.7
0.8
0.9
Ages (years)
0
20
40
60
80
A
B
C
D
E
F
G
H
I
J
K
FDR (Age associated divergence) =0.0857
p(Scalp)=0.00948
p(Dura)=0.00931
Scalp
Dura

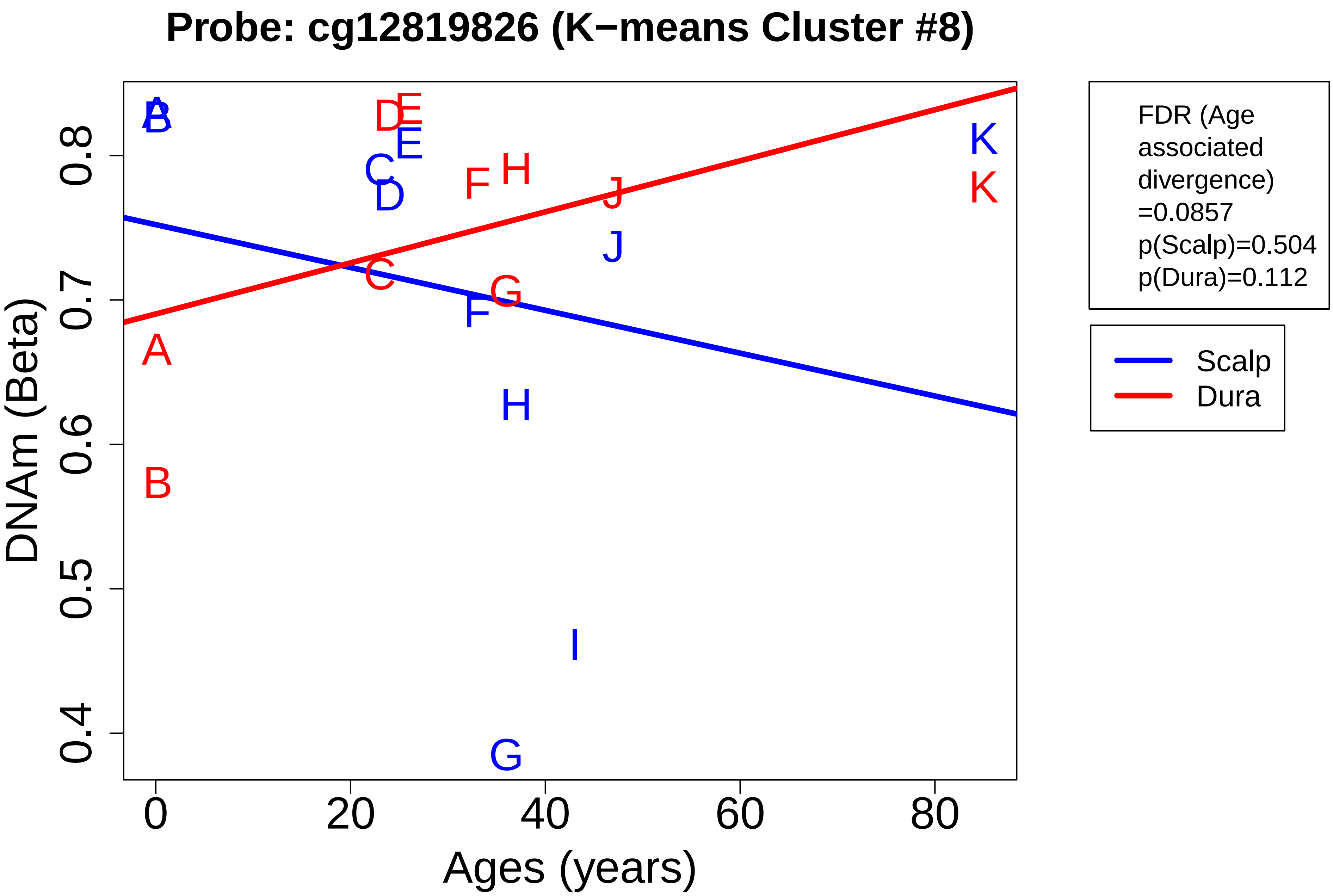

Probe: cg12819826 (K−means Cluster #8)
DNAm (Beta)
Ages (years)
0.4
0.5
0.6
0.7
0.8
0
20
40
60
80
FDR (Age associated divergence) =0.0857
p(Scalp)=0.504
p(Dura)=0.112
Scalp
Dura

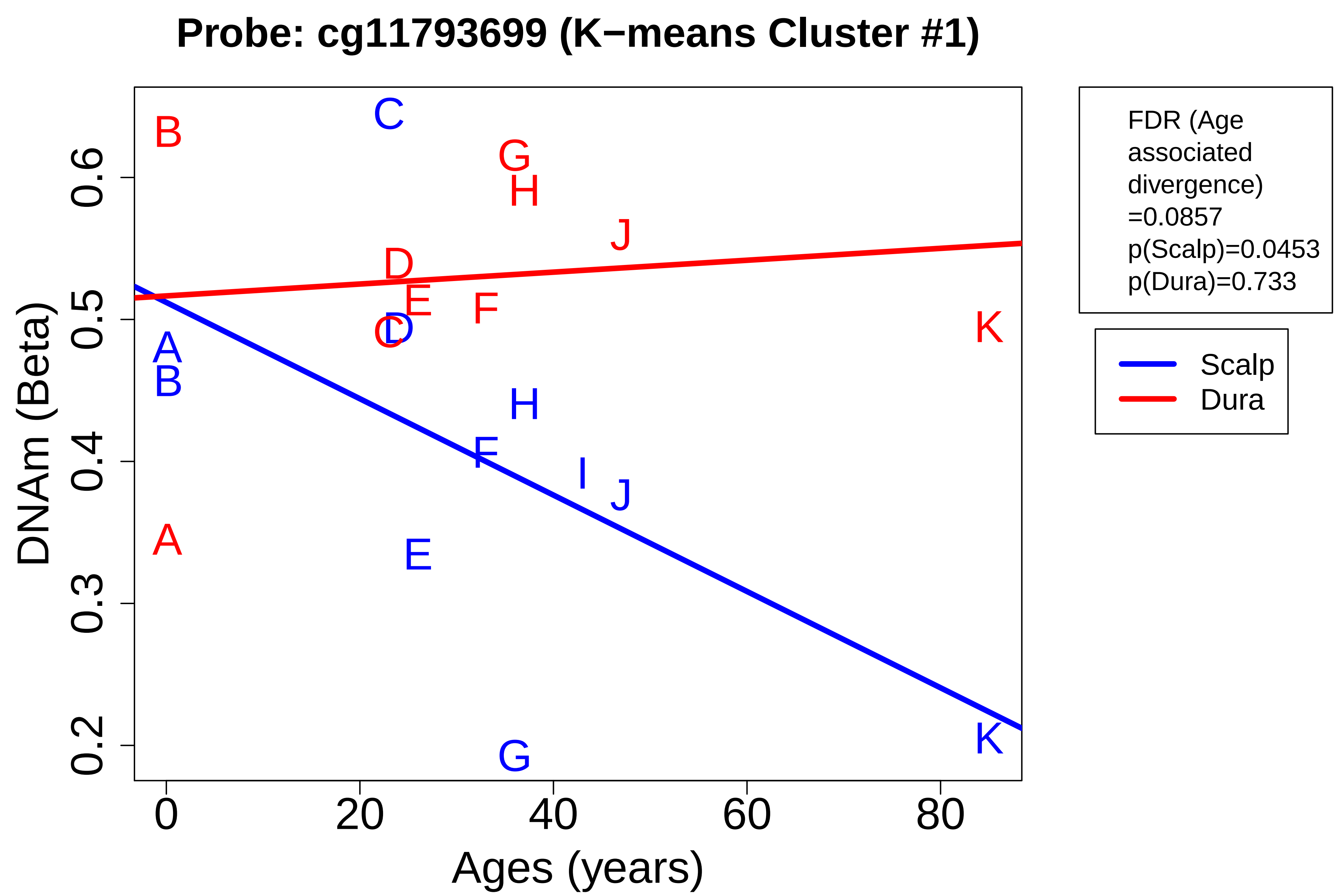

Probe: cg11793699 (K−means Cluster #1)
DNAm (Beta)
0.2
0.3
0.4
0.5
0.6
Ages (years)
0
20
40
60
80
FDR (Age associated divergence) =0.0857
p(Scalp)=0.0453
p(Dura)=0.733
Scalp
Dura
A
B
C
D
E
F
G
H
I
J
K

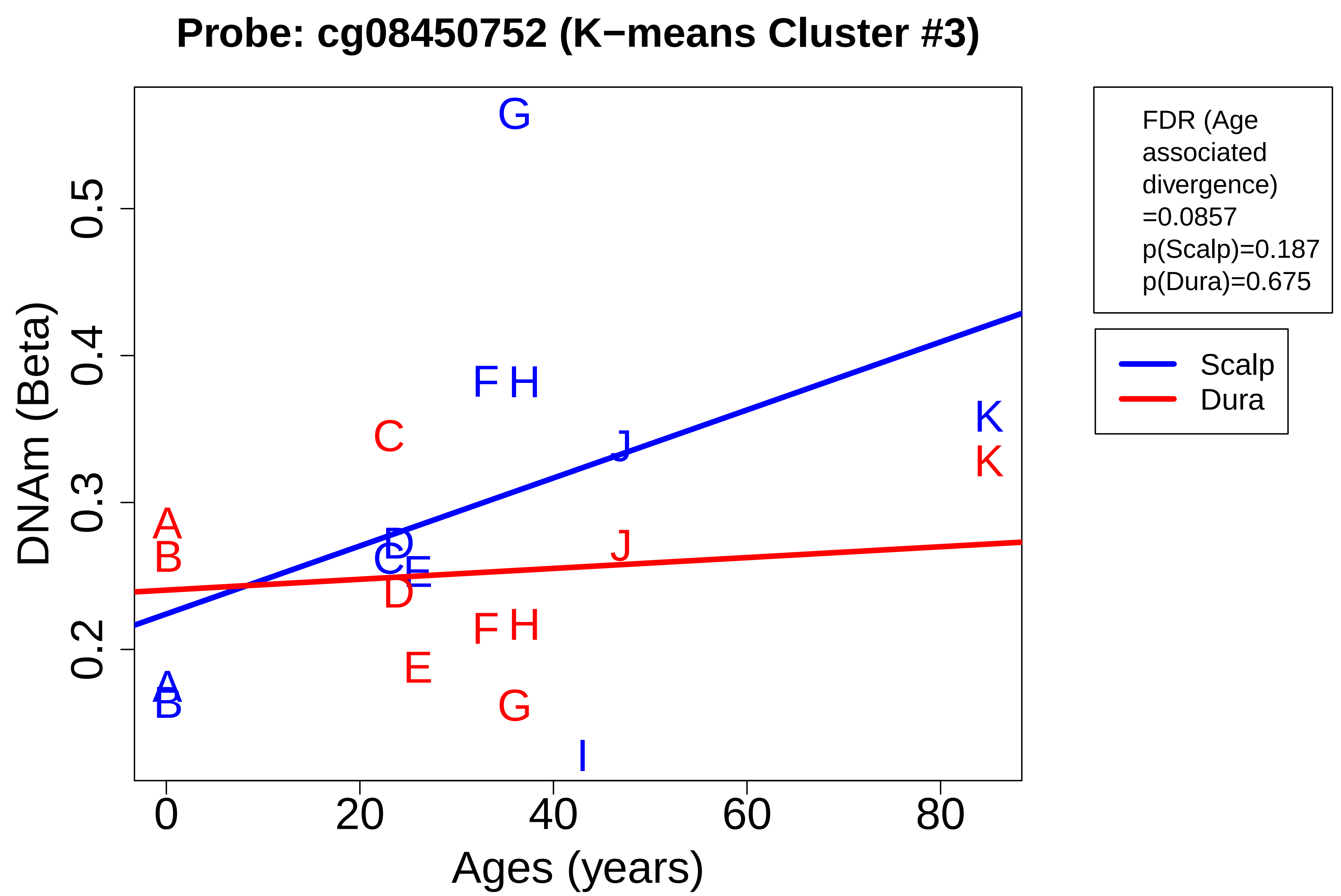

Probe: cg08450752 (K−means Cluster #3)
DNAm (Beta)
0.2
0.3
0.4
0.5
0
20
40
60
80
Ages (years)
FDR (Age associated divergence) =0.0857
p(Scalp)=0.187
p(Dura)=0.675
Scalp
Dura
A
B
C
D
E
F
G
H
I
J
K

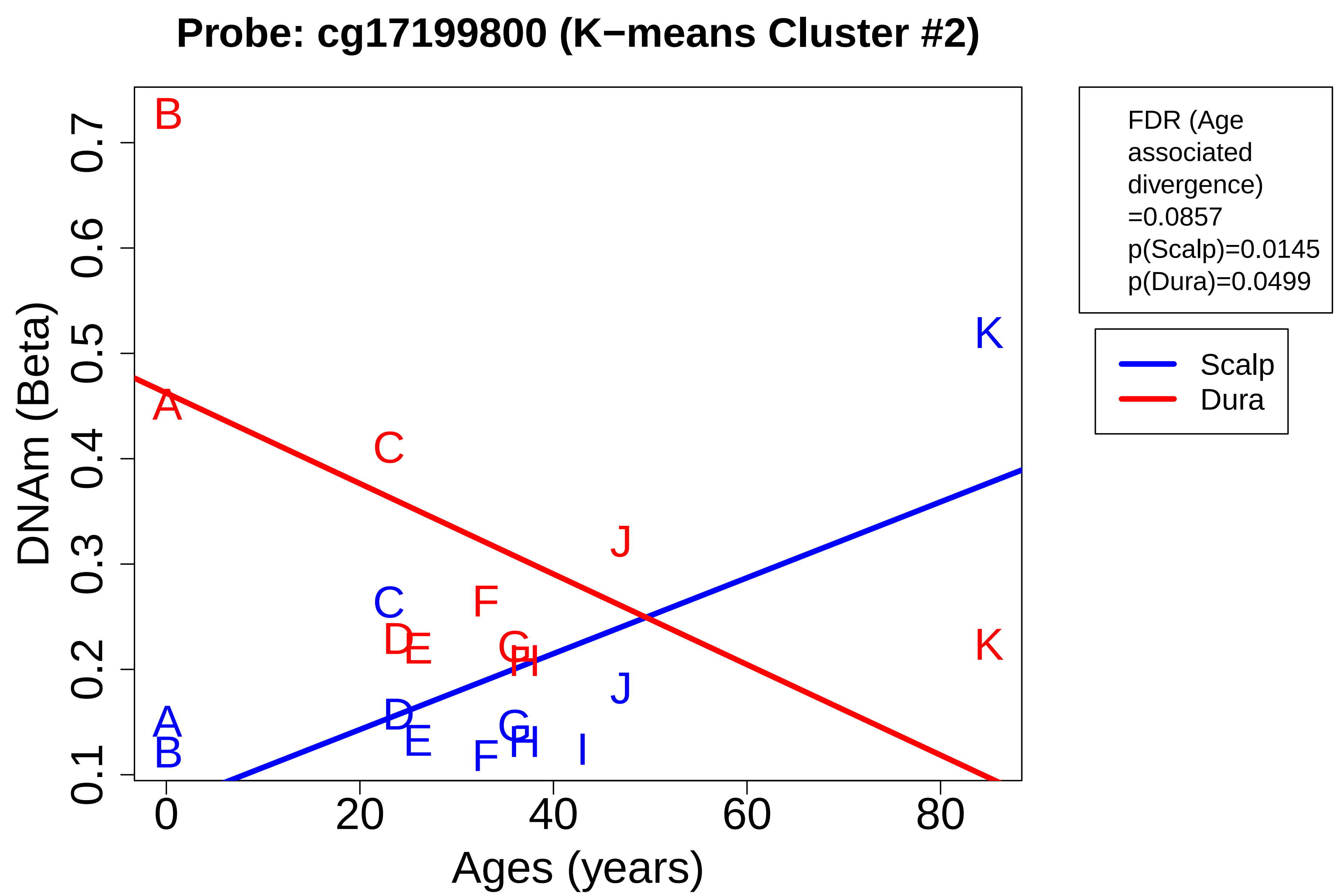

Probe: cg17199800 (K−means Cluster #2)
DNAm (Beta)
0.1
0.2
0.3
0.4
0.5
0.6
0.7
0
20
40
60
80
Ages (years)
FDR (Age associated divergence) =0.0857
p(Scalp)=0.0145
p(Dura)=0.0499
Scalp
Dura
A
B
C
D
E
F
G
H
I
J
K

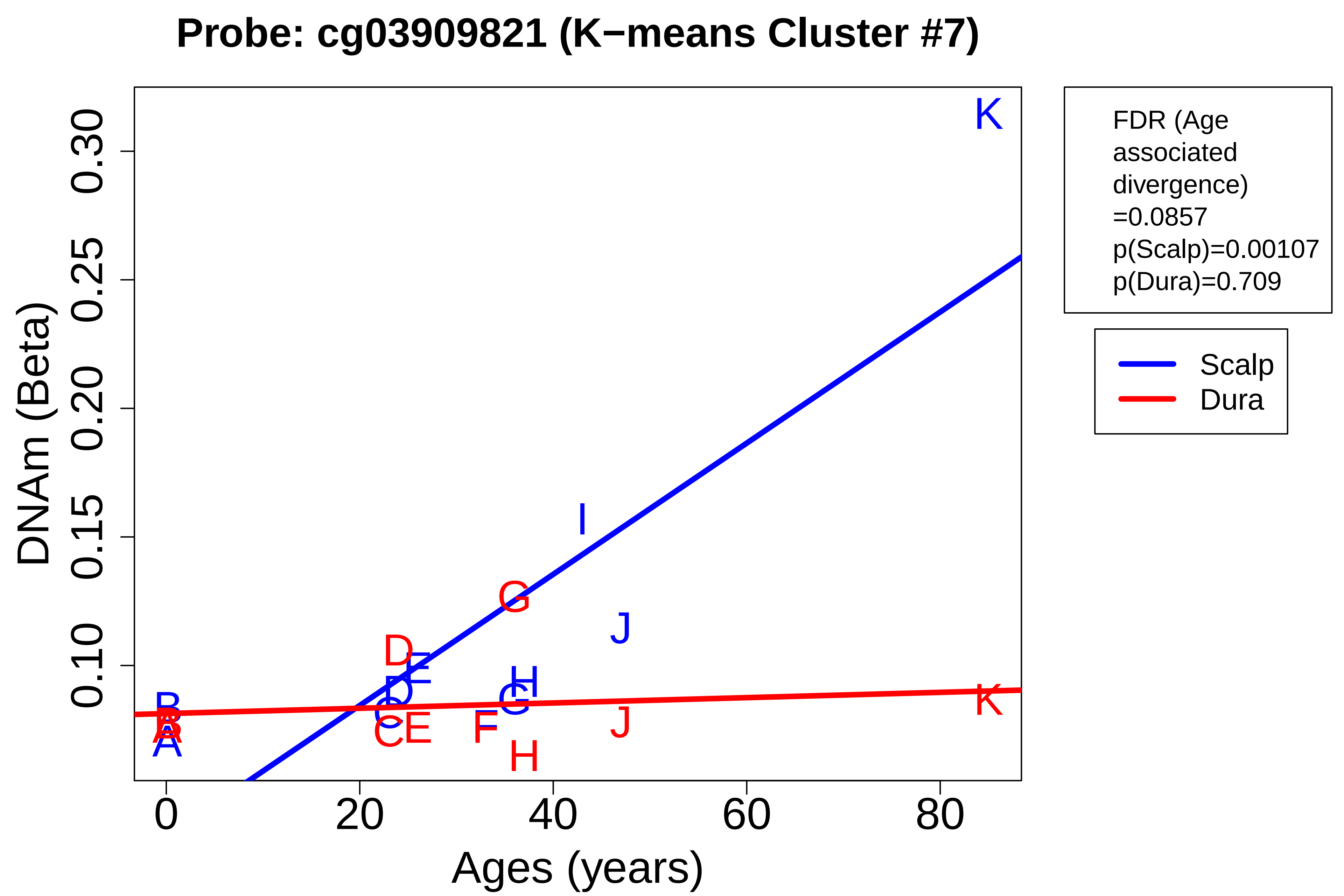

Probe: cg03909821 (K−means Cluster #7)
DNAm (Beta)
0.10
0.15
0.20
0.25
0.30
0
20
40
60
80
Ages (years)
FDR (Age associated divergence) =0.0857
p(Scalp)=0.00107
p(Dura)=0.709
Scalp
Dura

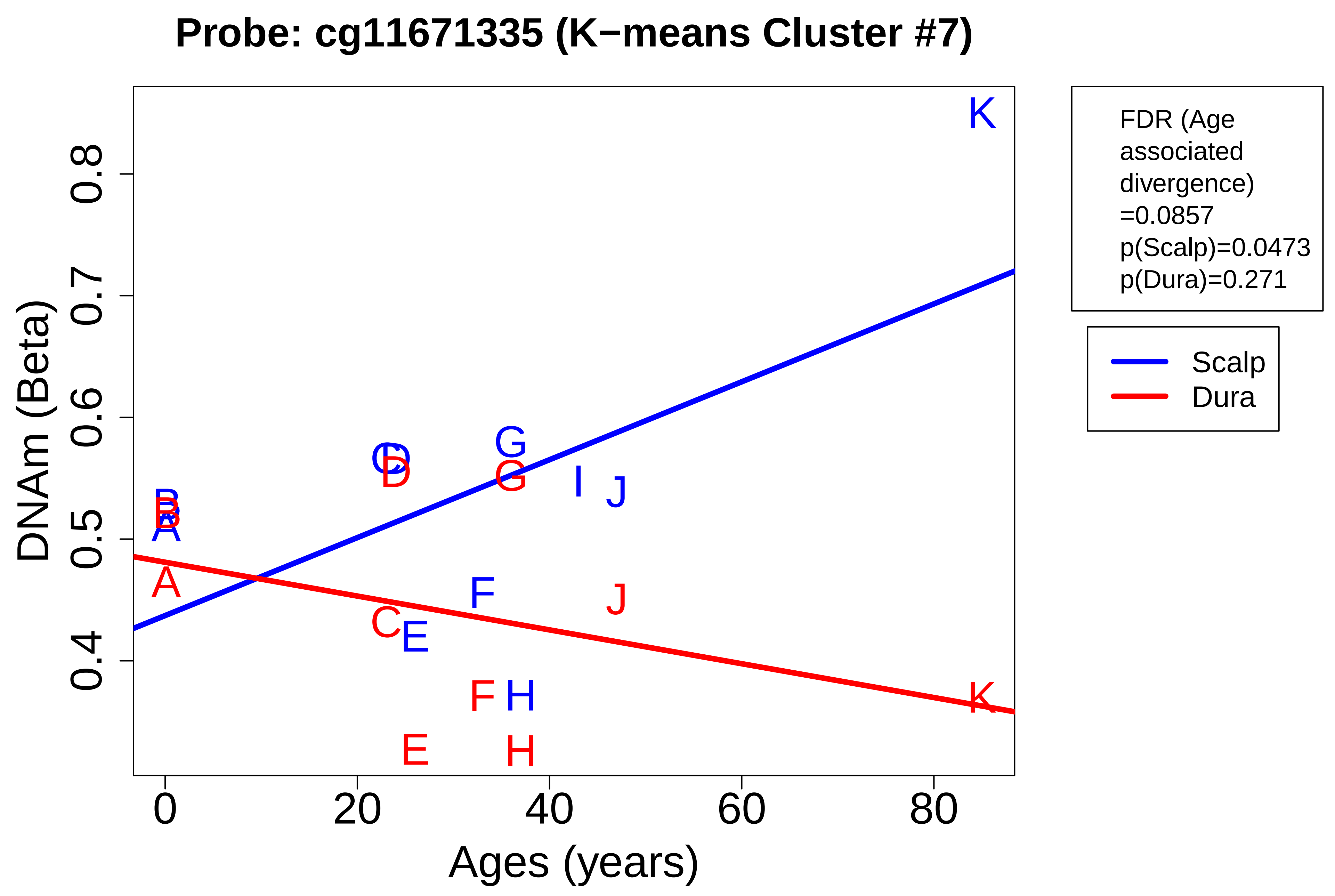

Probe: cg11671335 (K−means Cluster #7)
DNAm (Beta)
0.4
0.5
0.6
0.7
0.8
0
20
40
60
80
Ages (years)
FDR (Age associated divergence) =0.0857
p(Scalp)=0.0473
p(Dura)=0.271
Scalp
Dura

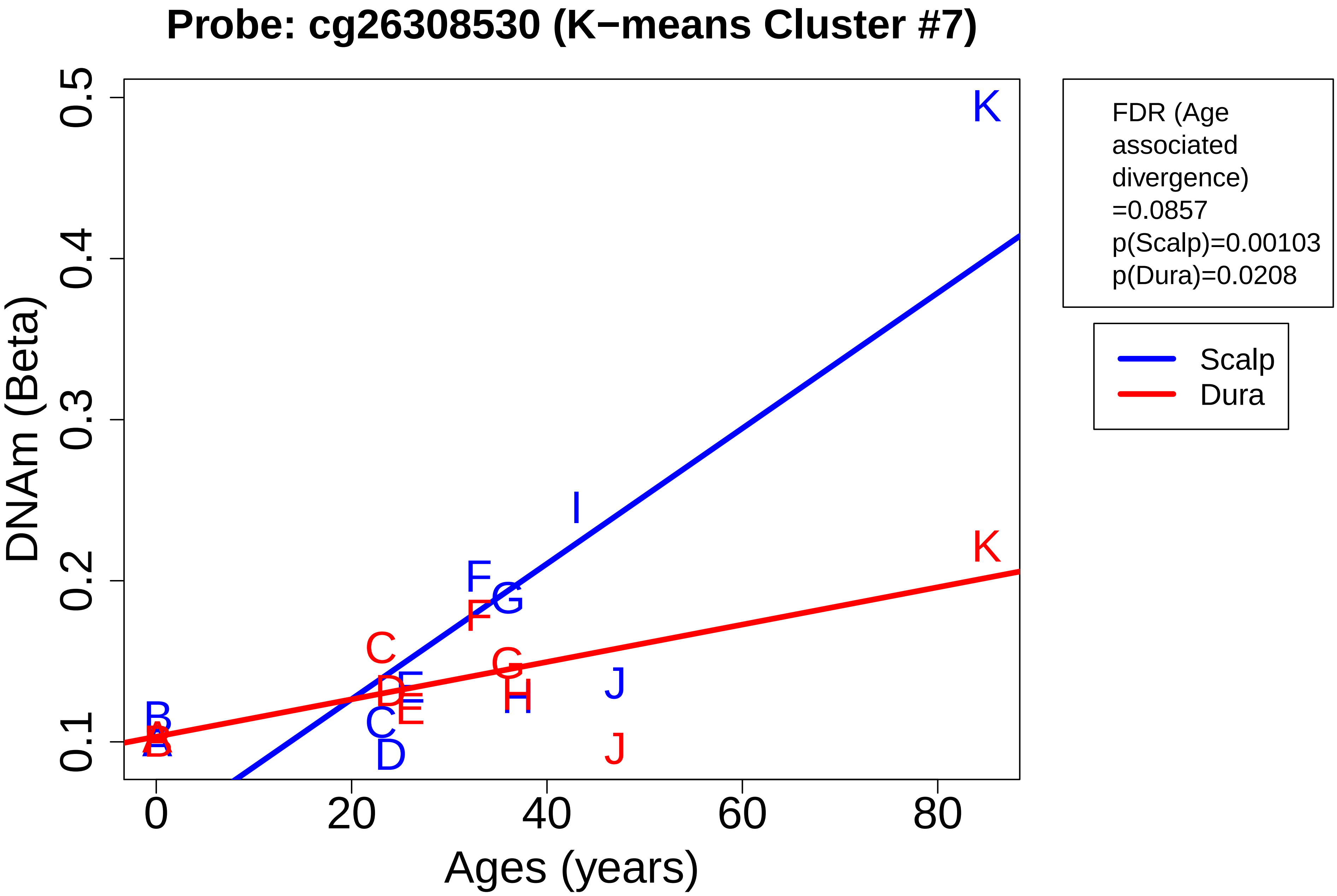

Probe: cg26308530 (K−means Cluster #7)
DNAm (Beta)
0.1
0.2
0.3
0.4
0.5
0
20
40
60
80
Ages (years)
FDR (Age associated divergence) =0.0857
p(Scalp)=0.00103
p(Dura)=0.0208
Scalp
Dura

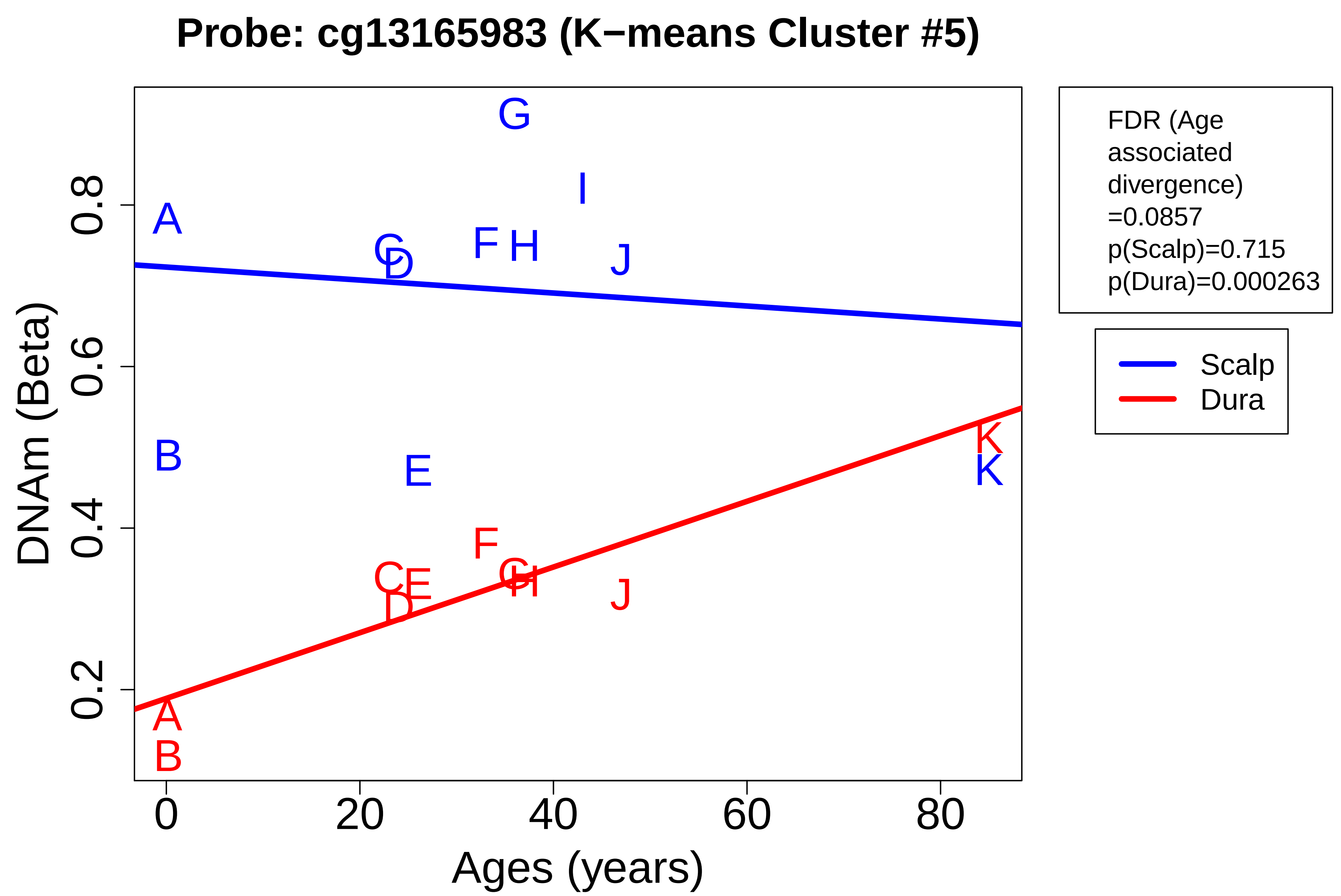

Probe: cg13165983 (K−means Cluster #5)
DNAm (Beta)
0.2
0.4
0.6
0.8
0
20
40
60
80
Ages (years)
A
B
C
D
E
F
G
H
I
J
K
FDR (Age associated divergence) =0.0857
p(Scalp)=0.715
p(Dura)=0.000263
Scalp
Dura

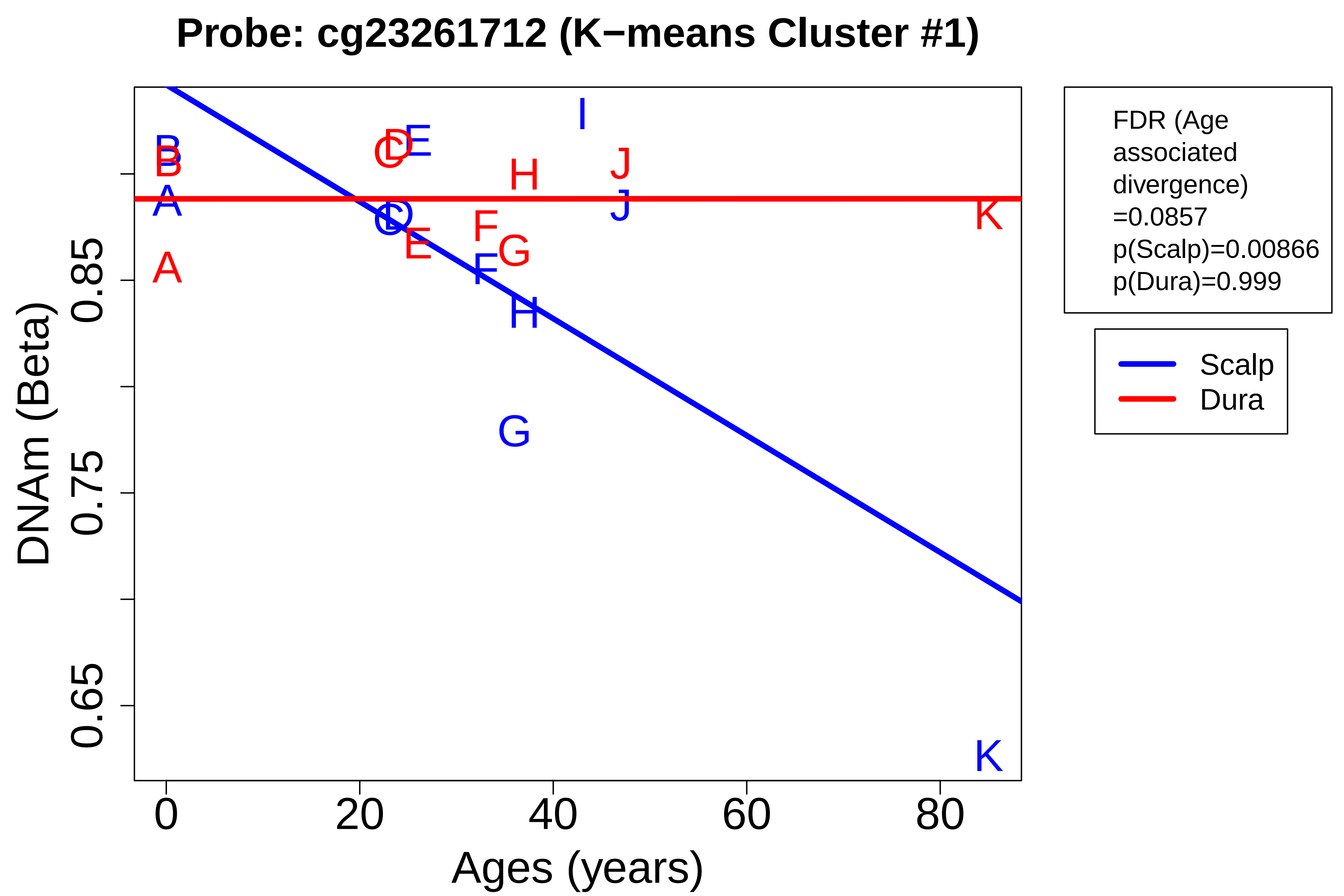

Probe: cg23261712 (K−means Cluster #1)
DNAm (Beta)
0.65
0.75
0.85
Ages (years)
0
20
40
60
80
A
B
C
D
E
F
G
H
I
J
K
FDR (Age associated divergence) =0.0857
p(Scalp)=0.00866
p(Dura)=0.999
Scalp
Dura

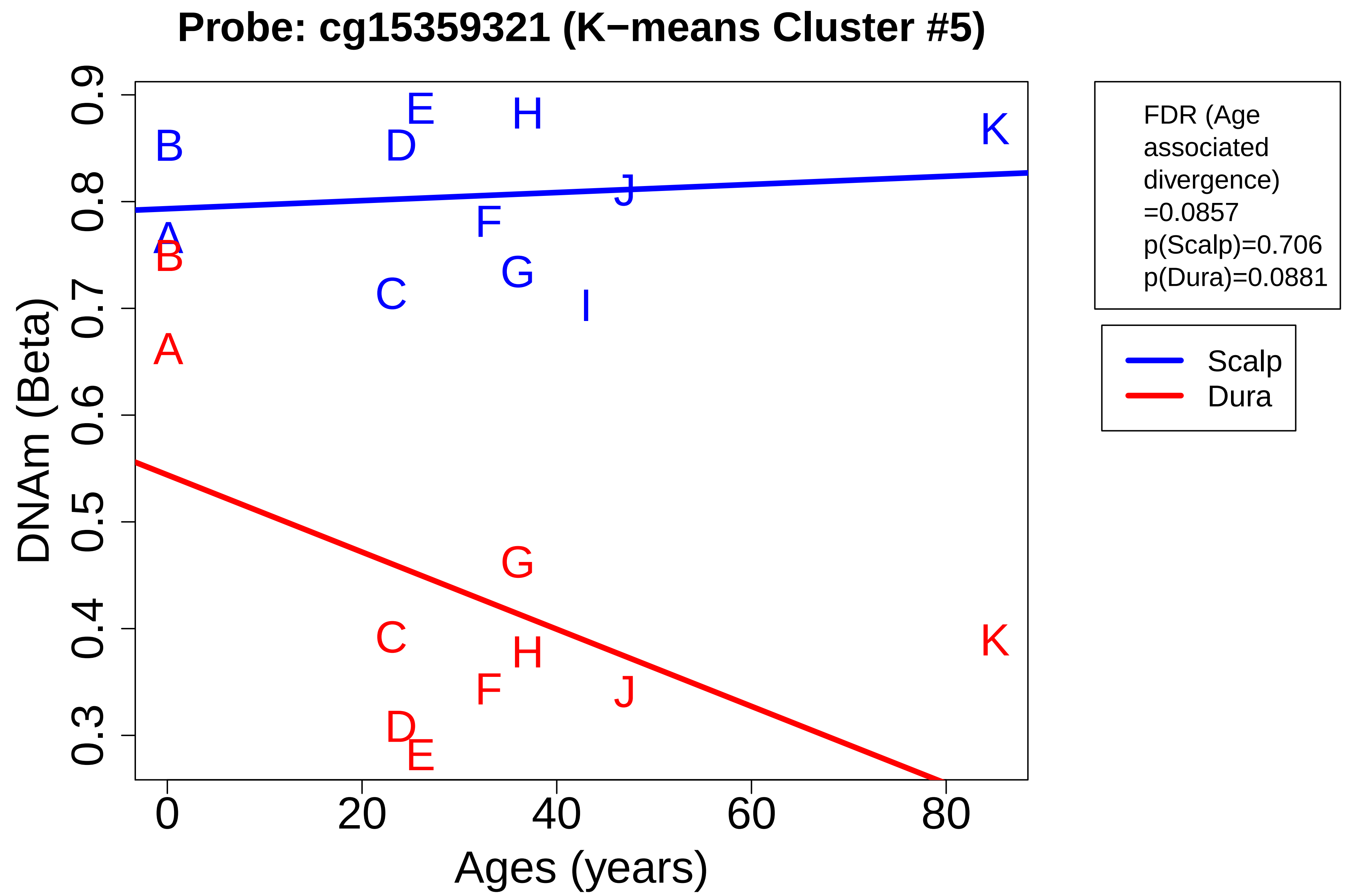

Probe: cg15359321 (K−means Cluster #5)
DNAm (Beta)
0.3
0.4
0.5
0.6
0.7
0.8
0.9
0
20
40
60
80
Ages (years)
A
B
C
D
E
F
G
H
I
J
K
FDR (Age associated divergence) =0.0857
p(Scalp)=0.706
p(Dura)=0.0881
Scalp
Dura

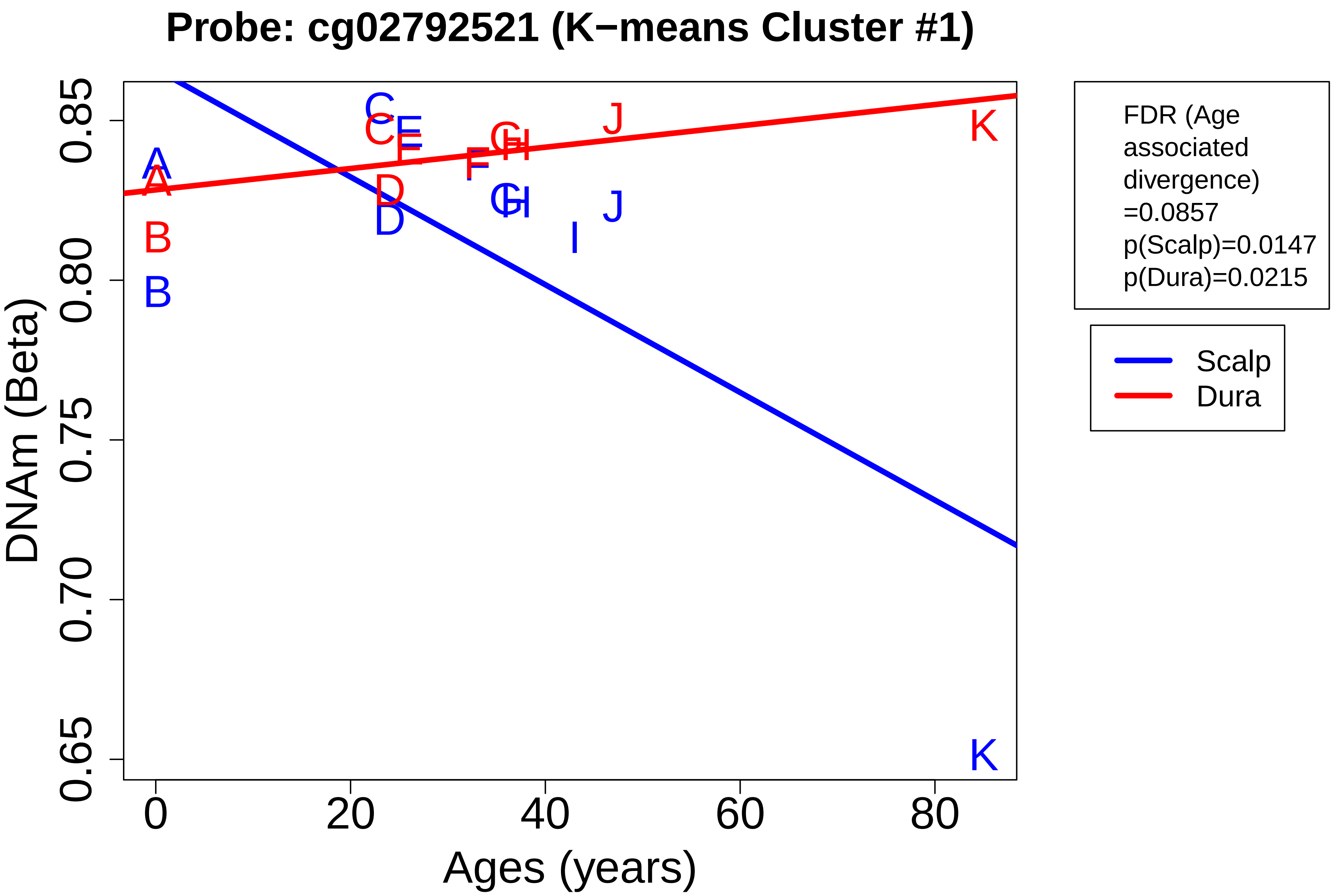

Probe: cg02792521 (K−means Cluster #1)
DNAm (Beta)
0.65
0.70
0.75
0.80
0.85
0
20
40
60
80
Ages (years)
A
B
C
D
E
F
G
H
I
J
K
FDR (Age associated divergence) =0.0857
p(Scalp)=0.0147
p(Dura)=0.0215
Scalp
Dura

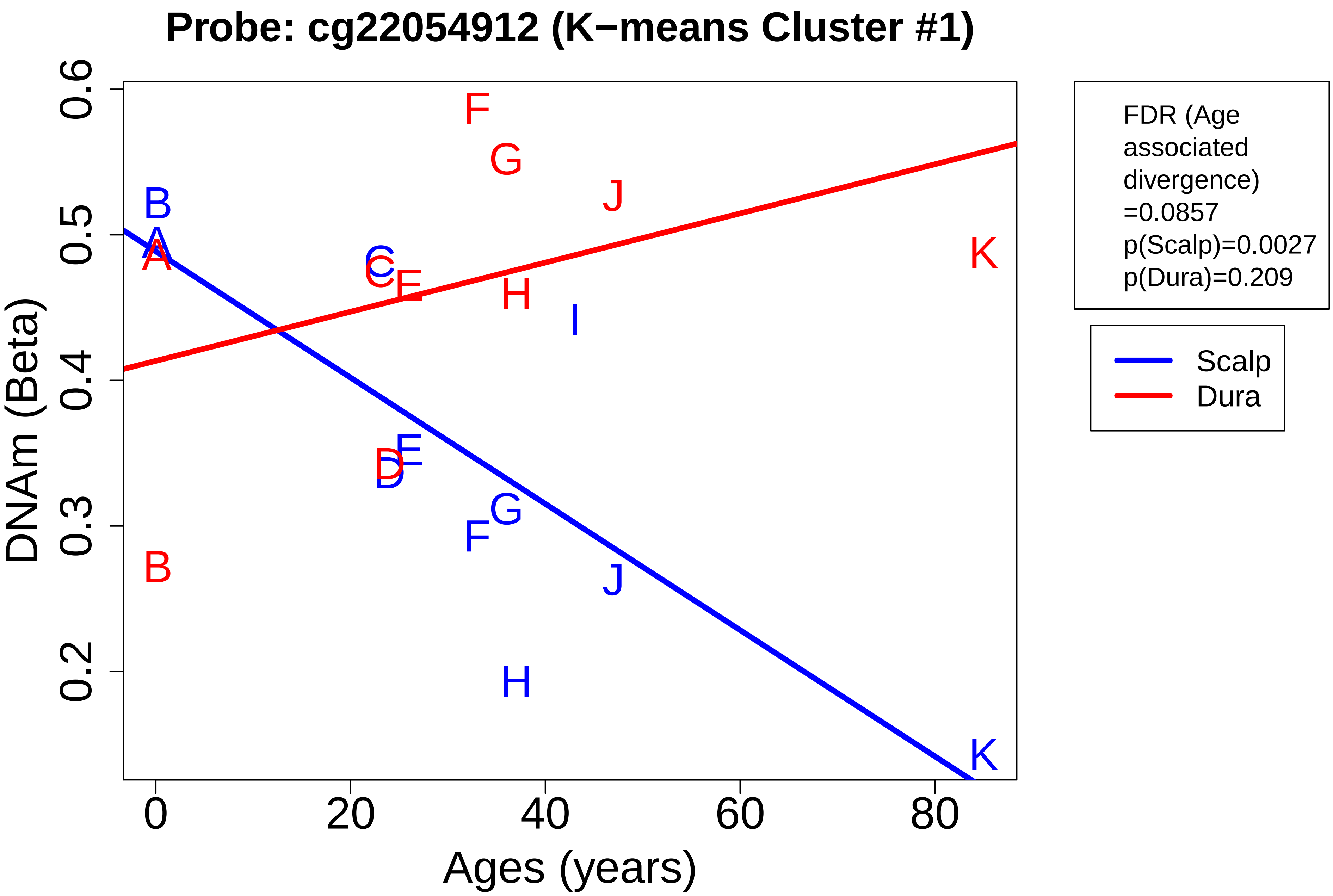
Probe: cg22054912 (K−means Cluster #1)
DNAm (Beta)
0.2
0.3
0.4
0.5
0.6
0
20
40
60
80
Ages (years)
A
B
C
D
E
F
G
H
I
J
K
A
B
C
D
E
F
G
H
J
K
FDR (Age associated divergence) =0.0857
p(Scalp)=0.0027
p(Dura)=0.209
Scalp
Dura

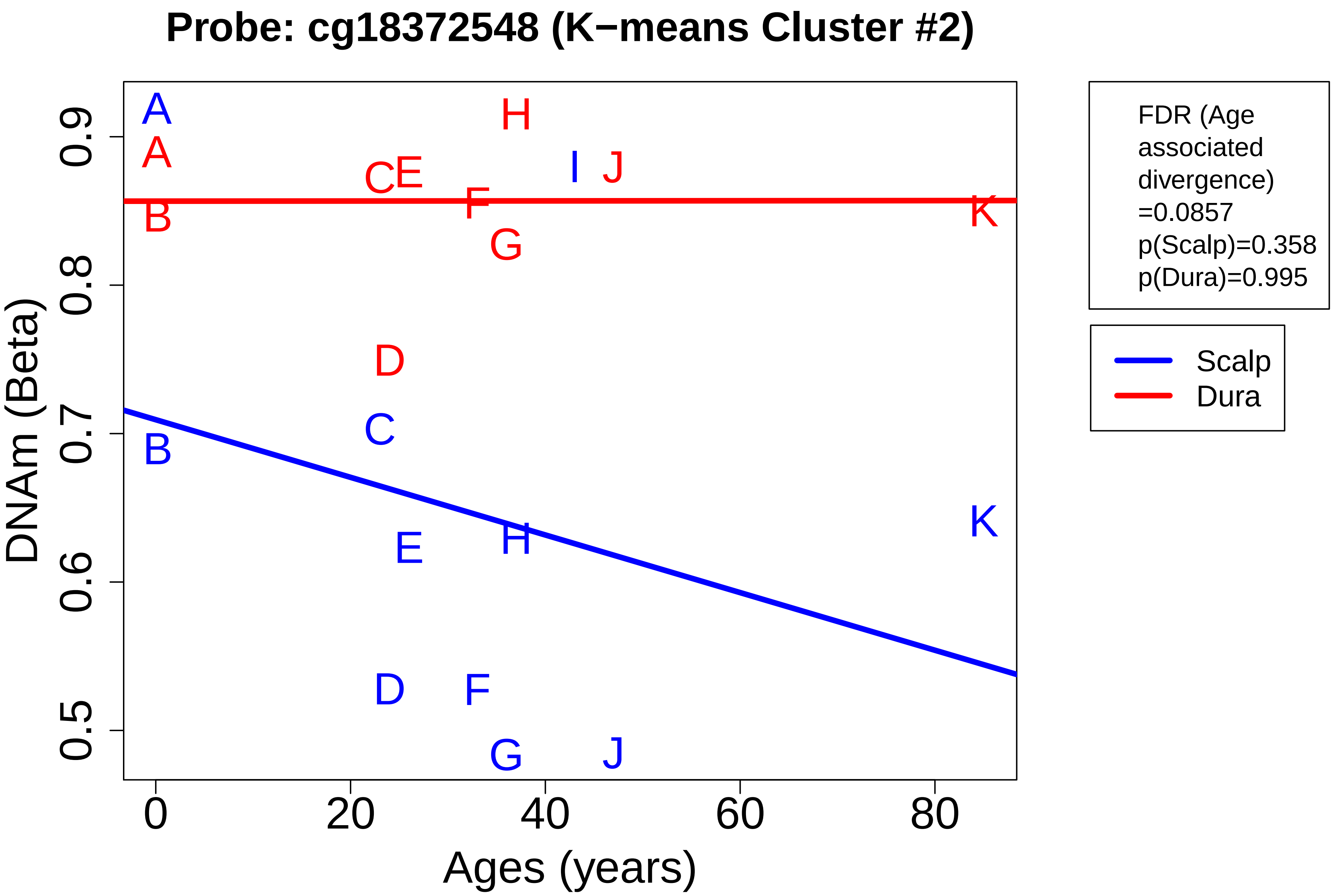

Probe: cg18372548 (K−means Cluster #2)
DNAm (Beta)
0.5
0.6
0.7
0.8
0.9
Ages (years)
0
20
40
60
80
A
A
H
I
J
C
E
F
B
K
G
D
C
B
K
E
H
D
F
G
J
FDR (Age associated divergence) =0.0857
p(Scalp)=0.358
p(Dura)=0.995
Scalp
Dura

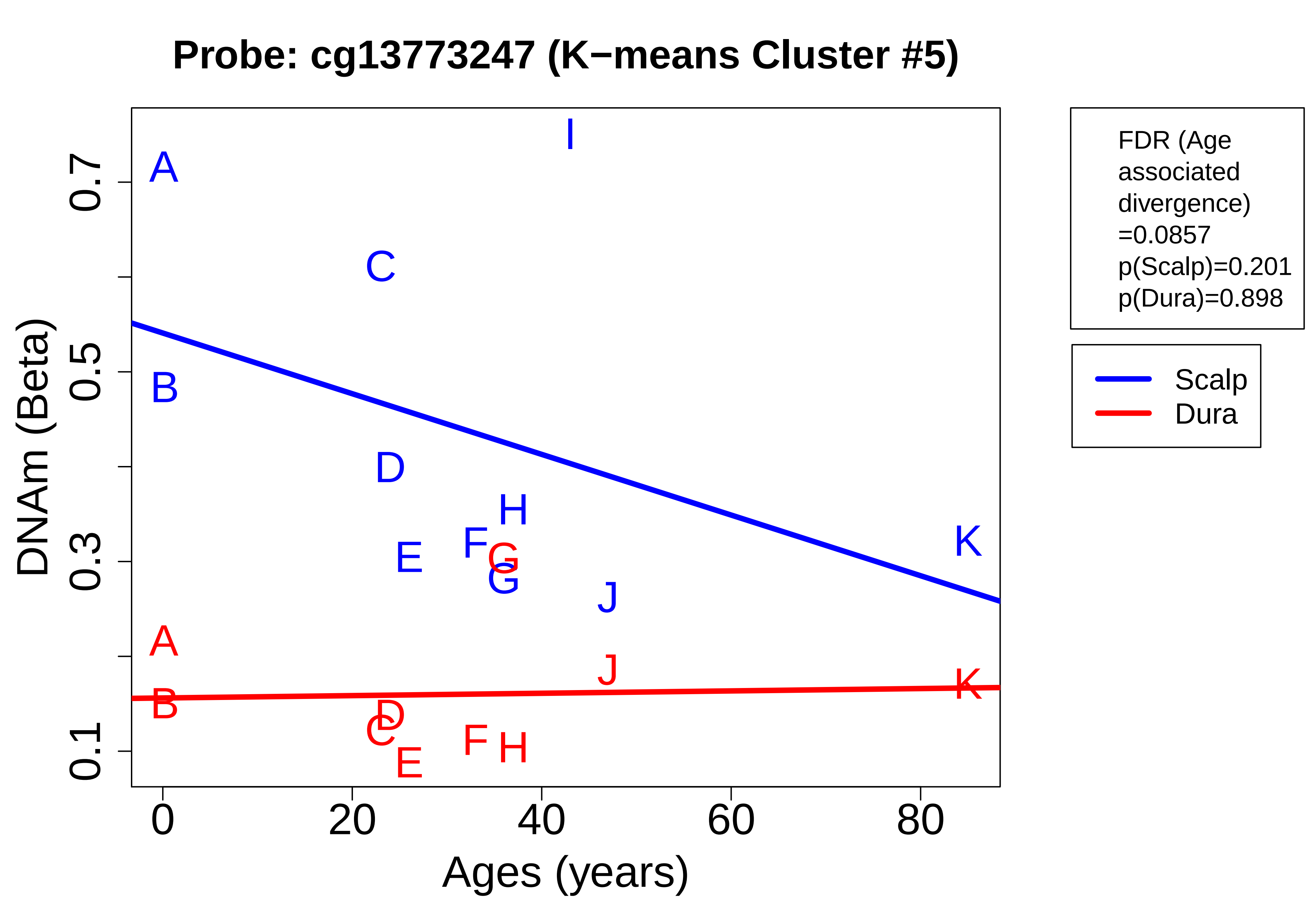

Probe: cg13773247 (K−means Cluster #5)
DNAm (Beta)
0.1
0.3
0.5
0.7
0
20
40
60
80
Ages (years)
A
B
C
D
E
F
G
H
I
J
K
A
B
C
D
E
F
G
H
J
K
FDR (Age associated divergence) =0.0857
p(Scalp)=0.201
p(Dura)=0.898
Scalp
Dura

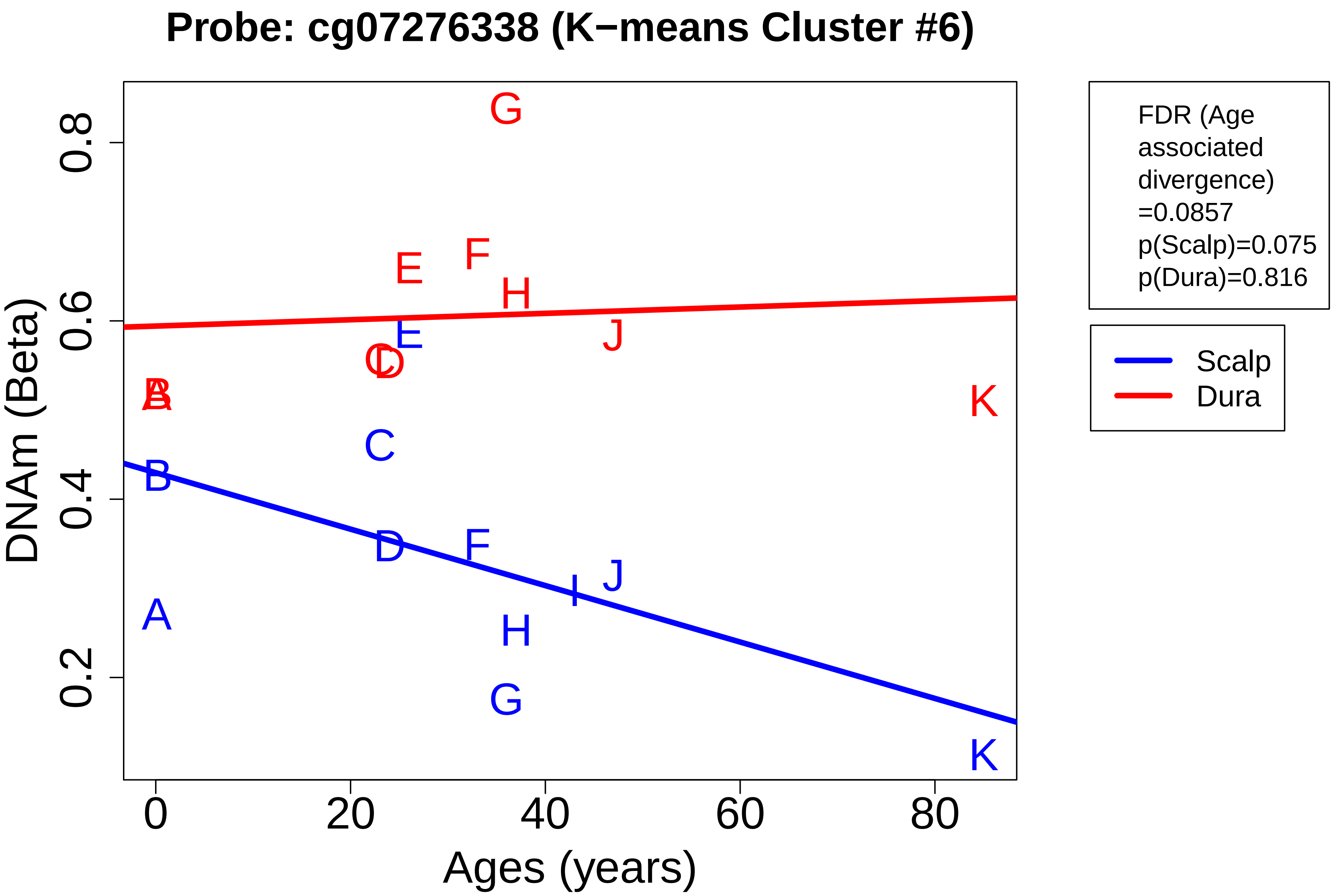

Probe: cg07276338 (K−means Cluster #6)
DNAm (Beta)
0.2
0.4
0.6
0.8
Ages (years)
0
20
40
60
80
FDR (Age associated divergence) =0.0857
p(Scalp)=0.075
p(Dura)=0.816
Scalp
Dura

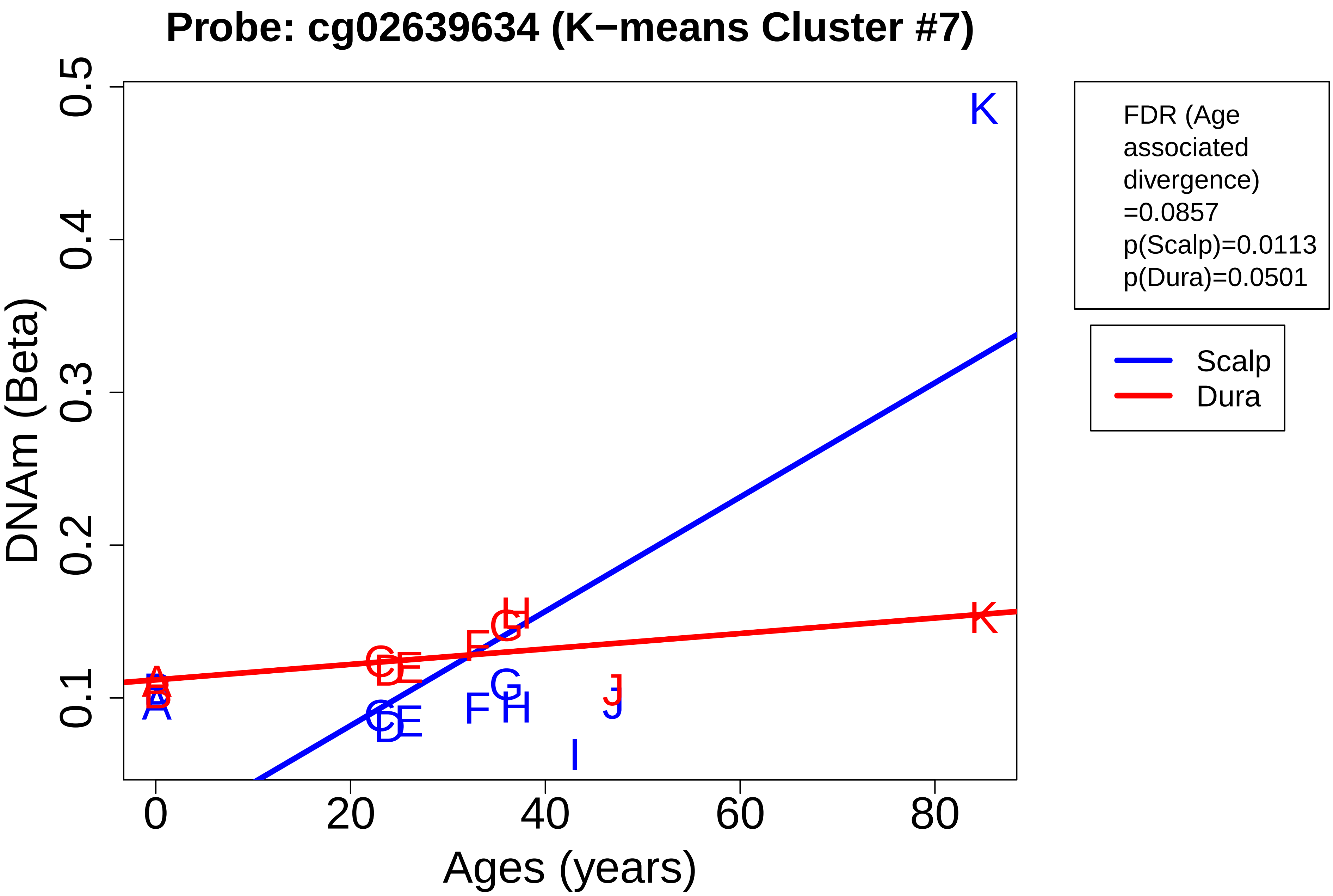
Probe: cg02639634 (K−means Cluster #7)
DNAm (Beta)
Ages (years)
0.1
0.2
0.3
0.4
0.5
0
20
40
60
80
A B C D E F G H I J K
FDR (Age associated divergence) =0.0857
p(Scalp)=0.0113
p(Dura)=0.0501
Scalp
Dura

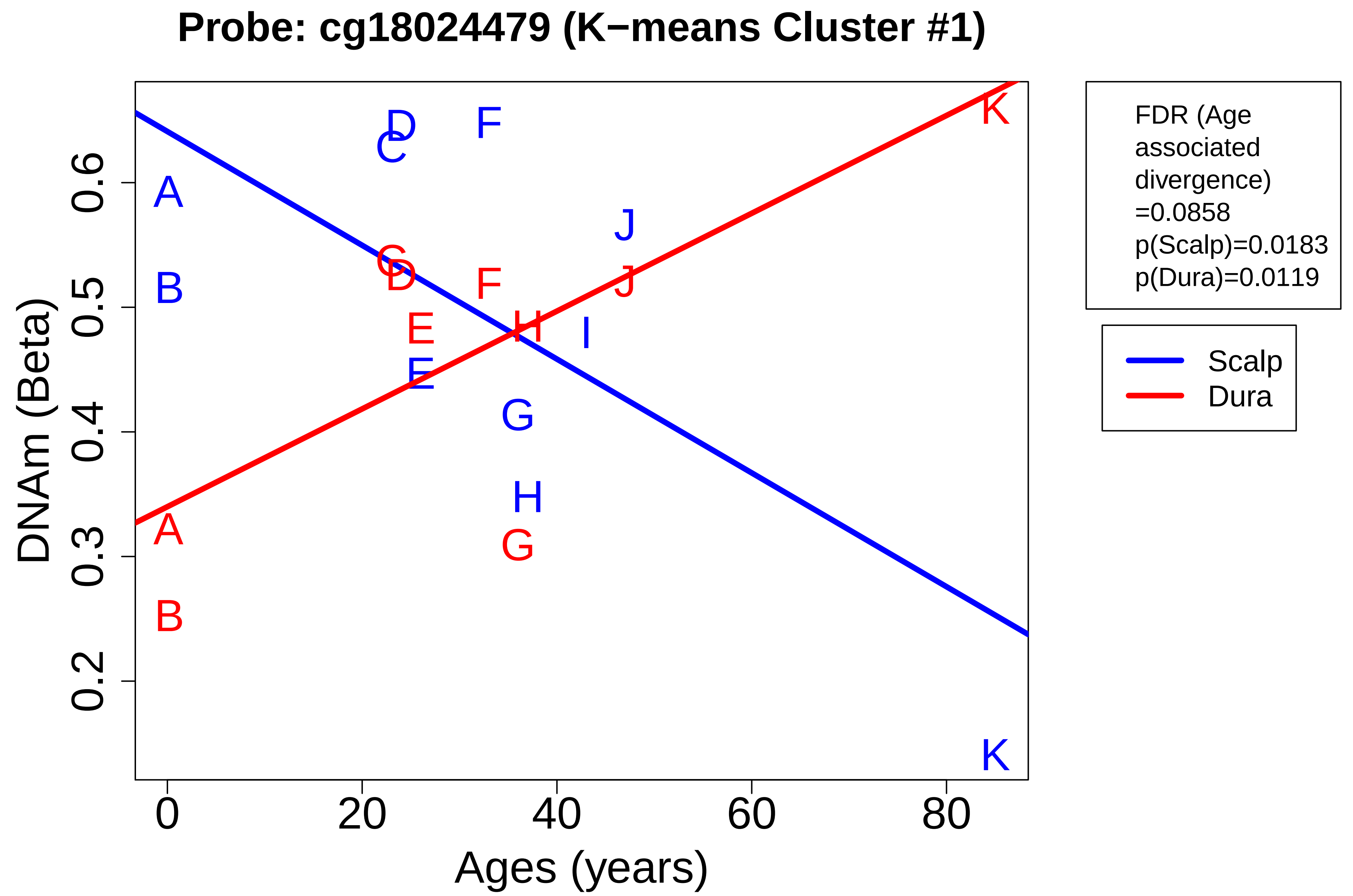

Probe: cg18024479 (K−means Cluster #1)
DNAm (Beta)
0.2
0.3
0.4
0.5
0.6
Ages (years)
0
20
40
60
80
FDR (Age associated divergence) =0.0858
p(Scalp)=0.0183
p(Dura)=0.0119
Scalp
Dura

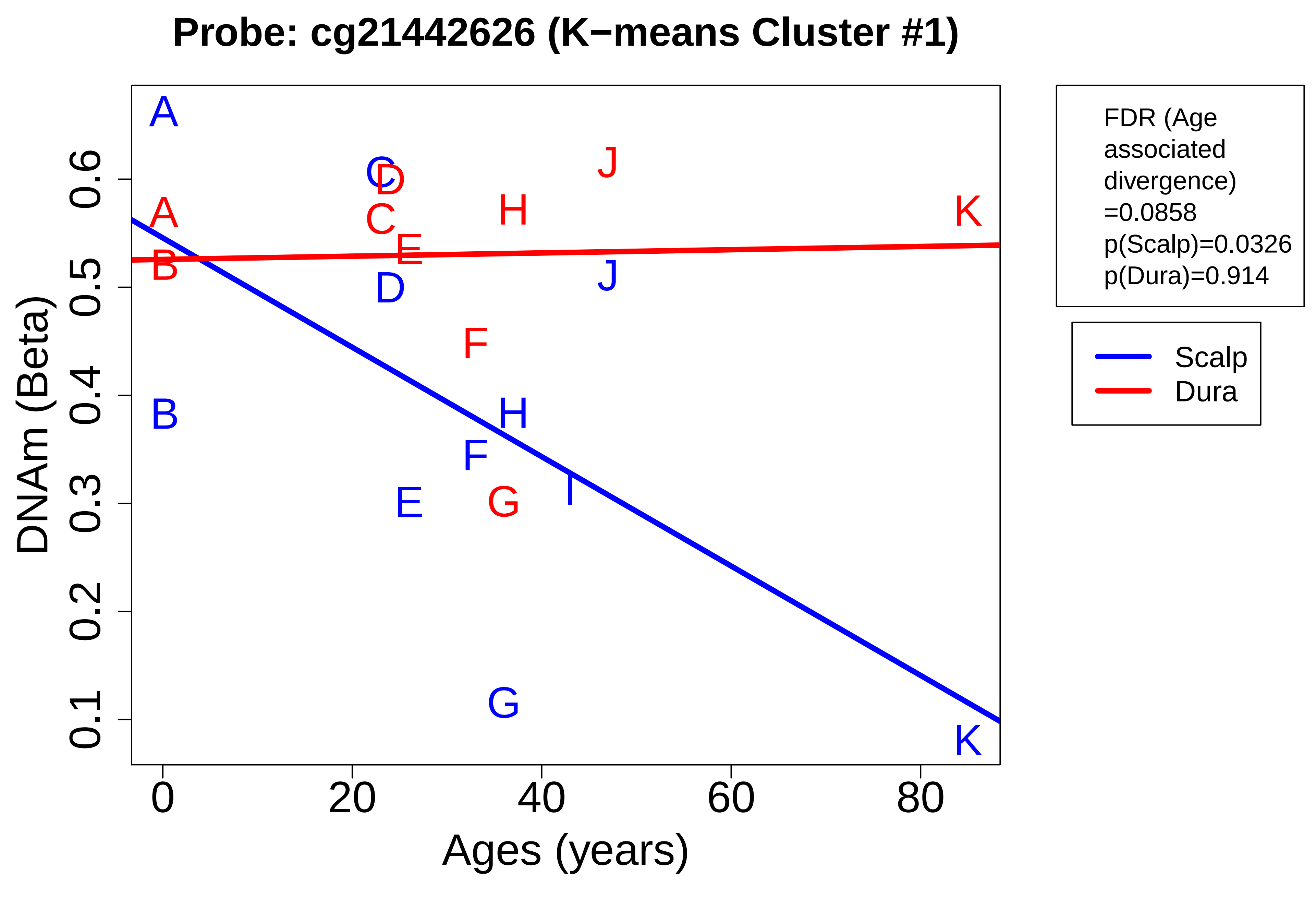

Probe: cg21442626 (K−means Cluster #1)
DNAm (Beta)
0.1
0.2
0.3
0.4
0.5
0.6
Ages (years)
0
20
40
60
80
A
B
C
D
E
F
G
H
I
J
K
FDR (Age associated divergence) =0.0858
p(Scalp)=0.0326
p(Dura)=0.914
Scalp
Dura

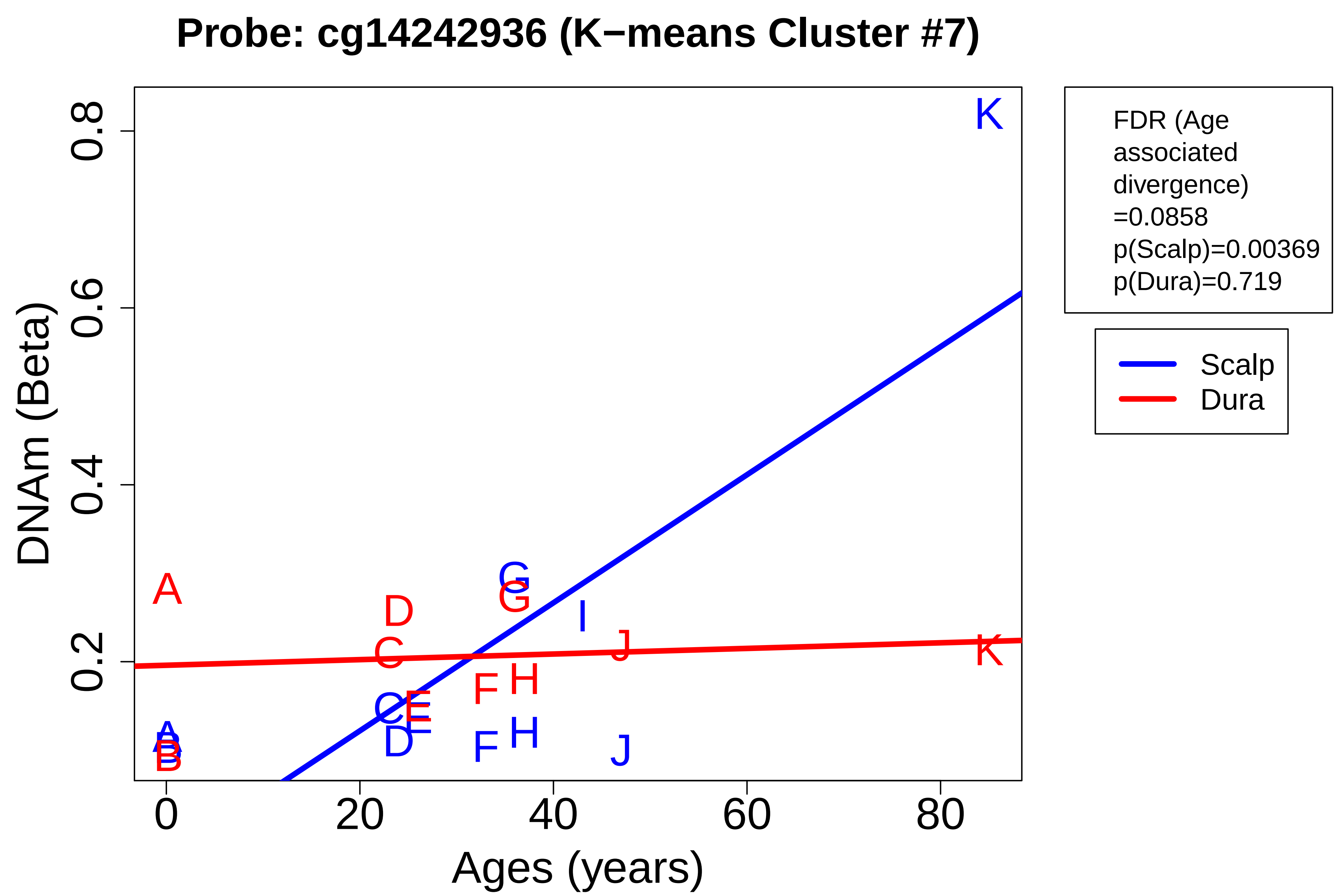

Probe: cg14242936 (K−means Cluster #7)
DNAm (Beta)
0.2
0.4
0.6
0.8
0
20
40
60
80
Ages (years)
FDR (Age associated divergence) =0.0858
p(Scalp)=0.00369
p(Dura)=0.719
Scalp
Dura
A
B
C
D
E
F
G
H
I
J
K

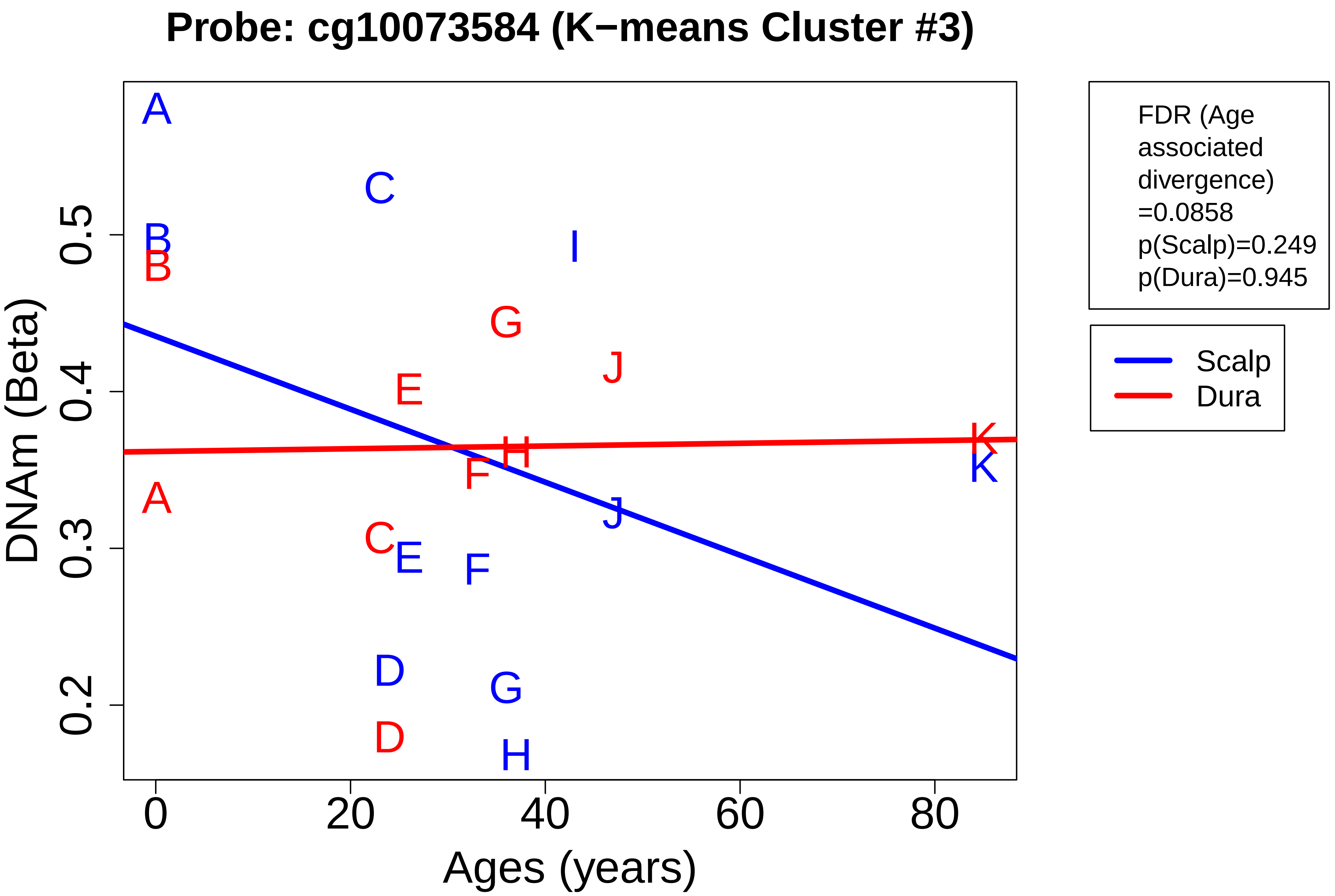

Probe: cg10073584 (K−means Cluster #3)
DNAm (Beta)
0.2
0.3
0.4
0.5
Ages (years)
0
20
40
60
80
FDR (Age associated divergence) =0.0858
p(Scalp)=0.249
p(Dura)=0.945
Scalp
Dura
A
B
C
D
E
F
G
H
I
J
K

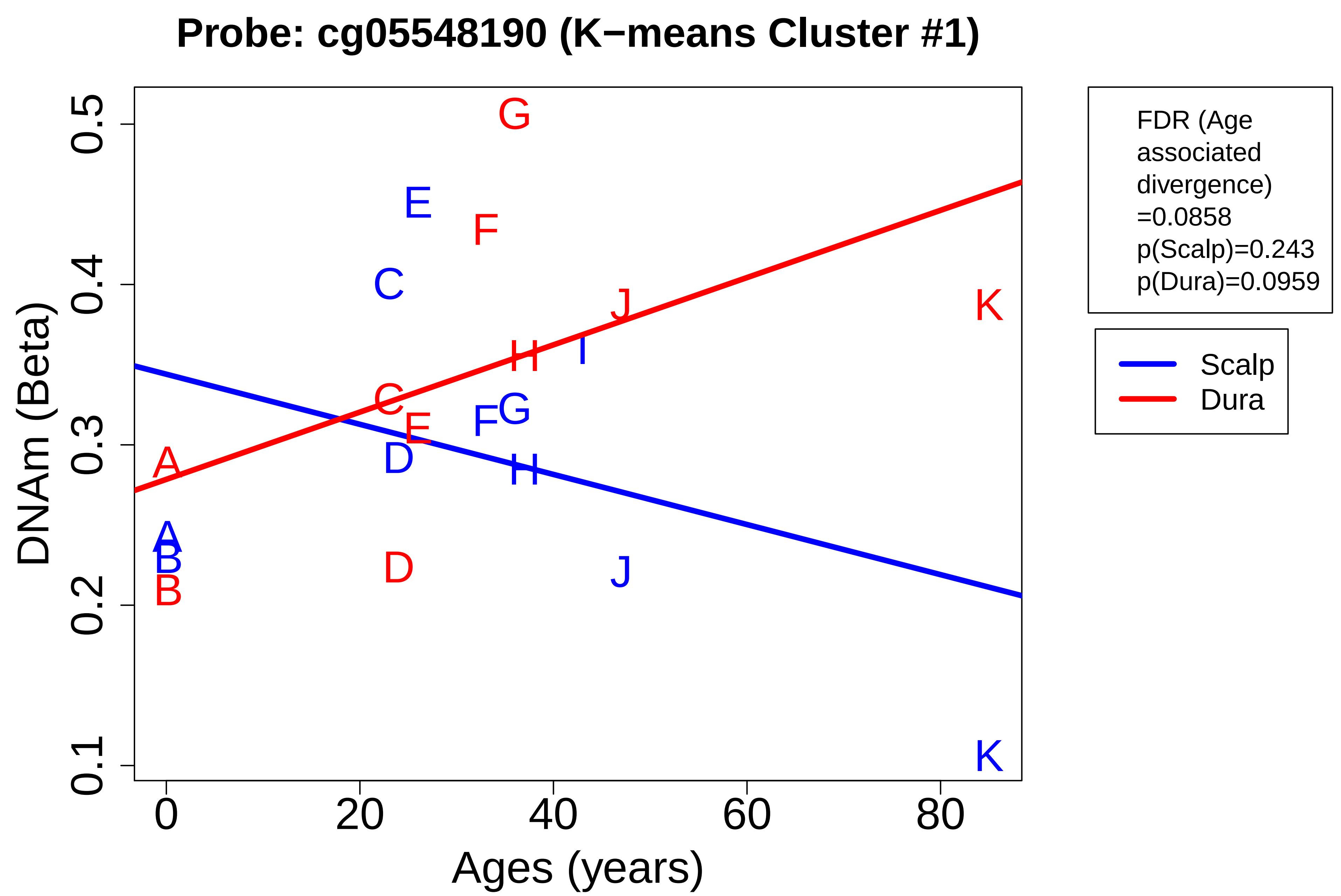

Probe: cg05548190 (K−means Cluster #1)
DNAm (Beta)
0.1
0.2
0.3
0.4
0.5
0
20
40
60
80
Ages (years)
A
B
C
D
E
F
G
H
I
J
K
FDR (Age associated divergence) =0.0858
p(Scalp)=0.243
p(Dura)=0.0959
Scalp
Dura

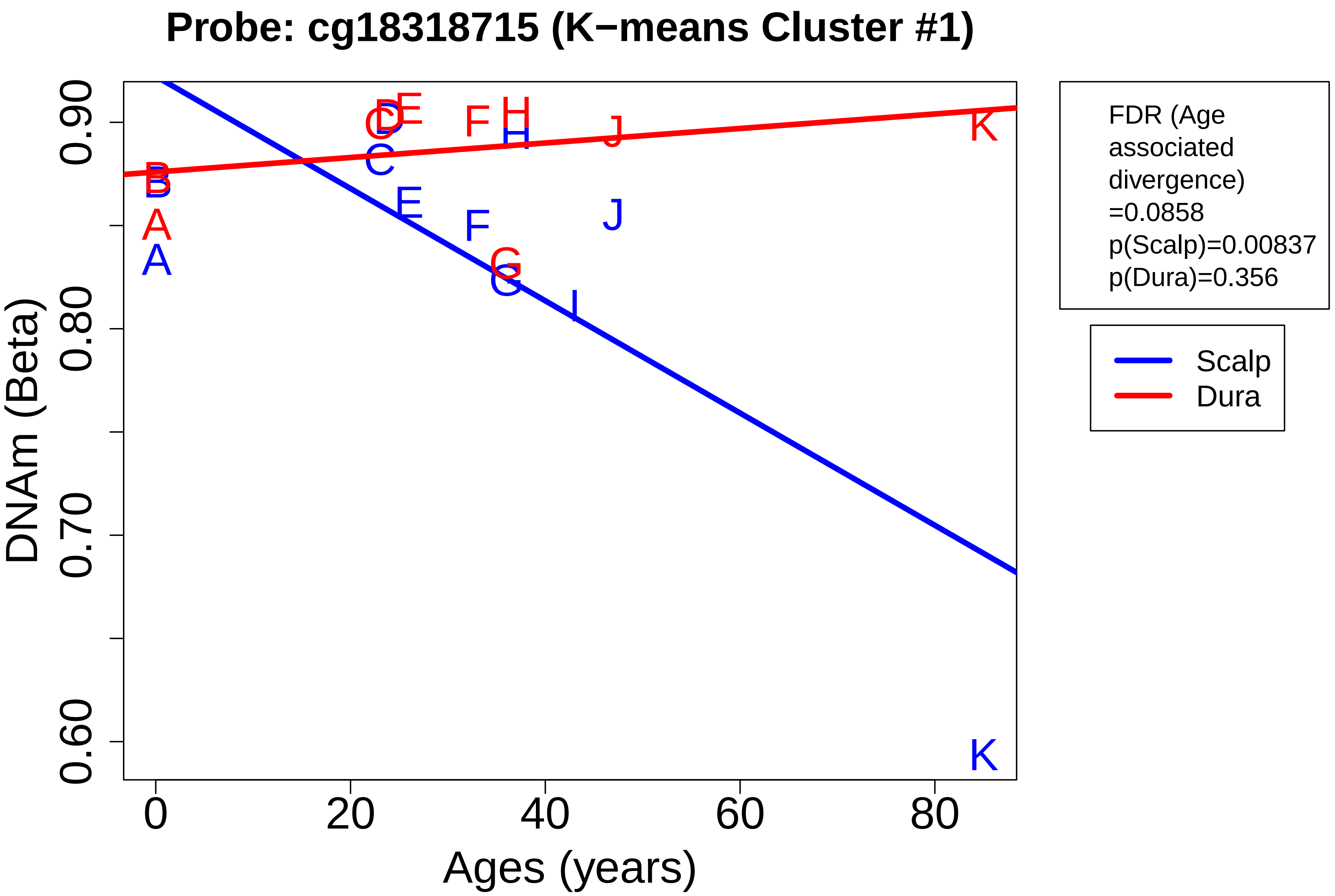

Probe: cg18318715 (K−means Cluster #1)
DNAm (Beta)
0.60
0.70
0.80
0.90
0
20
40
60
80
Ages (years)
FDR (Age associated divergence) =0.0858
p(Scalp)=0.00837
p(Dura)=0.356
Scalp
Dura
A
B
C
D
E
F
G
H
I
J
K

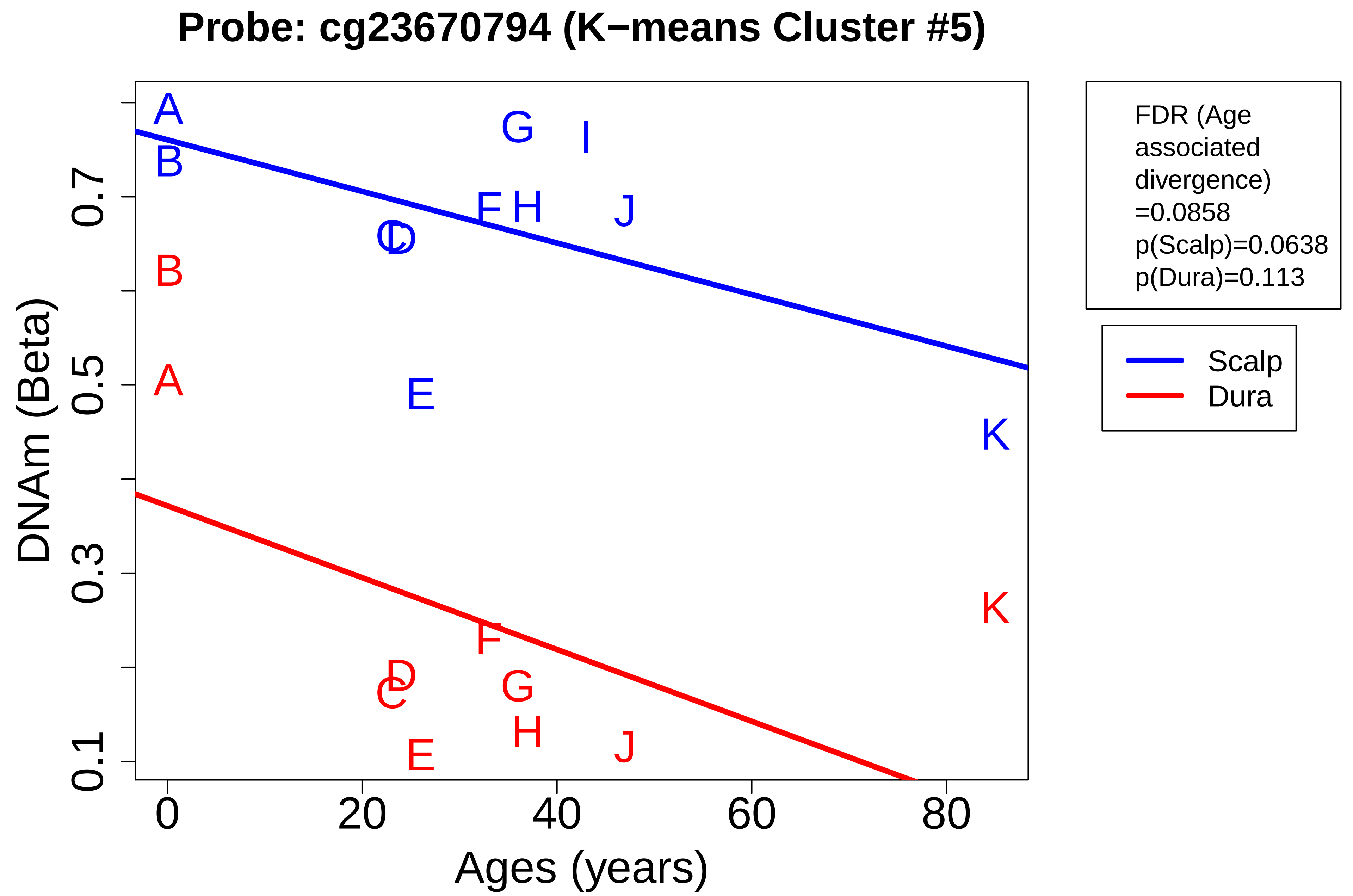

Probe: cg23670794 (K−means Cluster #5)
DNAm (Beta)
0.1
0.3
0.5
0.7
0
20
40
60
80
Ages (years)
A
B
G
I
F H
J
C D
E
K
B
A
K
F
D
C
G
H
J
E
FDR (Age associated divergence) =0.0858
p(Scalp)=0.0638
p(Dura)=0.113
Scalp
Dura

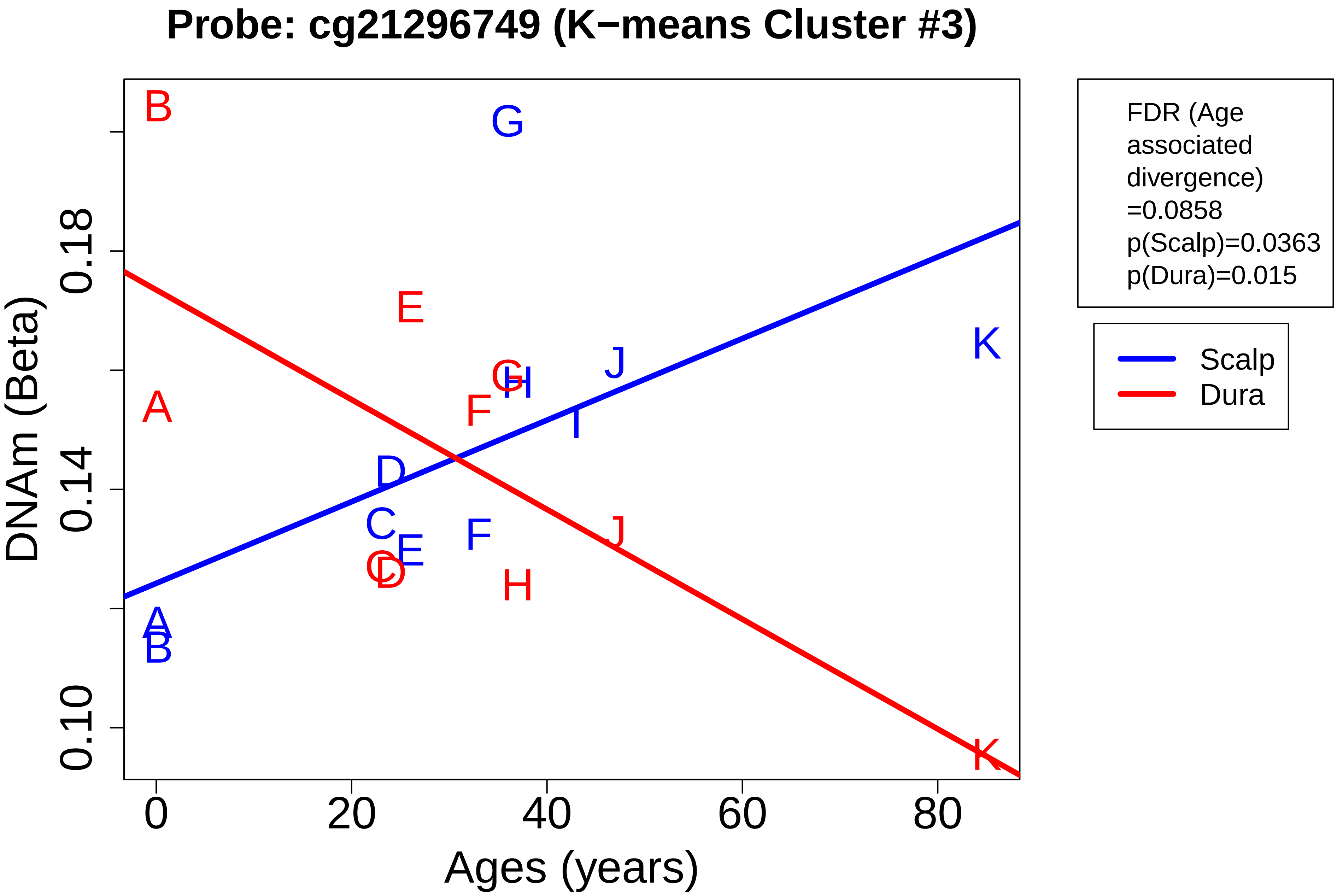

Probe: cg21296749 (K−means Cluster #3)
DNAm (Beta)
0.10
0.14
0.18
Ages (years)
0
20
40
60
80
FDR (Age associated divergence) =0.0858
p(Scalp)=0.0363
p(Dura)=0.015
Scalp
Dura

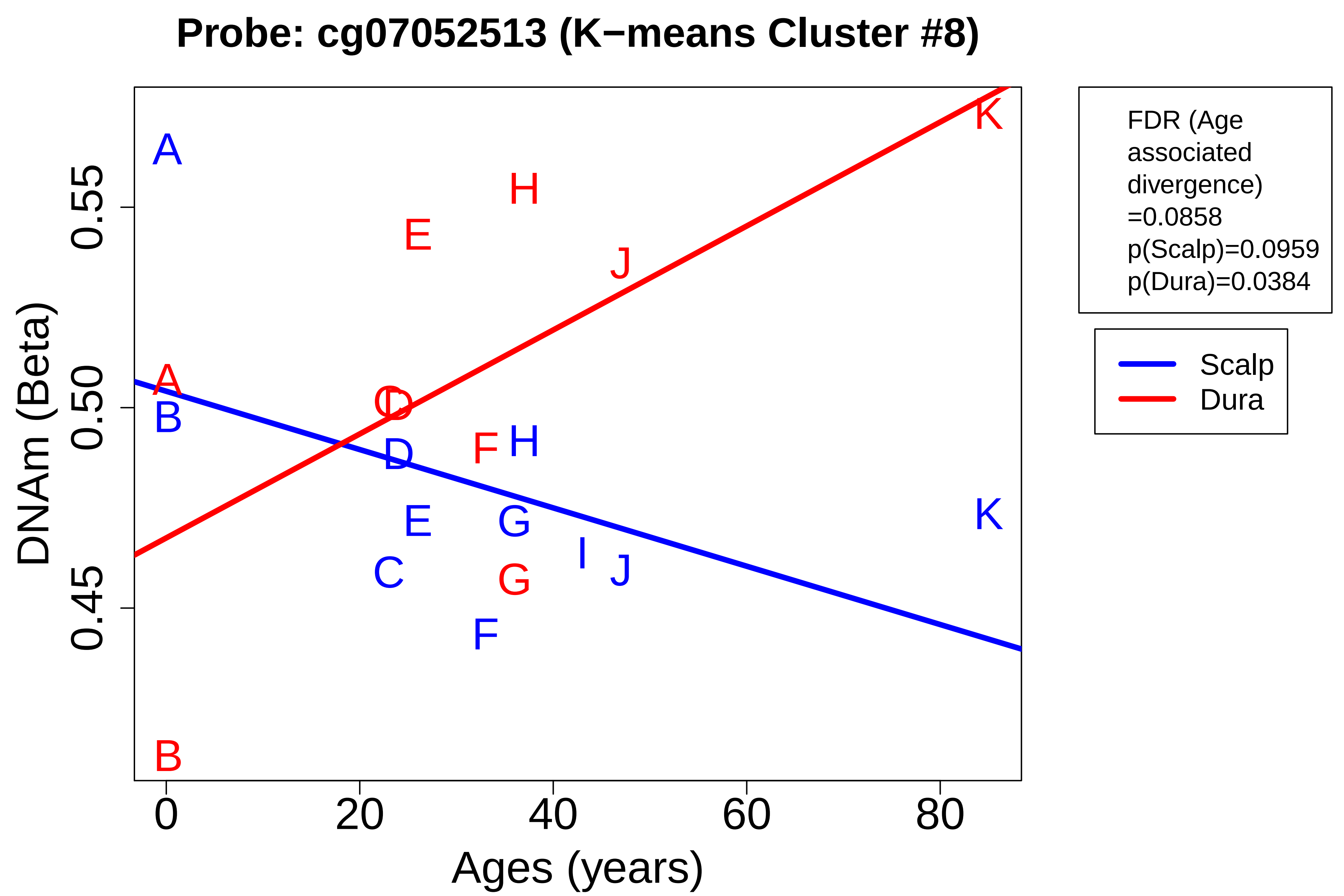

Probe: cg07052513 (K−means Cluster #8)
DNAm (Beta)
0.45
0.50
0.55
Ages (years)
0
20
40
60
80
FDR (Age
associated
divergence)
=0.0858
p(Scalp)=0.0959
p(Dura)=0.0384
Scalp
Dura
A
B
C
D
E
F
G
H
I
J
K

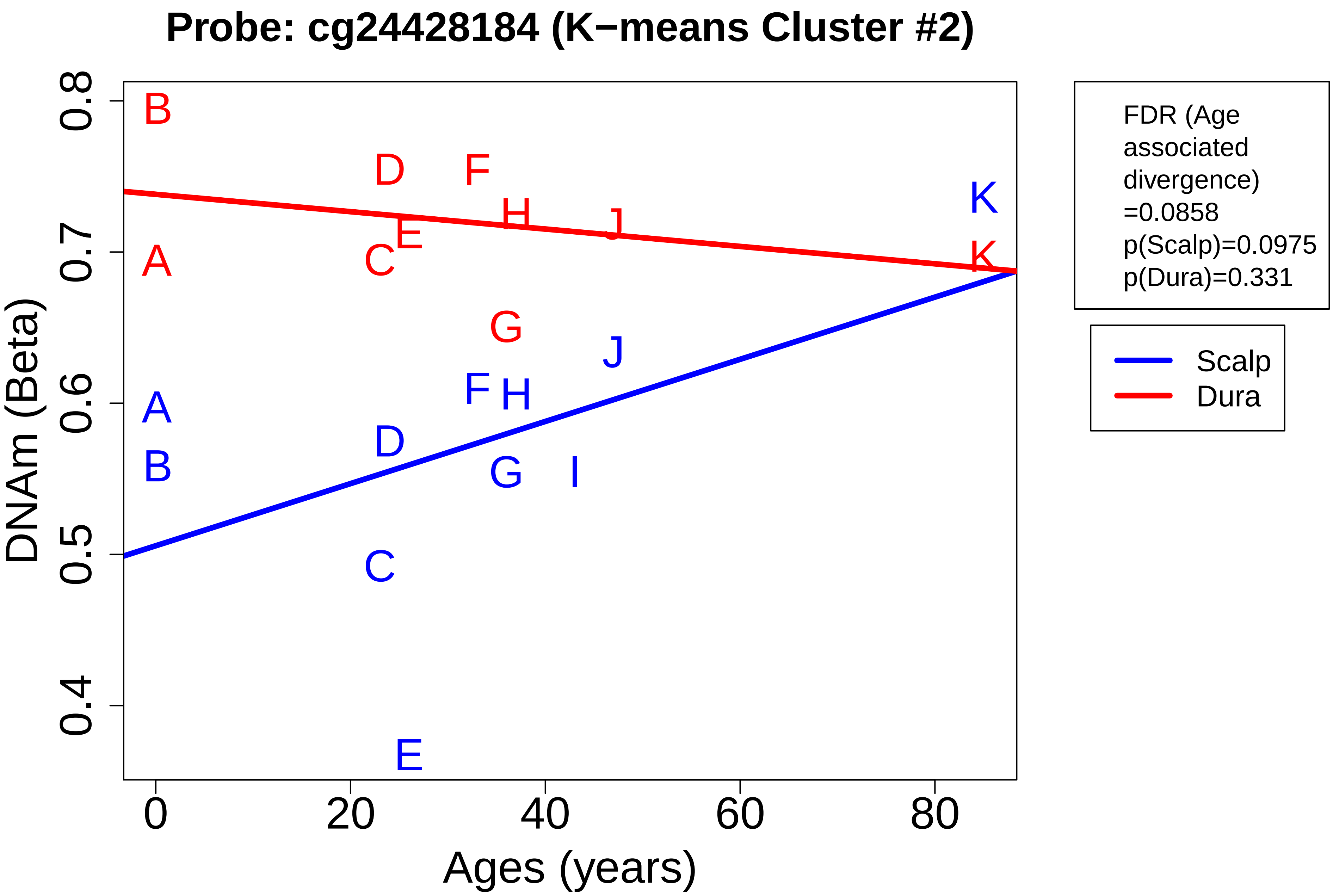

Probe: cg24428184 (K−means Cluster #2)
DNAm (Beta)
0.4
0.5
0.6
0.7
0.8
Ages (years)
0
20
40
60
80
FDR (Age associated divergence) =0.0858
p(Scalp)=0.0975
p(Dura)=0.331
Scalp
Dura

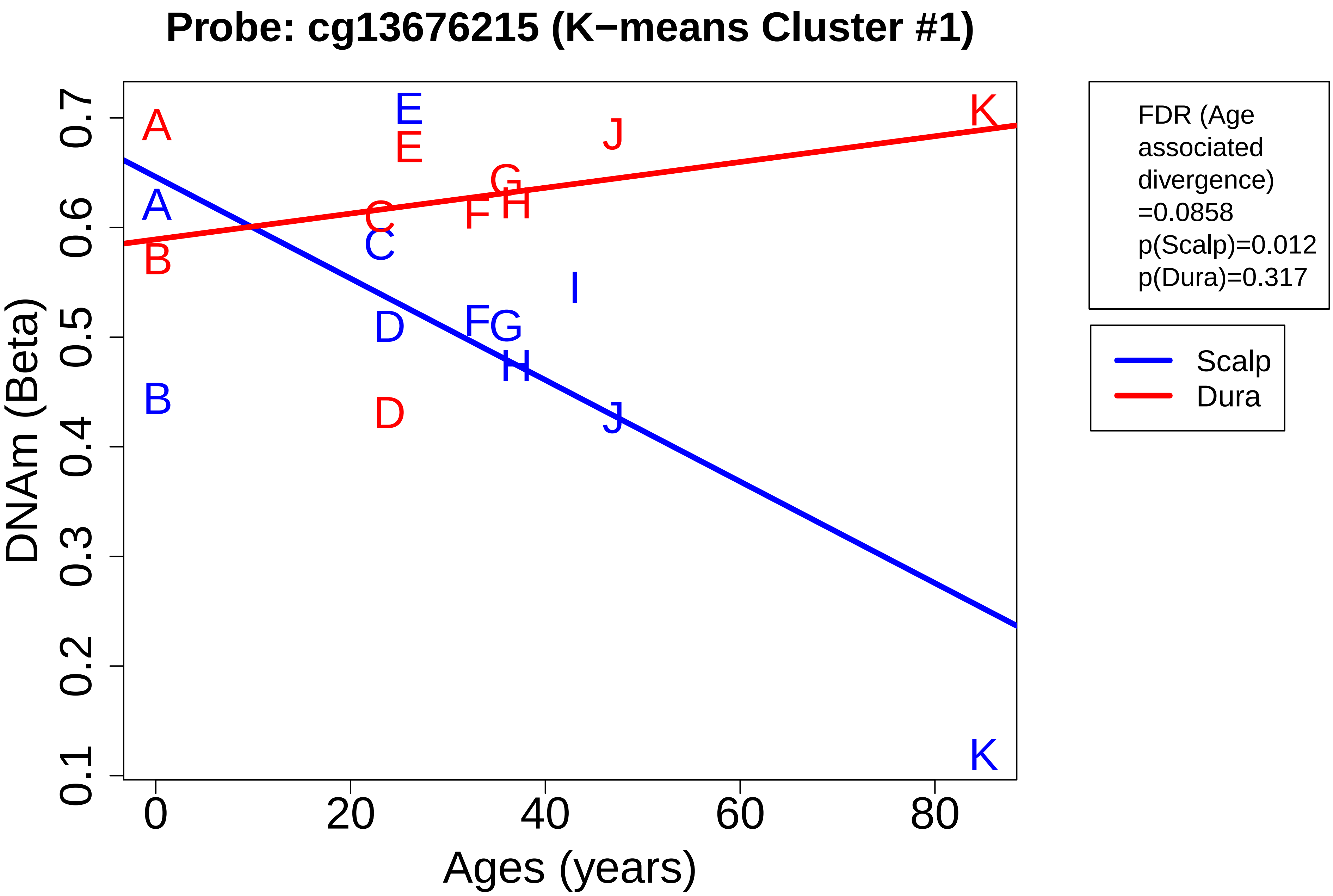

Probe: cg13676215 (K−means Cluster #1)
DNAm (Beta)
Ages (years)
0.1
0.2
0.3
0.4
0.5
0.6
0.7
0
20
40
60
80
FDR (Age associated divergence) =0.0858
p(Scalp)=0.012
p(Dura)=0.317
Scalp
Dura
A
B
C
D
E
F
G
H
I
J
K

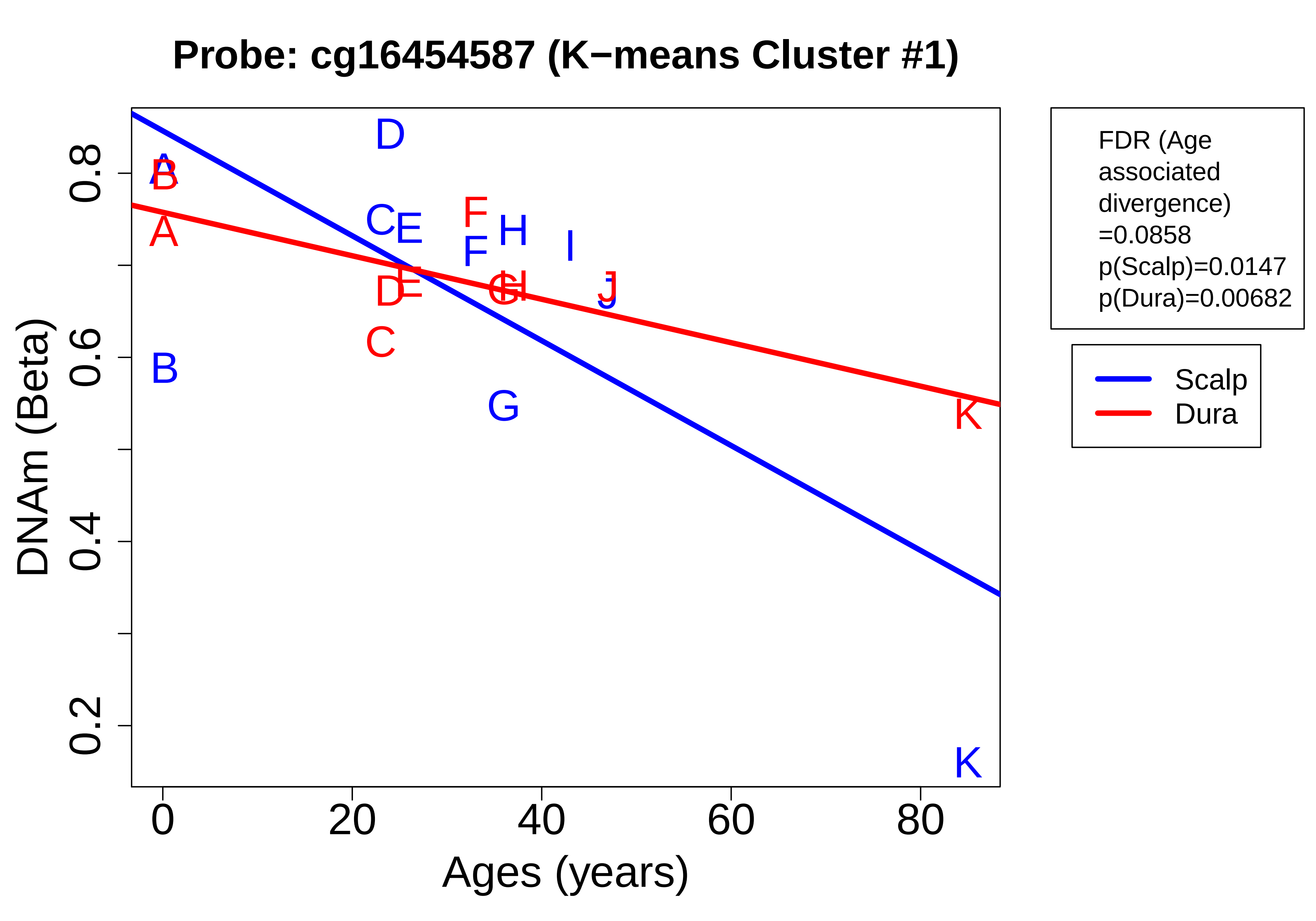

Probe: cg16454587 (K−means Cluster #1)
DNAm (Beta)
0.2
0.4
0.6
0.8
Ages (years)
0
20
40
60
80
FDR (Age associated divergence) =0.0858
p(Scalp)=0.0147
p(Dura)=0.00682
Scalp
Dura

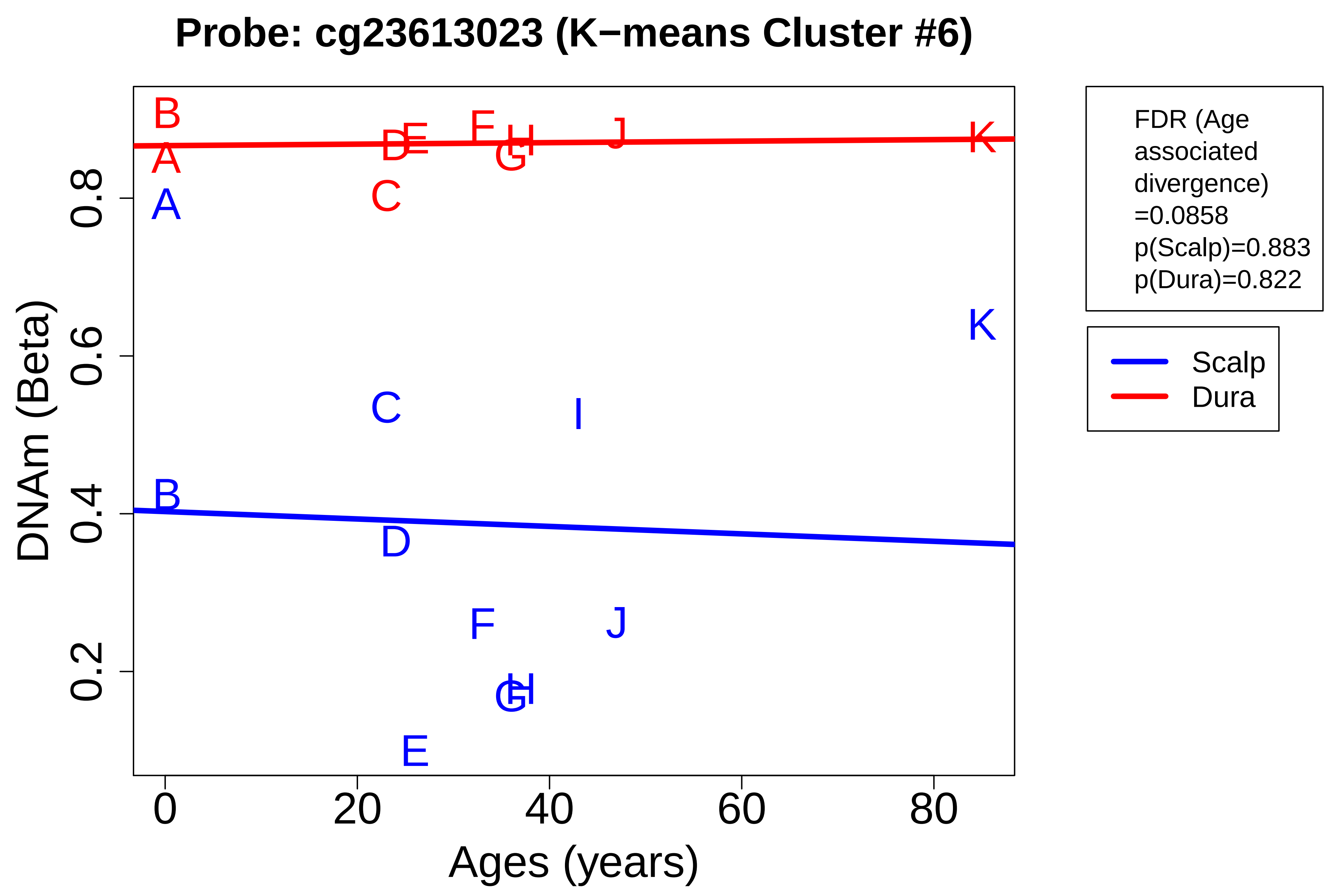

Probe: cg23613023 (K−means Cluster #6)
DNAm (Beta)
0.2
0.4
0.6
0.8
0
20
40
60
80
Ages (years)
FDR (Age associated divergence) =0.0858
p(Scalp)=0.883
p(Dura)=0.822
Scalp
Dura

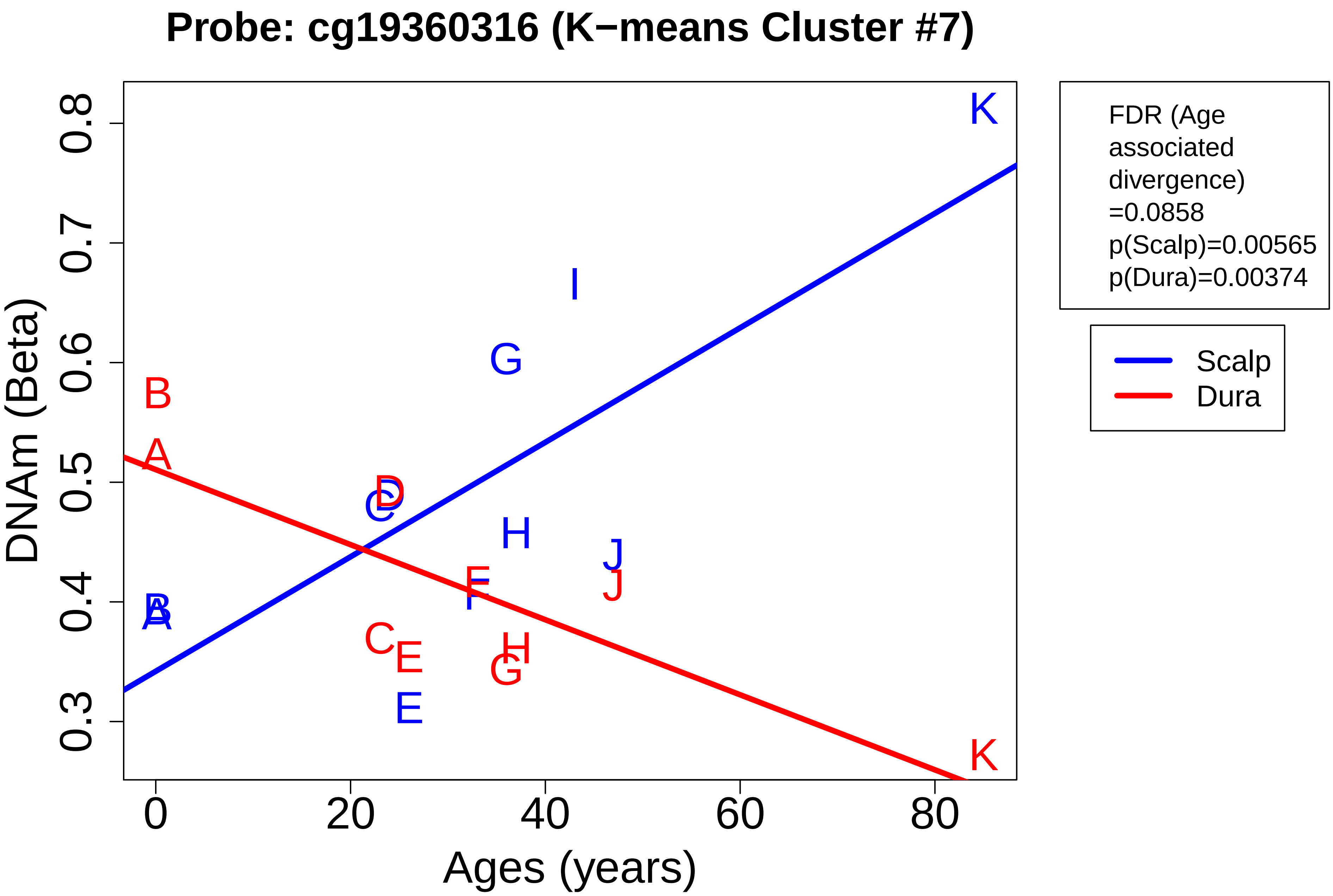

Probe: cg19360316 (K−means Cluster #7)
DNAm (Beta)
0.3
0.4
0.5
0.6
0.7
0.8
0
20
40
60
80
Ages (years)
K
I
G
B
A
D
C
H
J
F
E
K
FDR (Age associated divergence) =0.0858
p(Scalp)=0.00565
p(Dura)=0.00374
Scalp
Dura

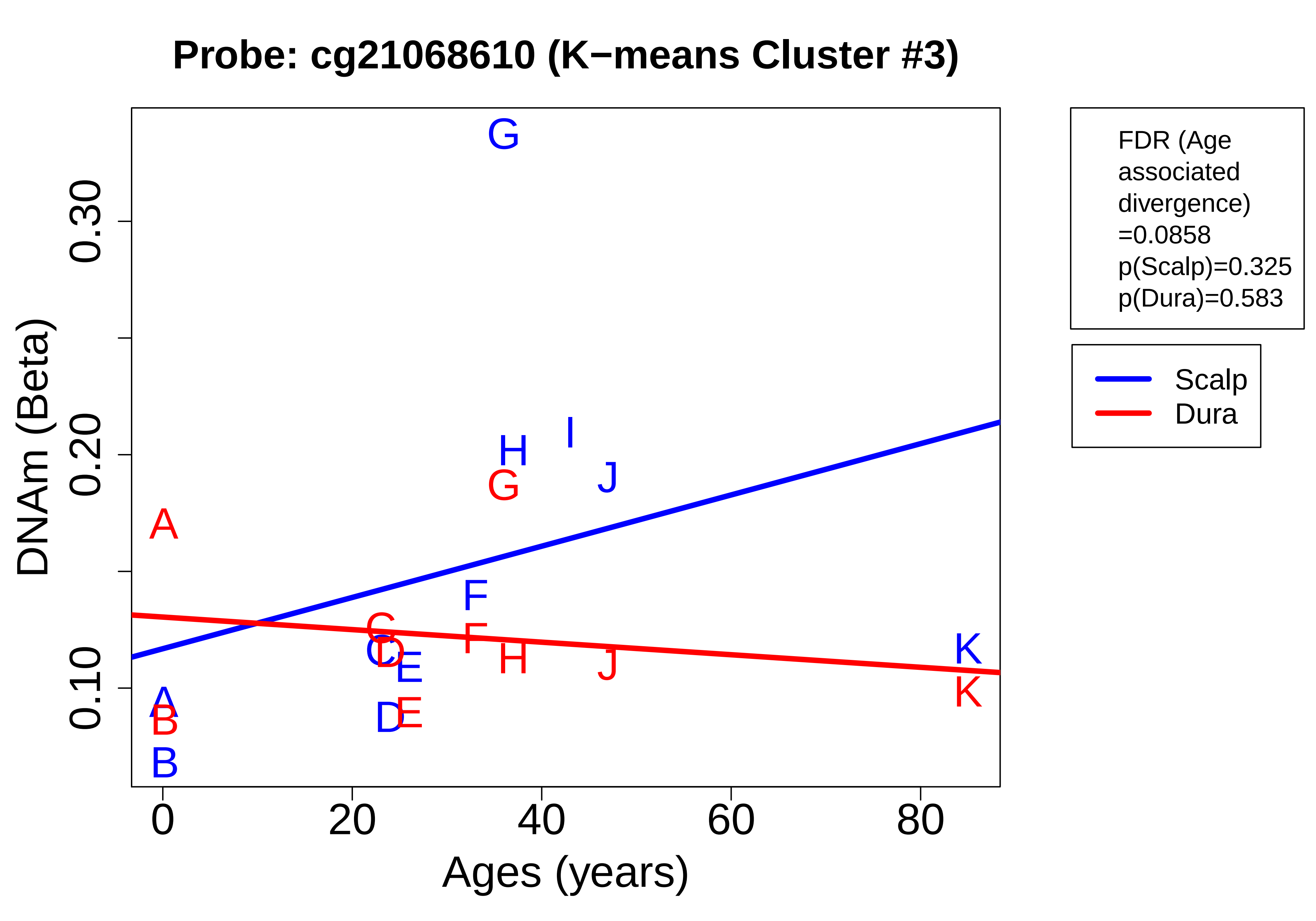
Probe: cg21068610 (K−means Cluster #3)
DNAm (Beta)
0.10
0.20
0.30
0
20
40
60
80
Ages (years)
FDR (Age associated divergence) =0.0858
p(Scalp)=0.325
p(Dura)=0.583
Scalp
Dura
A
B
C
D
E
F
G
H
I
J
K

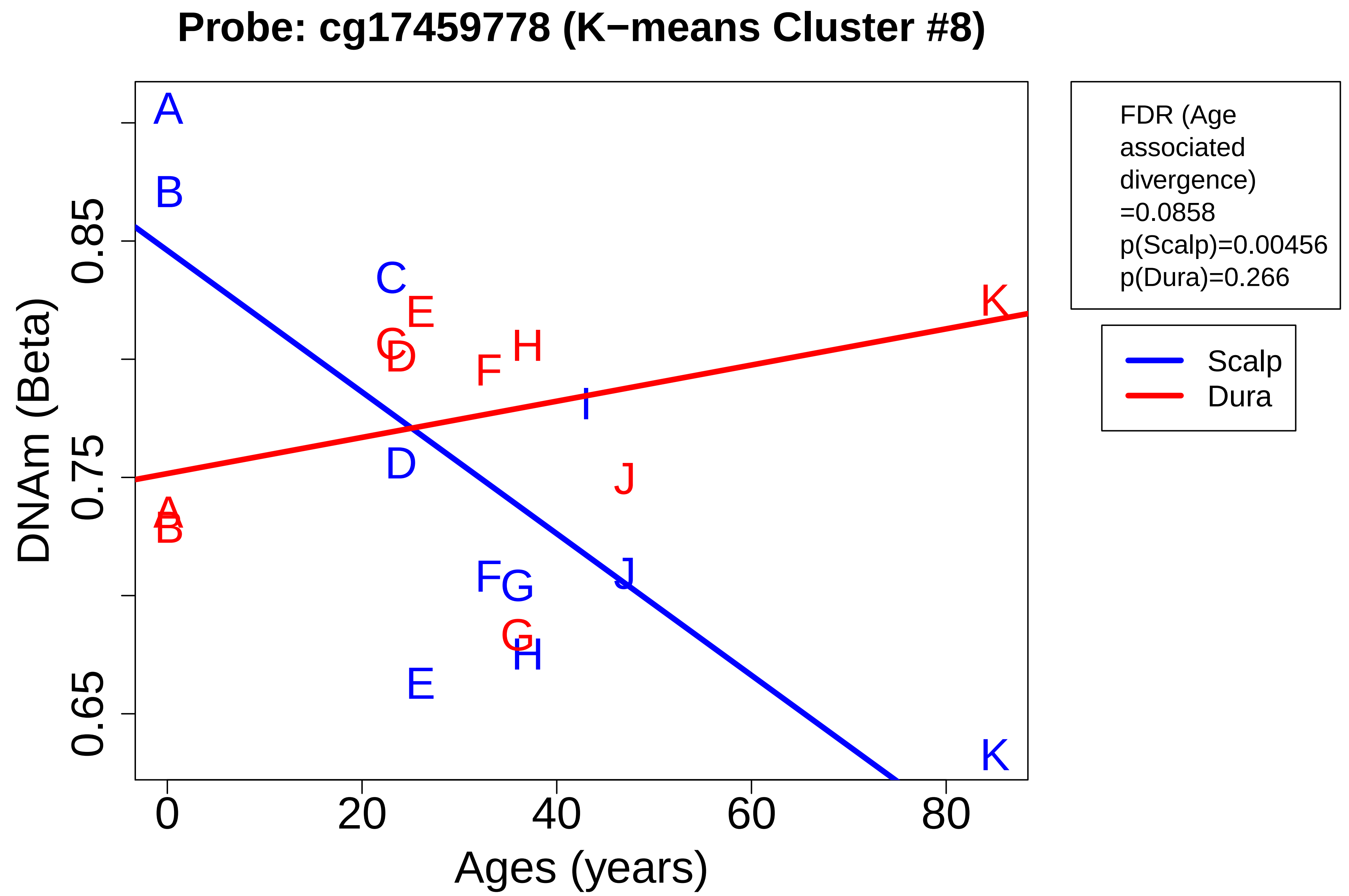
Probe: cg17459778 (K−means Cluster #8)
FDR (Age associated divergence) =0.0858
p(Scalp)=0.00456
p(Dura)=0.266
Scalp
Dura
DNAm (Beta)
0.65
0.75
0.85
Ages (years)
0
20
40
60
80
A
B
C
D
E
F
G
H
I
J
K

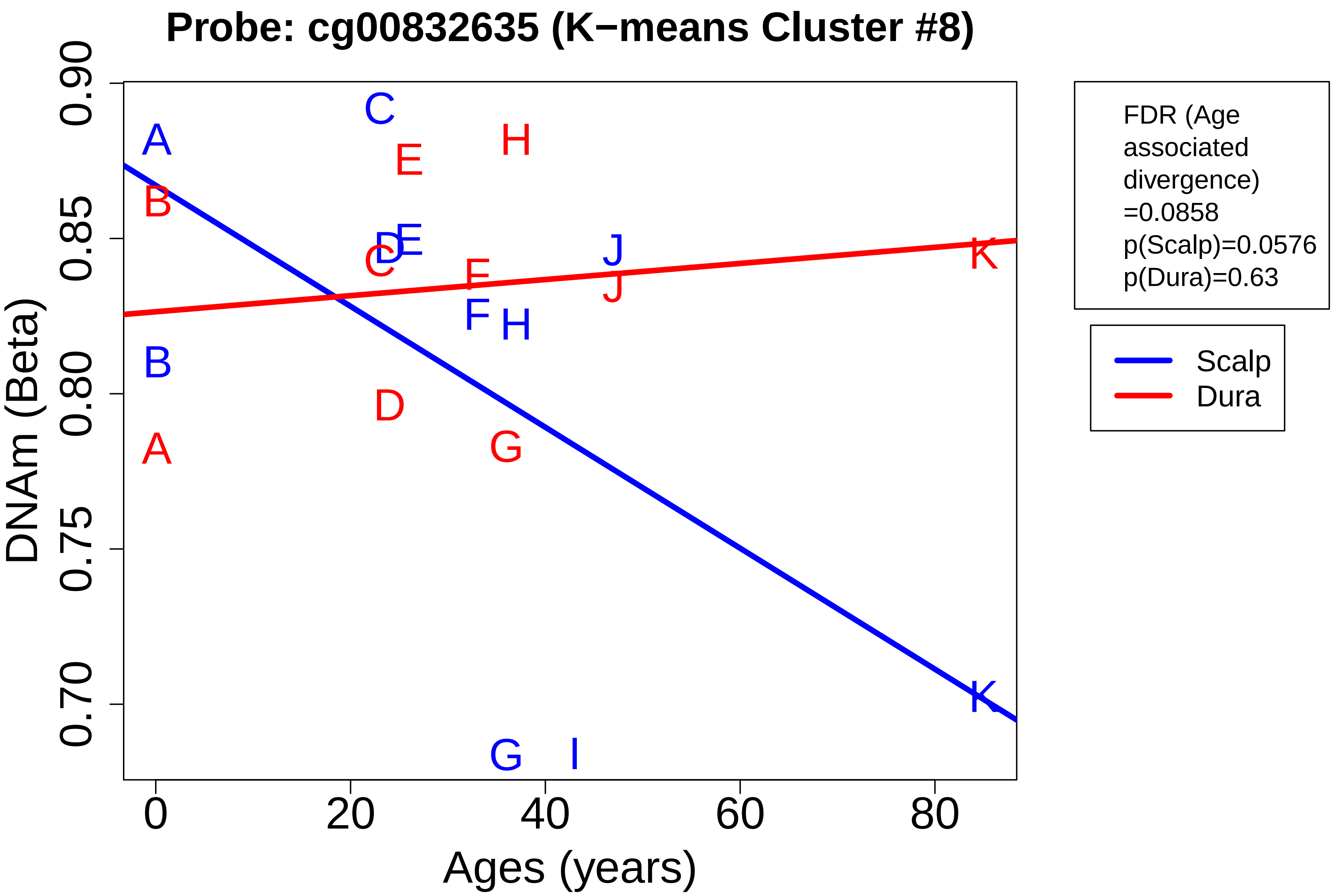
Probe: cg00832635 (K−means Cluster #8)
DNAm (Beta)
0.70
0.75
0.80
0.85
0.90
0
20
40
60
80
Ages (years)
A
B
C
D
E
F
G
H
I
J
K
FDR (Age associated divergence) =0.0858
p(Scalp)=0.0576
p(Dura)=0.63
Scalp
Dura

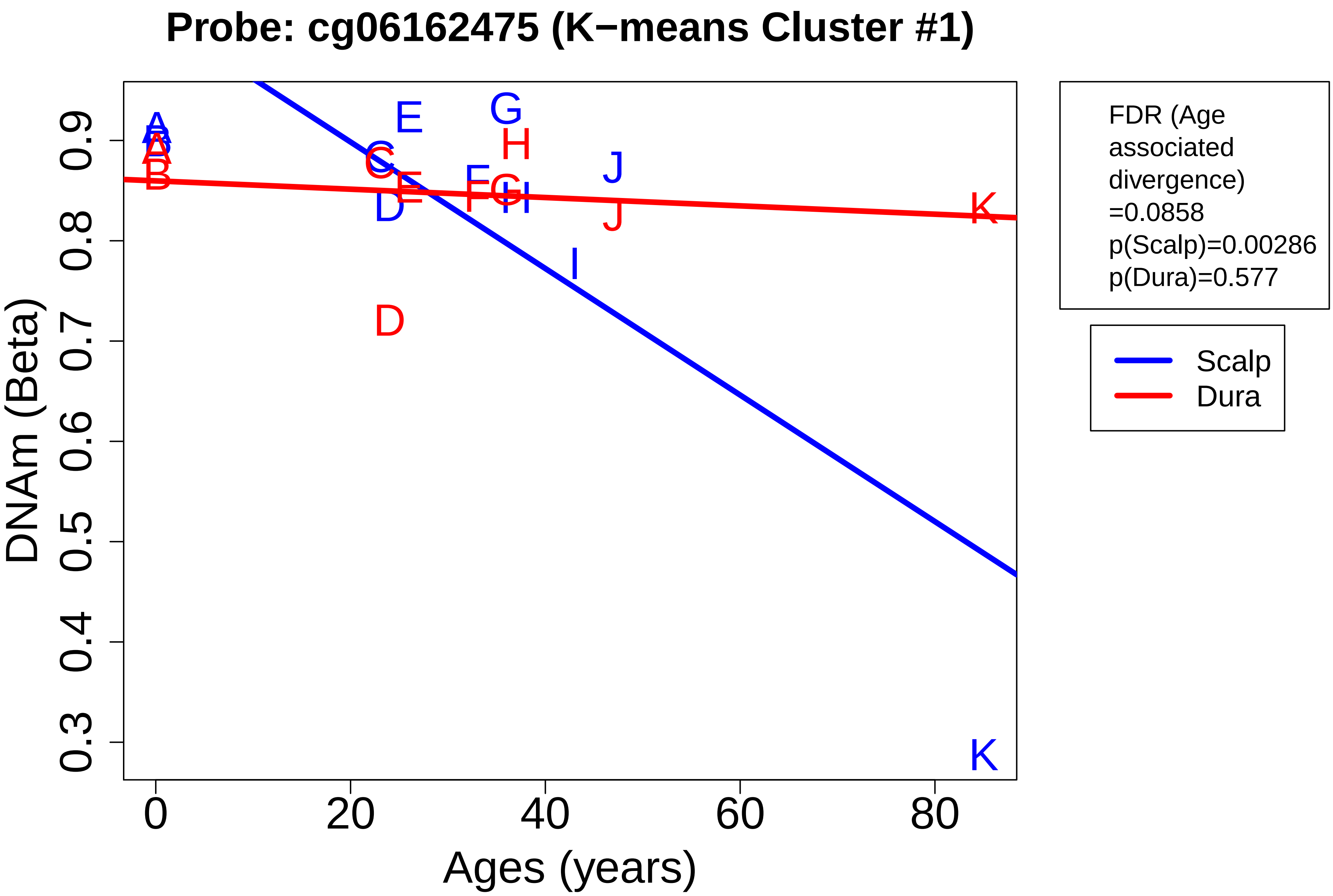

Probe: cg06162475 (K−means Cluster #1)
DNAm (Beta)
0.3
0.4
0.5
0.6
0.7
0.8
0.9
Ages (years)
0
20
40
60
80
A
B
C
D
E
F
G
H
I
J
K
FDR (Age associated divergence) =0.0858
p(Scalp)=0.00286
p(Dura)=0.577
Scalp
Dura

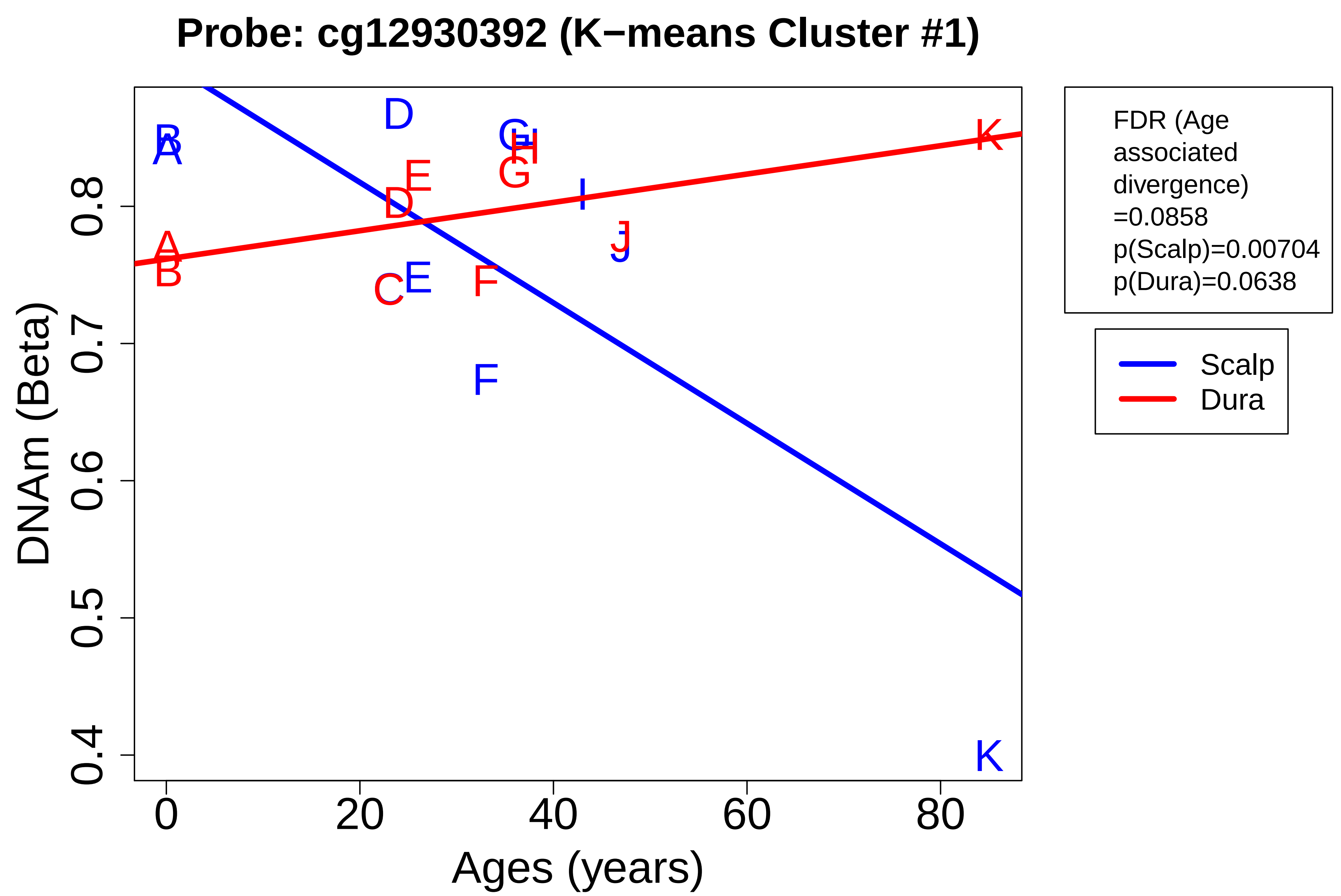

Probe: cg12930392 (K−means Cluster #1)
DNAm (Beta)
0.4
0.5
0.6
0.7
0.8
Ages (years)
0
20
40
60
80
FDR (Age associated divergence) =0.0858
p(Scalp)=0.00704
p(Dura)=0.0638
Scalp
Dura

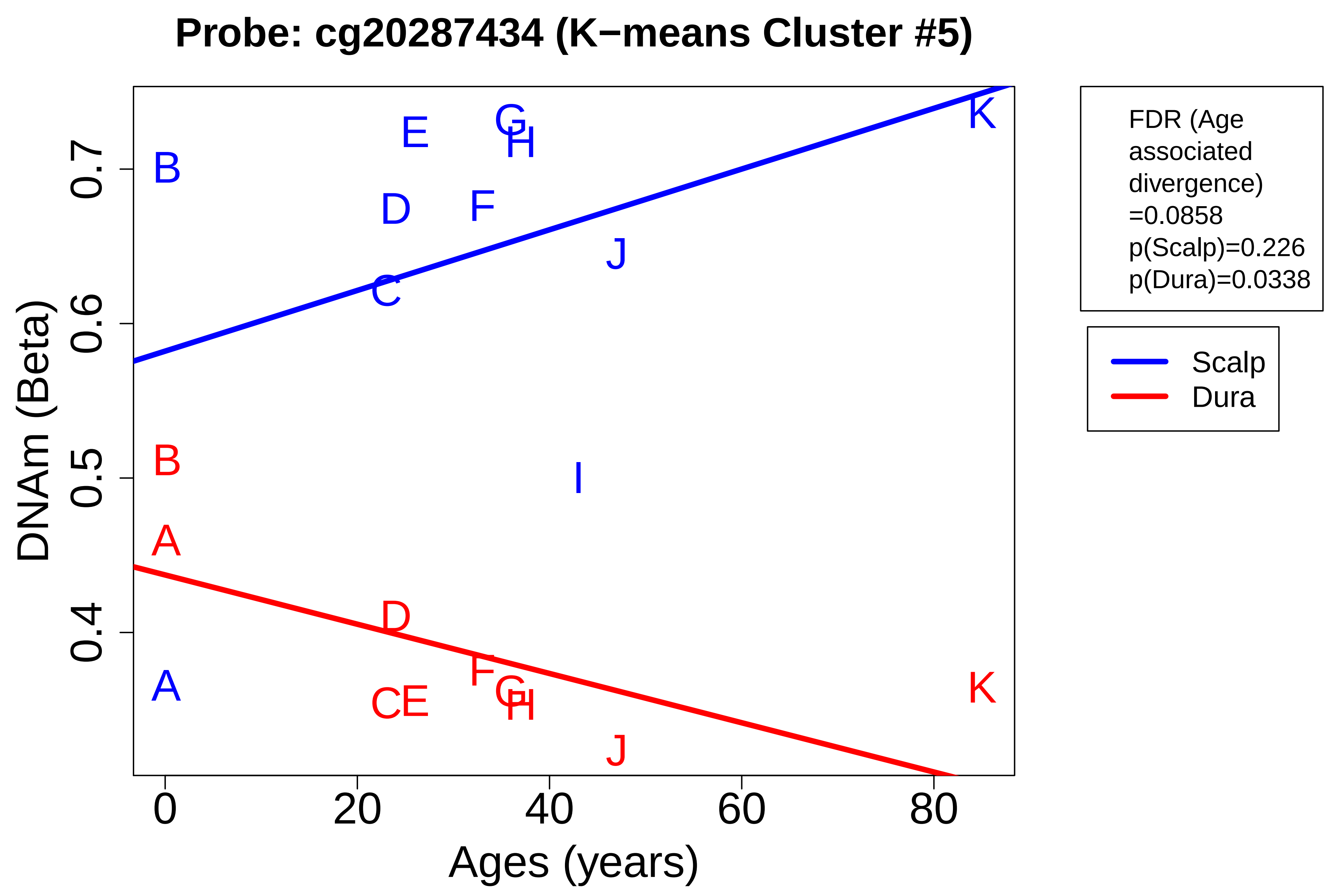

Probe: cg20287434 (K−means Cluster #5)
DNAm (Beta)
0.4
0.5
0.6
0.7
0
20
40
60
80
Ages (years)
A
B
C
D
E
F
G
H
I
J
K
FDR (Age associated divergence) =0.0858
p(Scalp)=0.226
p(Dura)=0.0338
Scalp
Dura

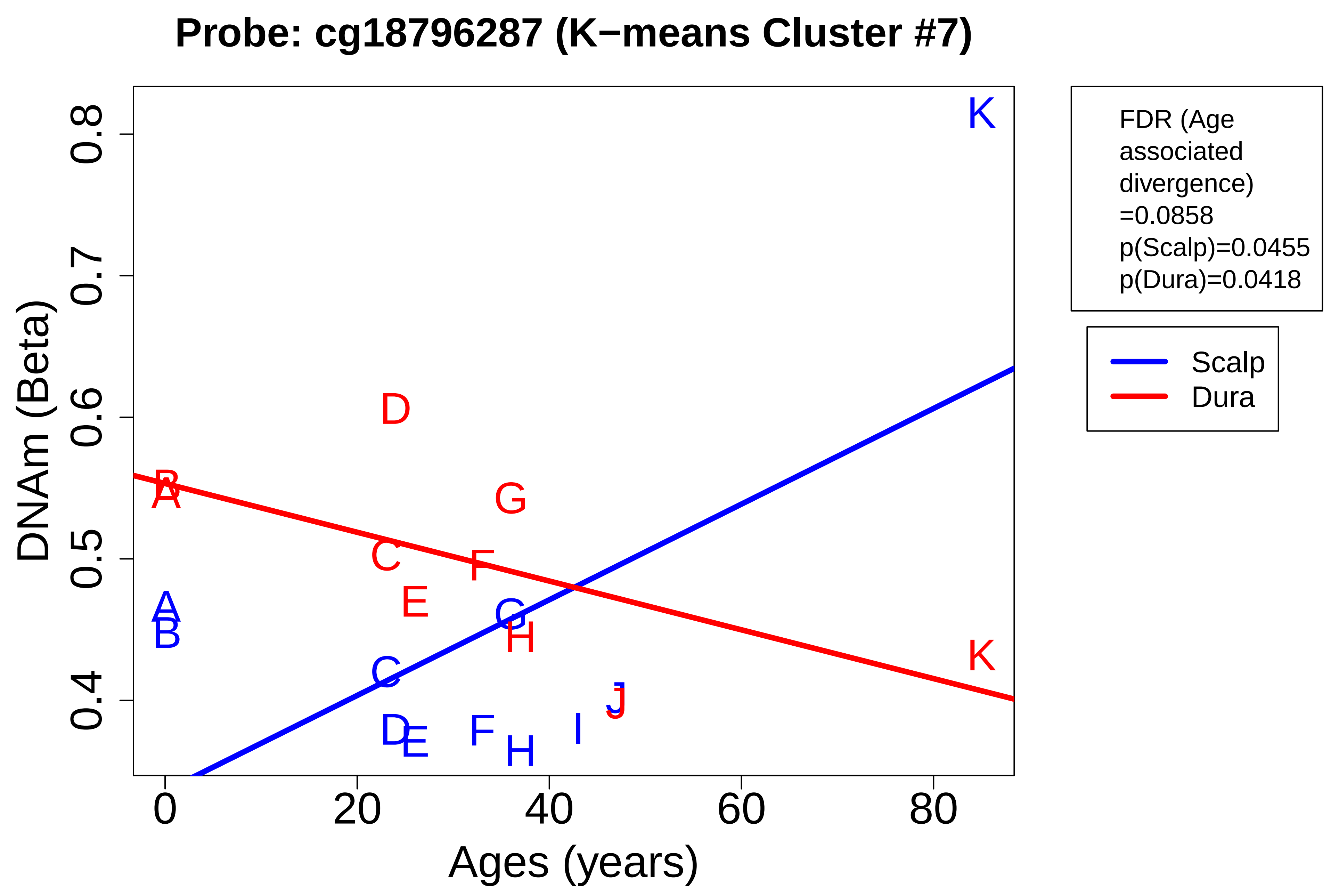

Probe: cg18796287 (K−means Cluster #7)
DNAm (Beta)
0.4
0.5
0.6
0.7
0.8
0
20
40
60
80
Ages (years)
FDR (Age associated divergence) =0.0858
p(Scalp)=0.0455
p(Dura)=0.0418
Scalp
Dura

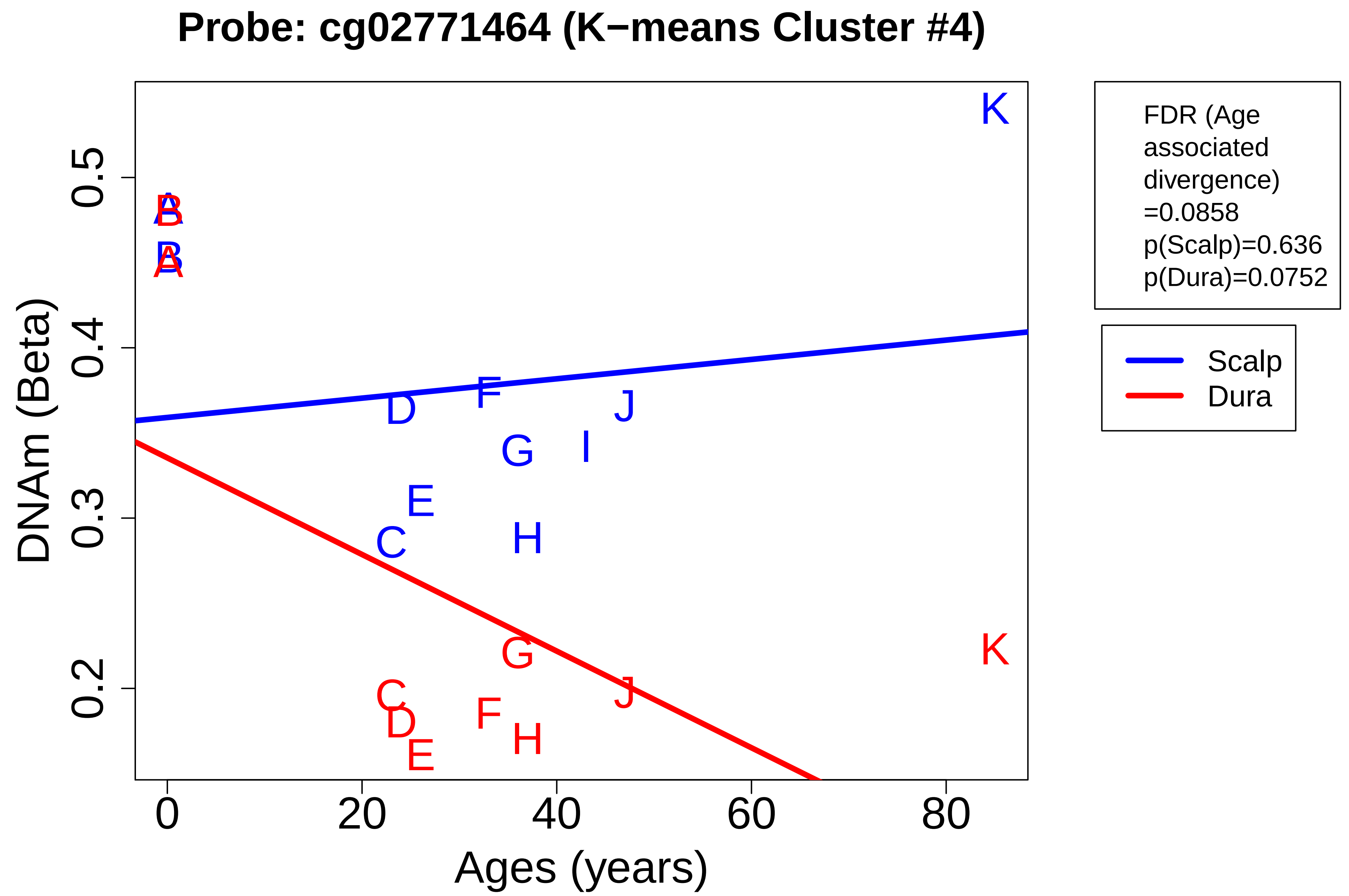

Probe: cg02771464 (K−means Cluster #4)
DNAm (Beta)
0.2
0.3
0.4
0.5
0
20
40
60
80
Ages (years)
A
B
C
D
E
F
G
H
I
J
K
FDR (Age associated divergence) =0.0858
p(Scalp)=0.636
p(Dura)=0.0752
Scalp
Dura

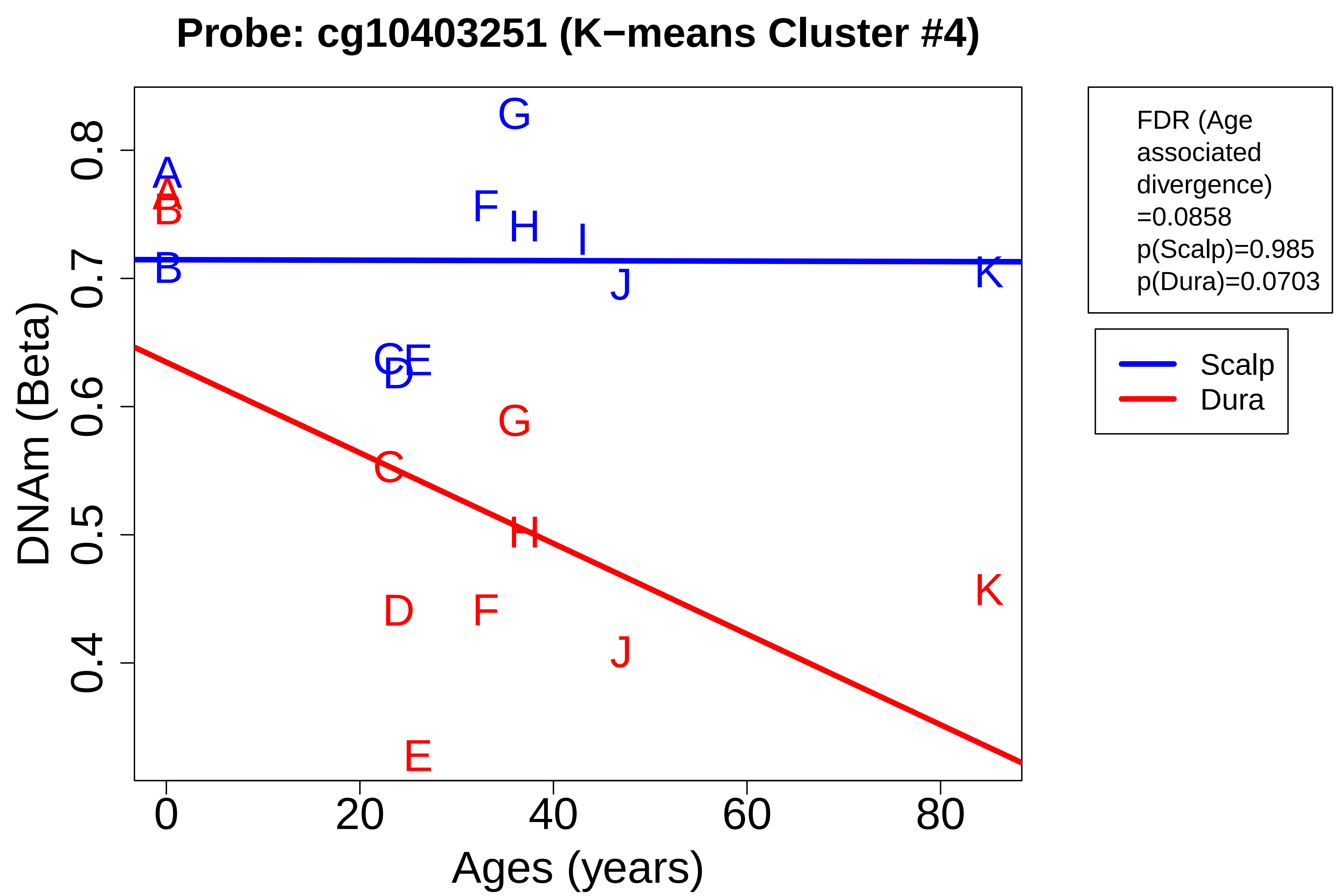

Probe: cg10403251 (K−means Cluster #4)
DNAm (Beta)
0.4
0.5
0.6
0.7
0.8
0
20
40
60
80
Ages (years)
A
B
C
D
E
F
G
H
I
J
K
FDR (Age associated divergence) =0.0858
p(Scalp)=0.985
p(Dura)=0.0703
Scalp
Dura

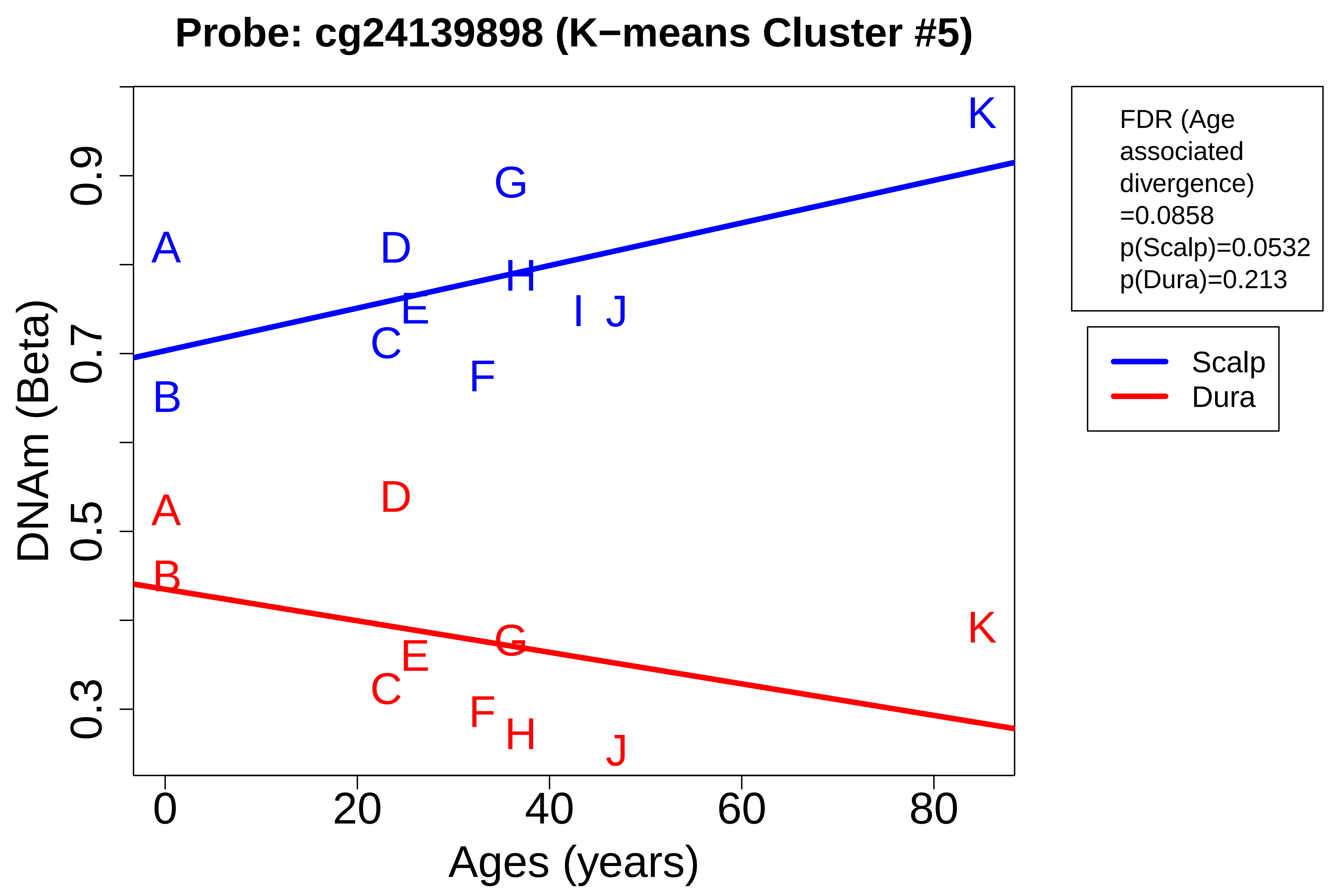

Probe: cg24139898 (K−means Cluster #5)
DNAm (Beta)
0.3
0.5
0.7
0.9
0
20
40
60
80
Ages (years)
A
B
C
D
E
F
G
H
I
J
K
FDR (Age associated divergence) =0.0858
p(Scalp)=0.0532
p(Dura)=0.213
Scalp
Dura

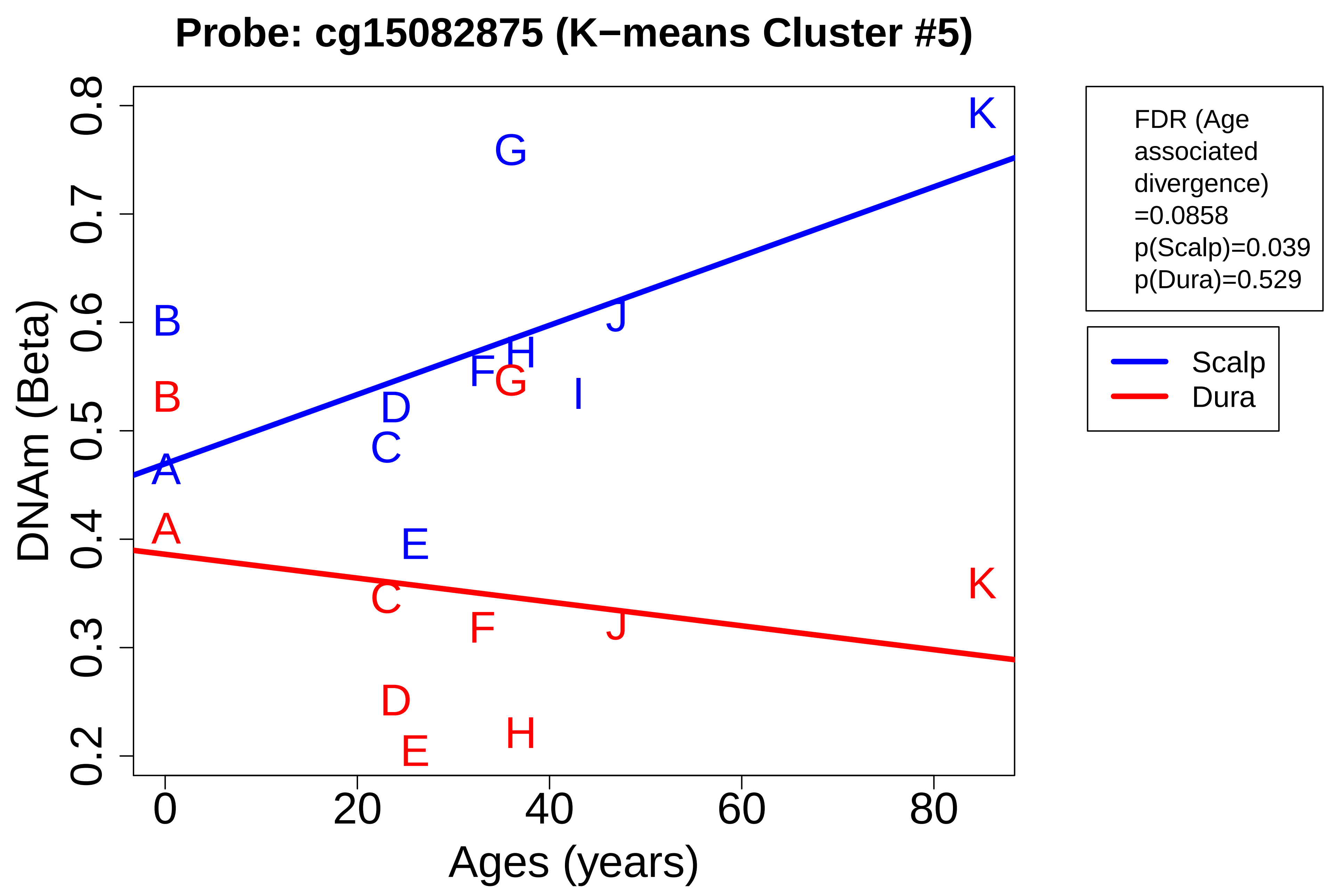

Probe: cg15082875 (K−means Cluster #5)
DNAm (Beta)
Ages (years)
0.2
0.3
0.4
0.5
0.6
0.7
0.8
0
20
40
60
80
FDR (Age associated divergence) =0.0858
p(Scalp)=0.039
p(Dura)=0.529
Scalp
Dura
A
B
C
D
E
F
G
H
I
J
K

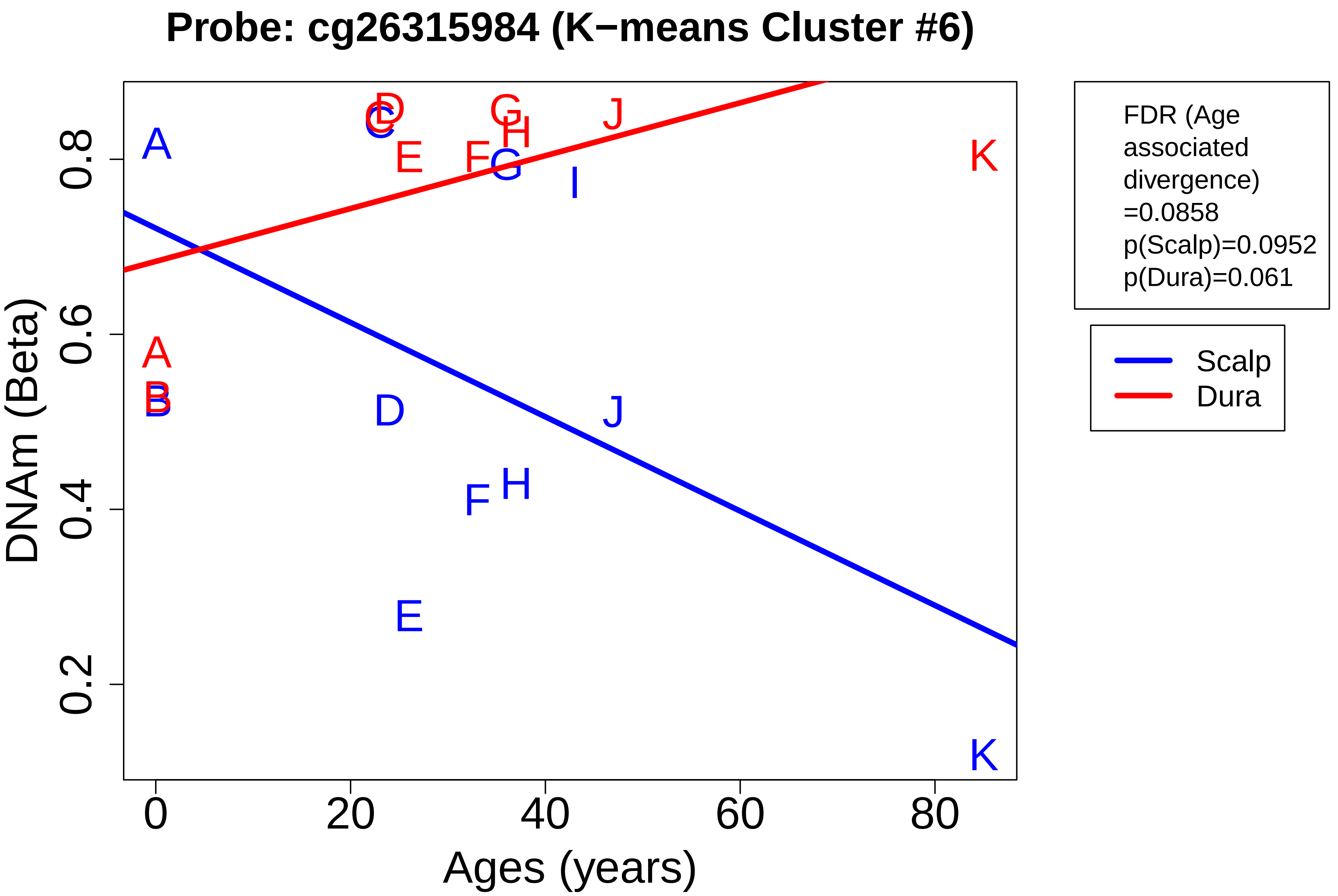

Probe: cg26315984 (K−means Cluster #6)
DNAm (Beta)
0.2
0.4
0.6
0.8
0
20
40
60
80
Ages (years)
FDR (Age associated divergence) =0.0858
p(Scalp)=0.0952
p(Dura)=0.061
Scalp
Dura

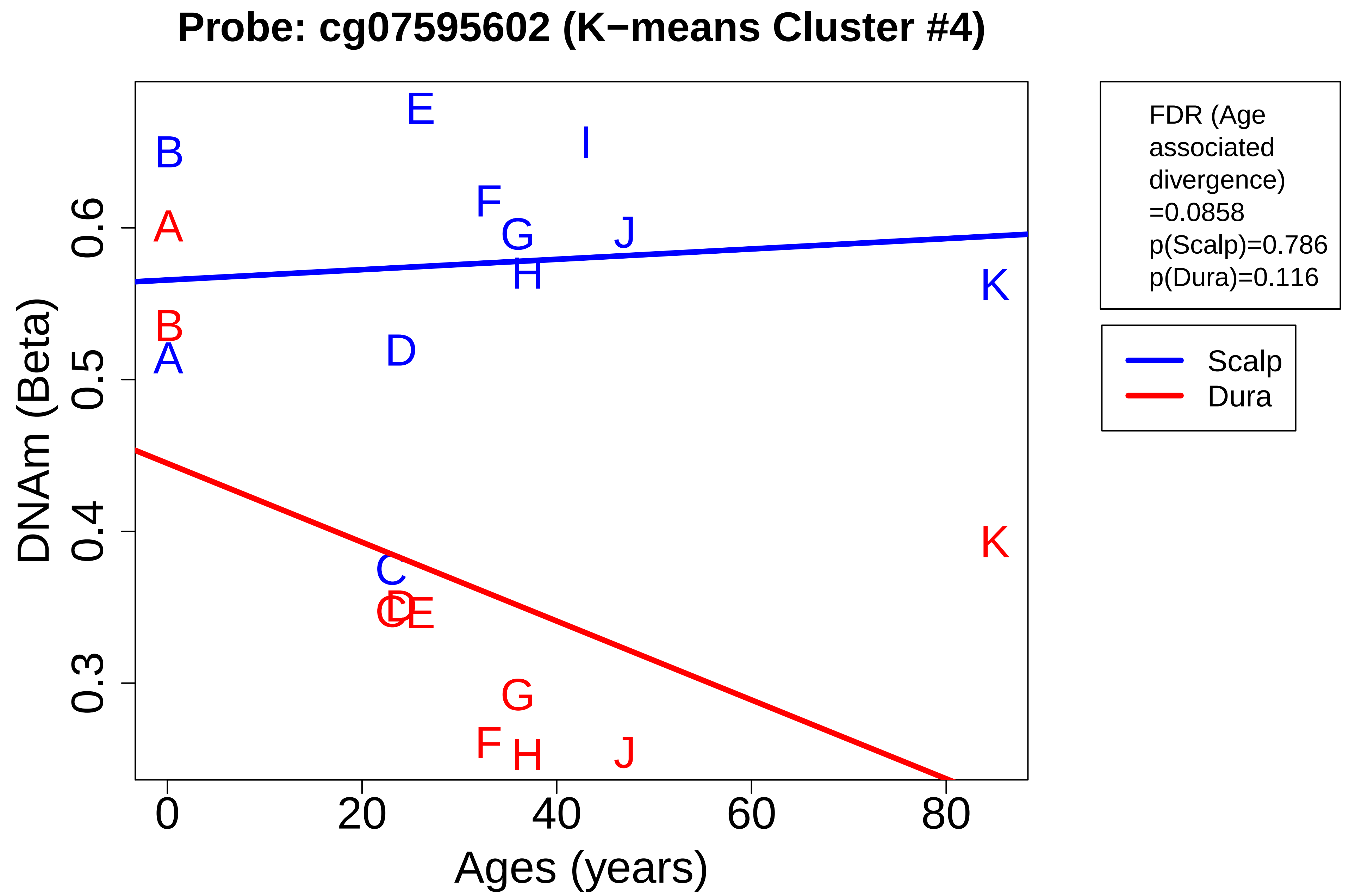

Probe: cg07595602 (K−means Cluster #4)
DNAm (Beta)
0.3
0.4
0.5
0.6
0
20
40
60
80
Ages (years)
A
B
C
D
E
F
G
H
I
J
K
FDR (Age associated divergence) =0.0858
p(Scalp)=0.786
p(Dura)=0.116
Scalp
Dura

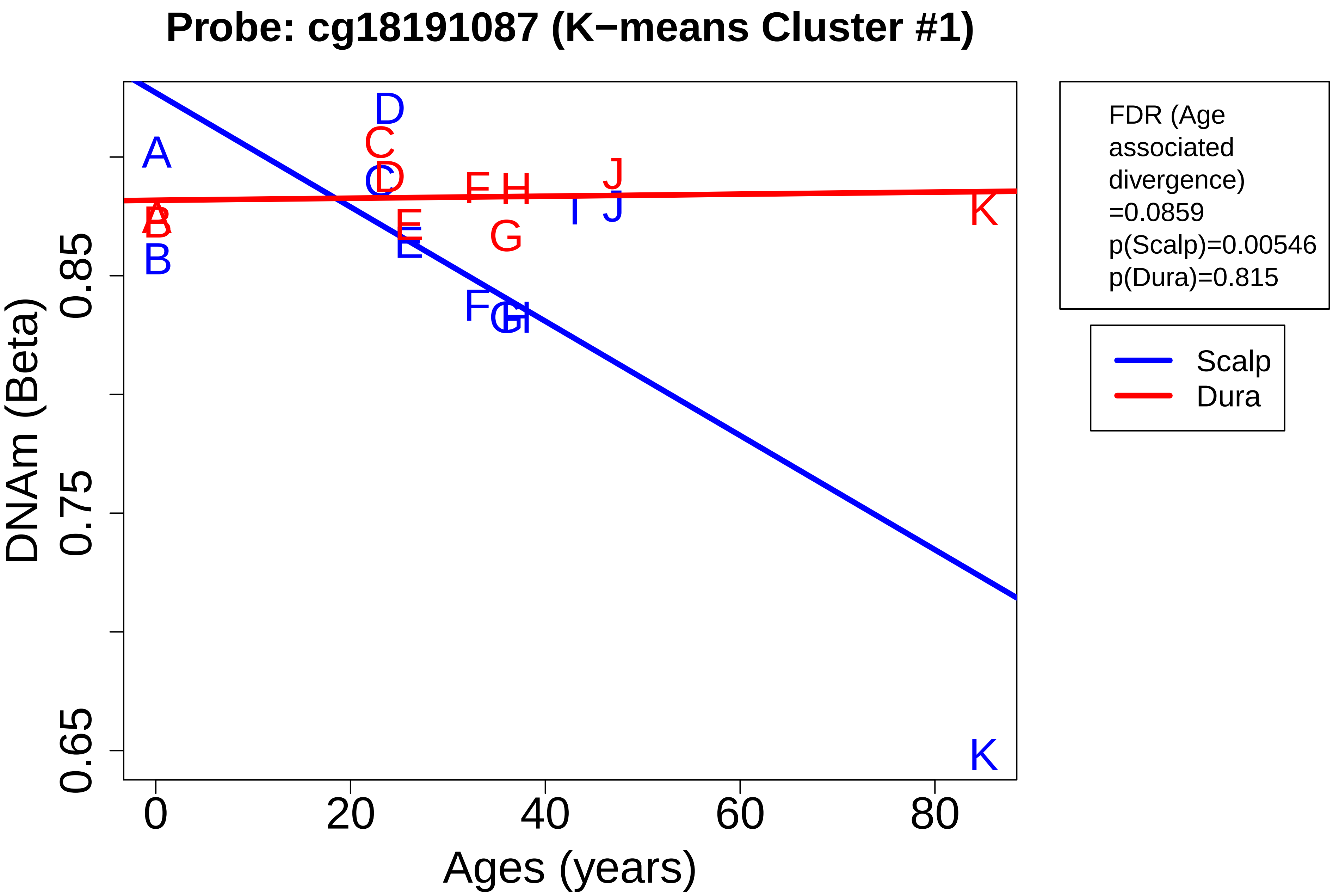

Probe: cg18191087 (K−means Cluster #1)
DNAm (Beta)
0.65
0.75
0.85
0
20
40
60
80
Ages (years)
FDR (Age associated divergence) =0.0859
p(Scalp)=0.00546
p(Dura)=0.815
Scalp
Dura

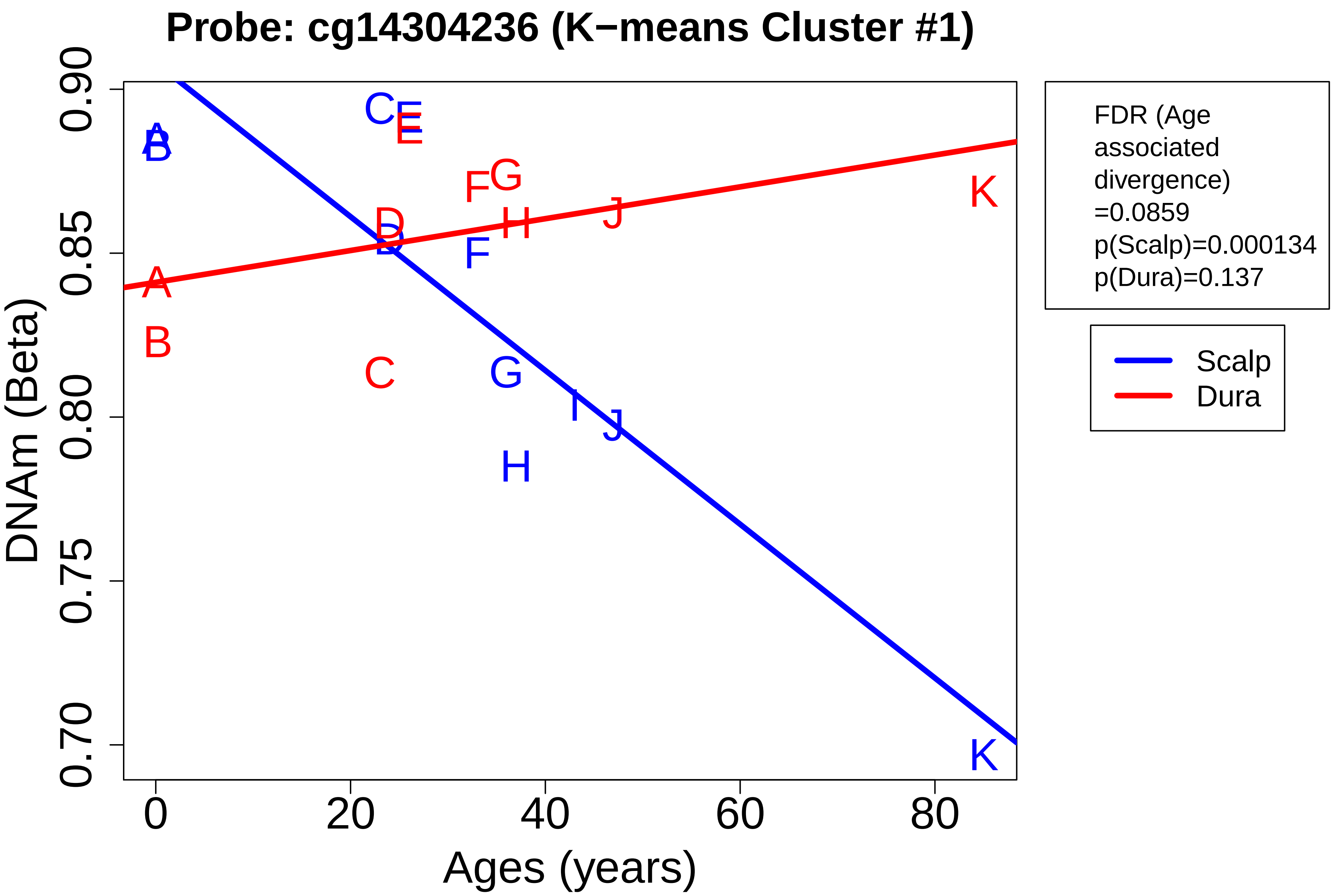

Probe: cg14304236 (K−means Cluster #1)
DNAm (Beta)
0.70
0.75
0.80
0.85
0.90
0
20
40
60
80
Ages (years)
FDR (Age associated divergence) =0.0859
p(Scalp)=0.000134
p(Dura)=0.137
Scalp
Dura

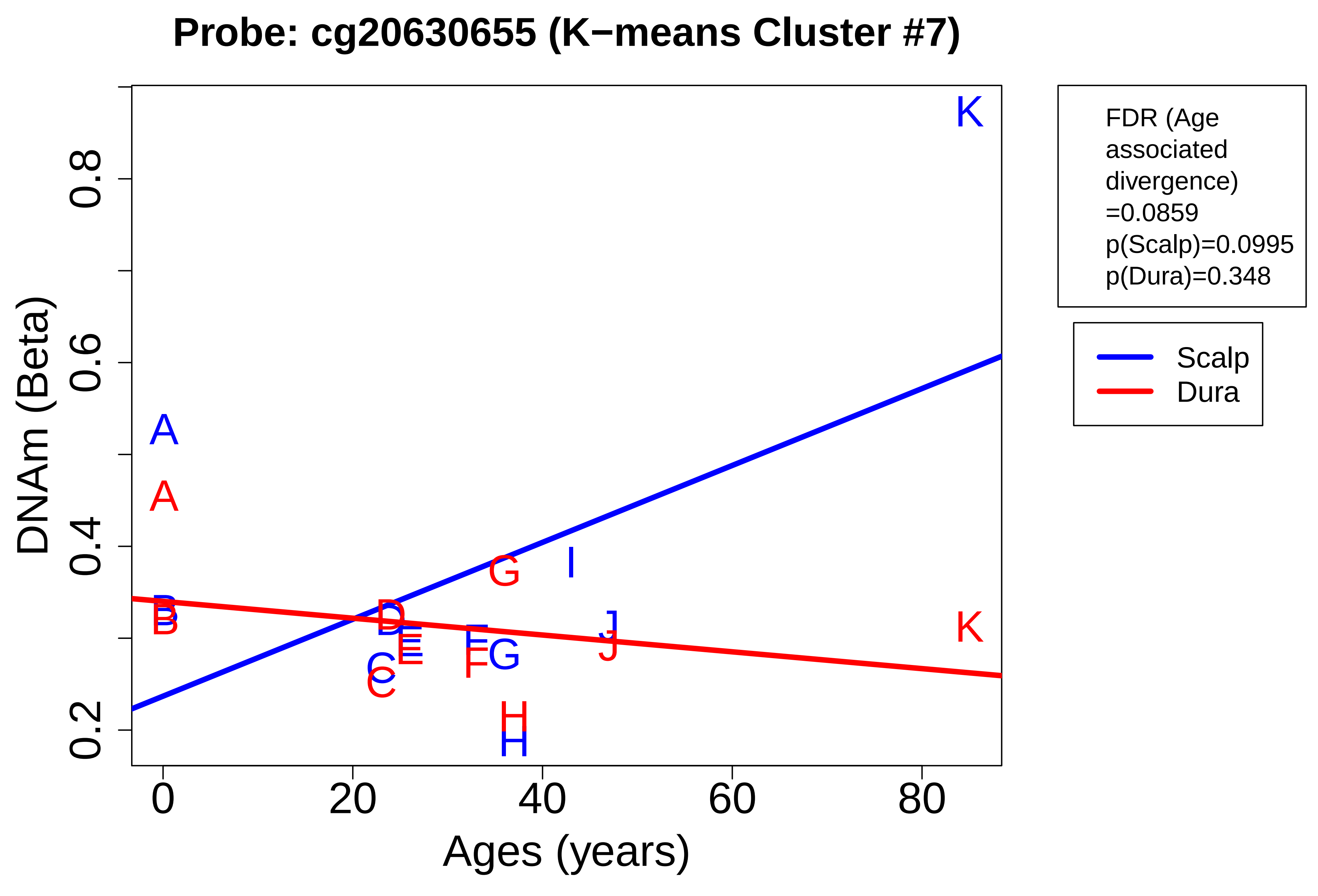
Probe: cg20630655 (K−means Cluster #7)
DNAm (Beta)
0.2
0.4
0.6
0.8
0
20
40
60
80
Ages (years)
FDR (Age associated divergence) =0.0859
p(Scalp)=0.0995
p(Dura)=0.348
Scalp
Dura
A
B
C
D
E
F
G
H
I
J
K

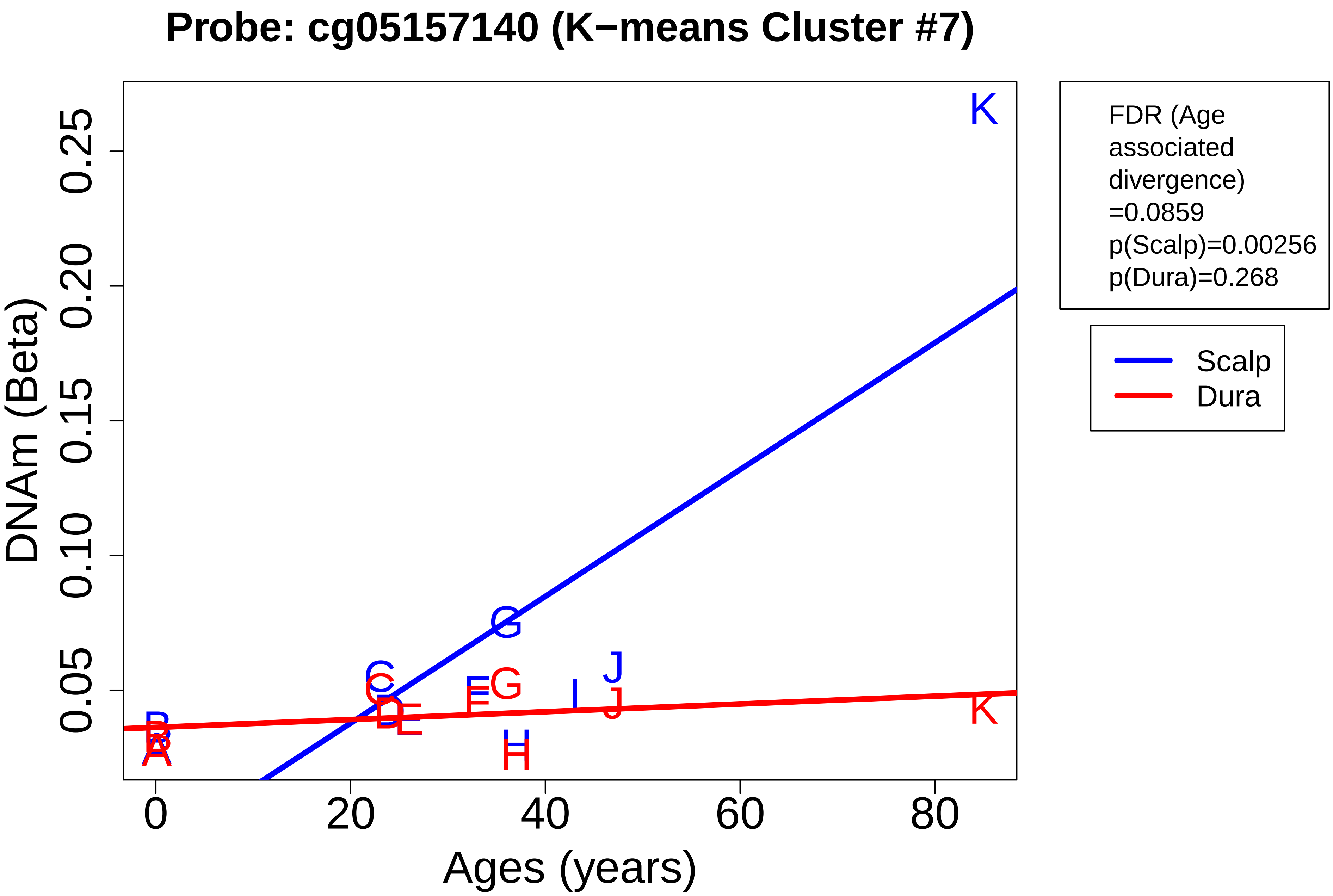
Probe: cg05157140 (K−means Cluster #7)
DNAm (Beta)
0.05
0.10
0.15
0.20
0.25
0
20
40
60
80
Ages (years)
FDR (Age associated divergence) =0.0859
p(Scalp)=0.00256
p(Dura)=0.268
Scalp
Dura
A
B
C
D
E
F
G
H
I
J
K

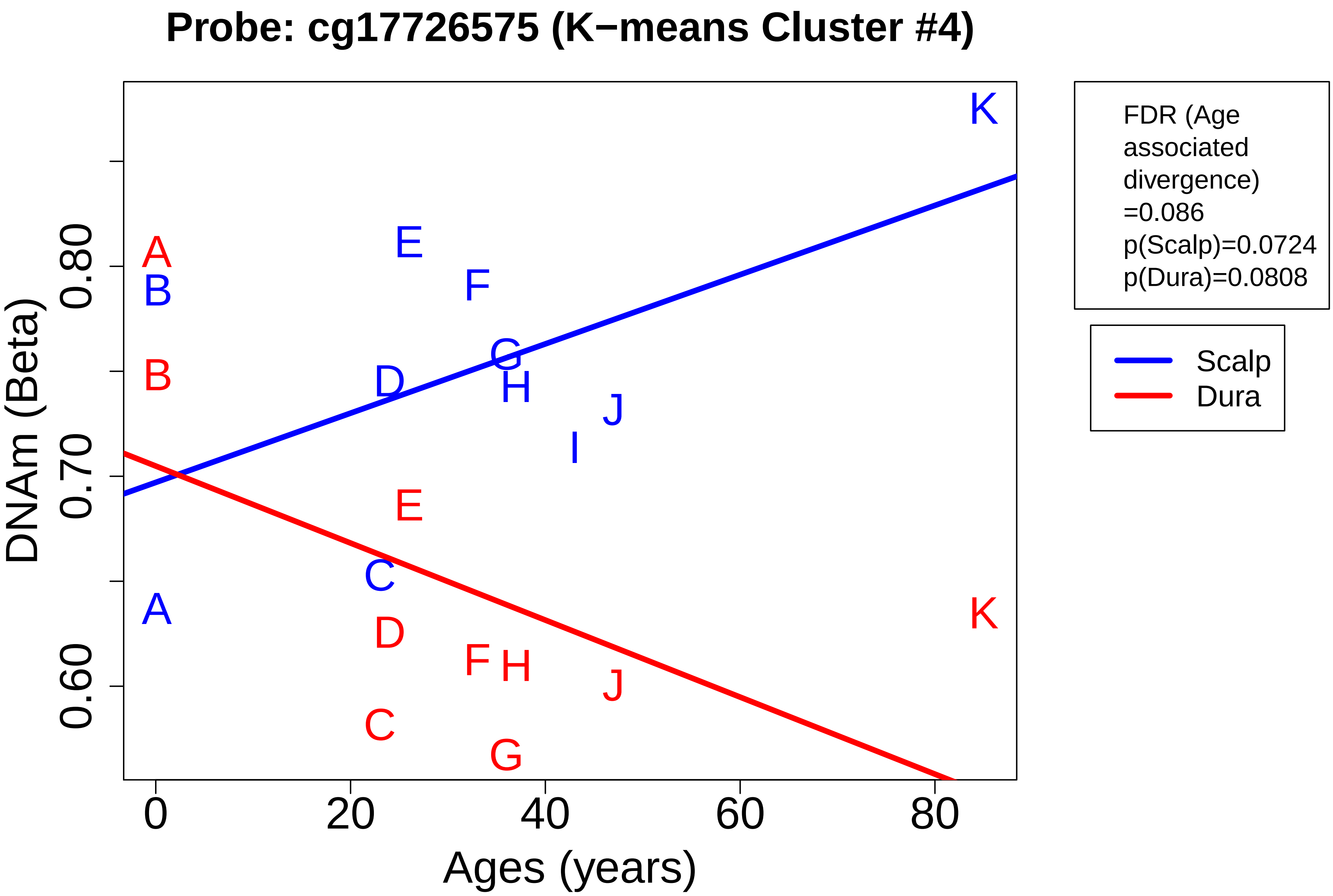
Probe: cg17726575 (K−means Cluster #4)
DNAm (Beta)
0.60
0.70
0.80
Ages (years)
0
20
40
60
80
FDR (Age associated divergence) =0.086
p(Scalp)=0.0724
p(Dura)=0.0808
Scalp
Dura
A
B
C
D
E
F
G
H
I
J
K

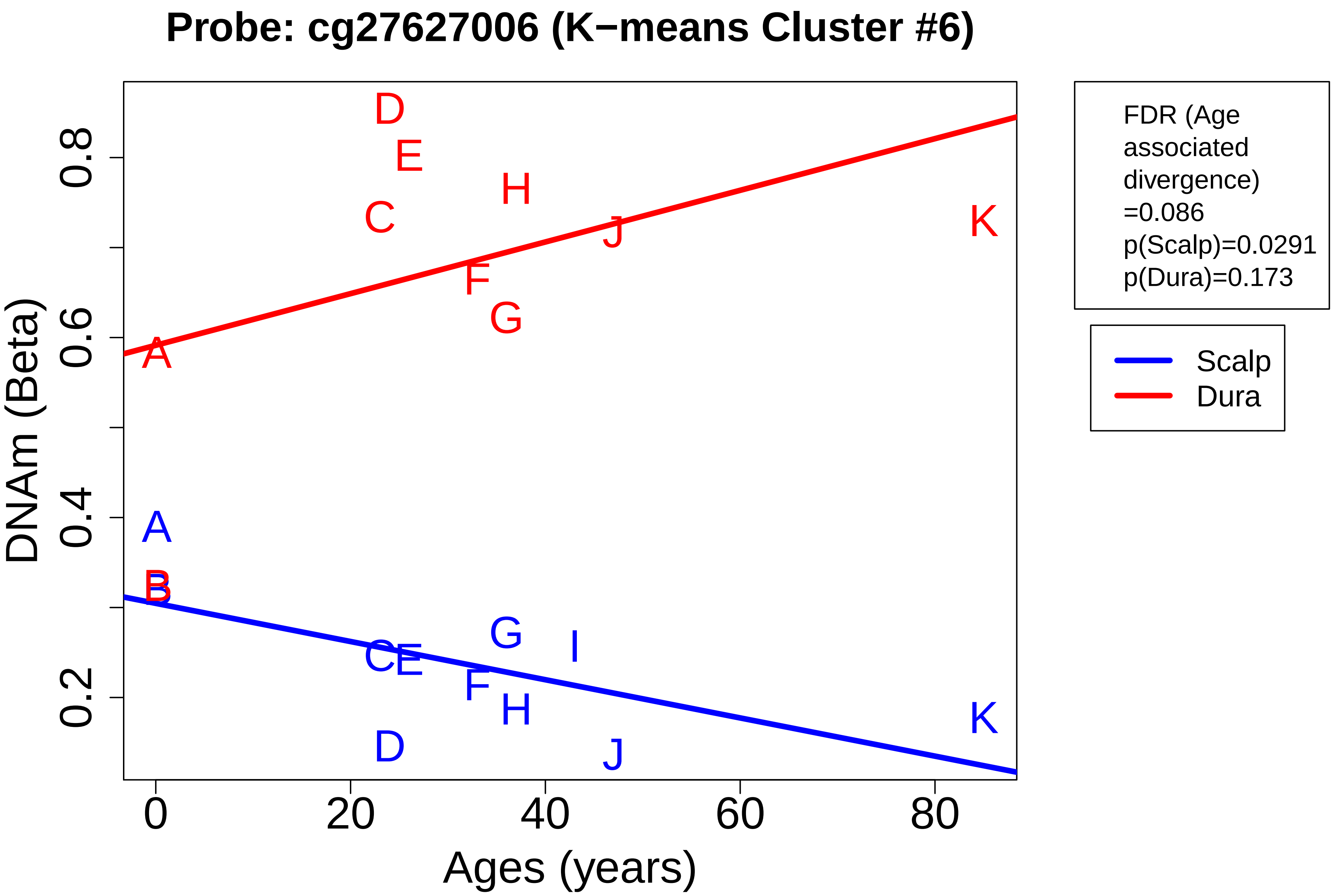

Probe: cg27627006 (K−means Cluster #6)
DNAm (Beta)
0.2
0.4
0.6
0.8
0
20
40
60
80
Ages (years)
FDR (Age associated divergence) =0.086
p(Scalp)=0.0291
p(Dura)=0.173
Scalp
Dura

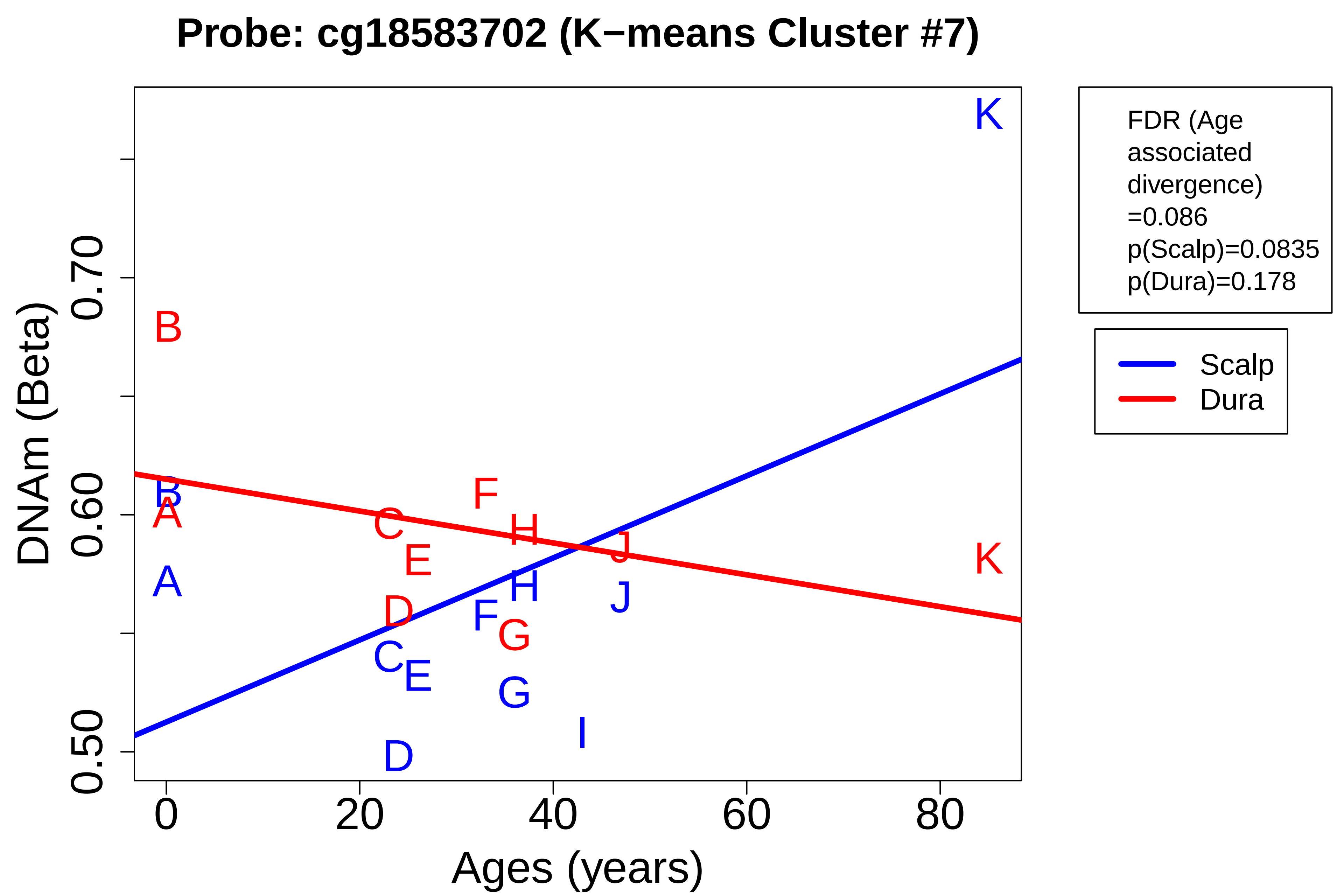

Probe: cg18583702 (K−means Cluster #7)
DNAm (Beta)
0.50
0.60
0.70
0
20
40
60
80
Ages (years)
FDR (Age associated divergence) =0.086
p(Scalp)=0.0835
p(Dura)=0.178
Scalp
Dura
A
B
C
D
E
F
G
H
I
J
K

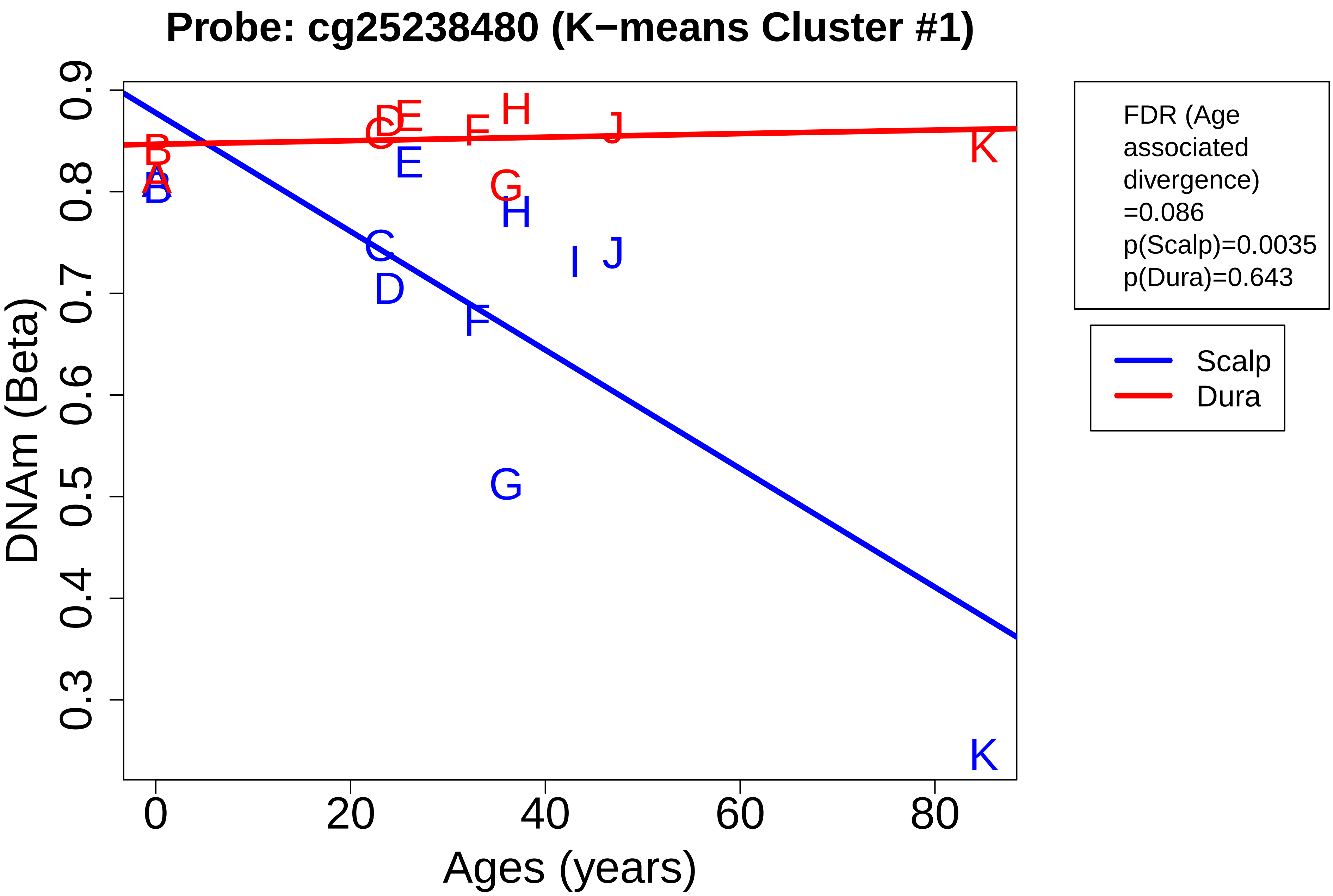

Probe: cg25238480 (K−means Cluster #1)
DNAm (Beta)
0.3
0.4
0.5
0.6
0.7
0.8
0.9
0
20
40
60
80
Ages (years)
FDR (Age associated divergence) =0.086
p(Scalp)=0.0035
p(Dura)=0.643
Scalp
Dura

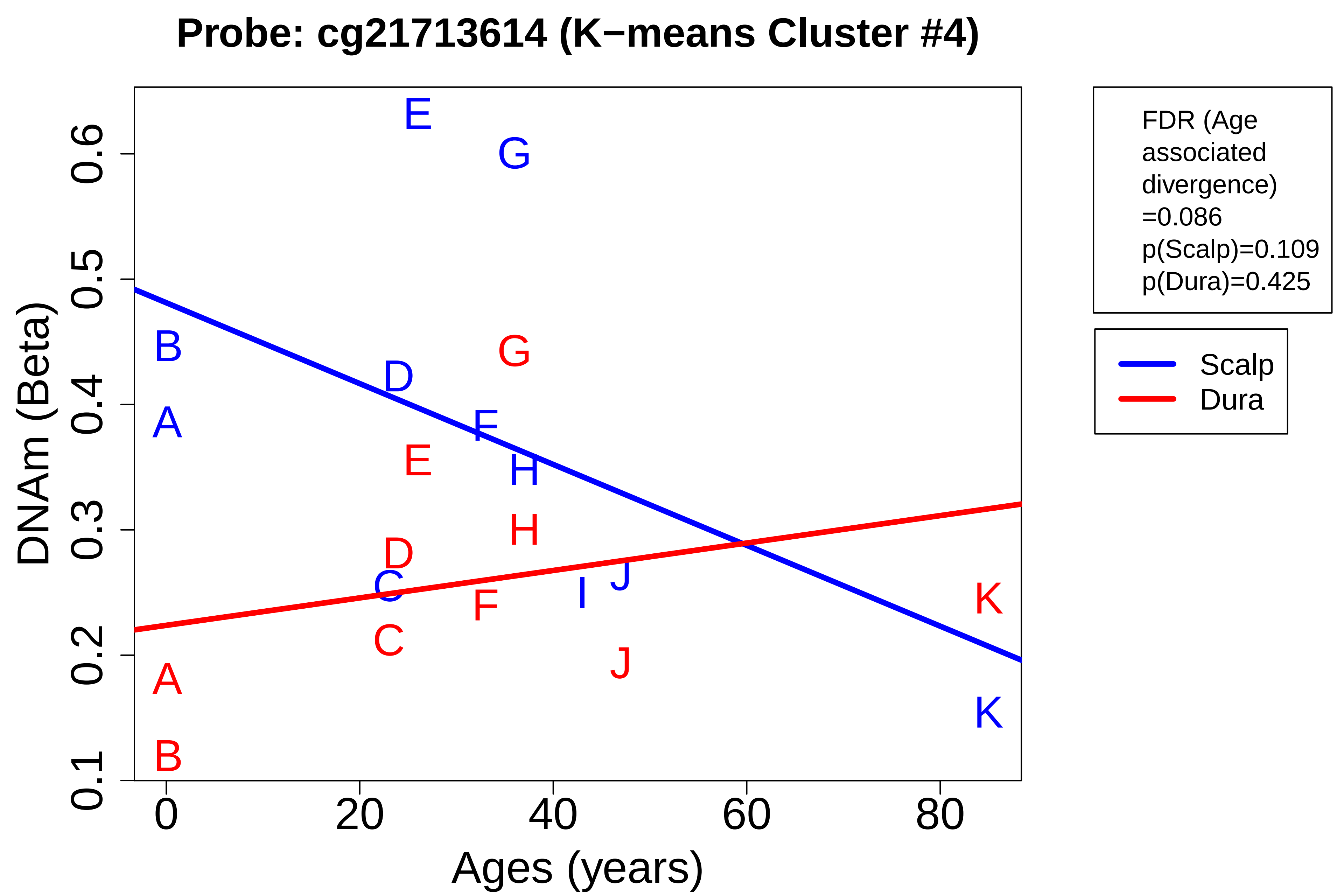

Probe: cg21713614 (K−means Cluster #4)
DNAm (Beta)
0.1
0.2
0.3
0.4
0.5
0.6
0
20
40
60
80
Ages (years)
FDR (Age associated divergence) =0.086
p(Scalp)=0.109
p(Dura)=0.425
Scalp
Dura

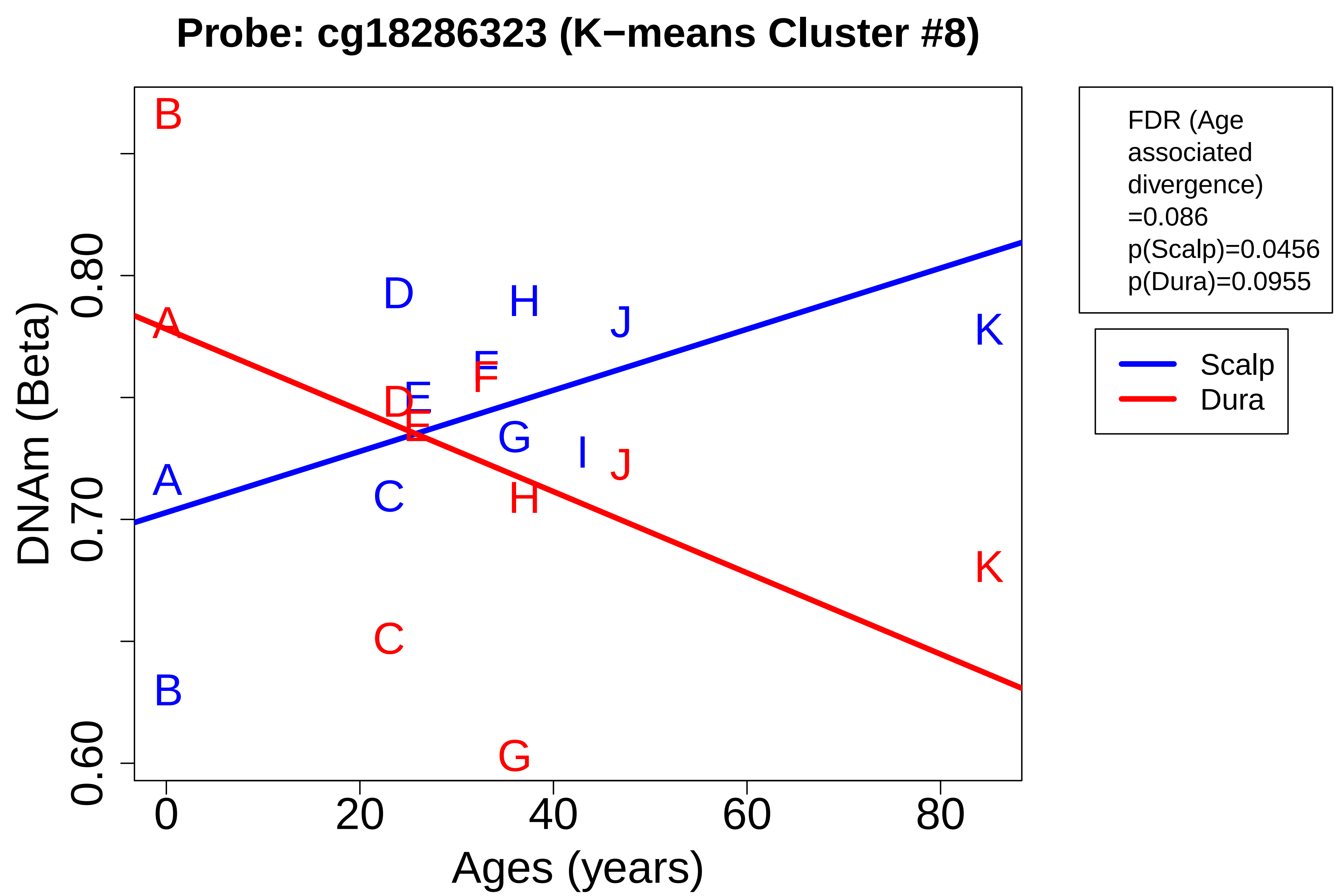

Probe: cg18286323 (K−means Cluster #8)
DNAm (Beta)
0.60
0.70
0.80
0
20
40
60
80
Ages (years)
FDR (Age associated divergence) =0.086
p(Scalp)=0.0456
p(Dura)=0.0955
Scalp
Dura

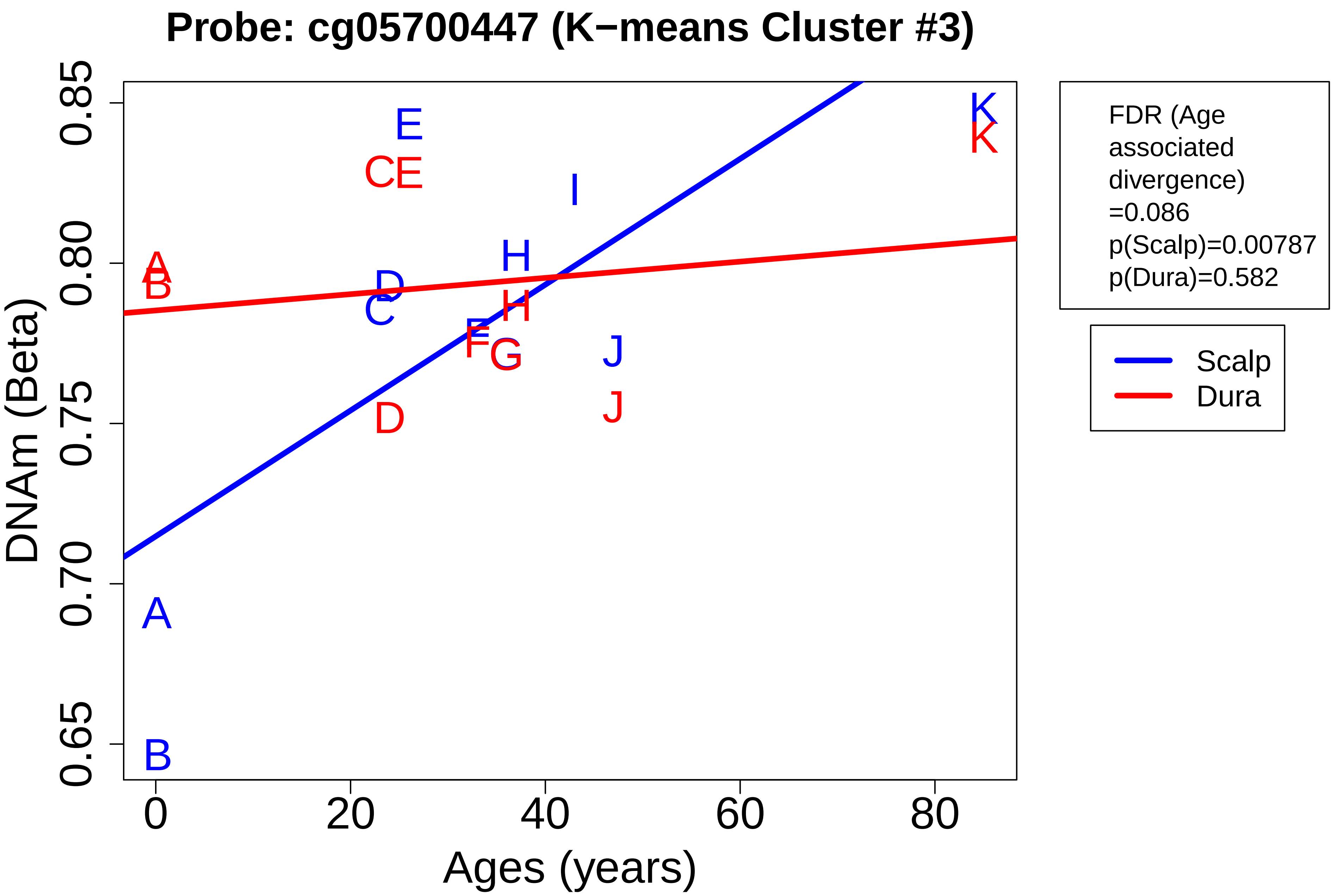

Probe: cg05700447 (K−means Cluster #3)
DNAm (Beta)
0.65
0.70
0.75
0.80
0.85
Ages (years)
0
20
40
60
80
FDR (Age associated divergence) =0.086
p(Scalp)=0.00787
p(Dura)=0.582
Scalp
Dura

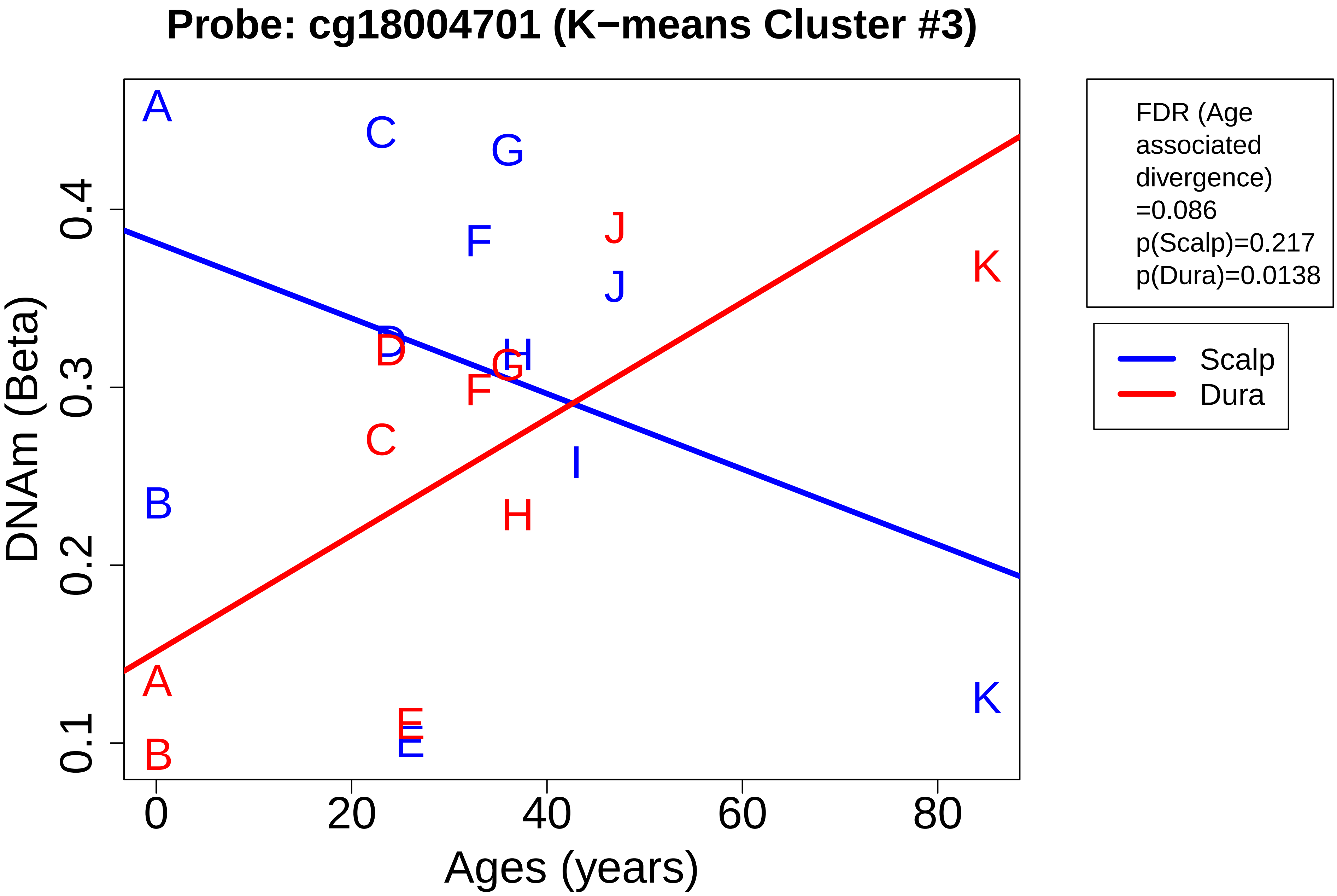
Probe: cg18004701 (K−means Cluster #3)
DNAm (Beta)
0.1
0.2
0.3
0.4
Ages (years)
0
20
40
60
80
A
B
C
D
E
F
G
H
I
J
K
FDR (Age associated divergence) =0.086
p(Scalp)=0.217
p(Dura)=0.0138
Scalp
Dura

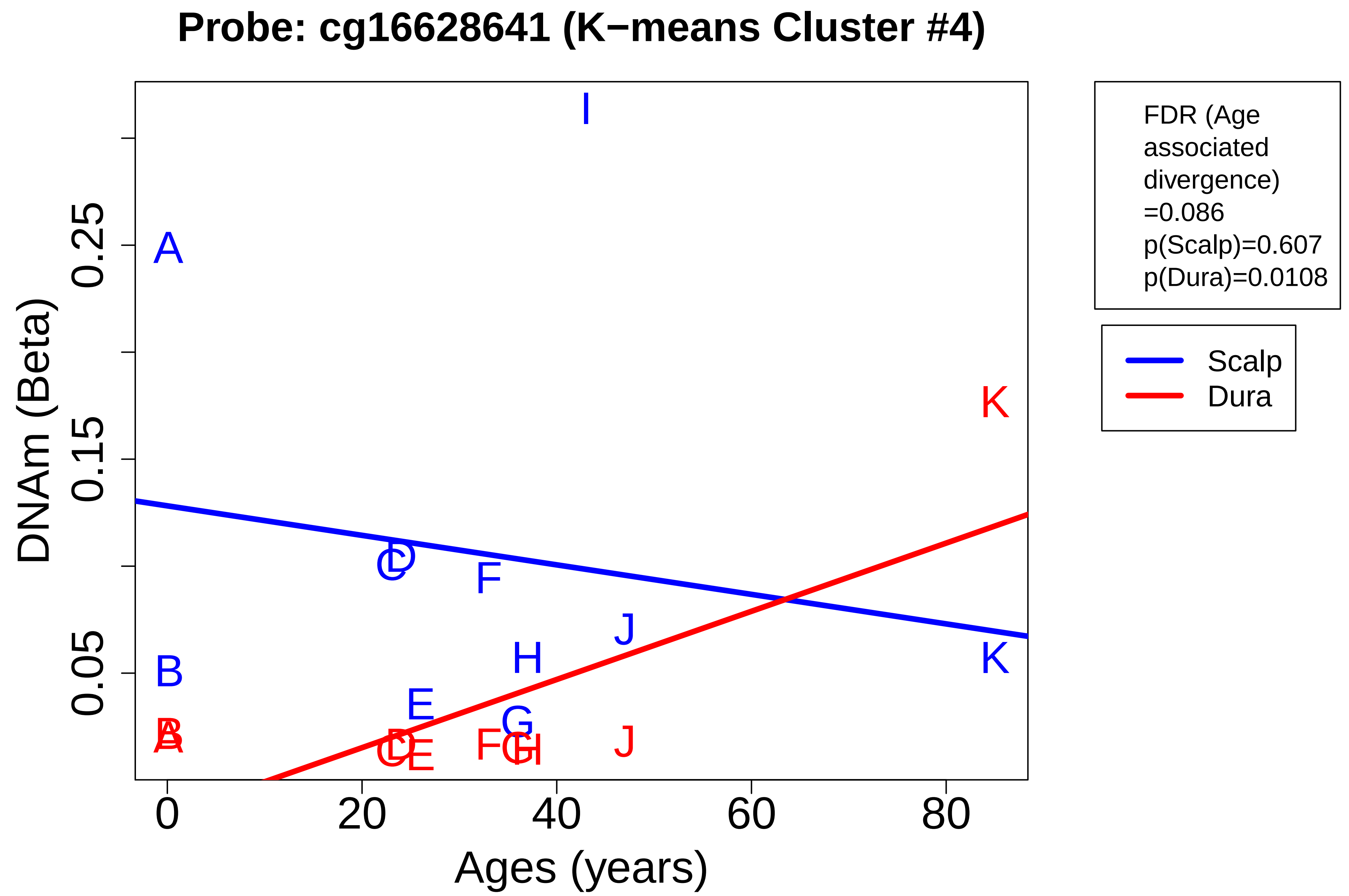

Probe: cg16628641 (K−means Cluster #4)
DNAm (Beta)
0.05
0.15
0.25
Ages (years)
0
20
40
60
80
FDR (Age associated divergence) =0.086
p(Scalp)=0.607
p(Dura)=0.0108
Scalp
Dura

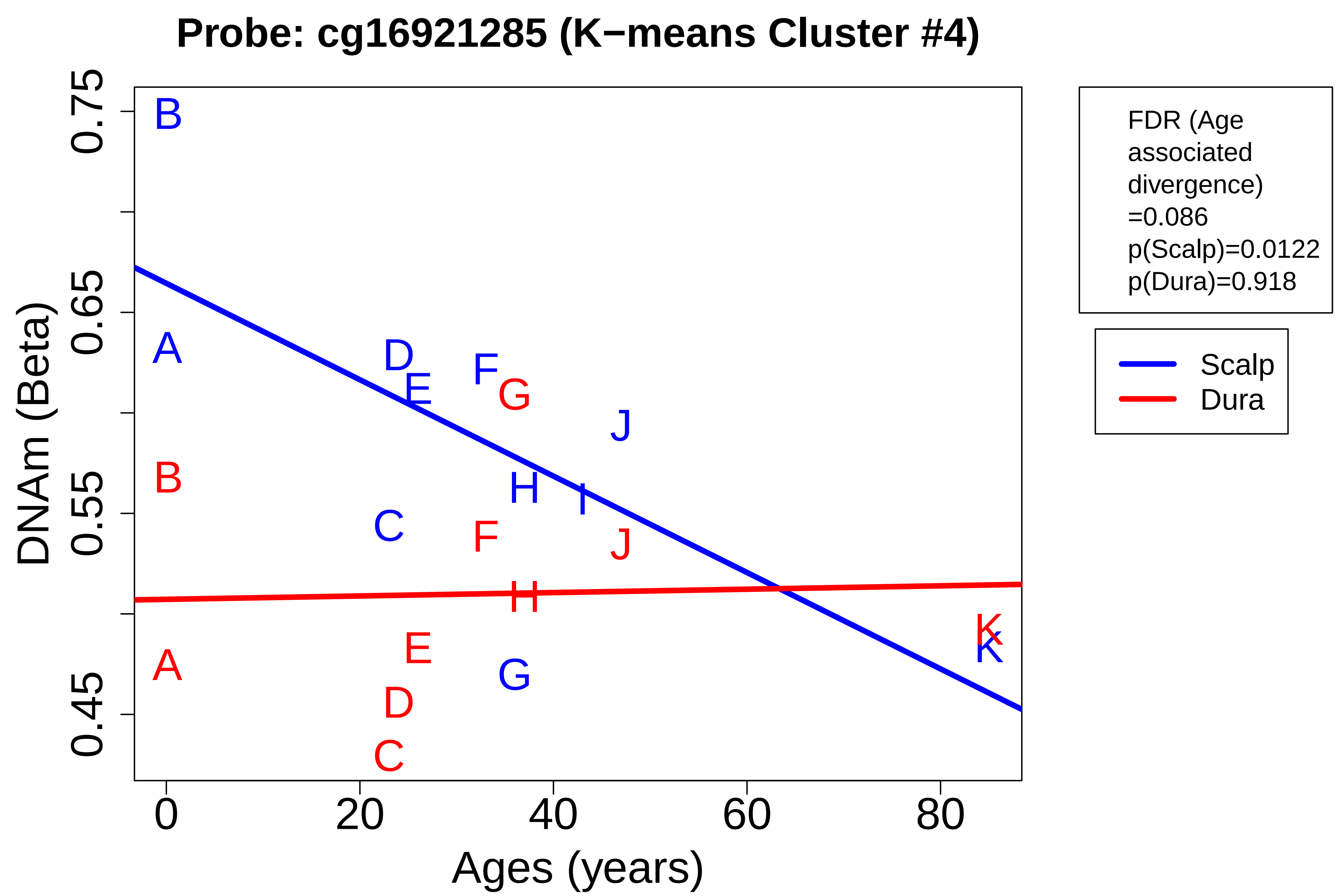
Probe: cg16921285 (K−means Cluster #4)
DNAm (Beta)
0.45
0.55
0.65
0.75
Ages (years)
0
20
40
60
80
A
B
C
D
E
F
G
H
I
J
K
FDR (Age associated divergence) =0.086
p(Scalp)=0.0122
p(Dura)=0.918
Scalp
Dura

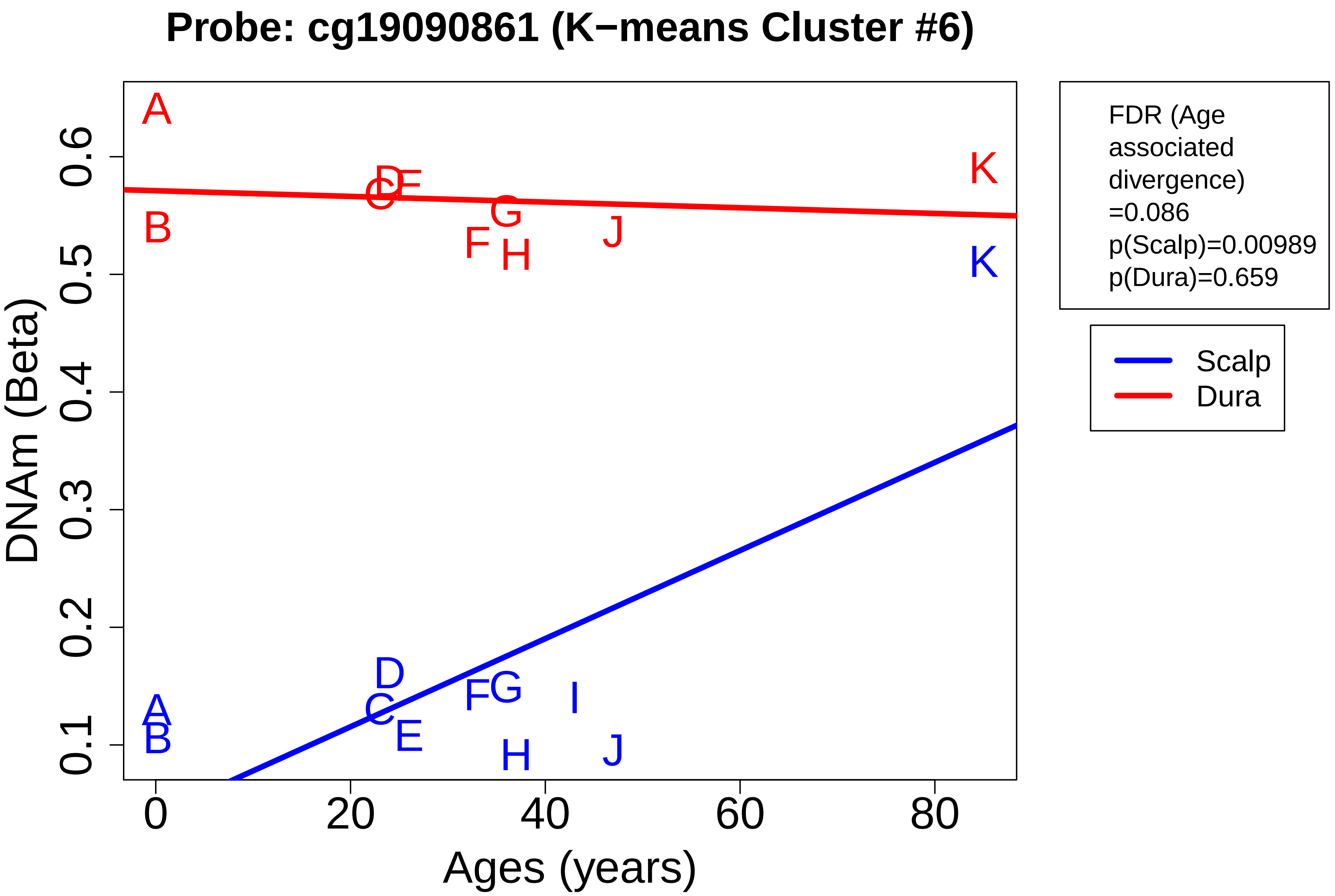

Probe: cg19090861 (K−means Cluster #6)
DNAm (Beta)
0.1
0.2
0.3
0.4
0.5
0.6
0
20
40
60
80
Ages (years)
FDR (Age associated divergence) =0.086
p(Scalp)=0.00989
p(Dura)=0.659
Scalp
Dura

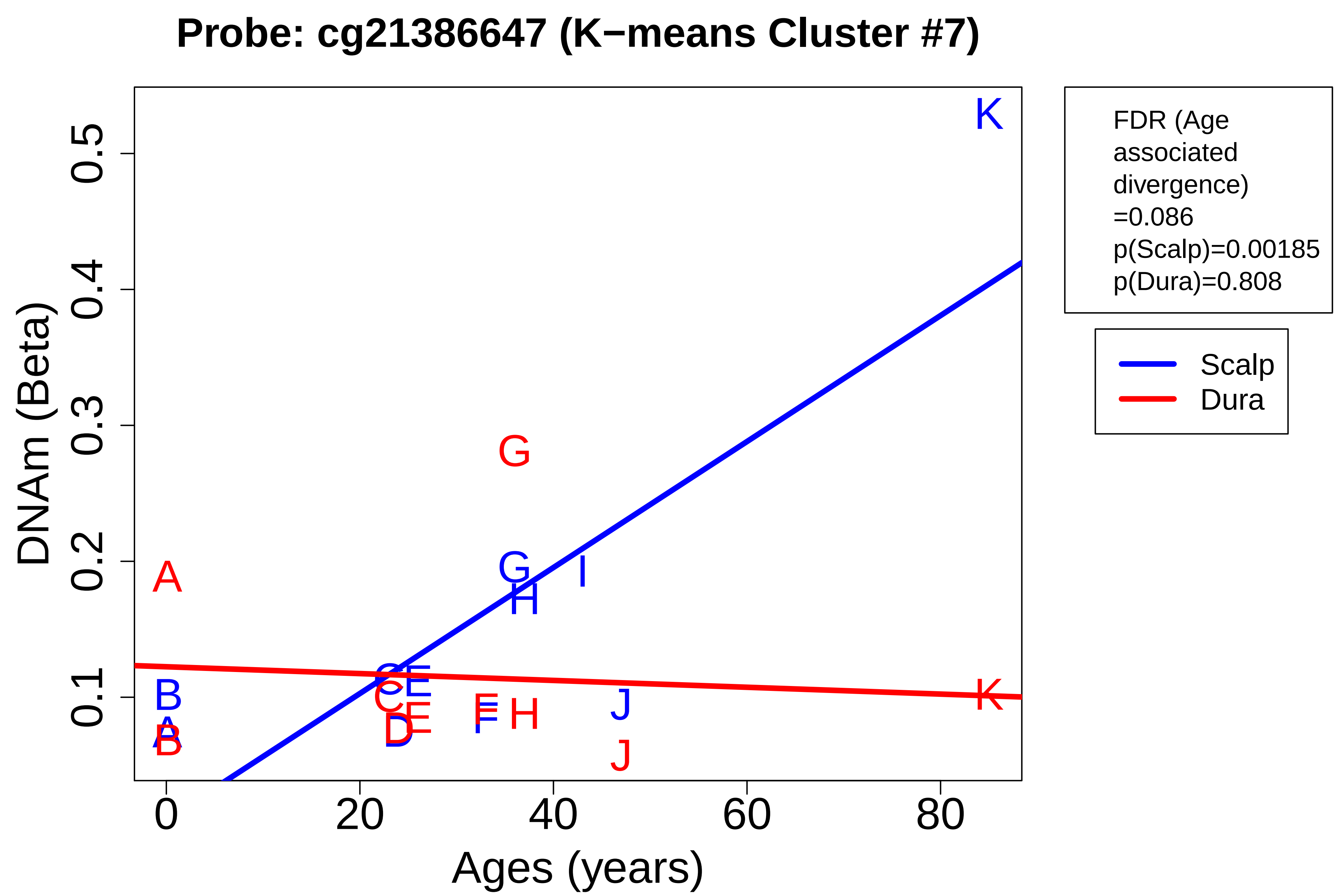

Probe: cg21386647 (K−means Cluster #7)
DNAm (Beta)
Ages (years)
0.1
0.2
0.3
0.4
0.5
0
20
40
60
80
FDR (Age associated divergence) =0.086
p(Scalp)=0.00185
p(Dura)=0.808
Scalp
Dura

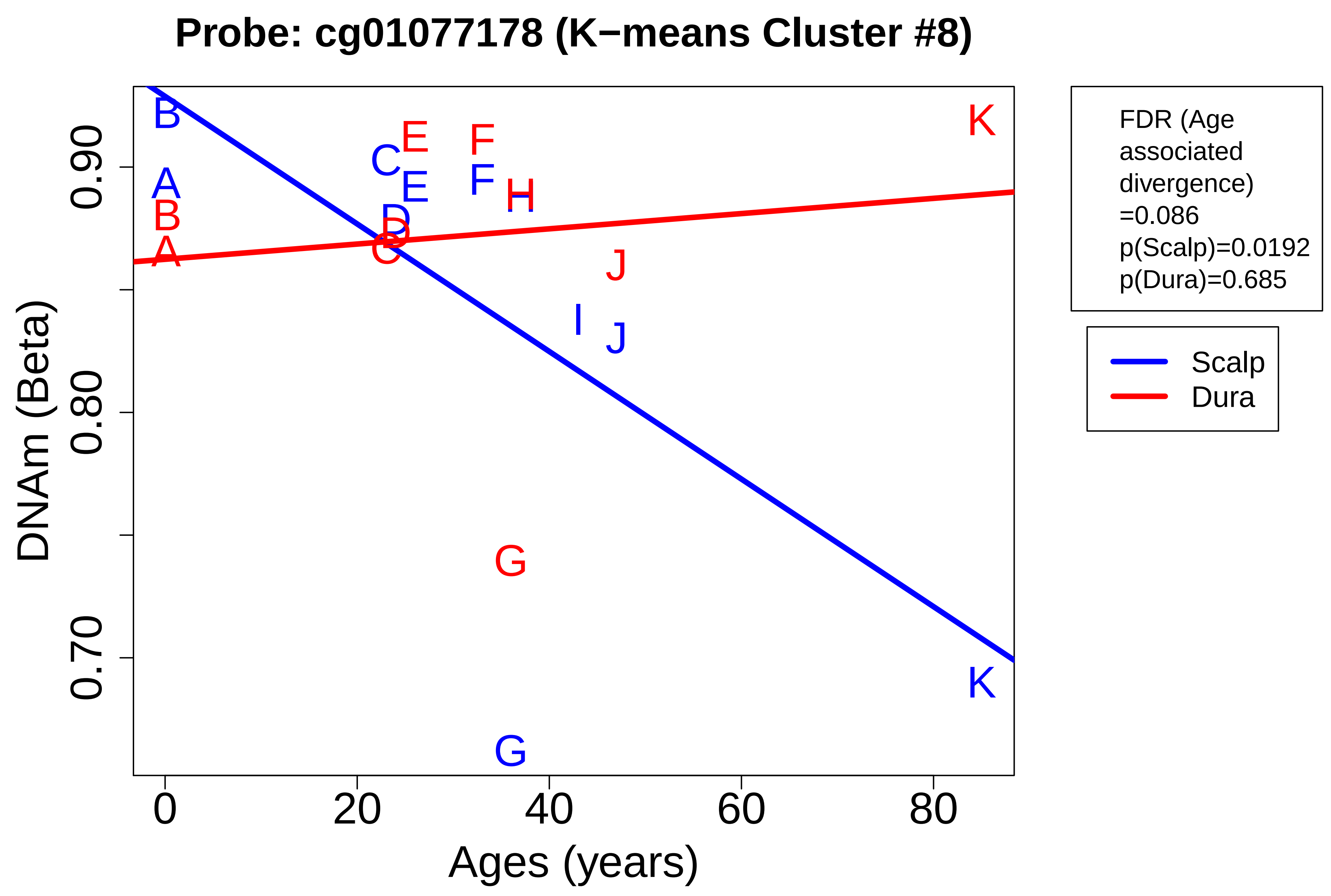

Probe: cg01077178 (K−means Cluster #8)
DNAm (Beta)
0.70
0.80
0.90
Ages (years)
0
20
40
60
80
FDR (Age associated divergence) =0.086
p(Scalp)=0.0192
p(Dura)=0.685
Scalp
Dura

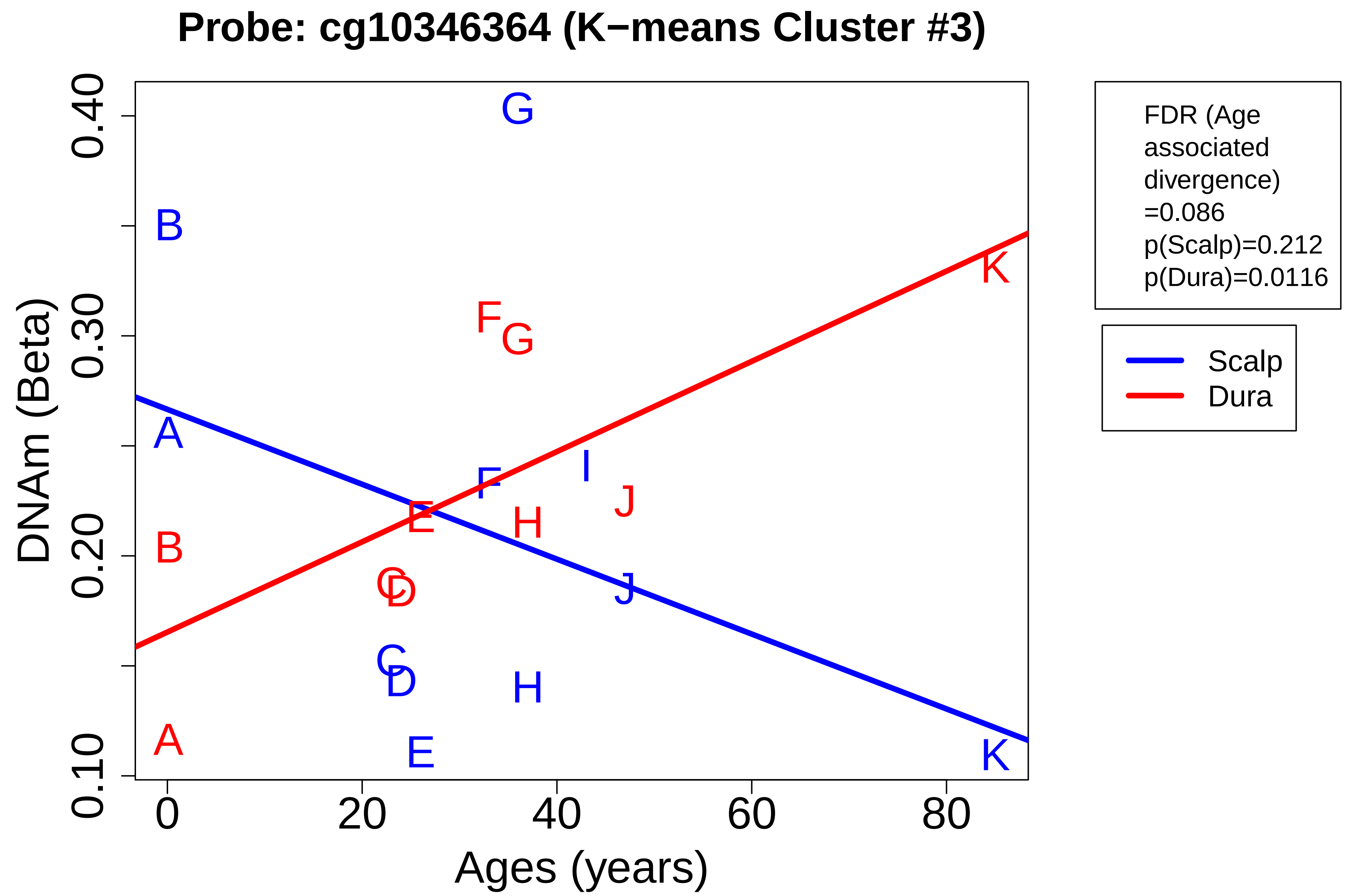
Probe: cg10346364 (K−means Cluster #3)
DNAm (Beta)
0.10
0.20
0.30
0.40
0
20
40
60
80
Ages (years)
FDR (Age associated divergence) =0.086
p(Scalp)=0.212
p(Dura)=0.0116
Scalp
Dura
A
B
C
D
E
F
G
H
I
J
K

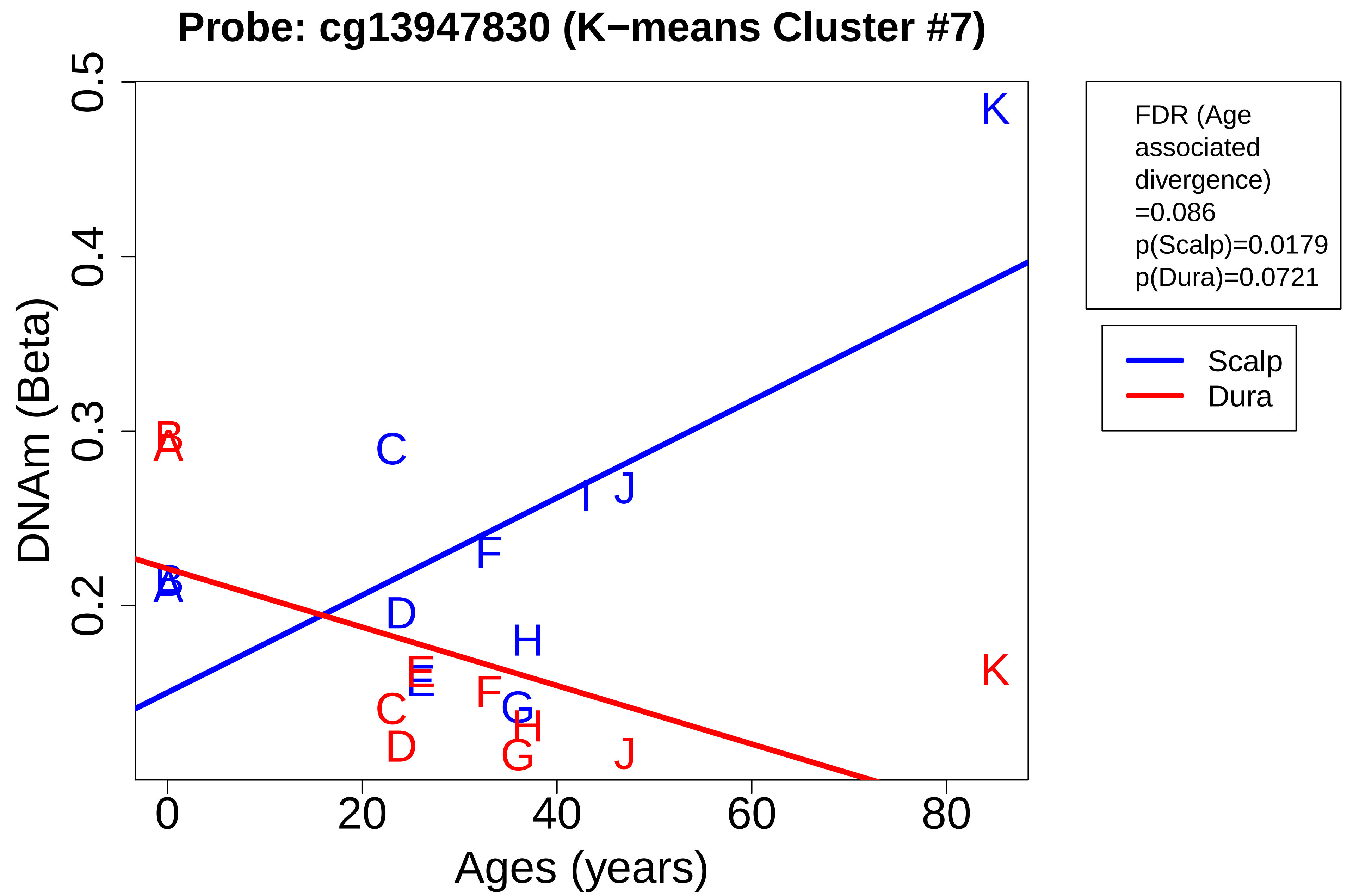

Probe: cg13947830 (K−means Cluster #7)
DNAm (Beta)
Ages (years)
0.2
0.3
0.4
0.5
0
20
40
60
80
FDR (Age associated divergence) =0.086
p(Scalp)=0.0179
p(Dura)=0.0721
Scalp
Dura
A
B
C
D
E
F
G
H
I
J
K

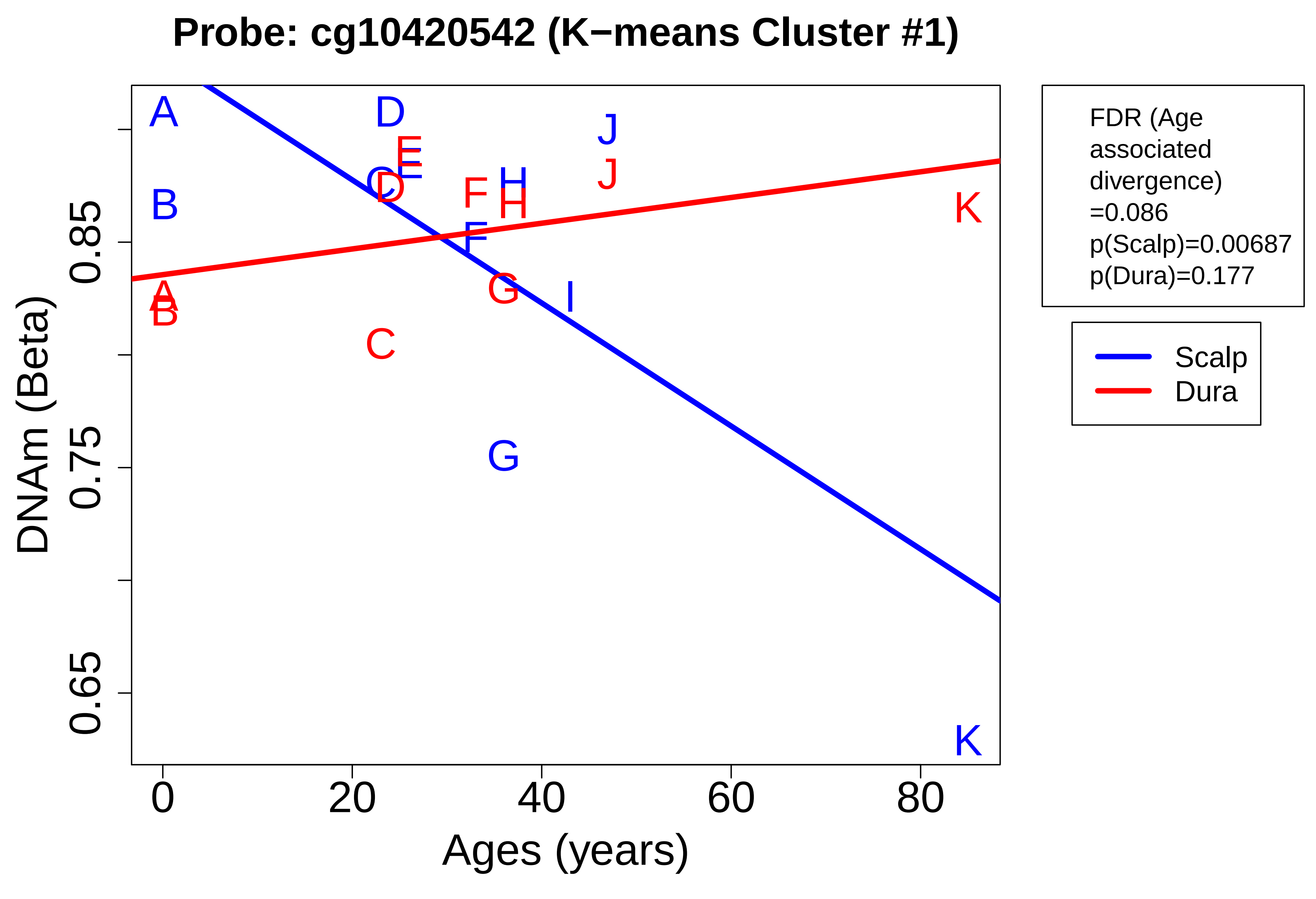

Probe: cg10420542 (K−means Cluster #1)
FDR (Age associated divergence) =0.086
p(Scalp)=0.00687
p(Dura)=0.177
Scalp
Dura
DNAm (Beta)
0.65
0.75
0.85
Ages (years)
0
20
40
60
80
A
B
C
D
E
F
G
H
I
J
K

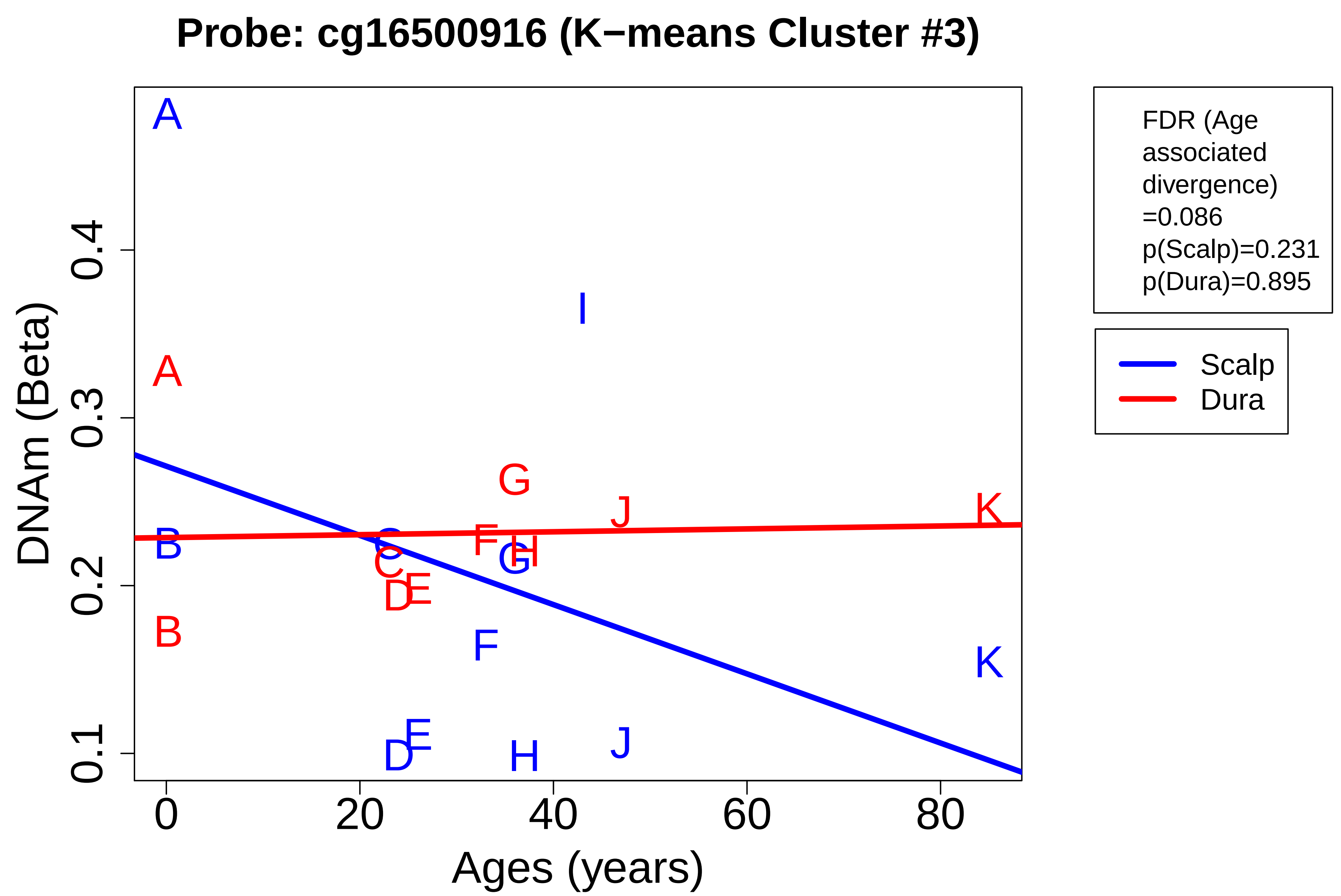

Probe: cg16500916 (K−means Cluster #3)
DNAm (Beta)
Ages (years)
0.1
0.2
0.3
0.4
0
20
40
60
80
A
B
C
D
E
F
G
H
I
J
K
FDR (Age associated divergence) =0.086
p(Scalp)=0.231
p(Dura)=0.895
Scalp
Dura

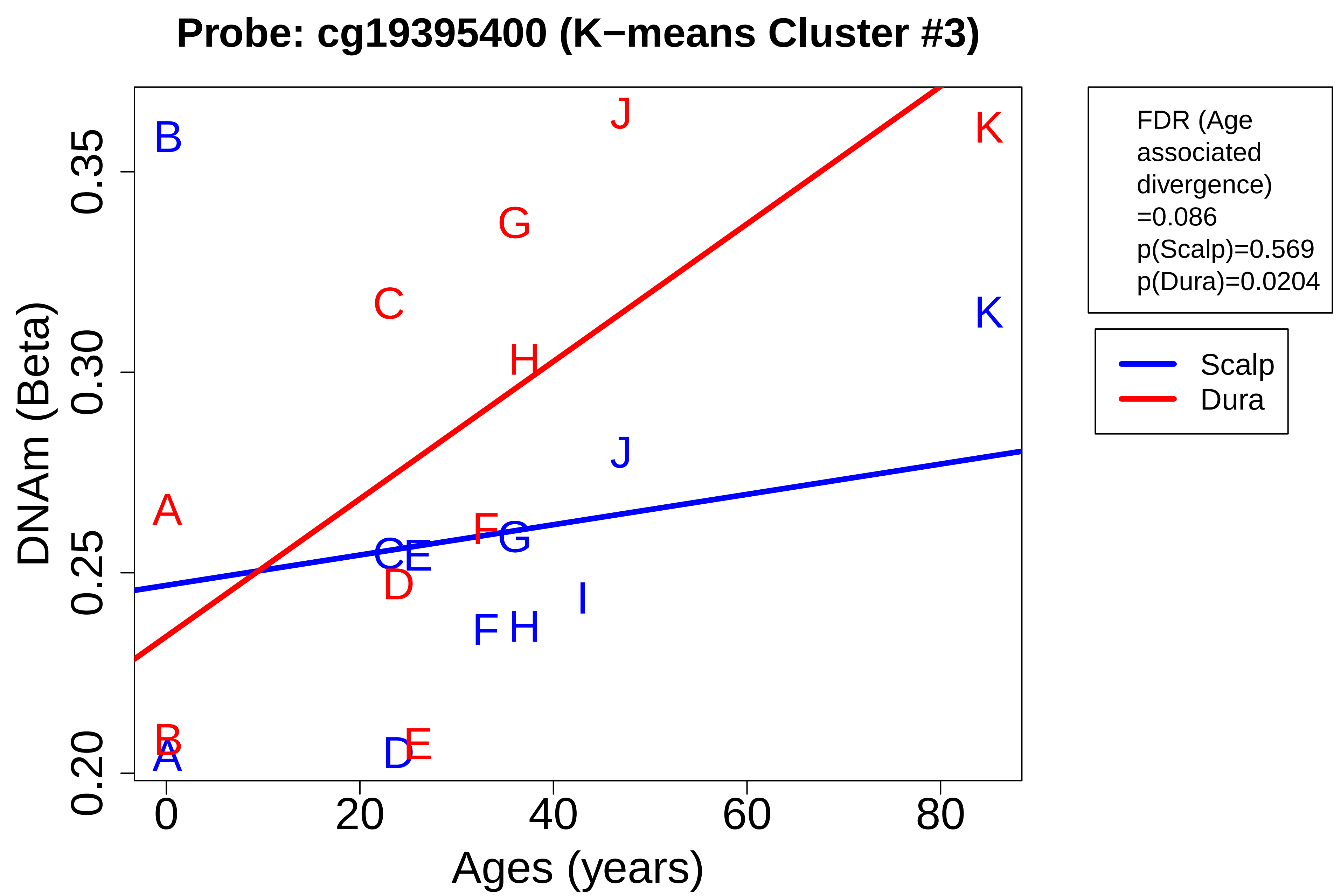

Probe: cg19395400 (K−means Cluster #3)
DNAm (Beta)
0.20
0.25
0.30
0.35
Ages (years)
0
20
40
60
80
FDR (Age associated divergence) =0.086
p(Scalp)=0.569
p(Dura)=0.0204
Scalp
Dura

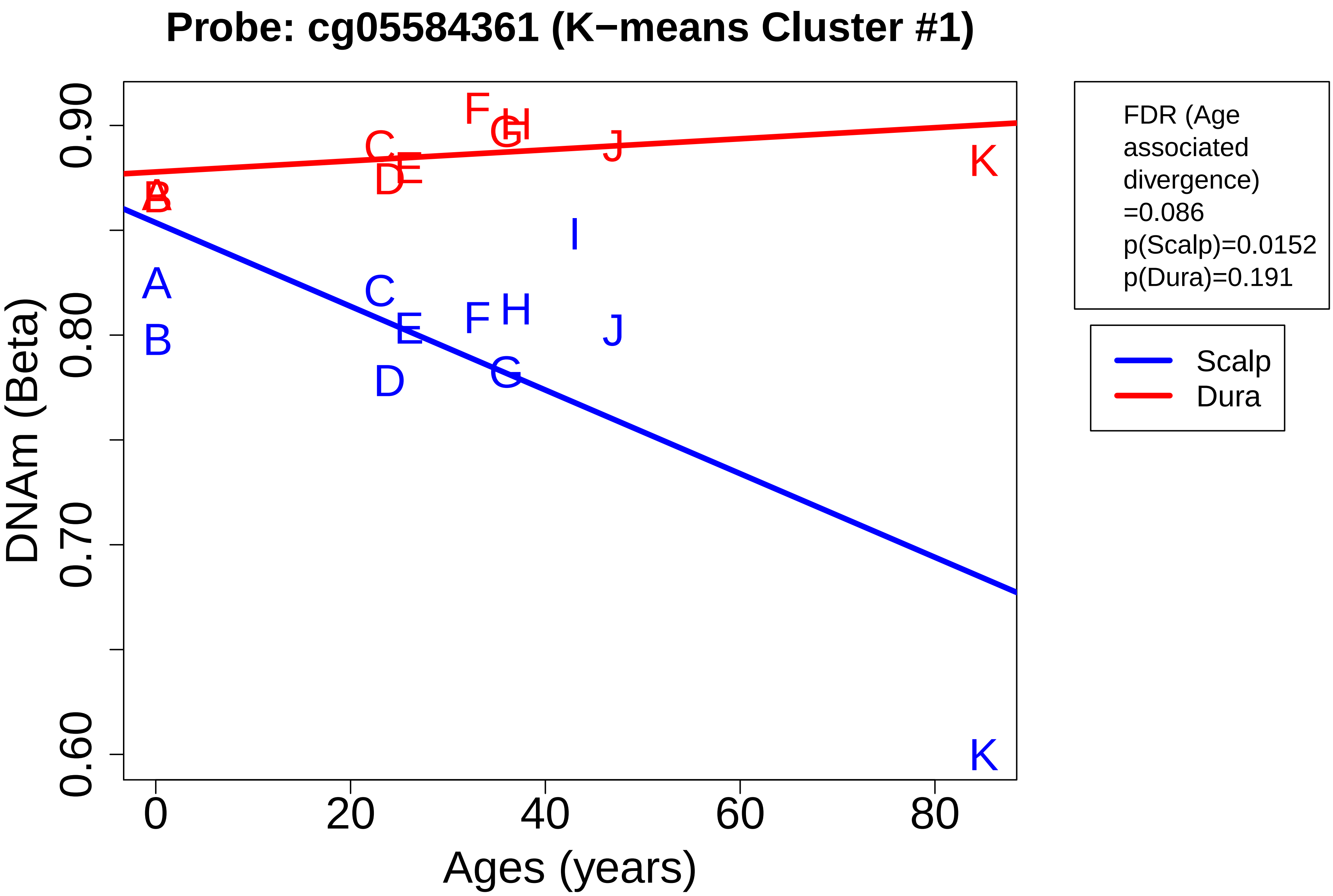

Probe: cg05584361 (K−means Cluster #1)
DNAm (Beta)
0.60
0.70
0.80
0.90
0
20
40
60
80
Ages (years)
FDR (Age associated divergence) =0.086
p(Scalp)=0.0152
p(Dura)=0.191
Scalp
Dura

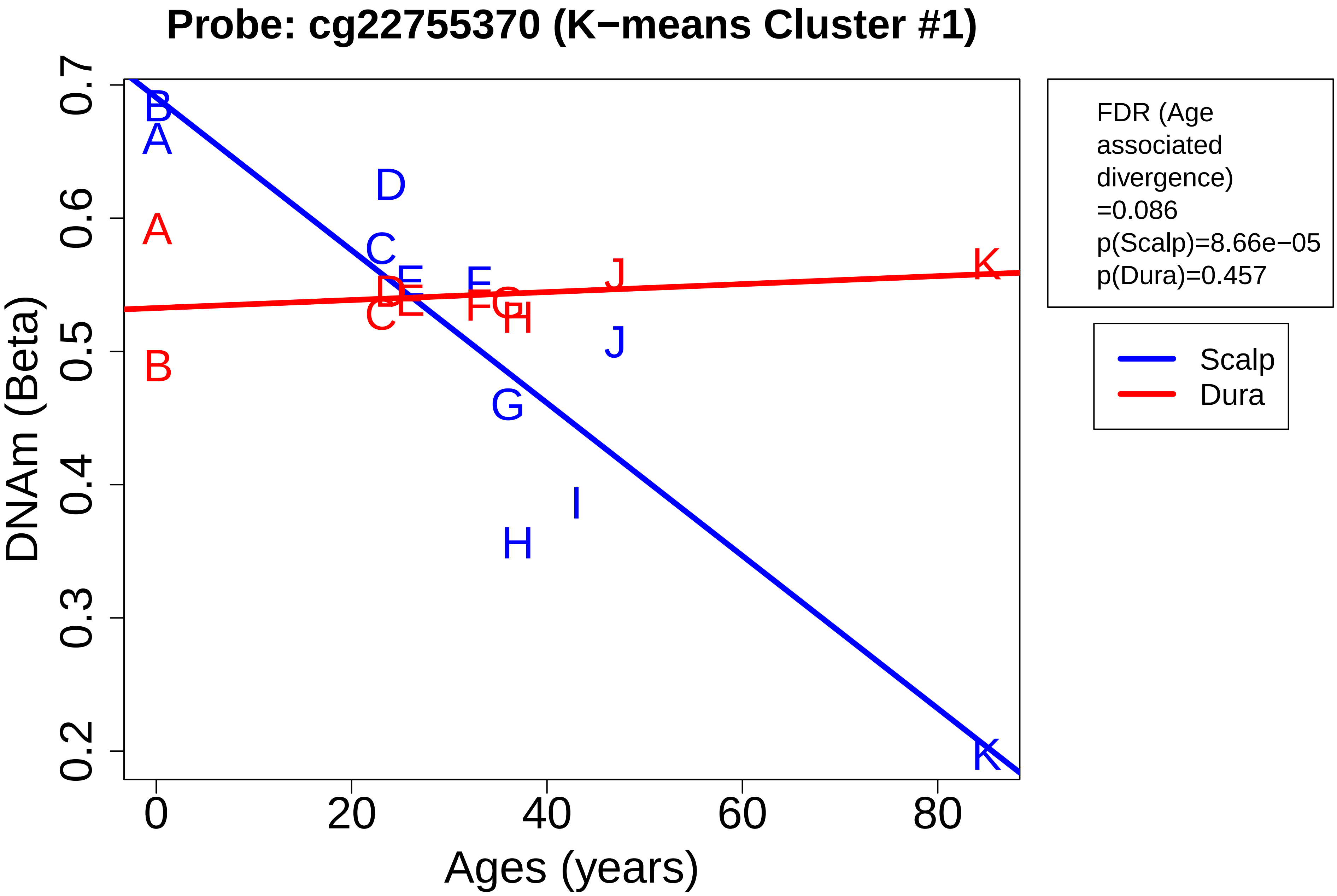

Probe: cg22755370 (K−means Cluster #1)
DNAm (Beta)
Ages (years)
0.2
0.3
0.4
0.5
0.6
0.7
0
20
40
60
80
FDR (Age associated divergence) =0.086
p(Scalp)=8.66e−05
p(Dura)=0.457
Scalp
Dura

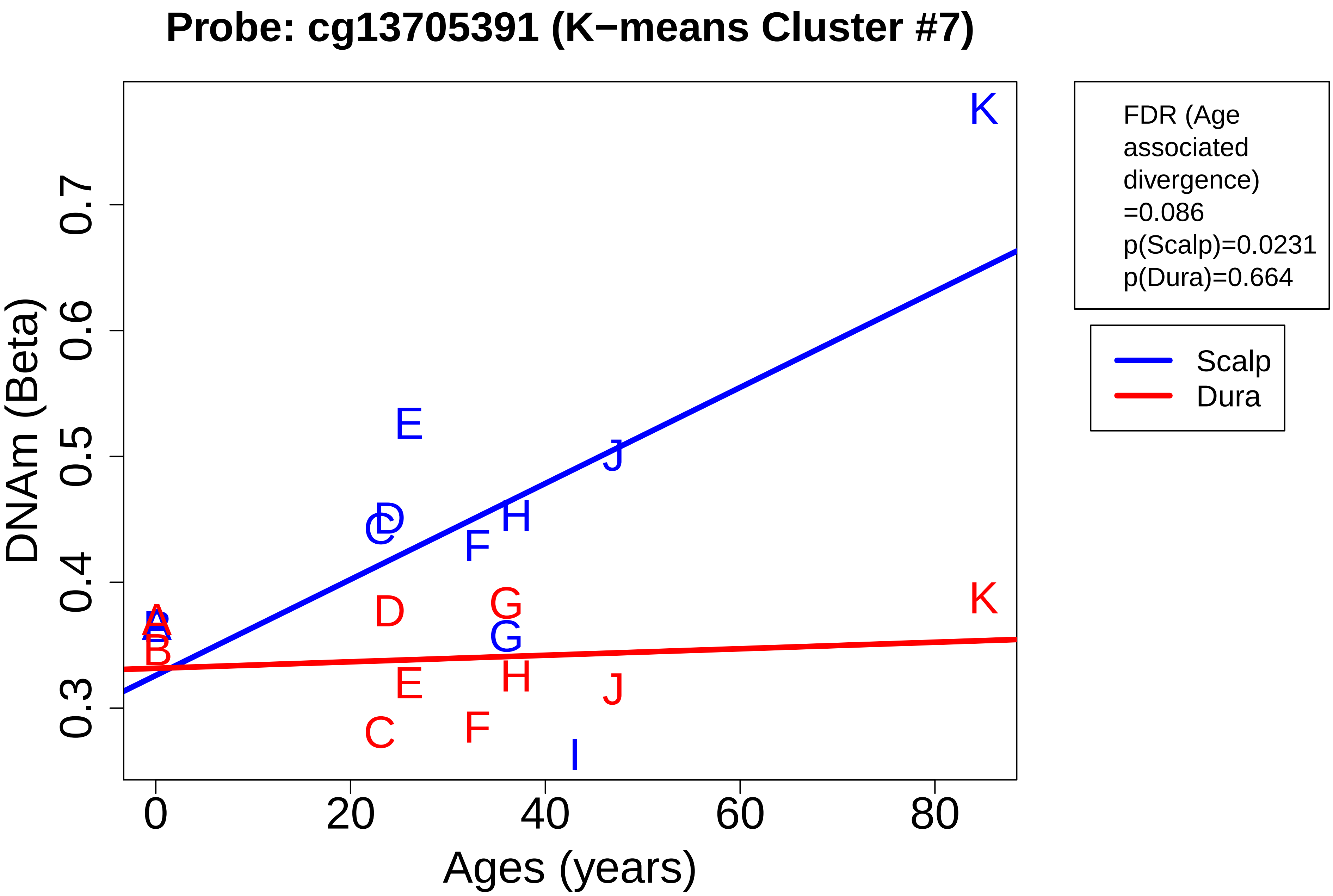

Probe: cg13705391 (K−means Cluster #7)
DNAm (Beta)
0.3
0.4
0.5
0.6
0.7
0
20
40
60
80
Ages (years)
FDR (Age associated divergence) =0.086
p(Scalp)=0.0231
p(Dura)=0.664
Scalp
Dura

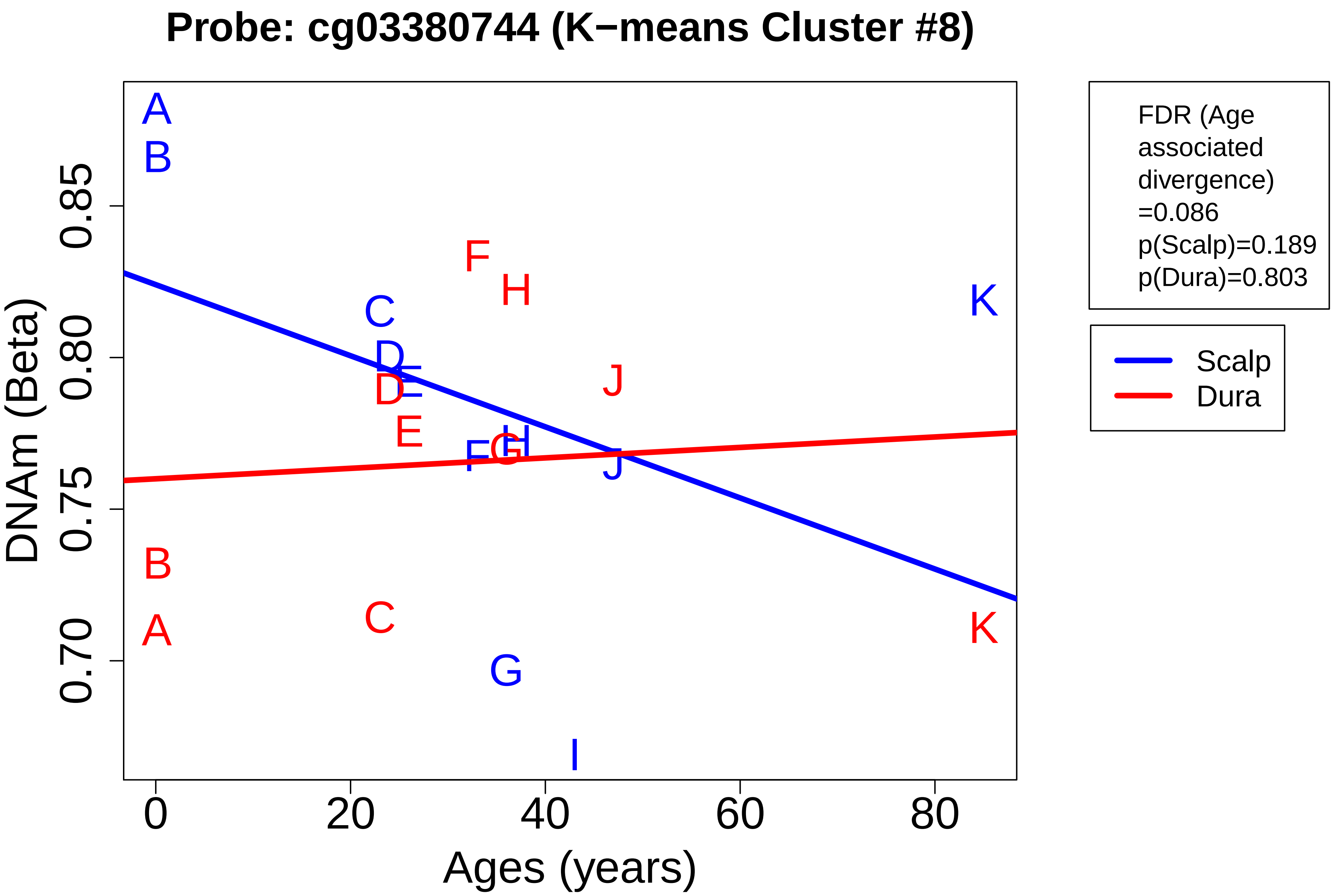
Probe: cg03380744 (K−means Cluster #8)
DNAm (Beta)
0.70
0.75
0.80
0.85
Ages (years)
0
20
40
60
80
FDR (Age associated divergence) =0.086
p(Scalp)=0.189
p(Dura)=0.803
Scalp
Dura
A
B
C
D
E
F
G
H
I
J
K

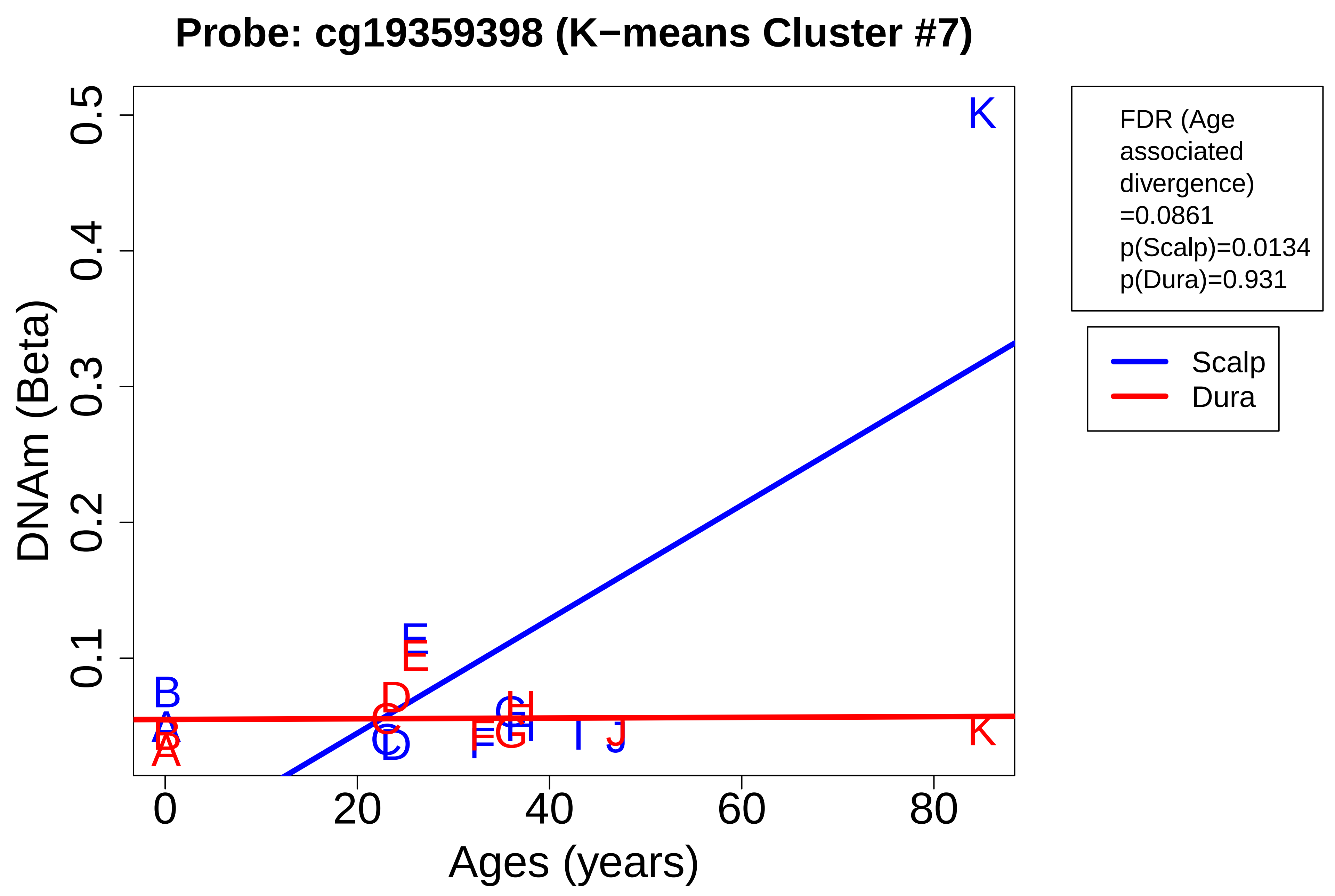

Probe: cg19359398 (K−means Cluster #7)
DNAm (Beta)
0.1
0.2
0.3
0.4
0.5
0
20
40
60
80
Ages (years)
FDR (Age associated divergence) =0.0861
p(Scalp)=0.0134
p(Dura)=0.931
Scalp
Dura

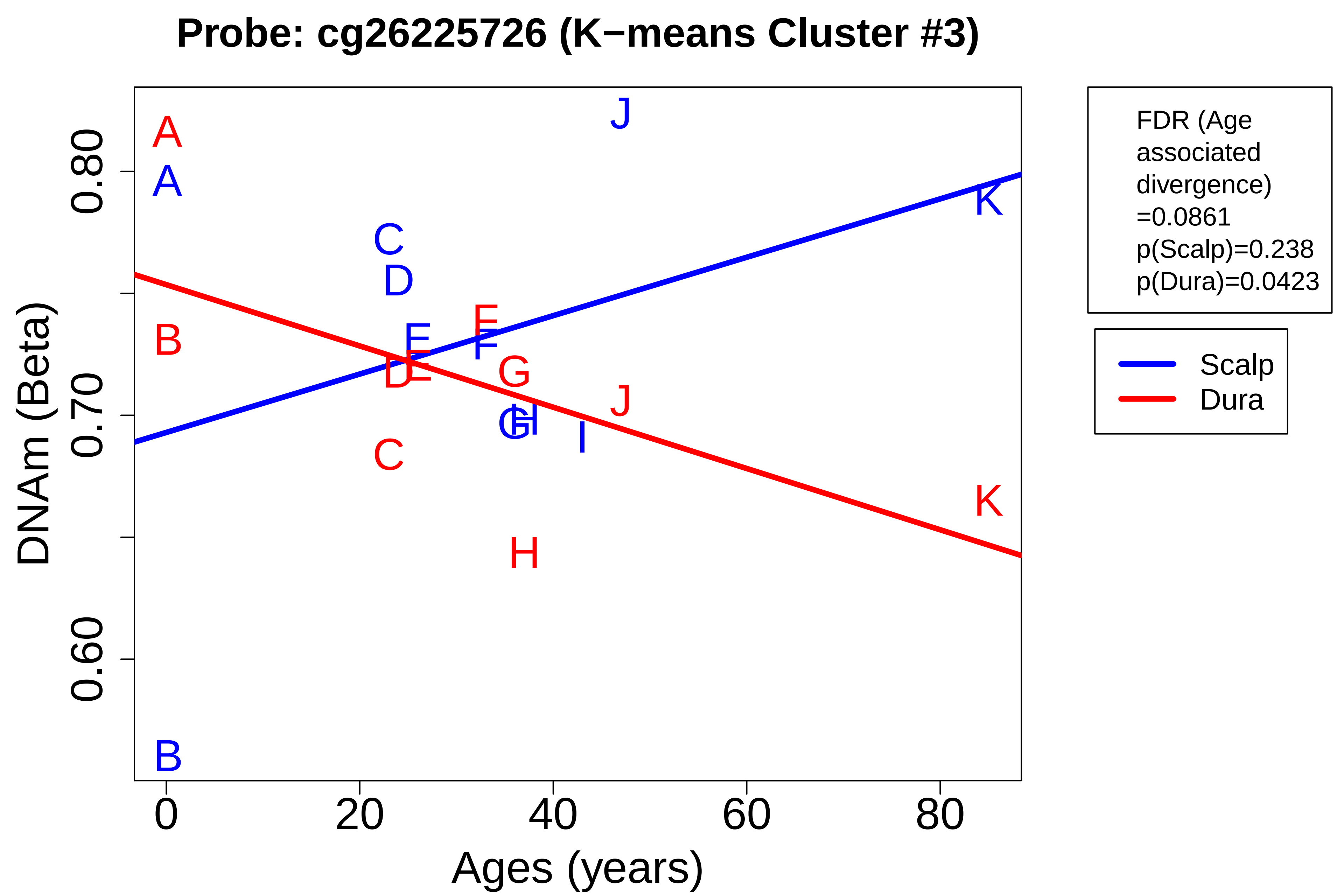
Probe: cg26225726 (K−means Cluster #3)
DNAm (Beta)
0.60
0.70
0.80
Ages (years)
0
20
40
60
80
FDR (Age associated divergence) =0.0861
p(Scalp)=0.238
p(Dura)=0.0423
Scalp
Dura
A
B
C
D
E
F
G
H
I
J
K

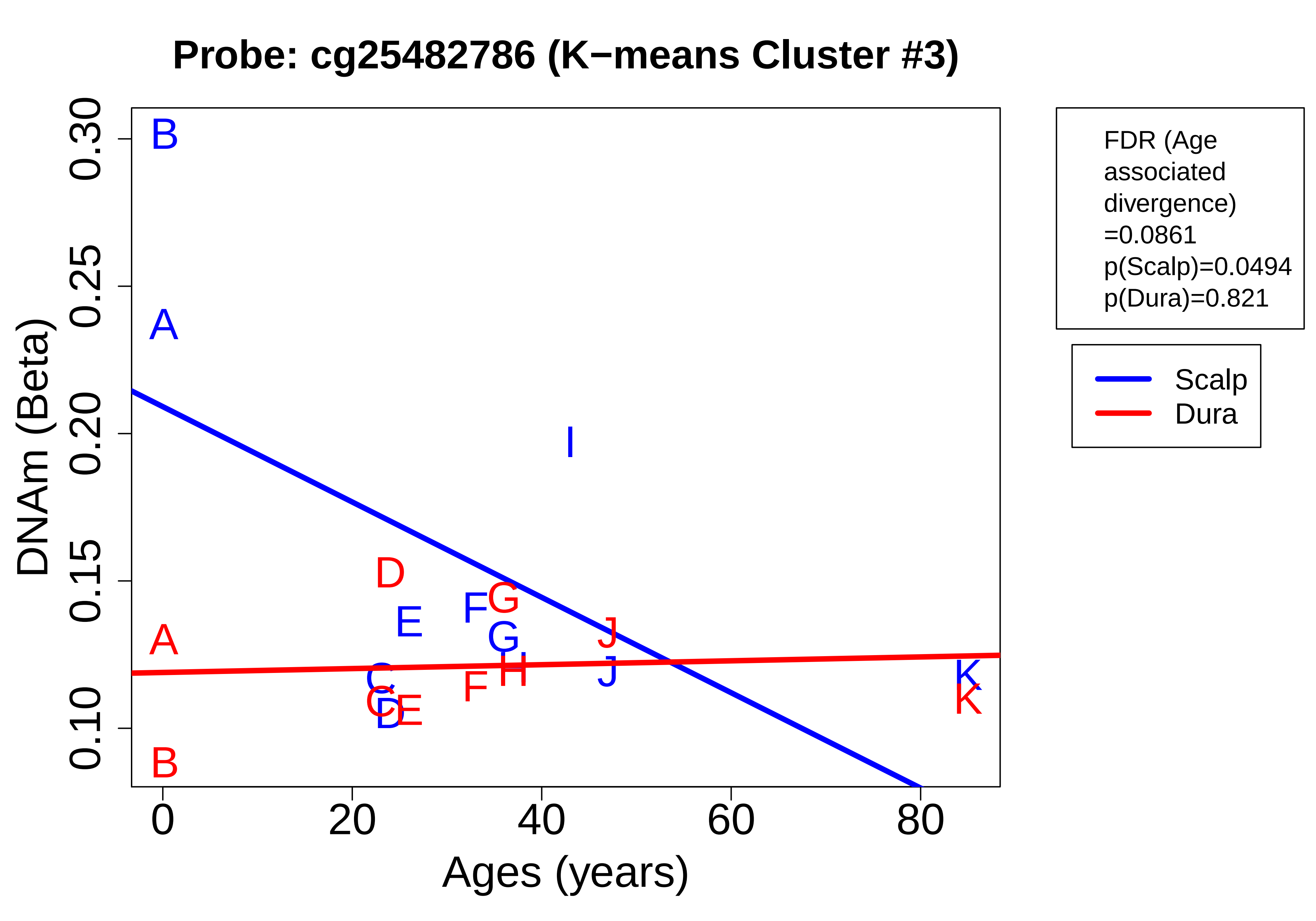
Probe: cg25482786 (K−means Cluster #3)
DNAm (Beta)
0.10
0.15
0.20
0.25
0.30
Ages (years)
0
20
40
60
80
FDR (Age associated divergence) =0.0861
p(Scalp)=0.0494
p(Dura)=0.821
Scalp
Dura

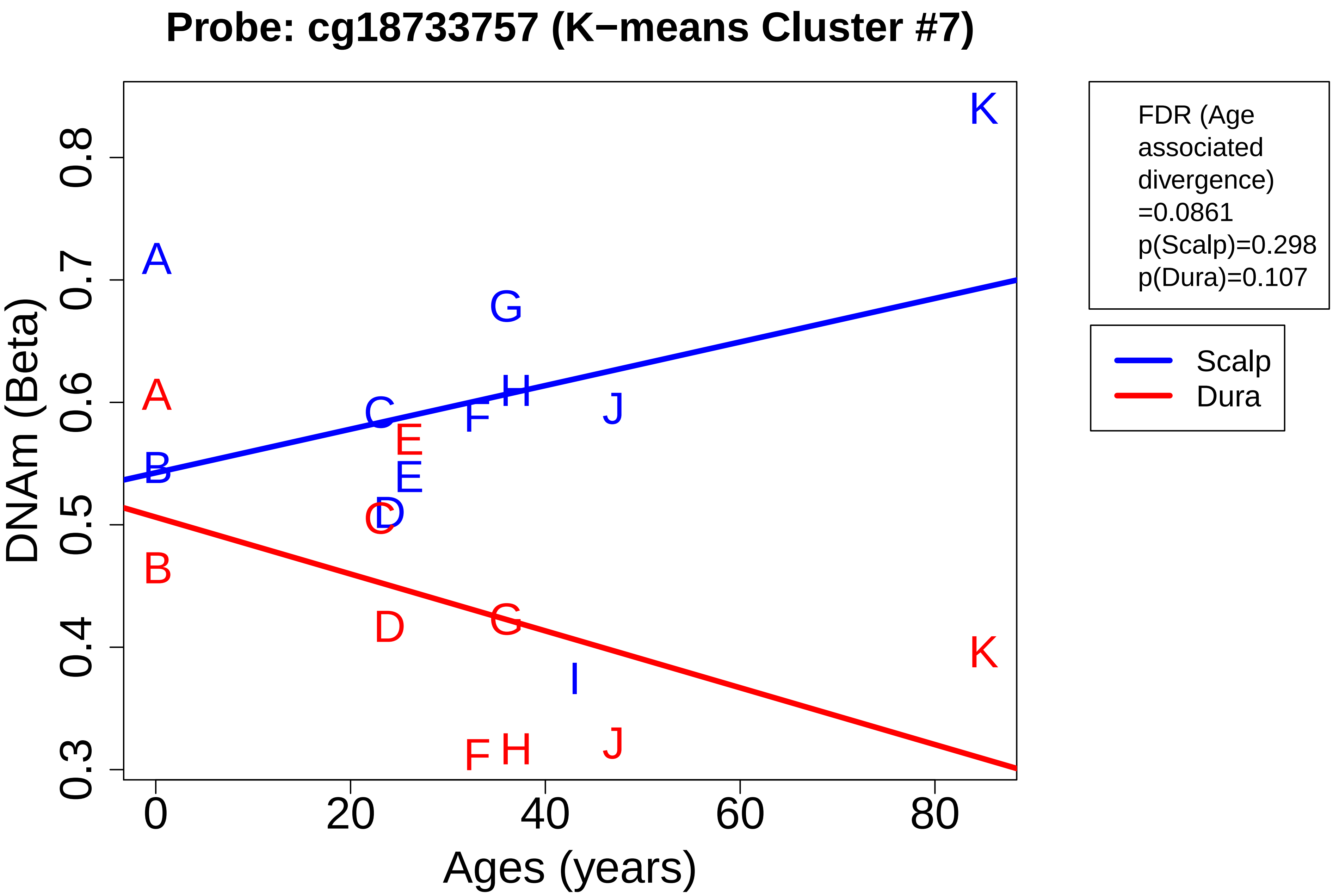

Probe: cg18733757 (K−means Cluster #7)
DNAm (Beta)
0.3
0.4
0.5
0.6
0.7
0.8
0
20
40
60
80
Ages (years)
A
B
C
D
E
F
G
H
I
J
K
FDR (Age associated divergence) =0.0861
p(Scalp)=0.298
p(Dura)=0.107
Scalp
Dura

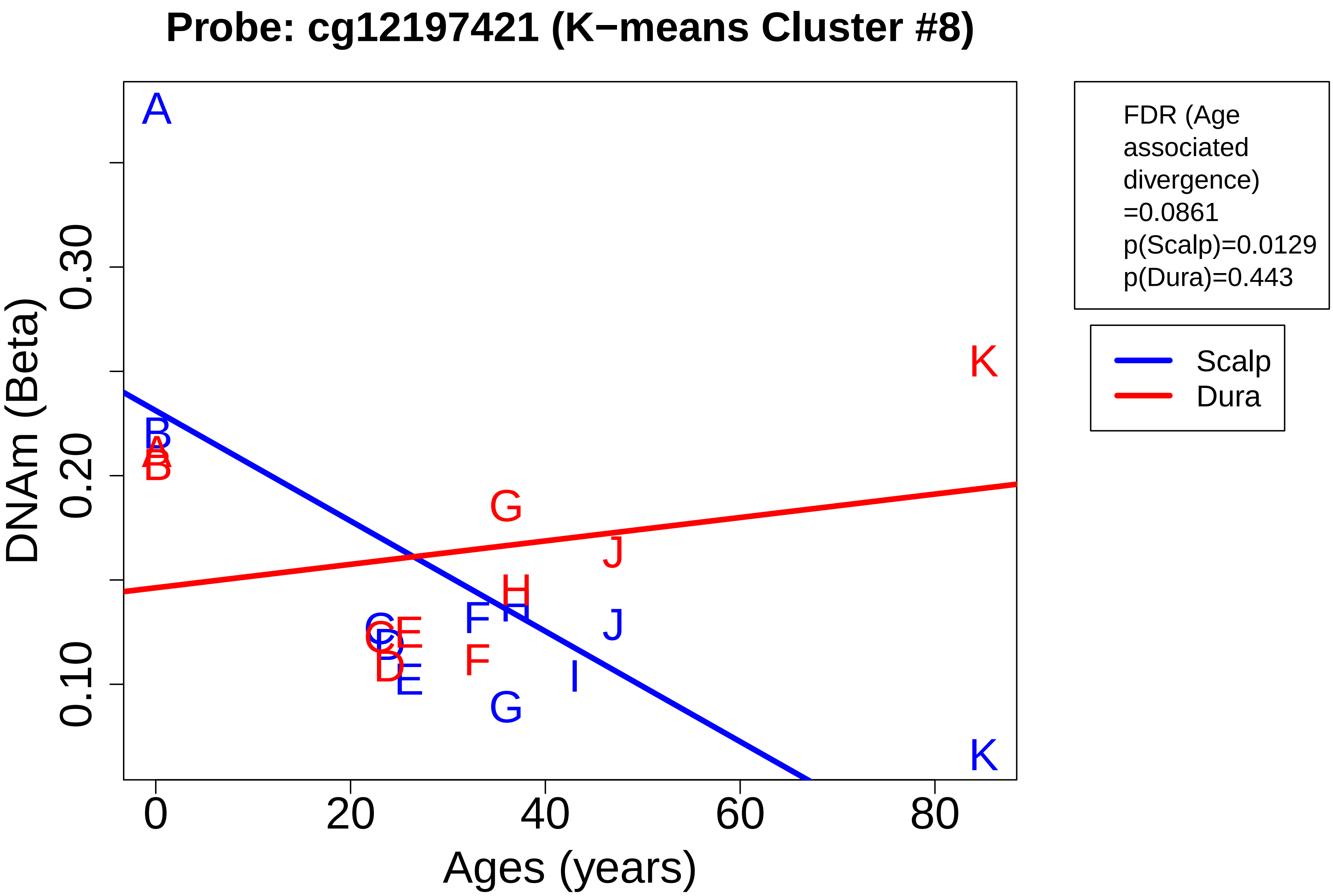

Probe: cg12197421 (K−means Cluster #8)
FDR (Age associated divergence) =0.0861
p(Scalp)=0.0129
p(Dura)=0.443
Scalp
Dura
DNAm (Beta)
0.10
0.20
0.30
0
20
40
60
80
Ages (years)
A
B
C
D
E
F
G
H
I
J
K

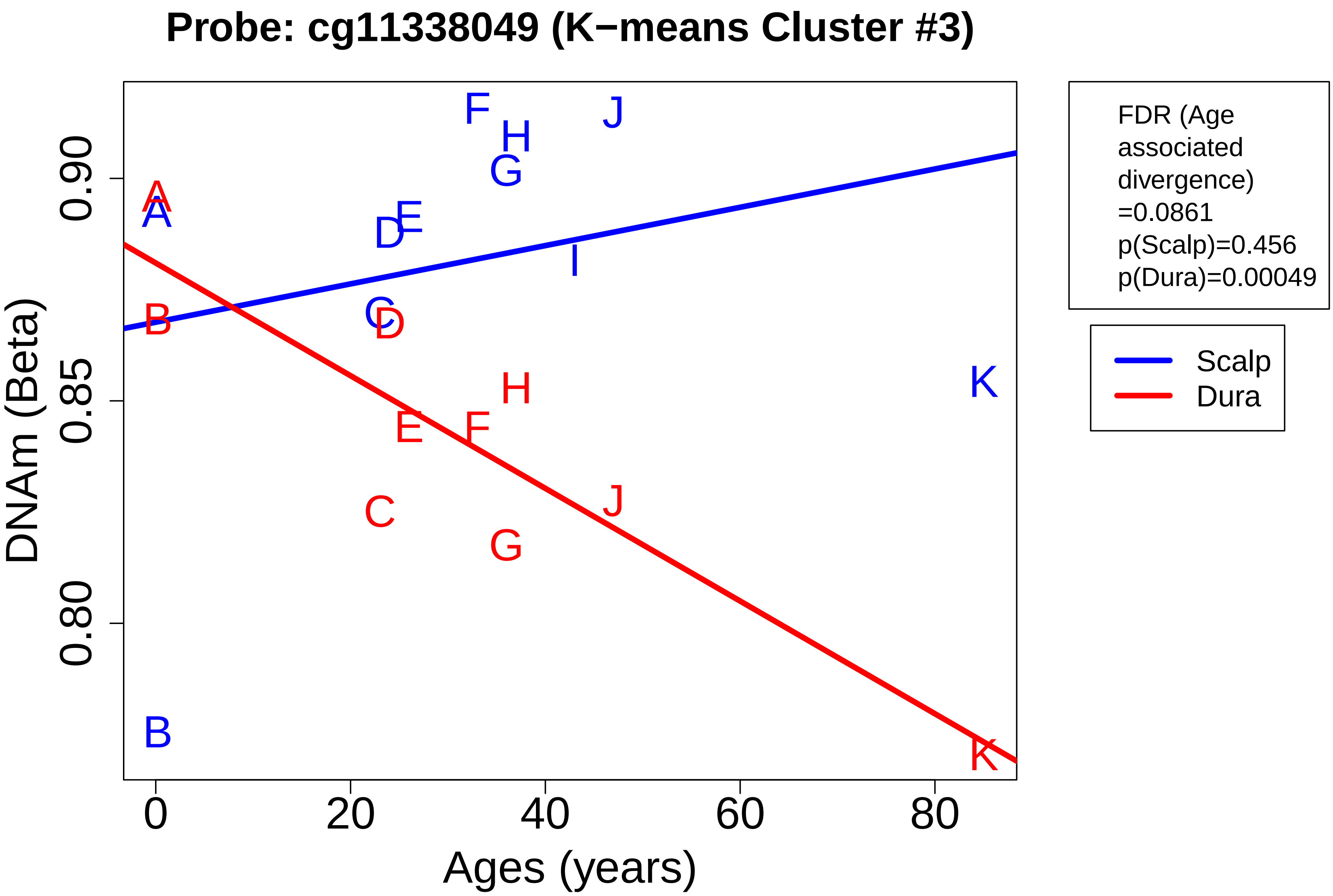

Probe: cg11338049 (K−means Cluster #3)
DNAm (Beta)
0.80
0.85
0.90
Ages (years)
0
20
40
60
80
A
B
C
D
E
F
G
H
I
J
K
FDR (Age associated divergence) =0.0861
p(Scalp)=0.456
p(Dura)=0.00049
Scalp
Dura

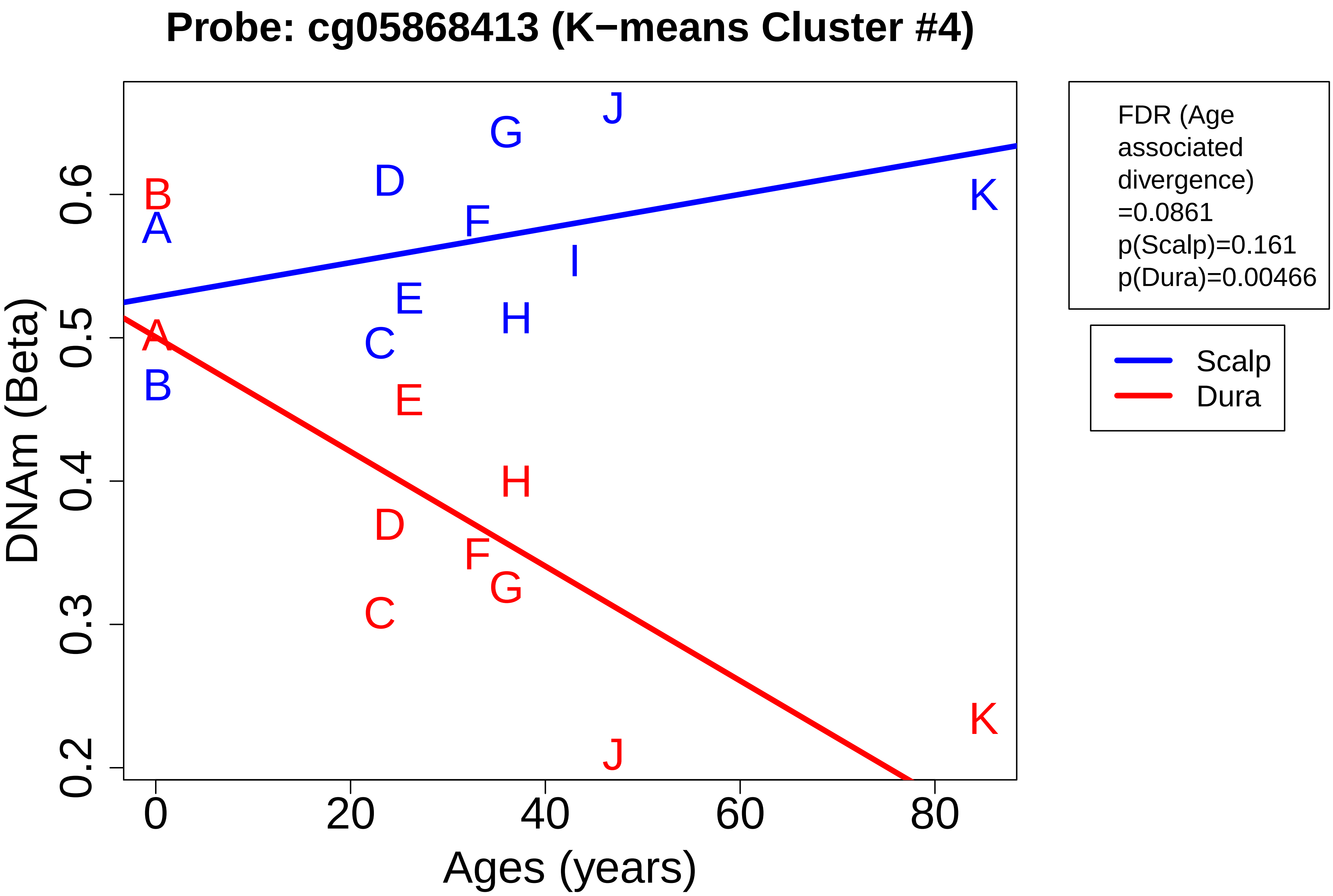
Probe: cg05868413 (K−means Cluster #4)
DNAm (Beta)
0.2
0.3
0.4
0.5
0.6
Ages (years)
0
20
40
60
80
FDR (Age associated divergence) =0.0861
p(Scalp)=0.161
p(Dura)=0.00466
Scalp
Dura
A
B
C
D
E
F
G
H
I
J
K

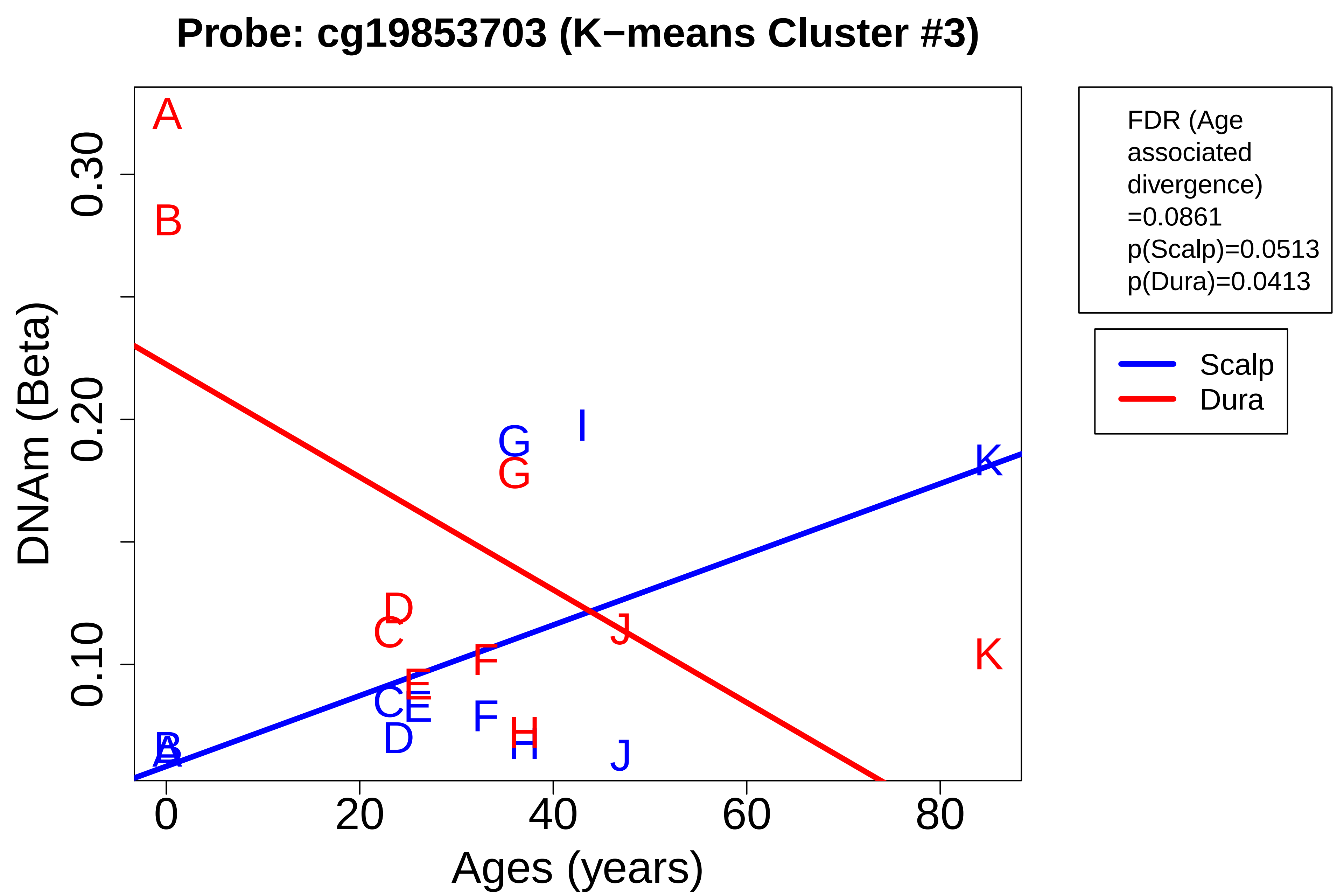

Probe: cg19853703 (K−means Cluster #3)
DNAm (Beta)
0.10
0.20
0.30
Ages (years)
0
20
40
60
80
FDR (Age associated divergence) =0.0861
p(Scalp)=0.0513
p(Dura)=0.0413
Scalp
Dura
A
B
C
D
E
F
G
H
I
J
K

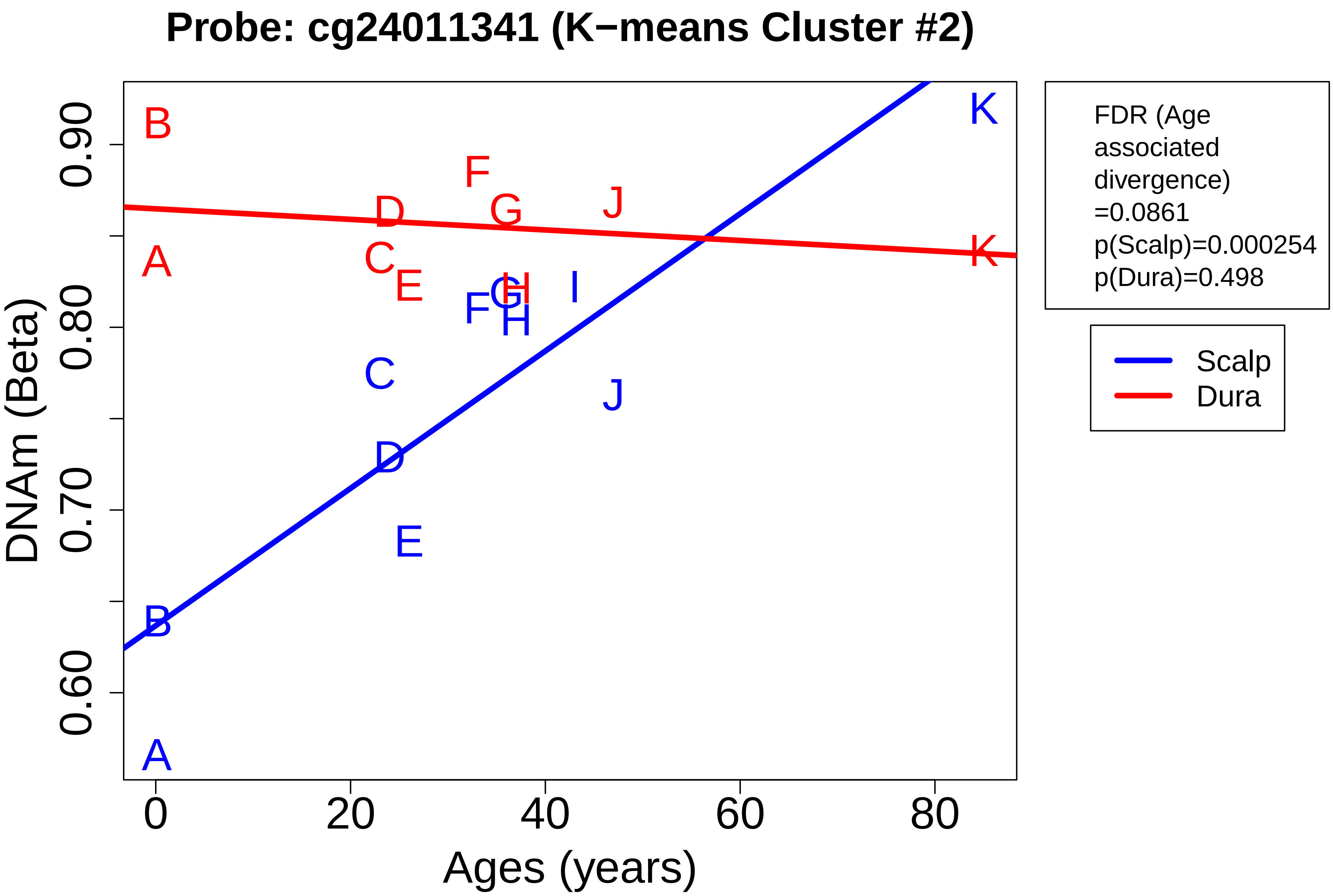

Probe: cg24011341 (K−means Cluster #2)
DNAm (Beta)
0.60
0.70
0.80
0.90
0
20
40
60
80
Ages (years)
A
B
C
D
E
F
G
H
I
J
K
FDR (Age associated divergence) =0.0861
p(Scalp)=0.000254
p(Dura)=0.498
Scalp
Dura

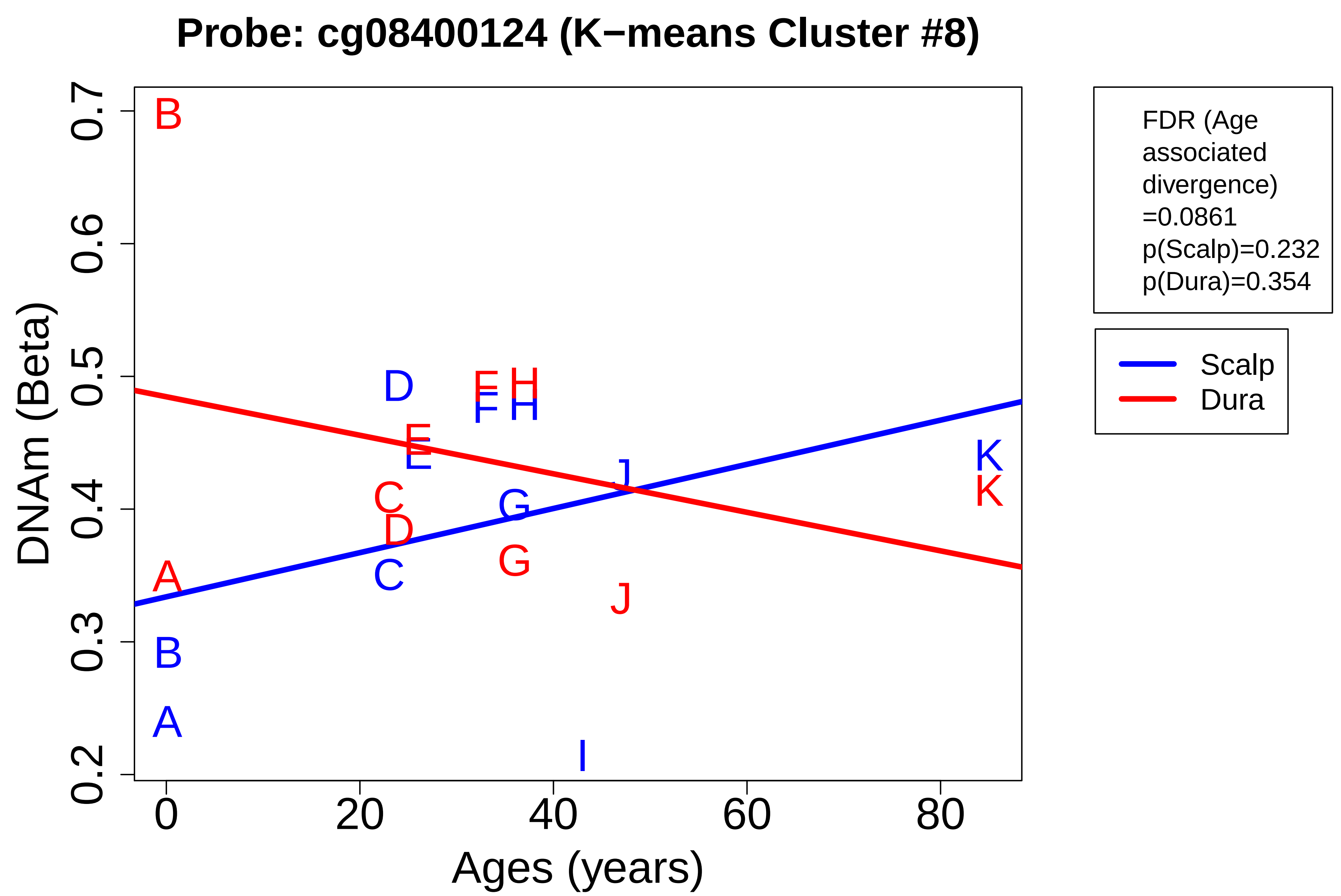

Probe: cg08400124 (K−means Cluster #8)
DNAm (Beta)
0.2
0.3
0.4
0.5
0.6
0.7
Ages (years)
0
20
40
60
80
FDR (Age associated divergence) =0.0861
p(Scalp)=0.232
p(Dura)=0.354
Scalp
Dura

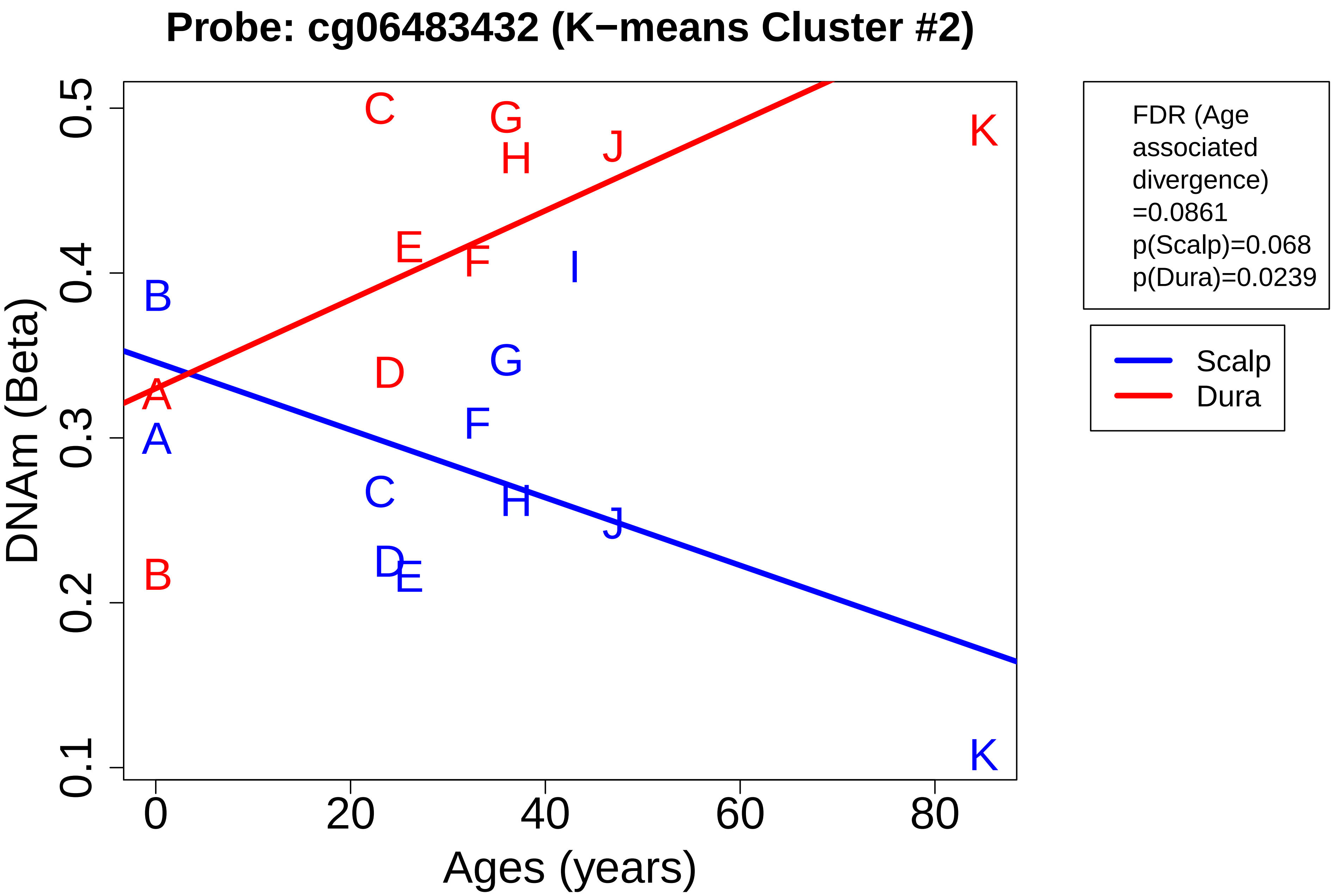

Probe: cg06483432 (K−means Cluster #2)
DNAm (Beta)
0.1
0.2
0.3
0.4
0.5
0
20
40
60
80
Ages (years)
FDR (Age associated divergence) =0.0861
p(Scalp)=0.068
p(Dura)=0.0239
Scalp
Dura
A
B
C
D
E
F
G
H
I
J
K

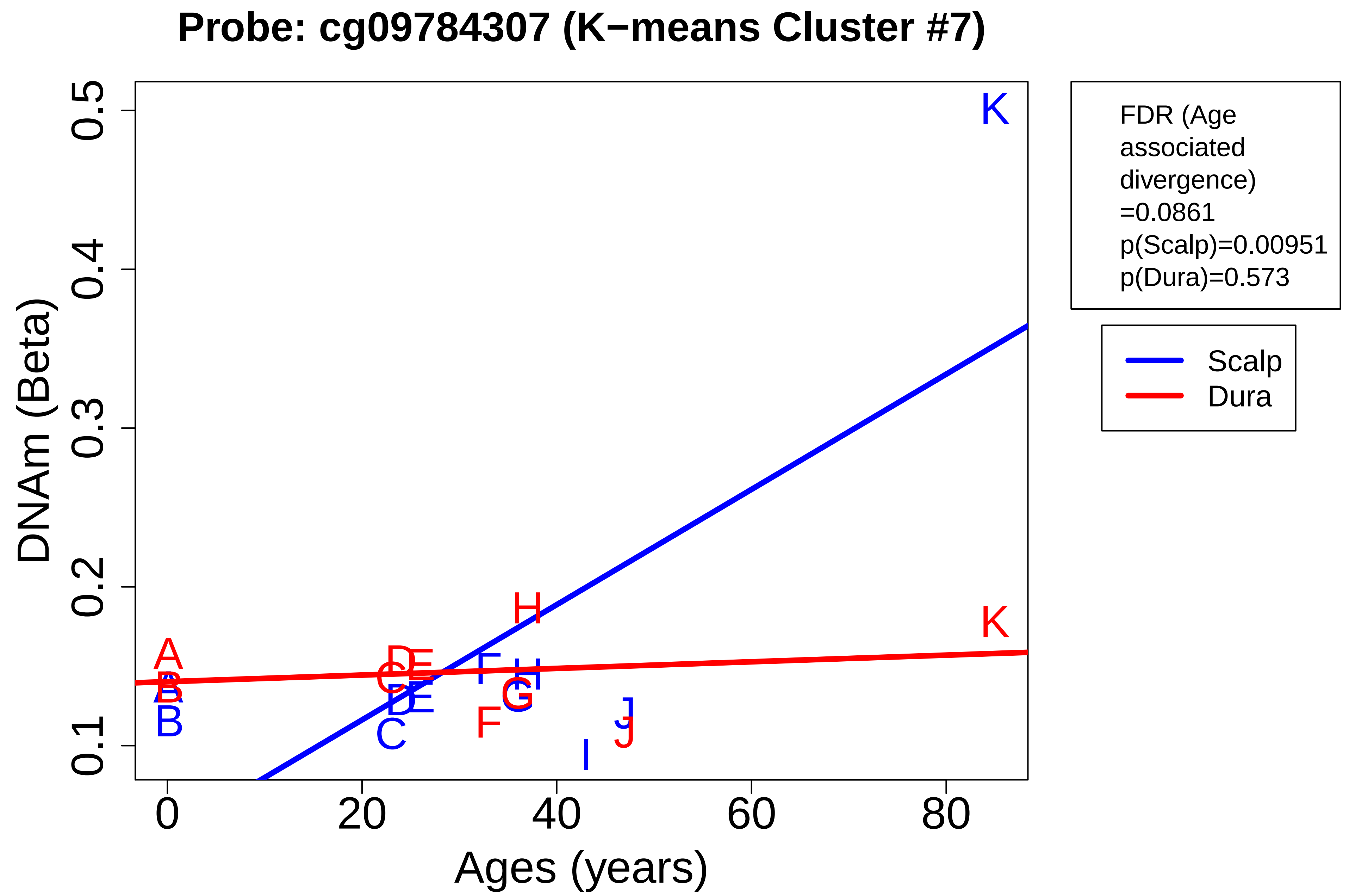

Probe: cg09784307 (K−means Cluster #7)
DNAm (Beta)
Ages (years)
0.1
0.2
0.3
0.4
0.5
0
20
40
60
80
FDR (Age associated divergence) =0.0861
p(Scalp)=0.00951
p(Dura)=0.573
Scalp
Dura

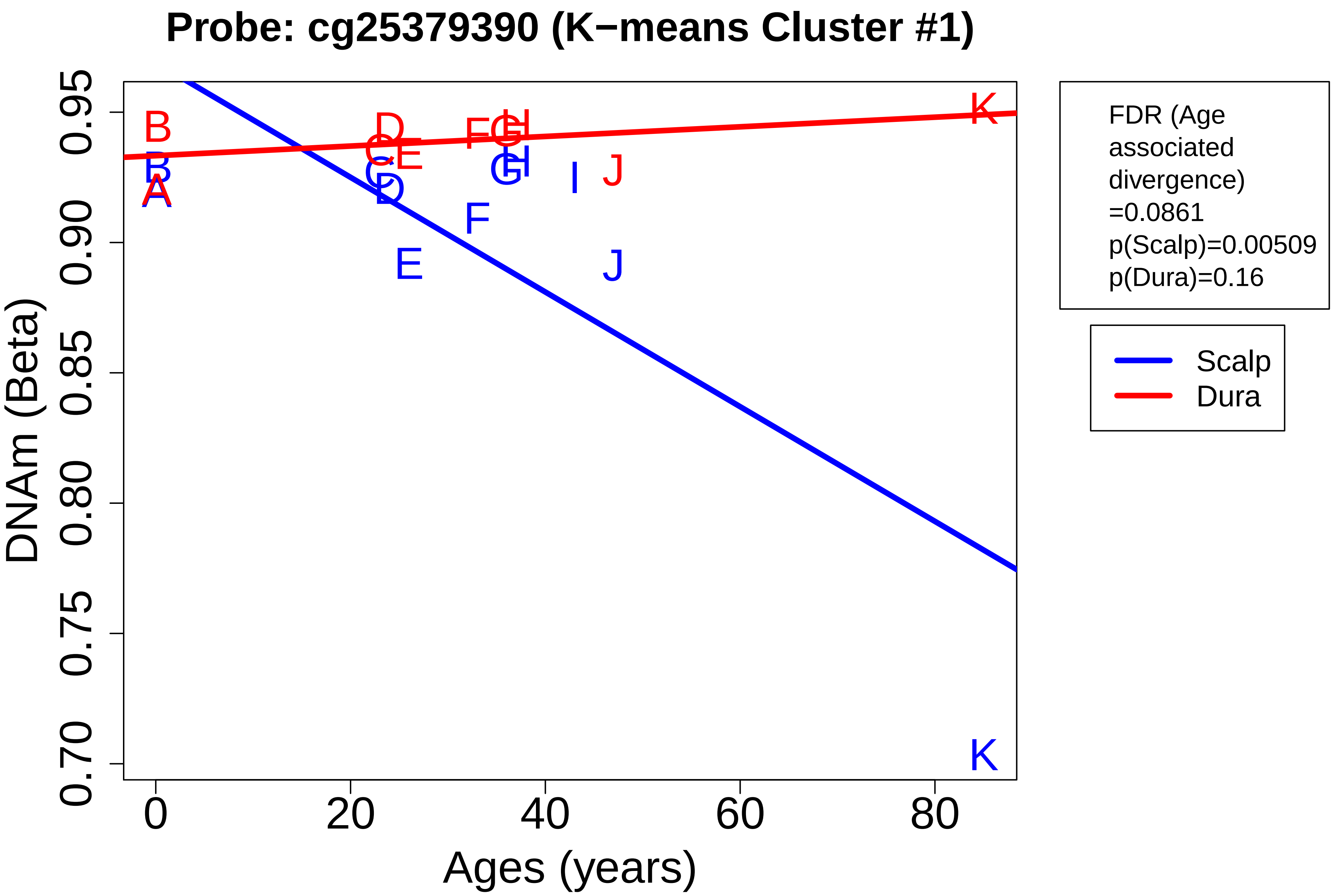

Probe: cg25379390 (K−means Cluster #1)
DNAm (Beta)
0.70
0.75
0.80
0.85
0.90
0.95
0
20
40
60
80
Ages (years)
FDR (Age associated divergence) =0.0861
p(Scalp)=0.00509
p(Dura)=0.16
Scalp
Dura

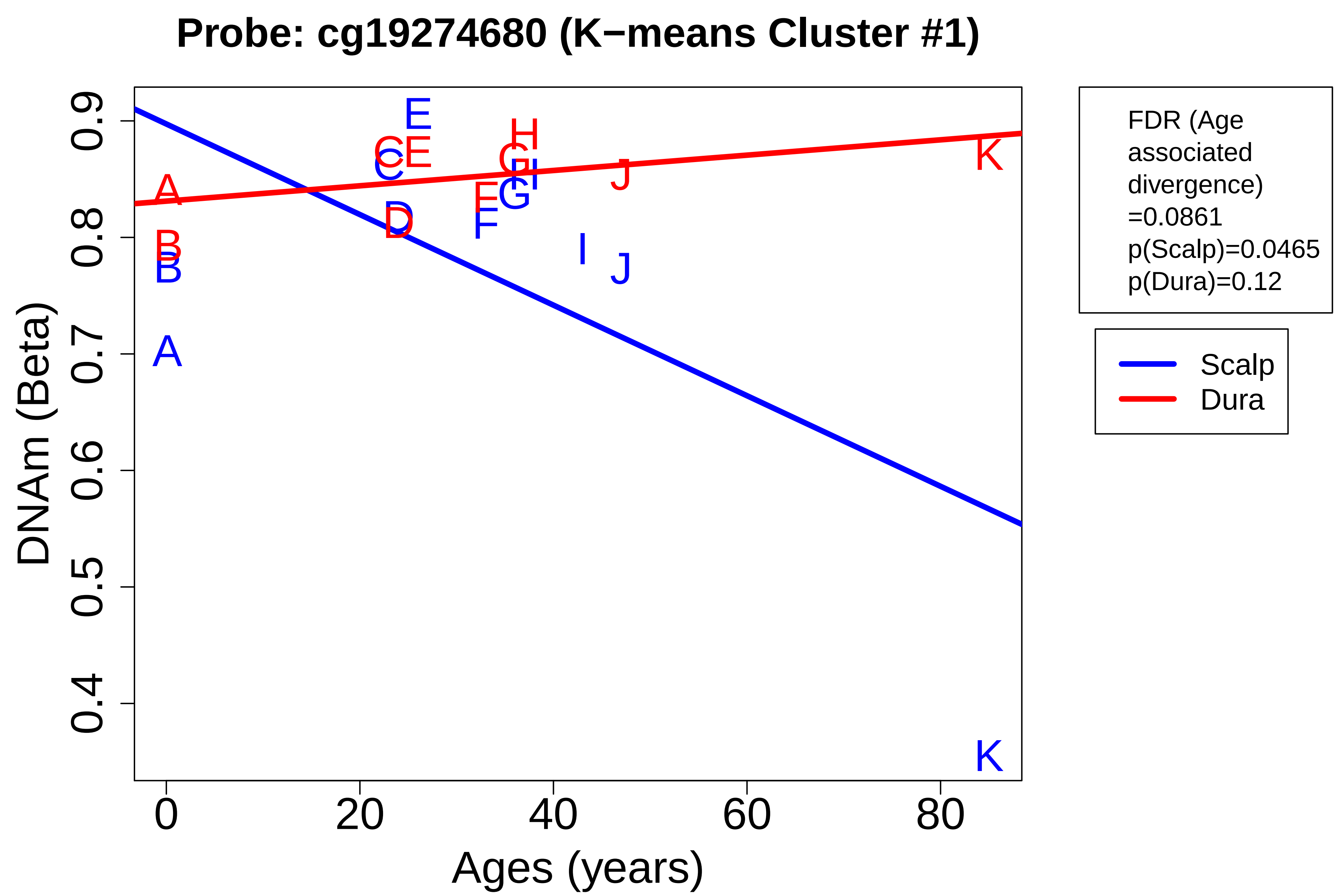

Probe: cg19274680 (K−means Cluster #1)
DNAm (Beta)
0.4
0.5
0.6
0.7
0.8
0.9
0
20
40
60
80
Ages (years)
A
B
C
D
E
F
G
H
I
J
K
FDR (Age associated divergence) =0.0861
p(Scalp)=0.0465
p(Dura)=0.12
Scalp
Dura

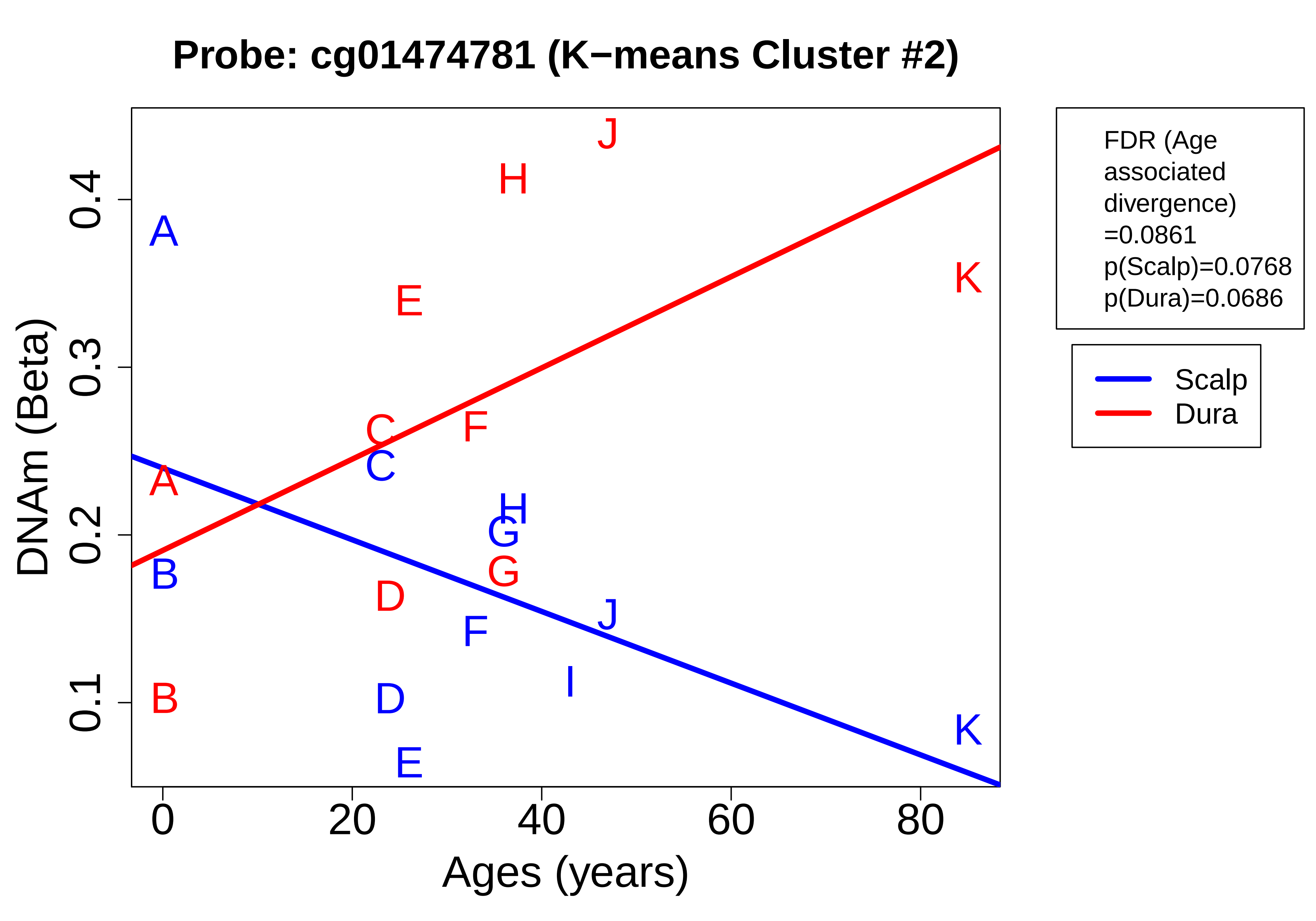

Probe: cg01474781 (K−means Cluster #2)
DNAm (Beta)
0.1
0.2
0.3
0.4
0
20
40
60
80
Ages (years)
FDR (Age associated divergence) =0.0861
p(Scalp)=0.0768
p(Dura)=0.0686
Scalp
Dura

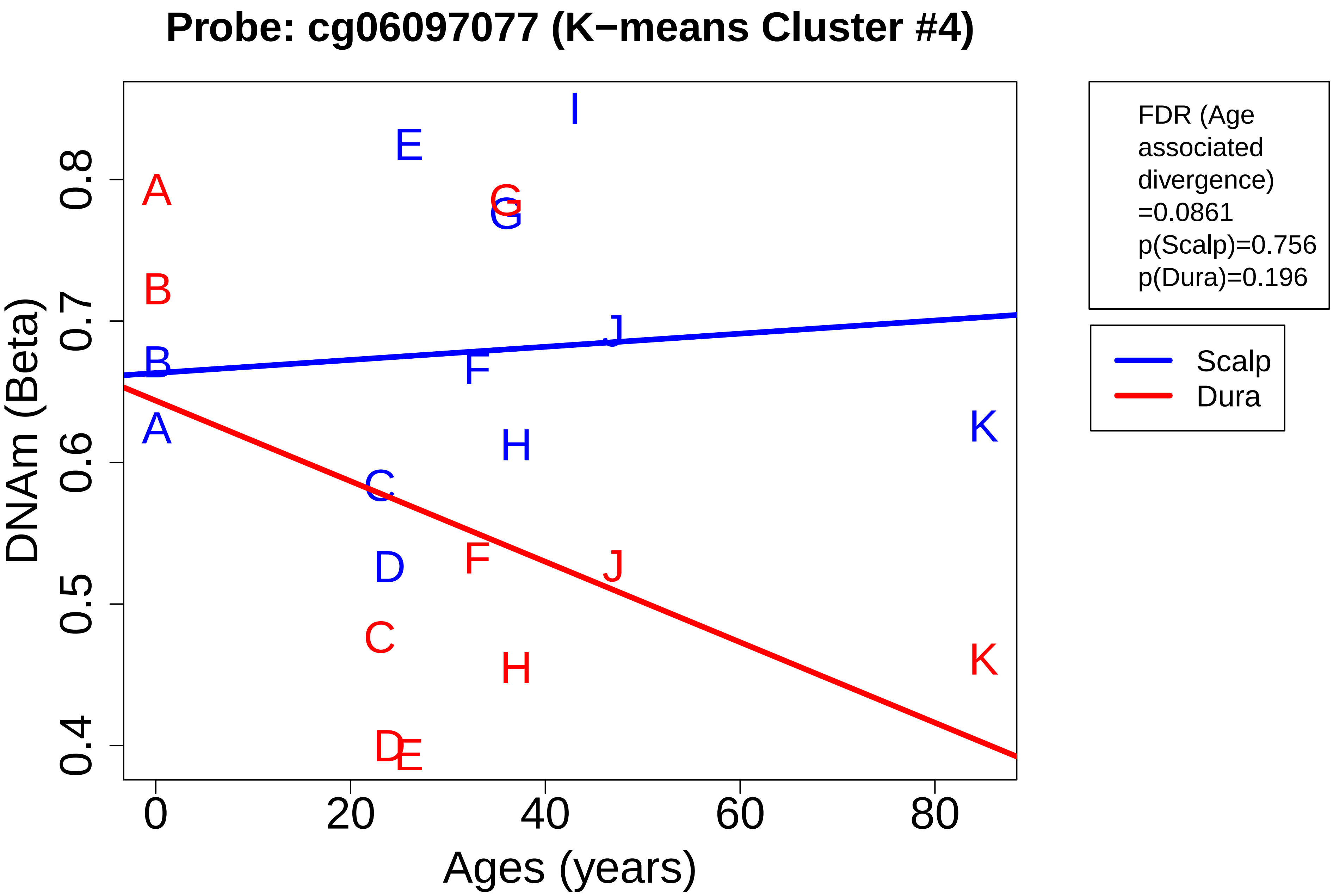

Probe: cg06097077 (K−means Cluster #4)
DNAm (Beta)
0.4
0.5
0.6
0.7
0.8
0
20
40
60
80
Ages (years)
FDR (Age associated divergence) =0.0861
p(Scalp)=0.756
p(Dura)=0.196
Scalp
Dura

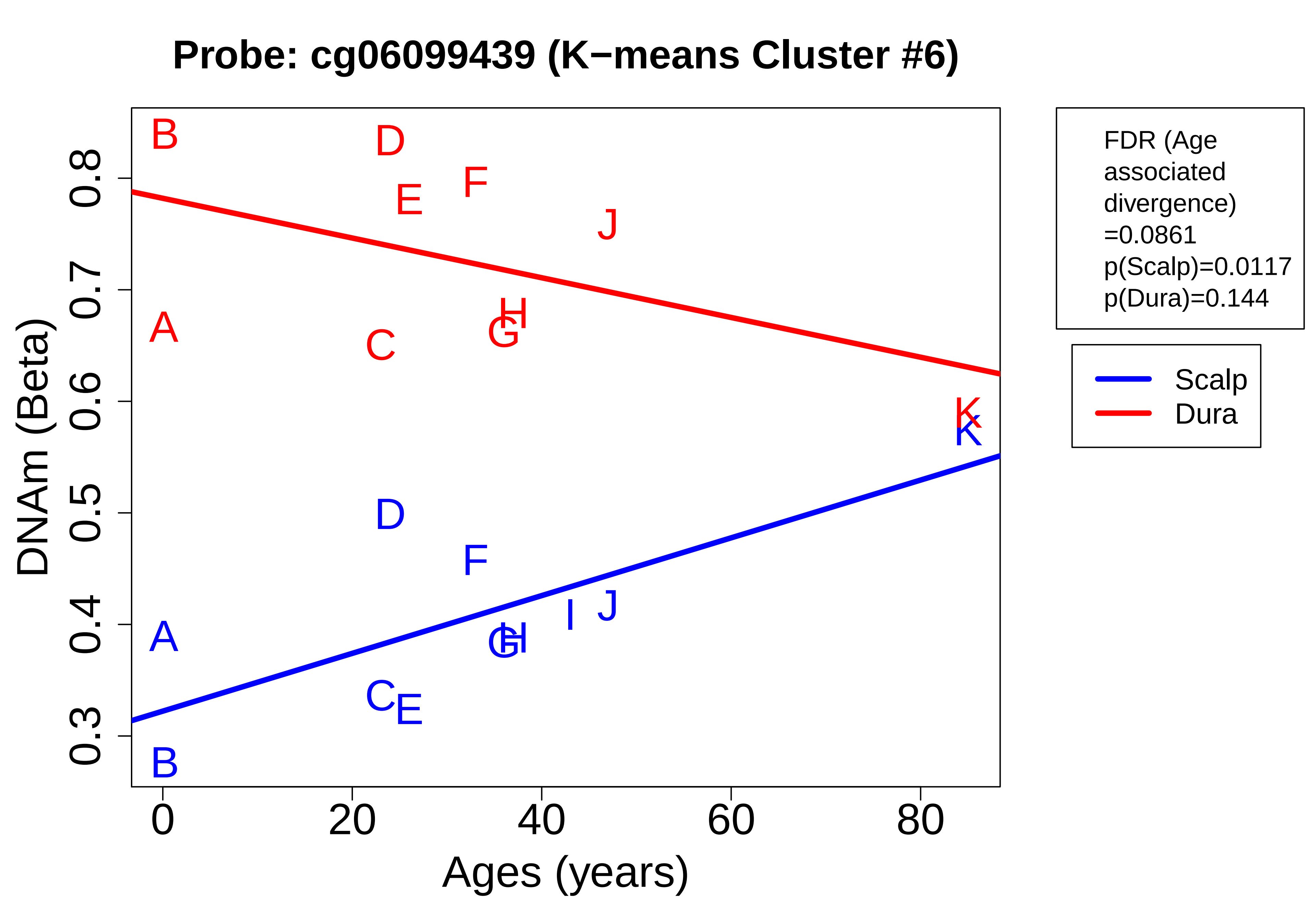

Probe: cg06099439 (K−means Cluster #6)
FDR (Age associated divergence) =0.0861
p(Scalp)=0.0117
p(Dura)=0.144
Scalp
Dura
DNAm (Beta)
Ages (years)
0.3
0.4
0.5
0.6
0.7
0.8
0
20
40
60
80

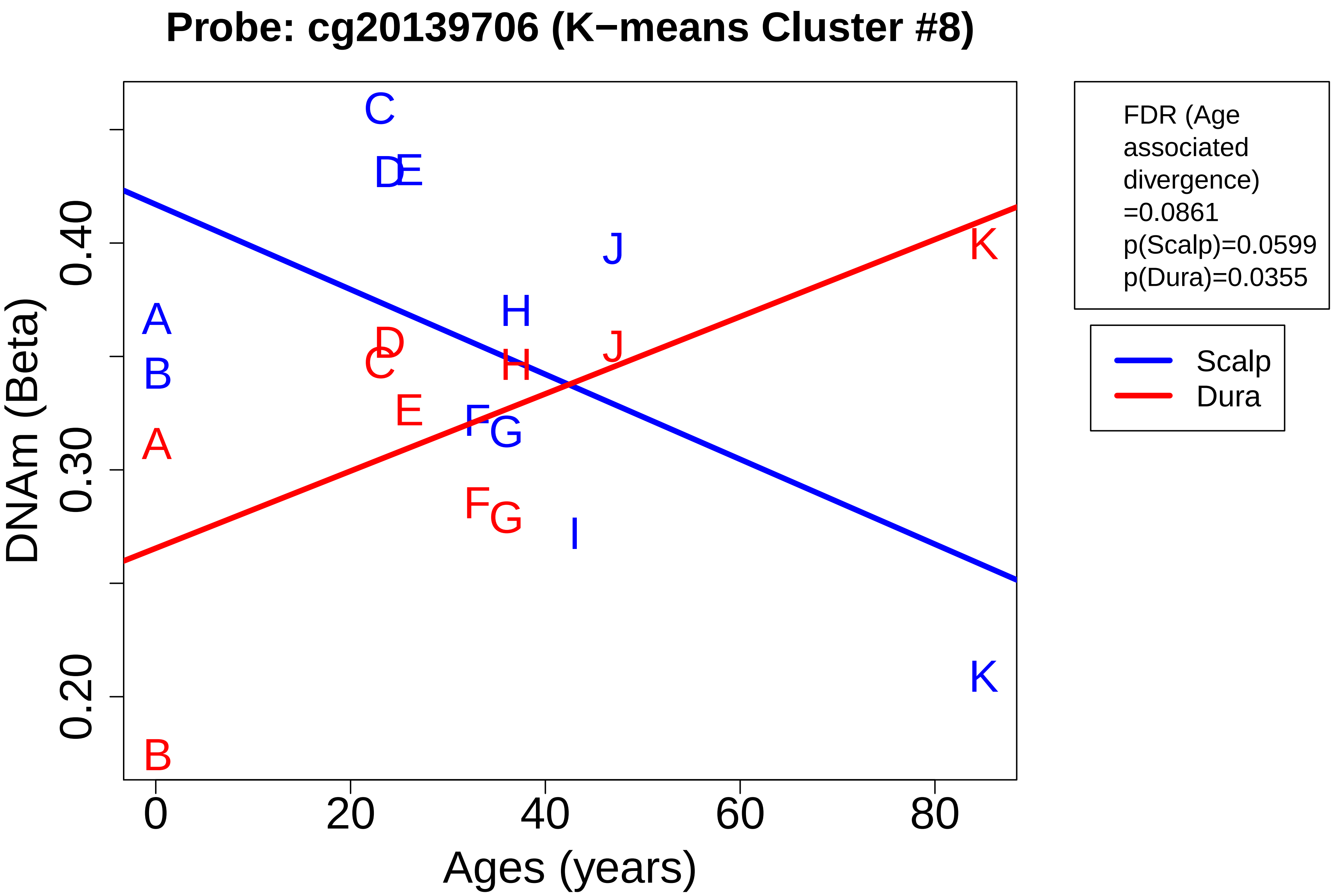

Probe: cg20139706 (K−means Cluster #8)
DNAm (Beta)
0.20
0.30
0.40
Ages (years)
0
20
40
60
80
A
B
C
D
E
F
G
H
I
J
K
FDR (Age associated divergence) =0.0861
p(Scalp)=0.0599
p(Dura)=0.0355
Scalp
Dura

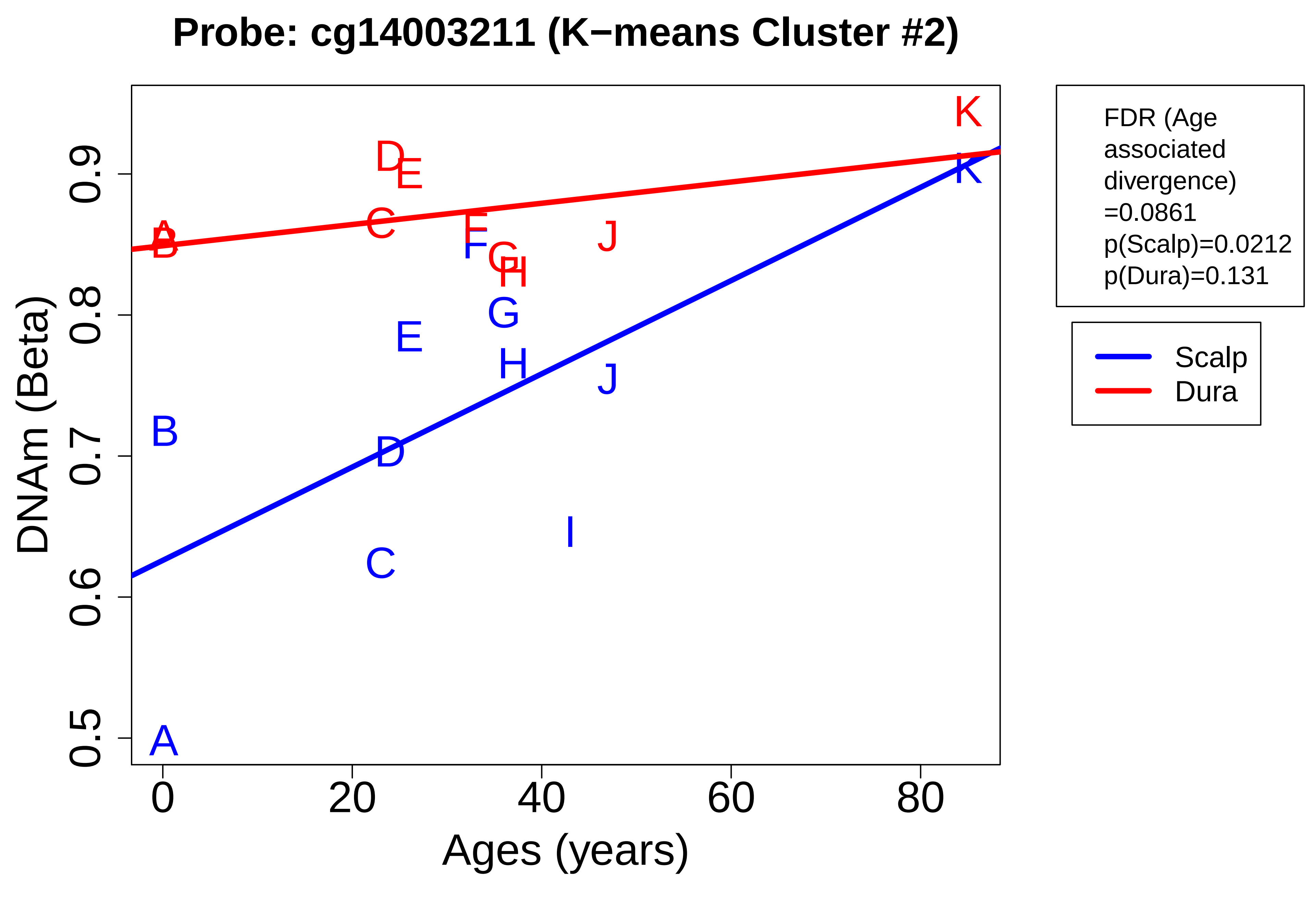

Probe: cg14003211 (K−means Cluster #2)
DNAm (Beta)
0.5
0.6
0.7
0.8
0.9
Ages (years)
0
20
40
60
80
A
B
C
D
E
F
G
H
I
J
K
FDR (Age associated divergence) =0.0861
p(Scalp)=0.0212
p(Dura)=0.131
Scalp
Dura

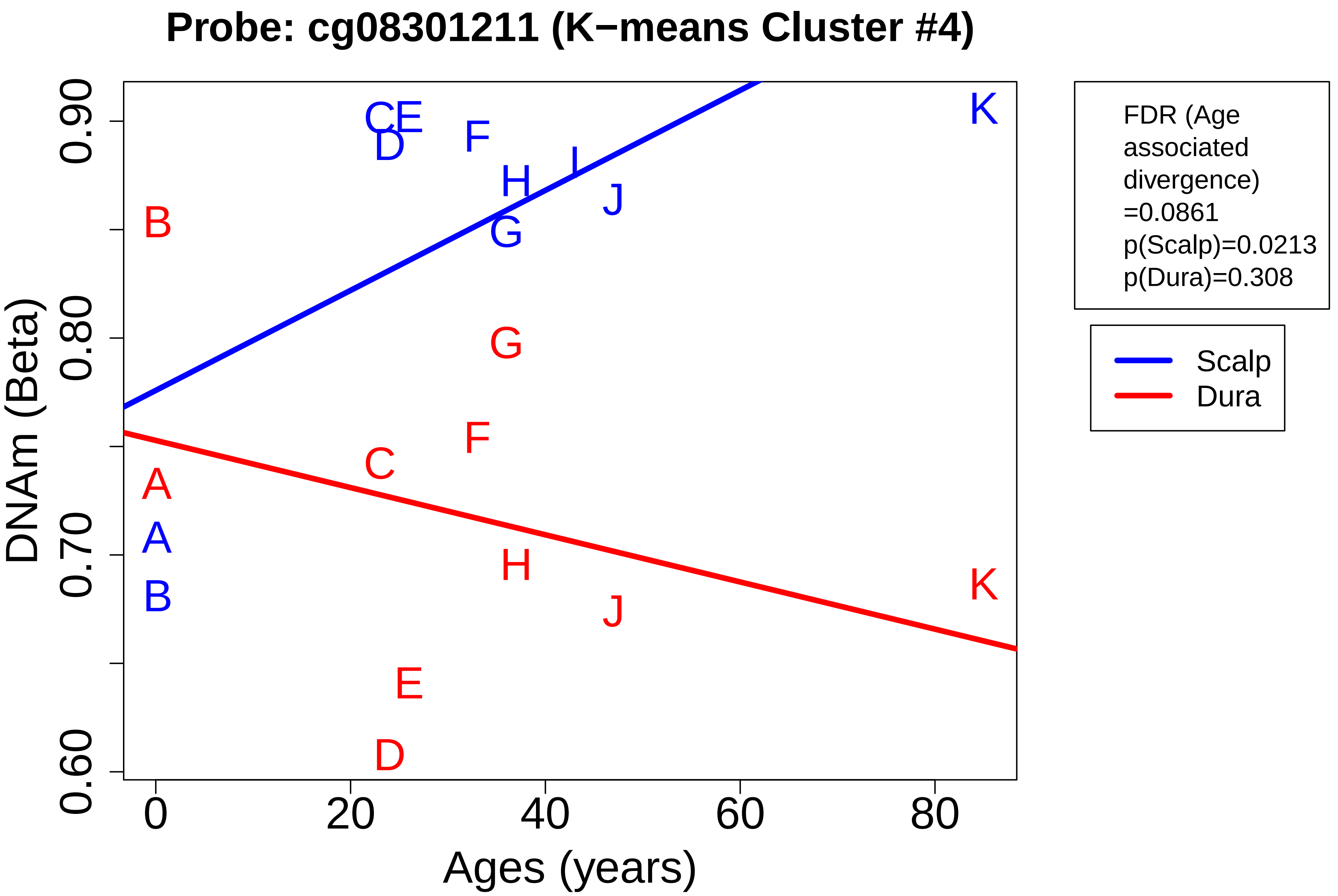
Probe: cg08301211 (K−means Cluster #4)
DNAm (Beta)
0.60
0.70
0.80
0.90
Ages (years)
0
20
40
60
80
FDR (Age associated divergence) =0.0861
p(Scalp)=0.0213
p(Dura)=0.308
Scalp
Dura

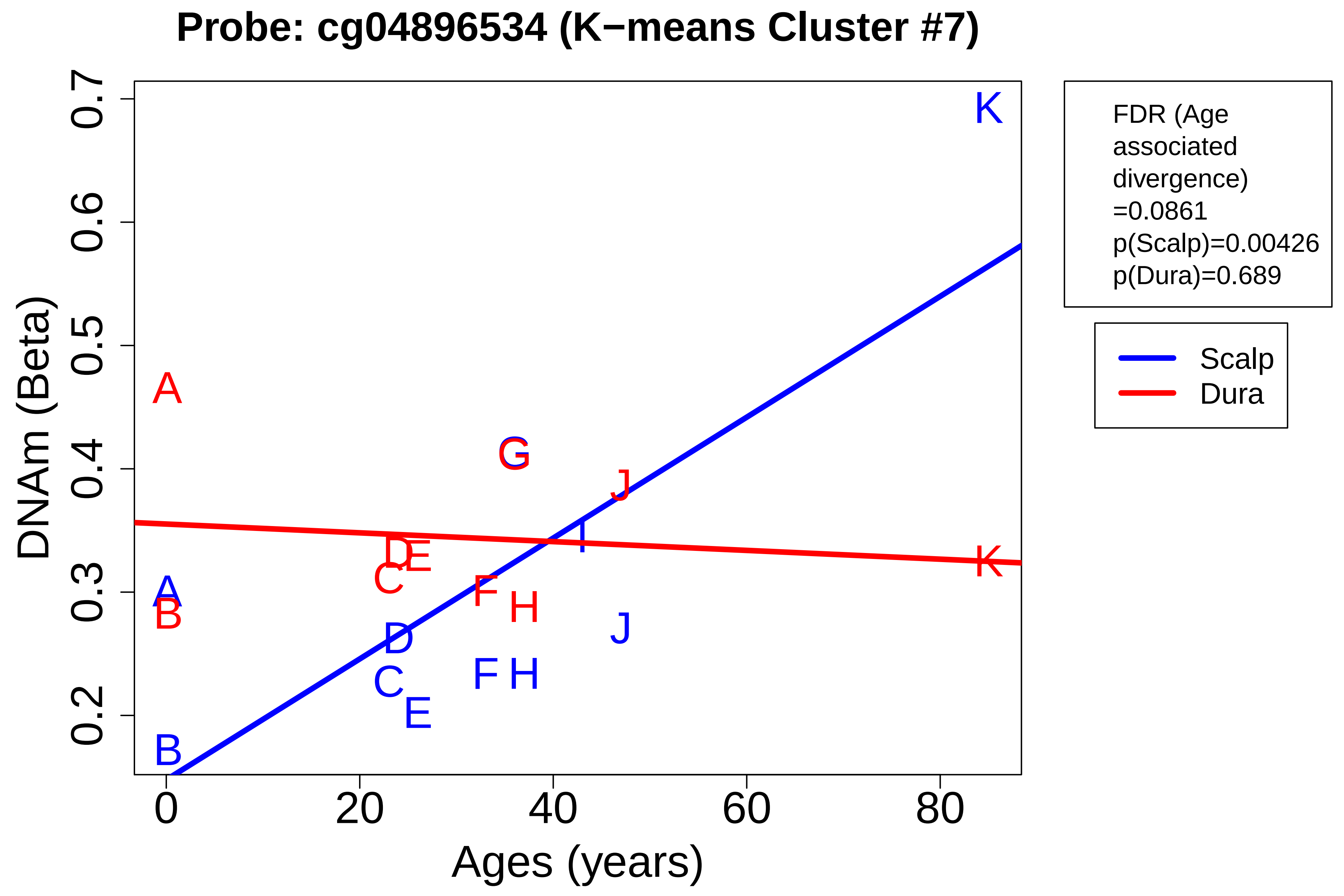

Probe: cg04896534 (K−means Cluster #7)
DNAm (Beta)
0.2
0.3
0.4
0.5
0.6
0.7
Ages (years)
0
20
40
60
80
A
B
C
D
E
F
G
H
I
J
K
FDR (Age associated divergence) =0.0861
p(Scalp)=0.00426
p(Dura)=0.689
Scalp
Dura

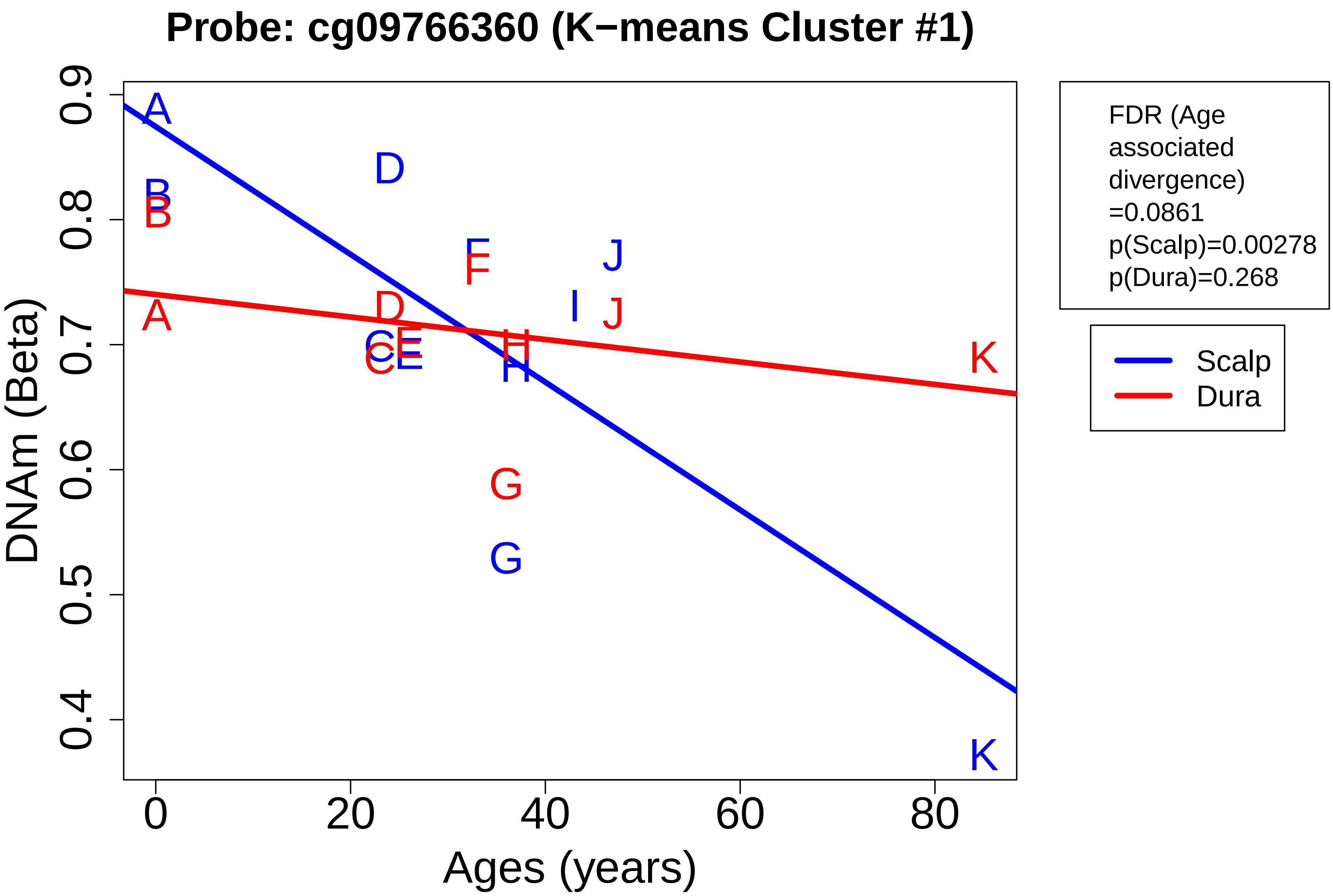

Probe: cg09766360 (K−means Cluster #1)
DNAm (Beta)
0.4
0.5
0.6
0.7
0.8
0.9
Ages (years)
0
20
40
60
80
A
B
C
D
E
F
G
H
I
J
K
FDR (Age associated divergence) =0.0861
p(Scalp)=0.00278
p(Dura)=0.268
Scalp
Dura

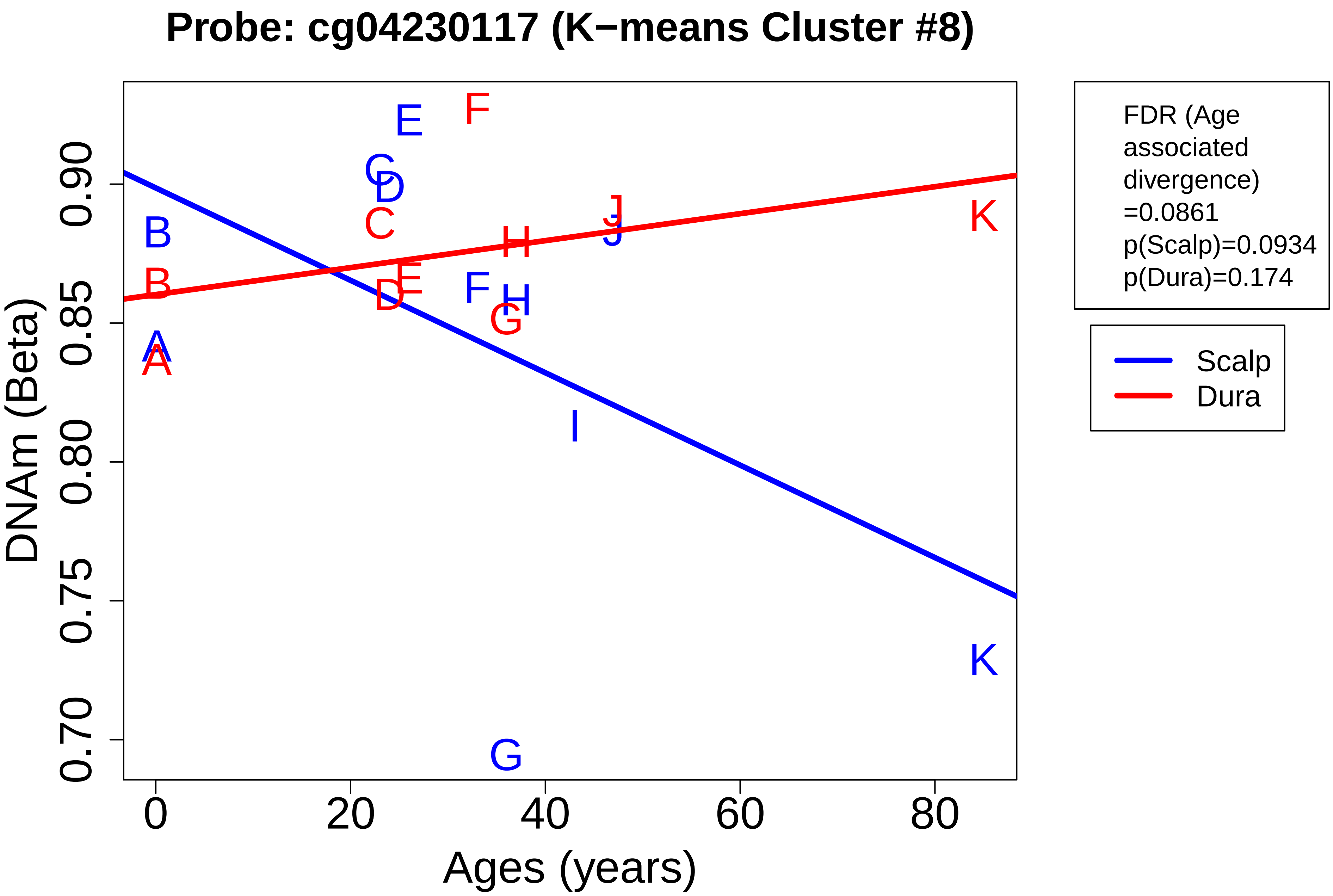

Probe: cg04230117 (K−means Cluster #8)
DNAm (Beta)
0.70
0.75
0.80
0.85
0.90
0
20
40
60
80
Ages (years)
FDR (Age associated divergence) =0.0861
p(Scalp)=0.0934
p(Dura)=0.174
Scalp
Dura

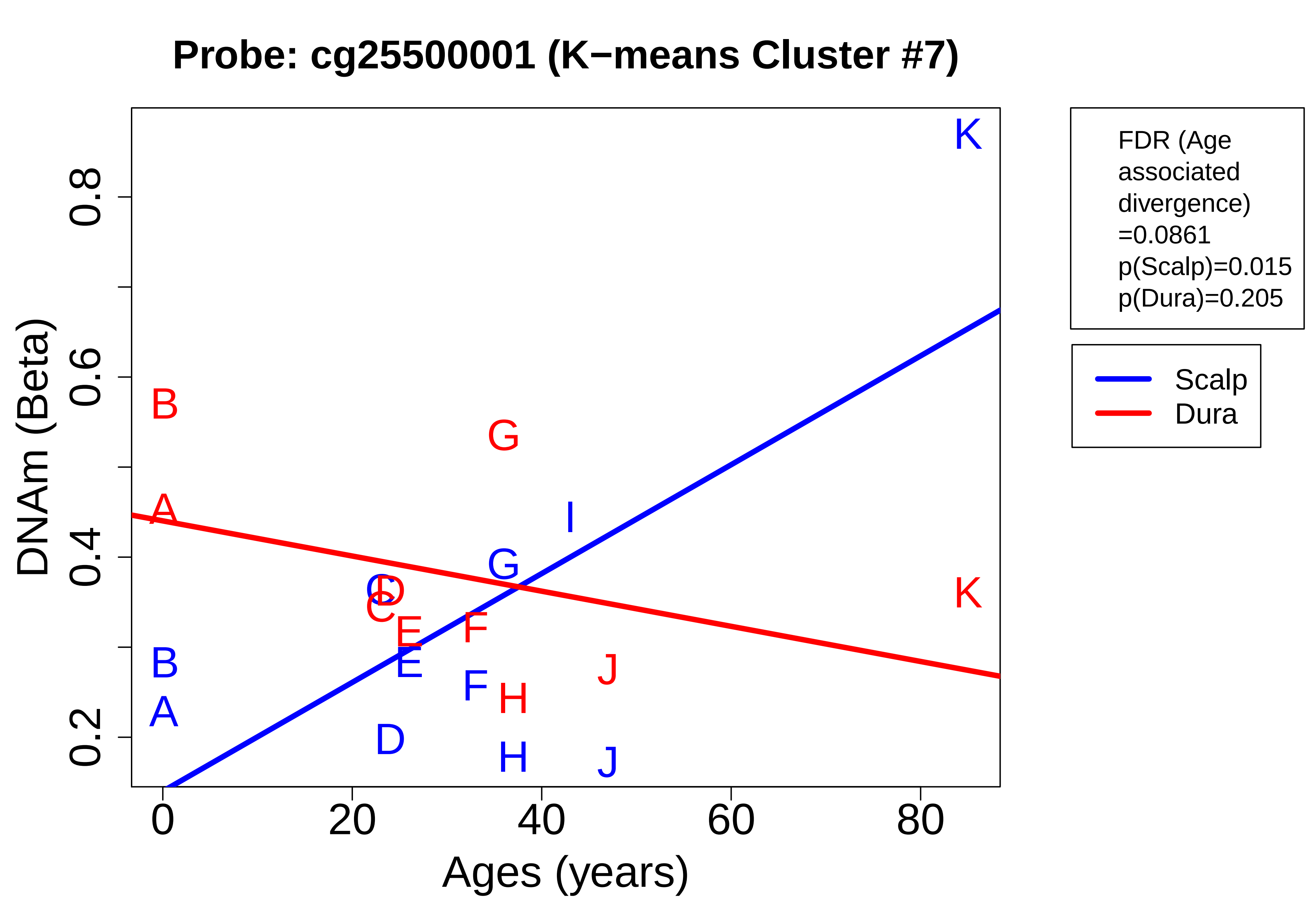

Probe: cg25500001 (K−means Cluster #7)
DNAm (Beta)
0.2
0.4
0.6
0.8
0
20
40
60
80
Ages (years)
FDR (Age associated divergence) =0.0861
p(Scalp)=0.015
p(Dura)=0.205
Scalp
Dura
A
B
C
D
E
F
G
H
I
J
K

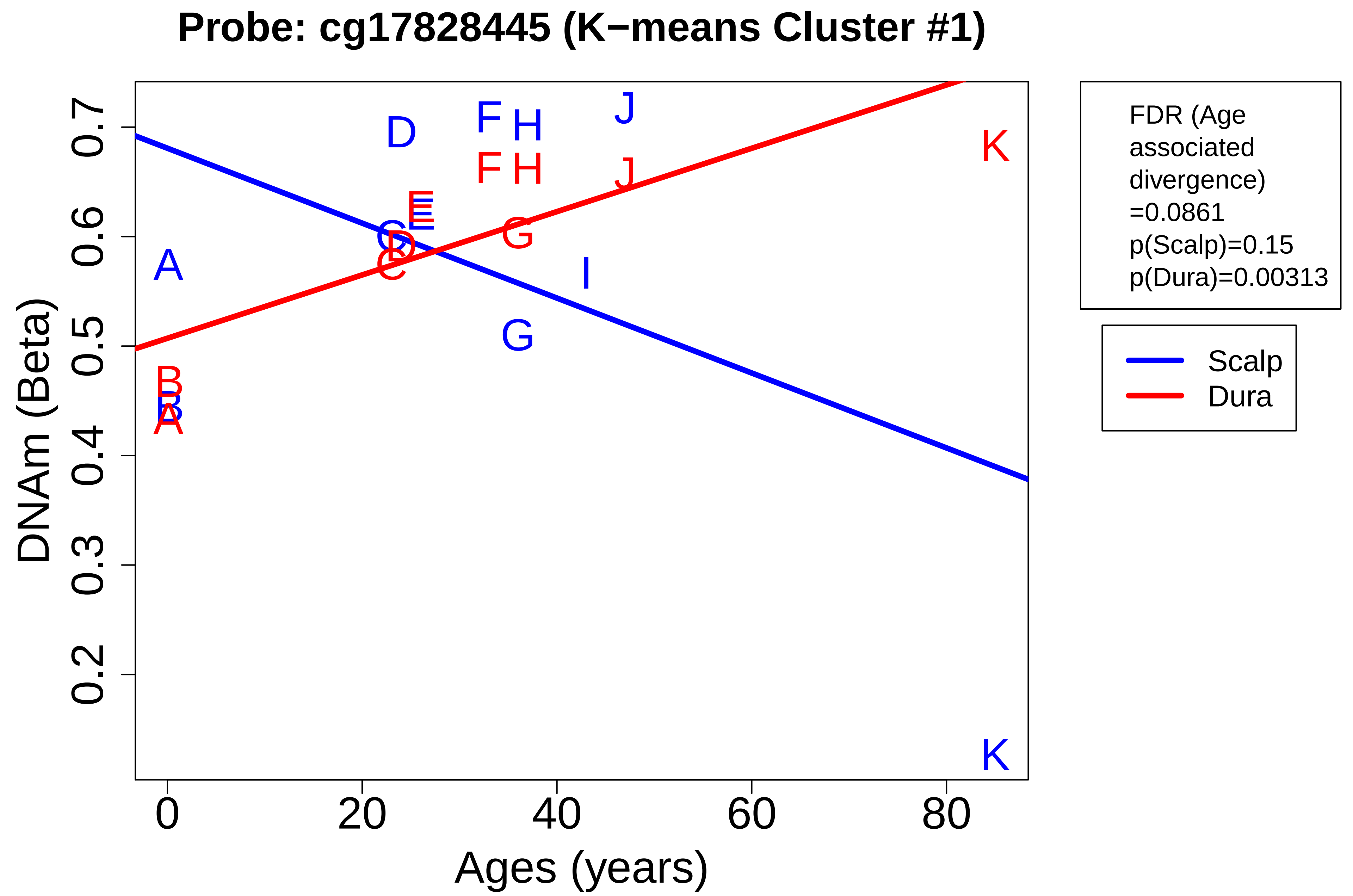

Probe: cg17828445 (K−means Cluster #1)
DNAm (Beta)
0.2
0.3
0.4
0.5
0.6
0.7
0
20
40
60
80
Ages (years)
A
B
C
D
E
F
G
H
I
J
K
FDR (Age associated divergence) =0.0861
p(Scalp)=0.15
p(Dura)=0.00313
Scalp
Dura

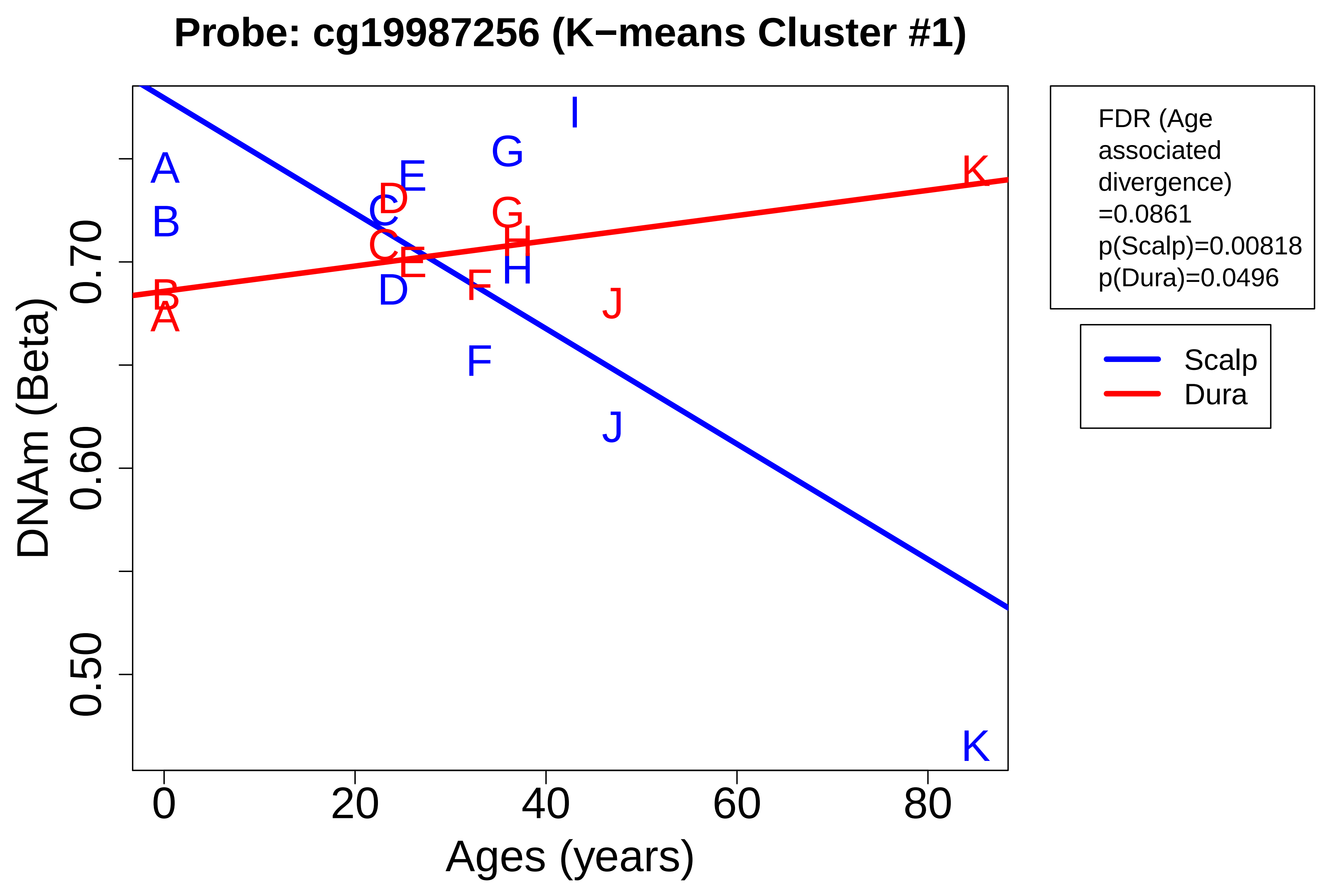

Probe: cg19987256 (K−means Cluster #1)
DNAm (Beta)
0.50
0.60
0.70
0
20
40
60
80
Ages (years)
A
B
C
D
E
F
G
H
I
J
K
FDR (Age associated divergence) =0.0861
p(Scalp)=0.00818
p(Dura)=0.0496
Scalp
Dura

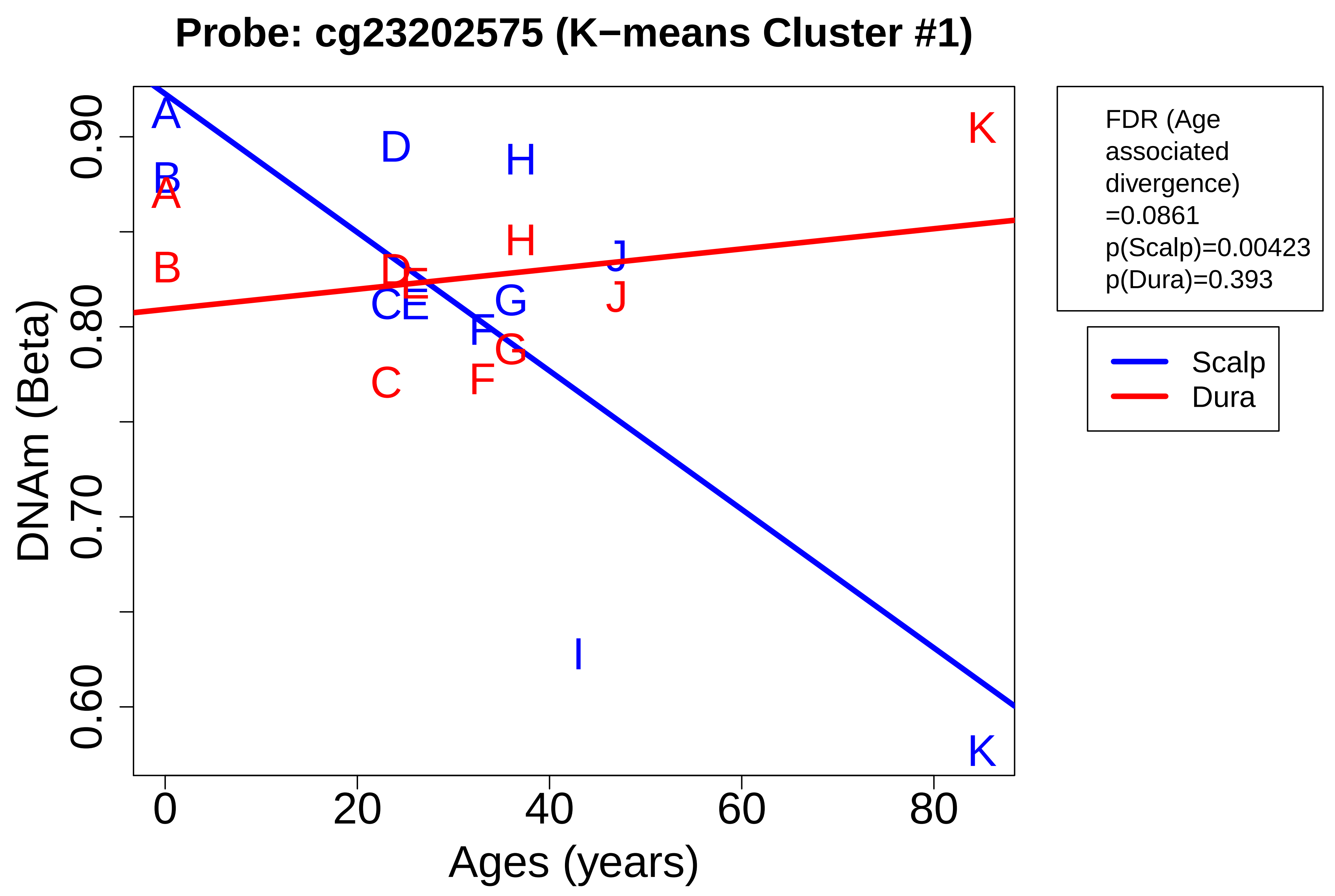

Probe: cg23202575 (K−means Cluster #1)
DNAm (Beta)
0.60
0.70
0.80
0.90
0
20
40
60
80
Ages (years)
A
B
C
D
E
F
G
H
I
J
K
FDR (Age associated divergence) =0.0861
p(Scalp)=0.00423
p(Dura)=0.393
Scalp
Dura

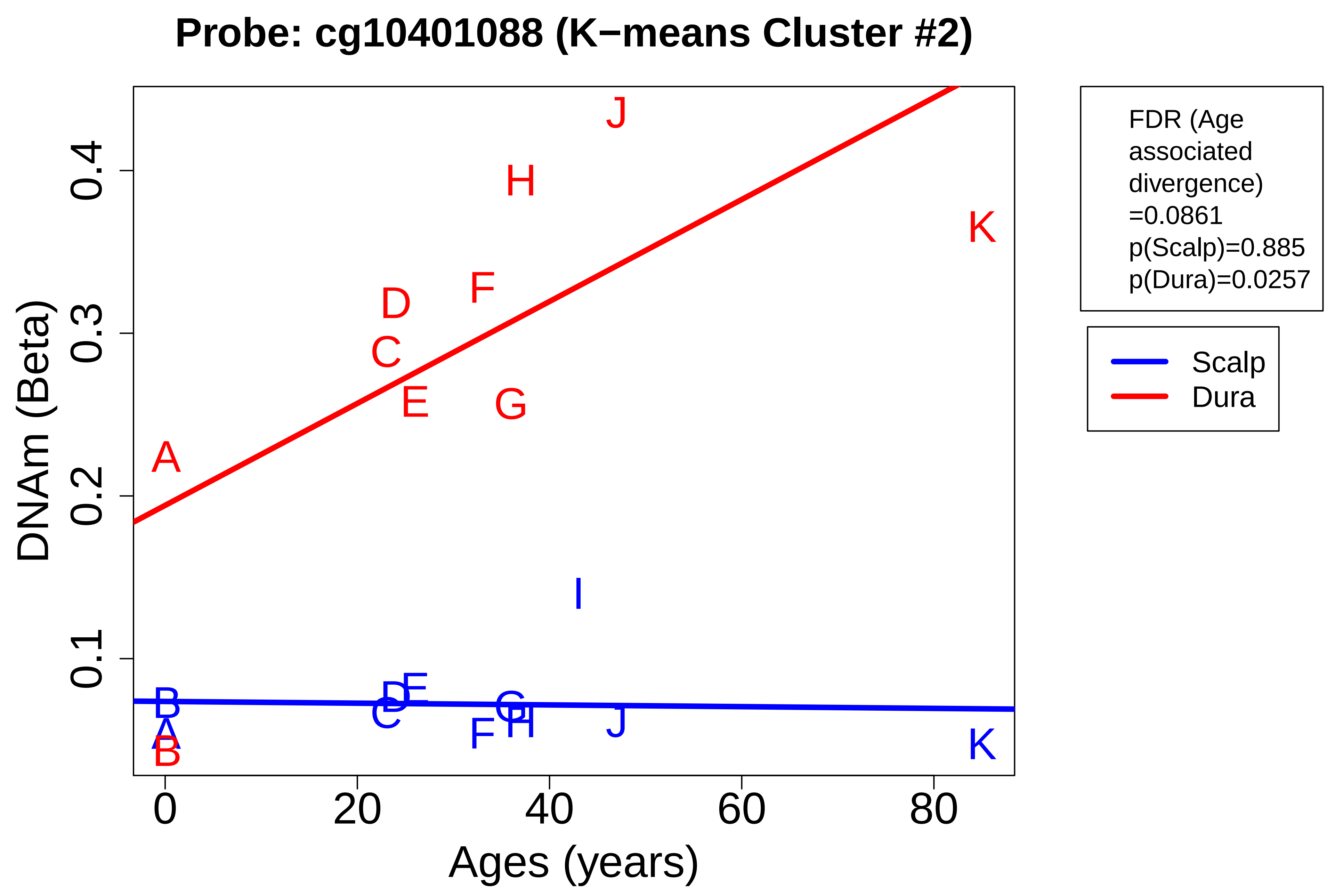

Probe: cg10401088 (K−means Cluster #2)
DNAm (Beta)
0.1
0.2
0.3
0.4
Ages (years)
0
20
40
60
80
FDR (Age associated divergence) =0.0861
p(Scalp)=0.885
p(Dura)=0.0257
Scalp
Dura
A
B
C
D
E
F
G
H
I
J
K

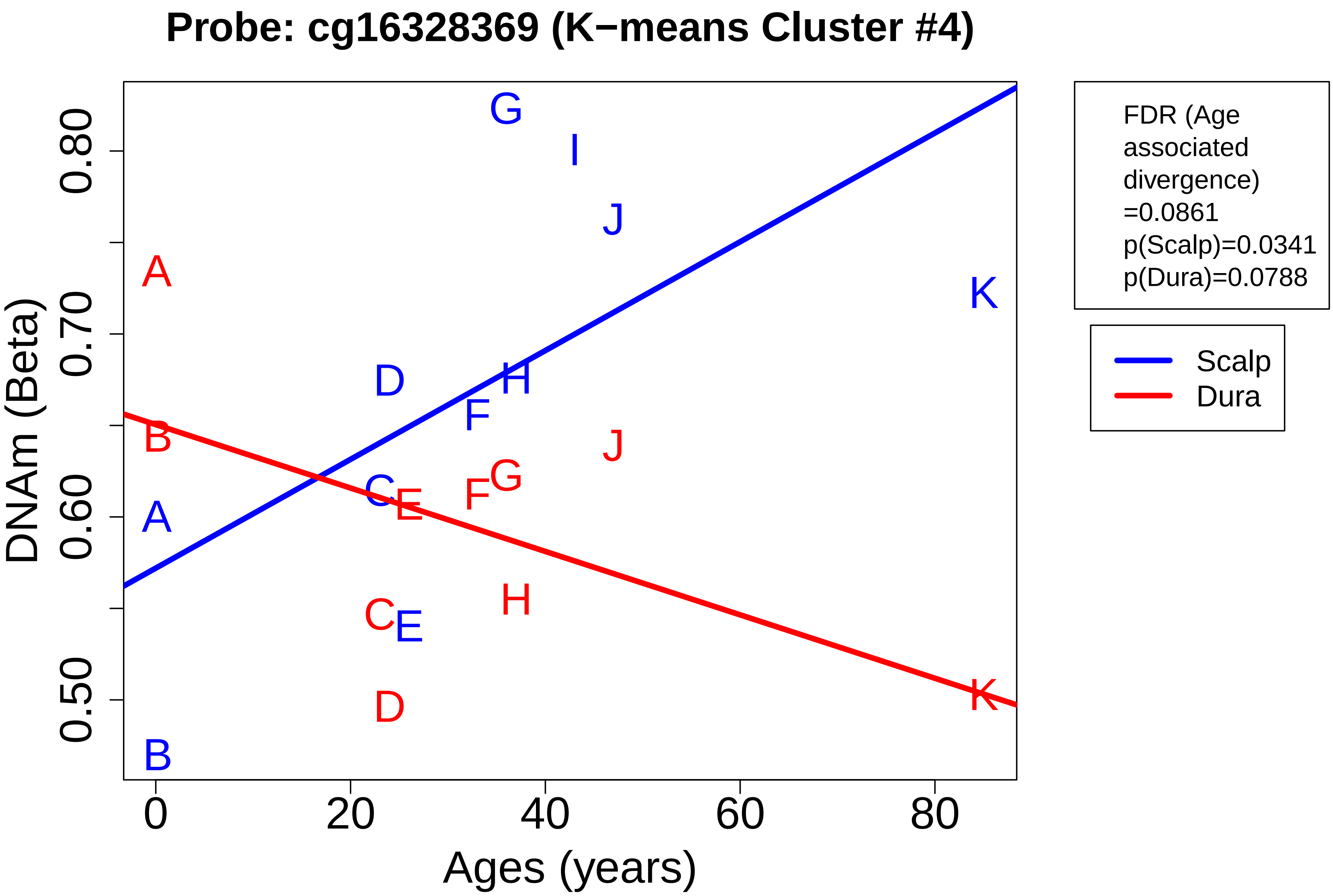

Probe: cg16328369 (K−means Cluster #4)
FDR (Age associated divergence) =0.0861
p(Scalp)=0.0341
p(Dura)=0.0788
Scalp
Dura
DNAm (Beta)
0.50
0.60
0.70
0.80
Ages (years)
0
20
40
60
80
A
B
C
D
E
F
G
H
I
J
K

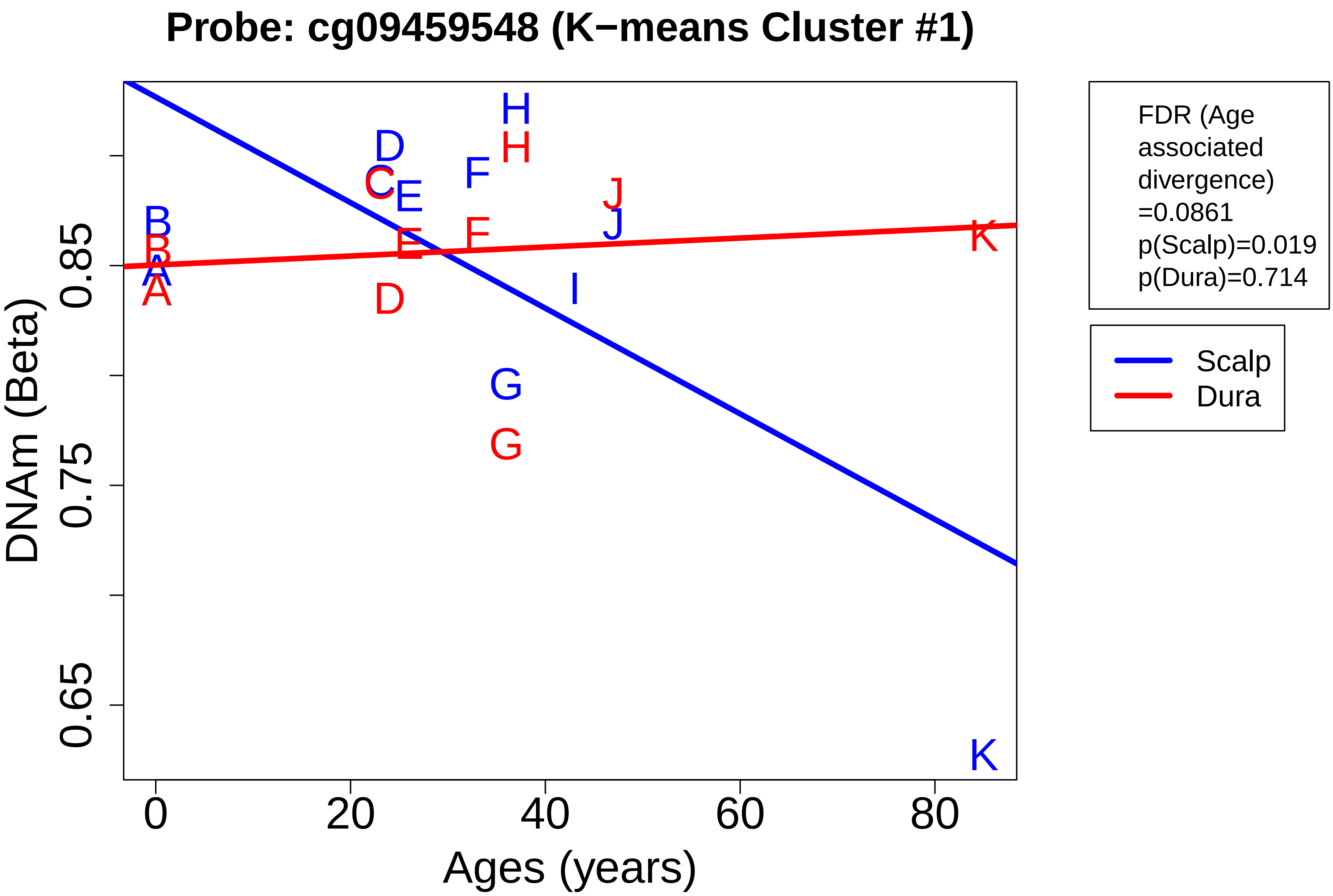

Probe: cg09459548 (K−means Cluster #1)
DNAm (Beta)
0.65
0.75
0.85
Ages (years)
0
20
40
60
80
FDR (Age associated divergence) =0.0861
p(Scalp)=0.019
p(Dura)=0.714
Scalp
Dura
A
B
C
D
E
F
G
H
I
J
K

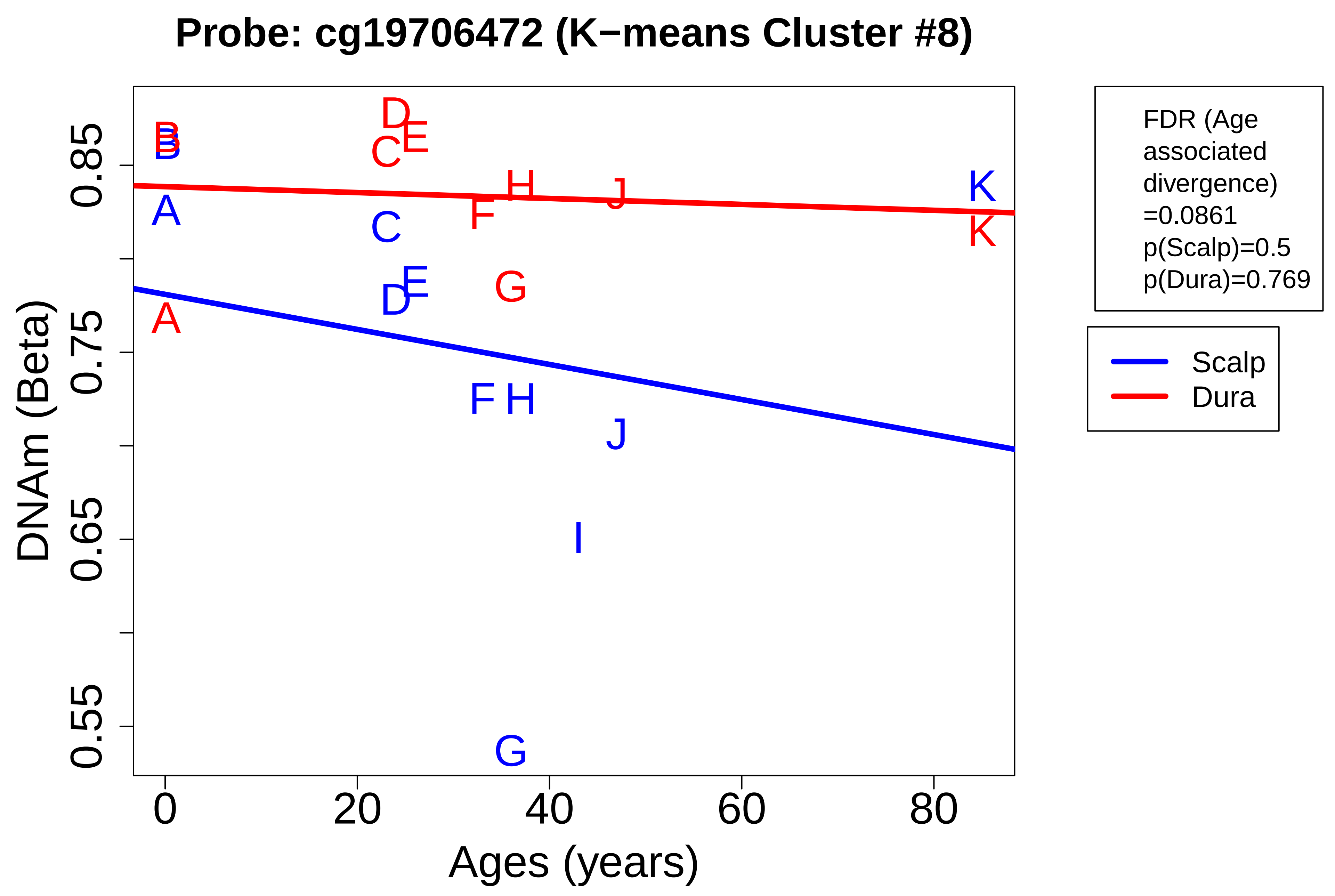

Probe: cg19706472 (K−means Cluster #8)
DNAm (Beta)
0.55
0.65
0.75
0.85
0
20
40
60
80
Ages (years)
A
B
C
D
E
F
G
H
I
J
K
FDR (Age associated divergence) =0.0861
p(Scalp)=0.5
p(Dura)=0.769
Scalp
Dura

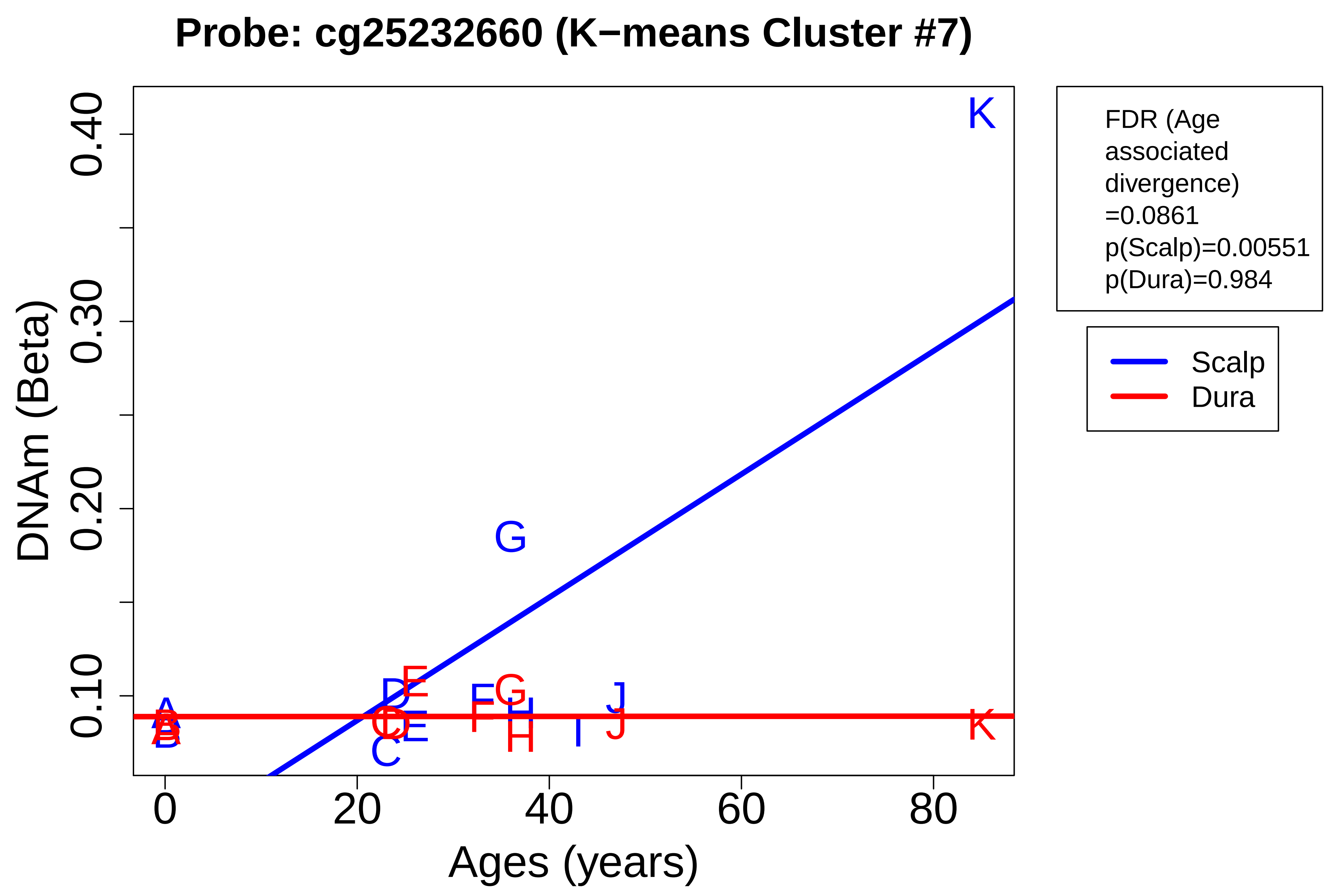

Probe: cg25232660 (K−means Cluster #7)
DNAm (Beta)
0.10
0.20
0.30
0.40
Ages (years)
0
20
40
60
80
FDR (Age associated divergence) =0.0861
p(Scalp)=0.00551
p(Dura)=0.984
Scalp
Dura

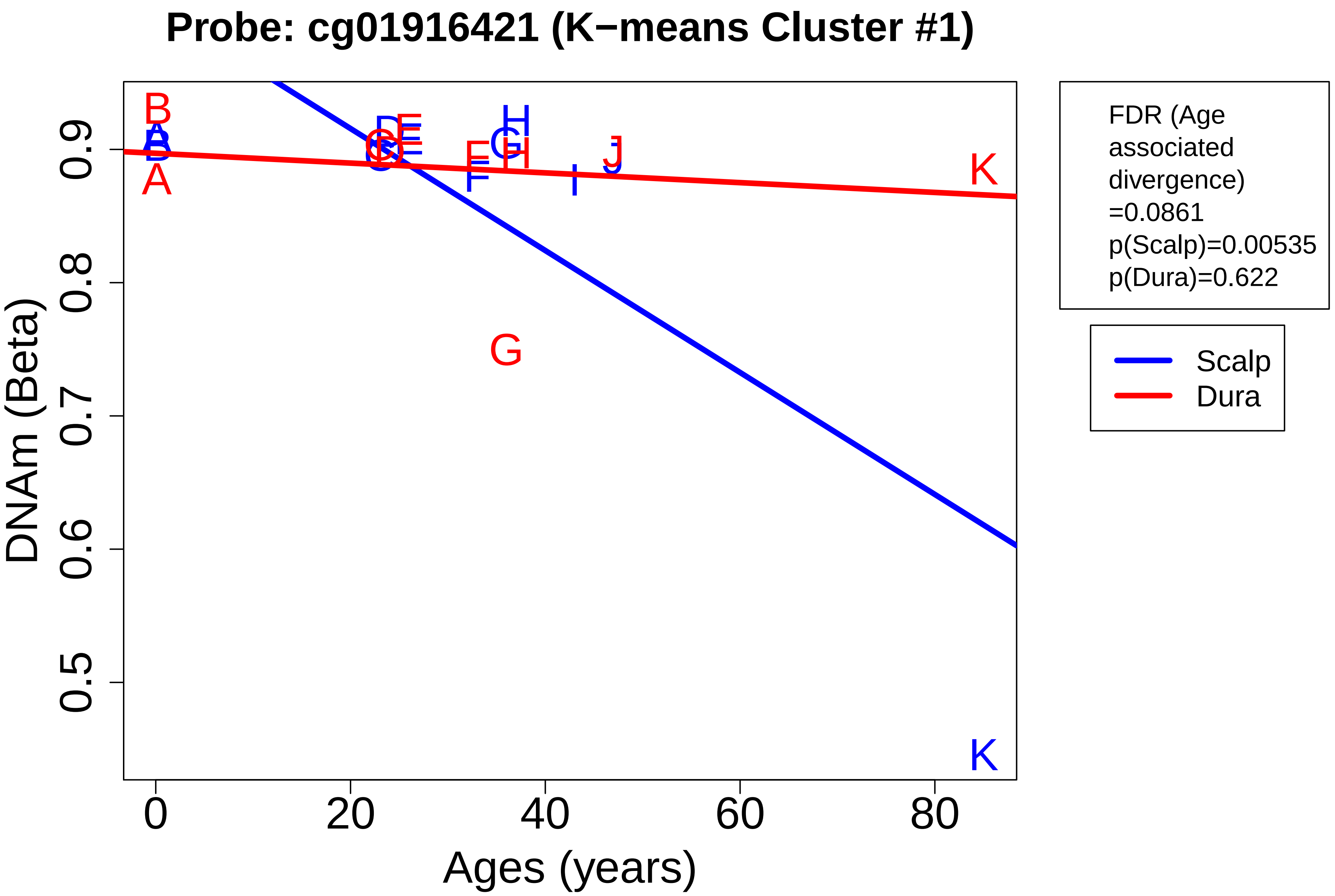

Probe: cg01916421 (K−means Cluster #1)
DNAm (Beta)
0.5
0.6
0.7
0.8
0.9
Ages (years)
0
20
40
60
80
FDR (Age associated divergence) =0.0861
p(Scalp)=0.00535
p(Dura)=0.622
Scalp
Dura

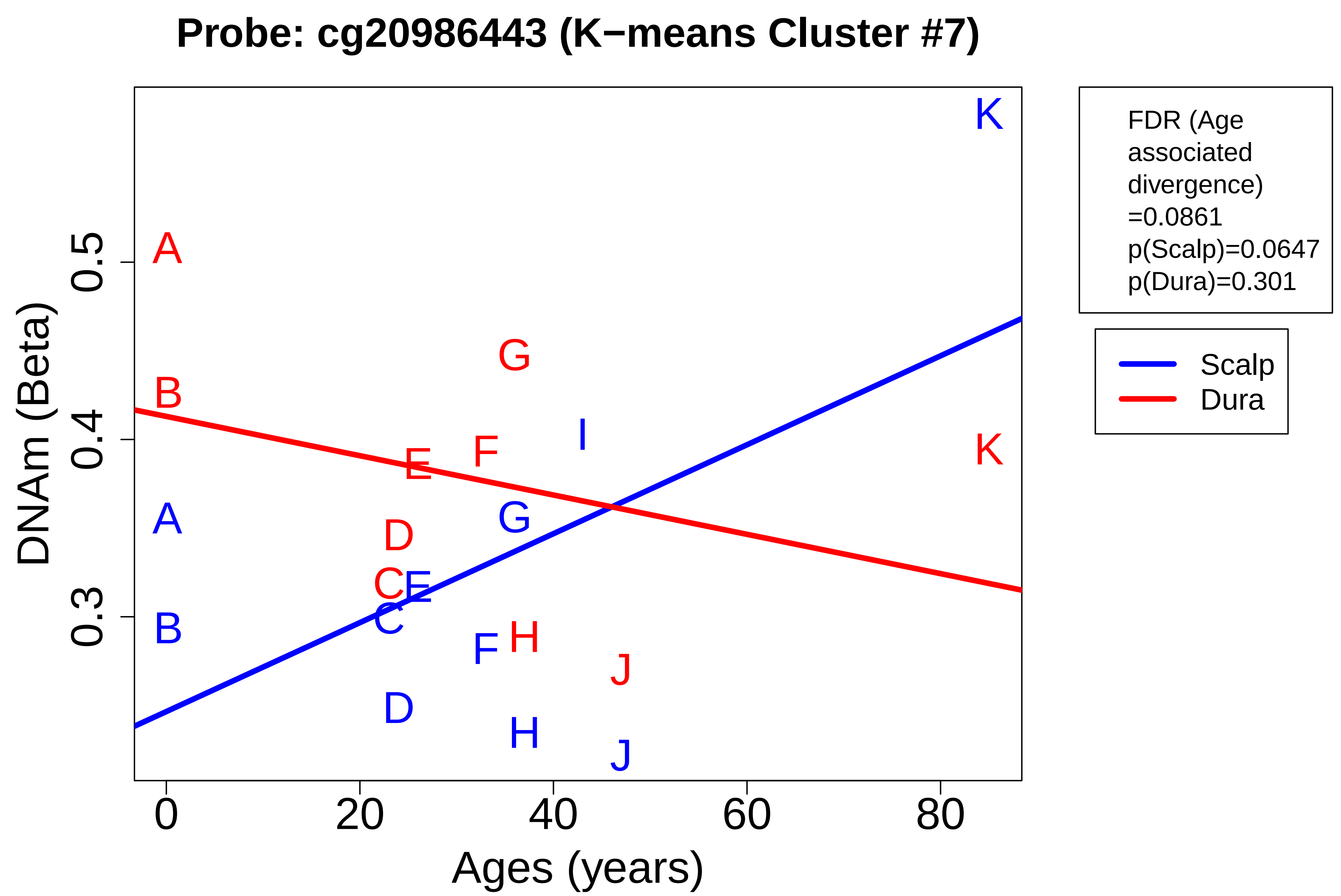

Probe: cg20986443 (K−means Cluster #7)
DNAm (Beta)
0.3
0.4
0.5
Ages (years)
0
20
40
60
80
A
B
C
D
E
F
G
H
I
J
K
FDR (Age associated divergence) =0.0861
p(Scalp)=0.0647
p(Dura)=0.301
Scalp
Dura

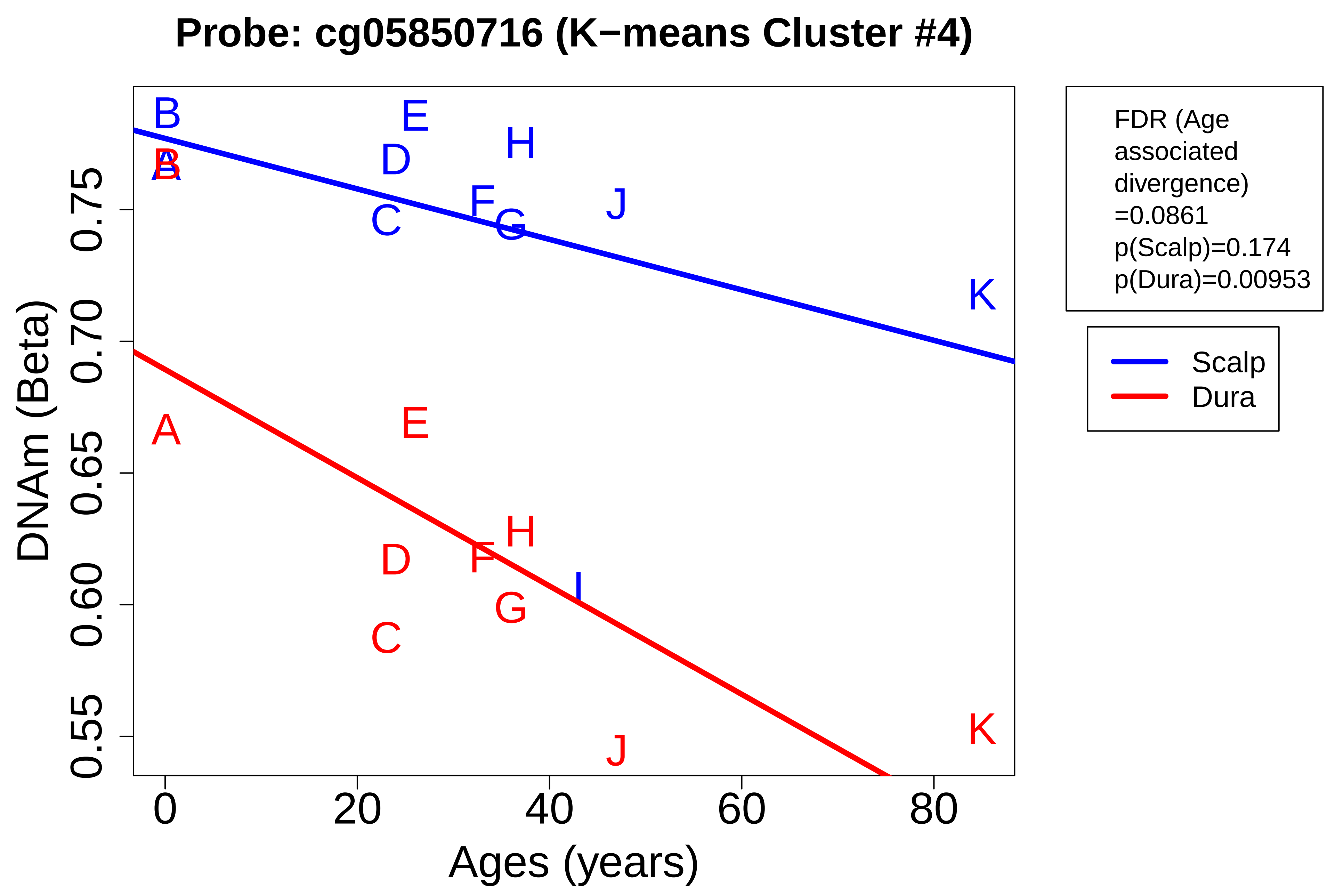

Probe: cg05850716 (K−means Cluster #4)
DNAm (Beta)
0.55
0.60
0.65
0.70
0.75
0
20
40
60
80
Ages (years)
FDR (Age associated divergence) =0.0861
p(Scalp)=0.174
p(Dura)=0.00953
Scalp
Dura

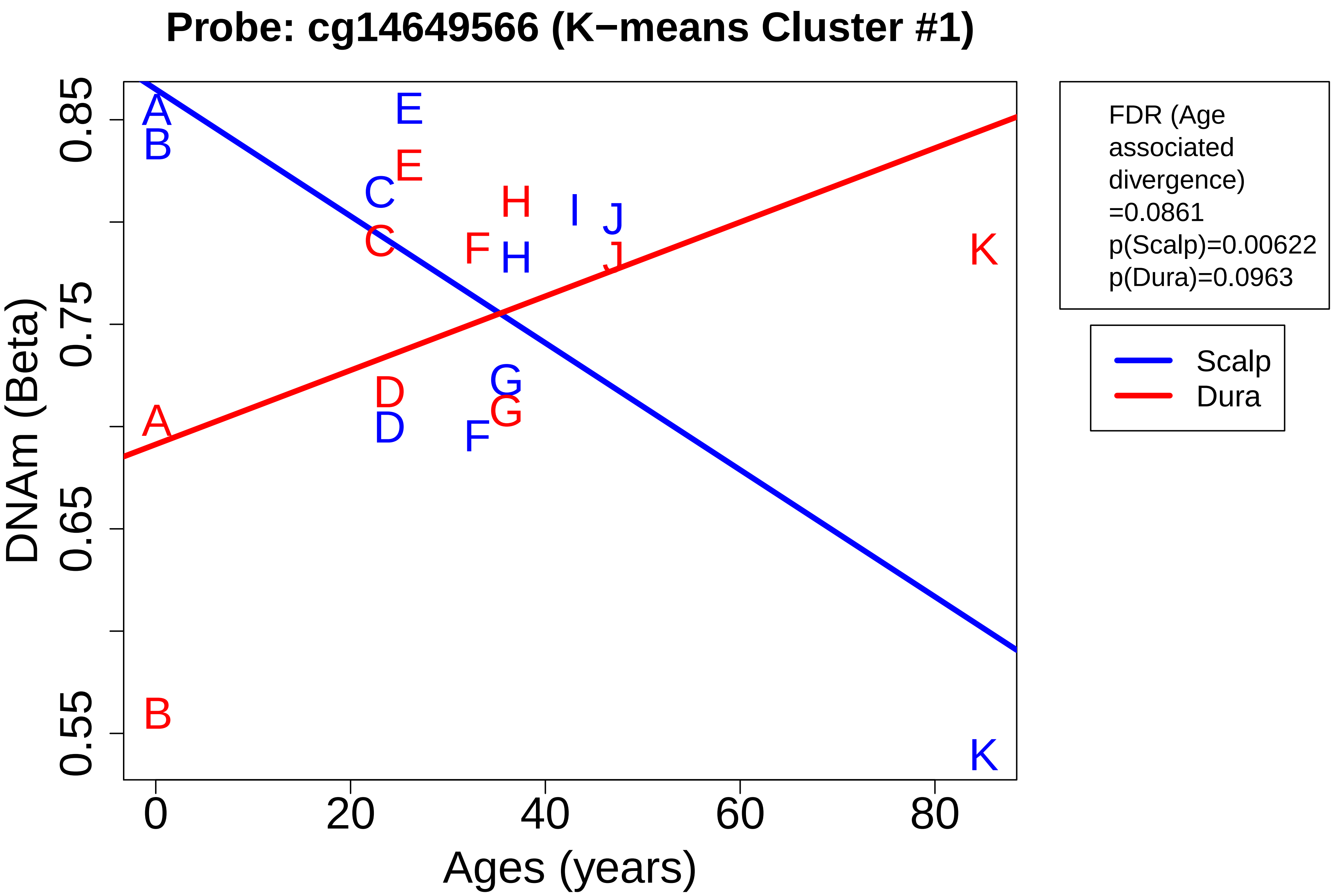
Probe: cg14649566 (K−means Cluster #1)
DNAm (Beta)
0.55
0.65
0.75
0.85
Ages (years)
0
20
40
60
80
FDR (Age associated divergence) =0.0861
p(Scalp)=0.00622
p(Dura)=0.0963
Scalp
Dura

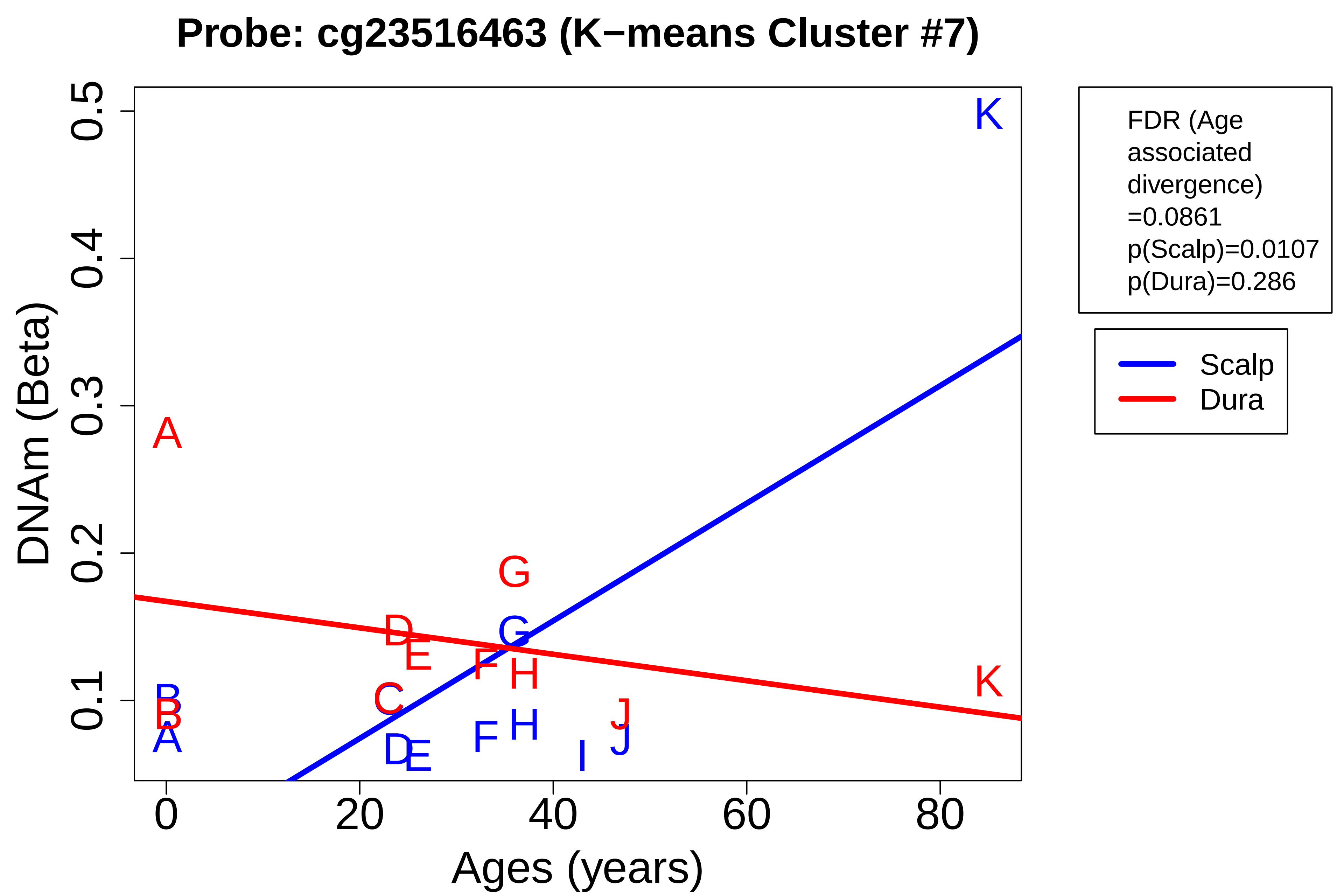

Probe: cg23516463 (K−means Cluster #7)
DNAm (Beta)
0.1
0.2
0.3
0.4
0.5
0
20
40
60
80
Ages (years)
FDR (Age associated divergence) =0.0861
p(Scalp)=0.0107
p(Dura)=0.286
Scalp
Dura
A
B
C
D
E
F
G
H
I
J
K

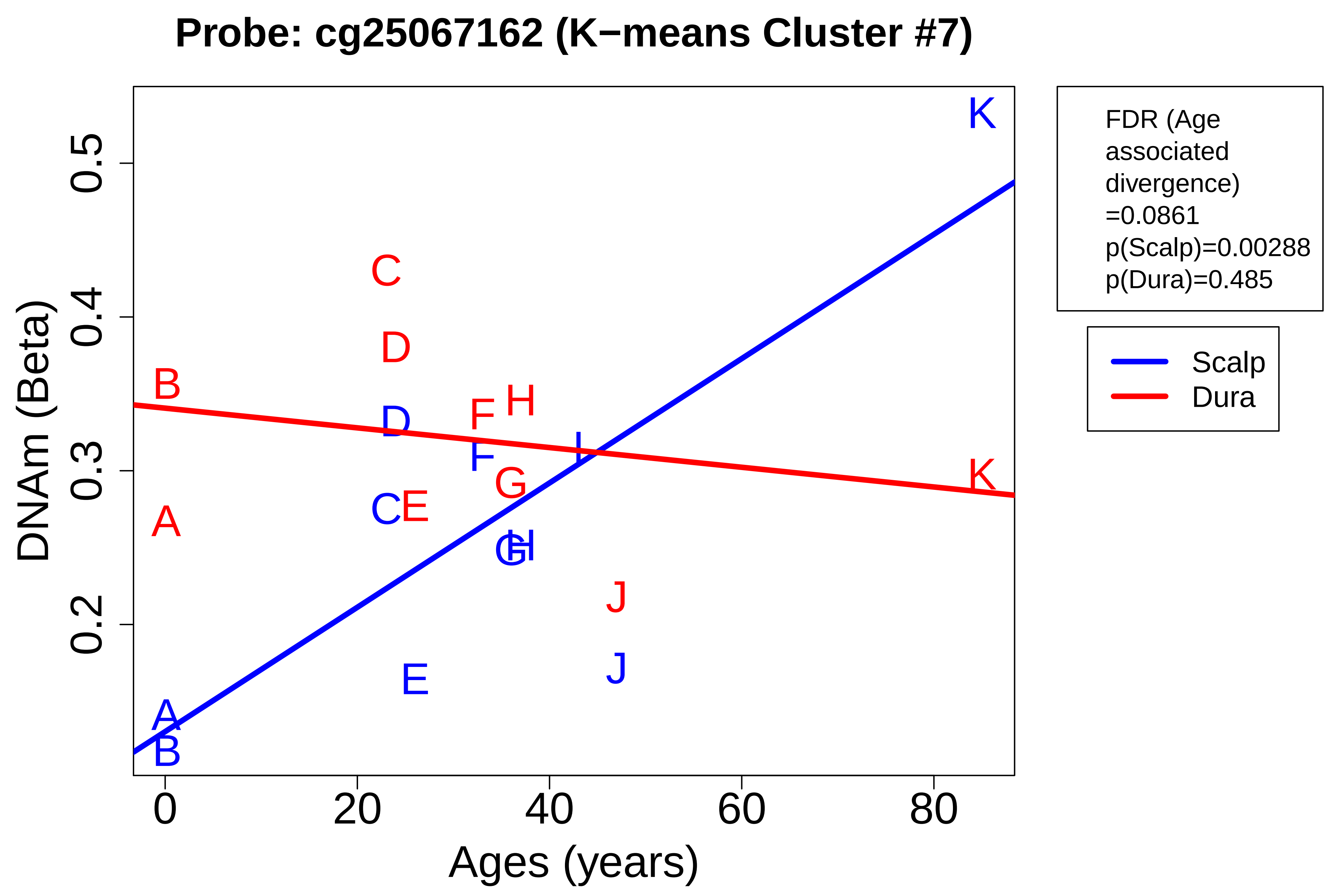

Probe: cg25067162 (K−means Cluster #7)
DNAm (Beta)
Ages (years)
0.2
0.3
0.4
0.5
0
20
40
60
80
FDR (Age associated divergence) =0.0861
p(Scalp)=0.00288
p(Dura)=0.485
Scalp
Dura
A
B
C
D
E
F
G
H
I
J
K

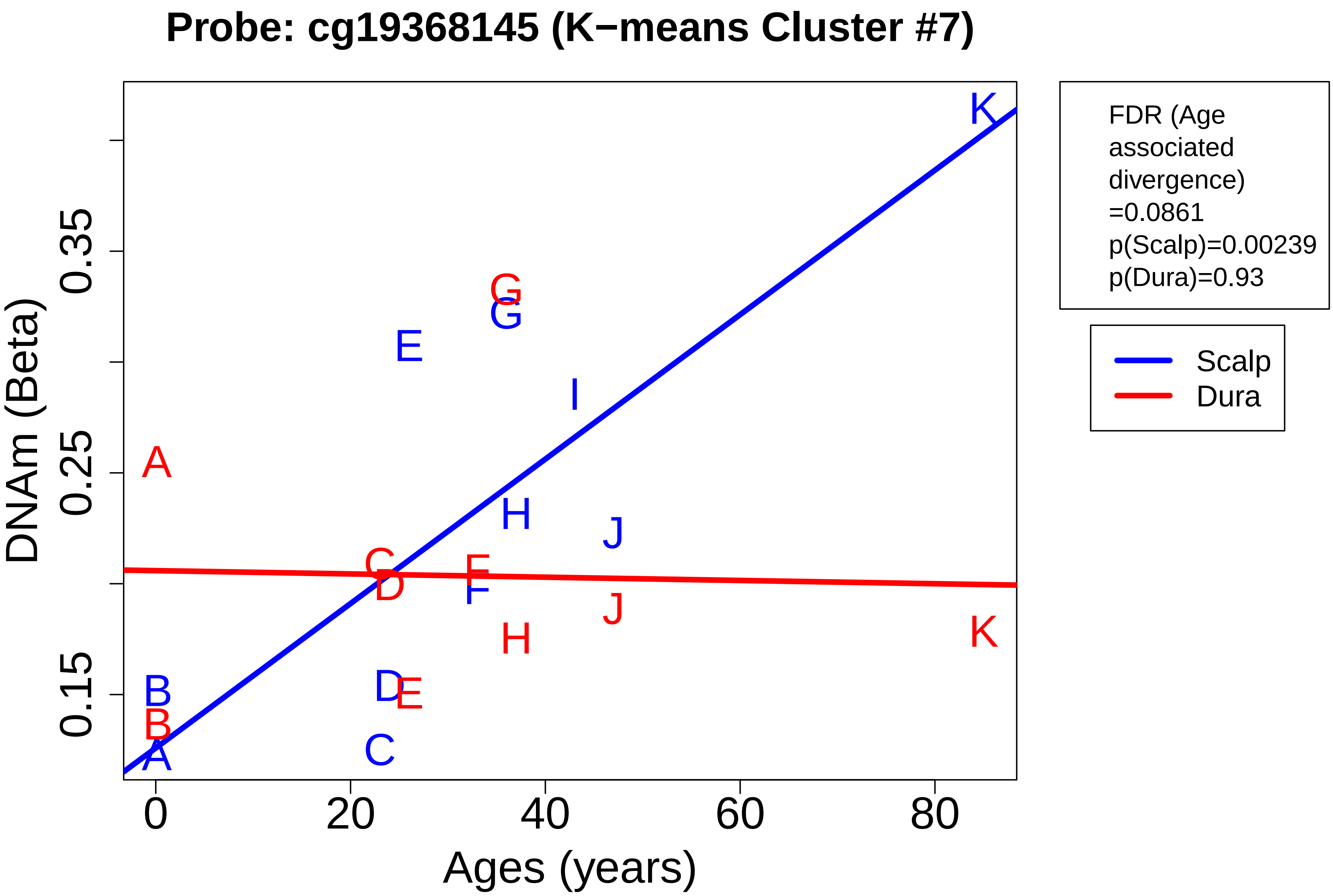

Probe: cg19368145 (K−means Cluster #7)
FDR (Age associated divergence) =0.0861
p(Scalp)=0.00239
p(Dura)=0.93
Scalp
Dura
DNAm (Beta)
0.15
0.25
0.35
0
20
40
60
80
Ages (years)
A
B
C
D
E
F
G
H
I
J
K

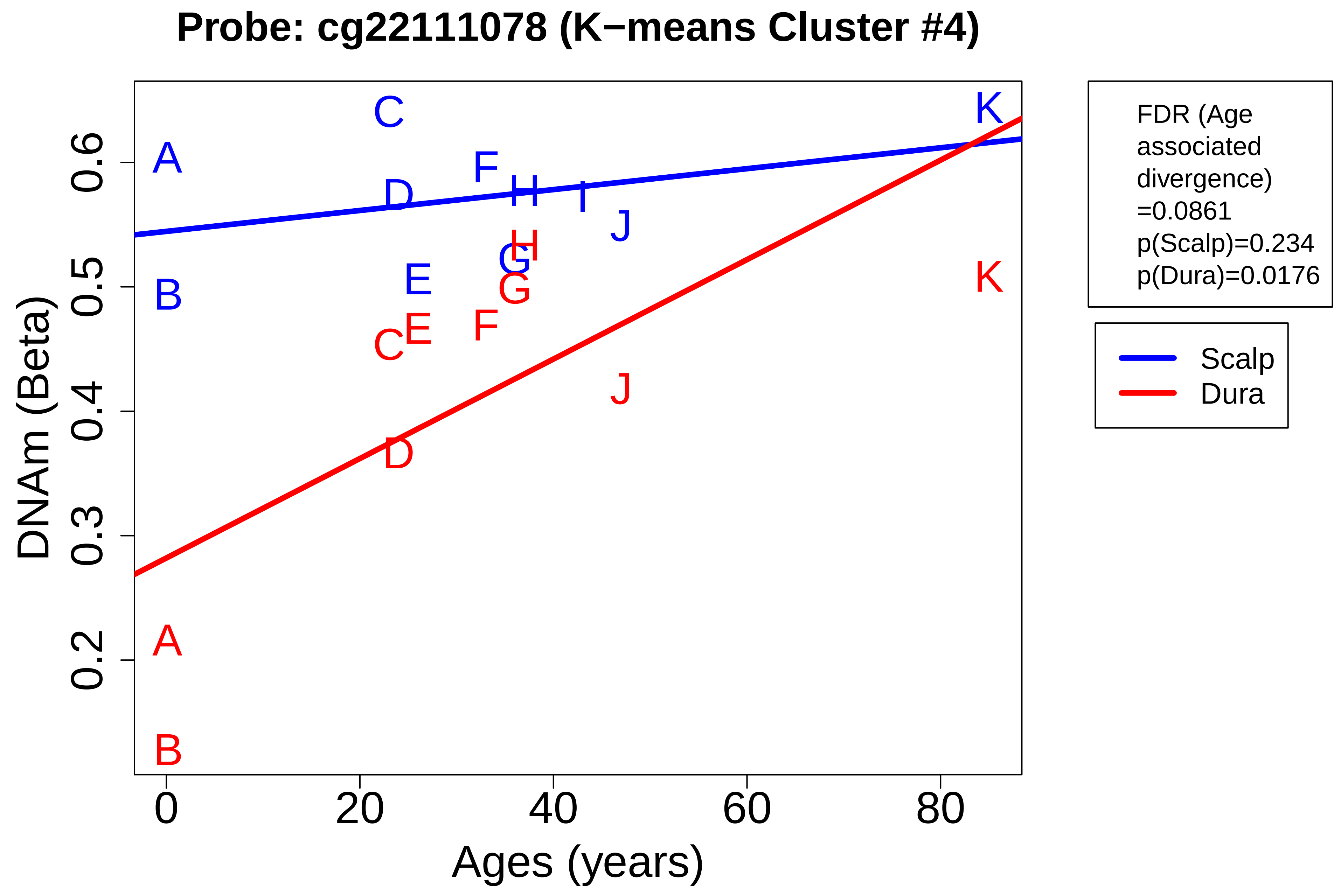

Probe: cg22111078 (K−means Cluster #4)
DNAm (Beta)
0.2
0.3
0.4
0.5
0.6
0
20
40
60
80
Ages (years)
FDR (Age associated divergence) =0.0861
p(Scalp)=0.234
p(Dura)=0.0176
Scalp
Dura

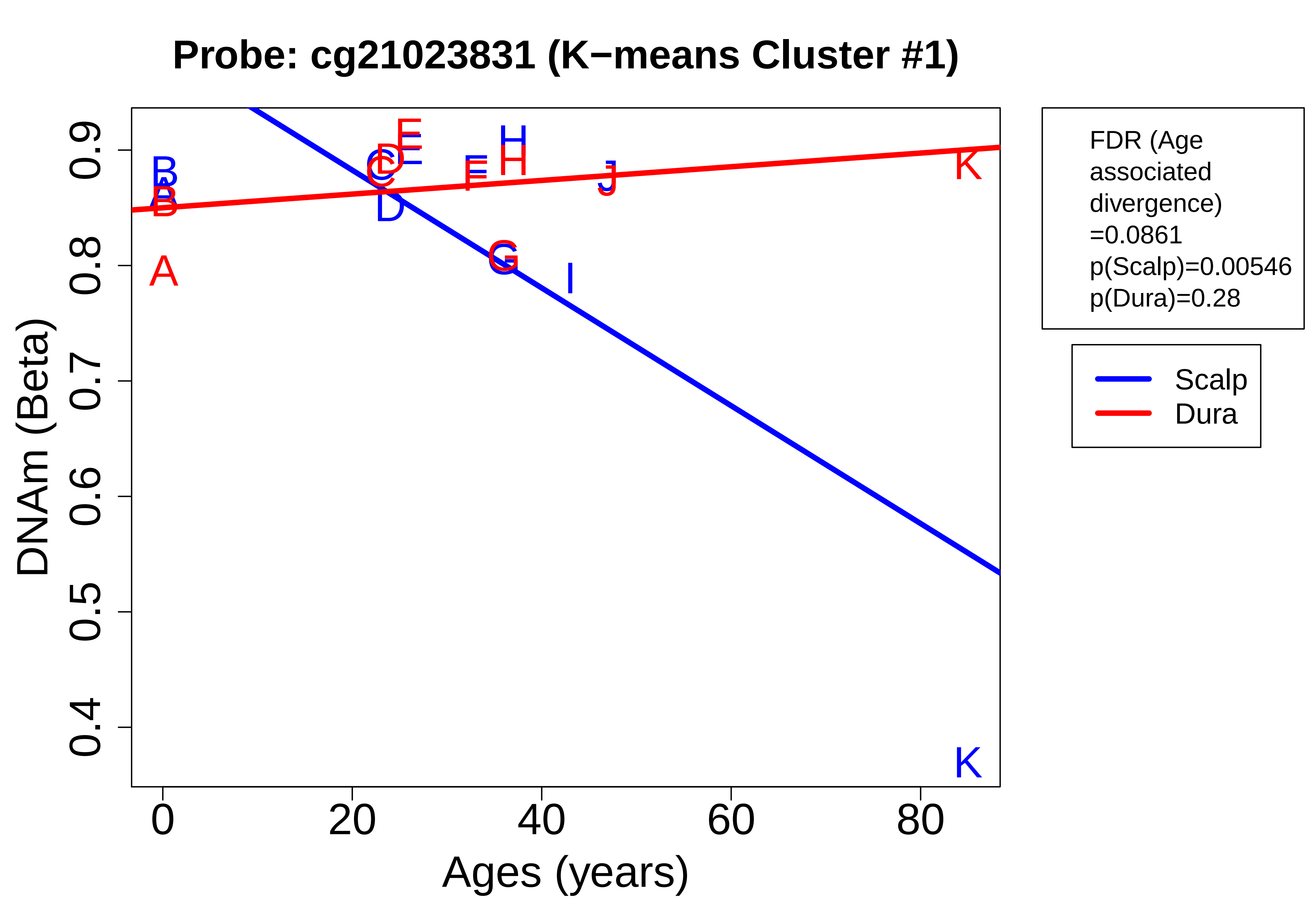

Probe: cg21023831 (K−means Cluster #1)
DNAm (Beta)
0.4
0.5
0.6
0.7
0.8
0.9
0
20
40
60
80
Ages (years)
A
B
C
D
E
F
G
H
I
J
K
FDR (Age associated divergence) =0.0861
p(Scalp)=0.00546
p(Dura)=0.28
Scalp
Dura

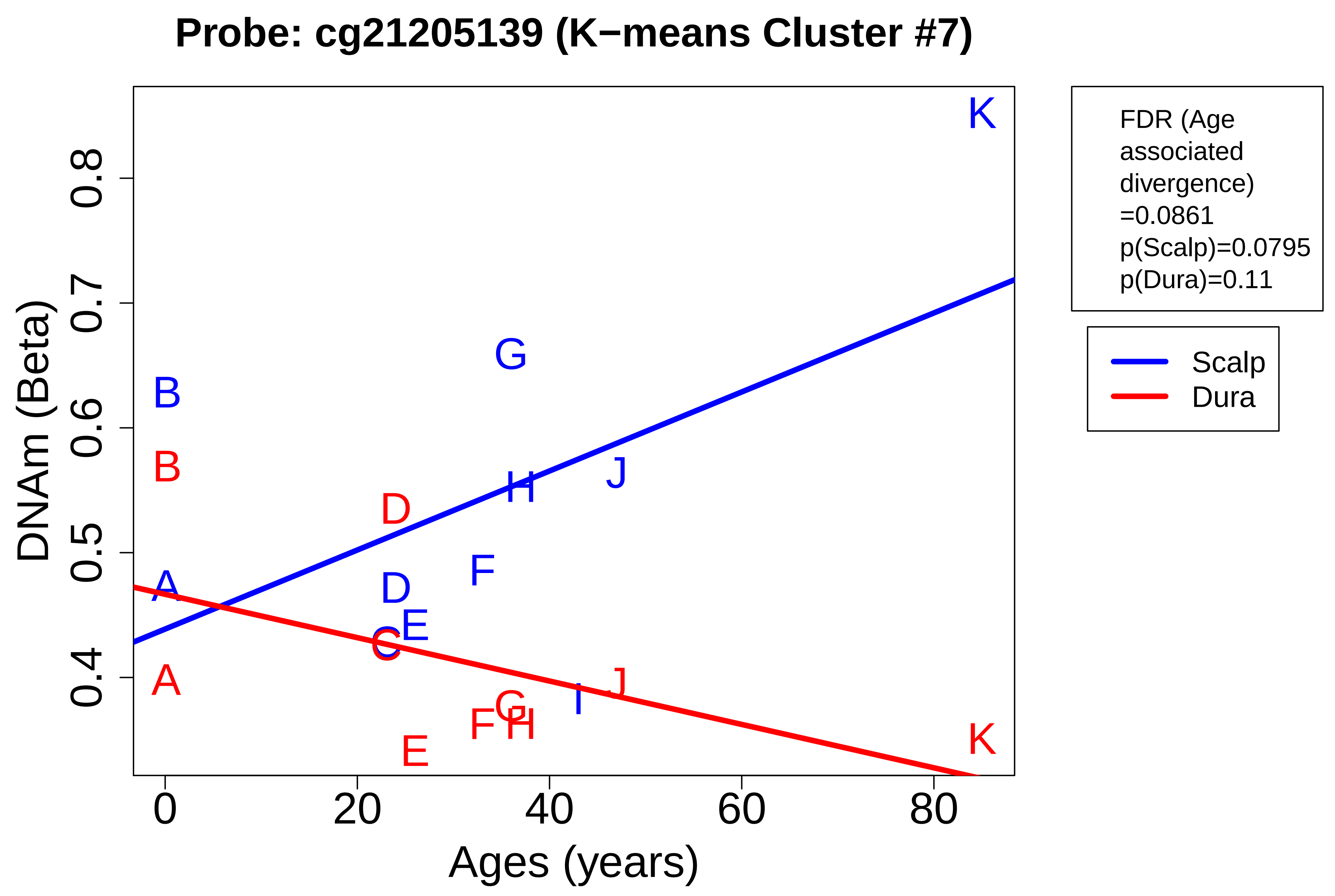

Probe: cg21205139 (K−means Cluster #7)
FDR (Age associated divergence) =0.0861
p(Scalp)=0.0795
p(Dura)=0.11
Scalp
Dura
DNAm (Beta)
Ages (years)
0.4
0.5
0.6
0.7
0.8
0
20
40
60
80

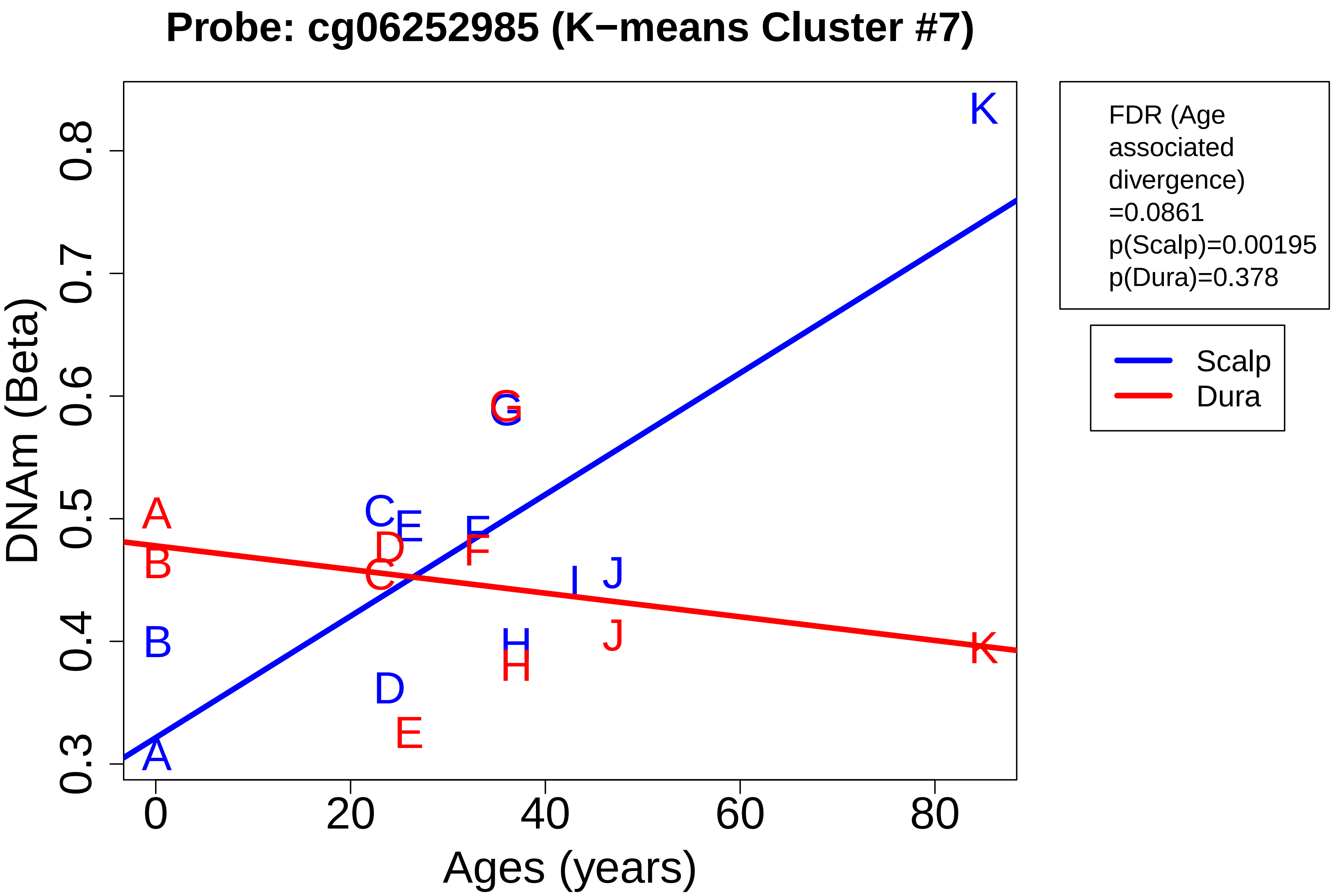
Probe: cg06252985 (K−means Cluster #7)
DNAm (Beta)
Ages (years)
0.3
0.4
0.5
0.6
0.7
0.8
0
20
40
60
80
FDR (Age associated divergence) =0.0861
p(Scalp)=0.00195
p(Dura)=0.378
Scalp
Dura
A
B
C
D
E
F
G
H
I
J
K

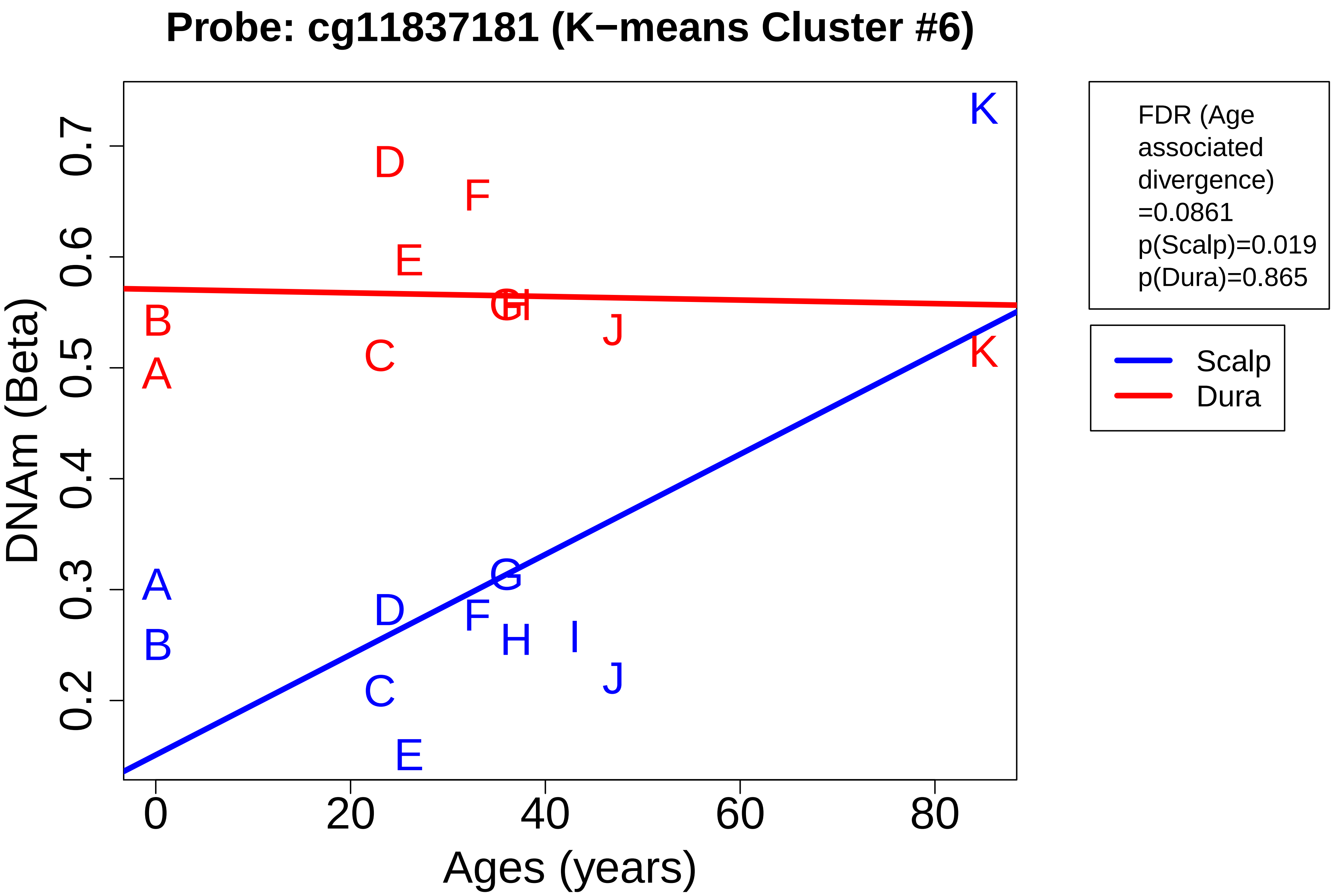
Probe: cg11837181 (K−means Cluster #6)
DNAm (Beta)
Ages (years)
0.2
0.3
0.4
0.5
0.6
0.7
0
20
40
60
80
FDR (Age associated divergence) =0.0861
p(Scalp)=0.019
p(Dura)=0.865
Scalp
Dura
A
B
C
D
E
F
G
H
I
J
K

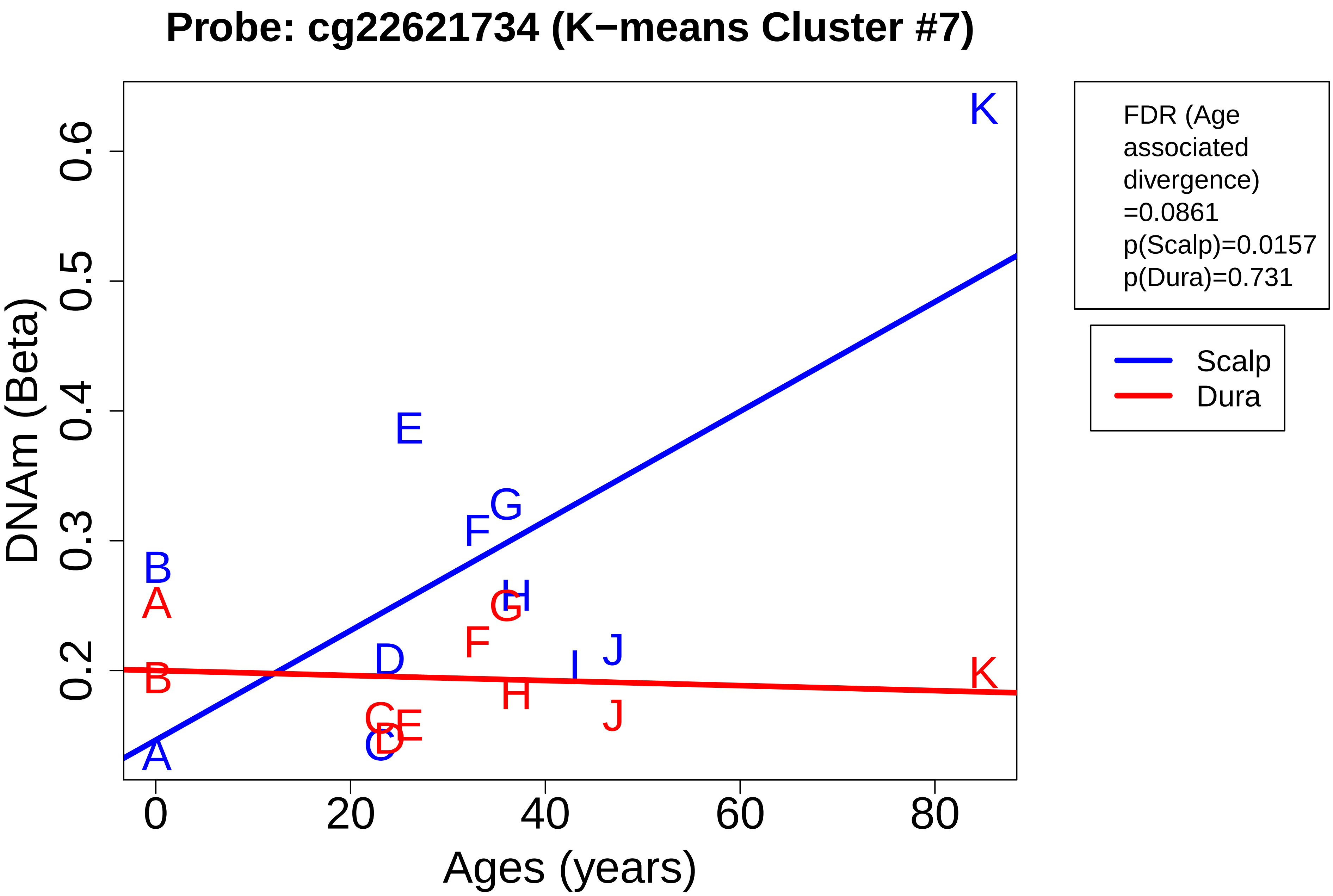

Probe: cg22621734 (K−means Cluster #7)
DNAm (Beta)
0.2
0.3
0.4
0.5
0.6
Ages (years)
0
20
40
60
80
FDR (Age associated divergence) =0.0861
p(Scalp)=0.0157
p(Dura)=0.731
Scalp
Dura
A
B
C
D
E
F
G
H
I
J
K

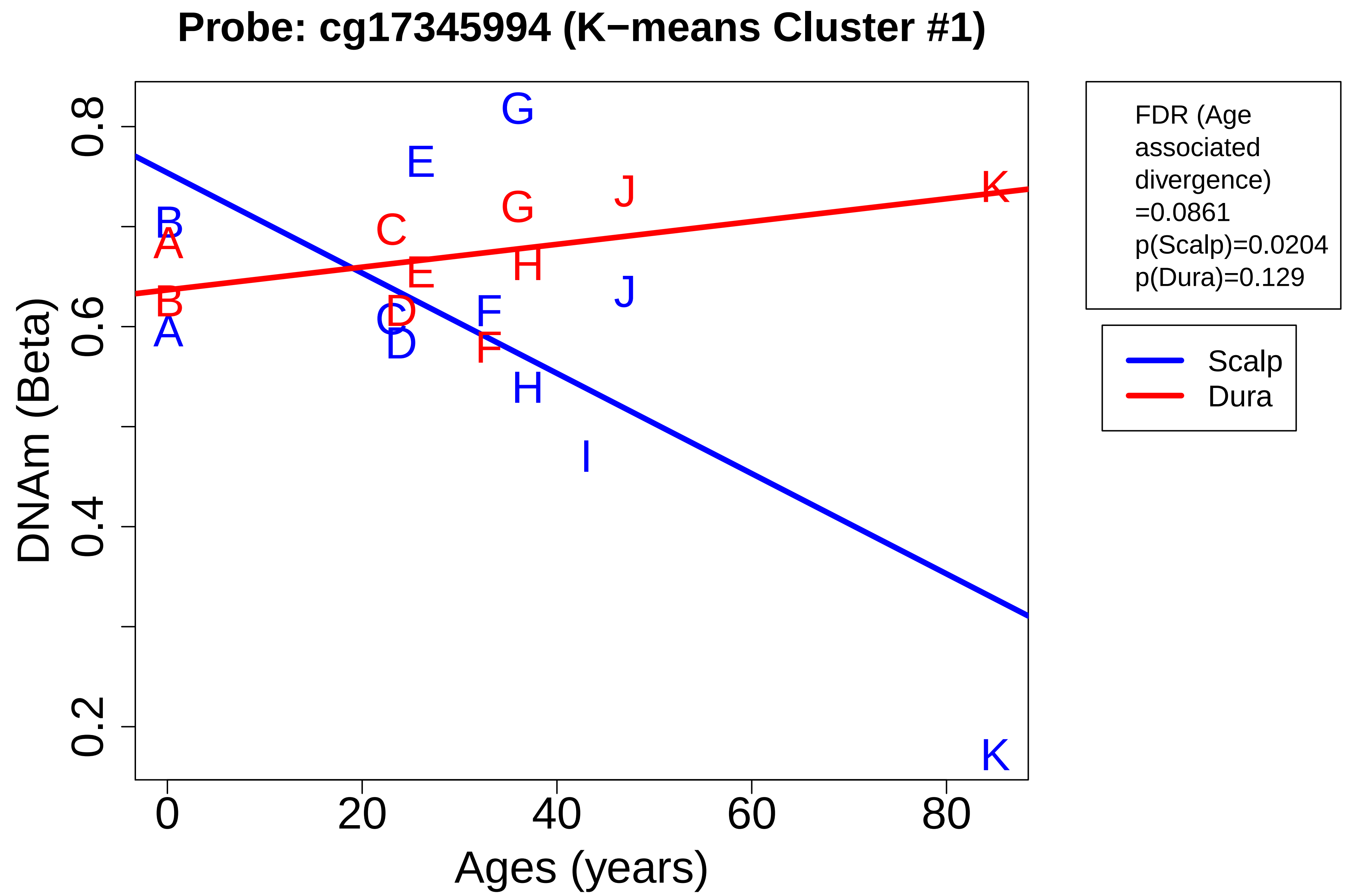

Probe: cg17345994 (K−means Cluster #1)
DNAm (Beta)
0.2
0.4
0.6
0.8
0
20
40
60
80
Ages (years)
FDR (Age associated divergence) =0.0861
p(Scalp)=0.0204
p(Dura)=0.129
Scalp
Dura

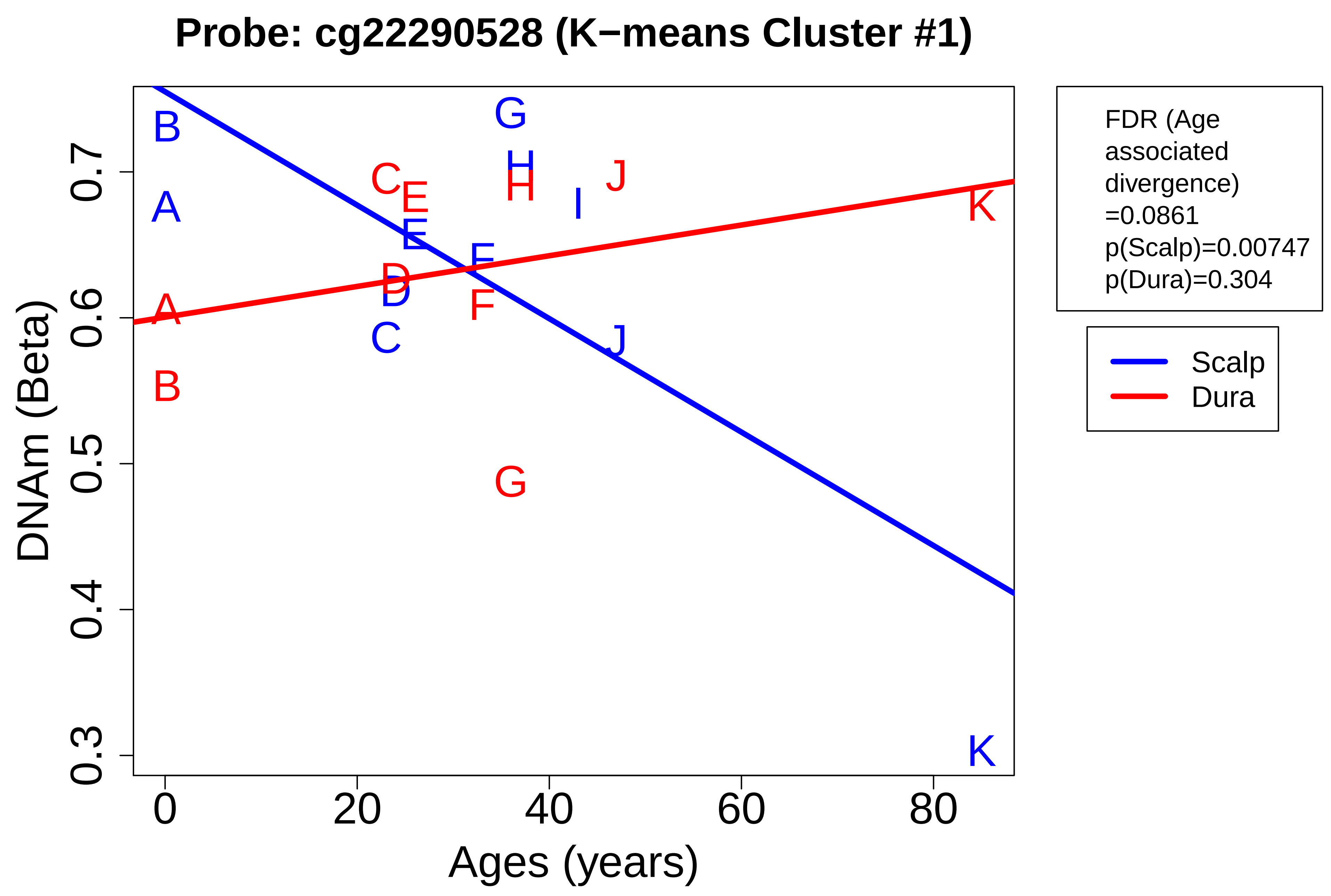
Probe: cg22290528 (K−means Cluster #1)
DNAm (Beta)
0.3
0.4
0.5
0.6
0.7
0
20
40
60
80
Ages (years)
A
B
C
D
E
F
G
H
I
J
K
FDR (Age associated divergence) =0.0861
p(Scalp)=0.00747
p(Dura)=0.304
Scalp
Dura

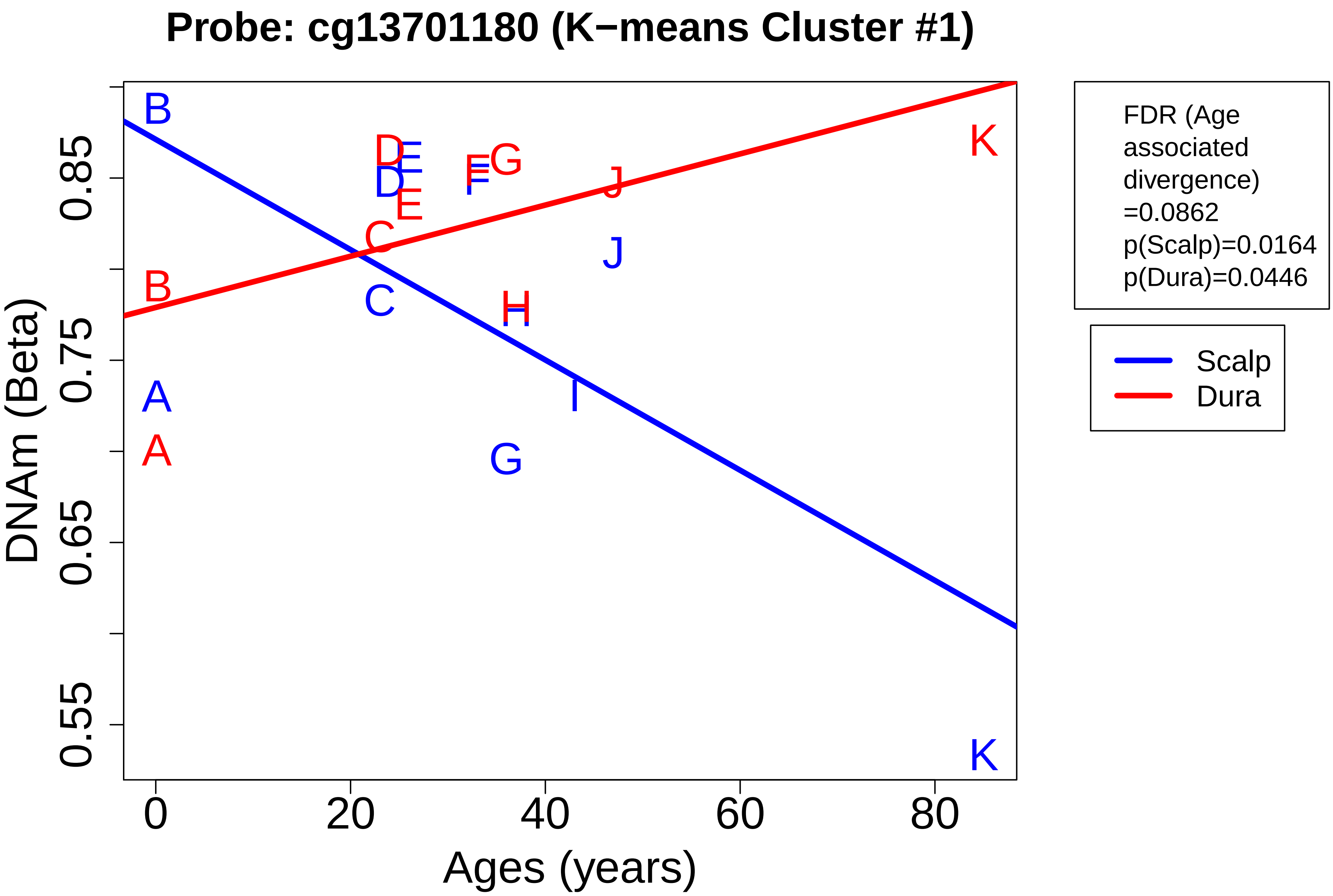

Probe: cg13701180 (K−means Cluster #1)
DNAm (Beta)
0.55
0.65
0.75
0.85
0
20
40
60
80
Ages (years)
B
D
E
F
G
J
K
C
H
A
I
FDR (Age associated divergence) =0.0862
p(Scalp)=0.0164
p(Dura)=0.0446
Scalp
Dura

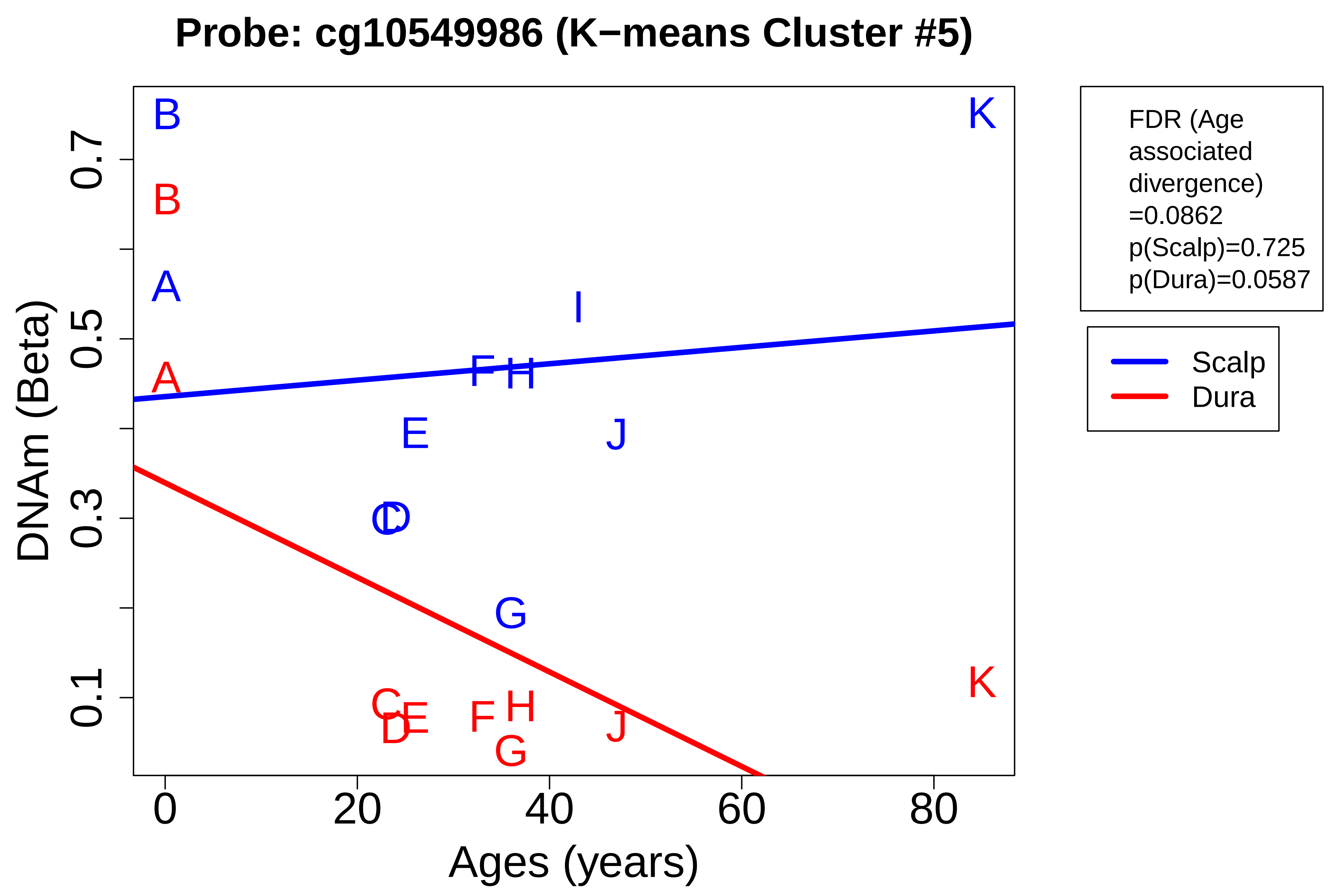

Probe: cg10549986 (K−means Cluster #5)
DNAm (Beta)
0.1
0.3
0.5
0.7
0
20
40
60
80
Ages (years)
FDR (Age associated divergence) =0.0862
p(Scalp)=0.725
p(Dura)=0.0587
Scalp
Dura

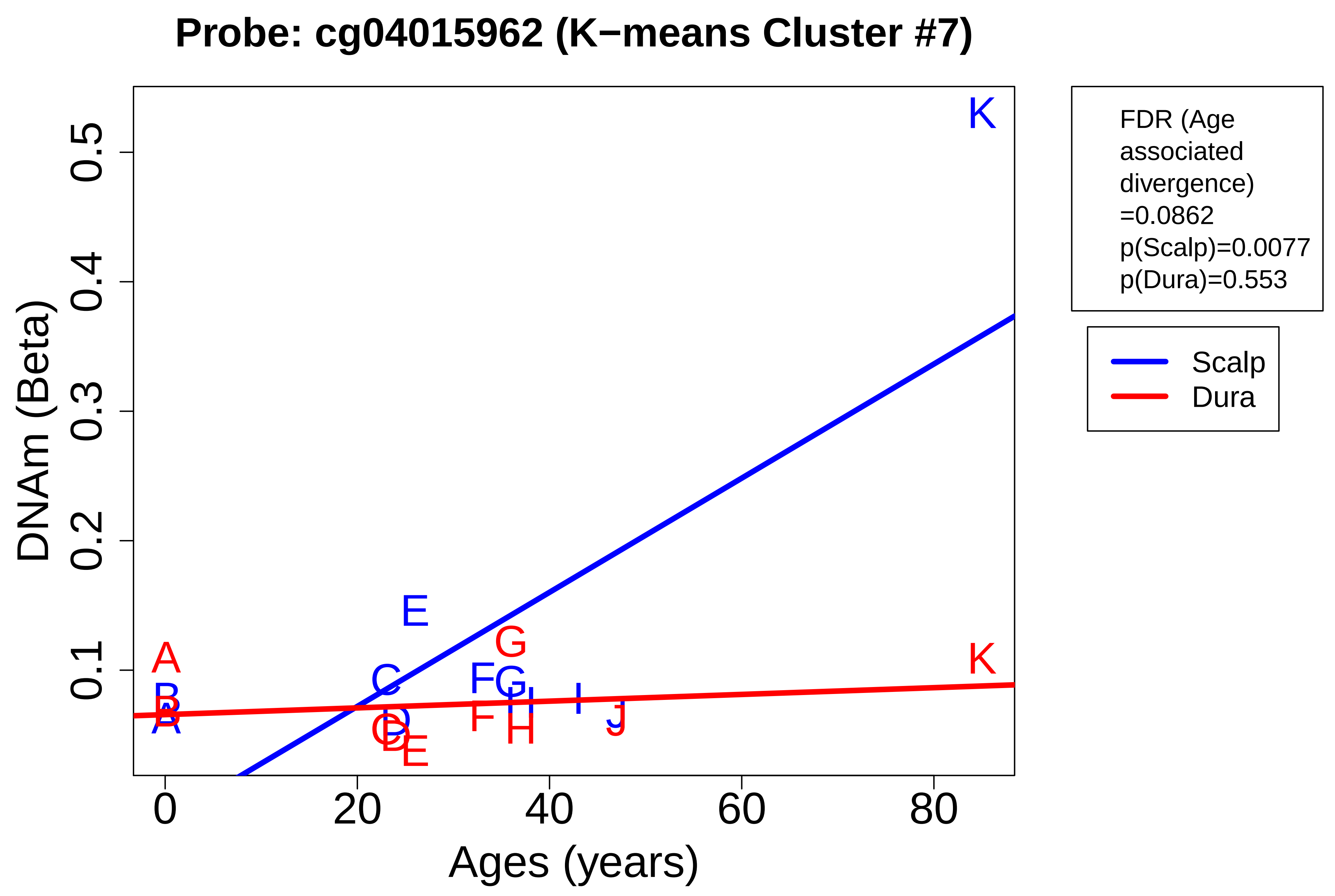

Probe: cg04015962 (K−means Cluster #7)
DNAm (Beta)
Ages (years)
0.1
0.2
0.3
0.4
0.5
0
20
40
60
80
FDR (Age associated divergence) =0.0862
p(Scalp)=0.0077
p(Dura)=0.553
Scalp
Dura

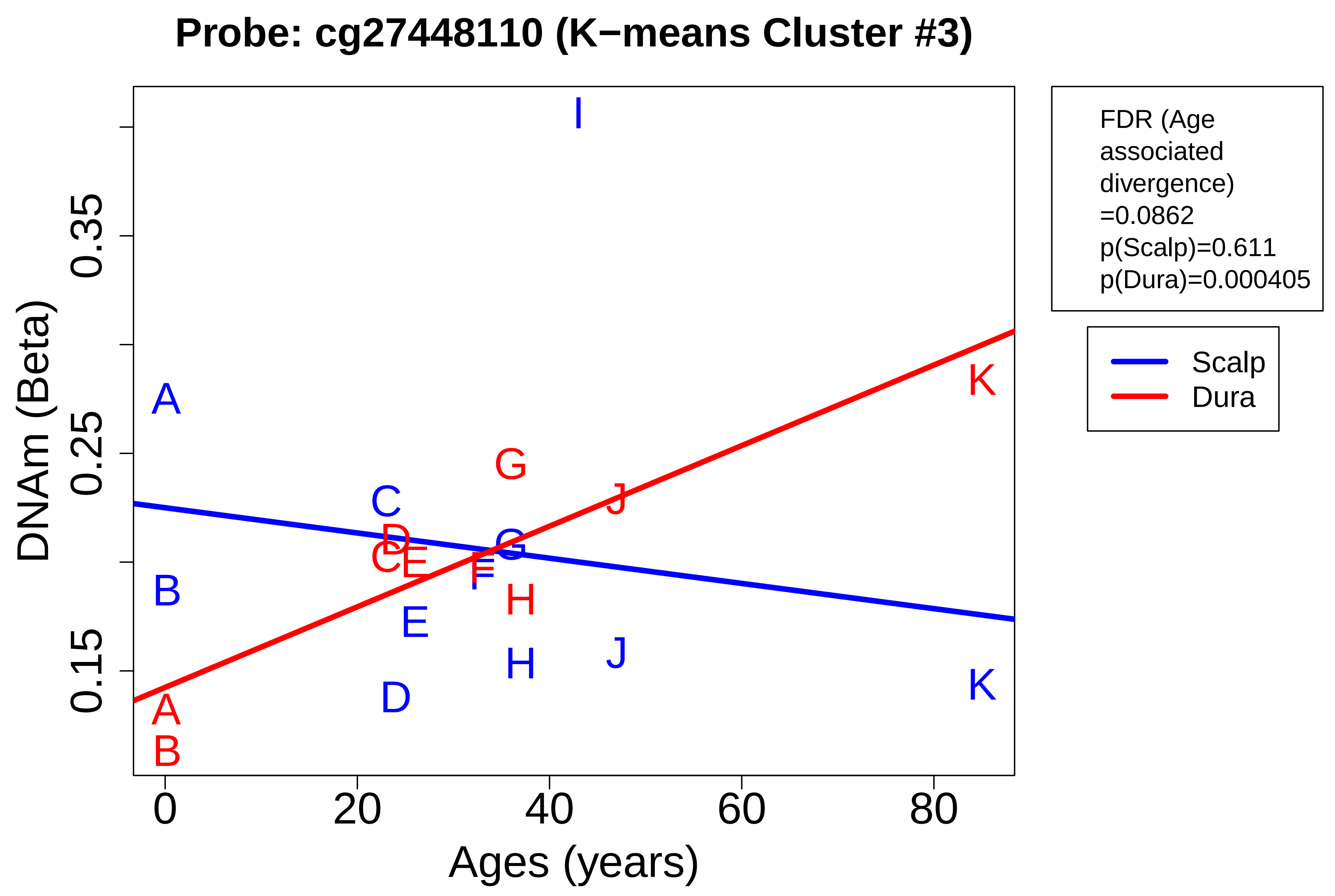
Probe: cg27448110 (K−means Cluster #3)
DNAm (Beta)
0.15
0.25
0.35
0
20
40
60
80
Ages (years)
A
B
C
D
E
F
G
H
I
J
K
FDR (Age associated divergence) =0.0862
p(Scalp)=0.611
p(Dura)=0.000405
Scalp
Dura

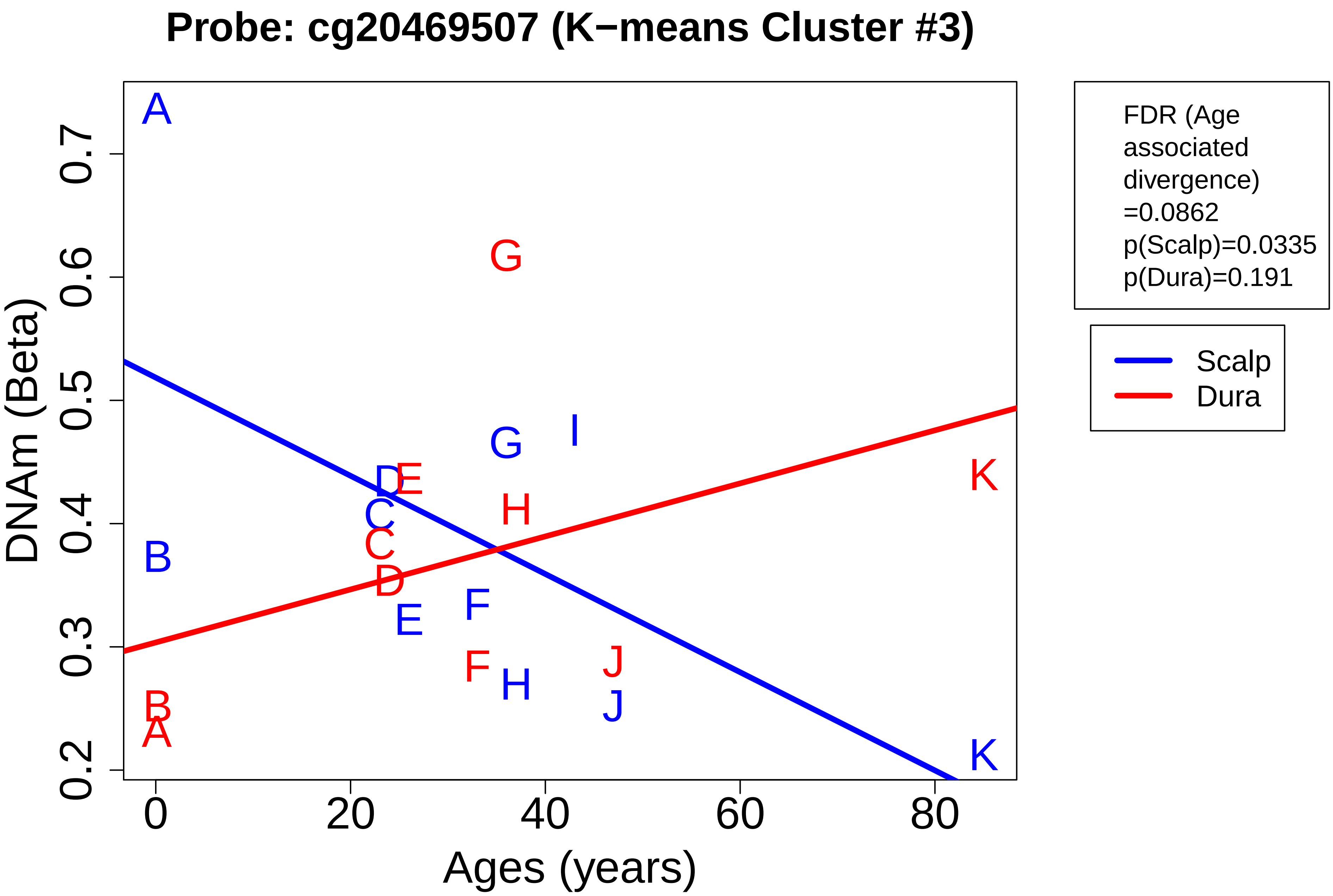

Probe: cg20469507 (K−means Cluster #3)
DNAm (Beta)
Ages (years)
0.2
0.3
0.4
0.5
0.6
0.7
0
20
40
60
80
A
B
C
D
E
F
G
H
I
J
K
A
B
C
D
E
F
G
H
J
K
FDR (Age associated divergence) =0.0862
p(Scalp)=0.0335
p(Dura)=0.191
Scalp
Dura

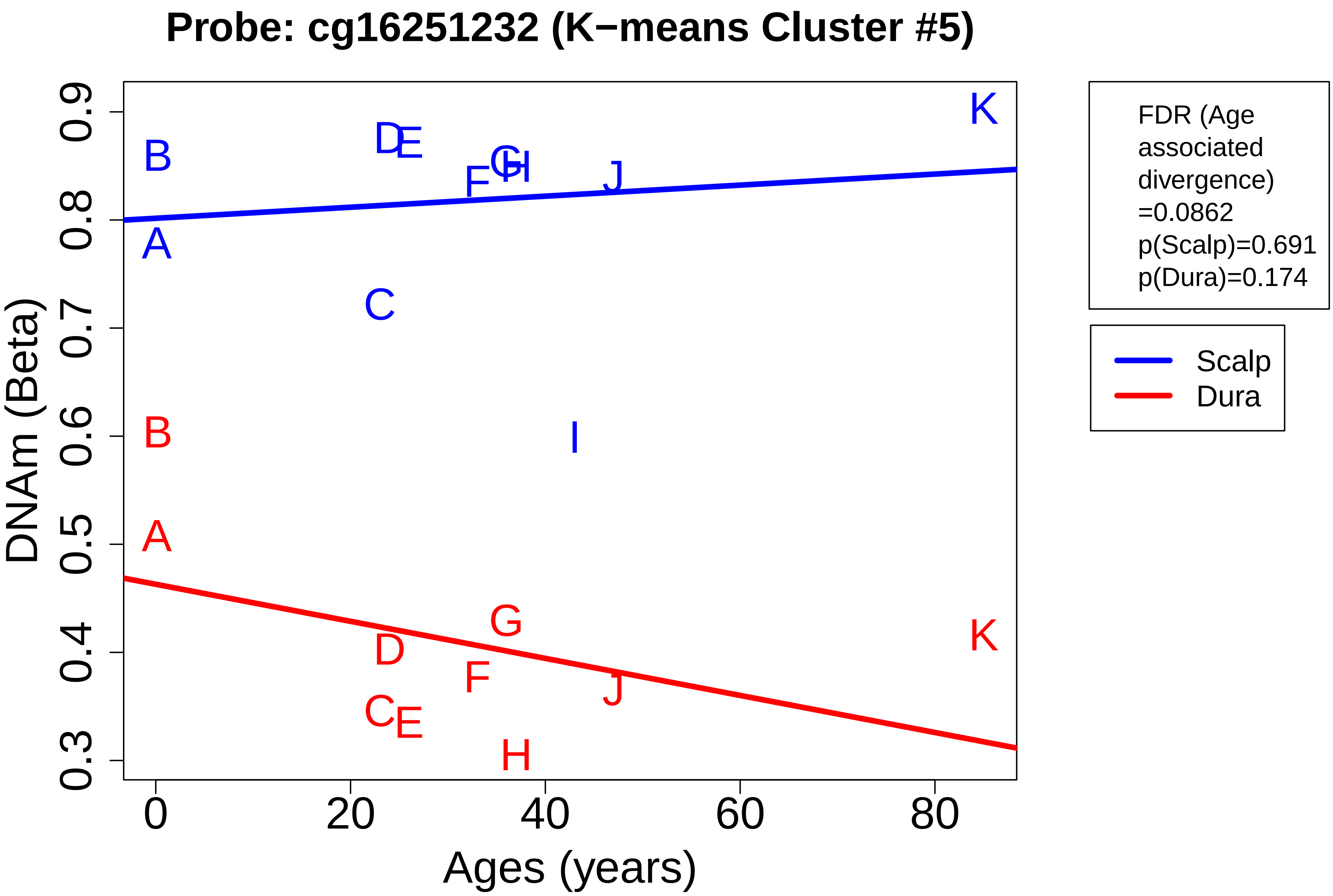

Probe: cg16251232 (K−means Cluster #5)
DNAm (Beta)
Ages (years)
0.3
0.4
0.5
0.6
0.7
0.8
0.9
0
20
40
60
80
A
B
C
D
E
F
G
H
I
J
K
FDR (Age associated divergence) =0.0862
p(Scalp)=0.691
p(Dura)=0.174
Scalp
Dura

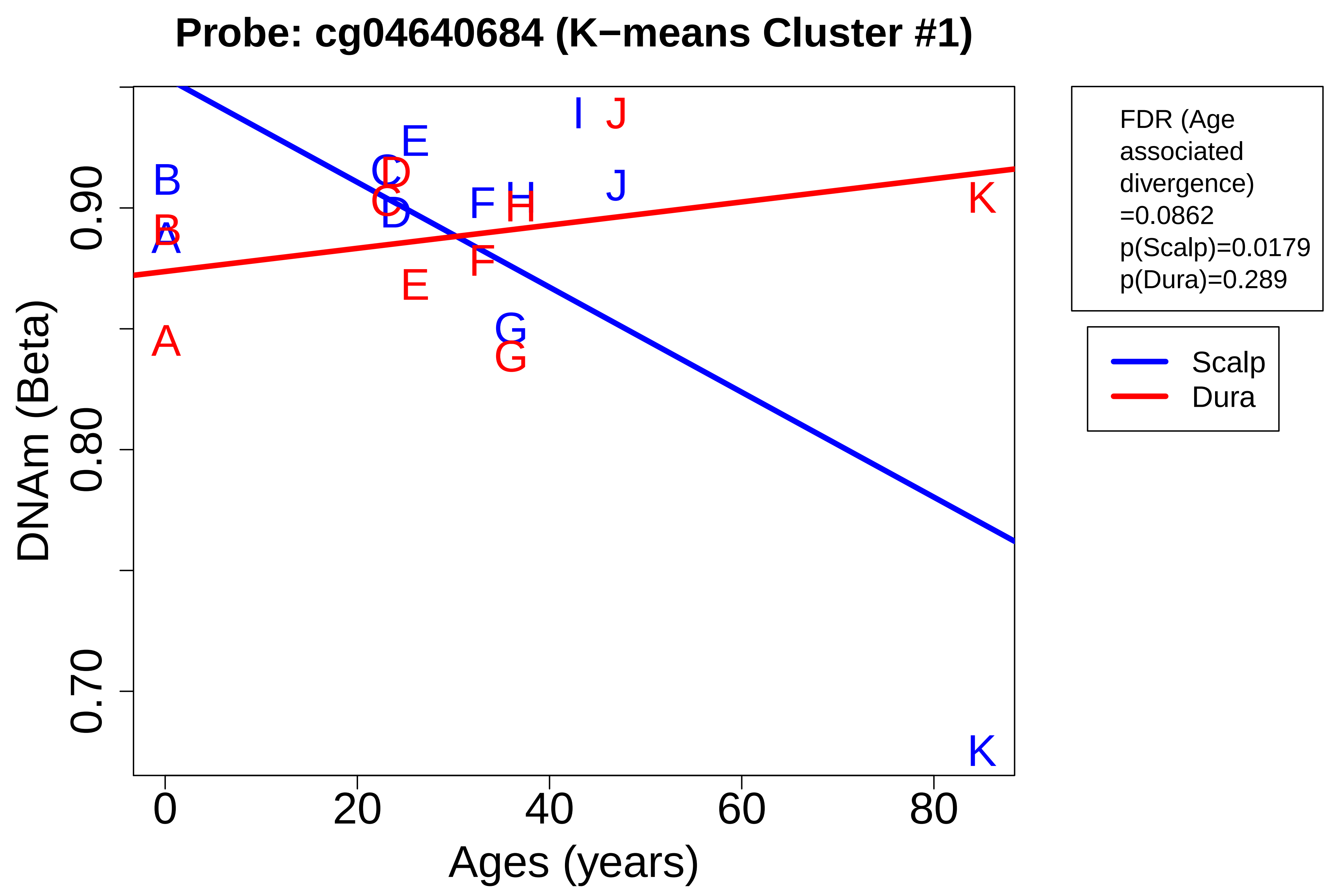

Probe: cg04640684 (K−means Cluster #1)
DNAm (Beta)
0.70
0.80
0.90
Ages (years)
0
20
40
60
80
FDR (Age associated divergence) =0.0862
p(Scalp)=0.0179
p(Dura)=0.289
Scalp
Dura
A B C D E F G H I J K

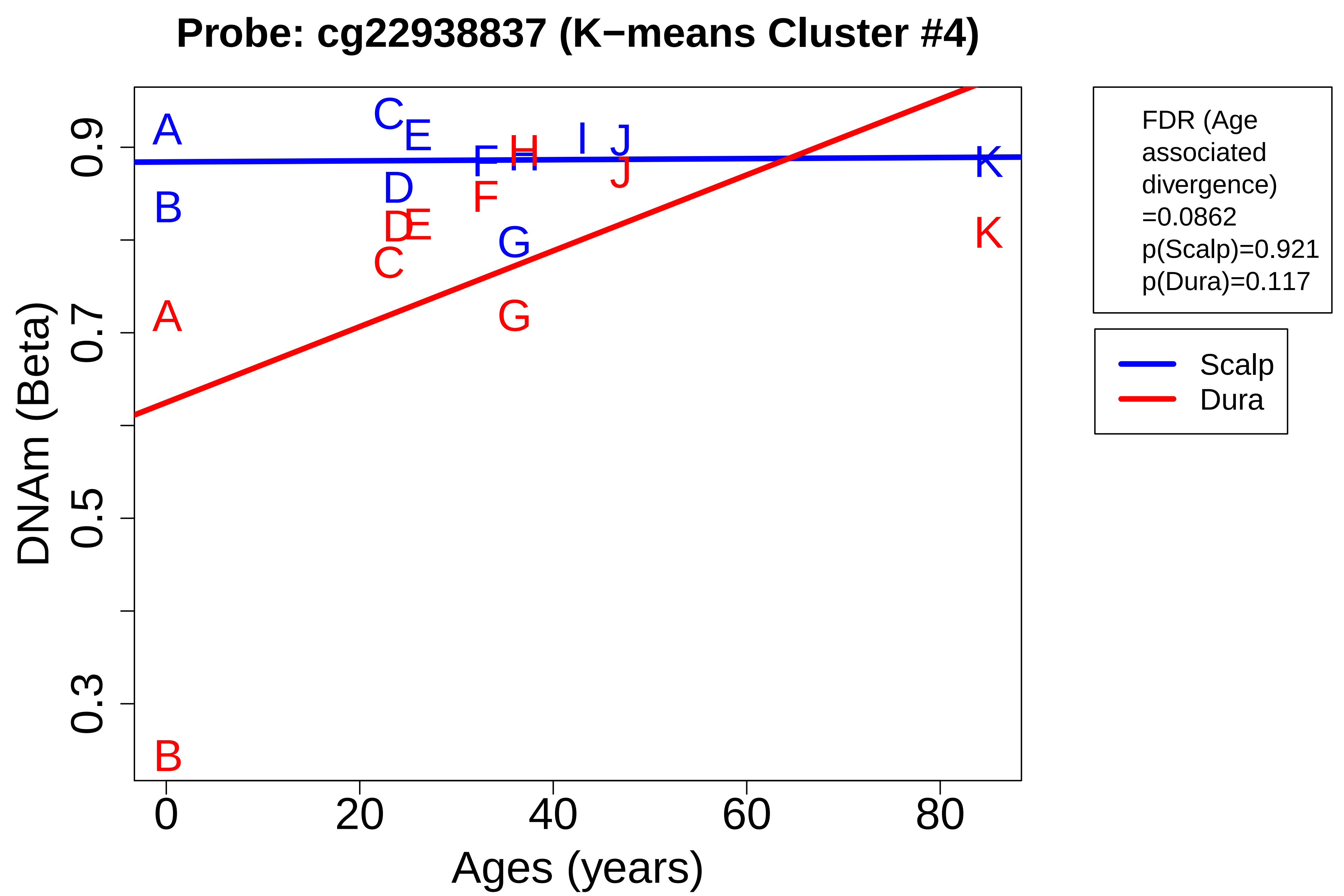

Probe: cg22938837 (K−means Cluster #4)
DNAm (Beta)
0.3
0.5
0.7
0.9
Ages (years)
0
20
40
60
80
A
B
C
D
E
F
G
H
I
J
K
FDR (Age associated divergence) =0.0862
p(Scalp)=0.921
p(Dura)=0.117
Scalp
Dura

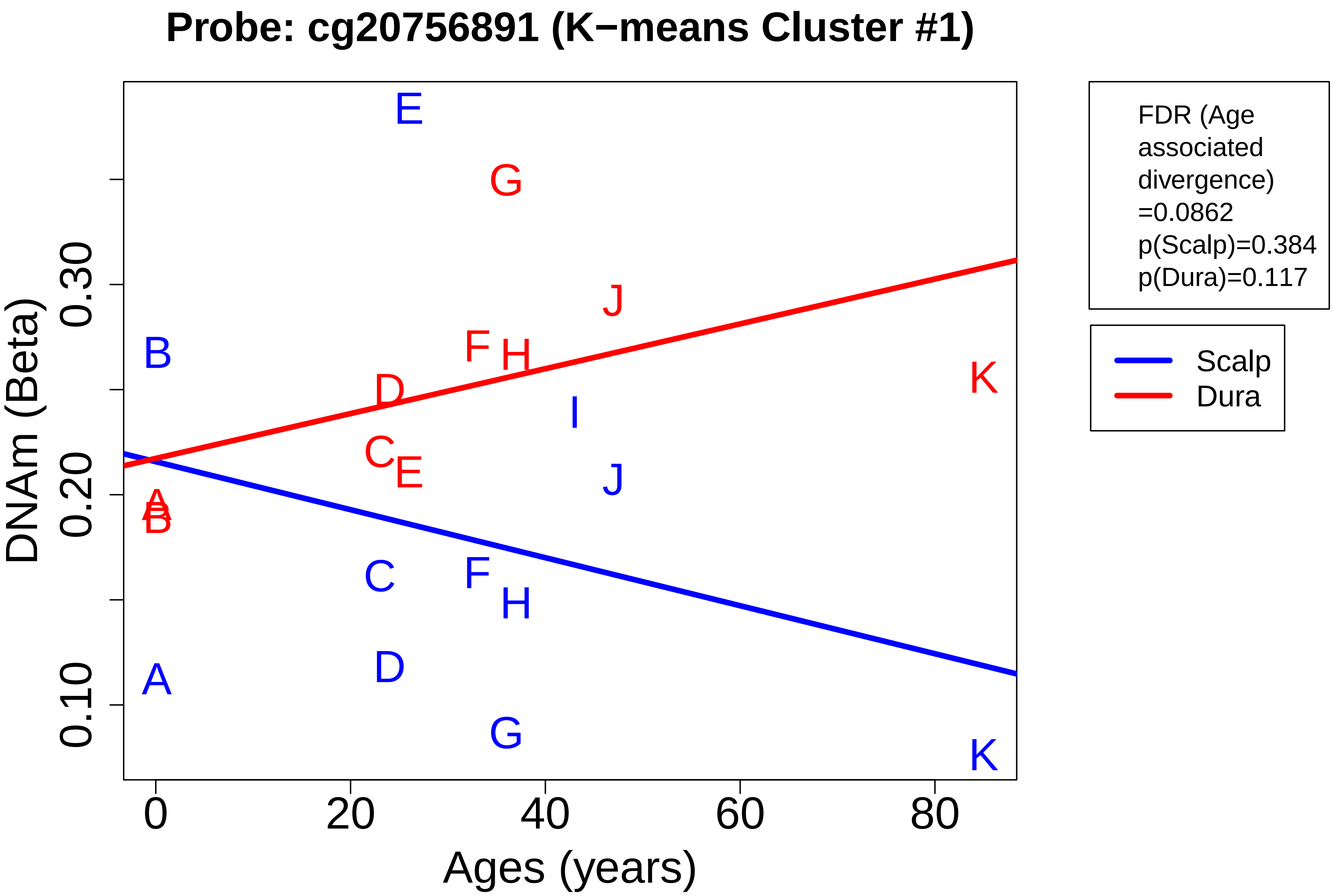

Probe: cg20756891 (K−means Cluster #1)
DNAm (Beta)
0.10
0.20
0.30
Ages (years)
0
20
40
60
80
FDR (Age associated divergence) =0.0862
p(Scalp)=0.384
p(Dura)=0.117
Scalp
Dura
A
B
C
D
E
F
G
H
I
J
K

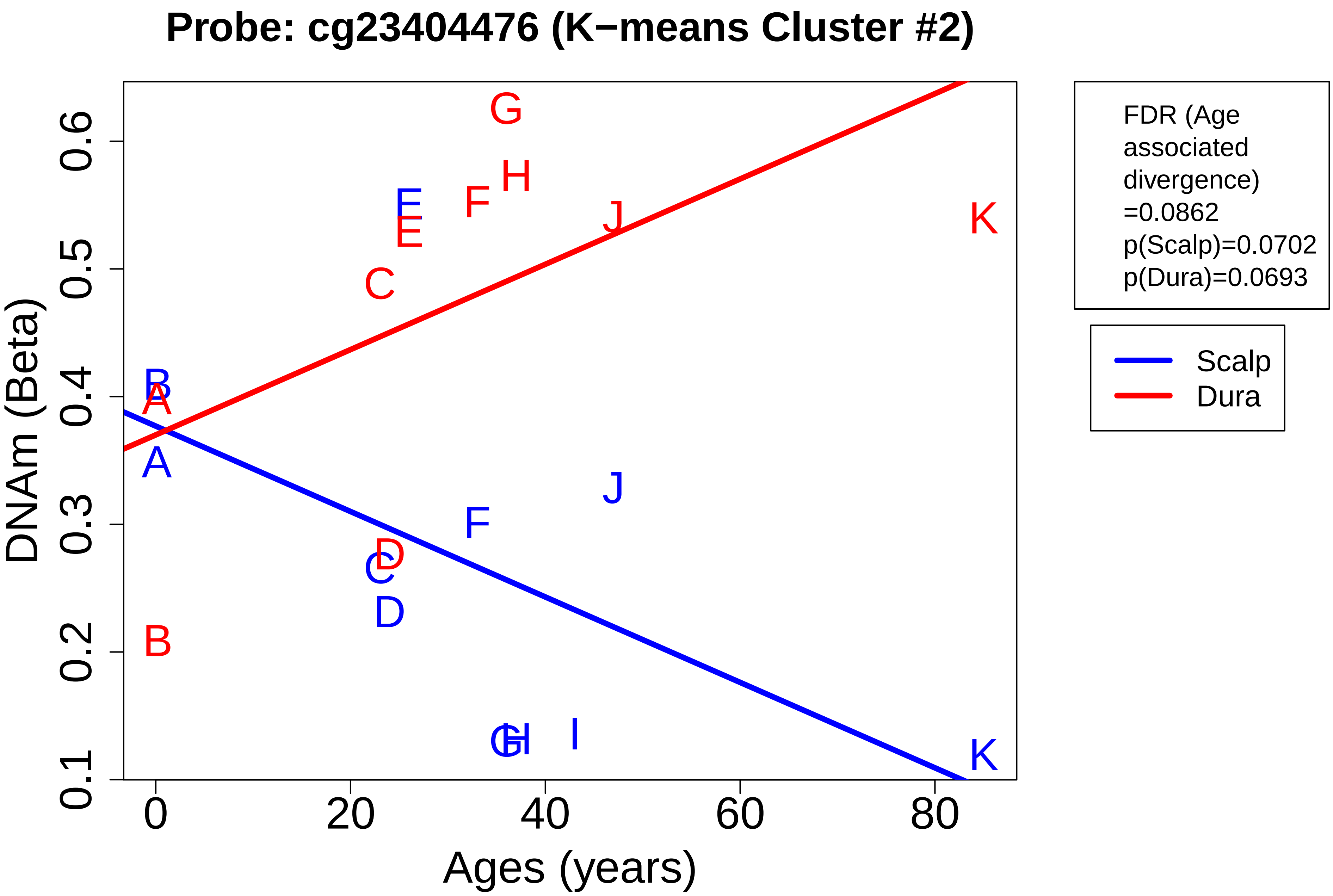

Probe: cg23404476 (K−means Cluster #2)
DNAm (Beta)
0.1
0.2
0.3
0.4
0.5
0.6
0
20
40
60
80
Ages (years)
FDR (Age associated divergence) =0.0862
p(Scalp)=0.0702
p(Dura)=0.0693
Scalp
Dura

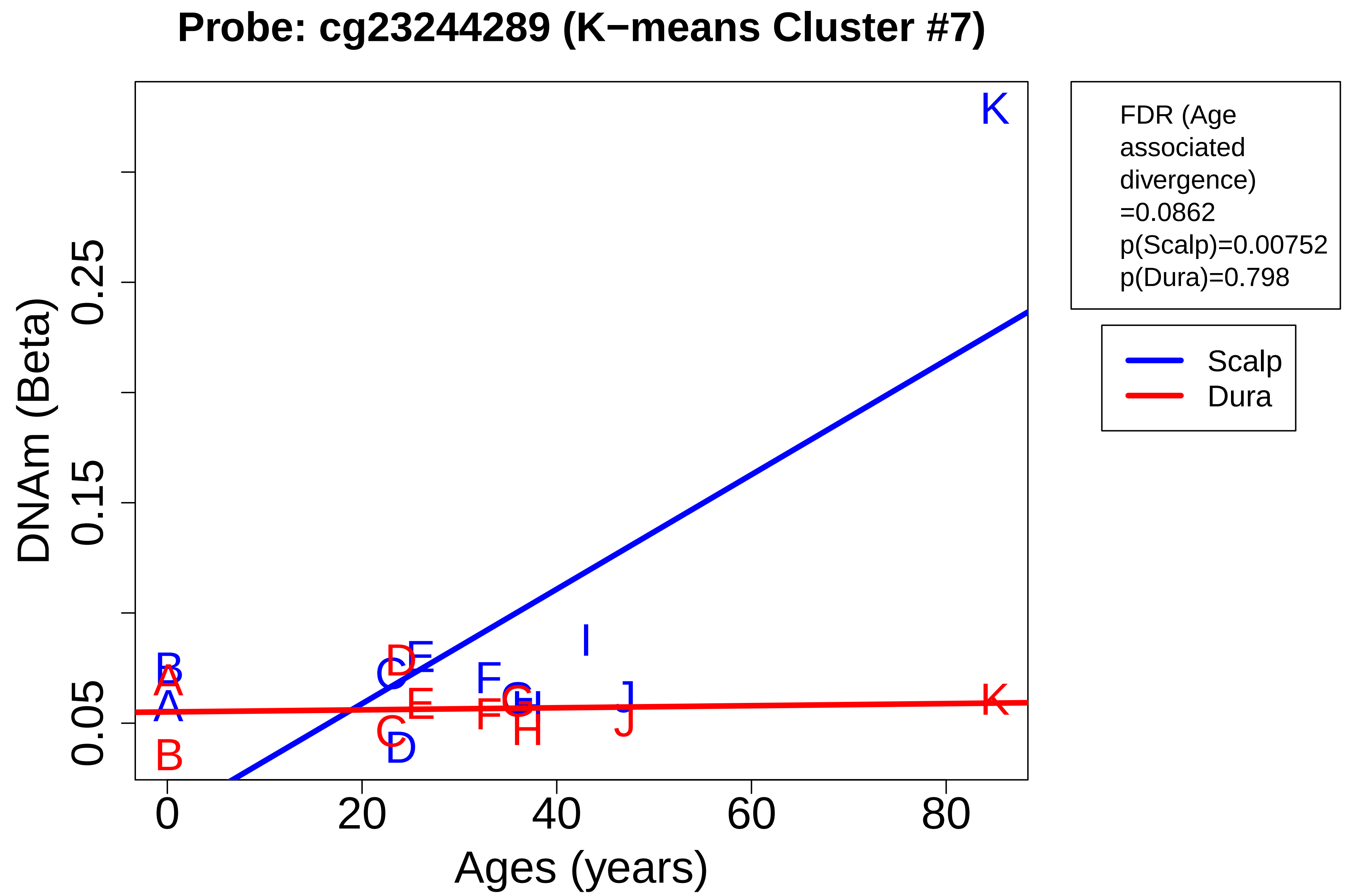

Probe: cg23244289 (K−means Cluster #7)
DNAm (Beta)
0.05
0.15
0.25
Ages (years)
0
20
40
60
80
FDR (Age associated divergence) =0.0862
p(Scalp)=0.00752
p(Dura)=0.798
Scalp
Dura

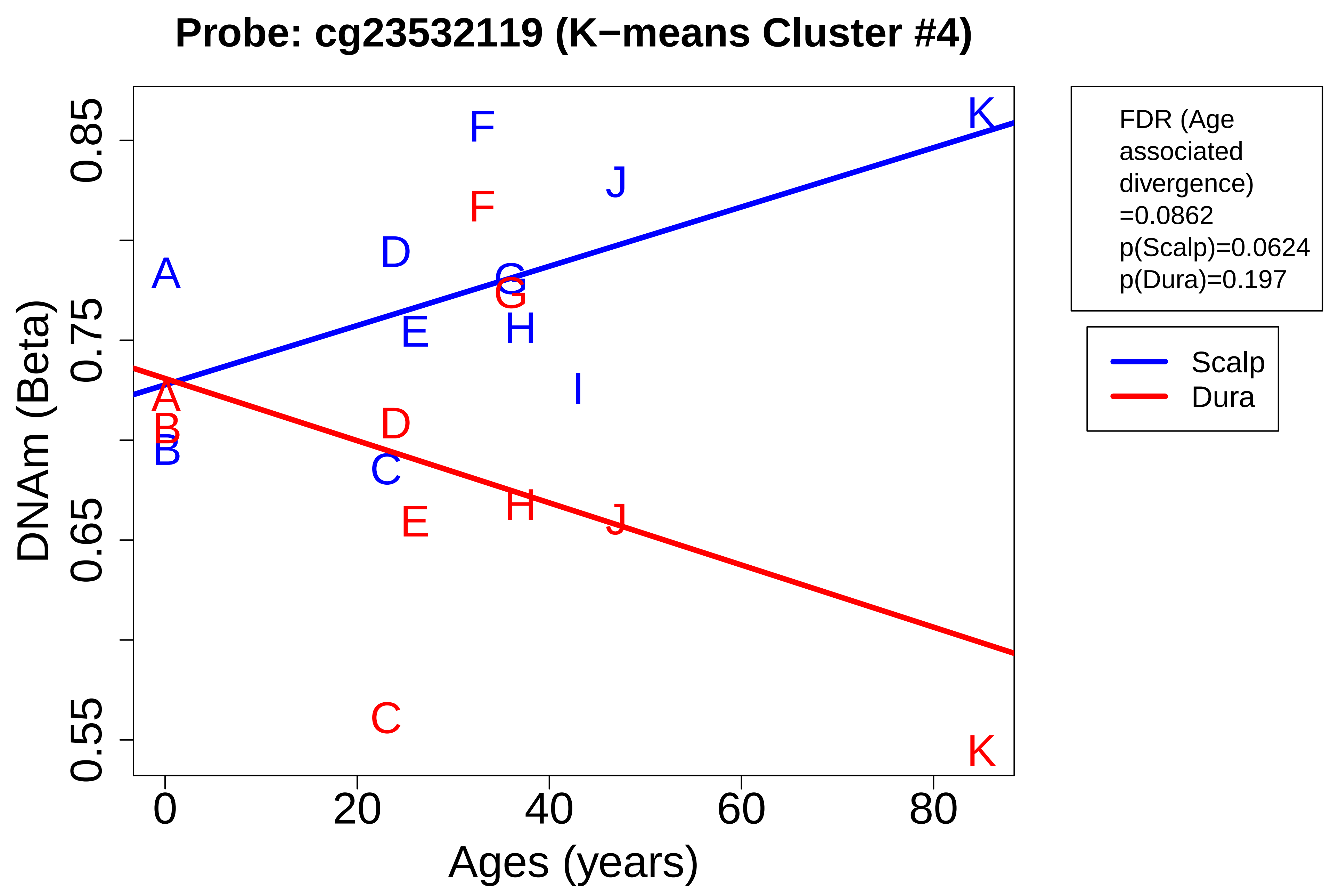

Probe: cg23532119 (K−means Cluster #4)
DNAm (Beta)
0.55
0.65
0.75
0.85
0
20
40
60
80
Ages (years)
A
B
C
D
E
F
G
H
I
J
K
A
B
C
D
E
F
G
H
J
K
FDR (Age associated divergence) =0.0862
p(Scalp)=0.0624
p(Dura)=0.197
Scalp
Dura

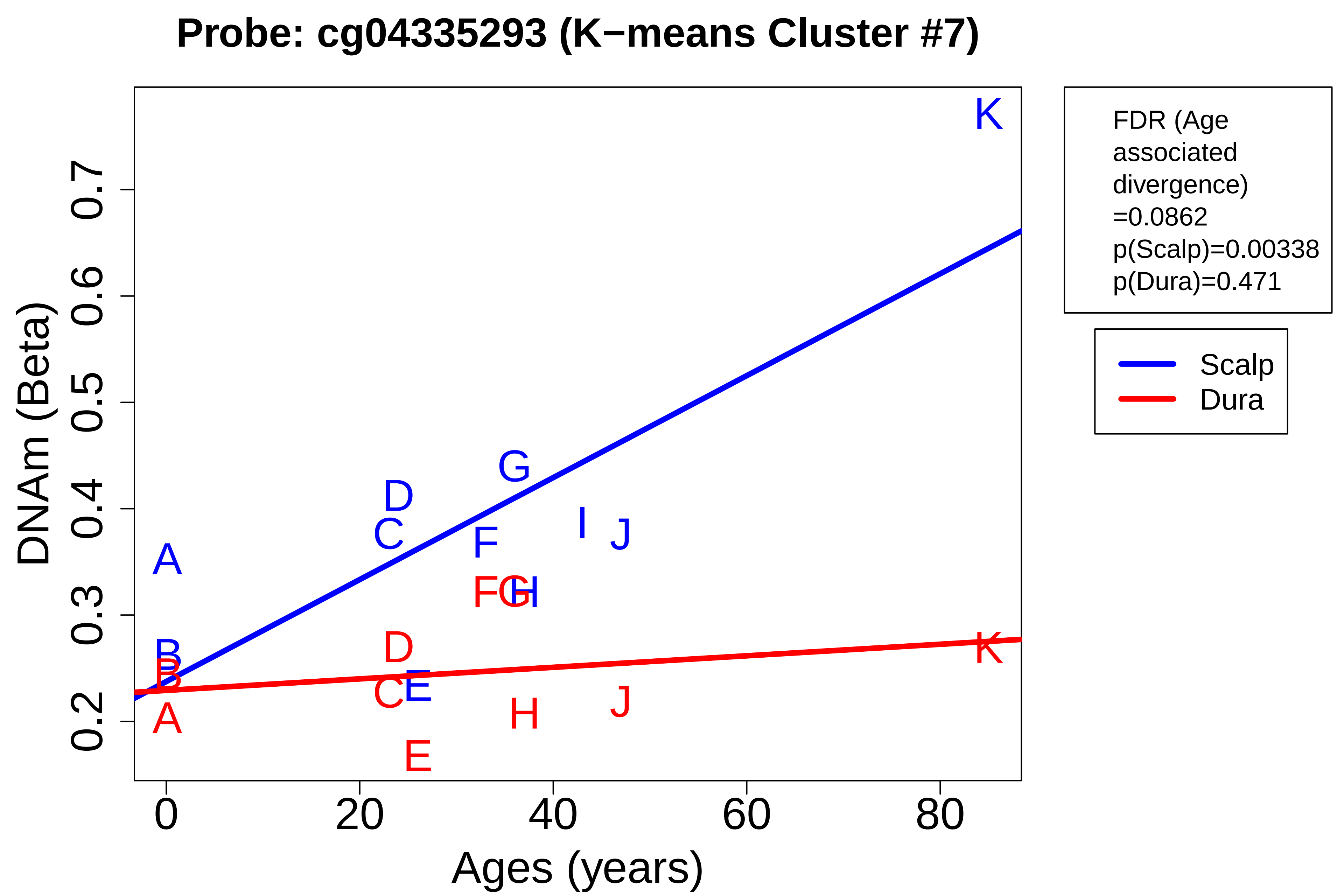

Probe: cg04335293 (K−means Cluster #7)
FDR (Age associated divergence) =0.0862
p(Scalp)=0.00338
p(Dura)=0.471
Scalp
Dura
DNAm (Beta)
0.2
0.3
0.4
0.5
0.6
0.7
0
20
40
60
80
Ages (years)
A
B
C
D
E
F
G
H
I
J
K

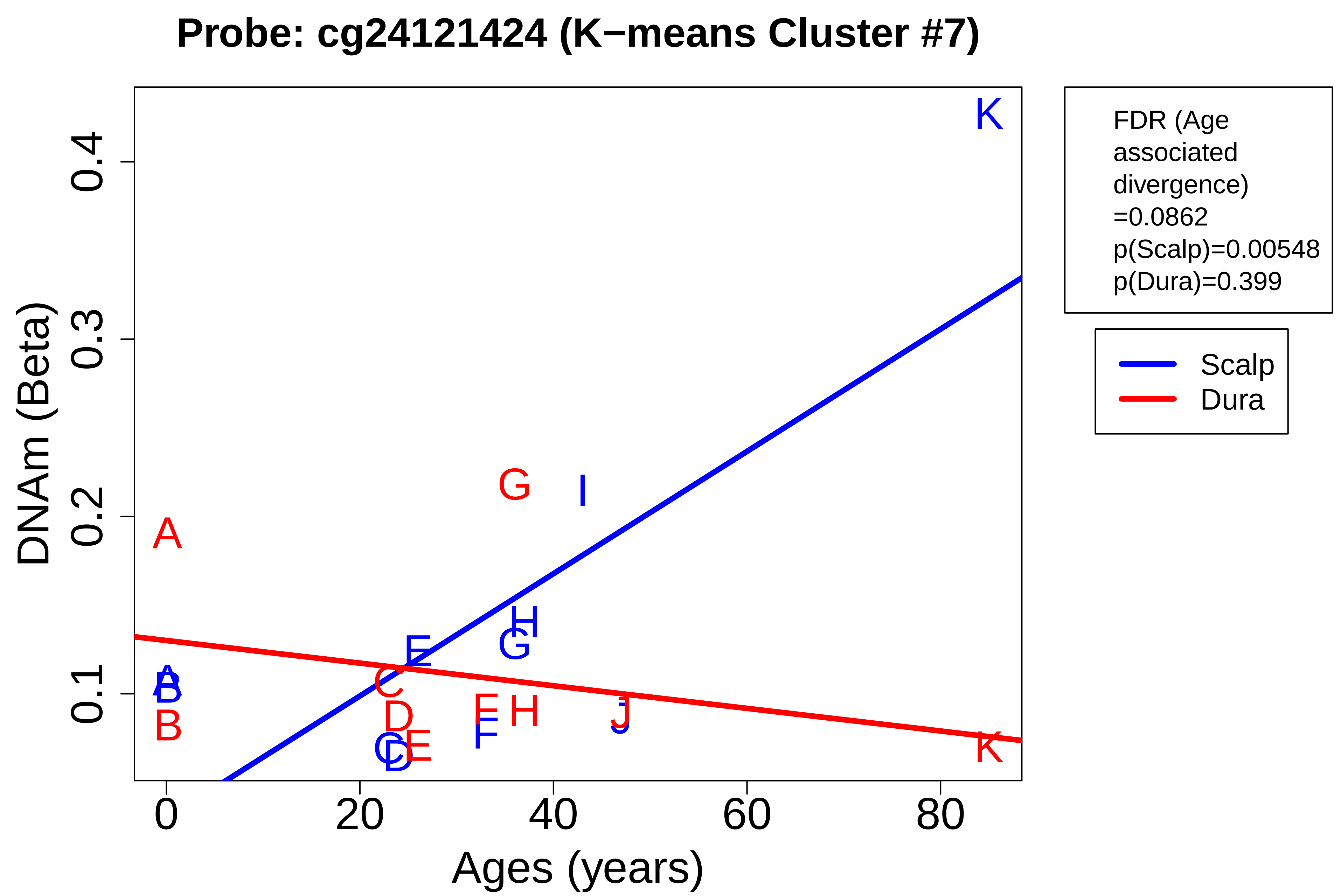

Probe: cg24121424 (K−means Cluster #7)
DNAm (Beta)
0.1
0.2
0.3
0.4
Ages (years)
0
20
40
60
80
FDR (Age associated divergence) =0.0862
p(Scalp)=0.00548
p(Dura)=0.399
Scalp
Dura
A
B
C
D
E
F
G
H
I
J
K

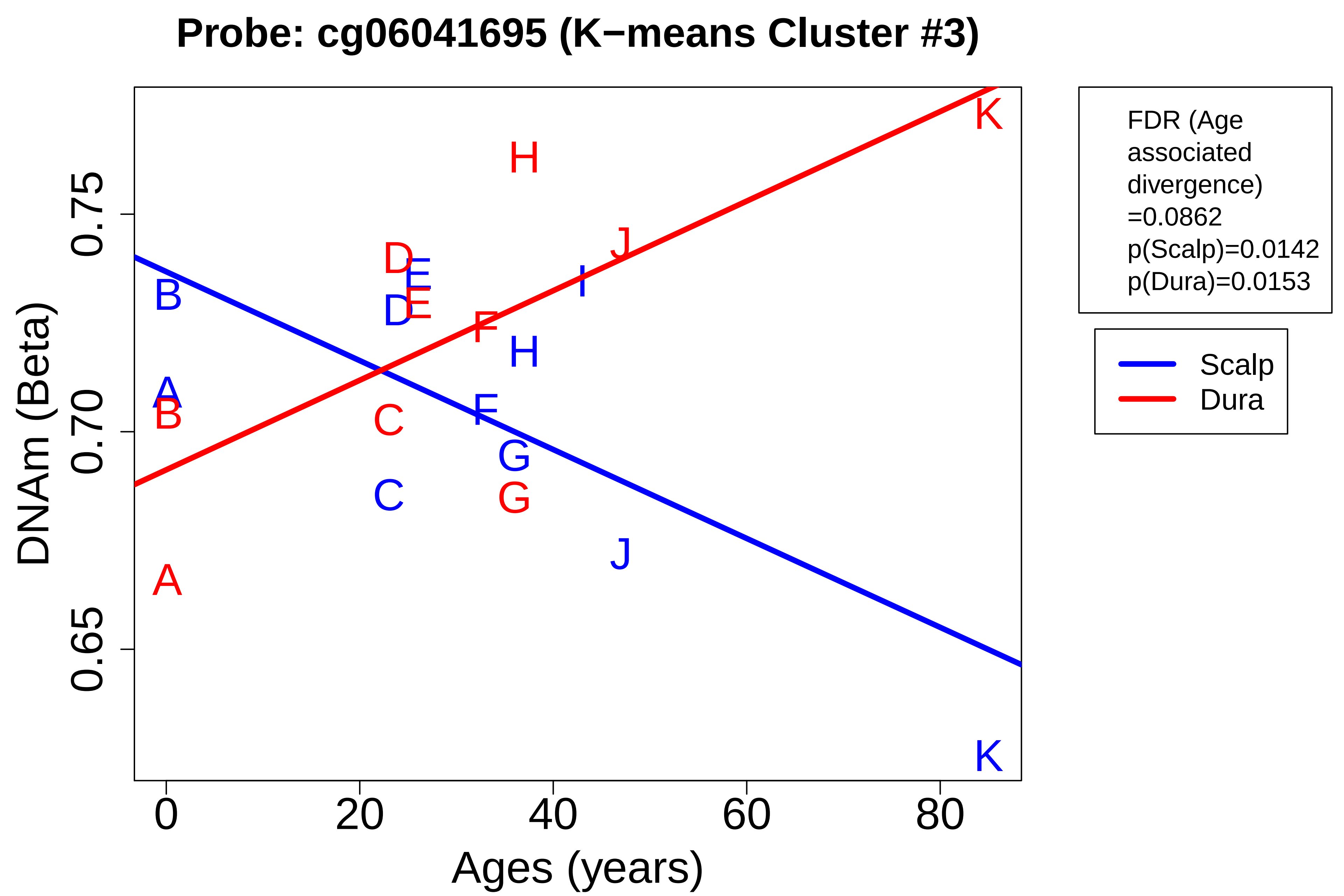

Probe: cg06041695 (K−means Cluster #3)
DNAm (Beta)
0.65
0.70
0.75
Ages (years)
0
20
40
60
80
FDR (Age associated divergence) =0.0862
p(Scalp)=0.0142
p(Dura)=0.0153
Scalp
Dura

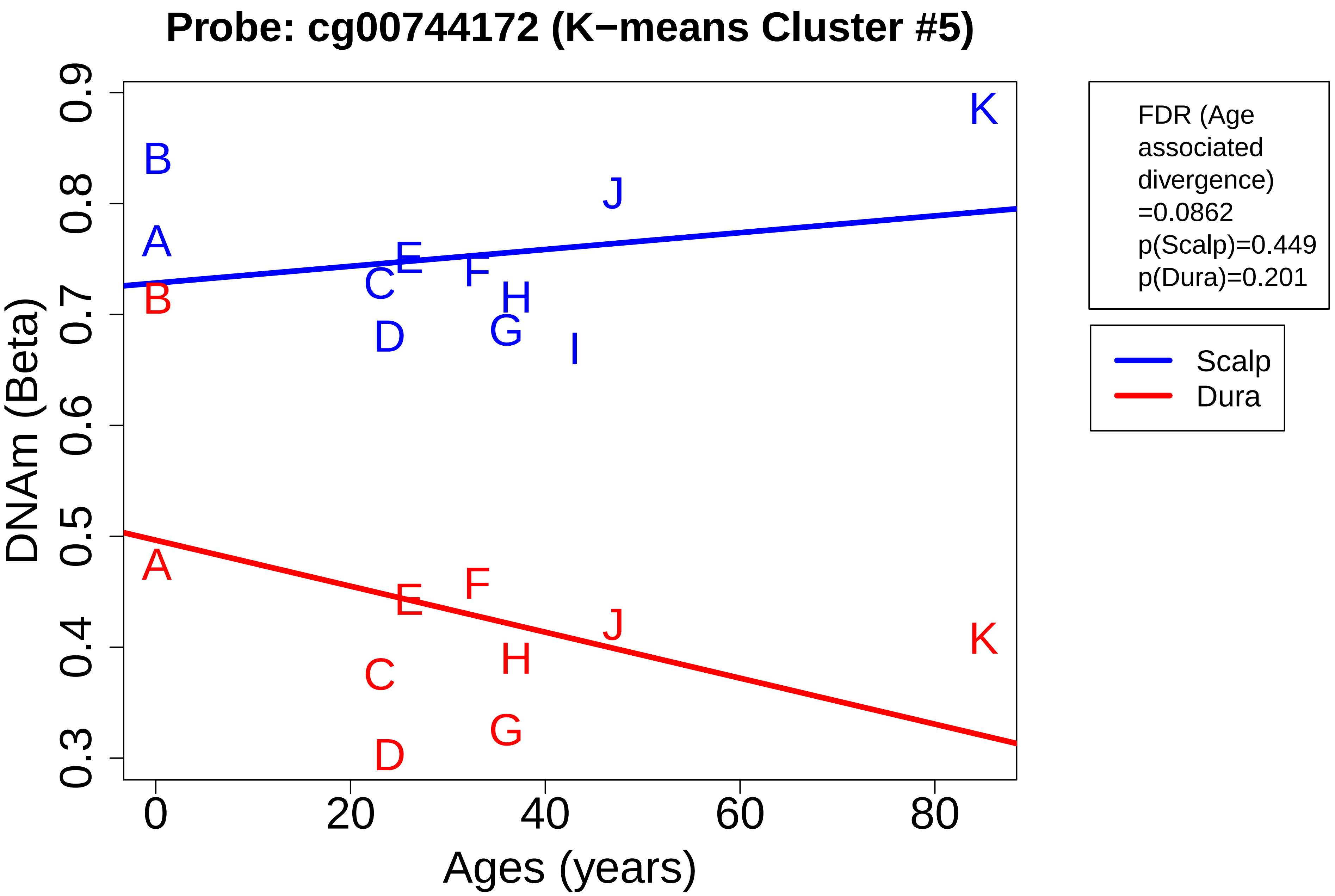
Probe: cg00744172 (K−means Cluster #5)
DNAm (Beta)
0.3
0.4
0.5
0.6
0.7
0.8
0.9
0
20
40
60
80
Ages (years)
A
B
C
D
E
F
G
H
I
J
K
A
B
C
D
E
F
G
H
J
K
FDR (Age associated divergence) =0.0862
p(Scalp)=0.449
p(Dura)=0.201
Scalp
Dura

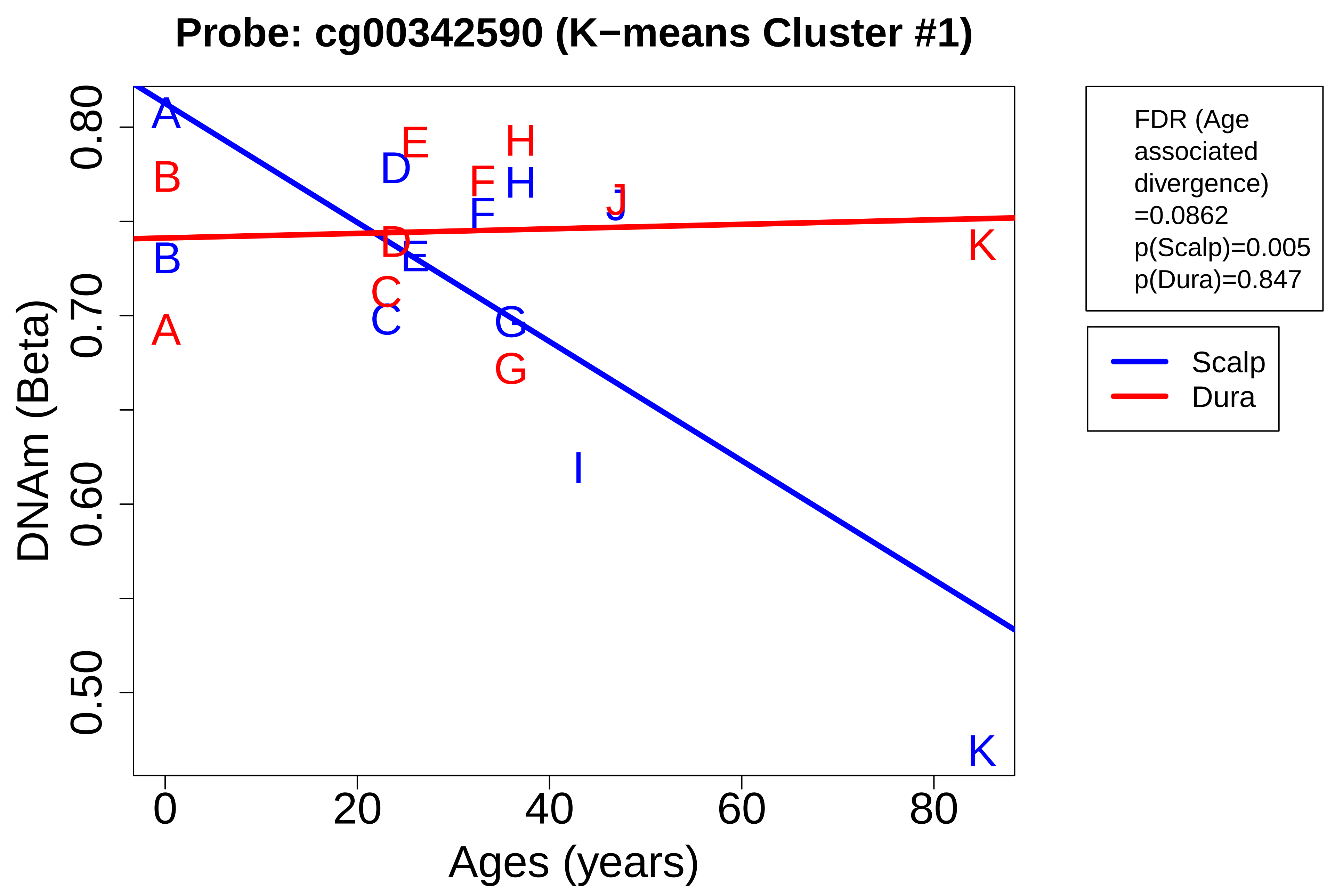

Probe: cg00342590 (K−means Cluster #1)
DNAm (Beta)
0.50
0.60
0.70
0.80
0
20
40
60
80
Ages (years)
A
B
C
D
E
F
G
H
I
J
K
FDR (Age associated divergence) =0.0862
p(Scalp)=0.005
p(Dura)=0.847
Scalp
Dura

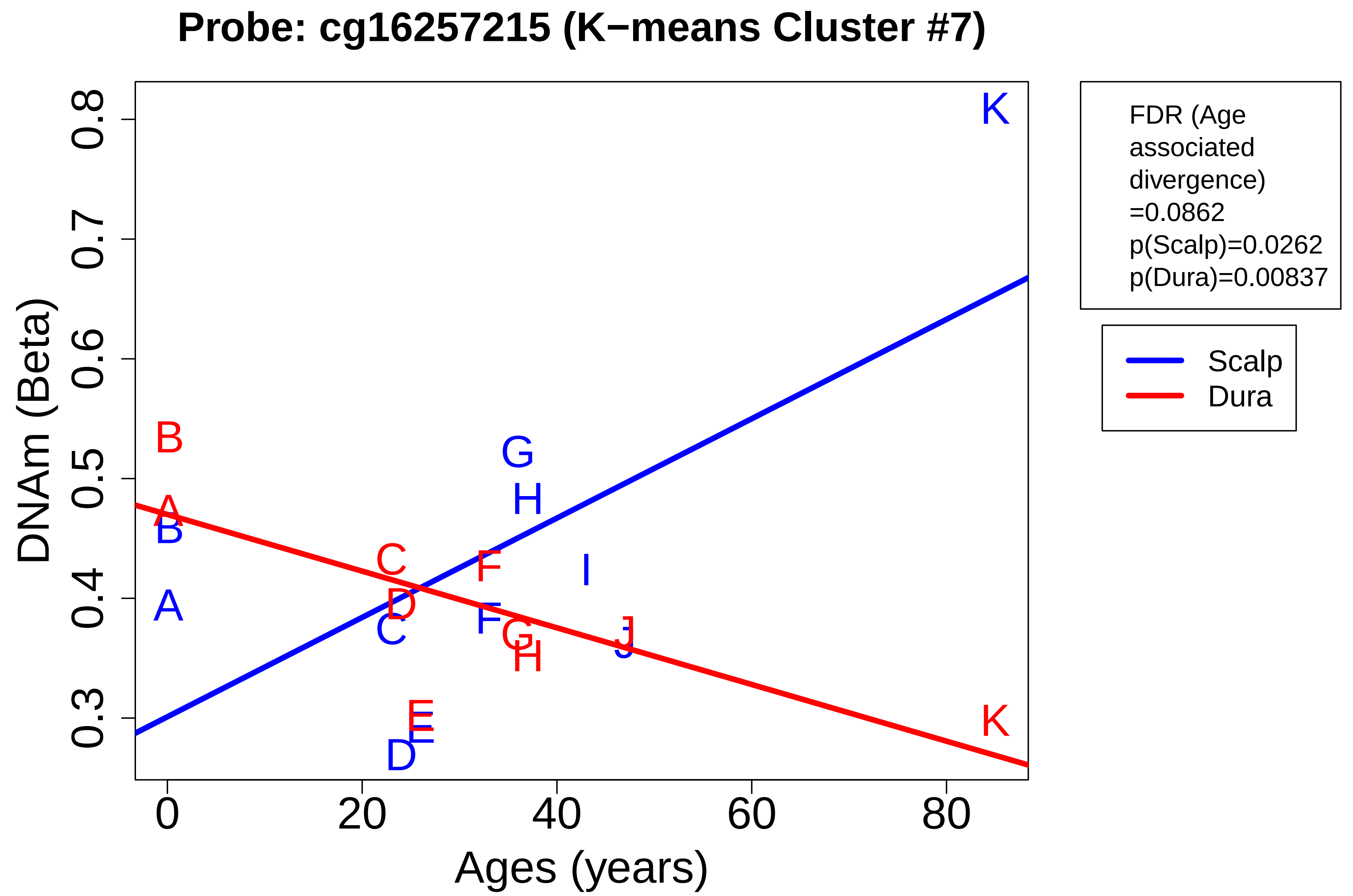

Probe: cg16257215 (K−means Cluster #7)
DNAm (Beta)
0.3
0.4
0.5
0.6
0.7
0.8
0
20
40
60
80
Ages (years)
FDR (Age associated divergence) =0.0862
p(Scalp)=0.0262
p(Dura)=0.00837
Scalp
Dura

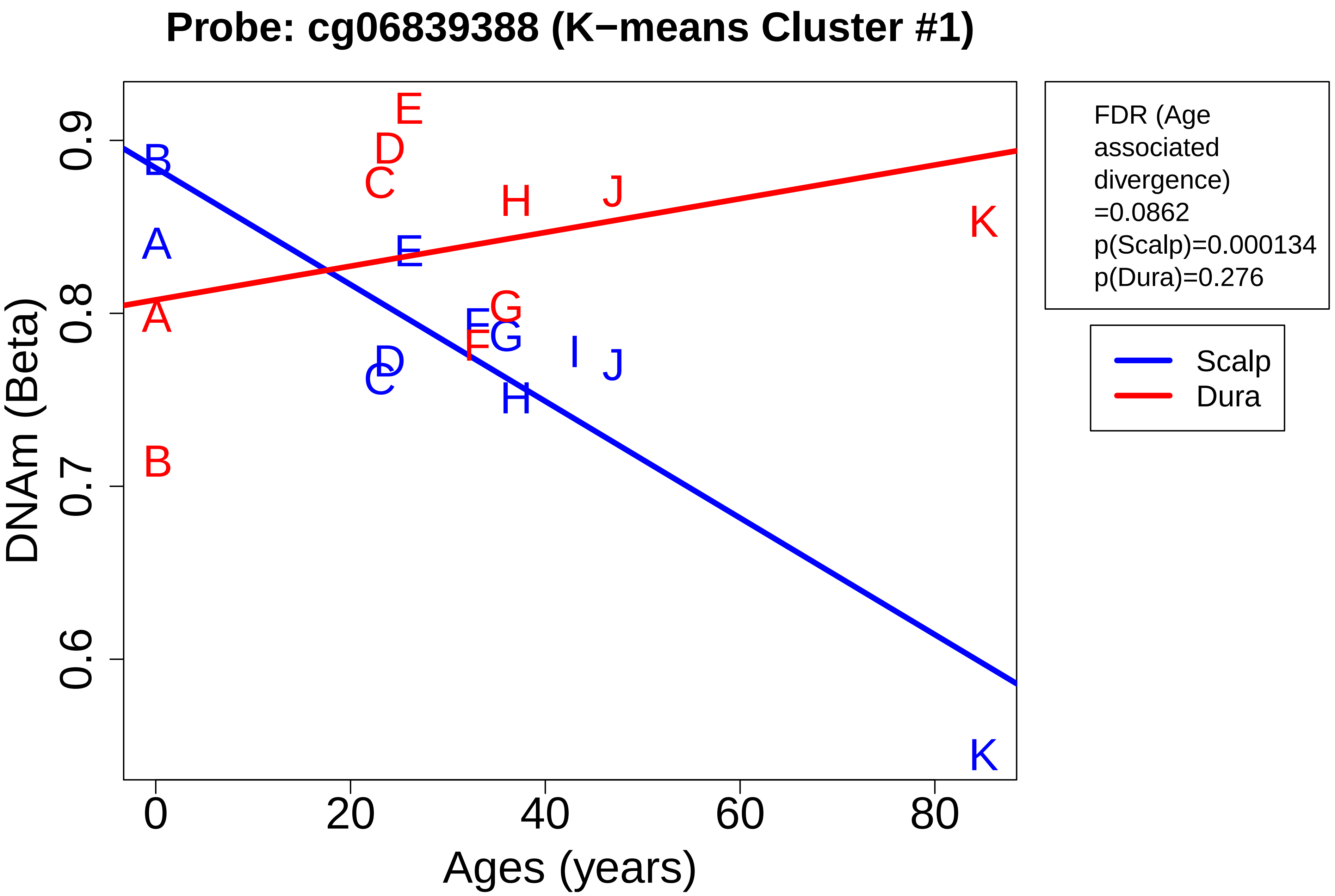

Probe: cg06839388 (K−means Cluster #1)
FDR (Age associated divergence) =0.0862
p(Scalp)=0.000134
p(Dura)=0.276
Scalp
Dura
DNAm (Beta)
0.6
0.7
0.8
0.9
0
20
40
60
80
Ages (years)

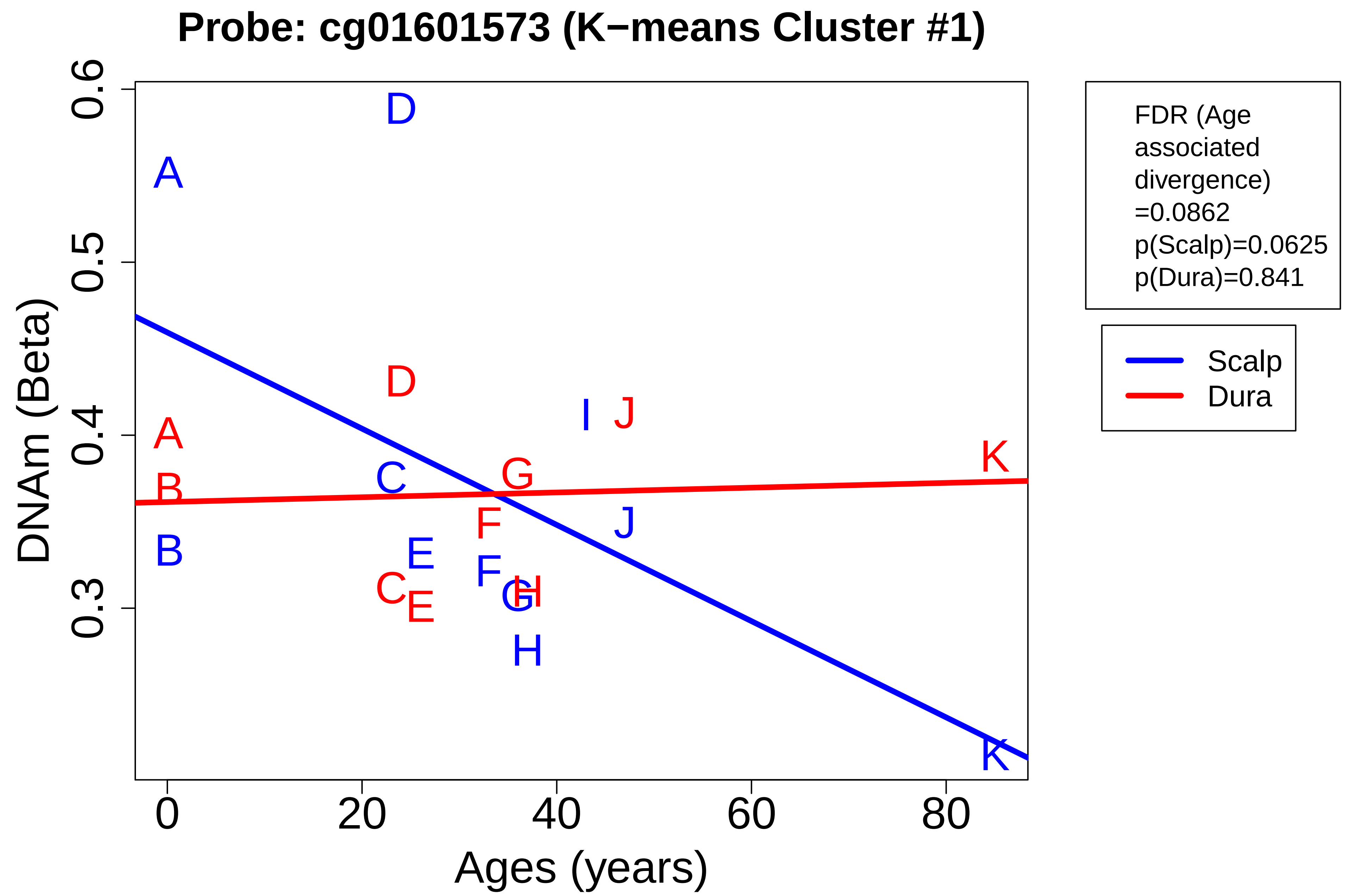
Probe: cg01601573 (K−means Cluster #1)
DNAm (Beta)
0.3
0.4
0.5
0.6
0
20
40
60
80
Ages (years)
A
B
C
D
E
F
G
H
I
J
K
A
B
C
D
E
F
G
H
J
K
FDR (Age associated divergence) =0.0862
p(Scalp)=0.0625
p(Dura)=0.841
Scalp
Dura

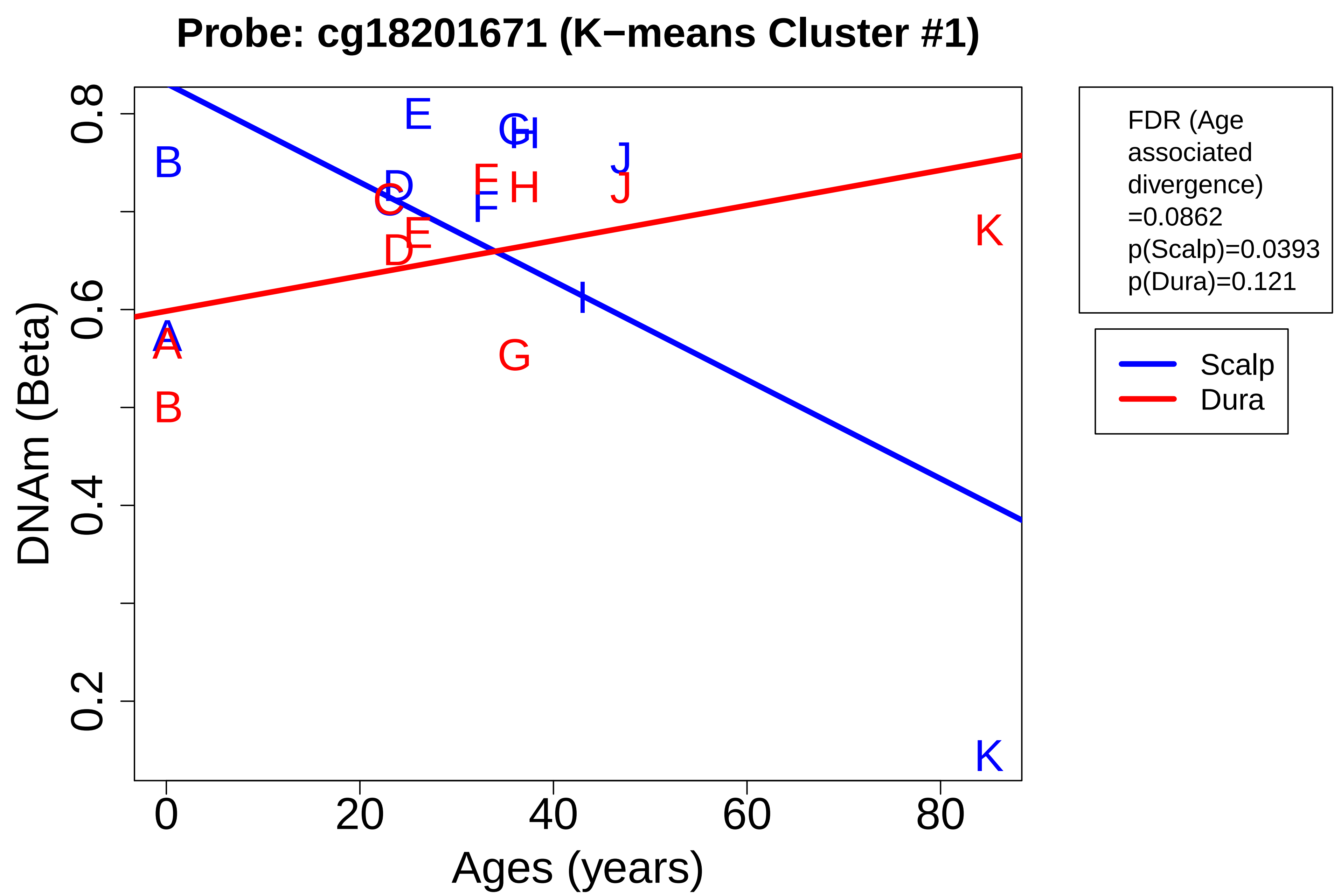
Probe: cg18201671 (K−means Cluster #1)
DNAm (Beta)
0.2
0.4
0.6
0.8
0
20
40
60
80
Ages (years)
FDR (Age associated divergence) =0.0862
p(Scalp)=0.0393
p(Dura)=0.121
Scalp
Dura
A
B
C
D
E
F
G
H
I
J
K

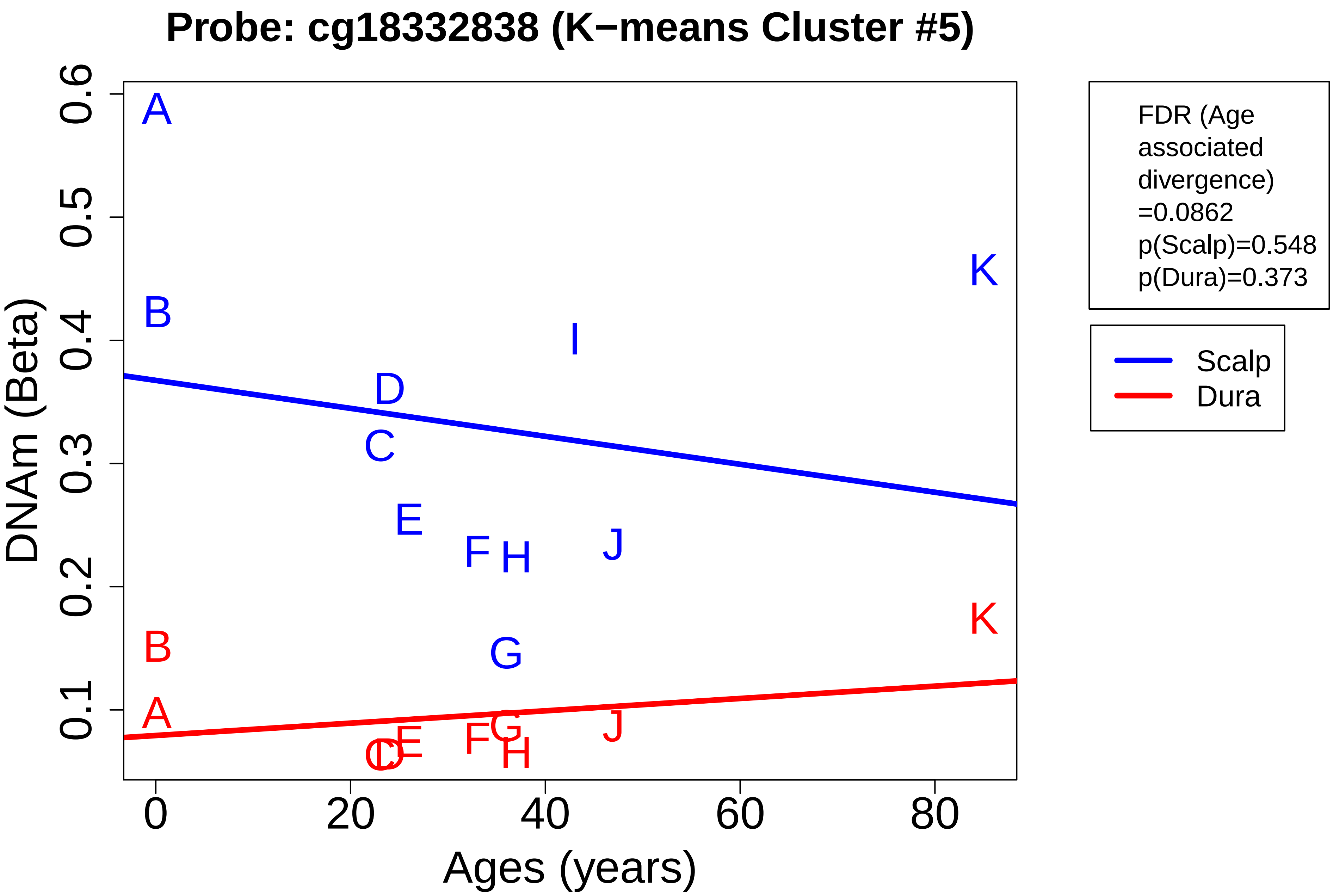

Probe: cg18332838 (K−means Cluster #5)
DNAm (Beta)
0.1
0.2
0.3
0.4
0.5
0.6
0
20
40
60
80
Ages (years)
A
B
C
D
E
F
G
H
I
J
K
FDR (Age associated divergence) =0.0862
p(Scalp)=0.548
p(Dura)=0.373
Scalp
Dura

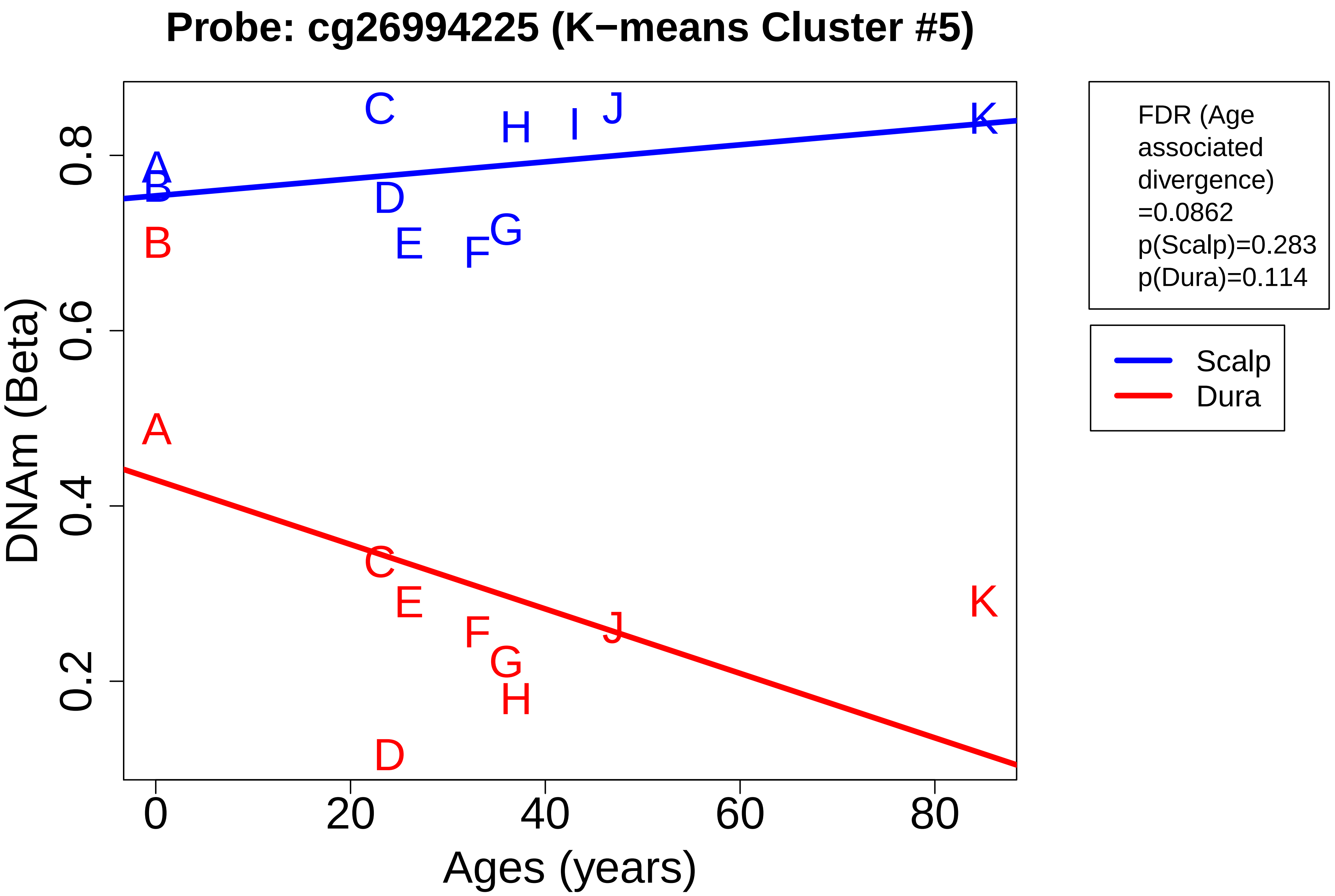

Probe: cg26994225 (K−means Cluster #5)
DNAm (Beta)
0.2
0.4
0.6
0.8
0
20
40
60
80
Ages (years)
A
B
C
D
E
F
G
H
I
J
K
A
B
C
D
E
F
G
H
J
K
FDR (Age associated divergence) =0.0862
p(Scalp)=0.283
p(Dura)=0.114
Scalp
Dura

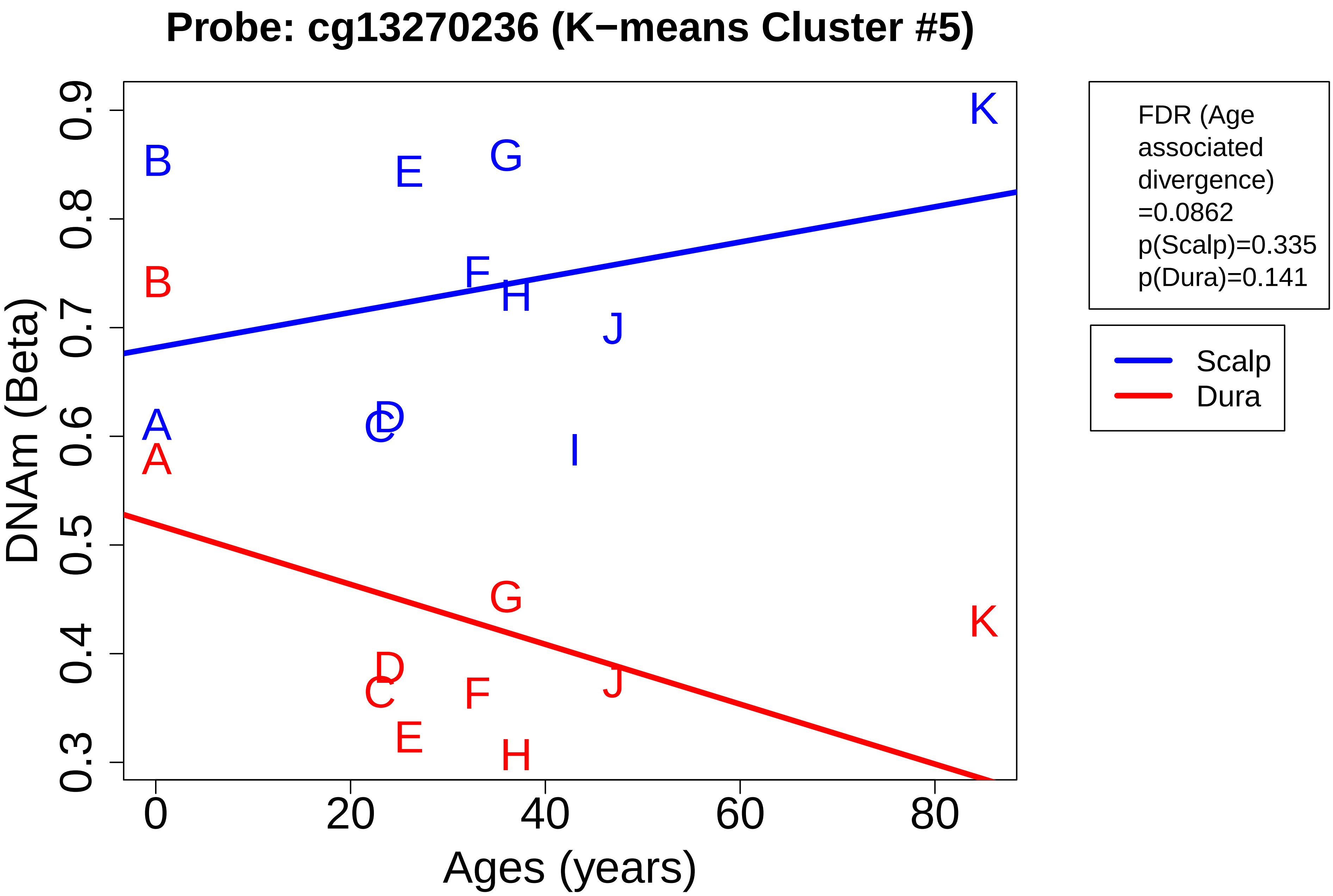

Probe: cg13270236 (K−means Cluster #5)
DNAm (Beta)
0.3
0.4
0.5
0.6
0.7
0.8
0.9
0
20
40
60
80
Ages (years)
FDR (Age associated divergence) =0.0862
p(Scalp)=0.335
p(Dura)=0.141
Scalp
Dura

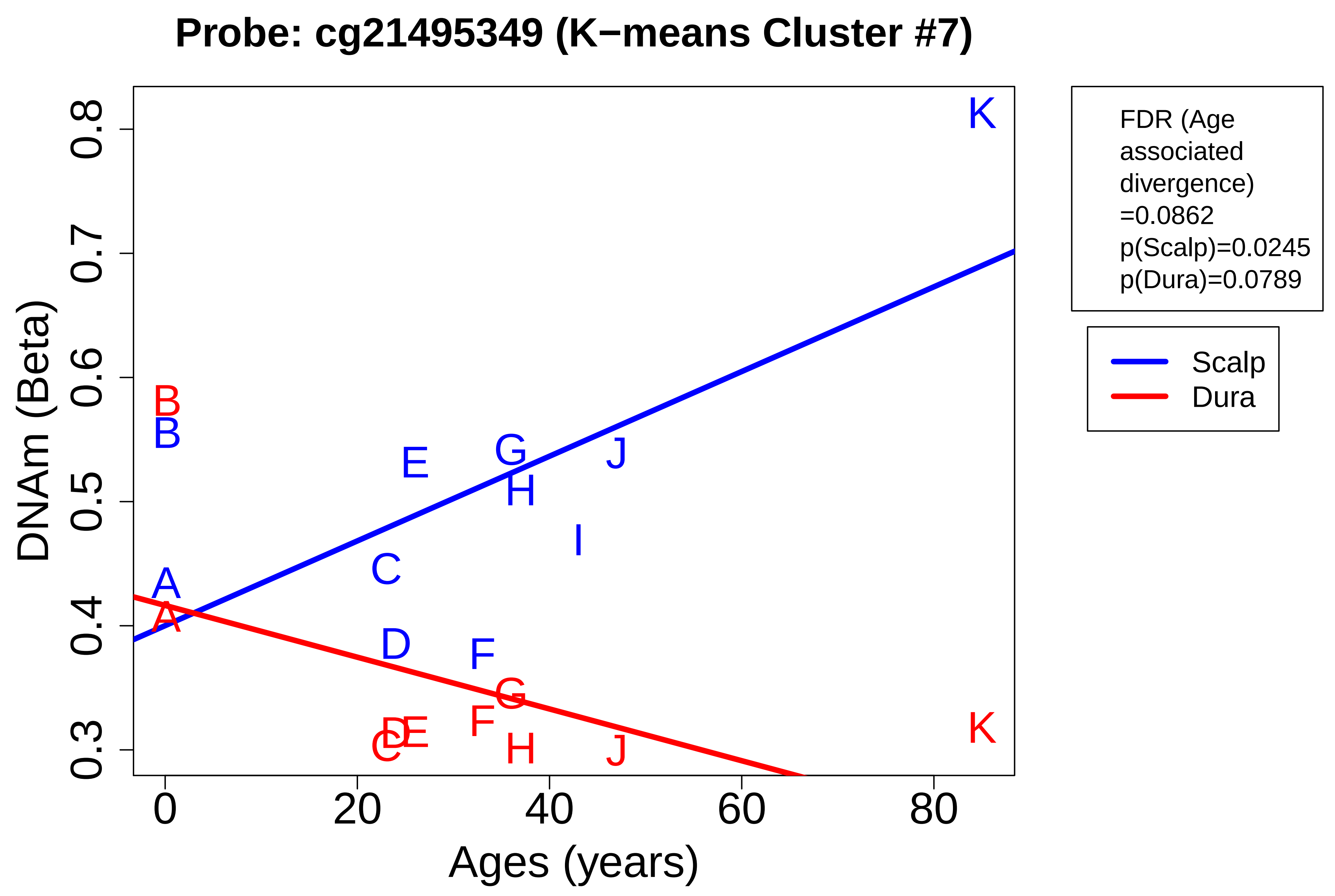

Probe: cg21495349 (K−means Cluster #7)
DNAm (Beta)
0.3
0.4
0.5
0.6
0.7
0.8
Ages (years)
0
20
40
60
80
FDR (Age associated divergence) =0.0862
p(Scalp)=0.0245
p(Dura)=0.0789
Scalp
Dura
A
B
C
D
E
F
G
H
I
J
K

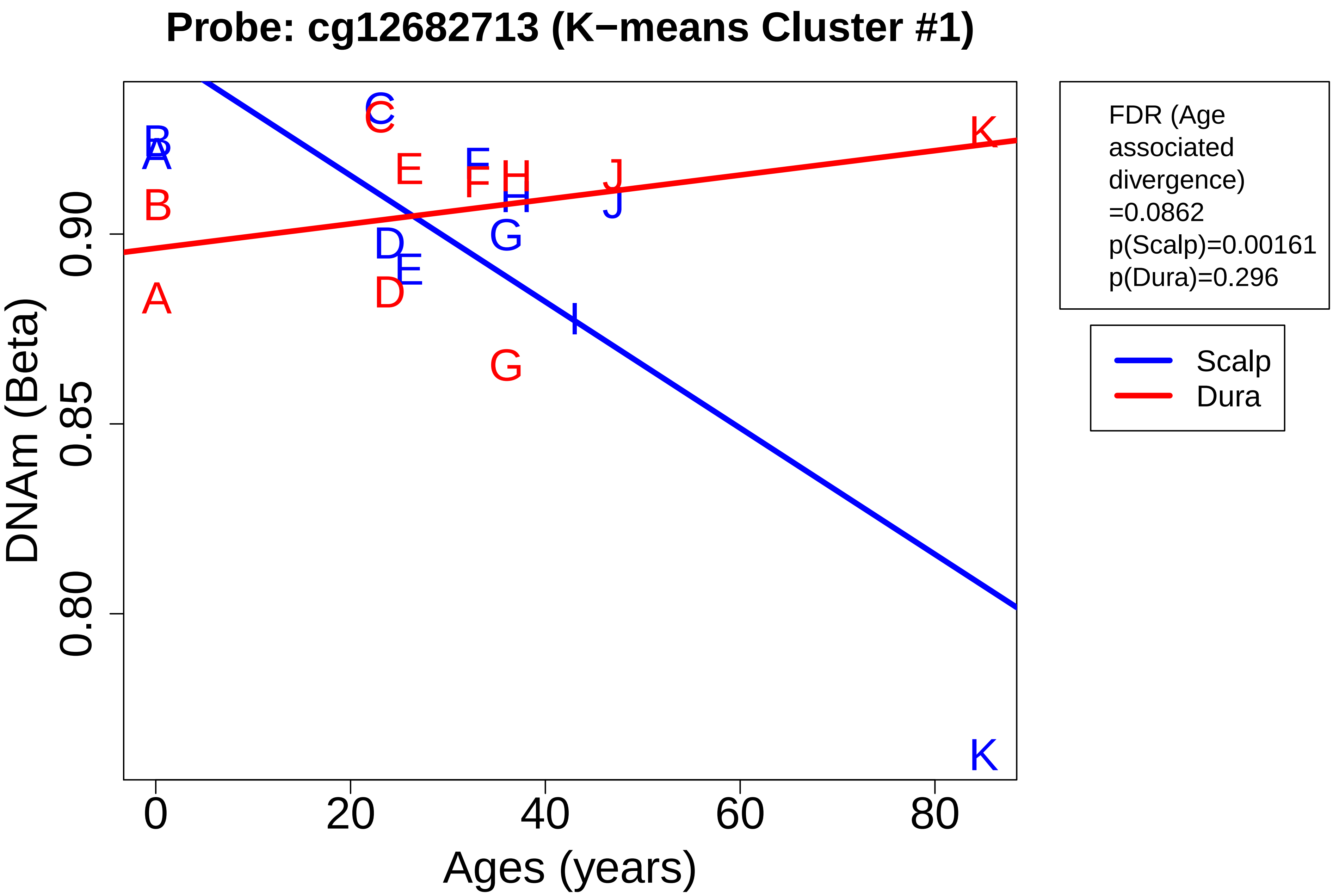

Probe: cg12682713 (K−means Cluster #1)
DNAm (Beta)
0.80
0.85
0.90
0
20
40
60
80
Ages (years)
FDR (Age associated divergence) =0.0862
p(Scalp)=0.00161
p(Dura)=0.296
Scalp
Dura

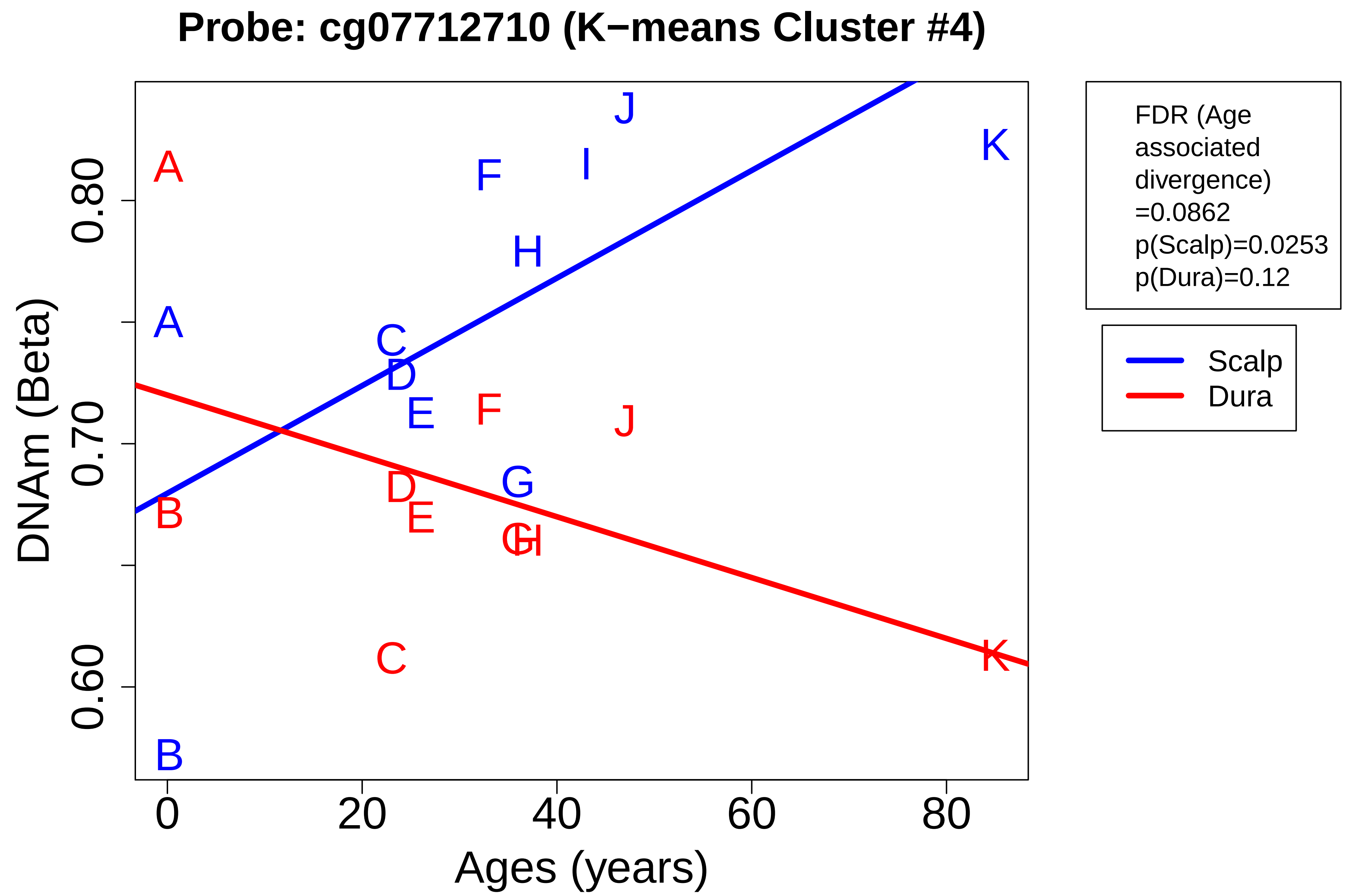

Probe: cg07712710 (K−means Cluster #4)
DNAm (Beta)
0.60
0.70
0.80
0
20
40
60
80
Ages (years)
A
B
C
D
E
F
G
H
I
J
K
FDR (Age associated divergence) =0.0862
p(Scalp)=0.0253
p(Dura)=0.12
Scalp
Dura

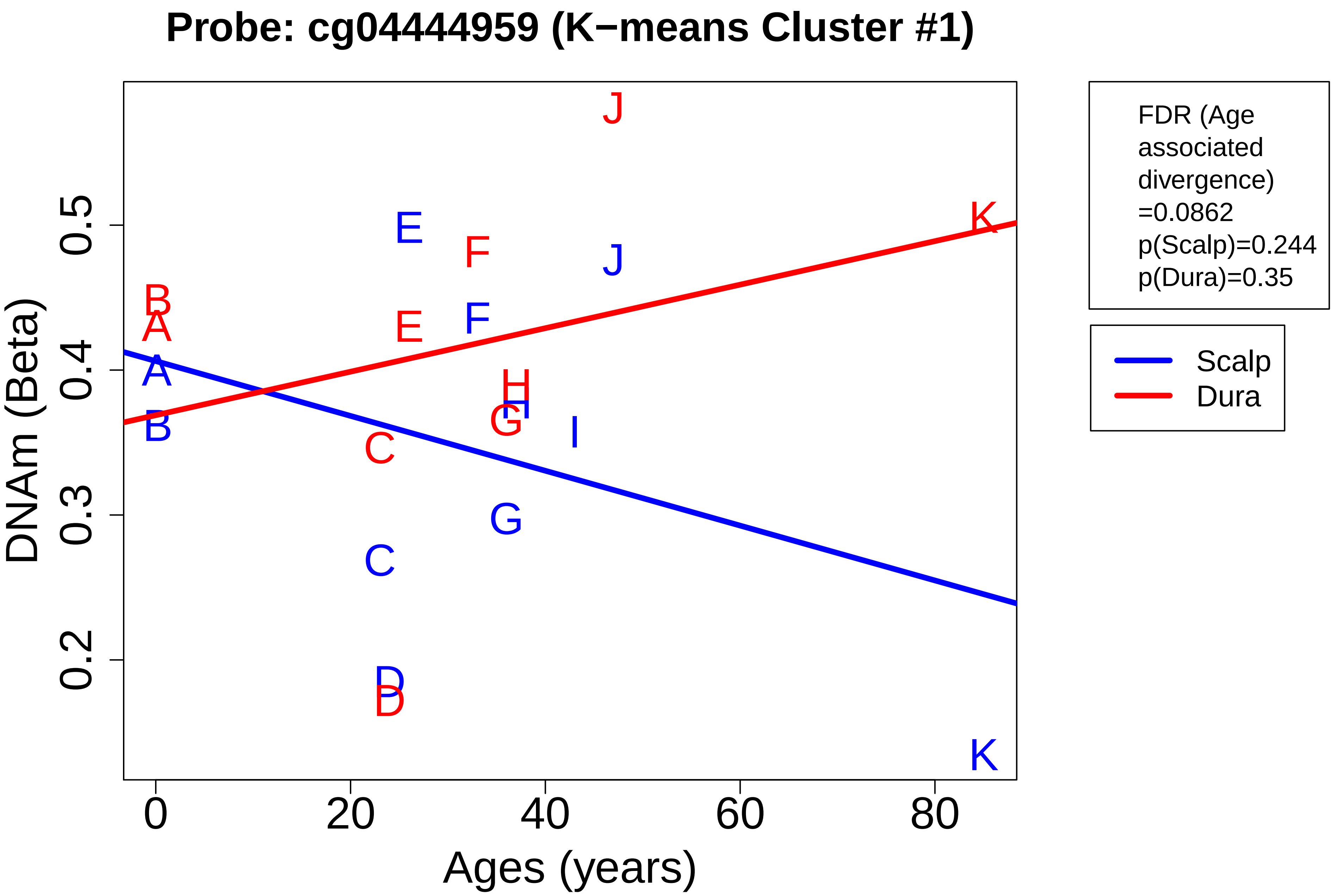

Probe: cg04444959 (K−means Cluster #1)
DNAm (Beta)
0.2
0.3
0.4
0.5
0
20
40
60
80
Ages (years)
FDR (Age associated divergence) =0.0862
p(Scalp)=0.244
p(Dura)=0.35
Scalp
Dura

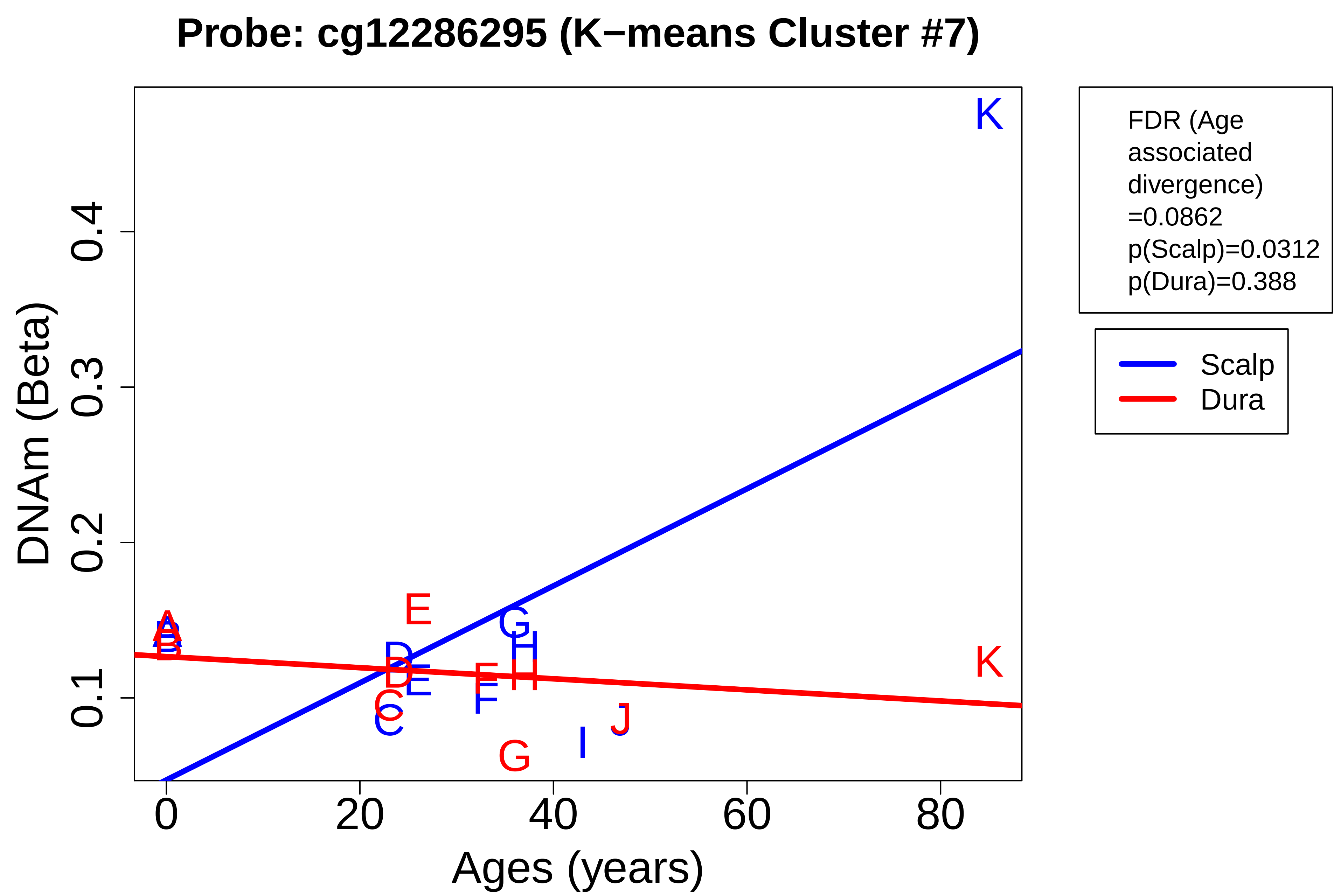

Probe: cg12286295 (K−means Cluster #7)
DNAm (Beta)
0.1
0.2
0.3
0.4
0
20
40
60
80
Ages (years)
FDR (Age associated divergence) =0.0862
p(Scalp)=0.0312
p(Dura)=0.388
Scalp
Dura
A
B
C
D
E
F
G
H
I
J
K

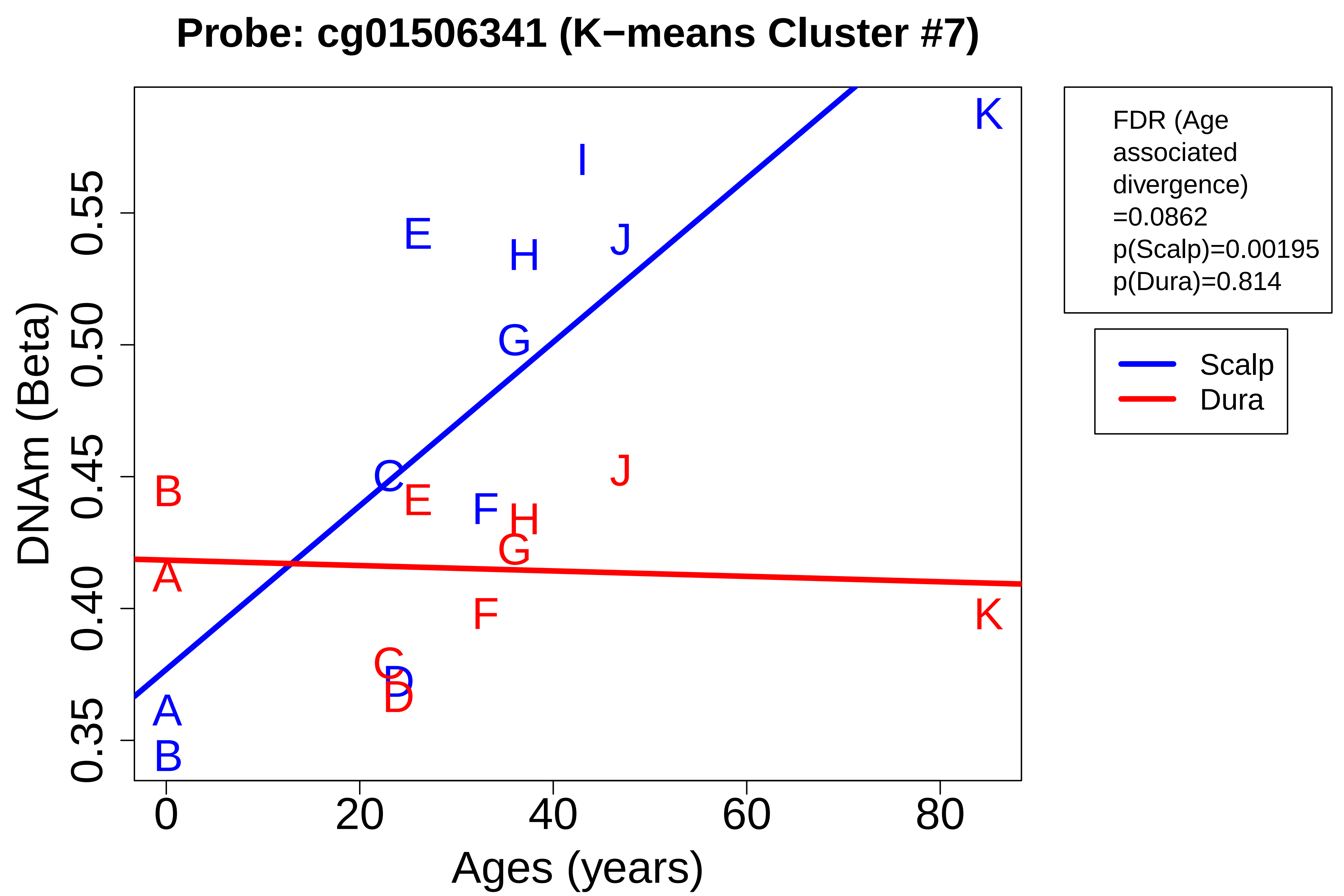

Probe: cg01506341 (K−means Cluster #7)
FDR (Age associated divergence) =0.0862
p(Scalp)=0.00195
p(Dura)=0.814
Scalp
Dura
DNAm (Beta)
0.35
0.40
0.45
0.50
0.55
0
20
40
60
80
Ages (years)

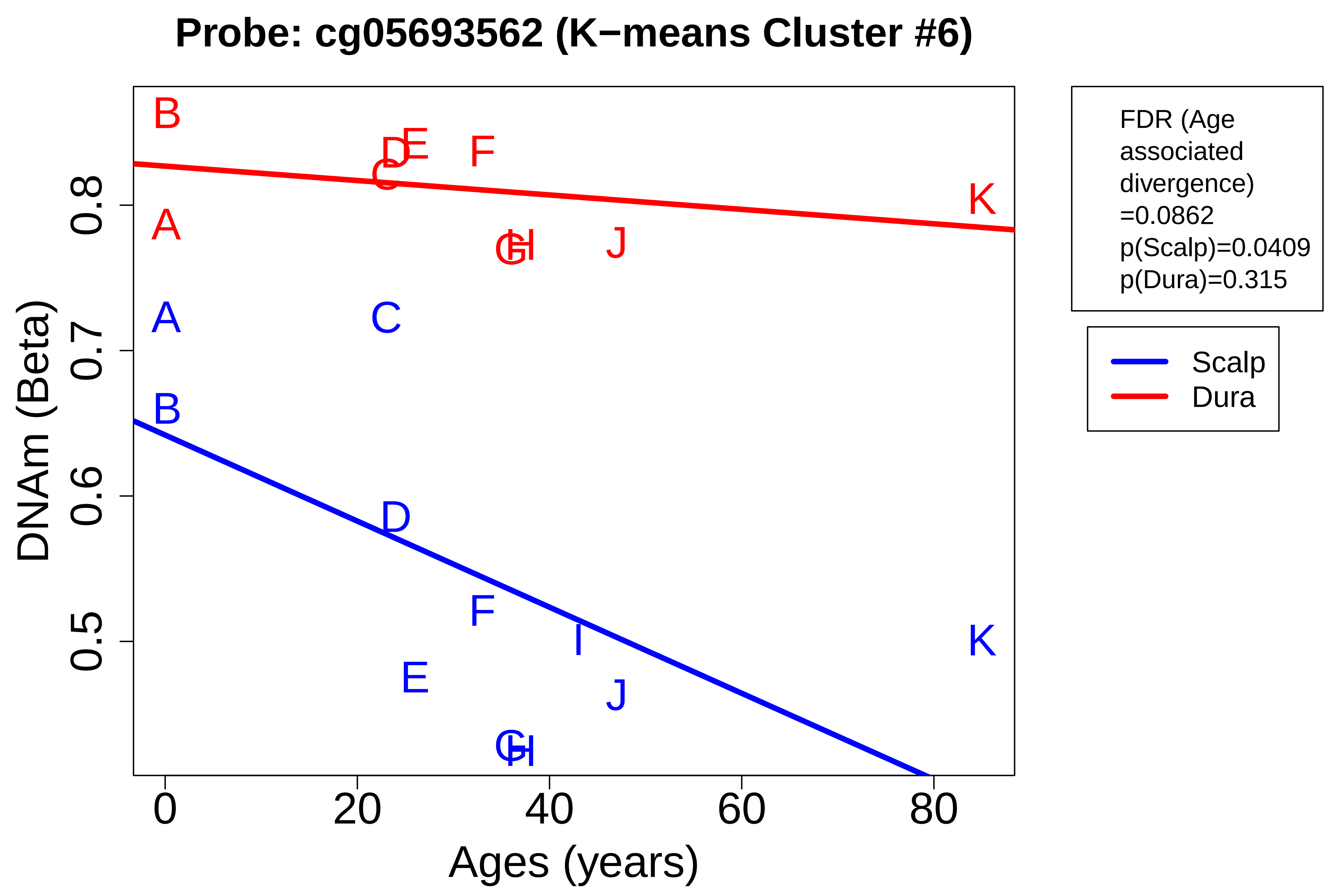

Probe: cg05693562 (K−means Cluster #6)
DNAm (Beta)
0.5
0.6
0.7
0.8
0
20
40
60
80
Ages (years)
B
D
E
F
C
K
A
G
H
J
A
C
B
D
F
I
K
E
J
G
H
FDR (Age associated divergence) =0.0862
p(Scalp)=0.0409
p(Dura)=0.315
Scalp
Dura

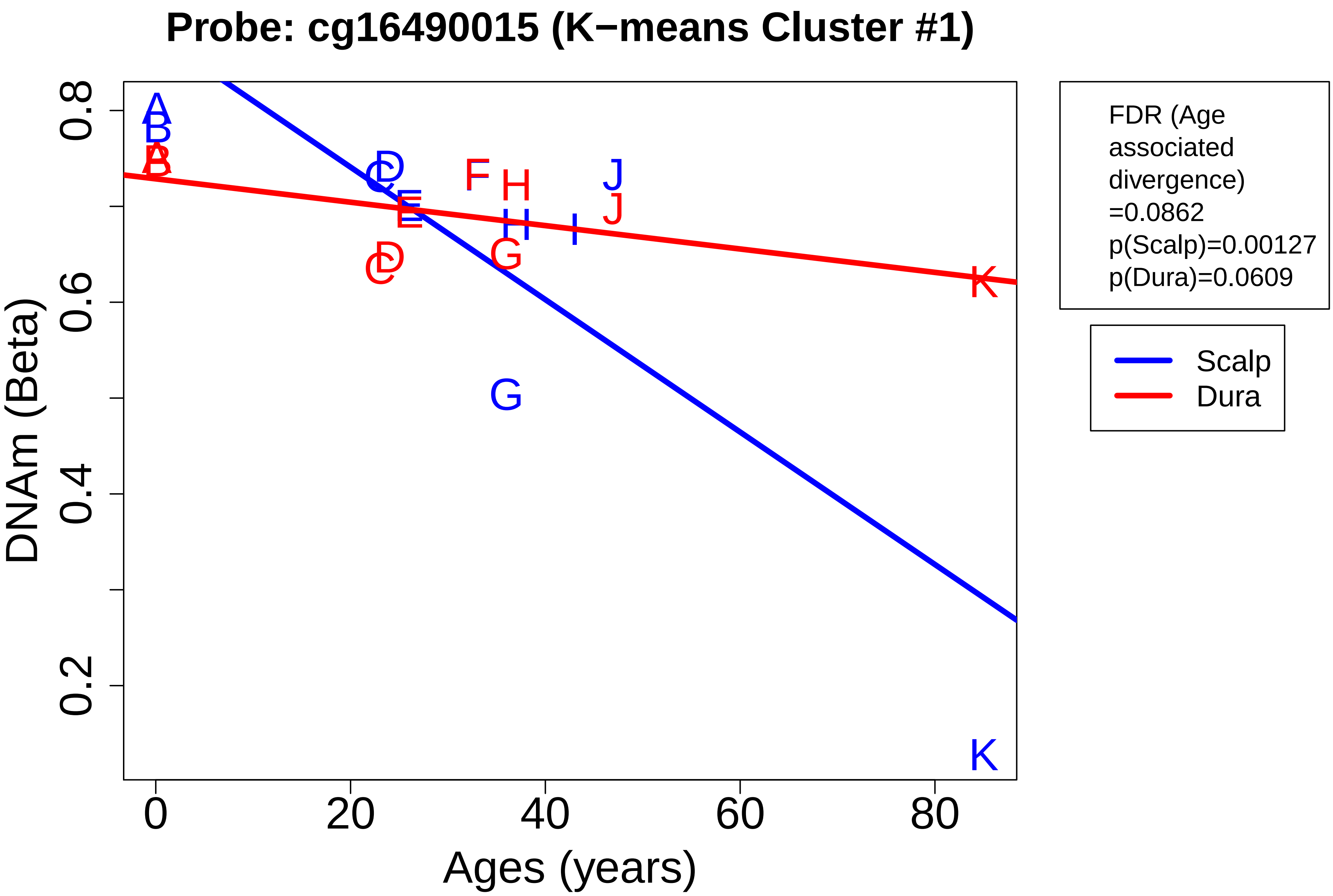

Probe: cg16490015 (K−means Cluster #1)
DNAm (Beta)
0.2
0.4
0.6
0.8
0
20
40
60
80
Ages (years)
FDR (Age associated divergence) =0.0862
p(Scalp)=0.00127
p(Dura)=0.0609
Scalp
Dura

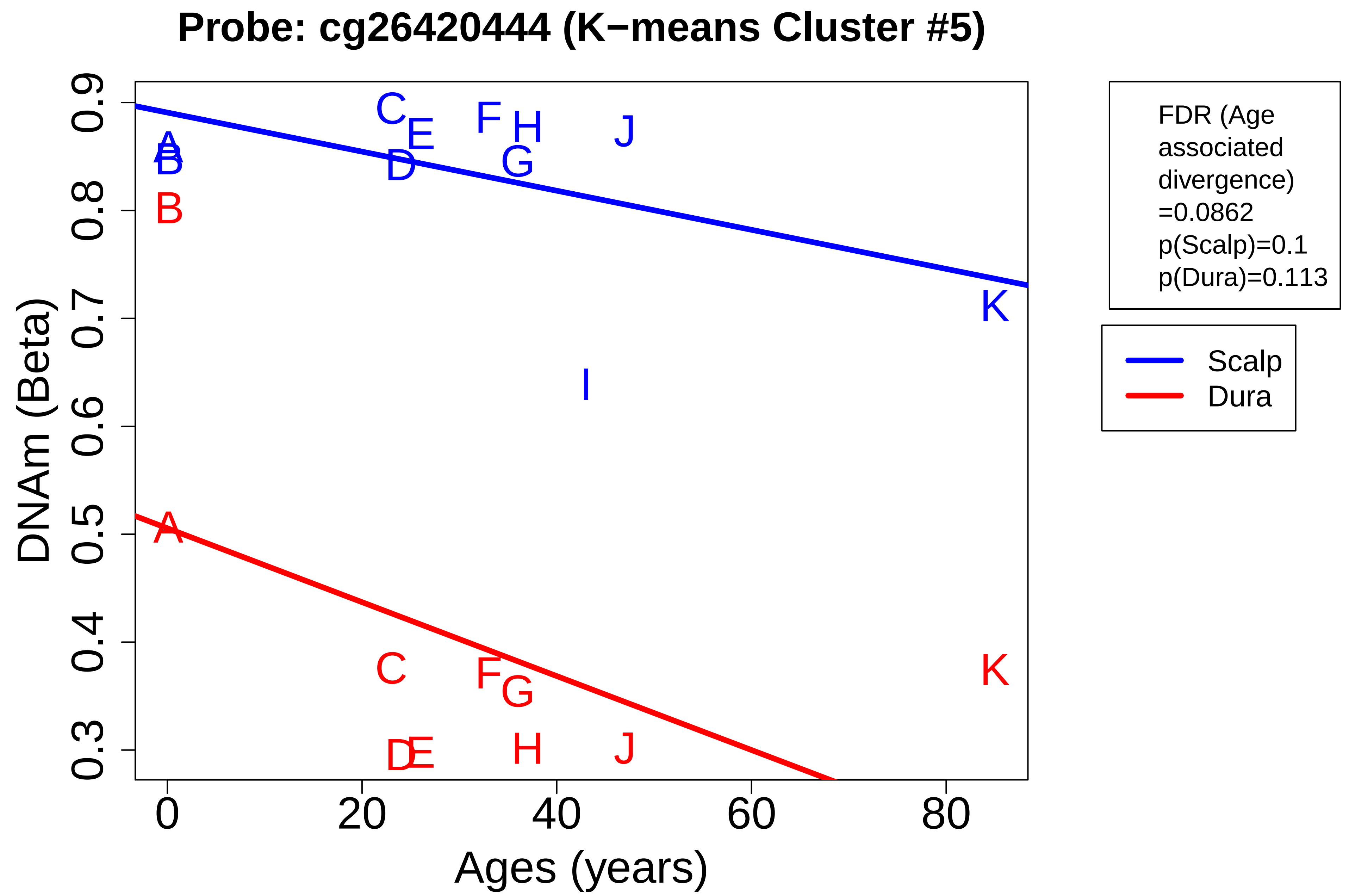

Probe: cg26420444 (K−means Cluster #5)
DNAm (Beta)
0.3
0.4
0.5
0.6
0.7
0.8
0.9
0
20
40
60
80
Ages (years)
A
B
C
D
E
F
G
H
I
J
K
A
B
C
D
E
F
G
H
J
K
FDR (Age associated divergence) =0.0862
p(Scalp)=0.1
p(Dura)=0.113
Scalp
Dura

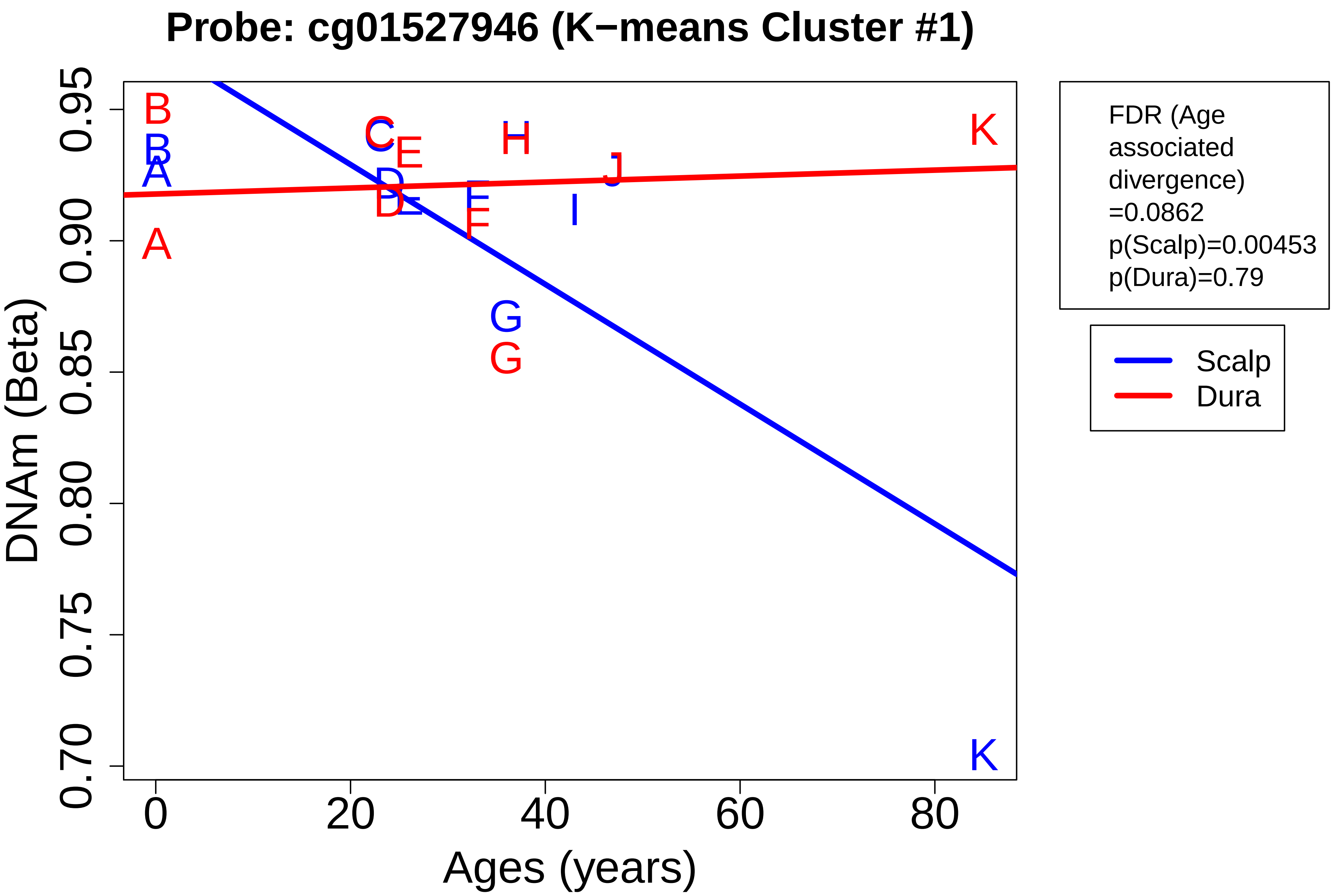

Probe: cg01527946 (K−means Cluster #1)
DNAm (Beta)
0.70
0.75
0.80
0.85
0.90
0.95
0
20
40
60
80
Ages (years)
FDR (Age associated divergence) =0.0862
p(Scalp)=0.00453
p(Dura)=0.79
Scalp
Dura
A
B
C
D
E
F
G
H
I
J
K

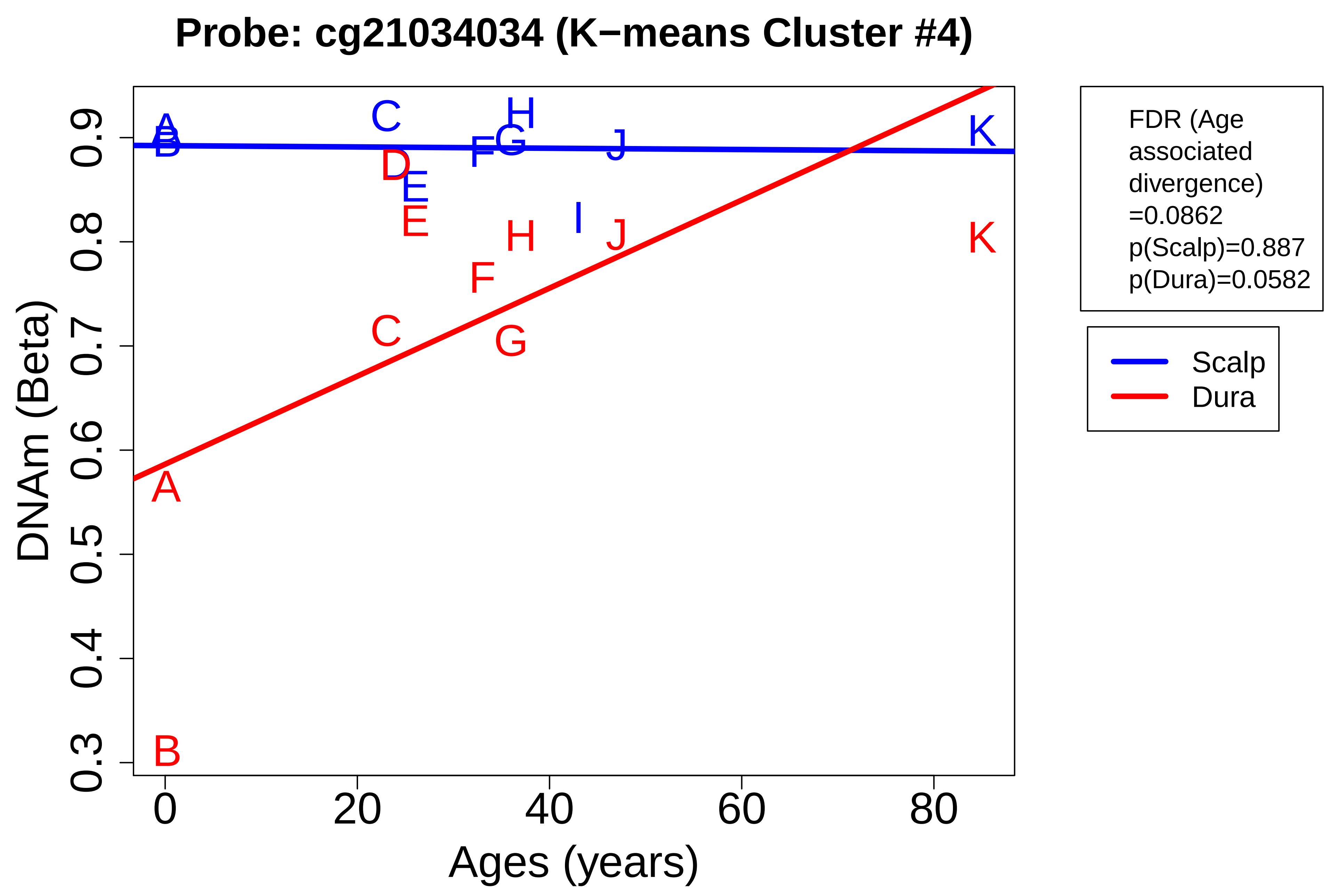

Probe: cg21034034 (K−means Cluster #4)
DNAm (Beta)
0.3
0.4
0.5
0.6
0.7
0.8
0.9
0
20
40
60
80
Ages (years)
FDR (Age associated divergence) =0.0862
p(Scalp)=0.887
p(Dura)=0.0582
Scalp
Dura

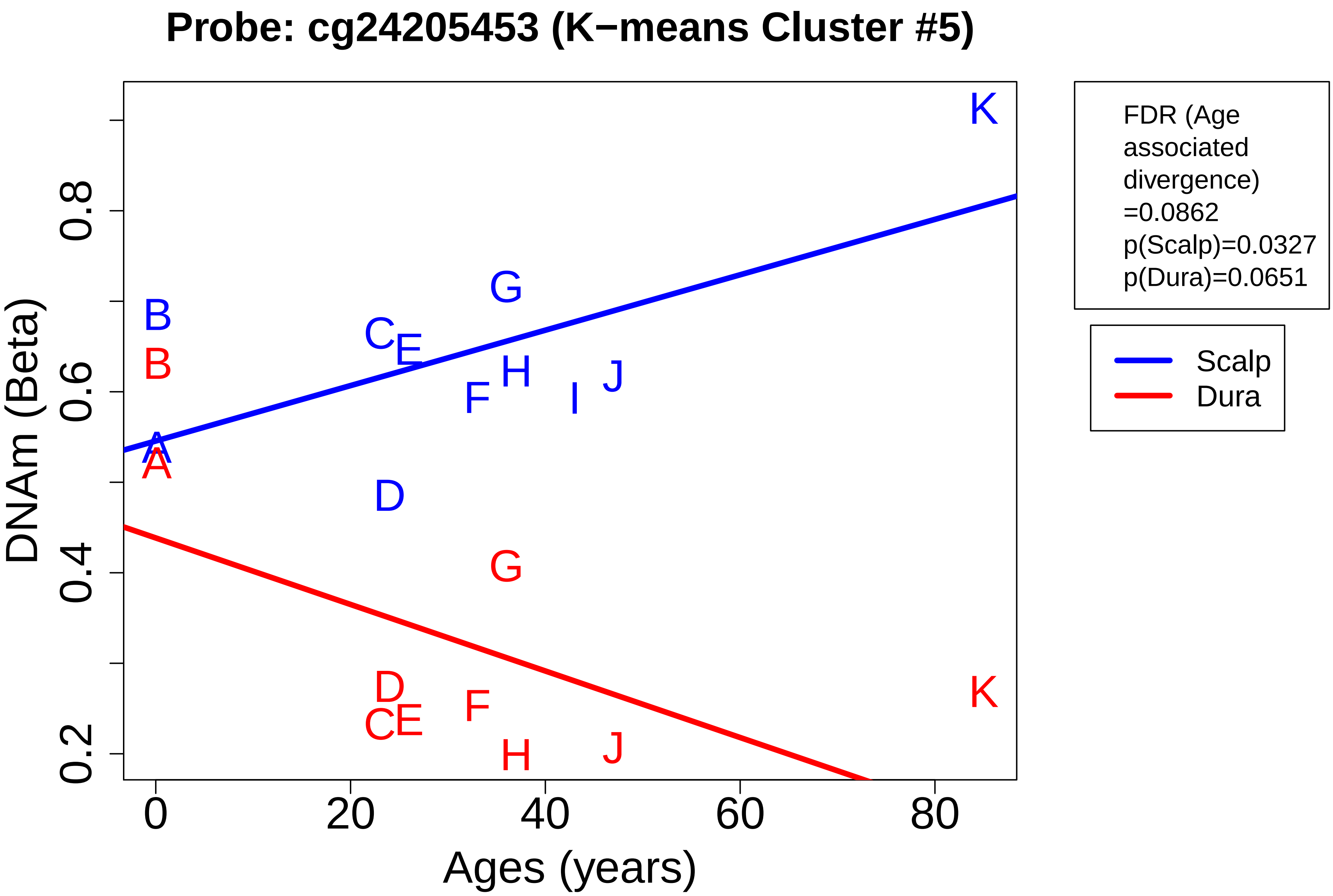

Probe: cg24205453 (K−means Cluster #5)
FDR (Age associated divergence) =0.0862
p(Scalp)=0.0327
p(Dura)=0.0651
Scalp
Dura
DNAm (Beta)
0.2
0.4
0.6
0.8
0
20
40
60
80
Ages (years)
A B C D E F G H I J K
A B C D E F G H J K

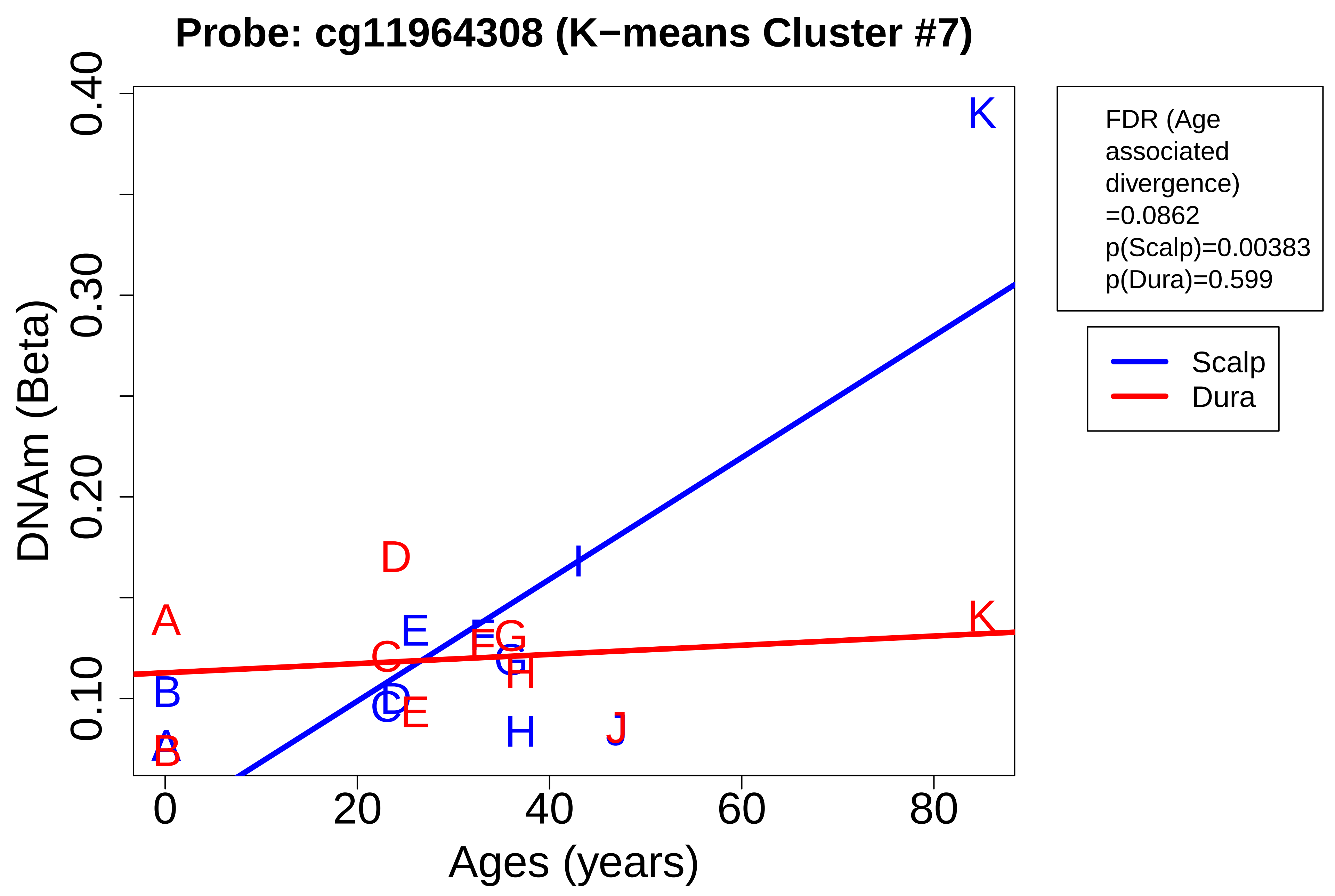

Probe: cg11964308 (K−means Cluster #7)
DNAm (Beta)
0.10
0.20
0.30
0.40
0
20
40
60
80
Ages (years)
FDR (Age associated divergence) =0.0862
p(Scalp)=0.00383
p(Dura)=0.599
Scalp
Dura
A
B
C
D
E
F
G
H
I
J
K

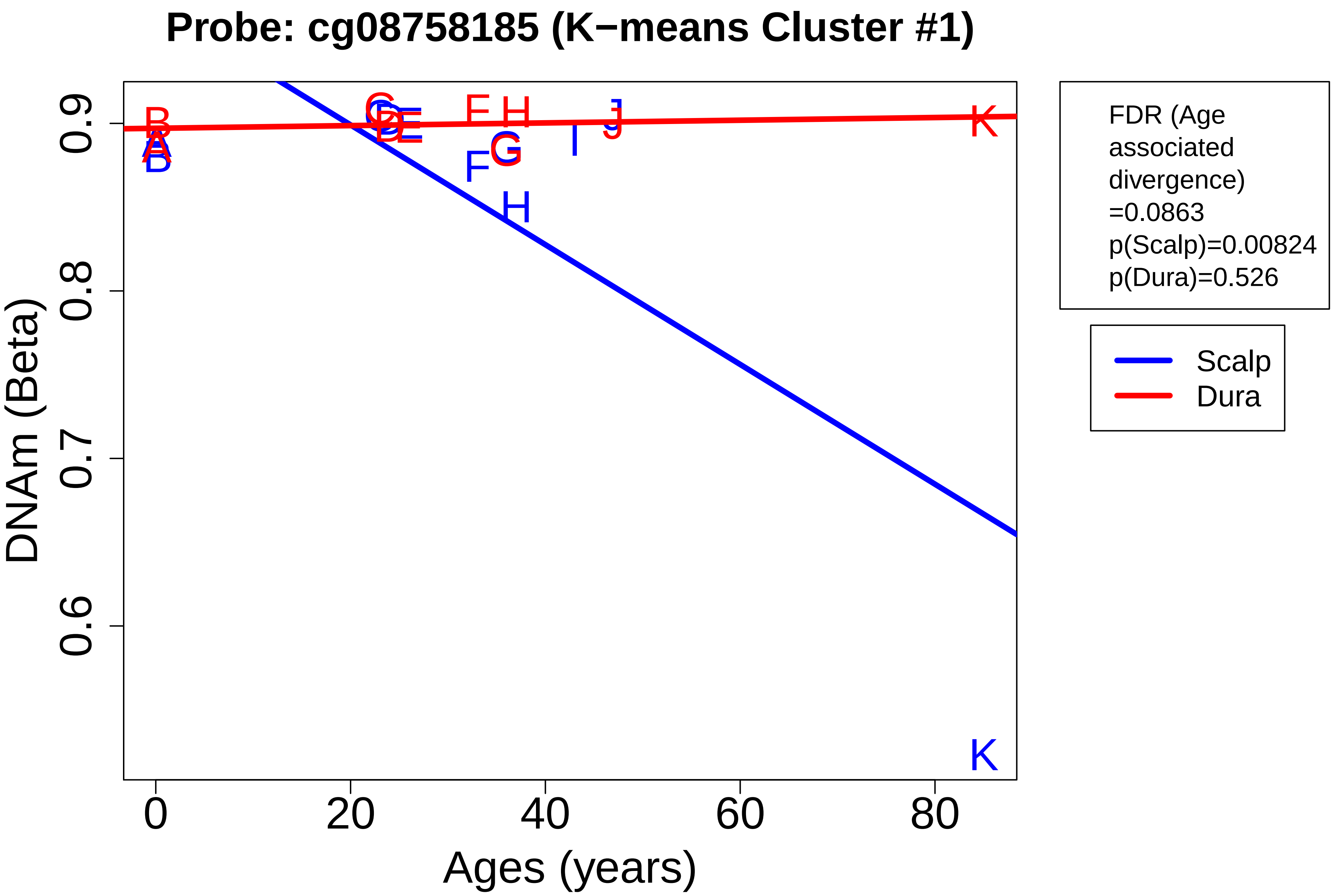
Probe: cg08758185 (K−means Cluster #1)
DNAm (Beta)
0.6
0.7
0.8
0.9
0
20
40
60
80
Ages (years)
FDR (Age associated divergence) =0.0863
p(Scalp)=0.00824
p(Dura)=0.526
Scalp
Dura

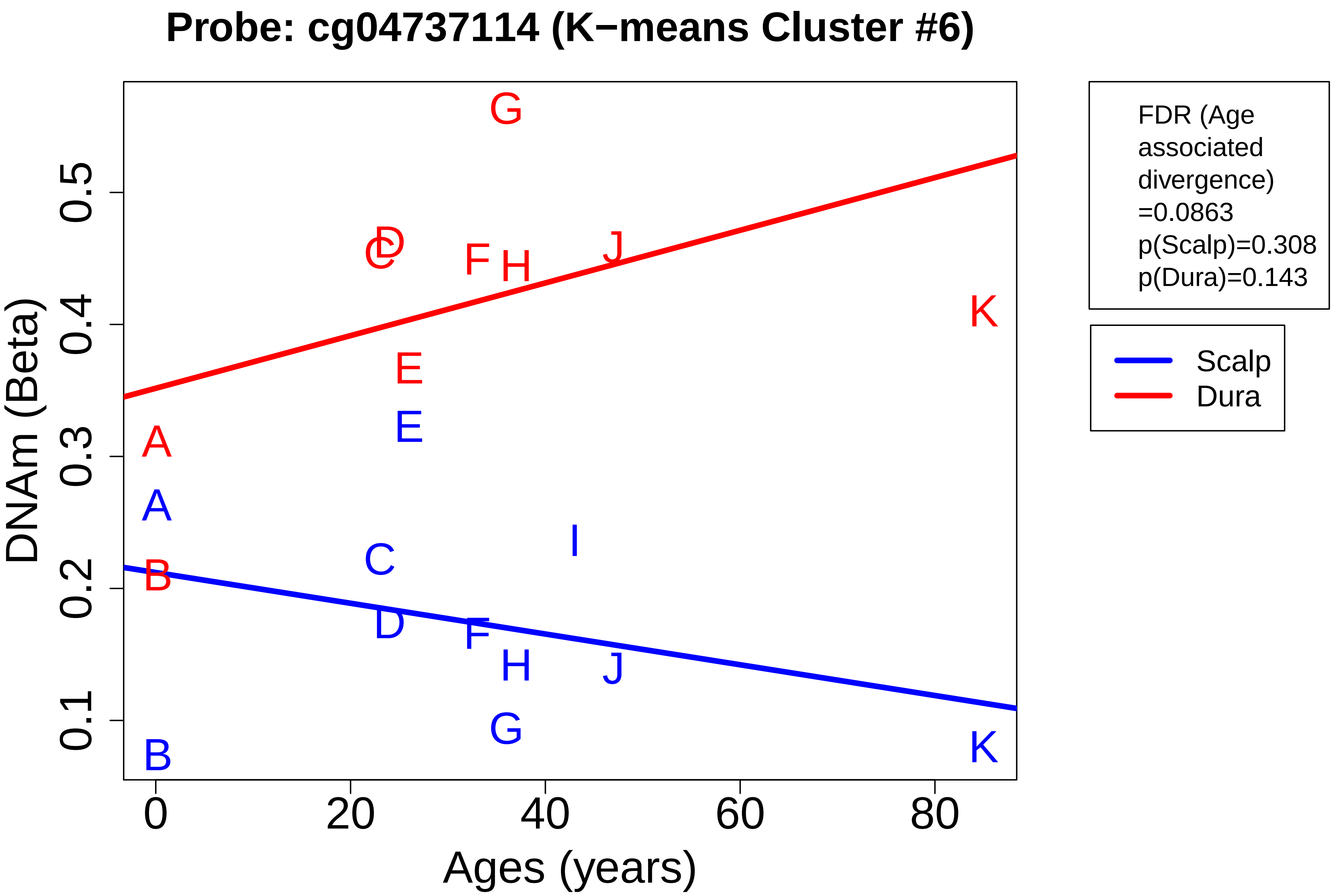

Probe: cg04737114 (K−means Cluster #6)
DNAm (Beta)
0.1
0.2
0.3
0.4
0.5
0
20
40
60
80
Ages (years)
FDR (Age associated divergence) =0.0863
p(Scalp)=0.308
p(Dura)=0.143
Scalp
Dura

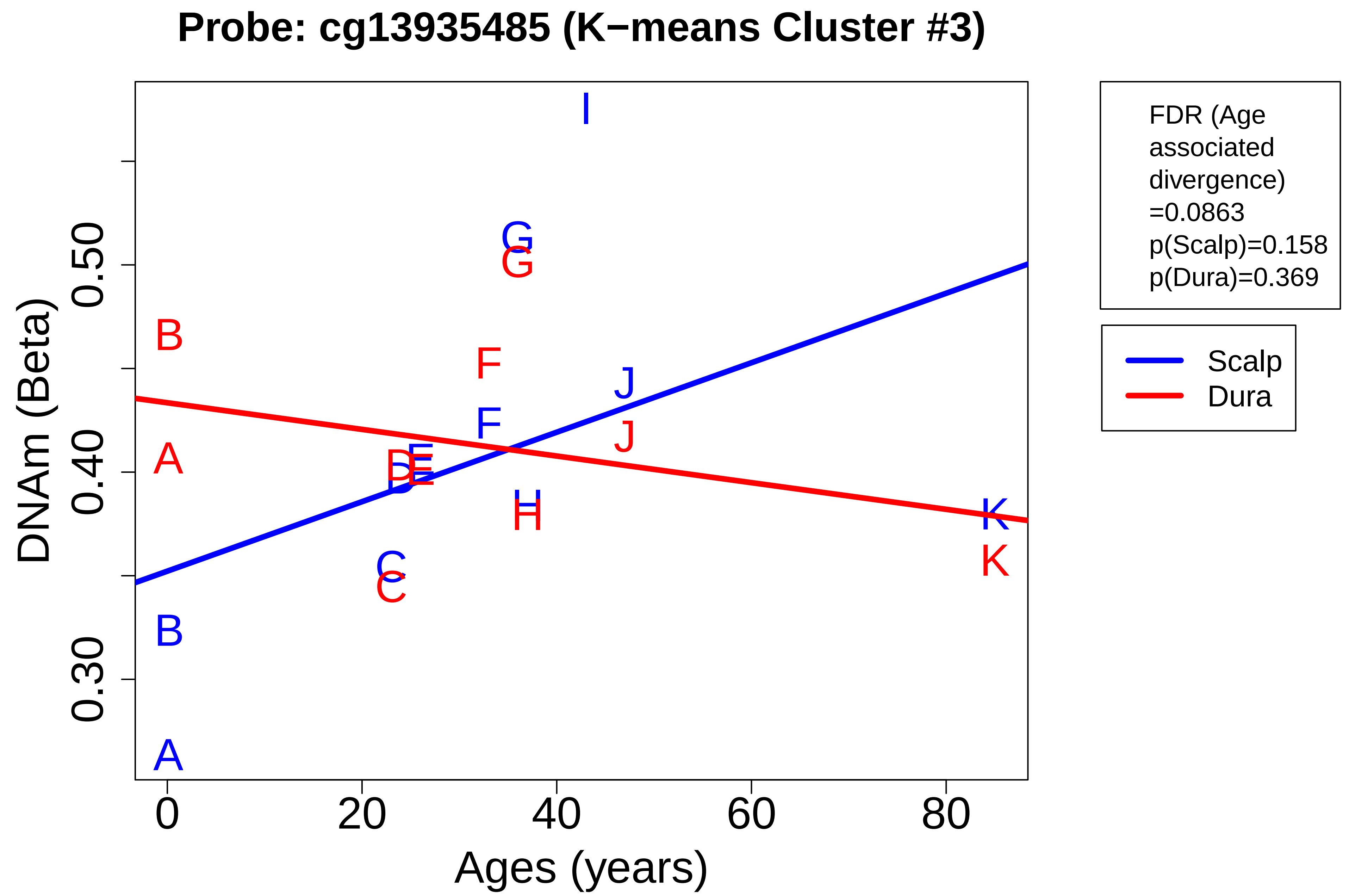

Probe: cg13935485 (K−means Cluster #3)
DNAm (Beta)
0.30
0.40
0.50
Ages (years)
0
20
40
60
80
FDR (Age associated divergence) =0.0863
p(Scalp)=0.158
p(Dura)=0.369
Scalp
Dura
A
B
C
D
E
F
G
H
I
J
K

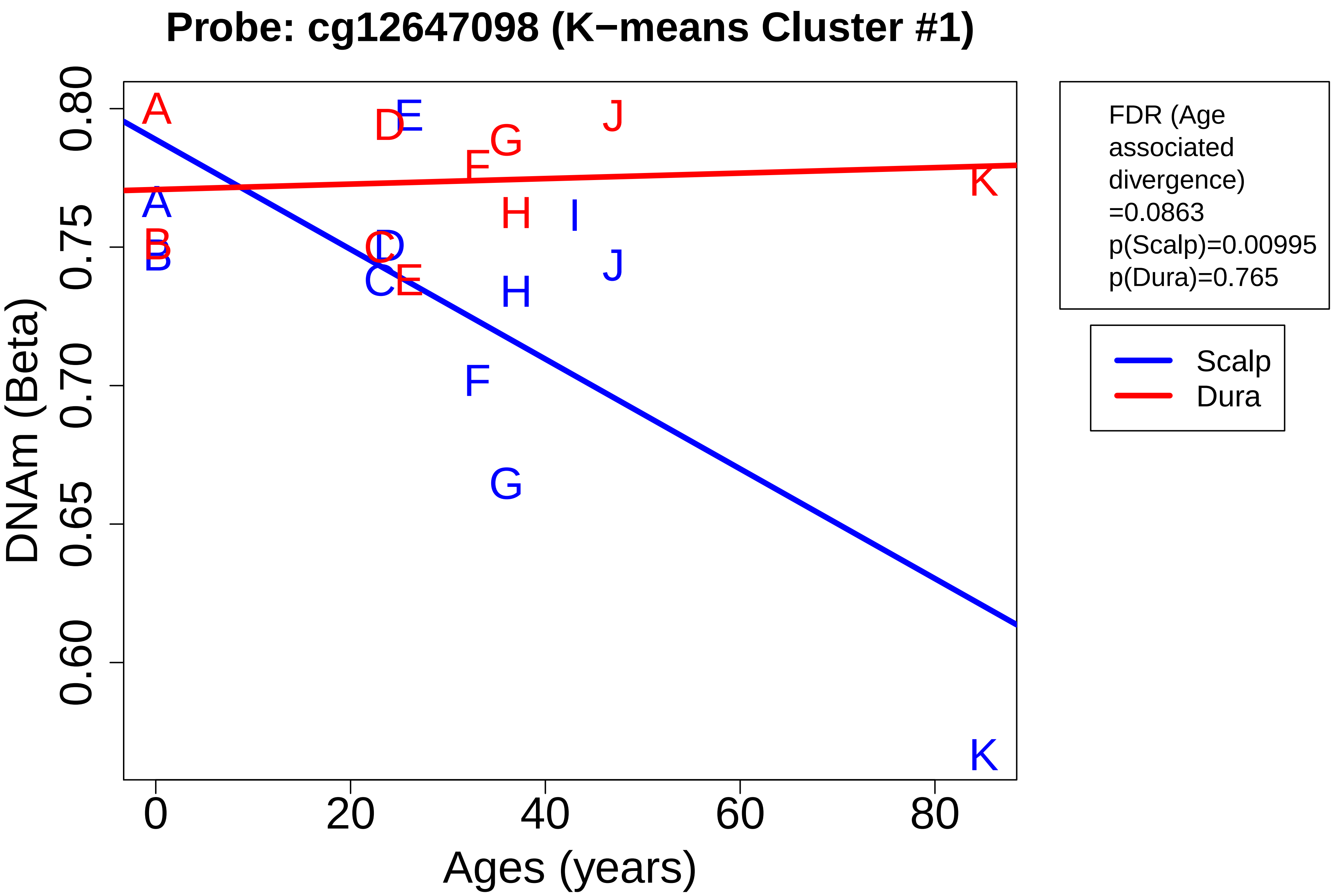

Probe: cg12647098 (K−means Cluster #1)
DNAm (Beta)
0.60
0.65
0.70
0.75
0.80
0
20
40
60
80
Ages (years)
A
B
C
D
E
F
G
H
I
J
K
FDR (Age associated divergence) =0.0863
p(Scalp)=0.00995
p(Dura)=0.765
Scalp
Dura

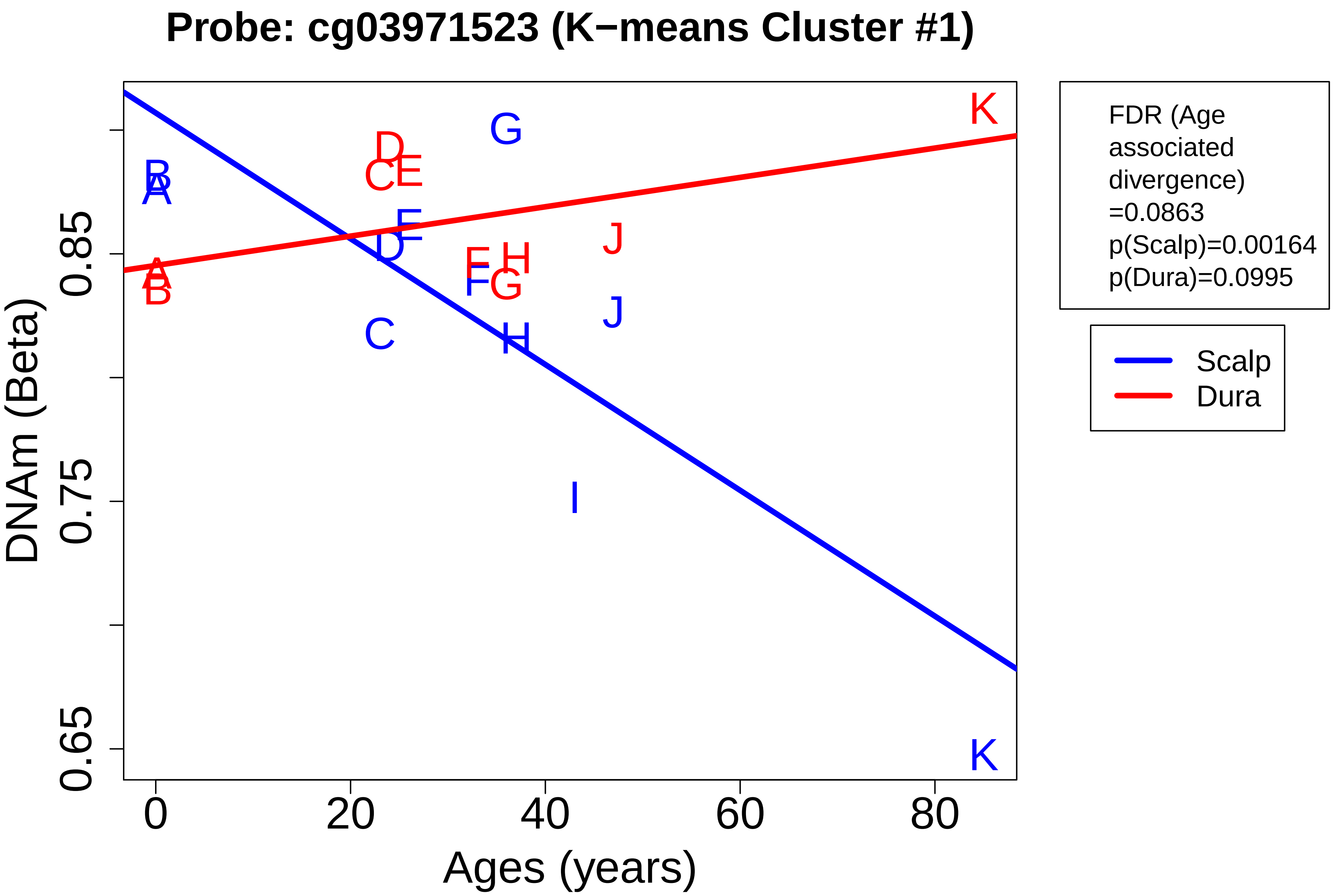

Probe: cg03971523 (K−means Cluster #1)
DNAm (Beta)
0.65
0.75
0.85
0
20
40
60
80
Ages (years)
A
B
C
D
E
F
G
H
I
J
K
FDR (Age associated divergence) =0.0863
p(Scalp)=0.00164
p(Dura)=0.0995
Scalp
Dura

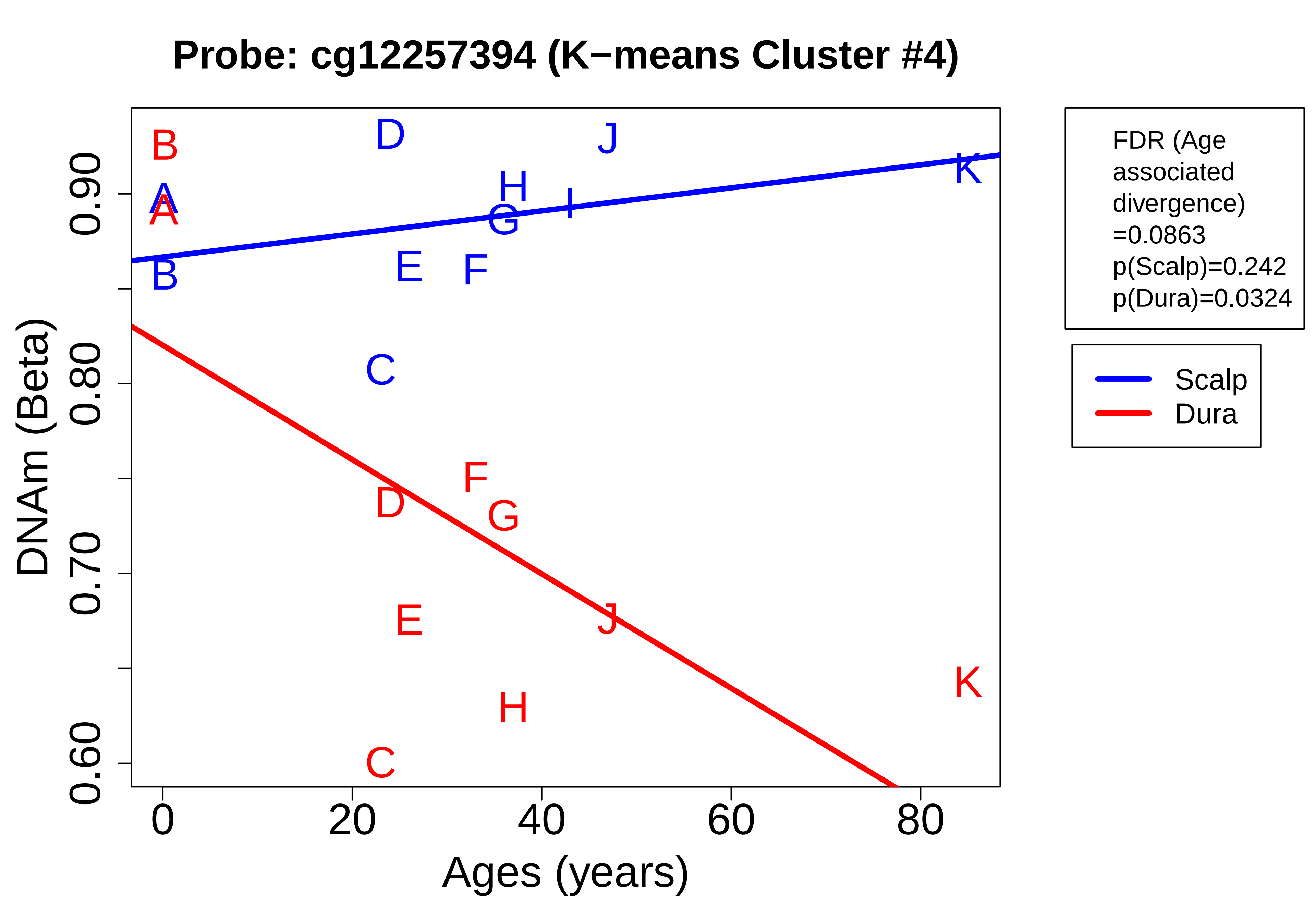

Probe: cg12257394 (K−means Cluster #4)
DNAm (Beta)
0.60
0.70
0.80
0.90
Ages (years)
0
20
40
60
80
A
B
C
D
E
F
G
H
I
J
K
FDR (Age associated divergence) =0.0863
p(Scalp)=0.242
p(Dura)=0.0324
Scalp
Dura

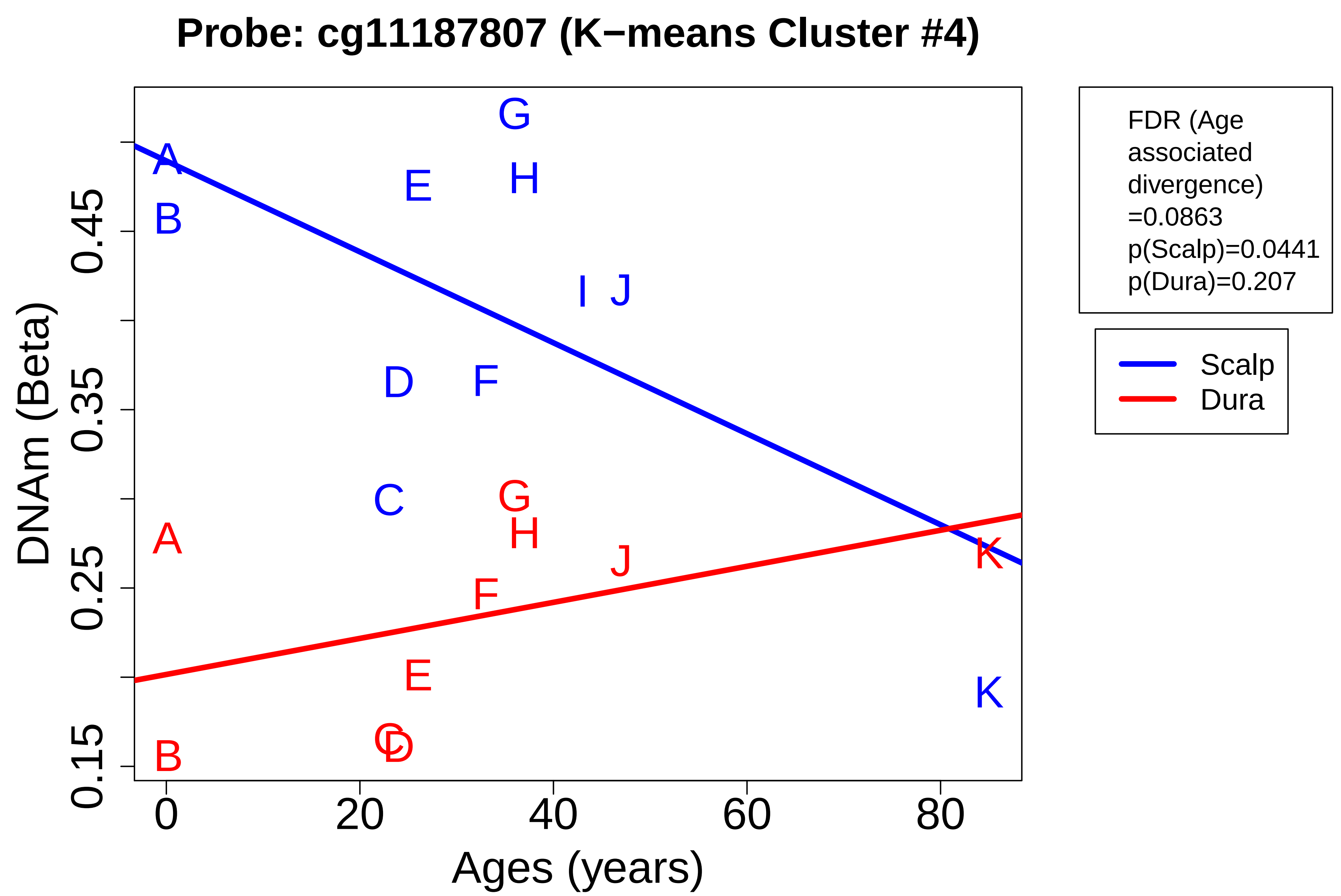

Probe: cg11187807 (K−means Cluster #4)
DNAm (Beta)
0.15
0.25
0.35
0.45
0
20
40
60
80
Ages (years)
A
B
C
D
E
F
G
H
I
J
K
FDR (Age associated divergence) =0.0863
p(Scalp)=0.0441
p(Dura)=0.207
Scalp
Dura

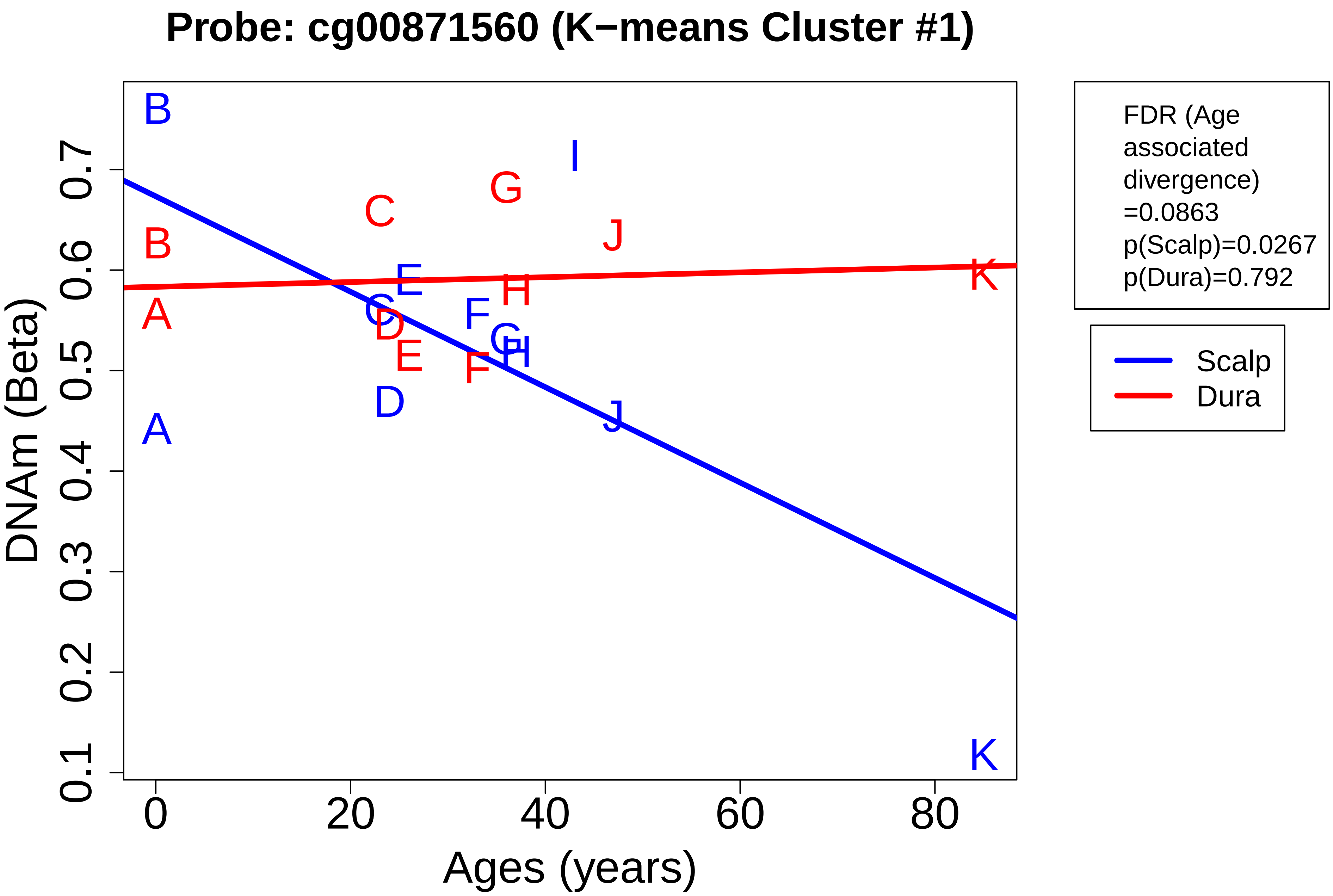

Probe: cg00871560 (K−means Cluster #1)
DNAm (Beta)
0.1
0.2
0.3
0.4
0.5
0.6
0.7
0
20
40
60
80
Ages (years)
FDR (Age associated divergence) =0.0863
p(Scalp)=0.0267
p(Dura)=0.792
Scalp
Dura

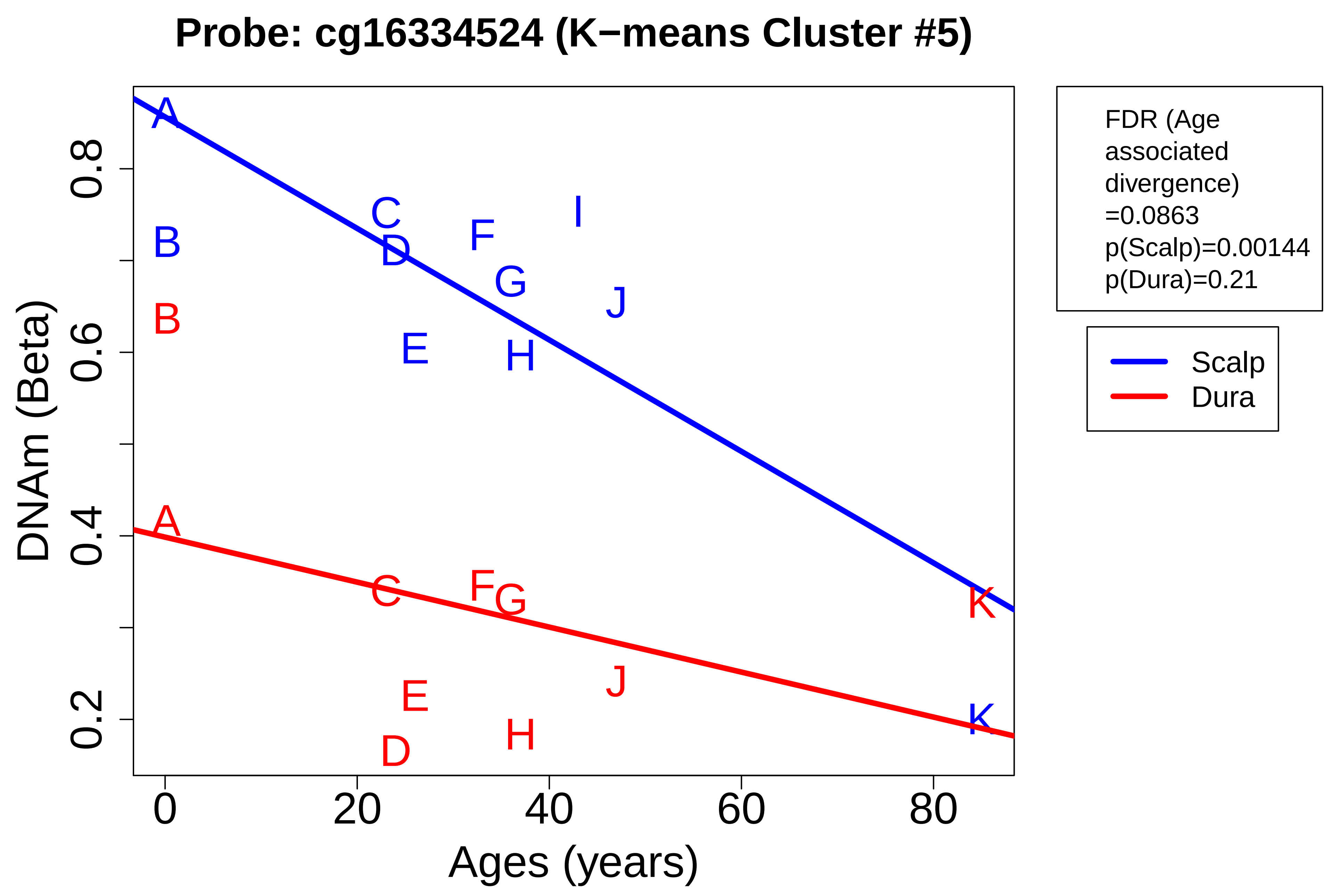

Probe: cg16334524 (K−means Cluster #5)
DNAm (Beta)
0.2
0.4
0.6
0.8
0
20
40
60
80
Ages (years)
A
B
C
D
E
F
G
H
I
J
K
FDR (Age associated divergence) =0.0863
p(Scalp)=0.00144
p(Dura)=0.21
Scalp
Dura

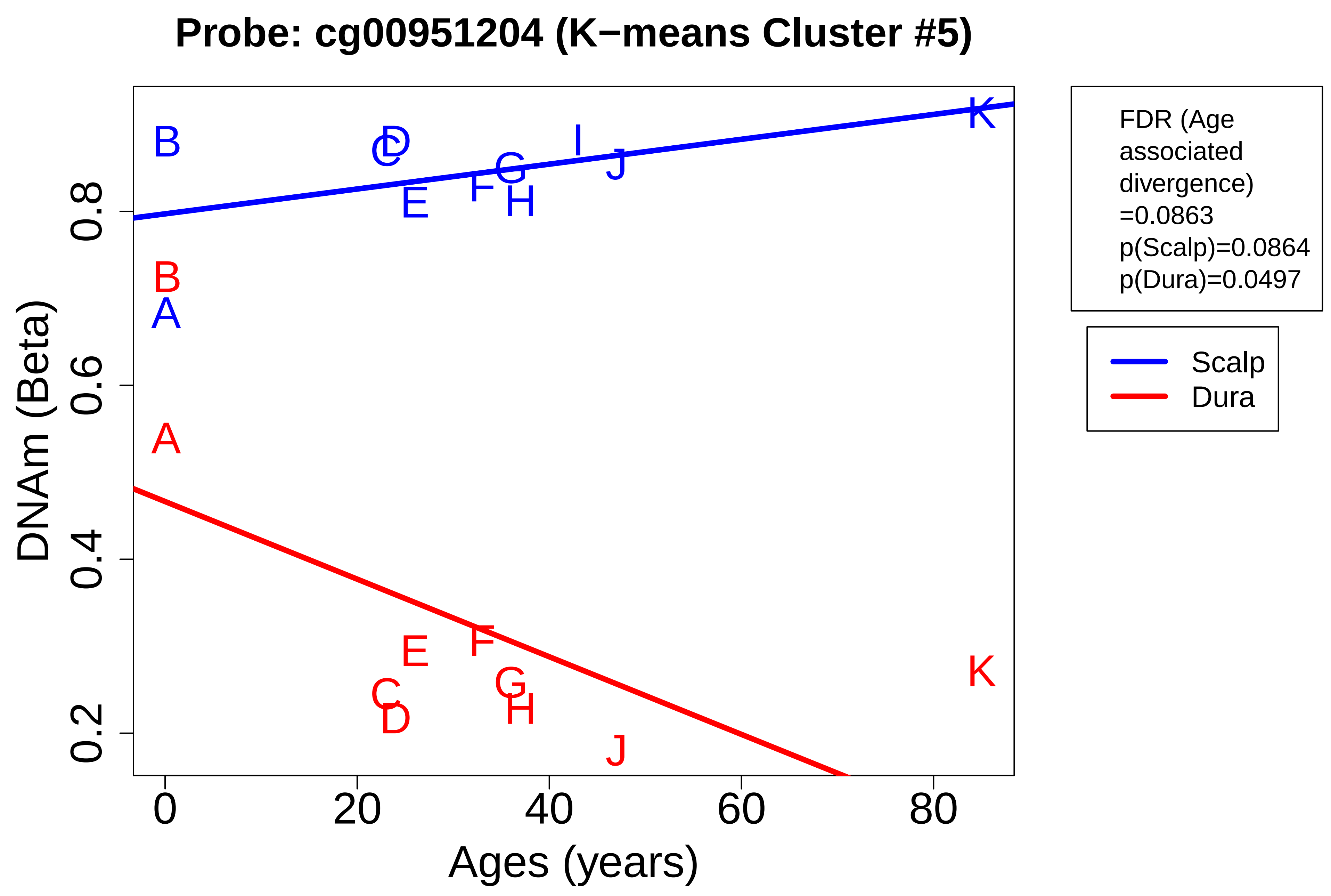

Probe: cg00951204 (K−means Cluster #5)
DNAm (Beta)
0.2
0.4
0.6
0.8
0
20
40
60
80
Ages (years)
A
B
C
D
E
F
G
H
I
J
K
A
B
C
D
E
F
G
H
J
K
FDR (Age associated divergence) =0.0863
p(Scalp)=0.0864
p(Dura)=0.0497
Scalp
Dura

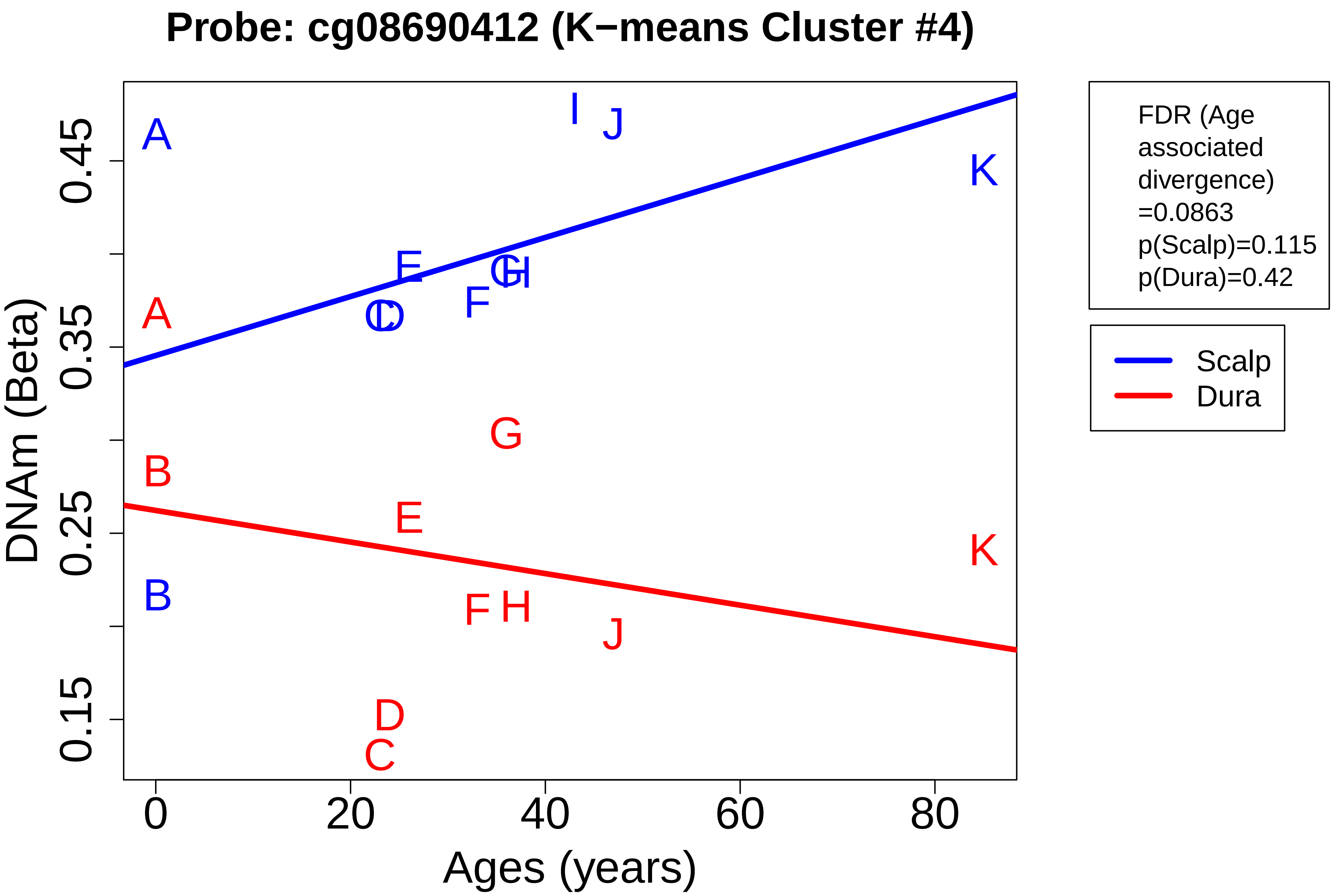

Probe: cg08690412 (K−means Cluster #4)
FDR (Age associated divergence) =0.0863
p(Scalp)=0.115
p(Dura)=0.42
Scalp
Dura
DNAm (Beta)
0.15
0.25
0.35
0.45
0
20
40
60
80
Ages (years)
A
B
C
D
E
F
G
H
I
J
K

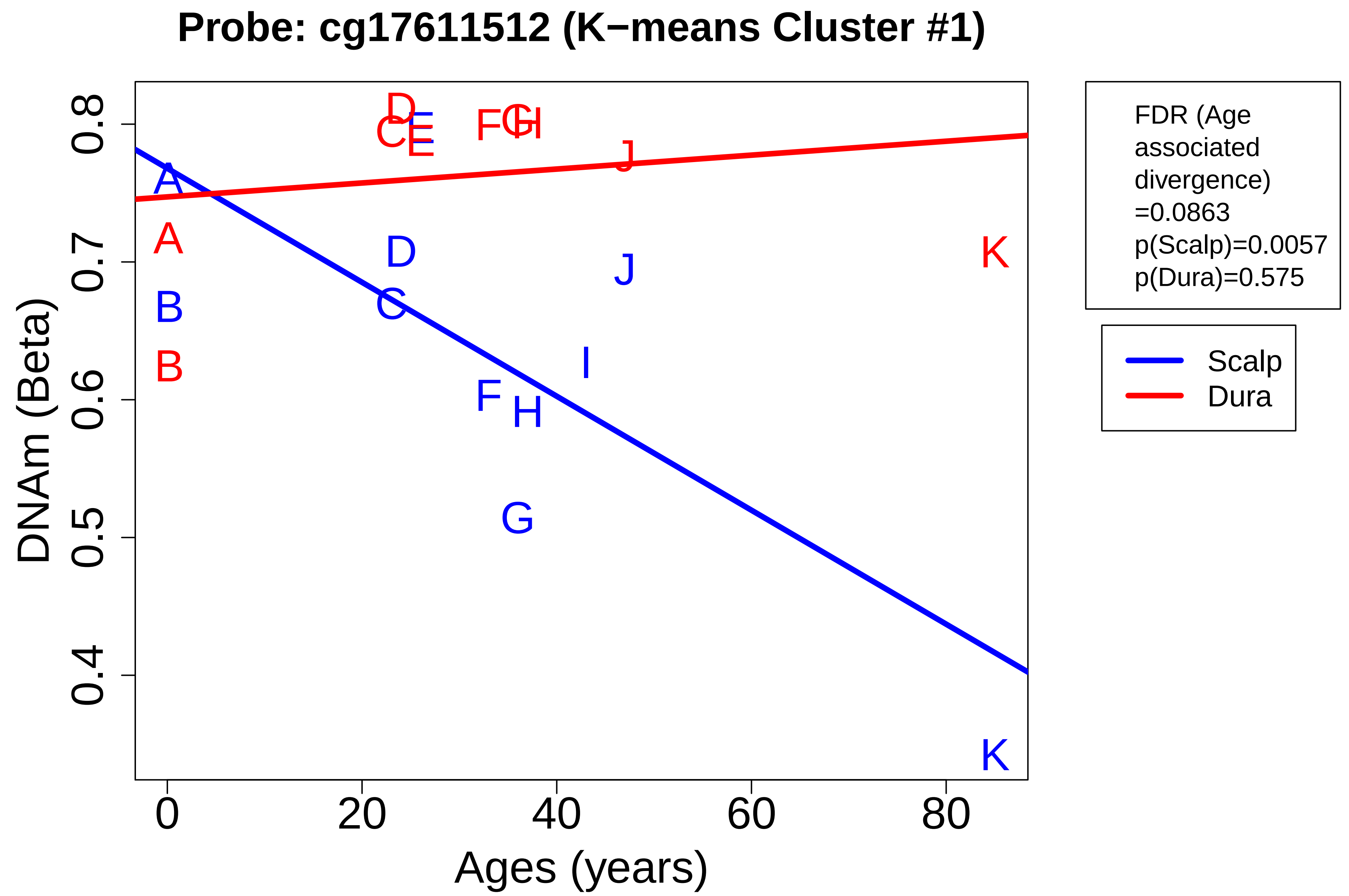

Probe: cg17611512 (K−means Cluster #1)
DNAm (Beta)
0.4
0.5
0.6
0.7
0.8
0
20
40
60
80
Ages (years)
FDR (Age associated divergence) =0.0863
p(Scalp)=0.0057
p(Dura)=0.575
Scalp
Dura

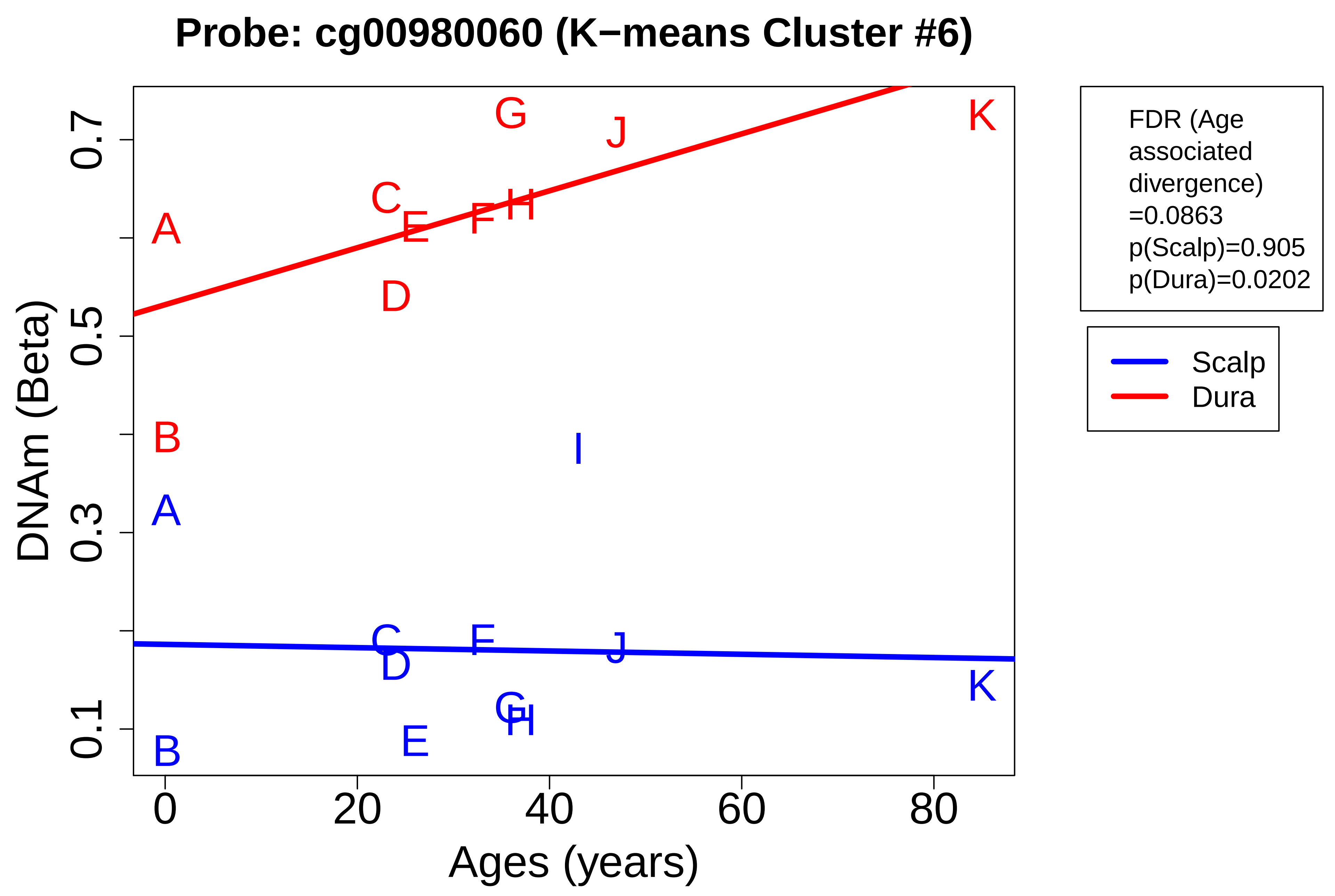

Probe: cg00980060 (K−means Cluster #6)
DNAm (Beta)
0.1
0.3
0.5
0.7
Ages (years)
0
20
40
60
80
A
B
C
D
E
F
G
H
I
J
K
FDR (Age associated divergence) =0.0863
p(Scalp)=0.905
p(Dura)=0.0202
Scalp
Dura

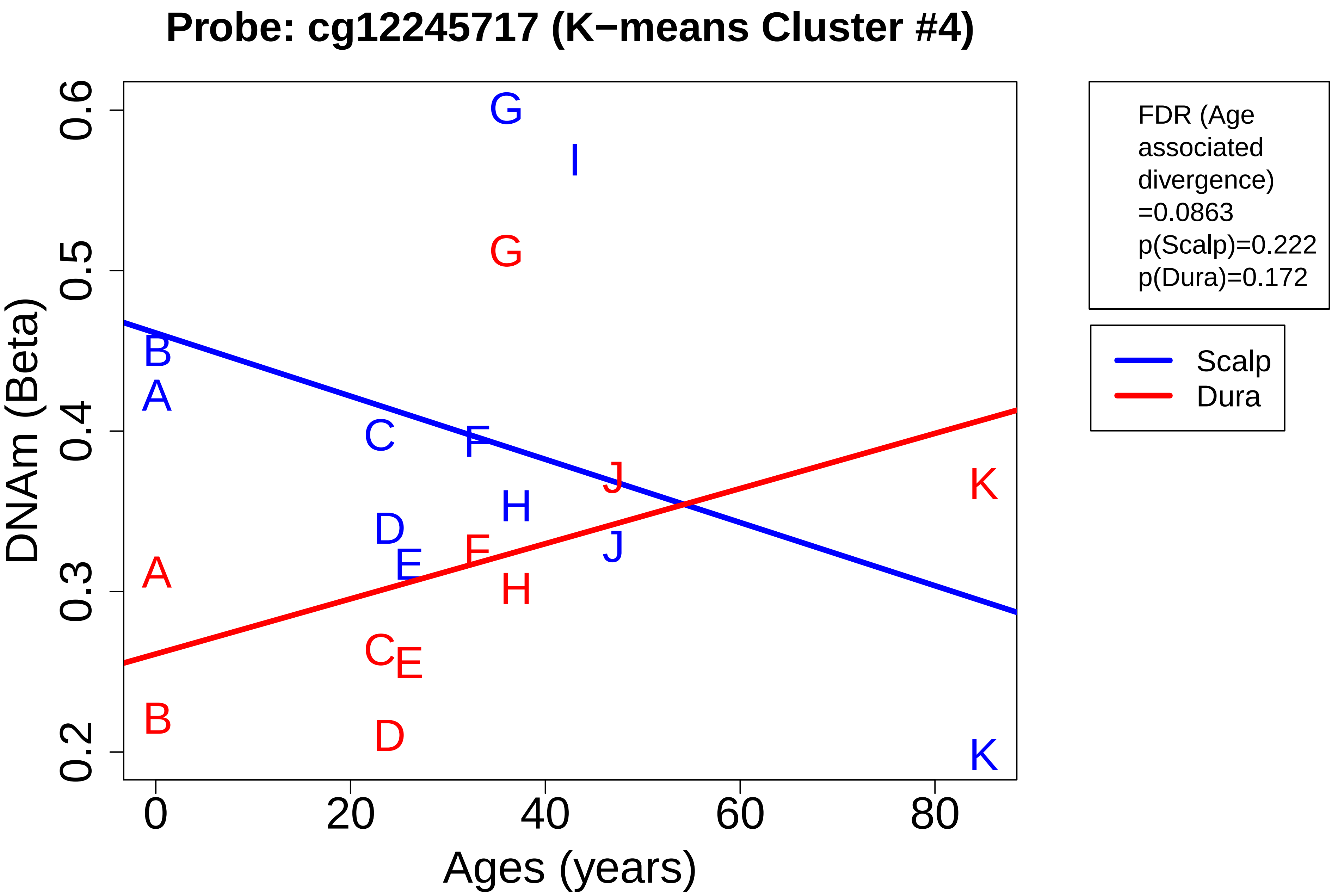

Probe: cg12245717 (K−means Cluster #4)
DNAm (Beta)
Ages (years)
0.2
0.3
0.4
0.5
0.6
0
20
40
60
80
FDR (Age associated divergence) =0.0863
p(Scalp)=0.222
p(Dura)=0.172
Scalp
Dura

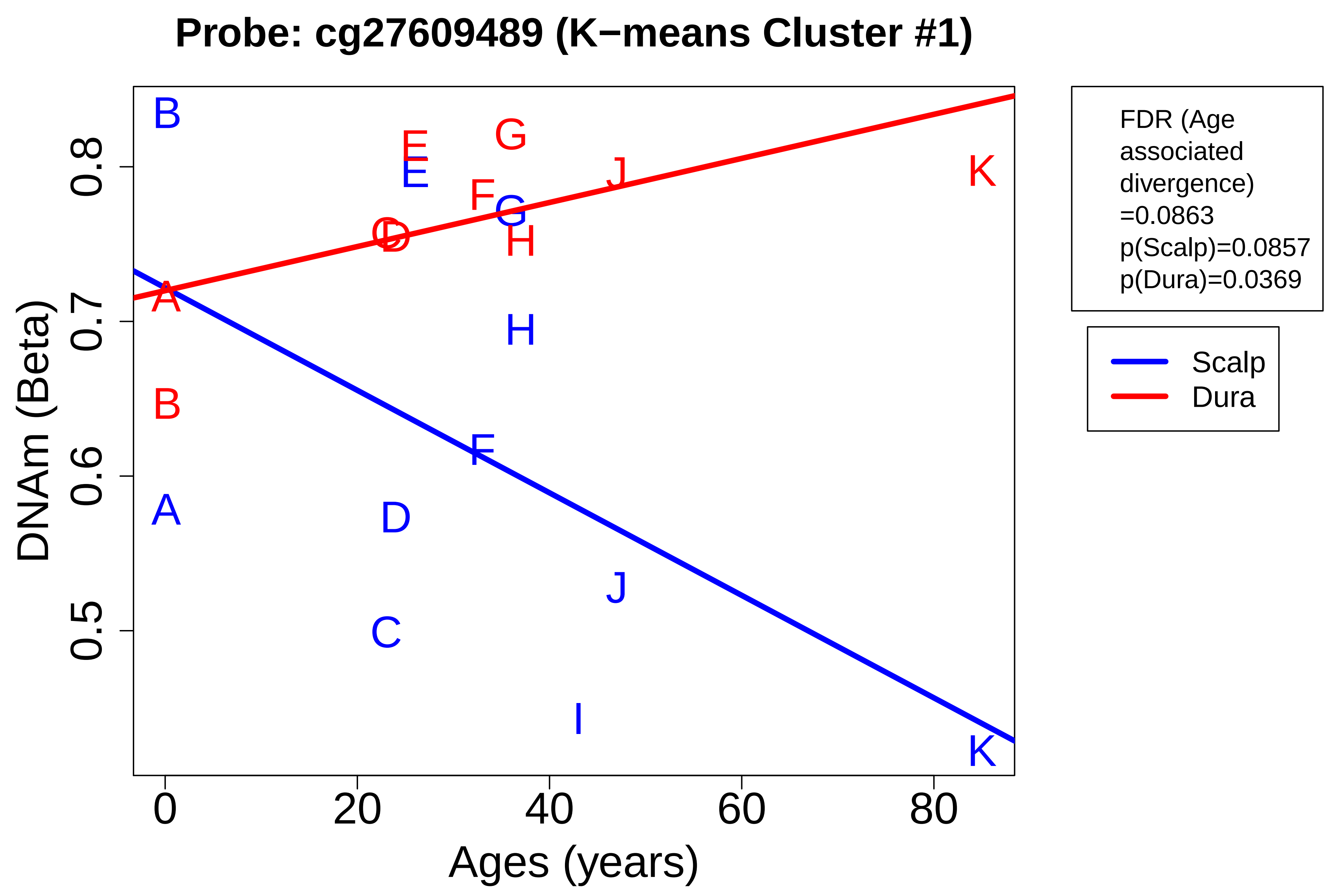
Probe: cg27609489 (K−means Cluster #1)
DNAm (Beta)
0.5
0.6
0.7
0.8
Ages (years)
0
20
40
60
80
FDR (Age associated divergence) =0.0863
p(Scalp)=0.0857
p(Dura)=0.0369
Scalp
Dura
A
B
C
D
E
F
G
H
I
J
K

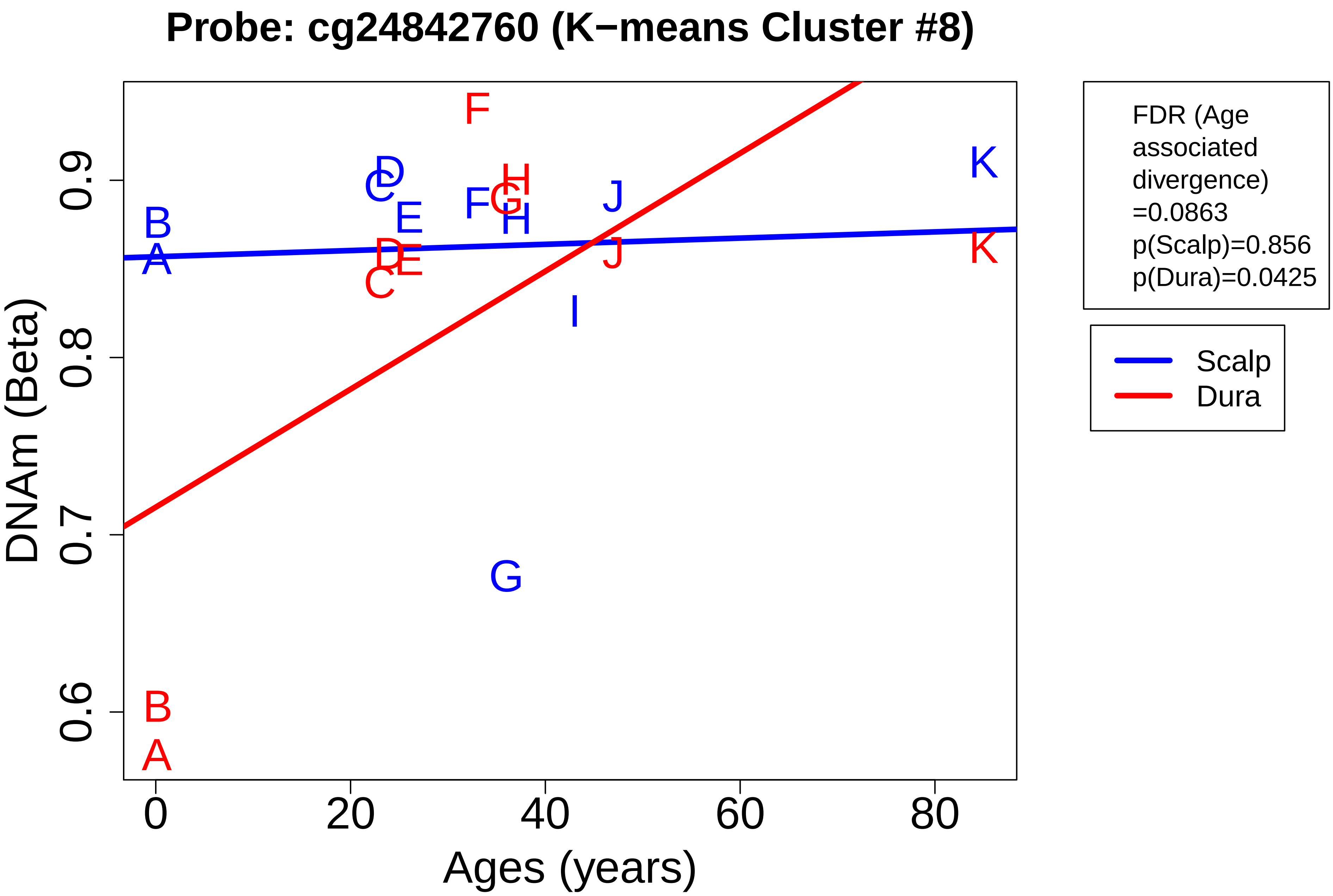

Probe: cg24842760 (K−means Cluster #8)
DNAm (Beta)
Ages (years)
0.6
0.7
0.8
0.9
0
20
40
60
80
FDR (Age associated divergence) =0.0863
p(Scalp)=0.856
p(Dura)=0.0425
Scalp
Dura
A
B
C
D
E
F
G
H
I
J
K

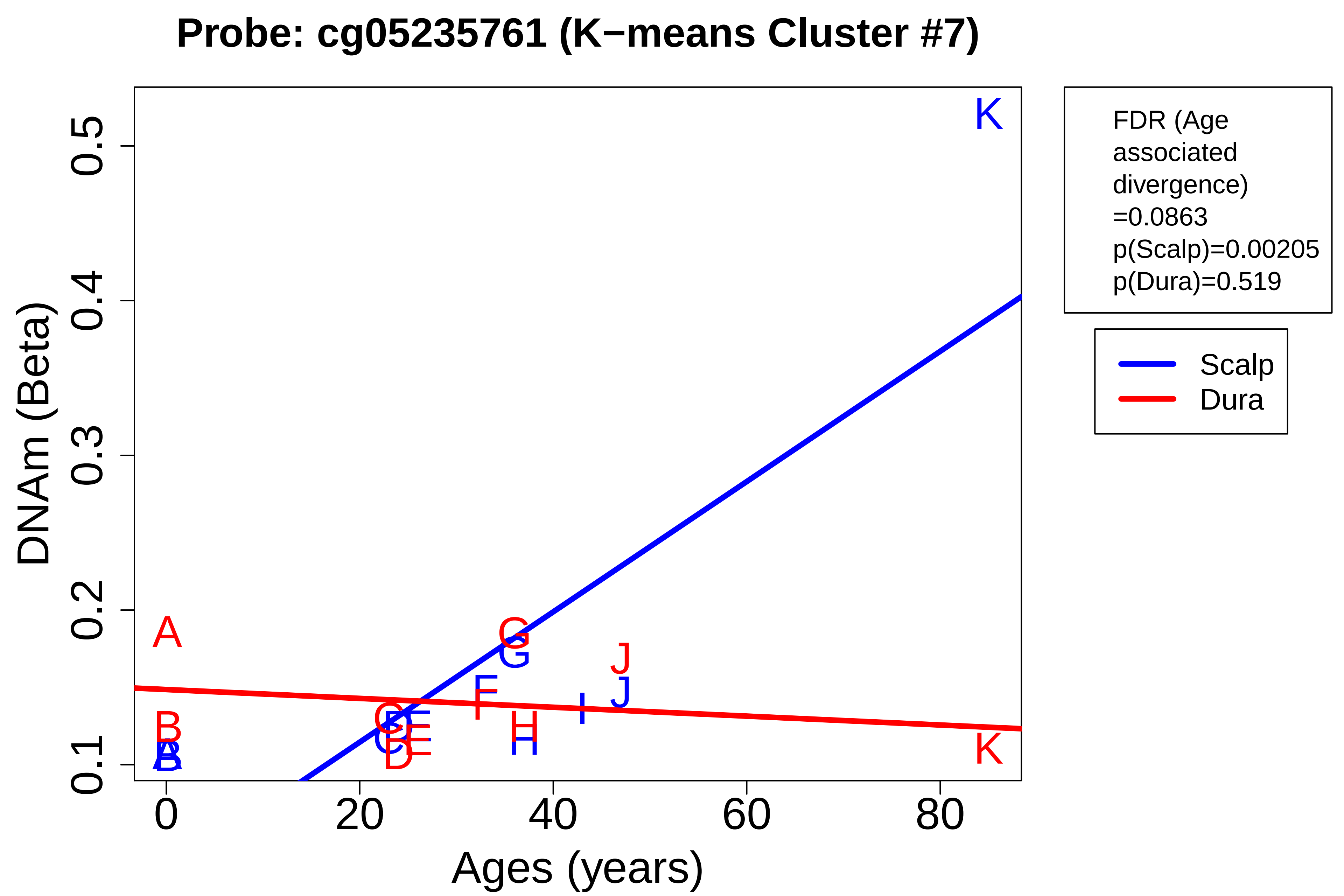

Probe: cg05235761 (K−means Cluster #7)
DNAm (Beta)
Ages (years)
0.1
0.2
0.3
0.4
0.5
0
20
40
60
80
FDR (Age associated divergence) =0.0863
p(Scalp)=0.00205
p(Dura)=0.519
Scalp
Dura

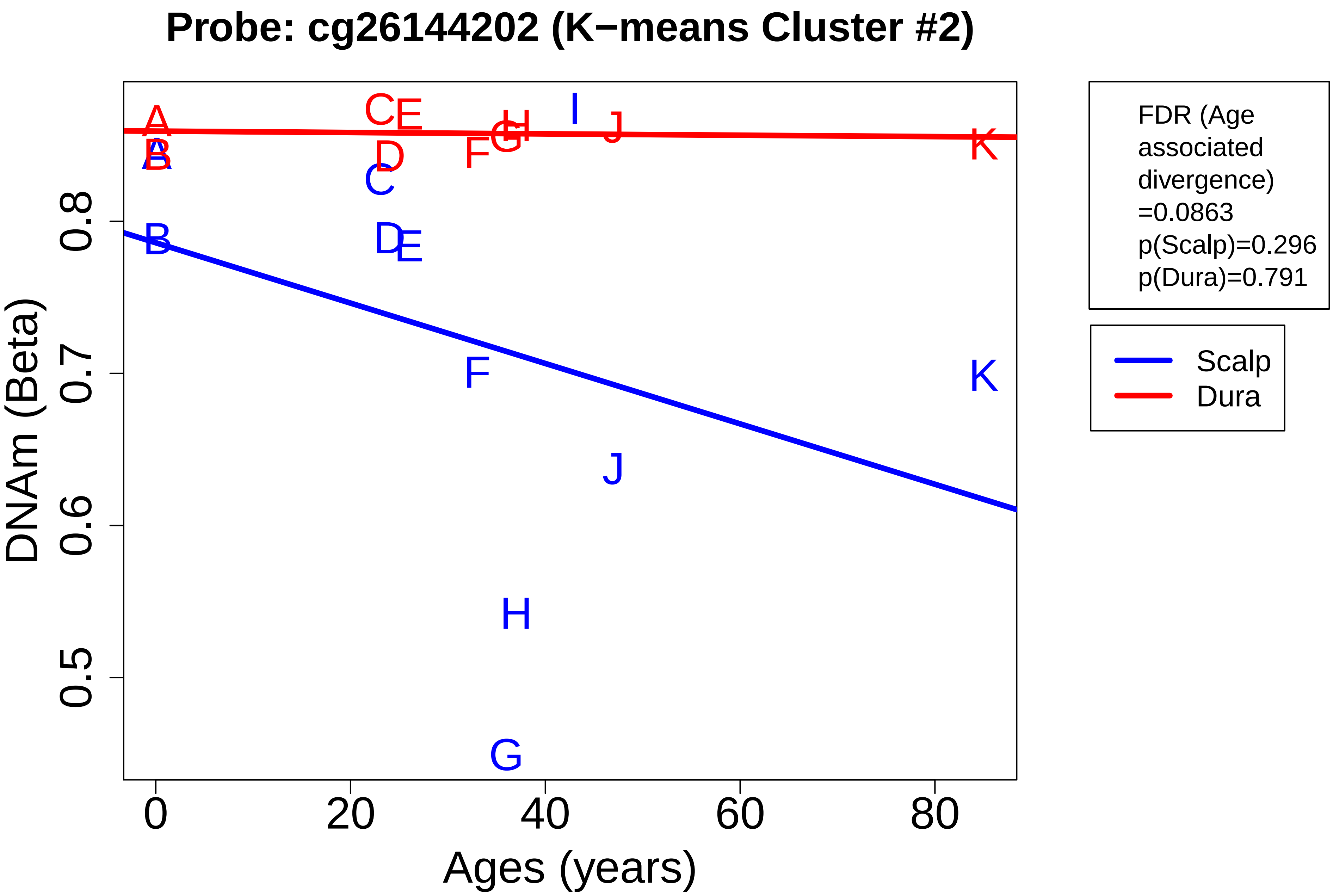

Probe: cg26144202 (K−means Cluster #2)
DNAm (Beta)
0.5
0.6
0.7
0.8
0
20
40
60
80
Ages (years)
FDR (Age associated divergence) =0.0863
p(Scalp)=0.296
p(Dura)=0.791
Scalp
Dura
A
B
C
D
E
F
G
H
I
J
K

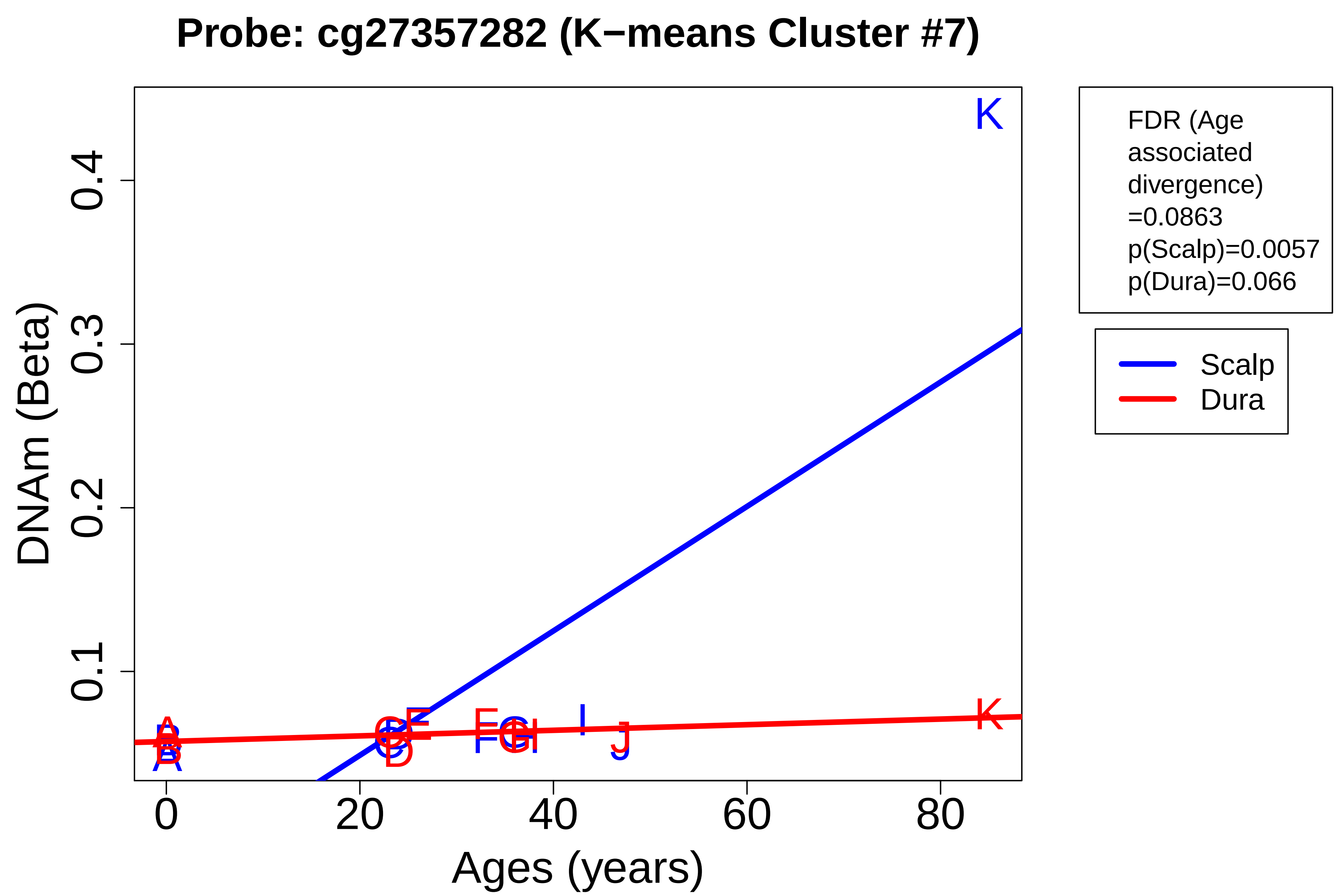

Probe: cg27357282 (K−means Cluster #7)
DNAm (Beta)
0.1
0.2
0.3
0.4
Ages (years)
0
20
40
60
80
FDR (Age associated divergence) =0.0863
p(Scalp)=0.0057
p(Dura)=0.066
Scalp
Dura

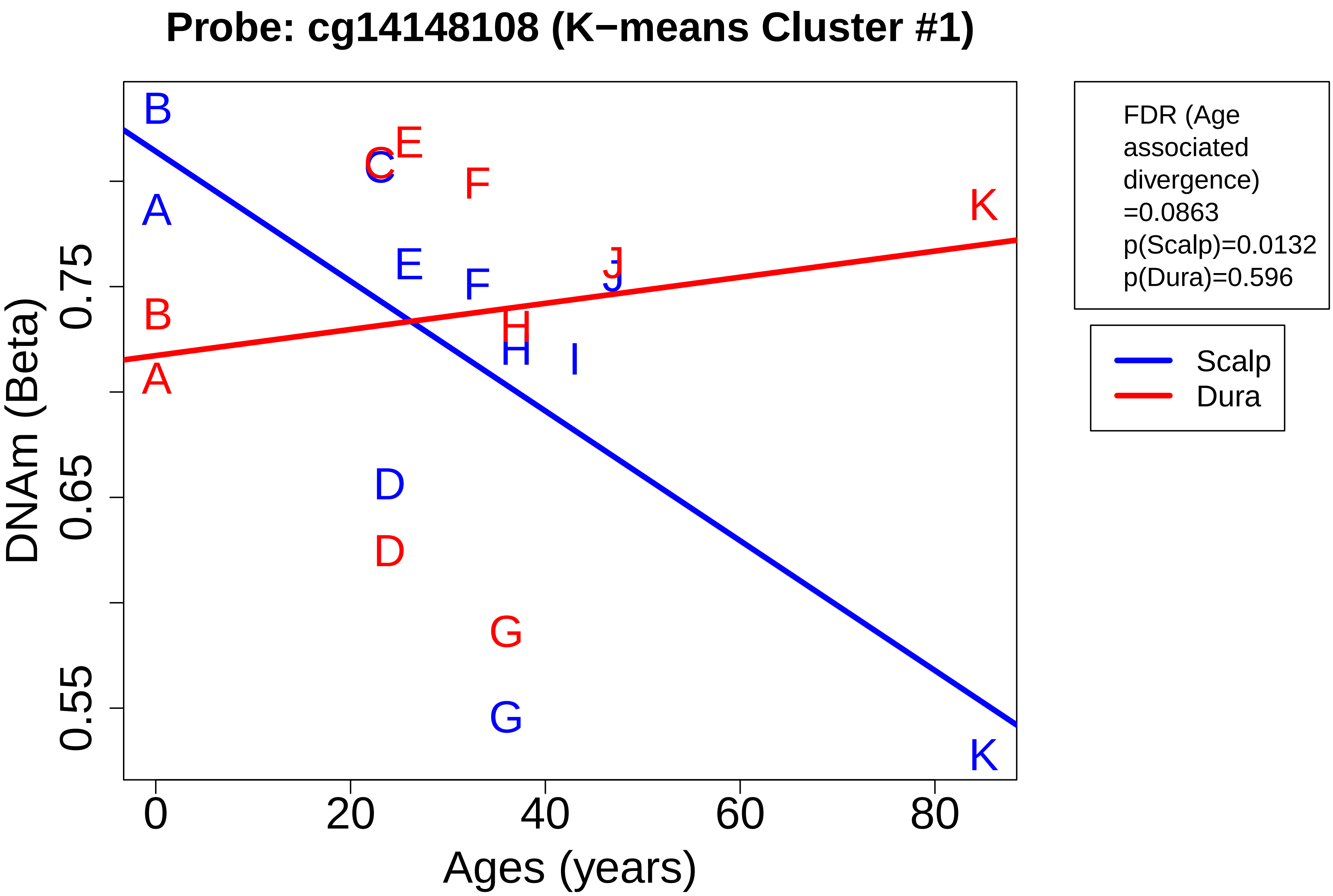

Probe: cg14148108 (K−means Cluster #1)
DNAm (Beta)
0.55
0.65
0.75
0
20
40
60
80
Ages (years)
FDR (Age associated divergence) =0.0863
p(Scalp)=0.0132
p(Dura)=0.596
Scalp
Dura

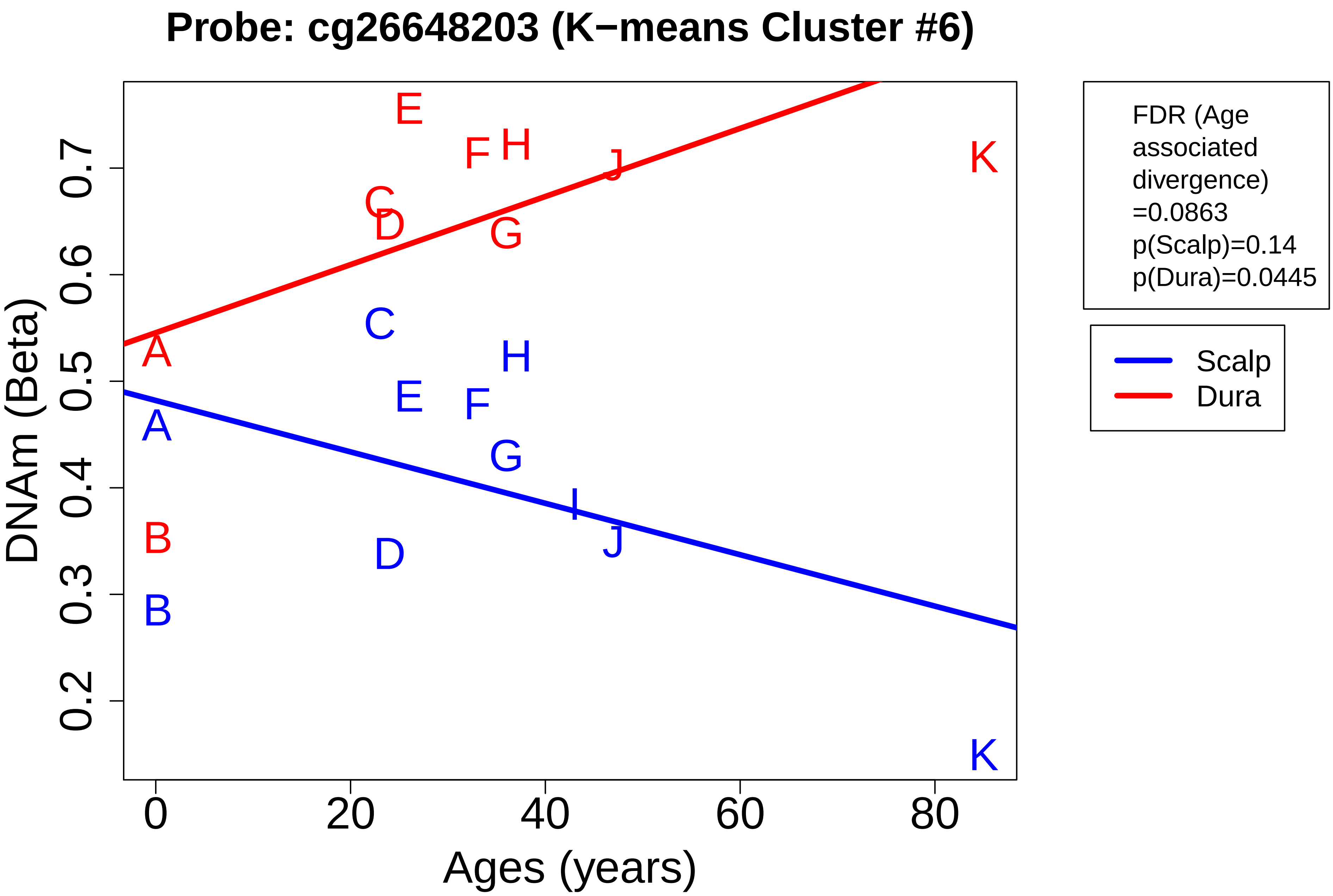

Probe: cg26648203 (K−means Cluster #6)
DNAm (Beta)
Ages (years)
0.2
0.3
0.4
0.5
0.6
0.7
0
20
40
60
80
FDR (Age associated divergence) =0.0863
p(Scalp)=0.14
p(Dura)=0.0445
Scalp
Dura

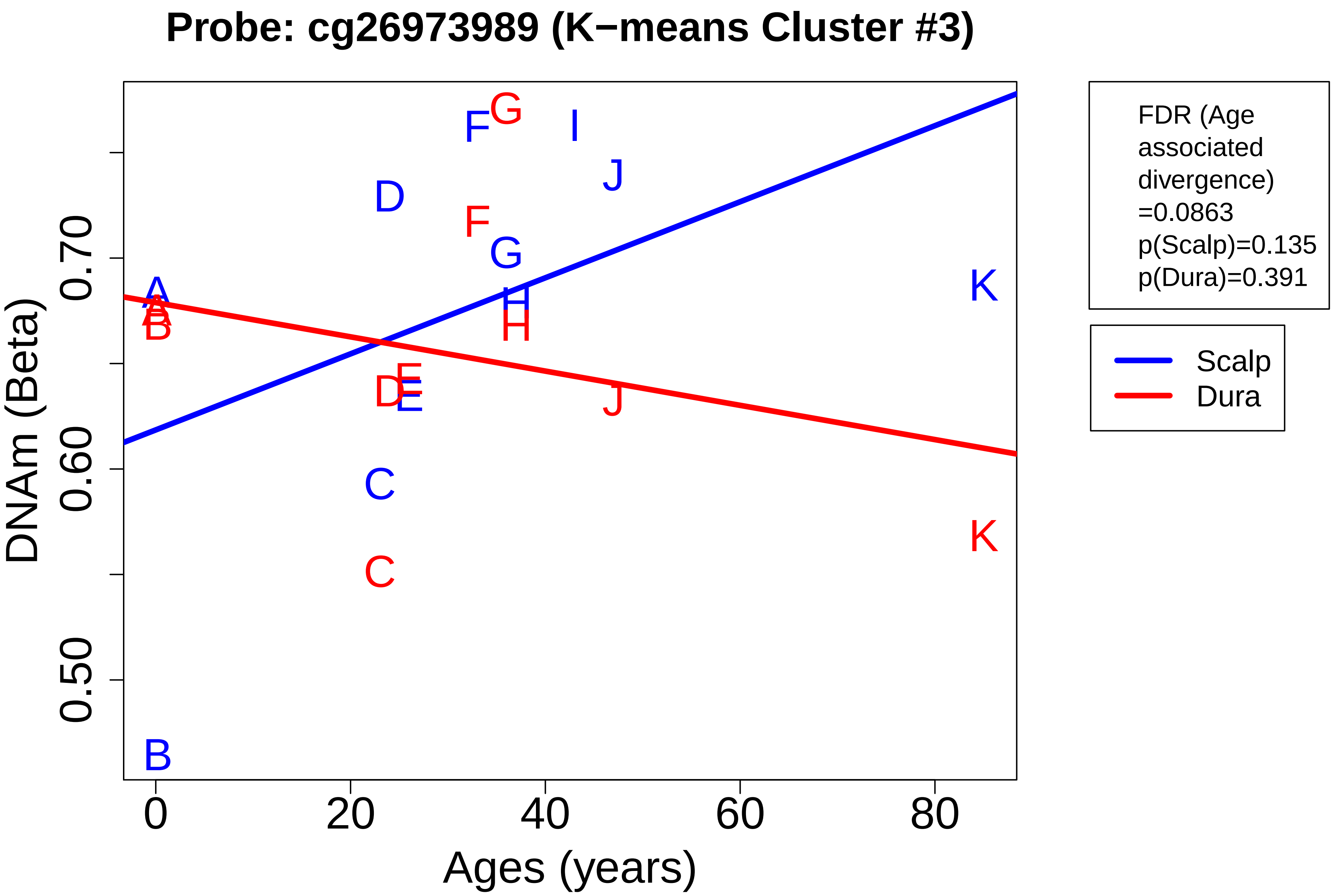

Probe: cg26973989 (K−means Cluster #3)
DNAm (Beta)
0.50
0.60
0.70
0
20
40
60
80
Ages (years)
A B C D E F G H I J K
A B C D E F G H J K
FDR (Age associated divergence) =0.0863
p(Scalp)=0.135
p(Dura)=0.391
Scalp
Dura

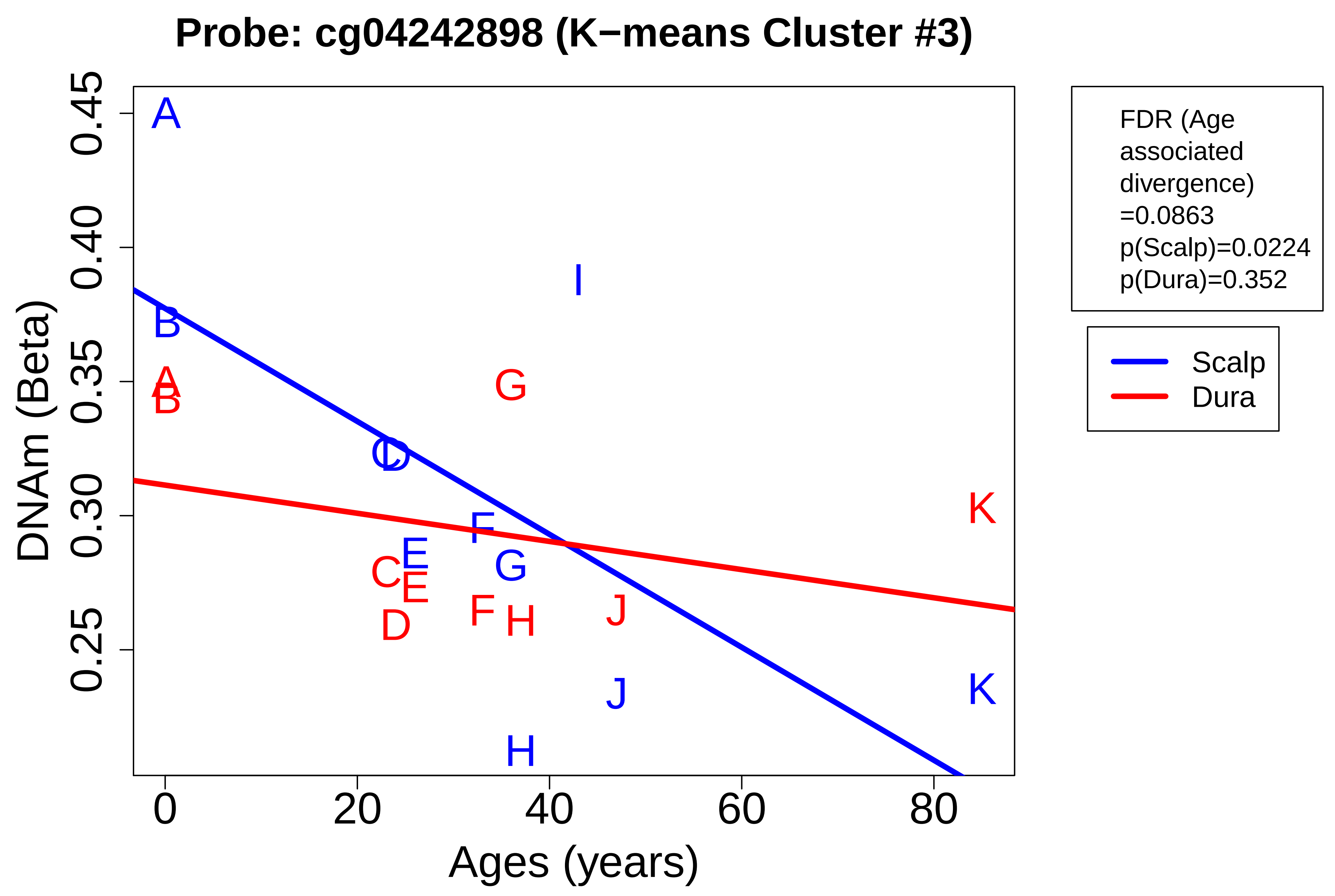

Probe: cg04242898 (K−means Cluster #3)
DNAm (Beta)
0.25
0.30
0.35
0.40
0.45
Ages (years)
0
20
40
60
80
A
B
C
D
E
F
G
H
I
J
K
FDR (Age associated divergence) =0.0863
p(Scalp)=0.0224
p(Dura)=0.352
Scalp
Dura

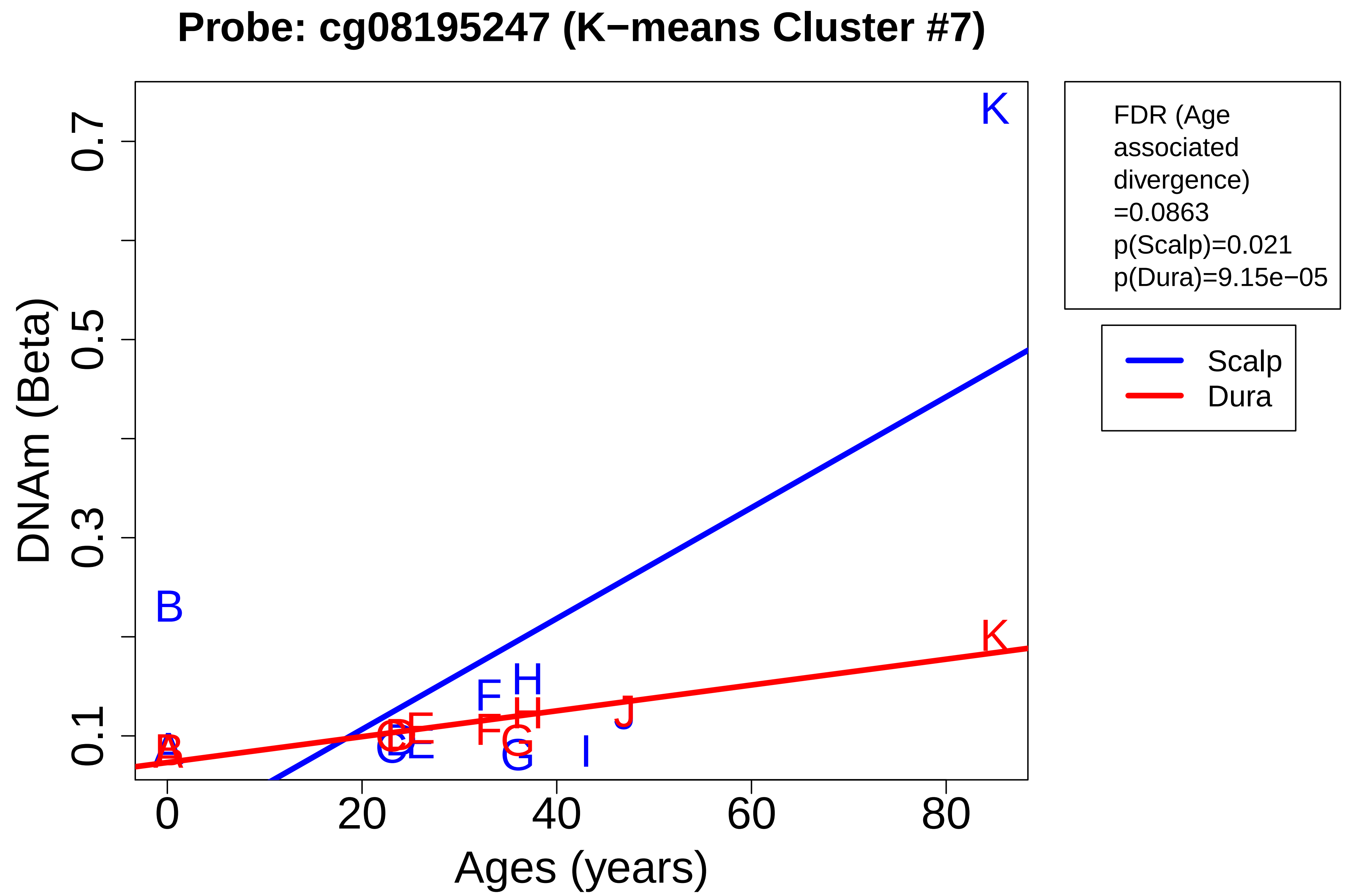

Probe: cg08195247 (K−means Cluster #7)
DNAm (Beta)
0.1
0.3
0.5
0.7
0
20
40
60
80
Ages (years)
FDR (Age associated divergence) =0.0863
p(Scalp)=0.021
p(Dura)=9.15e−05
Scalp
Dura

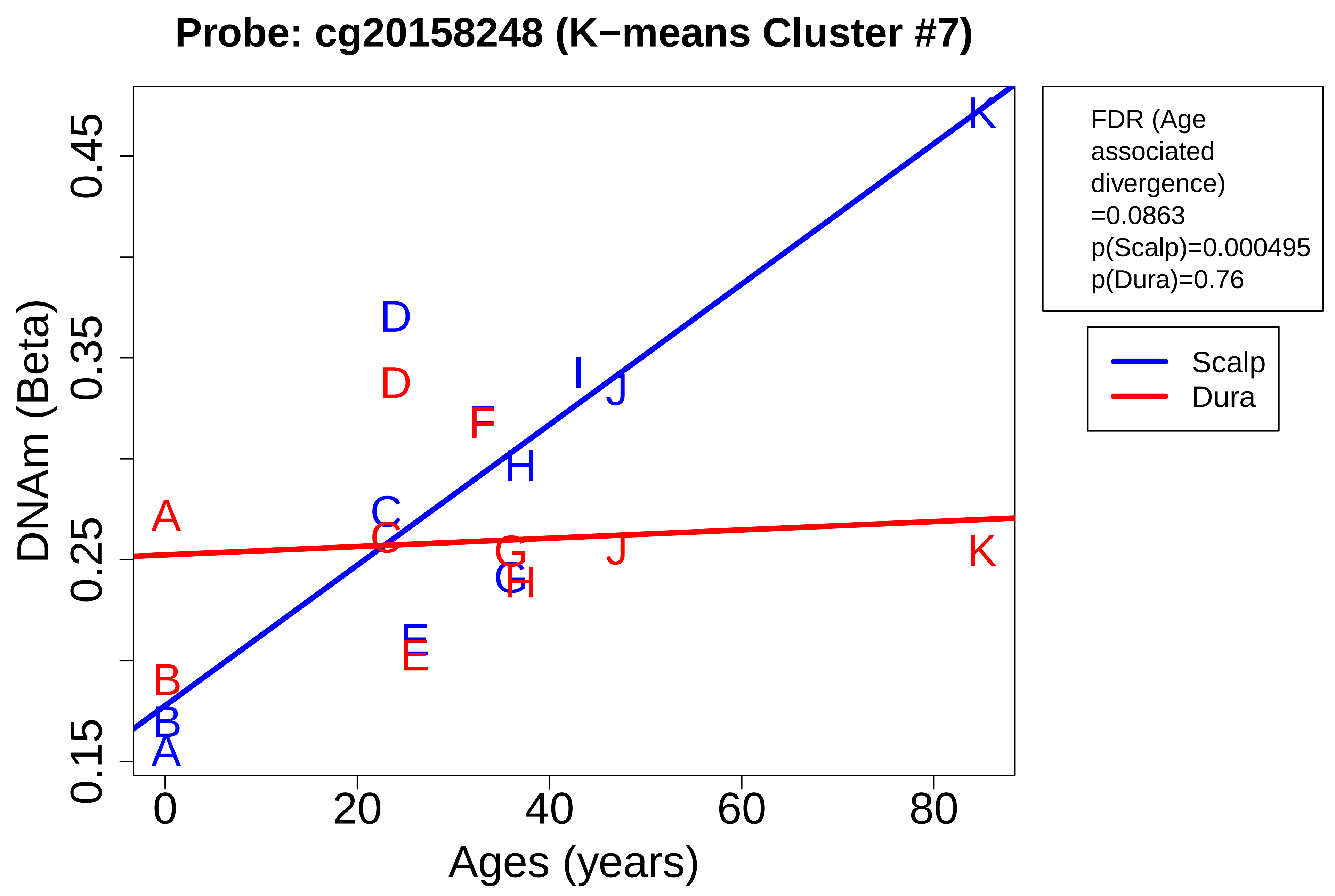

Probe: cg20158248 (K−means Cluster #7)
DNAm (Beta)
0.15
0.25
0.35
0.45
Ages (years)
0
20
40
60
80
A
B
C
D
E
F
G
H
I
J
K
FDR (Age associated divergence) =0.0863
p(Scalp)=0.000495
p(Dura)=0.76
Scalp
Dura

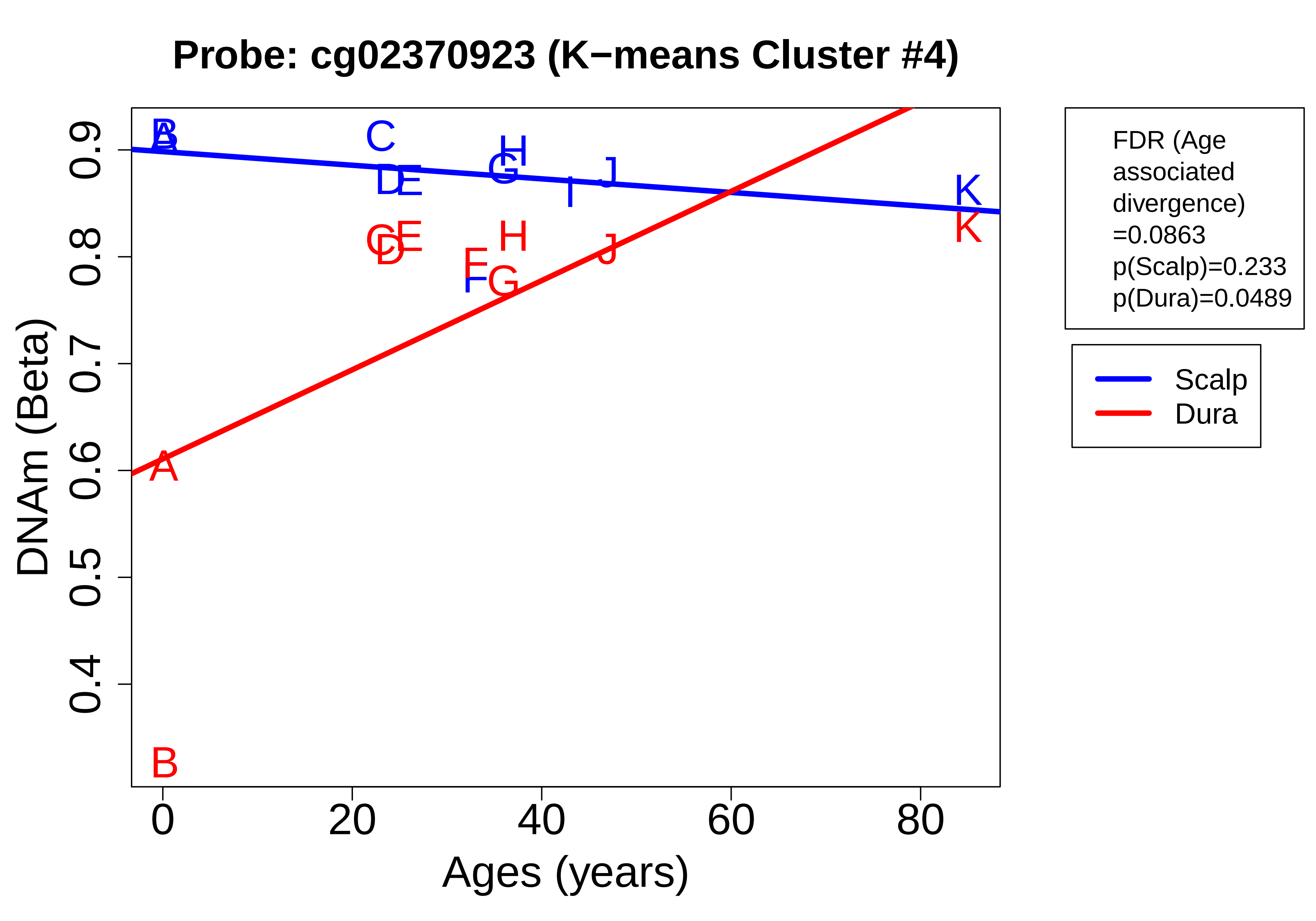

Probe: cg02370923 (K−means Cluster #4)
DNAm (Beta)
0.4
0.5
0.6
0.7
0.8
0.9
0
20
40
60
80
Ages (years)
A
B
C
D
E
F
G
H
I
J
K
FDR (Age associated divergence) =0.0863
p(Scalp)=0.233
p(Dura)=0.0489
Scalp
Dura

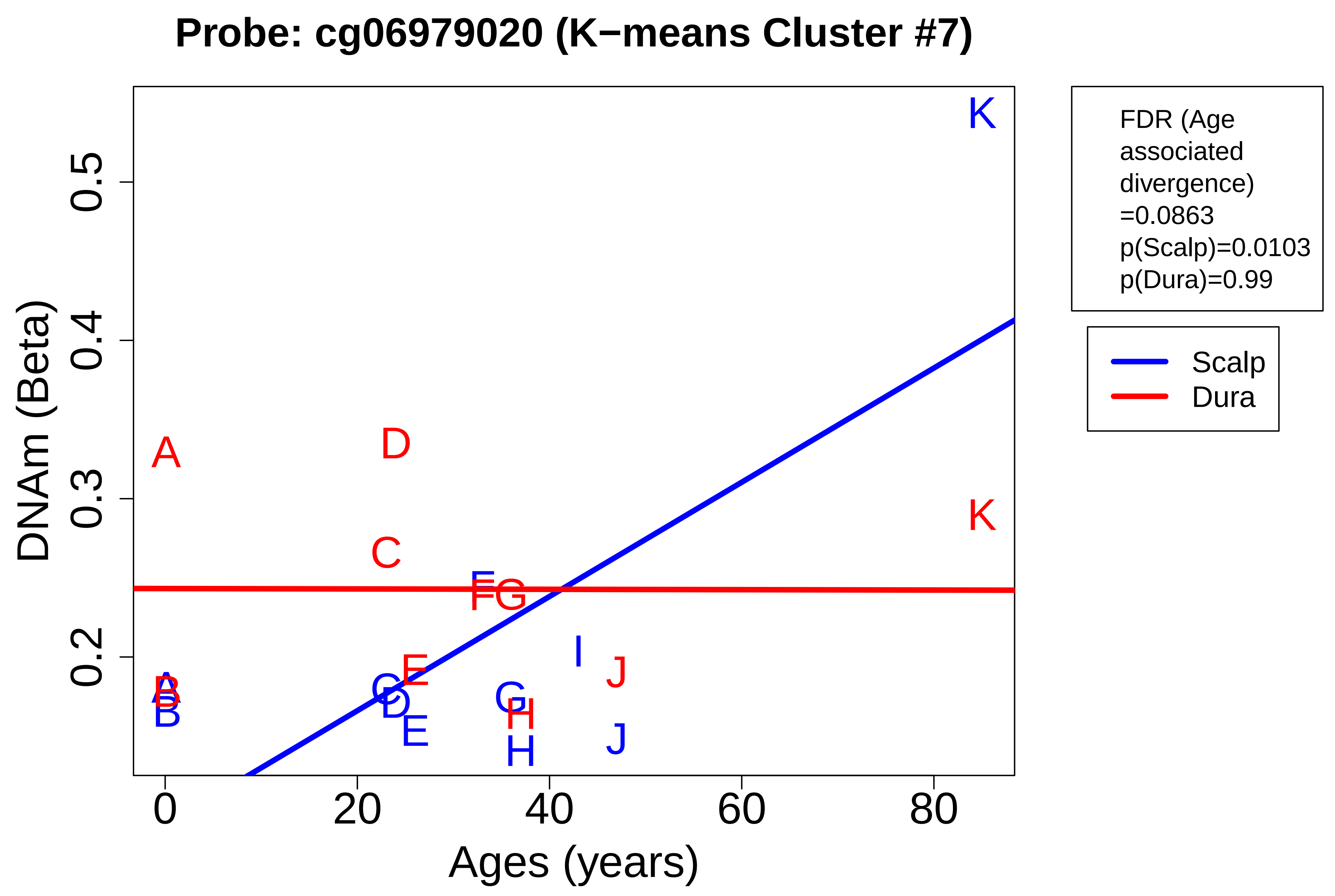

Probe: cg06979020 (K−means Cluster #7)
DNAm (Beta)
0.2
0.3
0.4
0.5
Ages (years)
0
20
40
60
80
FDR (Age associated divergence) =0.0863
p(Scalp)=0.0103
p(Dura)=0.99
Scalp
Dura
A
B
C
D
E
F
G
H
I
J
K

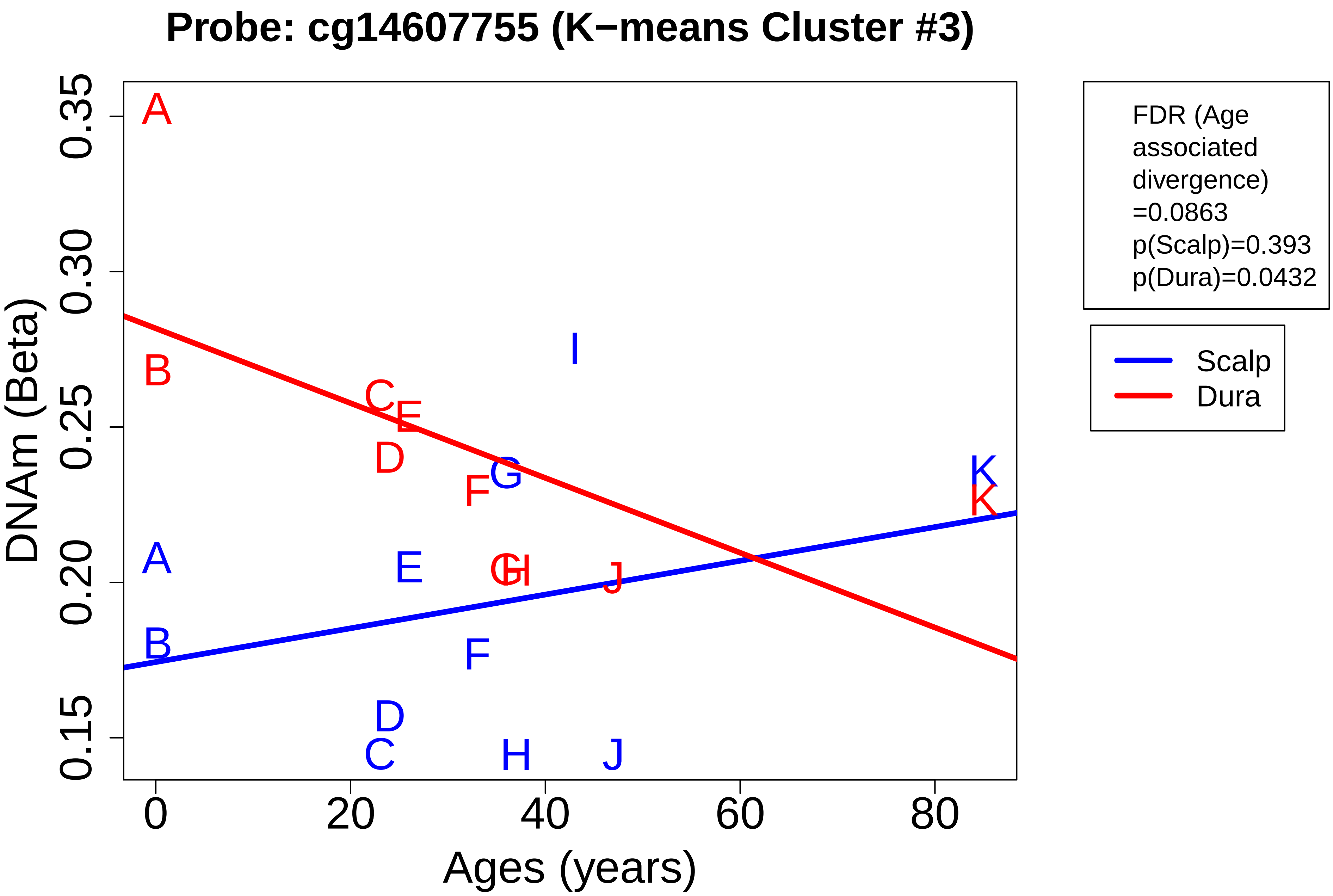

Probe: cg14607755 (K−means Cluster #3)
DNAm (Beta)
0.15
0.20
0.25
0.30
0.35
Ages (years)
0
20
40
60
80
FDR (Age associated divergence) =0.0863
p(Scalp)=0.393
p(Dura)=0.0432
Scalp
Dura
A
B
C
D
E
F
G
H
I
J
K

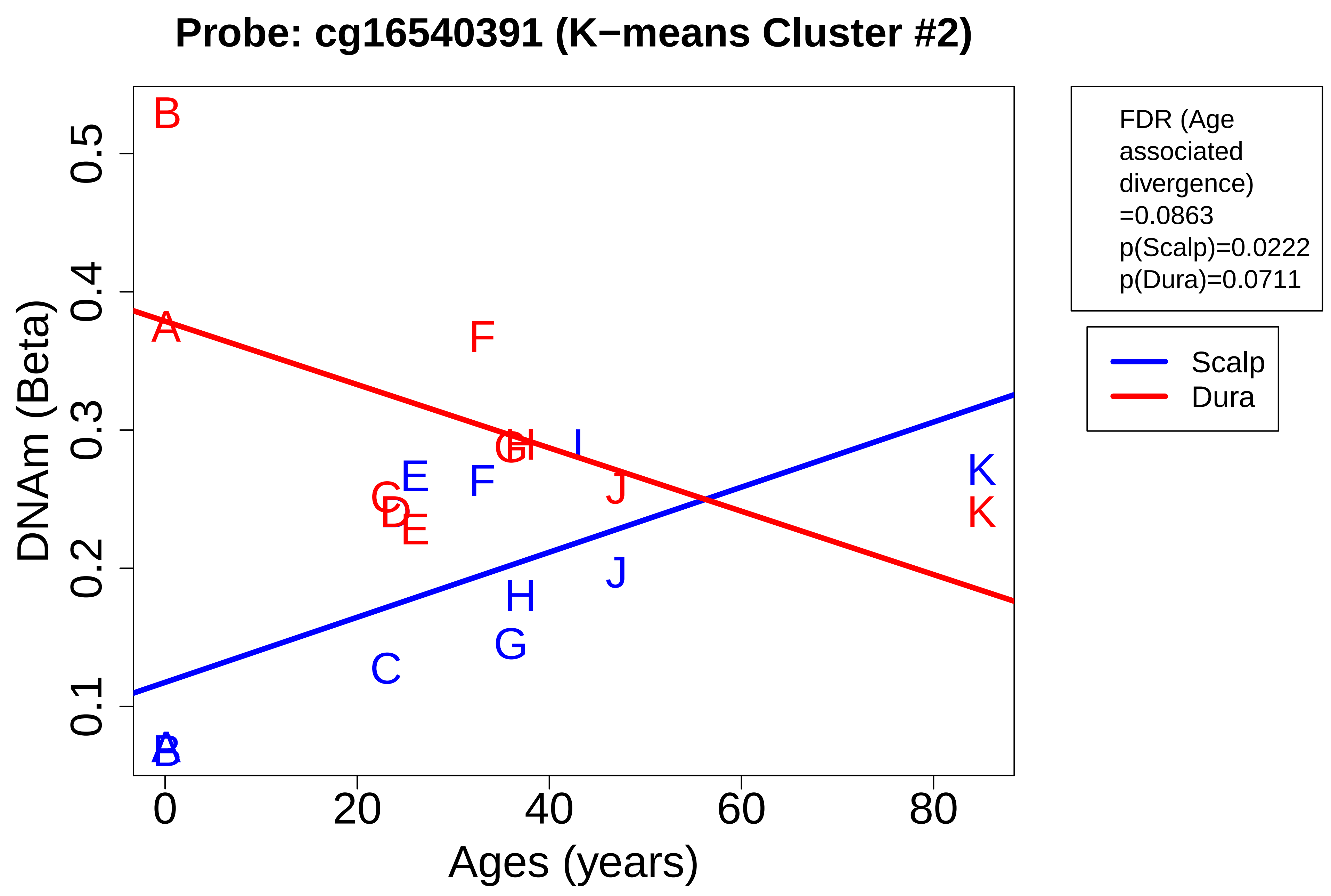

Probe: cg16540391 (K−means Cluster #2)
DNAm (Beta)
0.1
0.2
0.3
0.4
0.5
0
20
40
60
80
Ages (years)
FDR (Age associated divergence) =0.0863
p(Scalp)=0.0222
p(Dura)=0.0711
Scalp
Dura

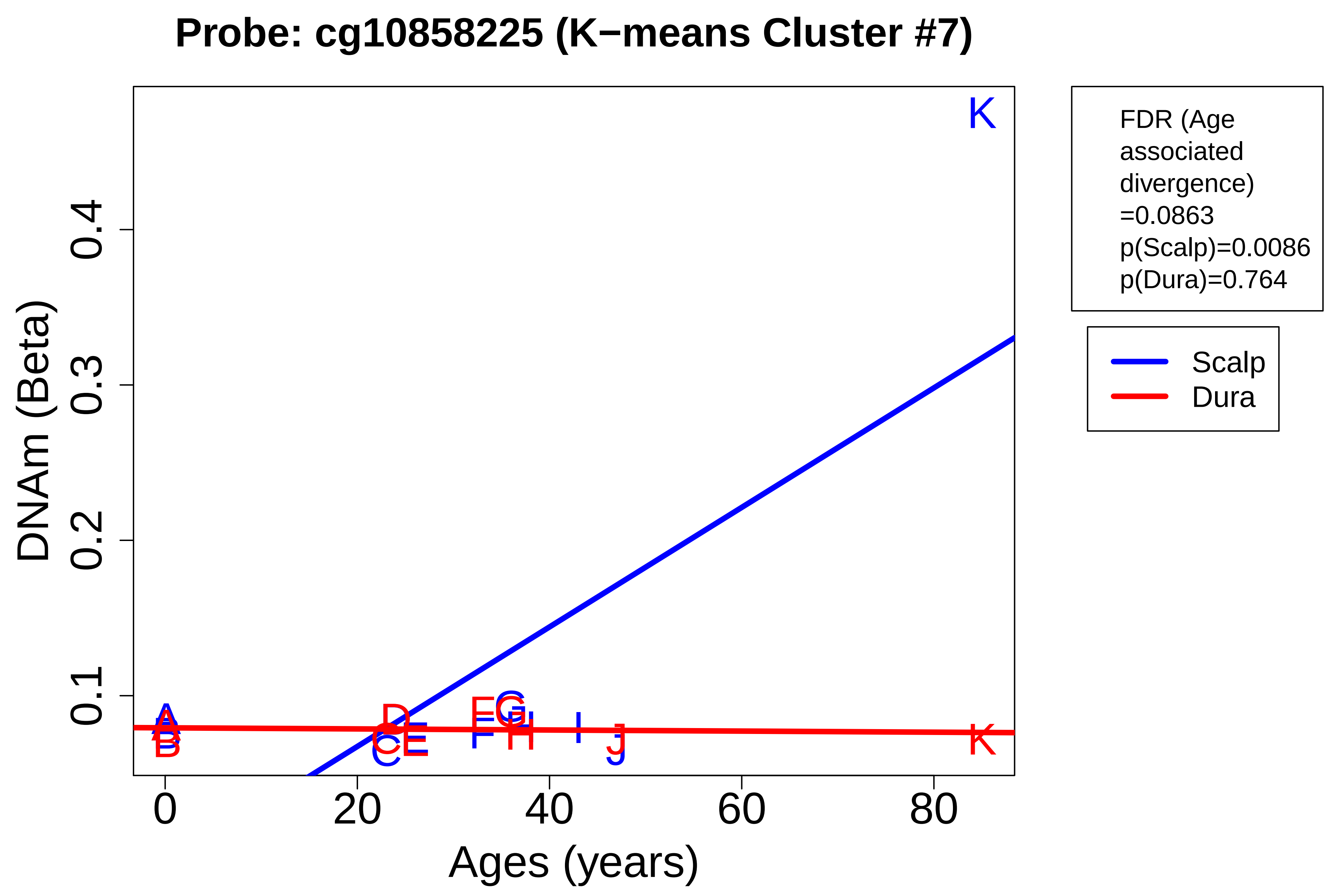

Probe: cg10858225 (K−means Cluster #7)
DNAm (Beta)
0.1
0.2
0.3
0.4
Ages (years)
0
20
40
60
80
FDR (Age associated divergence) =0.0863
p(Scalp)=0.0086
p(Dura)=0.764
Scalp
Dura

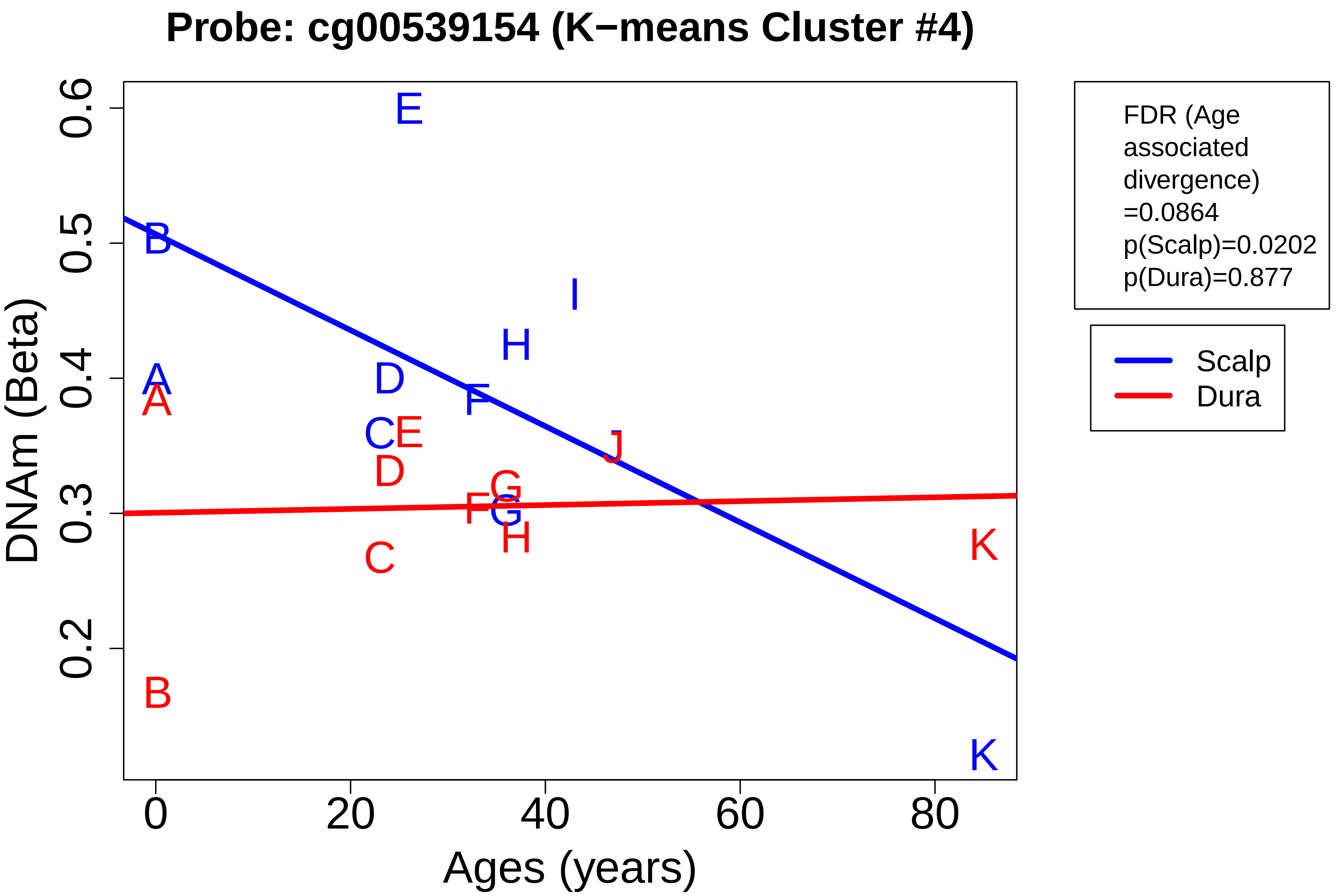

Probe: cg00539154 (K−means Cluster #4)
DNAm (Beta)
0.2
0.3
0.4
0.5
0.6
0
20
40
60
80
Ages (years)
FDR (Age associated divergence) =0.0864
p(Scalp)=0.0202
p(Dura)=0.877
Scalp
Dura
A
B
C
D
E
F
G
H
I
J
K

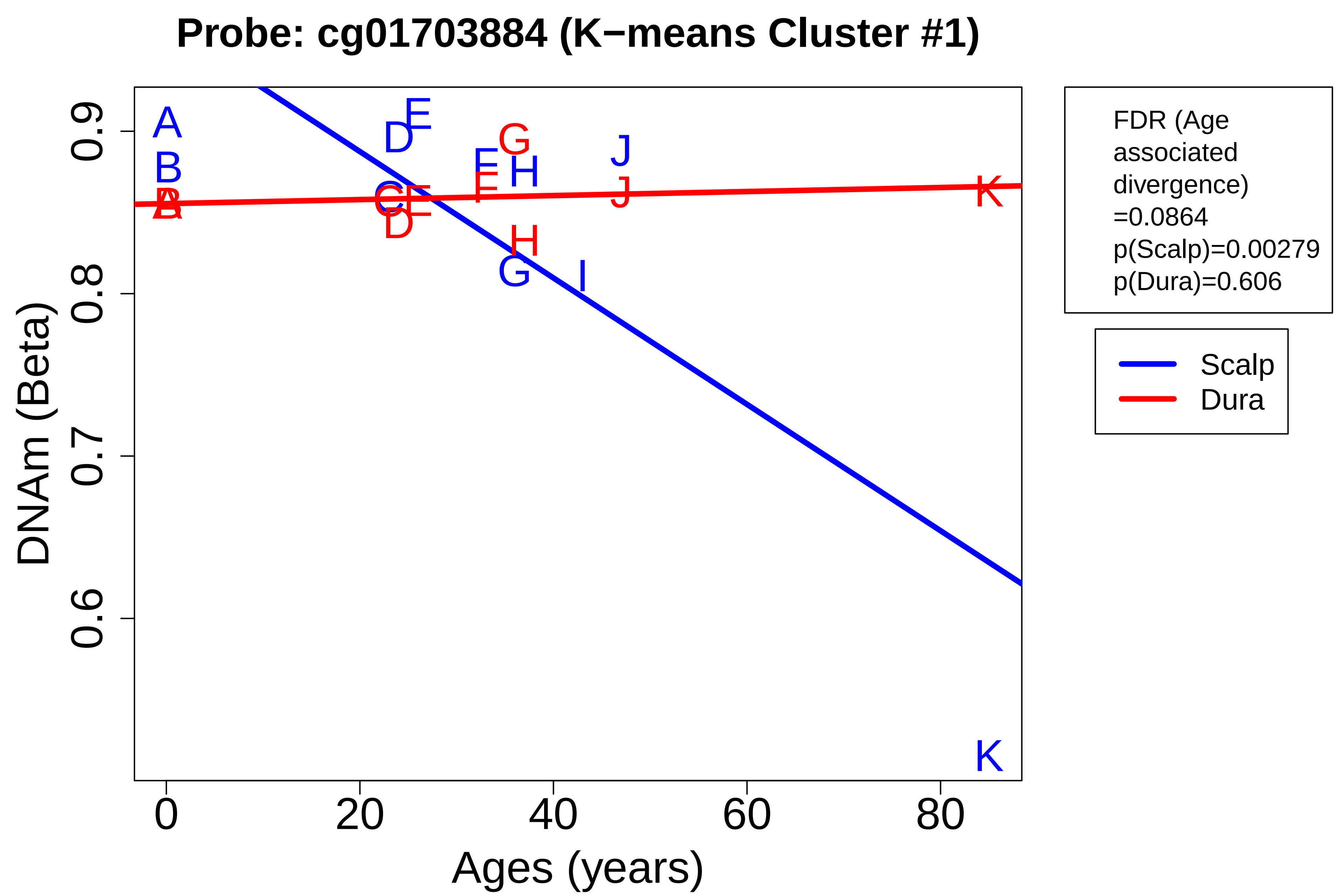

Probe: cg01703884 (K−means Cluster #1)
DNAm (Beta)
0.6
0.7
0.8
0.9
Ages (years)
0
20
40
60
80
FDR (Age associated divergence) =0.0864
p(Scalp)=0.00279
p(Dura)=0.606
Scalp
Dura

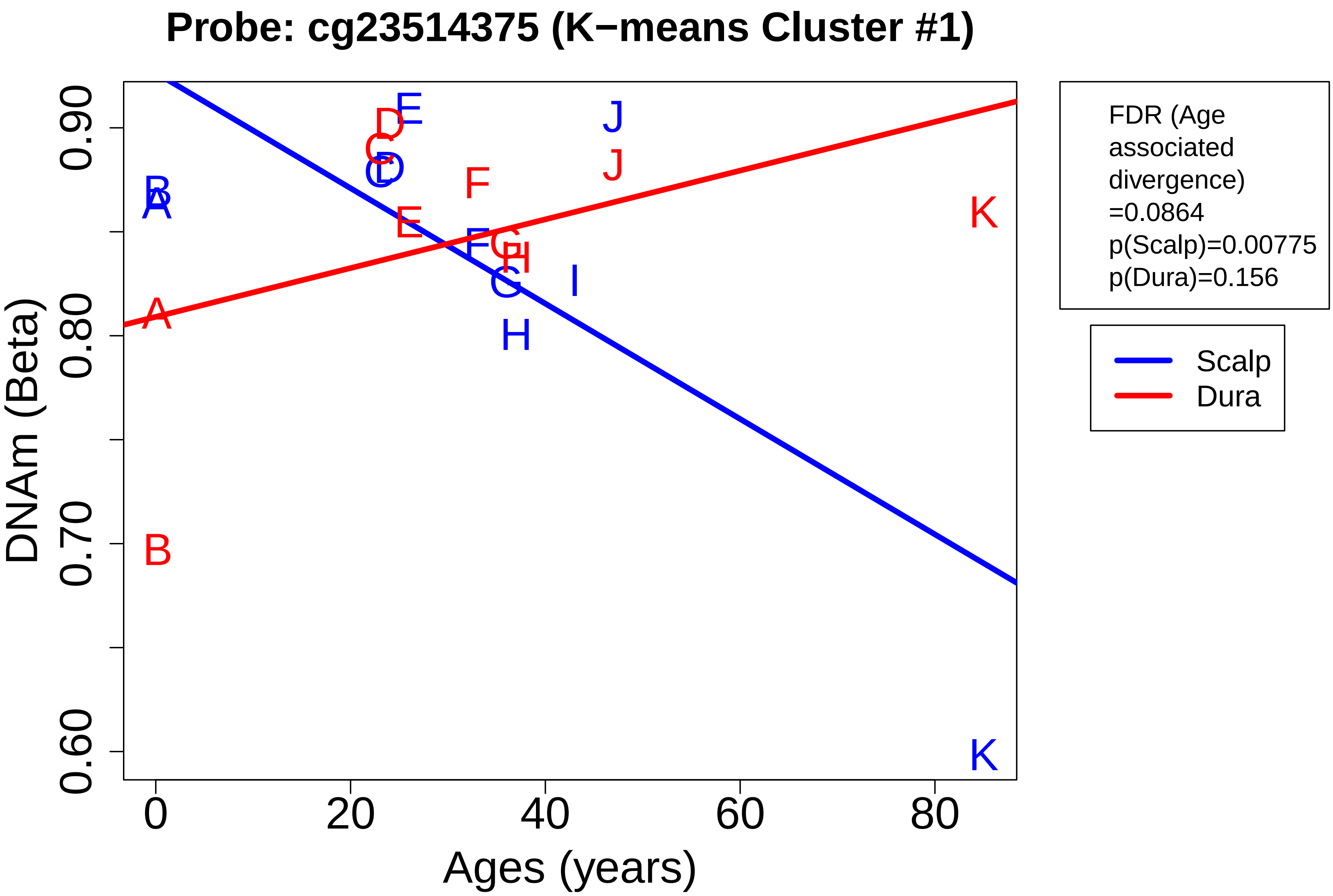

Probe: cg23514375 (K−means Cluster #1)
DNAm (Beta)
0.60
0.70
0.80
0.90
Ages (years)
0
20
40
60
80
FDR (Age associated divergence) =0.0864
p(Scalp)=0.00775
p(Dura)=0.156
Scalp
Dura

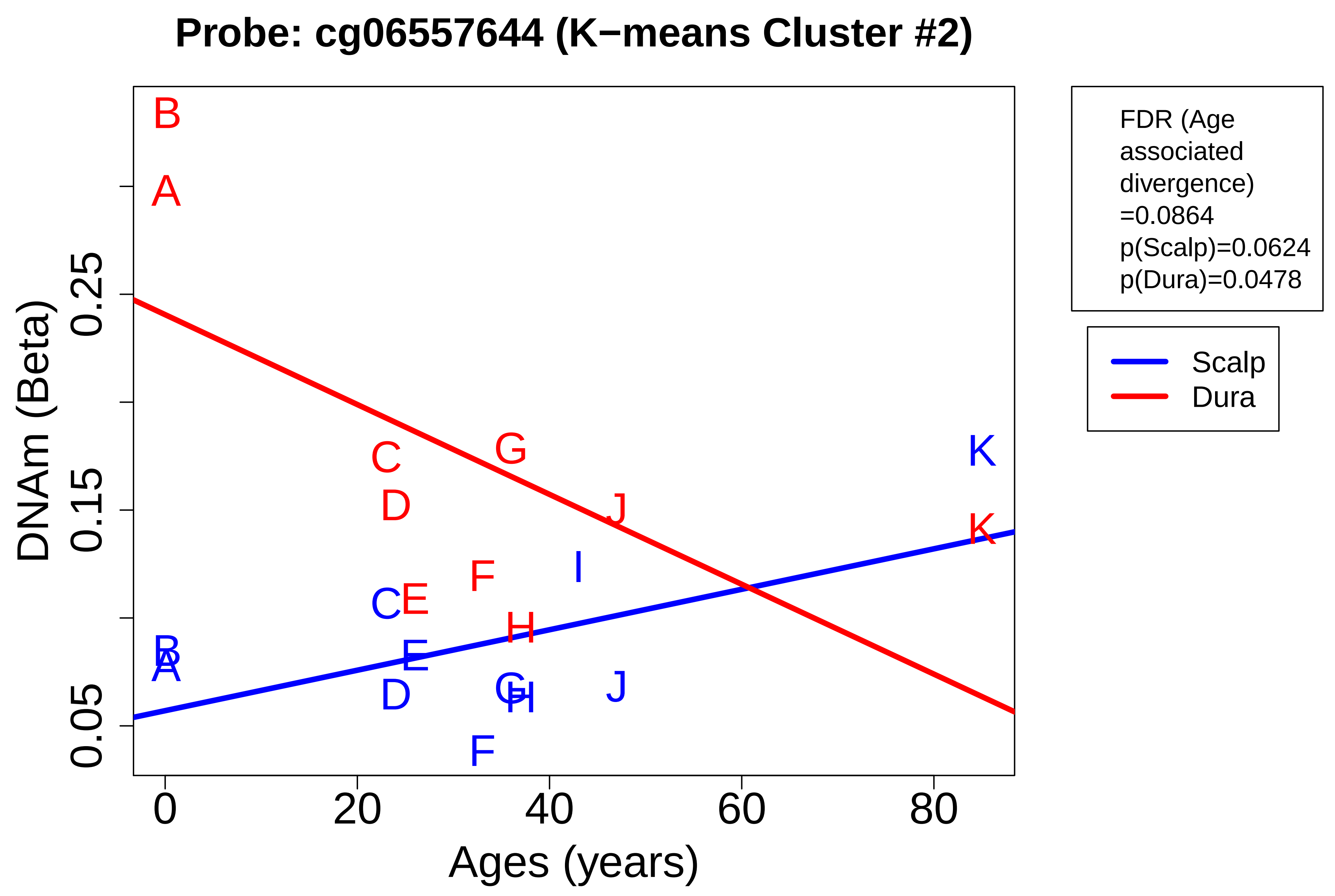

Probe: cg06557644 (K−means Cluster #2)
DNAm (Beta)
0.05
0.15
0.25
0
20
40
60
80
Ages (years)
FDR (Age associated divergence) =0.0864
p(Scalp)=0.0624
p(Dura)=0.0478
Scalp
Dura

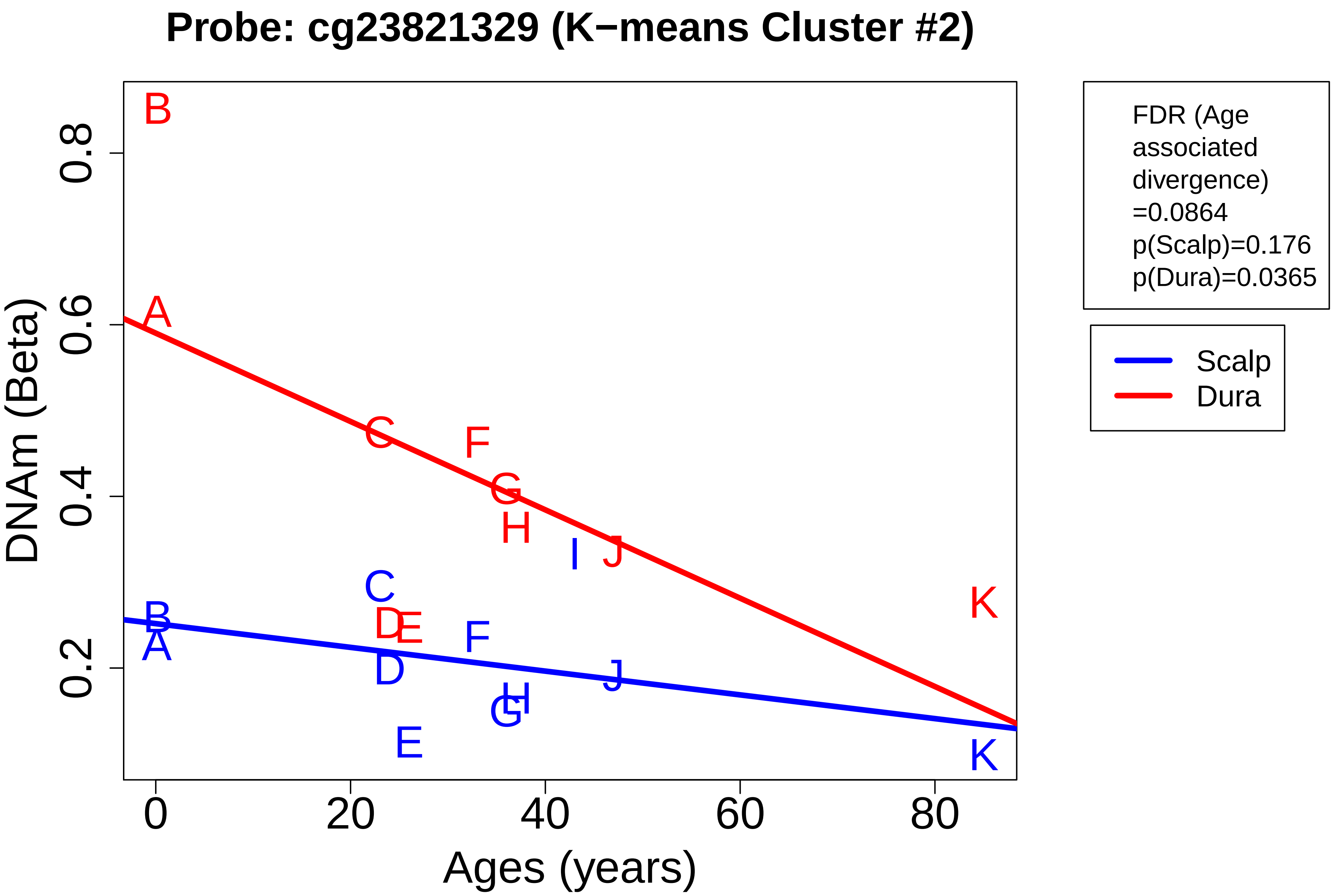
Probe: cg23821329 (K−means Cluster #2)
DNAm (Beta)
0.2
0.4
0.6
0.8
0
20
40
60
80
Ages (years)
FDR (Age associated divergence) =0.0864
p(Scalp)=0.176
p(Dura)=0.0365
Scalp
Dura
A
B
C
D
E
F
G
H
I
J
K

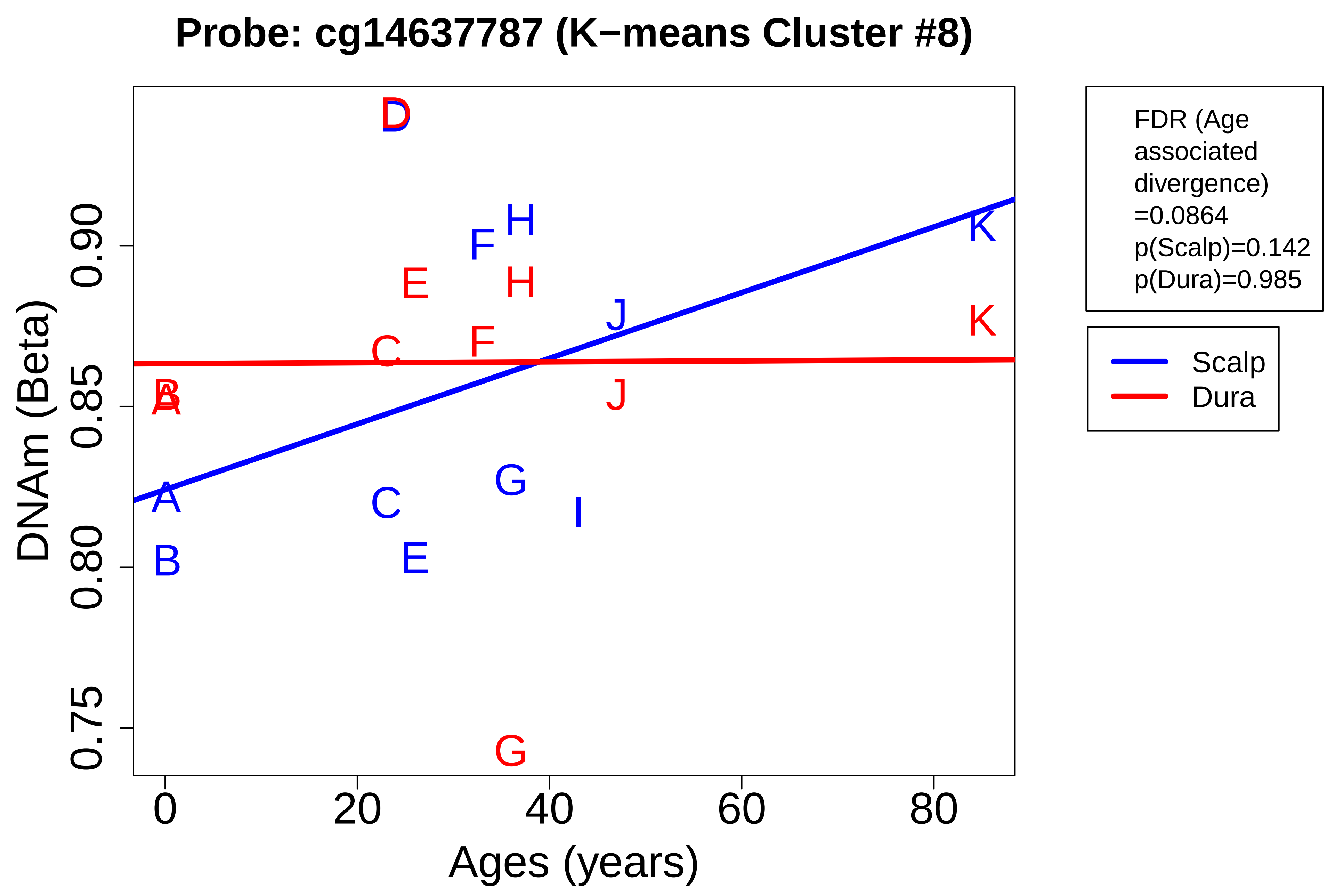

Probe: cg14637787 (K−means Cluster #8)
DNAm (Beta)
0.75
0.80
0.85
0.90
0
20
40
60
80
Ages (years)
A
B
C
D
E
F
G
H
I
J
K
FDR (Age associated divergence) =0.0864
p(Scalp)=0.142
p(Dura)=0.985
Scalp
Dura

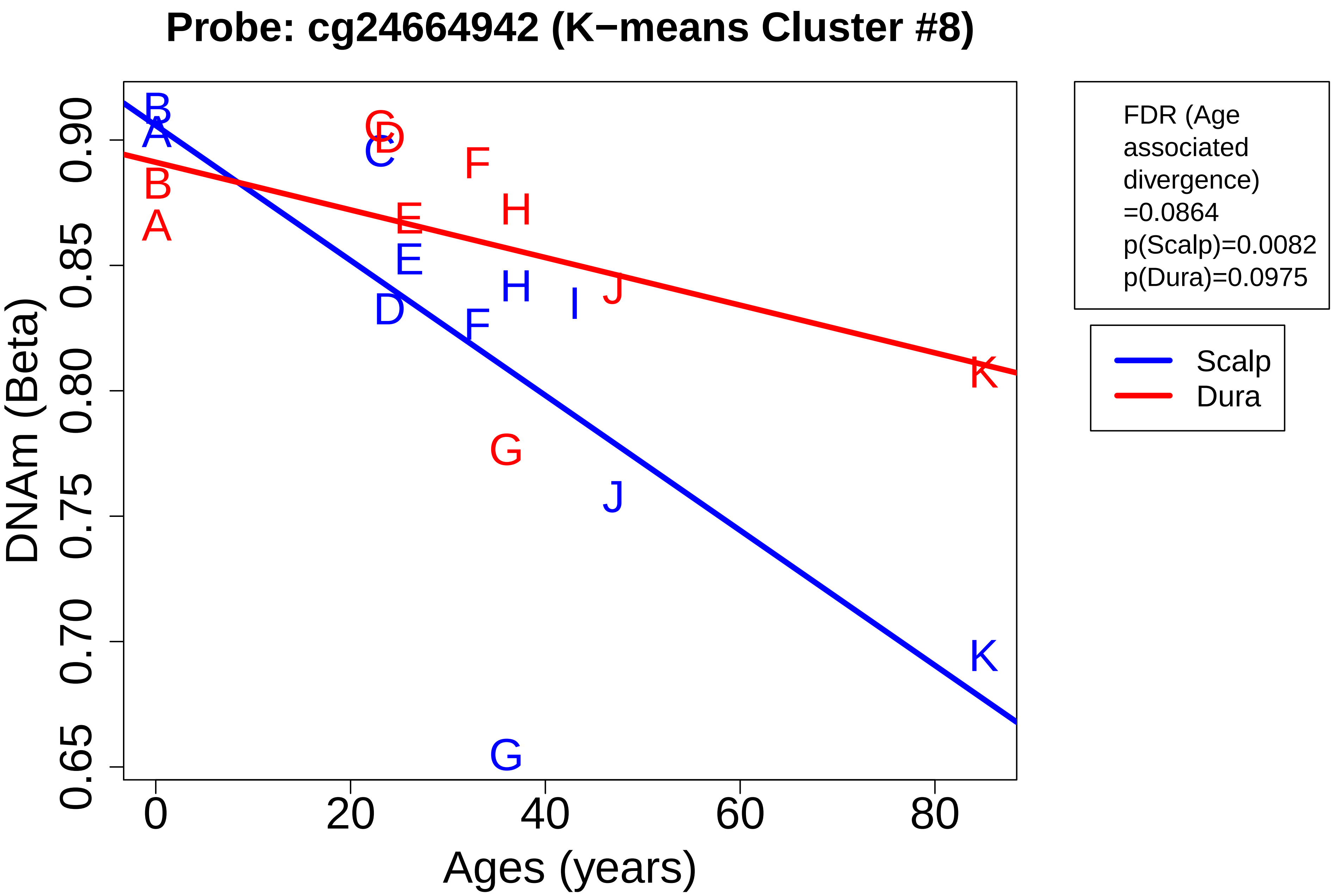

Probe: cg24664942 (K−means Cluster #8)
DNAm (Beta)
0.65
0.70
0.75
0.80
0.85
0.90
0
20
40
60
80
Ages (years)
FDR (Age associated divergence) =0.0864
p(Scalp)=0.0082
p(Dura)=0.0975
Scalp
Dura

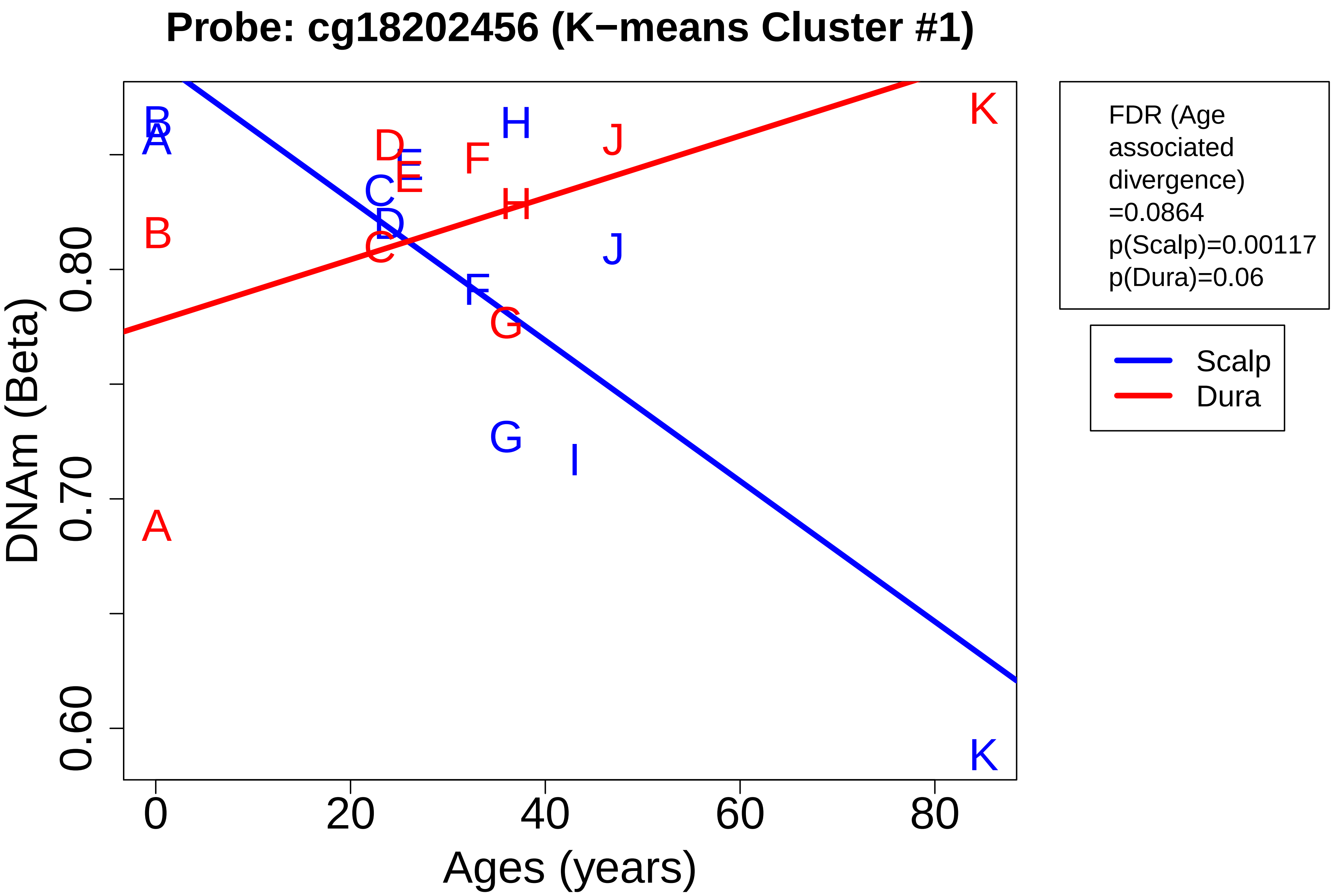

Probe: cg18202456 (K−means Cluster #1)
DNAm (Beta)
0.60
0.70
0.80
Ages (years)
0
20
40
60
80
FDR (Age associated divergence) =0.0864
p(Scalp)=0.00117
p(Dura)=0.06
Scalp
Dura

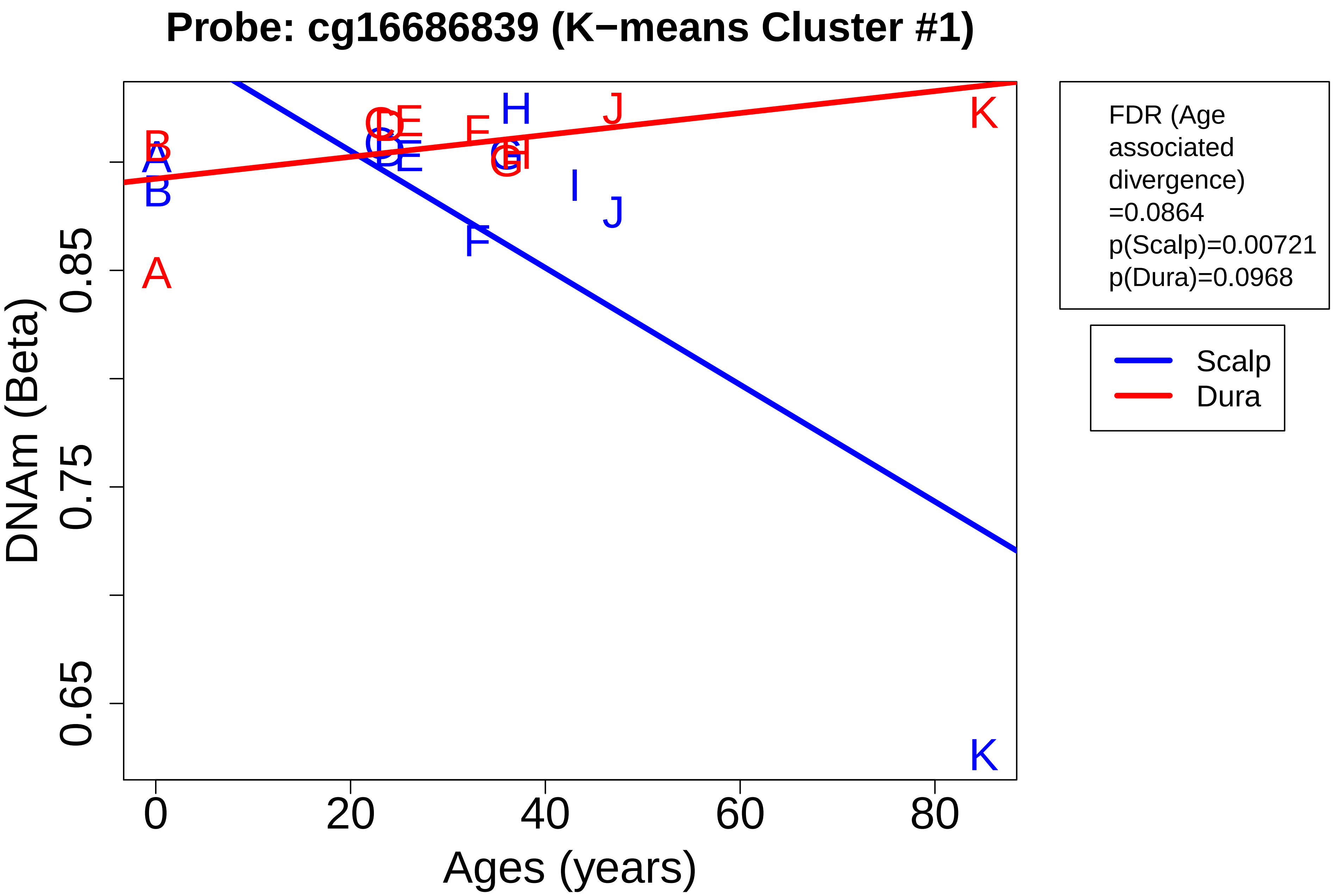

Probe: cg16686839 (K−means Cluster #1)
DNAm (Beta)
0.65
0.75
0.85
0
20
40
60
80
Ages (years)
A
B
C
D
E
F
G
H
I
J
K
FDR (Age associated divergence) =0.0864
p(Scalp)=0.00721
p(Dura)=0.0968
Scalp
Dura

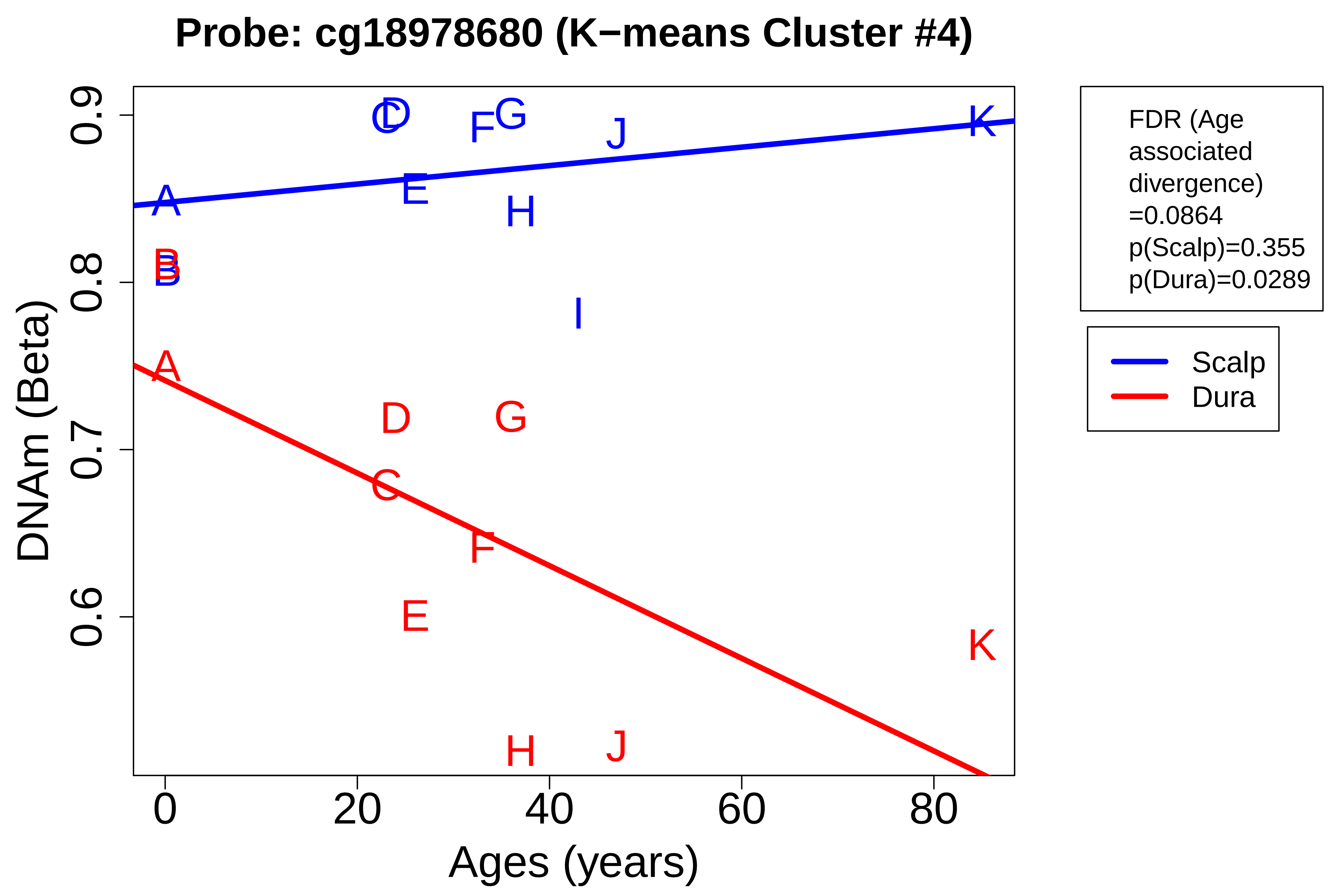

Probe: cg18978680 (K−means Cluster #4)
DNAm (Beta)
0.6
0.7
0.8
0.9
Ages (years)
0
20
40
60
80
A
B
C
D
E
F
G
H
I
J
K
FDR (Age associated divergence) =0.0864
p(Scalp)=0.355
p(Dura)=0.0289
Scalp
Dura

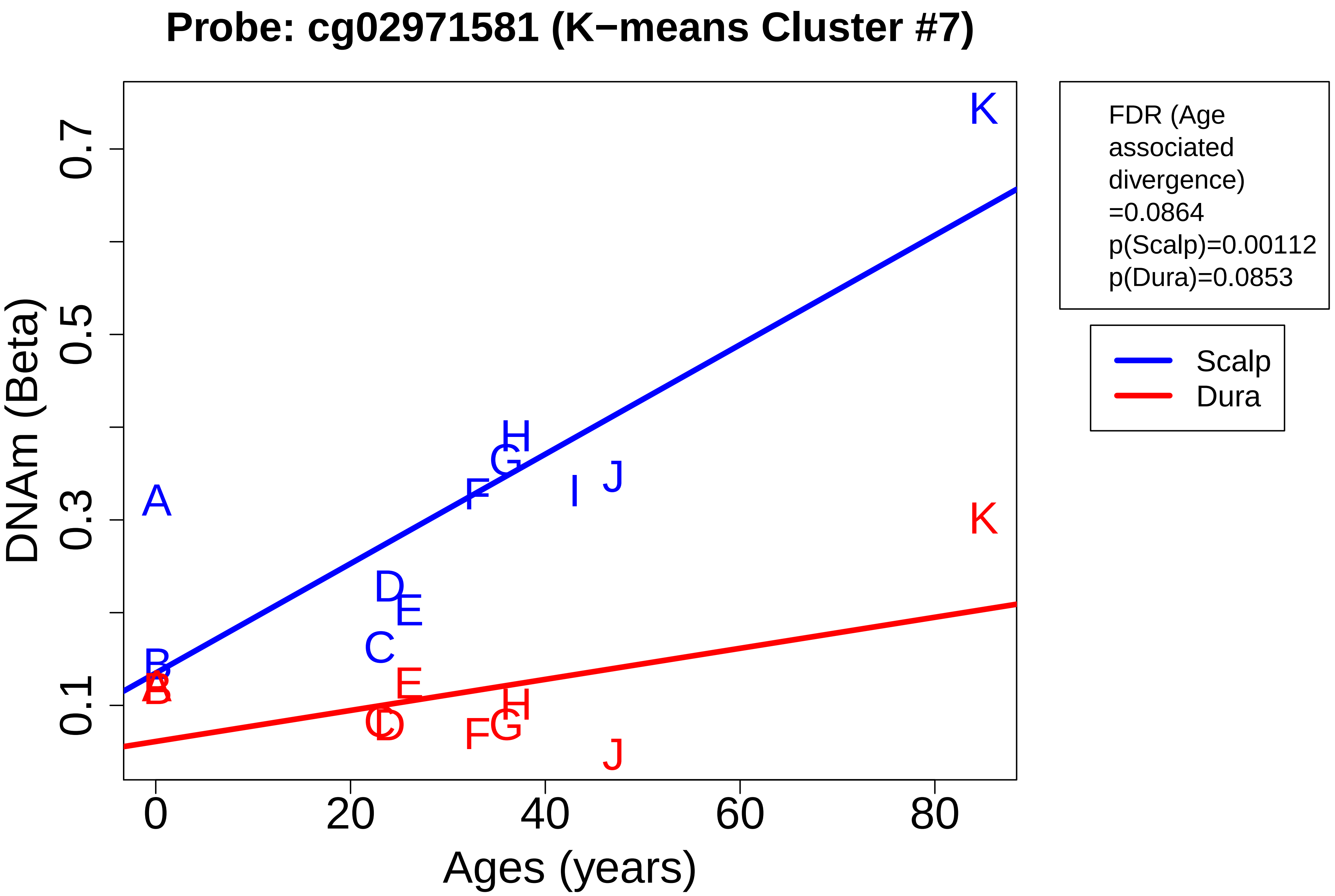

Probe: cg02971581 (K−means Cluster #7)
DNAm (Beta)
0.1
0.3
0.5
0.7
0
20
40
60
80
Ages (years)
FDR (Age associated divergence) =0.0864
p(Scalp)=0.00112
p(Dura)=0.0853
Scalp
Dura

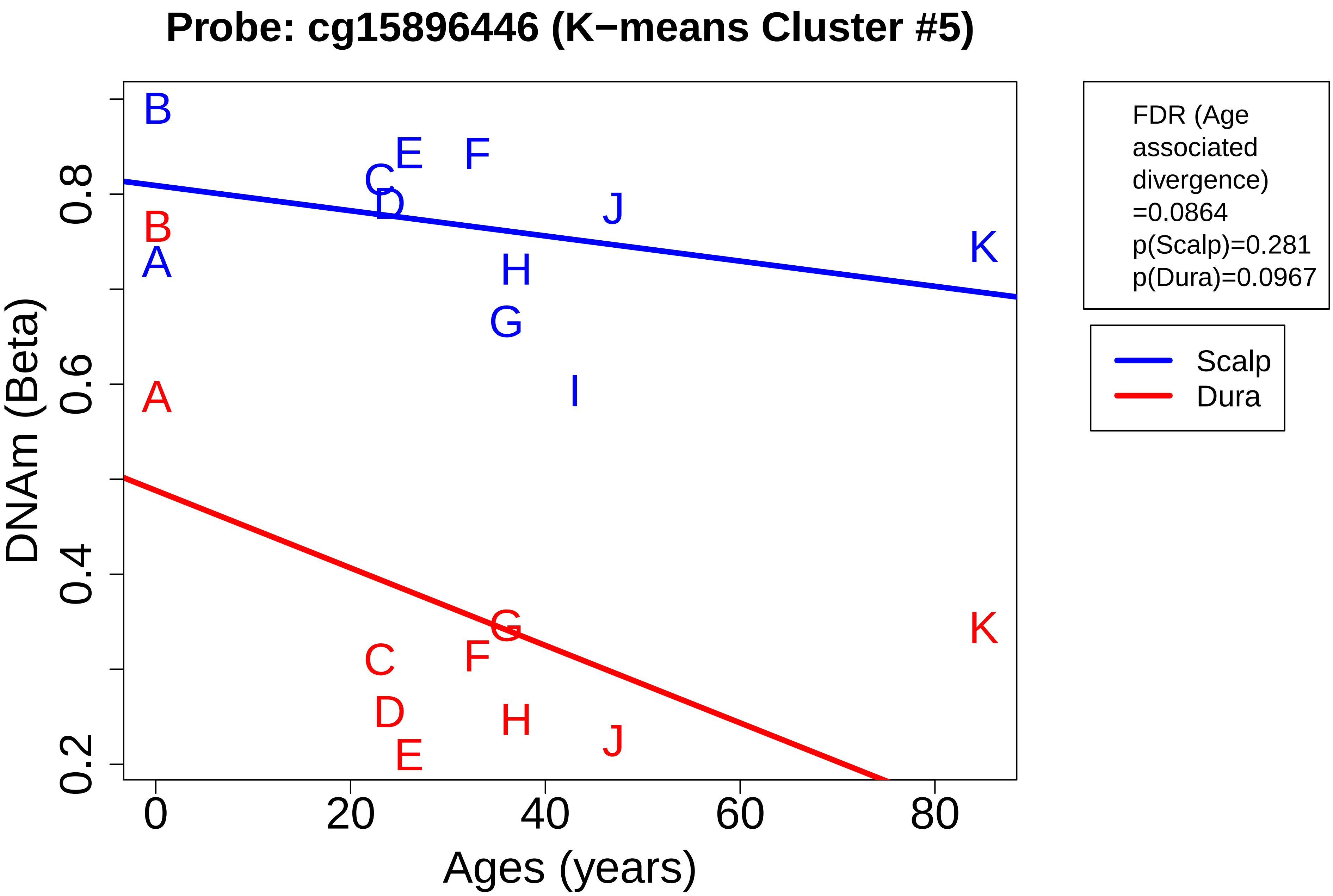

Probe: cg15896446 (K−means Cluster #5)
DNAm (Beta)
0.2
0.4
0.6
0.8
0
20
40
60
80
Ages (years)
A
B
C
D
E
F
G
H
I
J
K
FDR (Age associated divergence) =0.0864
p(Scalp)=0.281
p(Dura)=0.0967
Scalp
Dura

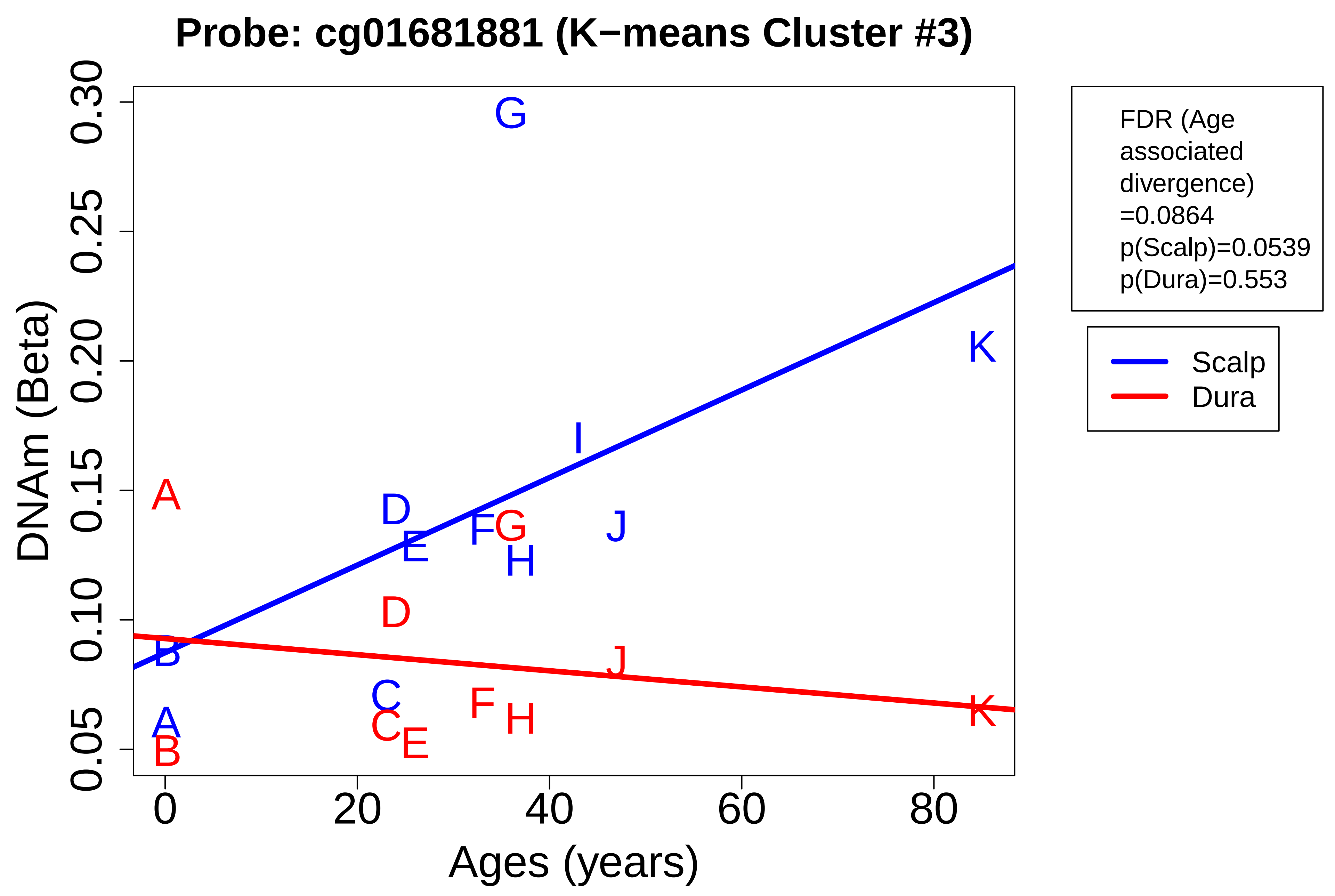

Probe: cg01681881 (K−means Cluster #3)
DNAm (Beta)
0.05
0.10
0.15
0.20
0.25
0.30
0
20
40
60
80
Ages (years)
FDR (Age associated divergence) =0.0864
p(Scalp)=0.0539
p(Dura)=0.553
Scalp
Dura
A B C D E F G H I J K

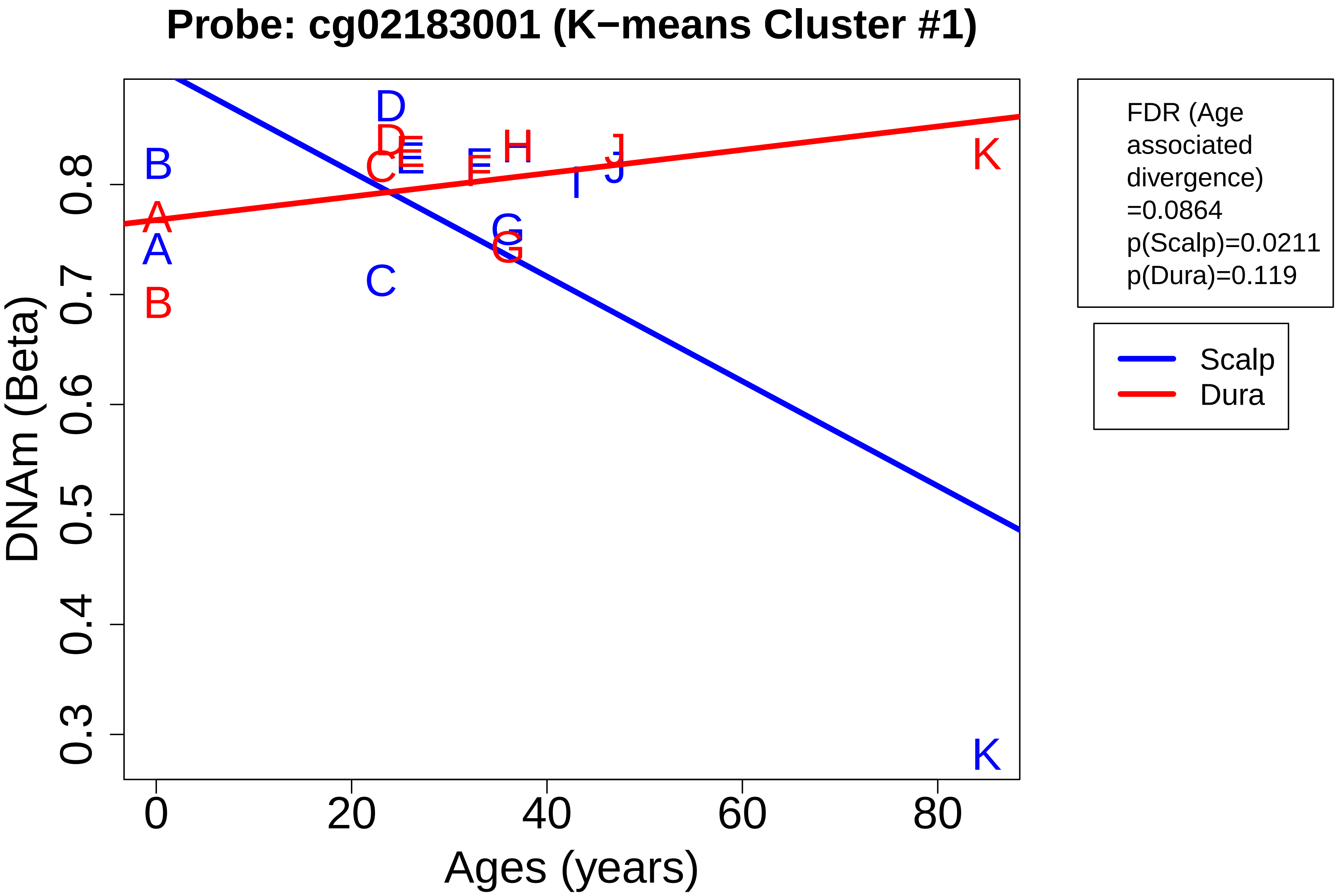

Probe: cg02183001 (K−means Cluster #1)
DNAm (Beta)
0.3
0.4
0.5
0.6
0.7
0.8
Ages (years)
0
20
40
60
80
A
B
C
D
E
F
G
H
I
J
K
FDR (Age associated divergence) =0.0864
p(Scalp)=0.0211
p(Dura)=0.119
Scalp
Dura

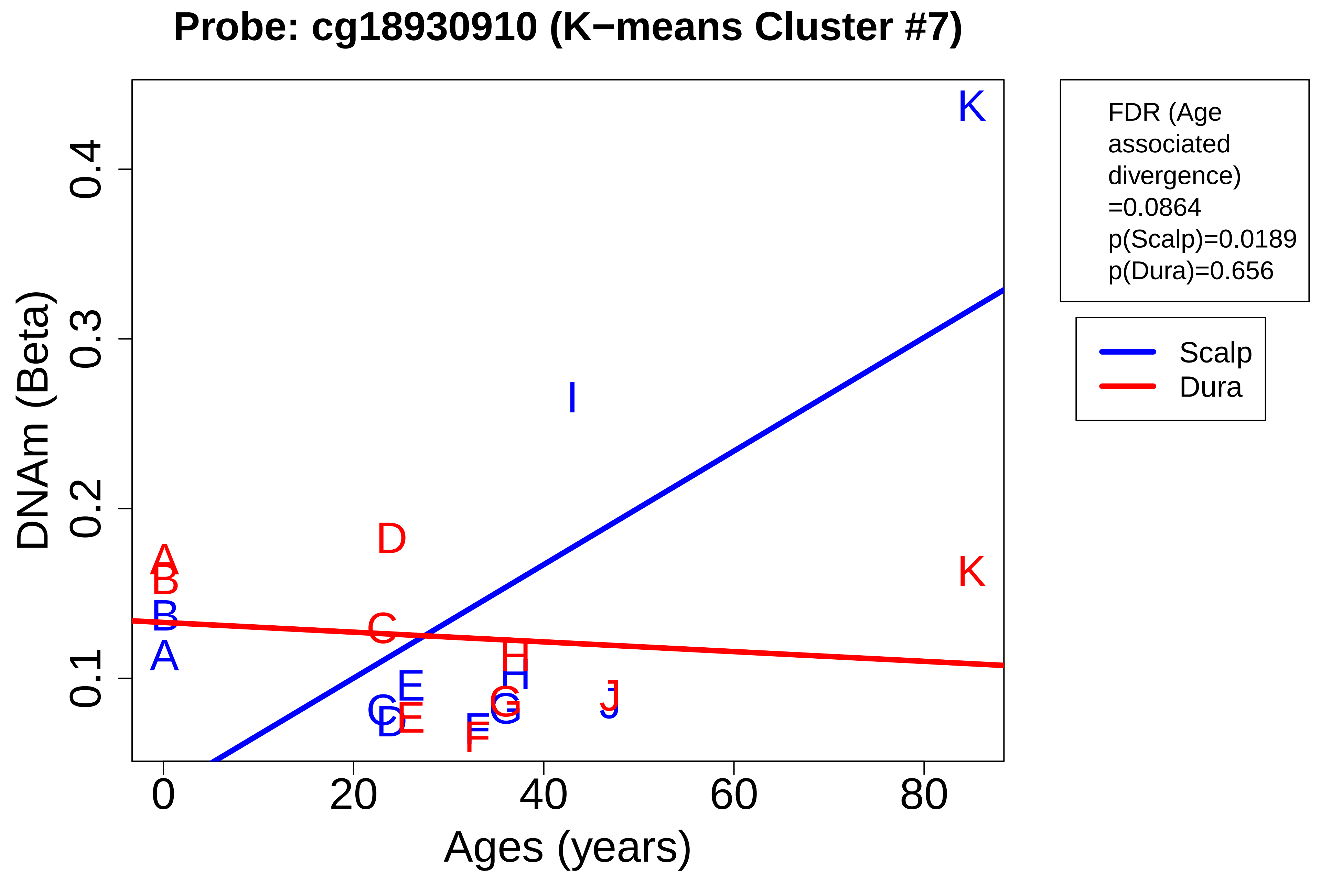
Probe: cg18930910 (K−means Cluster #7)
DNAm (Beta)
0.1
0.2
0.3
0.4
Ages (years)
0
20
40
60
80
FDR (Age associated divergence) =0.0864
p(Scalp)=0.0189
p(Dura)=0.656
Scalp
Dura
A
B
C
D
E
F
G
H
I
J
K

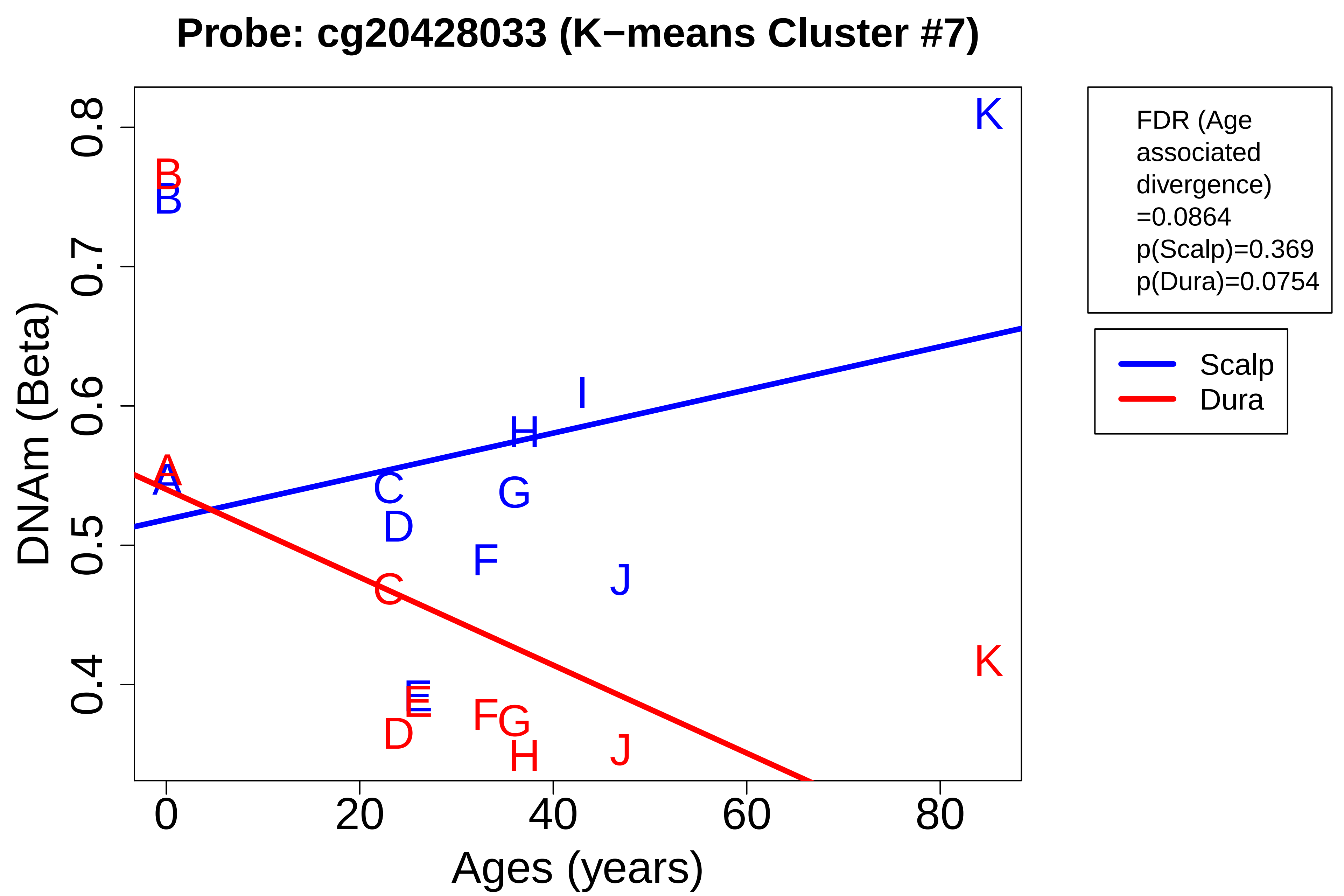

Probe: cg20428033 (K−means Cluster #7)
DNAm (Beta)
0.4
0.5
0.6
0.7
0.8
0
20
40
60
80
Ages (years)
A
B
C
D
E
F
G
H
I
J
K
A
B
C
D
E
F
G
H
J
K
FDR (Age associated divergence) =0.0864
p(Scalp)=0.369
p(Dura)=0.0754
Scalp
Dura

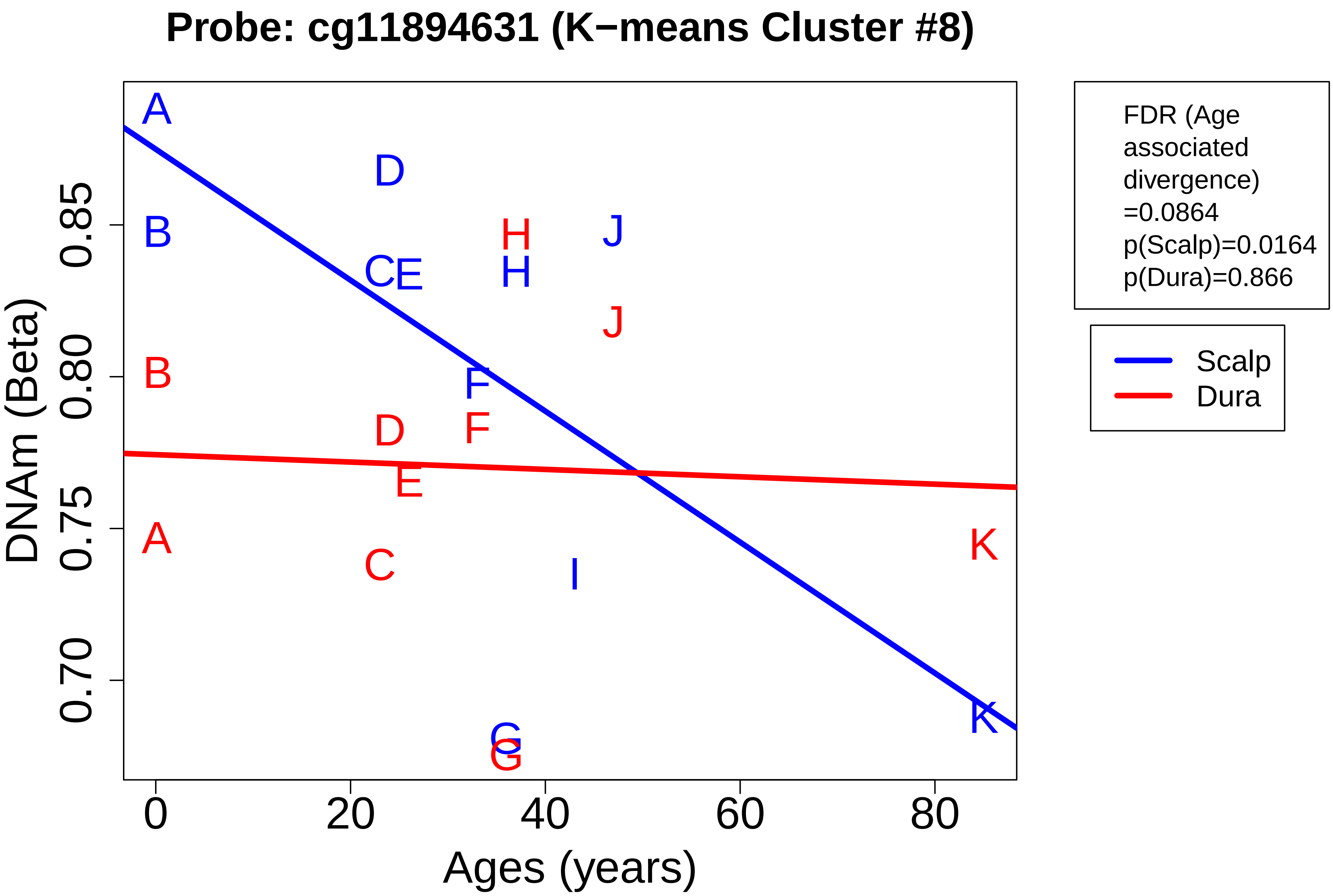

Probe: cg11894631 (K−means Cluster #8)
DNAm (Beta)
0.70
0.75
0.80
0.85
0
20
40
60
80
Ages (years)
A
B
C
D
E
F
G
H
I
J
K
FDR (Age associated divergence) =0.0864
p(Scalp)=0.0164
p(Dura)=0.866
Scalp
Dura

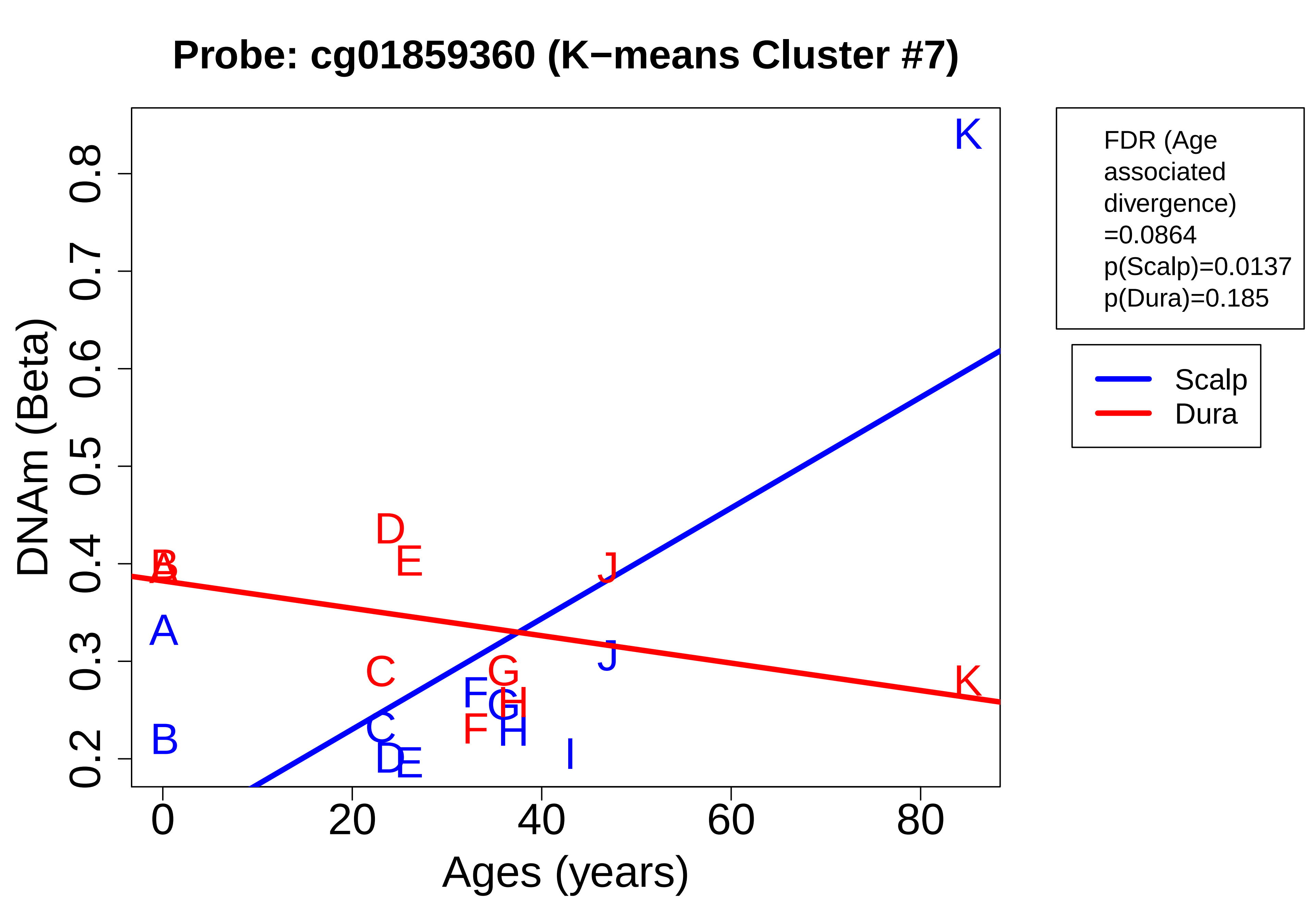

Probe: cg01859360 (K−means Cluster #7)
DNAm (Beta)
0.2
0.3
0.4
0.5
0.6
0.7
0.8
0
20
40
60
80
Ages (years)
FDR (Age associated divergence) =0.0864
p(Scalp)=0.0137
p(Dura)=0.185
Scalp
Dura
A
B
C
D
E
F
G
H
I
J
K

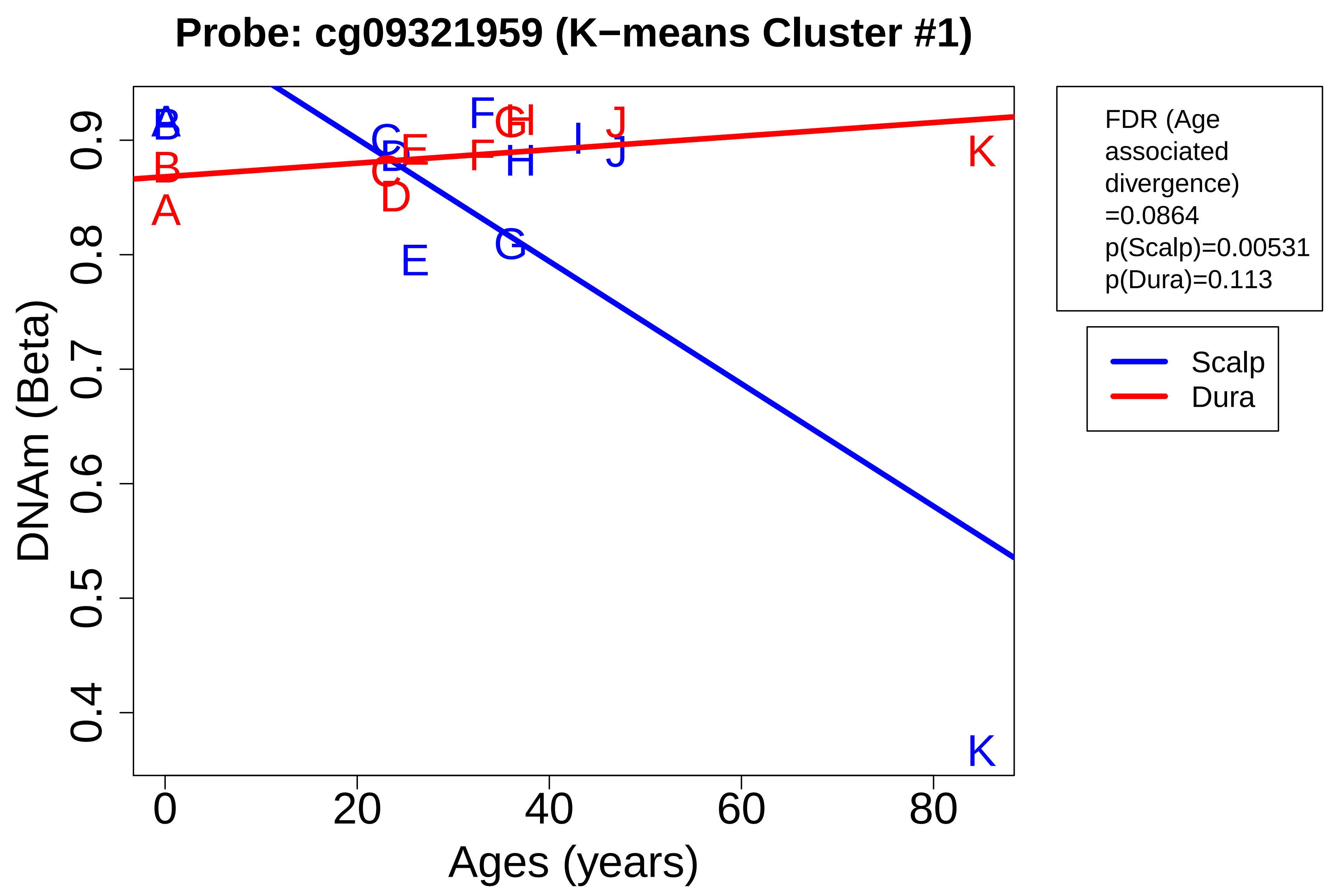
Probe: cg09321959 (K−means Cluster #1)
DNAm (Beta)
Ages (years)
0.4
0.5
0.6
0.7
0.8
0.9
0
20
40
60
80
FDR (Age associated divergence) =0.0864
p(Scalp)=0.00531
p(Dura)=0.113
Scalp
Dura

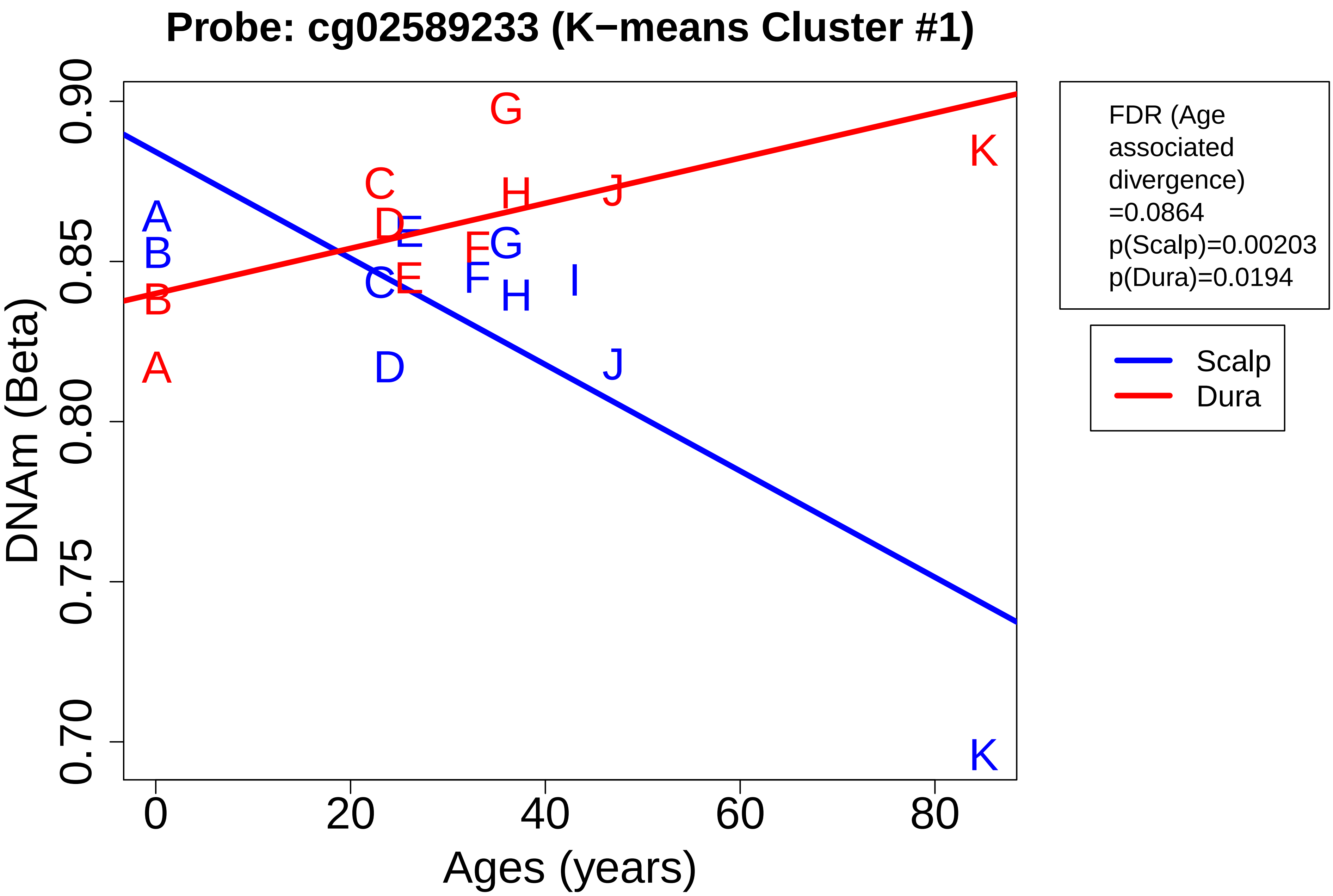

Probe: cg02589233 (K−means Cluster #1)
DNAm (Beta)
0.70
0.75
0.80
0.85
0.90
Ages (years)
0
20
40
60
80
FDR (Age associated divergence) =0.0864
p(Scalp)=0.00203
p(Dura)=0.0194
Scalp
Dura

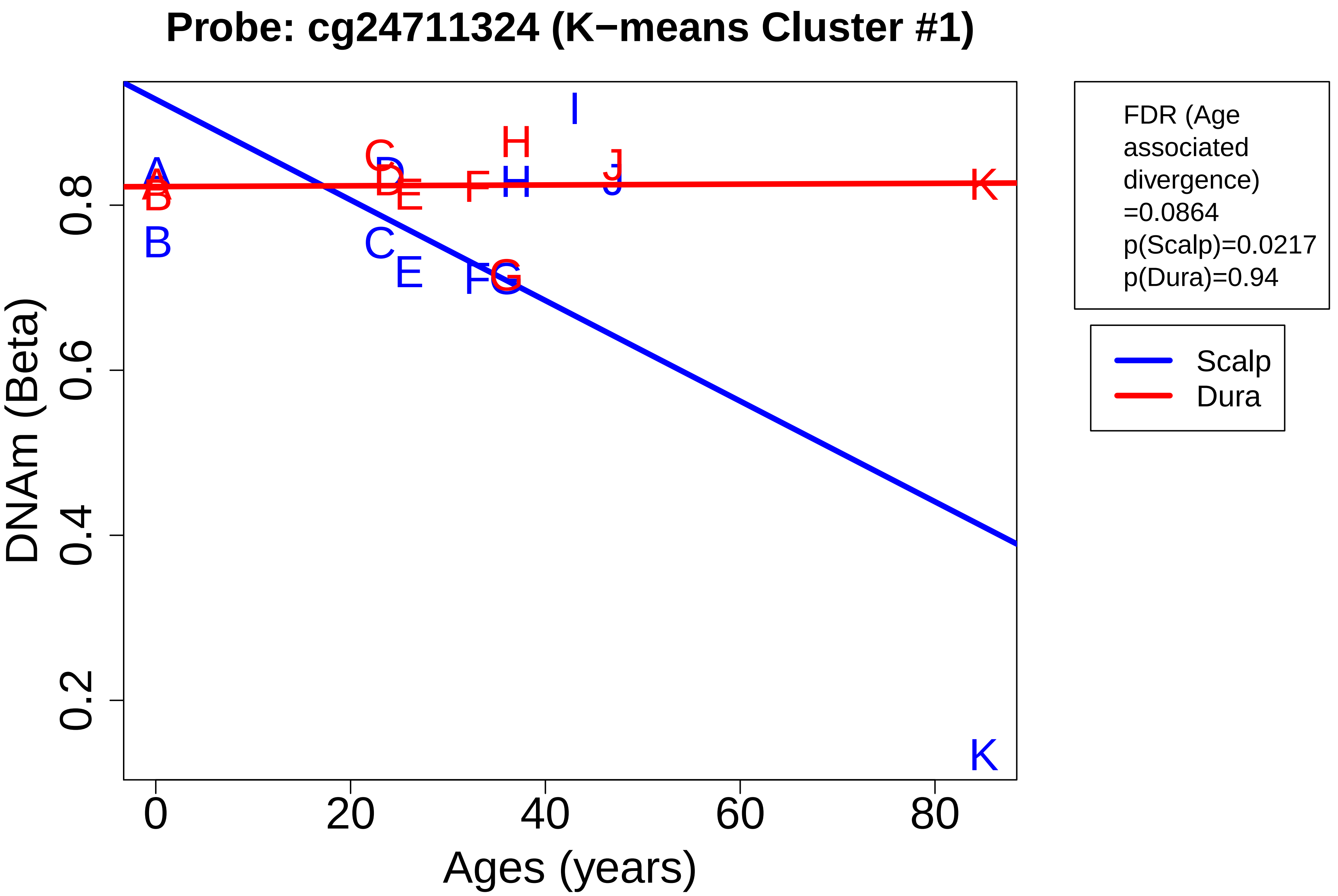

Probe: cg24711324 (K−means Cluster #1)
DNAm (Beta)
Ages (years)
0.2
0.4
0.6
0.8
0
20
40
60
80
FDR (Age associated divergence) =0.0864
p(Scalp)=0.0217
p(Dura)=0.94
Scalp
Dura

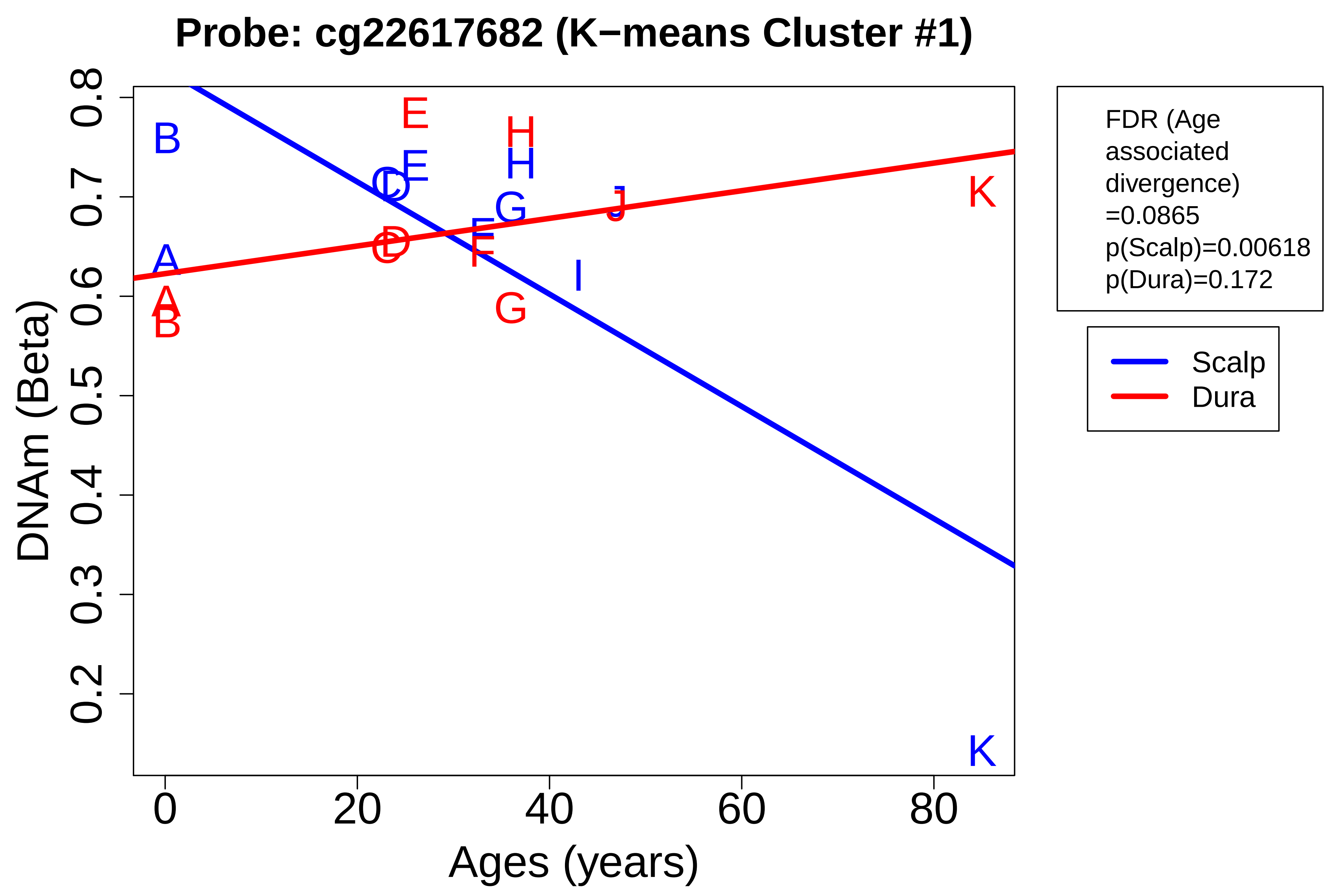

Probe: cg22617682 (K−means Cluster #1)
DNAm (Beta)
Ages (years)
0.2
0.3
0.4
0.5
0.6
0.7
0.8
0
20
40
60
80
FDR (Age associated divergence) =0.0865
p(Scalp)=0.00618
p(Dura)=0.172
Scalp
Dura

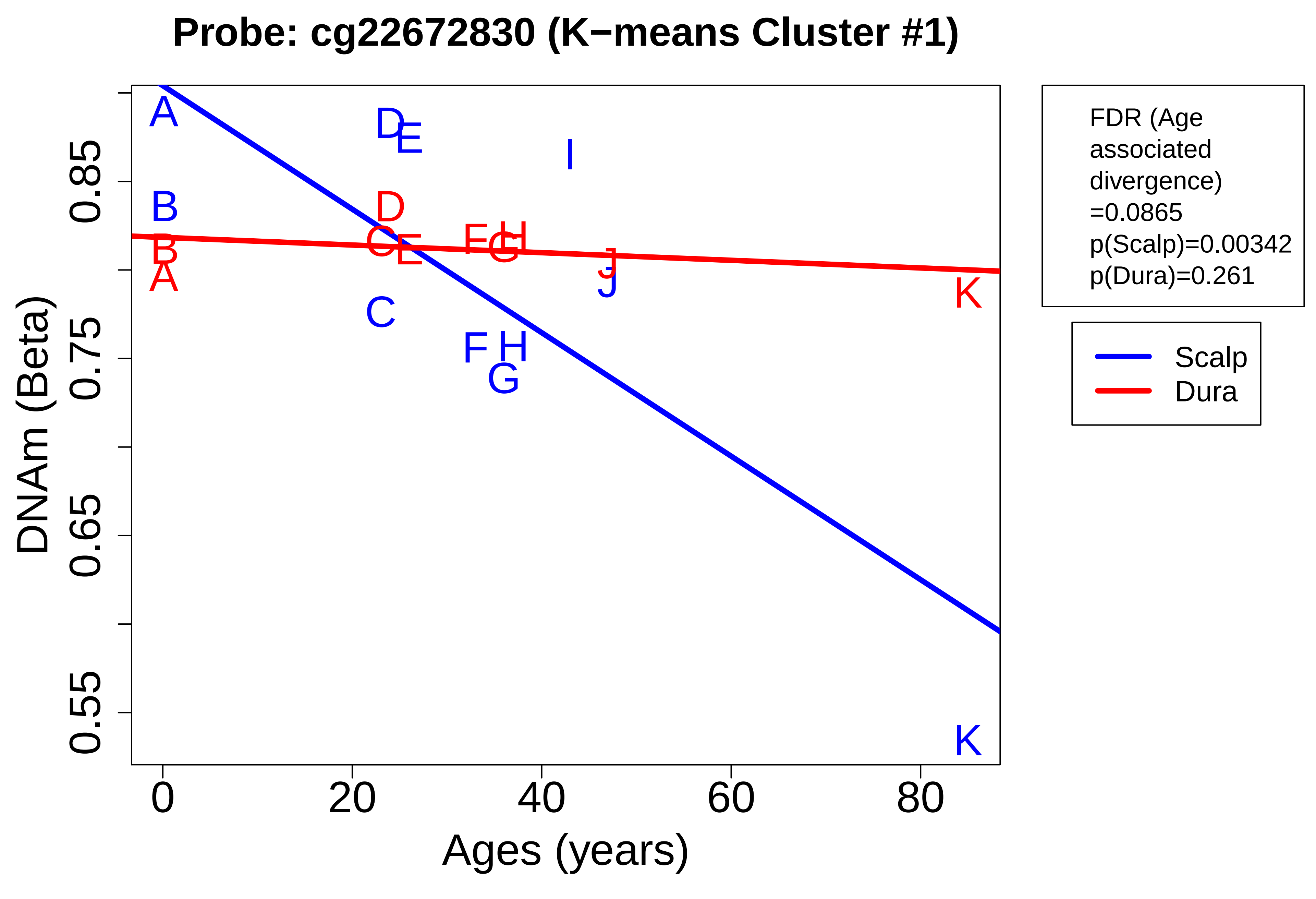

Probe: cg22672830 (K−means Cluster #1)
DNAm (Beta)
0.55
0.65
0.75
0.85
Ages (years)
0
20
40
60
80
FDR (Age associated divergence) =0.0865
p(Scalp)=0.00342
p(Dura)=0.261
Scalp
Dura

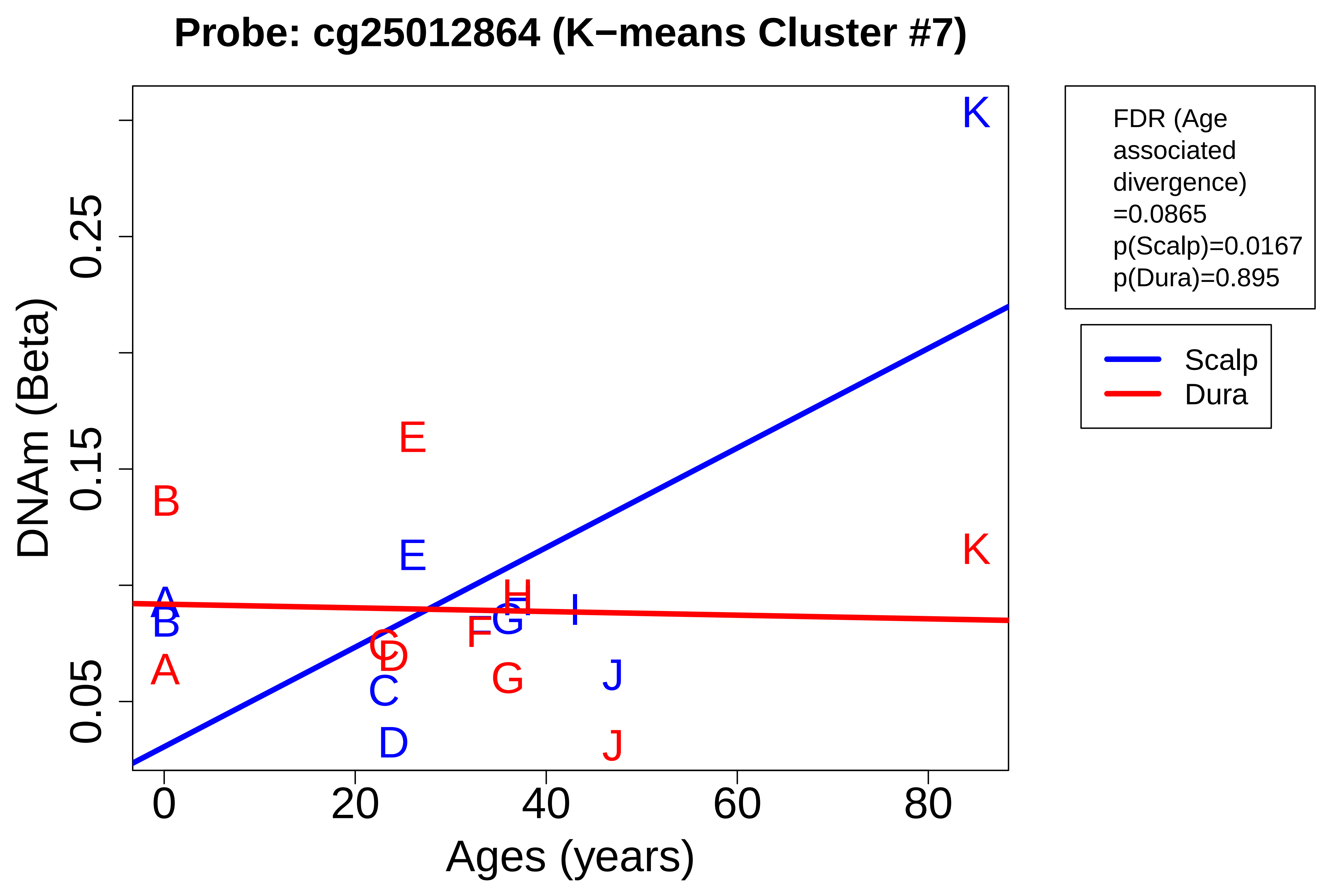
Probe: cg25012864 (K−means Cluster #7)
DNAm (Beta)
0.05
0.15
0.25
0
20
40
60
80
Ages (years)
FDR (Age associated divergence) =0.0865
p(Scalp)=0.0167
p(Dura)=0.895
Scalp
Dura
A
B
C
D
E
F
G
H
I
J
K

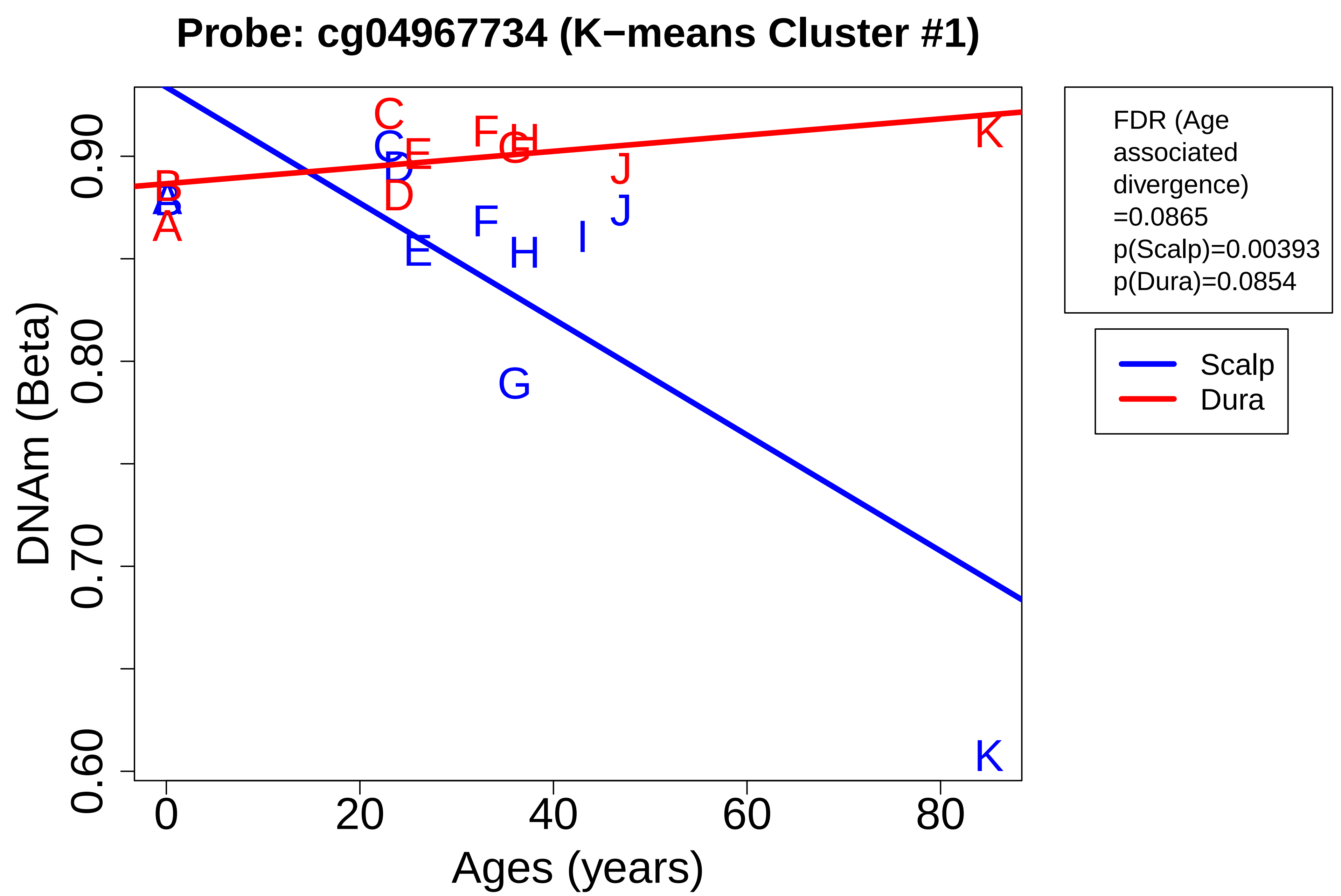

Probe: cg04967734 (K−means Cluster #1)
DNAm (Beta)
0.60
0.70
0.80
0.90
0
20
40
60
80
Ages (years)
FDR (Age associated divergence) =0.0865
p(Scalp)=0.00393
p(Dura)=0.0854
Scalp
Dura
A
B
C
D
E
F
G
H
I
J
K

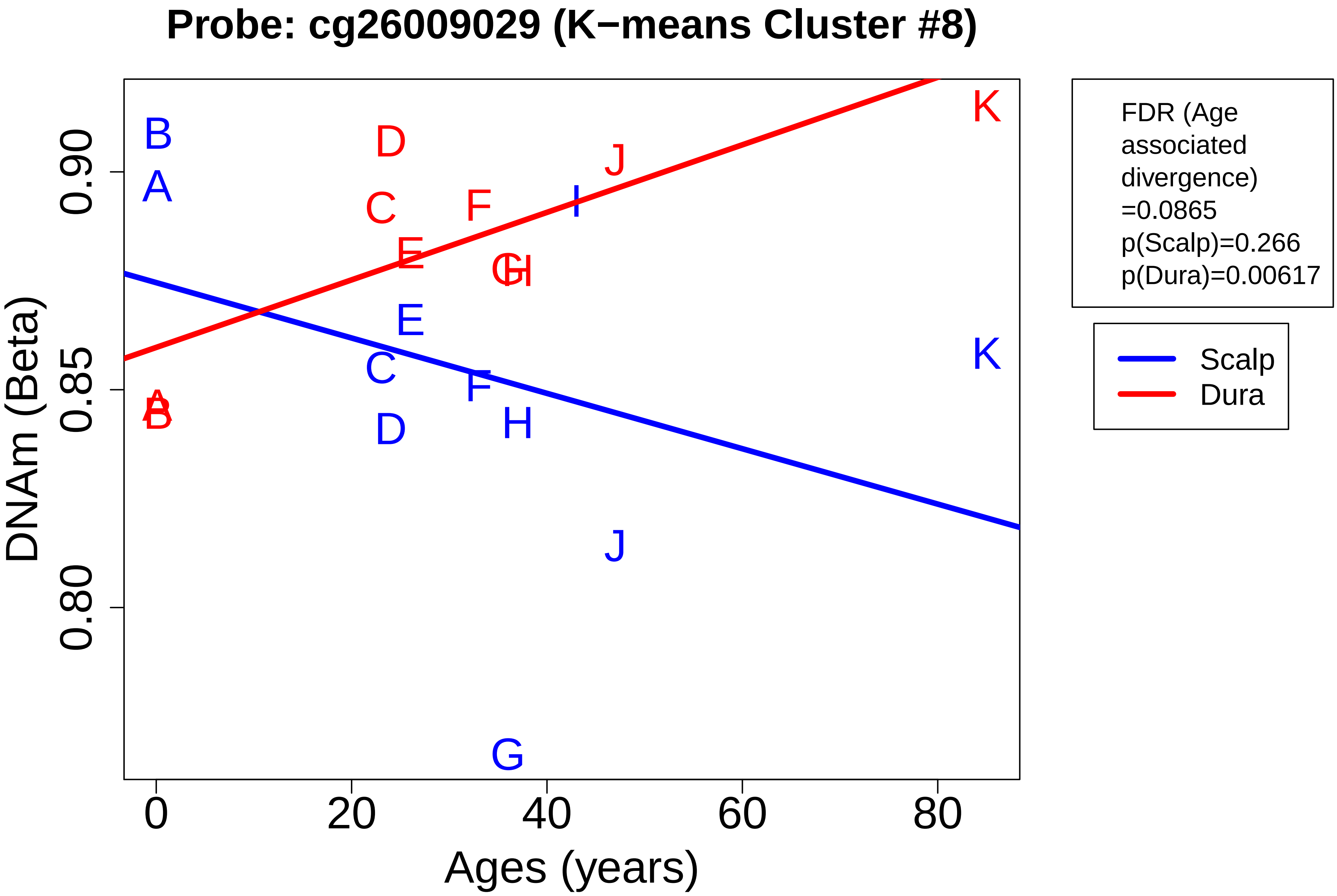

Probe: cg26009029 (K−means Cluster #8)
DNAm (Beta)
0.80
0.85
0.90
Ages (years)
0
20
40
60
80
FDR (Age associated divergence) =0.0865
p(Scalp)=0.266
p(Dura)=0.00617
Scalp
Dura

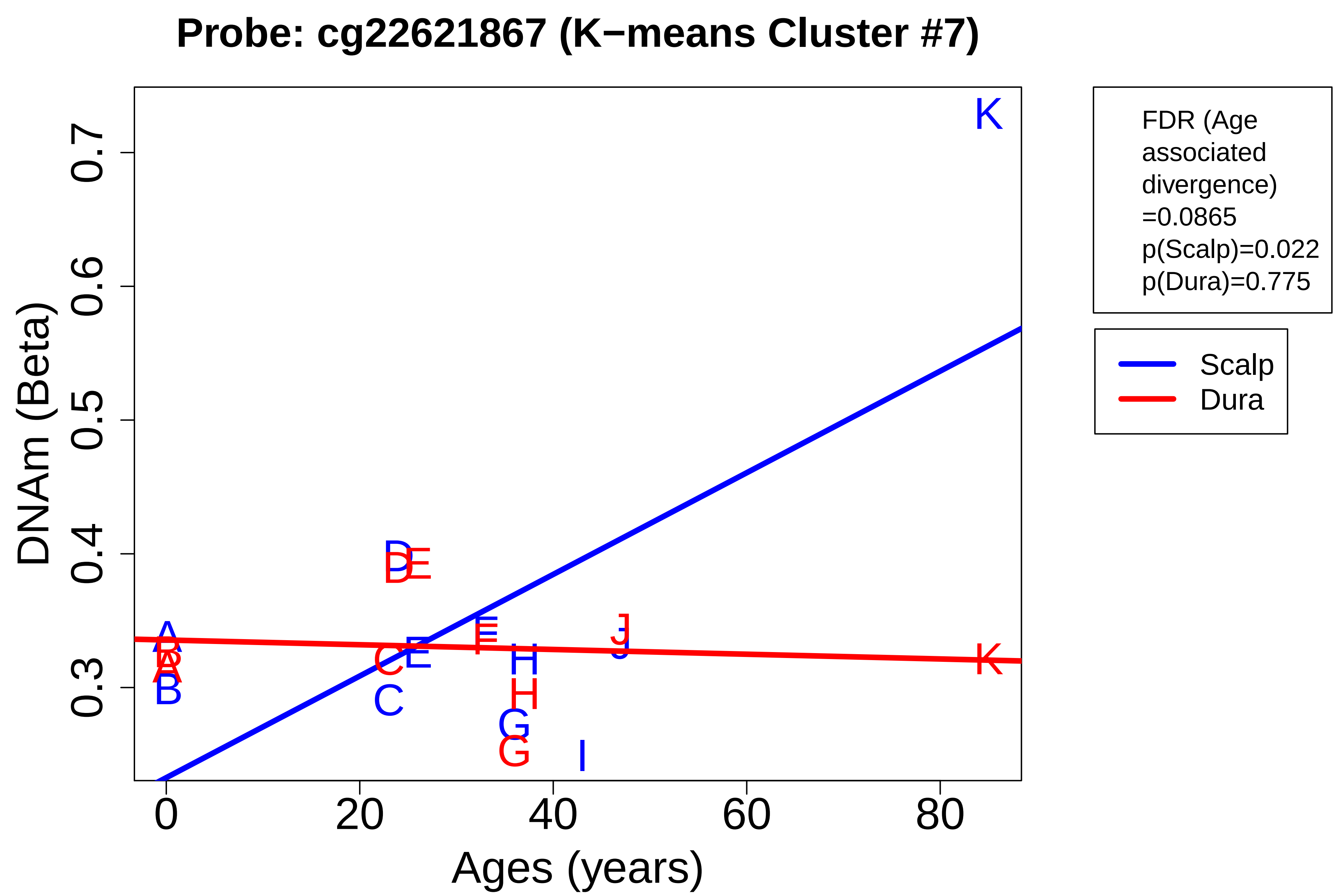

Probe: cg22621867 (K−means Cluster #7)
DNAm (Beta)
0.3
0.4
0.5
0.6
0.7
0
20
40
60
80
Ages (years)
A
B
C
D
E
F
G
H
I
J
K
FDR (Age associated divergence) =0.0865
p(Scalp)=0.022
p(Dura)=0.775
Scalp
Dura

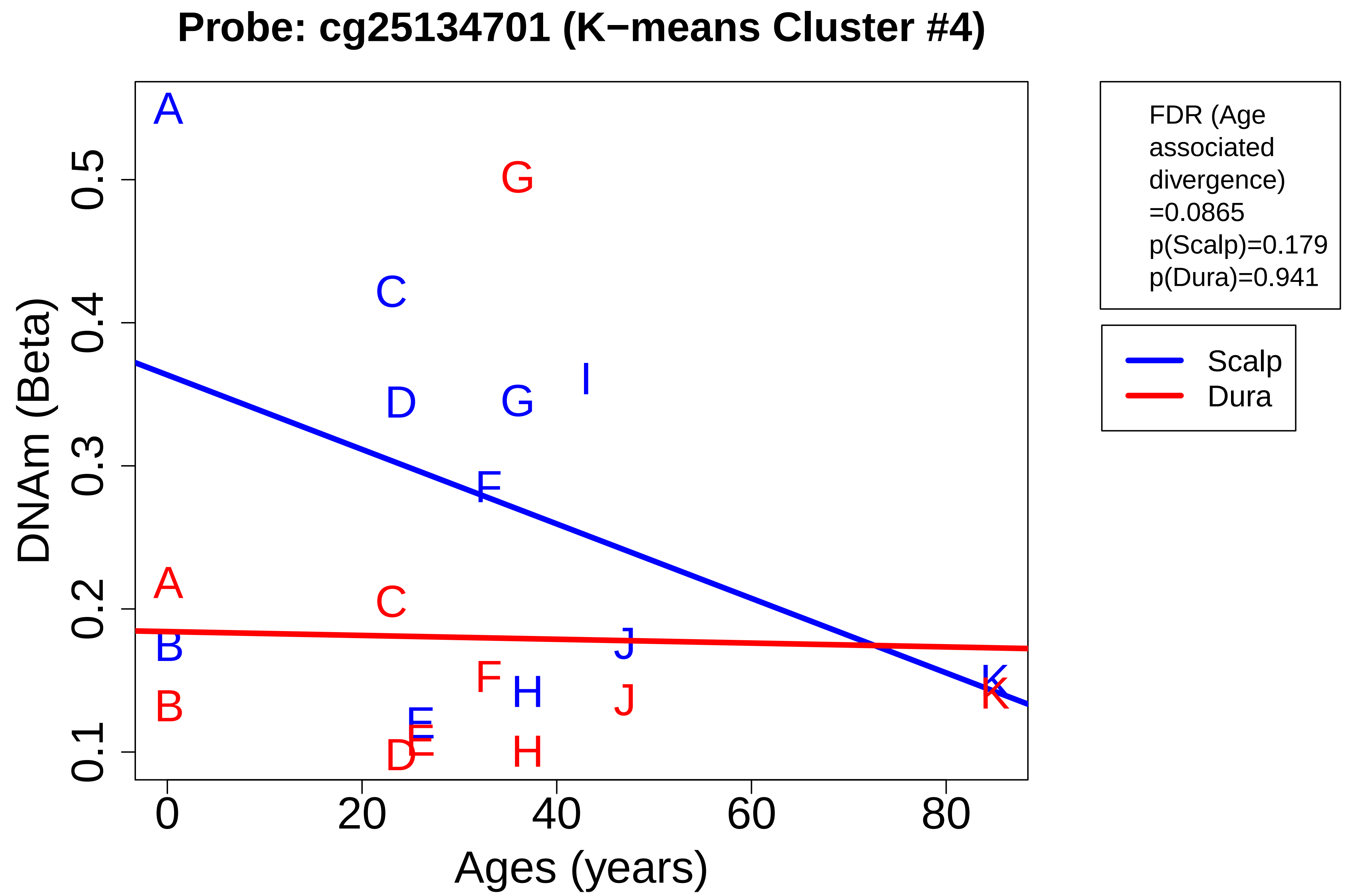

Probe: cg25134701 (K−means Cluster #4)
DNAm (Beta)
0.1
0.2
0.3
0.4
0.5
0
20
40
60
80
Ages (years)
A
B
C
D
E
F
G
H
I
J
K
FDR (Age associated divergence) =0.0865
p(Scalp)=0.179
p(Dura)=0.941
Scalp
Dura

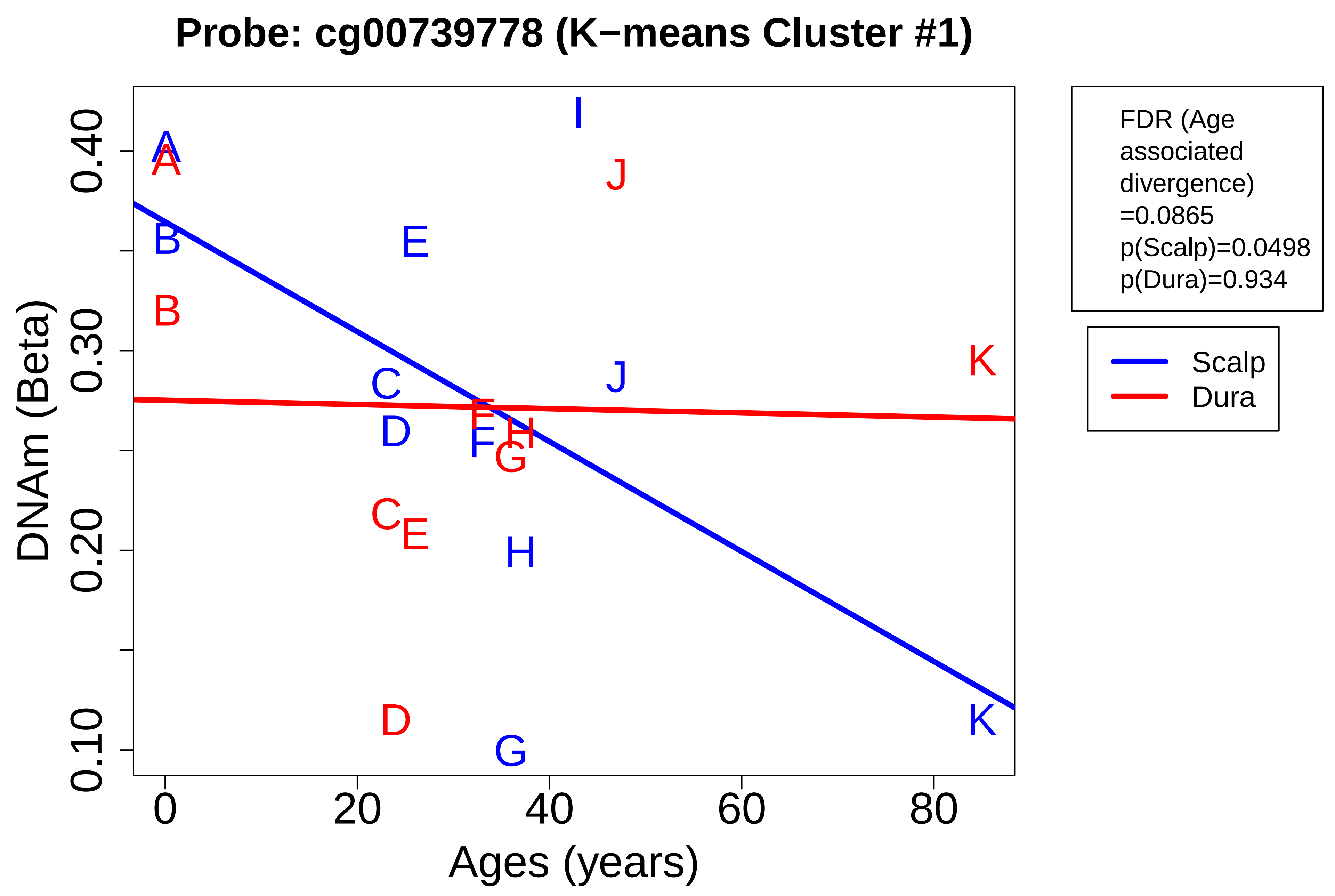

Probe: cg00739778 (K−means Cluster #1)
DNAm (Beta)
0.10
0.20
0.30
0.40
0
20
40
60
80
Ages (years)
A
B
C
D
E
F
G
H
I
J
K
FDR (Age associated divergence) =0.0865
p(Scalp)=0.0498
p(Dura)=0.934
Scalp
Dura

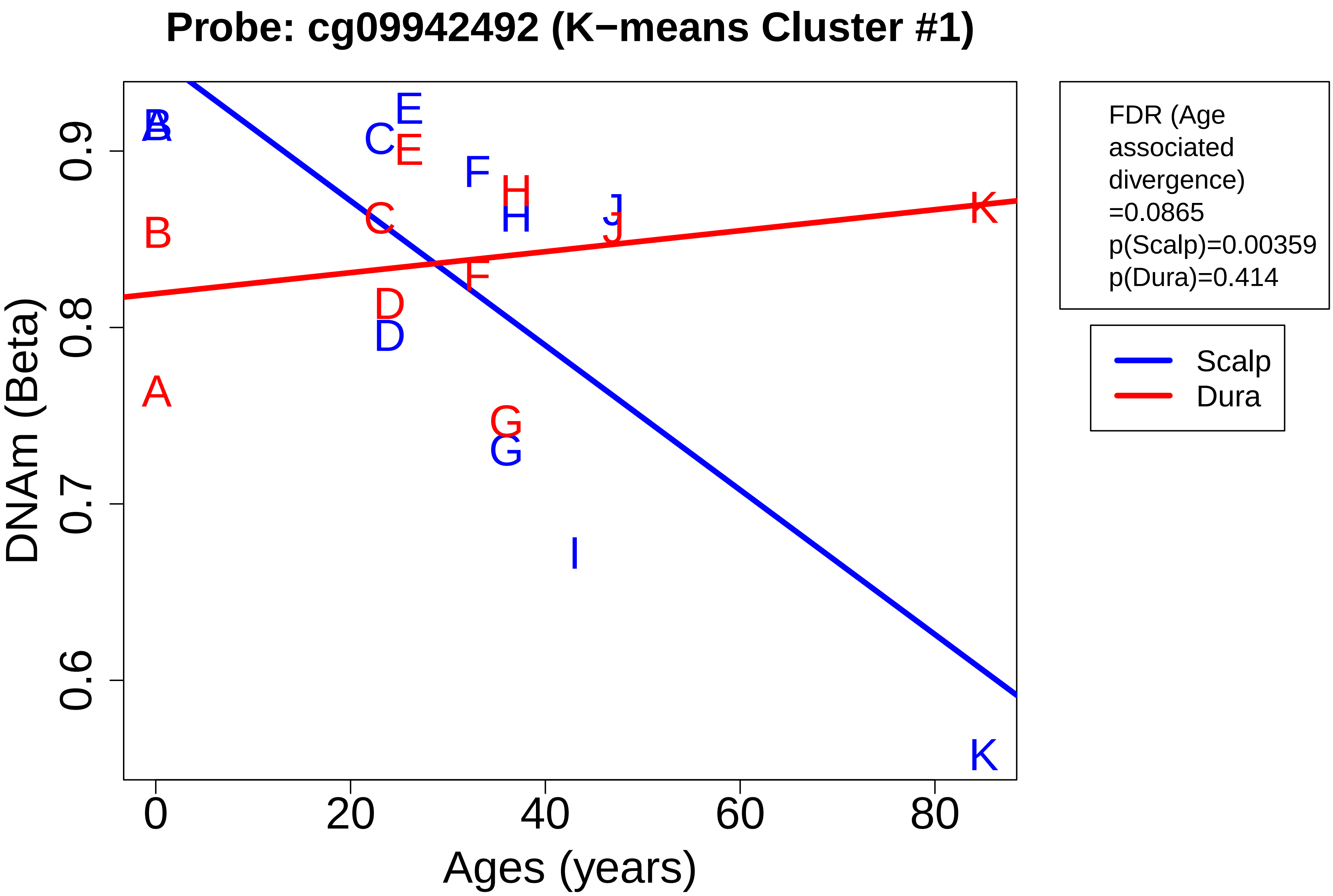
Probe: cg09942492 (K−means Cluster #1)
DNAm (Beta)
0.6
0.7
0.8
0.9
0
20
40
60
80
Ages (years)
A
B
C
D
E
F
G
H
I
J
K
FDR (Age
associated
divergence)
=0.0865
p(Scalp)=0.00359
p(Dura)=0.414
Scalp
Dura

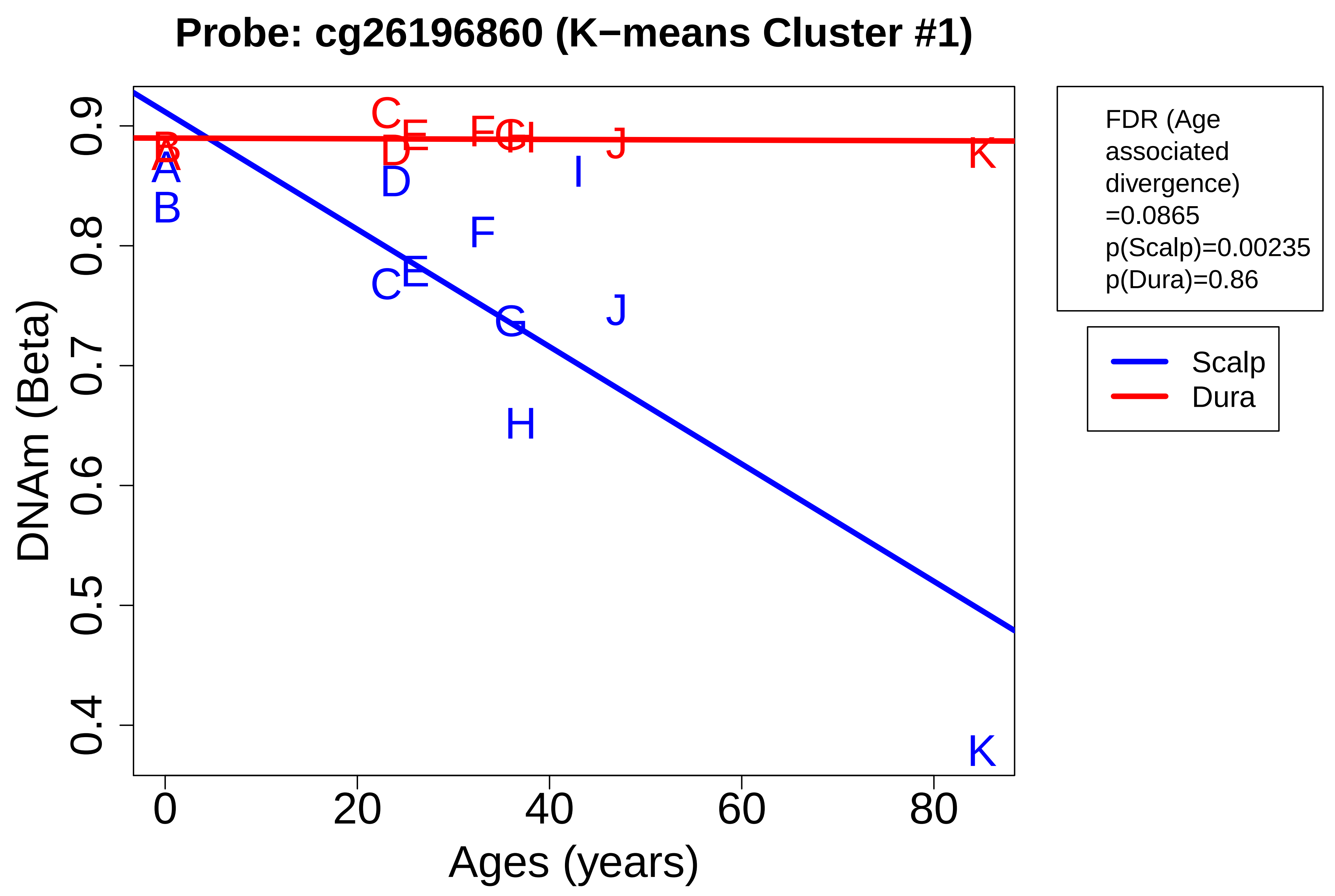

Probe: cg26196860 (K−means Cluster #1)
DNAm (Beta)
0.4
0.5
0.6
0.7
0.8
0.9
Ages (years)
0
20
40
60
80
FDR (Age associated divergence) =0.0865
p(Scalp)=0.00235
p(Dura)=0.86
Scalp
Dura

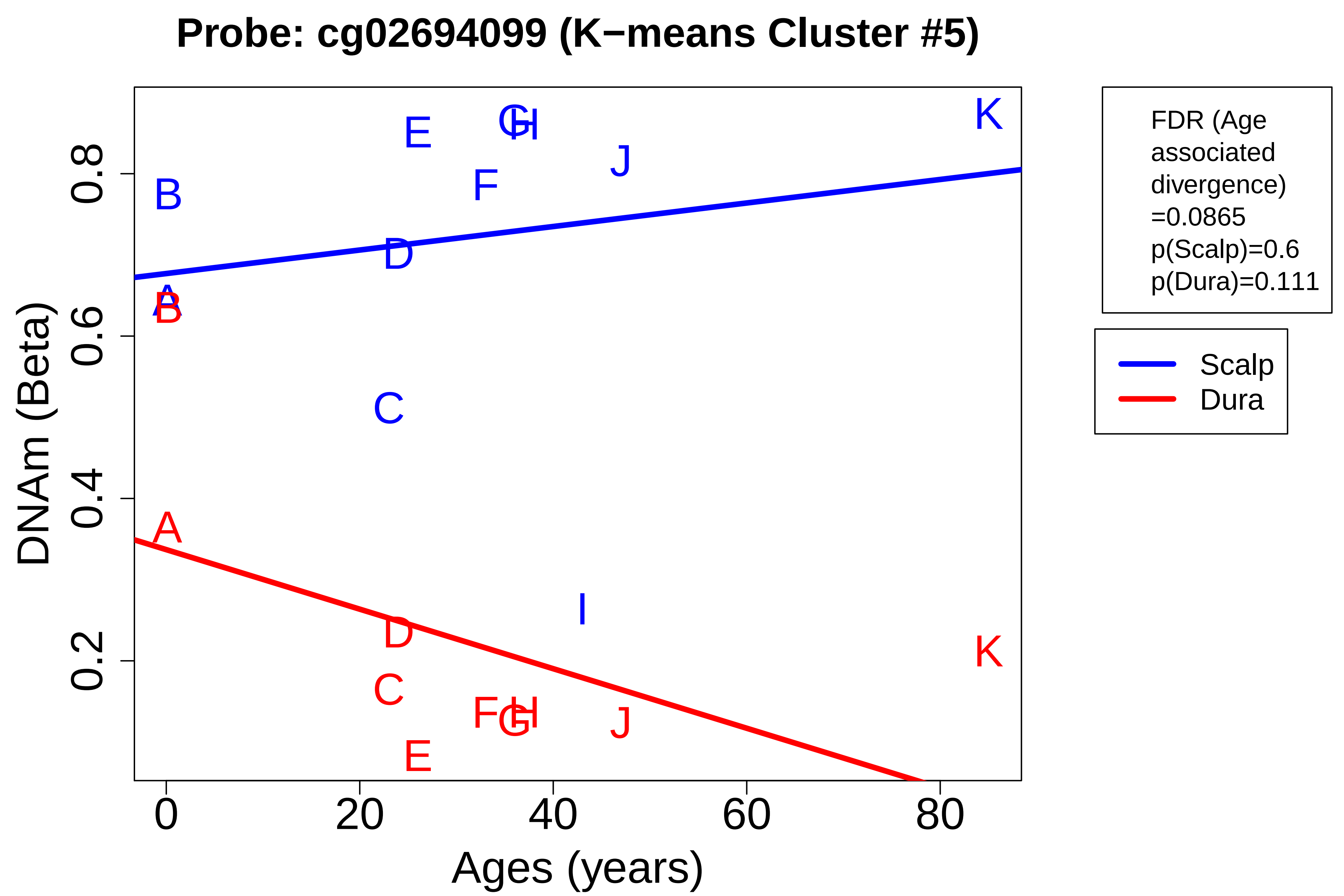

Probe: cg02694099 (K−means Cluster #5)
DNAm (Beta)
0.2
0.4
0.6
0.8
0
20
40
60
80
Ages (years)
FDR (Age associated divergence) =0.0865
p(Scalp)=0.6
p(Dura)=0.111
Scalp
Dura

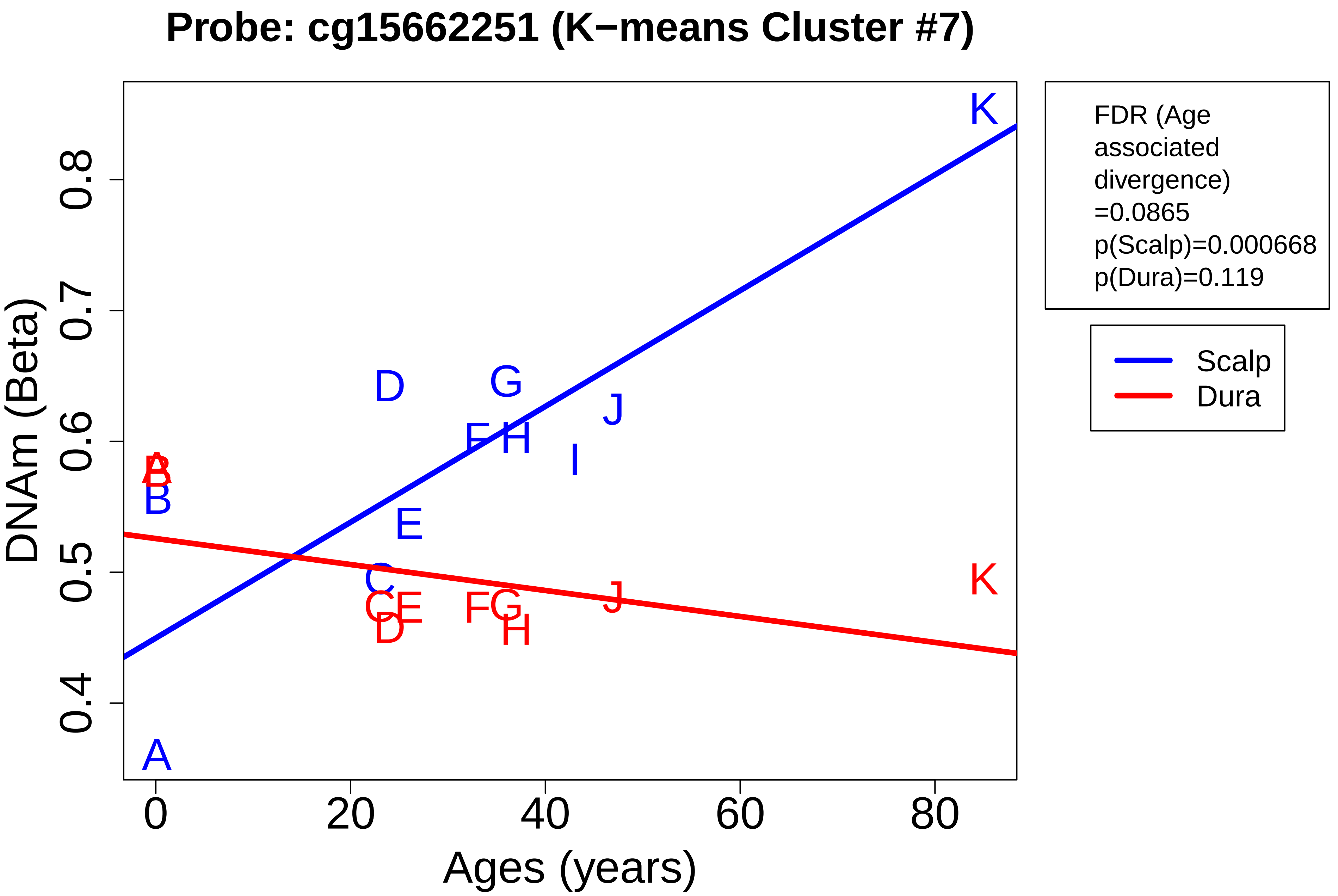

Probe: cg15662251 (K−means Cluster #7)
DNAm (Beta)
0.4
0.5
0.6
0.7
0.8
0
20
40
60
80
Ages (years)
FDR (Age associated divergence) =0.0865
p(Scalp)=0.000668
p(Dura)=0.119
Scalp
Dura

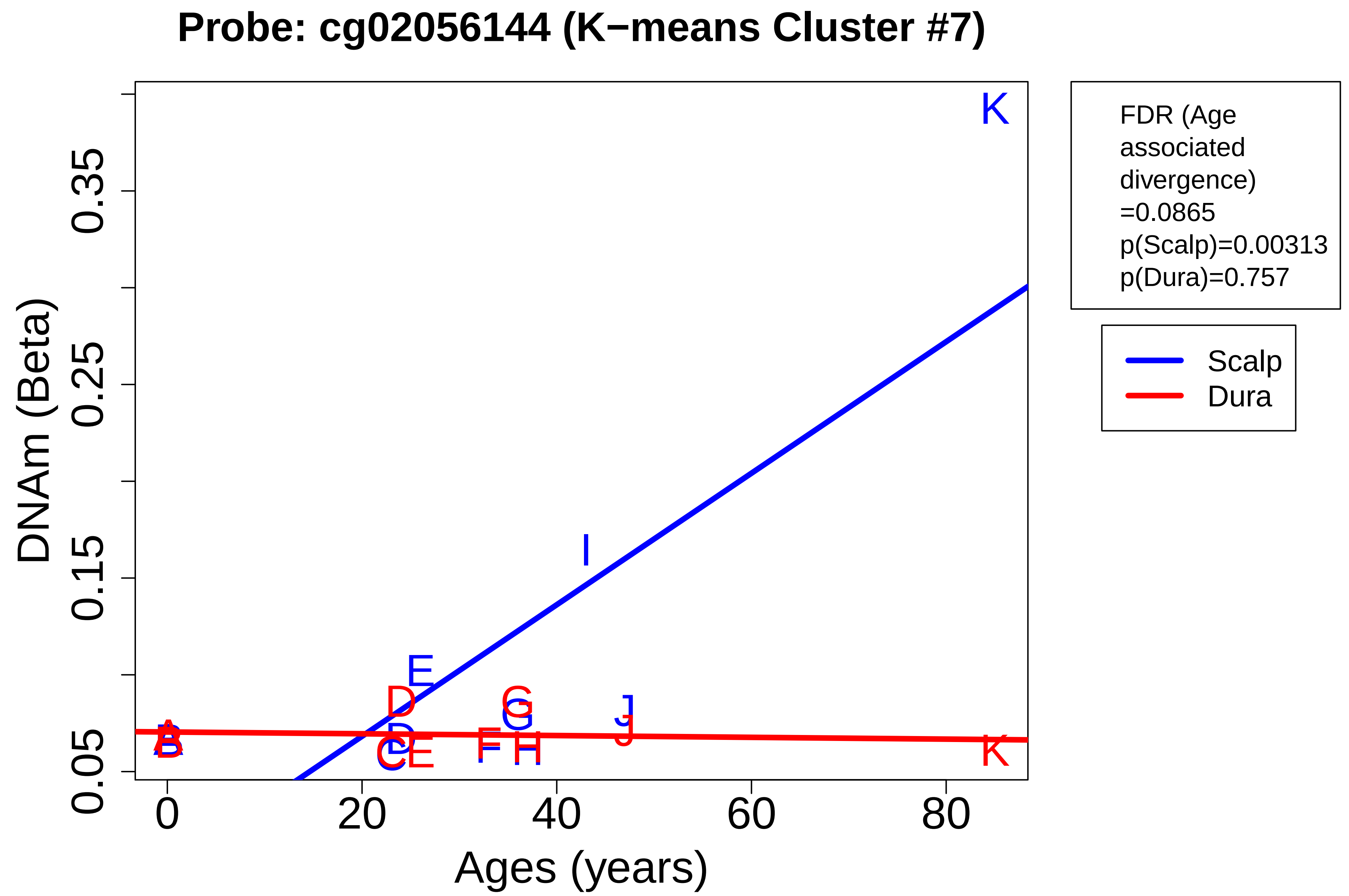

Probe: cg02056144 (K−means Cluster #7)
DNAm (Beta)
0.05
0.15
0.25
0.35
0
20
40
60
80
Ages (years)
FDR (Age associated divergence) =0.0865
p(Scalp)=0.00313
p(Dura)=0.757
Scalp
Dura

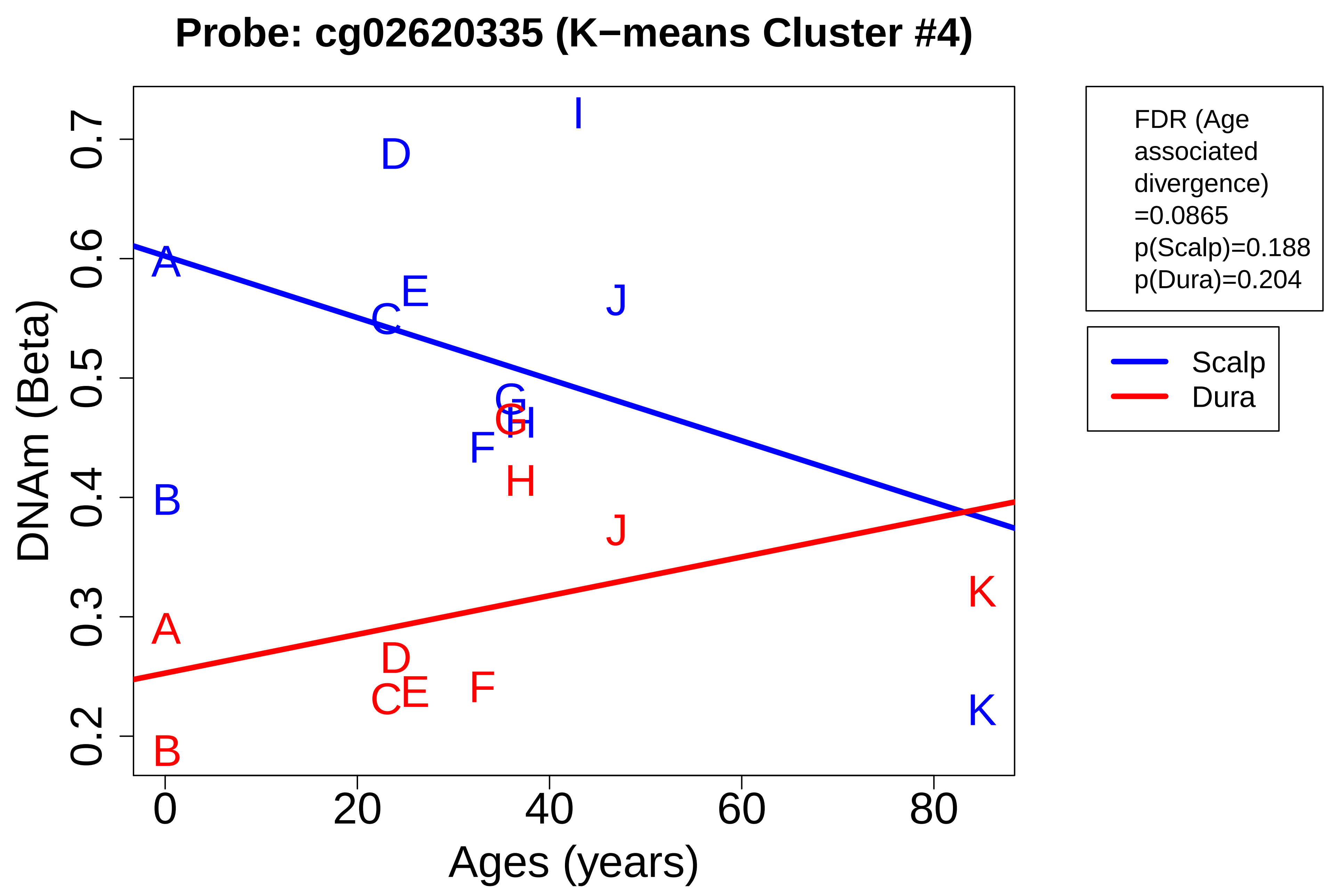

Probe: cg02620335 (K−means Cluster #4)
DNAm (Beta)
0.2
0.3
0.4
0.5
0.6
0.7
0
20
40
60
80
Ages (years)
A
B
C
D
E
F
G
H
I
J
K
FDR (Age associated divergence) =0.0865
p(Scalp)=0.188
p(Dura)=0.204
Scalp
Dura

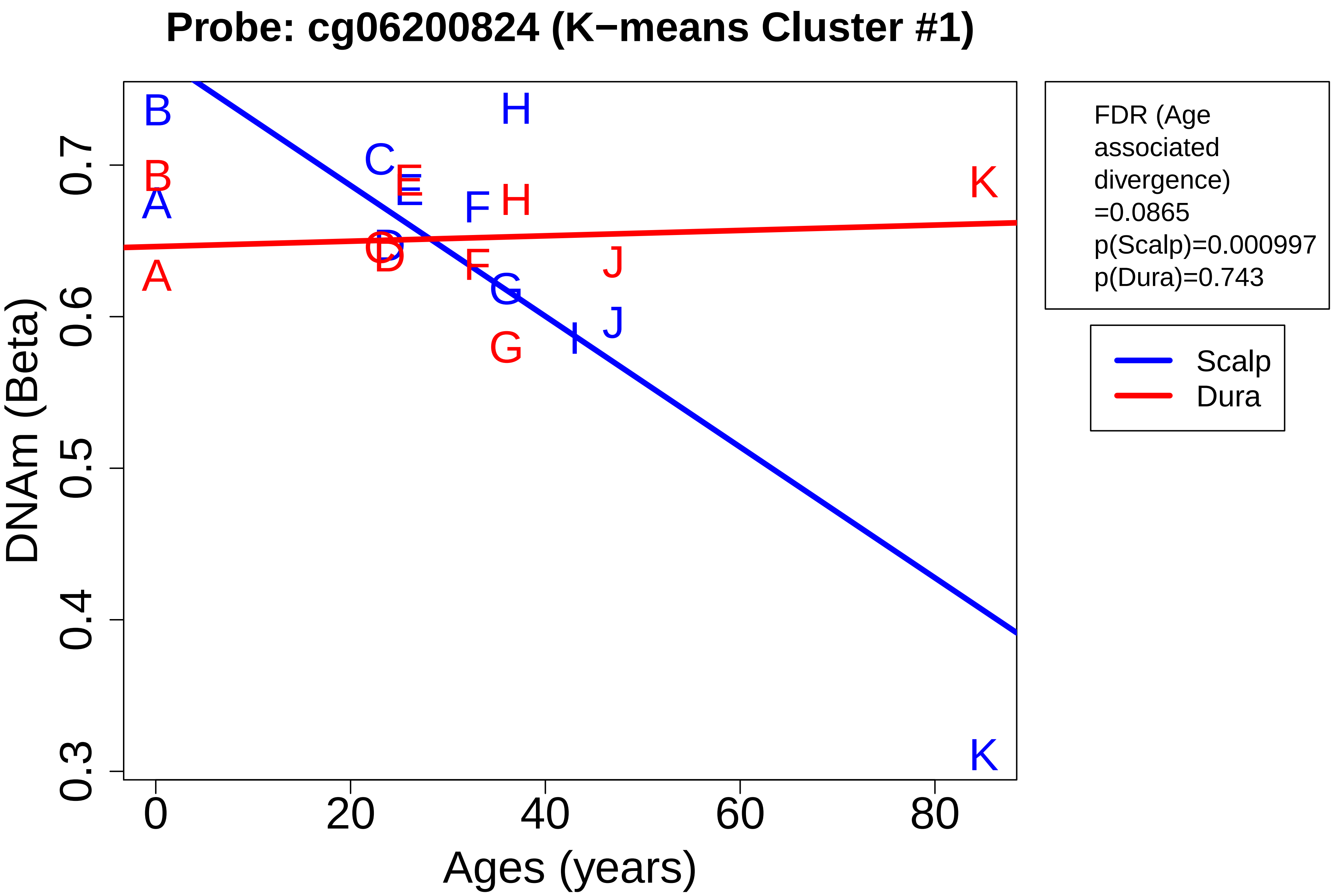

Probe: cg06200824 (K−means Cluster #1)
FDR (Age associated divergence) =0.0865
p(Scalp)=0.000997
p(Dura)=0.743
Scalp
Dura
DNAm (Beta)
0.3
0.4
0.5
0.6
0.7
Ages (years)
0
20
40
60
80
A
B
C
D
E
F
G
H
I
J
K

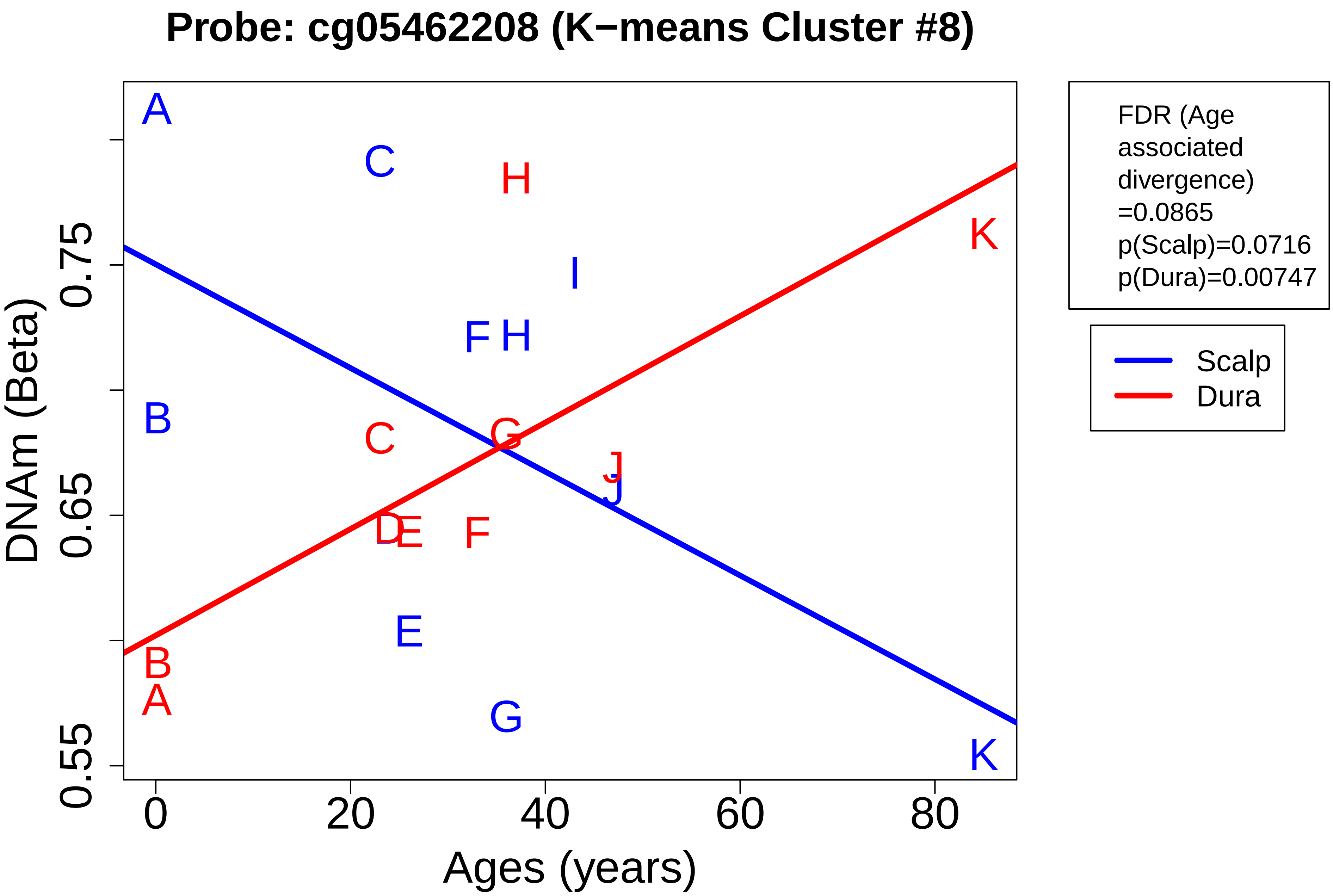
Probe: cg05462208 (K−means Cluster #8)
DNAm (Beta)
0.55
0.65
0.75
Ages (years)
0
20
40
60
80
A
C
H
K
I
F
H
B
C
G
J
J
D
E
F
E
B
A
G
K
FDR (Age associated divergence) =0.0865
p(Scalp)=0.0716
p(Dura)=0.00747
Scalp
Dura

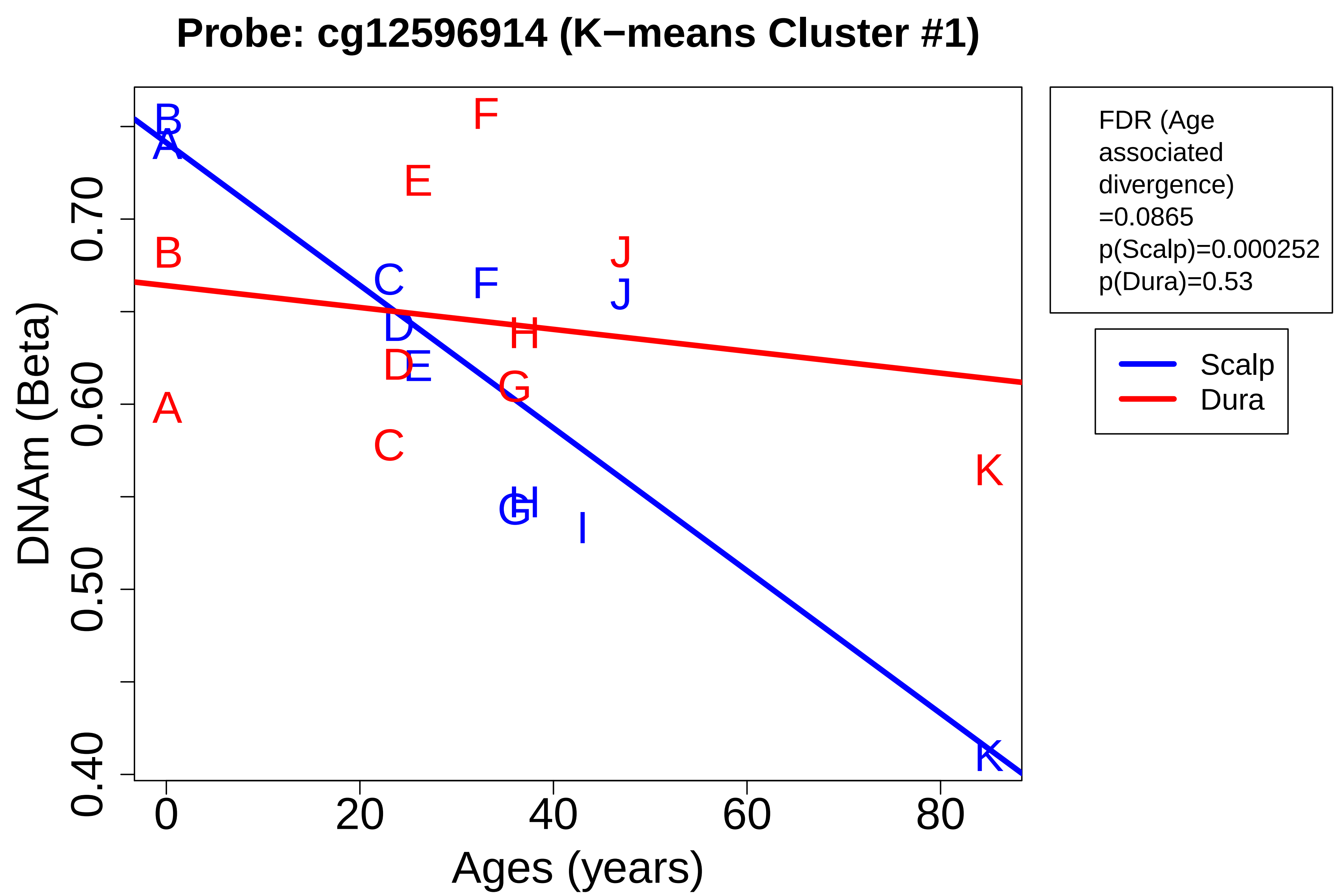

Probe: cg12596914 (K−means Cluster #1)
DNAm (Beta)
0.40
0.50
0.60
0.70
Ages (years)
0
20
40
60
80
FDR (Age associated divergence) =0.0865
p(Scalp)=0.000252
p(Dura)=0.53
Scalp
Dura
A
B
C
D
E
F
G
H
I
J
K

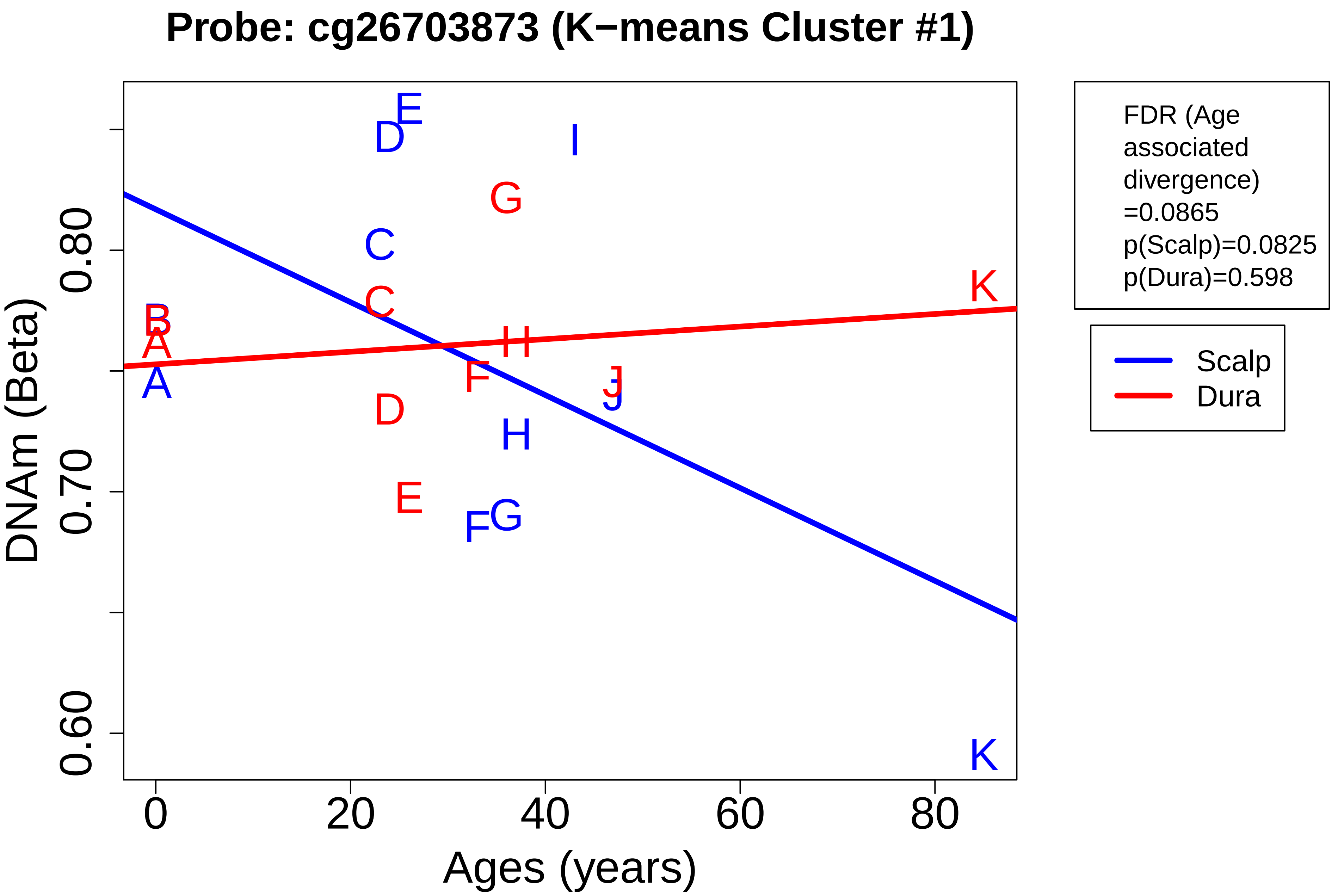

Probe: cg26703873 (K−means Cluster #1)
DNAm (Beta)
0.60
0.70
0.80
0
20
40
60
80
Ages (years)
FDR (Age associated divergence) =0.0865
p(Scalp)=0.0825
p(Dura)=0.598
Scalp
Dura

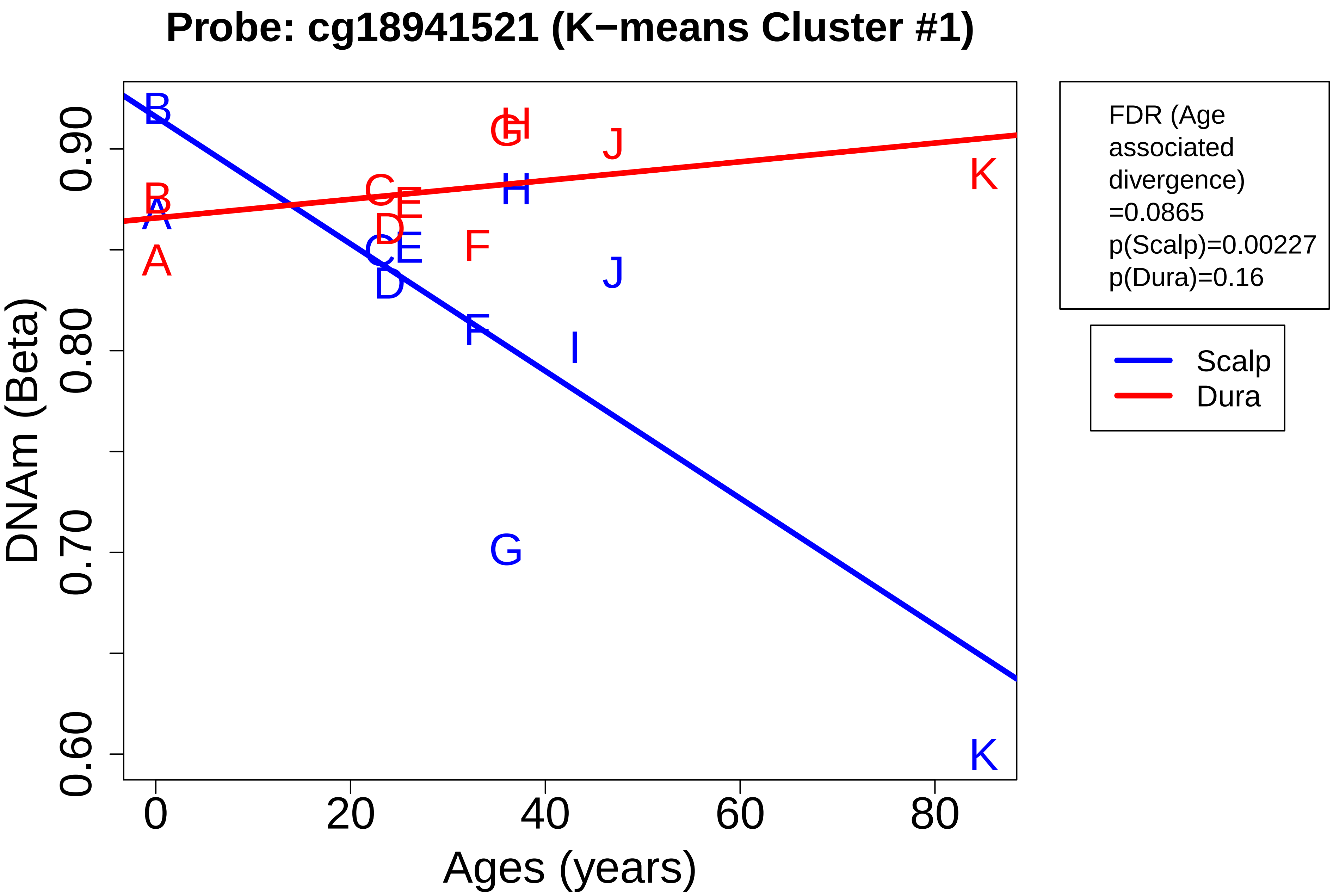

Probe: cg18941521 (K−means Cluster #1)
DNAm (Beta)
0.60
0.70
0.80
0.90
0
20
40
60
80
Ages (years)
FDR (Age associated divergence) =0.0865
p(Scalp)=0.00227
p(Dura)=0.16
Scalp
Dura

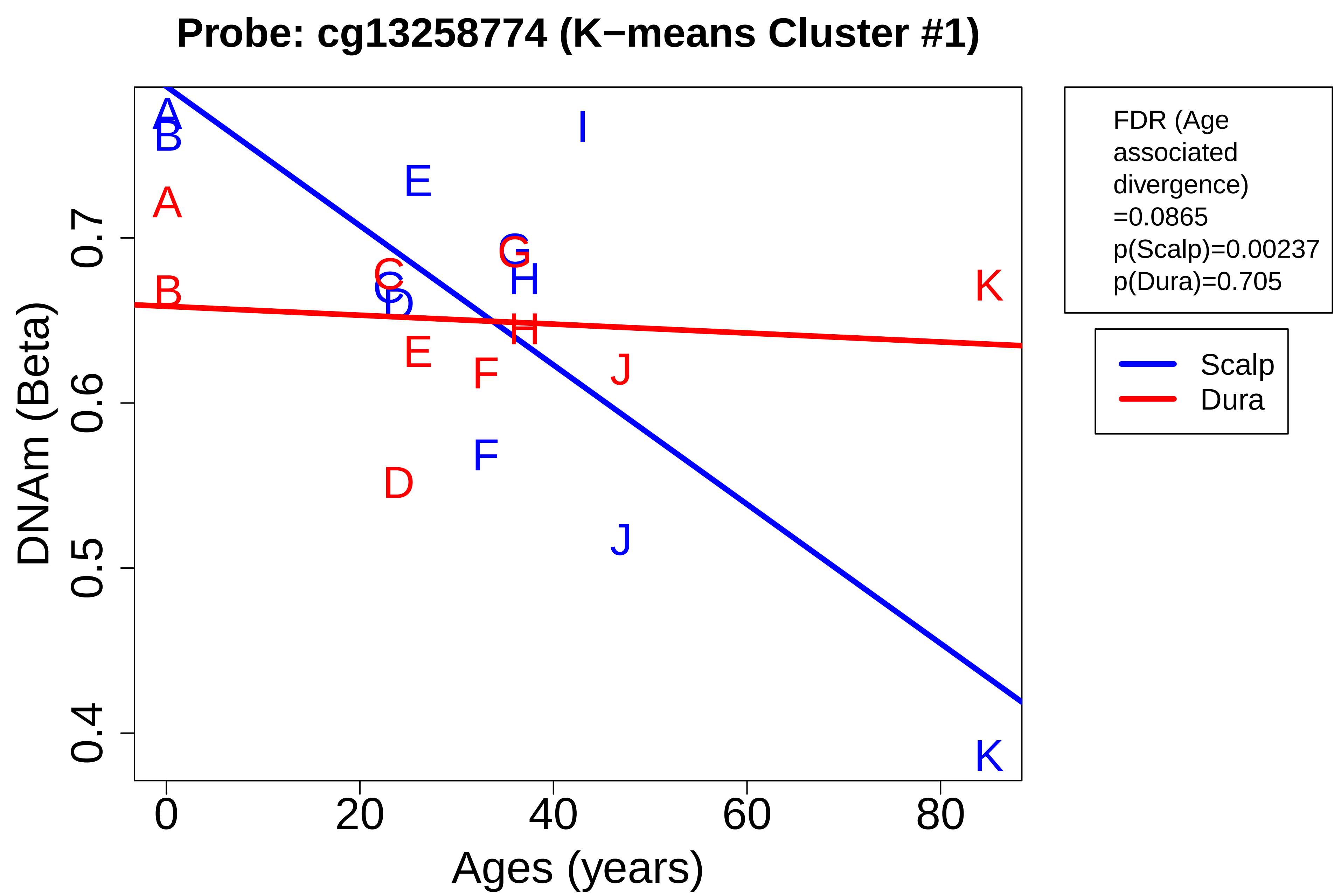

Probe: cg13258774 (K−means Cluster #1)
DNAm (Beta)
0.4
0.5
0.6
0.7
Ages (years)
0
20
40
60
80
A
B
C
D
E
F
G
H
I
J
K
FDR (Age associated divergence) =0.0865
p(Scalp)=0.00237
p(Dura)=0.705
Scalp
Dura

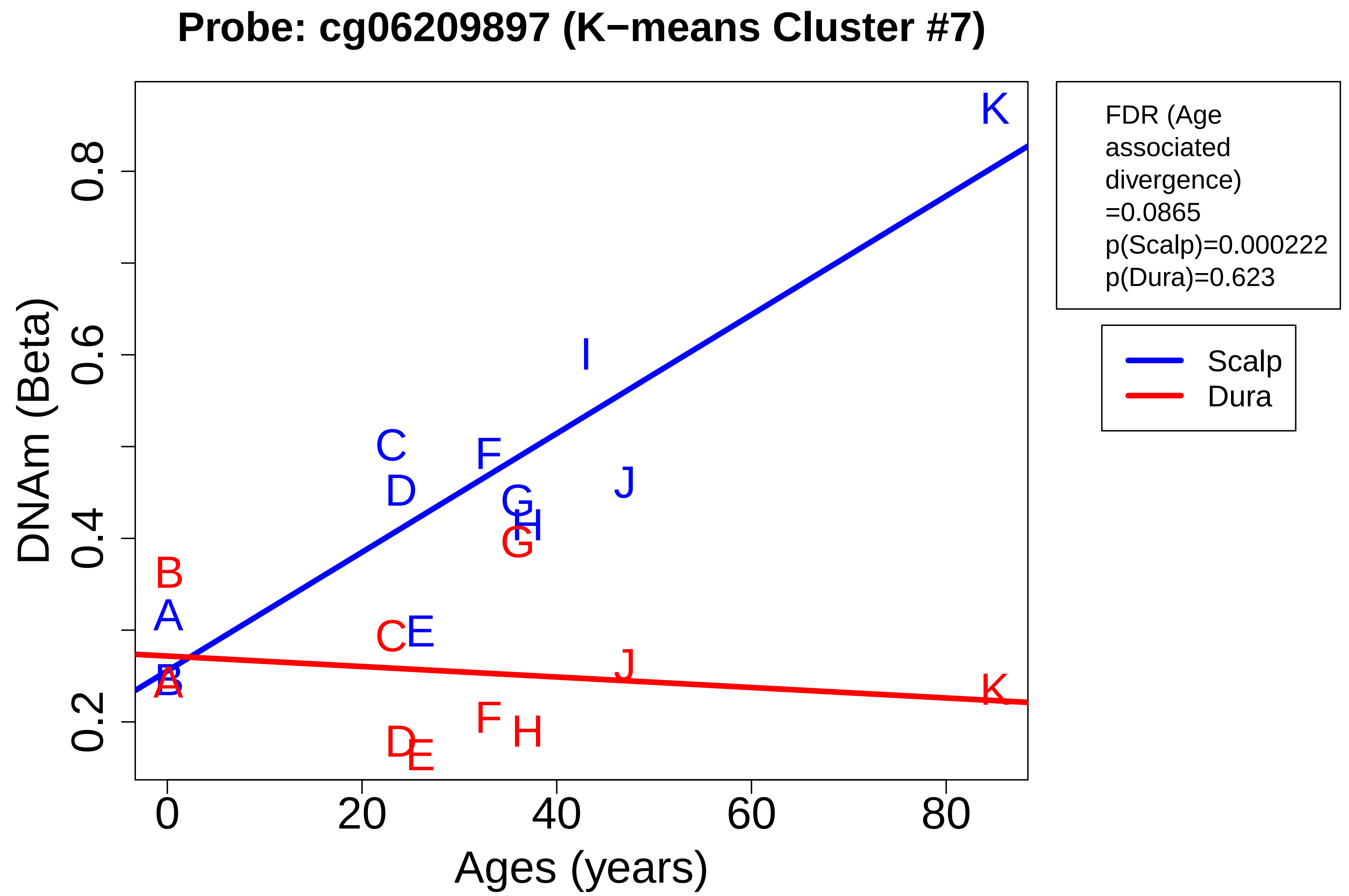

Probe: cg06209897 (K−means Cluster #7)
DNAm (Beta)
0.2
0.4
0.6
0.8
0
20
40
60
80
Ages (years)
FDR (Age associated divergence) =0.0865
p(Scalp)=0.000222
p(Dura)=0.623
Scalp
Dura
A B C D E F G H I J K

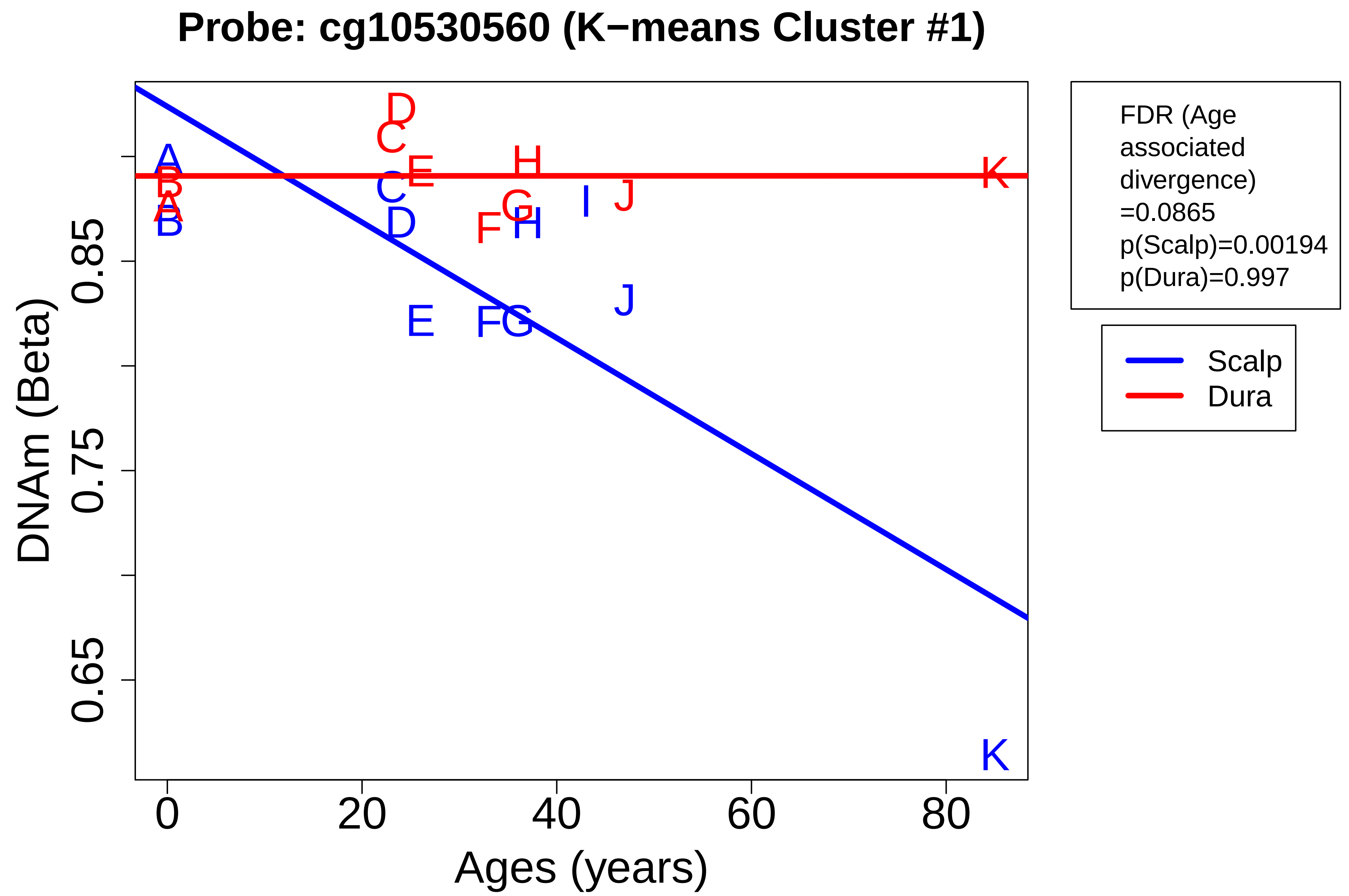

Probe: cg10530560 (K−means Cluster #1)
DNAm (Beta)
0.65
0.75
0.85
Ages (years)
0
20
40
60
80
A
B
C
D
E
F
G
H
I
J
K
A
B
C
D
E
F
G
H
J
K
FDR (Age associated divergence) =0.0865
p(Scalp)=0.00194
p(Dura)=0.997
Scalp
Dura

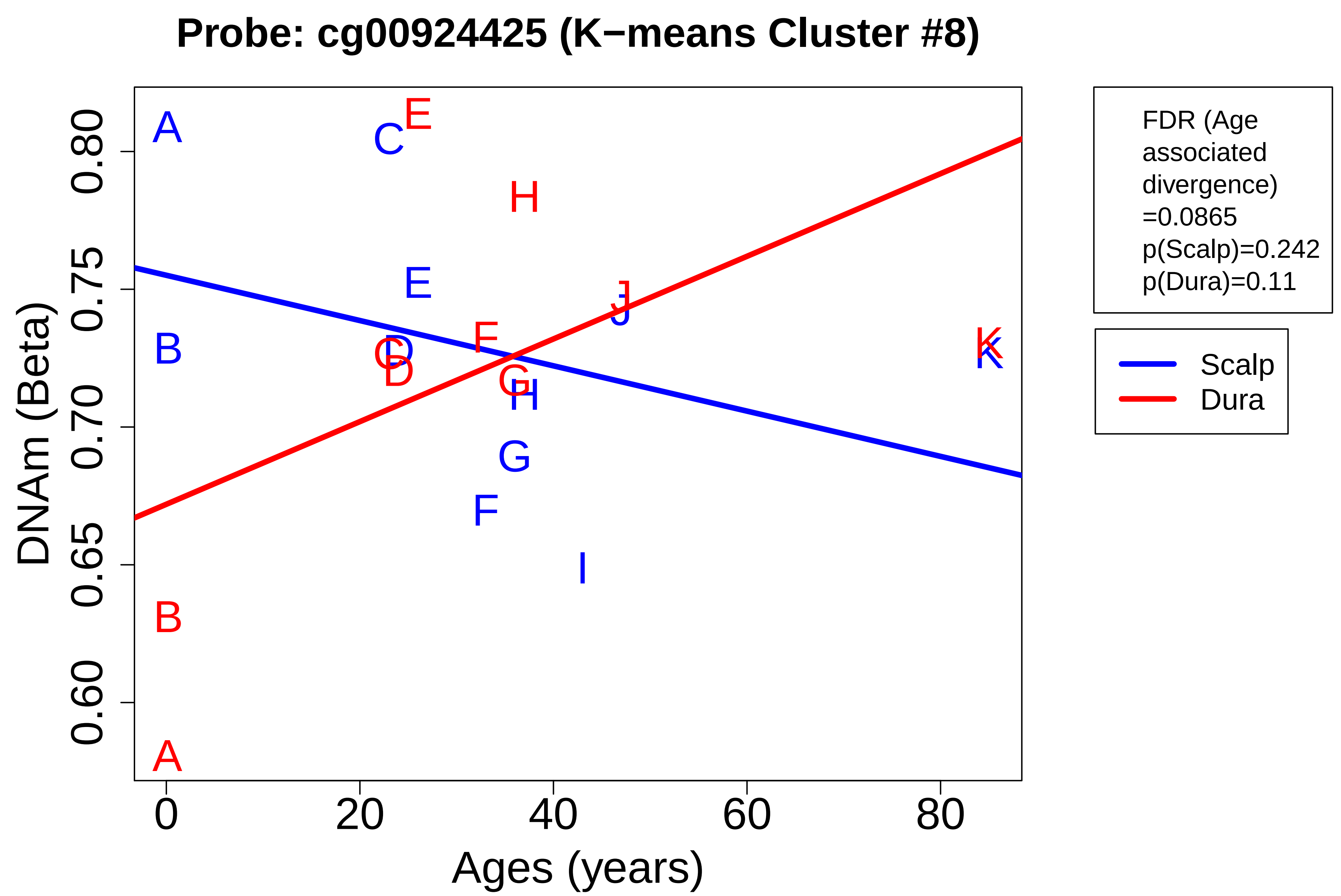

Probe: cg00924425 (K−means Cluster #8)
DNAm (Beta)
0.60
0.65
0.70
0.75
0.80
0
20
40
60
80
Ages (years)
A
B
C
D
E
F
G
H
I
J
K
FDR (Age associated divergence) =0.0865
p(Scalp)=0.242
p(Dura)=0.11
Scalp
Dura

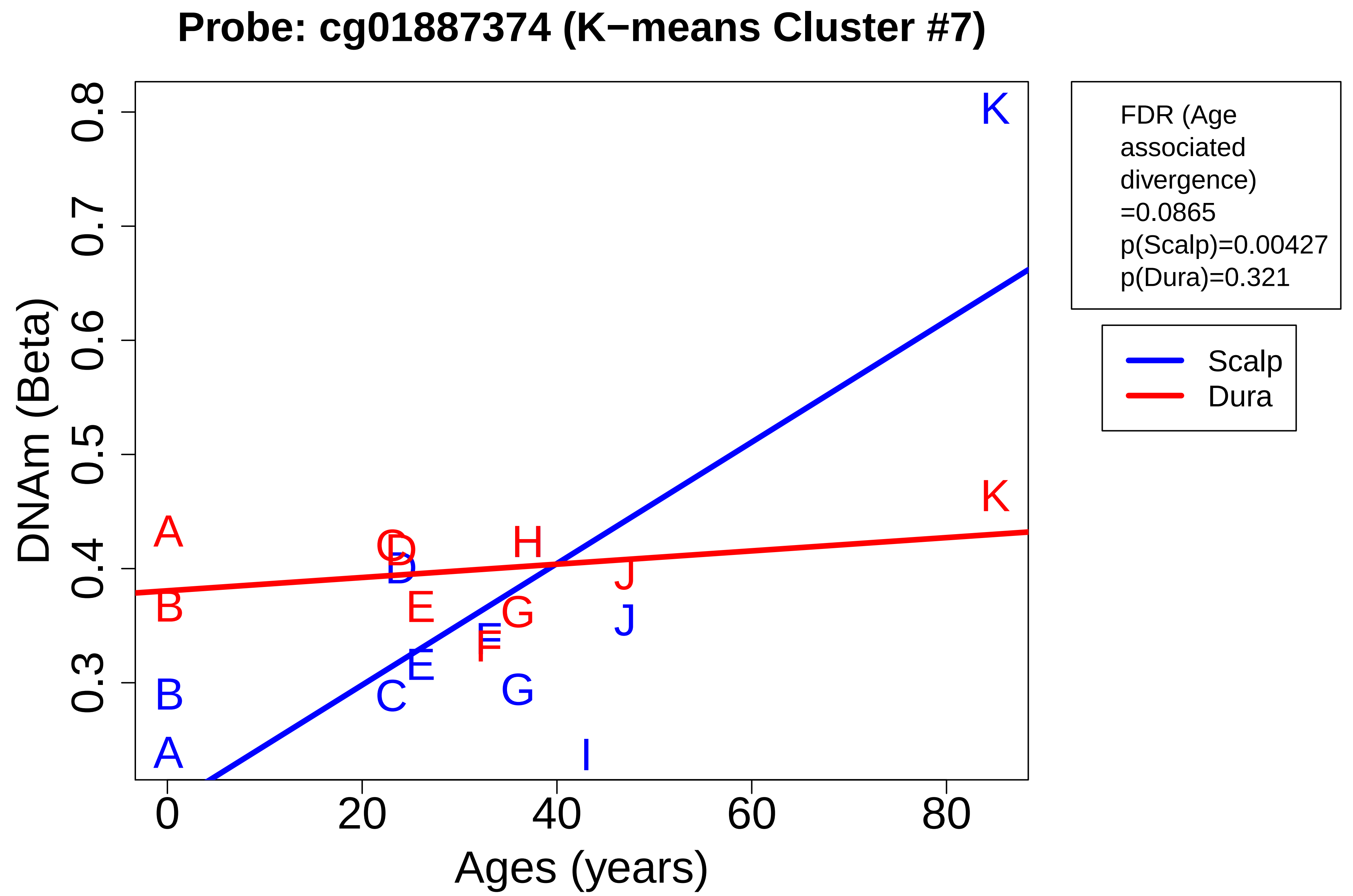

Probe: cg01887374 (K−means Cluster #7)
DNAm (Beta)
0.3
0.4
0.5
0.6
0.7
0.8
0
20
40
60
80
Ages (years)
A
B
C
D
E
F
G
H
I
J
K
FDR (Age associated divergence) =0.0865
p(Scalp)=0.00427
p(Dura)=0.321
Scalp
Dura

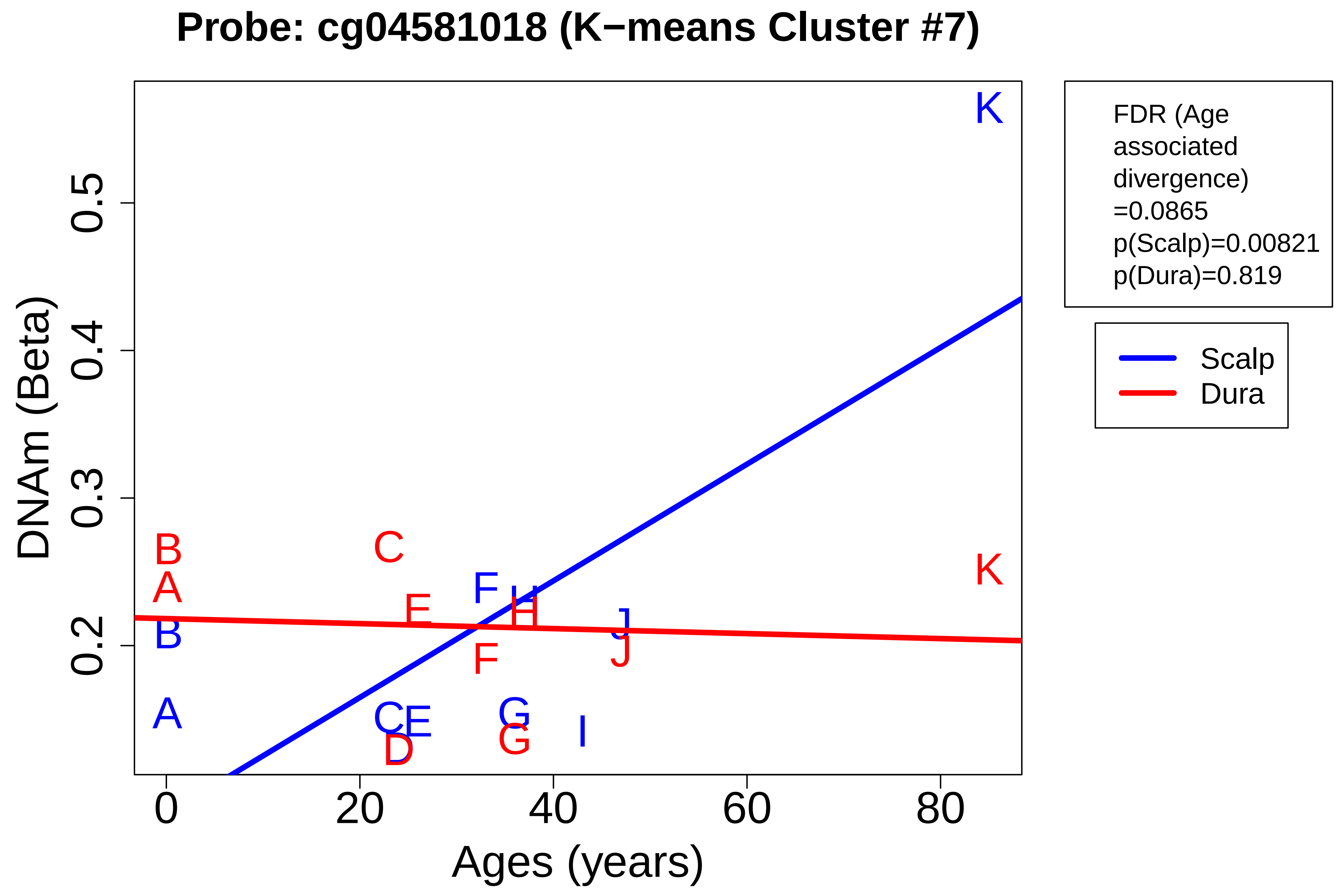

Probe: cg04581018 (K−means Cluster #7)
DNAm (Beta)
0.2
0.3
0.4
0.5
0
20
40
60
80
Ages (years)
FDR (Age associated divergence) =0.0865
p(Scalp)=0.00821
p(Dura)=0.819
Scalp
Dura

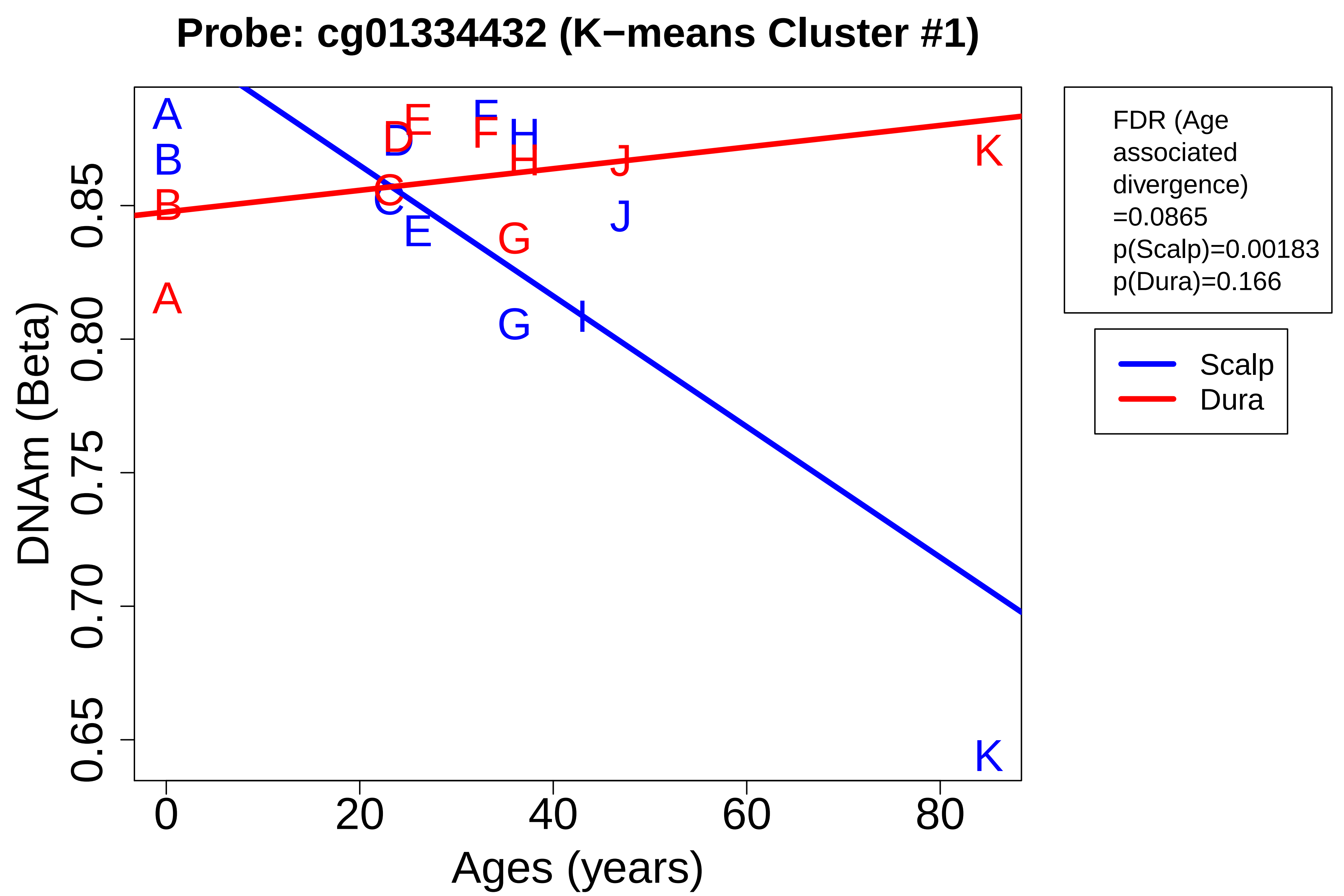

Probe: cg01334432 (K−means Cluster #1)
DNAm (Beta)
0.65
0.70
0.75
0.80
0.85
0
20
40
60
80
Ages (years)
A
B
C
D
E
F
G
H
I
J
K
FDR (Age associated divergence) =0.0865
p(Scalp)=0.00183
p(Dura)=0.166
Scalp
Dura

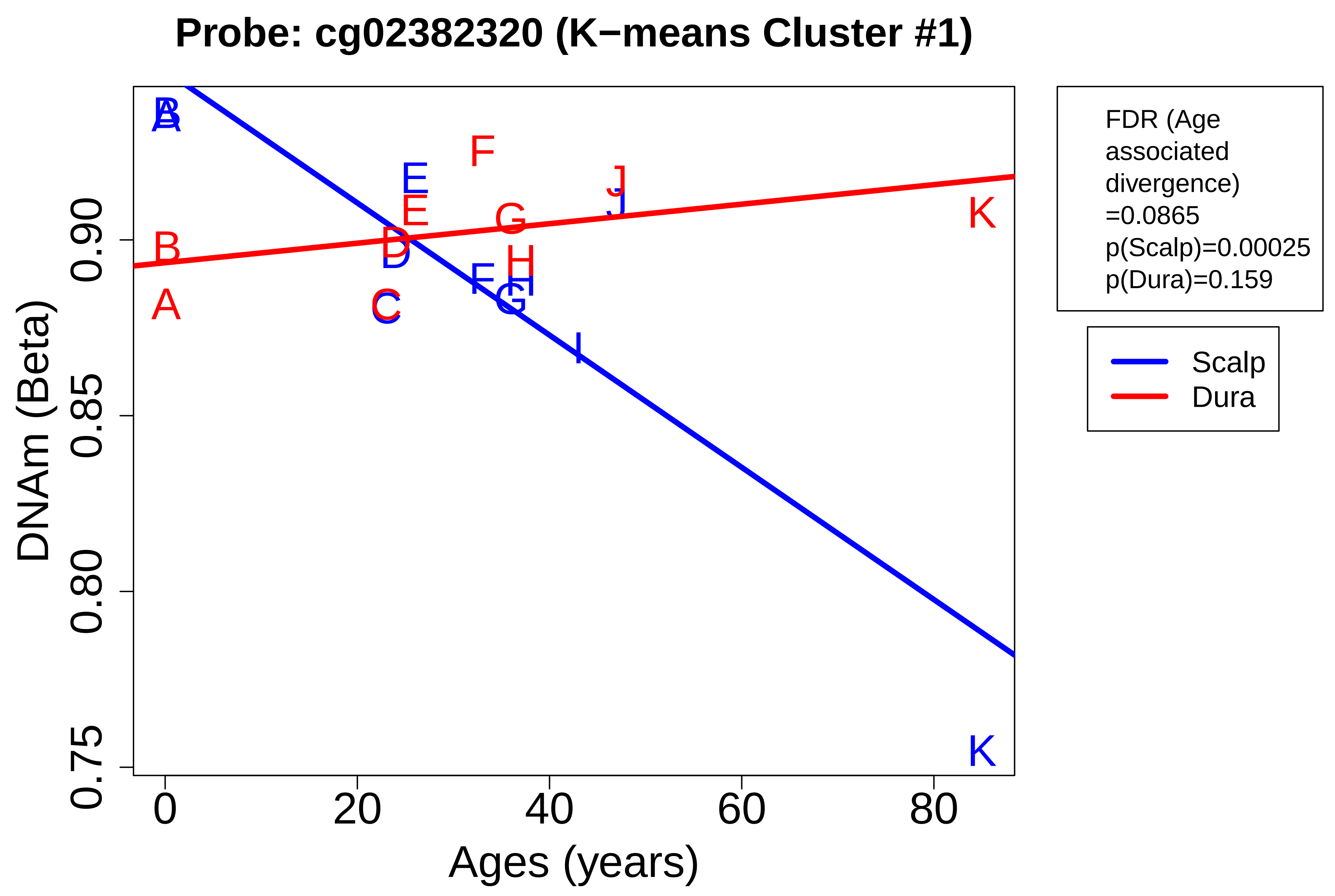

Probe: cg02382320 (K−means Cluster #1)
DNAm (Beta)
0.75
0.80
0.85
0.90
0
20
40
60
80
Ages (years)
FDR (Age associated divergence) =0.0865
p(Scalp)=0.00025
p(Dura)=0.159
Scalp
Dura

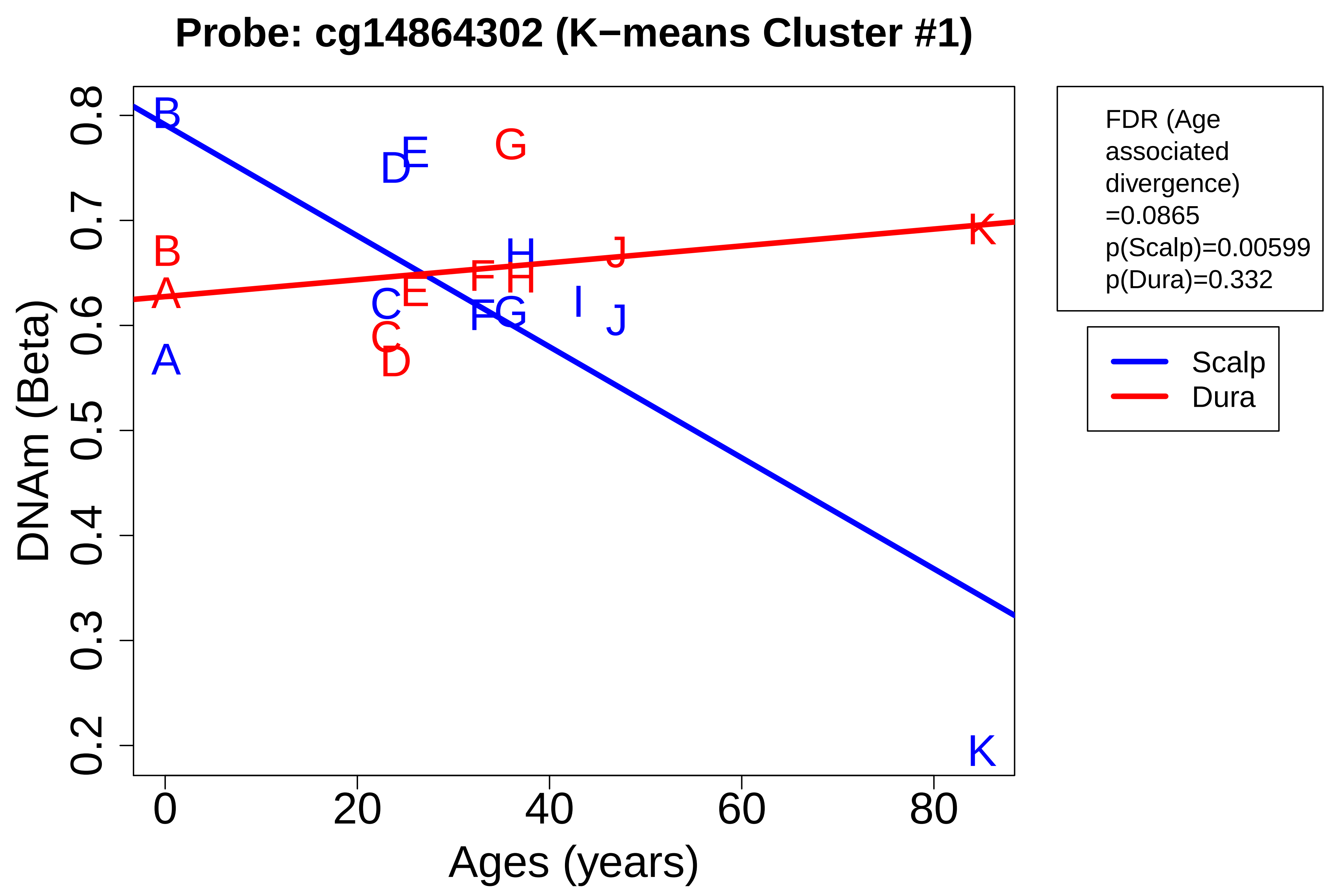

Probe: cg14864302 (K−means Cluster #1)
FDR (Age associated divergence) =0.0865
p(Scalp)=0.00599
p(Dura)=0.332
Scalp
Dura
DNAm (Beta)
0.2
0.3
0.4
0.5
0.6
0.7
0.8
Ages (years)
0
20
40
60
80

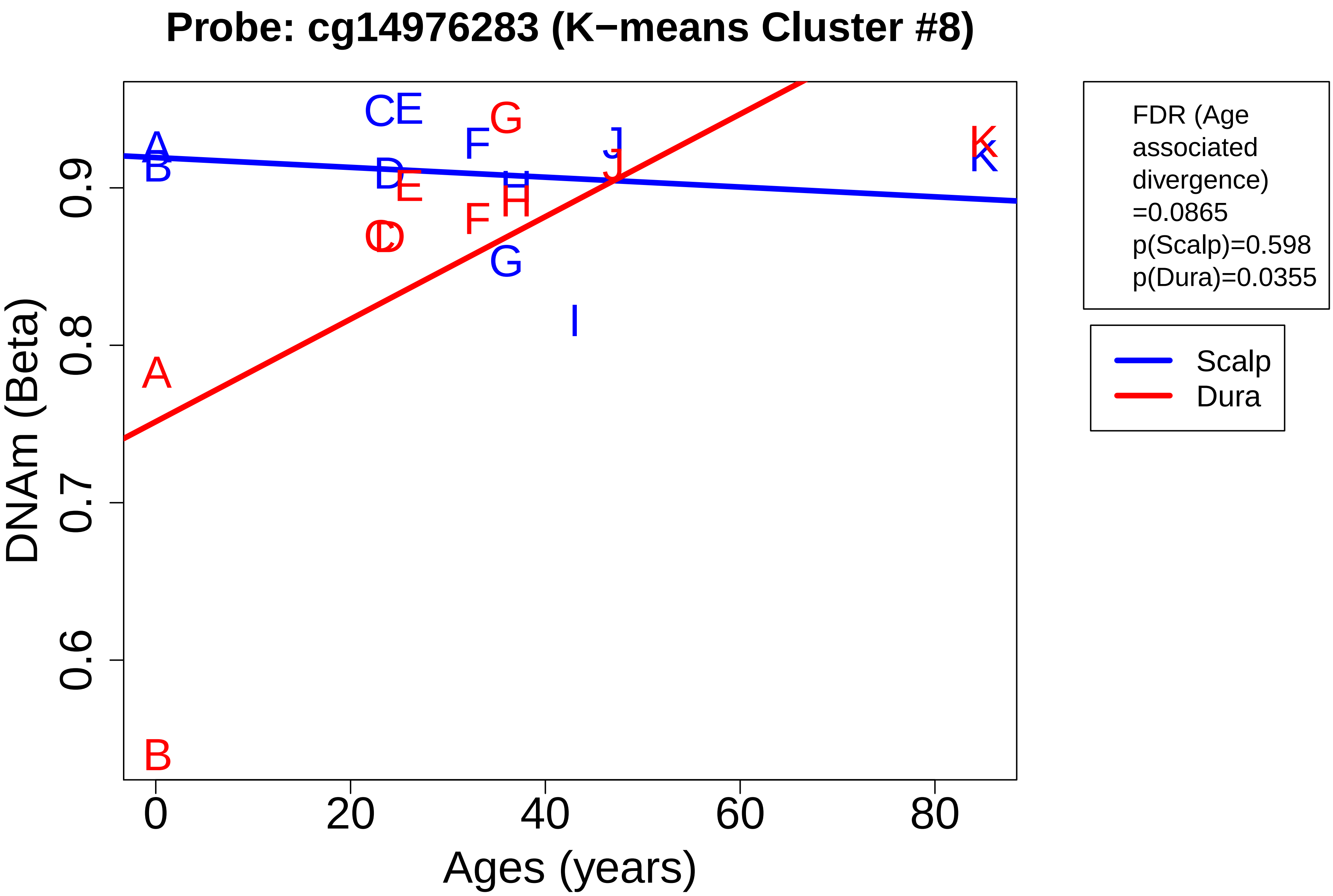

Probe: cg14976283 (K−means Cluster #8)
DNAm (Beta)
0.6
0.7
0.8
0.9
Ages (years)
0
20
40
60
80
A
B
C
D
E
F
G
H
I
J
K
FDR (Age associated divergence) =0.0865
p(Scalp)=0.598
p(Dura)=0.0355
Scalp
Dura

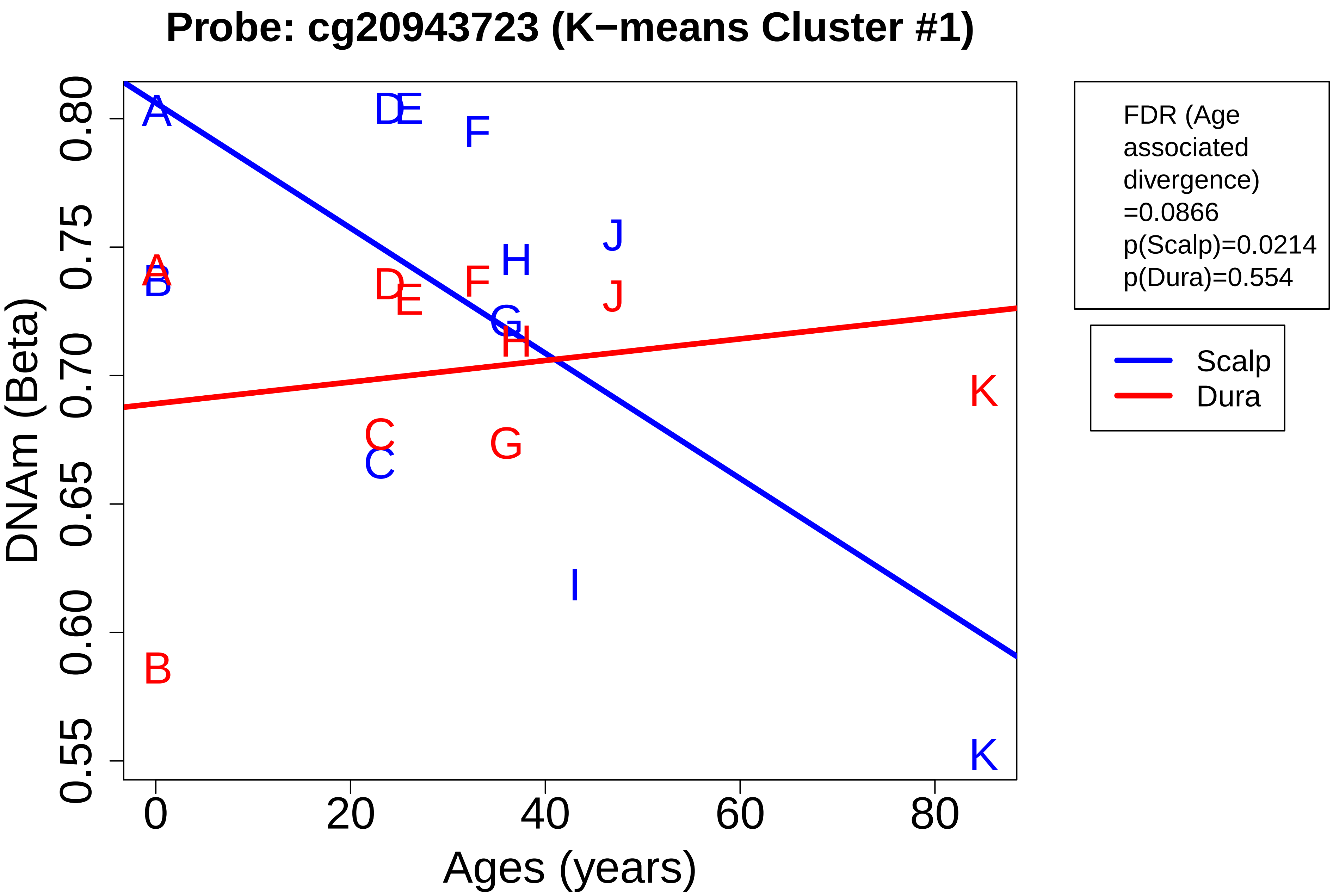

Probe: cg20943723 (K−means Cluster #1)
DNAm (Beta)
0.55
0.60
0.65
0.70
0.75
0.80
Ages (years)
0
20
40
60
80
A
B
C
D
E
F
G
H
I
J
K
FDR (Age associated divergence) =0.0866
p(Scalp)=0.0214
p(Dura)=0.554
Scalp
Dura

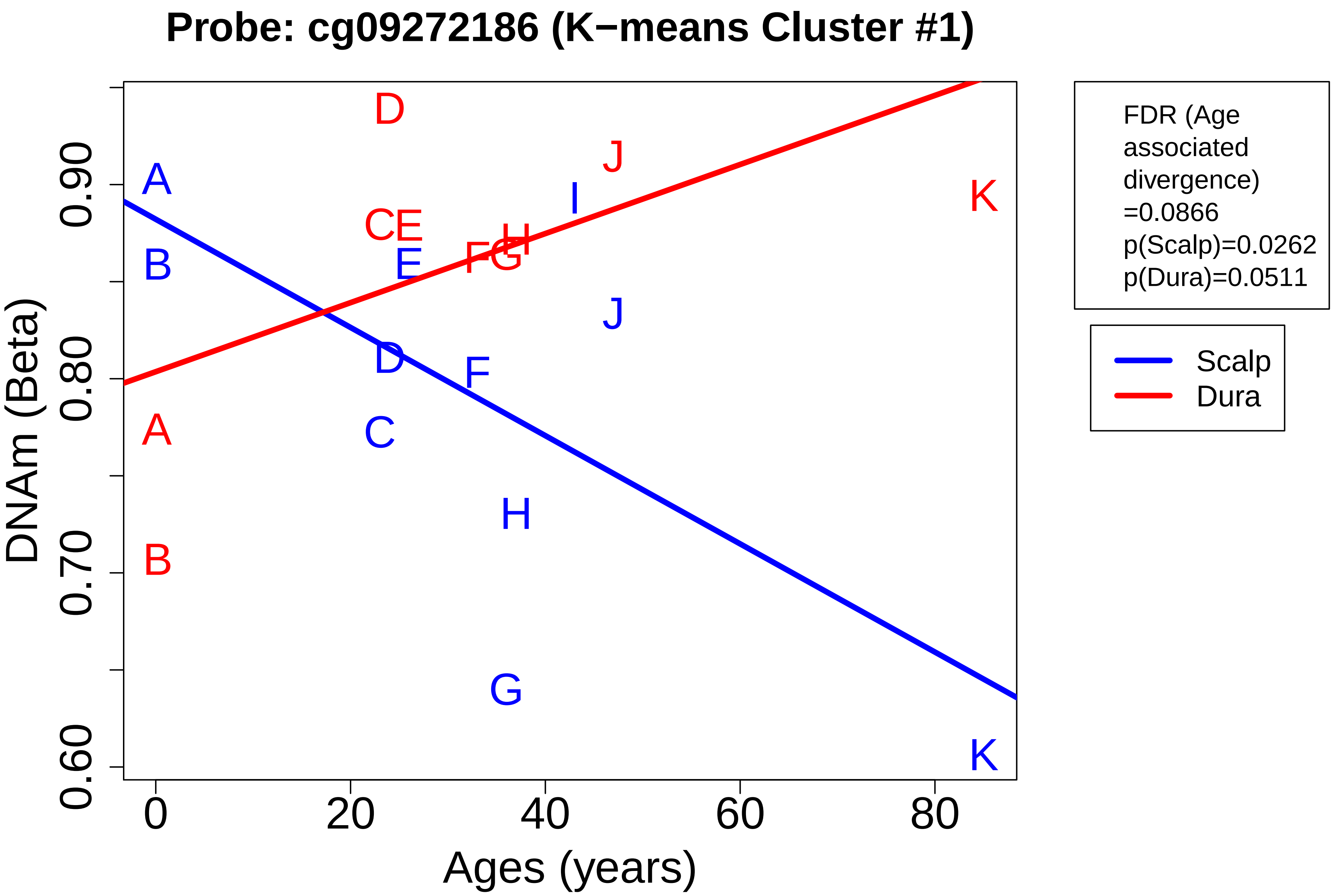

Probe: cg09272186 (K−means Cluster #1)
DNAm (Beta)
0.60
0.70
0.80
0.90
0
20
40
60
80
Ages (years)
FDR (Age associated divergence) =0.0866
p(Scalp)=0.0262
p(Dura)=0.0511
Scalp
Dura

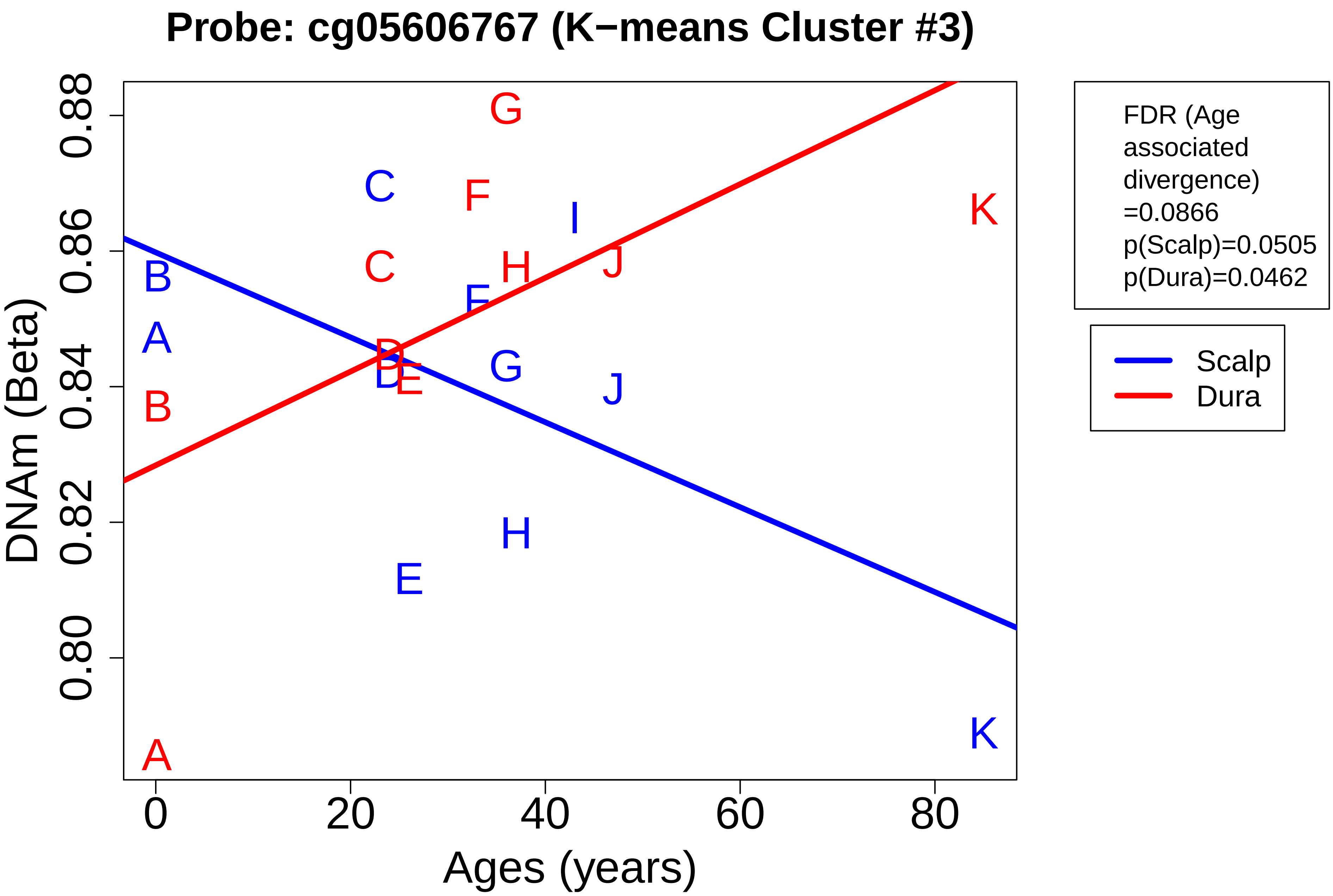

Probe: cg05606767 (K−means Cluster #3)
DNAm (Beta)
0.80
0.82
0.84
0.86
0.88
0
20
40
60
80
Ages (years)
FDR (Age associated divergence) =0.0866
p(Scalp)=0.0505
p(Dura)=0.0462
Scalp
Dura
A
B
C
D
E
F
G
H
I
J
K

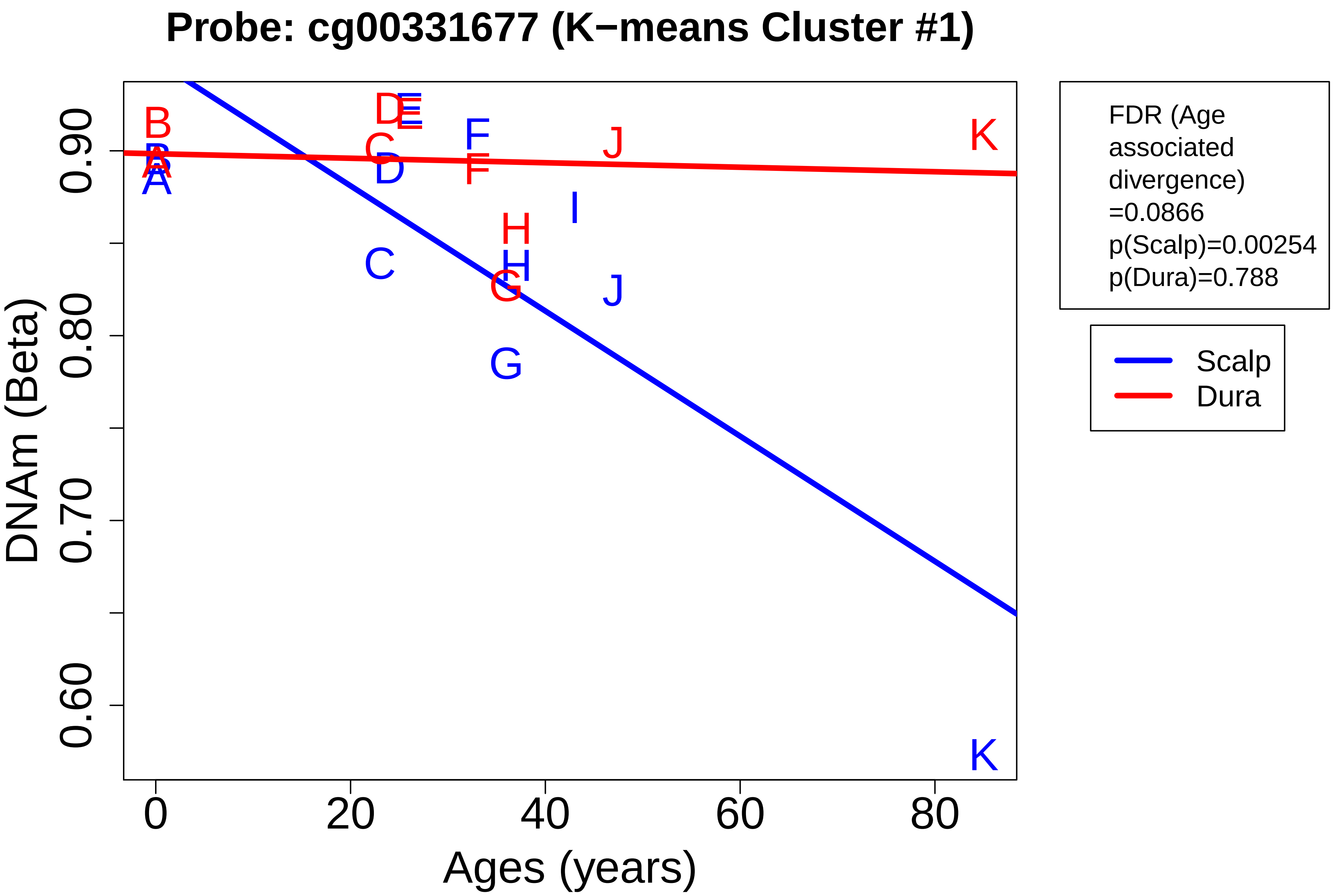
Probe: cg00331677 (K−means Cluster #1)
DNAm (Beta)
0.60
0.70
0.80
0.90
0
20
40
60
80
Ages (years)
FDR (Age associated divergence) =0.0866
p(Scalp)=0.00254
p(Dura)=0.788
Scalp
Dura

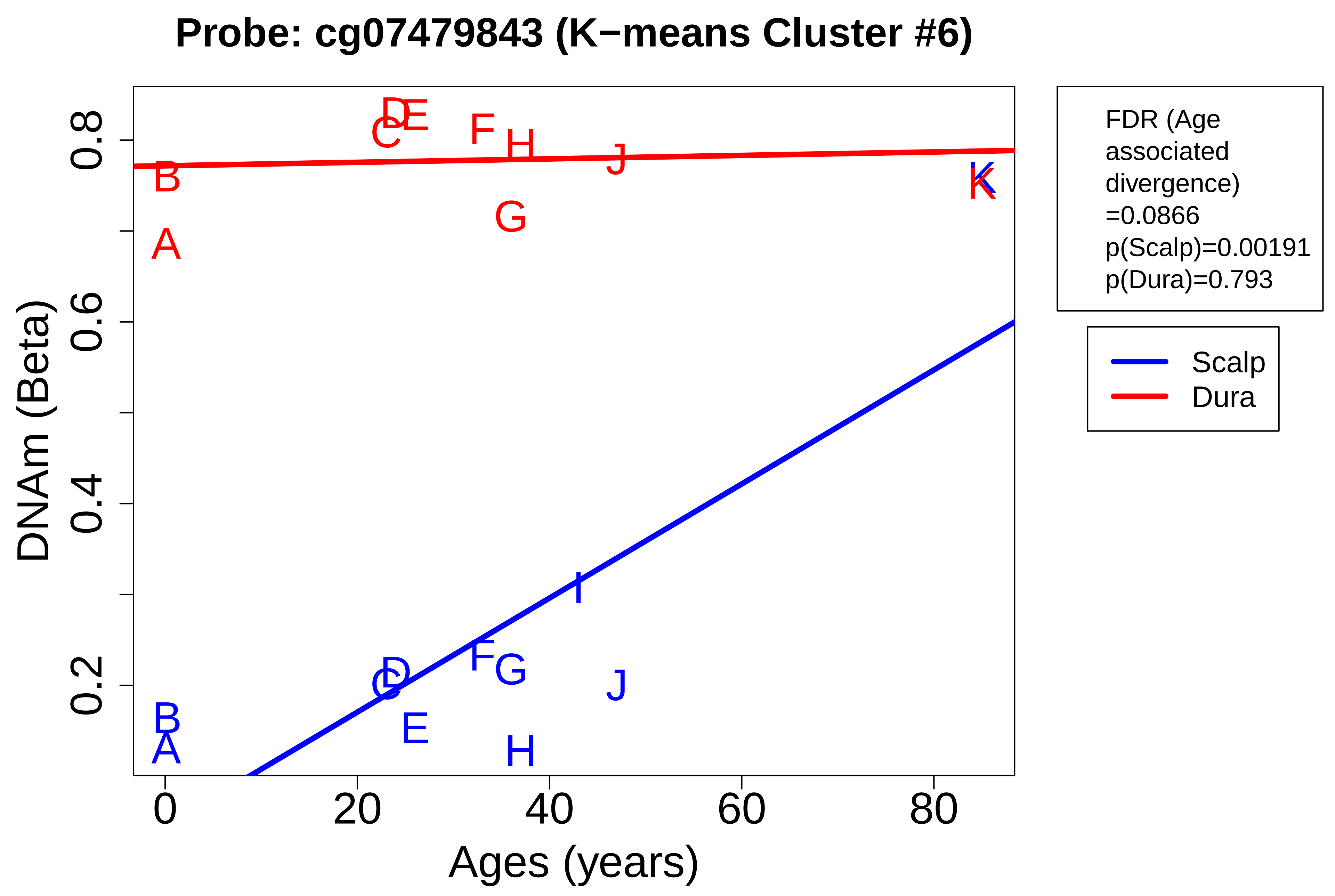

Probe: cg07479843 (K−means Cluster #6)
DNAm (Beta)
0.2
0.4
0.6
0.8
0
20
40
60
80
Ages (years)
FDR (Age associated divergence) =0.0866
p(Scalp)=0.00191
p(Dura)=0.793
Scalp
Dura
A
B
C
D
E
F
G
H
I
J
K

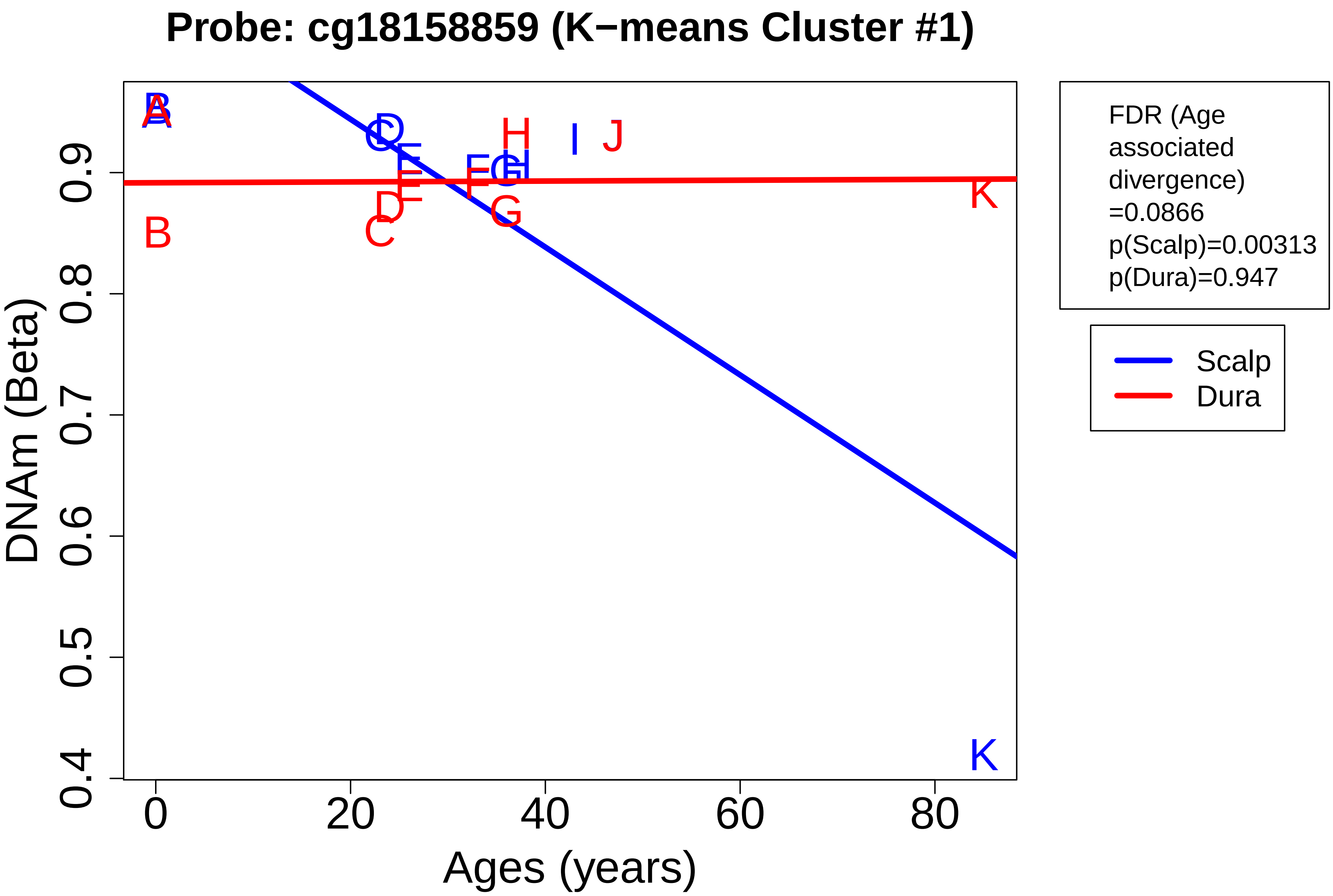

Probe: cg18158859 (K−means Cluster #1)
DNAm (Beta)
0.4
0.5
0.6
0.7
0.8
0.9
0
20
40
60
80
Ages (years)
FDR (Age associated divergence) =0.0866
p(Scalp)=0.00313
p(Dura)=0.947
Scalp
Dura

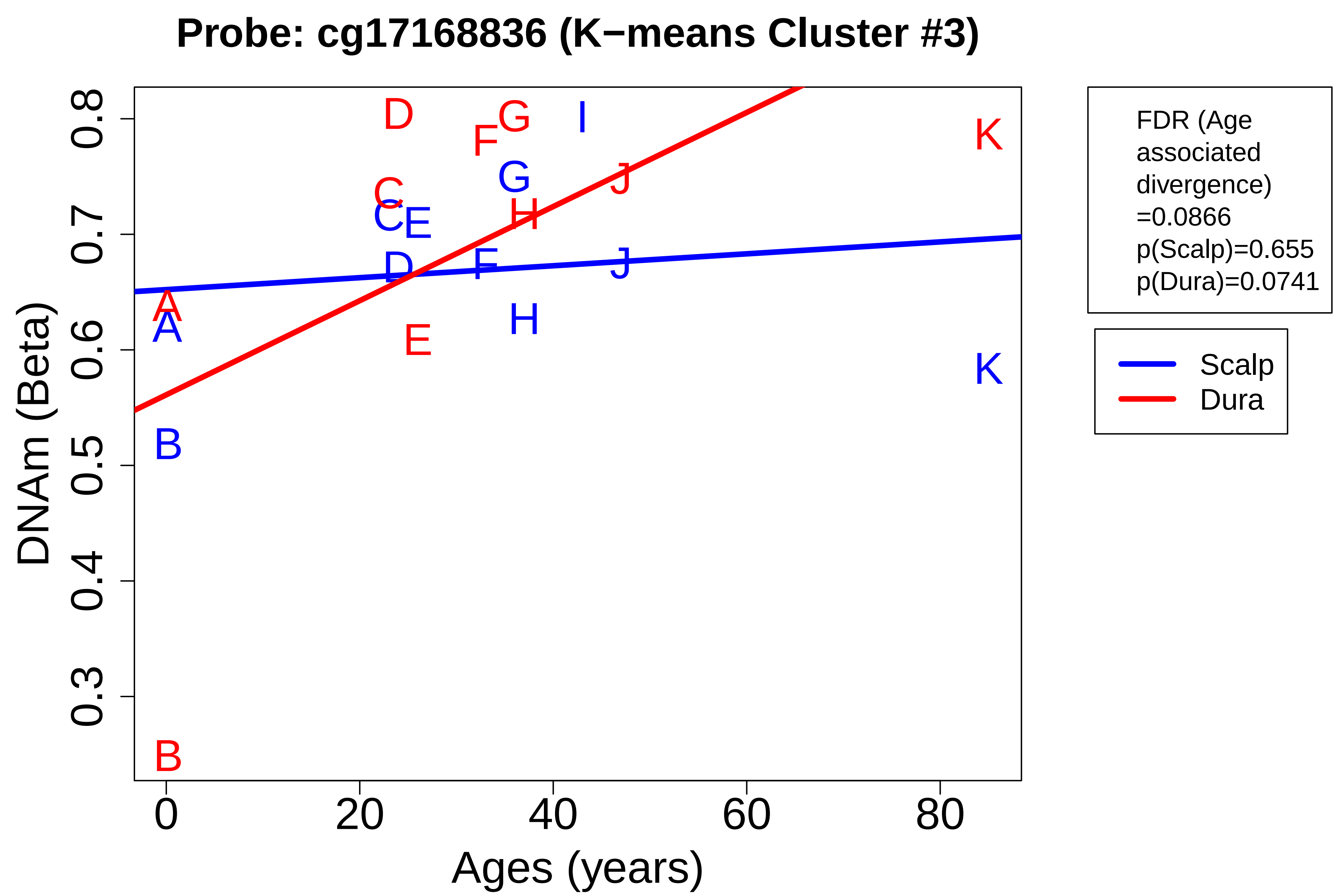
Probe: cg17168836 (K−means Cluster #3)
DNAm (Beta)
0.3
0.4
0.5
0.6
0.7
0.8
0
20
40
60
80
Ages (years)
FDR (Age associated divergence) =0.0866
p(Scalp)=0.655
p(Dura)=0.0741
Scalp
Dura
A
B
C
D
E
F
G
H
I
J
K

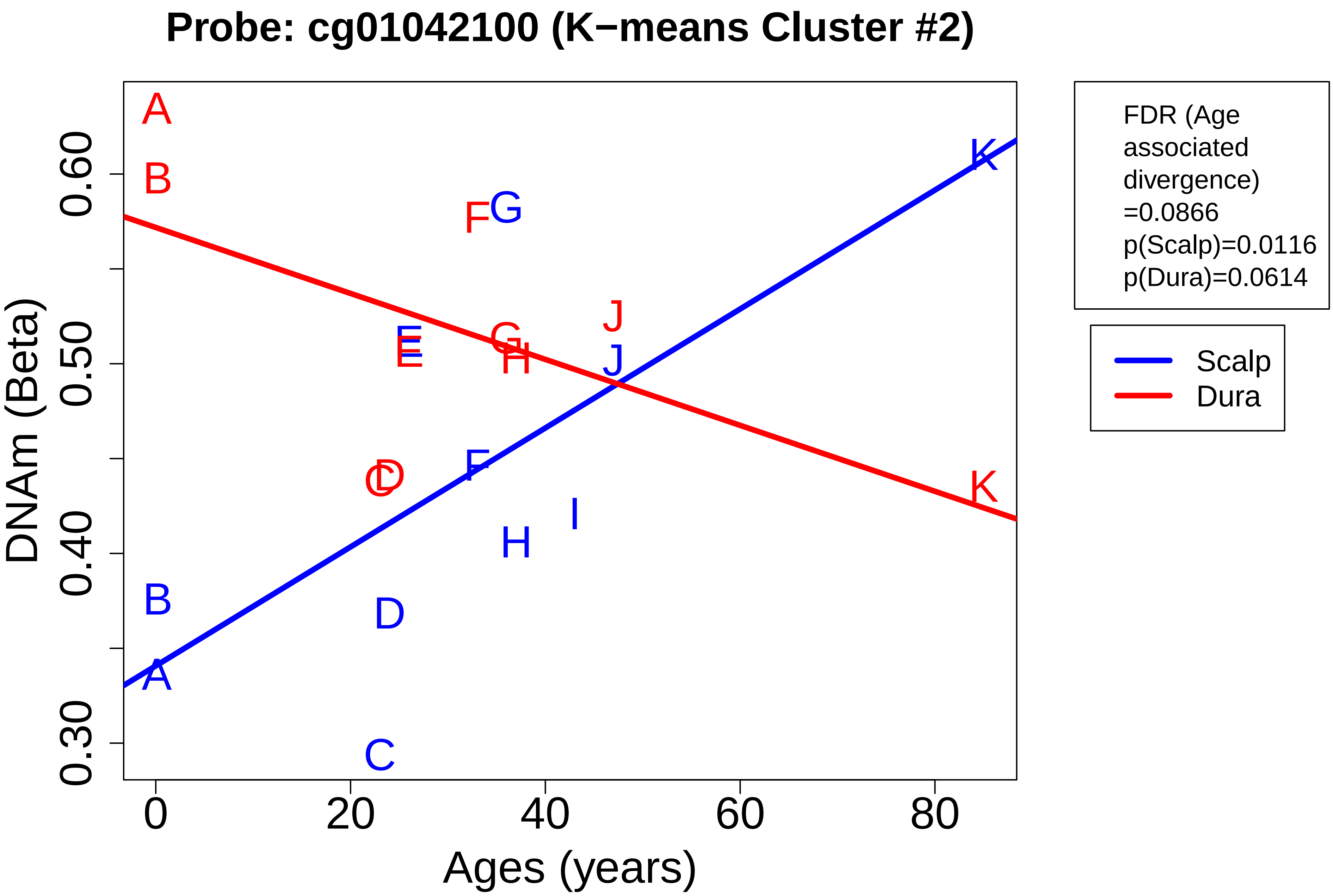

Probe: cg01042100 (K−means Cluster #2)
DNAm (Beta)
0.30
0.40
0.50
0.60
Ages (years)
0
20
40
60
80
A
B
C
D
E
F
G
H
I
J
K
FDR (Age associated divergence) =0.0866
p(Scalp)=0.0116
p(Dura)=0.0614
Scalp
Dura

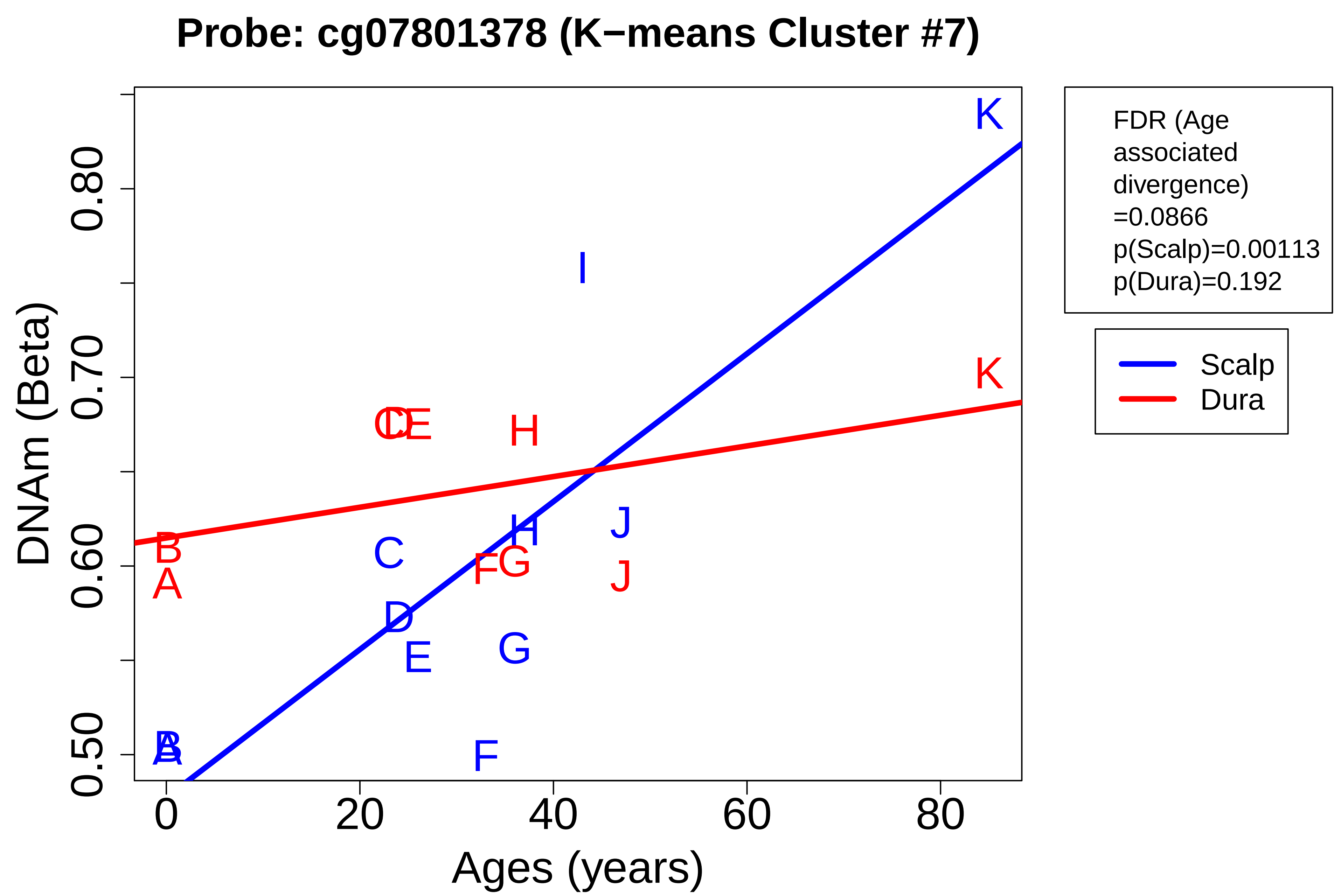

Probe: cg07801378 (K−means Cluster #7)
DNAm (Beta)
0.50
0.60
0.70
0.80
0
20
40
60
80
Ages (years)
FDR (Age associated divergence) =0.0866
p(Scalp)=0.00113
p(Dura)=0.192
Scalp
Dura

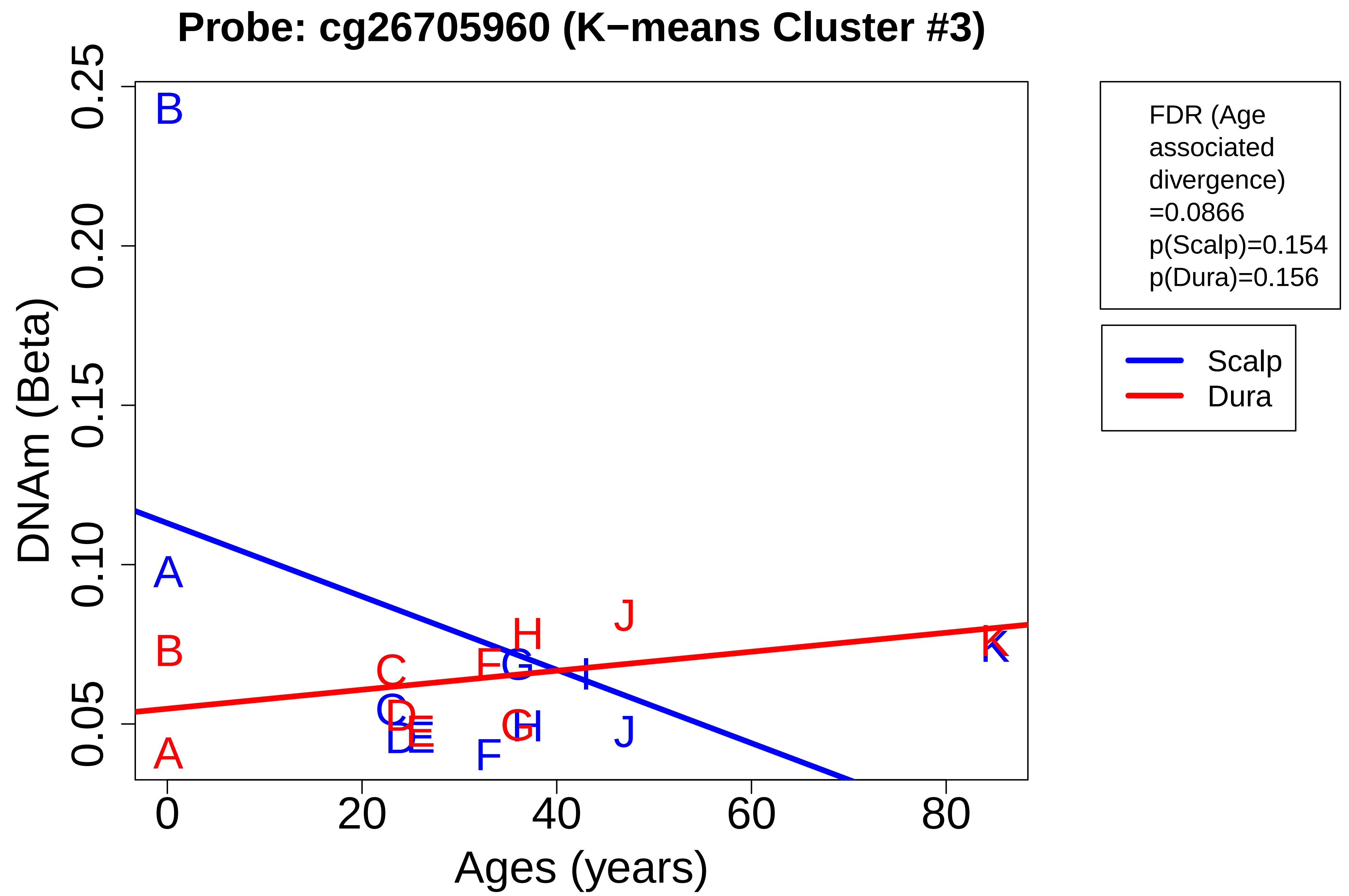

Probe: cg26705960 (K−means Cluster #3)
DNAm (Beta)
0.05
0.10
0.15
0.20
0.25
Ages (years)
0
20
40
60
80
FDR (Age associated divergence) =0.0866
p(Scalp)=0.154
p(Dura)=0.156
Scalp
Dura

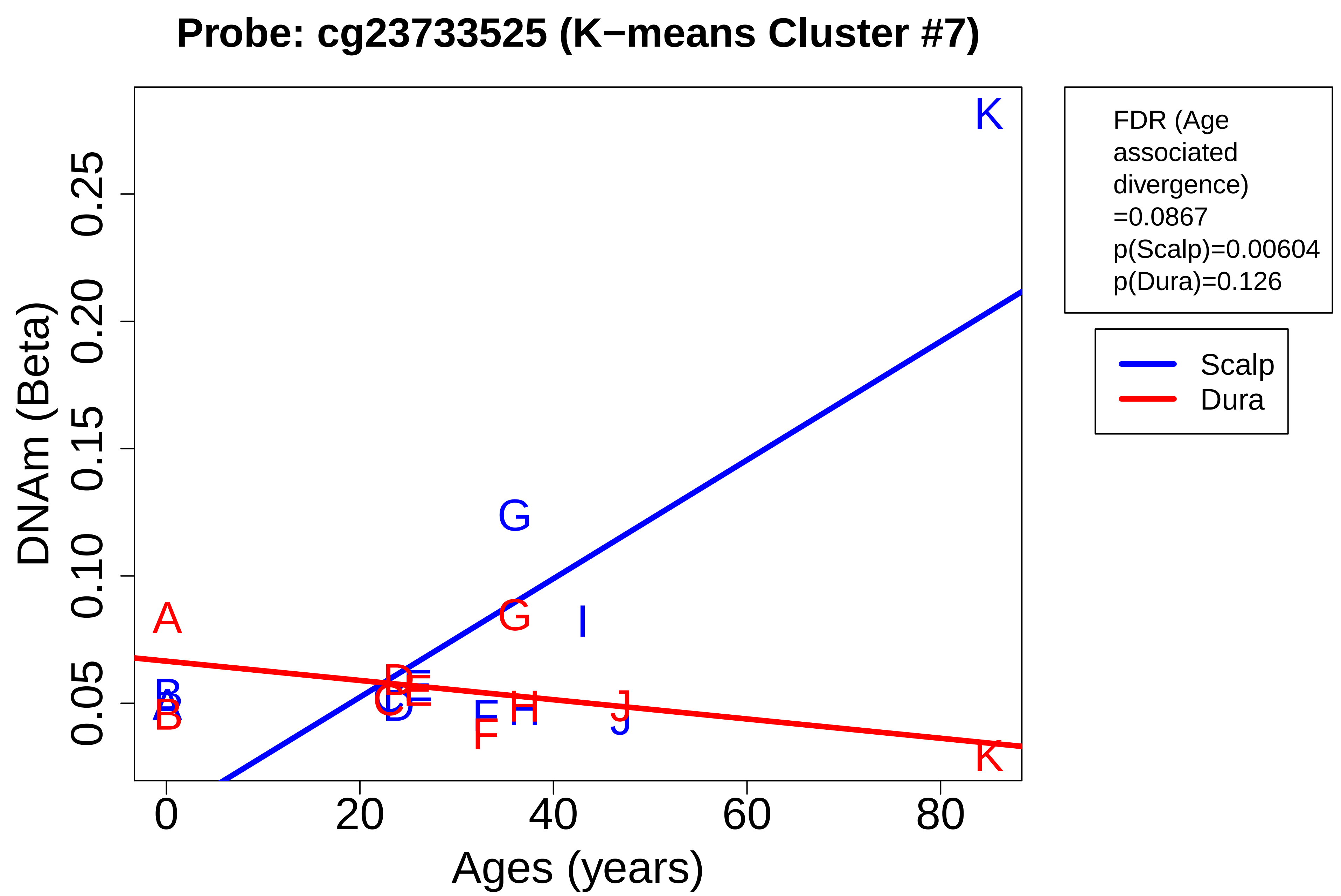

Probe: cg23733525 (K−means Cluster #7)
FDR (Age associated divergence) =0.0867
p(Scalp)=0.00604
p(Dura)=0.126
Scalp
Dura
DNAm (Beta)
0.05
0.10
0.15
0.20
0.25
0
20
40
60
80
Ages (years)

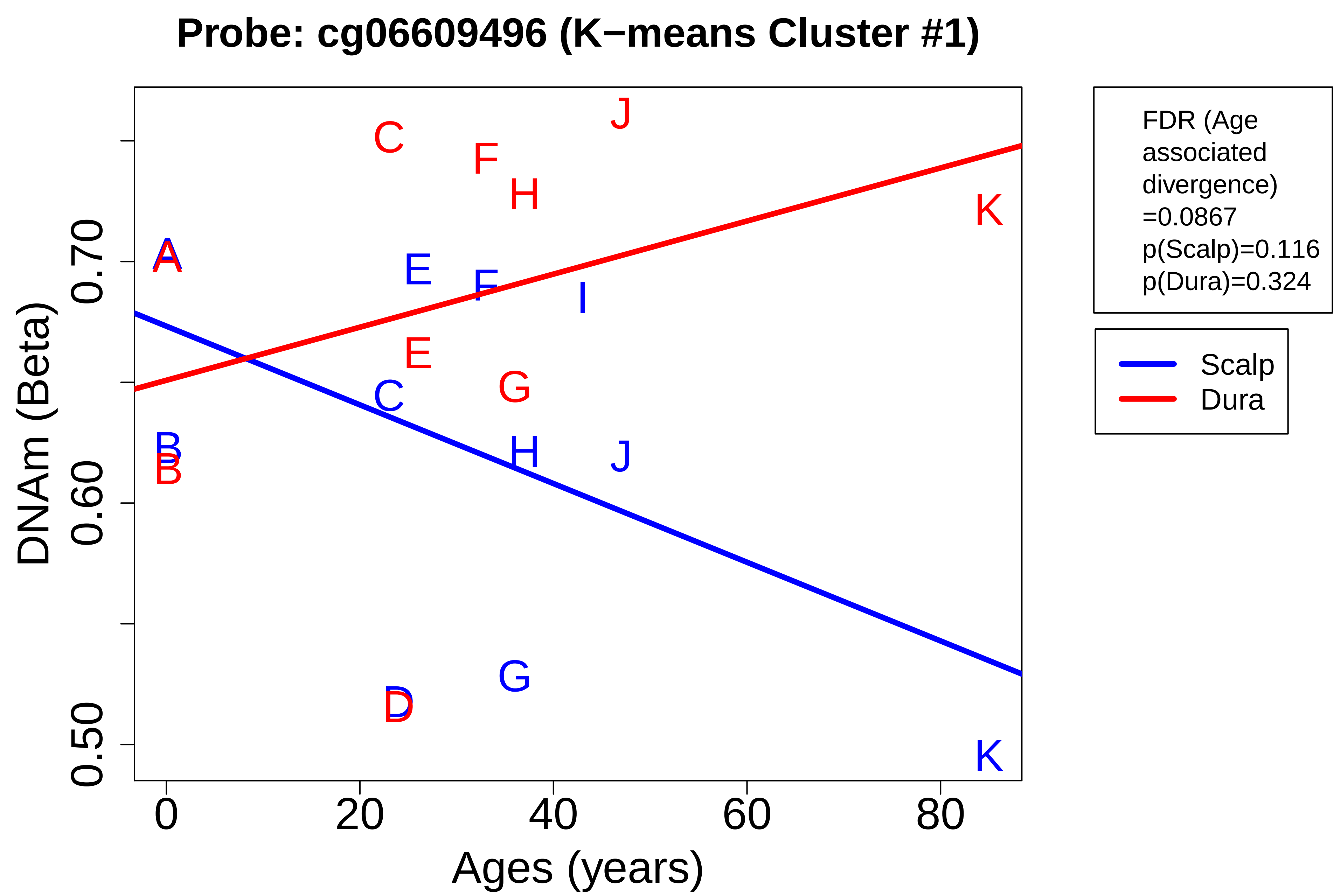

Probe: cg06609496 (K−means Cluster #1)
DNAm (Beta)
0.50
0.60
0.70
0
20
40
60
80
Ages (years)
FDR (Age
associated
divergence)
=0.0867
p(Scalp)=0.116
p(Dura)=0.324
Scalp
Dura

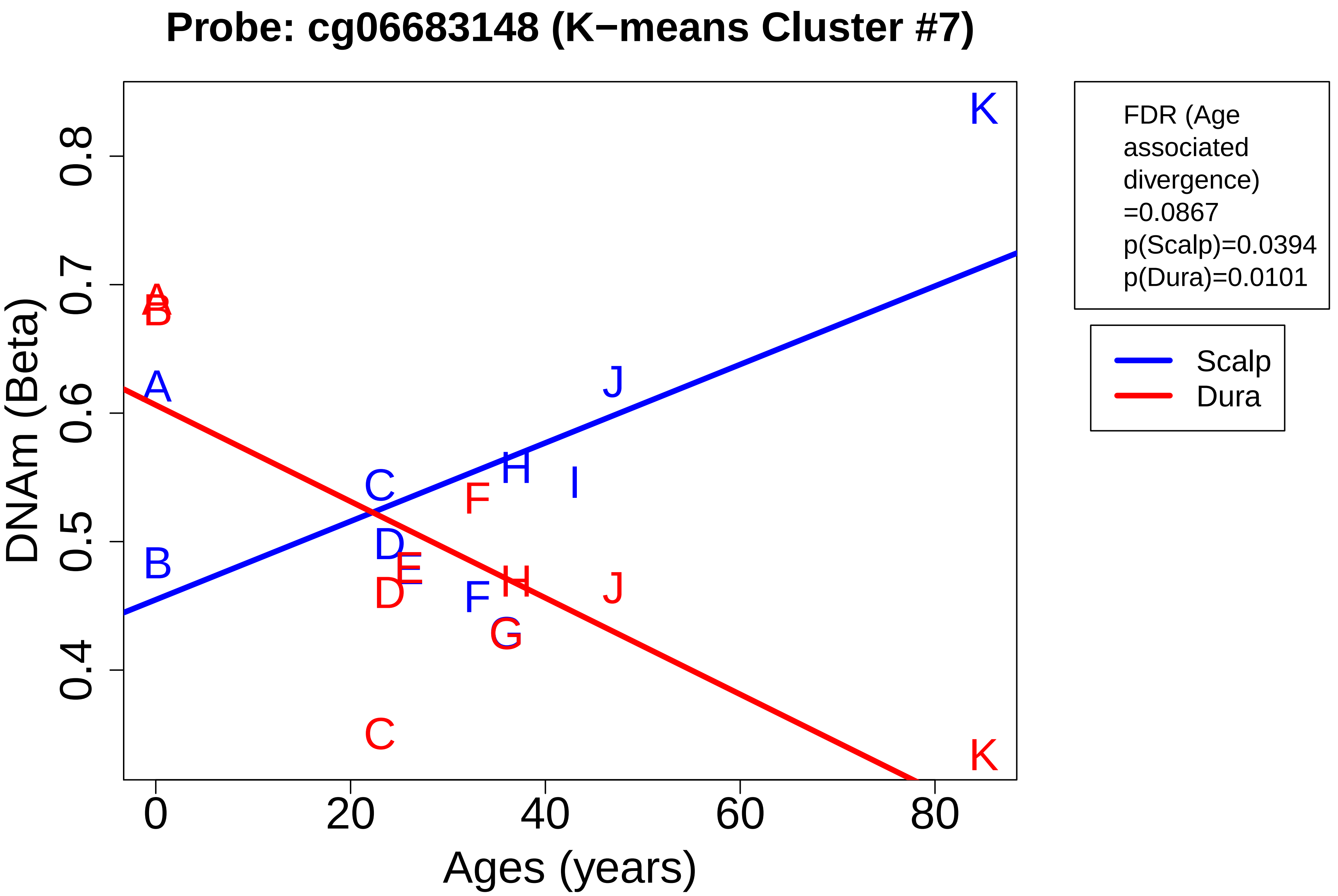

Probe: cg06683148 (K−means Cluster #7)
FDR (Age associated divergence) =0.0867
p(Scalp)=0.0394
p(Dura)=0.0101
Scalp
Dura
DNAm (Beta)
0.4
0.5
0.6
0.7
0.8
Ages (years)
0
20
40
60
80
A
B
C
D
E
F
G
H
I
J
K

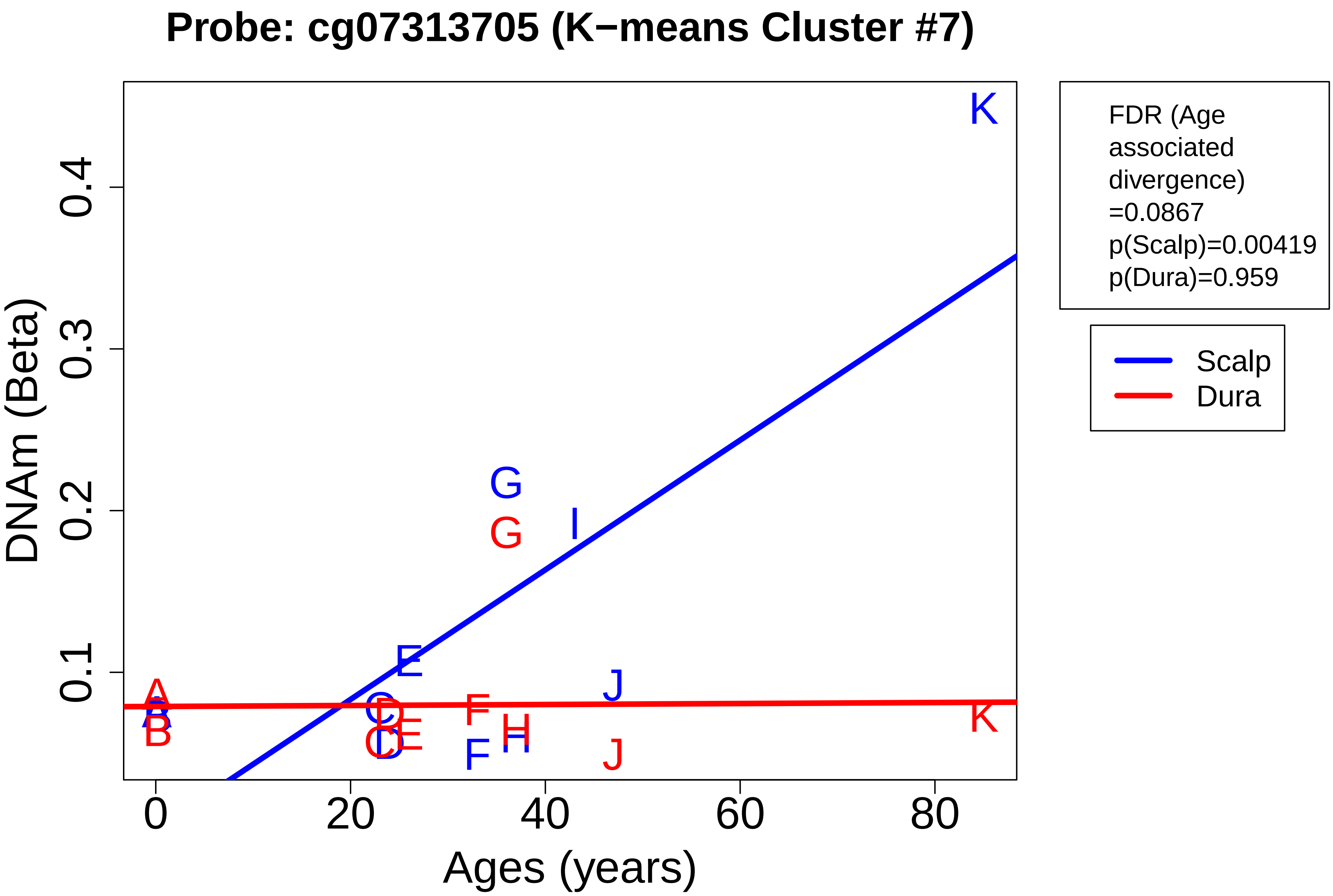

Probe: cg07313705 (K−means Cluster #7)
DNAm (Beta)
0.1
0.2
0.3
0.4
Ages (years)
0
20
40
60
80
FDR (Age associated divergence) =0.0867
p(Scalp)=0.00419
p(Dura)=0.959
Scalp
Dura

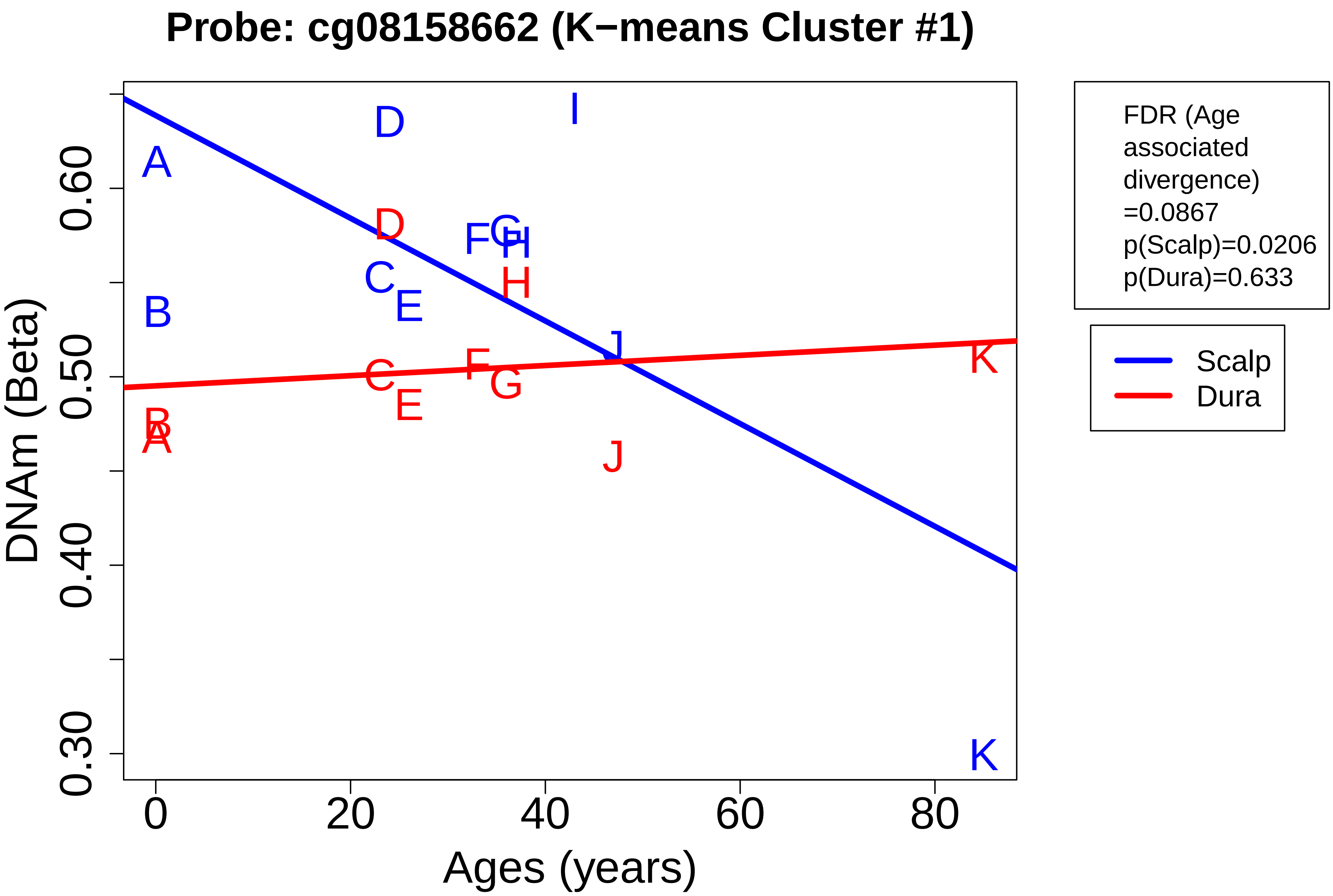

Probe: cg08158662 (K−means Cluster #1)
DNAm (Beta)
0.30
0.40
0.50
0.60
Ages (years)
0
20
40
60
80
A
B
C
D
E
F
G
H
I
J
K
FDR (Age associated divergence) =0.0867
p(Scalp)=0.0206
p(Dura)=0.633
Scalp
Dura

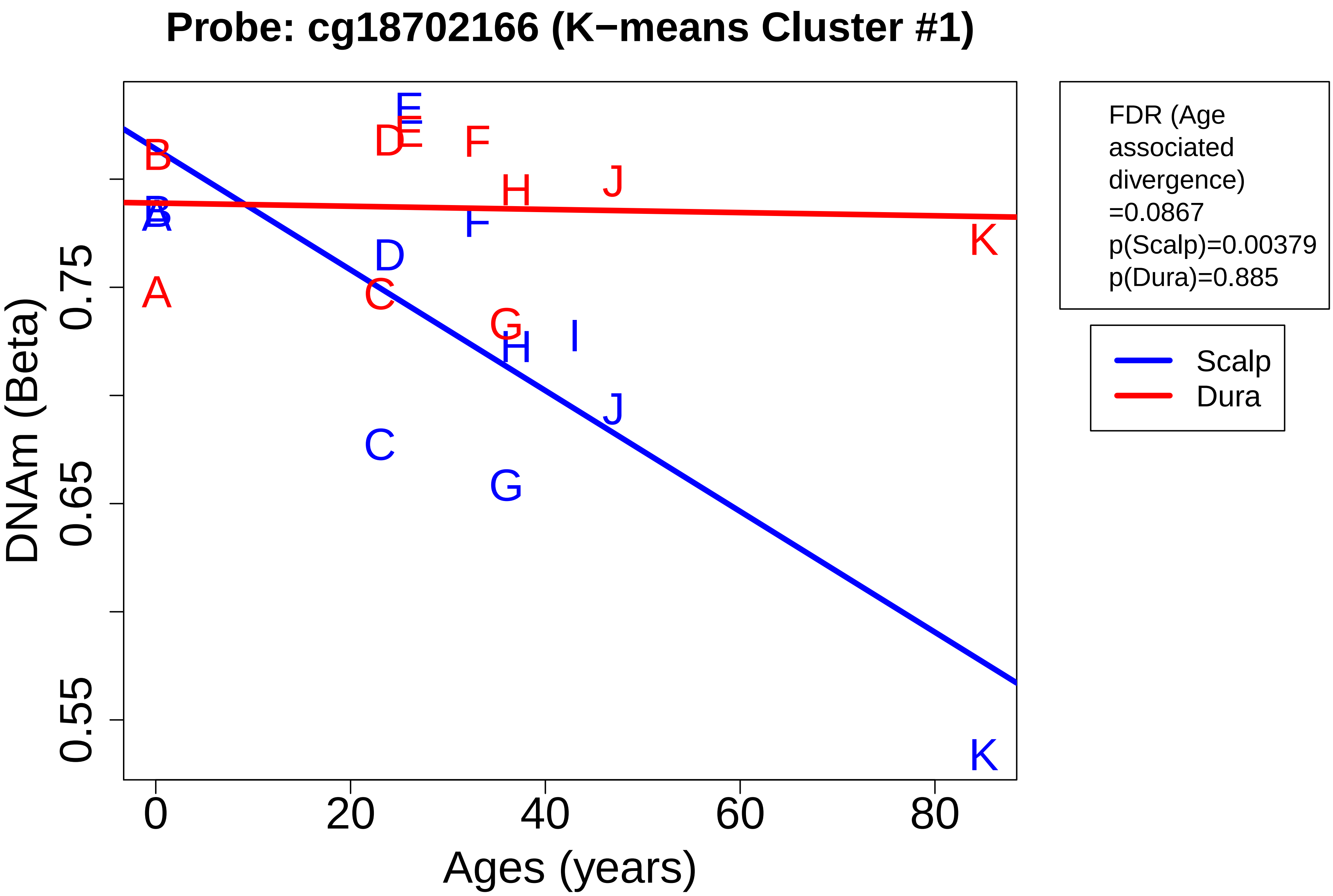

Probe: cg18702166 (K−means Cluster #1)
DNAm (Beta)
0.55
0.65
0.75
0
20
40
60
80
Ages (years)
FDR (Age associated divergence) =0.0867
p(Scalp)=0.00379
p(Dura)=0.885
Scalp
Dura

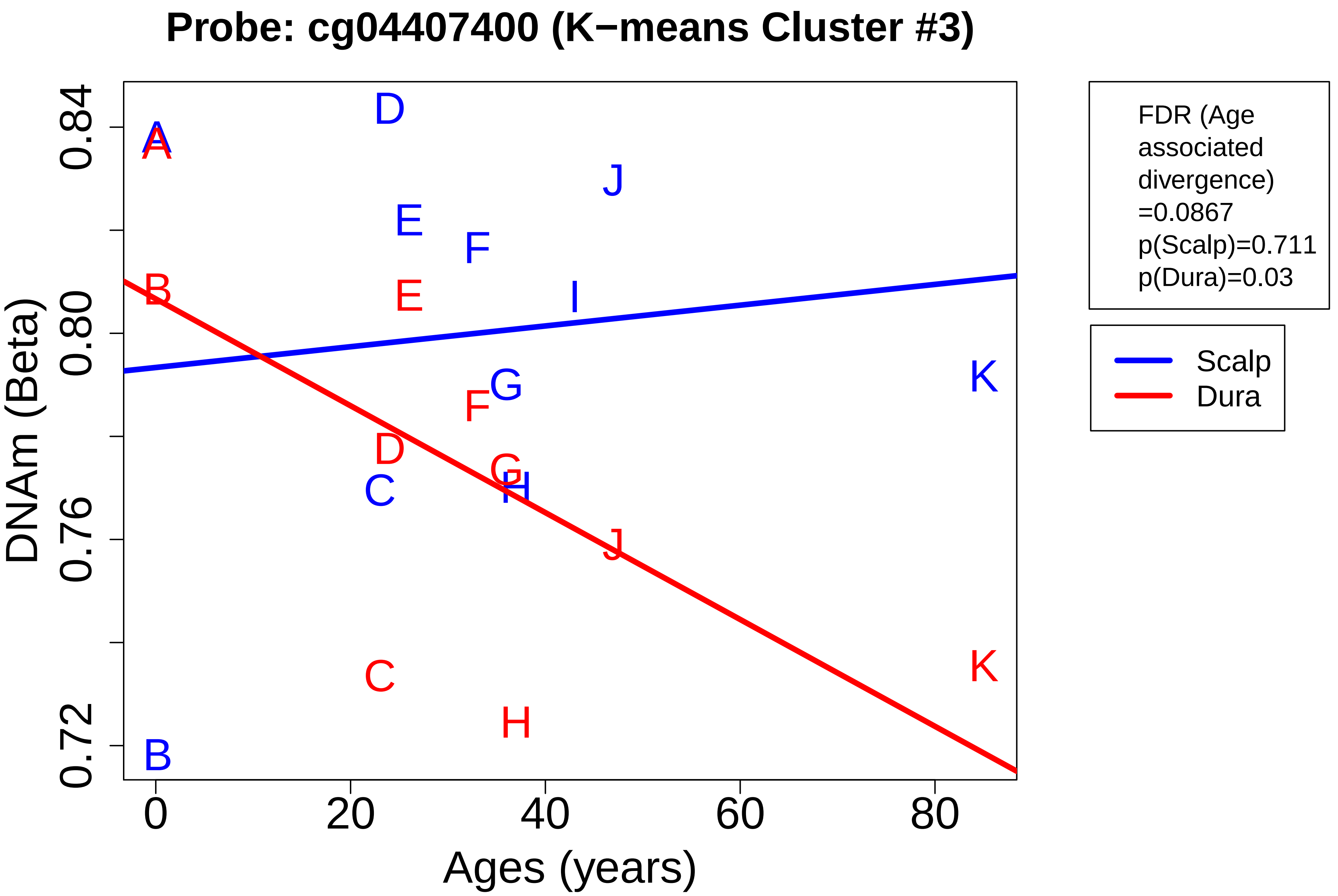

Probe: cg04407400 (K−means Cluster #3)
DNAm (Beta)
0.72
0.76
0.80
0.84
0
20
40
60
80
Ages (years)
FDR (Age associated divergence) =0.0867
p(Scalp)=0.711
p(Dura)=0.03
Scalp
Dura

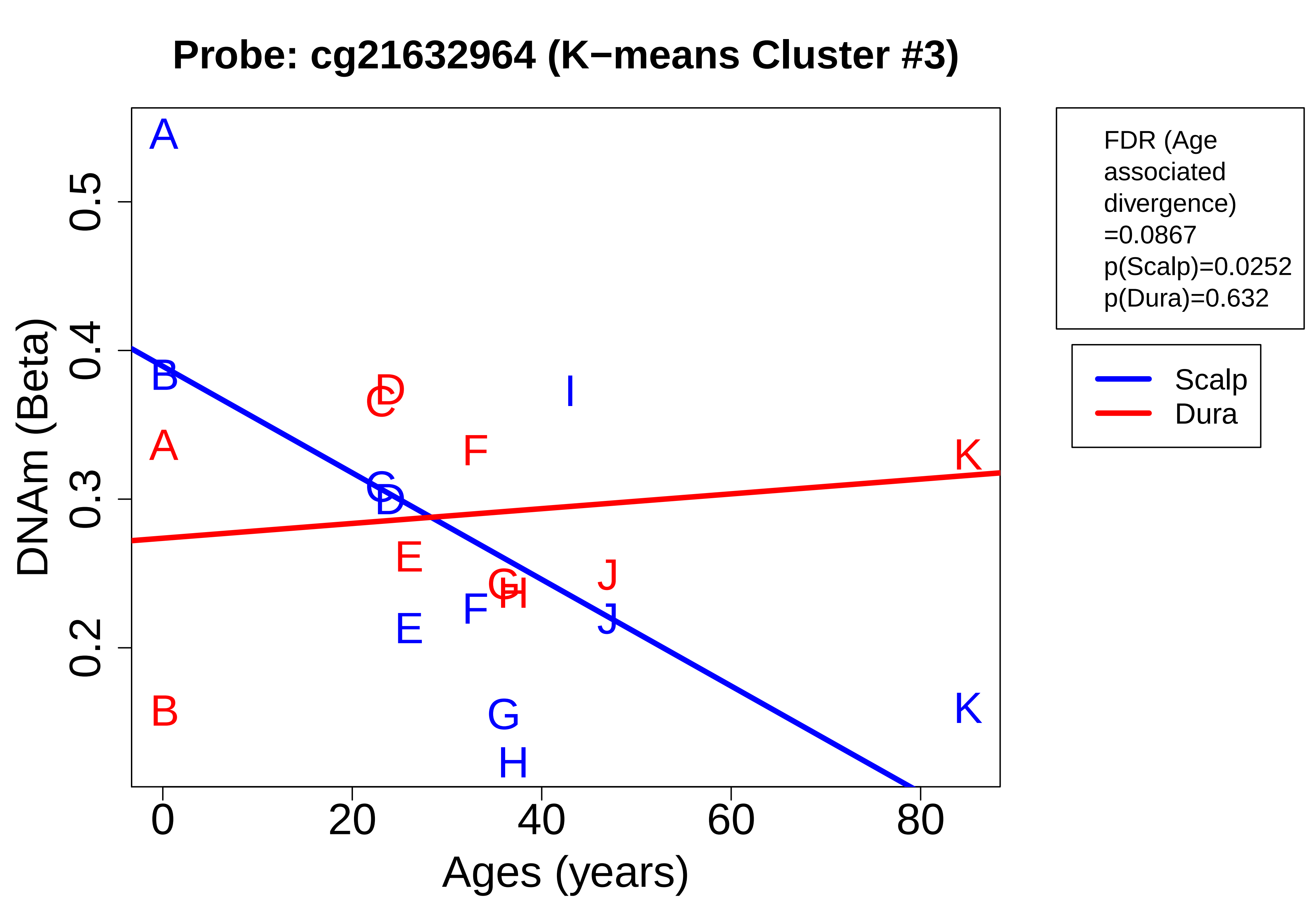

Probe: cg21632964 (K−means Cluster #3)
FDR (Age associated divergence) =0.0867
p(Scalp)=0.0252
p(Dura)=0.632
Scalp
Dura
DNAm (Beta)
0.2
0.3
0.4
0.5
0
20
40
60
80
Ages (years)
A
B
C
D
E
F
G
H
I
J
K

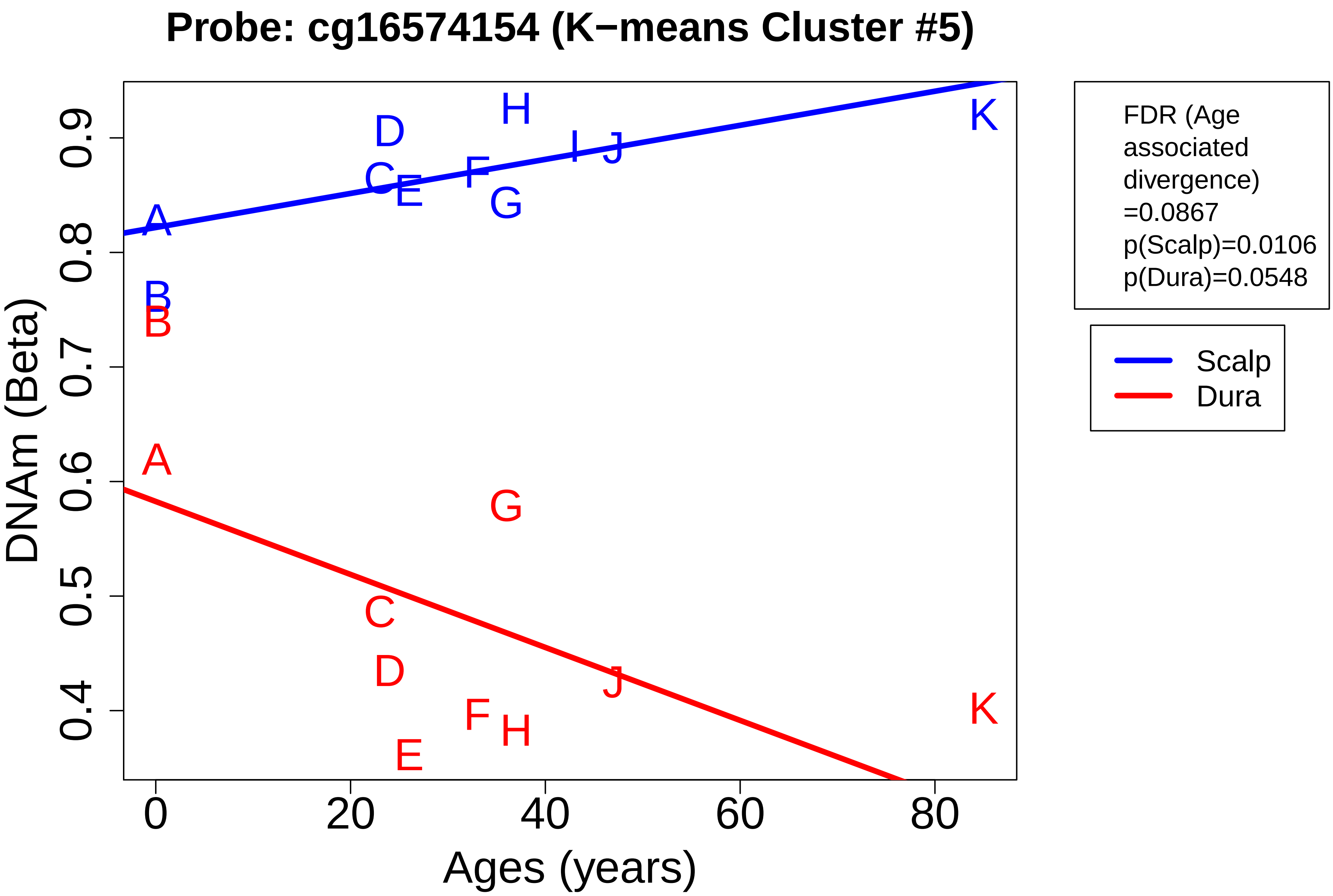

Probe: cg16574154 (K−means Cluster #5)
DNAm (Beta)
0.4
0.5
0.6
0.7
0.8
0.9
0
20
40
60
80
Ages (years)
A
B
C
D
E
F
G
H
I
J
K
FDR (Age associated divergence) =0.0867
p(Scalp)=0.0106
p(Dura)=0.0548
Scalp
Dura

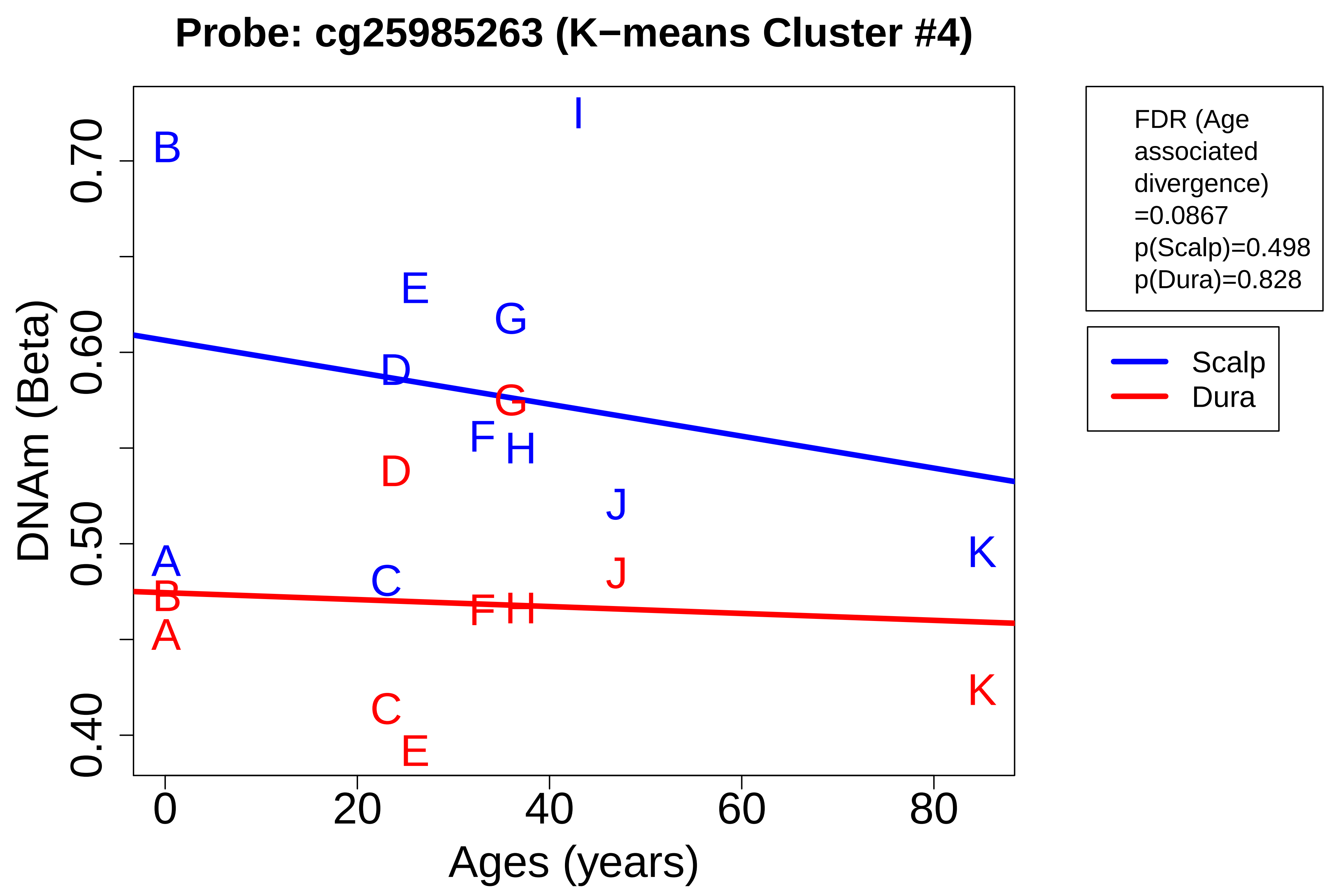

Probe: cg25985263 (K−means Cluster #4)
DNAm (Beta)
0.40
0.50
0.60
0.70
0
20
40
60
80
Ages (years)
FDR (Age associated divergence) =0.0867
p(Scalp)=0.498
p(Dura)=0.828
Scalp
Dura
A
B
C
D
E
F
G
H
I
J
K
A
B
C
D
E
F
G
H
J
K

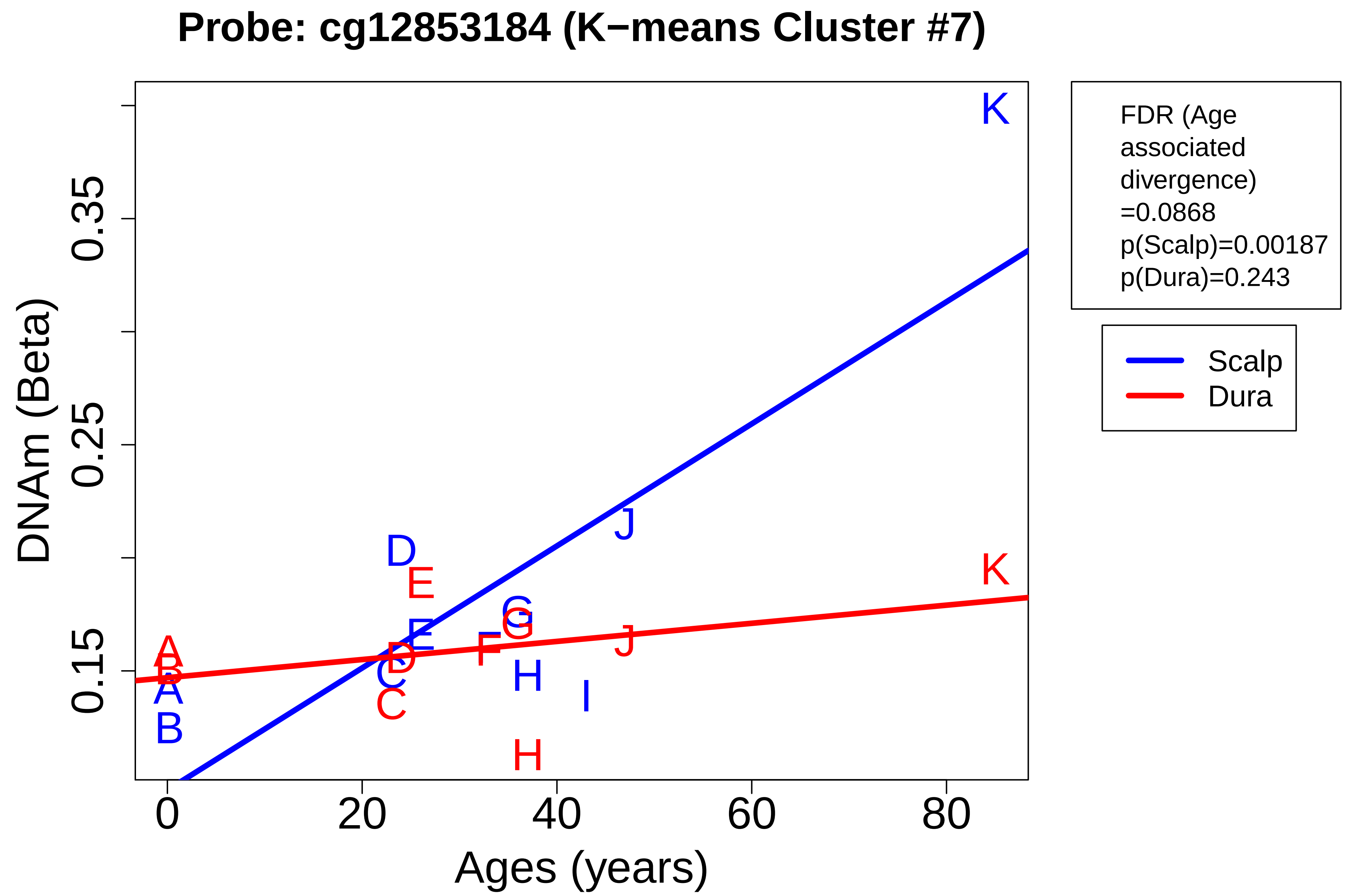
Probe: cg12853184 (K−means Cluster #7)
DNAm (Beta)
0.15
0.25
0.35
0
20
40
60
80
Ages (years)
A
B
C
D
E
F
G
H
I
J
K
FDR (Age associated divergence) =0.0868
p(Scalp)=0.00187
p(Dura)=0.243
Scalp
Dura

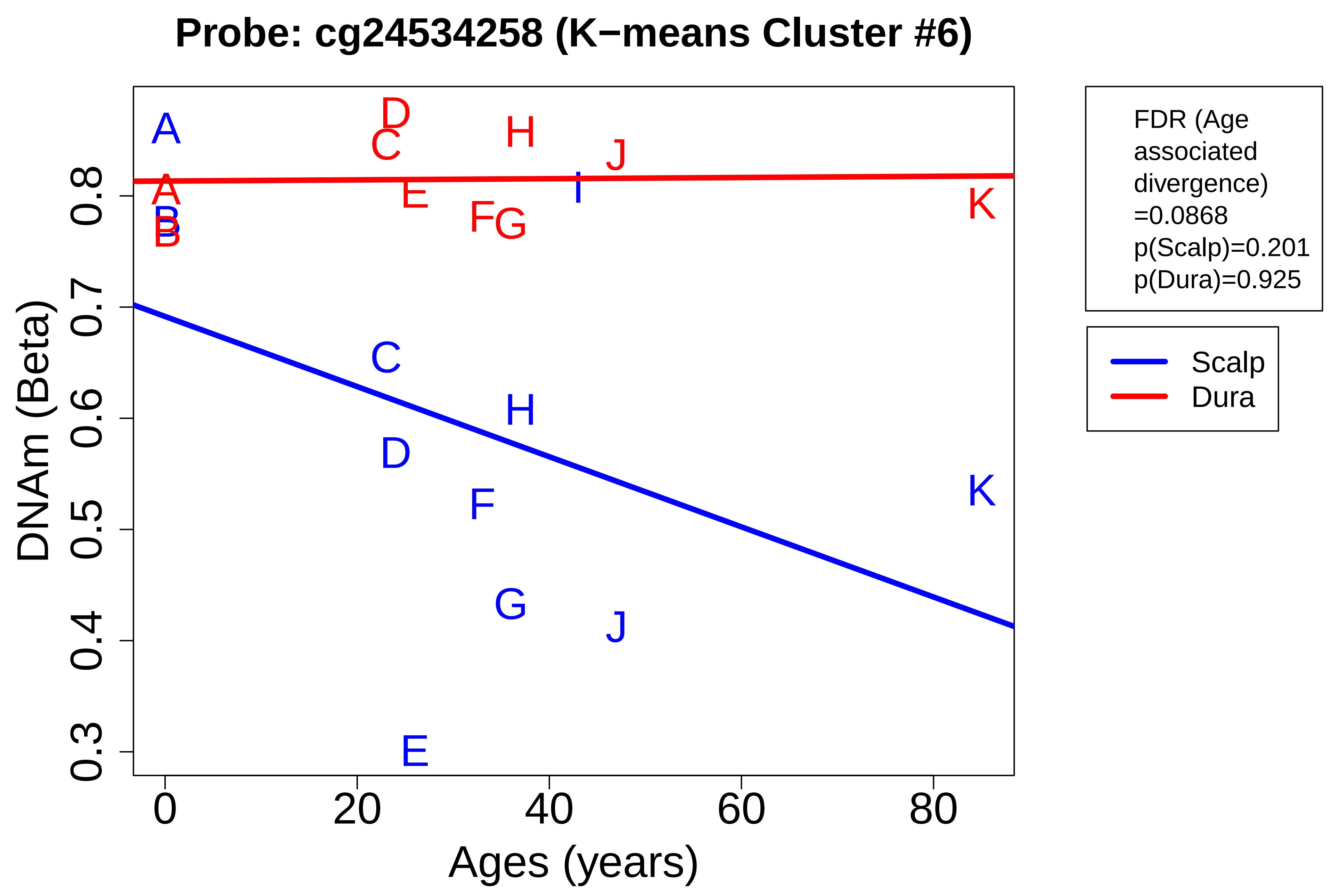
Probe: cg24534258 (K−means Cluster #6)
DNAm (Beta)
0.3
0.4
0.5
0.6
0.7
0.8
0
20
40
60
80
Ages (years)
FDR (Age associated divergence) =0.0868
p(Scalp)=0.201
p(Dura)=0.925
Scalp
Dura

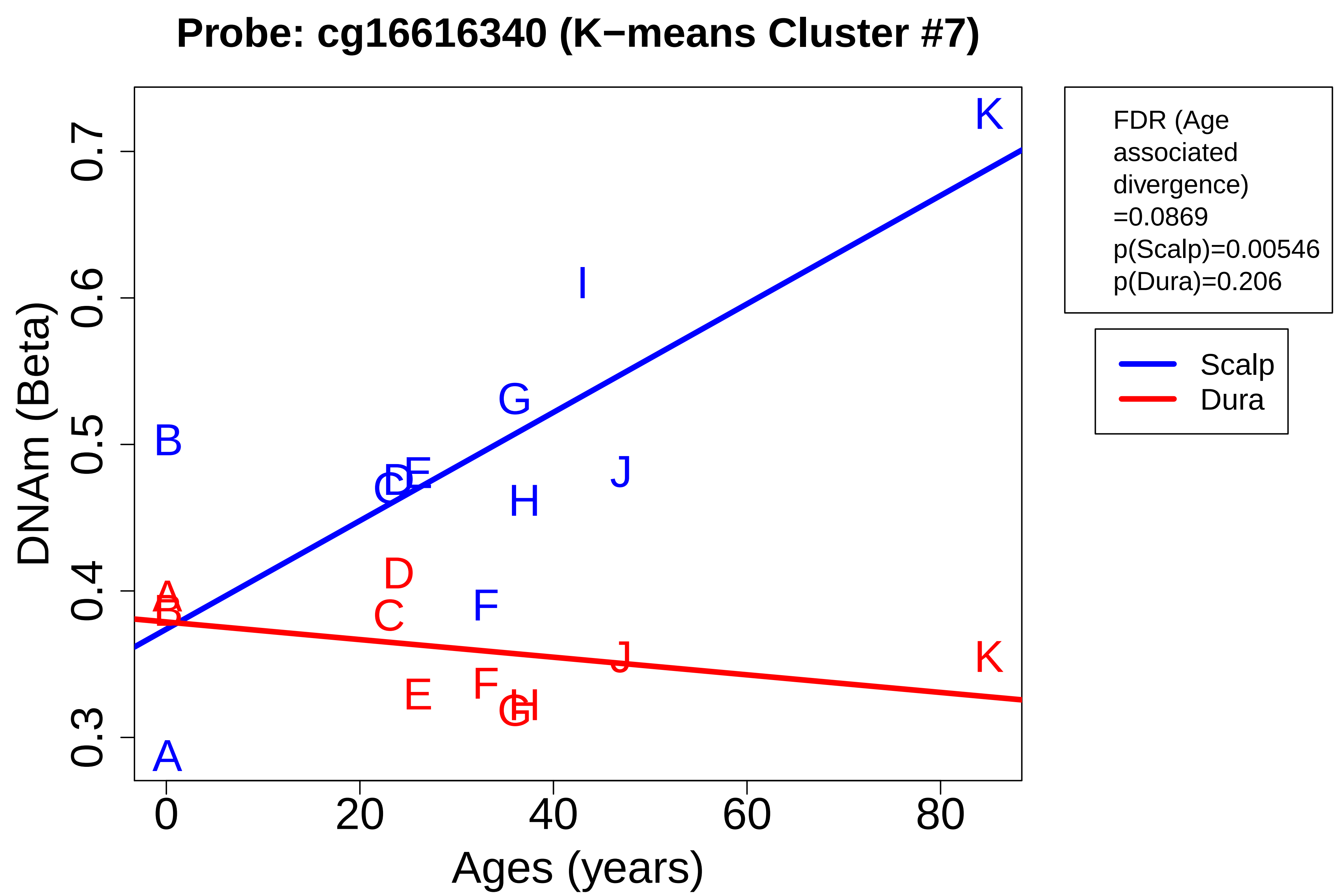
Probe: cg16616340 (K−means Cluster #7)
DNAm (Beta)
Ages (years)
0.3
0.4
0.5
0.6
0.7
0
20
40
60
80
FDR (Age associated divergence) =0.0869
p(Scalp)=0.00546
p(Dura)=0.206
Scalp
Dura

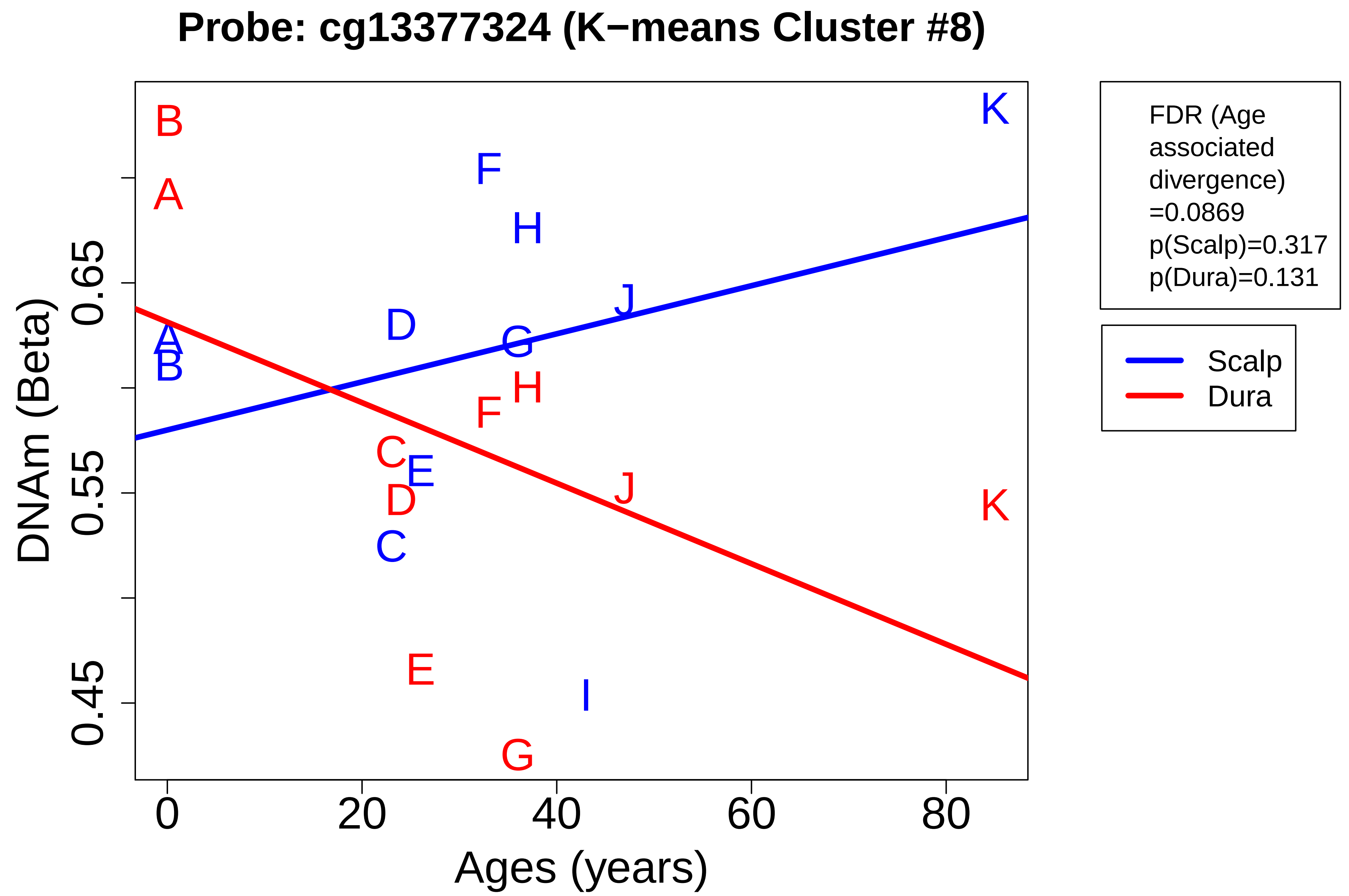

Probe: cg13377324 (K−means Cluster #8)
DNAm (Beta)
0.45
0.55
0.65
Ages (years)
0
20
40
60
80
FDR (Age associated divergence) =0.0869
p(Scalp)=0.317
p(Dura)=0.131
Scalp
Dura
A
B
C
D
E
F
G
H
I
J
K

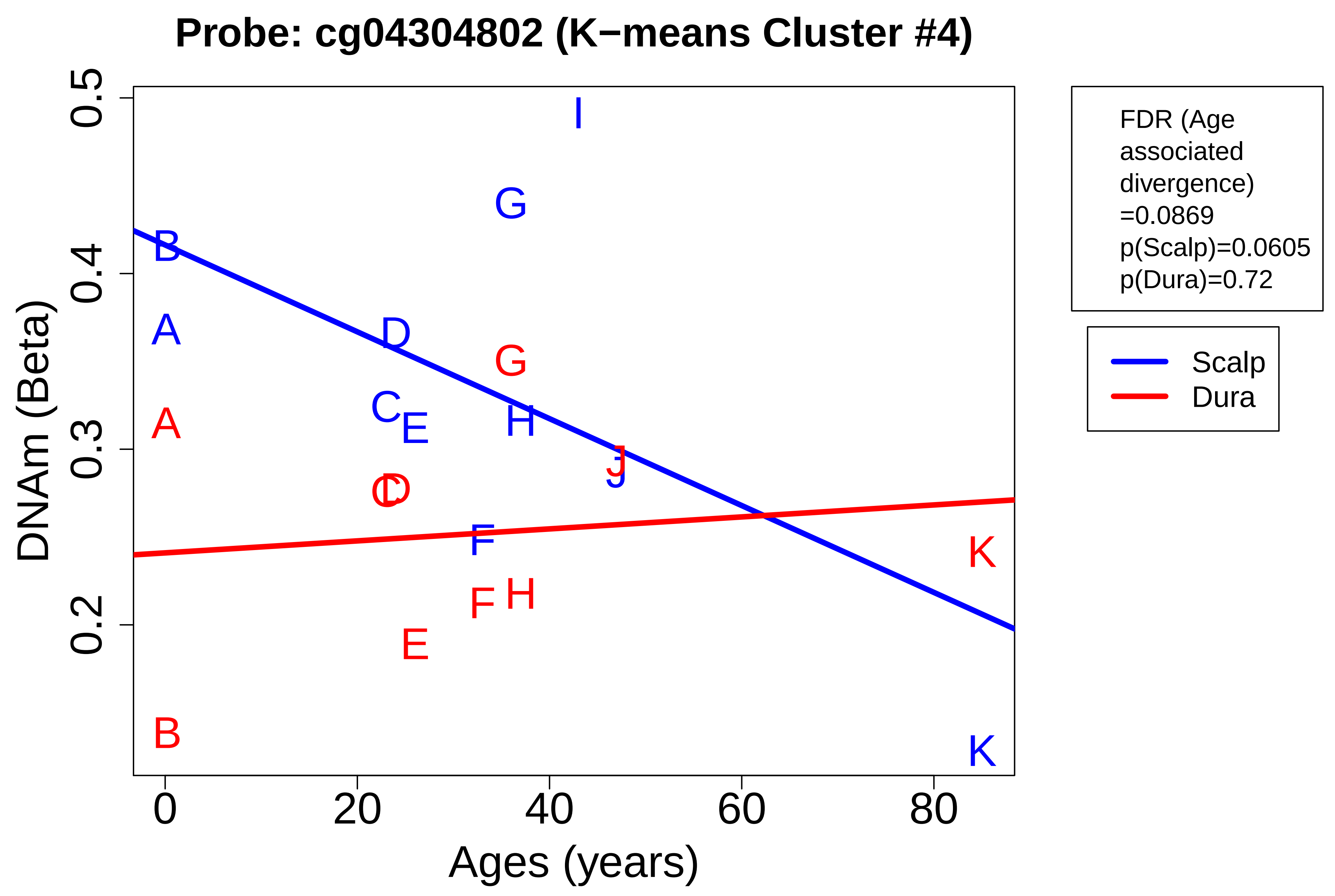

Probe: cg04304802 (K−means Cluster #4)
DNAm (Beta)
0.2
0.3
0.4
0.5
Ages (years)
0
20
40
60
80
FDR (Age associated divergence) =0.0869
p(Scalp)=0.0605
p(Dura)=0.72
Scalp
Dura
A
B
C
D
E
F
G
H
I
J
K

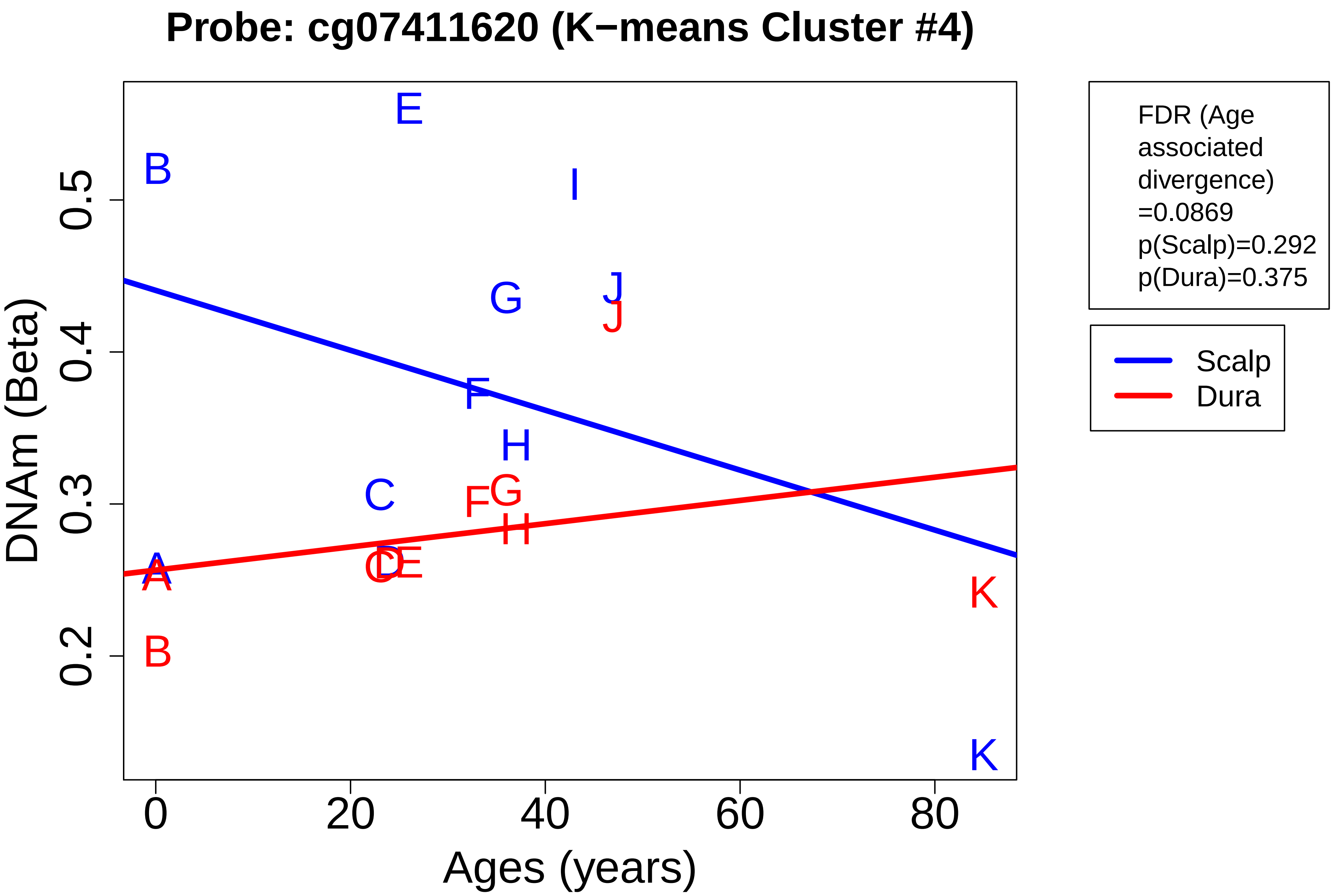

Probe: cg07411620 (K−means Cluster #4)
DNAm (Beta)
0.2
0.3
0.4
0.5
0
20
40
60
80
Ages (years)
FDR (Age associated divergence) =0.0869
p(Scalp)=0.292
p(Dura)=0.375
Scalp
Dura

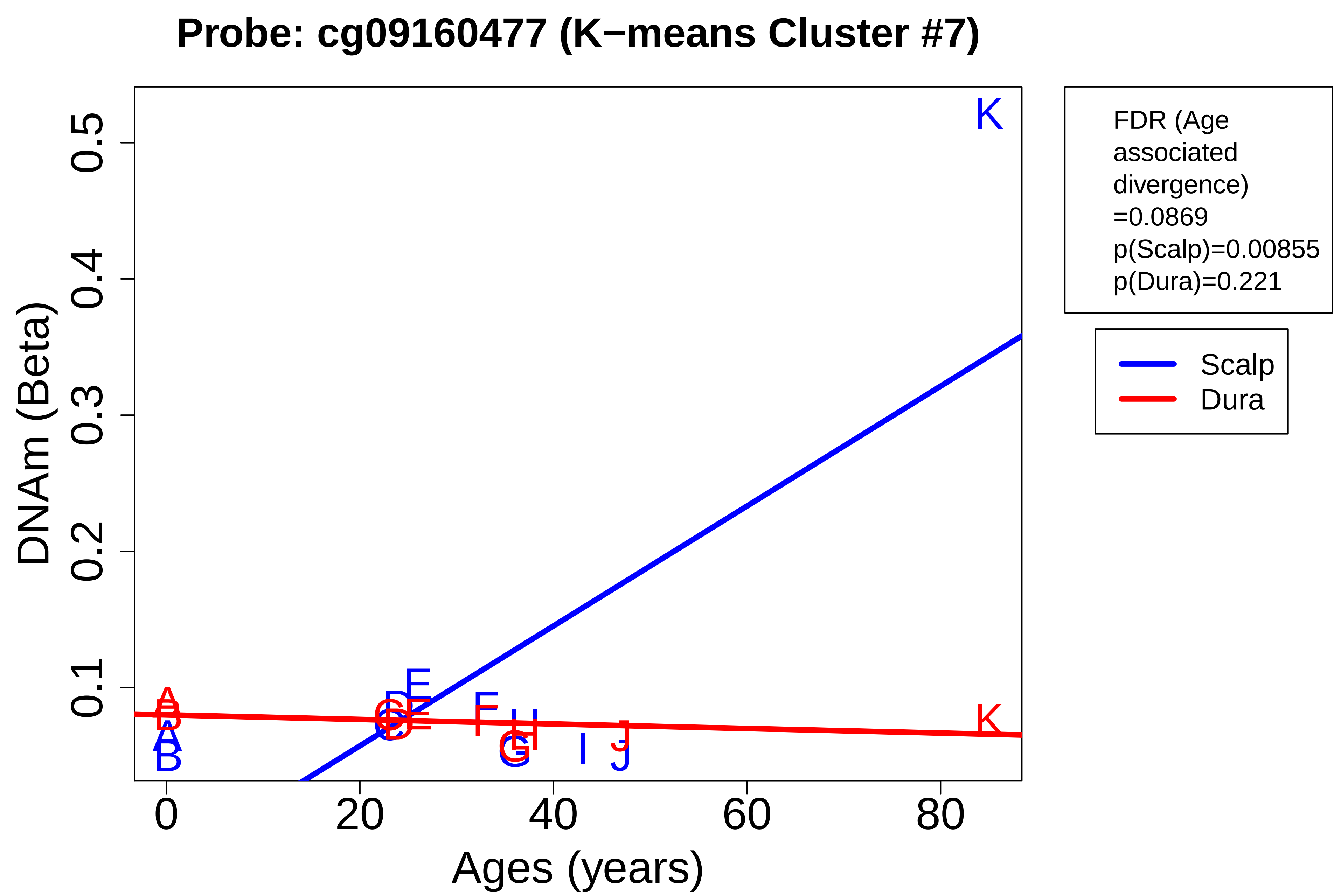
Probe: cg09160477 (K−means Cluster #7)
DNAm (Beta)
0.1
0.2
0.3
0.4
0.5
Ages (years)
0
20
40
60
80
FDR (Age associated divergence) =0.0869
p(Scalp)=0.00855
p(Dura)=0.221
Scalp
Dura

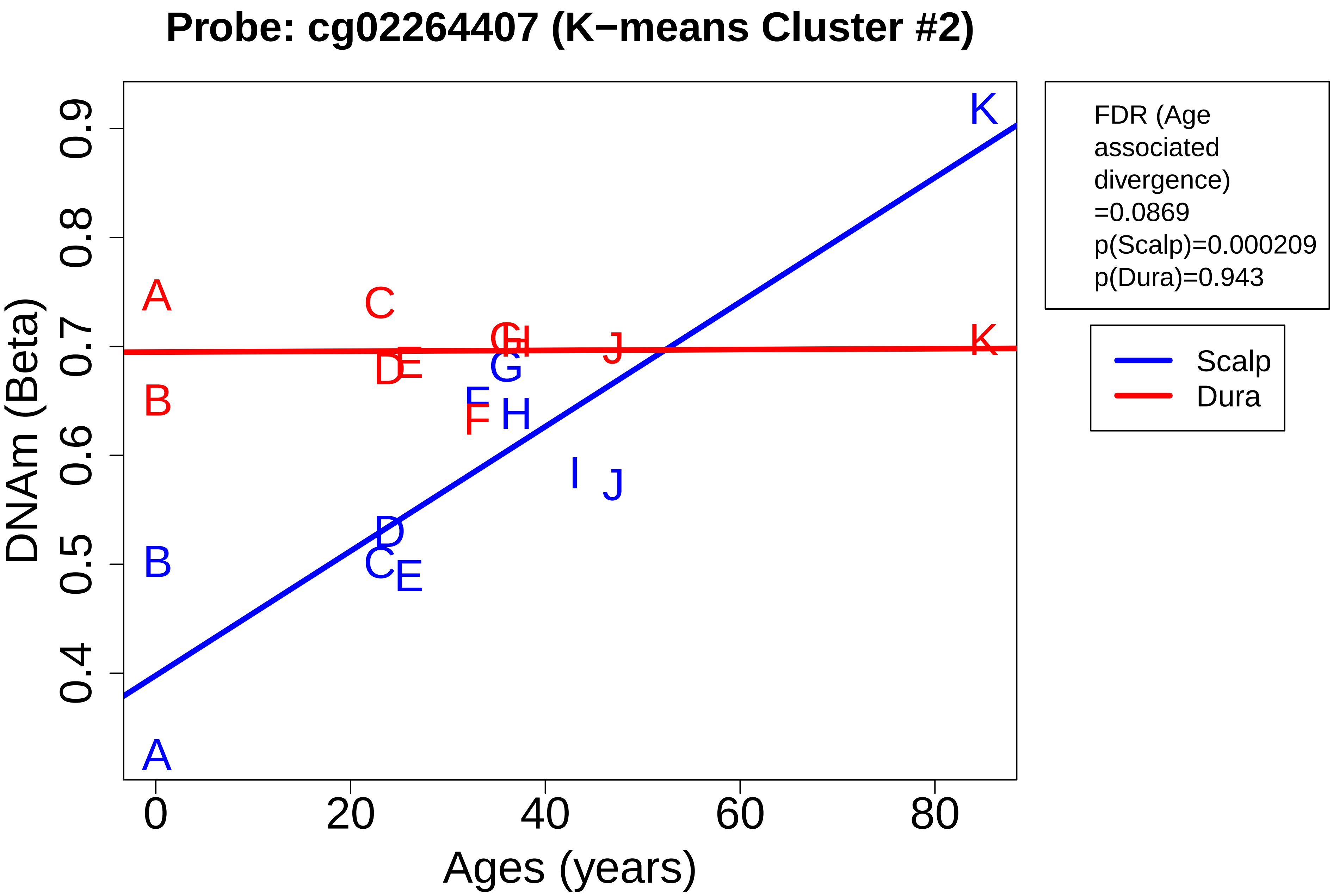

Probe: cg02264407 (K−means Cluster #2)
DNAm (Beta)
0.4
0.5
0.6
0.7
0.8
0.9
0
20
40
60
80
Ages (years)
FDR (Age associated divergence) =0.0869
p(Scalp)=0.000209
p(Dura)=0.943
Scalp
Dura

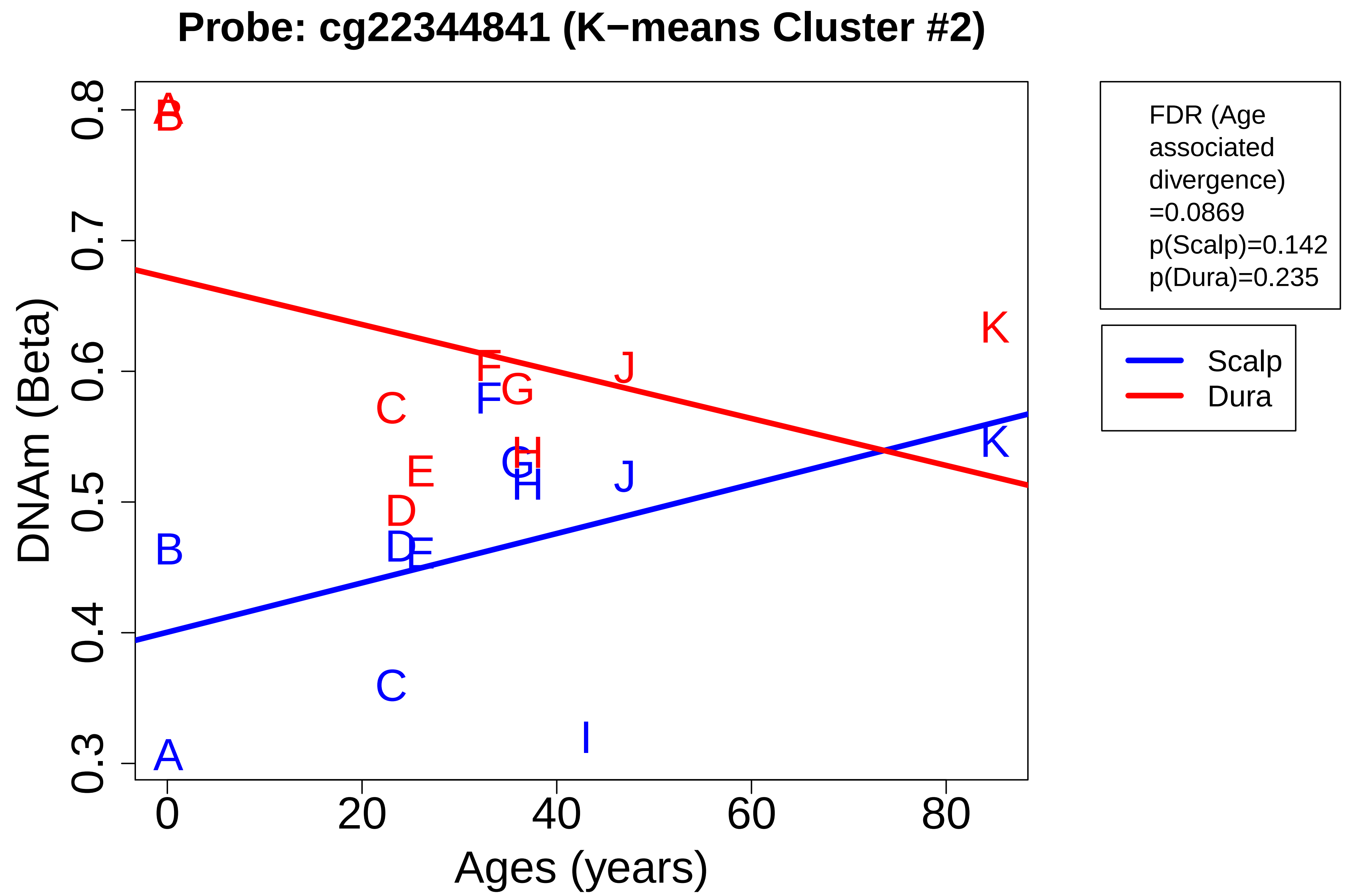

Probe: cg22344841 (K−means Cluster #2)
DNAm (Beta)
0.3
0.4
0.5
0.6
0.7
0.8
0
20
40
60
80
Ages (years)
A
B
C
D
E
F
G
H
I
J
K
FDR (Age associated divergence) =0.0869
p(Scalp)=0.142
p(Dura)=0.235
Scalp
Dura

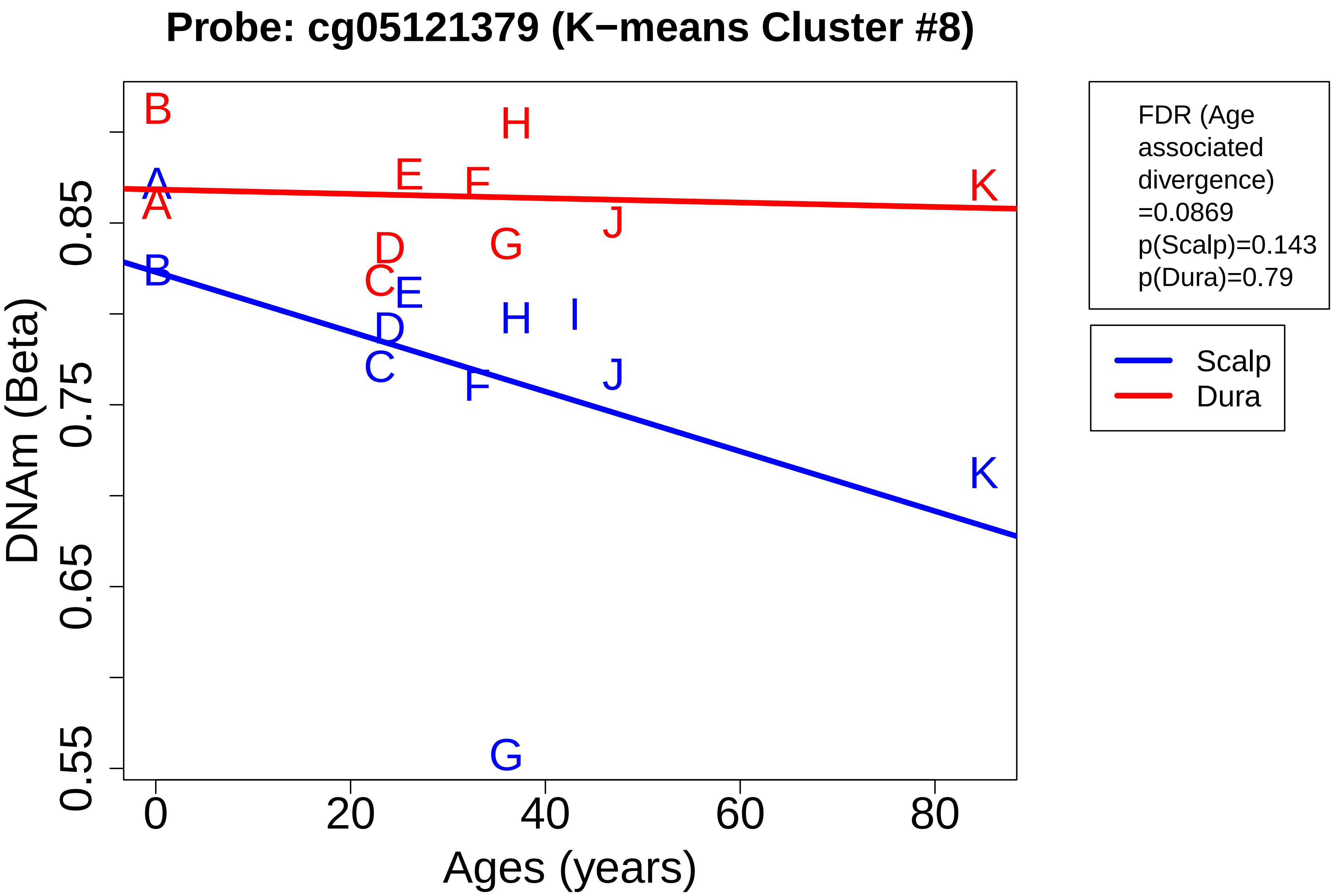
Probe: cg05121379 (K−means Cluster #8)
DNAm (Beta)
0.55
0.65
0.75
0.85
0
20
40
60
80
Ages (years)
FDR (Age associated divergence) =0.0869
p(Scalp)=0.143
p(Dura)=0.79
Scalp
Dura

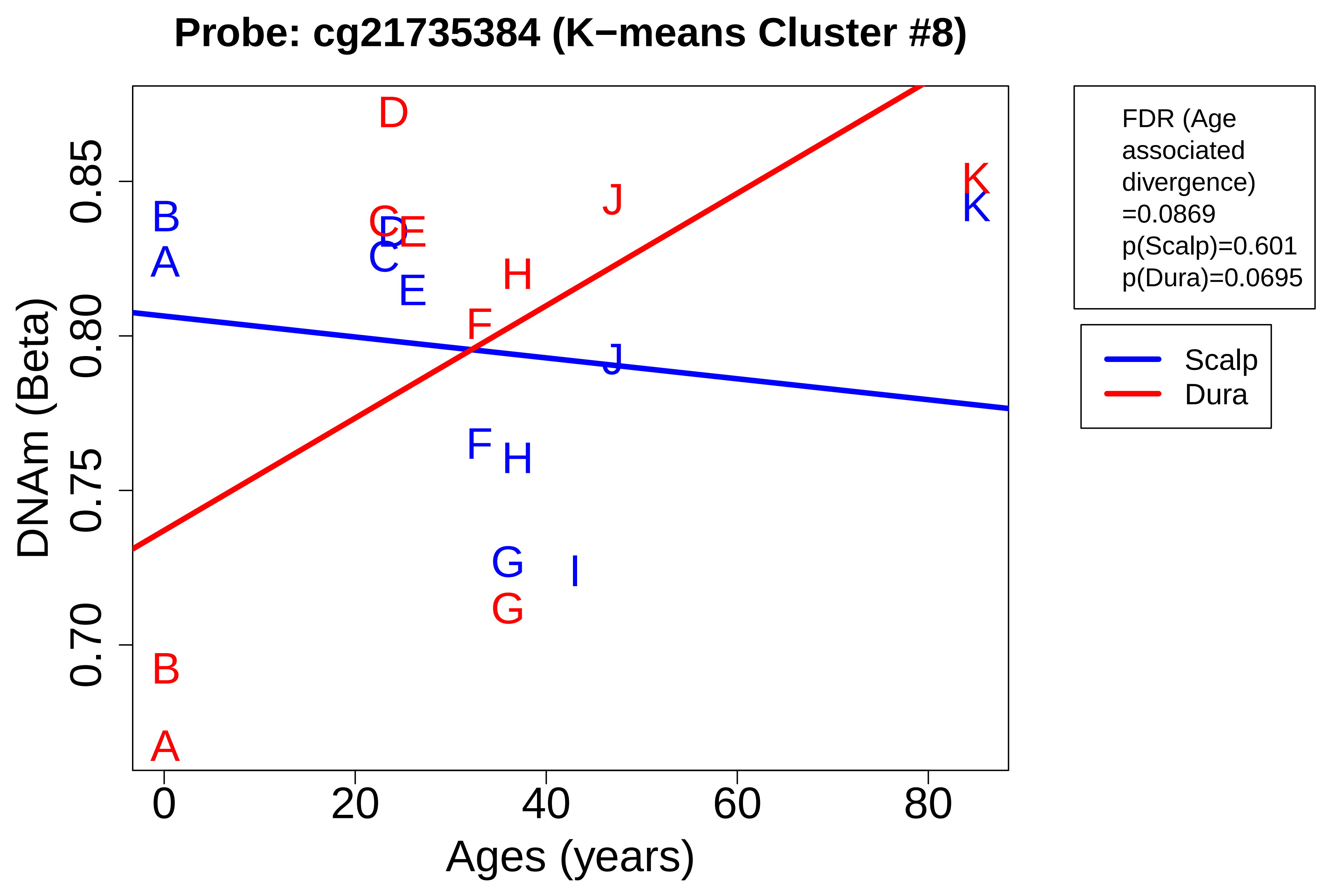

Probe: cg21735384 (K−means Cluster #8)
DNAm (Beta)
0.70
0.75
0.80
0.85
0
20
40
60
80
Ages (years)
FDR (Age associated divergence) =0.0869
p(Scalp)=0.601
p(Dura)=0.0695
Scalp
Dura
A
B
C
D
E
F
G
H
I
J
K

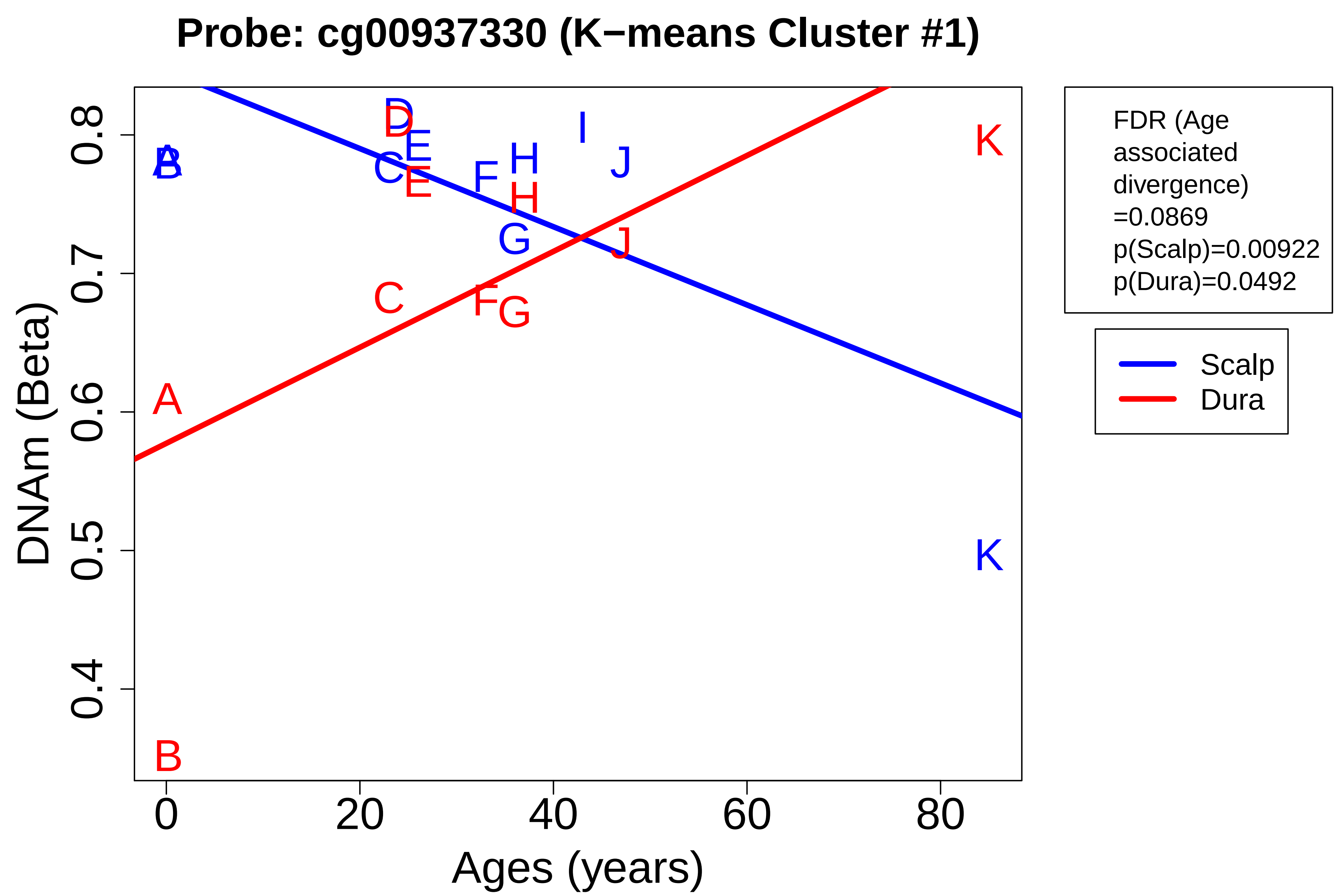

Probe: cg00937330 (K−means Cluster #1)
FDR (Age associated divergence) =0.0869
p(Scalp)=0.00922
p(Dura)=0.0492
Scalp
Dura
DNAm (Beta)
0.4
0.5
0.6
0.7
0.8
Ages (years)
0
20
40
60
80
A
B
C
D
E
F
G
H
I
J
K

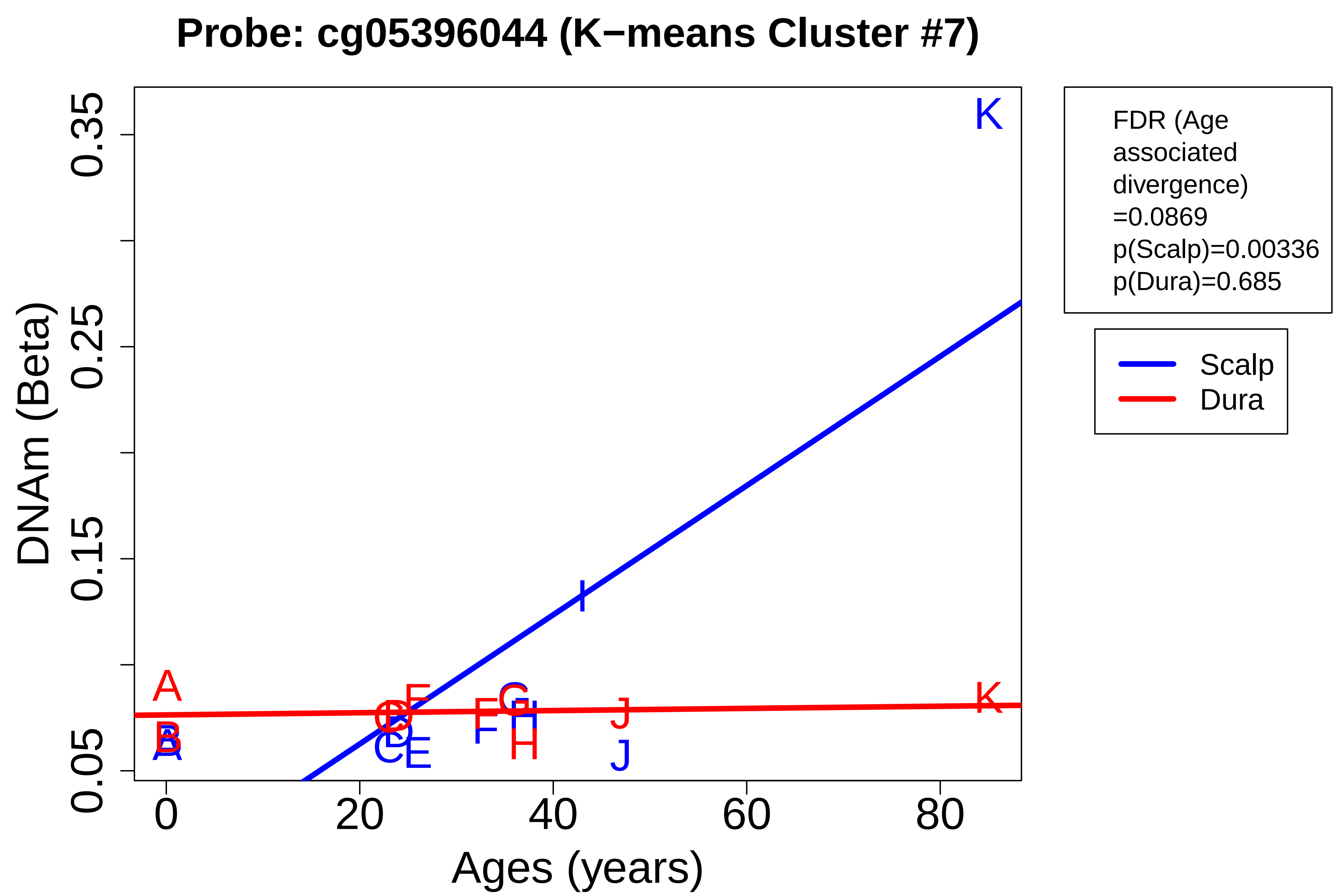

Probe: cg05396044 (K−means Cluster #7)
DNAm (Beta)
0.05
0.15
0.25
0.35
Ages (years)
0
20
40
60
80
FDR (Age associated divergence) =0.0869
p(Scalp)=0.00336
p(Dura)=0.685
Scalp
Dura

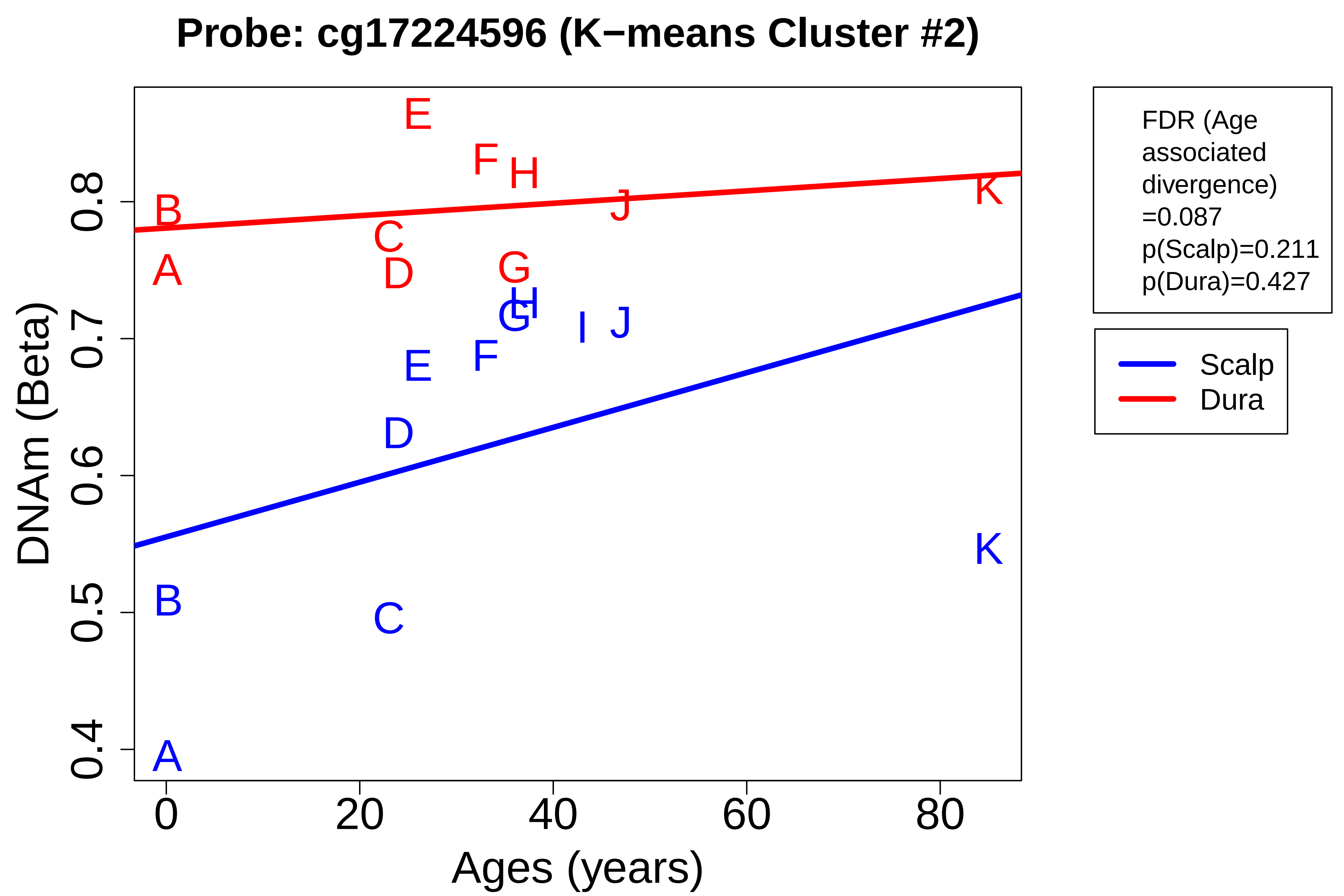

Probe: cg17224596 (K−means Cluster #2)
DNAm (Beta)
0.4
0.5
0.6
0.7
0.8
0
20
40
60
80
Ages (years)
A
B
C
D
E
F
G
H
I
J
K
FDR (Age associated divergence) =0.087
p(Scalp)=0.211
p(Dura)=0.427
Scalp
Dura

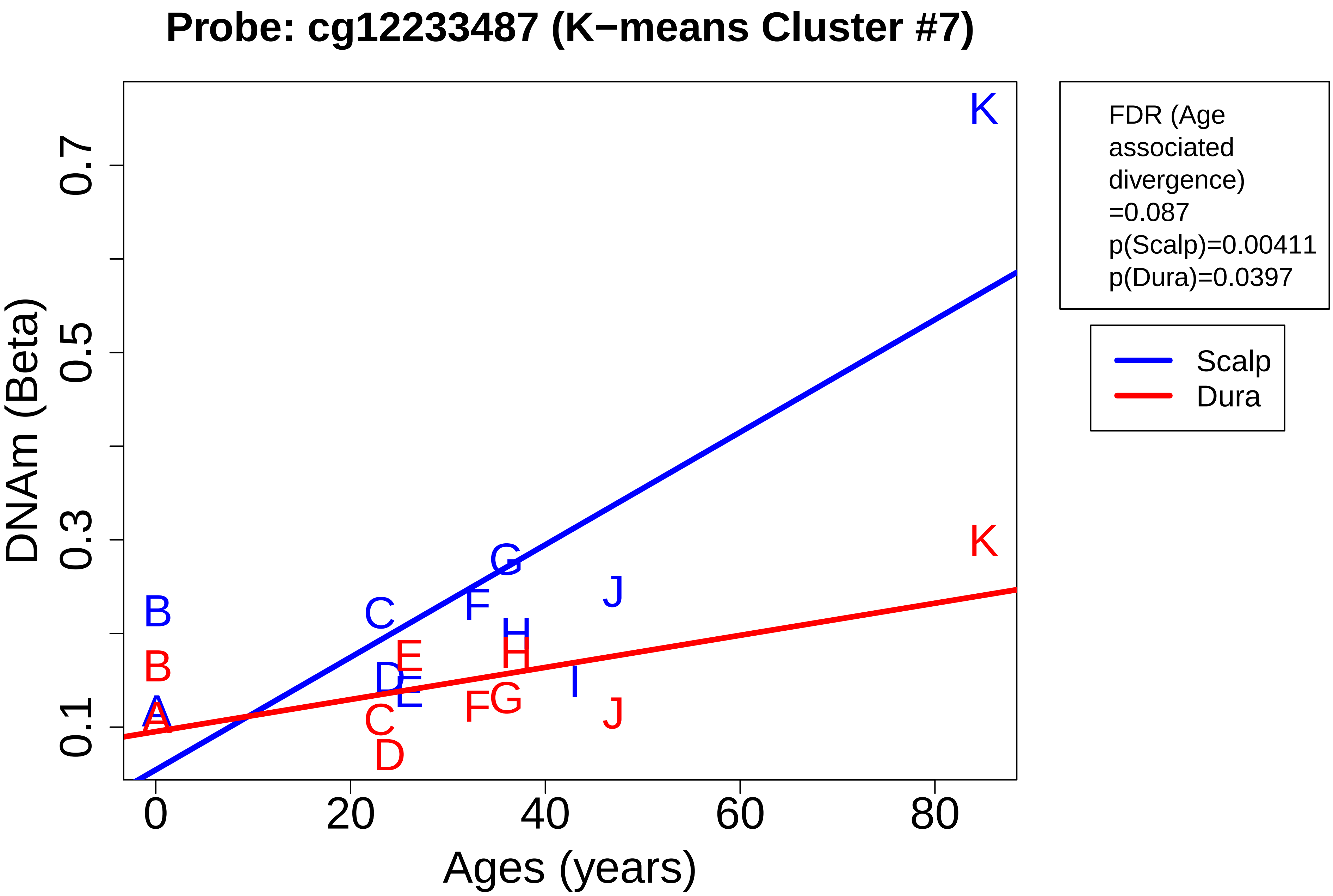

Probe: cg12233487 (K−means Cluster #7)
DNAm (Beta)
0.1
0.3
0.5
0.7
0
20
40
60
80
Ages (years)
FDR (Age associated divergence) =0.087
p(Scalp)=0.00411
p(Dura)=0.0397
Scalp
Dura
A B C D E F G H I J K
A B C D E F G H J K

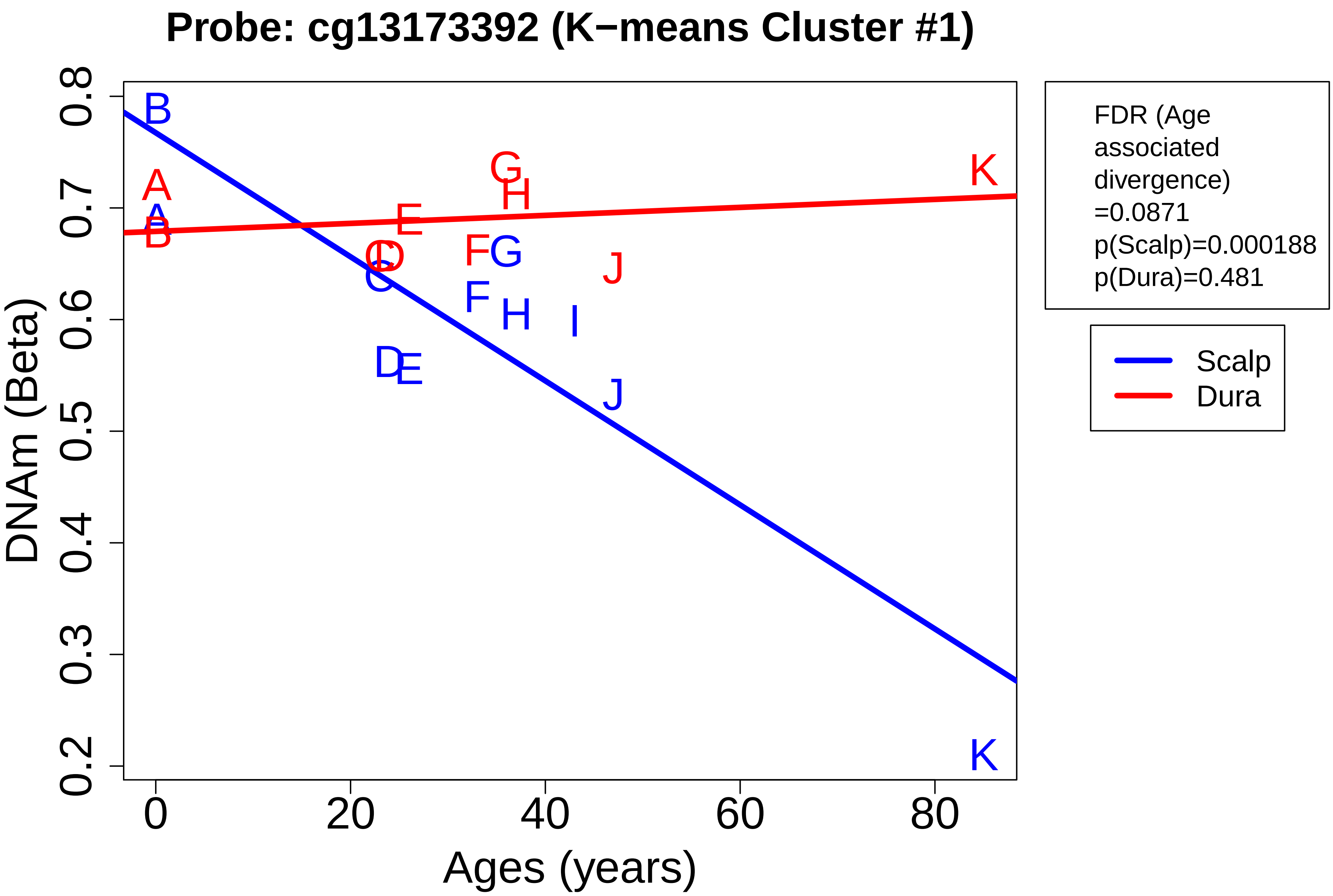

Probe: cg13173392 (K−means Cluster #1)
DNAm (Beta)
0.2
0.3
0.4
0.5
0.6
0.7
0.8
0
20
40
60
80
Ages (years)
FDR (Age associated divergence) =0.0871
p(Scalp)=0.000188
p(Dura)=0.481
Scalp
Dura

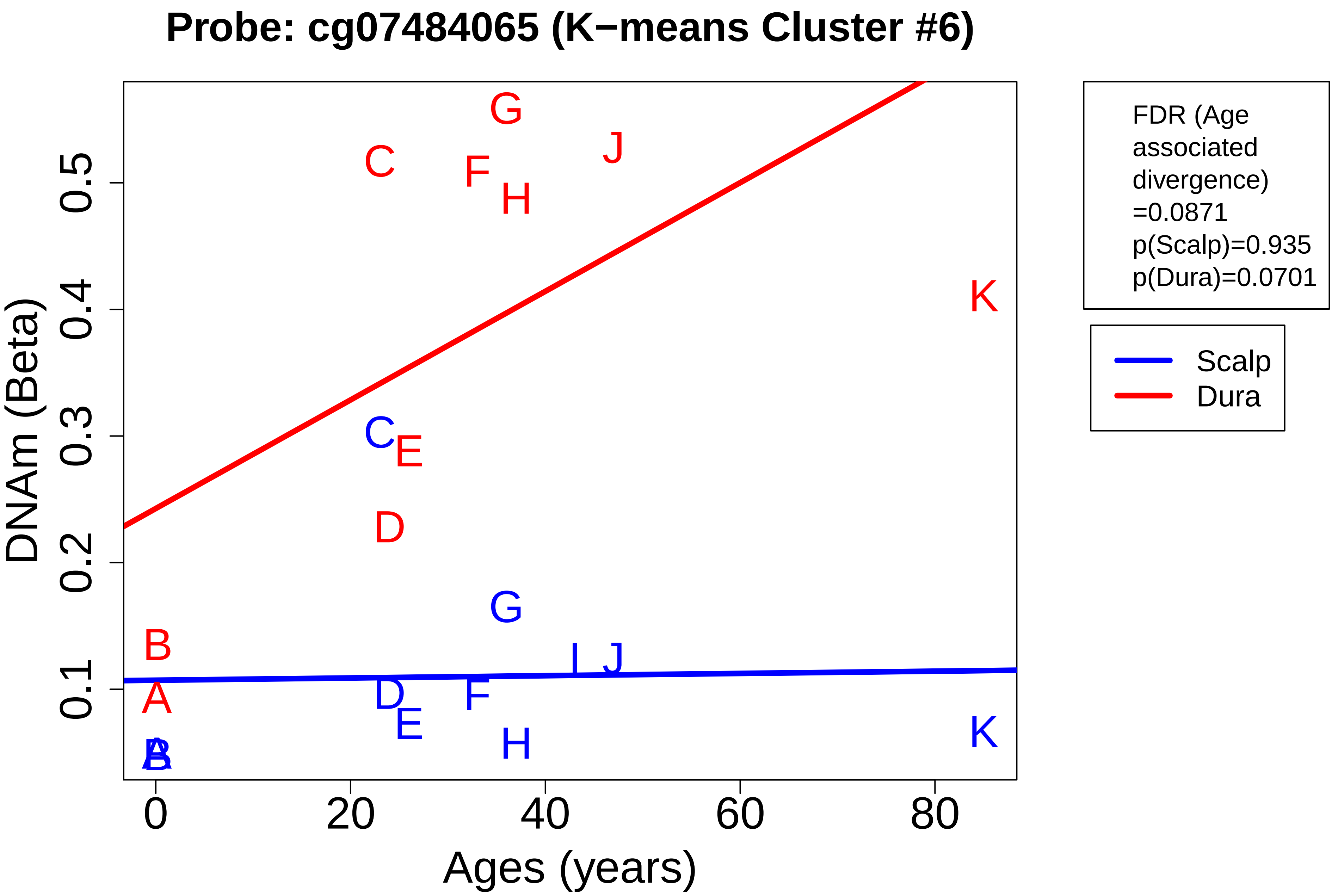

Probe: cg07484065 (K−means Cluster #6)
DNAm (Beta)
0.1
0.2
0.3
0.4
0.5
0
20
40
60
80
Ages (years)
FDR (Age associated divergence) =0.0871
p(Scalp)=0.935
p(Dura)=0.0701
Scalp
Dura

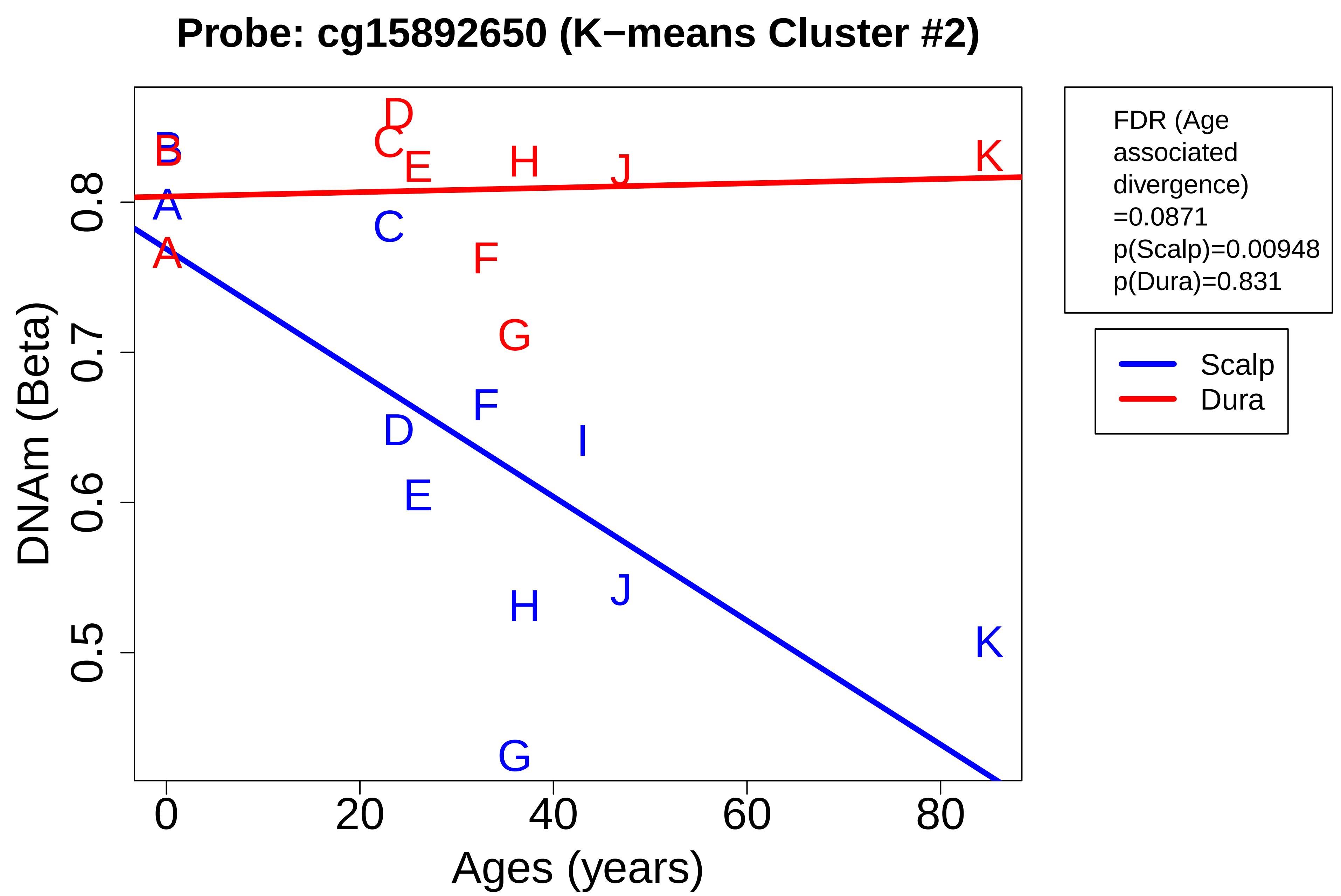

Probe: cg15892650 (K−means Cluster #2)
DNAm (Beta)
0.5
0.6
0.7
0.8
0
20
40
60
80
Ages (years)
A B C D E F G H I J K
FDR (Age associated divergence) =0.0871
p(Scalp)=0.00948
p(Dura)=0.831
Scalp
Dura

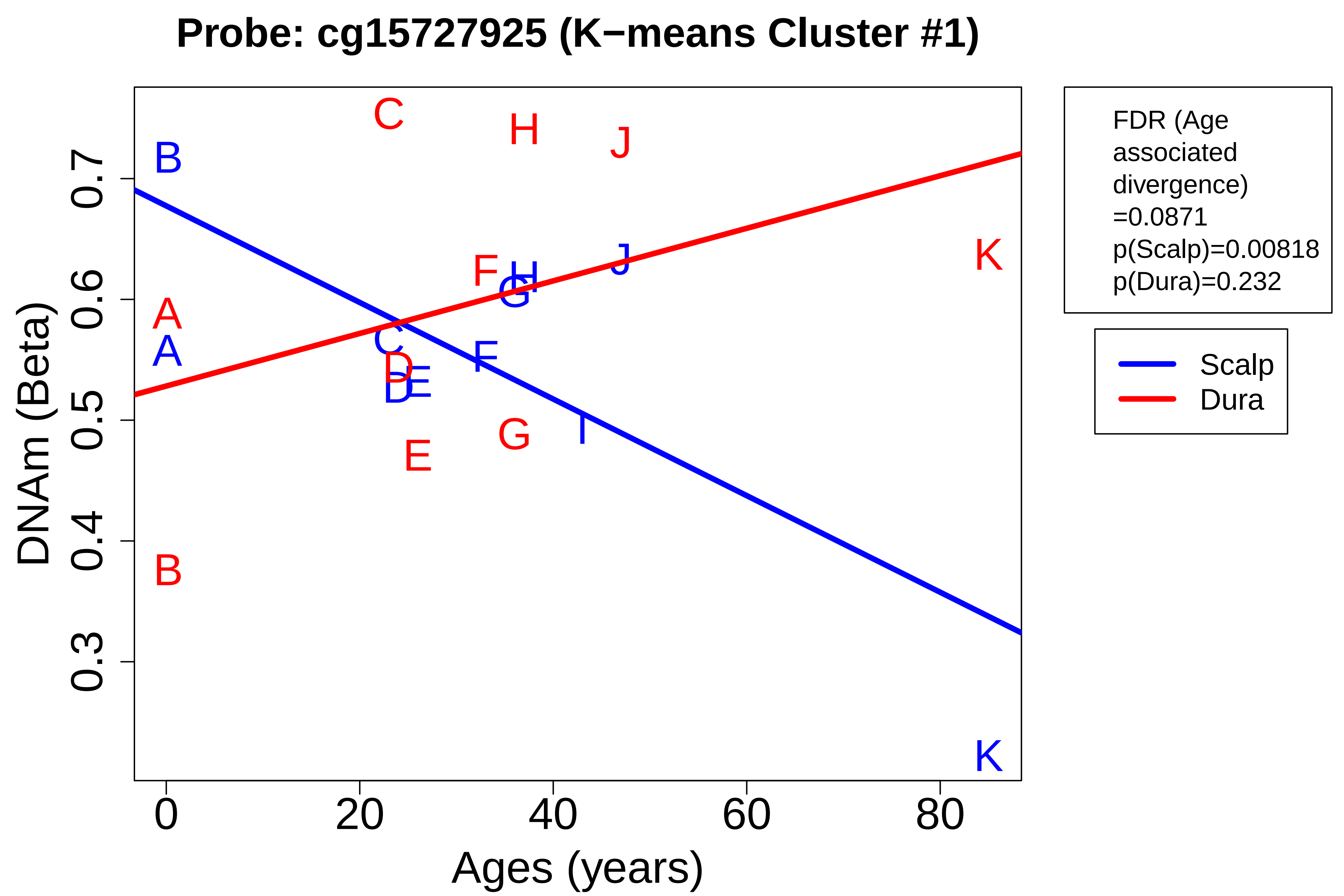

Probe: cg15727925 (K−means Cluster #1)
DNAm (Beta)
Ages (years)
0.3
0.4
0.5
0.6
0.7
0
20
40
60
80
FDR (Age associated divergence) =0.0871
p(Scalp)=0.00818
p(Dura)=0.232
Scalp
Dura

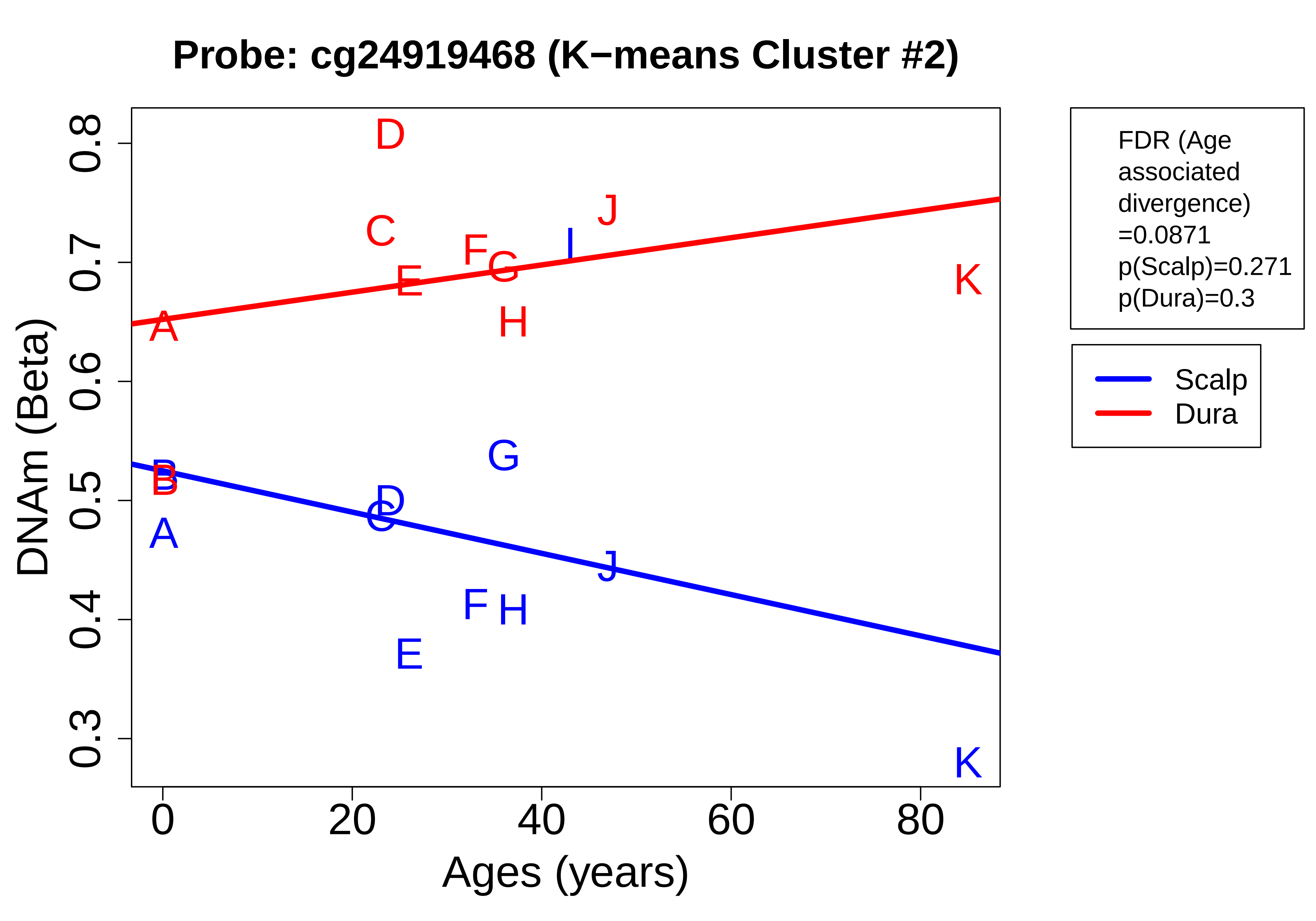
Probe: cg24919468 (K−means Cluster #2)
DNAm (Beta)
0.3
0.4
0.5
0.6
0.7
0.8
Ages (years)
0
20
40
60
80
FDR (Age associated divergence) =0.0871
p(Scalp)=0.271
p(Dura)=0.3
Scalp
Dura

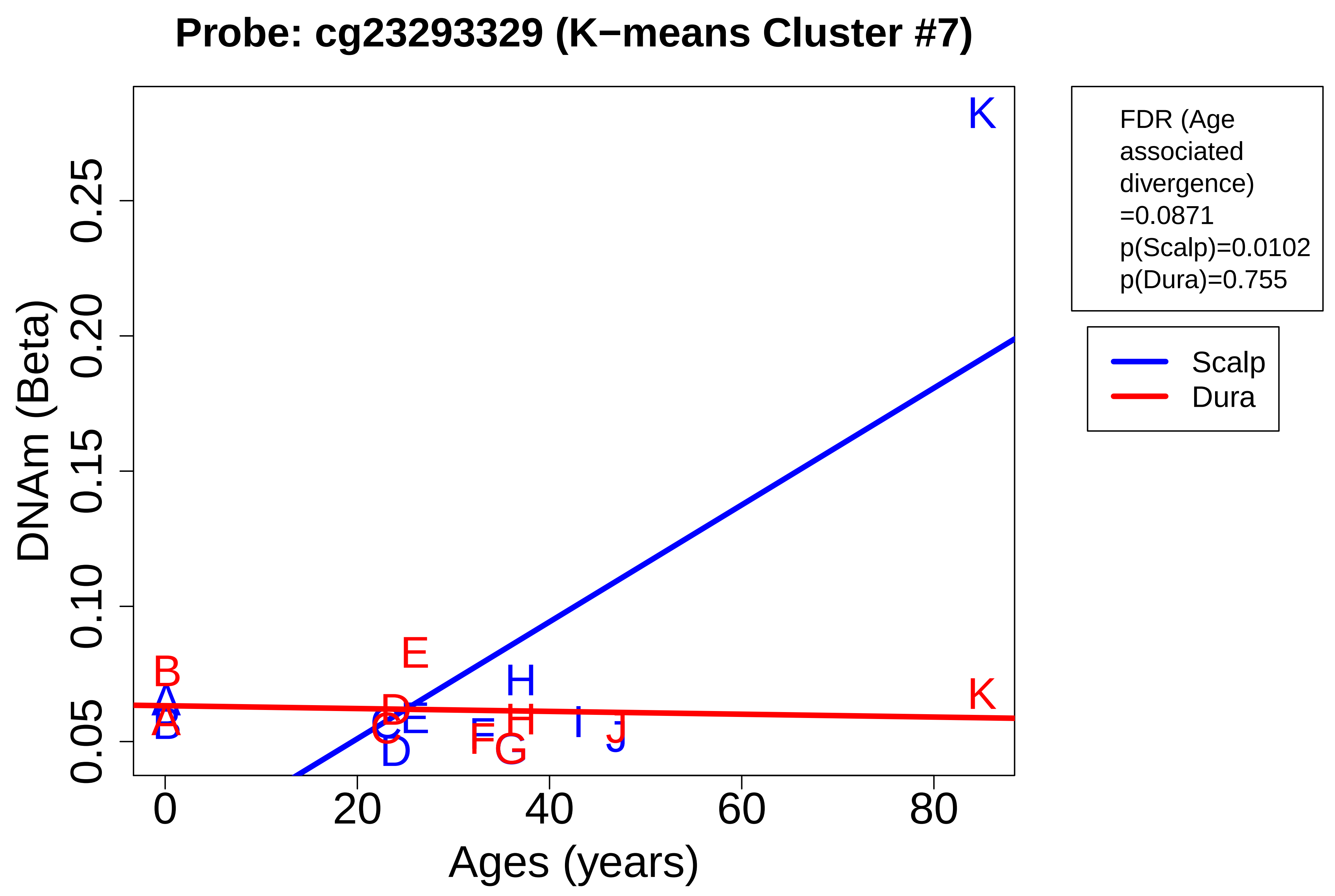

Probe: cg23293329 (K−means Cluster #7)
DNAm (Beta)
0.05
0.10
0.15
0.20
0.25
0
20
40
60
80
Ages (years)
FDR (Age associated divergence) =0.0871
p(Scalp)=0.0102
p(Dura)=0.755
Scalp
Dura

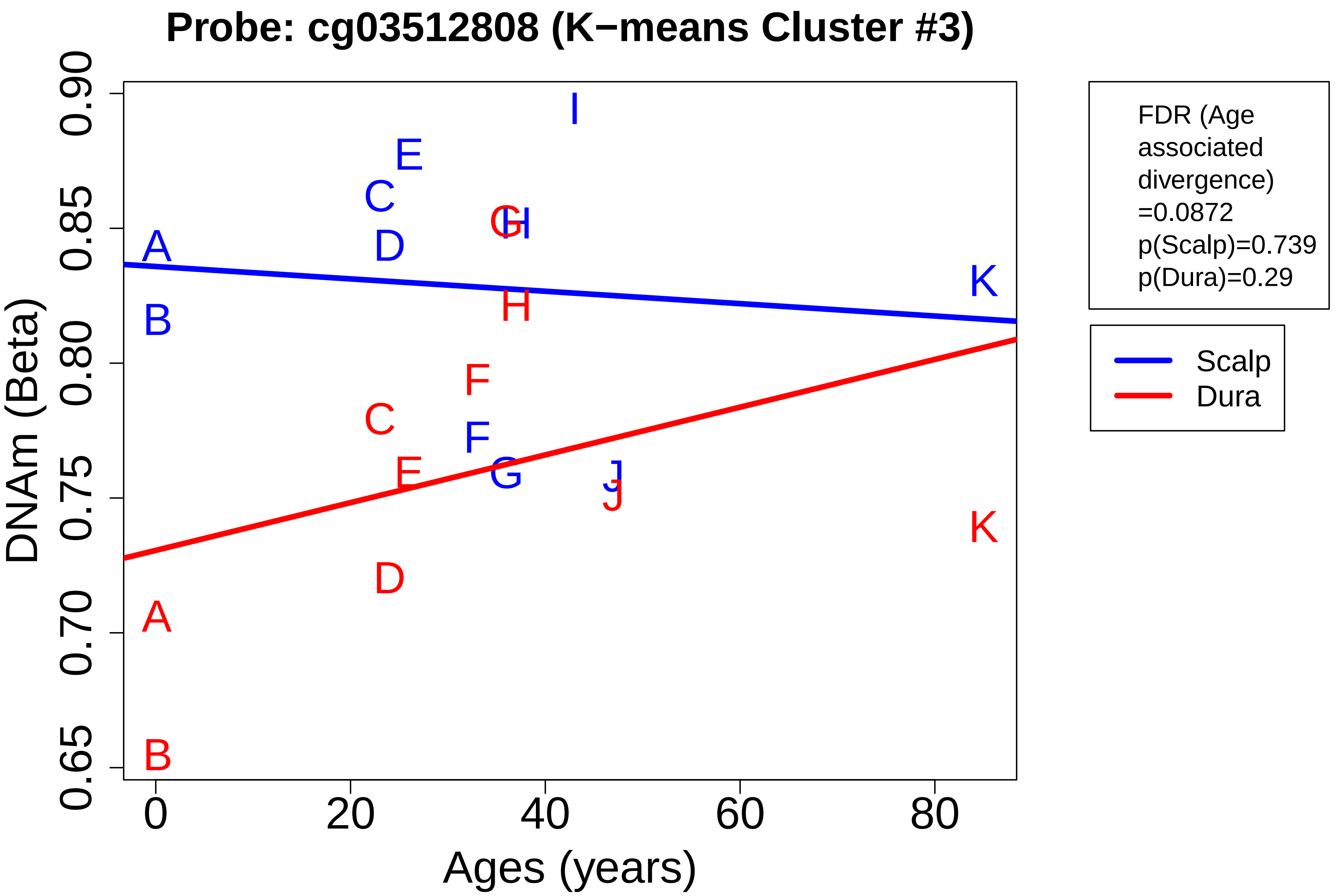
Probe: cg03512808 (K−means Cluster #3)
DNAm (Beta)
0.65
0.70
0.75
0.80
0.85
0.90
0
20
40
60
80
Ages (years)
FDR (Age associated divergence) =0.0872
p(Scalp)=0.739
p(Dura)=0.29
Scalp
Dura

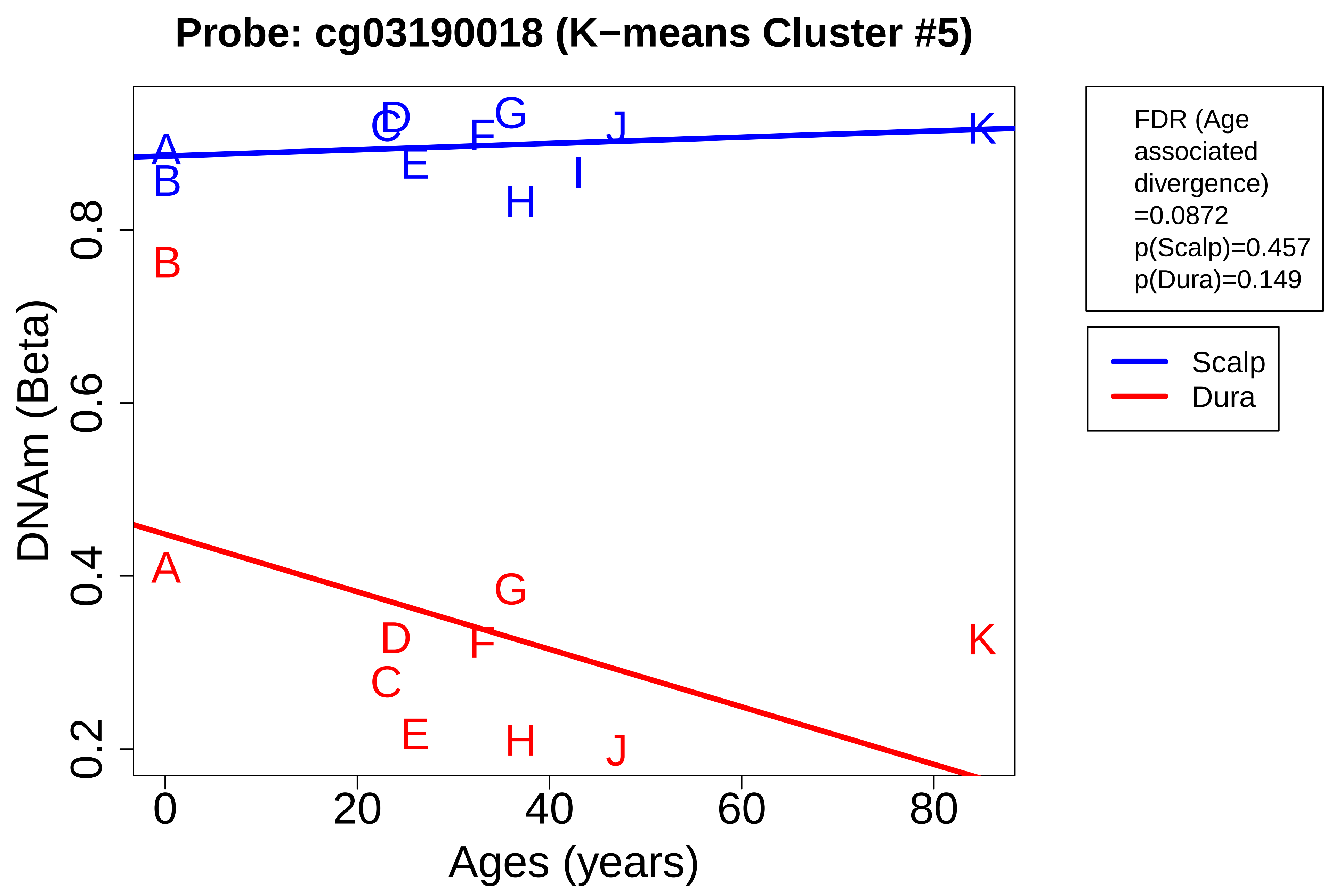

Probe: cg03190018 (K−means Cluster #5)
DNAm (Beta)
0.2
0.4
0.6
0.8
0
20
40
60
80
Ages (years)
A
B
C
D
E
F
G
H
I
J
K
FDR (Age associated divergence) =0.0872
p(Scalp)=0.457
p(Dura)=0.149
Scalp
Dura

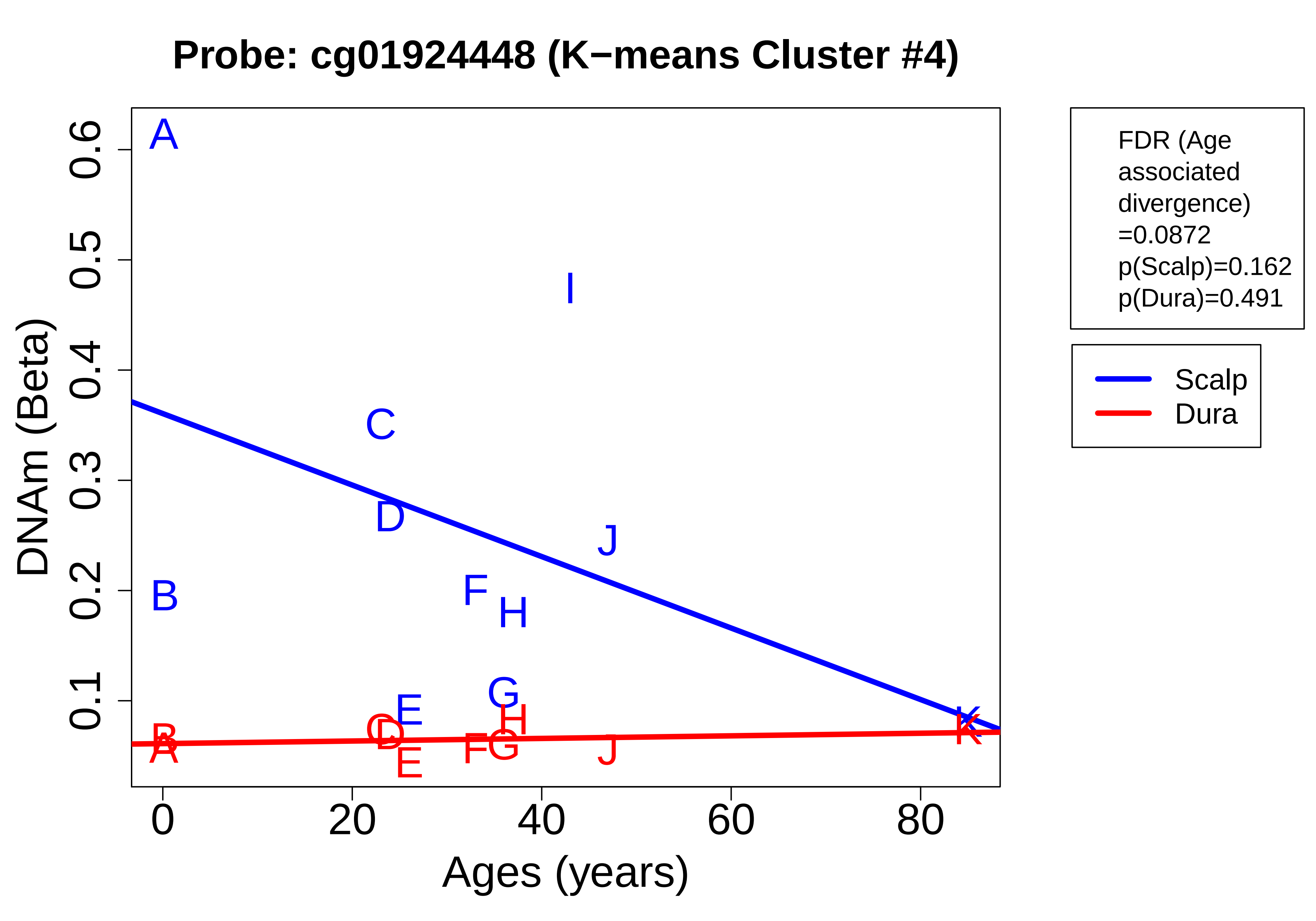

Probe: cg01924448 (K−means Cluster #4)
DNAm (Beta)
Ages (years)
0.1
0.2
0.3
0.4
0.5
0.6
0
20
40
60
80
FDR (Age associated divergence) =0.0872
p(Scalp)=0.162
p(Dura)=0.491
Scalp
Dura

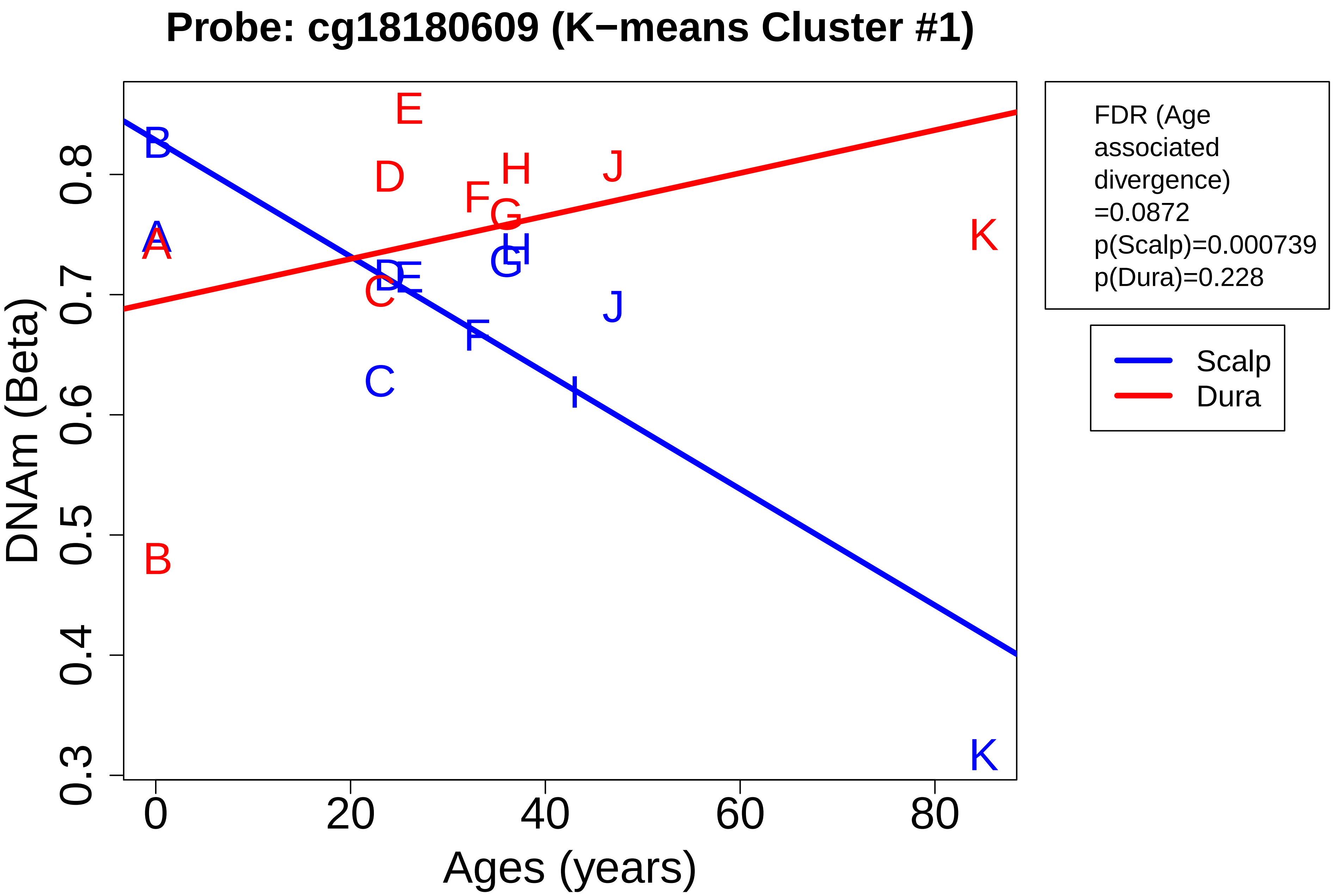

Probe: cg18180609 (K−means Cluster #1)
FDR (Age associated divergence) =0.0872
p(Scalp)=0.000739
p(Dura)=0.228
Scalp
Dura
DNAm (Beta)
Ages (years)
0.3
0.4
0.5
0.6
0.7
0.8
0
20
40
60
80

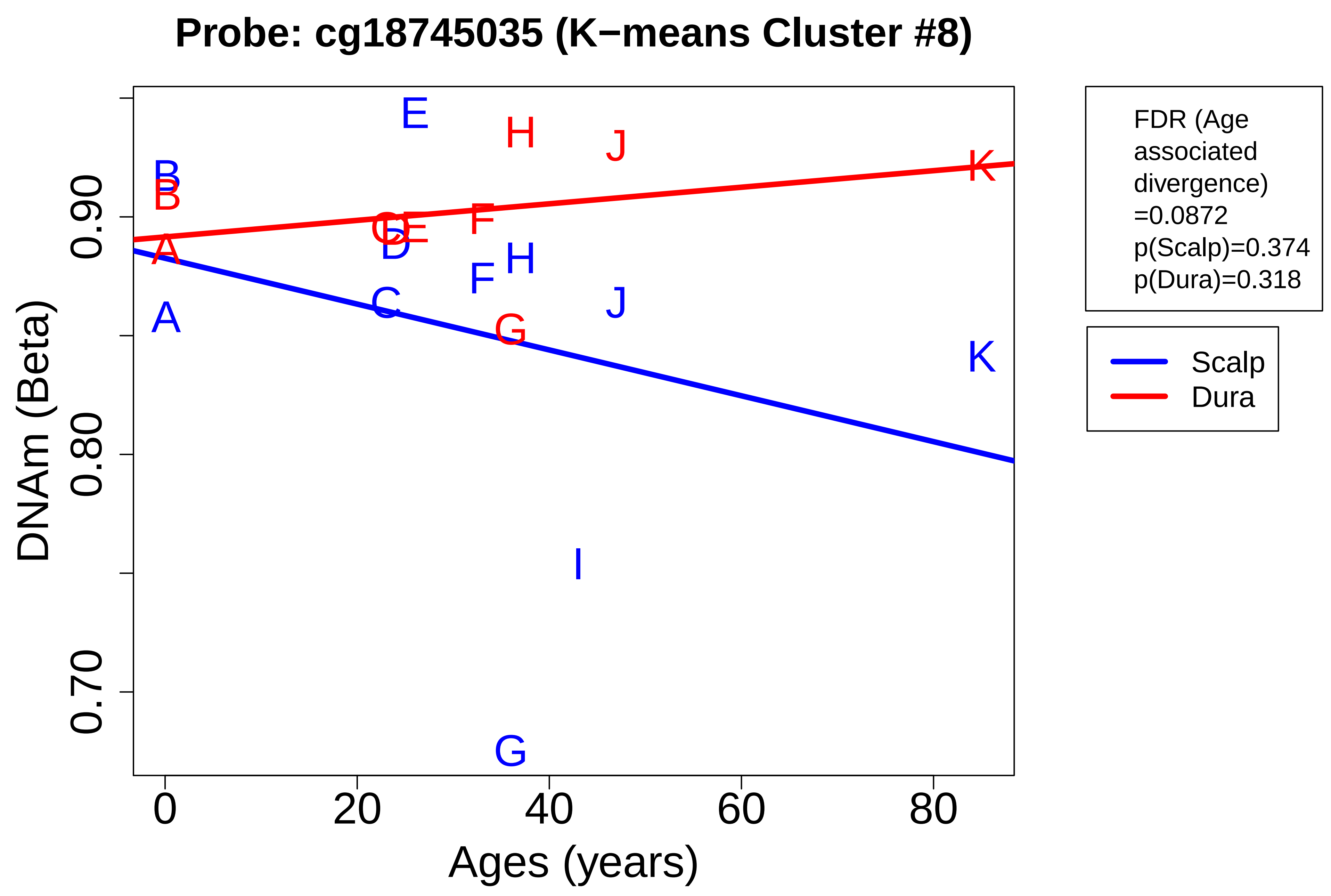

Probe: cg18745035 (K−means Cluster #8)
DNAm (Beta)
0.70
0.80
0.90
0
20
40
60
80
Ages (years)
FDR (Age associated divergence) =0.0872
p(Scalp)=0.374
p(Dura)=0.318
Scalp
Dura

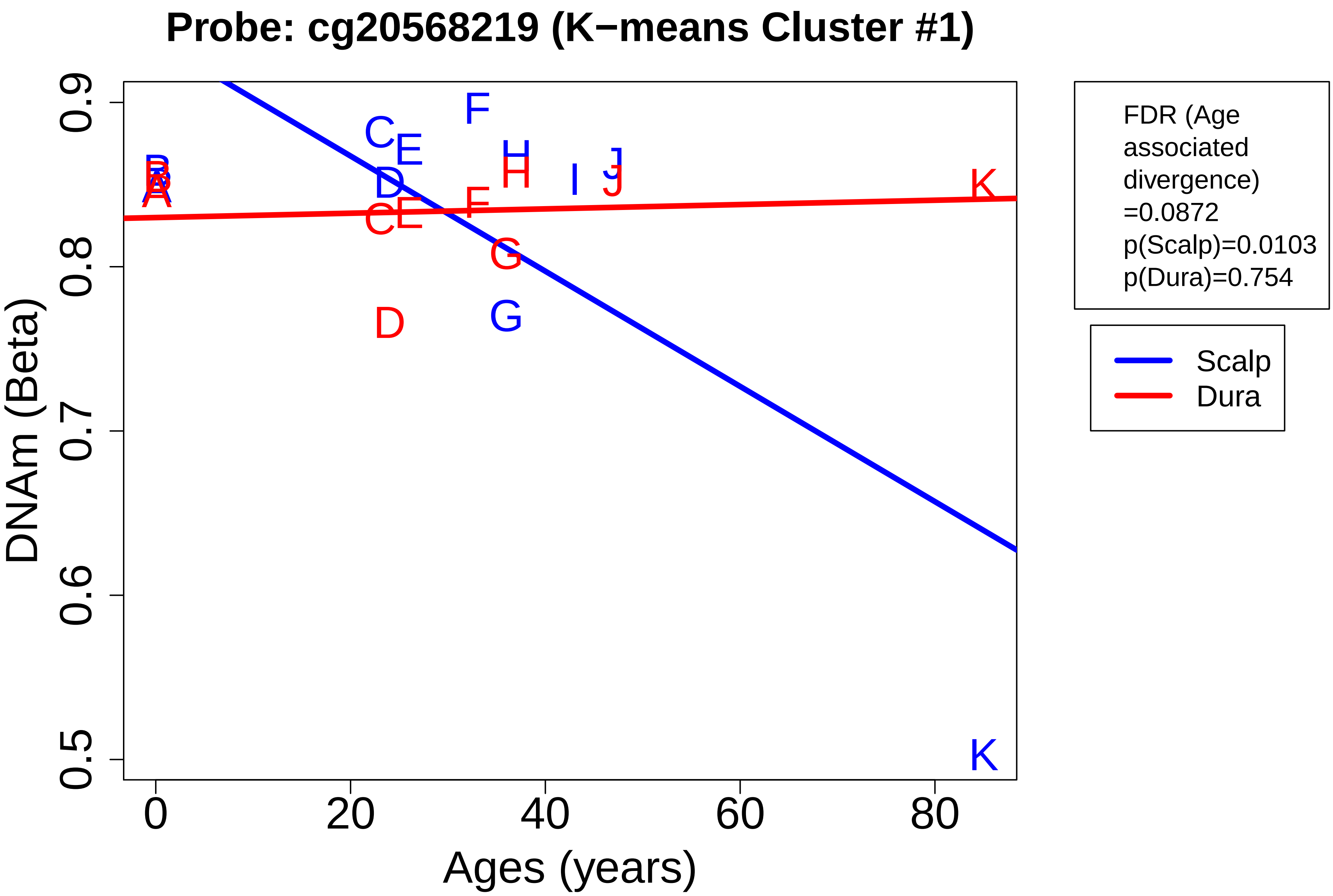

Probe: cg20568219 (K−means Cluster #1)
DNAm (Beta)
0.5
0.6
0.7
0.8
0.9
Ages (years)
0
20
40
60
80
FDR (Age associated divergence) =0.0872
p(Scalp)=0.0103
p(Dura)=0.754
Scalp
Dura

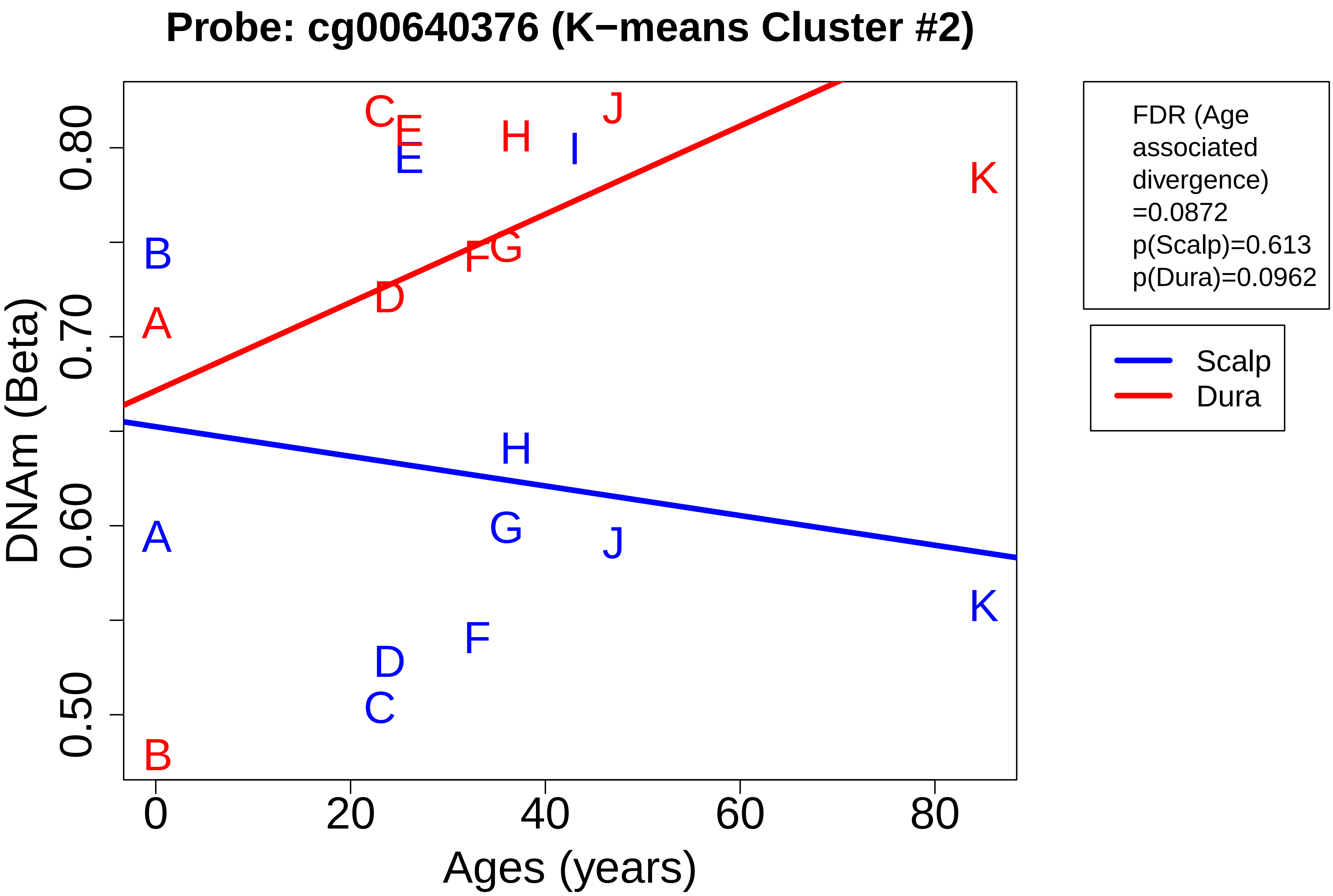
Probe: cg00640376 (K−means Cluster #2)
DNAm (Beta)
0.50
0.60
0.70
0.80
Ages (years)
0
20
40
60
80
FDR (Age associated divergence) =0.0872
p(Scalp)=0.613
p(Dura)=0.0962
Scalp
Dura

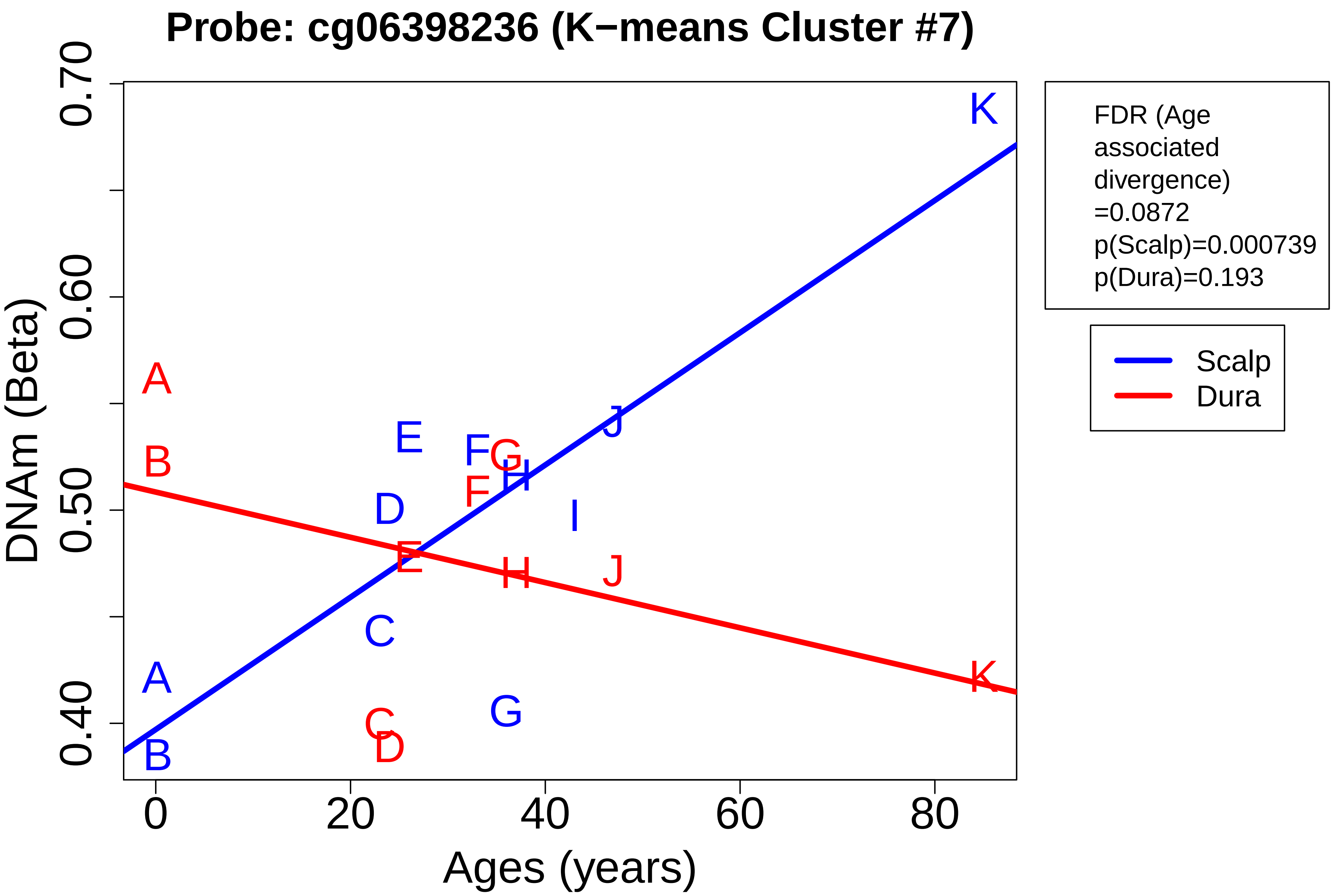

Probe: cg06398236 (K−means Cluster #7)
DNAm (Beta)
0.40
0.50
0.60
0.70
Ages (years)
0
20
40
60
80
FDR (Age associated divergence) =0.0872
p(Scalp)=0.000739
p(Dura)=0.193
Scalp
Dura
A
B
C
D
E
F
G
H
I
J
K

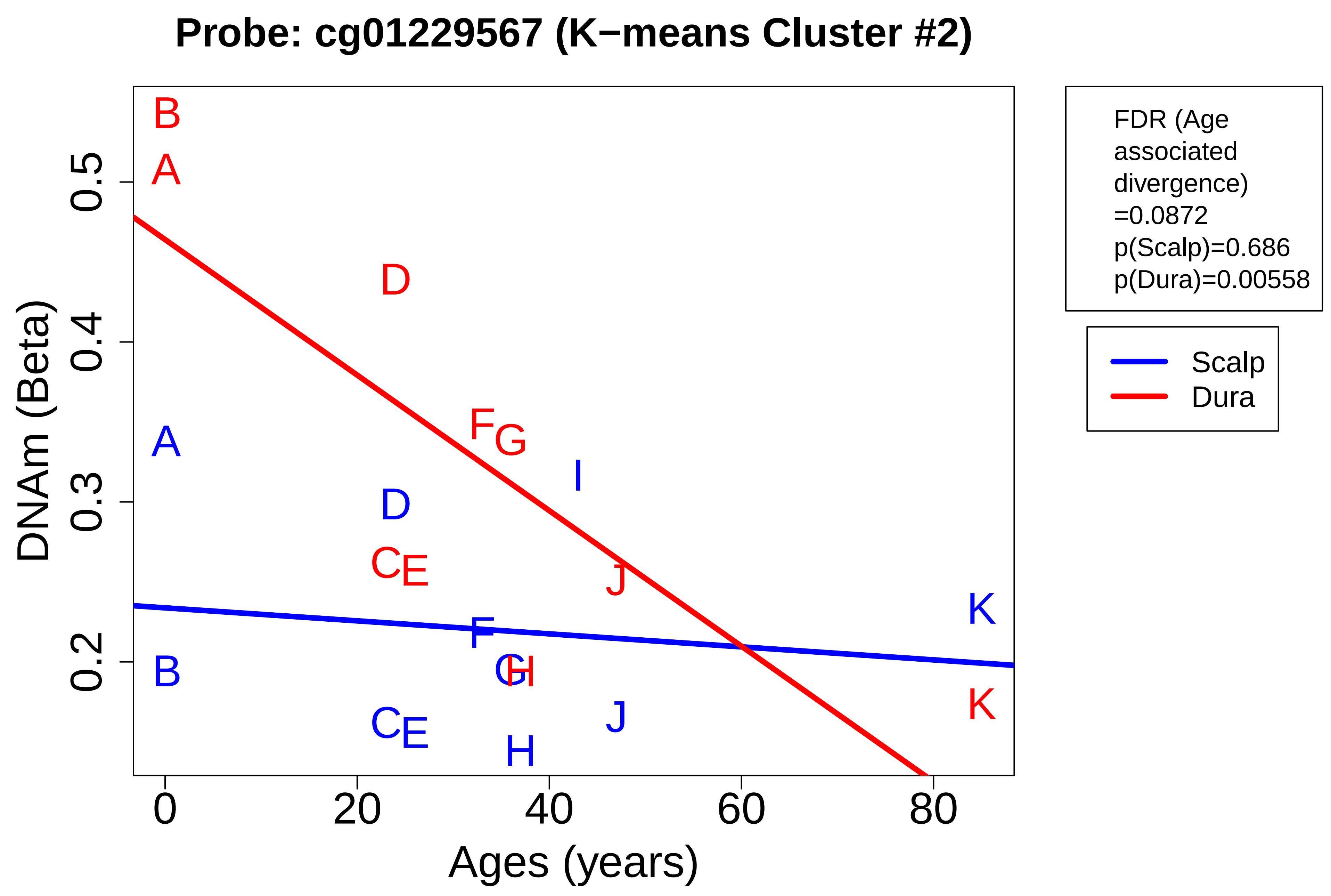

Probe: cg01229567 (K−means Cluster #2)
DNAm (Beta)
0.2
0.3
0.4
0.5
Ages (years)
0
20
40
60
80
FDR (Age associated divergence) =0.0872
p(Scalp)=0.686
p(Dura)=0.00558
Scalp
Dura
A
B
C
D
E
F
G
H
I
J
K

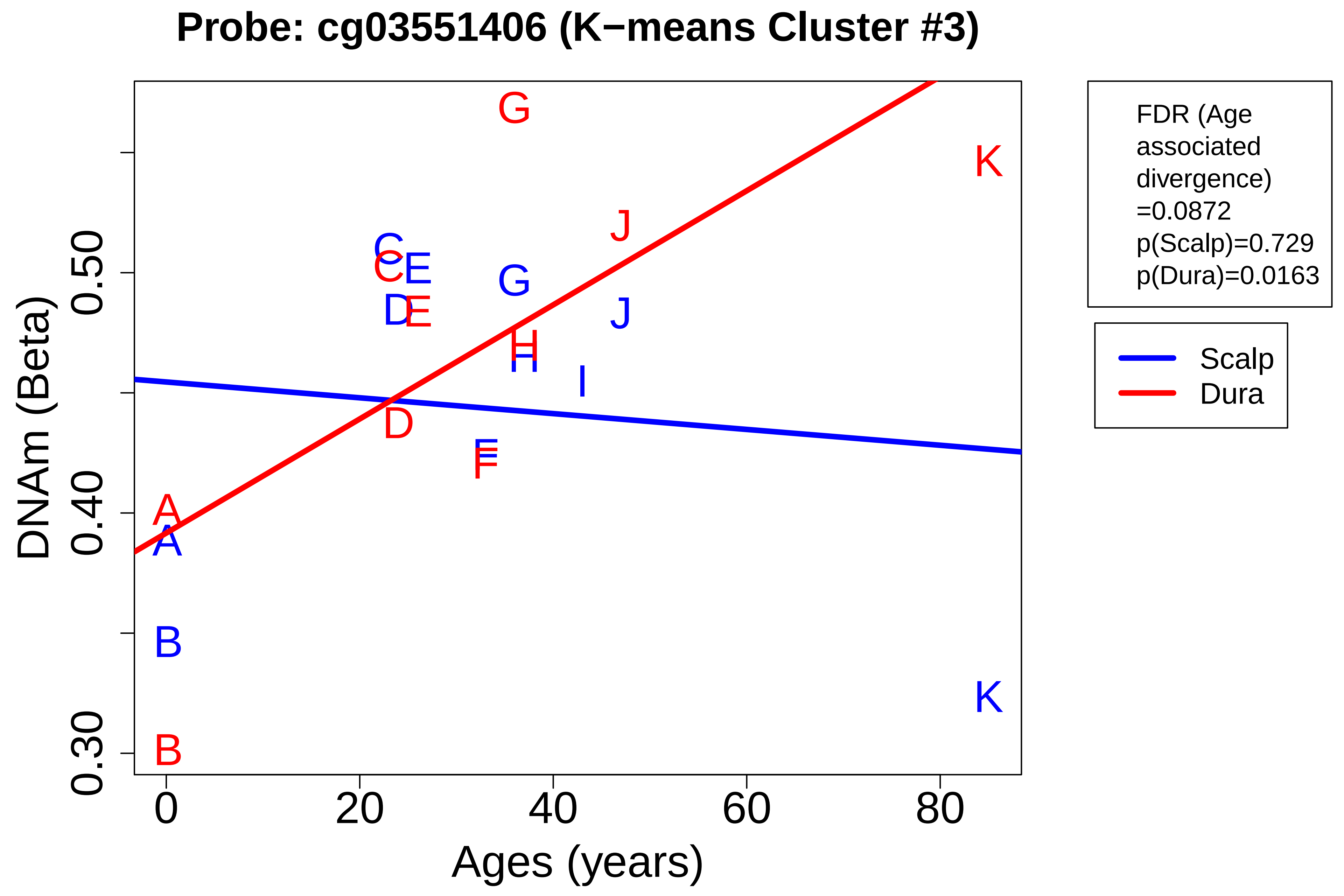
Probe: cg03551406 (K−means Cluster #3)
DNAm (Beta)
0.30
0.40
0.50
Ages (years)
0
20
40
60
80
FDR (Age associated divergence) =0.0872
p(Scalp)=0.729
p(Dura)=0.0163
Scalp
Dura

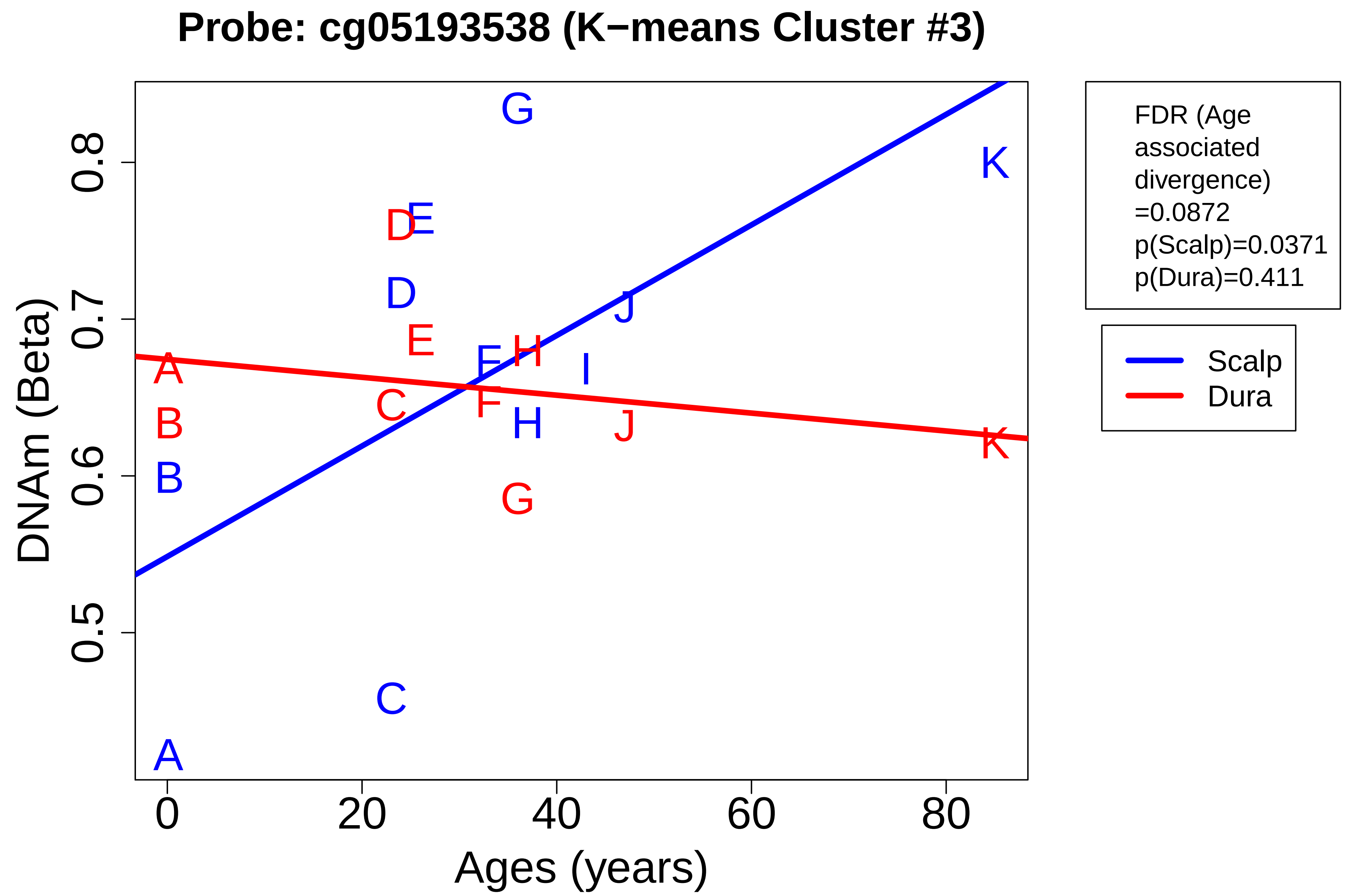

Probe: cg05193538 (K−means Cluster #3)
DNAm (Beta)
Ages (years)
0.5
0.6
0.7
0.8
0
20
40
60
80
FDR (Age associated divergence) =0.0872
p(Scalp)=0.0371
p(Dura)=0.411
Scalp
Dura
A
B
C
D
E
F
G
H
I
J
K

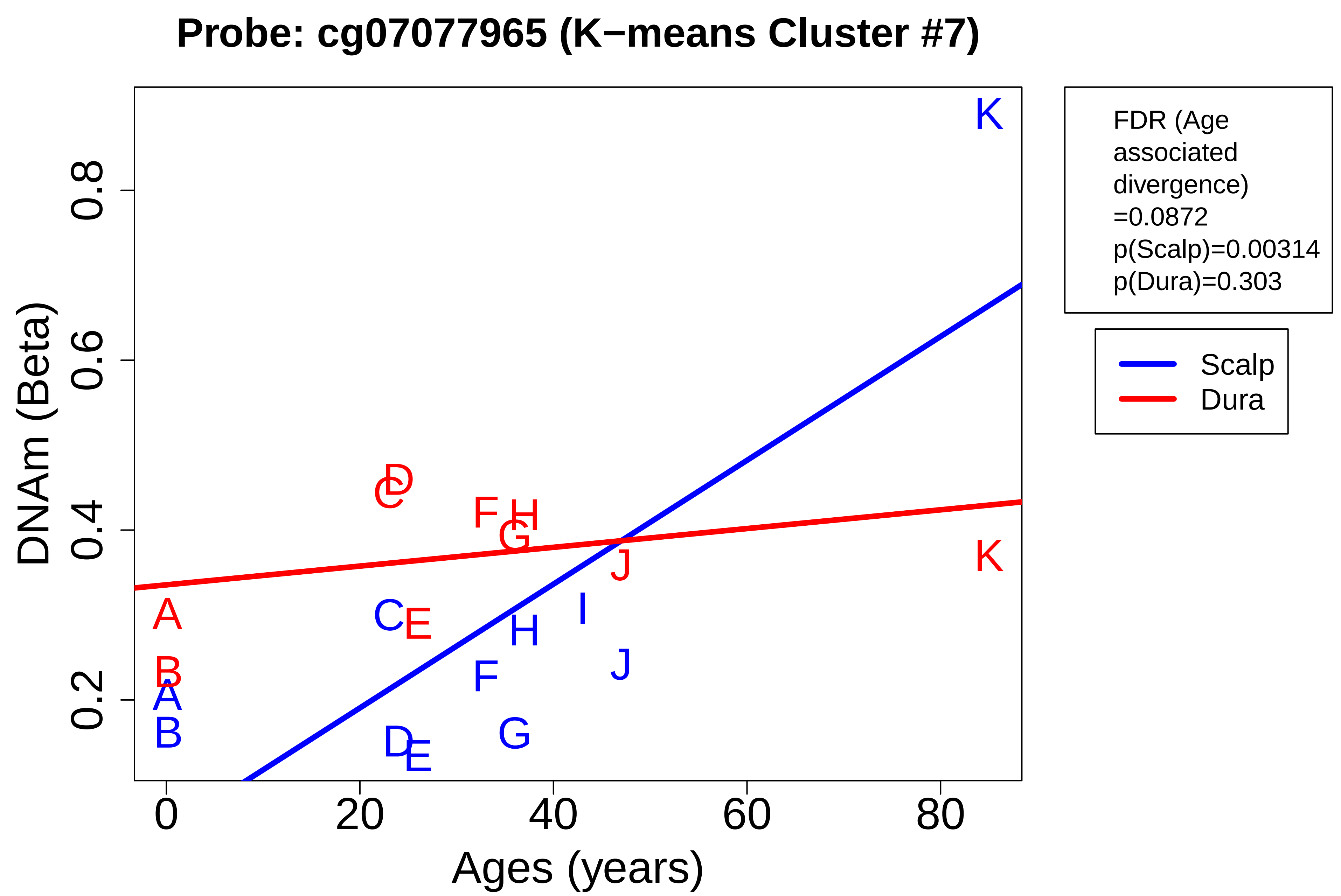

Probe: cg07077965 (K−means Cluster #7)
DNAm (Beta)
0.2
0.4
0.6
0.8
0
20
40
60
80
Ages (years)
A
B
C
D
E
F
G
H
I
J
K
FDR (Age associated divergence) =0.0872
p(Scalp)=0.00314
p(Dura)=0.303
Scalp
Dura

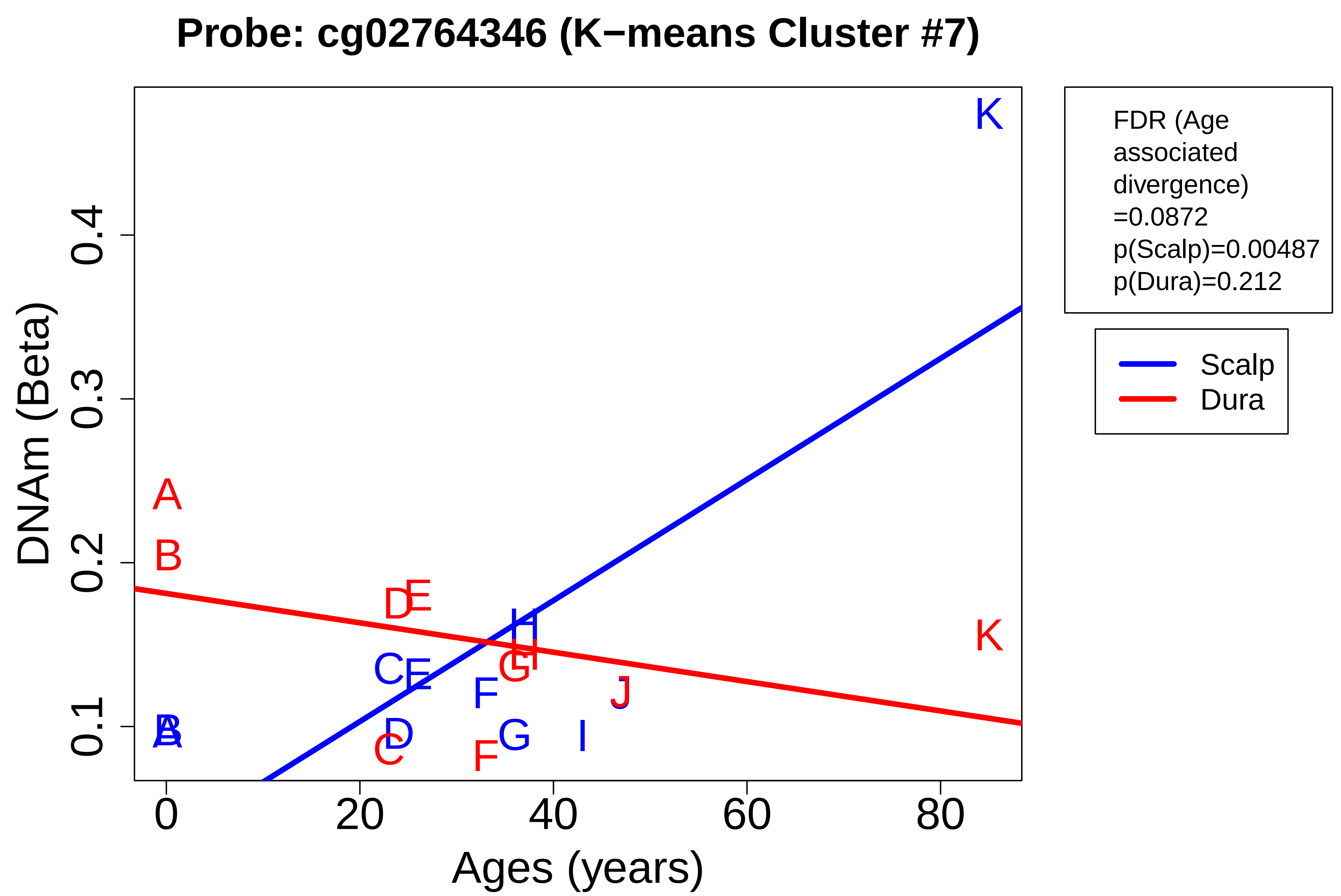

Probe: cg02764346 (K−means Cluster #7)
DNAm (Beta)
0.1
0.2
0.3
0.4
0
20
40
60
80
Ages (years)
FDR (Age associated divergence) =0.0872
p(Scalp)=0.00487
p(Dura)=0.212
Scalp
Dura

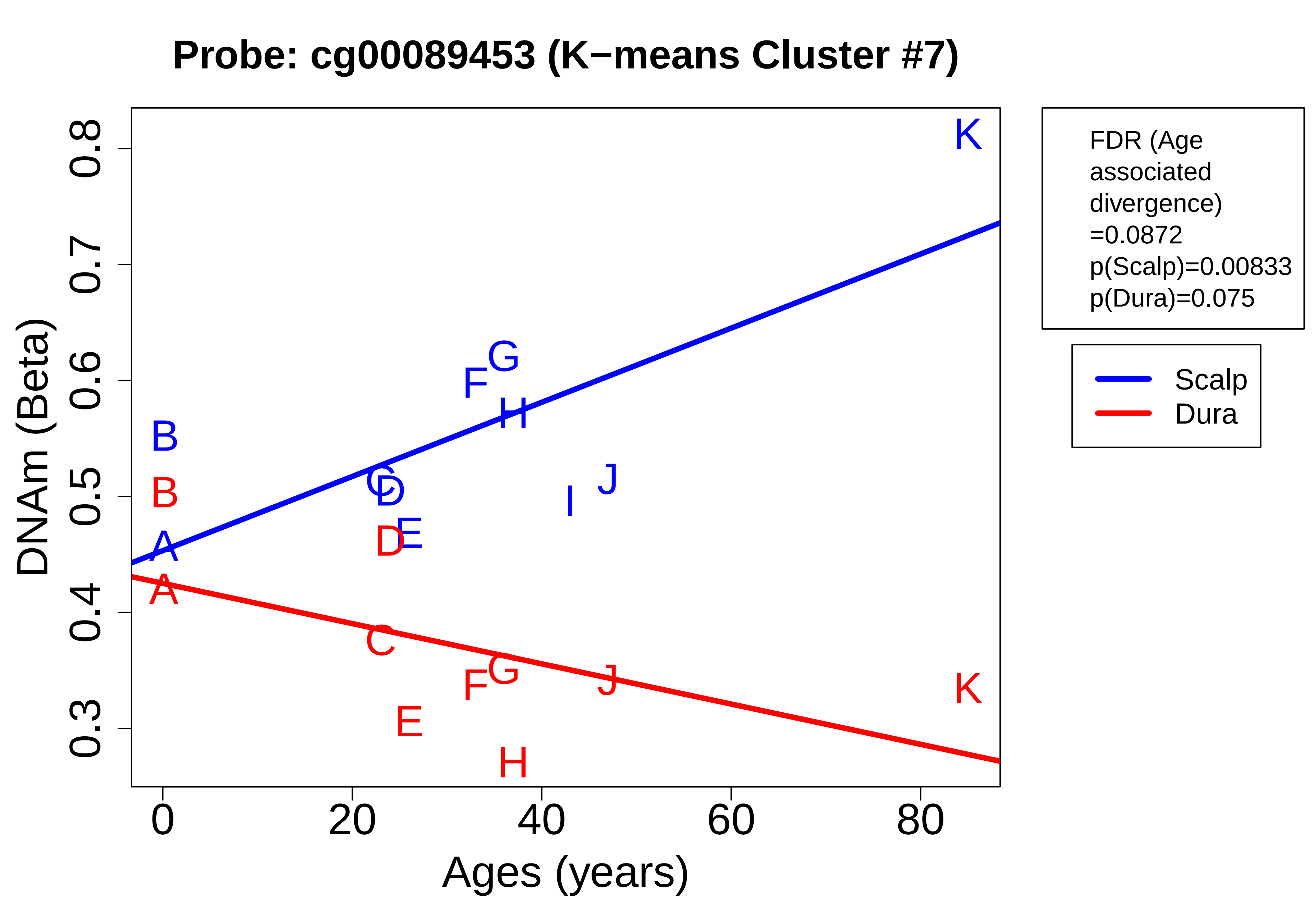

Probe: cg00089453 (K−means Cluster #7)
DNAm (Beta)
0.3
0.4
0.5
0.6
0.7
0.8
0
20
40
60
80
Ages (years)
A
B
C
D
E
F
G
H
I
J
K
FDR (Age associated divergence) =0.0872
p(Scalp)=0.00833
p(Dura)=0.075
Scalp
Dura

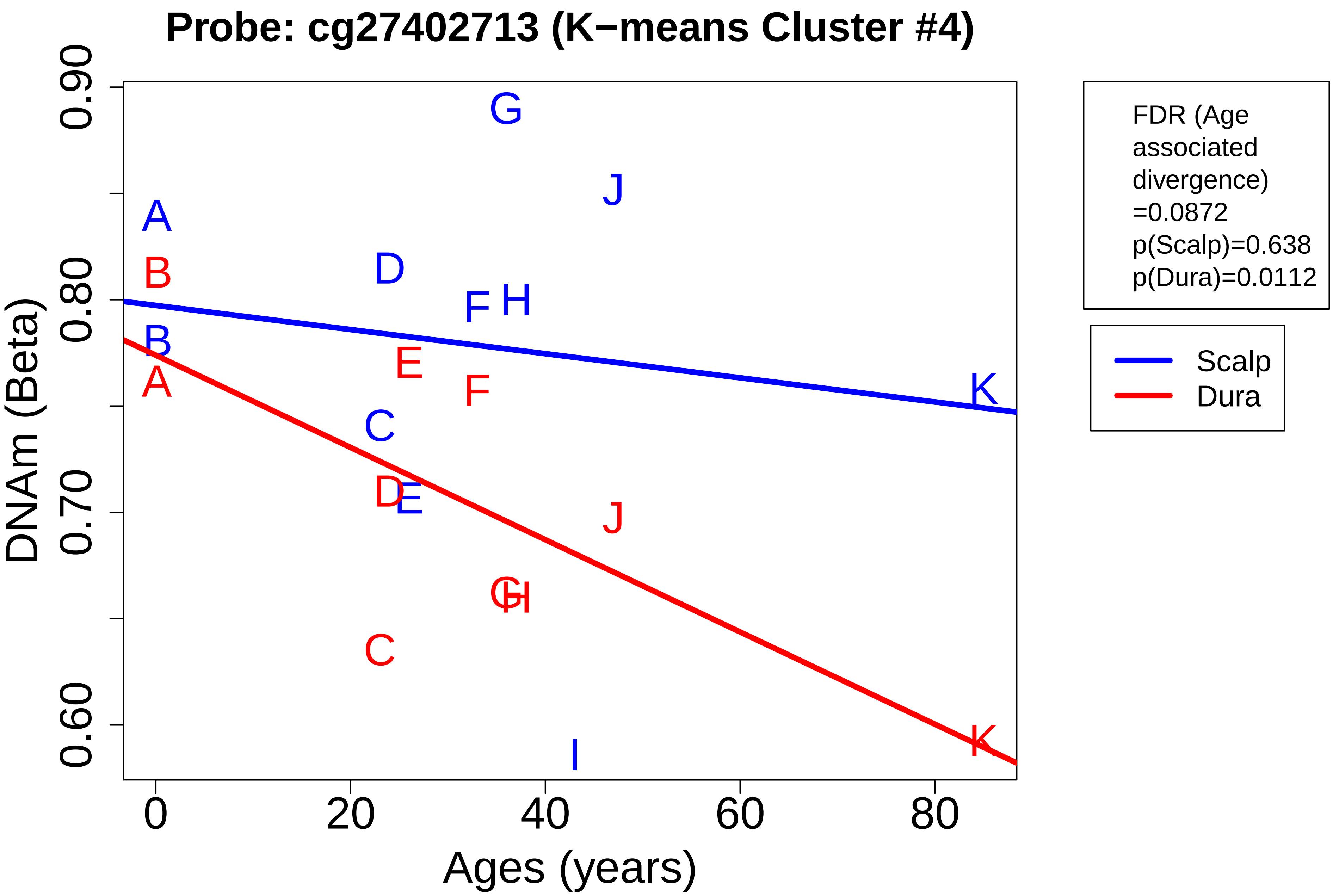

Probe: cg27402713 (K−means Cluster #4)
DNAm (Beta)
0.60
0.70
0.80
0.90
0
20
40
60
80
Ages (years)
A
B
C
D
E
F
G
H
I
J
K
FDR (Age associated divergence) =0.0872
p(Scalp)=0.638
p(Dura)=0.0112
Scalp
Dura

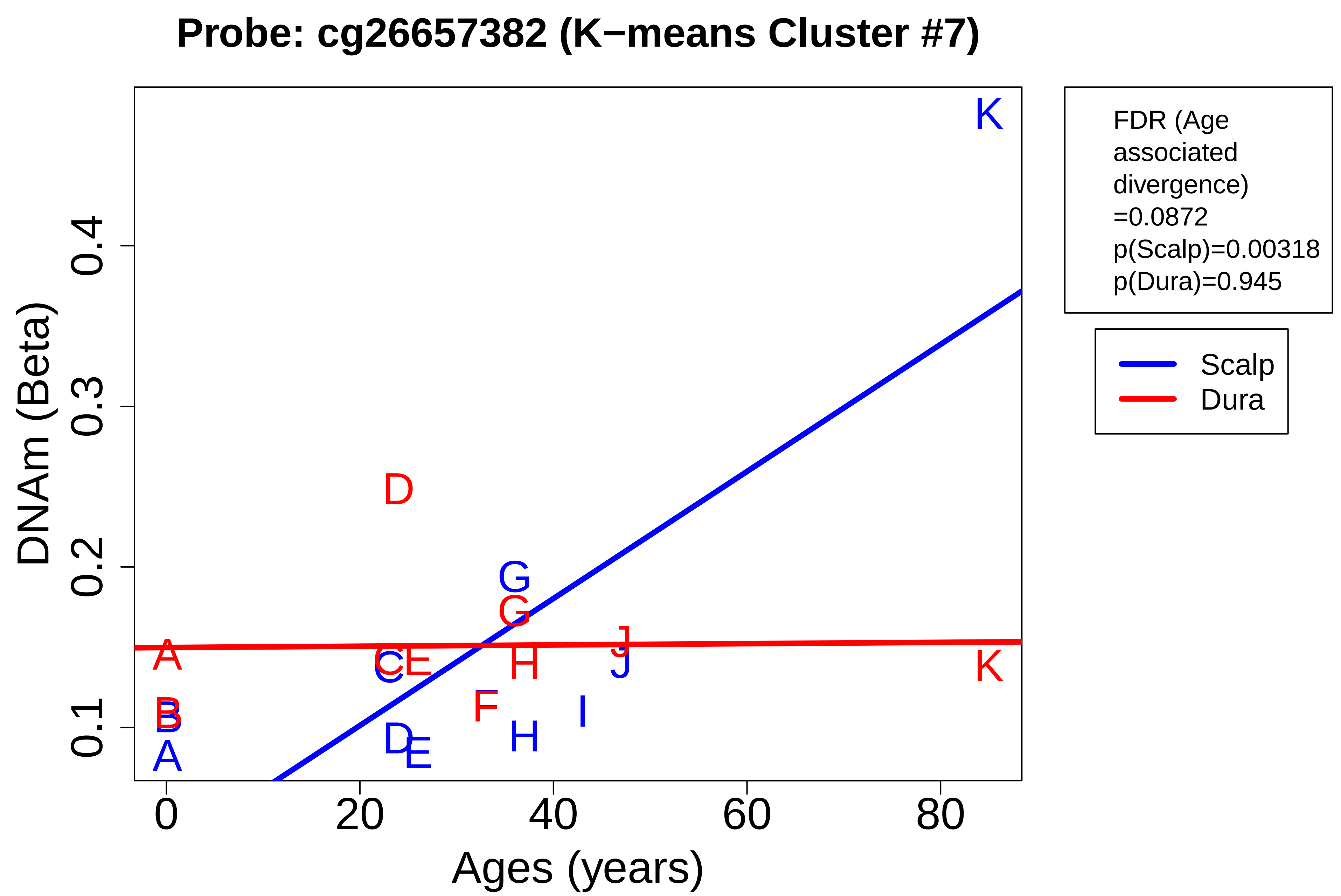
Probe: cg26657382 (K−means Cluster #7)
DNAm (Beta)
0.1
0.2
0.3
0.4
0
20
40
60
80
Ages (years)
FDR (Age associated divergence) =0.0872
p(Scalp)=0.00318
p(Dura)=0.945
Scalp
Dura

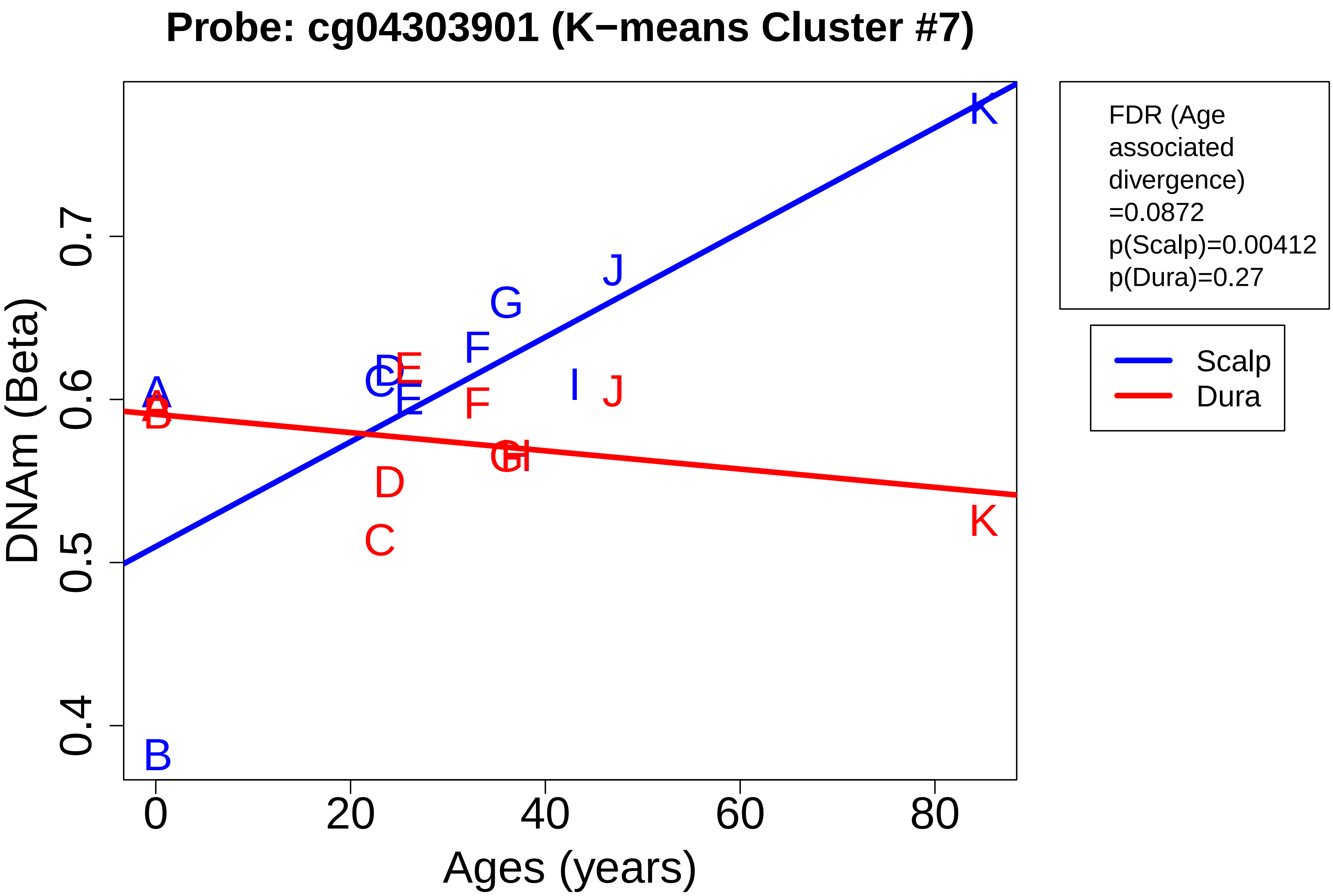

Probe: cg04303901 (K−means Cluster #7)
DNAm (Beta)
0.4
0.5
0.6
0.7
Ages (years)
0
20
40
60
80
FDR (Age associated divergence) =0.0872
p(Scalp)=0.00412
p(Dura)=0.27
Scalp
Dura
A
B
C
D
E
F
G
H
I
J
K

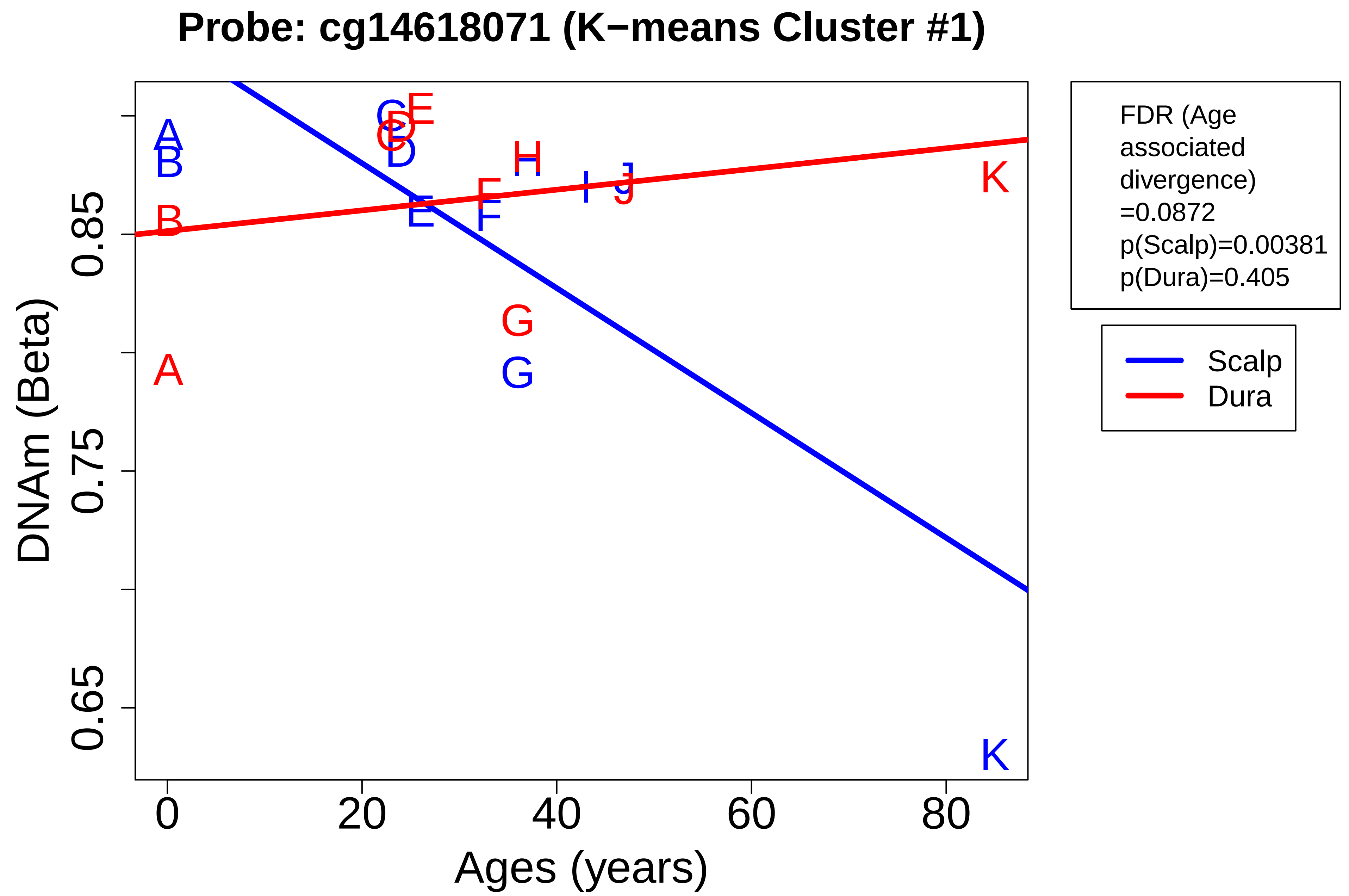

Probe: cg14618071 (K−means Cluster #1)
DNAm (Beta)
0.65
0.75
0.85
Ages (years)
0
20
40
60
80
A
B
C
D
E
F
G
H
I
J
K
FDR (Age associated divergence) =0.0872
p(Scalp)=0.00381
p(Dura)=0.405
Scalp
Dura

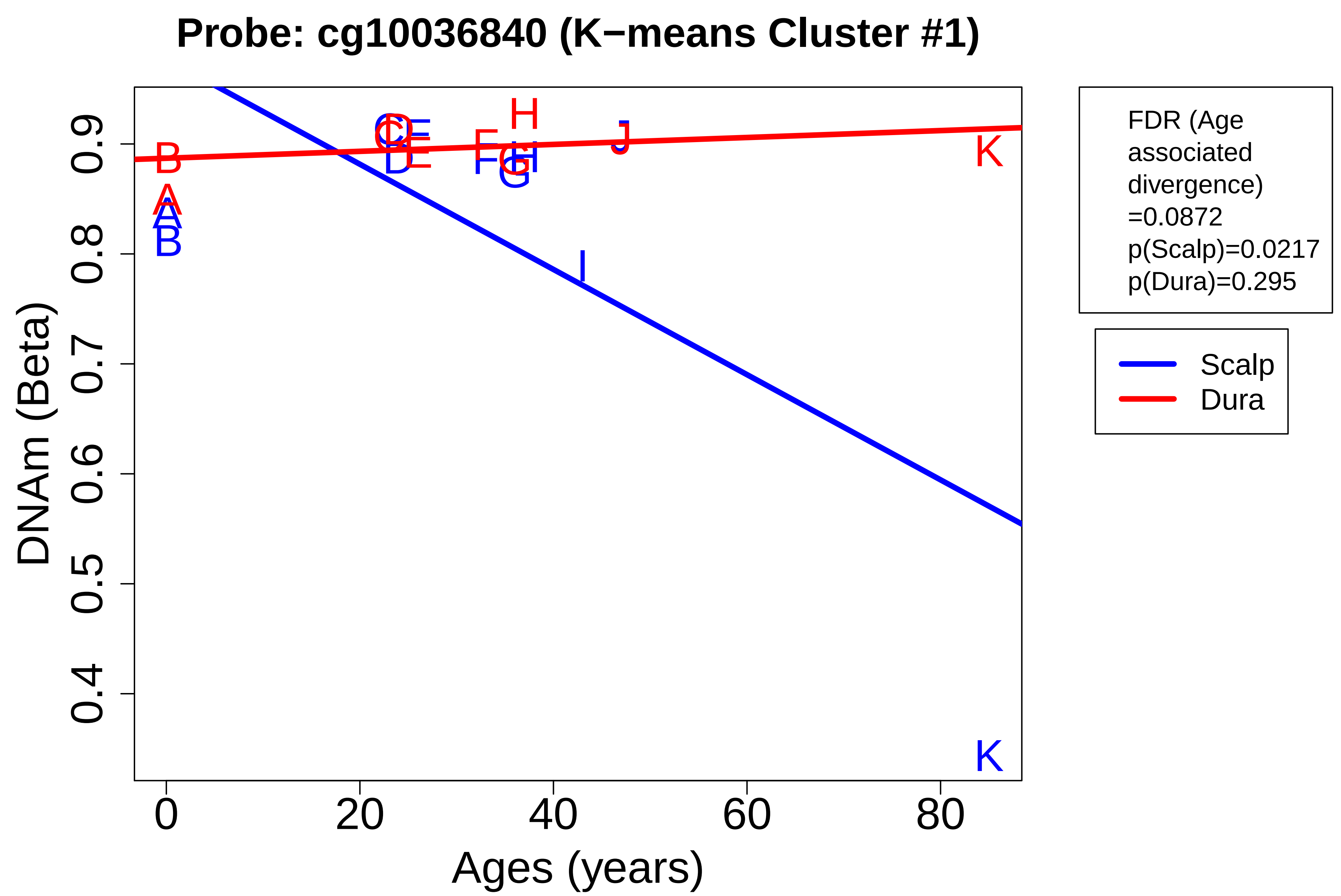

Probe: cg10036840 (K−means Cluster #1)
DNAm (Beta)
0.4
0.5
0.6
0.7
0.8
0.9
0
20
40
60
80
Ages (years)
FDR (Age associated divergence) =0.0872
p(Scalp)=0.0217
p(Dura)=0.295
Scalp
Dura

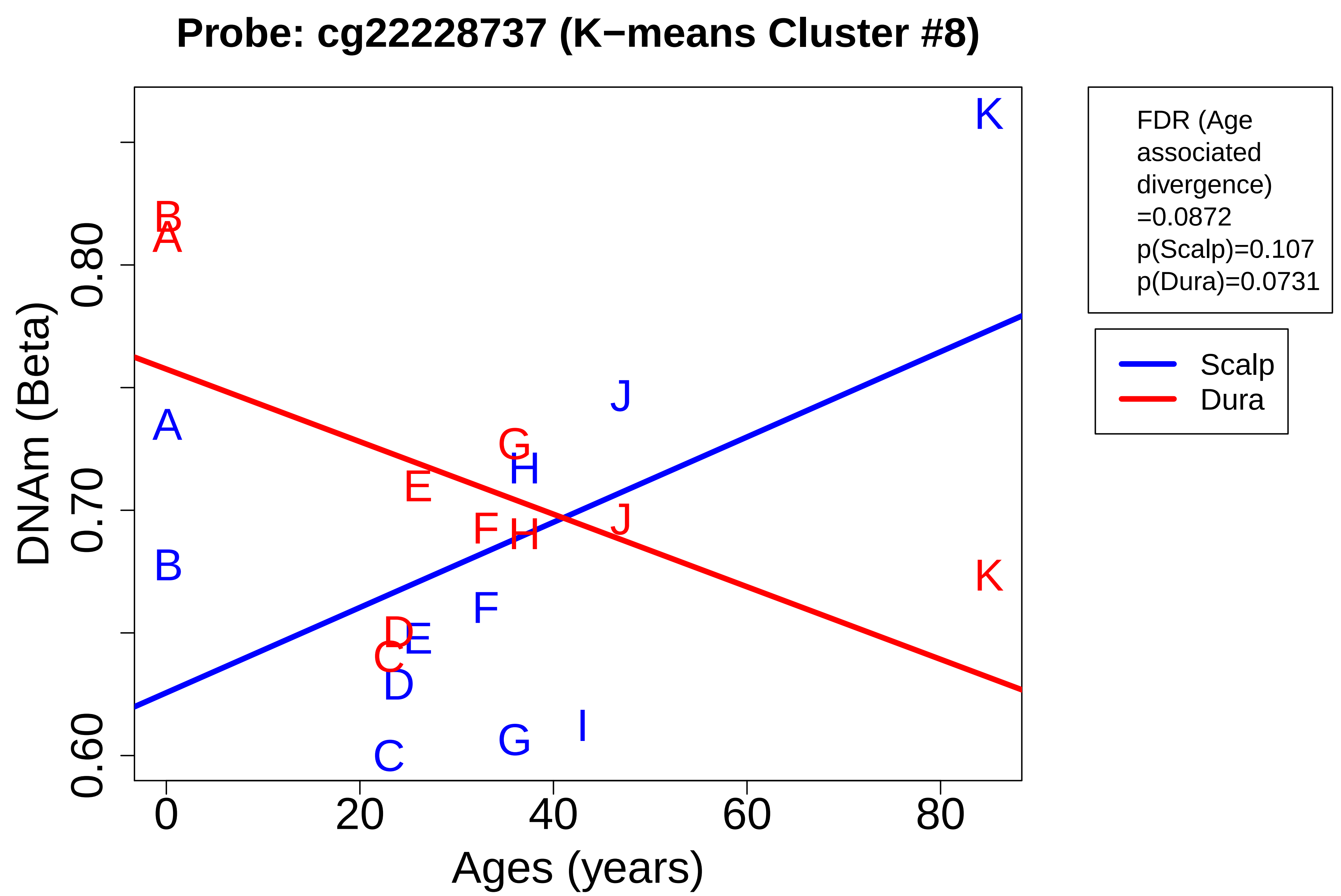

Probe: cg22228737 (K−means Cluster #8)
DNAm (Beta)
0.60
0.70
0.80
0
20
40
60
80
Ages (years)
K
B
A
J
A
G
H
E
F
H
J
B
K
F
D
E
C
D
C
G
I
FDR (Age associated divergence) =0.0872
p(Scalp)=0.107
p(Dura)=0.0731
Scalp
Dura

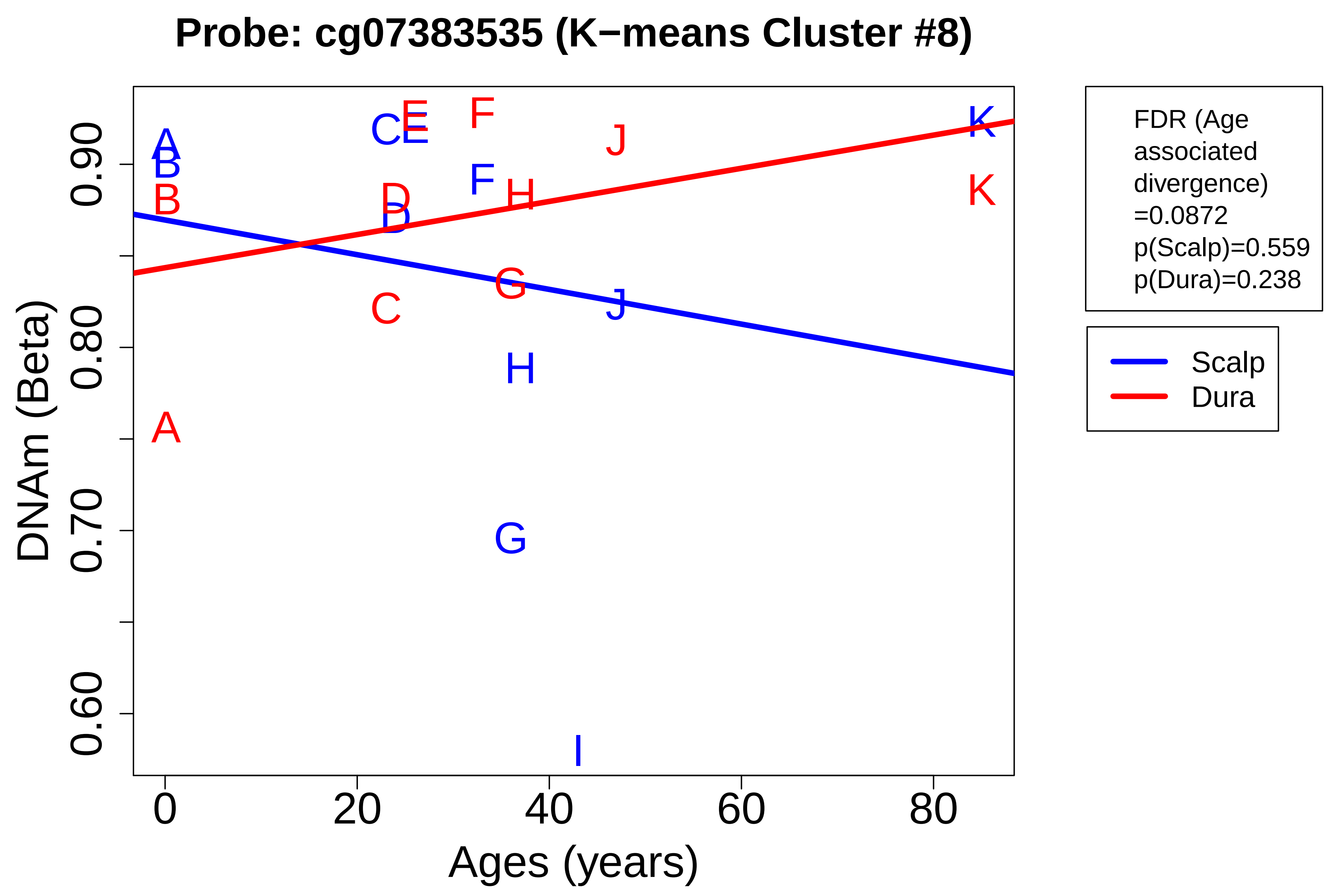

Probe: cg07383535 (K−means Cluster #8)
DNAm (Beta)
0.60
0.70
0.80
0.90
0
20
40
60
80
Ages (years)
FDR (Age associated divergence) =0.0872
p(Scalp)=0.559
p(Dura)=0.238
Scalp
Dura
A
B
C
D
E
F
G
H
I
J
K

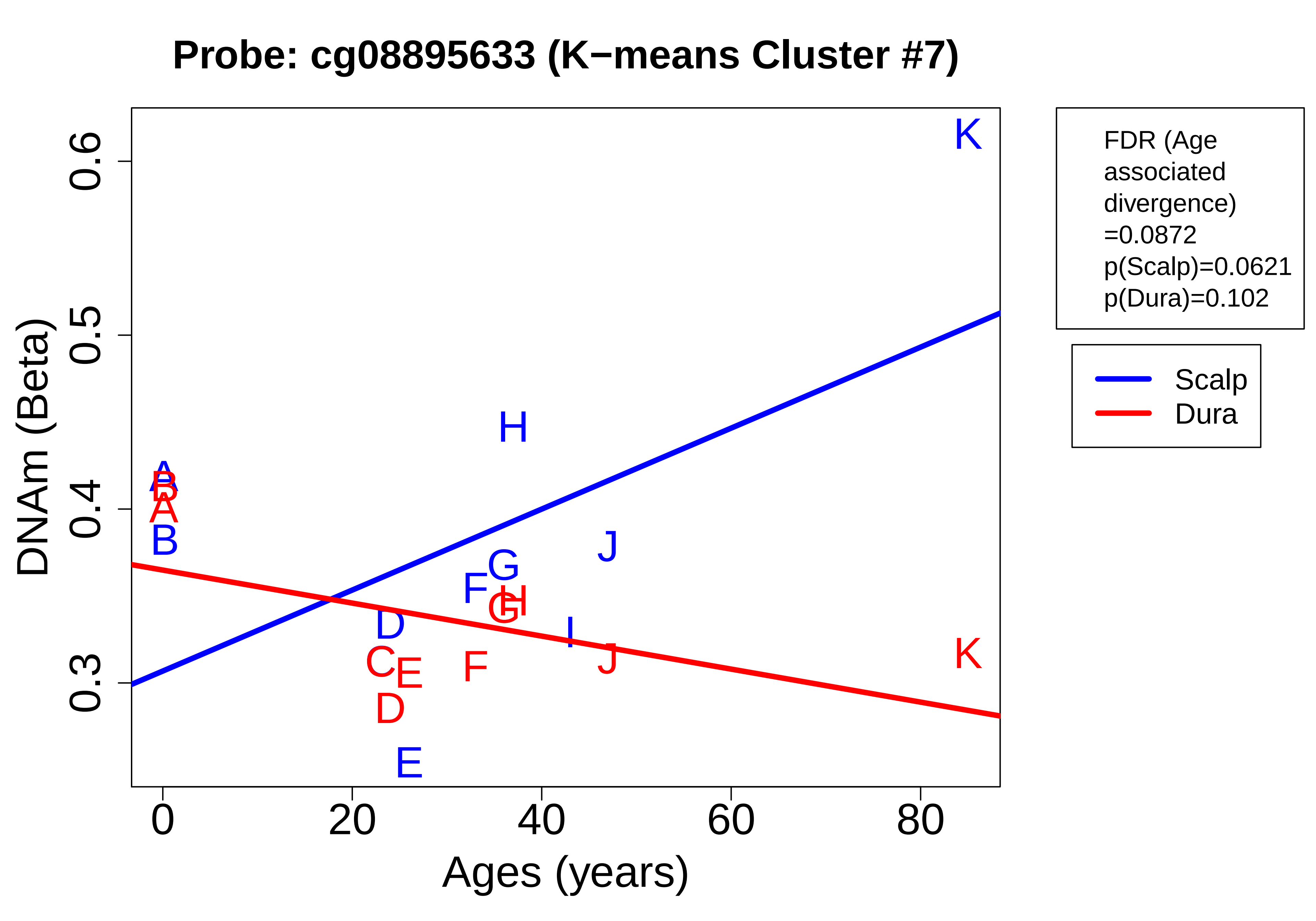

Probe: cg08895633 (K−means Cluster #7)
DNAm (Beta)
0.3
0.4
0.5
0.6
0
20
40
60
80
Ages (years)
FDR (Age associated divergence) =0.0872
p(Scalp)=0.0621
p(Dura)=0.102
Scalp
Dura
A
B
C
D
E
F
G
H
I
J
K

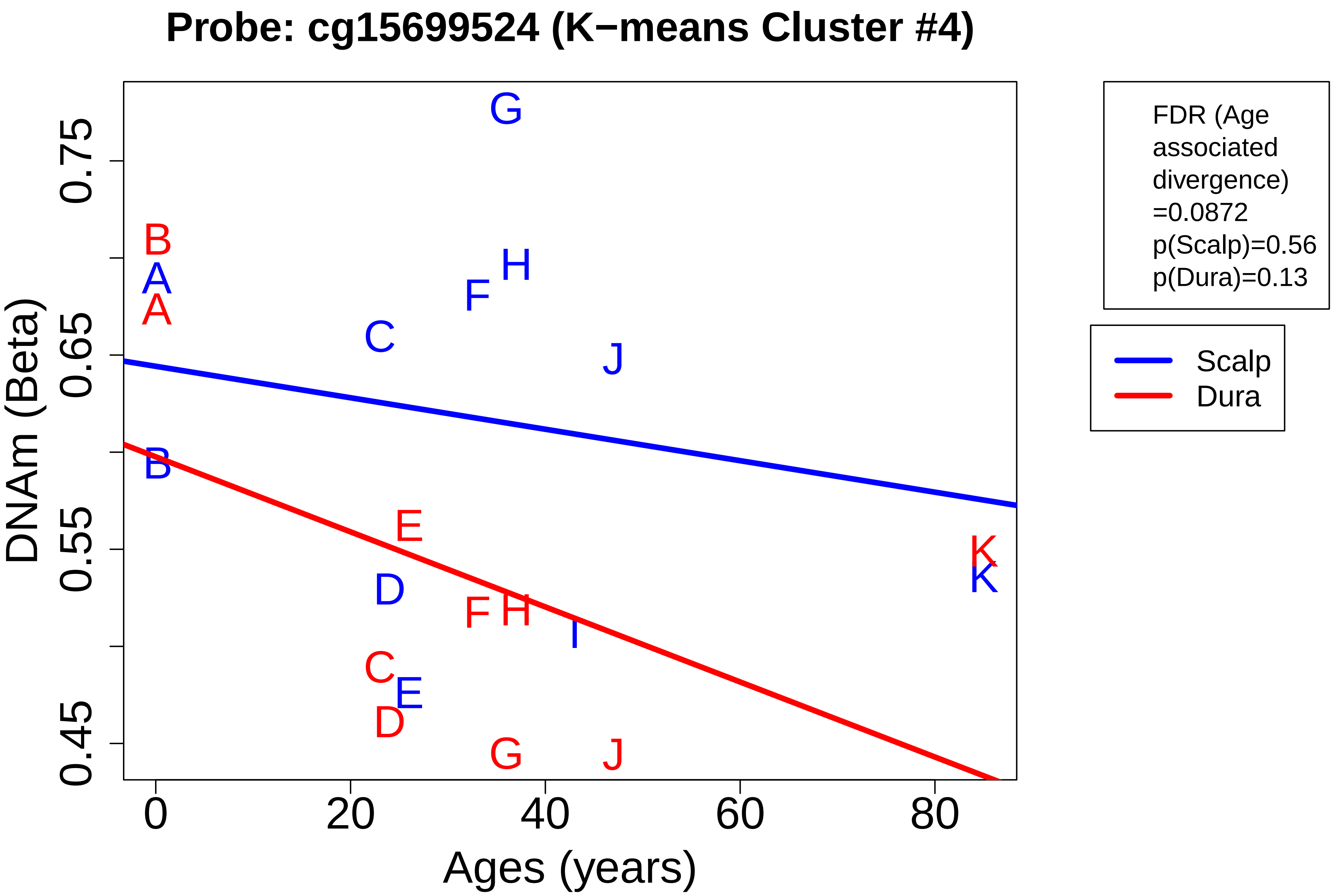

Probe: cg15699524 (K−means Cluster #4)
DNAm (Beta)
0.45
0.55
0.65
0.75
Ages (years)
0
20
40
60
80
A
B
C
D
E
F
G
H
I
J
K
FDR (Age associated divergence) =0.0872
p(Scalp)=0.56
p(Dura)=0.13
Scalp
Dura

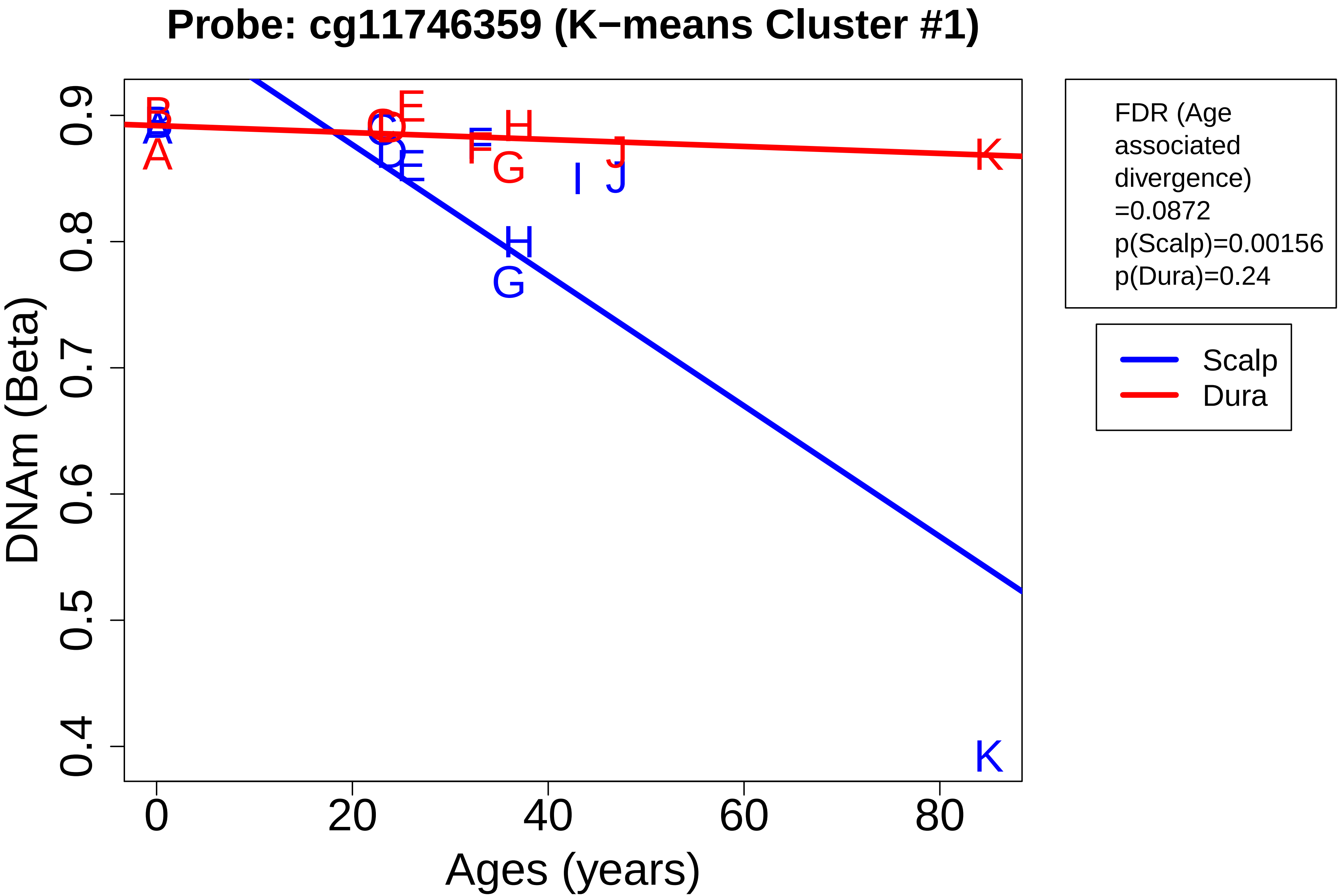

Probe: cg11746359 (K−means Cluster #1)
DNAm (Beta)
0.4
0.5
0.6
0.7
0.8
0.9
Ages (years)
0
20
40
60
80
FDR (Age associated divergence) =0.0872
p(Scalp)=0.00156
p(Dura)=0.24
Scalp
Dura

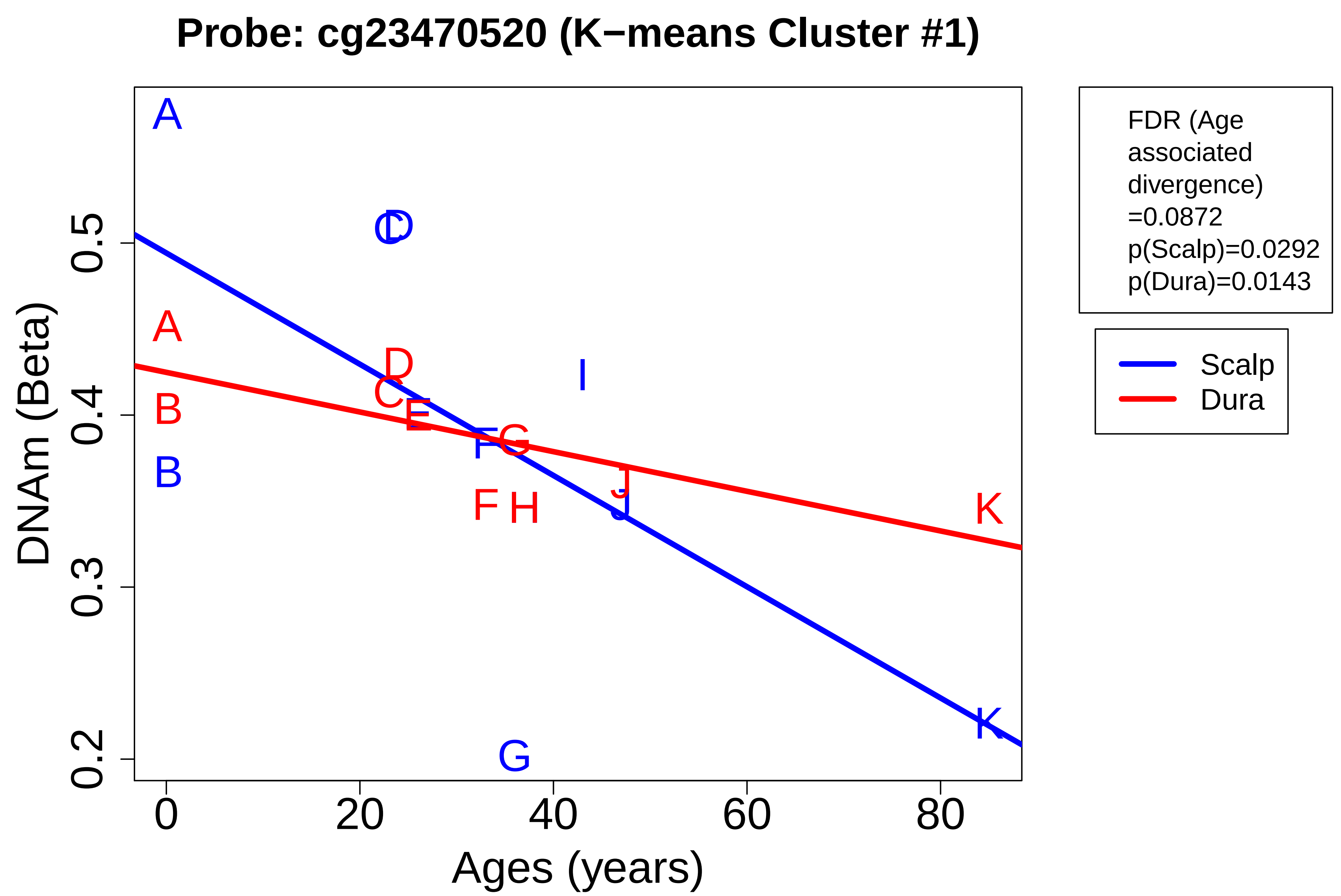

Probe: cg23470520 (K−means Cluster #1)
FDR (Age associated divergence) =0.0872
p(Scalp)=0.0292
p(Dura)=0.0143
Scalp
Dura
DNAm (Beta)
Ages (years)
0.2
0.3
0.4
0.5
0
20
40
60
80
A
B
C
D
E
F
G
H
I
J
K

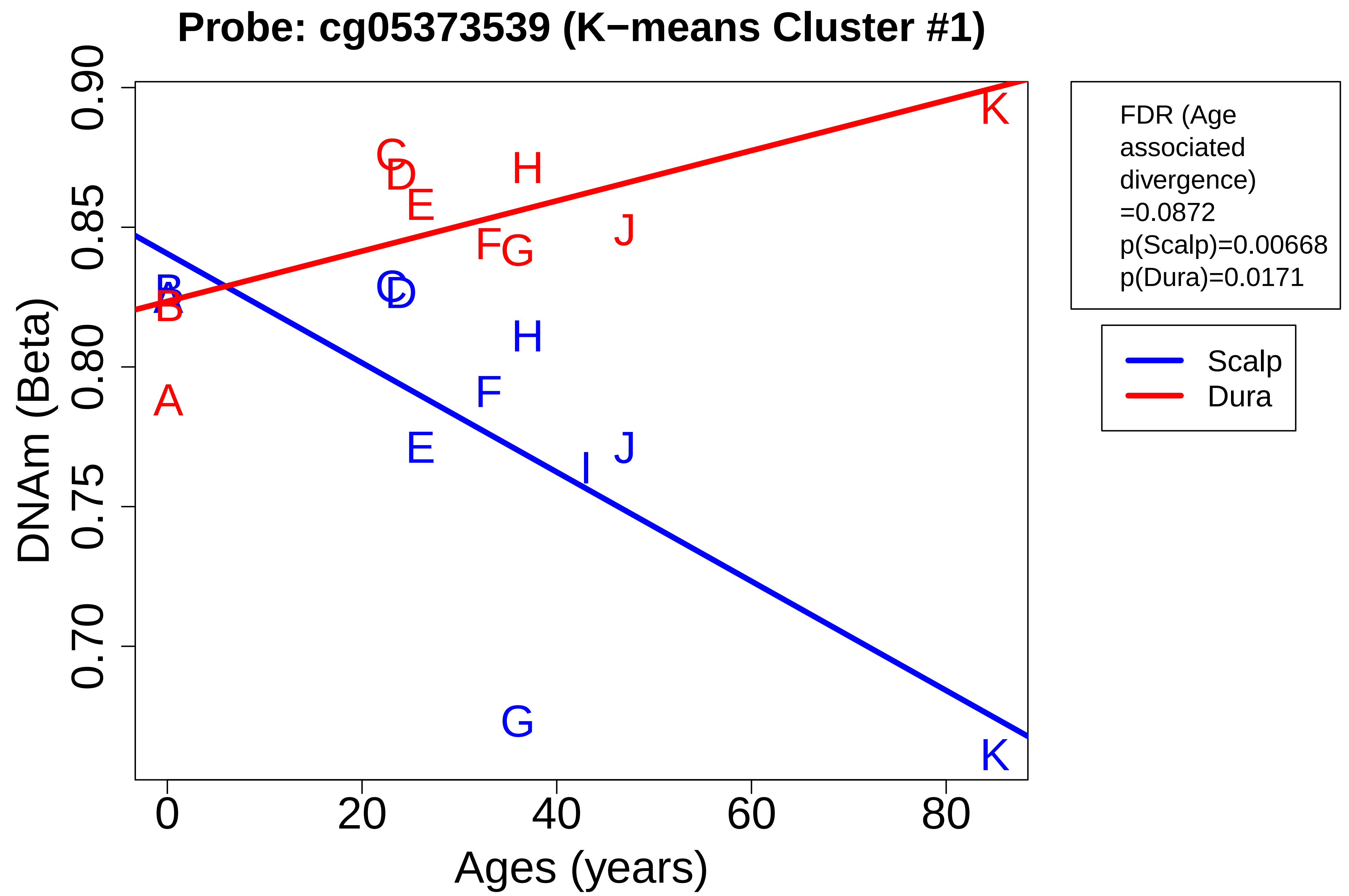

Probe: cg05373539 (K−means Cluster #1)
DNAm (Beta)
0.70
0.75
0.80
0.85
0.90
0
20
40
60
80
Ages (years)
FDR (Age associated divergence) =0.0872
p(Scalp)=0.00668
p(Dura)=0.0171
Scalp
Dura

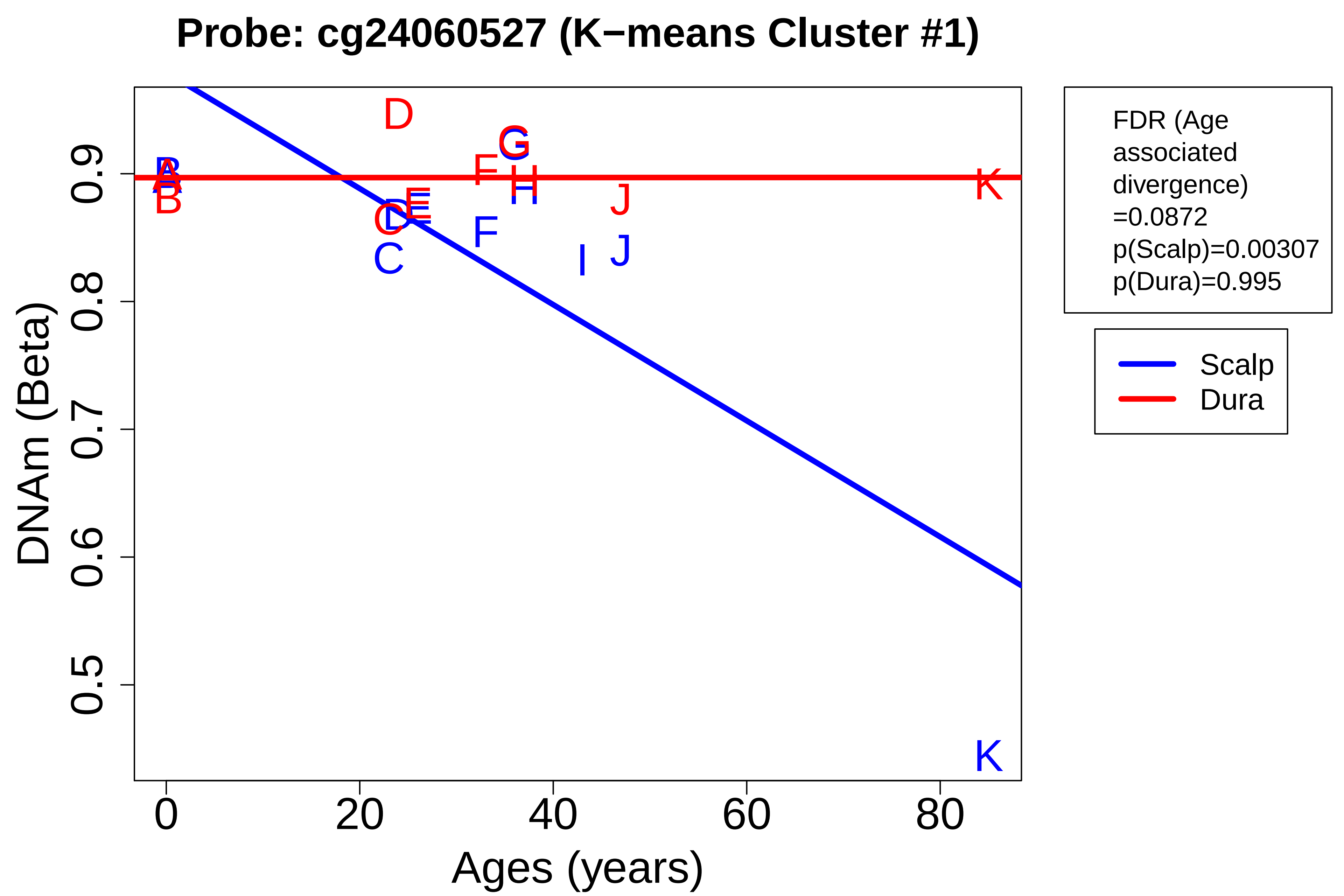
Probe: cg24060527 (K−means Cluster #1)
DNAm (Beta)
0.5
0.6
0.7
0.8
0.9
Ages (years)
0
20
40
60
80
FDR (Age associated divergence) =0.0872
p(Scalp)=0.00307
p(Dura)=0.995
Scalp
Dura

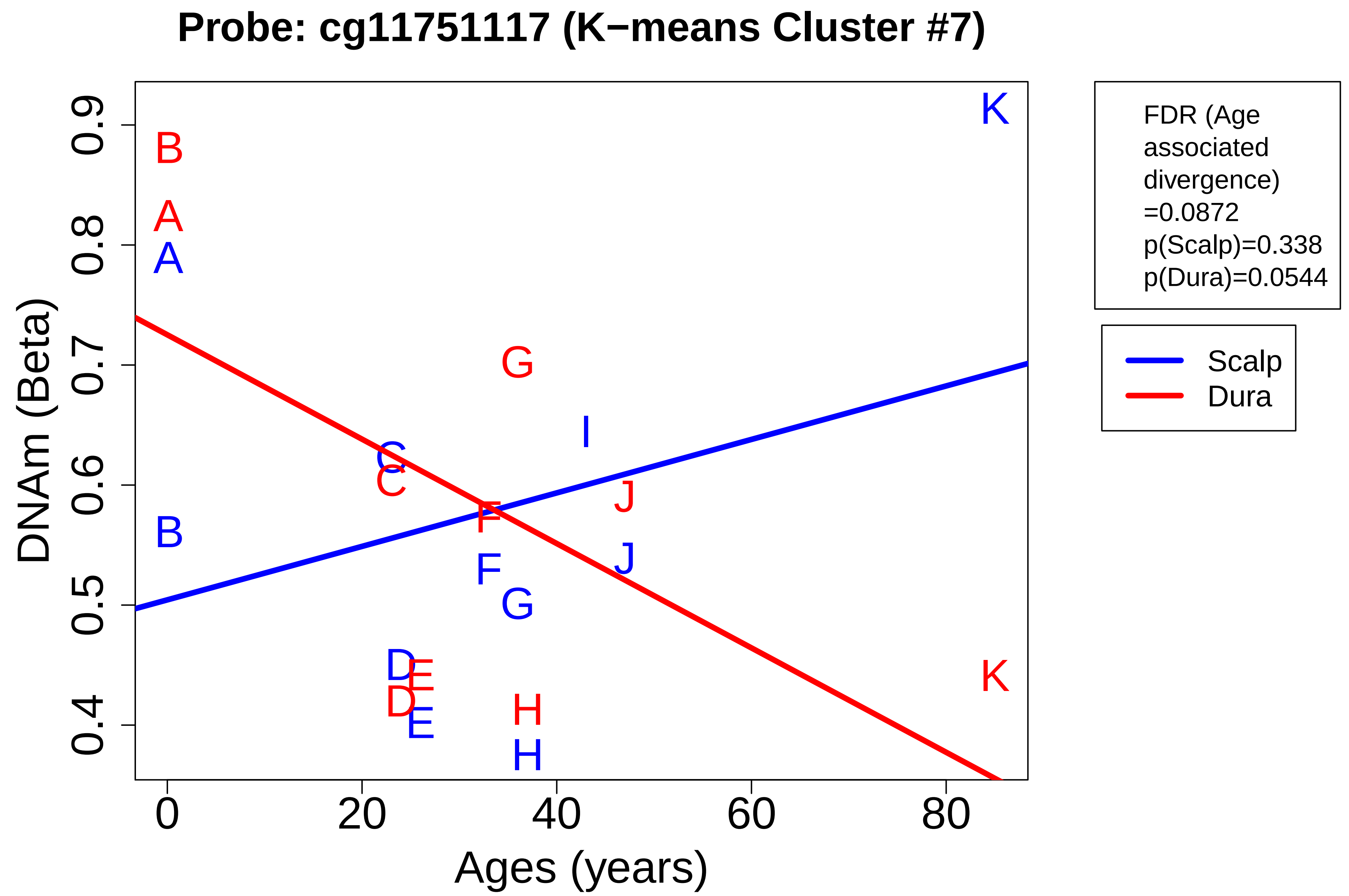
Probe: cg11751117 (K−means Cluster #7)
DNAm (Beta)
0.4
0.5
0.6
0.7
0.8
0.9
0
20
40
60
80
Ages (years)
FDR (Age associated divergence) =0.0872
p(Scalp)=0.338
p(Dura)=0.0544
Scalp
Dura

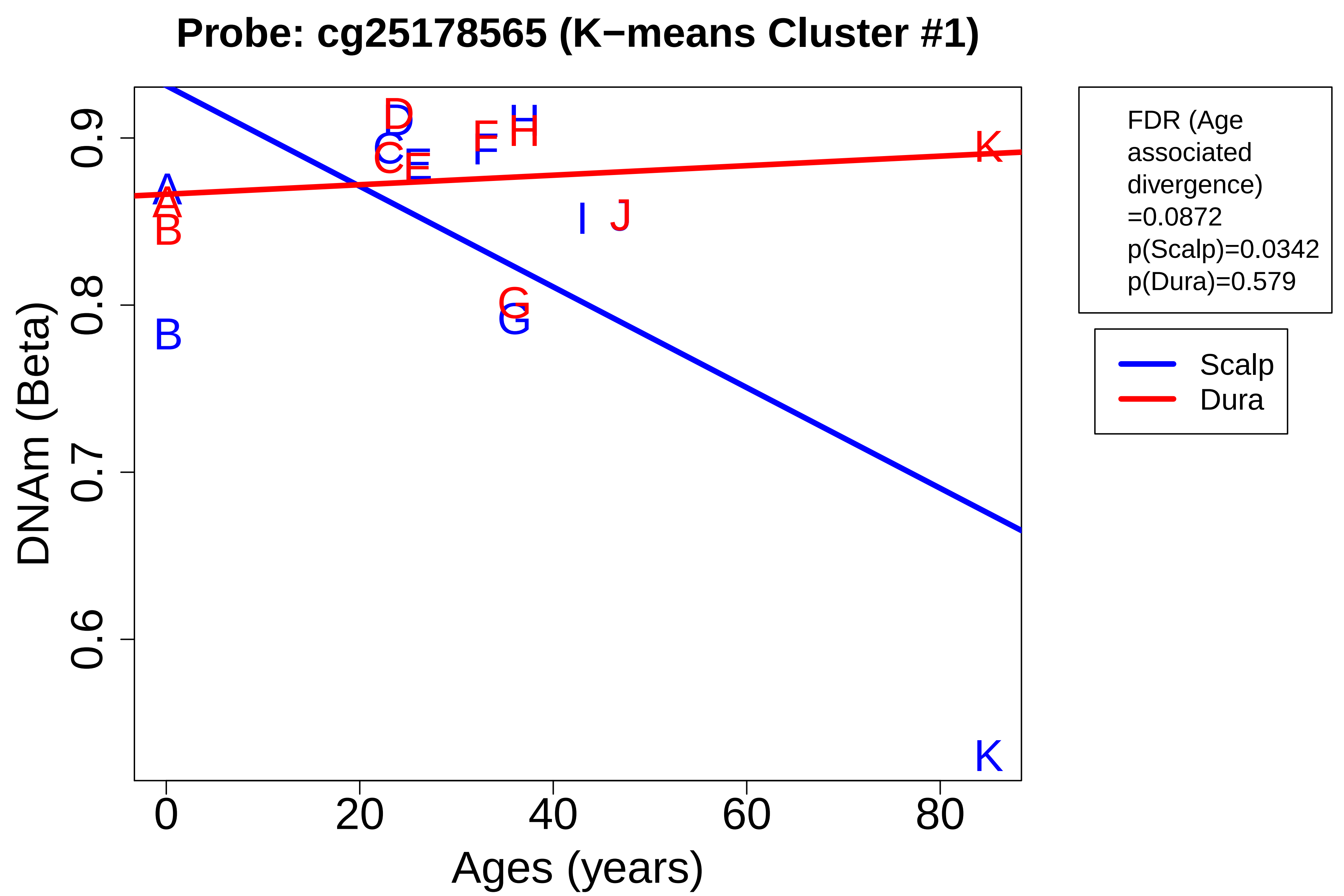

Probe: cg25178565 (K−means Cluster #1)
DNAm (Beta)
0.6
0.7
0.8
0.9
0
20
40
60
80
Ages (years)
A
B
C
D
E
F
G
H
I
J
K
FDR (Age associated divergence) =0.0872
p(Scalp)=0.0342
p(Dura)=0.579
Scalp
Dura

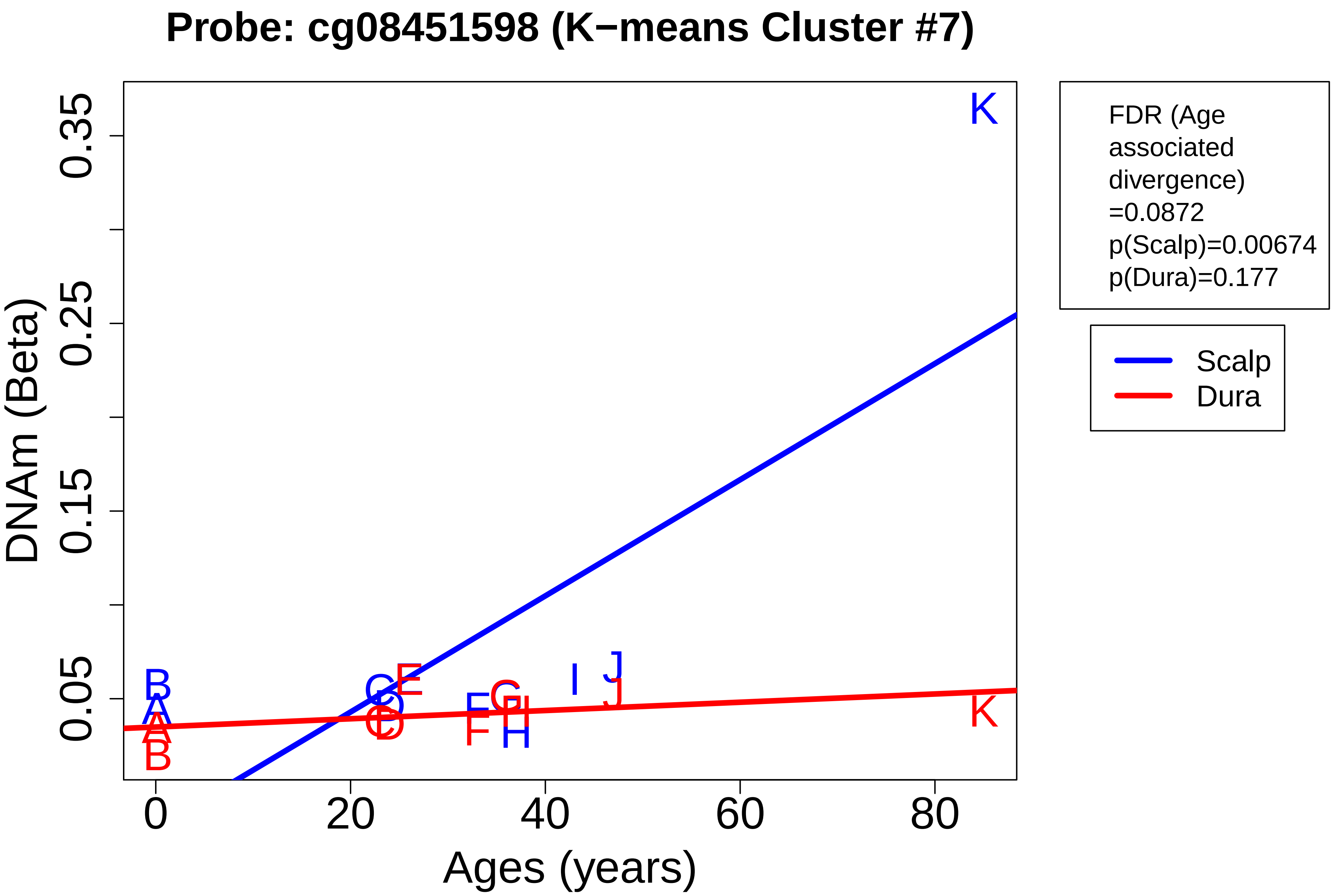

Probe: cg08451598 (K−means Cluster #7)
DNAm (Beta)
0.05
0.15
0.25
0.35
0
20
40
60
80
Ages (years)
FDR (Age associated divergence) =0.0872
p(Scalp)=0.00674
p(Dura)=0.177
Scalp
Dura

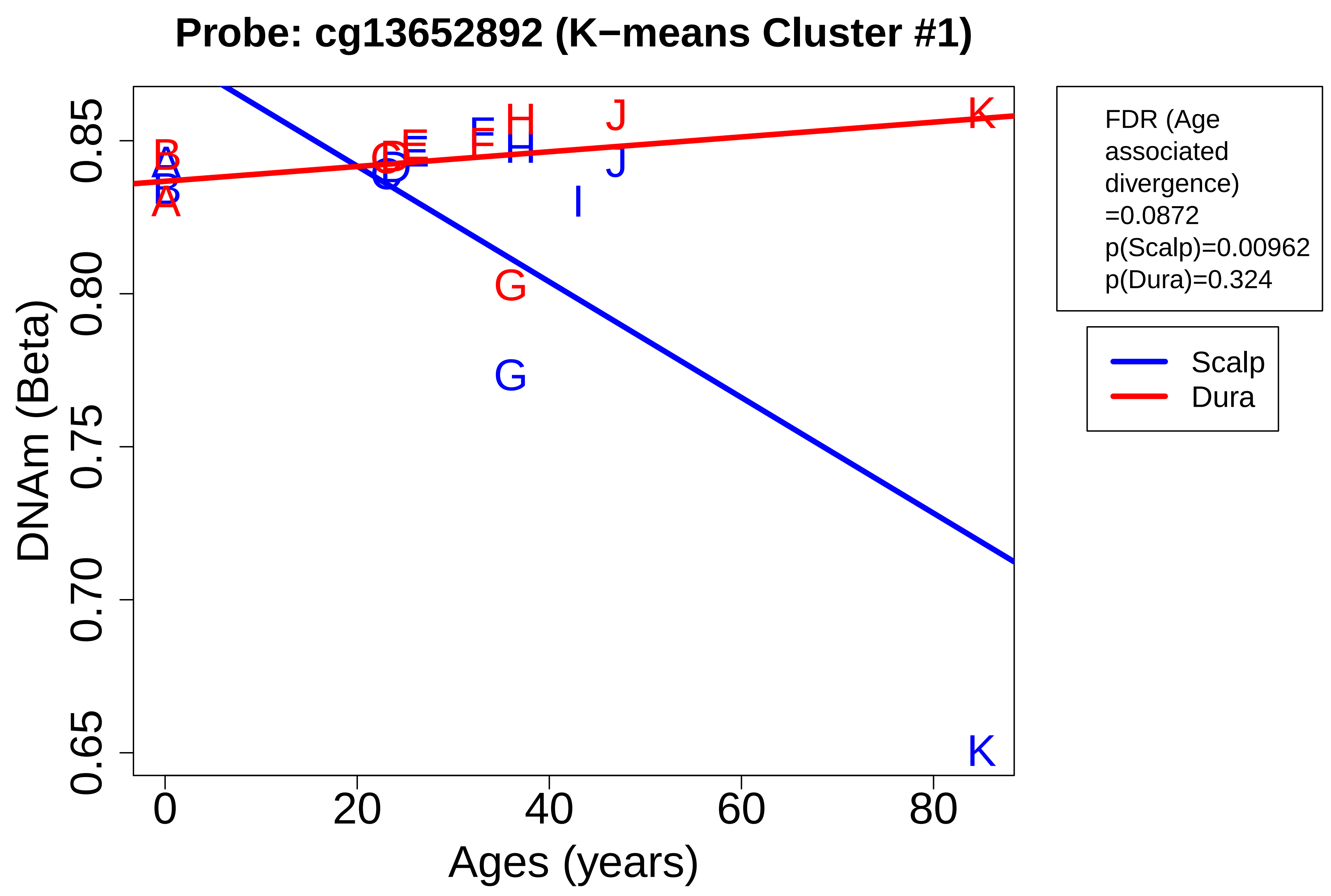

Probe: cg13652892 (K−means Cluster #1)
DNAm (Beta)
0.65
0.70
0.75
0.80
0.85
Ages (years)
0
20
40
60
80
FDR (Age associated divergence) =0.0872
p(Scalp)=0.00962
p(Dura)=0.324
Scalp
Dura

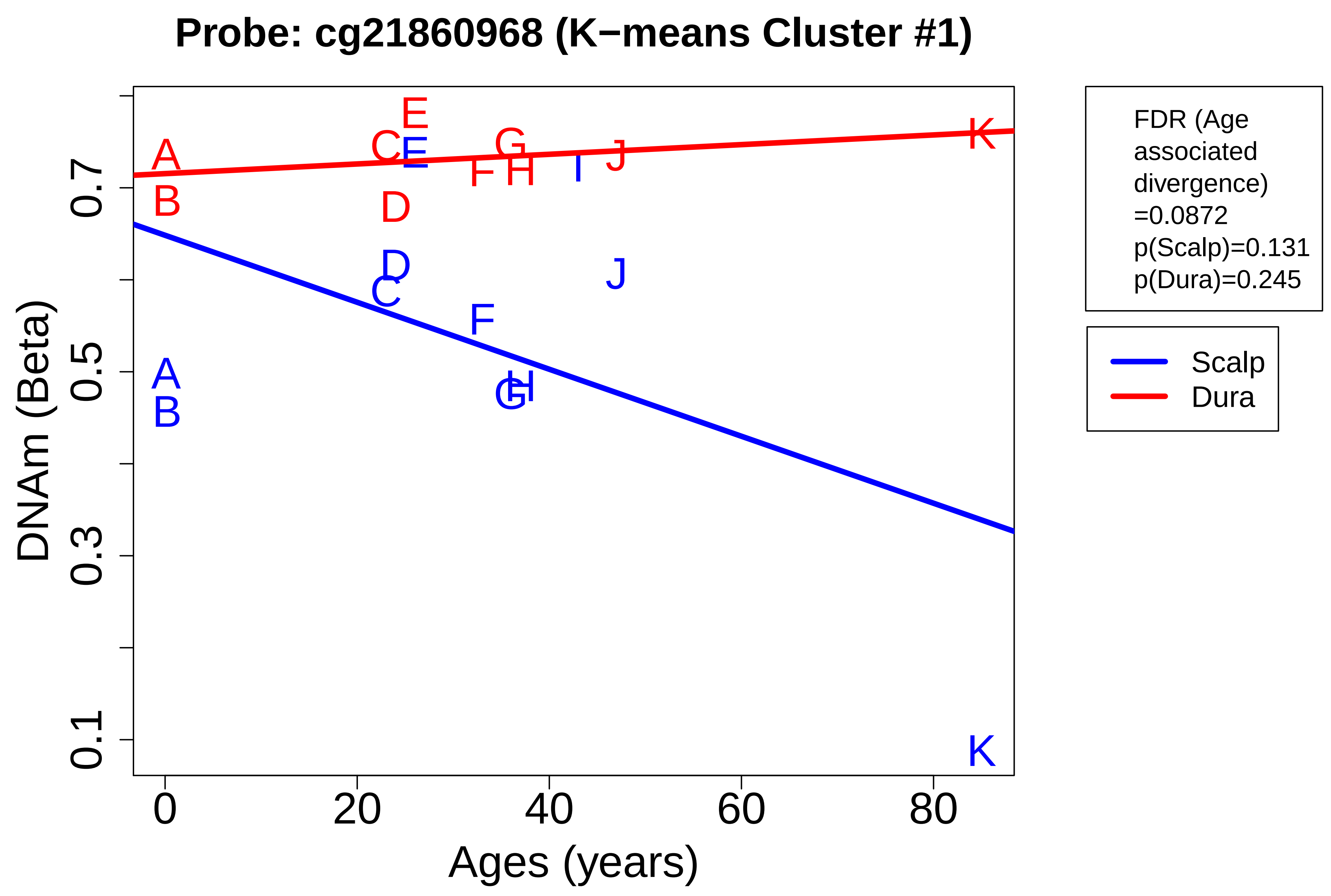

Probe: cg21860968 (K−means Cluster #1)
DNAm (Beta)
0.1
0.3
0.5
0.7
0
20
40
60
80
Ages (years)
FDR (Age associated divergence) =0.0872
p(Scalp)=0.131
p(Dura)=0.245
Scalp
Dura
A
B
C
D
E
F
G
H
I
J
K

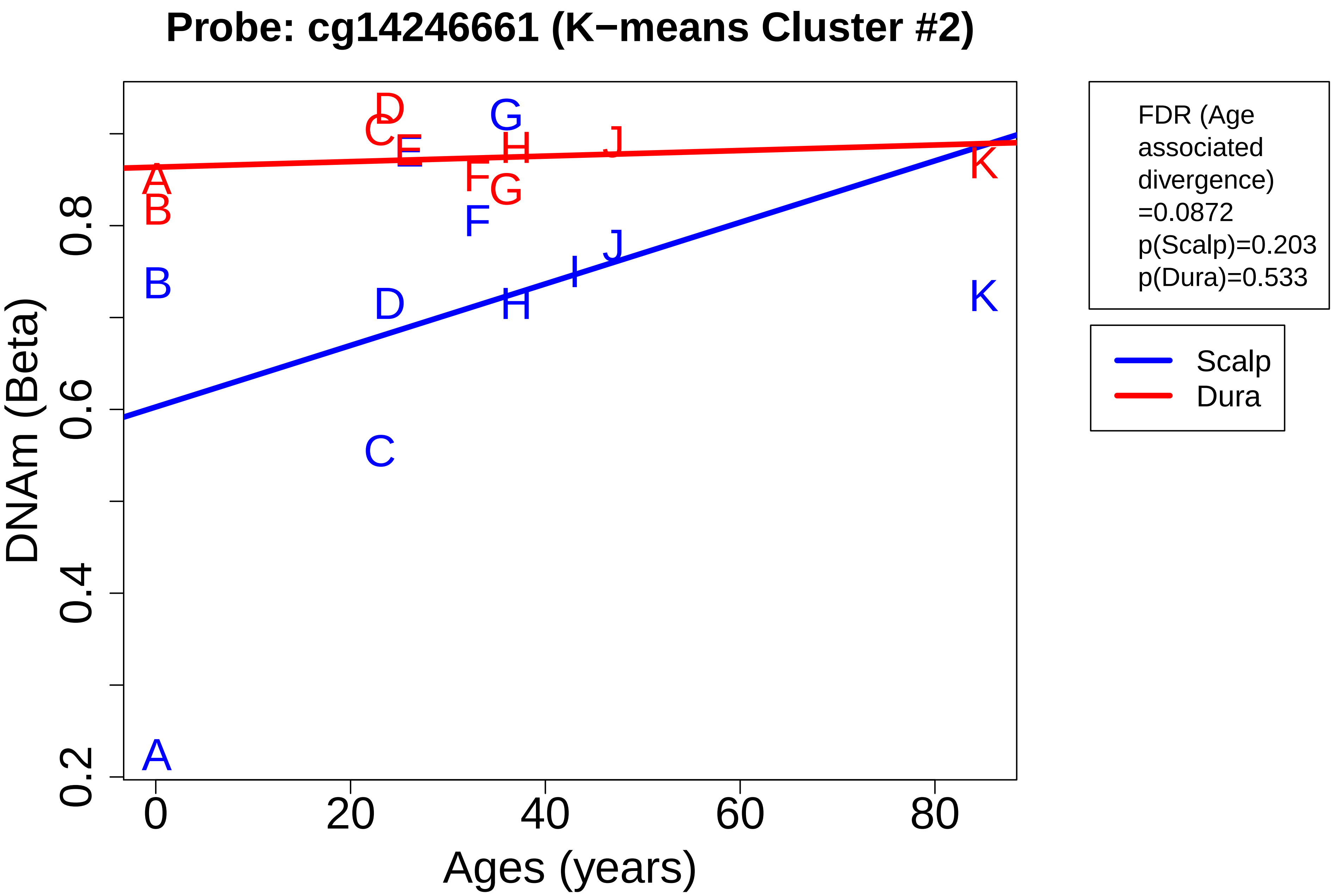

Probe: cg14246661 (K−means Cluster #2)
DNAm (Beta)
0.2
0.4
0.6
0.8
0
20
40
60
80
Ages (years)
FDR (Age associated divergence) =0.0872
p(Scalp)=0.203
p(Dura)=0.533
Scalp
Dura

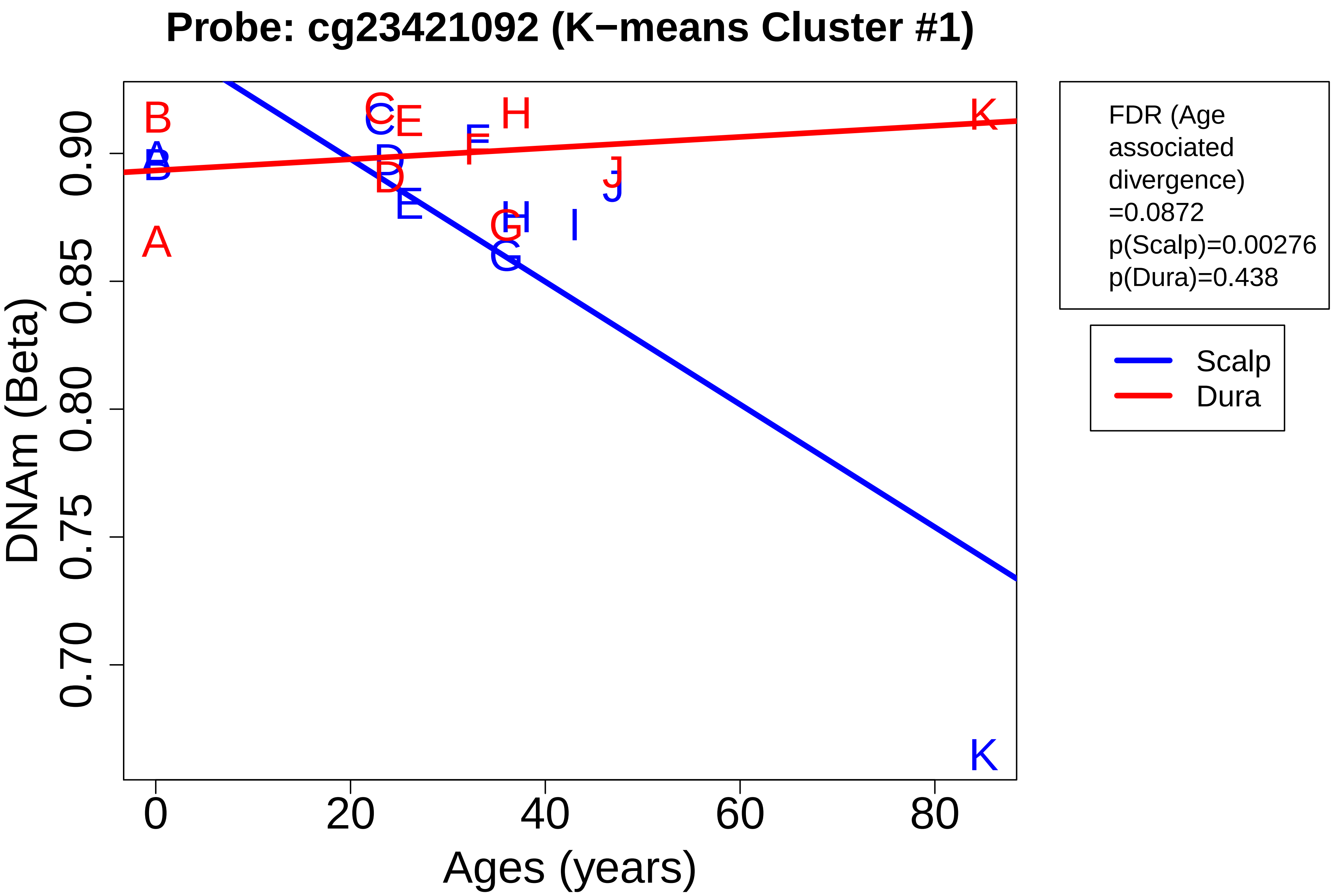

Probe: cg23421092 (K−means Cluster #1)
DNAm (Beta)
0.70
0.75
0.80
0.85
0.90
0
20
40
60
80
Ages (years)
FDR (Age associated divergence) =0.0872
p(Scalp)=0.00276
p(Dura)=0.438
Scalp
Dura

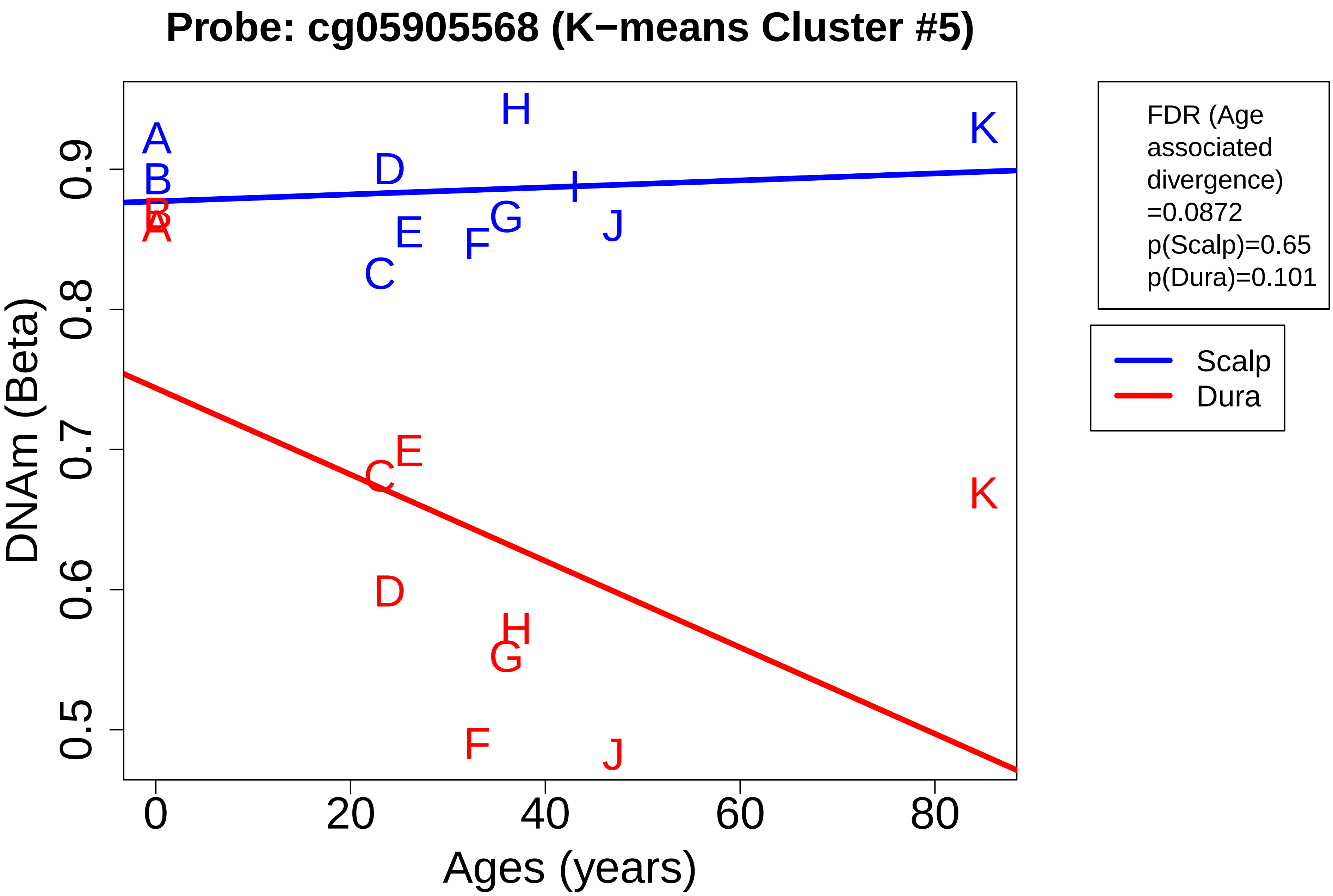

Probe: cg05905568 (K−means Cluster #5)
FDR (Age associated divergence) =0.0872
p(Scalp)=0.65
p(Dura)=0.101
Scalp
Dura
DNAm (Beta)
Ages (years)
0.5
0.6
0.7
0.8
0.9
0
20
40
60
80
A
B
C
D
E
F
G
H
J
K

Probe: cg02770857 (K−means Cluster #5)

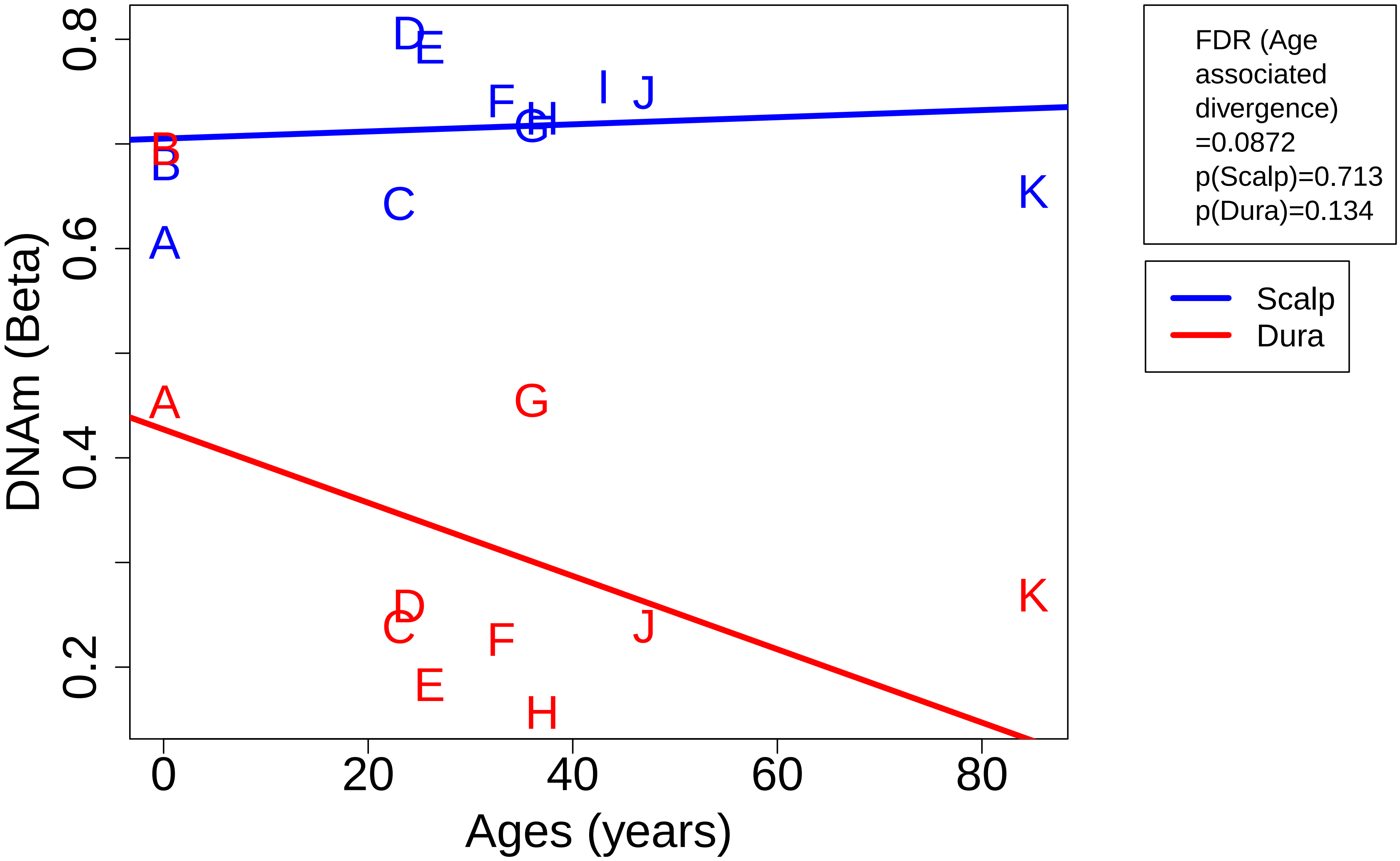

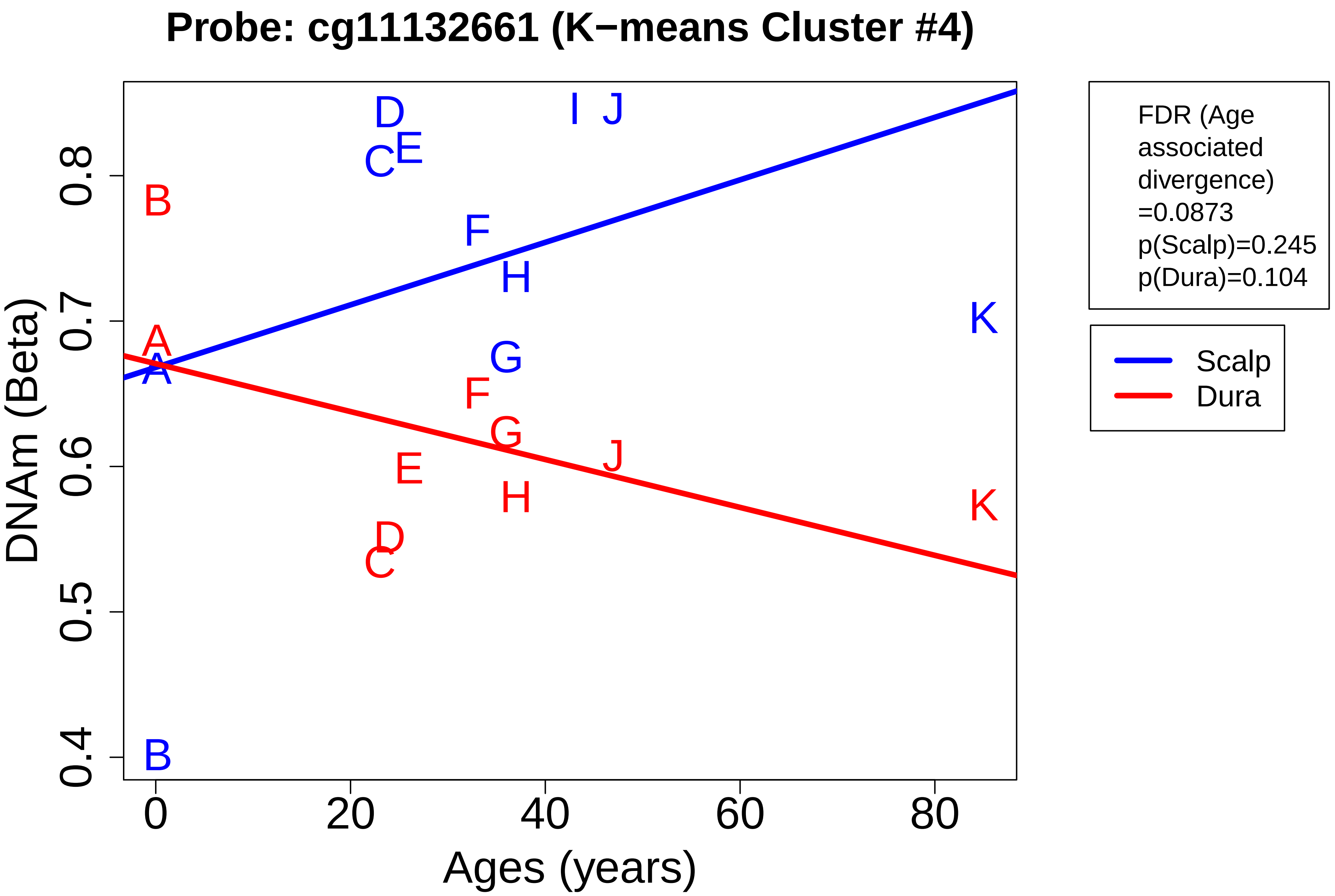
Probe: cg11132661 (K−means Cluster #4)
DNAm (Beta)
0.4
0.5
0.6
0.7
0.8
0
20
40
60
80
Ages (years)
FDR (Age associated divergence) =0.0873
p(Scalp)=0.245
p(Dura)=0.104
Scalp
Dura
A
B
C
D
E
F
G
H
I
J
K

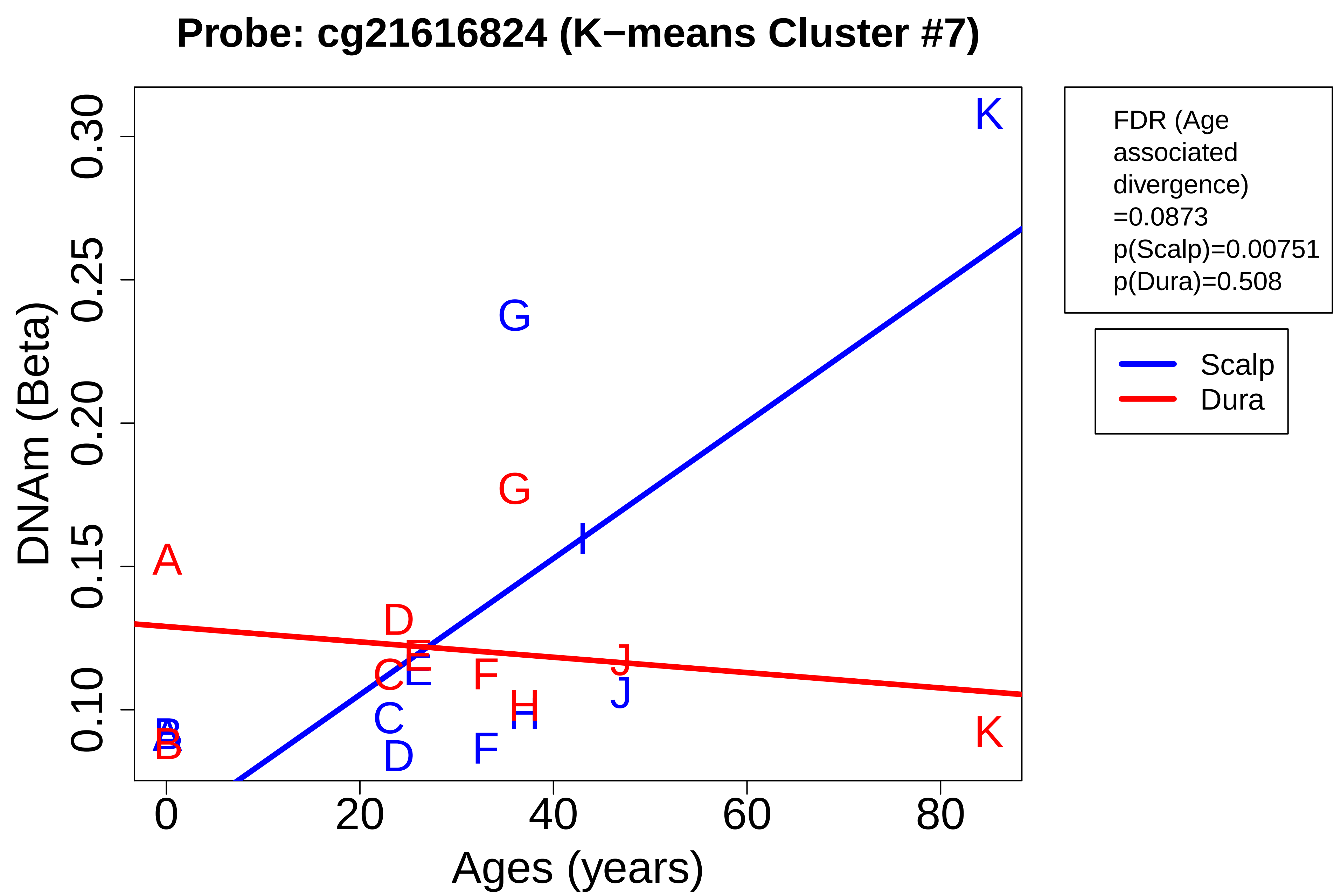

Probe: cg21616824 (K−means Cluster #7)
DNAm (Beta)
0.10
0.15
0.20
0.25
0.30
0
20
40
60
80
Ages (years)
FDR (Age associated divergence) =0.0873
p(Scalp)=0.00751
p(Dura)=0.508
Scalp
Dura

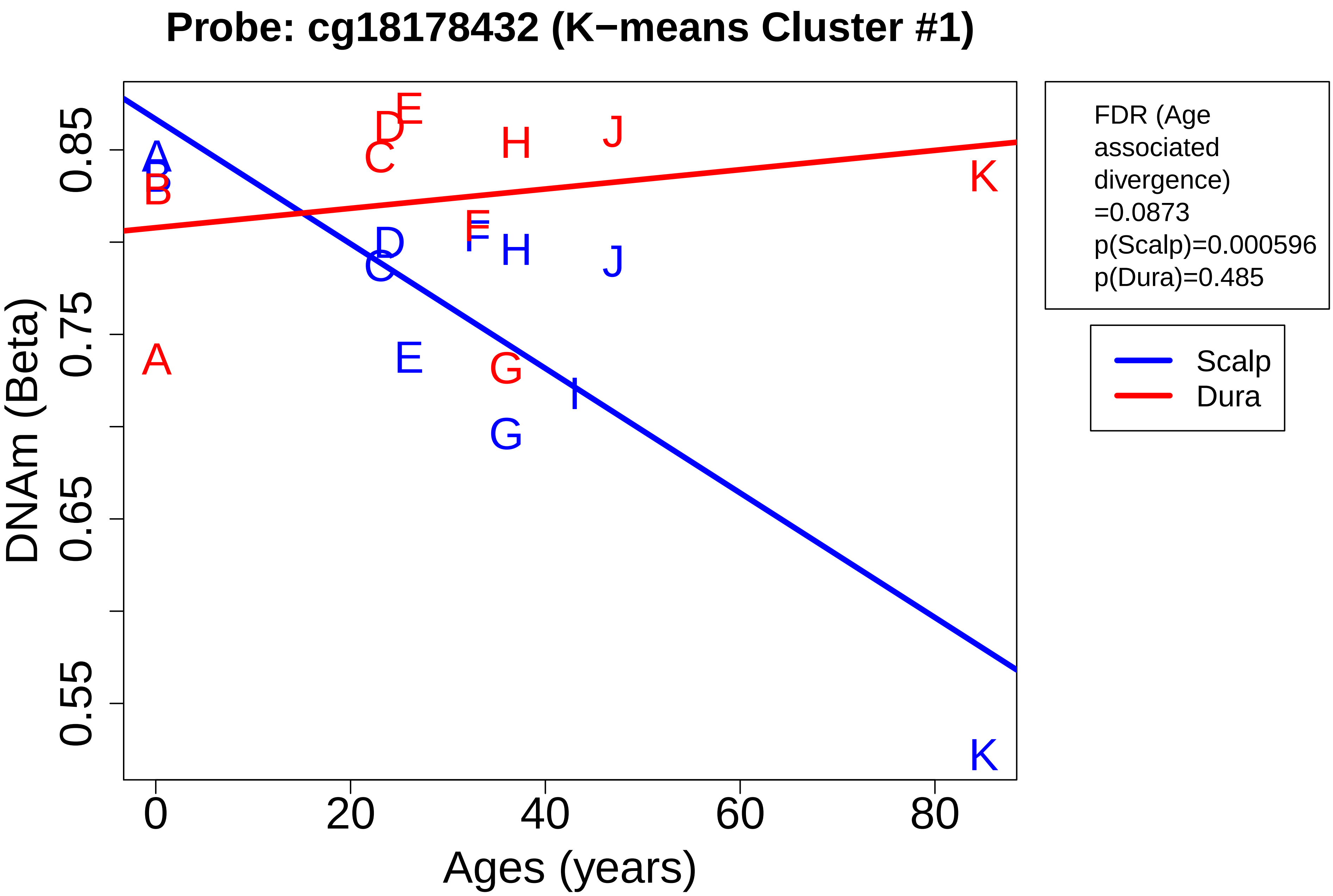

Probe: cg18178432 (K−means Cluster #1)
DNAm (Beta)
0.55
0.65
0.75
0.85
Ages (years)
0
20
40
60
80
FDR (Age associated divergence) =0.0873
p(Scalp)=0.000596
p(Dura)=0.485
Scalp
Dura

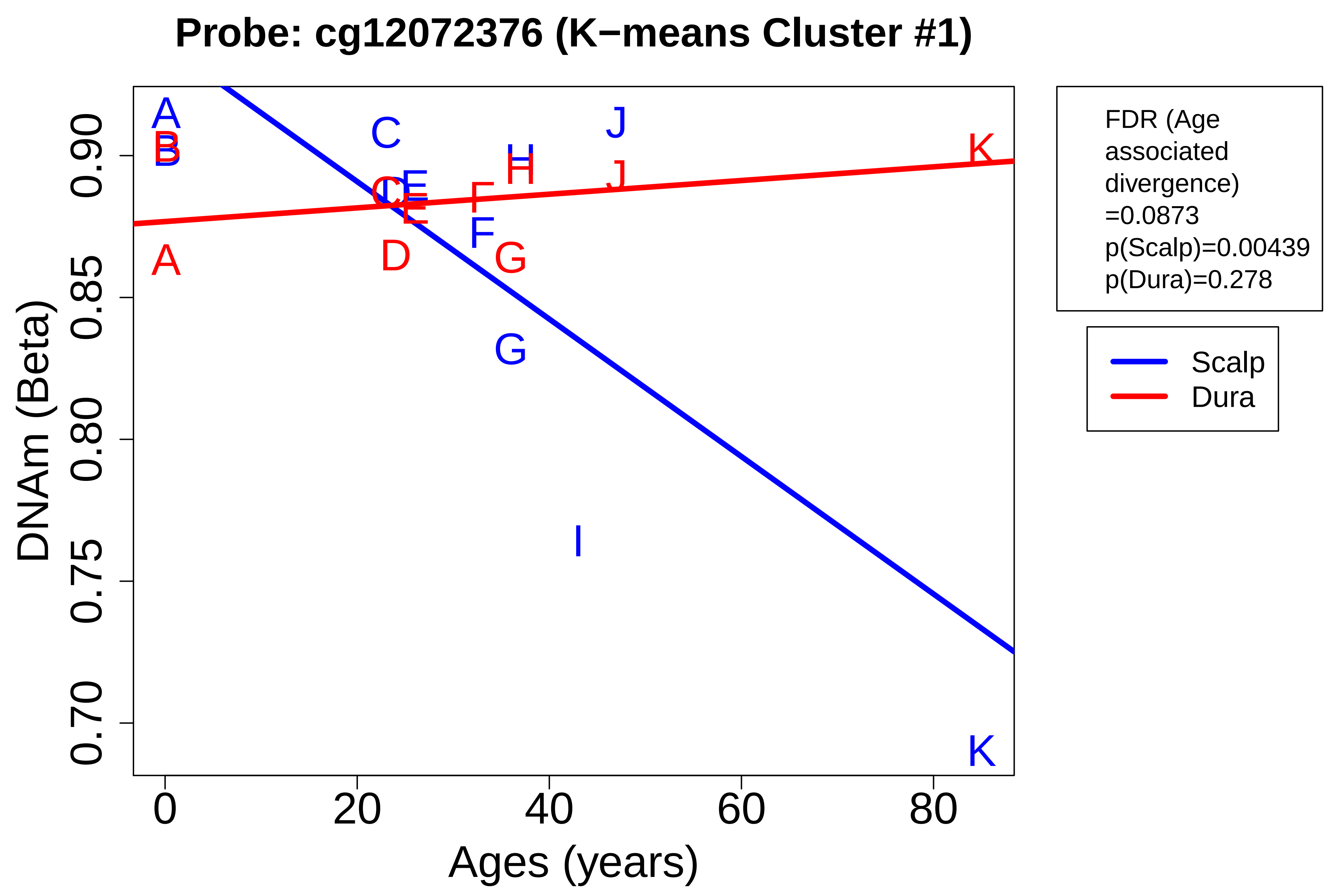

Probe: cg12072376 (K−means Cluster #1)
DNAm (Beta)
0.70
0.75
0.80
0.85
0.90
Ages (years)
0
20
40
60
80
A
B
C
D
E
F
G
H
I
J
K
FDR (Age associated divergence) =0.0873
p(Scalp)=0.00439
p(Dura)=0.278
Scalp
Dura

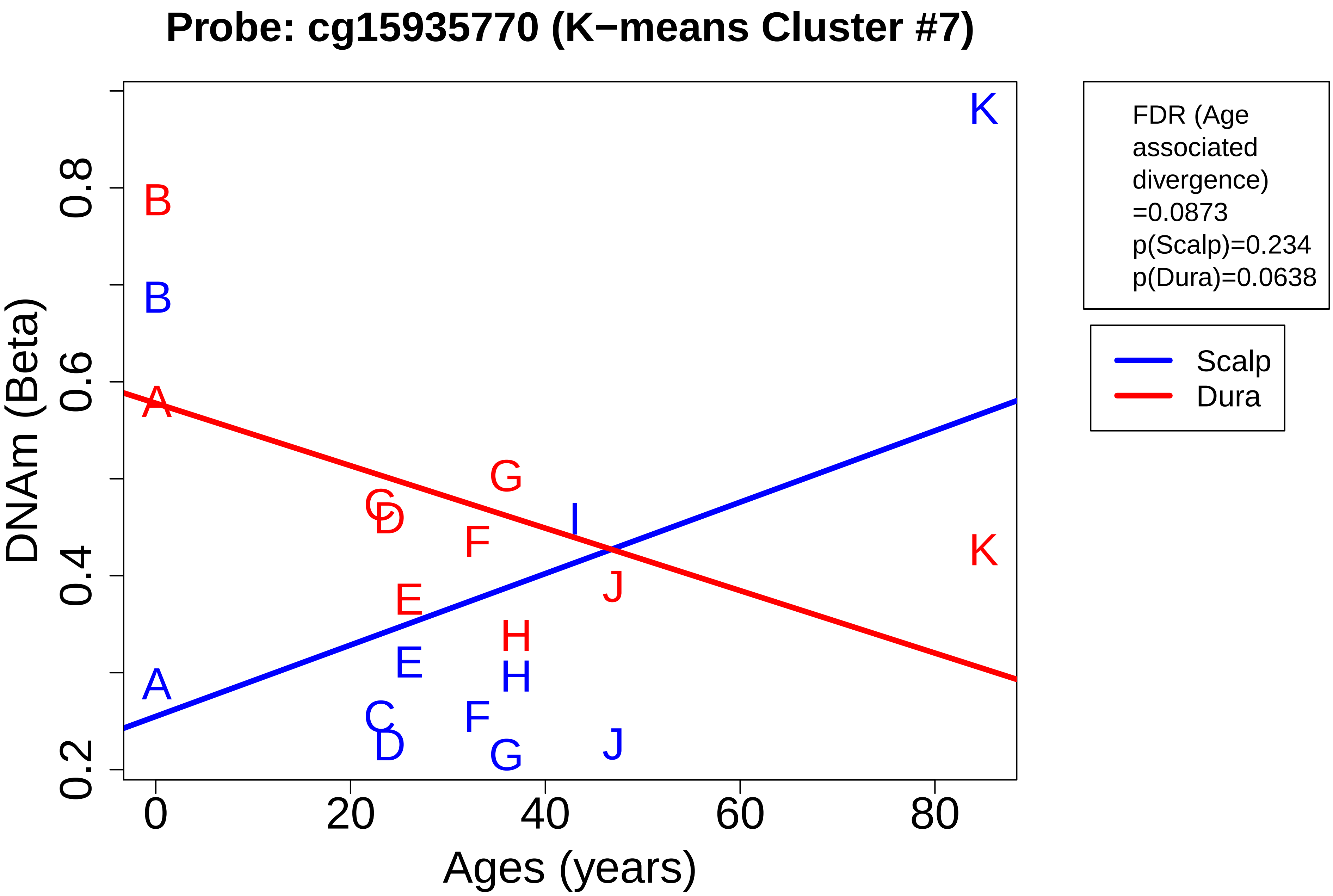

Probe: cg15935770 (K−means Cluster #7)
FDR (Age associated divergence) =0.0873
p(Scalp)=0.234
p(Dura)=0.0638
Scalp
Dura
DNAm (Beta)
Ages (years)
0.2
0.4
0.6
0.8
0
20
40
60
80

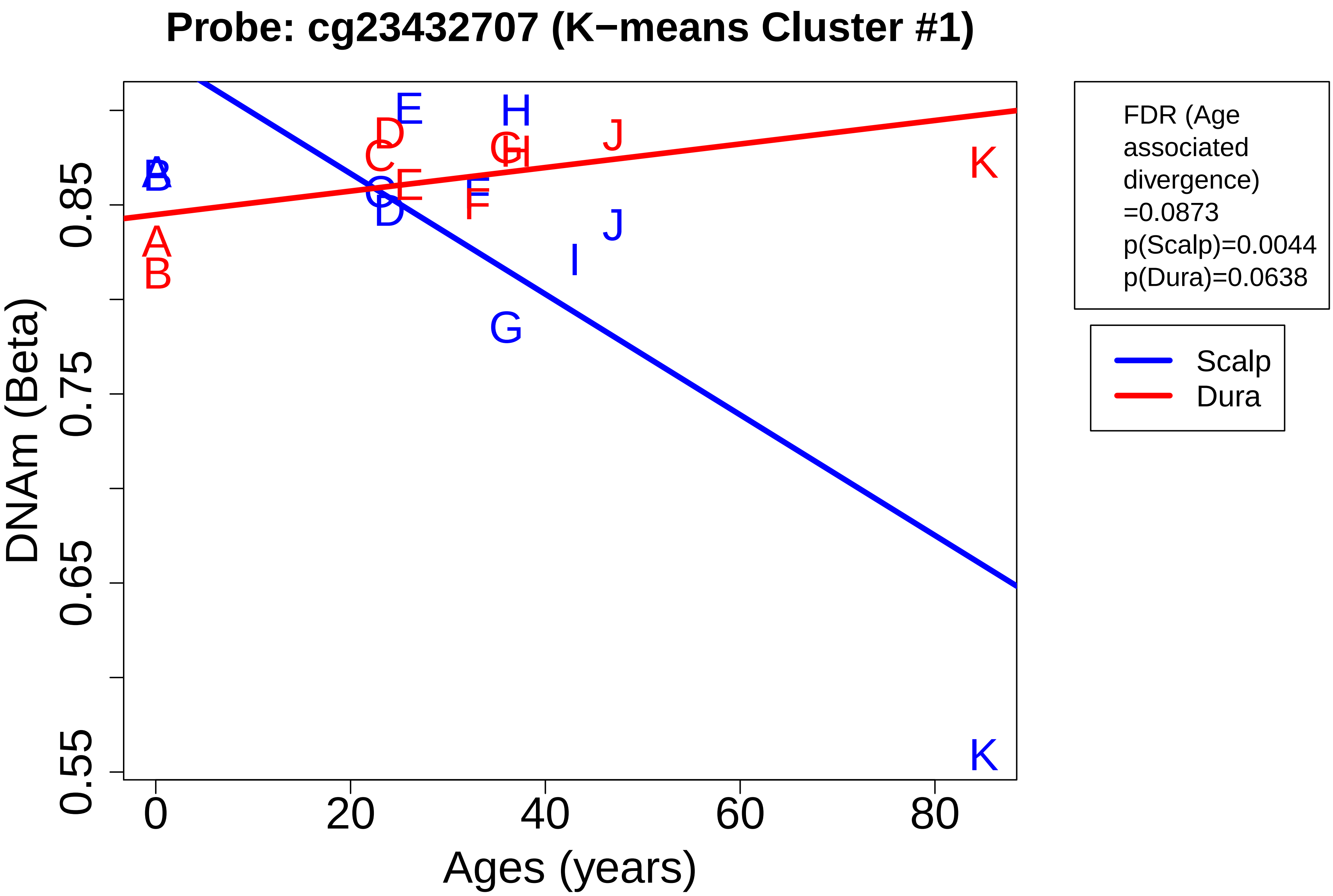

Probe: cg23432707 (K−means Cluster #1)
DNAm (Beta)
0.55
0.65
0.75
0.85
Ages (years)
0
20
40
60
80
FDR (Age associated divergence) =0.0873
p(Scalp)=0.0044
p(Dura)=0.0638
Scalp
Dura

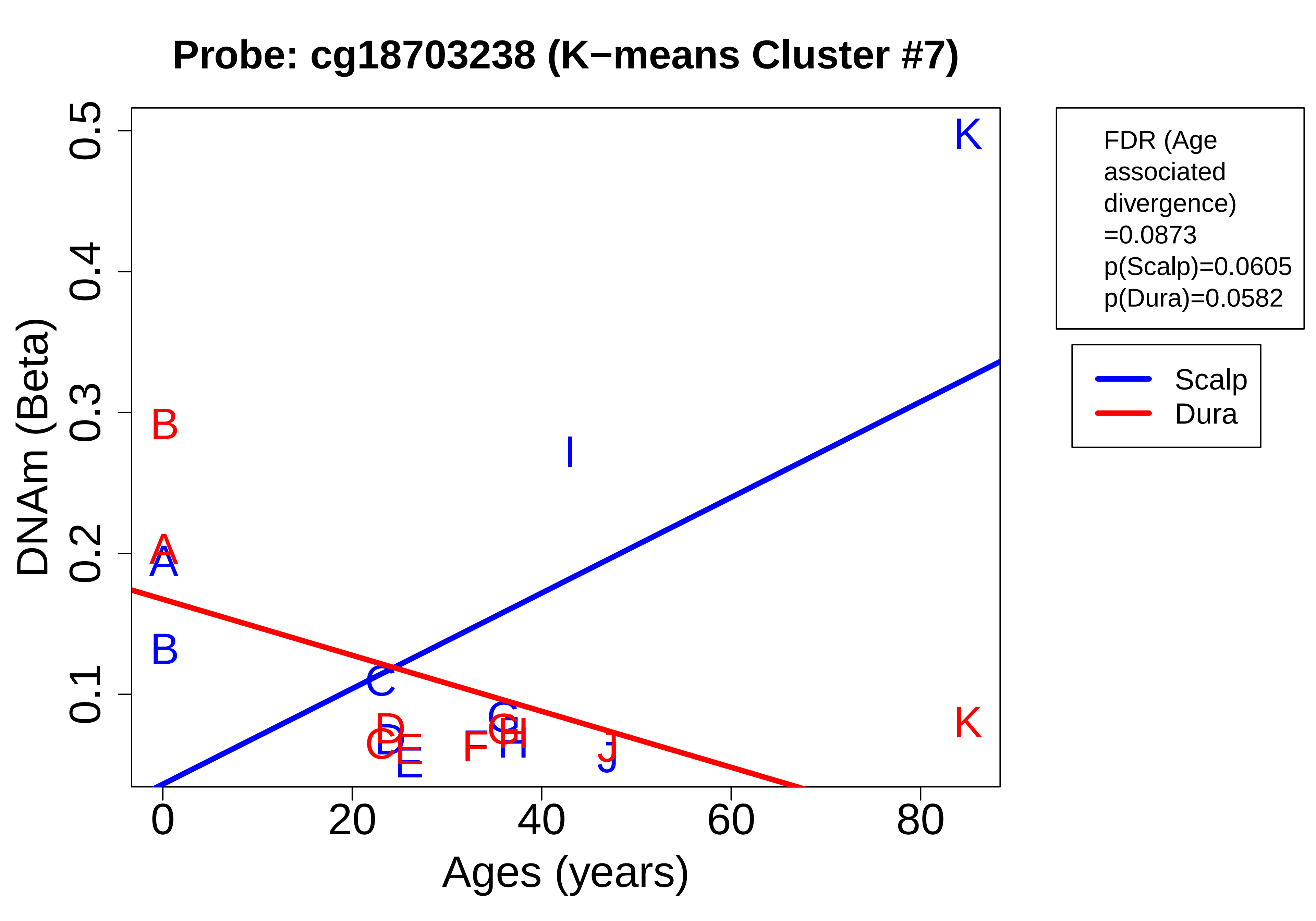

Probe: cg18703238 (K−means Cluster #7)
DNAm (Beta)
0.1
0.2
0.3
0.4
0.5
Ages (years)
0
20
40
60
80
FDR (Age associated divergence) =0.0873
p(Scalp)=0.0605
p(Dura)=0.0582
Scalp
Dura

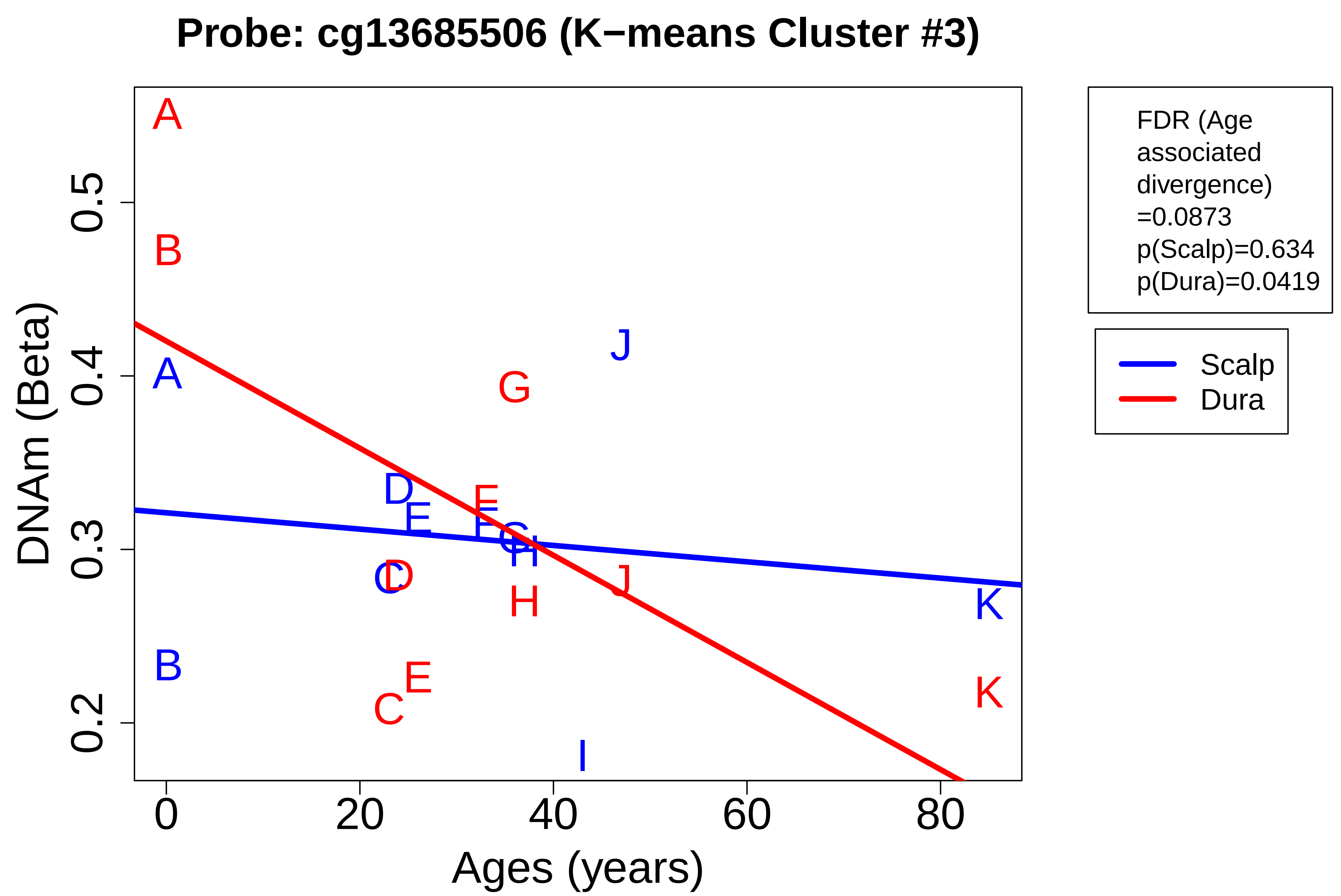

Probe: cg13685506 (K−means Cluster #3)
DNAm (Beta)
0.2
0.3
0.4
0.5
Ages (years)
0
20
40
60
80
FDR (Age associated divergence) =0.0873
p(Scalp)=0.634
p(Dura)=0.0419
Scalp
Dura

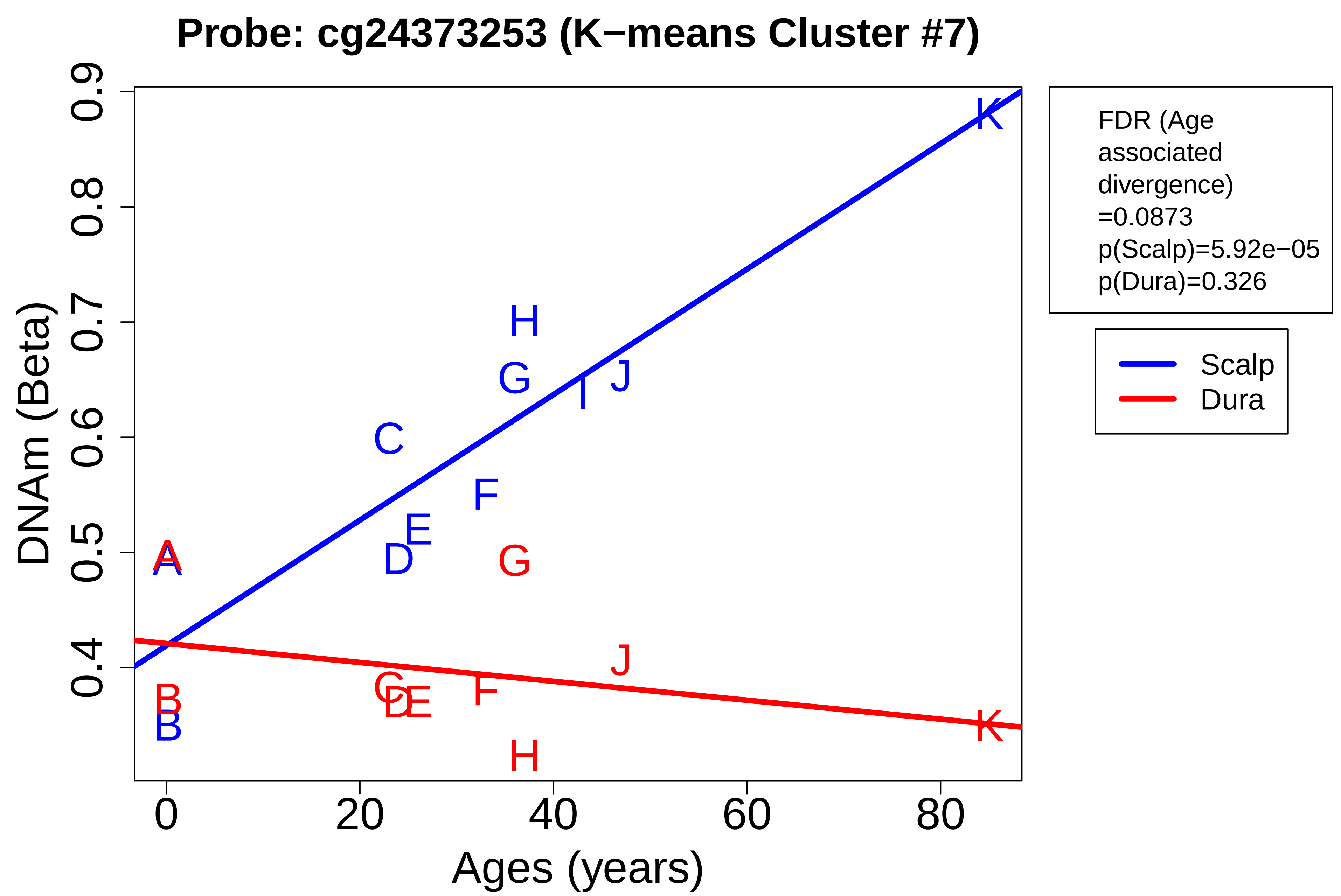

Probe: cg24373253 (K−means Cluster #7)
DNAm (Beta)
0.4
0.5
0.6
0.7
0.8
0.9
0
20
40
60
80
Ages (years)
FDR (Age associated divergence) =0.0873
p(Scalp)=5.92e−05
p(Dura)=0.326
Scalp
Dura
A B C D E F G H I J K

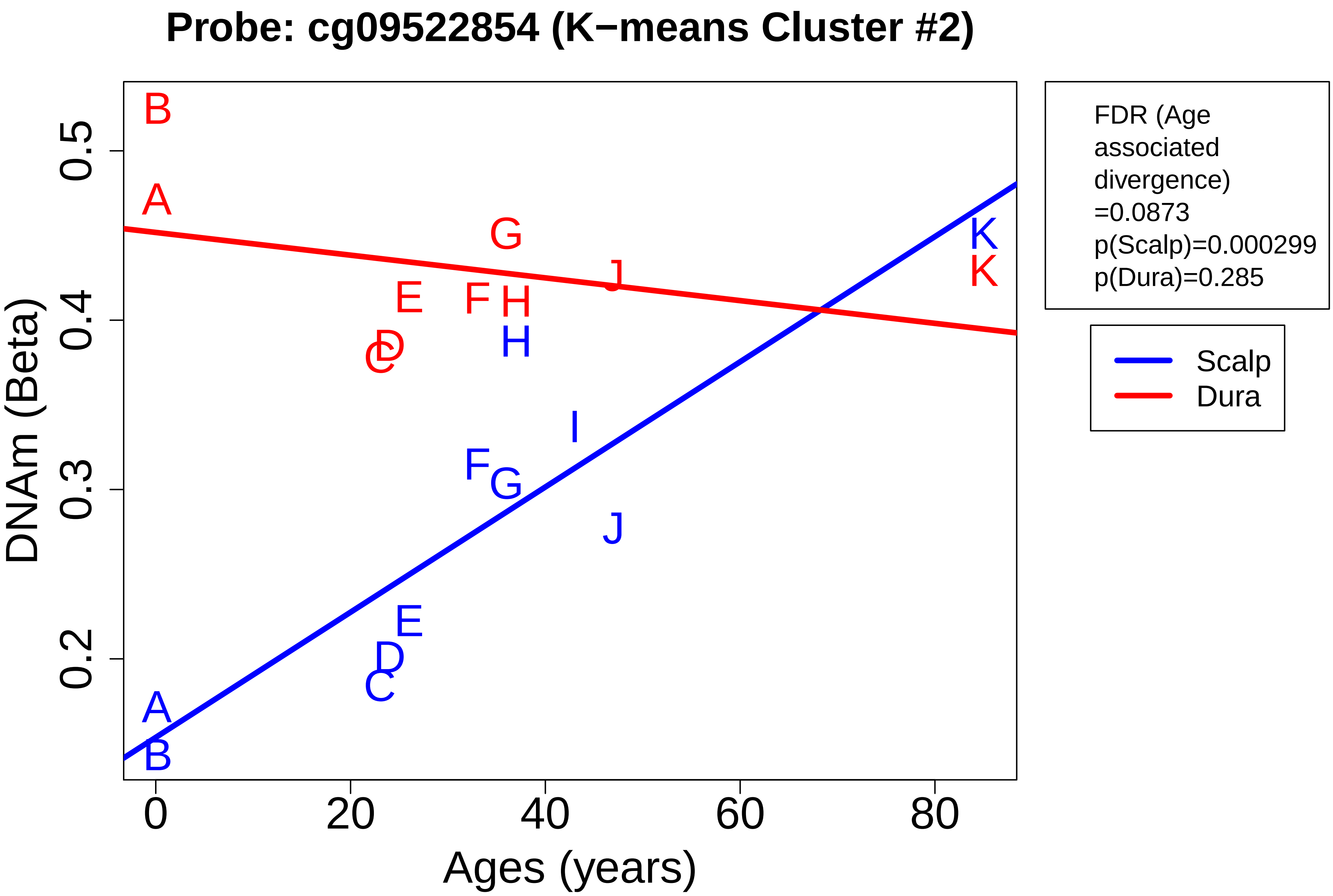

Probe: cg09522854 (K−means Cluster #2)
DNAm (Beta)
0.2
0.3
0.4
0.5
0
20
40
60
80
Ages (years)
FDR (Age associated divergence) =0.0873
p(Scalp)=0.000299
p(Dura)=0.285
Scalp
Dura
A
B
C
D
E
F
G
H
I
J
K

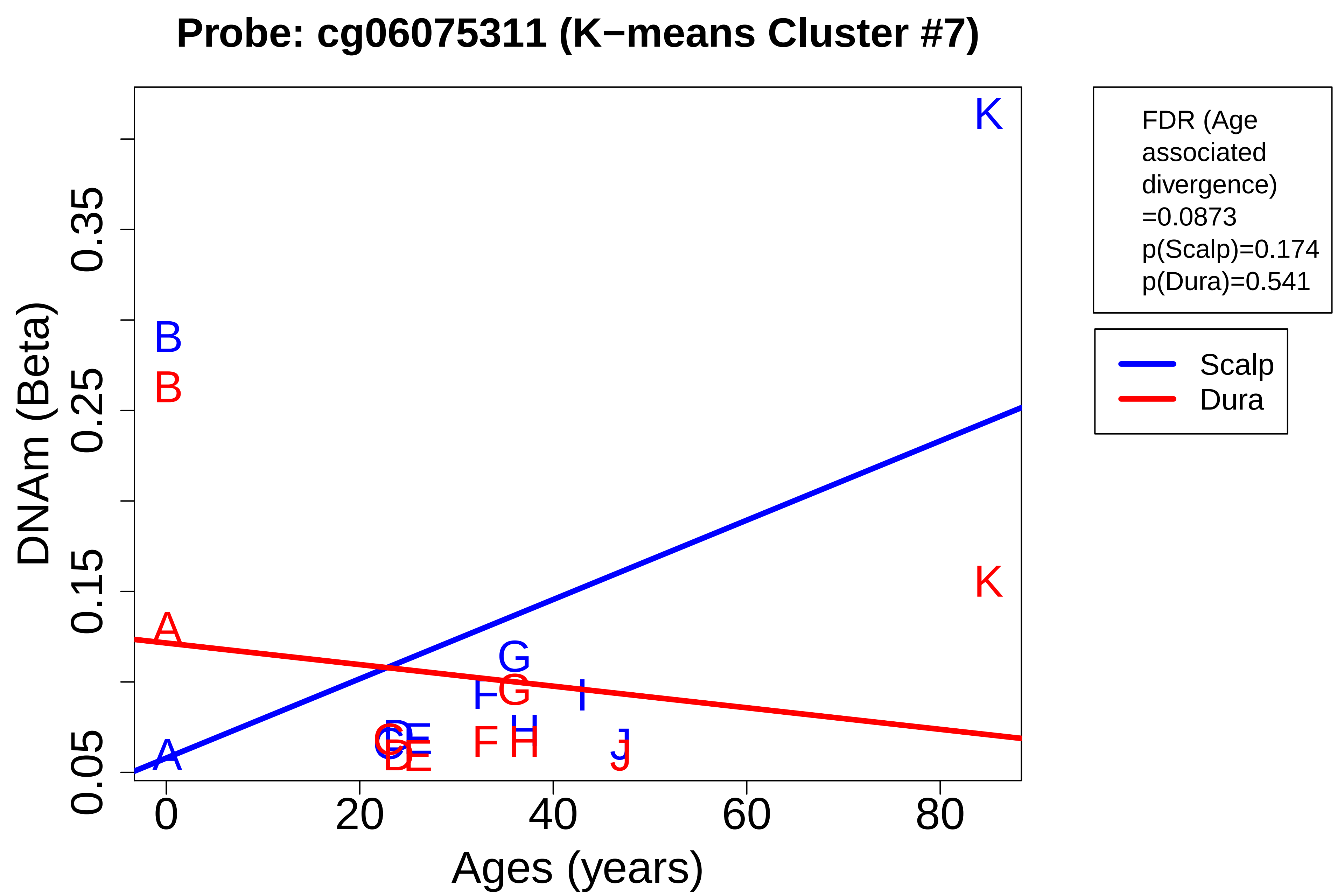

Probe: cg06075311 (K−means Cluster #7)
DNAm (Beta)
0.05
0.15
0.25
0.35
0
20
40
60
80
Ages (years)
FDR (Age associated divergence) =0.0873
p(Scalp)=0.174
p(Dura)=0.541
Scalp
Dura
A
B
C
D
E
F
G
H
I
J
K

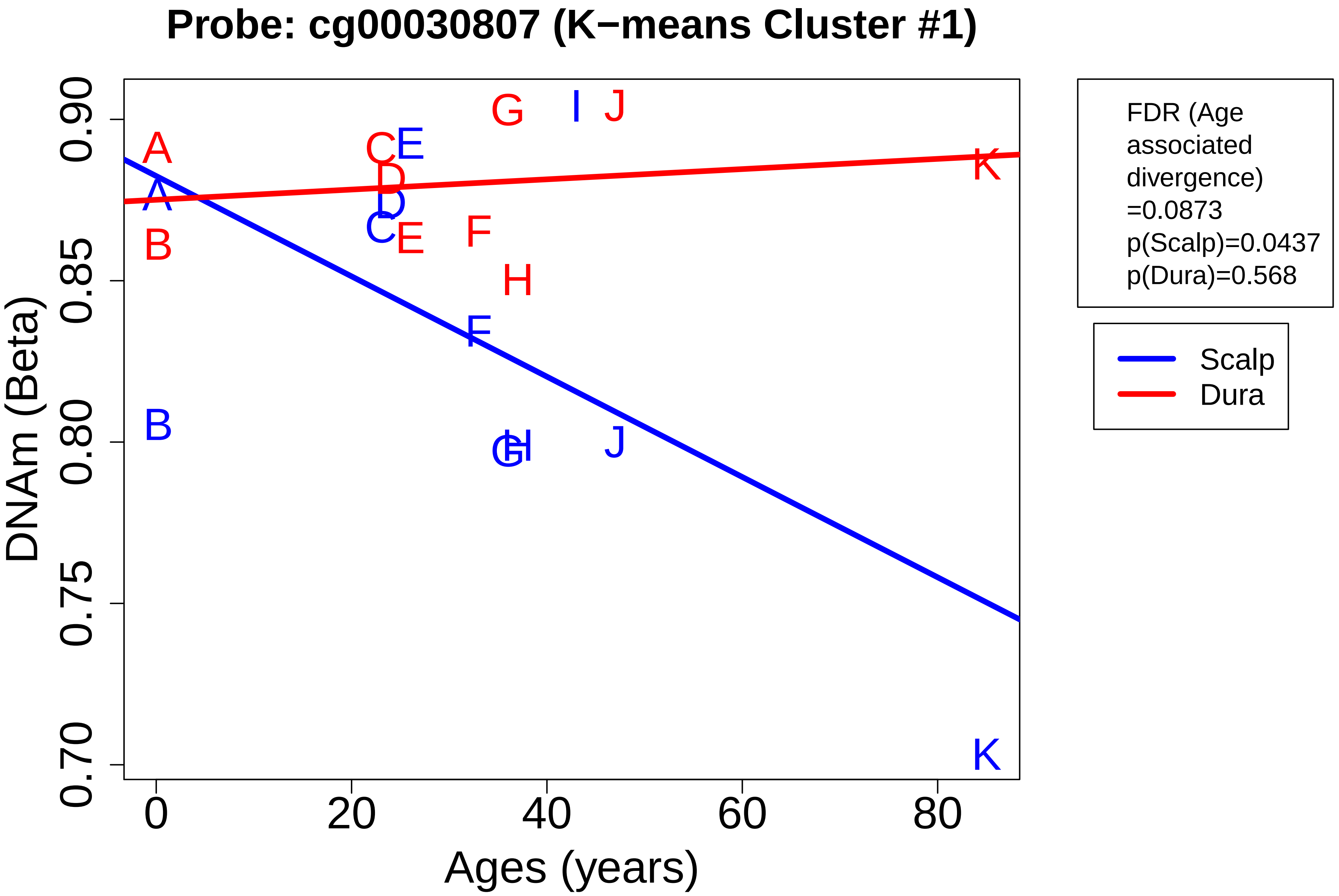

Probe: cg00030807 (K−means Cluster #1)
DNAm (Beta)
0.70
0.75
0.80
0.85
0.90
0
20
40
60
80
Ages (years)
FDR (Age associated divergence) =0.0873
p(Scalp)=0.0437
p(Dura)=0.568
Scalp
Dura

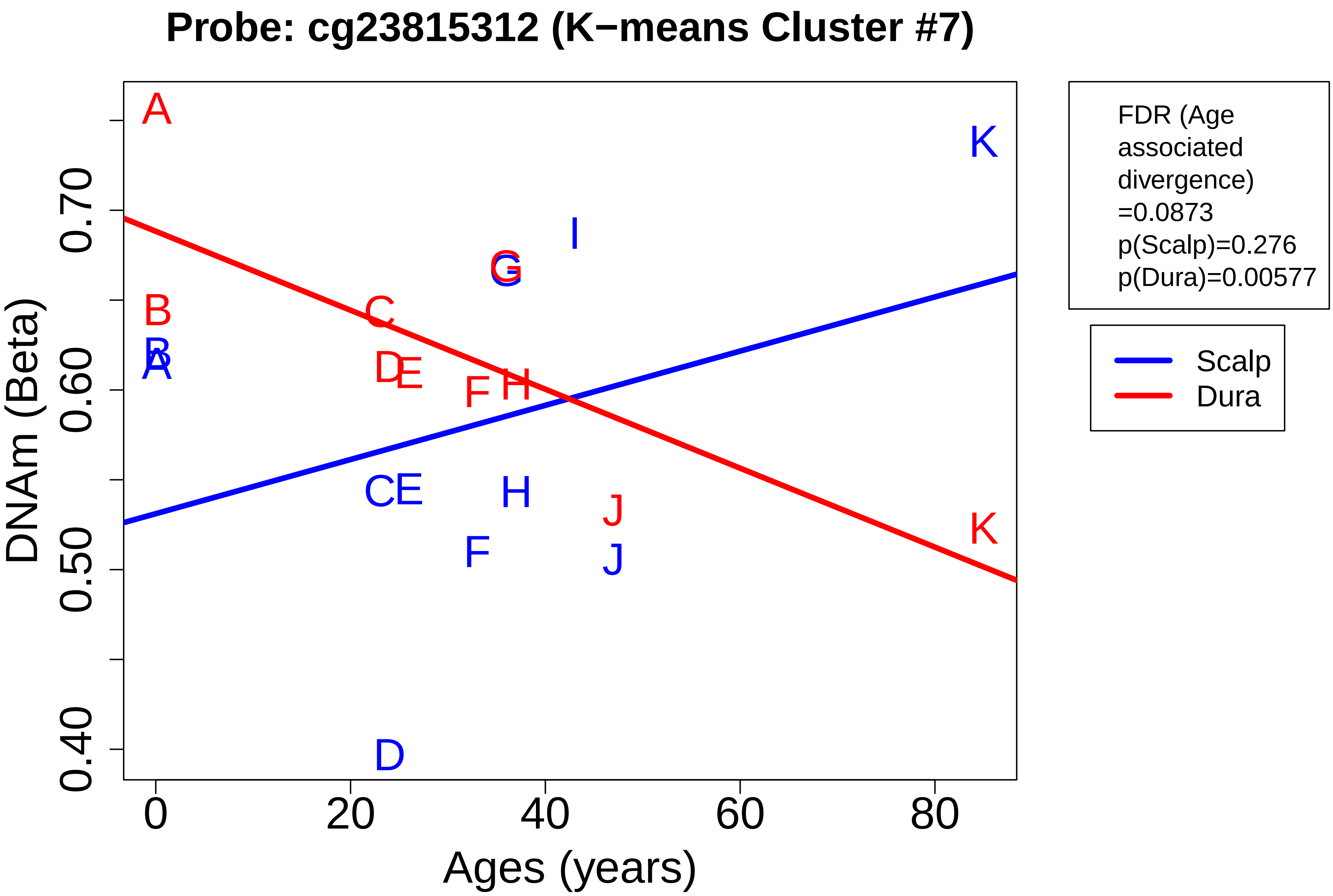

Probe: cg23815312 (K−means Cluster #7)
DNAm (Beta)
0.40
0.50
0.60
0.70
Ages (years)
0
20
40
60
80
A
B
C
D
E
F
G
H
I
J
K
FDR (Age associated divergence) =0.0873
p(Scalp)=0.276
p(Dura)=0.00577
Scalp
Dura

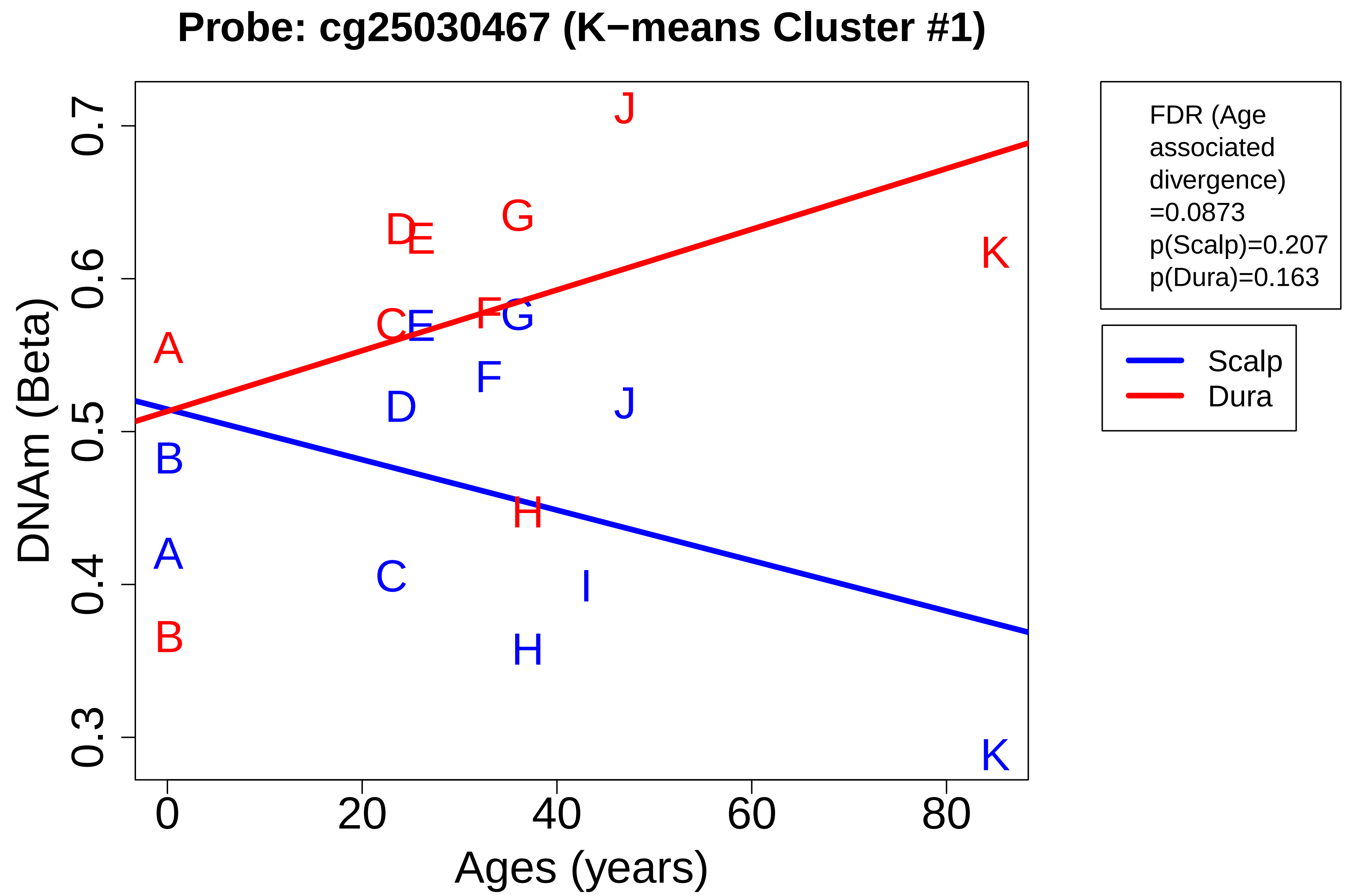

Probe: cg25030467 (K−means Cluster #1)
DNAm (Beta)
0.3
0.4
0.5
0.6
0.7
Ages (years)
0
20
40
60
80
J
G
D
E
K
C
E
F
G
A
F
D
J
B
H
A
C
I
B
H
K
FDR (Age associated divergence) =0.0873
p(Scalp)=0.207
p(Dura)=0.163
Scalp
Dura

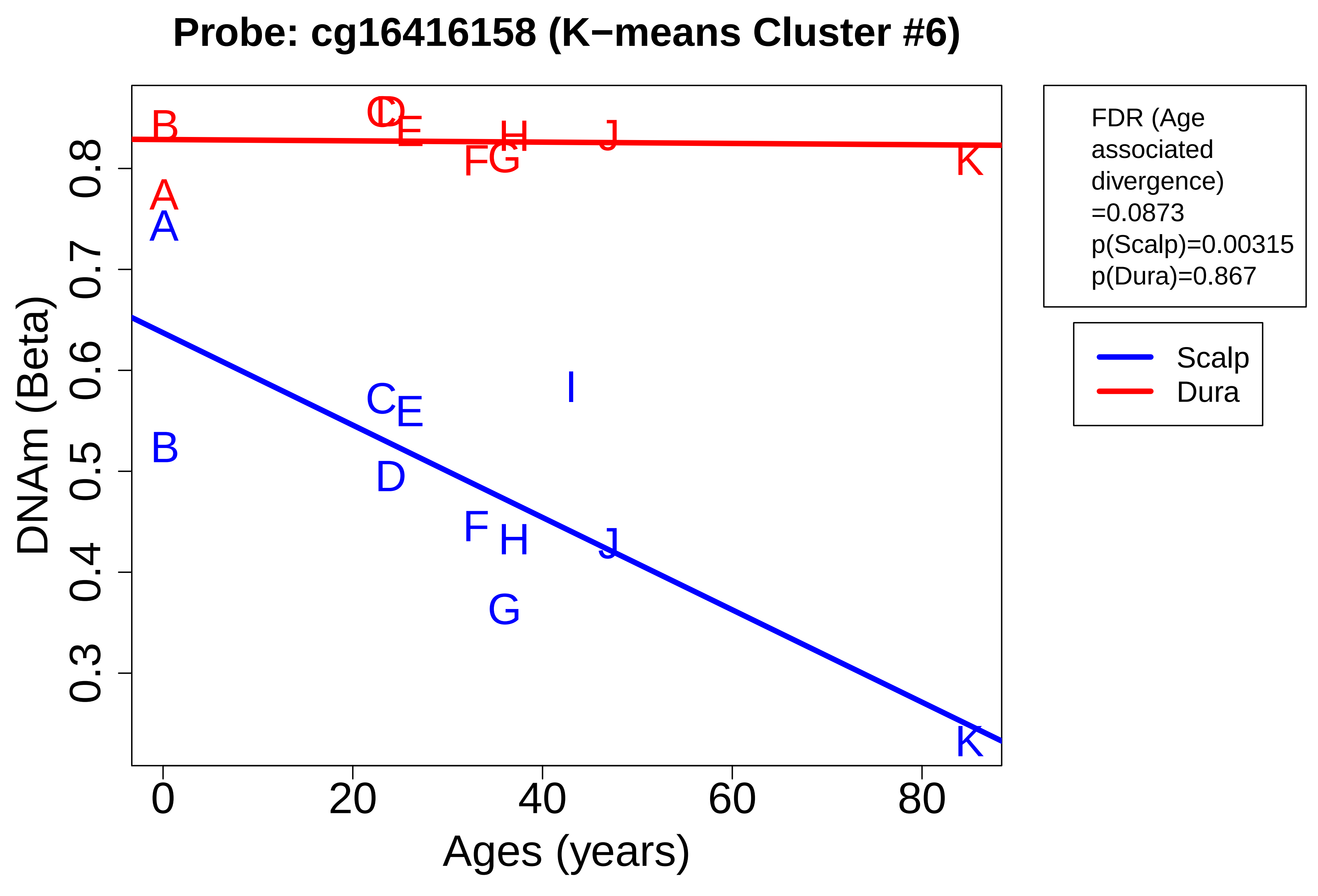

Probe: cg16416158 (K−means Cluster #6)
DNAm (Beta)
0.3
0.4
0.5
0.6
0.7
0.8
Ages (years)
0
20
40
60
80
FDR (Age associated divergence) =0.0873
p(Scalp)=0.00315
p(Dura)=0.867
Scalp
Dura

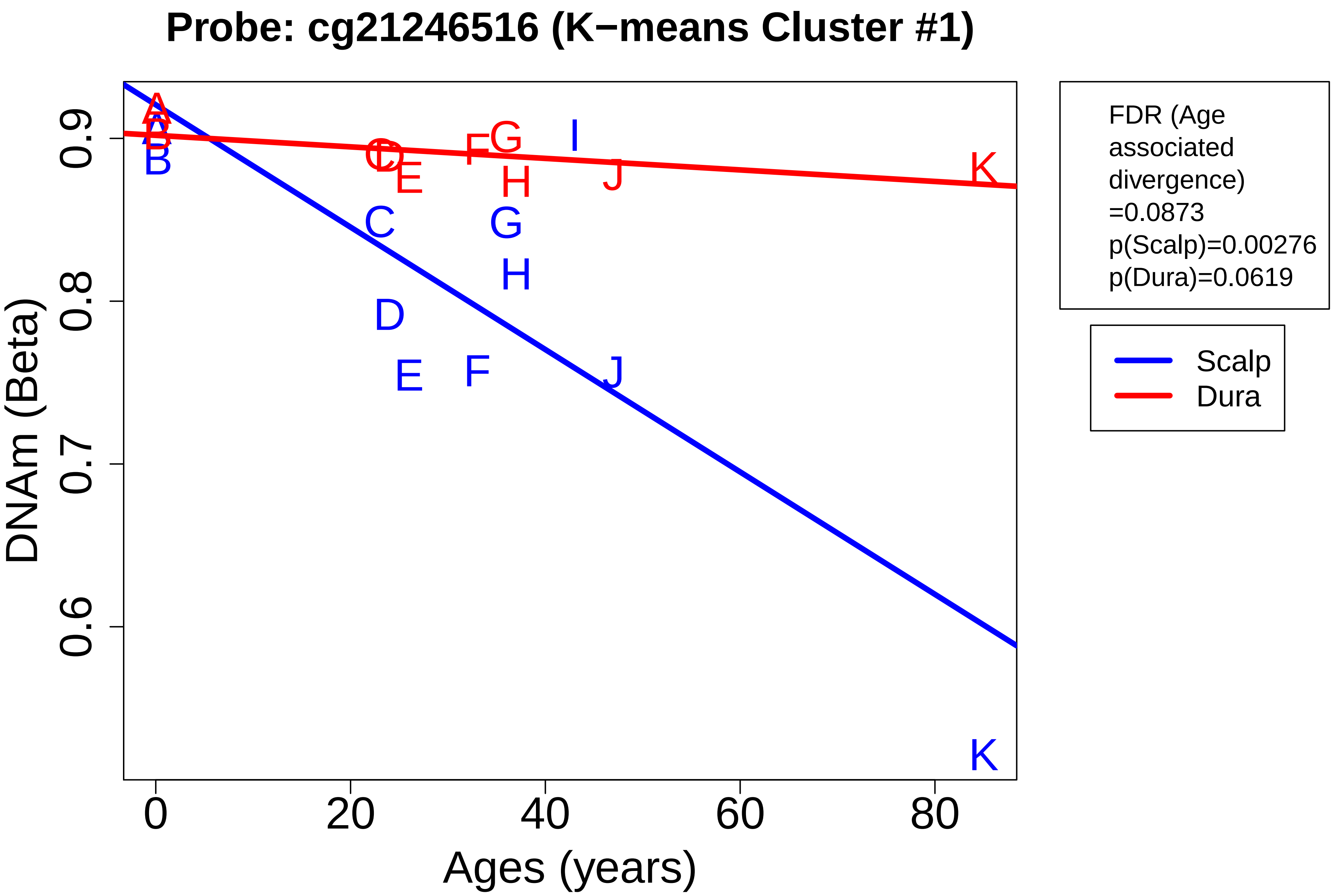

Probe: cg21246516 (K−means Cluster #1)
DNAm (Beta)
0.6
0.7
0.8
0.9
Ages (years)
0
20
40
60
80
FDR (Age associated divergence) =0.0873
p(Scalp)=0.00276
p(Dura)=0.0619
Scalp
Dura

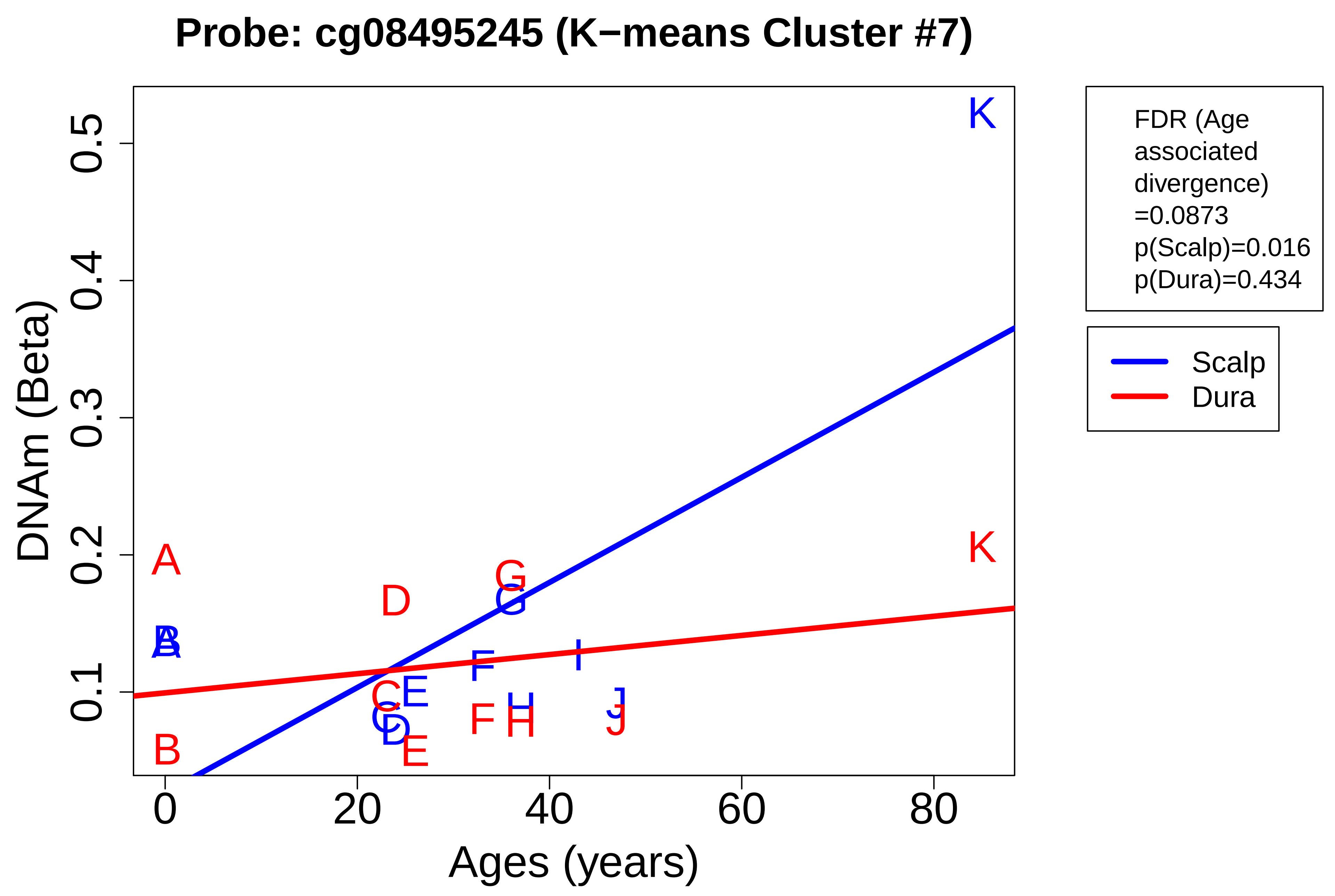

Probe: cg08495245 (K−means Cluster #7)
DNAm (Beta)
0.1
0.2
0.3
0.4
0.5
Ages (years)
0
20
40
60
80
A
B
C
D
E
F
G
H
I
J
K
FDR (Age associated divergence) =0.0873
p(Scalp)=0.016
p(Dura)=0.434
Scalp
Dura

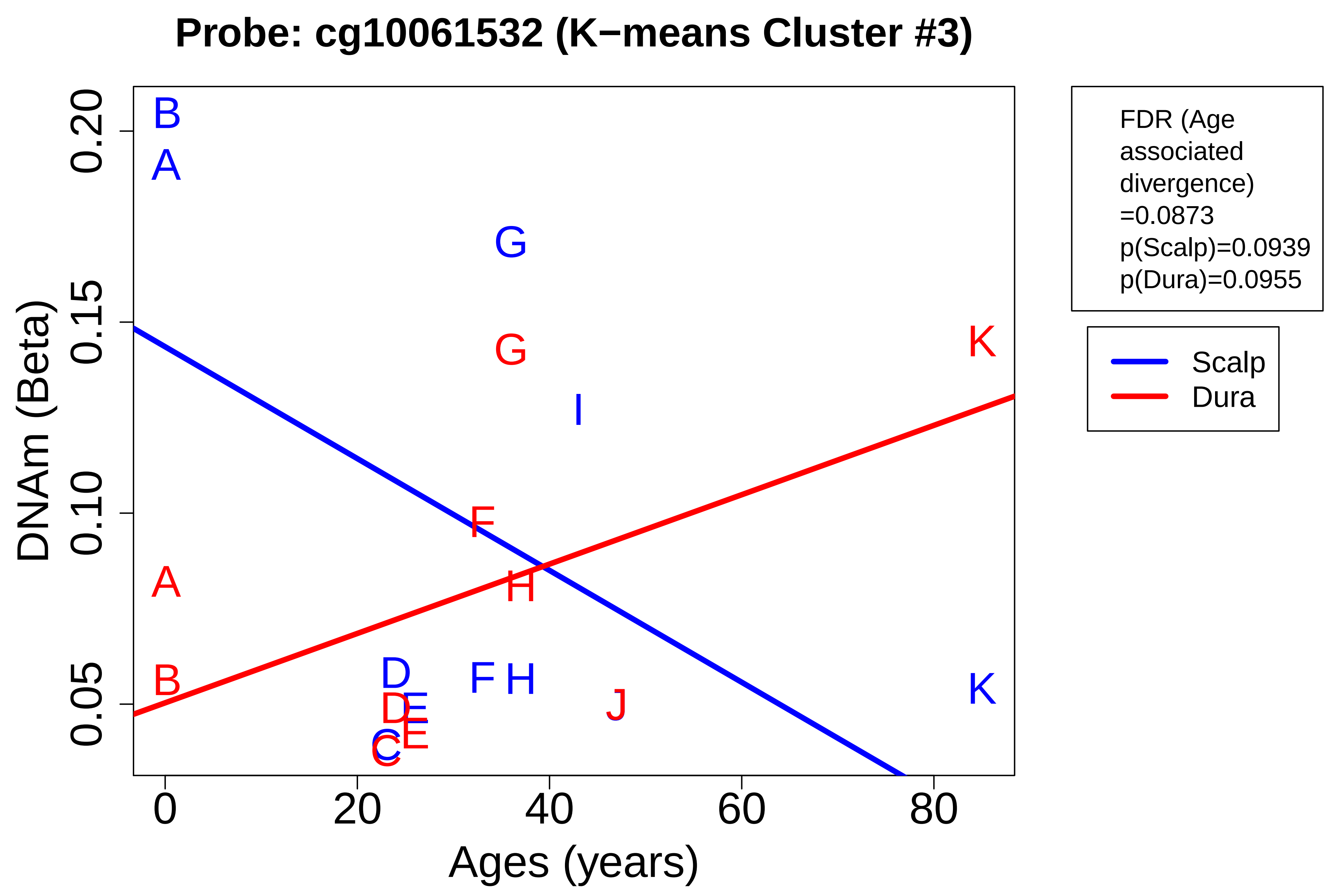

Probe: cg10061532 (K−means Cluster #3)
DNAm (Beta)
0.05
0.10
0.15
0.20
Ages (years)
0
20
40
60
80
B
A
G
G
K
I
F
A
H
B
D
F
H
D
E
E
C
C
J
K
FDR (Age associated divergence) =0.0873
p(Scalp)=0.0939
p(Dura)=0.0955
Scalp
Dura

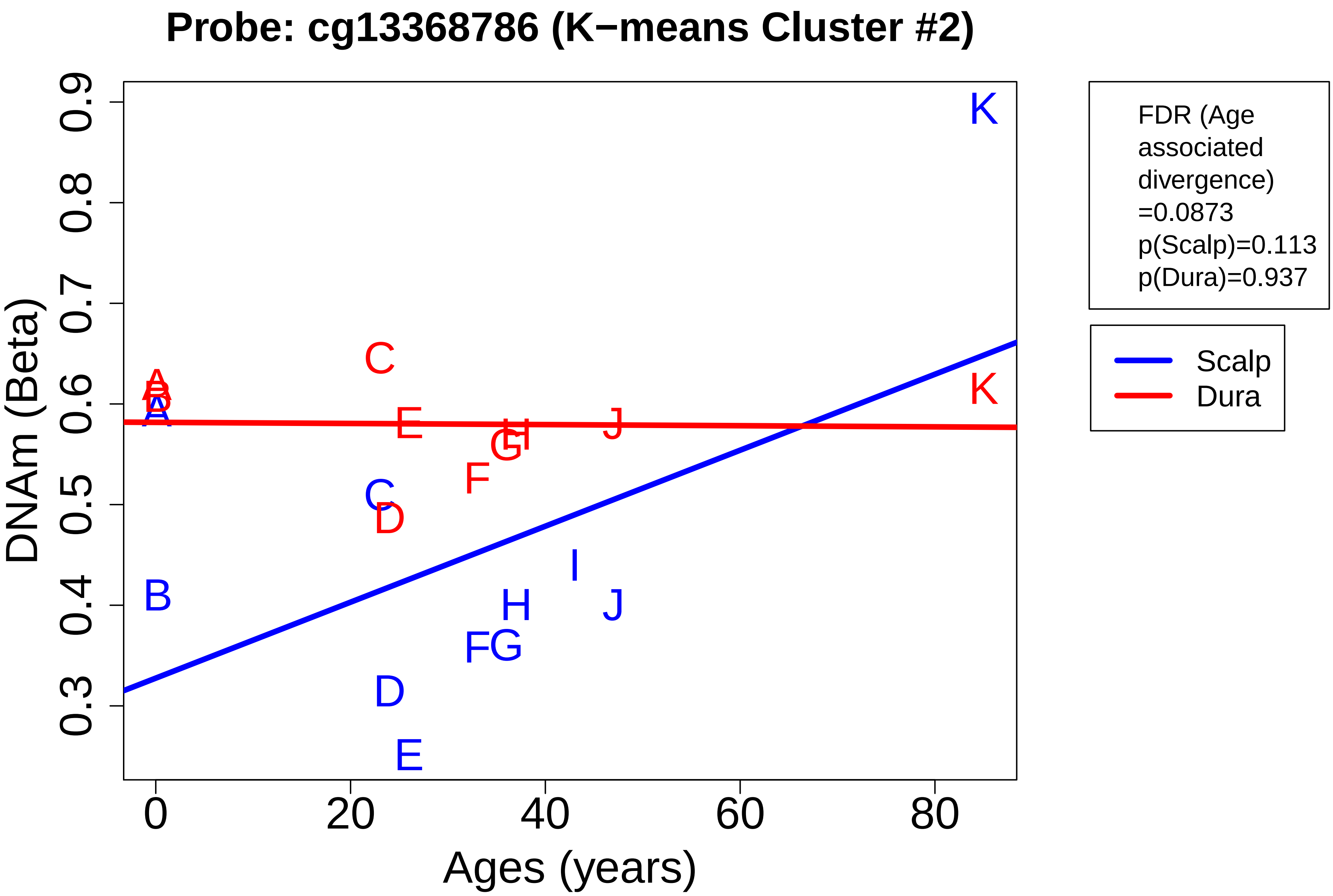

Probe: cg13368786 (K−means Cluster #2)
DNAm (Beta)
Ages (years)
0.3
0.4
0.5
0.6
0.7
0.8
0.9
0
20
40
60
80
FDR (Age associated divergence) =0.0873
p(Scalp)=0.113
p(Dura)=0.937
Scalp
Dura

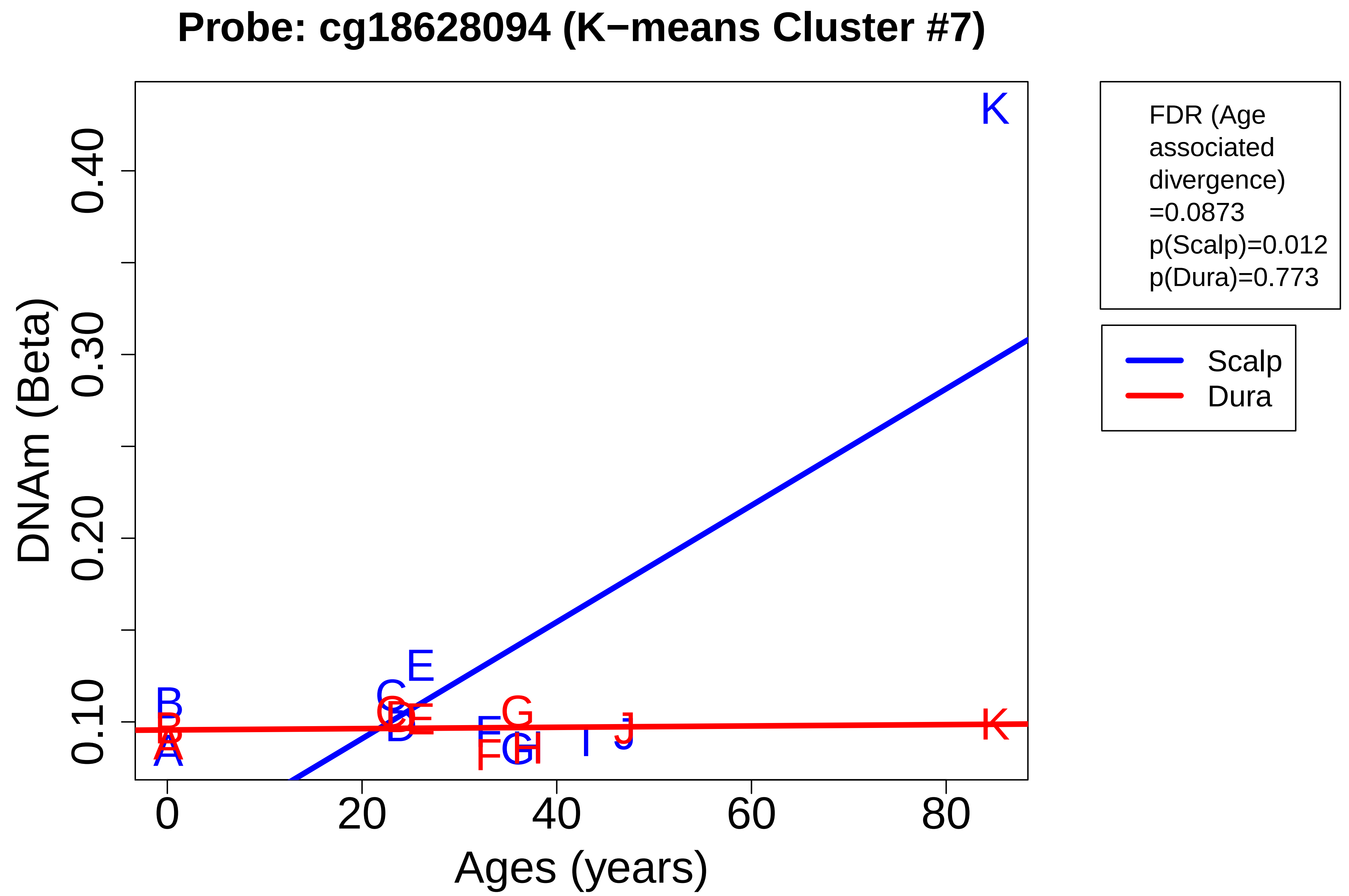

Probe: cg18628094 (K−means Cluster #7)
DNAm (Beta)
0.10
0.20
0.30
0.40
Ages (years)
0
20
40
60
80
FDR (Age associated divergence) =0.0873
p(Scalp)=0.012
p(Dura)=0.773
Scalp
Dura

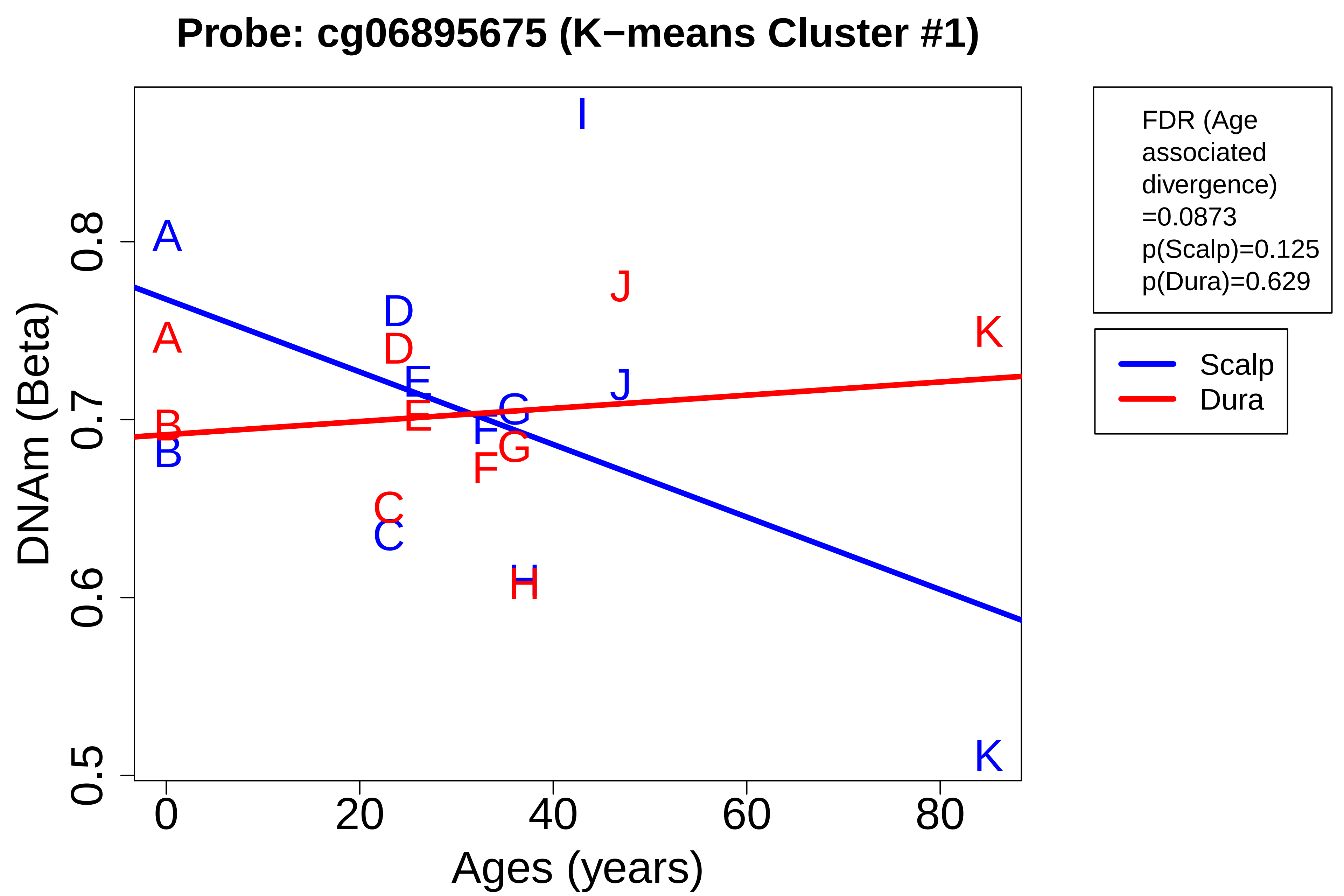

Probe: cg06895675 (K−means Cluster #1)
DNAm (Beta)
0.5
0.6
0.7
0.8
0
20
40
60
80
Ages (years)
FDR (Age associated divergence) =0.0873
p(Scalp)=0.125
p(Dura)=0.629
Scalp
Dura

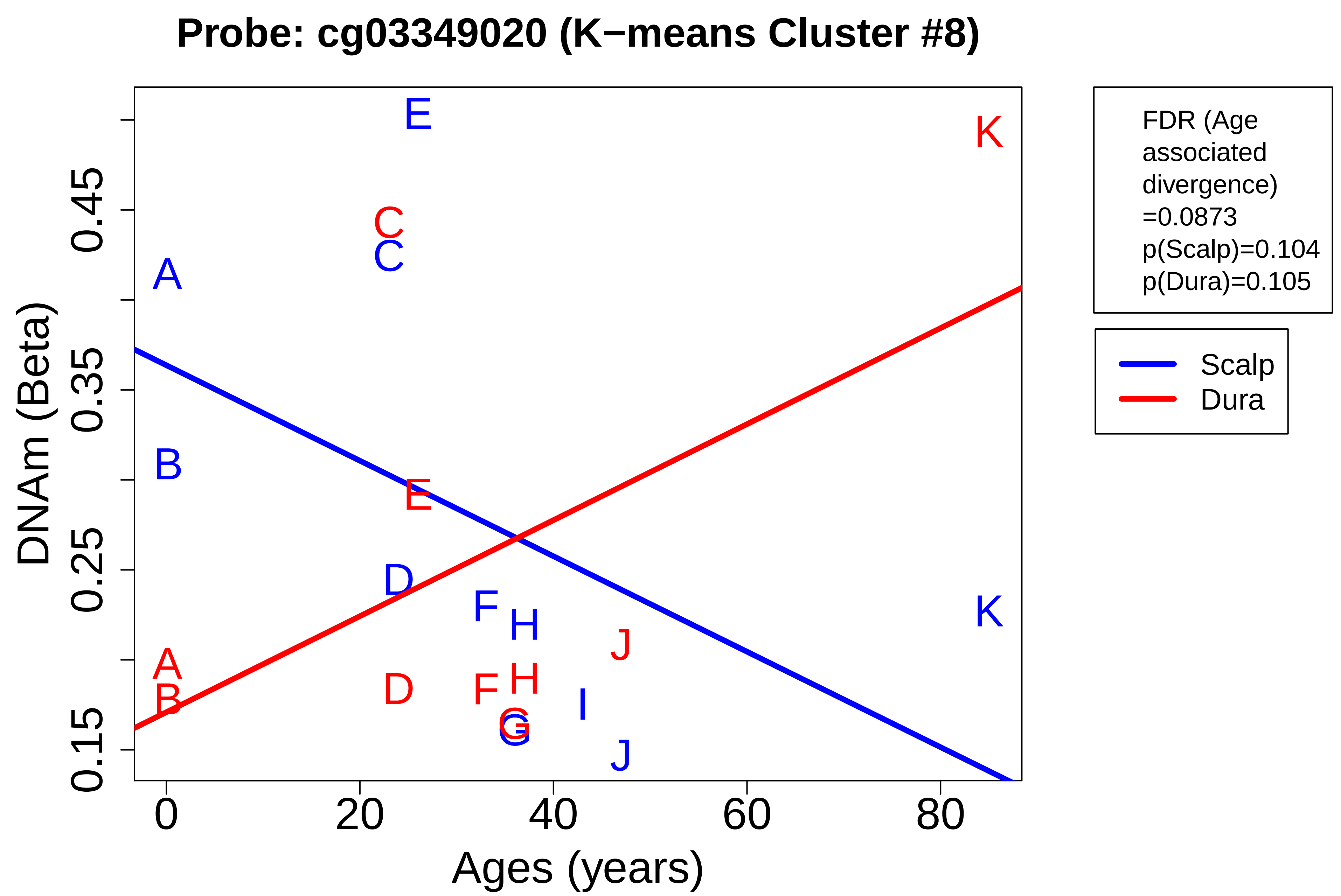

Probe: cg03349020 (K−means Cluster #8)
FDR (Age associated divergence) =0.0873
p(Scalp)=0.104
p(Dura)=0.105
Scalp
Dura
DNAm (Beta)
0.15
0.25
0.35
0.45
Ages (years)
0
20
40
60
80
A
B
C
D
E
F
G
H
I
J
K

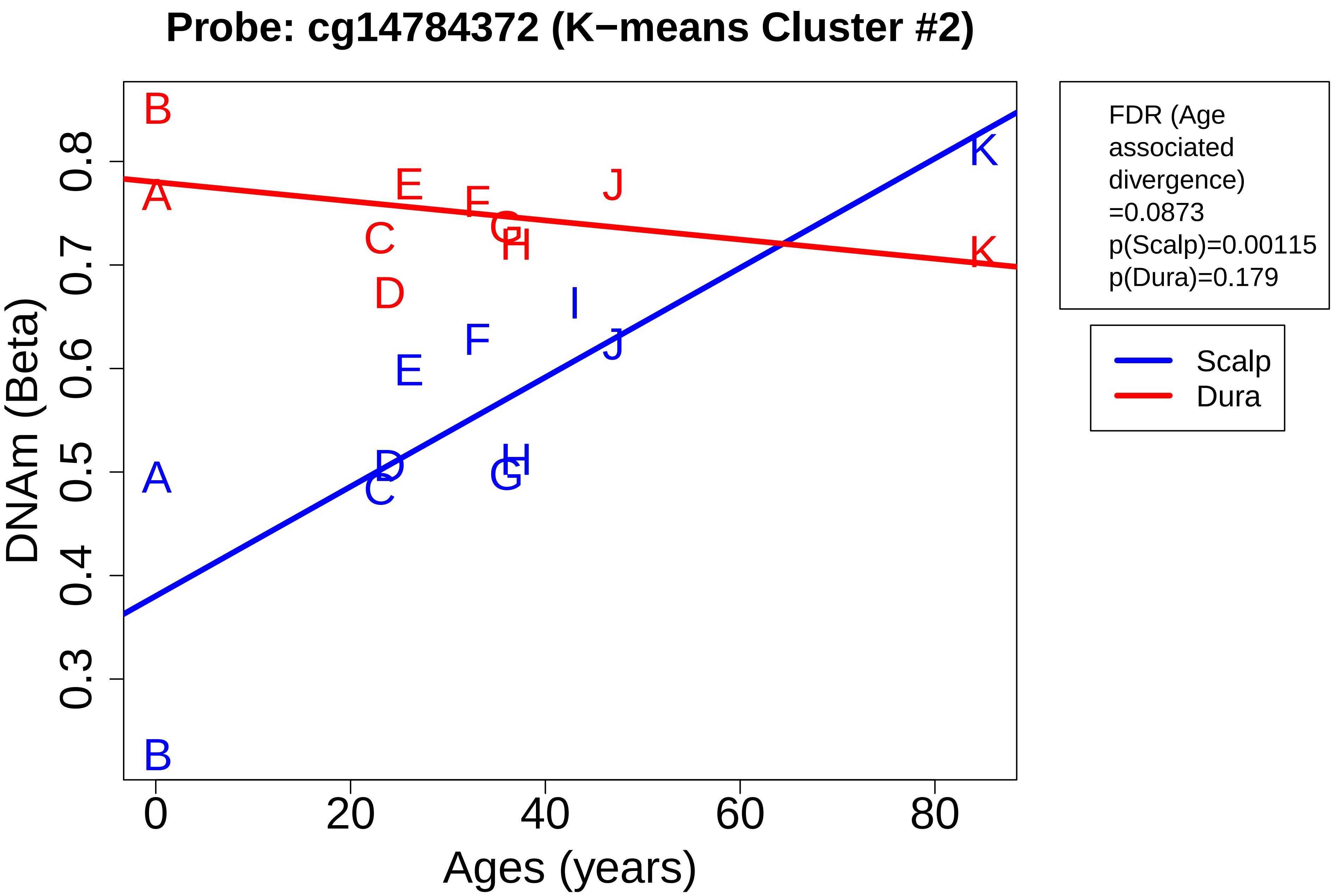

Probe: cg14784372 (K−means Cluster #2)
DNAm (Beta)
0.3
0.4
0.5
0.6
0.7
0.8
0
20
40
60
80
Ages (years)
FDR (Age associated divergence) =0.0873
p(Scalp)=0.00115
p(Dura)=0.179
Scalp
Dura

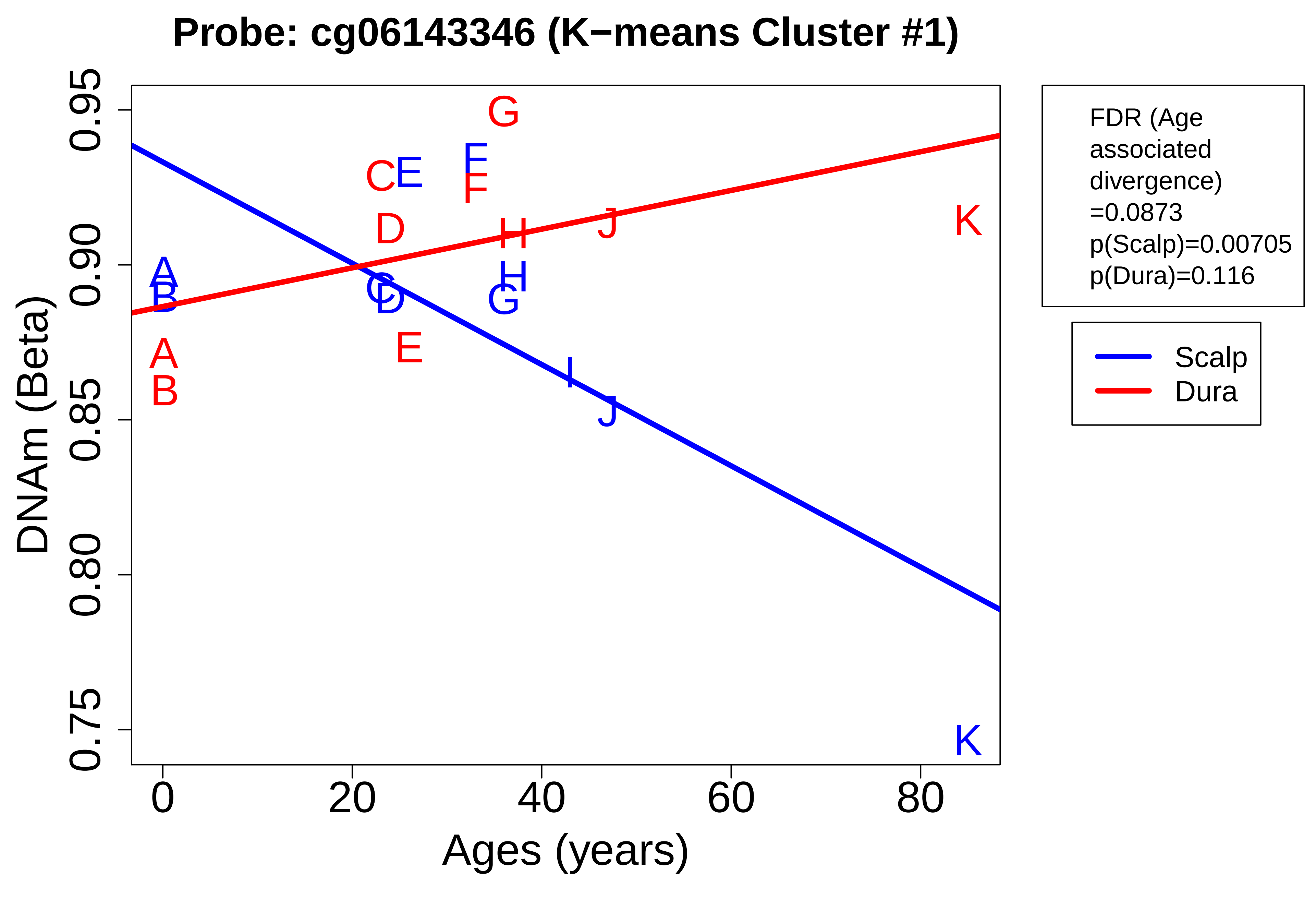

Probe: cg06143346 (K−means Cluster #1)
DNAm (Beta)
Ages (years)
0.75
0.80
0.85
0.90
0.95
0
20
40
60
80
FDR (Age associated divergence) =0.0873
p(Scalp)=0.00705
p(Dura)=0.116
Scalp
Dura

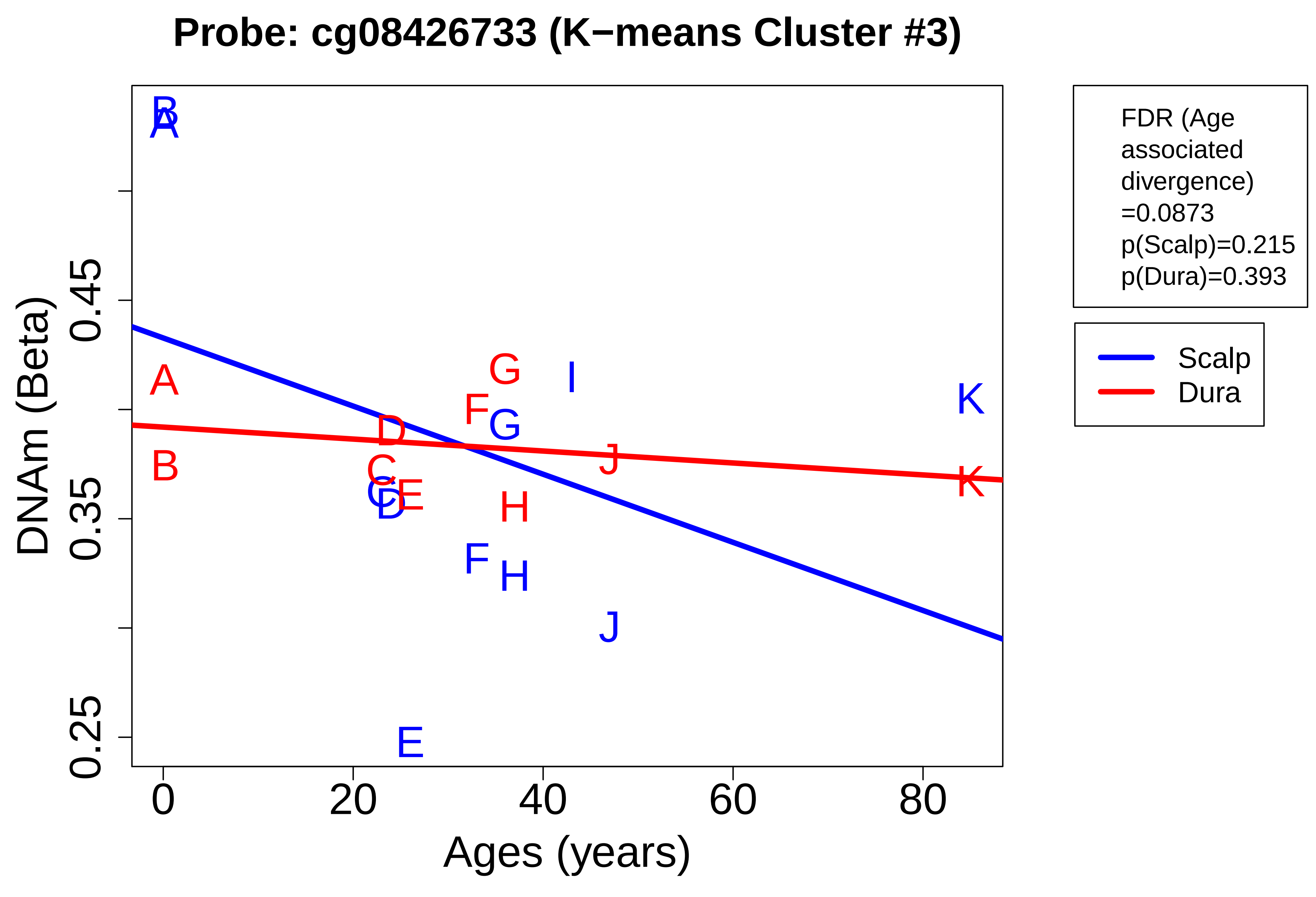

Probe: cg08426733 (K−means Cluster #3)
DNAm (Beta)
0.25
0.35
0.45
0
20
40
60
80
Ages (years)
FDR (Age associated divergence) =0.0873
p(Scalp)=0.215
p(Dura)=0.393
Scalp
Dura
A
B
C
D
E
F
G
H
I
J
K

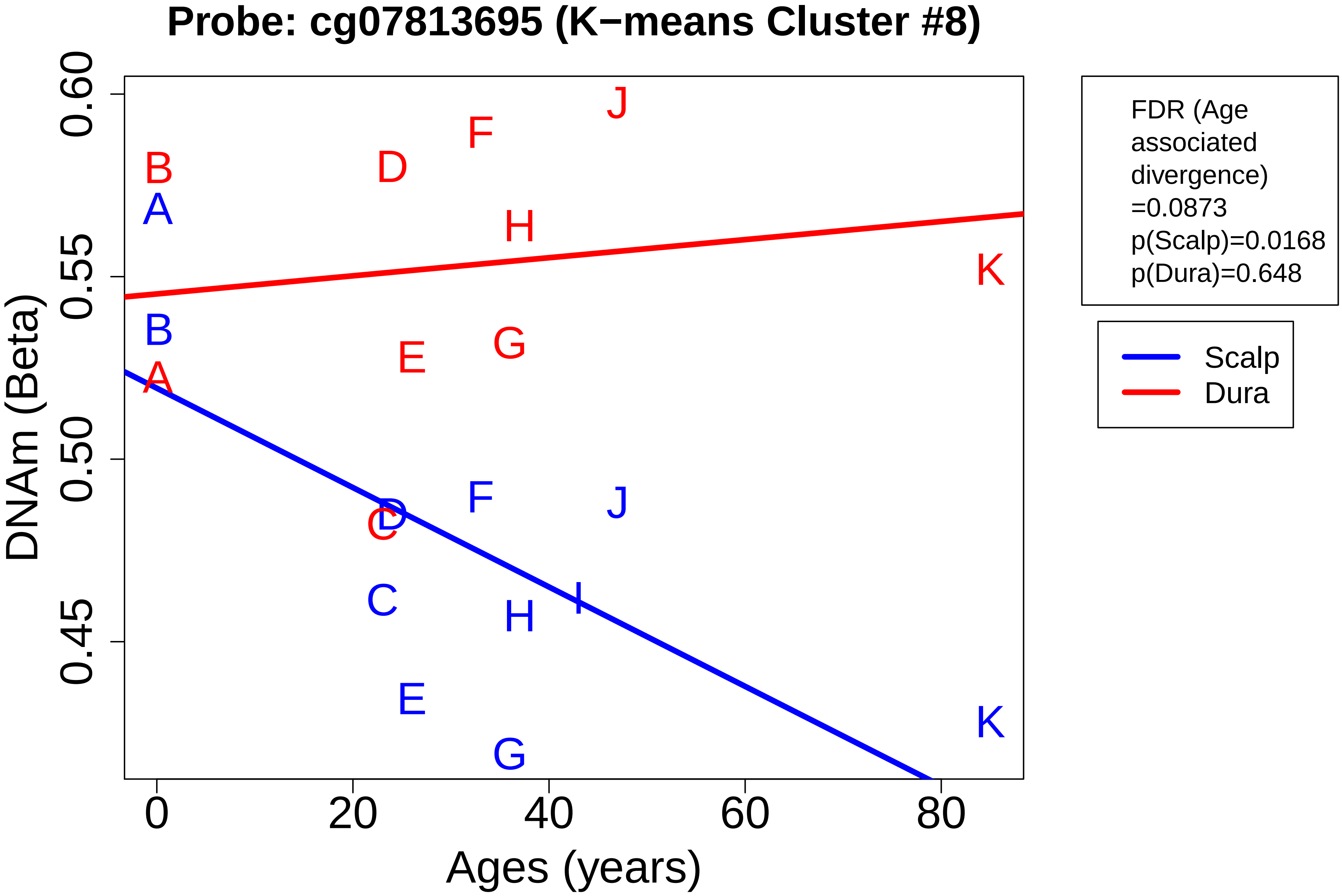

Probe: cg07813695 (K−means Cluster #8)
DNAm (Beta)
0.60
0.55
0.50
0.45
0
20
40
60
80
Ages (years)
FDR (Age associated divergence) =0.0873
p(Scalp)=0.0168
p(Dura)=0.648
Scalp
Dura

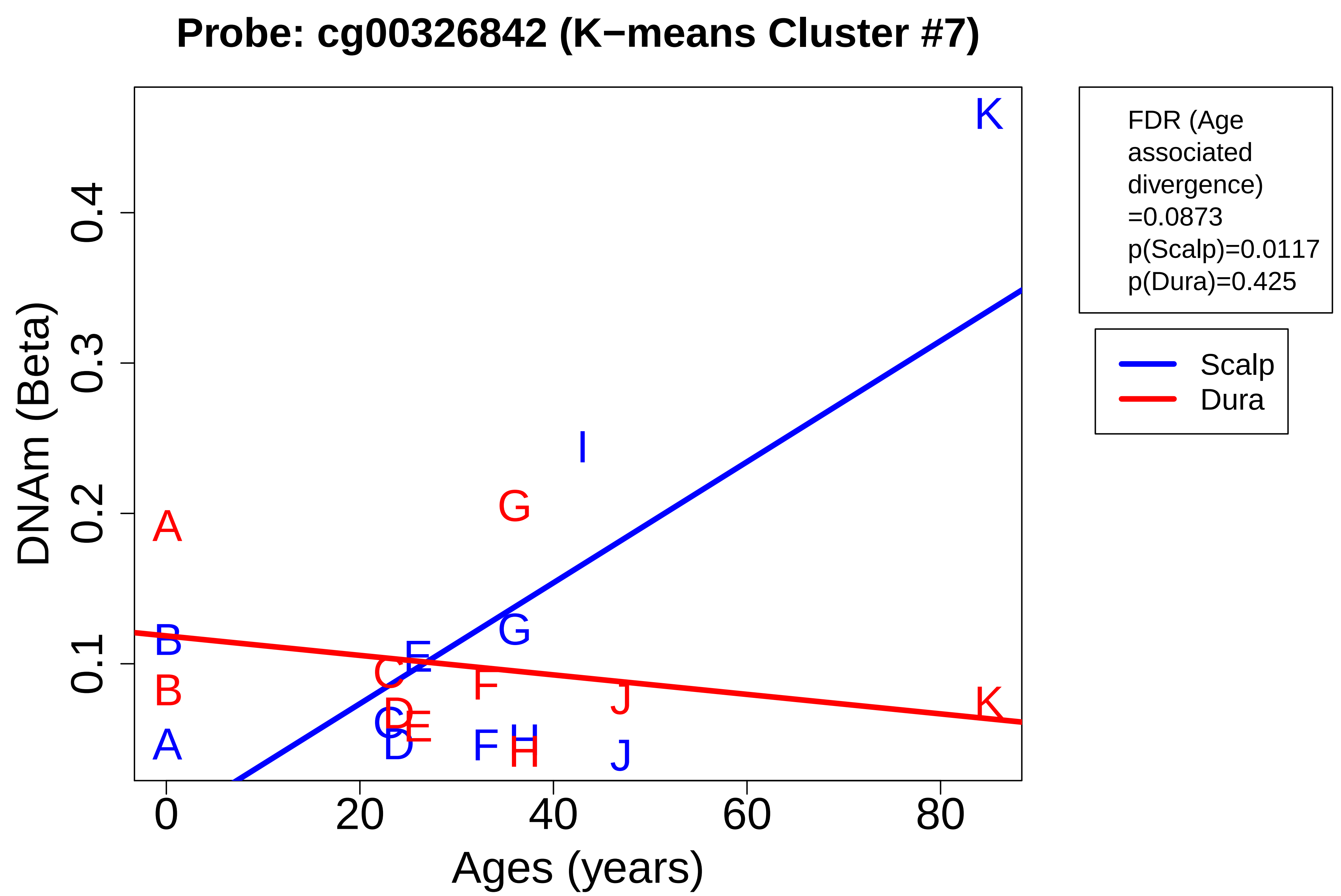

Probe: cg00326842 (K−means Cluster #7)
DNAm (Beta)
0.1
0.2
0.3
0.4
0
20
40
60
80
Ages (years)
FDR (Age associated divergence) =0.0873
p(Scalp)=0.0117
p(Dura)=0.425
Scalp
Dura
A
B
C
D
E
F
G
H
I
J
K

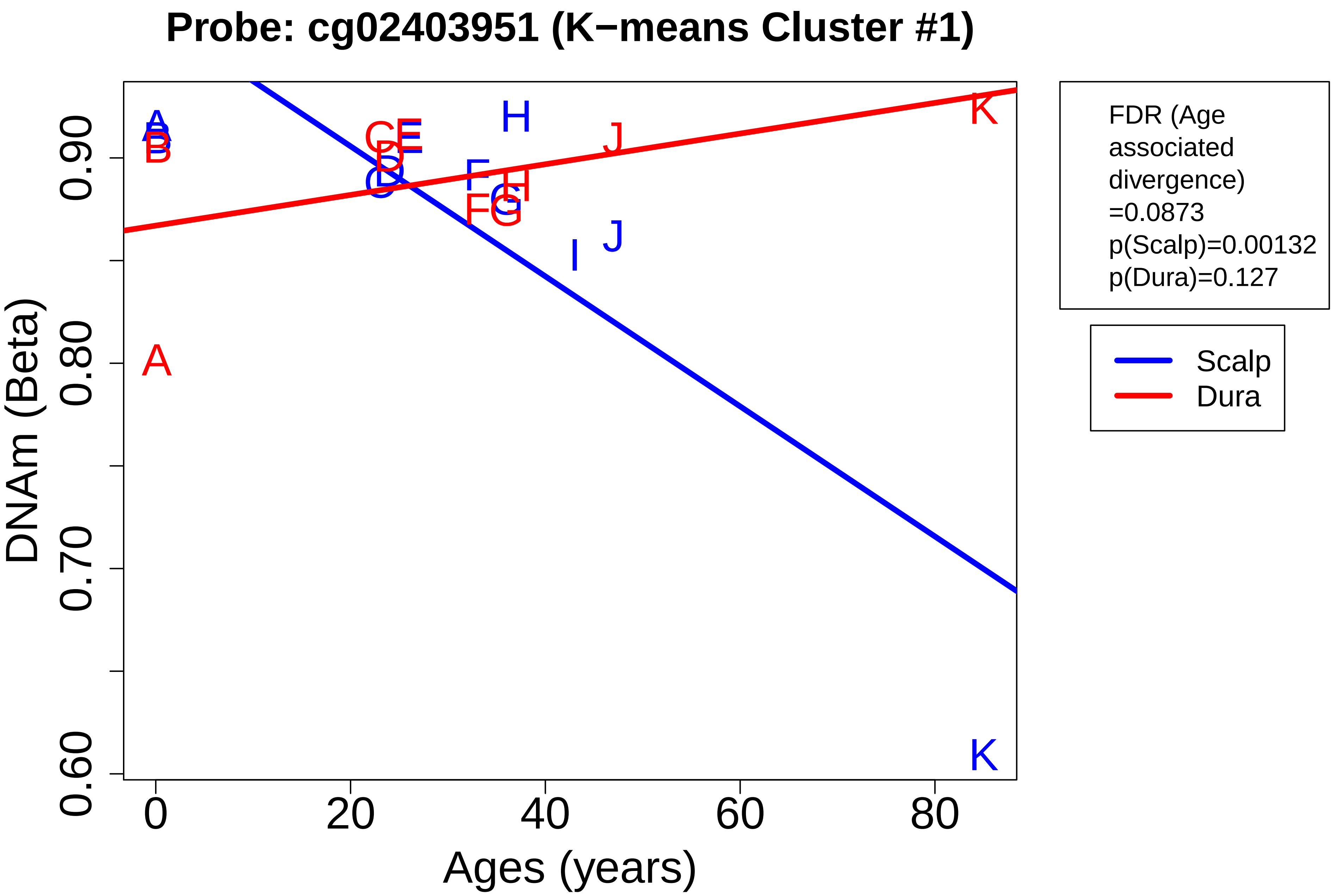

Probe: cg02403951 (K−means Cluster #1)
DNAm (Beta)
0.60
0.70
0.80
0.90
Ages (years)
0
20
40
60
80
A
B
C
D
E
F
G
H
I
J
K
FDR (Age associated divergence) =0.0873
p(Scalp)=0.00132
p(Dura)=0.127
Scalp
Dura

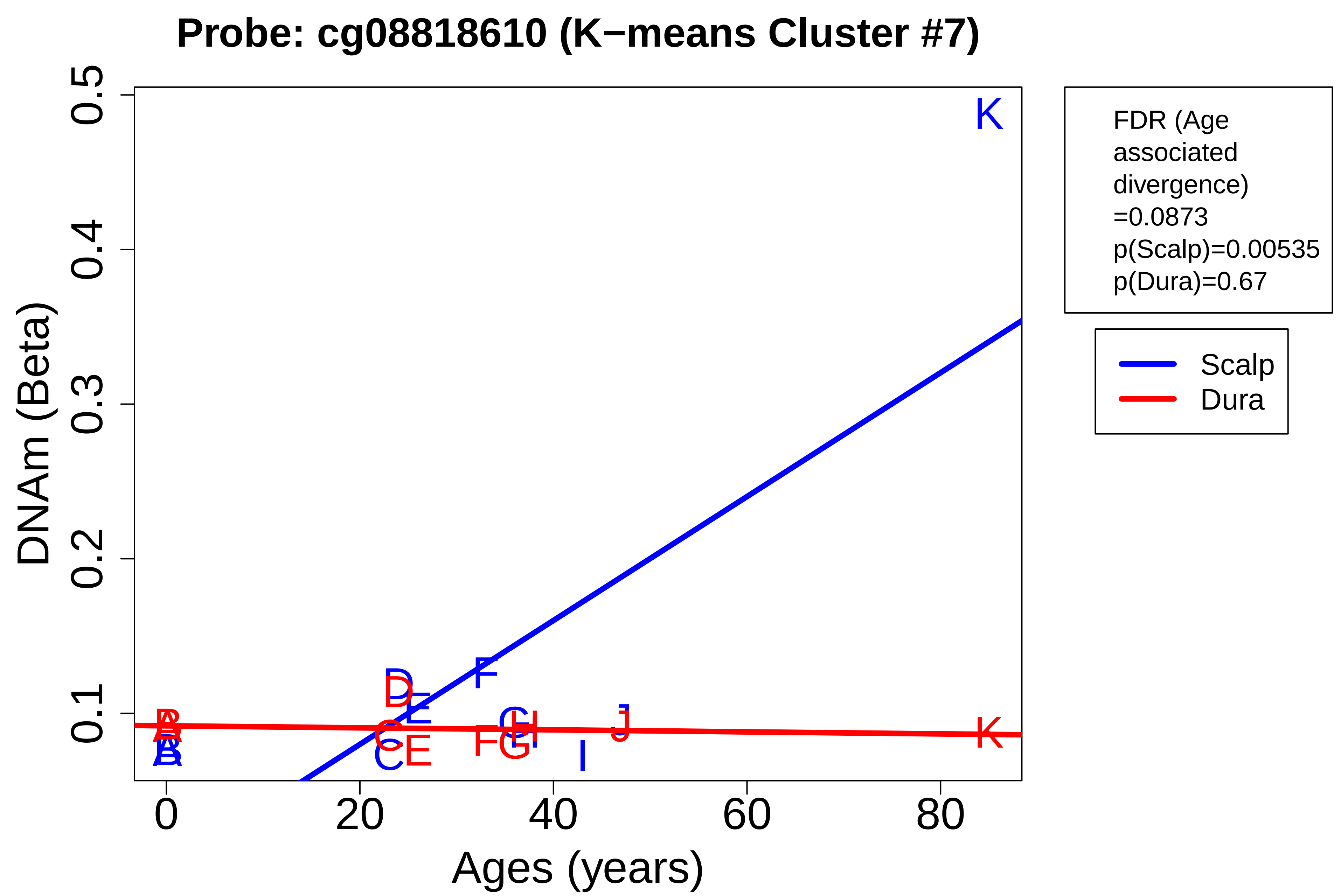

Probe: cg08818610 (K−means Cluster #7)
DNAm (Beta)
0.1
0.2
0.3
0.4
0.5
0
20
40
60
80
Ages (years)
FDR (Age associated divergence) =0.0873
p(Scalp)=0.00535
p(Dura)=0.67
Scalp
Dura

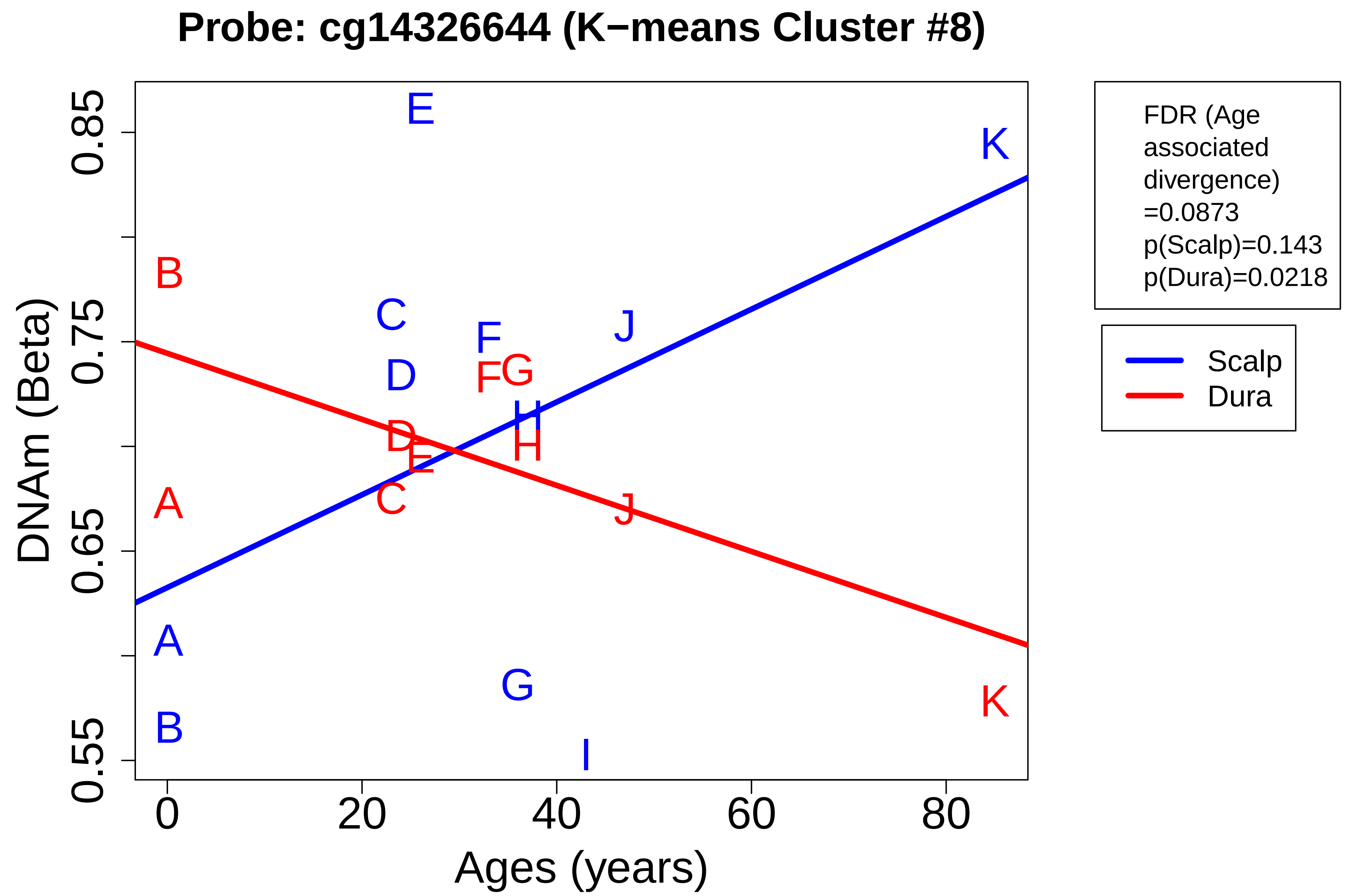

Probe: cg14326644 (K−means Cluster #8)
DNAm (Beta)
0.55
0.65
0.75
0.85
0
20
40
60
80
Ages (years)
A
B
C
D
E
F
G
H
I
J
K
FDR (Age associated divergence) =0.0873
p(Scalp)=0.143
p(Dura)=0.0218
Scalp
Dura

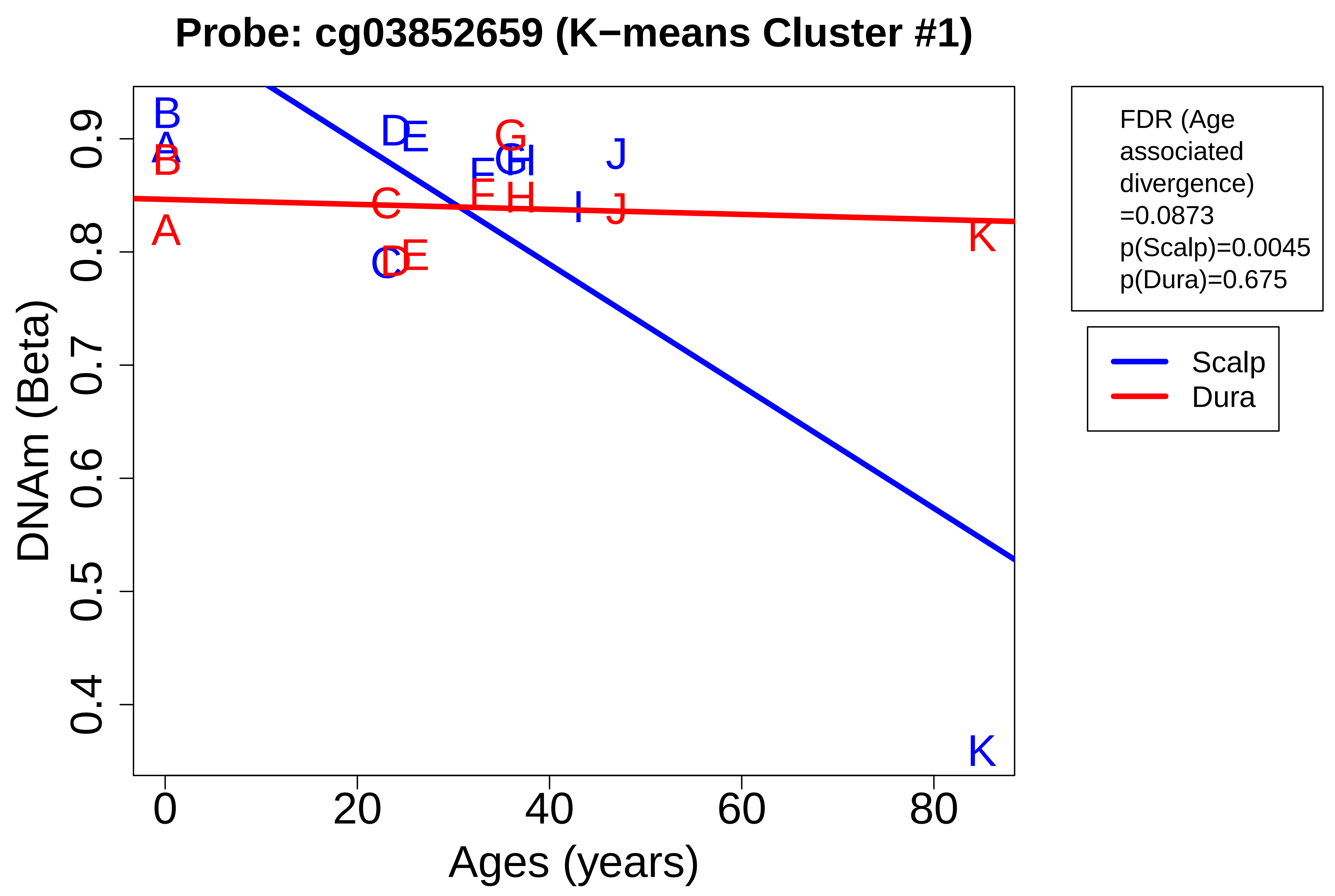

Probe: cg03852659 (K−means Cluster #1)
DNAm (Beta)
0.4
0.5
0.6
0.7
0.8
0.9
0
20
40
60
80
Ages (years)
FDR (Age associated divergence) =0.0873
p(Scalp)=0.0045
p(Dura)=0.675
Scalp
Dura

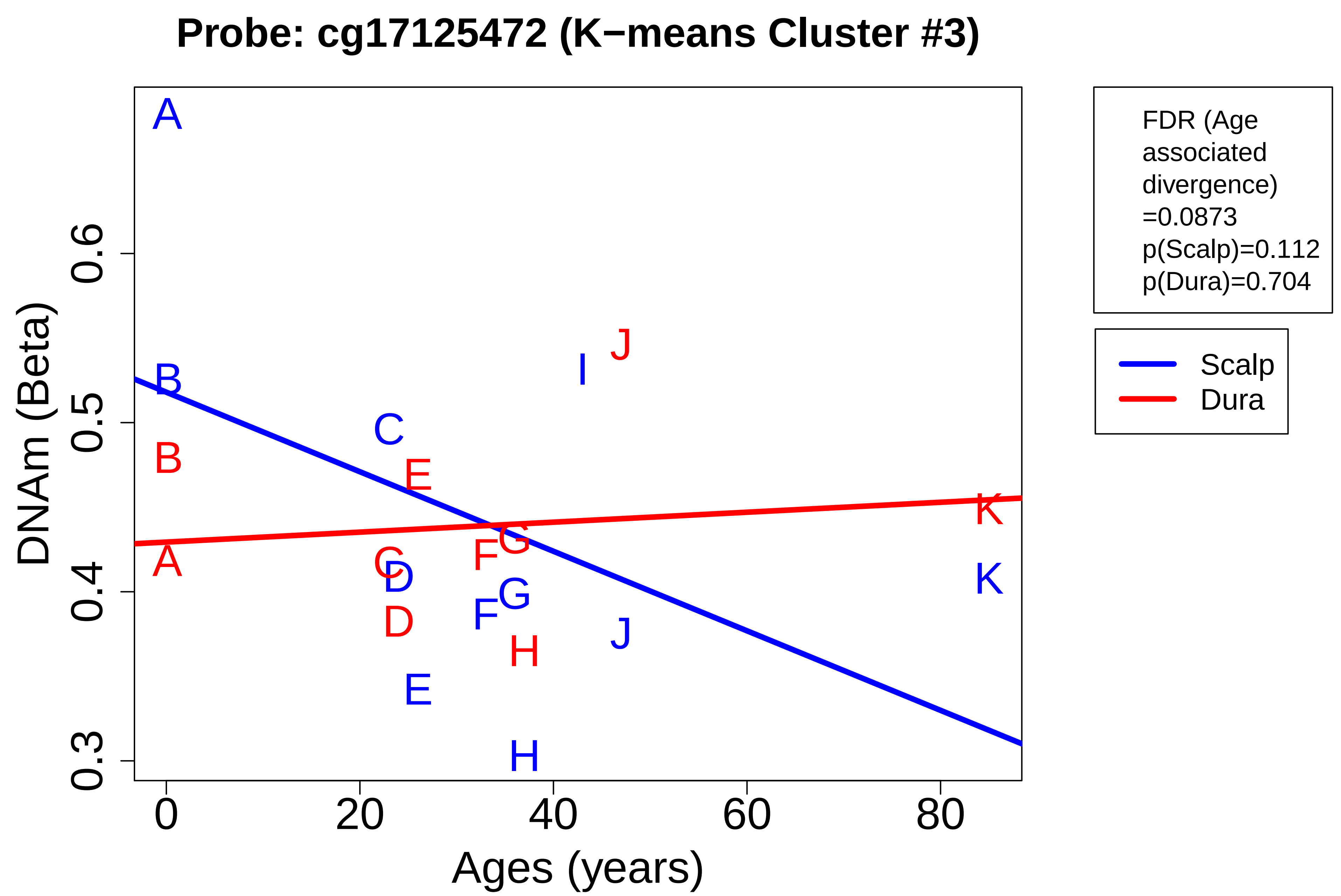

Probe: cg17125472 (K−means Cluster #3)
DNAm (Beta)
0.3
0.4
0.5
0.6
0
20
40
60
80
Ages (years)
FDR (Age associated divergence) =0.0873
p(Scalp)=0.112
p(Dura)=0.704
Scalp
Dura
A
B
C
D
E
F
G
H
I
J
K

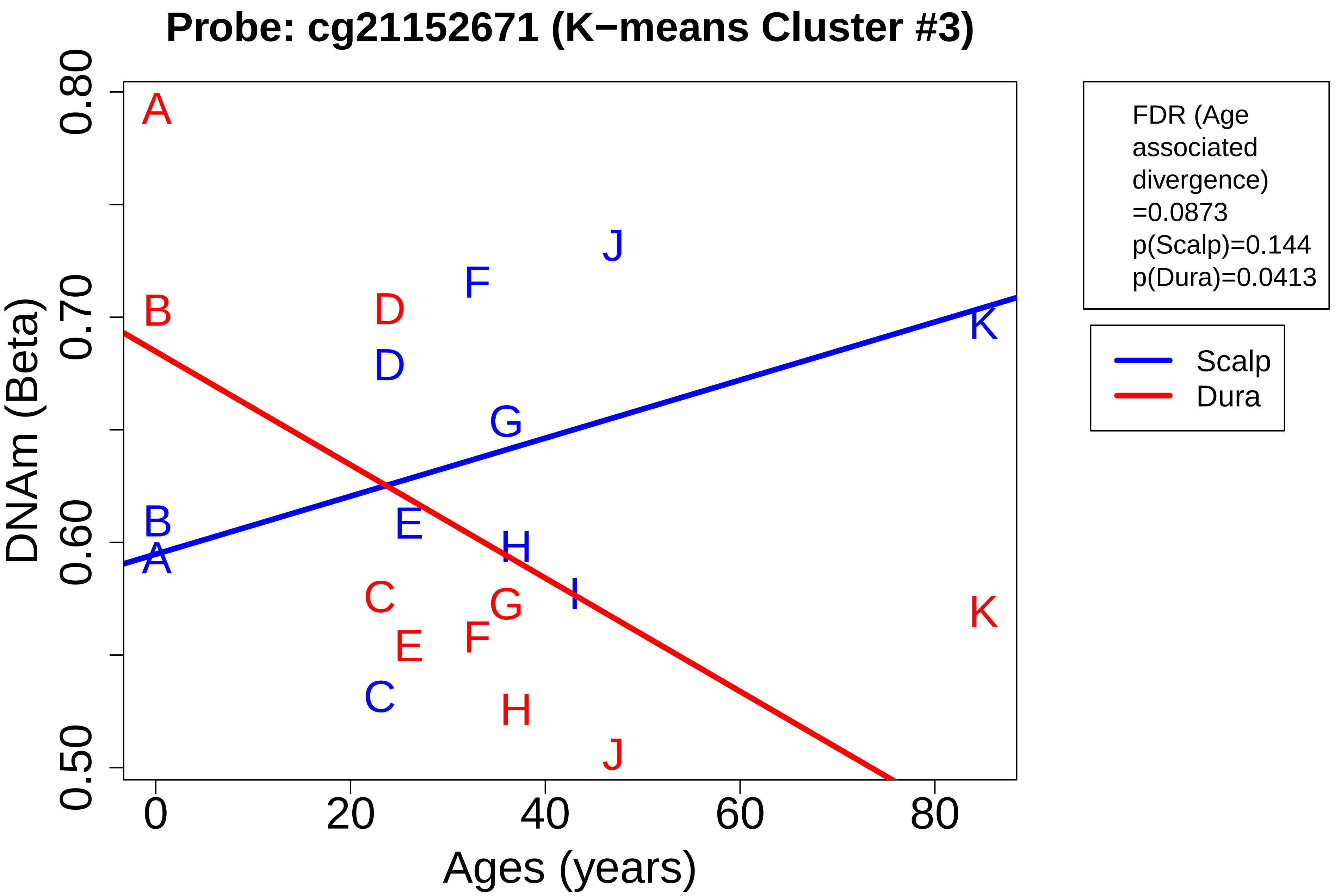

Probe: cg21152671 (K−means Cluster #3)
DNAm (Beta)
0.50
0.60
0.70
0.80
Ages (years)
0
20
40
60
80
A
B
C
D
E
F
G
H
I
J
K
FDR (Age associated divergence) =0.0873
p(Scalp)=0.144
p(Dura)=0.0413
Scalp
Dura

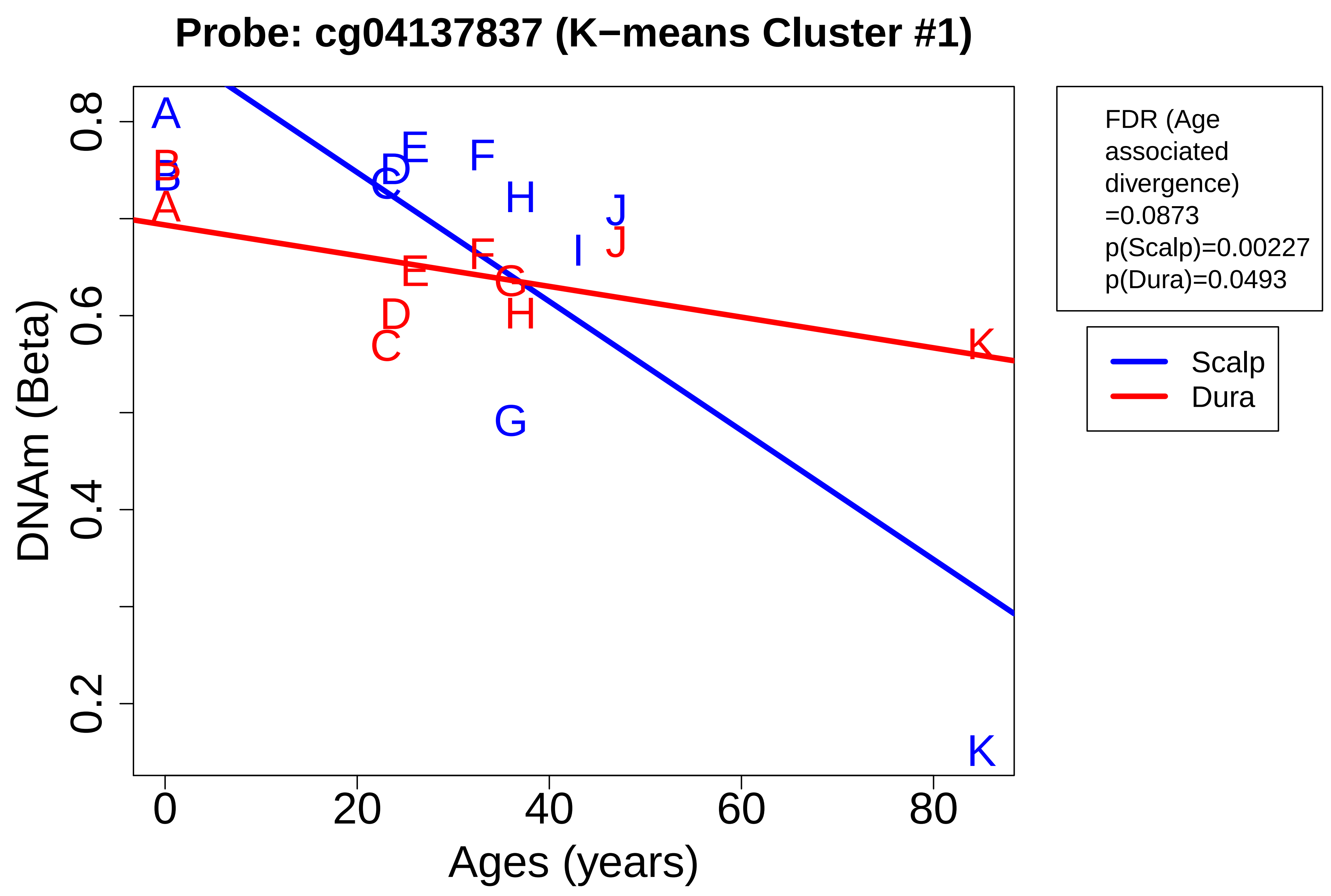

Probe: cg04137837 (K−means Cluster #1)
DNAm (Beta)
0.2
0.4
0.6
0.8
Ages (years)
0
20
40
60
80
A
B
C
D
E
F
G
H
I
J
K
FDR (Age associated divergence) =0.0873
p(Scalp)=0.00227
p(Dura)=0.0493
Scalp
Dura

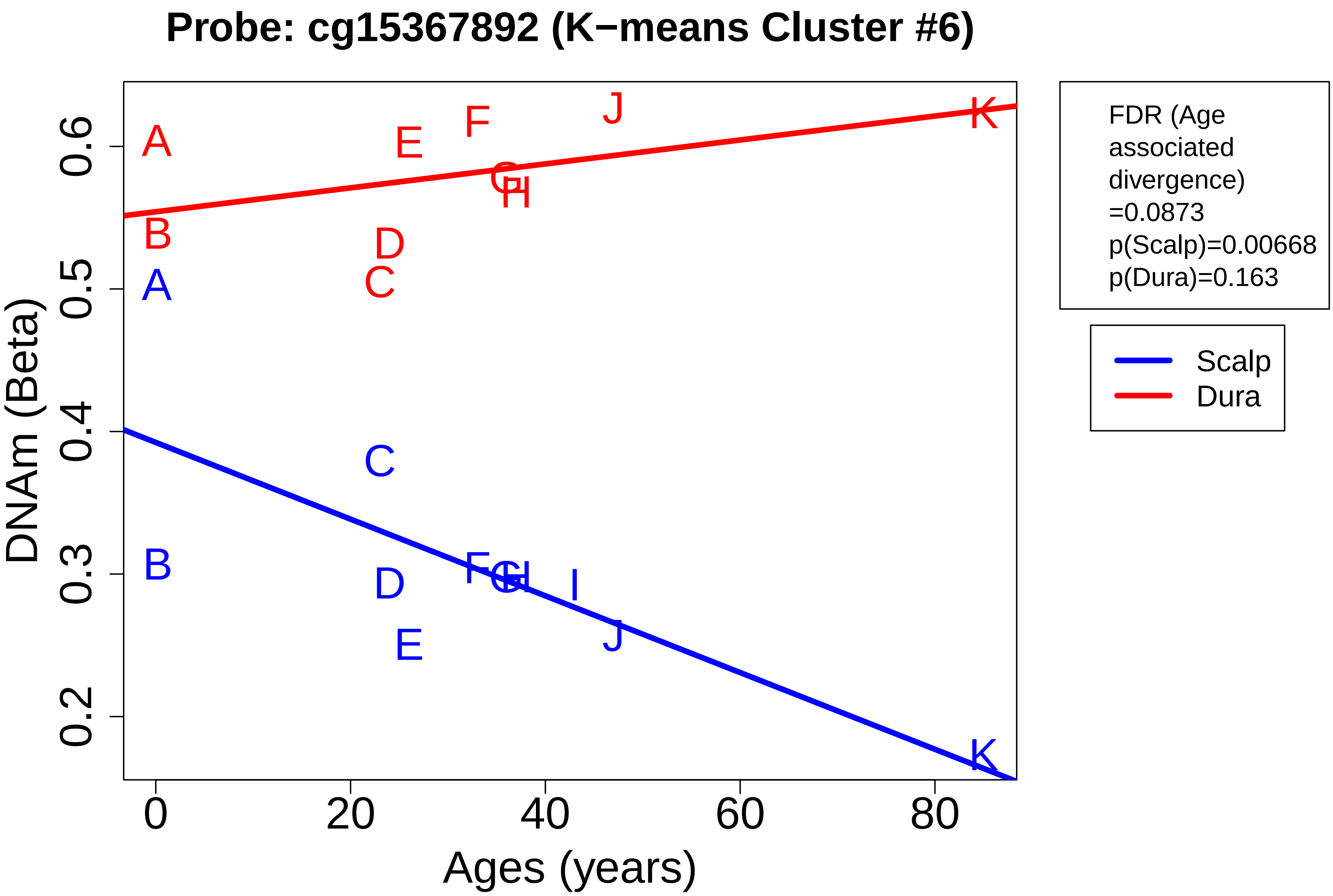

Probe: cg15367892 (K−means Cluster #6)
DNAm (Beta)
0.2
0.3
0.4
0.5
0.6
Ages (years)
0
20
40
60
80
FDR (Age associated divergence) =0.0873
p(Scalp)=0.00668
p(Dura)=0.163
Scalp
Dura
A
B
C
D
E
F
G
H
I
J
K

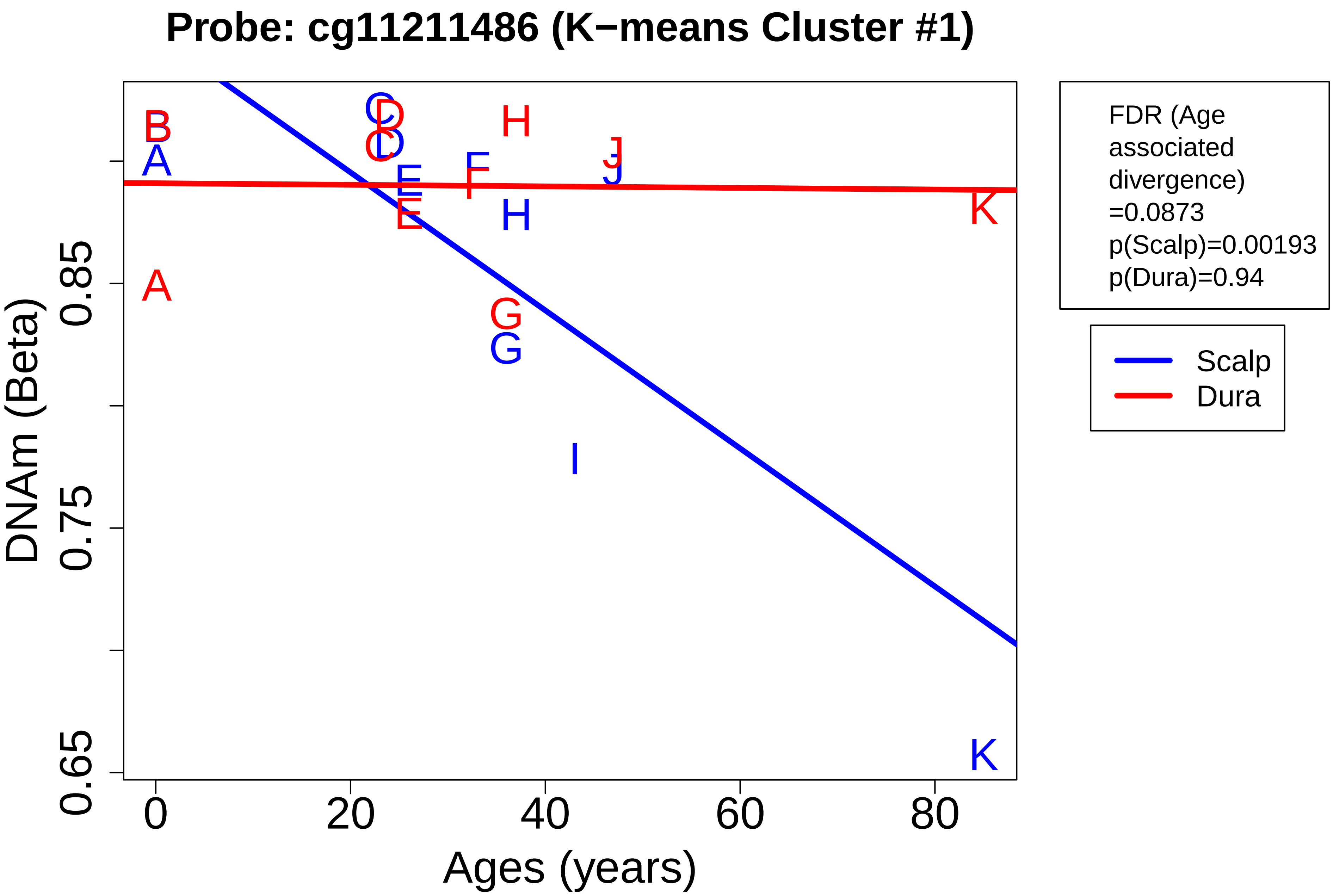
Probe: cg11211486 (K−means Cluster #1)
DNAm (Beta)
0.65
0.75
0.85
0
20
40
60
80
Ages (years)
FDR (Age associated divergence) =0.0873
p(Scalp)=0.00193
p(Dura)=0.94
Scalp
Dura

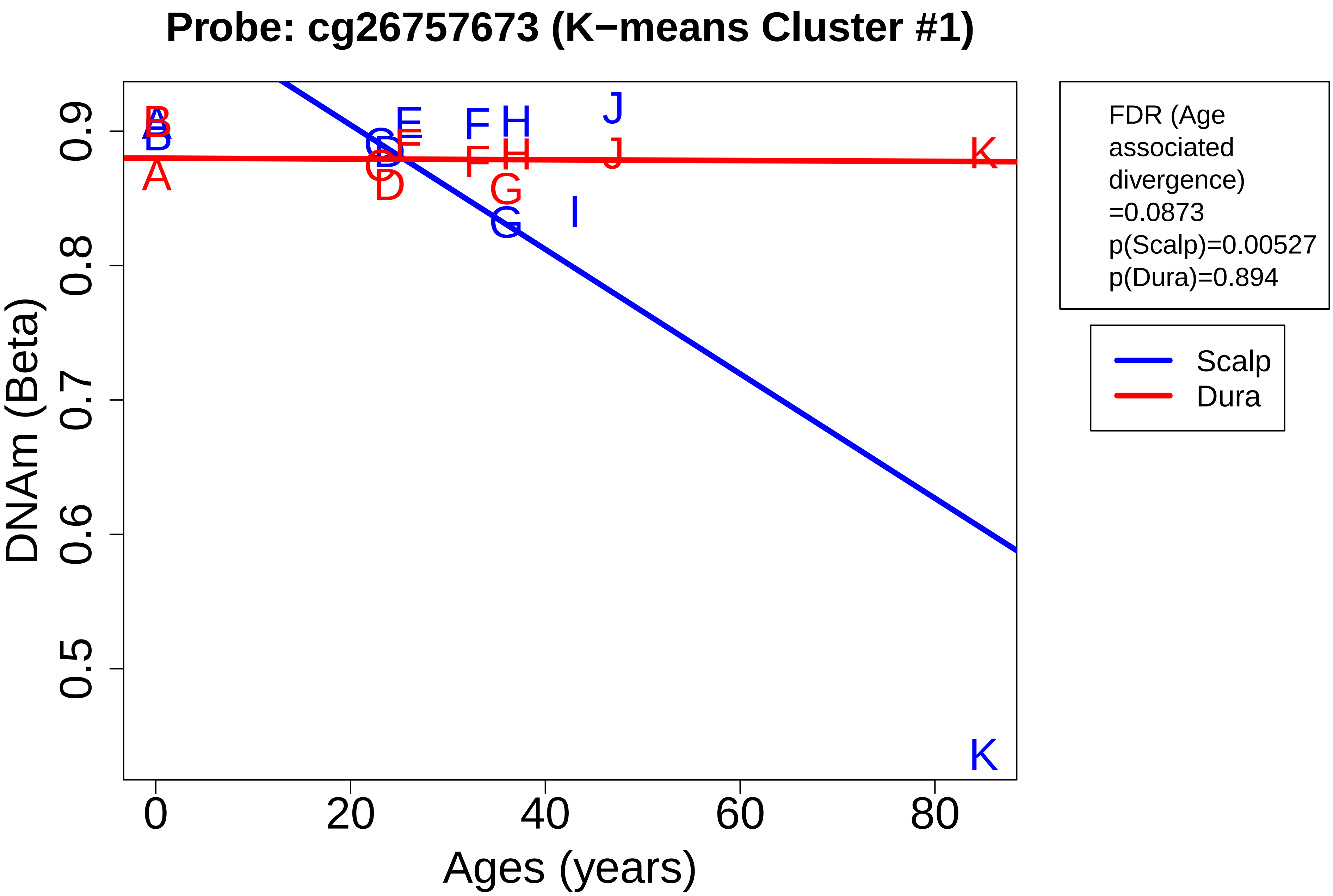

Probe: cg26757673 (K−means Cluster #1)
DNAm (Beta)
0.5
0.6
0.7
0.8
0.9
0
20
40
60
80
Ages (years)
FDR (Age associated divergence) =0.0873
p(Scalp)=0.00527
p(Dura)=0.894
Scalp
Dura
A
B
C
D
E
F
G
H
I
J
K

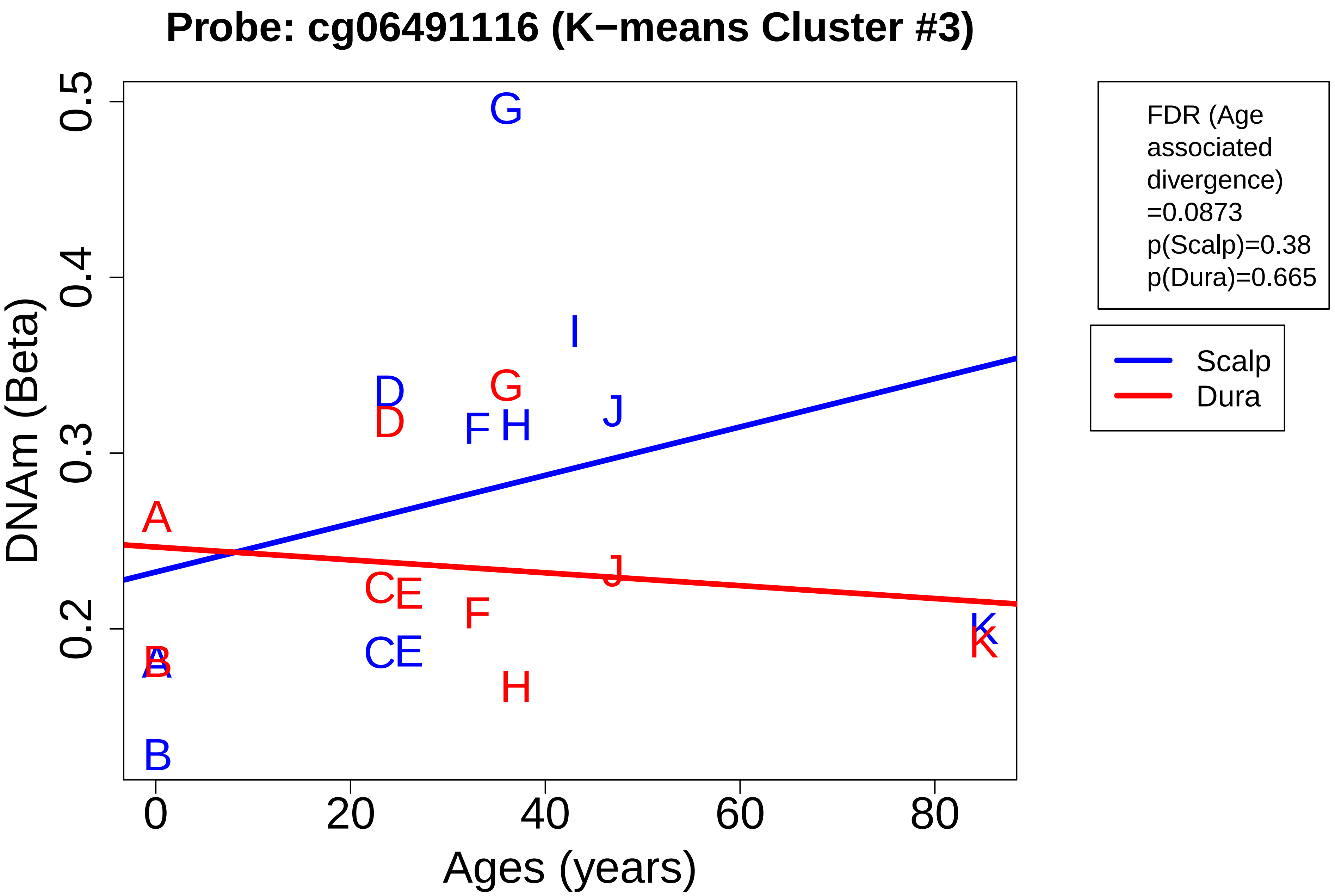

Probe: cg06491116 (K−means Cluster #3)
DNAm (Beta)
0.2
0.3
0.4
0.5
Ages (years)
0
20
40
60
80
FDR (Age associated divergence) =0.0873
p(Scalp)=0.38
p(Dura)=0.665
Scalp
Dura
A
B
C
D
E
F
G
H
I
J
K

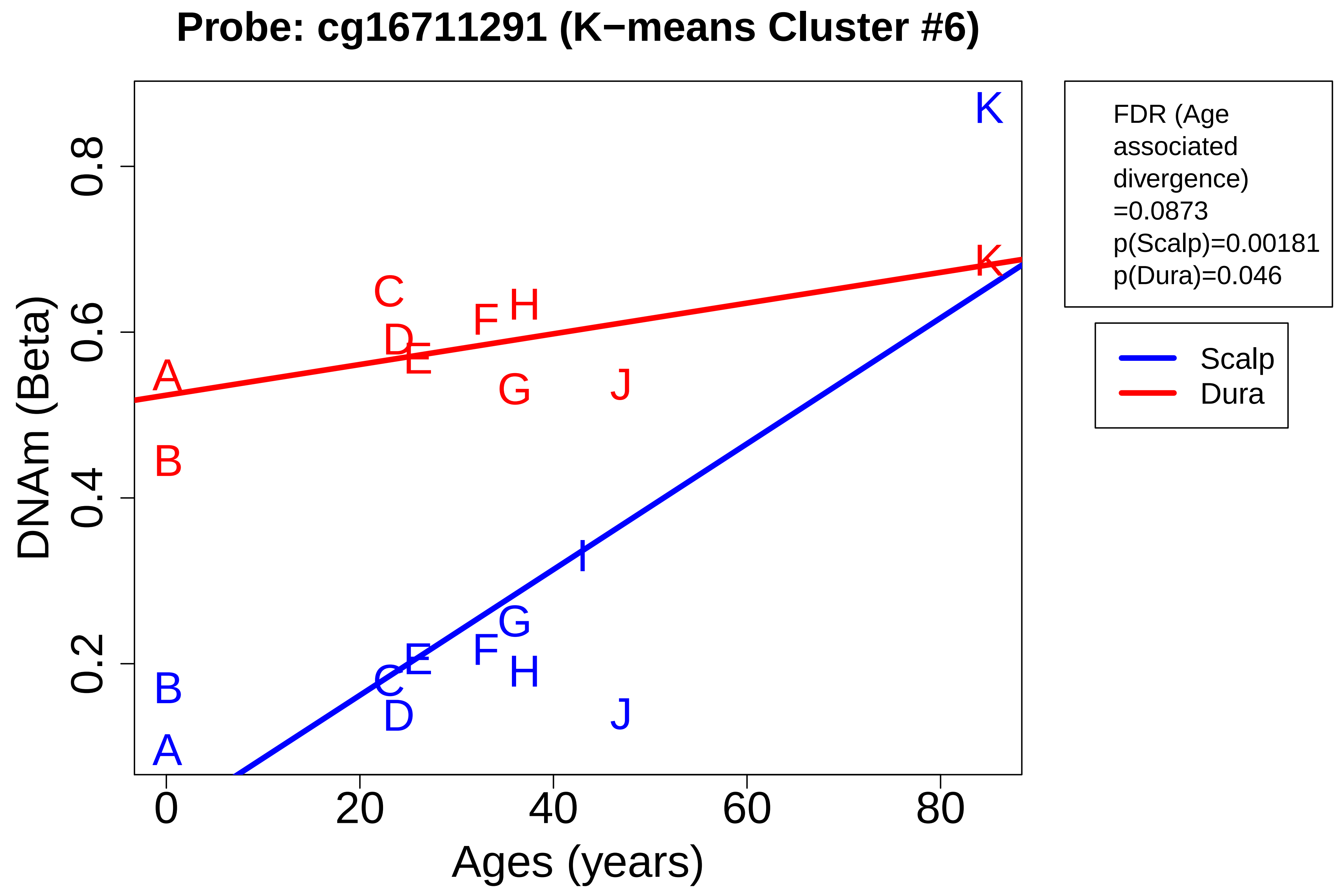

Probe: cg16711291 (K−means Cluster #6)
DNAm (Beta)
Ages (years)
0.2
0.4
0.6
0.8
0
20
40
60
80
FDR (Age associated divergence) =0.0873
p(Scalp)=0.00181
p(Dura)=0.046
Scalp
Dura
A
B
C
D
E
F
G
H
I
J
K

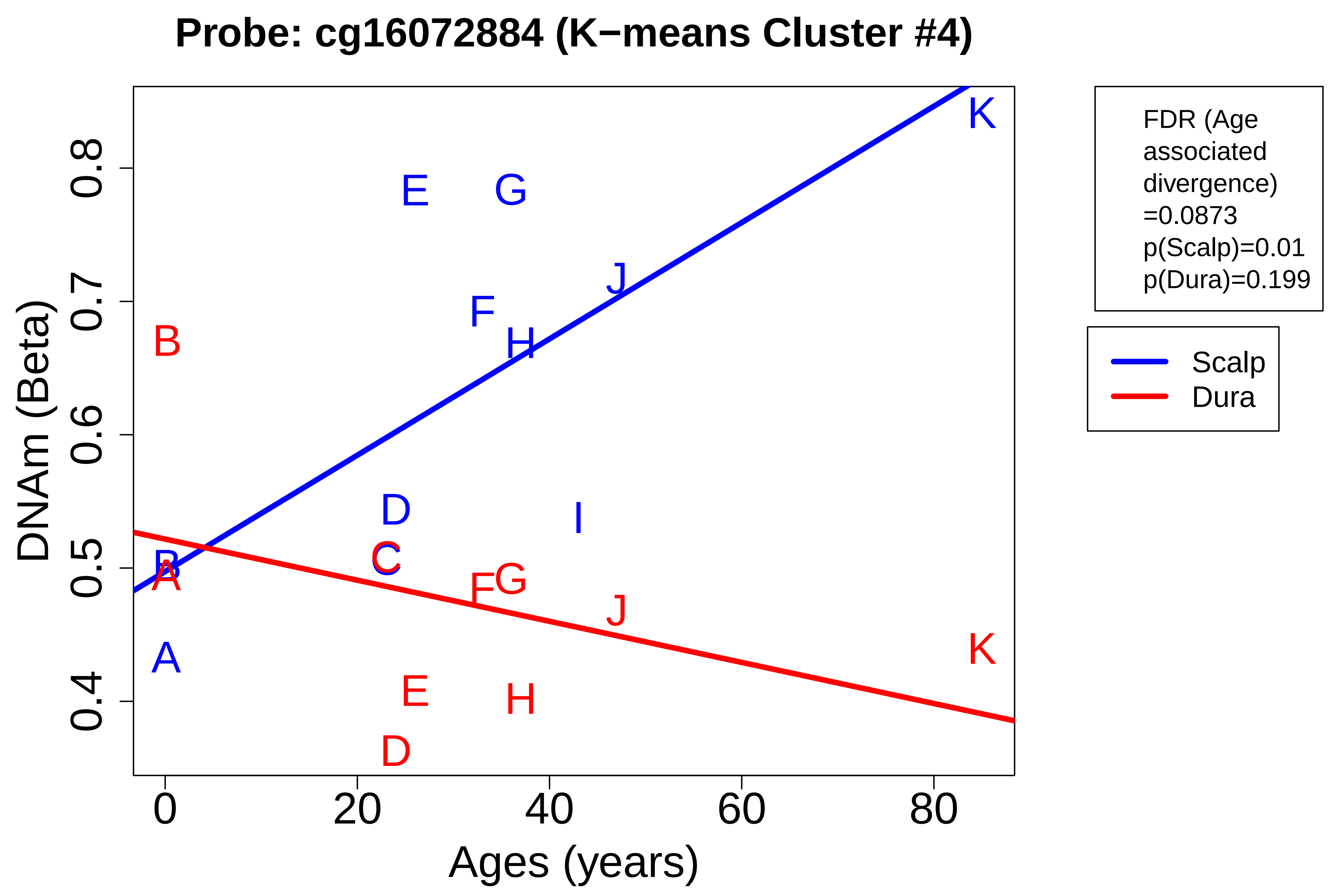

Probe: cg16072884 (K−means Cluster #4)
DNAm (Beta)
0.4
0.5
0.6
0.7
0.8
0
20
40
60
80
Ages (years)
A
B
C
D
E
F
G
H
I
J
K
FDR (Age associated divergence) =0.0873
p(Scalp)=0.01
p(Dura)=0.199
Scalp
Dura

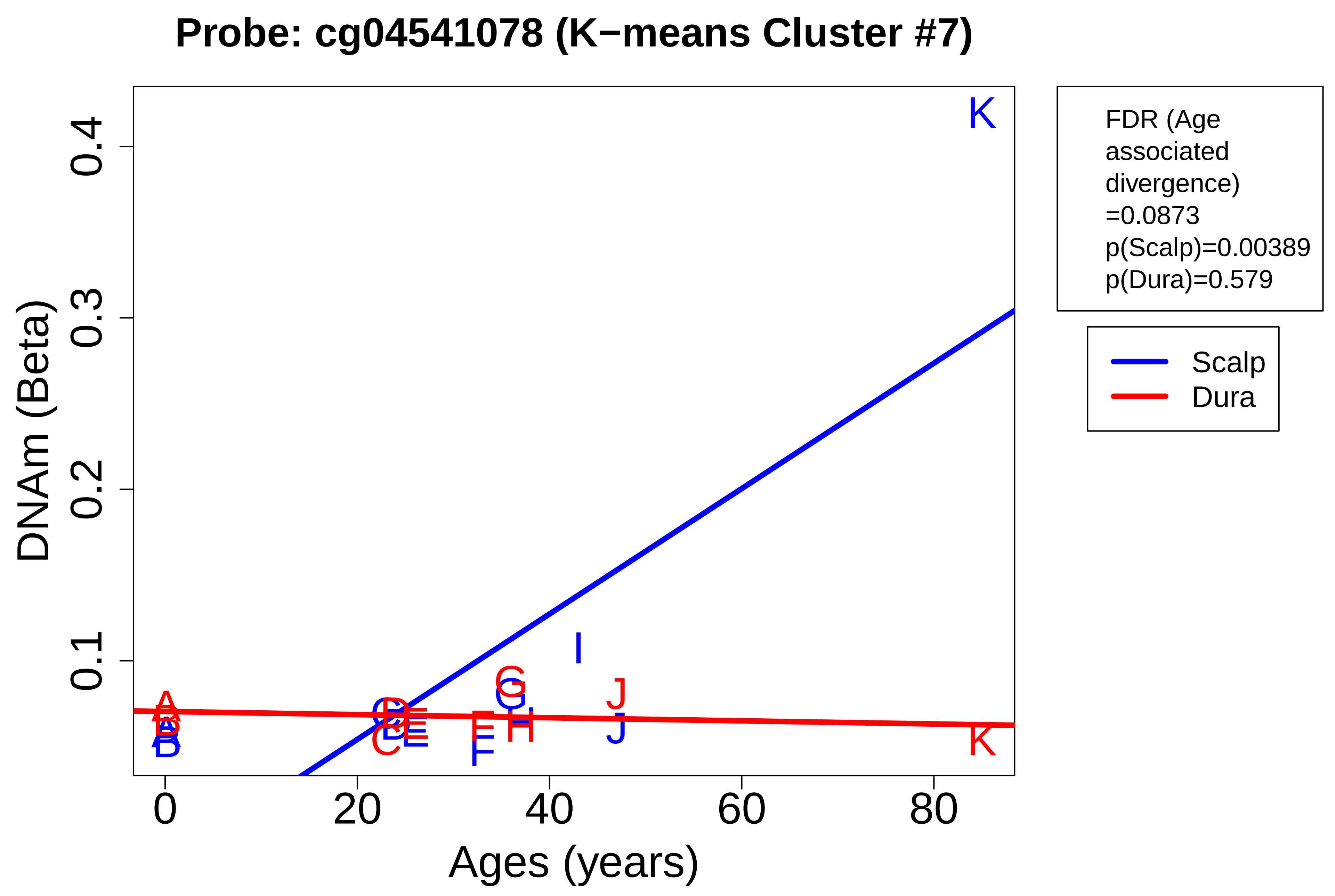

Probe: cg04541078 (K−means Cluster #7)
DNAm (Beta)
0.1
0.2
0.3
0.4
Ages (years)
0
20
40
60
80
FDR (Age associated divergence) =0.0873
p(Scalp)=0.00389
p(Dura)=0.579
Scalp
Dura

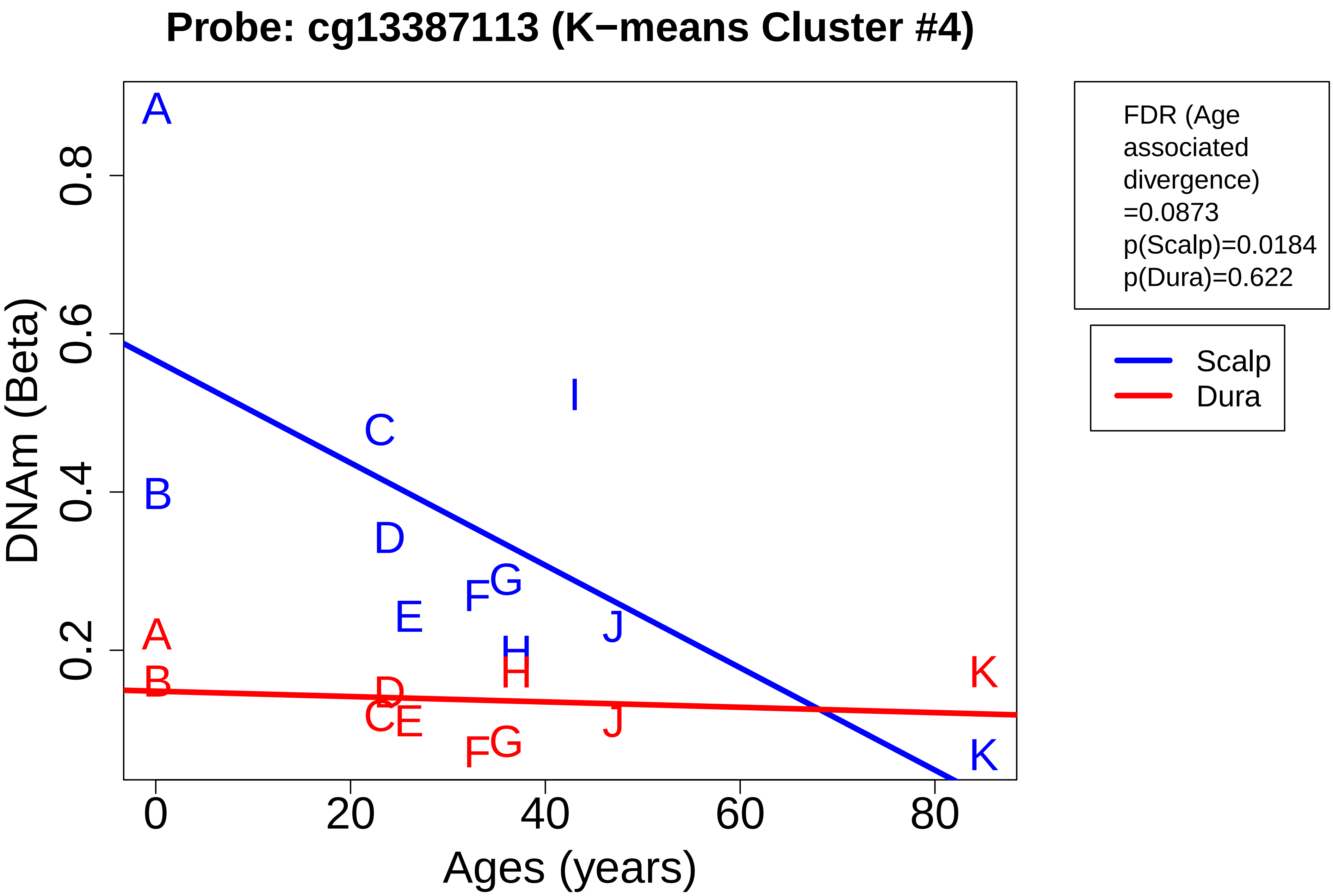

Probe: cg13387113 (K−means Cluster #4)
DNAm (Beta)
0.2
0.4
0.6
0.8
0
20
40
60
80
Ages (years)
FDR (Age associated divergence) =0.0873
p(Scalp)=0.0184
p(Dura)=0.622
Scalp
Dura
A
B
C
D
E
F
G
H
I
J
K

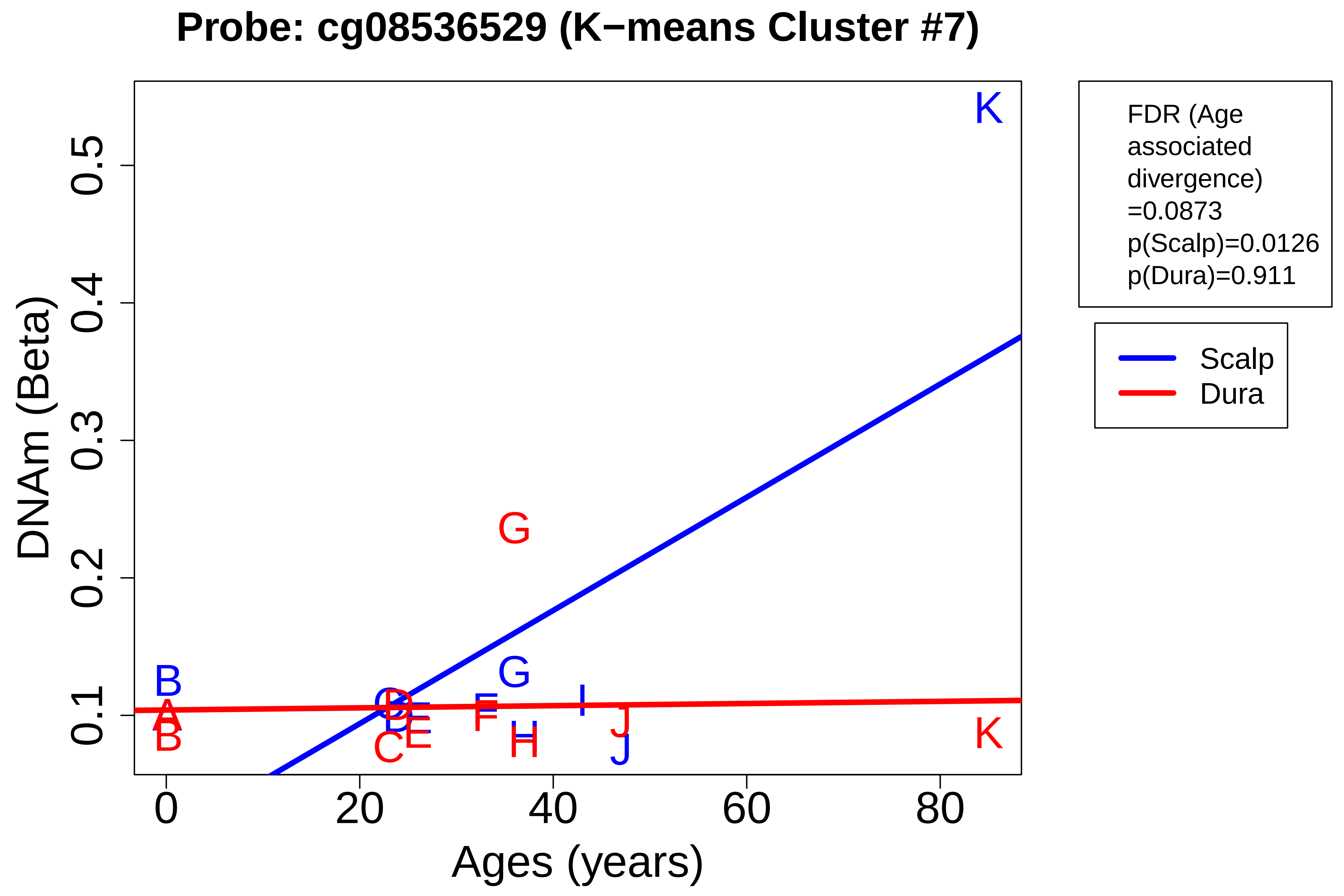
Probe: cg08536529 (K−means Cluster #7)
DNAm (Beta)
0.1
0.2
0.3
0.4
0.5
0
20
40
60
80
Ages (years)
FDR (Age associated divergence) =0.0873
p(Scalp)=0.0126
p(Dura)=0.911
Scalp
Dura

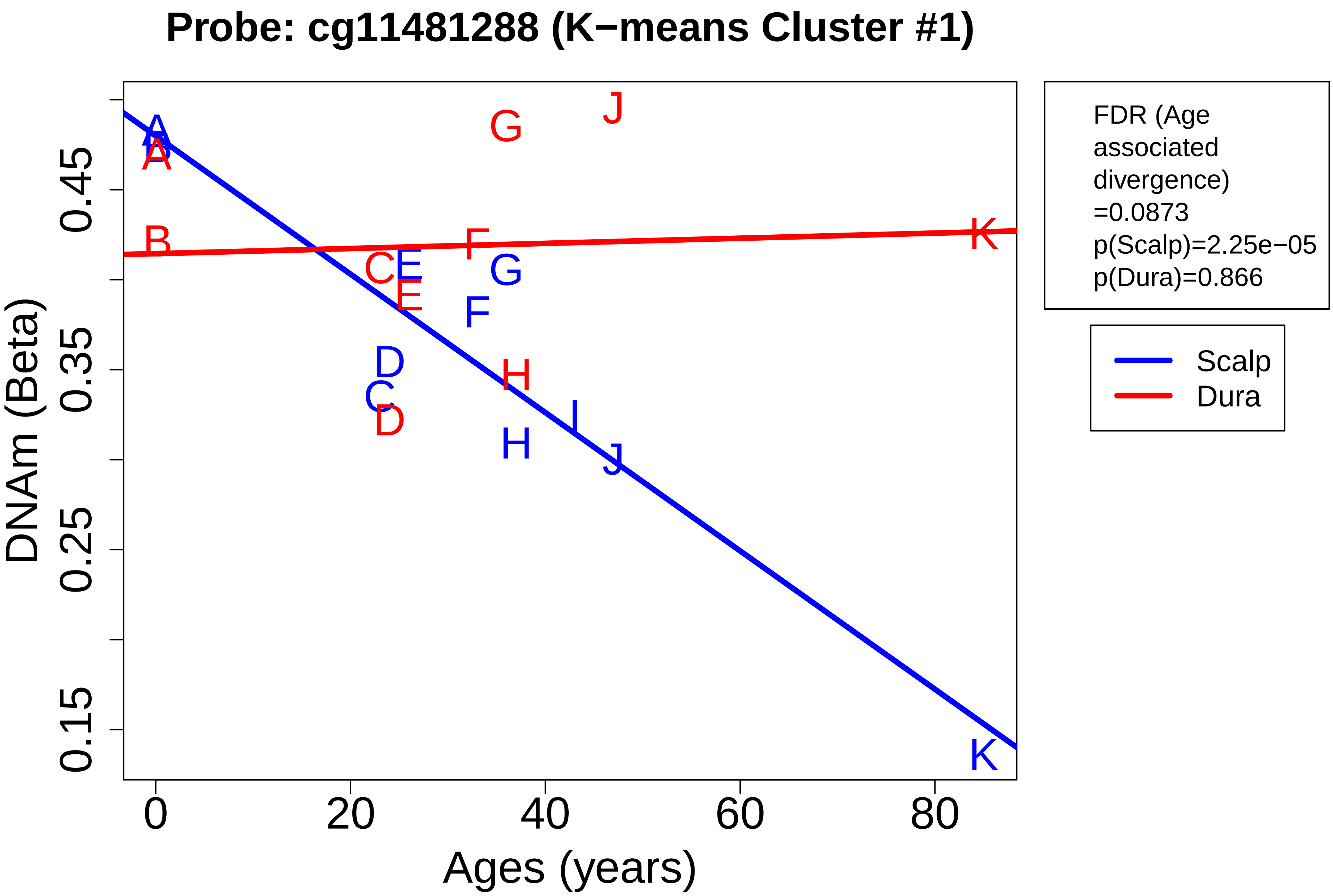
Probe: cg11481288 (K−means Cluster #1)
DNAm (Beta)
0.15
0.25
0.35
0.45
0
20
40
60
80
Ages (years)
FDR (Age associated divergence) =0.0873
p(Scalp)=2.25e−05
p(Dura)=0.866
Scalp
Dura
A
B
C
D
E
F
G
H
I
J
K

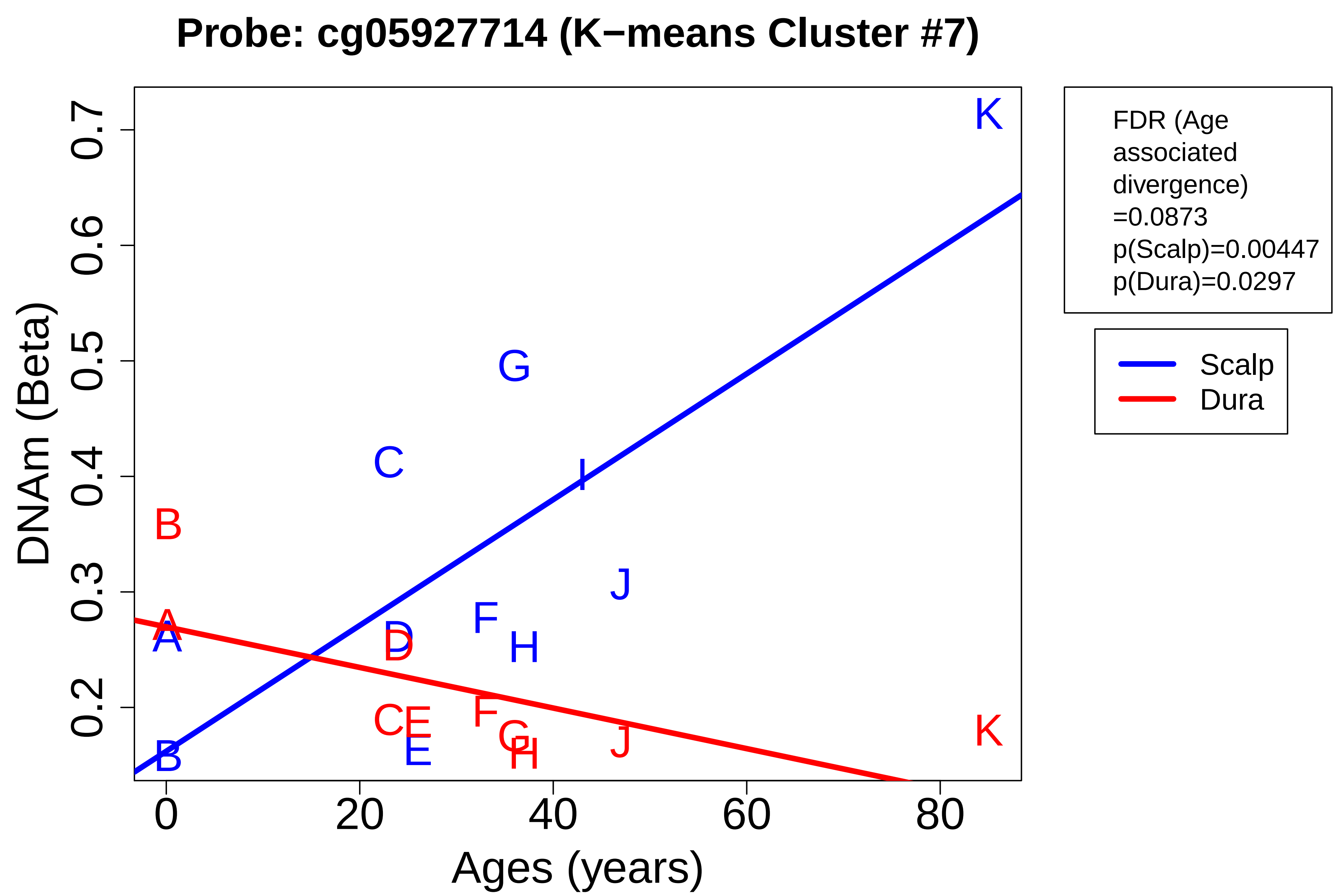

Probe: cg05927714 (K−means Cluster #7)
DNAm (Beta)
0.2
0.3
0.4
0.5
0.6
0.7
0
20
40
60
80
Ages (years)
FDR (Age associated divergence) =0.0873
p(Scalp)=0.00447
p(Dura)=0.0297
Scalp
Dura
A
B
C
D
E
F
G
H
I
J
K

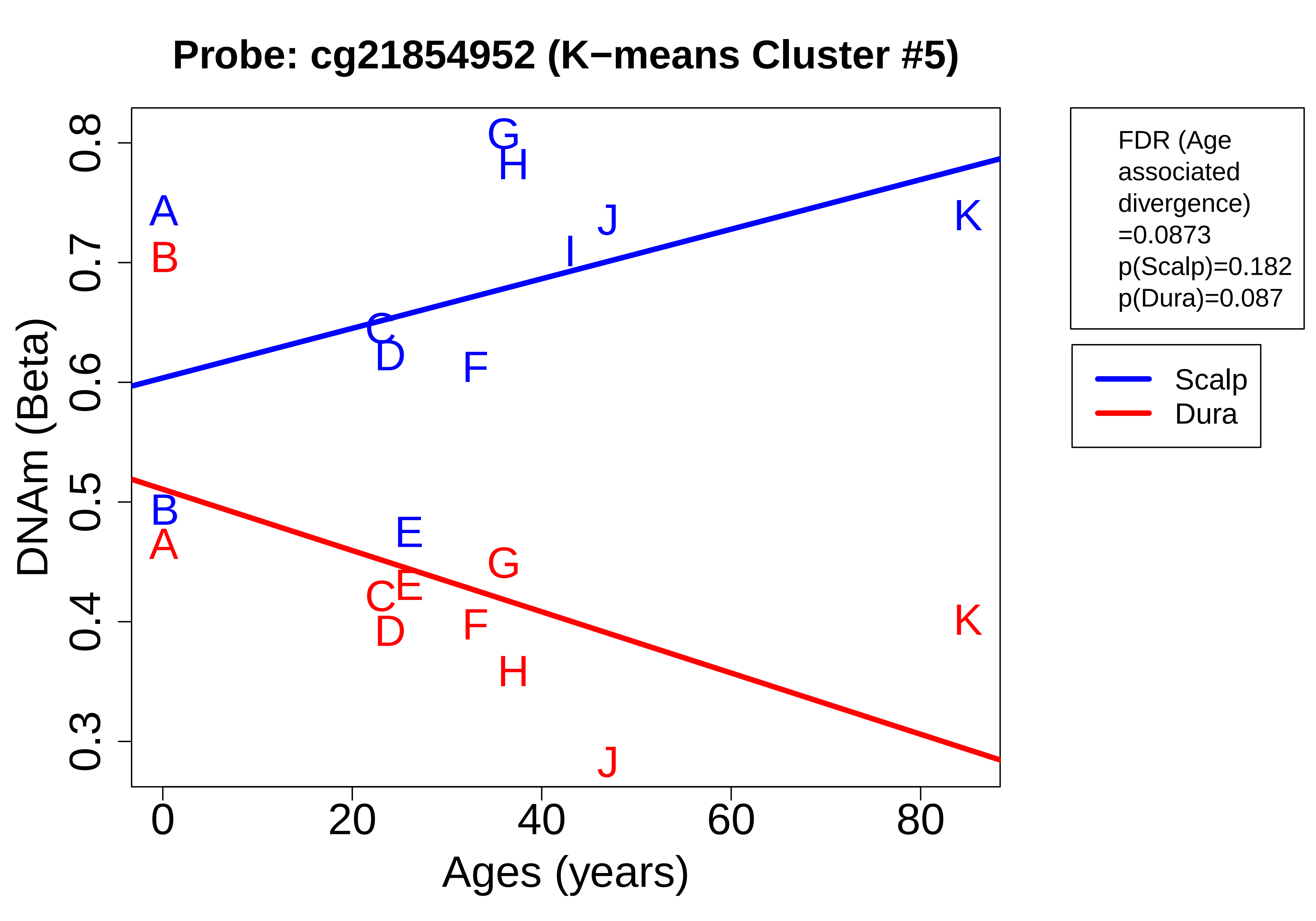

Probe: cg21854952 (K−means Cluster #5)
DNAm (Beta)
0.3
0.4
0.5
0.6
0.7
0.8
Ages (years)
0
20
40
60
80
FDR (Age associated divergence) =0.0873
p(Scalp)=0.182
p(Dura)=0.087
Scalp
Dura
A
B
C
D
E
F
G
H
I
J
K

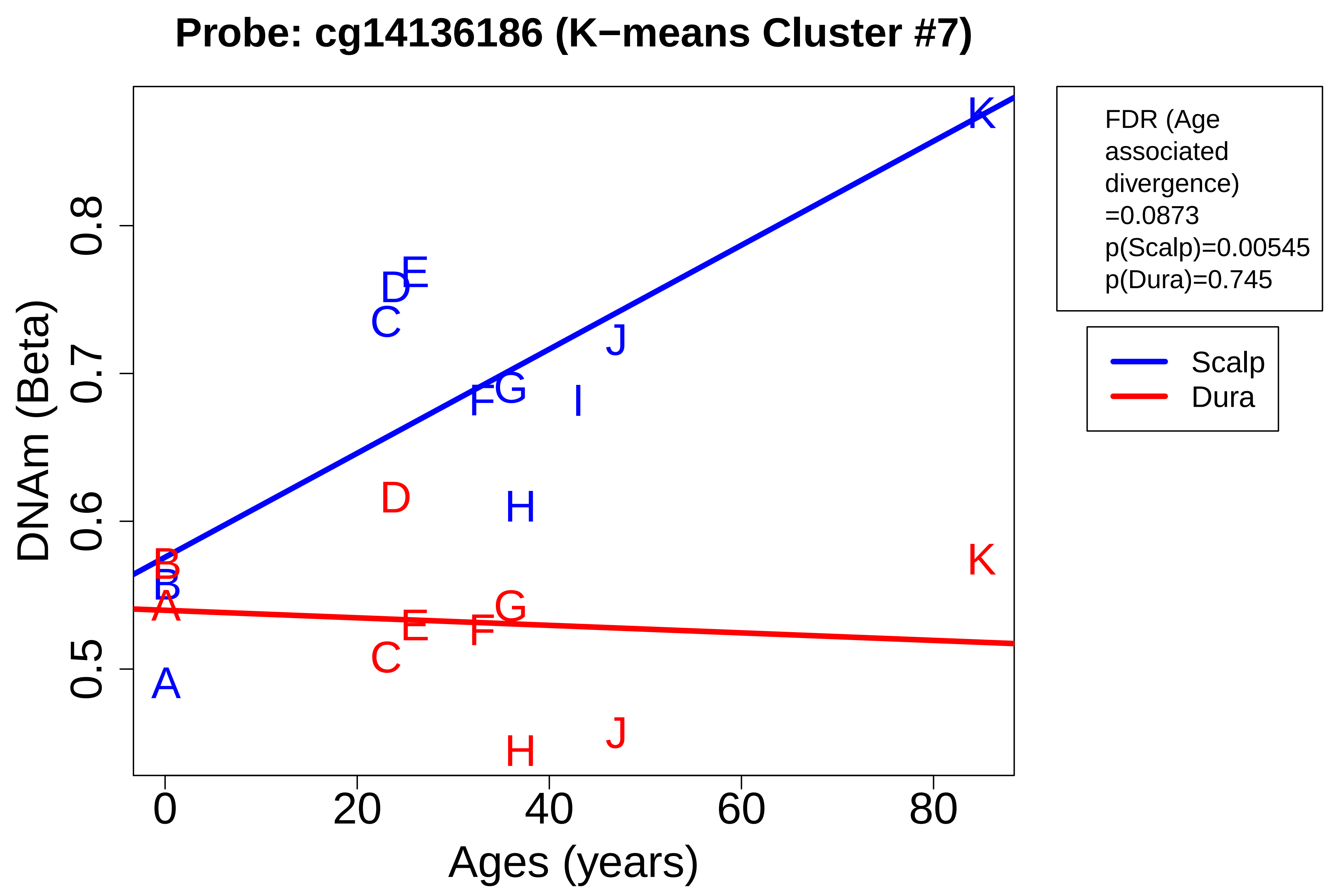
Probe: cg14136186 (K−means Cluster #7)
FDR (Age associated divergence) =0.0873
p(Scalp)=0.00545
p(Dura)=0.745
Scalp
Dura
DNAm (Beta)
0.5
0.6
0.7
0.8
0
20
40
60
80
Ages (years)
A
B
C
D
E
F
G
H
I
J
K

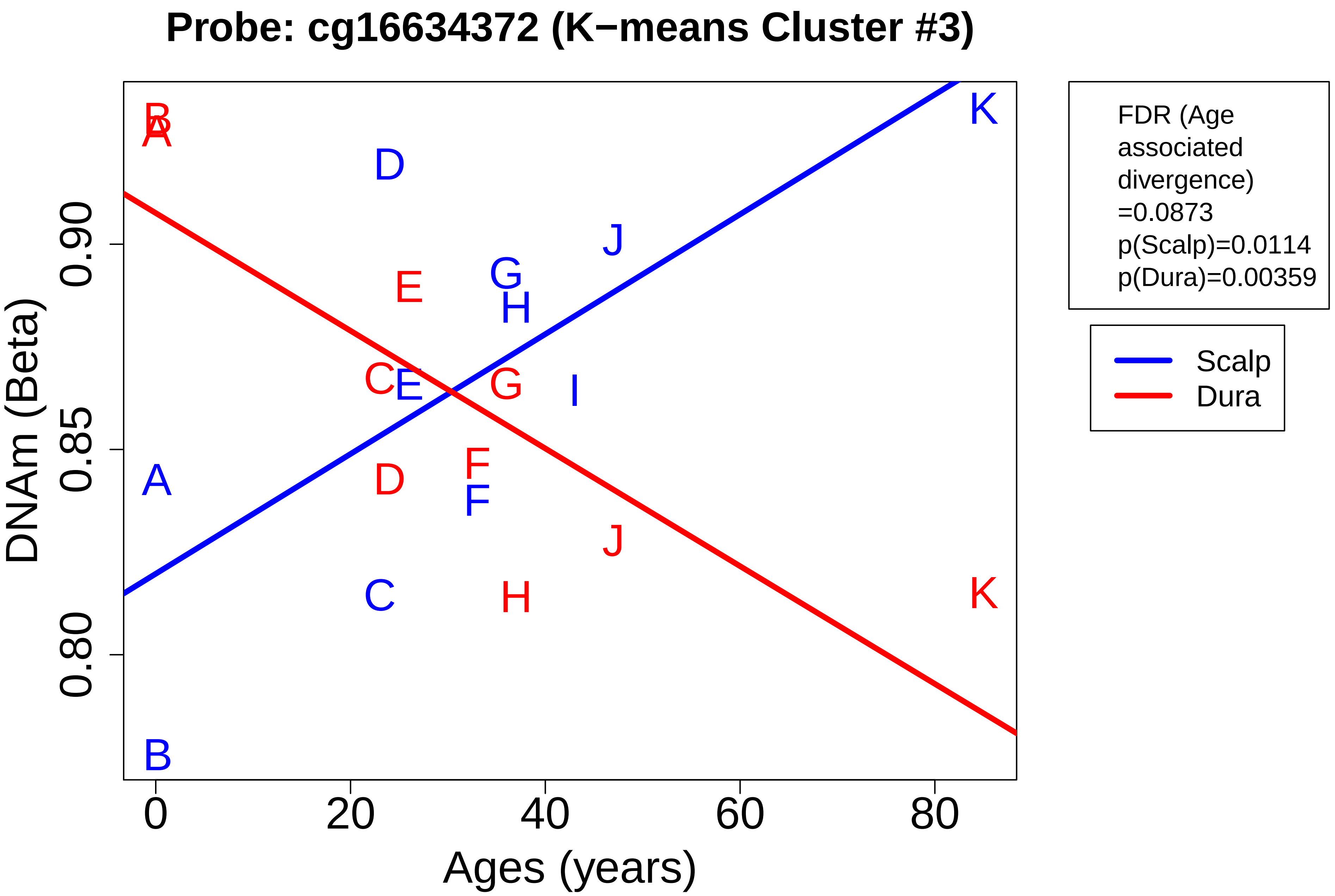

Probe: cg16634372 (K−means Cluster #3)
DNAm (Beta)
0.80
0.85
0.90
Ages (years)
0
20
40
60
80
FDR (Age associated divergence) =0.0873
p(Scalp)=0.0114
p(Dura)=0.00359
Scalp
Dura

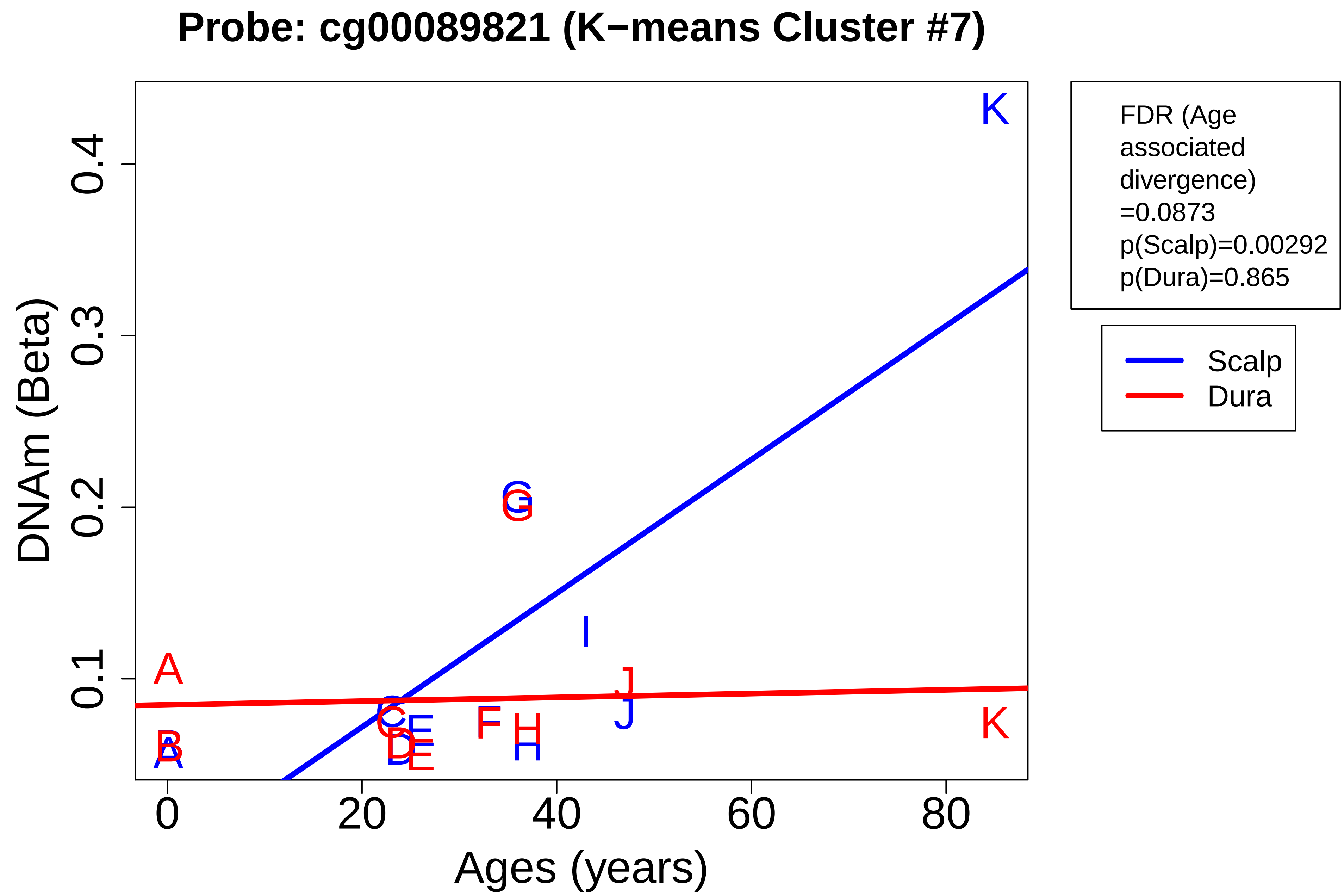

Probe: cg00089821 (K−means Cluster #7)
FDR (Age associated divergence) =0.0873
p(Scalp)=0.00292
p(Dura)=0.865
Scalp
Dura
DNAm (Beta)
0.1
0.2
0.3
0.4
0
20
40
60
80
Ages (years)
A
B
C
D
E
F
G
H
I
J
K

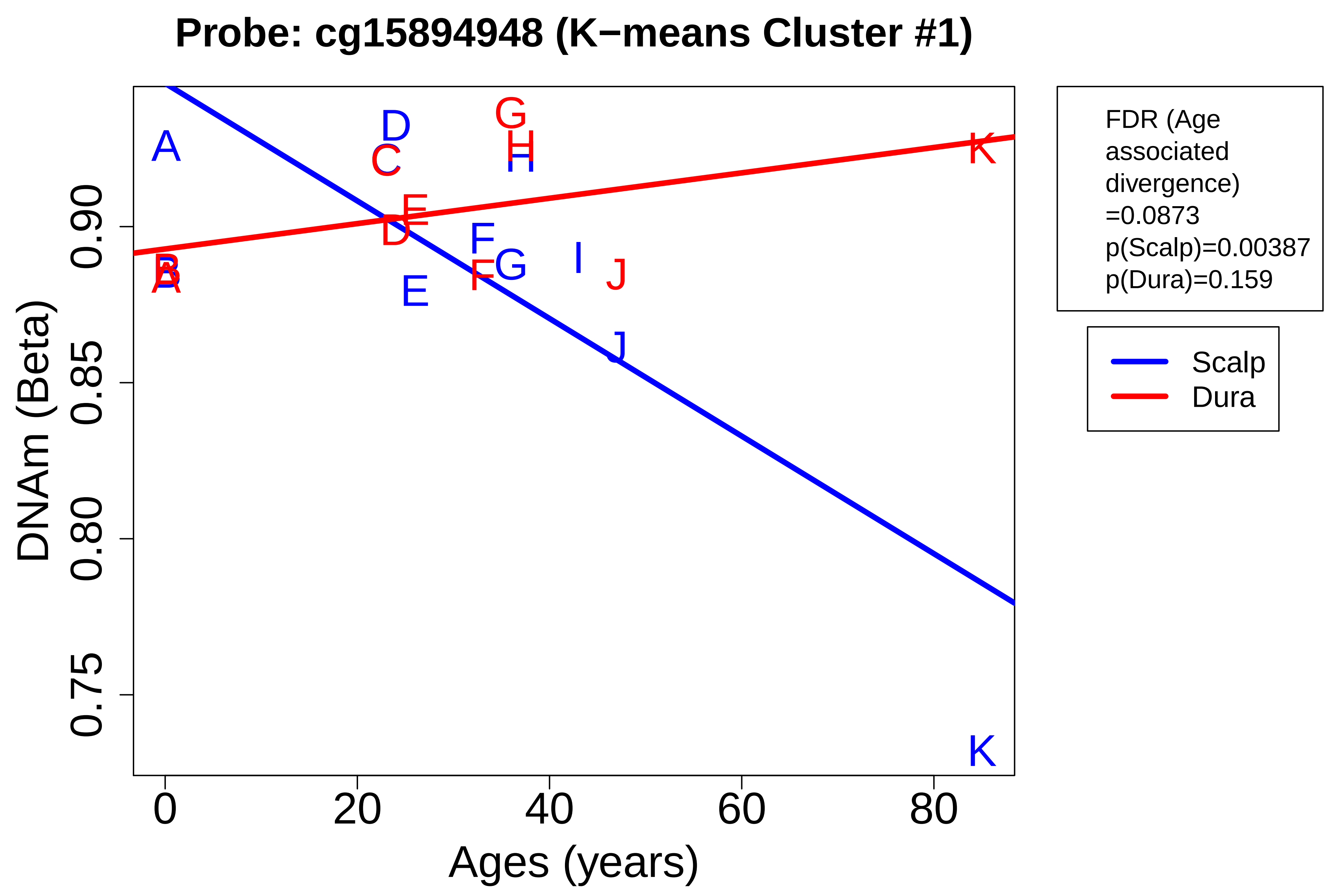

Probe: cg15894948 (K−means Cluster #1)
DNAm (Beta)
0.75
0.80
0.85
0.90
Ages (years)
0
20
40
60
80
FDR (Age associated divergence) =0.0873
p(Scalp)=0.00387
p(Dura)=0.159
Scalp
Dura

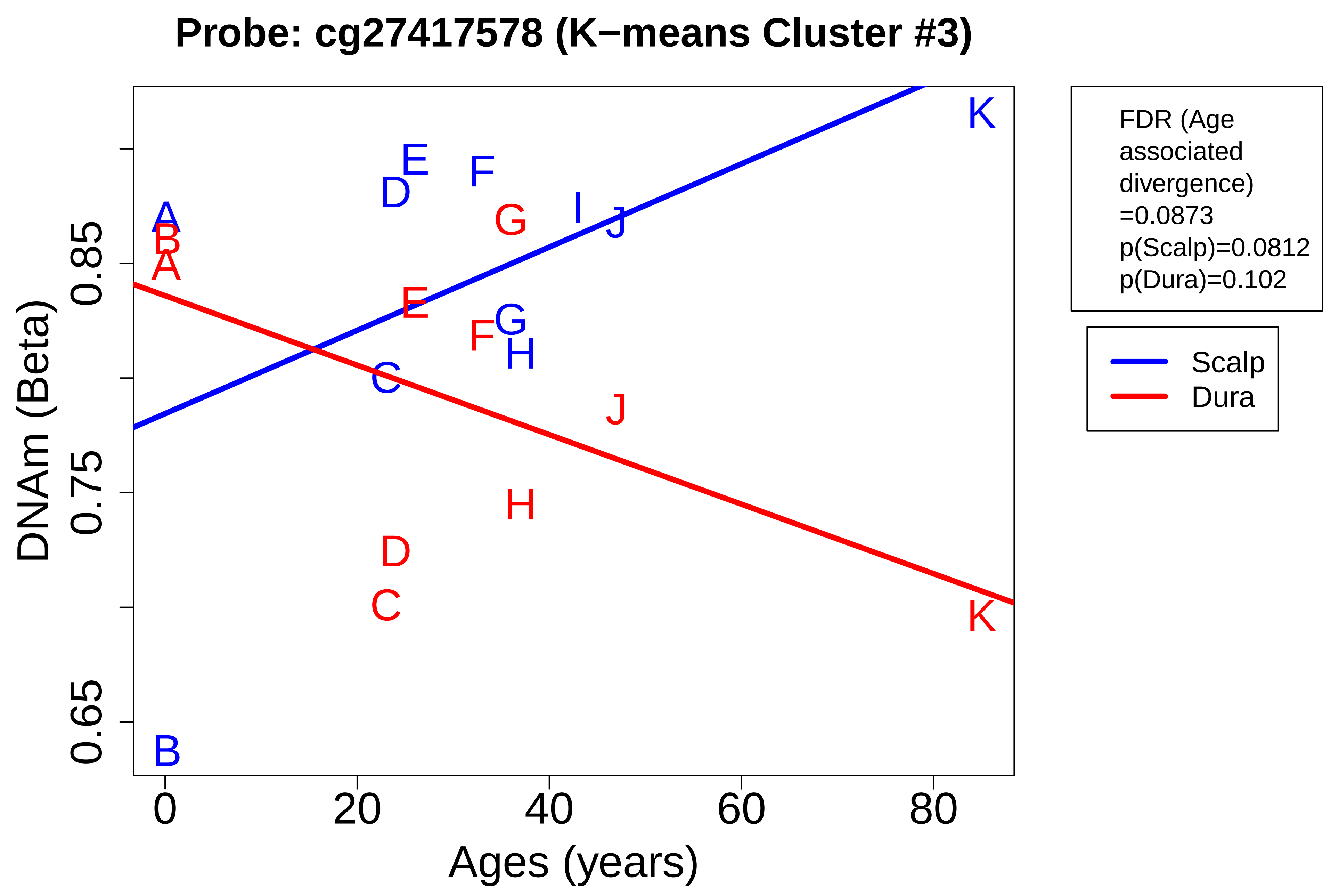

Probe: cg27417578 (K−means Cluster #3)
FDR (Age associated divergence) =0.0873
p(Scalp)=0.0812
p(Dura)=0.102
Scalp
Dura
DNAm (Beta)
0.65
0.75
0.85
0
20
40
60
80
Ages (years)
A
B
C
D
E
F
G
H
I
J
K

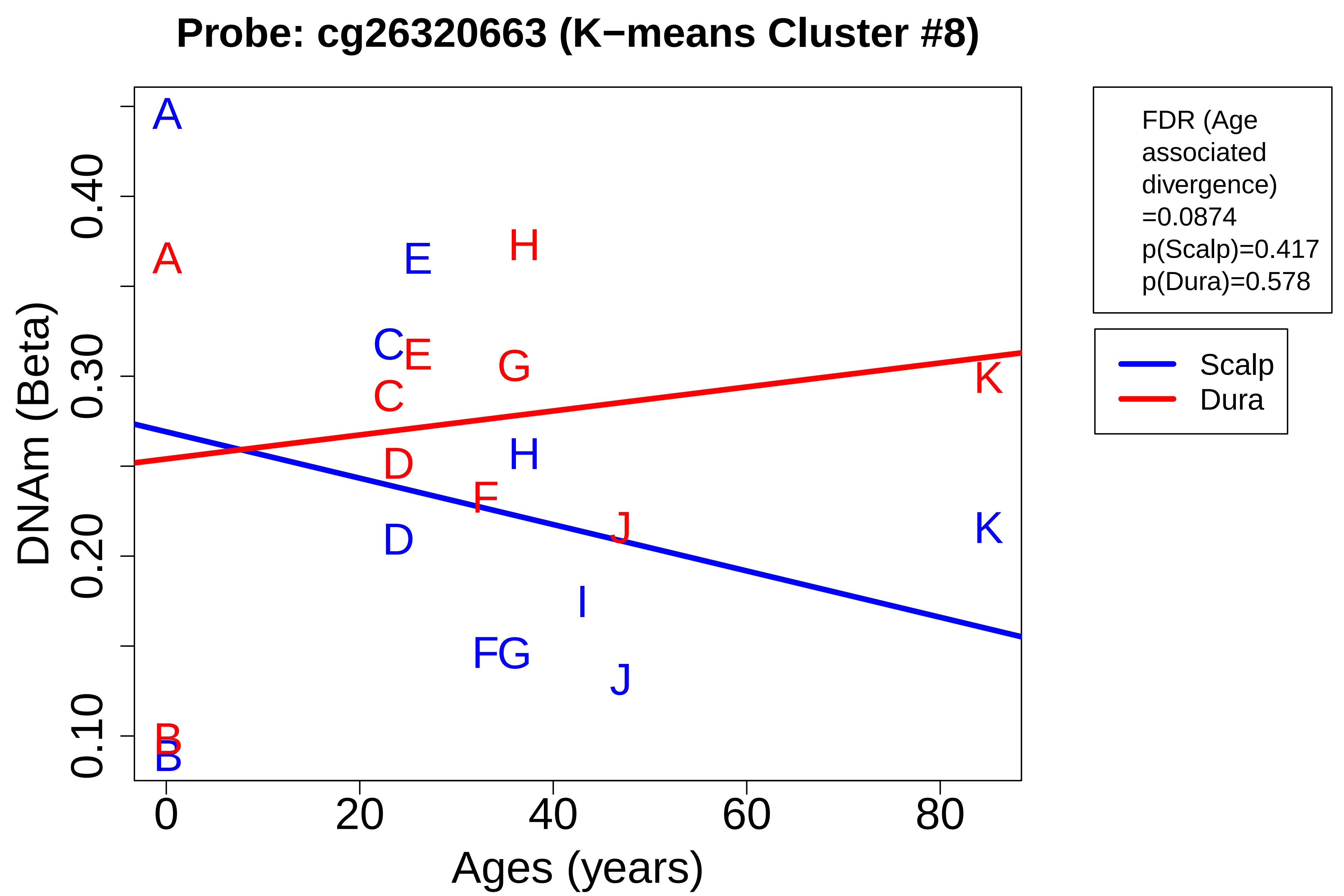
Probe: cg26320663 (K−means Cluster #8)
DNAm (Beta)
0.10
0.20
0.30
0.40
0
20
40
60
80
Ages (years)
FDR (Age associated divergence) =0.0874
p(Scalp)=0.417
p(Dura)=0.578
Scalp
Dura
A
B
C
D
E
F
G
H
I
J
K

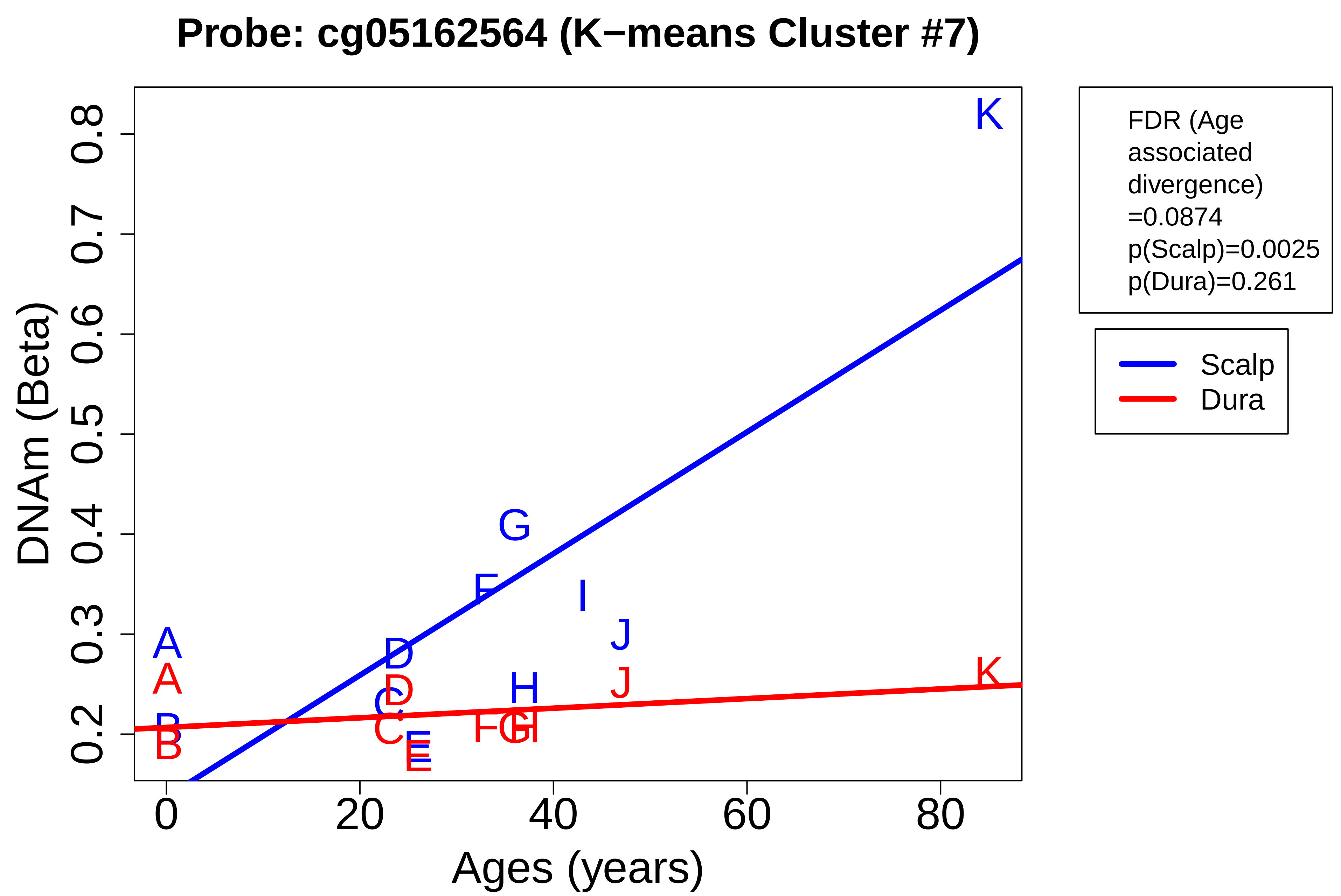

Probe: cg05162564 (K−means Cluster #7)
DNAm (Beta)
0.2
0.3
0.4
0.5
0.6
0.7
0.8
0
20
40
60
80
Ages (years)
A B C D E F G H I J K
A B C D E F G H J K
FDR (Age associated divergence) =0.0874
p(Scalp)=0.0025
p(Dura)=0.261
Scalp
Dura

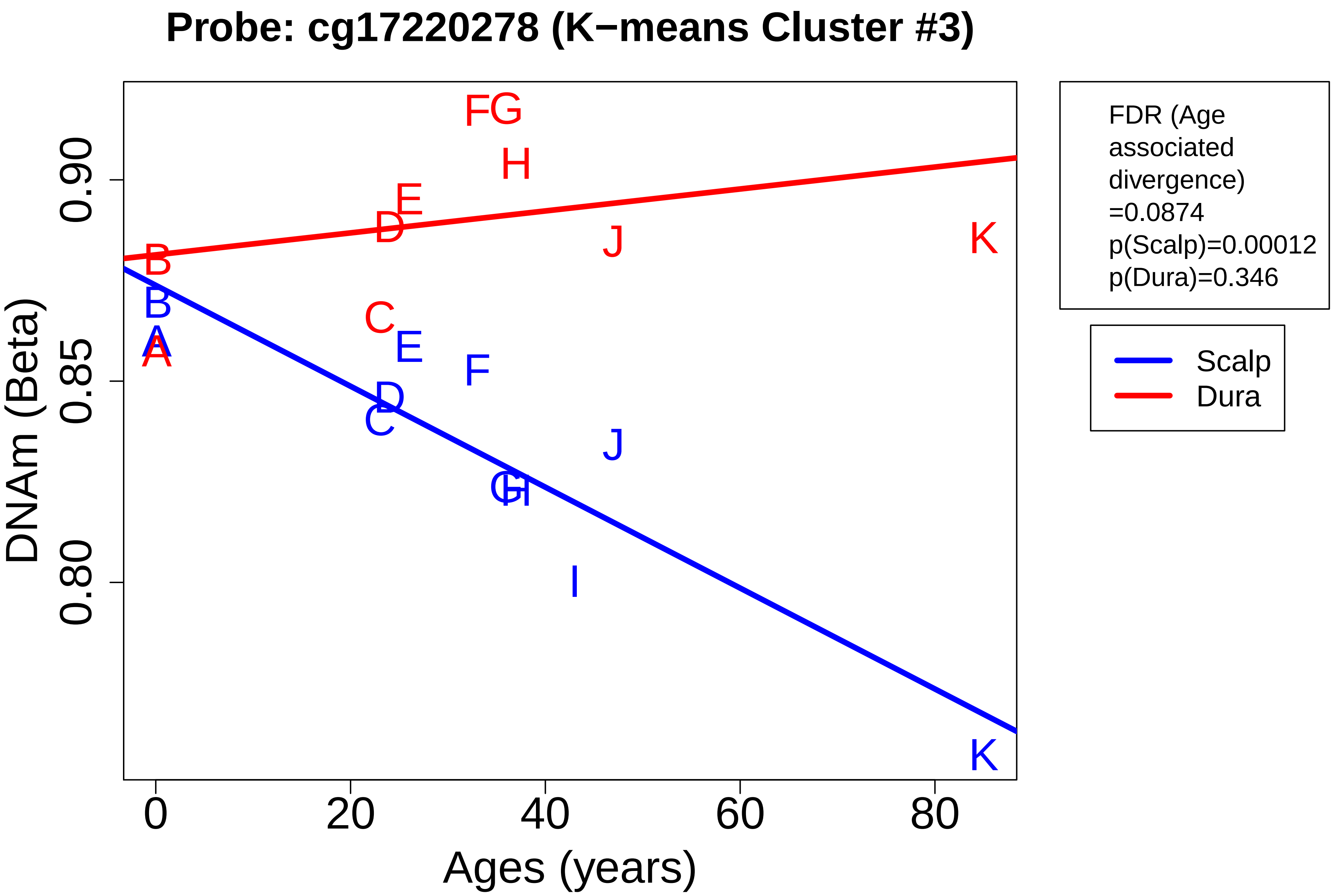
Probe: cg17220278 (K−means Cluster #3)
DNAm (Beta)
0.80
0.85
0.90
Ages (years)
0
20
40
60
80
FG
H
E
D
J
K
B
B
A
A
C
E
F
D
C
J
GH
I
K
FDR (Age associated divergence) =0.0874
p(Scalp)=0.00012
p(Dura)=0.346
Scalp
Dura

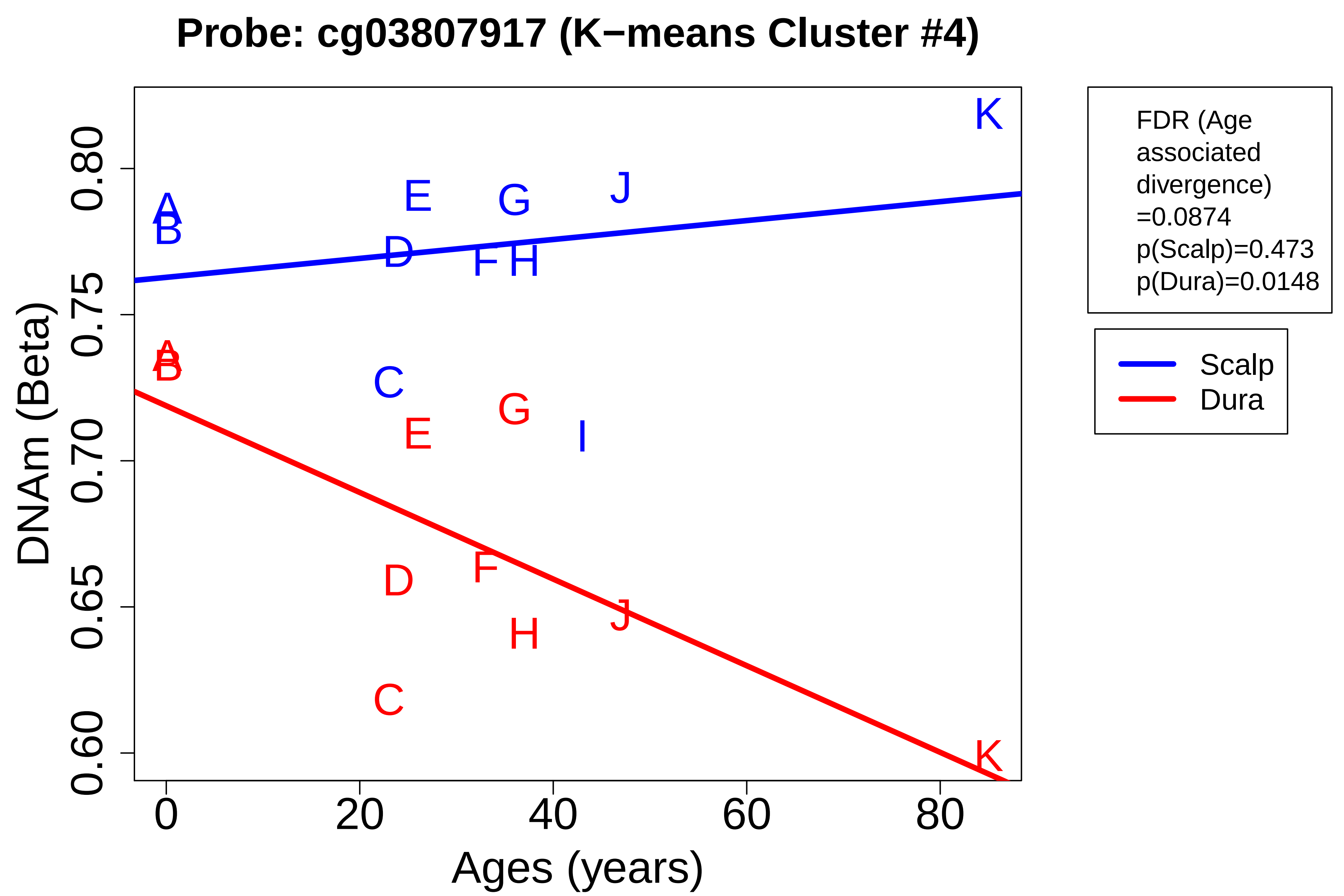

Probe: cg03807917 (K−means Cluster #4)
DNAm (Beta)
0.60
0.65
0.70
0.75
0.80
0
20
40
60
80
Ages (years)
FDR (Age associated divergence) =0.0874
p(Scalp)=0.473
p(Dura)=0.0148
Scalp
Dura
A
B
C
D
E
F
G
H
I
J
K

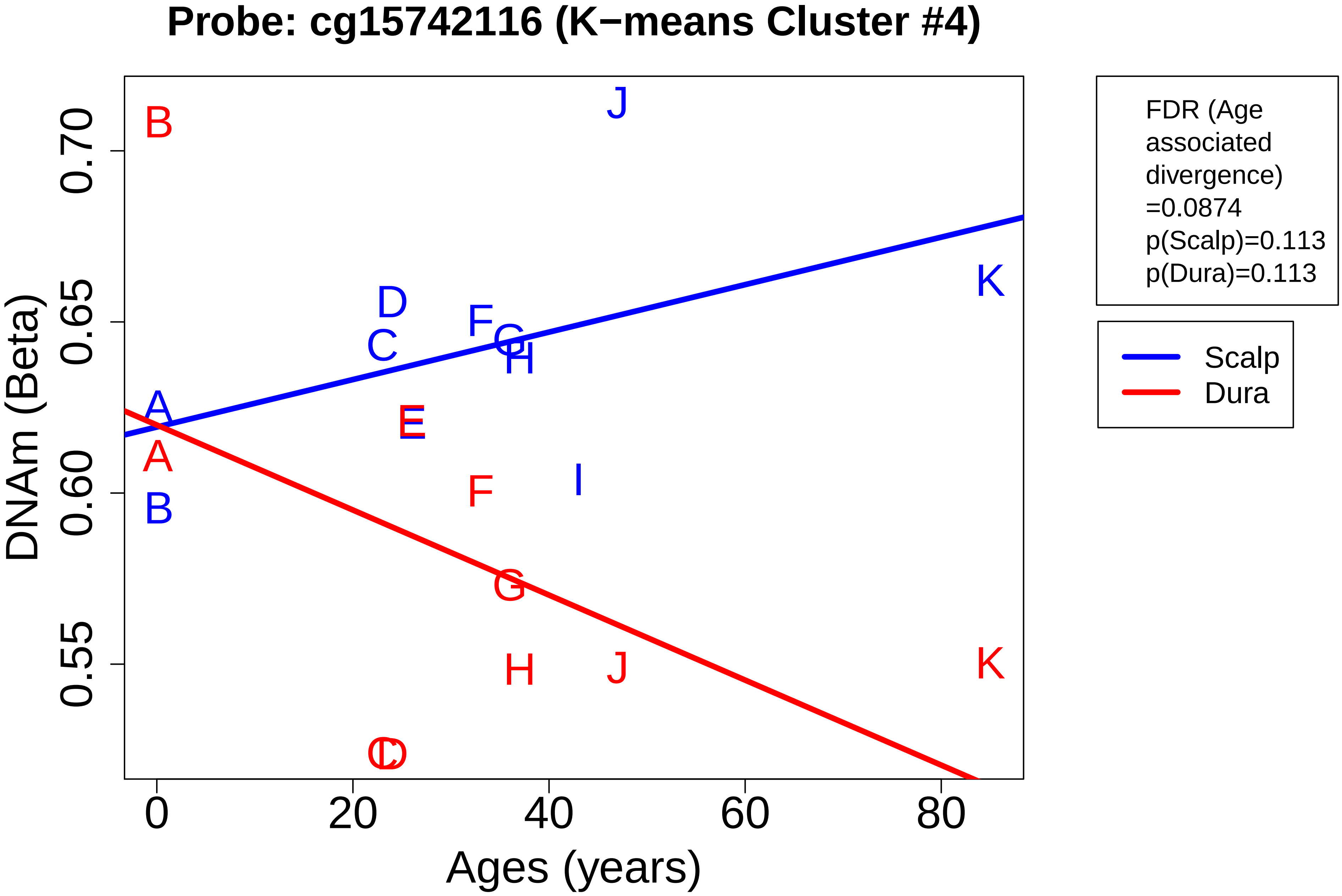

Probe: cg15742116 (K−means Cluster #4)
DNAm (Beta)
0.55
0.60
0.65
0.70
0
20
40
60
80
Ages (years)
FDR (Age associated divergence) =0.0874
p(Scalp)=0.113
p(Dura)=0.113
Scalp
Dura

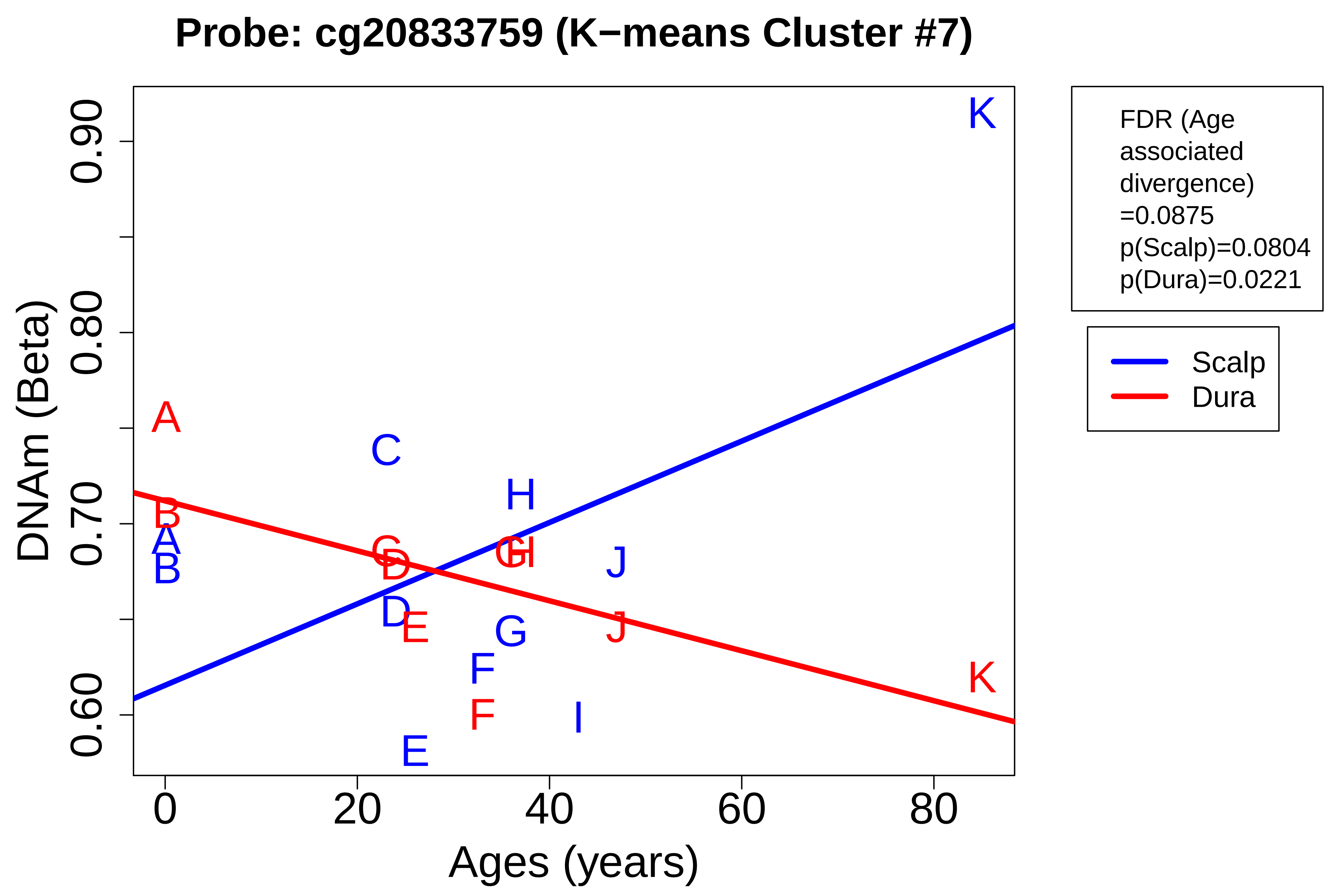

Probe: cg20833759 (K−means Cluster #7)
DNAm (Beta)
0.60
0.70
0.80
0.90
0
20
40
60
80
Ages (years)
A
B
C
D
E
F
G
H
I
J
K
FDR (Age associated divergence) =0.0875
p(Scalp)=0.0804
p(Dura)=0.0221
Scalp
Dura

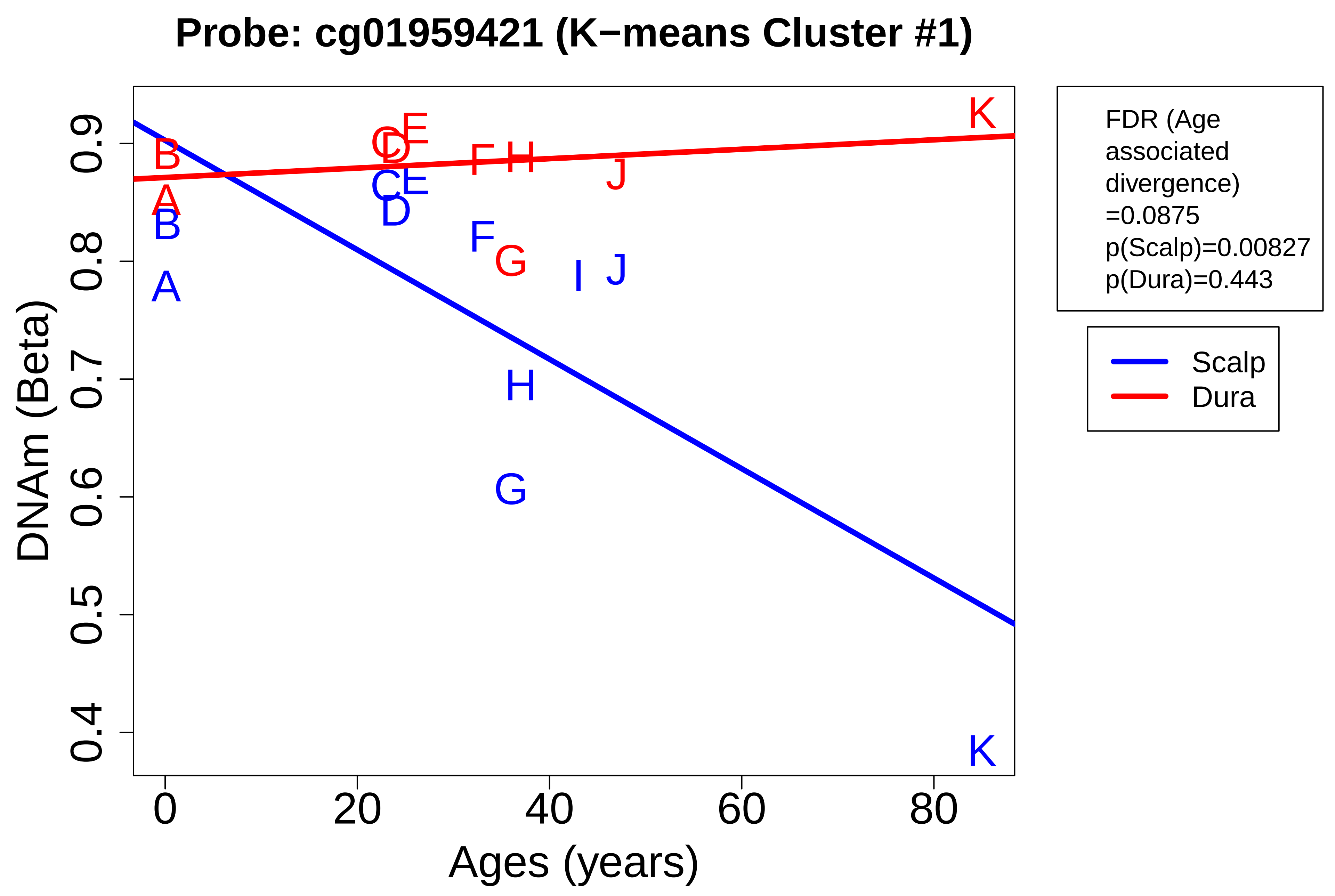

Probe: cg01959421 (K−means Cluster #1)
FDR (Age associated divergence) =0.0875
p(Scalp)=0.00827
p(Dura)=0.443
Scalp
Dura
DNAm (Beta)
Ages (years)
0.4
0.5
0.6
0.7
0.8
0.9
0
20
40
60
80

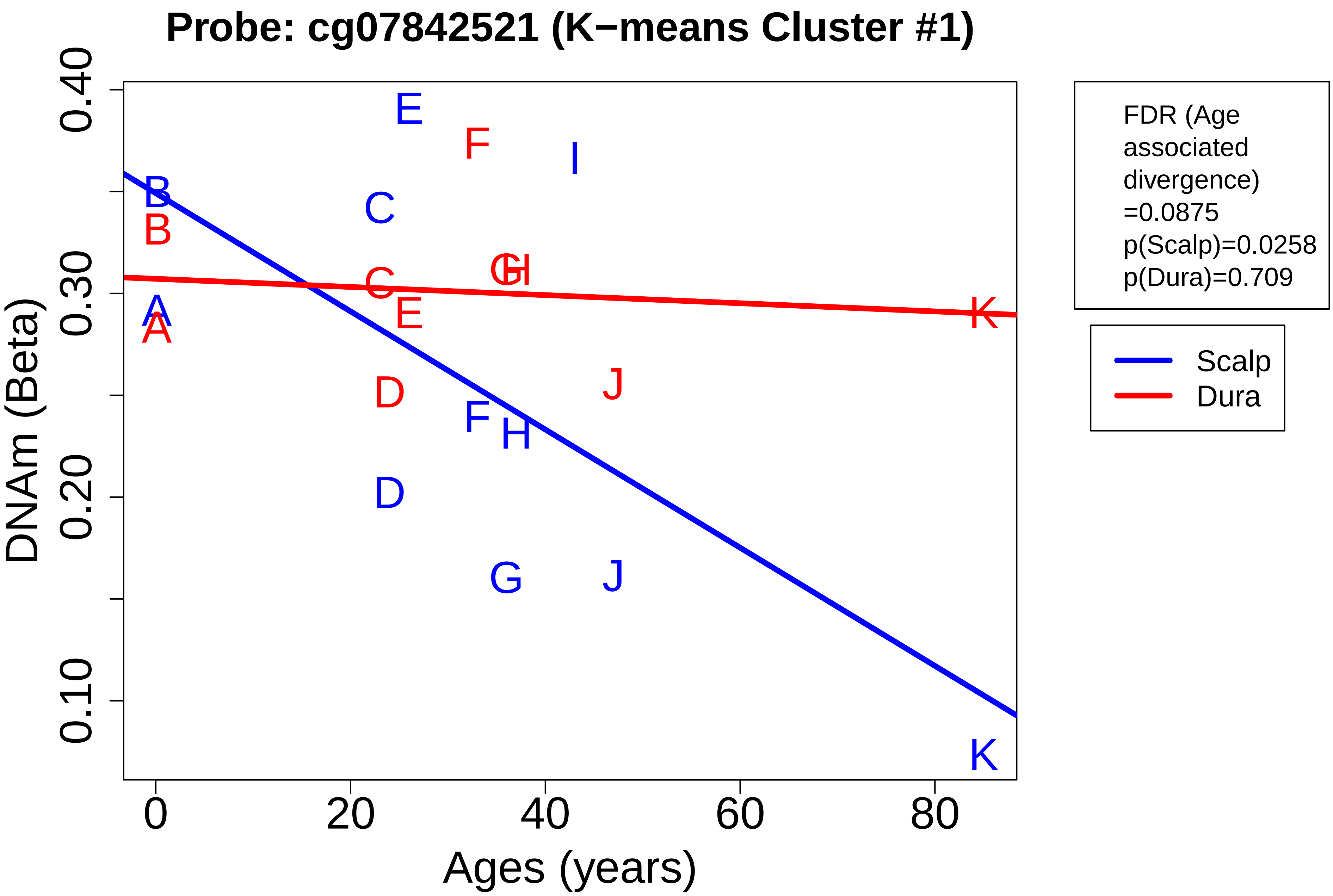
Probe: cg07842521 (K−means Cluster #1)
DNAm (Beta)
0.10
0.20
0.30
0.40
Ages (years)
0
20
40
60
80
A
B
C
D
E
F
G
H
I
J
K
FDR (Age associated divergence) =0.0875
p(Scalp)=0.0258
p(Dura)=0.709
Scalp
Dura

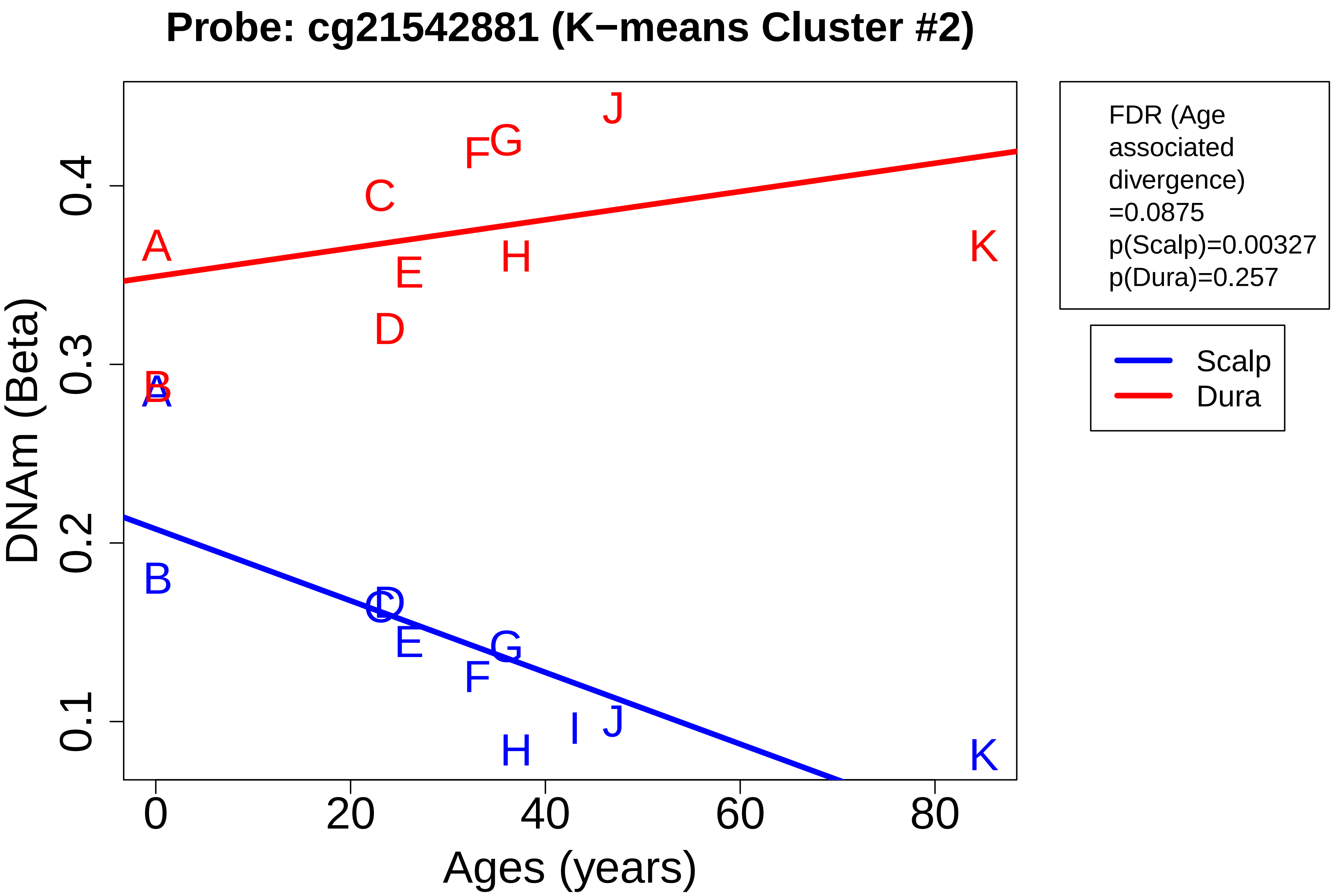

Probe: cg21542881 (K−means Cluster #2)
DNAm (Beta)
0.1
0.2
0.3
0.4
Ages (years)
0
20
40
60
80
FDR (Age associated divergence) =0.0875
p(Scalp)=0.00327
p(Dura)=0.257
Scalp
Dura
A
B
C
D
E
F
G
H
I
J
K

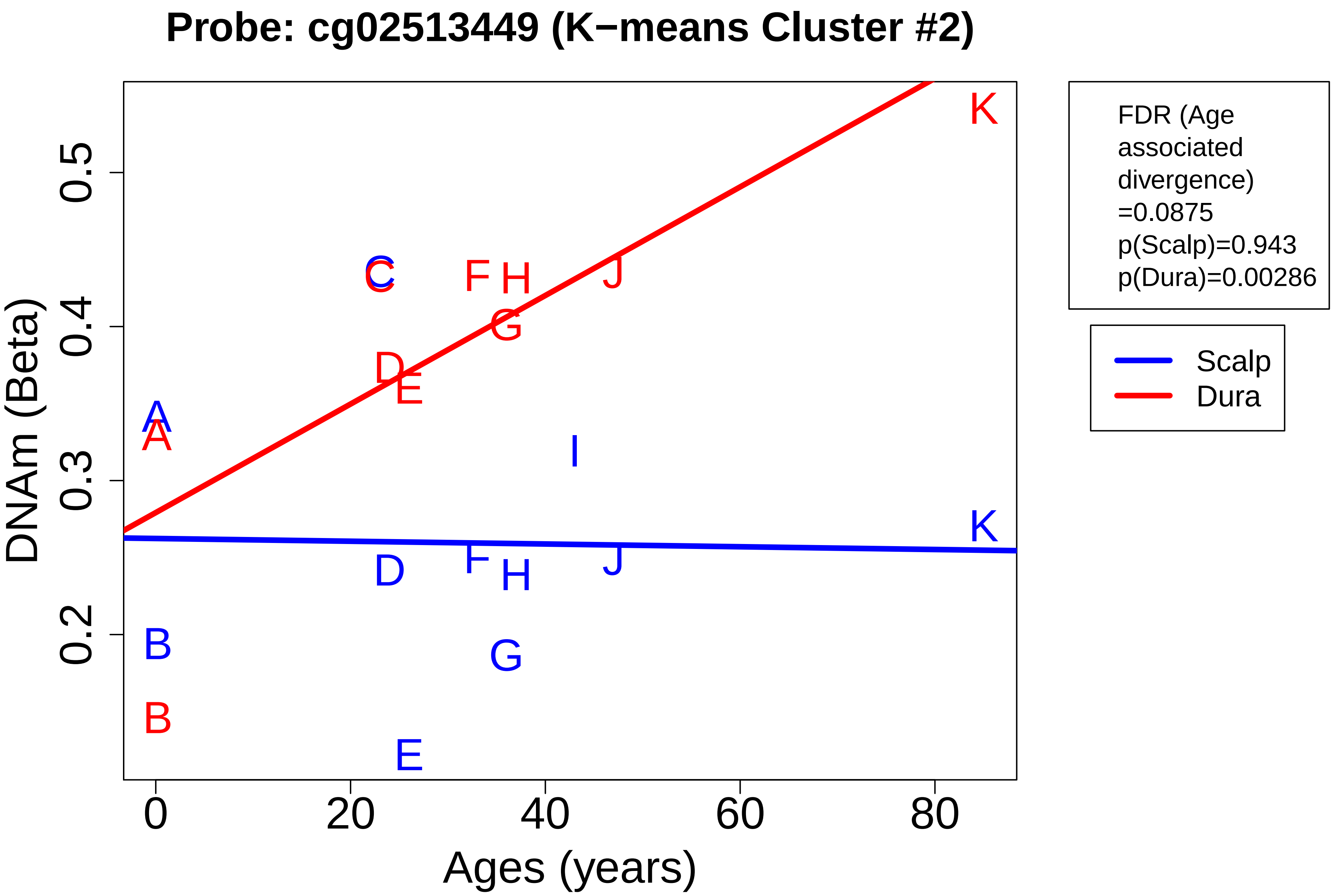
Probe: cg02513449 (K−means Cluster #2)
DNAm (Beta)
Ages (years)
0.2
0.3
0.4
0.5
0
20
40
60
80
FDR (Age associated divergence) =0.0875
p(Scalp)=0.943
p(Dura)=0.00286
Scalp
Dura

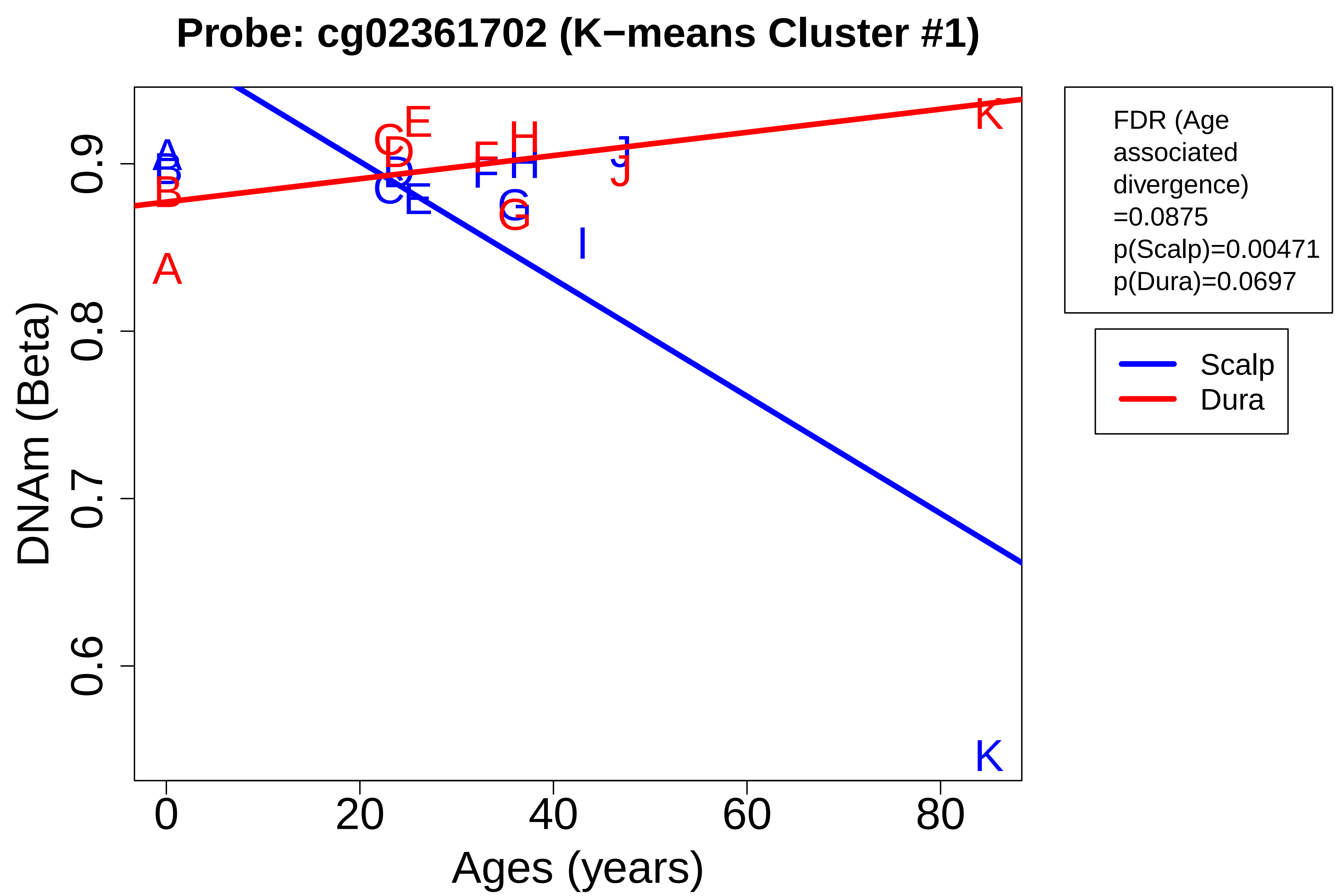

Probe: cg02361702 (K−means Cluster #1)
DNAm (Beta)
0.6
0.7
0.8
0.9
Ages (years)
0
20
40
60
80
A
B
C
D
E
F
G
H
I
J
K
FDR (Age associated divergence) =0.0875
p(Scalp)=0.00471
p(Dura)=0.0697
Scalp
Dura

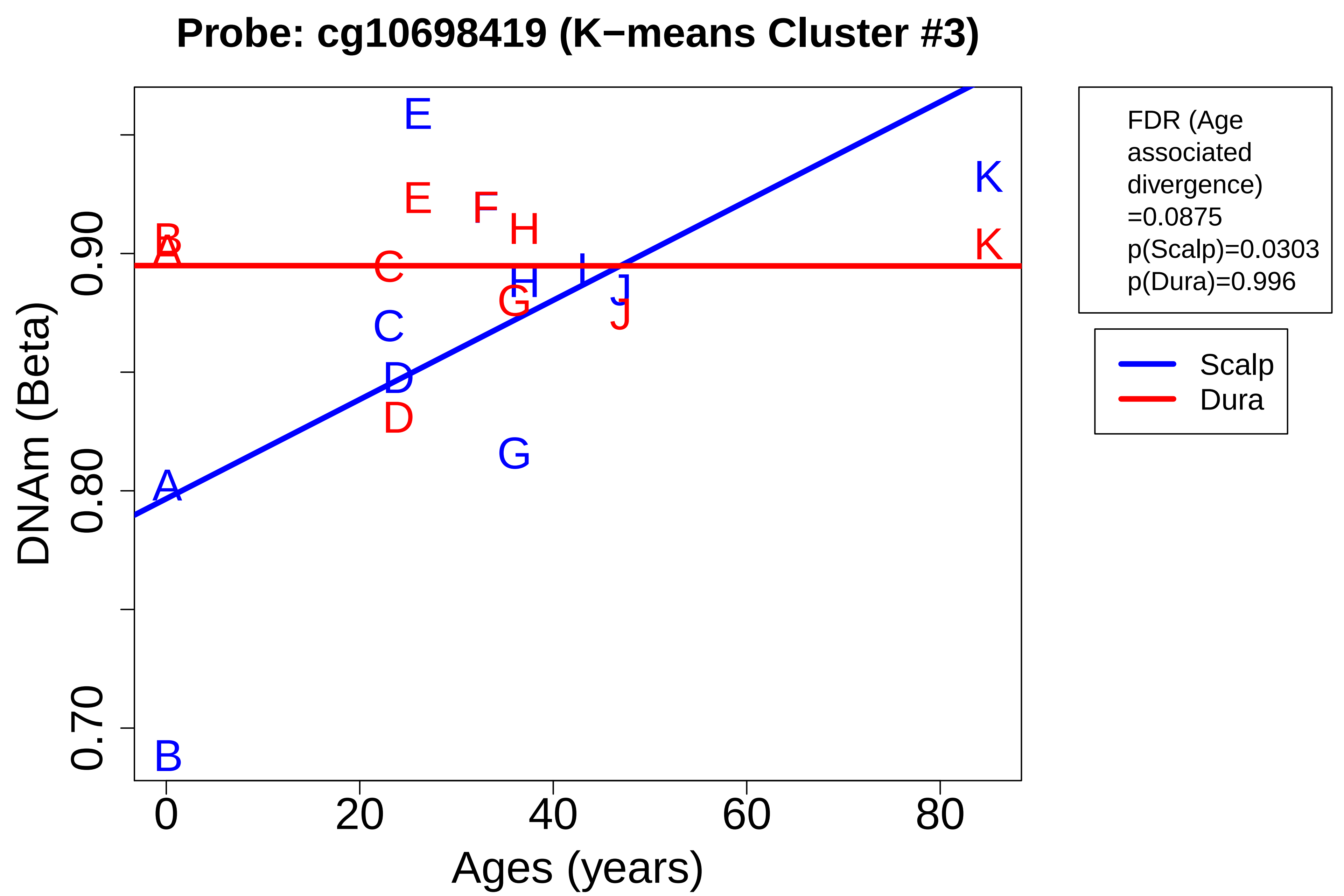
Probe: cg10698419 (K−means Cluster #3)
DNAm (Beta)
Ages (years)
0.70
0.80
0.90
0
20
40
60
80
FDR (Age associated divergence) =0.0875
p(Scalp)=0.0303
p(Dura)=0.996
Scalp
Dura

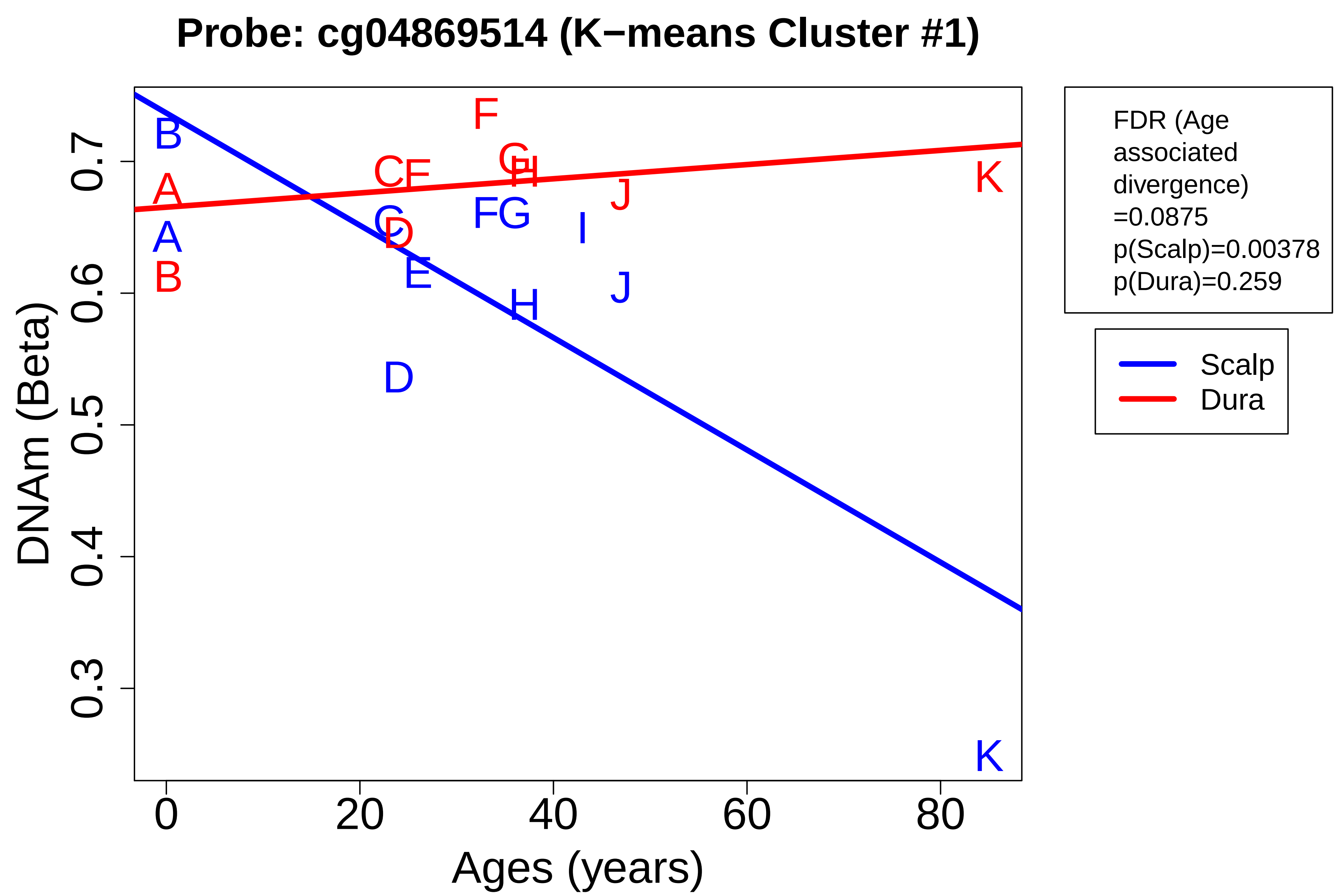
Probe: cg04869514 (K−means Cluster #1)
FDR (Age associated divergence) =0.0875
p(Scalp)=0.00378
p(Dura)=0.259
Scalp
Dura
DNAm (Beta)
0.3
0.4
0.5
0.6
0.7
0
20
40
60
80
Ages (years)

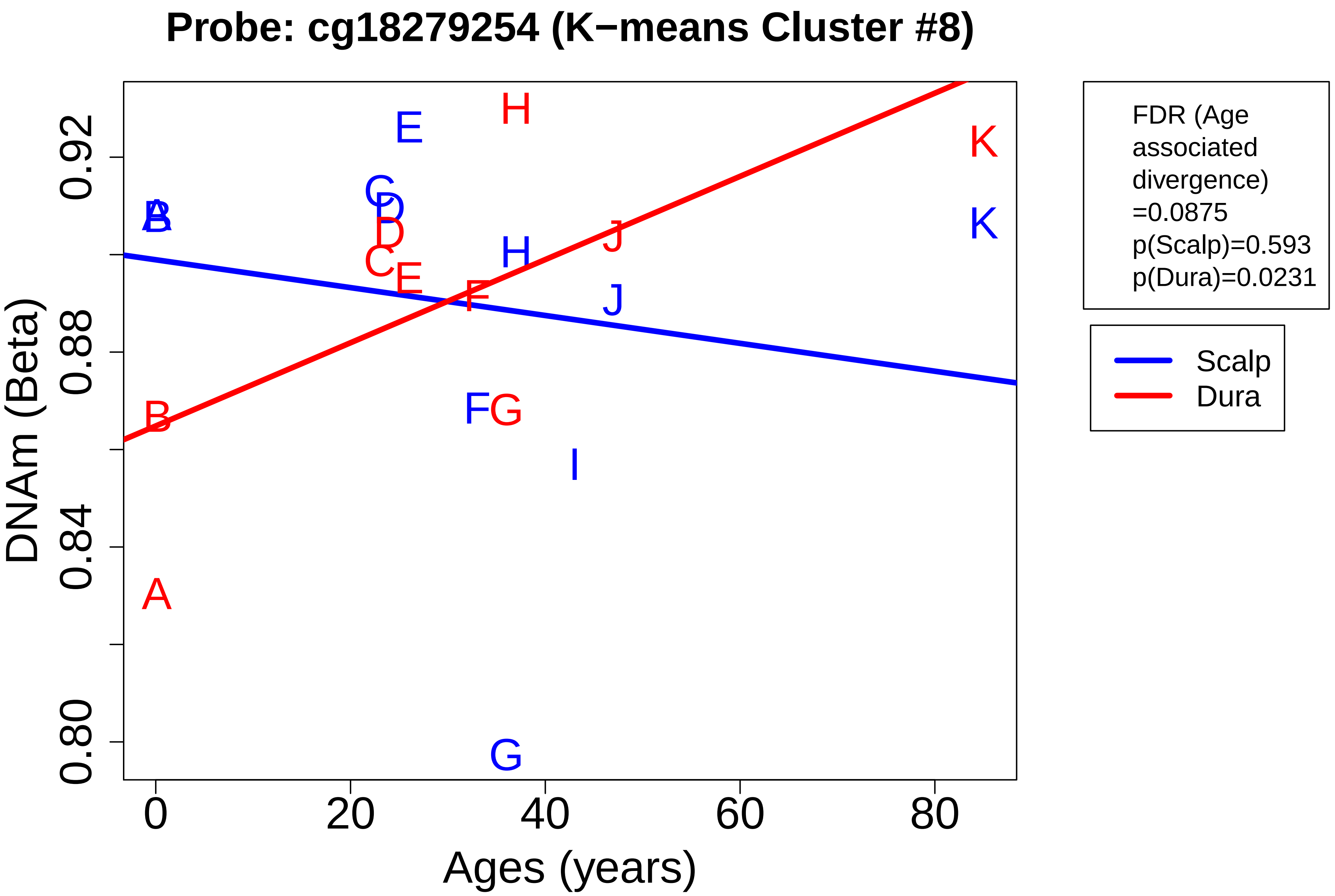

Probe: cg18279254 (K−means Cluster #8)
FDR (Age associated divergence) =0.0875
p(Scalp)=0.593
p(Dura)=0.0231
Scalp
Dura
DNAm (Beta)
0.80
0.84
0.88
0.92
0
20
40
60
80
Ages (years)

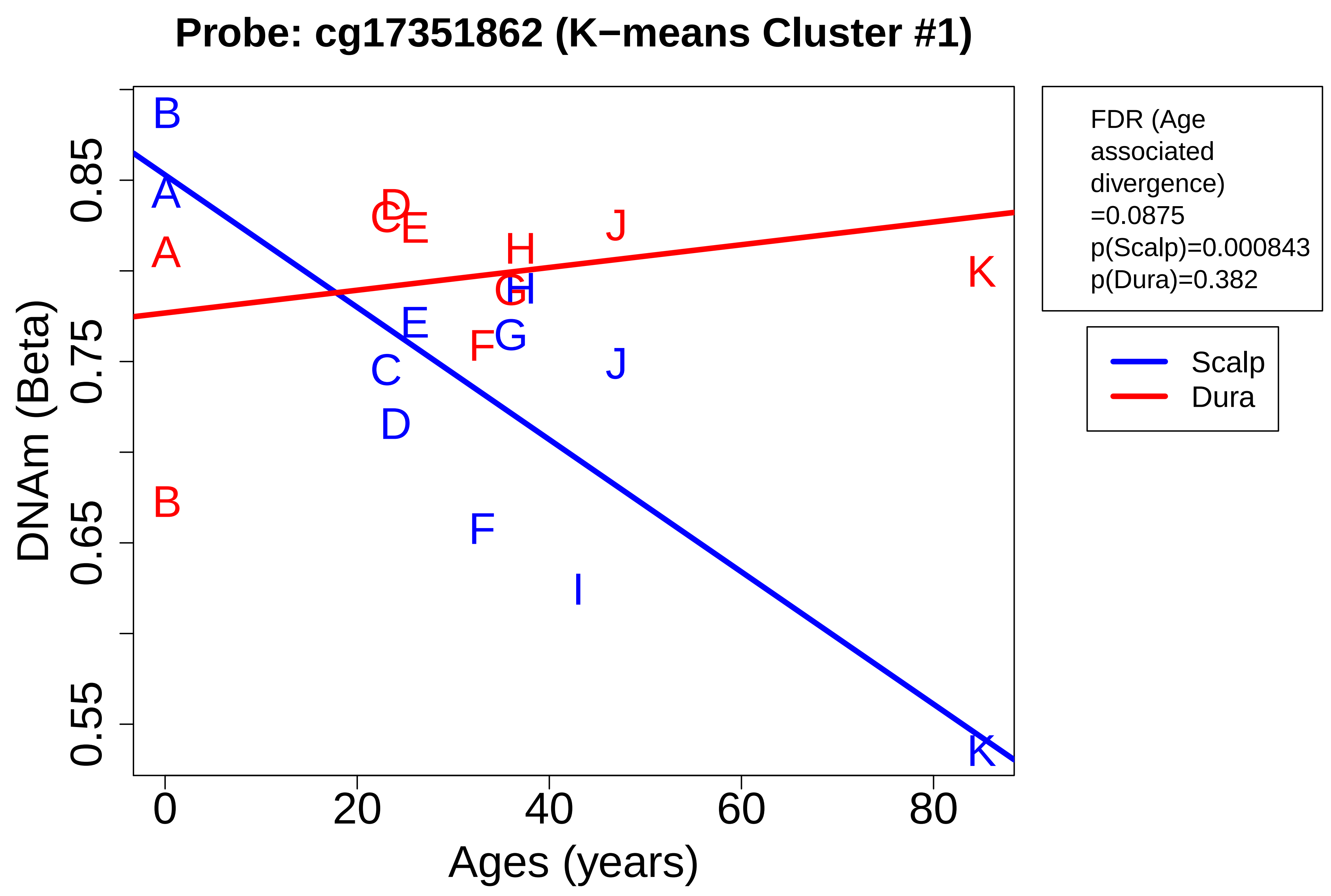

Probe: cg17351862 (K−means Cluster #1)
DNAm (Beta)
0.55
0.65
0.75
0.85
0
20
40
60
80
Ages (years)
B
A
A
D
C
E
J
H
K
G
H
E
F
G
C
J
D
B
F
I
K
FDR (Age associated divergence) =0.0875
p(Scalp)=0.000843
p(Dura)=0.382
Scalp
Dura

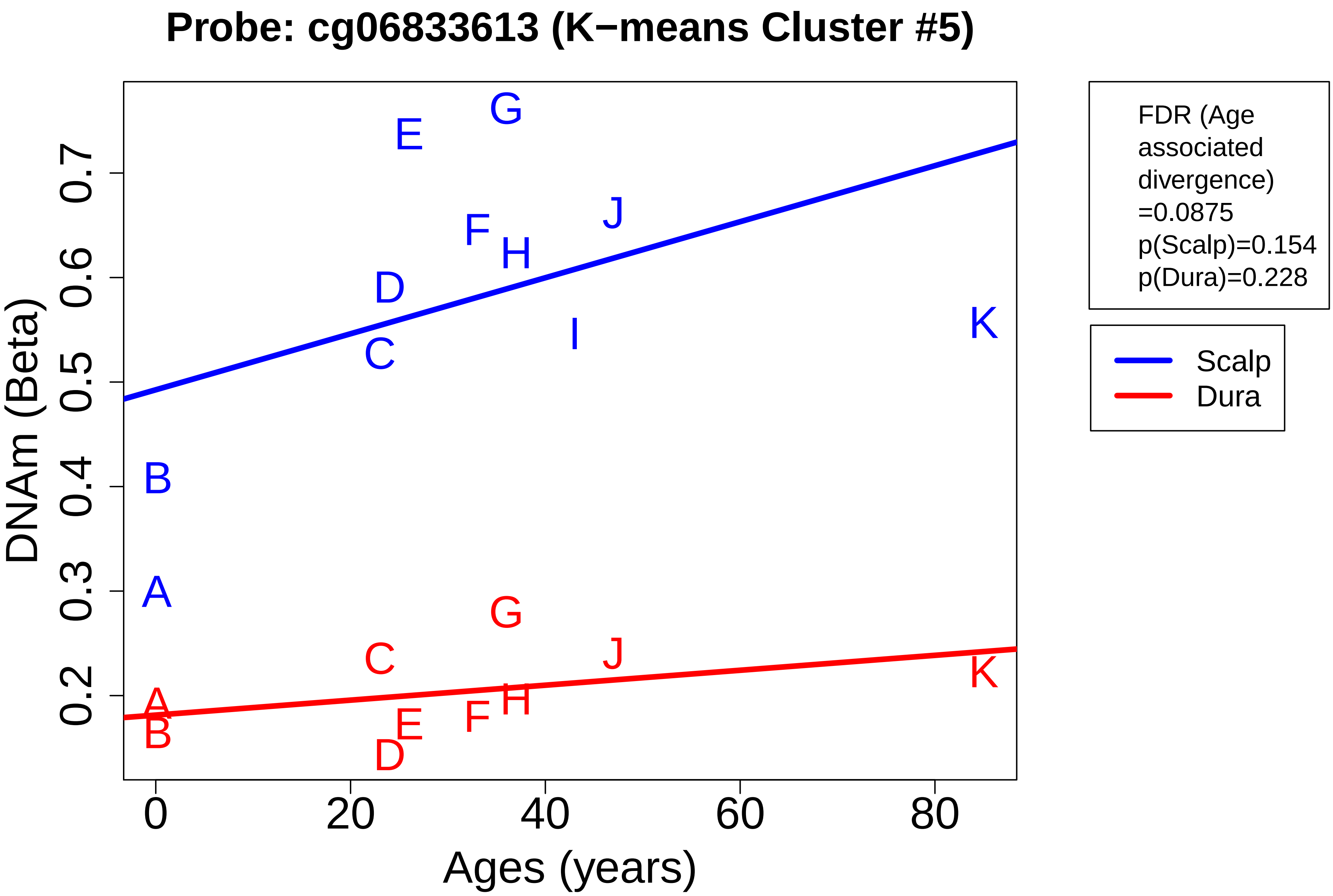

Probe: cg06833613 (K−means Cluster #5)
DNAm (Beta)
0.2
0.3
0.4
0.5
0.6
0.7
0
20
40
60
80
Ages (years)
FDR (Age associated divergence) =0.0875
p(Scalp)=0.154
p(Dura)=0.228
Scalp
Dura

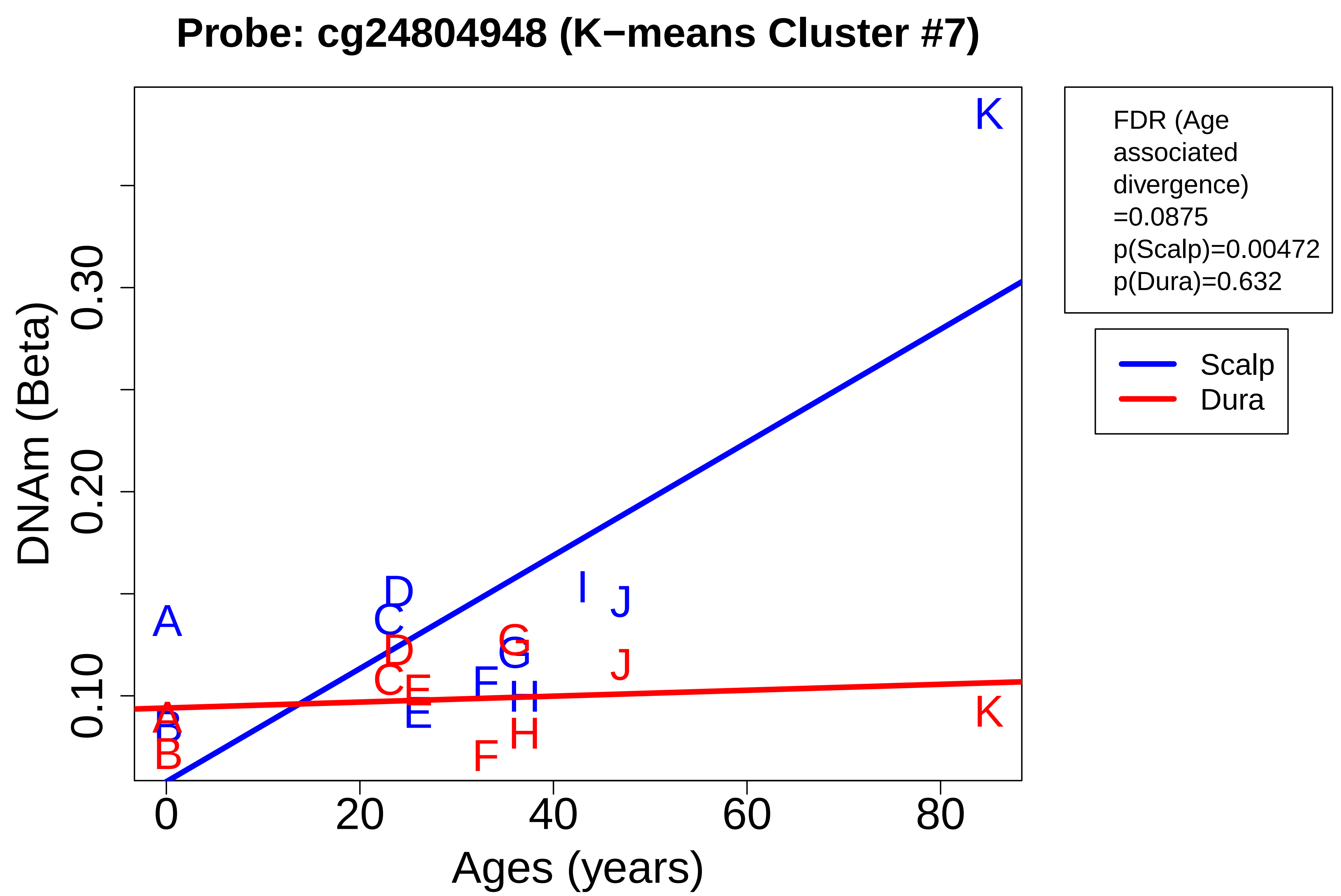
Probe: cg24804948 (K−means Cluster #7)
DNAm (Beta)
0.10
0.20
0.30
0
20
40
60
80
Ages (years)
FDR (Age associated divergence) =0.0875
p(Scalp)=0.00472
p(Dura)=0.632
Scalp
Dura
A
B
C
D
E
F
G
H
I
J
K

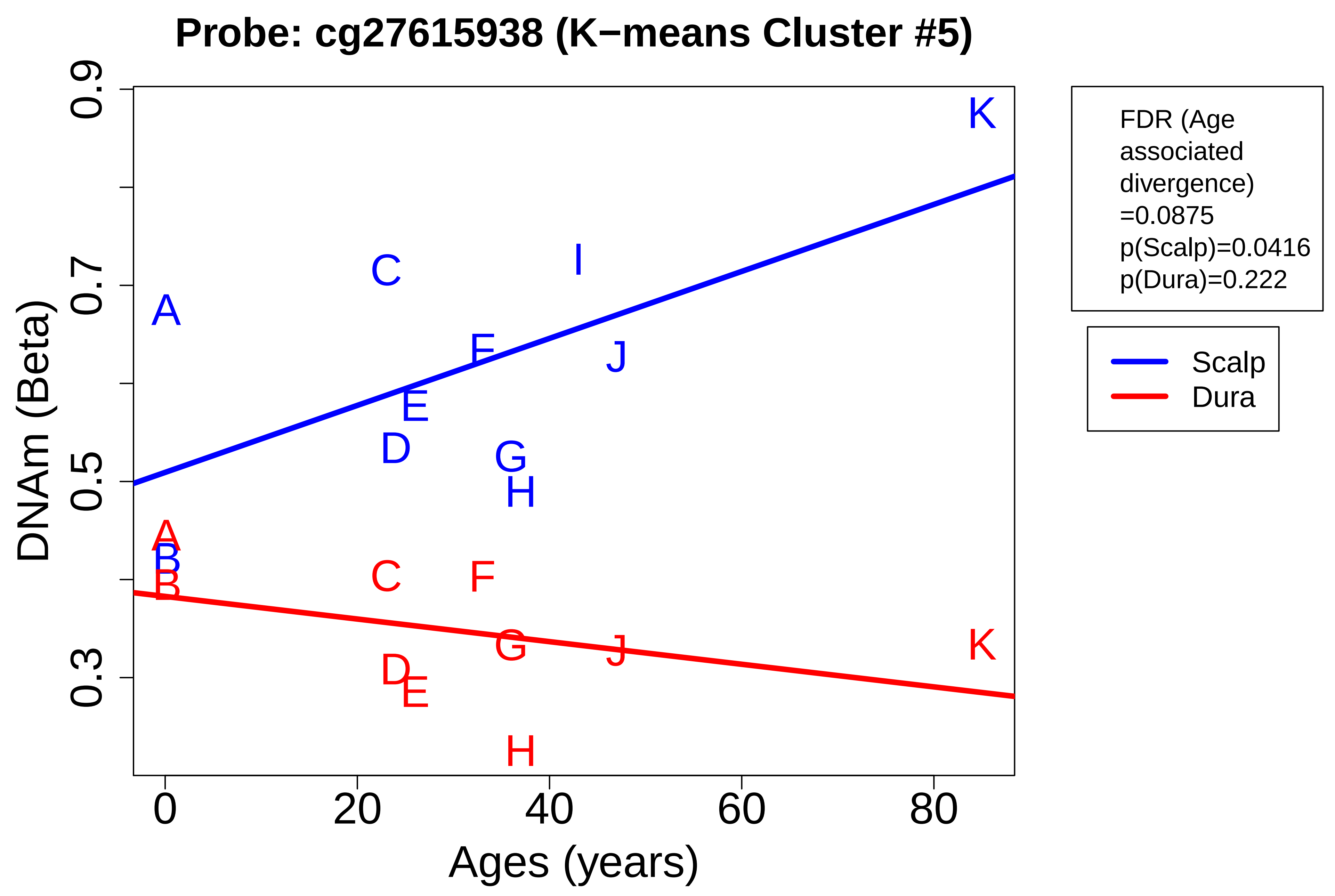

Probe: cg27615938 (K−means Cluster #5)
DNAm (Beta)
0.3
0.5
0.7
0.9
Ages (years)
0
20
40
60
80
A
B
C
D
E
F
G
H
I
J
K
FDR (Age associated divergence) =0.0875
p(Scalp)=0.0416
p(Dura)=0.222
Scalp
Dura

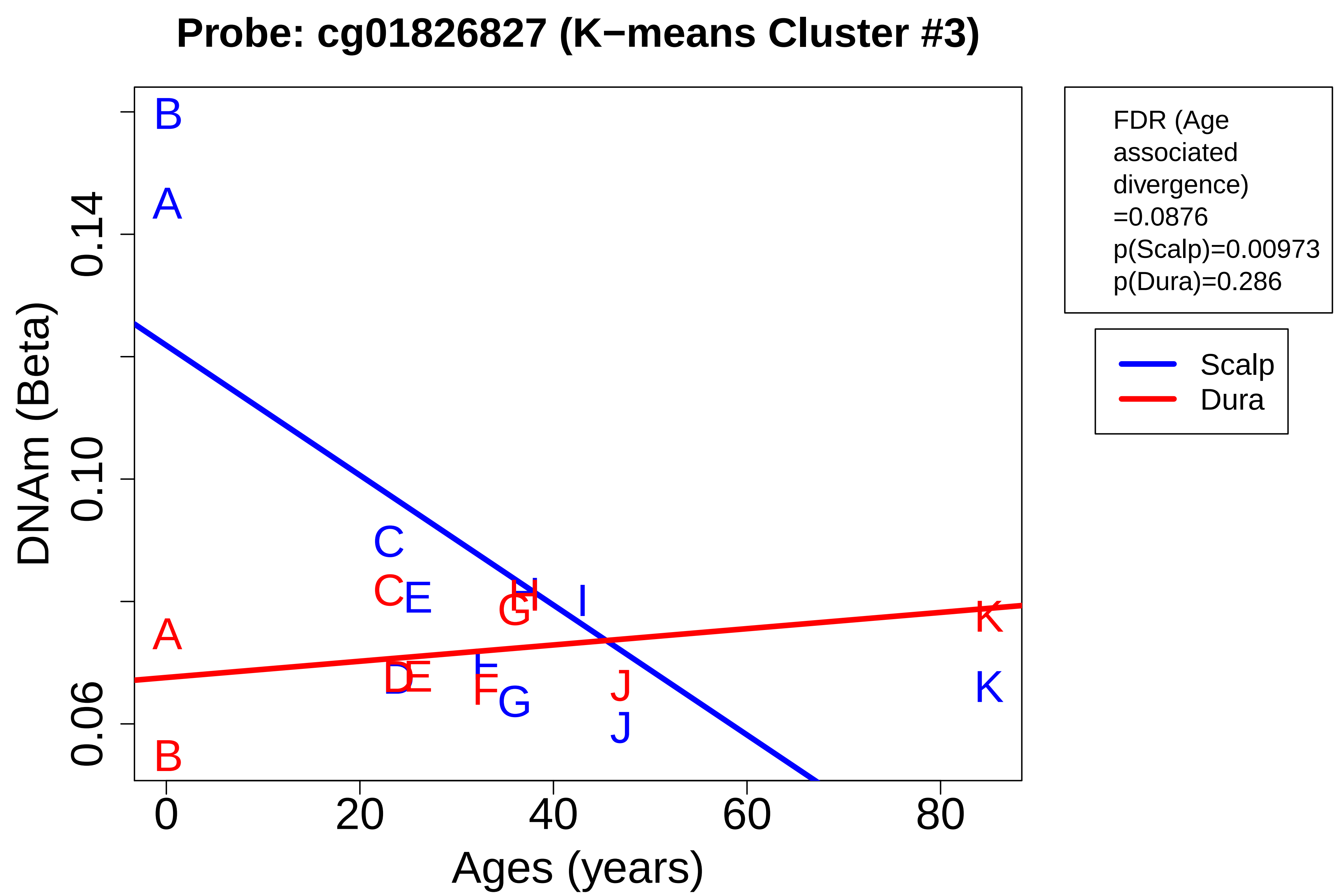

Probe: cg01826827 (K−means Cluster #3)
DNAm (Beta)
0.06
0.10
0.14
Ages (years)
0
20
40
60
80
FDR (Age associated divergence) =0.0876
p(Scalp)=0.00973
p(Dura)=0.286
Scalp
Dura

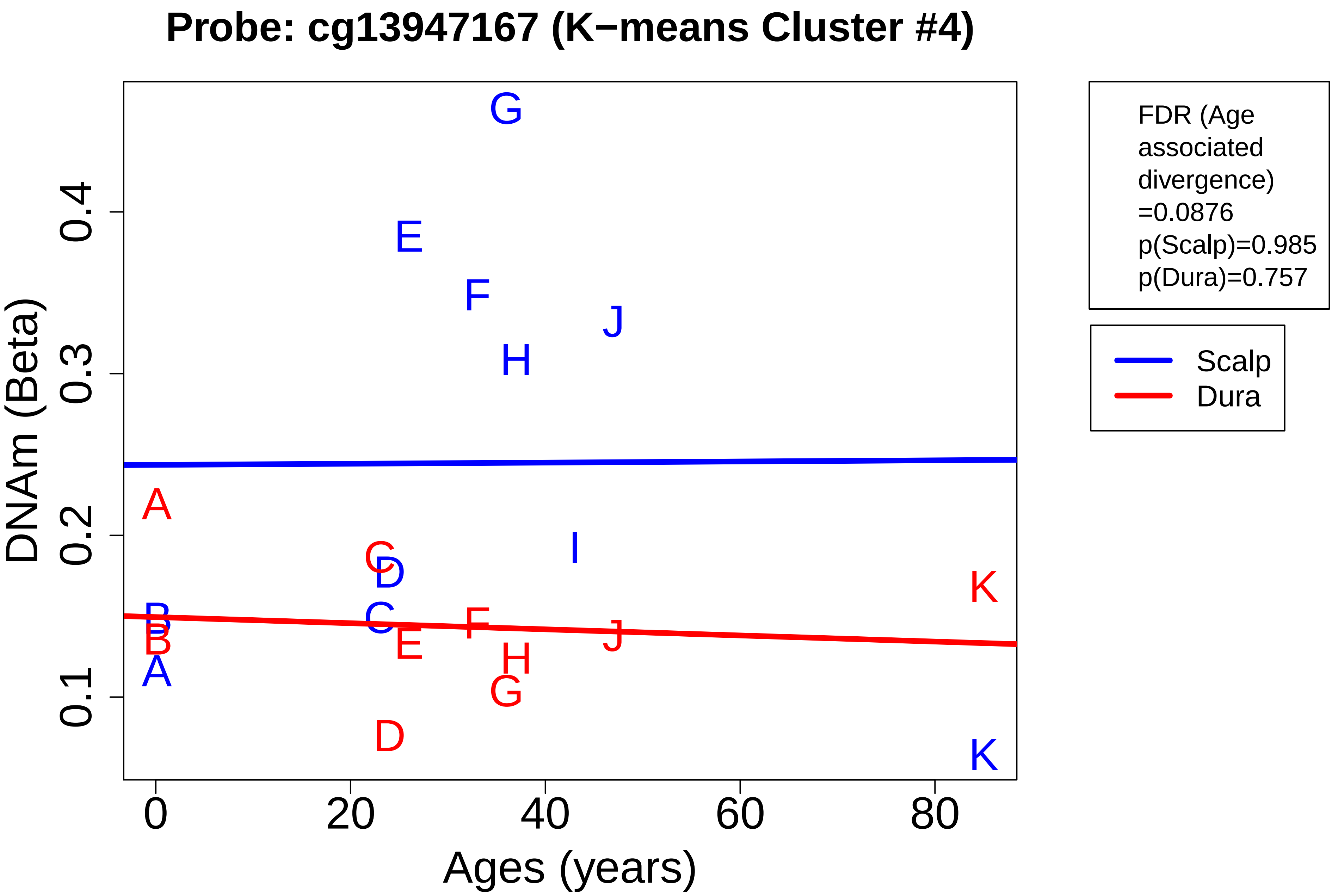

Probe: cg13947167 (K−means Cluster #4)
DNAm (Beta)
0.1
0.2
0.3
0.4
Ages (years)
0
20
40
60
80
FDR (Age associated divergence) =0.0876
p(Scalp)=0.985
p(Dura)=0.757
Scalp
Dura
A
B
C
D
E
F
G
H
I
J
K

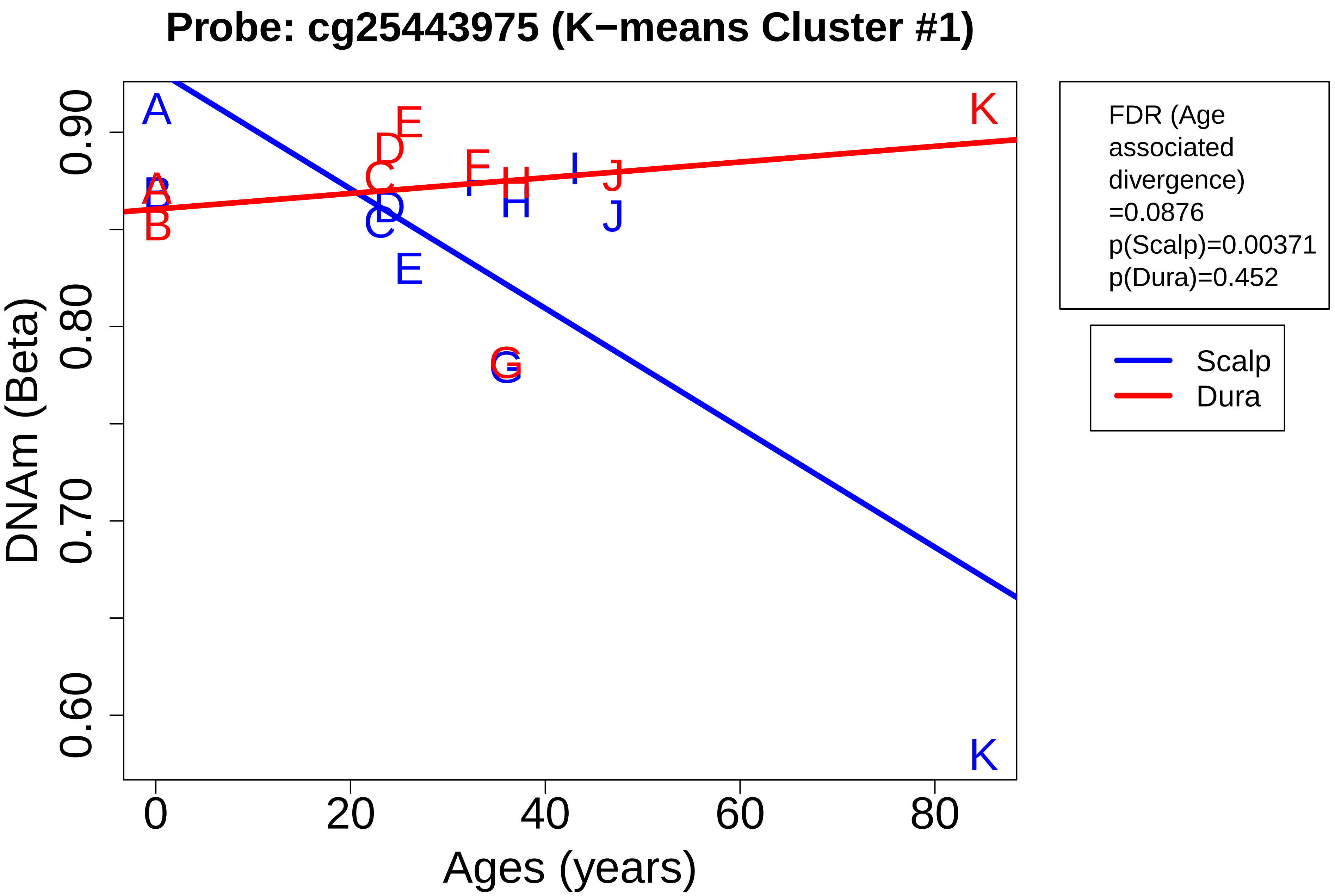

Probe: cg25443975 (K−means Cluster #1)
DNAm (Beta)
0.60
0.70
0.80
0.90
0
20
40
60
80
Ages (years)
A
B
C
D
E
F
G
H
I
J
K
FDR (Age associated divergence) =0.0876
p(Scalp)=0.00371
p(Dura)=0.452
Scalp
Dura

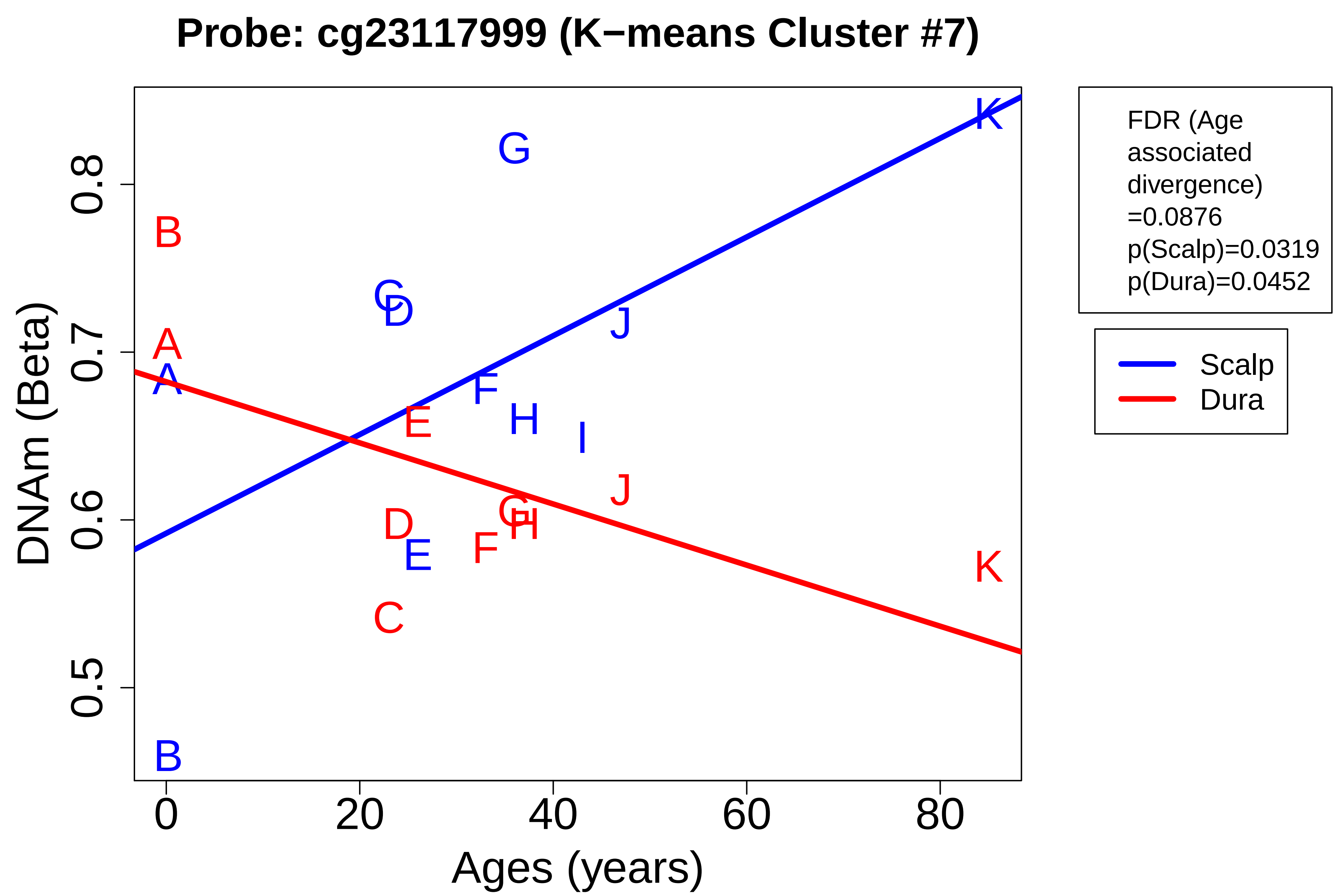

Probe: cg23117999 (K−means Cluster #7)
DNAm (Beta)
0.5
0.6
0.7
0.8
0
20
40
60
80
Ages (years)
FDR (Age associated divergence) =0.0876
p(Scalp)=0.0319
p(Dura)=0.0452
Scalp
Dura

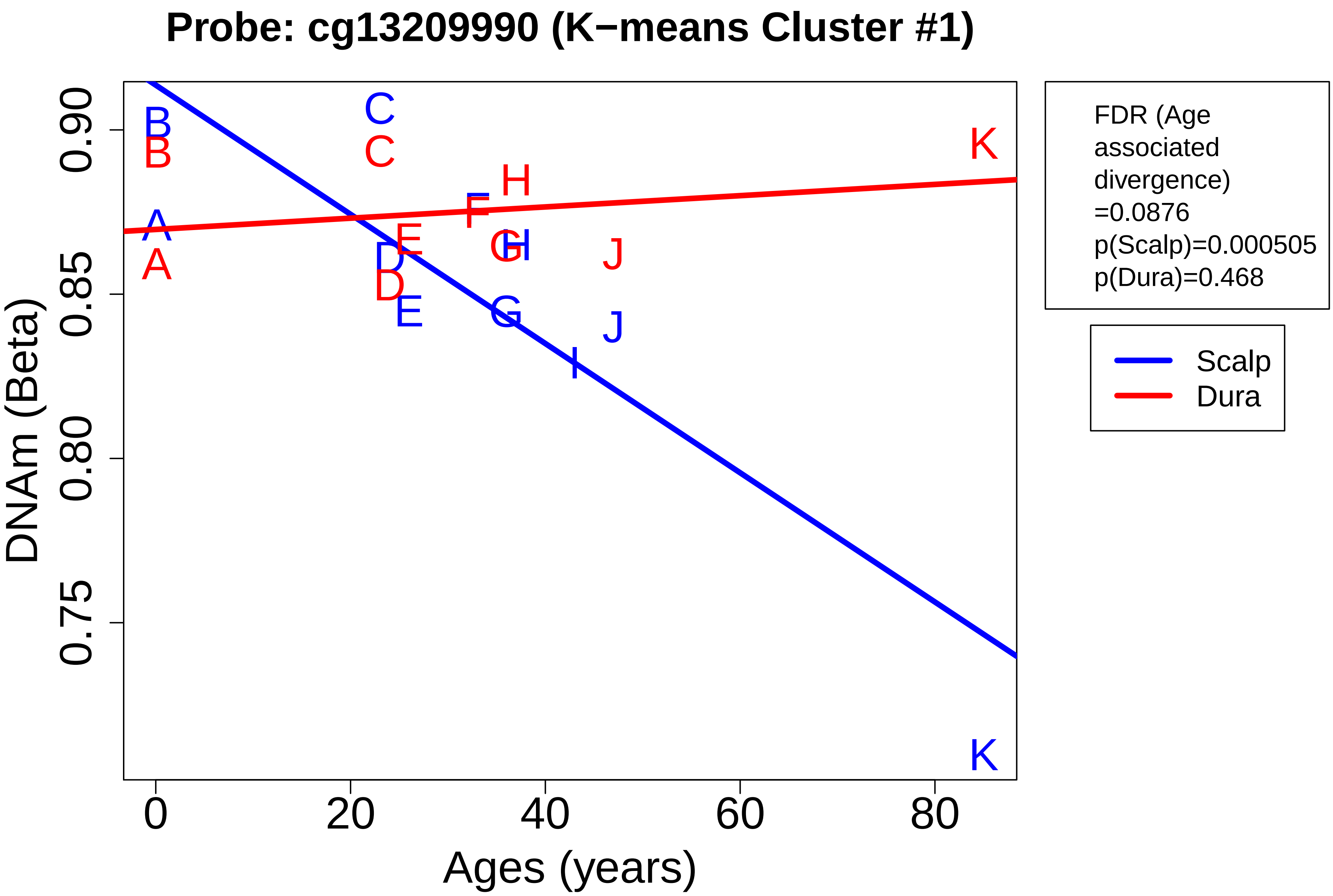

Probe: cg13209990 (K−means Cluster #1)
DNAm (Beta)
0.75
0.80
0.85
0.90
0
20
40
60
80
Ages (years)
FDR (Age associated divergence) =0.0876
p(Scalp)=0.000505
p(Dura)=0.468
Scalp
Dura
A
B
C
D
E
F
G
H
I
J
K

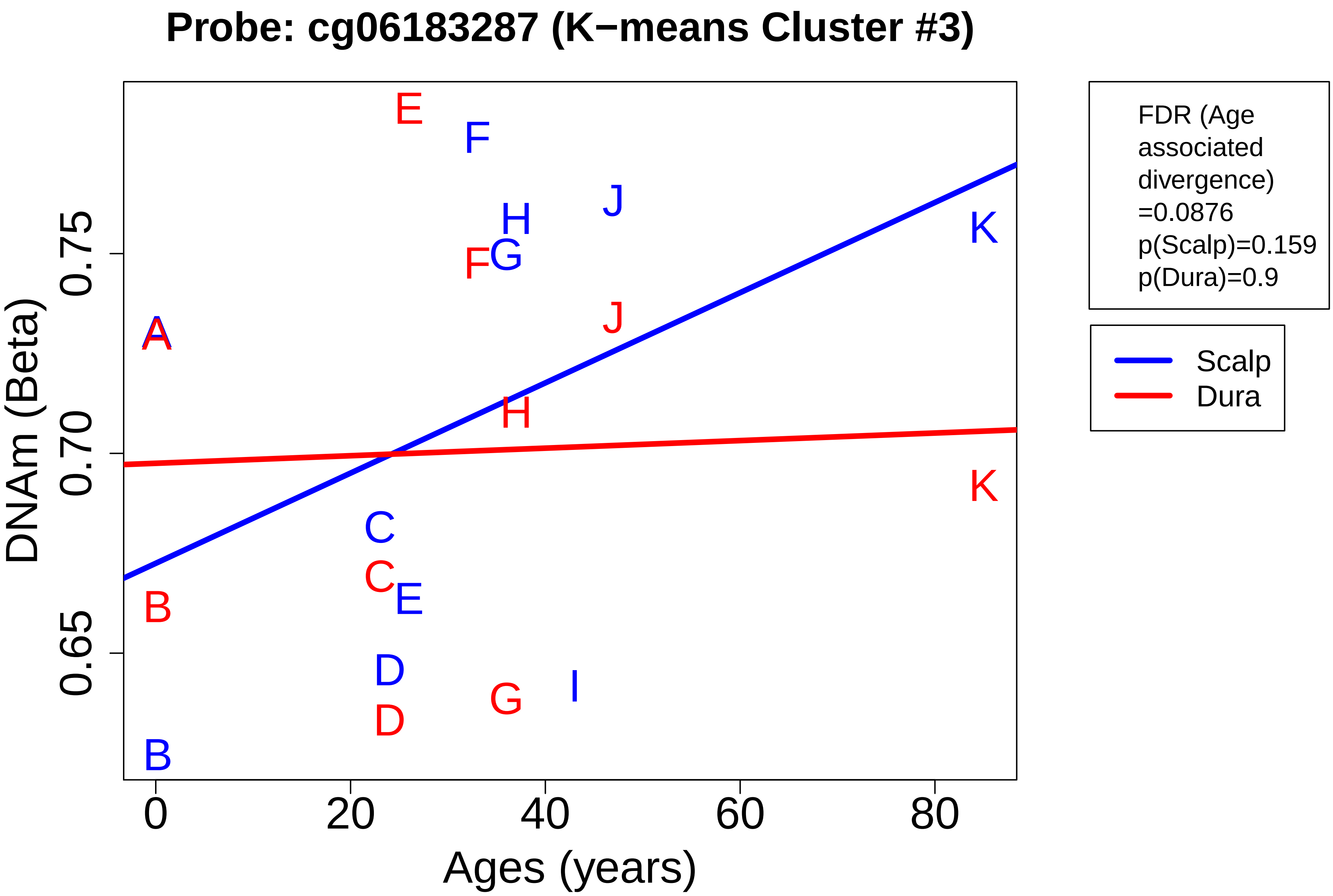
Probe: cg06183287 (K−means Cluster #3)
DNAm (Beta)
0.65
0.70
0.75
Ages (years)
0
20
40
60
80
FDR (Age associated divergence) =0.0876
p(Scalp)=0.159
p(Dura)=0.9
Scalp
Dura

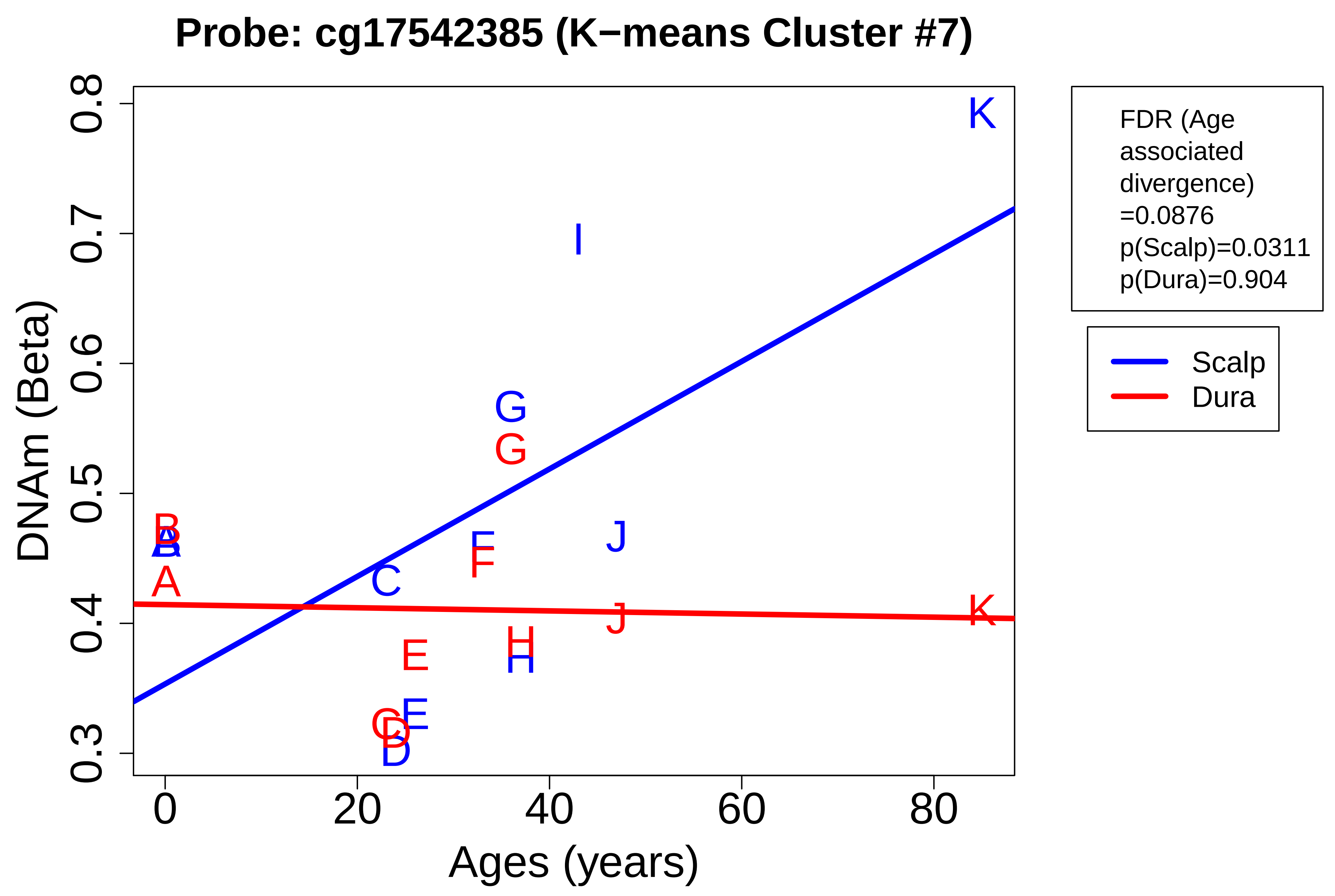
Probe: cg17542385 (K−means Cluster #7)
DNAm (Beta)
0.3
0.4
0.5
0.6
0.7
0.8
Ages (years)
0
20
40
60
80
K
I
G
G
B
A
A
C
F
F
J
J
K
E
H
H
C
D
D
E
FDR (Age associated divergence) =0.0876
p(Scalp)=0.0311
p(Dura)=0.904
Scalp
Dura

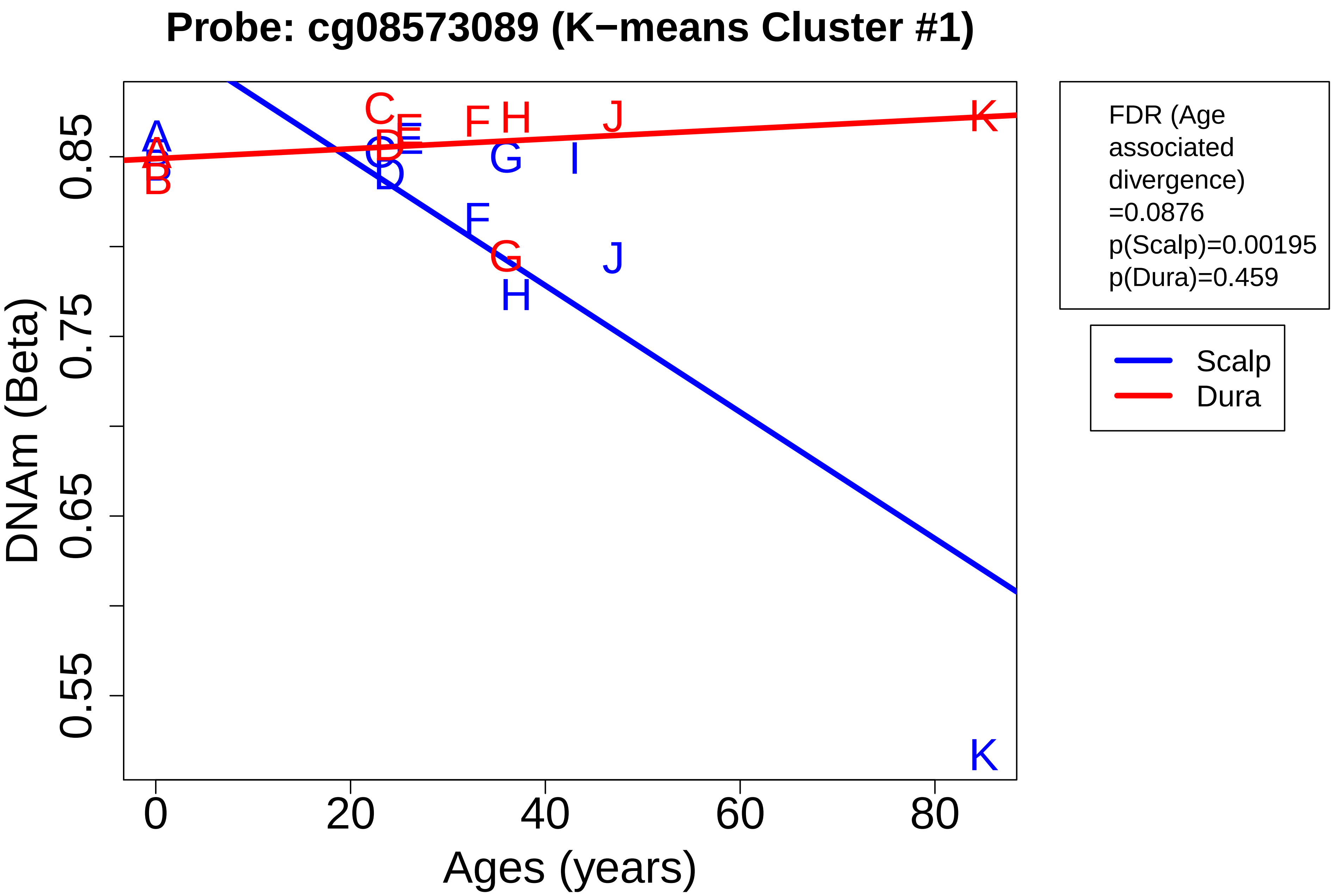
Probe: cg08573089 (K−means Cluster #1)
DNAm (Beta)
0.55
0.65
0.75
0.85
Ages (years)
0
20
40
60
80
A
B
C
D
E
F
G
H
I
J
K
FDR (Age associated divergence) =0.0876
p(Scalp)=0.00195
p(Dura)=0.459
Scalp
Dura

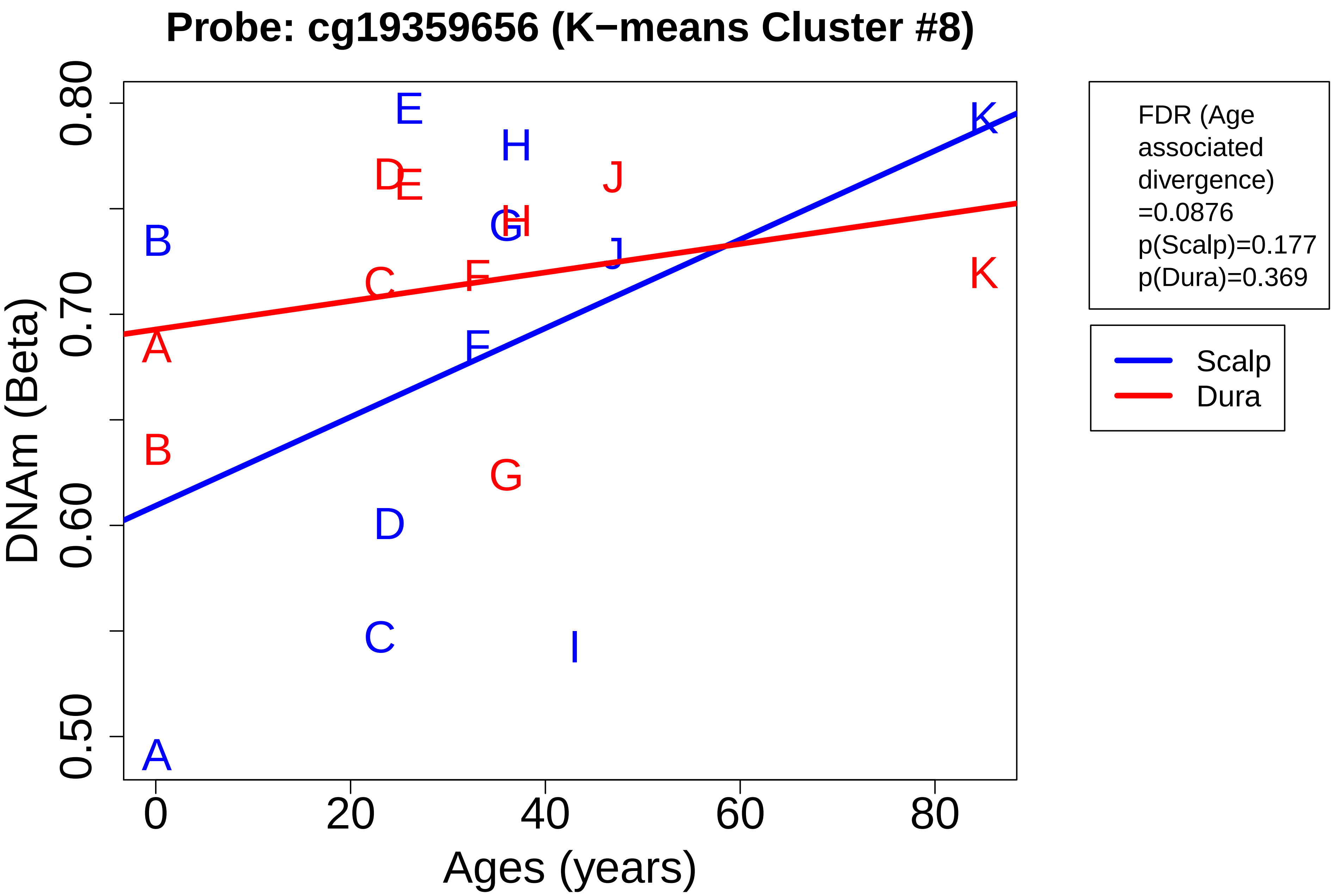

Probe: cg19359656 (K−means Cluster #8)
DNAm (Beta)
0.50
0.60
0.70
0.80
Ages (years)
0
20
40
60
80
FDR (Age associated divergence) =0.0876
p(Scalp)=0.177
p(Dura)=0.369
Scalp
Dura

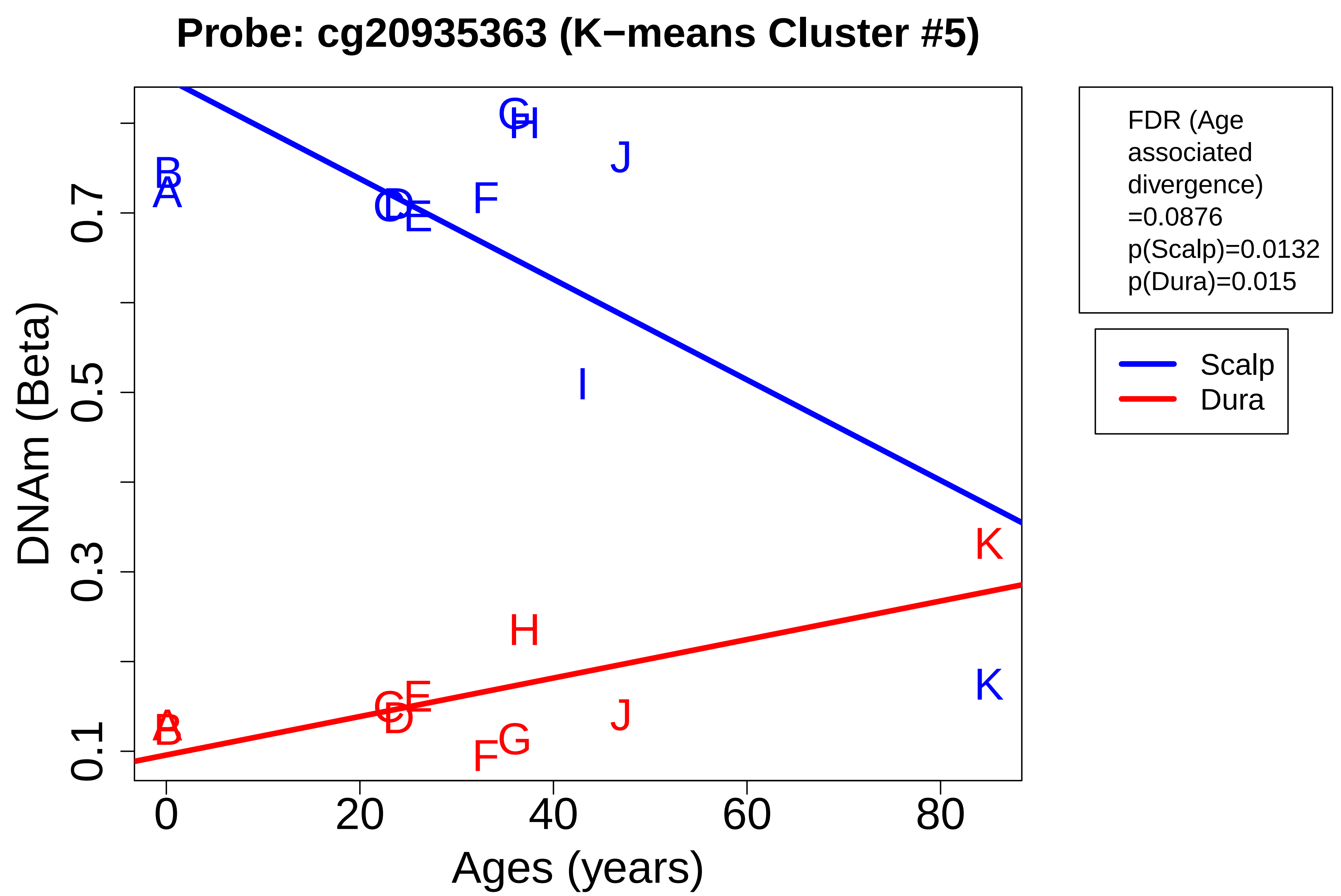
Probe: cg20935363 (K−means Cluster #5)
DNAm (Beta)
0.1
0.3
0.5
0.7
Ages (years)
0
20
40
60
80
FDR (Age associated divergence) =0.0876
p(Scalp)=0.0132
p(Dura)=0.015
Scalp
Dura
A
B
C
D
E
F
G
H
I
J
K

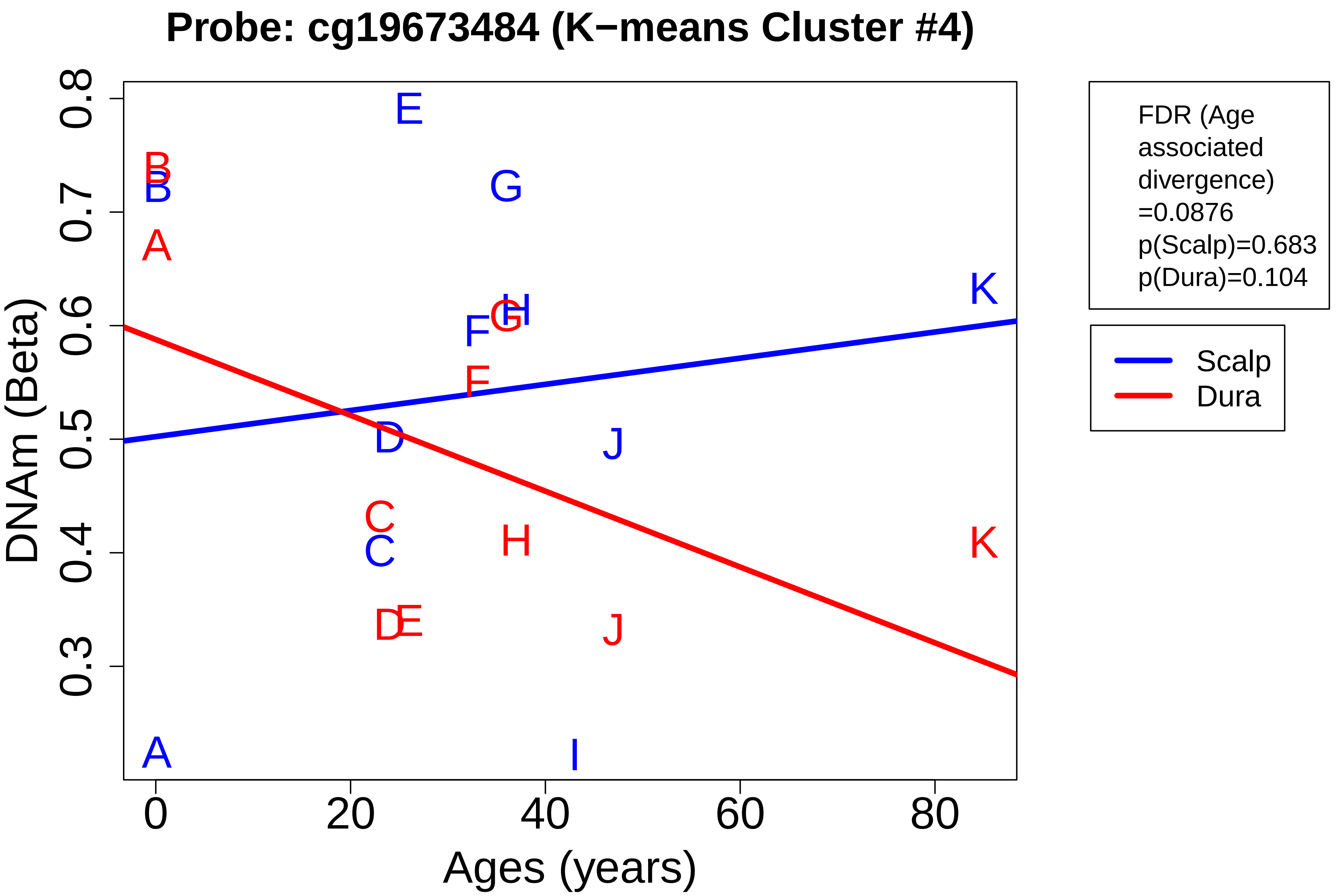

Probe: cg19673484 (K−means Cluster #4)
DNAm (Beta)
Ages (years)
0.3
0.4
0.5
0.6
0.7
0.8
0
20
40
60
80
FDR (Age associated divergence) =0.0876
p(Scalp)=0.683
p(Dura)=0.104
Scalp
Dura

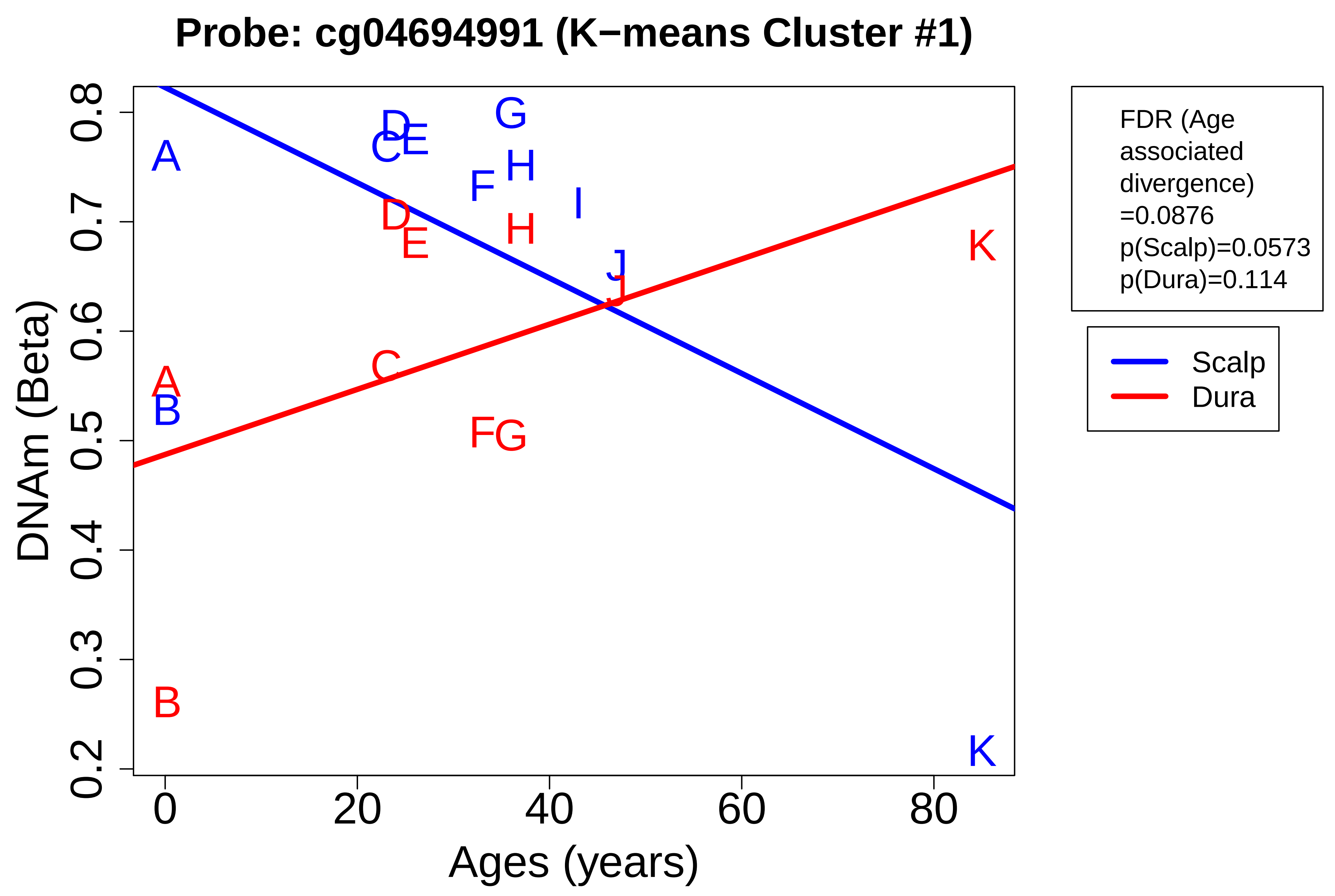
Probe: cg04694991 (K−means Cluster #1)
DNAm (Beta)
0.2
0.3
0.4
0.5
0.6
0.7
0.8
Ages (years)
0
20
40
60
80
A
B
C
D
E
F
G
H
I
J
K
FDR (Age associated divergence) =0.0876
p(Scalp)=0.0573
p(Dura)=0.114
Scalp
Dura

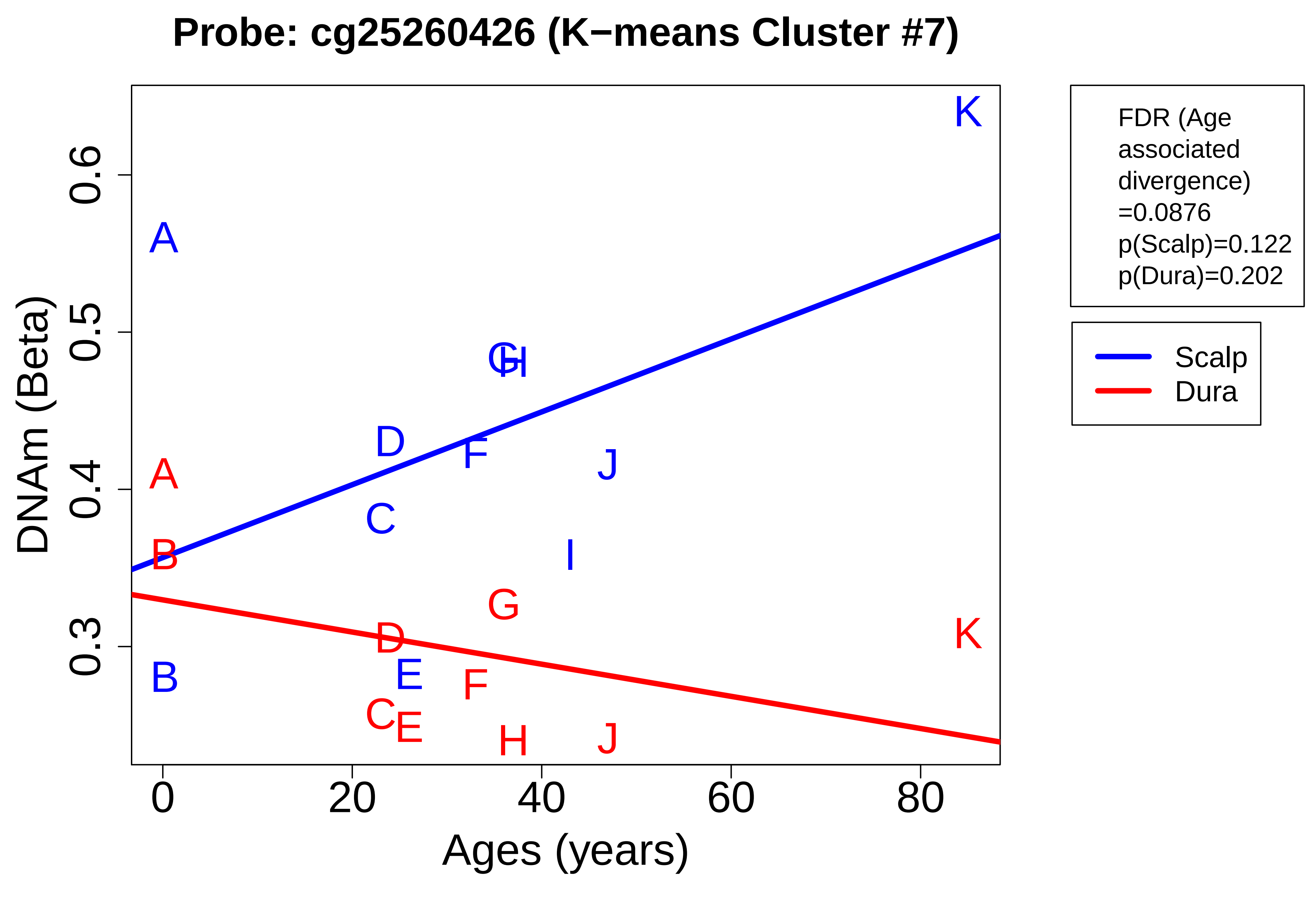
Probe: cg25260426 (K−means Cluster #7)
FDR (Age associated divergence) =0.0876
p(Scalp)=0.122
p(Dura)=0.202
Scalp
Dura
DNAm (Beta)
Ages (years)
0.3
0.4
0.5
0.6
0
20
40
60
80
A
B
C
D
E
F
G
H
I
J
K

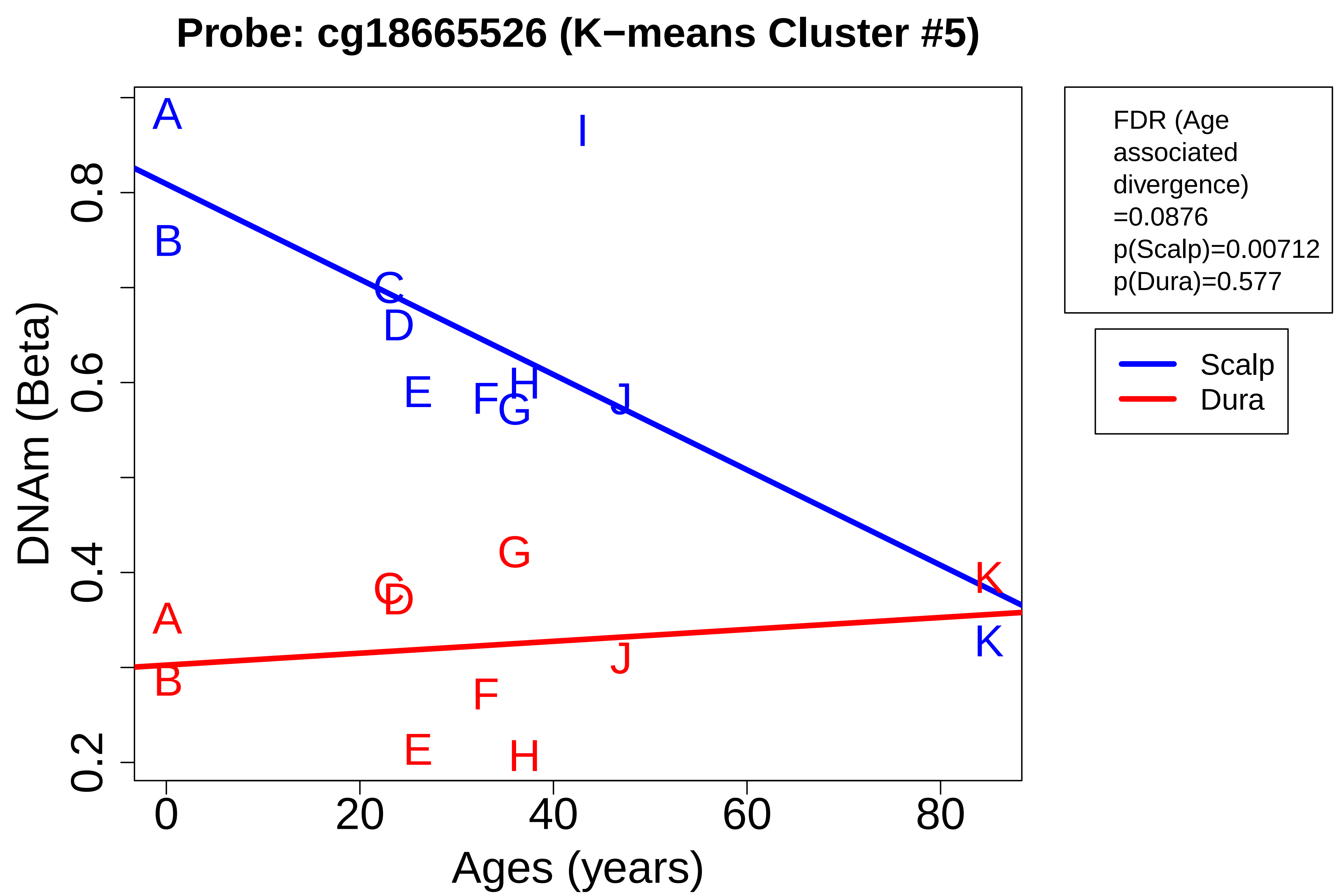

Probe: cg18665526 (K−means Cluster #5)
DNAm (Beta)
0.2
0.4
0.6
0.8
0
20
40
60
80
Ages (years)
A
B
C
D
E
F
G
H
I
J
K
FDR (Age associated divergence) =0.0876
p(Scalp)=0.00712
p(Dura)=0.577
Scalp
Dura

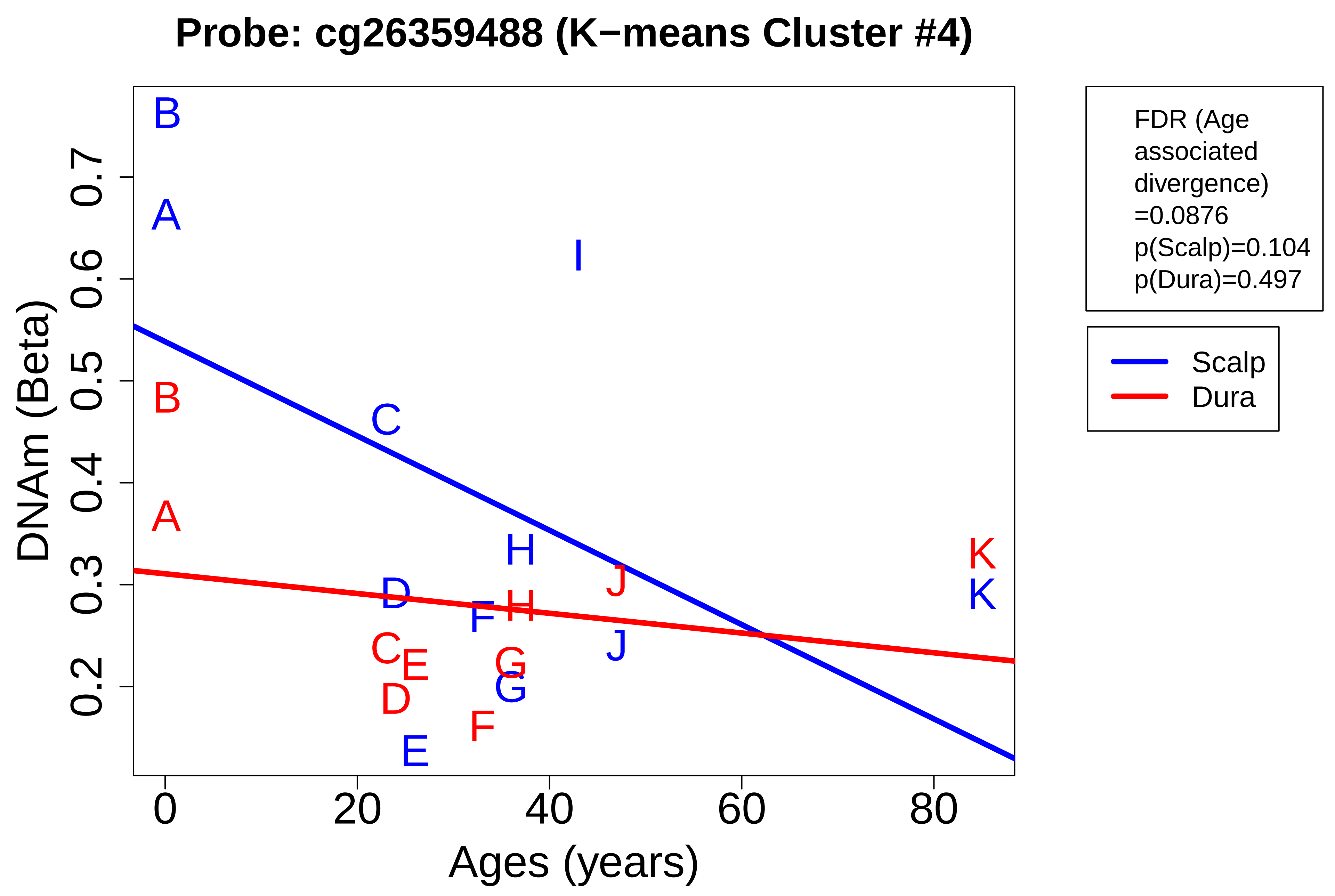

Probe: cg26359488 (K−means Cluster #4)
FDR (Age associated divergence) =0.0876
p(Scalp)=0.104
p(Dura)=0.497
Scalp
Dura
DNAm (Beta)
0.2
0.3
0.4
0.5
0.6
0.7
Ages (years)
0
20
40
60
80
A
B
C
D
E
F
G
H
I
J
K

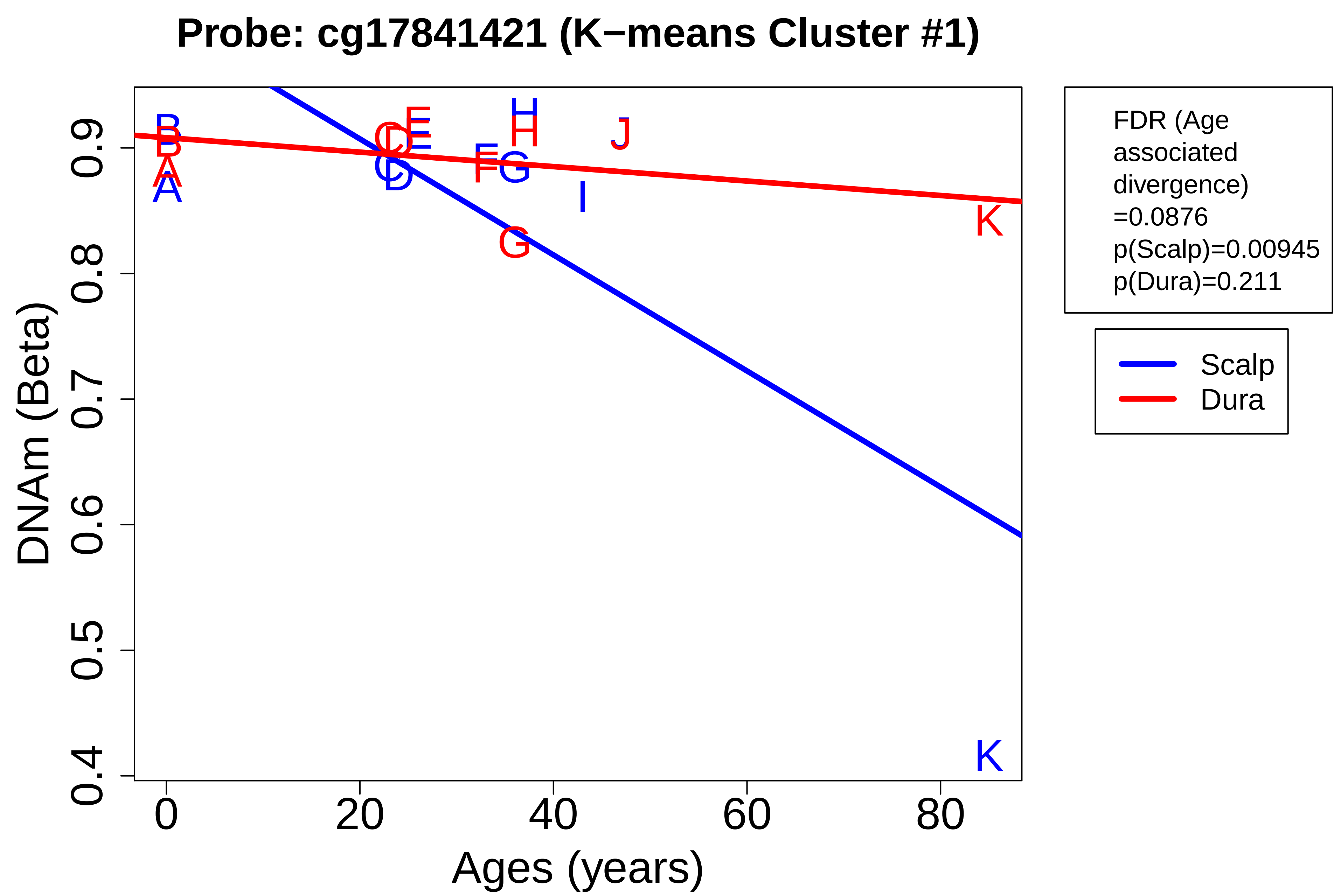

Probe: cg17841421 (K−means Cluster #1)
DNAm (Beta)
0.4
0.5
0.6
0.7
0.8
0.9
0
20
40
60
80
Ages (years)
A
B
C
D
E
F
G
H
I
J
K
FDR (Age associated divergence) =0.0876
p(Scalp)=0.00945
p(Dura)=0.211
Scalp
Dura

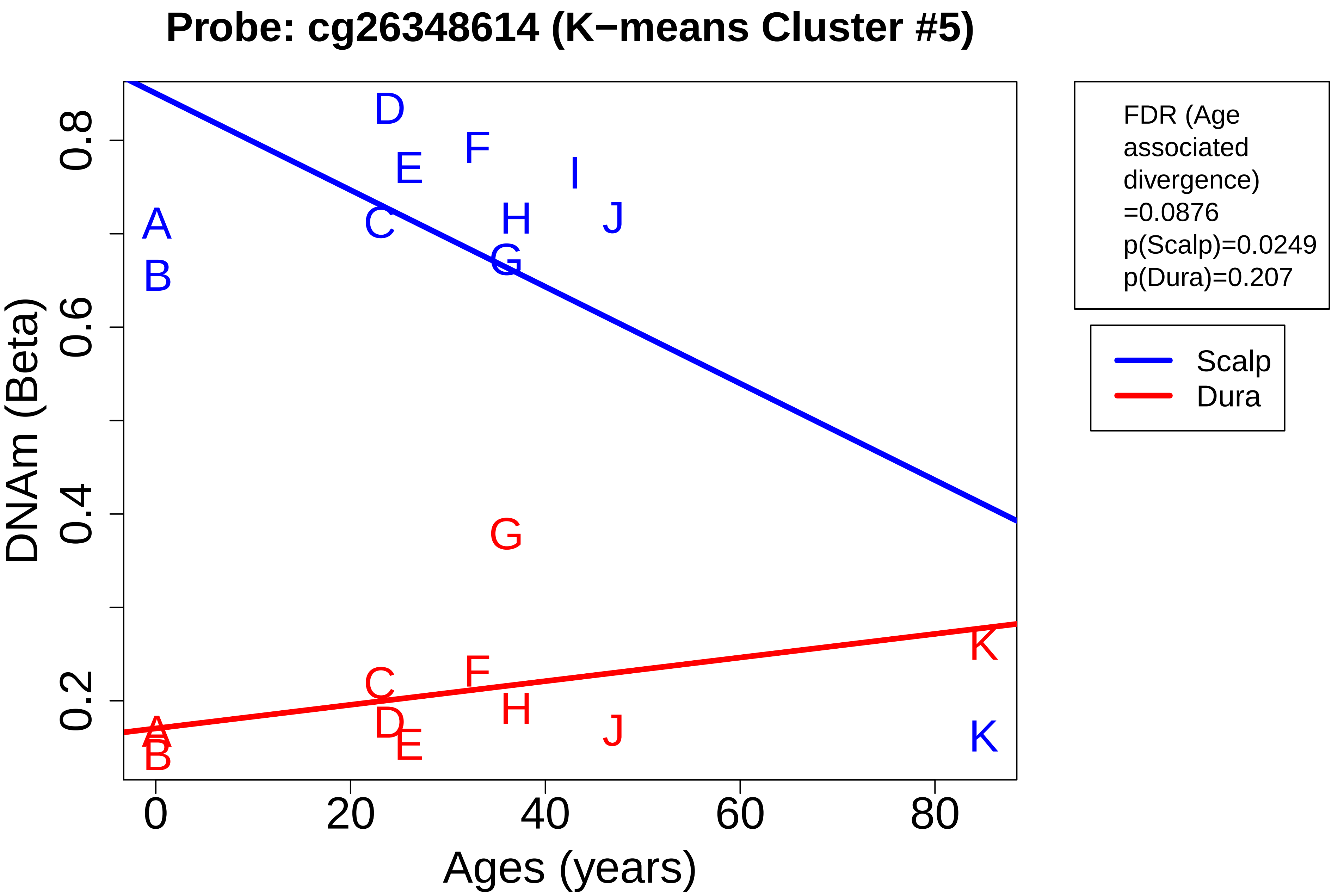

Probe: cg26348614 (K−means Cluster #5)
DNAm (Beta)
0.2
0.4
0.6
0.8
Ages (years)
0
20
40
60
80
FDR (Age associated divergence) =0.0876
p(Scalp)=0.0249
p(Dura)=0.207
Scalp
Dura
A
B
C
D
E
F
G
H
I
J
K

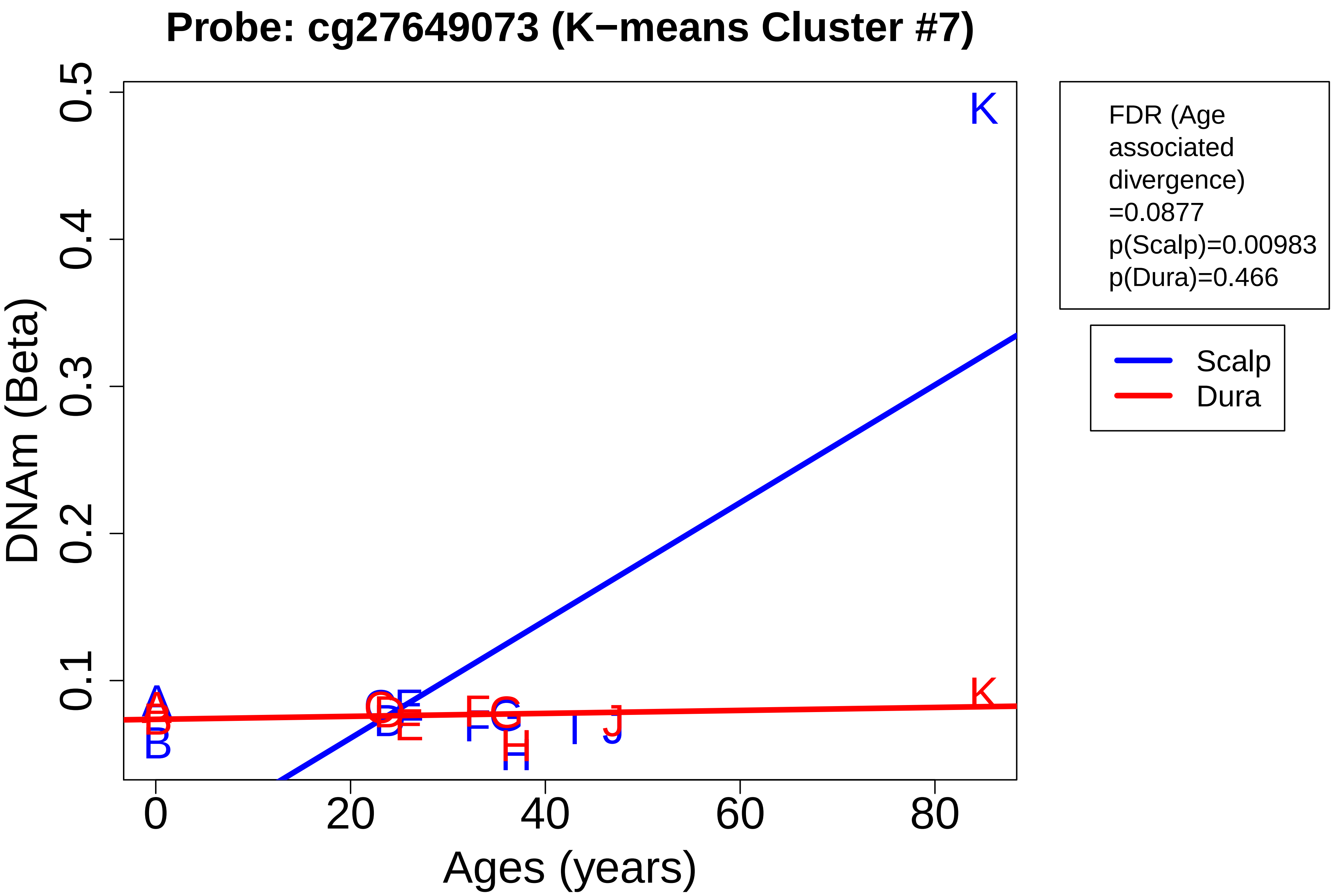

Probe: cg27649073 (K−means Cluster #7)
DNAm (Beta)
0.1
0.2
0.3
0.4
0.5
Ages (years)
0
20
40
60
80
FDR (Age associated divergence) =0.0877
p(Scalp)=0.00983
p(Dura)=0.466
Scalp
Dura

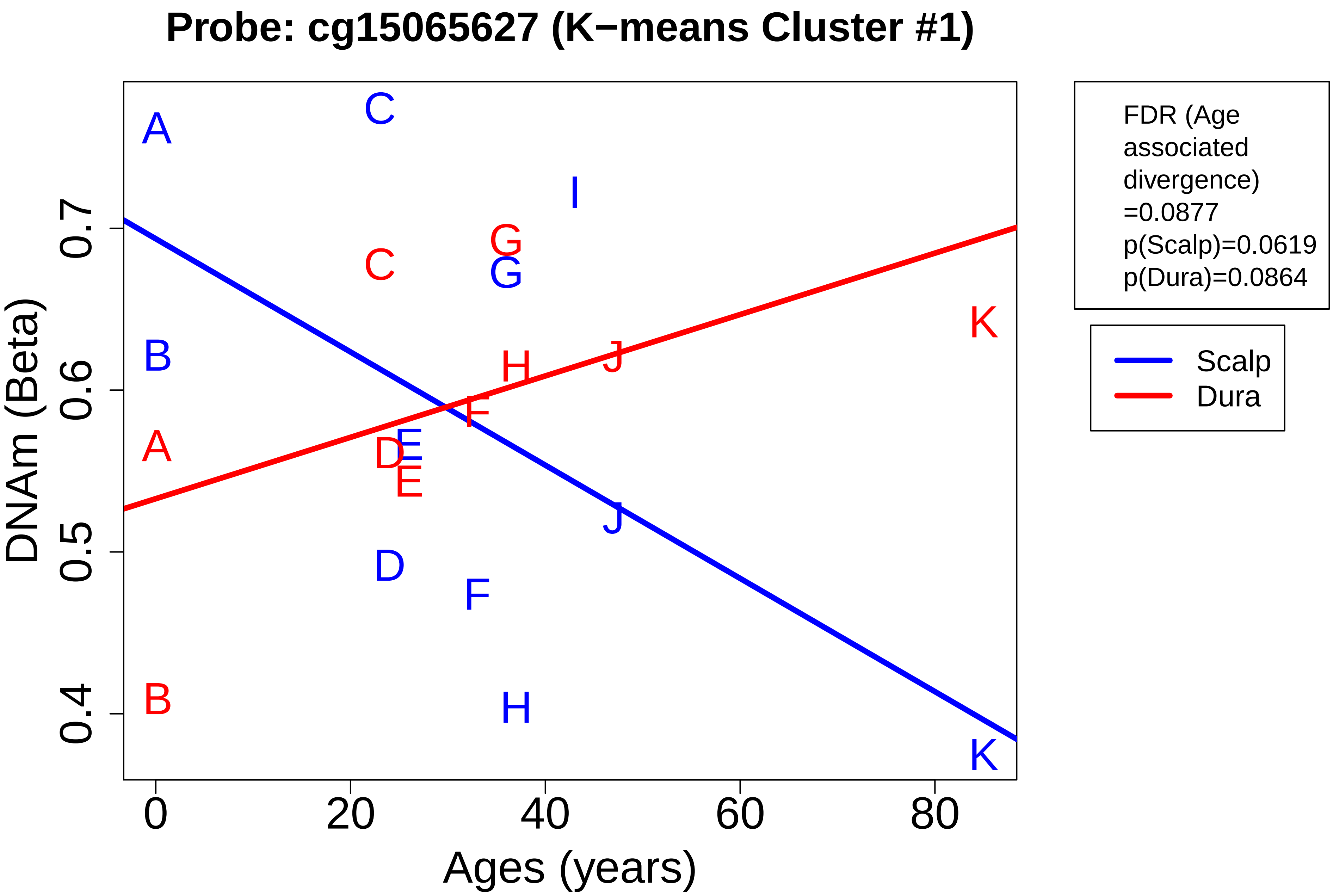

Probe: cg15065627 (K−means Cluster #1)
DNAm (Beta)
0.4
0.5
0.6
0.7
0
20
40
60
80
Ages (years)
A
B
C
D
E
F
G
H
I
J
K
FDR (Age associated divergence) =0.0877
p(Scalp)=0.0619
p(Dura)=0.0864
Scalp
Dura

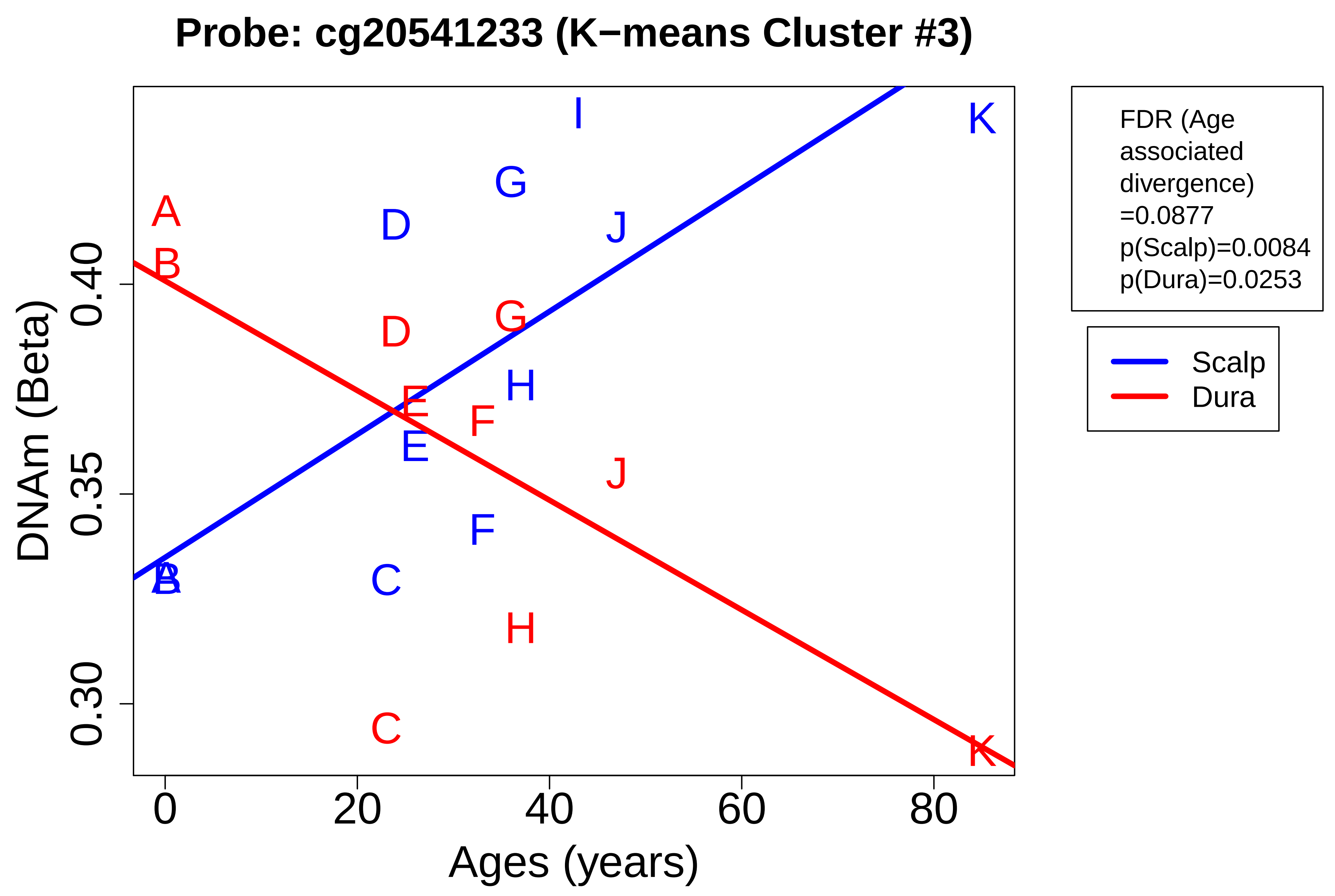

Probe: cg20541233 (K−means Cluster #3)
DNAm (Beta)
0.30
0.35
0.40
Ages (years)
0
20
40
60
80
FDR (Age associated divergence) =0.0877
p(Scalp)=0.0084
p(Dura)=0.0253
Scalp
Dura

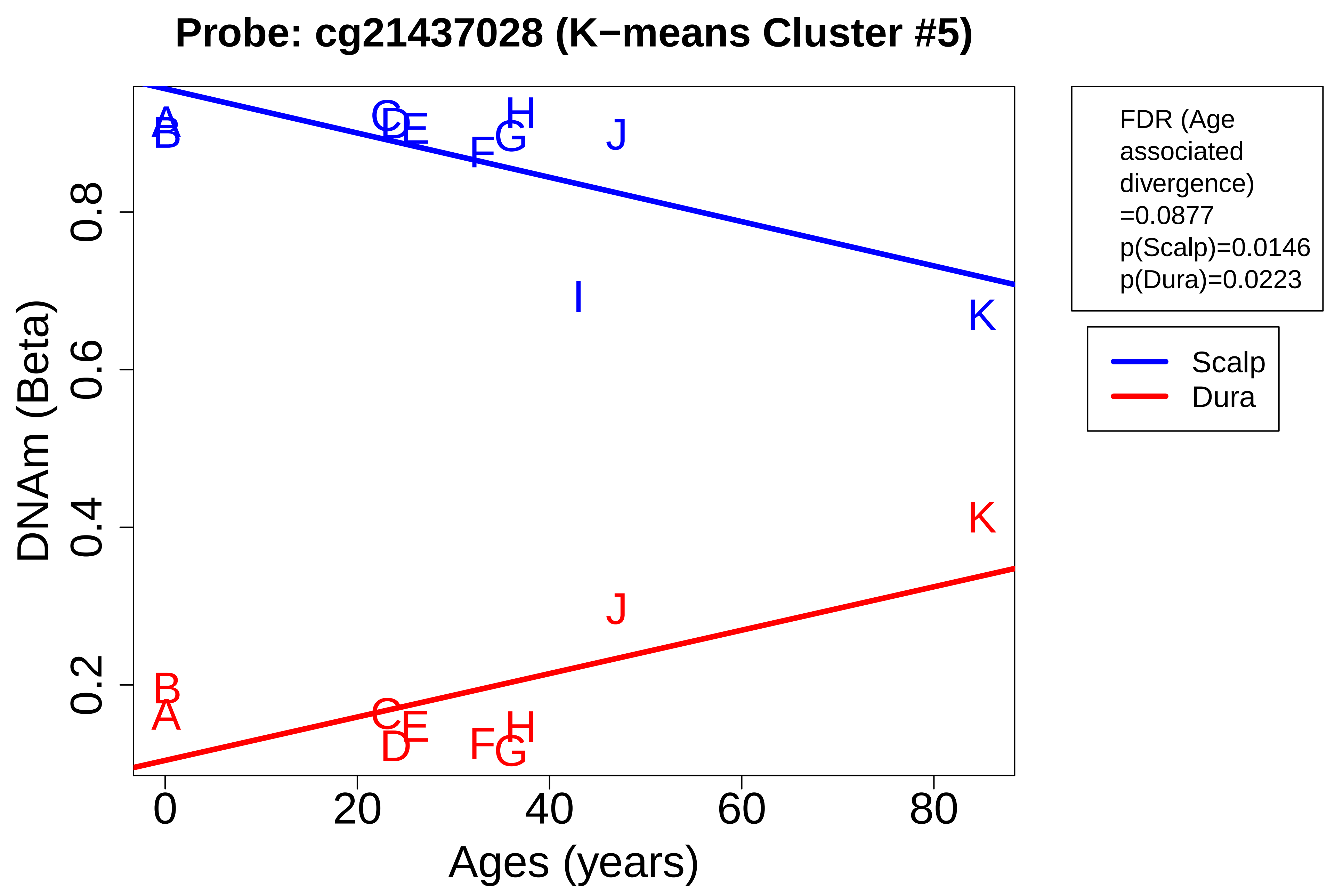

Probe: cg21437028 (K−means Cluster #5)
DNAm (Beta)
0.2
0.4
0.6
0.8
Ages (years)
0
20
40
60
80
FDR (Age associated divergence) =0.0877
p(Scalp)=0.0146
p(Dura)=0.0223
Scalp
Dura
A
B
C
D
E
F
G
H
I
J
K

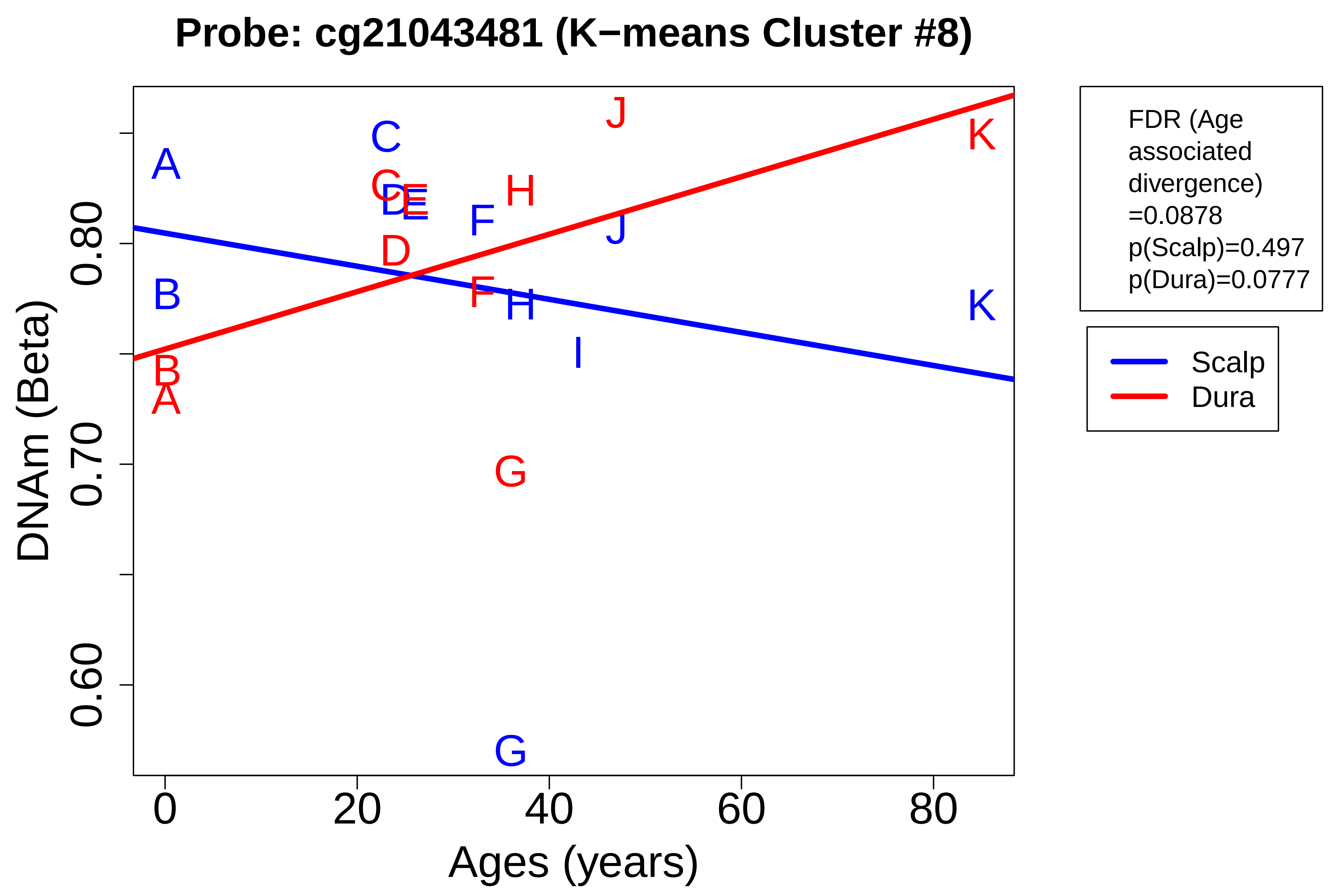

Probe: cg21043481 (K−means Cluster #8)
DNAm (Beta)
0.60
0.70
0.80
Ages (years)
0
20
40
60
80
A
B
C
D
E
F
G
H
I
J
K
FDR (Age associated divergence) =0.0878
p(Scalp)=0.497
p(Dura)=0.0777
Scalp
Dura

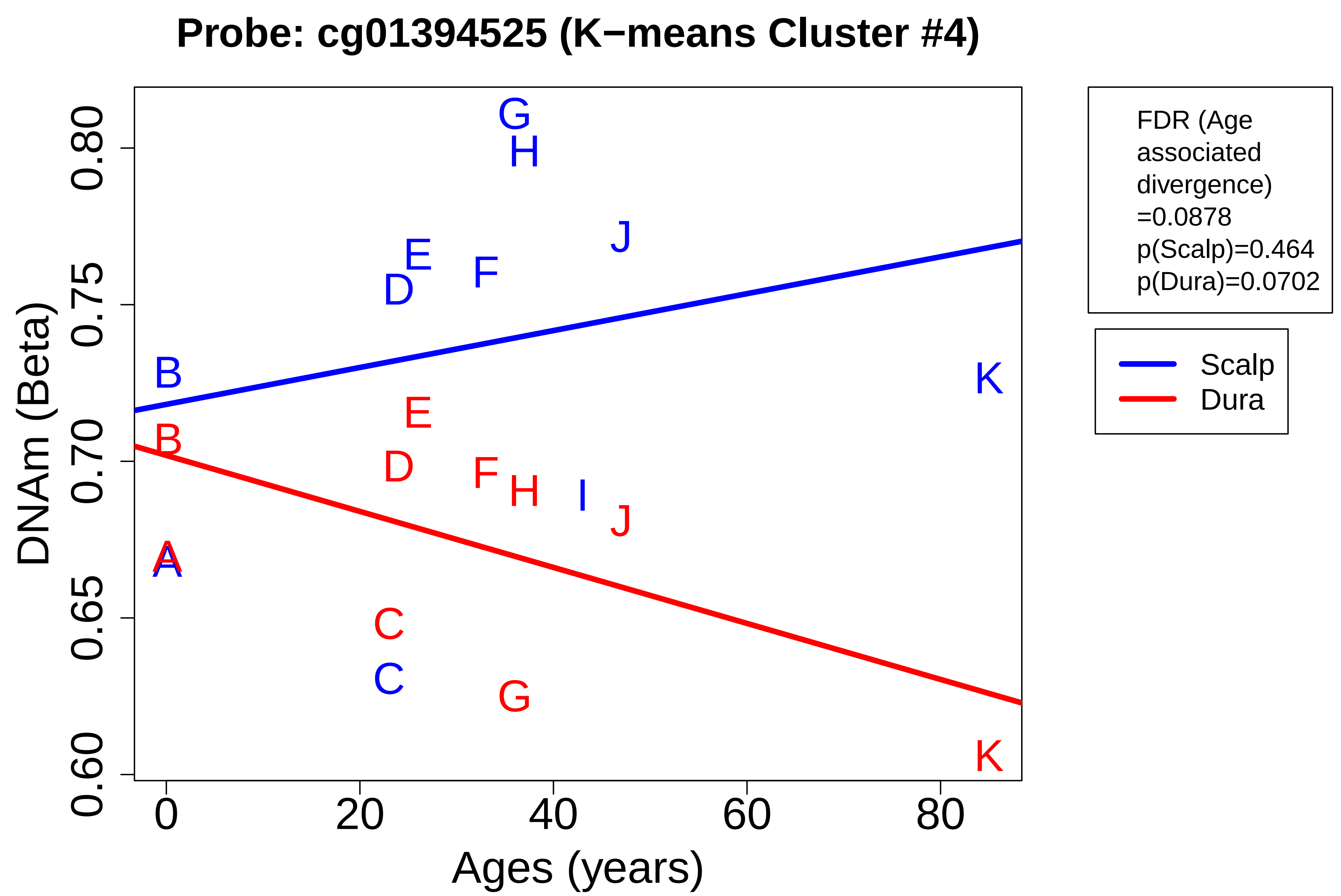

Probe: cg01394525 (K−means Cluster #4)
DNAm (Beta)
0.60
0.65
0.70
0.75
0.80
0
20
40
60
80
Ages (years)
FDR (Age associated divergence) =0.0878
p(Scalp)=0.464
p(Dura)=0.0702
Scalp
Dura
A
B
C
D
E
F
G
H
I
J
K

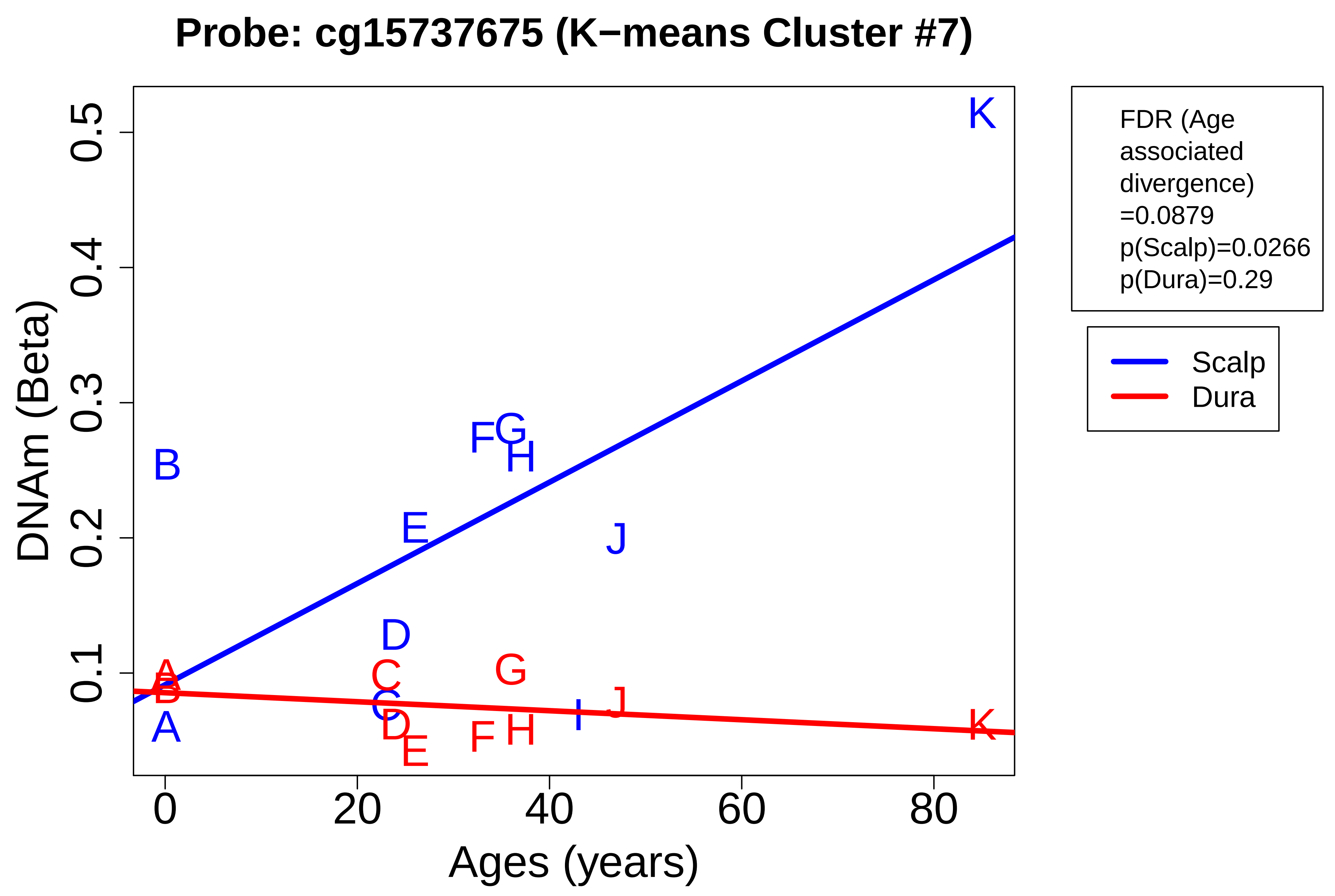
Probe: cg15737675 (K−means Cluster #7)
DNAm (Beta)
0.1
0.2
0.3
0.4
0.5
0
20
40
60
80
Ages (years)
FDR (Age associated divergence) =0.0879
p(Scalp)=0.0266
p(Dura)=0.29
Scalp
Dura

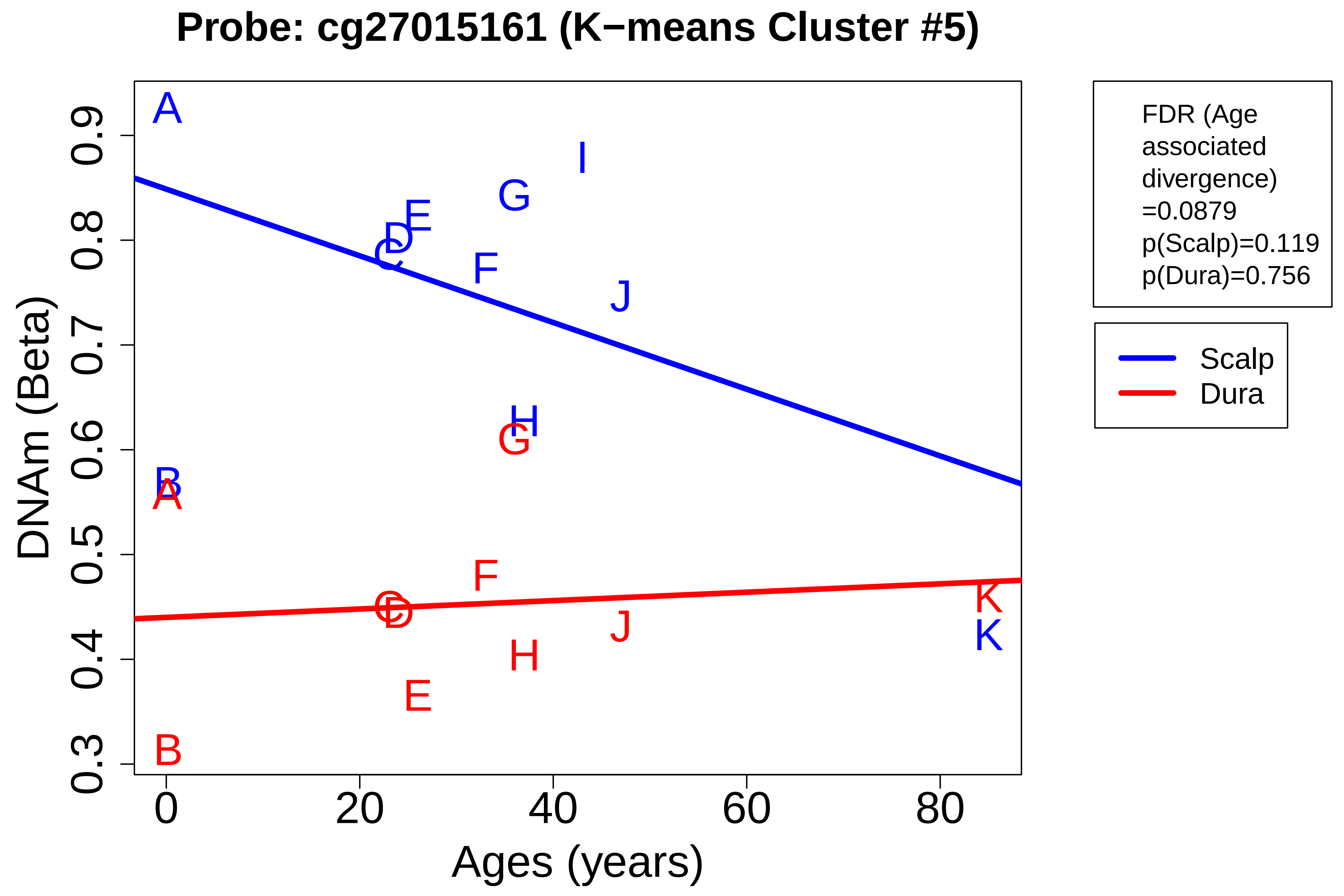

Probe: cg27015161 (K−means Cluster #5)
DNAm (Beta)
Ages (years)
FDR (Age associated divergence) =0.0879
p(Scalp)=0.119
p(Dura)=0.756
Scalp
Dura

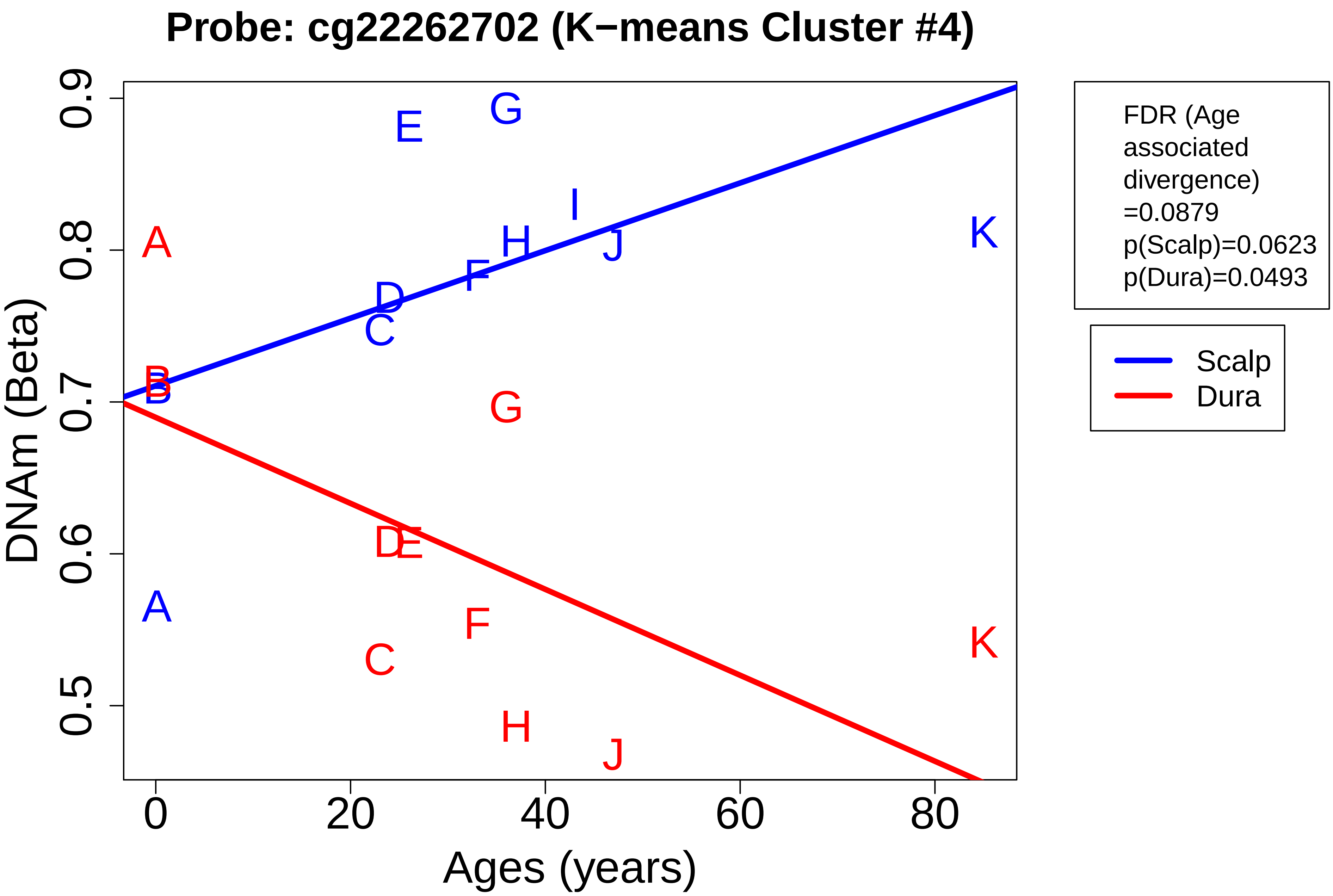

Probe: cg22262702 (K−means Cluster #4)
DNAm (Beta)
0.5
0.6
0.7
0.8
0.9
0
20
40
60
80
Ages (years)
FDR (Age associated divergence) =0.0879
p(Scalp)=0.0623
p(Dura)=0.0493
Scalp
Dura
A
B
C
D
E
F
G
H
I
J
K

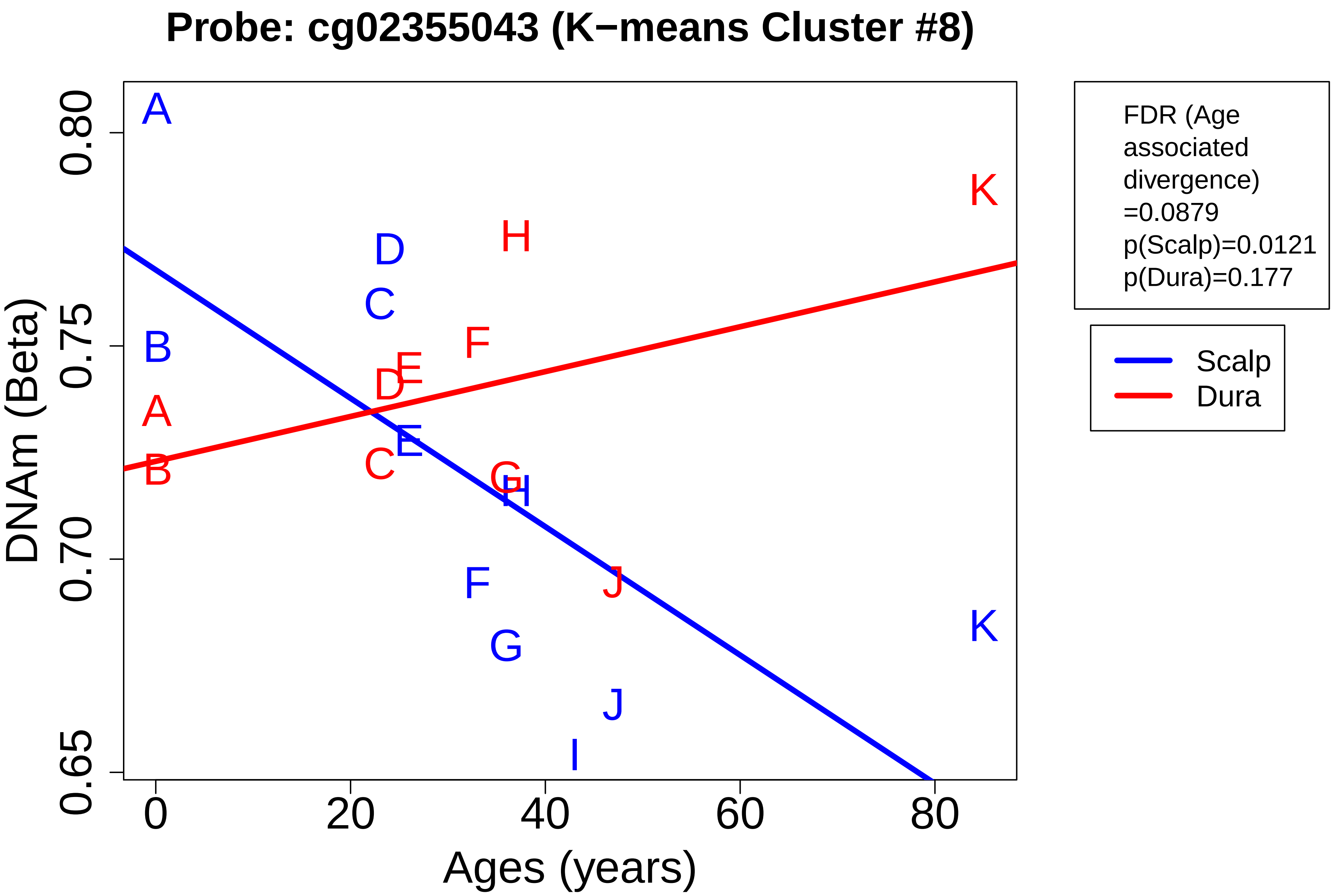

Probe: cg02355043 (K−means Cluster #8)
DNAm (Beta)
0.65
0.70
0.75
0.80
0
20
40
60
80
Ages (years)
FDR (Age associated divergence) =0.0879
p(Scalp)=0.0121
p(Dura)=0.177
Scalp
Dura
A
B
C
D
E
F
G
H
I
J
K

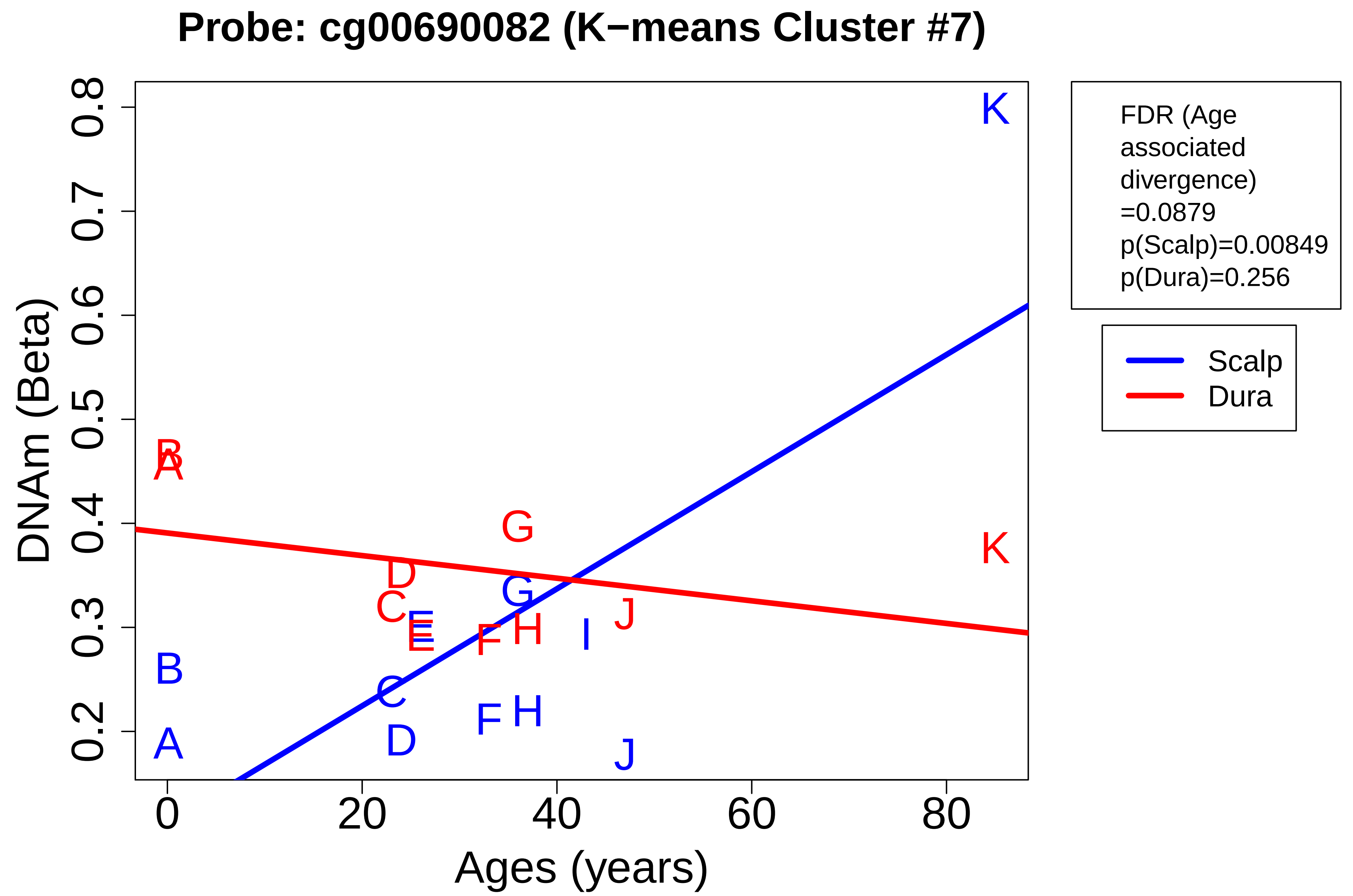

Probe: cg00690082 (K−means Cluster #7)
DNAm (Beta)
0.2
0.3
0.4
0.5
0.6
0.7
0.8
0
20
40
60
80
Ages (years)
A
B
C
D
E
F
G
H
I
J
K
FDR (Age associated divergence) =0.0879
p(Scalp)=0.00849
p(Dura)=0.256
Scalp
Dura

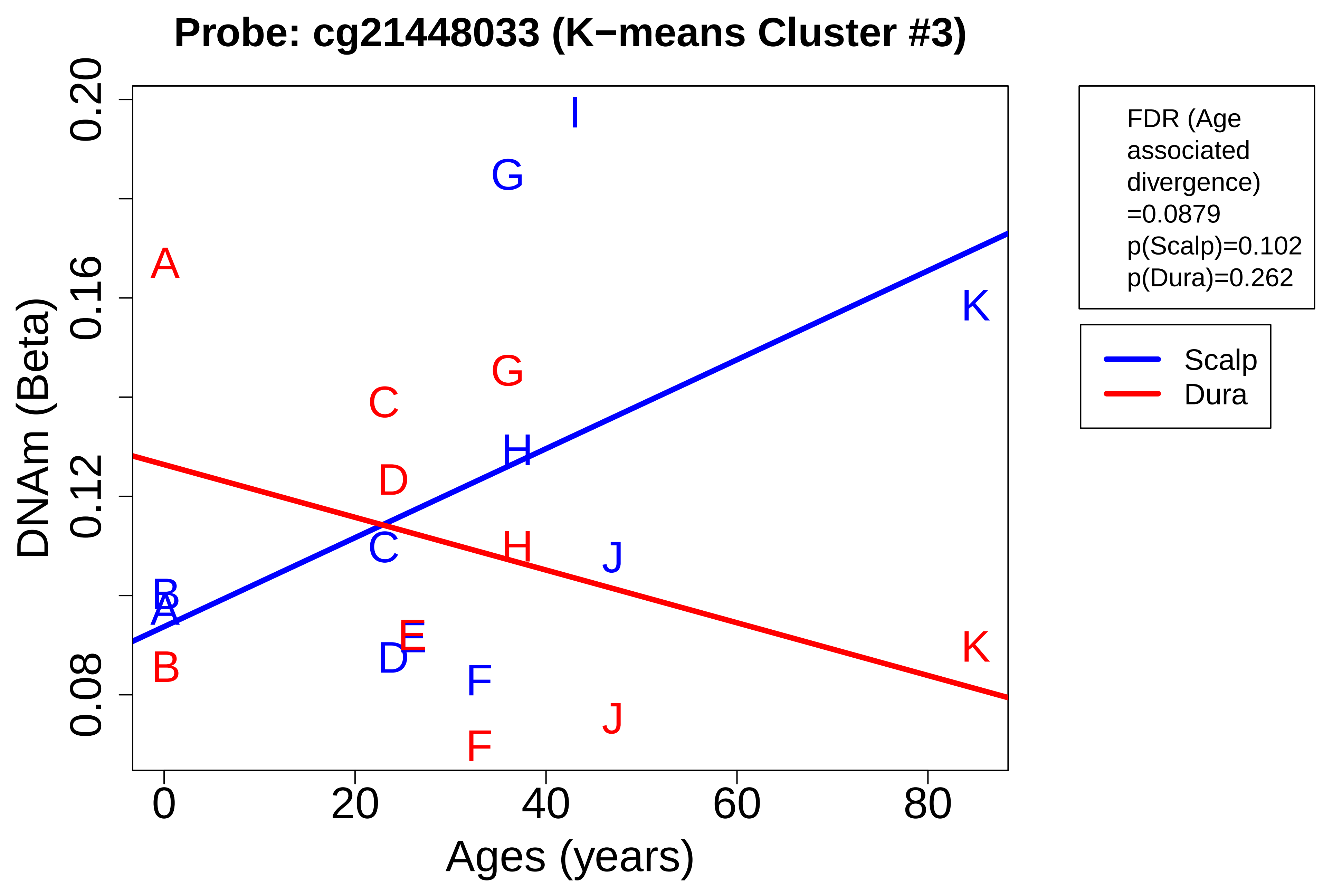

Probe: cg21448033 (K−means Cluster #3)
DNAm (Beta)
0.08
0.12
0.16
0.20
Ages (years)
0
20
40
60
80
I
G
A
K
G
C
H
D
C
H
J
B
A
E
D
B
F
K
J
F
FDR (Age associated divergence) =0.0879
p(Scalp)=0.102
p(Dura)=0.262
Scalp
Dura

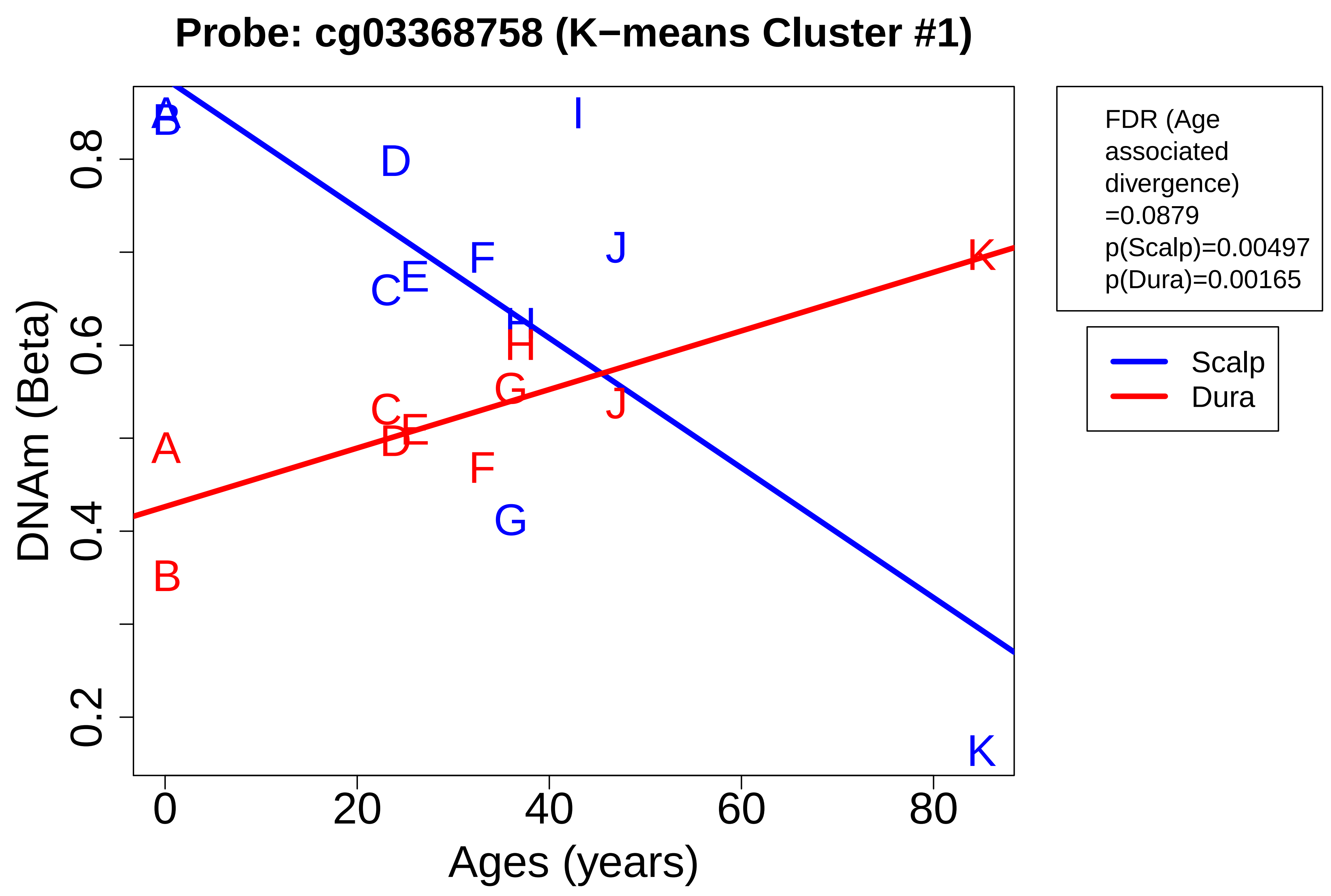

Probe: cg03368758 (K−means Cluster #1)
DNAm (Beta)
0.2
0.4
0.6
0.8
0
20
40
60
80
Ages (years)
A
B
C
D
E
F
G
H
I
J
K
FDR (Age associated divergence) =0.0879
p(Scalp)=0.00497
p(Dura)=0.00165
Scalp
Dura

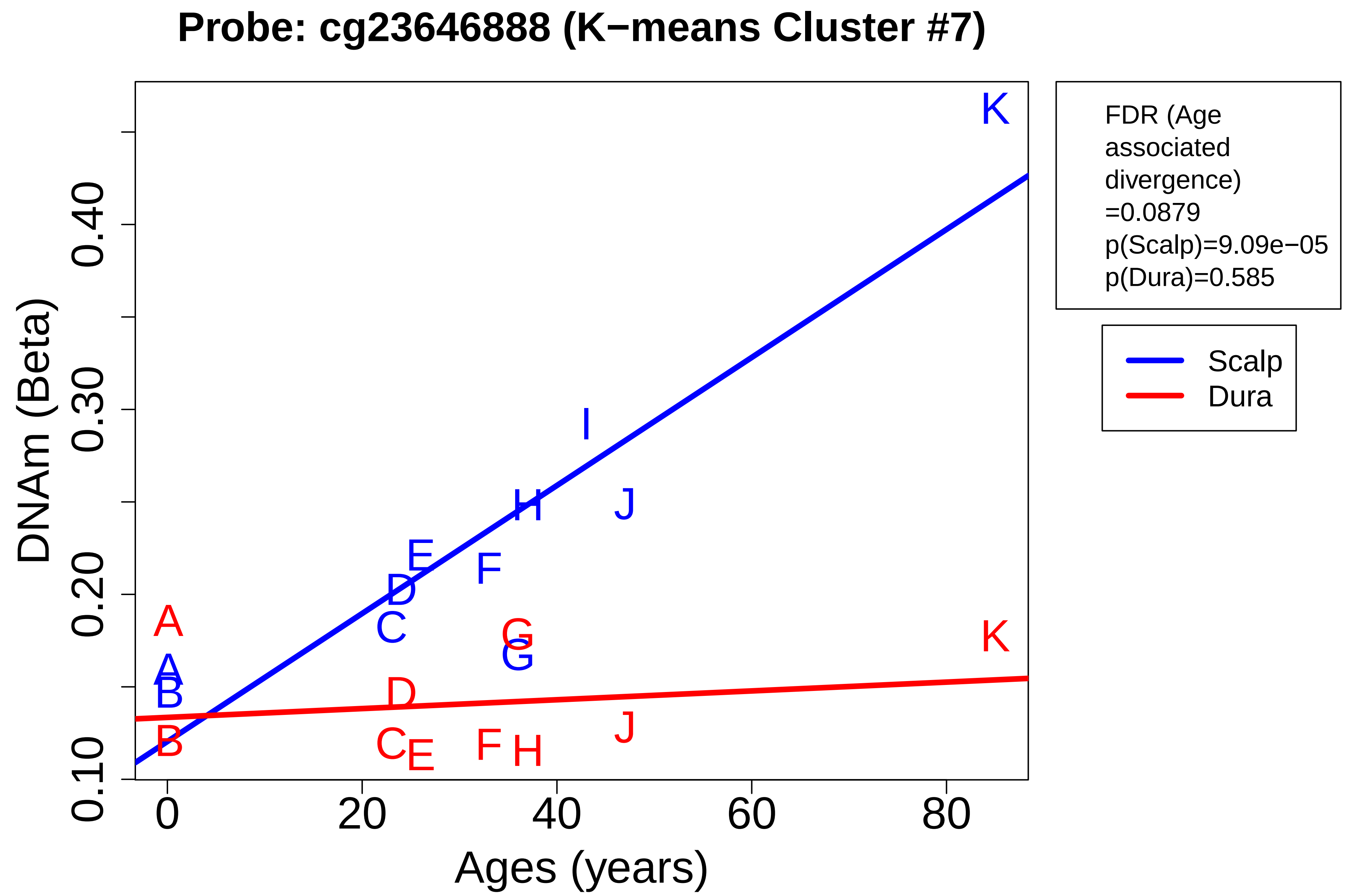

Probe: cg23646888 (K−means Cluster #7)
DNAm (Beta)
0.10
0.20
0.30
0.40
0
20
40
60
80
Ages (years)
A
B
C
D
E
F
G
H
I
J
K
FDR (Age associated divergence) =0.0879
p(Scalp)=9.09e−05
p(Dura)=0.585
Scalp
Dura

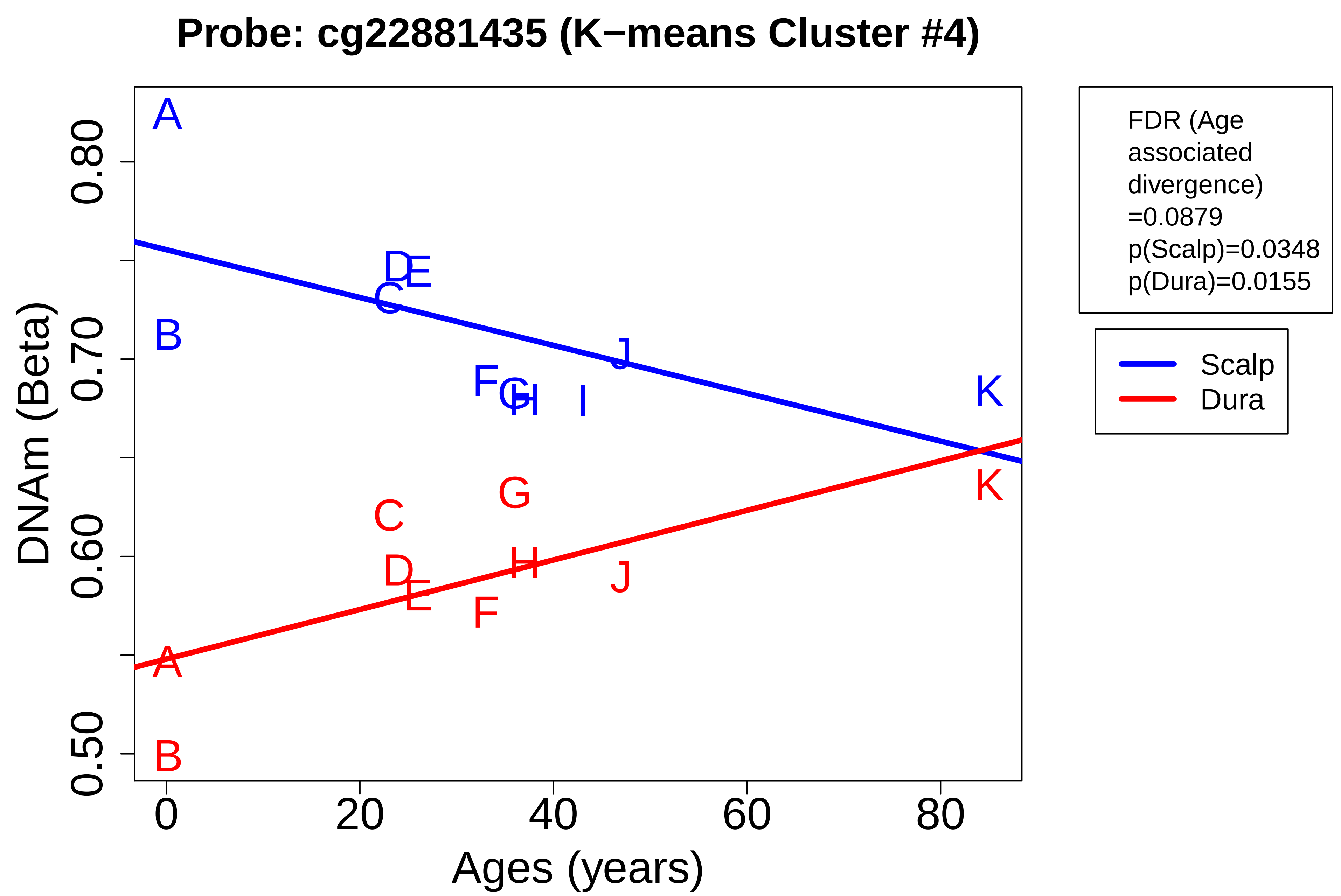

Probe: cg22881435 (K−means Cluster #4)
DNAm (Beta)
0.50
0.60
0.70
0.80
0
20
40
60
80
Ages (years)
FDR (Age associated divergence) =0.0879
p(Scalp)=0.0348
p(Dura)=0.0155
Scalp
Dura
A
B
C
D
E
F
G
H
I
J
K

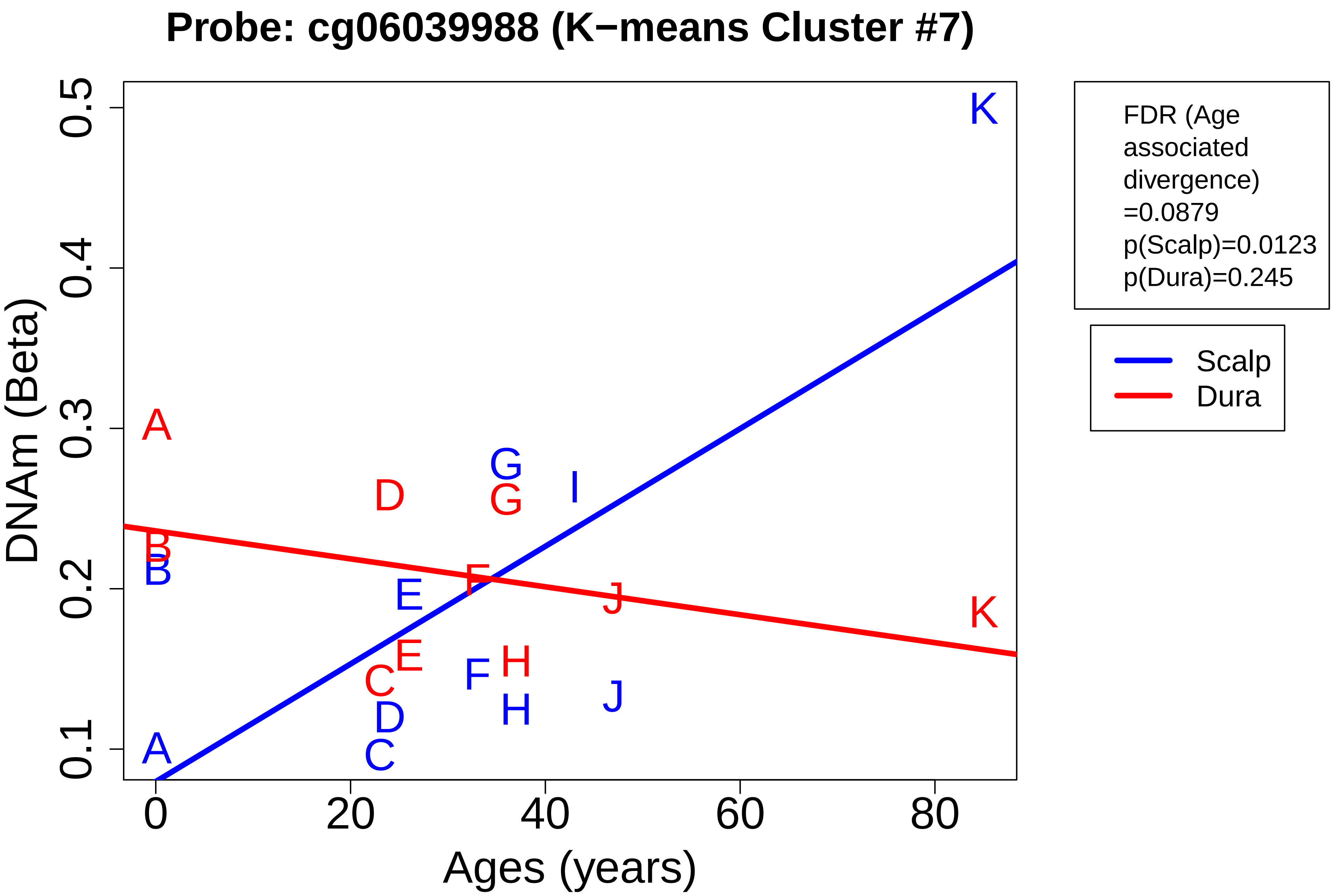
Probe: cg06039988 (K−means Cluster #7)
DNAm (Beta)
Ages (years)
0.1
0.2
0.3
0.4
0.5
0
20
40
60
80
FDR (Age associated divergence) =0.0879
p(Scalp)=0.0123
p(Dura)=0.245
Scalp
Dura
A
B
C
D
E
F
G
H
I
J
K

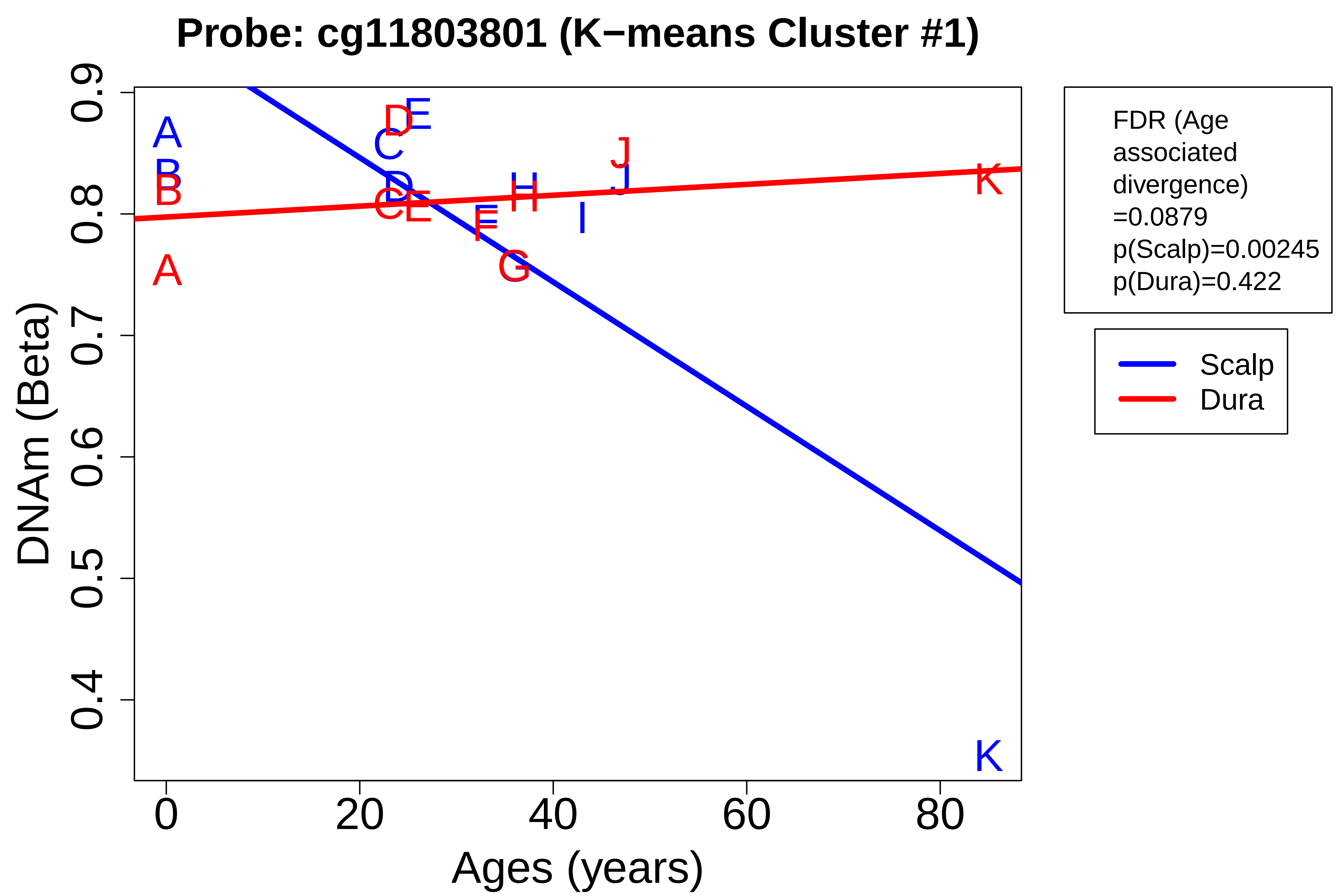

Probe: cg11803801 (K−means Cluster #1)
DNAm (Beta)
0.4
0.5
0.6
0.7
0.8
0.9
0
20
40
60
80
Ages (years)
A
B
C
D
E
F
G
H
I
J
K
FDR (Age
associated
divergence)
=0.0879
p(Scalp)=0.00245
p(Dura)=0.422
Scalp
Dura

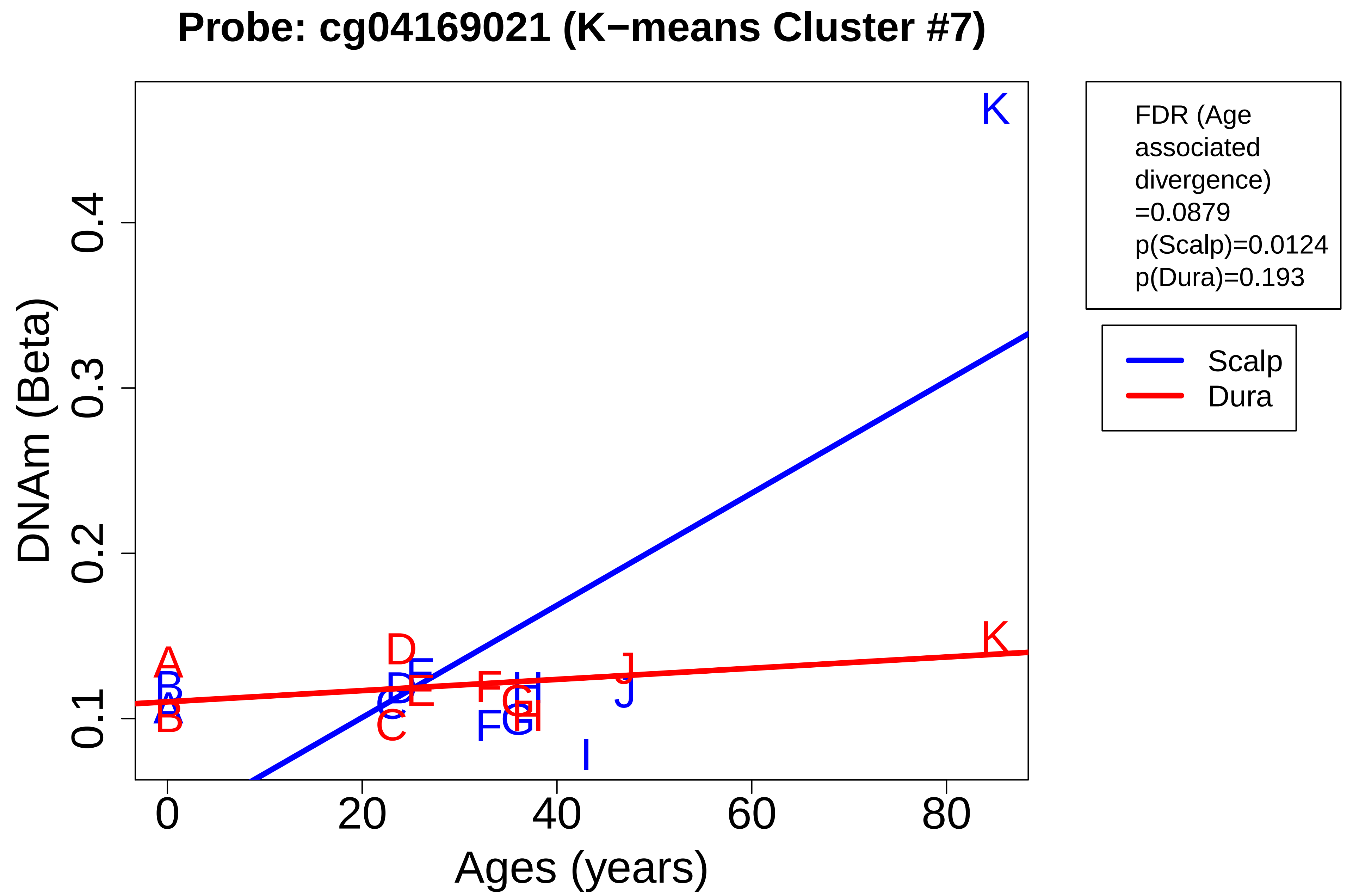

Probe: cg04169021 (K−means Cluster #7)
DNAm (Beta)
0.1
0.2
0.3
0.4
Ages (years)
0
20
40
60
80
FDR (Age associated divergence) =0.0879
p(Scalp)=0.0124
p(Dura)=0.193
Scalp
Dura

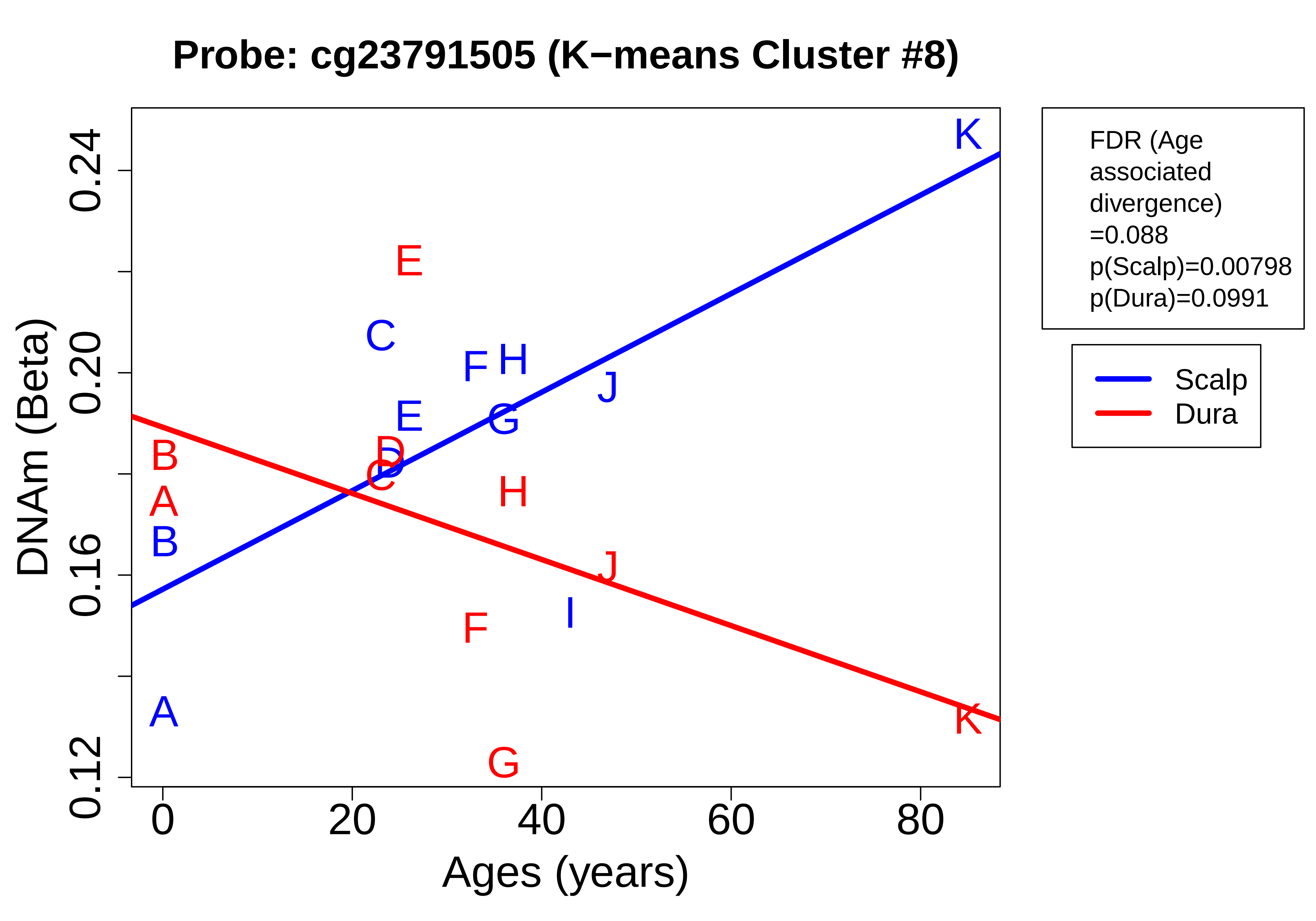
Probe: cg23791505 (K−means Cluster #8)
FDR (Age associated divergence) =0.088
p(Scalp)=0.00798
p(Dura)=0.0991
Scalp
Dura
DNAm (Beta)
0.12
0.16
0.20
0.24
0
20
40
60
80
Ages (years)
A
B
C
D
E
F
G
H
I
J
K

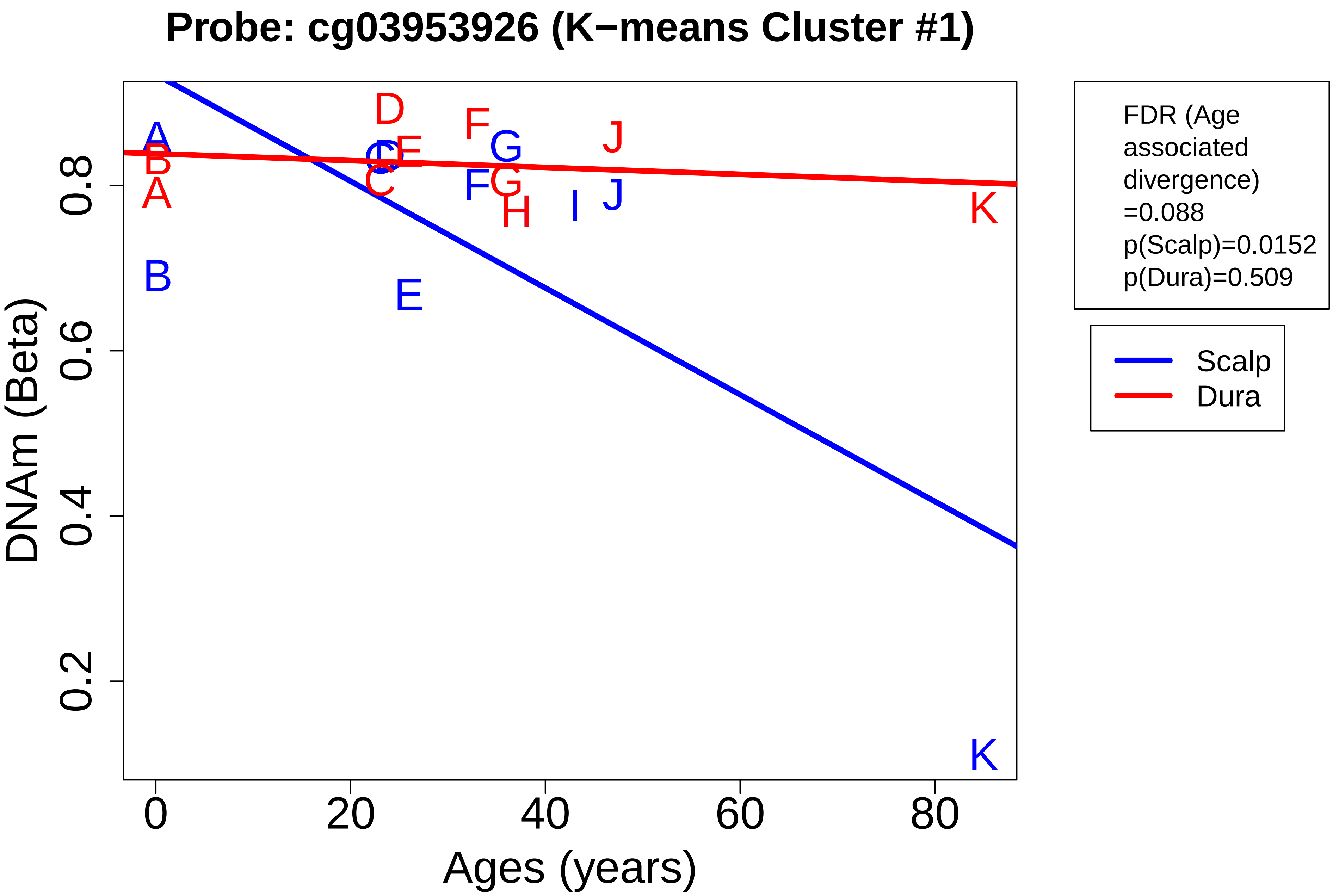
Probe: cg03953926 (K−means Cluster #1)
DNAm (Beta)
0.2
0.4
0.6
0.8
0
20
40
60
80
Ages (years)
FDR (Age associated divergence) =0.088
p(Scalp)=0.0152
p(Dura)=0.509
Scalp
Dura

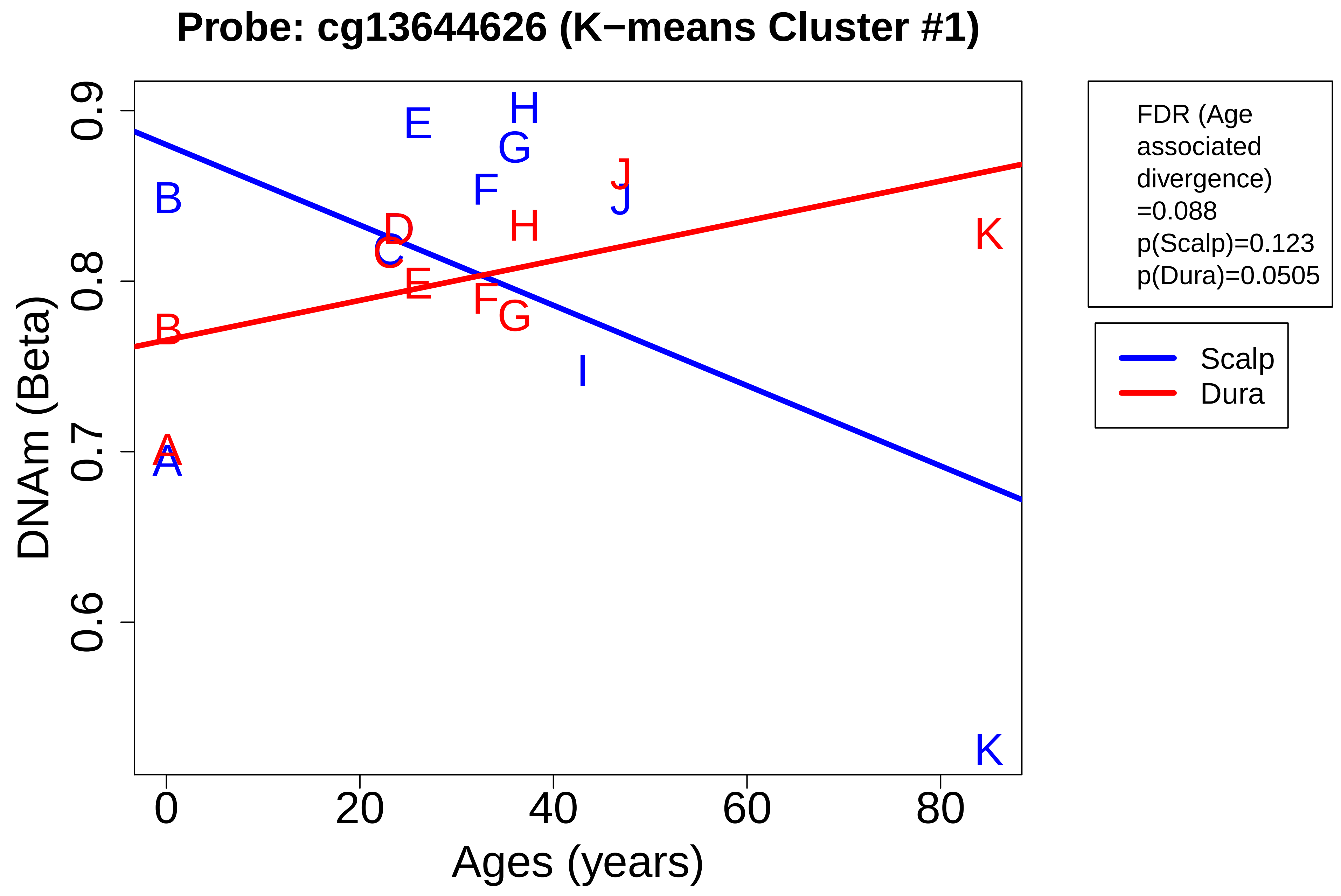
Probe: cg13644626 (K−means Cluster #1)
DNAm (Beta)
0.6
0.7
0.8
0.9
0
20
40
60
80
Ages (years)
A
B
C
D
E
F
G
H
I
J
K
FDR (Age associated divergence) =0.088
p(Scalp)=0.123
p(Dura)=0.0505
Scalp
Dura

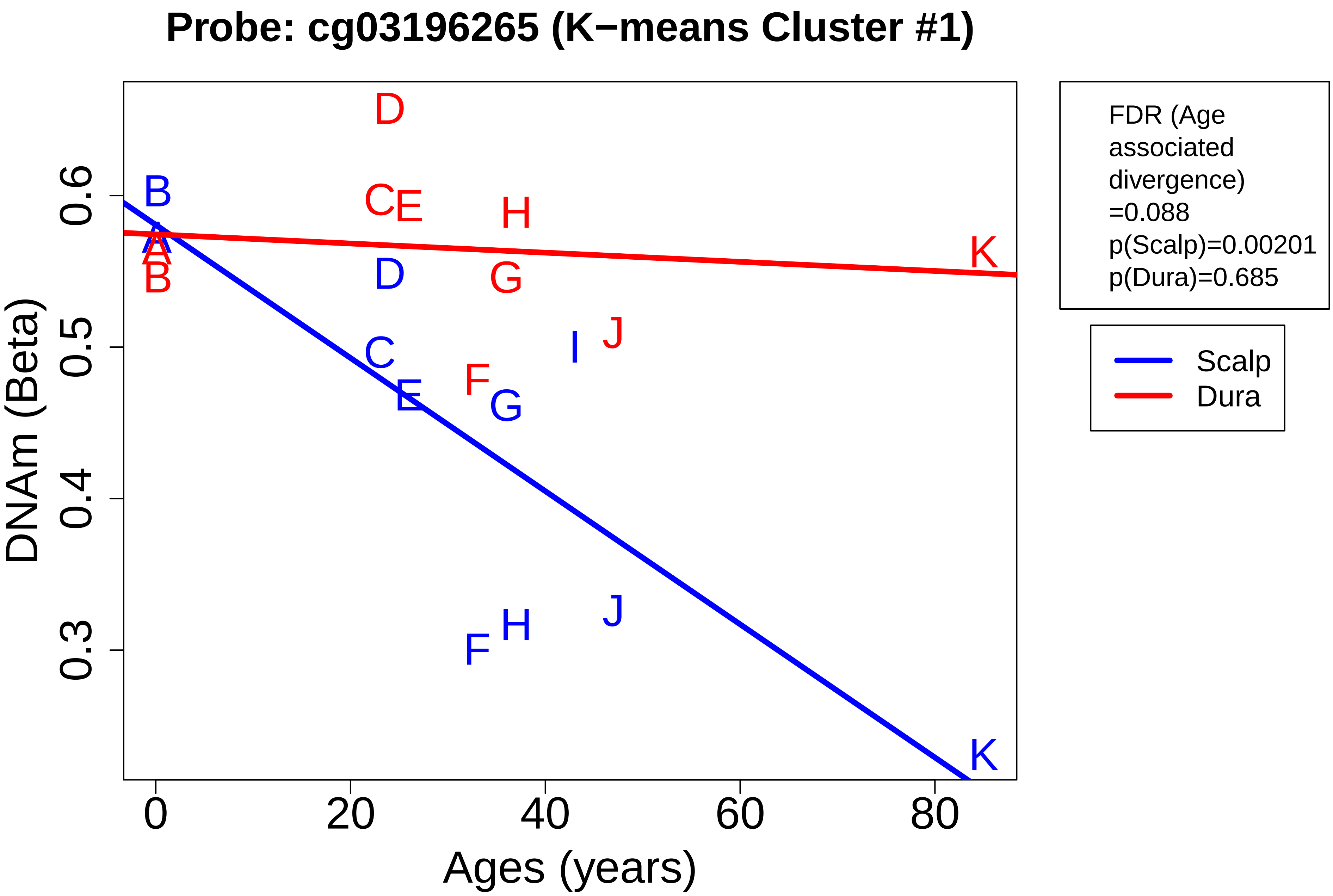

Probe: cg03196265 (K−means Cluster #1)
FDR (Age associated divergence) =0.088
p(Scalp)=0.00201
p(Dura)=0.685
Scalp
Dura
DNAm (Beta)
0.3
0.4
0.5
0.6
0
20
40
60
80
Ages (years)

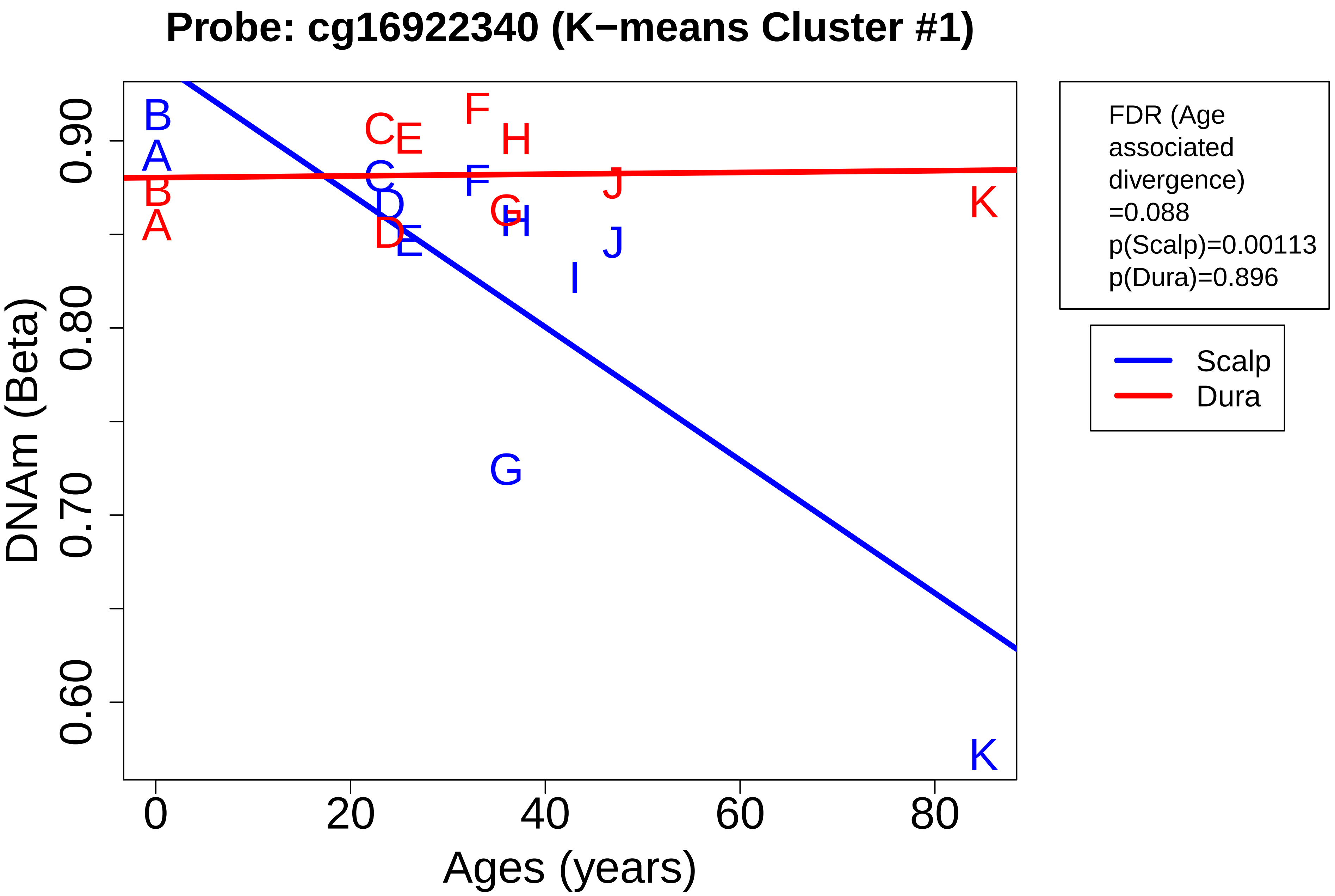

Probe: cg16922340 (K−means Cluster #1)
DNAm (Beta)
0.60
0.70
0.80
0.90
0
20
40
60
80
Ages (years)
A B C D E F G H I J K
FDR (Age associated divergence) =0.088
p(Scalp)=0.00113
p(Dura)=0.896
Scalp
Dura

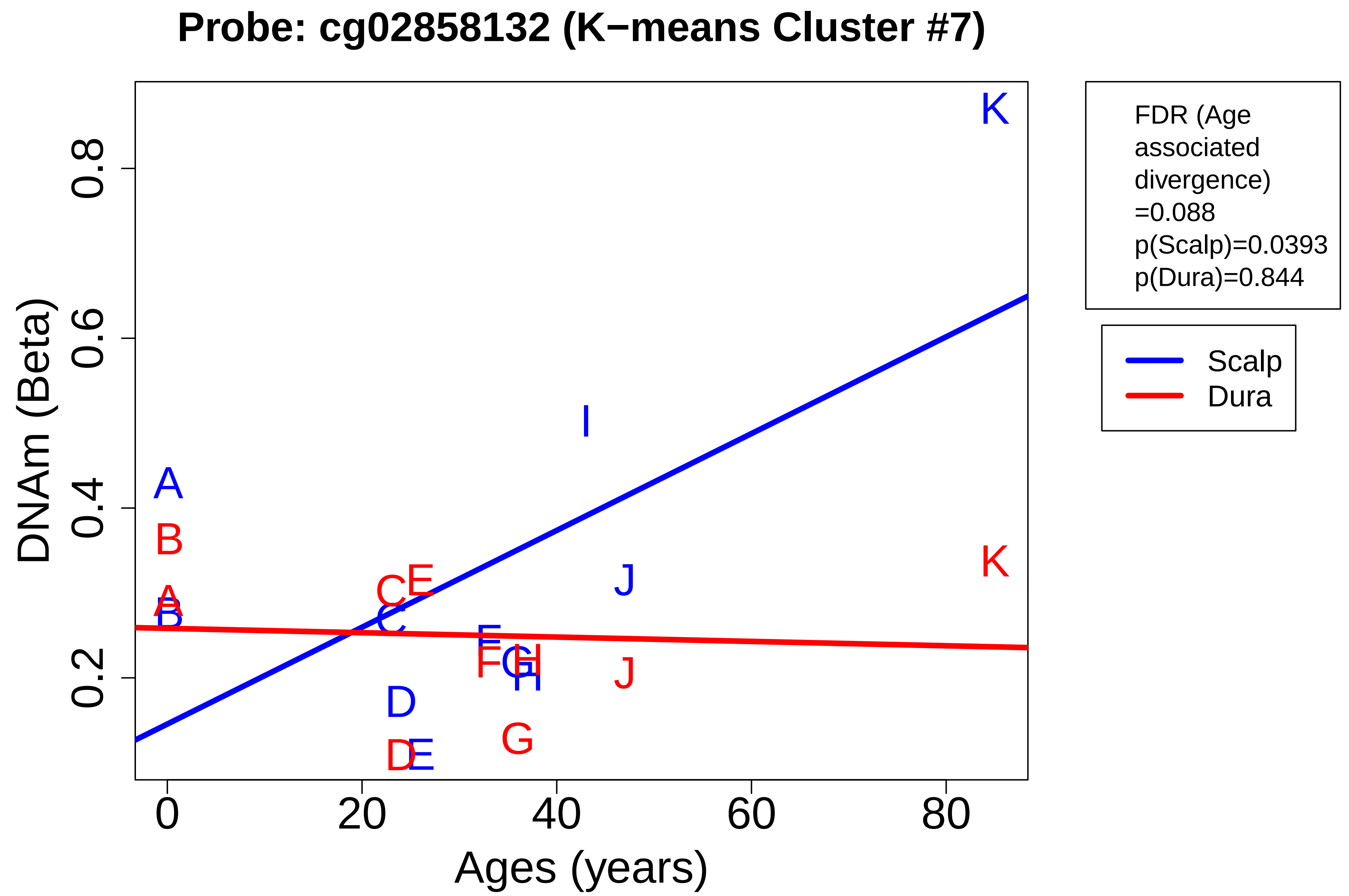

Probe: cg02858132 (K−means Cluster #7)
DNAm (Beta)
0.2
0.4
0.6
0.8
Ages (years)
0
20
40
60
80
FDR (Age associated divergence) =0.088
p(Scalp)=0.0393
p(Dura)=0.844
Scalp
Dura

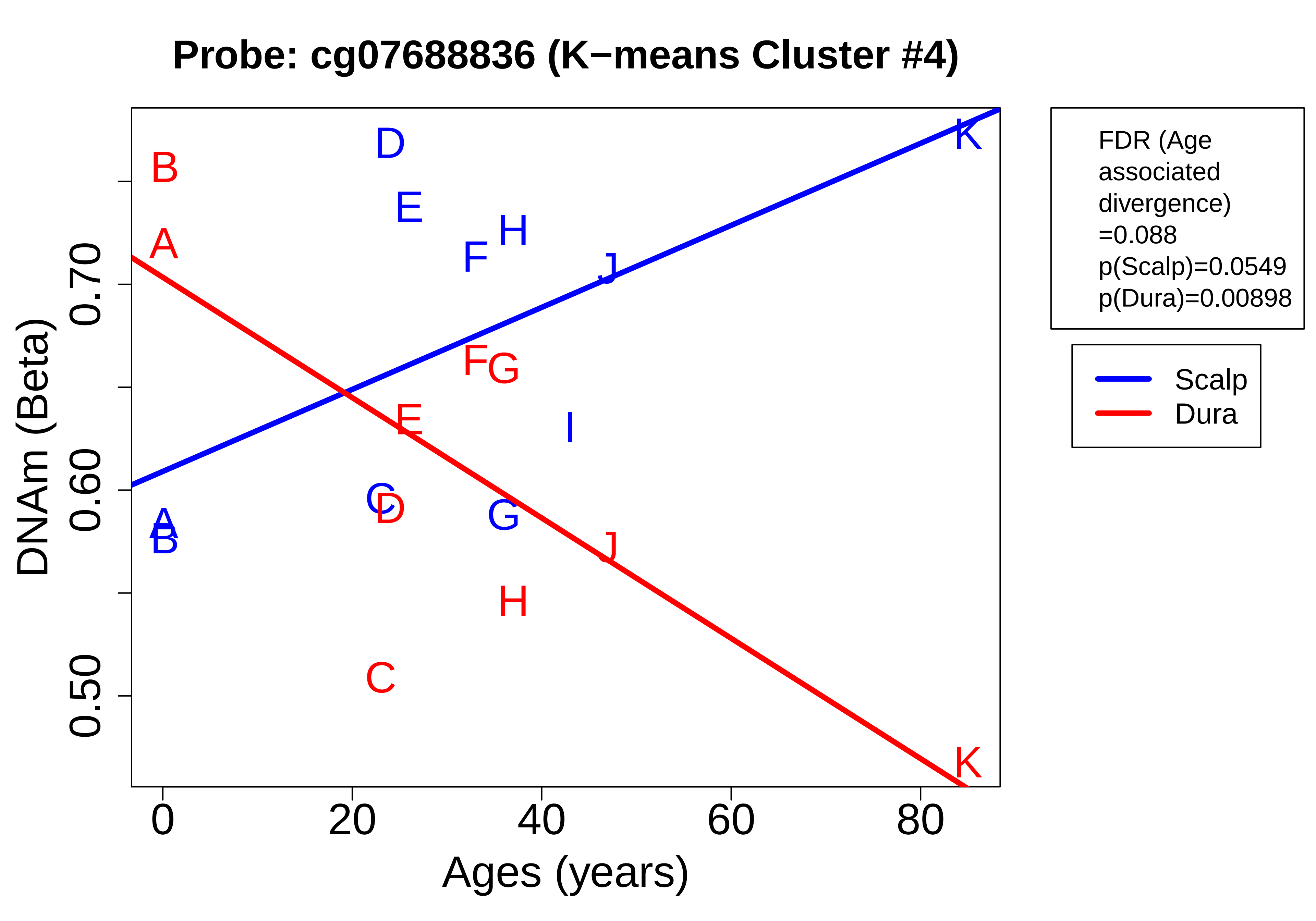

Probe: cg07688836 (K−means Cluster #4)
DNAm (Beta)
0.50
0.60
0.70
Ages (years)
0
20
40
60
80
FDR (Age associated divergence) =0.088
p(Scalp)=0.0549
p(Dura)=0.00898
Scalp
Dura

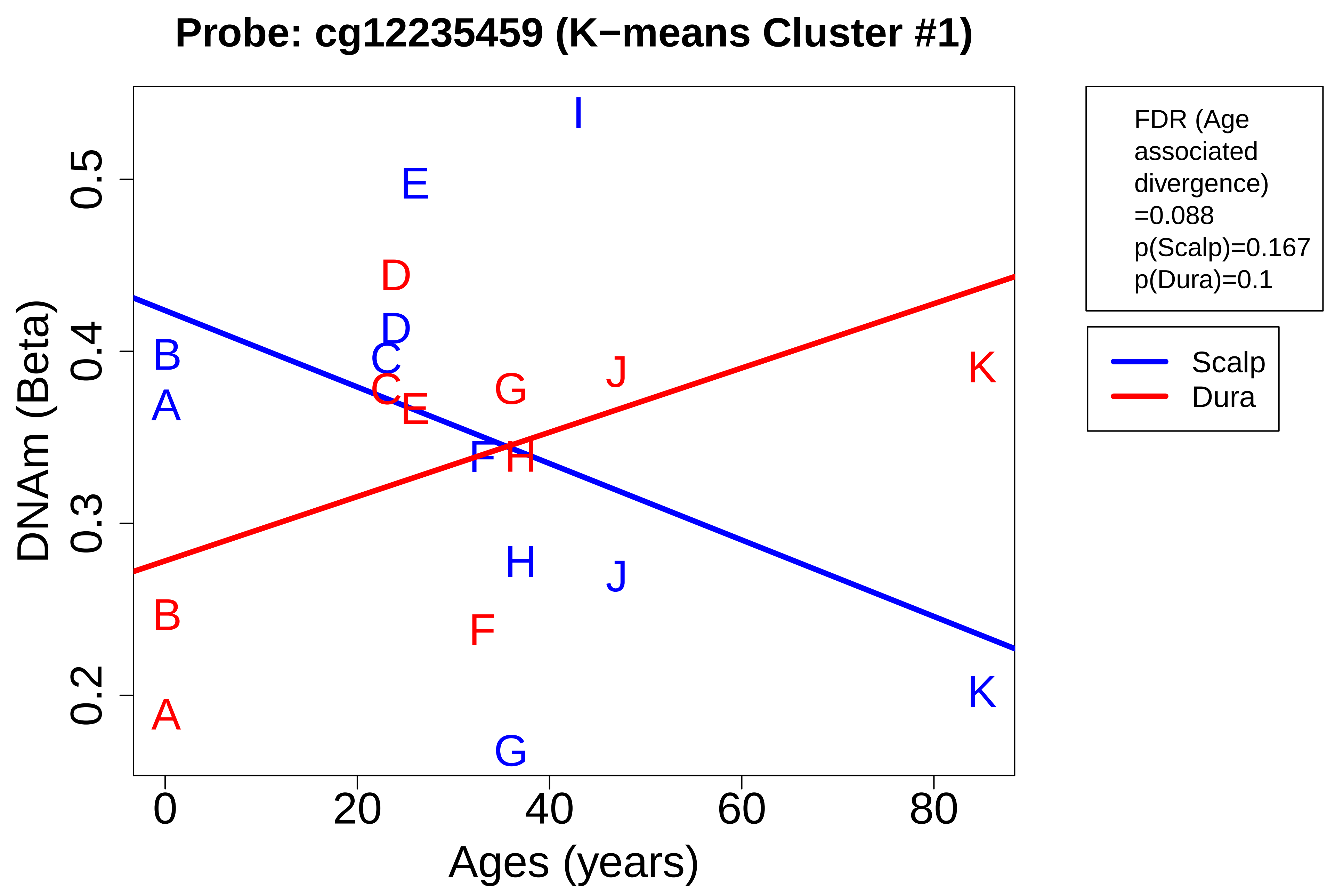

Probe: cg12235459 (K−means Cluster #1)
FDR (Age associated divergence) =0.088
p(Scalp)=0.167
p(Dura)=0.1
Scalp
Dura
DNAm (Beta)
Ages (years)
0.2
0.3
0.4
0.5
0
20
40
60
80

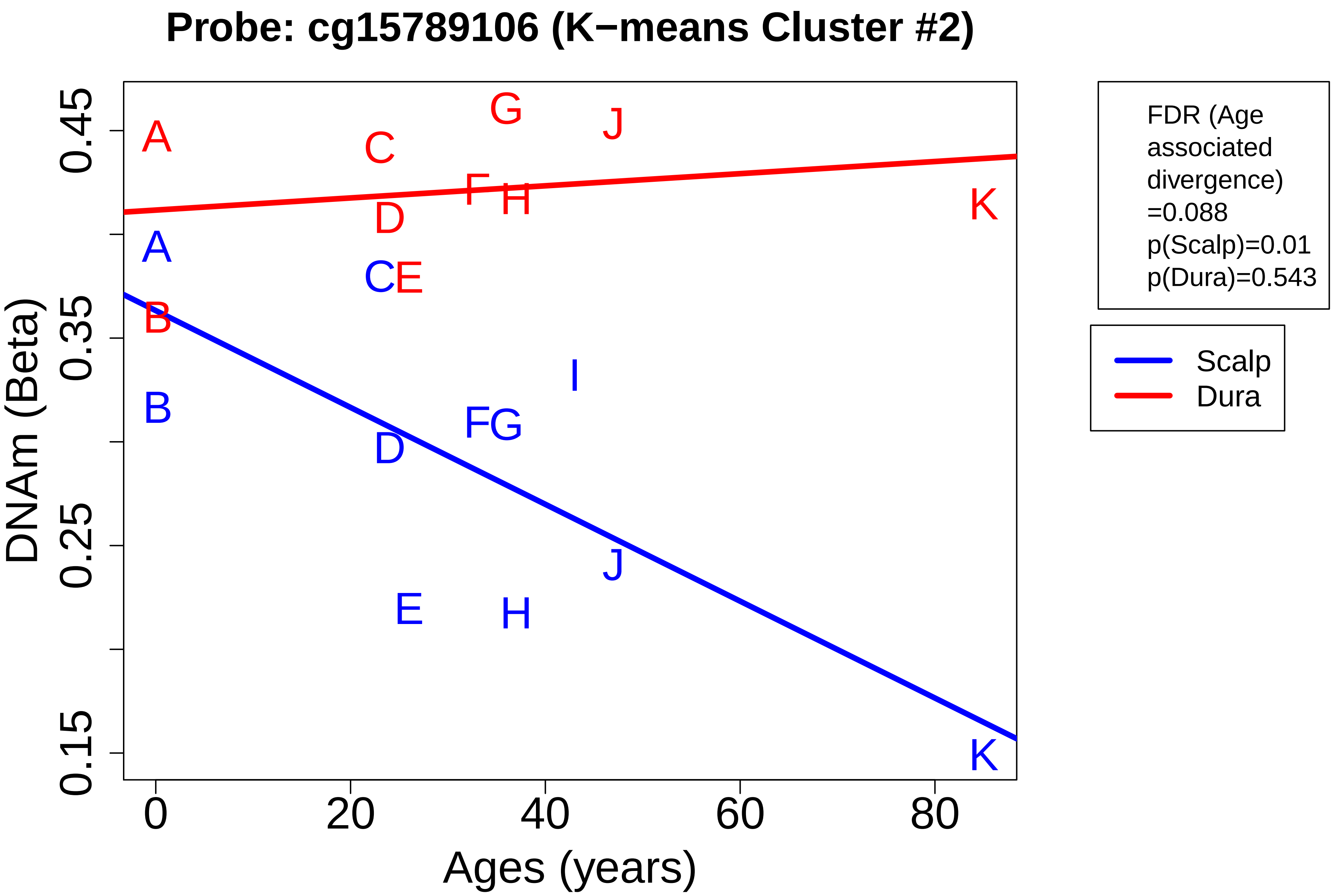

Probe: cg15789106 (K−means Cluster #2)
DNAm (Beta)
0.15
0.25
0.35
0.45
0
20
40
60
80
Ages (years)
A
B
C
D
E
F
G
H
I
J
K
FDR (Age associated divergence) =0.088
p(Scalp)=0.01
p(Dura)=0.543
Scalp
Dura

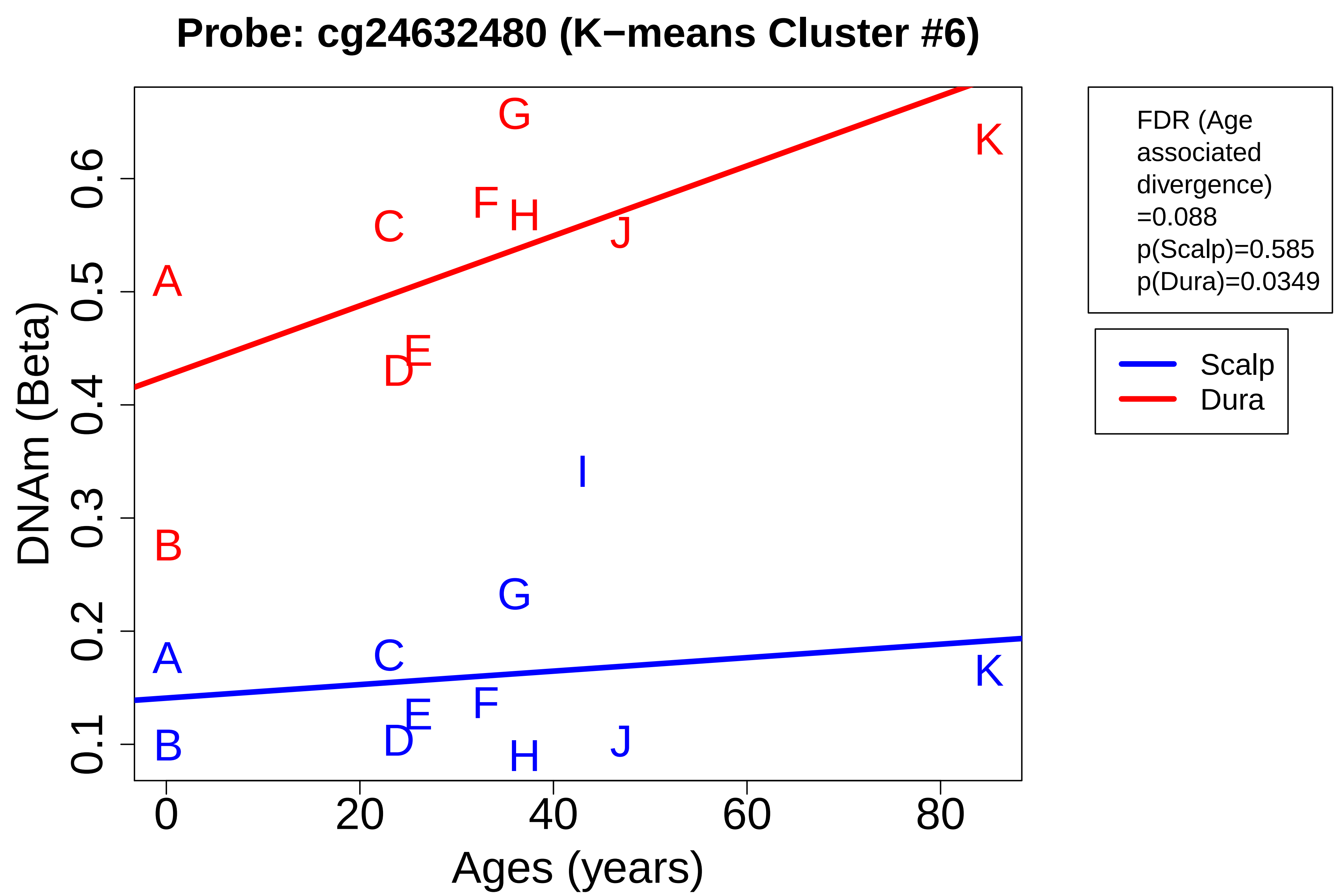

Probe: cg24632480 (K−means Cluster #6)
DNAm (Beta)
0.1
0.2
0.3
0.4
0.5
0.6
0
20
40
60
80
Ages (years)
FDR (Age associated divergence) =0.088
p(Scalp)=0.585
p(Dura)=0.0349
Scalp
Dura
A
B
C
D
E
F
G
H
I
J
K

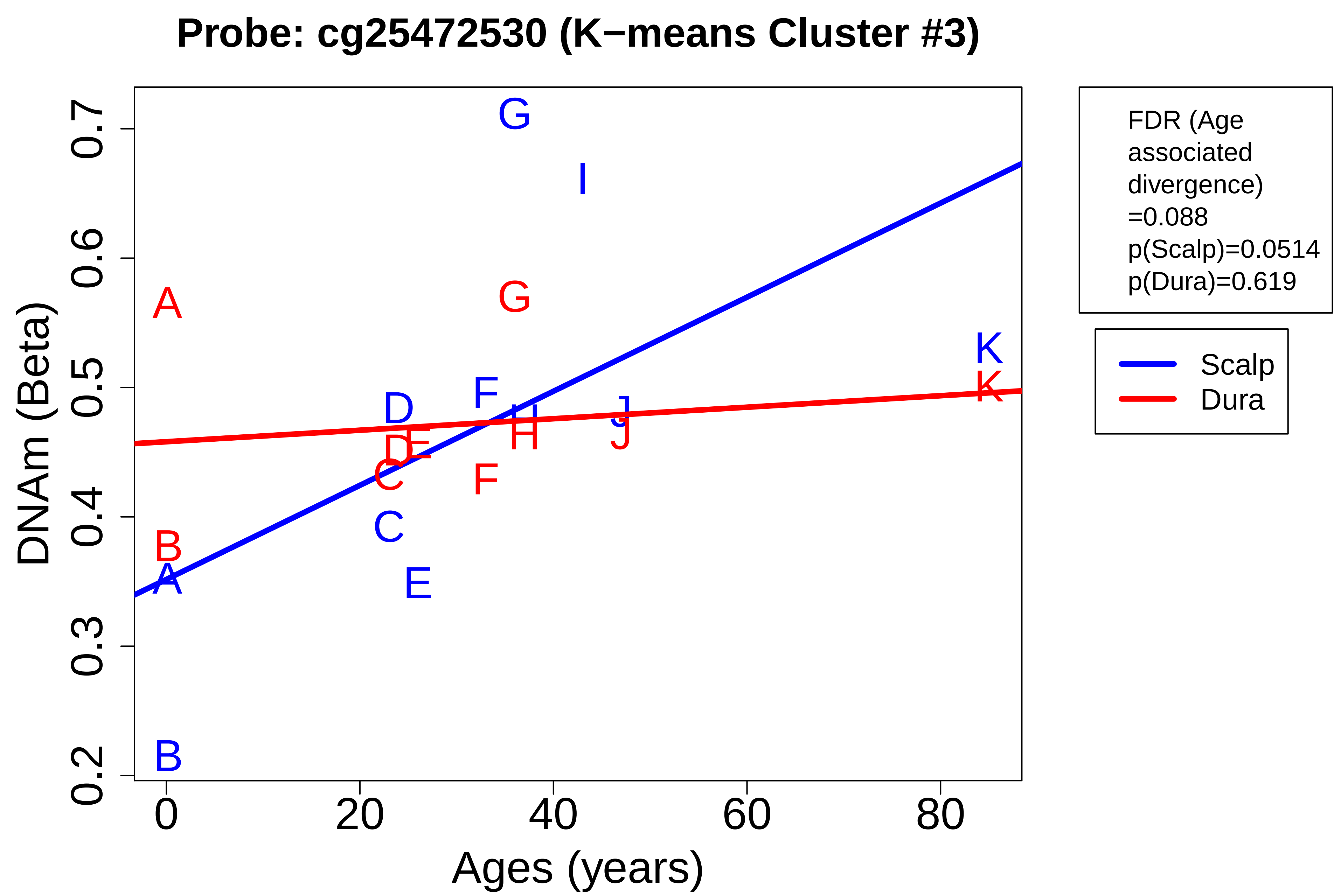

Probe: cg25472530 (K−means Cluster #3)
DNAm (Beta)
Ages (years)
0.2
0.3
0.4
0.5
0.6
0.7
0
20
40
60
80
FDR (Age associated divergence) =0.088
p(Scalp)=0.0514
p(Dura)=0.619
Scalp
Dura

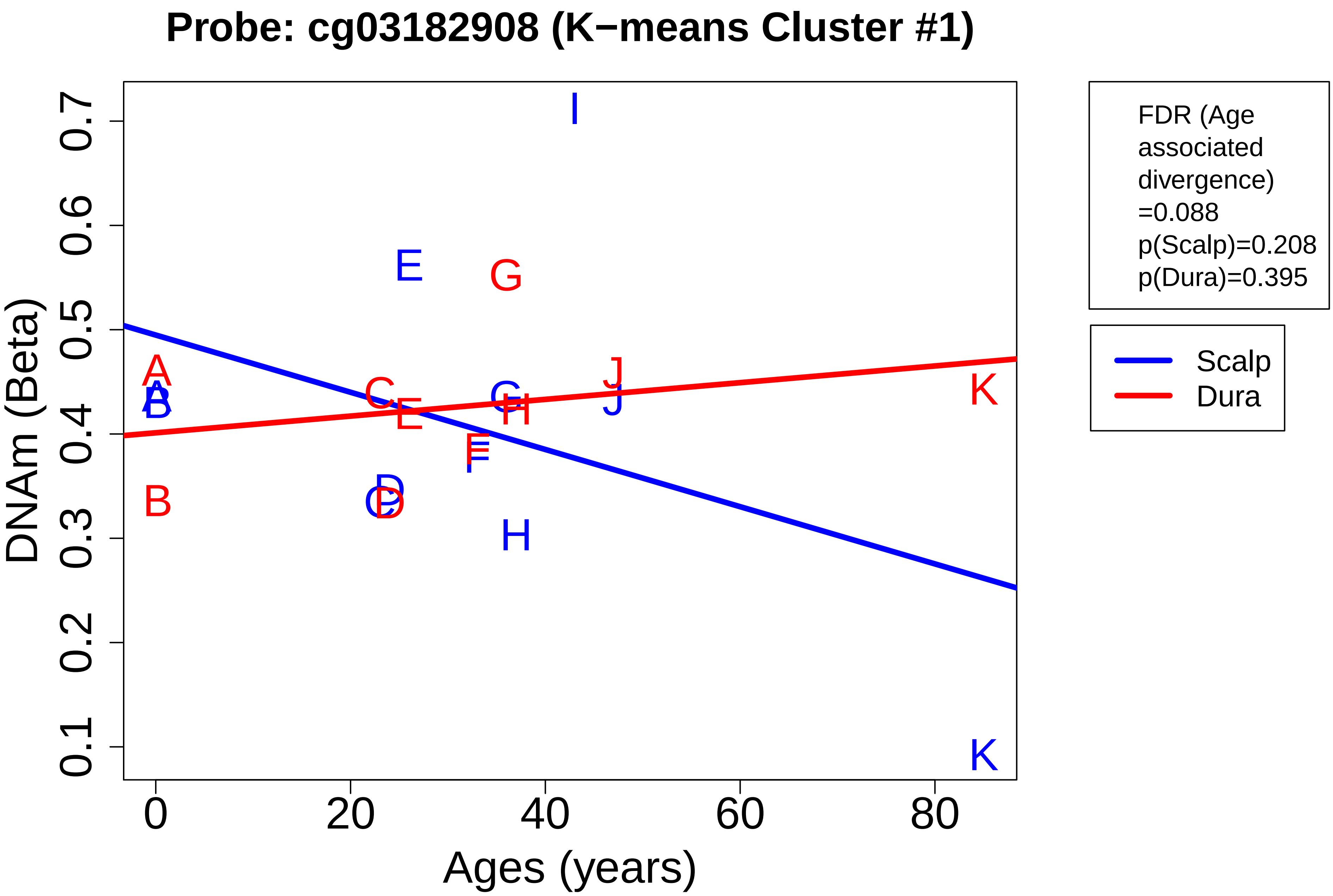

Probe: cg03182908 (K−means Cluster #1)
DNAm (Beta)
0.1
0.2
0.3
0.4
0.5
0.6
0.7
0
20
40
60
80
Ages (years)
FDR (Age associated divergence) =0.088
p(Scalp)=0.208
p(Dura)=0.395
Scalp
Dura

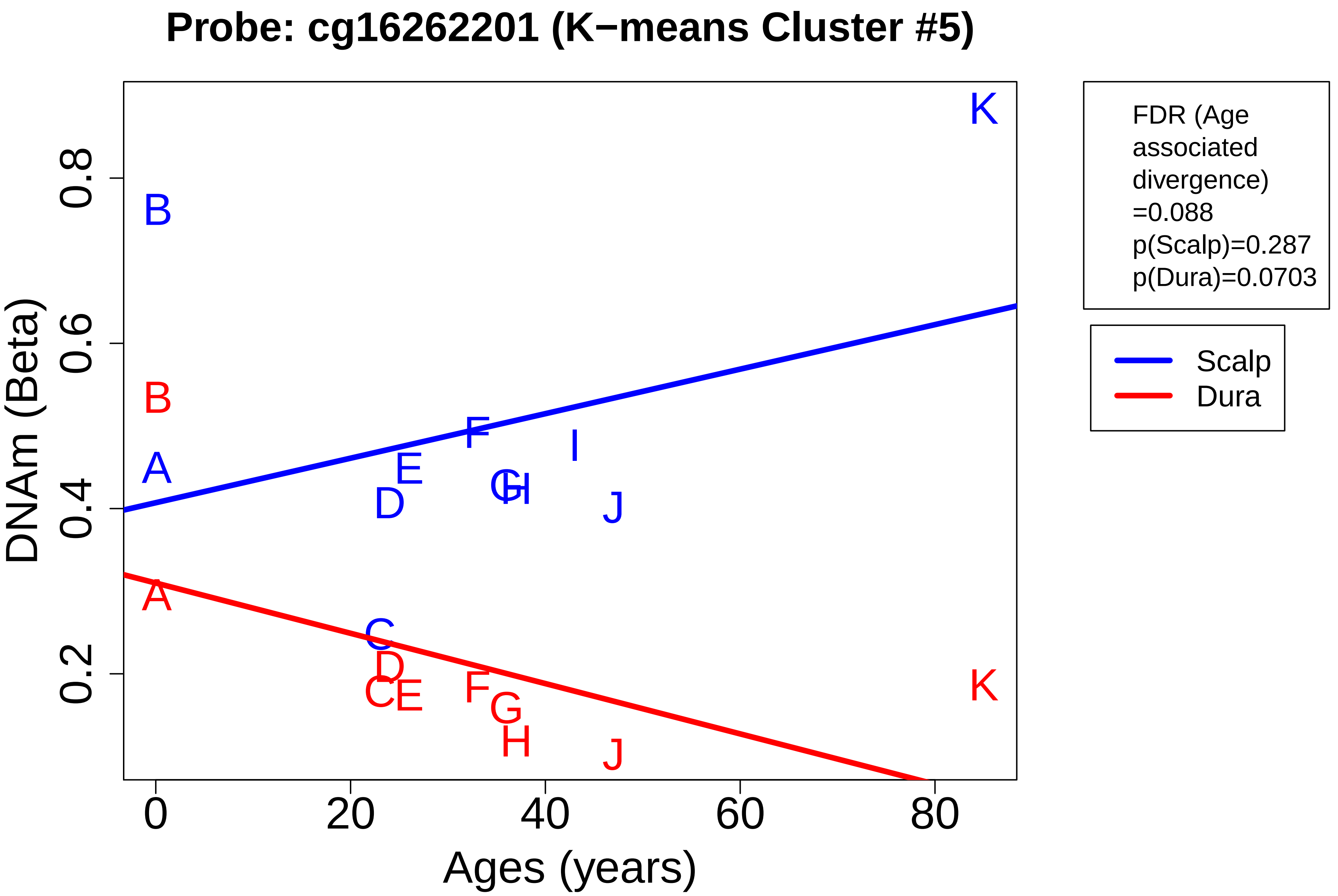
Probe: cg16262201 (K−means Cluster #5)
FDR (Age associated divergence) =0.088
p(Scalp)=0.287
p(Dura)=0.0703
Scalp
Dura
DNAm (Beta)
Ages (years)
0.2
0.4
0.6
0.8
0
20
40
60
80
A
B
C
D
E
F
G
H
I
J
K

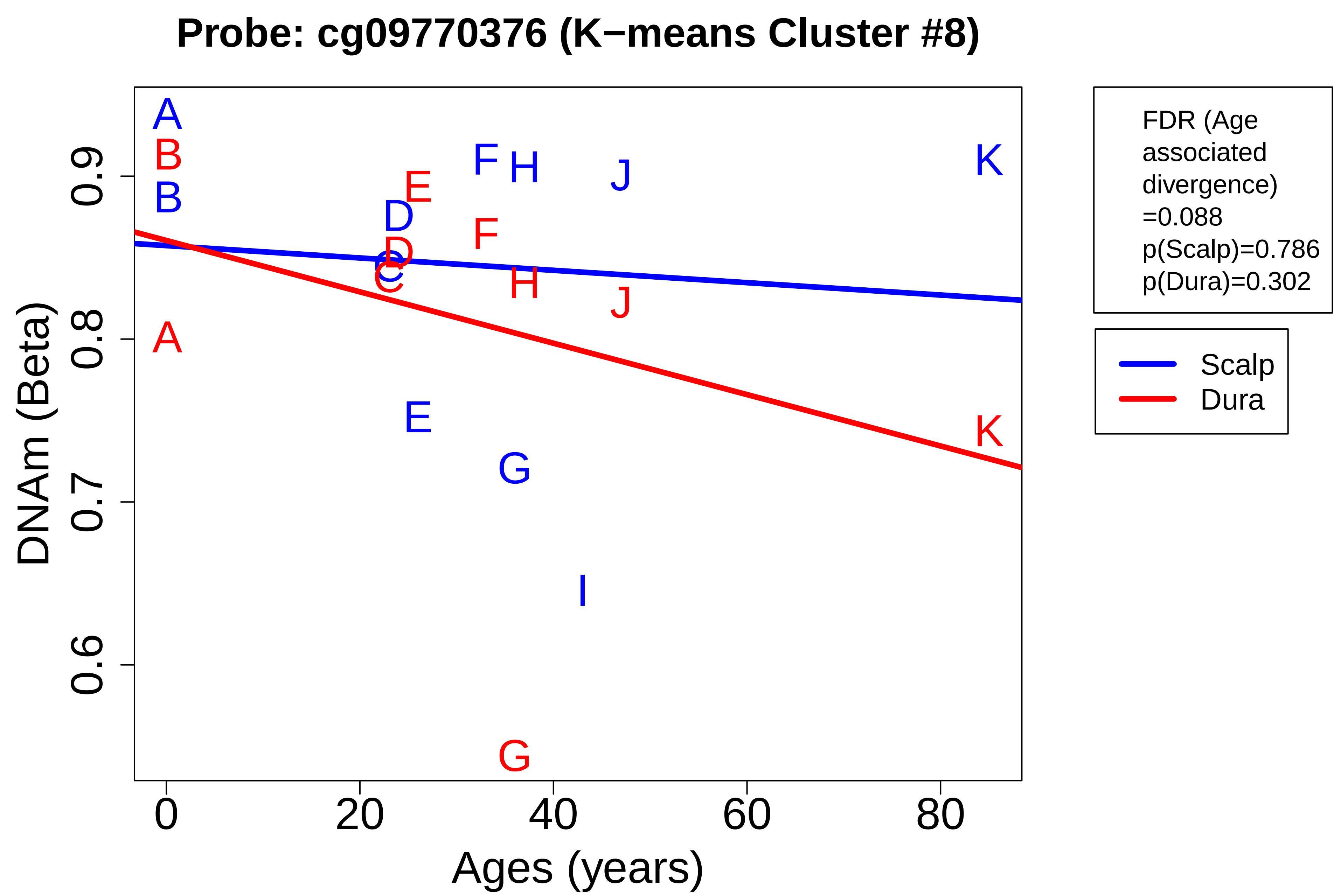

Probe: cg09770376 (K−means Cluster #8)
DNAm (Beta)
Ages (years)
0.6
0.7
0.8
0.9
0
20
40
60
80
A
B
B
A
C
C
D
D
E
E
F
F
G
G
H
H
I
J
J
K
K
FDR (Age associated divergence) =0.088
p(Scalp)=0.786
p(Dura)=0.302
Scalp
Dura

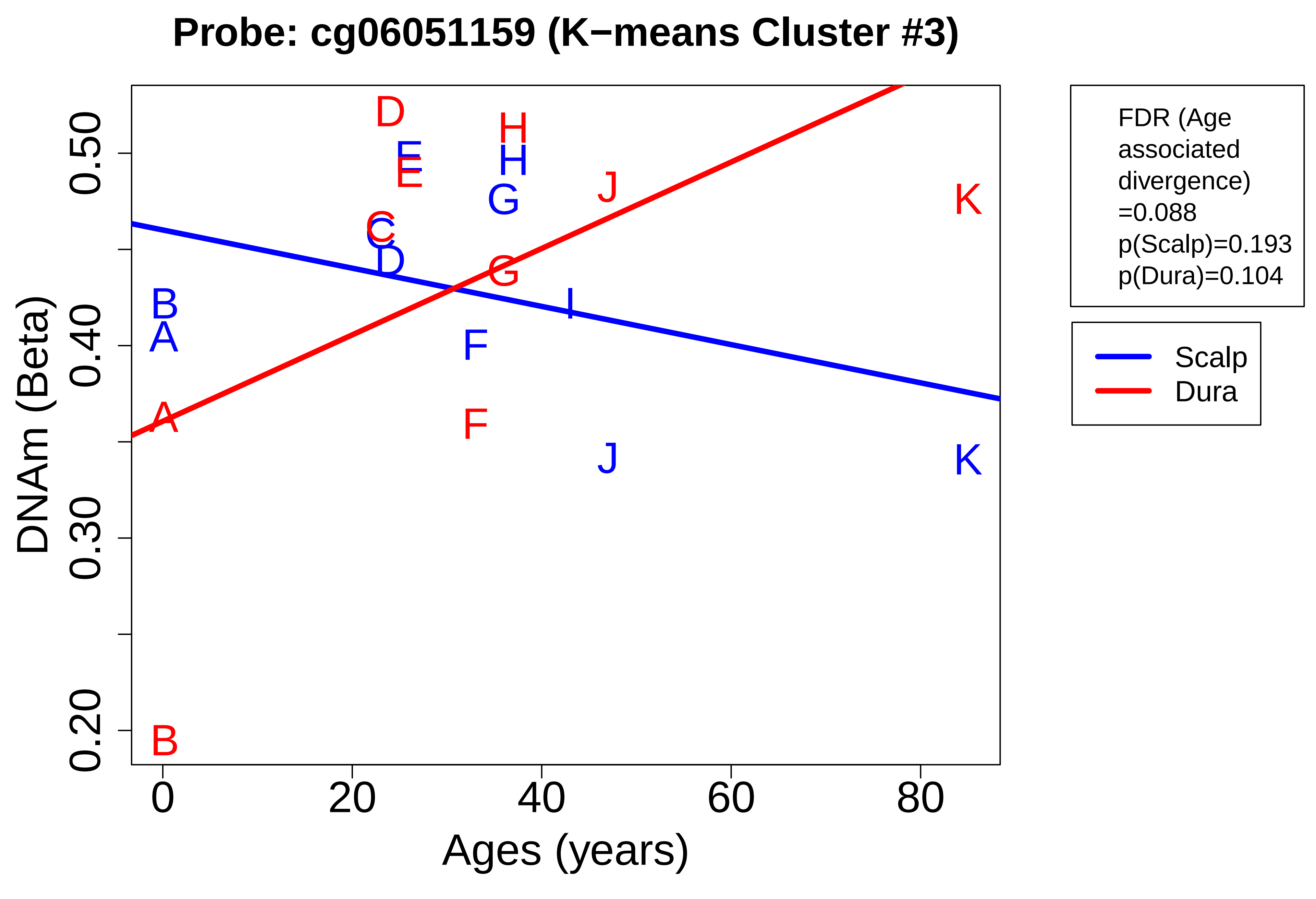
Probe: cg06051159 (K−means Cluster #3)
DNAm (Beta)
0.20
0.30
0.40
0.50
0
20
40
60
80
Ages (years)
FDR (Age associated divergence) =0.088
p(Scalp)=0.193
p(Dura)=0.104
Scalp
Dura
A
B
C
D
E
F
G
H
I
J
K

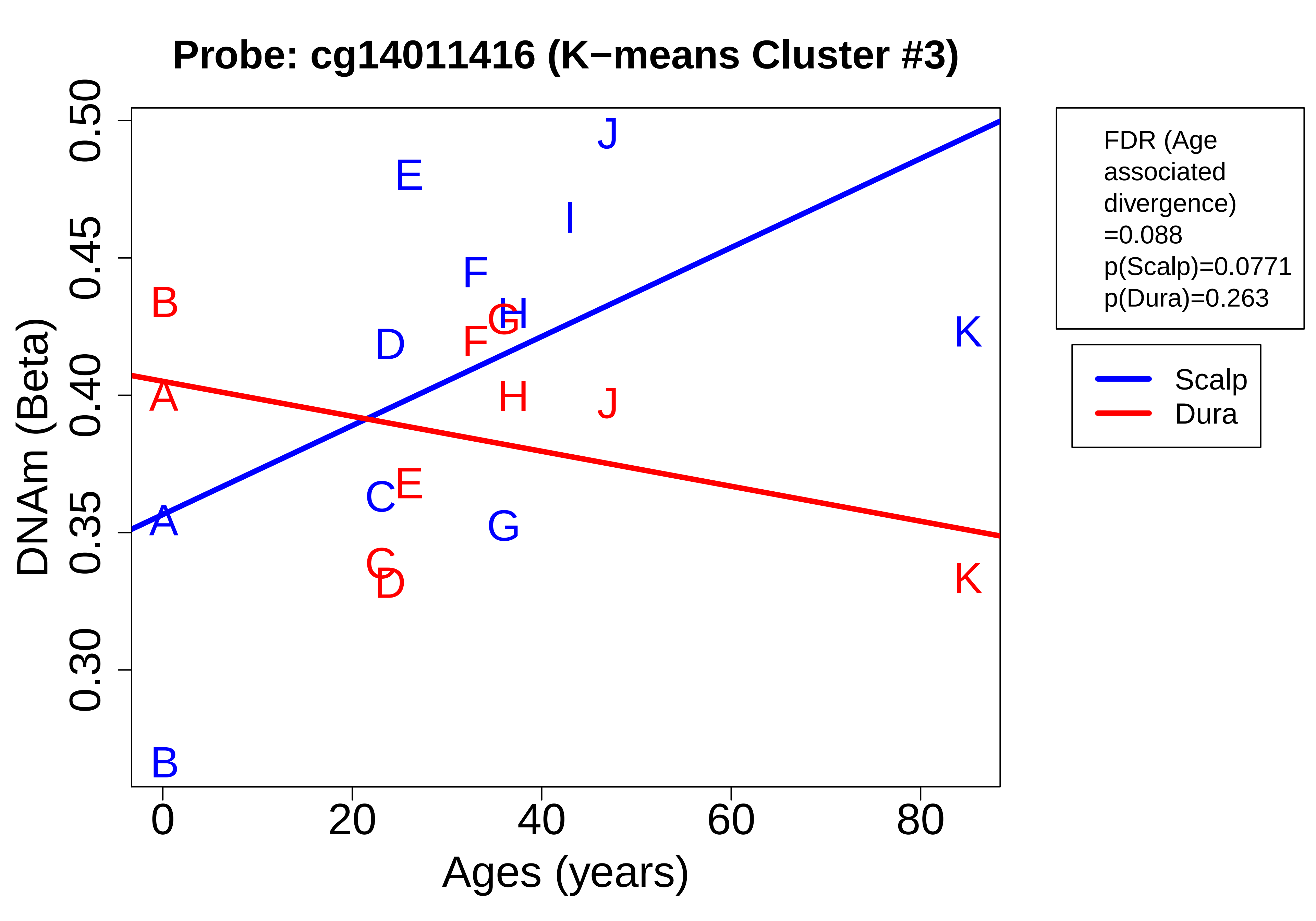

Probe: cg14011416 (K−means Cluster #3)
DNAm (Beta)
0.30
0.35
0.40
0.45
0.50
0
20
40
60
80
Ages (years)
FDR (Age associated divergence) =0.088
p(Scalp)=0.0771
p(Dura)=0.263
Scalp
Dura

Probe: cg10179300 (K−means Cluster #1)

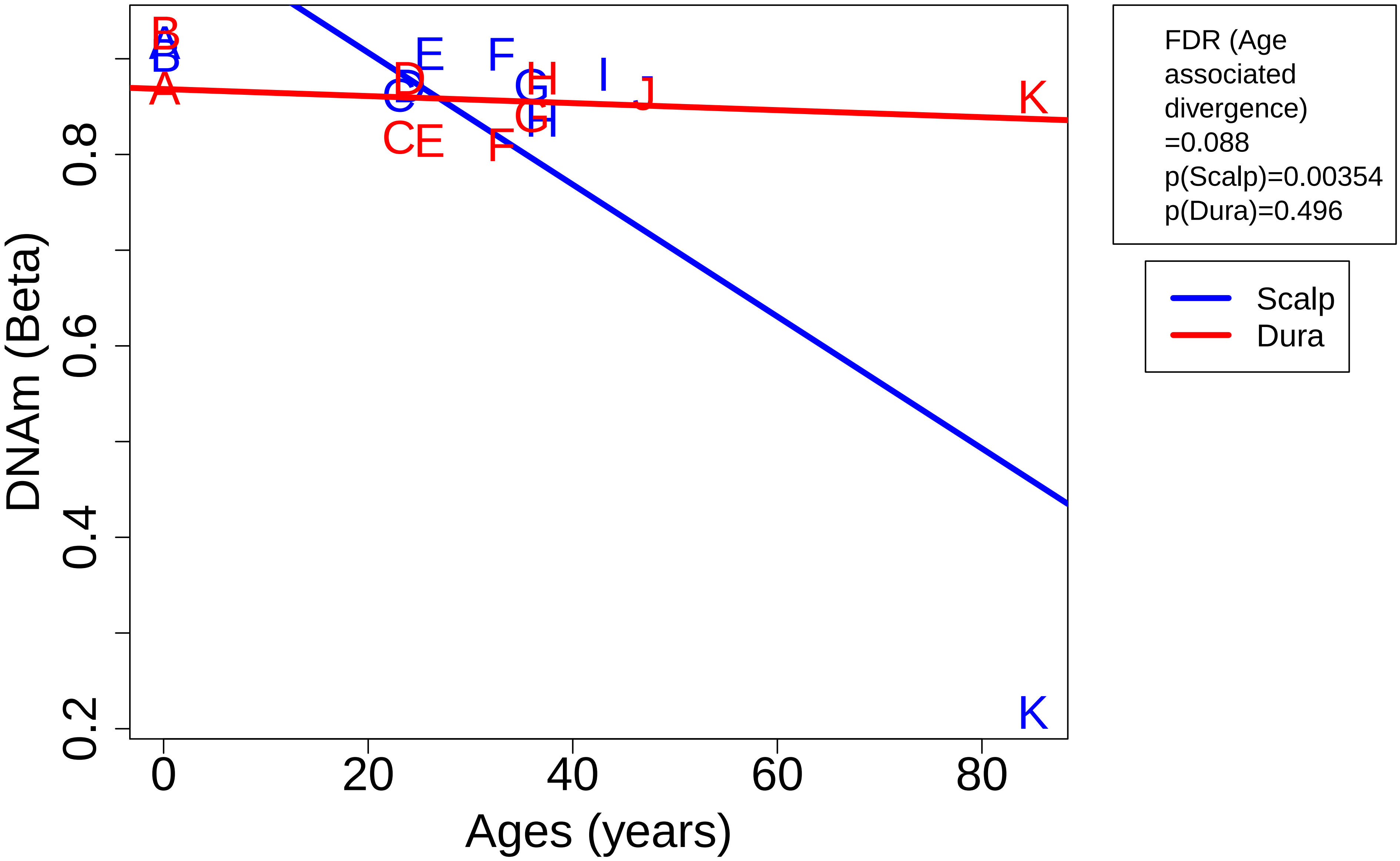

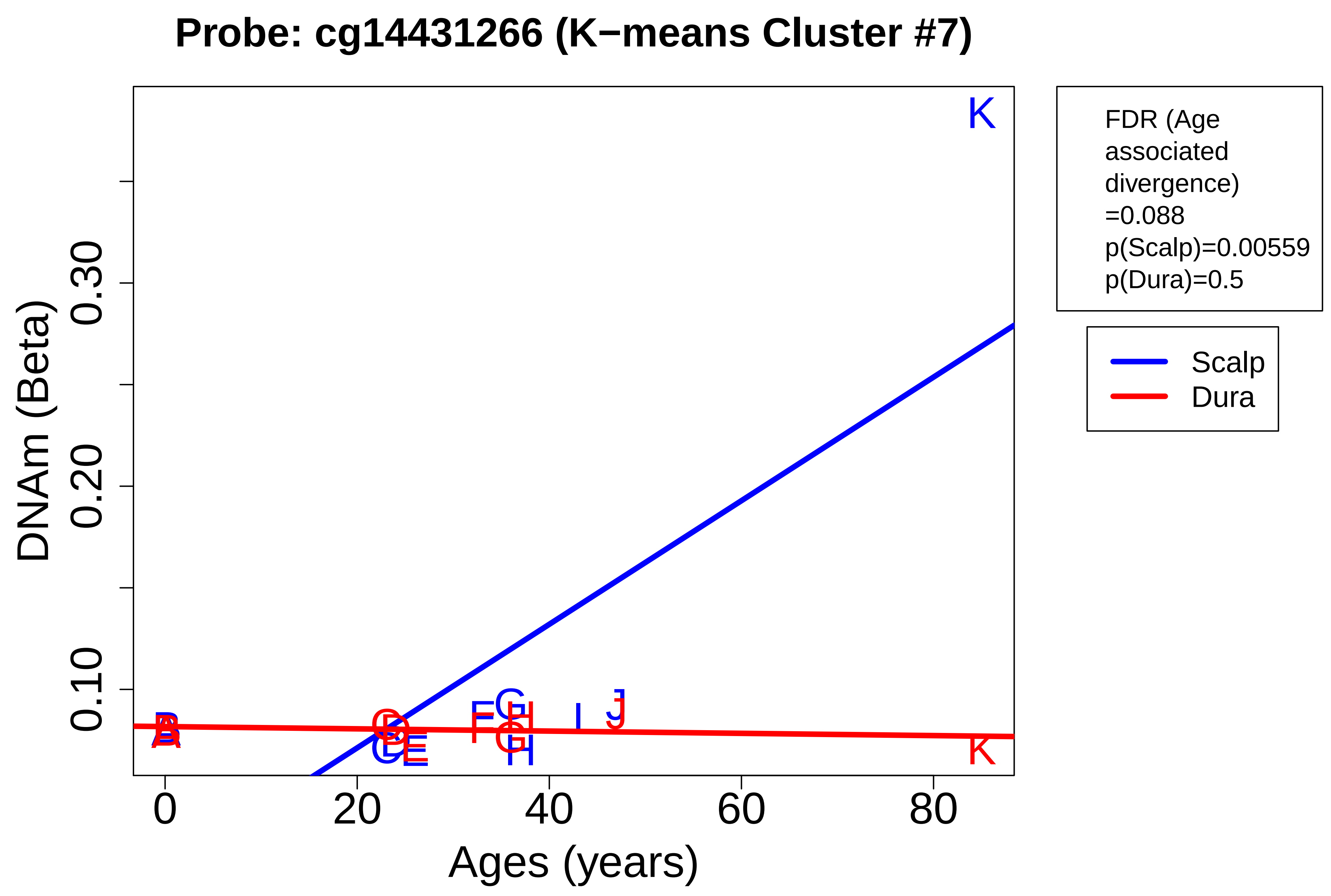

Probe: cg14431266 (K−means Cluster #7)
DNAm (Beta)
0.10
0.20
0.30
0
20
40
60
80
Ages (years)
FDR (Age associated divergence) =0.088
p(Scalp)=0.00559
p(Dura)=0.5
Scalp
Dura

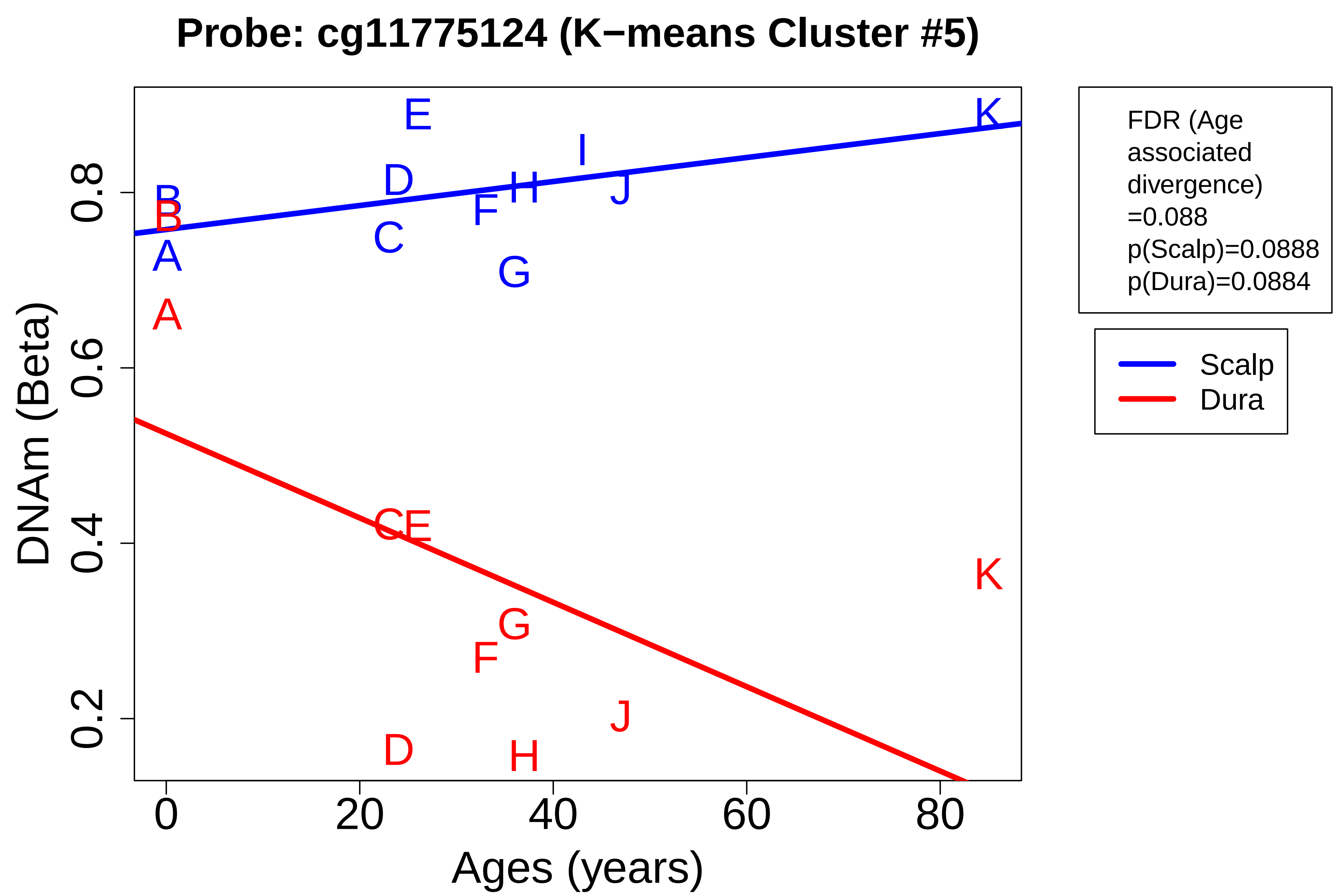

Probe: cg11775124 (K−means Cluster #5)
DNAm (Beta)
0.2
0.4
0.6
0.8
0
20
40
60
80
Ages (years)
A
B
C
D
E
F
G
H
I
J
K
A
B
CE
D
F
G
H
J
K
FDR (Age associated divergence) =0.088
p(Scalp)=0.0888
p(Dura)=0.0884
Scalp
Dura

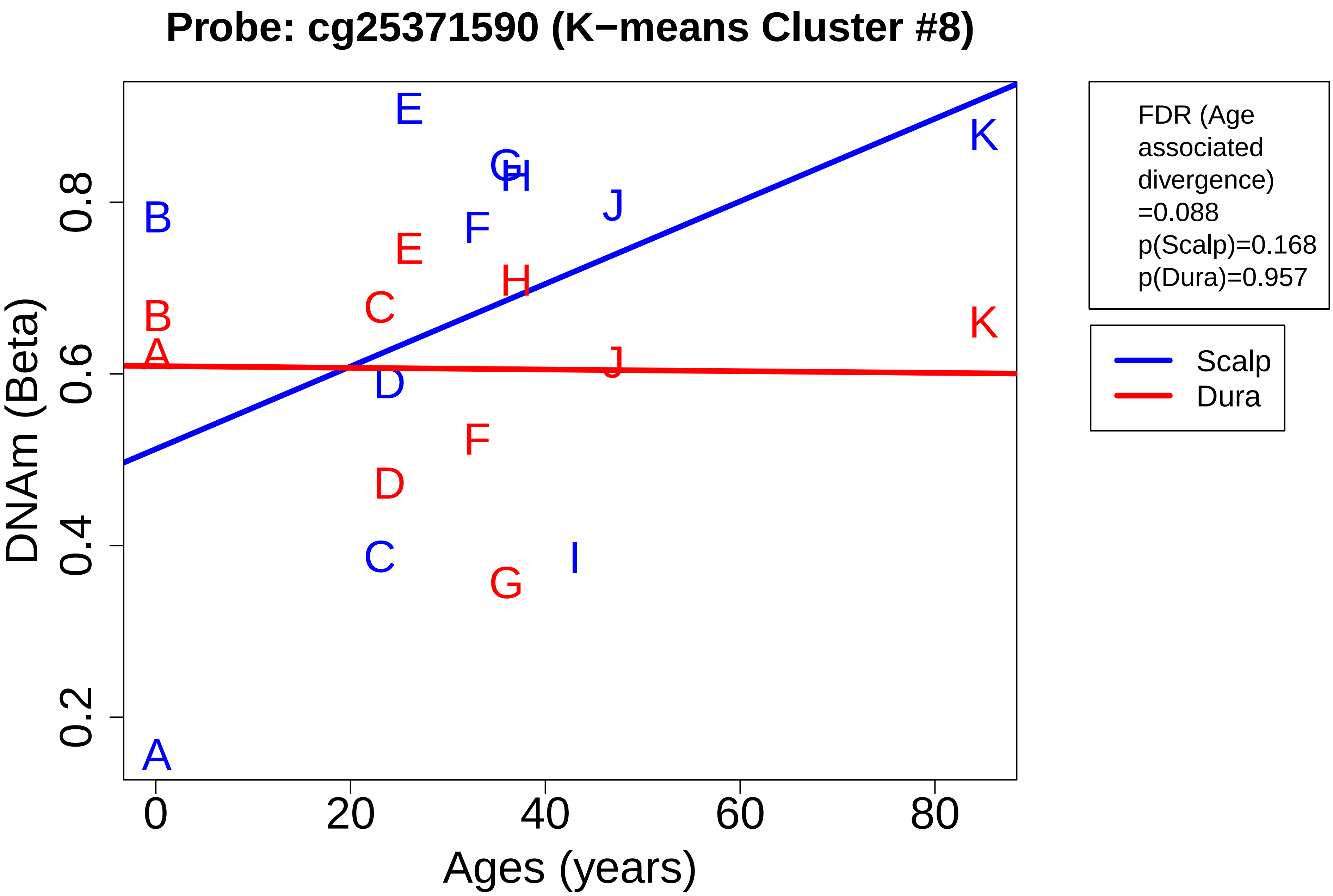

Probe: cg25371590 (K−means Cluster #8)
DNAm (Beta)
0.2
0.4
0.6
0.8
Ages (years)
0
20
40
60
80
FDR (Age associated divergence) =0.088
p(Scalp)=0.168
p(Dura)=0.957
Scalp
Dura
A
B
C
D
E
F
G
H
I
J
K

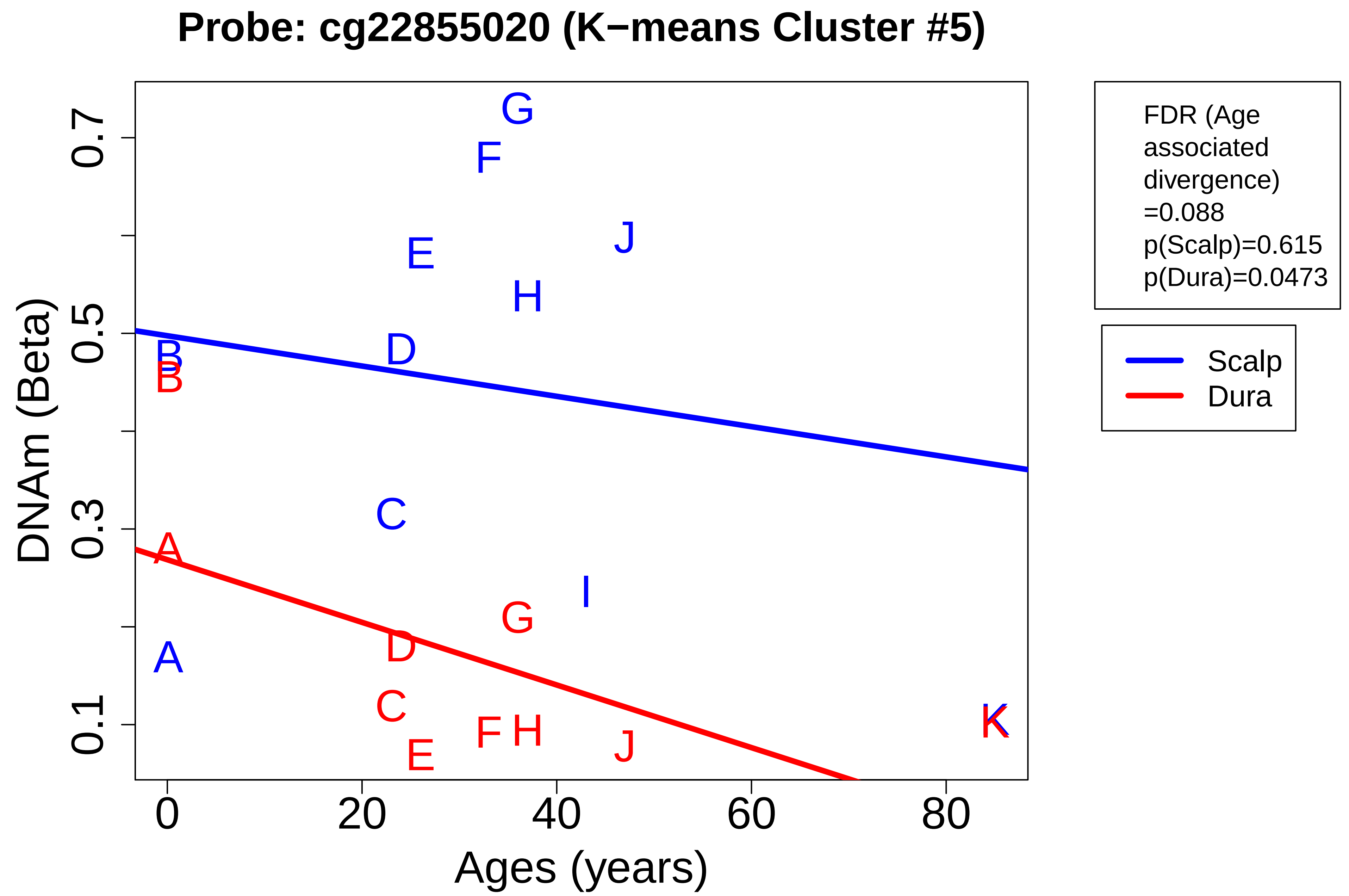

Probe: cg22855020 (K−means Cluster #5)
DNAm (Beta)
0.1
0.3
0.5
0.7
Ages (years)
0
20
40
60
80
FDR (Age associated divergence) =0.088
p(Scalp)=0.615
p(Dura)=0.0473
Scalp
Dura

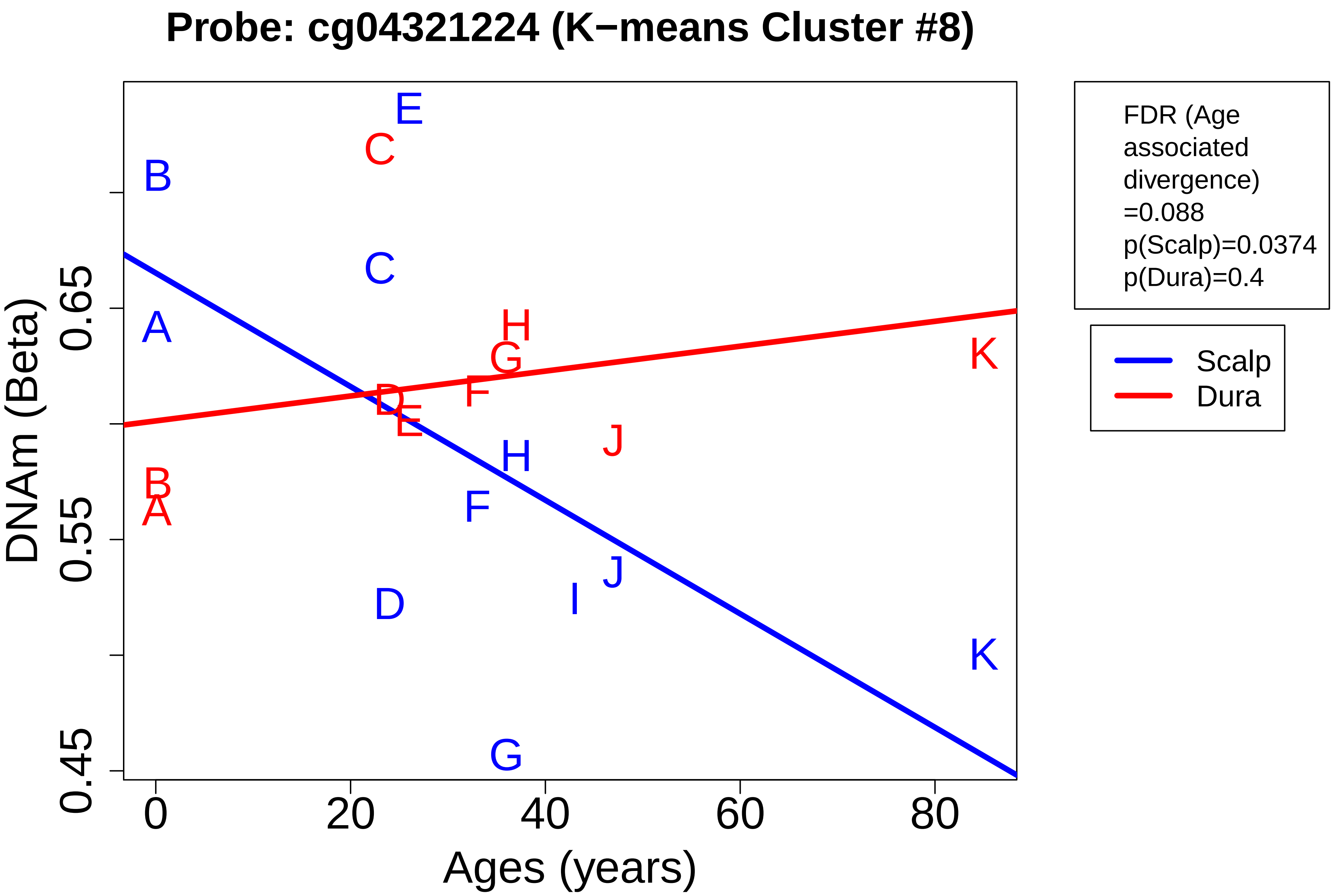

Probe: cg04321224 (K−means Cluster #8)
DNAm (Beta)
0.45
0.55
0.65
Ages (years)
0
20
40
60
80
FDR (Age associated divergence) =0.088
p(Scalp)=0.0374
p(Dura)=0.4
Scalp
Dura

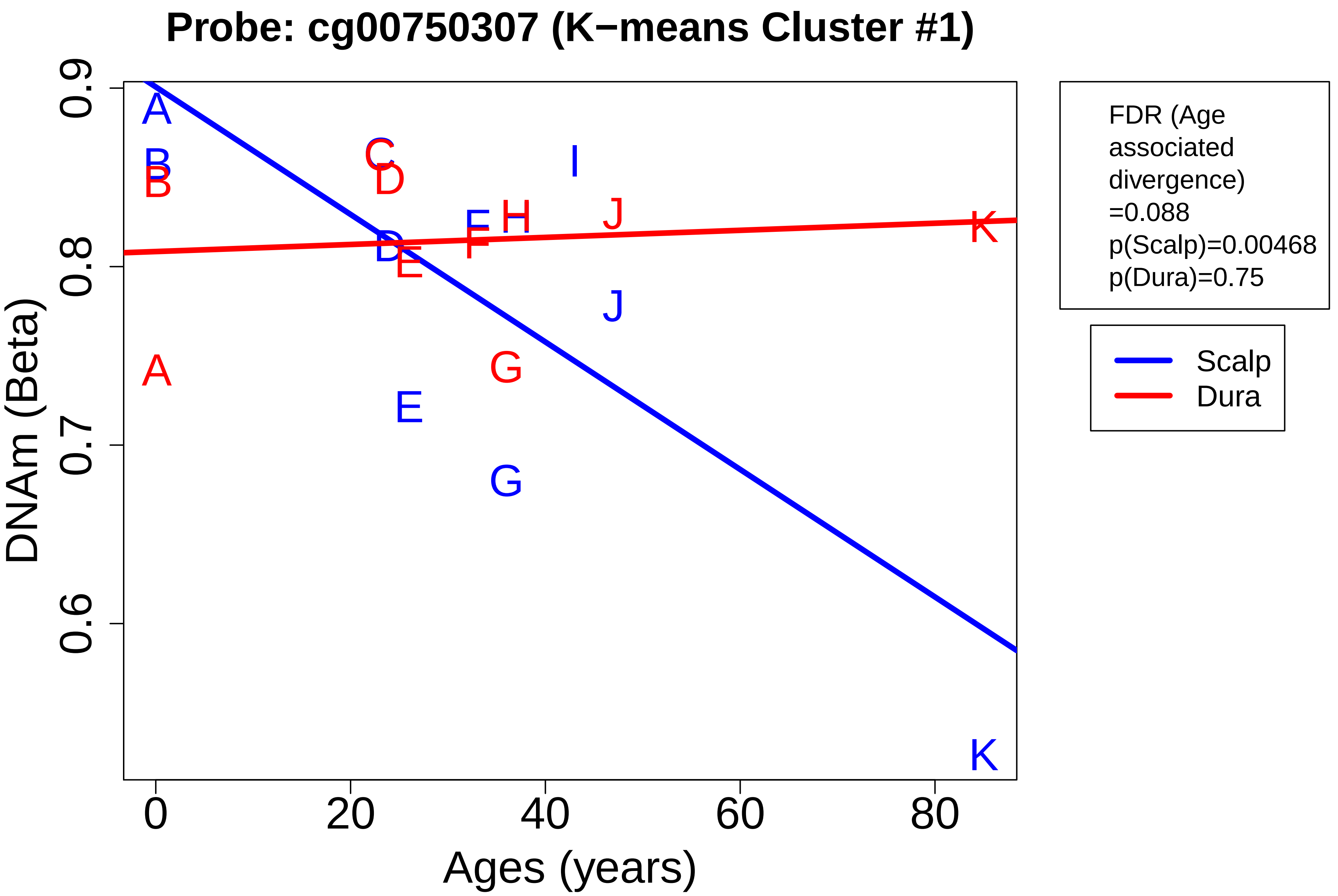

Probe: cg00750307 (K−means Cluster #1)
DNAm (Beta)
0.6
0.7
0.8
0.9
Ages (years)
0
20
40
60
80
FDR (Age associated divergence) =0.088
p(Scalp)=0.00468
p(Dura)=0.75
Scalp
Dura
A
B
C
D
E
F
G
H
I
J
K

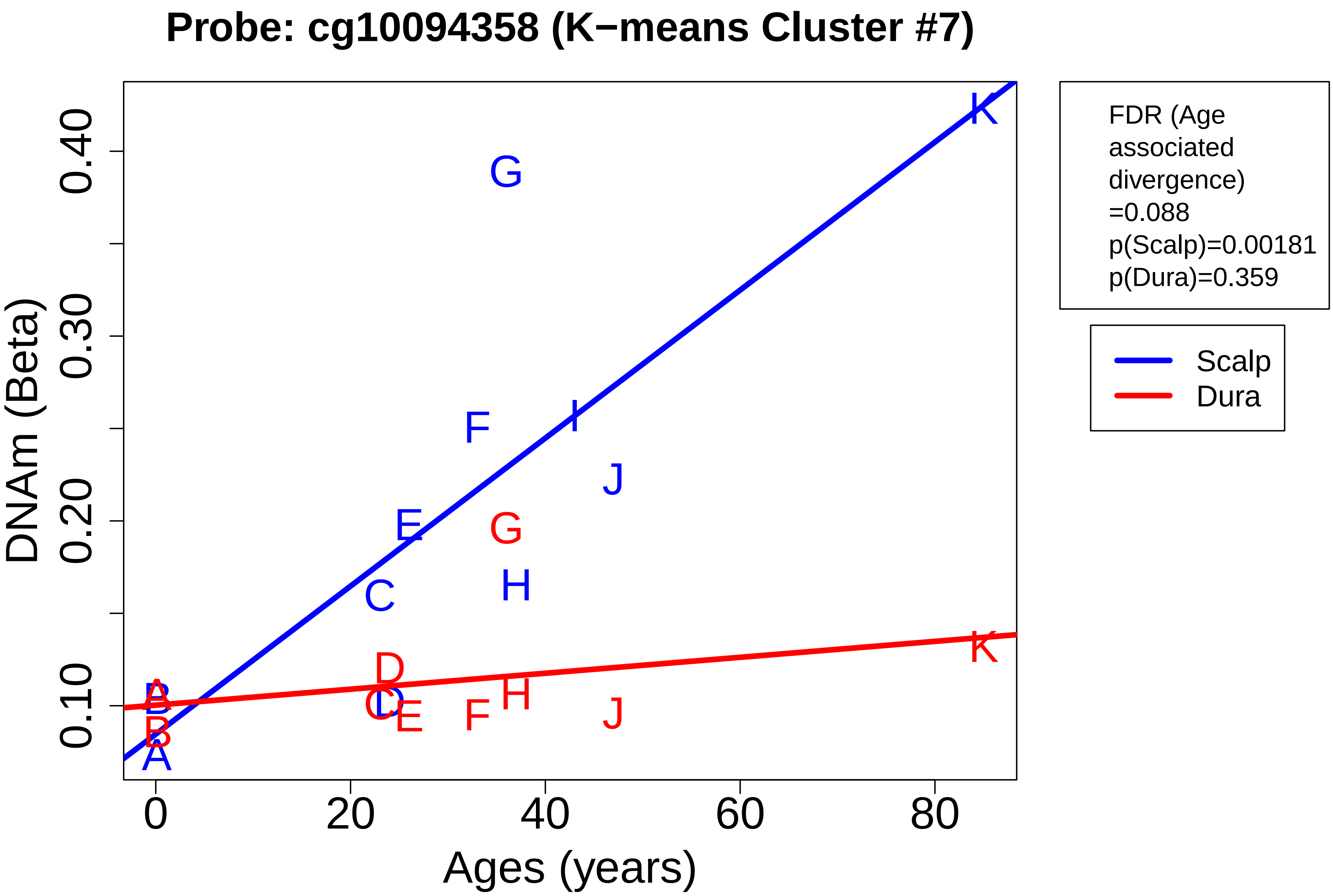
Probe: cg10094358 (K−means Cluster #7)
DNAm (Beta)
0.10
0.20
0.30
0.40
Ages (years)
0
20
40
60
80
FDR (Age associated divergence) =0.088
p(Scalp)=0.00181
p(Dura)=0.359
Scalp
Dura
A
B
C
D
E
F
G
H
I
J
K

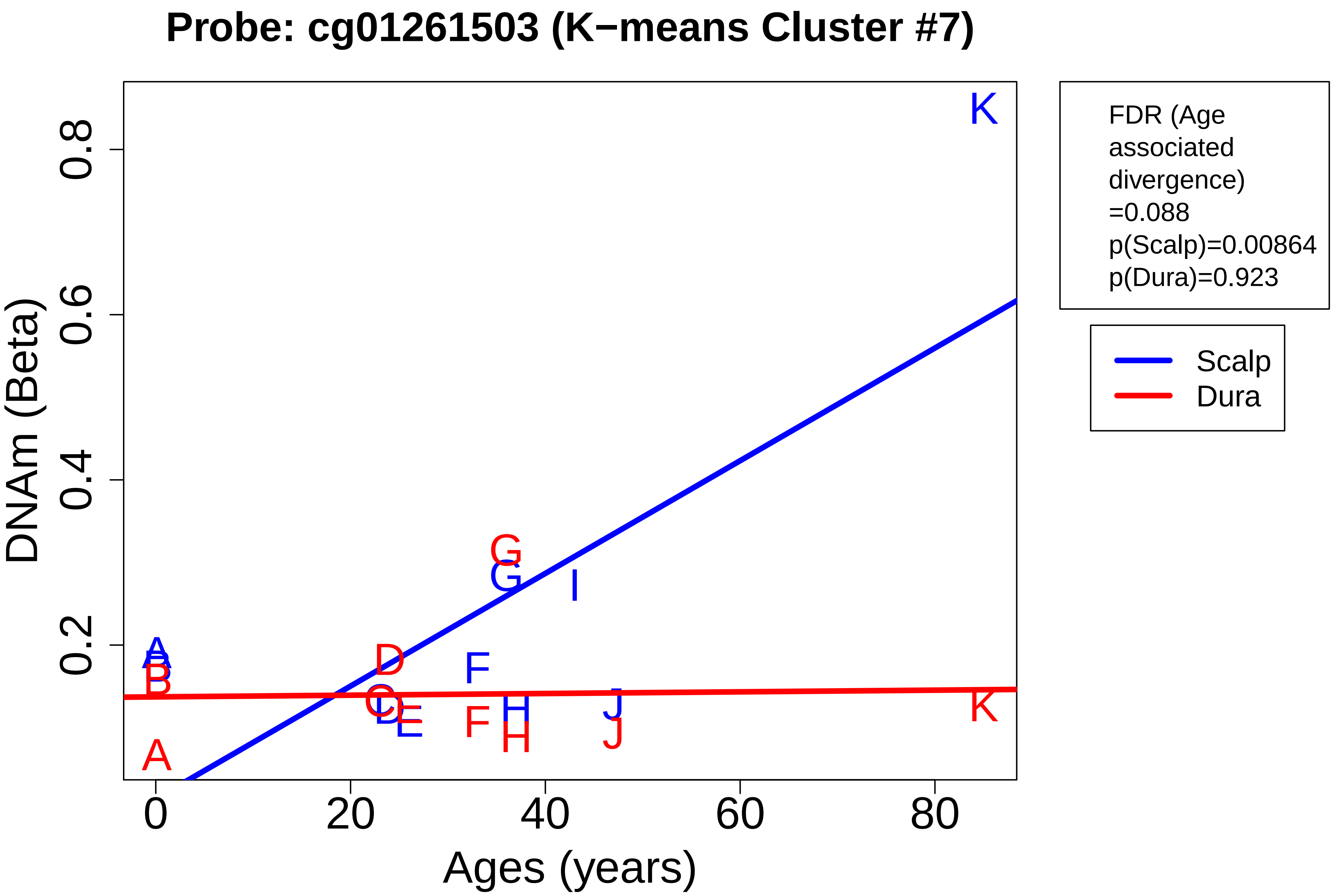

Probe: cg01261503 (K−means Cluster #7)
DNAm (Beta)
0.2
0.4
0.6
0.8
0
20
40
60
80
Ages (years)
A
B
C
D
E
F
G
H
I
J
K
FDR (Age associated divergence) =0.088
p(Scalp)=0.00864
p(Dura)=0.923
Scalp
Dura

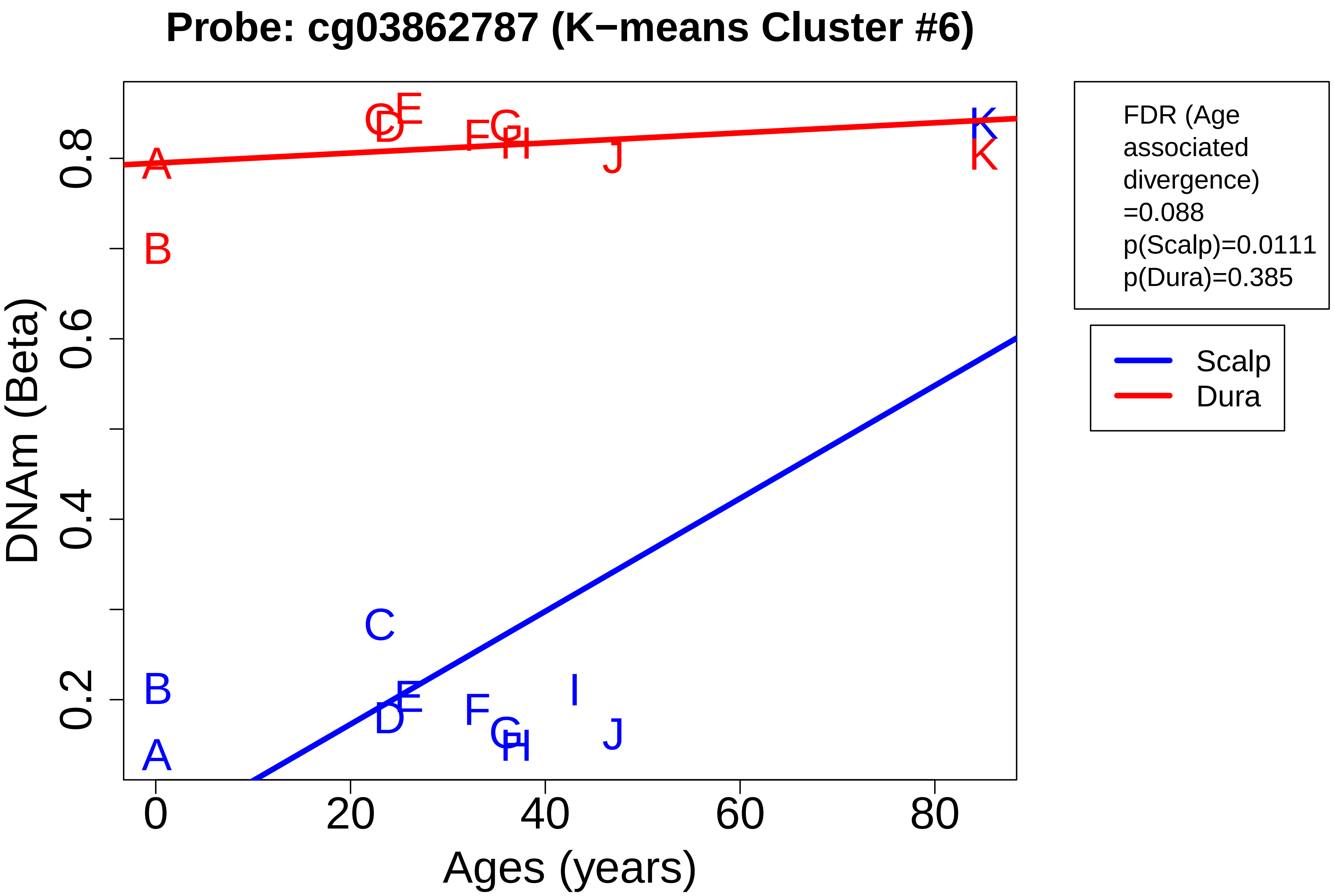

Probe: cg03862787 (K−means Cluster #6)
DNAm (Beta)
0.2
0.4
0.6
0.8
0
20
40
60
80
Ages (years)
FDR (Age associated divergence) =0.088
p(Scalp)=0.0111
p(Dura)=0.385
Scalp
Dura
A
B
C
D
E
F
G
H
I
J
K

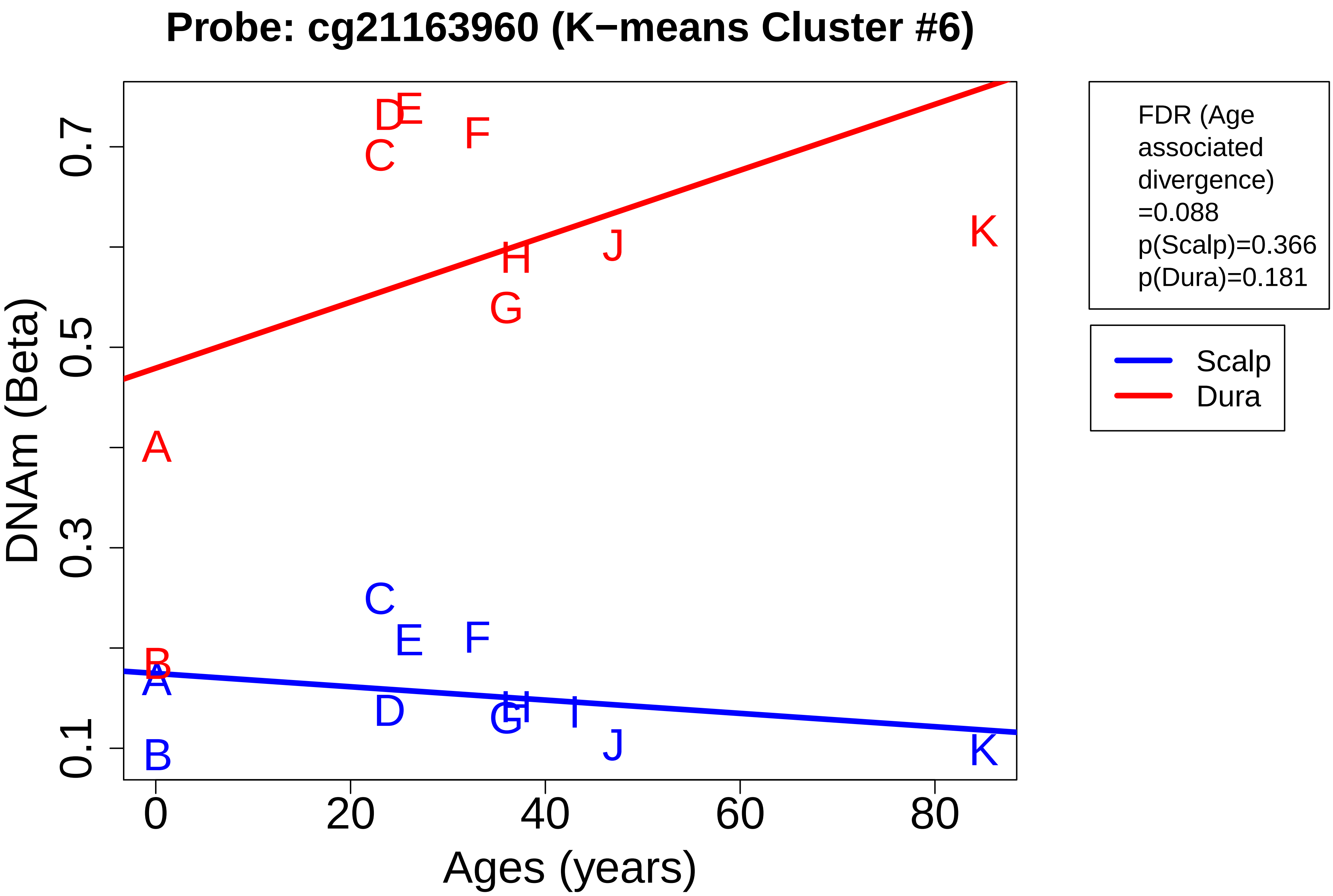

Probe: cg21163960 (K−means Cluster #6)
DNAm (Beta)
0.1
0.3
0.5
0.7
0
20
40
60
80
Ages (years)
FDR (Age associated divergence) =0.088
p(Scalp)=0.366
p(Dura)=0.181
Scalp
Dura
A
B
C
D
E
F
G
H
I
J
K

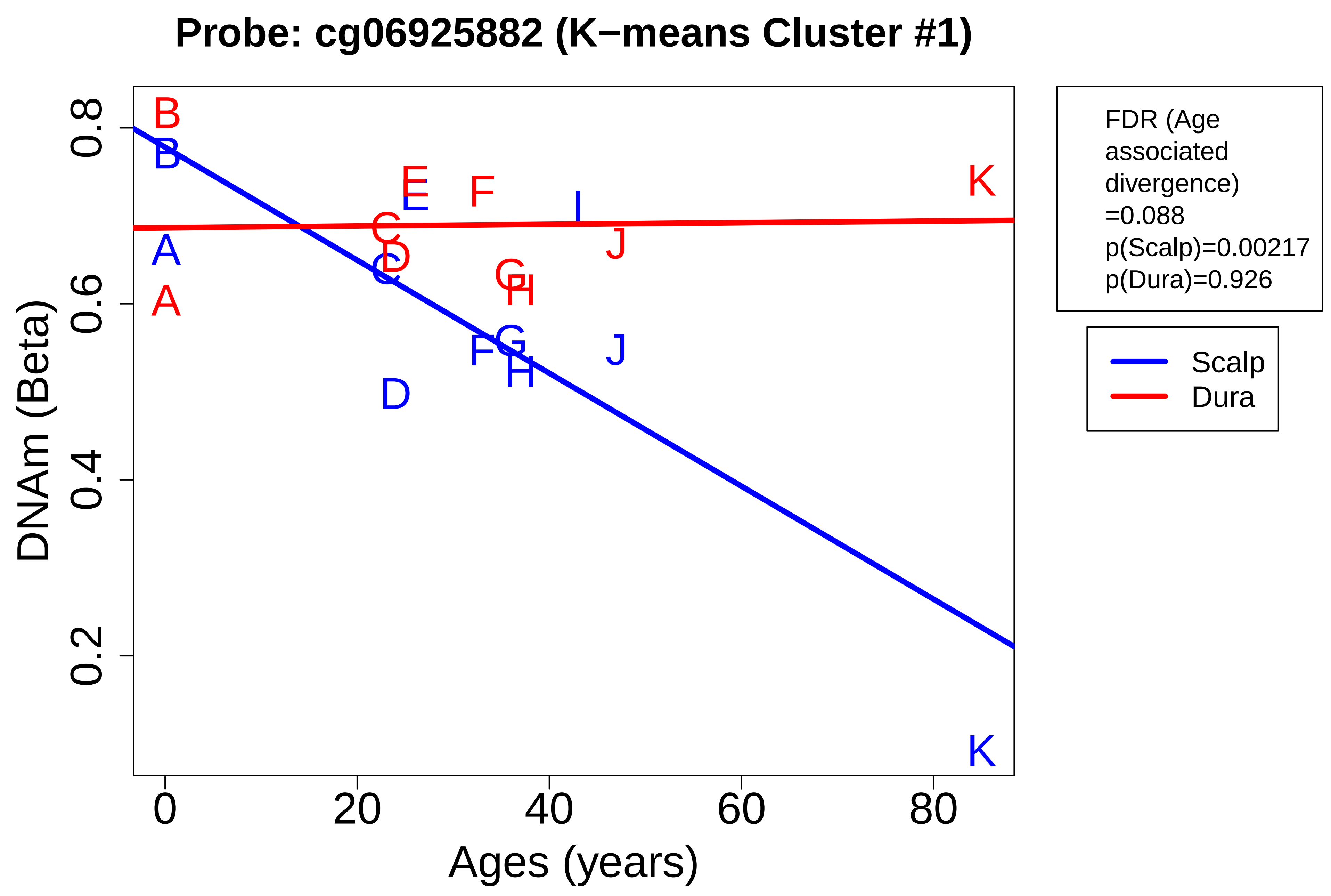

Probe: cg06925882 (K−means Cluster #1)
DNAm (Beta)
0.2
0.4
0.6
0.8
Ages (years)
0
20
40
60
80
A
B
C
D
E
F
G
H
I
J
K
FDR (Age associated divergence) =0.088
p(Scalp)=0.00217
p(Dura)=0.926
Scalp
Dura

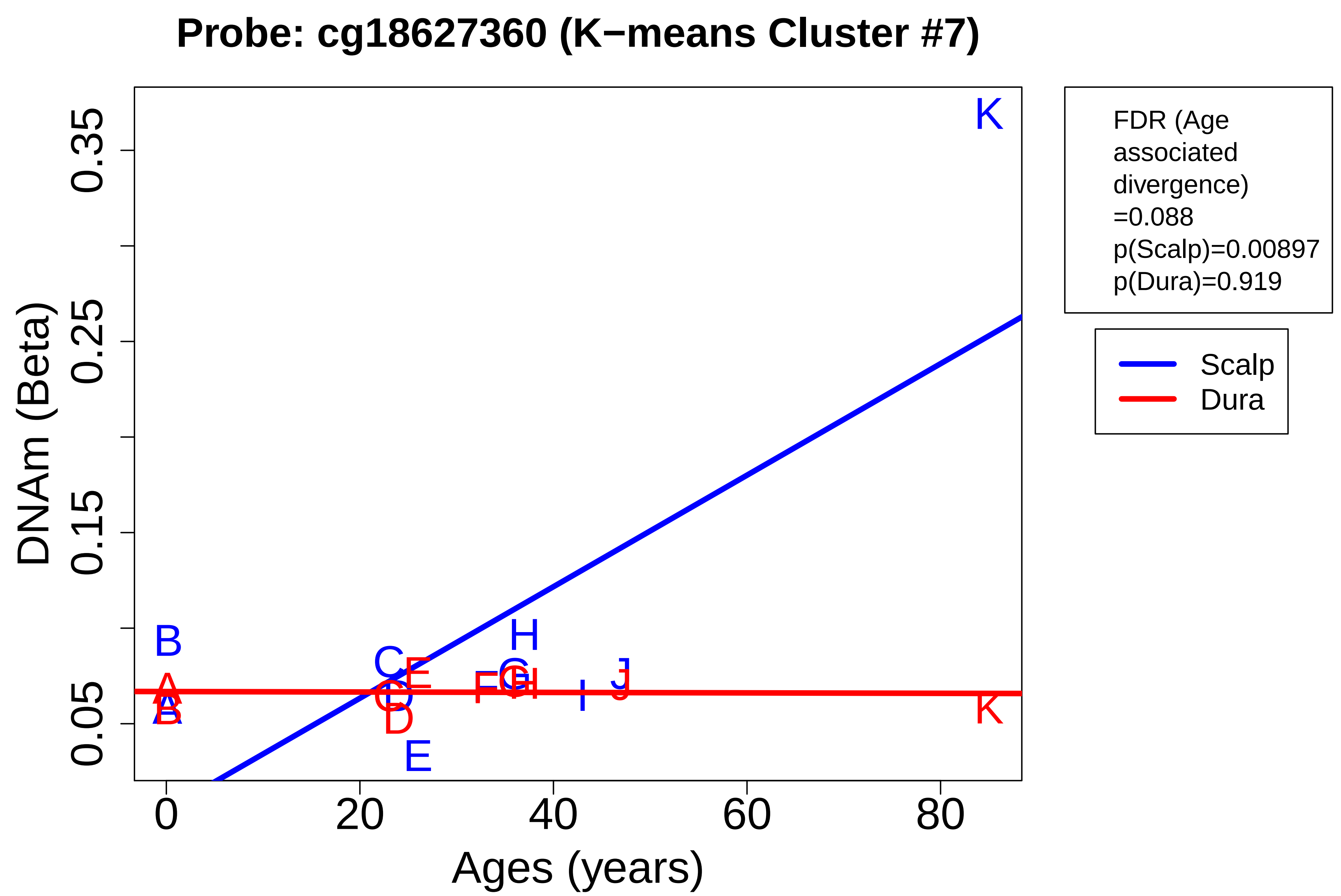
Probe: cg18627360 (K−means Cluster #7)
FDR (Age associated divergence) =0.088
p(Scalp)=0.00897
p(Dura)=0.919
Scalp
Dura
DNAm (Beta)
0.05
0.15
0.25
0.35
0
20
40
60
80
Ages (years)
A
B
C
D
E
F
G
H
I
J
K

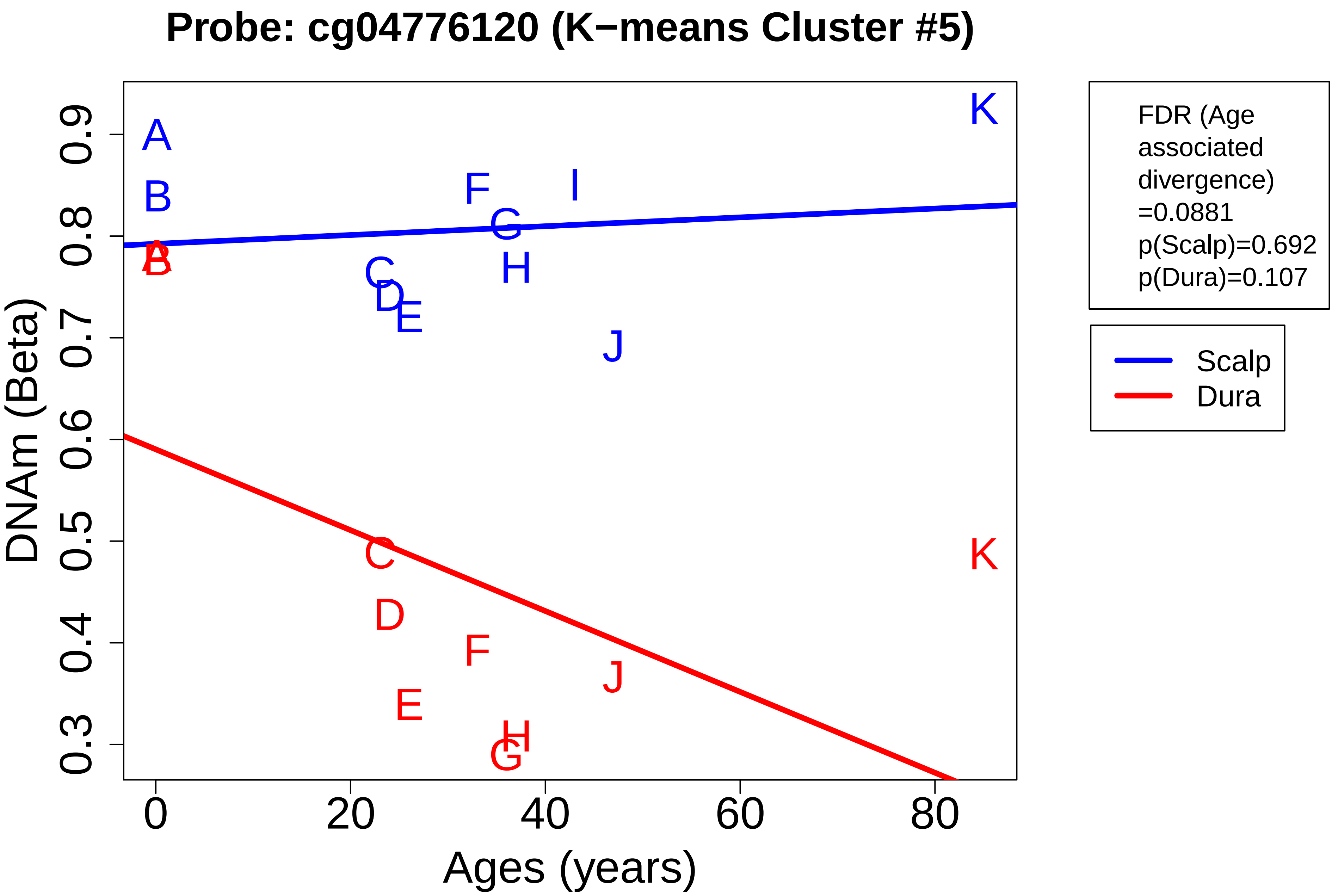

Probe: cg04776120 (K−means Cluster #5)
DNAm (Beta)
0.3
0.4
0.5
0.6
0.7
0.8
0.9
0
20
40
60
80
Ages (years)
A
B
C
D
E
F
G
H
I
J
K
FDR (Age associated divergence) =0.0881
p(Scalp)=0.692
p(Dura)=0.107
Scalp
Dura

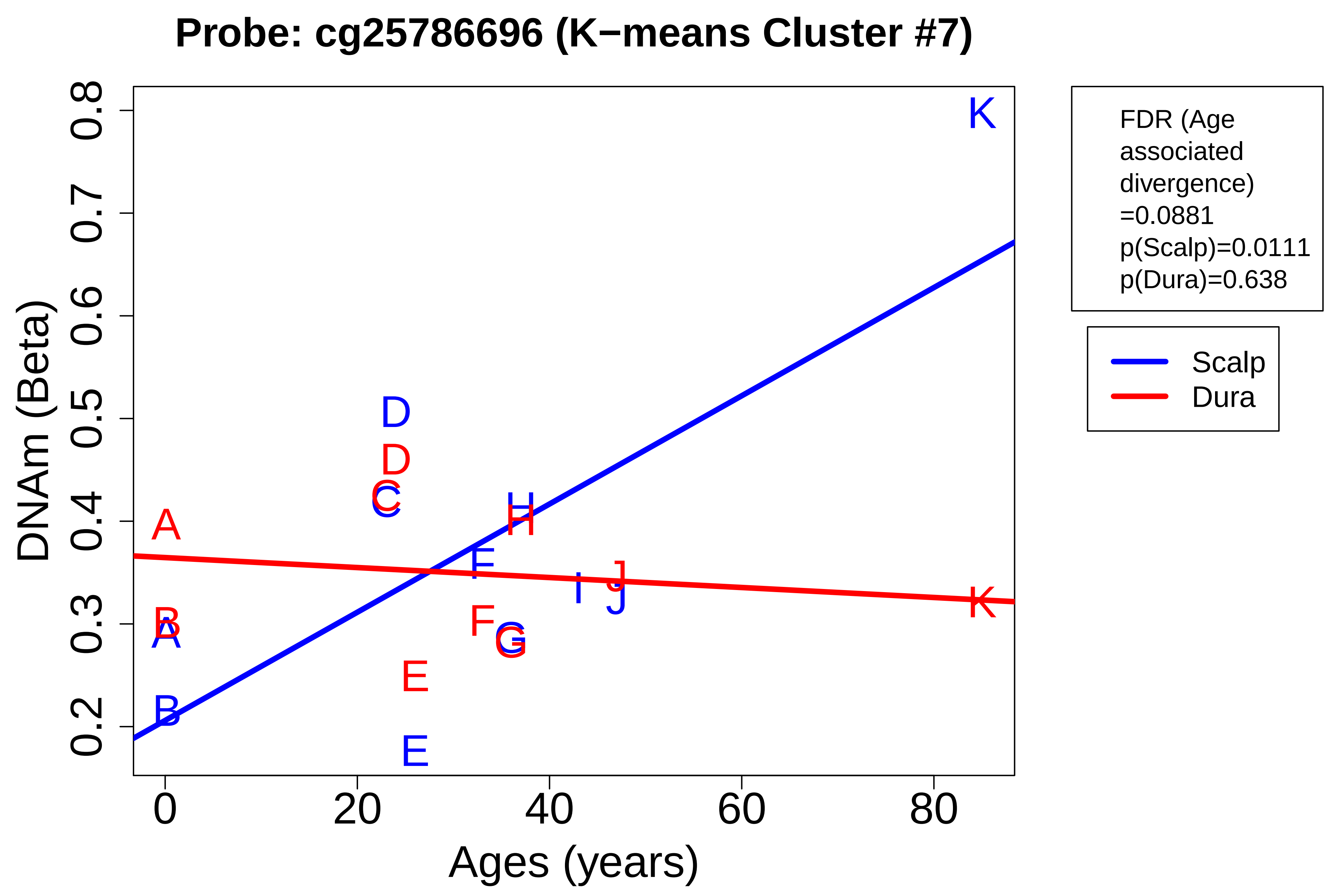
Probe: cg25786696 (K−means Cluster #7)
DNAm (Beta)
Ages (years)
0.2
0.3
0.4
0.5
0.6
0.7
0.8
0
20
40
60
80
A
B
C
D
E
F
G
H
I
J
K
FDR (Age associated divergence) =0.0881
p(Scalp)=0.0111
p(Dura)=0.638
Scalp
Dura

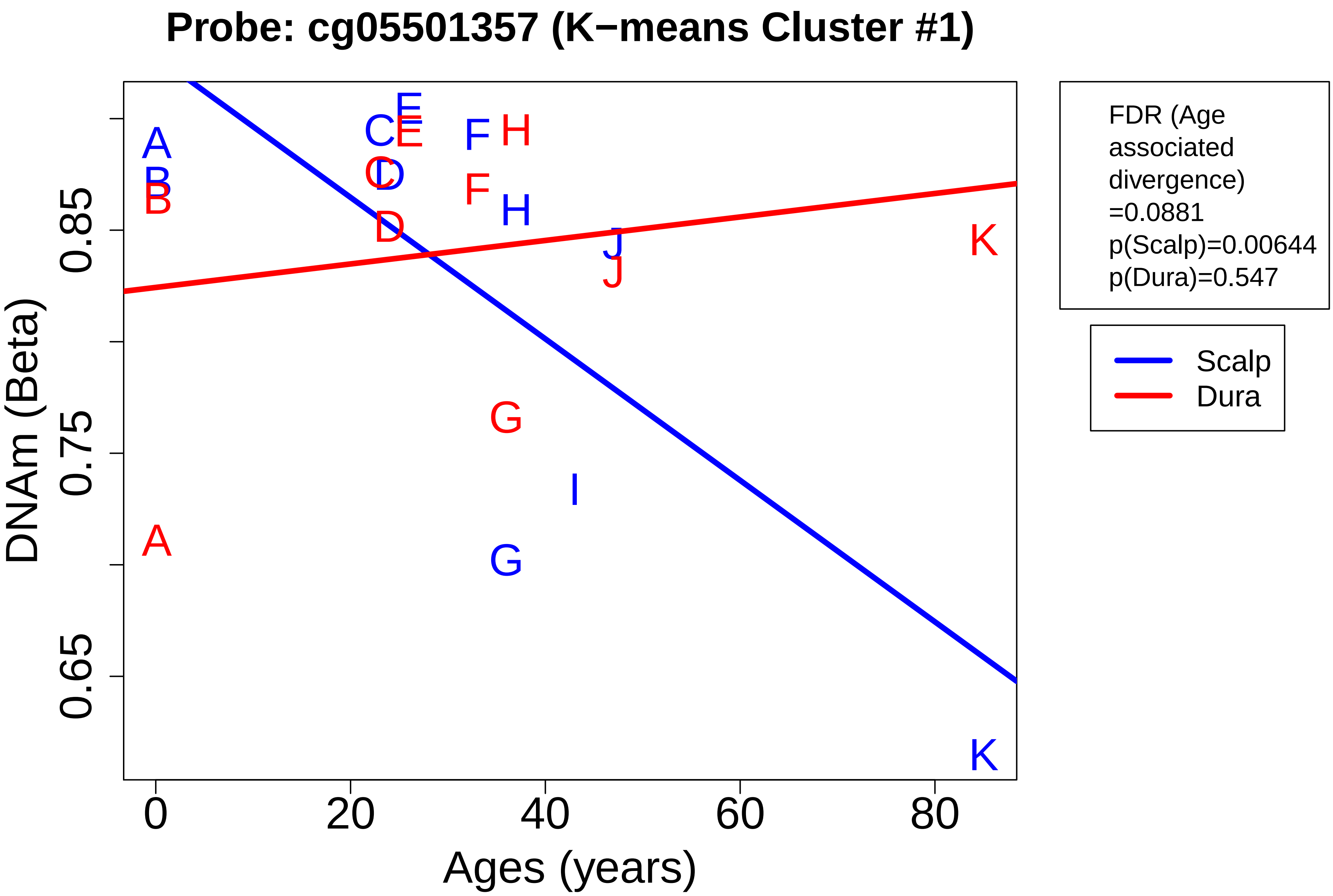

Probe: cg05501357 (K−means Cluster #1)
FDR (Age associated divergence) =0.0881
p(Scalp)=0.00644
p(Dura)=0.547
Scalp
Dura
DNAm (Beta)
0.65
0.75
0.85
0
20
40
60
80
Ages (years)
A
B
C
D
E
F
G
H
I
J
K

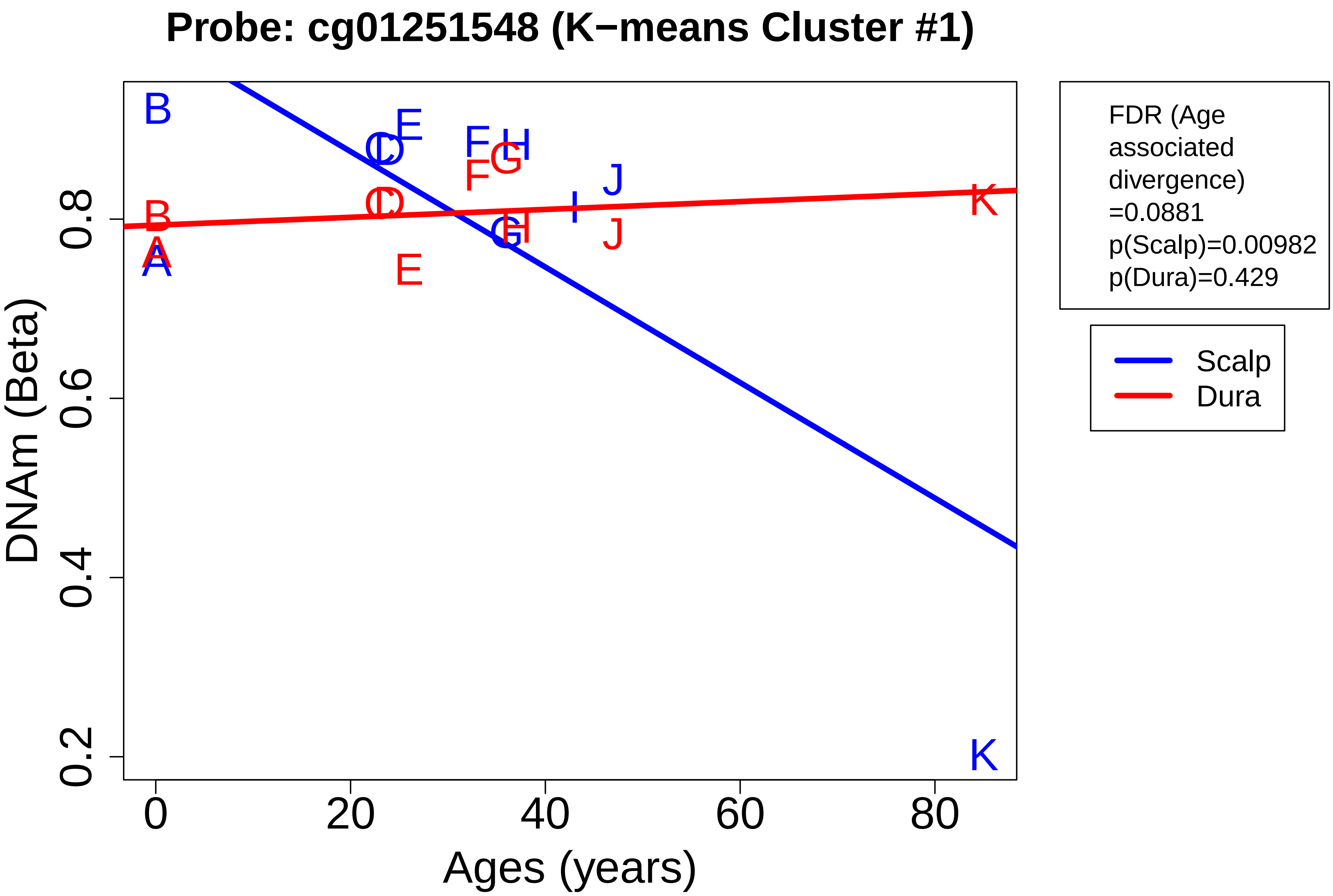

Probe: cg01251548 (K−means Cluster #1)
DNAm (Beta)
0.2
0.4
0.6
0.8
0
20
40
60
80
Ages (years)
FDR (Age associated divergence) =0.0881
p(Scalp)=0.00982
p(Dura)=0.429
Scalp
Dura

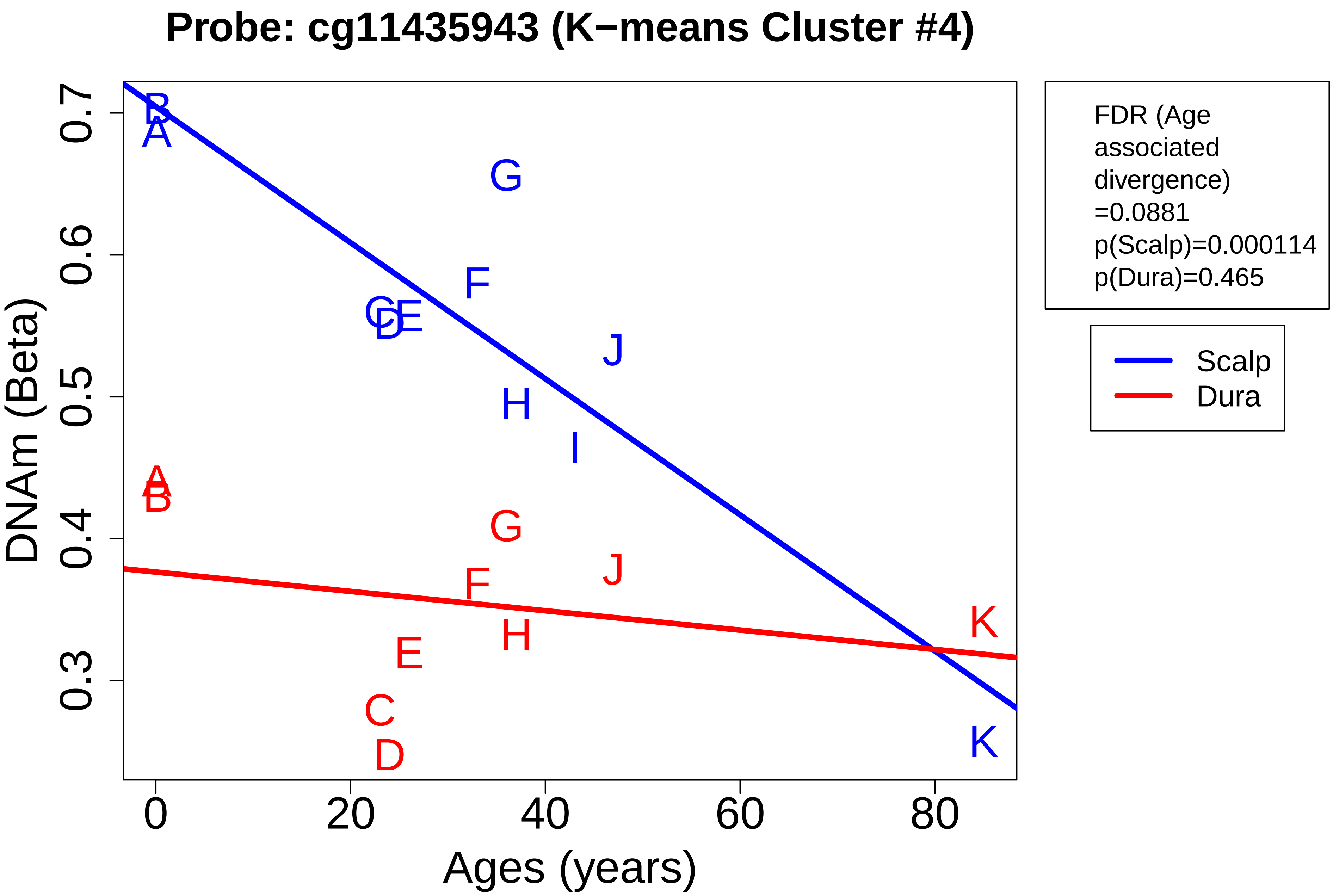

Probe: cg11435943 (K−means Cluster #4)
DNAm (Beta)
0.3
0.4
0.5
0.6
0.7
Ages (years)
0
20
40
60
80
A
B
C
D
E
F
G
H
I
J
K
FDR (Age associated divergence) =0.0881
p(Scalp)=0.000114
p(Dura)=0.465
Scalp
Dura

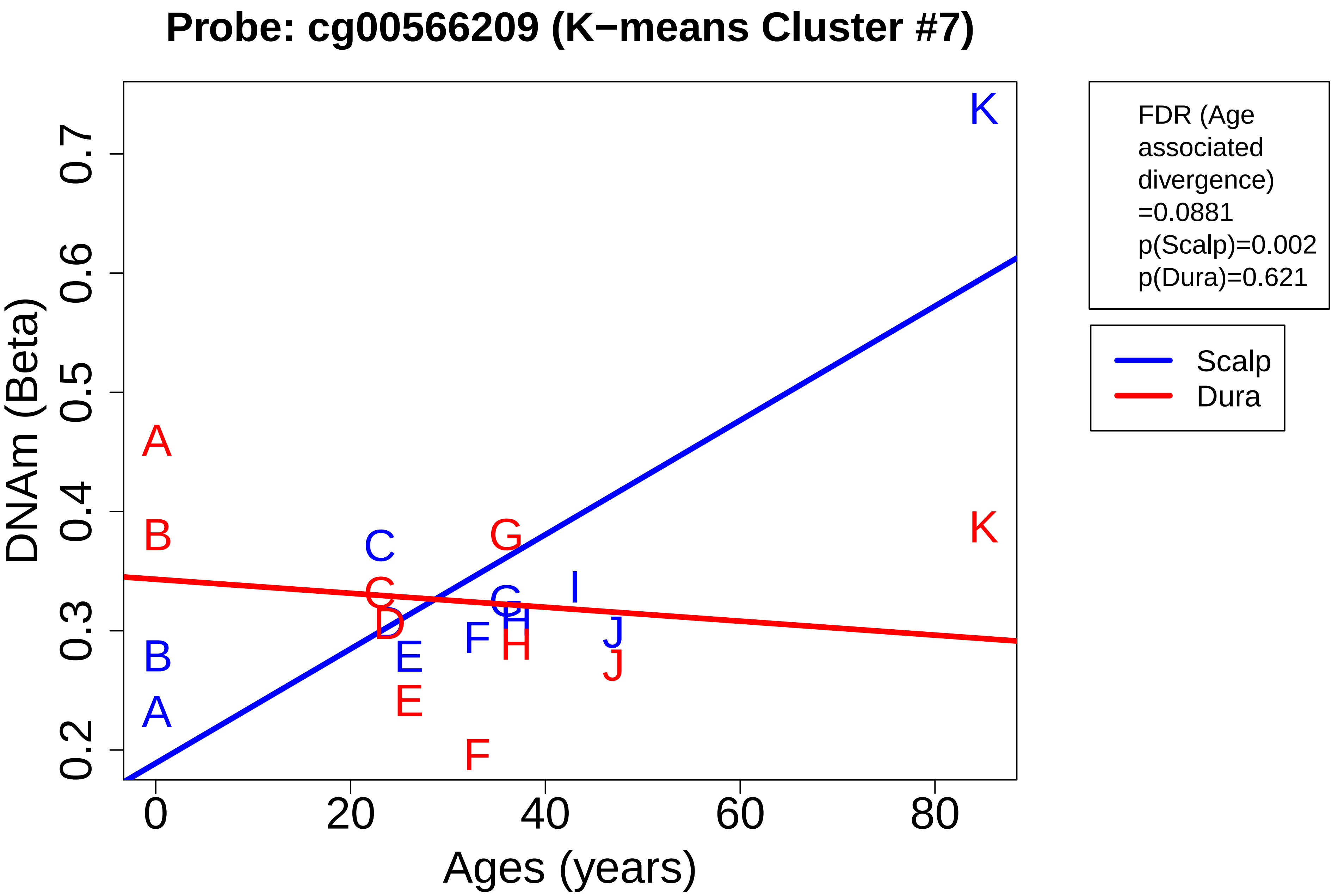

Probe: cg00566209 (K−means Cluster #7)
DNAm (Beta)
0.2
0.3
0.4
0.5
0.6
0.7
Ages (years)
0
20
40
60
80
A
B
C
D
E
F
G
H
I
J
K
FDR (Age associated divergence) =0.0881
p(Scalp)=0.002
p(Dura)=0.621
Scalp
Dura

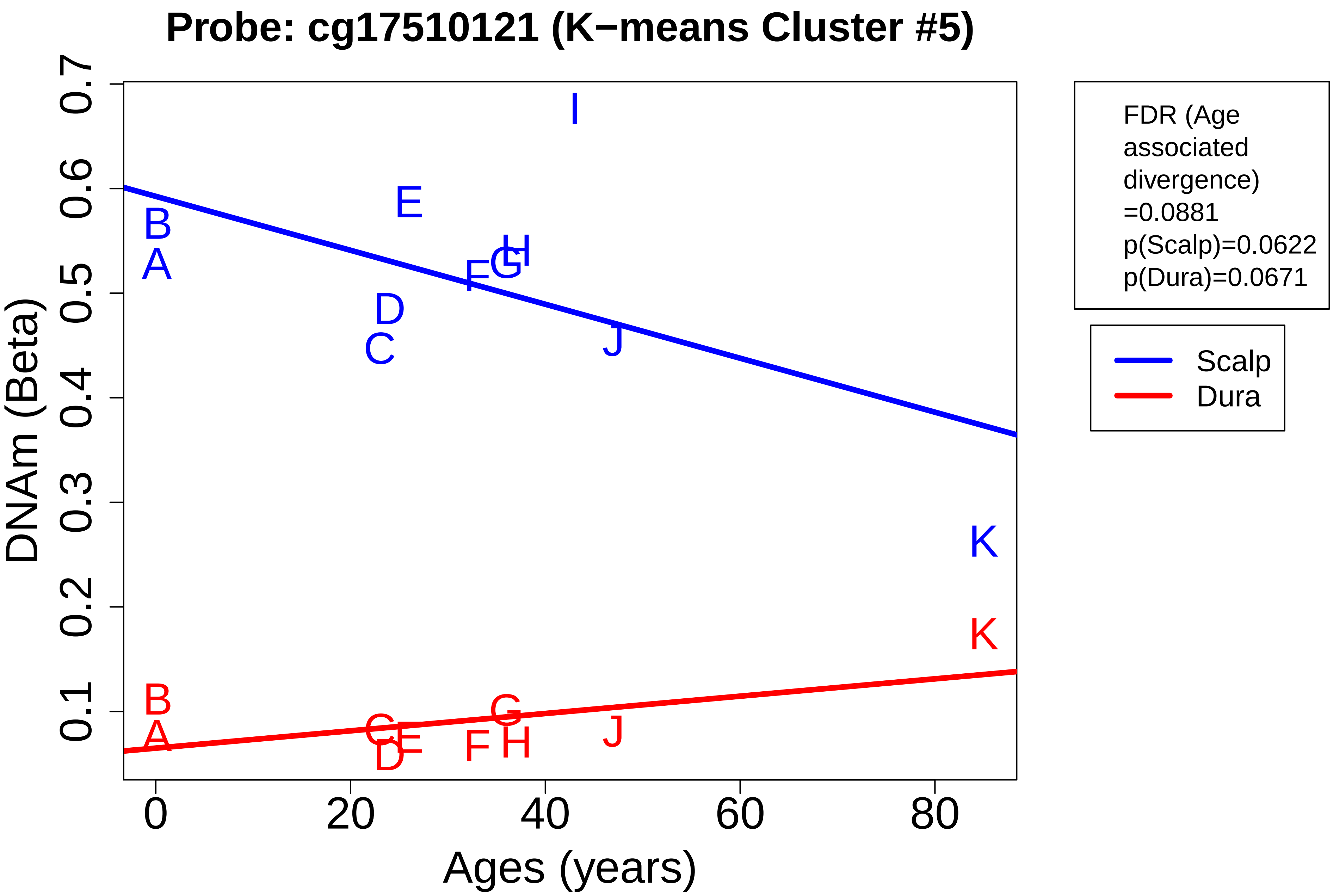

Probe: cg17510121 (K−means Cluster #5)
DNAm (Beta)
0.1
0.2
0.3
0.4
0.5
0.6
0.7
0
20
40
60
80
Ages (years)
FDR (Age associated divergence) =0.0881
p(Scalp)=0.0622
p(Dura)=0.0671
Scalp
Dura

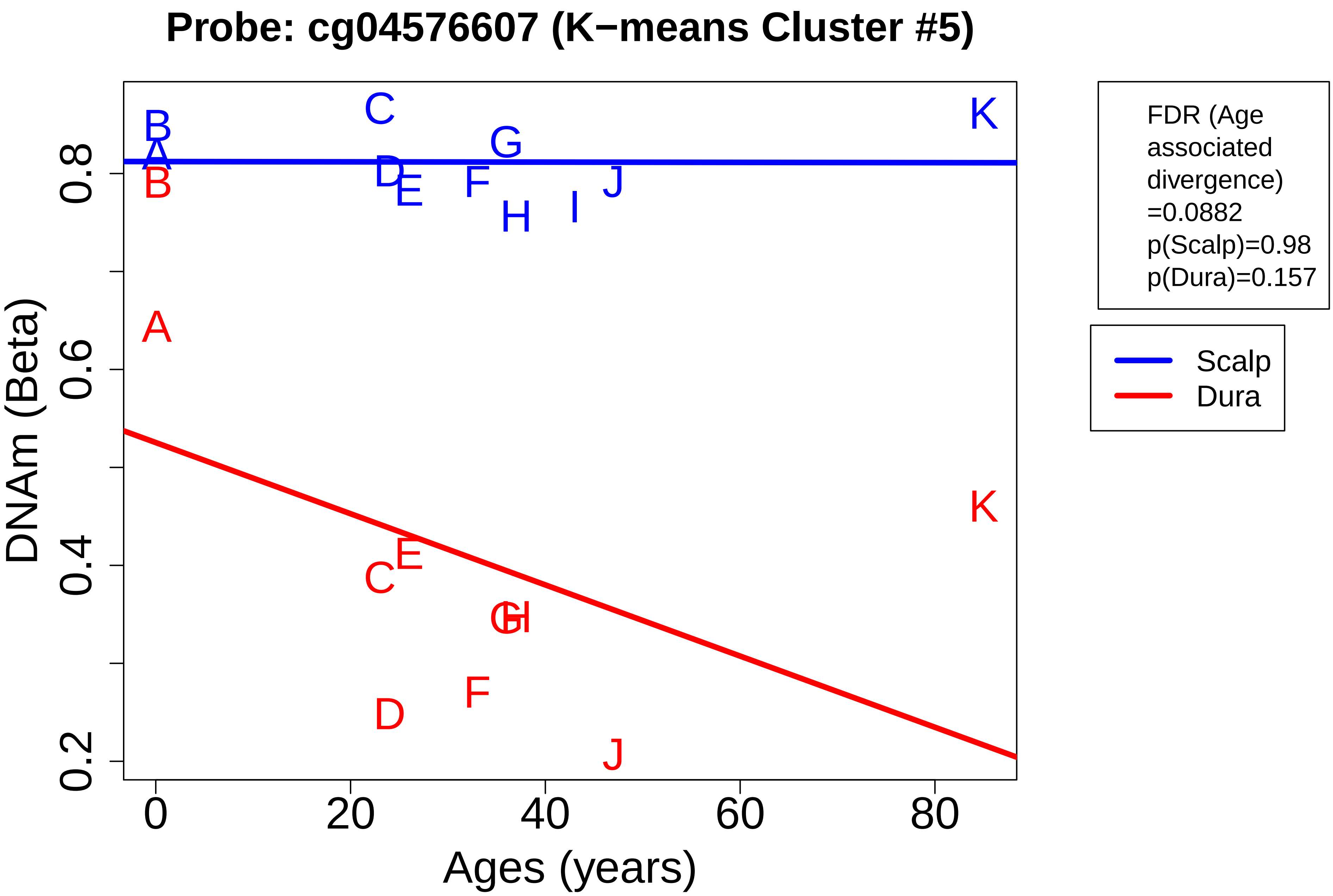
Probe: cg04576607 (K−means Cluster #5)
DNAm (Beta)
0.2
0.4
0.6
0.8
0
20
40
60
80
Ages (years)
A B C D E F G H I J K
FDR (Age associated divergence) =0.0882
p(Scalp)=0.98
p(Dura)=0.157
Scalp
Dura

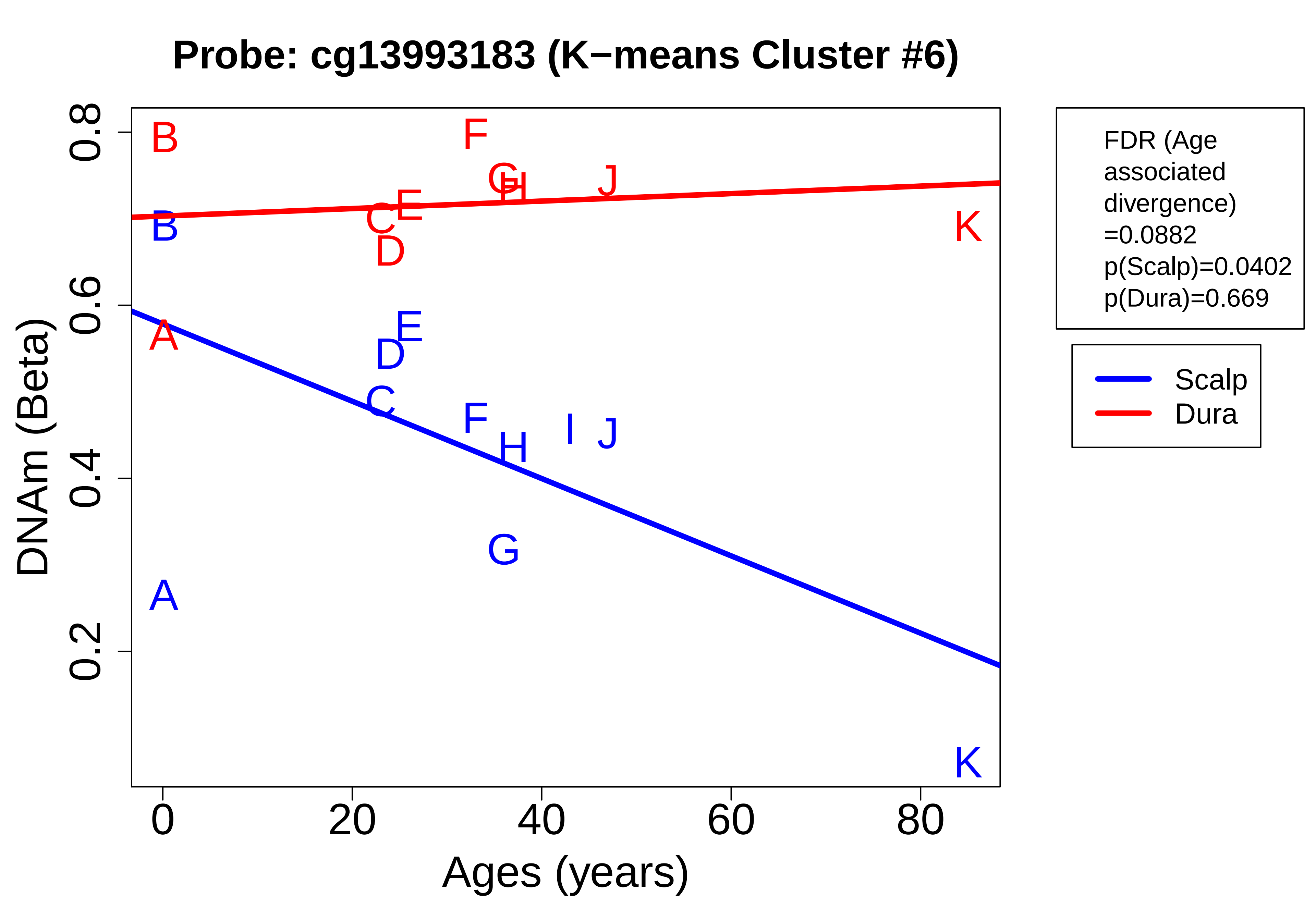

Probe: cg13993183 (K−means Cluster #6)
DNAm (Beta)
0.2
0.4
0.6
0.8
0
20
40
60
80
Ages (years)
A
B
C
D
E
F
G
H
I
J
K
FDR (Age associated divergence) =0.0882
p(Scalp)=0.0402
p(Dura)=0.669
Scalp
Dura

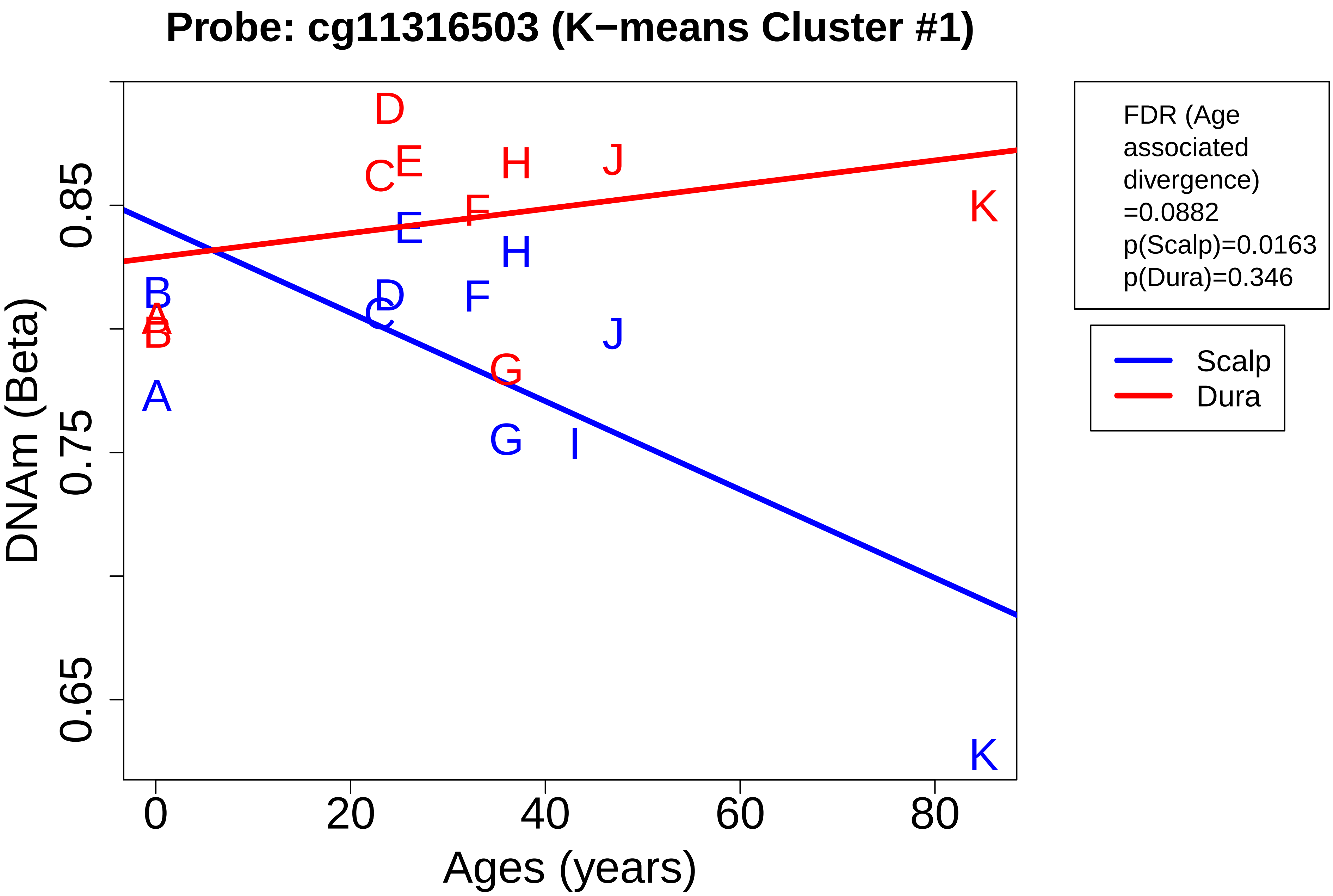

Probe: cg11316503 (K−means Cluster #1)
DNAm (Beta)
0.65
0.75
0.85
0
20
40
60
80
Ages (years)
FDR (Age associated divergence) =0.0882
p(Scalp)=0.0163
p(Dura)=0.346
Scalp
Dura

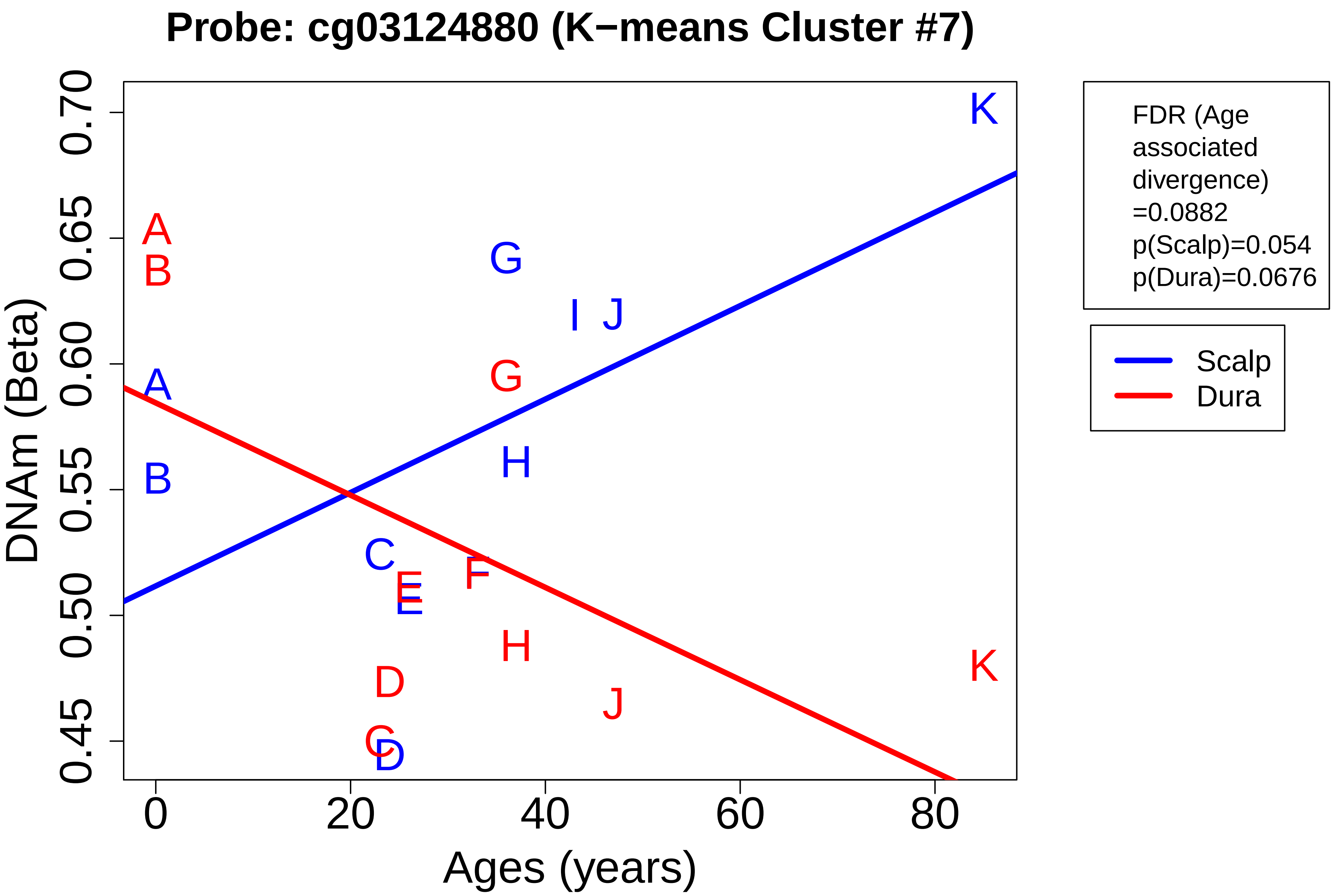
Probe: cg03124880 (K−means Cluster #7)
DNAm (Beta)
0.45
0.50
0.55
0.60
0.65
0.70
0
20
40
60
80
Ages (years)
A
B
C
D
E
F
G
H
I
J
K
FDR (Age associated divergence) =0.0882
p(Scalp)=0.054
p(Dura)=0.0676
Scalp
Dura

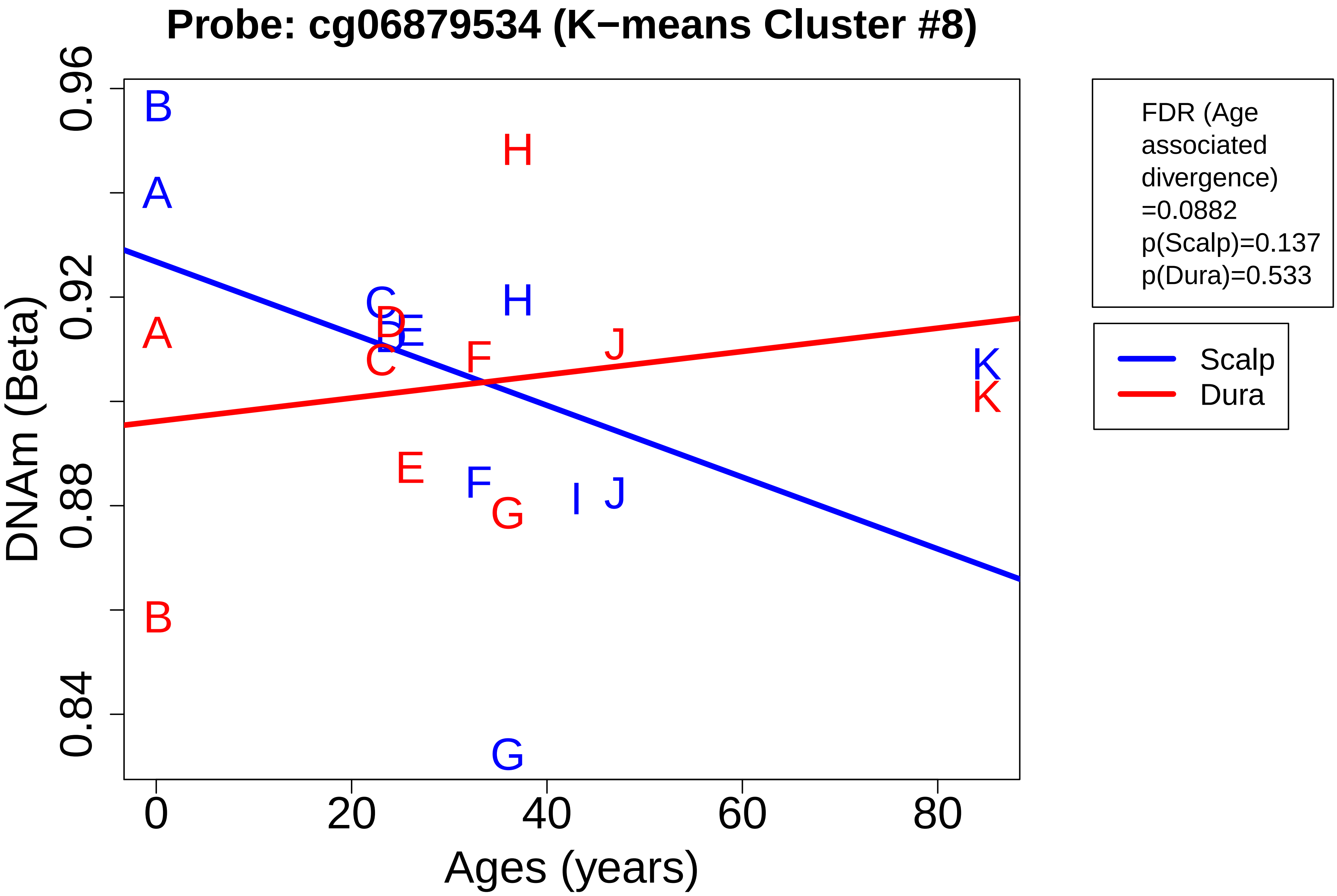

Probe: cg06879534 (K−means Cluster #8)
DNAm (Beta)
0.84
0.88
0.92
0.96
0
20
40
60
80
Ages (years)
FDR (Age associated divergence) =0.0882
p(Scalp)=0.137
p(Dura)=0.533
Scalp
Dura

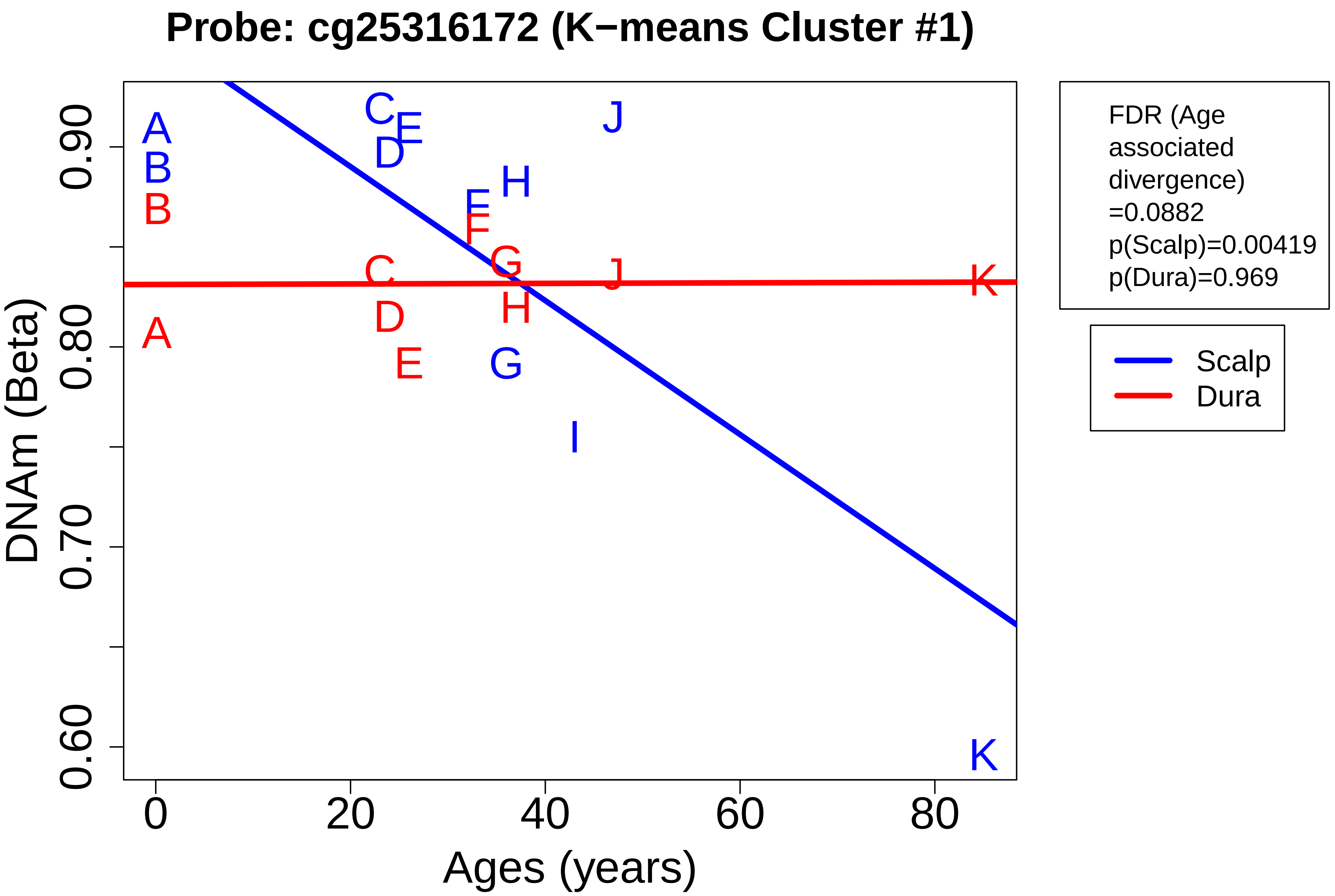

Probe: cg25316172 (K−means Cluster #1)
DNAm (Beta)
0.60
0.70
0.80
0.90
0
20
40
60
80
Ages (years)
FDR (Age associated divergence) =0.0882
p(Scalp)=0.00419
p(Dura)=0.969
Scalp
Dura

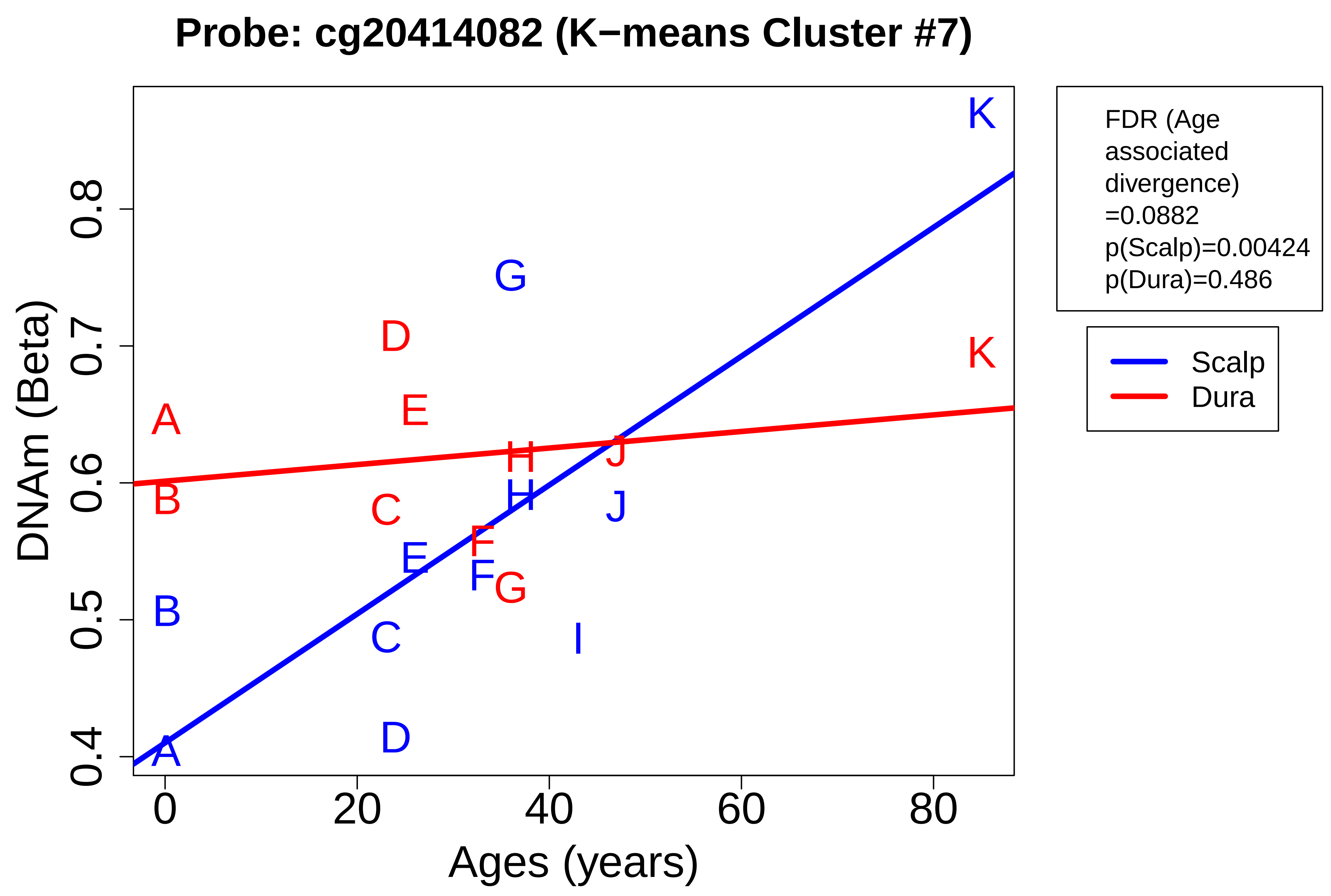

Probe: cg20414082 (K−means Cluster #7)
DNAm (Beta)
0.4
0.5
0.6
0.7
0.8
Ages (years)
0
20
40
60
80
A
B
C
D
E
F
G
H
I
J
K
FDR (Age associated divergence) =0.0882
p(Scalp)=0.00424
p(Dura)=0.486
Scalp
Dura

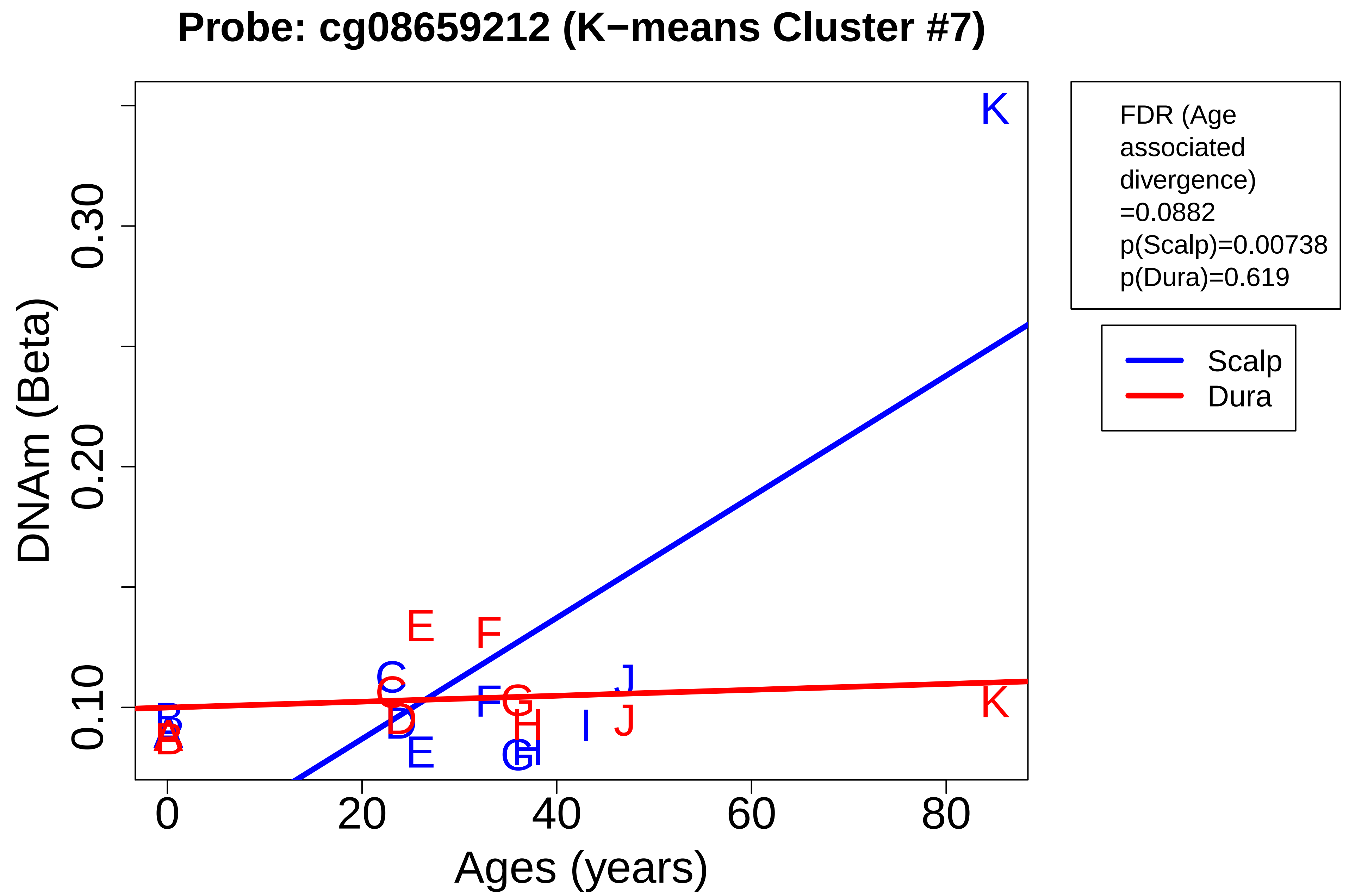
Probe: cg08659212 (K−means Cluster #7)
DNAm (Beta)
0.10
0.20
0.30
Ages (years)
0
20
40
60
80
FDR (Age associated divergence) =0.0882
p(Scalp)=0.00738
p(Dura)=0.619
Scalp
Dura

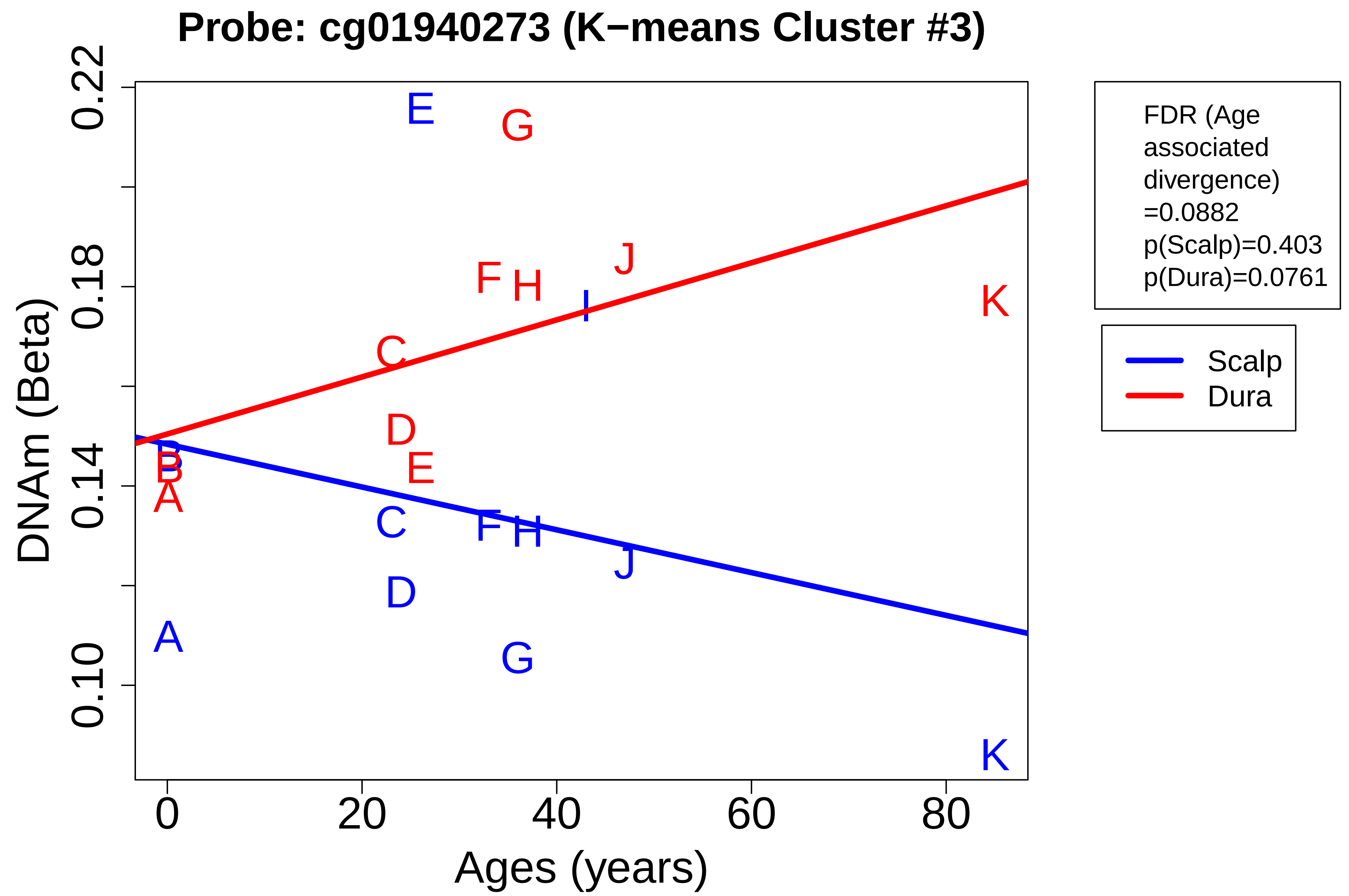

Probe: cg01940273 (K−means Cluster #3)
DNAm (Beta)
0.10
0.14
0.18
0.22
Ages (years)
0
20
40
60
80
FDR (Age associated divergence) =0.0882
p(Scalp)=0.403
p(Dura)=0.0761
Scalp
Dura

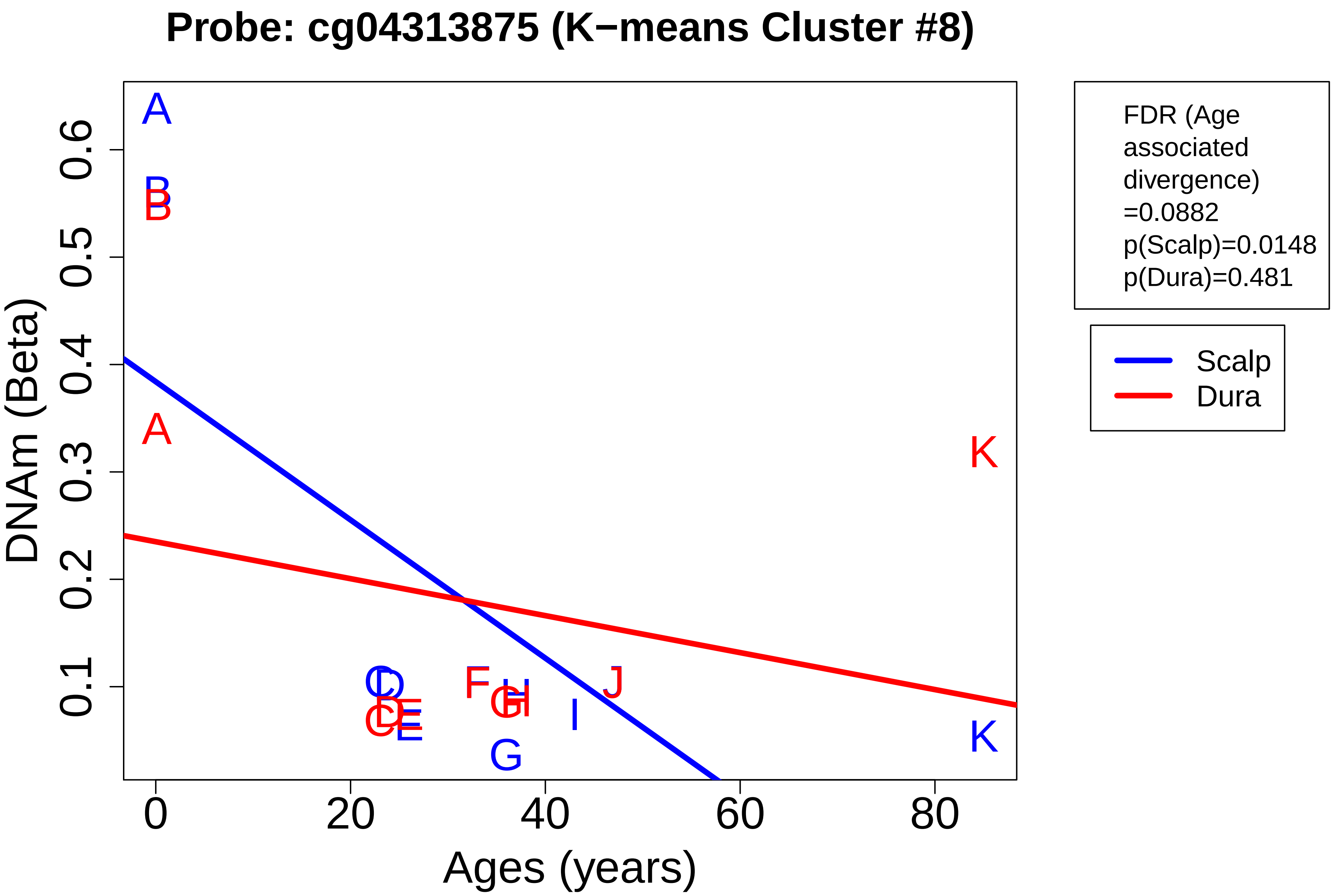

Probe: cg04313875 (K−means Cluster #8)
DNAm (Beta)
0.1
0.2
0.3
0.4
0.5
0.6
0
20
40
60
80
Ages (years)
A
B
B
A
C
D
E
C
D
E
F
G
H
G
H
I
J
K
K
FDR (Age associated divergence) =0.0882
p(Scalp)=0.0148
p(Dura)=0.481
Scalp
Dura

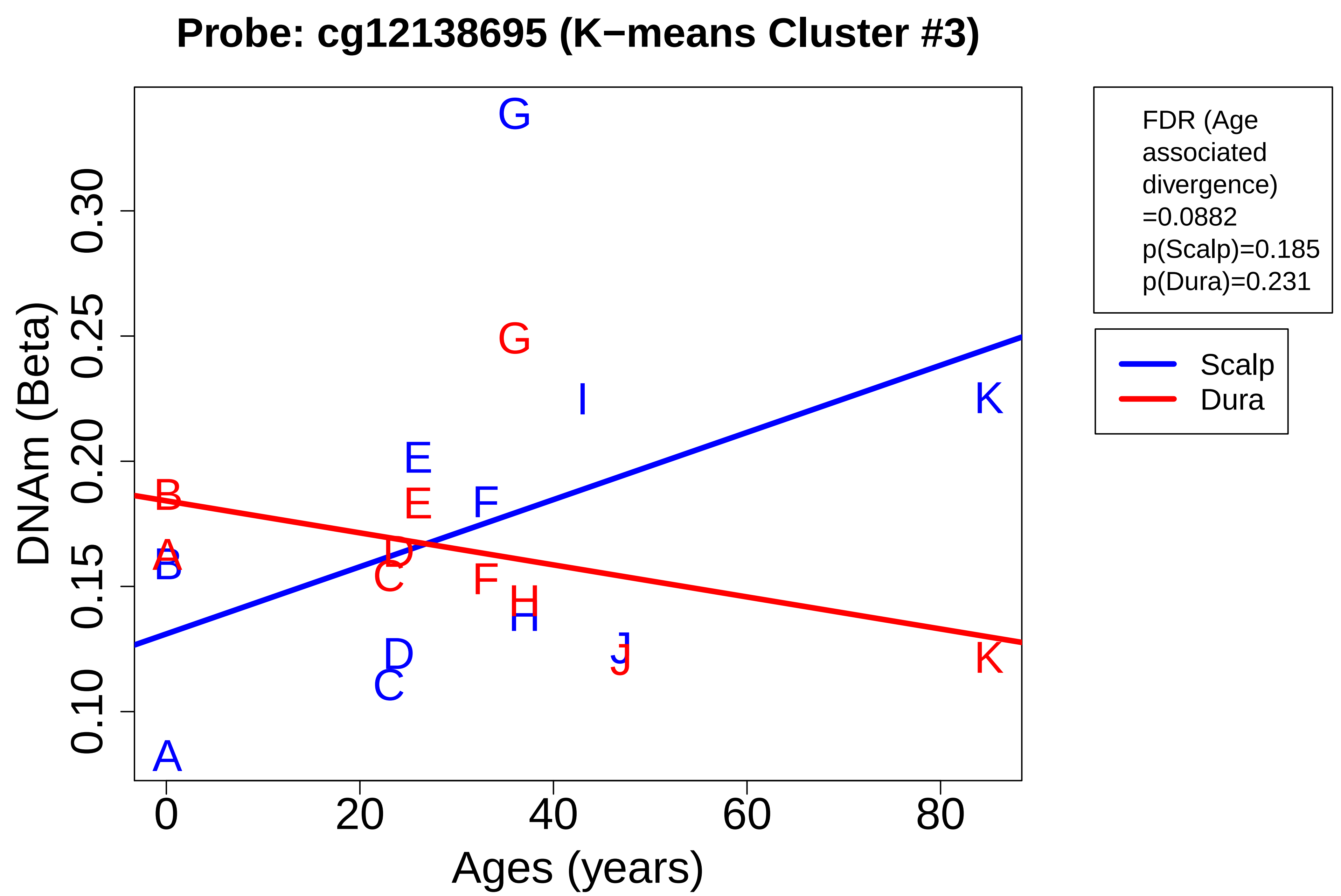
Probe: cg12138695 (K−means Cluster #3)
DNAm (Beta)
0.10
0.15
0.20
0.25
0.30
Ages (years)
0
20
40
60
80
FDR (Age associated divergence) =0.0882
p(Scalp)=0.185
p(Dura)=0.231
Scalp
Dura
A
B
C
D
E
F
G
H
I
J
K

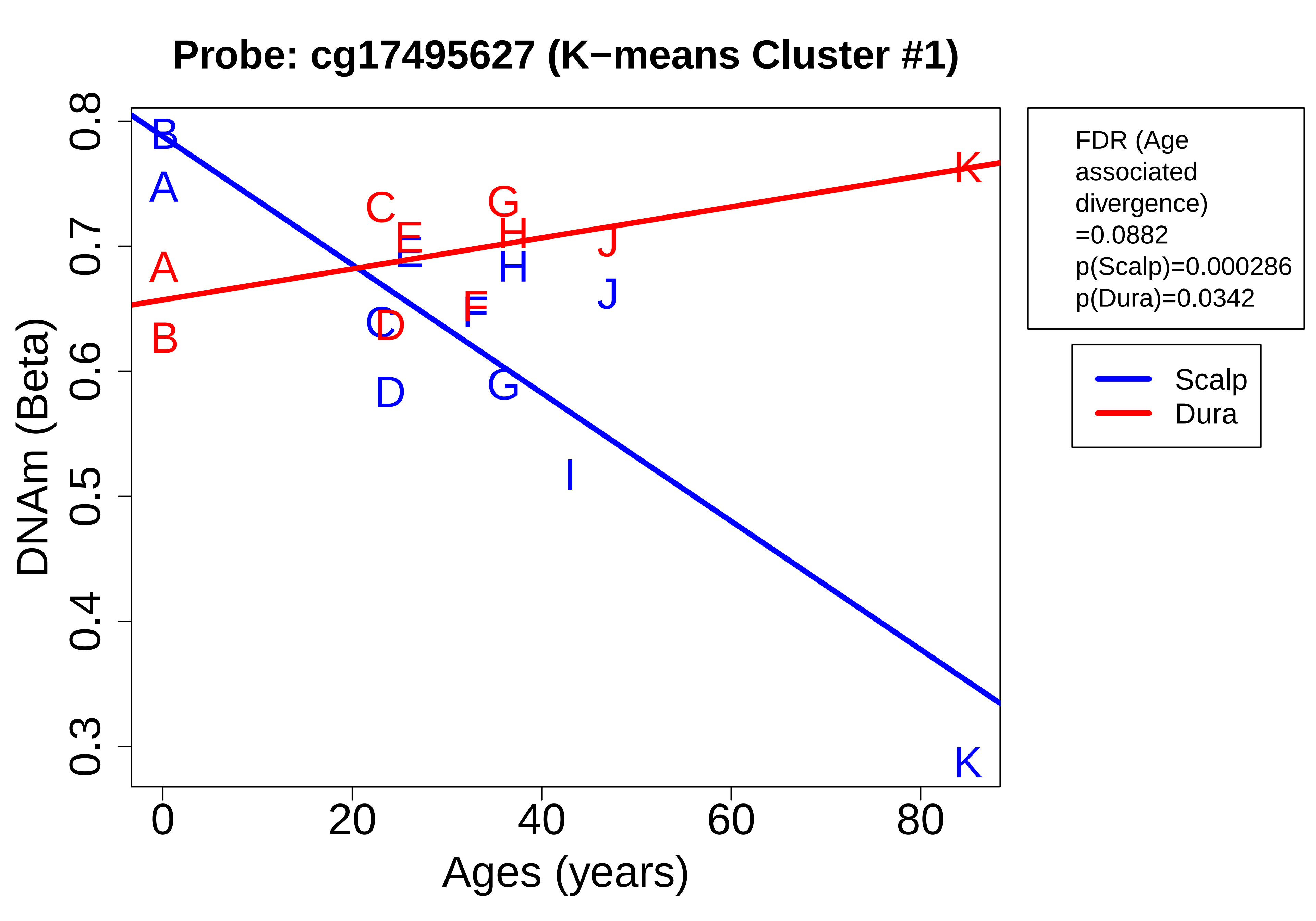
Probe: cg17495627 (K−means Cluster #1)
DNAm (Beta)
Ages (years)
0.3
0.4
0.5
0.6
0.7
0.8
0
20
40
60
80
FDR (Age associated divergence) =0.0882
p(Scalp)=0.000286
p(Dura)=0.0342
Scalp
Dura

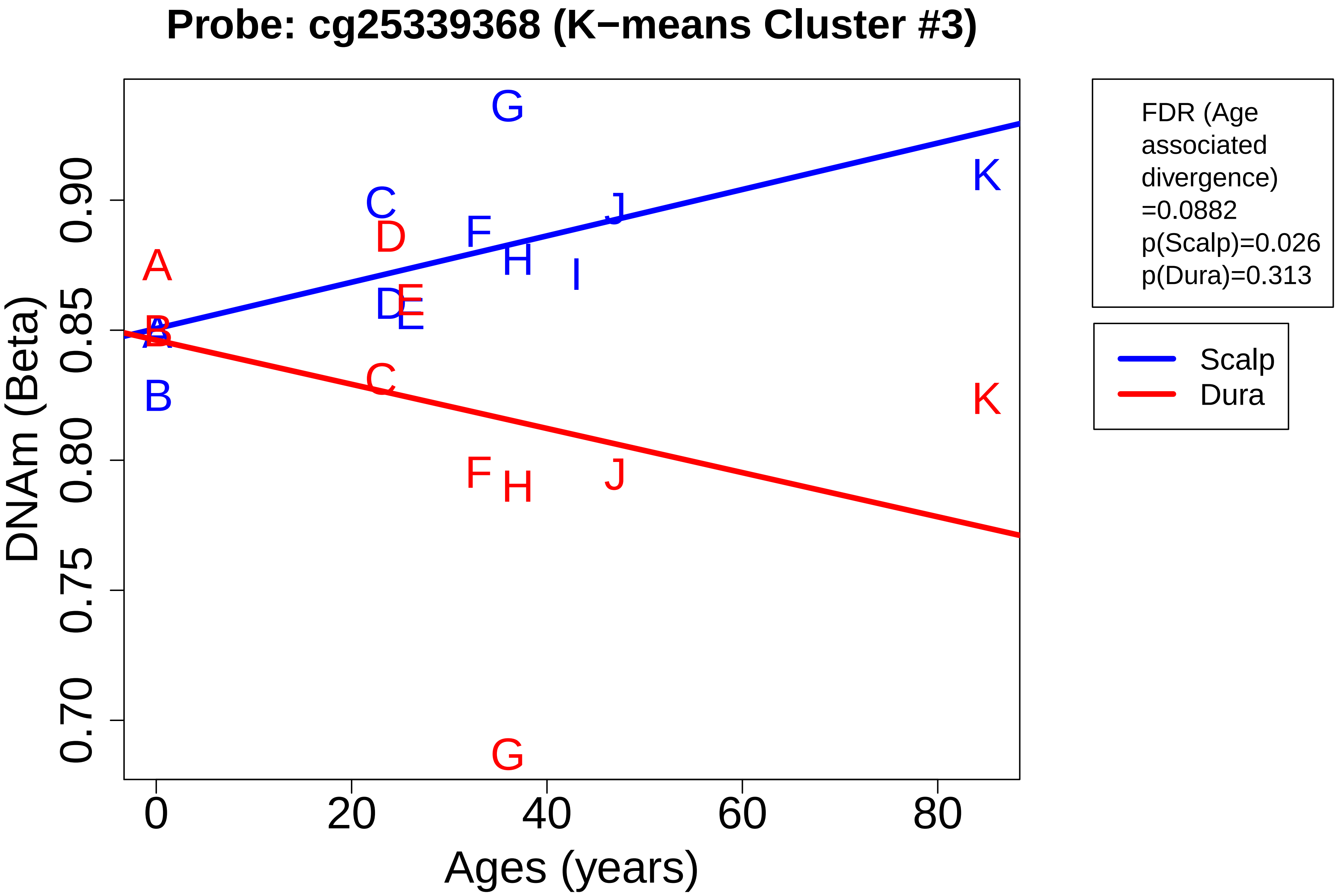

Probe: cg25339368 (K−means Cluster #3)
DNAm (Beta)
0.70
0.75
0.80
0.85
0.90
0
20
40
60
80
Ages (years)
FDR (Age associated divergence) =0.0882
p(Scalp)=0.026
p(Dura)=0.313
Scalp
Dura

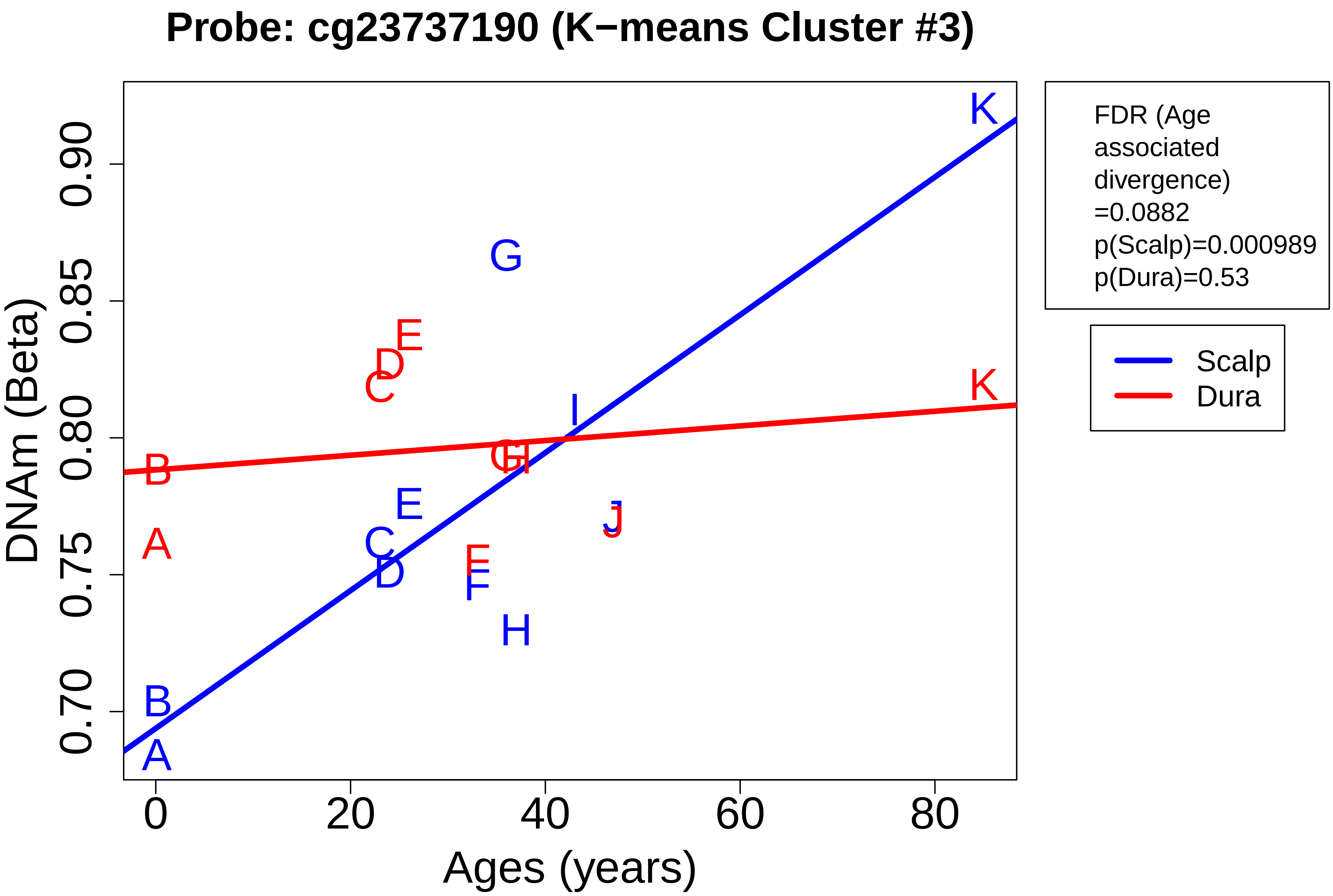

Probe: cg23737190 (K−means Cluster #3)
DNAm (Beta)
0.70
0.75
0.80
0.85
0.90
Ages (years)
0
20
40
60
80
FDR (Age associated divergence) =0.0882
p(Scalp)=0.000989
p(Dura)=0.53
Scalp
Dura
A
B
C
D
E
F
G
H
I
J
K

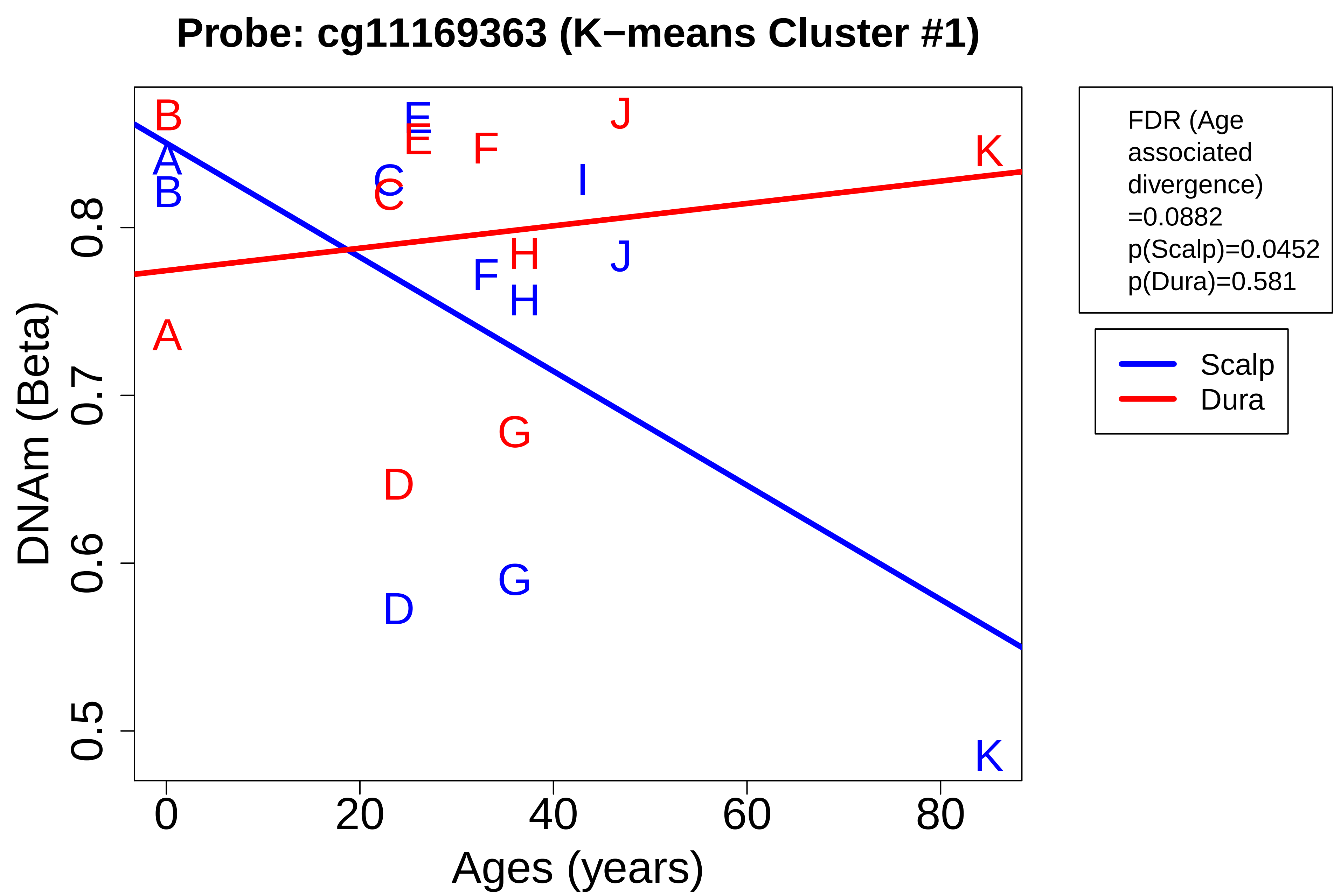

Probe: cg11169363 (K−means Cluster #1)
FDR (Age associated divergence) =0.0882
p(Scalp)=0.0452
p(Dura)=0.581
Scalp
Dura
DNAm (Beta)
0.5
0.6
0.7
0.8
Ages (years)
0
20
40
60
80
A
B
C
D
E
F
G
H
I
J
K

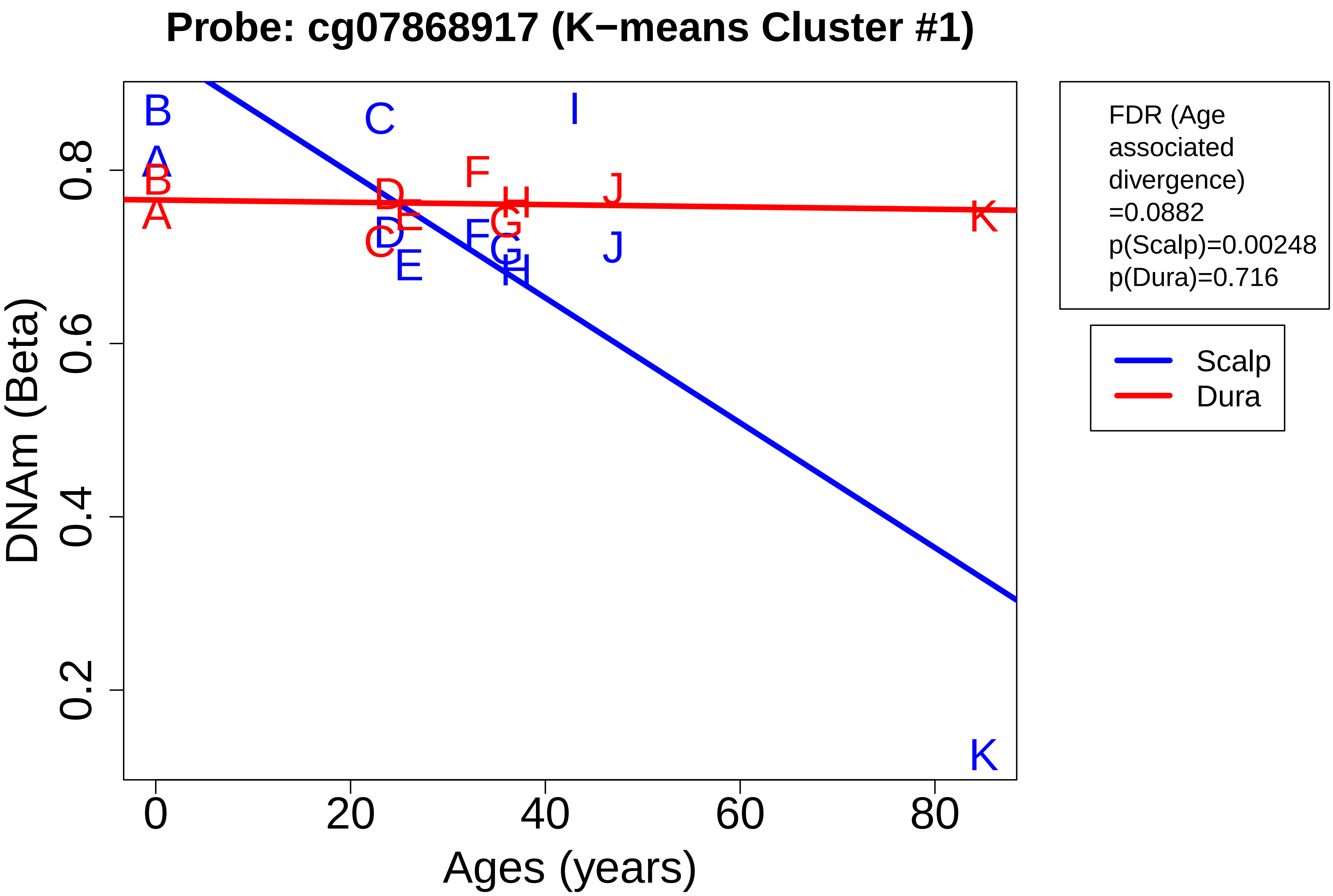

Probe: cg07868917 (K−means Cluster #1)
DNAm (Beta)
0.2
0.4
0.6
0.8
0
20
40
60
80
Ages (years)
FDR (Age associated divergence) =0.0882
p(Scalp)=0.00248
p(Dura)=0.716
Scalp
Dura

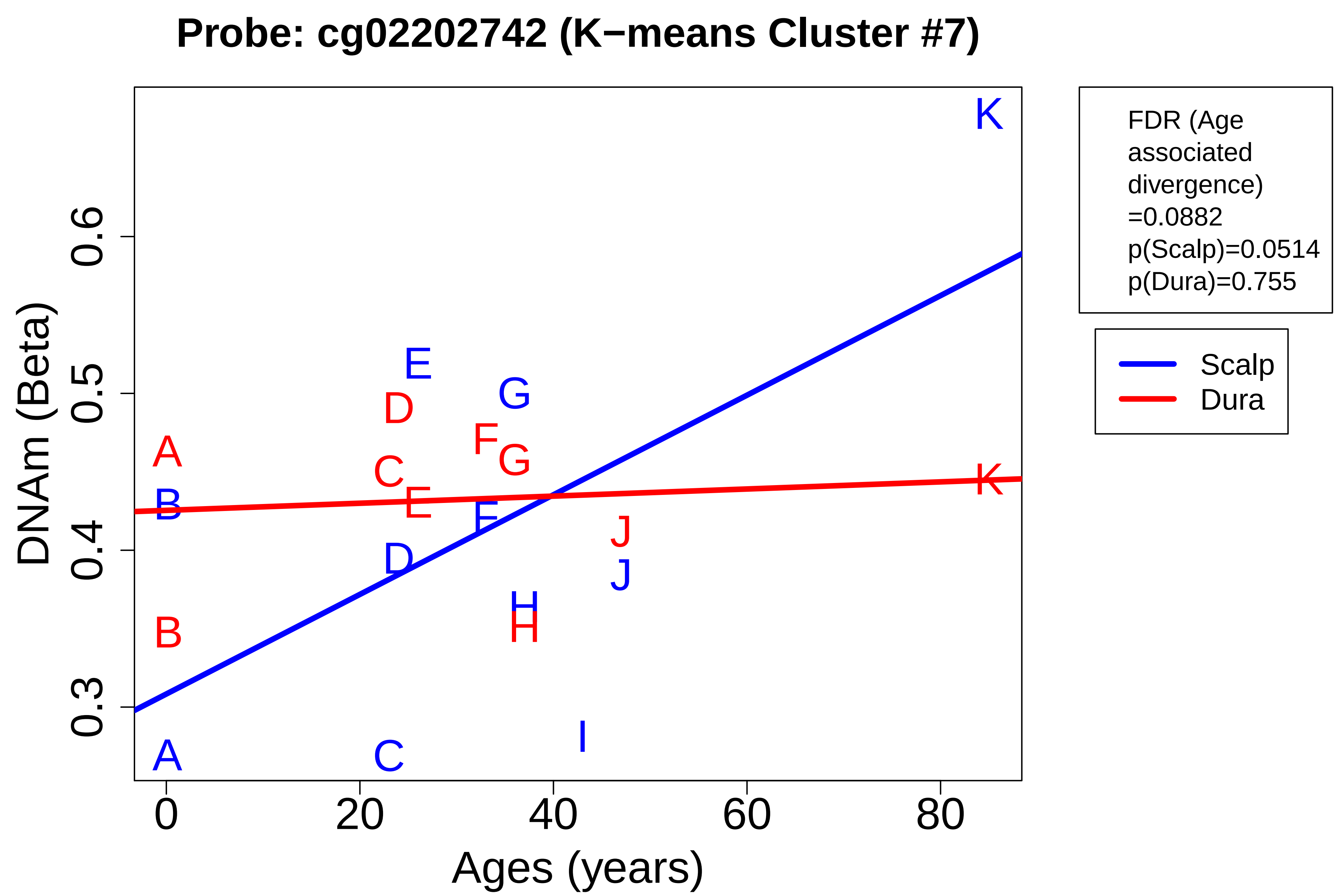

Probe: cg02202742 (K−means Cluster #7)
DNAm (Beta)
0.3
0.4
0.5
0.6
0
20
40
60
80
Ages (years)
A
B
C
D
E
F
G
H
I
J
K
FDR (Age associated divergence) =0.0882
p(Scalp)=0.0514
p(Dura)=0.755
Scalp
Dura

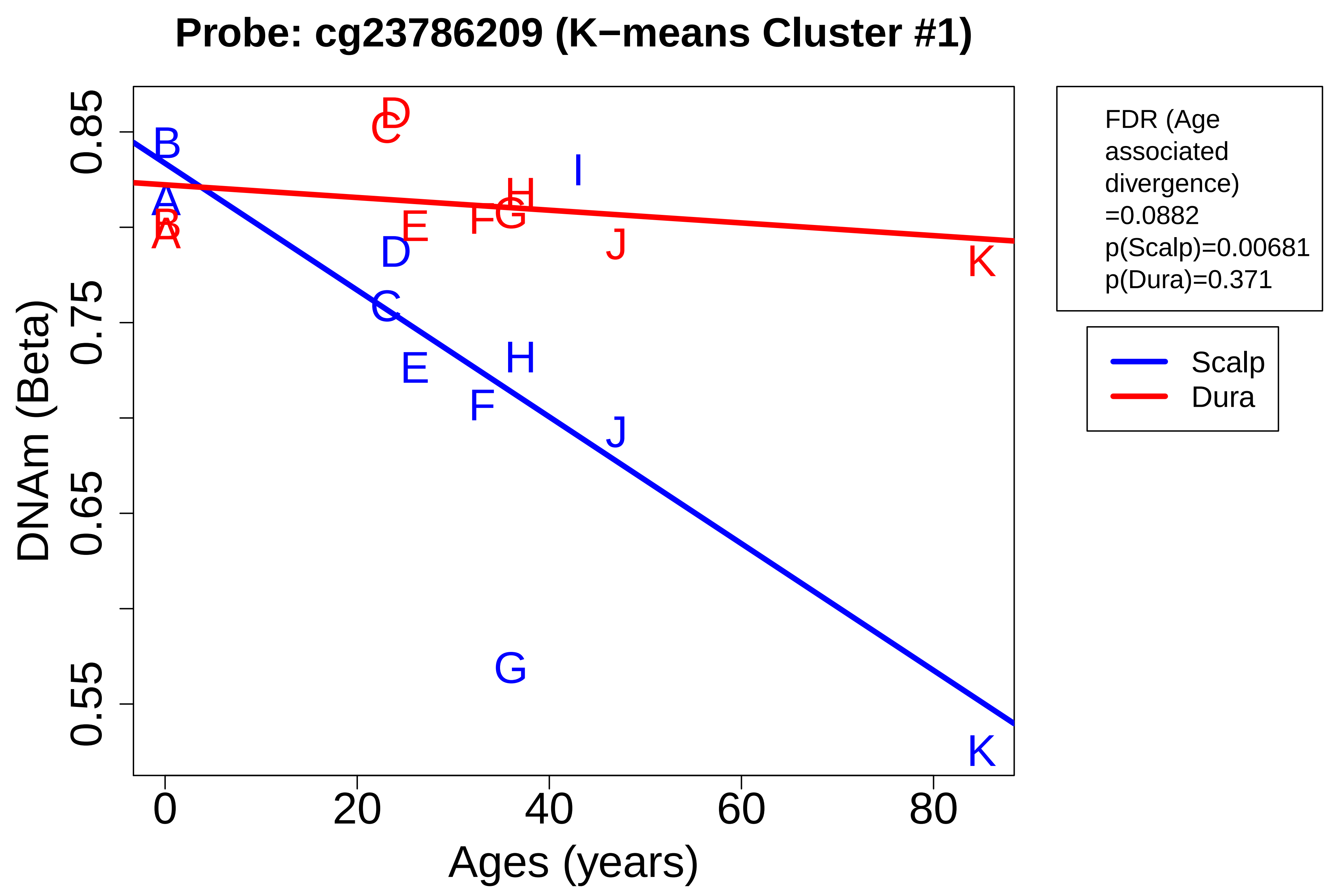

Probe: cg23786209 (K−means Cluster #1)
DNAm (Beta)
0.55
0.65
0.75
0.85
0
20
40
60
80
Ages (years)
FDR (Age associated divergence) =0.0882
p(Scalp)=0.00681
p(Dura)=0.371
Scalp
Dura

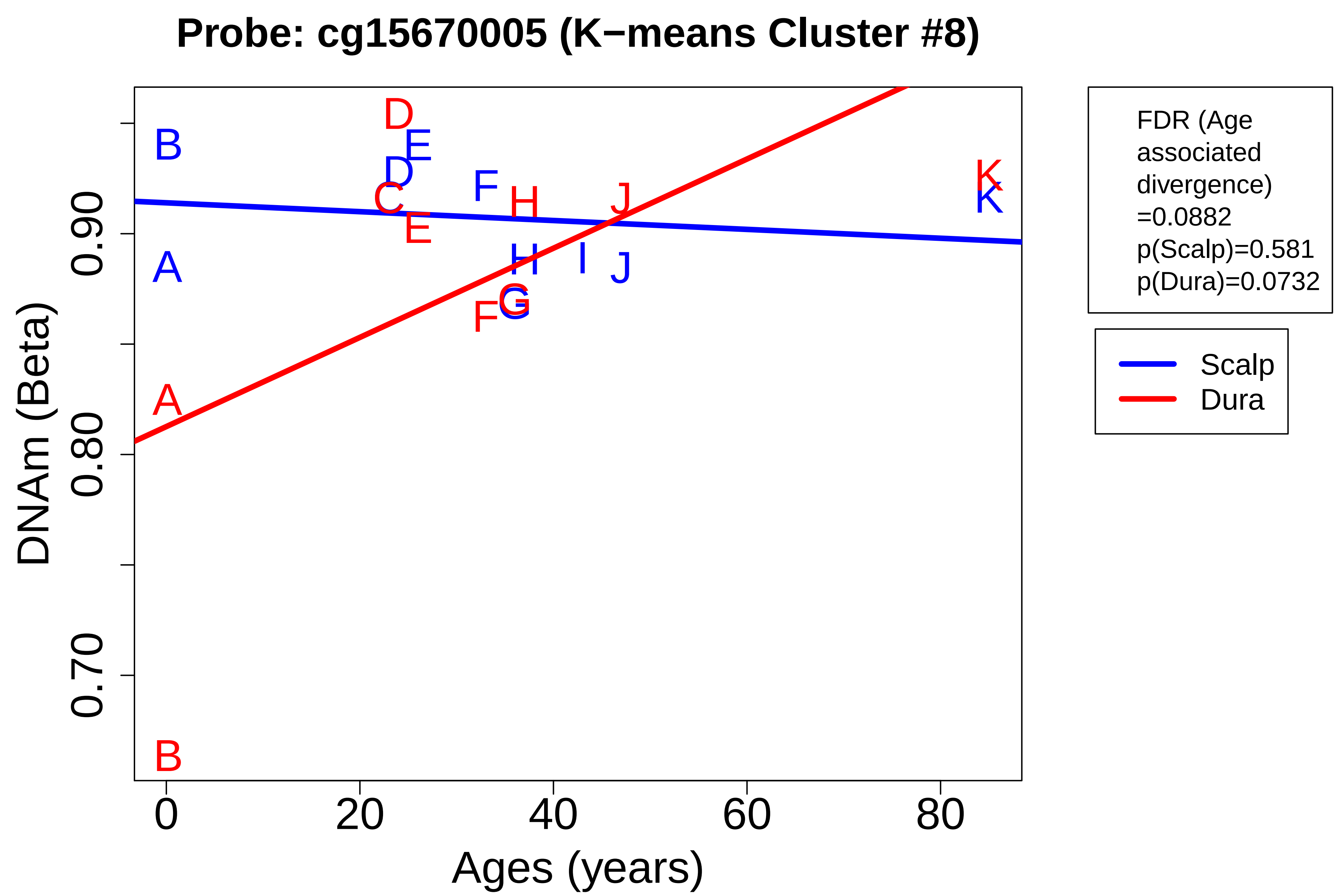

Probe: cg15670005 (K−means Cluster #8)
FDR (Age associated divergence) =0.0882
p(Scalp)=0.581
p(Dura)=0.0732
Scalp
Dura
DNAm (Beta)
0.70
0.80
0.90
0
20
40
60
80
Ages (years)
A
B
C
D
E
F
G
H
I
J
K

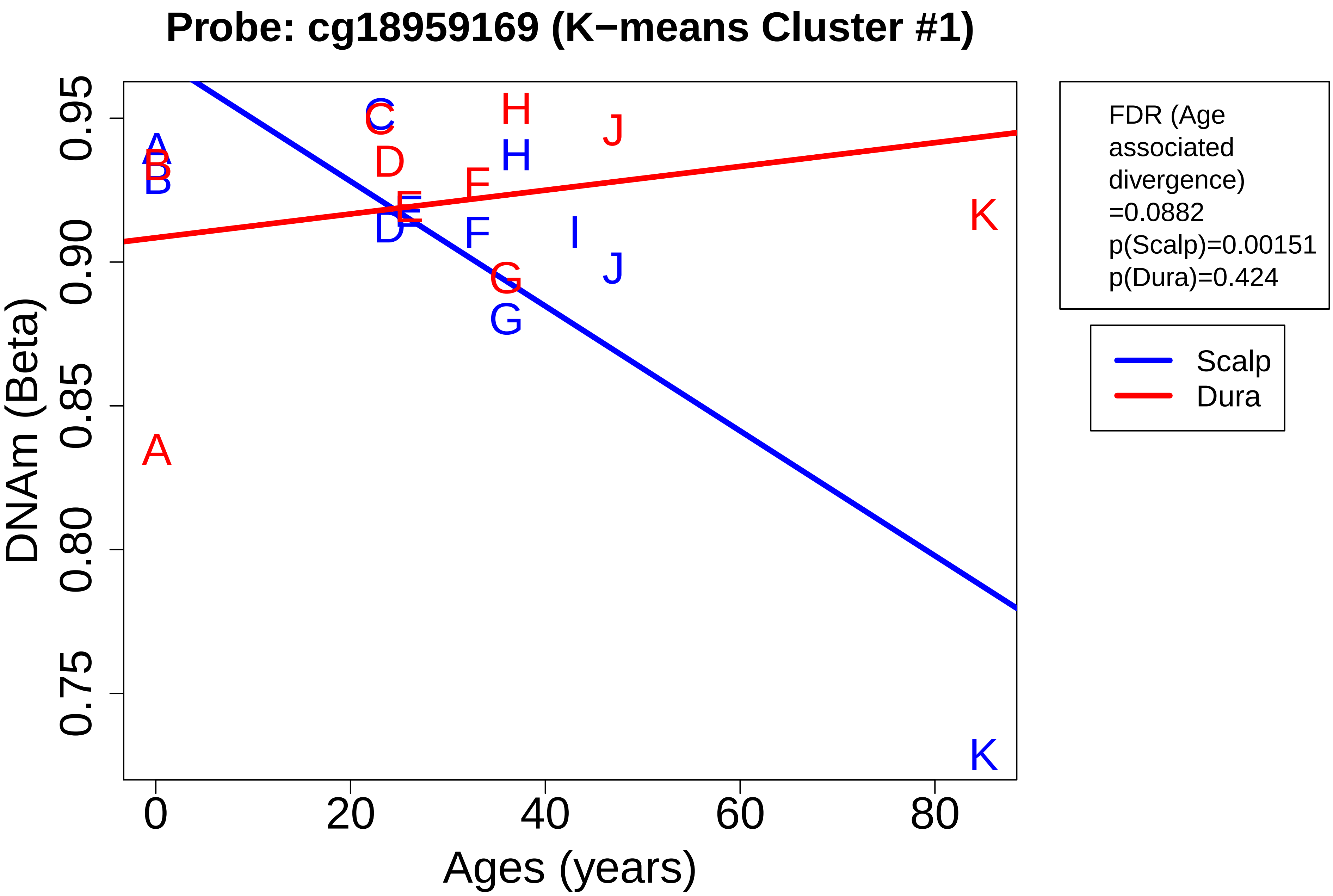

Probe: cg18959169 (K−means Cluster #1)
DNAm (Beta)
0.75
0.80
0.85
0.90
0.95
0
20
40
60
80
Ages (years)
FDR (Age associated divergence) =0.0882
p(Scalp)=0.00151
p(Dura)=0.424
Scalp
Dura
A
B
C
D
E
F
G
H
I
J
K

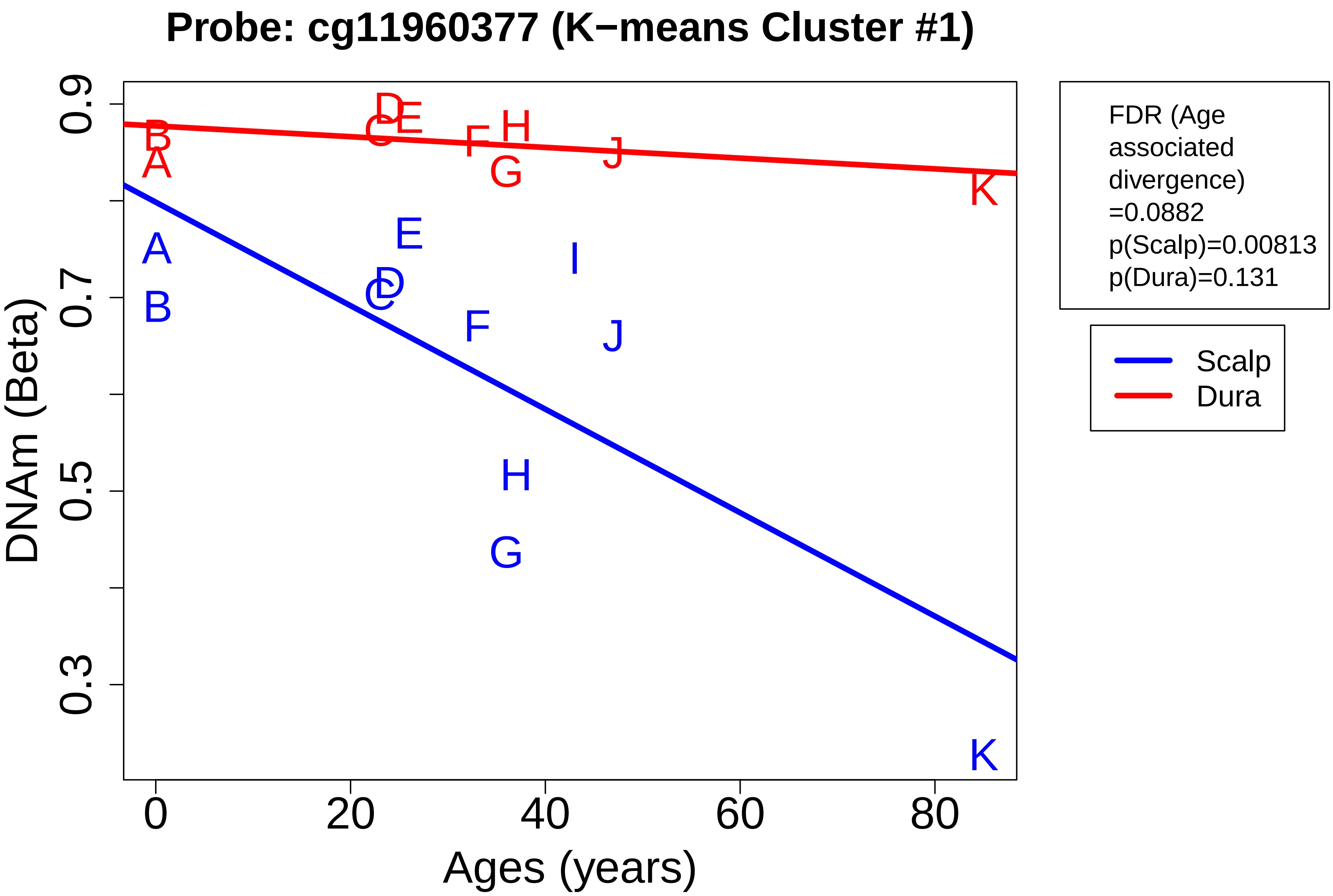
Probe: cg11960377 (K−means Cluster #1)
DNAm (Beta)
0.9
0.7
0.5
0.3
0
20
40
60
80
Ages (years)
A
B
C
D
E
F
G
H
I
J
K
FDR (Age associated divergence) =0.0882
p(Scalp)=0.00813
p(Dura)=0.131
Scalp
Dura

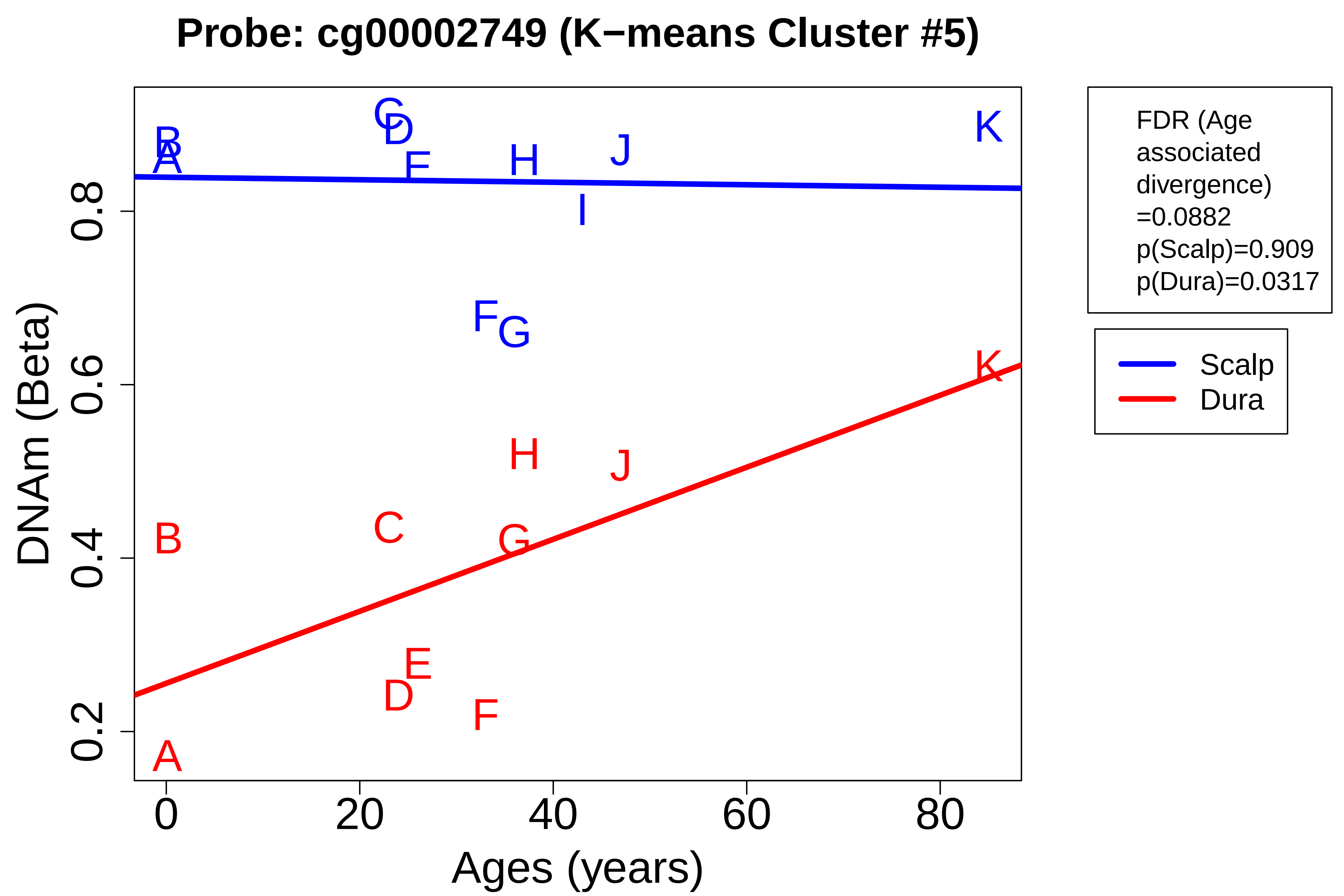

Probe: cg00002749 (K−means Cluster #5)
DNAm (Beta)
0.2
0.4
0.6
0.8
0
20
40
60
80
Ages (years)
A
B
C
D
E
F
G
H
I
J
K
FDR (Age associated divergence) =0.0882
p(Scalp)=0.909
p(Dura)=0.0317
Scalp
Dura

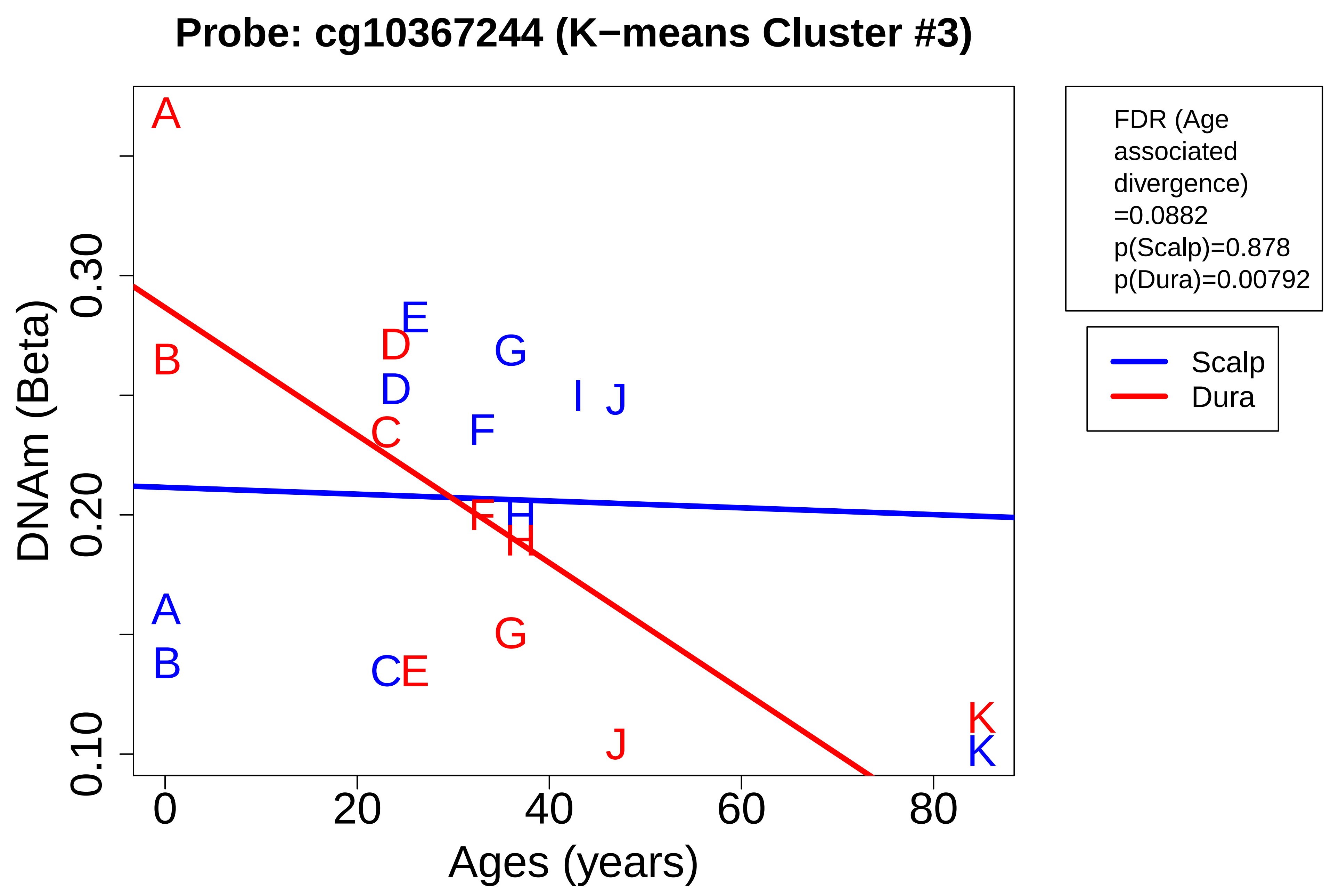

Probe: cg10367244 (K−means Cluster #3)
DNAm (Beta)
0.10
0.20
0.30
Ages (years)
0
20
40
60
80
A
B
C
D
E
F
G
H
I
J
K
FDR (Age associated divergence) =0.0882
p(Scalp)=0.878
p(Dura)=0.00792
Scalp
Dura

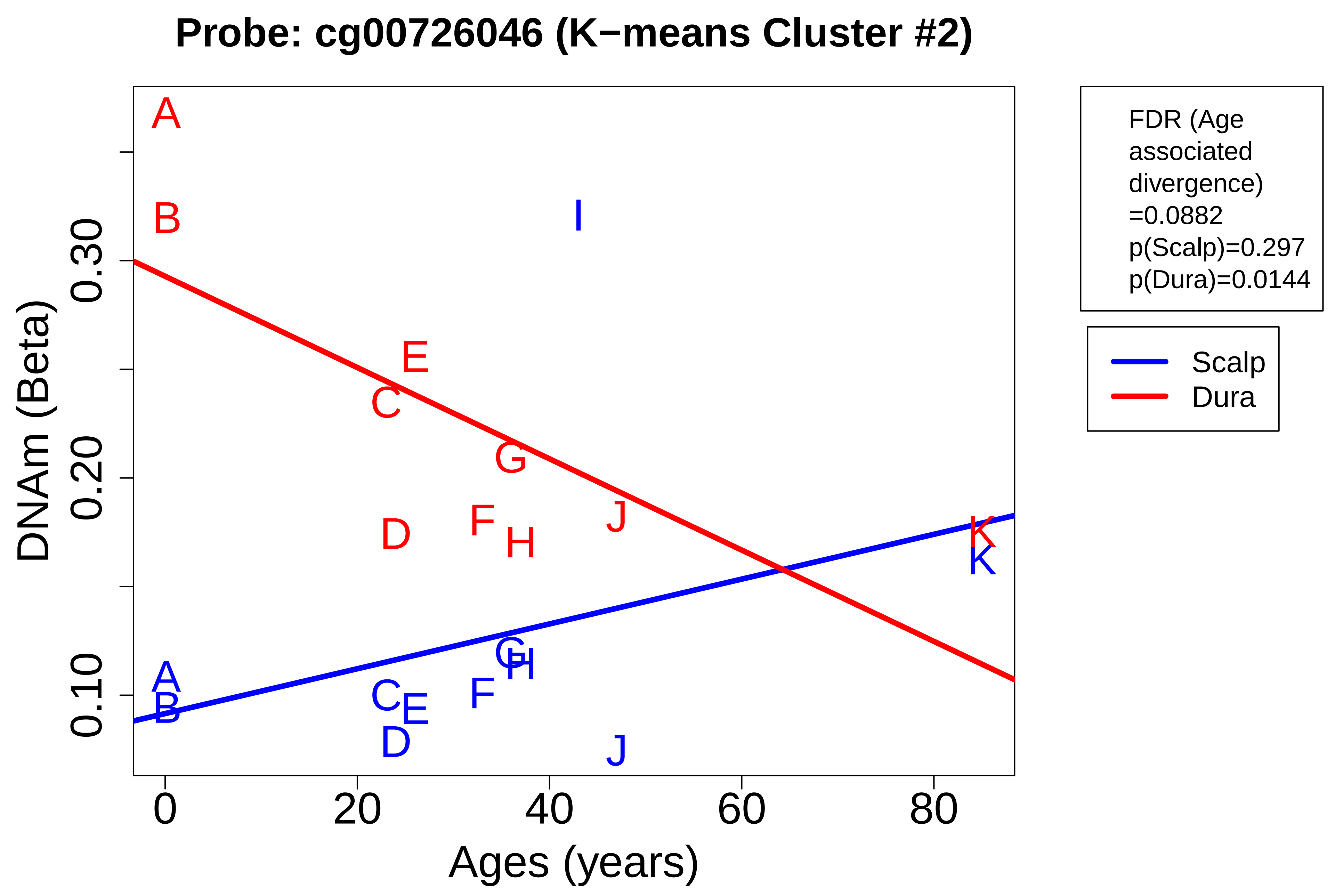

Probe: cg00726046 (K−means Cluster #2)
FDR (Age associated divergence) =0.0882
p(Scalp)=0.297
p(Dura)=0.0144
Scalp
Dura
DNAm (Beta)
0.10
0.20
0.30
0
20
40
60
80
Ages (years)
A
B
C
D
E
F
G
H
I
J
K

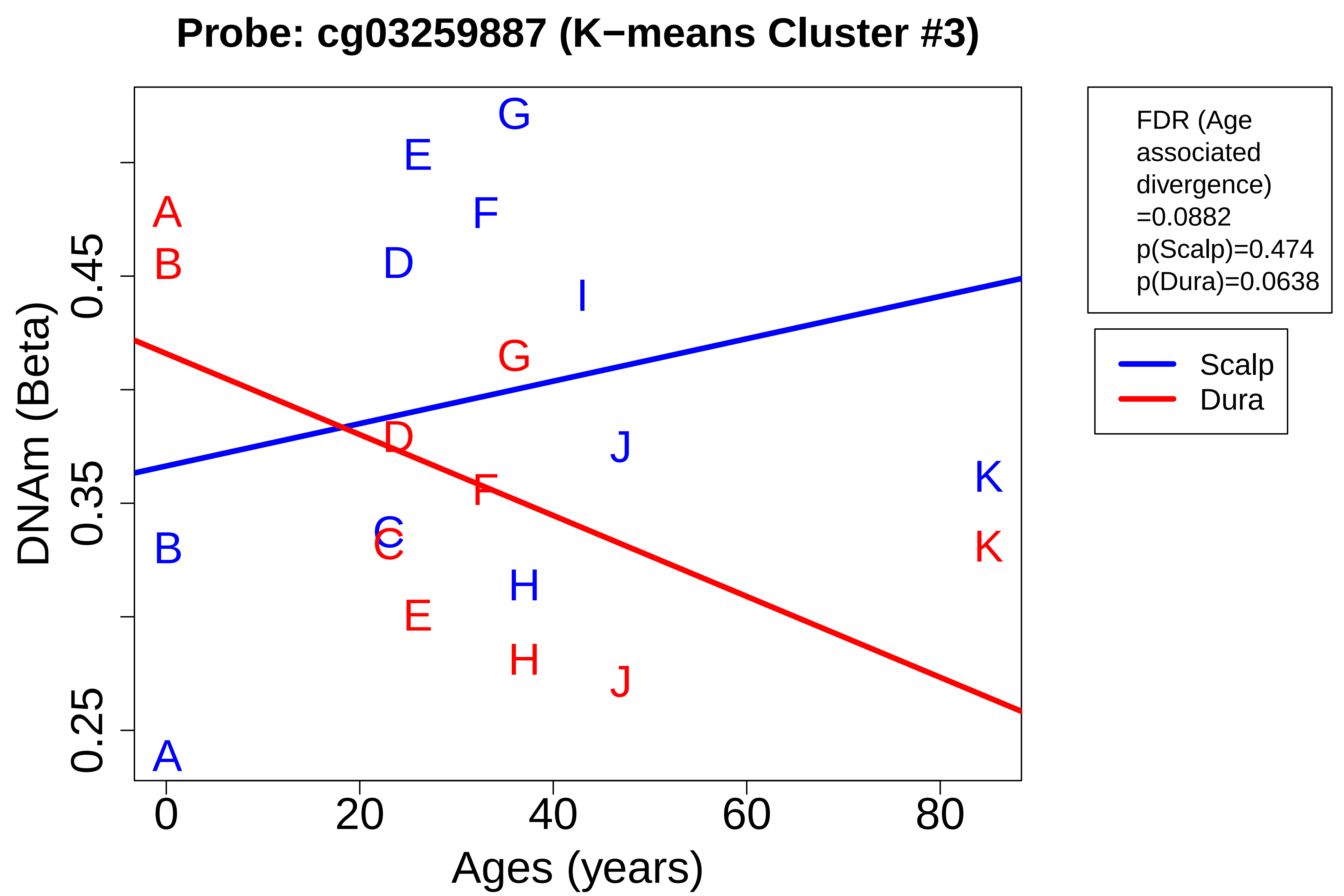

Probe: cg03259887 (K−means Cluster #3)
DNAm (Beta)
0.25
0.35
0.45
0
20
40
60
80
Ages (years)
A
B
C
D
E
F
G
H
I
J
K
A
B
C
D
E
F
G
H
J
K
FDR (Age associated divergence) =0.0882
p(Scalp)=0.474
p(Dura)=0.0638
Scalp
Dura

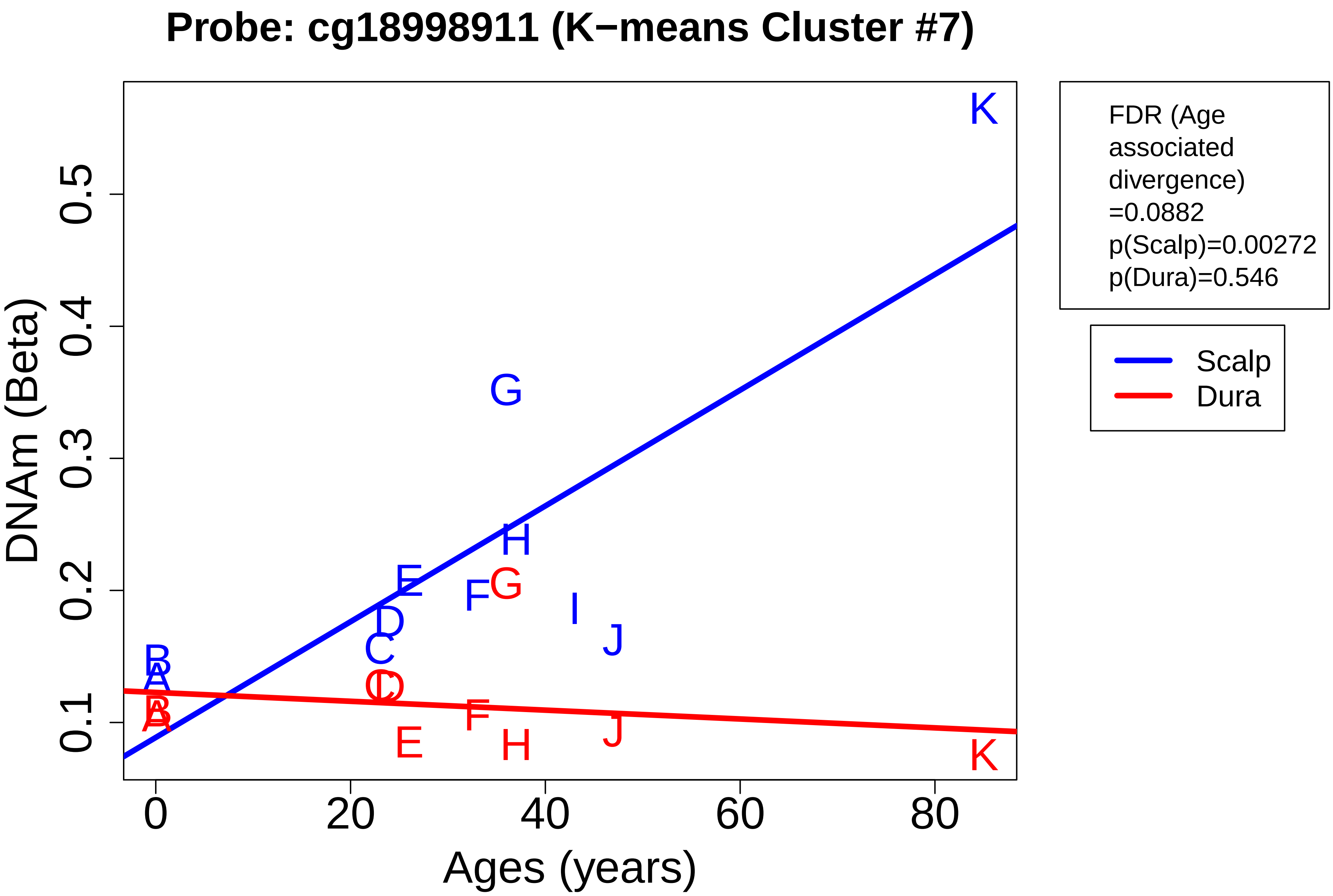

Probe: cg18998911 (K−means Cluster #7)
DNAm (Beta)
0.1
0.2
0.3
0.4
0.5
0
20
40
60
80
Ages (years)
FDR (Age associated divergence) =0.0882
p(Scalp)=0.00272
p(Dura)=0.546
Scalp
Dura

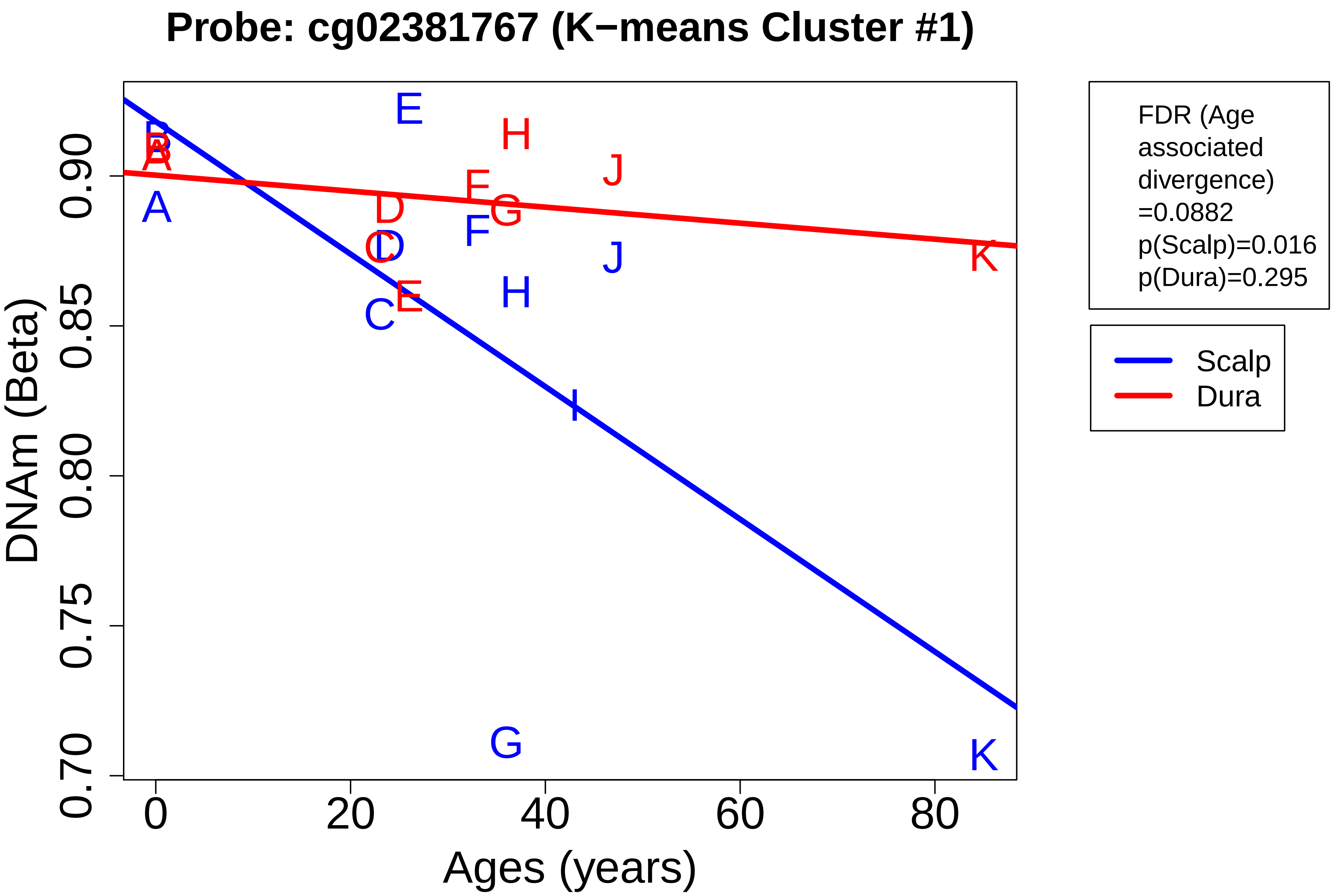

Probe: cg02381767 (K−means Cluster #1)
DNAm (Beta)
0.70
0.75
0.80
0.85
0.90
0
20
40
60
80
Ages (years)
FDR (Age associated divergence) =0.0882
p(Scalp)=0.016
p(Dura)=0.295
Scalp
Dura

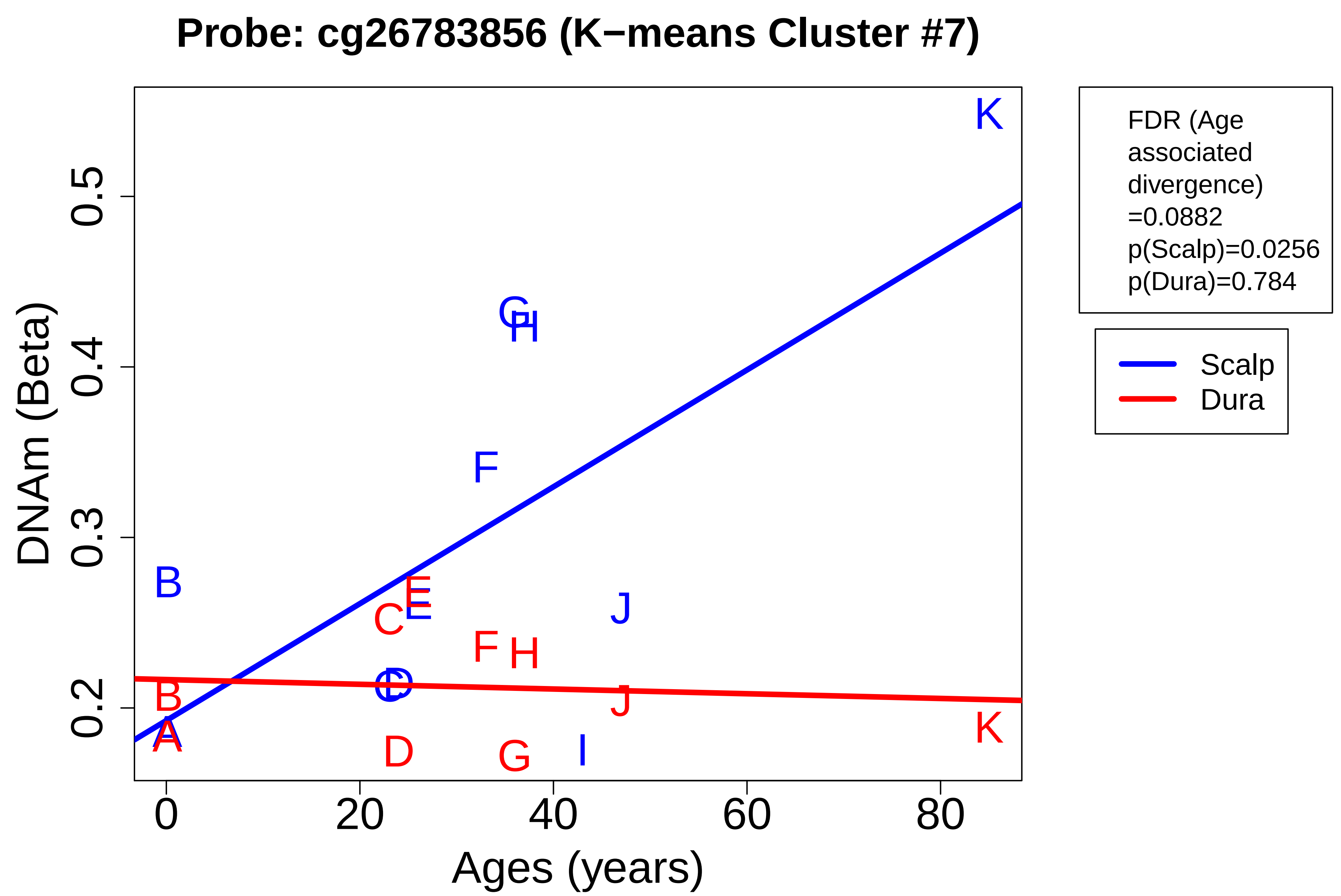

Probe: cg26783856 (K−means Cluster #7)
DNAm (Beta)
0.2
0.3
0.4
0.5
0
20
40
60
80
Ages (years)
FDR (Age associated divergence) =0.0882
p(Scalp)=0.0256
p(Dura)=0.784
Scalp
Dura

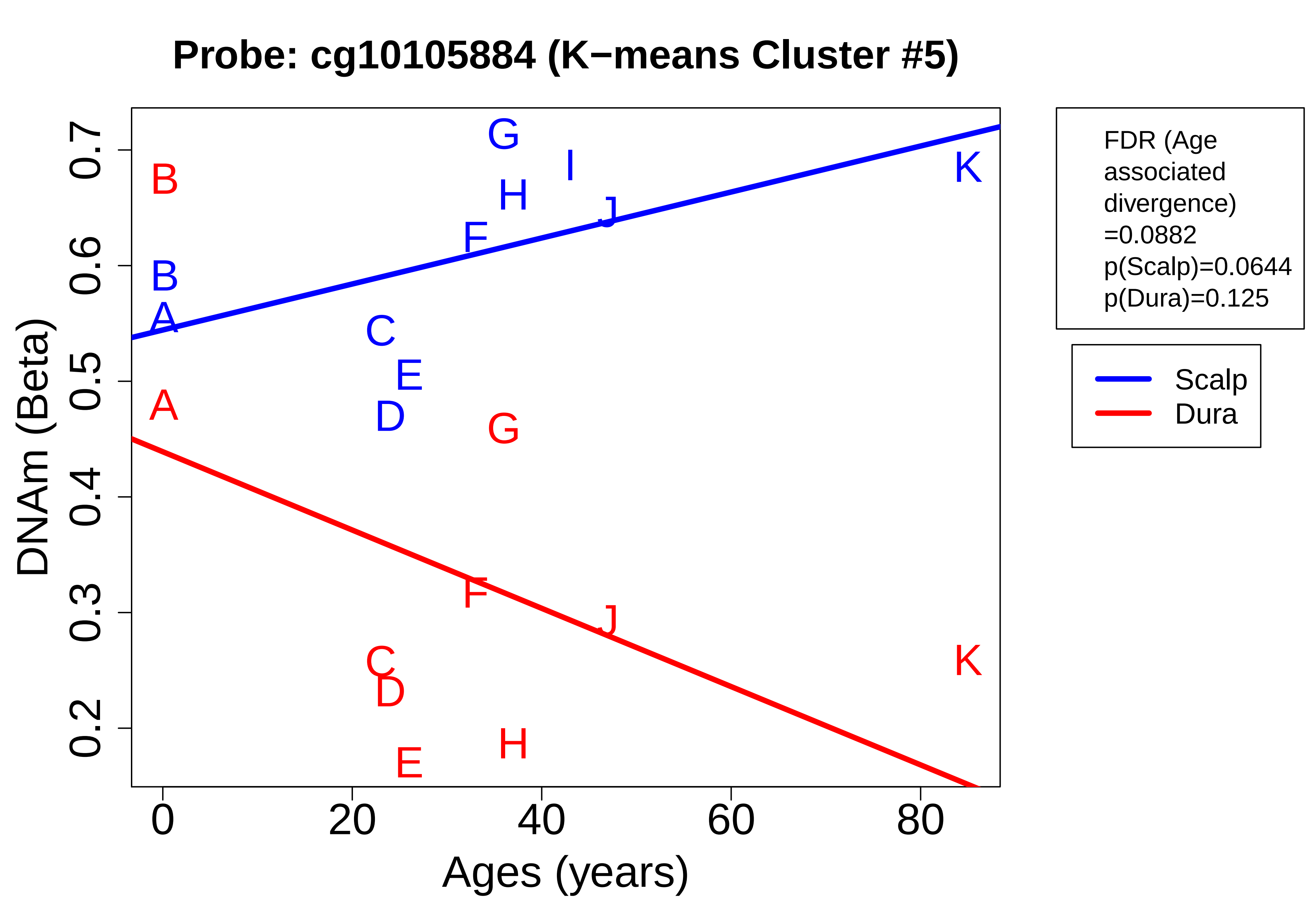

Probe: cg10105884 (K−means Cluster #5)
DNAm (Beta)
0.2
0.3
0.4
0.5
0.6
0.7
Ages (years)
0
20
40
60
80
FDR (Age associated divergence) =0.0882
p(Scalp)=0.0644
p(Dura)=0.125
Scalp
Dura

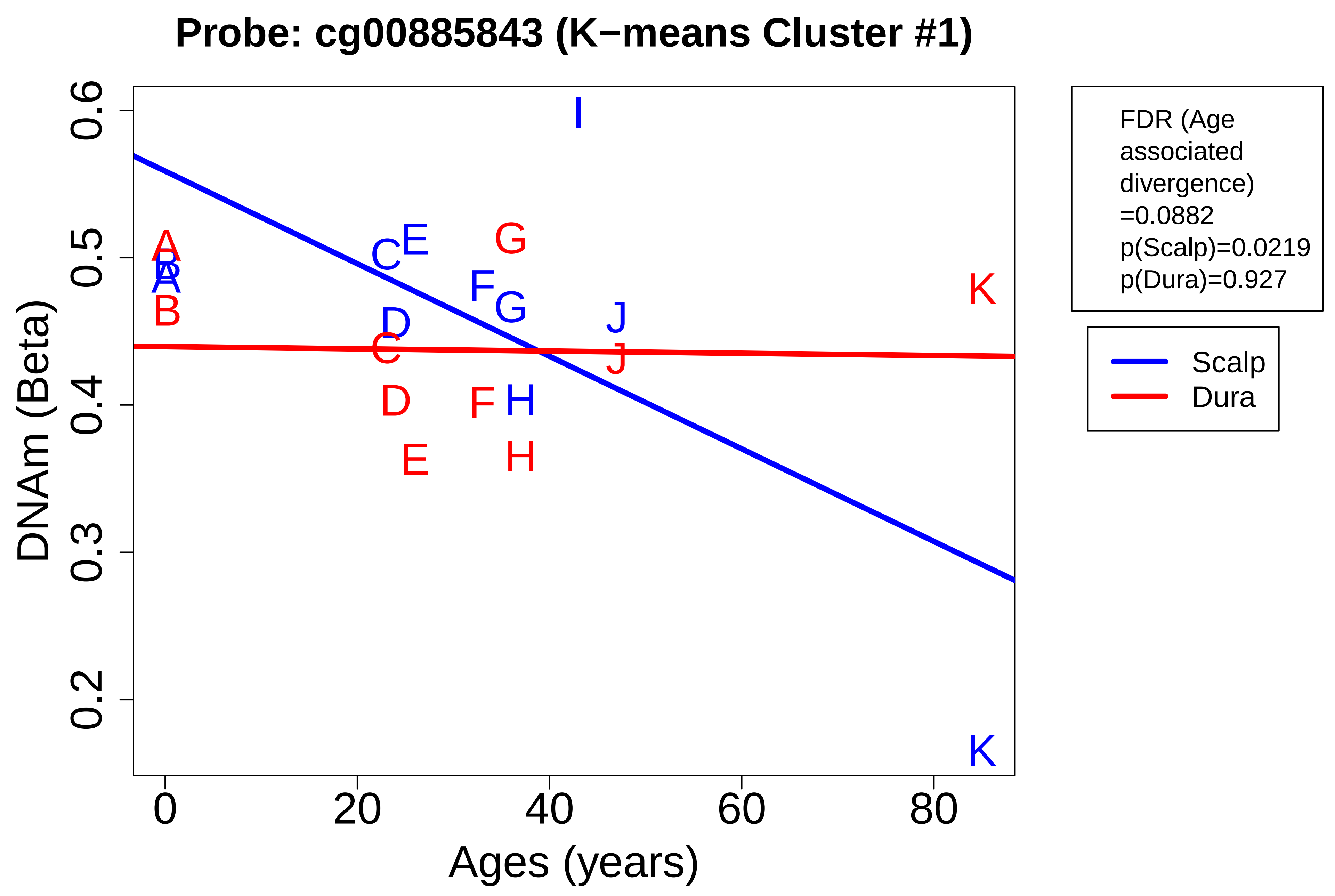

Probe: cg00885843 (K−means Cluster #1)
DNAm (Beta)
0.2
0.3
0.4
0.5
0.6
0
20
40
60
80
Ages (years)
FDR (Age associated divergence) =0.0882
p(Scalp)=0.0219
p(Dura)=0.927
Scalp
Dura
A
B
C
D
E
F
G
H
I
J
K

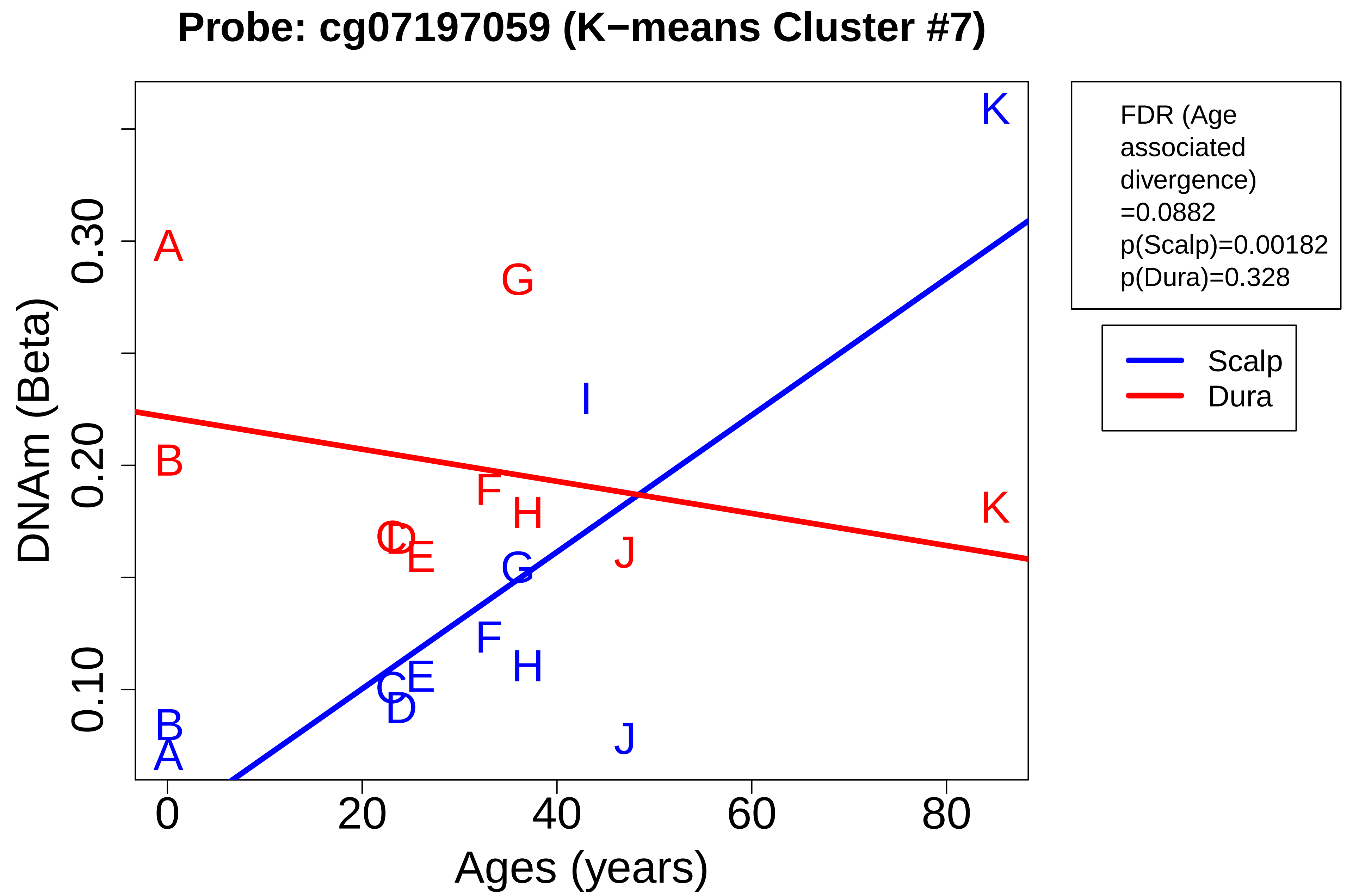

Probe: cg07197059 (K−means Cluster #7)
DNAm (Beta)
0.10
0.20
0.30
0
20
40
60
80
Ages (years)
A
B
C
D
E
F
G
H
I
J
K
FDR (Age associated divergence) =0.0882
p(Scalp)=0.00182
p(Dura)=0.328
Scalp
Dura

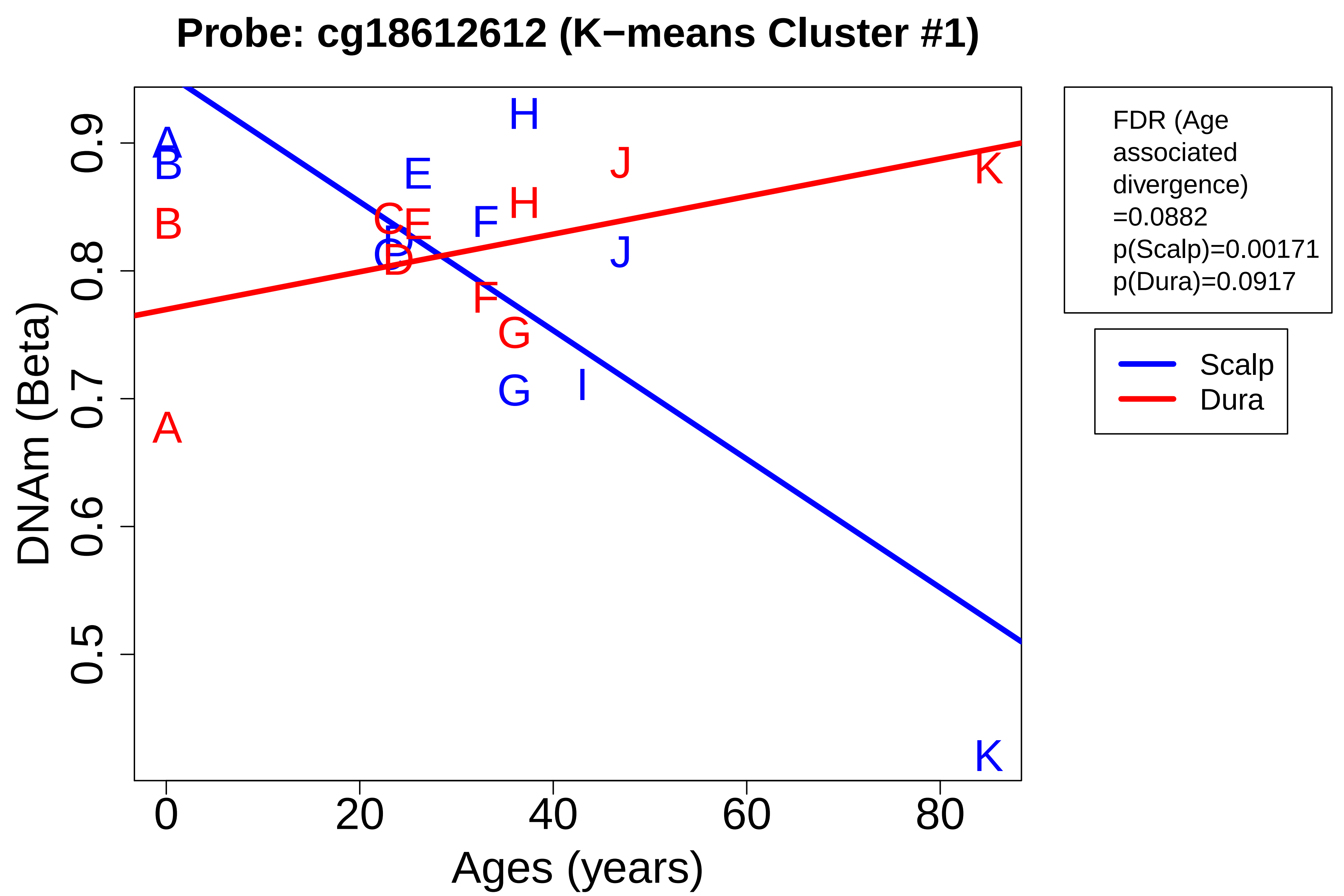

Probe: cg18612612 (K−means Cluster #1)
FDR (Age associated divergence) =0.0882
p(Scalp)=0.00171
p(Dura)=0.0917
Scalp
Dura
DNAm (Beta)
Ages (years)
0.5
0.6
0.7
0.8
0.9
0
20
40
60
80

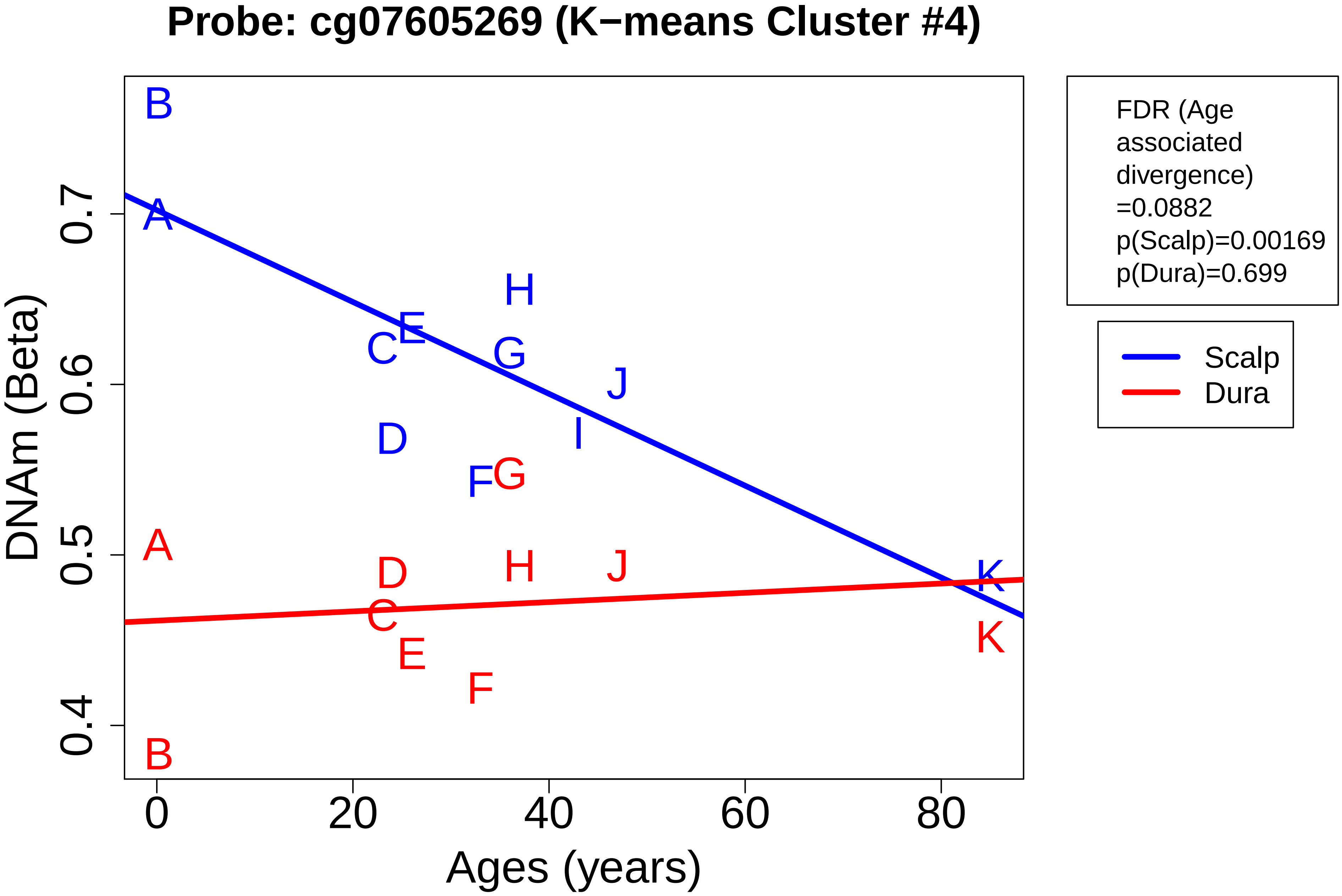

Probe: cg07605269 (K−means Cluster #4)
DNAm (Beta)
0.4
0.5
0.6
0.7
Ages (years)
0
20
40
60
80
FDR (Age associated divergence) =0.0882
p(Scalp)=0.00169
p(Dura)=0.699
Scalp
Dura

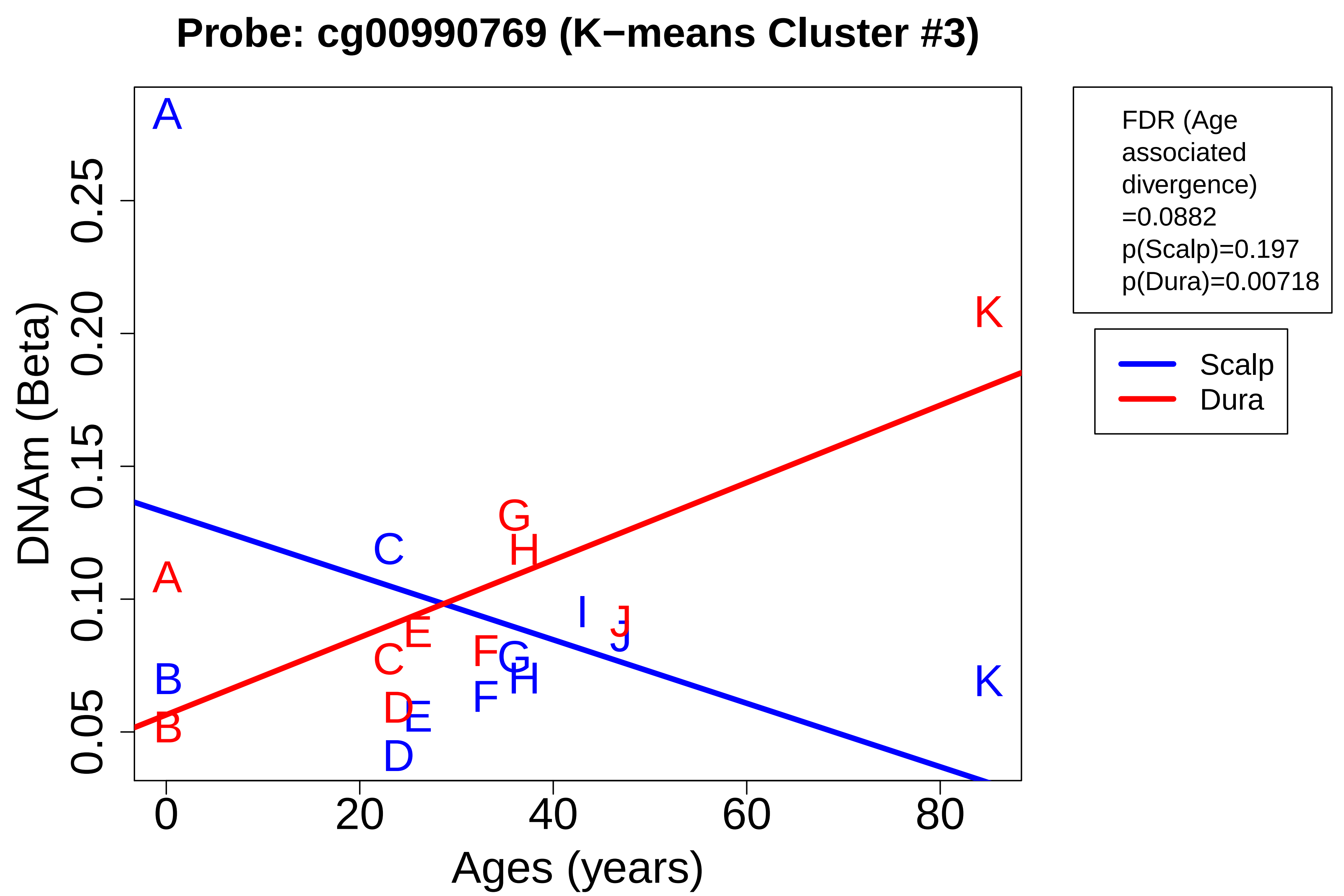

Probe: cg00990769 (K−means Cluster #3)
FDR (Age associated divergence) =0.0882
p(Scalp)=0.197
p(Dura)=0.00718
Scalp
Dura
DNAm (Beta)
0.05
0.10
0.15
0.20
0.25
0
20
40
60
80
Ages (years)
A
B
C
D
E
F
G
H
I
J
K

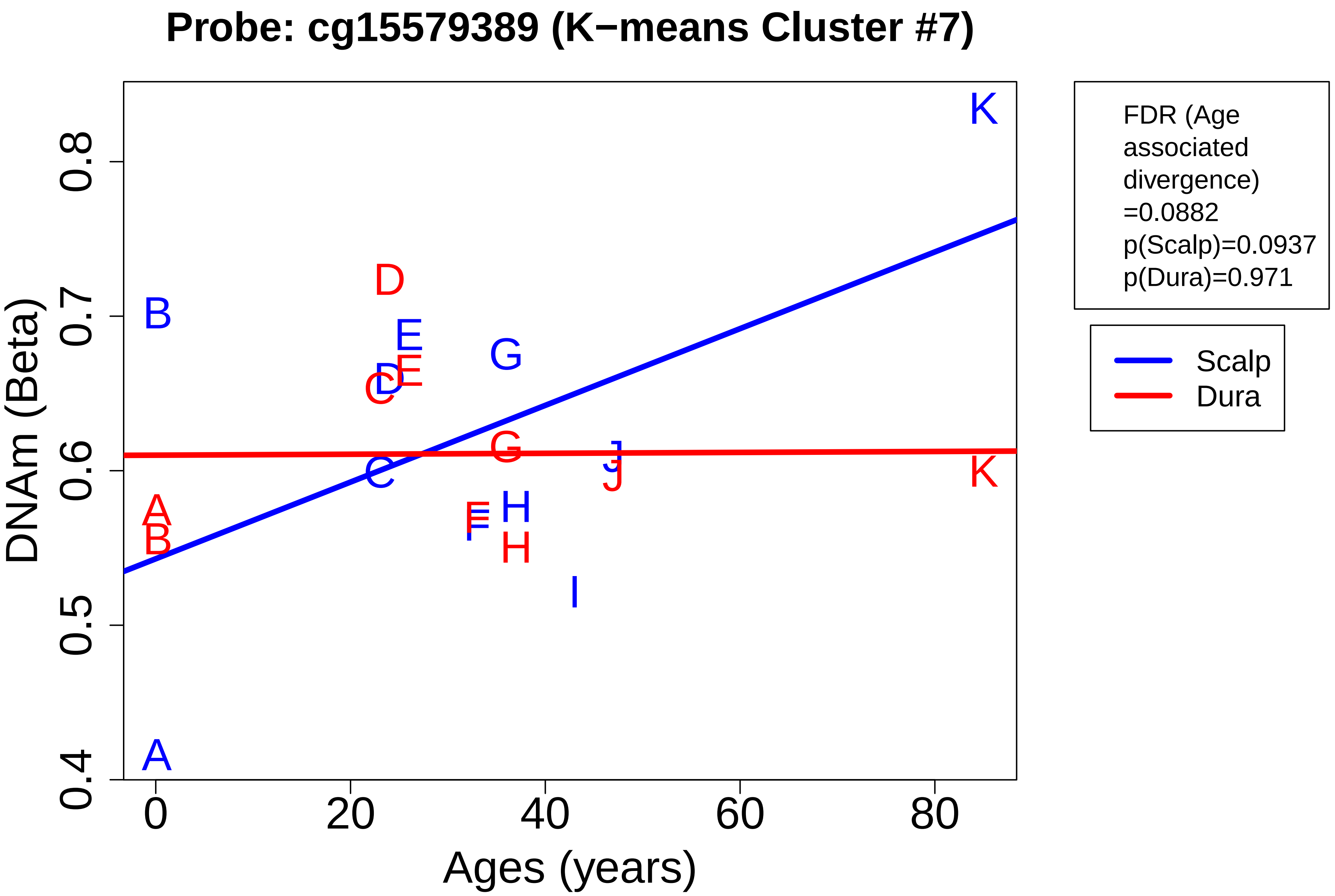

Probe: cg15579389 (K−means Cluster #7)
DNAm (Beta)
0.4
0.5
0.6
0.7
0.8
Ages (years)
0
20
40
60
80
A
B
C
D
E
F
G
H
I
J
K
FDR (Age associated divergence) =0.0882
p(Scalp)=0.0937
p(Dura)=0.971
Scalp
Dura

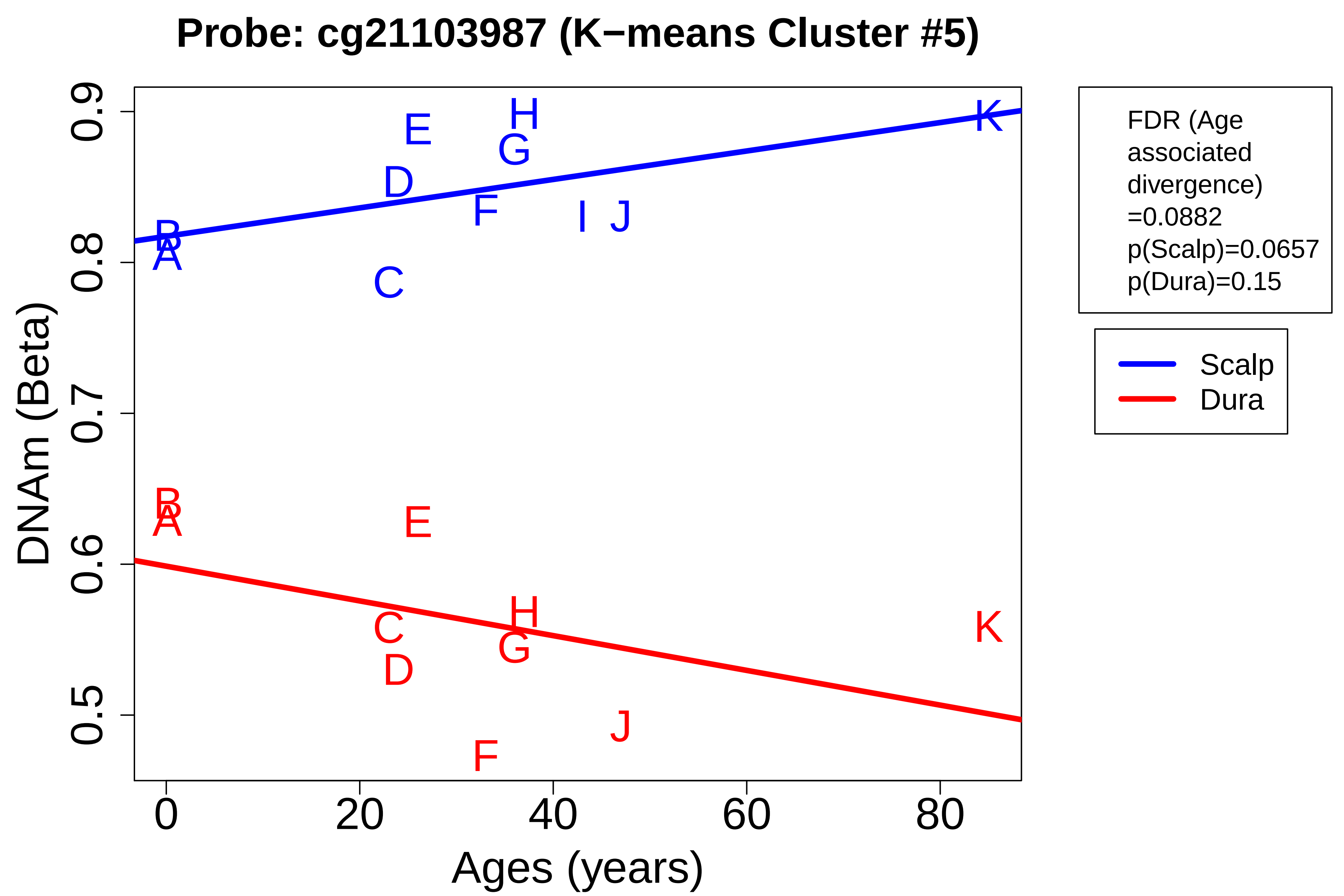
Probe: cg21103987 (K−means Cluster #5)
DNAm (Beta)
0.5
0.6
0.7
0.8
0.9
0
20
40
60
80
Ages (years)
E
H
K
G
D
F
I
J
B
A
C
B
A
E
H
C
G
D
J
F
K
FDR (Age associated divergence) =0.0882
p(Scalp)=0.0657
p(Dura)=0.15
Scalp
Dura

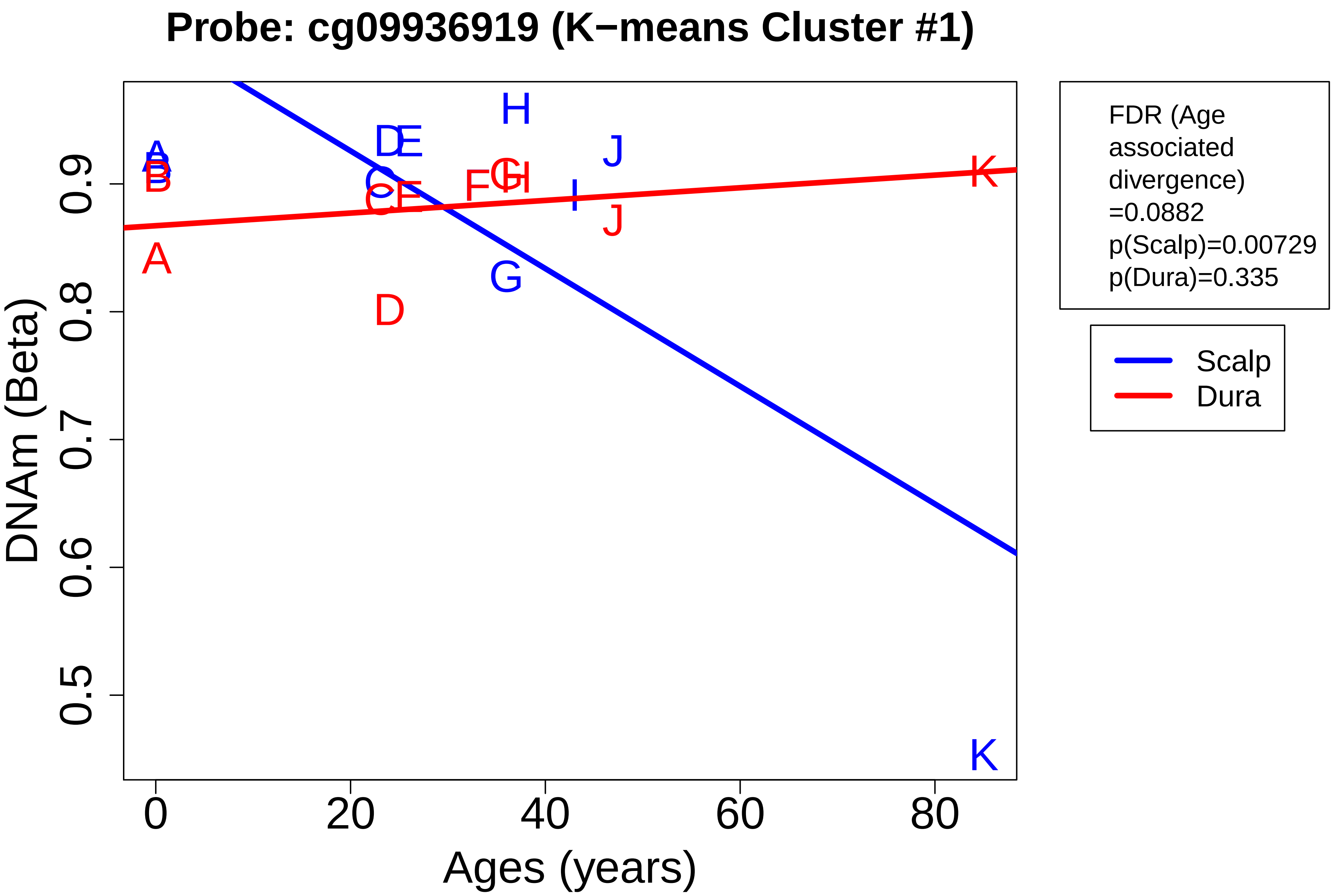

Probe: cg09936919 (K−means Cluster #1)
DNAm (Beta)
0.5
0.6
0.7
0.8
0.9
0
20
40
60
80
Ages (years)
FDR (Age associated divergence) =0.0882
p(Scalp)=0.00729
p(Dura)=0.335
Scalp
Dura

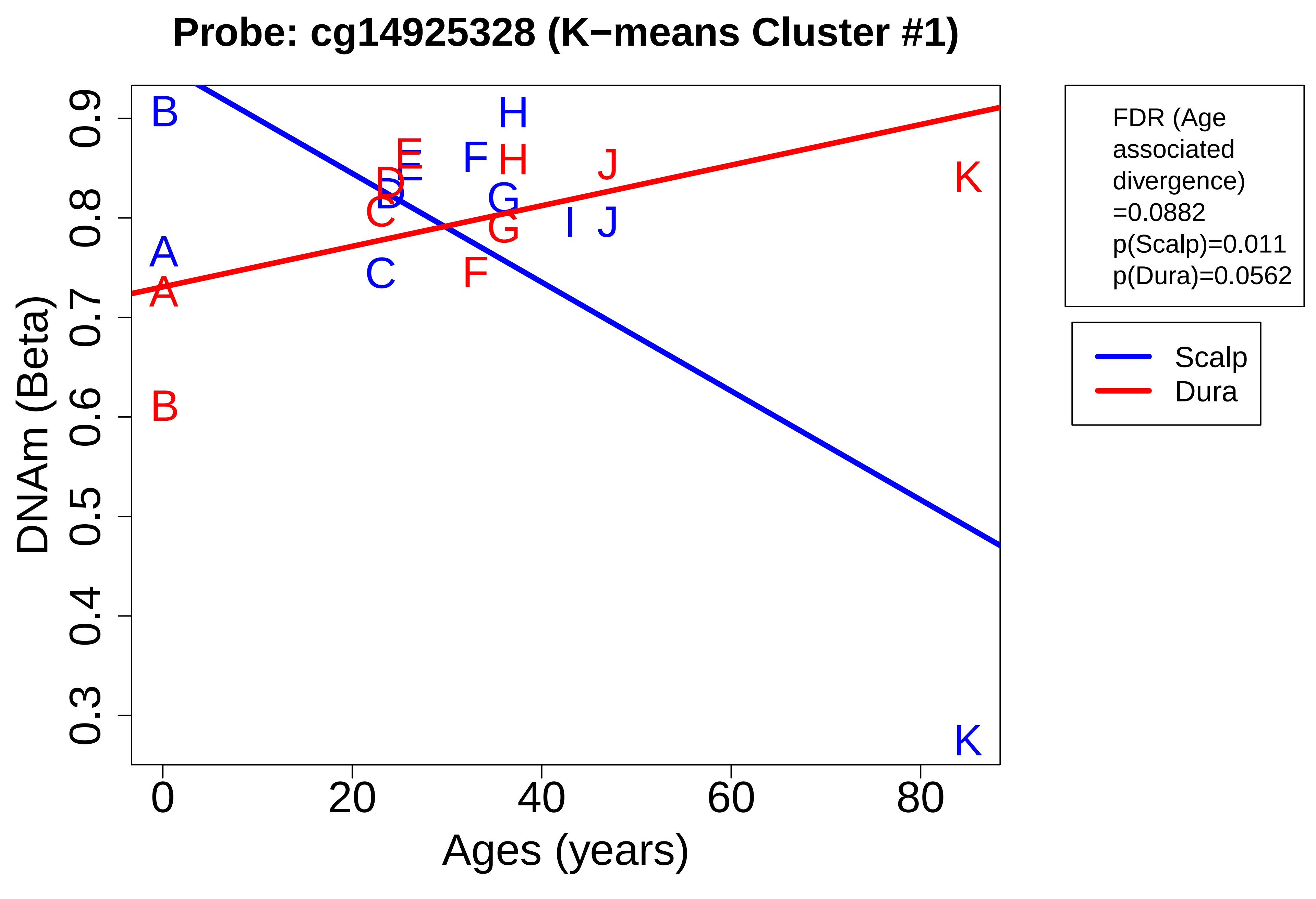

Probe: cg14925328 (K−means Cluster #1)
DNAm (Beta)
0.3
0.4
0.5
0.6
0.7
0.8
0.9
0
20
40
60
80
Ages (years)
FDR (Age associated divergence) =0.0882
p(Scalp)=0.011
p(Dura)=0.0562
Scalp
Dura

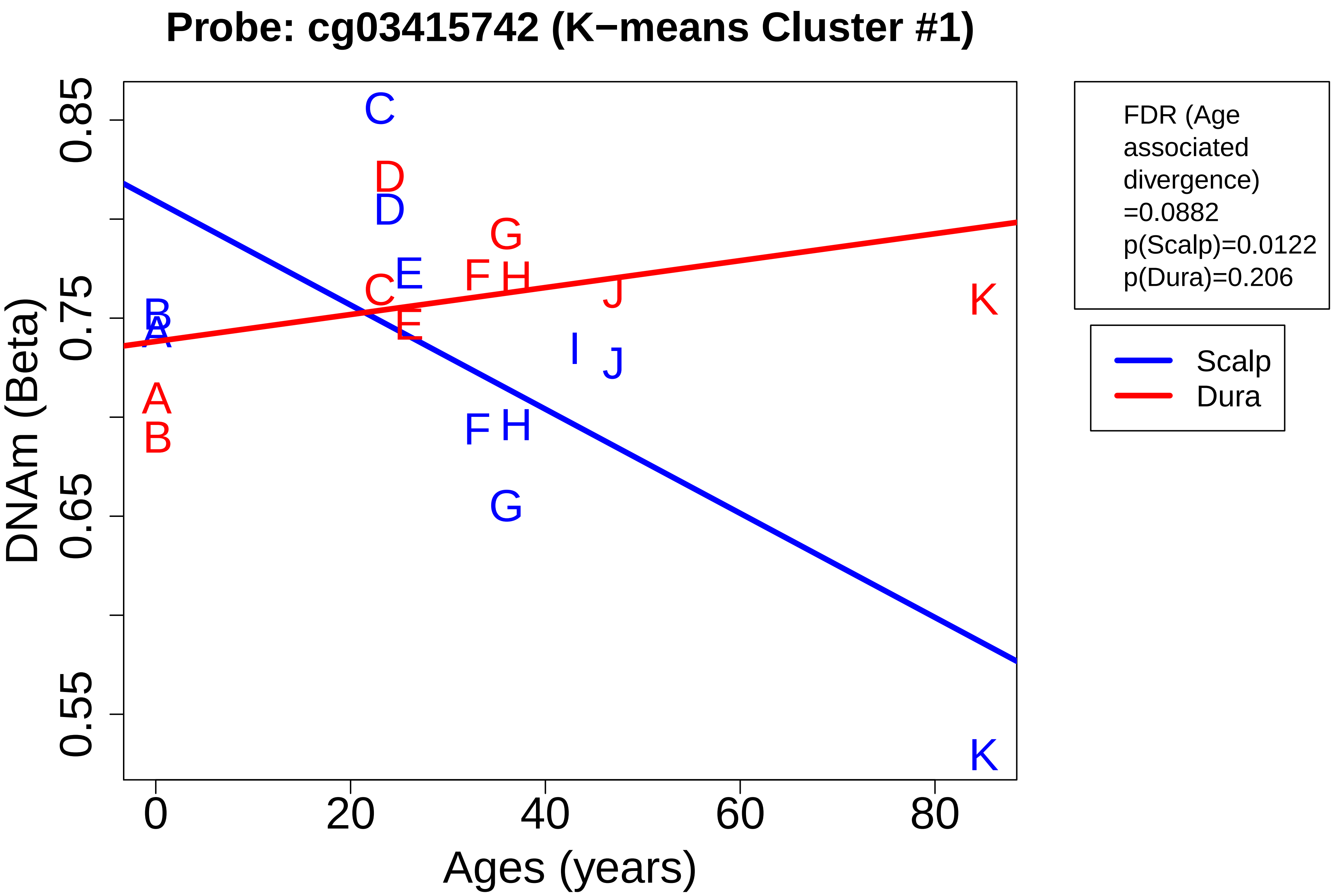

Probe: cg03415742 (K−means Cluster #1)
FDR (Age associated divergence) =0.0882
p(Scalp)=0.0122
p(Dura)=0.206
Scalp
Dura
DNAm (Beta)
0.55
0.65
0.75
0.85
Ages (years)
0
20
40
60
80

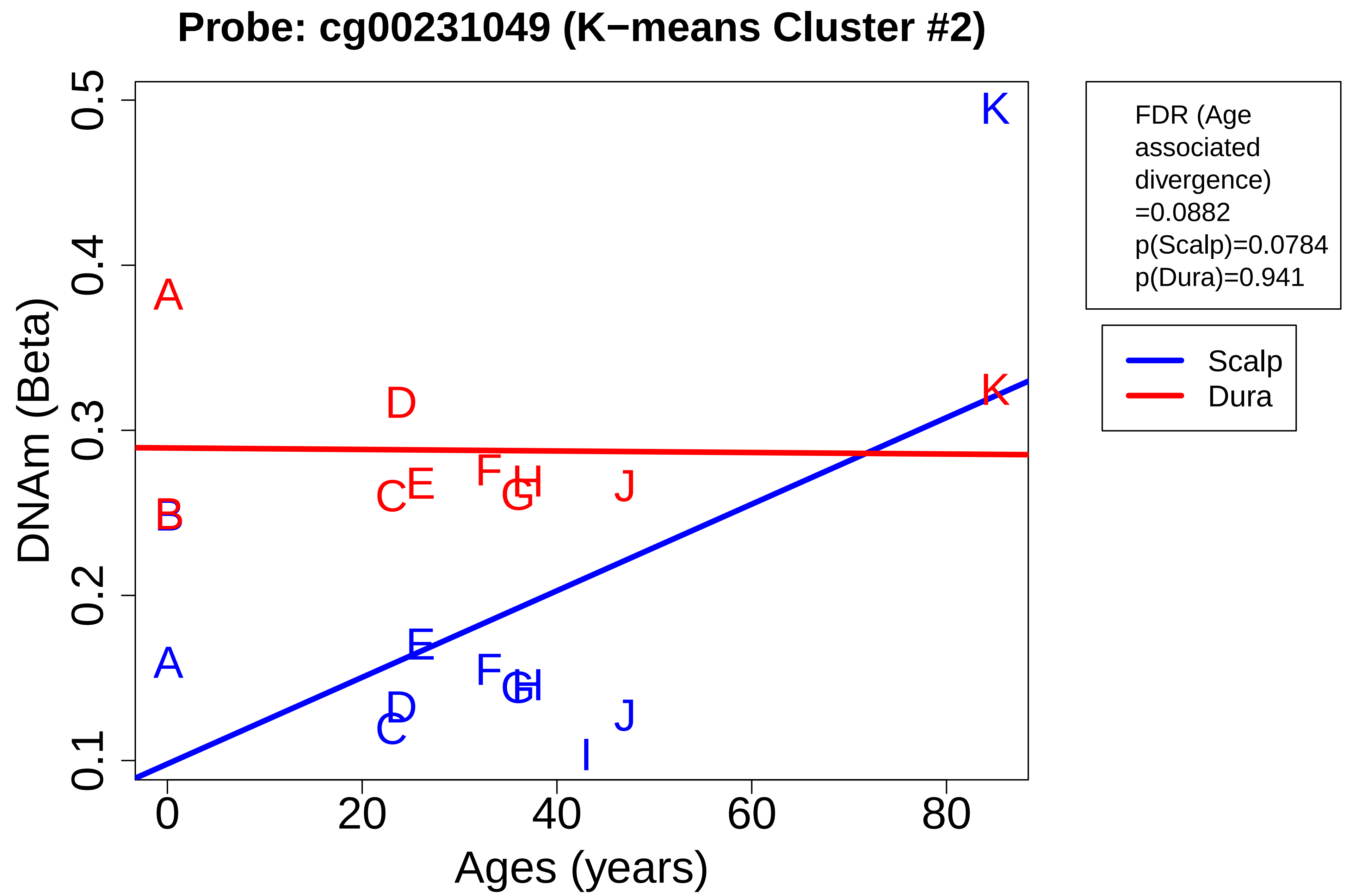
Probe: cg00231049 (K−means Cluster #2)
DNAm (Beta)
Ages (years)
0.1
0.2
0.3
0.4
0.5
0
20
40
60
80
FDR (Age associated divergence) =0.0882
p(Scalp)=0.0784
p(Dura)=0.941
Scalp
Dura
A
B
C
D
E
F
G
H
I
J
K

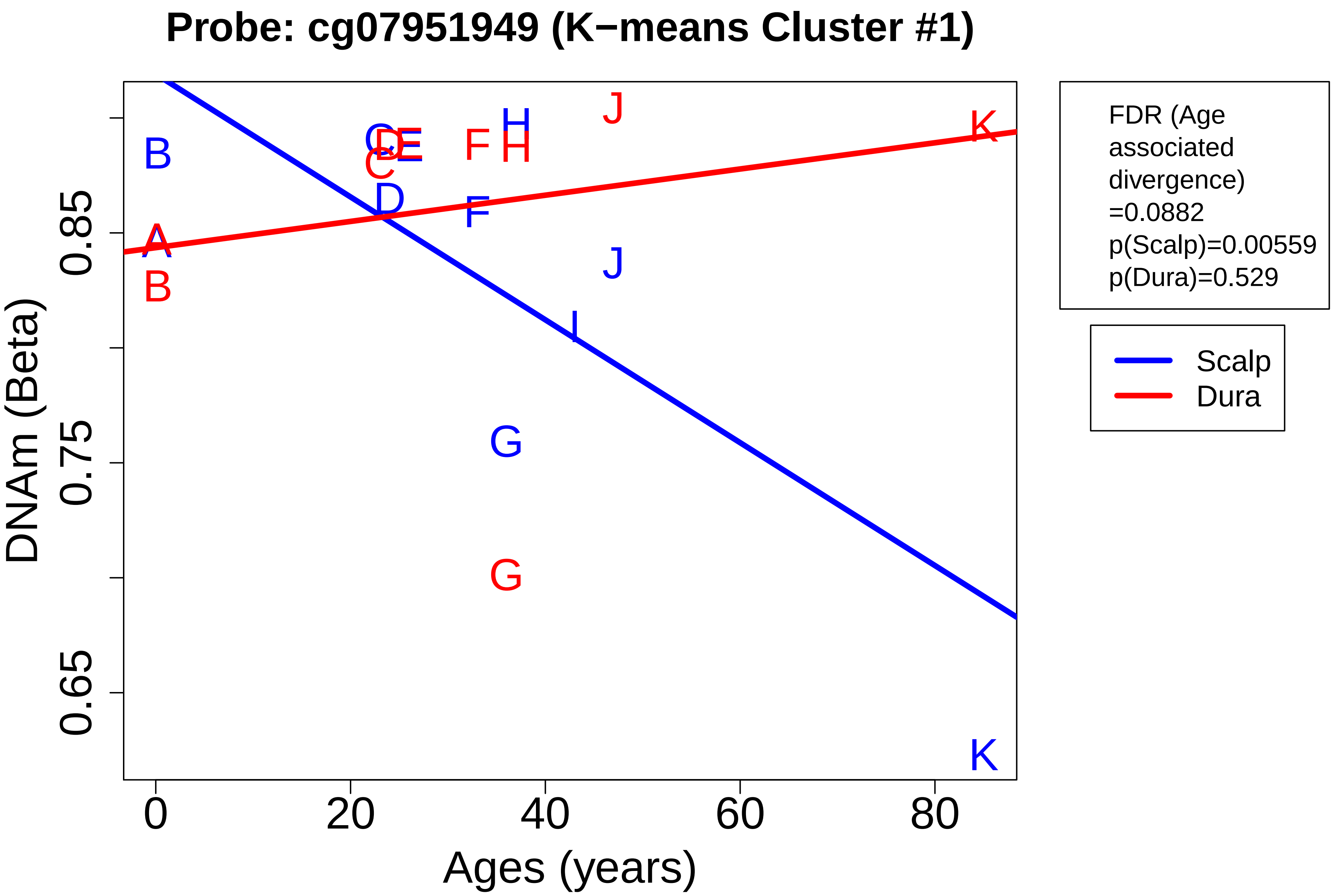

Probe: cg07951949 (K−means Cluster #1)
DNAm (Beta)
0.65
0.75
0.85
Ages (years)
0
20
40
60
80
A
B
C
D
E
F
G
H
I
J
K
FDR (Age associated divergence) =0.0882
p(Scalp)=0.00559
p(Dura)=0.529
Scalp
Dura

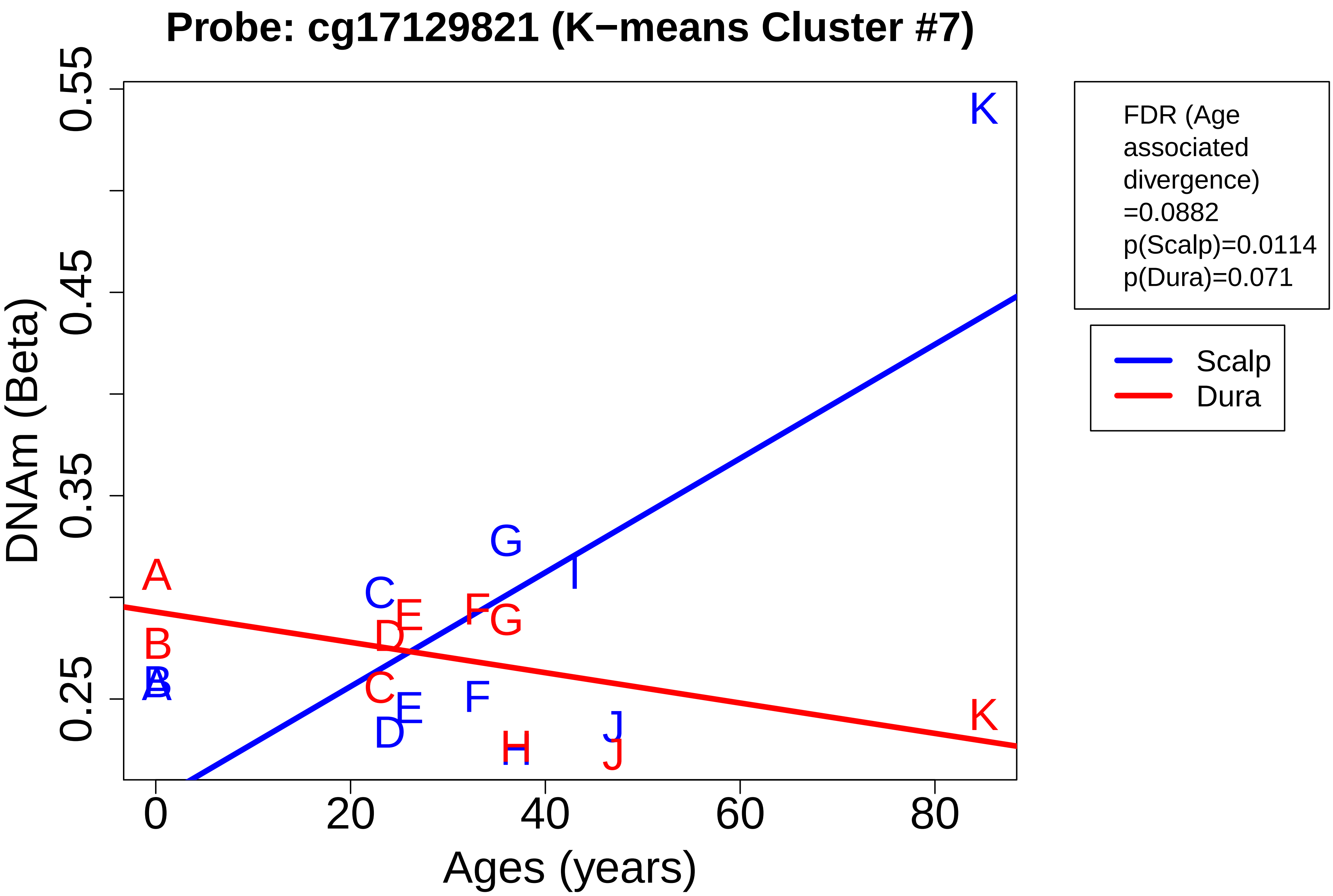

Probe: cg17129821 (K−means Cluster #7)
DNAm (Beta)
0.25
0.35
0.45
0.55
0
20
40
60
80
Ages (years)
K
G
I
A
C
E
F
G
D
B
A
C
E
F
D
H
J
J
K
FDR (Age associated divergence) =0.0882
p(Scalp)=0.0114
p(Dura)=0.071
Scalp
Dura

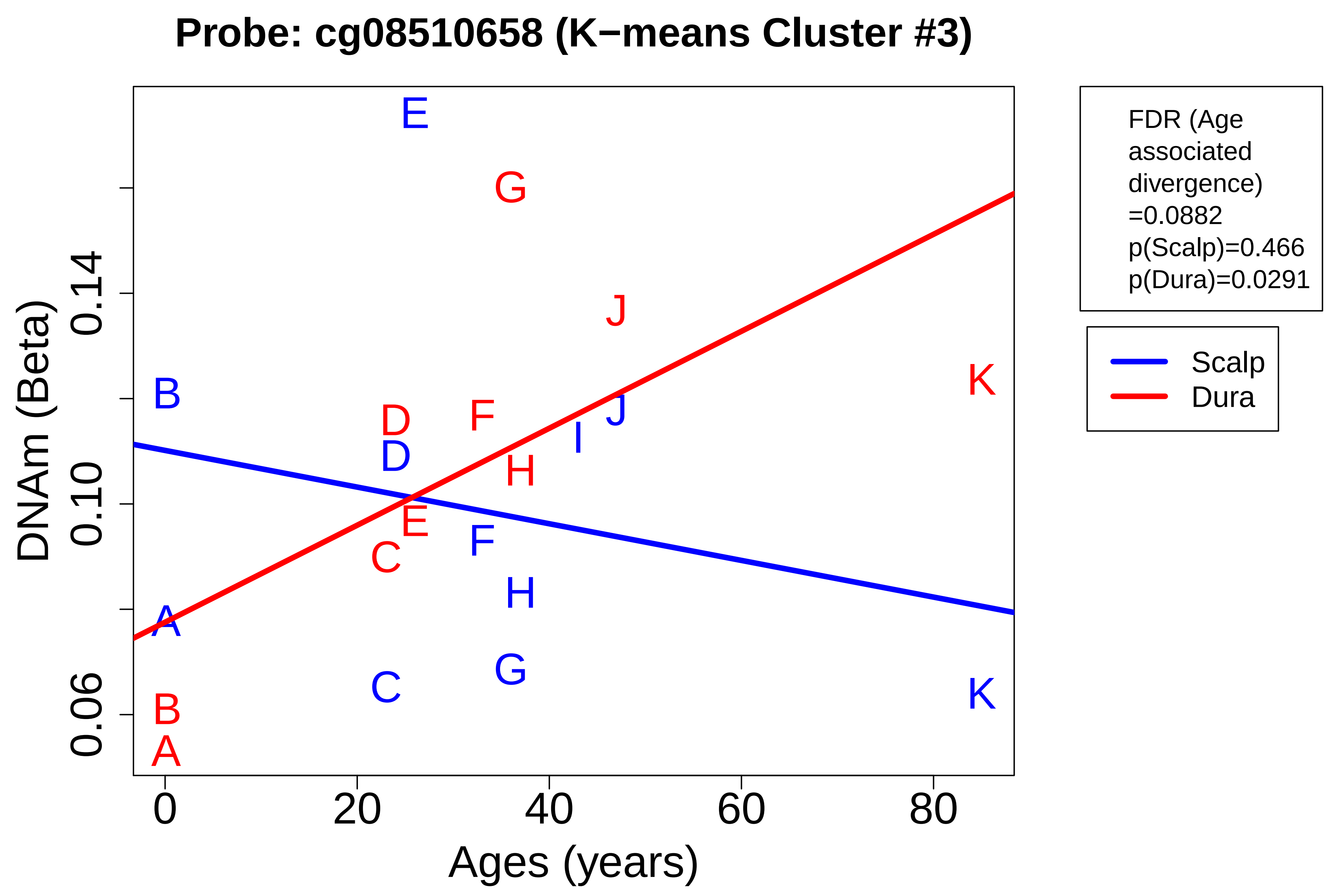

Probe: cg08510658 (K−means Cluster #3)
DNAm (Beta)
0.06
0.10
0.14
Ages (years)
0
20
40
60
80
FDR (Age associated divergence) =0.0882
p(Scalp)=0.466
p(Dura)=0.0291
Scalp
Dura

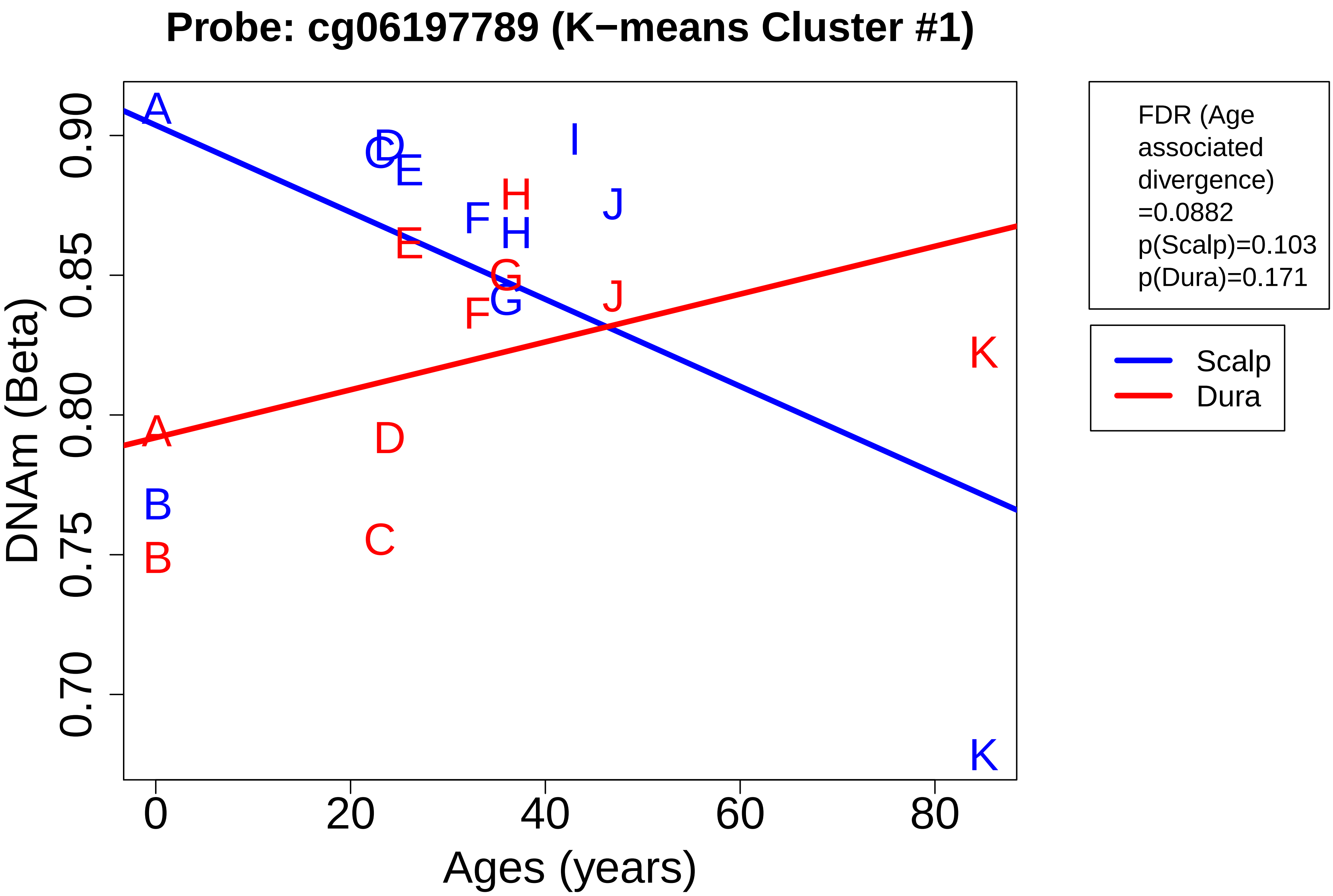

Probe: cg06197789 (K−means Cluster #1)
FDR (Age associated divergence) =0.0882
p(Scalp)=0.103
p(Dura)=0.171
Scalp
Dura
DNAm (Beta)
0.70
0.75
0.80
0.85
0.90
0
20
40
60
80
Ages (years)
A
B
C
D
E
F
G
H
I
J
K

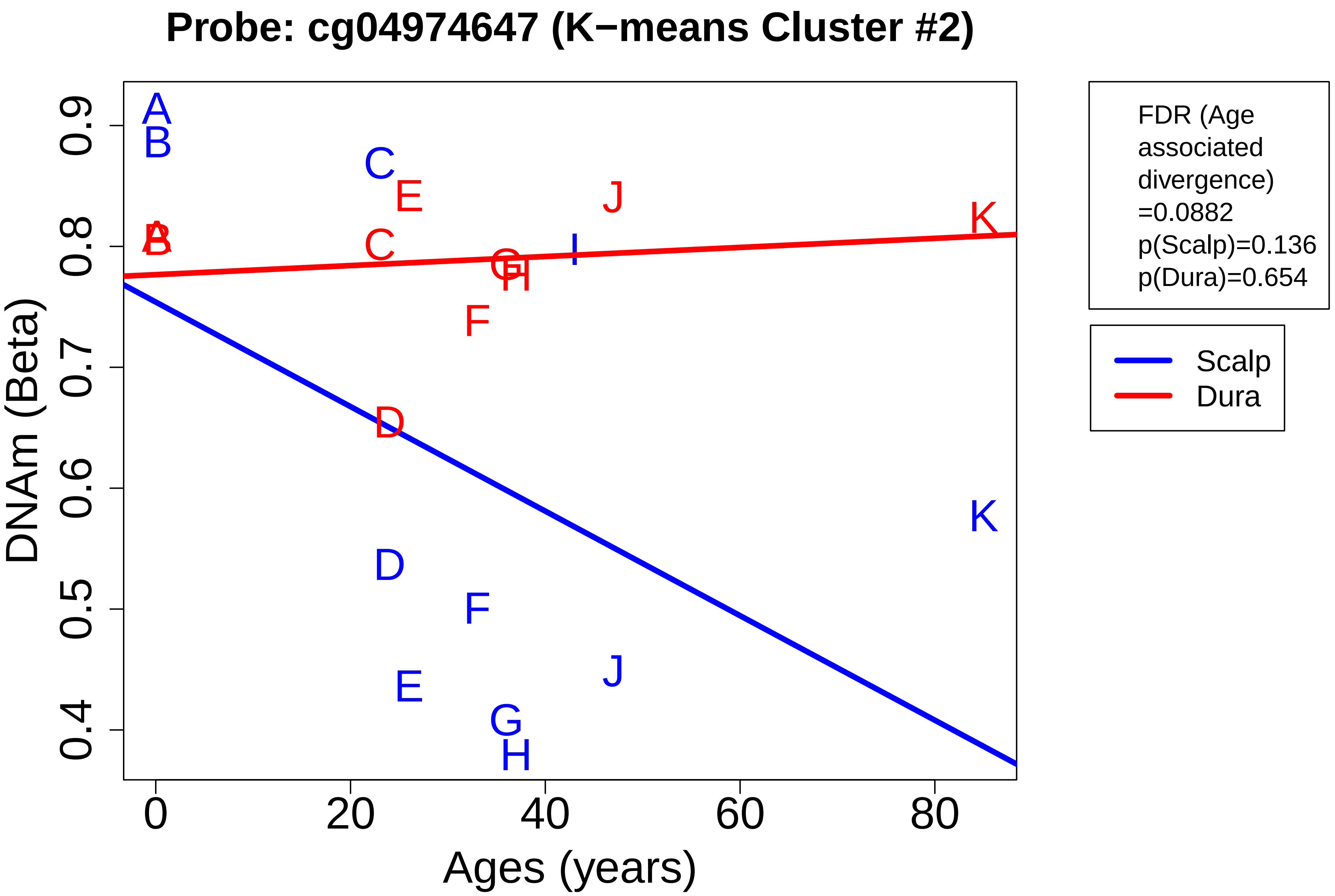
Probe: cg04974647 (K−means Cluster #2)
DNAm (Beta)
0.4
0.5
0.6
0.7
0.8
0.9
Ages (years)
0
20
40
60
80
A
B
C
D
E
F
G
H
I
J
K
FDR (Age associated divergence) =0.0882
p(Scalp)=0.136
p(Dura)=0.654
Scalp
Dura

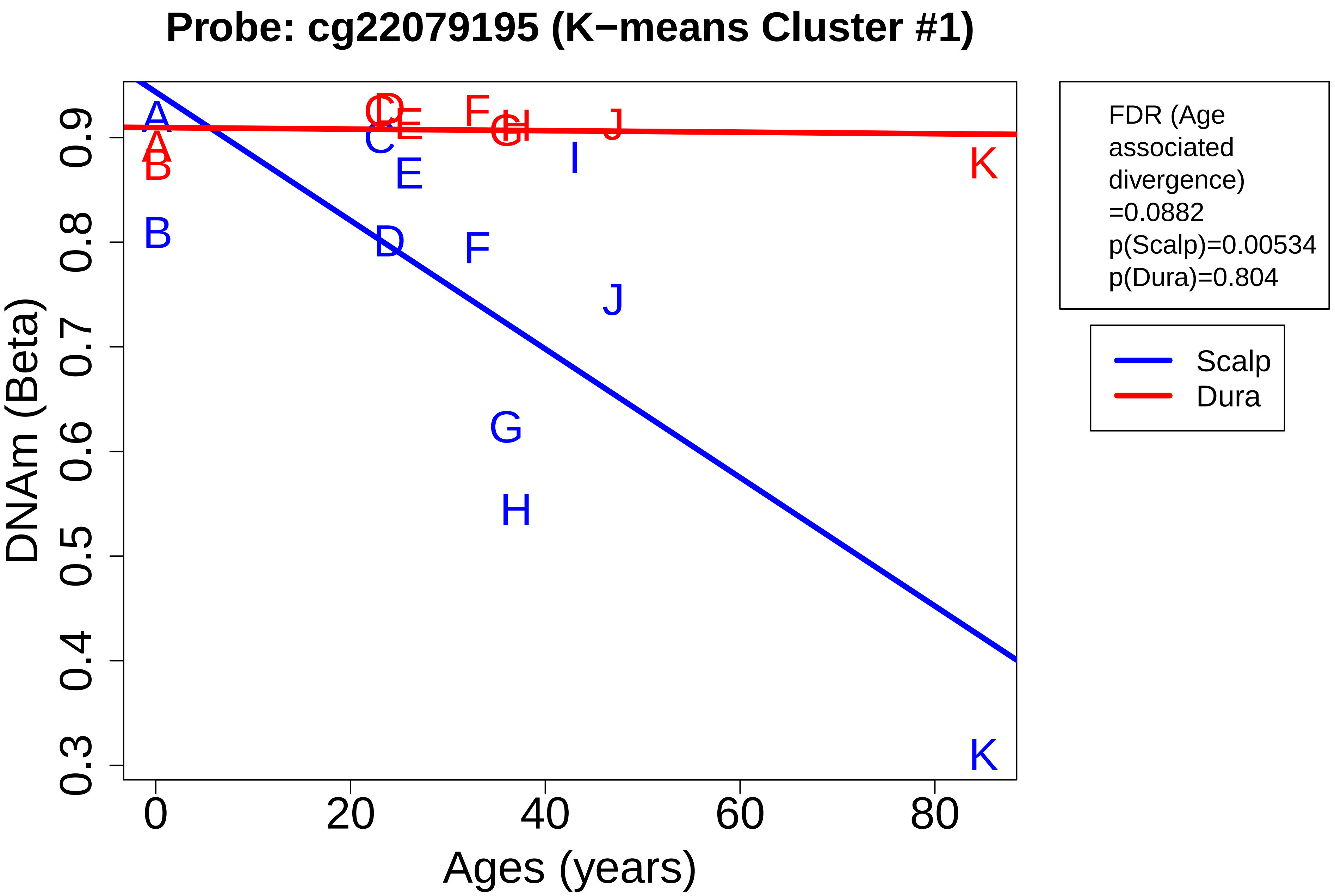

Probe: cg22079195 (K−means Cluster #1)
DNAm (Beta)
Ages (years)
0.3
0.4
0.5
0.6
0.7
0.8
0.9
0
20
40
60
80
FDR (Age associated divergence) =0.0882
p(Scalp)=0.00534
p(Dura)=0.804
Scalp
Dura

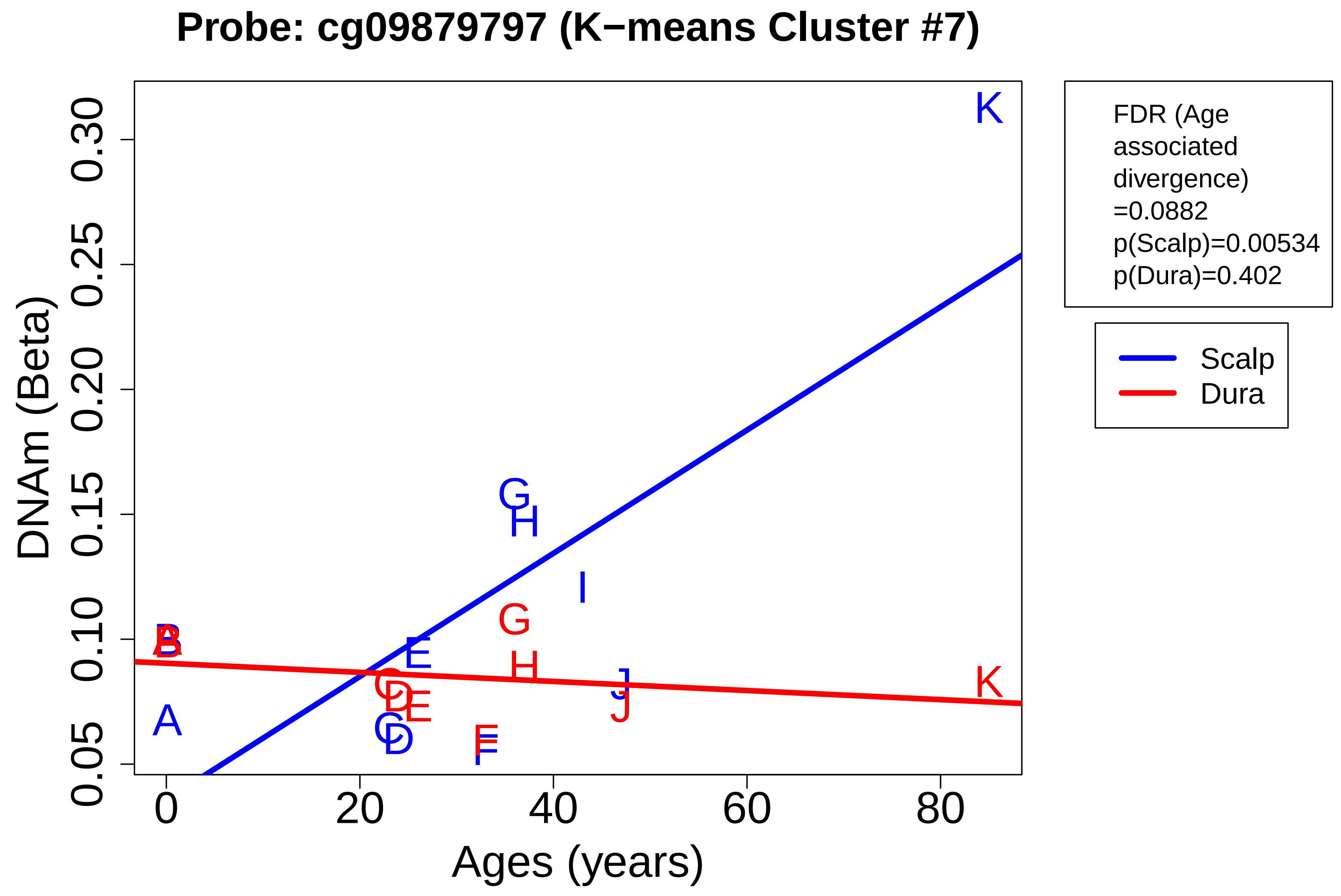

Probe: cg09879797 (K−means Cluster #7)
DNAm (Beta)
0.05
0.10
0.15
0.20
0.25
0.30
0
20
40
60
80
Ages (years)
FDR (Age associated divergence) =0.0882
p(Scalp)=0.00534
p(Dura)=0.402
Scalp
Dura

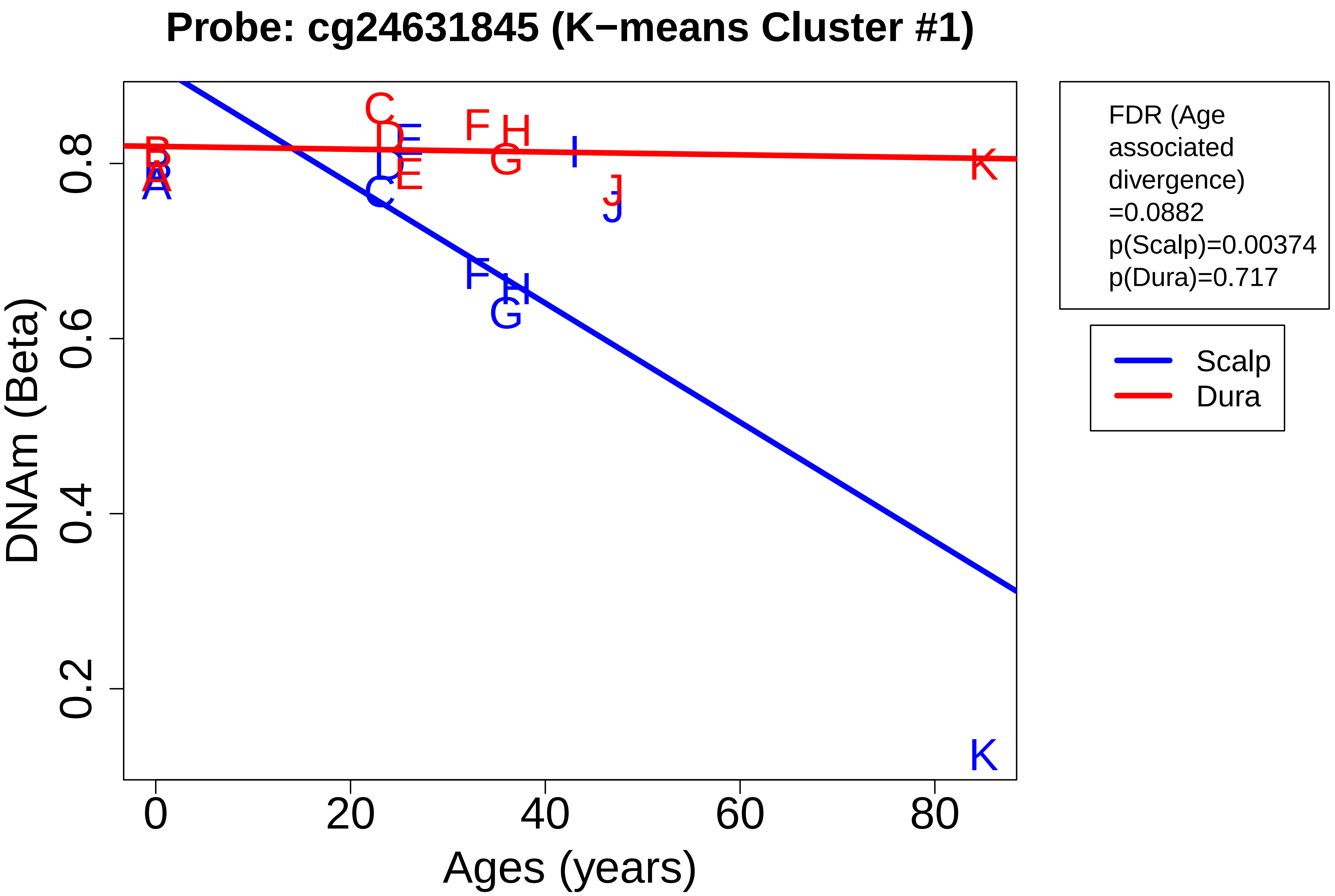

Probe: cg24631845 (K−means Cluster #1)
DNAm (Beta)
0.2
0.4
0.6
0.8
0
20
40
60
80
Ages (years)
FDR (Age associated divergence) =0.0882
p(Scalp)=0.00374
p(Dura)=0.717
Scalp
Dura
A
B
C
D
E
F
G
H
I
J
K

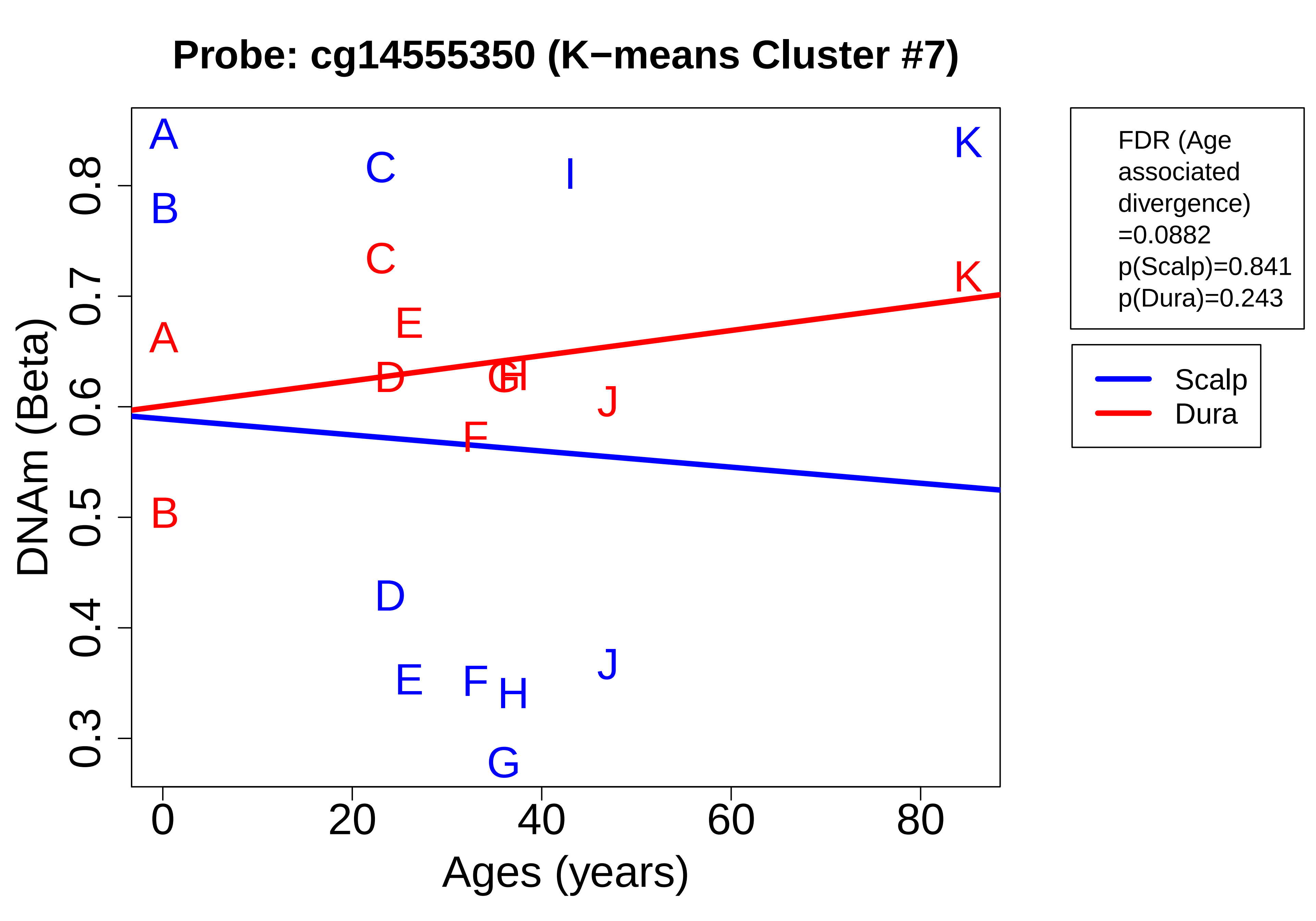

Probe: cg14555350 (K−means Cluster #7)
DNAm (Beta)
0.3
0.4
0.5
0.6
0.7
0.8
0
20
40
60
80
Ages (years)
FDR (Age associated divergence) =0.0882
p(Scalp)=0.841
p(Dura)=0.243
Scalp
Dura
A
B
C
D
E
F
G
H
I
J
K

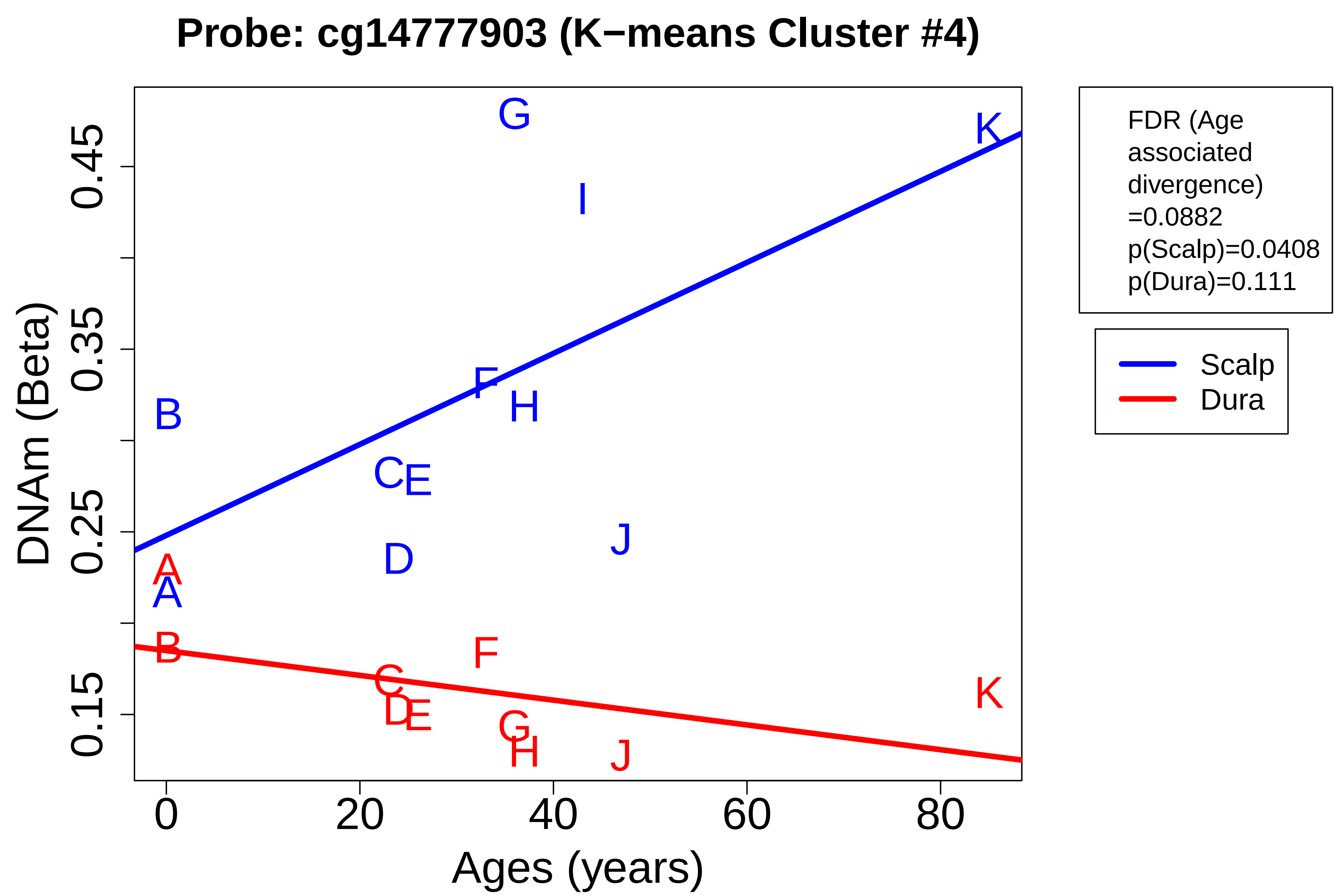

Probe: cg14777903 (K−means Cluster #4)
DNAm (Beta)
0.15
0.25
0.35
0.45
Ages (years)
0
20
40
60
80
FDR (Age associated divergence) =0.0882
p(Scalp)=0.0408
p(Dura)=0.111
Scalp
Dura

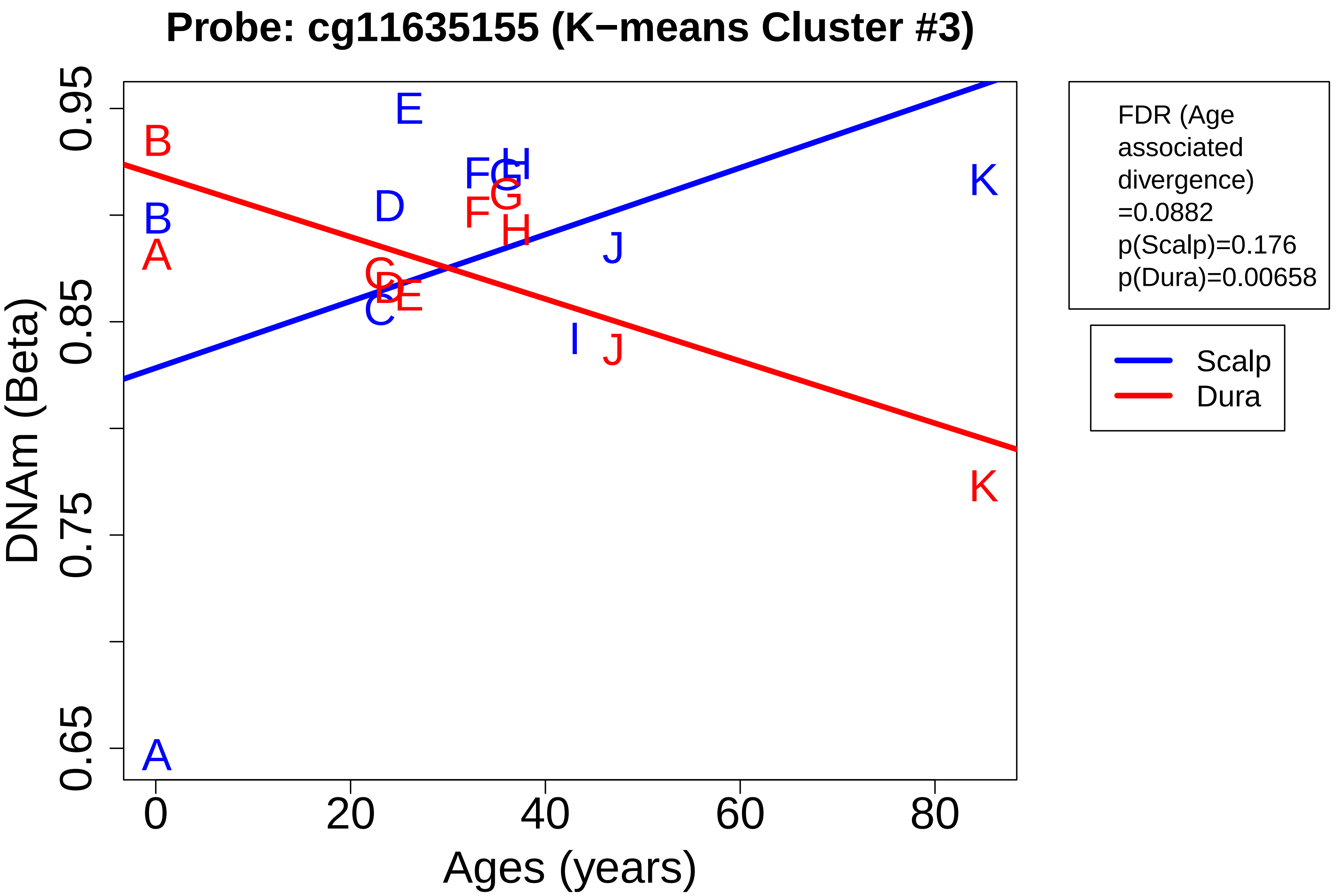

Probe: cg11635155 (K−means Cluster #3)
DNAm (Beta)
0.65
0.75
0.85
0.95
0
20
40
60
80
Ages (years)
FDR (Age associated divergence) =0.0882
p(Scalp)=0.176
p(Dura)=0.00658
Scalp
Dura

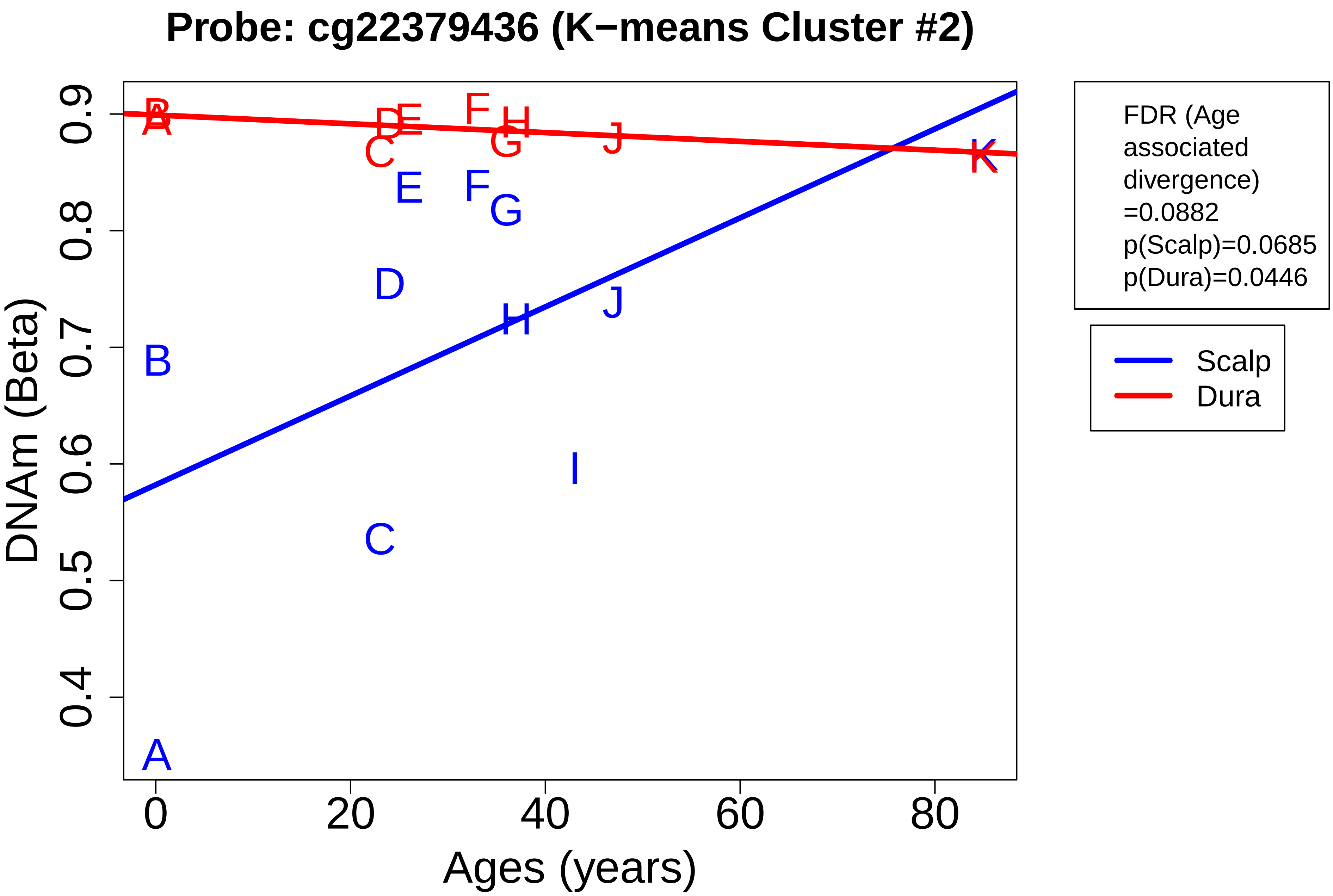

Probe: cg22379436 (K−means Cluster #2)
DNAm (Beta)
0.4
0.5
0.6
0.7
0.8
0.9
0
20
40
60
80
Ages (years)
FDR (Age associated divergence) =0.0882
p(Scalp)=0.0685
p(Dura)=0.0446
Scalp
Dura

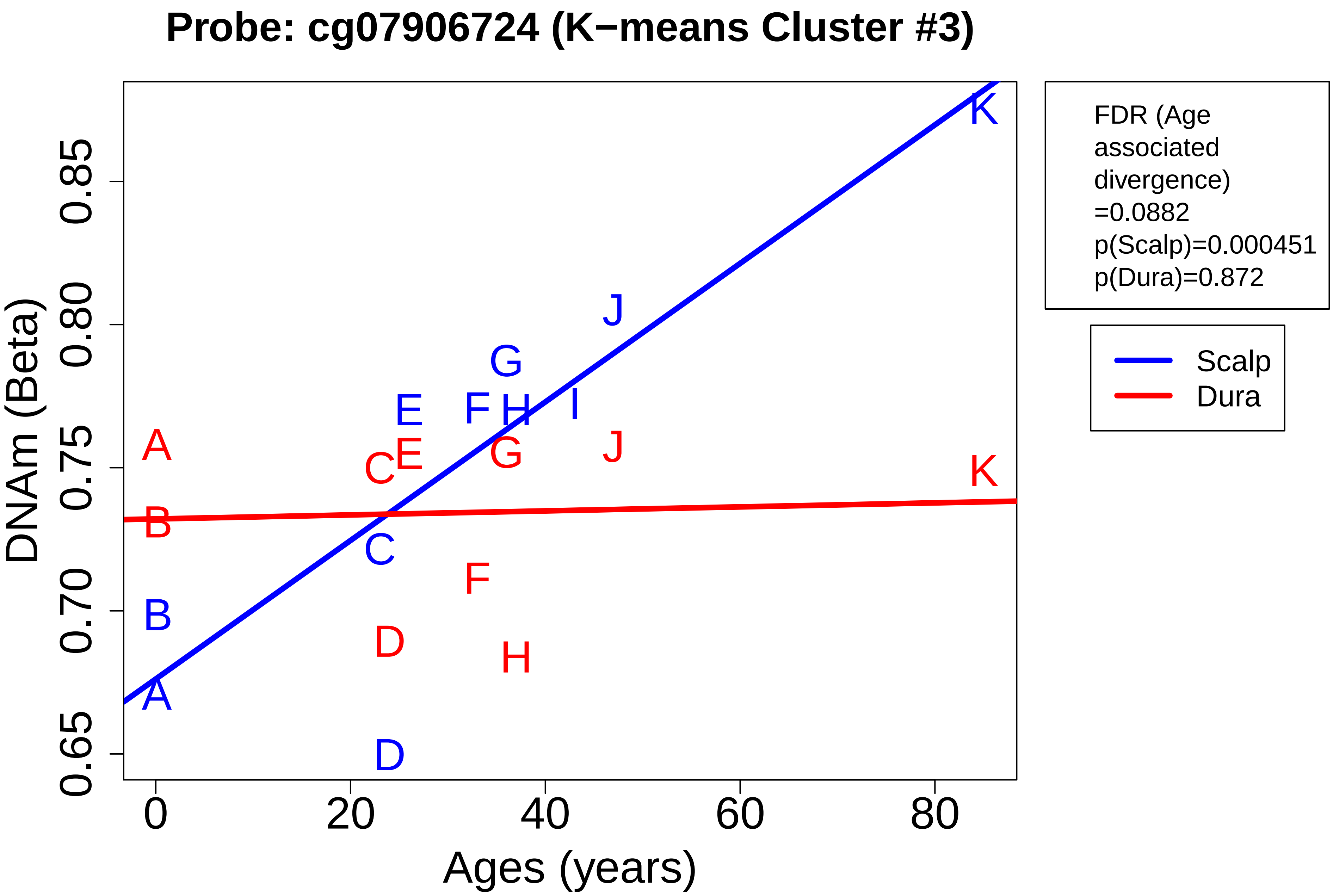

Probe: cg07906724 (K−means Cluster #3)
DNAm (Beta)
0.65
0.70
0.75
0.80
0.85
Ages (years)
0
20
40
60
80
A
B
C
D
E
F
G
H
I
J
K
A
B
C
D
E
F
G
H
J
K
FDR (Age associated divergence) =0.0882
p(Scalp)=0.000451
p(Dura)=0.872
Scalp
Dura

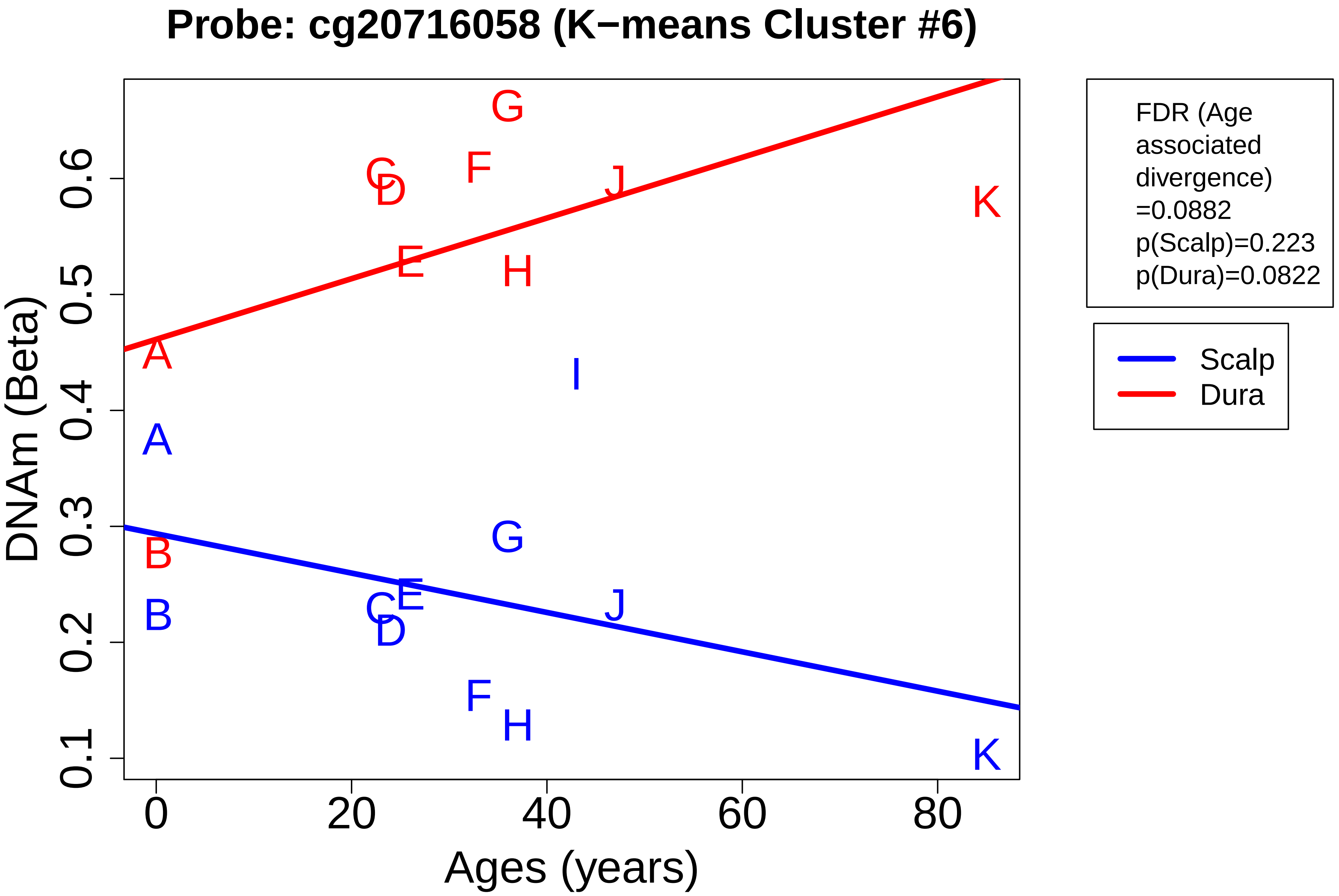

Probe: cg20716058 (K−means Cluster #6)
FDR (Age associated divergence) =0.0882
p(Scalp)=0.223
p(Dura)=0.0822
Scalp
Dura
DNAm (Beta)
Ages (years)
0.1
0.2
0.3
0.4
0.5
0.6
0
20
40
60
80

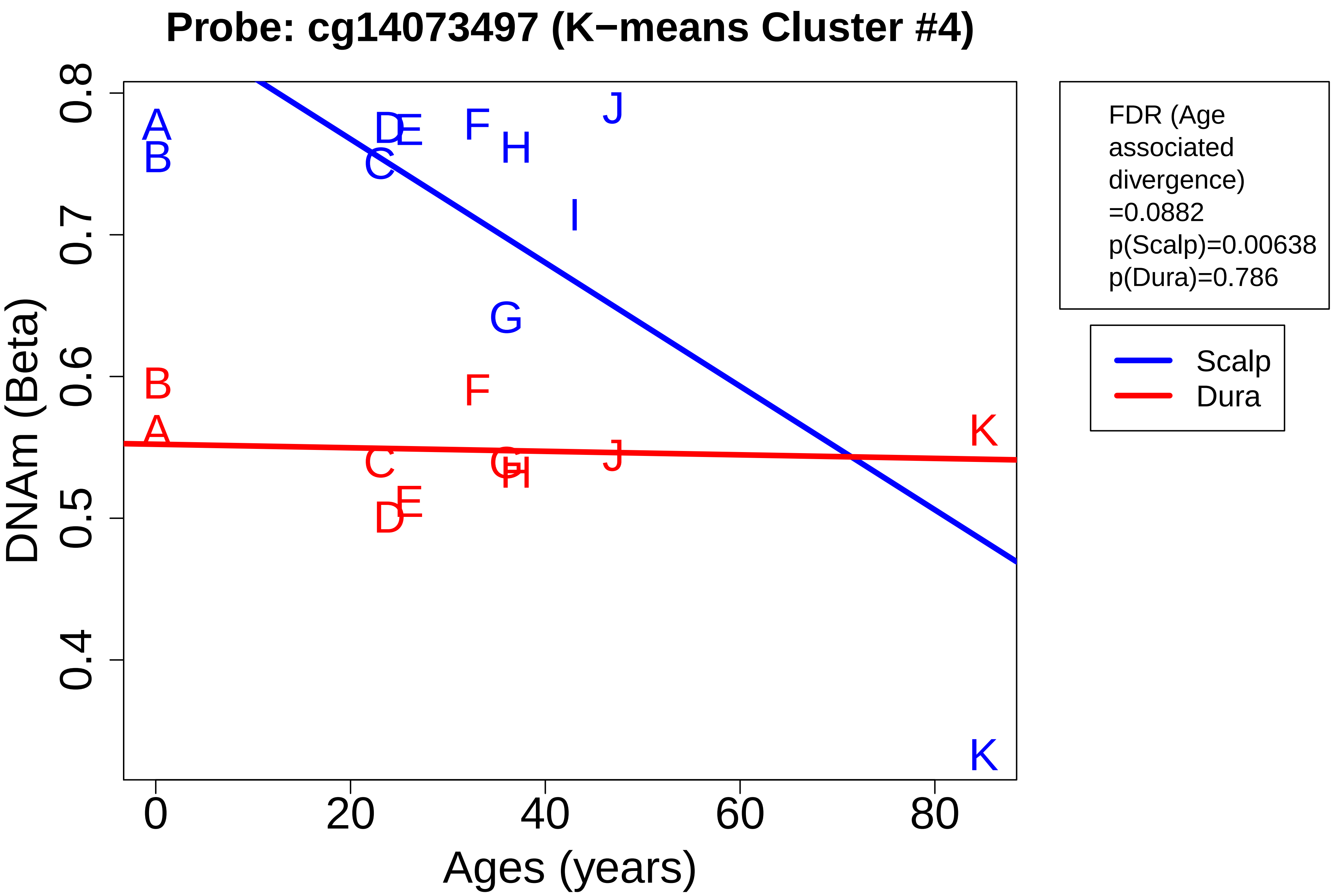

Probe: cg14073497 (K−means Cluster #4)
DNAm (Beta)
0.4
0.5
0.6
0.7
0.8
Ages (years)
0
20
40
60
80
FDR (Age associated divergence) =0.0882
p(Scalp)=0.00638
p(Dura)=0.786
Scalp
Dura

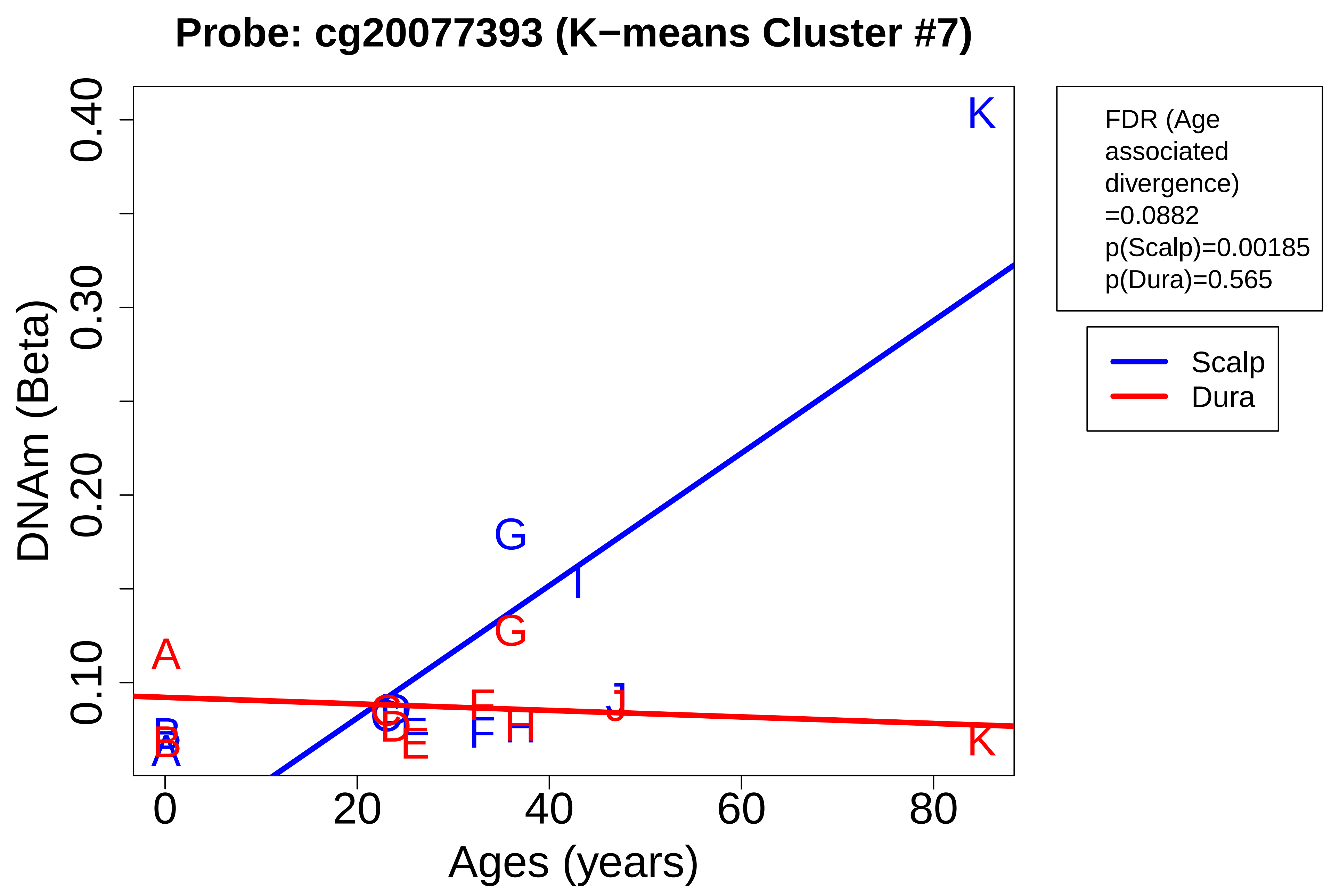

Probe: cg20077393 (K−means Cluster #7)
FDR (Age associated divergence) =0.0882
p(Scalp)=0.00185
p(Dura)=0.565
Scalp
Dura
DNAm (Beta)
0.10
0.20
0.30
0.40
0
20
40
60
80
Ages (years)
A
B
C
D
E
F
G
H
I
J
K

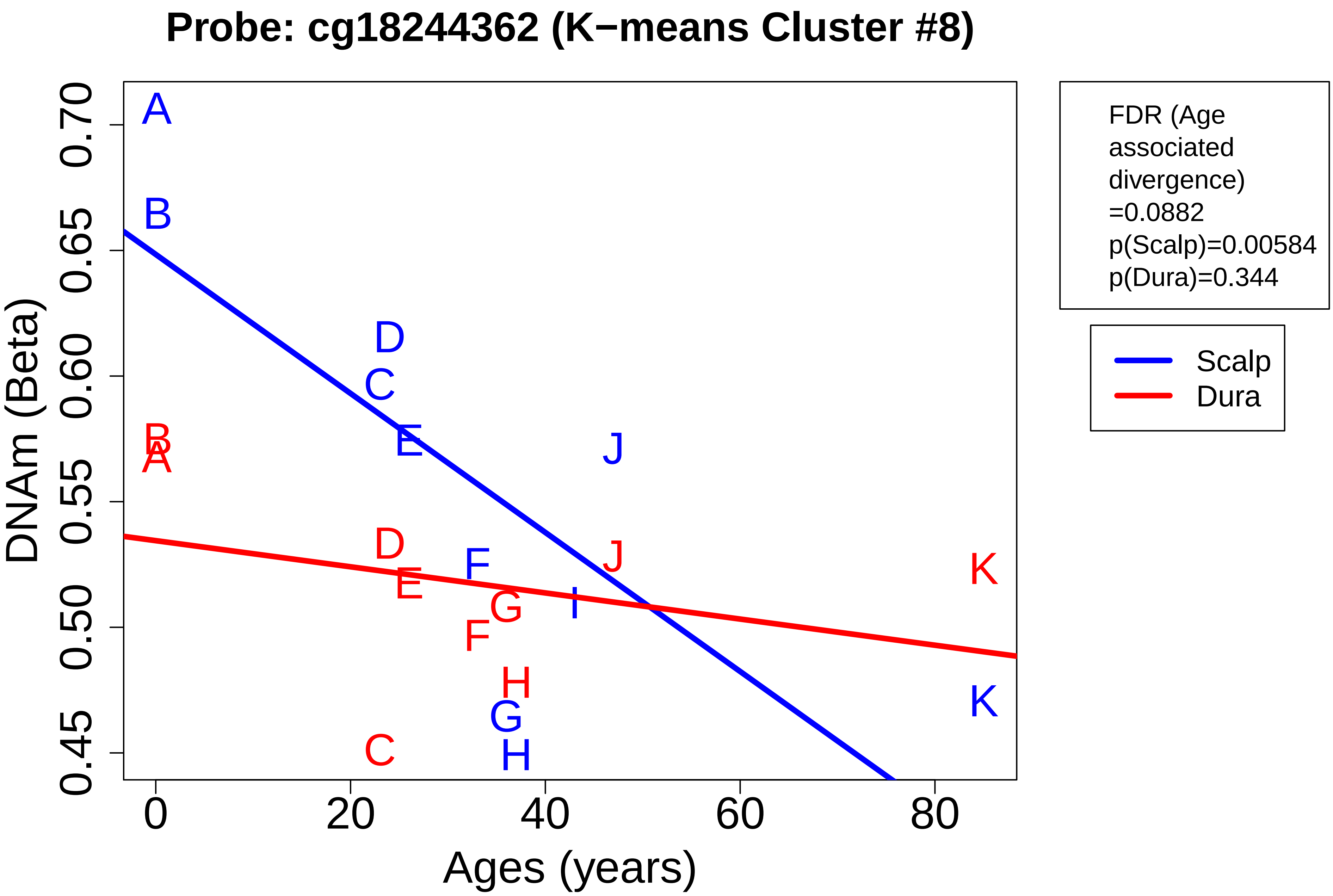
Probe: cg18244362 (K−means Cluster #8)
DNAm (Beta)
Ages (years)
FDR (Age associated divergence) =0.0882
p(Scalp)=0.00584
p(Dura)=0.344
Scalp
Dura

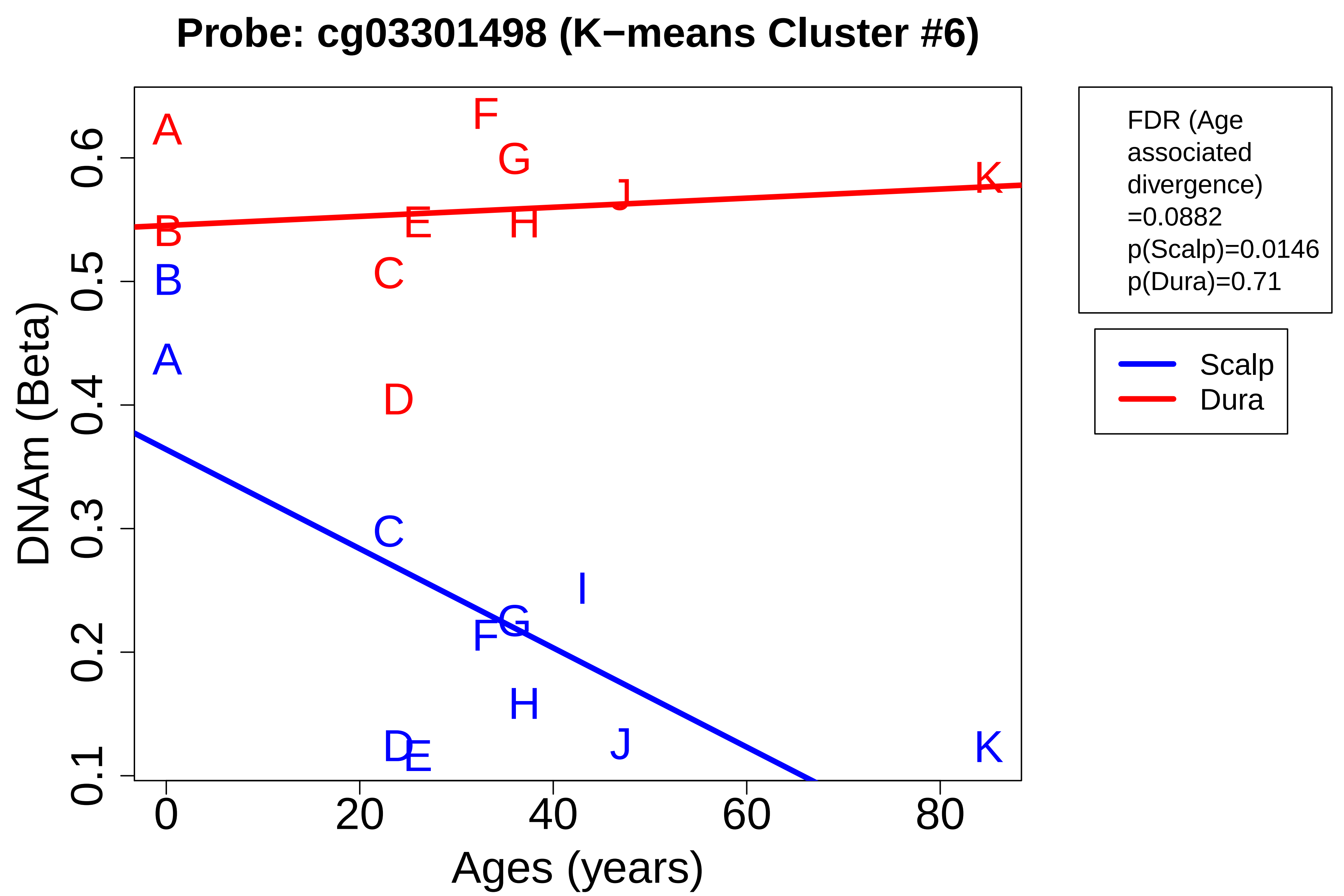

Probe: cg03301498 (K−means Cluster #6)
DNAm (Beta)
0.1
0.2
0.3
0.4
0.5
0.6
0
20
40
60
80
Ages (years)
FDR (Age associated divergence) =0.0882
p(Scalp)=0.0146
p(Dura)=0.71
Scalp
Dura
A
B
C
D
E
F
G
H
I
J
K

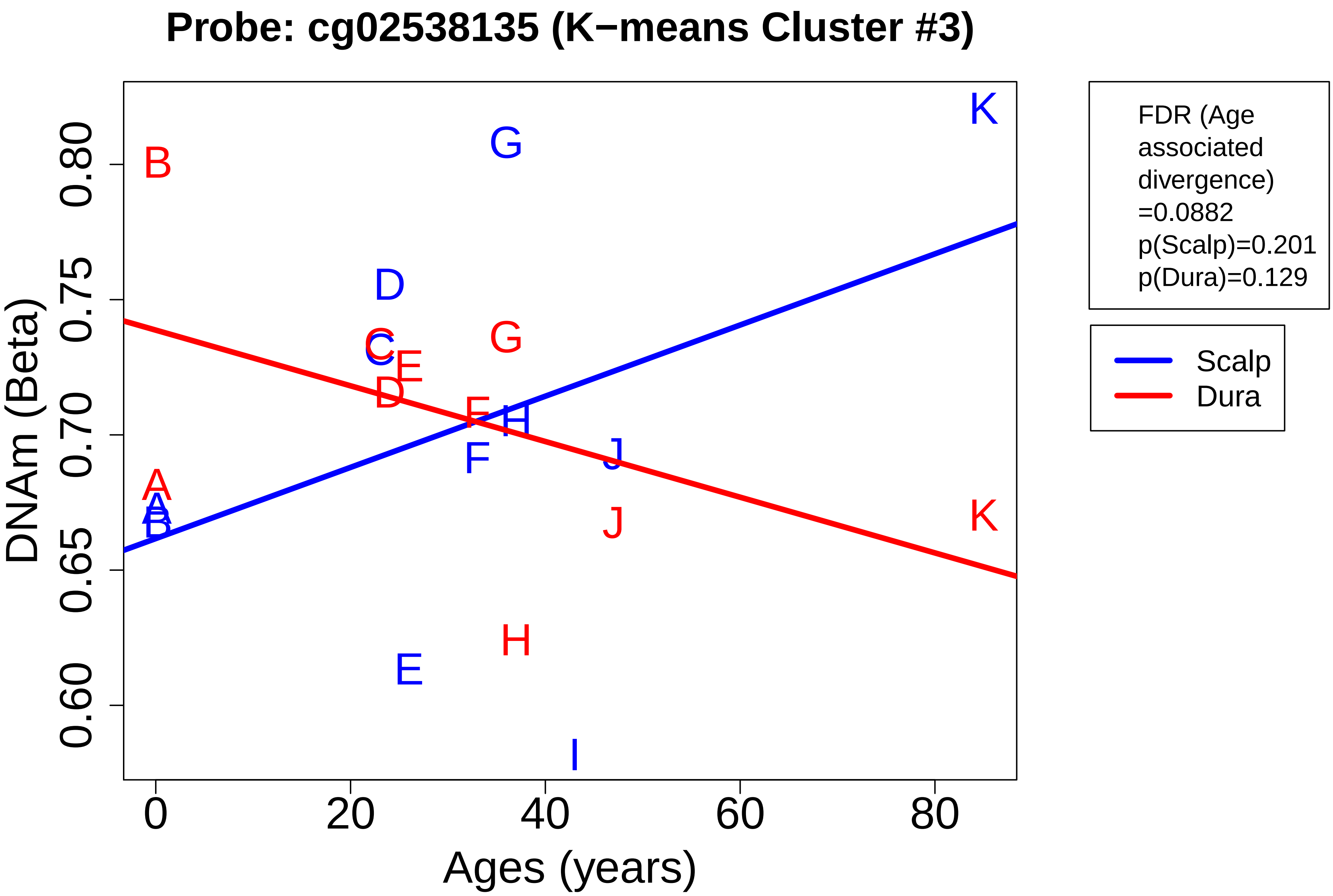

Probe: cg02538135 (K−means Cluster #3)
DNAm (Beta)
0.60
0.65
0.70
0.75
0.80
Ages (years)
0
20
40
60
80
FDR (Age associated divergence) =0.0882
p(Scalp)=0.201
p(Dura)=0.129
Scalp
Dura

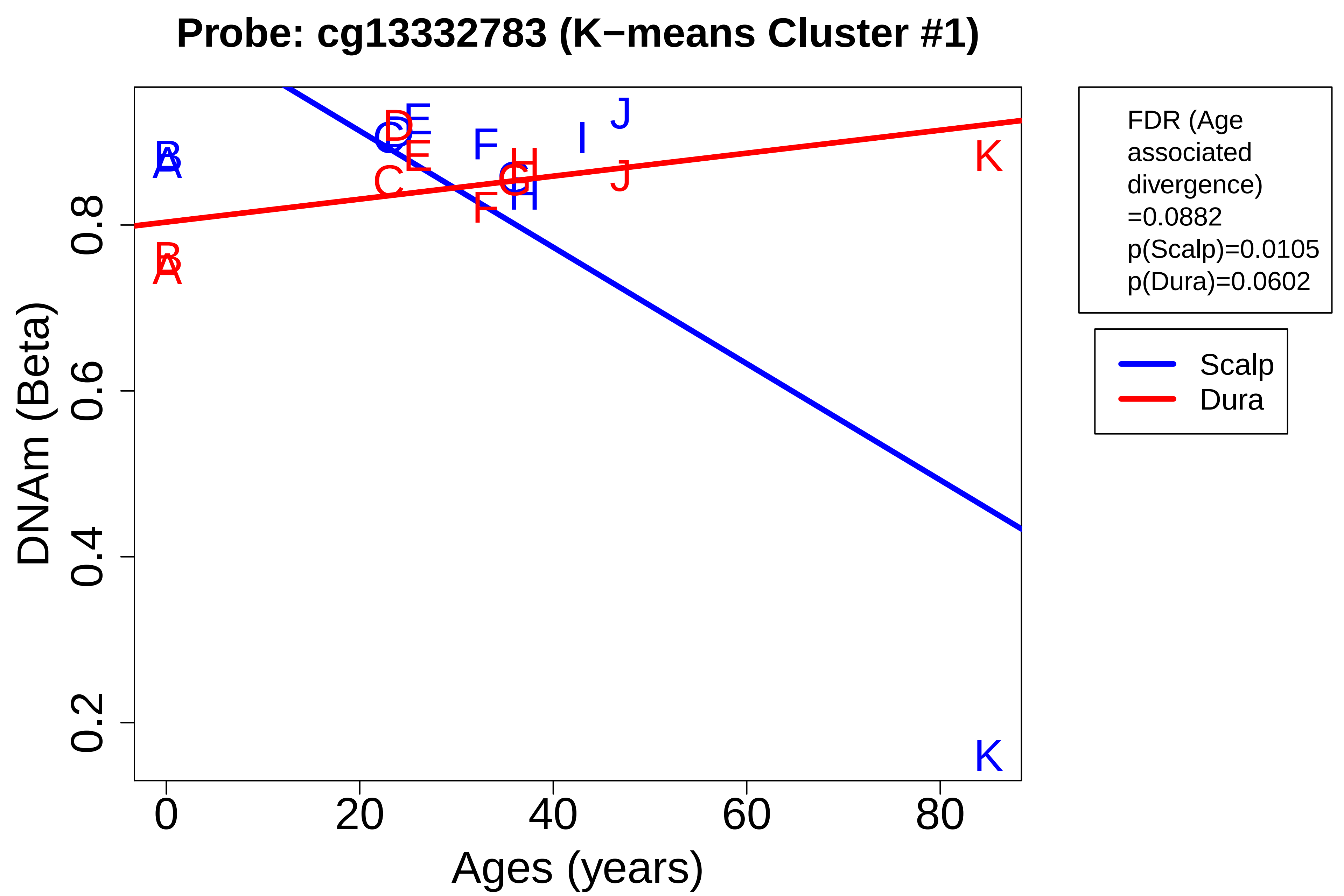

Probe: cg13332783 (K−means Cluster #1)
FDR (Age associated divergence) =0.0882
p(Scalp)=0.0105
p(Dura)=0.0602
Scalp
Dura
DNAm (Beta)
0.2
0.4
0.6
0.8
Ages (years)
0
20
40
60
80

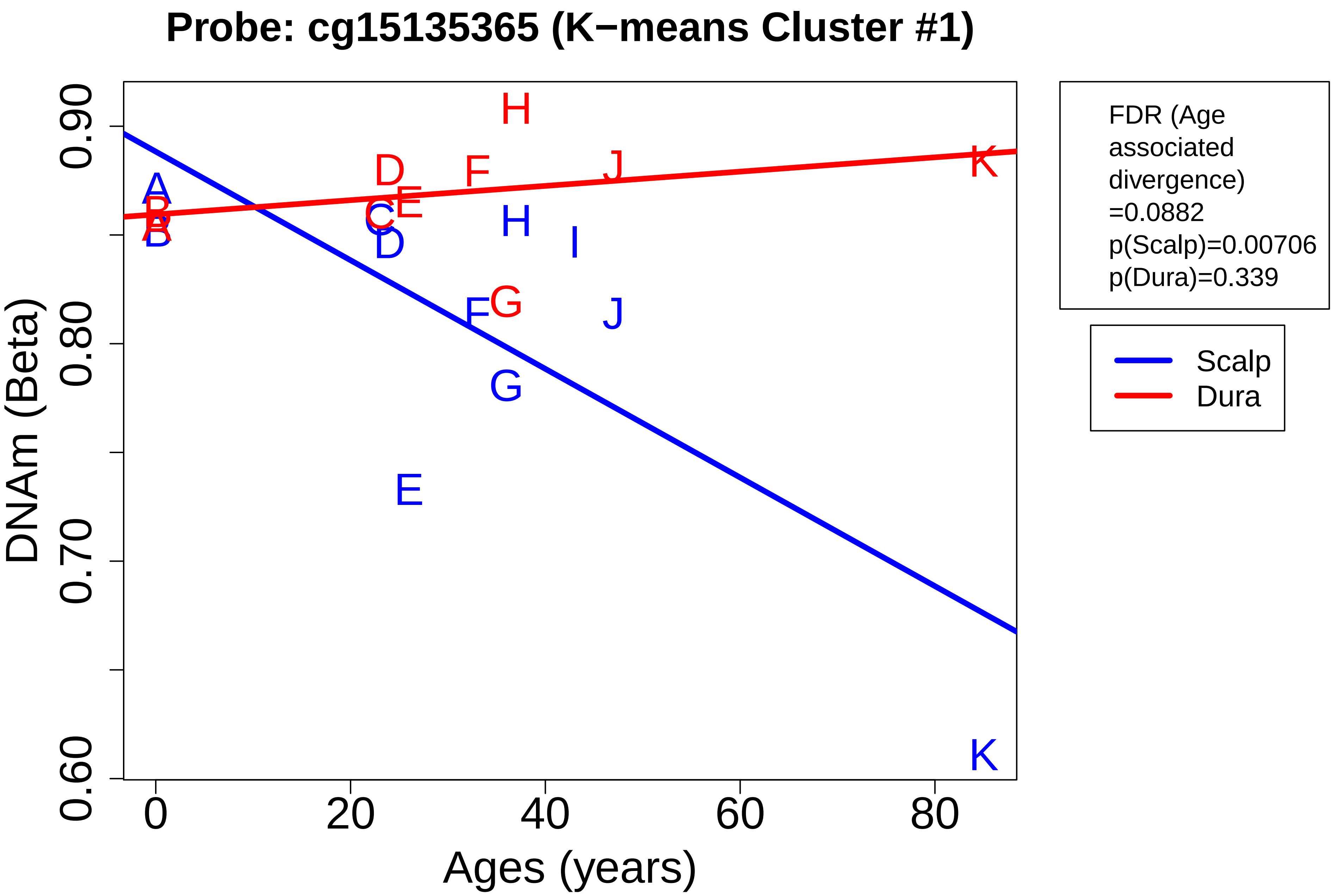

Probe: cg15135365 (K−means Cluster #1)
DNAm (Beta)
0.60
0.70
0.80
0.90
0
20
40
60
80
Ages (years)
A
B
C
D
E
F
G
H
I
J
K
FDR (Age associated divergence) =0.0882
p(Scalp)=0.00706
p(Dura)=0.339
Scalp
Dura

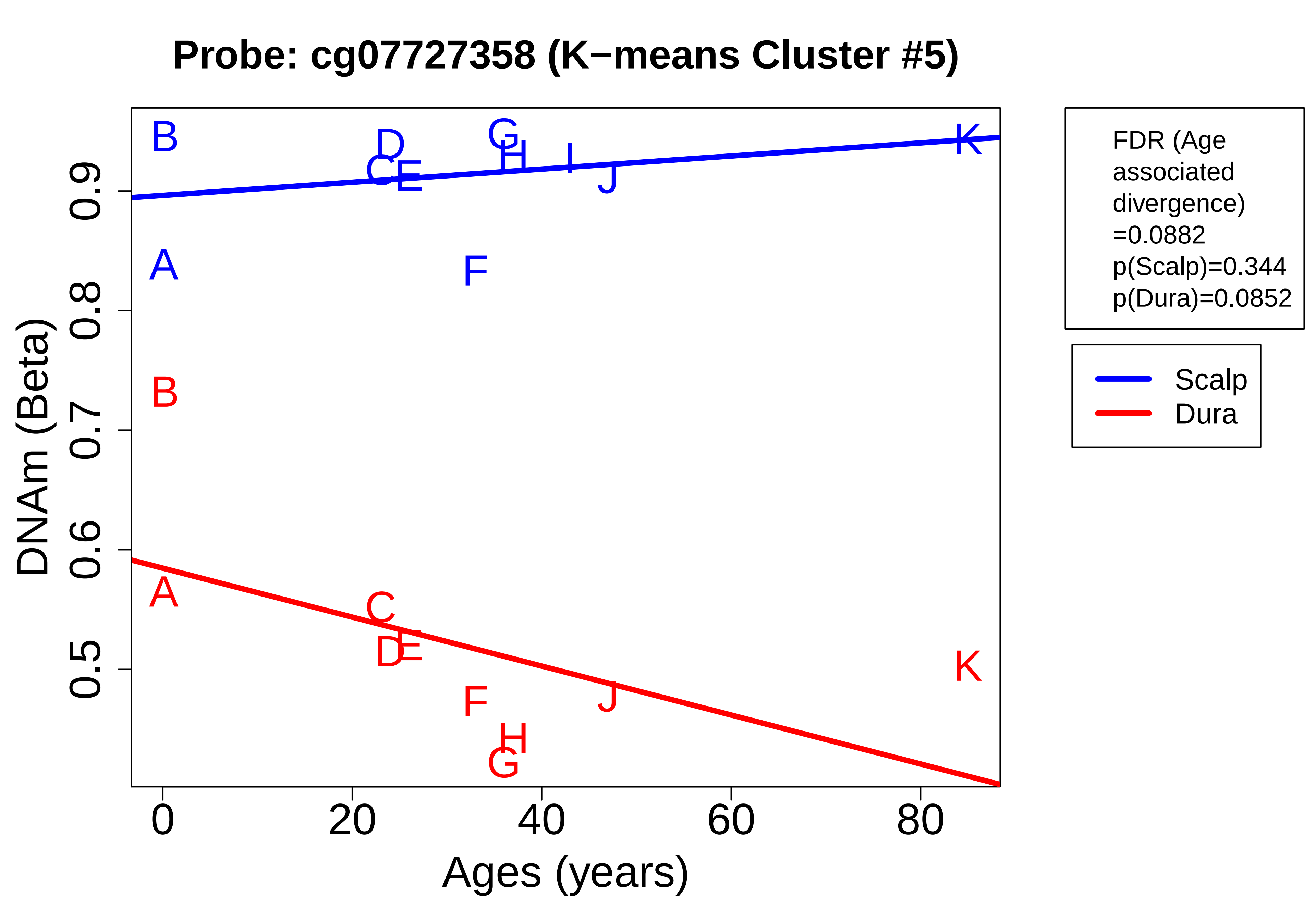

Probe: cg07727358 (K−means Cluster #5)
DNAm (Beta)
0.5
0.6
0.7
0.8
0.9
Ages (years)
0
20
40
60
80
A
B
C
D
E
F
G
H
I
J
K
FDR (Age associated divergence) =0.0882
p(Scalp)=0.344
p(Dura)=0.0852
Scalp
Dura

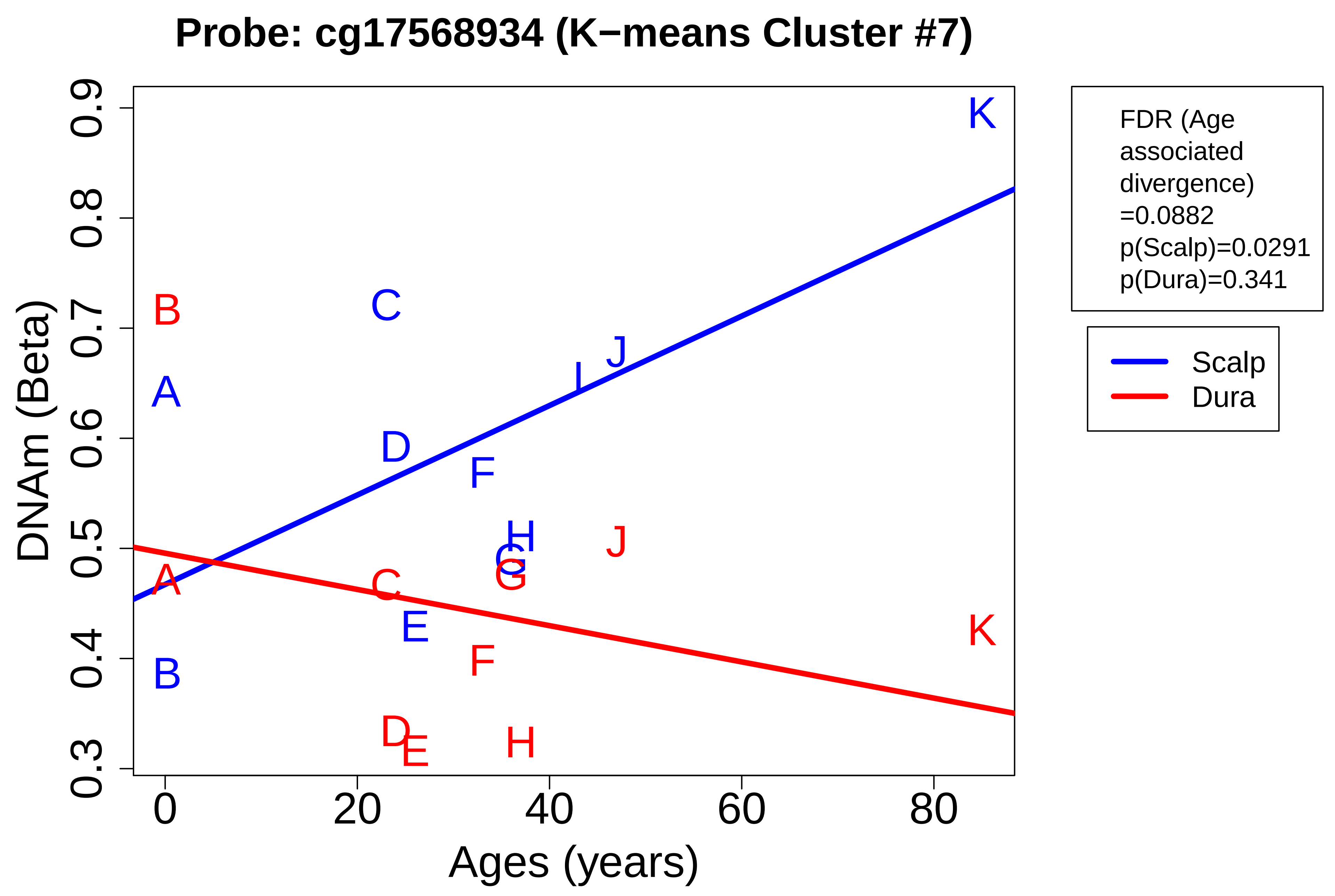

Probe: cg17568934 (K−means Cluster #7)
DNAm (Beta)
Ages (years)
0.3
0.4
0.5
0.6
0.7
0.8
0.9
0
20
40
60
80
FDR (Age associated divergence) =0.0882
p(Scalp)=0.0291
p(Dura)=0.341
Scalp
Dura
A
B
C
D
E
F
G
H
I
J
K

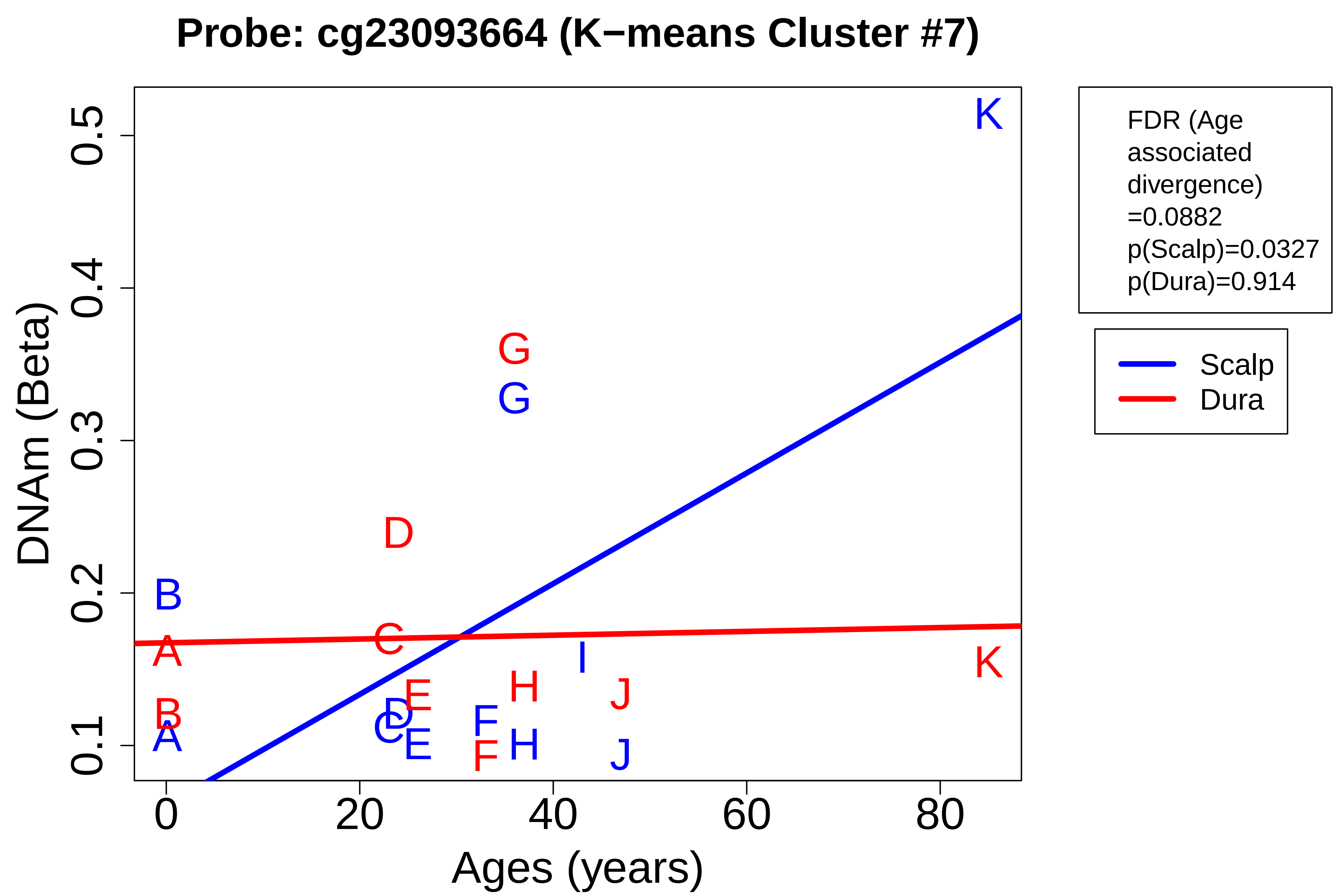

Probe: cg23093664 (K−means Cluster #7)
DNAm (Beta)
0.1
0.2
0.3
0.4
0.5
Ages (years)
0
20
40
60
80
FDR (Age associated divergence) =0.0882
p(Scalp)=0.0327
p(Dura)=0.914
Scalp
Dura
A B C D E F G H I J K

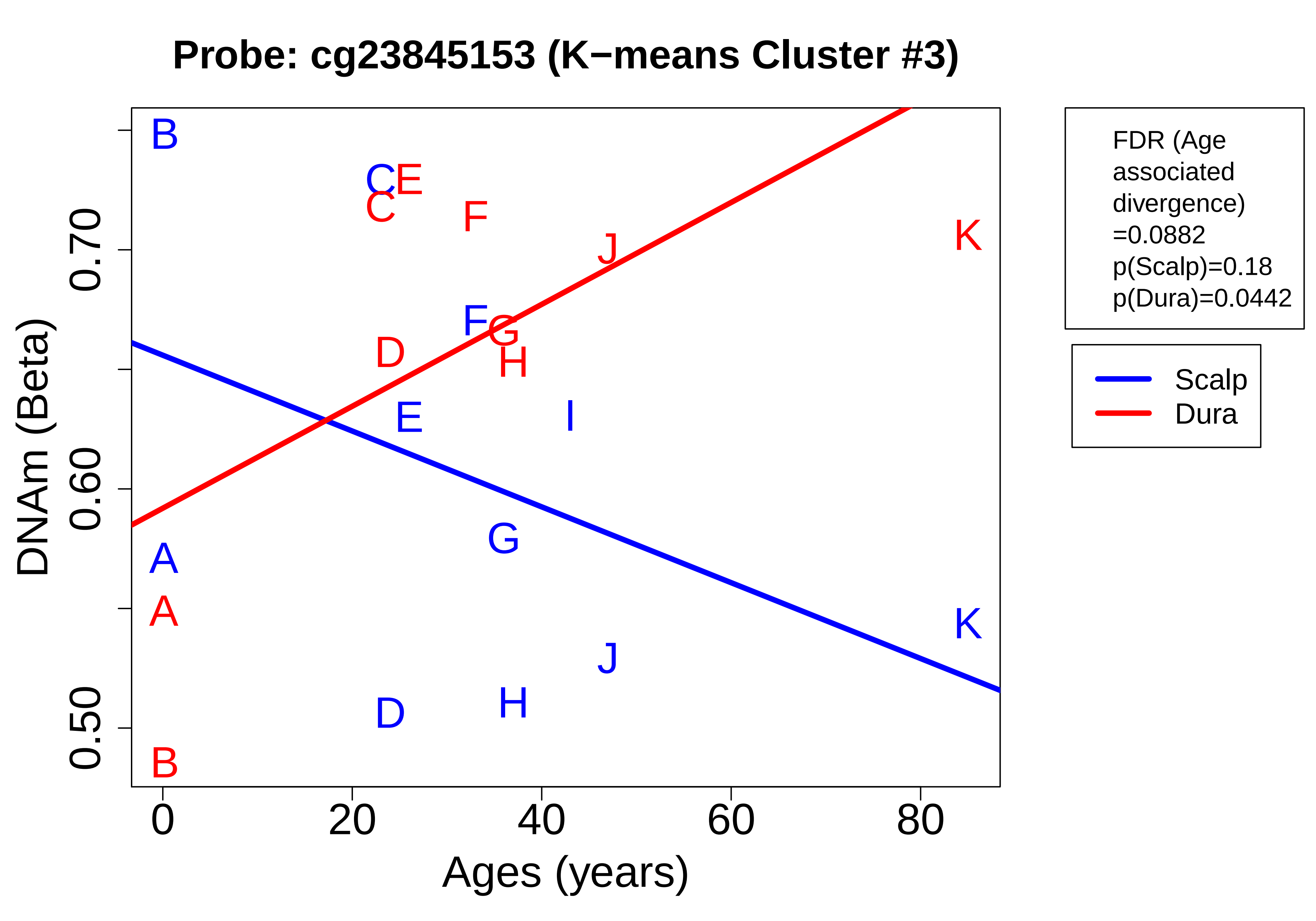

Probe: cg23845153 (K−means Cluster #3)
DNAm (Beta)
0.50
0.60
0.70
Ages (years)
0
20
40
60
80
A
B
C
D
E
F
G
H
I
J
K
FDR (Age associated divergence) =0.0882
p(Scalp)=0.18
p(Dura)=0.0442
Scalp
Dura

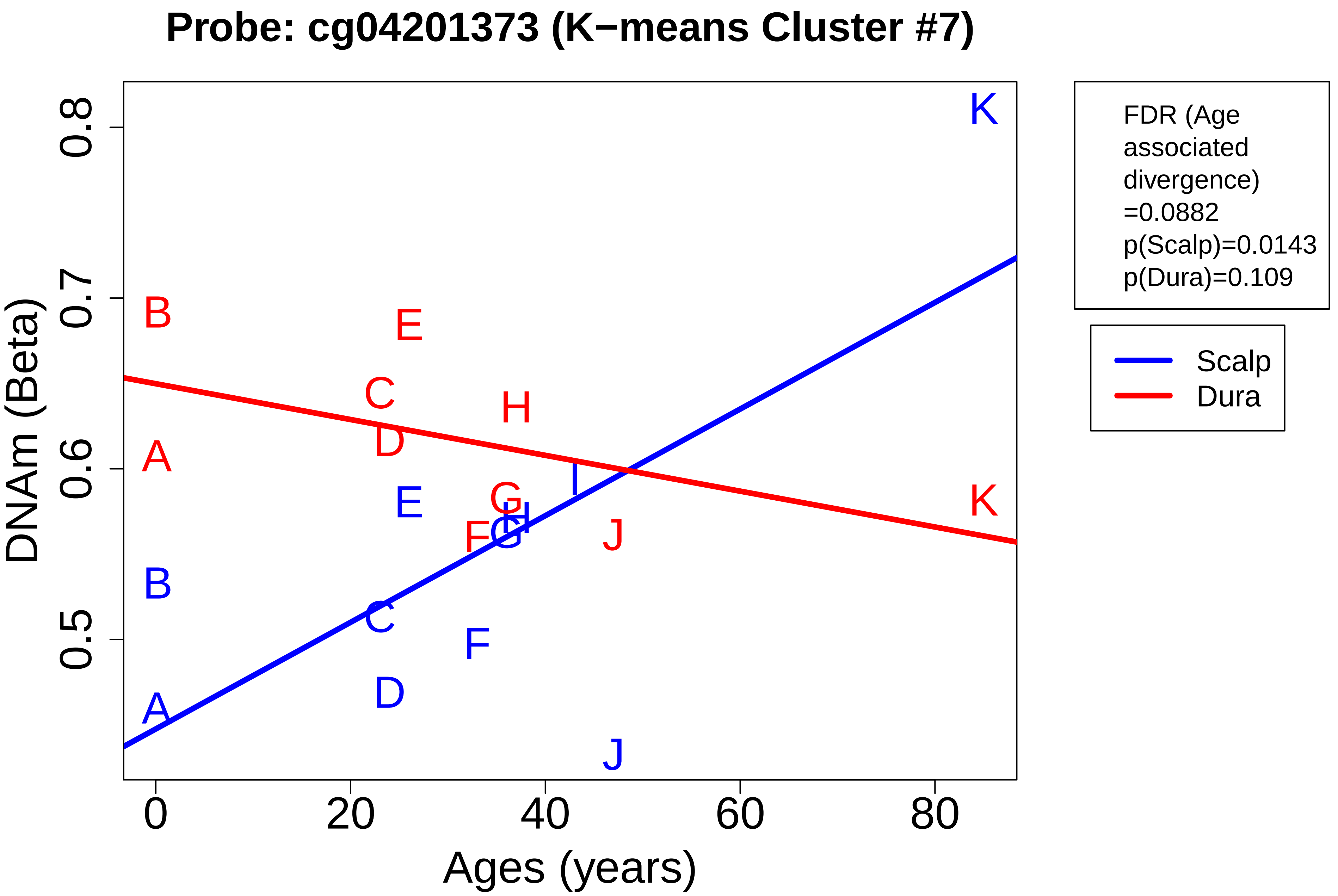
Probe: cg04201373 (K−means Cluster #7)
DNAm (Beta)
0.5
0.6
0.7
0.8
Ages (years)
0
20
40
60
80
FDR (Age associated divergence) =0.0882
p(Scalp)=0.0143
p(Dura)=0.109
Scalp
Dura
A
B
C
D
E
F
G
H
I
J
K

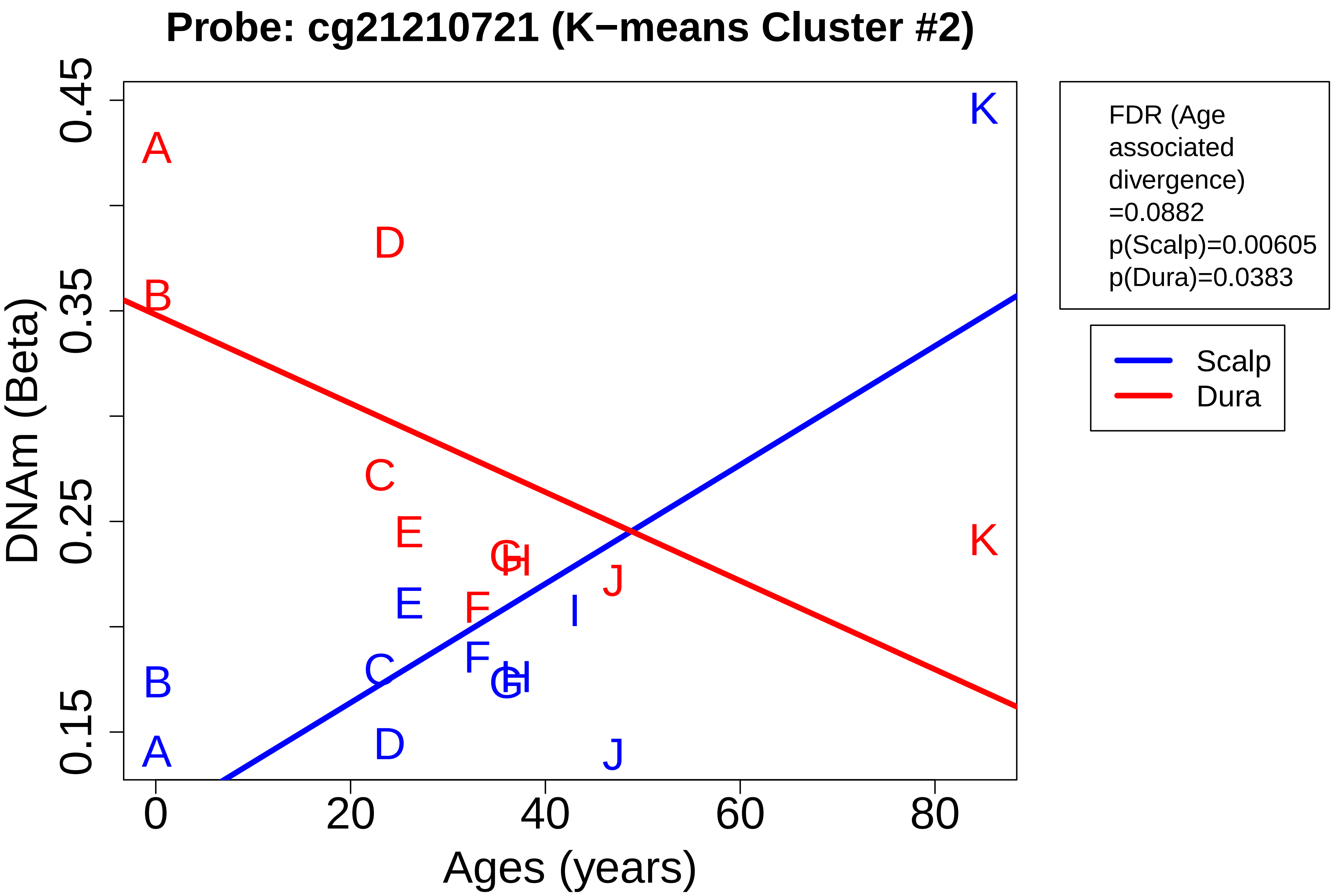

Probe: cg21210721 (K−means Cluster #2)
DNAm (Beta)
0.15
0.25
0.35
0.45
Ages (years)
0
20
40
60
80
FDR (Age associated divergence) =0.0882
p(Scalp)=0.00605
p(Dura)=0.0383
Scalp
Dura

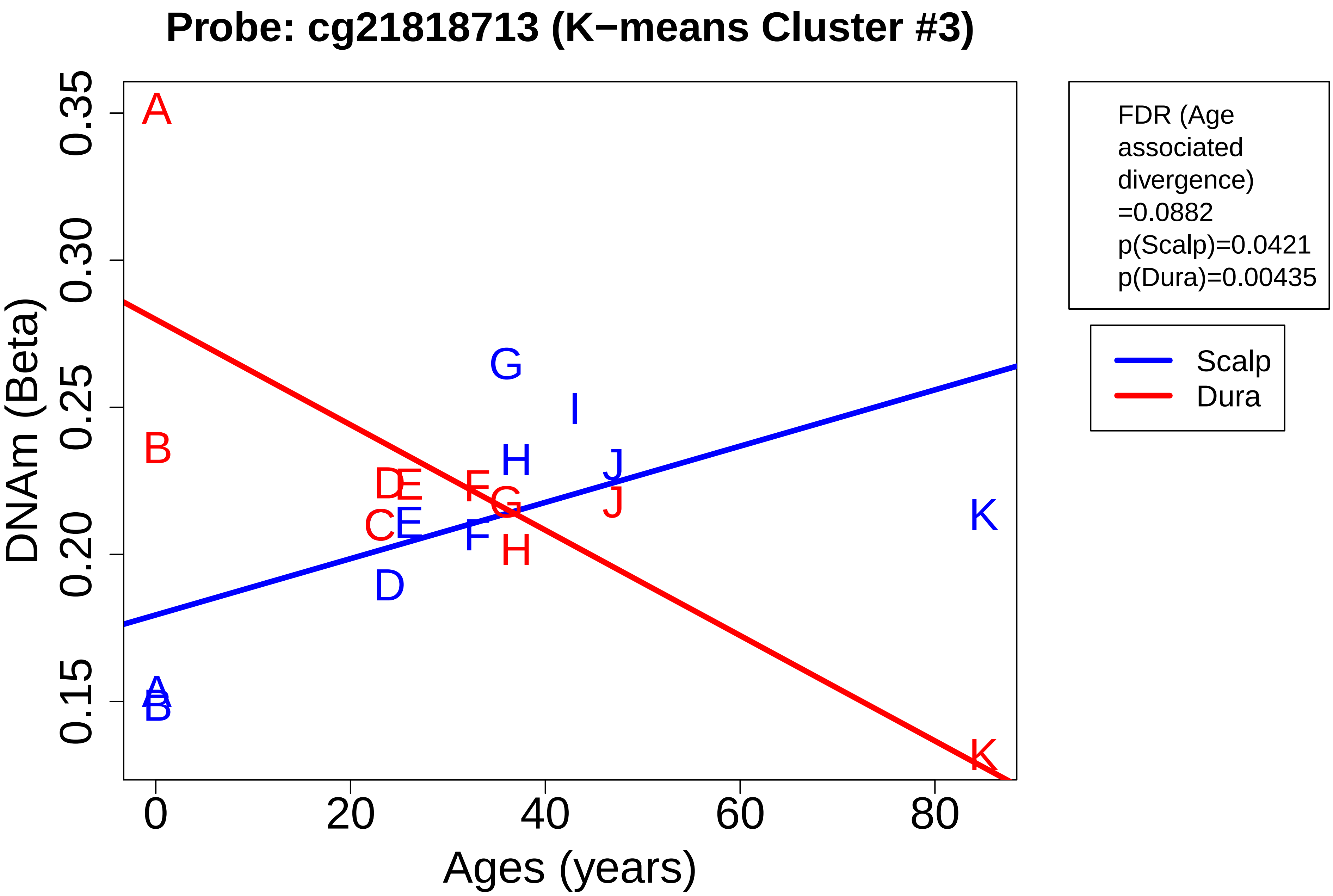

Probe: cg21818713 (K−means Cluster #3)
DNAm (Beta)
0.15
0.20
0.25
0.30
0.35
Ages (years)
0
20
40
60
80
FDR (Age associated divergence) =0.0882
p(Scalp)=0.0421
p(Dura)=0.00435
Scalp
Dura

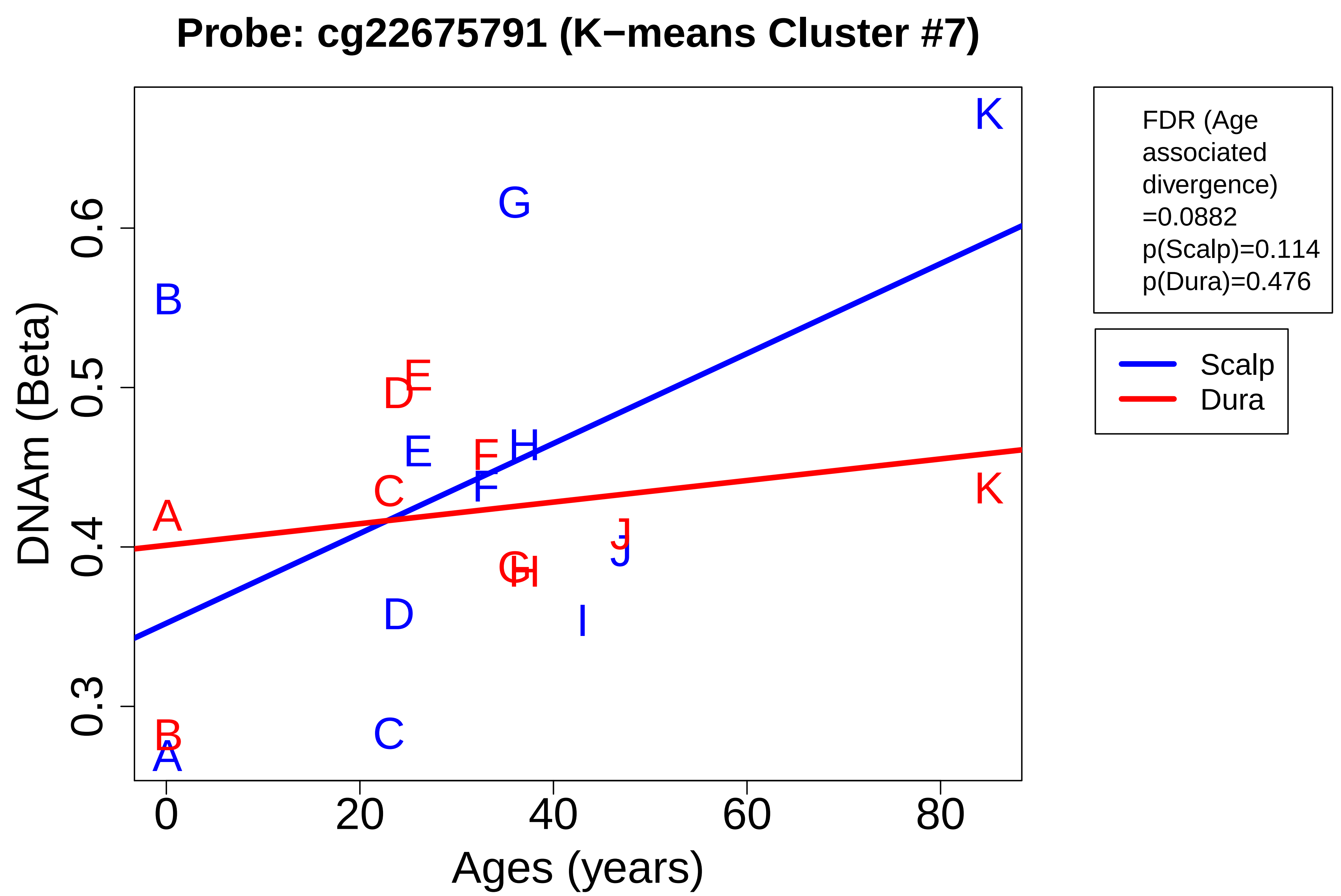
Probe: cg22675791 (K−means Cluster #7)
DNAm (Beta)
0.3
0.4
0.5
0.6
0
20
40
60
80
Ages (years)
FDR (Age associated divergence) =0.0882
p(Scalp)=0.114
p(Dura)=0.476
Scalp
Dura

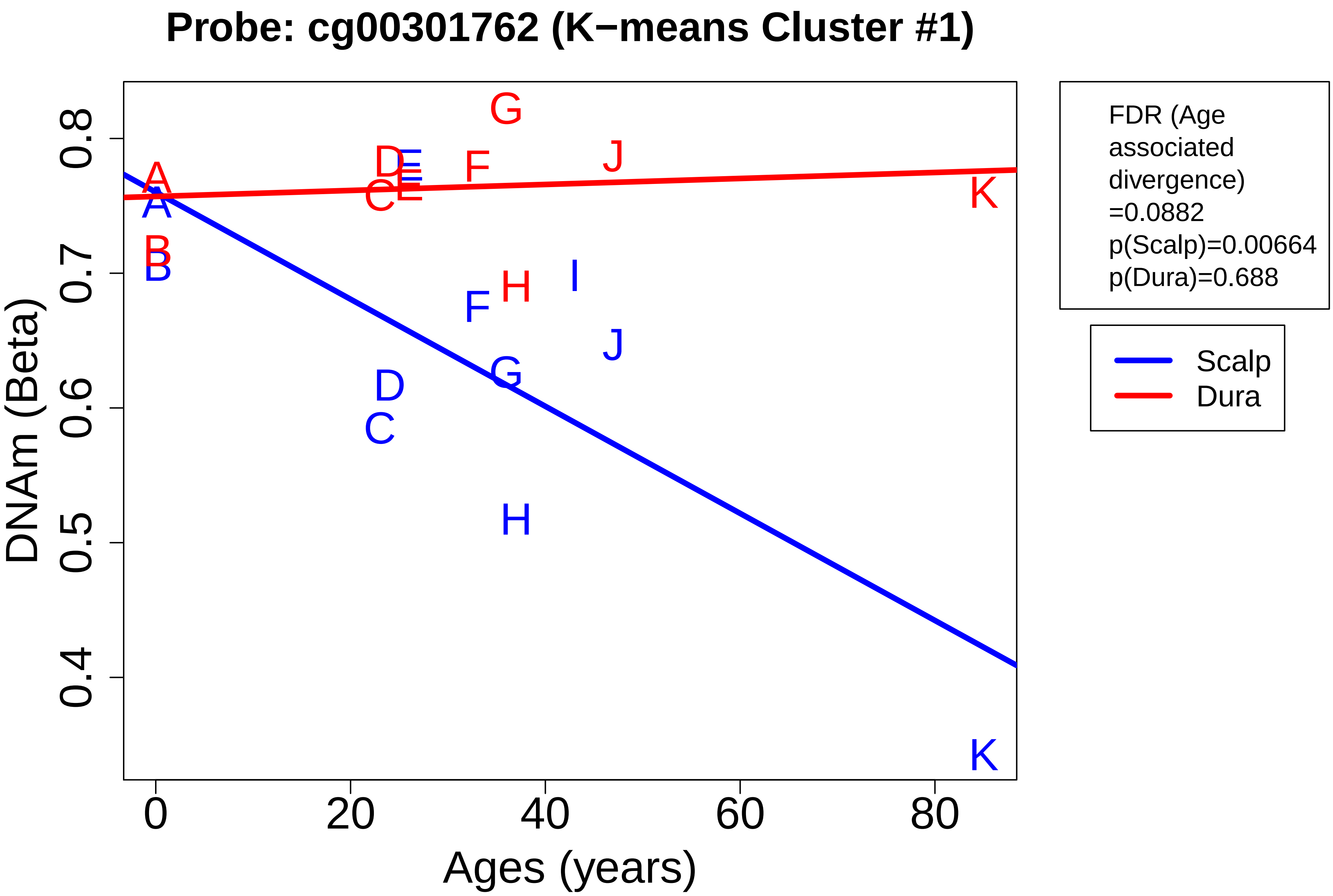

Probe: cg00301762 (K−means Cluster #1)
DNAm (Beta)
Ages (years)
0.4
0.5
0.6
0.7
0.8
0
20
40
60
80
FDR (Age associated divergence) =0.0882
p(Scalp)=0.00664
p(Dura)=0.688
Scalp
Dura

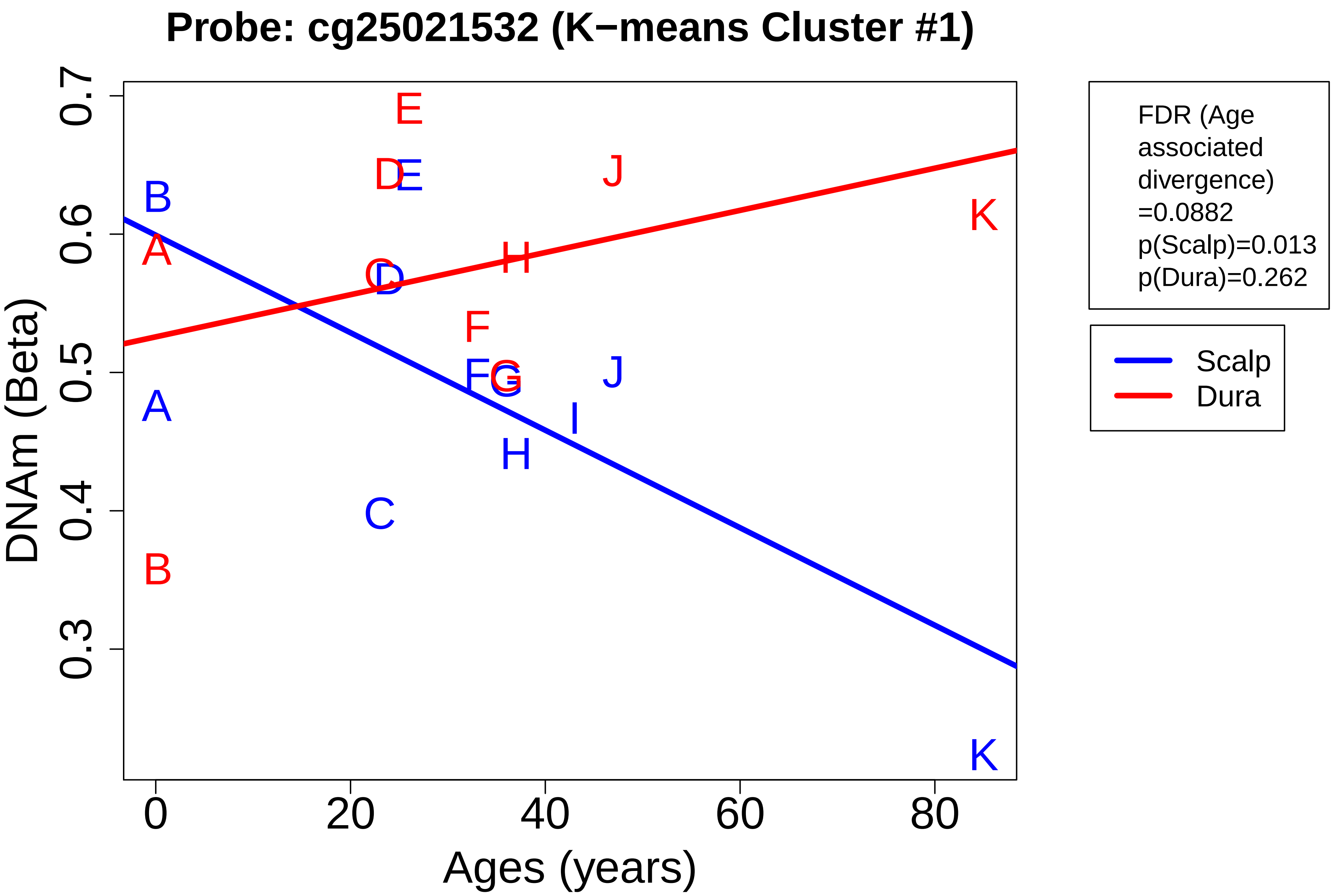

Probe: cg25021532 (K−means Cluster #1)
DNAm (Beta)
0.3
0.4
0.5
0.6
0.7
0
20
40
60
80
Ages (years)
E
D
E
J
B
K
A
C
D
H
F
F
G
J
A
I
H
C
B
K
FDR (Age associated divergence) =0.0882
p(Scalp)=0.013
p(Dura)=0.262
Scalp
Dura

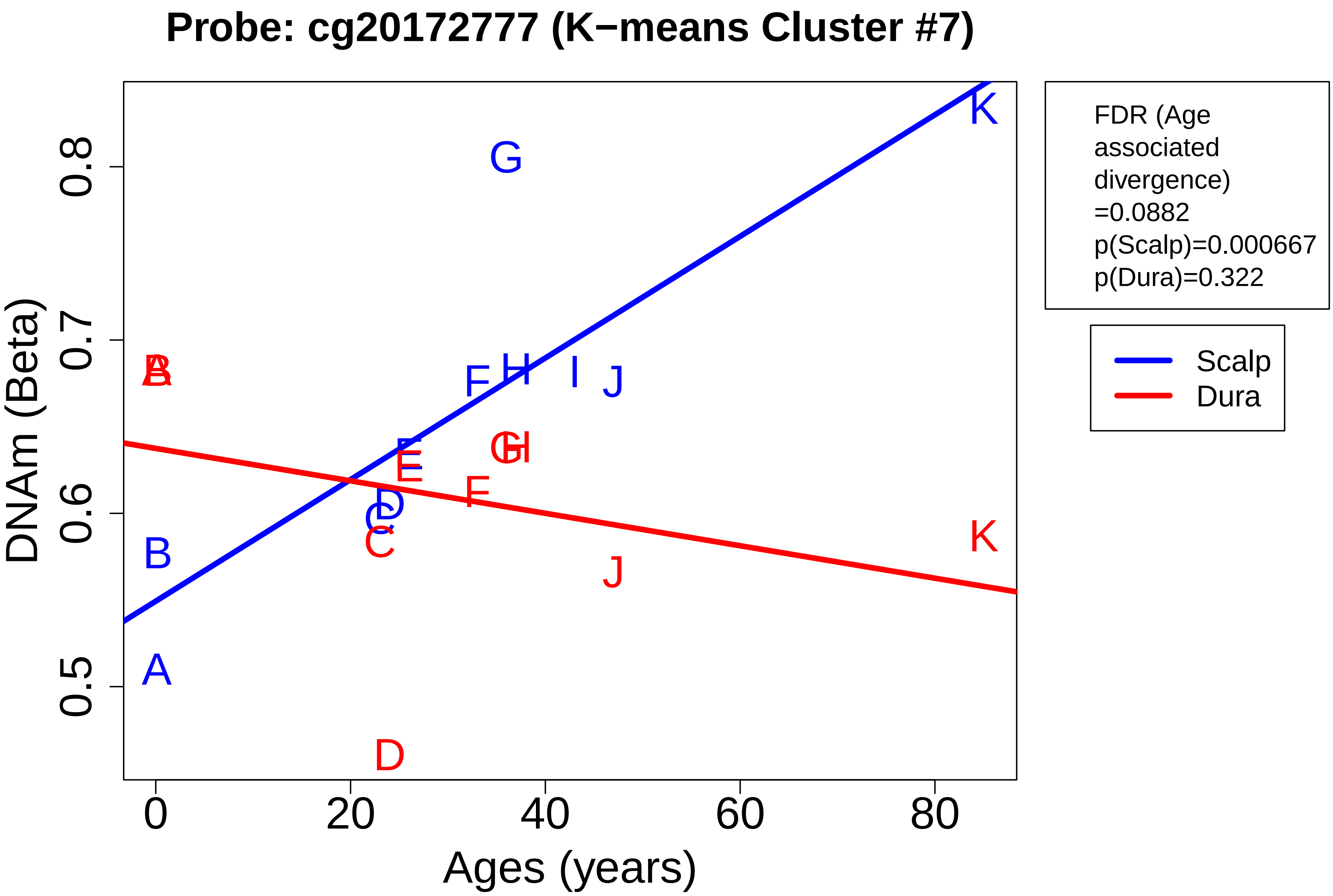

Probe: cg20172777 (K−means Cluster #7)
DNAm (Beta)
0.5
0.6
0.7
0.8
Ages (years)
0
20
40
60
80
FDR (Age associated divergence) =0.0882
p(Scalp)=0.000667
p(Dura)=0.322
Scalp
Dura

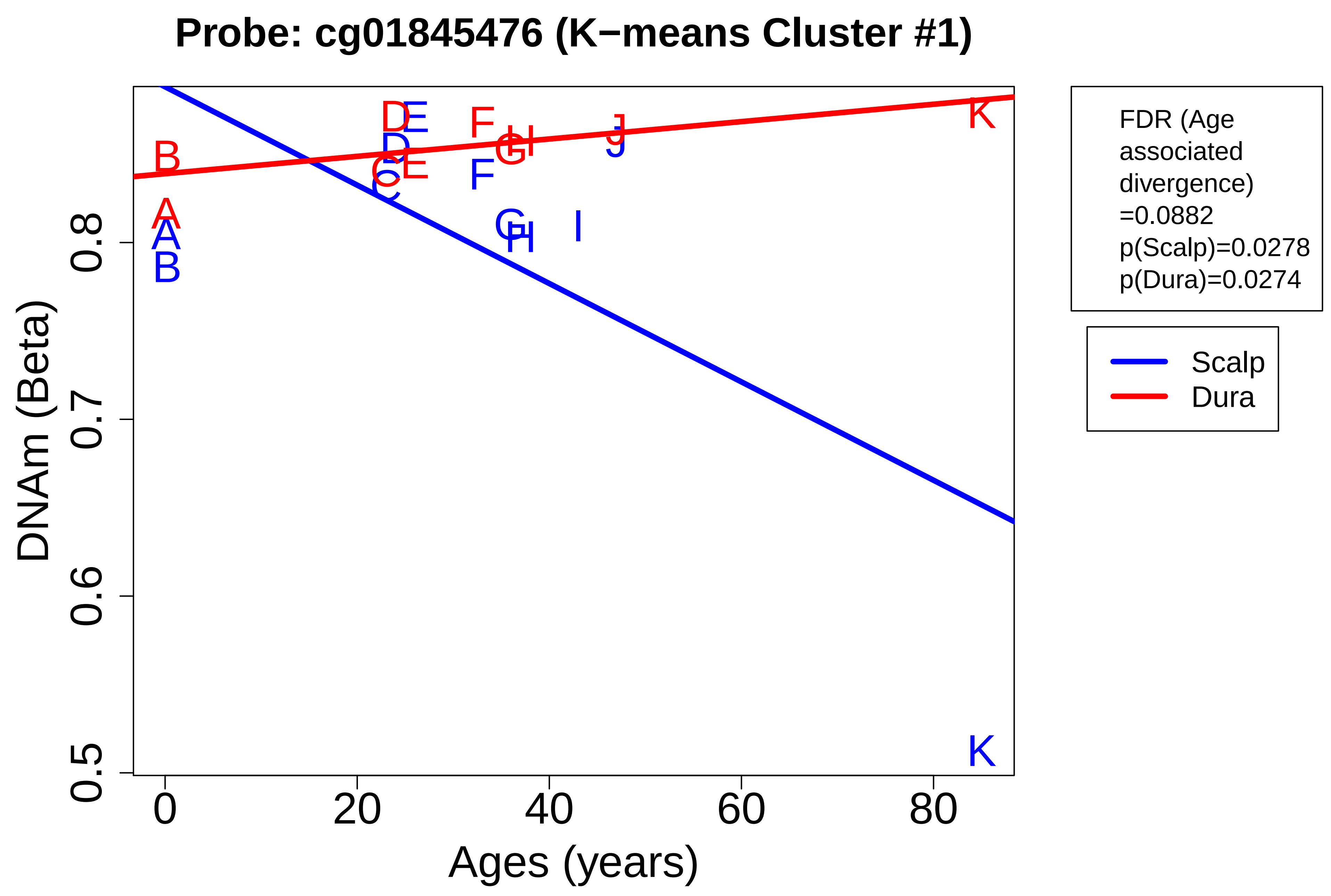
Probe: cg01845476 (K−means Cluster #1)
DNAm (Beta)
0.5
0.6
0.7
0.8
0
20
40
60
80
Ages (years)
FDR (Age associated divergence) =0.0882
p(Scalp)=0.0278
p(Dura)=0.0274
Scalp
Dura

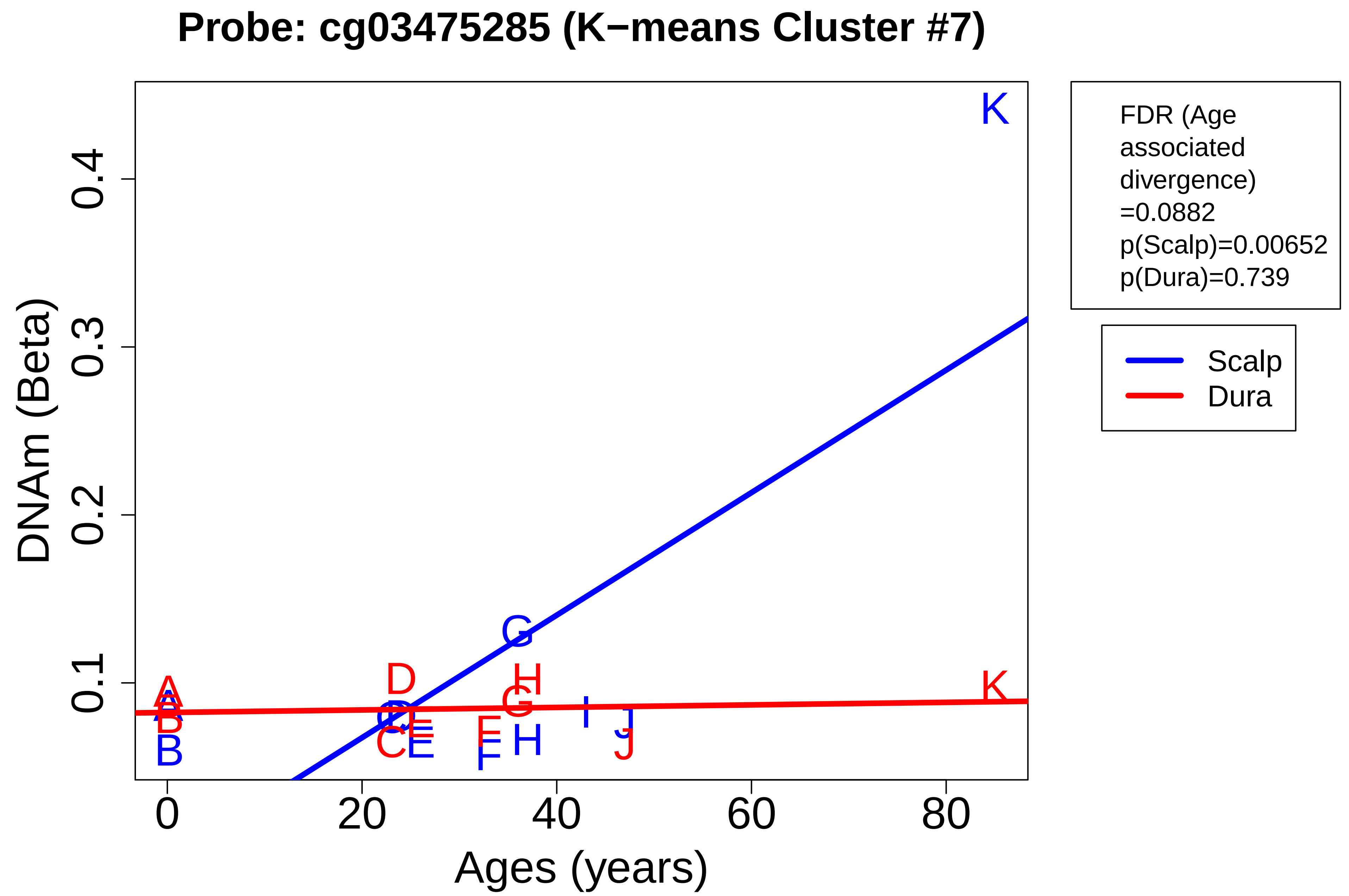

Probe: cg03475285 (K−means Cluster #7)
DNAm (Beta)
0.1
0.2
0.3
0.4
Ages (years)
0
20
40
60
80
FDR (Age associated divergence) =0.0882
p(Scalp)=0.00652
p(Dura)=0.739
Scalp
Dura
A
B
C
D
E
F
G
H
I
J
K

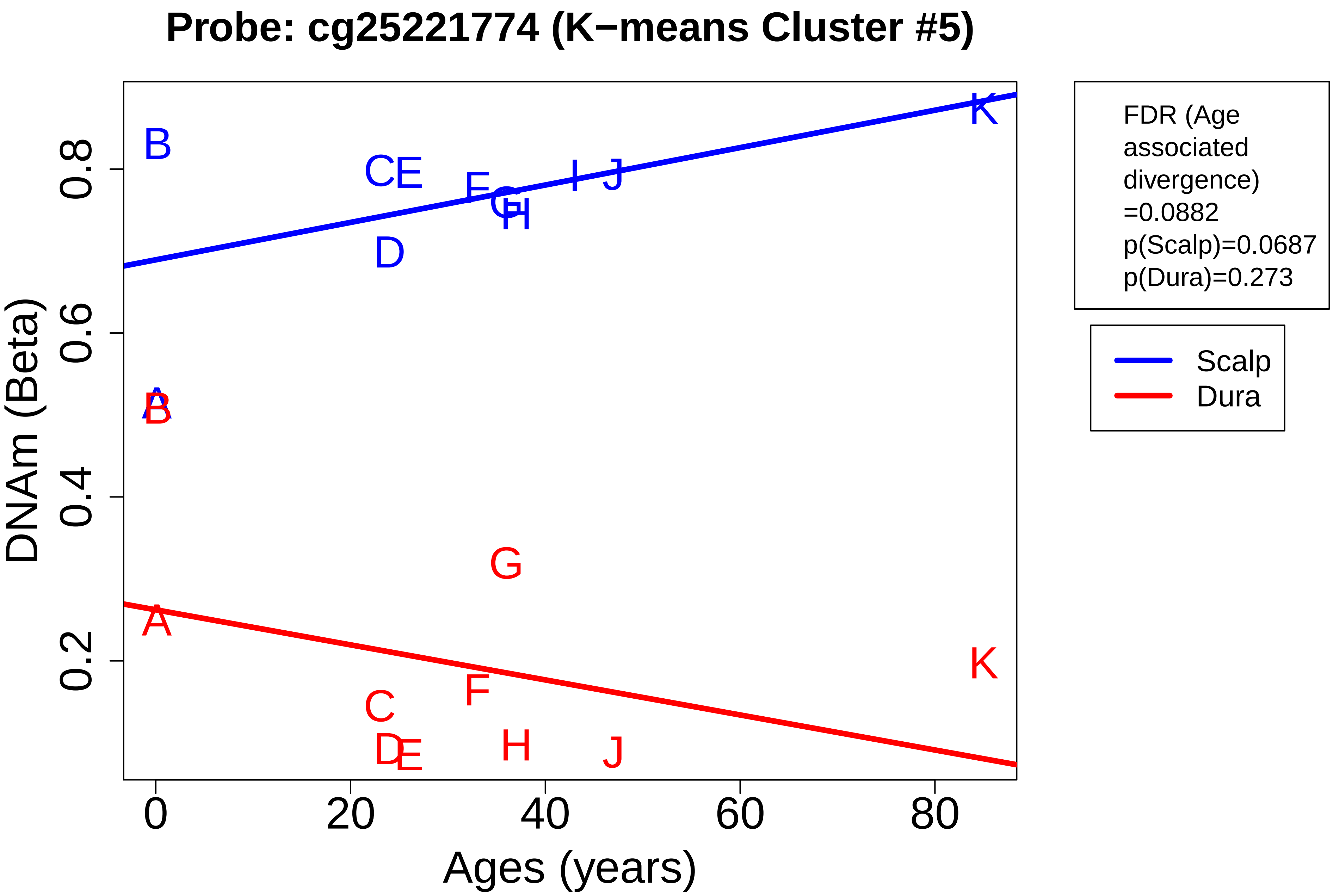

Probe: cg25221774 (K−means Cluster #5)
FDR (Age associated divergence) =0.0882
p(Scalp)=0.0687
p(Dura)=0.273
Scalp
Dura
DNAm (Beta)
0.2
0.4
0.6
0.8
0
20
40
60
80
Ages (years)

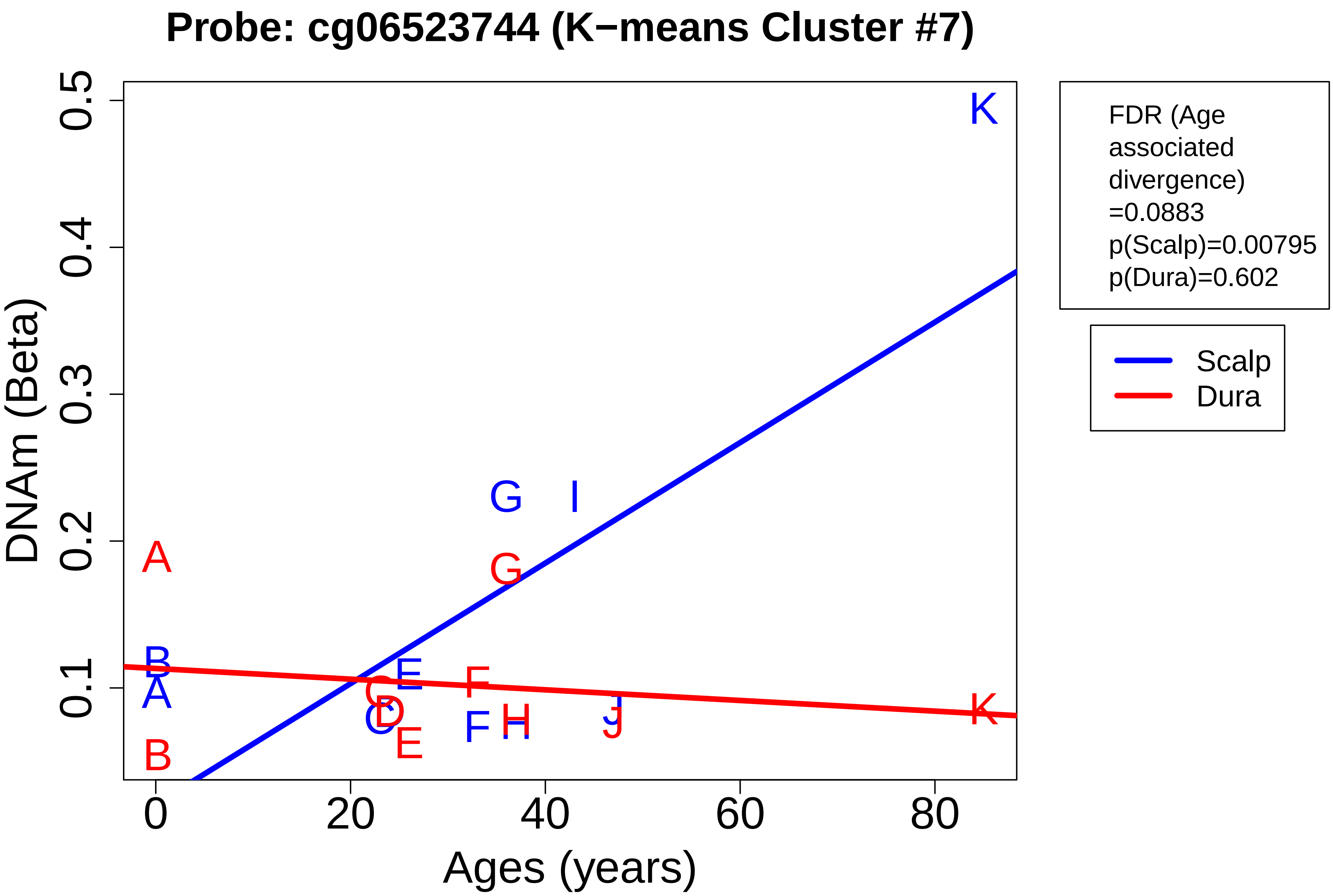

Probe: cg06523744 (K−means Cluster #7)
DNAm (Beta)
0.1
0.2
0.3
0.4
0.5
0
20
40
60
80
Ages (years)
FDR (Age
associated
divergence)
=0.0883
p(Scalp)=0.00795
p(Dura)=0.602
Scalp
Dura

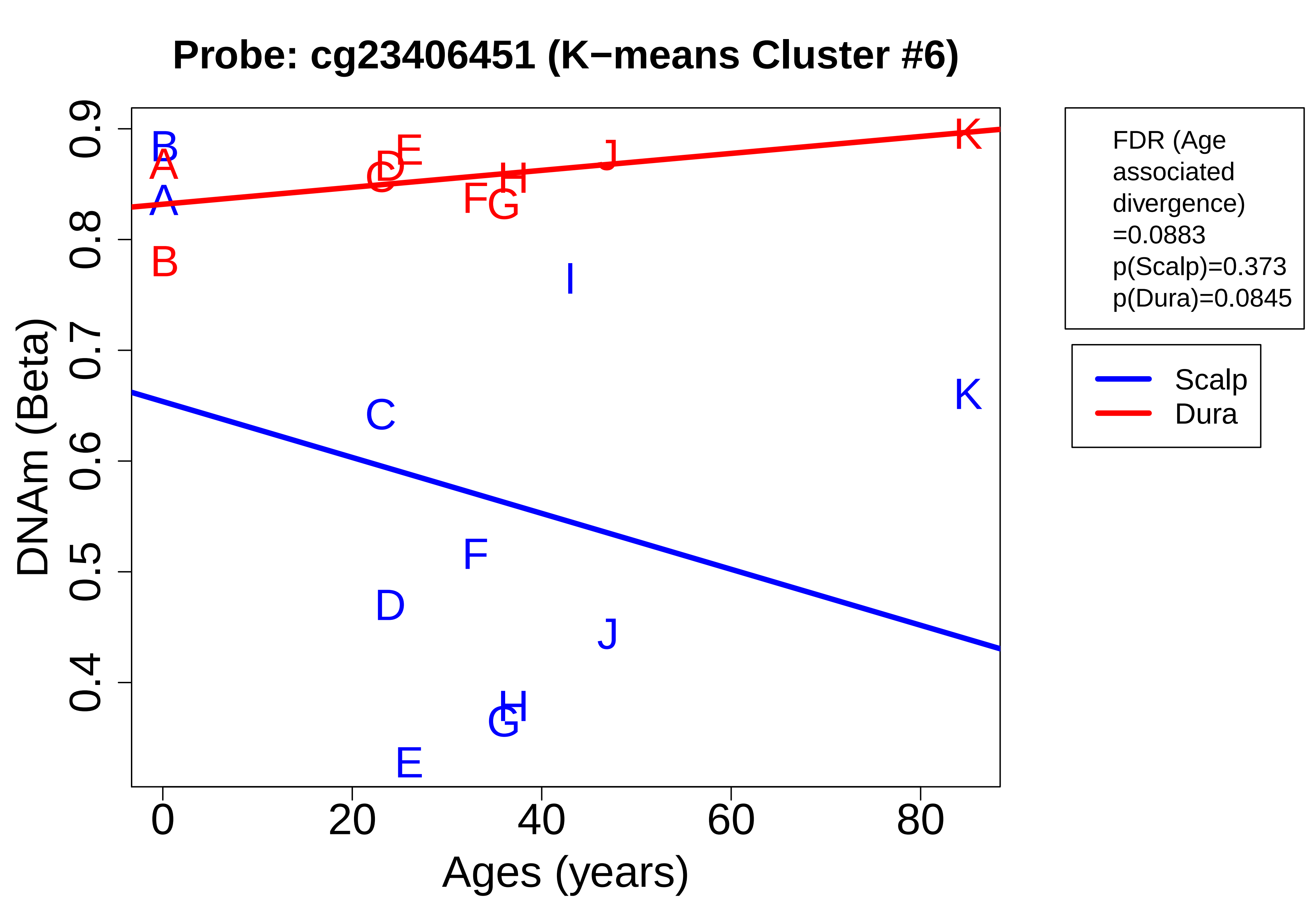

Probe: cg23406451 (K−means Cluster #6)
DNAm (Beta)
0.4
0.5
0.6
0.7
0.8
0.9
0
20
40
60
80
Ages (years)
FDR (Age associated divergence) =0.0883
p(Scalp)=0.373
p(Dura)=0.0845
Scalp
Dura
A
B
C
D
E
F
G
H
I
J
K

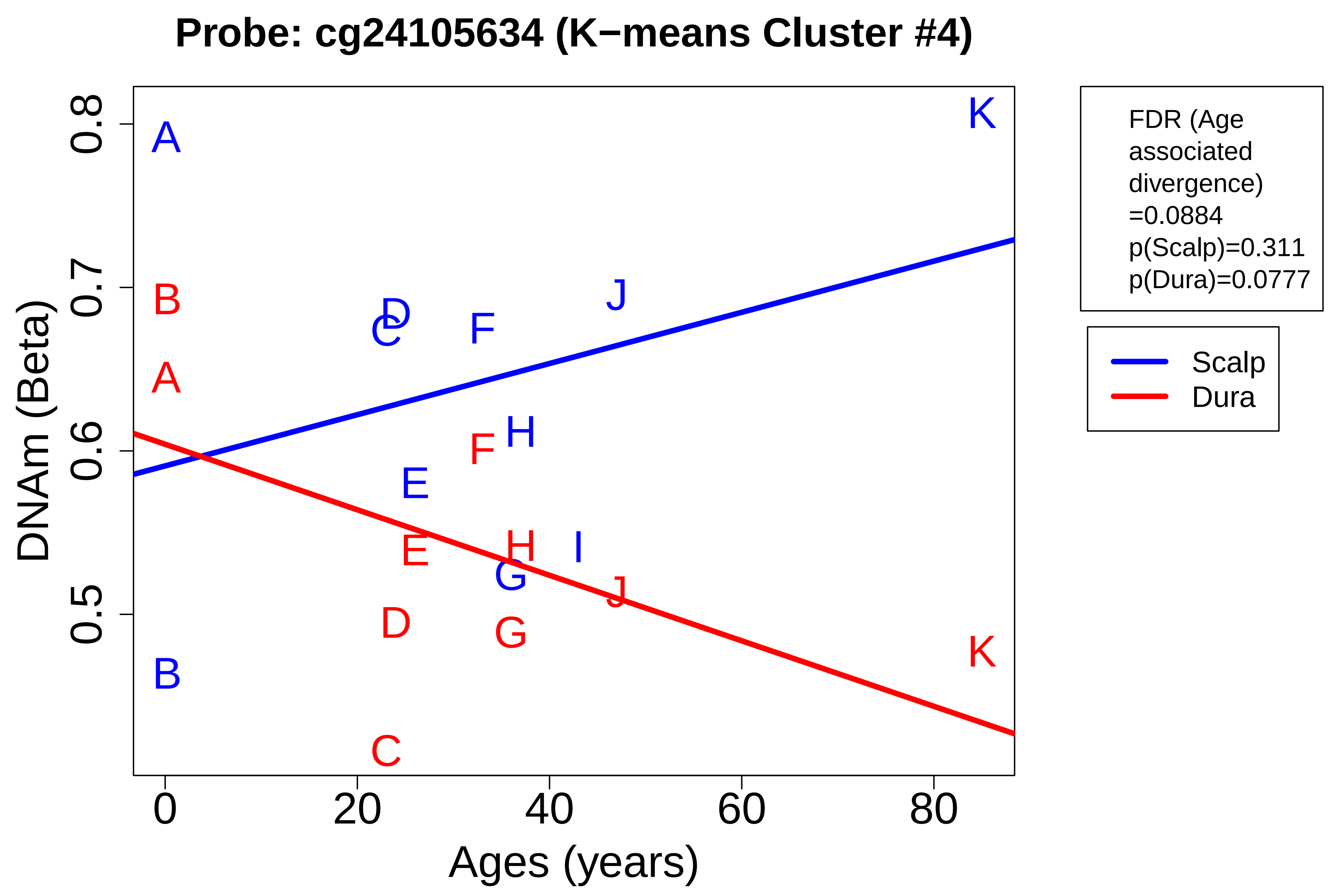

Probe: cg24105634 (K−means Cluster #4)
DNAm (Beta)
0.5
0.6
0.7
0.8
Ages (years)
0
20
40
60
80
FDR (Age associated divergence) =0.0884
p(Scalp)=0.311
p(Dura)=0.0777
Scalp
Dura
A
B
C
D
E
F
G
H
I
J
K

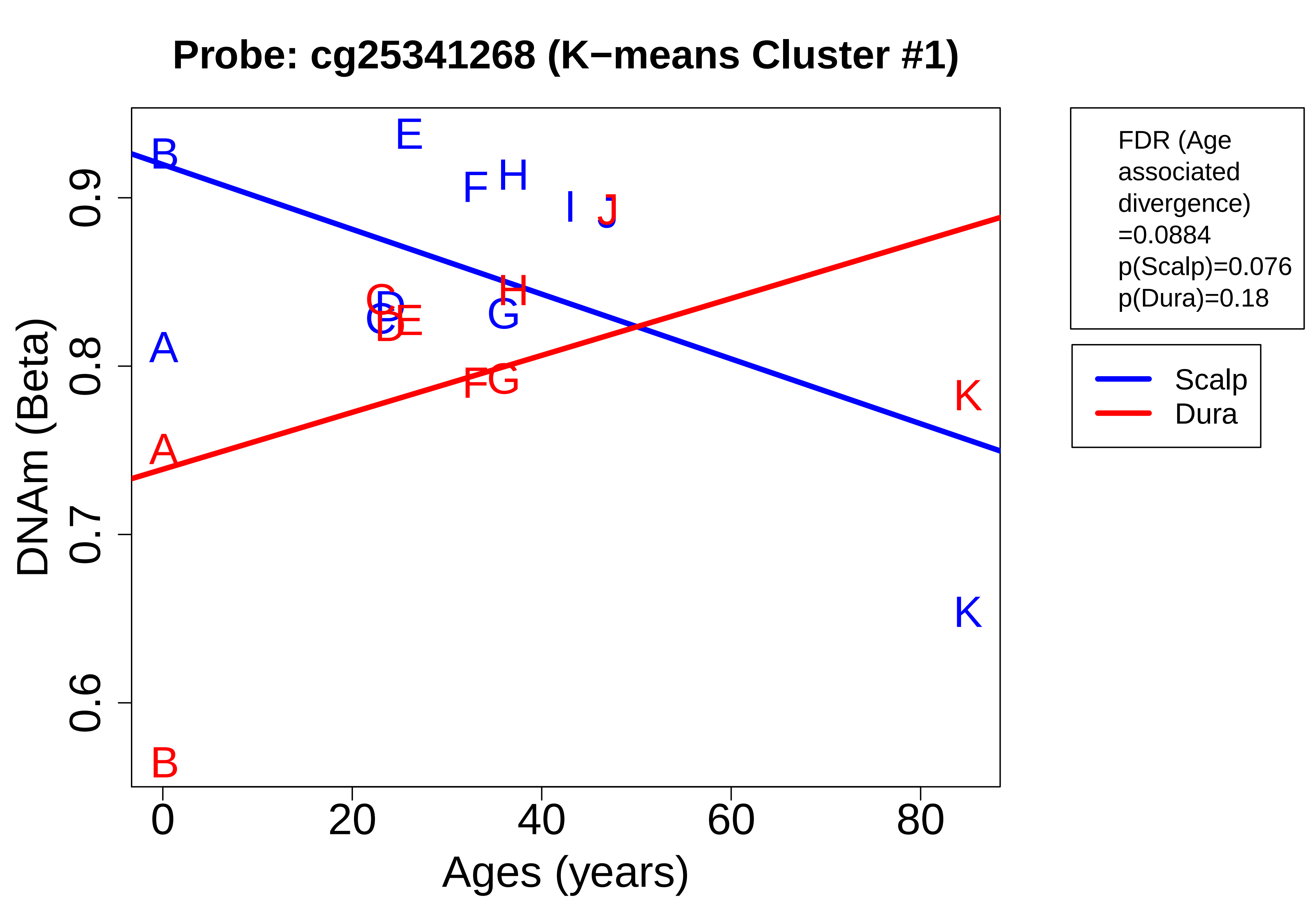
Probe: cg25341268 (K−means Cluster #1)
DNAm (Beta)
0.6
0.7
0.8
0.9
Ages (years)
0
20
40
60
80
FDR (Age associated divergence) =0.0884
p(Scalp)=0.076
p(Dura)=0.18
Scalp
Dura
A
B
C
D
E
F
G
H
I
J
K

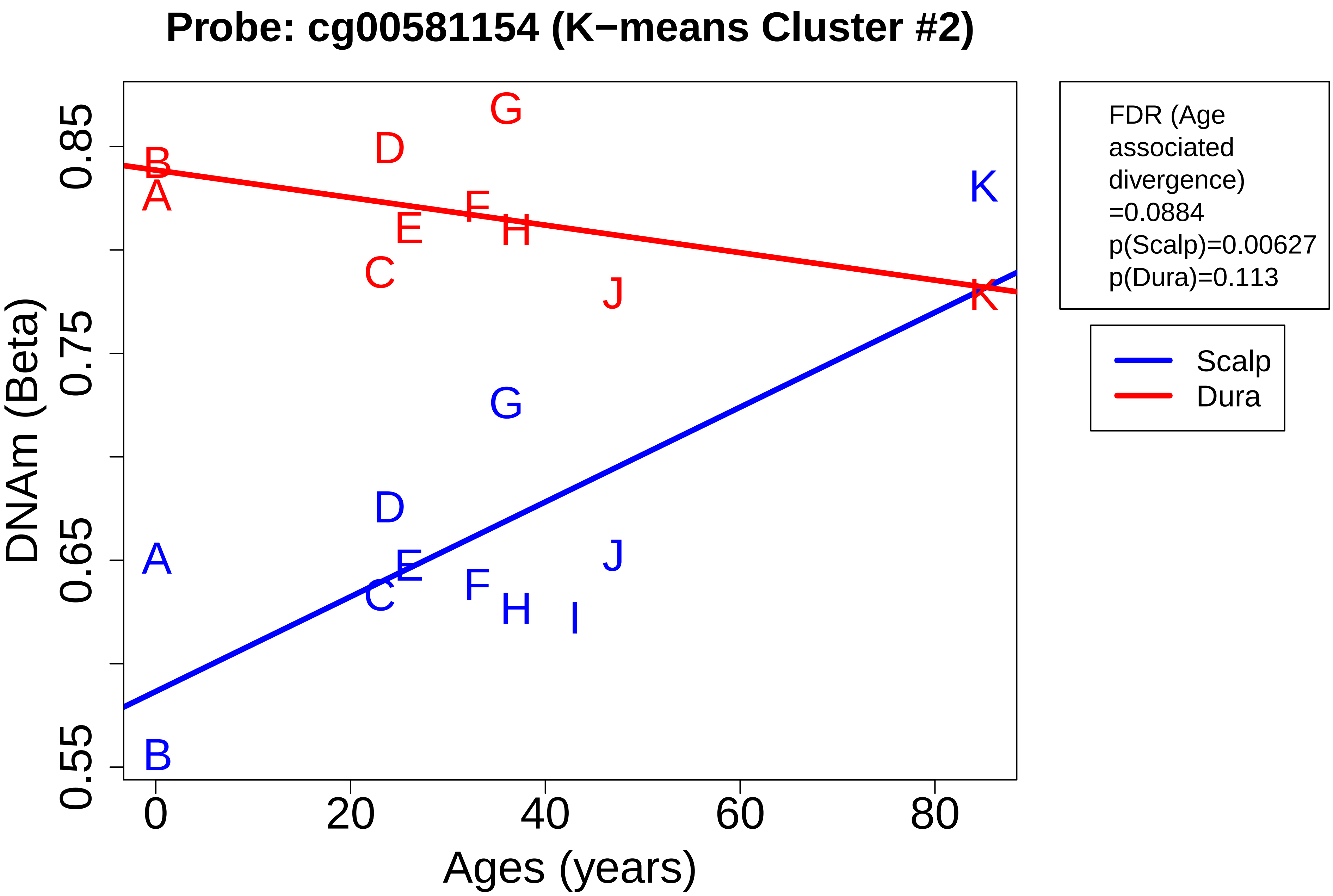

Probe: cg00581154 (K−means Cluster #2)
DNAm (Beta)
0.55
0.65
0.75
0.85
0
20
40
60
80
Ages (years)
FDR (Age associated divergence) =0.0884
p(Scalp)=0.00627
p(Dura)=0.113
Scalp
Dura

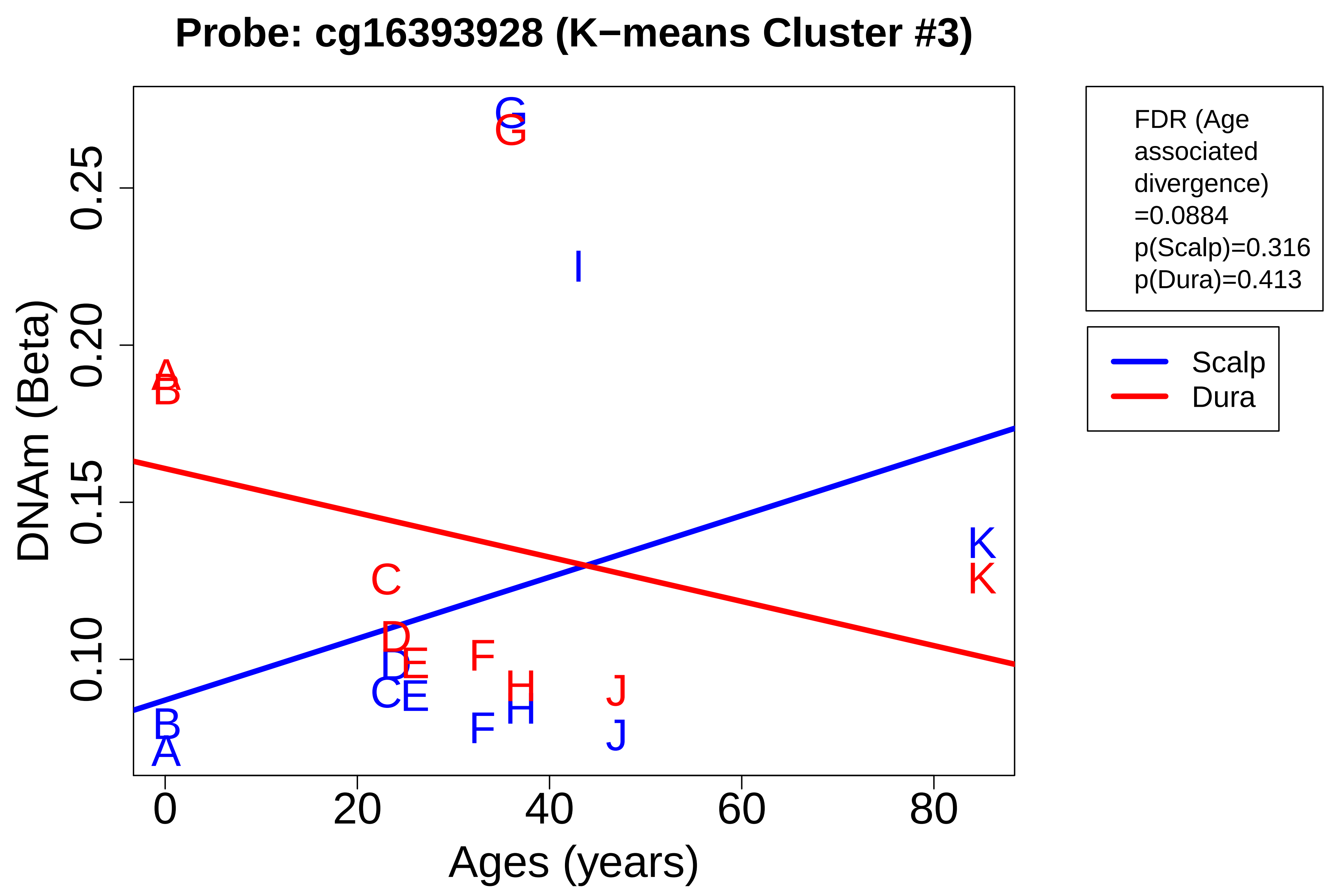

Probe: cg16393928 (K−means Cluster #3)
DNAm (Beta)
0.10
0.15
0.20
0.25
0
20
40
60
80
Ages (years)
FDR (Age associated divergence) =0.0884
p(Scalp)=0.316
p(Dura)=0.413
Scalp
Dura

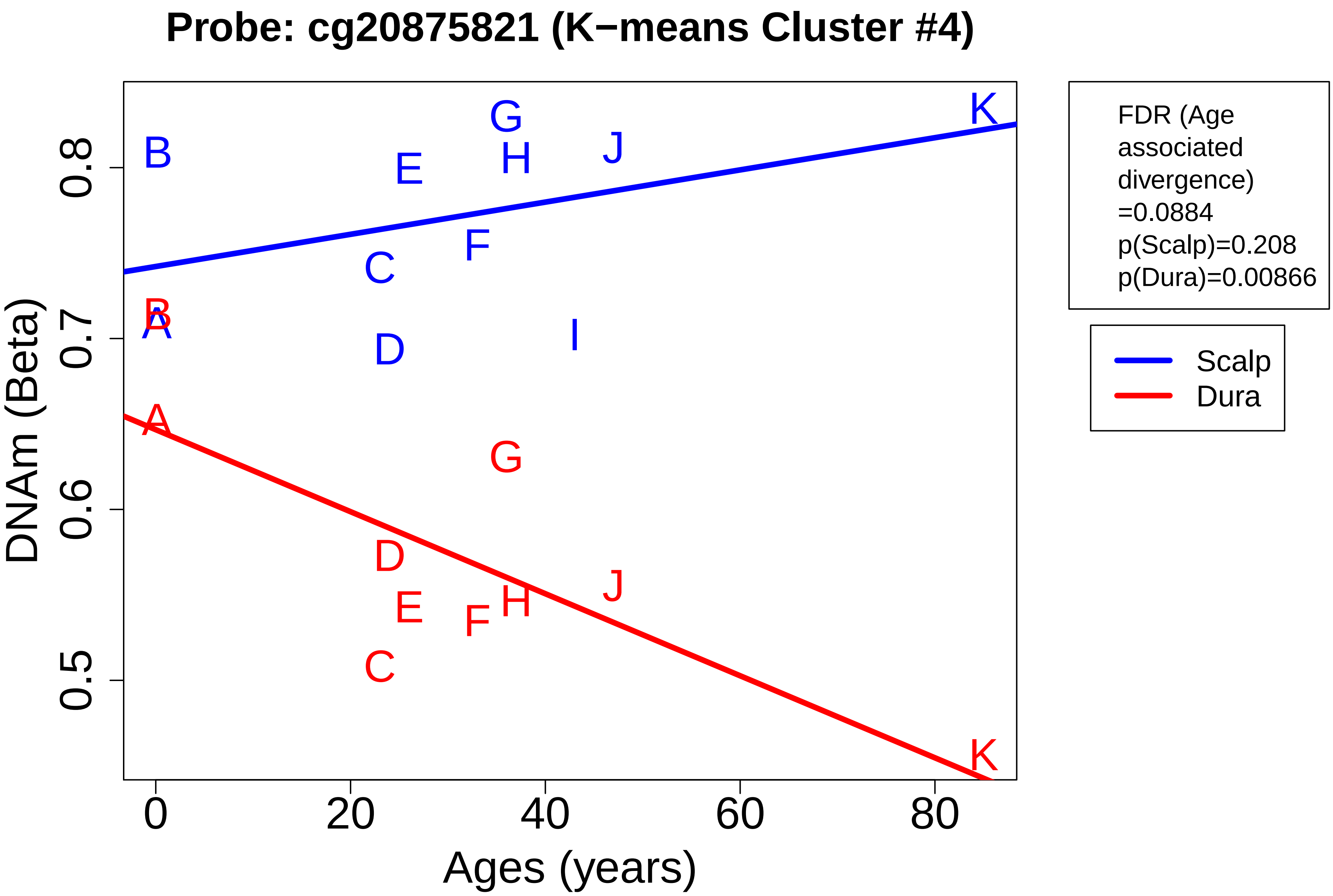

Probe: cg20875821 (K−means Cluster #4)
FDR (Age associated divergence) =0.0884
p(Scalp)=0.208
p(Dura)=0.00866
Scalp
Dura
DNAm (Beta)
0.5
0.6
0.7
0.8
0
20
40
60
80
Ages (years)
A
B
C
D
E
F
G
H
I
J
K

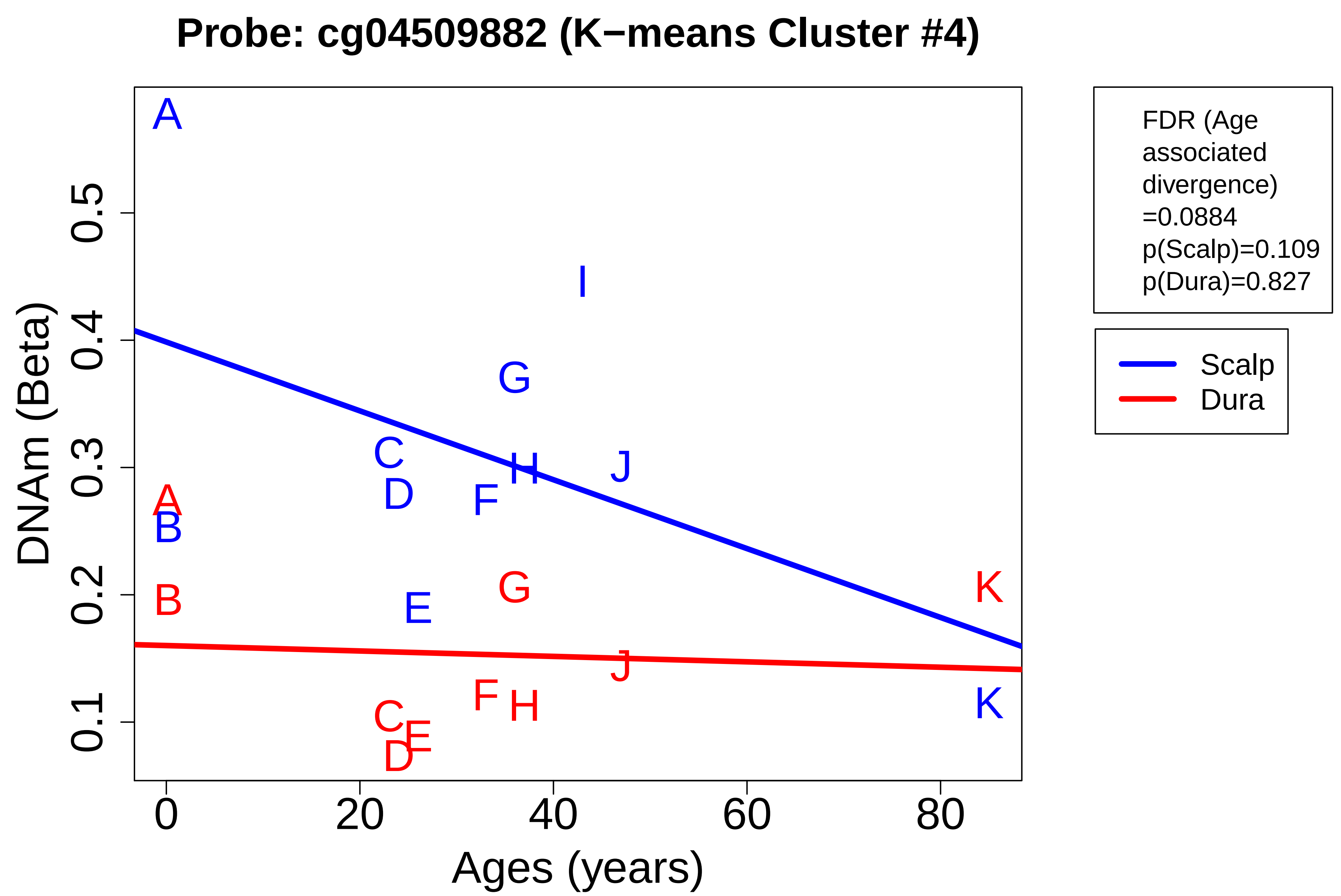

Probe: cg04509882 (K−means Cluster #4)
FDR (Age associated divergence) =0.0884
p(Scalp)=0.109
p(Dura)=0.827
Scalp
Dura
DNAm (Beta)
0.1
0.2
0.3
0.4
0.5
0
20
40
60
80
Ages (years)
A
B
C
D
E
F
G
H
I
J
K

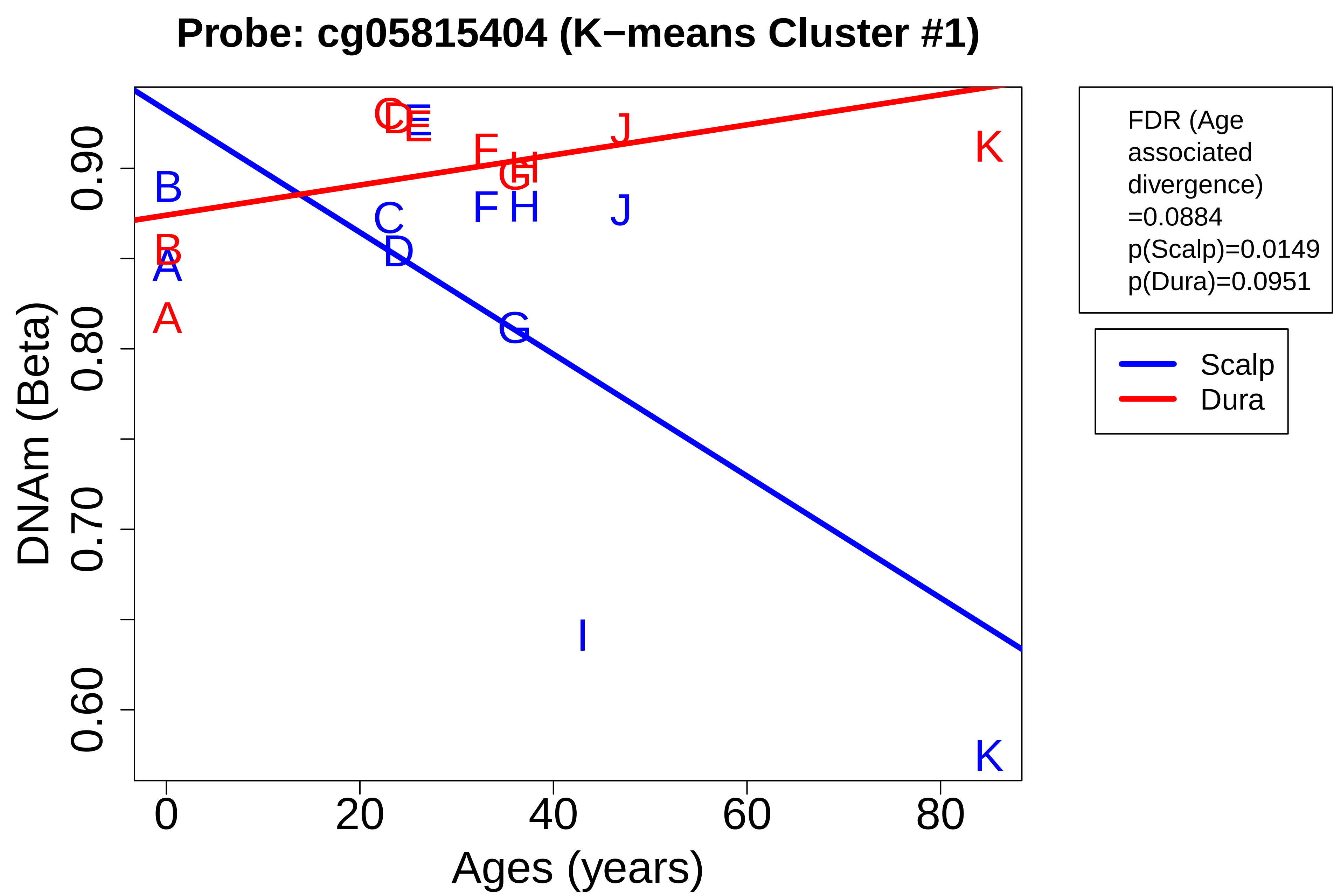

Probe: cg05815404 (K−means Cluster #1)
DNAm (Beta)
0.60
0.70
0.80
0.90
0
20
40
60
80
Ages (years)
FDR (Age associated divergence) =0.0884
p(Scalp)=0.0149
p(Dura)=0.0951
Scalp
Dura

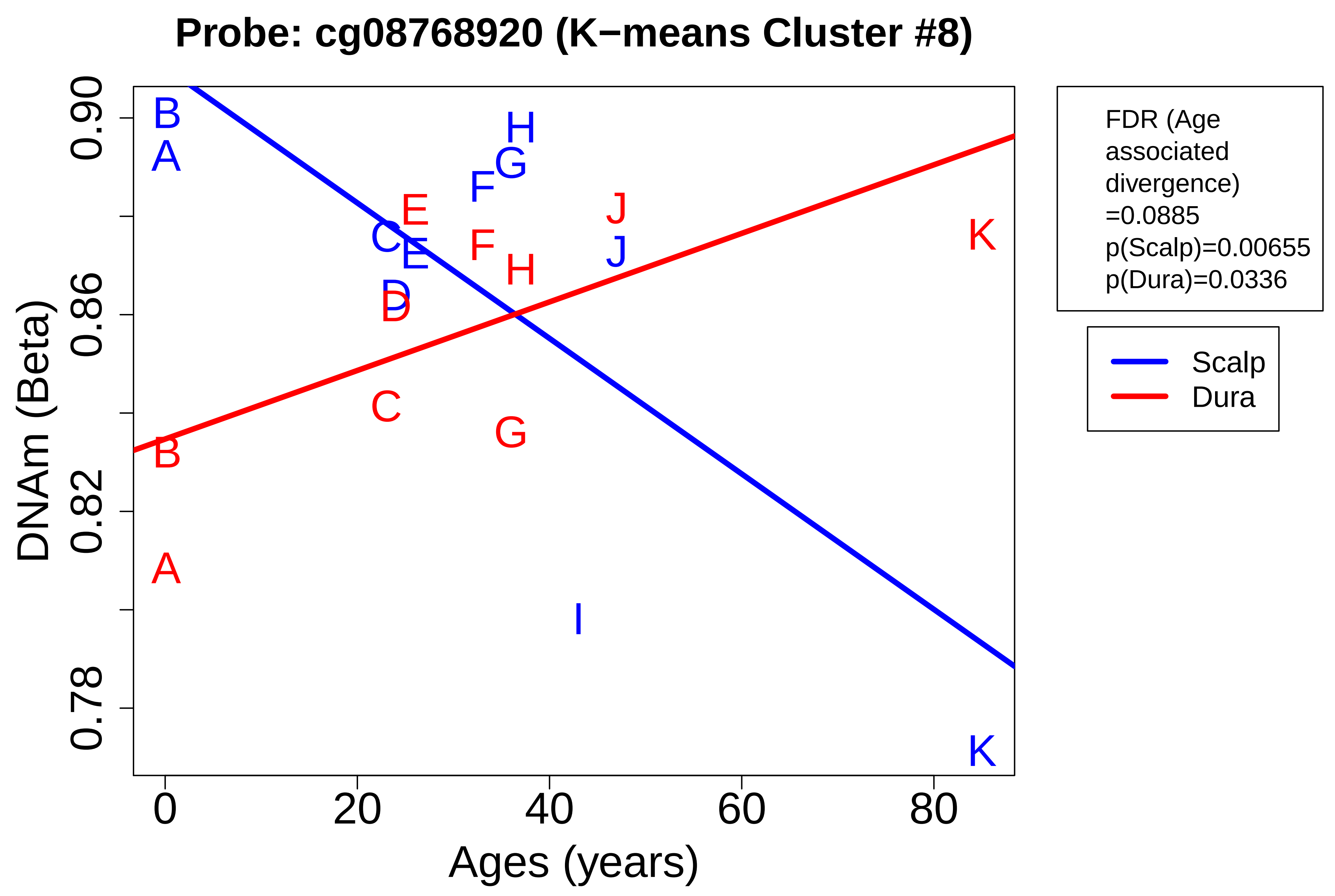

Probe: cg08768920 (K−means Cluster #8)
DNAm (Beta)
0.78
0.82
0.86
0.90
Ages (years)
0
20
40
60
80
FDR (Age associated divergence) =0.0885
p(Scalp)=0.00655
p(Dura)=0.0336
Scalp
Dura
A
B
C
D
E
F
G
H
I
J
K

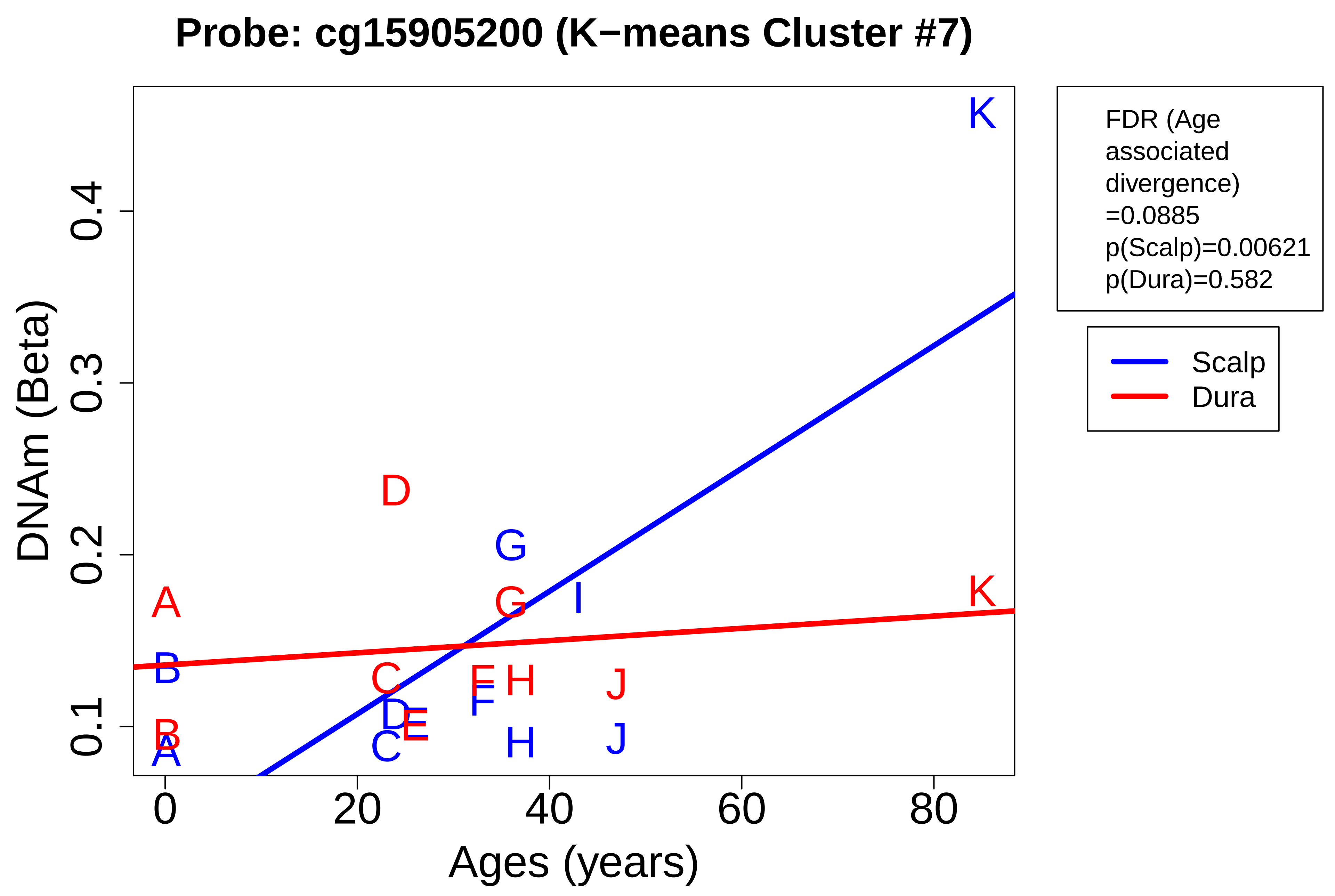

Probe: cg15905200 (K−means Cluster #7)
DNAm (Beta)
0.1
0.2
0.3
0.4
0
20
40
60
80
Ages (years)
FDR (Age associated divergence) =0.0885
p(Scalp)=0.00621
p(Dura)=0.582
Scalp
Dura
A
B
C
D
E
F
G
H
I
J
K

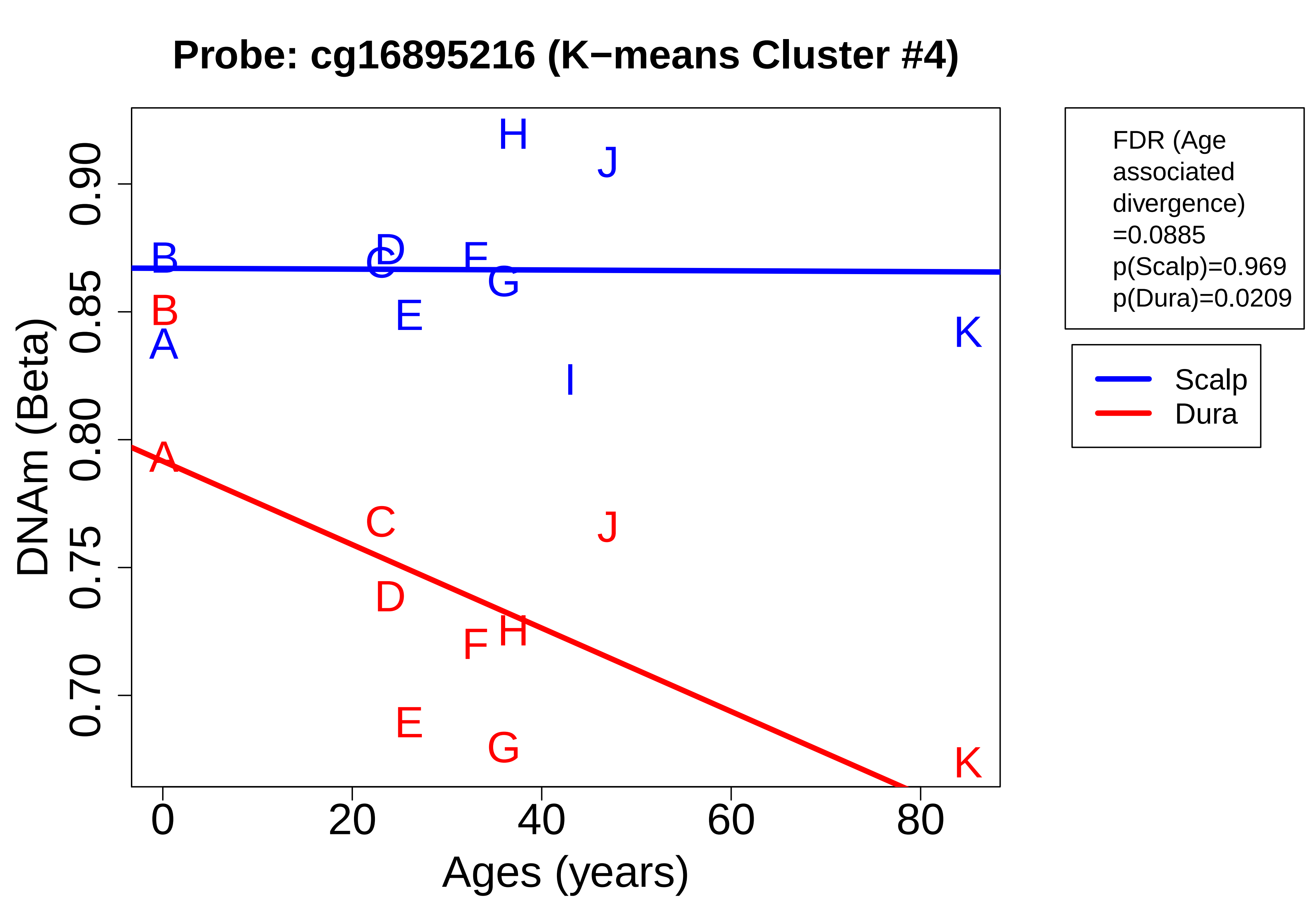

Probe: cg16895216 (K−means Cluster #4)
DNAm (Beta)
0.70
0.75
0.80
0.85
0.90
Ages (years)
0
20
40
60
80
A
B
C
D
E
F
G
H
I
J
K
FDR (Age associated divergence) =0.0885
p(Scalp)=0.969
p(Dura)=0.0209
Scalp
Dura

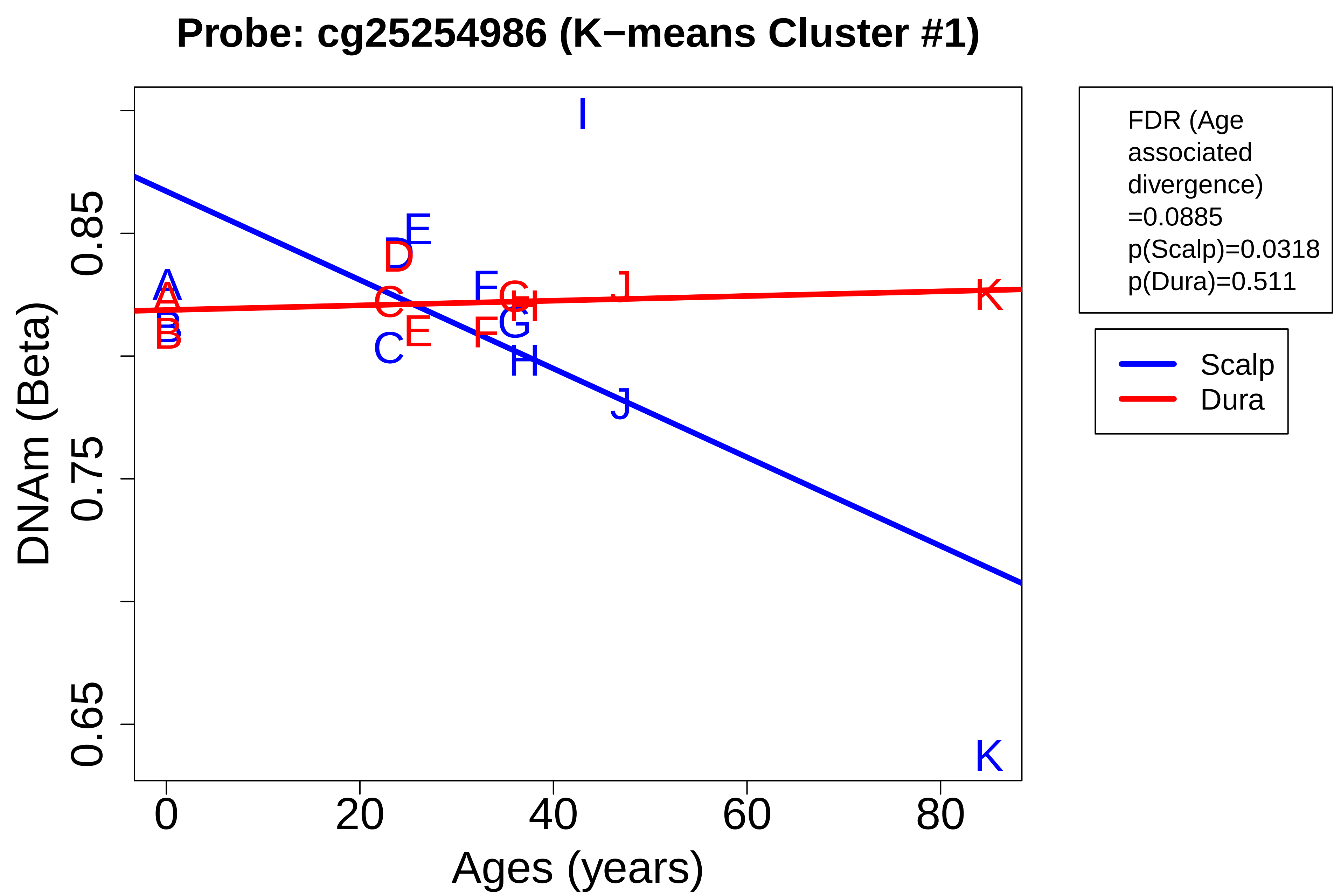

Probe: cg25254986 (K−means Cluster #1)
DNAm (Beta)
0.65
0.75
0.85
Ages (years)
0
20
40
60
80
FDR (Age associated divergence) =0.0885
p(Scalp)=0.0318
p(Dura)=0.511
Scalp
Dura

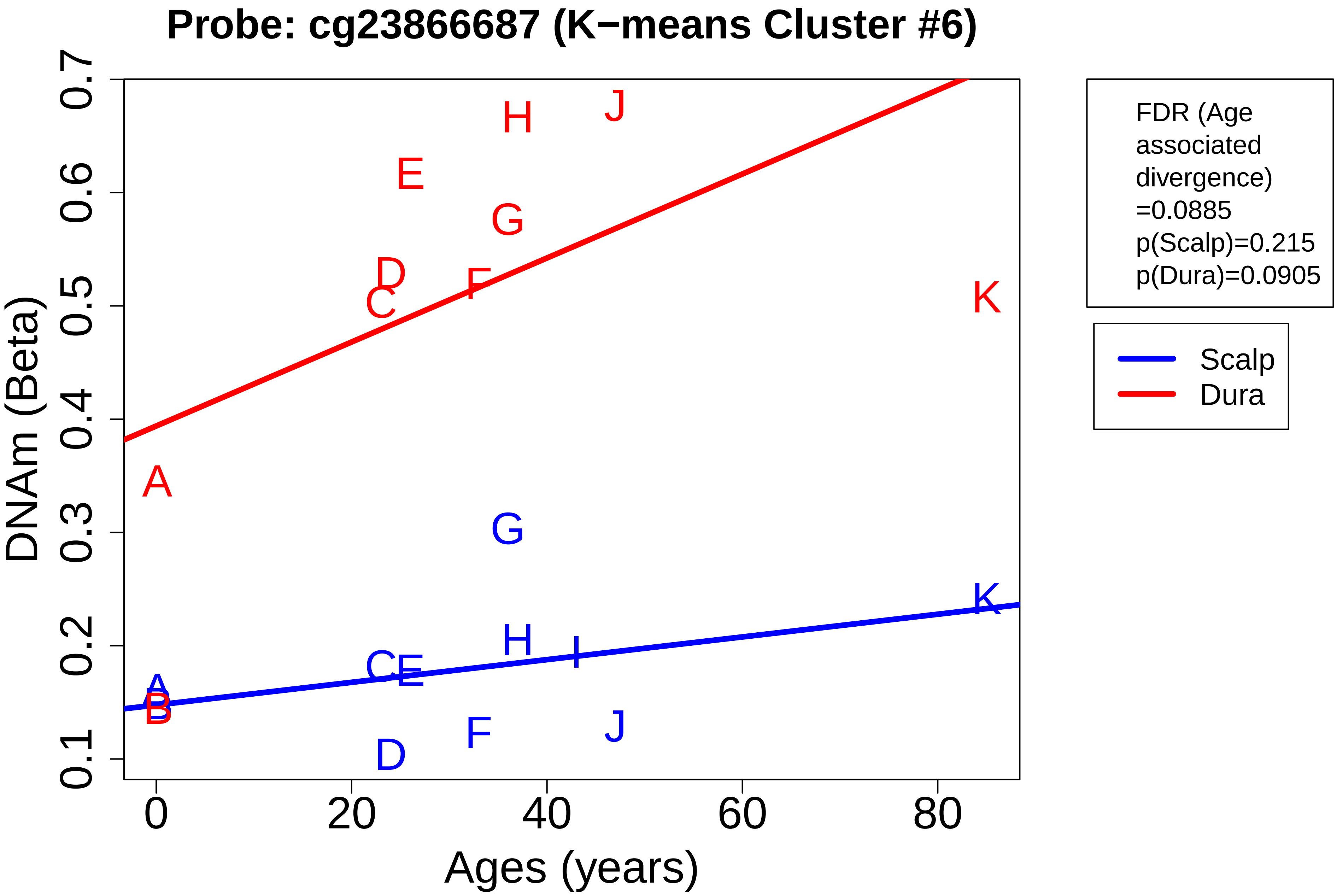

Probe: cg23866687 (K−means Cluster #6)
DNAm (Beta)
0.1
0.2
0.3
0.4
0.5
0.6
0.7
Ages (years)
0
20
40
60
80
FDR (Age associated divergence) =0.0885
p(Scalp)=0.215
p(Dura)=0.0905
Scalp
Dura

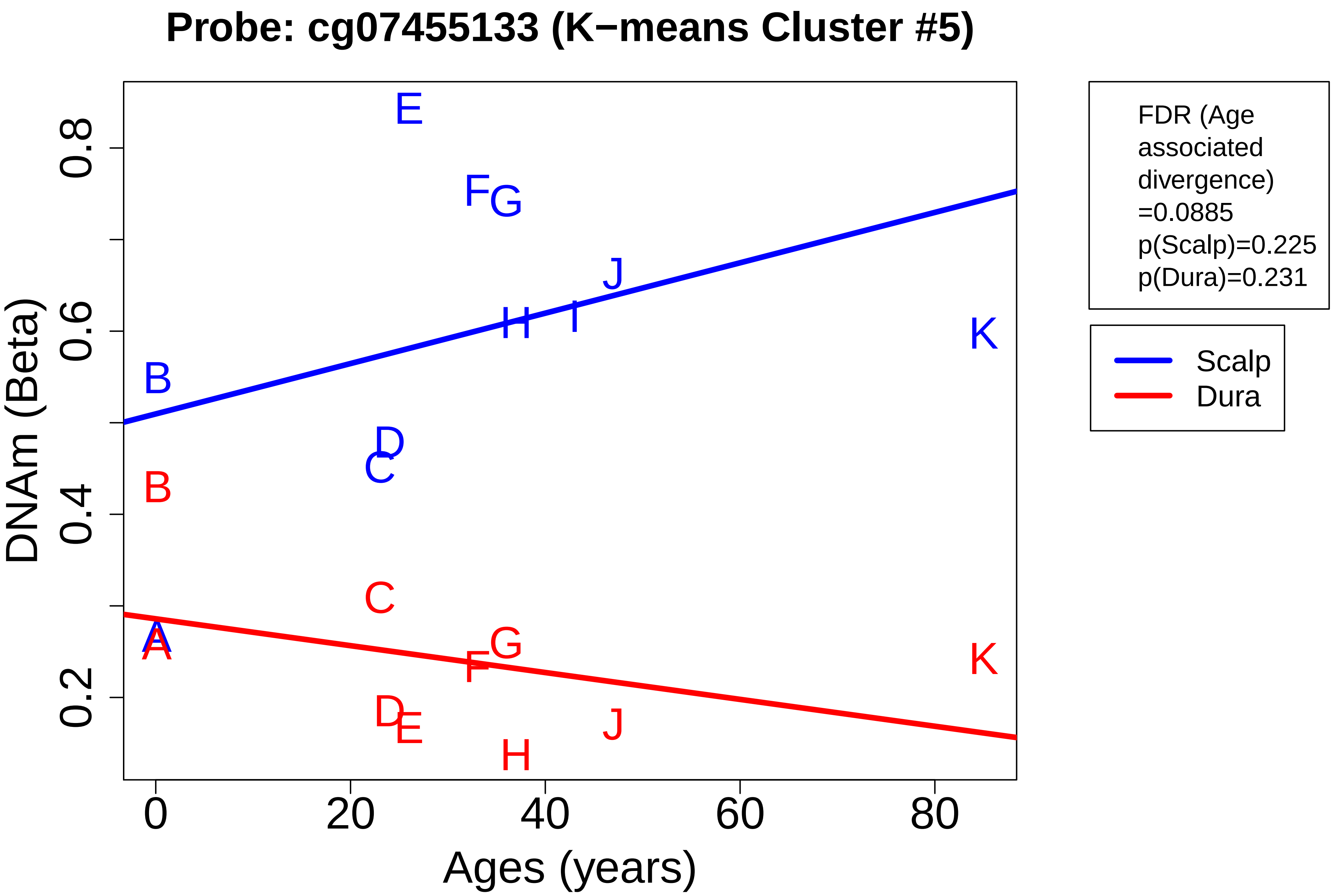

Probe: cg07455133 (K−means Cluster #5)
DNAm (Beta)
0.2
0.4
0.6
0.8
0
20
40
60
80
Ages (years)
FDR (Age associated divergence) =0.0885
p(Scalp)=0.225
p(Dura)=0.231
Scalp
Dura
A
B
C
D
E
F
G
H
I
J
K

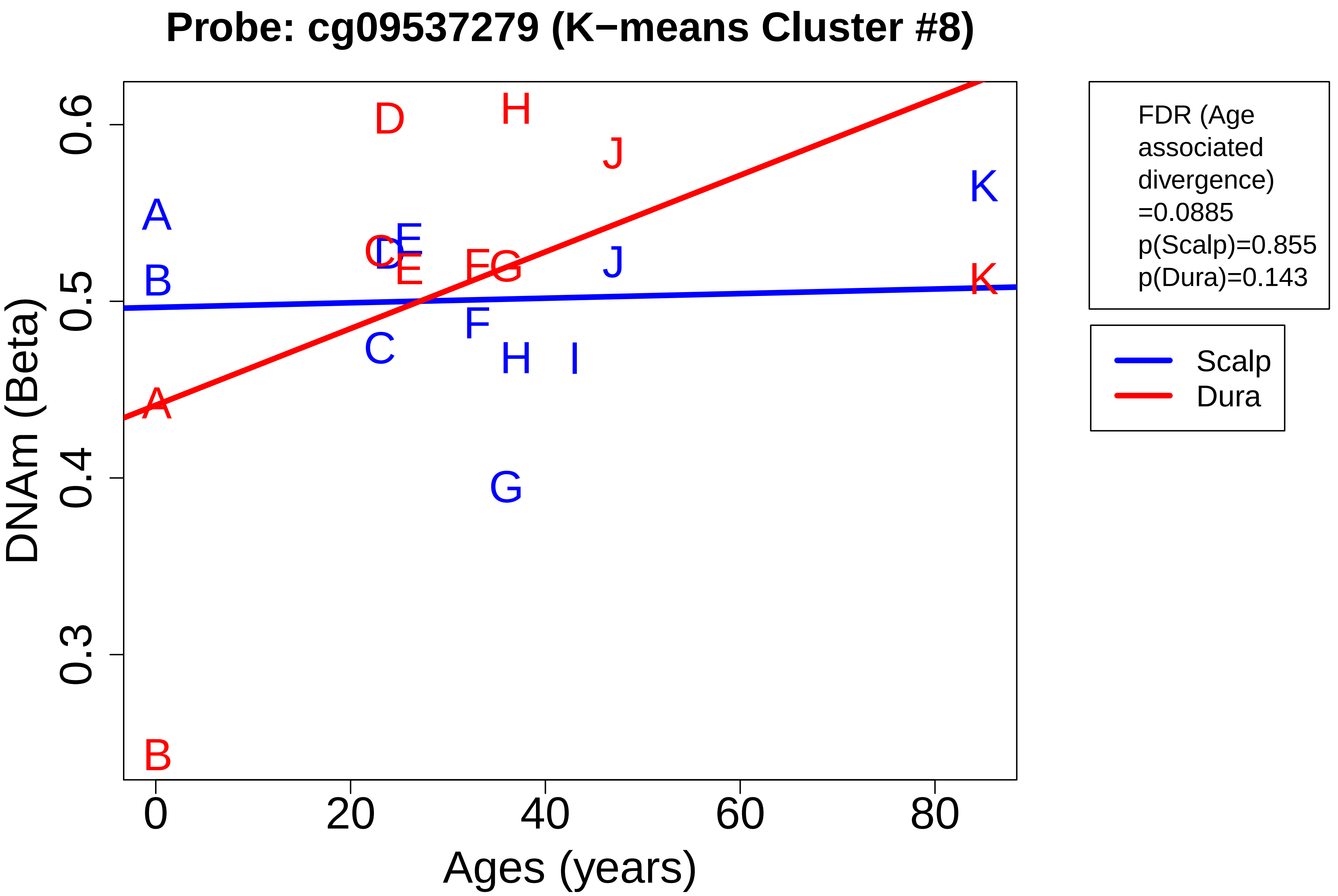
Probe: cg09537279 (K−means Cluster #8)
DNAm (Beta)
0.3
0.4
0.5
0.6
0
20
40
60
80
Ages (years)
A
B
C
D
E
F
G
H
I
J
K
FDR (Age associated divergence) =0.0885
p(Scalp)=0.855
p(Dura)=0.143
Scalp
Dura

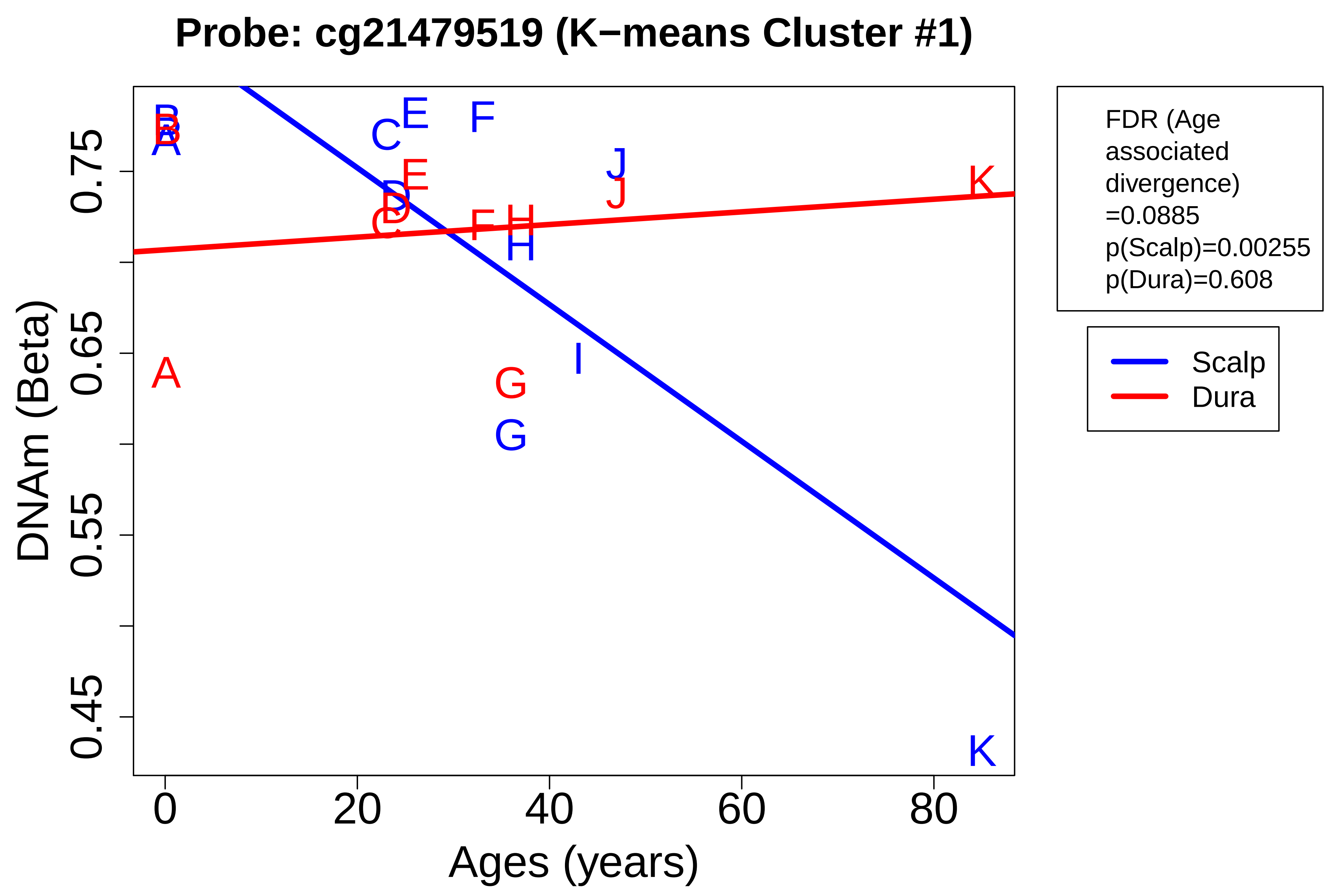

Probe: cg21479519 (K−means Cluster #1)
DNAm (Beta)
0.45
0.55
0.65
0.75
Ages (years)
0
20
40
60
80
FDR (Age associated divergence) =0.0885
p(Scalp)=0.00255
p(Dura)=0.608
Scalp
Dura
A
B
C
D
E
F
G
H
I
J
K

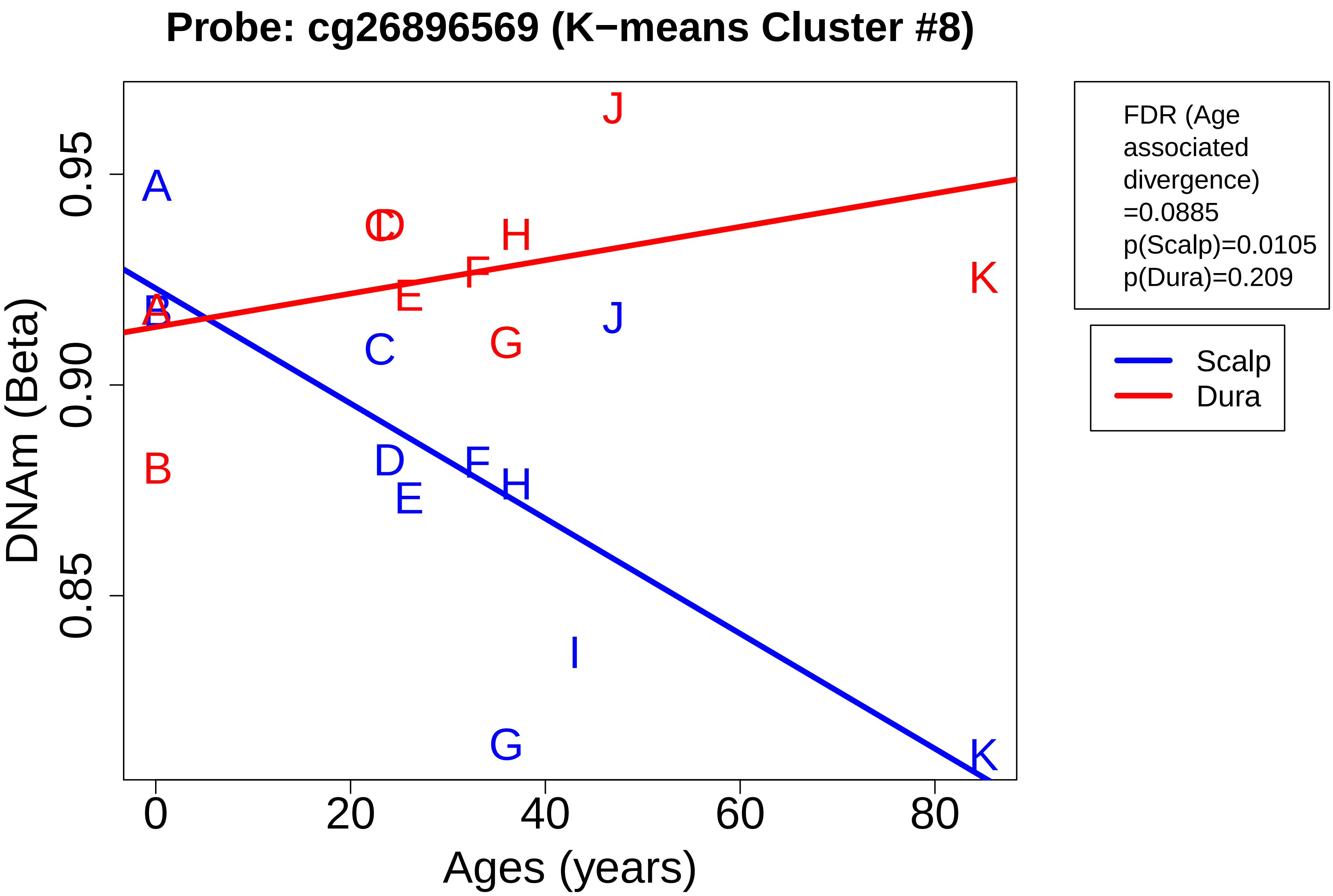

Probe: cg26896569 (K−means Cluster #8)
DNAm (Beta)
0.85
0.90
0.95
Ages (years)
0
20
40
60
80
FDR (Age associated divergence) =0.0885
p(Scalp)=0.0105
p(Dura)=0.209
Scalp
Dura
A
B
C
D
E
F
G
H
I
J
K

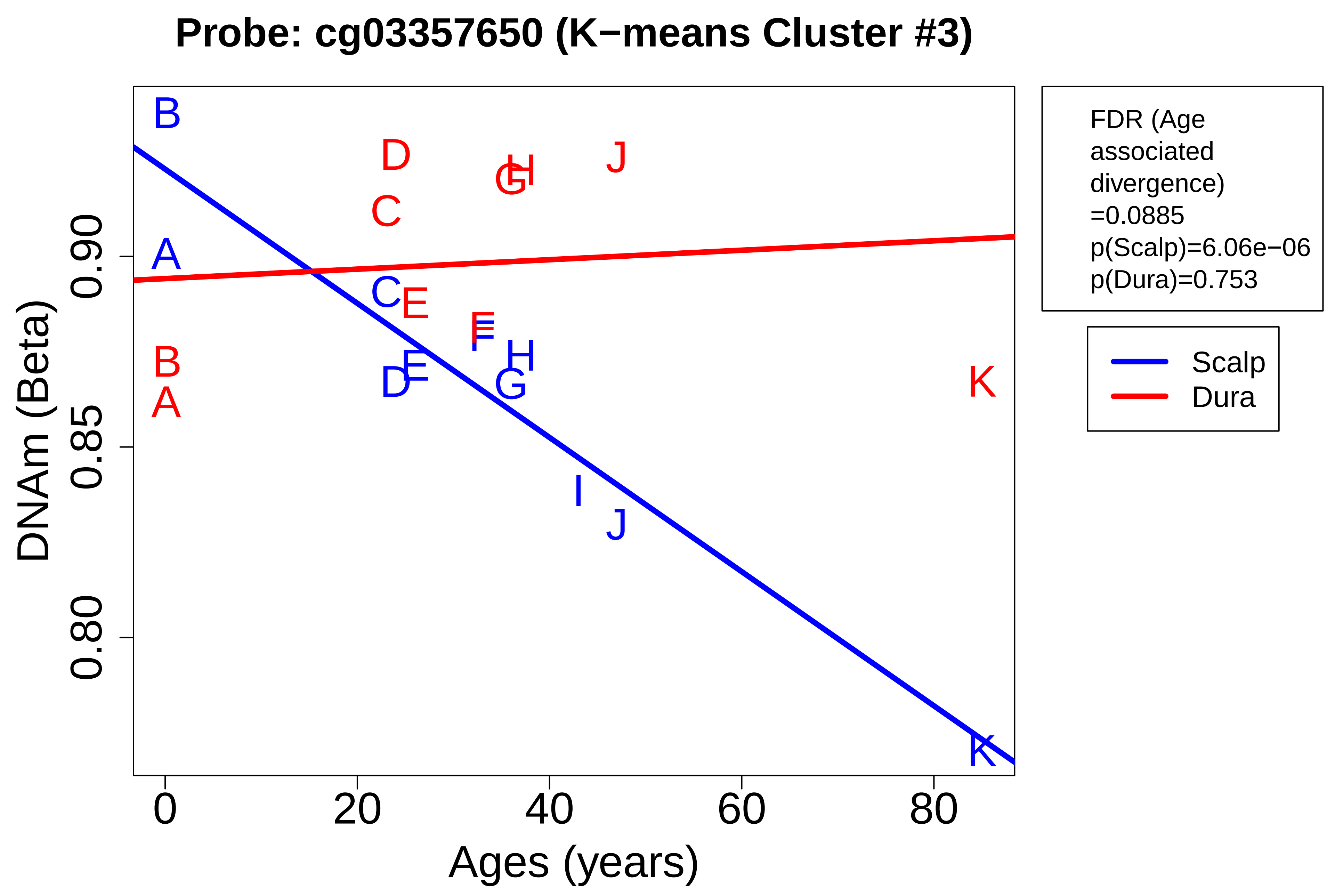
Probe: cg03357650 (K−means Cluster #3)
DNAm (Beta)
0.80
0.85
0.90
Ages (years)
0
20
40
60
80
FDR (Age associated divergence) =0.0885
p(Scalp)=6.06e−06
p(Dura)=0.753
Scalp
Dura
A
B
C
D
E
F
G
H
I
J
K

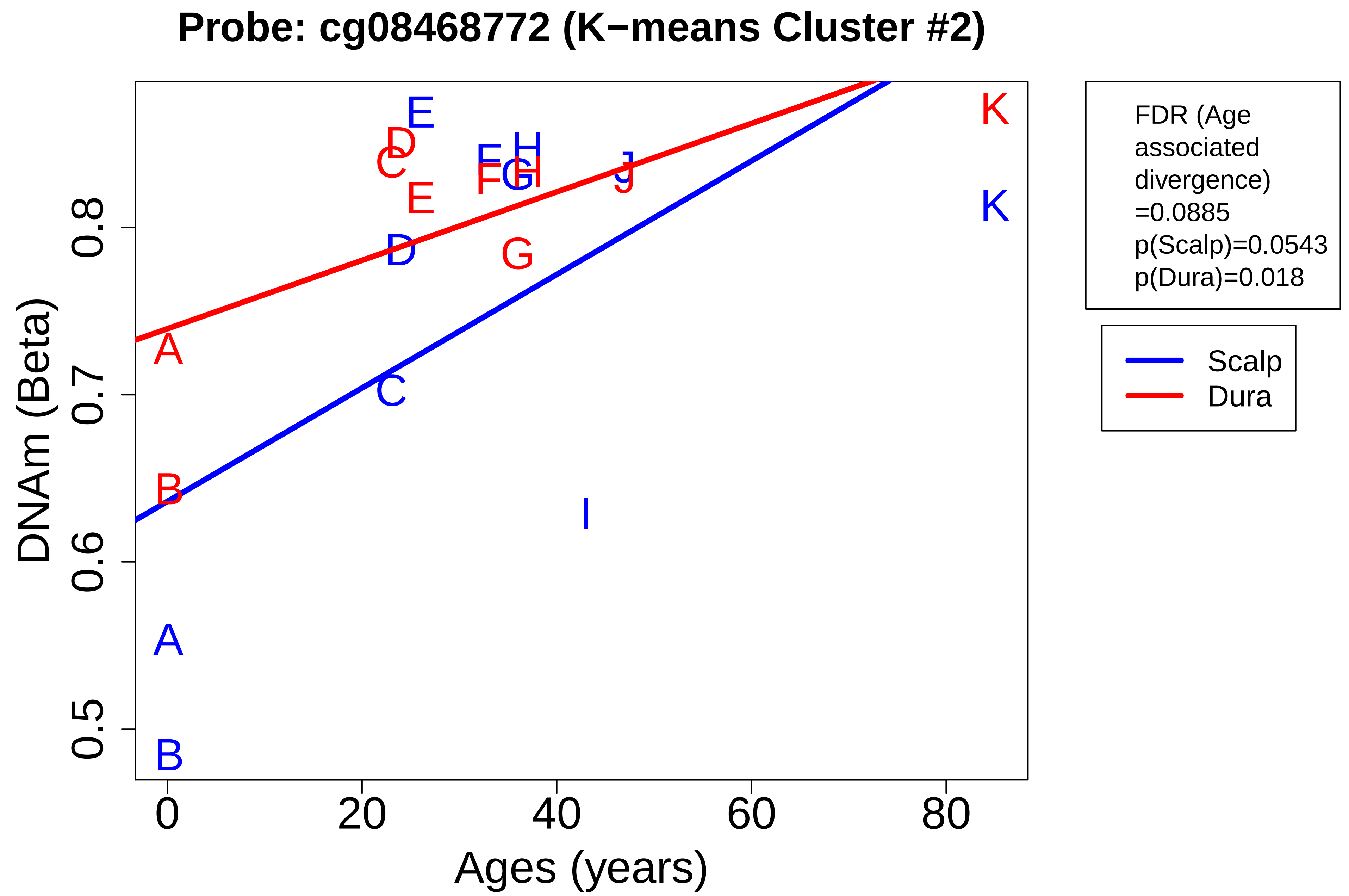

Probe: cg08468772 (K−means Cluster #2)
DNAm (Beta)
0.5
0.6
0.7
0.8
0
20
40
60
80
Ages (years)
FDR (Age associated divergence) =0.0885
p(Scalp)=0.0543
p(Dura)=0.018
Scalp
Dura

Probe: cg25075504 (K−means Cluster #4)

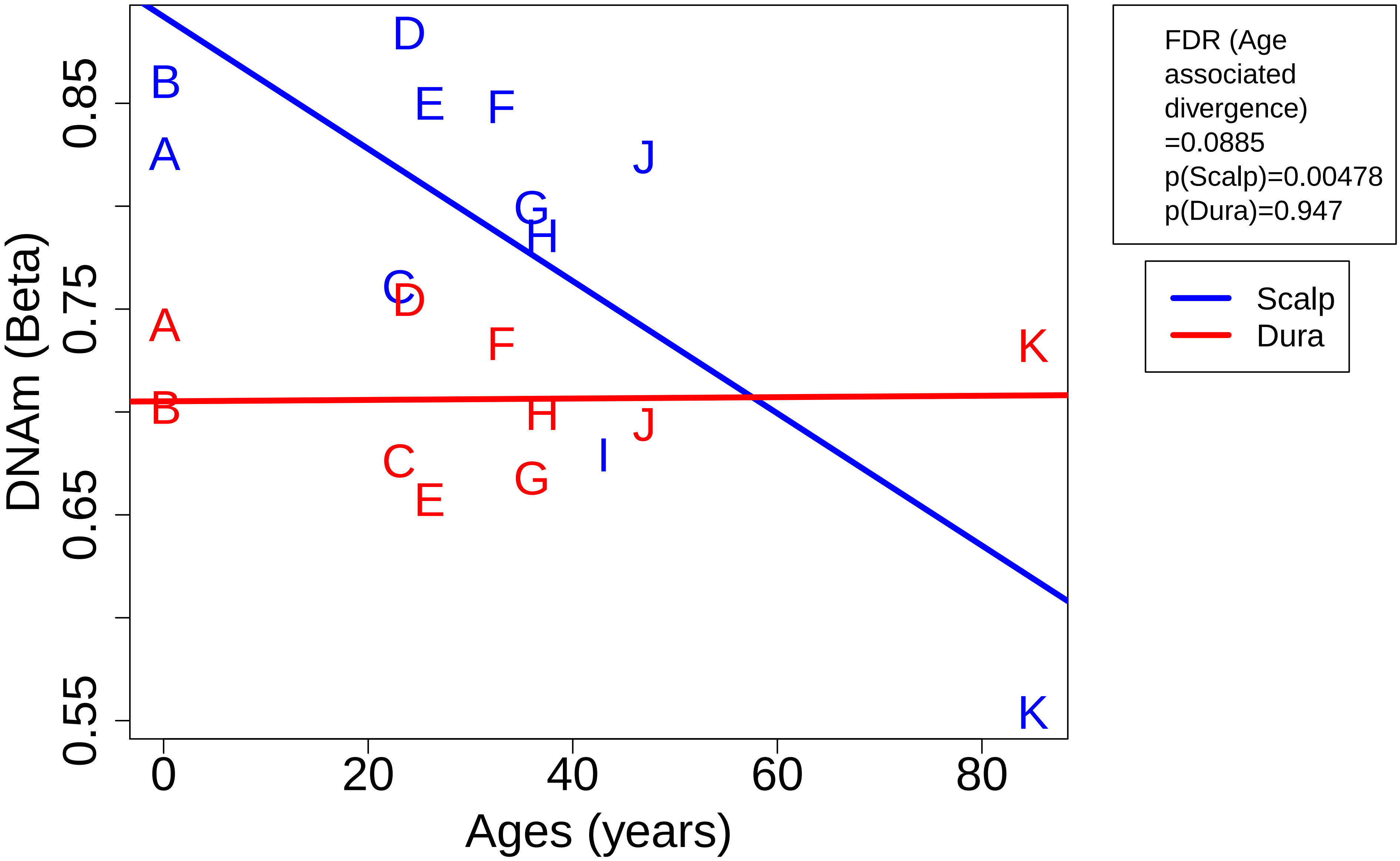

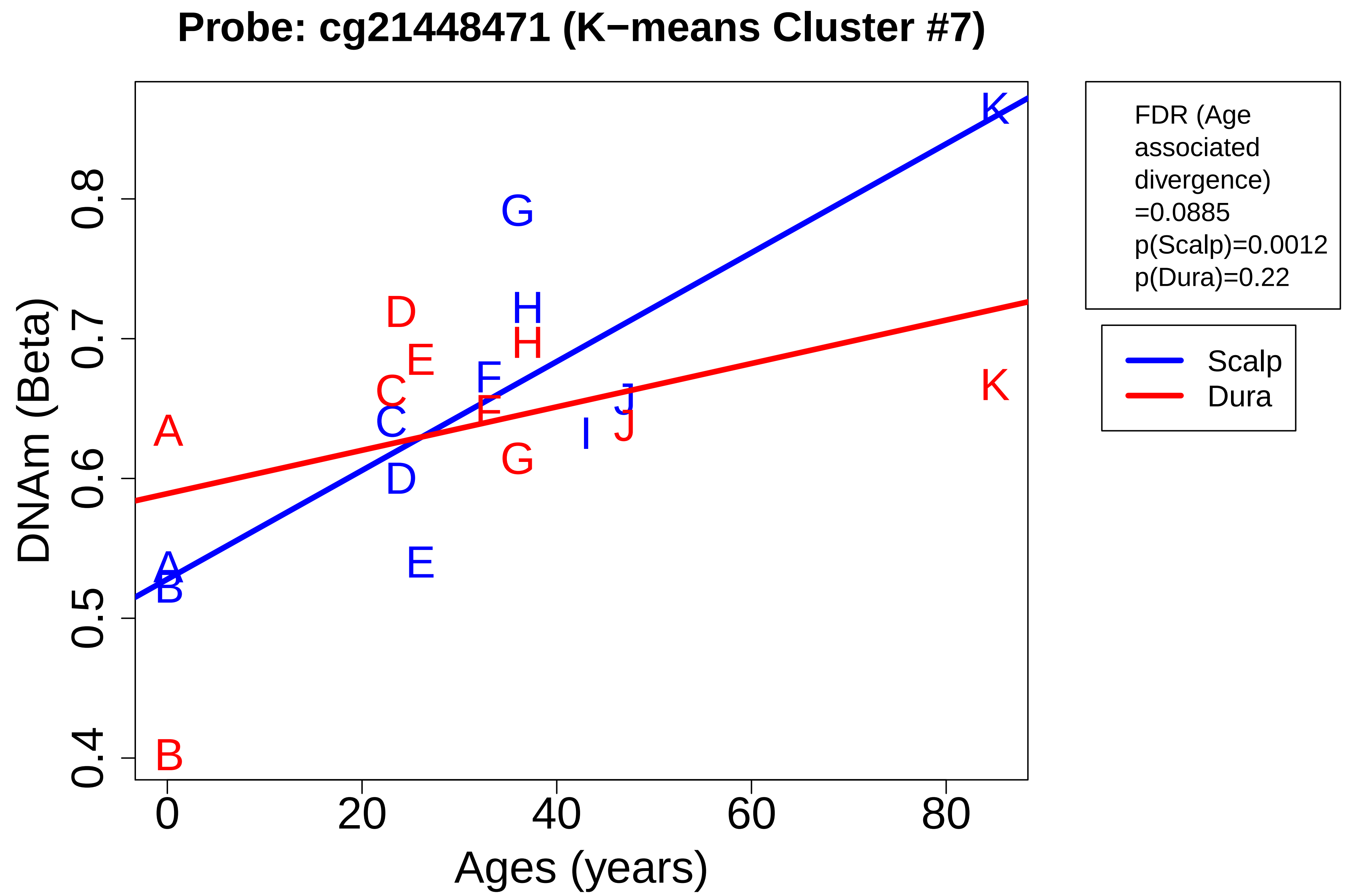

Probe: cg21448471 (K−means Cluster #7)
DNAm (Beta)
0.4
0.5
0.6
0.7
0.8
0
20
40
60
80
Ages (years)
FDR (Age associated divergence) =0.0885
p(Scalp)=0.0012
p(Dura)=0.22
Scalp
Dura
A
B
C
D
E
F
G
H
I
J
K

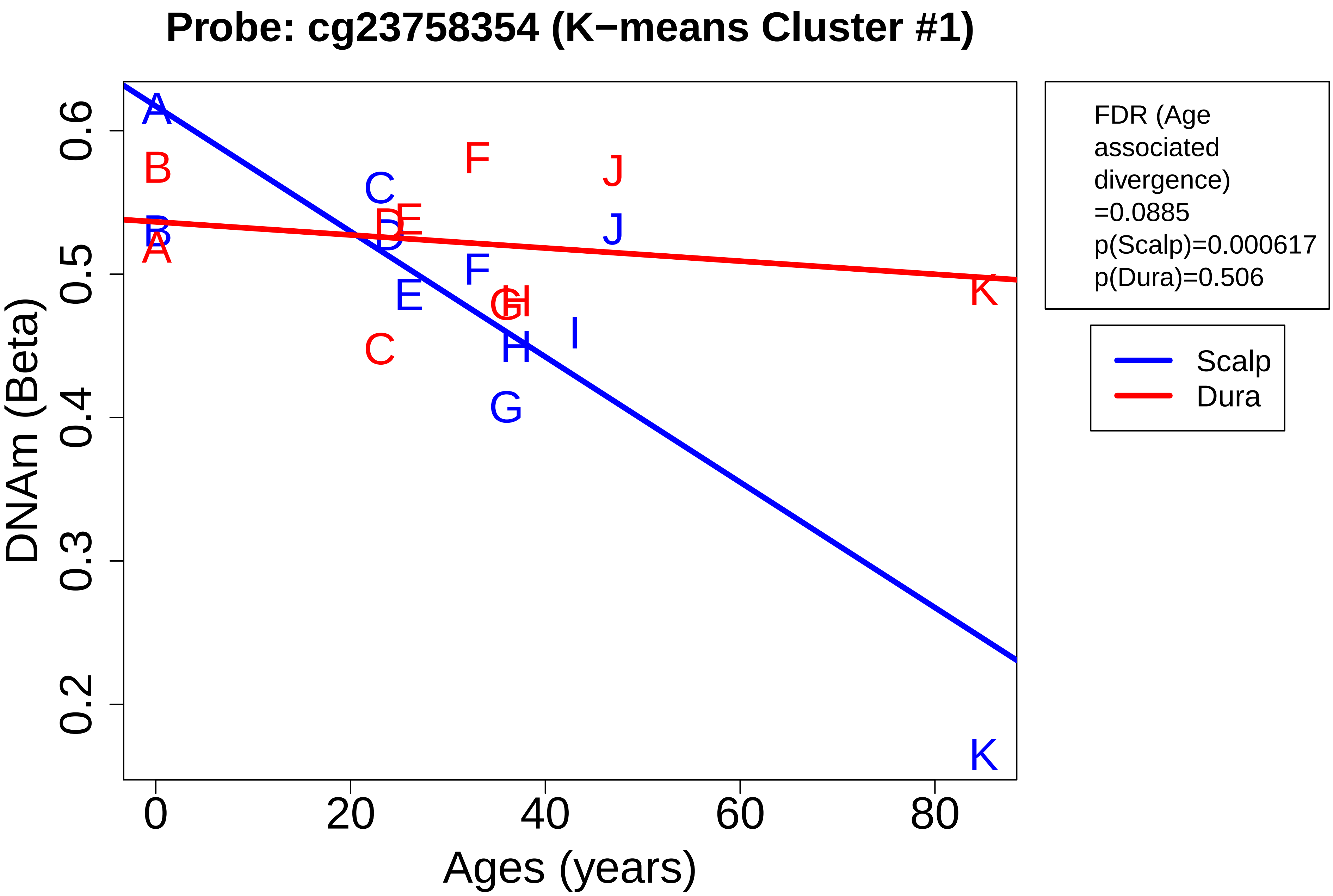

Probe: cg23758354 (K−means Cluster #1)
DNAm (Beta)
0.2
0.3
0.4
0.5
0.6
0
20
40
60
80
Ages (years)
FDR (Age associated divergence) =0.0885
p(Scalp)=0.000617
p(Dura)=0.506
Scalp
Dura

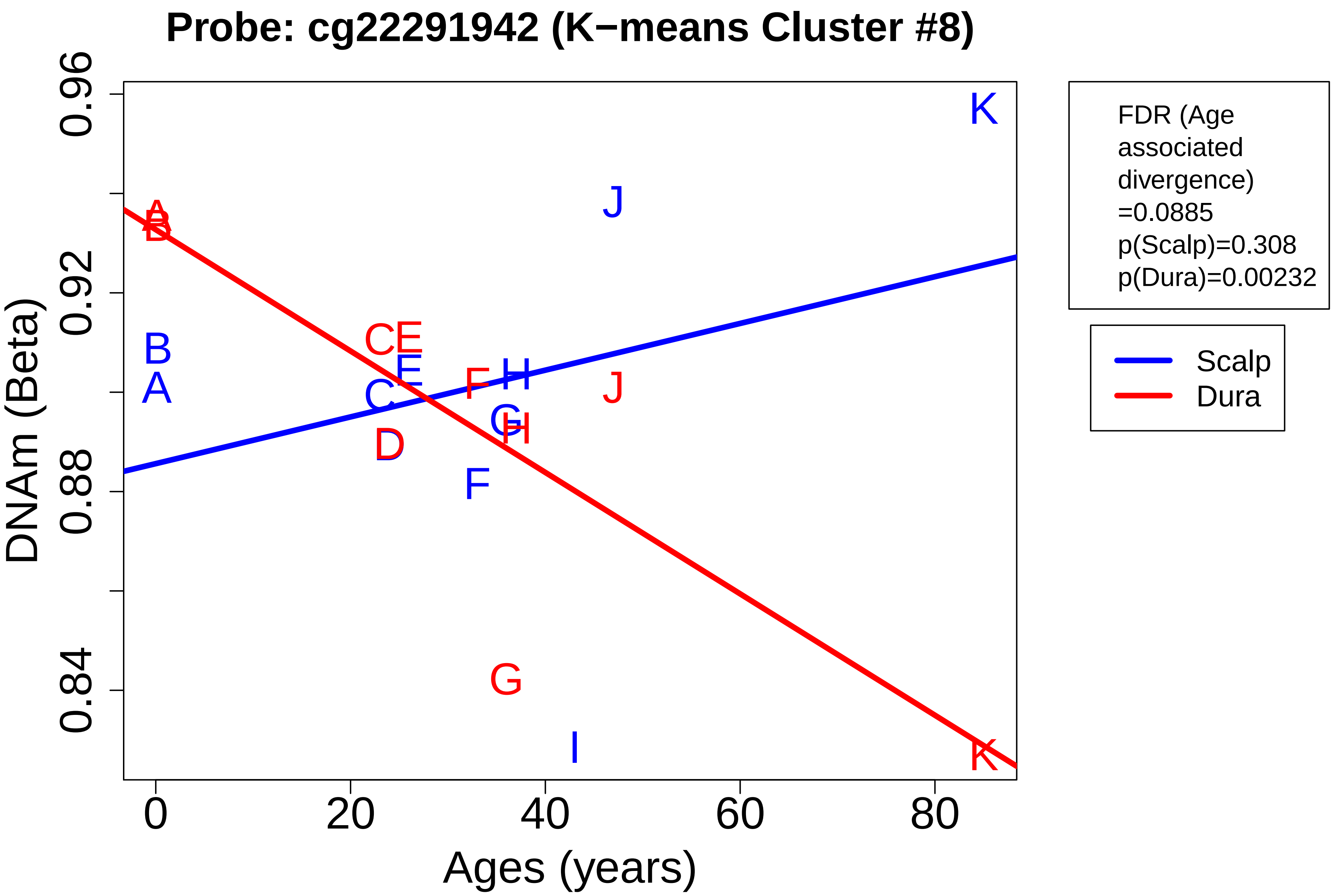
Probe: cg22291942 (K−means Cluster #8)
DNAm (Beta)
0.96
0.92
0.88
0.84
0
20
40
60
80
Ages (years)
FDR (Age associated divergence) =0.0885
p(Scalp)=0.308
p(Dura)=0.00232
Scalp
Dura
A
B
C
D
E
F
G
H
I
J
K

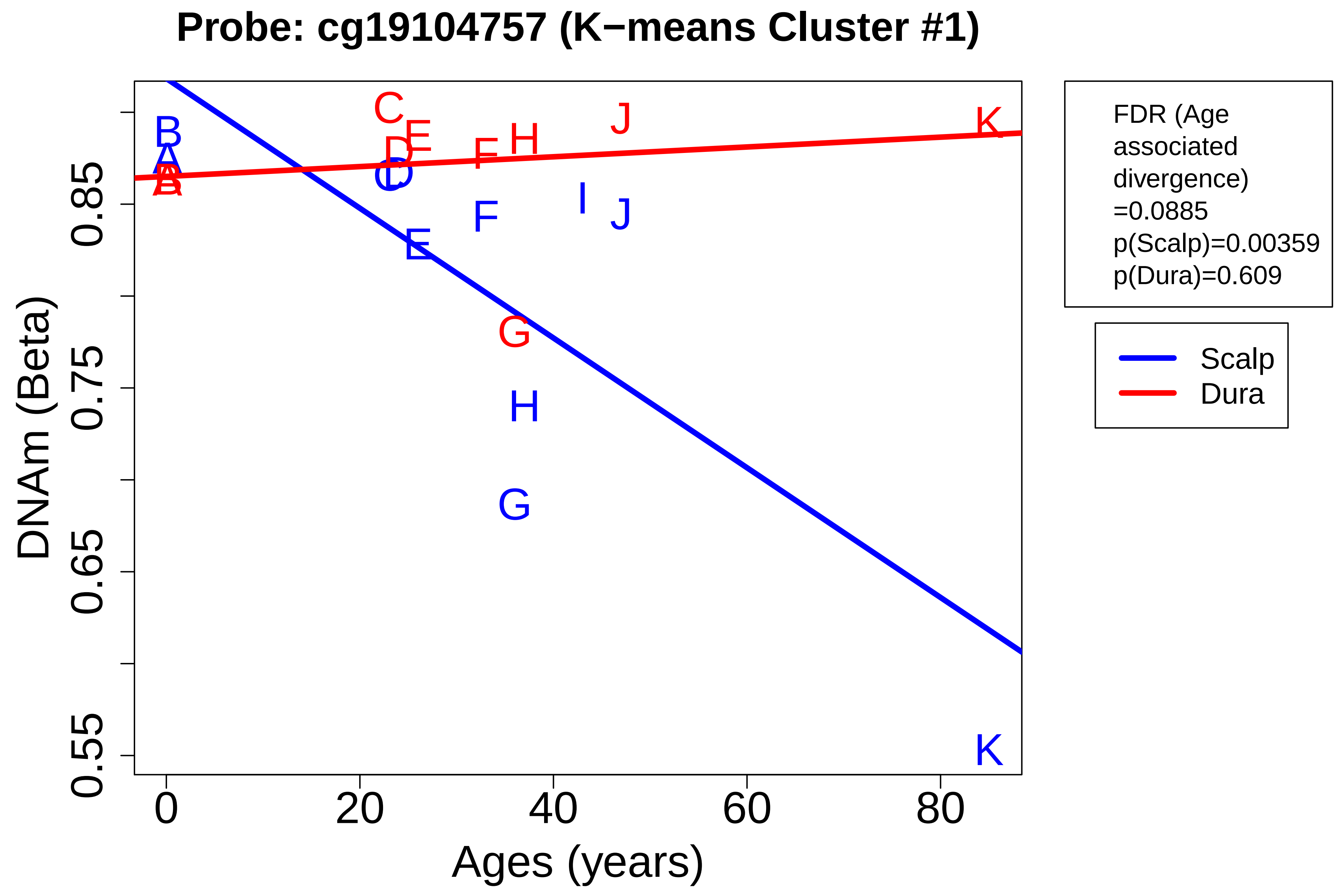

Probe: cg19104757 (K−means Cluster #1)
DNAm (Beta)
0.55
0.65
0.75
0.85
0
20
40
60
80
Ages (years)
FDR (Age associated divergence) =0.0885
p(Scalp)=0.00359
p(Dura)=0.609
Scalp
Dura

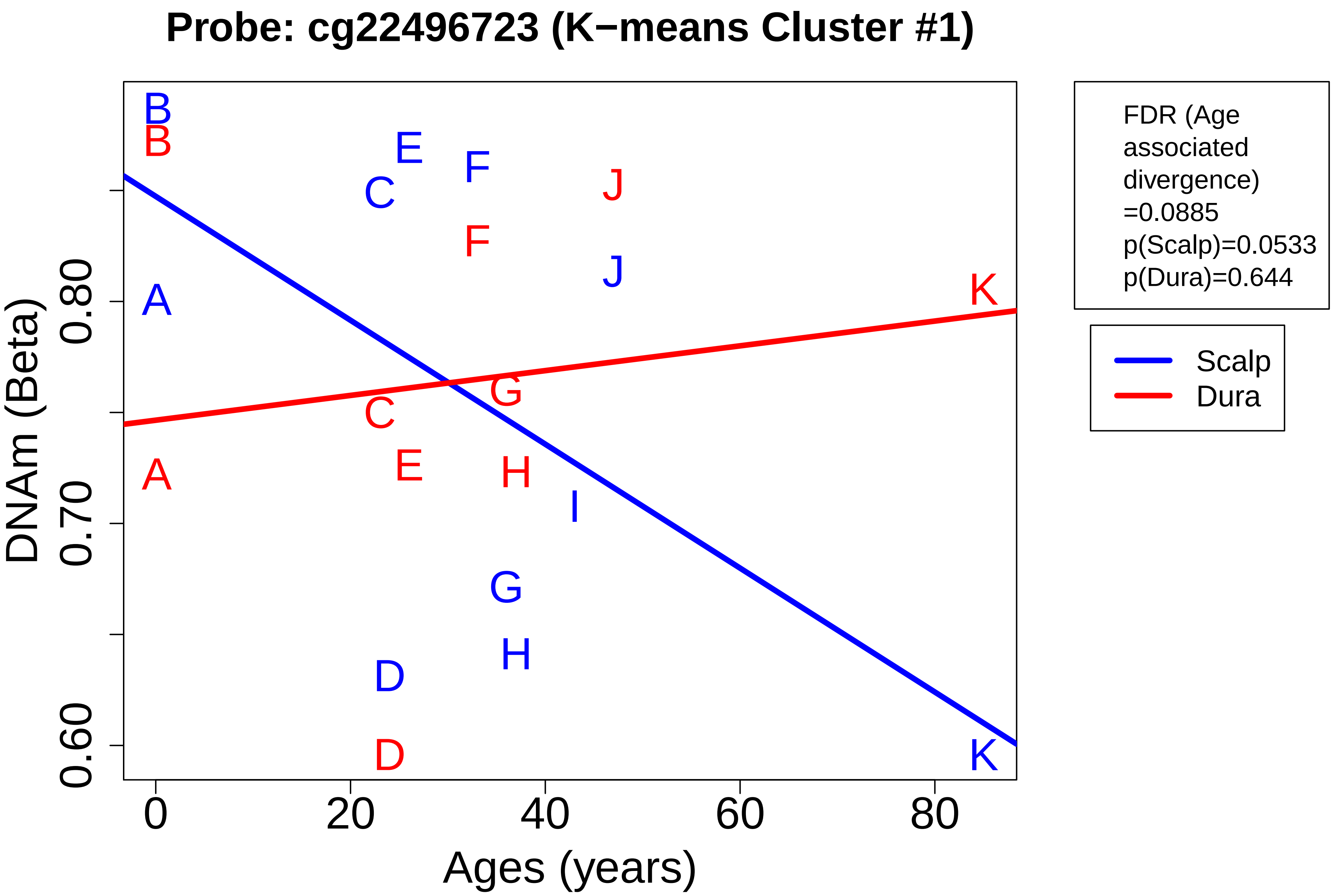

Probe: cg22496723 (K−means Cluster #1)
DNAm (Beta)
0.60
0.70
0.80
Ages (years)
0
20
40
60
80
B
B
E
F
C
J
F
J
A
K
G
C
E
H
A
I
G
H
D
D
K
FDR (Age associated divergence) =0.0885
p(Scalp)=0.0533
p(Dura)=0.644
Scalp
Dura

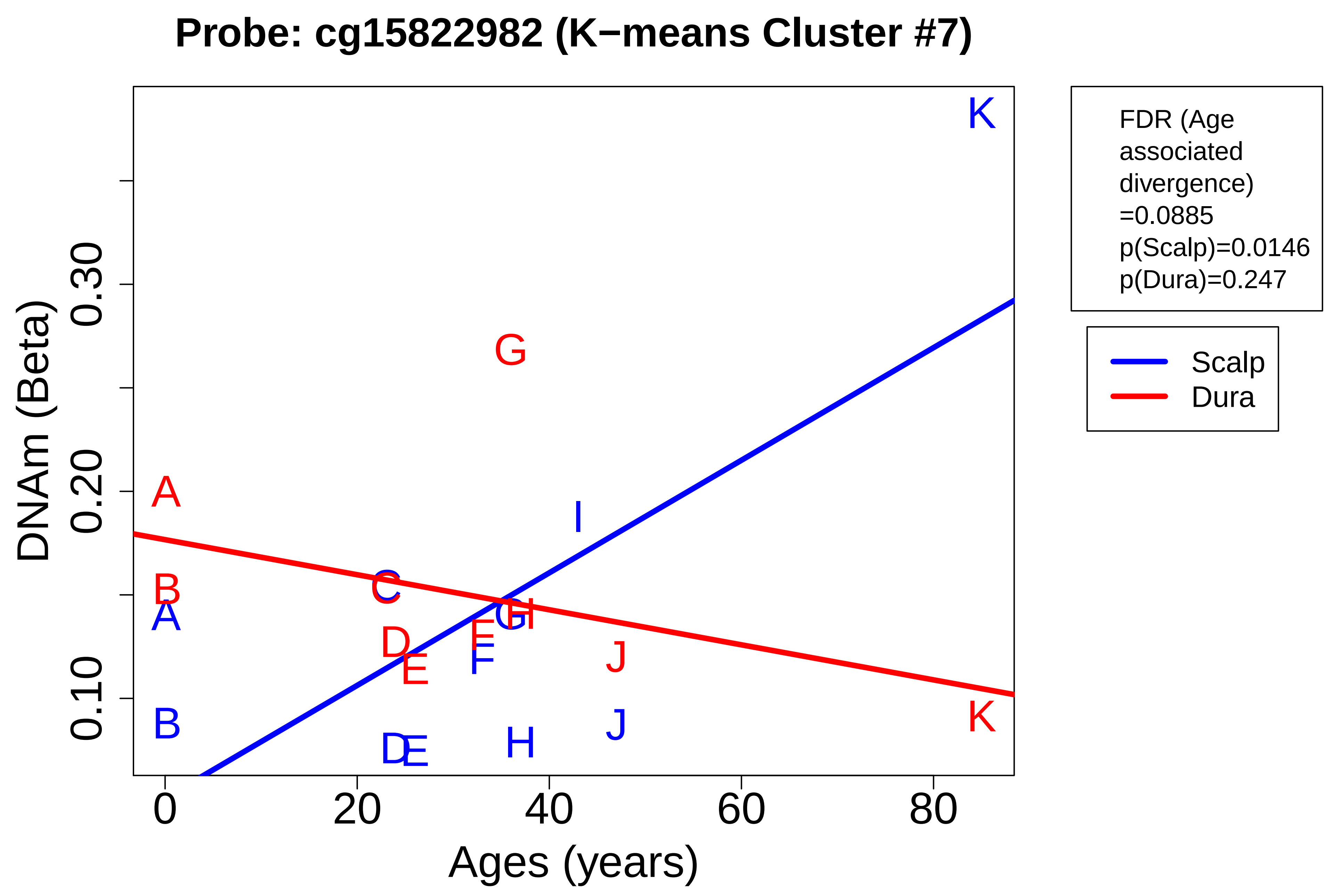
Probe: cg15822982 (K−means Cluster #7)
DNAm (Beta)
0.10
0.20
0.30
Ages (years)
0
20
40
60
80
A
B
C
D
E
F
G
H
I
J
K
FDR (Age associated divergence) =0.0885
p(Scalp)=0.0146
p(Dura)=0.247
Scalp
Dura

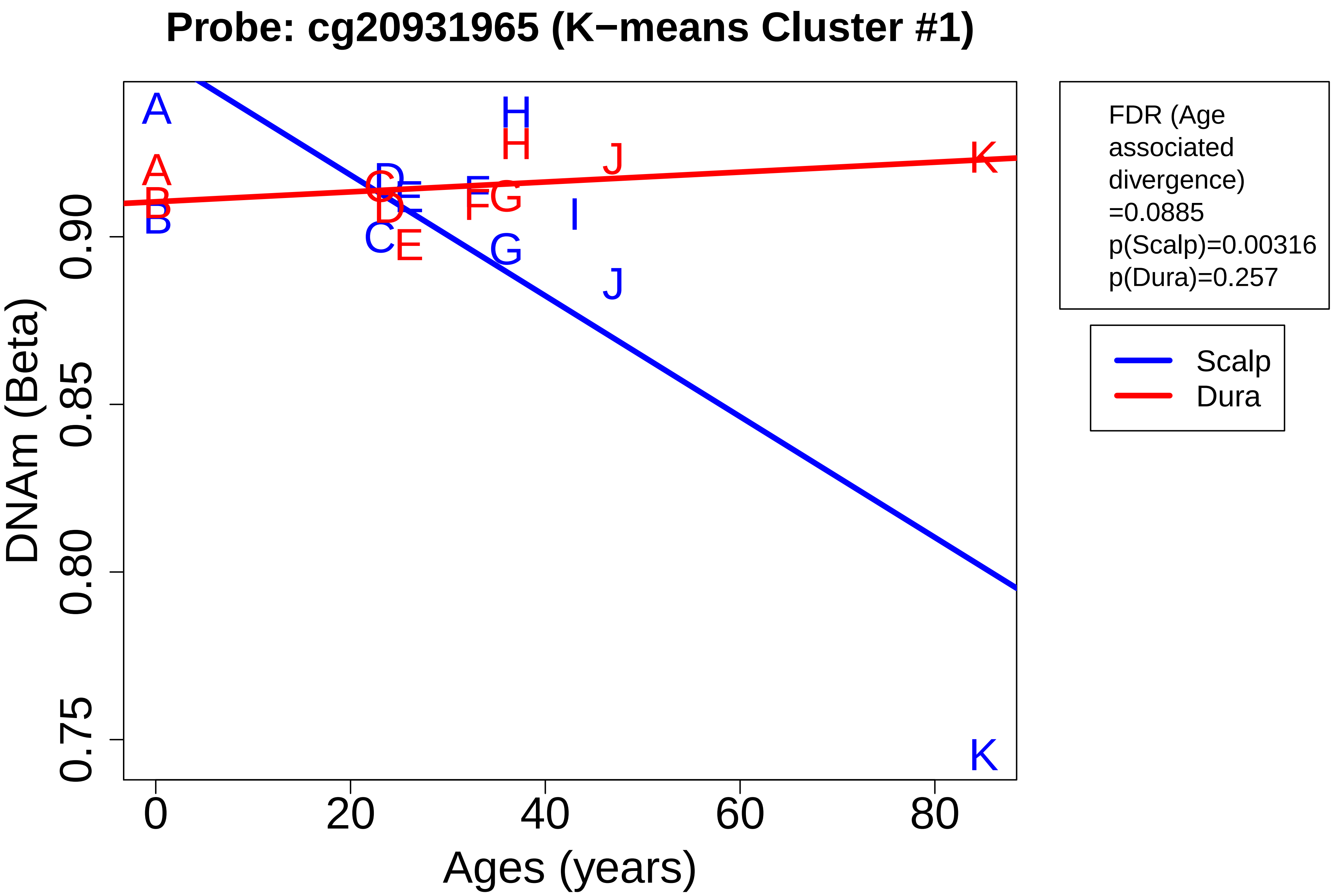

Probe: cg20931965 (K−means Cluster #1)
DNAm (Beta)
0.75
0.80
0.85
0.90
0
20
40
60
80
Ages (years)
A
B
C
D
E
F
G
H
I
J
K
FDR (Age associated divergence) =0.0885
p(Scalp)=0.00316
p(Dura)=0.257
Scalp
Dura

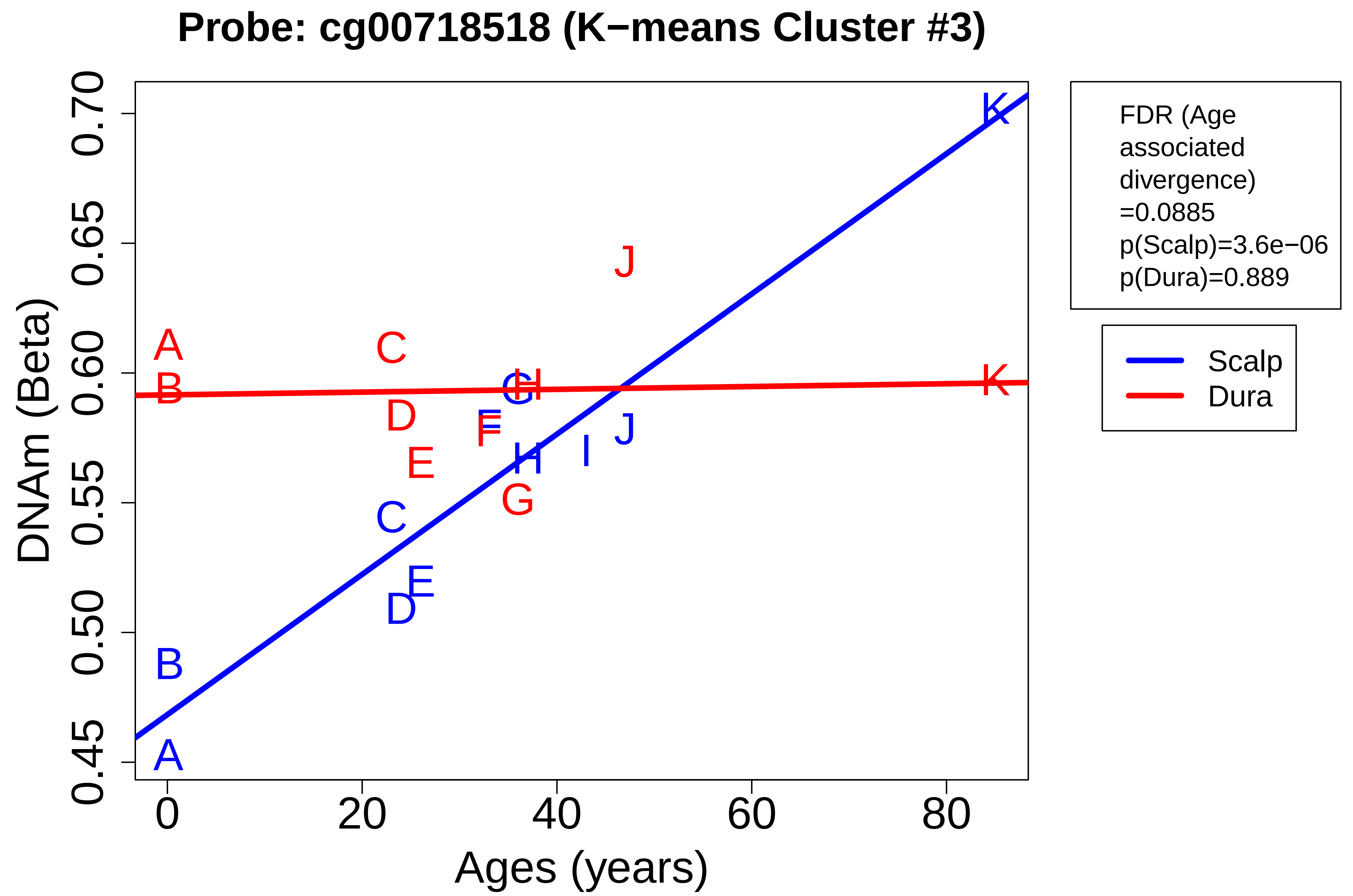

Probe: cg00718518 (K−means Cluster #3)
DNAm (Beta)
0.45
0.50
0.55
0.60
0.65
0.70
0
20
40
60
80
Ages (years)
FDR (Age associated divergence) =0.0885
p(Scalp)=3.6e−06
p(Dura)=0.889
Scalp
Dura

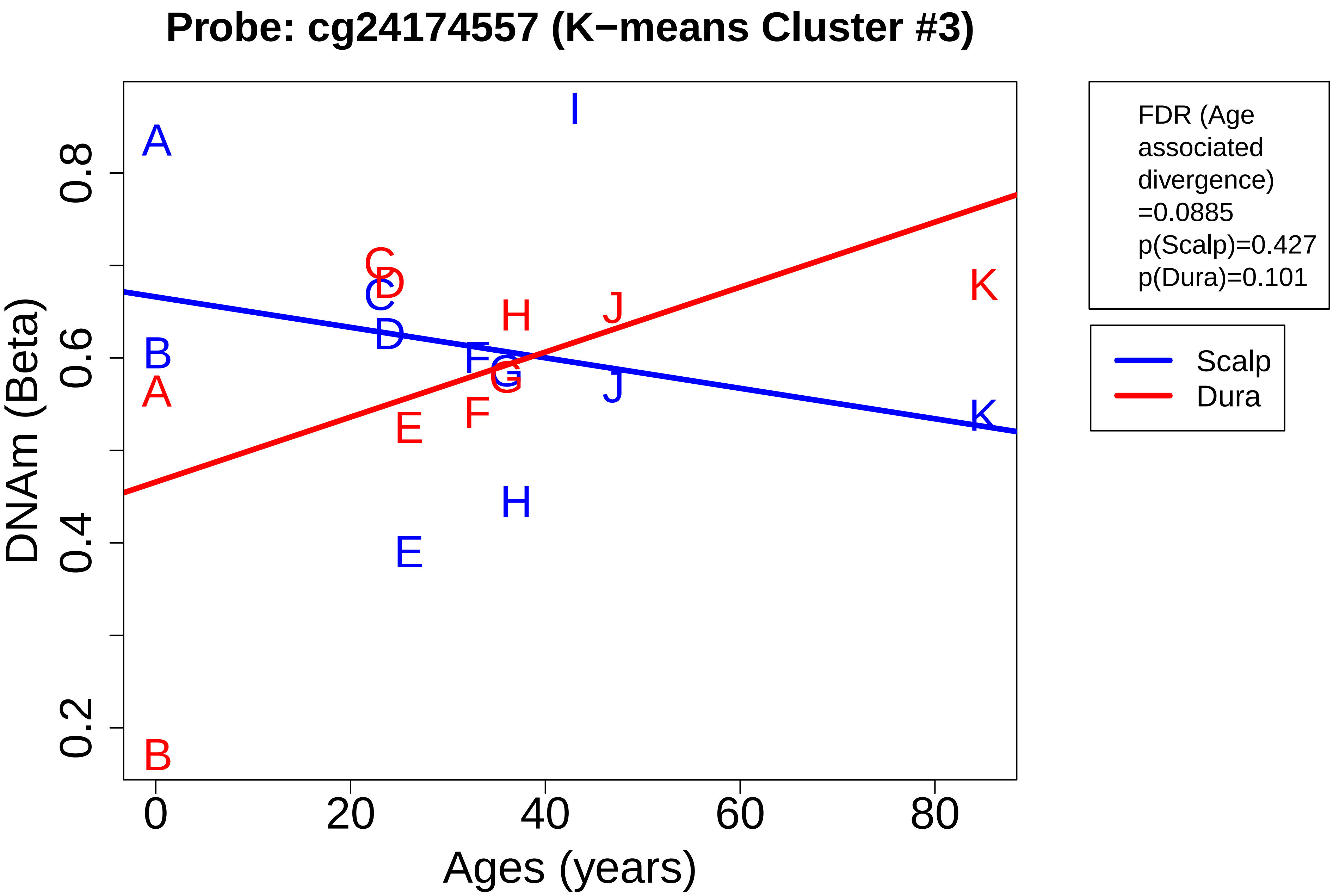

Probe: cg24174557 (K−means Cluster #3)
DNAm (Beta)
0.2
0.4
0.6
0.8
Ages (years)
0
20
40
60
80
FDR (Age associated divergence) =0.0885
p(Scalp)=0.427
p(Dura)=0.101
Scalp
Dura
A
B
C
D
E
F
G
H
I
J
K

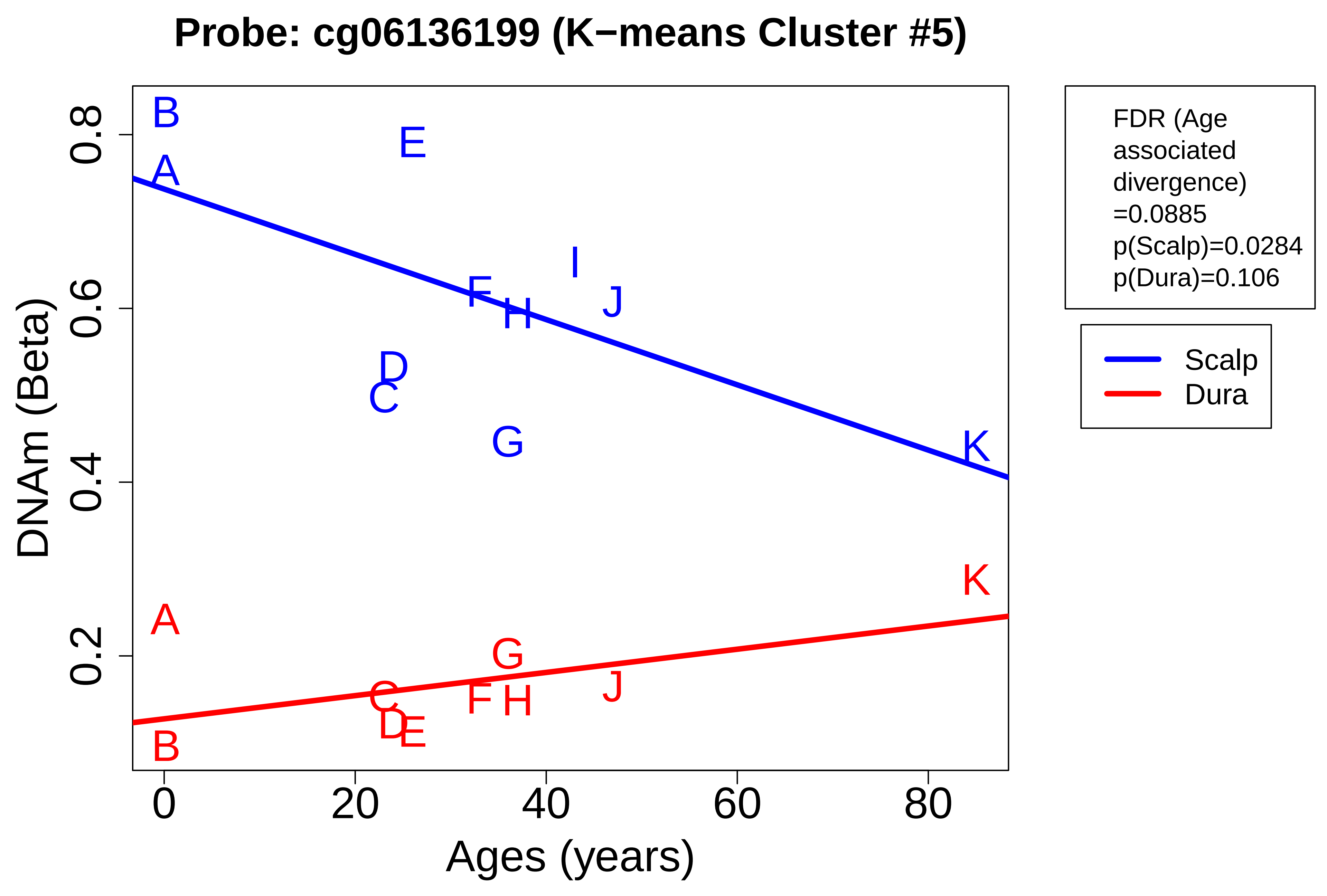
Probe: cg06136199 (K−means Cluster #5)
DNAm (Beta)
0.2
0.4
0.6
0.8
0
20
40
60
80
Ages (years)
B
E
A
I
F
J
H
D
C
G
K
K
A
G
J
C
F
H
D
E
B
FDR (Age associated divergence) =0.0885
p(Scalp)=0.0284
p(Dura)=0.106
Scalp
Dura

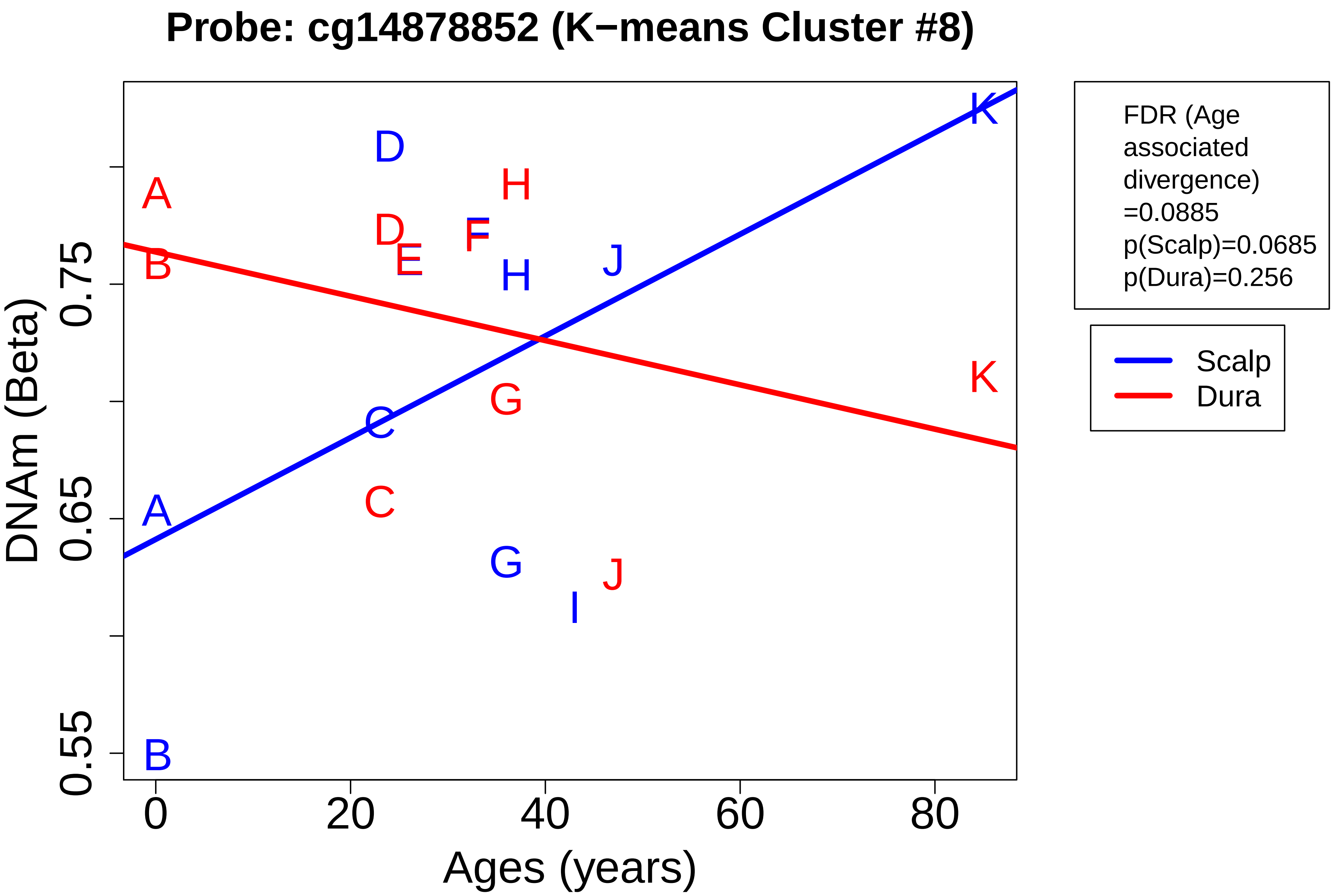

Probe: cg14878852 (K−means Cluster #8)
DNAm (Beta)
0.55
0.65
0.75
Ages (years)
0
20
40
60
80
A
B
C
D
E
F
G
H
I
J
K
FDR (Age associated divergence) =0.0885
p(Scalp)=0.0685
p(Dura)=0.256
Scalp
Dura

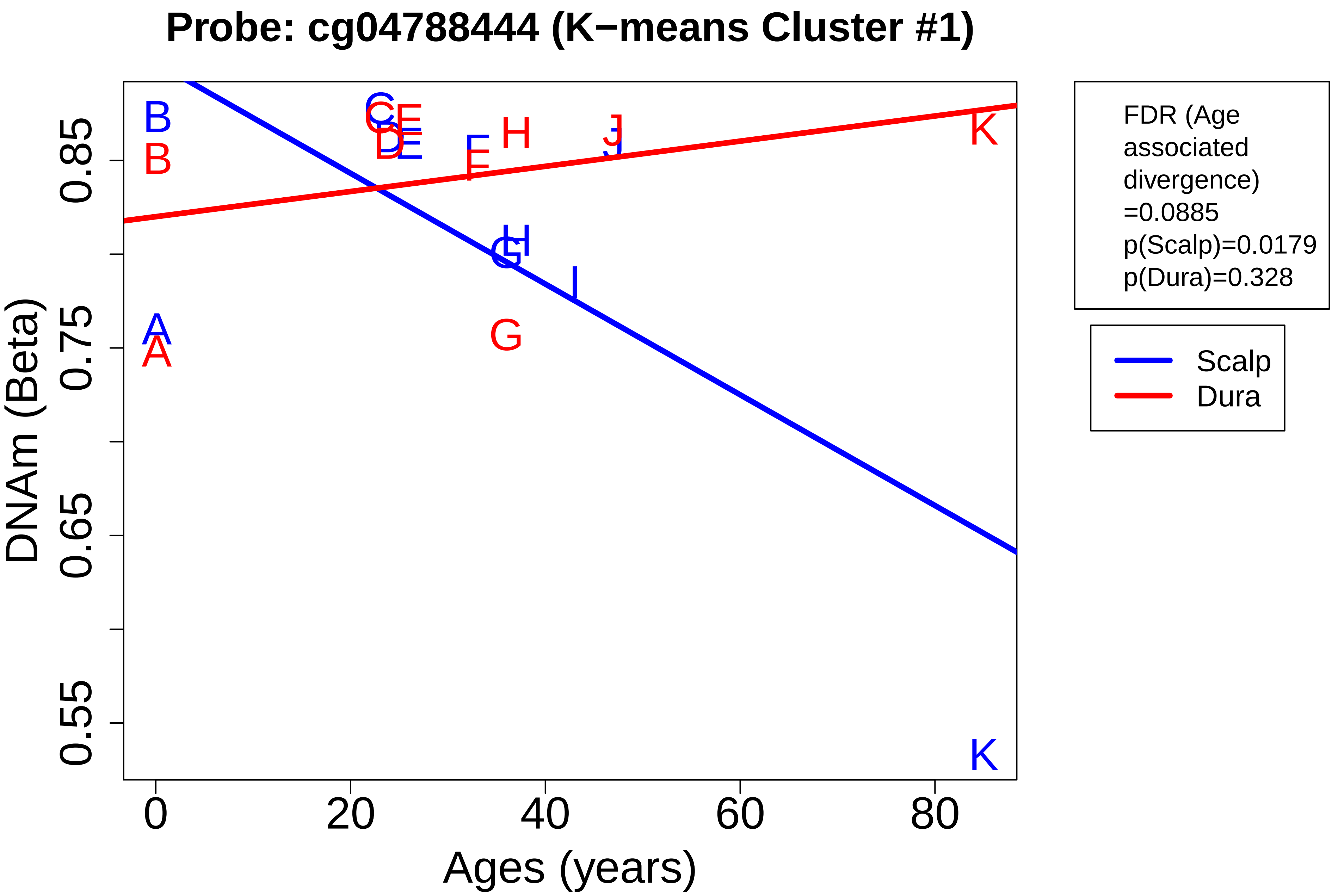

Probe: cg04788444 (K−means Cluster #1)
DNAm (Beta)
0.55
0.65
0.75
0.85
Ages (years)
0
20
40
60
80
FDR (Age associated divergence) =0.0885
p(Scalp)=0.0179
p(Dura)=0.328
Scalp
Dura

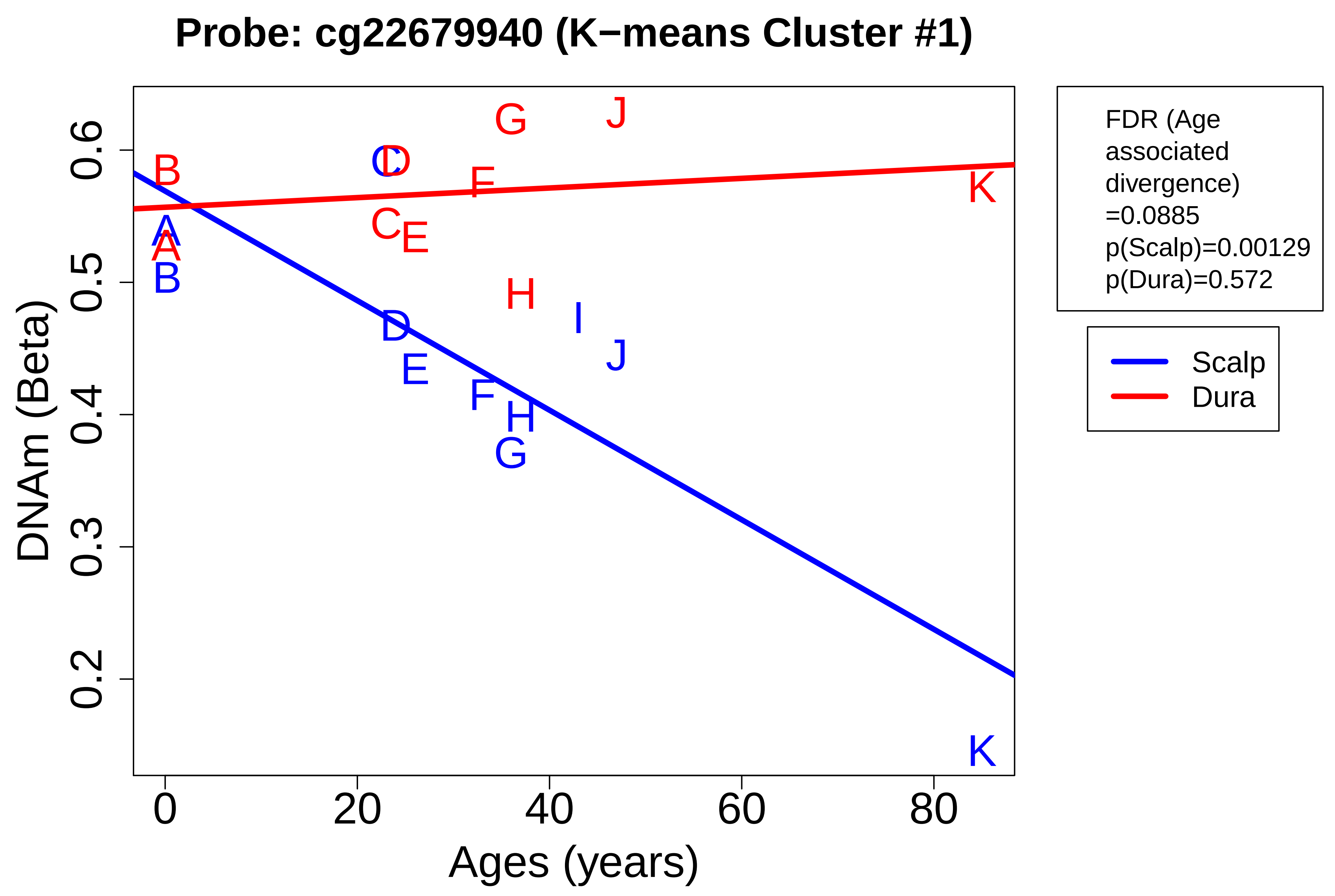

Probe: cg22679940 (K−means Cluster #1)
DNAm (Beta)
0.2
0.3
0.4
0.5
0.6
Ages (years)
0
20
40
60
80
FDR (Age associated divergence) =0.0885
p(Scalp)=0.00129
p(Dura)=0.572
Scalp
Dura

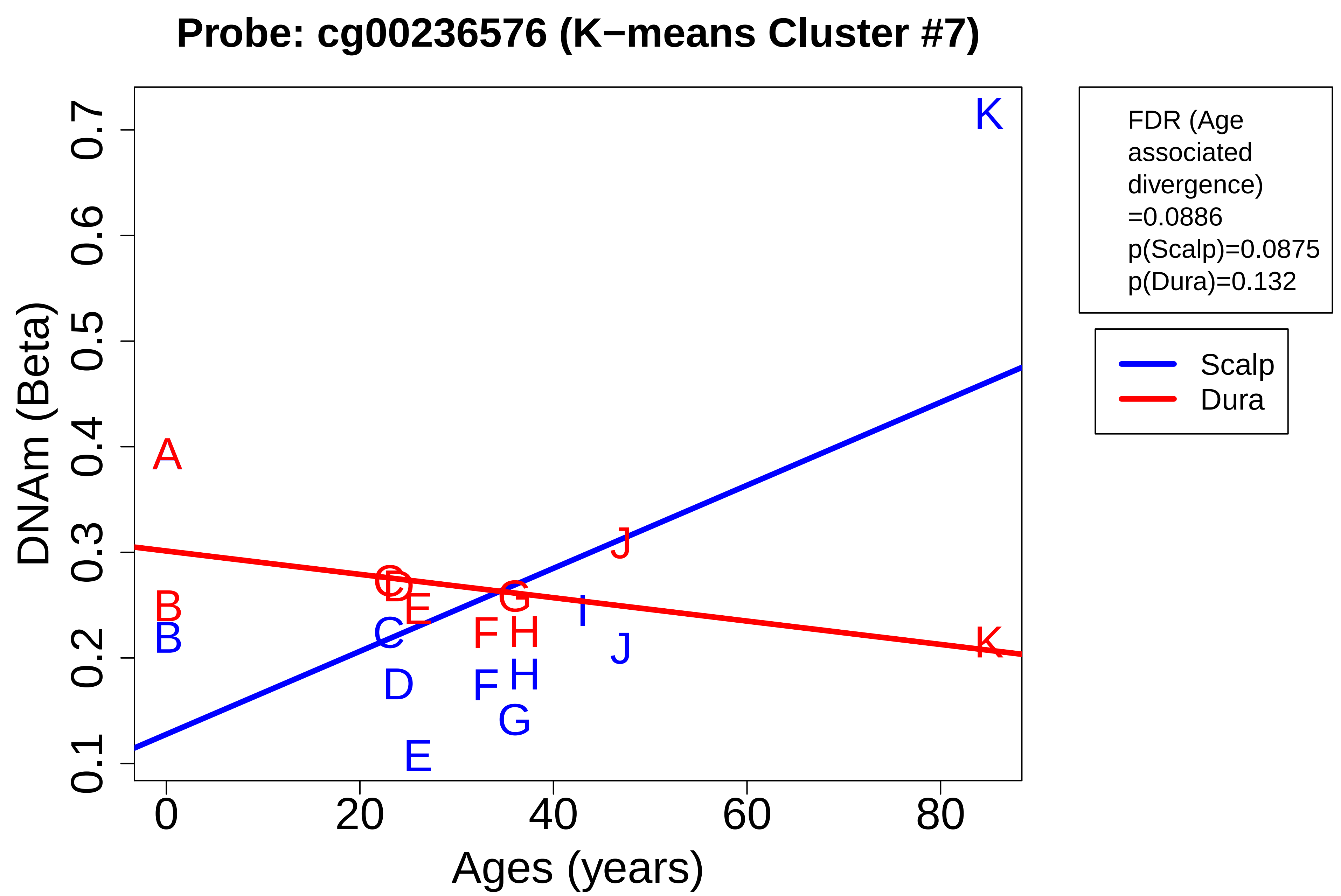

Probe: cg00236576 (K−means Cluster #7)
DNAm (Beta)
0.1
0.2
0.3
0.4
0.5
0.6
0.7
Ages (years)
0
20
40
60
80
A
B
B
C
C
D
D
E
E
F
F
G
G
H
H
I
J
J
K
K
FDR (Age associated divergence) =0.0886
p(Scalp)=0.0875
p(Dura)=0.132
Scalp
Dura

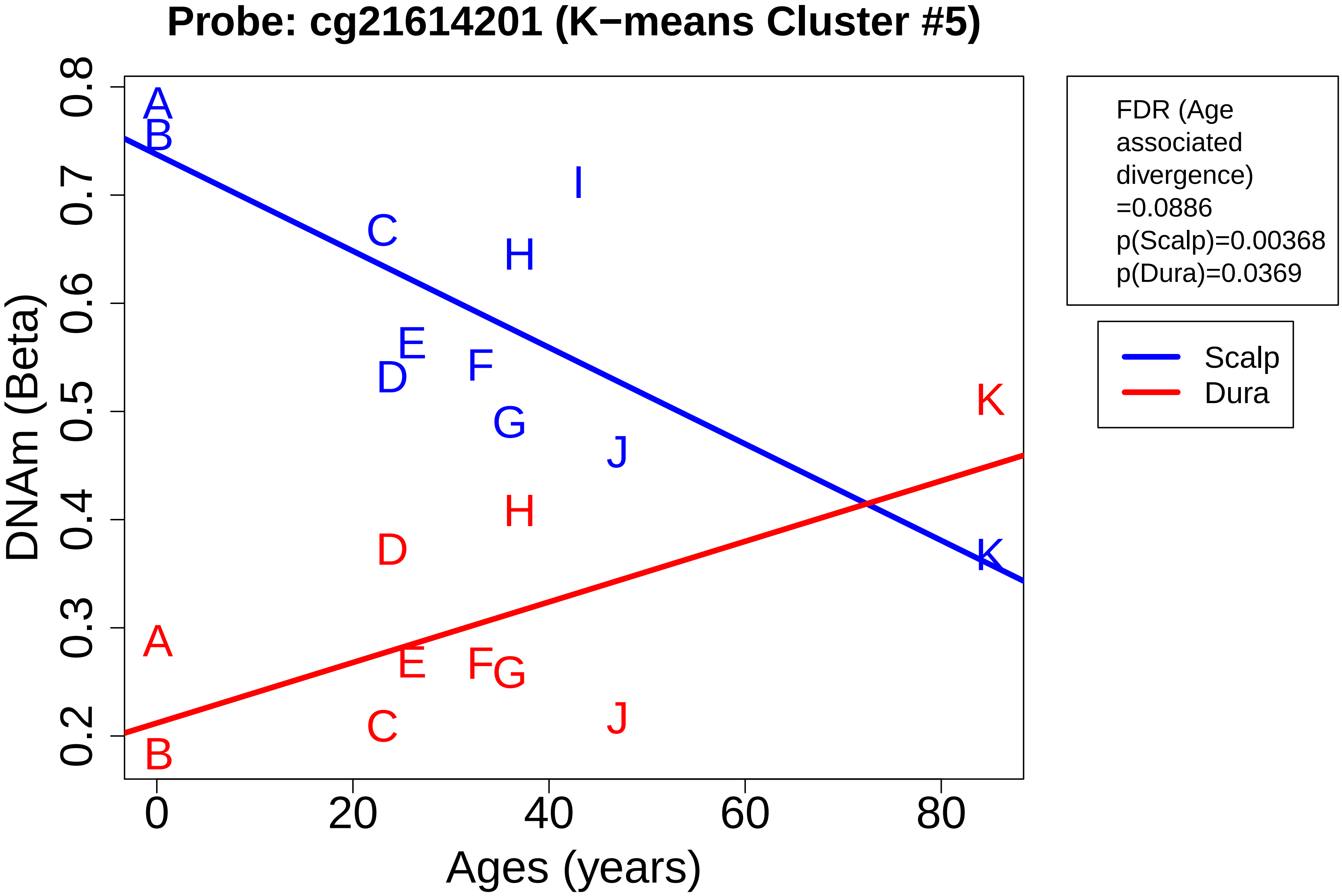

Probe: cg21614201 (K−means Cluster #5)
DNAm (Beta)
0.2
0.3
0.4
0.5
0.6
0.7
0.8
0
20
40
60
80
Ages (years)
FDR (Age associated divergence) =0.0886
p(Scalp)=0.00368
p(Dura)=0.0369
Scalp
Dura
A
B
C
D
E
F
G
H
I
J
K

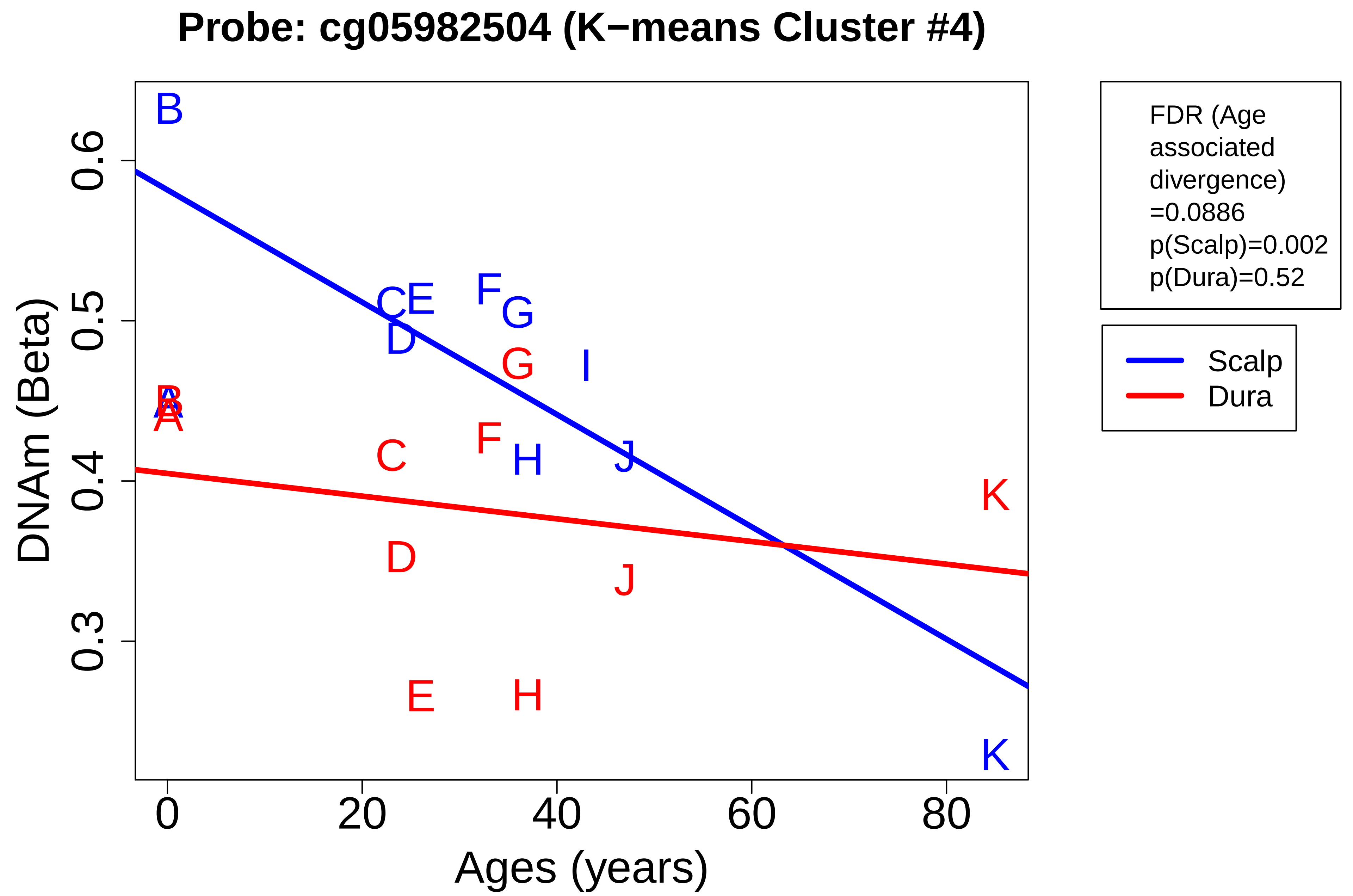

Probe: cg05982504 (K−means Cluster #4)
DNAm (Beta)
0.3
0.4
0.5
0.6
Ages (years)
0
20
40
60
80
FDR (Age associated divergence) =0.0886
p(Scalp)=0.002
p(Dura)=0.52
Scalp
Dura

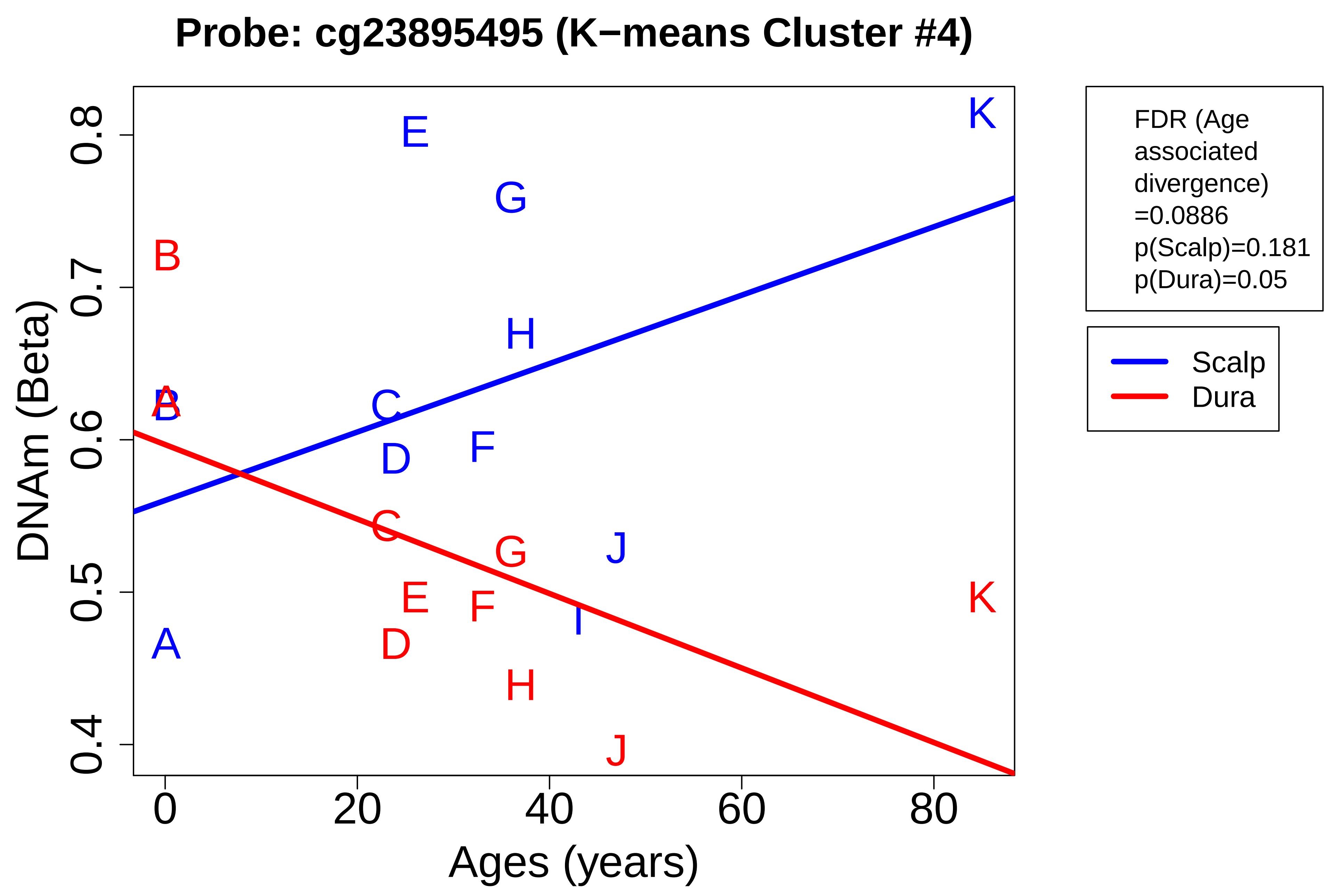

Probe: cg23895495 (K−means Cluster #4)
DNAm (Beta)
0.4
0.5
0.6
0.7
0.8
0
20
40
60
80
Ages (years)
FDR (Age associated divergence) =0.0886
p(Scalp)=0.181
p(Dura)=0.05
Scalp
Dura
A
B
C
D
E
F
G
H
I
J
K

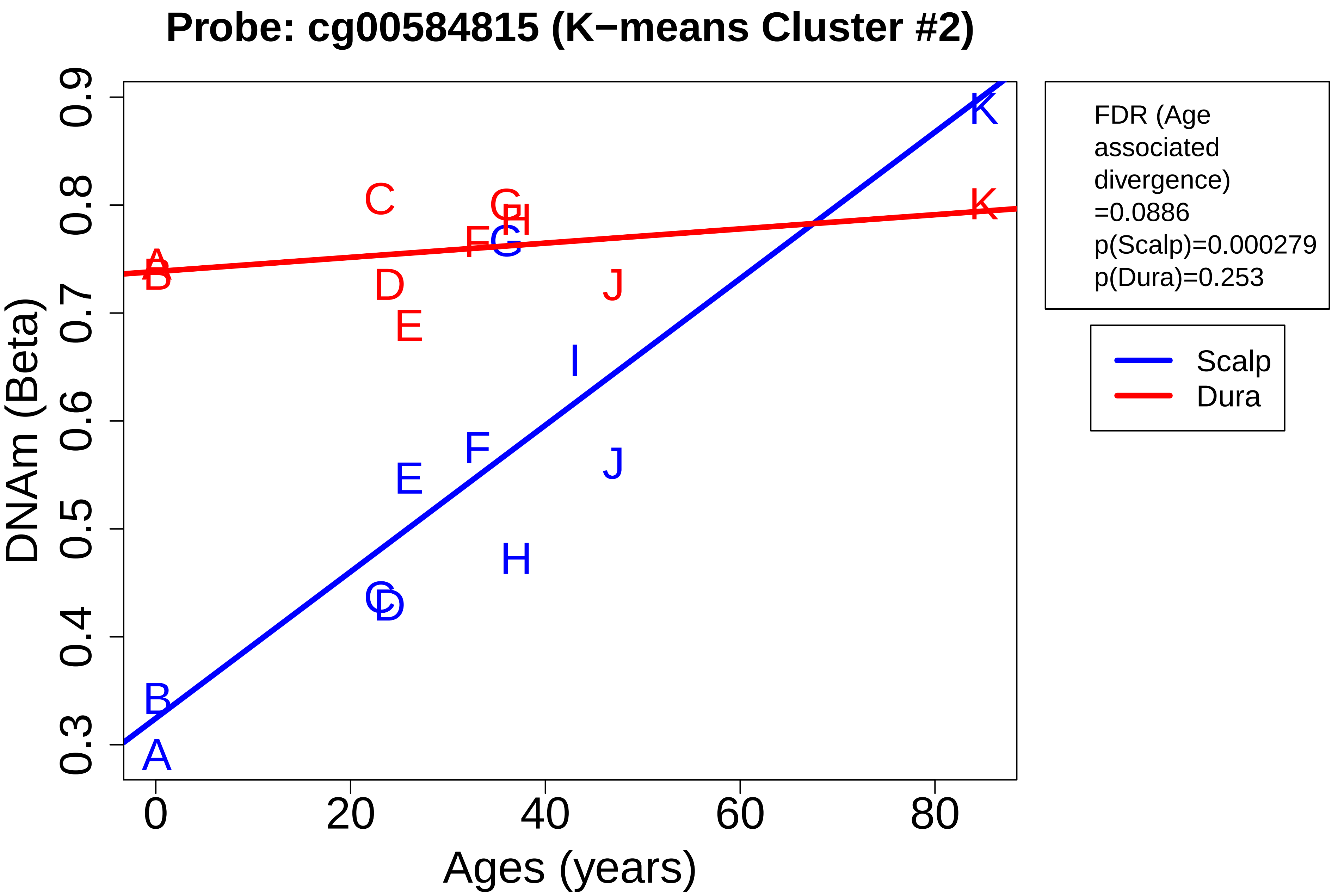

Probe: cg00584815 (K−means Cluster #2)
DNAm (Beta)
0.3
0.4
0.5
0.6
0.7
0.8
0.9
Ages (years)
0
20
40
60
80
FDR (Age associated divergence) =0.0886
p(Scalp)=0.000279
p(Dura)=0.253
Scalp
Dura

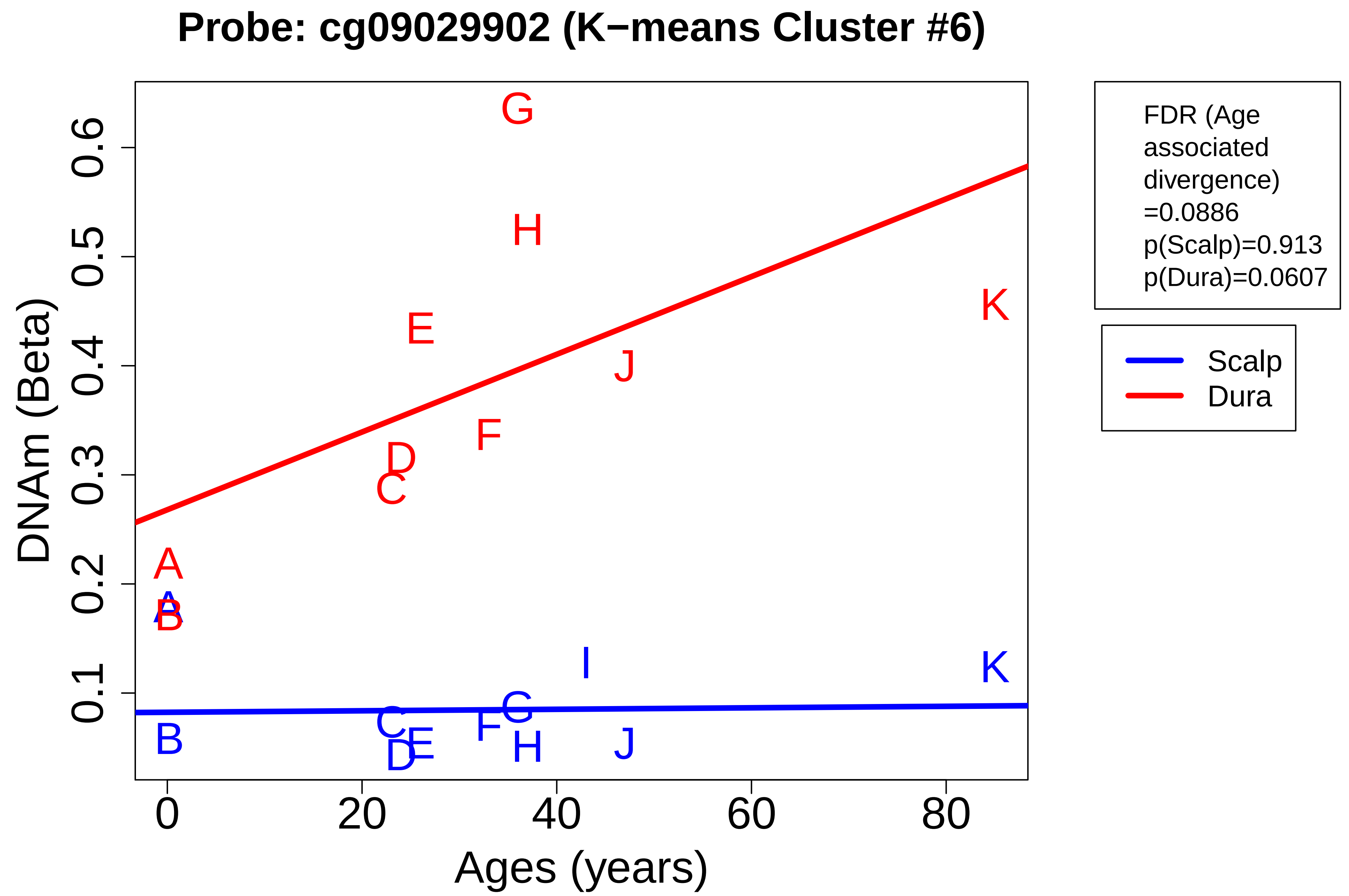

Probe: cg09029902 (K−means Cluster #6)
DNAm (Beta)
0.1
0.2
0.3
0.4
0.5
0.6
Ages (years)
0
20
40
60
80
FDR (Age associated divergence) =0.0886
p(Scalp)=0.913
p(Dura)=0.0607
Scalp
Dura

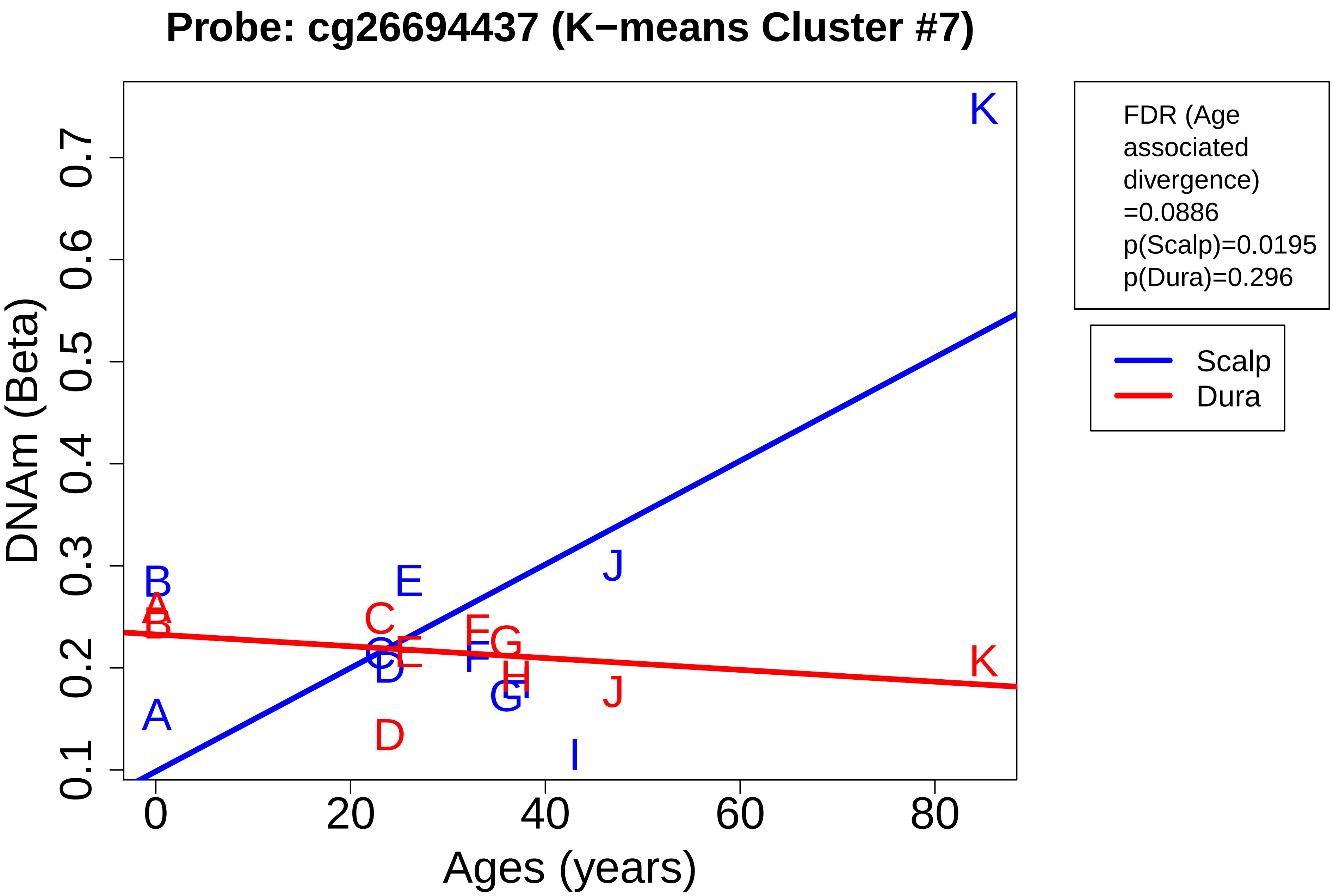

Probe: cg26694437 (K−means Cluster #7)
DNAm (Beta)
0.1
0.2
0.3
0.4
0.5
0.6
0.7
0
20
40
60
80
Ages (years)
FDR (Age associated divergence) =0.0886
p(Scalp)=0.0195
p(Dura)=0.296
Scalp
Dura

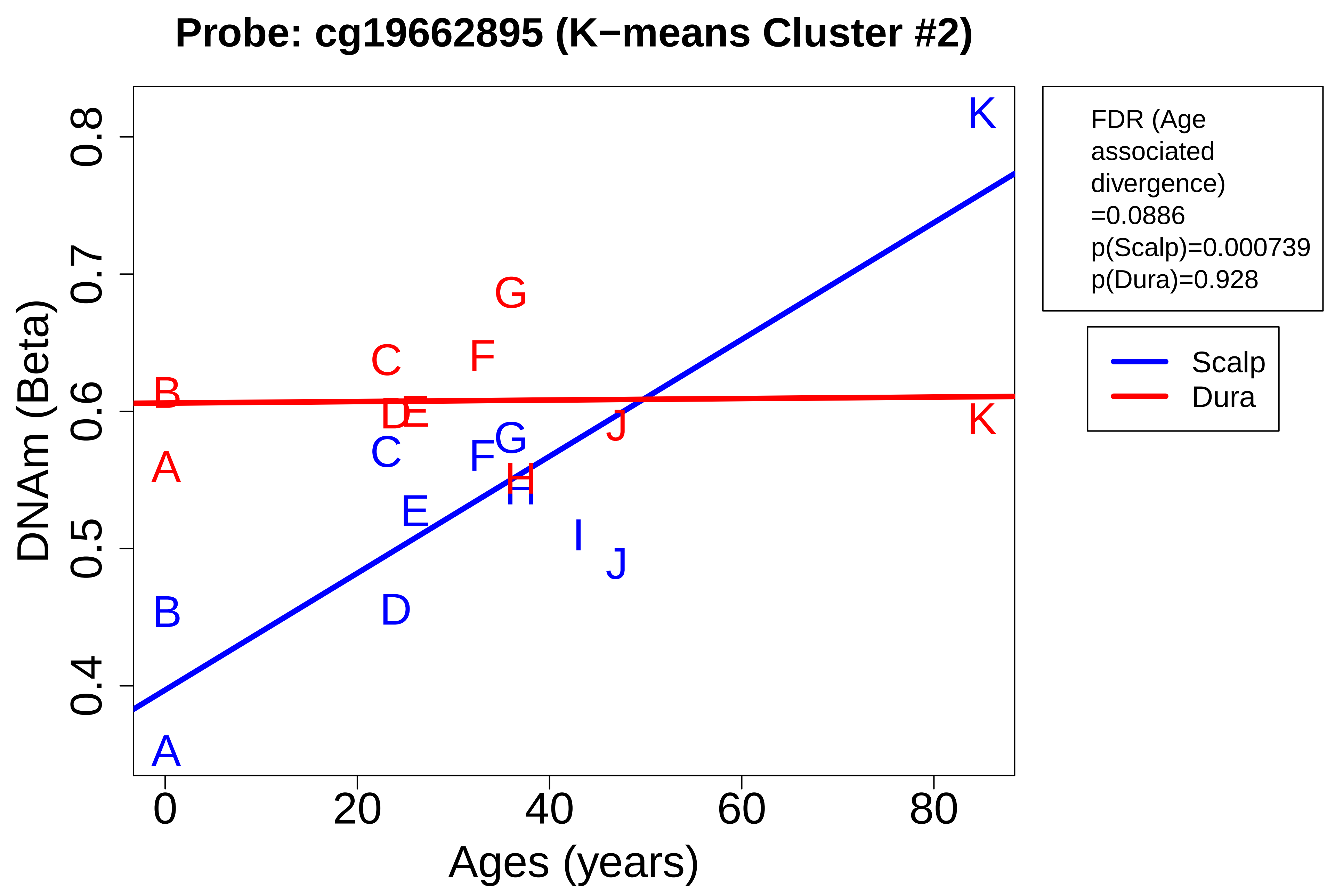

Probe: cg19662895 (K−means Cluster #2)
DNAm (Beta)
0.4
0.5
0.6
0.7
0.8
0
20
40
60
80
Ages (years)
FDR (Age associated divergence) =0.0886
p(Scalp)=0.000739
p(Dura)=0.928
Scalp
Dura
A
B
C
D
E
F
G
H
I
J
K

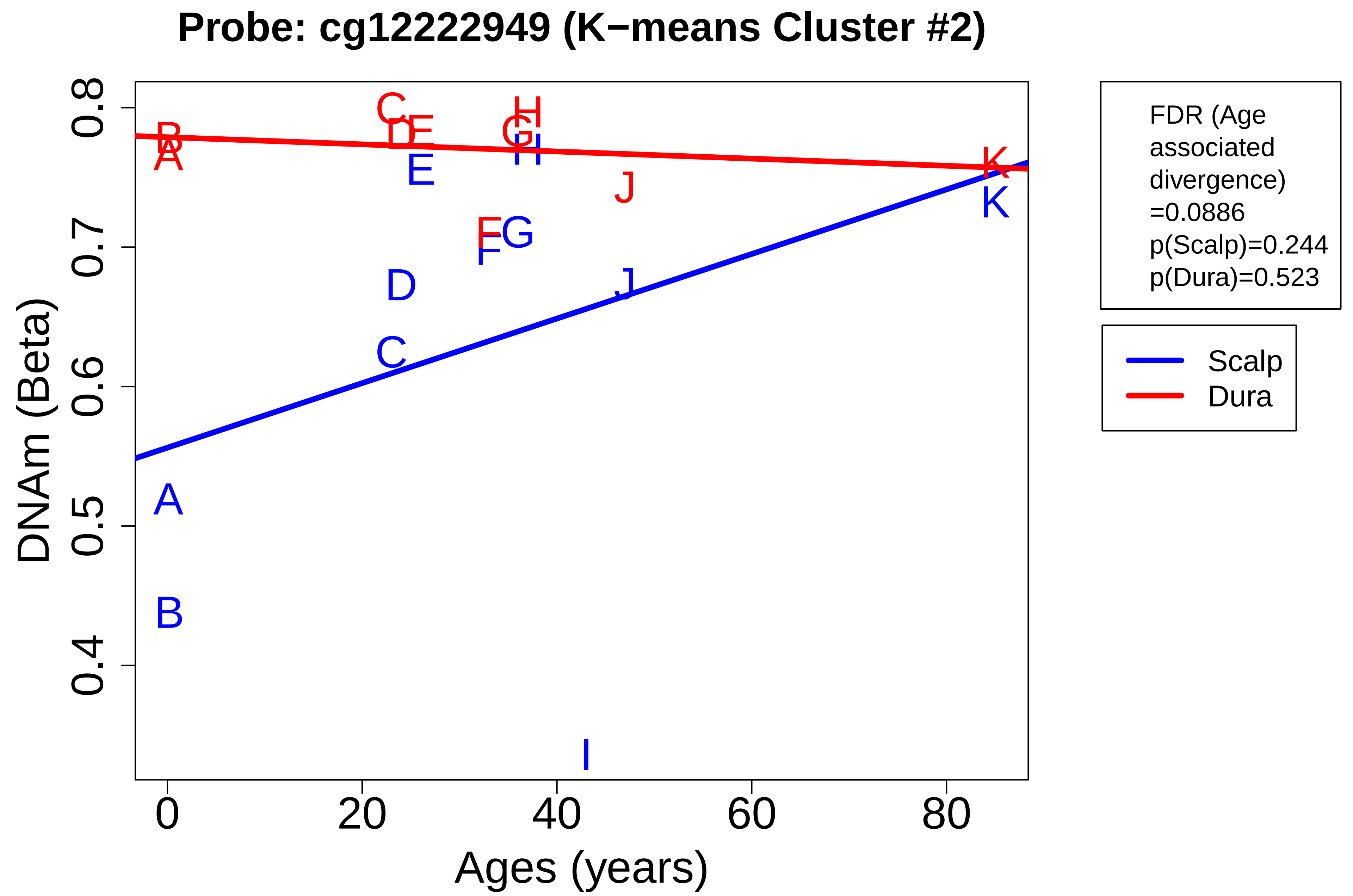

Probe: cg12222949 (K−means Cluster #2)
DNAm (Beta)
0.4
0.5
0.6
0.7
0.8
Ages (years)
0
20
40
60
80
A
B
C
D
E
F
G
H
I
J
K
FDR (Age associated divergence) =0.0886
p(Scalp)=0.244
p(Dura)=0.523
Scalp
Dura

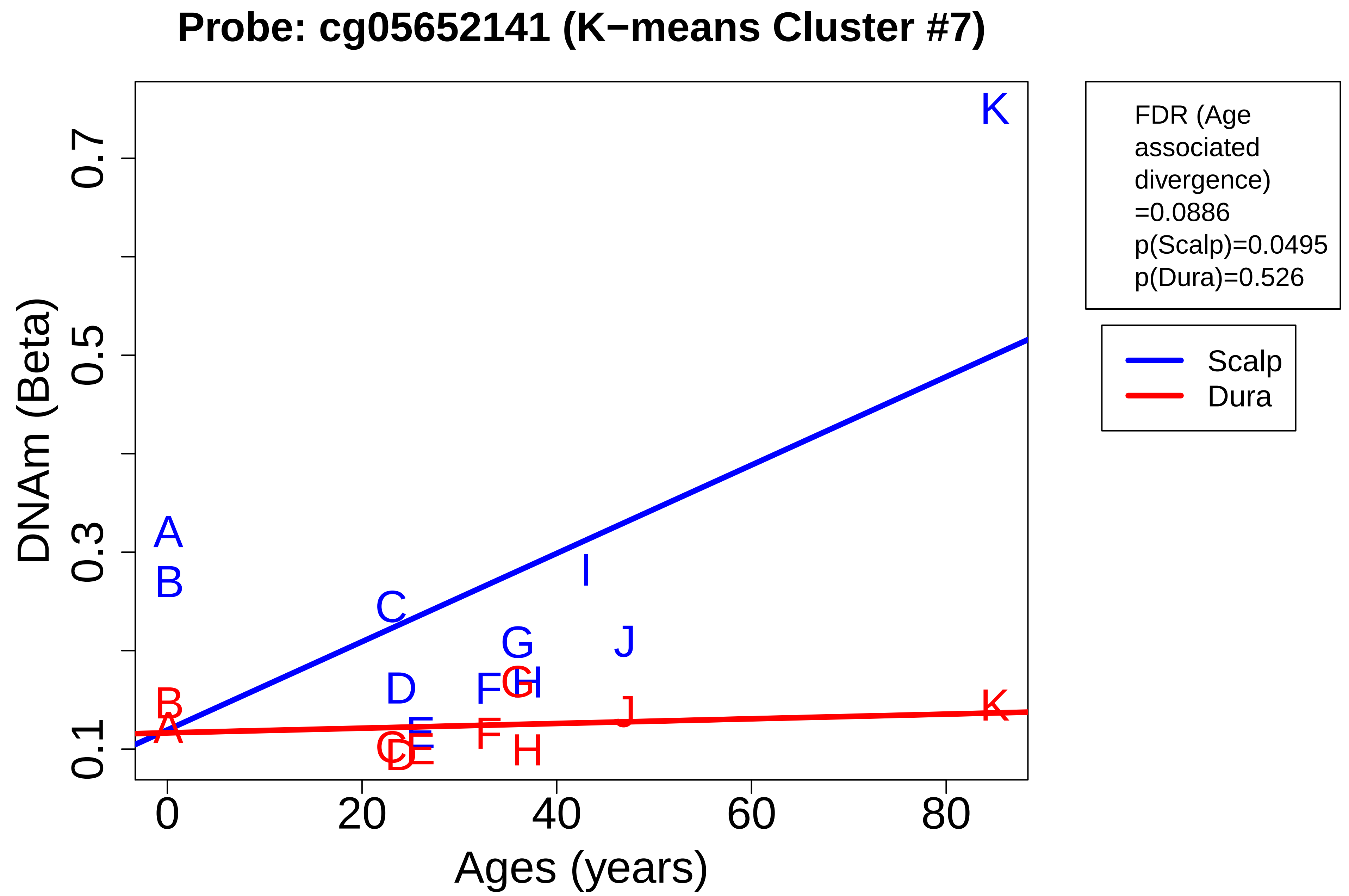

Probe: cg05652141 (K−means Cluster #7)
DNAm (Beta)
0.1
0.3
0.5
0.7
0
20
40
60
80
Ages (years)
FDR (Age associated divergence) =0.0886
p(Scalp)=0.0495
p(Dura)=0.526
Scalp
Dura

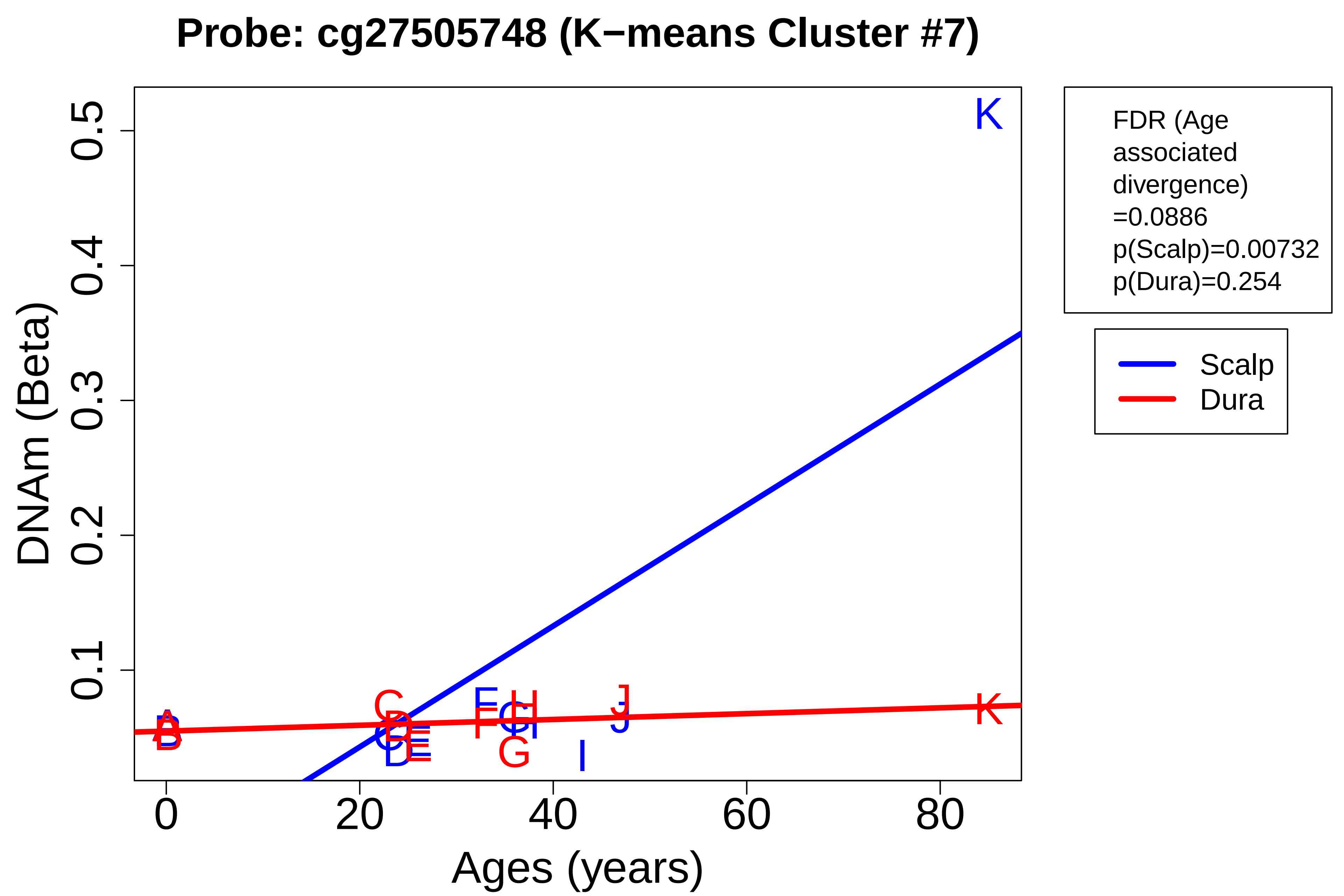

Probe: cg27505748 (K−means Cluster #7)
DNAm (Beta)
0.1
0.2
0.3
0.4
0.5
Ages (years)
0
20
40
60
80
FDR (Age associated divergence) =0.0886
p(Scalp)=0.00732
p(Dura)=0.254
Scalp
Dura

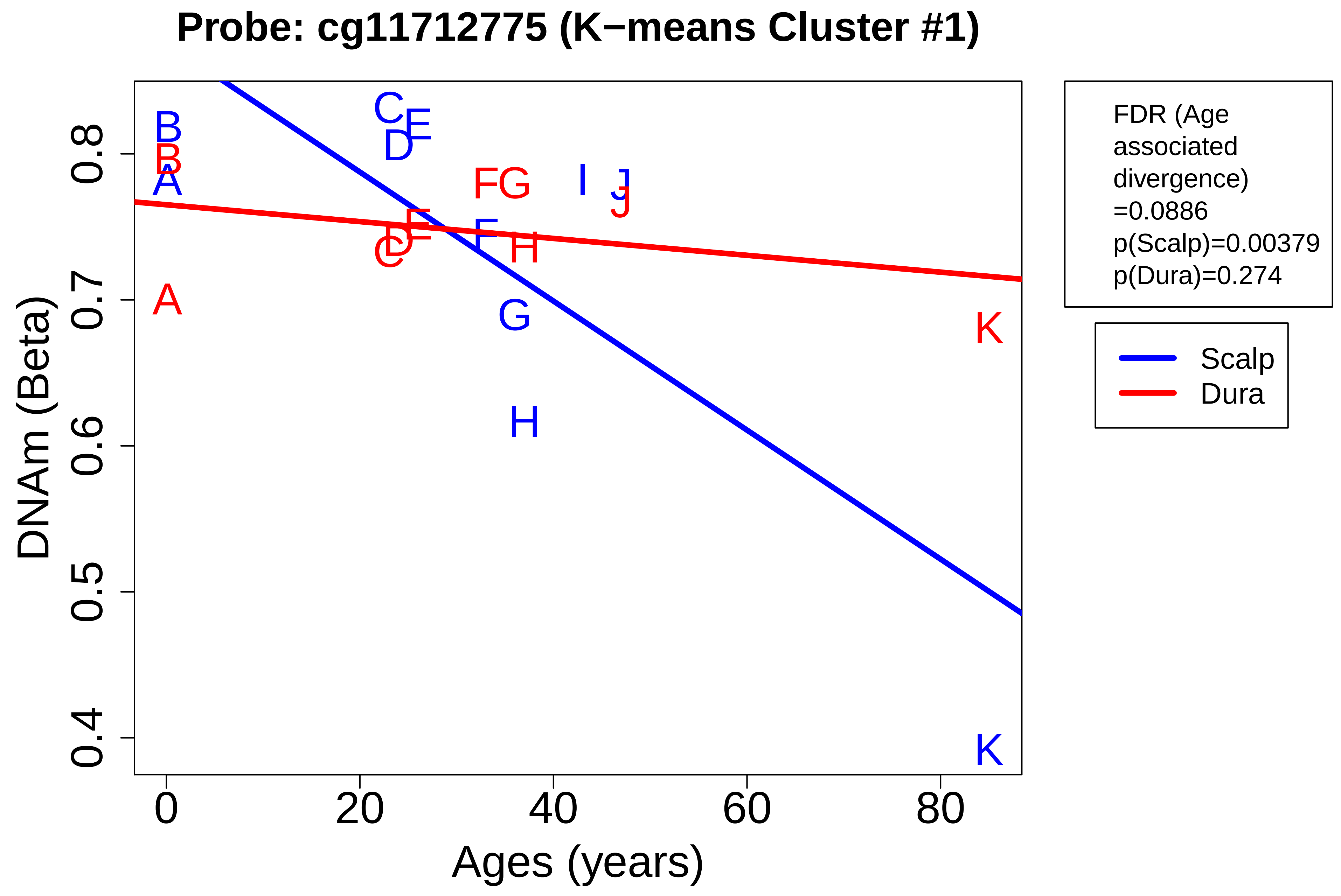

Probe: cg11712775 (K−means Cluster #1)
DNAm (Beta)
0.4
0.5
0.6
0.7
0.8
0
20
40
60
80
Ages (years)
A
B
C
D
E
F
G
H
I
J
K
FDR (Age associated divergence) =0.0886
p(Scalp)=0.00379
p(Dura)=0.274
Scalp
Dura

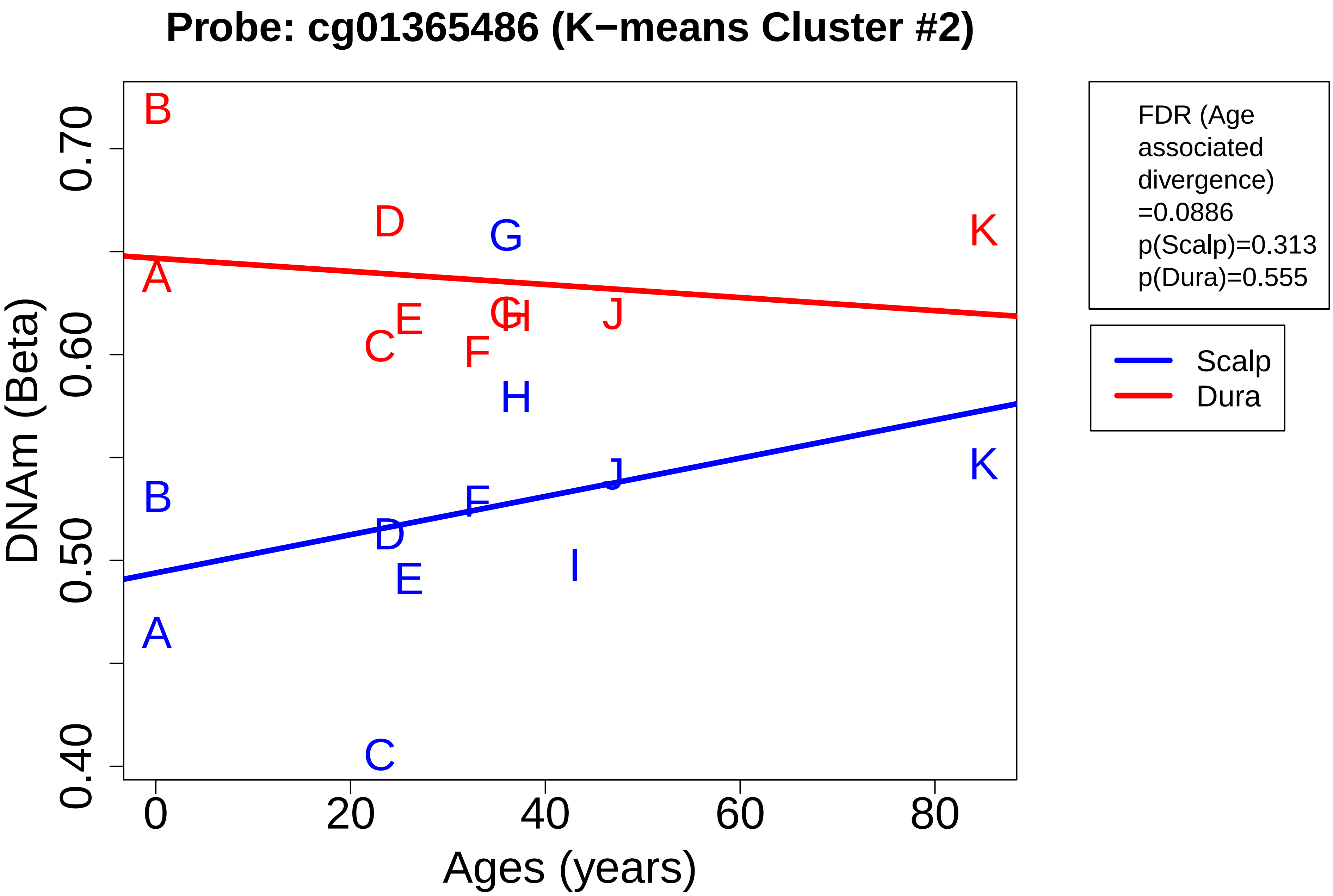

Probe: cg01365486 (K−means Cluster #2)
DNAm (Beta)
0.40
0.50
0.60
0.70
Ages (years)
0
20
40
60
80
FDR (Age associated divergence) =0.0886
p(Scalp)=0.313
p(Dura)=0.555
Scalp
Dura

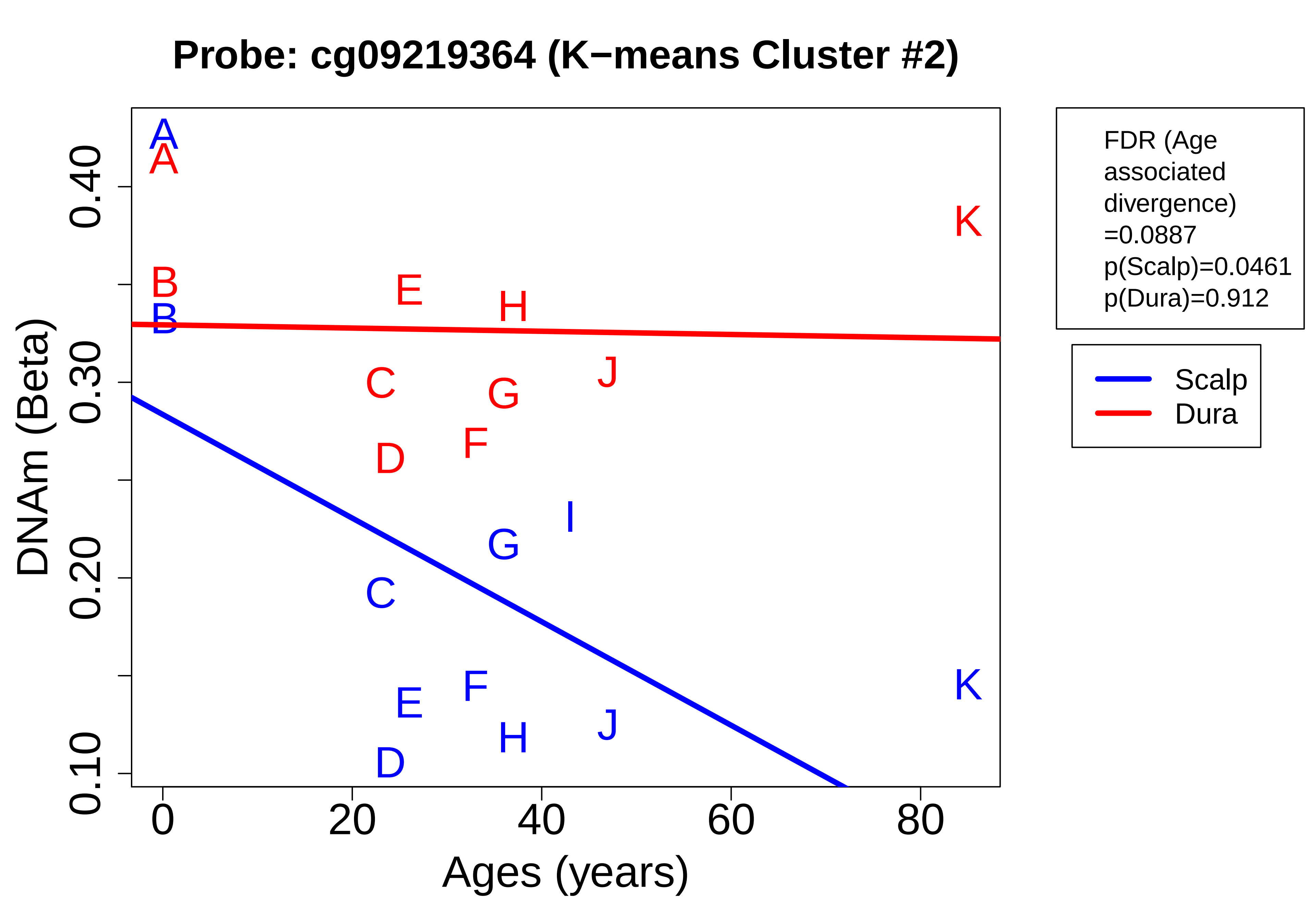
Probe: cg09219364 (K−means Cluster #2)
DNAm (Beta)
0.10
0.20
0.30
0.40
0
20
40
60
80
Ages (years)
A
A
B
B
C
C
D
D
E
E
F
F
G
G
H
H
I
J
J
K
K
FDR (Age associated divergence) =0.0887
p(Scalp)=0.0461
p(Dura)=0.912
Scalp
Dura

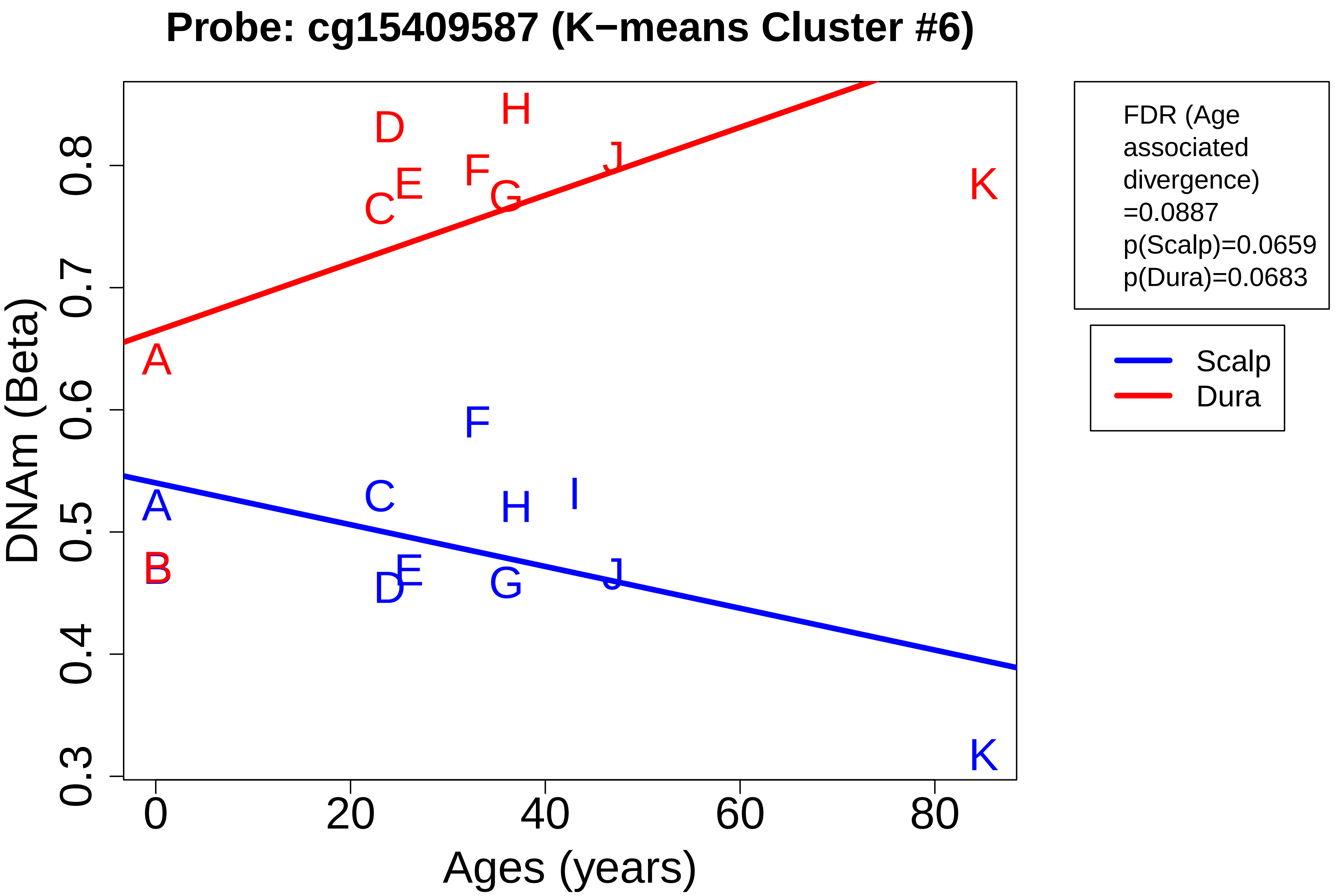

Probe: cg15409587 (K−means Cluster #6)
DNAm (Beta)
0.3
0.4
0.5
0.6
0.7
0.8
0
20
40
60
80
Ages (years)
FDR (Age associated divergence) =0.0887
p(Scalp)=0.0659
p(Dura)=0.0683
Scalp
Dura
A
B
C
D
E
F
G
H
I
J
K

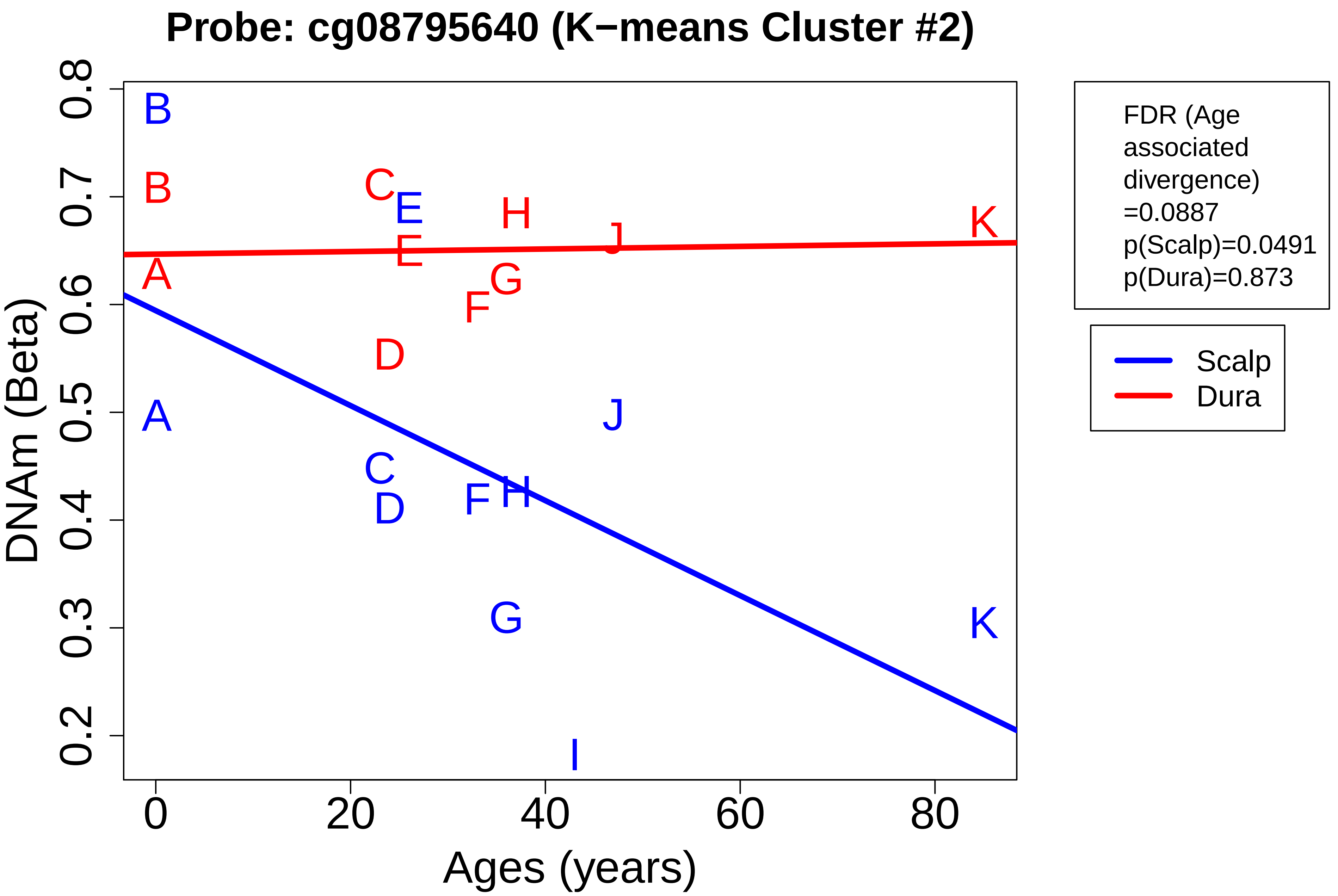

Probe: cg08795640 (K−means Cluster #2)
DNAm (Beta)
0.2
0.3
0.4
0.5
0.6
0.7
0.8
Ages (years)
0
20
40
60
80
FDR (Age associated divergence) =0.0887
p(Scalp)=0.0491
p(Dura)=0.873
Scalp
Dura
A
B
C
D
E
F
G
H
I
J
K

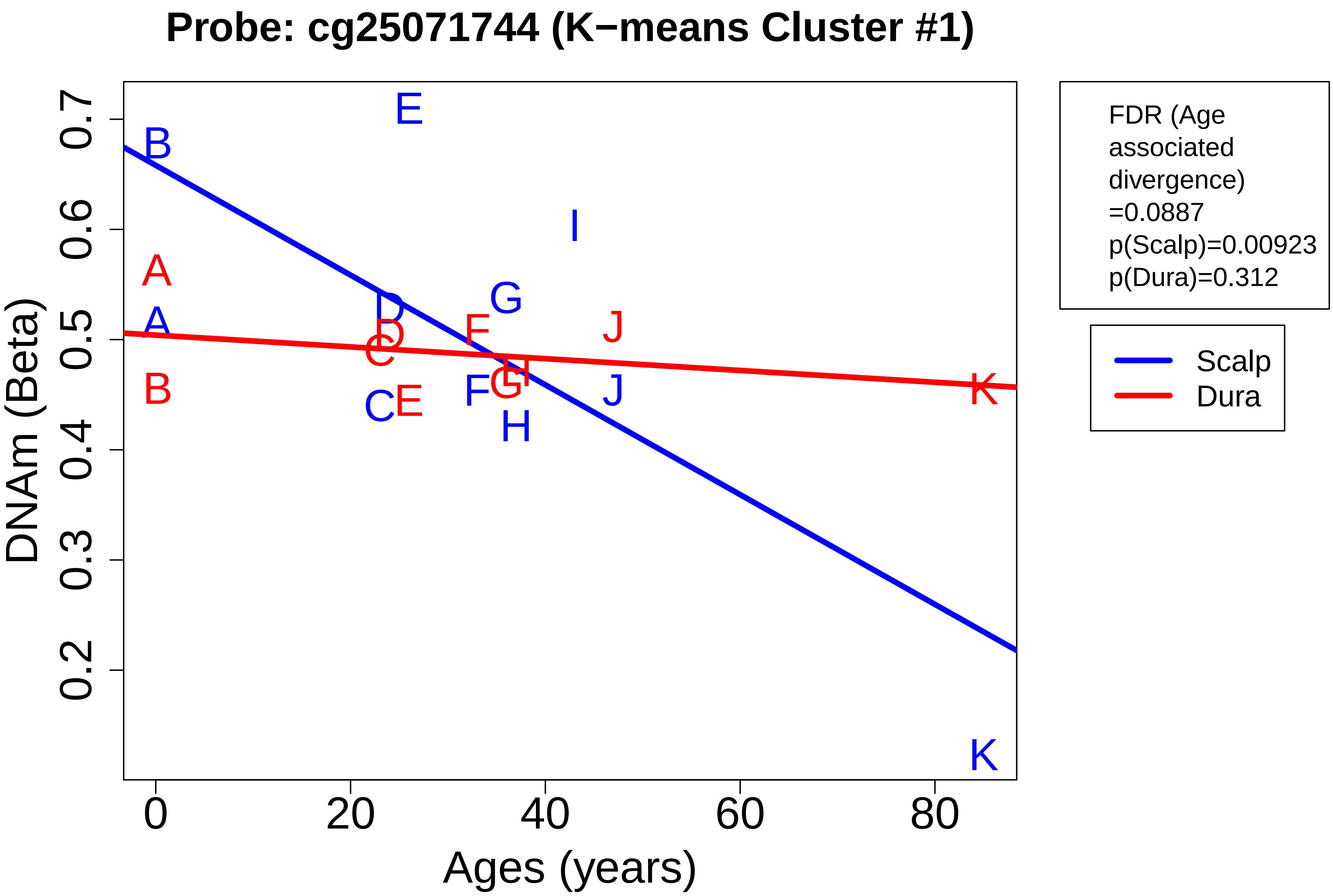

Probe: cg25071744 (K−means Cluster #1)
DNAm (Beta)
0.2
0.3
0.4
0.5
0.6
0.7
Ages (years)
0
20
40
60
80
FDR (Age associated divergence) =0.0887
p(Scalp)=0.00923
p(Dura)=0.312
Scalp
Dura

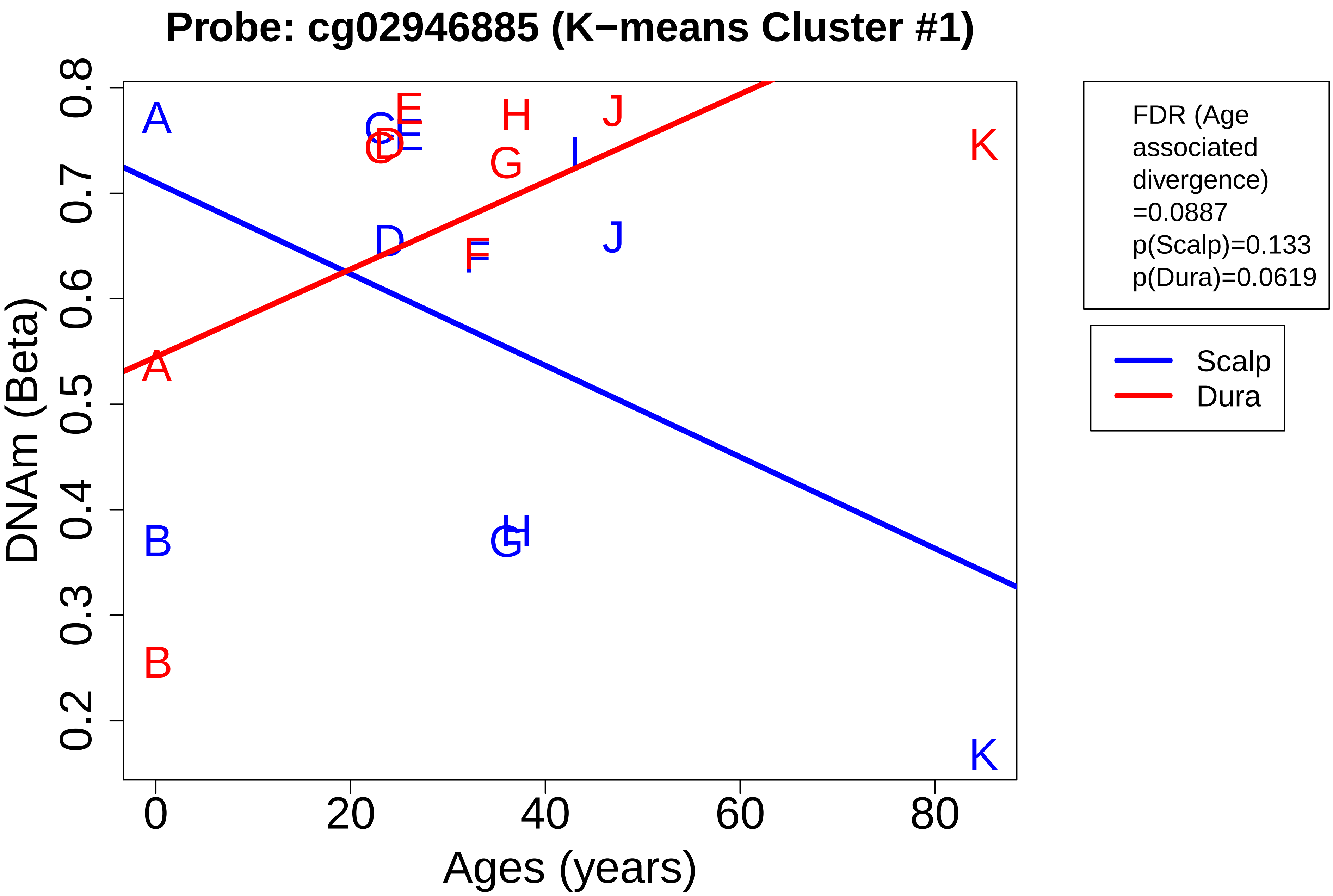

Probe: cg02946885 (K−means Cluster #1)
DNAm (Beta)
Ages (years)
0.2
0.3
0.4
0.5
0.6
0.7
0.8
0
20
40
60
80
FDR (Age associated divergence) =0.0887
p(Scalp)=0.133
p(Dura)=0.0619
Scalp
Dura

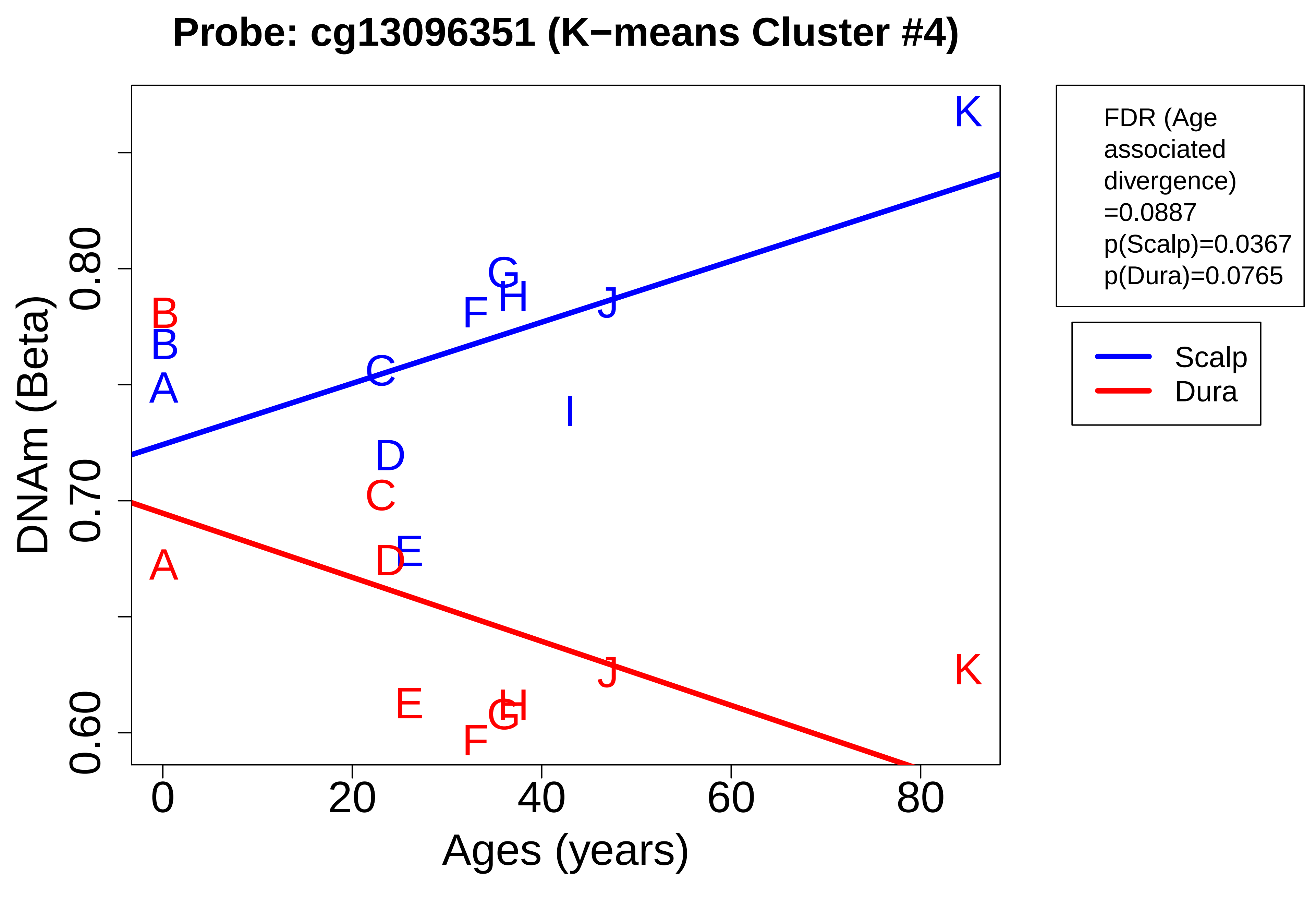

Probe: cg13096351 (K−means Cluster #4)
DNAm (Beta)
0.60
0.70
0.80
0
20
40
60
80
Ages (years)
A
B
C
D
E
F
G
H
I
J
K
FDR (Age associated divergence) =0.0887
p(Scalp)=0.0367
p(Dura)=0.0765
Scalp
Dura

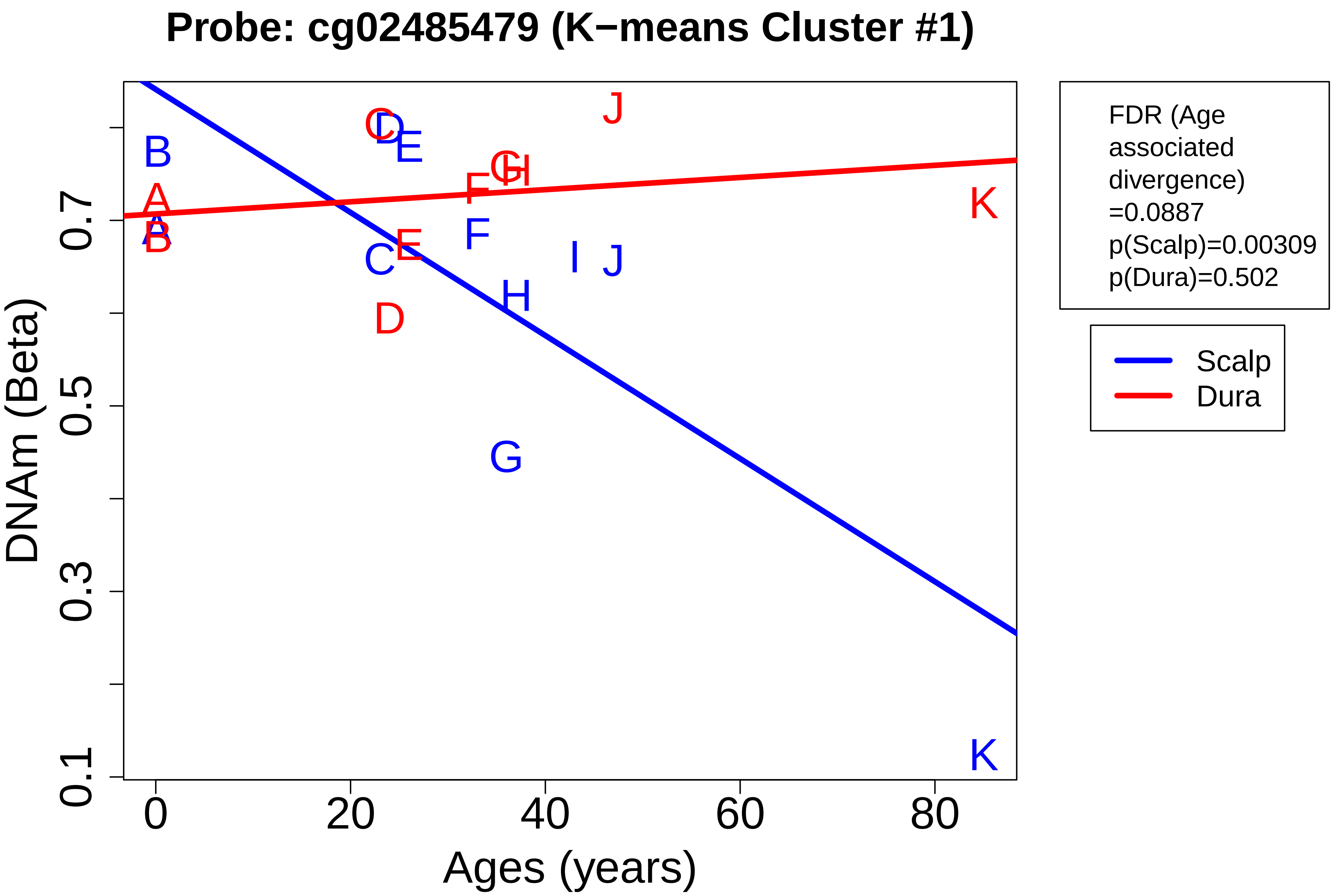
Probe: cg02485479 (K−means Cluster #1)
DNAm (Beta)
0.1
0.3
0.5
0.7
0
20
40
60
80
Ages (years)
FDR (Age associated divergence) =0.0887
p(Scalp)=0.00309
p(Dura)=0.502
Scalp
Dura

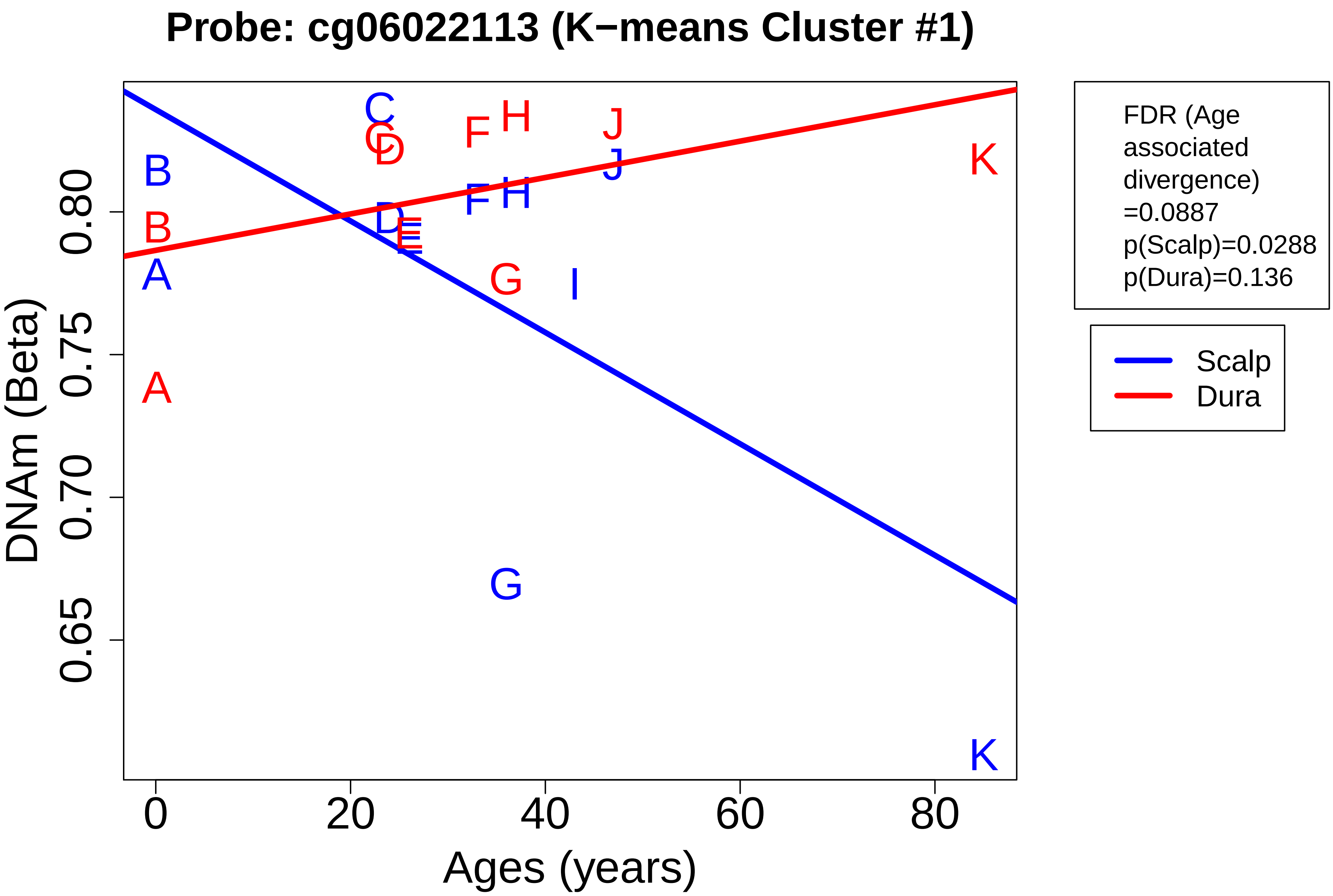

Probe: cg06022113 (K−means Cluster #1)
DNAm (Beta)
0.65
0.70
0.75
0.80
0
20
40
60
80
Ages (years)
FDR (Age associated divergence) =0.0887
p(Scalp)=0.0288
p(Dura)=0.136
Scalp
Dura

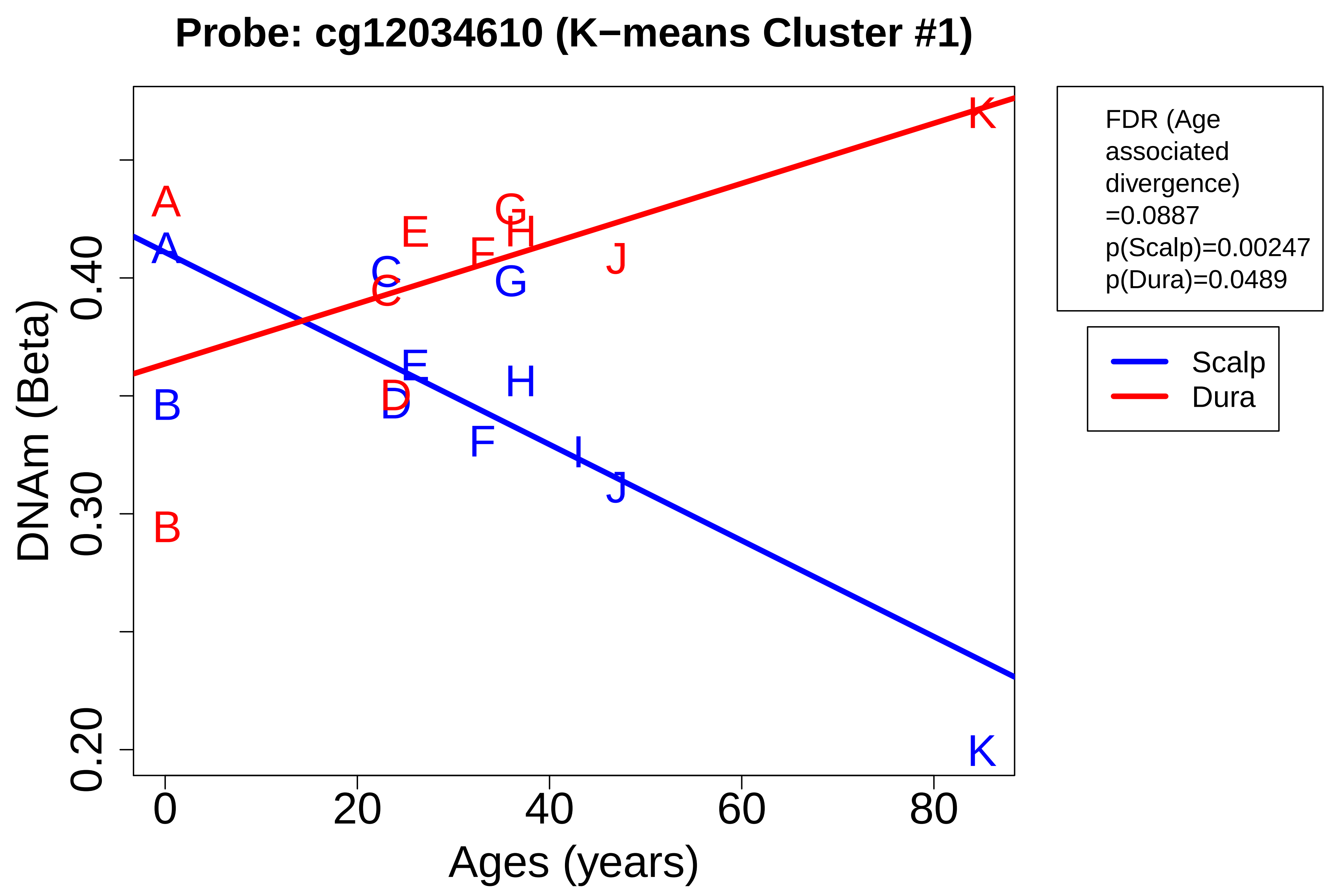

Probe: cg12034610 (K−means Cluster #1)
FDR (Age associated divergence) =0.0887
p(Scalp)=0.00247
p(Dura)=0.0489
Scalp
Dura
DNAm (Beta)
0.20
0.30
0.40
0
20
40
60
80
Ages (years)
A
B
C
D
E
F
G
H
I
J
K

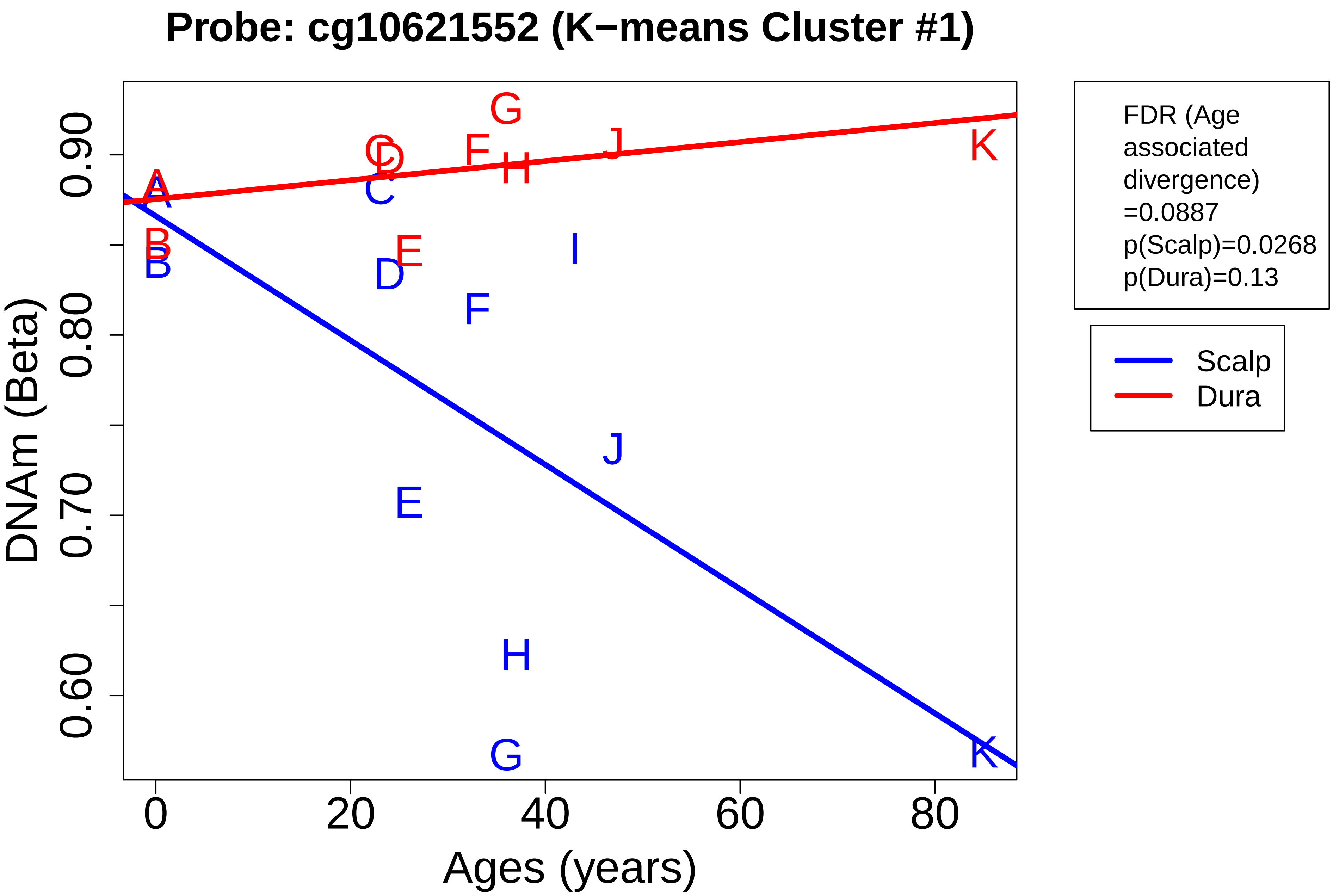

Probe: cg10621552 (K−means Cluster #1)
DNAm (Beta)
0.60
0.70
0.80
0.90
Ages (years)
0
20
40
60
80
FDR (Age associated divergence) =0.0887
p(Scalp)=0.0268
p(Dura)=0.13
Scalp
Dura

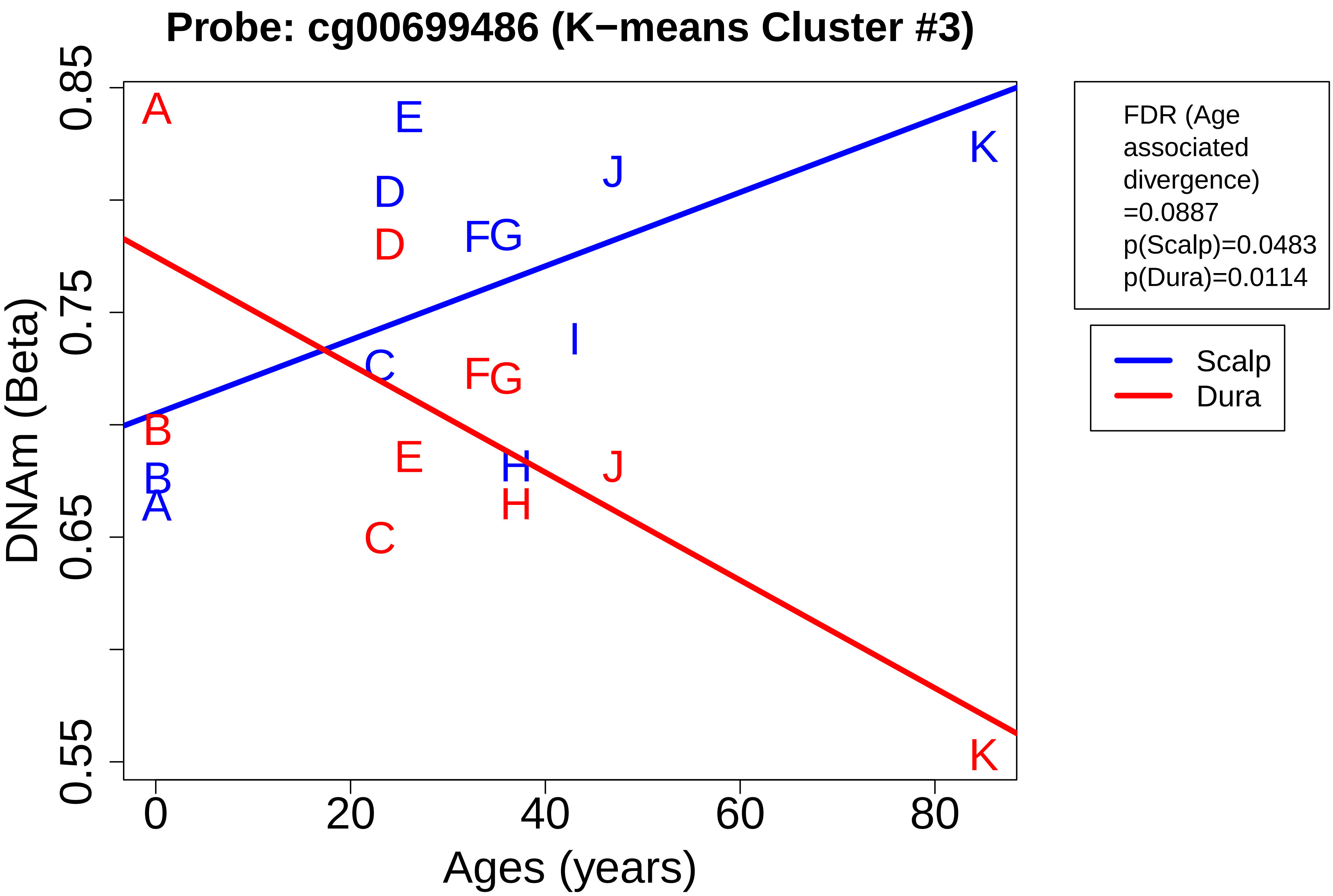

Probe: cg00699486 (K−means Cluster #3)
DNAm (Beta)
0.85
0.75
0.65
0.55
Ages (years)
0
20
40
60
80
A
E
K
J
D
D
FG
FG
I
C
B
E
H
J
B
A
H
C
K
FDR (Age associated divergence) =0.0887
p(Scalp)=0.0483
p(Dura)=0.0114
Scalp
Dura

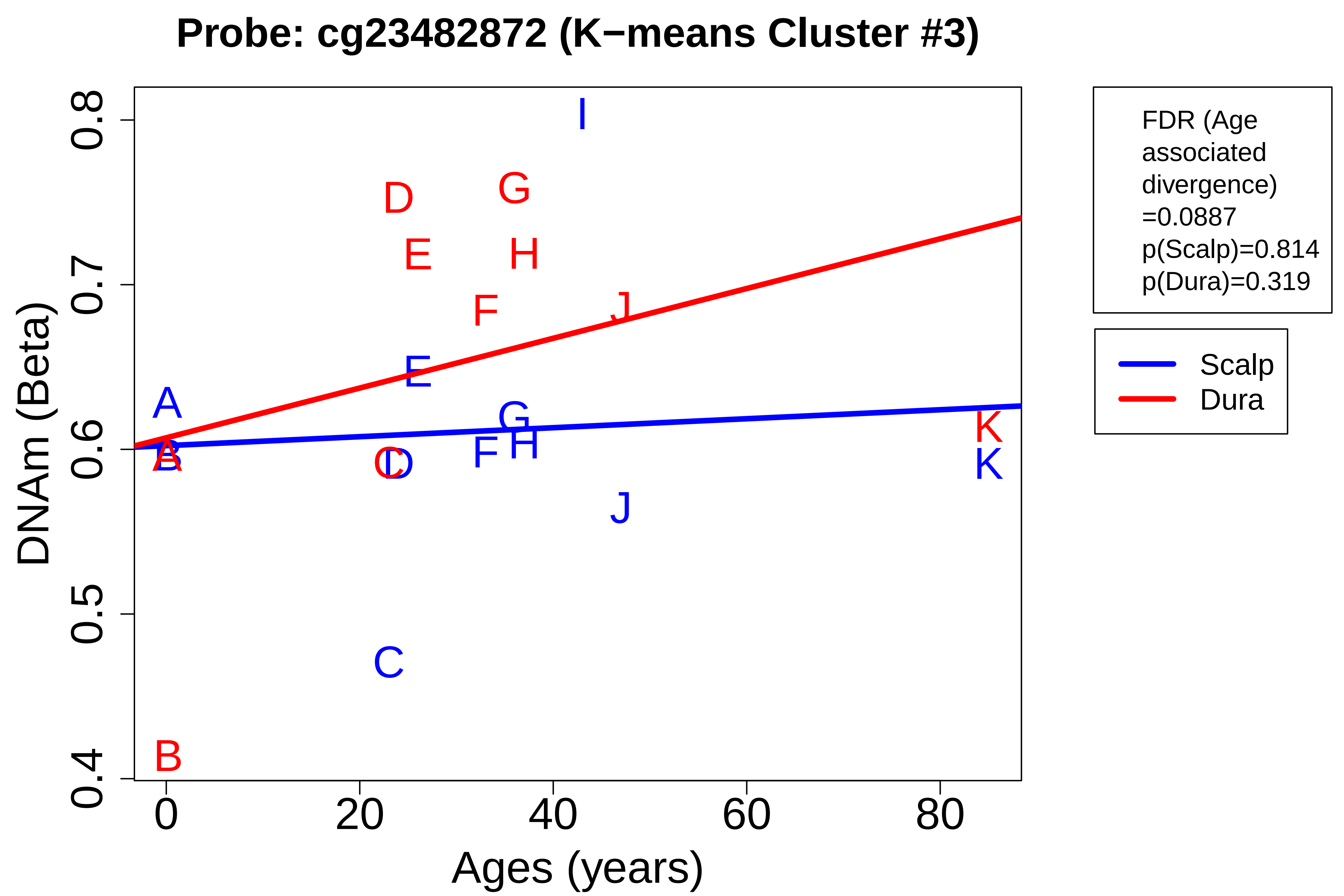

Probe: cg23482872 (K−means Cluster #3)
DNAm (Beta)
0.4
0.5
0.6
0.7
0.8
0
20
40
60
80
Ages (years)
FDR (Age associated divergence) =0.0887
p(Scalp)=0.814
p(Dura)=0.319
Scalp
Dura

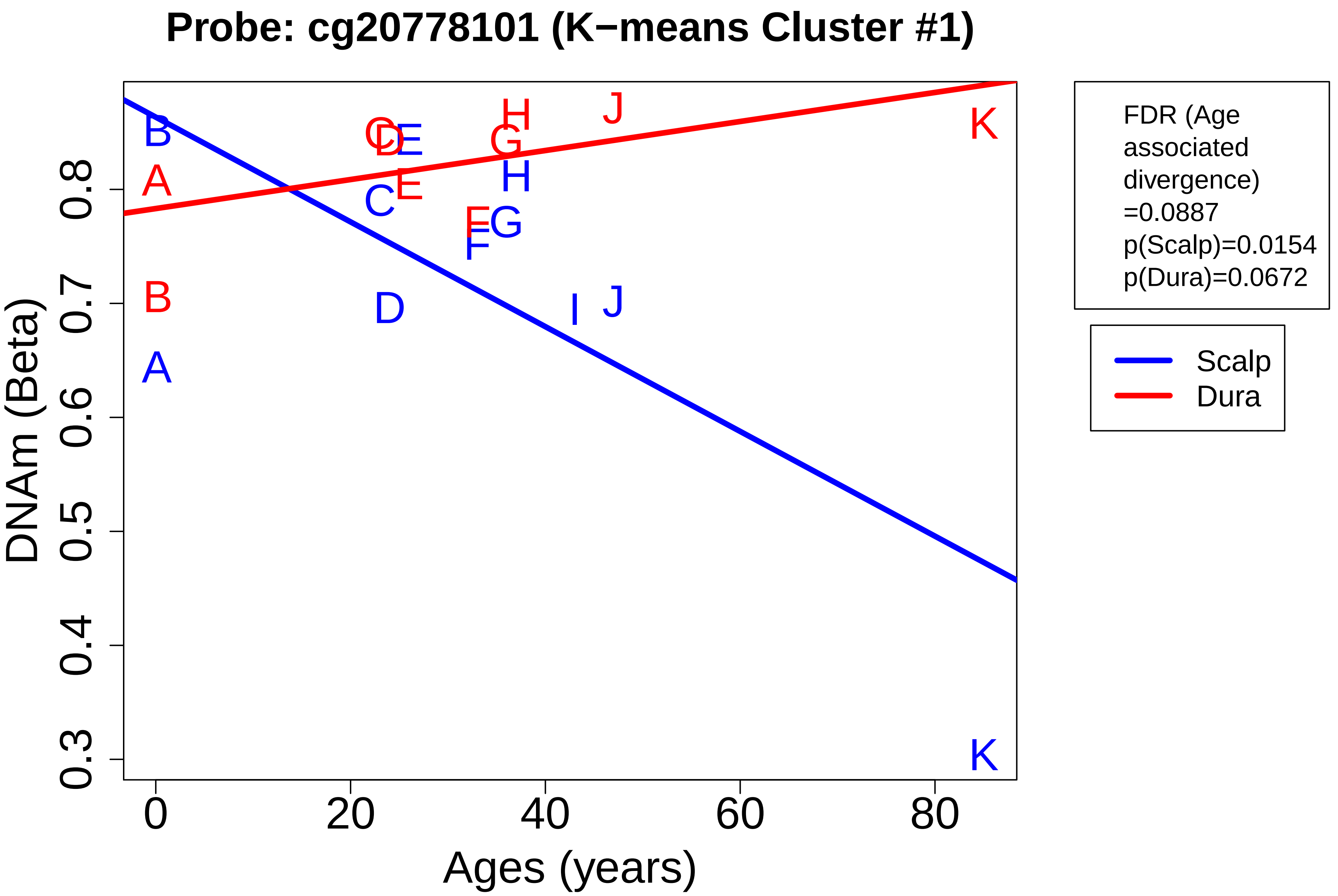

Probe: cg20778101 (K−means Cluster #1)
DNAm (Beta)
0.3
0.4
0.5
0.6
0.7
0.8
0
20
40
60
80
Ages (years)
FDR (Age associated divergence) =0.0887
p(Scalp)=0.0154
p(Dura)=0.0672
Scalp
Dura

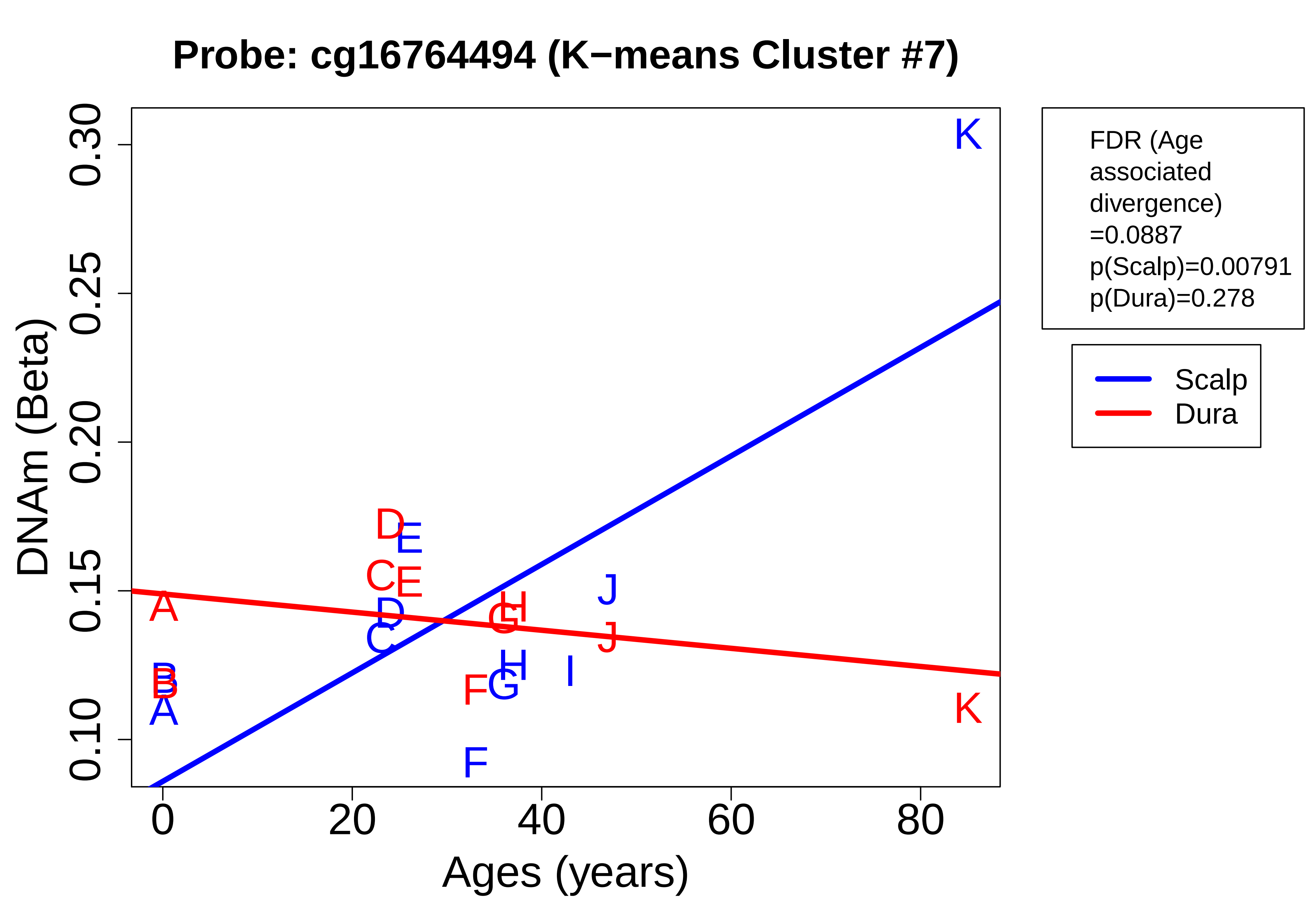

Probe: cg16764494 (K−means Cluster #7)
DNAm (Beta)
0.10
0.15
0.20
0.25
0.30
Ages (years)
0
20
40
60
80
FDR (Age associated divergence) =0.0887
p(Scalp)=0.00791
p(Dura)=0.278
Scalp
Dura

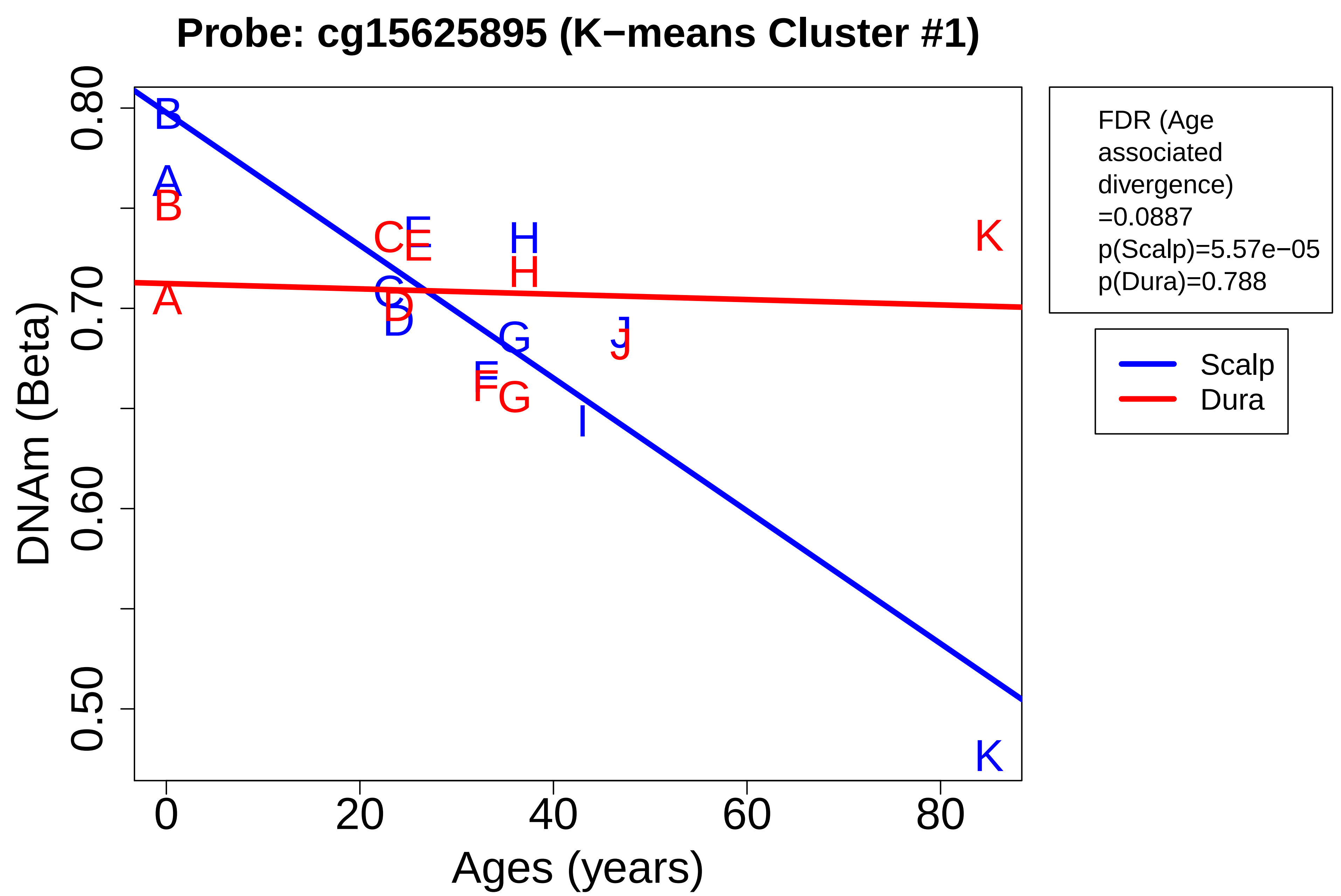

Probe: cg15625895 (K−means Cluster #1)
DNAm (Beta)
0.50
0.60
0.70
0.80
Ages (years)
0
20
40
60
80
A
B
C
D
E
F
G
H
I
J
K
FDR (Age associated divergence) =0.0887
p(Scalp)=5.57e−05
p(Dura)=0.788
Scalp
Dura

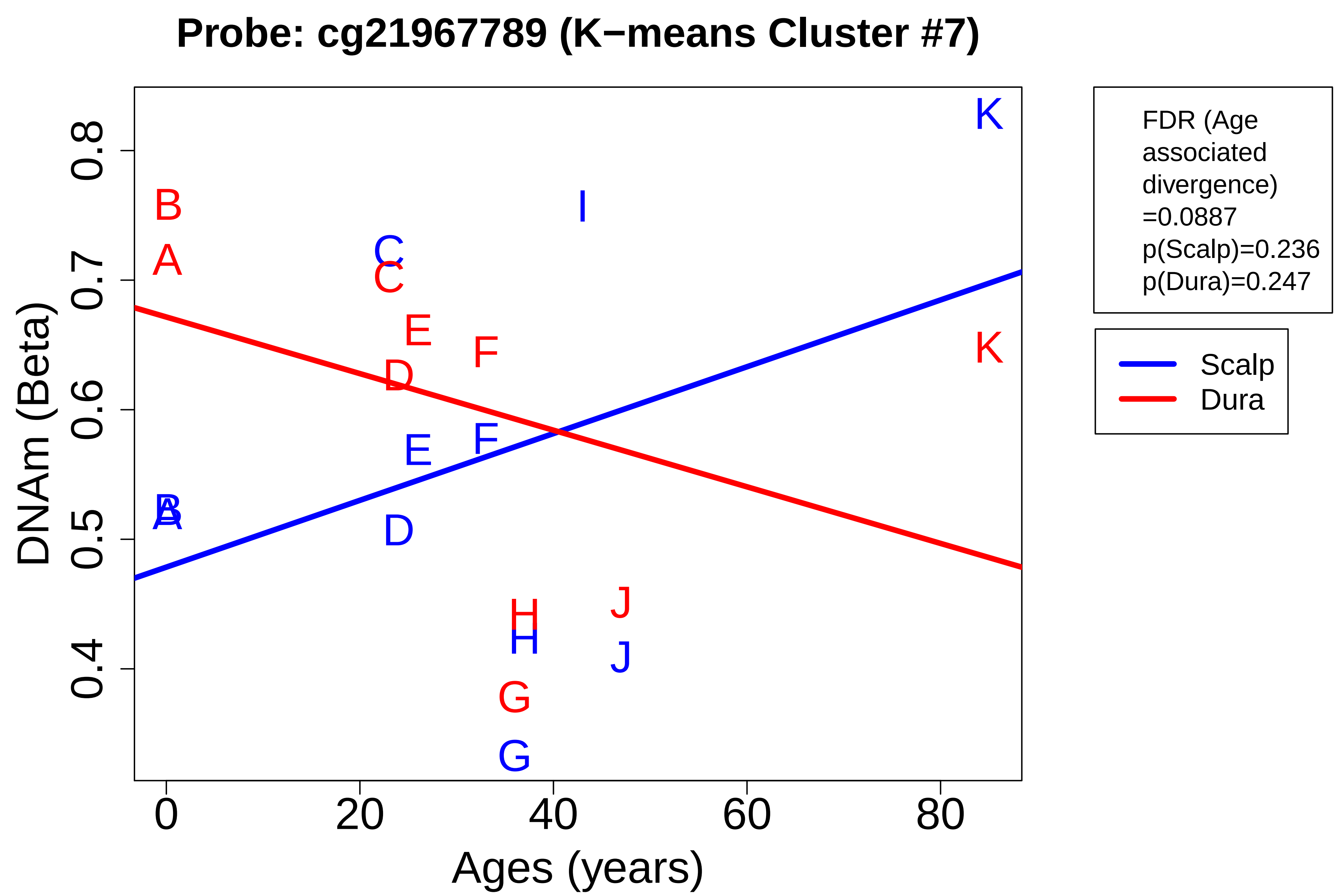

Probe: cg21967789 (K−means Cluster #7)
DNAm (Beta)
0.4
0.5
0.6
0.7
0.8
0
20
40
60
80
Ages (years)
FDR (Age associated divergence) =0.0887
p(Scalp)=0.236
p(Dura)=0.247
Scalp
Dura

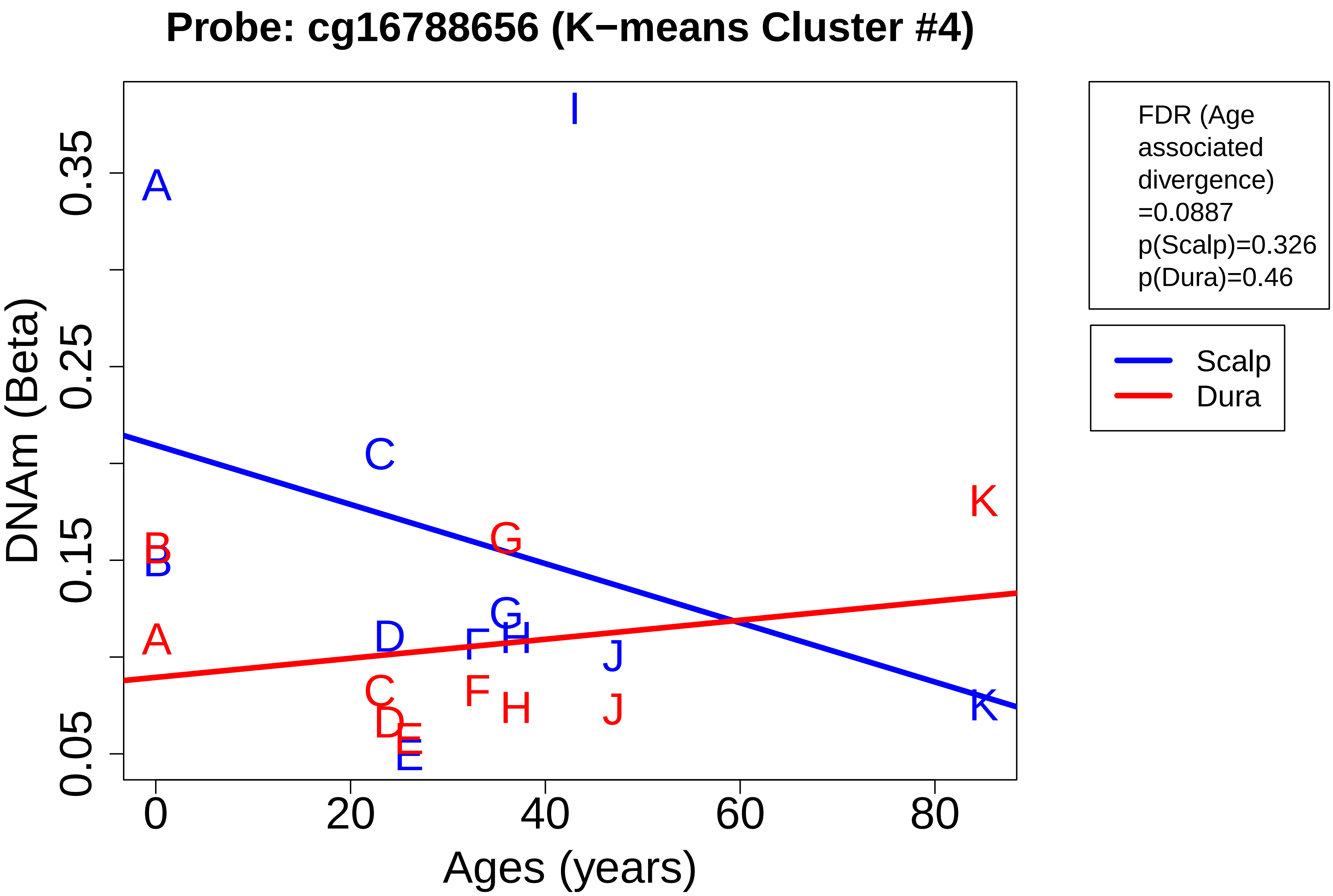

Probe: cg16788656 (K−means Cluster #4)
FDR (Age associated divergence) =0.0887
p(Scalp)=0.326
p(Dura)=0.46
Scalp
Dura
DNAm (Beta)
0.05
0.15
0.25
0.35
0
20
40
60
80
Ages (years)
A
B
C
D
E
F
G
H
I
J
K

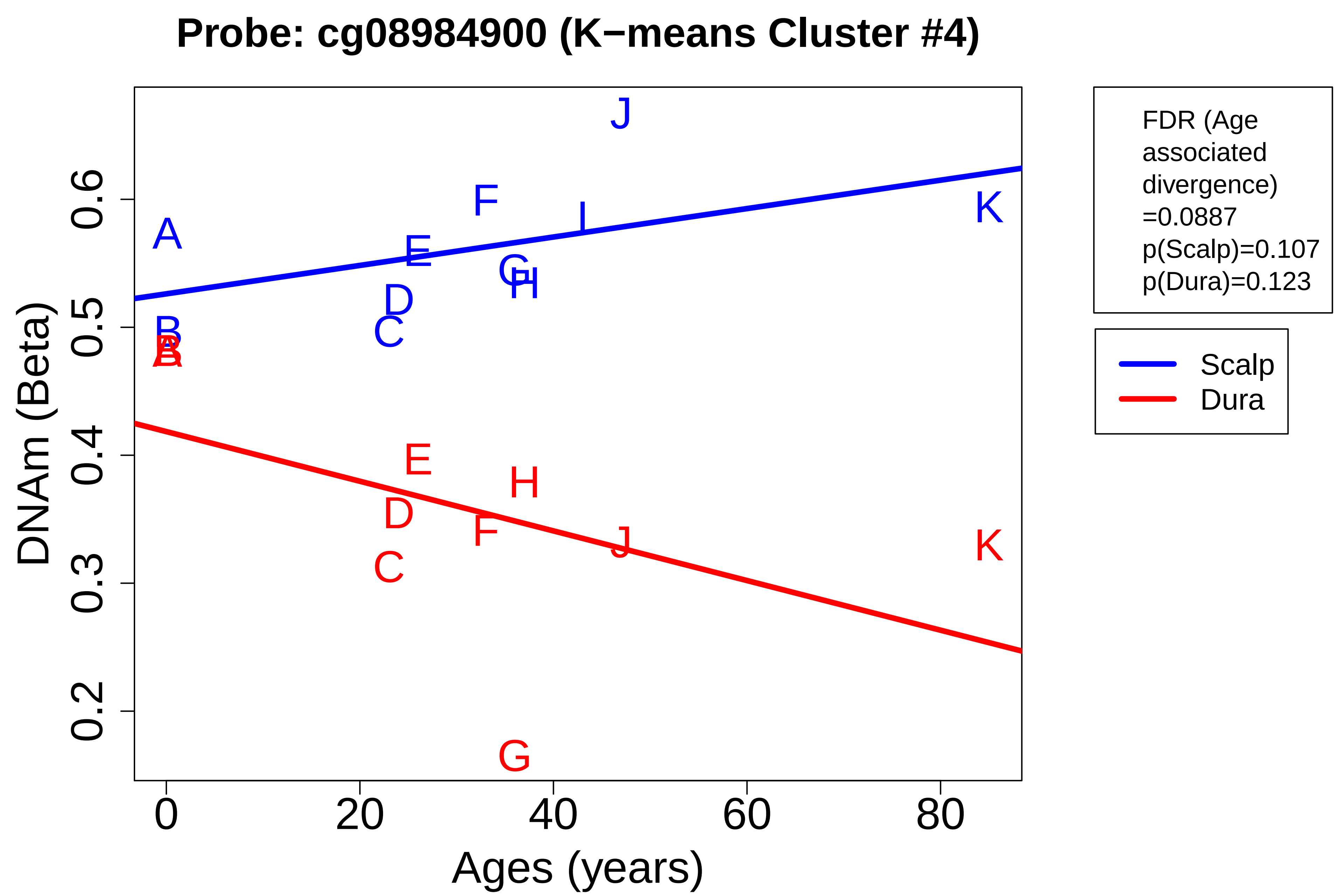

Probe: cg08984900 (K−means Cluster #4)
DNAm (Beta)
0.2
0.3
0.4
0.5
0.6
0
20
40
60
80
Ages (years)
FDR (Age associated divergence) =0.0887
p(Scalp)=0.107
p(Dura)=0.123
Scalp
Dura

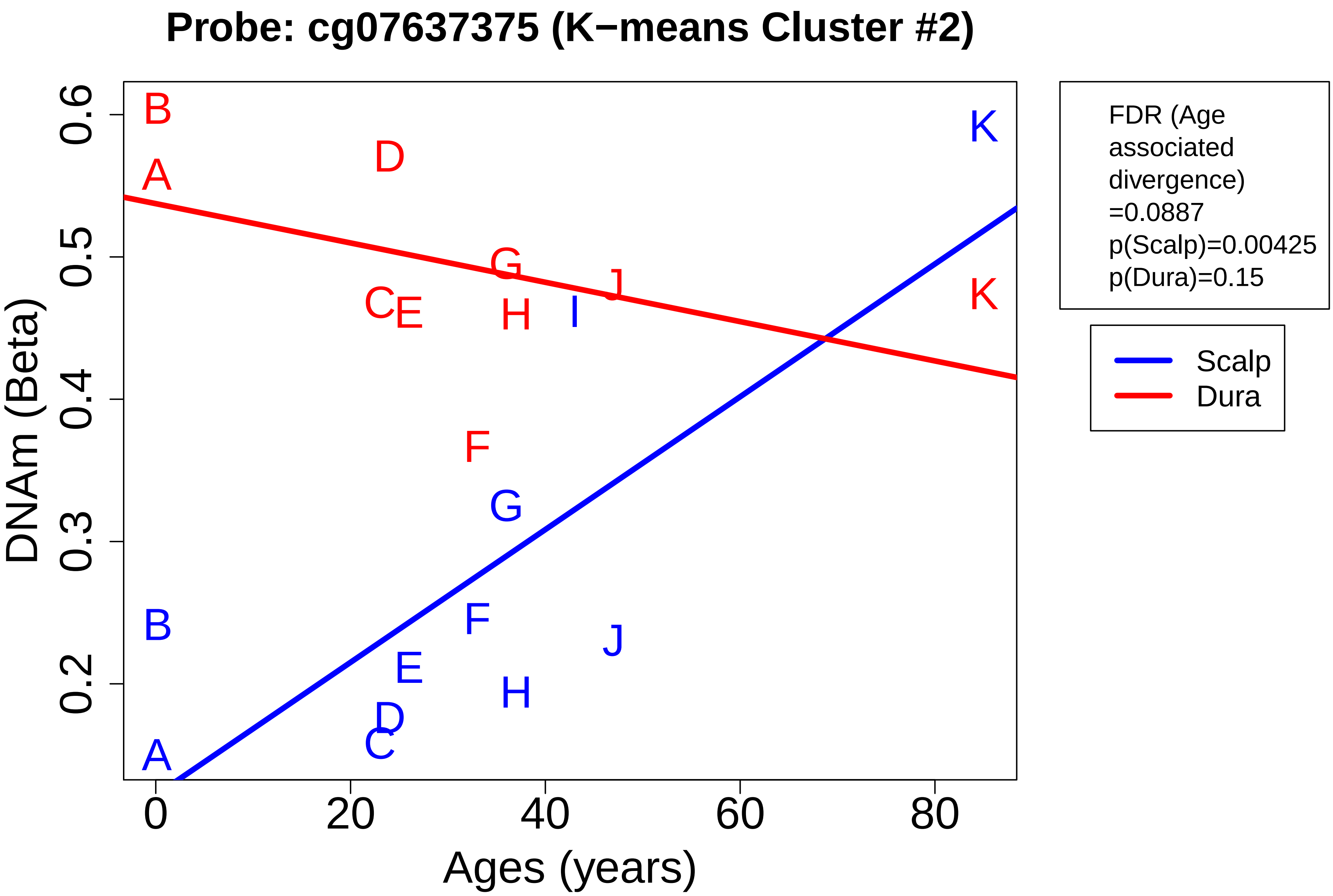
Probe: cg07637375 (K−means Cluster #2)
DNAm (Beta)
Ages (years)
0.2
0.3
0.4
0.5
0.6
0
20
40
60
80
FDR (Age associated divergence) =0.0887
p(Scalp)=0.00425
p(Dura)=0.15
Scalp
Dura

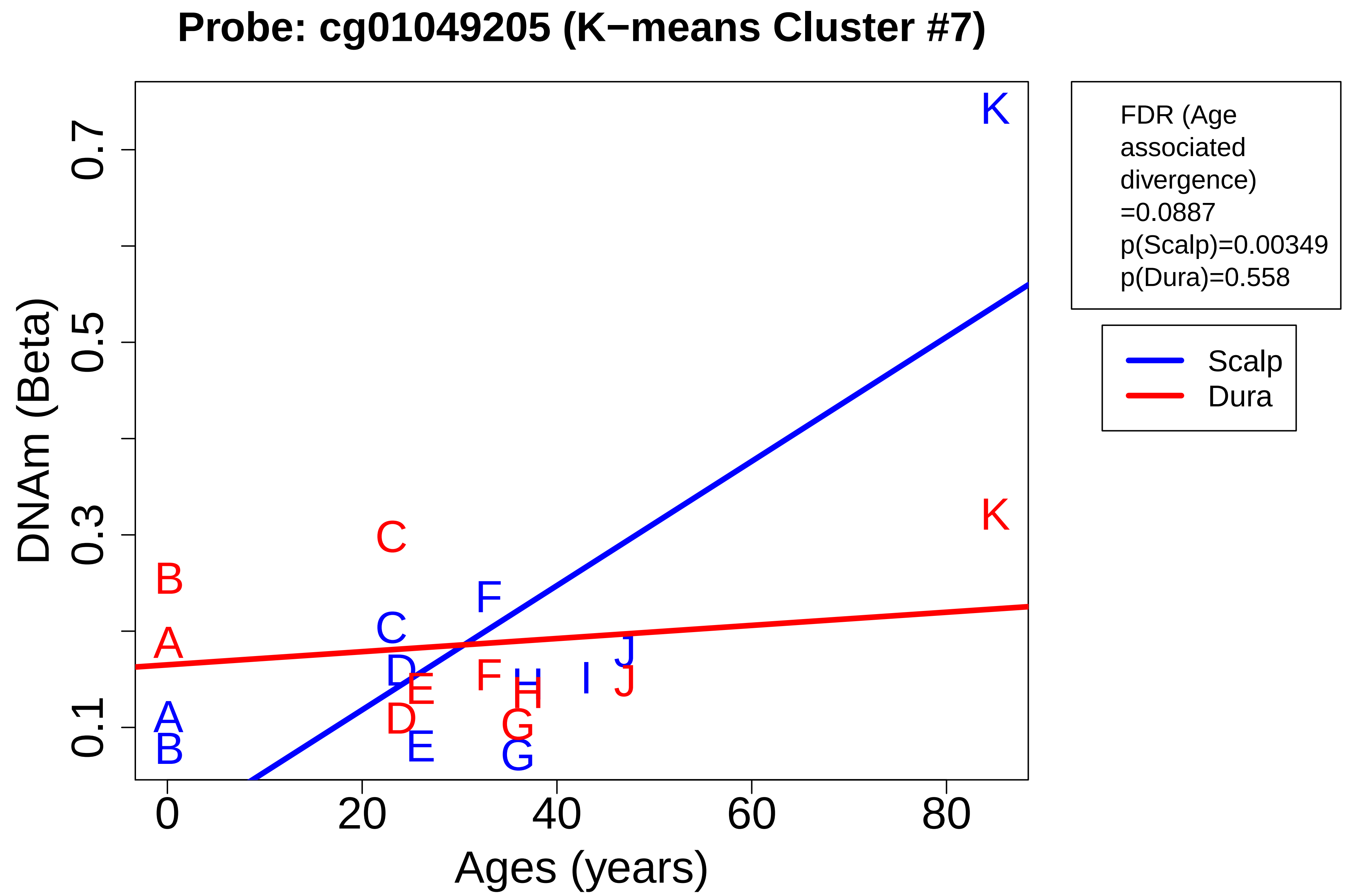

Probe: cg01049205 (K−means Cluster #7)
DNAm (Beta)
0.1
0.3
0.5
0.7
0
20
40
60
80
Ages (years)
A
B
C
D
E
F
G
H
I
J
K
FDR (Age associated divergence) =0.0887
p(Scalp)=0.00349
p(Dura)=0.558
Scalp
Dura

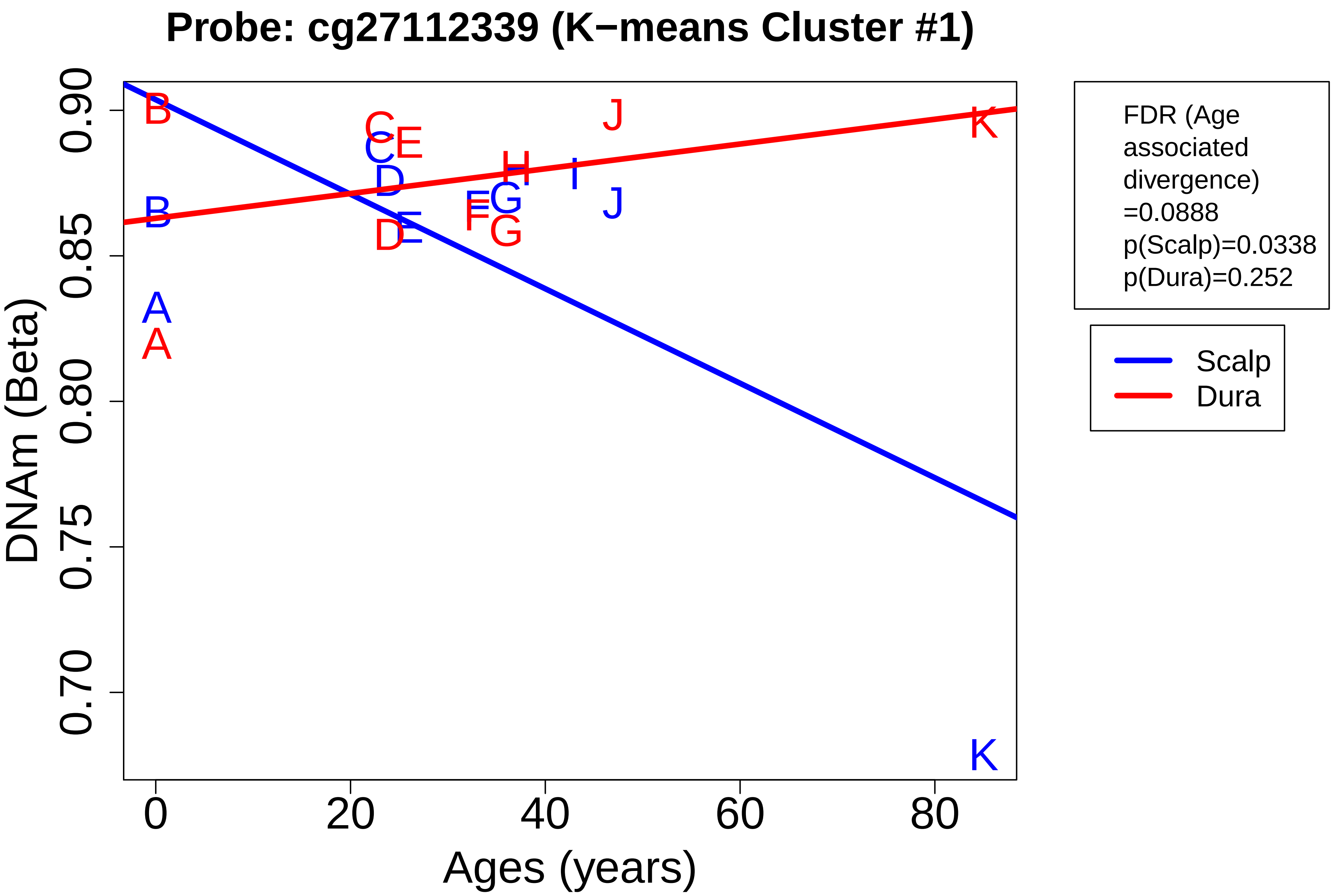

Probe: cg27112339 (K−means Cluster #1)
DNAm (Beta)
0.70
0.75
0.80
0.85
0.90
Ages (years)
0
20
40
60
80
FDR (Age associated divergence) =0.0888
p(Scalp)=0.0338
p(Dura)=0.252
Scalp
Dura

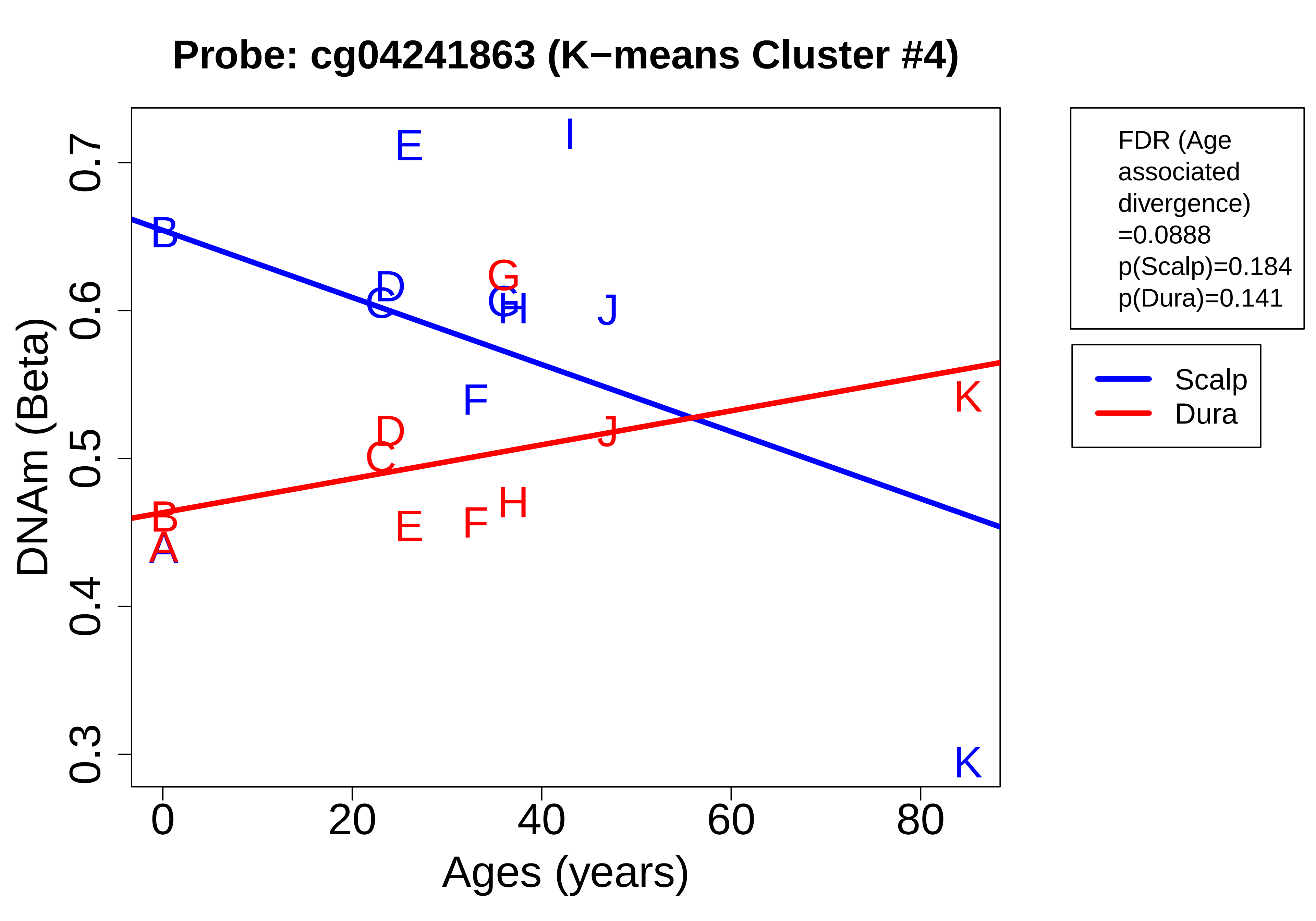

Probe: cg04241863 (K−means Cluster #4)
DNAm (Beta)
Ages (years)
0.3
0.4
0.5
0.6
0.7
0
20
40
60
80
FDR (Age associated divergence) =0.0888
p(Scalp)=0.184
p(Dura)=0.141
Scalp
Dura
A
B
C
D
E
F
G
H
I
J
K

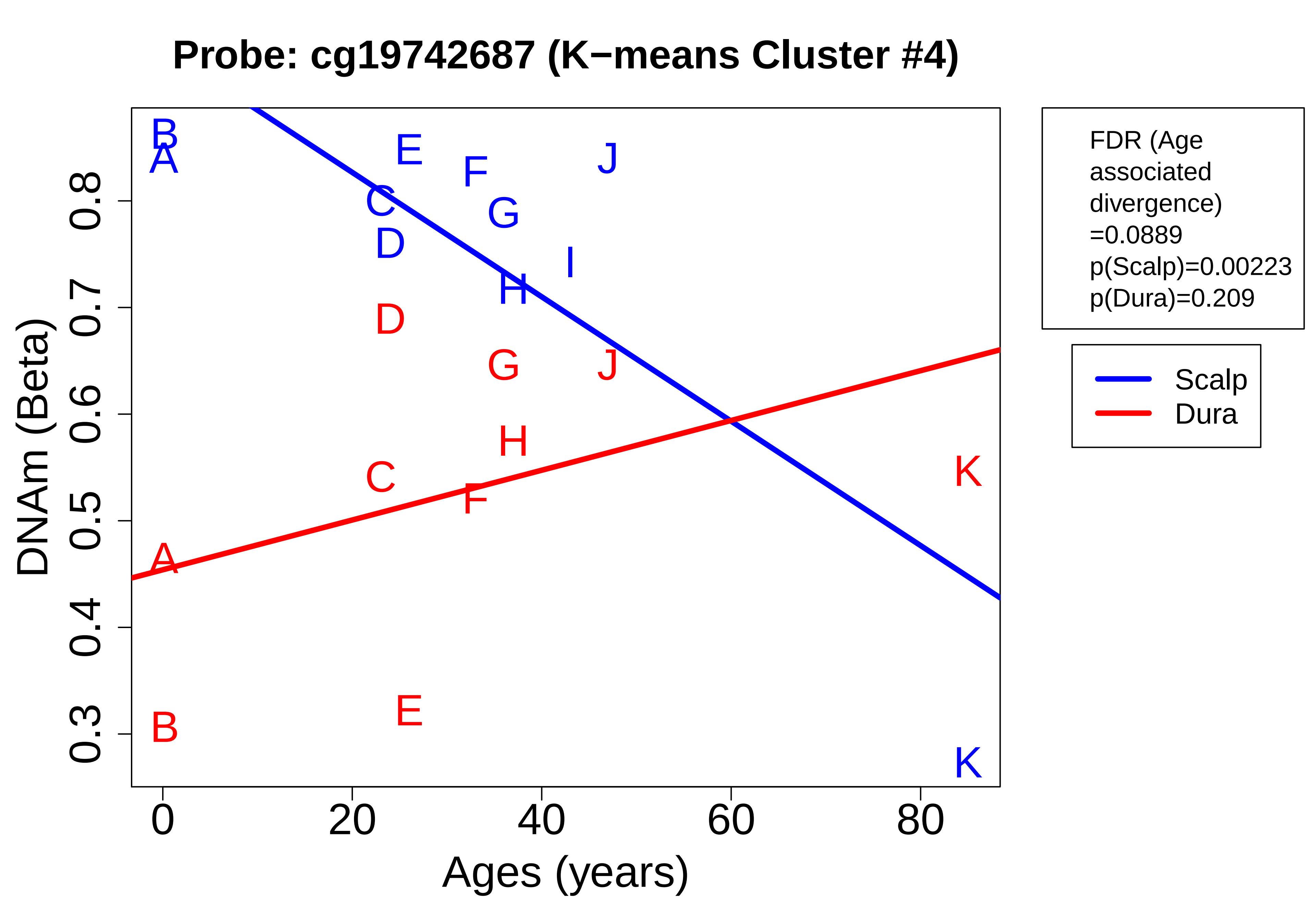
Probe: cg19742687 (K−means Cluster #4)
DNAm (Beta)
Ages (years)
0.3
0.4
0.5
0.6
0.7
0.8
0
20
40
60
80
FDR (Age associated divergence) =0.0889
p(Scalp)=0.00223
p(Dura)=0.209
Scalp
Dura

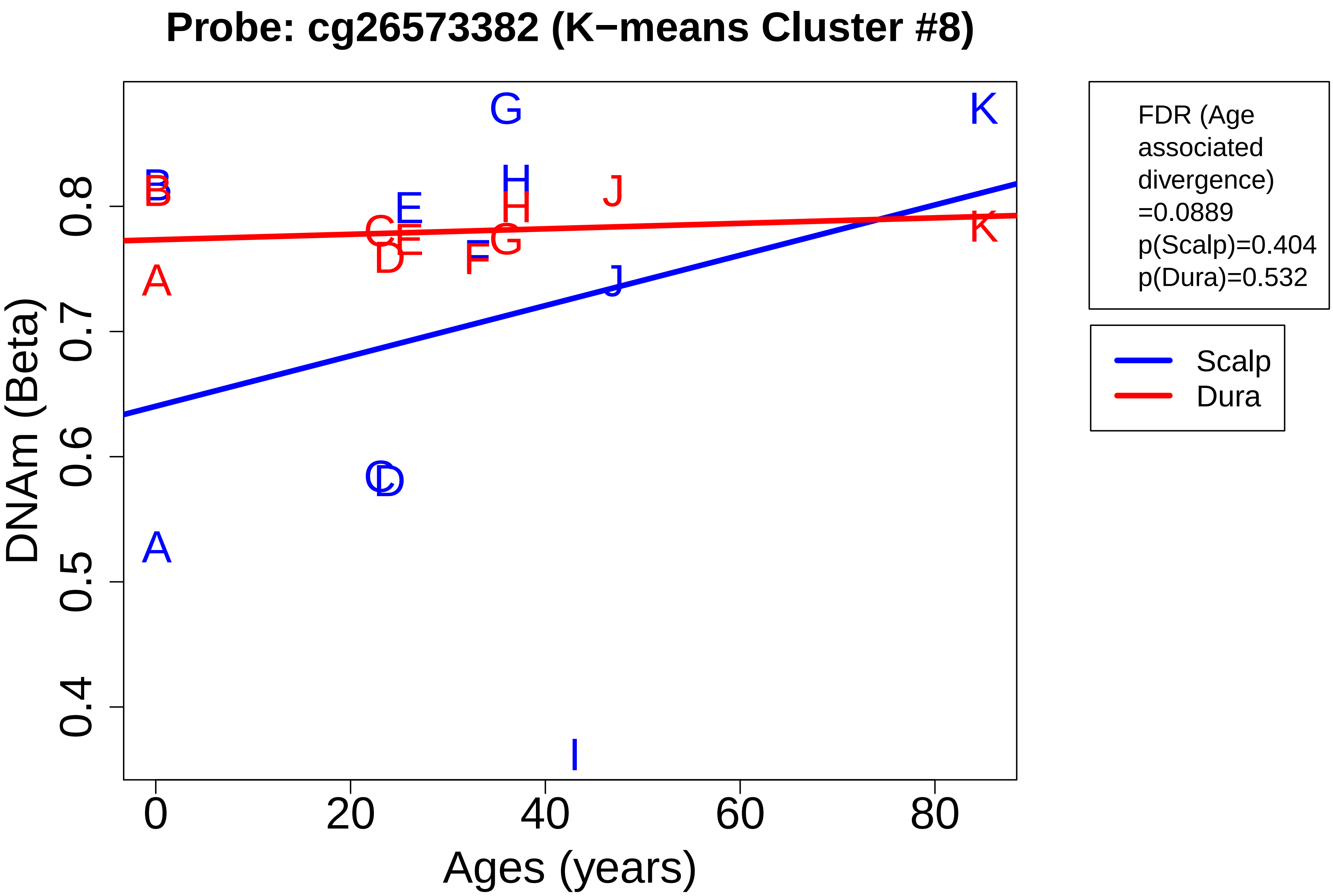

Probe: cg26573382 (K−means Cluster #8)
DNAm (Beta)
Ages (years)
0.4
0.5
0.6
0.7
0.8
0
20
40
60
80
FDR (Age associated divergence) =0.0889
p(Scalp)=0.404
p(Dura)=0.532
Scalp
Dura

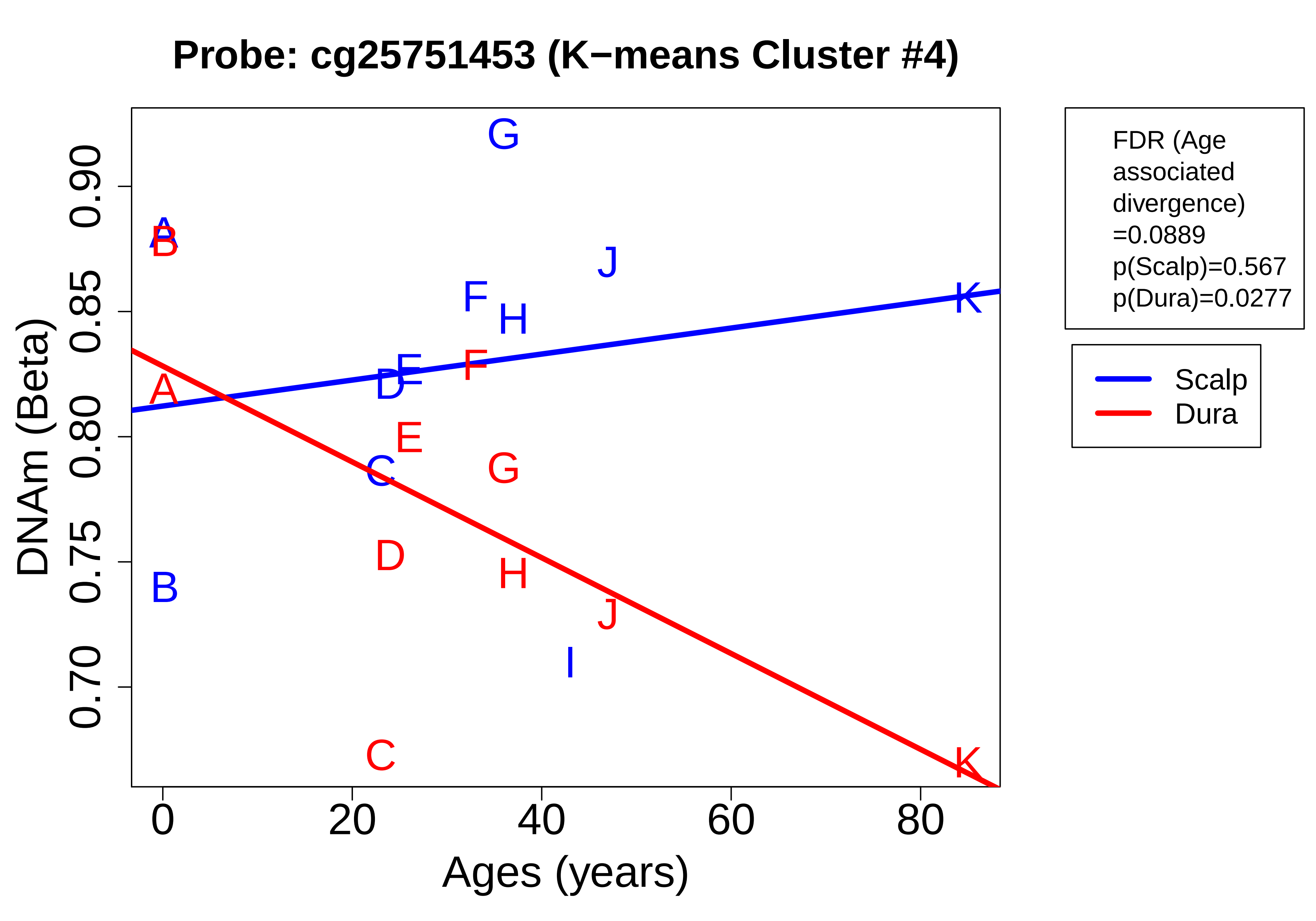
Probe: cg25751453 (K−means Cluster #4)
DNAm (Beta)
0.70
0.75
0.80
0.85
0.90
0
20
40
60
80
Ages (years)
FDR (Age associated divergence) =0.0889
p(Scalp)=0.567
p(Dura)=0.0277
Scalp
Dura
A
B
C
D
E
F
G
H
I
J
K

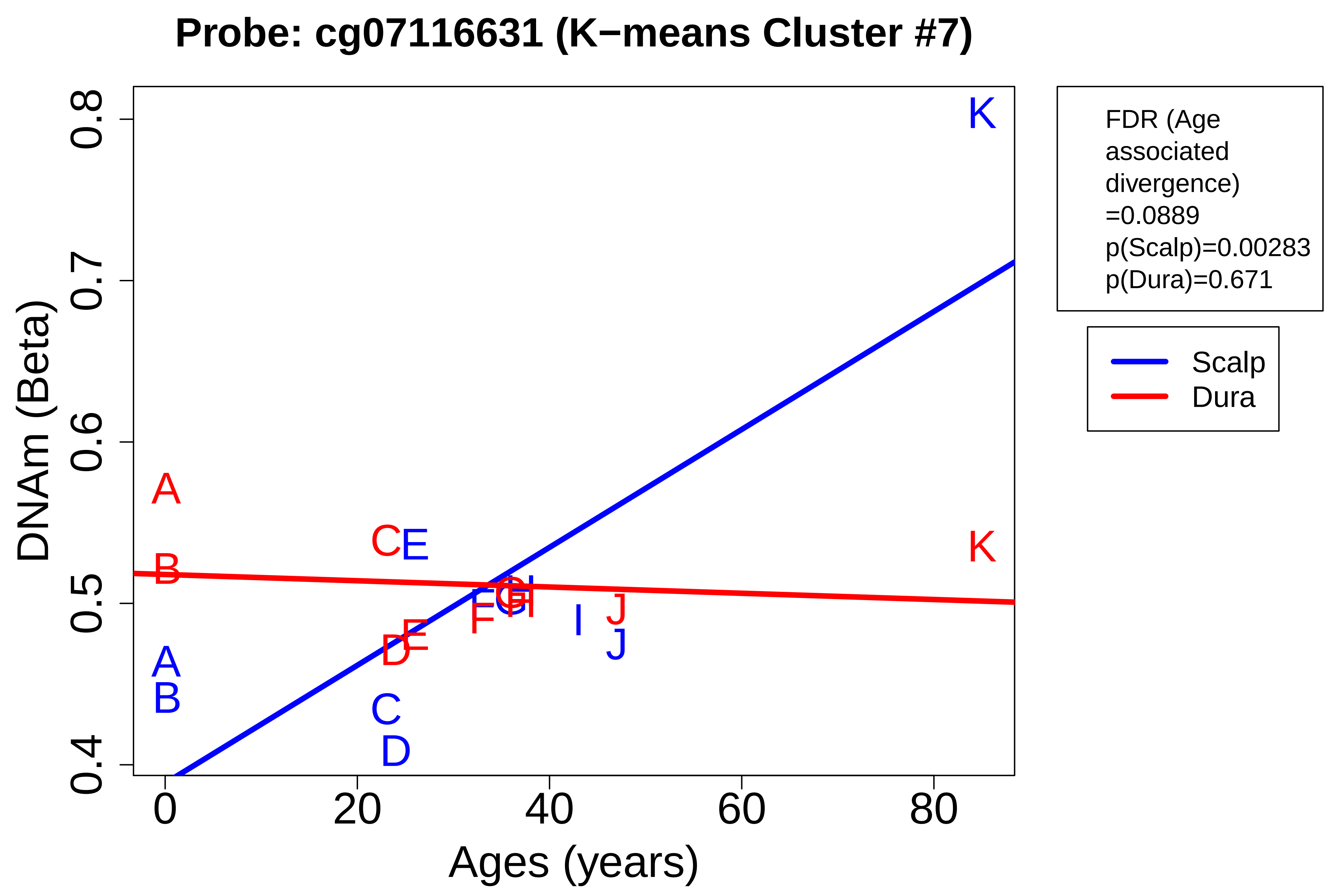

Probe: cg07116631 (K−means Cluster #7)
DNAm (Beta)
0.4
0.5
0.6
0.7
0.8
0
20
40
60
80
Ages (years)
FDR (Age associated divergence) =0.0889
p(Scalp)=0.00283
p(Dura)=0.671
Scalp
Dura

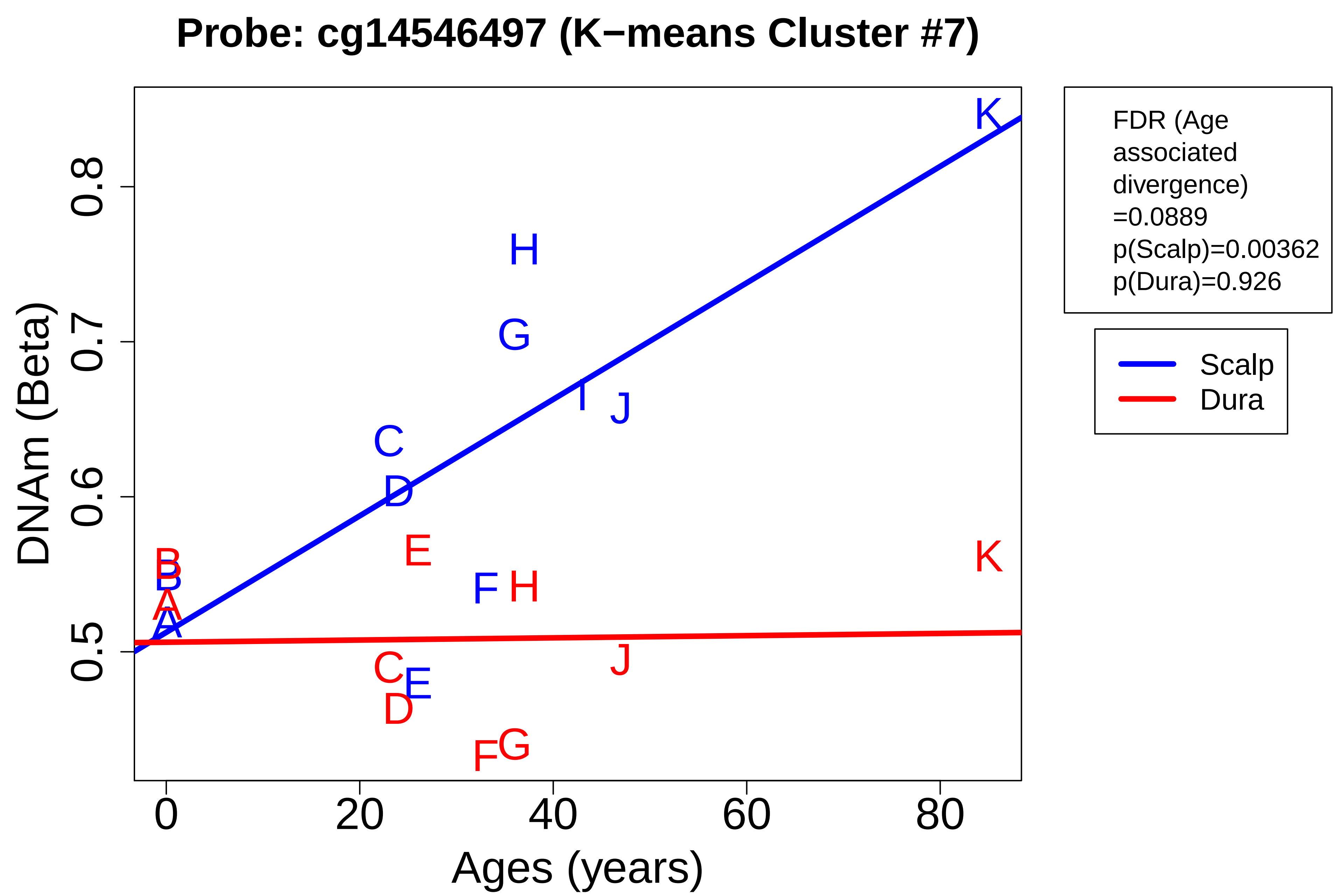
Probe: cg14546497 (K−means Cluster #7)
DNAm (Beta)
0.5
0.6
0.7
0.8
Ages (years)
0
20
40
60
80
A
B
C
D
E
F
G
H
I
J
K
FDR (Age associated divergence) =0.0889
p(Scalp)=0.00362
p(Dura)=0.926
Scalp
Dura

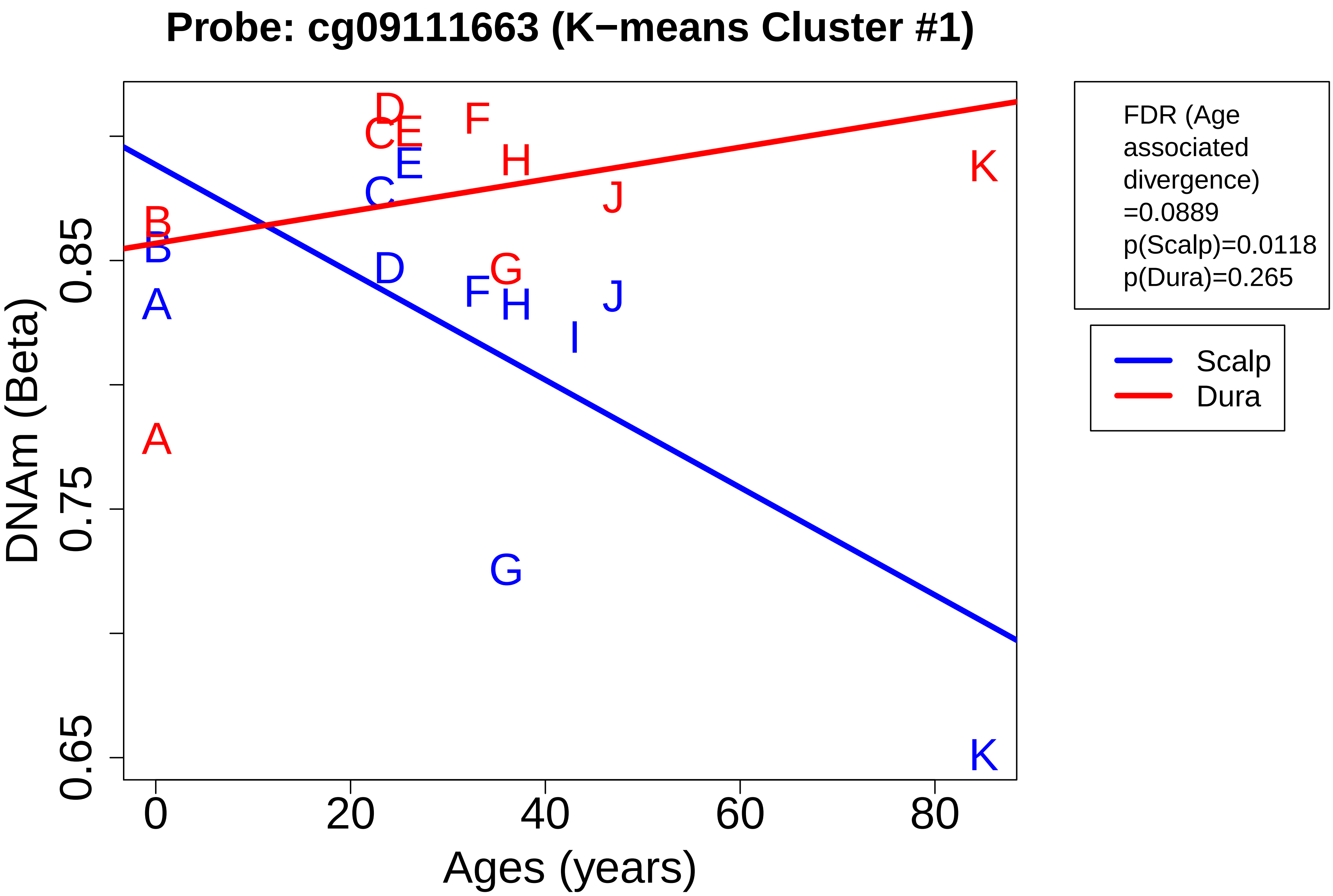
Probe: cg09111663 (K−means Cluster #1)
DNAm (Beta)
0.65
0.75
0.85
Ages (years)
0
20
40
60
80
A
B
C
D
E
F
G
H
I
J
K
FDR (Age associated divergence) =0.0889
p(Scalp)=0.0118
p(Dura)=0.265
Scalp
Dura

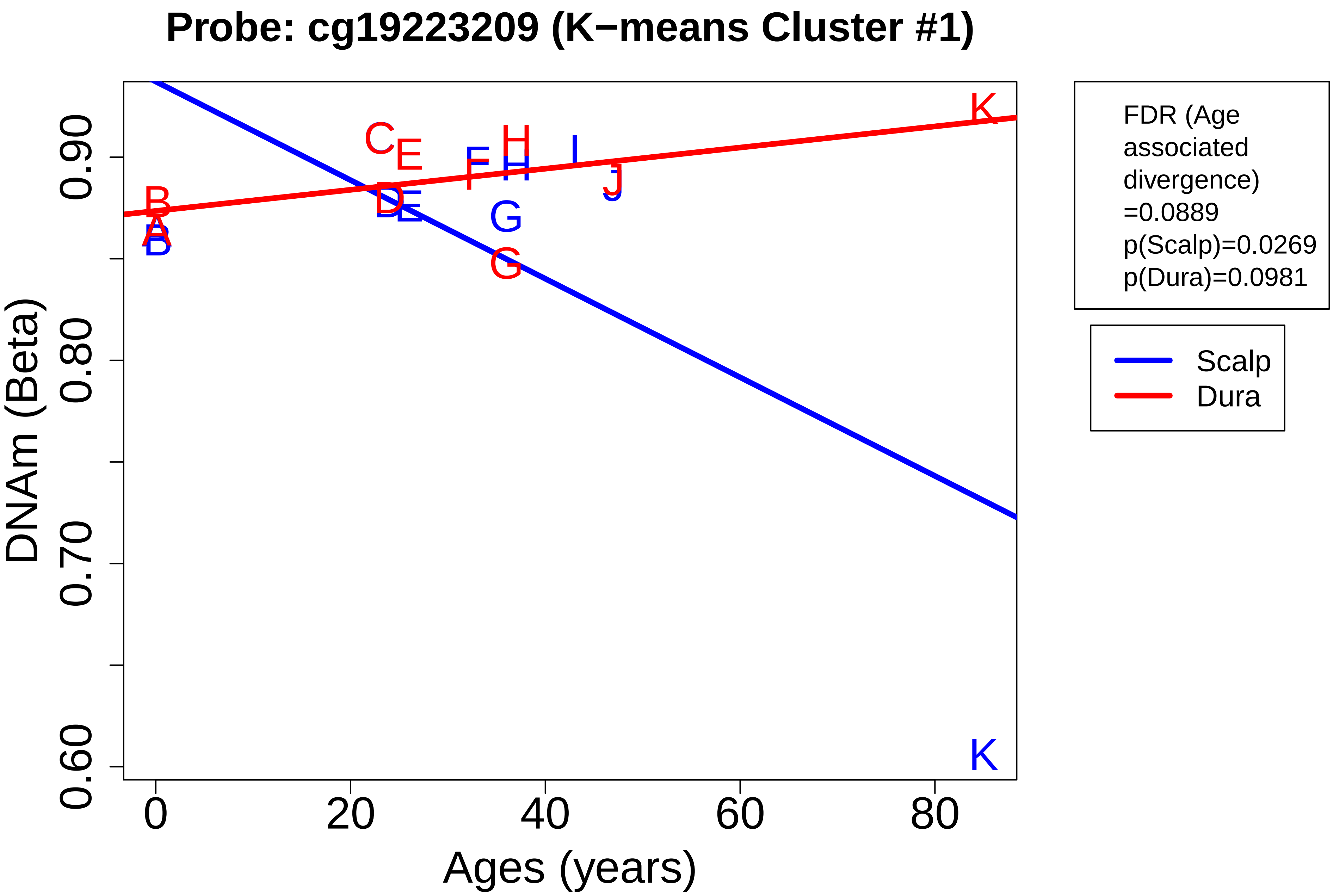

Probe: cg19223209 (K−means Cluster #1)
DNAm (Beta)
0.60
0.70
0.80
0.90
0
20
40
60
80
Ages (years)
FDR (Age associated divergence) =0.0889
p(Scalp)=0.0269
p(Dura)=0.0981
Scalp
Dura

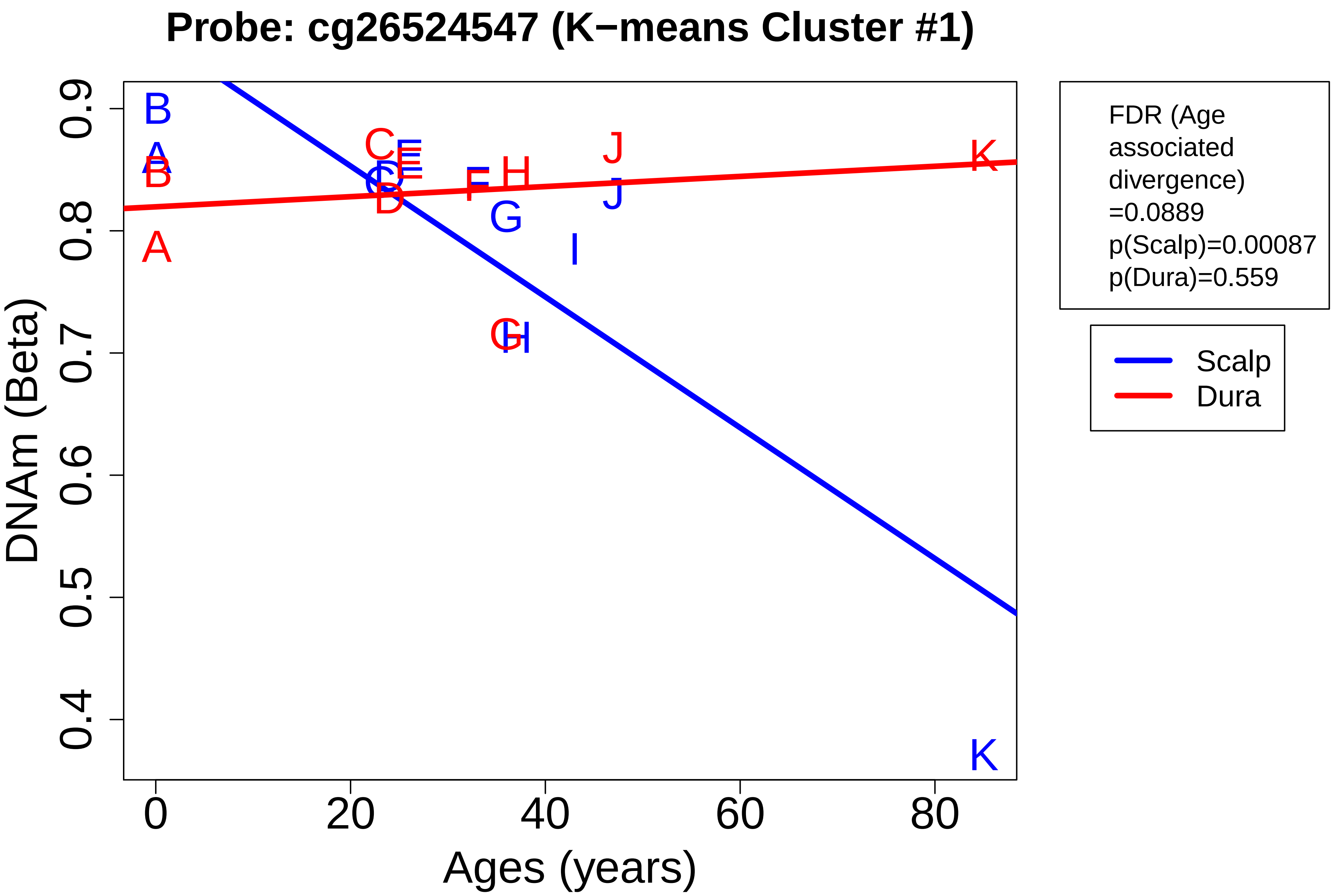

Probe: cg26524547 (K−means Cluster #1)
DNAm (Beta)
0.4
0.5
0.6
0.7
0.8
0.9
0
20
40
60
80
Ages (years)
A
B
C
D
E
F
G
H
I
J
K
FDR (Age associated divergence) =0.0889
p(Scalp)=0.00087
p(Dura)=0.559
Scalp
Dura

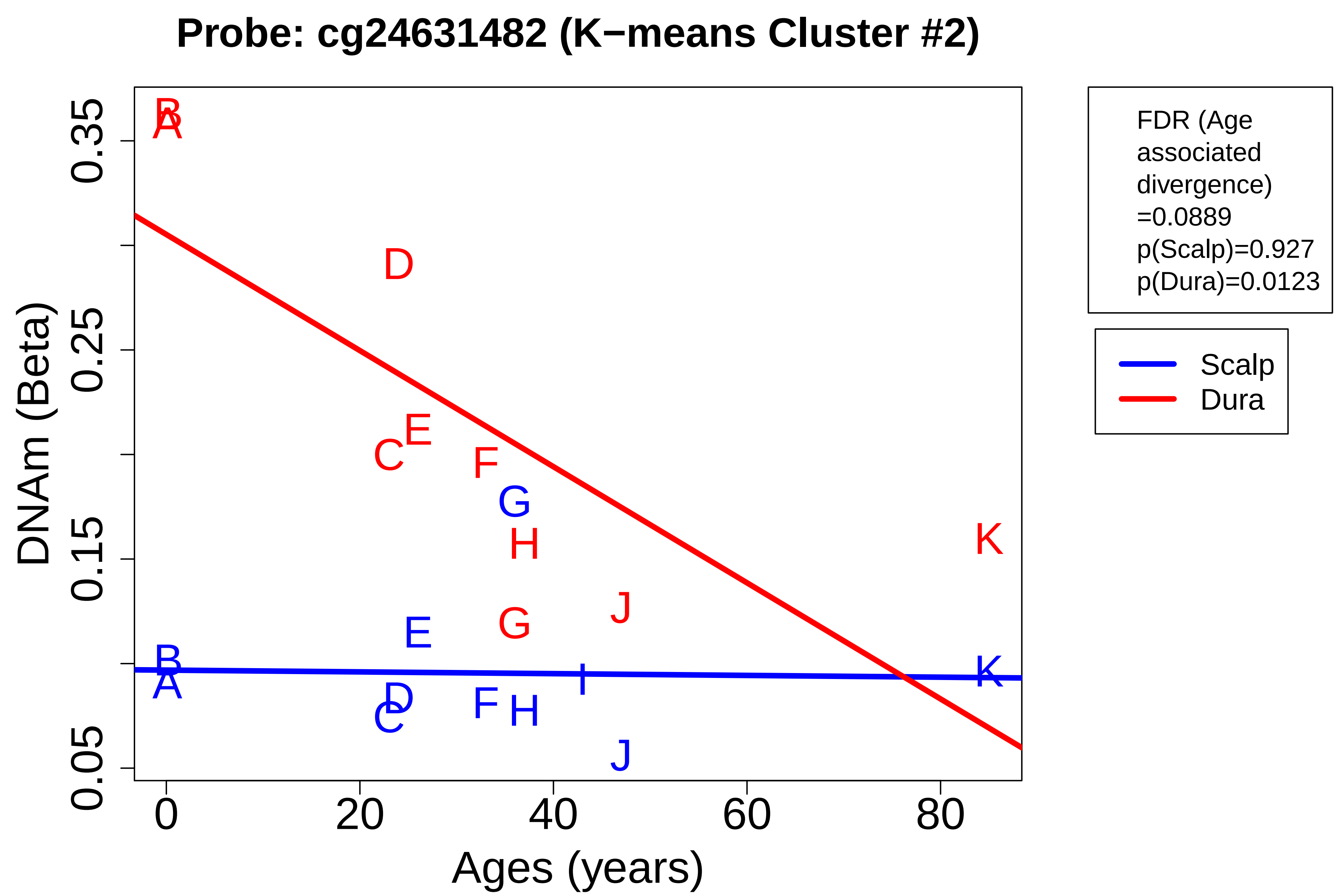
Probe: cg24631482 (K−means Cluster #2)
DNAm (Beta)
0.05
0.15
0.25
0.35
Ages (years)
0
20
40
60
80
A
B
C
D
E
F
G
H
I
J
K
FDR (Age associated divergence) =0.0889
p(Scalp)=0.927
p(Dura)=0.0123
Scalp
Dura

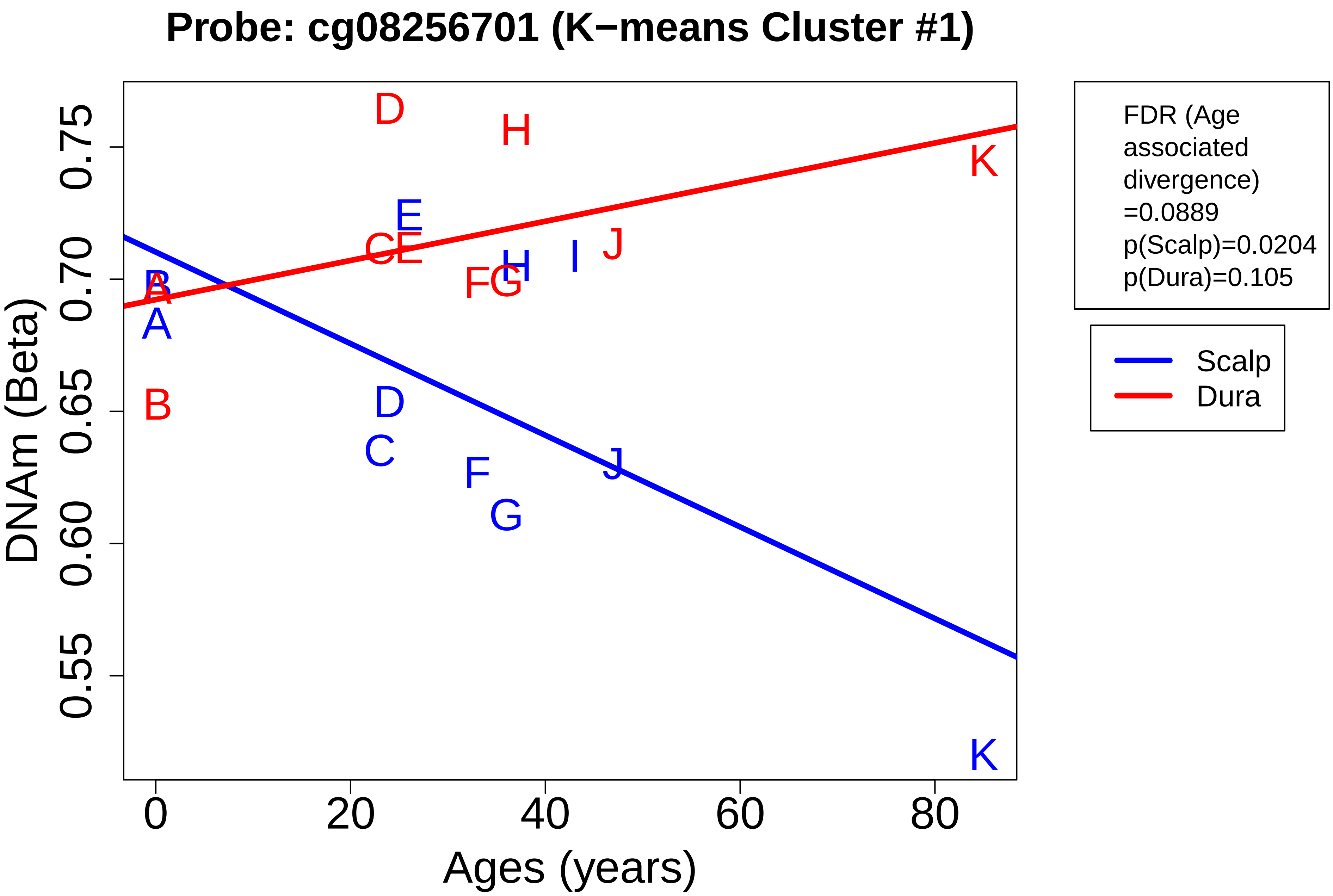

Probe: cg08256701 (K−means Cluster #1)
DNAm (Beta)
0.55
0.60
0.65
0.70
0.75
0
20
40
60
80
Ages (years)
FDR (Age associated divergence) =0.0889
p(Scalp)=0.0204
p(Dura)=0.105
Scalp
Dura

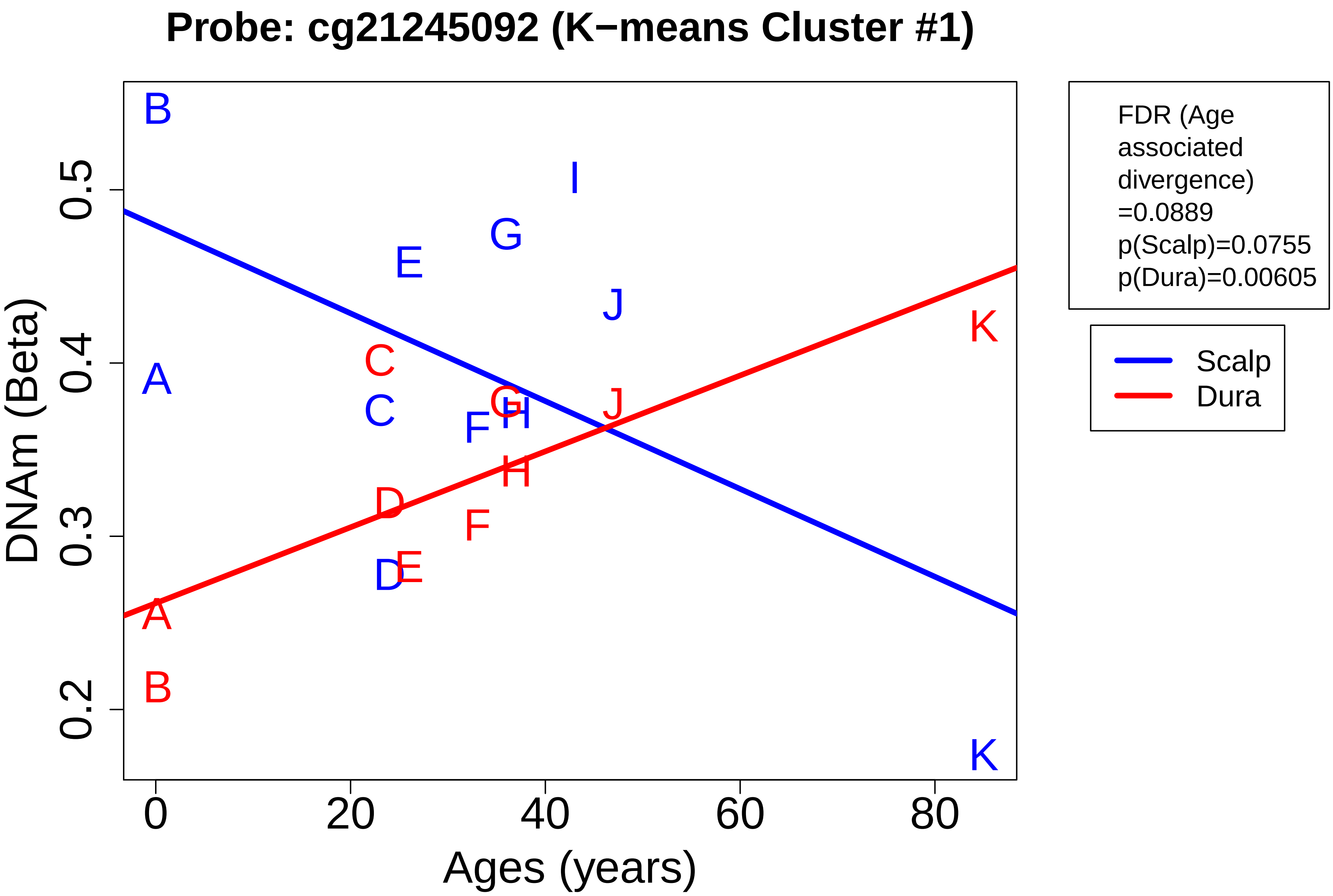

Probe: cg21245092 (K−means Cluster #1)
DNAm (Beta)
0.2
0.3
0.4
0.5
0
20
40
60
80
Ages (years)
FDR (Age associated divergence) =0.0889
p(Scalp)=0.0755
p(Dura)=0.00605
Scalp
Dura

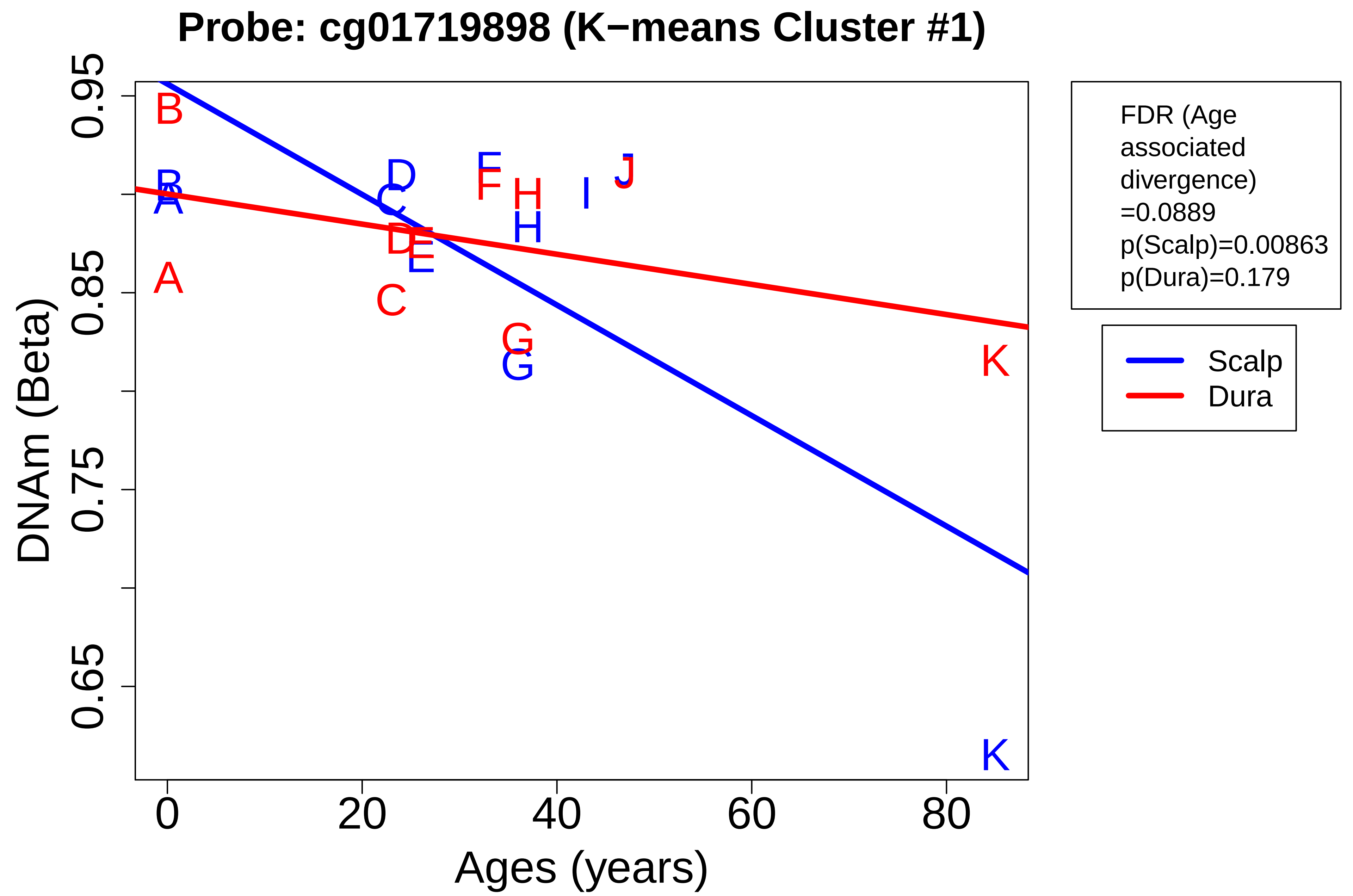

Probe: cg01719898 (K−means Cluster #1)
DNAm (Beta)
0.65
0.75
0.85
0.95
Ages (years)
0
20
40
60
80
A
B
C
D
E
F
G
H
I
J
K
FDR (Age associated divergence) =0.0889
p(Scalp)=0.00863
p(Dura)=0.179
Scalp
Dura

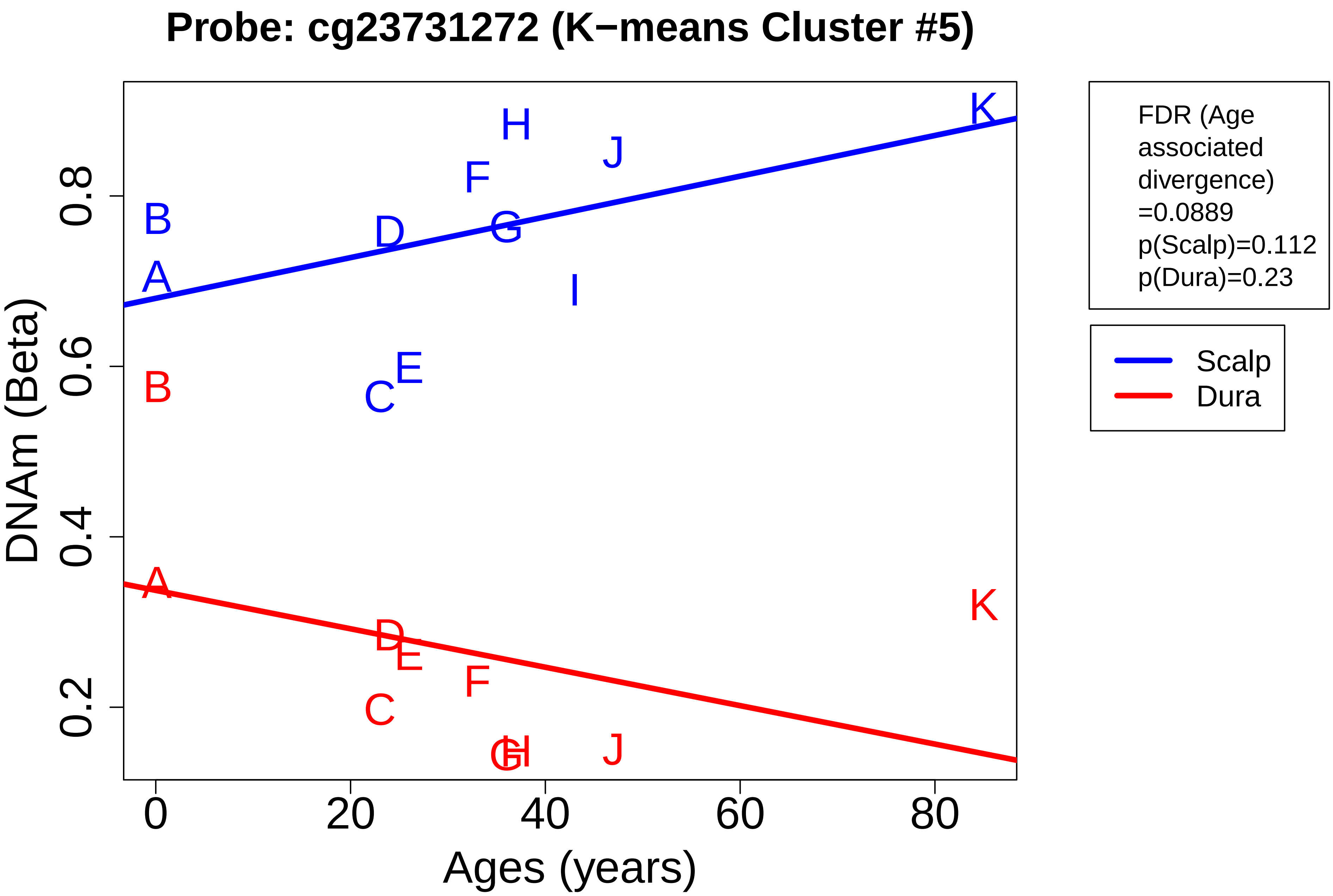

Probe: cg23731272 (K−means Cluster #5)
DNAm (Beta)
0.2
0.4
0.6
0.8
0
20
40
60
80
Ages (years)
A
B
C
D
E
F
G
H
I
J
K
FDR (Age associated divergence) =0.0889
p(Scalp)=0.112
p(Dura)=0.23
Scalp
Dura

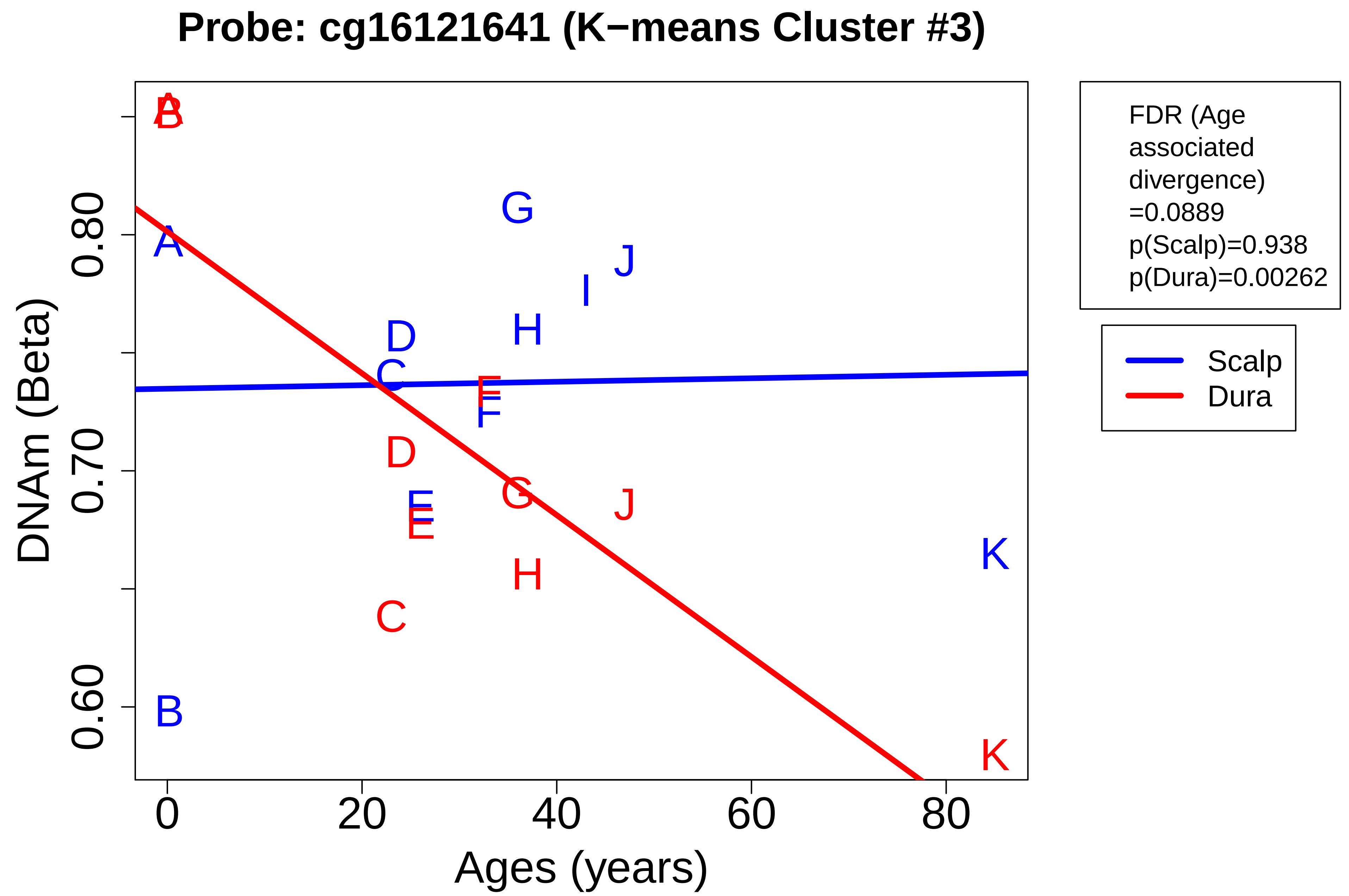

Probe: cg16121641 (K−means Cluster #3)
DNAm (Beta)
0.60
0.70
0.80
Ages (years)
0
20
40
60
80
FDR (Age associated divergence) =0.0889
p(Scalp)=0.938
p(Dura)=0.00262
Scalp
Dura

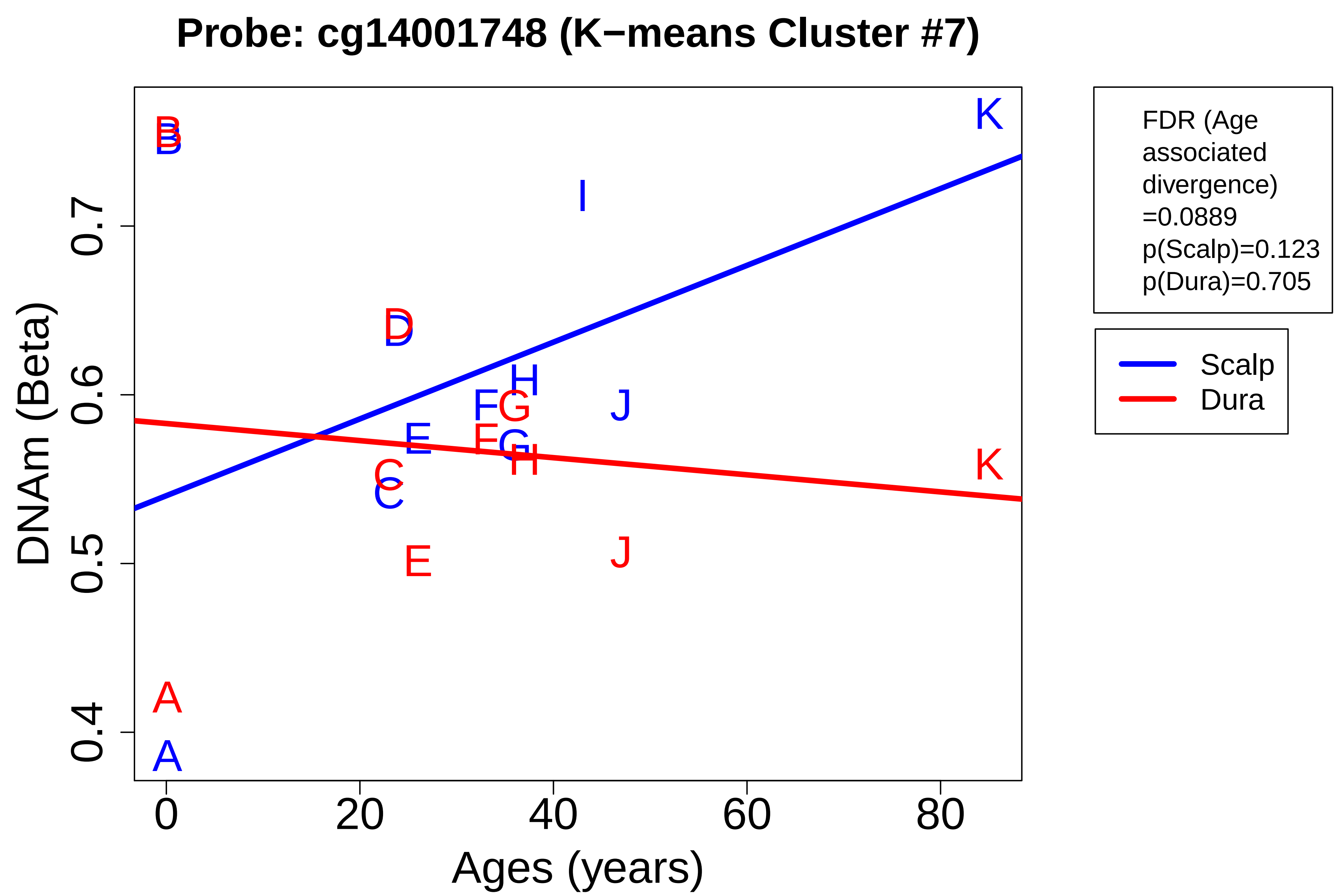

Probe: cg14001748 (K−means Cluster #7)
DNAm (Beta)
0.4
0.5
0.6
0.7
0
20
40
60
80
Ages (years)
FDR (Age associated divergence) =0.0889
p(Scalp)=0.123
p(Dura)=0.705
Scalp
Dura

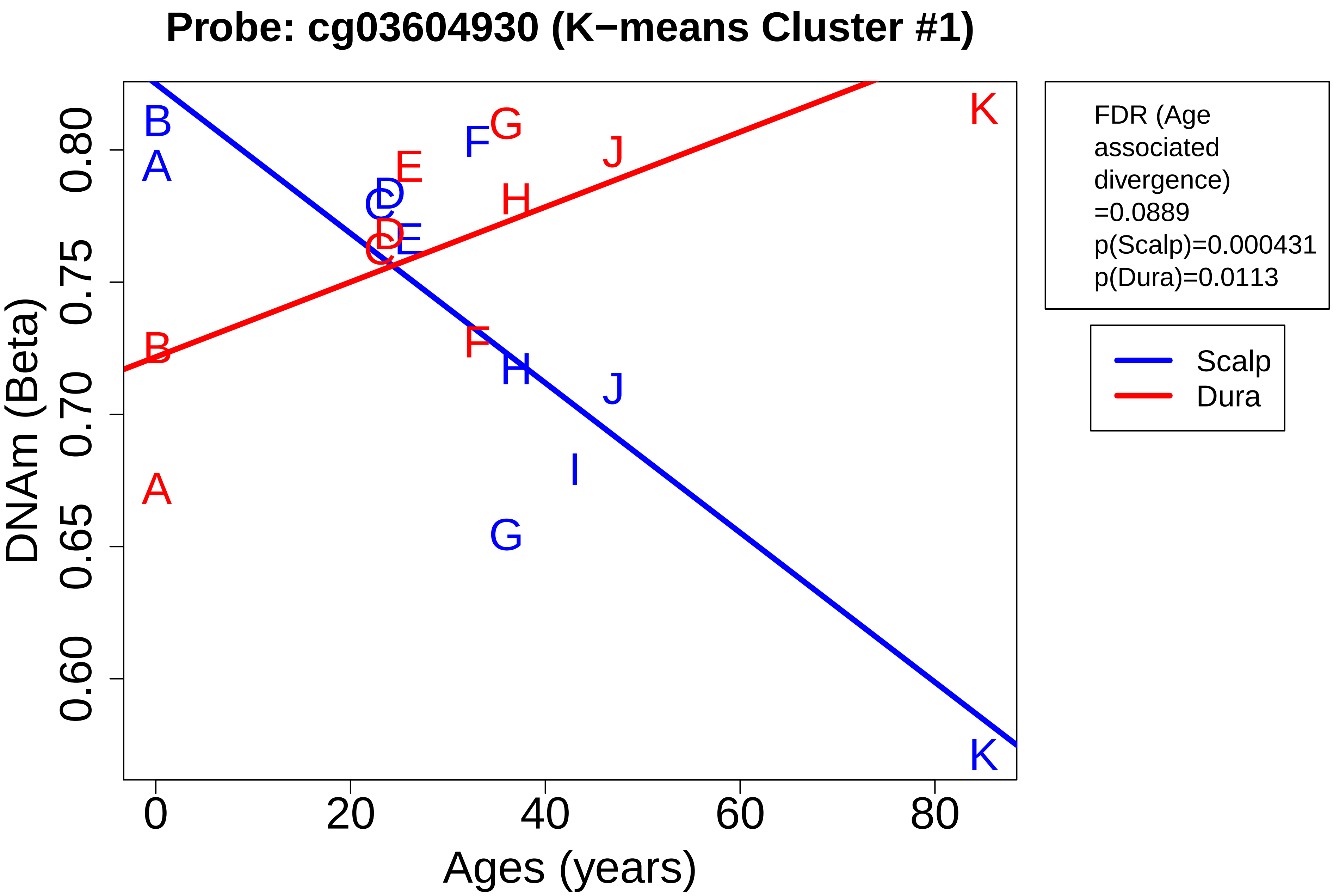

Probe: cg03604930 (K−means Cluster #1)
DNAm (Beta)
0.60
0.65
0.70
0.75
0.80
0
20
40
60
80
Ages (years)
FDR (Age associated divergence) =0.0889
p(Scalp)=0.000431
p(Dura)=0.0113
Scalp
Dura

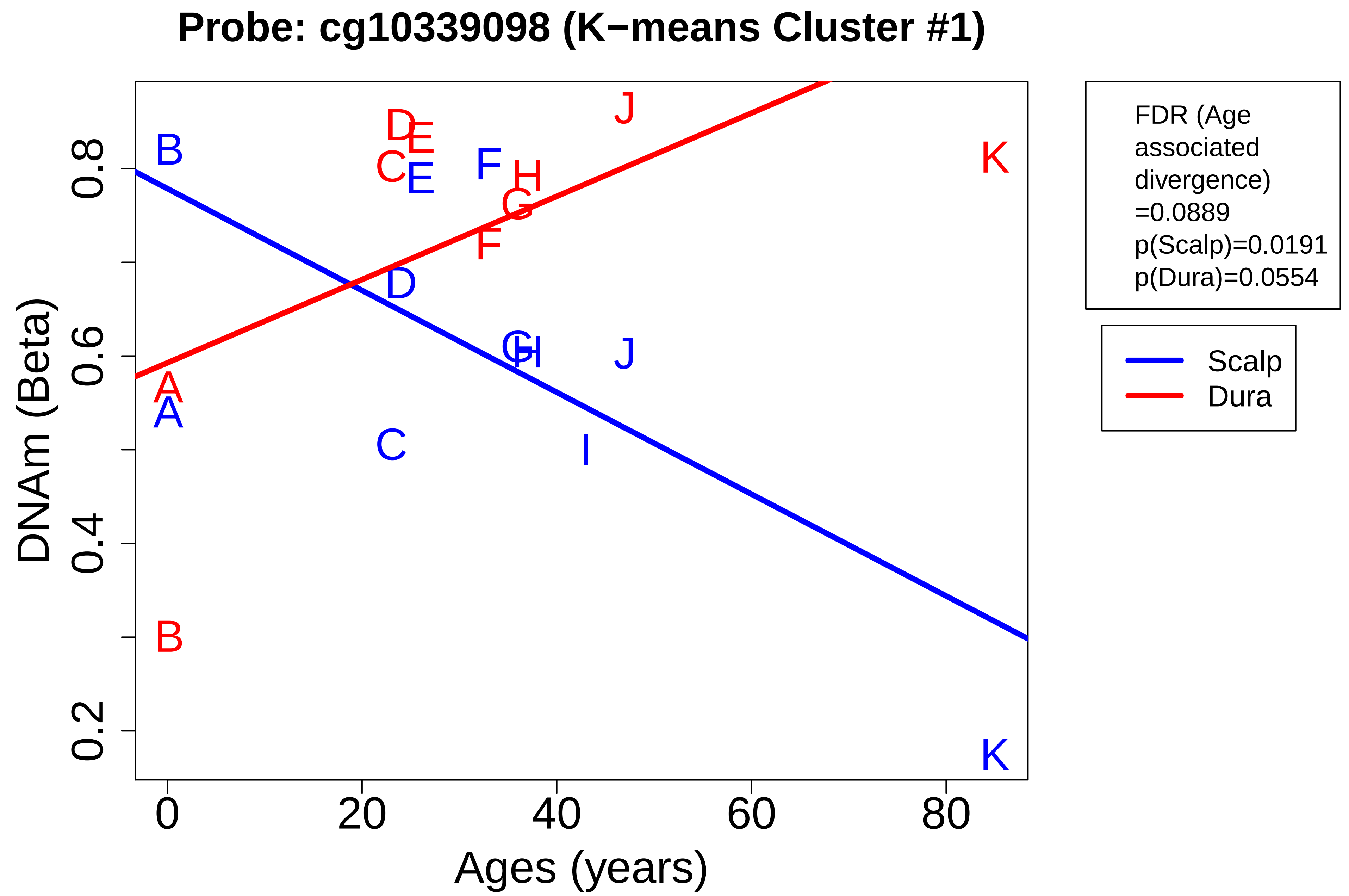

Probe: cg10339098 (K−means Cluster #1)
DNAm (Beta)
0.2
0.4
0.6
0.8
0
20
40
60
80
Ages (years)
FDR (Age associated divergence) =0.0889
p(Scalp)=0.0191
p(Dura)=0.0554
Scalp
Dura

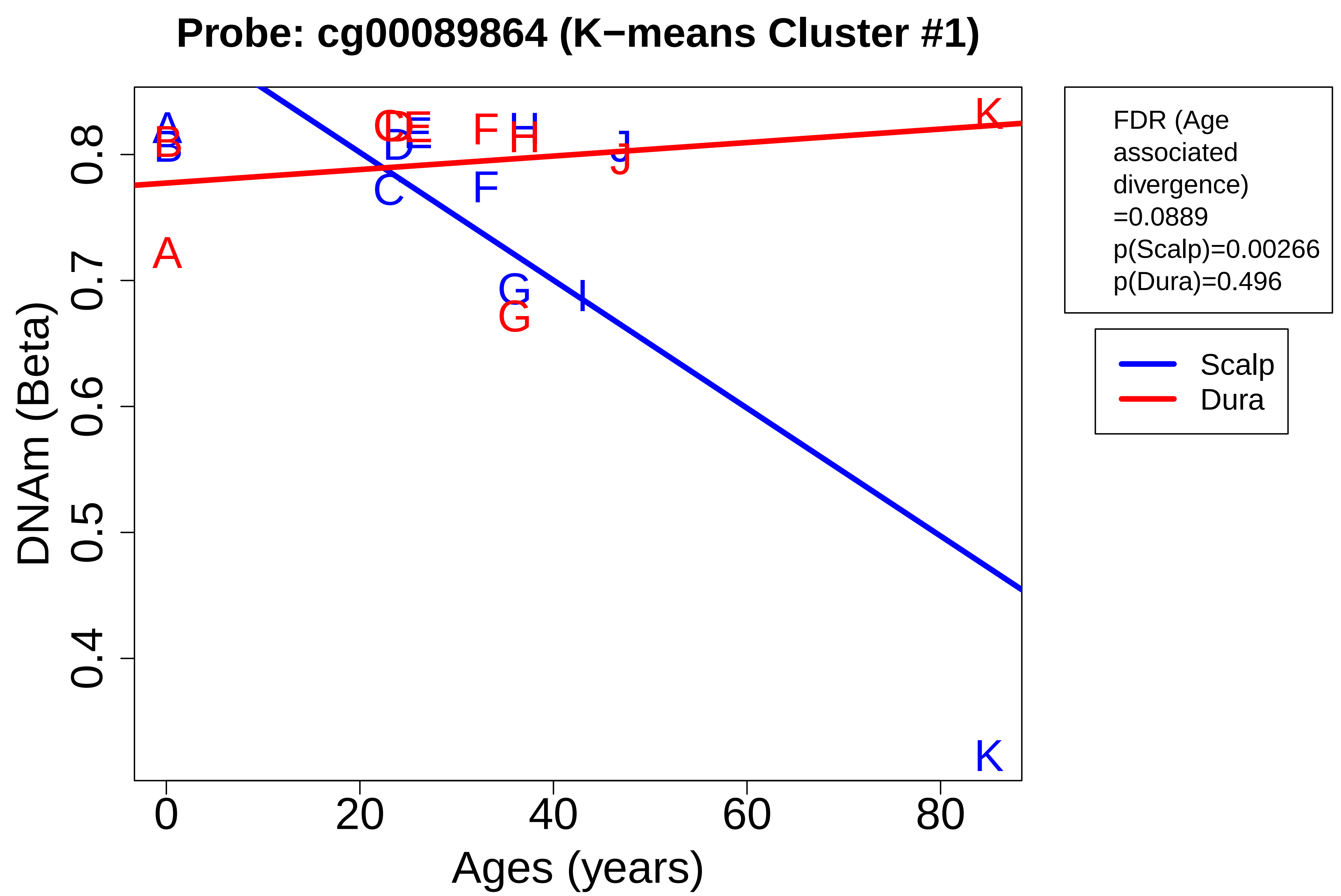

Probe: cg00089864 (K−means Cluster #1)
DNAm (Beta)
0.4
0.5
0.6
0.7
0.8
0
20
40
60
80
Ages (years)
FDR (Age associated divergence) =0.0889
p(Scalp)=0.00266
p(Dura)=0.496
Scalp
Dura

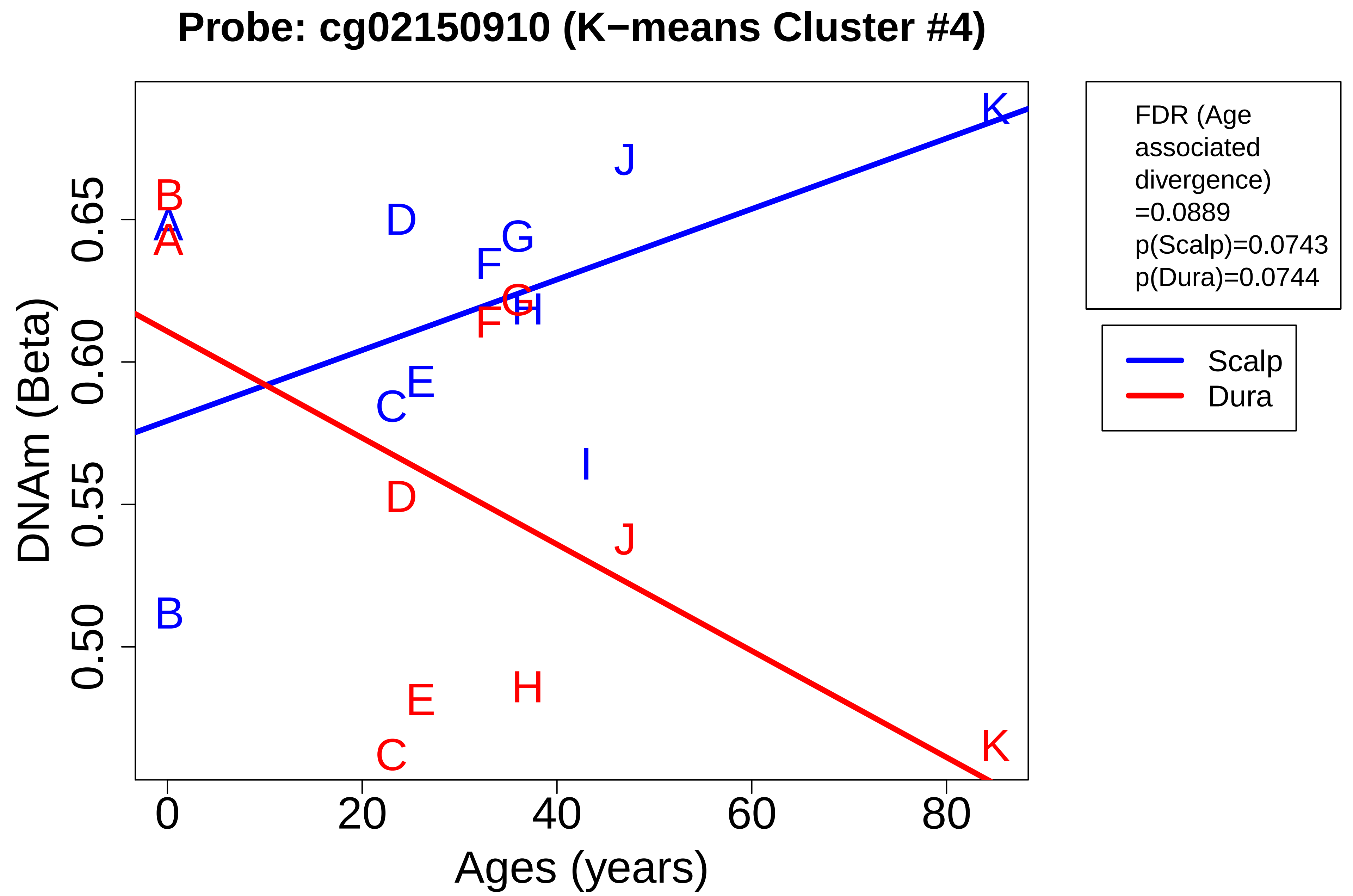

Probe: cg02150910 (K−means Cluster #4)
DNAm (Beta)
0.50
0.55
0.60
0.65
0
20
40
60
80
Ages (years)
A
B
C
D
E
F
G
H
I
J
K
FDR (Age associated divergence) =0.0889
p(Scalp)=0.0743
p(Dura)=0.0744
Scalp
Dura

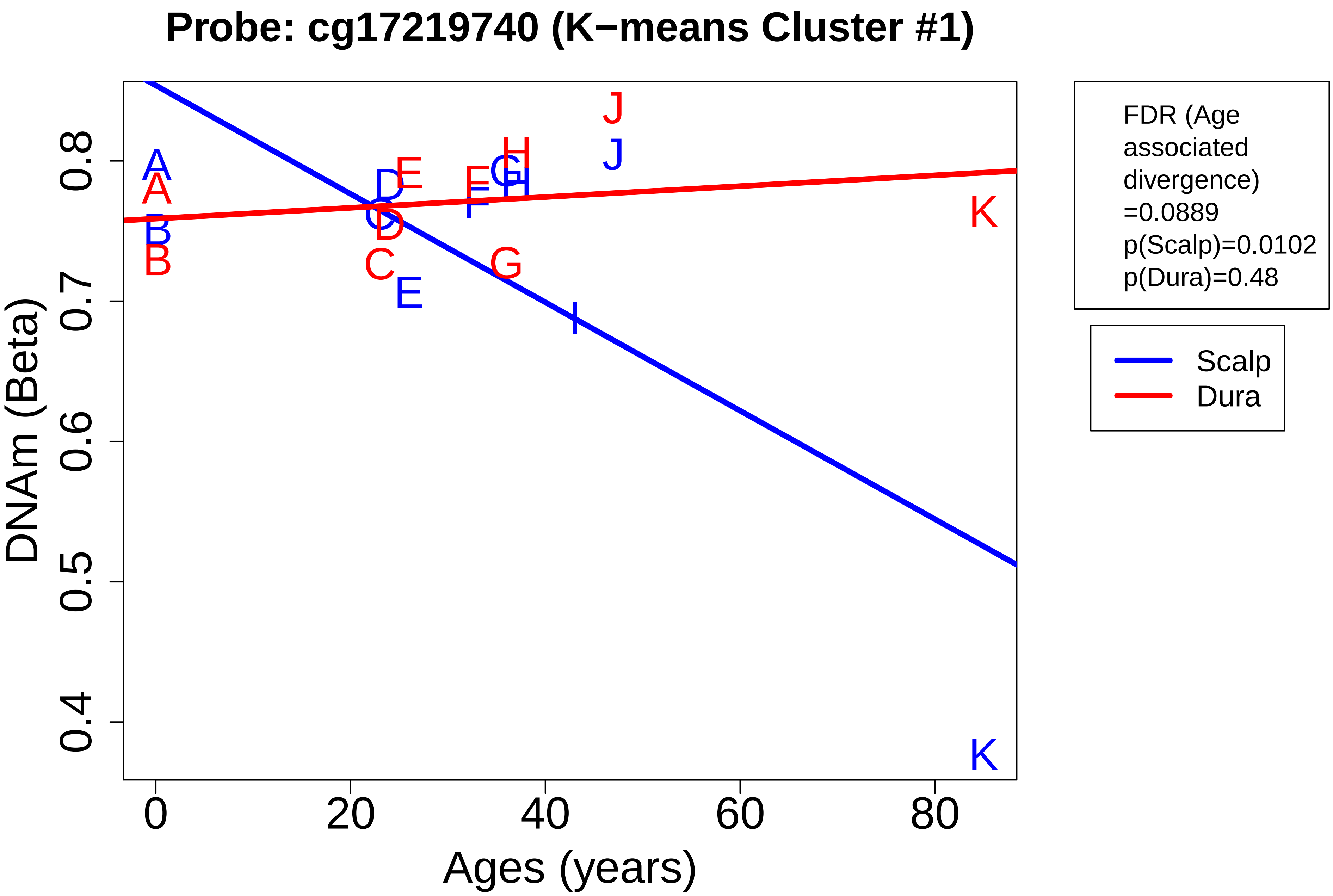

Probe: cg17219740 (K−means Cluster #1)
DNAm (Beta)
0.4
0.5
0.6
0.7
0.8
0
20
40
60
80
Ages (years)
A
B
C
D
E
F
G
H
I
J
K
FDR (Age associated divergence) =0.0889
p(Scalp)=0.0102
p(Dura)=0.48
Scalp
Dura

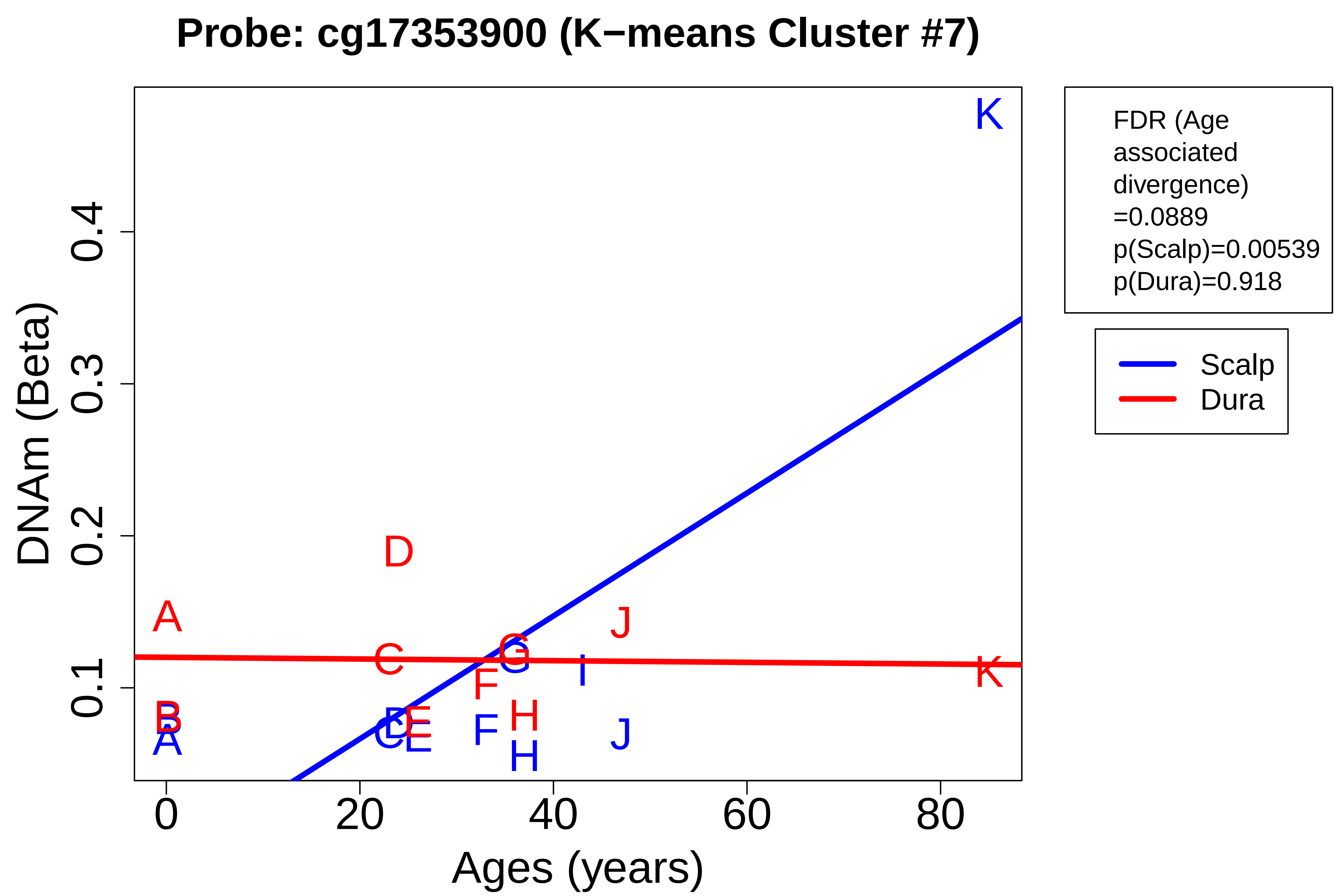

Probe: cg17353900 (K−means Cluster #7)
DNAm (Beta)
0.1
0.2
0.3
0.4
0
20
40
60
80
Ages (years)
FDR (Age associated divergence) =0.0889
p(Scalp)=0.00539
p(Dura)=0.918
Scalp
Dura

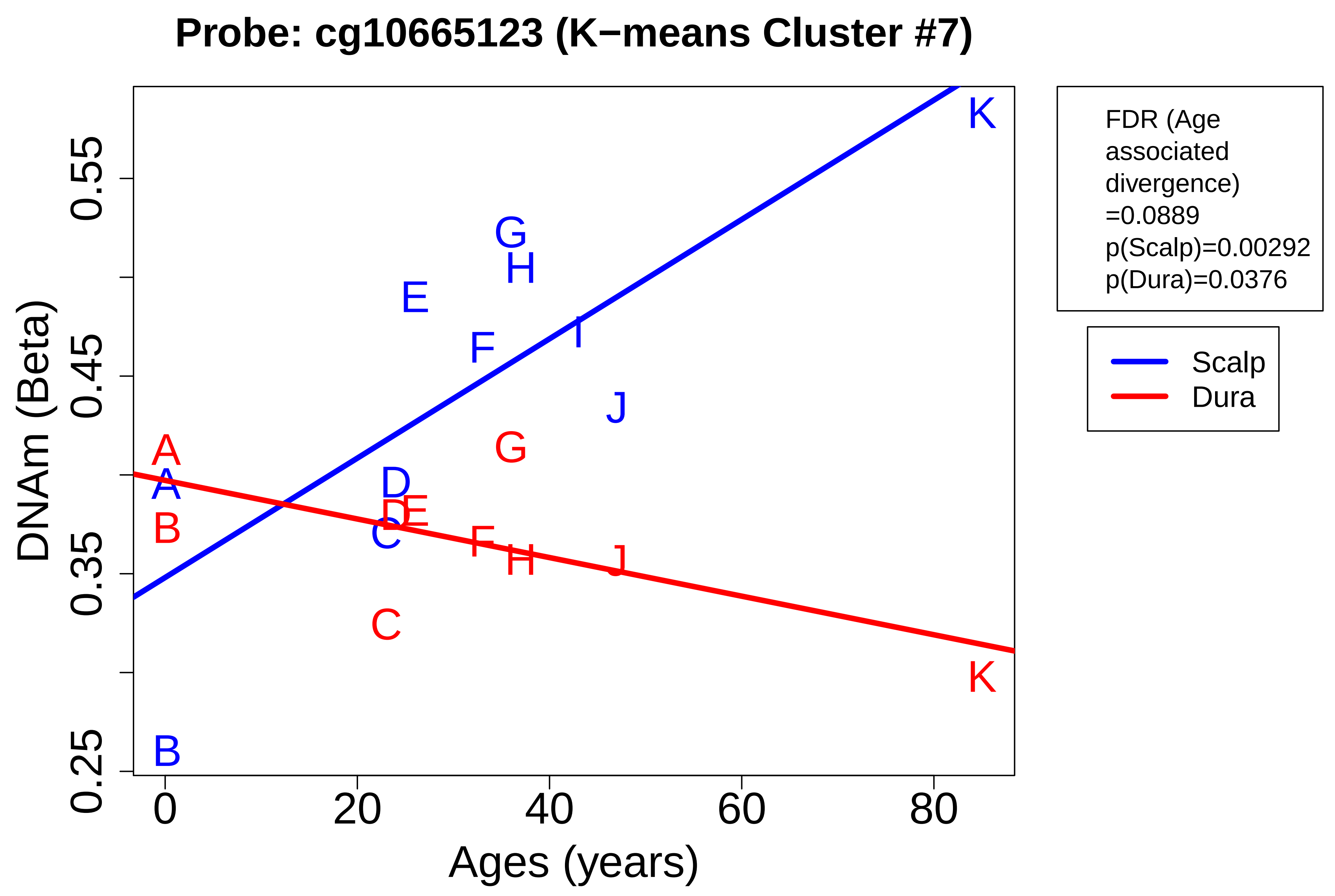

Probe: cg10665123 (K−means Cluster #7)
DNAm (Beta)
0.25
0.35
0.45
0.55
0
20
40
60
80
Ages (years)
A
B
C
D
E
F
G
H
I
J
K
FDR (Age associated divergence) =0.0889
p(Scalp)=0.00292
p(Dura)=0.0376
Scalp
Dura

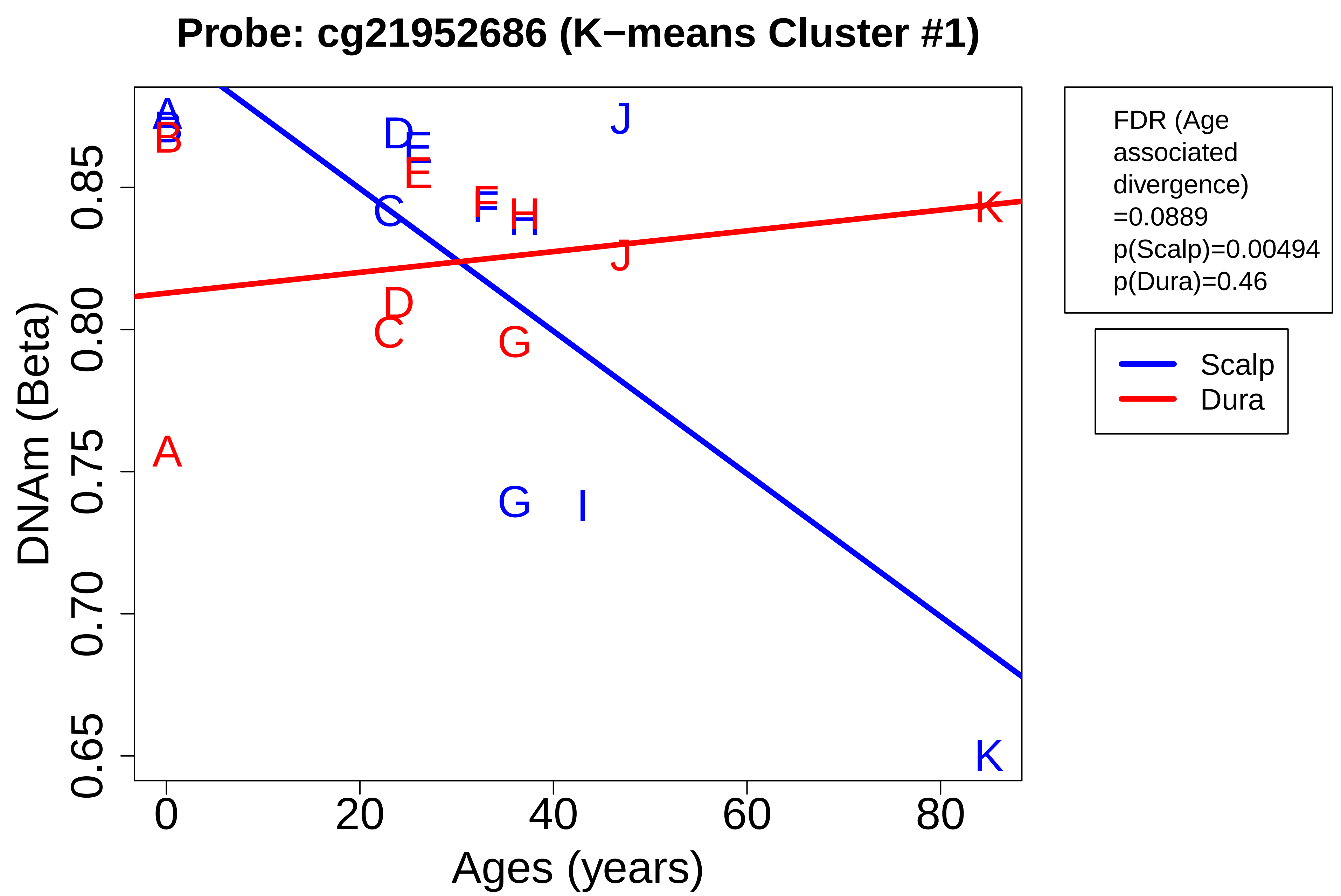

Probe: cg21952686 (K−means Cluster #1)
DNAm (Beta)
0.65
0.70
0.75
0.80
0.85
0
20
40
60
80
Ages (years)
A
B
C
D
E
F
G
H
I
J
K
FDR (Age associated divergence) =0.0889
p(Scalp)=0.00494
p(Dura)=0.46
Scalp
Dura

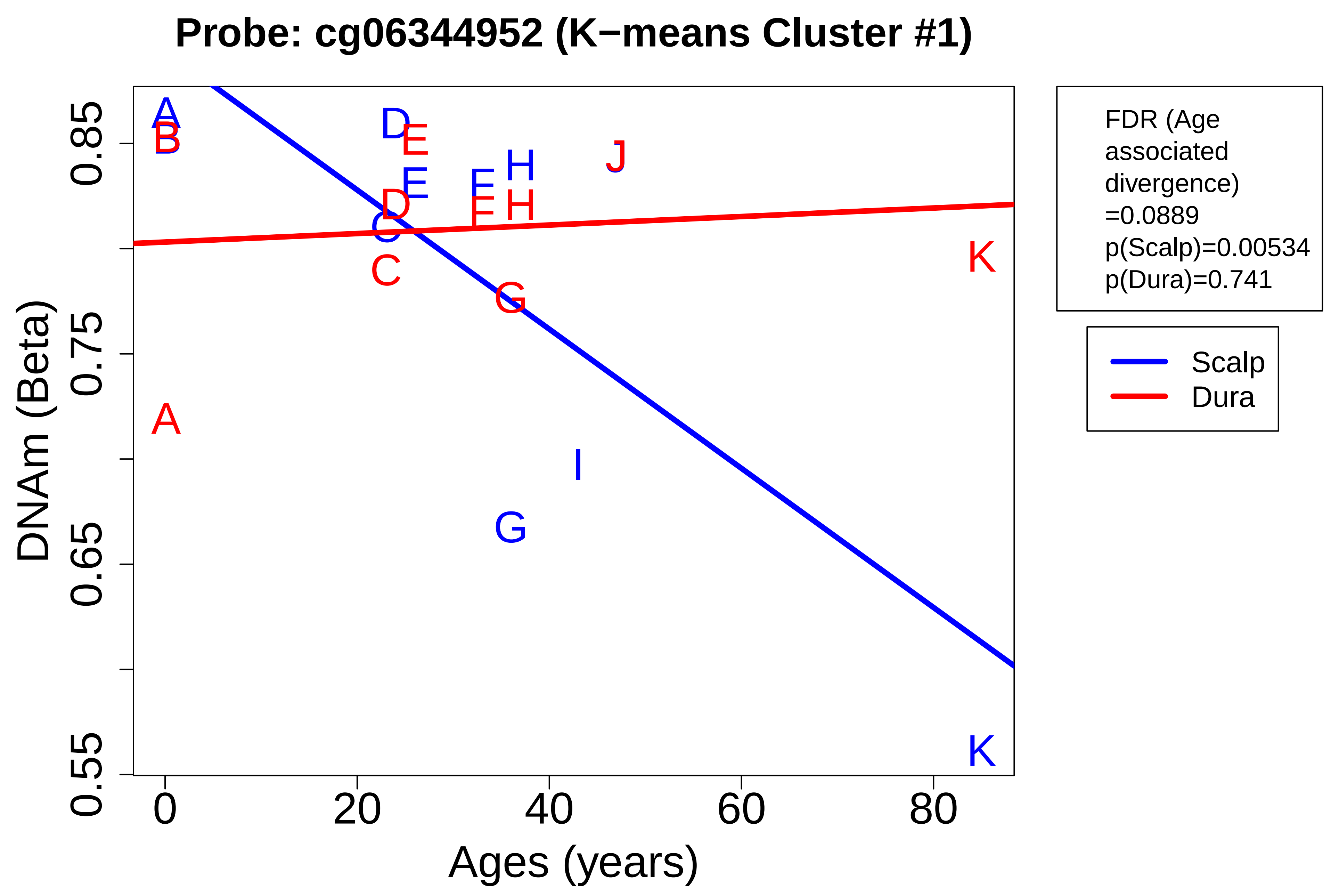

Probe: cg06344952 (K−means Cluster #1)
DNAm (Beta)
0.55
0.65
0.75
0.85
0
20
40
60
80
Ages (years)
A
B
C
D
E
F
G
H
I
J
K
FDR (Age associated divergence) =0.0889
p(Scalp)=0.00534
p(Dura)=0.741
Scalp
Dura

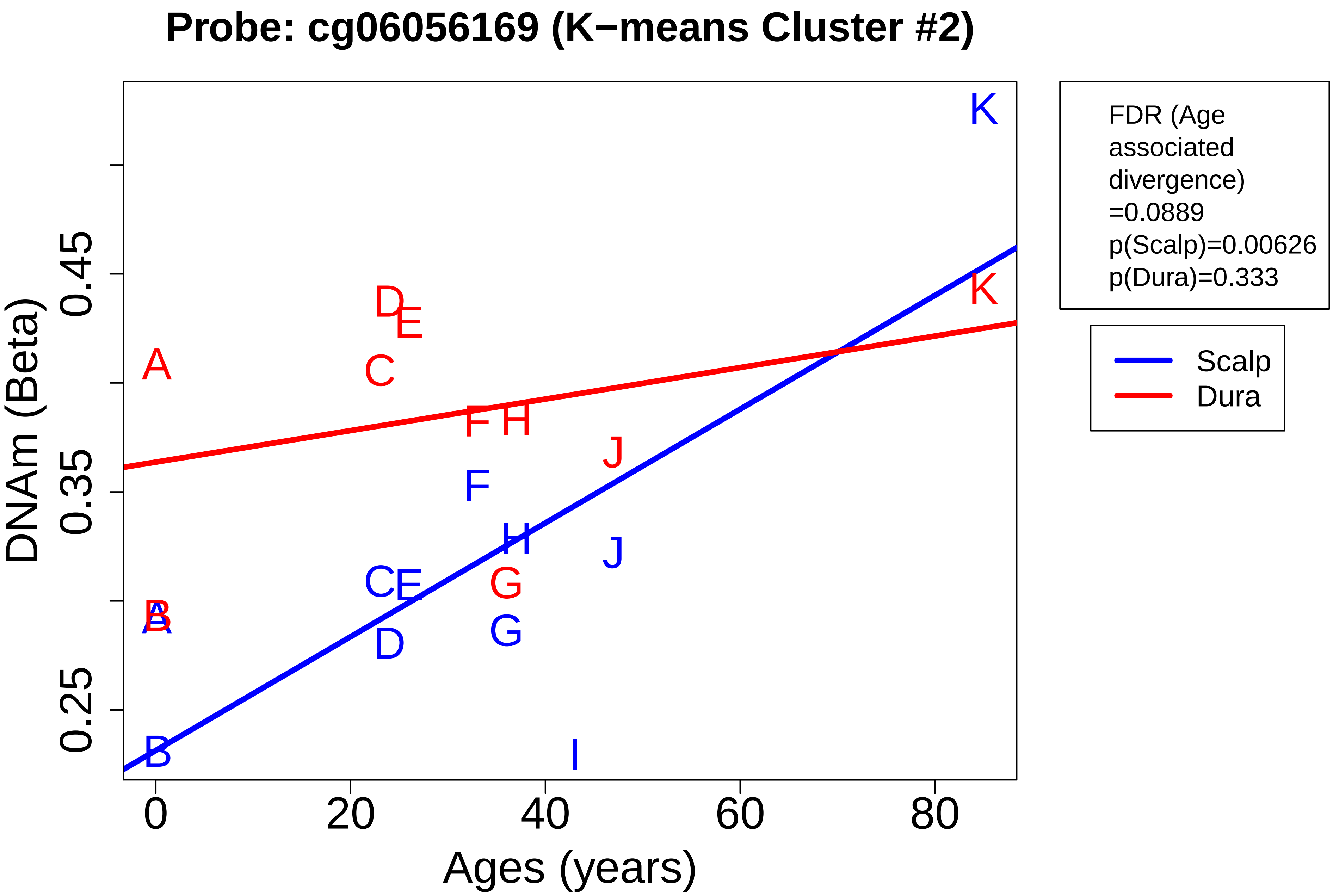
Probe: cg06056169 (K−means Cluster #2)
DNAm (Beta)
0.25
0.35
0.45
Ages (years)
0
20
40
60
80
FDR (Age associated divergence) =0.0889
p(Scalp)=0.00626
p(Dura)=0.333
Scalp
Dura
A
B
C
D
E
F
G
H
I
J
K

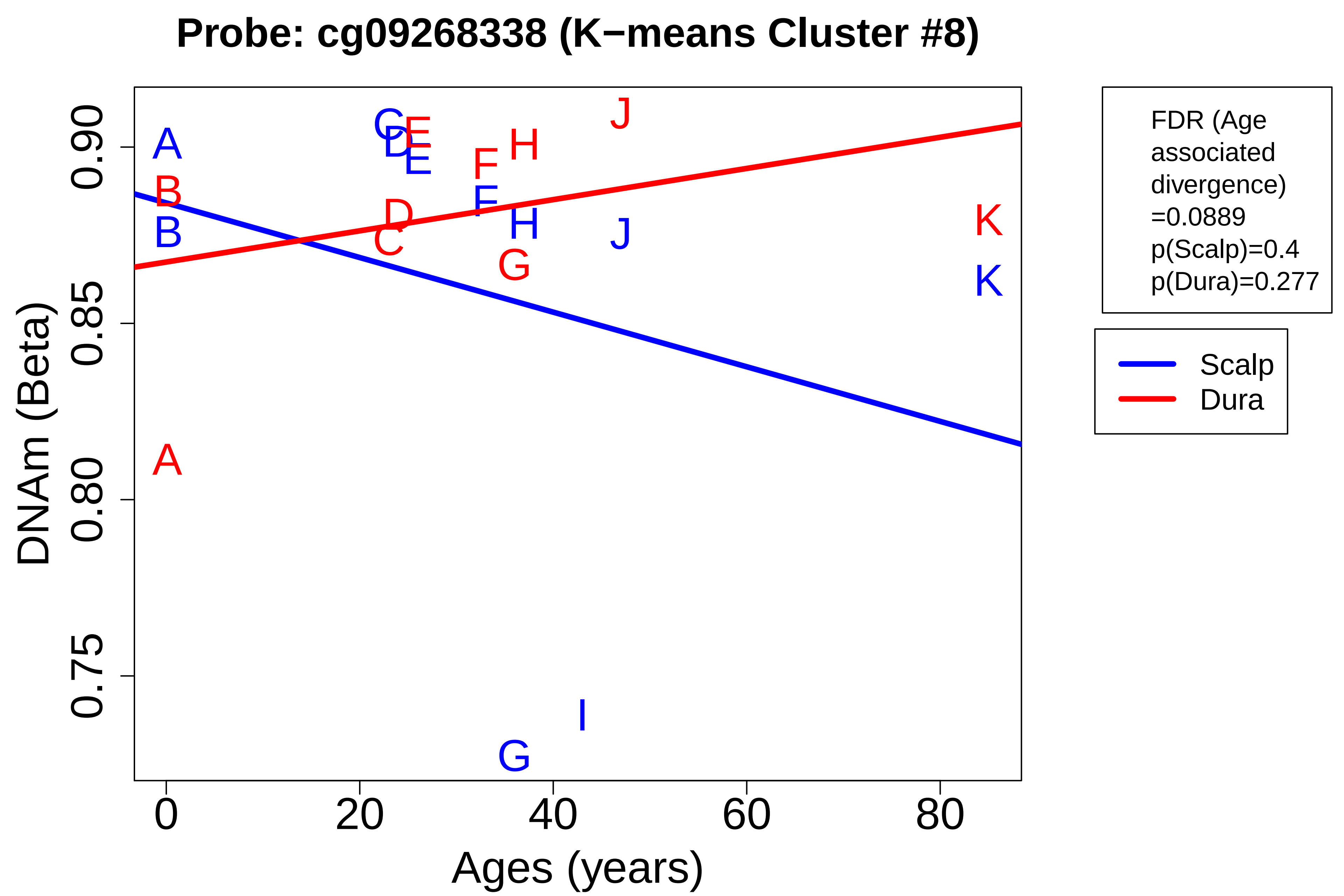

Probe: cg09268338 (K−means Cluster #8)
DNAm (Beta)
0.75
0.80
0.85
0.90
Ages (years)
0
20
40
60
80
A
B
C
D
E
F
G
H
I
J
K
FDR (Age associated divergence) =0.0889
p(Scalp)=0.4
p(Dura)=0.277
Scalp
Dura

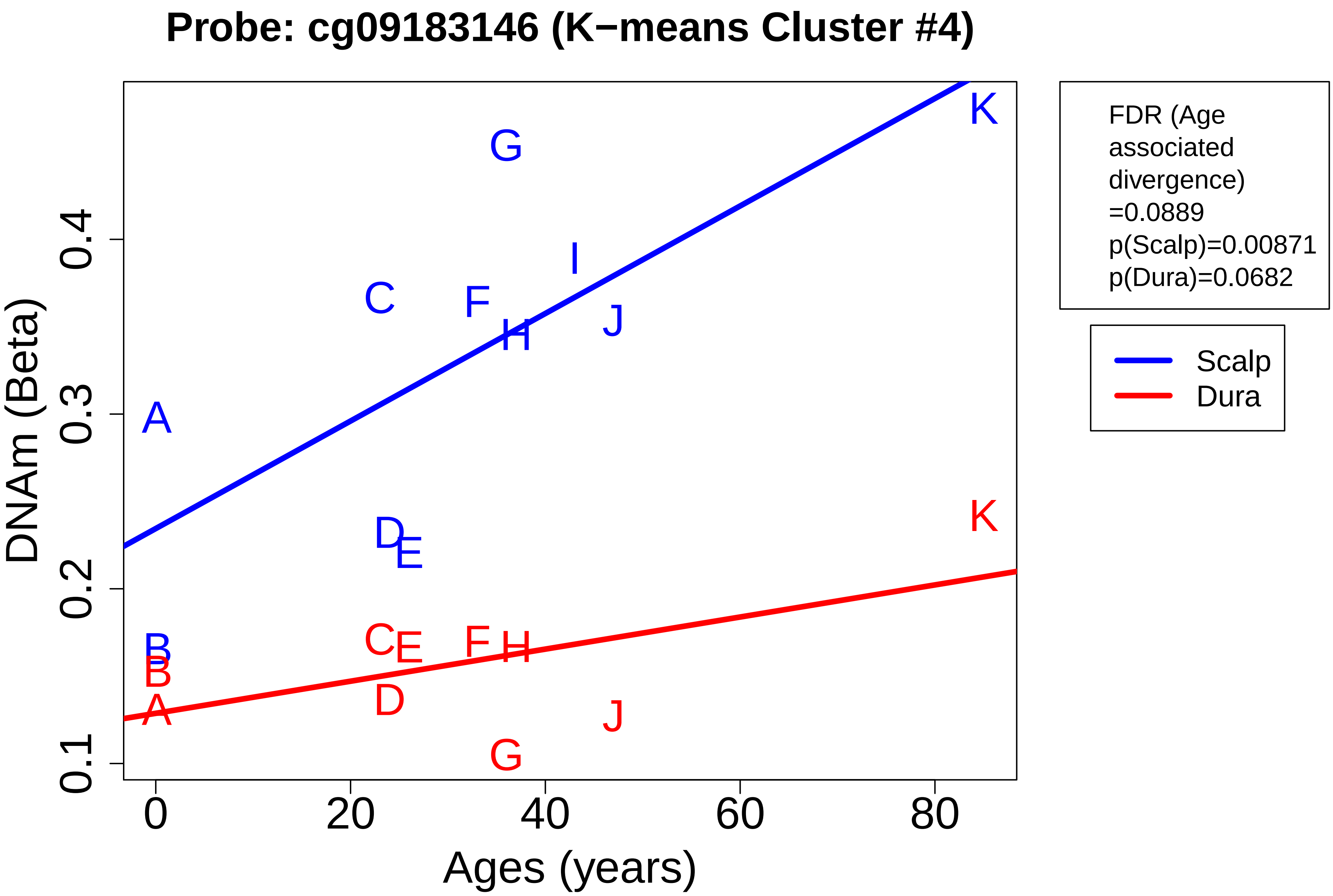

Probe: cg09183146 (K−means Cluster #4)
DNAm (Beta)
0.1
0.2
0.3
0.4
0
20
40
60
80
Ages (years)
A
B
C
D
E
F
G
H
I
J
K
FDR (Age associated divergence) =0.0889
p(Scalp)=0.00871
p(Dura)=0.0682
Scalp
Dura

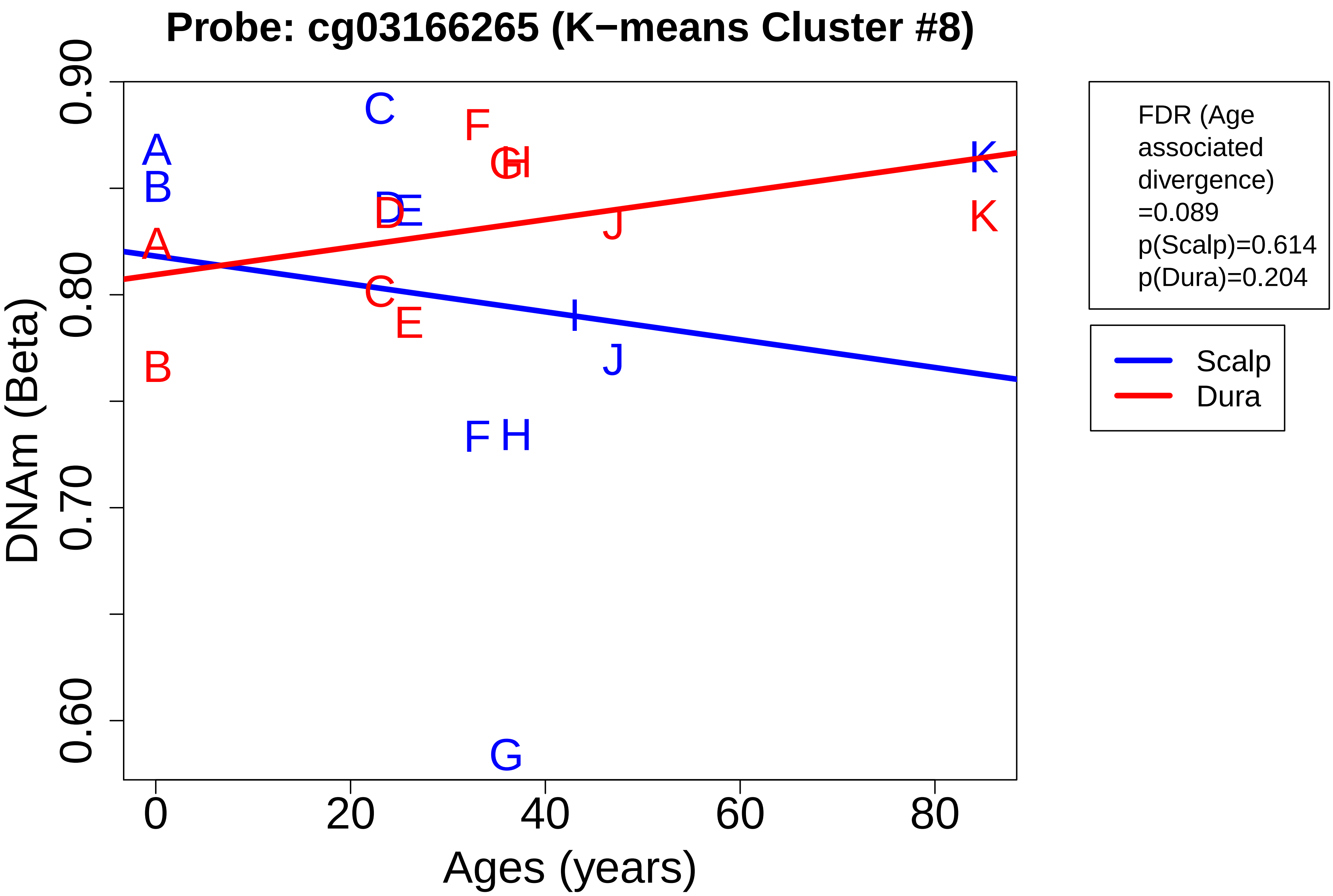

Probe: cg03166265 (K−means Cluster #8)
DNAm (Beta)
0.60
0.70
0.80
0.90
Ages (years)
0
20
40
60
80
FDR (Age associated divergence) =0.089
p(Scalp)=0.614
p(Dura)=0.204
Scalp
Dura

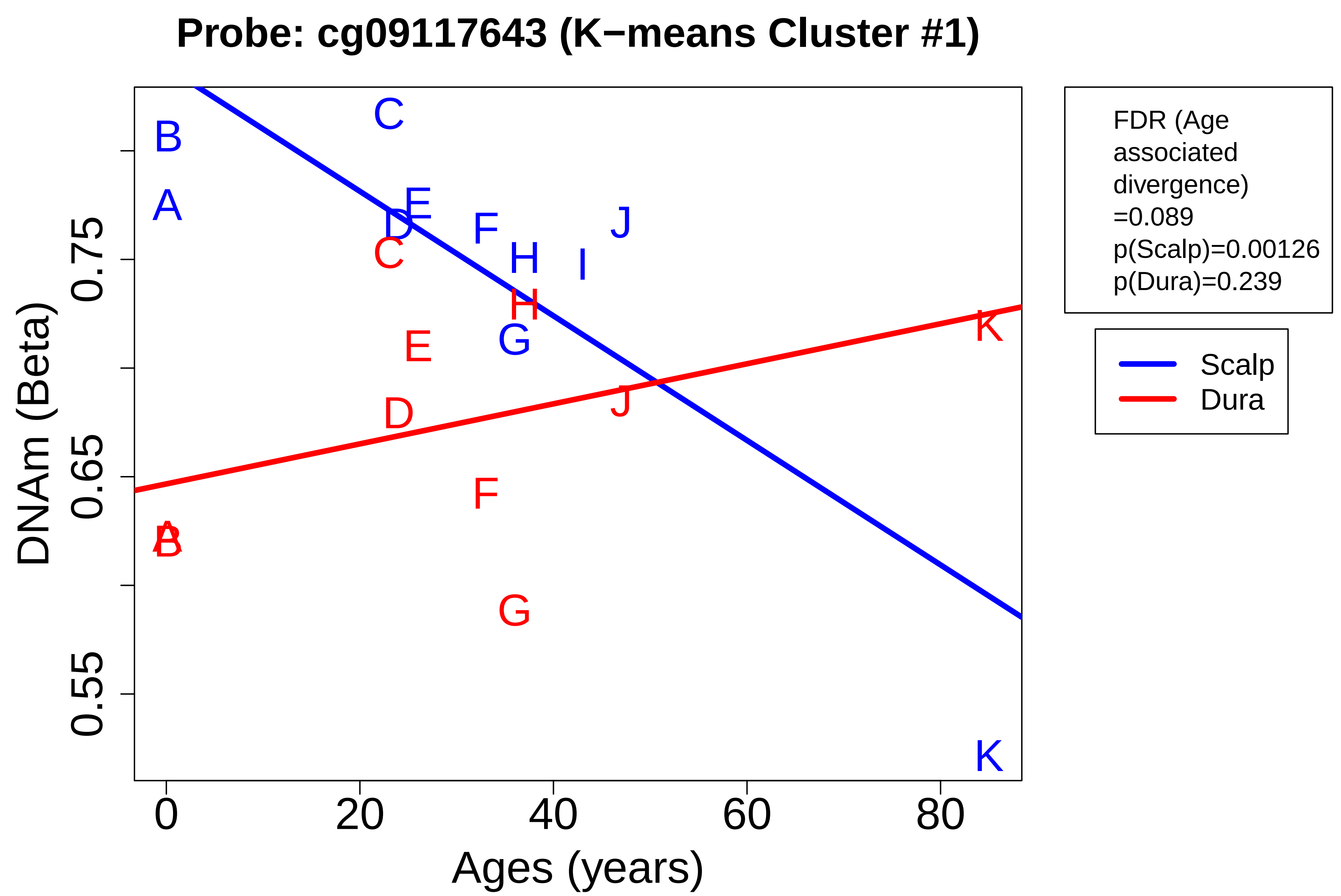

Probe: cg09117643 (K−means Cluster #1)
DNAm (Beta)
0.55
0.65
0.75
0
20
40
60
80
Ages (years)
FDR (Age associated divergence) =0.089
p(Scalp)=0.00126
p(Dura)=0.239
Scalp
Dura

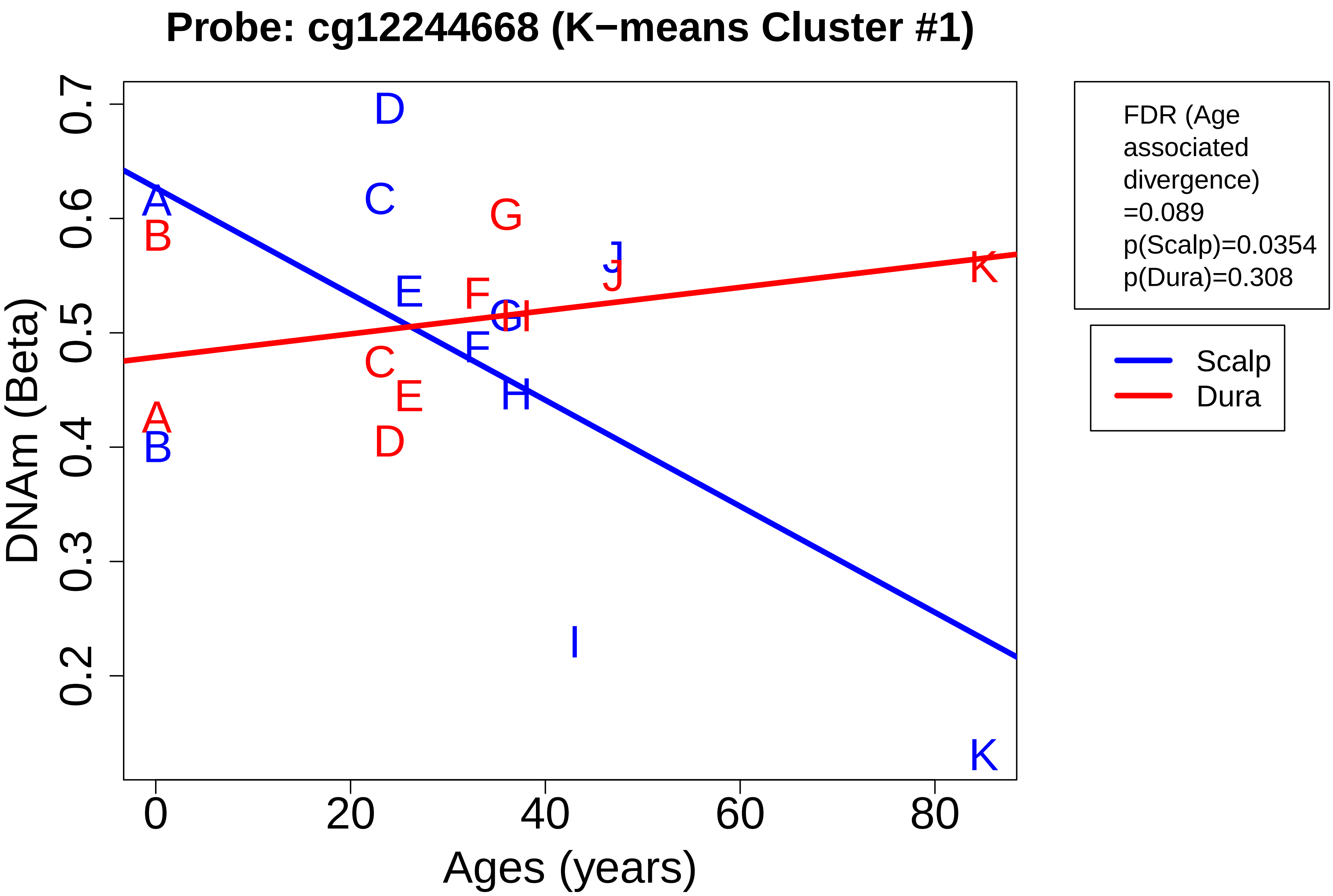

Probe: cg12244668 (K−means Cluster #1)
DNAm (Beta)
0.2
0.3
0.4
0.5
0.6
0.7
Ages (years)
0
20
40
60
80
FDR (Age associated divergence) =0.089
p(Scalp)=0.0354
p(Dura)=0.308
Scalp
Dura

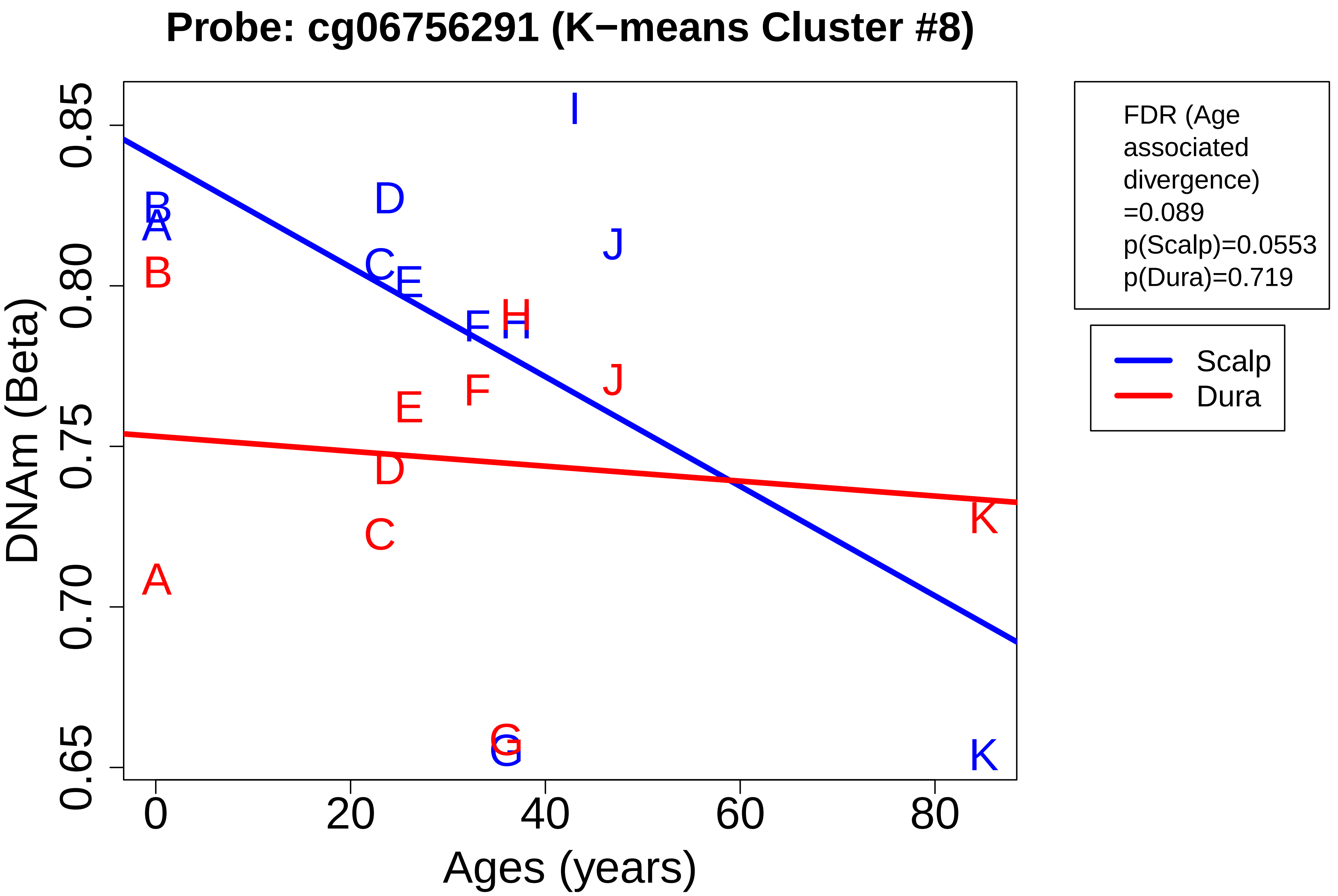
Probe: cg06756291 (K−means Cluster #8)
DNAm (Beta)
Ages (years)
0.65
0.70
0.75
0.80
0.85
0
20
40
60
80
FDR (Age associated divergence) =0.089
p(Scalp)=0.0553
p(Dura)=0.719
Scalp
Dura
A
B
C
D
E
F
G
H
I
J
K

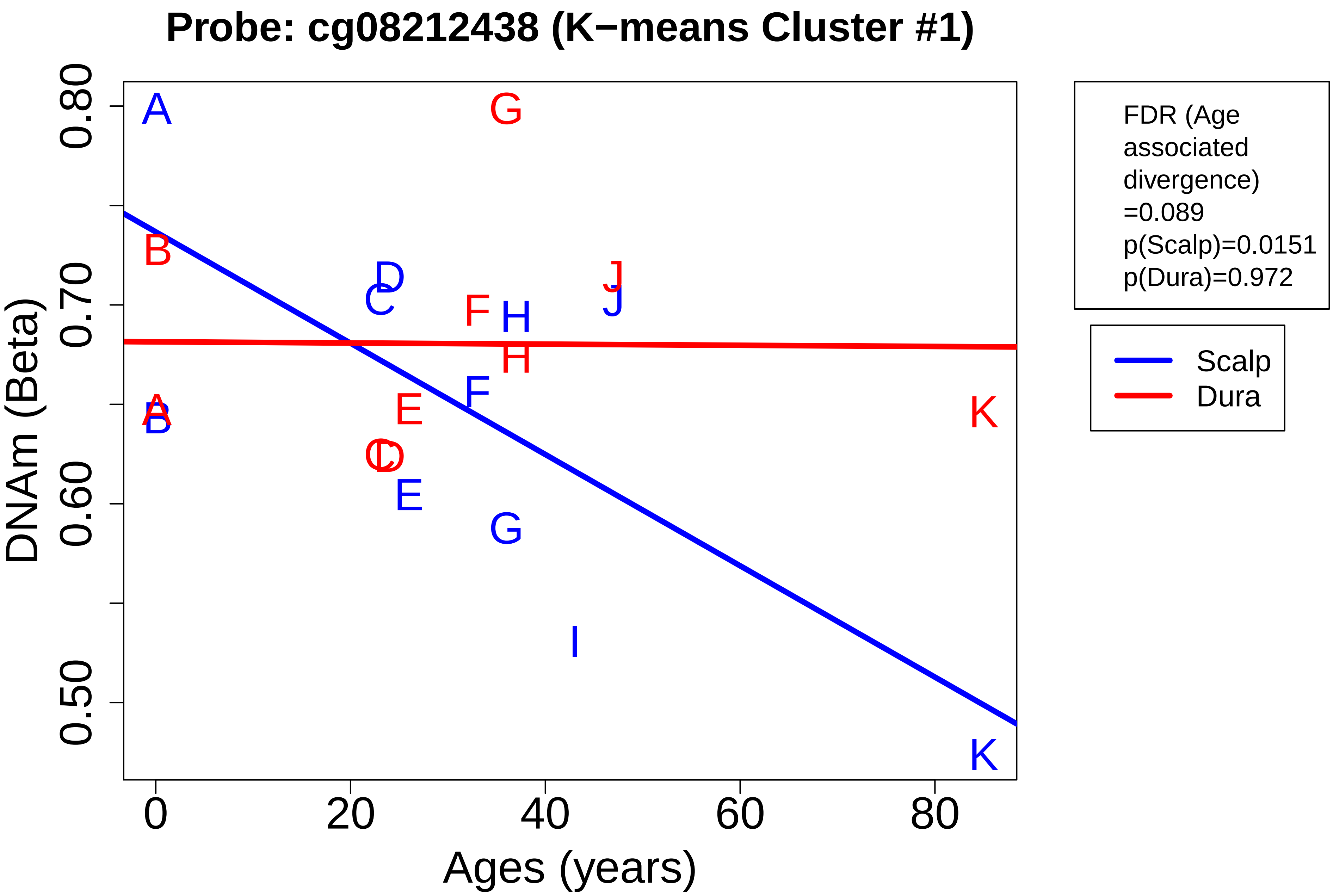

Probe: cg08212438 (K−means Cluster #1)
DNAm (Beta)
0.50
0.60
0.70
0.80
Ages (years)
0
20
40
60
80
A
B
C
D
E
F
G
H
I
J
K
FDR (Age associated divergence) =0.089
p(Scalp)=0.0151
p(Dura)=0.972
Scalp
Dura

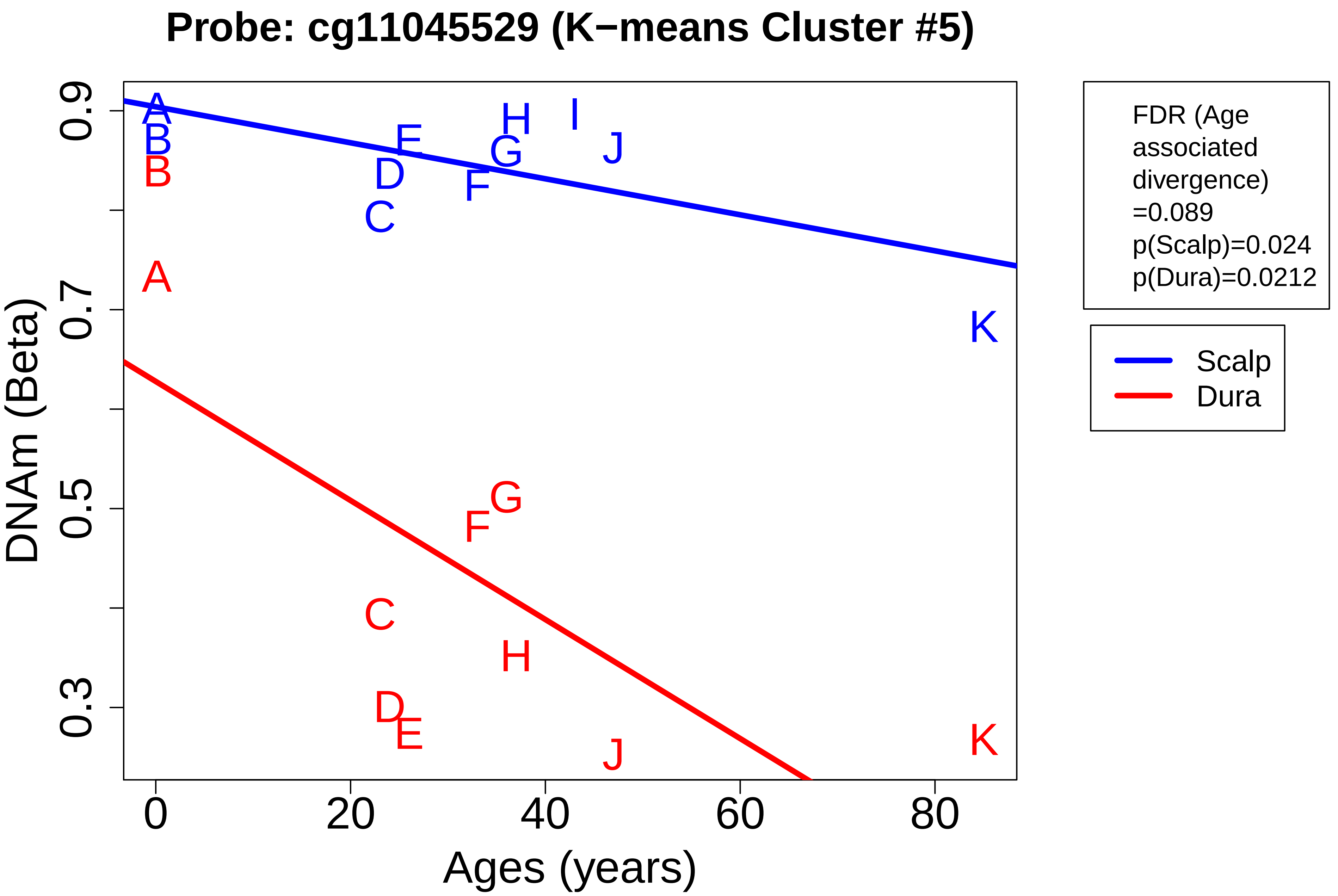

Probe: cg11045529 (K−means Cluster #5)
DNAm (Beta)
0.9
0.7
0.5
0.3
0
20
40
60
80
Ages (years)
A
B
C
D
E
F
G
H
I
J
K
FDR (Age associated divergence) =0.089
p(Scalp)=0.024
p(Dura)=0.0212
Scalp
Dura

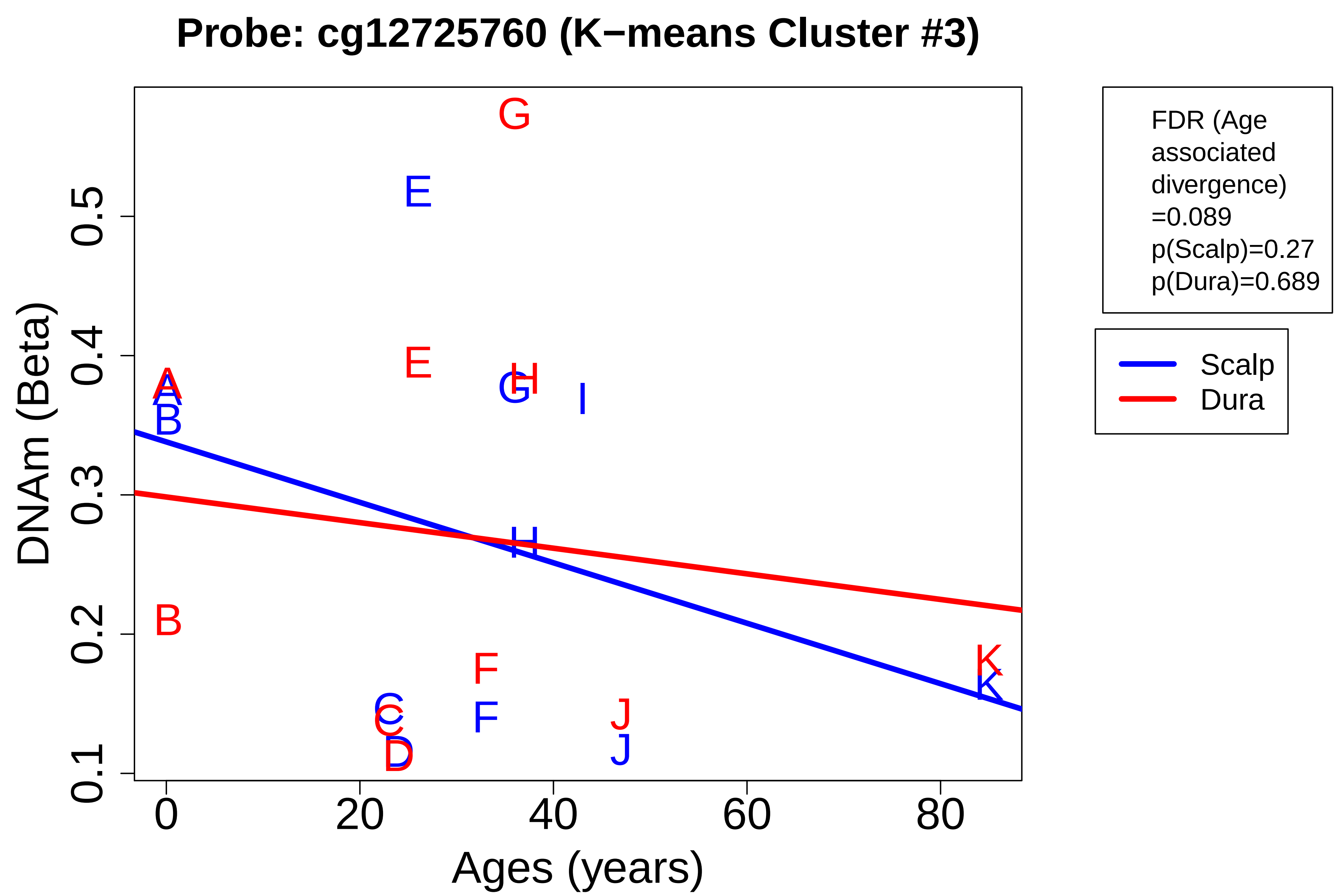

Probe: cg12725760 (K−means Cluster #3)
DNAm (Beta)
0.1
0.2
0.3
0.4
0.5
Ages (years)
0
20
40
60
80
FDR (Age associated divergence) =0.089
p(Scalp)=0.27
p(Dura)=0.689
Scalp
Dura

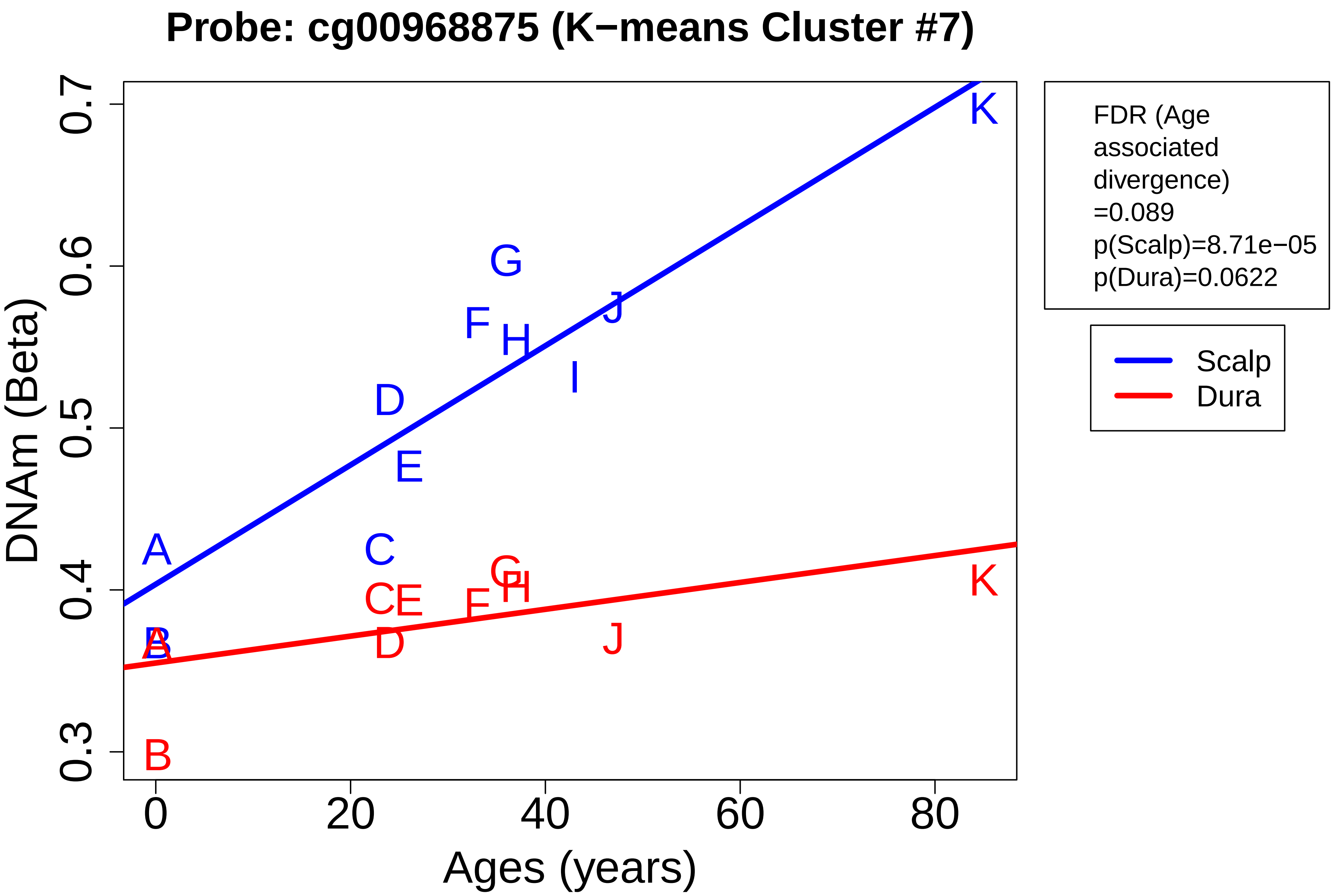

Probe: cg00968875 (K−means Cluster #7)
DNAm (Beta)
Ages (years)
0.3
0.4
0.5
0.6
0.7
0
20
40
60
80
FDR (Age associated divergence) =0.089
p(Scalp)=8.71e−05
p(Dura)=0.0622
Scalp
Dura

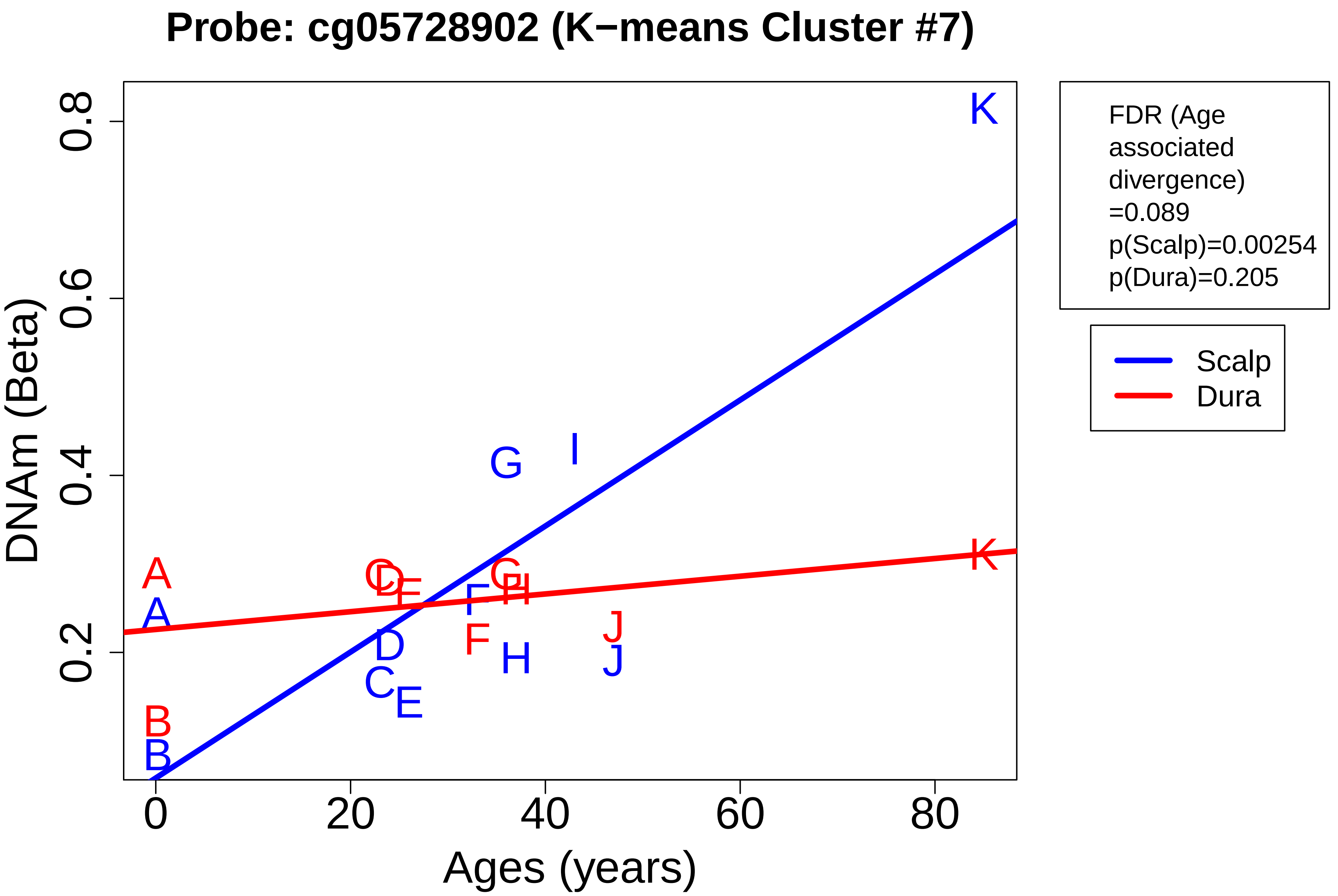
Probe: cg05728902 (K−means Cluster #7)
FDR (Age associated divergence) =0.089
p(Scalp)=0.00254
p(Dura)=0.205
Scalp
Dura
DNAm (Beta)
Ages (years)
0.2
0.4
0.6
0.8
0
20
40
60
80

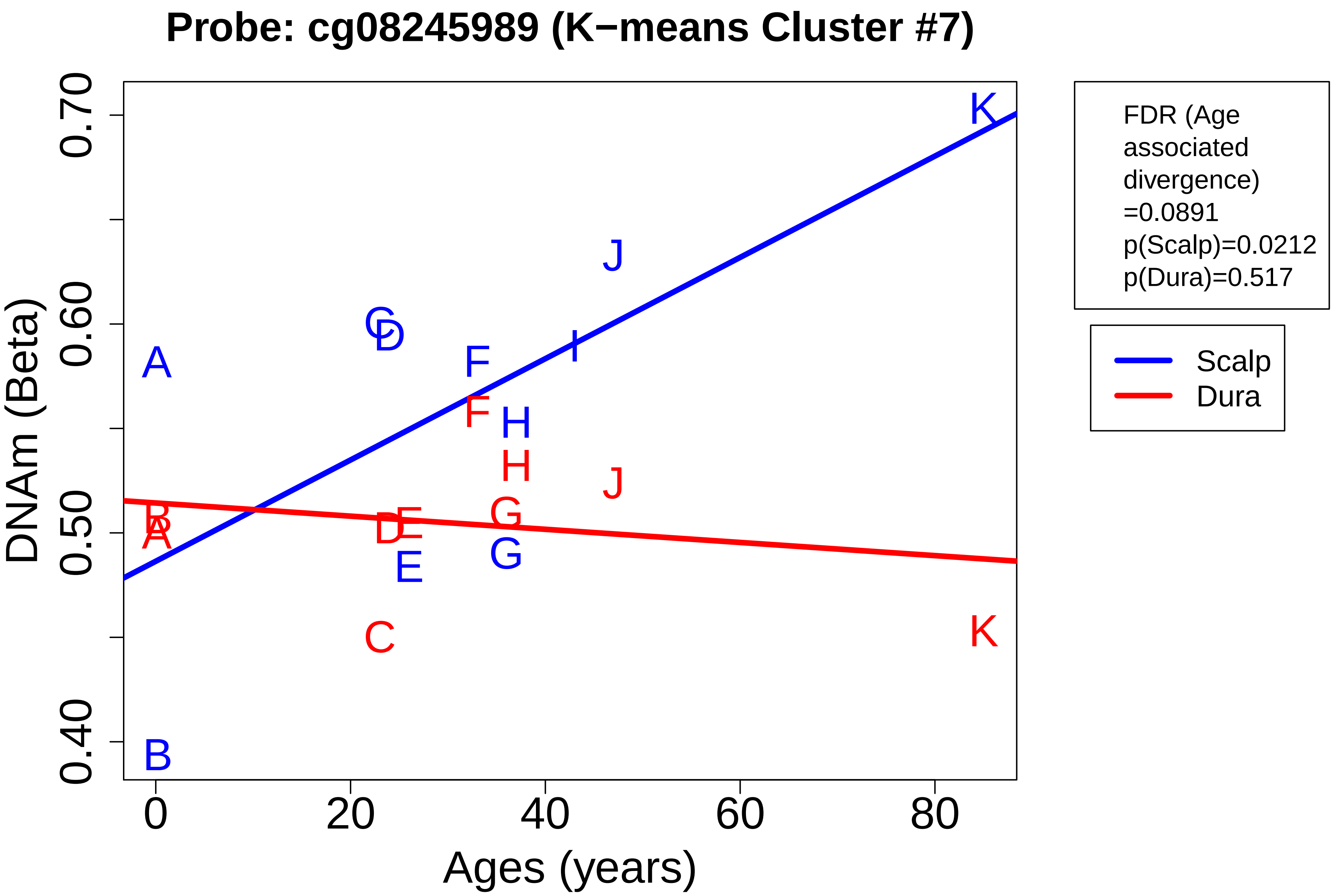

Probe: cg08245989 (K−means Cluster #7)
DNAm (Beta)
0.40
0.50
0.60
0.70
Ages (years)
0
20
40
60
80
FDR (Age associated divergence) =0.0891
p(Scalp)=0.0212
p(Dura)=0.517
Scalp
Dura
A
B
C
D
E
F
G
H
I
J
K

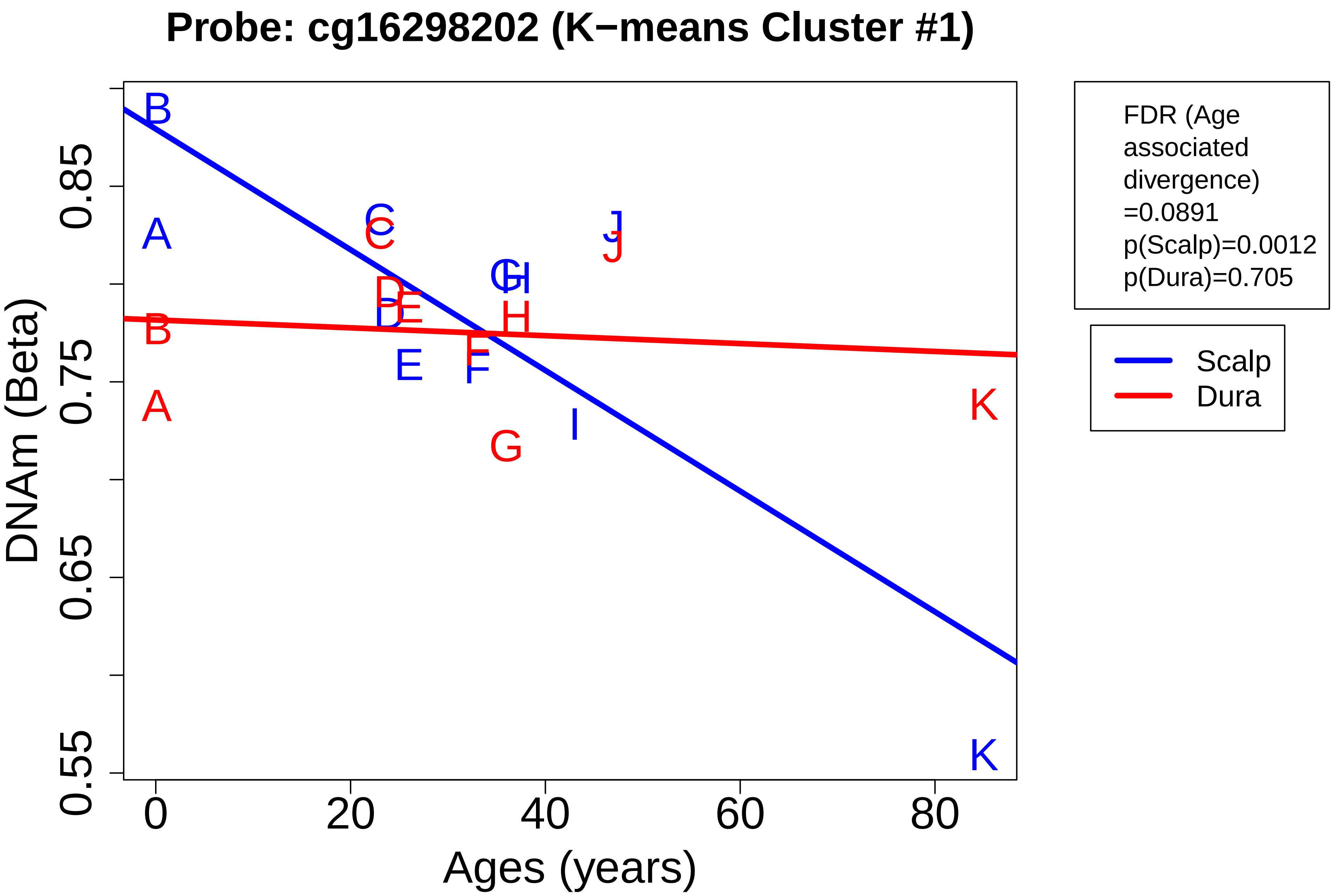
Probe: cg16298202 (K−means Cluster #1)
DNAm (Beta)
0.55
0.65
0.75
0.85
Ages (years)
0
20
40
60
80
FDR (Age associated divergence) =0.0891
p(Scalp)=0.0012
p(Dura)=0.705
Scalp
Dura
A
B
C
D
E
F
G
H
I
J
K

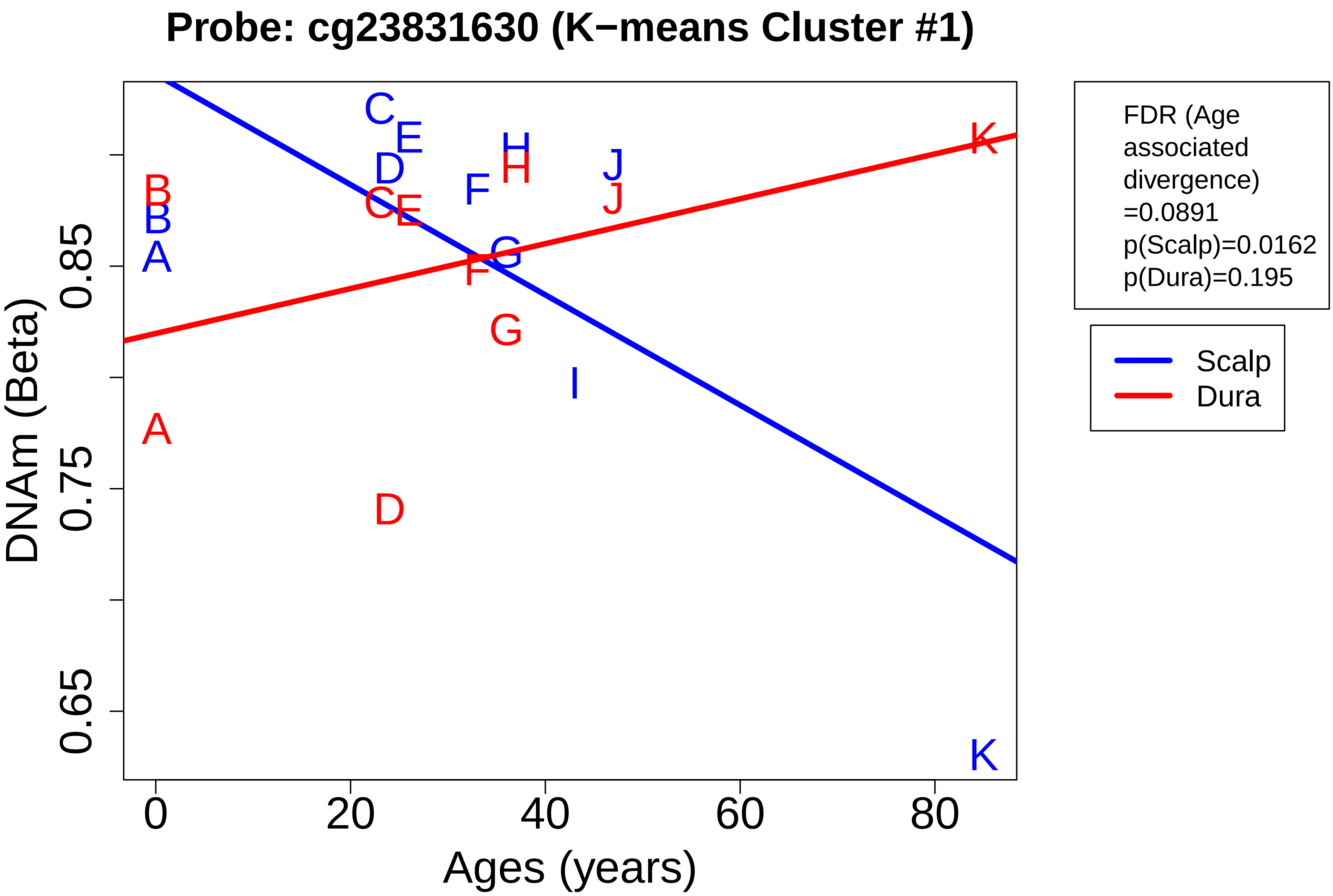

Probe: cg23831630 (K−means Cluster #1)
FDR (Age associated divergence) =0.0891
p(Scalp)=0.0162
p(Dura)=0.195
Scalp
Dura
DNAm (Beta)
0.65
0.75
0.85
Ages (years)
0
20
40
60
80

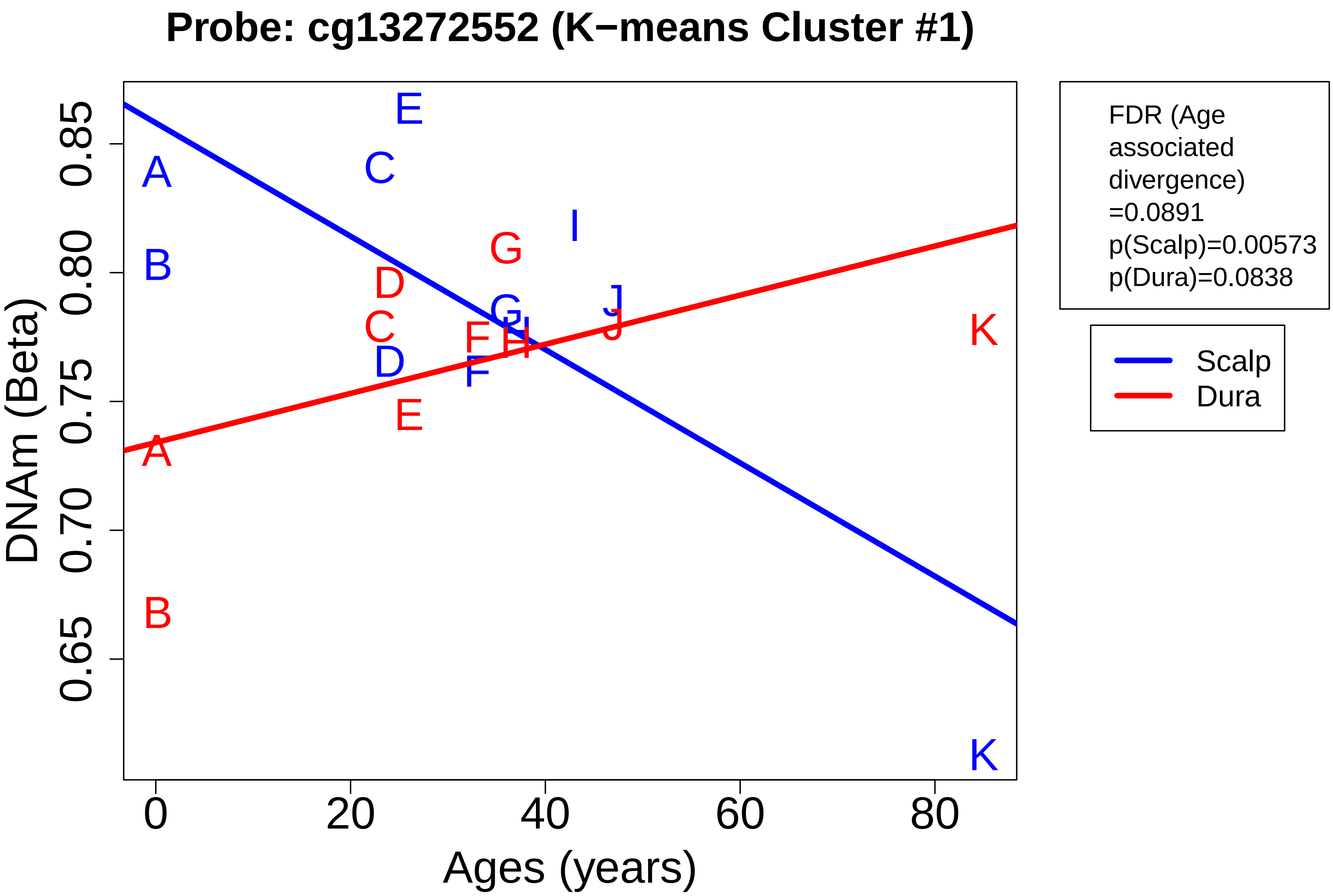

Probe: cg13272552 (K−means Cluster #1)
DNAm (Beta)
0.65
0.70
0.75
0.80
0.85
Ages (years)
0
20
40
60
80
FDR (Age associated divergence) =0.0891
p(Scalp)=0.00573
p(Dura)=0.0838
Scalp
Dura
A
B
C
D
E
F
G
H
I
J
K

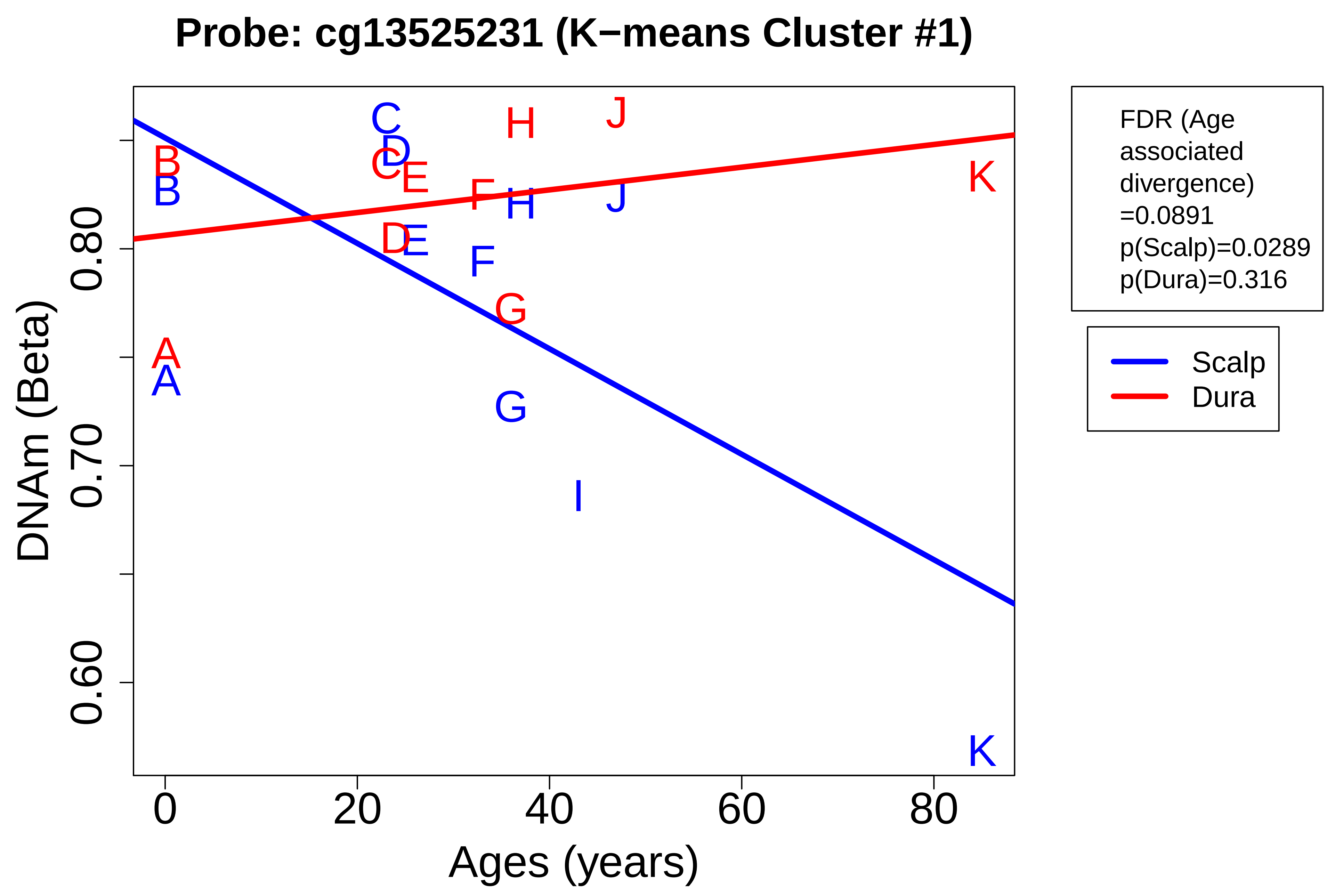

Probe: cg13525231 (K−means Cluster #1)
DNAm (Beta)
0.60
0.70
0.80
0
20
40
60
80
Ages (years)
A
B
C
D
E
F
G
H
I
J
K
A
B
C
D
E
F
G
H
J
K
FDR (Age associated divergence) =0.0891
p(Scalp)=0.0289
p(Dura)=0.316
Scalp
Dura

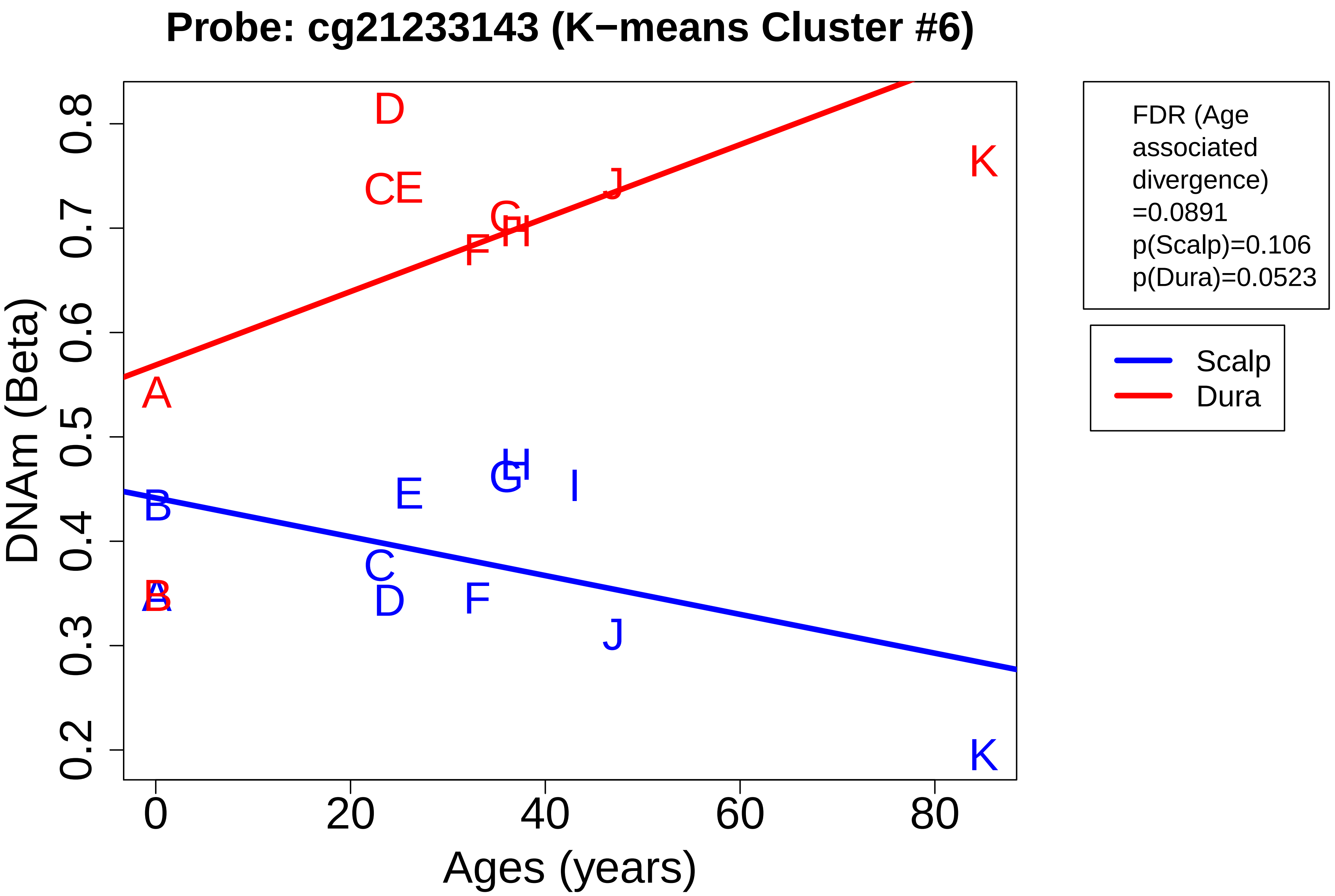
Probe: cg21233143 (K−means Cluster #6)
DNAm (Beta)
Ages (years)
0.2
0.3
0.4
0.5
0.6
0.7
0.8
0
20
40
60
80
FDR (Age associated divergence) =0.0891
p(Scalp)=0.106
p(Dura)=0.0523
Scalp
Dura
A
B
C
D
E
F
G
H
I
J
K
CE

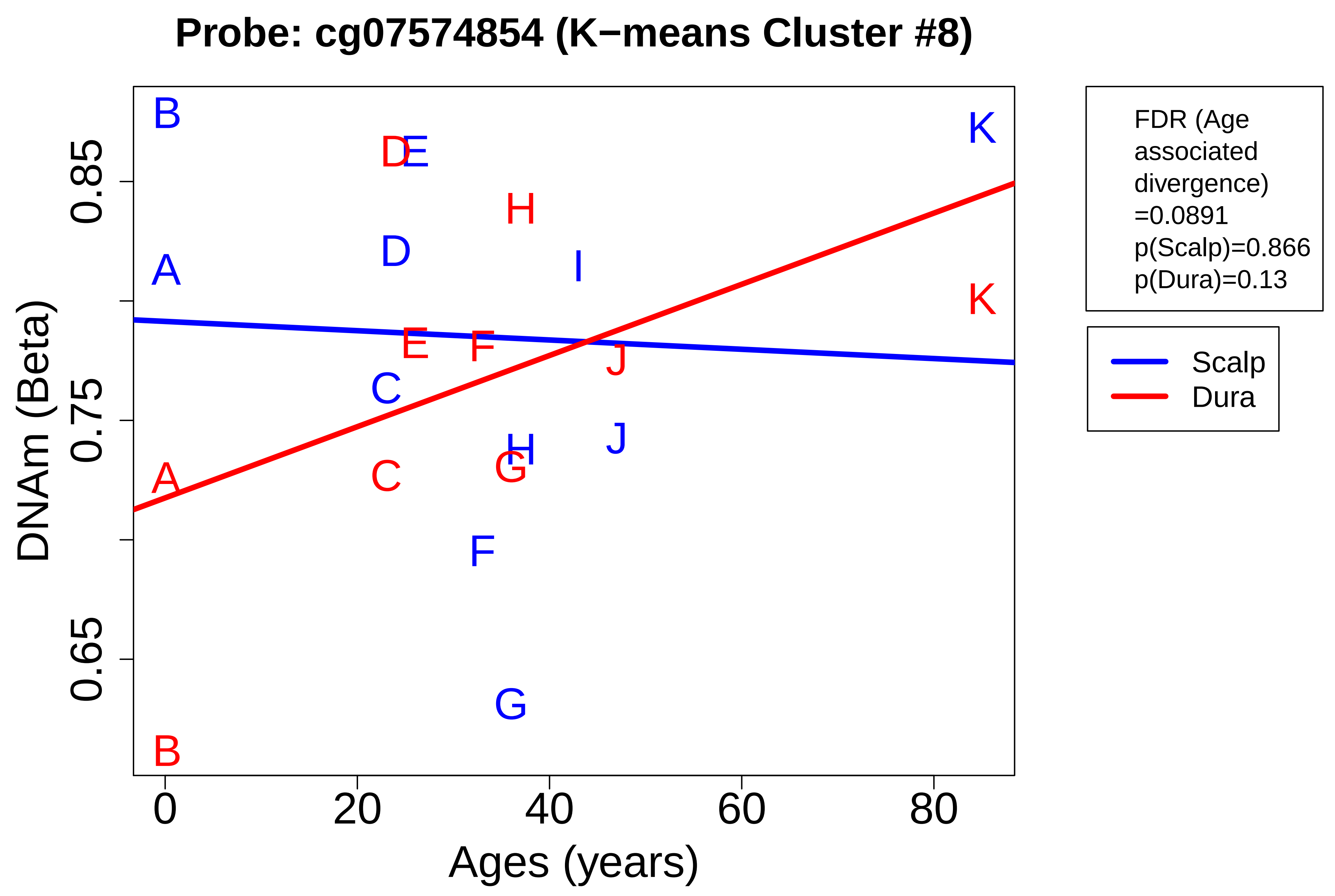

Probe: cg07574854 (K−means Cluster #8)
FDR (Age associated divergence) =0.0891
p(Scalp)=0.866
p(Dura)=0.13
Scalp
Dura
DNAm (Beta)
0.65
0.75
0.85
Ages (years)
0
20
40
60
80
A
B
C
D
E
F
G
H
I
J
K

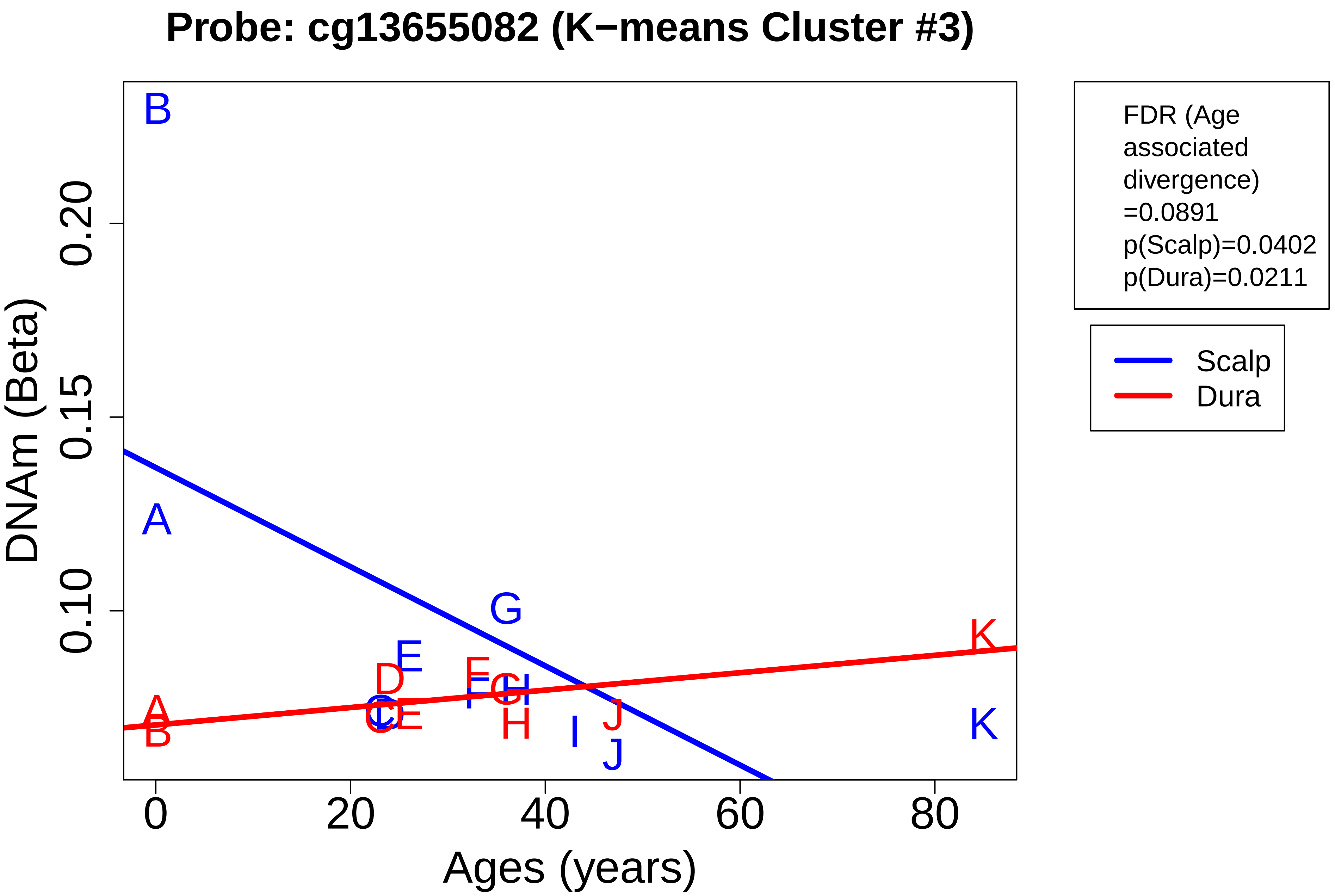

Probe: cg13655082 (K−means Cluster #3)
FDR (Age associated divergence) =0.0891
p(Scalp)=0.0402
p(Dura)=0.0211
Scalp
Dura
DNAm (Beta)
0.10
0.15
0.20
Ages (years)
0
20
40
60
80

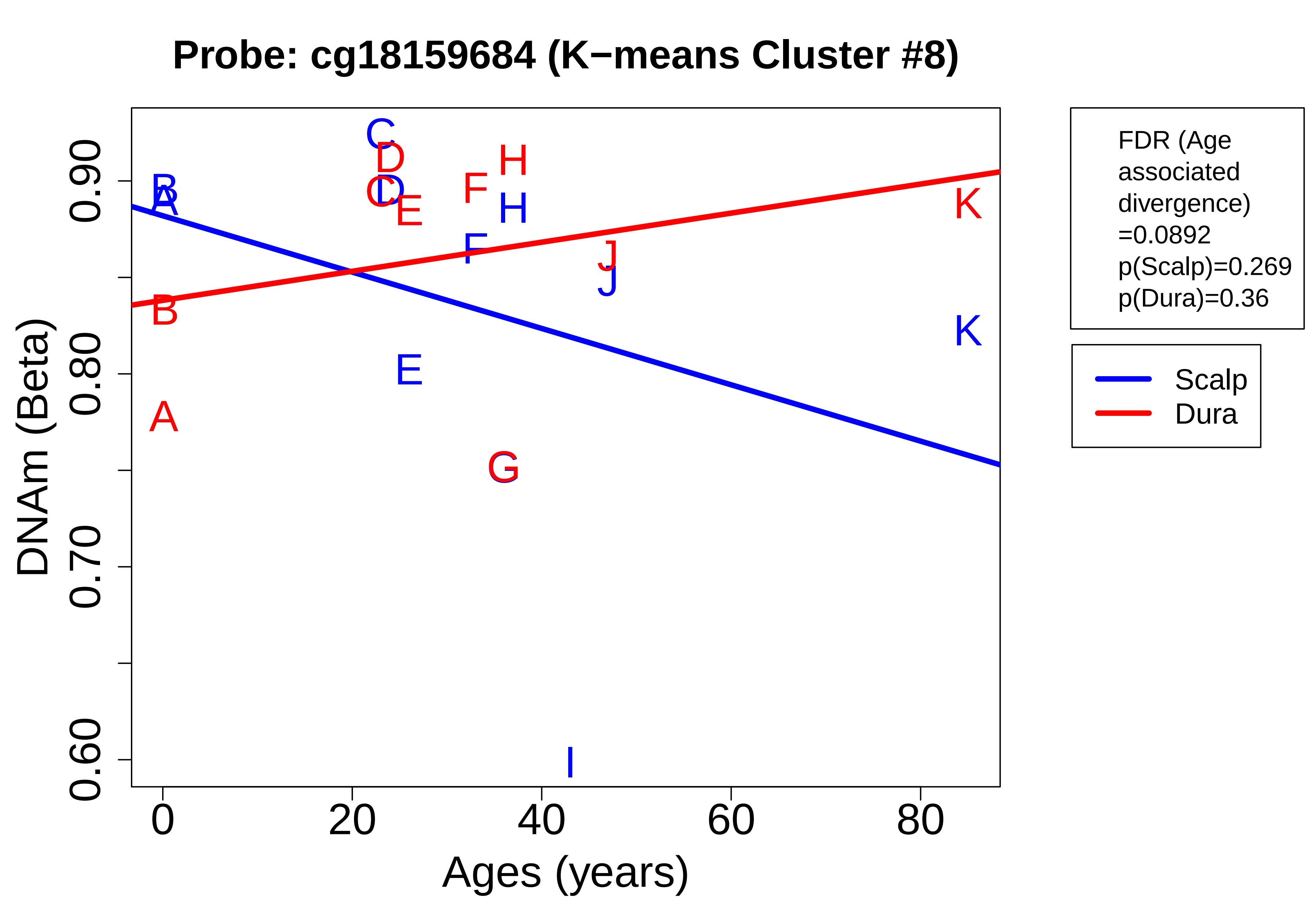
Probe: cg18159684 (K−means Cluster #8)
DNAm (Beta)
0.60
0.70
0.80
0.90
Ages (years)
0
20
40
60
80
A
B
C
D
E
F
G
H
I
J
K
FDR (Age associated divergence) =0.0892
p(Scalp)=0.269
p(Dura)=0.36
Scalp
Dura

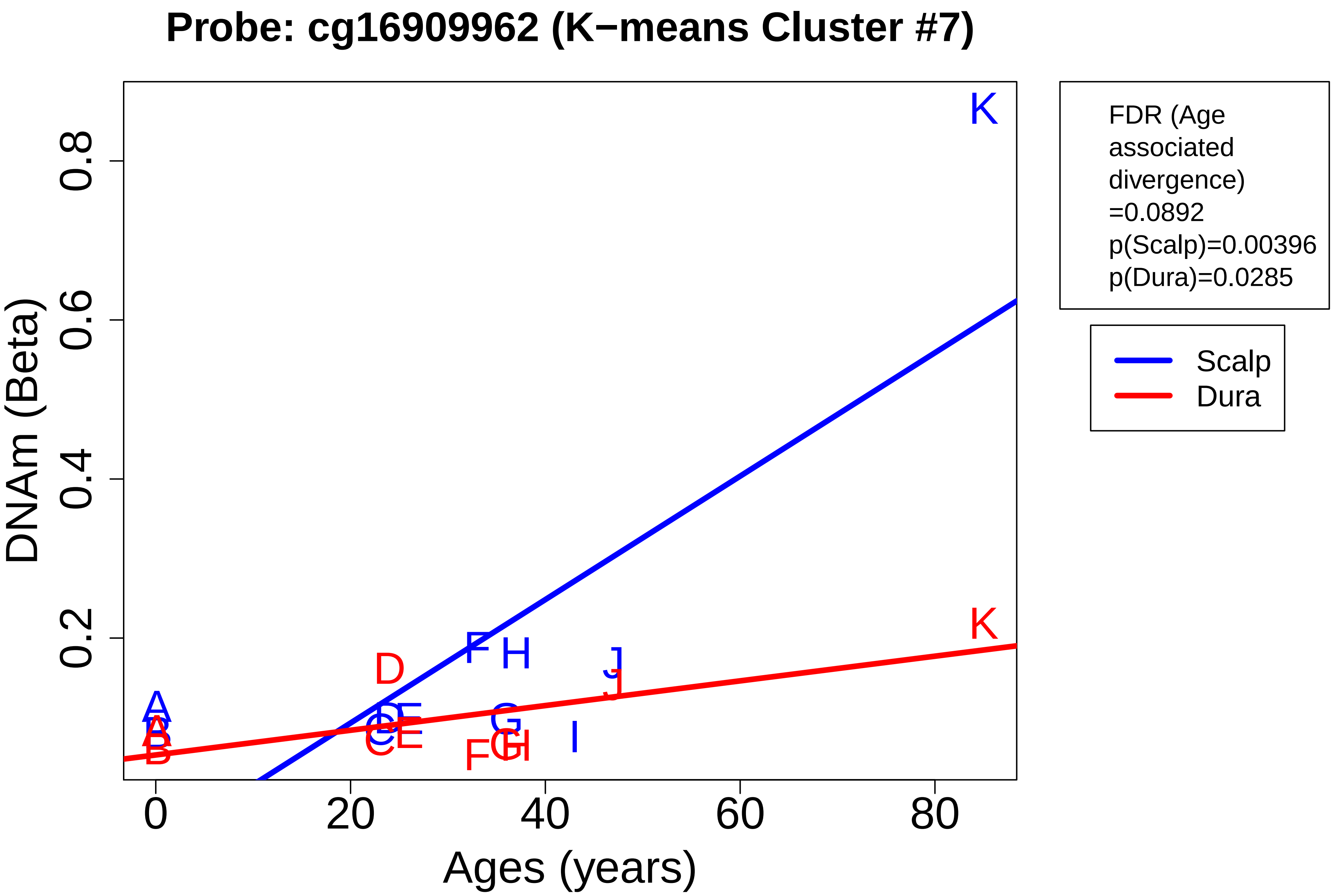
Probe: cg16909962 (K−means Cluster #7)
DNAm (Beta)
0.2
0.4
0.6
0.8
Ages (years)
0
20
40
60
80
FDR (Age associated divergence) =0.0892
p(Scalp)=0.00396
p(Dura)=0.0285
Scalp
Dura

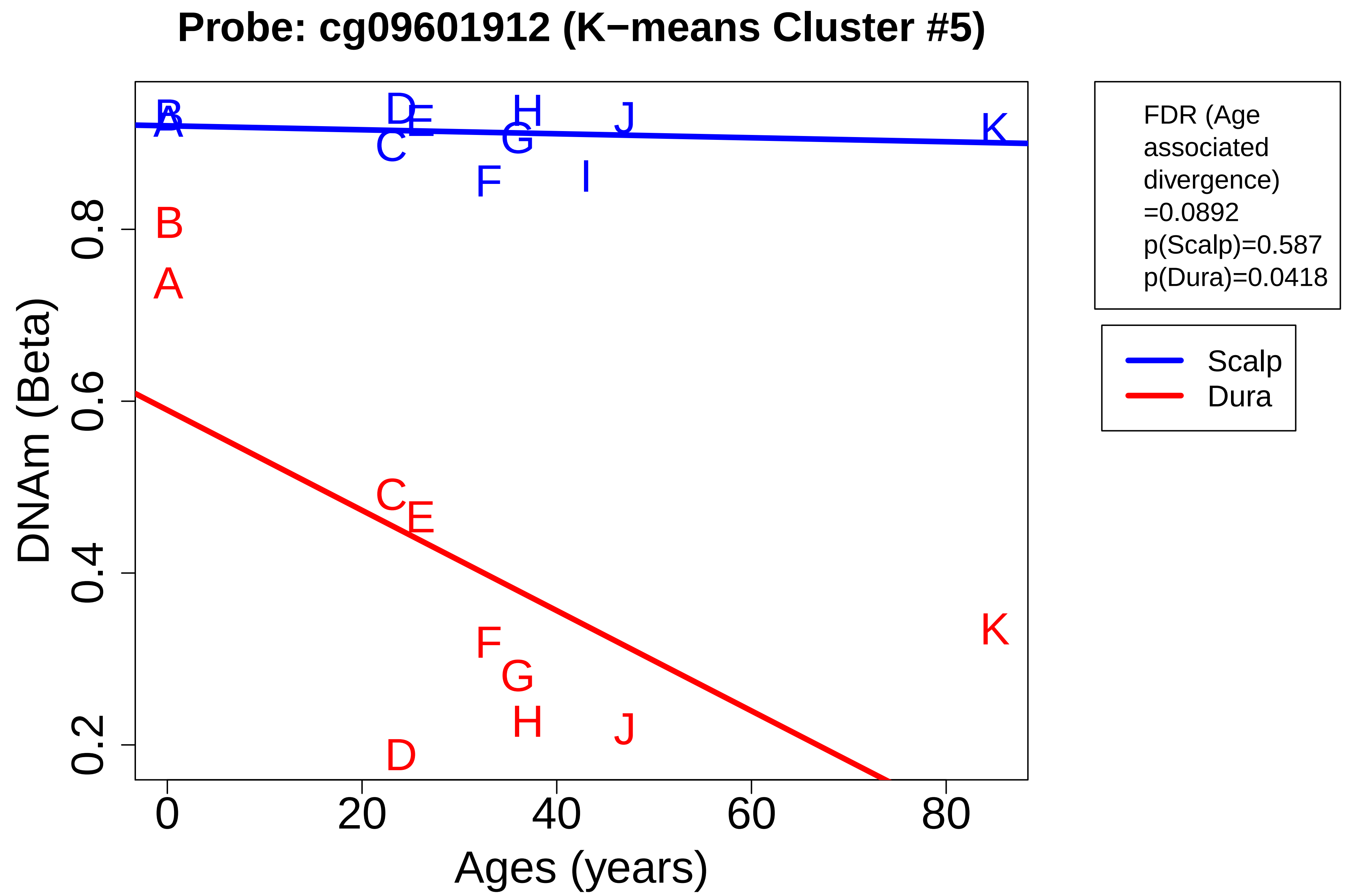

Probe: cg09601912 (K−means Cluster #5)
DNAm (Beta)
0.2
0.4
0.6
0.8
0
20
40
60
80
Ages (years)
FDR (Age associated divergence) =0.0892
p(Scalp)=0.587
p(Dura)=0.0418
Scalp
Dura

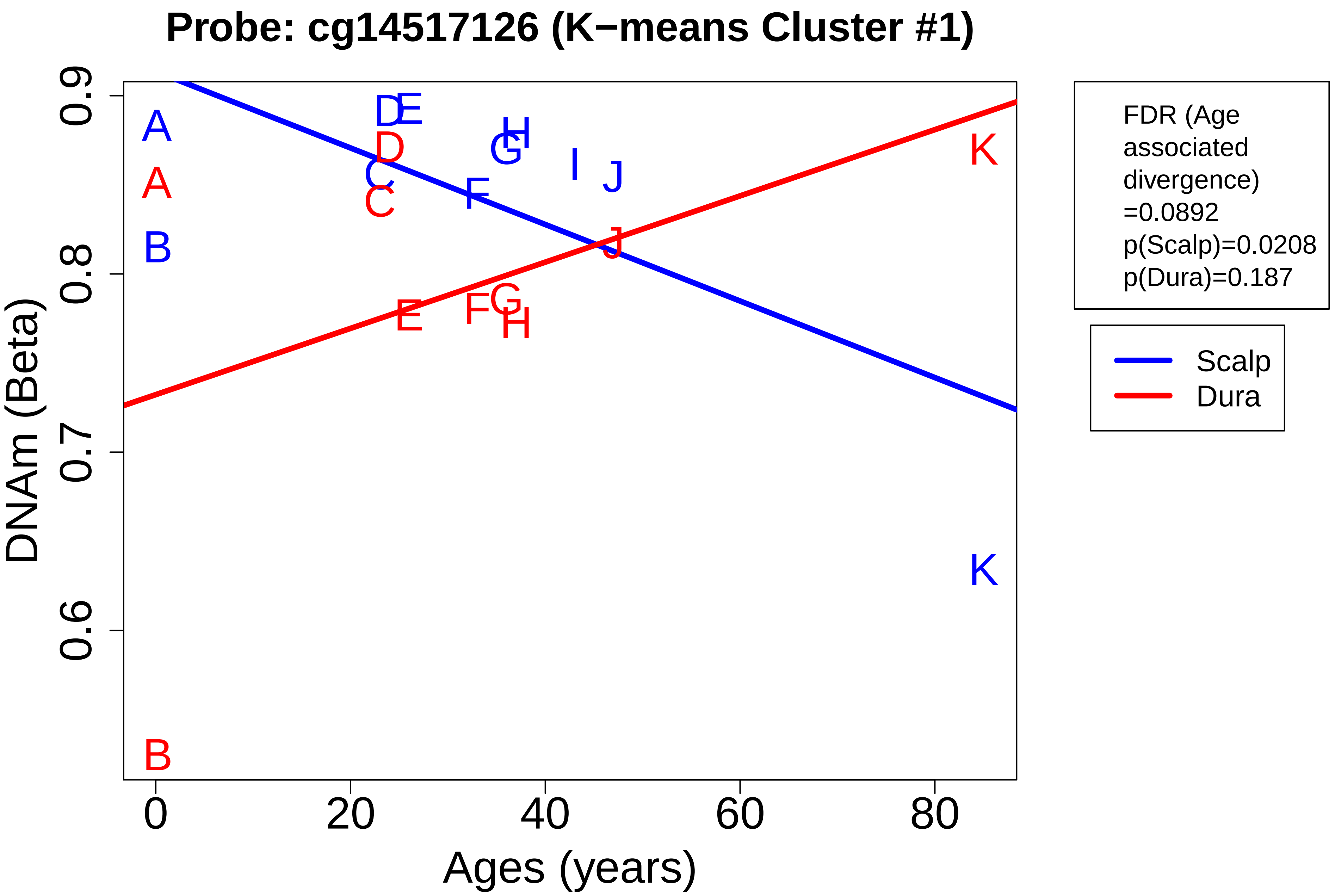
Probe: cg14517126 (K−means Cluster #1)
DNAm (Beta)
0.6
0.7
0.8
0.9
Ages (years)
0
20
40
60
80
A
B
C
D
E
F
G
H
I
J
K
FDR (Age associated divergence) =0.0892
p(Scalp)=0.0208
p(Dura)=0.187
Scalp
Dura

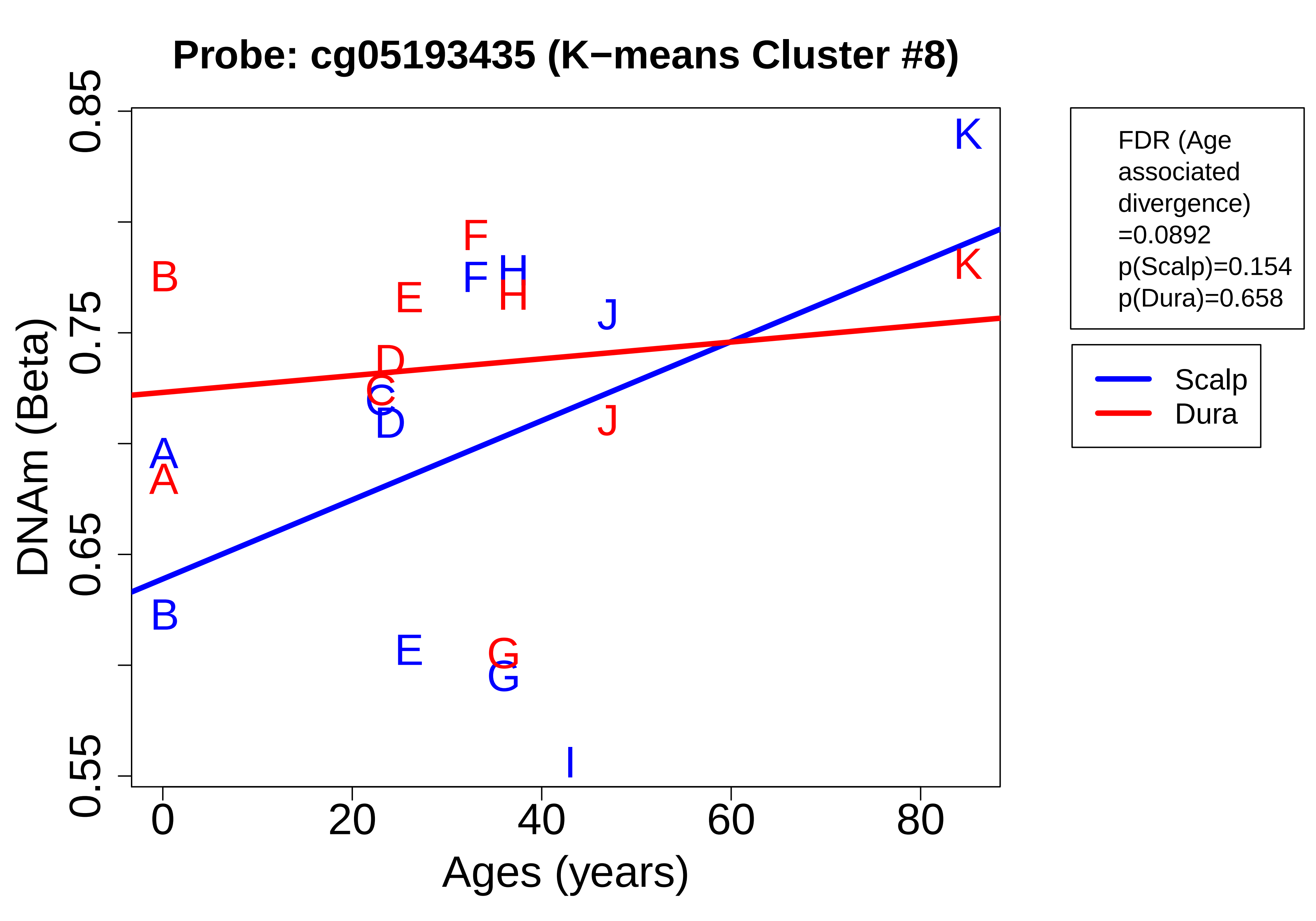
Probe: cg05193435 (K−means Cluster #8)
DNAm (Beta)
0.55
0.65
0.75
0.85
Ages (years)
0
20
40
60
80
A
B
C
D
E
F
G
H
I
J
K
FDR (Age associated divergence) =0.0892
p(Scalp)=0.154
p(Dura)=0.658
Scalp
Dura

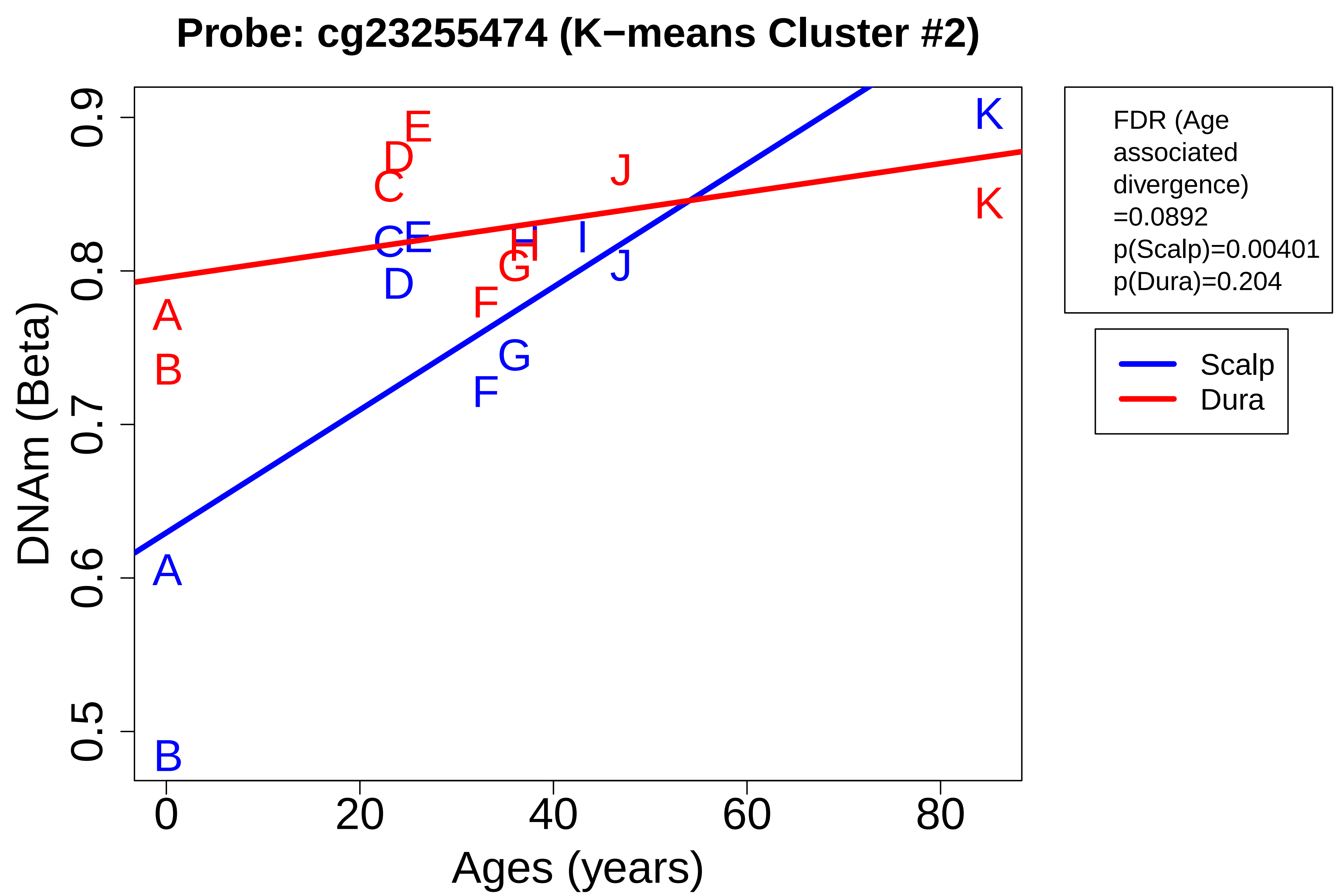

Probe: cg23255474 (K−means Cluster #2)
DNAm (Beta)
0.5
0.6
0.7
0.8
0.9
0
20
40
60
80
Ages (years)
A
B
C
D
E
F
G
H
I
J
K
FDR (Age associated divergence) =0.0892
p(Scalp)=0.00401
p(Dura)=0.204
Scalp
Dura

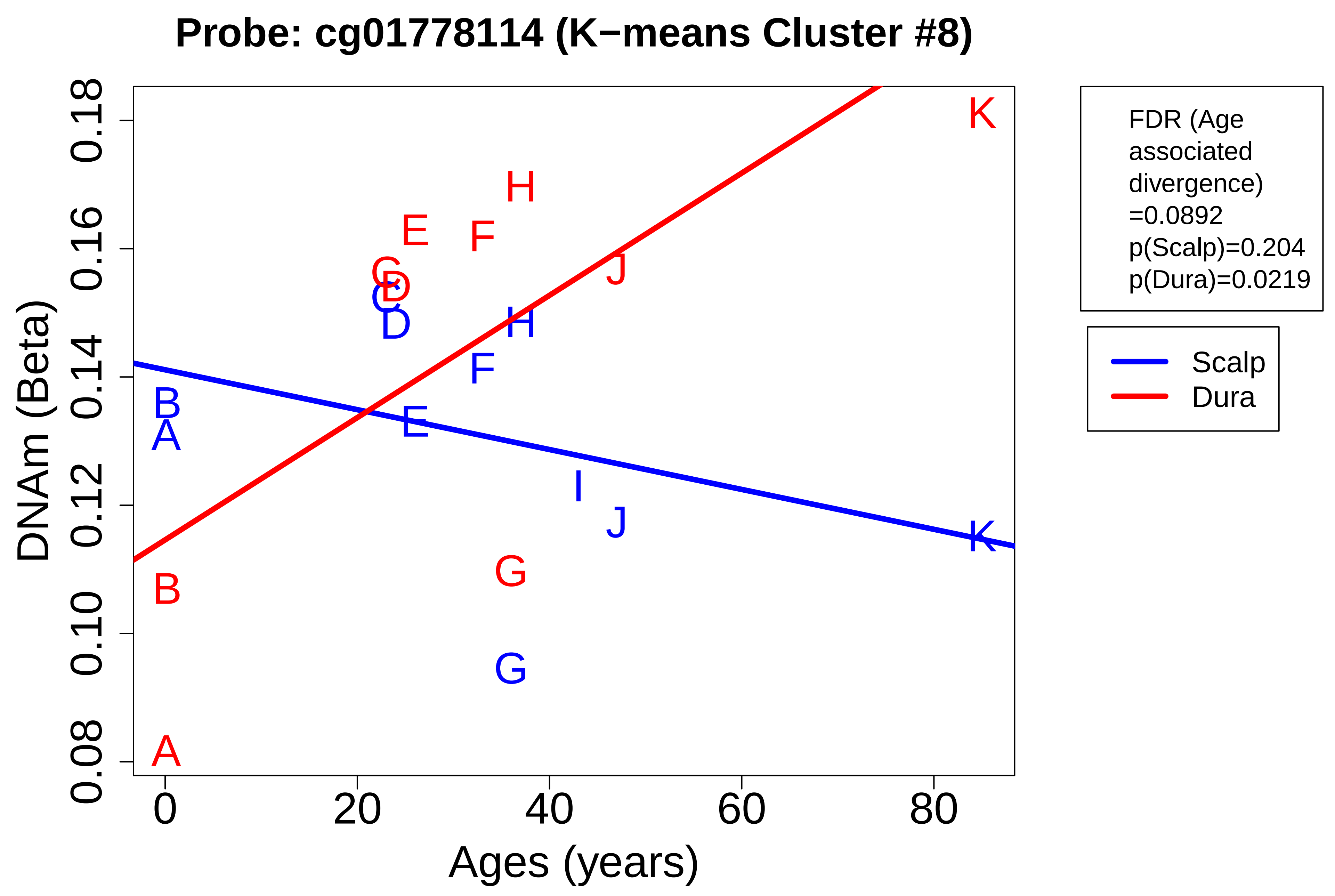

Probe: cg01778114 (K−means Cluster #8)
DNAm (Beta)
0.08
0.10
0.12
0.14
0.16
0.18
Ages (years)
0
20
40
60
80
FDR (Age associated divergence) =0.0892
p(Scalp)=0.204
p(Dura)=0.0219
Scalp
Dura

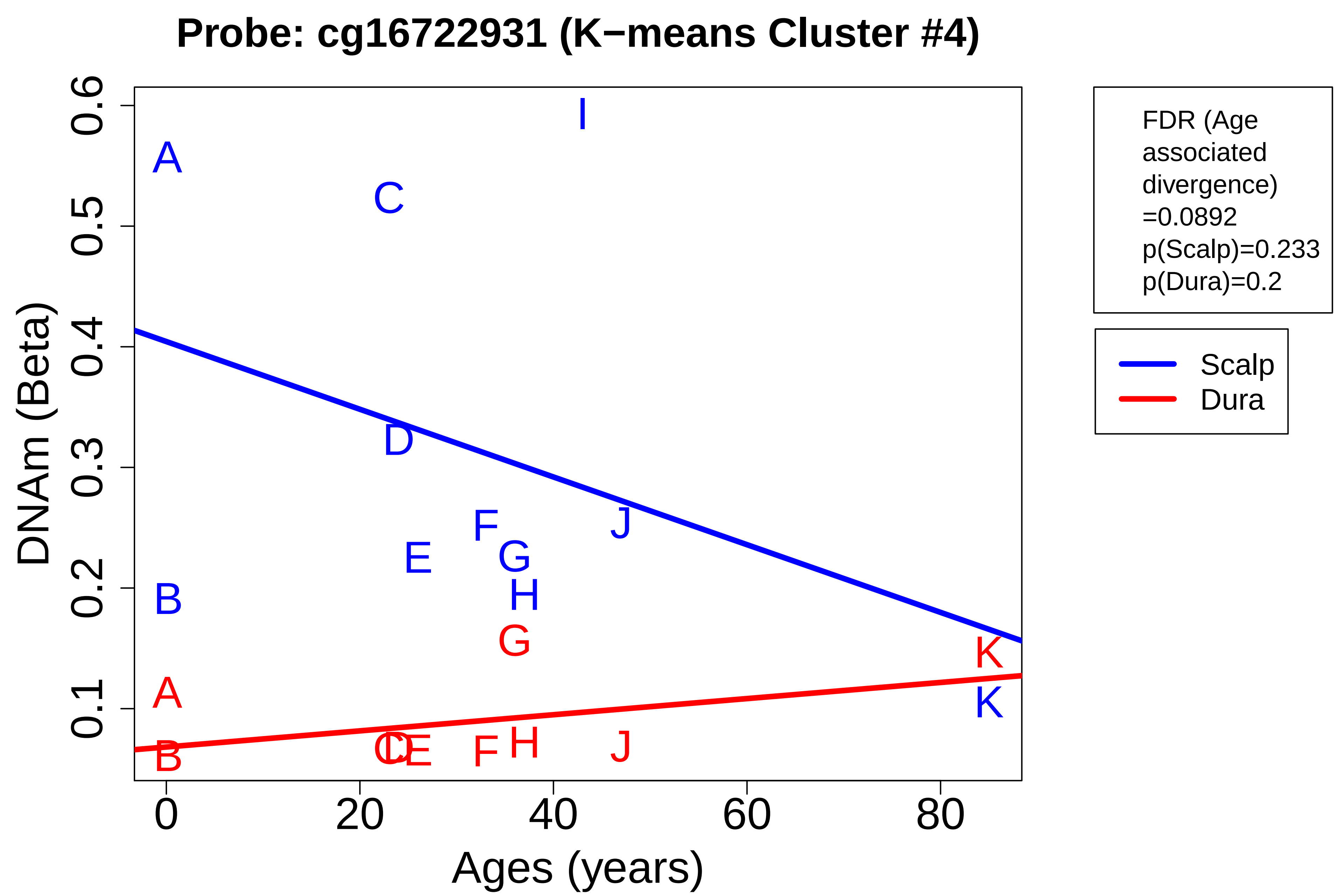

Probe: cg16722931 (K−means Cluster #4)
DNAm (Beta)
0.1
0.2
0.3
0.4
0.5
0.6
Ages (years)
0
20
40
60
80
A
B
C
D
E
F
G
H
I
J
K
FDR (Age associated divergence) =0.0892
p(Scalp)=0.233
p(Dura)=0.2
Scalp
Dura

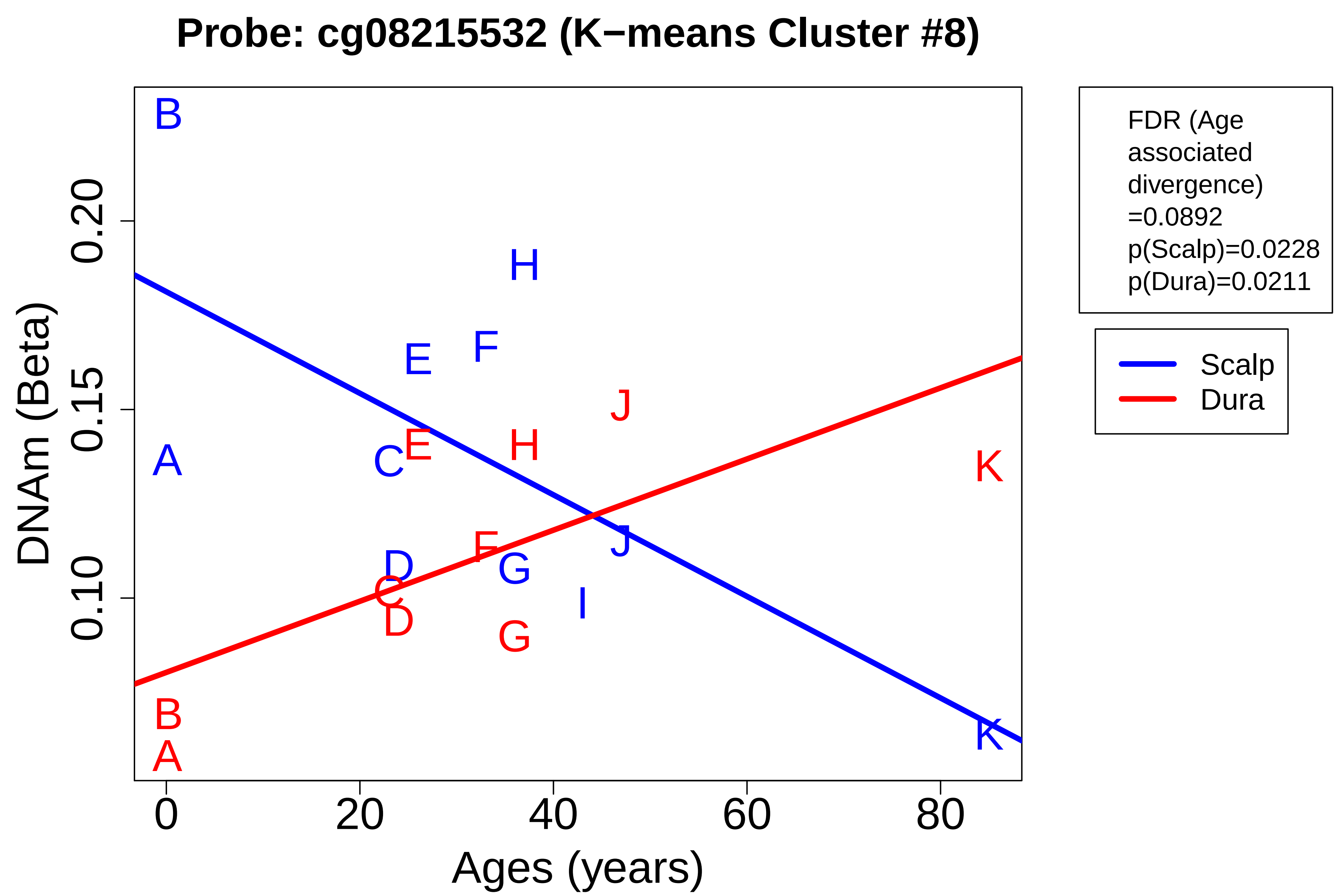
Probe: cg08215532 (K−means Cluster #8)
DNAm (Beta)
0.10
0.15
0.20
Ages (years)
0
20
40
60
80
FDR (Age associated divergence) =0.0892
p(Scalp)=0.0228
p(Dura)=0.0211
Scalp
Dura
A
B
C
D
E
F
G
H
I
J
K

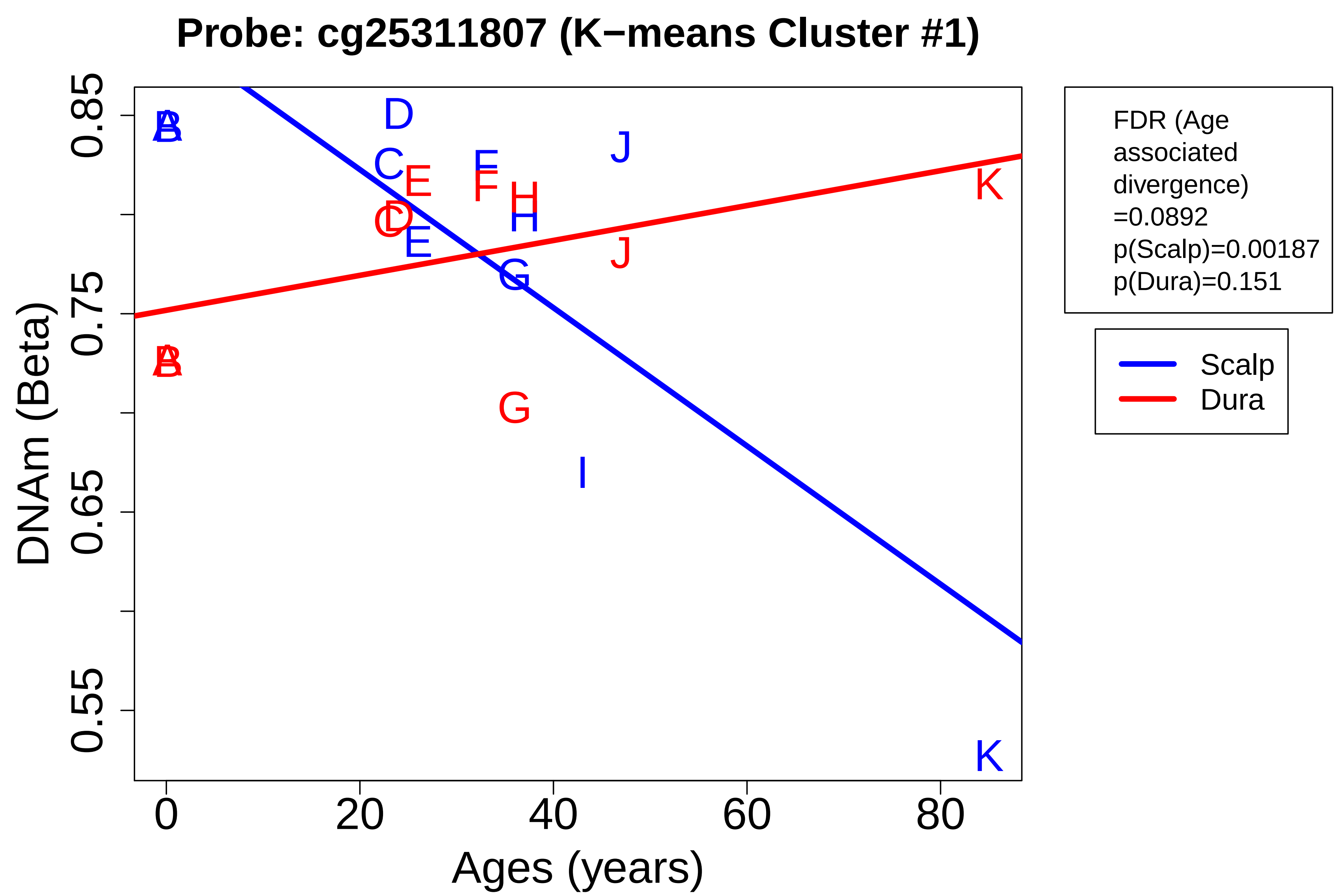
Probe: cg25311807 (K−means Cluster #1)
DNAm (Beta)
0.55
0.65
0.75
0.85
0
20
40
60
80
Ages (years)
FDR (Age associated divergence) =0.0892
p(Scalp)=0.00187
p(Dura)=0.151
Scalp
Dura

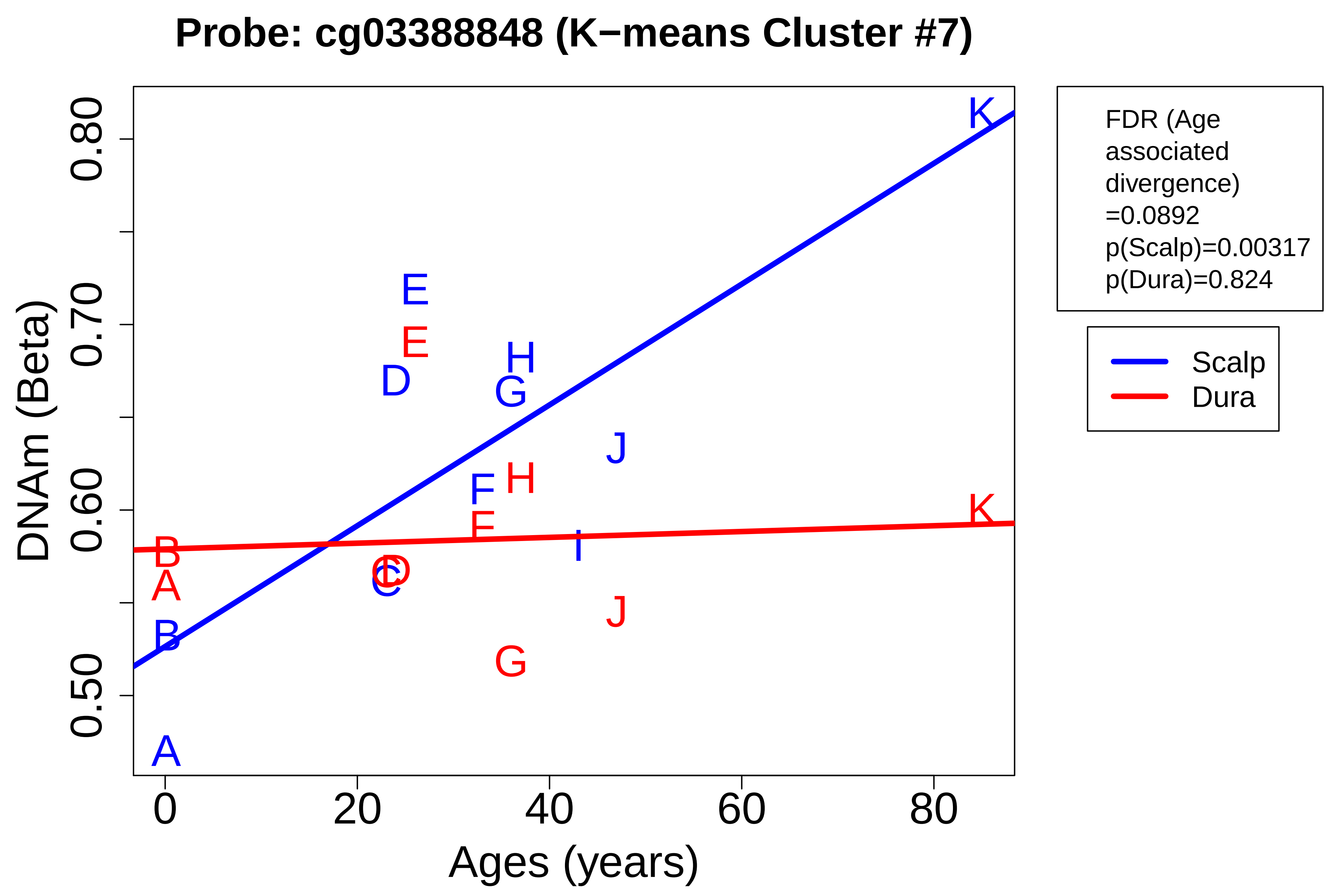
Probe: cg03388848 (K−means Cluster #7)
DNAm (Beta)
0.50
0.60
0.70
0.80
0
20
40
60
80
Ages (years)
FDR (Age associated divergence) =0.0892
p(Scalp)=0.00317
p(Dura)=0.824
Scalp
Dura
A
B
C
D
E
F
G
H
I
J
K

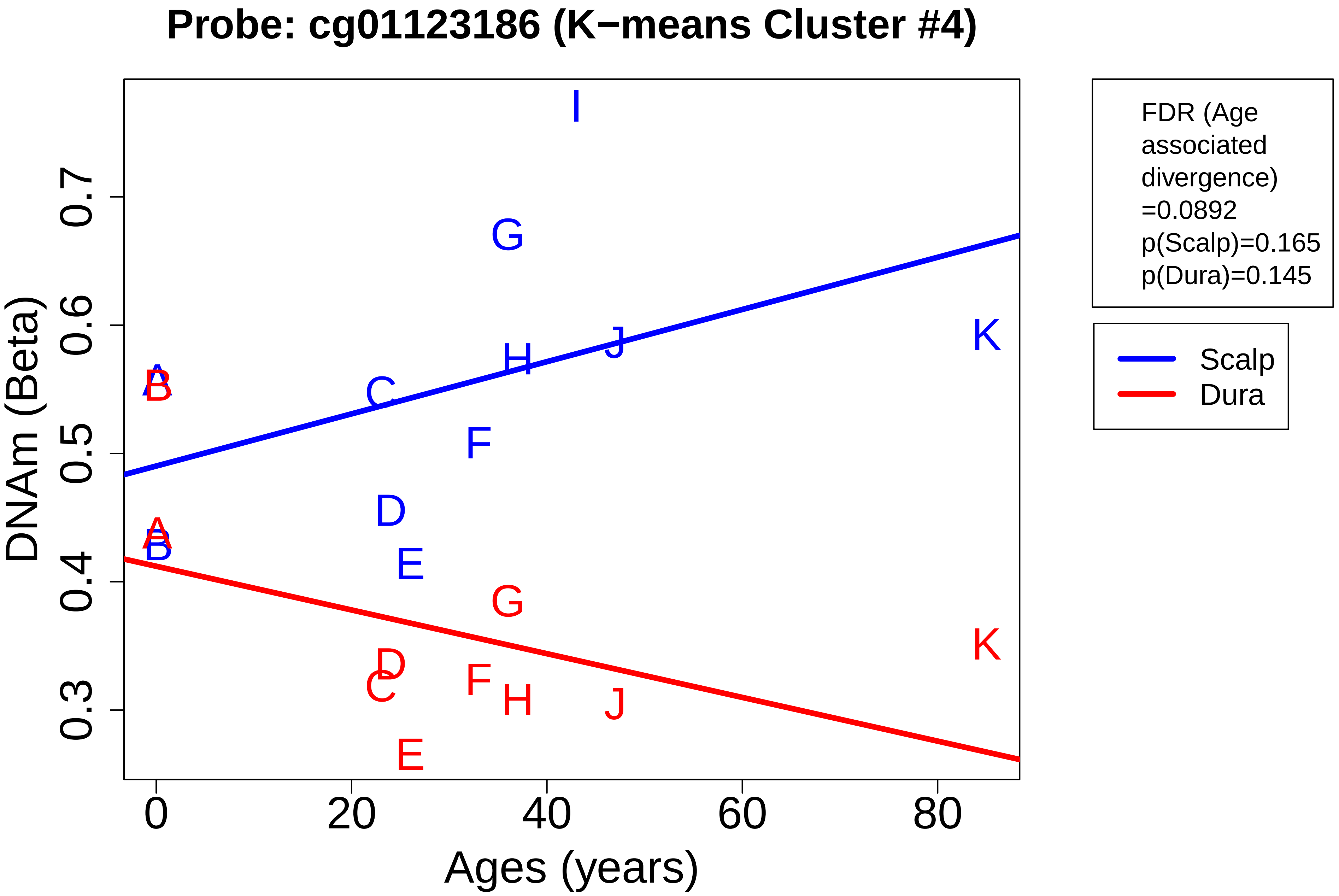

Probe: cg01123186 (K−means Cluster #4)
DNAm (Beta)
0.3
0.4
0.5
0.6
0.7
0
20
40
60
80
Ages (years)
FDR (Age associated divergence) =0.0892
p(Scalp)=0.165
p(Dura)=0.145
Scalp
Dura
A
B
C
D
E
F
G
H
I
J
K

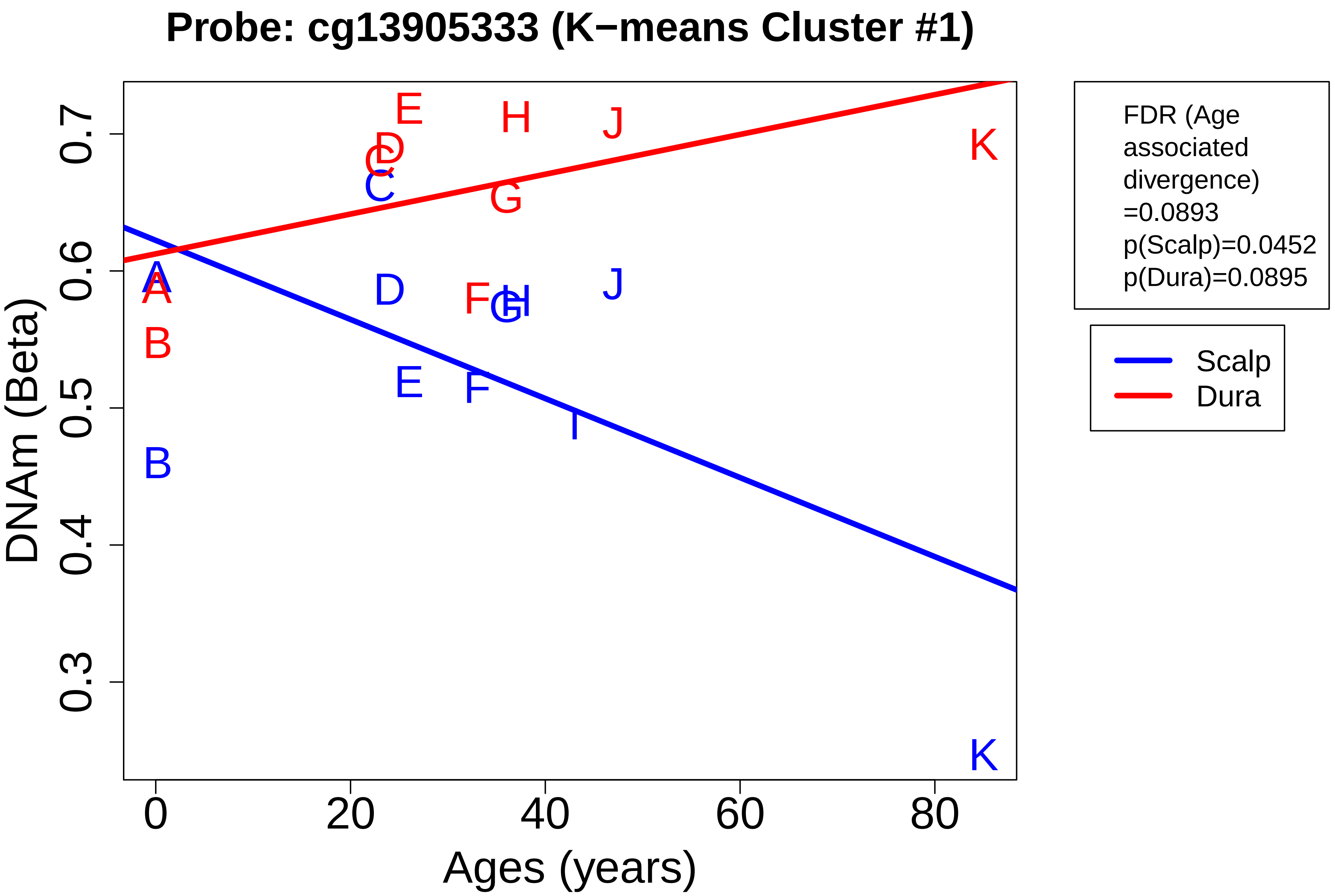

Probe: cg13905333 (K−means Cluster #1)
DNAm (Beta)
0.3
0.4
0.5
0.6
0.7
Ages (years)
0
20
40
60
80
FDR (Age associated divergence) =0.0893
p(Scalp)=0.0452
p(Dura)=0.0895
Scalp
Dura

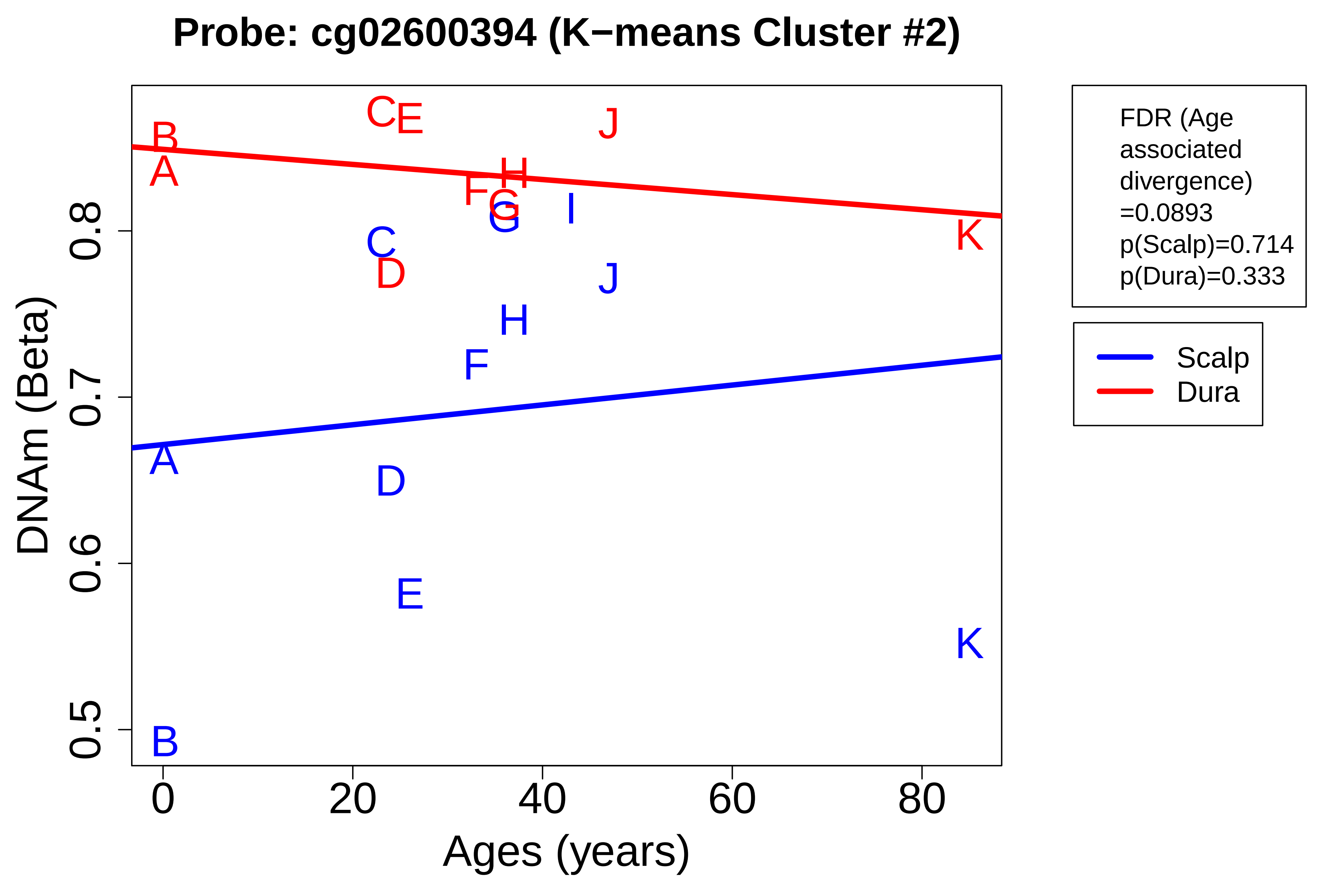

Probe: cg02600394 (K−means Cluster #2)
DNAm (Beta)
0.5
0.6
0.7
0.8
Ages (years)
0
20
40
60
80
FDR (Age associated divergence) =0.0893
p(Scalp)=0.714
p(Dura)=0.333
Scalp
Dura

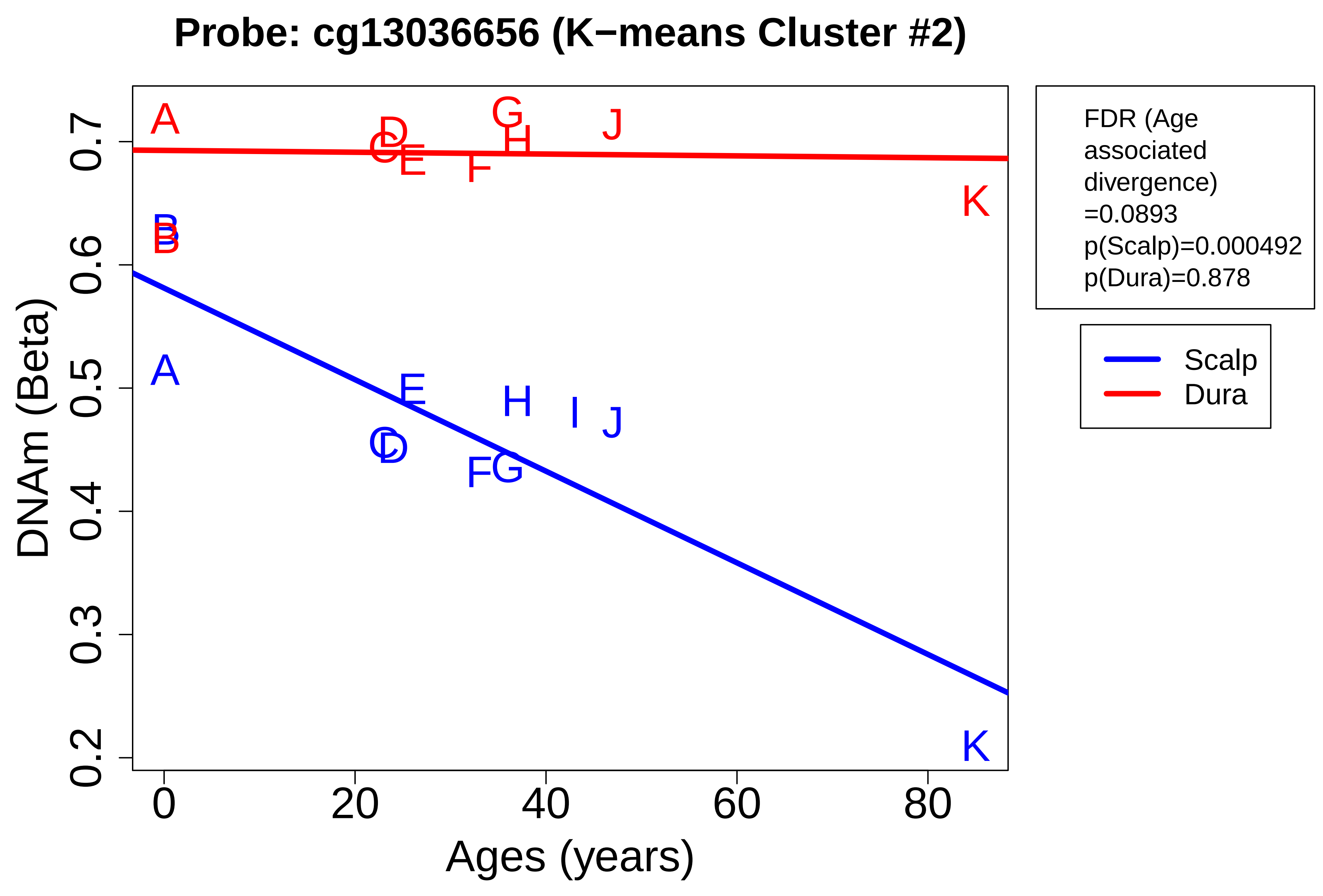

Probe: cg13036656 (K−means Cluster #2)
FDR (Age associated divergence) =0.0893
p(Scalp)=0.000492
p(Dura)=0.878
Scalp
Dura
DNAm (Beta)
0.2
0.3
0.4
0.5
0.6
0.7
0
20
40
60
80
Ages (years)
A
B
C
D
E
F
G
H
I
J
K

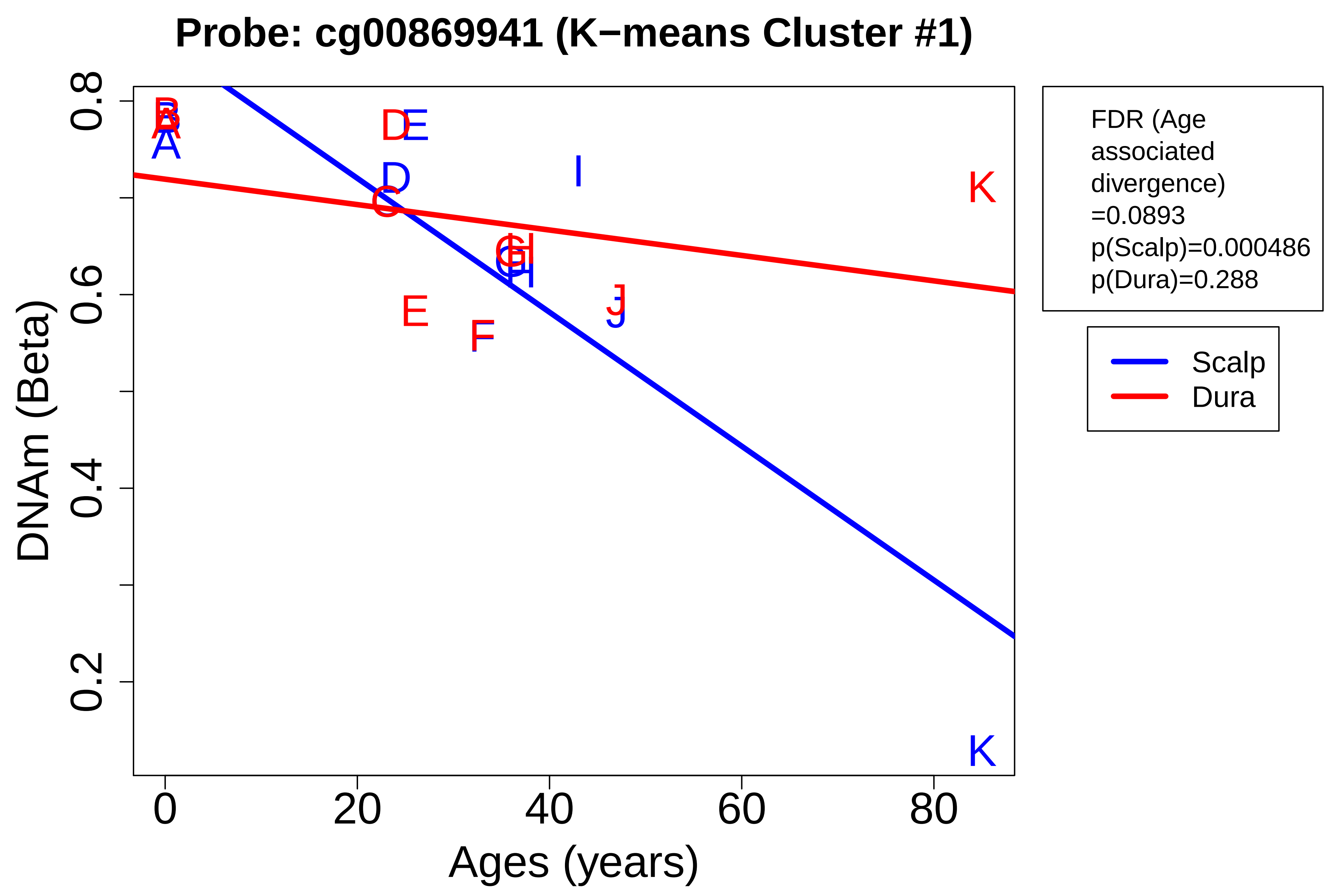
Probe: cg00869941 (K−means Cluster #1)
DNAm (Beta)
0.2
0.4
0.6
0.8
0
20
40
60
80
Ages (years)
A
B
C
D
E
F
G
H
I
J
K
FDR (Age associated divergence) =0.0893
p(Scalp)=0.000486
p(Dura)=0.288
Scalp
Dura

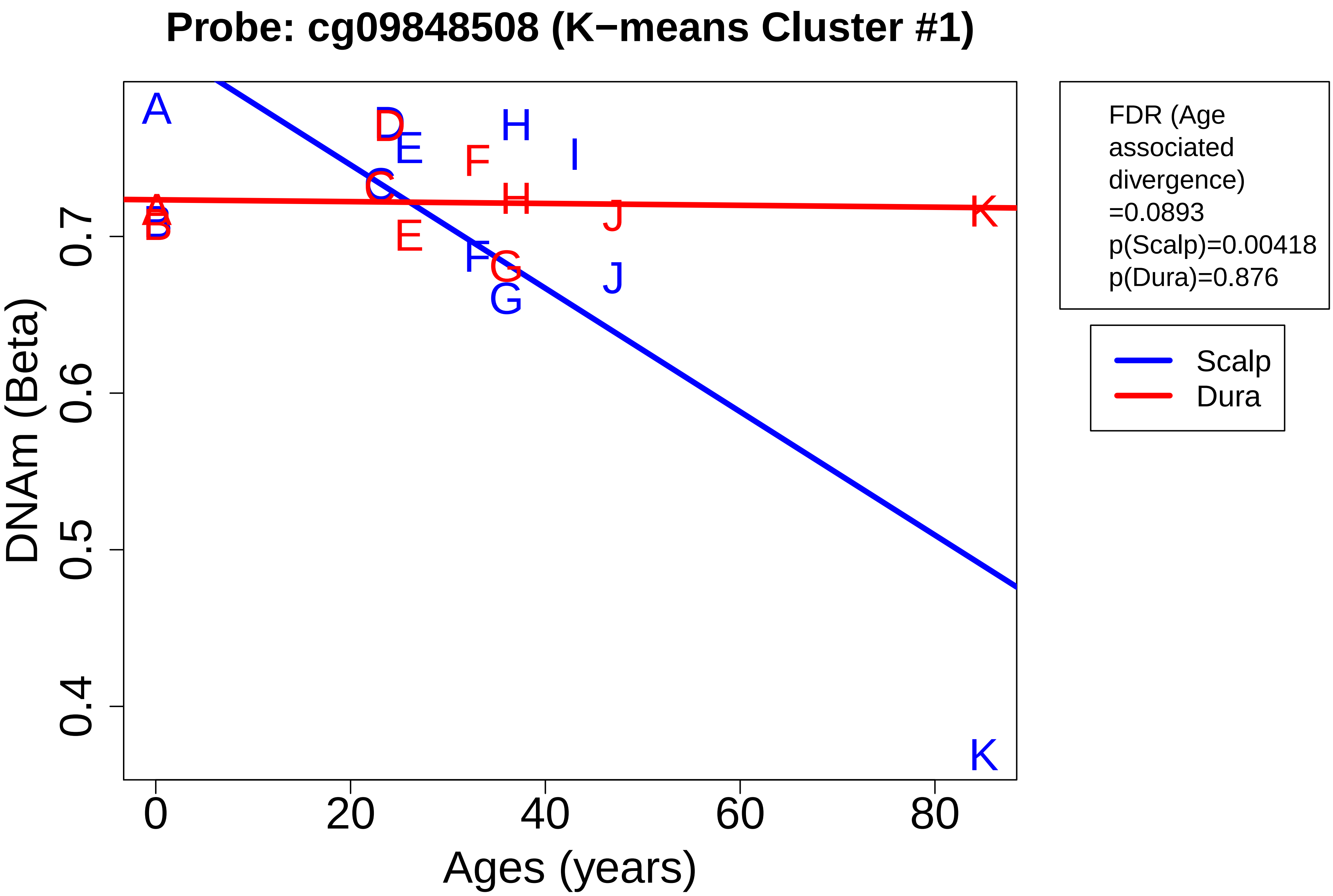

Probe: cg09848508 (K−means Cluster #1)
DNAm (Beta)
0.4
0.5
0.6
0.7
0
20
40
60
80
Ages (years)
FDR (Age associated divergence) =0.0893
p(Scalp)=0.00418
p(Dura)=0.876
Scalp
Dura

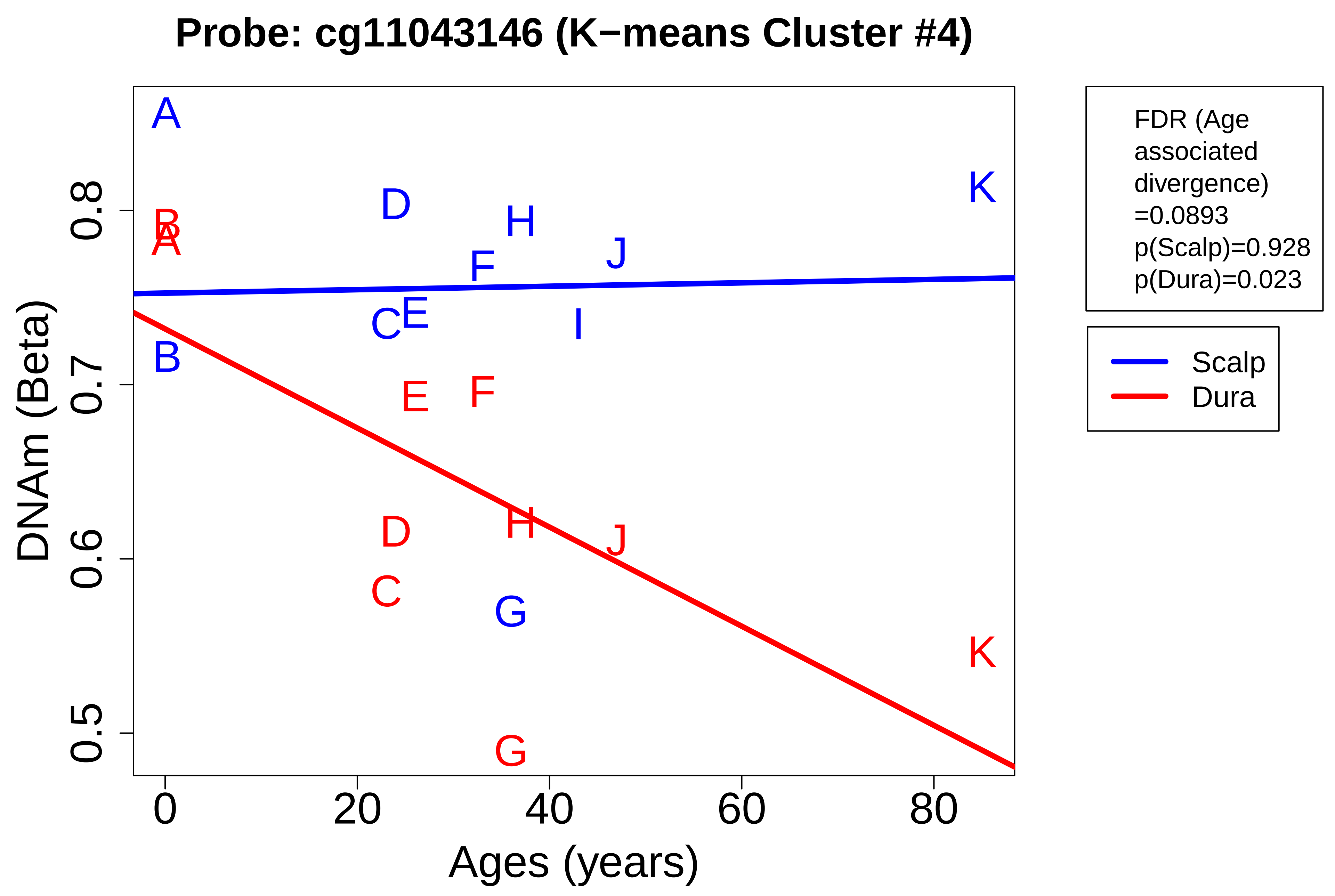

Probe: cg11043146 (K−means Cluster #4)
DNAm (Beta)
0.5
0.6
0.7
0.8
Ages (years)
0
20
40
60
80
A
B
C
D
E
F
G
H
I
J
K
FDR (Age associated divergence) =0.0893
p(Scalp)=0.928
p(Dura)=0.023
Scalp
Dura

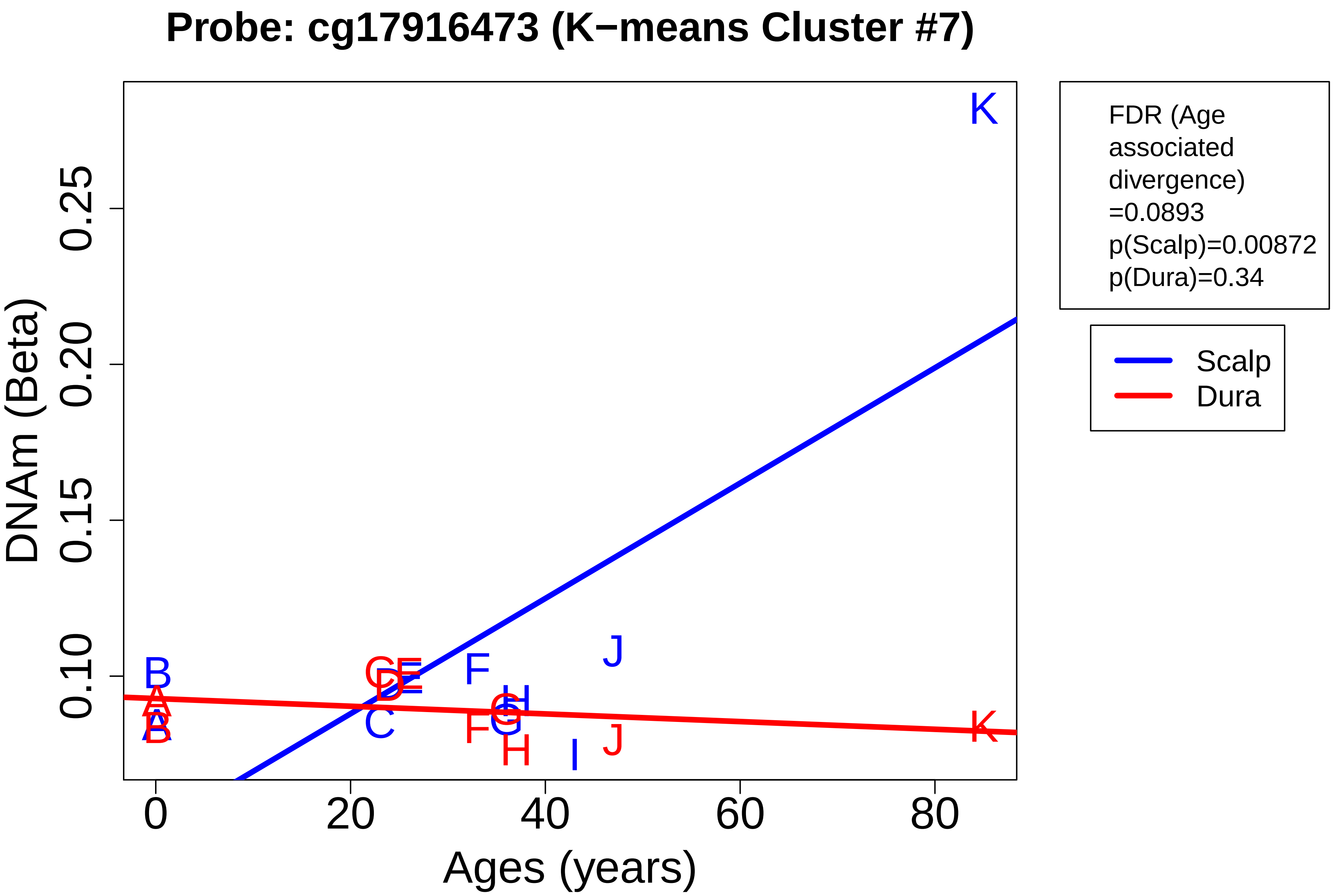

Probe: cg17916473 (K−means Cluster #7)
DNAm (Beta)
0.10
0.15
0.20
0.25
Ages (years)
0
20
40
60
80
FDR (Age associated divergence) =0.0893
p(Scalp)=0.00872
p(Dura)=0.34
Scalp
Dura
A
B
C
D
E
F
G
H
I
J
K

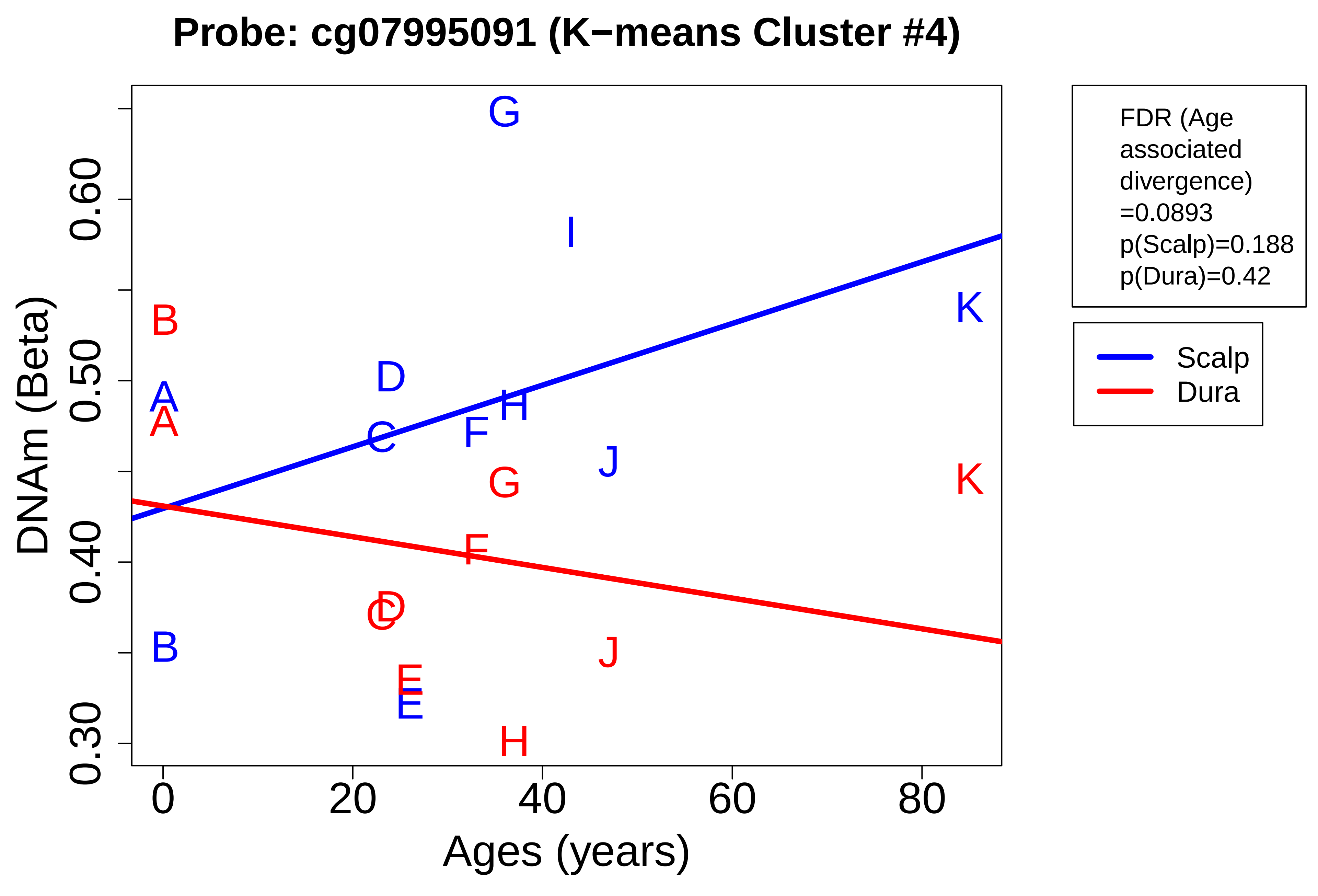

Probe: cg07995091 (K−means Cluster #4)
DNAm (Beta)
0.30
0.40
0.50
0.60
0
20
40
60
80
Ages (years)
FDR (Age associated divergence) =0.0893
p(Scalp)=0.188
p(Dura)=0.42
Scalp
Dura
A
B
C
D
E
F
G
H
I
J
K

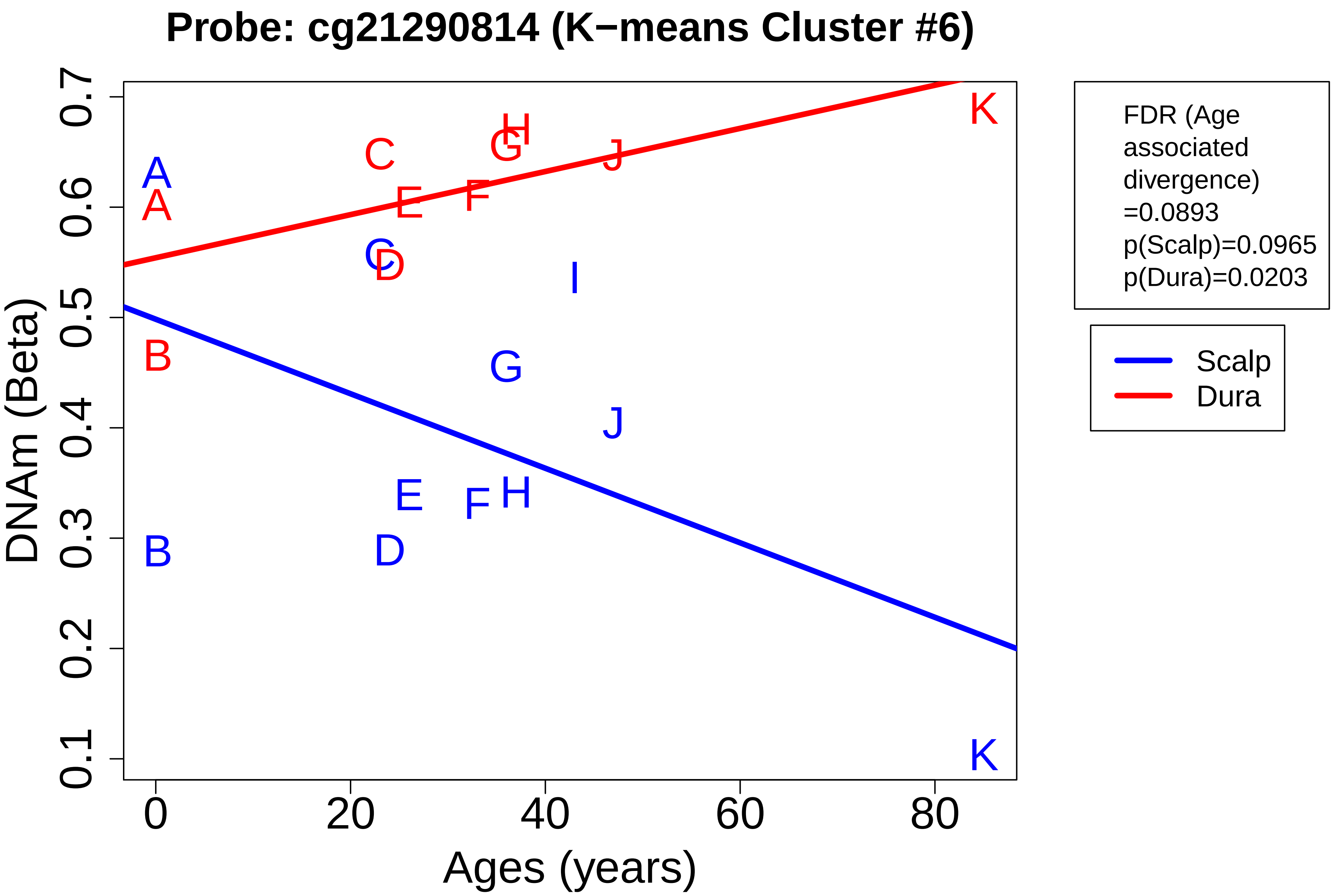
Probe: cg21290814 (K−means Cluster #6)
DNAm (Beta)
Ages (years)
0.1
0.2
0.3
0.4
0.5
0.6
0.7
0
20
40
60
80
A
B
C
D
E
F
G
H
I
J
K
FDR (Age associated divergence) =0.0893
p(Scalp)=0.0965
p(Dura)=0.0203
Scalp
Dura

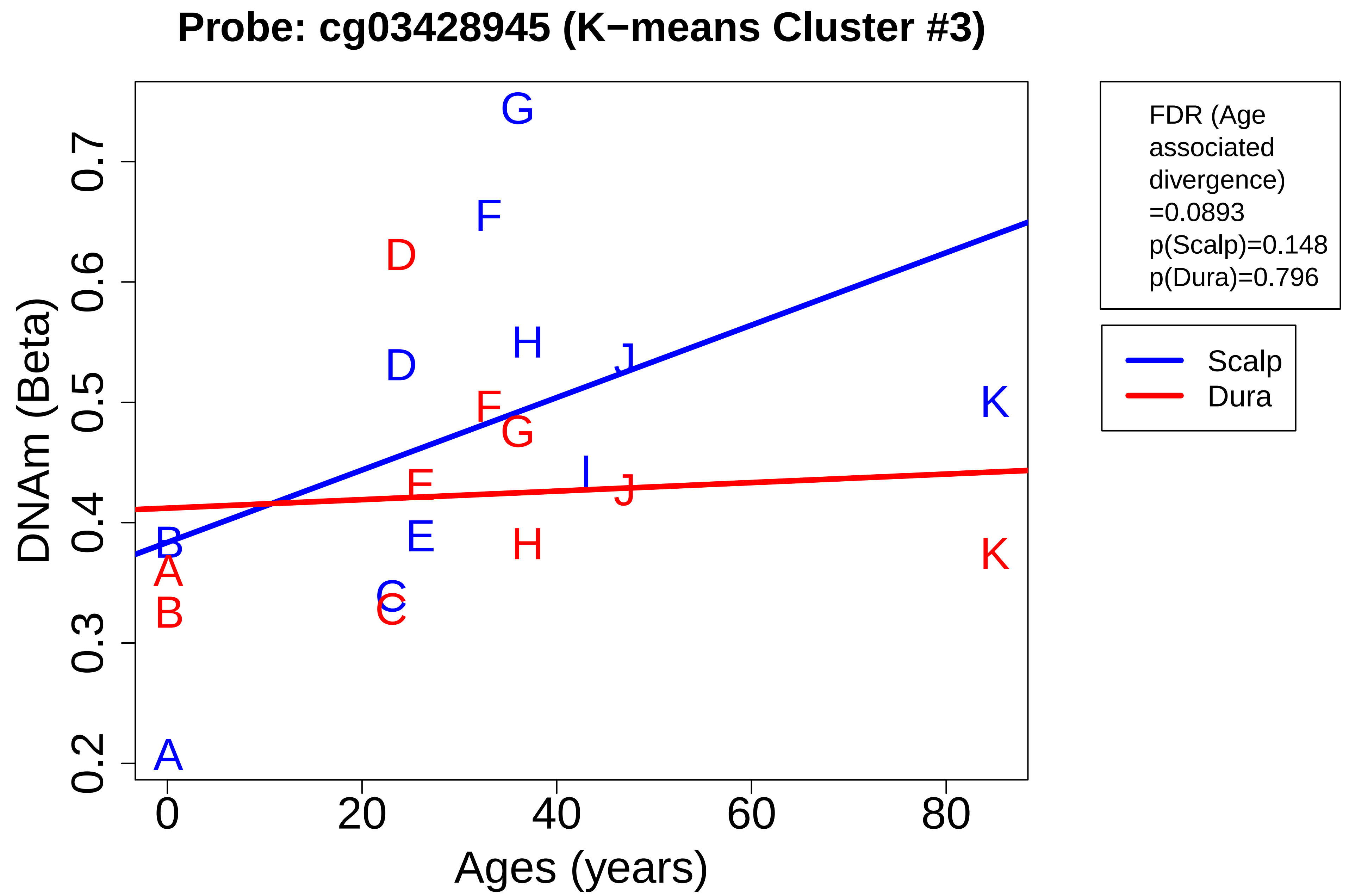

Probe: cg03428945 (K−means Cluster #3)
DNAm (Beta)
Ages (years)
0.2
0.3
0.4
0.5
0.6
0.7
0
20
40
60
80
FDR (Age associated divergence) =0.0893
p(Scalp)=0.148
p(Dura)=0.796
Scalp
Dura

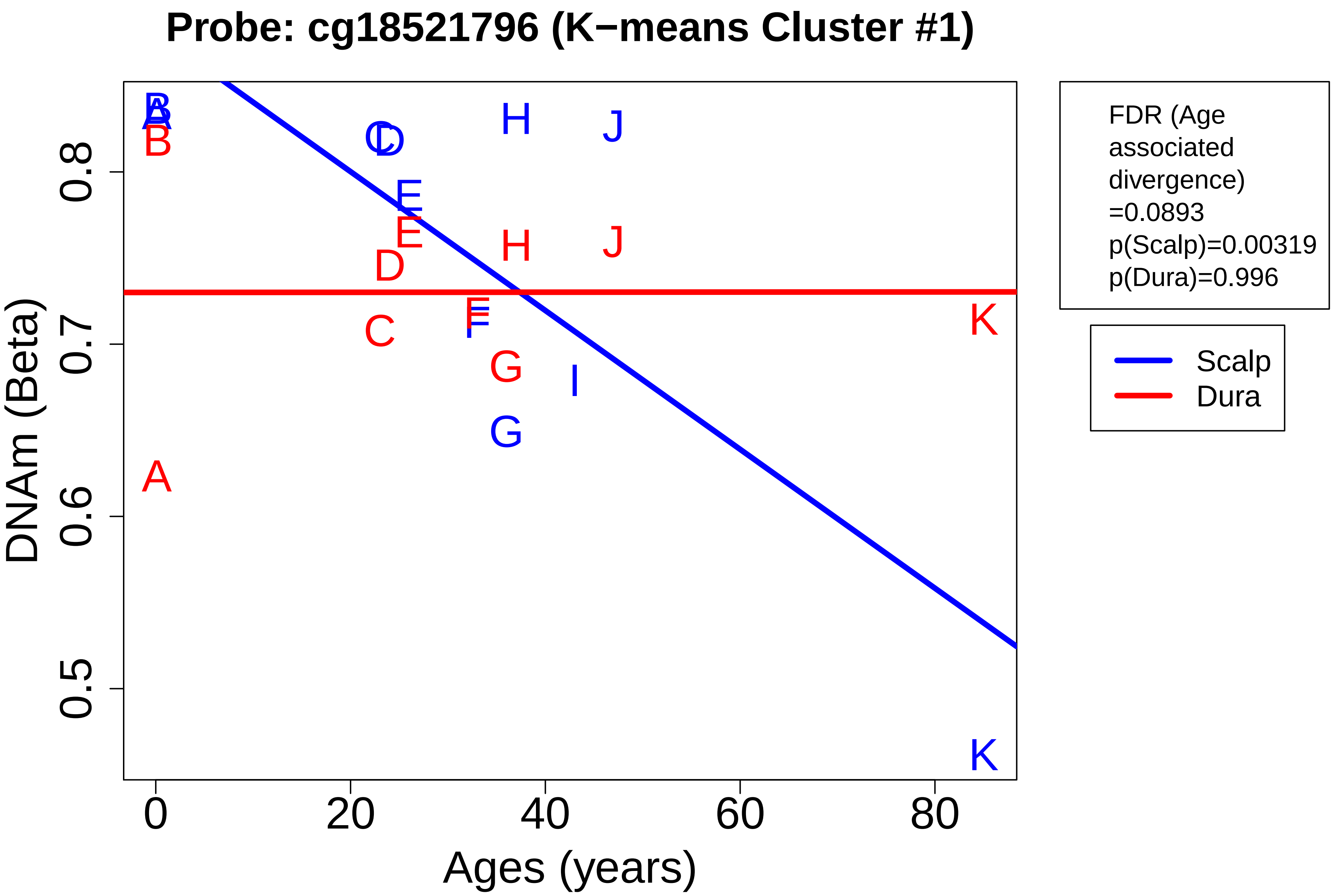

Probe: cg18521796 (K−means Cluster #1)
FDR (Age associated divergence) =0.0893
p(Scalp)=0.00319
p(Dura)=0.996
Scalp
Dura
DNAm (Beta)
0.5
0.6
0.7
0.8
0
20
40
60
80
Ages (years)

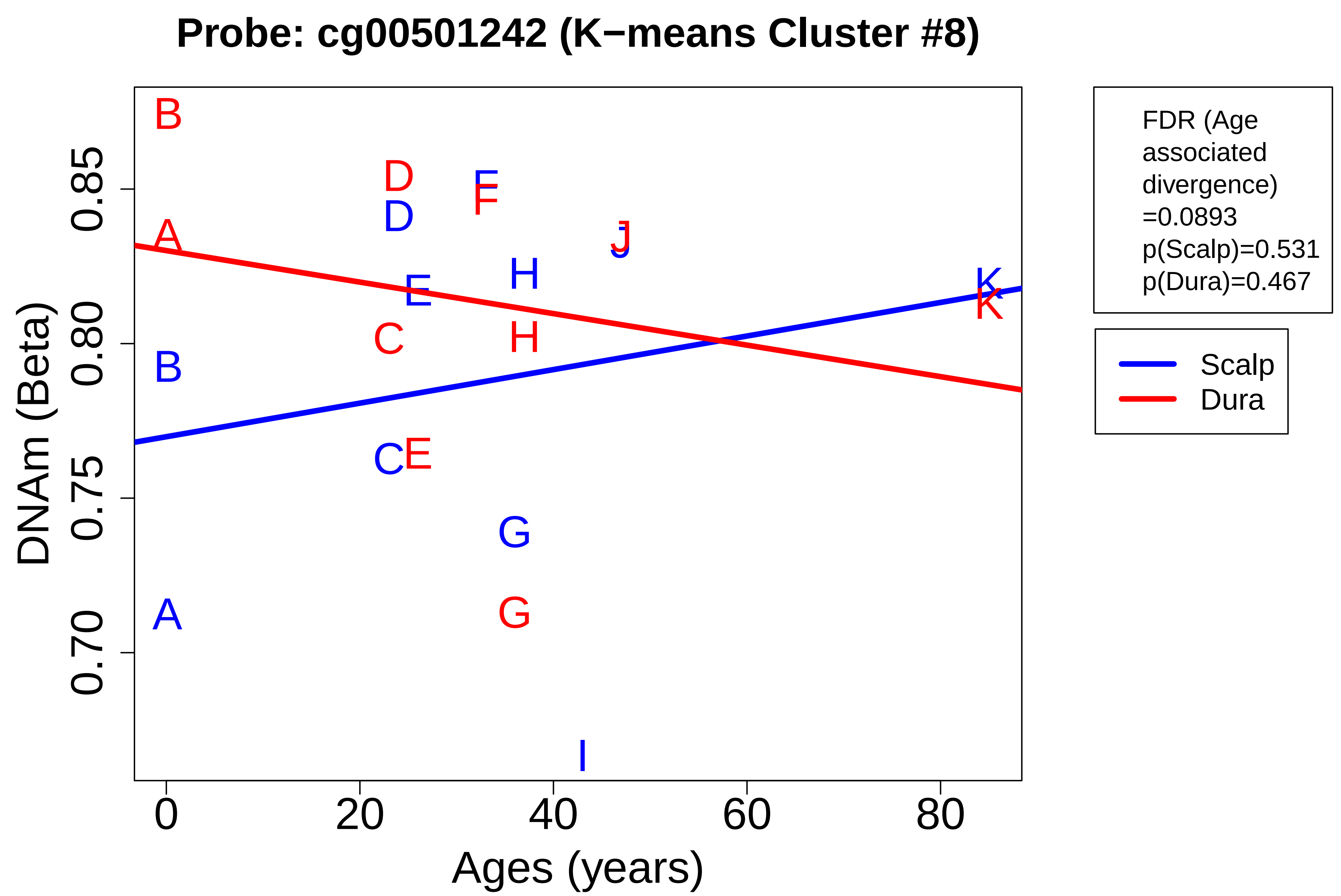

Probe: cg00501242 (K−means Cluster #8)
DNAm (Beta)
0.70
0.75
0.80
0.85
0
20
40
60
80
Ages (years)
FDR (Age associated divergence) =0.0893
p(Scalp)=0.531
p(Dura)=0.467
Scalp
Dura

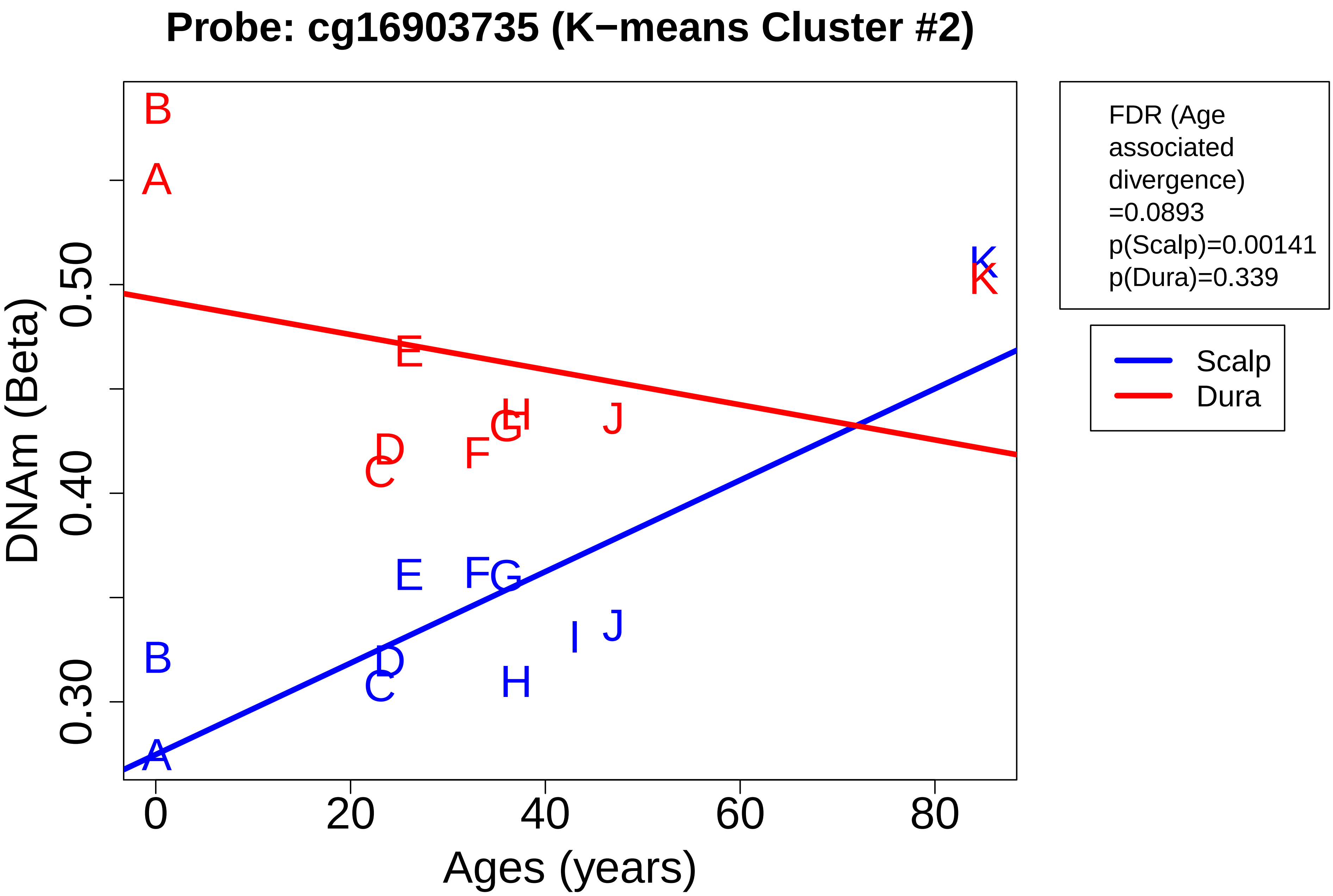
Probe: cg16903735 (K−means Cluster #2)
DNAm (Beta)
0.30
0.40
0.50
0
20
40
60
80
Ages (years)
FDR (Age associated divergence) =0.0893
p(Scalp)=0.00141
p(Dura)=0.339
Scalp
Dura
A
B
C
D
E
F
G
H
I
J
K

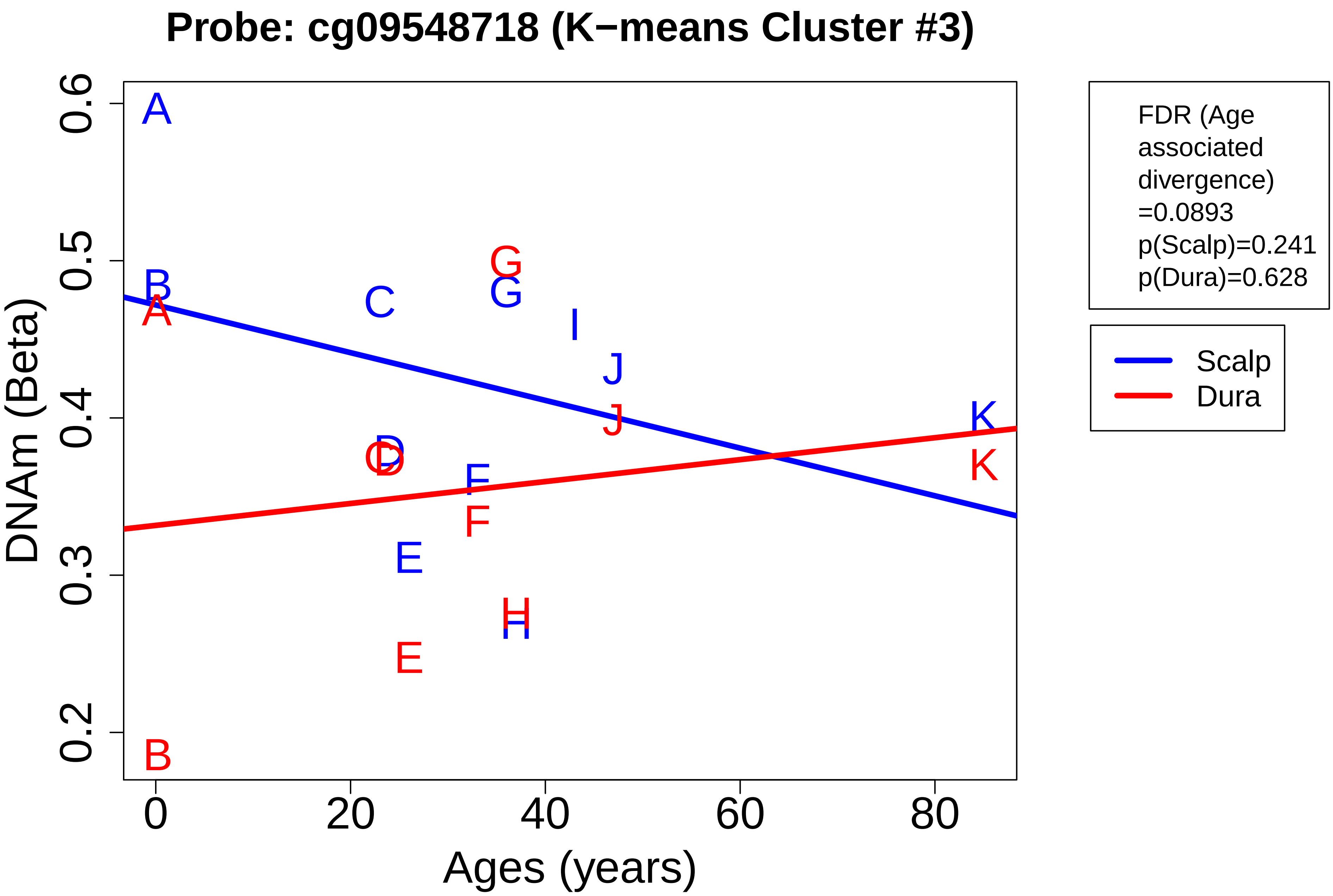

Probe: cg09548718 (K−means Cluster #3)
DNAm (Beta)
0.2
0.3
0.4
0.5
0.6
Ages (years)
0
20
40
60
80
A
B
A
C
G
G
I
J
J
K
D
D
F
F
E
H
H
E
B
K
FDR (Age associated divergence) =0.0893
p(Scalp)=0.241
p(Dura)=0.628
Scalp
Dura

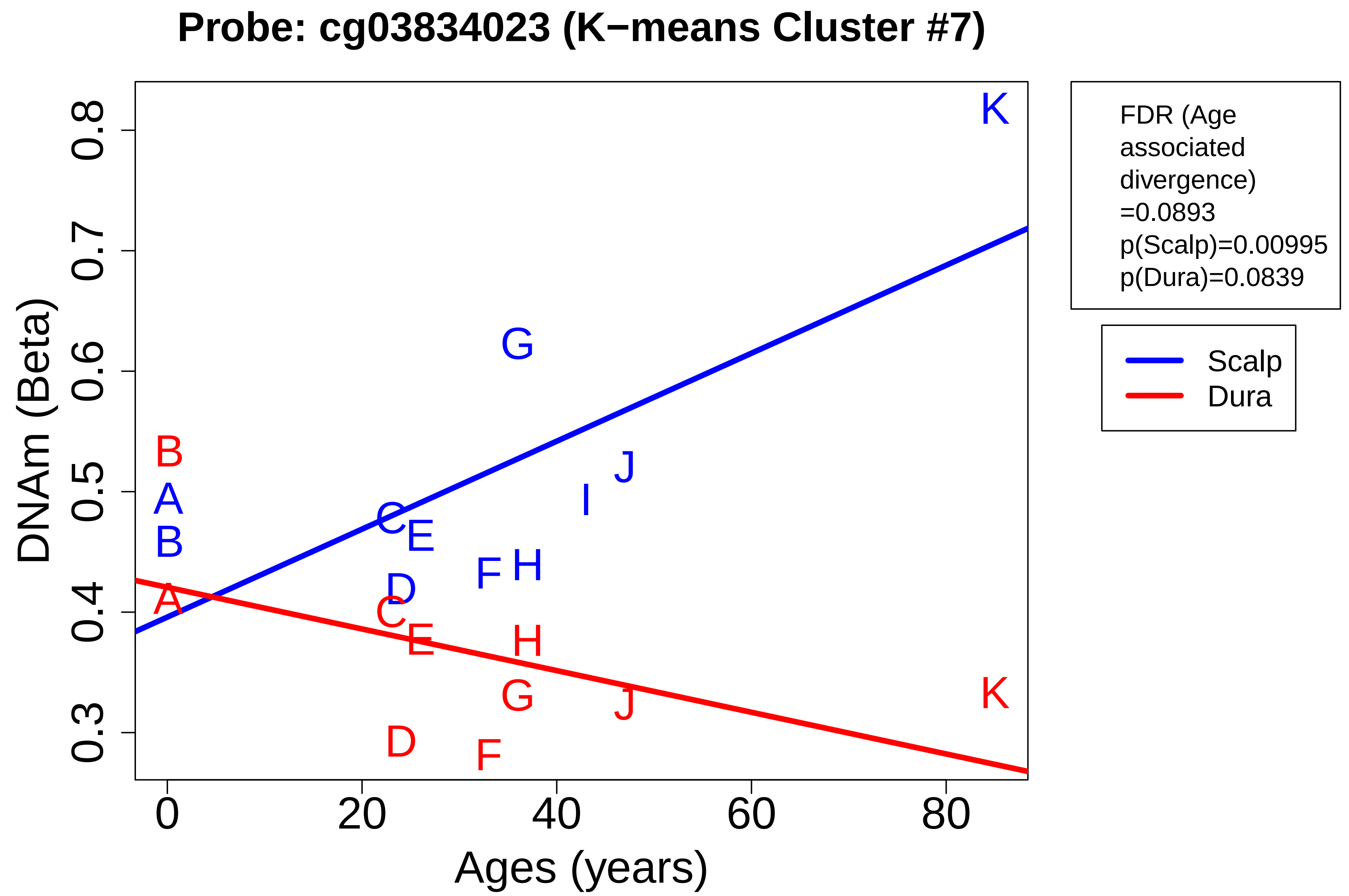

Probe: cg03834023 (K−means Cluster #7)
DNAm (Beta)
0.3
0.4
0.5
0.6
0.7
0.8
0
20
40
60
80
Ages (years)
FDR (Age associated divergence) =0.0893
p(Scalp)=0.00995
p(Dura)=0.0839
Scalp
Dura
A
B
C
D
E
F
G
H
I
J
K

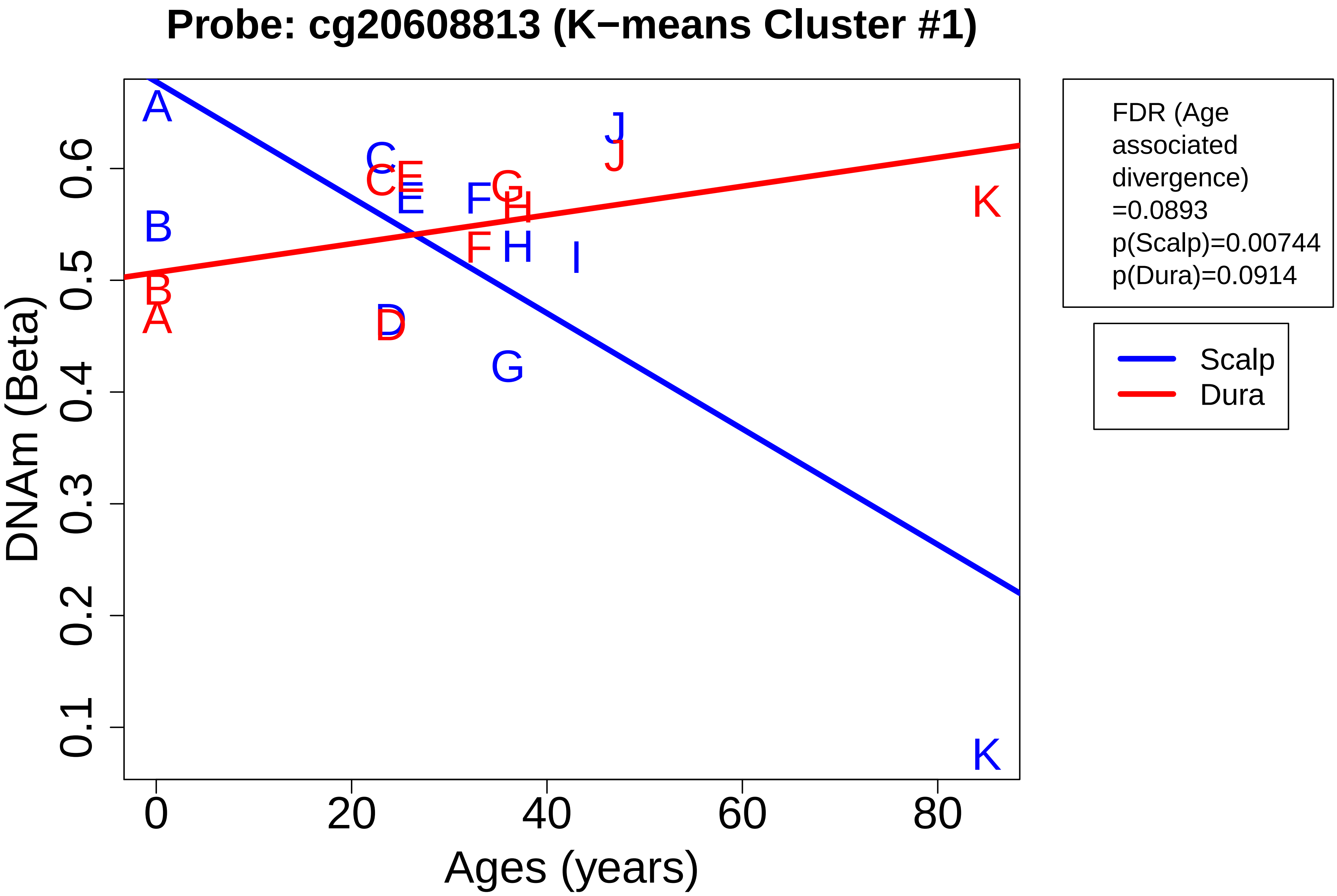

Probe: cg20608813 (K−means Cluster #1)
DNAm (Beta)
0.1
0.2
0.3
0.4
0.5
0.6
Ages (years)
0
20
40
60
80
A
B
C
D
E
F
G
H
I
J
K
FDR (Age associated divergence) =0.0893
p(Scalp)=0.00744
p(Dura)=0.0914
Scalp
Dura

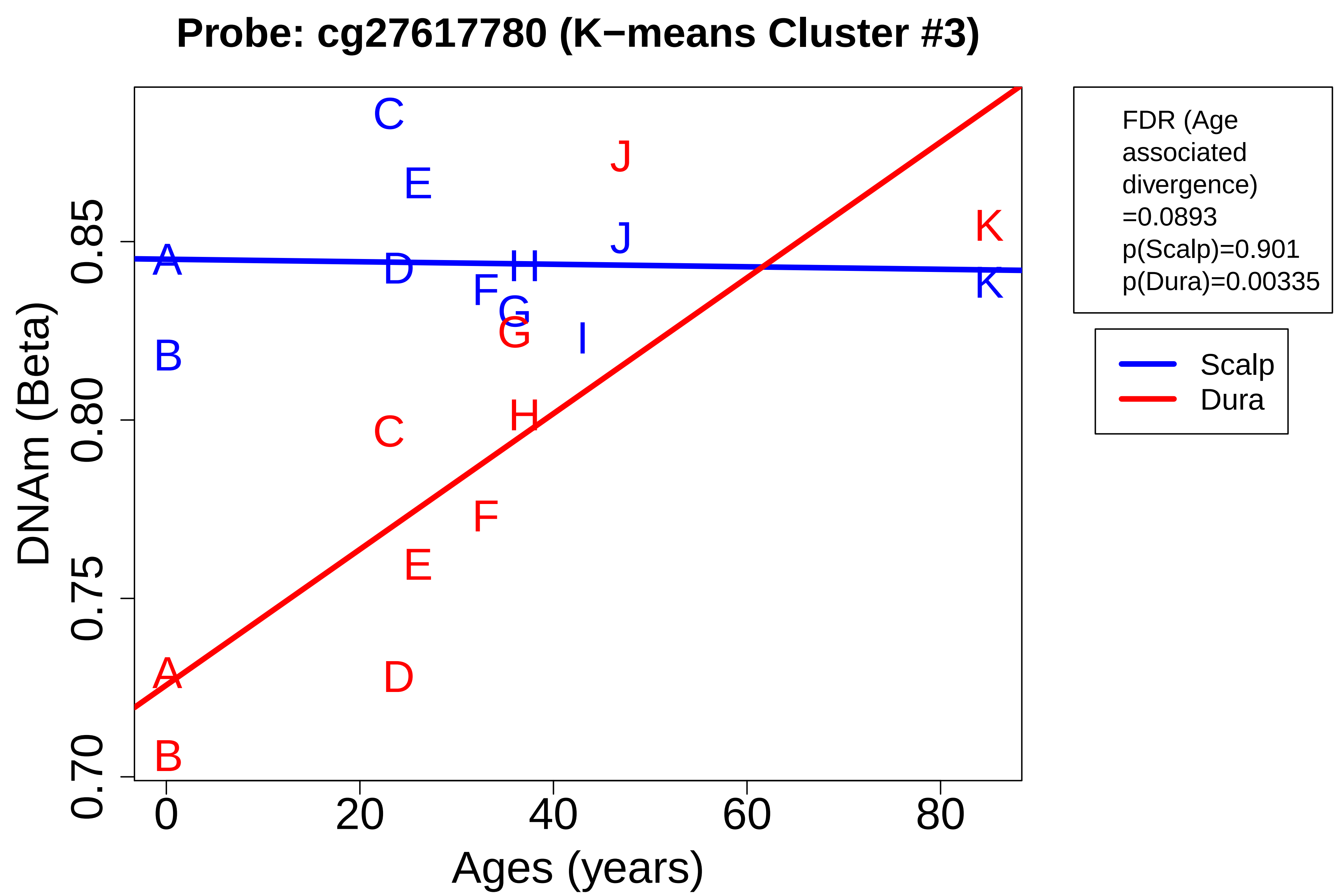

Probe: cg27617780 (K−means Cluster #3)
DNAm (Beta)
0.70
0.75
0.80
0.85
0
20
40
60
80
Ages (years)
A
B
C
D
E
F
G
H
I
J
K
FDR (Age associated divergence) =0.0893
p(Scalp)=0.901
p(Dura)=0.00335
Scalp
Dura

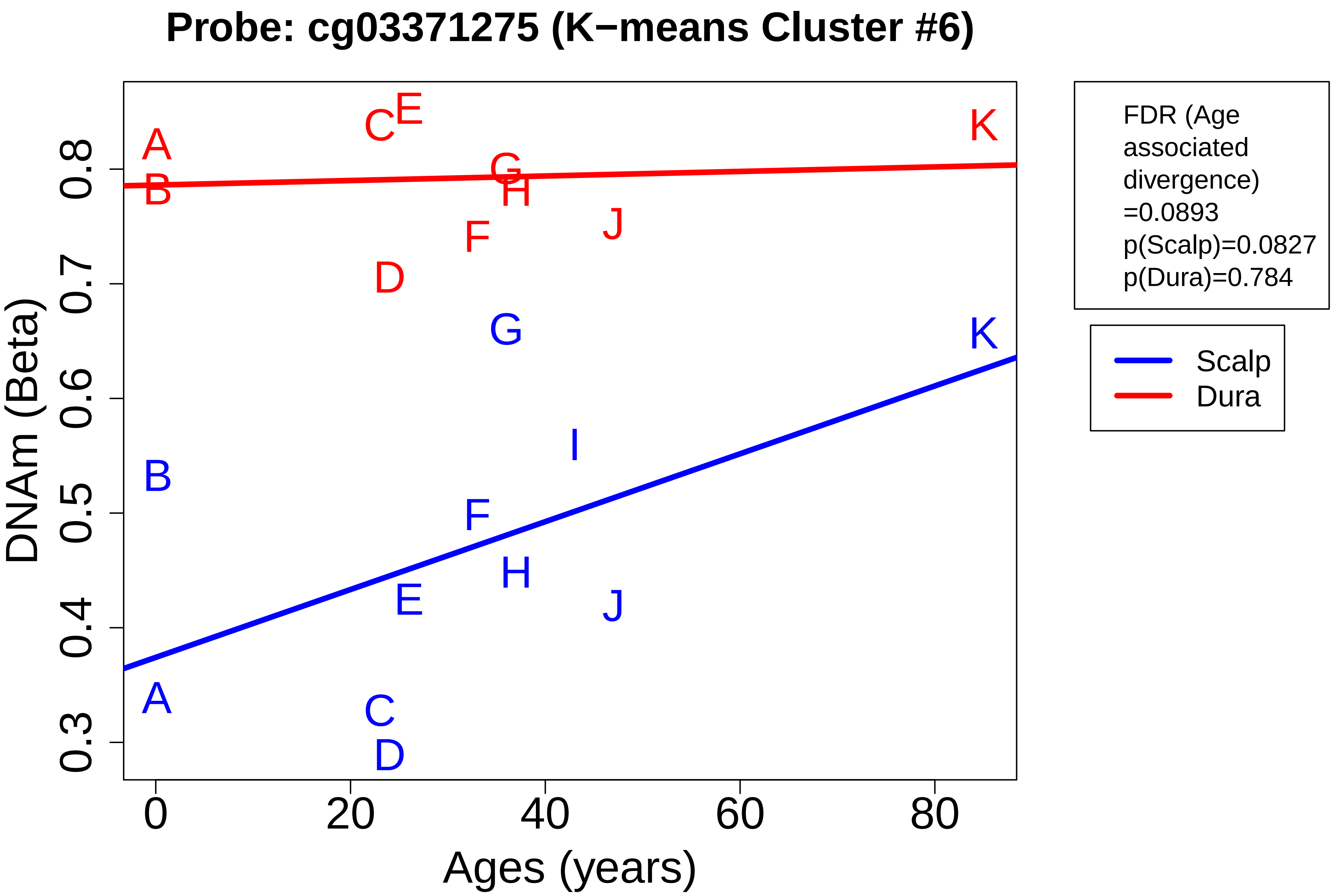
Probe: cg03371275 (K−means Cluster #6)
DNAm (Beta)
Ages (years)
0.3
0.4
0.5
0.6
0.7
0.8
0
20
40
60
80
FDR (Age associated divergence) =0.0893
p(Scalp)=0.0827
p(Dura)=0.784
Scalp
Dura
A
B
C
D
E
F
G
H
I
J
K

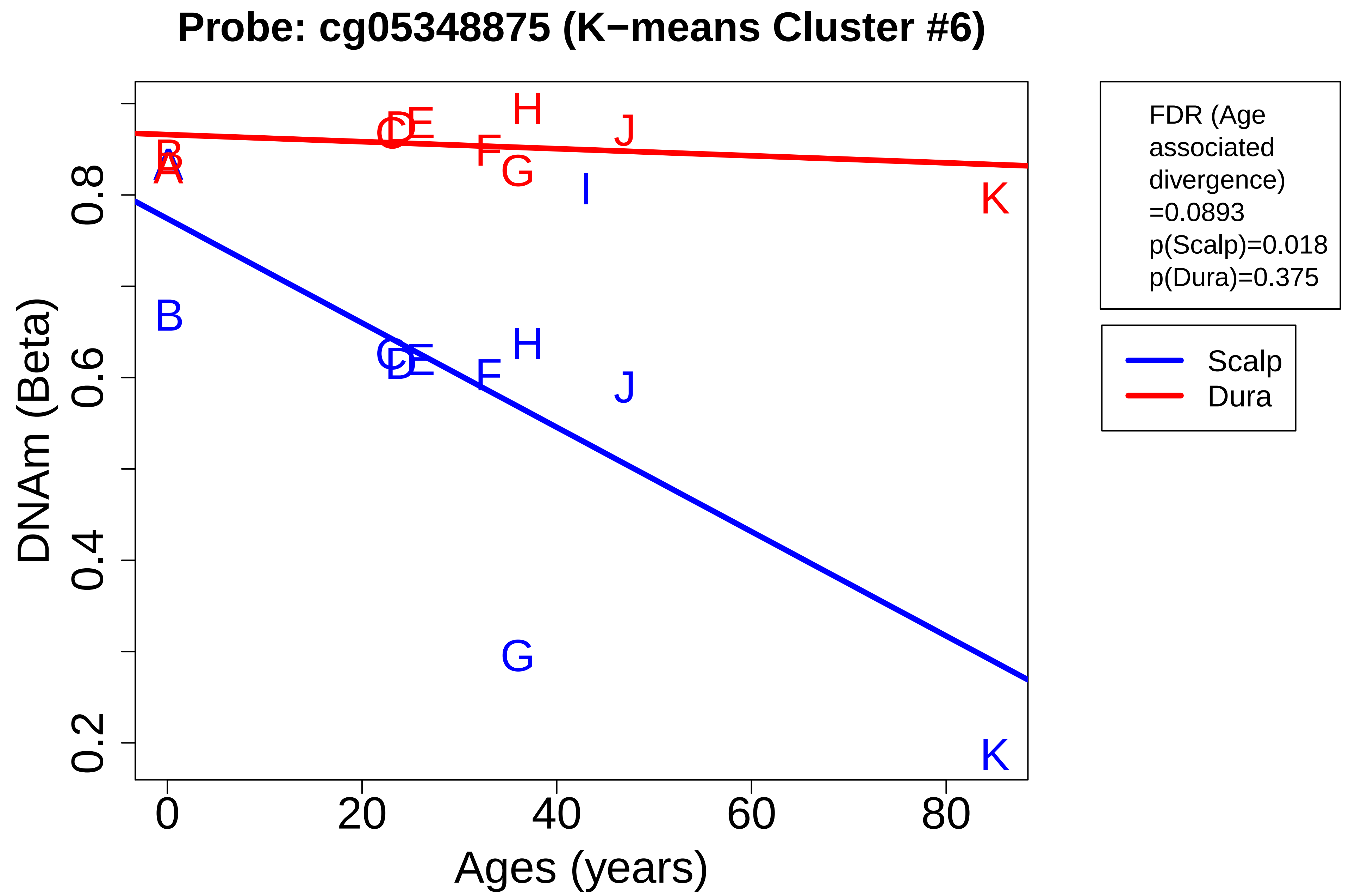

Probe: cg05348875 (K−means Cluster #6)
DNAm (Beta)
0.2
0.4
0.6
0.8
0
20
40
60
80
Ages (years)
FDR (Age associated divergence) =0.0893
p(Scalp)=0.018
p(Dura)=0.375
Scalp
Dura

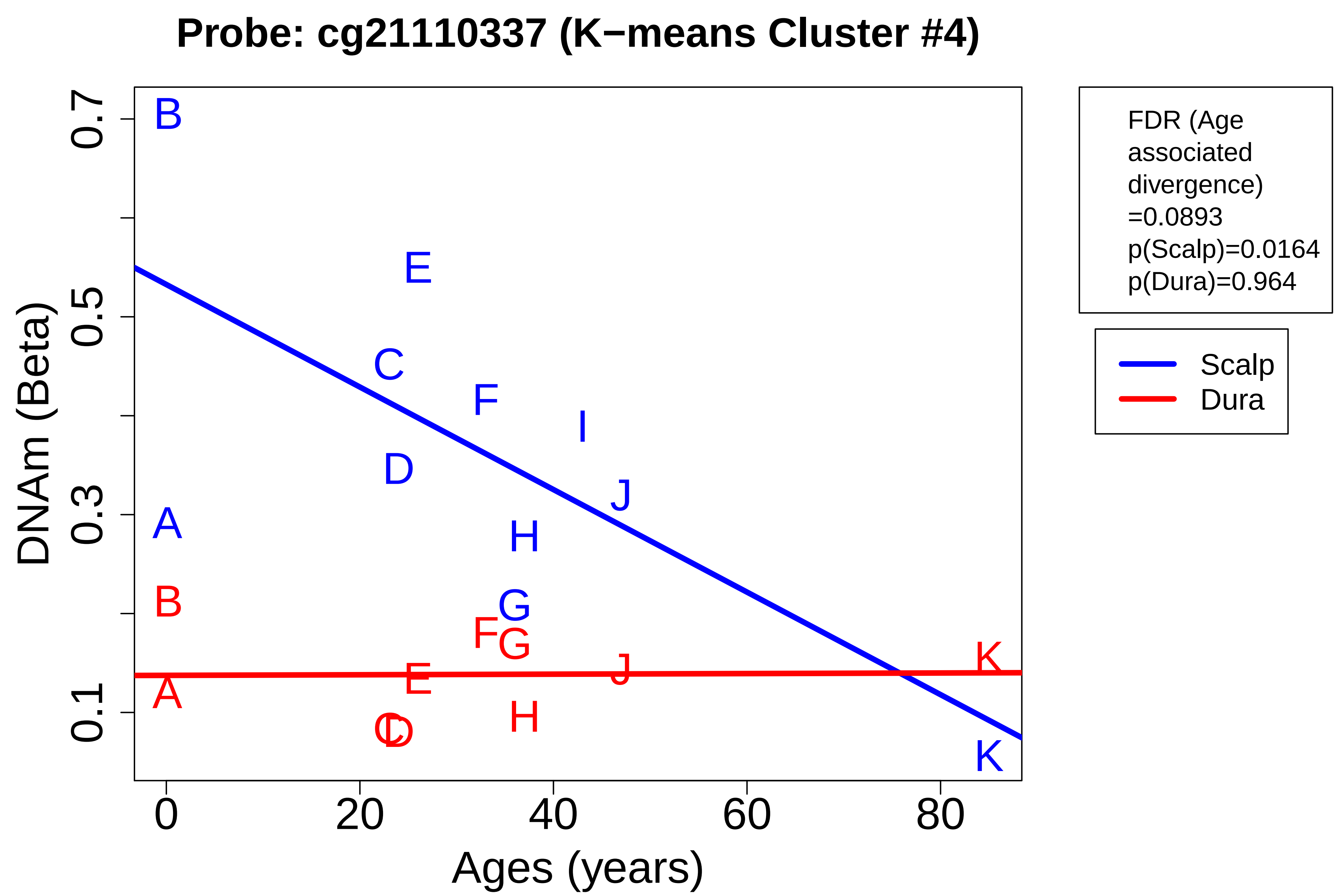

Probe: cg21110337 (K−means Cluster #4)
DNAm (Beta)
0.1
0.3
0.5
0.7
0
20
40
60
80
Ages (years)
FDR (Age associated divergence) =0.0893
p(Scalp)=0.0164
p(Dura)=0.964
Scalp
Dura

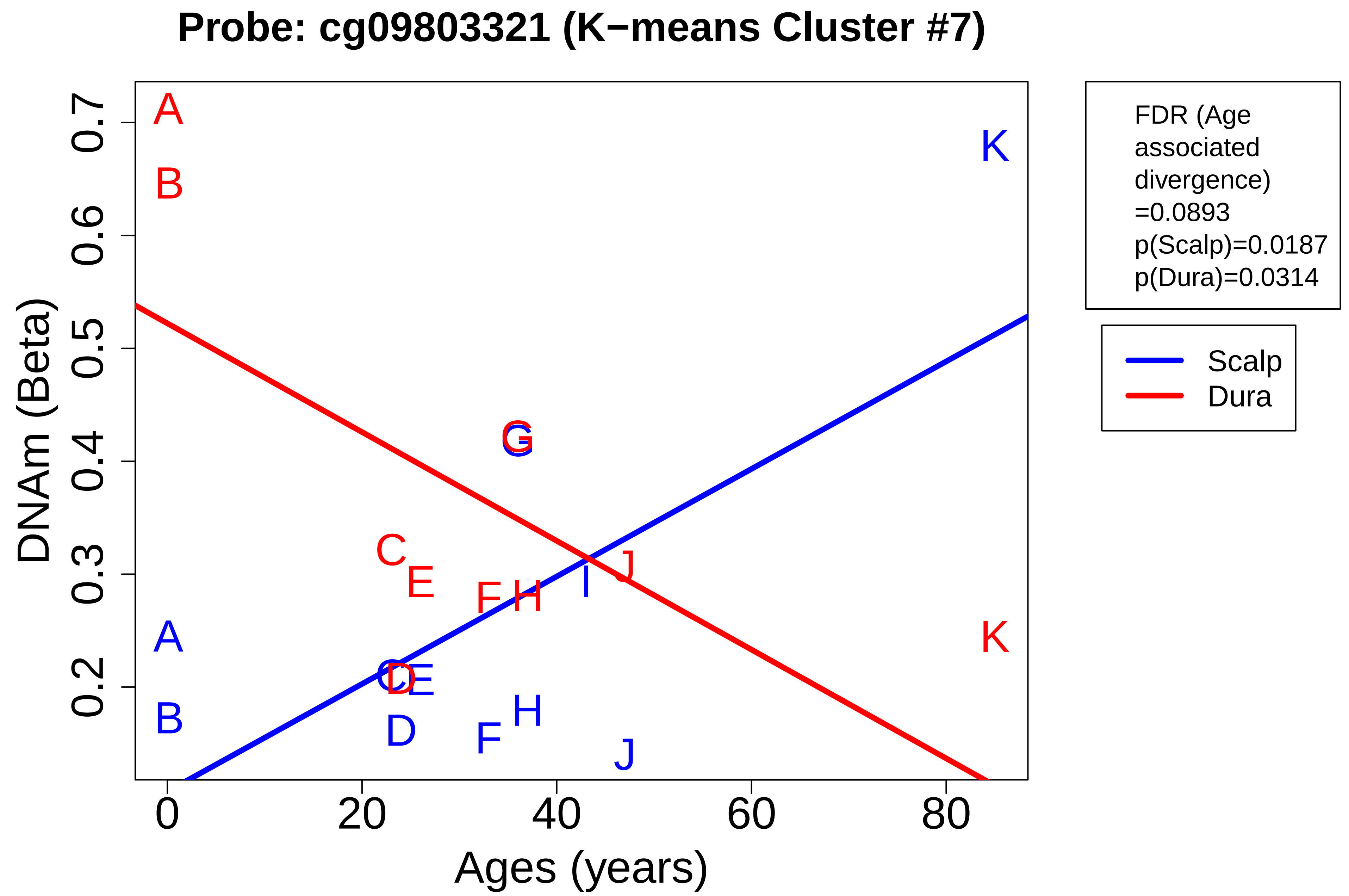

Probe: cg09803321 (K−means Cluster #7)
DNAm (Beta)
0.2
0.3
0.4
0.5
0.6
0.7
0
20
40
60
80
Ages (years)
FDR (Age associated divergence) =0.0893
p(Scalp)=0.0187
p(Dura)=0.0314
Scalp
Dura

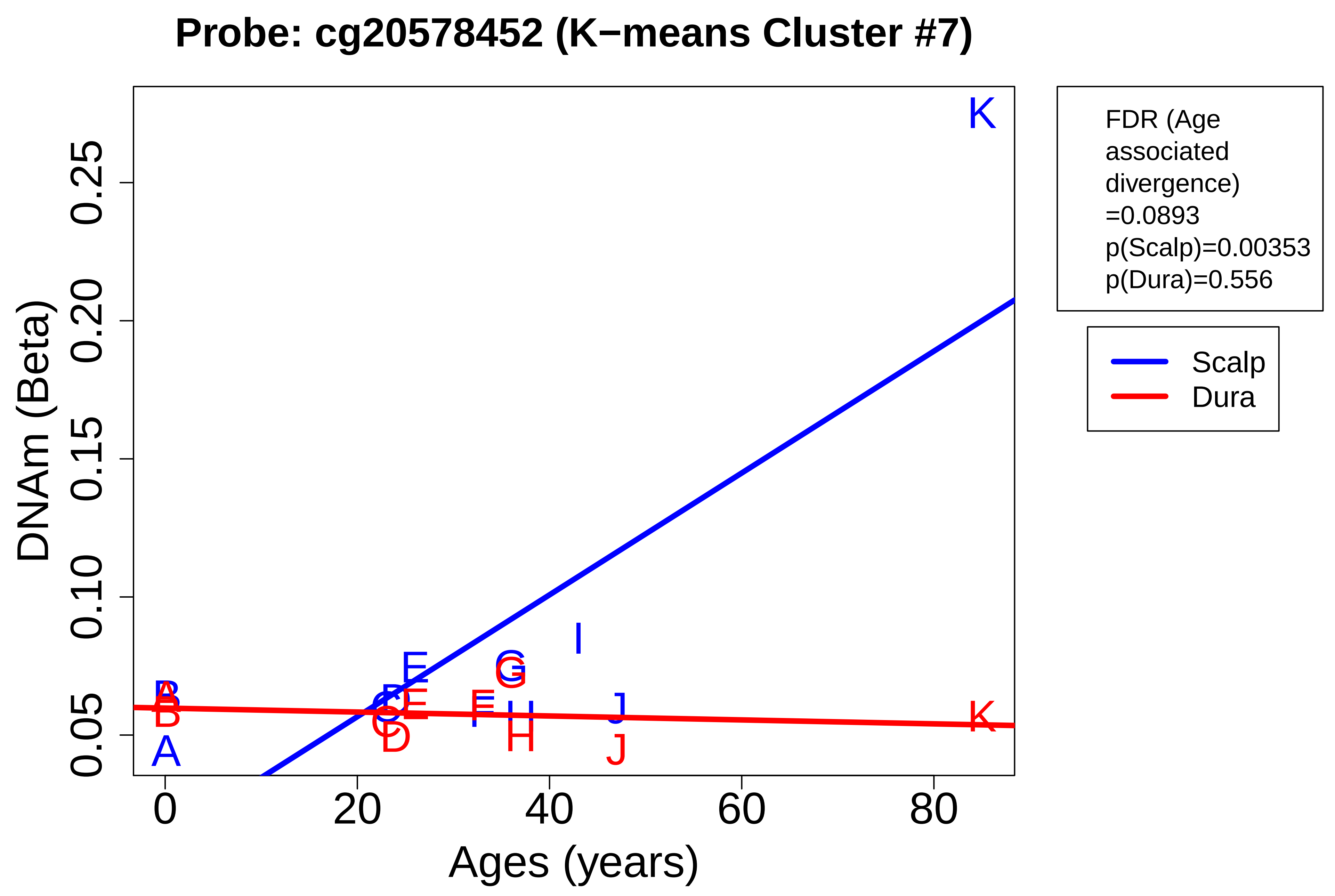

Probe: cg20578452 (K−means Cluster #7)
DNAm (Beta)
0.05
0.10
0.15
0.20
0.25
Ages (years)
0
20
40
60
80
FDR (Age associated divergence) =0.0893
p(Scalp)=0.00353
p(Dura)=0.556
Scalp
Dura
A
B
C
D
E
F
G
H
I
J
K

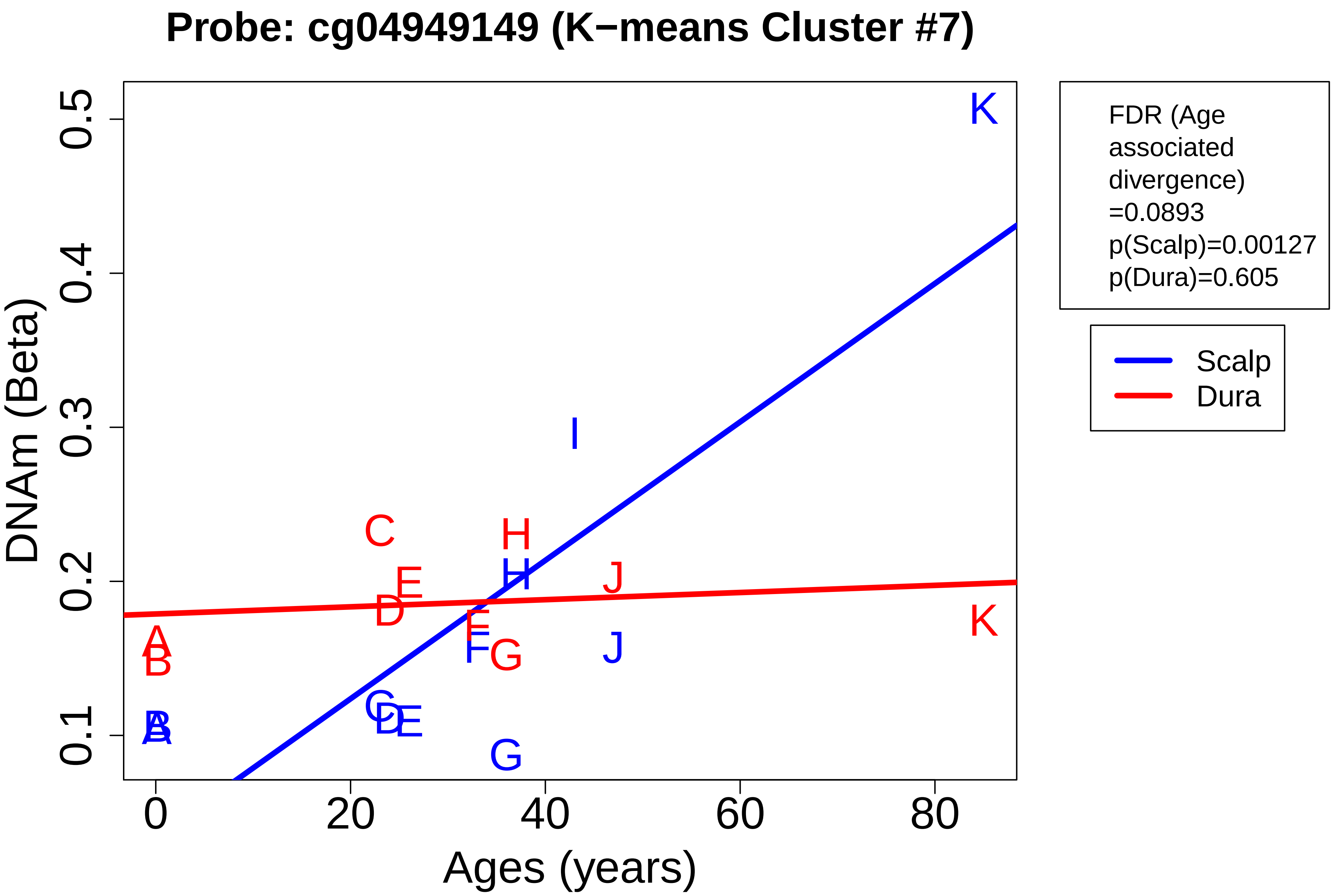
Probe: cg04949149 (K−means Cluster #7)
DNAm (Beta)
Ages (years)
FDR (Age associated divergence) =0.0893
p(Scalp)=0.00127
p(Dura)=0.605
Scalp
Dura
0.1
0.2
0.3
0.4
0.5
0
20
40
60
80

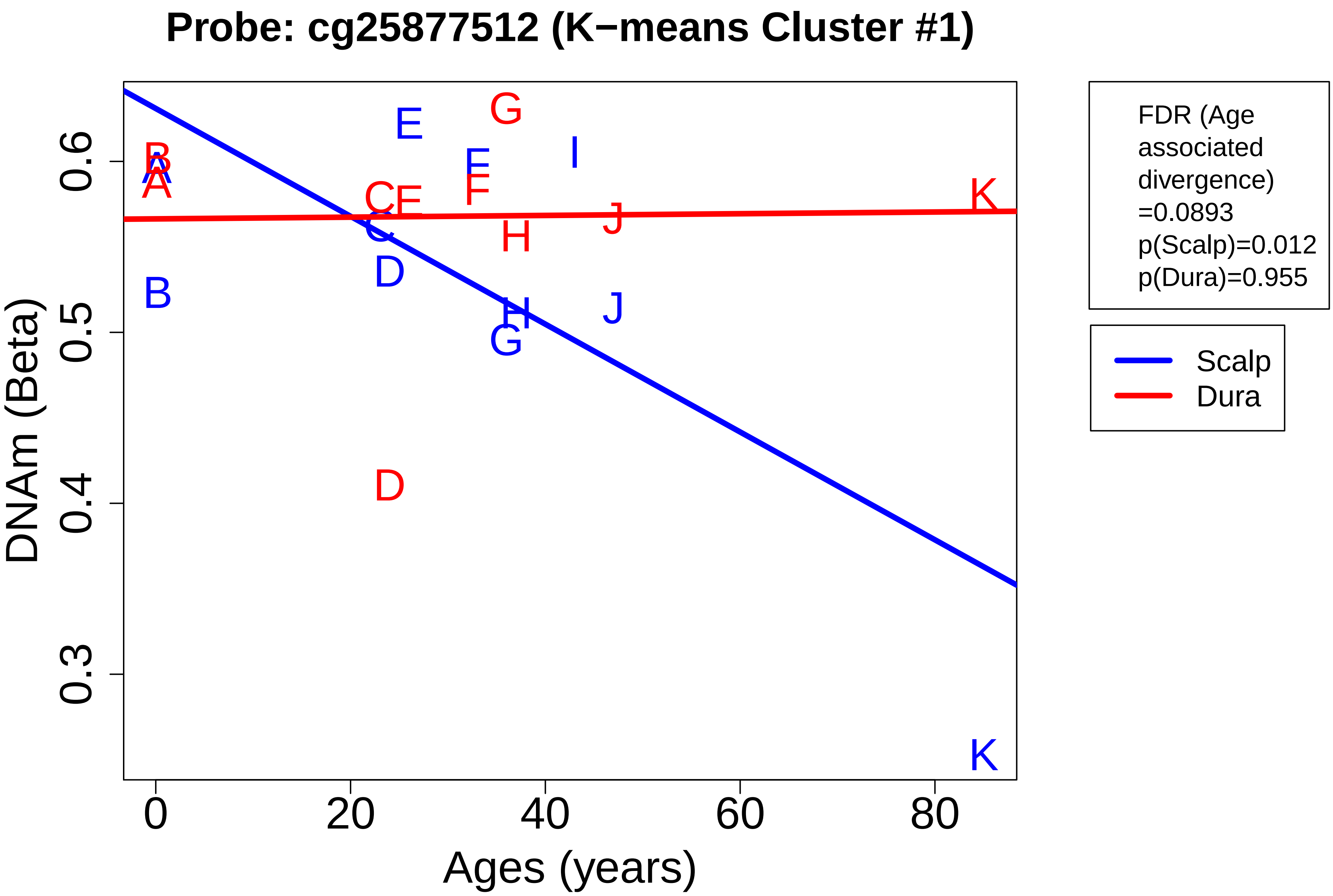

Probe: cg25877512 (K−means Cluster #1)
DNAm (Beta)
0.3
0.4
0.5
0.6
0
20
40
60
80
Ages (years)
A B C D E F G H I J K
FDR (Age associated divergence) =0.0893
p(Scalp)=0.012
p(Dura)=0.955
Scalp
Dura

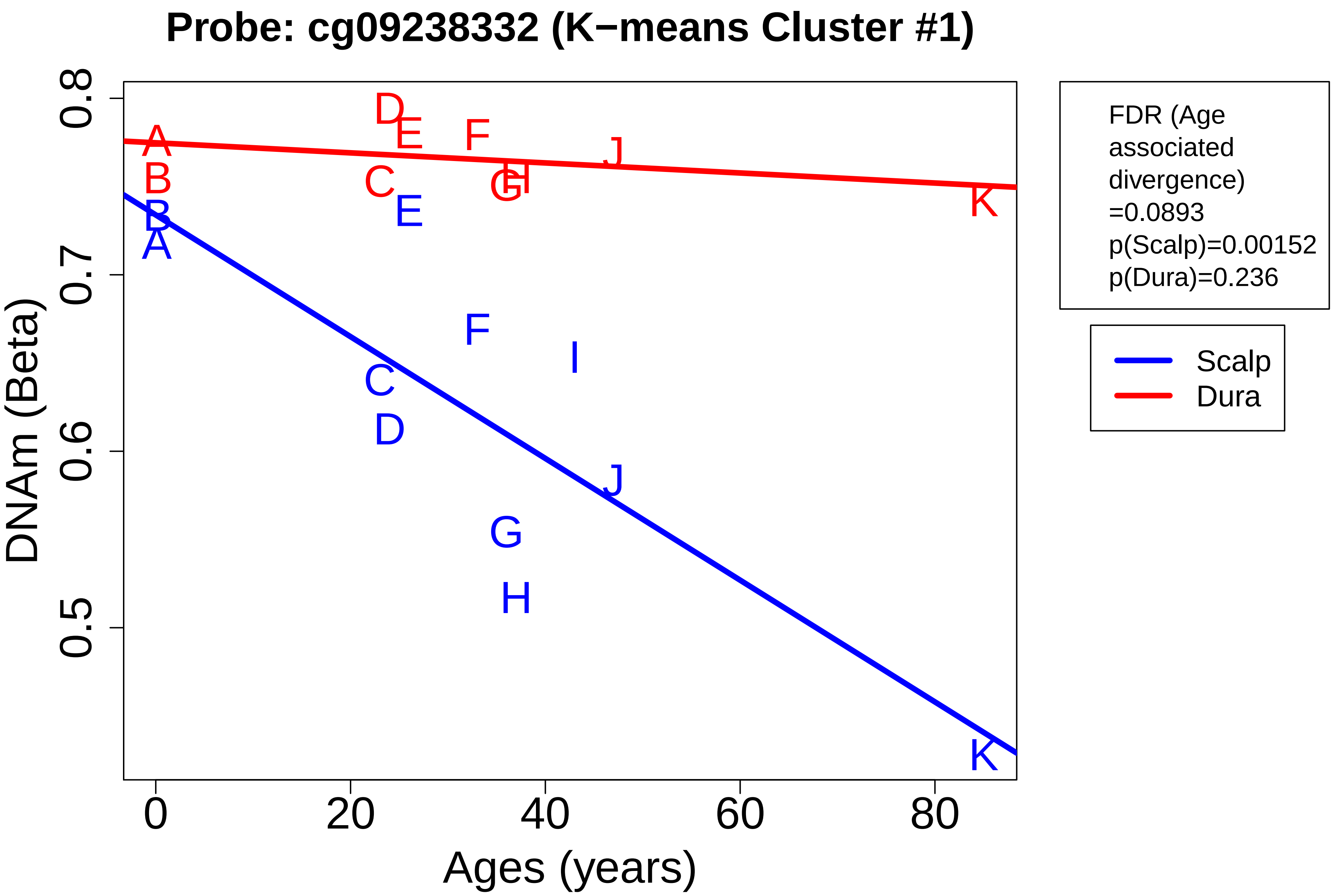

Probe: cg09238332 (K−means Cluster #1)
DNAm (Beta)
0.5
0.6
0.7
0.8
0
20
40
60
80
Ages (years)
A
B
C
D
E
F
G
H
I
J
K
FDR (Age associated divergence) =0.0893
p(Scalp)=0.00152
p(Dura)=0.236
Scalp
Dura

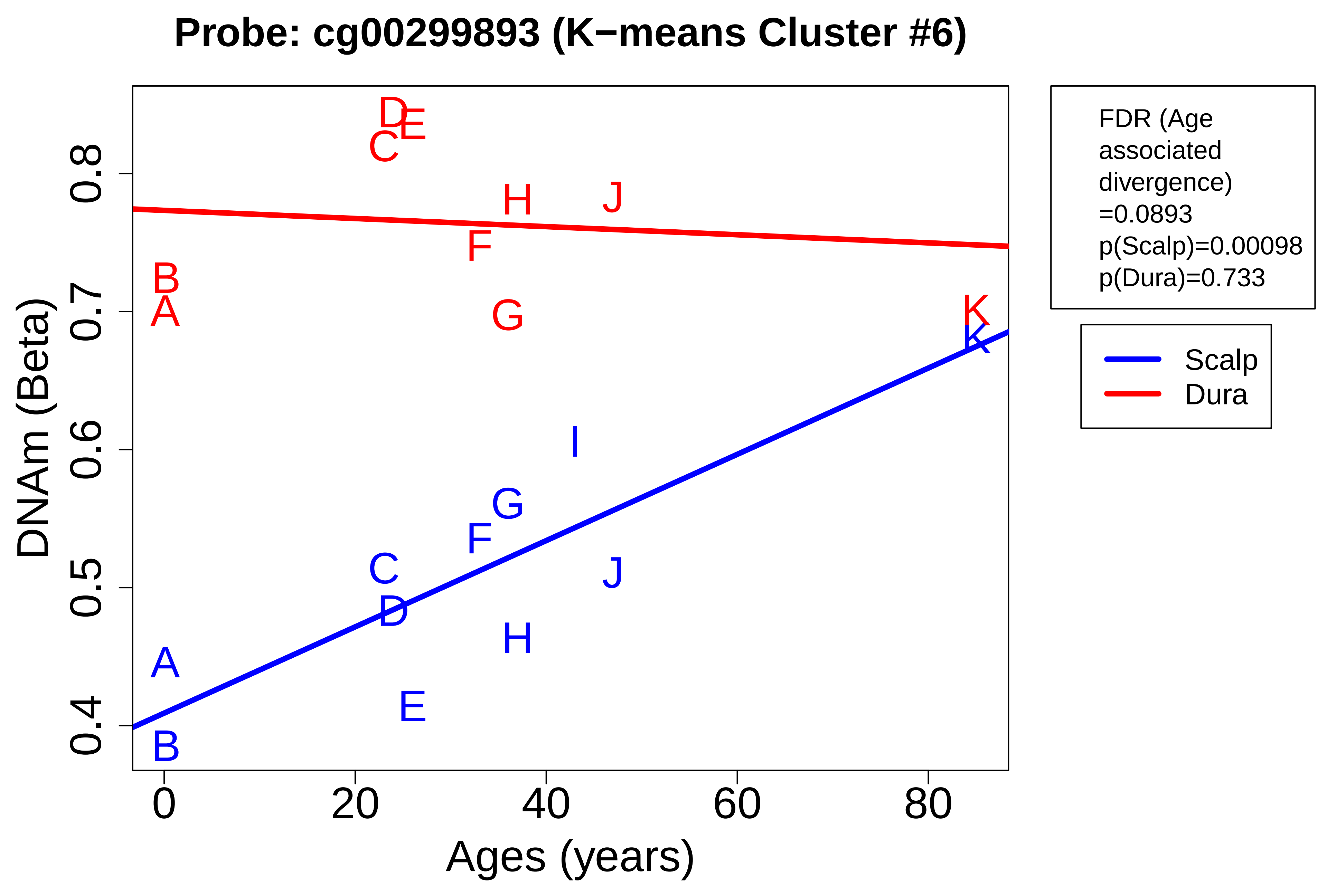

Probe: cg00299893 (K−means Cluster #6)
DNAm (Beta)
Ages (years)
0.4
0.5
0.6
0.7
0.8
0
20
40
60
80
FDR (Age associated divergence) =0.0893
p(Scalp)=0.00098
p(Dura)=0.733
Scalp
Dura
A
B
C
D
E
F
G
H
I
J
K

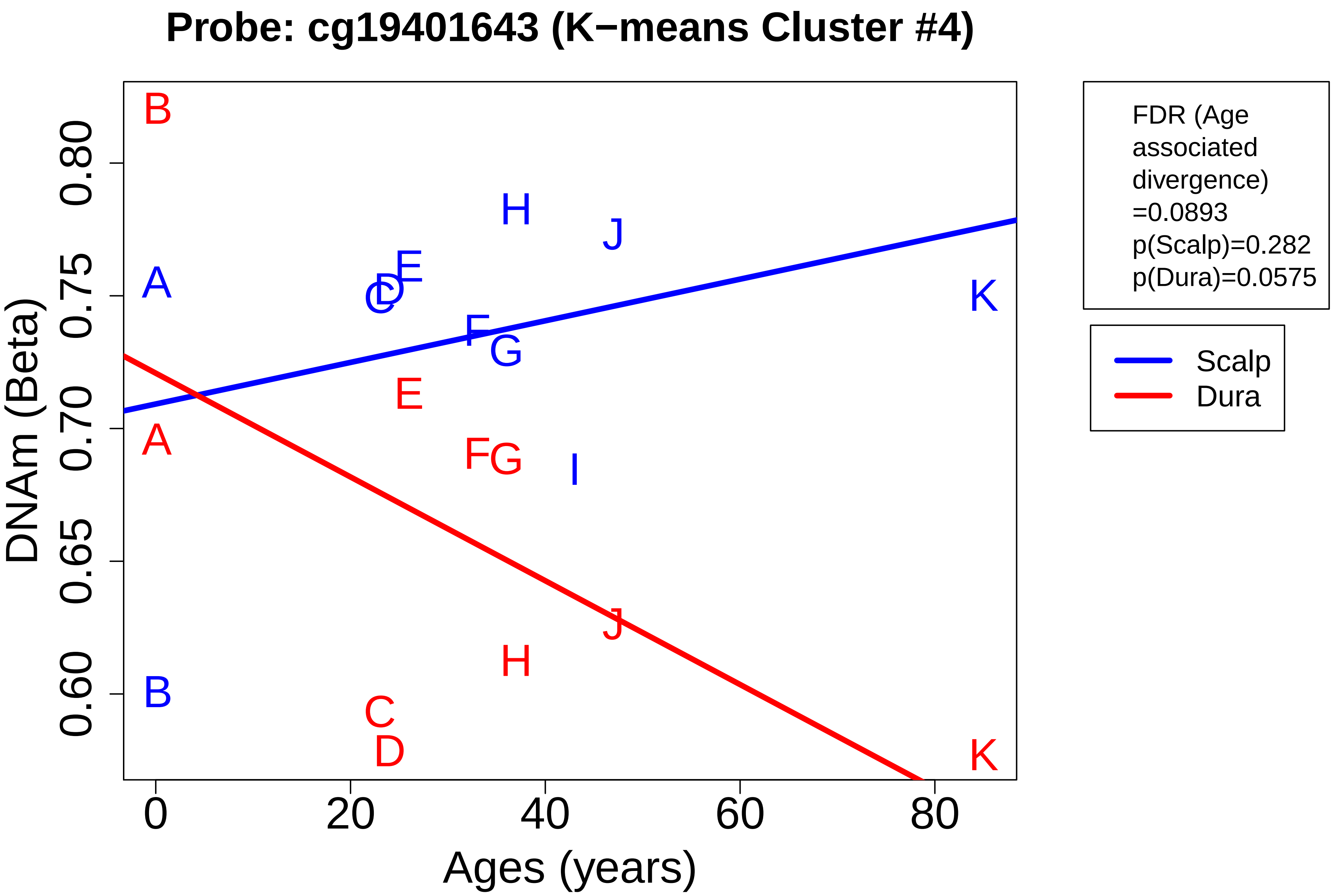

Probe: cg19401643 (K−means Cluster #4)
DNAm (Beta)
0.60
0.65
0.70
0.75
0.80
Ages (years)
0
20
40
60
80
FDR (Age associated divergence) =0.0893
p(Scalp)=0.282
p(Dura)=0.0575
Scalp
Dura

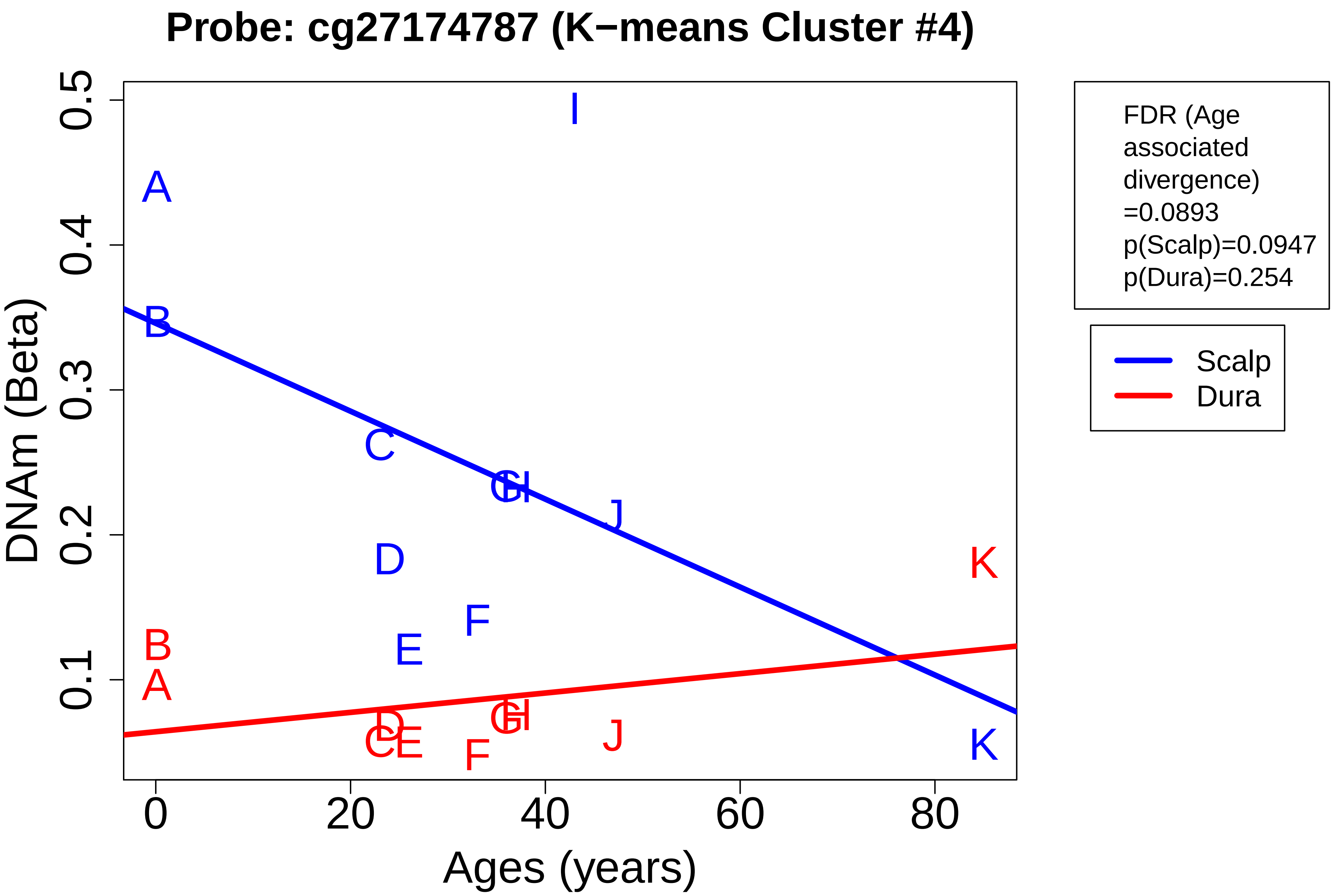

Probe: cg27174787 (K−means Cluster #4)
DNAm (Beta)
0.1
0.2
0.3
0.4
0.5
0
20
40
60
80
Ages (years)
A
B
C
D
E
F
G
H
I
J
K
FDR (Age associated divergence) =0.0893
p(Scalp)=0.0947
p(Dura)=0.254
Scalp
Dura

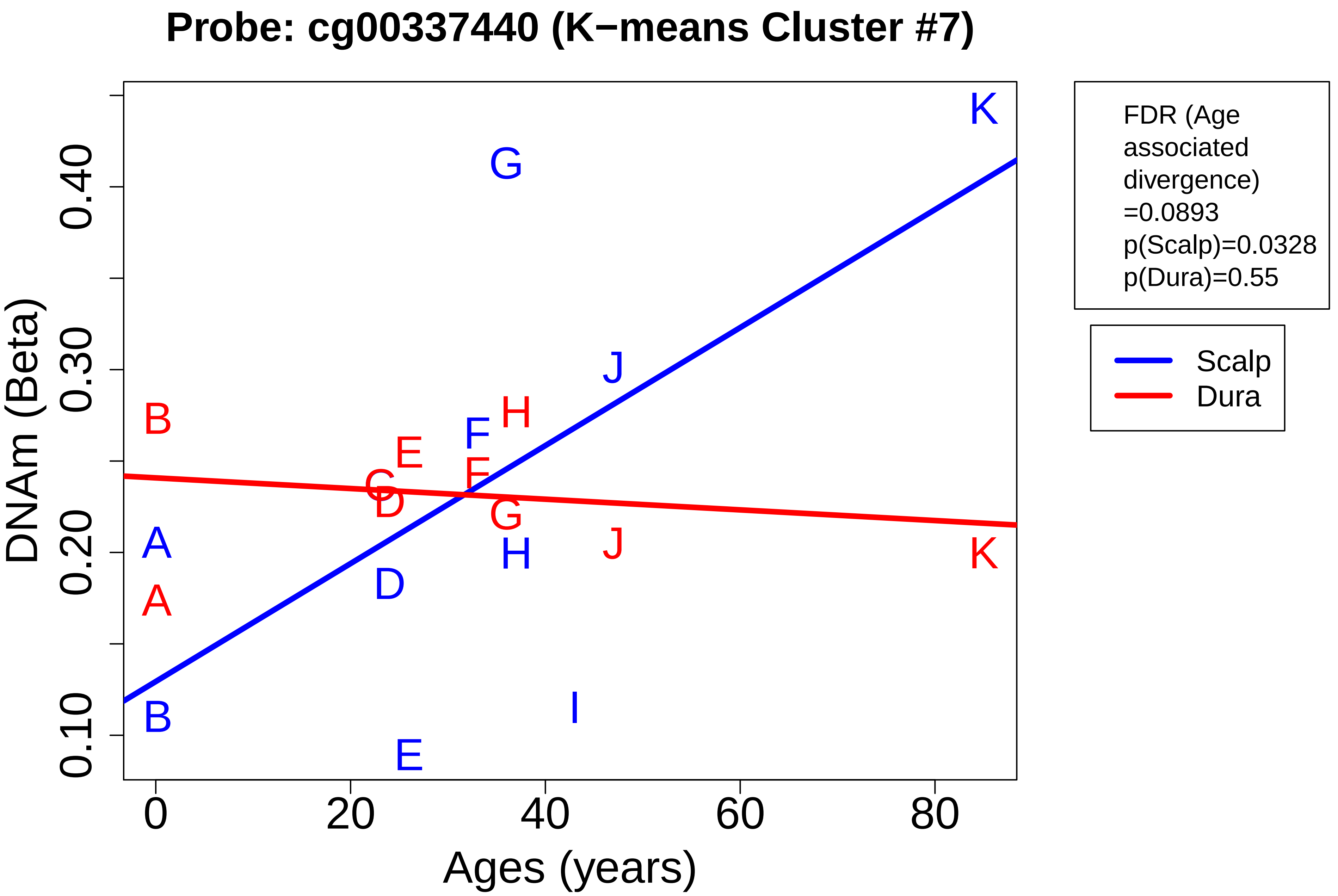

Probe: cg00337440 (K−means Cluster #7)
DNAm (Beta)
0.10
0.20
0.30
0.40
0
20
40
60
80
Ages (years)
FDR (Age associated divergence) =0.0893
p(Scalp)=0.0328
p(Dura)=0.55
Scalp
Dura

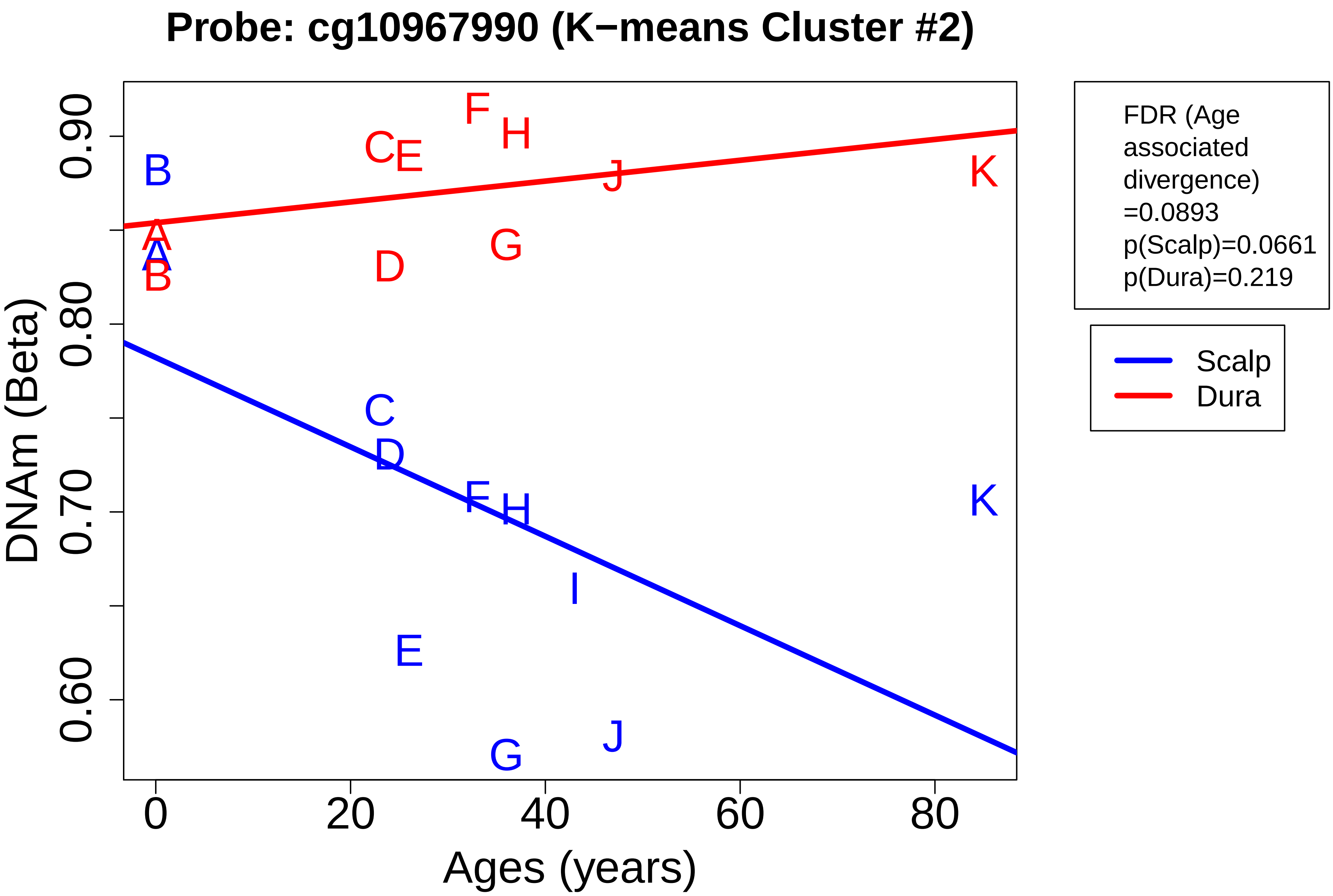

Probe: cg10967990 (K−means Cluster #2)
DNAm (Beta)
0.60
0.70
0.80
0.90
0
20
40
60
80
Ages (years)
FDR (Age associated divergence) =0.0893
p(Scalp)=0.0661
p(Dura)=0.219
Scalp
Dura
A
B
C
D
E
F
G
H
I
J
K

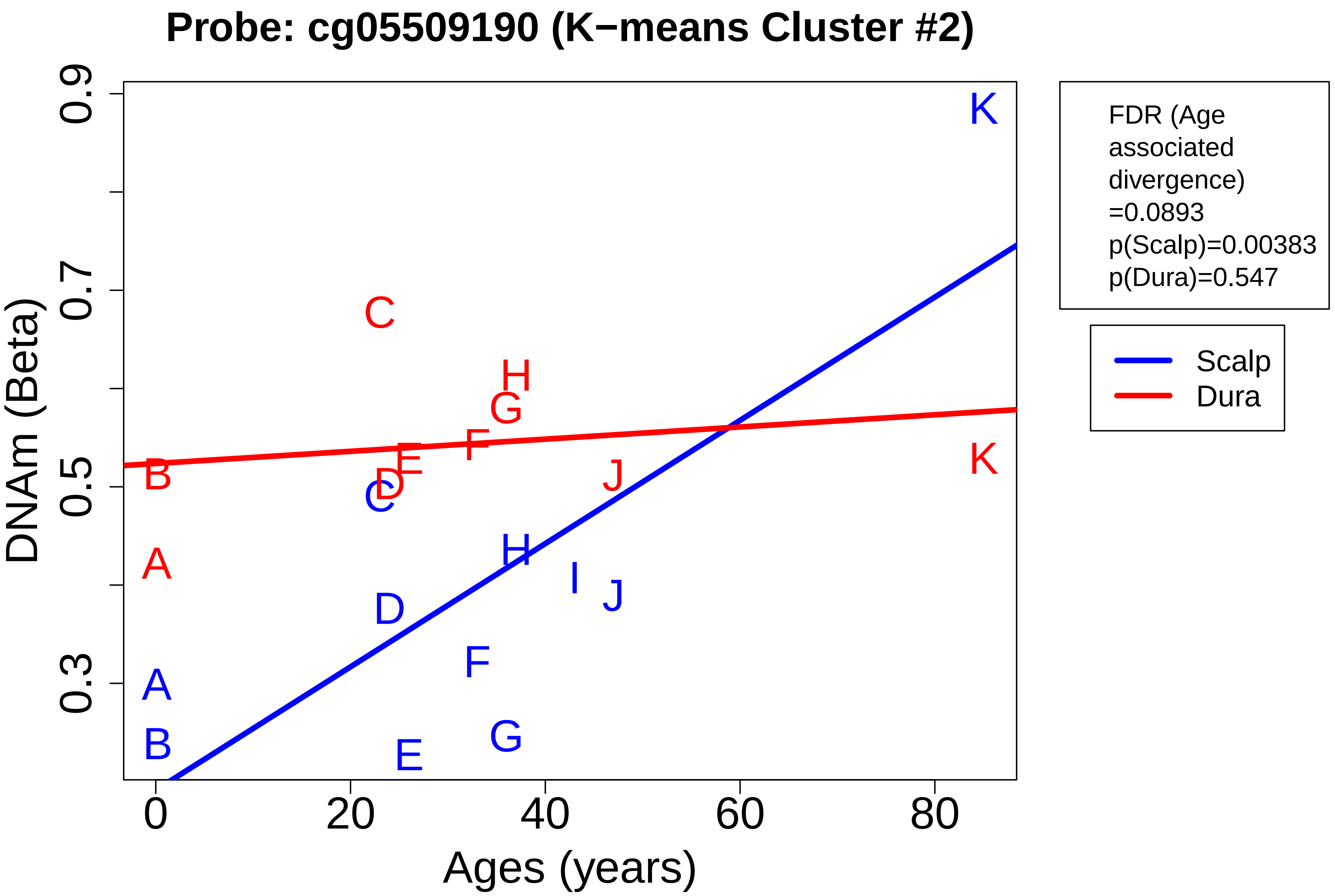

Probe: cg05509190 (K−means Cluster #2)
DNAm (Beta)
0.3
0.5
0.7
0.9
0
20
40
60
80
Ages (years)
A
B
C
D
E
F
G
H
I
J
K
FDR (Age associated divergence) =0.0893
p(Scalp)=0.00383
p(Dura)=0.547
Scalp
Dura

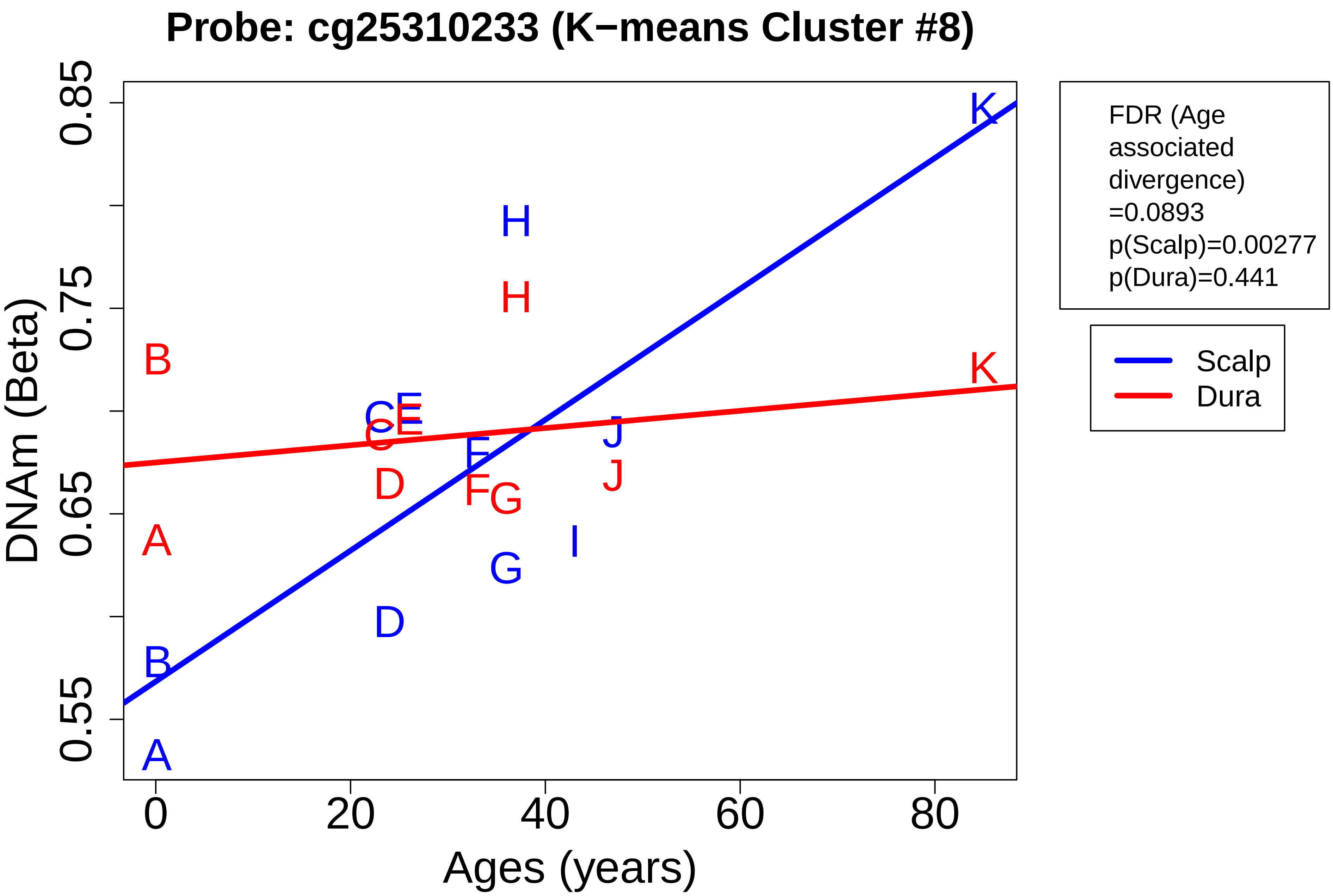

Probe: cg25310233 (K−means Cluster #8)
DNAm (Beta)
0.55
0.65
0.75
0.85
0
20
40
60
80
Ages (years)
FDR (Age associated divergence) =0.0893
p(Scalp)=0.00277
p(Dura)=0.441
Scalp
Dura
A
B
C
D
E
F
G
H
I
J
K

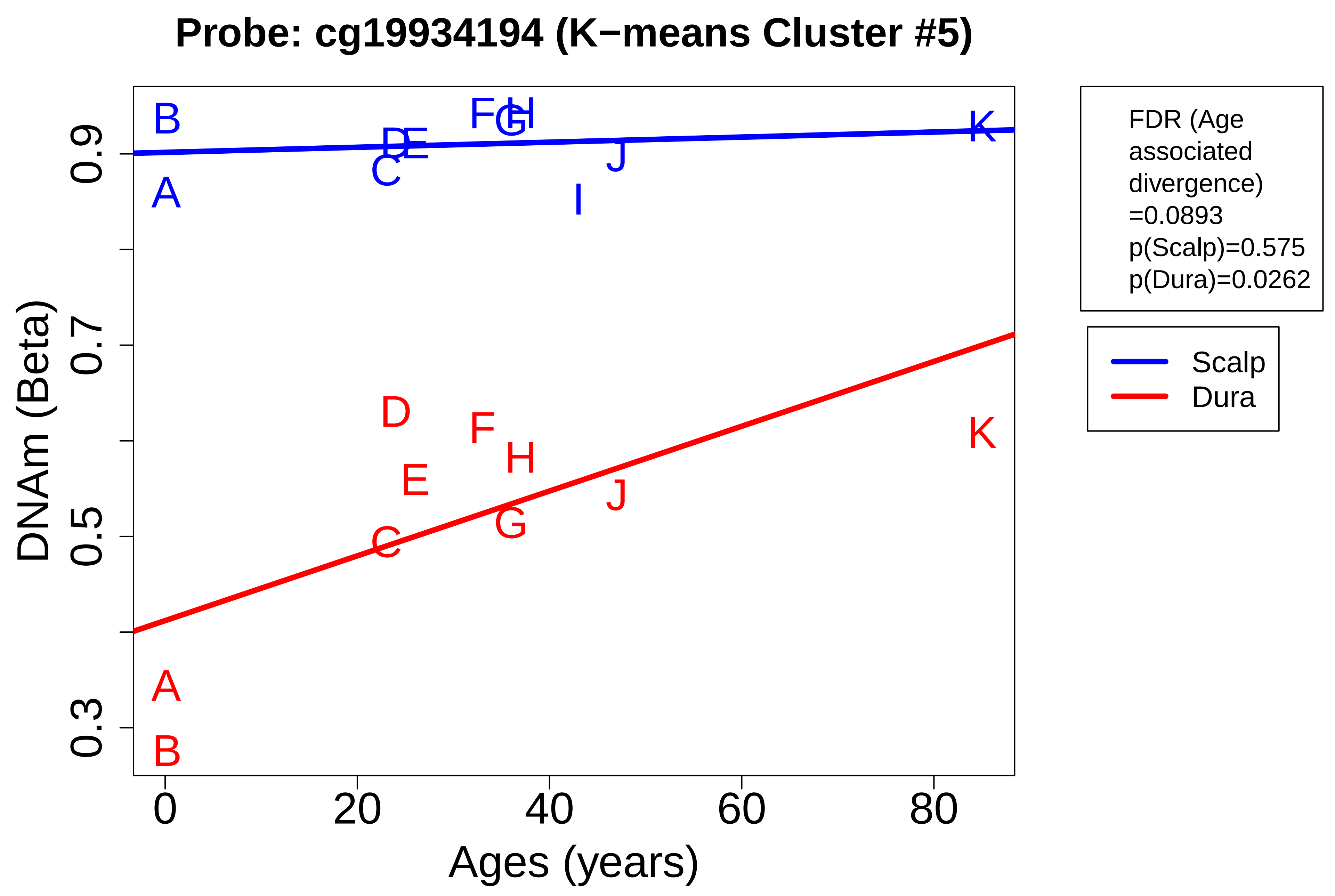

Probe: cg19934194 (K−means Cluster #5)
DNAm (Beta)
0.3
0.5
0.7
0.9
0
20
40
60
80
Ages (years)
FDR (Age associated divergence) =0.0893
p(Scalp)=0.575
p(Dura)=0.0262
Scalp
Dura

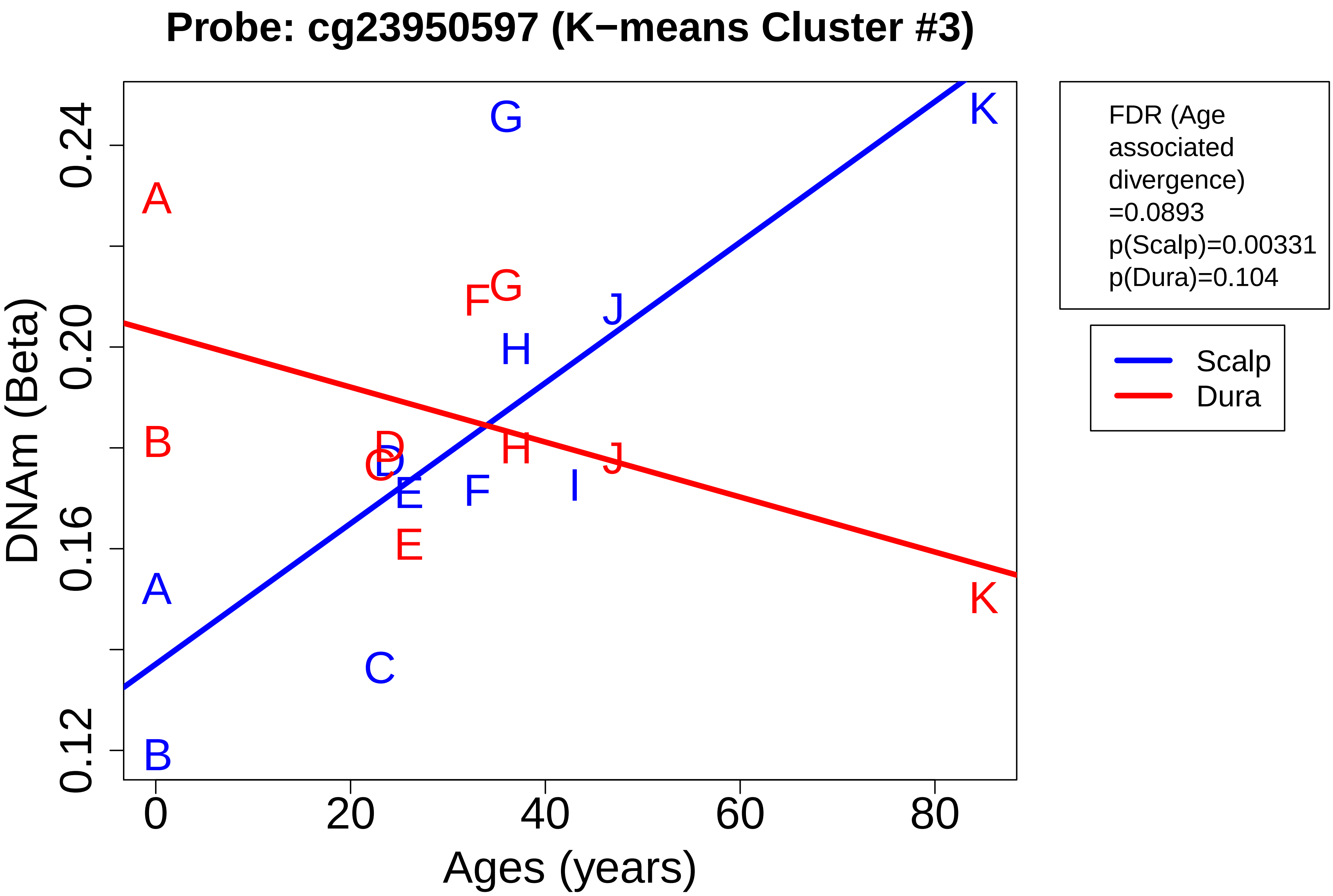

Probe: cg23950597 (K−means Cluster #3)
DNAm (Beta)
0.12
0.16
0.20
0.24
Ages (years)
0
20
40
60
80
FDR (Age associated divergence) =0.0893
p(Scalp)=0.00331
p(Dura)=0.104
Scalp
Dura
A
B
C
D
E
F
G
H
I
J
K

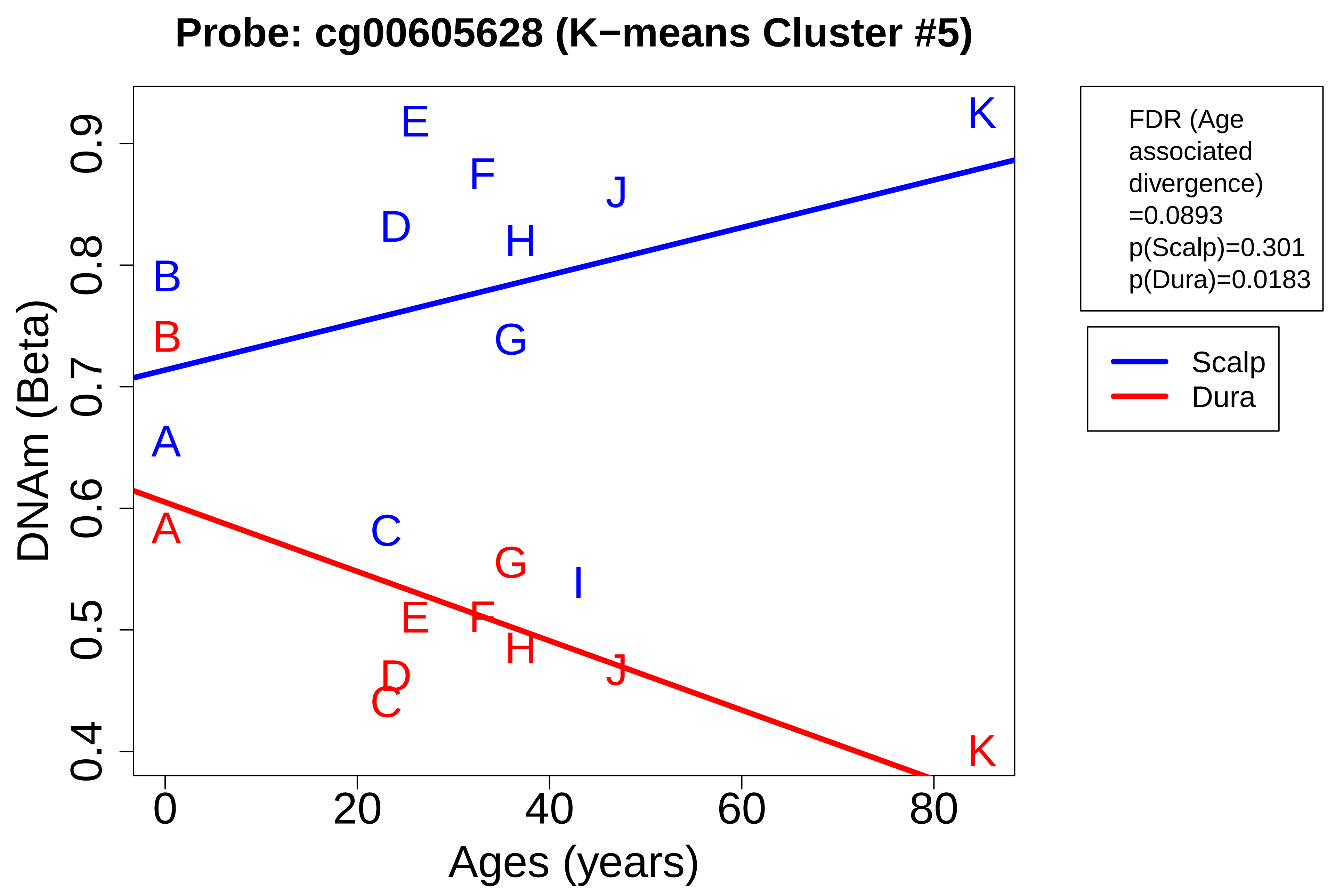

Probe: cg00605628 (K−means Cluster #5)
DNAm (Beta)
0.4
0.5
0.6
0.7
0.8
0.9
0
20
40
60
80
Ages (years)
A
B
C
D
E
F
G
H
I
J
K
A
B
C
D
E
F
G
H
J
K
FDR (Age associated divergence) =0.0893
p(Scalp)=0.301
p(Dura)=0.0183
Scalp
Dura

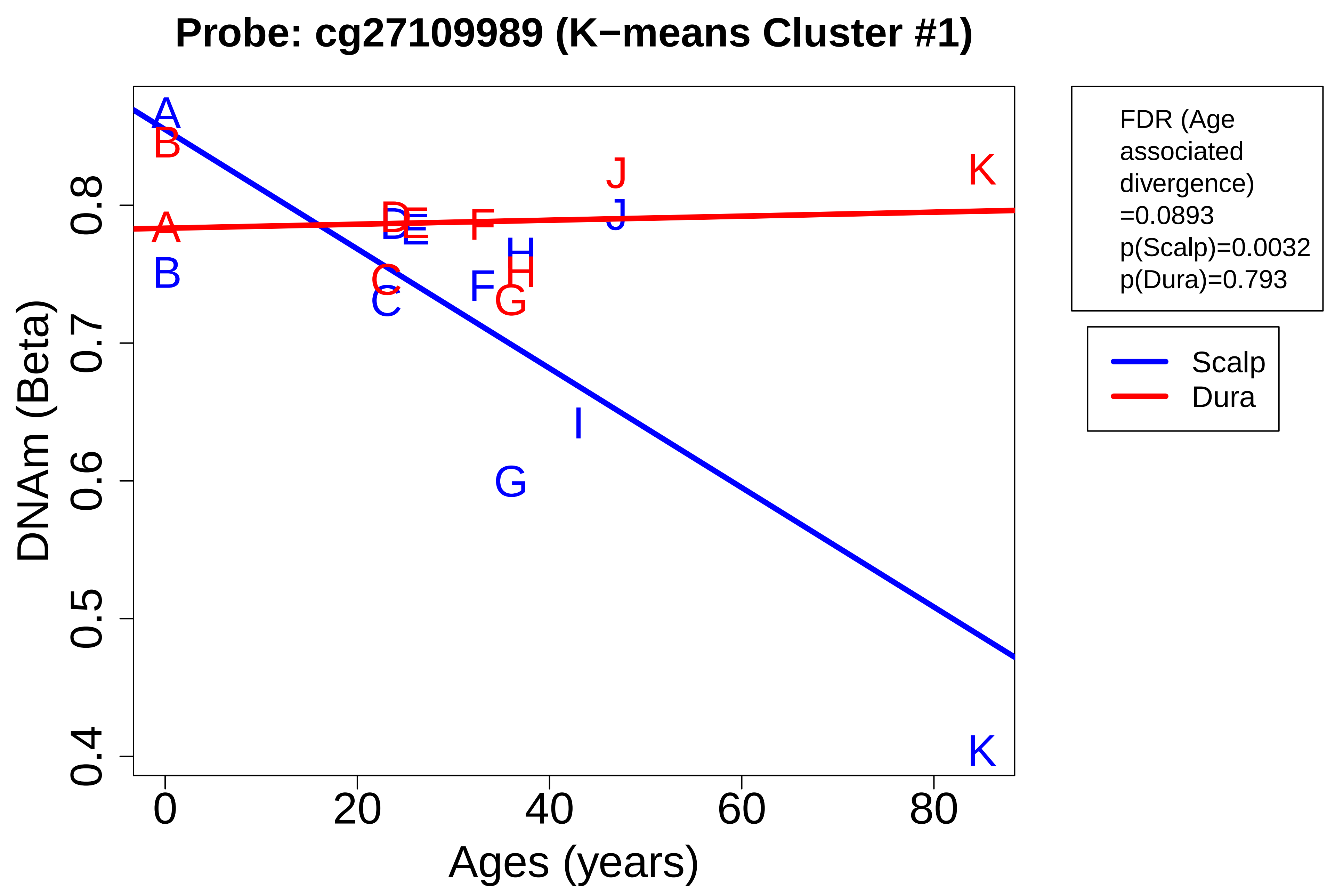

Probe: cg27109989 (K−means Cluster #1)
DNAm (Beta)
0.4
0.5
0.6
0.7
0.8
Ages (years)
0
20
40
60
80
A
B
C
D
E
F
G
H
I
J
K
FDR (Age associated divergence) =0.0893
p(Scalp)=0.0032
p(Dura)=0.793
Scalp
Dura

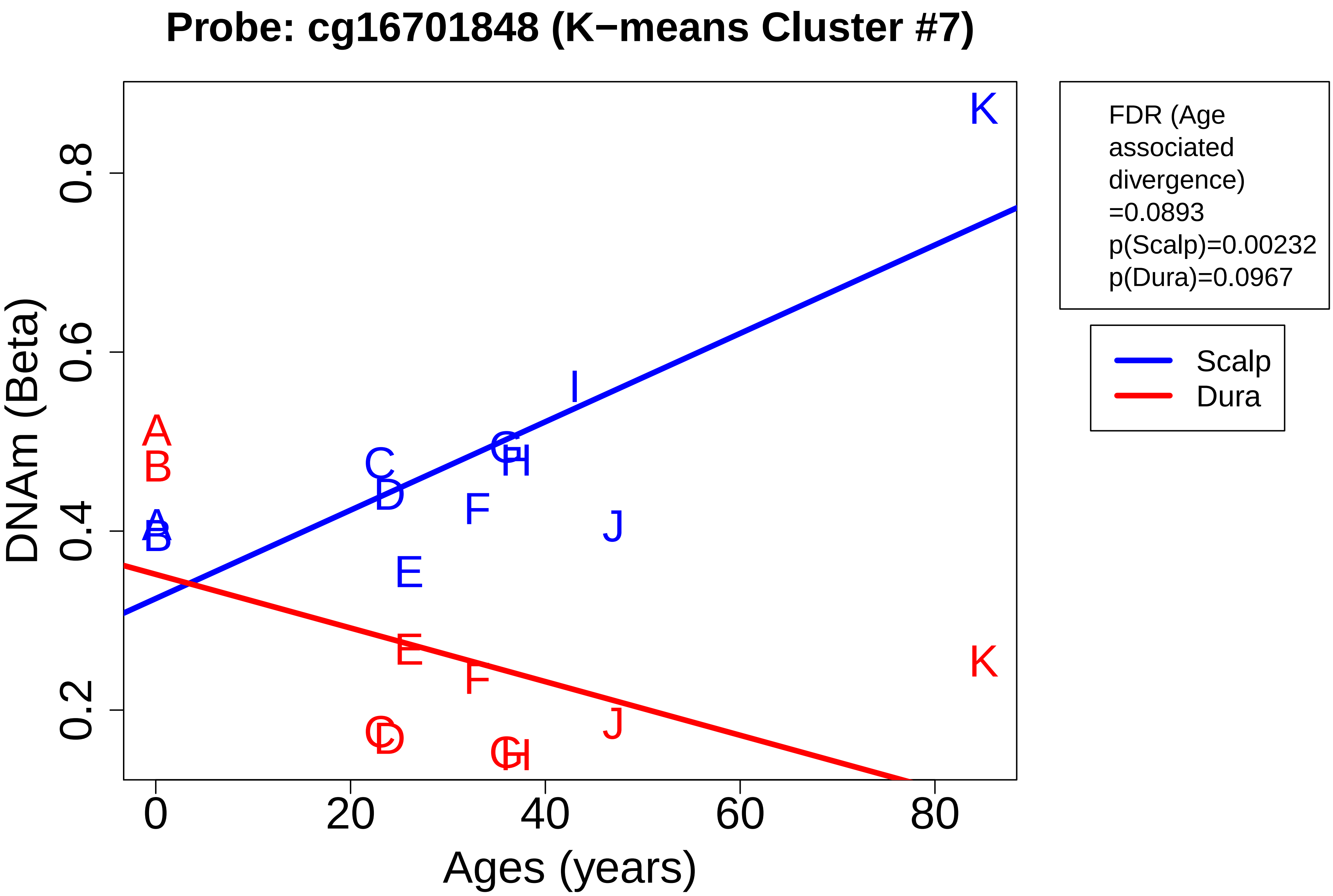
Probe: cg16701848 (K−means Cluster #7)
FDR (Age associated divergence) =0.0893
p(Scalp)=0.00232
p(Dura)=0.0967
Scalp
Dura
DNAm (Beta)
Ages (years)
0.2
0.4
0.6
0.8
0
20
40
60
80
A
B
C
D
E
F
G
H
I
J
K

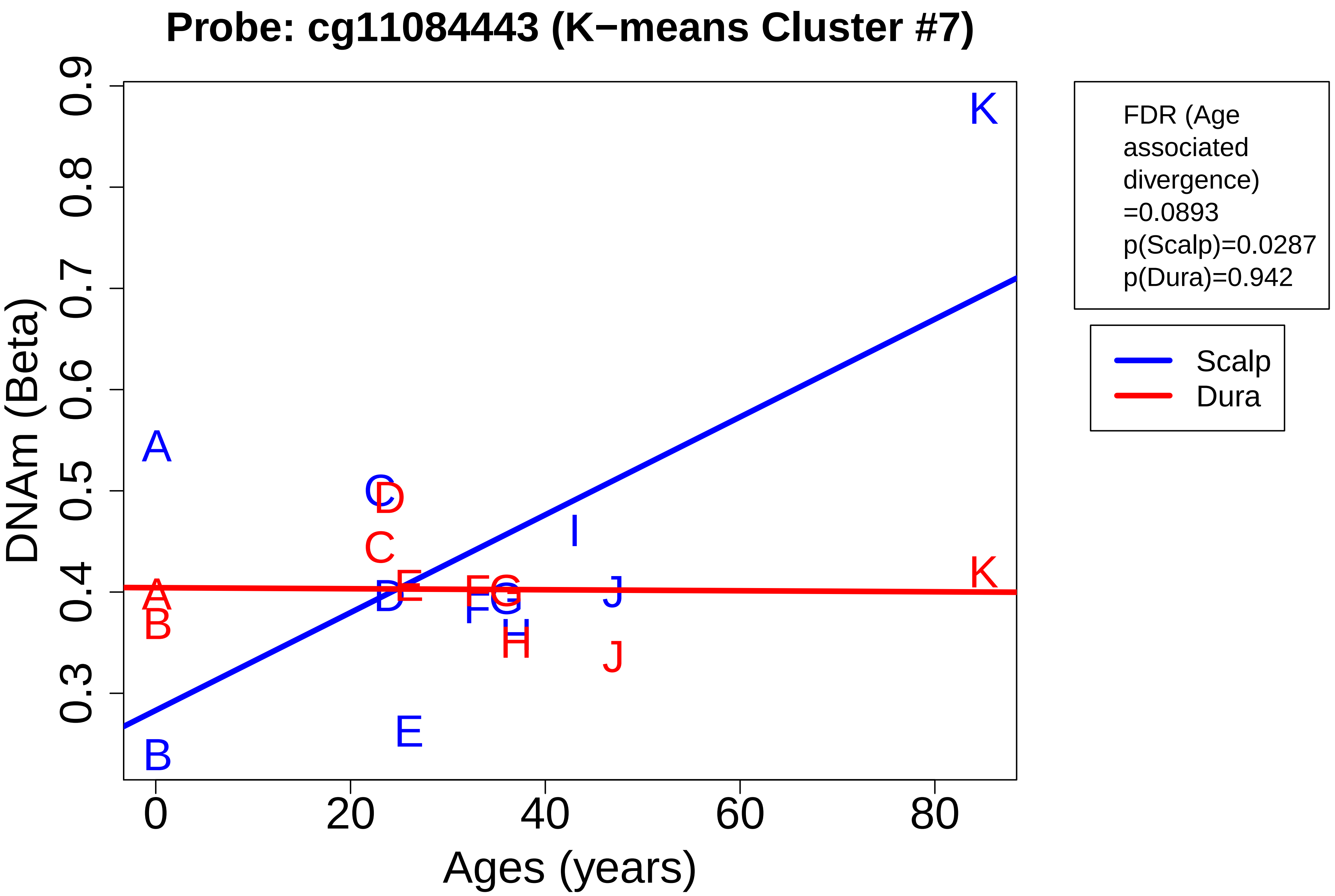

Probe: cg11084443 (K−means Cluster #7)
DNAm (Beta)
0.3
0.4
0.5
0.6
0.7
0.8
0.9
Ages (years)
0
20
40
60
80
FDR (Age associated divergence) =0.0893
p(Scalp)=0.0287
p(Dura)=0.942
Scalp
Dura
A
B
C
D
E
F
G
H
I
J
K

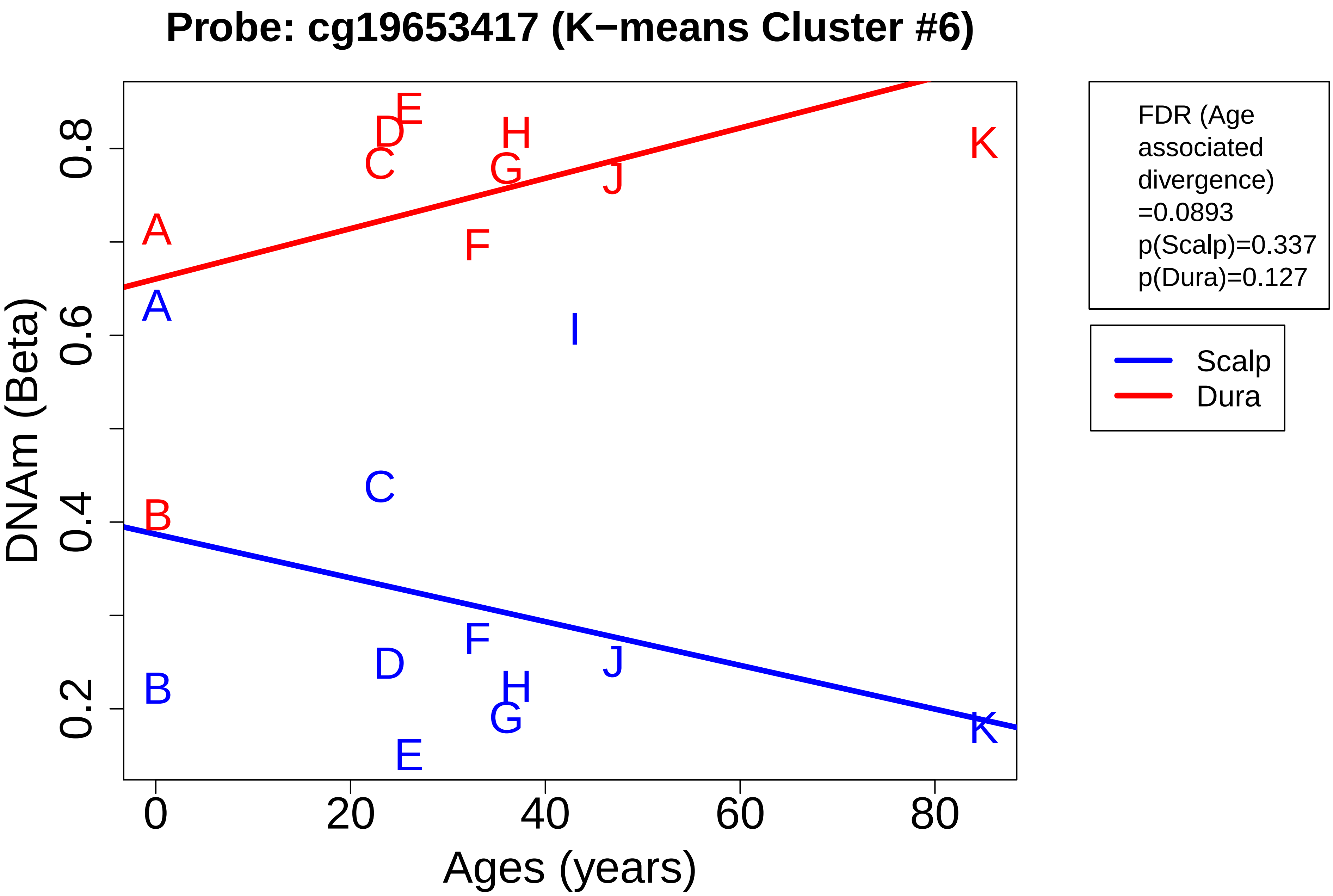
Probe: cg19653417 (K−means Cluster #6)
DNAm (Beta)
0.2
0.4
0.6
0.8
0
20
40
60
80
Ages (years)
A
B
C
D
E
F
G
H
I
J
K
FDR (Age associated divergence) =0.0893
p(Scalp)=0.337
p(Dura)=0.127
Scalp
Dura

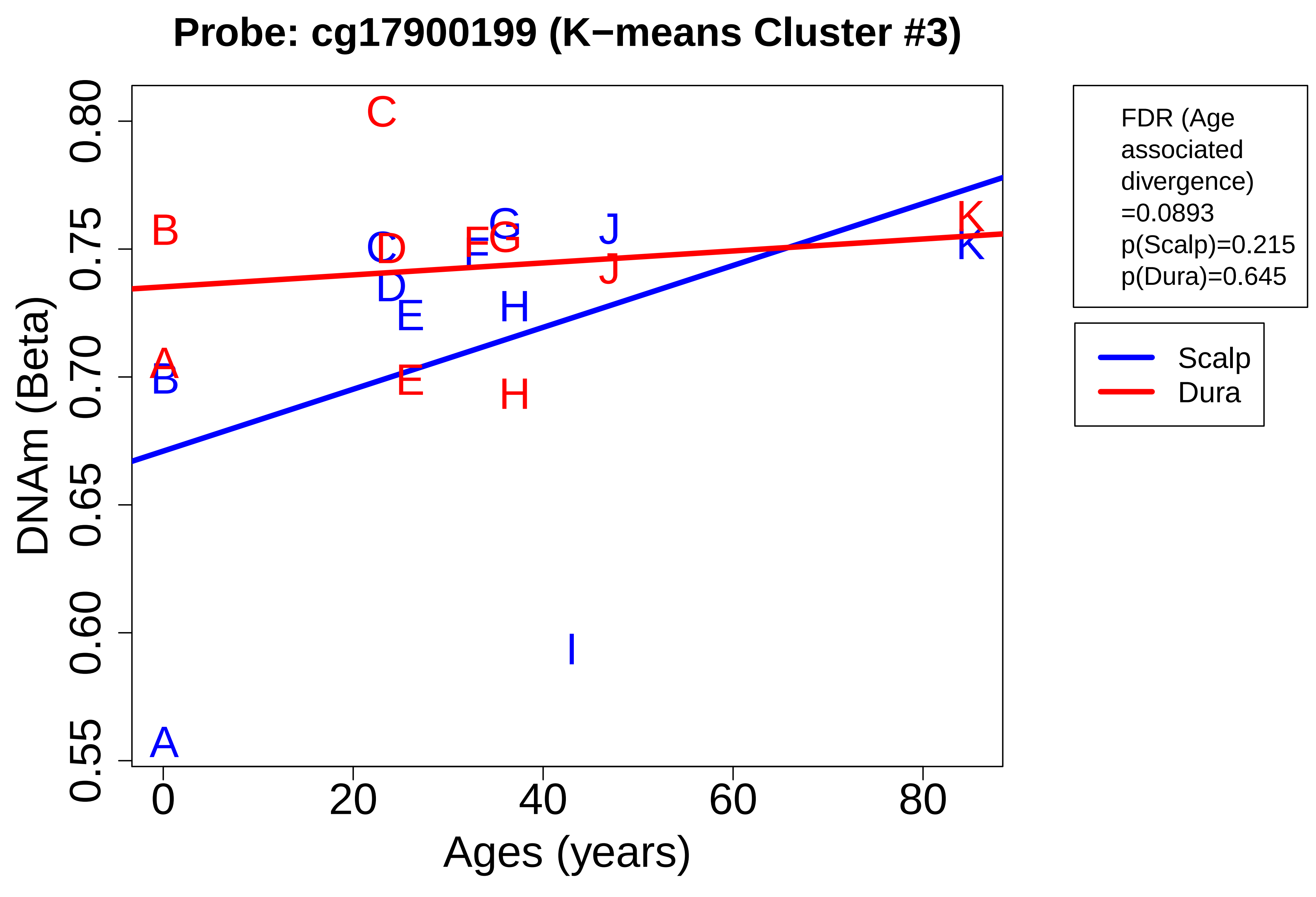

Probe: cg17900199 (K−means Cluster #3)
DNAm (Beta)
0.55
0.60
0.65
0.70
0.75
0.80
0
20
40
60
80
Ages (years)
A
B
C
D
E
F
G
H
I
J
K
FDR (Age associated divergence) =0.0893
p(Scalp)=0.215
p(Dura)=0.645
Scalp
Dura

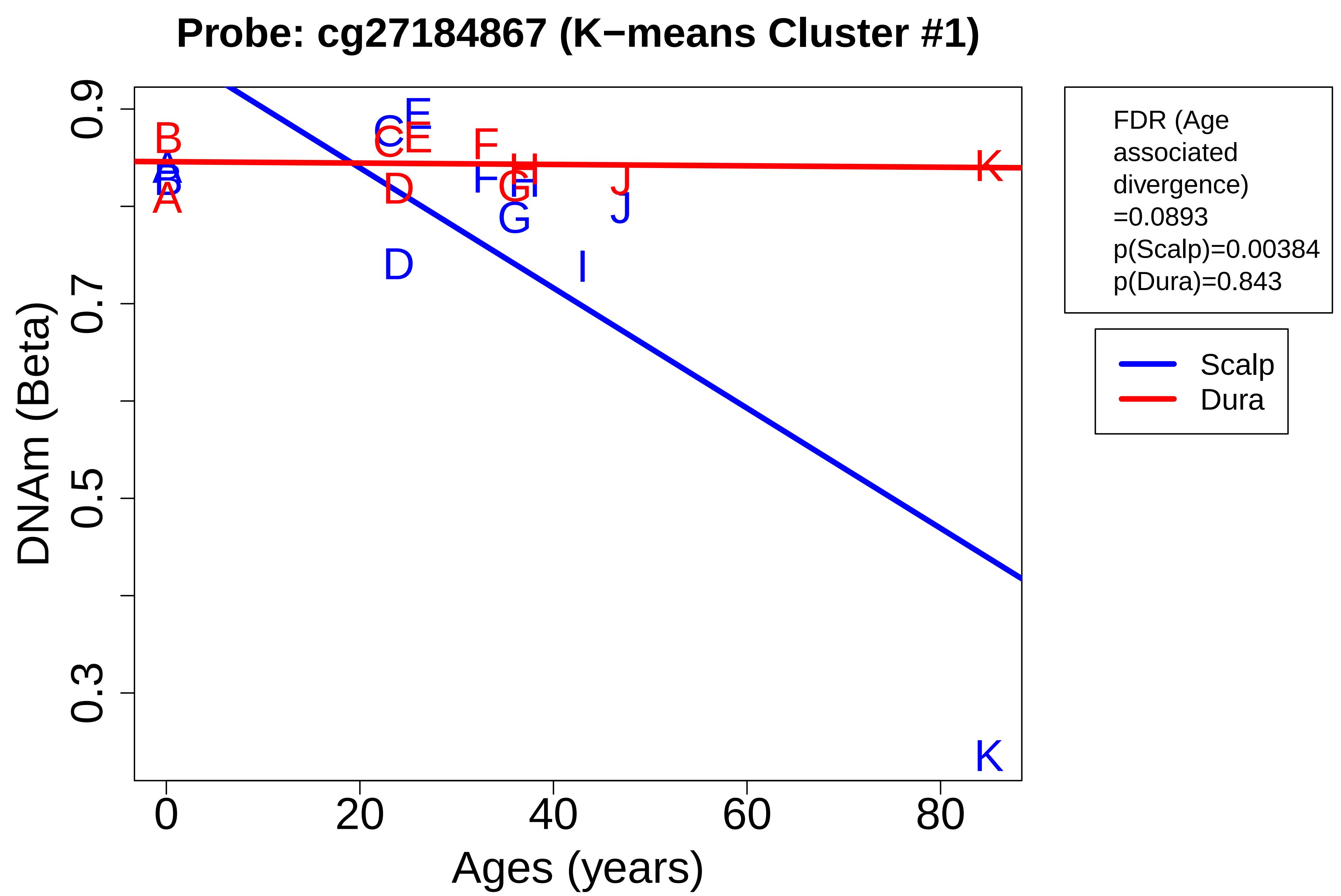

Probe: cg27184867 (K−means Cluster #1)
DNAm (Beta)
0.3
0.5
0.7
0.9
0
20
40
60
80
Ages (years)
FDR (Age associated divergence) =0.0893
p(Scalp)=0.00384
p(Dura)=0.843
Scalp
Dura

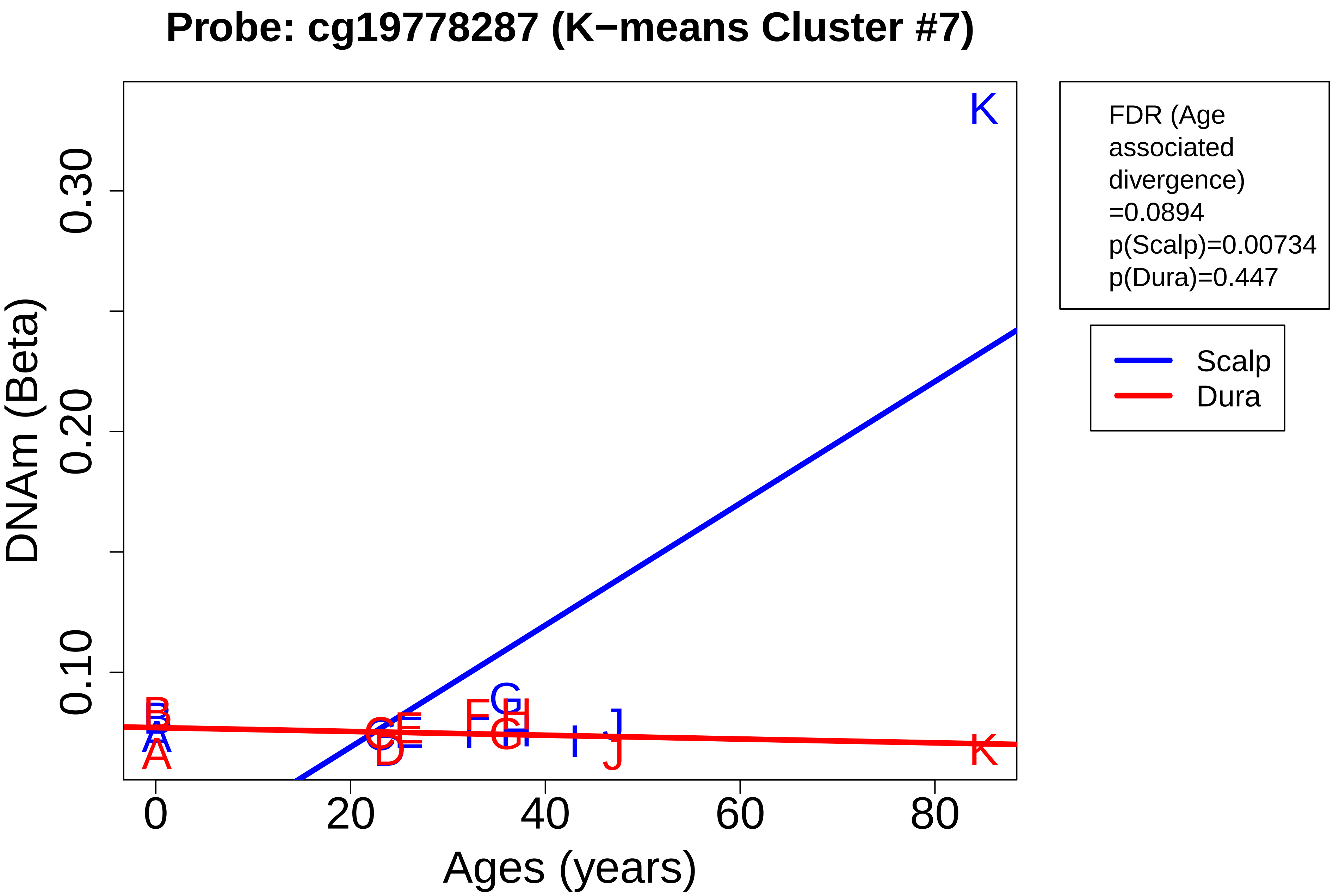

Probe: cg19778287 (K−means Cluster #7)
FDR (Age associated divergence) =0.0894
p(Scalp)=0.00734
p(Dura)=0.447
Scalp
Dura
DNAm (Beta)
0.10
0.20
0.30
Ages (years)
0
20
40
60
80

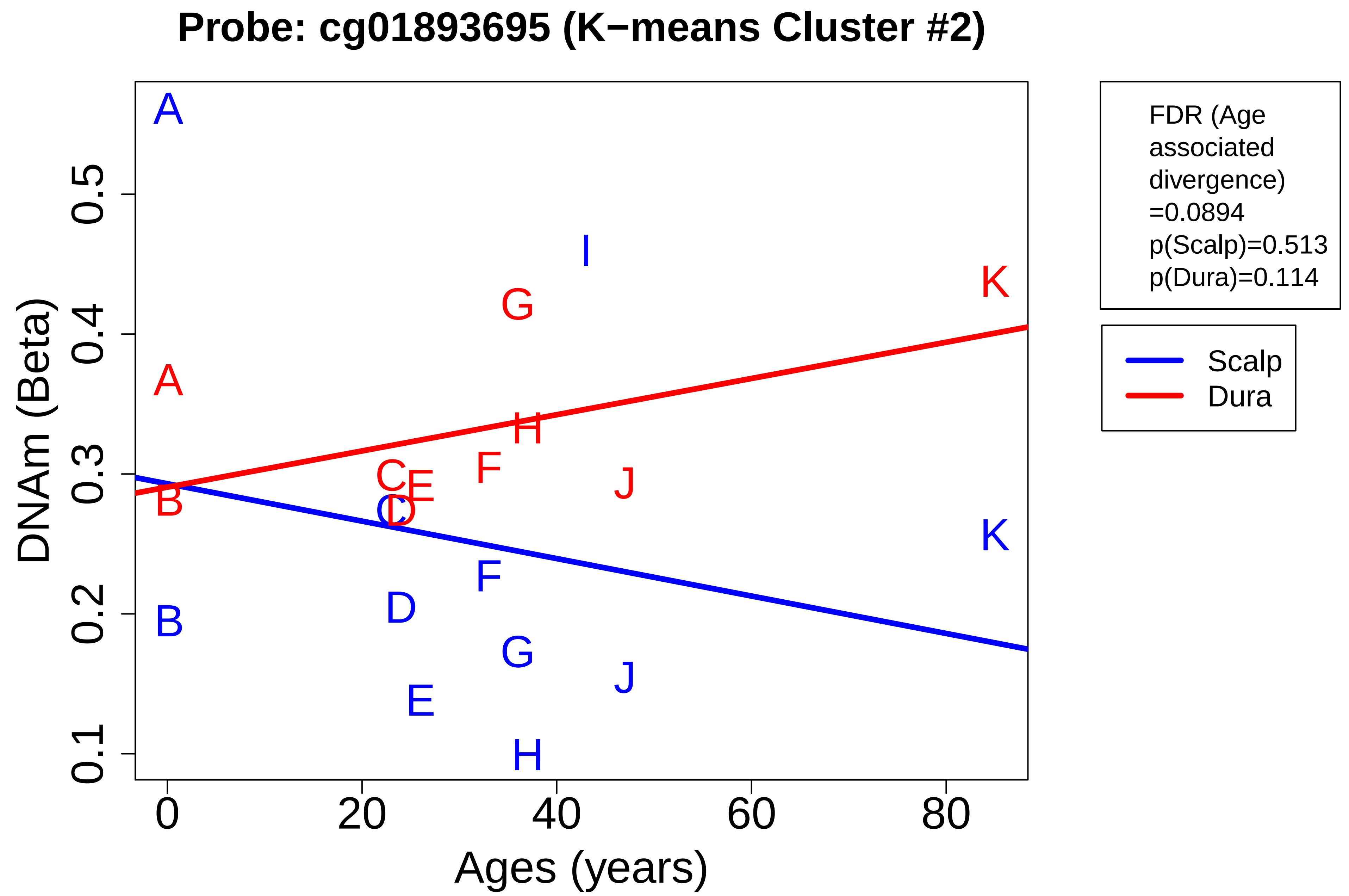

Probe: cg01893695 (K−means Cluster #2)
DNAm (Beta)
0.1
0.2
0.3
0.4
0.5
Ages (years)
0
20
40
60
80
A
B
C
D
E
F
G
H
I
J
K
FDR (Age associated divergence) =0.0894
p(Scalp)=0.513
p(Dura)=0.114
Scalp
Dura

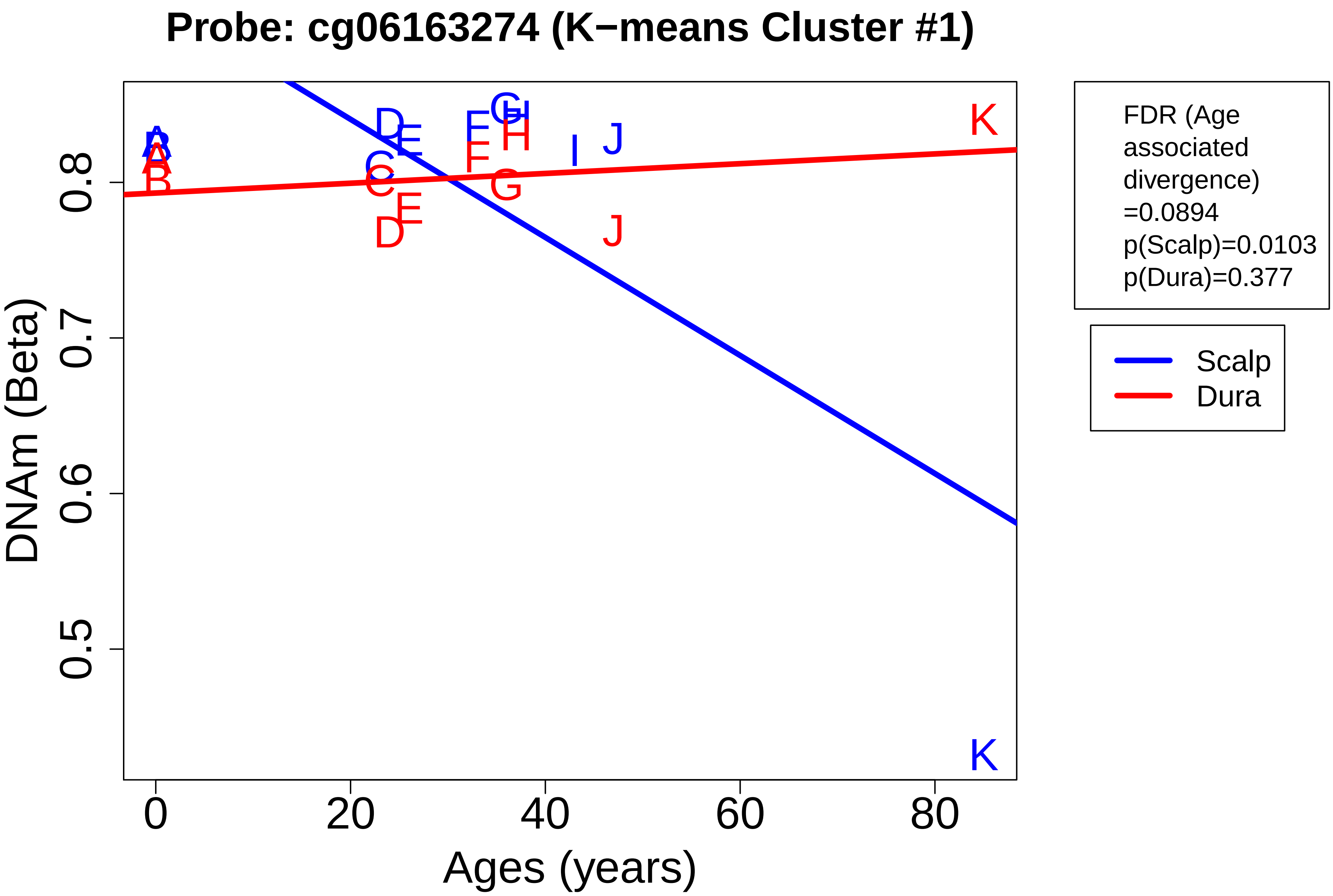

Probe: cg06163274 (K−means Cluster #1)
DNAm (Beta)
0.5
0.6
0.7
0.8
0
20
40
60
80
Ages (years)
FDR (Age associated divergence) =0.0894
p(Scalp)=0.0103
p(Dura)=0.377
Scalp
Dura

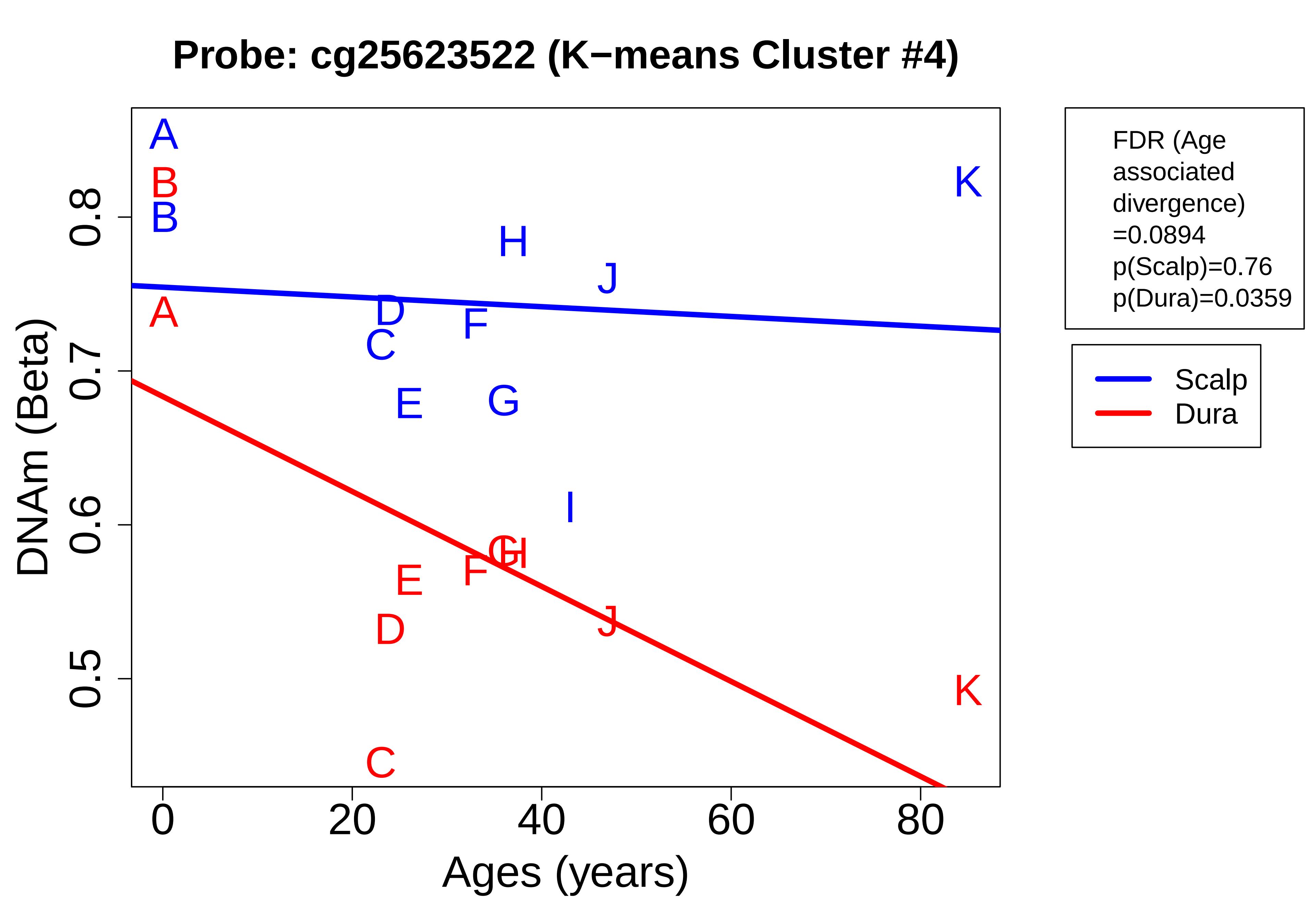
Probe: cg25623522 (K−means Cluster #4)
DNAm (Beta)
0.5
0.6
0.7
0.8
0
20
40
60
80
Ages (years)
A
B
C
D
E
F
G
H
I
J
K
FDR (Age associated divergence) =0.0894
p(Scalp)=0.76
p(Dura)=0.0359
Scalp
Dura

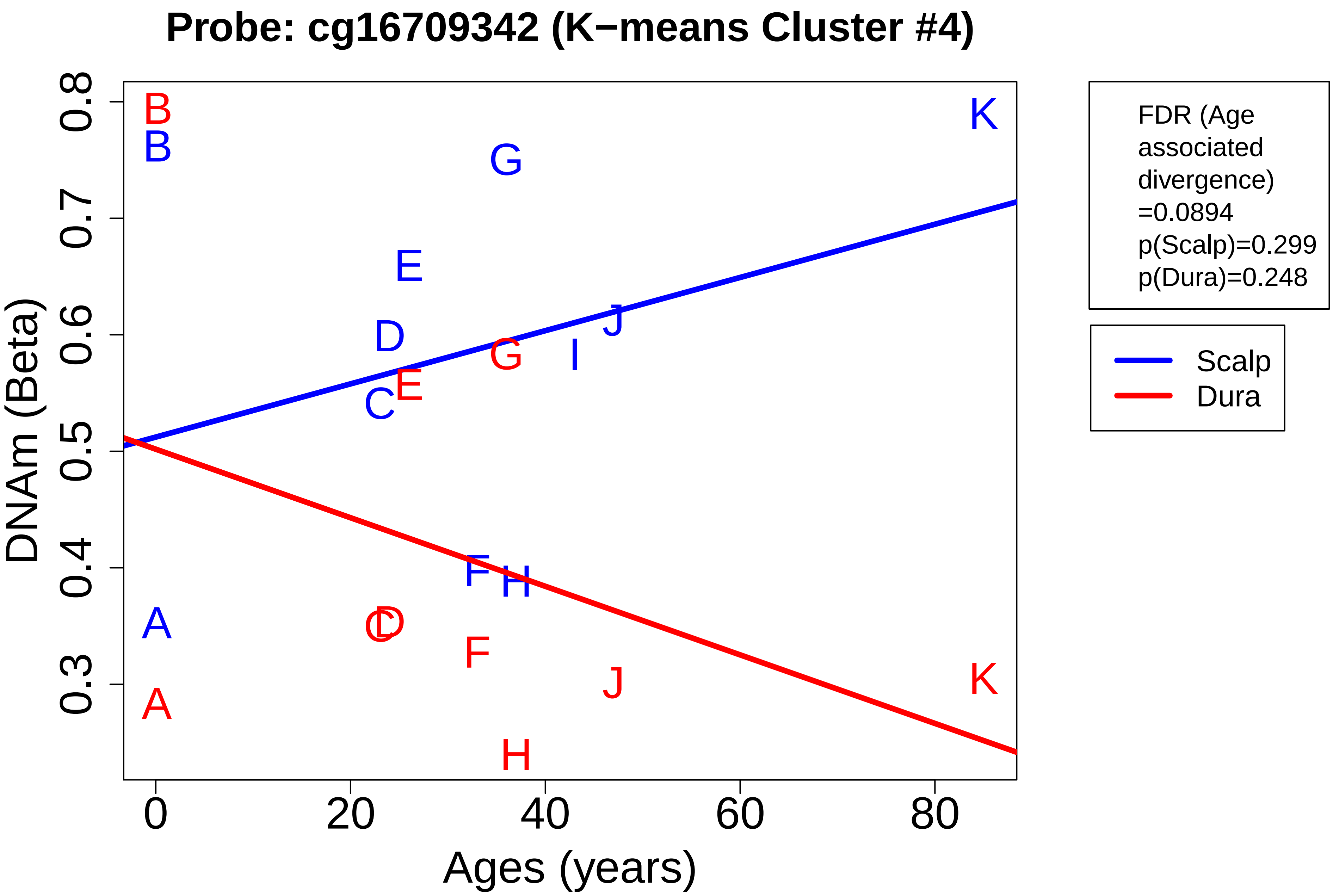

Probe: cg16709342 (K−means Cluster #4)
DNAm (Beta)
0.3
0.4
0.5
0.6
0.7
0.8
Ages (years)
0
20
40
60
80
B
B
K
G
E
J
D
G
I
E
C
F
H
A
C
D
F
J
K
A
H
FDR (Age associated divergence) =0.0894
p(Scalp)=0.299
p(Dura)=0.248
Scalp
Dura

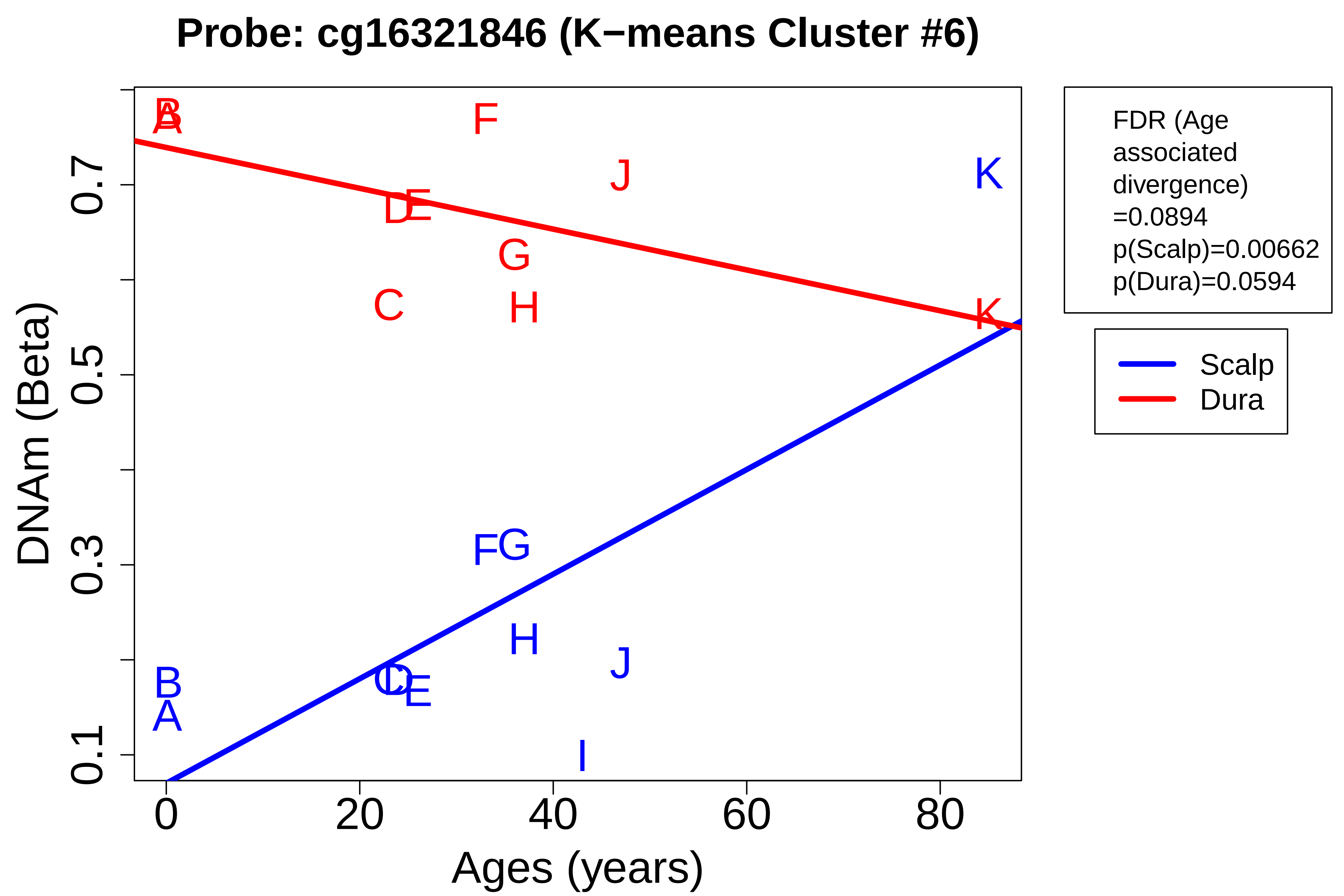

Probe: cg16321846 (K−means Cluster #6)
DNAm (Beta)
0.1
0.3
0.5
0.7
0
20
40
60
80
Ages (years)
FDR (Age associated divergence) =0.0894
p(Scalp)=0.00662
p(Dura)=0.0594
Scalp
Dura

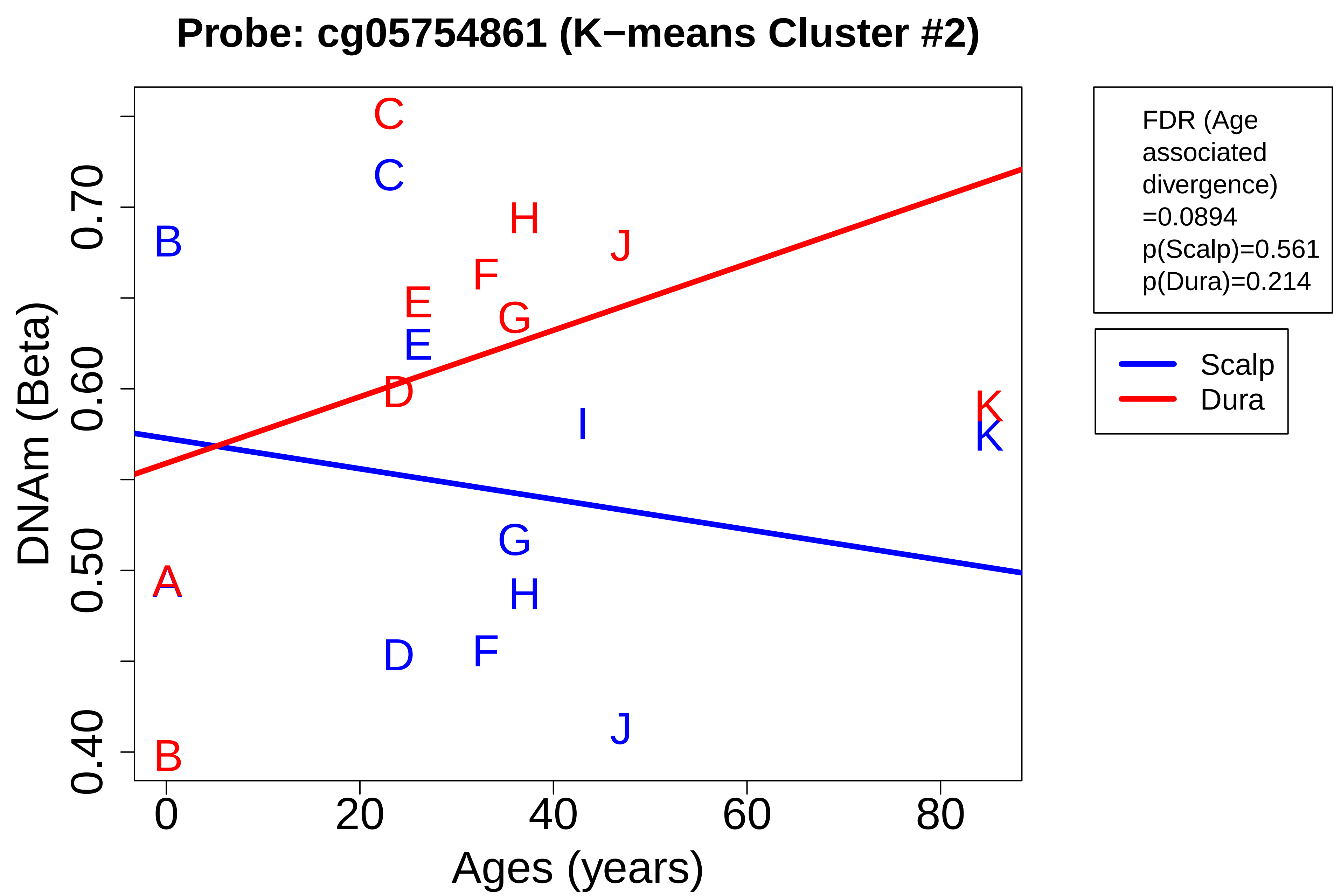

Probe: cg05754861 (K−means Cluster #2)
DNAm (Beta)
0.40
0.50
0.60
0.70
Ages (years)
0
20
40
60
80
A
B
C
D
E
F
G
H
I
J
K
FDR (Age associated divergence) =0.0894
p(Scalp)=0.561
p(Dura)=0.214
Scalp
Dura

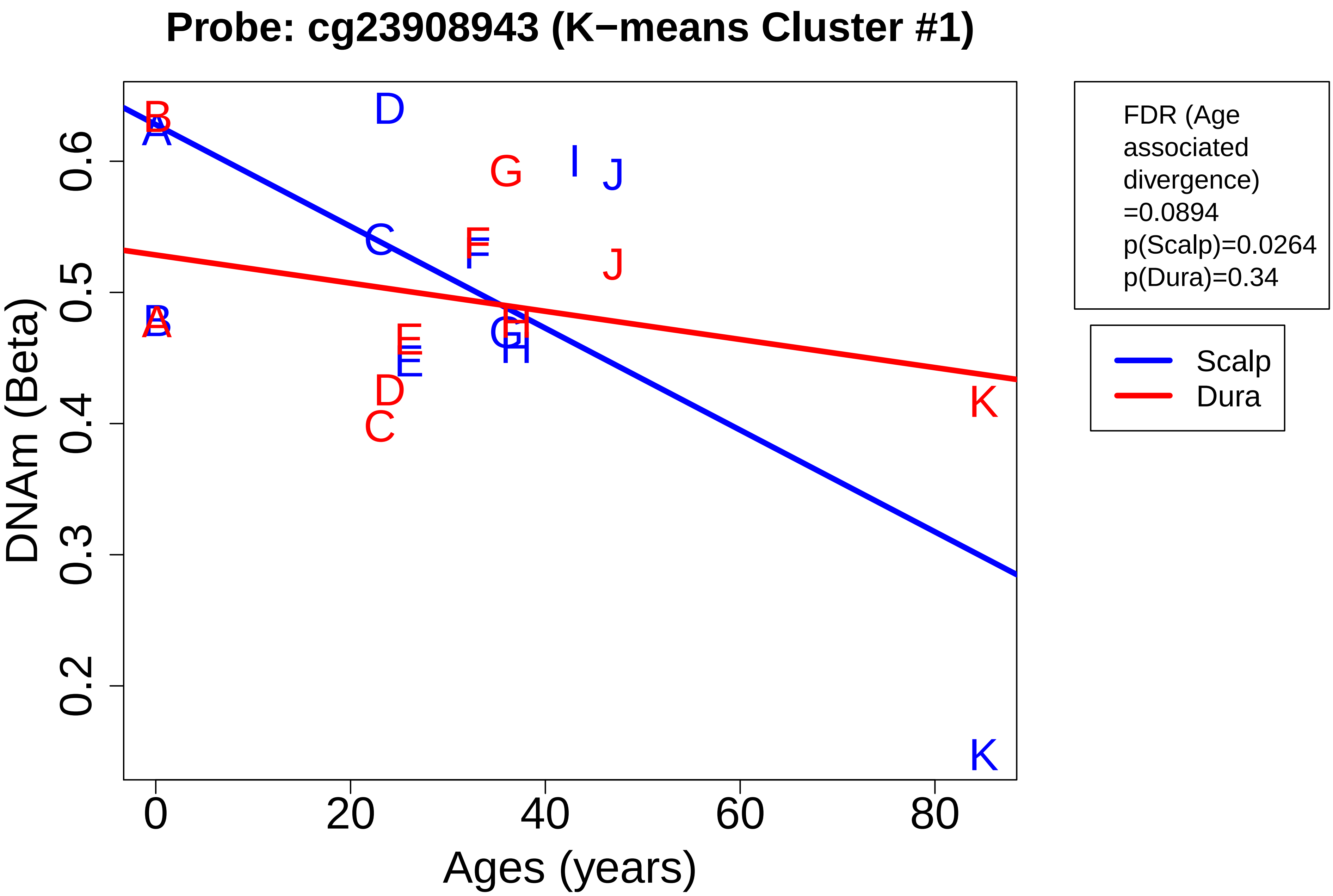

Probe: cg23908943 (K−means Cluster #1)
DNAm (Beta)
0.2
0.3
0.4
0.5
0.6
Ages (years)
0
20
40
60
80
FDR (Age associated divergence) =0.0894
p(Scalp)=0.0264
p(Dura)=0.34
Scalp
Dura
A
B
C
D
E
F
G
H
I
J
K

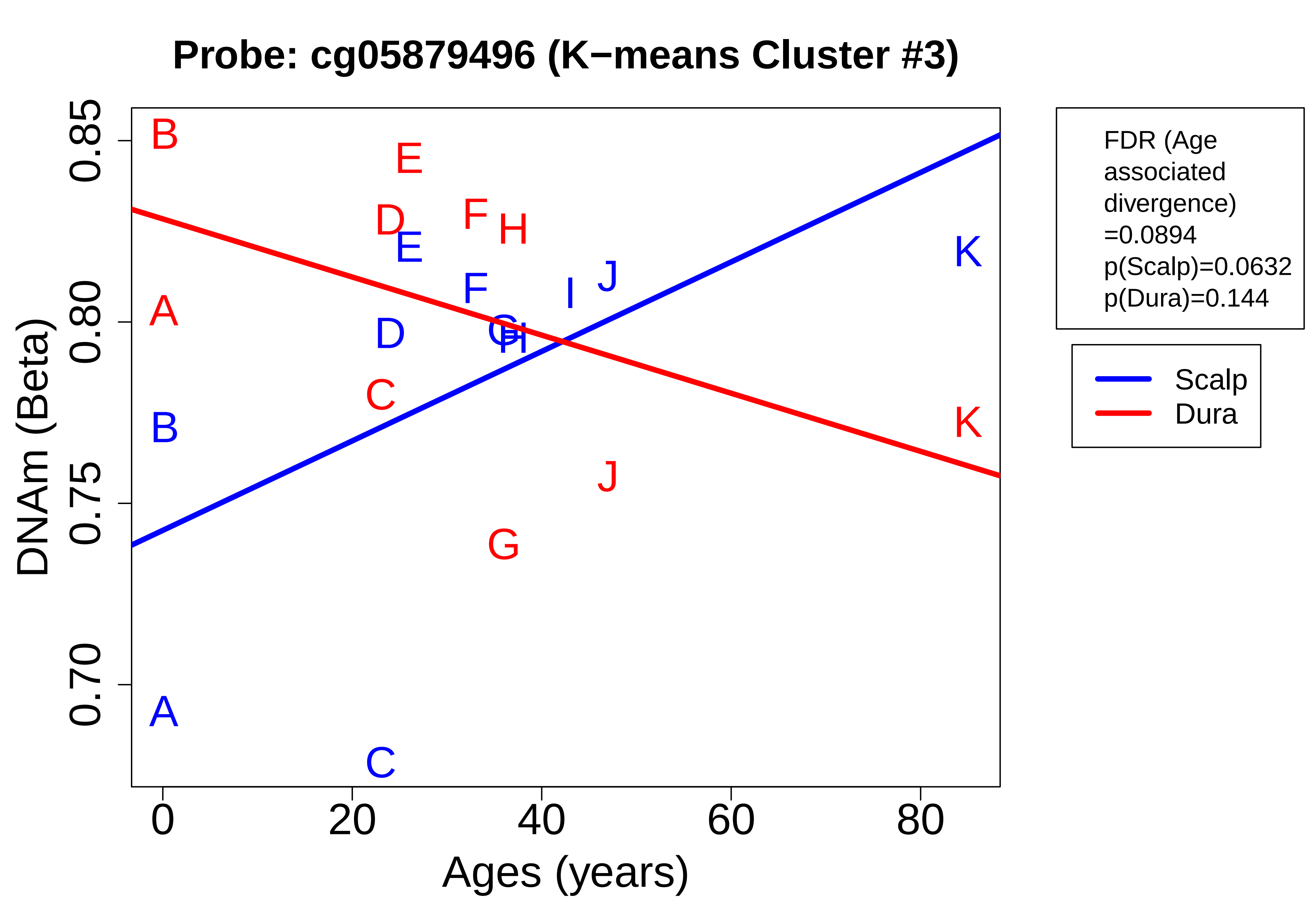
Probe: cg05879496 (K−means Cluster #3)
DNAm (Beta)
0.70
0.75
0.80
0.85
Ages (years)
0
20
40
60
80
FDR (Age associated divergence) =0.0894
p(Scalp)=0.0632
p(Dura)=0.144
Scalp
Dura

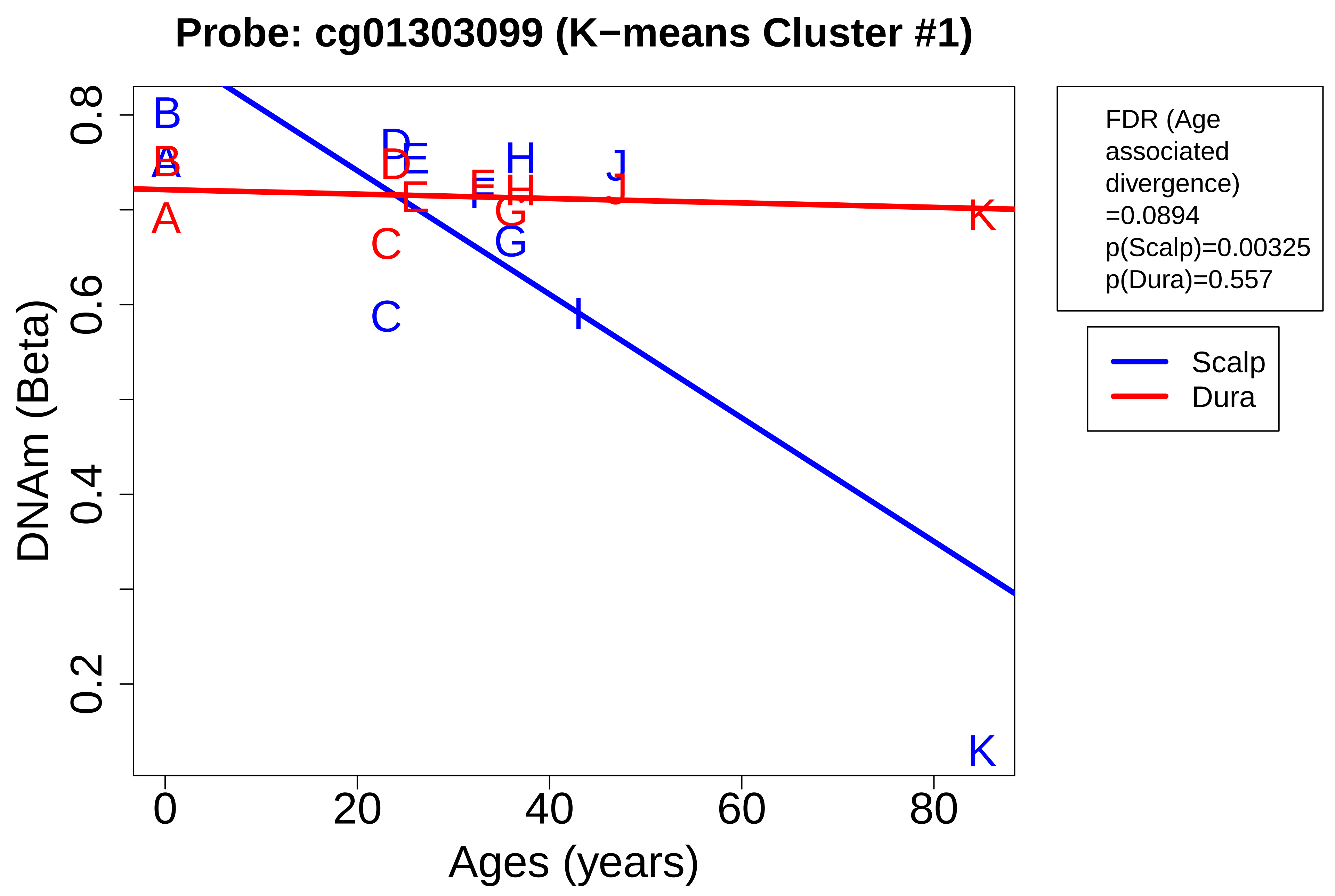

Probe: cg01303099 (K−means Cluster #1)
DNAm (Beta)
0.2
0.4
0.6
0.8
Ages (years)
0
20
40
60
80
FDR (Age associated divergence) =0.0894
p(Scalp)=0.00325
p(Dura)=0.557
Scalp
Dura

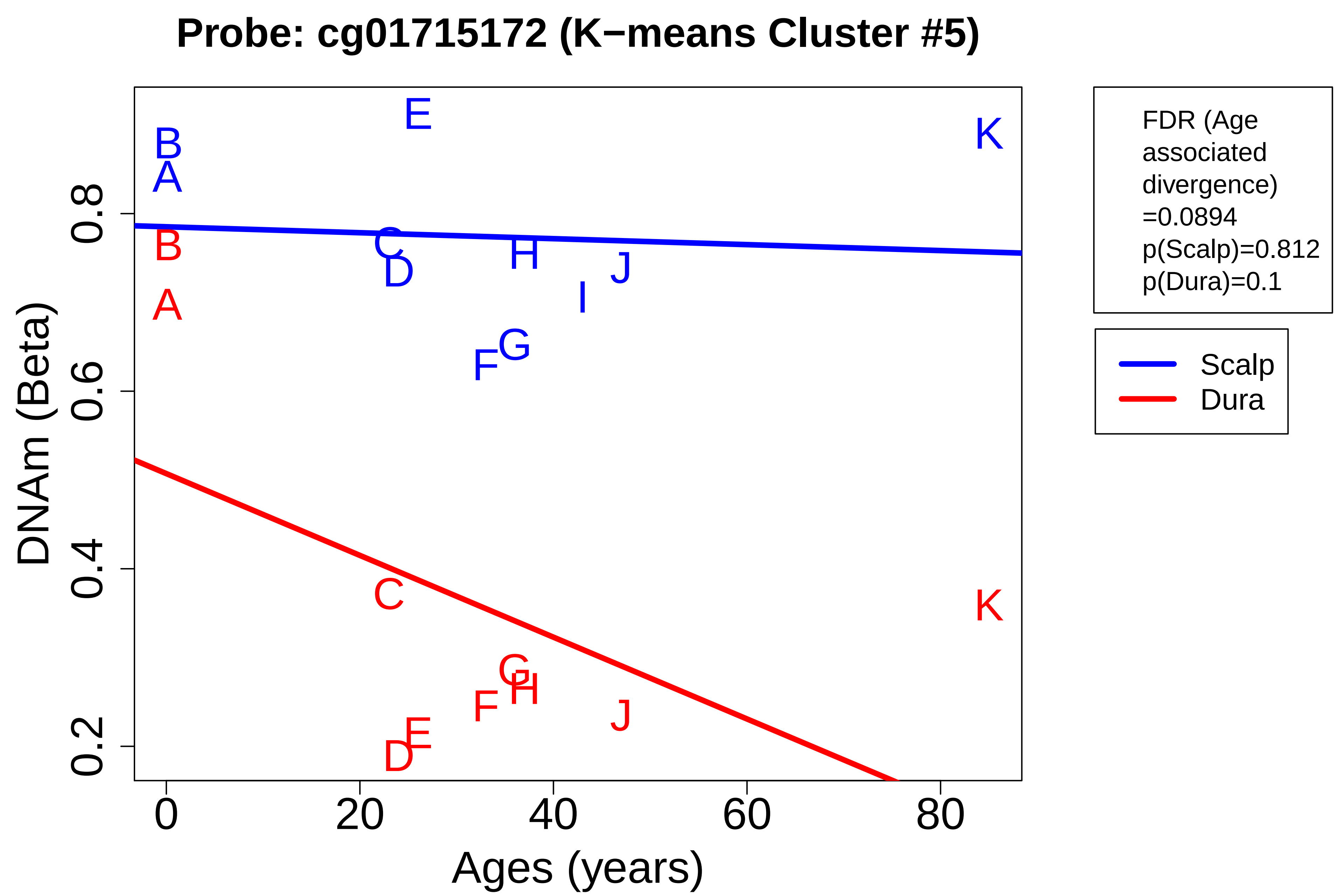

Probe: cg01715172 (K−means Cluster #5)
DNAm (Beta)
0.2
0.4
0.6
0.8
Ages (years)
0
20
40
60
80
A
B
C
D
E
F
G
H
I
J
K
FDR (Age associated divergence) =0.0894
p(Scalp)=0.812
p(Dura)=0.1
Scalp
Dura

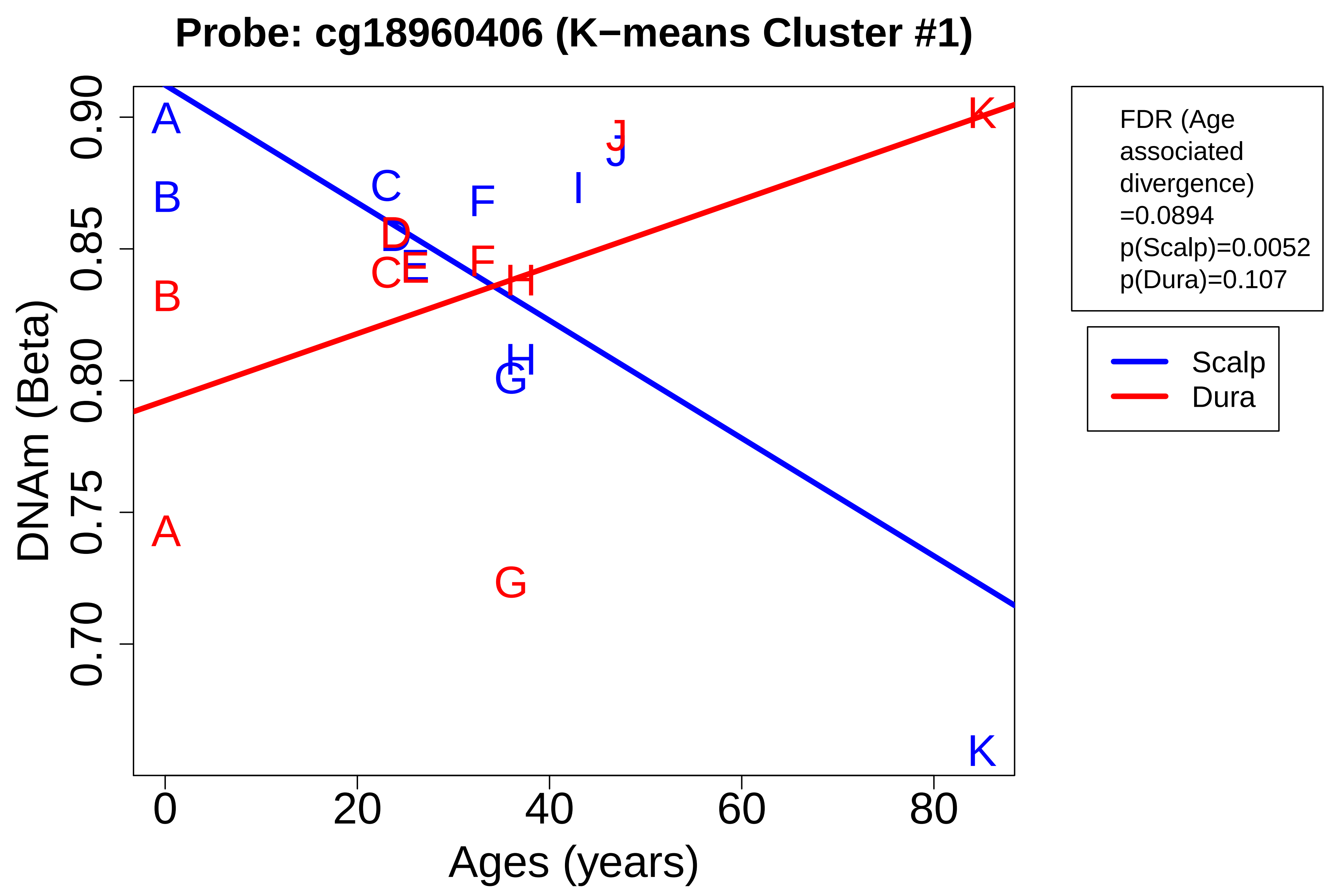

Probe: cg18960406 (K−means Cluster #1)
DNAm (Beta)
0.70
0.75
0.80
0.85
0.90
Ages (years)
0
20
40
60
80
FDR (Age associated divergence) =0.0894
p(Scalp)=0.0052
p(Dura)=0.107
Scalp
Dura
A
B
C
D
E
F
G
H
I
J
K

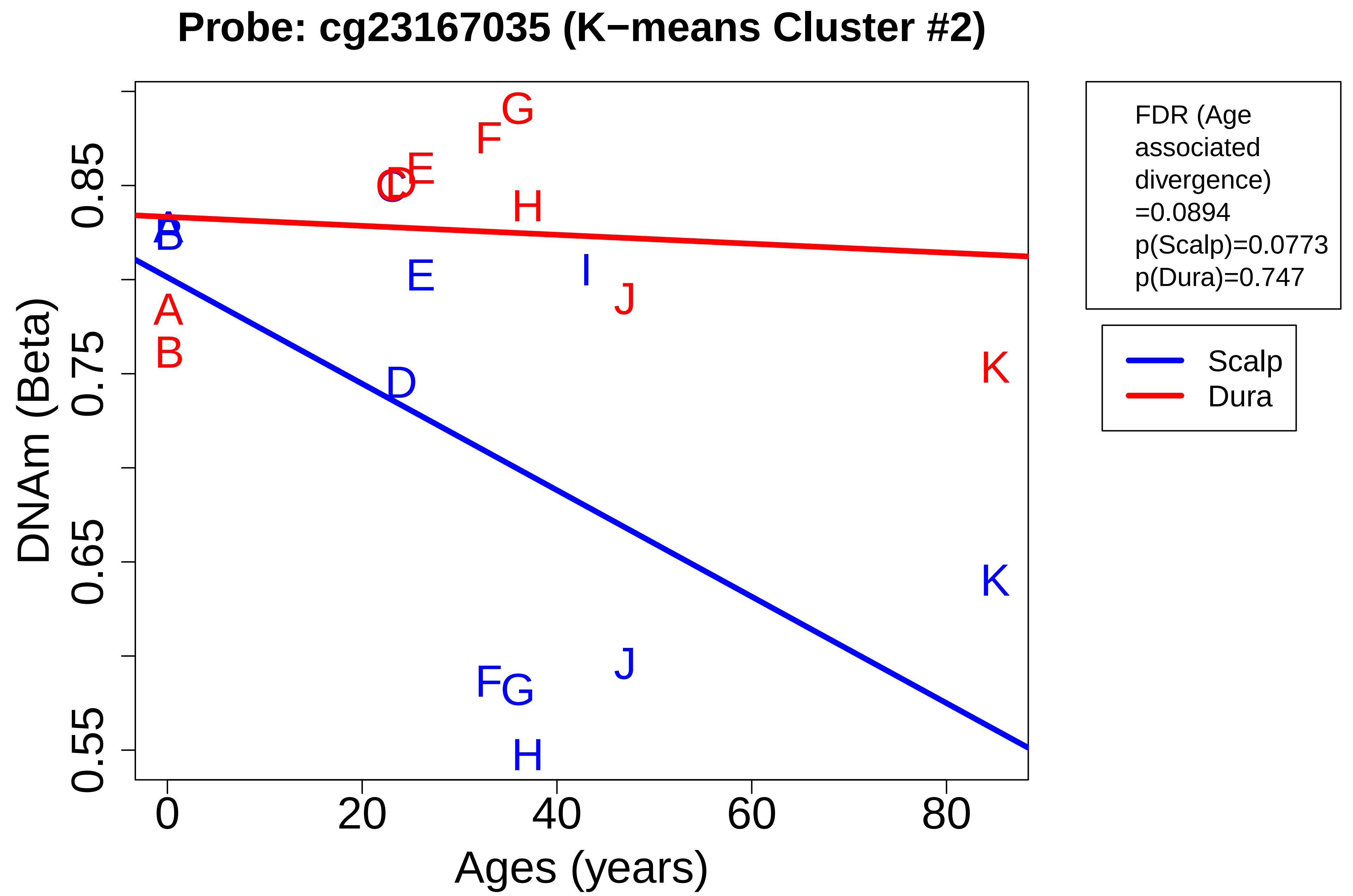

Probe: cg23167035 (K−means Cluster #2)
DNAm (Beta)
0.55
0.65
0.75
0.85
0
20
40
60
80
Ages (years)
FDR (Age associated divergence) =0.0894
p(Scalp)=0.0773
p(Dura)=0.747
Scalp
Dura

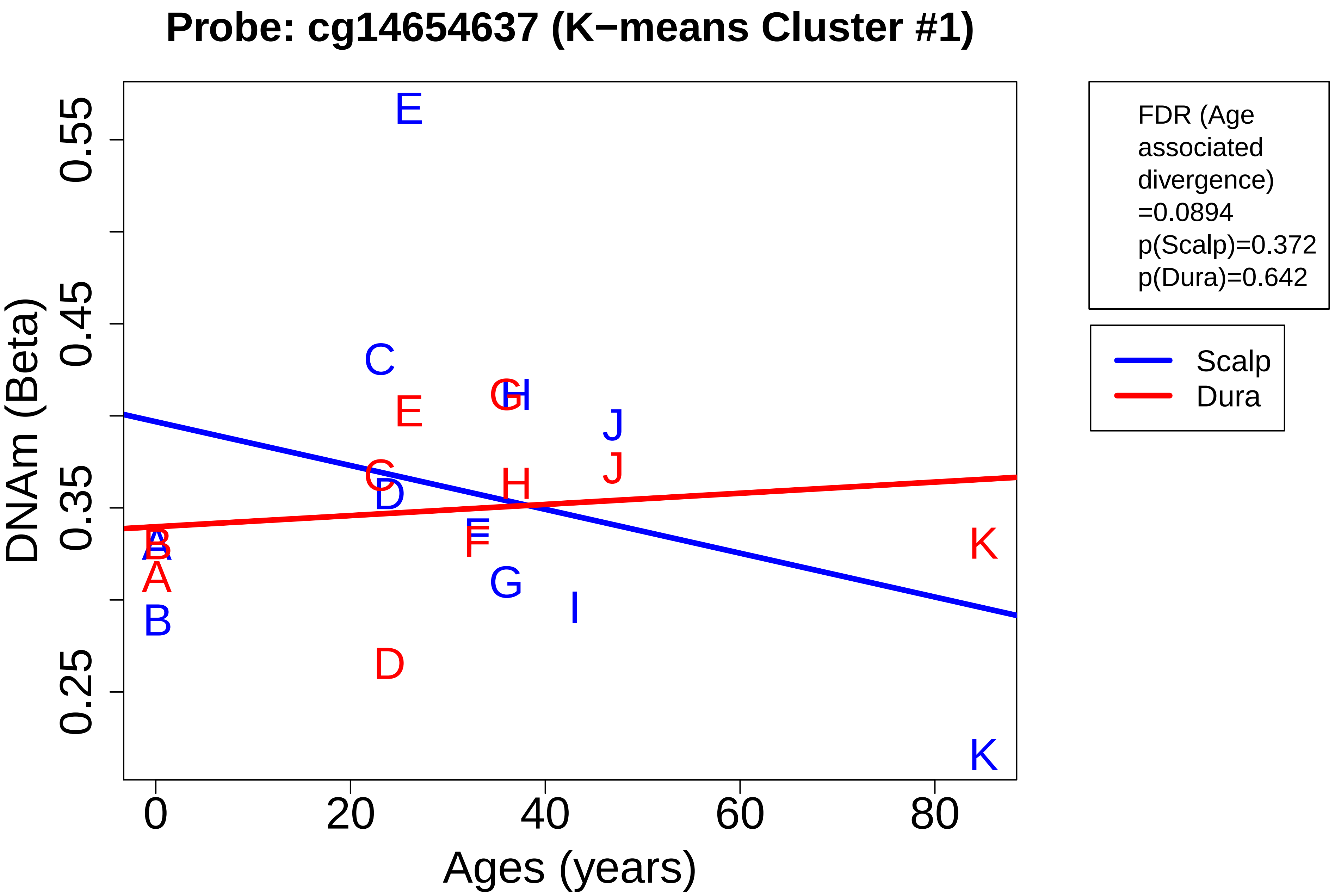
Probe: cg14654637 (K−means Cluster #1)
DNAm (Beta)
0.25
0.35
0.45
0.55
Ages (years)
0
20
40
60
80
FDR (Age associated divergence) =0.0894
p(Scalp)=0.372
p(Dura)=0.642
Scalp
Dura
A
B
C
D
E
F
G
H
I
J
K

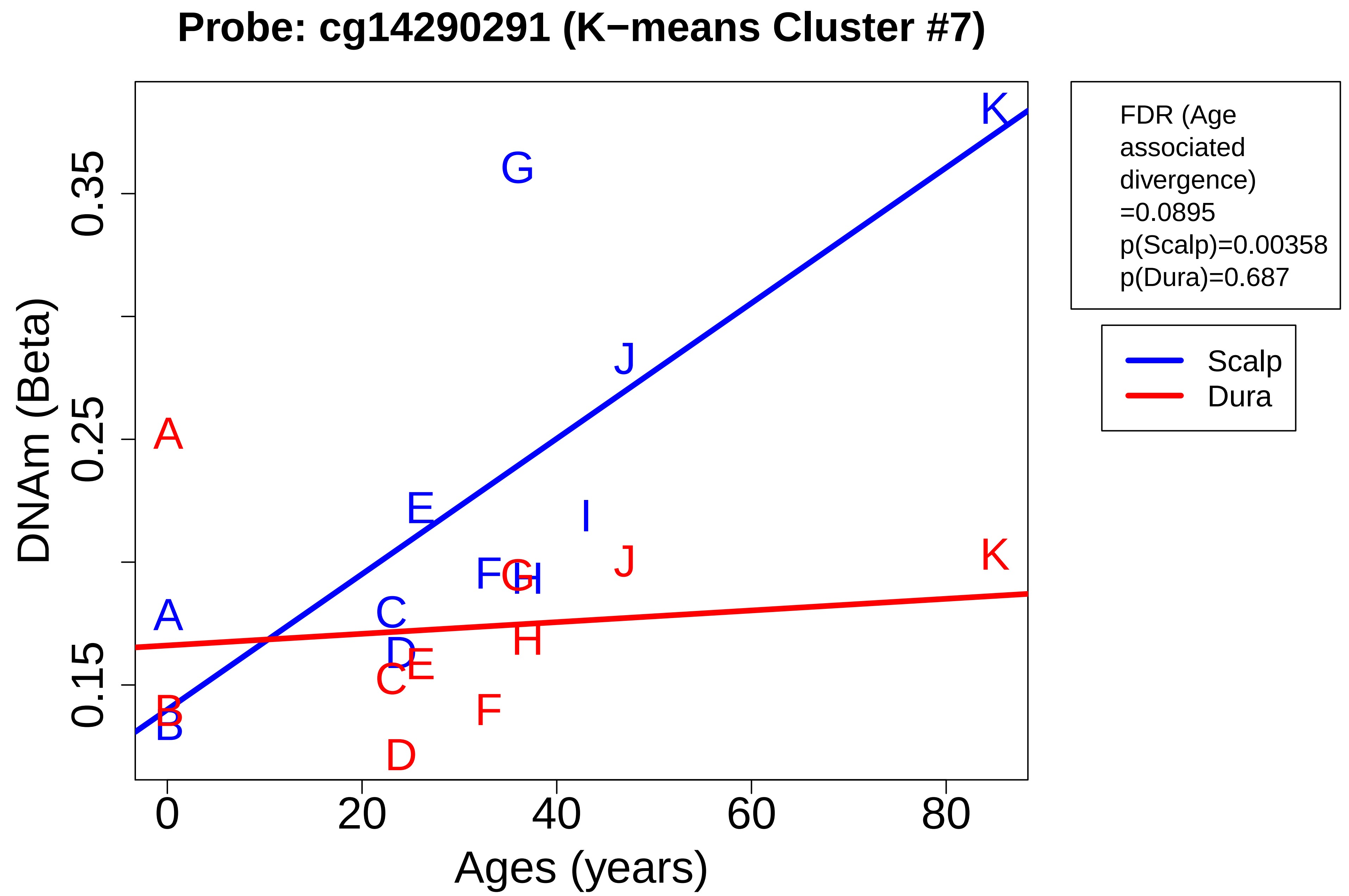

Probe: cg14290291 (K−means Cluster #7)
DNAm (Beta)
0.15
0.25
0.35
Ages (years)
0
20
40
60
80
A
B
C
D
E
F
G
H
I
J
K
FDR (Age associated divergence) =0.0895
p(Scalp)=0.00358
p(Dura)=0.687
Scalp
Dura

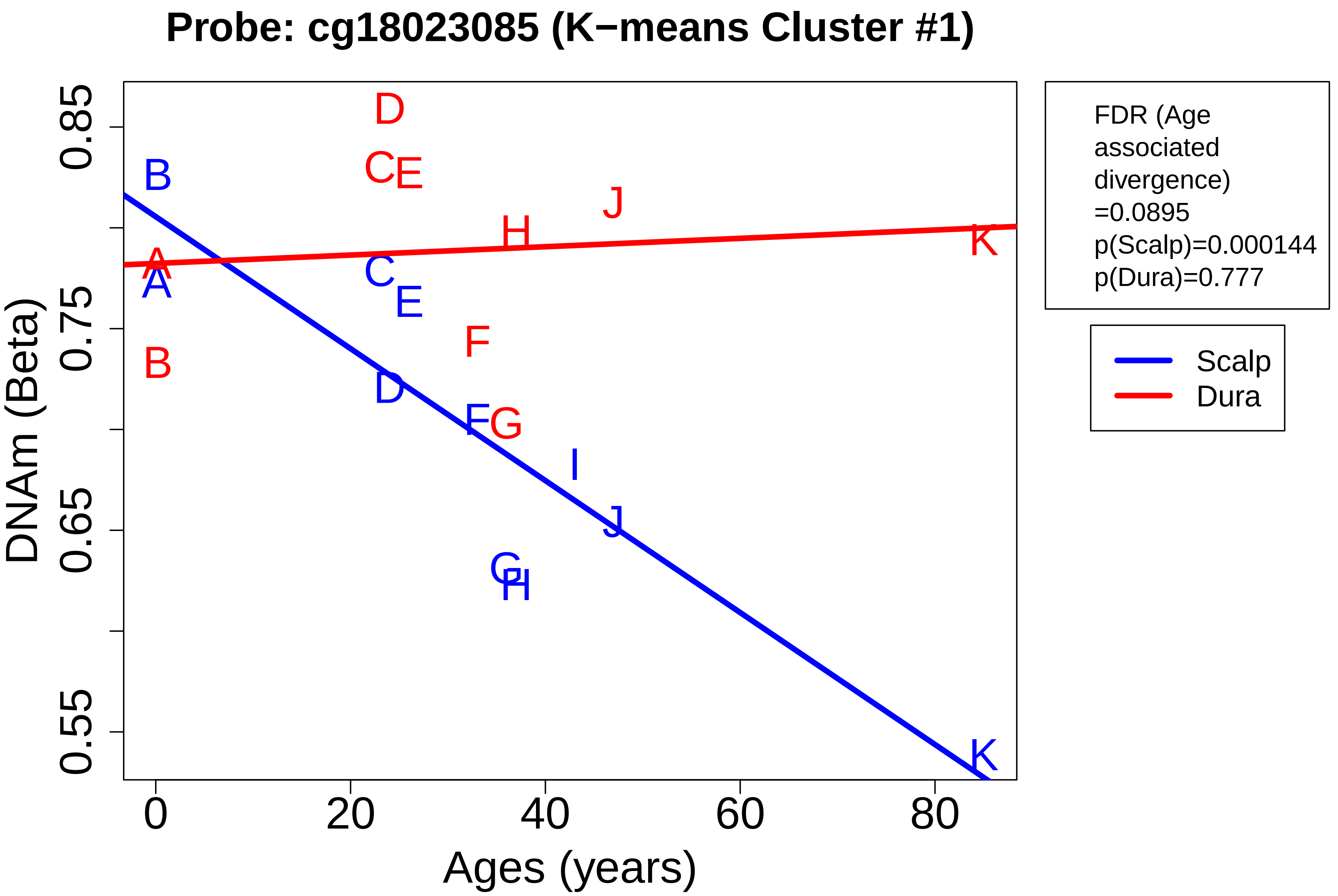

Probe: cg18023085 (K−means Cluster #1)
DNAm (Beta)
0.55
0.65
0.75
0.85
Ages (years)
0
20
40
60
80
FDR (Age associated divergence) =0.0895
p(Scalp)=0.000144
p(Dura)=0.777
Scalp
Dura

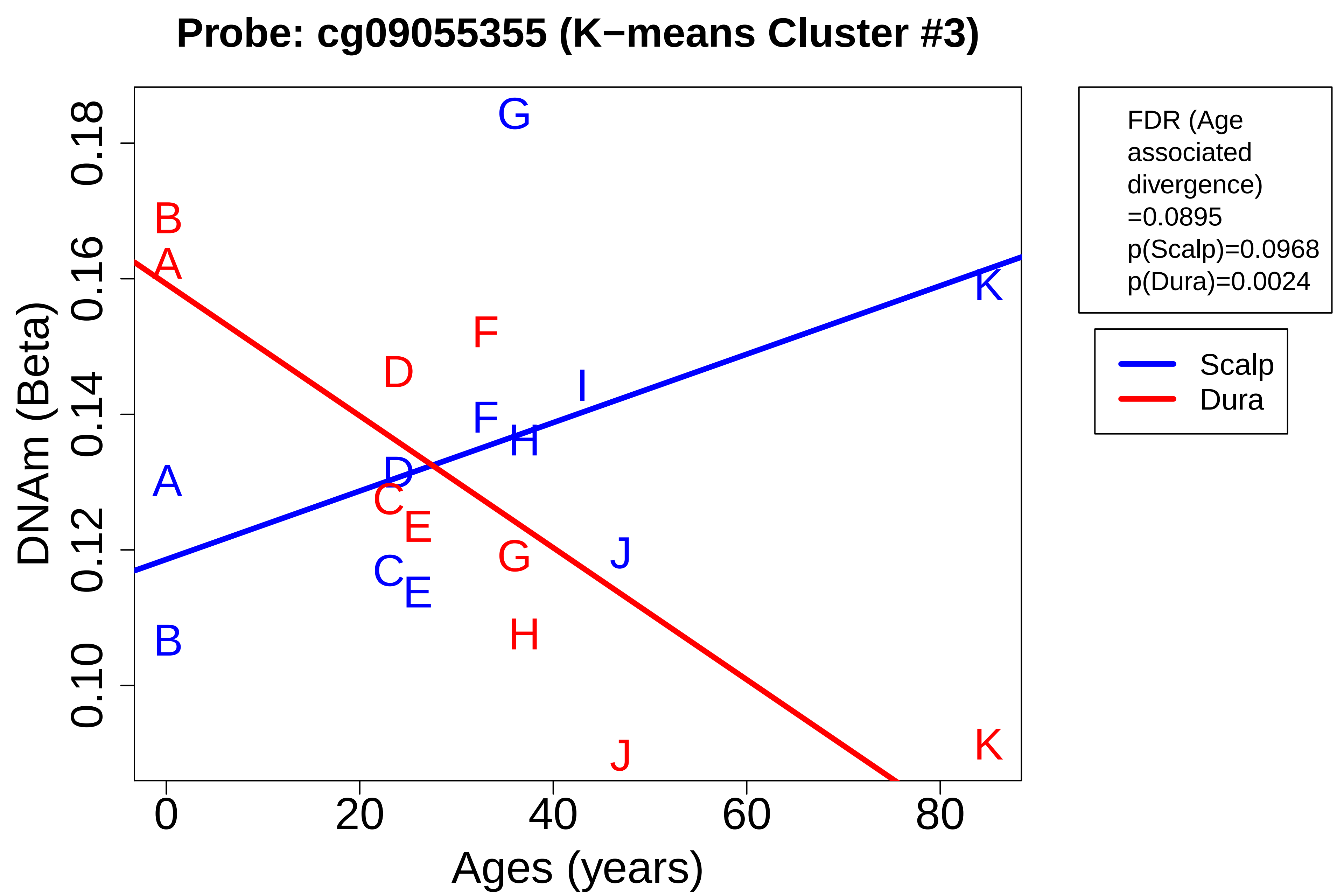

Probe: cg09055355 (K−means Cluster #3)
DNAm (Beta)
0.10
0.12
0.14
0.16
0.18
Ages (years)
0
20
40
60
80
FDR (Age associated divergence) =0.0895
p(Scalp)=0.0968
p(Dura)=0.0024
Scalp
Dura
A
B
C
D
E
F
G
H
I
J
K

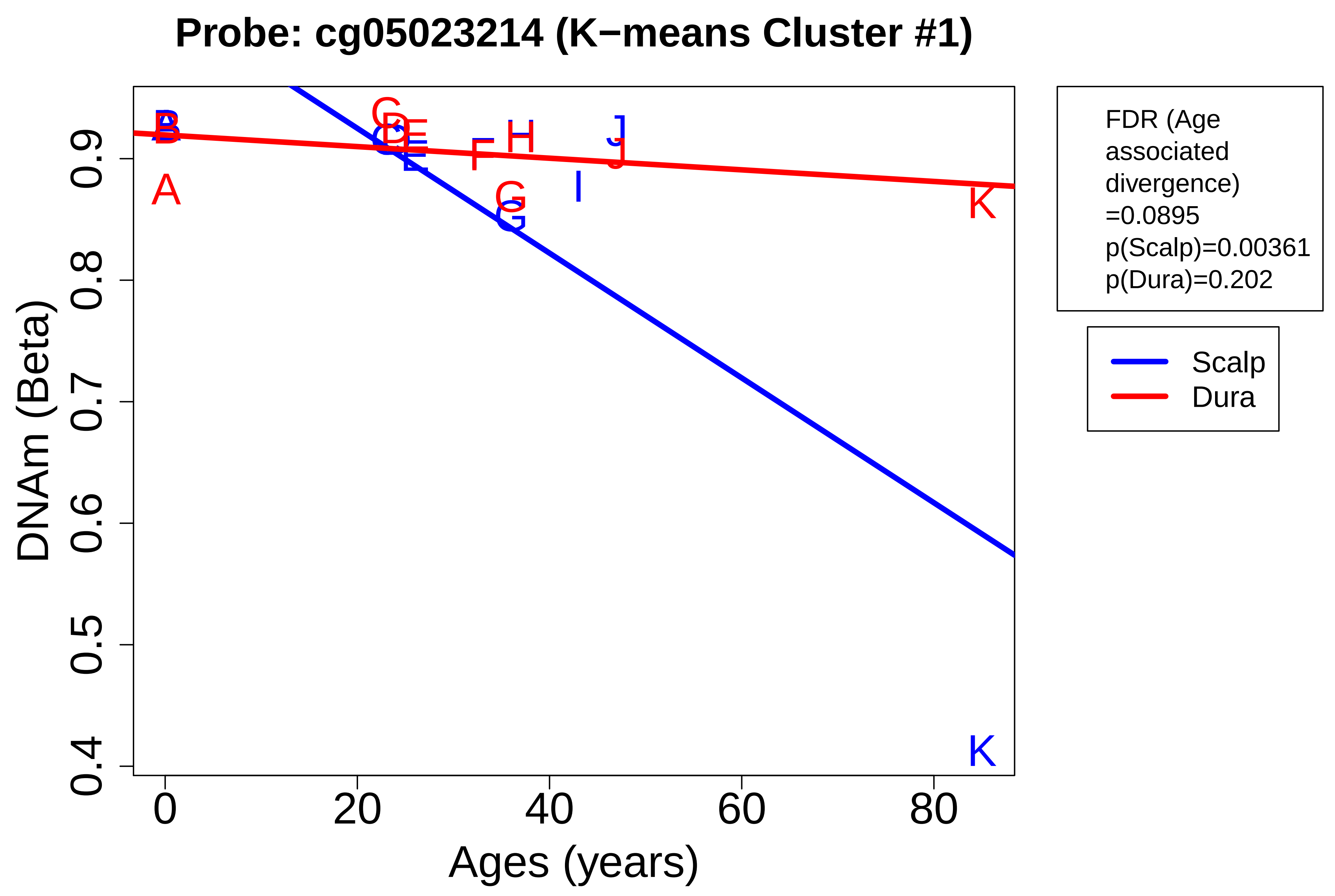

Probe: cg05023214 (K−means Cluster #1)
DNAm (Beta)
0.4
0.5
0.6
0.7
0.8
0.9
Ages (years)
0
20
40
60
80
FDR (Age associated divergence) =0.0895
p(Scalp)=0.00361
p(Dura)=0.202
Scalp
Dura
A
B
C
D
E
F
G
H
I
J
K

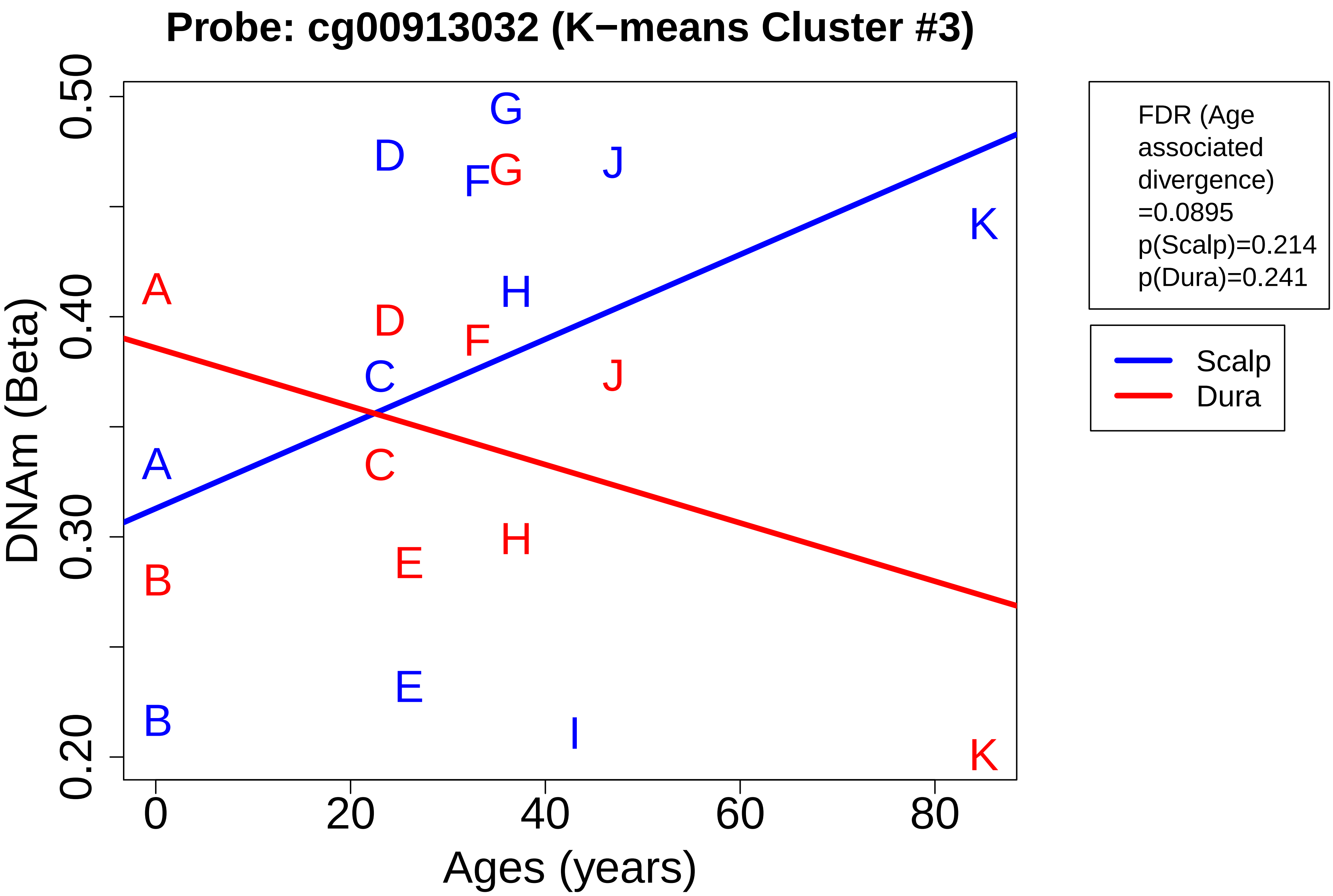

Probe: cg00913032 (K−means Cluster #3)
DNAm (Beta)
0.20
0.30
0.40
0.50
Ages (years)
0
20
40
60
80
FDR (Age associated divergence) =0.0895
p(Scalp)=0.214
p(Dura)=0.241
Scalp
Dura
A
B
C
D
E
F
G
H
I
J
K

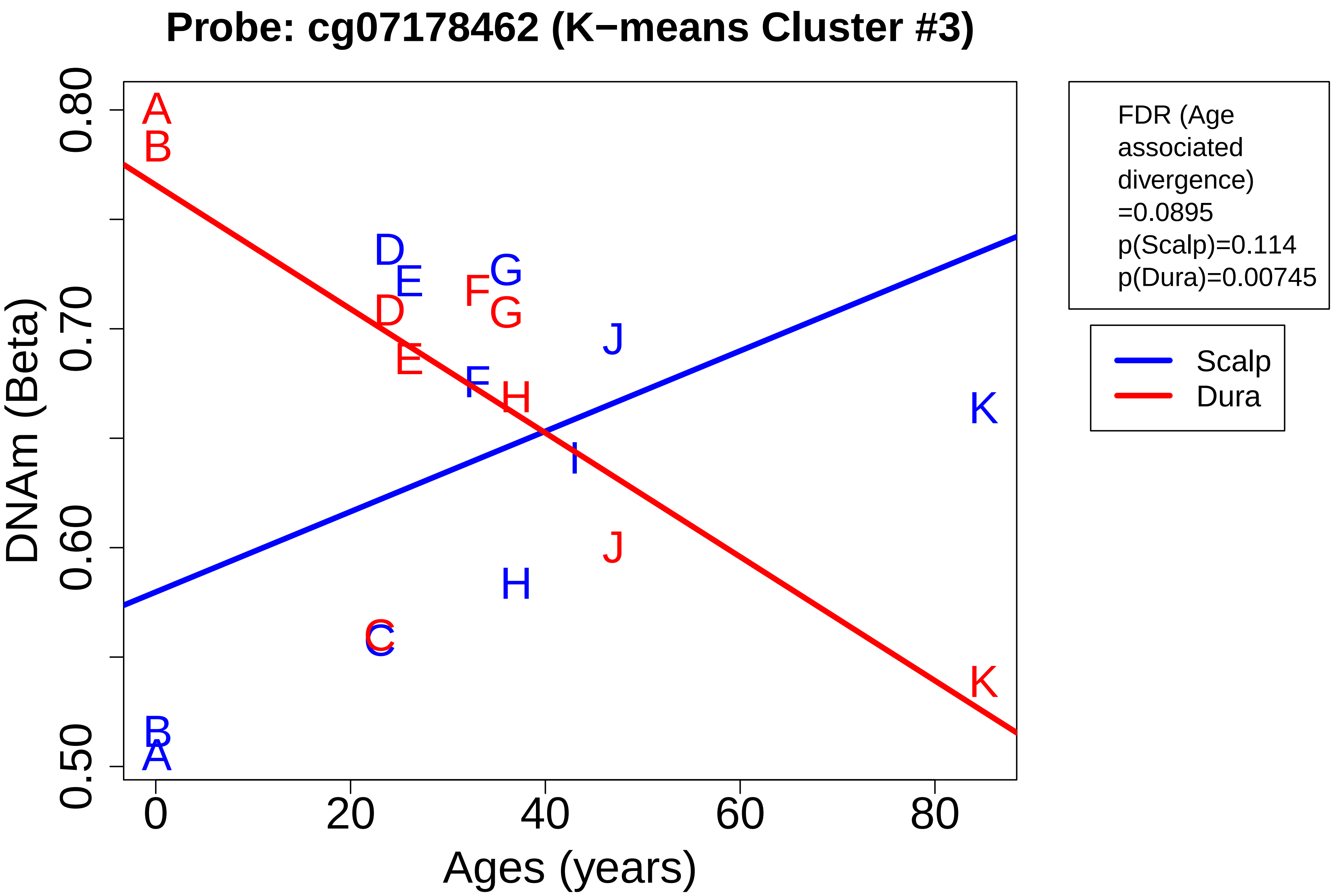

Probe: cg07178462 (K−means Cluster #3)
DNAm (Beta)
0.50
0.60
0.70
0.80
Ages (years)
0
20
40
60
80
FDR (Age associated divergence) =0.0895
p(Scalp)=0.114
p(Dura)=0.00745
Scalp
Dura

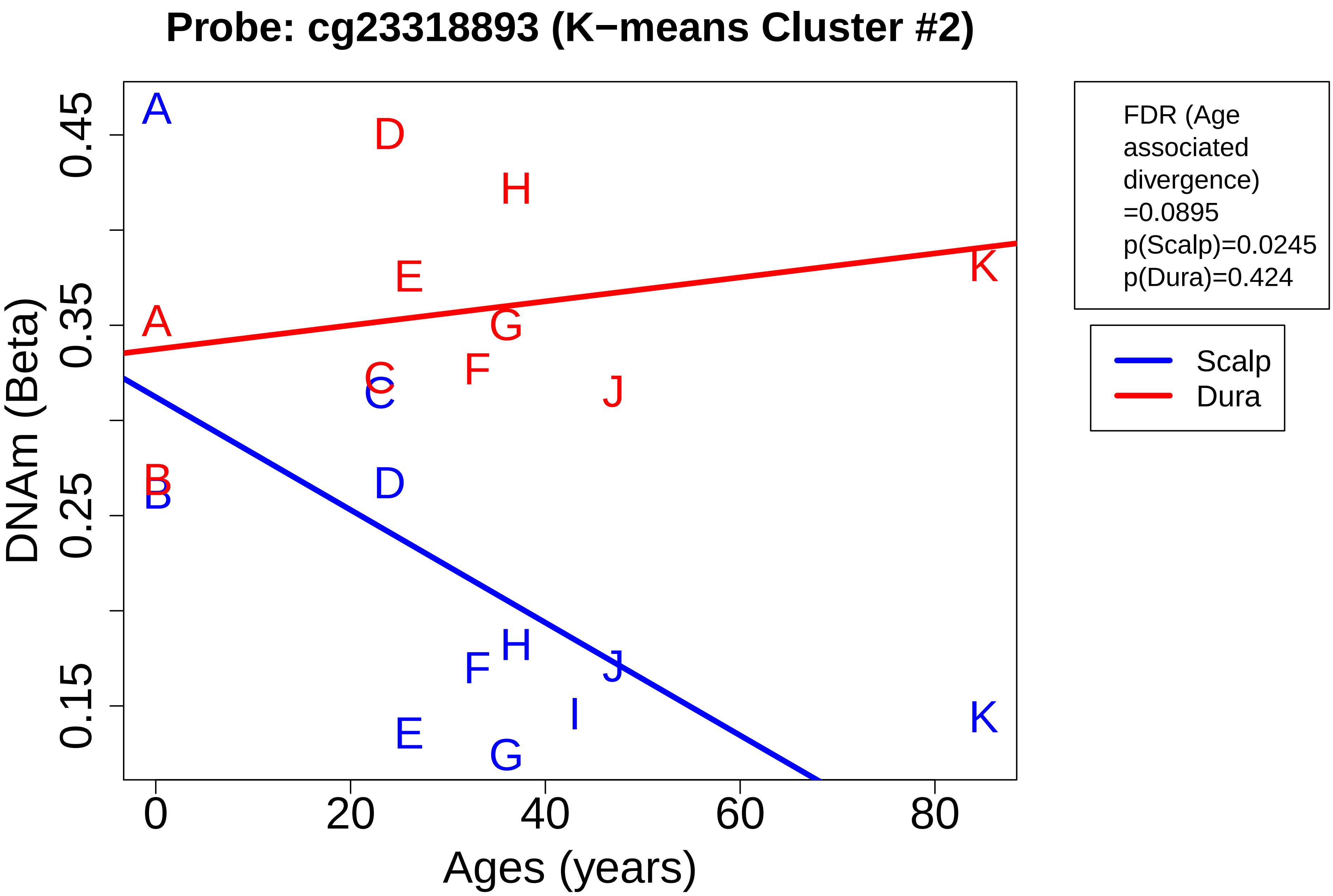

Probe: cg23318893 (K−means Cluster #2)
DNAm (Beta)
0.15
0.25
0.35
0.45
Ages (years)
0
20
40
60
80
A
D
H
E
K
A
G
C
F
C
J
B
B
D
H
F
J
I
K
E
G
FDR (Age associated divergence) =0.0895
p(Scalp)=0.0245
p(Dura)=0.424
Scalp
Dura

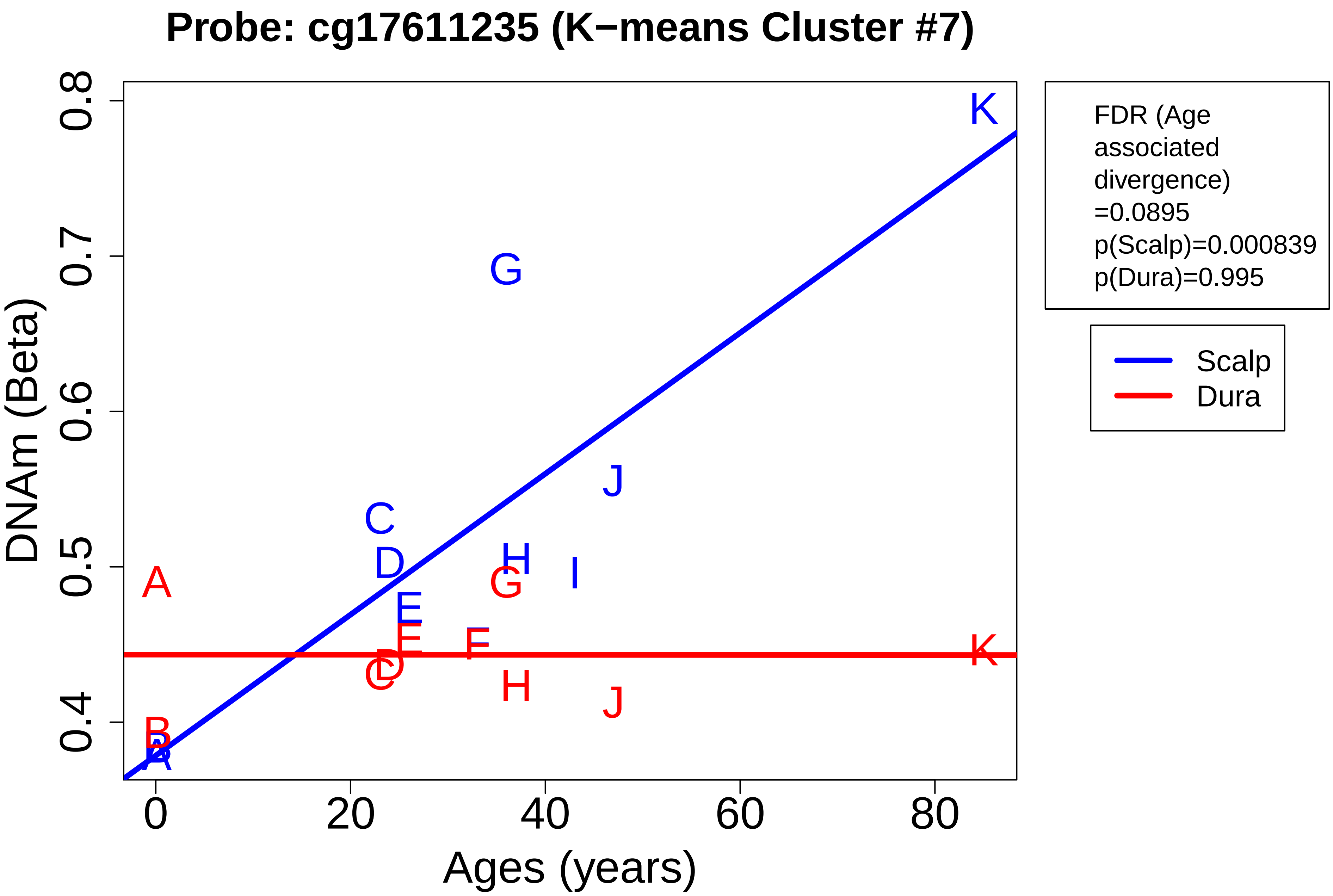
Probe: cg17611235 (K−means Cluster #7)
DNAm (Beta)
0.4
0.5
0.6
0.7
0.8
Ages (years)
0
20
40
60
80
FDR (Age associated divergence) =0.0895
p(Scalp)=0.000839
p(Dura)=0.995
Scalp
Dura

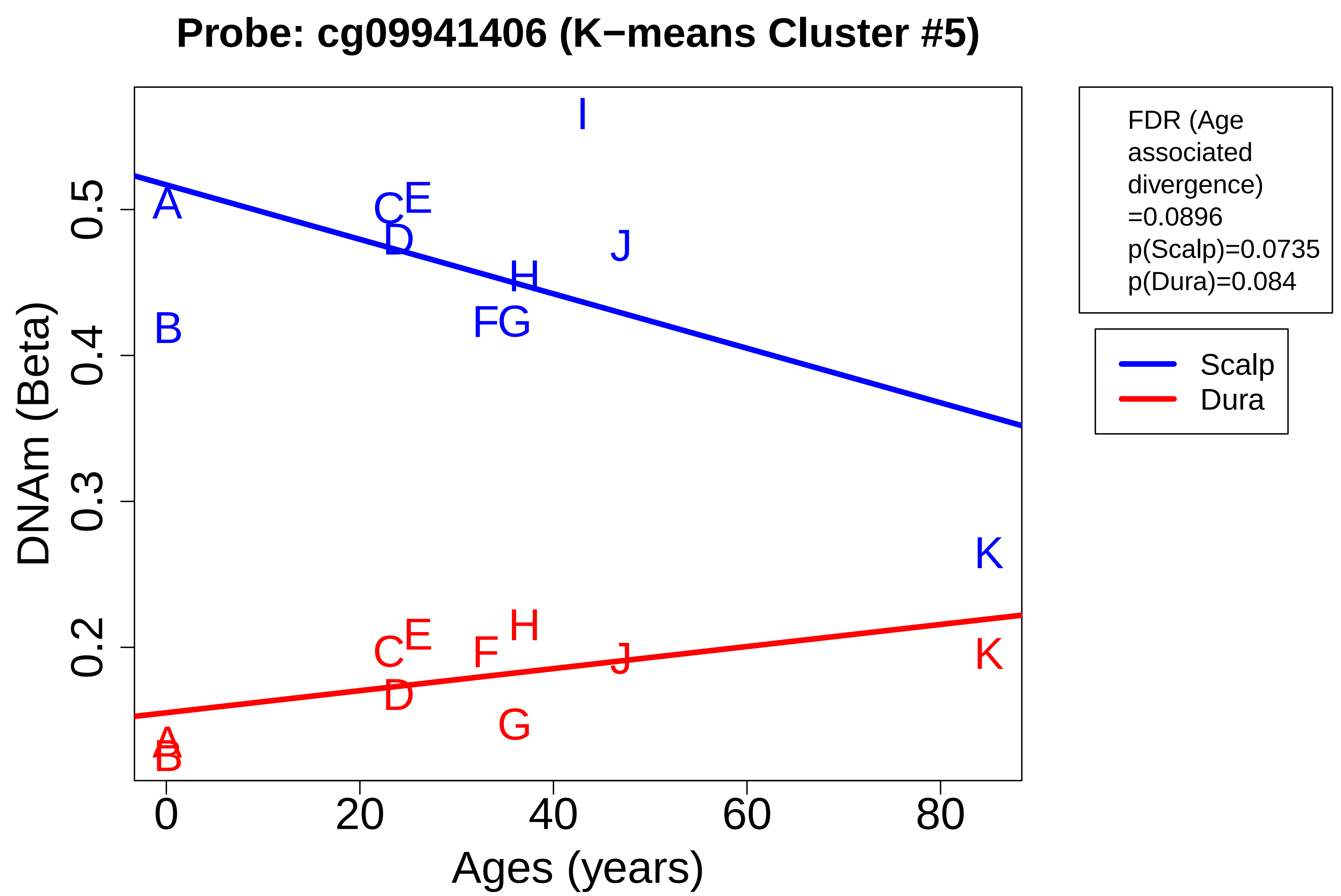

Probe: cg09941406 (K−means Cluster #5)
FDR (Age associated divergence) =0.0896
p(Scalp)=0.0735
p(Dura)=0.084
Scalp
Dura
DNAm (Beta)
0.2
0.3
0.4
0.5
Ages (years)
0
20
40
60
80
A
B
C
D
E
F
G
H
I
J
K

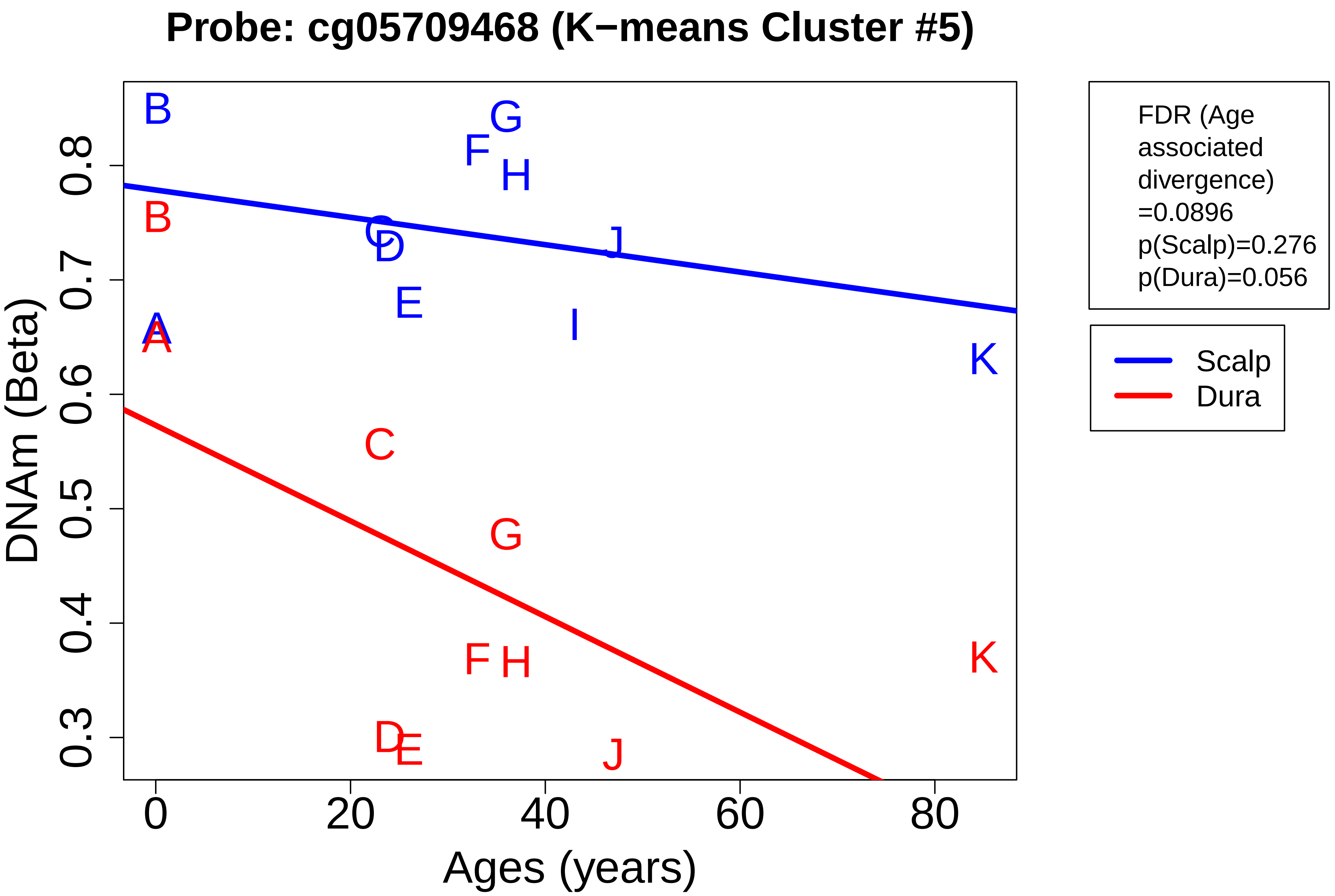

Probe: cg05709468 (K−means Cluster #5)
DNAm (Beta)
0.3
0.4
0.5
0.6
0.7
0.8
0
20
40
60
80
Ages (years)
B
G
F
H
B
C
D
J
E
A
A
I
K
C
G
F
H
K
D
E
J
FDR (Age associated divergence) =0.0896
p(Scalp)=0.276
p(Dura)=0.056
Scalp
Dura

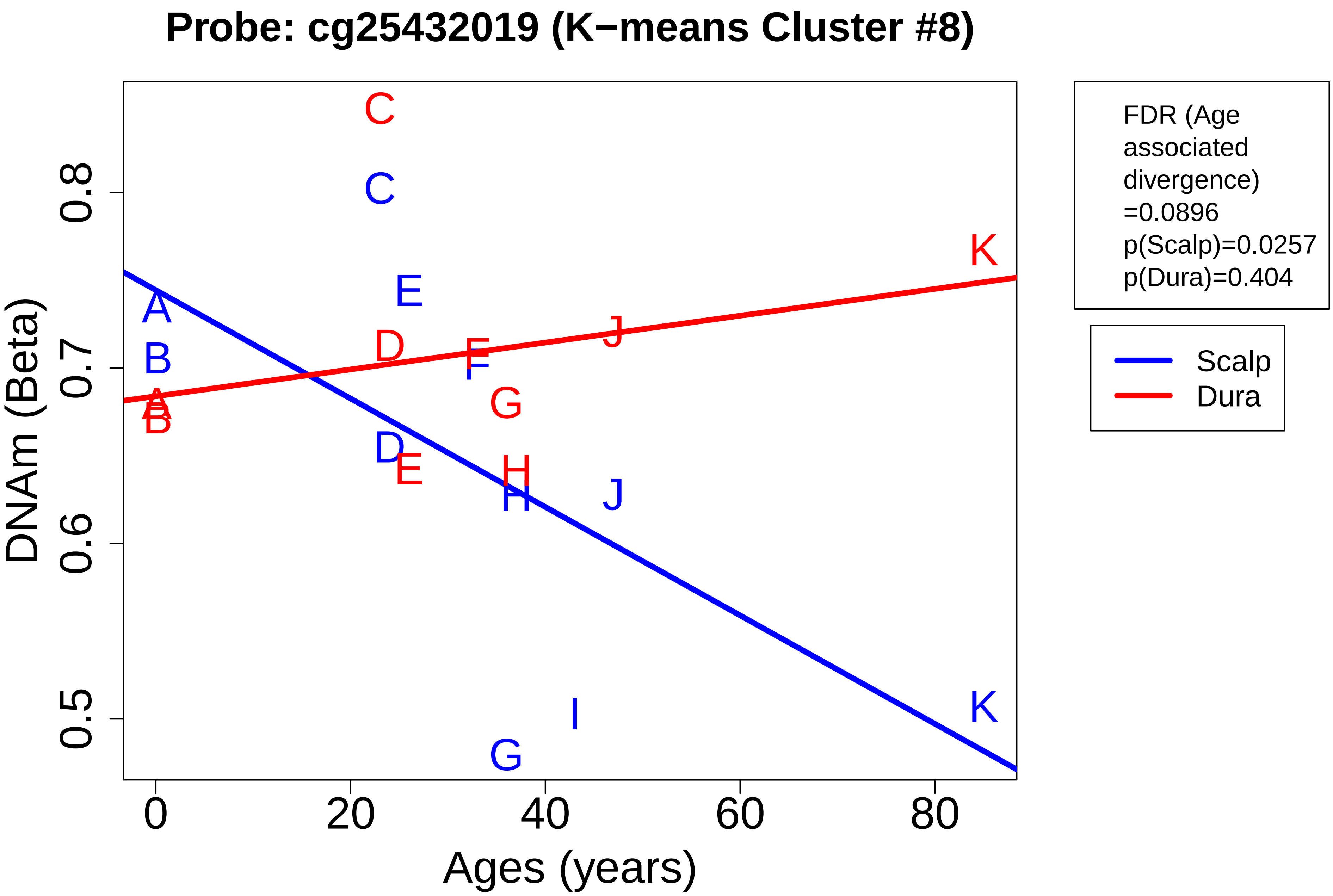

Probe: cg25432019 (K−means Cluster #8)
DNAm (Beta)
0.5
0.6
0.7
0.8
0
20
40
60
80
Ages (years)
FDR (Age associated divergence) =0.0896
p(Scalp)=0.0257
p(Dura)=0.404
Scalp
Dura
A
B
C
D
E
F
G
H
I
J
K

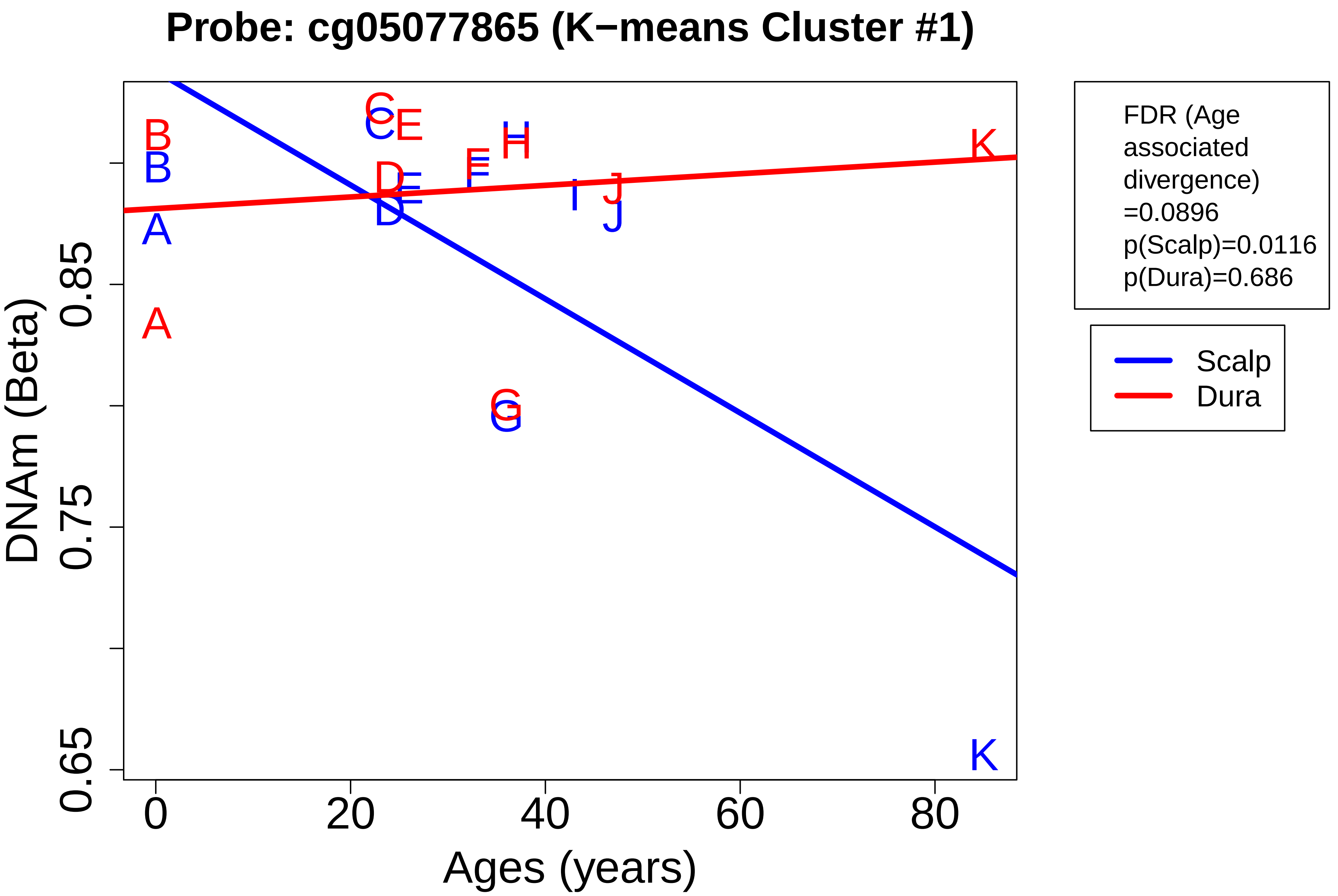

Probe: cg05077865 (K−means Cluster #1)
DNAm (Beta)
0.65
0.75
0.85
0
20
40
60
80
Ages (years)
FDR (Age associated divergence) =0.0896
p(Scalp)=0.0116
p(Dura)=0.686
Scalp
Dura

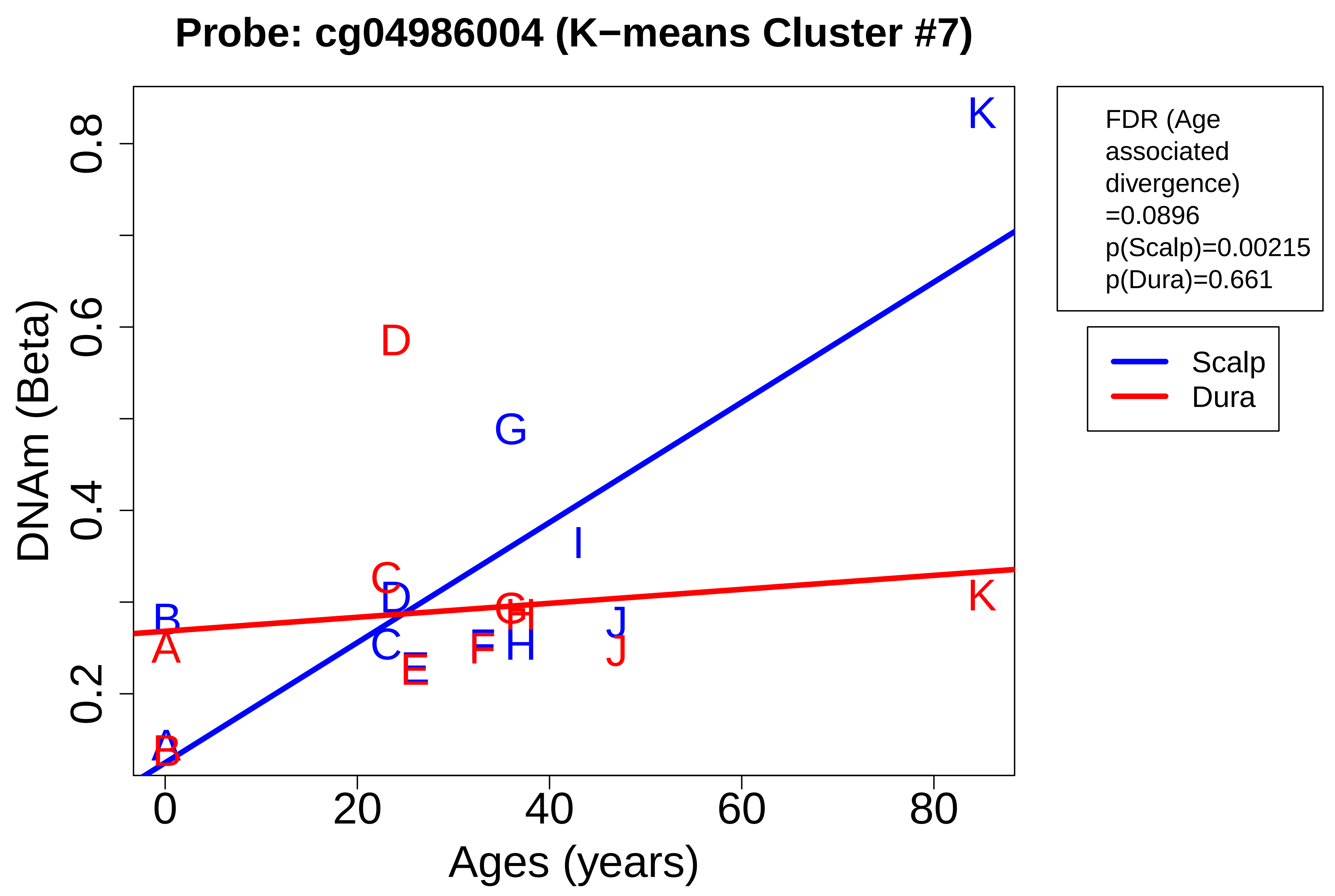

Probe: cg04986004 (K−means Cluster #7)
FDR (Age associated divergence) =0.0896
p(Scalp)=0.00215
p(Dura)=0.661
Scalp
Dura
DNAm (Beta)
0.2
0.4
0.6
0.8
Ages (years)
0
20
40
60
80
A
B
C
D
E
F
G
H
I
J
K

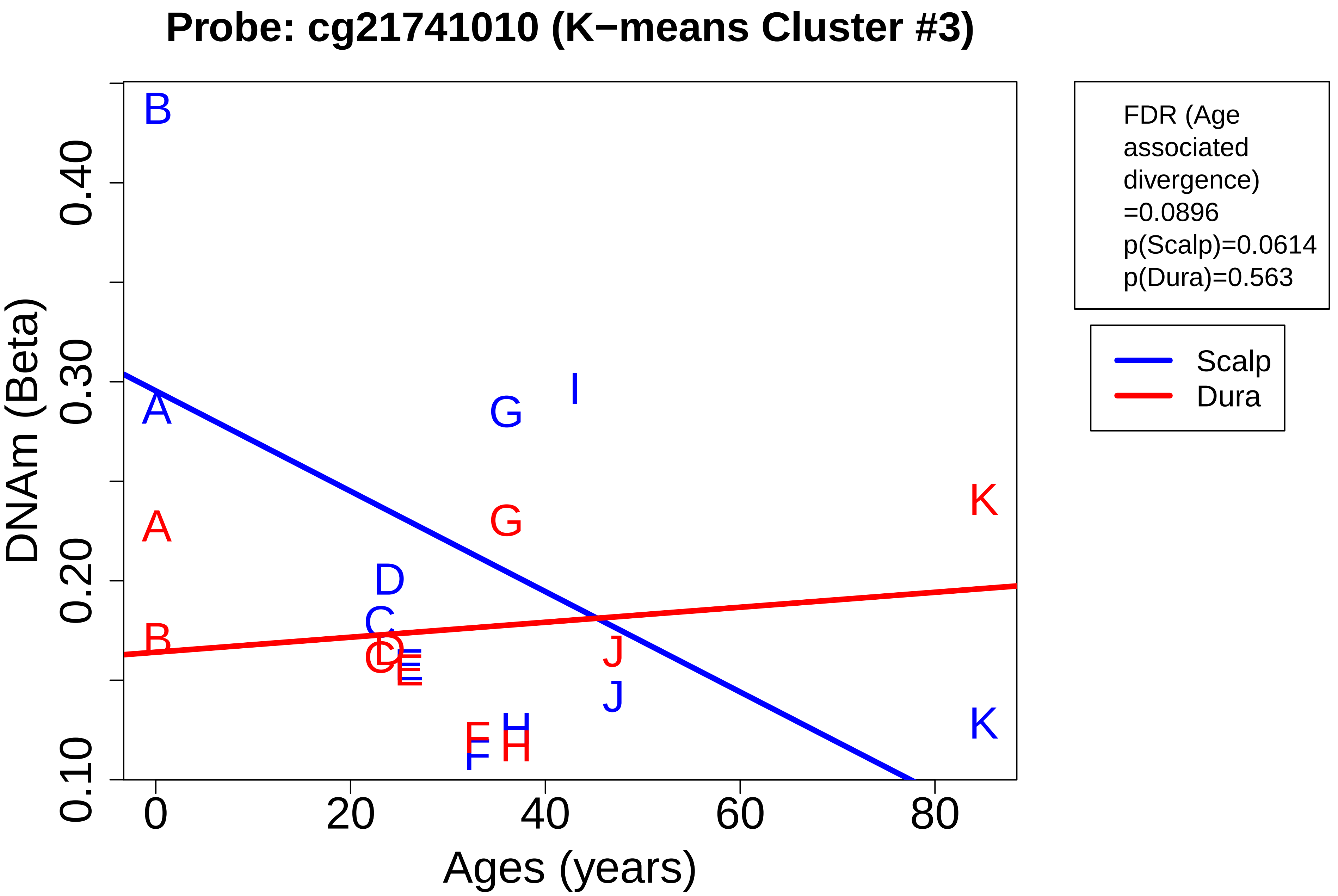

Probe: cg21741010 (K−means Cluster #3)
DNAm (Beta)
0.10
0.20
0.30
0.40
0
20
40
60
80
Ages (years)
FDR (Age associated divergence) =0.0896
p(Scalp)=0.0614
p(Dura)=0.563
Scalp
Dura

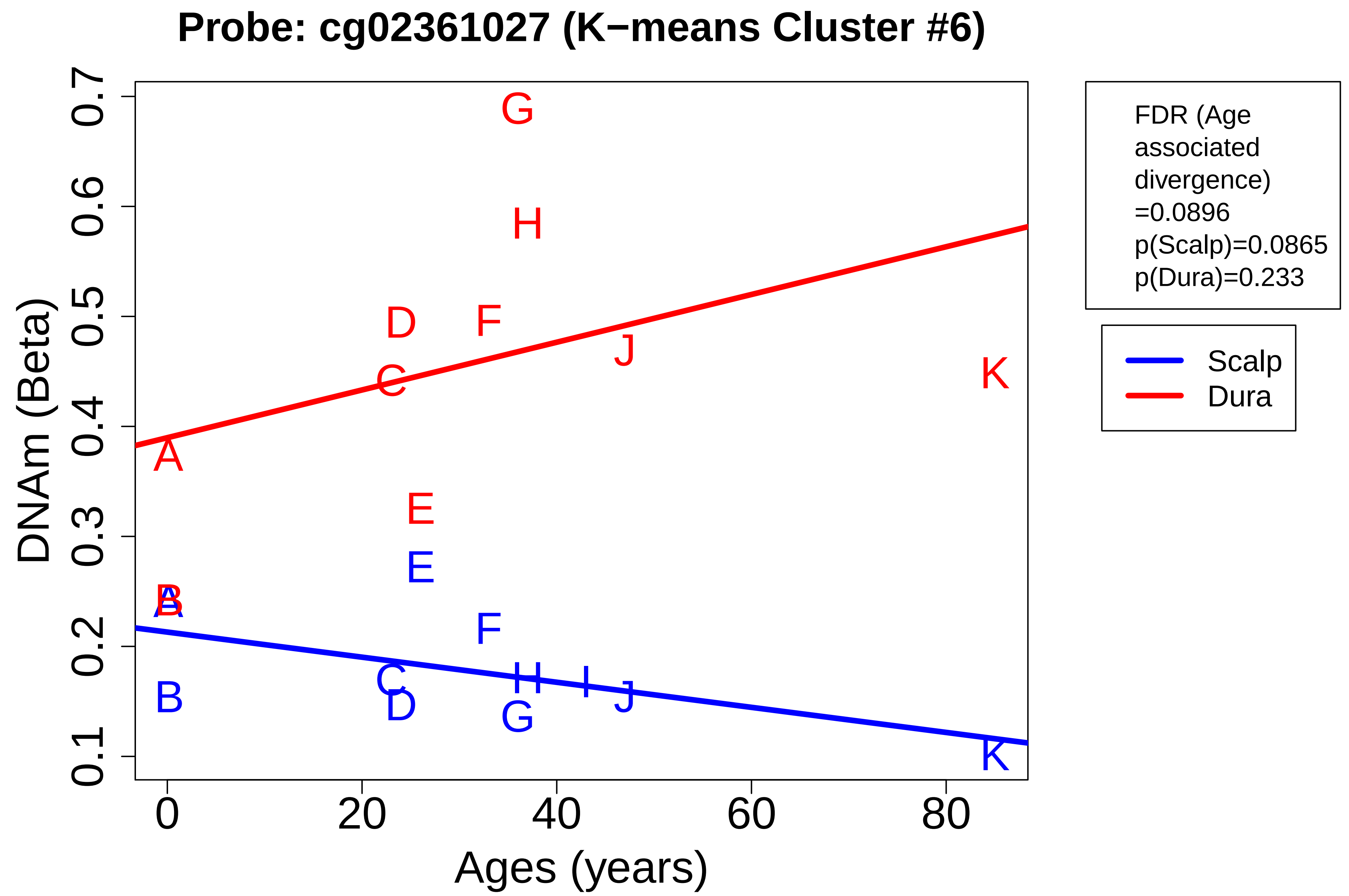

Probe: cg02361027 (K−means Cluster #6)
DNAm (Beta)
0.1
0.2
0.3
0.4
0.5
0.6
0.7
0
20
40
60
80
Ages (years)
FDR (Age associated divergence) =0.0896
p(Scalp)=0.0865
p(Dura)=0.233
Scalp
Dura

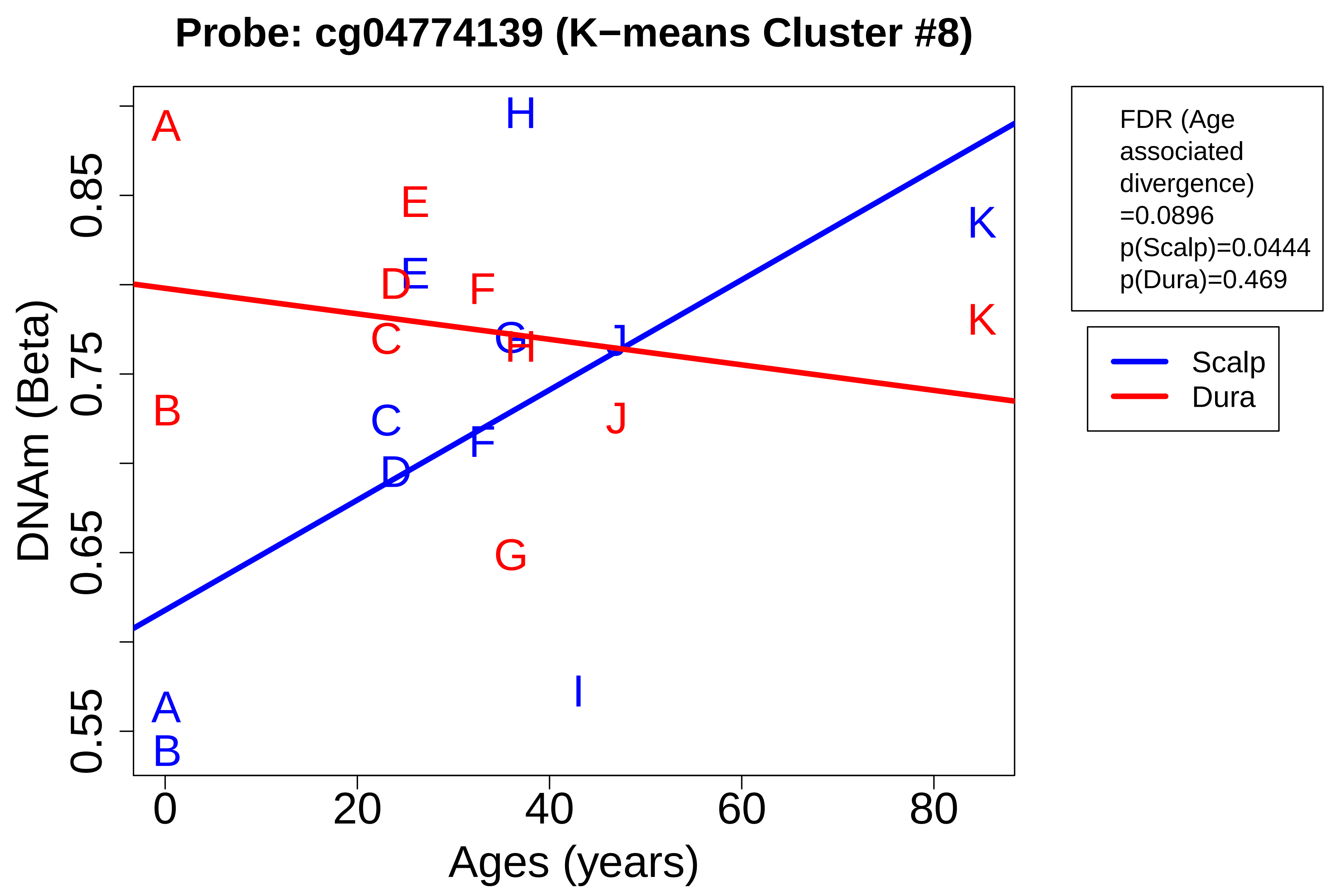
Probe: cg04774139 (K−means Cluster #8)
DNAm (Beta)
0.55
0.65
0.75
0.85
0
20
40
60
80
Ages (years)
A
B
C
D
E
F
G
H
I
J
K
FDR (Age associated divergence) =0.0896
p(Scalp)=0.0444
p(Dura)=0.469
Scalp
Dura

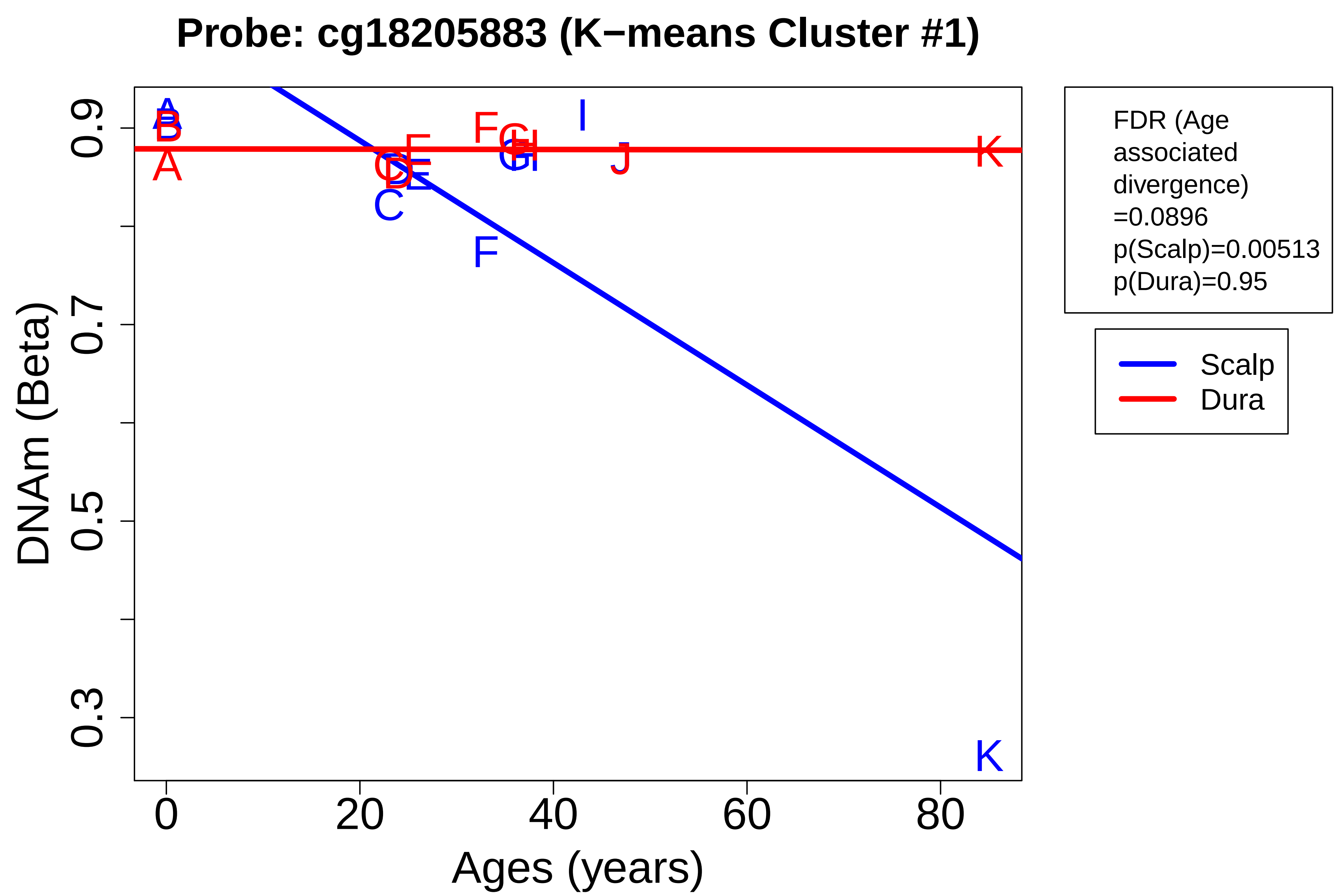

Probe: cg18205883 (K−means Cluster #1)
DNAm (Beta)
Ages (years)
FDR (Age associated divergence) =0.0896
p(Scalp)=0.00513
p(Dura)=0.95
Scalp
Dura

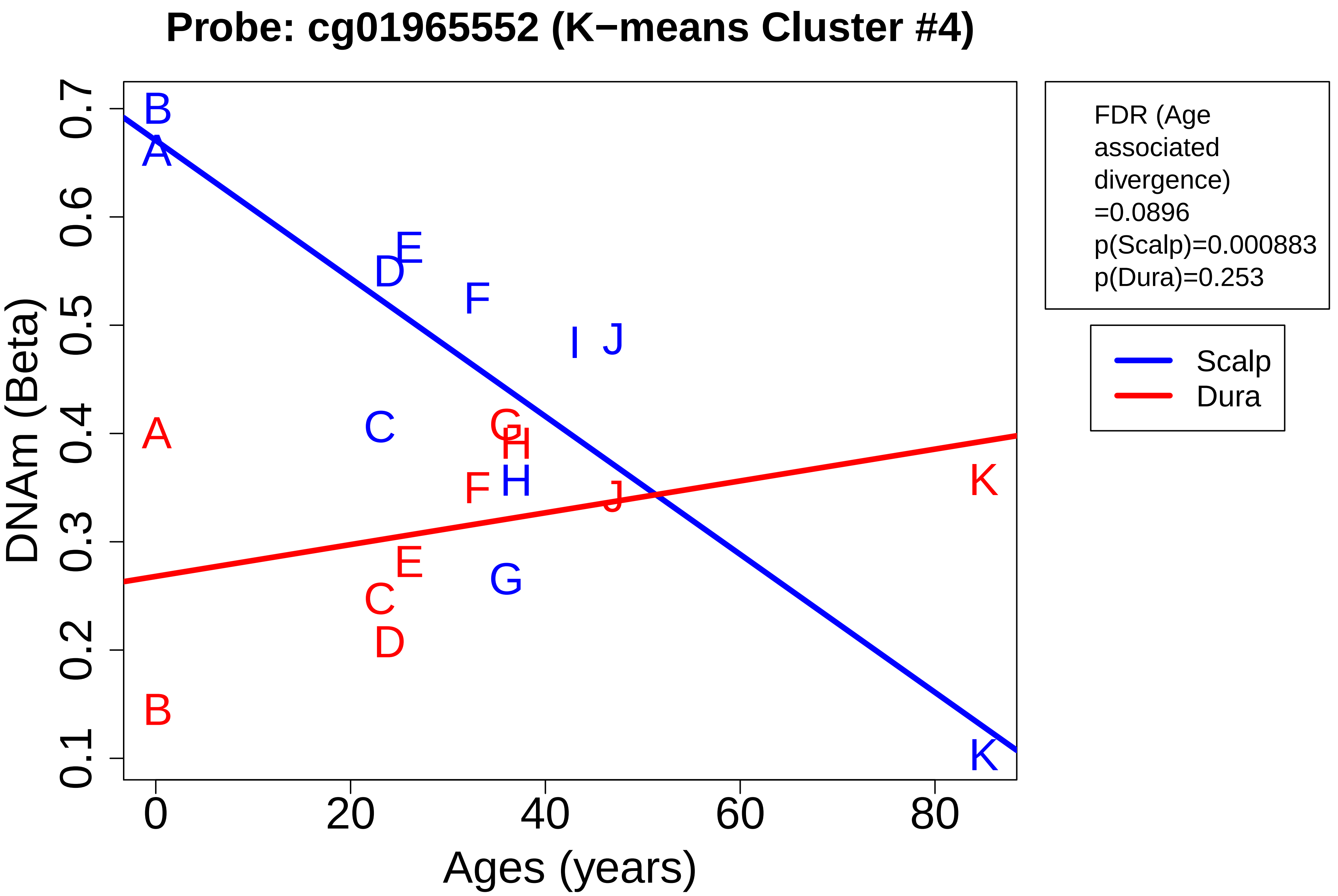

Probe: cg01965552 (K−means Cluster #4)
DNAm (Beta)
0.1
0.2
0.3
0.4
0.5
0.6
0.7
Ages (years)
0
20
40
60
80
FDR (Age associated divergence) =0.0896
p(Scalp)=0.000883
p(Dura)=0.253
Scalp
Dura

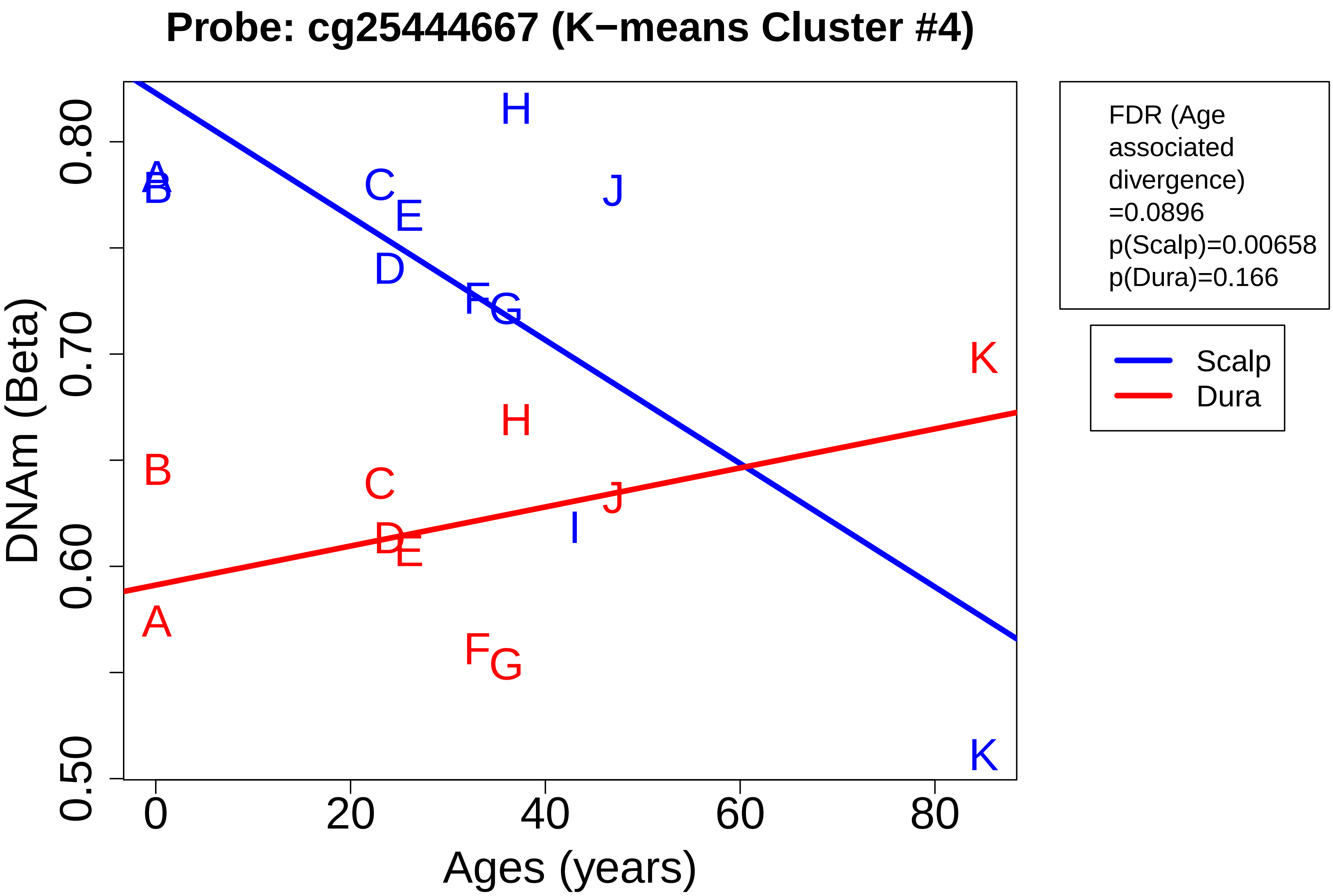
Probe: cg25444667 (K−means Cluster #4)
DNAm (Beta)
0.50
0.60
0.70
0.80
Ages (years)
0
20
40
60
80
A
B
C
D
E
F
G
H
I
J
K
FDR (Age associated divergence) =0.0896
p(Scalp)=0.00658
p(Dura)=0.166
Scalp
Dura

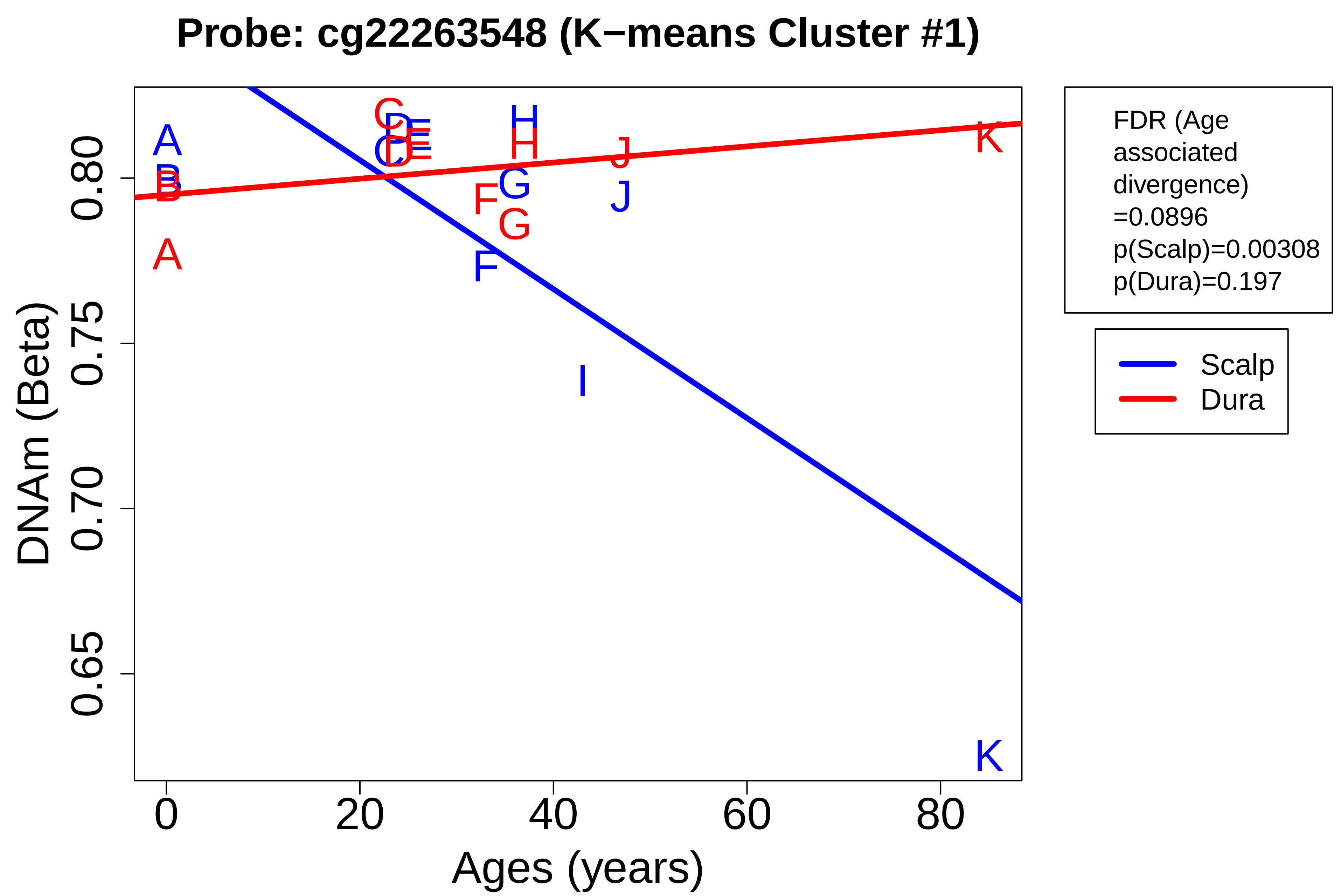

Probe: cg22263548 (K−means Cluster #1)
DNAm (Beta)
0.65
0.70
0.75
0.80
Ages (years)
0
20
40
60
80
FDR (Age associated divergence) =0.0896
p(Scalp)=0.00308
p(Dura)=0.197
Scalp
Dura

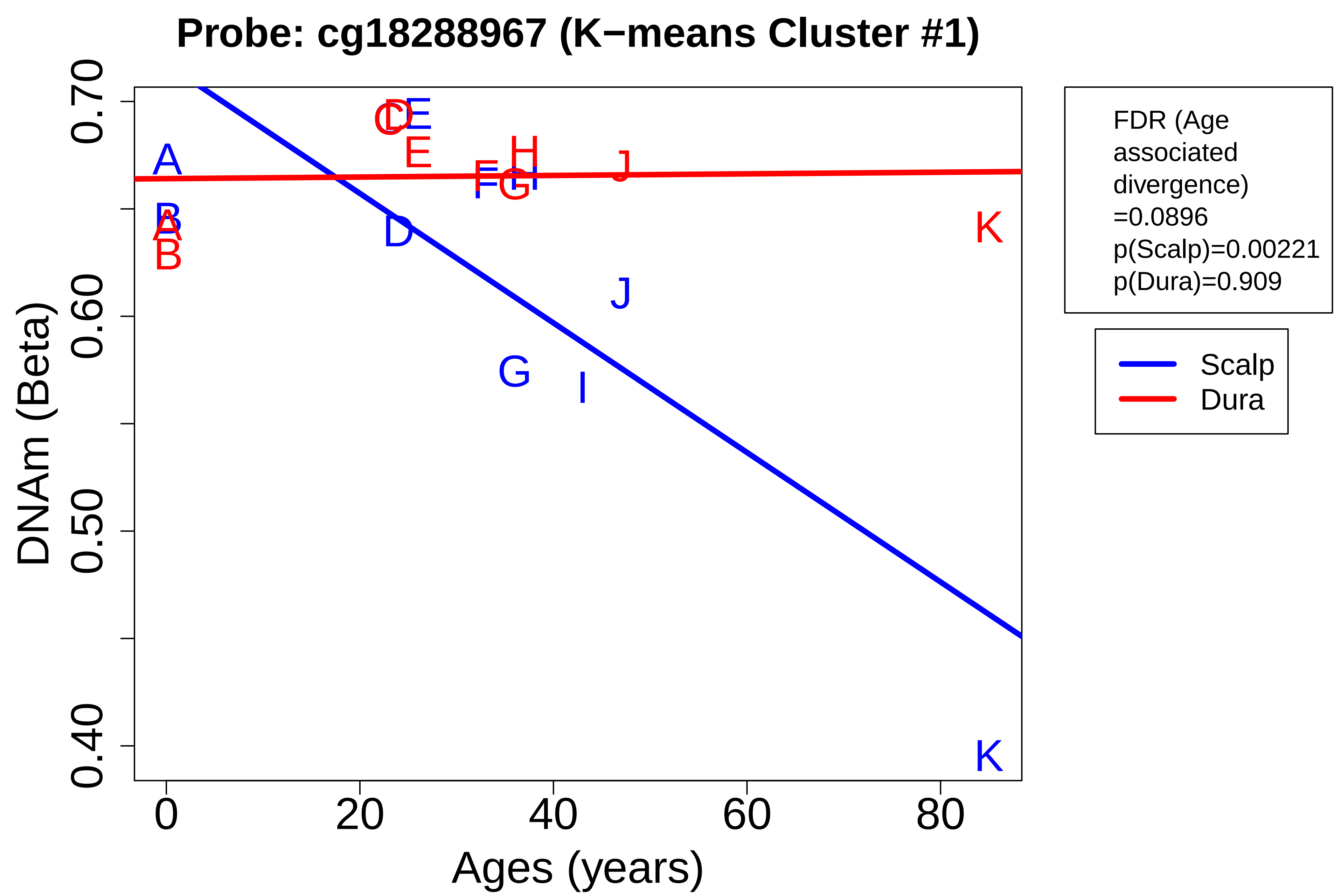

Probe: cg18288967 (K−means Cluster #1)
DNAm (Beta)
0.40
0.50
0.60
0.70
Ages (years)
0
20
40
60
80
A
B
C
D
E
F
G
H
I
J
K
FDR (Age associated divergence) =0.0896
p(Scalp)=0.00221
p(Dura)=0.909
Scalp
Dura

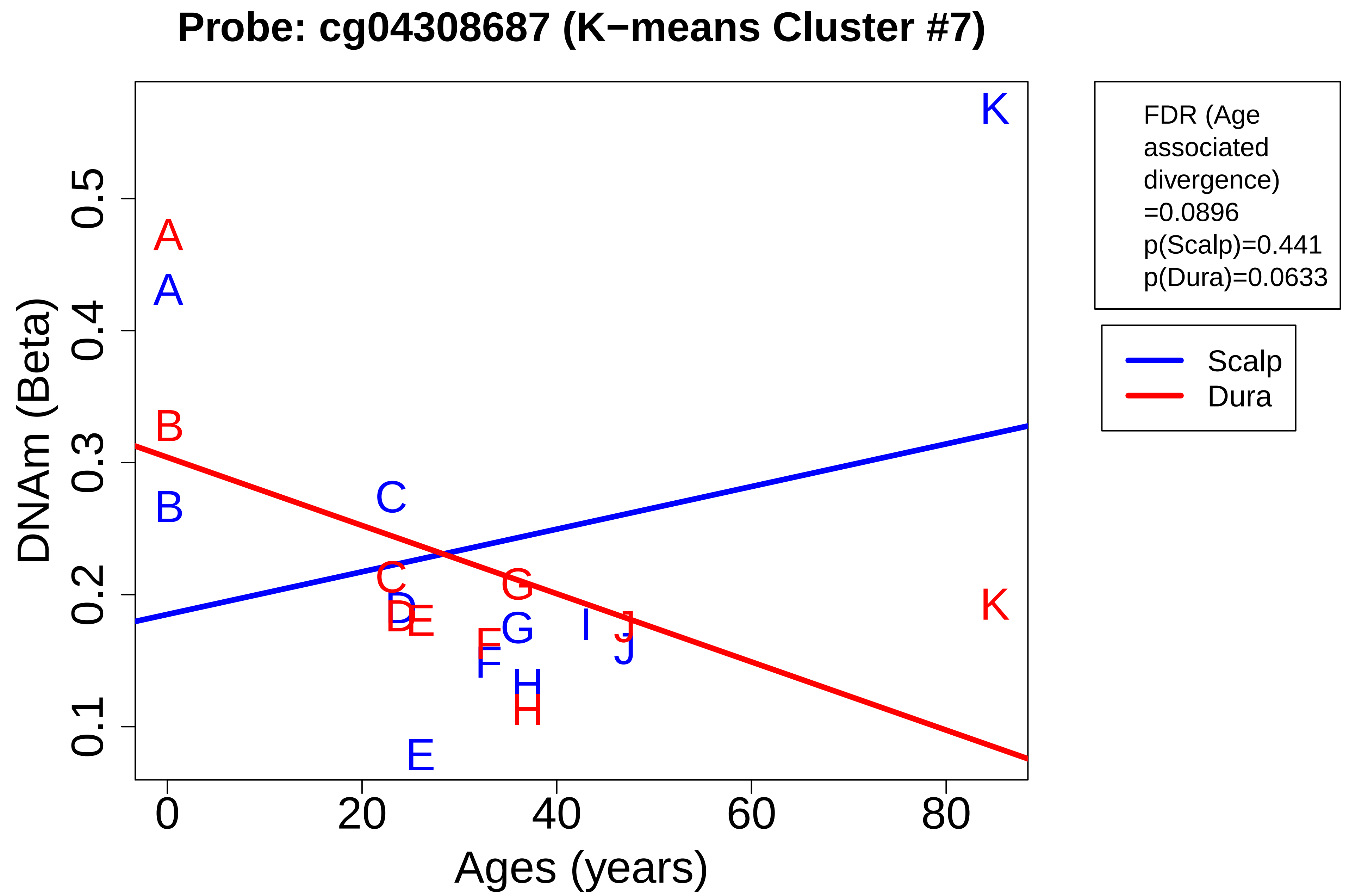

Probe: cg04308687 (K−means Cluster #7)
DNAm (Beta)
0.1
0.2
0.3
0.4
0.5
0
20
40
60
80
Ages (years)
A
B
C
D
E
F
G
H
I
J
K
FDR (Age associated divergence) =0.0896
p(Scalp)=0.441
p(Dura)=0.0633
Scalp
Dura

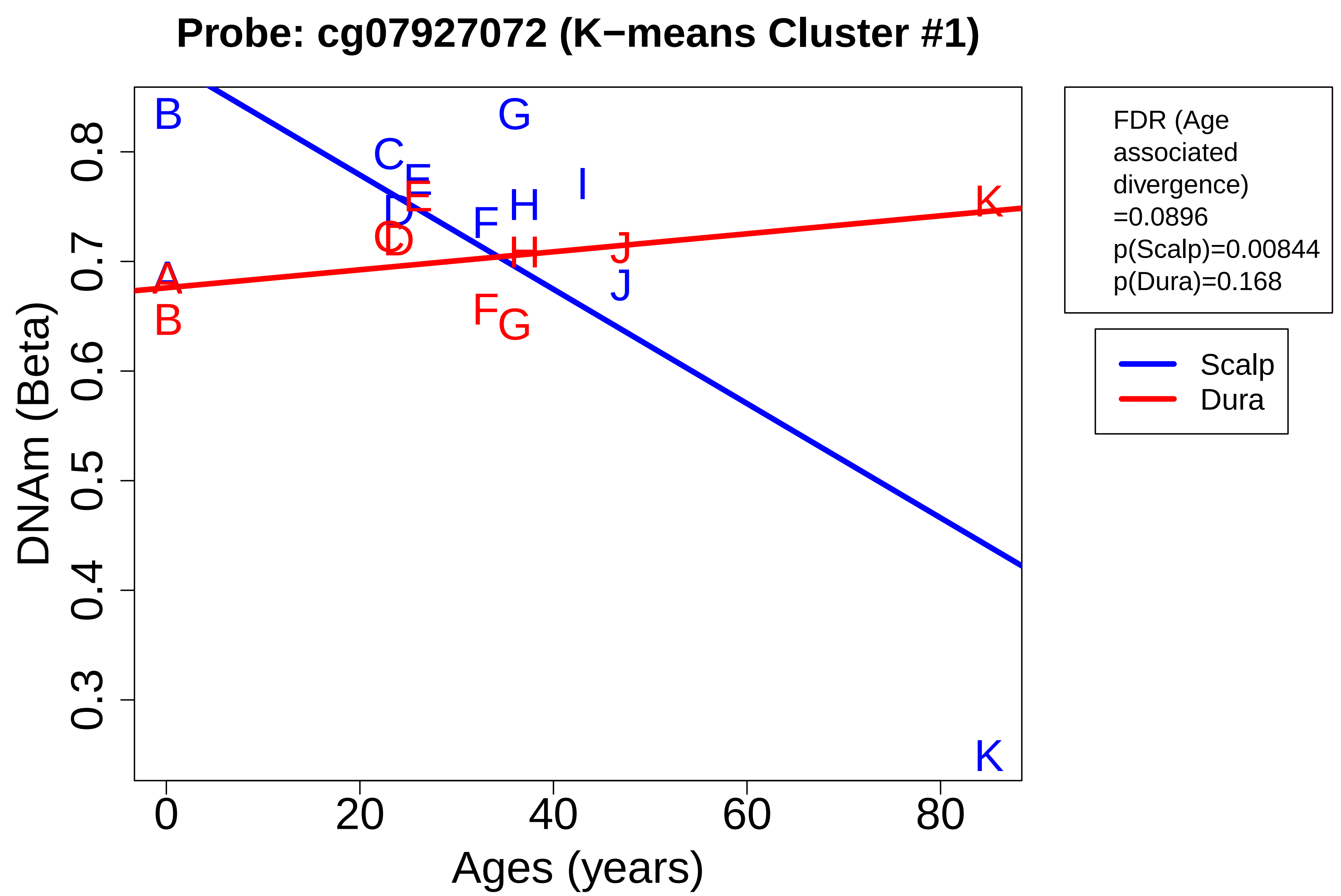

Probe: cg07927072 (K−means Cluster #1)
DNAm (Beta)
0.3
0.4
0.5
0.6
0.7
0.8
0
20
40
60
80
Ages (years)
A
B
C
D
E
F
G
H
I
J
K
FDR (Age associated divergence) =0.0896
p(Scalp)=0.00844
p(Dura)=0.168
Scalp
Dura

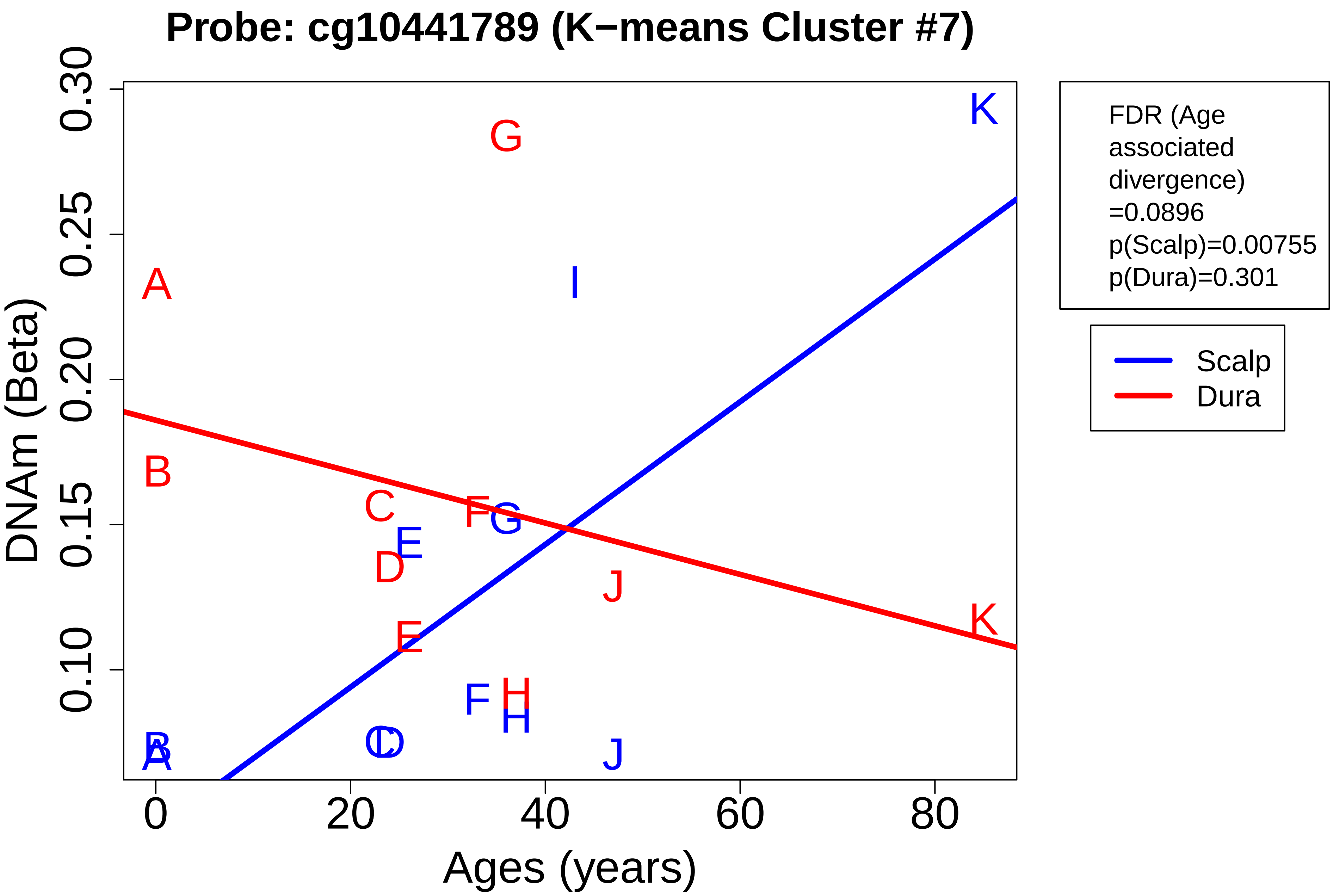
Probe: cg10441789 (K−means Cluster #7)
DNAm (Beta)
0.10
0.15
0.20
0.25
0.30
0
20
40
60
80
Ages (years)
FDR (Age associated divergence) =0.0896
p(Scalp)=0.00755
p(Dura)=0.301
Scalp
Dura

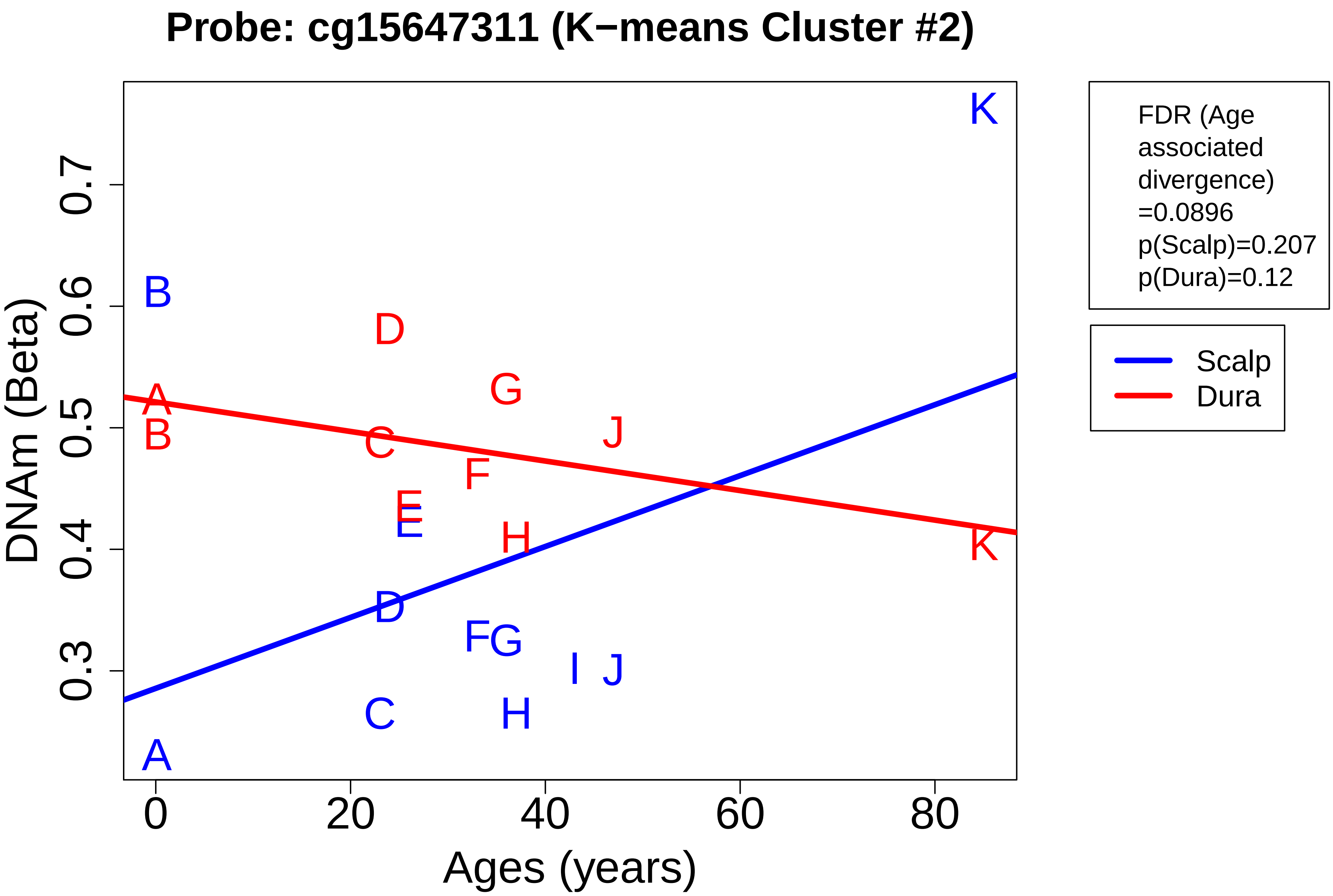

Probe: cg15647311 (K−means Cluster #2)
DNAm (Beta)
0.3
0.4
0.5
0.6
0.7
0
20
40
60
80
Ages (years)
FDR (Age associated divergence) =0.0896
p(Scalp)=0.207
p(Dura)=0.12
Scalp
Dura

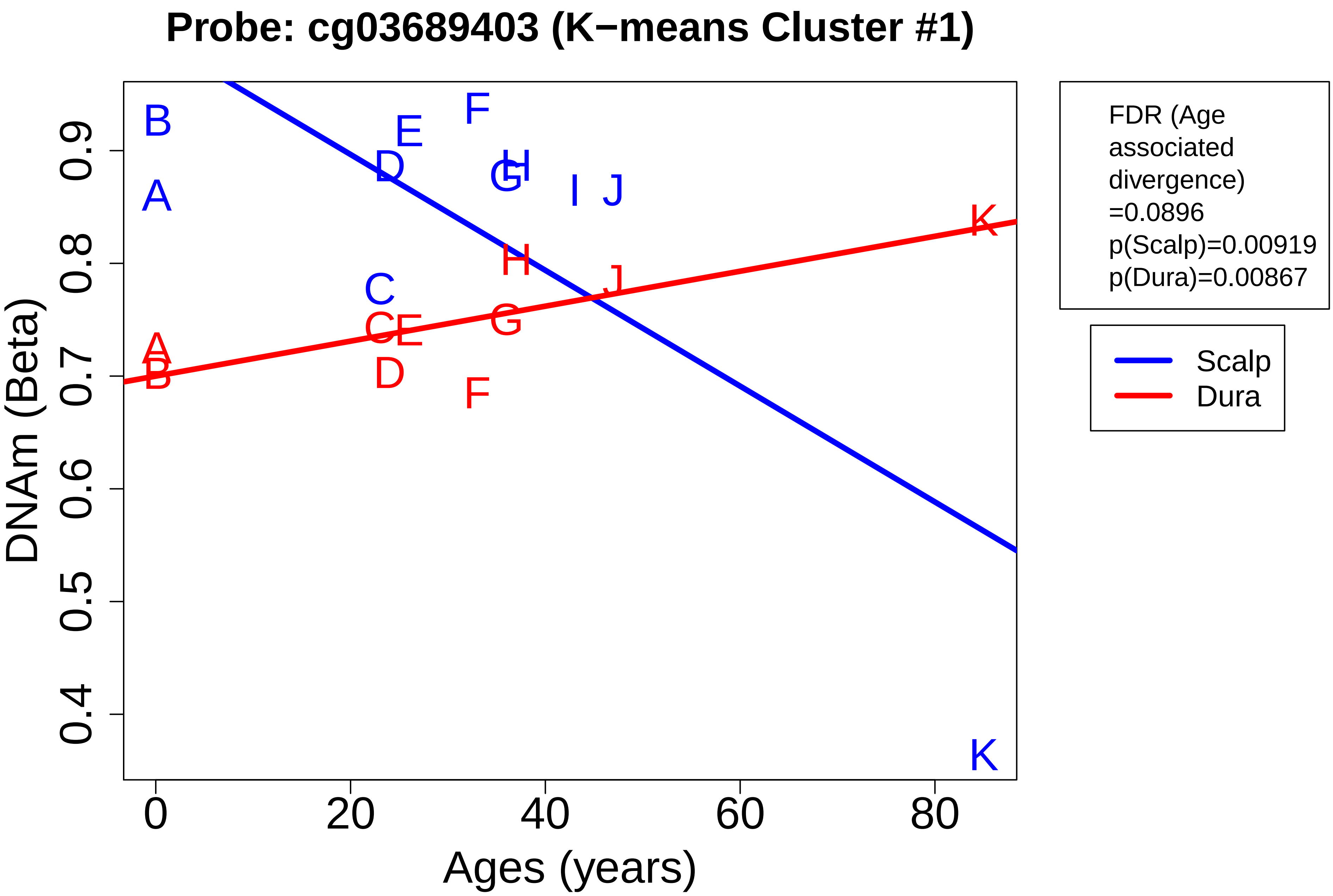

Probe: cg03689403 (K−means Cluster #1)
DNAm (Beta)
0.4
0.5
0.6
0.7
0.8
0.9
0
20
40
60
80
Ages (years)
FDR (Age associated divergence) =0.0896
p(Scalp)=0.00919
p(Dura)=0.00867
Scalp
Dura

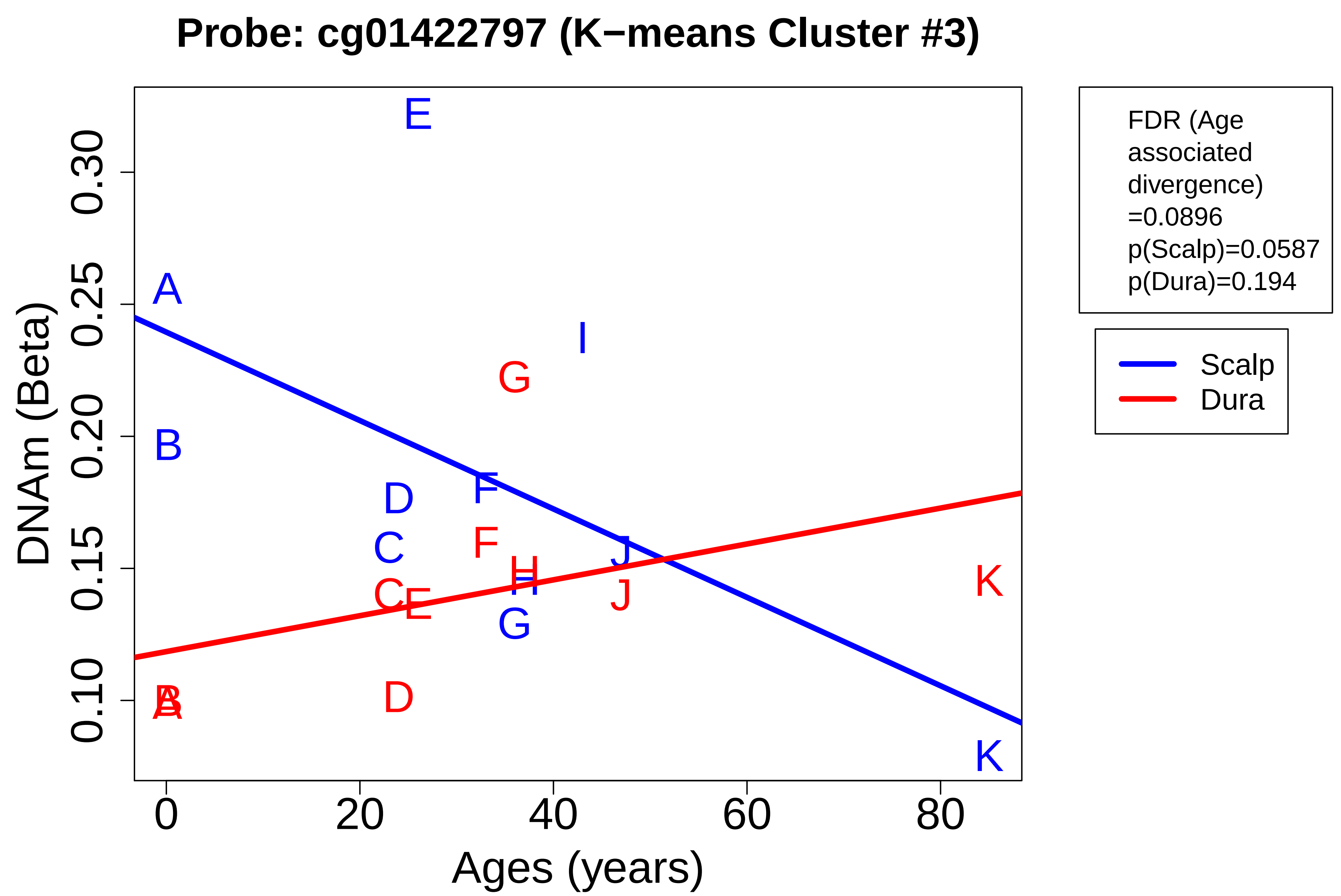

Probe: cg01422797 (K−means Cluster #3)
DNAm (Beta)
0.10
0.15
0.20
0.25
0.30
Ages (years)
0
20
40
60
80
A
B
C
D
E
F
G
H
I
J
K
FDR (Age associated divergence) =0.0896
p(Scalp)=0.0587
p(Dura)=0.194
Scalp
Dura

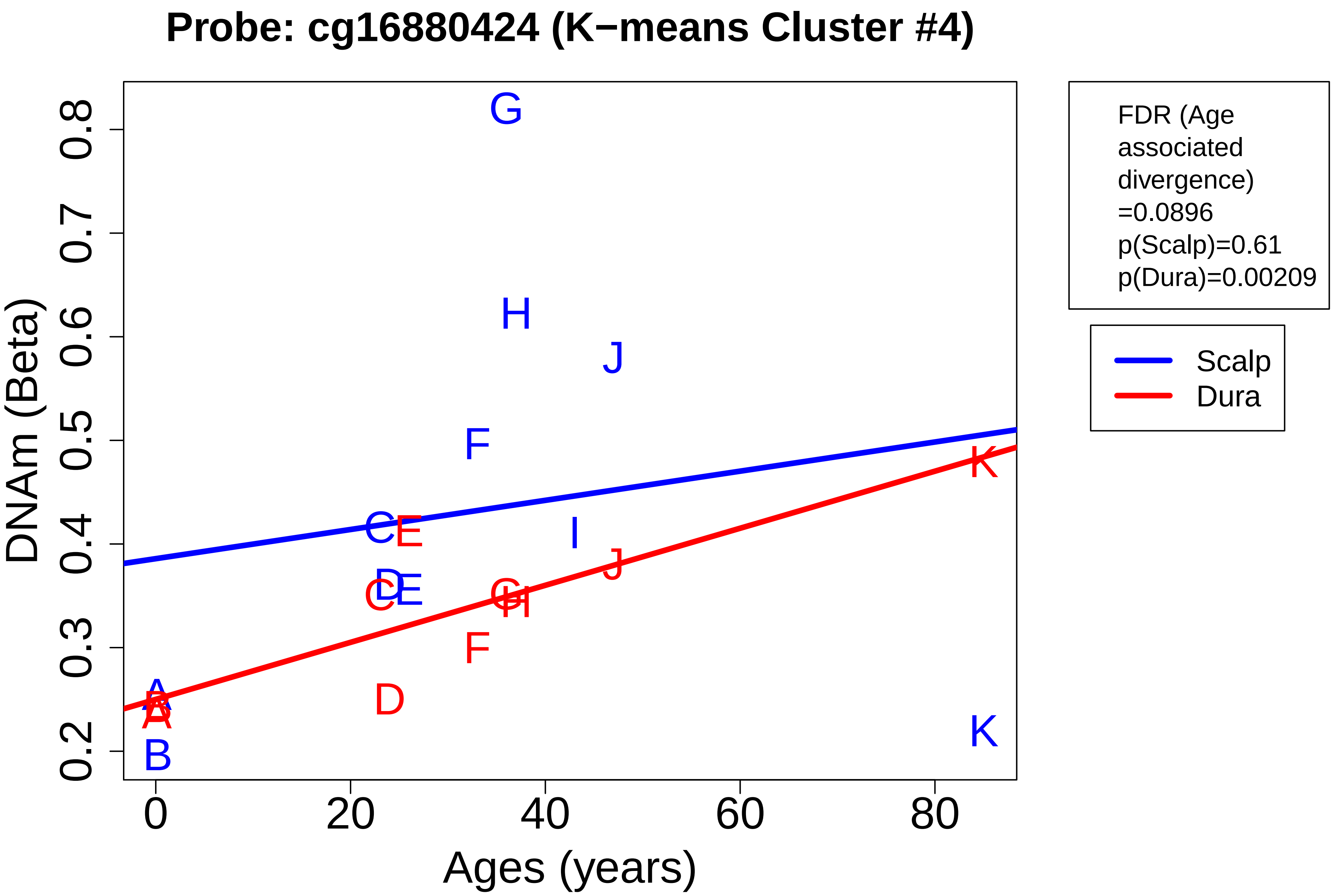

Probe: cg16880424 (K−means Cluster #4)
DNAm (Beta)
0.2
0.3
0.4
0.5
0.6
0.7
0.8
Ages (years)
0
20
40
60
80
FDR (Age associated divergence) =0.0896
p(Scalp)=0.61
p(Dura)=0.00209
Scalp
Dura

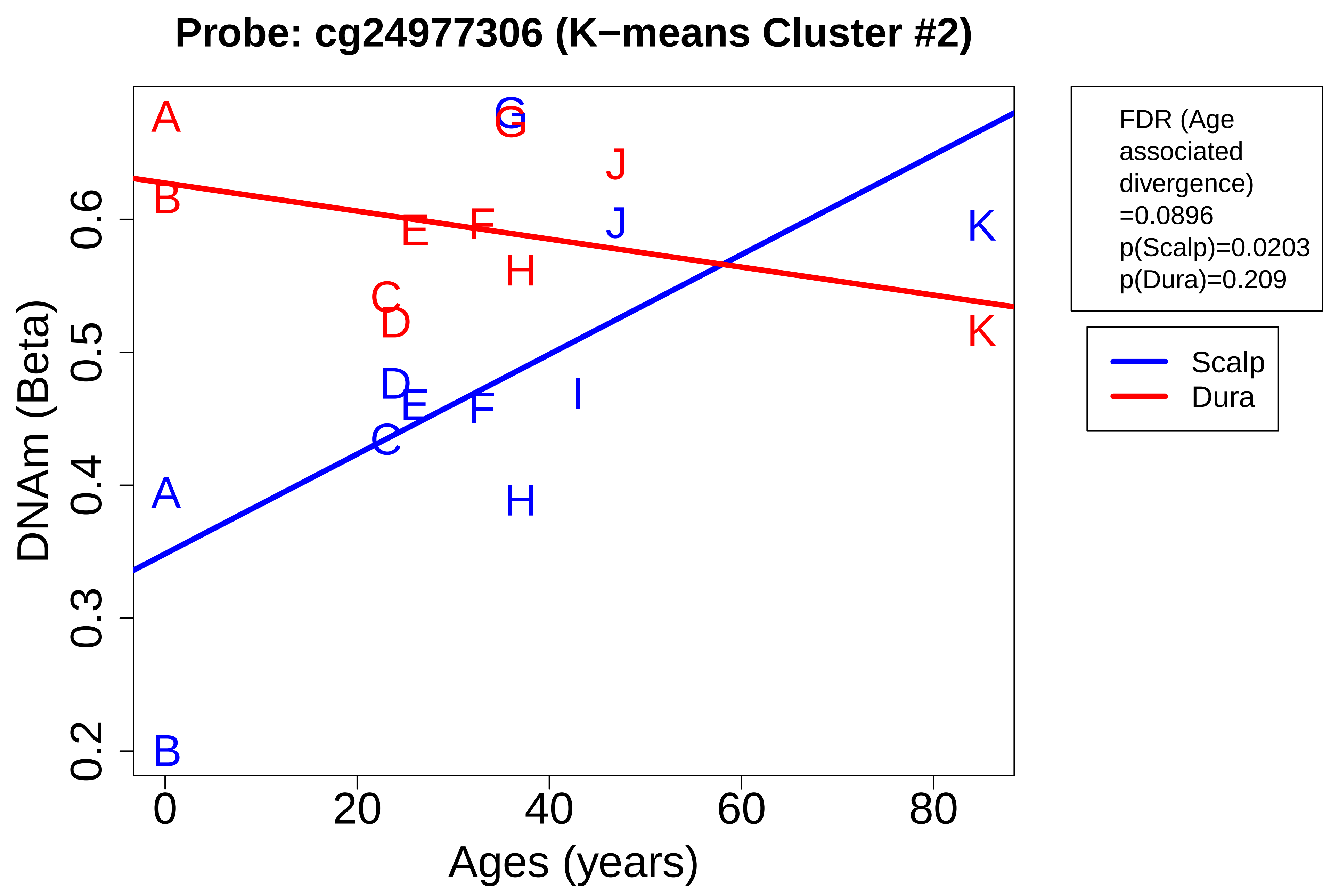

Probe: cg24977306 (K−means Cluster #2)
DNAm (Beta)
0.2
0.3
0.4
0.5
0.6
0
20
40
60
80
Ages (years)
A
B
C
D
E
F
G
H
I
J
K
FDR (Age associated divergence) =0.0896
p(Scalp)=0.0203
p(Dura)=0.209
Scalp
Dura

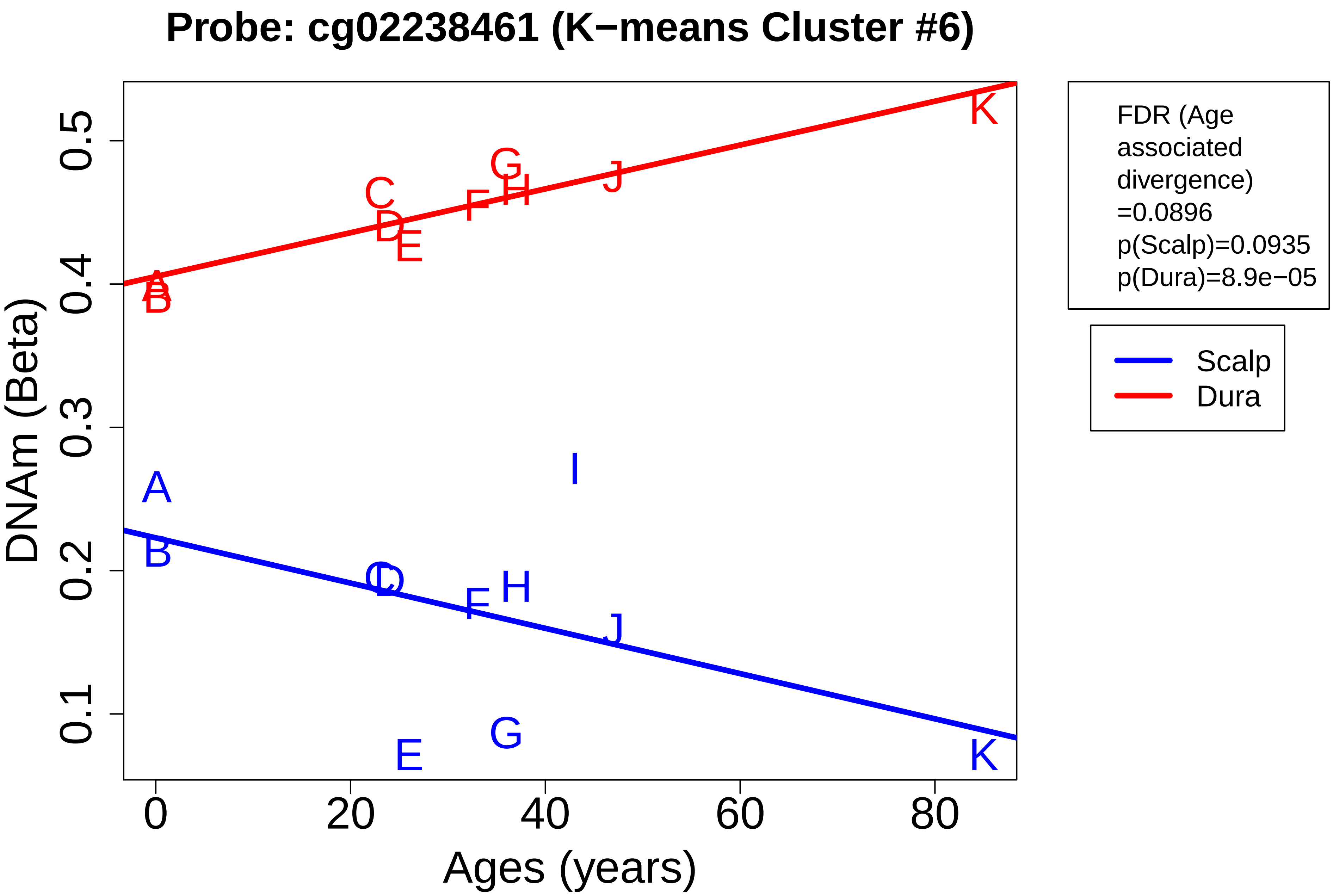

Probe: cg02238461 (K−means Cluster #6)
DNAm (Beta)
0.1
0.2
0.3
0.4
0.5
Ages (years)
0
20
40
60
80
FDR (Age associated divergence) =0.0896
p(Scalp)=0.0935
p(Dura)=8.9e−05
Scalp
Dura

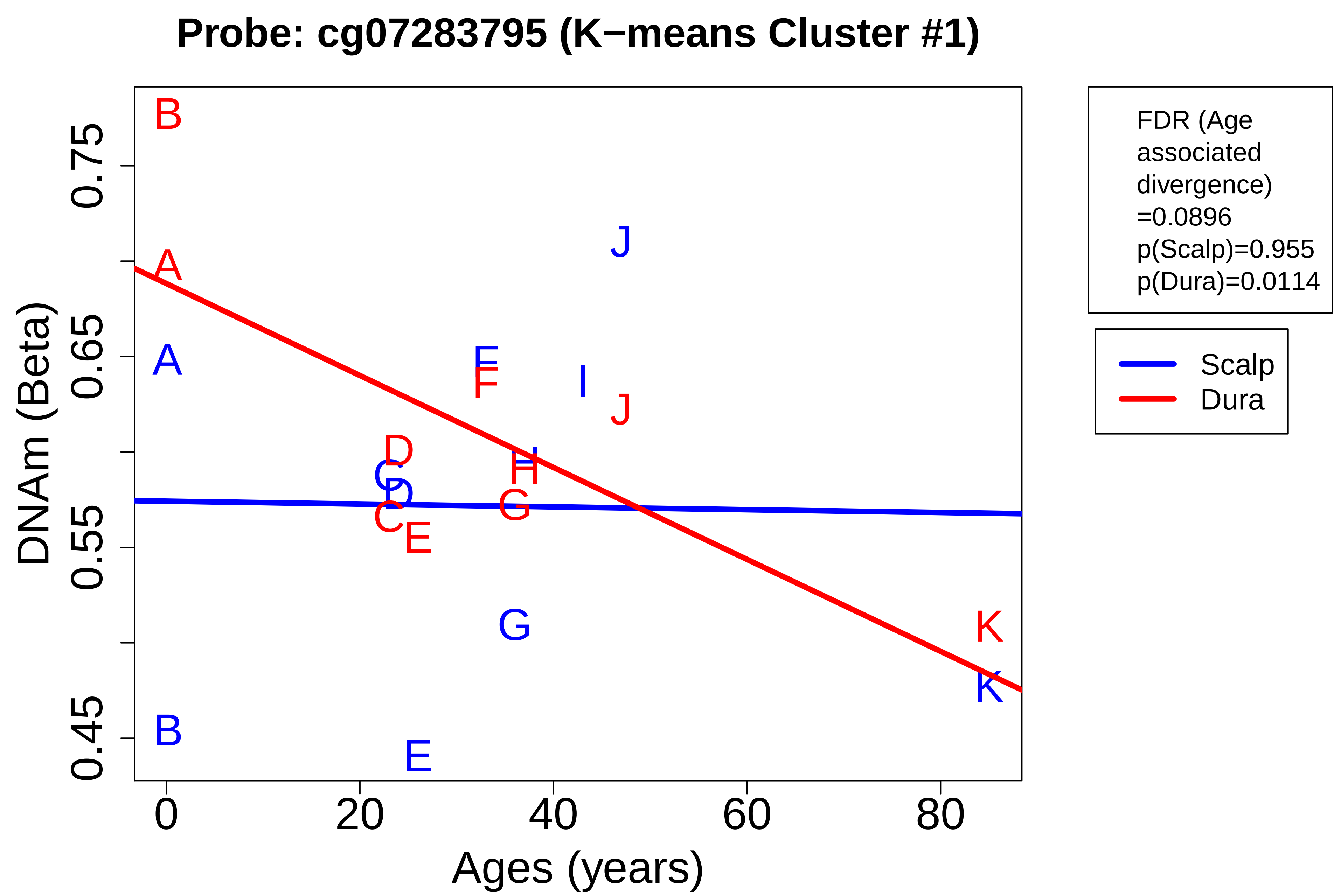

Probe: cg07283795 (K−means Cluster #1)
DNAm (Beta)
0.45
0.55
0.65
0.75
0
20
40
60
80
Ages (years)
FDR (Age associated divergence) =0.0896
p(Scalp)=0.955
p(Dura)=0.0114
Scalp
Dura

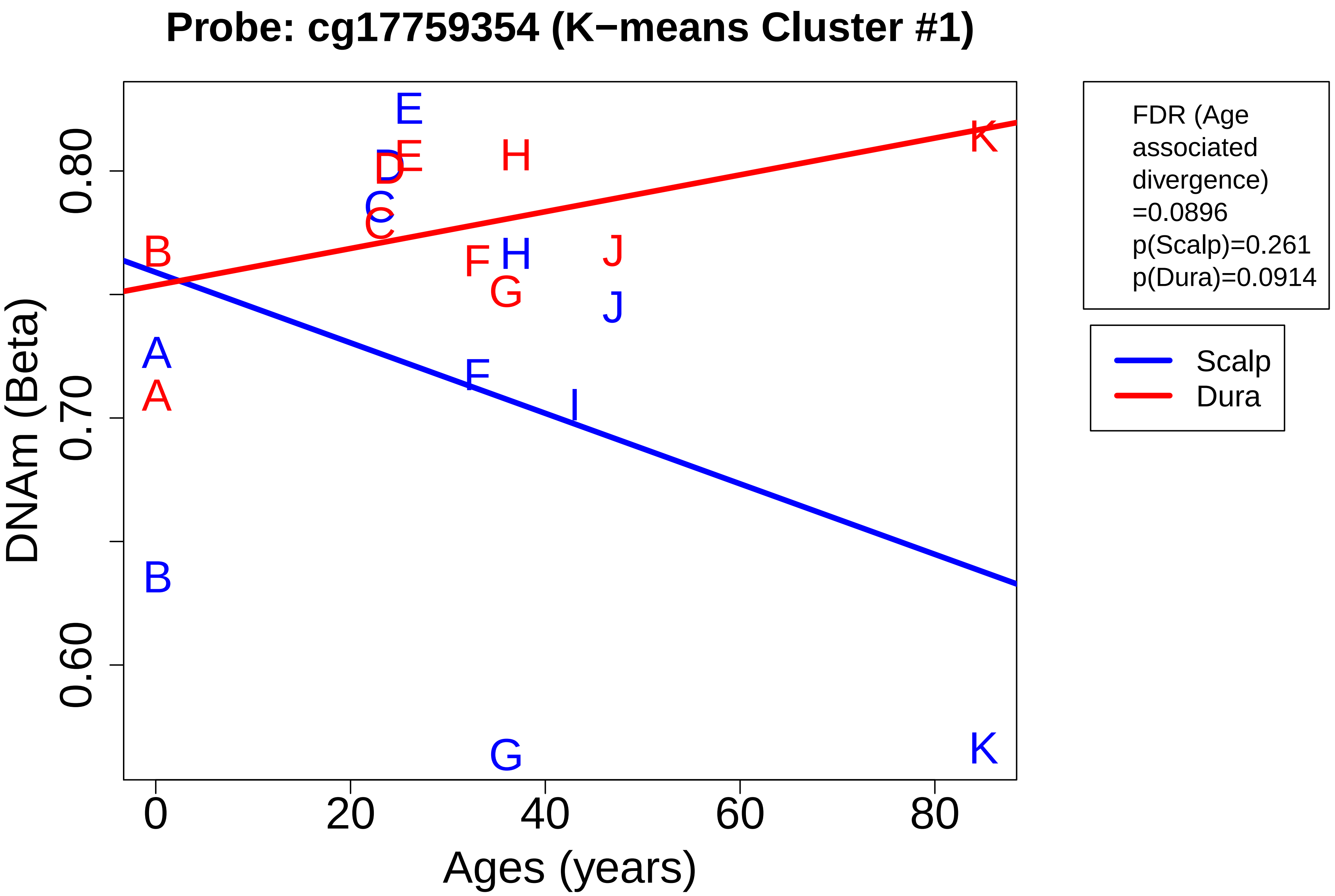

Probe: cg17759354 (K−means Cluster #1)
DNAm (Beta)
Ages (years)
0.60
0.70
0.80
0
20
40
60
80
FDR (Age associated divergence) =0.0896
p(Scalp)=0.261
p(Dura)=0.0914
Scalp
Dura

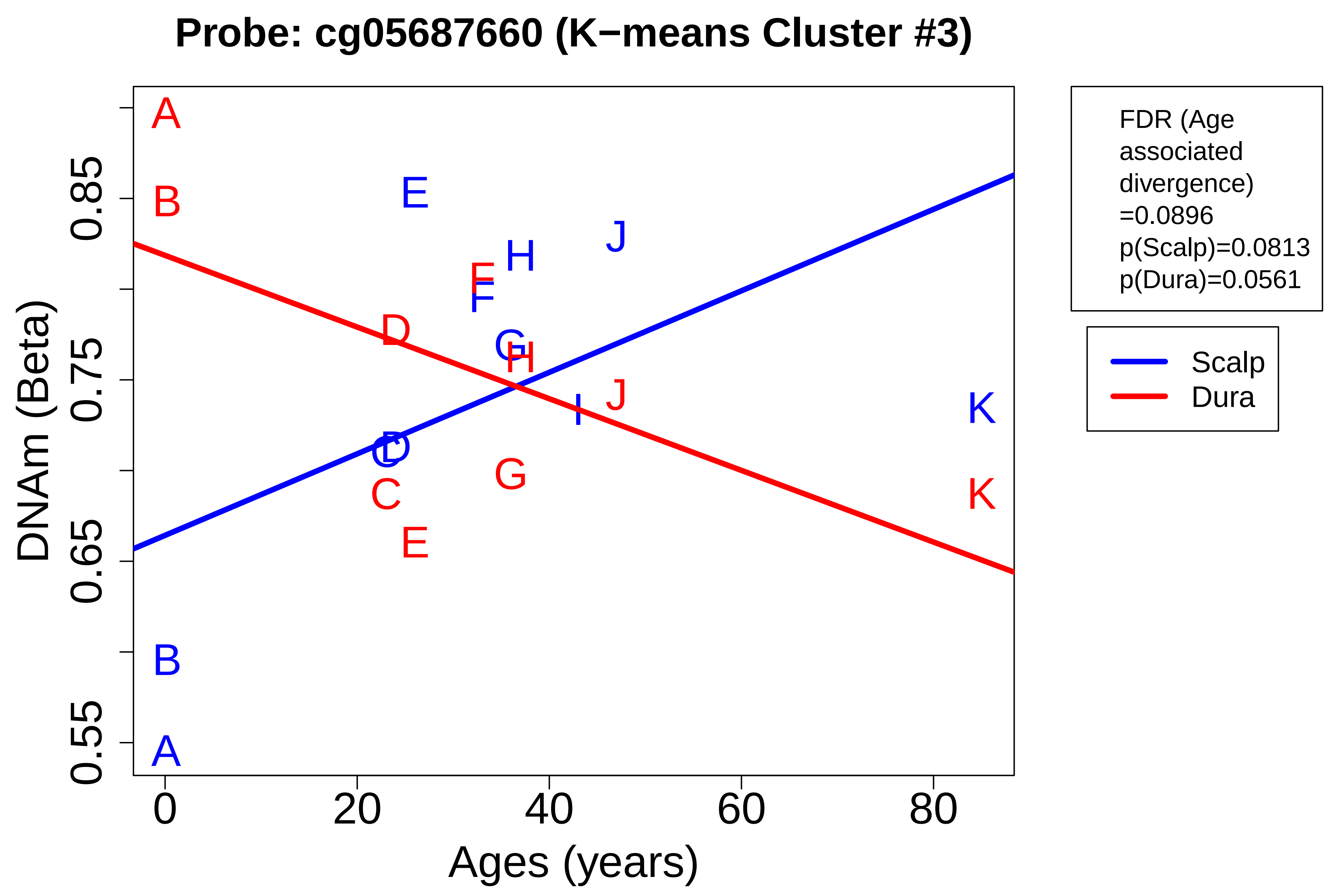

Probe: cg05687660 (K−means Cluster #3)
DNAm (Beta)
0.55
0.65
0.75
0.85
0
20
40
60
80
Ages (years)
FDR (Age associated divergence) =0.0896
p(Scalp)=0.0813
p(Dura)=0.0561
Scalp
Dura

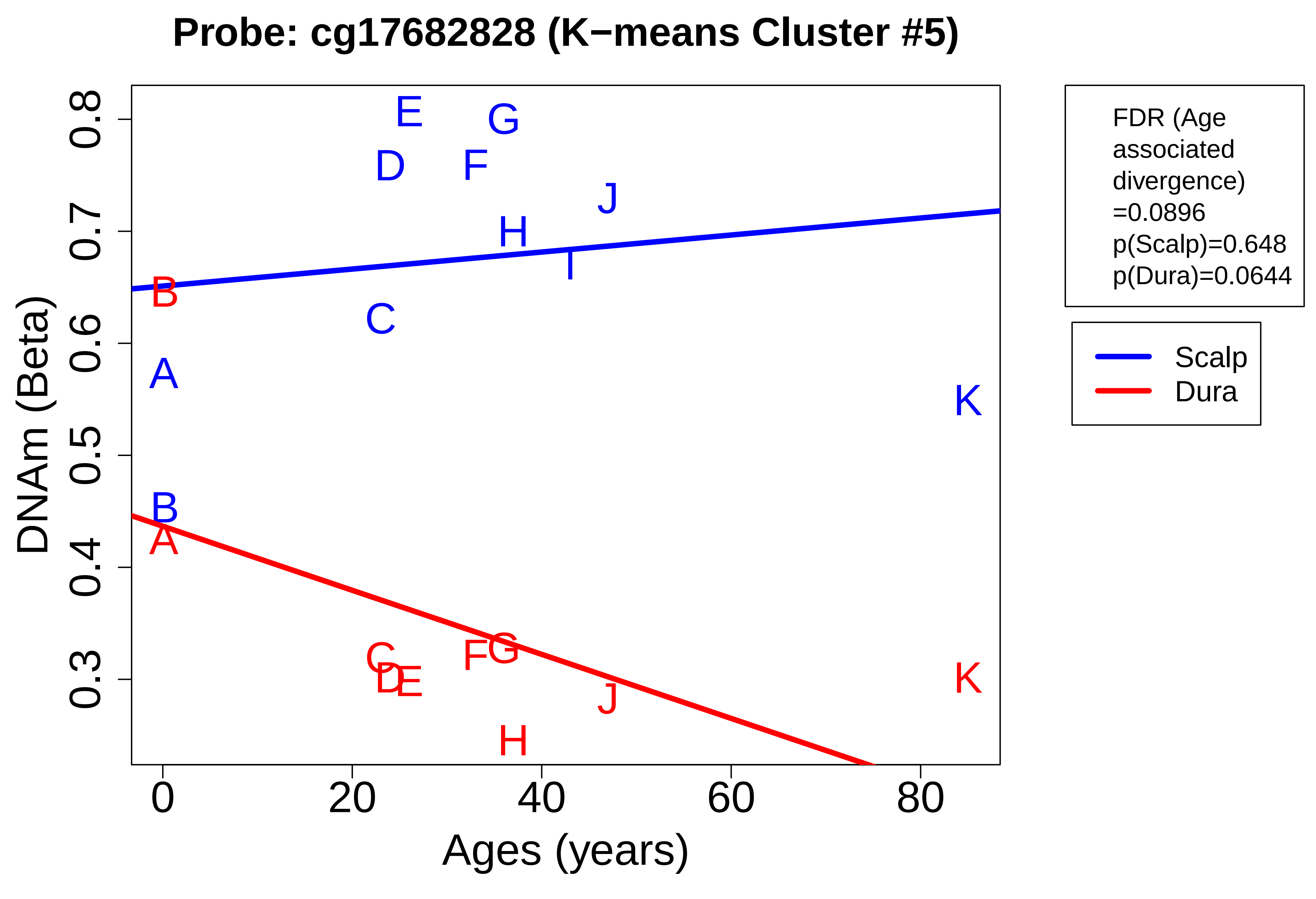

Probe: cg17682828 (K−means Cluster #5)
DNAm (Beta)
0.3
0.4
0.5
0.6
0.7
0.8
0
20
40
60
80
Ages (years)
FDR (Age associated divergence) =0.0896
p(Scalp)=0.648
p(Dura)=0.0644
Scalp
Dura
A B C D E F G H I J K

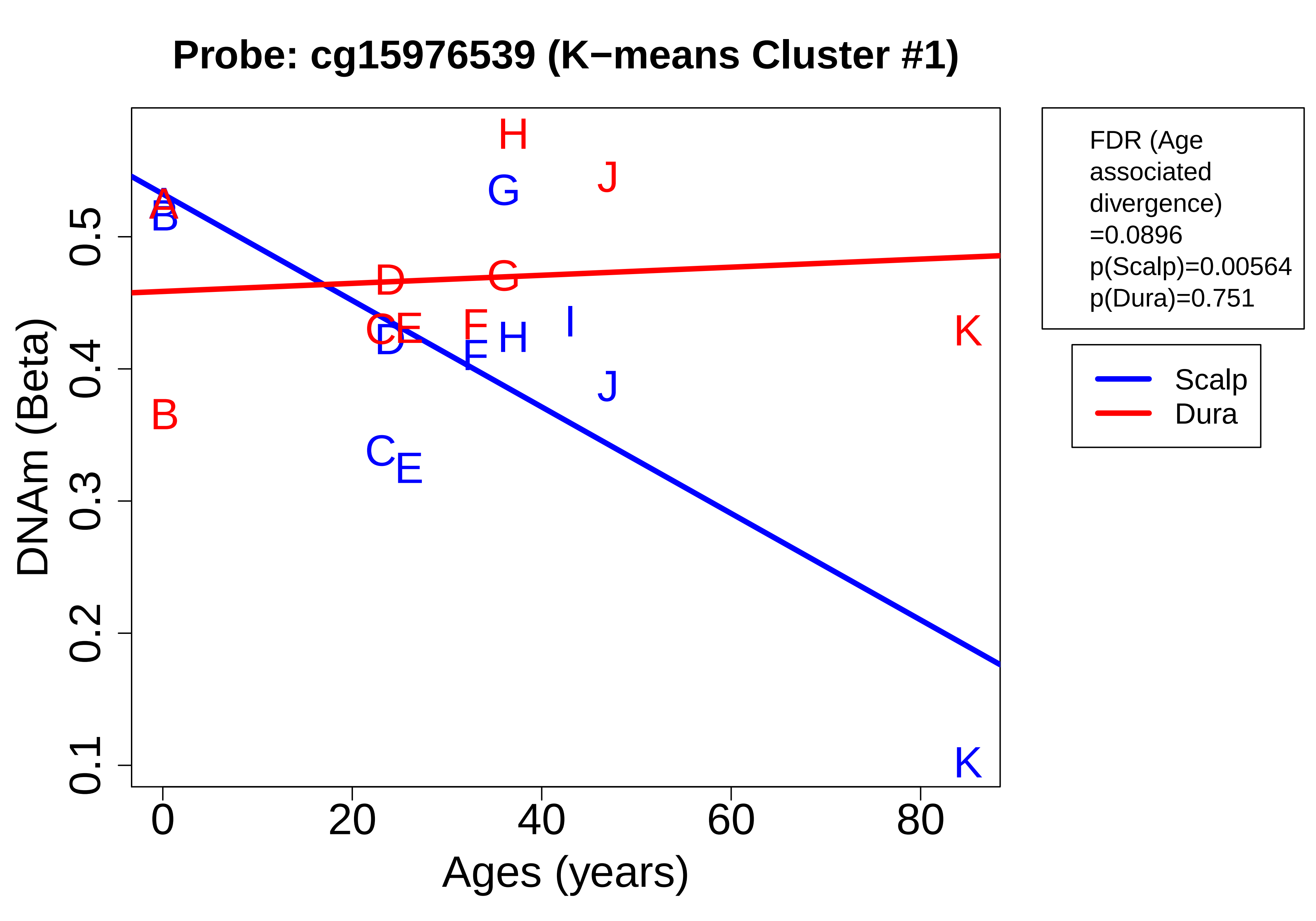

Probe: cg15976539 (K−means Cluster #1)
FDR (Age associated divergence) =0.0896
p(Scalp)=0.00564
p(Dura)=0.751
Scalp
Dura
DNAm (Beta)
0.1
0.2
0.3
0.4
0.5
0
20
40
60
80
Ages (years)
A
B
C
D
E
F
G
H
I
J
K

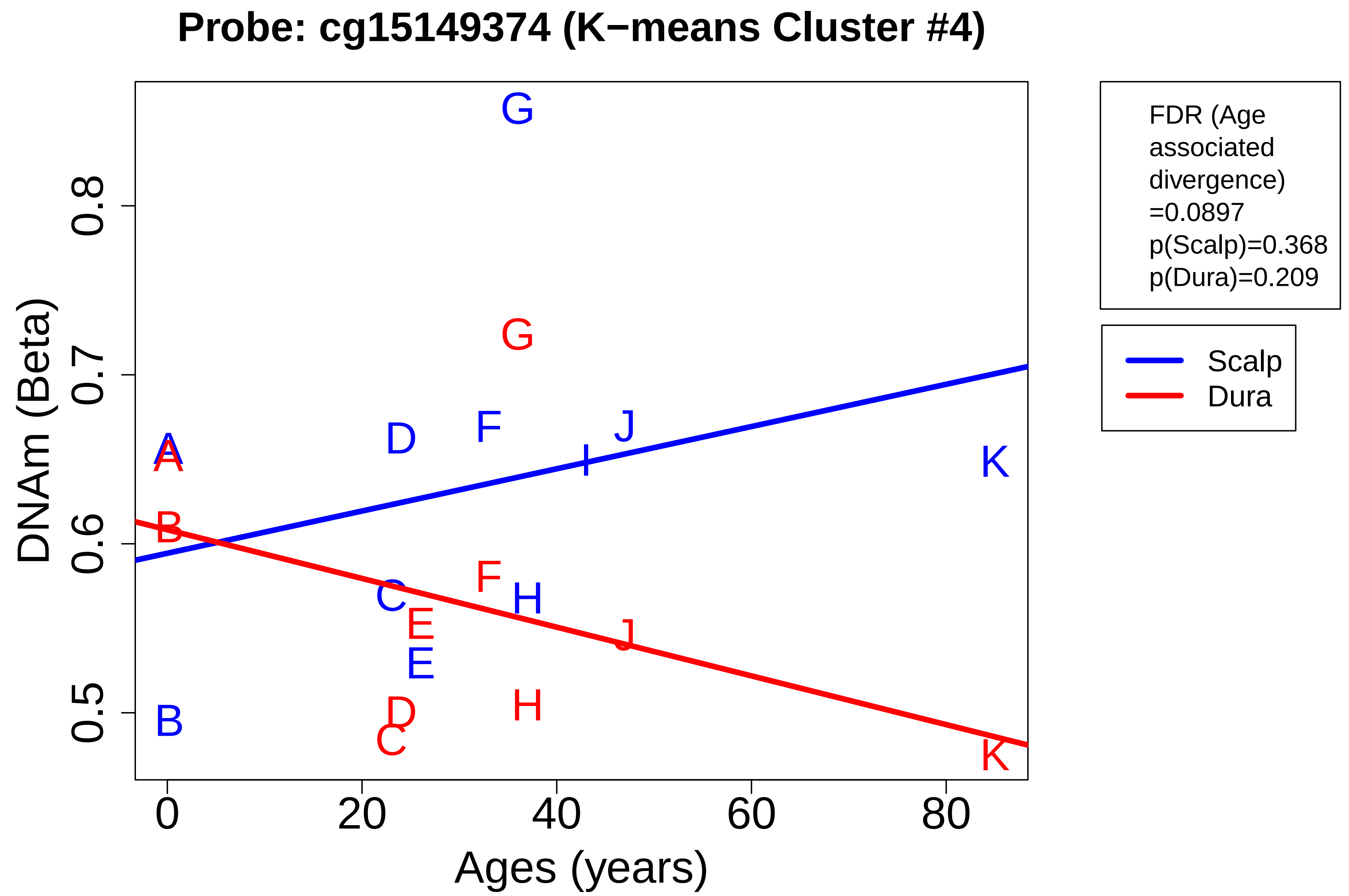
Probe: cg15149374 (K−means Cluster #4)
FDR (Age associated divergence) =0.0897
p(Scalp)=0.368
p(Dura)=0.209
Scalp
Dura
DNAm (Beta)
0.5
0.6
0.7
0.8
0
20
40
60
80
Ages (years)
A
B
C
D
E
F
G
H
I
J
K

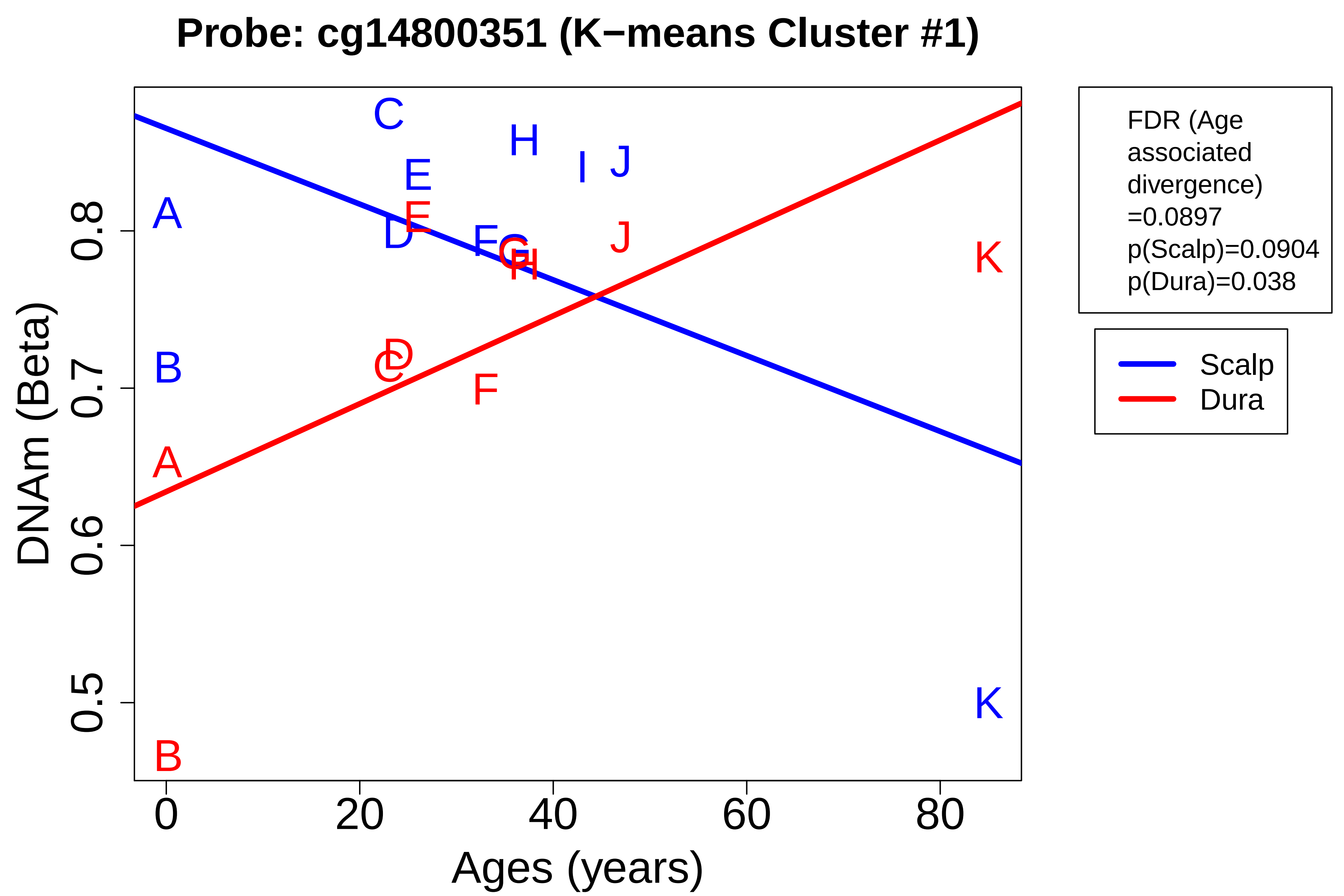

Probe: cg14800351 (K−means Cluster #1)
DNAm (Beta)
0.5
0.6
0.7
0.8
Ages (years)
0
20
40
60
80
FDR (Age associated divergence) =0.0897
p(Scalp)=0.0904
p(Dura)=0.038
Scalp
Dura
A
B
C
D
E
F
G
H
I
J
K

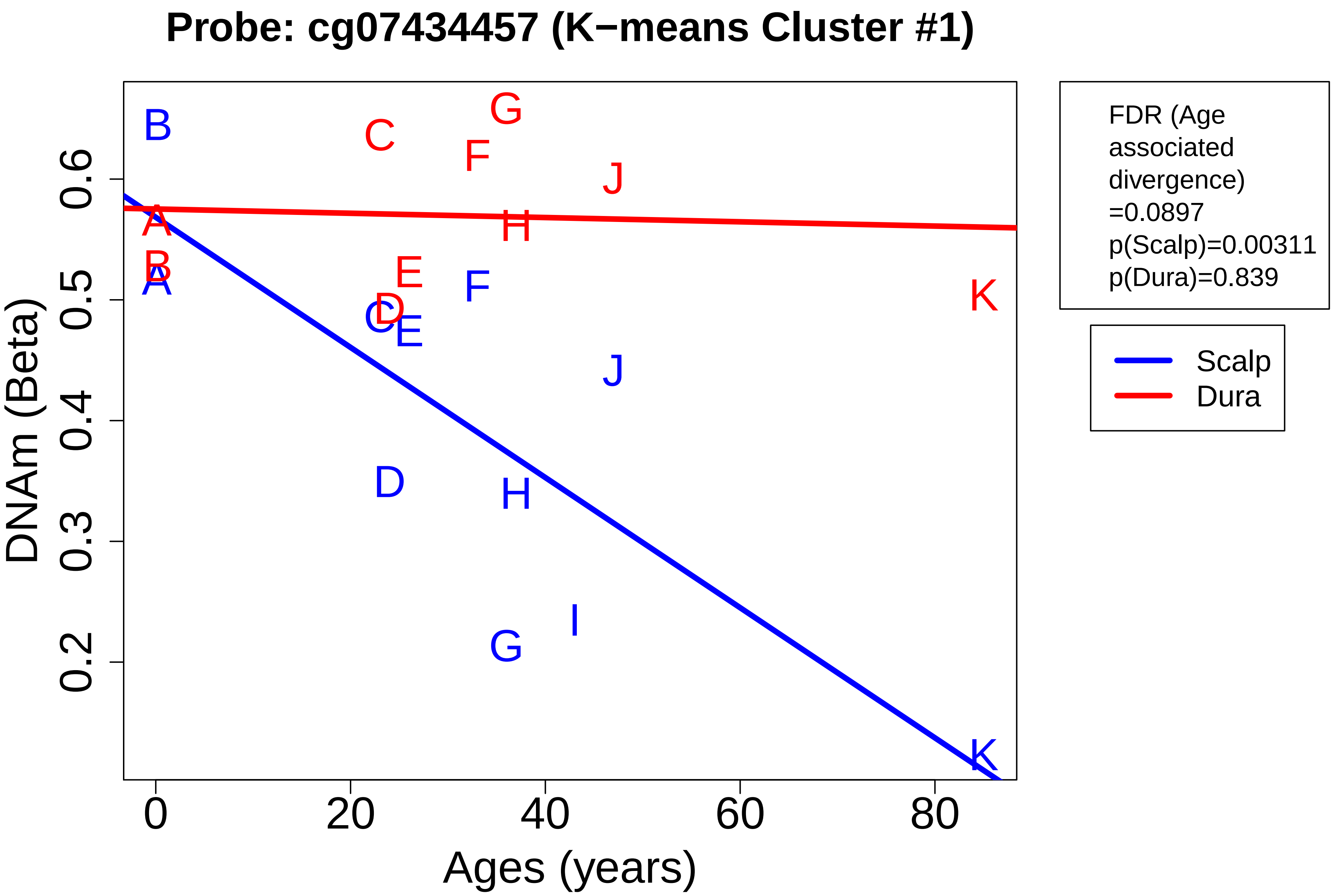

Probe: cg07434457 (K−means Cluster #1)
DNAm (Beta)
0.2
0.3
0.4
0.5
0.6
Ages (years)
0
20
40
60
80
B
C
G
F
J
A
H
B
A
E
F
K
C
D
E
J
D
H
G
I
K
FDR (Age associated divergence) =0.0897
p(Scalp)=0.00311
p(Dura)=0.839
Scalp
Dura

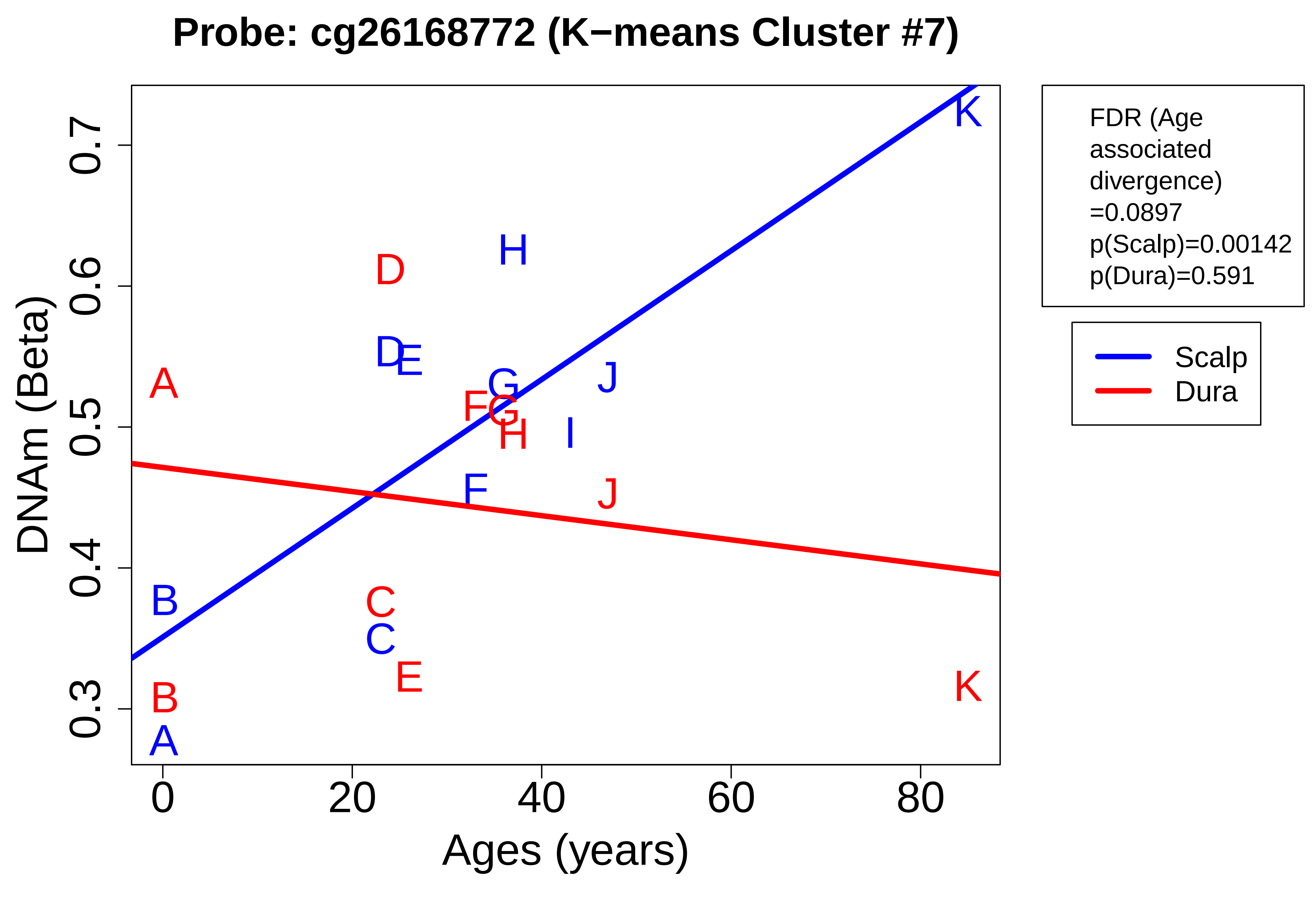

Probe: cg26168772 (K−means Cluster #7)
DNAm (Beta)
0.3
0.4
0.5
0.6
0.7
Ages (years)
0
20
40
60
80
FDR (Age associated divergence) =0.0897
p(Scalp)=0.00142
p(Dura)=0.591
Scalp
Dura

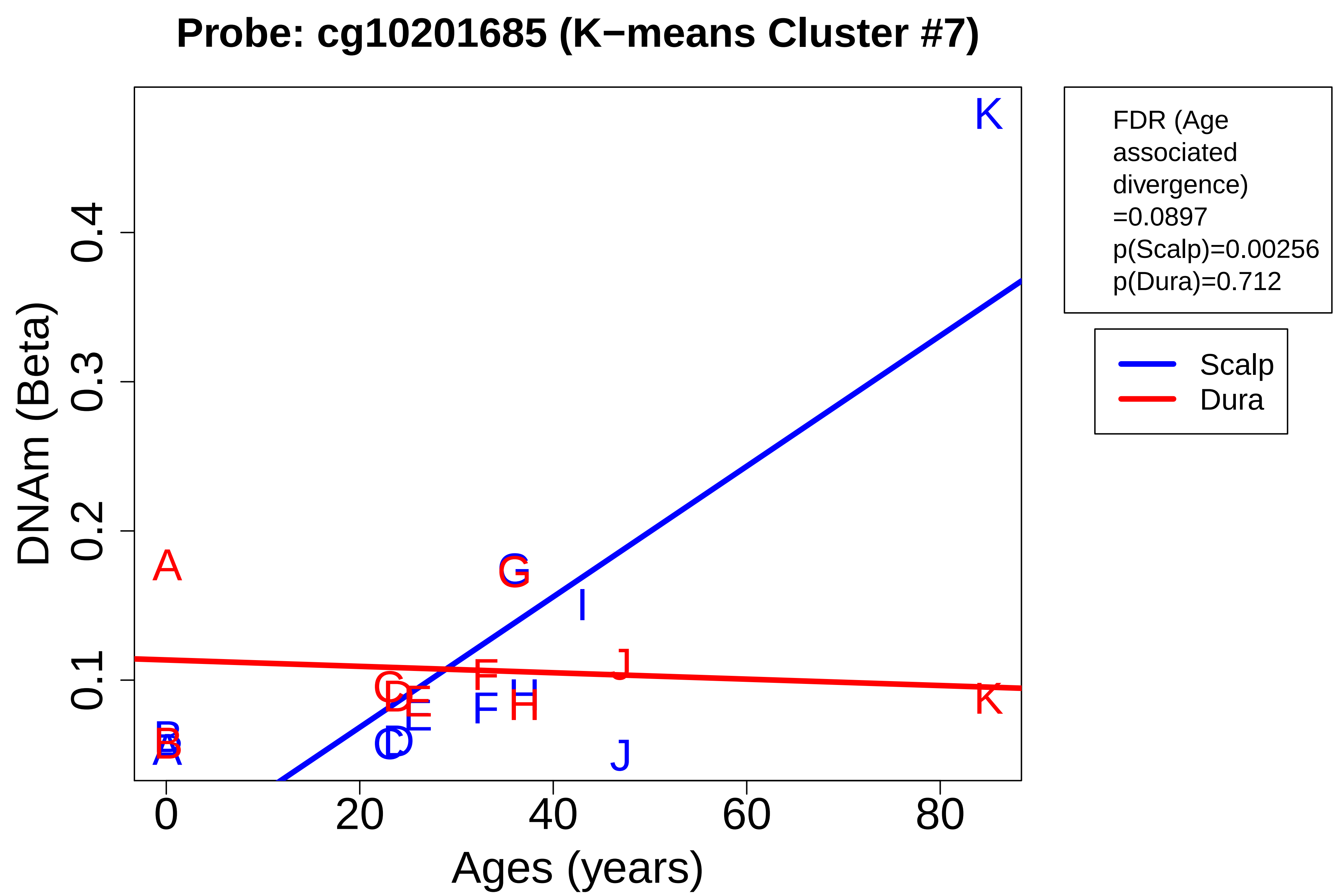

Probe: cg10201685 (K−means Cluster #7)
DNAm (Beta)
0.1
0.2
0.3
0.4
0
20
40
60
80
Ages (years)
FDR (Age associated divergence) =0.0897
p(Scalp)=0.00256
p(Dura)=0.712
Scalp
Dura

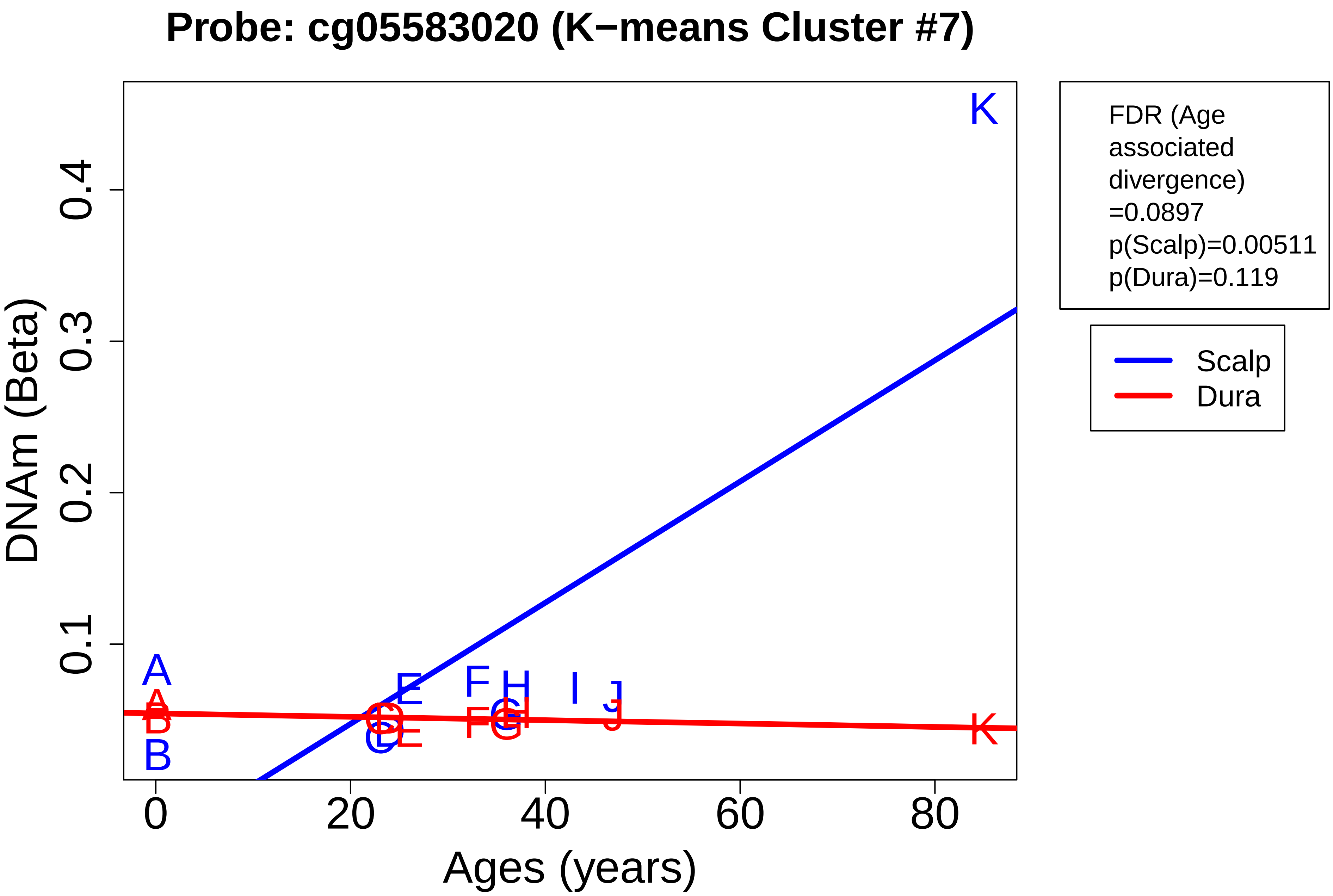
Probe: cg05583020 (K−means Cluster #7)
FDR (Age associated divergence) =0.0897
p(Scalp)=0.00511
p(Dura)=0.119
Scalp
Dura
DNAm (Beta)
Ages (years)
0.1
0.2
0.3
0.4
0
20
40
60
80

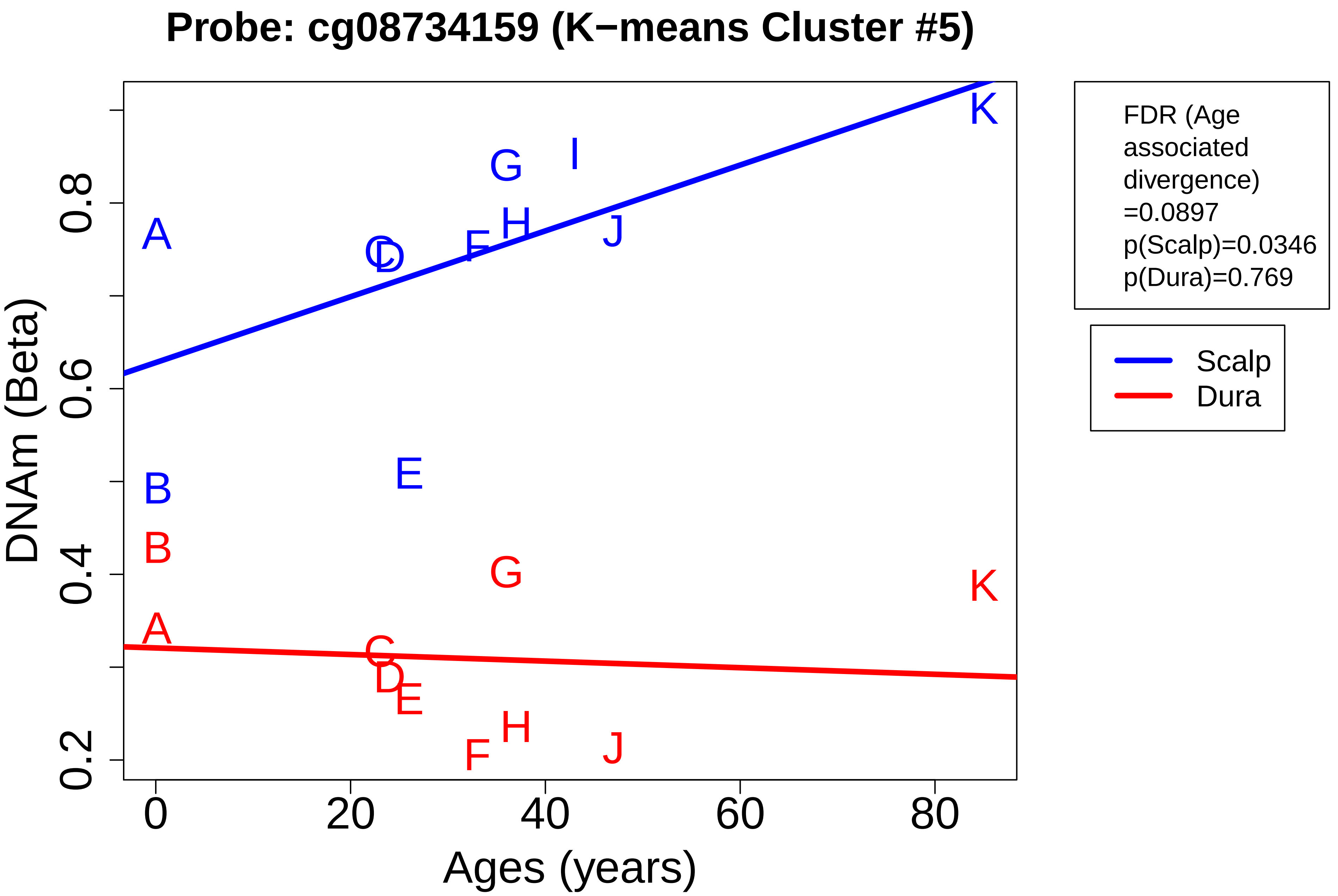

Probe: cg08734159 (K−means Cluster #5)
DNAm (Beta)
0.2
0.4
0.6
0.8
0
20
40
60
80
Ages (years)
FDR (Age associated divergence) =0.0897
p(Scalp)=0.0346
p(Dura)=0.769
Scalp
Dura
A
B
C
D
E
F
G
H
I
J
K

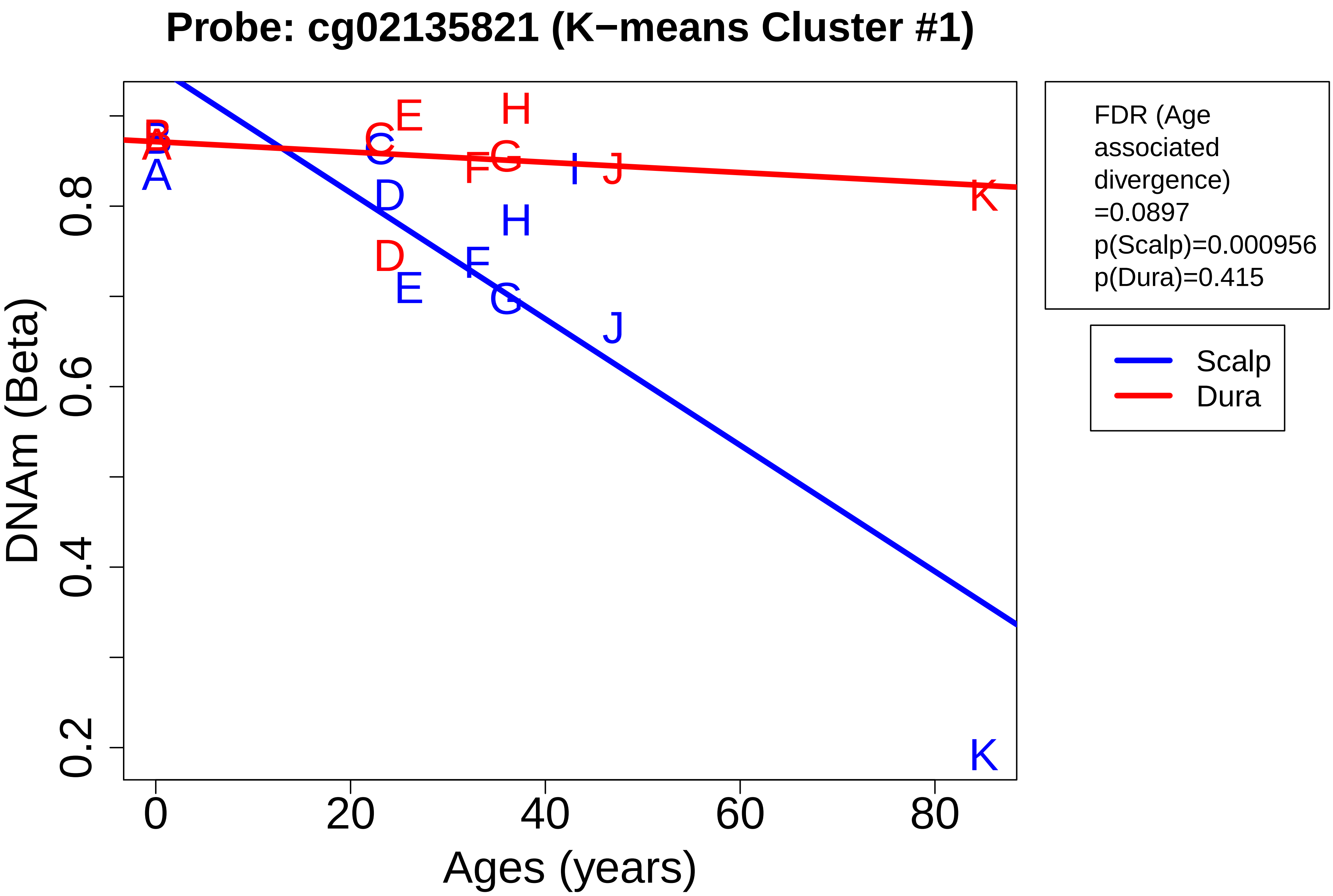
Probe: cg02135821 (K−means Cluster #1)
DNAm (Beta)
Ages (years)
0.2
0.4
0.6
0.8
0
20
40
60
80
FDR (Age associated divergence) =0.0897
p(Scalp)=0.000956
p(Dura)=0.415
Scalp
Dura
A
B
C
D
E
F
G
H
I
J
K

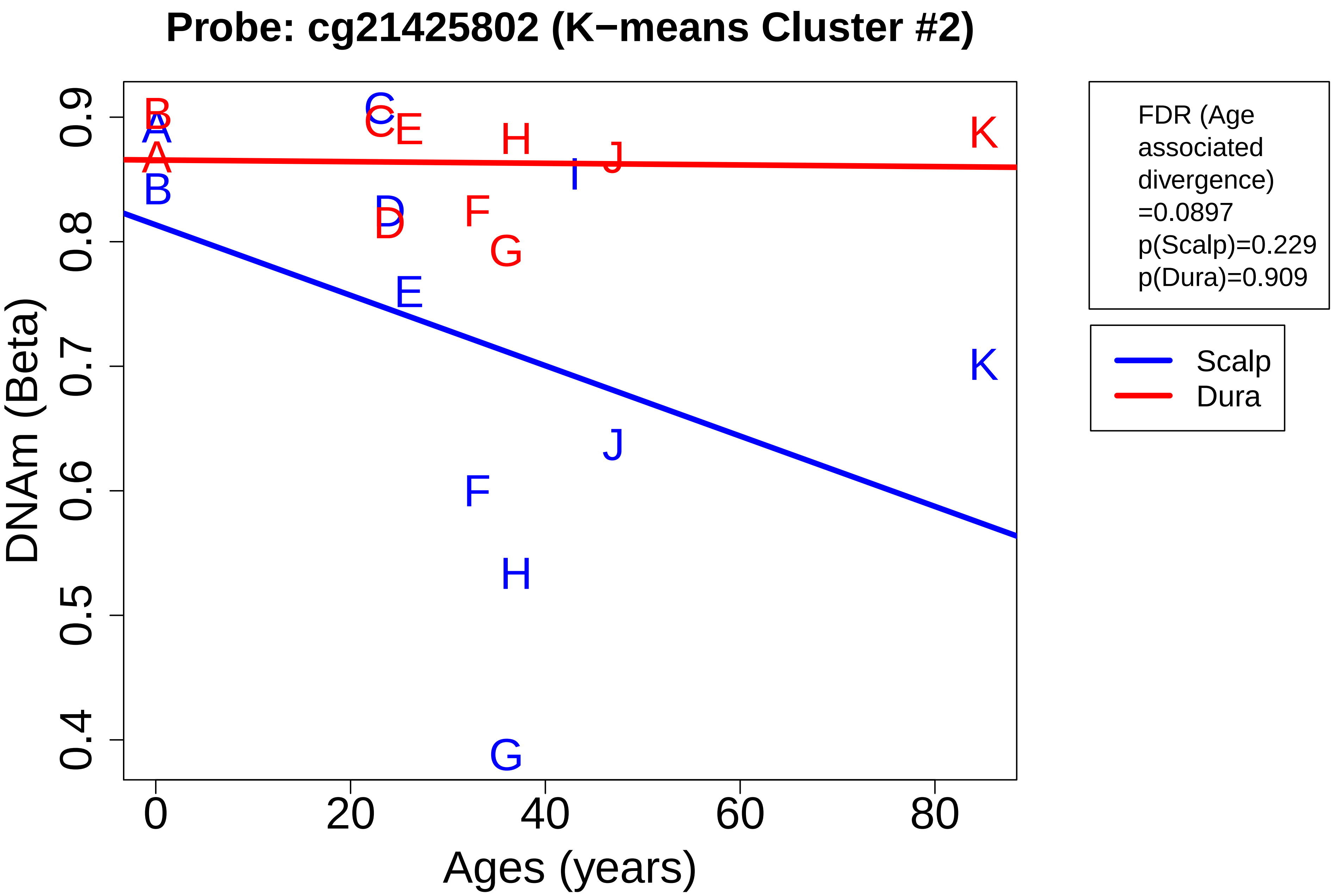

Probe: cg21425802 (K−means Cluster #2)
DNAm (Beta)
0.4
0.5
0.6
0.7
0.8
0.9
0
20
40
60
80
Ages (years)
FDR (Age associated divergence) =0.0897
p(Scalp)=0.229
p(Dura)=0.909
Scalp
Dura

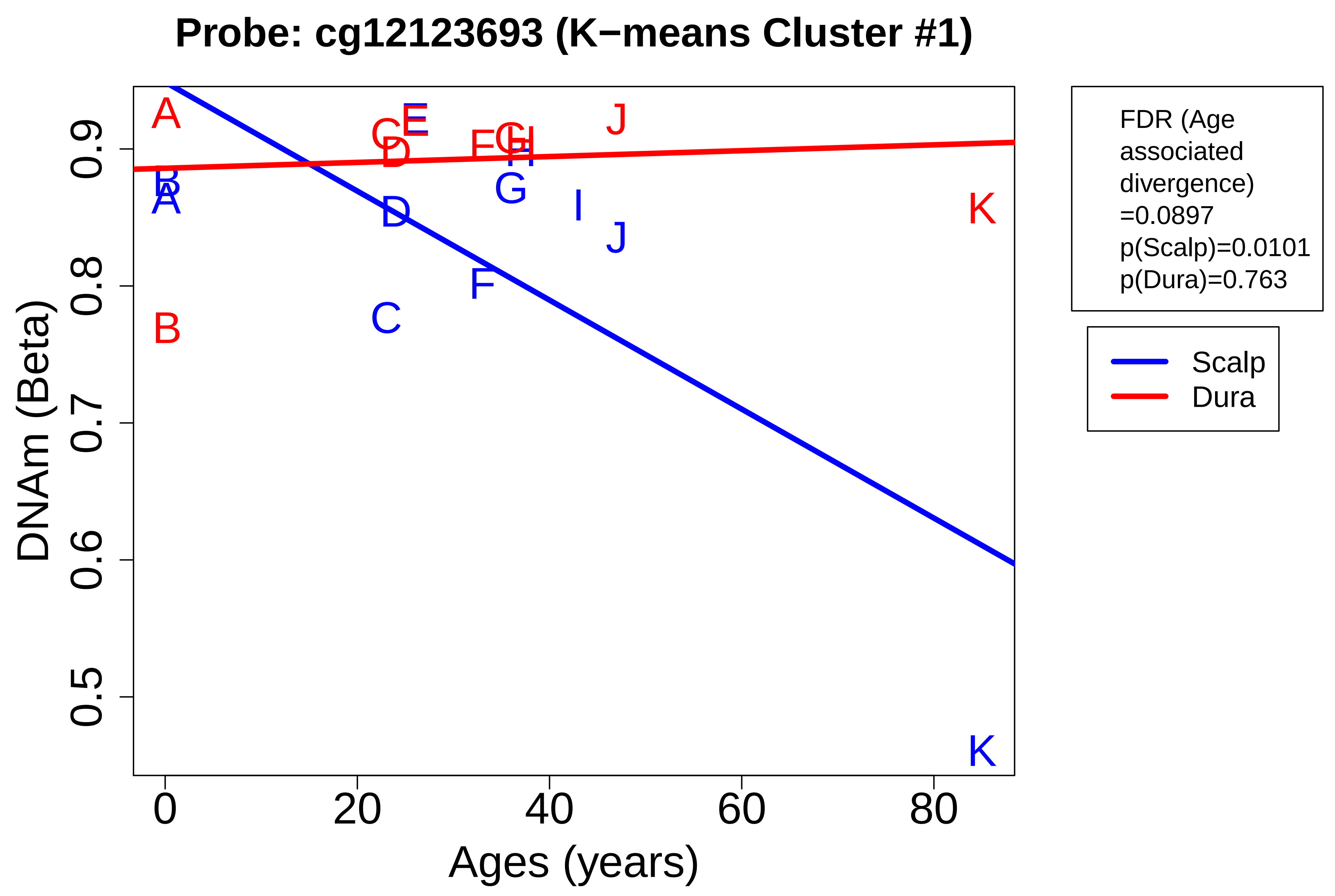

Probe: cg12123693 (K−means Cluster #1)
DNAm (Beta)
0.5
0.6
0.7
0.8
0.9
Ages (years)
0
20
40
60
80
FDR (Age associated divergence) =0.0897
p(Scalp)=0.0101
p(Dura)=0.763
Scalp
Dura

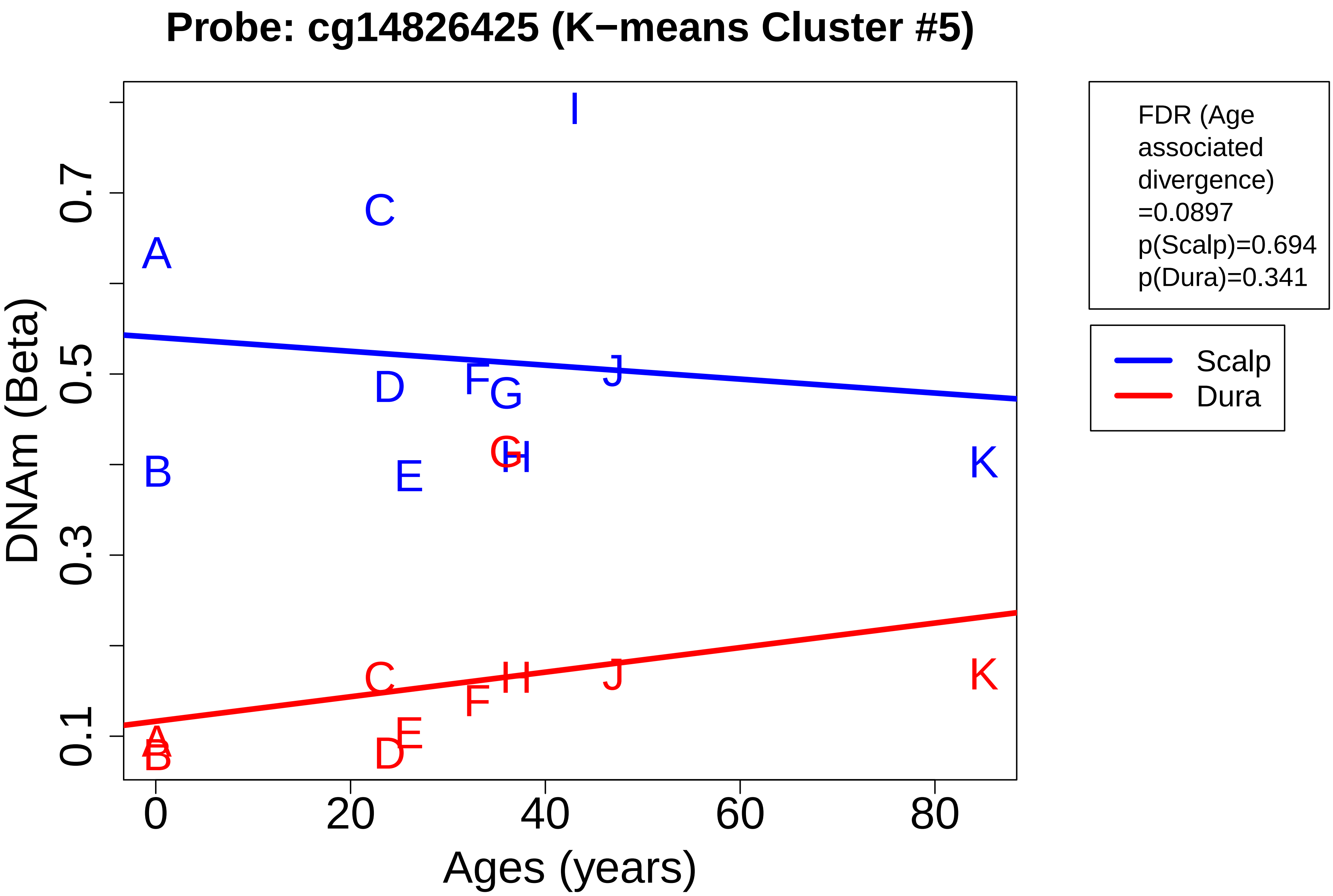
Probe: cg14826425 (K−means Cluster #5)
DNAm (Beta)
0.1
0.3
0.5
0.7
Ages (years)
0
20
40
60
80
FDR (Age associated divergence) =0.0897
p(Scalp)=0.694
p(Dura)=0.341
Scalp
Dura
A
B
C
D
E
F
G
H
I
J
K

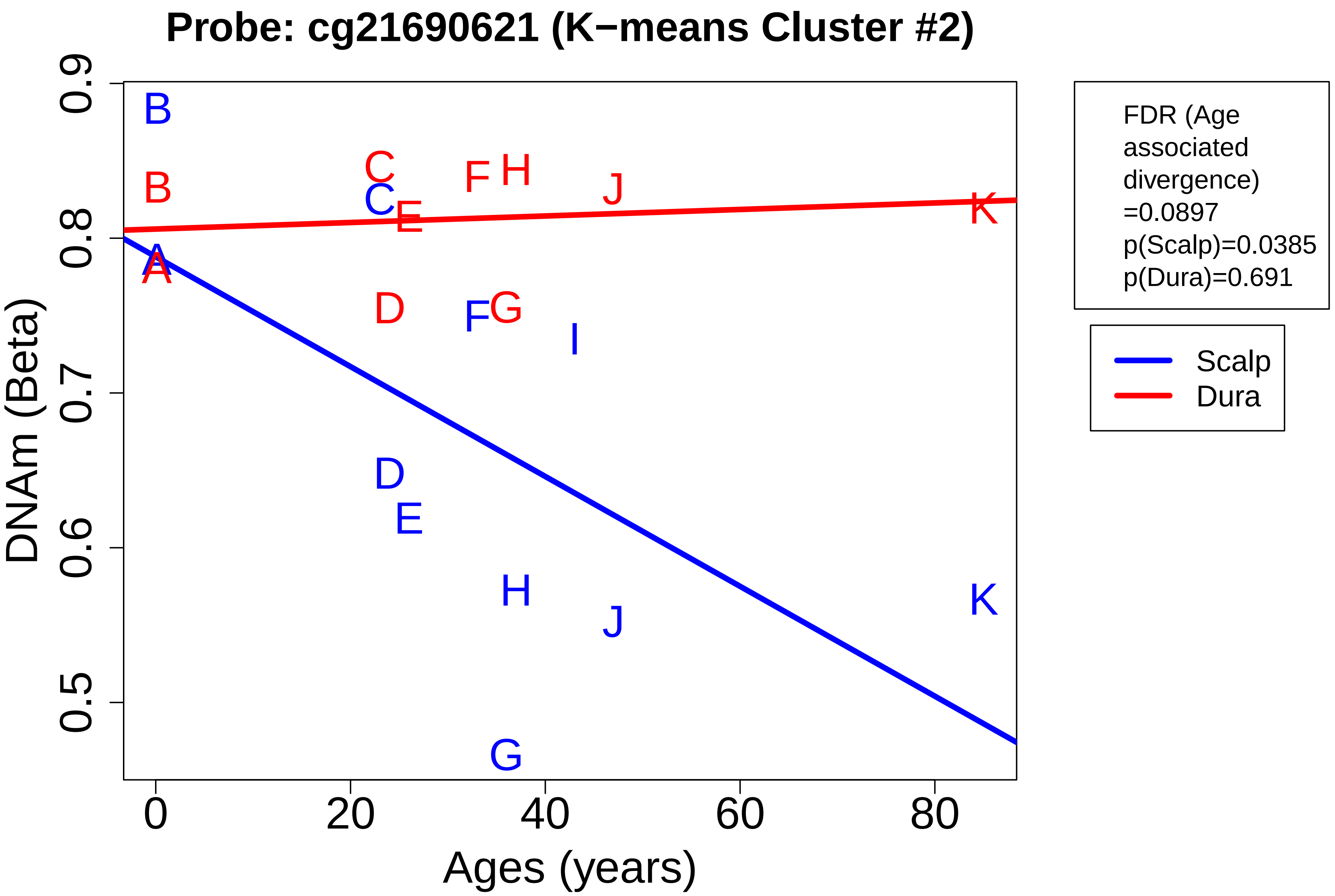
Probe: cg21690621 (K−means Cluster #2)
DNAm (Beta)
0.5
0.6
0.7
0.8
0.9
Ages (years)
0
20
40
60
80
FDR (Age associated divergence) =0.0897
p(Scalp)=0.0385
p(Dura)=0.691
Scalp
Dura

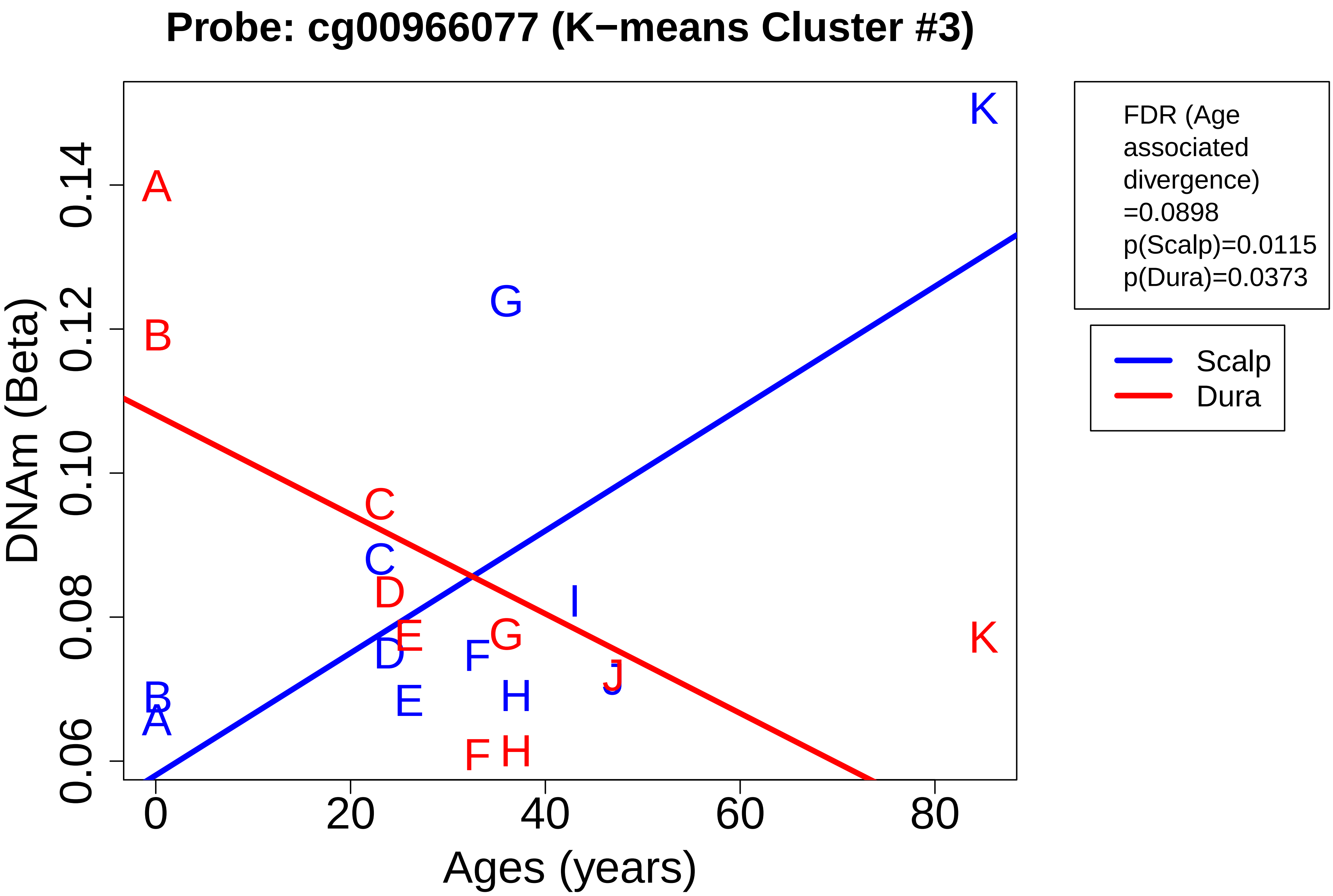

Probe: cg00966077 (K−means Cluster #3)
DNAm (Beta)
0.06
0.08
0.10
0.12
0.14
0
20
40
60
80
Ages (years)
FDR (Age associated divergence) =0.0898
p(Scalp)=0.0115
p(Dura)=0.0373
Scalp
Dura
A
B
C
D
E
F
G
H
I
J
K

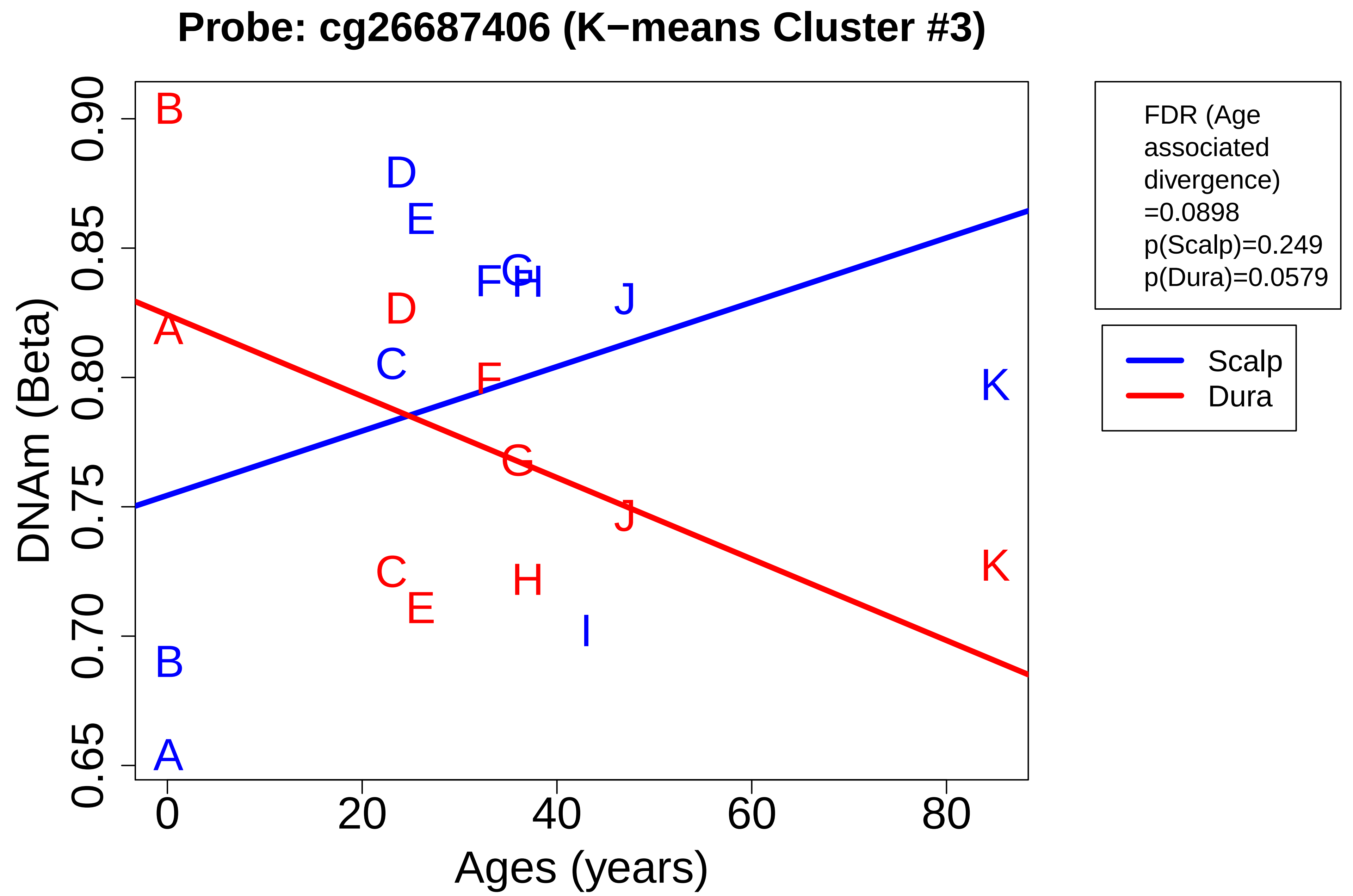

Probe: cg26687406 (K−means Cluster #3)
DNAm (Beta)
0.65
0.70
0.75
0.80
0.85
0.90
Ages (years)
0
20
40
60
80
A
B
C
D
E
F
G
H
I
J
K
FDR (Age associated divergence) =0.0898
p(Scalp)=0.249
p(Dura)=0.0579
Scalp
Dura

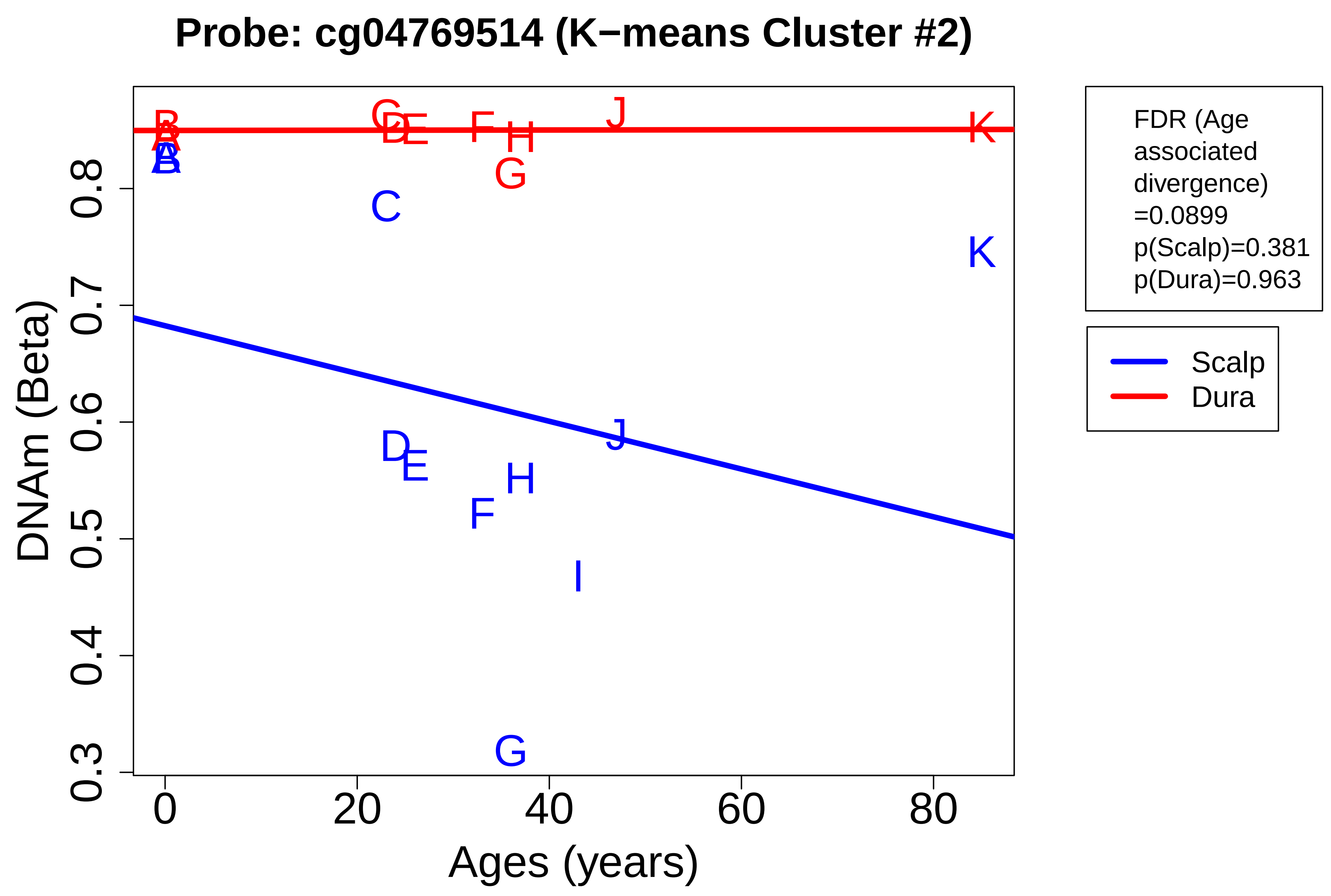
Probe: cg04769514 (K−means Cluster #2)
DNAm (Beta)
Ages (years)
0.3
0.4
0.5
0.6
0.7
0.8
0
20
40
60
80
FDR (Age associated divergence) =0.0899
p(Scalp)=0.381
p(Dura)=0.963
Scalp
Dura

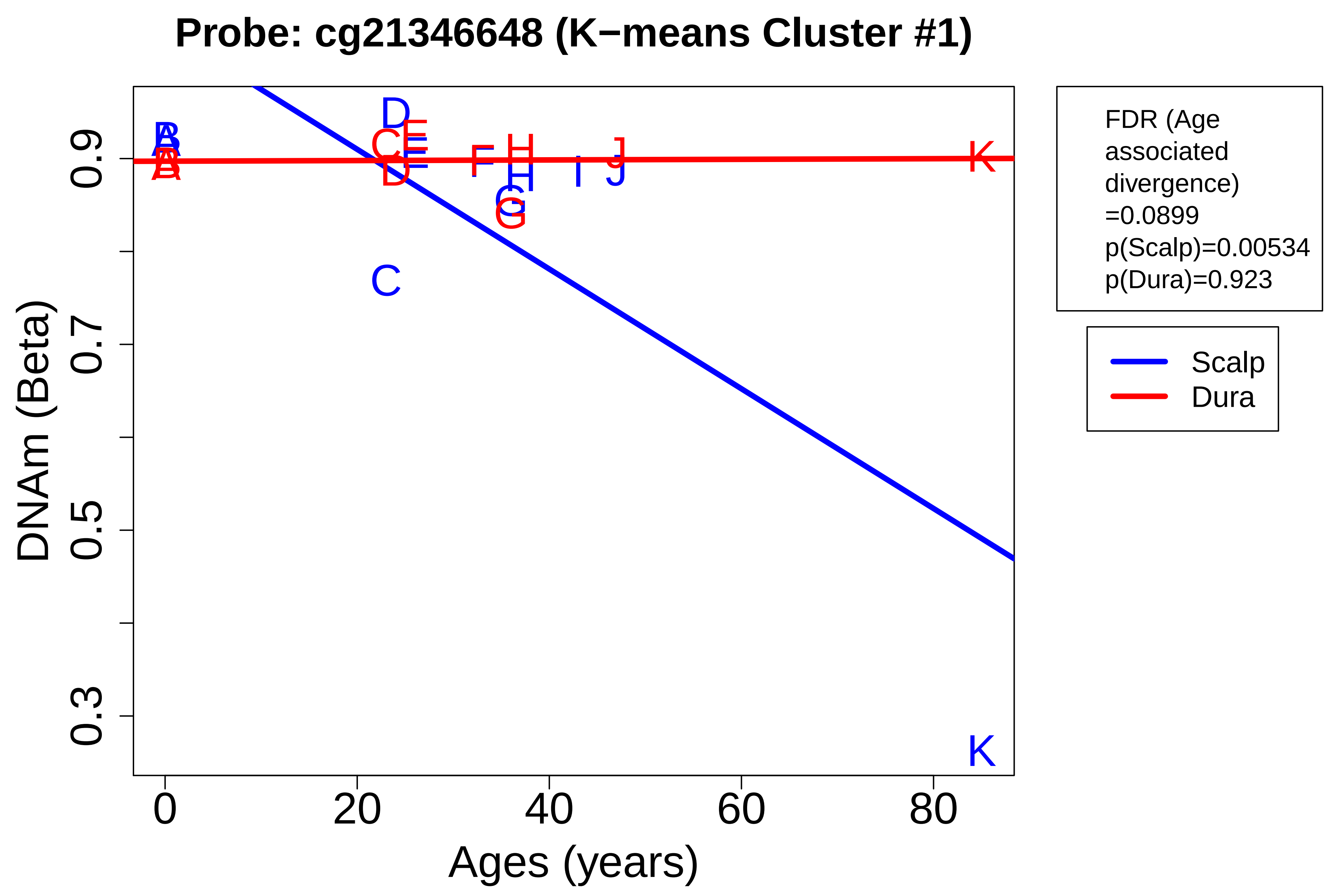

Probe: cg21346648 (K−means Cluster #1)
DNAm (Beta)
0.3
0.5
0.7
0.9
Ages (years)
0
20
40
60
80
FDR (Age
associated
divergence)
=0.0899
p(Scalp)=0.00534
p(Dura)=0.923
Scalp
Dura

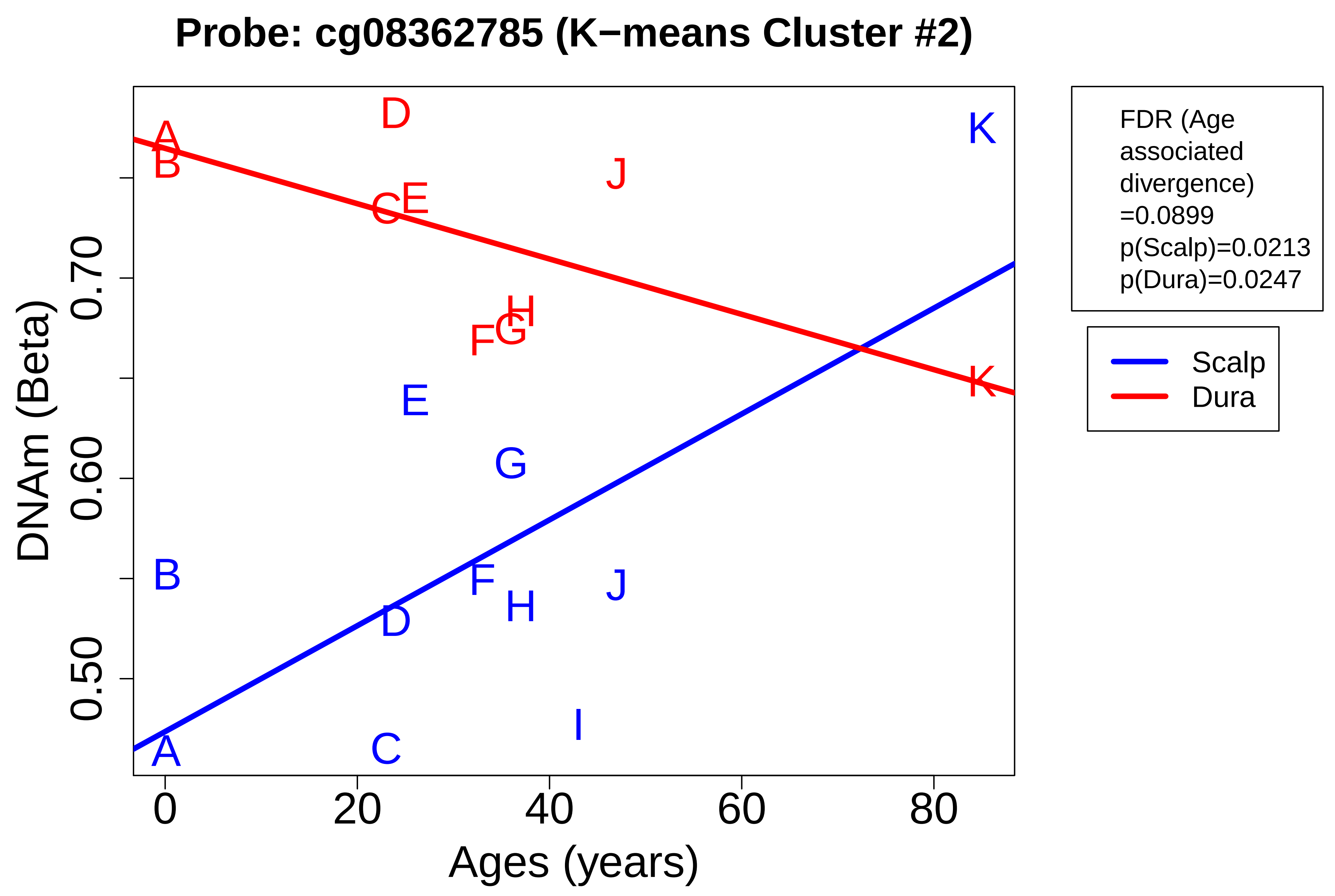

Probe: cg08362785 (K−means Cluster #2)
DNAm (Beta)
0.50
0.60
0.70
0
20
40
60
80
Ages (years)
FDR (Age associated divergence) =0.0899
p(Scalp)=0.0213
p(Dura)=0.0247
Scalp
Dura
A
B
C
D
E
F
G
H
I
J
K

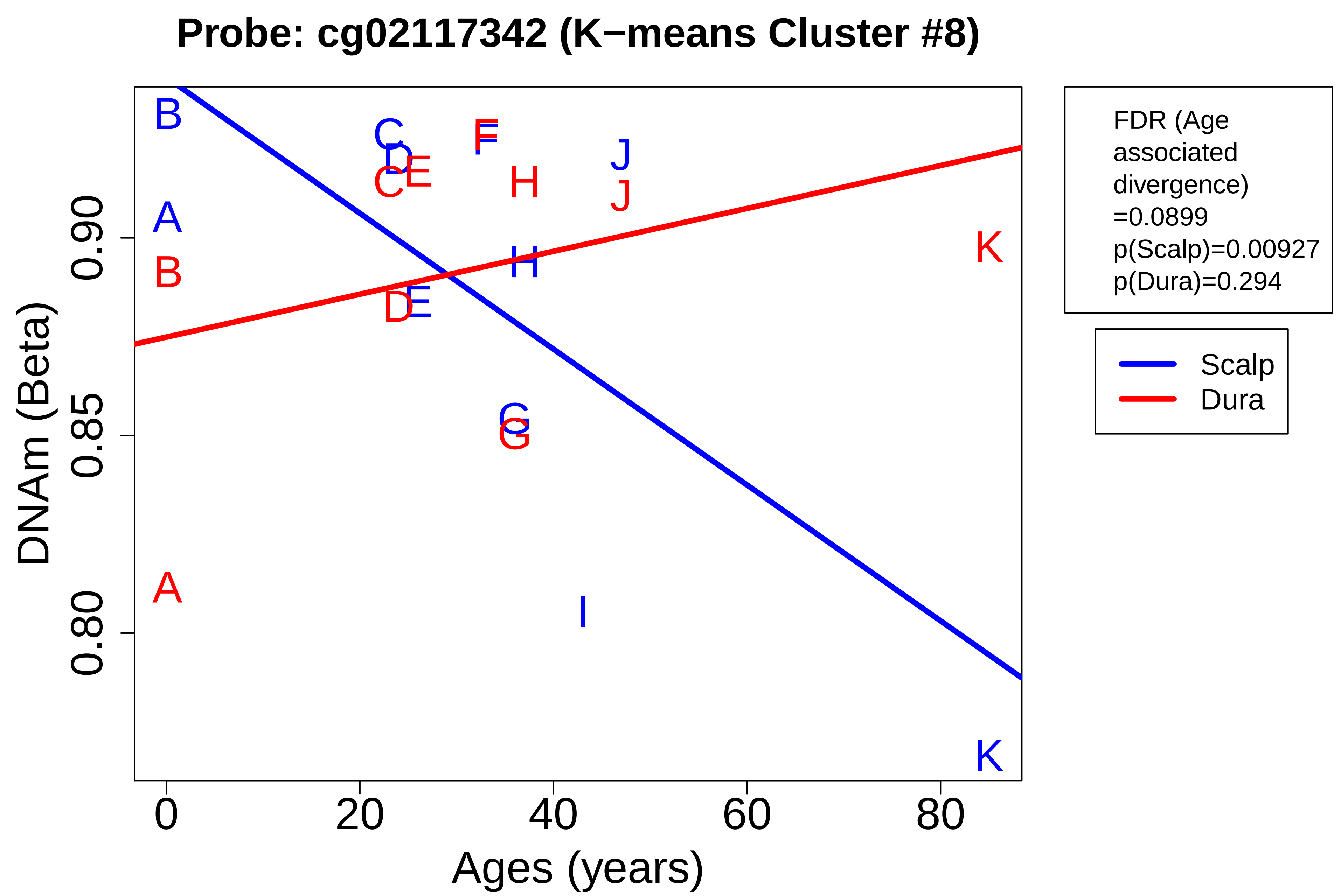

Probe: cg02117342 (K−means Cluster #8)
DNAm (Beta)
0.80
0.85
0.90
Ages (years)
0
20
40
60
80
A
B
C
D
E
F
G
H
I
J
K
FDR (Age associated divergence) =0.0899
p(Scalp)=0.00927
p(Dura)=0.294
Scalp
Dura

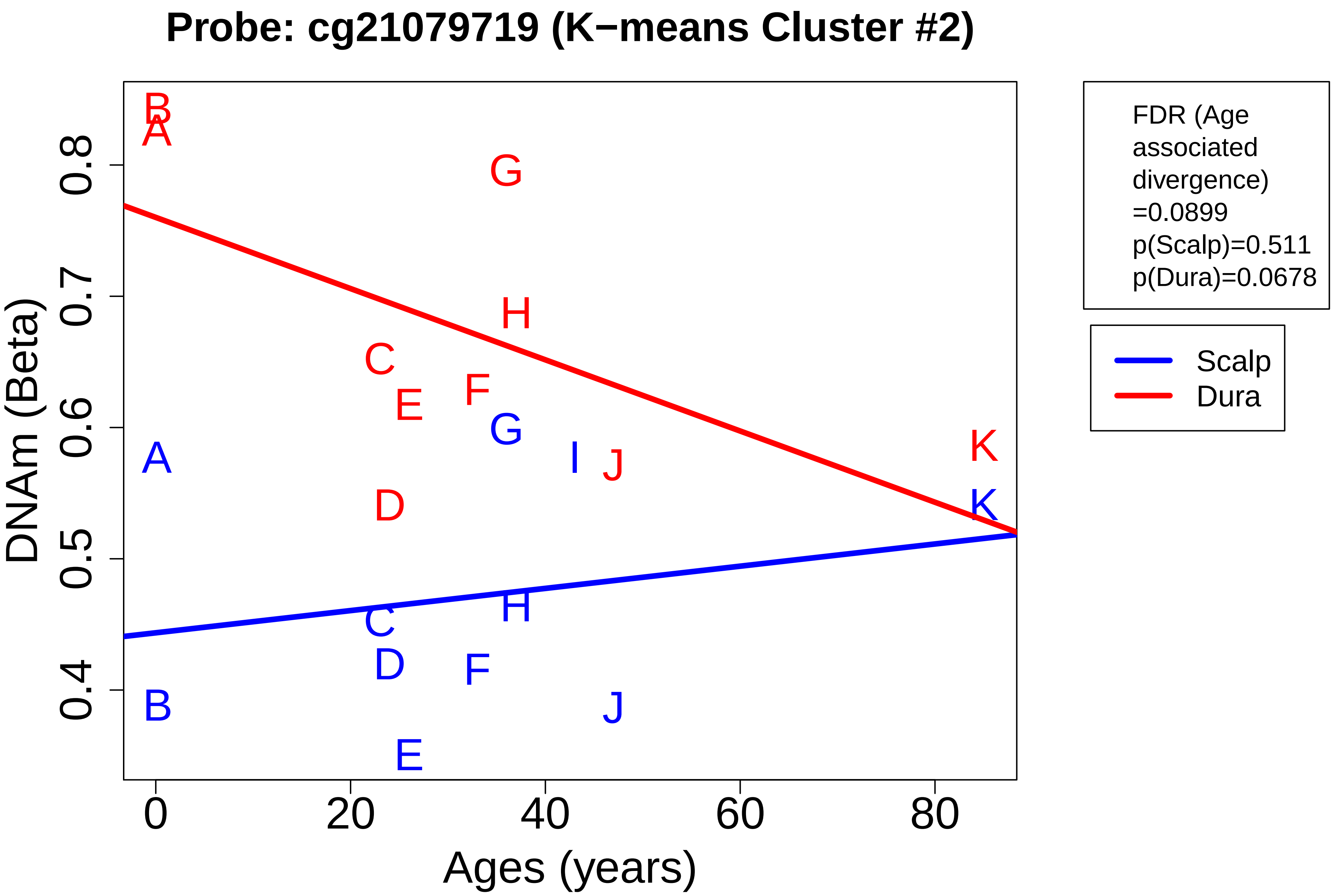

Probe: cg21079719 (K−means Cluster #2)
FDR (Age associated divergence) =0.0899
p(Scalp)=0.511
p(Dura)=0.0678
Scalp
Dura
DNAm (Beta)
0.4
0.5
0.6
0.7
0.8
0
20
40
60
80
Ages (years)
A B C D E F G H I J K

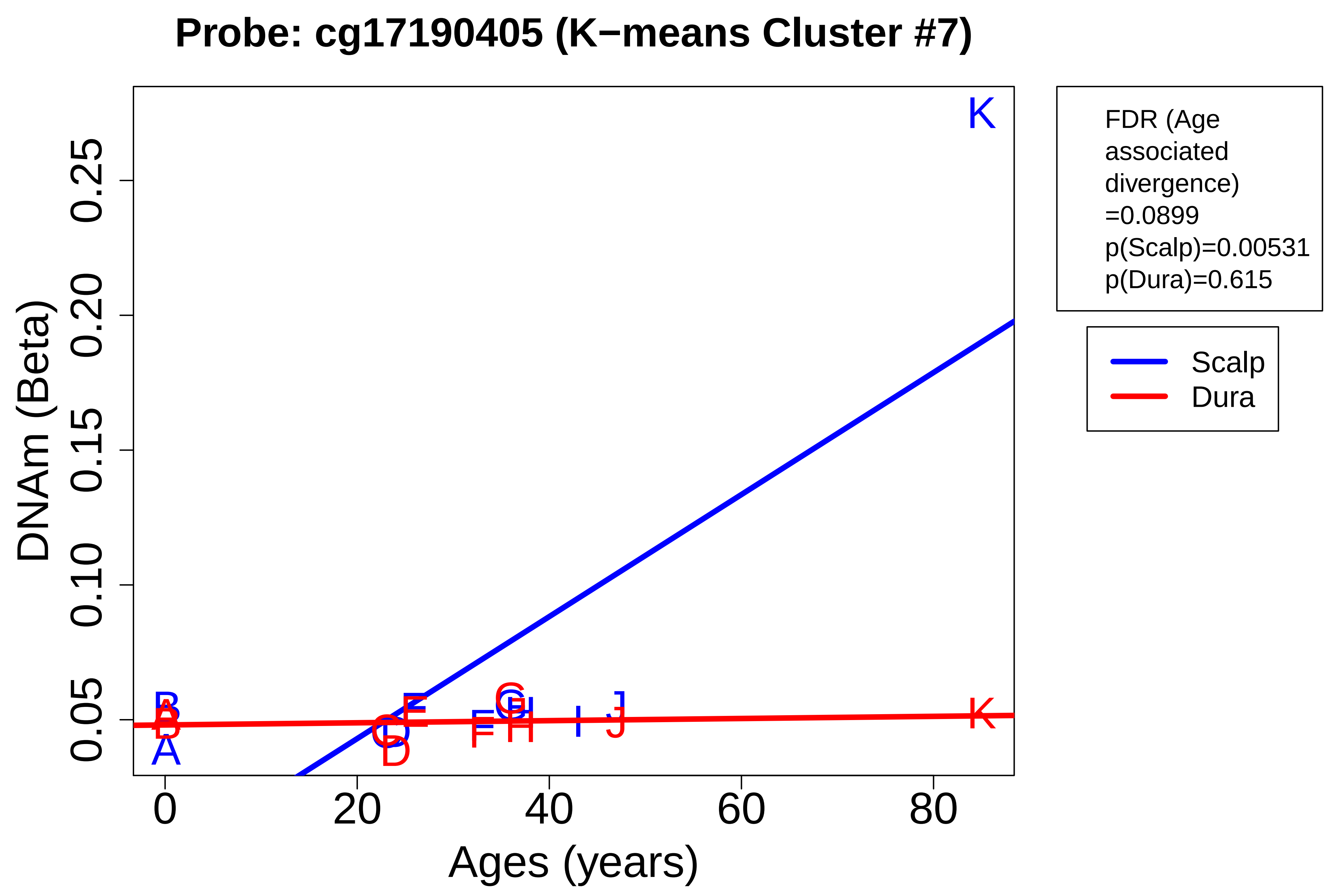
Probe: cg17190405 (K−means Cluster #7)
DNAm (Beta)
0.05
0.10
0.15
0.20
0.25
Ages (years)
0
20
40
60
80
A
B
C
D
E
F
G
H
I
J
K
FDR (Age associated divergence) =0.0899
p(Scalp)=0.00531
p(Dura)=0.615
Scalp
Dura

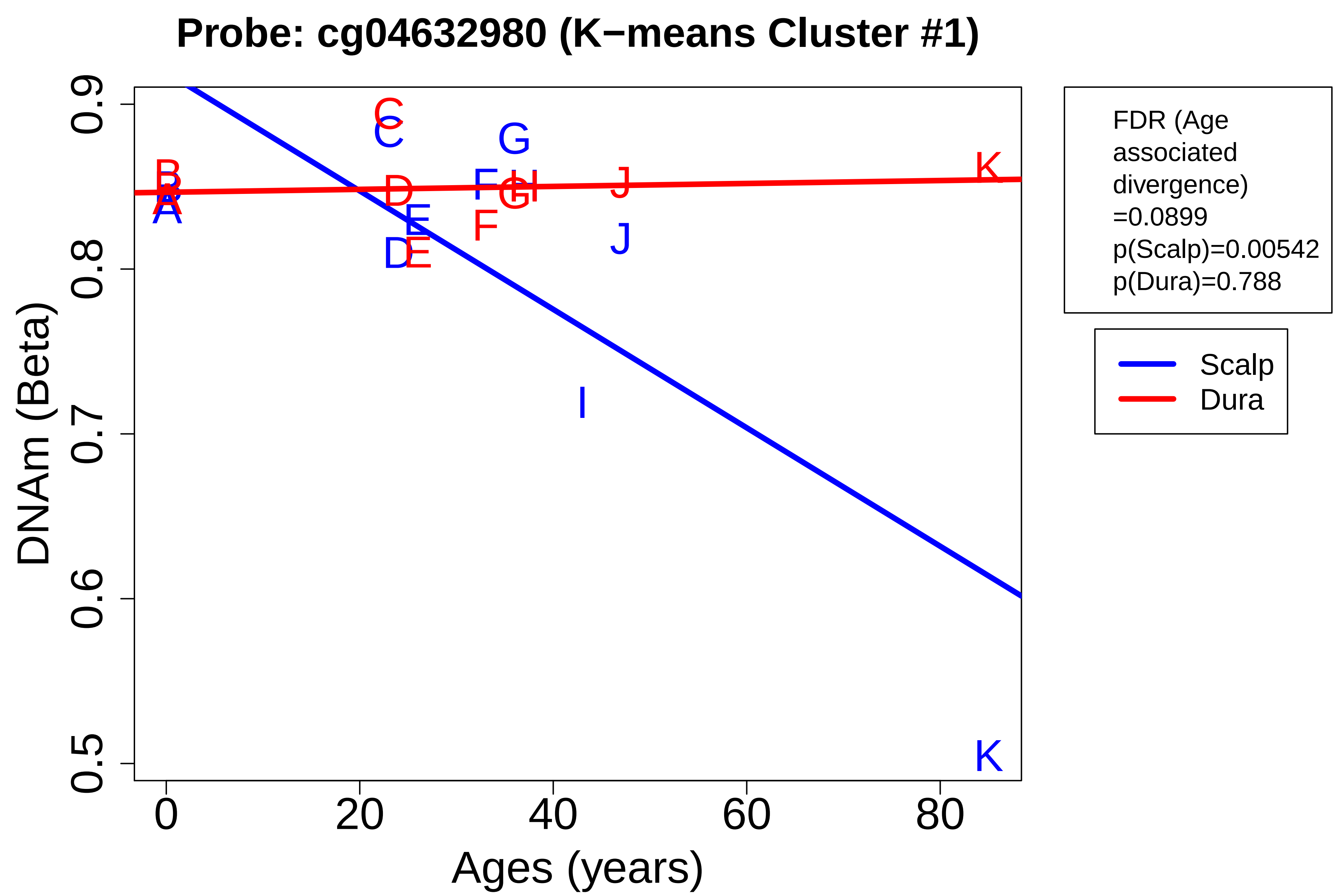

Probe: cg04632980 (K−means Cluster #1)
DNAm (Beta)
0.5
0.6
0.7
0.8
0.9
0
20
40
60
80
Ages (years)
FDR (Age associated divergence) =0.0899
p(Scalp)=0.00542
p(Dura)=0.788
Scalp
Dura

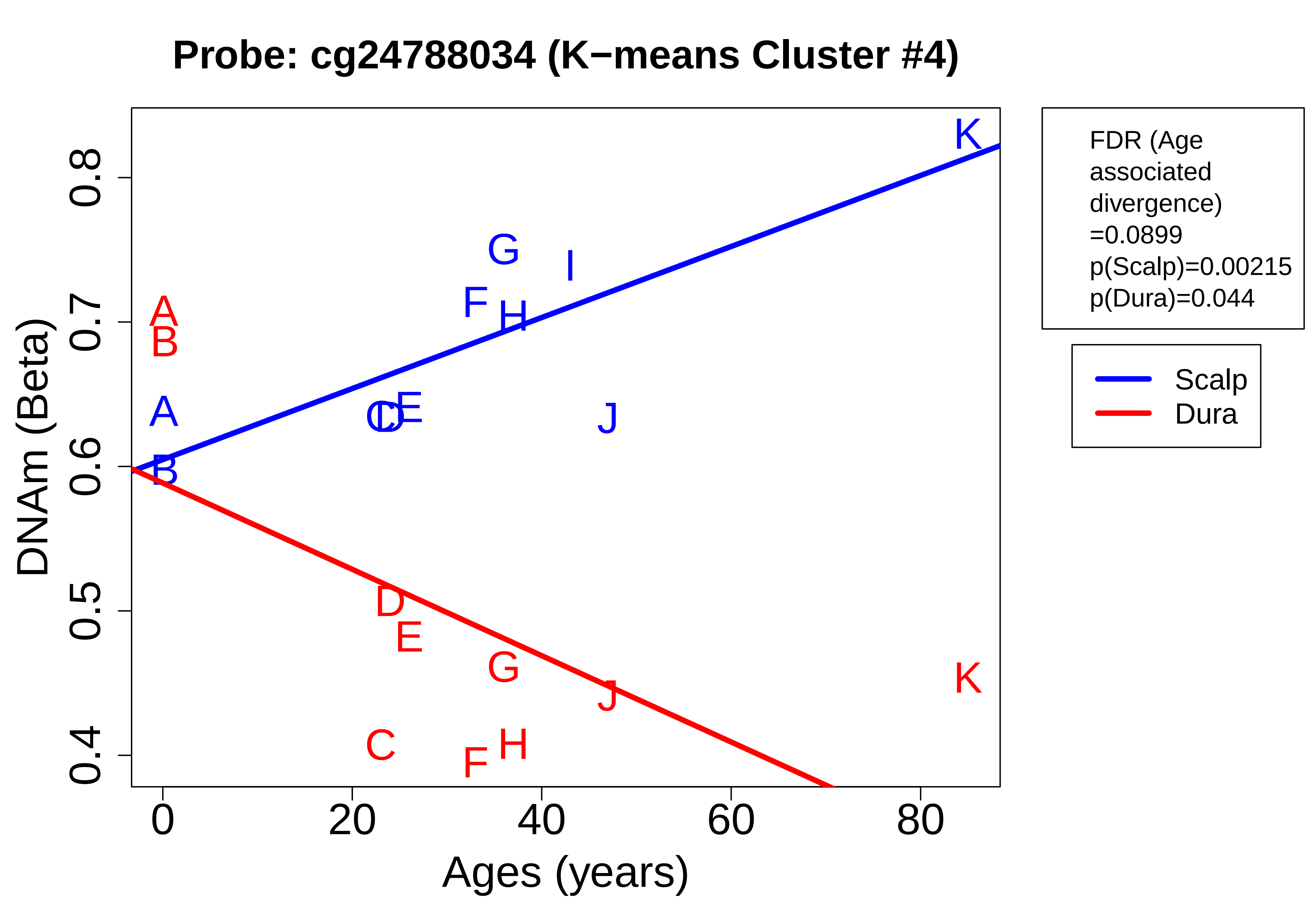

Probe: cg24788034 (K−means Cluster #4)
DNAm (Beta)
0.4
0.5
0.6
0.7
0.8
Ages (years)
0
20
40
60
80
FDR (Age associated divergence) =0.0899
p(Scalp)=0.00215
p(Dura)=0.044
Scalp
Dura

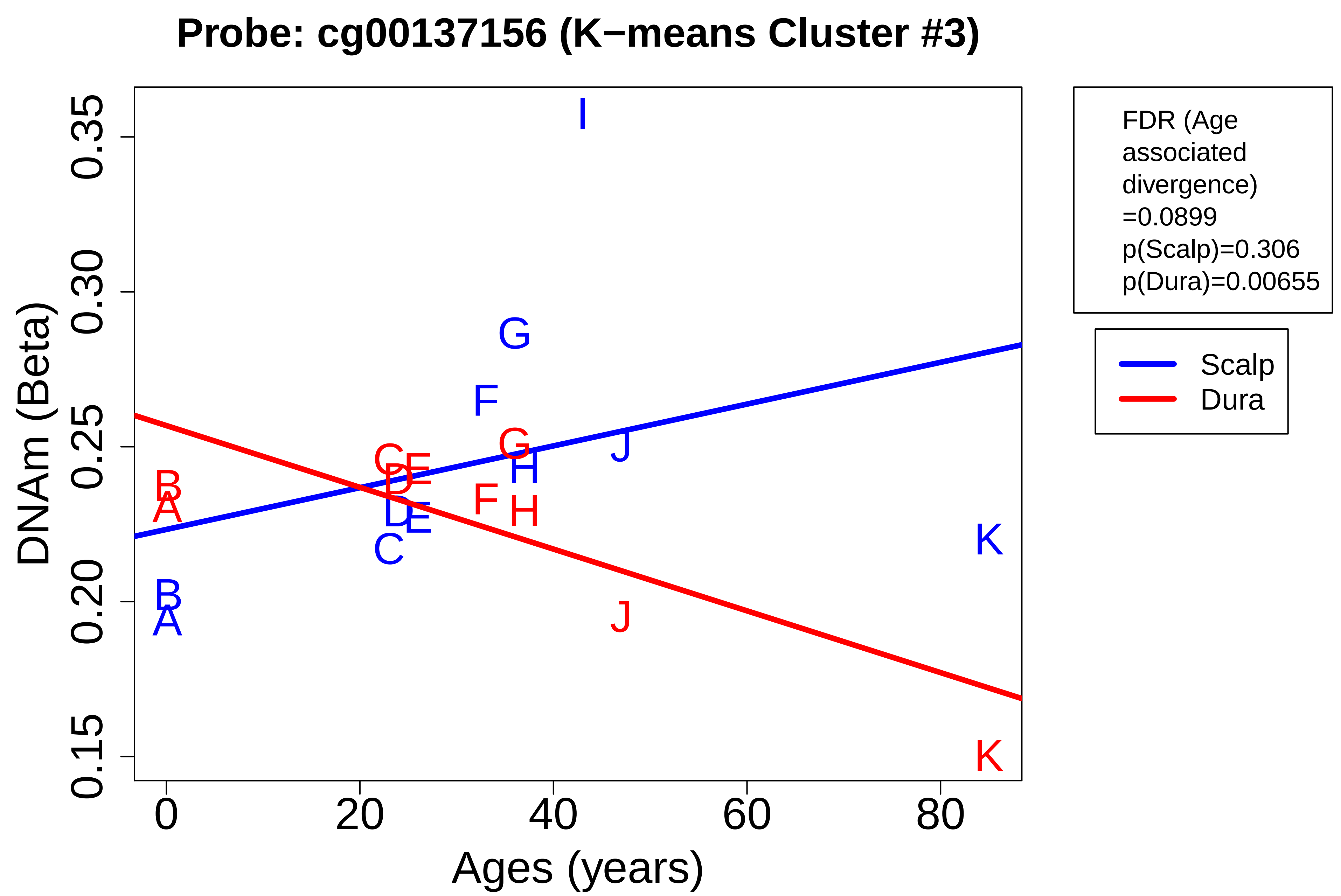

Probe: cg00137156 (K−means Cluster #3)
DNAm (Beta)
0.15
0.20
0.25
0.30
0.35
0
20
40
60
80
Ages (years)
FDR (Age associated divergence) =0.0899
p(Scalp)=0.306
p(Dura)=0.00655
Scalp
Dura
A
B
C
D
E
F
G
H
I
J
K

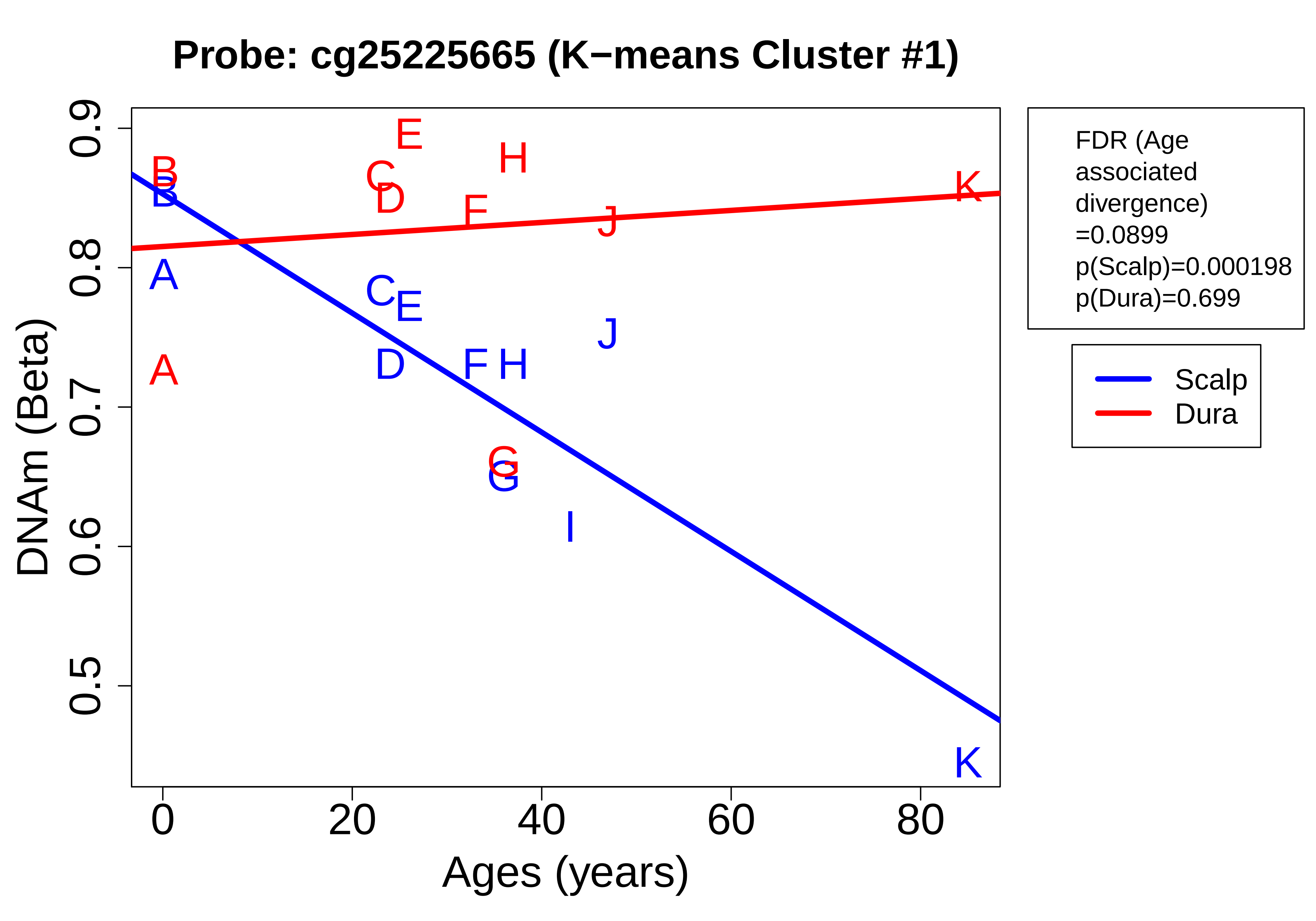

Probe: cg25225665 (K−means Cluster #1)
DNAm (Beta)
0.5
0.6
0.7
0.8
0.9
0
20
40
60
80
Ages (years)
FDR (Age associated divergence) =0.0899
p(Scalp)=0.000198
p(Dura)=0.699
Scalp
Dura
A
B
C
D
E
F
G
H
I
J
K

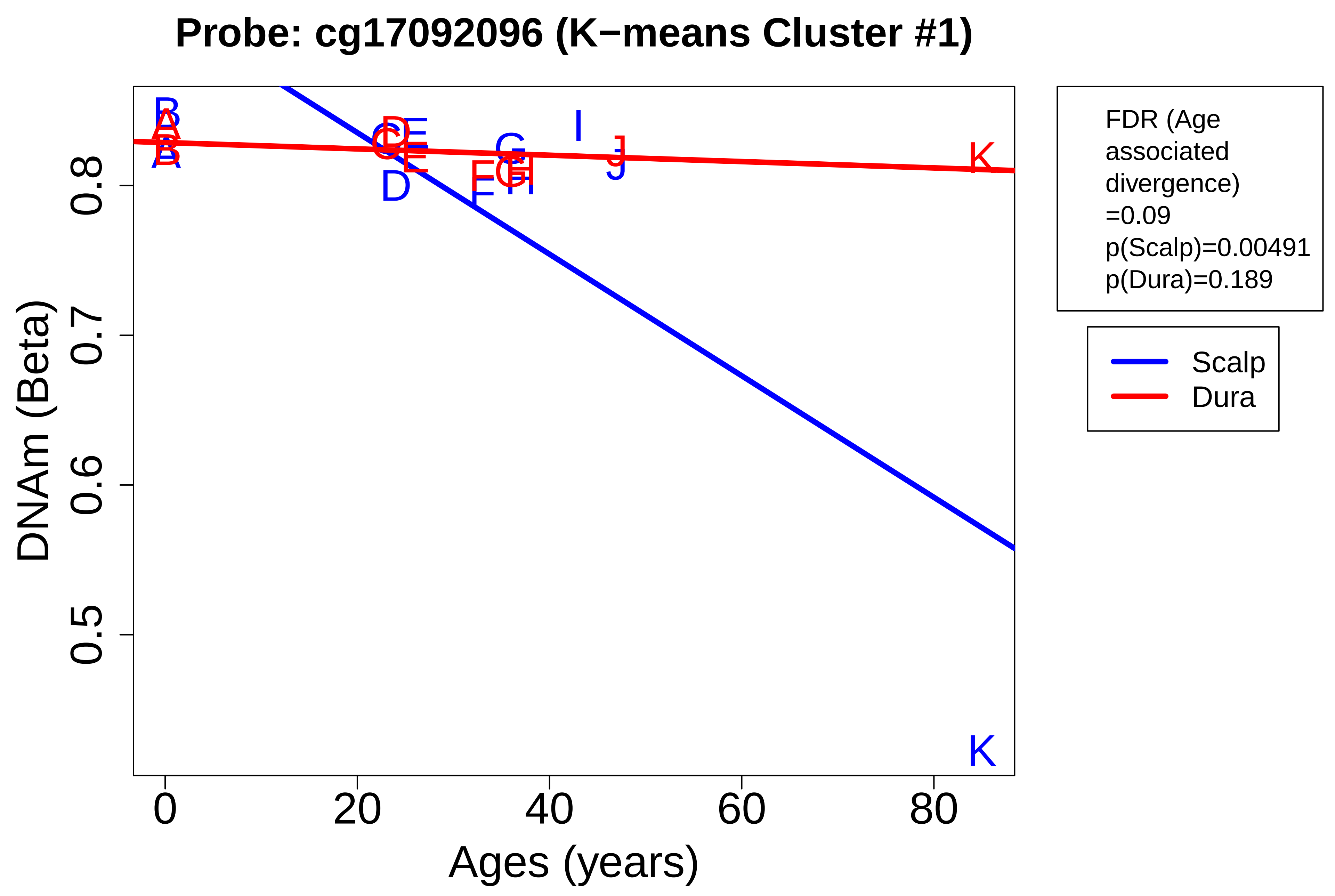
Probe: cg17092096 (K−means Cluster #1)
DNAm (Beta)
0.5
0.6
0.7
0.8
0
20
40
60
80
Ages (years)
FDR (Age associated divergence) =0.09
p(Scalp)=0.00491
p(Dura)=0.189
Scalp
Dura

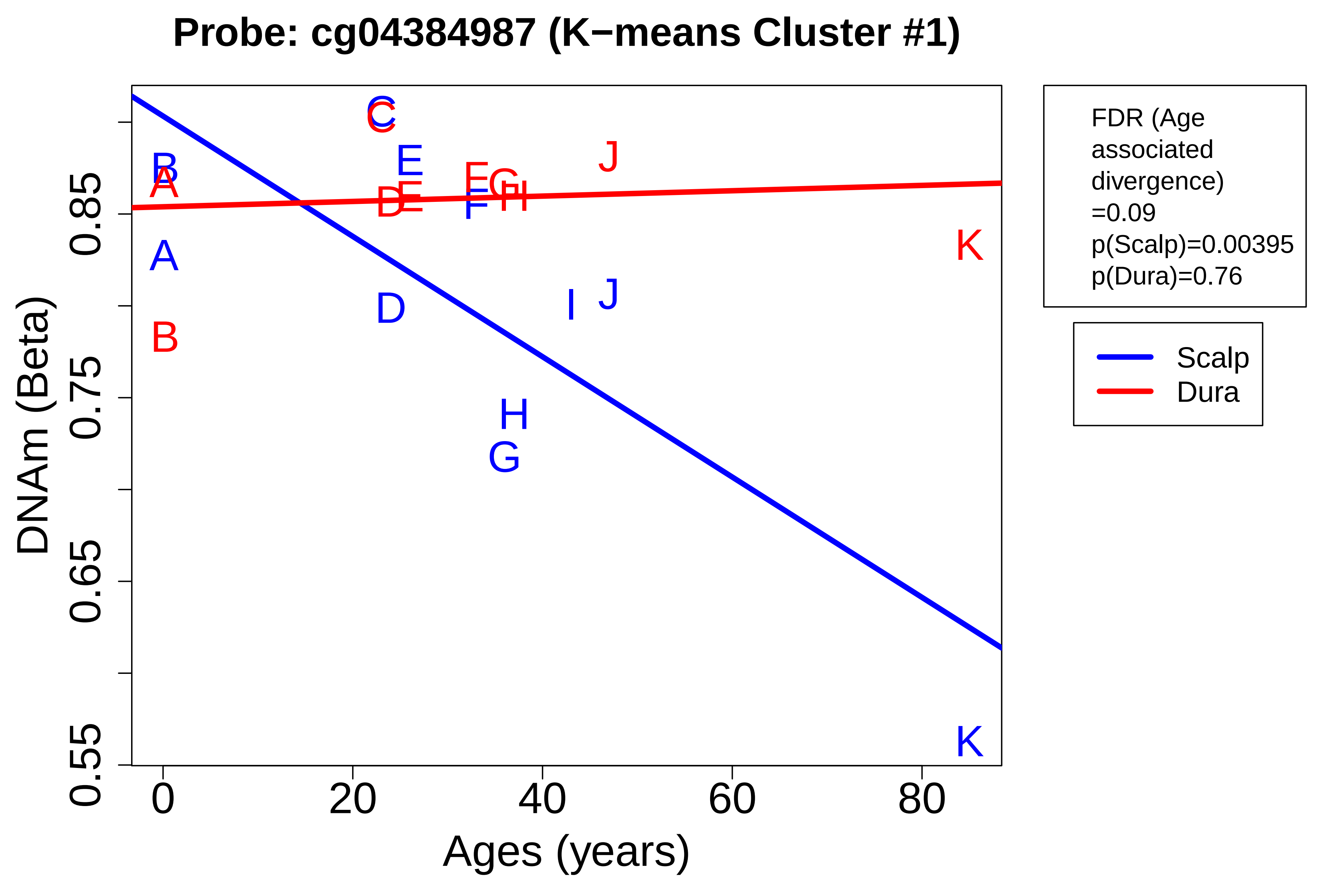
Probe: cg04384987 (K−means Cluster #1)
DNAm (Beta)
0.55
0.65
0.75
0.85
Ages (years)
0
20
40
60
80
FDR (Age associated divergence) =0.09
p(Scalp)=0.00395
p(Dura)=0.76
Scalp
Dura
A
B
C
D
E
F
G
H
I
J
K

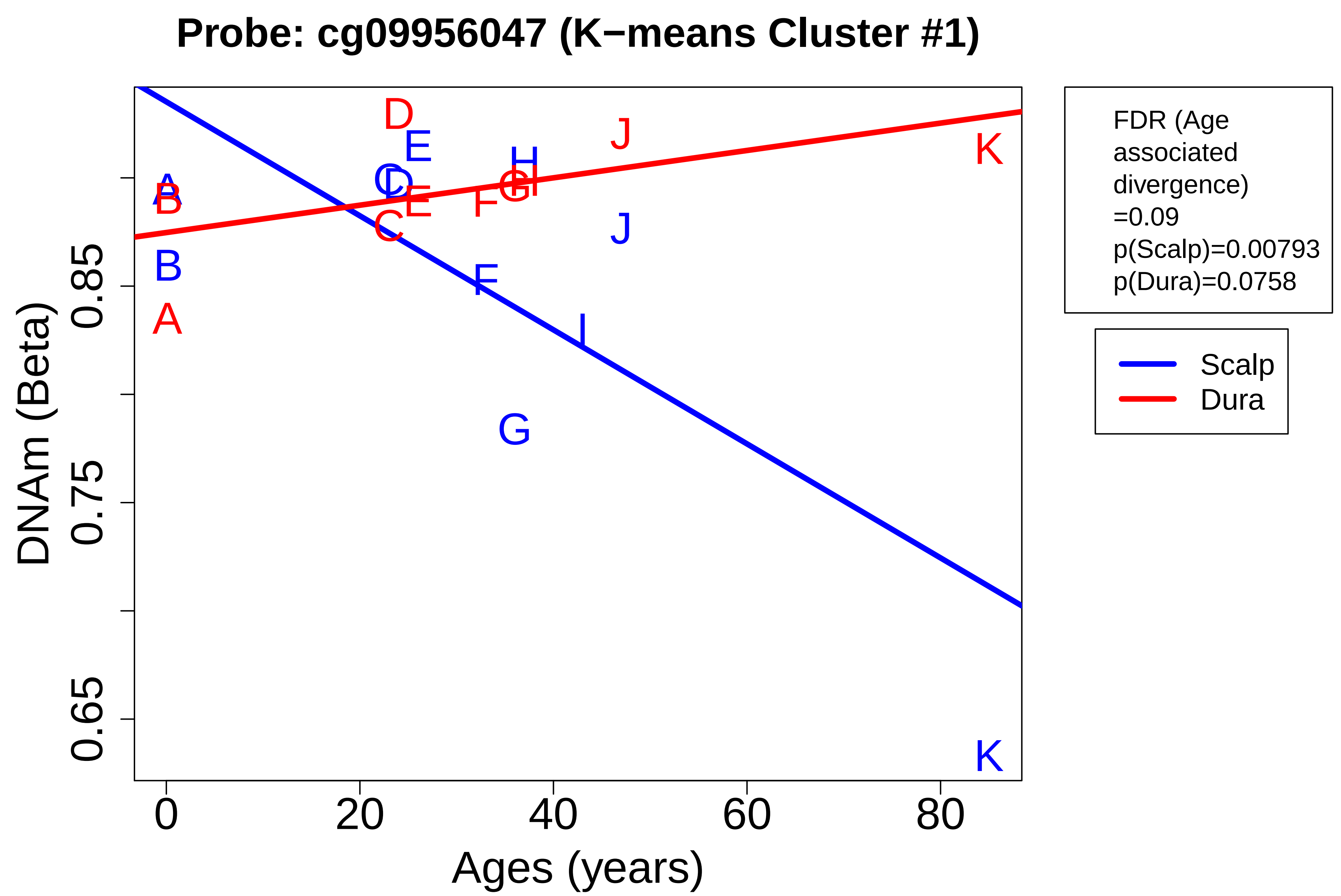

Probe: cg09956047 (K−means Cluster #1)
DNAm (Beta)
0.65
0.75
0.85
Ages (years)
0
20
40
60
80
FDR (Age associated divergence) =0.09
p(Scalp)=0.00793
p(Dura)=0.0758
Scalp
Dura

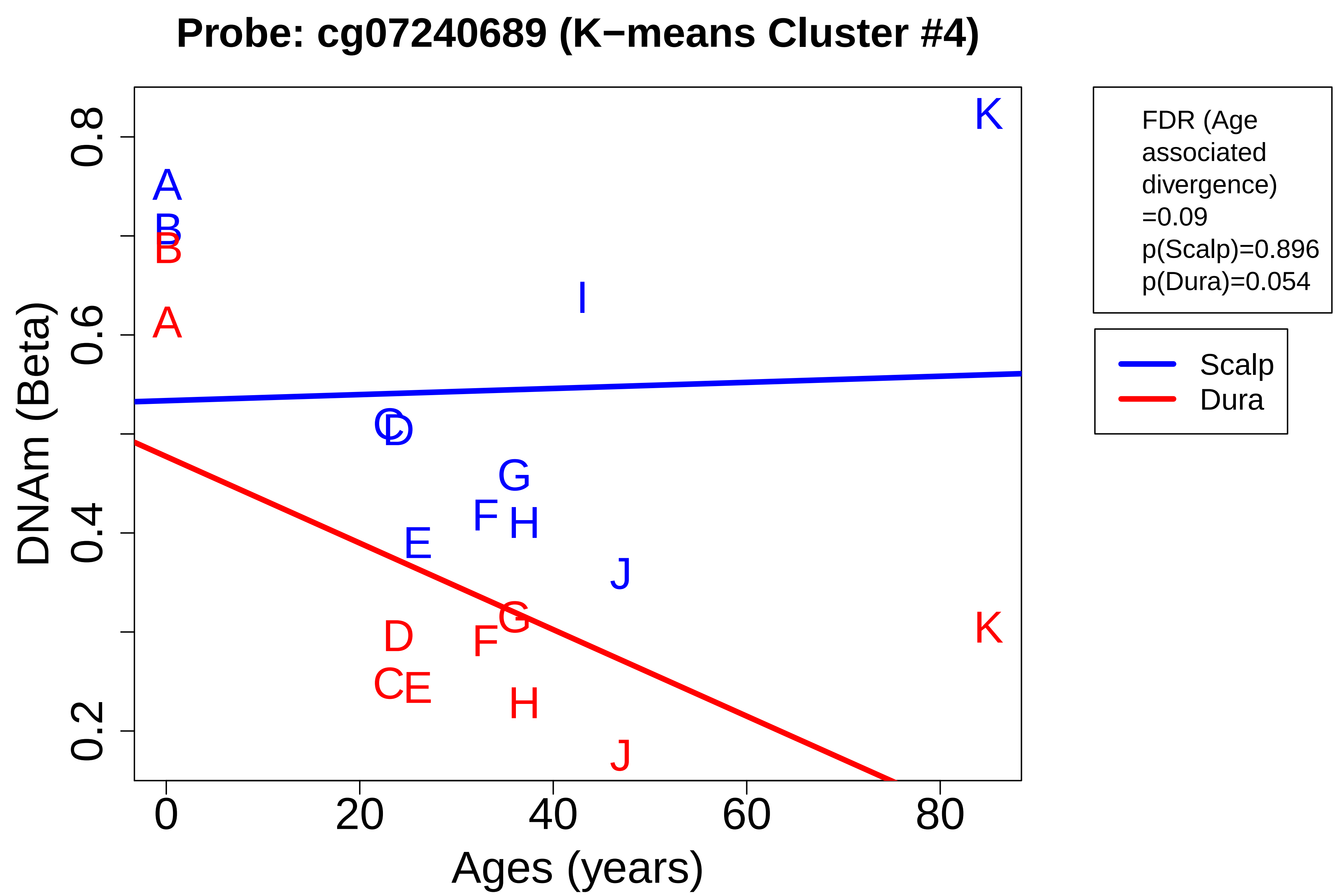

Probe: cg07240689 (K−means Cluster #4)
DNAm (Beta)
0.2
0.4
0.6
0.8
Ages (years)
0
20
40
60
80
A
B
C
D
E
F
G
H
I
J
K
FDR (Age associated divergence) =0.09
p(Scalp)=0.896
p(Dura)=0.054
Scalp
Dura

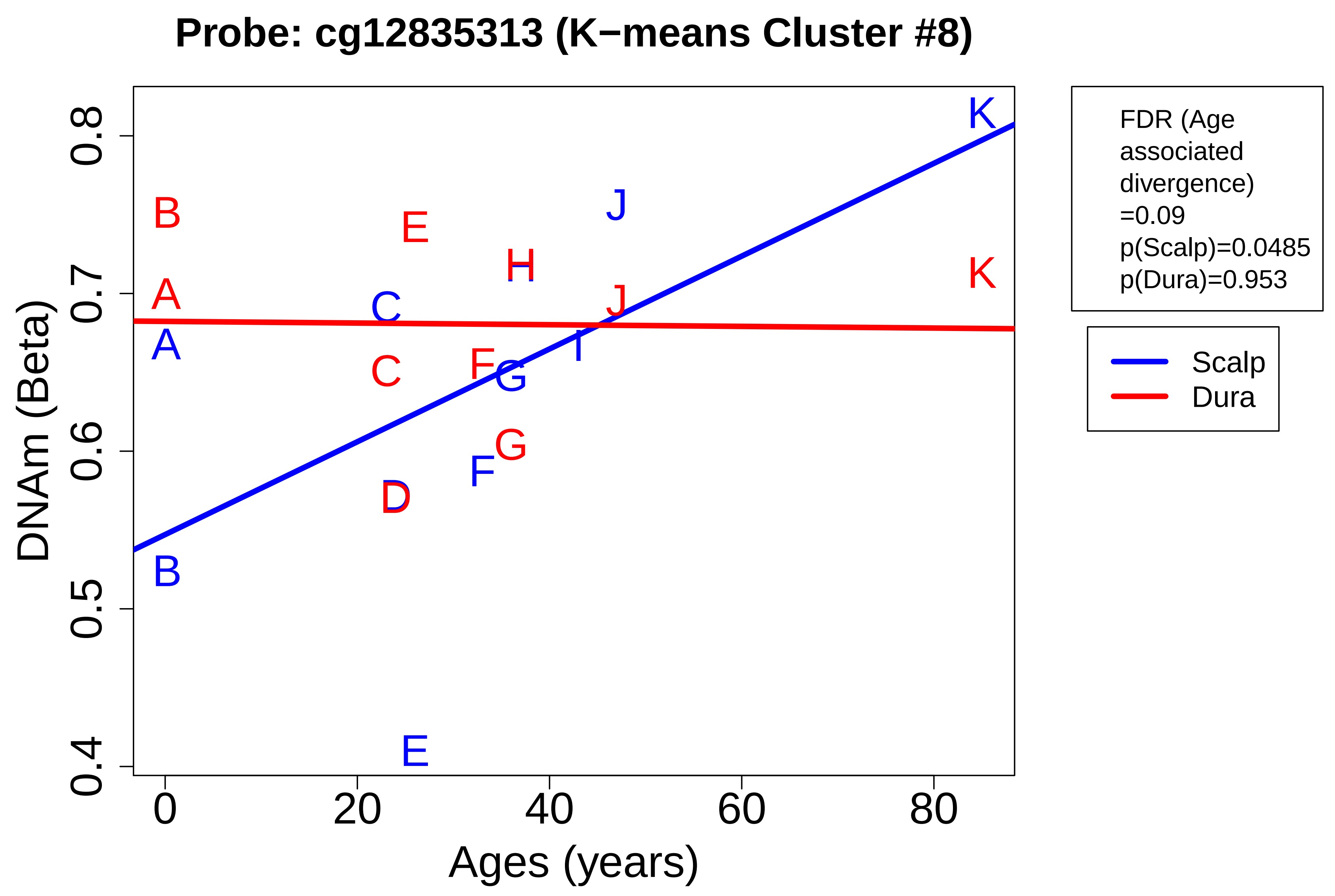

Probe: cg12835313 (K−means Cluster #8)
DNAm (Beta)
Ages (years)
0.4
0.5
0.6
0.7
0.8
0
20
40
60
80
FDR (Age associated divergence) =0.09
p(Scalp)=0.0485
p(Dura)=0.953
Scalp
Dura
A
B
C
D
E
F
G
H
I
J
K

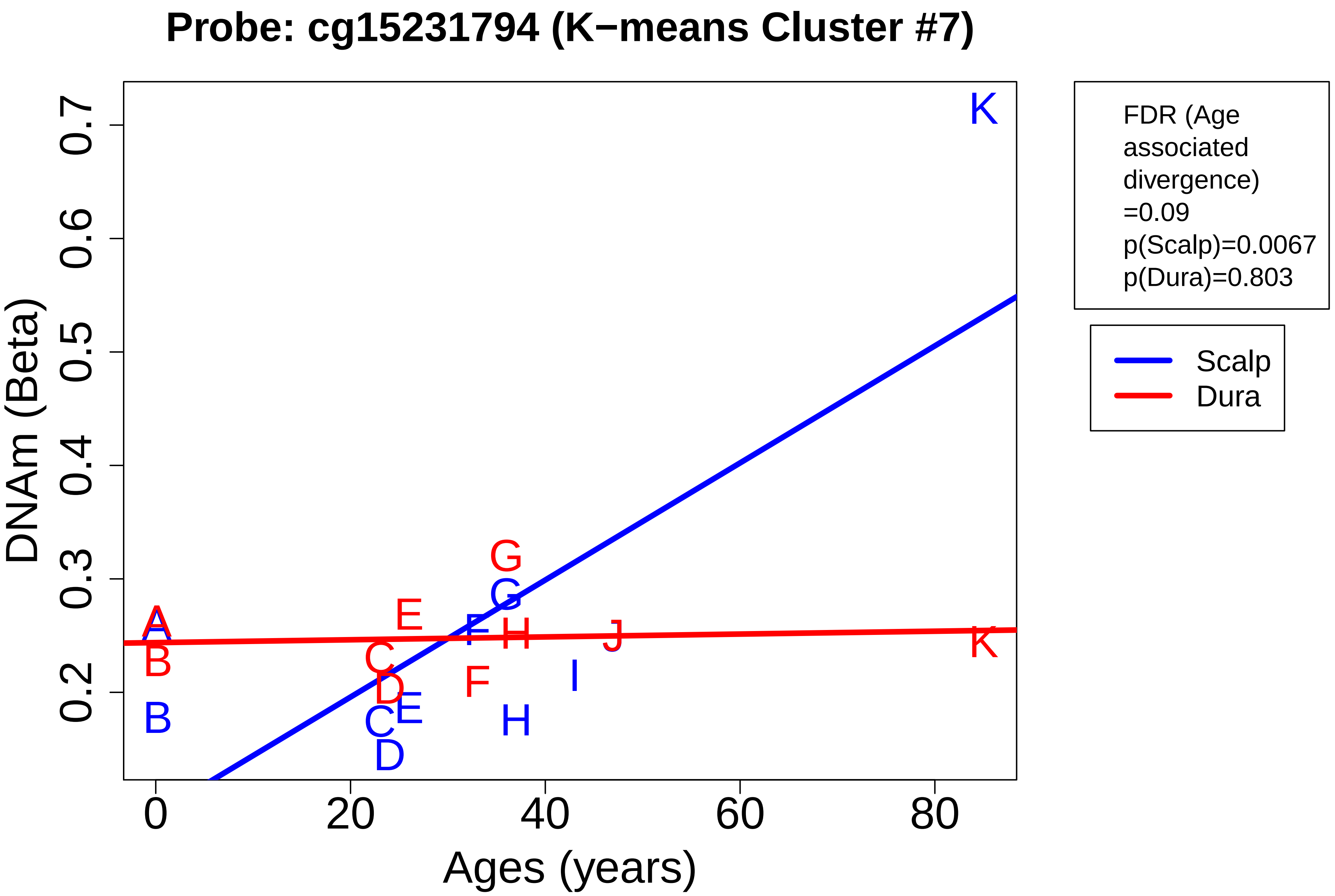

Probe: cg15231794 (K−means Cluster #7)
DNAm (Beta)
0.2
0.3
0.4
0.5
0.6
0.7
0
20
40
60
80
Ages (years)
FDR (Age associated divergence) =0.09
p(Scalp)=0.0067
p(Dura)=0.803
Scalp
Dura
A
B
C
D
E
F
G
H
I
J
K

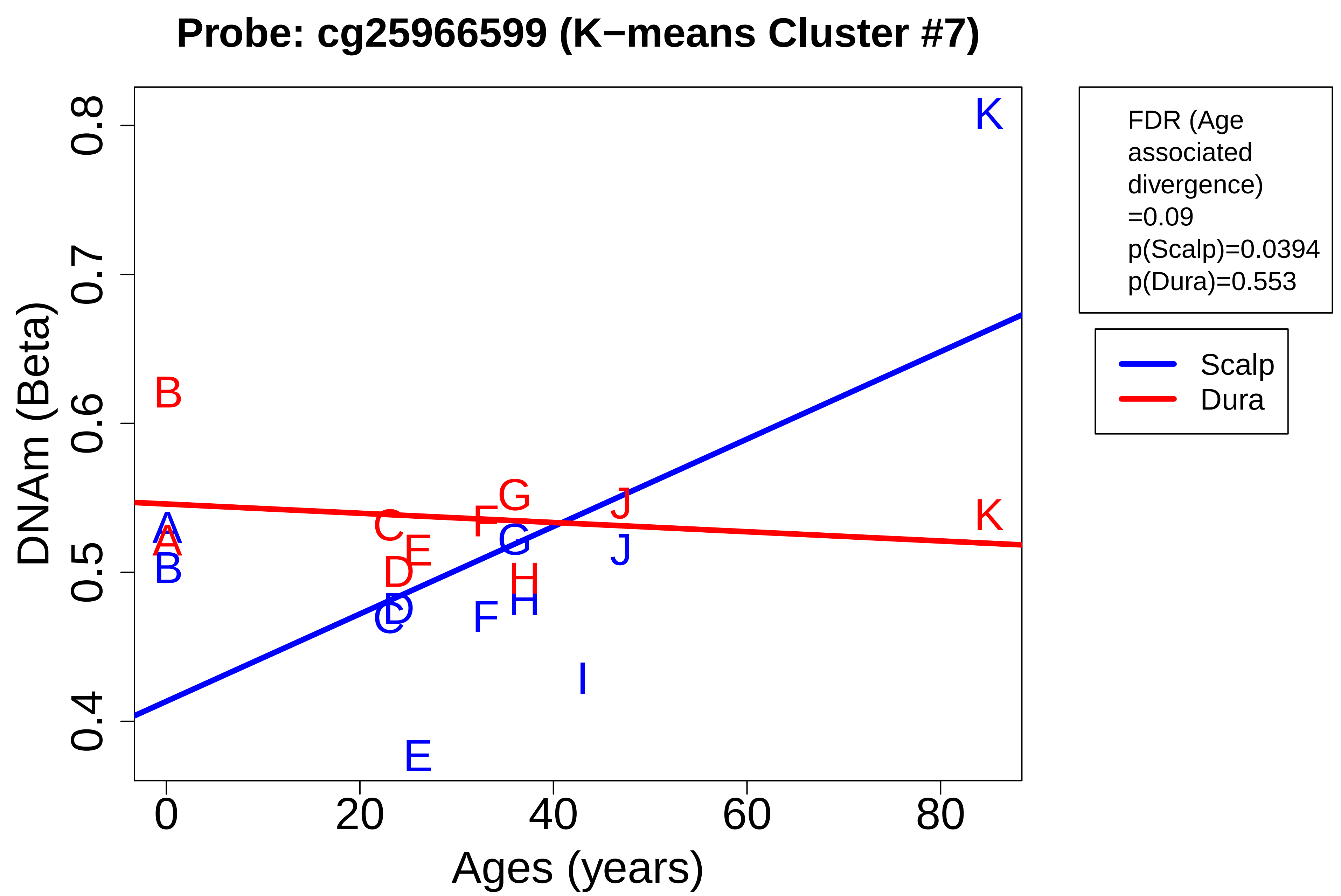
Probe: cg25966599 (K−means Cluster #7)
DNAm (Beta)
0.4
0.5
0.6
0.7
0.8
Ages (years)
0
20
40
60
80
FDR (Age associated divergence) =0.09
p(Scalp)=0.0394
p(Dura)=0.553
Scalp
Dura
A
B
C
D
E
F
G
H
I
J
K

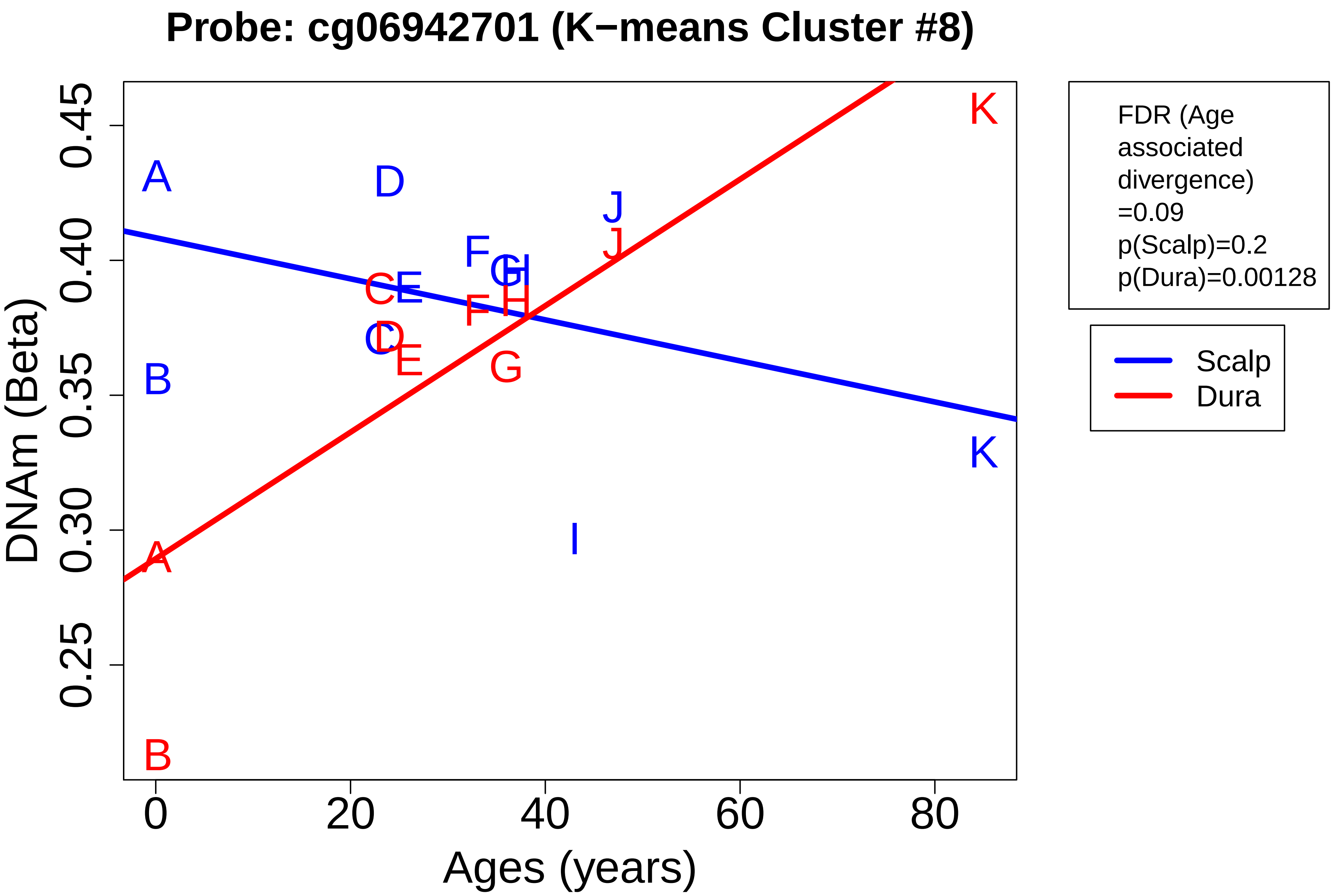

Probe: cg06942701 (K−means Cluster #8)
DNAm (Beta)
0.25
0.30
0.35
0.40
0.45
0
20
40
60
80
Ages (years)
FDR (Age associated divergence) =0.09
p(Scalp)=0.2
p(Dura)=0.00128
Scalp
Dura
A
B
C
D
E
F
G
H
I
J
K

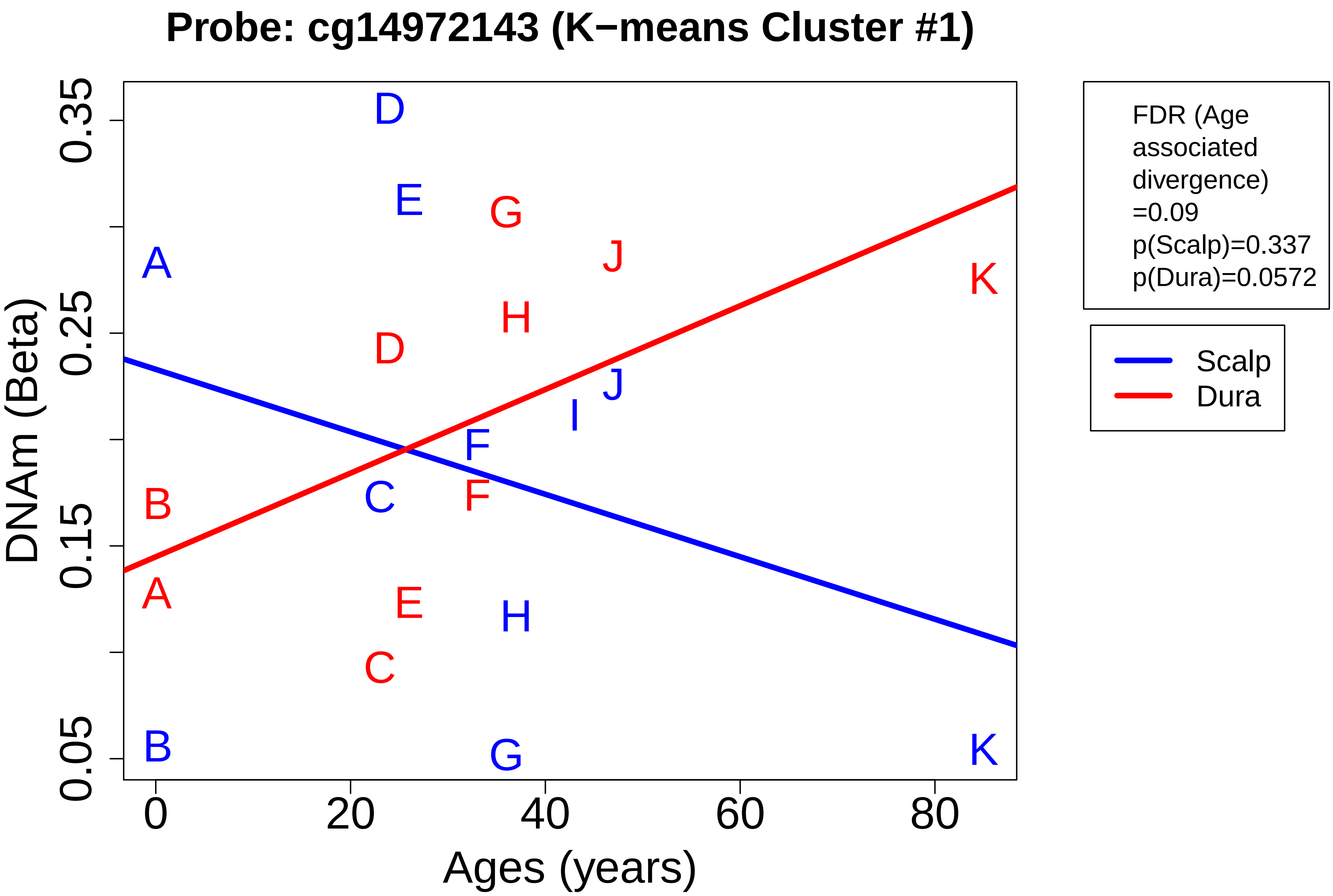

Probe: cg14972143 (K−means Cluster #1)
DNAm (Beta)
0.05
0.15
0.25
0.35
0
20
40
60
80
Ages (years)
A B C D E F G H I J K
A B C D E F G H J K
FDR (Age associated divergence) =0.09
p(Scalp)=0.337
p(Dura)=0.0572
Scalp
Dura

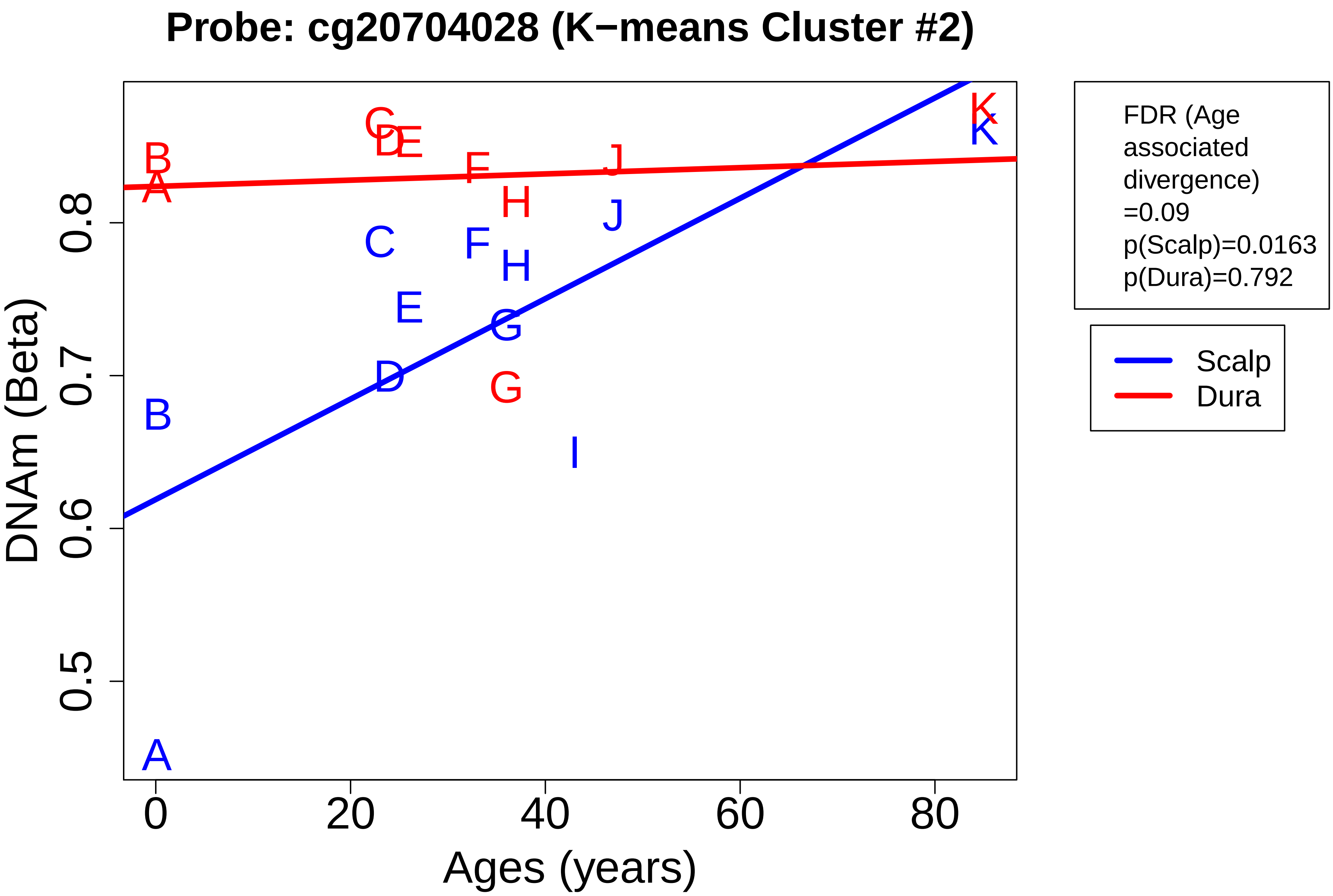

Probe: cg20704028 (K−means Cluster #2)
DNAm (Beta)
0.5
0.6
0.7
0.8
0
20
40
60
80
Ages (years)
A
B
C
D
E
F
G
H
I
J
K
FDR (Age associated divergence) =0.09
p(Scalp)=0.0163
p(Dura)=0.792
Scalp
Dura

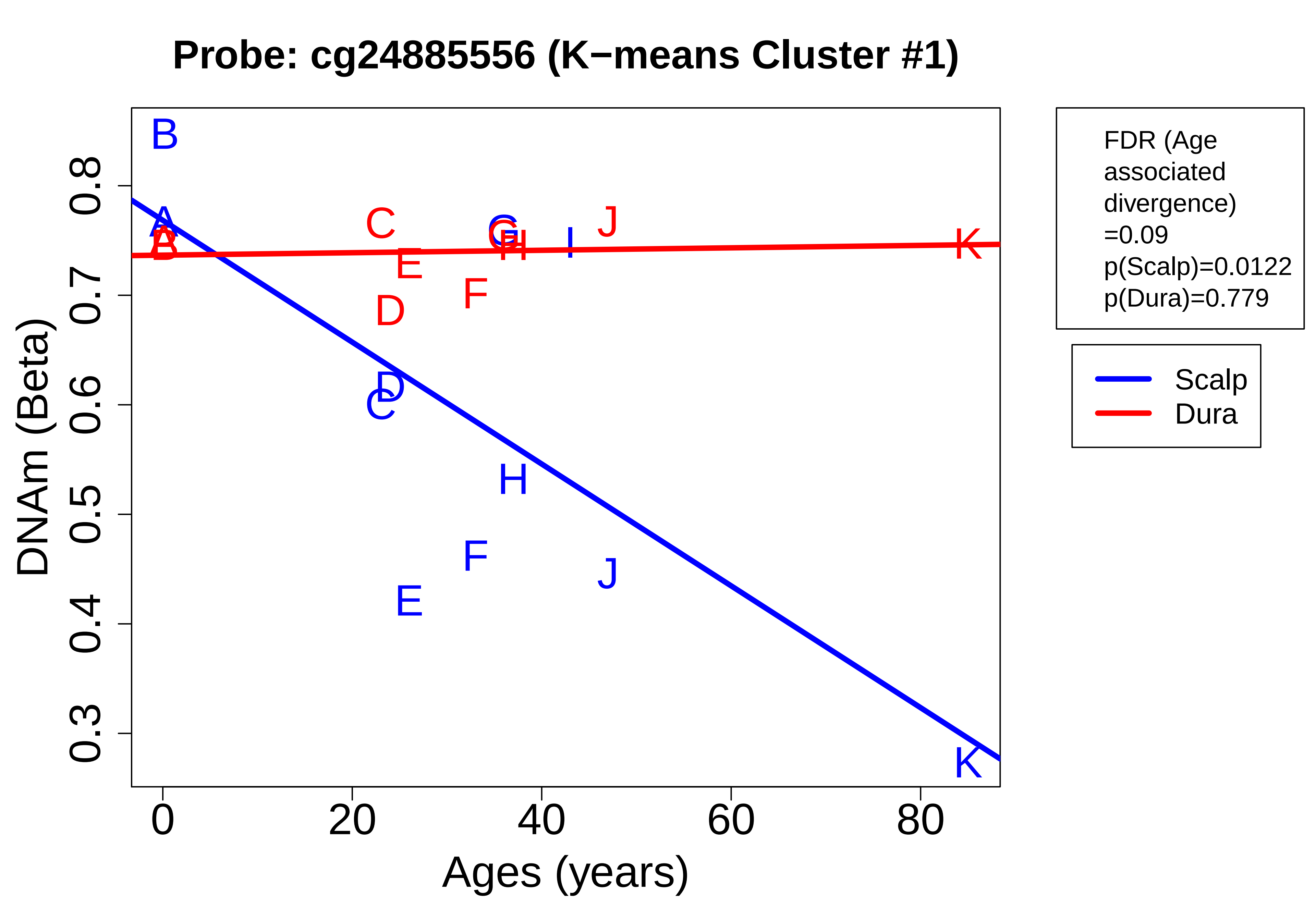

Probe: cg24885556 (K−means Cluster #1)
DNAm (Beta)
0.3
0.4
0.5
0.6
0.7
0.8
0
20
40
60
80
Ages (years)
FDR (Age associated divergence) =0.09
p(Scalp)=0.0122
p(Dura)=0.779
Scalp
Dura

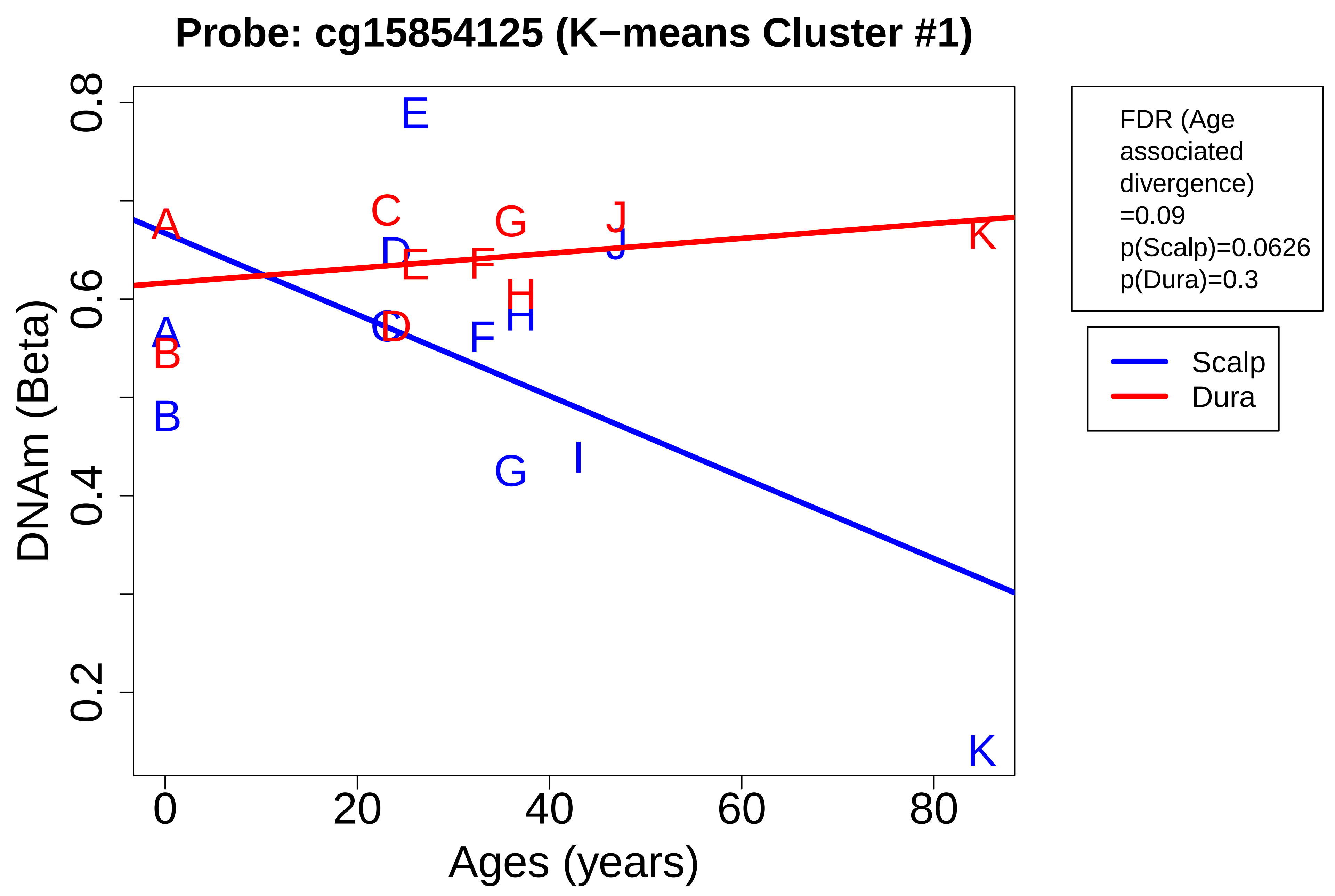

Probe: cg15854125 (K−means Cluster #1)
DNAm (Beta)
0.2
0.4
0.6
0.8
Ages (years)
0
20
40
60
80
FDR (Age associated divergence) =0.09
p(Scalp)=0.0626
p(Dura)=0.3
Scalp
Dura
A
B
C
D
E
F
G
H
I
J
K

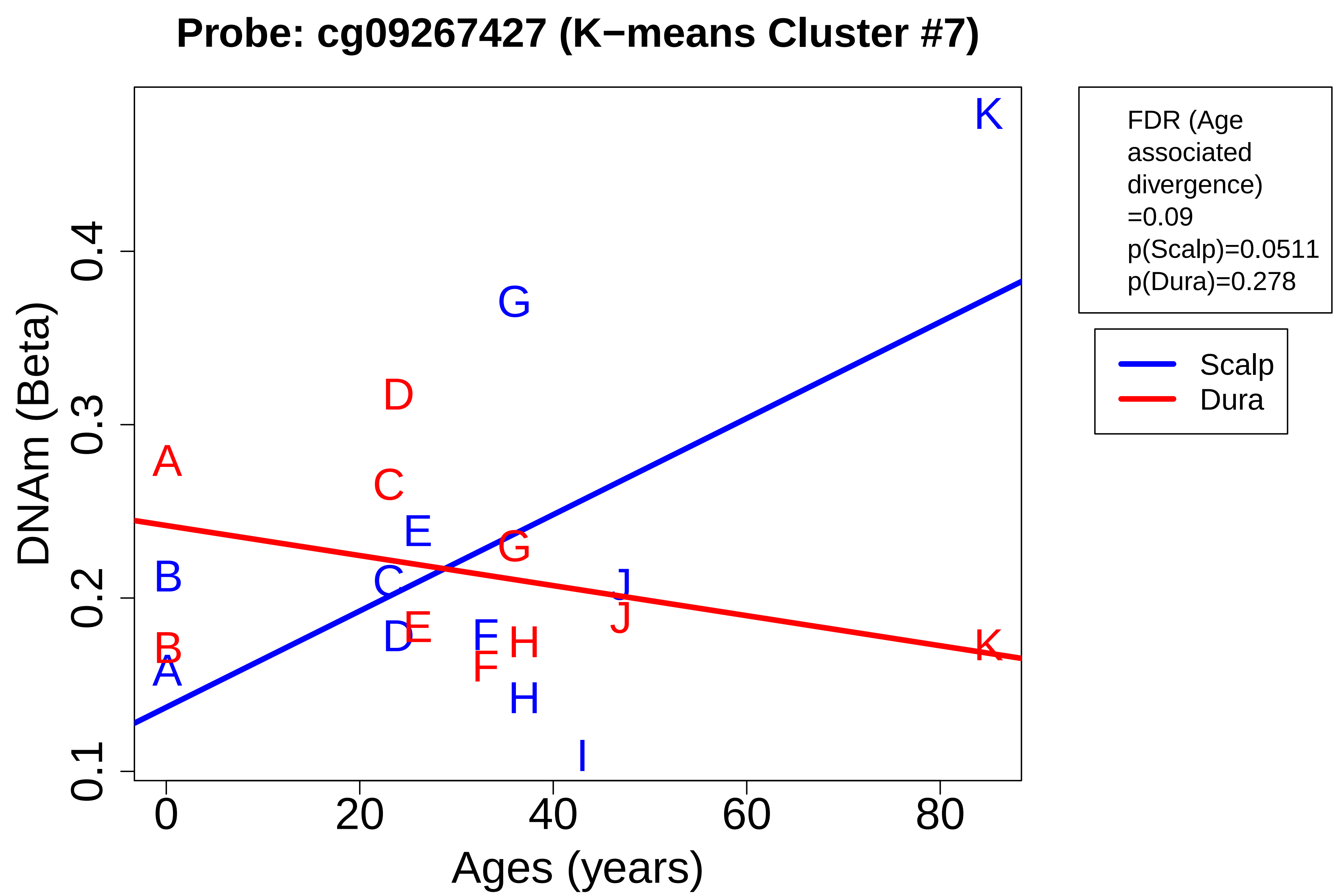

Probe: cg09267427 (K−means Cluster #7)
DNAm (Beta)
0.1
0.2
0.3
0.4
0
20
40
60
80
Ages (years)
FDR (Age associated divergence) =0.09
p(Scalp)=0.0511
p(Dura)=0.278
Scalp
Dura
A
B
C
D
E
F
G
H
I
J
K

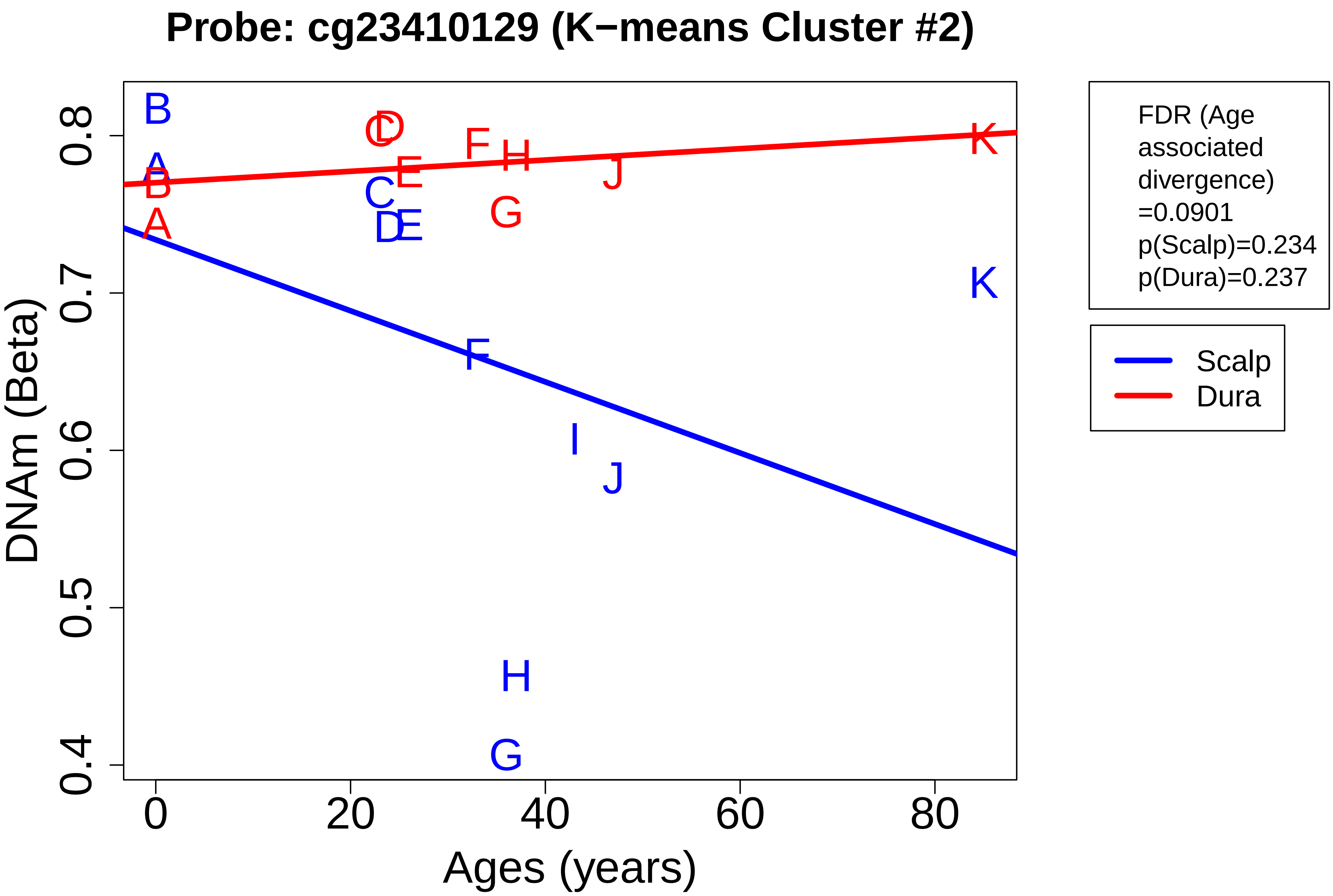

Probe: cg23410129 (K−means Cluster #2)
DNAm (Beta)
0.4
0.5
0.6
0.7
0.8
0
20
40
60
80
Ages (years)
FDR (Age associated divergence) =0.0901
p(Scalp)=0.234
p(Dura)=0.237
Scalp
Dura

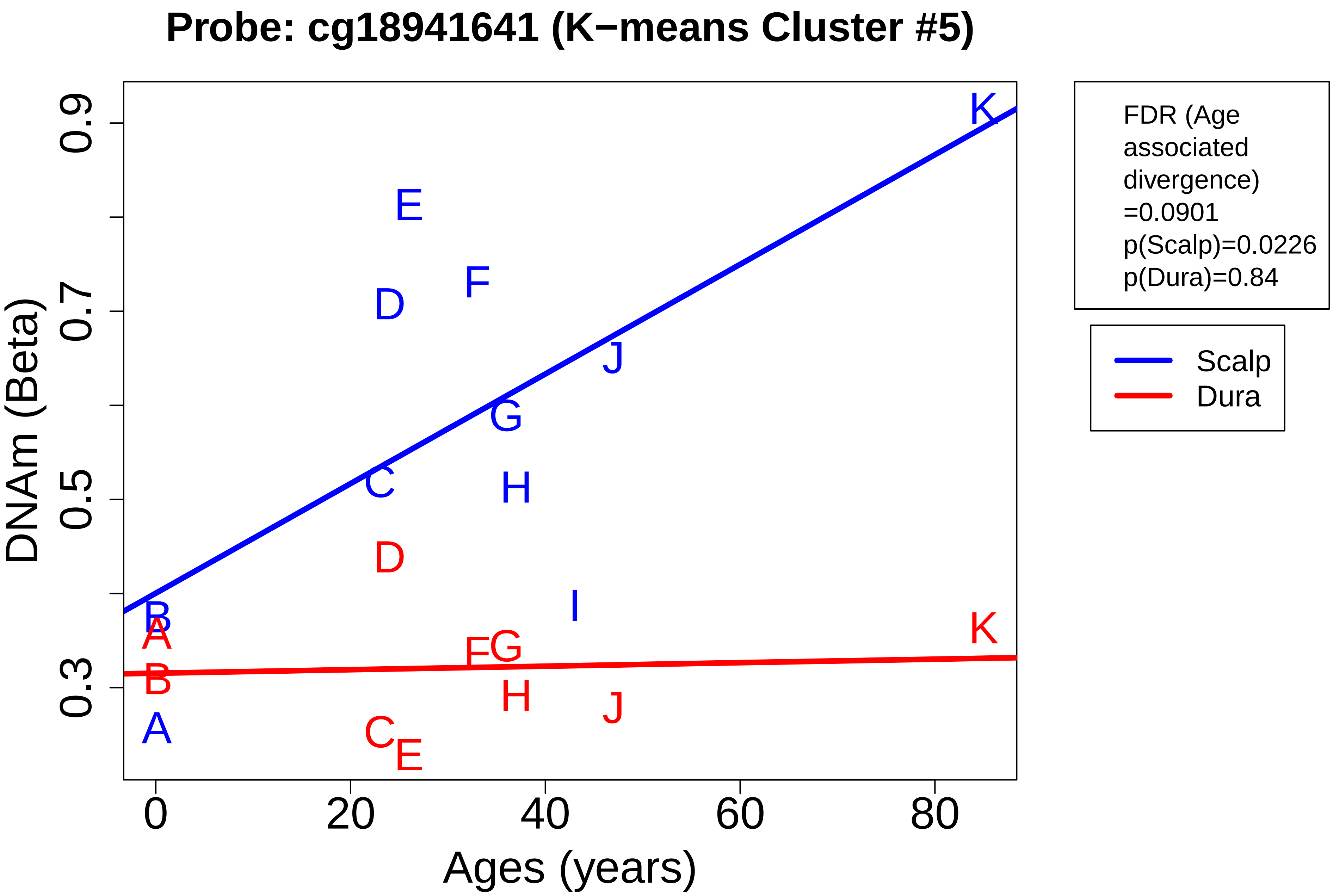
Probe: cg18941641 (K−means Cluster #5)
DNAm (Beta)
0.3
0.5
0.7
0.9
0
20
40
60
80
Ages (years)
FDR (Age associated divergence) =0.0901
p(Scalp)=0.0226
p(Dura)=0.84
Scalp
Dura

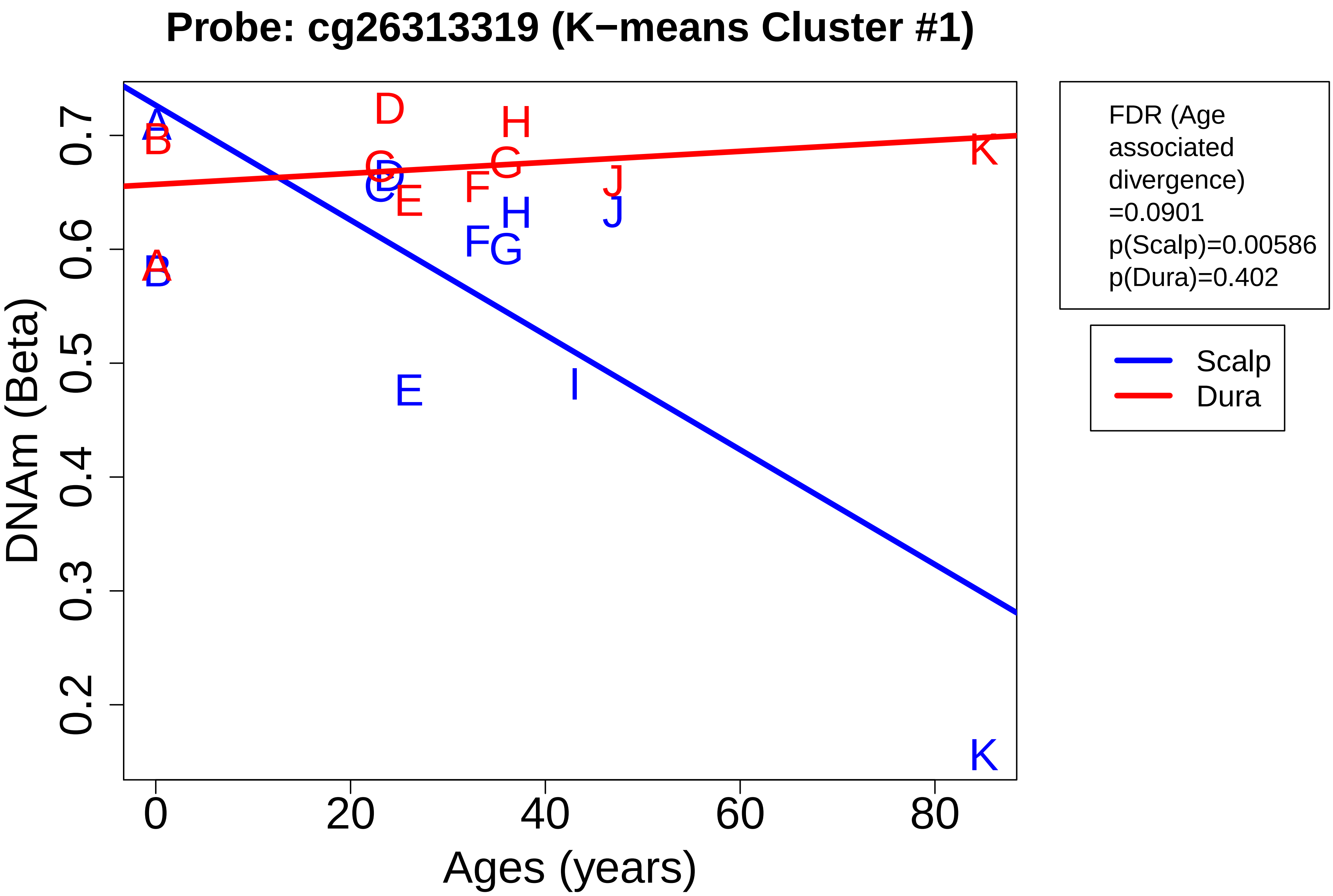

Probe: cg26313319 (K−means Cluster #1)
DNAm (Beta)
0.2
0.3
0.4
0.5
0.6
0.7
Ages (years)
0
20
40
60
80
FDR (Age associated divergence) =0.0901
p(Scalp)=0.00586
p(Dura)=0.402
Scalp
Dura

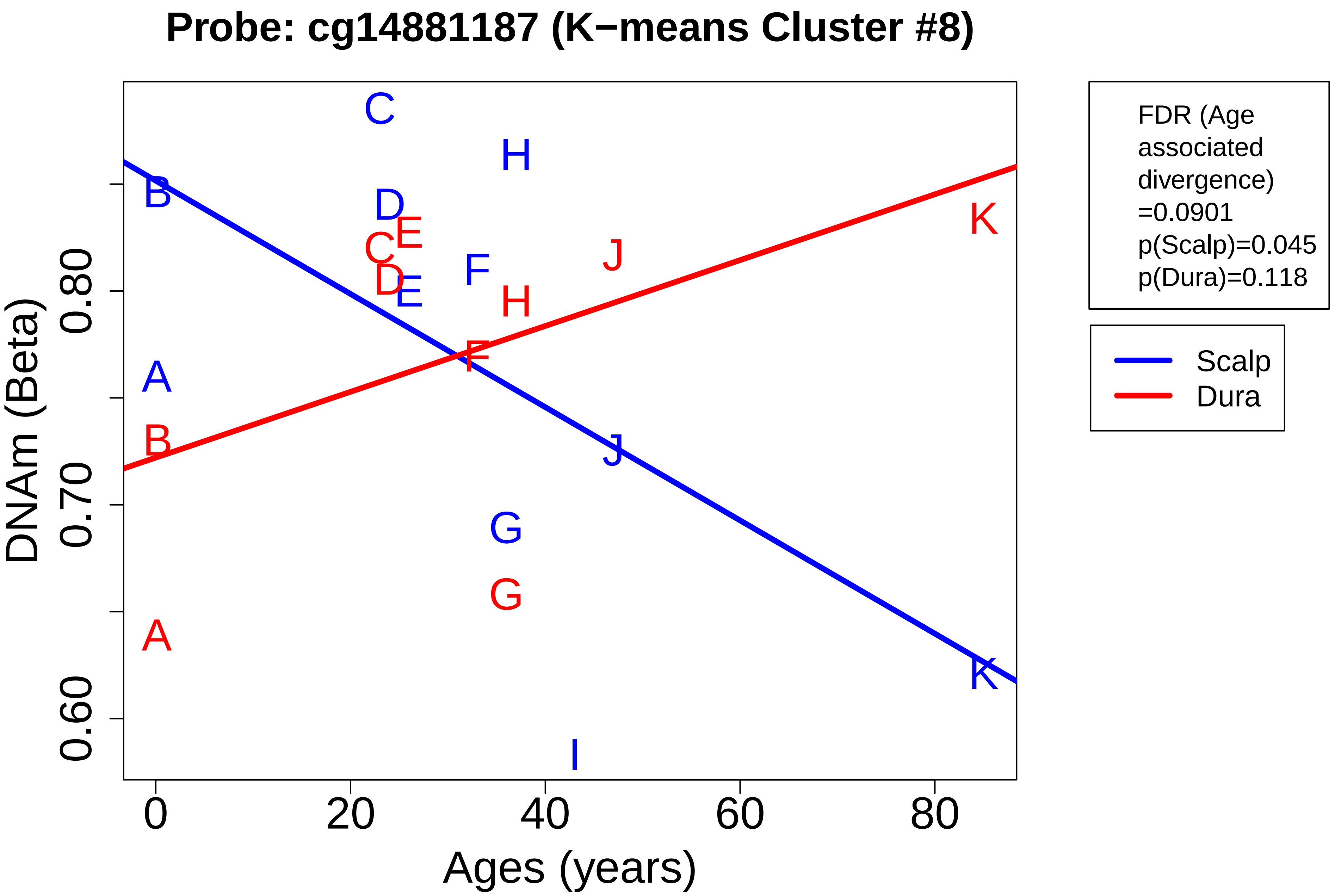
Probe: cg14881187 (K−means Cluster #8)
FDR (Age associated divergence) =0.0901
p(Scalp)=0.045
p(Dura)=0.118
Scalp
Dura
DNAm (Beta)
0.60
0.70
0.80
Ages (years)
0
20
40
60
80
A
B
C
D
E
F
G
H
I
J
K

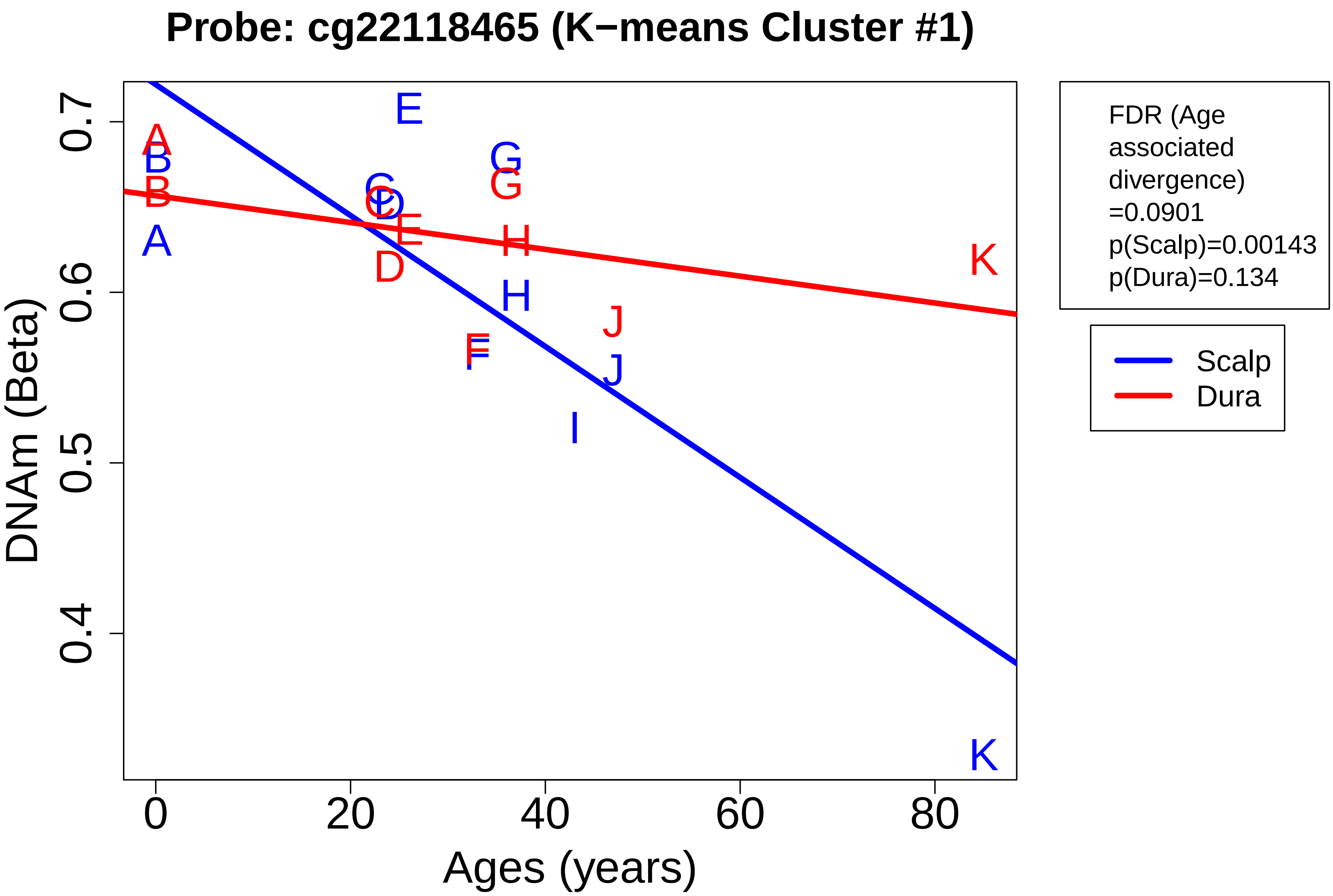

Probe: cg22118465 (K−means Cluster #1)
DNAm (Beta)
0.4
0.5
0.6
0.7
0
20
40
60
80
Ages (years)
A
B
C
D
E
F
G
H
I
J
K
FDR (Age associated divergence) =0.0901
p(Scalp)=0.00143
p(Dura)=0.134
Scalp
Dura

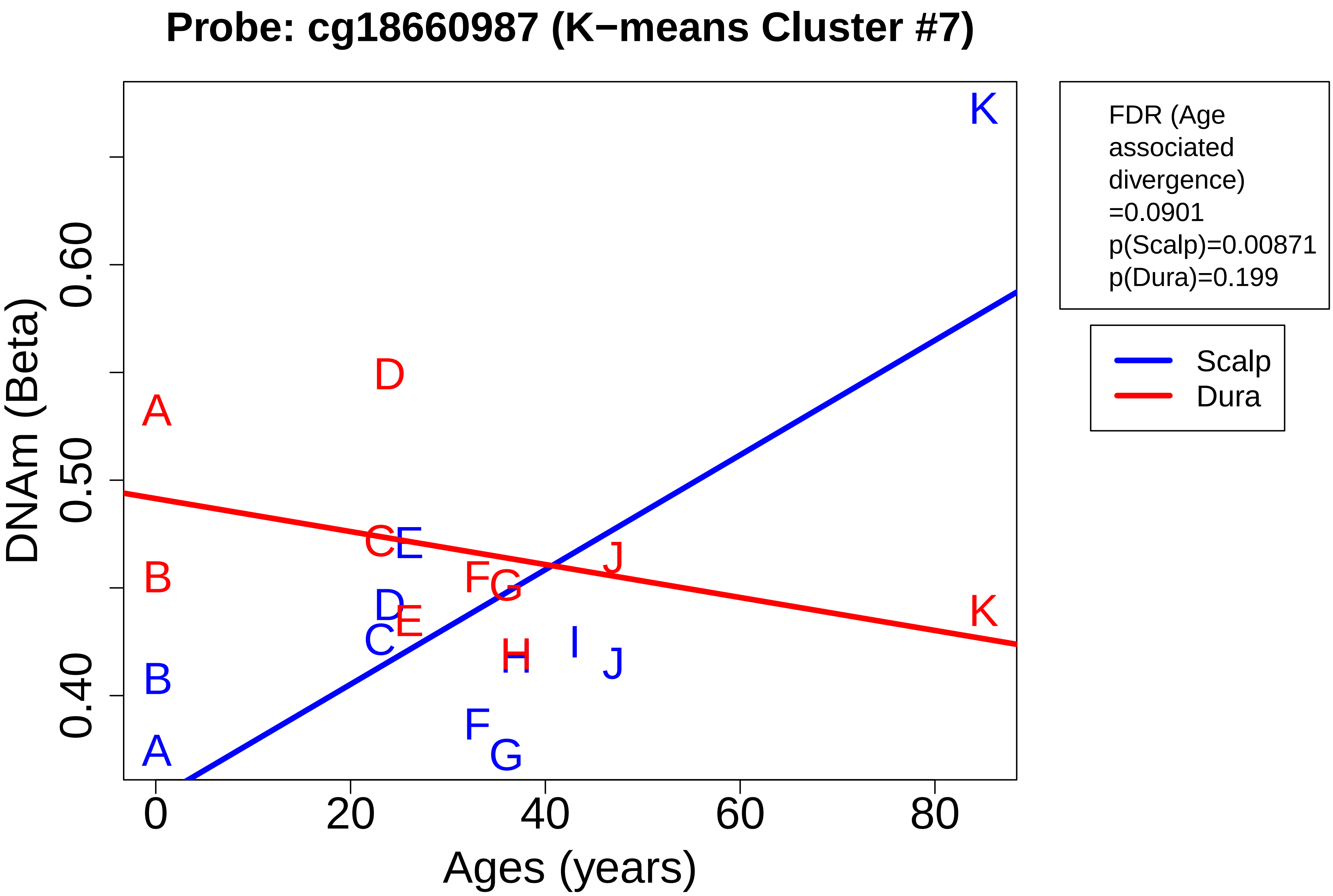

Probe: cg18660987 (K−means Cluster #7)
FDR (Age associated divergence) =0.0901
p(Scalp)=0.00871
p(Dura)=0.199
Scalp
Dura
DNAm (Beta)
0.40
0.50
0.60
Ages (years)
0
20
40
60
80
A
B
C
D
E
F
G
H
I
J
K

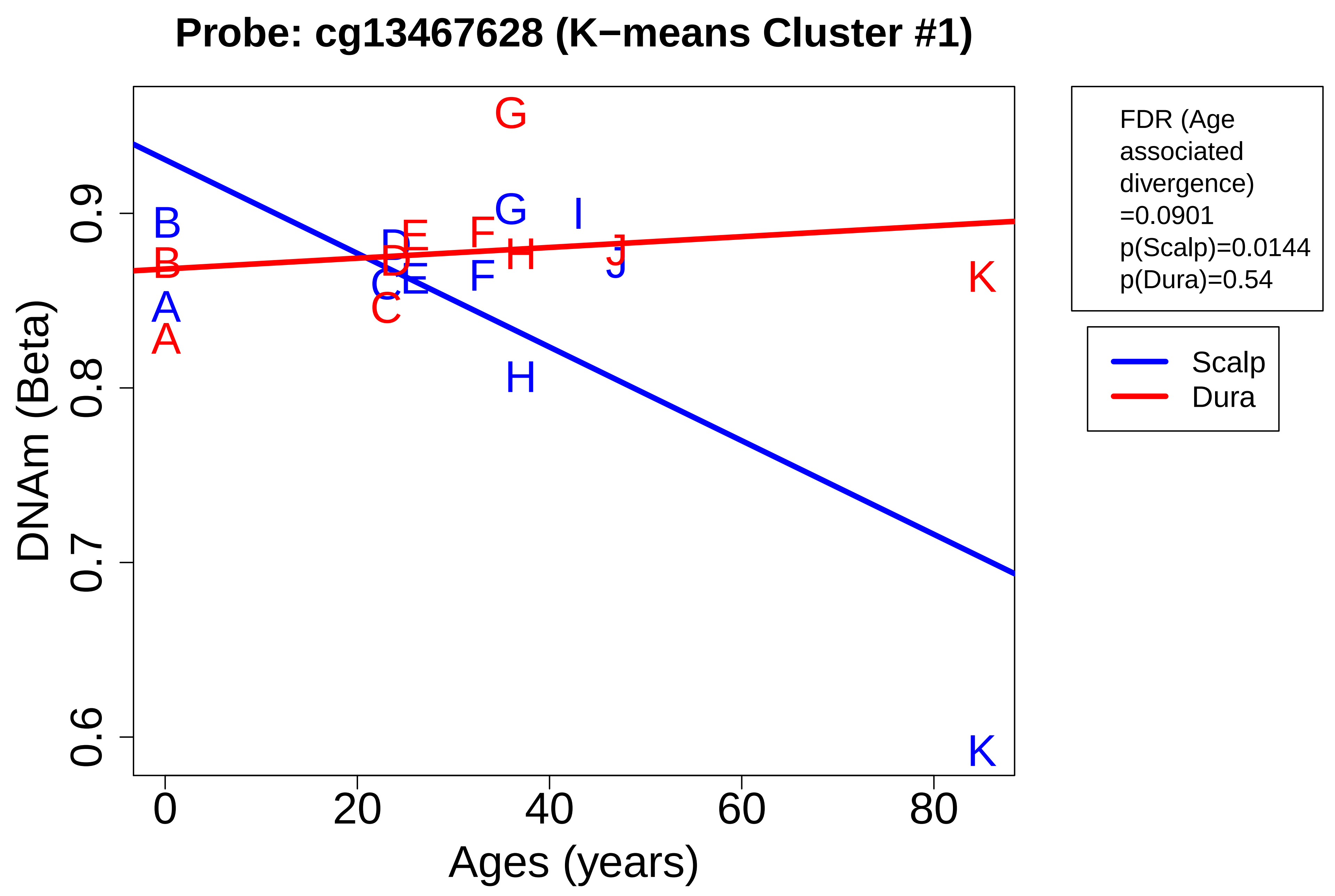

Probe: cg13467628 (K−means Cluster #1)
DNAm (Beta)
0.6
0.7
0.8
0.9
0
20
40
60
80
Ages (years)
FDR (Age associated divergence) =0.0901
p(Scalp)=0.0144
p(Dura)=0.54
Scalp
Dura

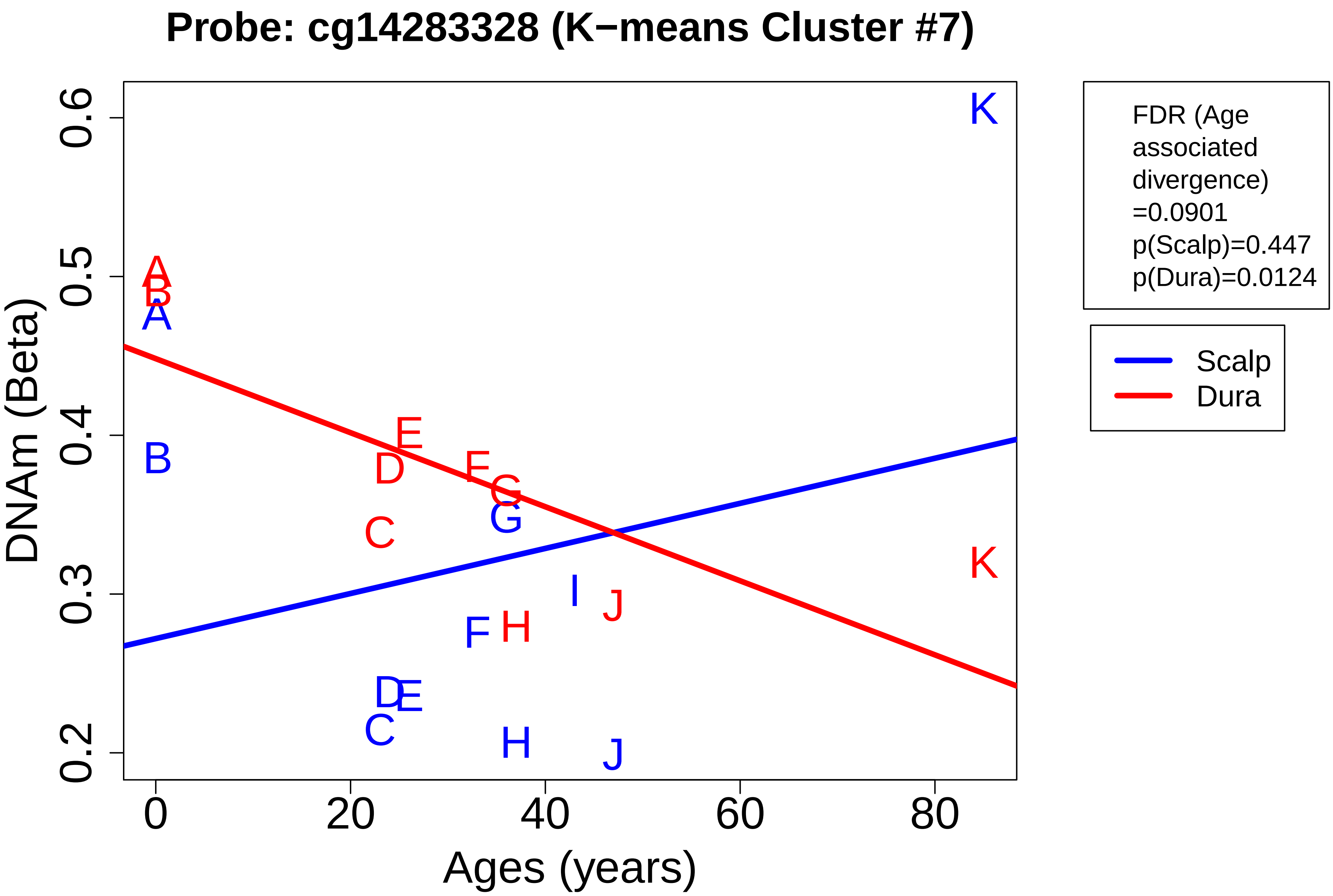

Probe: cg14283328 (K−means Cluster #7)
DNAm (Beta)
0.2
0.3
0.4
0.5
0.6
0
20
40
60
80
Ages (years)
A
B
C
D
E
F
G
H
I
J
K
FDR (Age associated divergence) =0.0901
p(Scalp)=0.447
p(Dura)=0.0124
Scalp
Dura

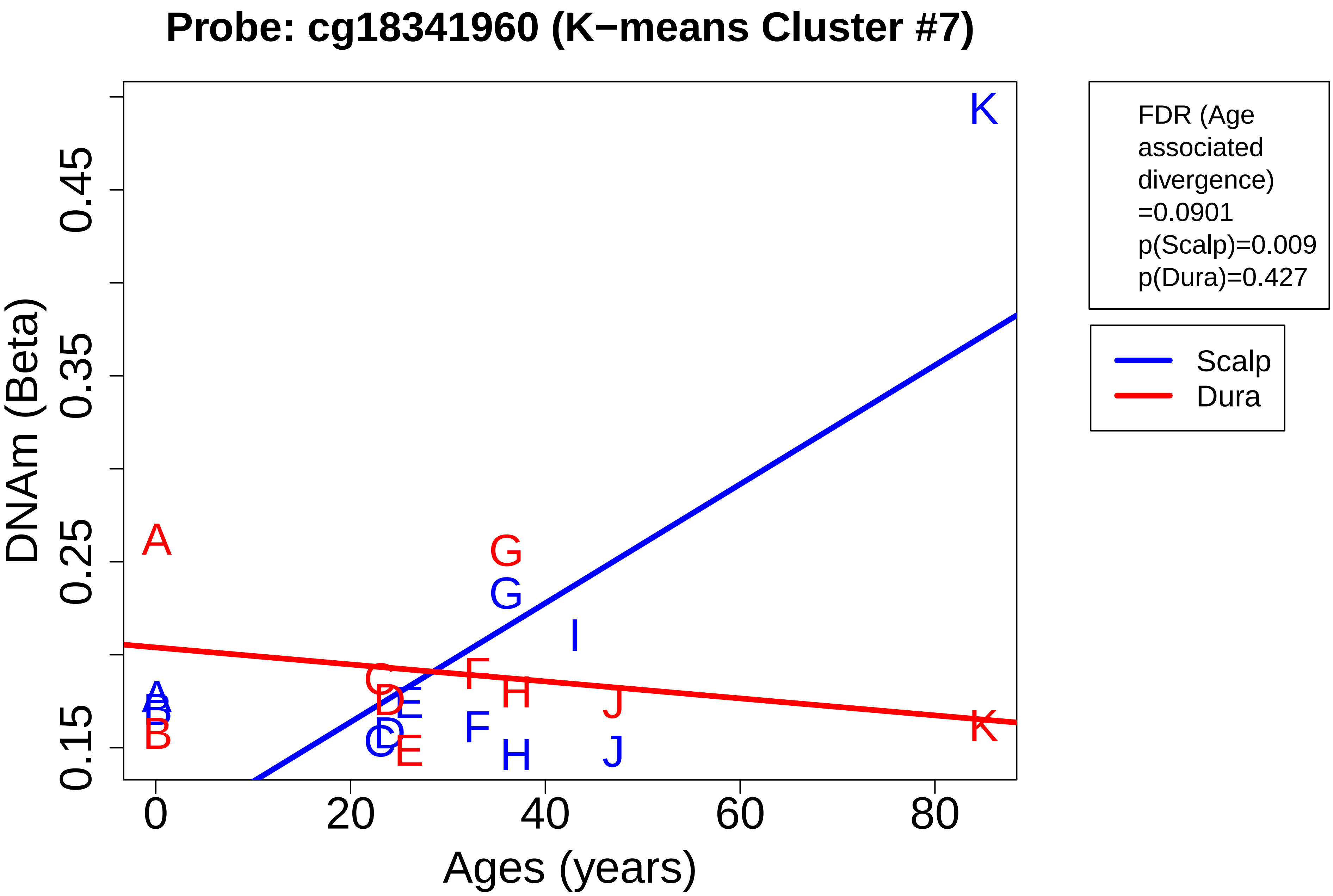

Probe: cg18341960 (K−means Cluster #7)
DNAm (Beta)
0.15
0.25
0.35
0.45
0
20
40
60
80
Ages (years)
FDR (Age associated divergence) =0.0901
p(Scalp)=0.009
p(Dura)=0.427
Scalp
Dura

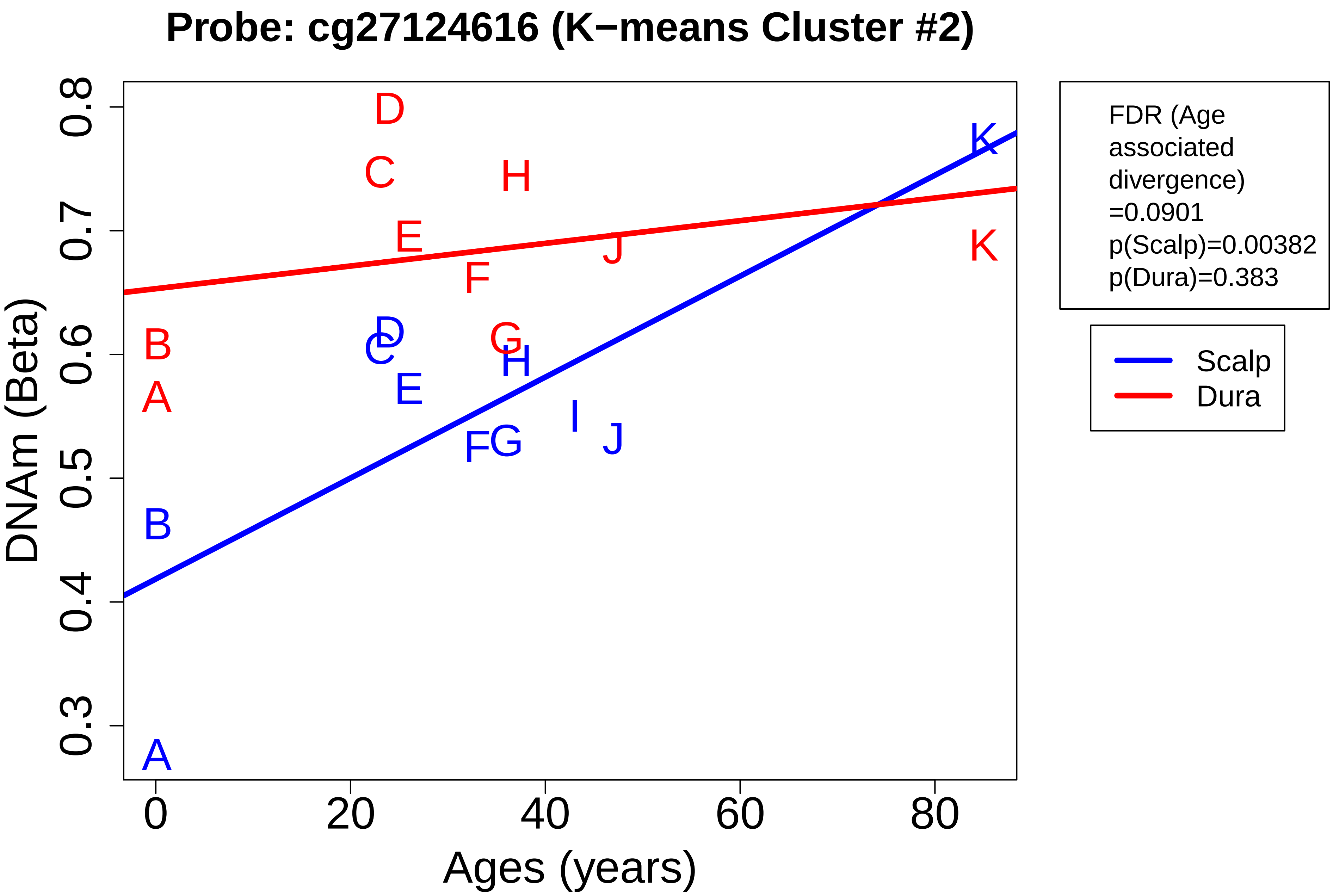

Probe: cg27124616 (K−means Cluster #2)
DNAm (Beta)
0.3
0.4
0.5
0.6
0.7
0.8
0
20
40
60
80
Ages (years)
FDR (Age associated divergence) =0.0901
p(Scalp)=0.00382
p(Dura)=0.383
Scalp
Dura
A
B
C
D
E
F
G
H
I
J
K

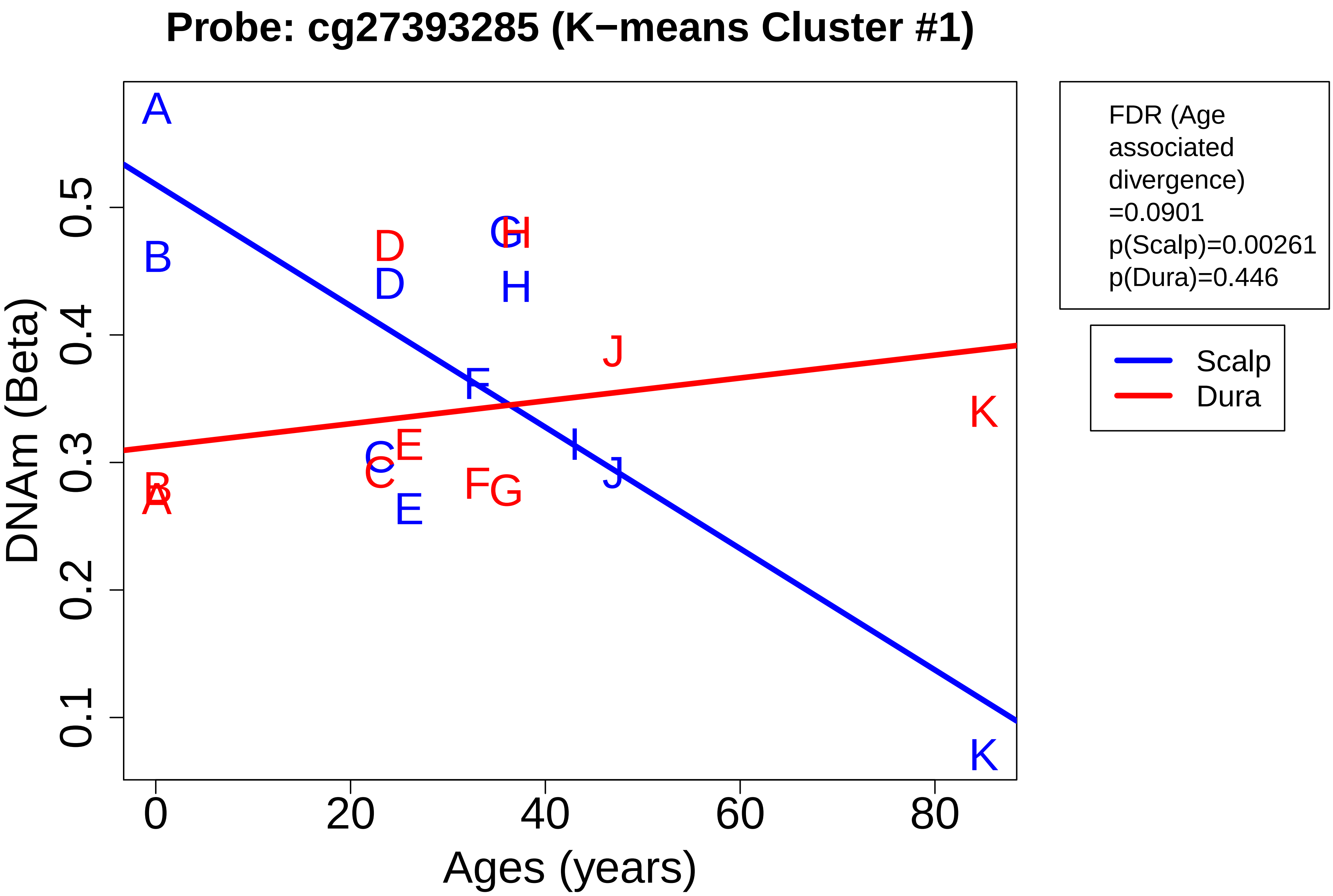

Probe: cg27393285 (K−means Cluster #1)
FDR (Age associated divergence) =0.0901
p(Scalp)=0.00261
p(Dura)=0.446
Scalp
Dura
DNAm (Beta)
Ages (years)
0.1
0.2
0.3
0.4
0.5
0
20
40
60
80

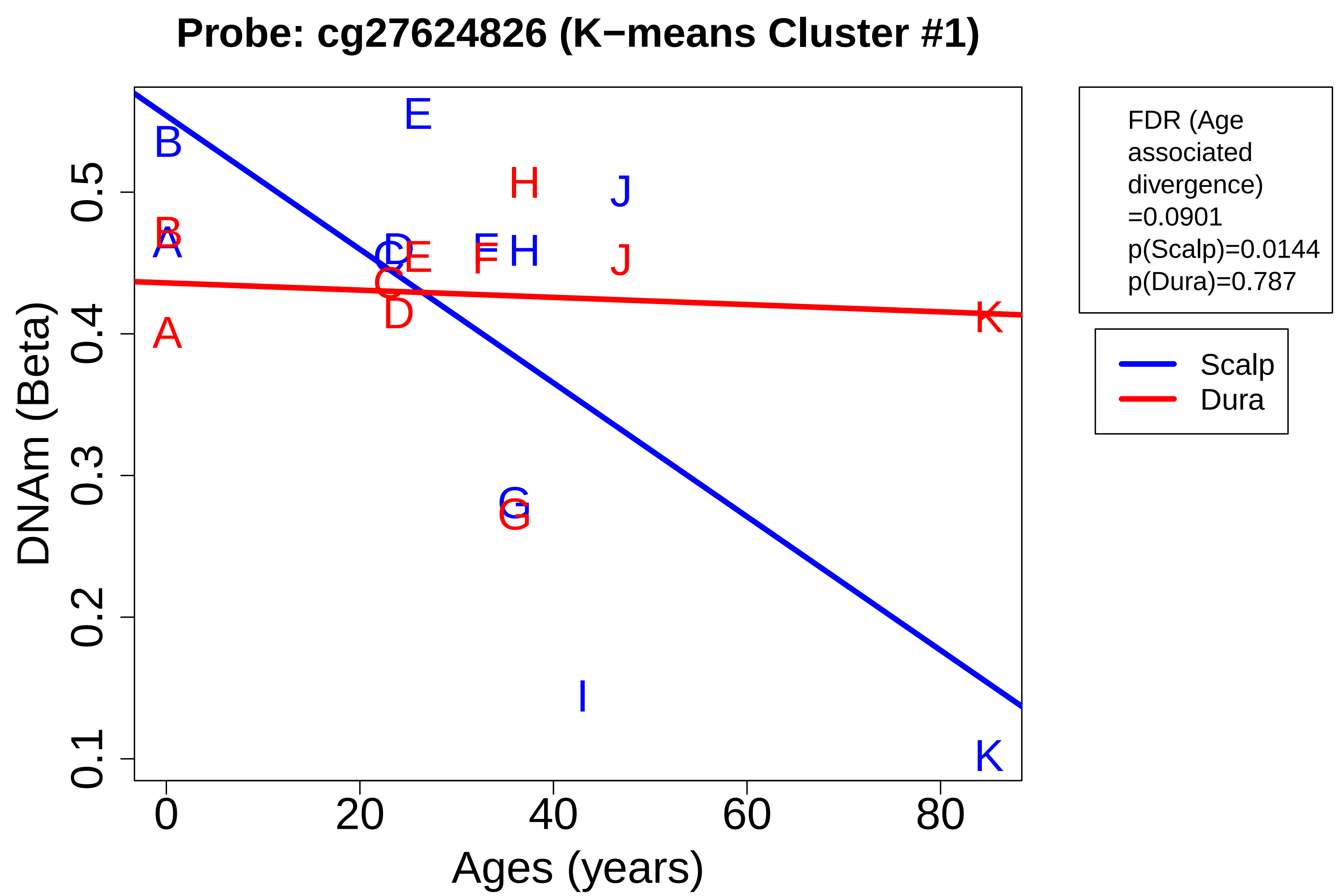

Probe: cg27624826 (K−means Cluster #1)
DNAm (Beta)
0.1
0.2
0.3
0.4
0.5
0
20
40
60
80
Ages (years)
FDR (Age associated divergence) =0.0901
p(Scalp)=0.0144
p(Dura)=0.787
Scalp
Dura

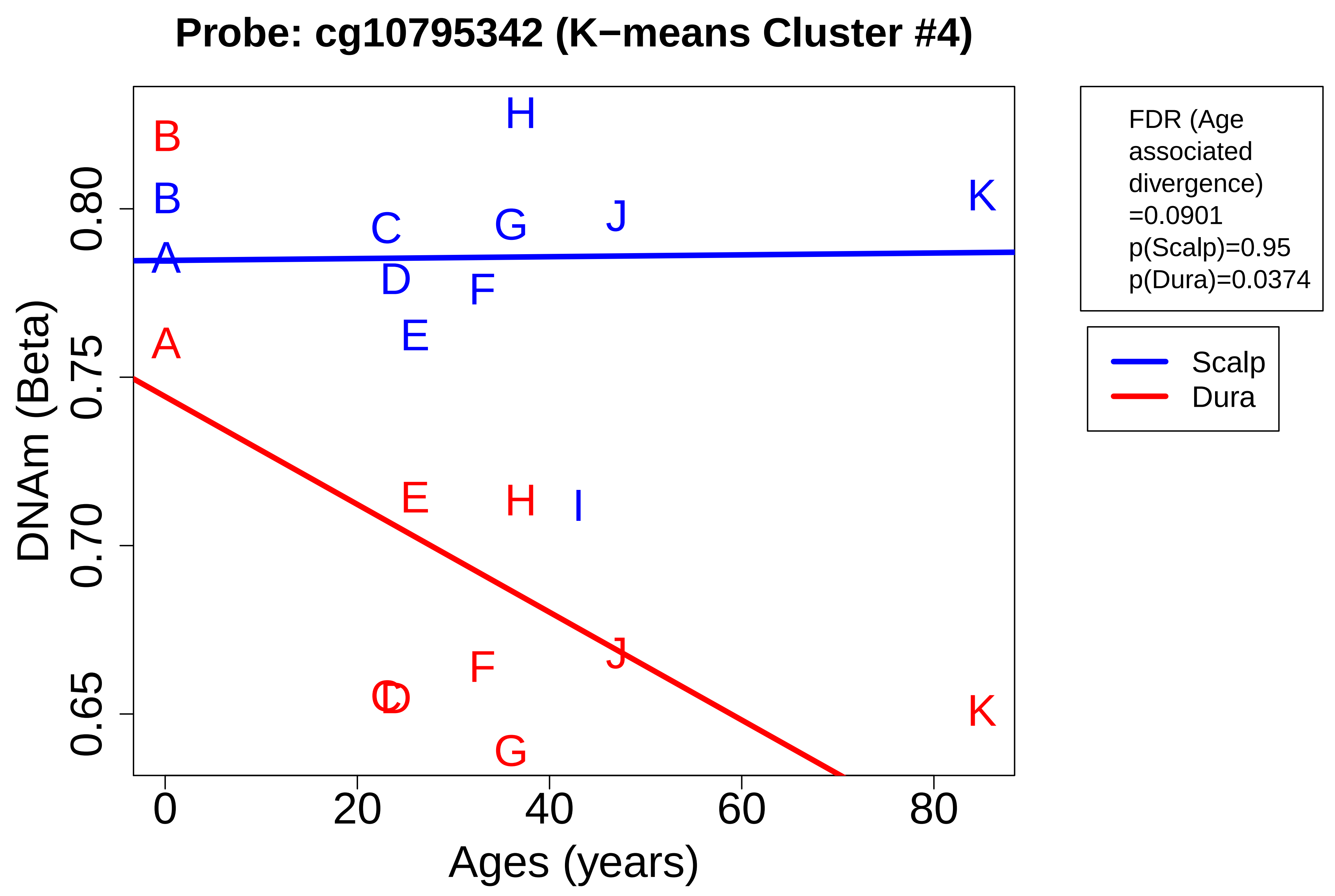

Probe: cg10795342 (K−means Cluster #4)
DNAm (Beta)
0.65
0.70
0.75
0.80
Ages (years)
0
20
40
60
80
FDR (Age associated divergence) =0.0901
p(Scalp)=0.95
p(Dura)=0.0374
Scalp
Dura
A
B
C
D
E
F
G
H
I
J
K

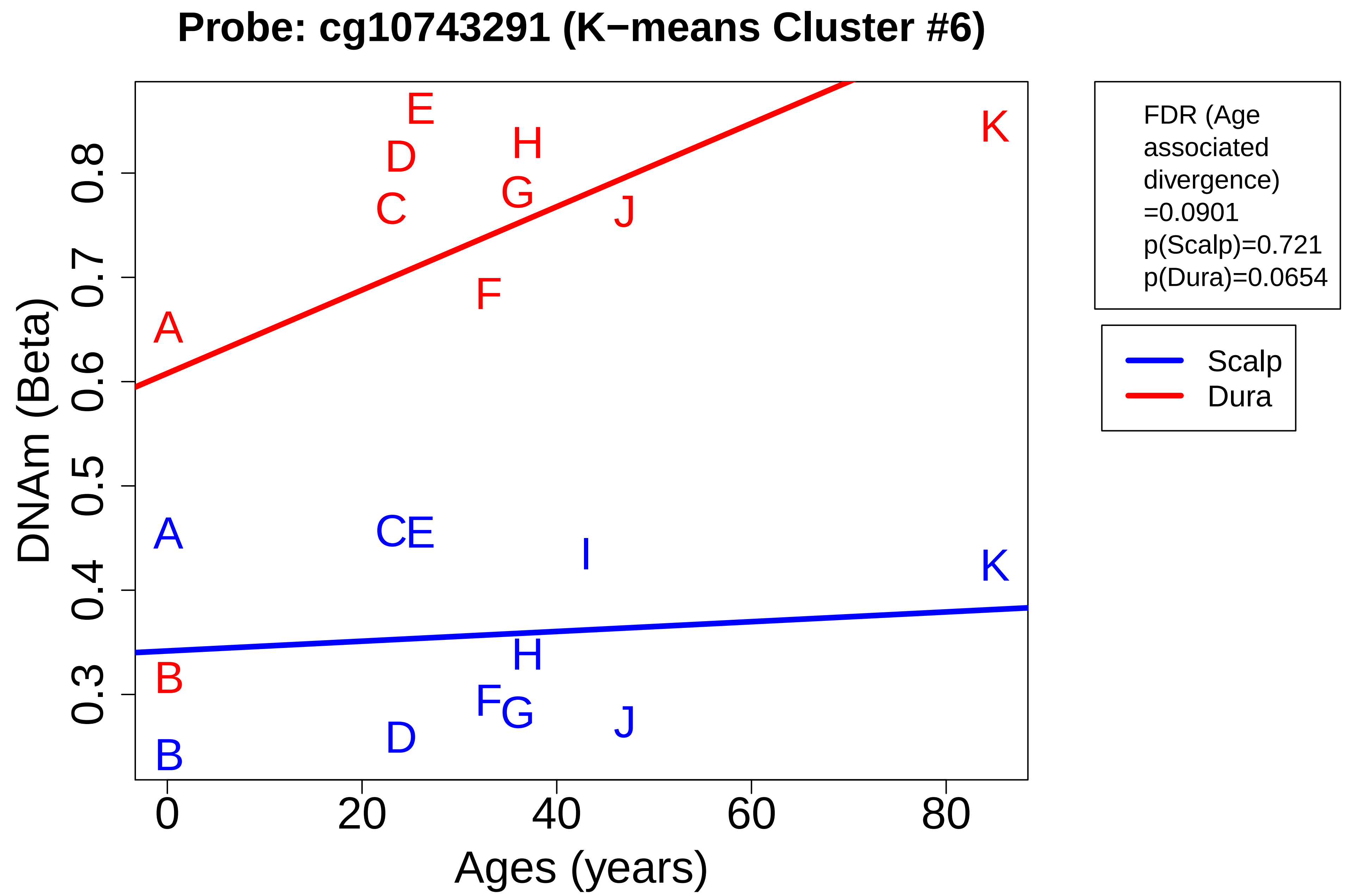

Probe: cg10743291 (K−means Cluster #6)
DNAm (Beta)
0.3
0.4
0.5
0.6
0.7
0.8
Ages (years)
0
20
40
60
80
E
D
H
K
G
C
J
F
A
A
CE
I
K
H
B
FG
J
D
B
FDR (Age associated divergence) =0.0901
p(Scalp)=0.721
p(Dura)=0.0654
Scalp
Dura

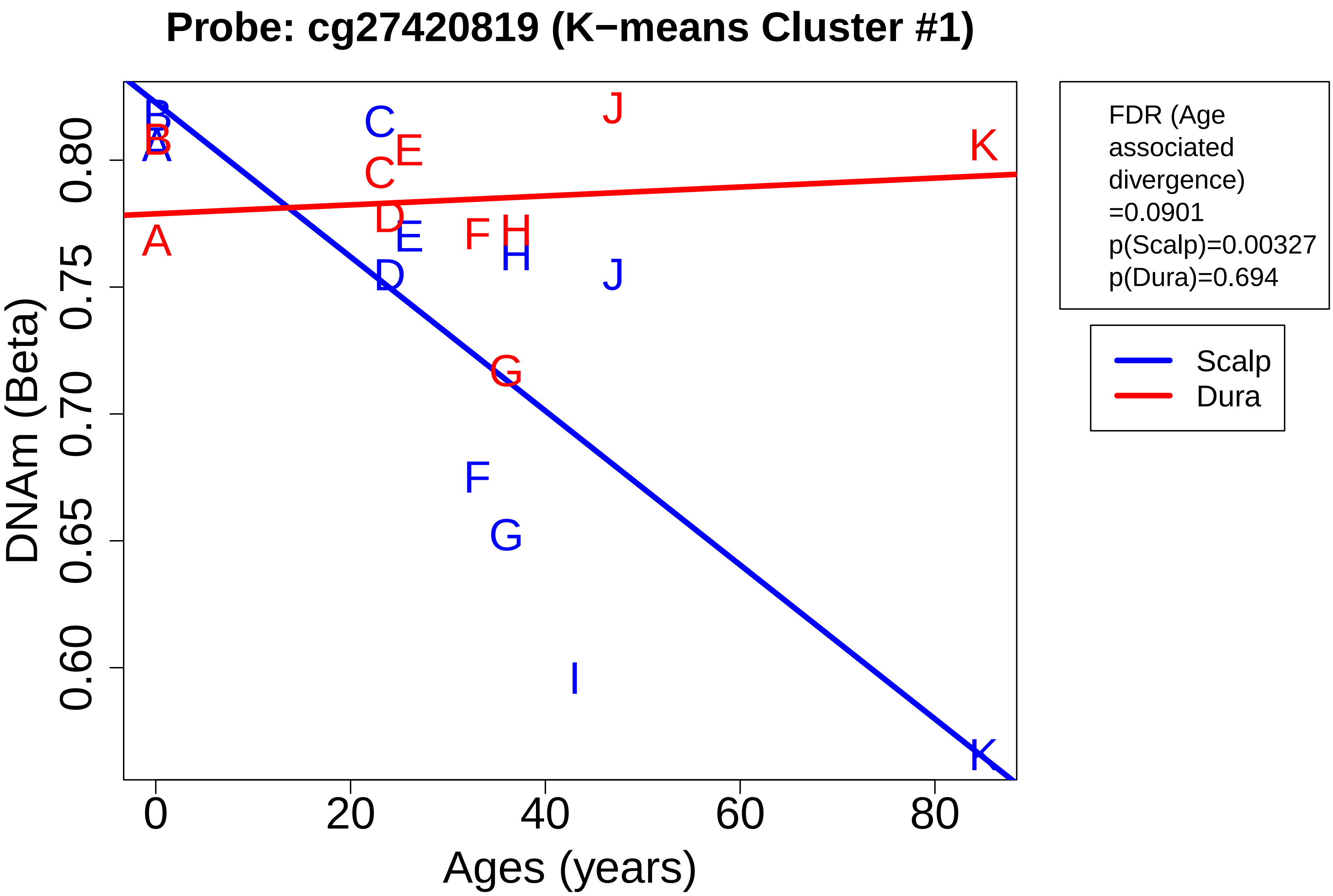
Probe: cg27420819 (K−means Cluster #1)
DNAm (Beta)
0.60
0.65
0.70
0.75
0.80
0
20
40
60
80
Ages (years)
A
B
C
D
E
F
G
H
I
J
K
FDR (Age associated divergence) =0.0901
p(Scalp)=0.00327
p(Dura)=0.694
Scalp
Dura

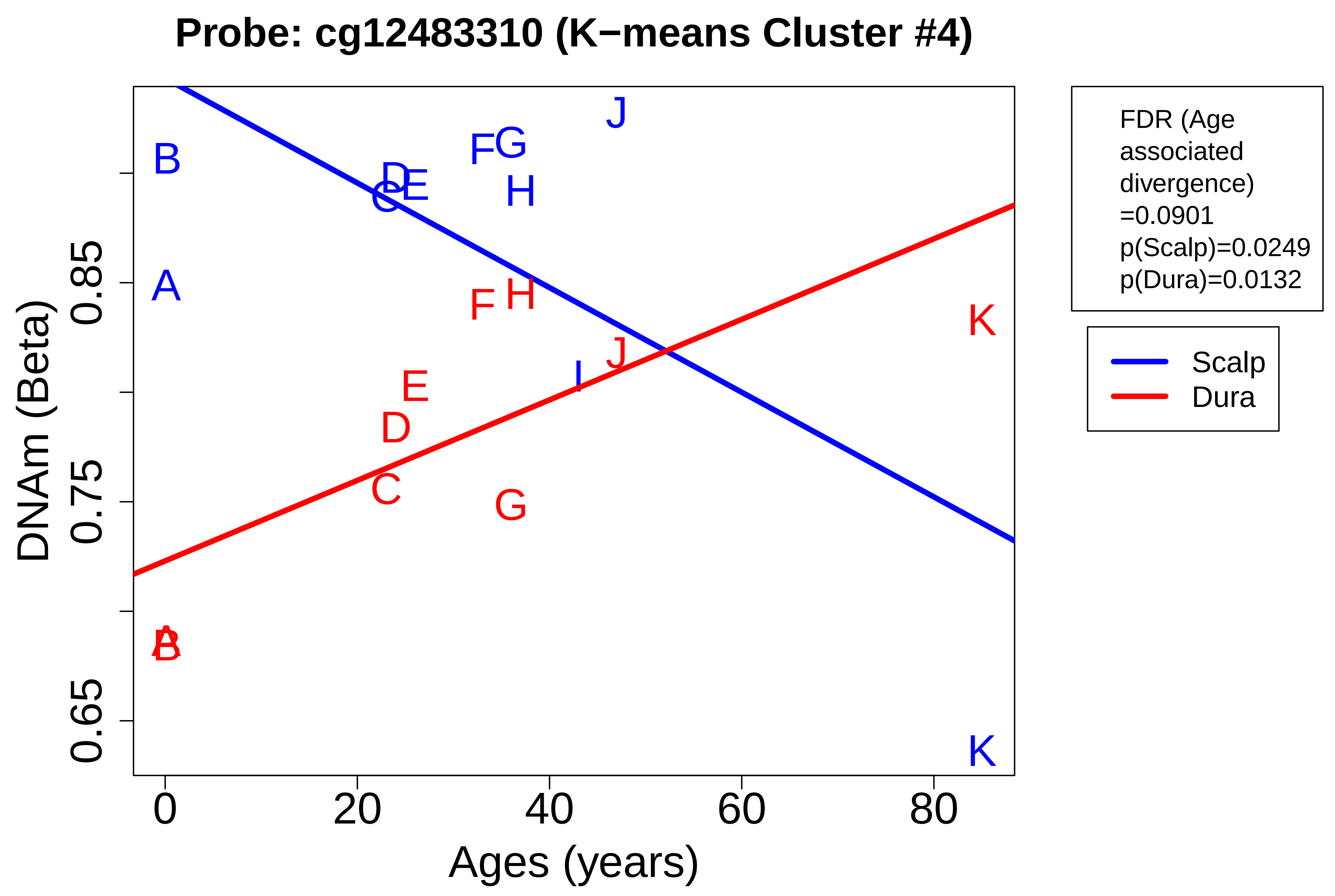
Probe: cg12483310 (K−means Cluster #4)
DNAm (Beta)
0.65
0.75
0.85
0
20
40
60
80
Ages (years)
FDR (Age associated divergence) =0.0901
p(Scalp)=0.0249
p(Dura)=0.0132
Scalp
Dura

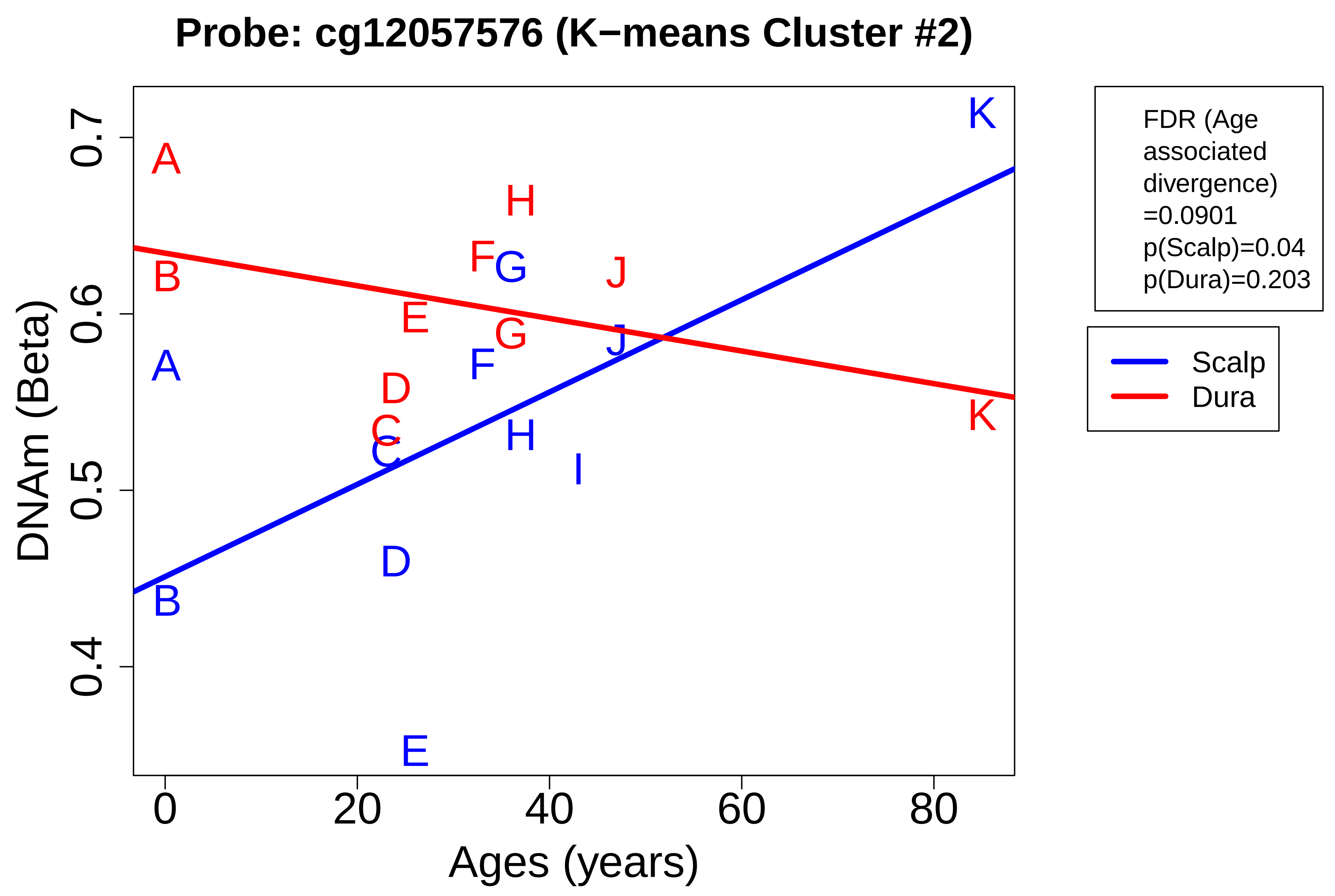

Probe: cg12057576 (K−means Cluster #2)
FDR (Age associated divergence) =0.0901
p(Scalp)=0.04
p(Dura)=0.203
Scalp
Dura
DNAm (Beta)
0.4
0.5
0.6
0.7
Ages (years)
0
20
40
60
80
A
B
C
D
E
F
G
H
I
J
K

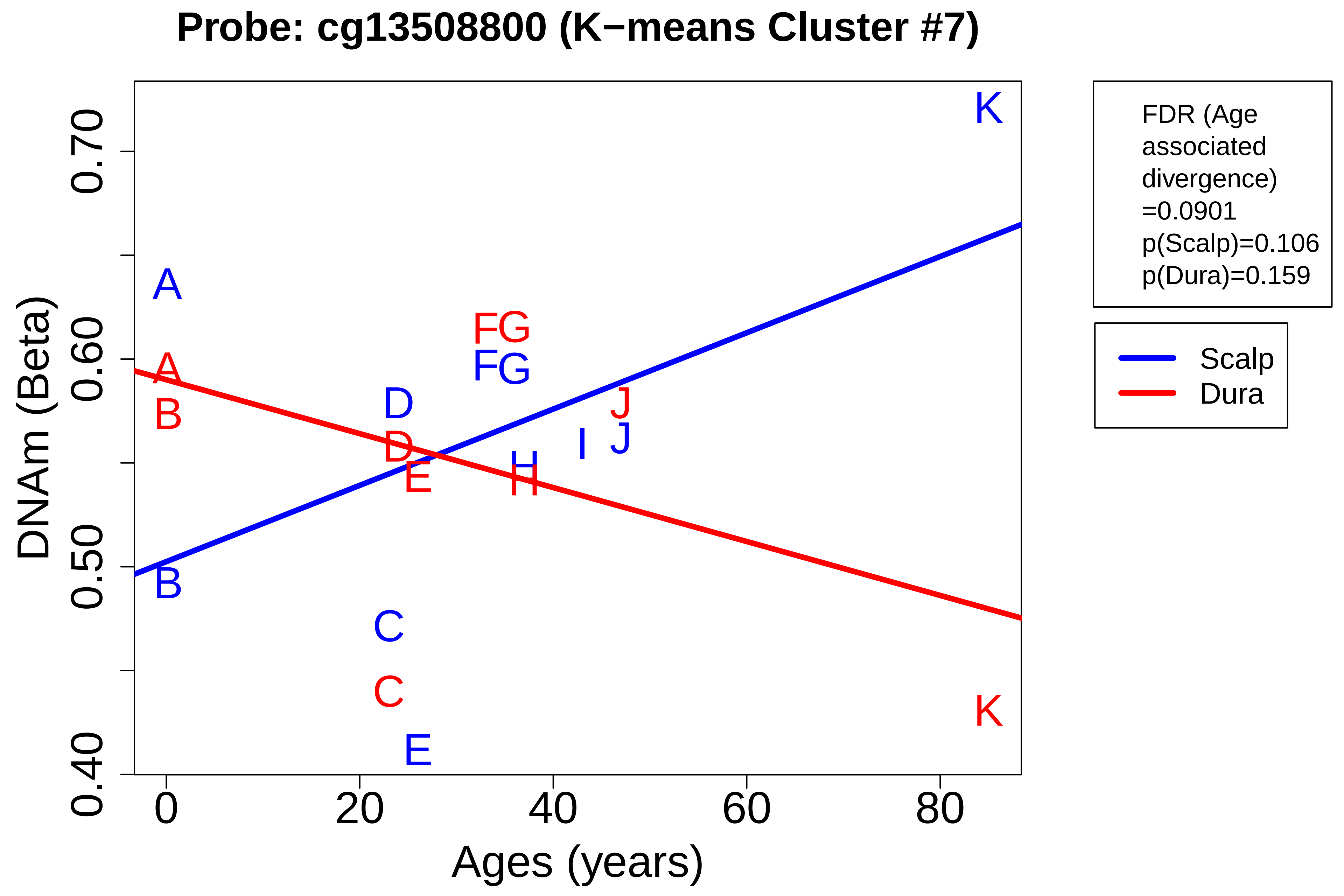

Probe: cg13508800 (K−means Cluster #7)
DNAm (Beta)
0.40
0.50
0.60
0.70
Ages (years)
0
20
40
60
80
A
B
C
D
E
FG
H
I
J
K
FDR (Age associated divergence) =0.0901
p(Scalp)=0.106
p(Dura)=0.159
Scalp
Dura

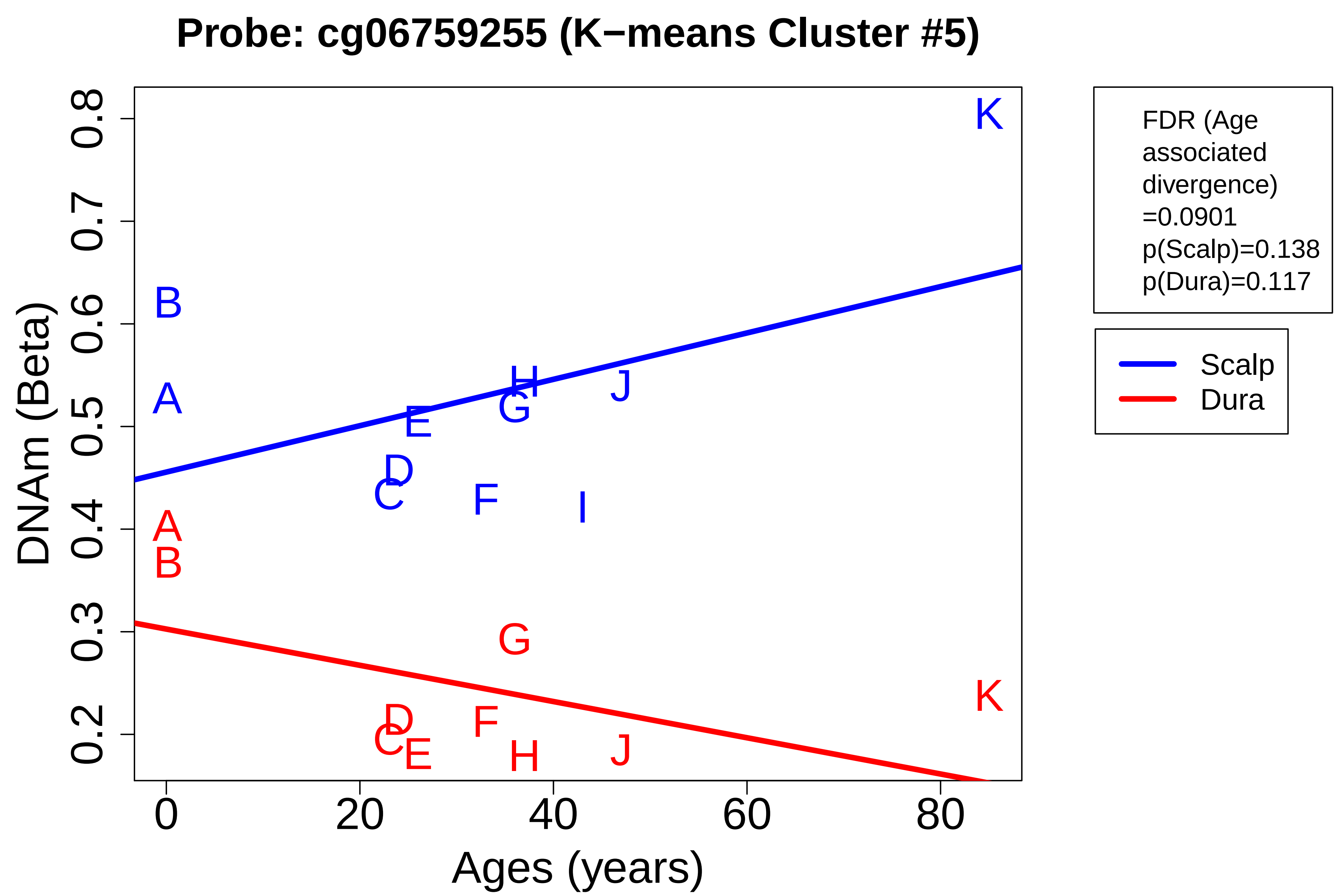
Probe: cg06759255 (K−means Cluster #5)
FDR (Age associated divergence) =0.0901
p(Scalp)=0.138
p(Dura)=0.117
Scalp
Dura
DNAm (Beta)
Ages (years)
0.2
0.3
0.4
0.5
0.6
0.7
0.8
0
20
40
60
80

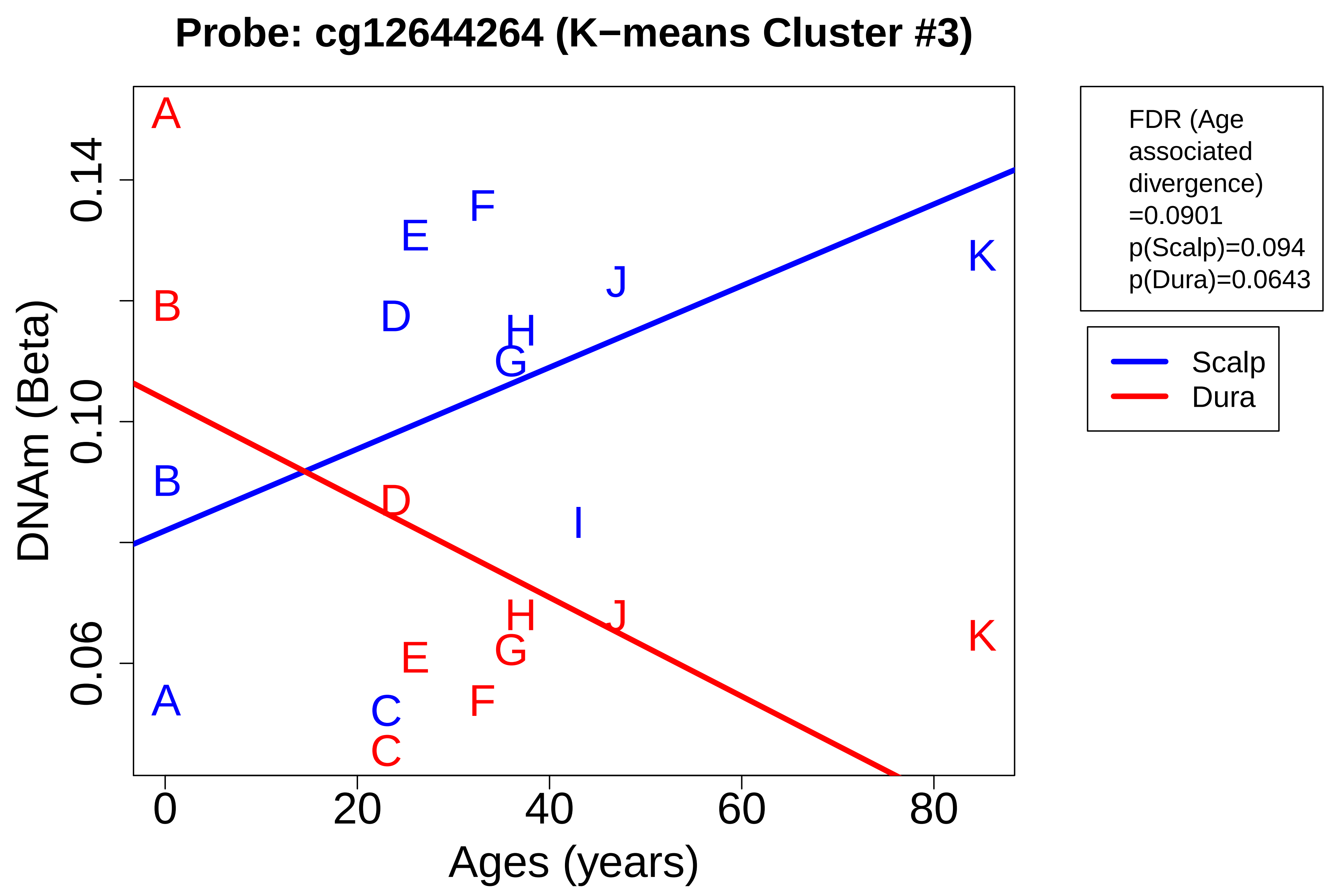

Probe: cg12644264 (K−means Cluster #3)
DNAm (Beta)
0.06
0.10
0.14
0
20
40
60
80
Ages (years)
A
B
C
D
E
F
G
H
I
J
K
FDR (Age associated divergence) =0.0901
p(Scalp)=0.094
p(Dura)=0.0643
Scalp
Dura

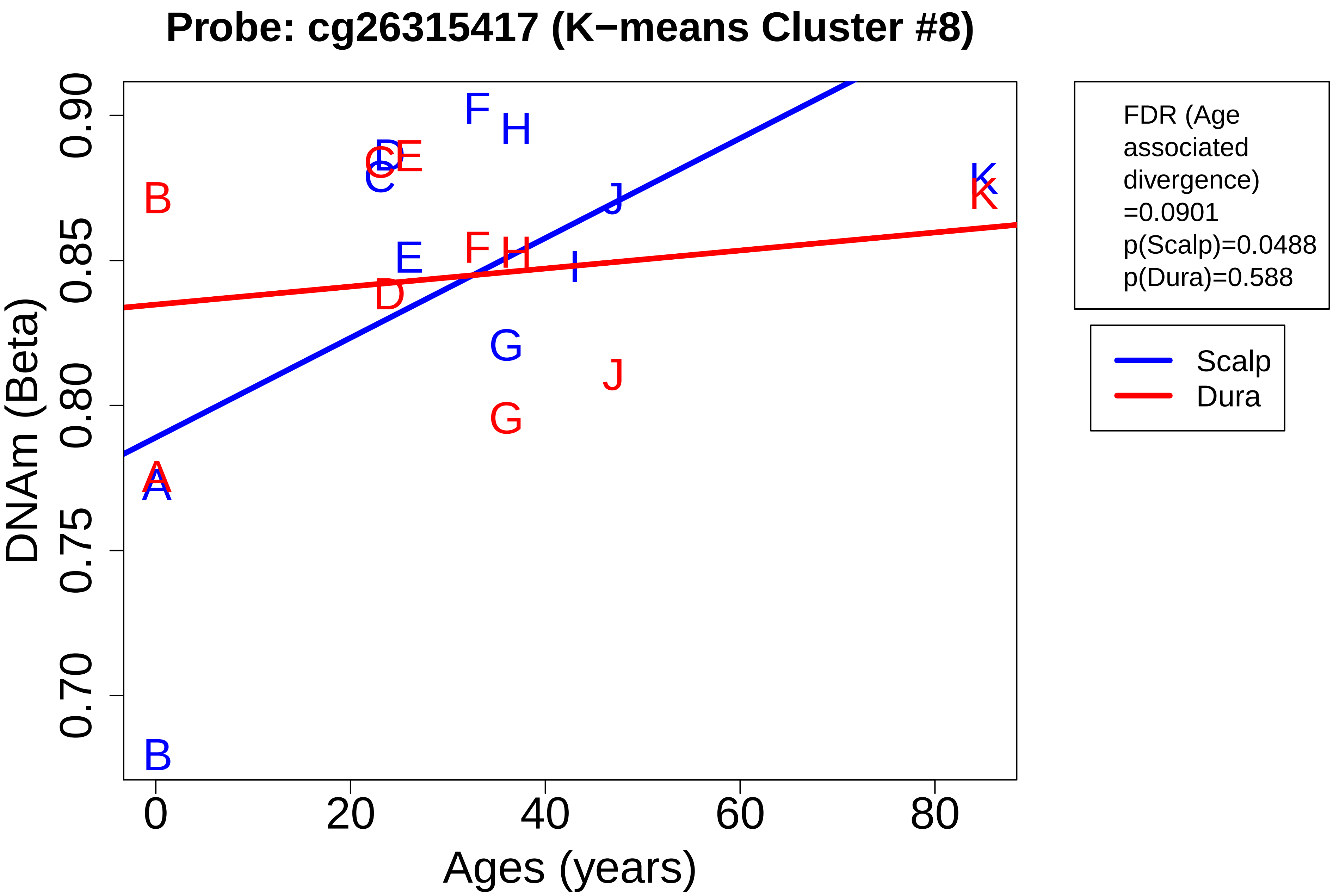

Probe: cg26315417 (K−means Cluster #8)
DNAm (Beta)
0.70
0.75
0.80
0.85
0.90
0
20
40
60
80
Ages (years)
FDR (Age associated divergence) =0.0901
p(Scalp)=0.0488
p(Dura)=0.588
Scalp
Dura

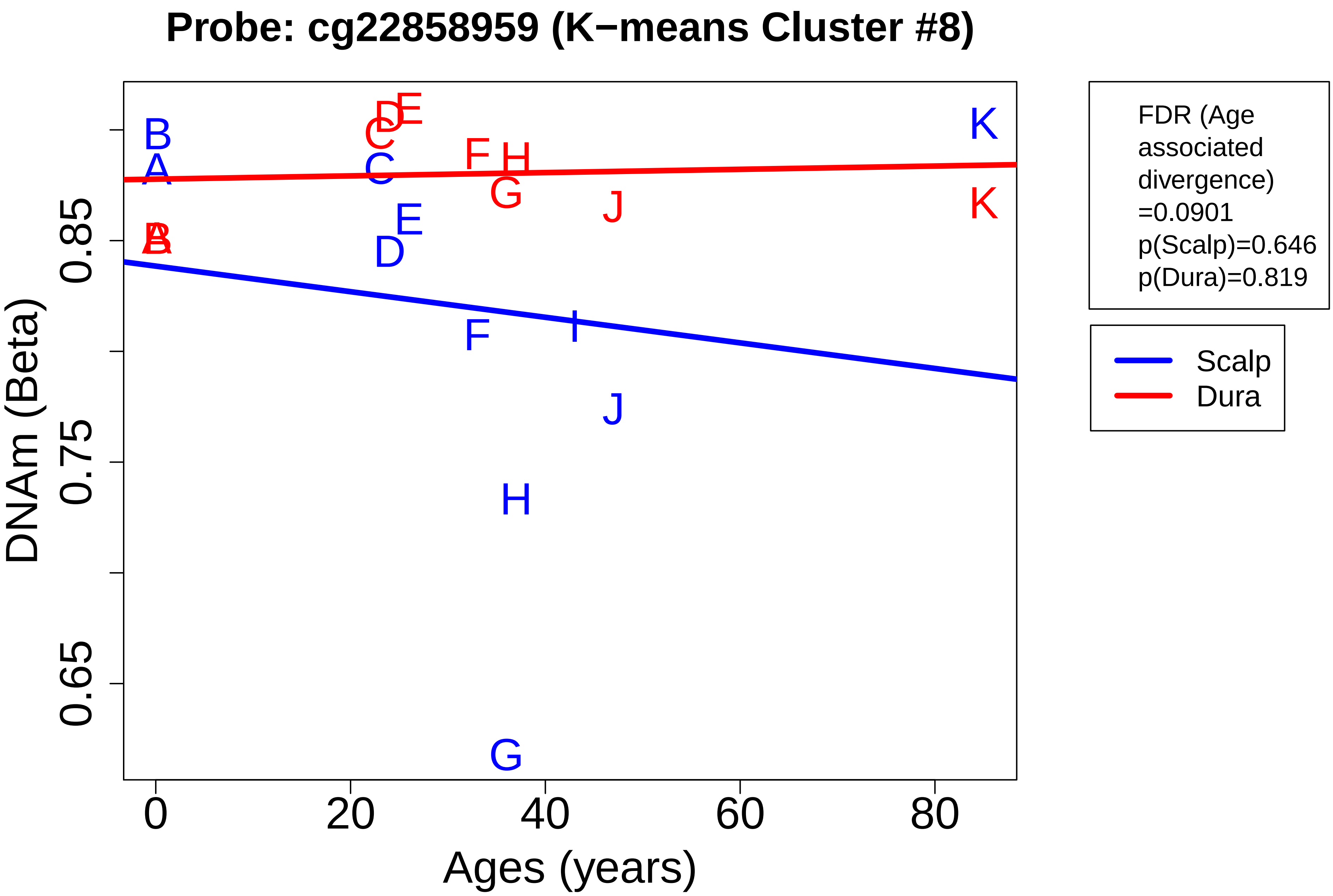

Probe: cg22858959 (K−means Cluster #8)
DNAm (Beta)
0.65
0.75
0.85
0
20
40
60
80
Ages (years)
FDR (Age associated divergence) =0.0901
p(Scalp)=0.646
p(Dura)=0.819
Scalp
Dura

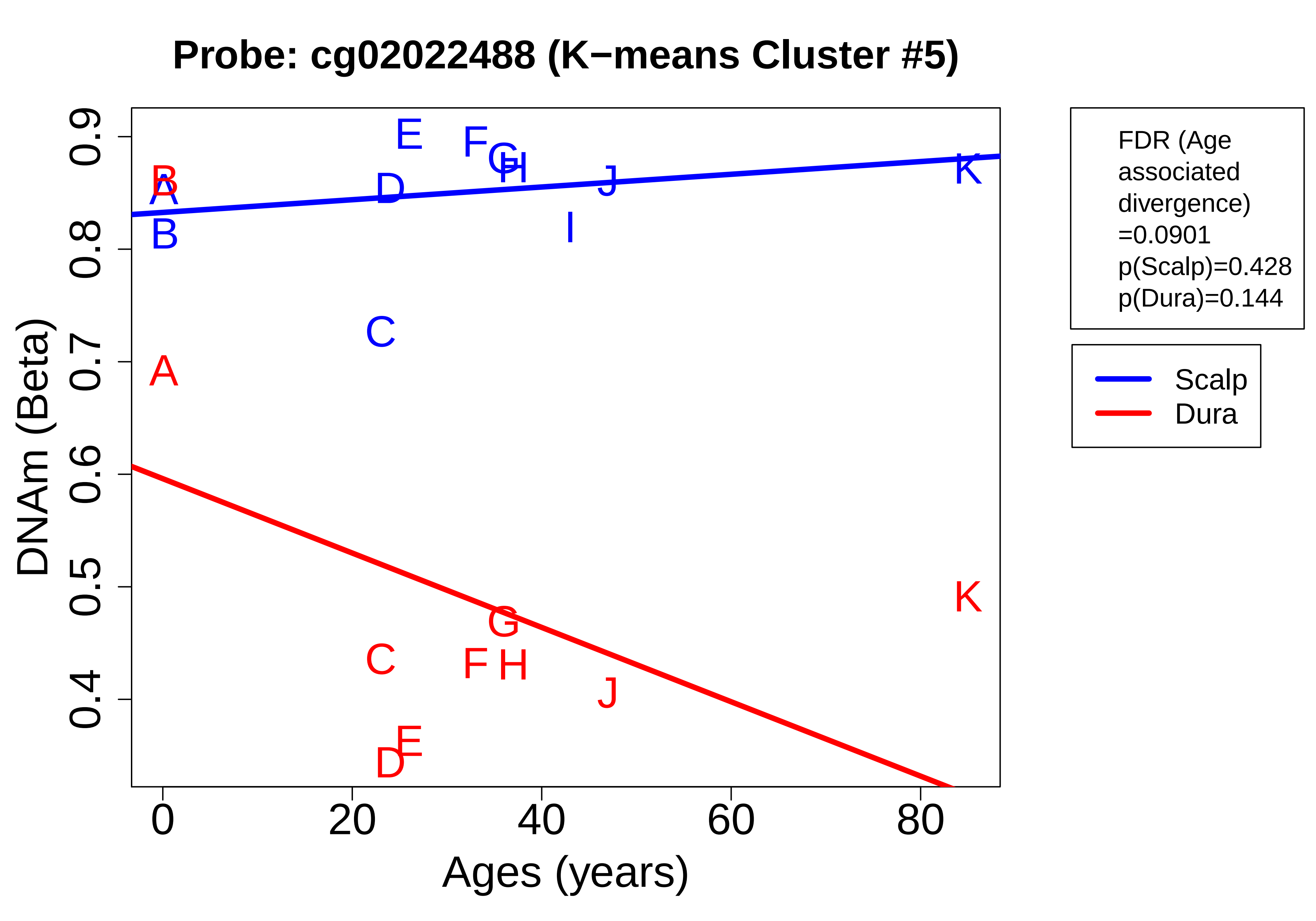

Probe: cg02022488 (K−means Cluster #5)
DNAm (Beta)
Ages (years)
0.4
0.5
0.6
0.7
0.8
0.9
0
20
40
60
80
FDR (Age associated divergence) =0.0901
p(Scalp)=0.428
p(Dura)=0.144
Scalp
Dura
A
B
C
D
E
F
G
H
I
J
K

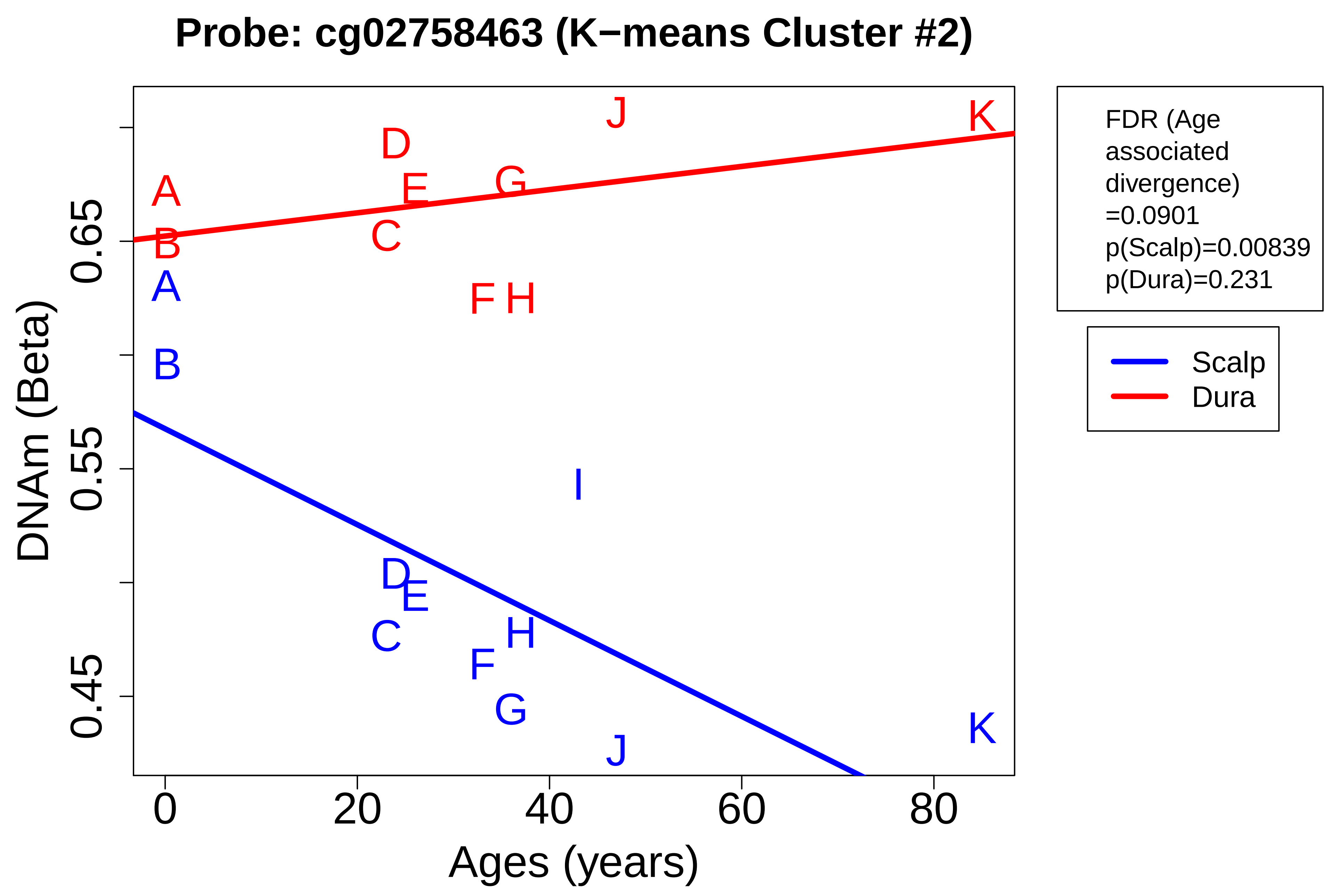
Probe: cg02758463 (K−means Cluster #2)
DNAm (Beta)
0.45
0.55
0.65
0
20
40
60
80
Ages (years)
A
B
C
D
E
F
G
H
I
J
K
FDR (Age associated divergence) =0.0901
p(Scalp)=0.00839
p(Dura)=0.231
Scalp
Dura

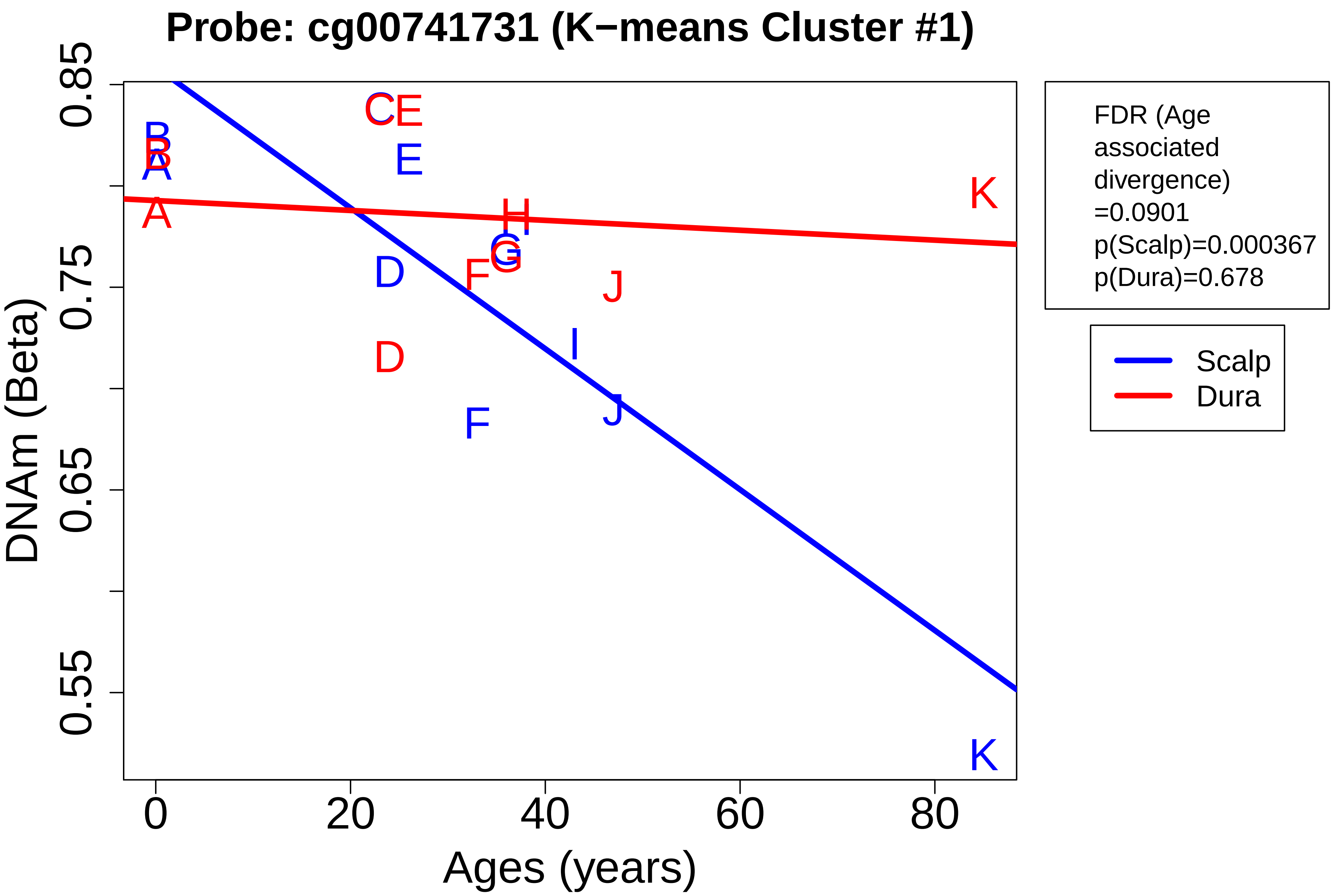

Probe: cg00741731 (K−means Cluster #1)
DNAm (Beta)
0.55
0.65
0.75
0.85
Ages (years)
0
20
40
60
80
FDR (Age associated divergence) =0.0901
p(Scalp)=0.000367
p(Dura)=0.678
Scalp
Dura

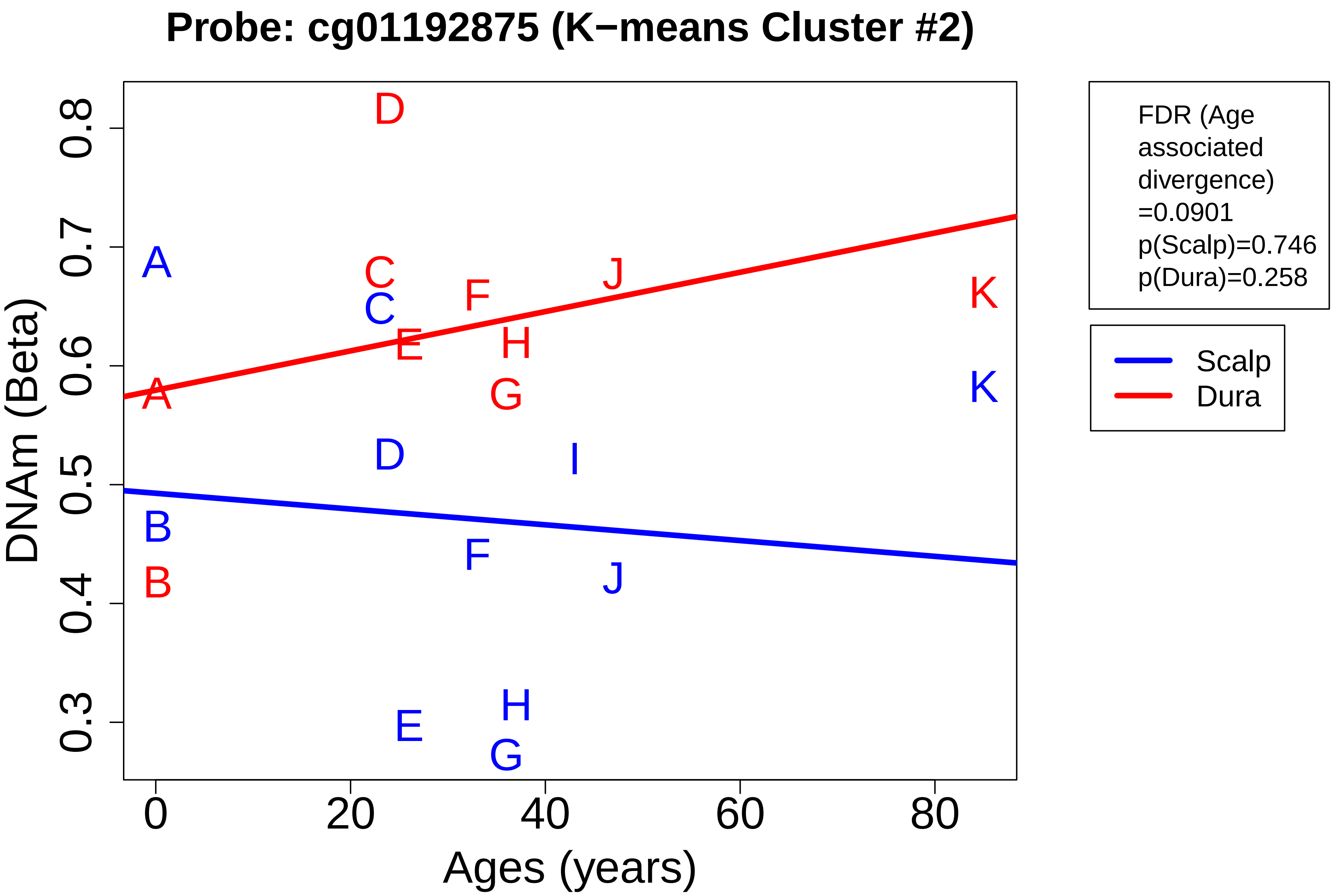

Probe: cg01192875 (K−means Cluster #2)
DNAm (Beta)
0.3
0.4
0.5
0.6
0.7
0.8
0
20
40
60
80
Ages (years)
FDR (Age associated divergence) =0.0901
p(Scalp)=0.746
p(Dura)=0.258
Scalp
Dura
A
B
C
D
E
F
G
H
I
J
K

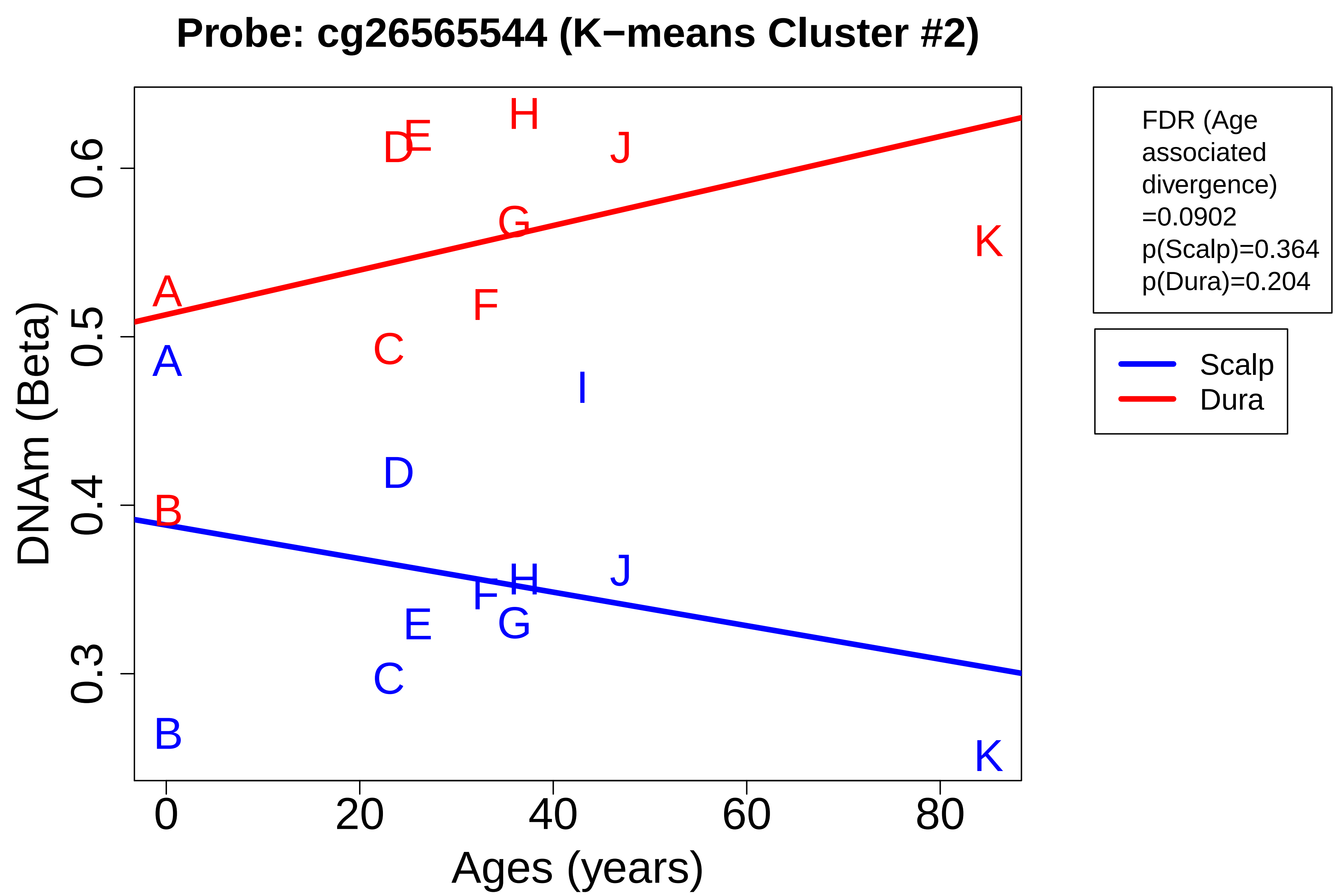

Probe: cg26565544 (K−means Cluster #2)
DNAm (Beta)
0.3
0.4
0.5
0.6
0
20
40
60
80
Ages (years)
A
B
C
D
E
F
G
H
I
J
K
FDR (Age associated divergence) =0.0902
p(Scalp)=0.364
p(Dura)=0.204
Scalp
Dura

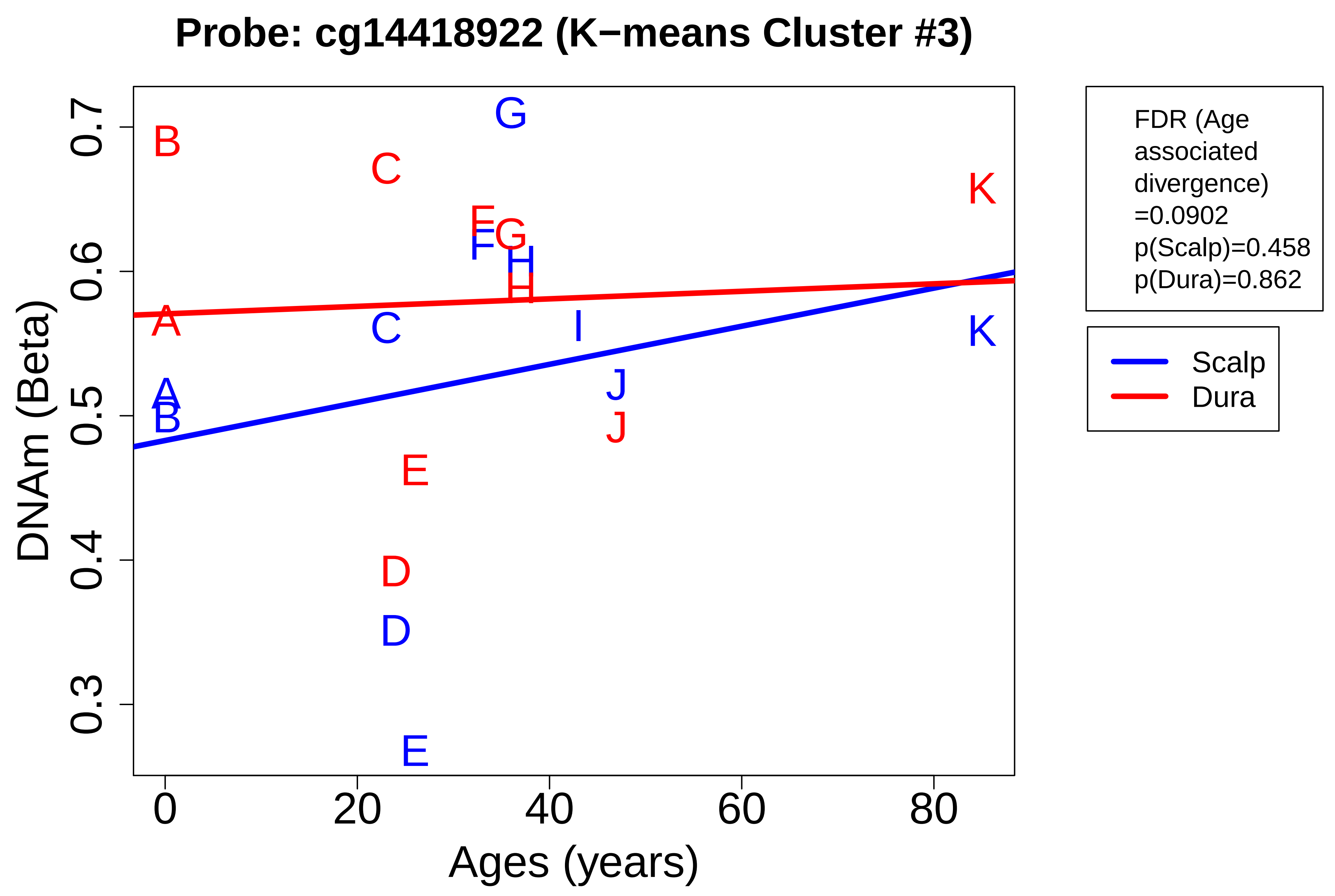
Probe: cg14418922 (K−means Cluster #3)
DNAm (Beta)
0.3
0.4
0.5
0.6
0.7
0
20
40
60
80
Ages (years)
A
B
C
D
E
F
G
H
I
J
K
FDR (Age associated divergence) =0.0902
p(Scalp)=0.458
p(Dura)=0.862
Scalp
Dura

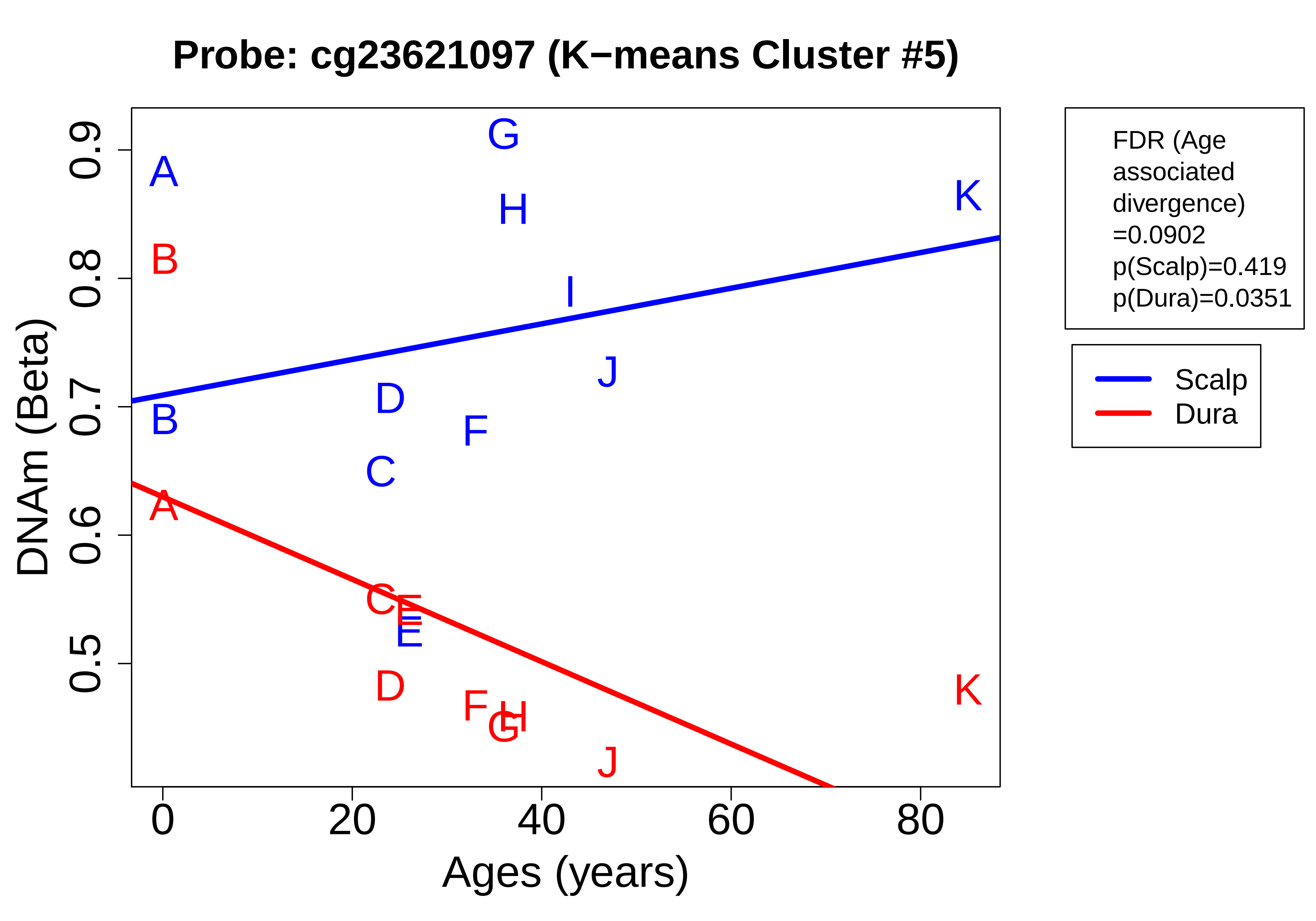

Probe: cg23621097 (K−means Cluster #5)
DNAm (Beta)
0.5
0.6
0.7
0.8
0.9
0
20
40
60
80
Ages (years)
A
B
C
D
E
F
G
H
I
J
K
FDR (Age associated divergence) =0.0902
p(Scalp)=0.419
p(Dura)=0.0351
Scalp
Dura

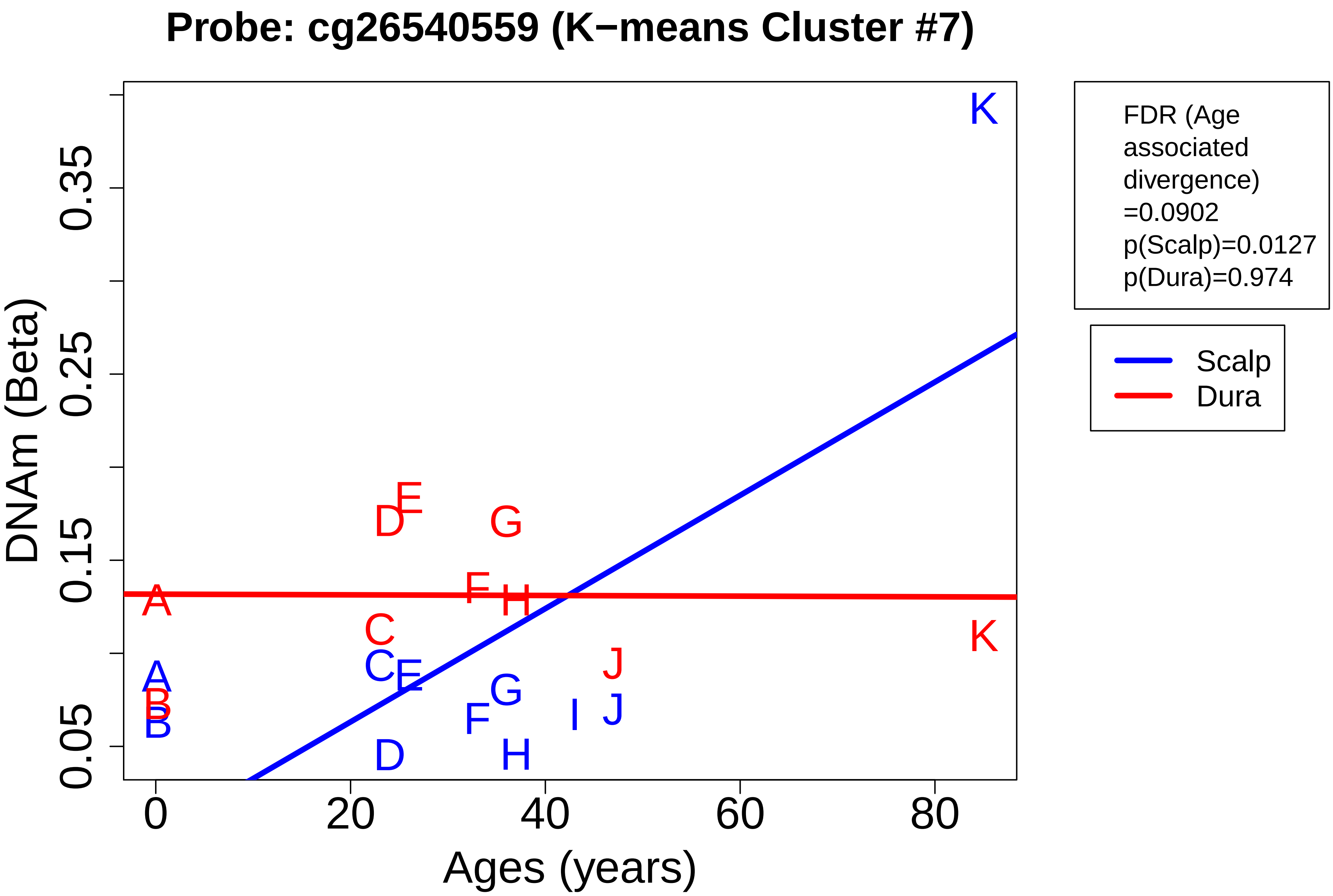

Probe: cg26540559 (K−means Cluster #7)
DNAm (Beta)
0.05
0.15
0.25
0.35
0
20
40
60
80
Ages (years)
FDR (Age associated divergence) =0.0902
p(Scalp)=0.0127
p(Dura)=0.974
Scalp
Dura
A
B
C
D
E
F
G
H
I
J
K

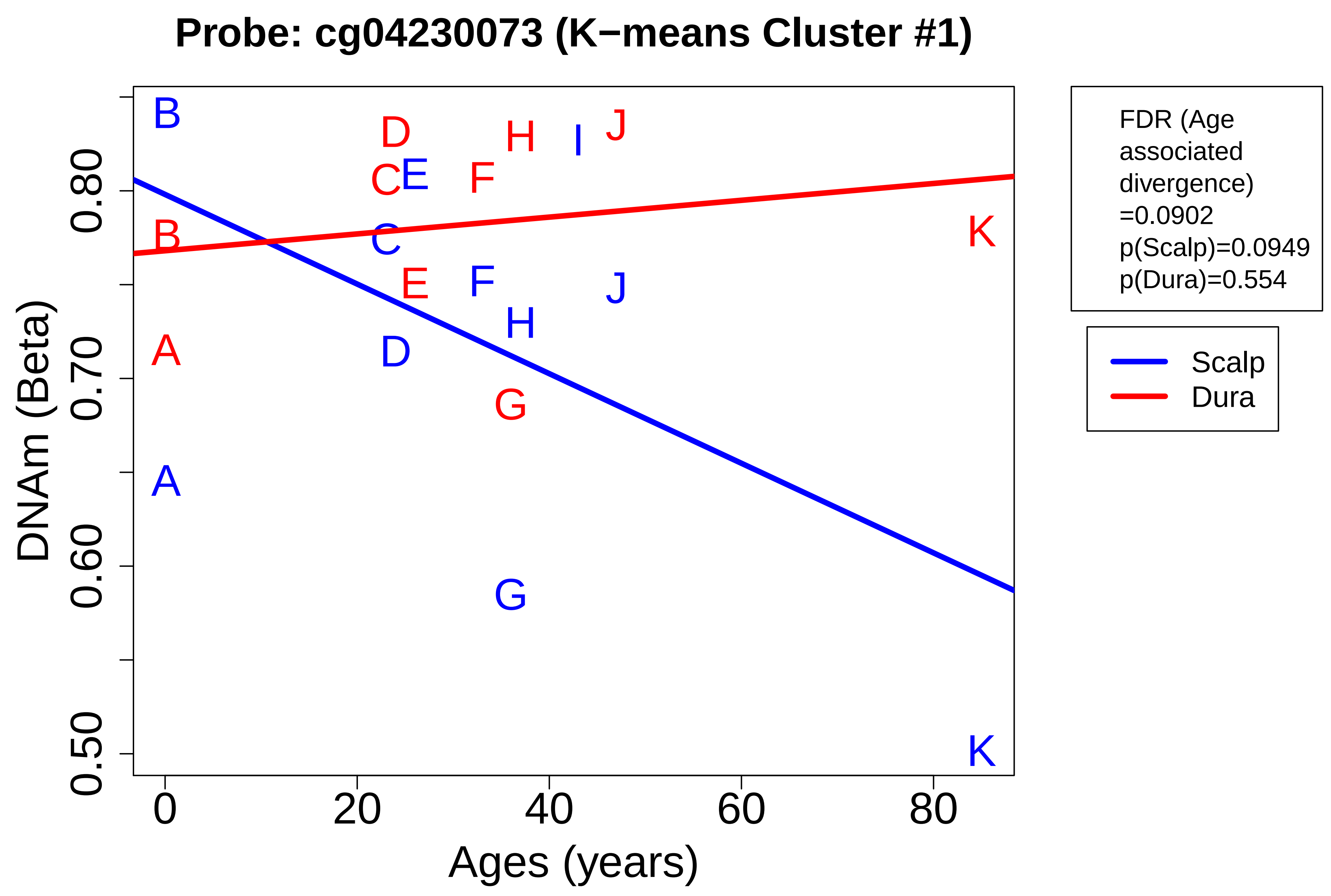
Probe: cg04230073 (K−means Cluster #1)
DNAm (Beta)
0.50
0.60
0.70
0.80
Ages (years)
0
20
40
60
80
FDR (Age associated divergence) =0.0902
p(Scalp)=0.0949
p(Dura)=0.554
Scalp
Dura

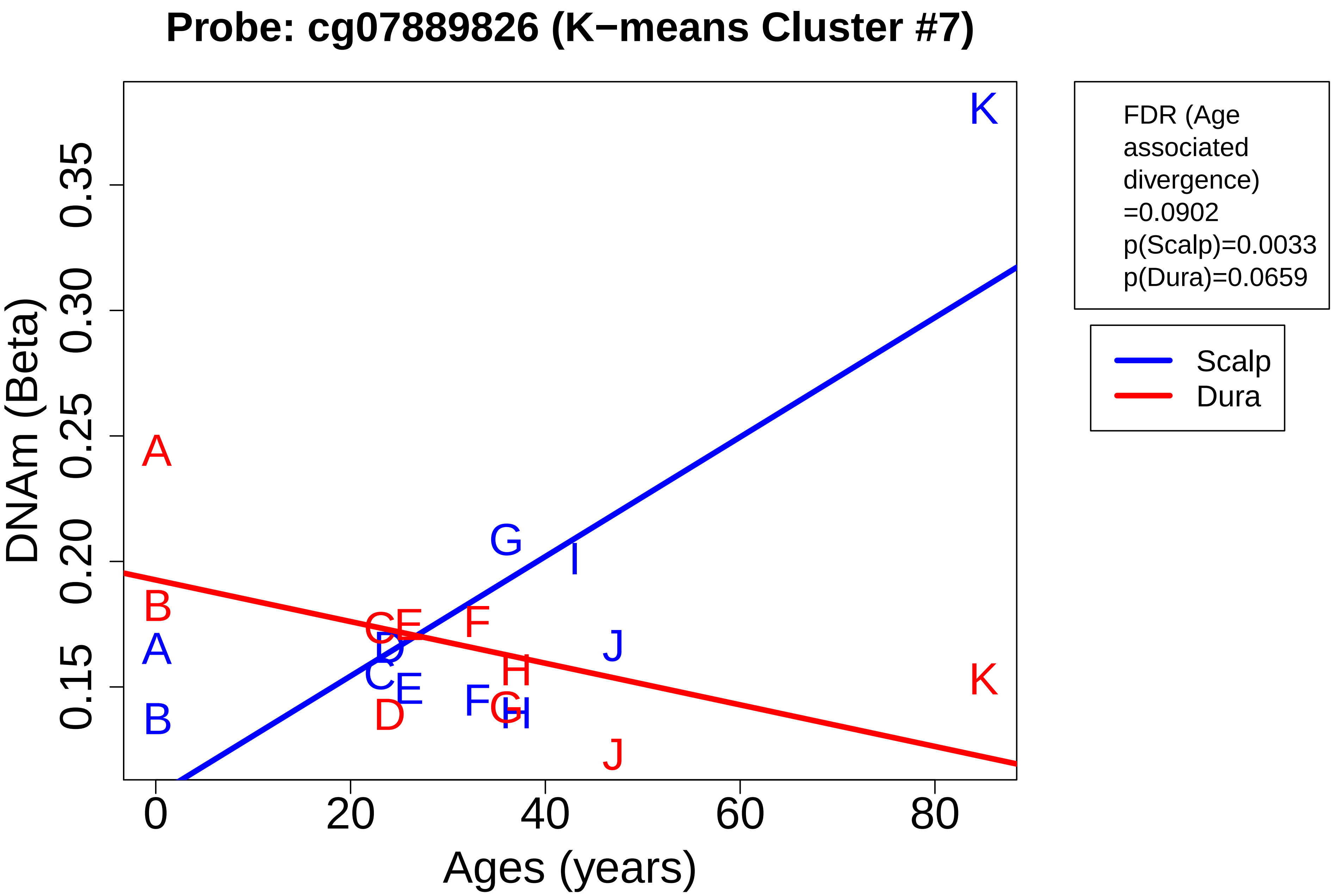

Probe: cg07889826 (K−means Cluster #7)
DNAm (Beta)
0.15
0.20
0.25
0.30
0.35
0
20
40
60
80
Ages (years)
FDR (Age associated divergence) =0.0902
p(Scalp)=0.0033
p(Dura)=0.0659
Scalp
Dura
A
B
C
D
E
F
G
H
I
J
K

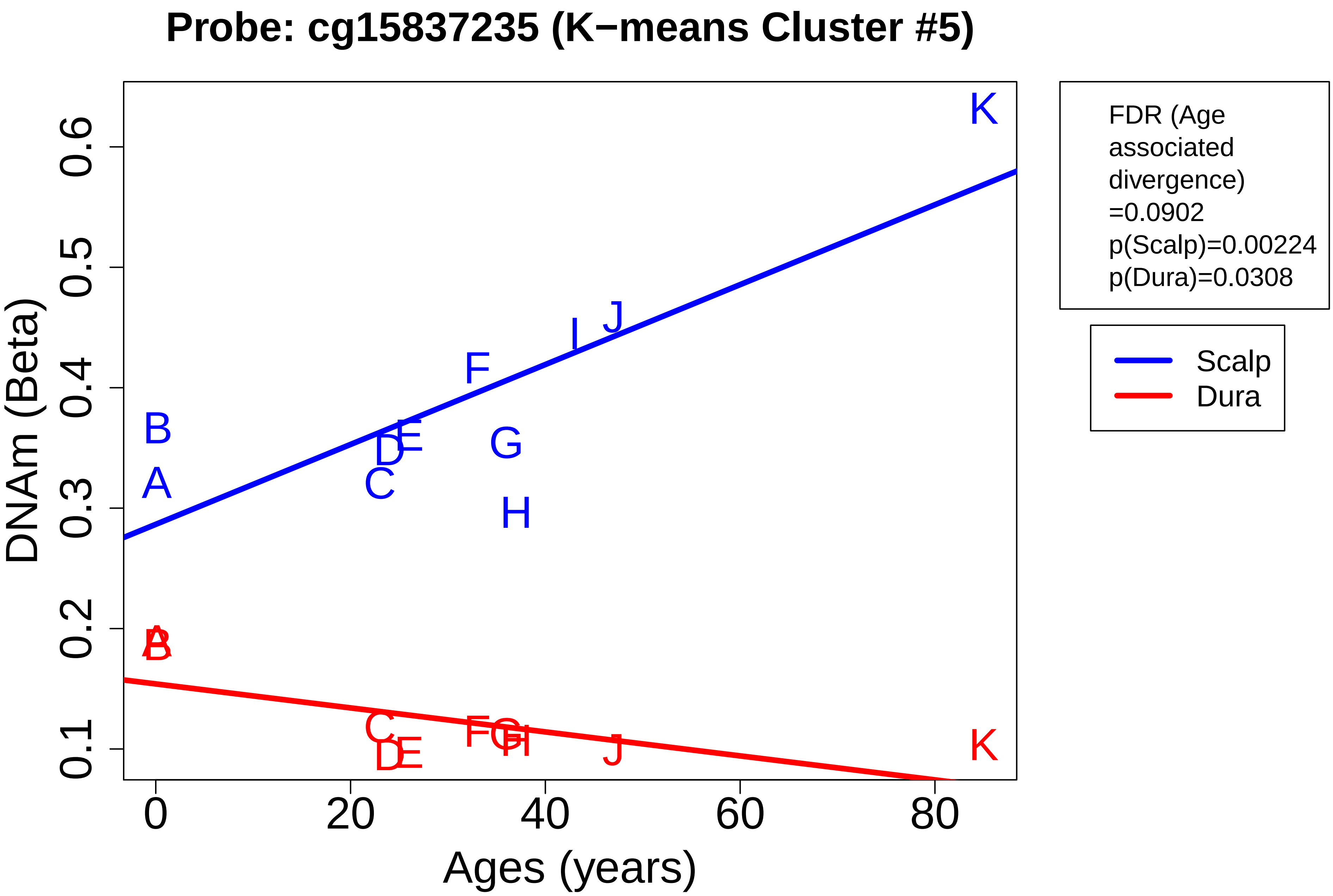

Probe: cg15837235 (K−means Cluster #5)
DNAm (Beta)
0.1
0.2
0.3
0.4
0.5
0.6
0
20
40
60
80
Ages (years)
FDR (Age associated divergence) =0.0902
p(Scalp)=0.00224
p(Dura)=0.0308
Scalp
Dura
A
B
C
D
E
F
G
H
I
J
K

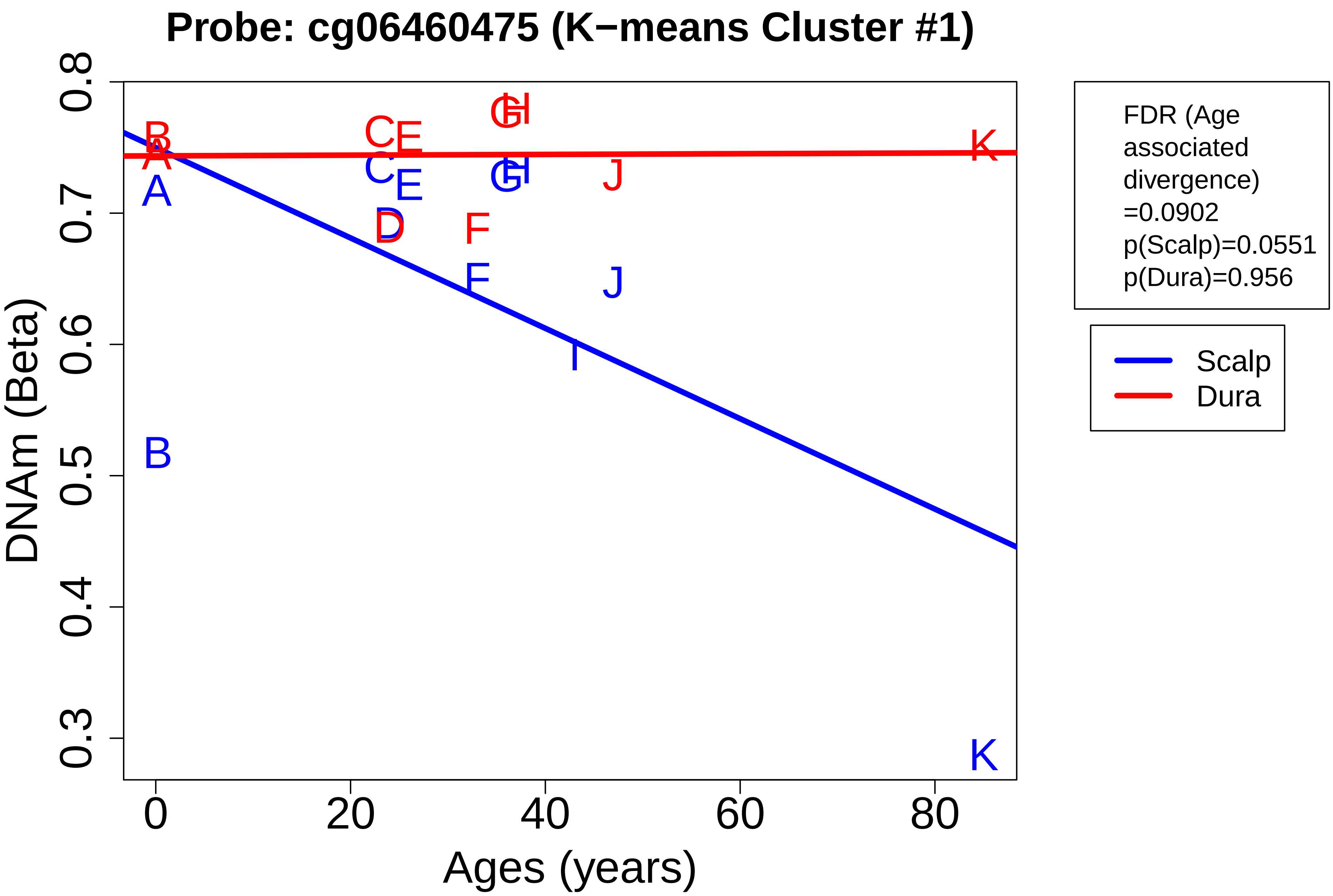

Probe: cg06460475 (K−means Cluster #1)
DNAm (Beta)
0.3
0.4
0.5
0.6
0.7
0.8
0
20
40
60
80
Ages (years)
FDR (Age associated divergence) =0.0902
p(Scalp)=0.0551
p(Dura)=0.956
Scalp
Dura

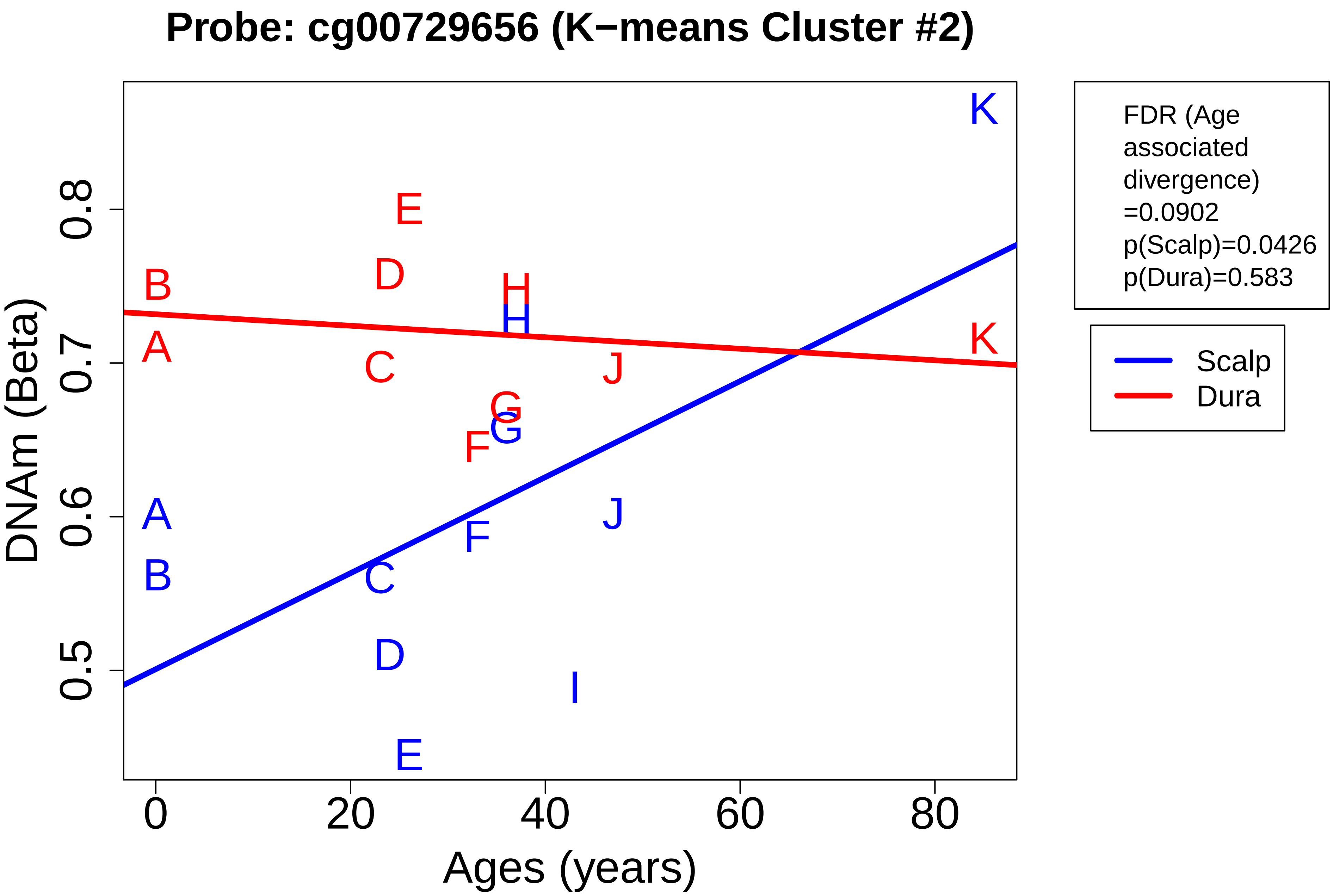

Probe: cg00729656 (K−means Cluster #2)
DNAm (Beta)
0.5
0.6
0.7
0.8
0
20
40
60
80
Ages (years)
FDR (Age associated divergence) =0.0902
p(Scalp)=0.0426
p(Dura)=0.583
Scalp
Dura

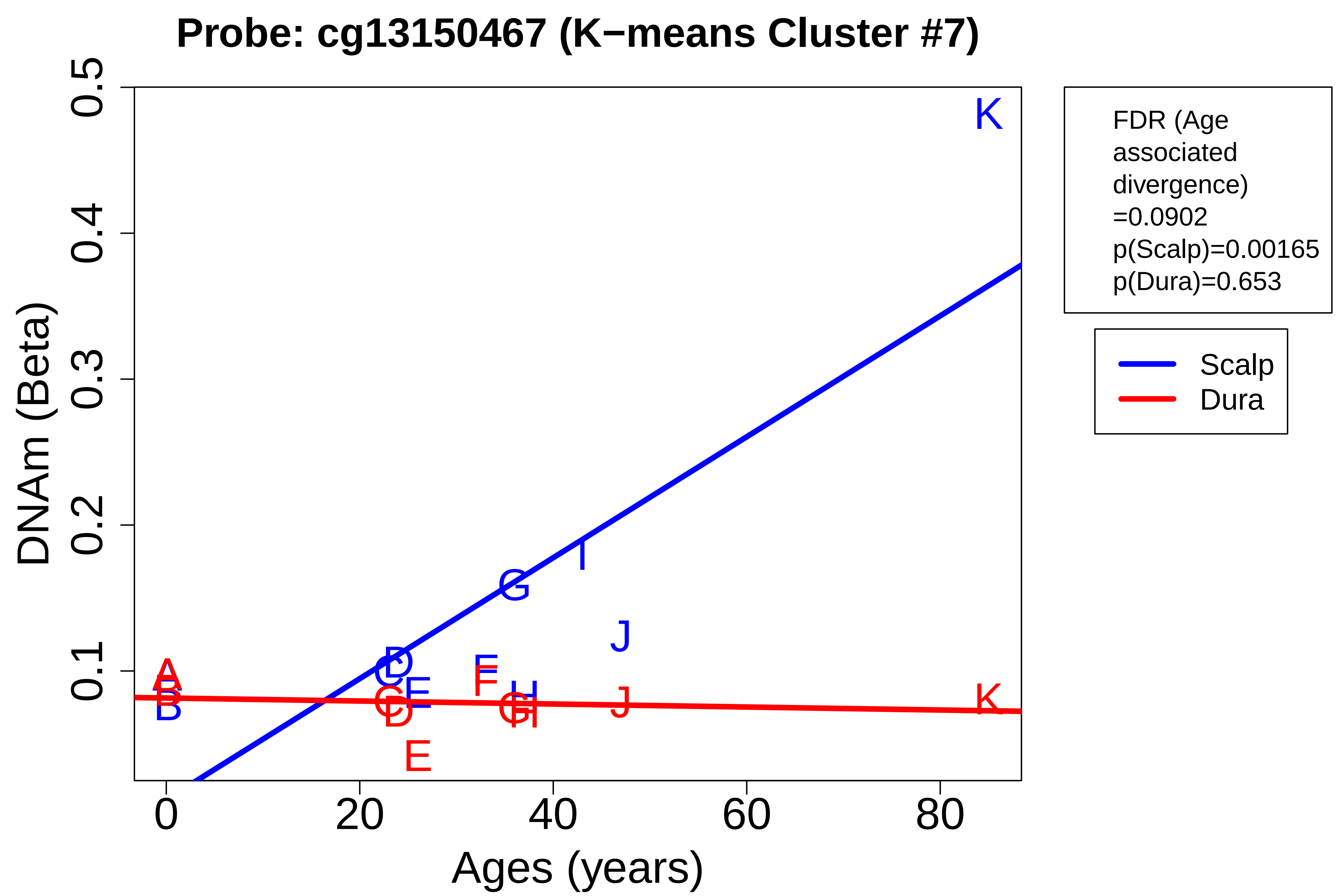

Probe: cg13150467 (K−means Cluster #7)
DNAm (Beta)
0.1
0.2
0.3
0.4
0.5
0
20
40
60
80
Ages (years)
FDR (Age associated divergence) =0.0902
p(Scalp)=0.00165
p(Dura)=0.653
Scalp
Dura

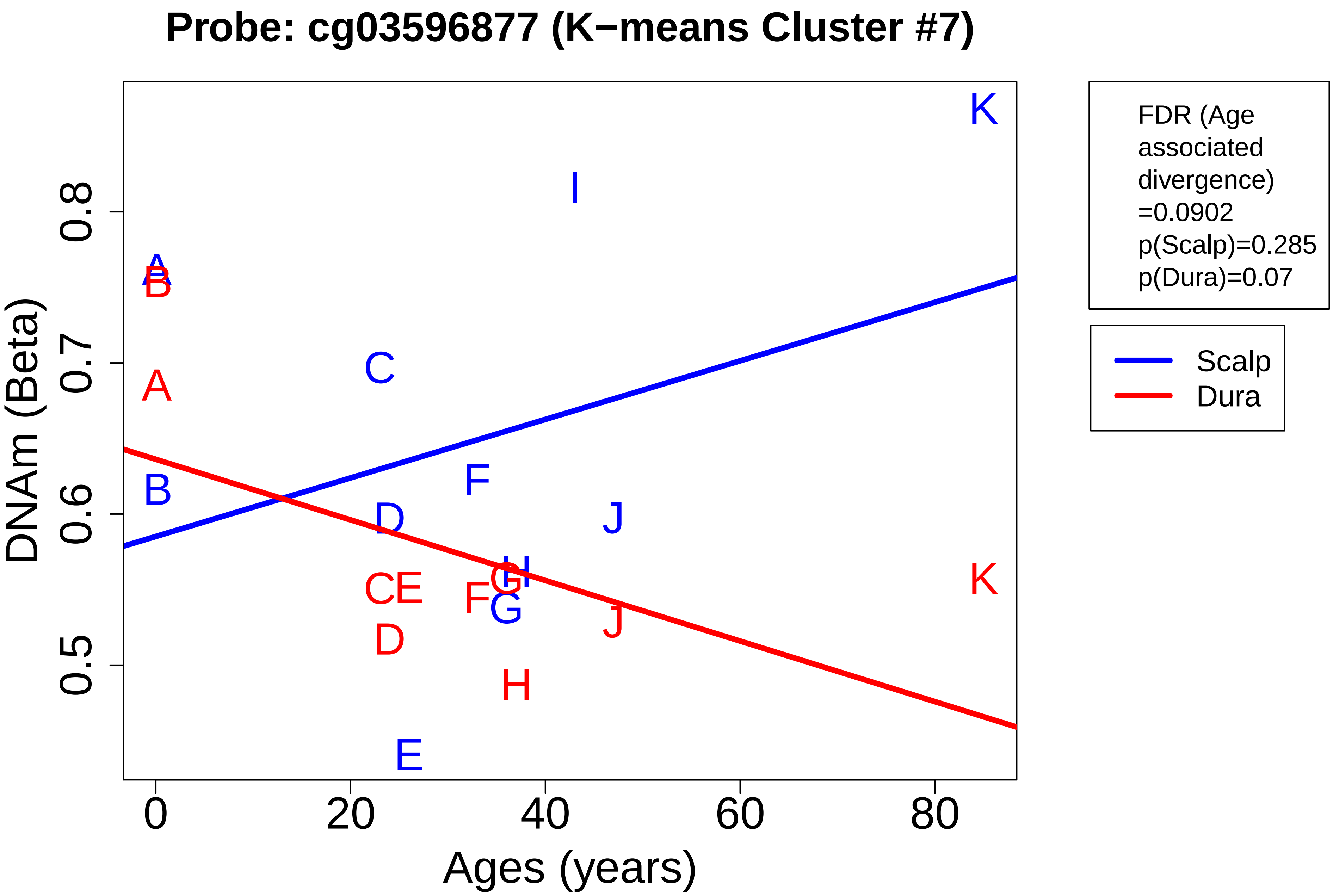

Probe: cg03596877 (K−means Cluster #7)
DNAm (Beta)
0.5
0.6
0.7
0.8
0
20
40
60
80
Ages (years)
FDR (Age associated divergence) =0.0902
p(Scalp)=0.285
p(Dura)=0.07
Scalp
Dura

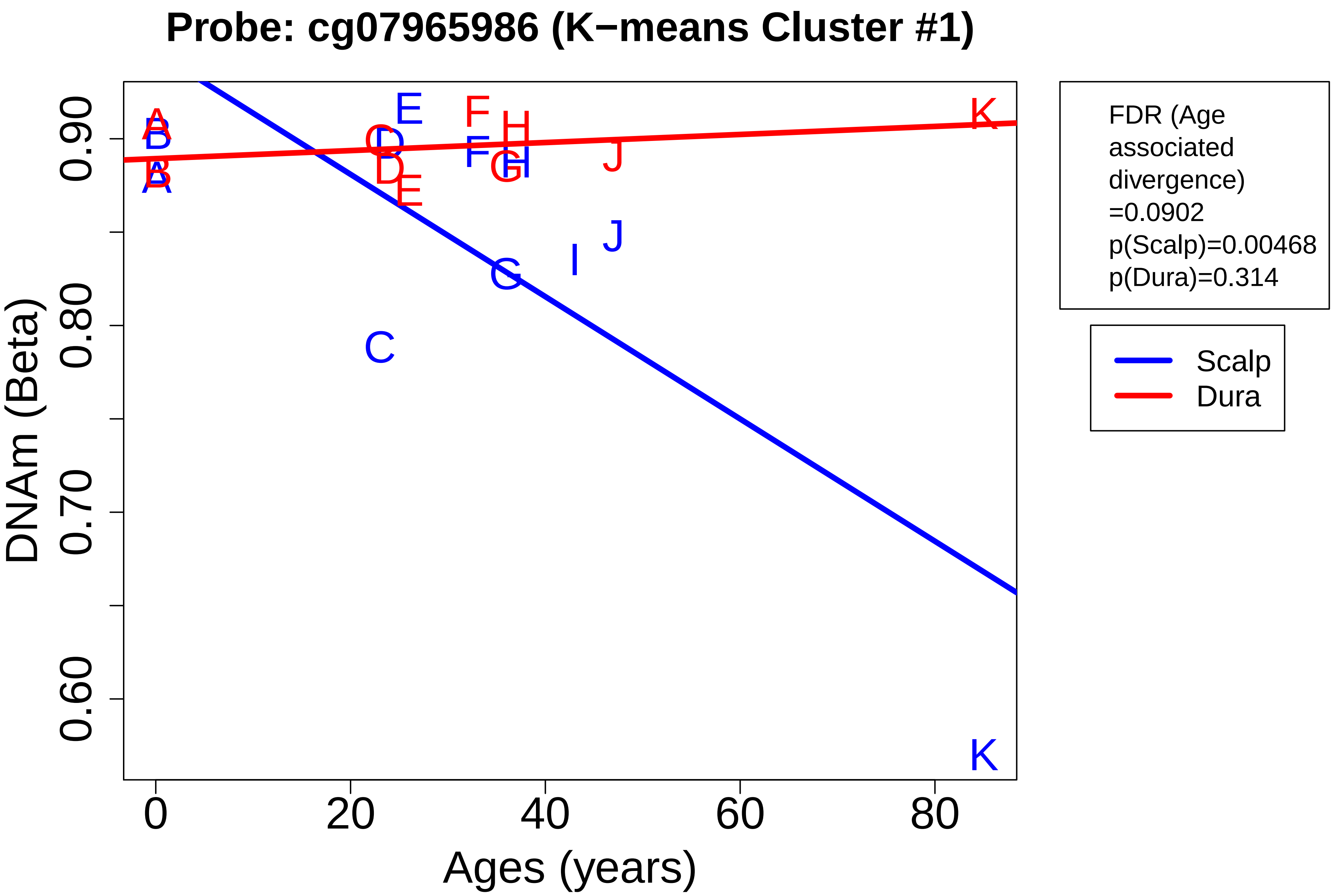

Probe: cg07965986 (K−means Cluster #1)
DNAm (Beta)
0.60
0.70
0.80
0.90
Ages (years)
0
20
40
60
80
FDR (Age associated divergence) =0.0902
p(Scalp)=0.00468
p(Dura)=0.314
Scalp
Dura

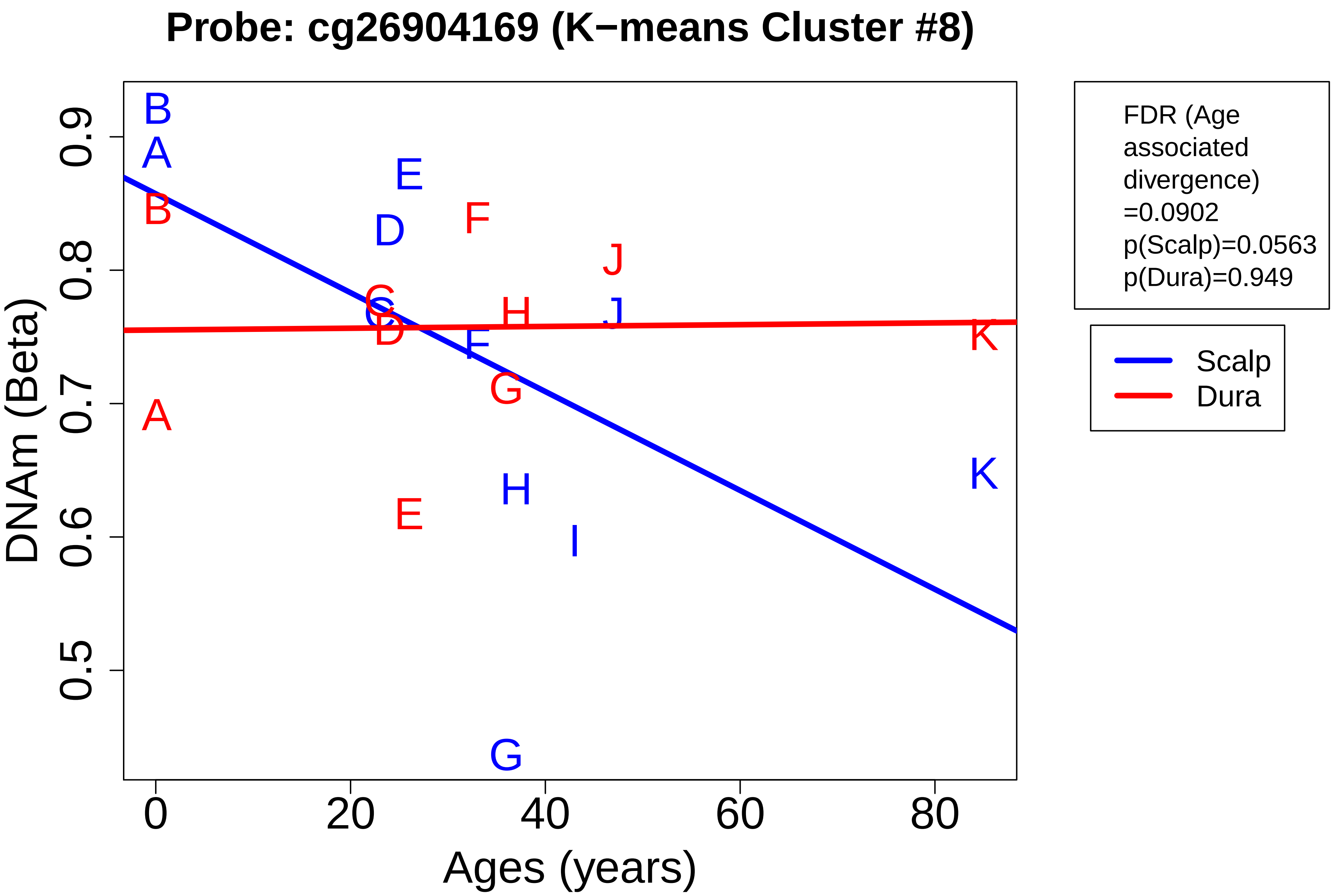

Probe: cg26904169 (K−means Cluster #8)
DNAm (Beta)
0.5
0.6
0.7
0.8
0.9
Ages (years)
0
20
40
60
80
B
A
B
E
D
F
J
C
C
D
H
J
E
K
G
A
K
H
E
I
G
FDR (Age associated divergence) =0.0902
p(Scalp)=0.0563
p(Dura)=0.949
Scalp
Dura

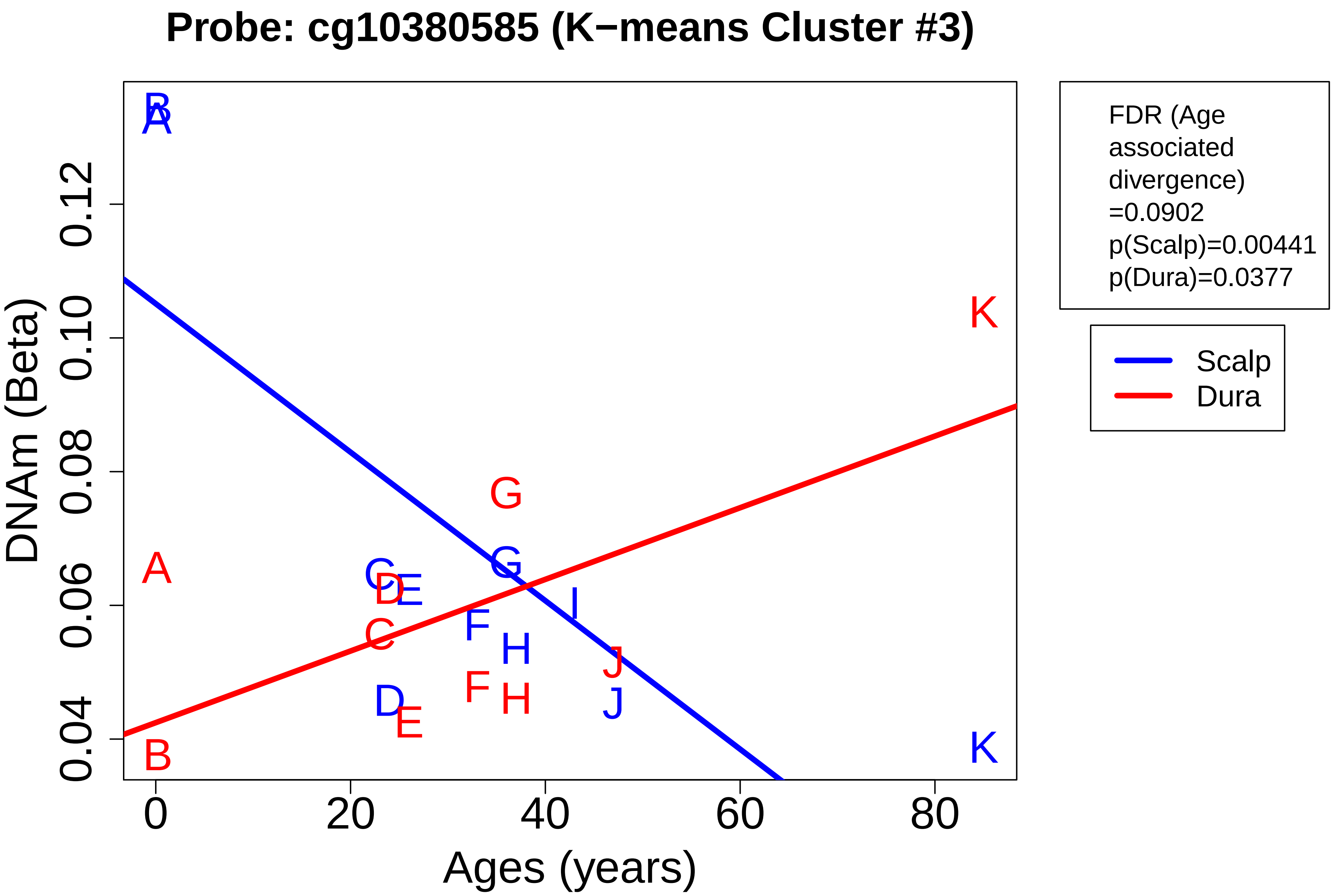

Probe: cg10380585 (K−means Cluster #3)
FDR (Age associated divergence) =0.0902
p(Scalp)=0.00441
p(Dura)=0.0377
Scalp
Dura
DNAm (Beta)
0.04
0.06
0.08
0.10
0.12
0
20
40
60
80
Ages (years)

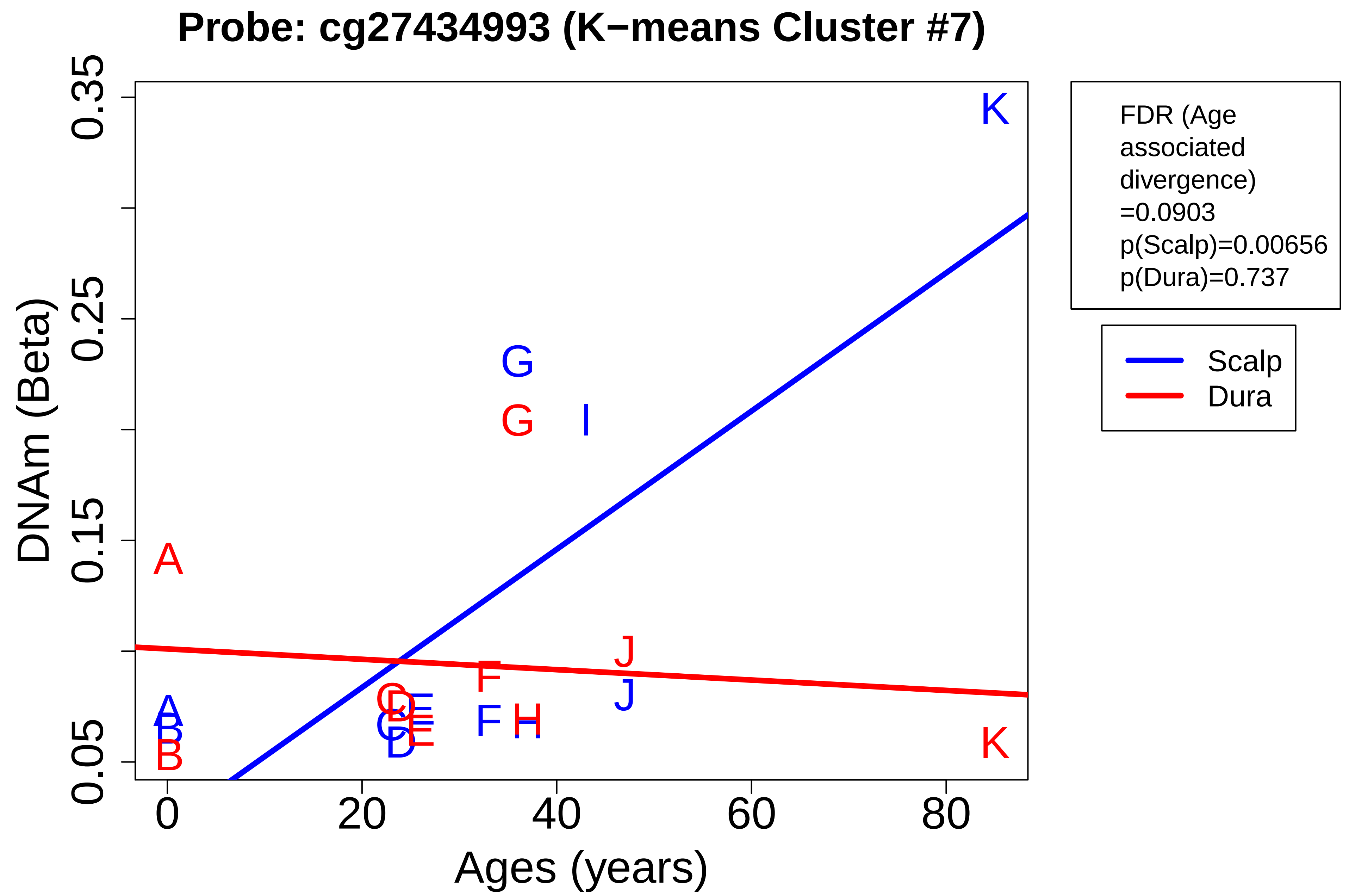

Probe: cg27434993 (K−means Cluster #7)
DNAm (Beta)
0.05
0.15
0.25
0.35
Ages (years)
0
20
40
60
80
K
G
G
I
A
J
F
J
A
B
B
C
D
E
C
D
E
F
H
H
K
FDR (Age associated divergence) =0.0903
p(Scalp)=0.00656
p(Dura)=0.737
Scalp
Dura

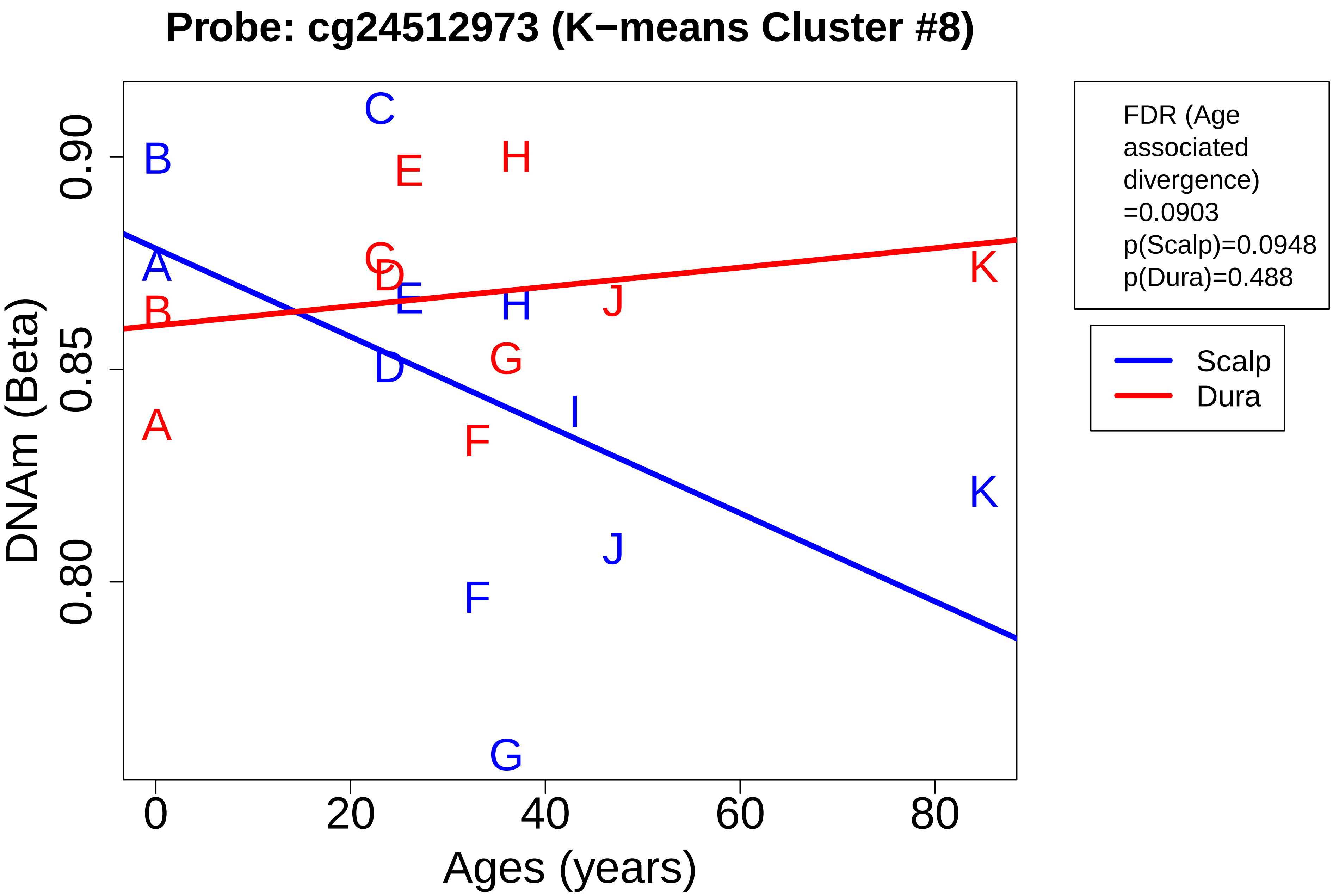

Probe: cg24512973 (K−means Cluster #8)
DNAm (Beta)
0.80
0.85
0.90
Ages (years)
0
20
40
60
80
FDR (Age associated divergence) =0.0903
p(Scalp)=0.0948
p(Dura)=0.488
Scalp
Dura
A
B
C
D
E
F
G
H
I
J
K

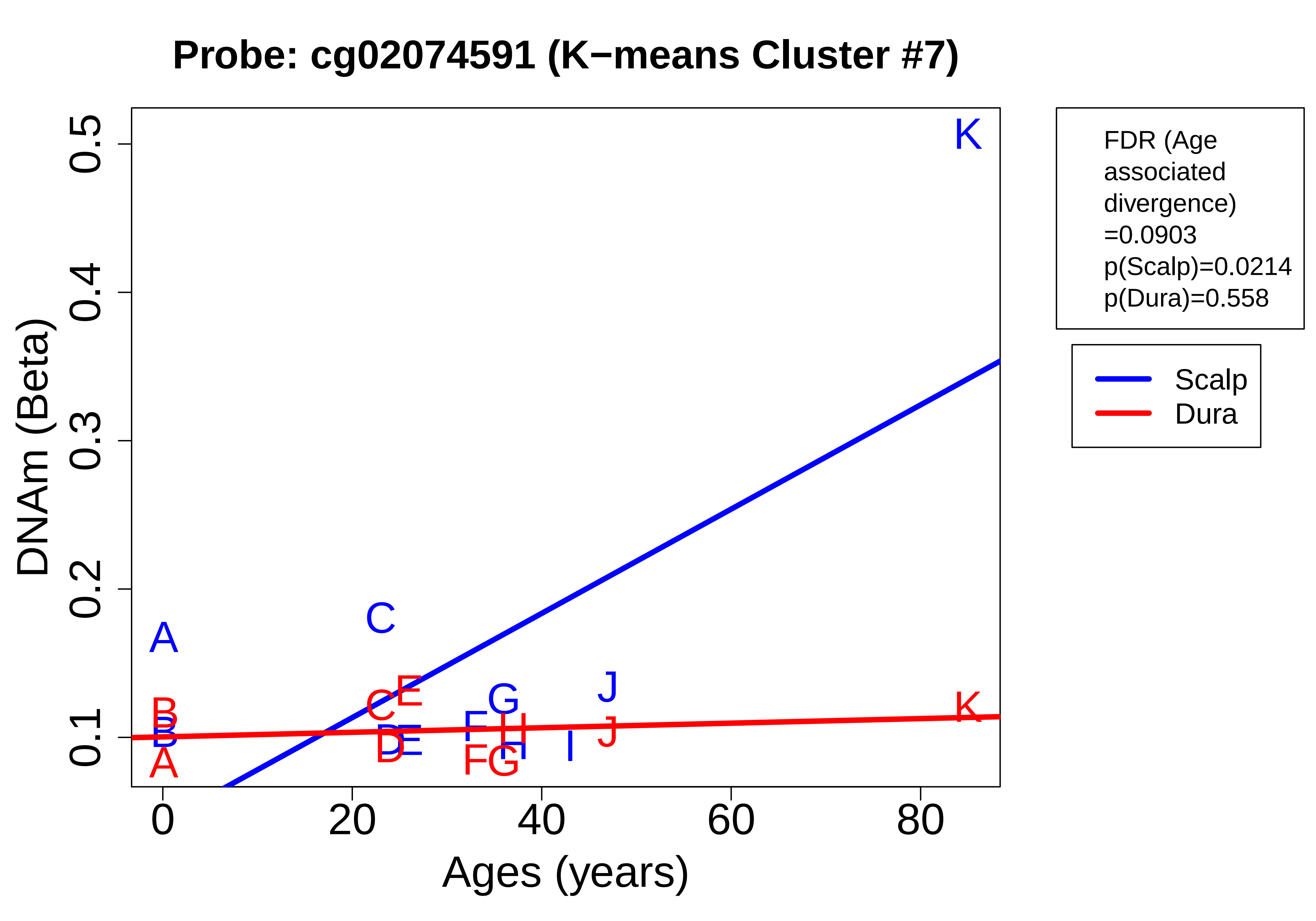

Probe: cg02074591 (K−means Cluster #7)
DNAm (Beta)
0.1
0.2
0.3
0.4
0.5
Ages (years)
0
20
40
60
80
FDR (Age associated divergence) =0.0903
p(Scalp)=0.0214
p(Dura)=0.558
Scalp
Dura

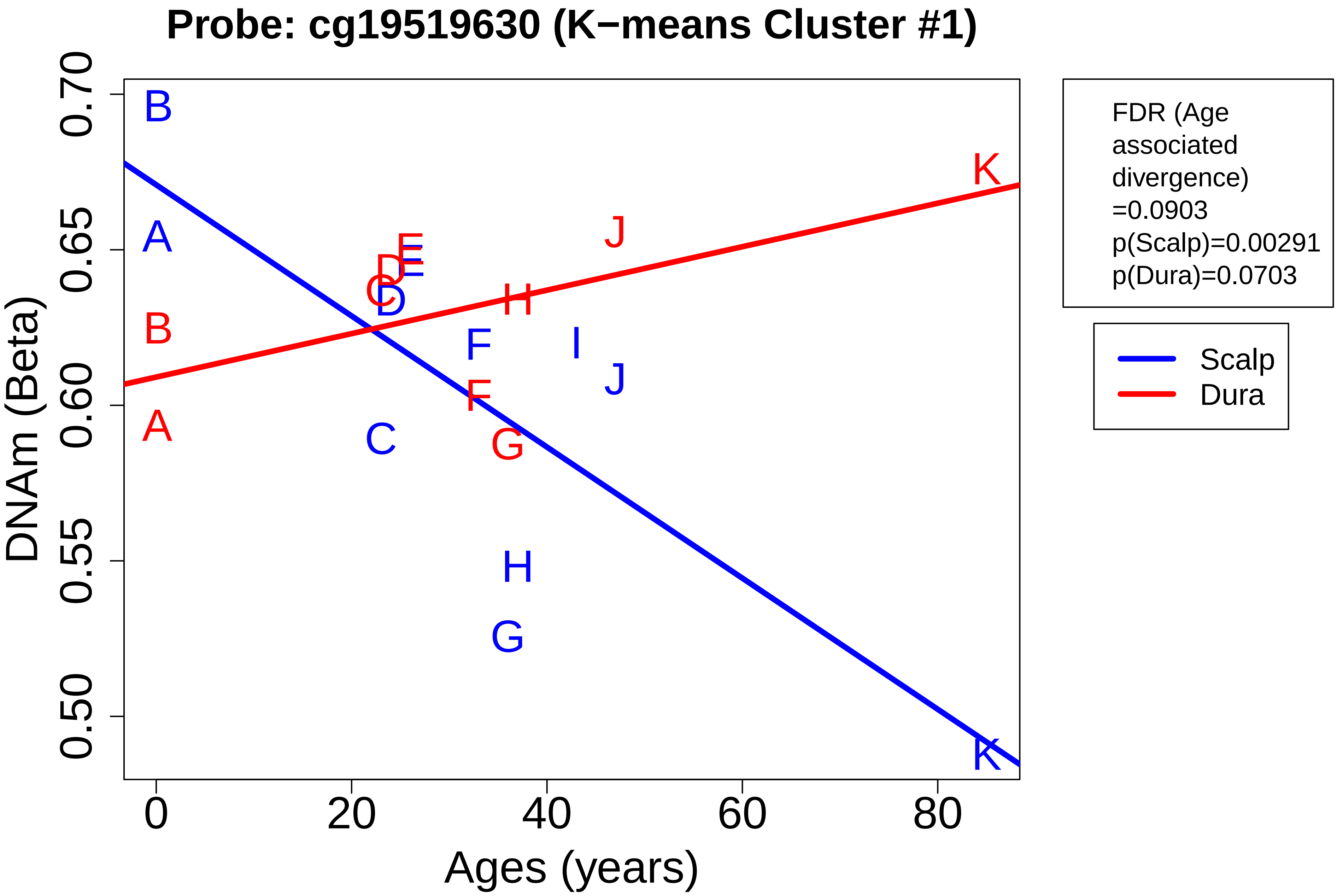
Probe: cg19519630 (K−means Cluster #1)
DNAm (Beta)
0.50
0.55
0.60
0.65
0.70
0
20
40
60
80
Ages (years)
FDR (Age associated divergence) =0.0903
p(Scalp)=0.00291
p(Dura)=0.0703
Scalp
Dura

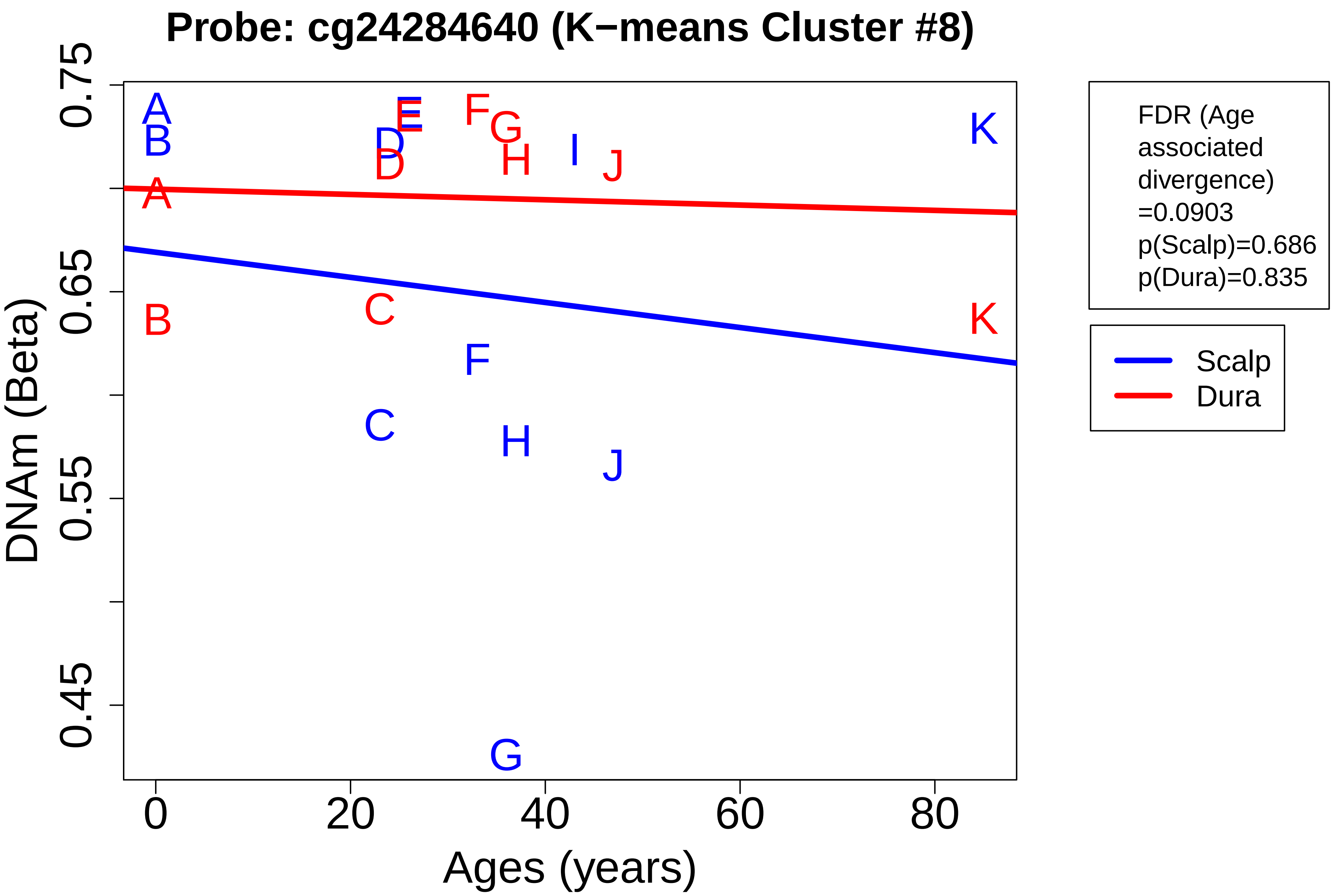

Probe: cg24284640 (K−means Cluster #8)
DNAm (Beta)
0.75
0.65
0.55
0.45
Ages (years)
0
20
40
60
80
A
B
C
D
E
F
G
H
I
J
K
FDR (Age associated divergence) =0.0903
p(Scalp)=0.686
p(Dura)=0.835
Scalp
Dura

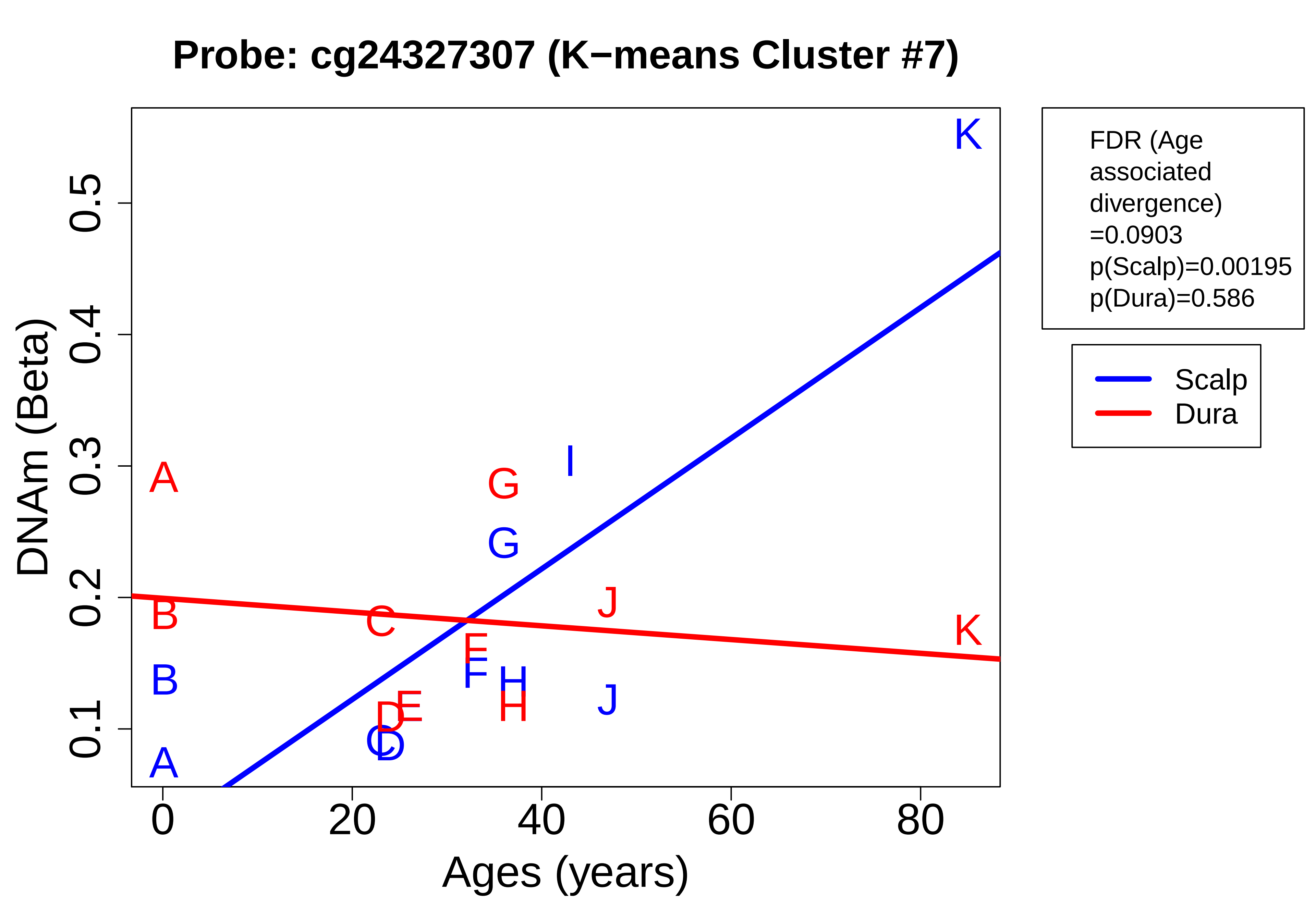

Probe: cg24327307 (K−means Cluster #7)
FDR (Age associated divergence) =0.0903
p(Scalp)=0.00195
p(Dura)=0.586
Scalp
Dura
DNAm (Beta)
0.1
0.2
0.3
0.4
0.5
0
20
40
60
80
Ages (years)
A
B
C
D
E
F
G
H
I
J
K

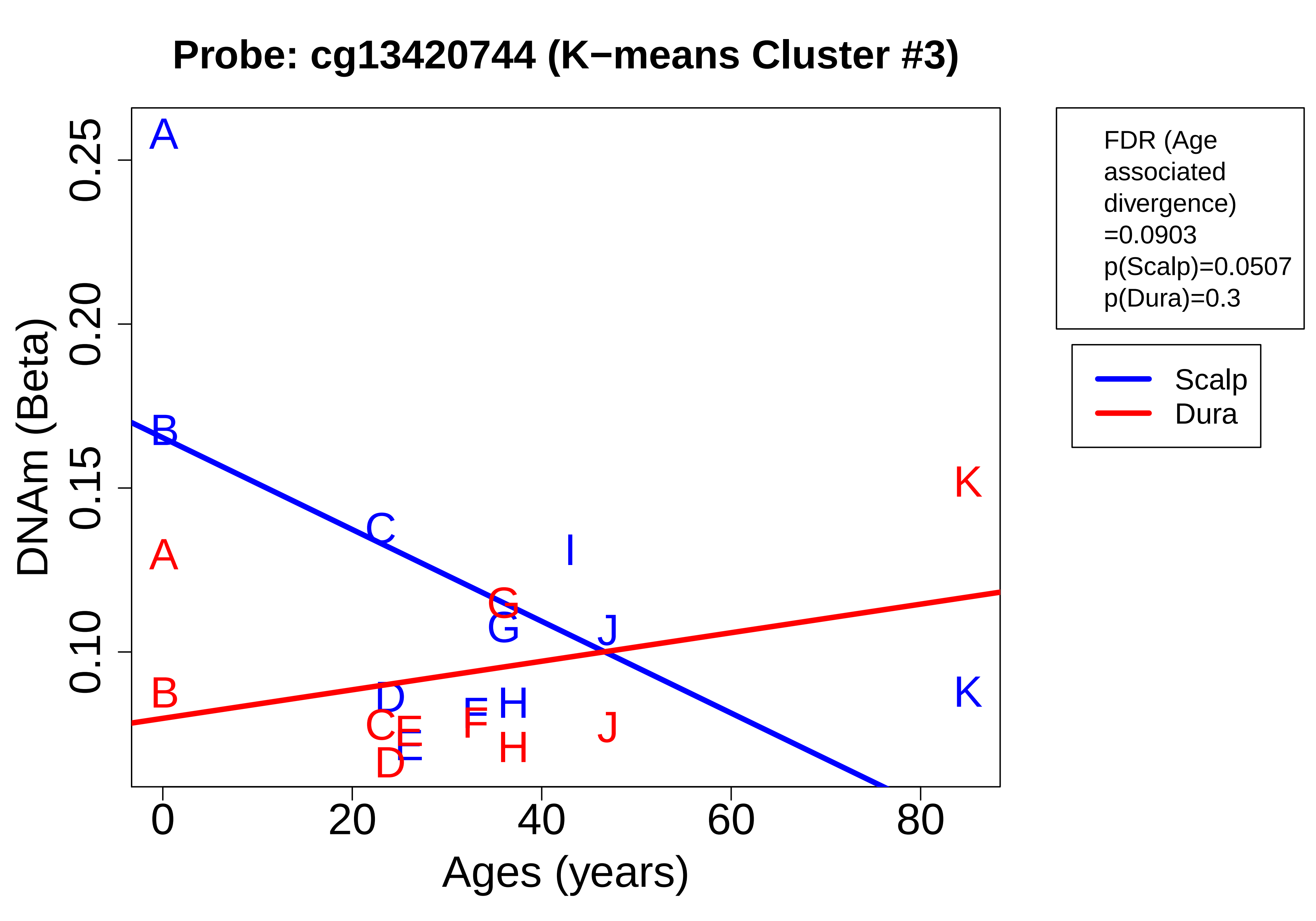

Probe: cg13420744 (K−means Cluster #3)
DNAm (Beta)
0.10
0.15
0.20
0.25
Ages (years)
0
20
40
60
80
A
B
C
D
E
F
G
H
I
J
K
FDR (Age associated divergence) =0.0903
p(Scalp)=0.0507
p(Dura)=0.3
Scalp
Dura

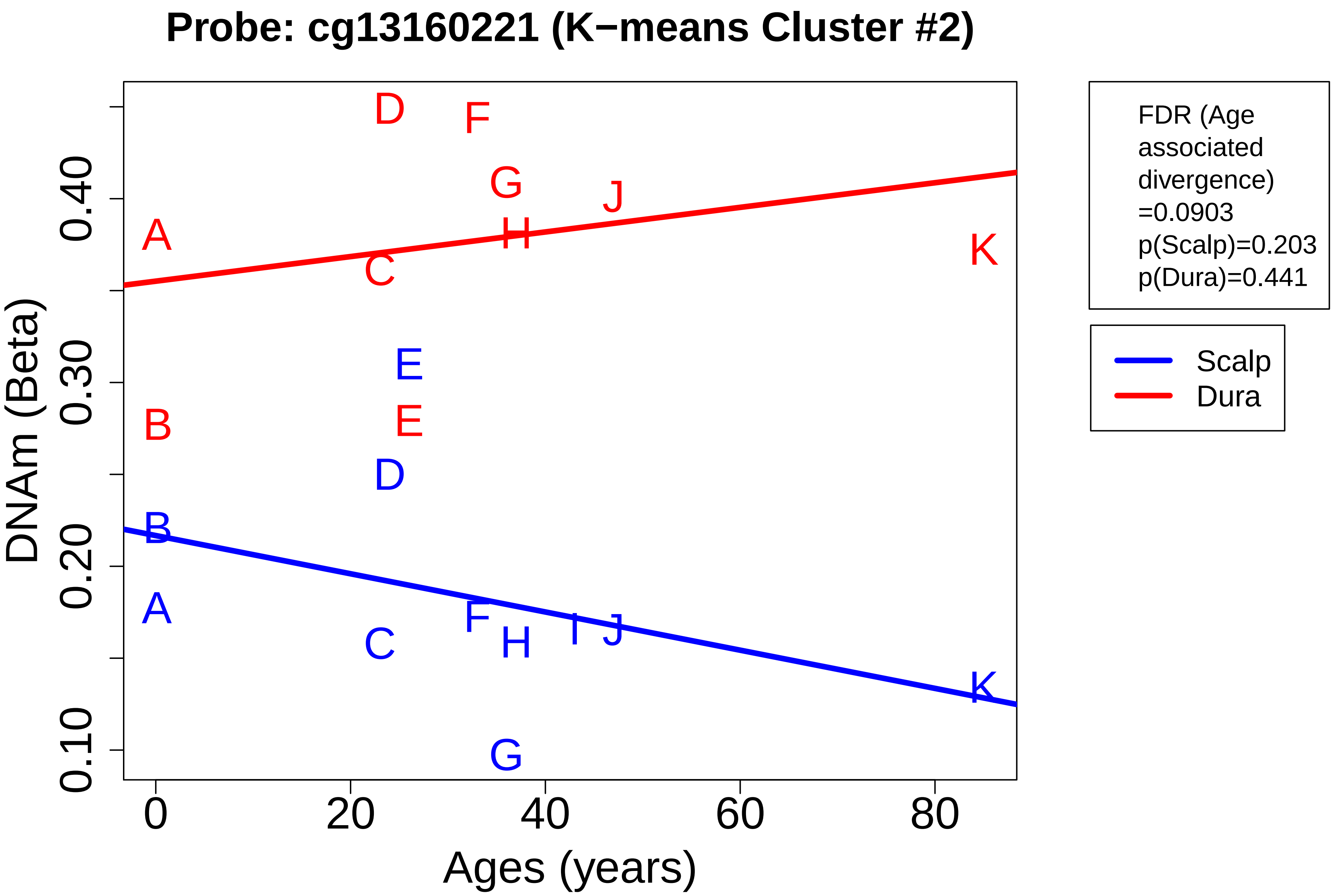
Probe: cg13160221 (K−means Cluster #2)
DNAm (Beta)
0.10
0.20
0.30
0.40
0
20
40
60
80
Ages (years)
FDR (Age associated divergence) =0.0903
p(Scalp)=0.203
p(Dura)=0.441
Scalp
Dura

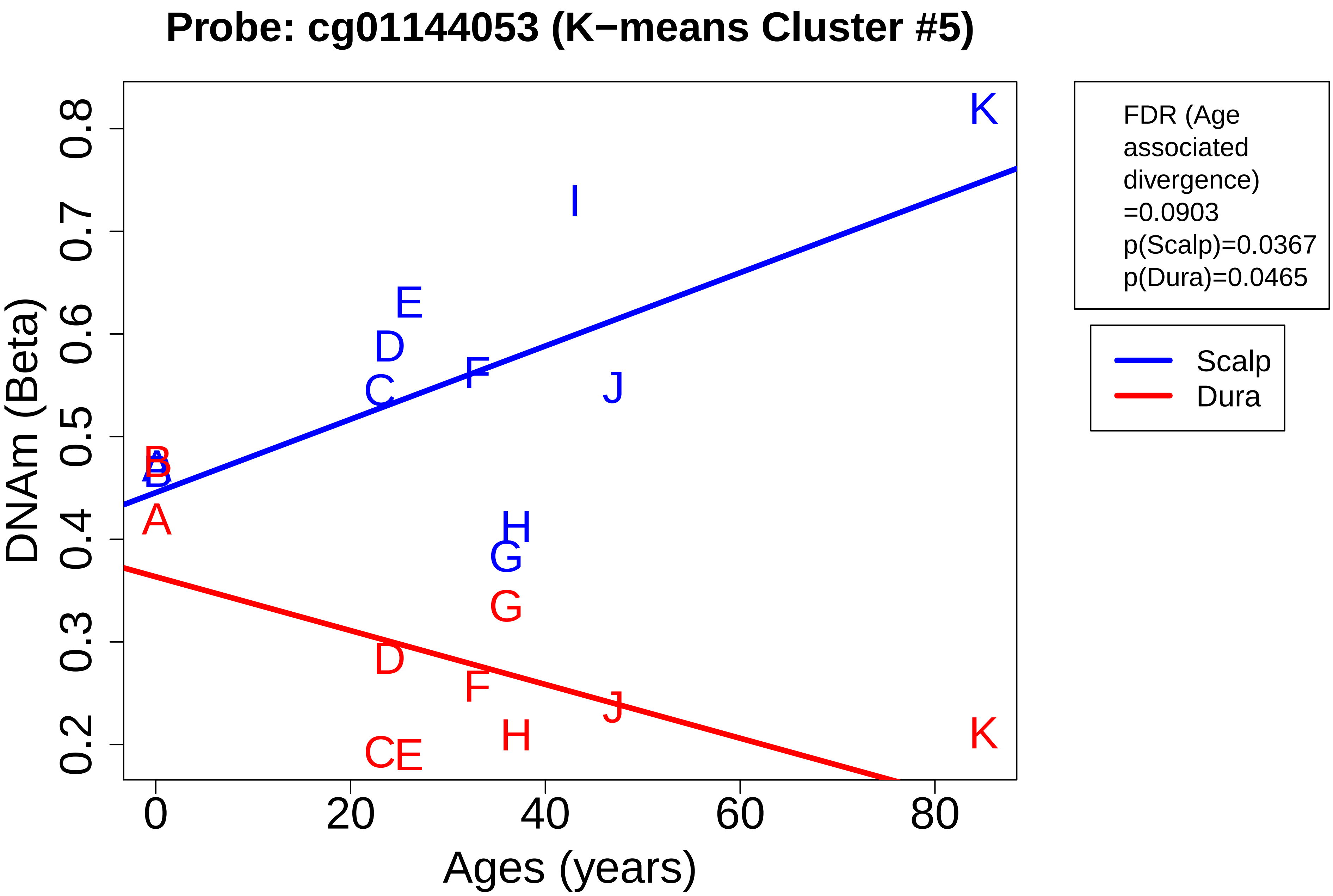

Probe: cg01144053 (K−means Cluster #5)
DNAm (Beta)
0.2
0.3
0.4
0.5
0.6
0.7
0.8
Ages (years)
0
20
40
60
80
FDR (Age associated divergence) =0.0903
p(Scalp)=0.0367
p(Dura)=0.0465
Scalp
Dura

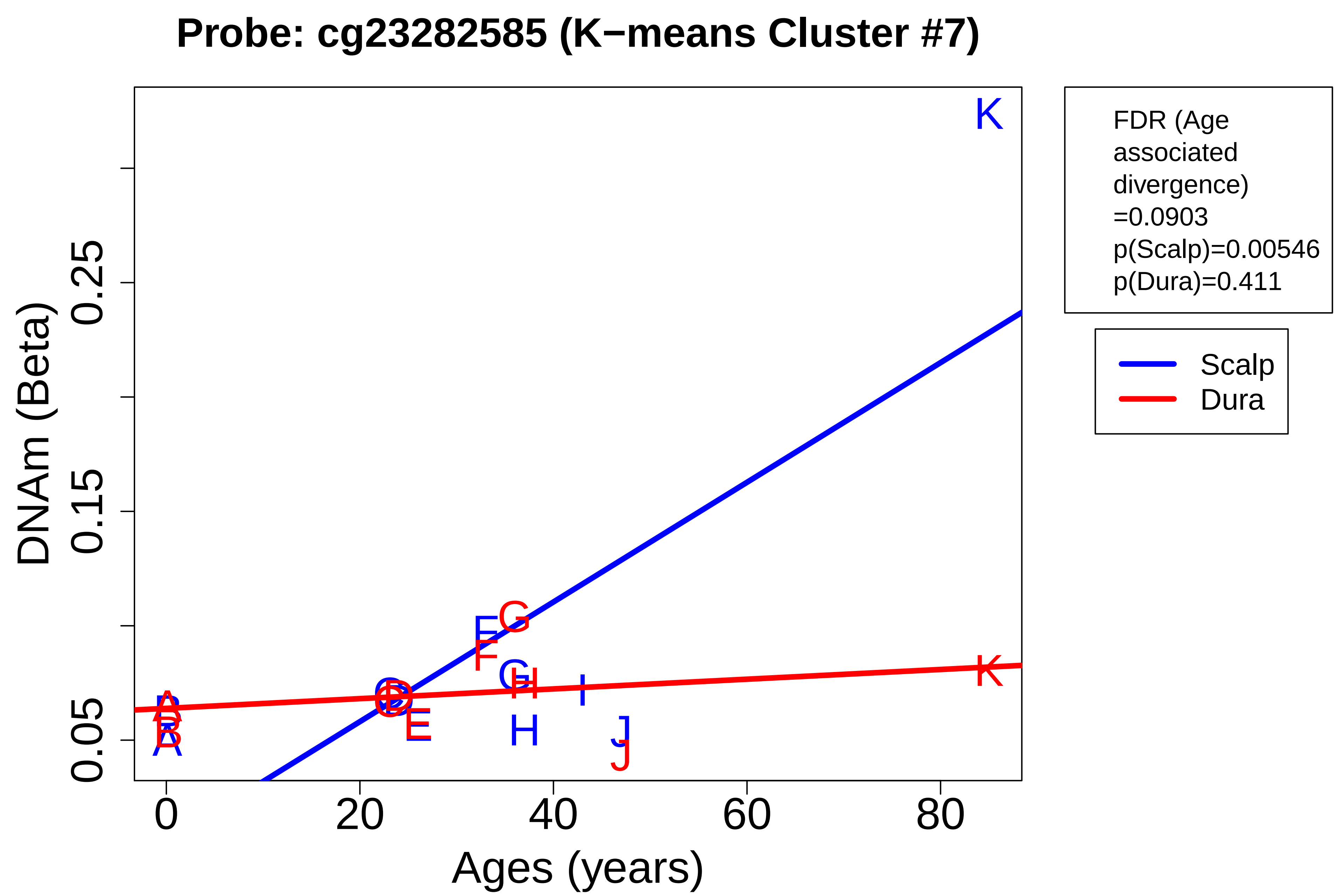

Probe: cg23282585 (K−means Cluster #7)
DNAm (Beta)
0.05
0.15
0.25
Ages (years)
0
20
40
60
80
A
B
C
D
E
F
G
H
I
J
K
FDR (Age associated divergence) =0.0903
p(Scalp)=0.00546
p(Dura)=0.411
Scalp
Dura

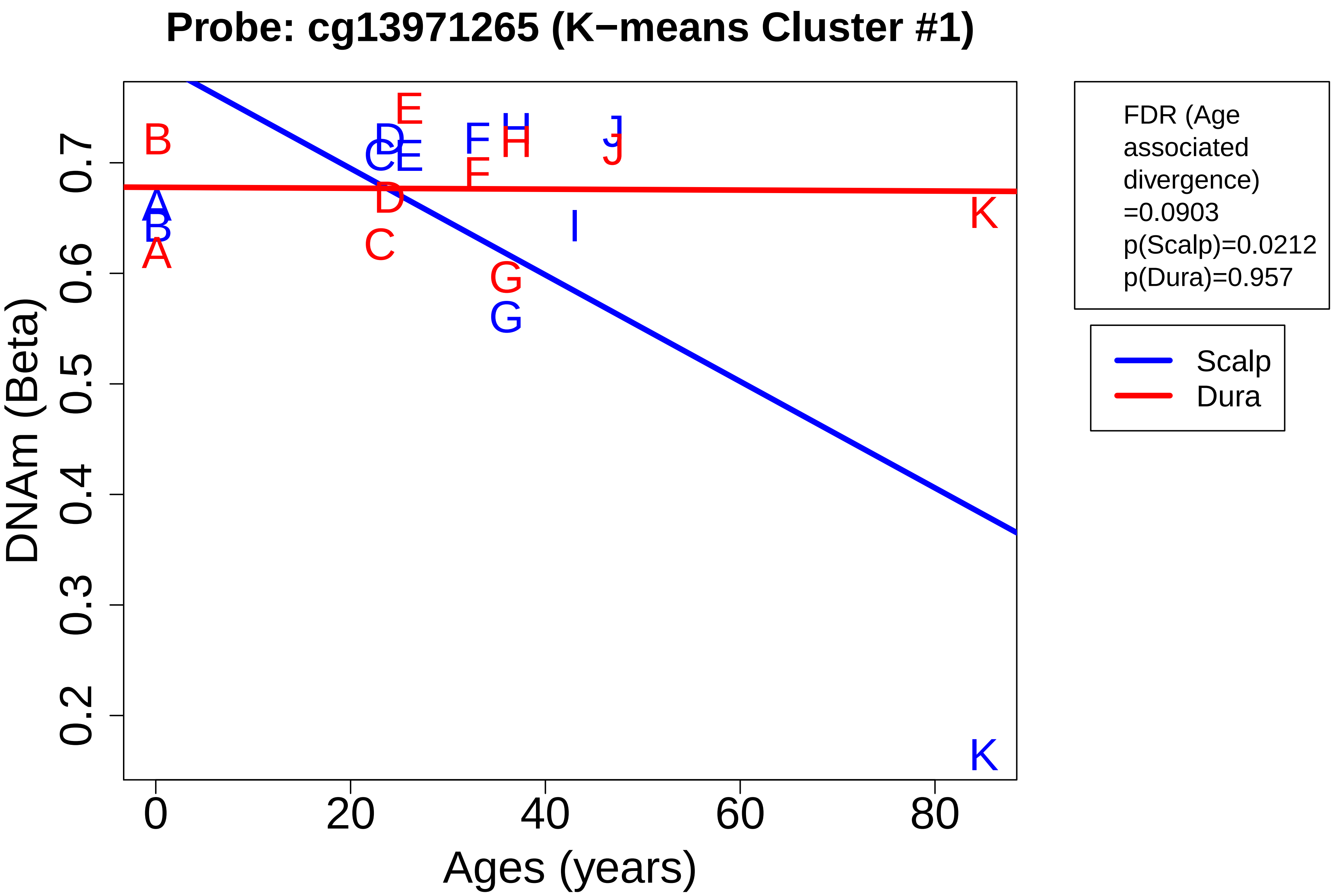
Probe: cg13971265 (K−means Cluster #1)
DNAm (Beta)
0.2
0.3
0.4
0.5
0.6
0.7
Ages (years)
0
20
40
60
80
FDR (Age associated divergence) =0.0903
p(Scalp)=0.0212
p(Dura)=0.957
Scalp
Dura

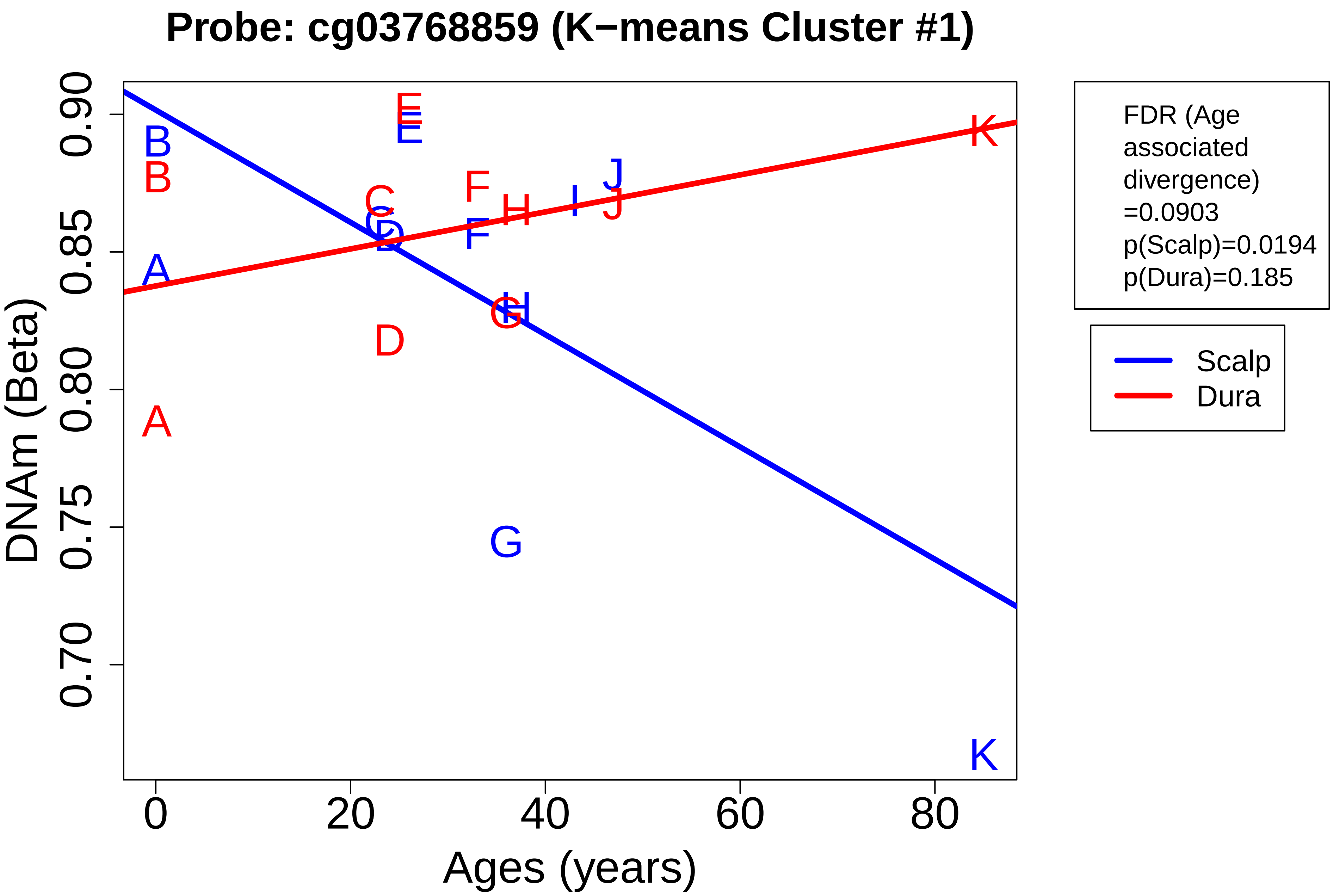

Probe: cg03768859 (K−means Cluster #1)
DNAm (Beta)
0.70
0.75
0.80
0.85
0.90
0
20
40
60
80
Ages (years)
FDR (Age associated divergence) =0.0903
p(Scalp)=0.0194
p(Dura)=0.185
Scalp
Dura

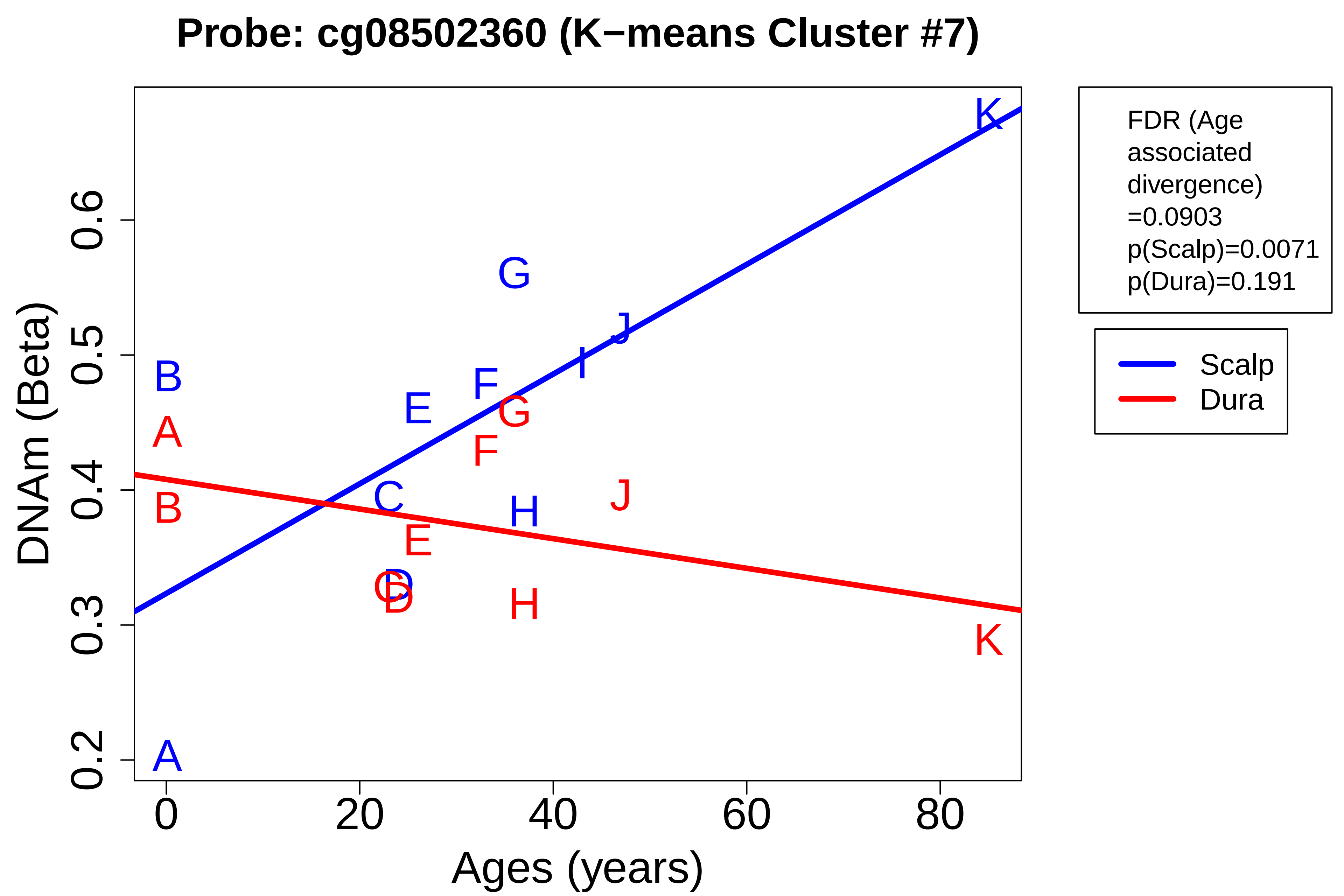

Probe: cg08502360 (K−means Cluster #7)
FDR (Age associated divergence) =0.0903
p(Scalp)=0.0071
p(Dura)=0.191
Scalp
Dura
DNAm (Beta)
0.2
0.3
0.4
0.5
0.6
Ages (years)
0
20
40
60
80
A
B
C
D
E
F
G
H
I
J
K

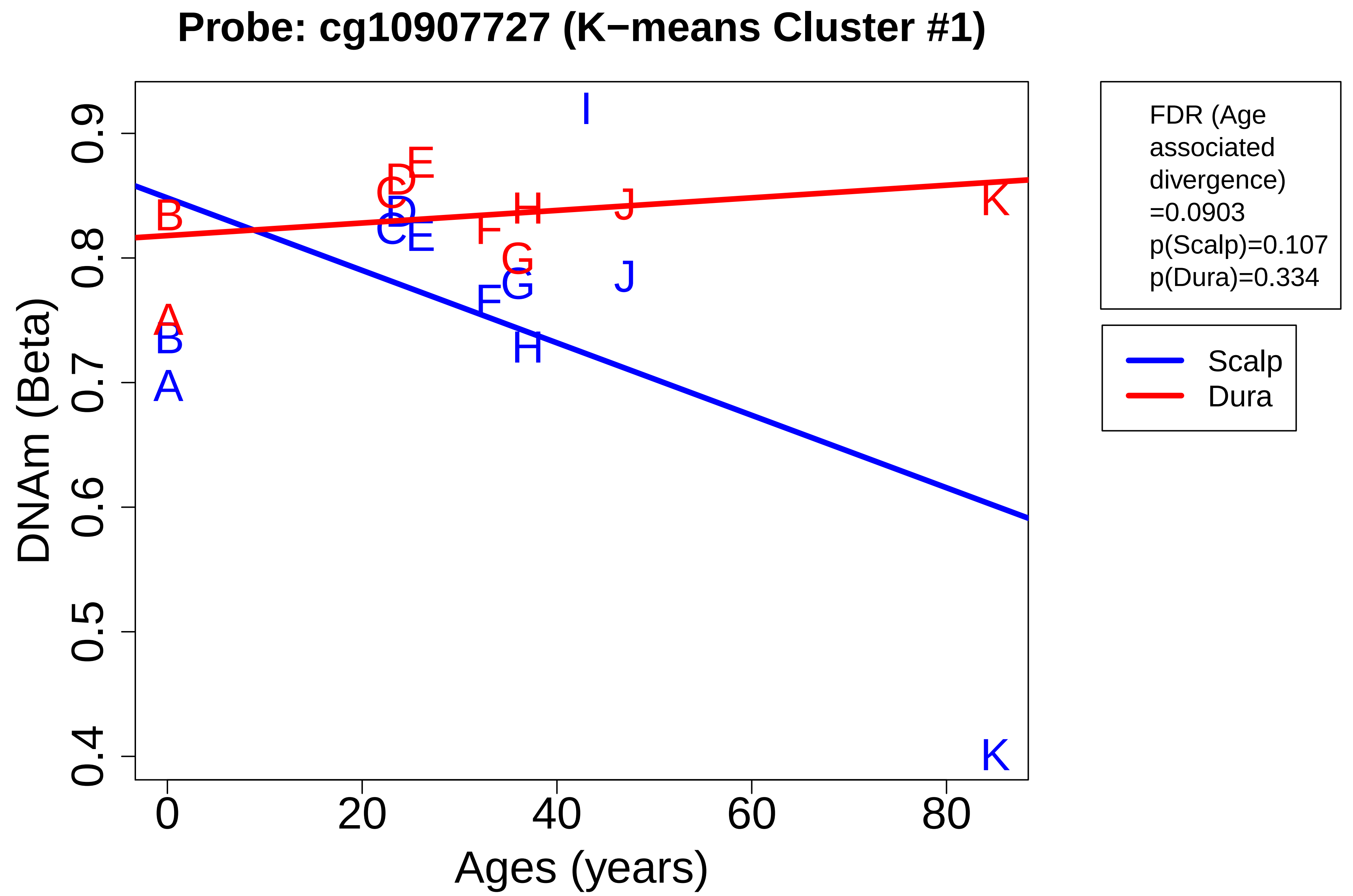

Probe: cg10907727 (K−means Cluster #1)
DNAm (Beta)
0.4
0.5
0.6
0.7
0.8
0.9
0
20
40
60
80
Ages (years)
FDR (Age associated divergence) =0.0903
p(Scalp)=0.107
p(Dura)=0.334
Scalp
Dura

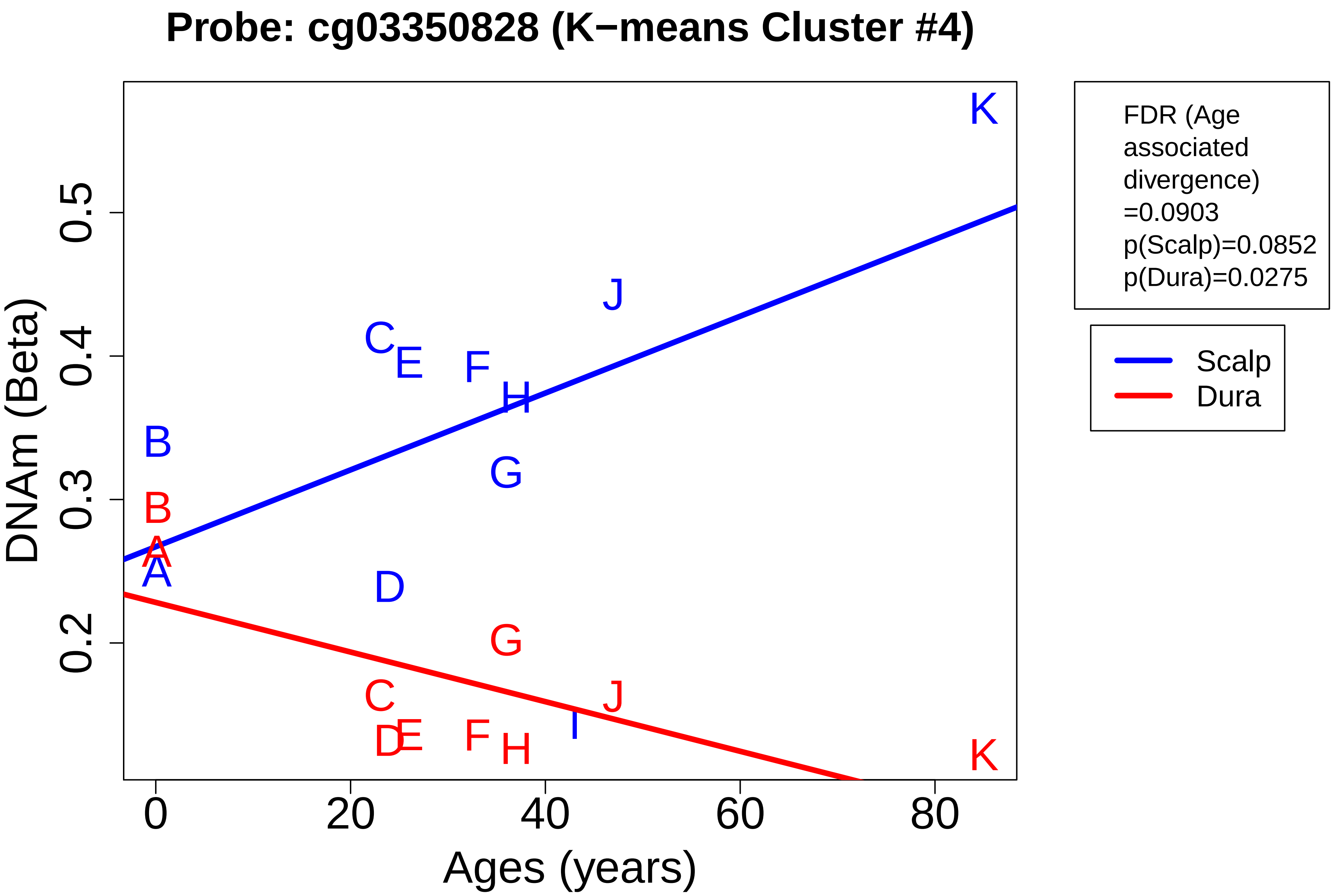

Probe: cg03350828 (K−means Cluster #4)
DNAm (Beta)
0.2
0.3
0.4
0.5
0
20
40
60
80
Ages (years)
FDR (Age associated divergence) =0.0903
p(Scalp)=0.0852
p(Dura)=0.0275
Scalp
Dura

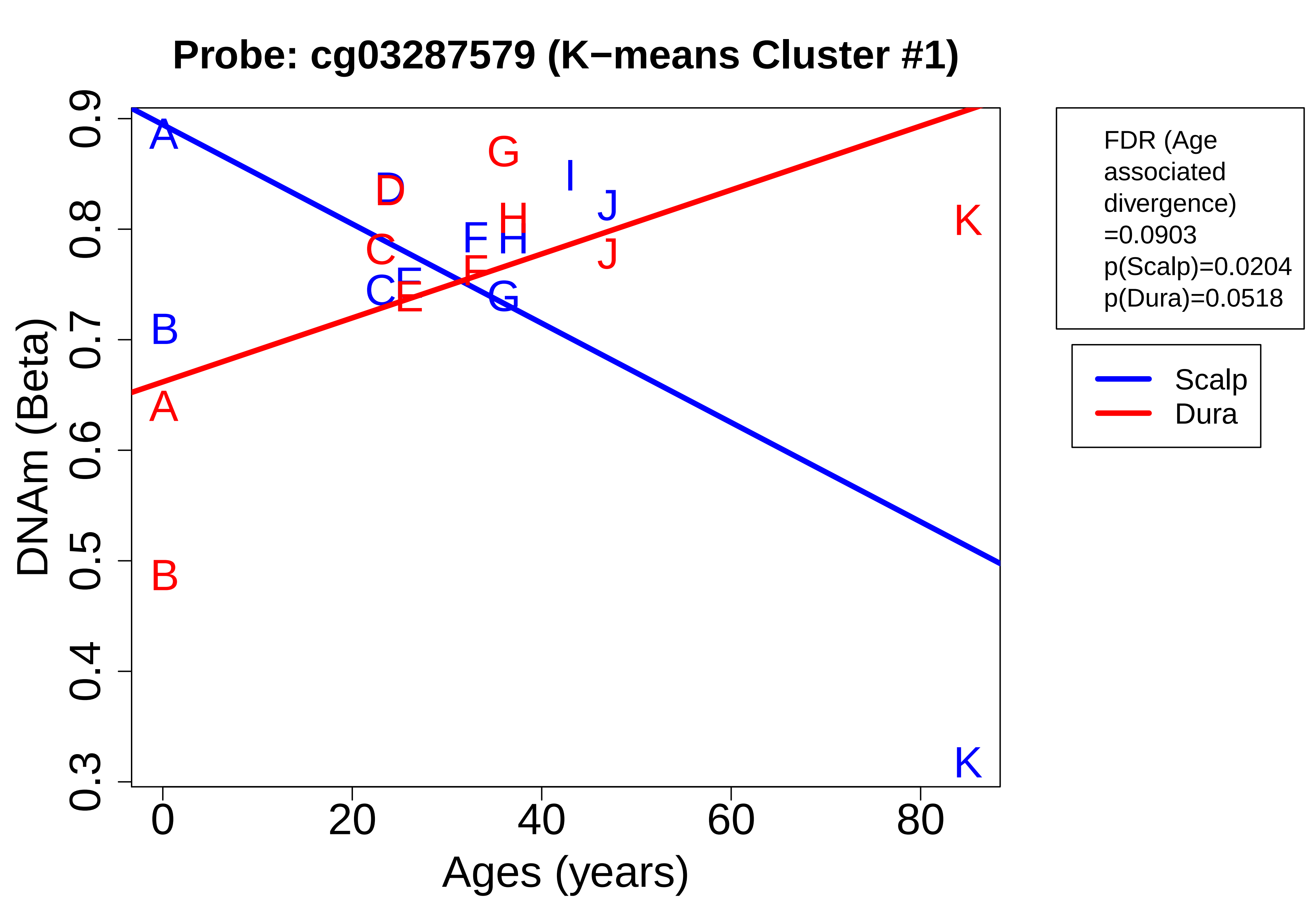

Probe: cg03287579 (K−means Cluster #1)
DNAm (Beta)
0.3
0.4
0.5
0.6
0.7
0.8
0.9
Ages (years)
0
20
40
60
80
FDR (Age associated divergence) =0.0903
p(Scalp)=0.0204
p(Dura)=0.0518
Scalp
Dura

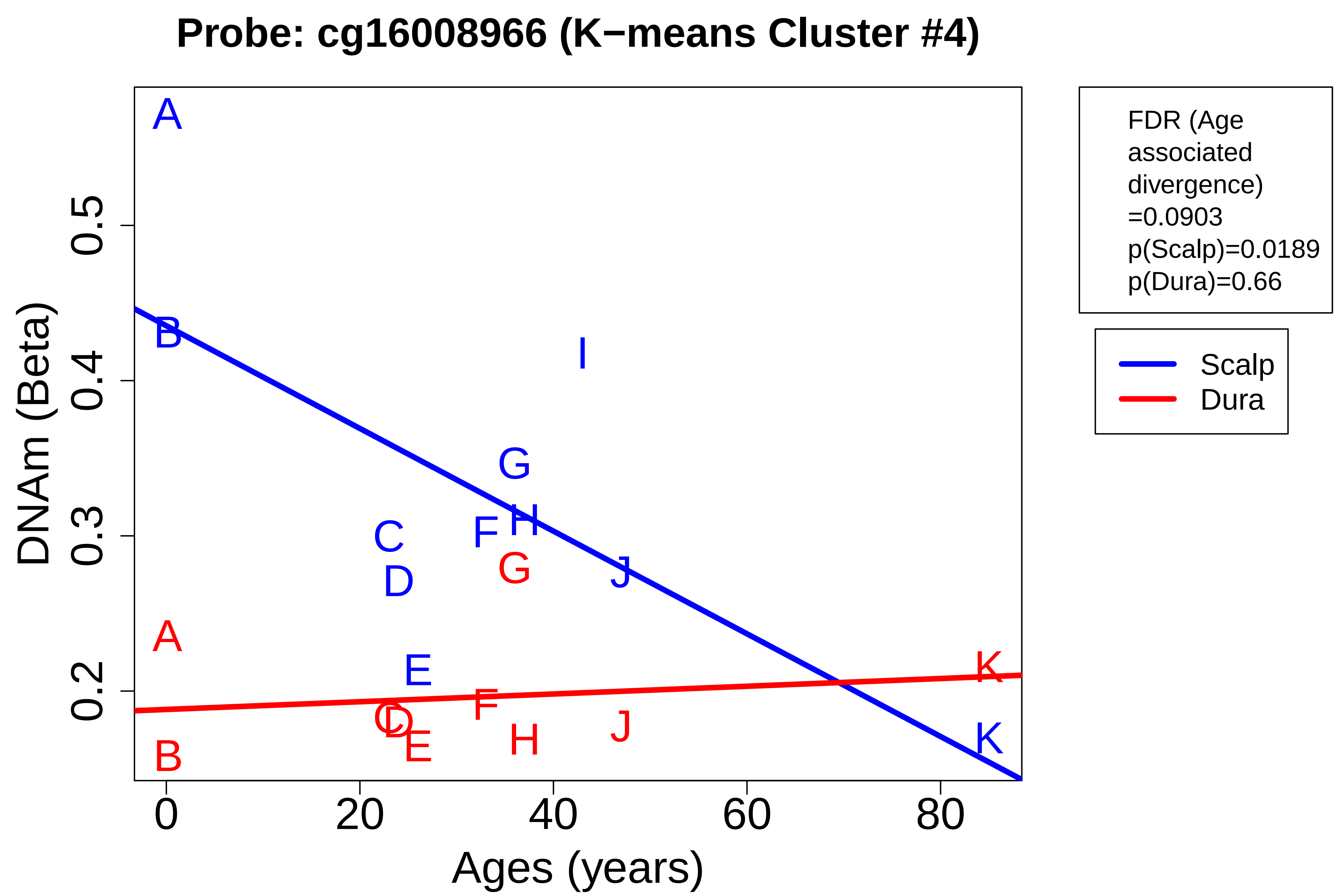

Probe: cg16008966 (K−means Cluster #4)
DNAm (Beta)
0.2
0.3
0.4
0.5
0
20
40
60
80
Ages (years)
FDR (Age associated divergence) =0.0903
p(Scalp)=0.0189
p(Dura)=0.66
Scalp
Dura
A
B
C
D
E
F
G
H
I
J
K

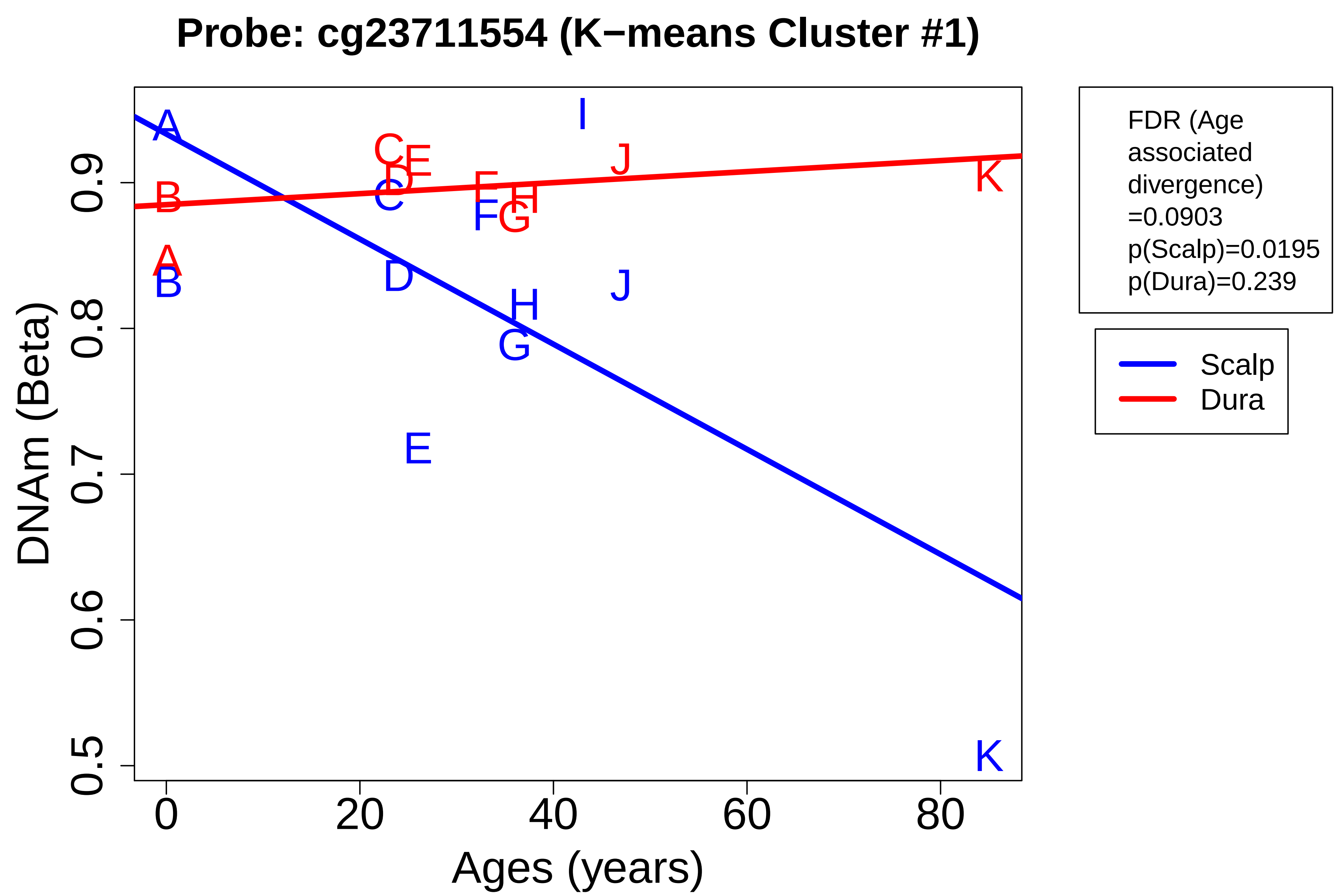

Probe: cg23711554 (K−means Cluster #1)
DNAm (Beta)
0.5
0.6
0.7
0.8
0.9
0
20
40
60
80
Ages (years)
FDR (Age associated divergence) =0.0903
p(Scalp)=0.0195
p(Dura)=0.239
Scalp
Dura
A
B
C
D
E
F
G
H
I
J
K

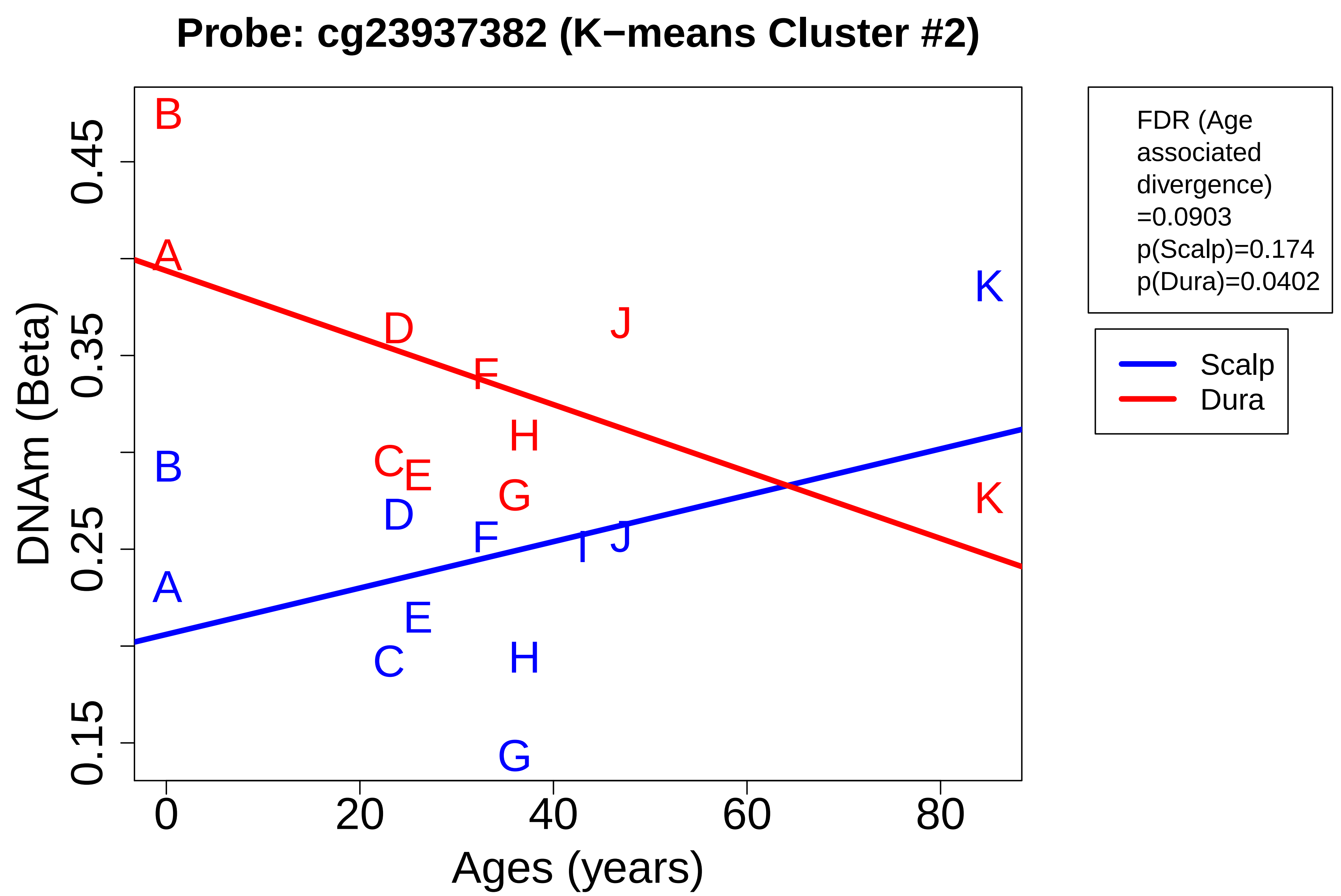
Probe: cg23937382 (K−means Cluster #2)
DNAm (Beta)
0.15
0.25
0.35
0.45
Ages (years)
0
20
40
60
80
FDR (Age associated divergence) =0.0903
p(Scalp)=0.174
p(Dura)=0.0402
Scalp
Dura

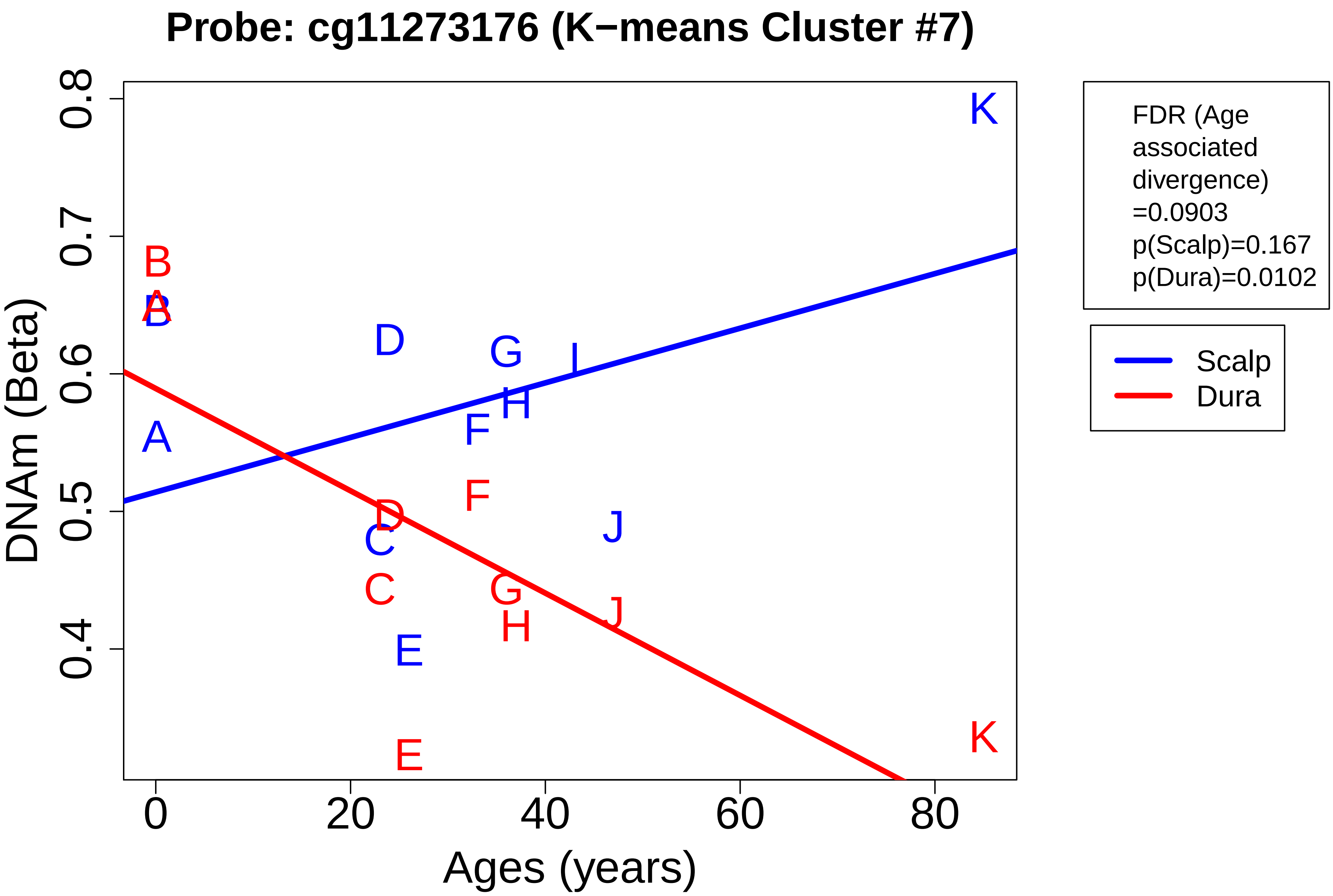

Probe: cg11273176 (K−means Cluster #7)
DNAm (Beta)
0.4
0.5
0.6
0.7
0.8
0
20
40
60
80
Ages (years)
FDR (Age associated divergence) =0.0903
p(Scalp)=0.167
p(Dura)=0.0102
Scalp
Dura
A
B
C
D
E
F
G
H
I
J
K

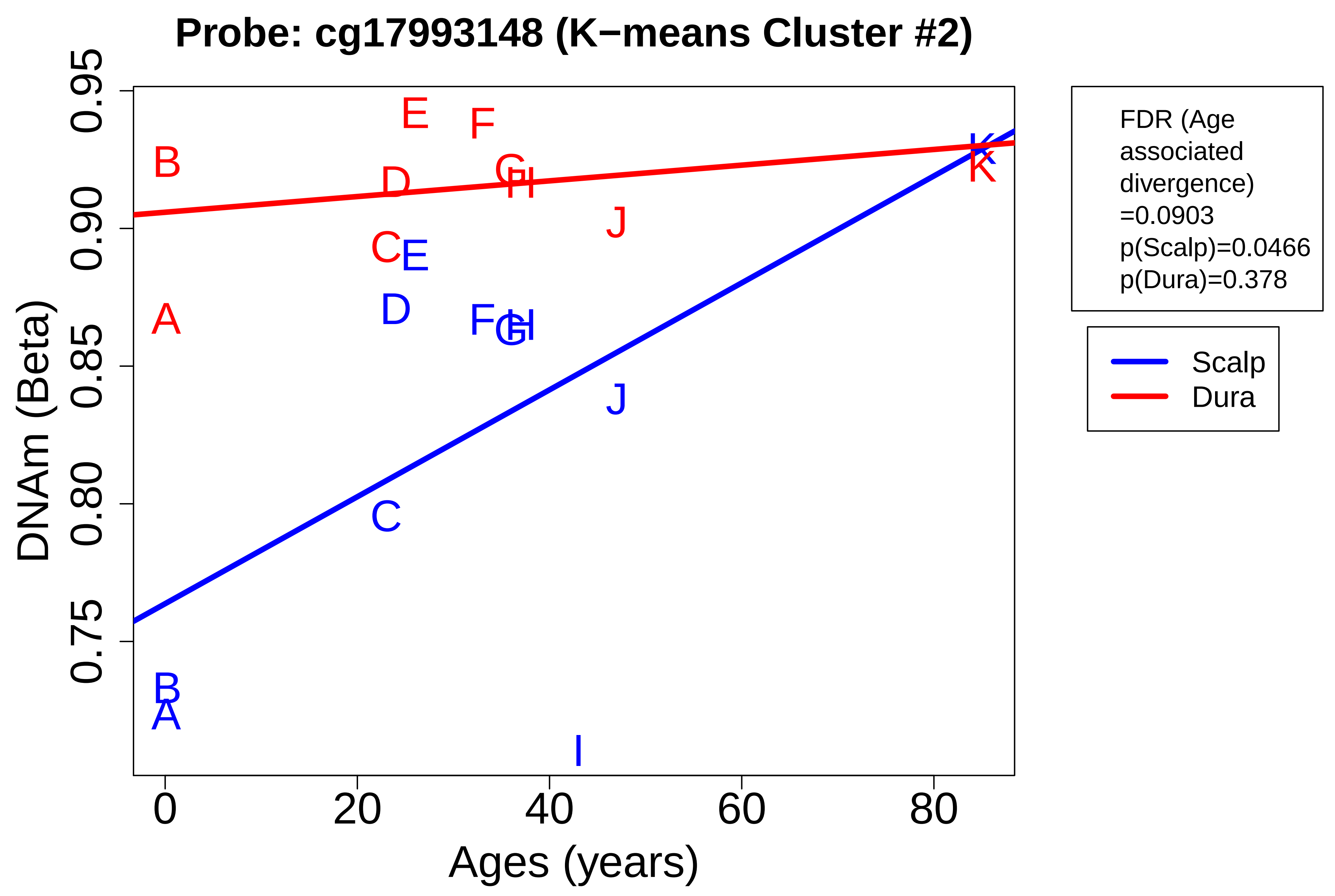
Probe: cg17993148 (K−means Cluster #2)
DNAm (Beta)
0.75
0.80
0.85
0.90
0.95
0
20
40
60
80
Ages (years)
FDR (Age associated divergence) =0.0903
p(Scalp)=0.0466
p(Dura)=0.378
Scalp
Dura

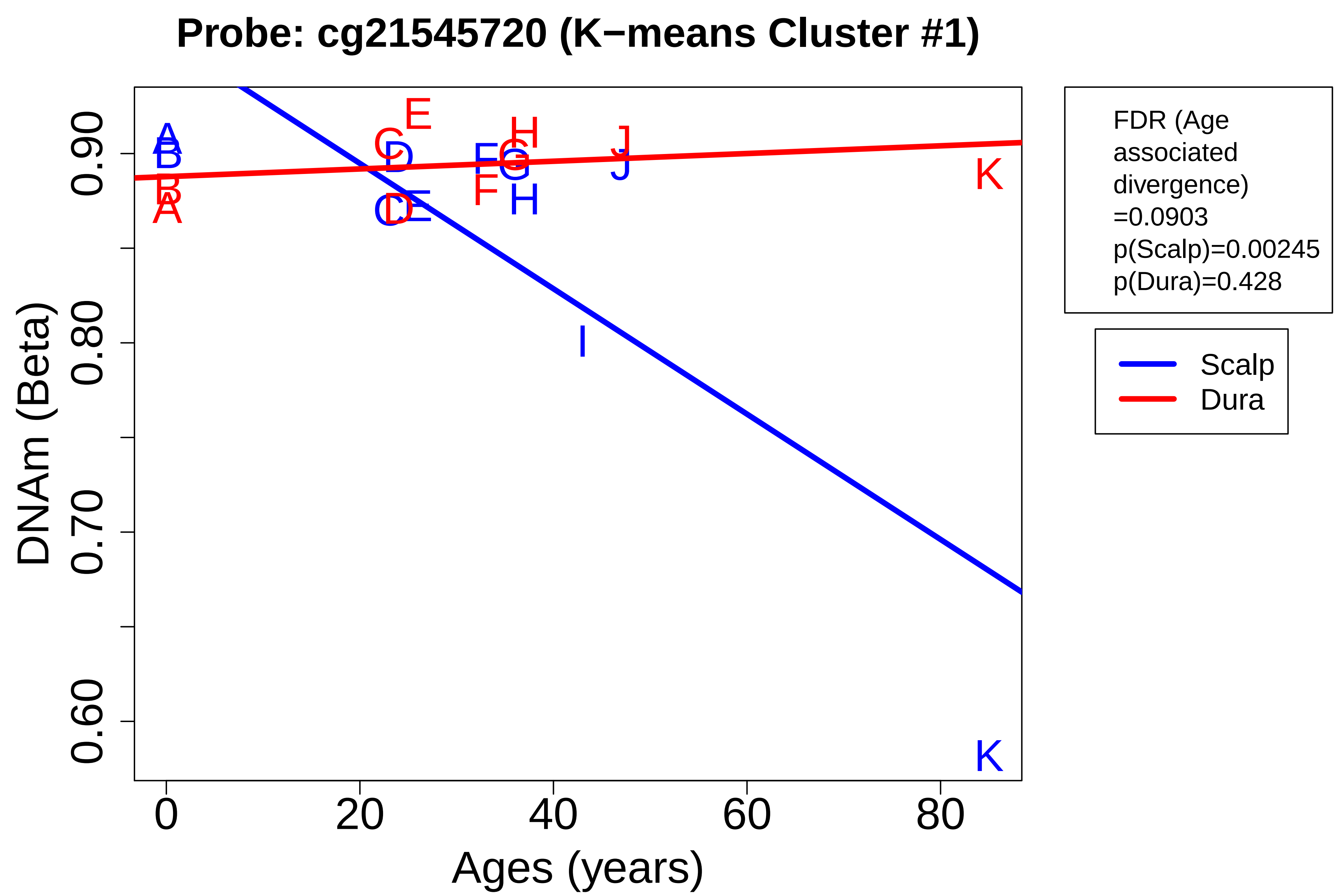

Probe: cg21545720 (K−means Cluster #1)
FDR (Age associated divergence) =0.0903
p(Scalp)=0.00245
p(Dura)=0.428
Scalp
Dura
DNAm (Beta)
0.60
0.70
0.80
0.90
Ages (years)
0
20
40
60
80

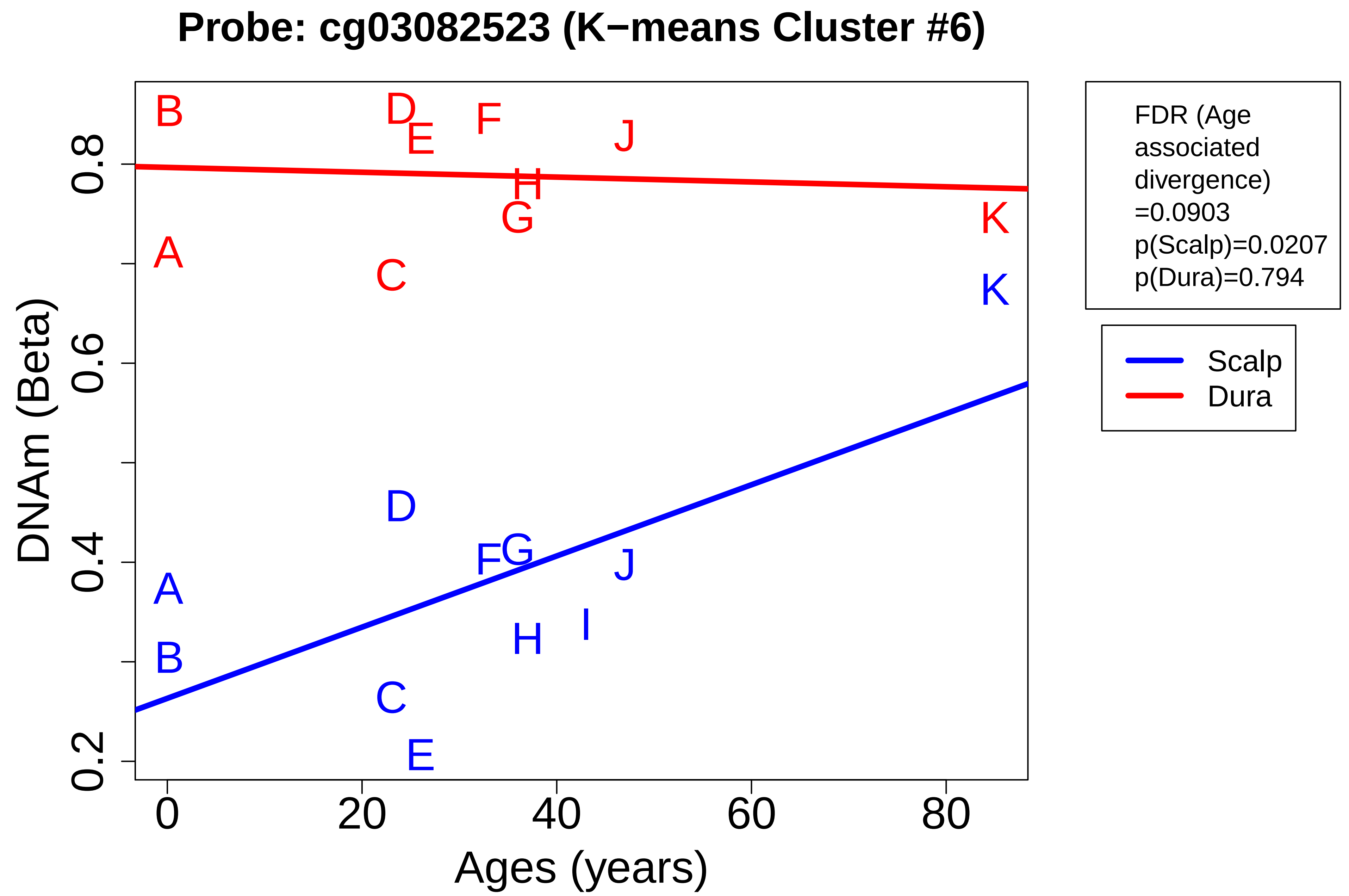

Probe: cg03082523 (K−means Cluster #6)
DNAm (Beta)
0.2
0.4
0.6
0.8
Ages (years)
0
20
40
60
80
FDR (Age associated divergence) =0.0903
p(Scalp)=0.0207
p(Dura)=0.794
Scalp
Dura
A
B
C
D
E
F
G
H
I
J
K

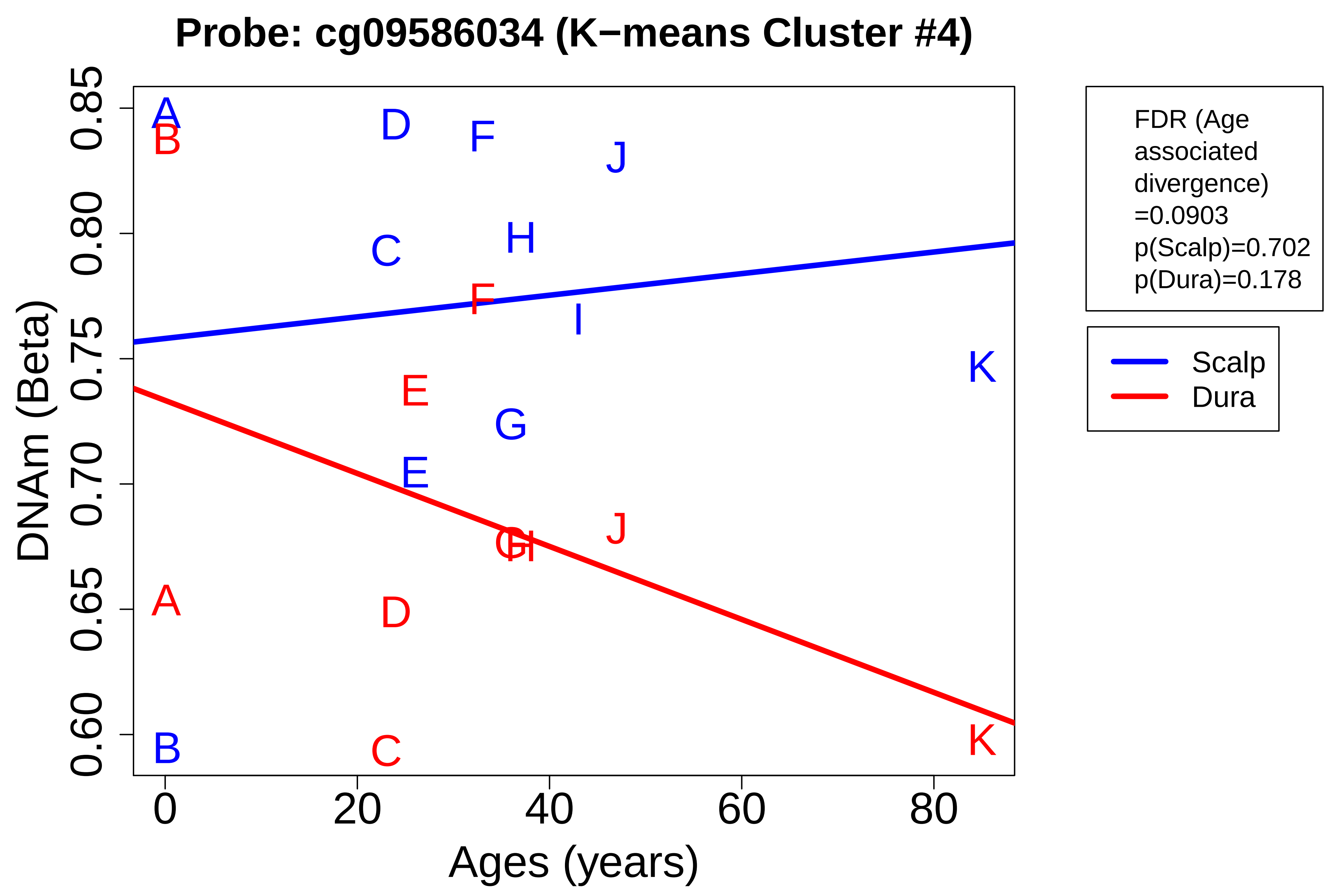

Probe: cg09586034 (K−means Cluster #4)
DNAm (Beta)
0.60
0.65
0.70
0.75
0.80
0.85
Ages (years)
0
20
40
60
80
FDR (Age associated divergence) =0.0903
p(Scalp)=0.702
p(Dura)=0.178
Scalp
Dura

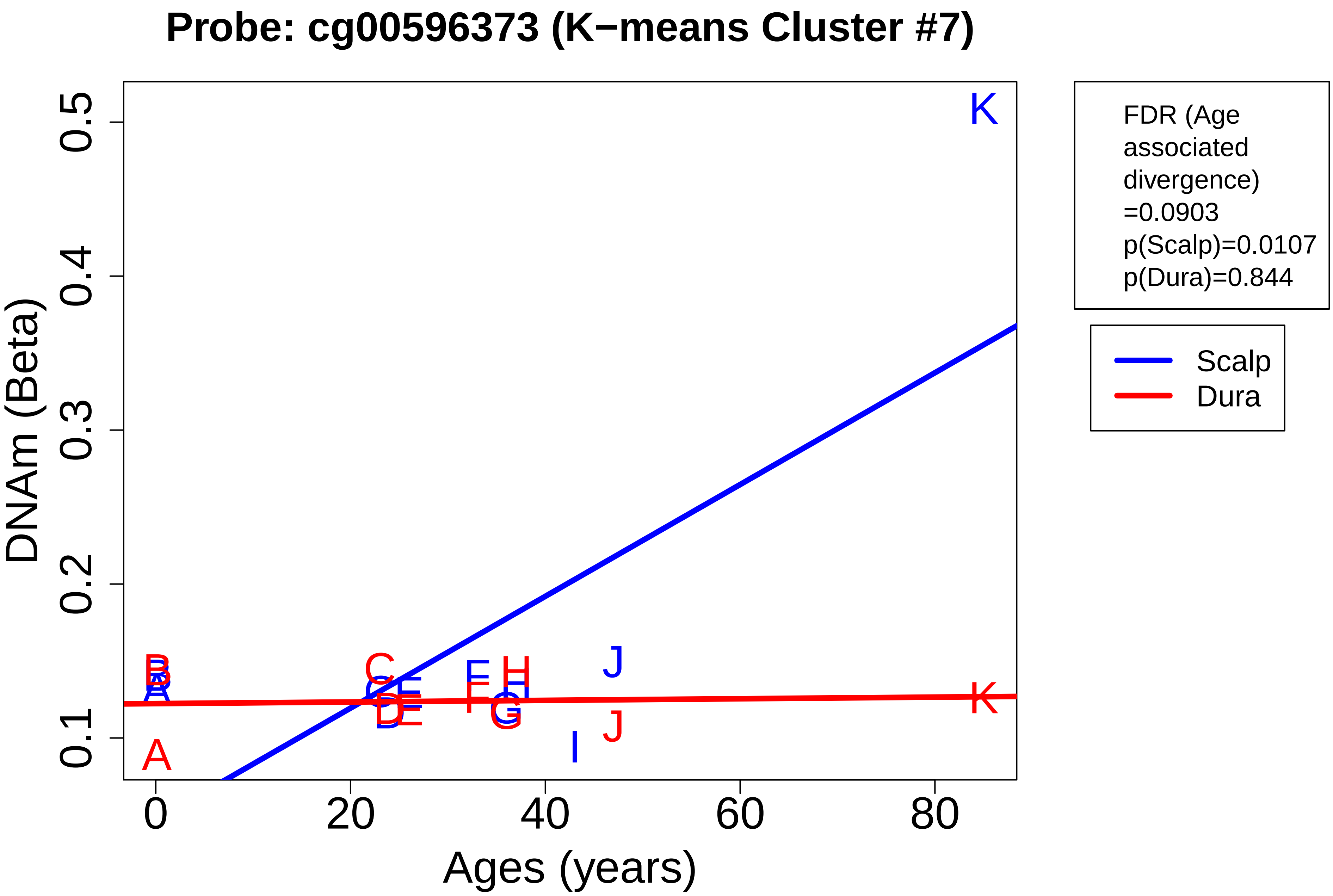

Probe: cg00596373 (K−means Cluster #7)
DNAm (Beta)
0.1
0.2
0.3
0.4
0.5
0
20
40
60
80
Ages (years)
A
B
C
D
E
F
G
H
I
J
K
FDR (Age associated divergence) =0.0903
p(Scalp)=0.0107
p(Dura)=0.844
Scalp
Dura

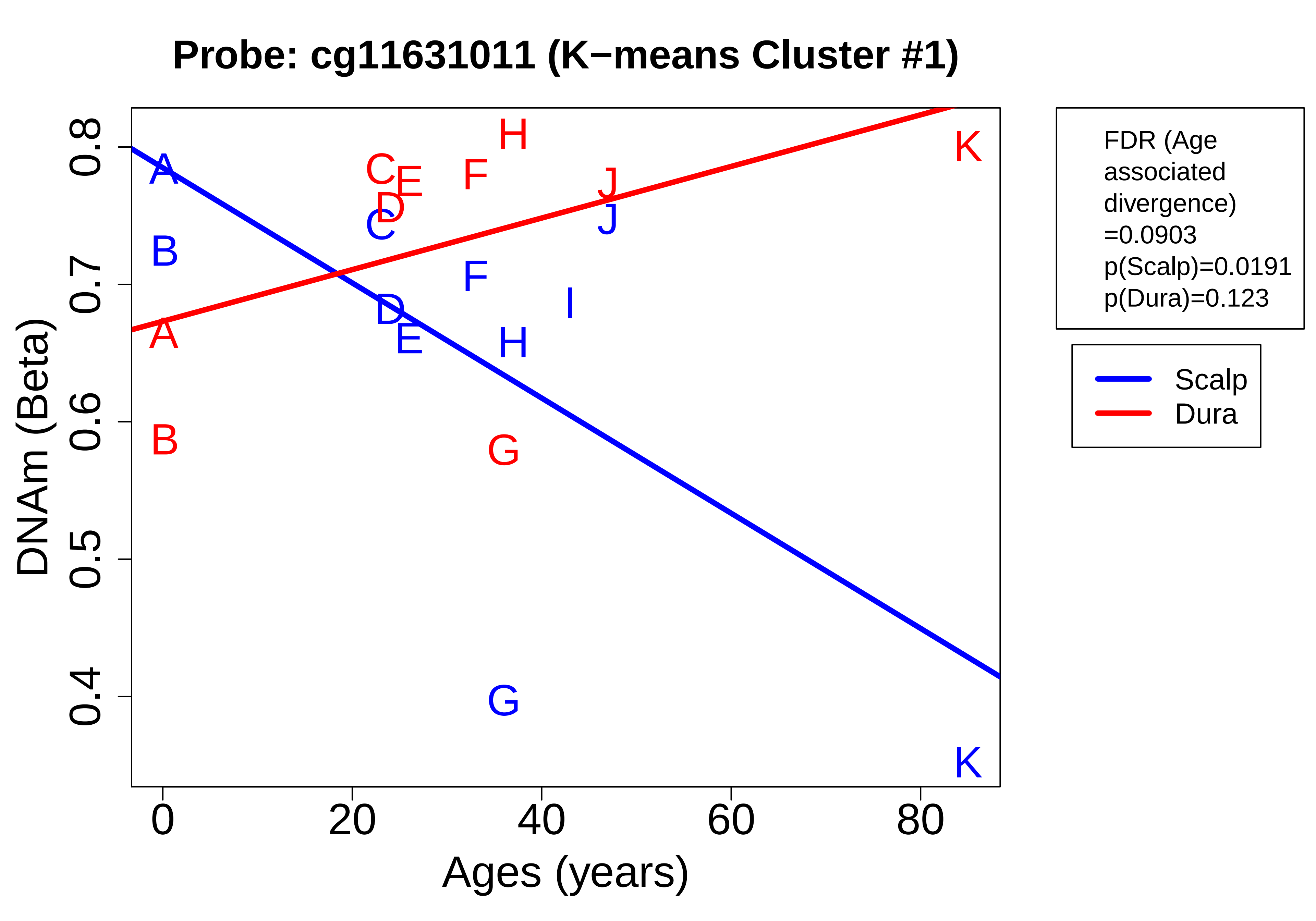

Probe: cg11631011 (K−means Cluster #1)
DNAm (Beta)
0.4
0.5
0.6
0.7
0.8
0
20
40
60
80
Ages (years)
A
B
C
D
E
F
G
H
I
J
K
FDR (Age associated divergence) =0.0903
p(Scalp)=0.0191
p(Dura)=0.123
Scalp
Dura

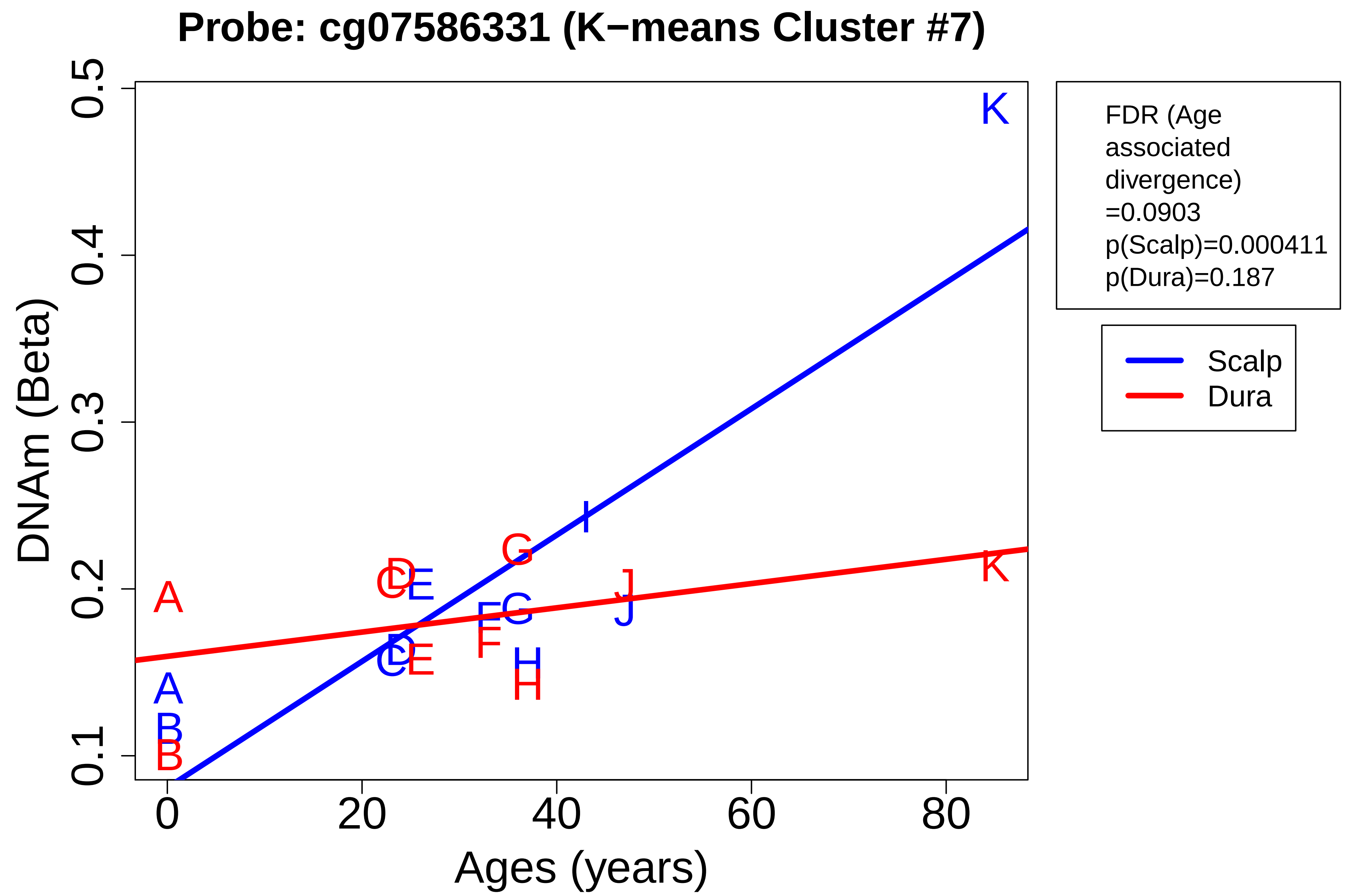

Probe: cg07586331 (K−means Cluster #7)
DNAm (Beta)
0.1
0.2
0.3
0.4
0.5
0
20
40
60
80
Ages (years)
FDR (Age associated divergence) =0.0903
p(Scalp)=0.000411
p(Dura)=0.187
Scalp
Dura

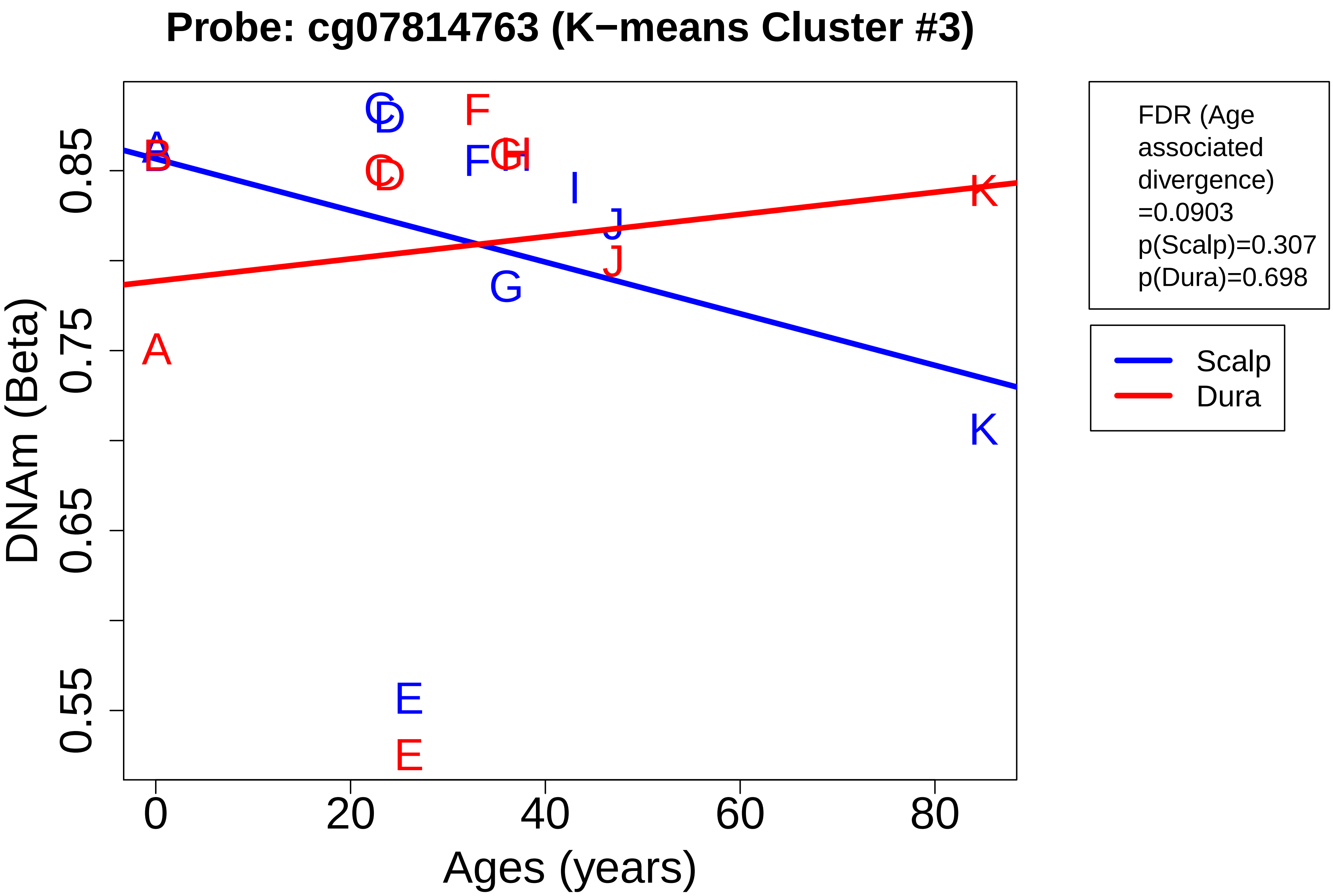
Probe: cg07814763 (K−means Cluster #3)
DNAm (Beta)
0.55
0.65
0.75
0.85
Ages (years)
0
20
40
60
80
A
B
C
D
E
F
G
H
I
J
K
FDR (Age associated divergence) =0.0903
p(Scalp)=0.307
p(Dura)=0.698
Scalp
Dura

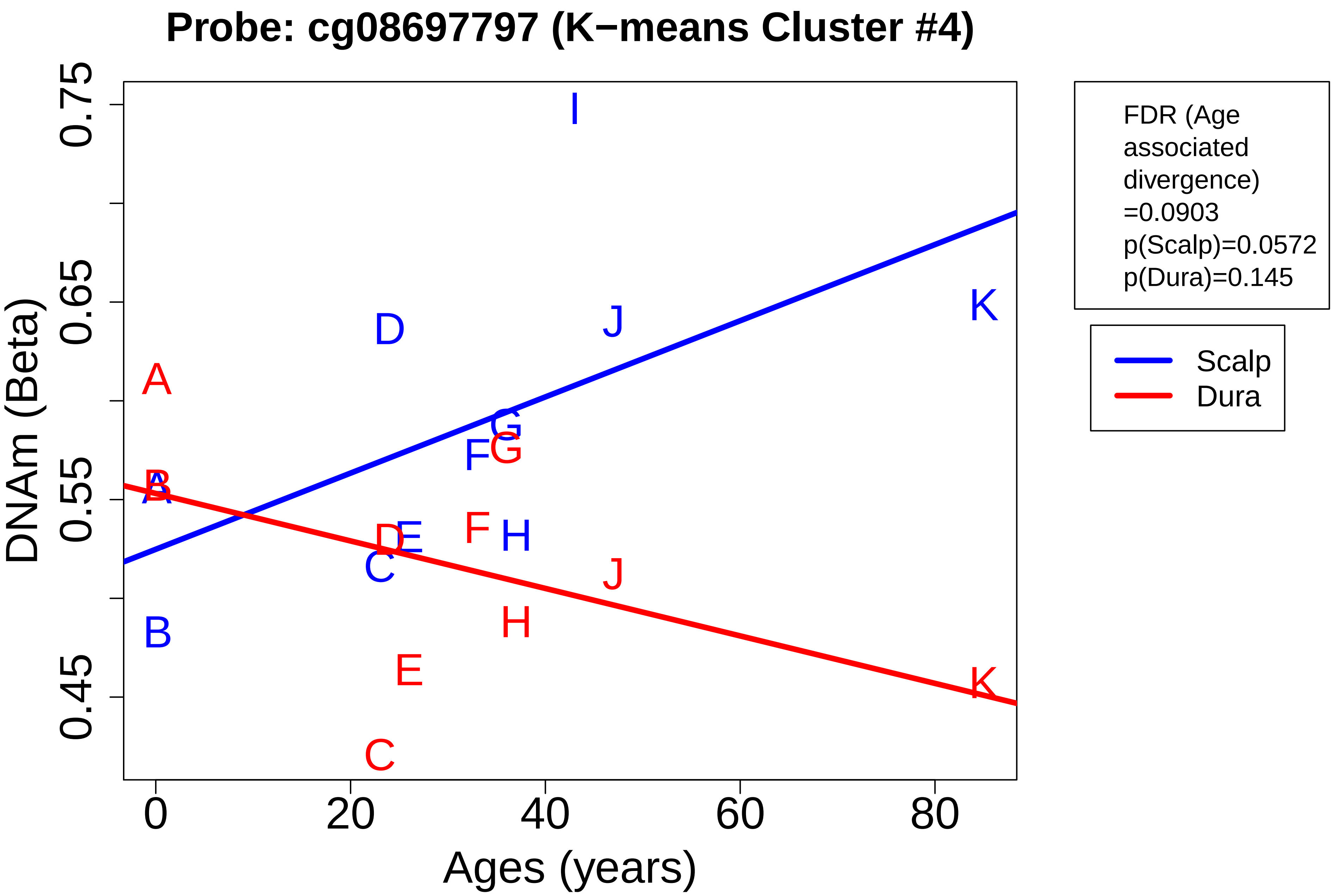

Probe: cg08697797 (K−means Cluster #4)
DNAm (Beta)
0.45
0.55
0.65
0.75
Ages (years)
0
20
40
60
80
FDR (Age associated divergence) =0.0903
p(Scalp)=0.0572
p(Dura)=0.145
Scalp
Dura
A
B
C
D
E
F
G
H
I
J
K

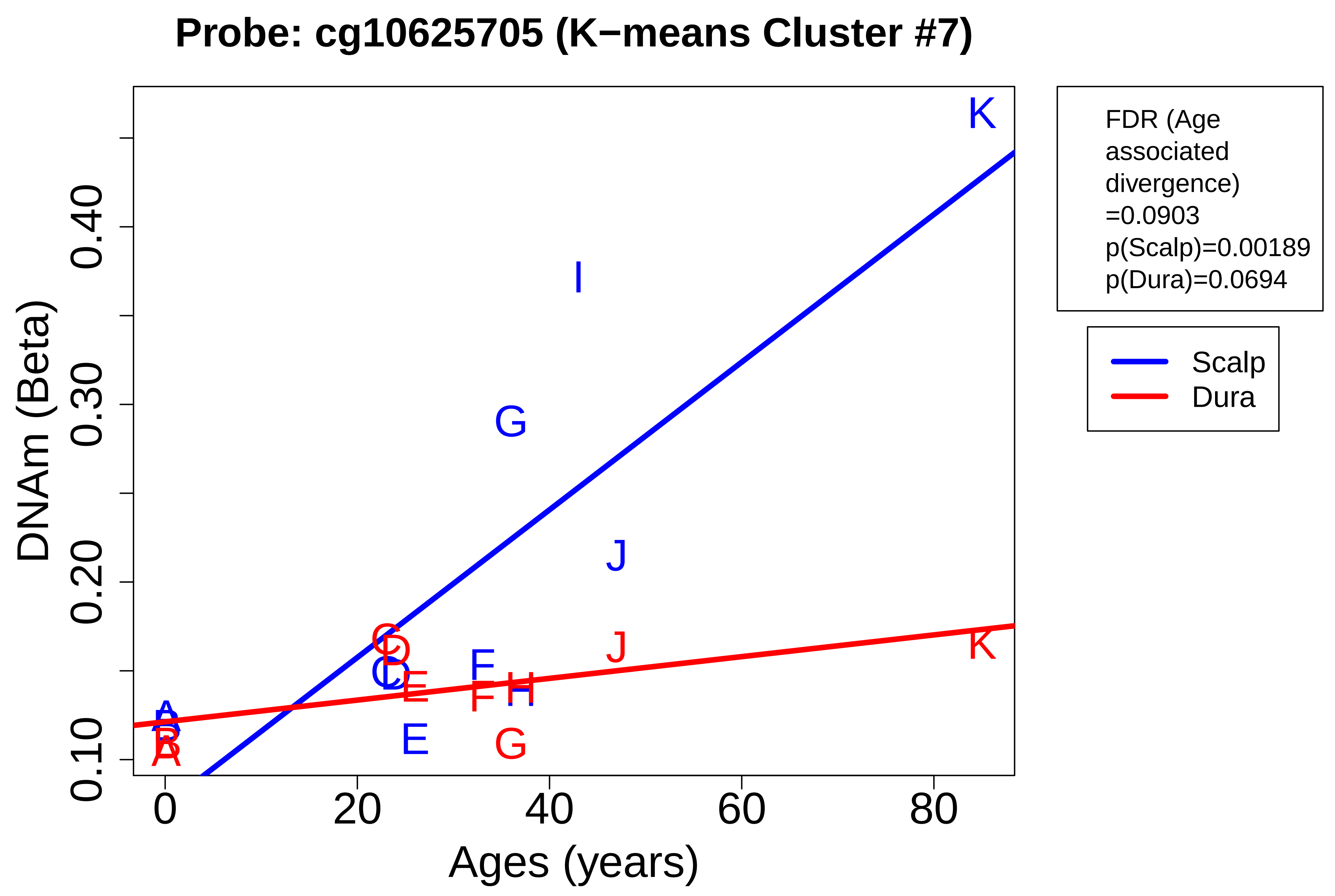

Probe: cg10625705 (K−means Cluster #7)
DNAm (Beta)
0.10
0.20
0.30
0.40
Ages (years)
0
20
40
60
80
FDR (Age associated divergence) =0.0903
p(Scalp)=0.00189
p(Dura)=0.0694
Scalp
Dura

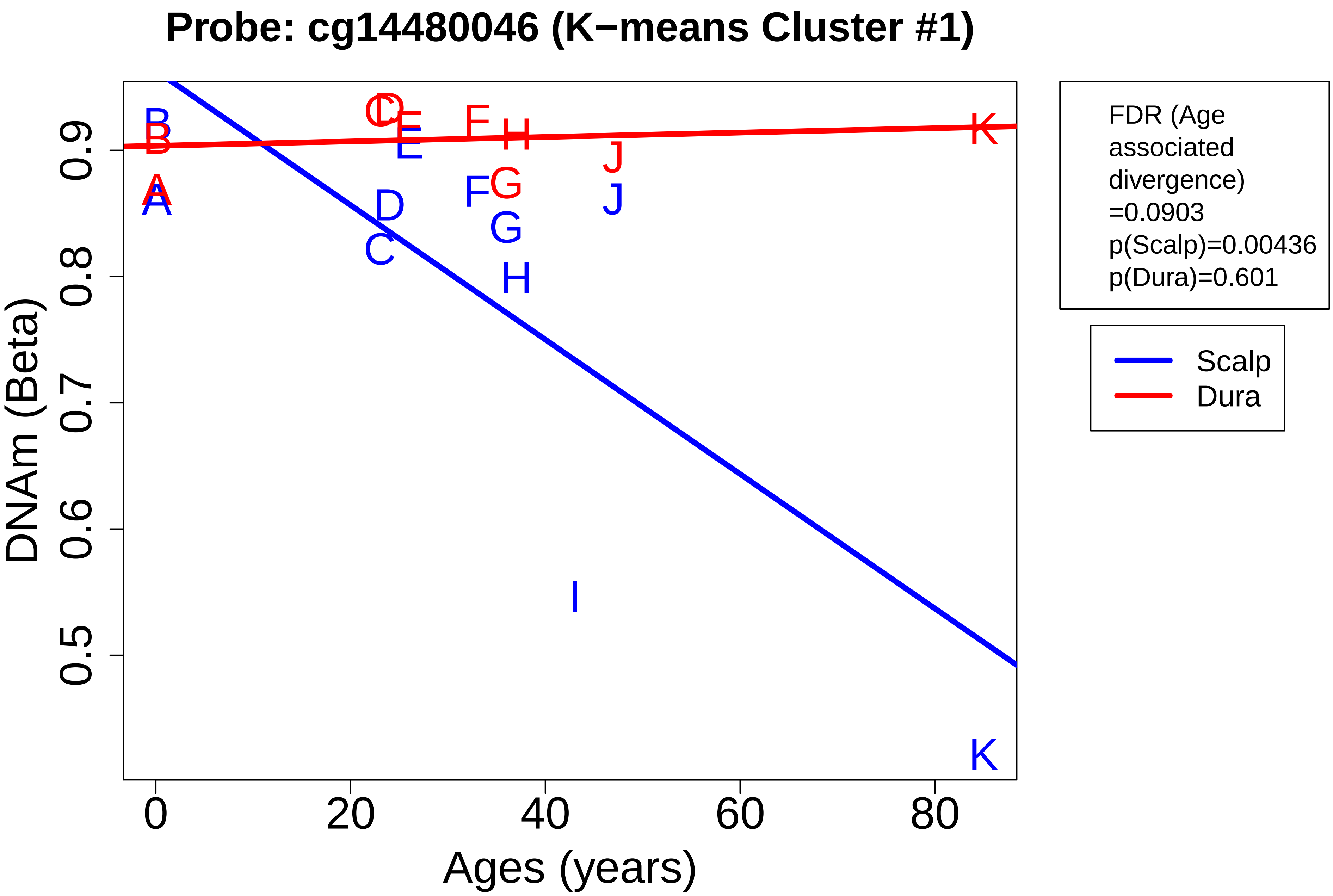
Probe: cg14480046 (K−means Cluster #1)
DNAm (Beta)
0.5
0.6
0.7
0.8
0.9
Ages (years)
0
20
40
60
80
A B C D E F G H I J K
FDR (Age associated divergence) =0.0903
p(Scalp)=0.00436
p(Dura)=0.601
Scalp
Dura

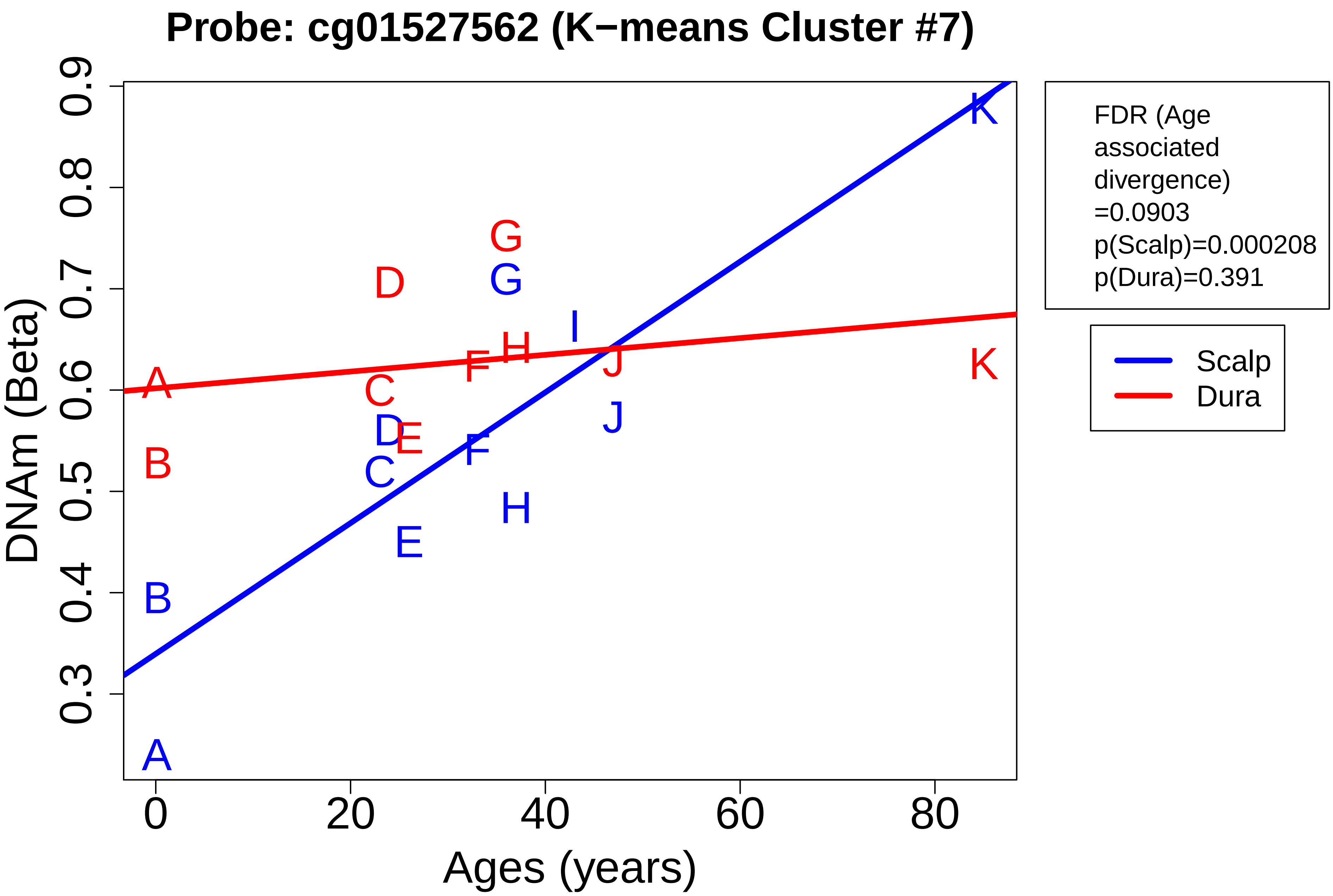

Probe: cg01527562 (K−means Cluster #7)
DNAm (Beta)
0.3
0.4
0.5
0.6
0.7
0.8
0.9
0
20
40
60
80
Ages (years)
A
B
C
D
E
F
G
H
I
J
K
FDR (Age associated divergence) =0.0903
p(Scalp)=0.000208
p(Dura)=0.391
Scalp
Dura

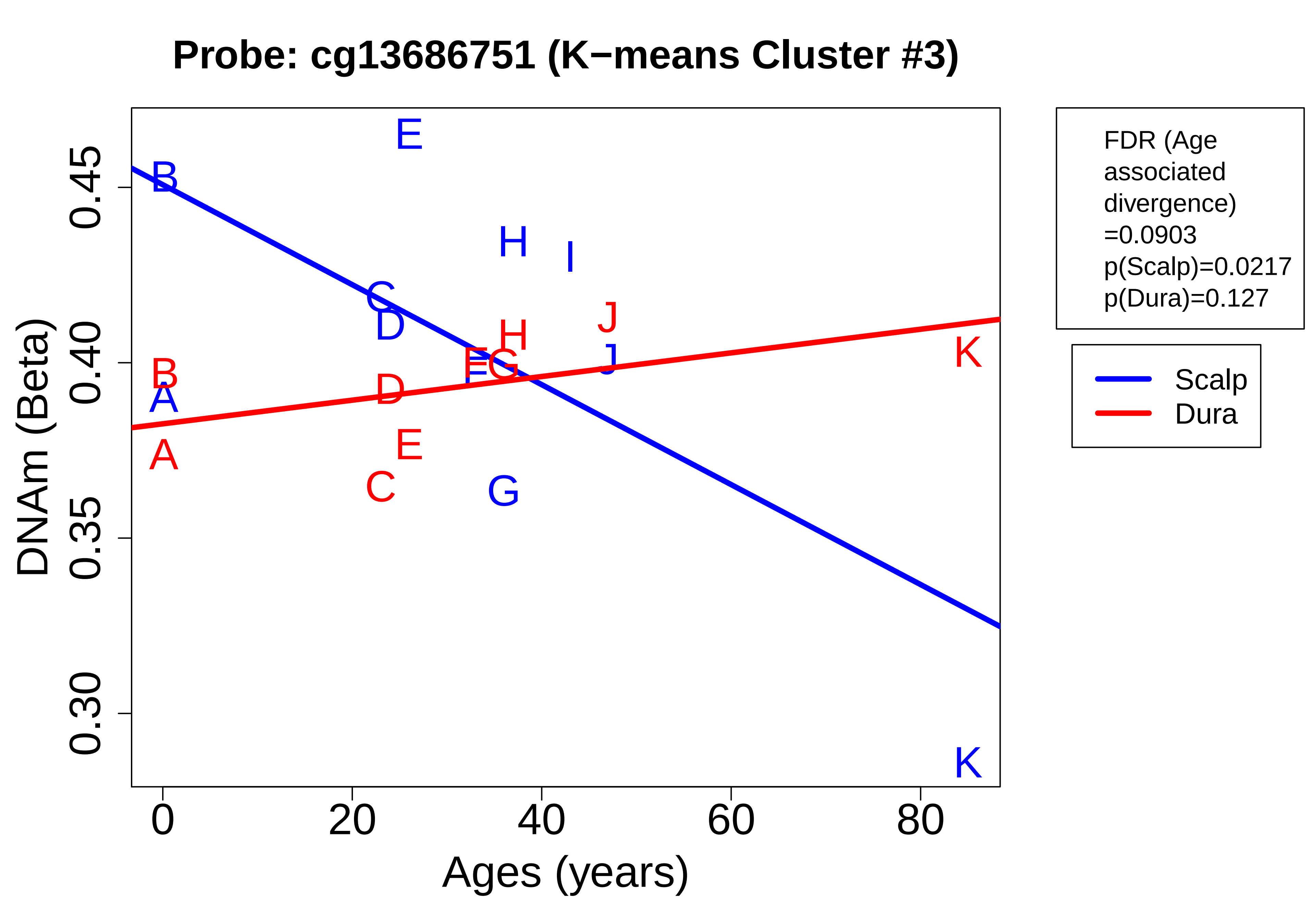

Probe: cg13686751 (K−means Cluster #3)
DNAm (Beta)
0.30
0.35
0.40
0.45
Ages (years)
0
20
40
60
80
FDR (Age associated divergence) =0.0903
p(Scalp)=0.0217
p(Dura)=0.127
Scalp
Dura

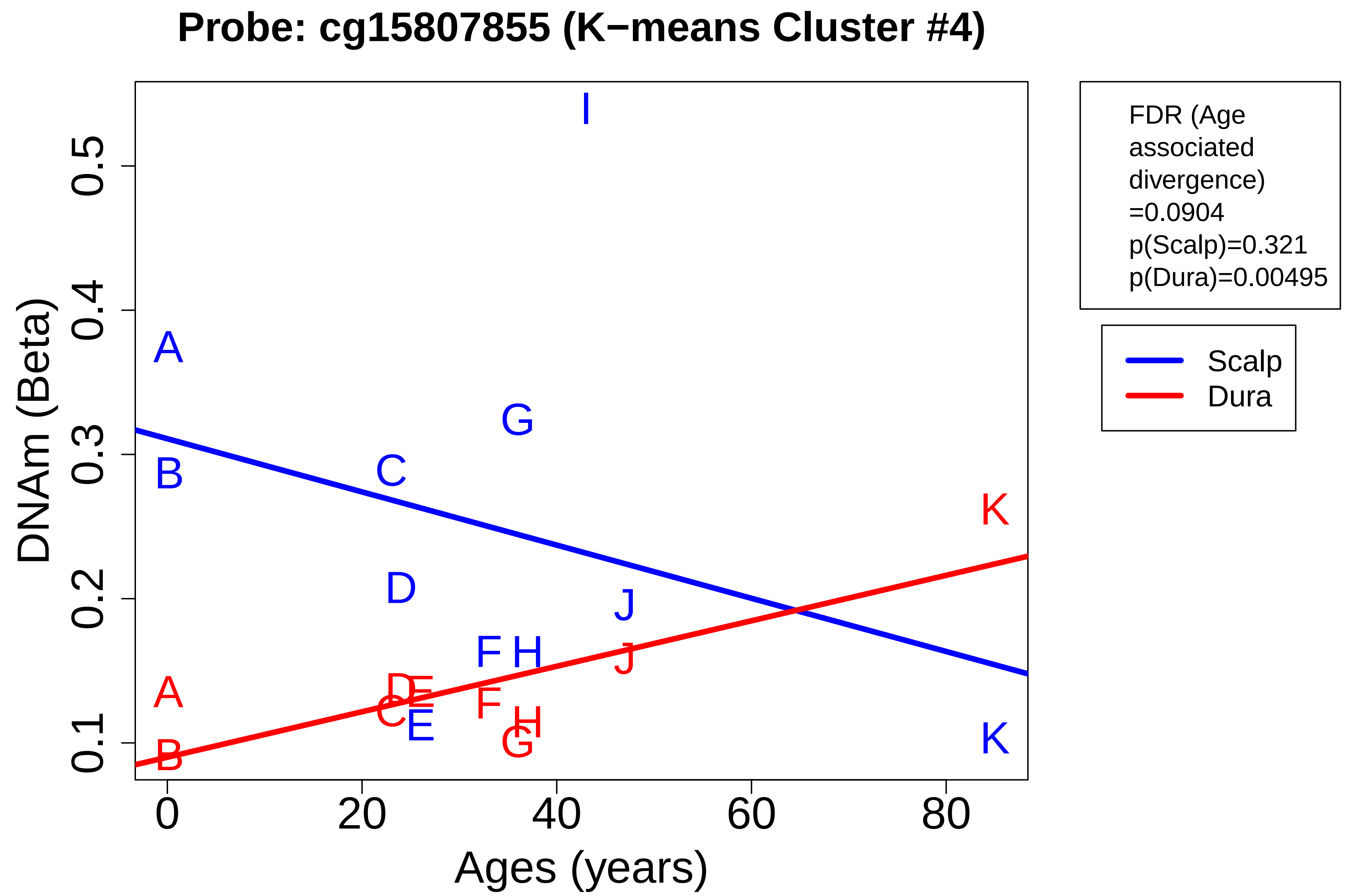

Probe: cg15807855 (K−means Cluster #4)
DNAm (Beta)
0.1
0.2
0.3
0.4
0.5
0
20
40
60
80
Ages (years)
A
B
C
D
E
F
G
H
I
J
K
FDR (Age associated divergence) =0.0904
p(Scalp)=0.321
p(Dura)=0.00495
Scalp
Dura

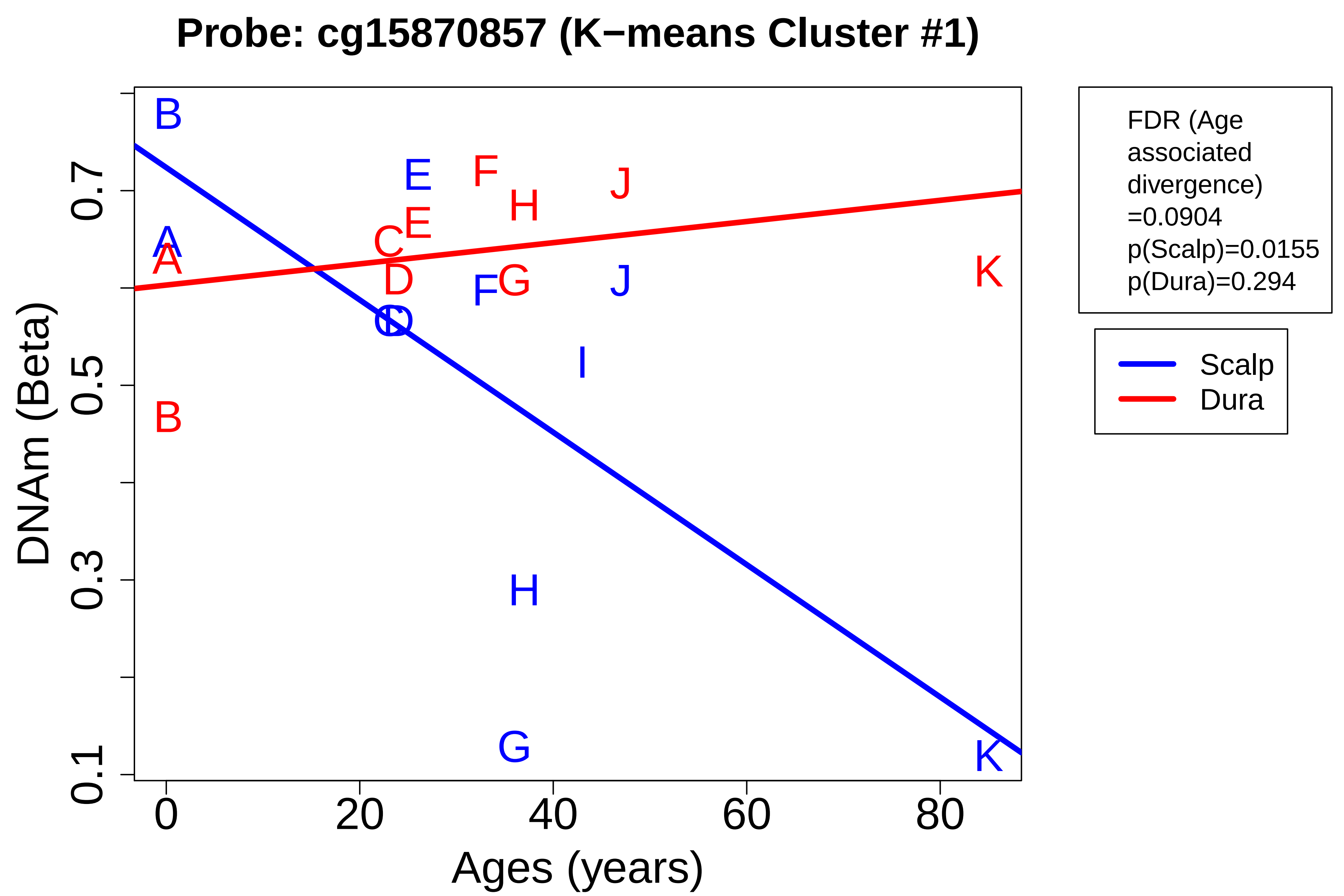

Probe: cg15870857 (K−means Cluster #1)
DNAm (Beta)
0.1
0.3
0.5
0.7
0
20
40
60
80
Ages (years)
FDR (Age associated divergence) =0.0904
p(Scalp)=0.0155
p(Dura)=0.294
Scalp
Dura

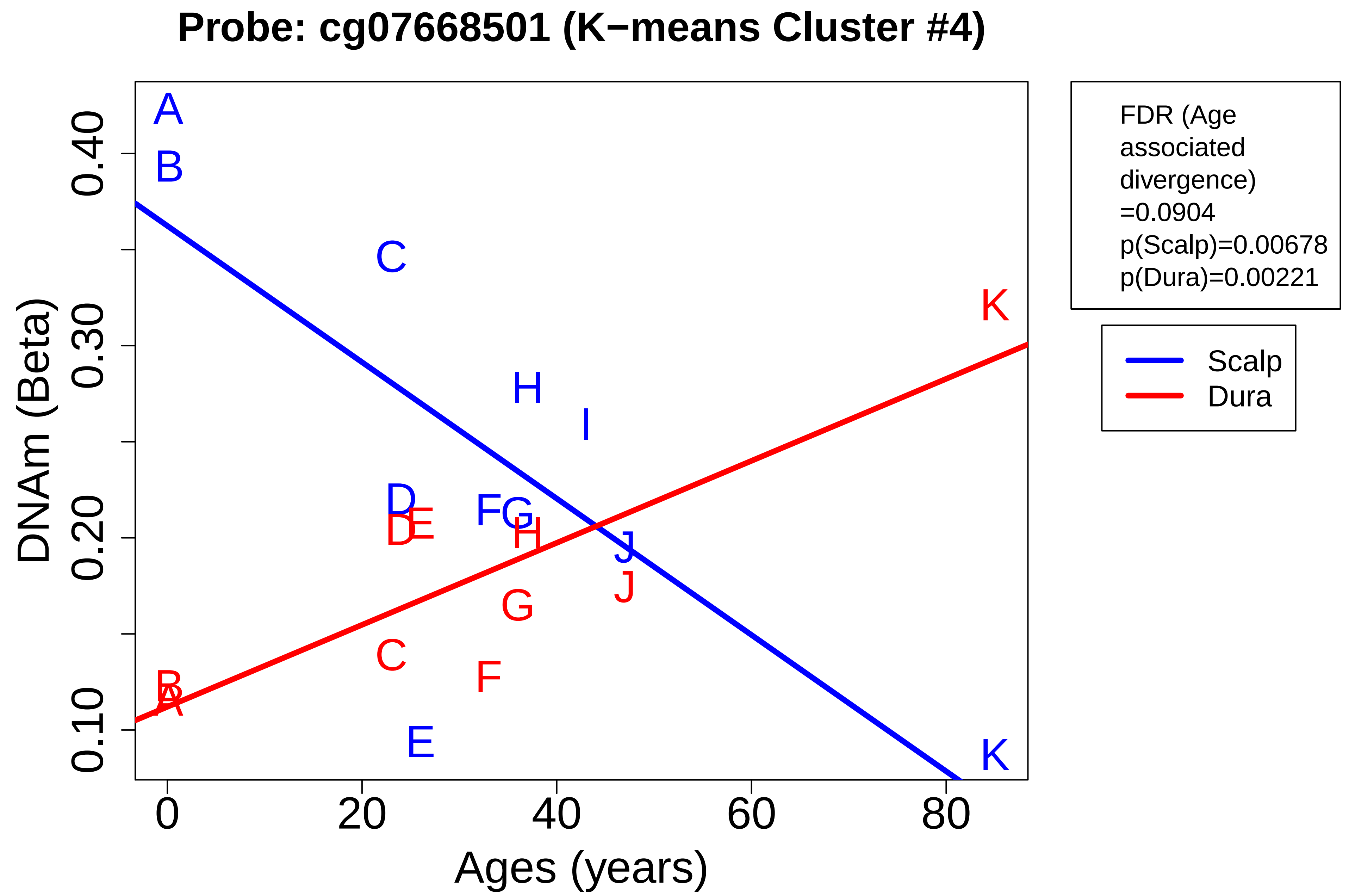

Probe: cg07668501 (K−means Cluster #4)
FDR (Age associated divergence) =0.0904
p(Scalp)=0.00678
p(Dura)=0.00221
Scalp
Dura
DNAm (Beta)
0.10
0.20
0.30
0.40
Ages (years)
0
20
40
60
80
A
B
C
D
E
F
G
H
I
J
K

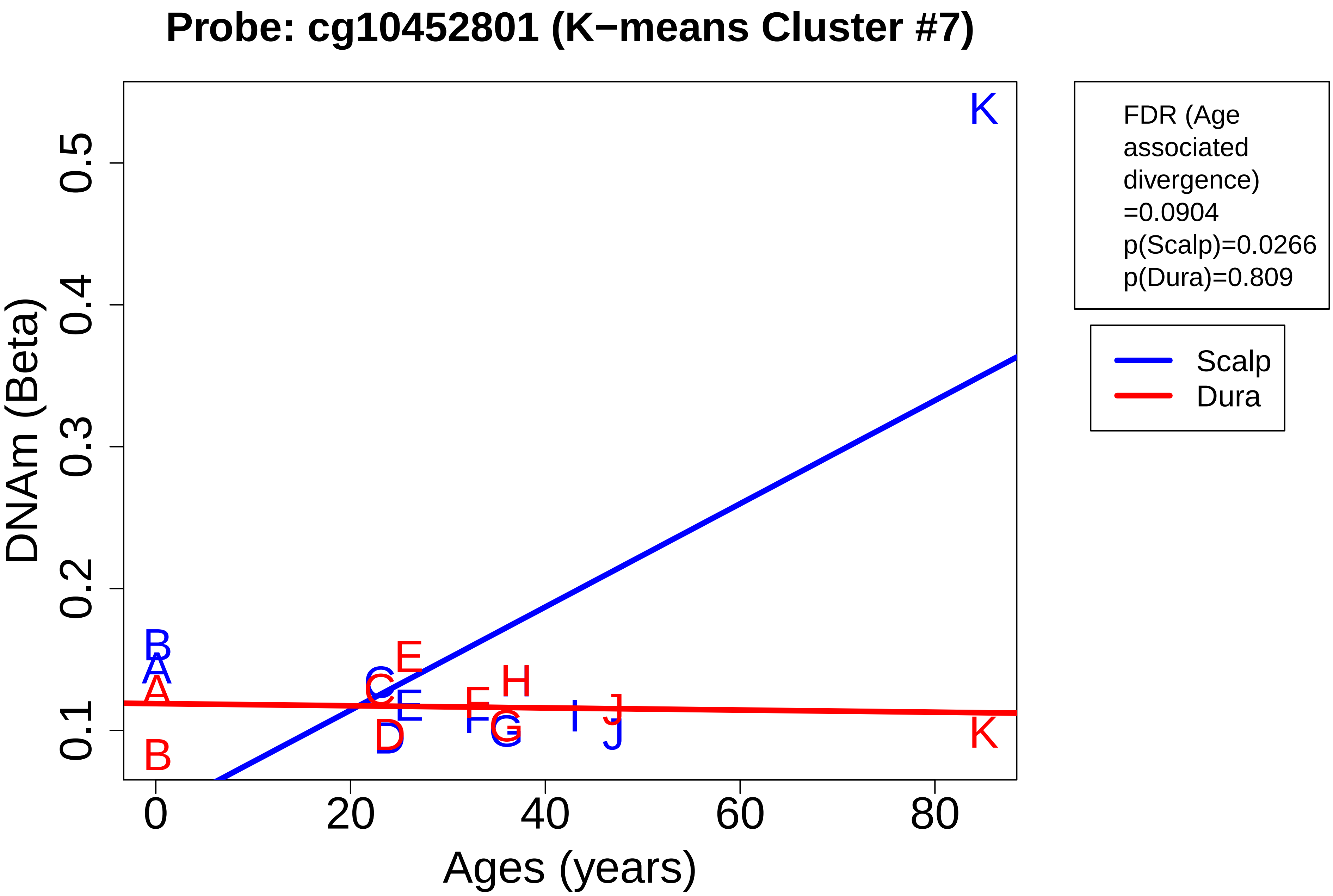

Probe: cg10452801 (K−means Cluster #7)
DNAm (Beta)
0.1
0.2
0.3
0.4
0.5
0
20
40
60
80
Ages (years)
FDR (Age associated divergence) =0.0904
p(Scalp)=0.0266
p(Dura)=0.809
Scalp
Dura

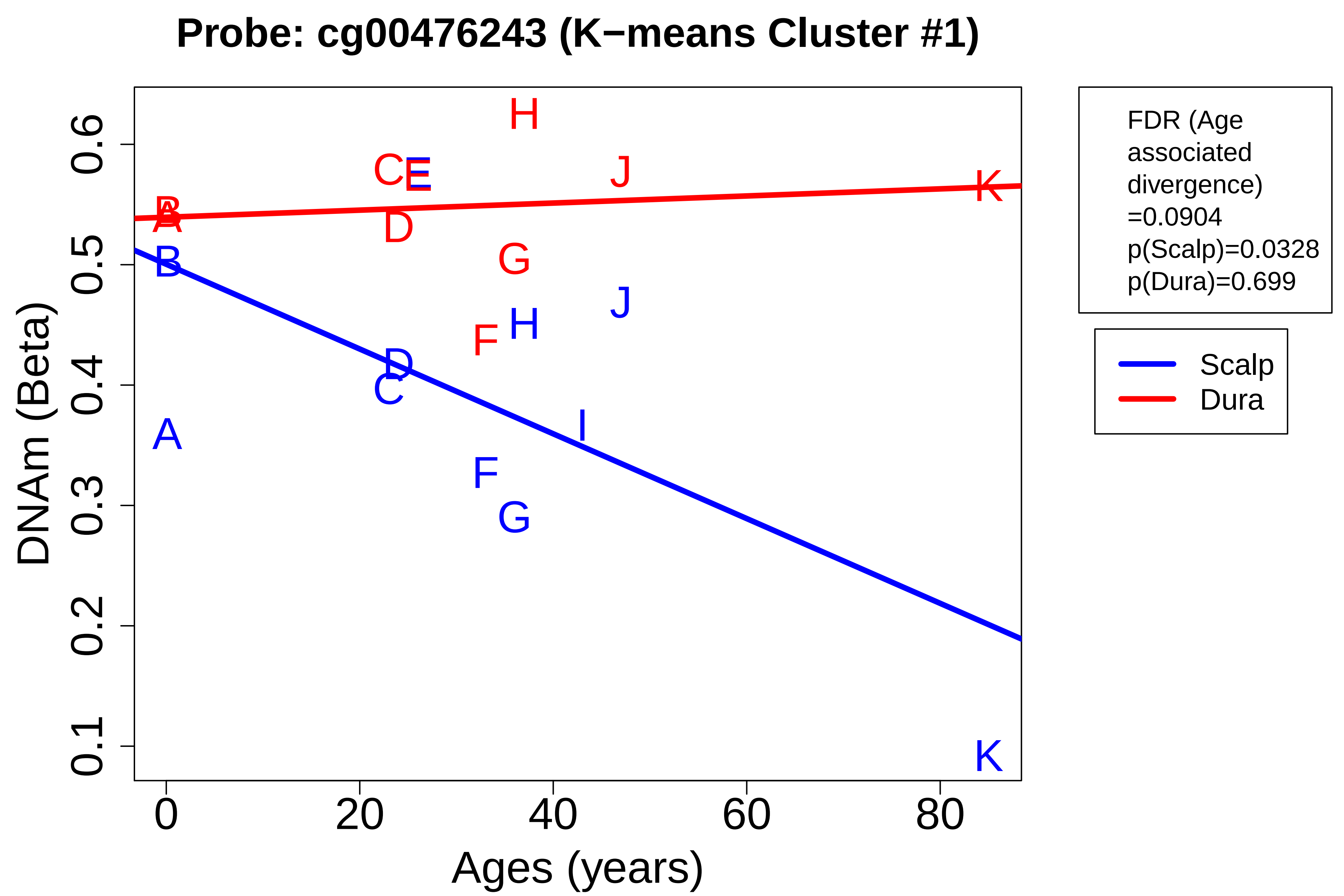

Probe: cg00476243 (K−means Cluster #1)
DNAm (Beta)
0.1
0.2
0.3
0.4
0.5
0.6
Ages (years)
0
20
40
60
80
FDR (Age associated divergence) =0.0904
p(Scalp)=0.0328
p(Dura)=0.699
Scalp
Dura

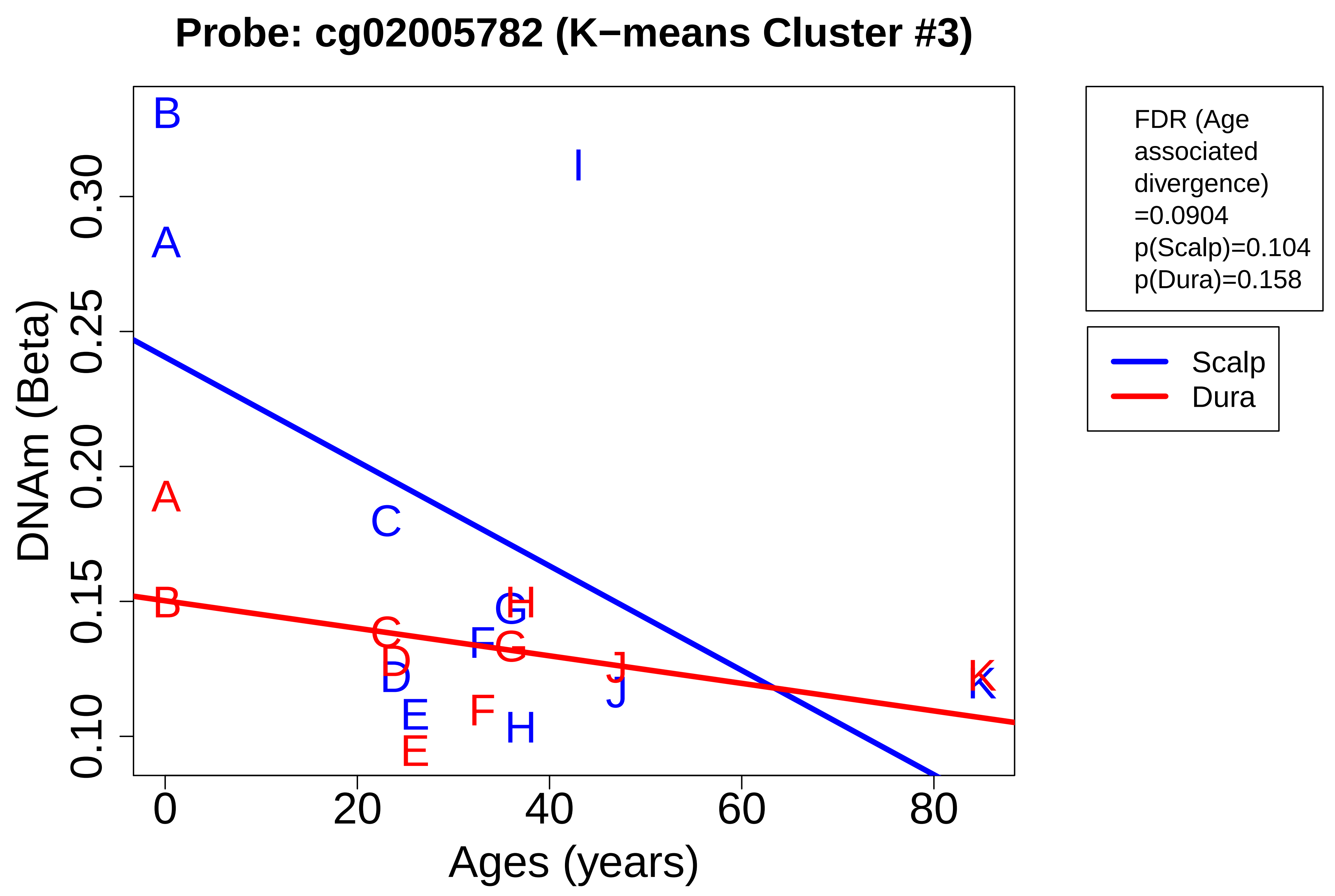

Probe: cg02005782 (K−means Cluster #3)
FDR (Age associated divergence) =0.0904
p(Scalp)=0.104
p(Dura)=0.158
Scalp
Dura
DNAm (Beta)
0.10
0.15
0.20
0.25
0.30
0
20
40
60
80
Ages (years)
A
B
C
D
E
F
G
H
I
J
K

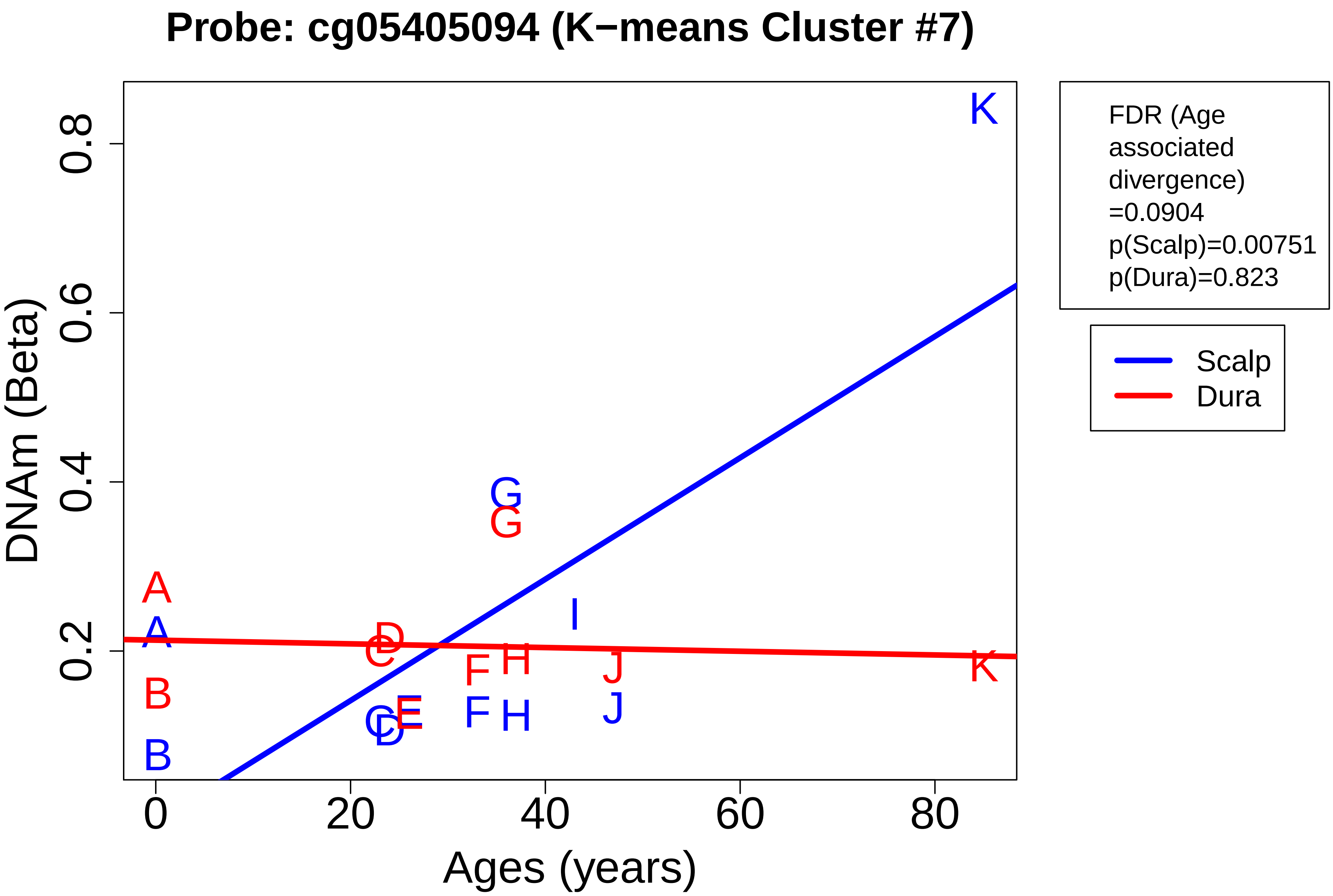

Probe: cg05405094 (K−means Cluster #7)
DNAm (Beta)
0.2
0.4
0.6
0.8
0
20
40
60
80
Ages (years)
FDR (Age associated divergence) =0.0904
p(Scalp)=0.00751
p(Dura)=0.823
Scalp
Dura
A
B
C
D
E
F
G
H
I
J
K

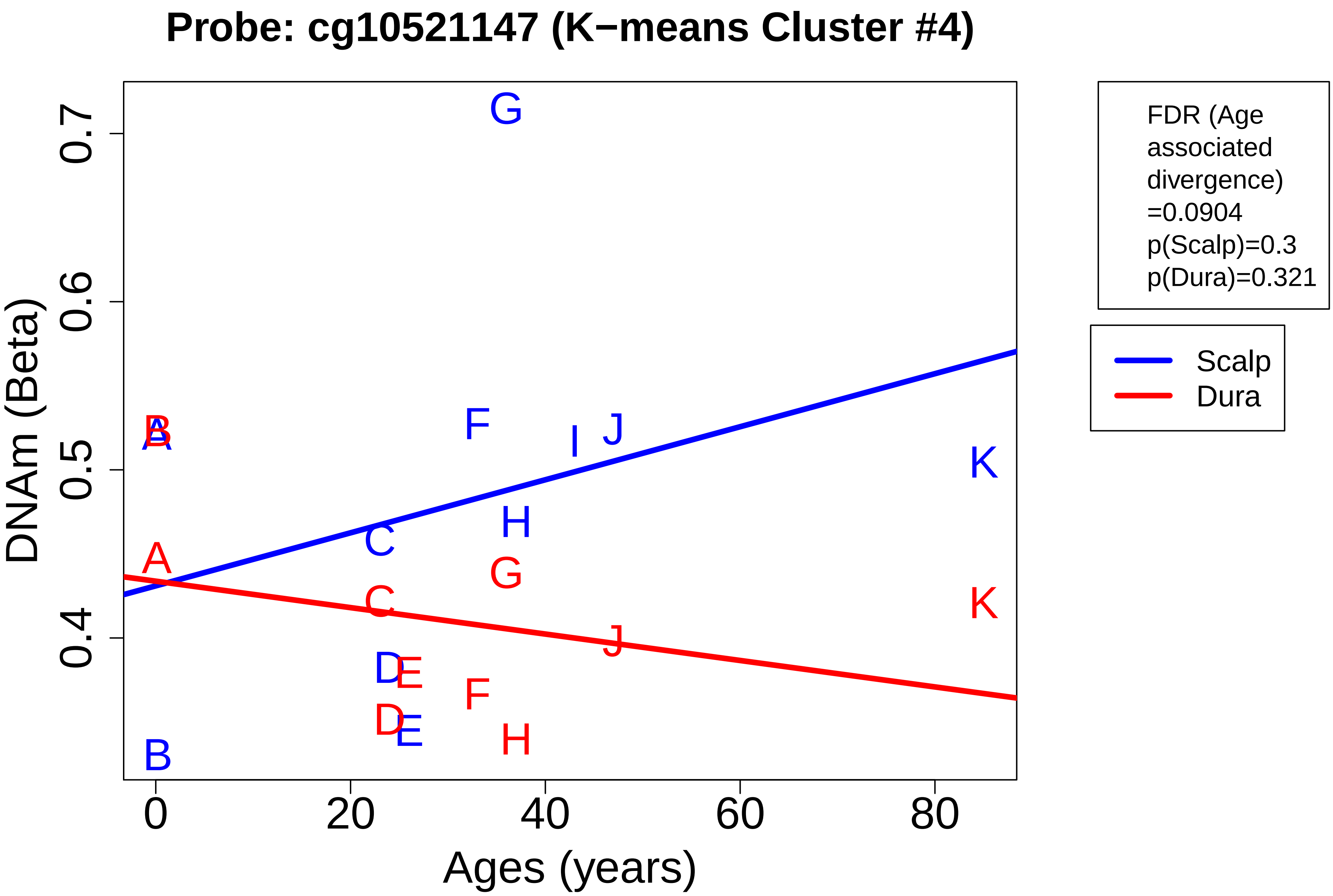

Probe: cg10521147 (K−means Cluster #4)
DNAm (Beta)
0.4
0.5
0.6
0.7
0
20
40
60
80
Ages (years)
FDR (Age associated divergence) =0.0904
p(Scalp)=0.3
p(Dura)=0.321
Scalp
Dura
A
B
C
D
E
F
G
H
I
J
K

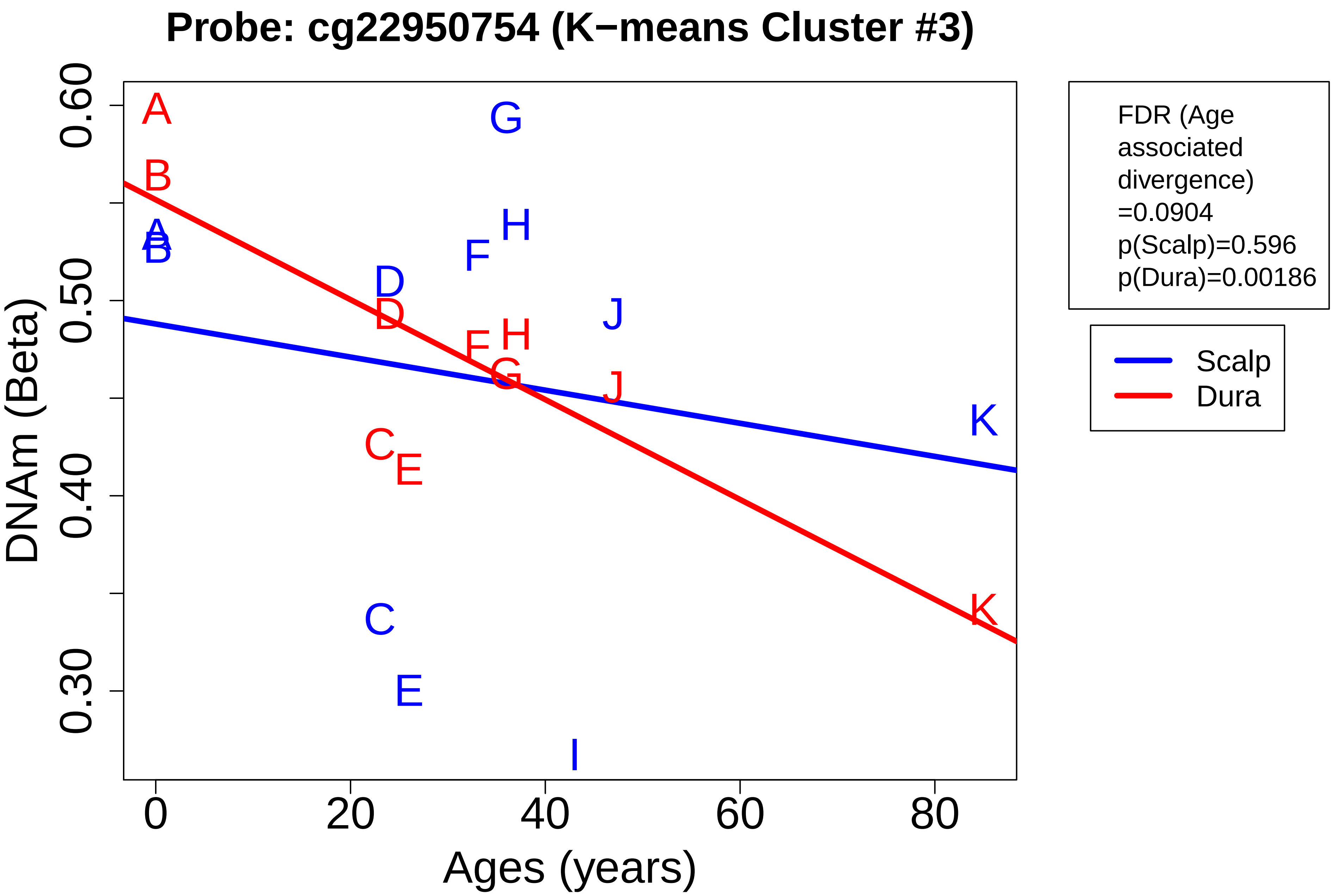
Probe: cg22950754 (K−means Cluster #3)
DNAm (Beta)
0.30
0.40
0.50
0.60
Ages (years)
0
20
40
60
80
FDR (Age associated divergence) =0.0904
p(Scalp)=0.596
p(Dura)=0.00186
Scalp
Dura
A
B
C
D
E
F
G
H
I
J
K

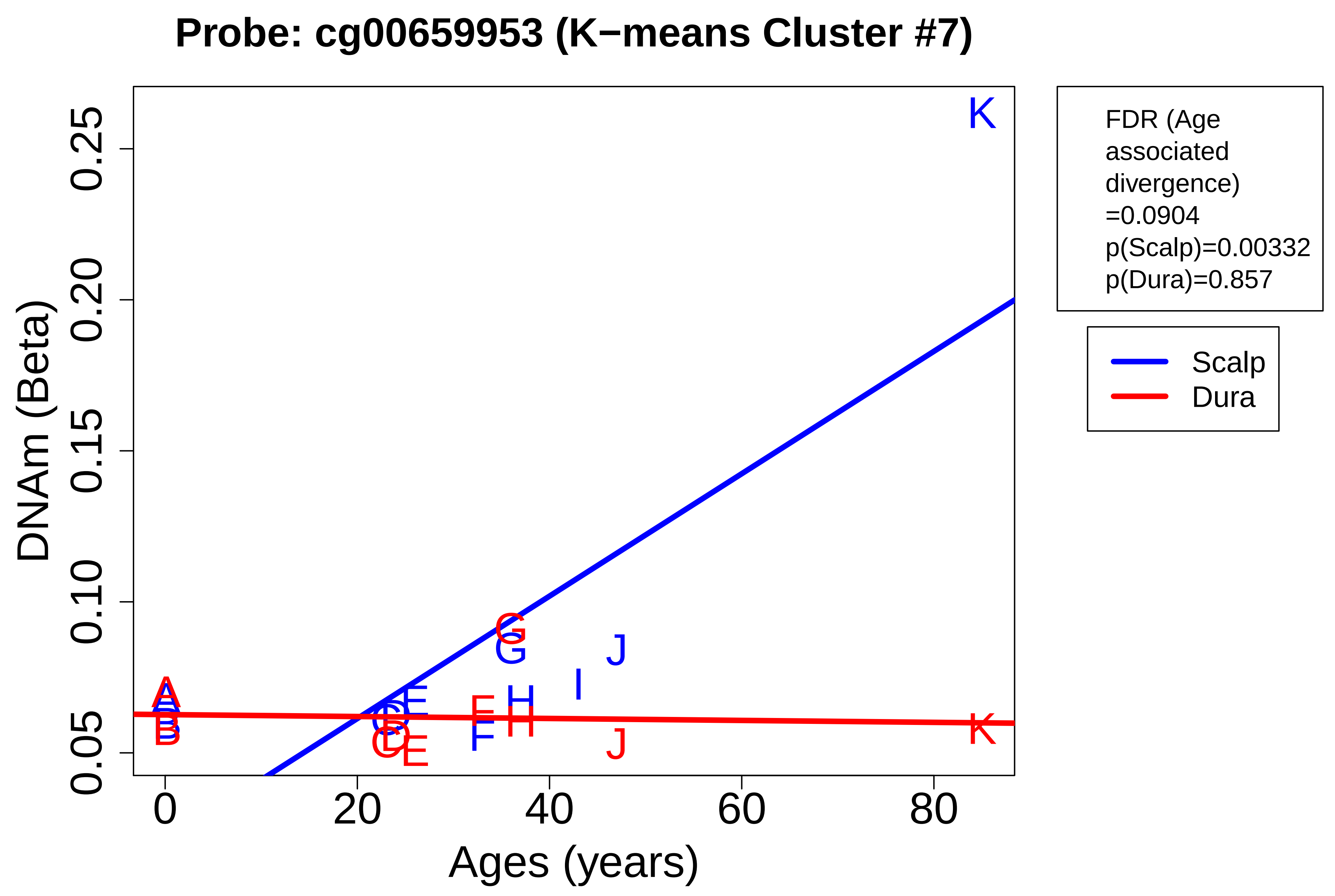

Probe: cg00659953 (K−means Cluster #7)
DNAm (Beta)
0.05
0.10
0.15
0.20
0.25
0
20
40
60
80
Ages (years)
A
B
C
D
E
F
G
H
I
J
K
FDR (Age associated divergence) =0.0904
p(Scalp)=0.00332
p(Dura)=0.857
Scalp
Dura

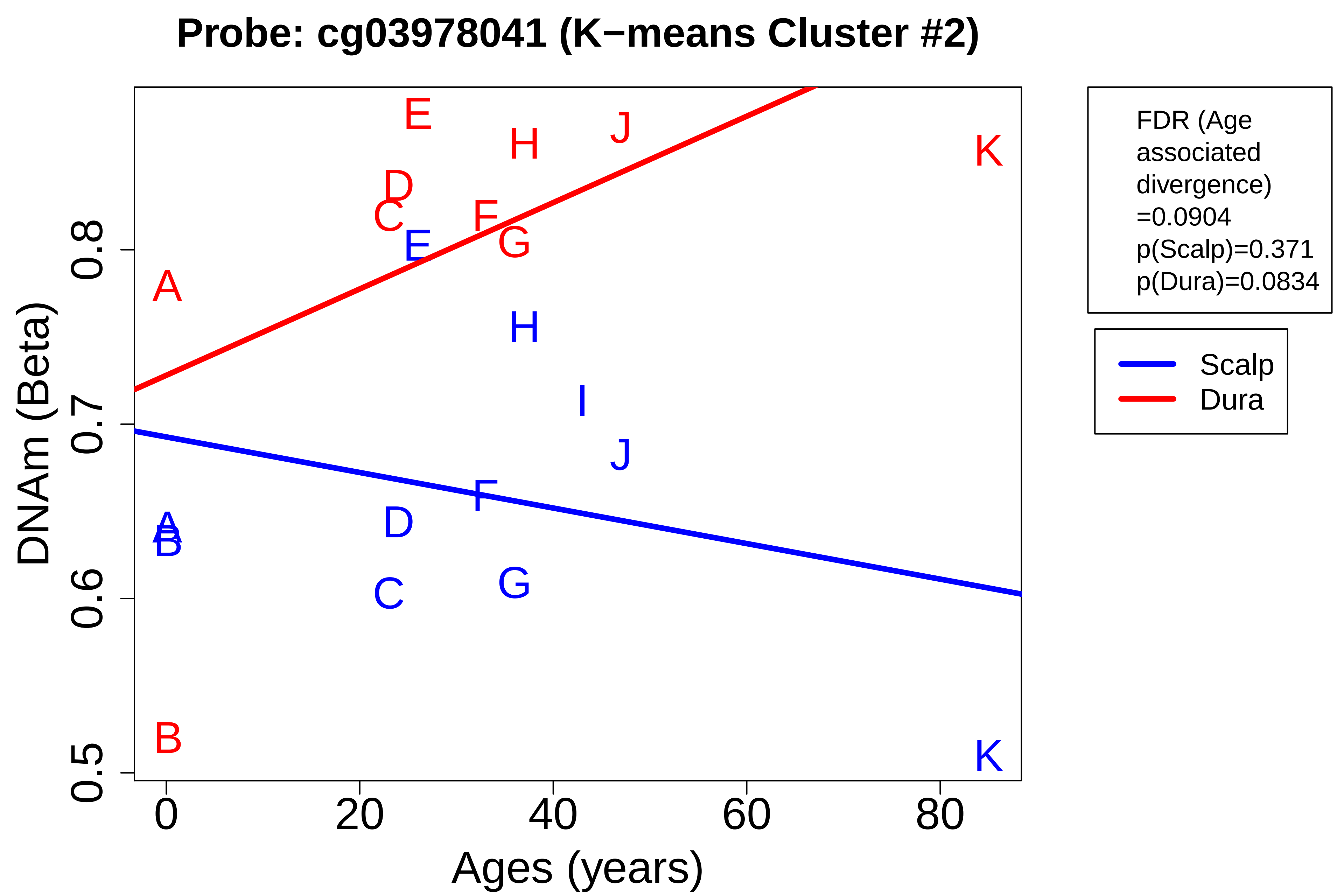

Probe: cg03978041 (K−means Cluster #2)
DNAm (Beta)
0.5
0.6
0.7
0.8
0
20
40
60
80
Ages (years)
FDR (Age associated divergence) =0.0904
p(Scalp)=0.371
p(Dura)=0.0834
Scalp
Dura
A
B
C
D
E
F
G
H
I
J
K

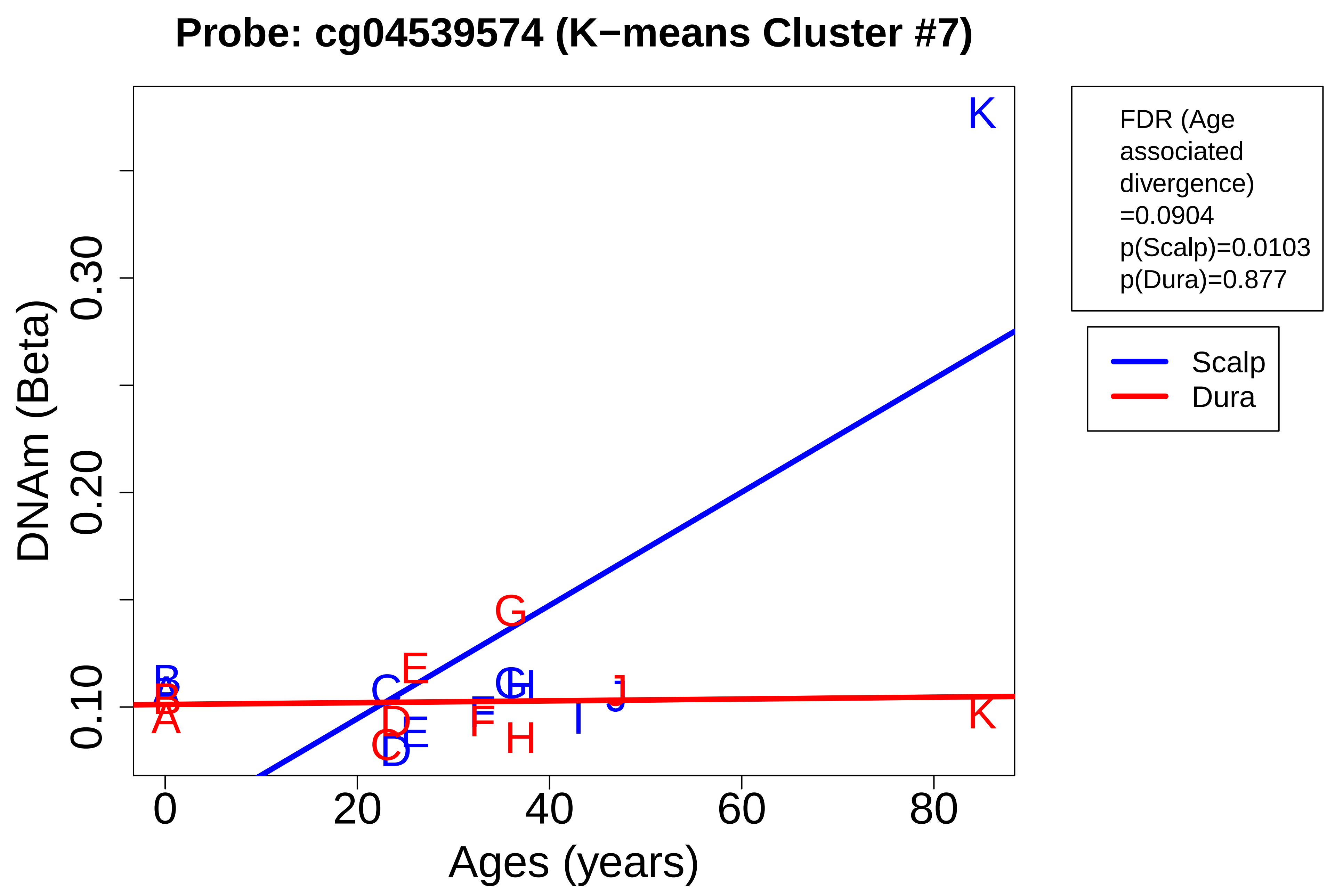

Probe: cg04539574 (K−means Cluster #7)
DNAm (Beta)
0.10
0.20
0.30
Ages (years)
0
20
40
60
80
FDR (Age associated divergence) =0.0904
p(Scalp)=0.0103
p(Dura)=0.877
Scalp
Dura

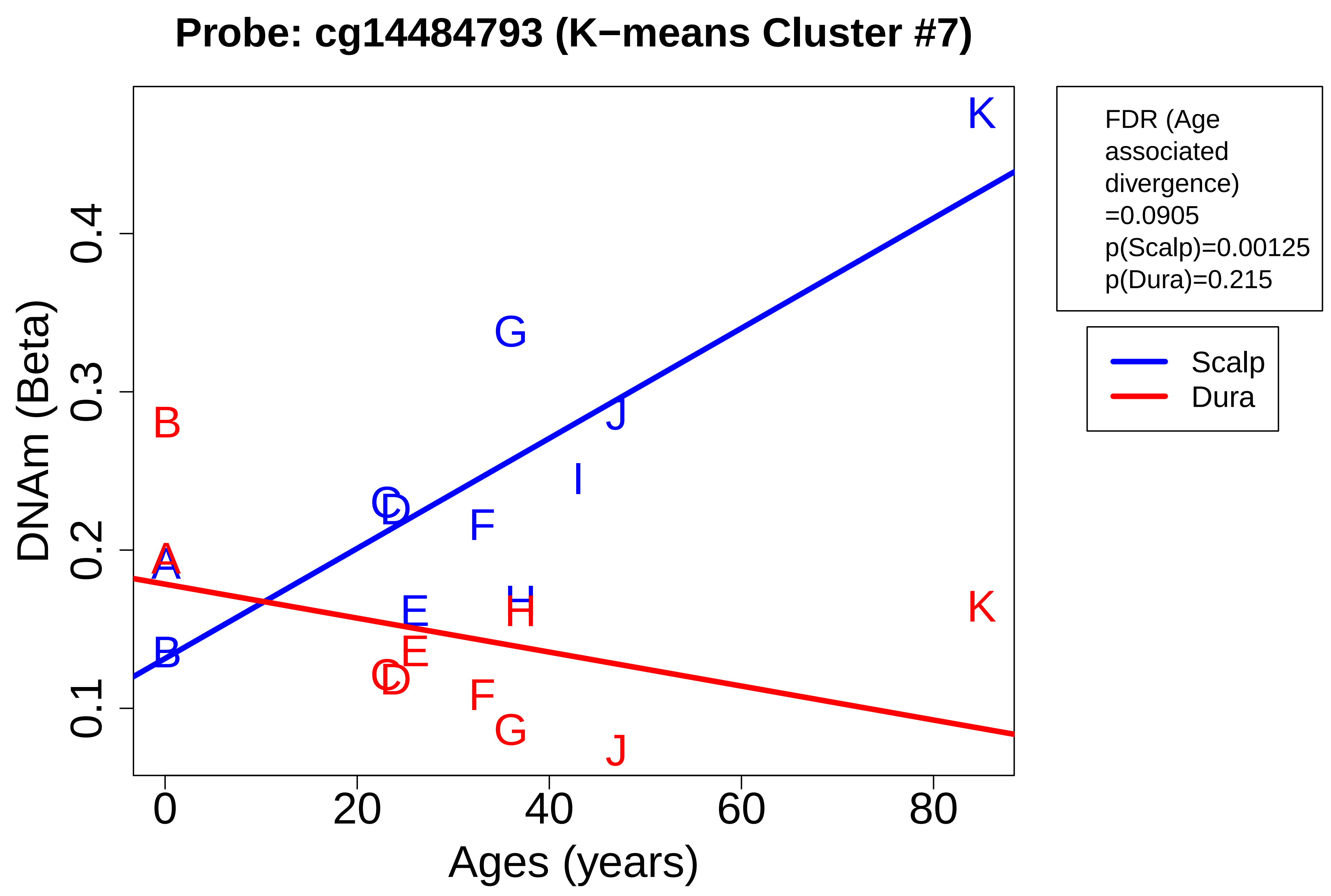

Probe: cg14484793 (K−means Cluster #7)
DNAm (Beta)
0.1
0.2
0.3
0.4
0
20
40
60
80
Ages (years)
A
B
C
D
E
F
G
H
I
J
K
FDR (Age associated divergence) =0.0905
p(Scalp)=0.00125
p(Dura)=0.215
Scalp
Dura

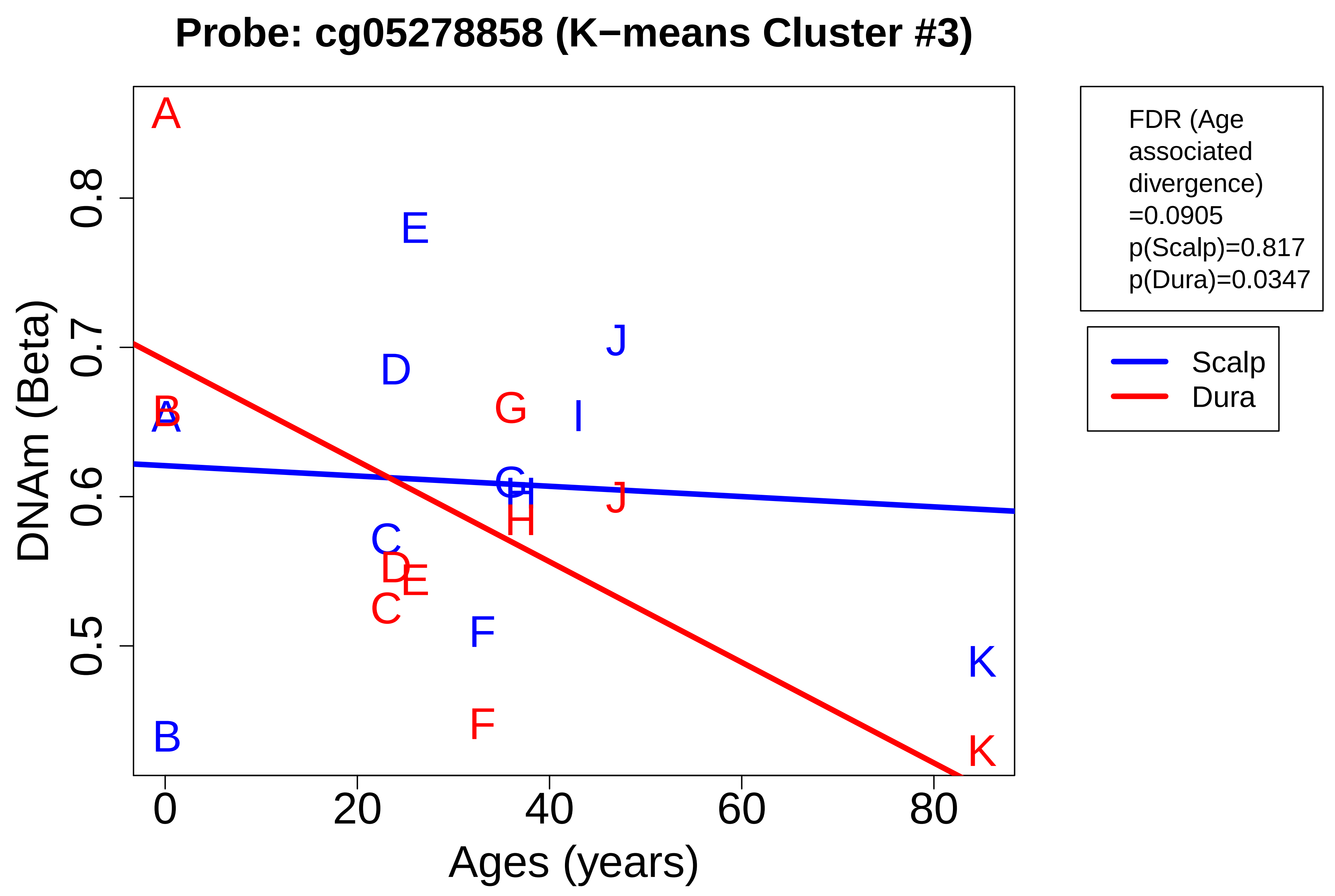
Probe: cg05278858 (K−means Cluster #3)
FDR (Age associated divergence) =0.0905
p(Scalp)=0.817
p(Dura)=0.0347
Scalp
Dura
DNAm (Beta)
Ages (years)
0.5
0.6
0.7
0.8
0
20
40
60
80
A
B
C
D
E
F
G
H
I
J
K

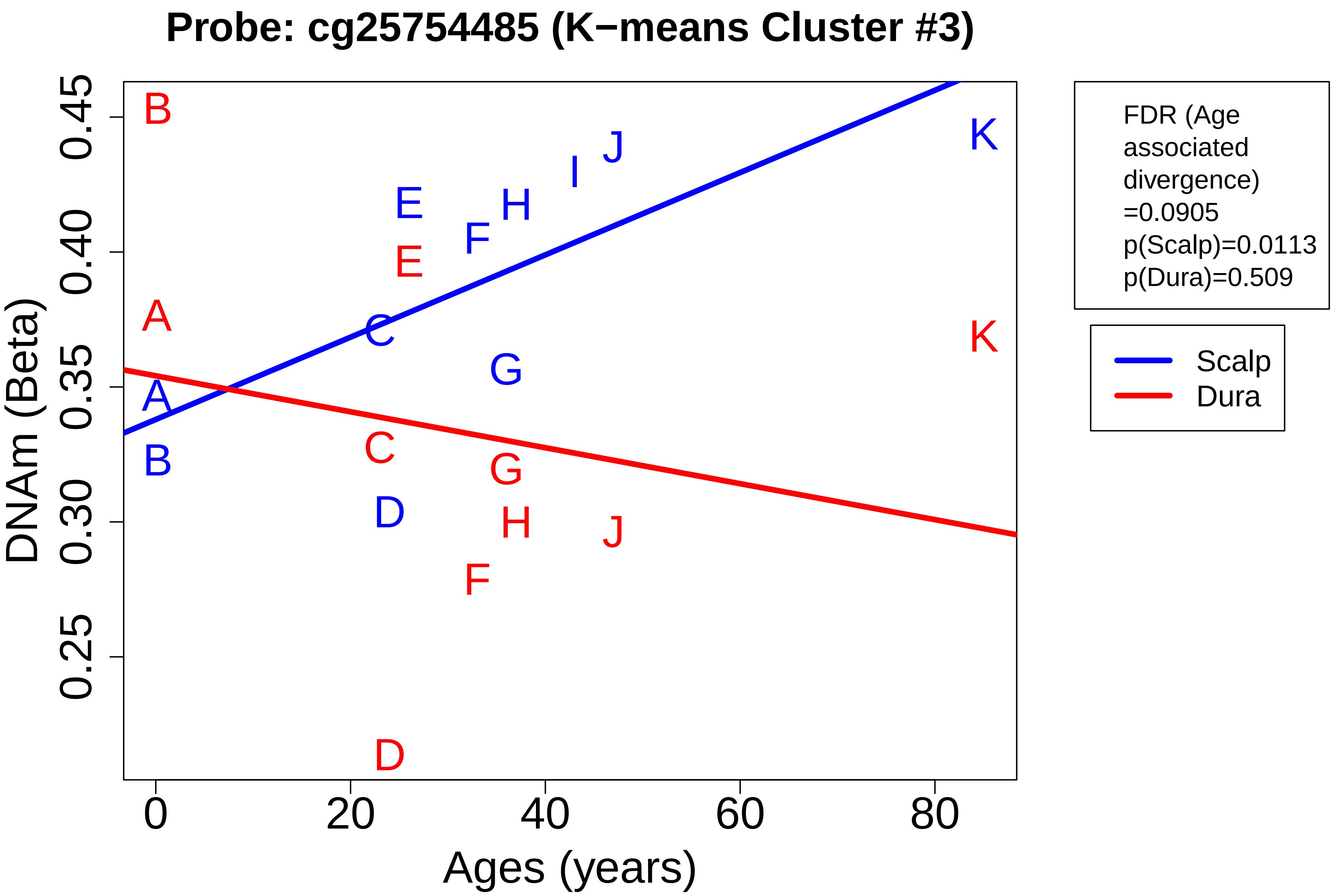

Probe: cg25754485 (K−means Cluster #3)
DNAm (Beta)
0.25
0.30
0.35
0.40
0.45
0
20
40
60
80
Ages (years)
A
B
C
D
E
F
G
H
I
J
K
FDR (Age associated divergence) =0.0905
p(Scalp)=0.0113
p(Dura)=0.509
Scalp
Dura

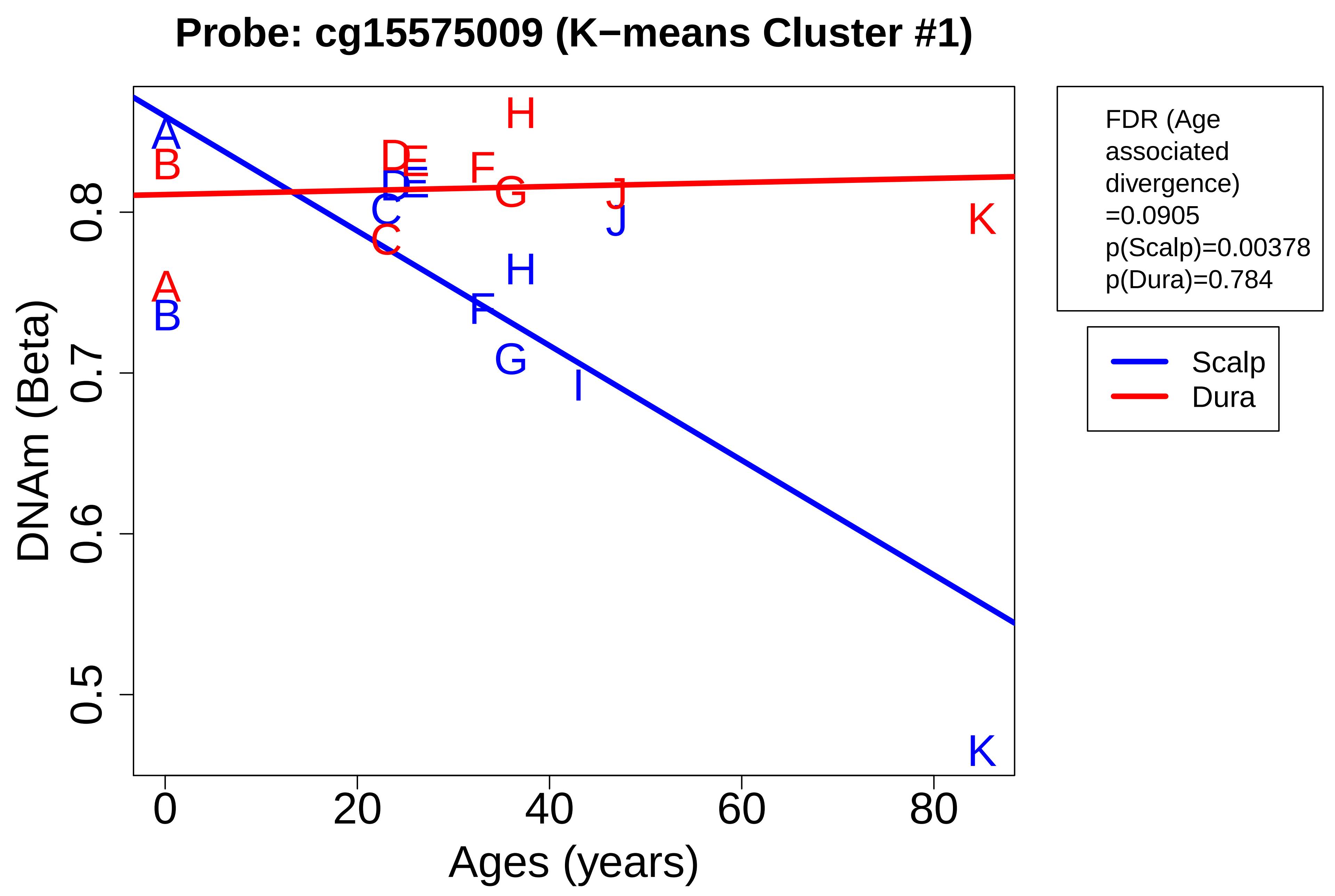

Probe: cg15575009 (K−means Cluster #1)
DNAm (Beta)
0.5
0.6
0.7
0.8
0
20
40
60
80
Ages (years)
A
B
C
D
E
F
G
H
I
J
K
FDR (Age associated divergence) =0.0905
p(Scalp)=0.00378
p(Dura)=0.784
Scalp
Dura

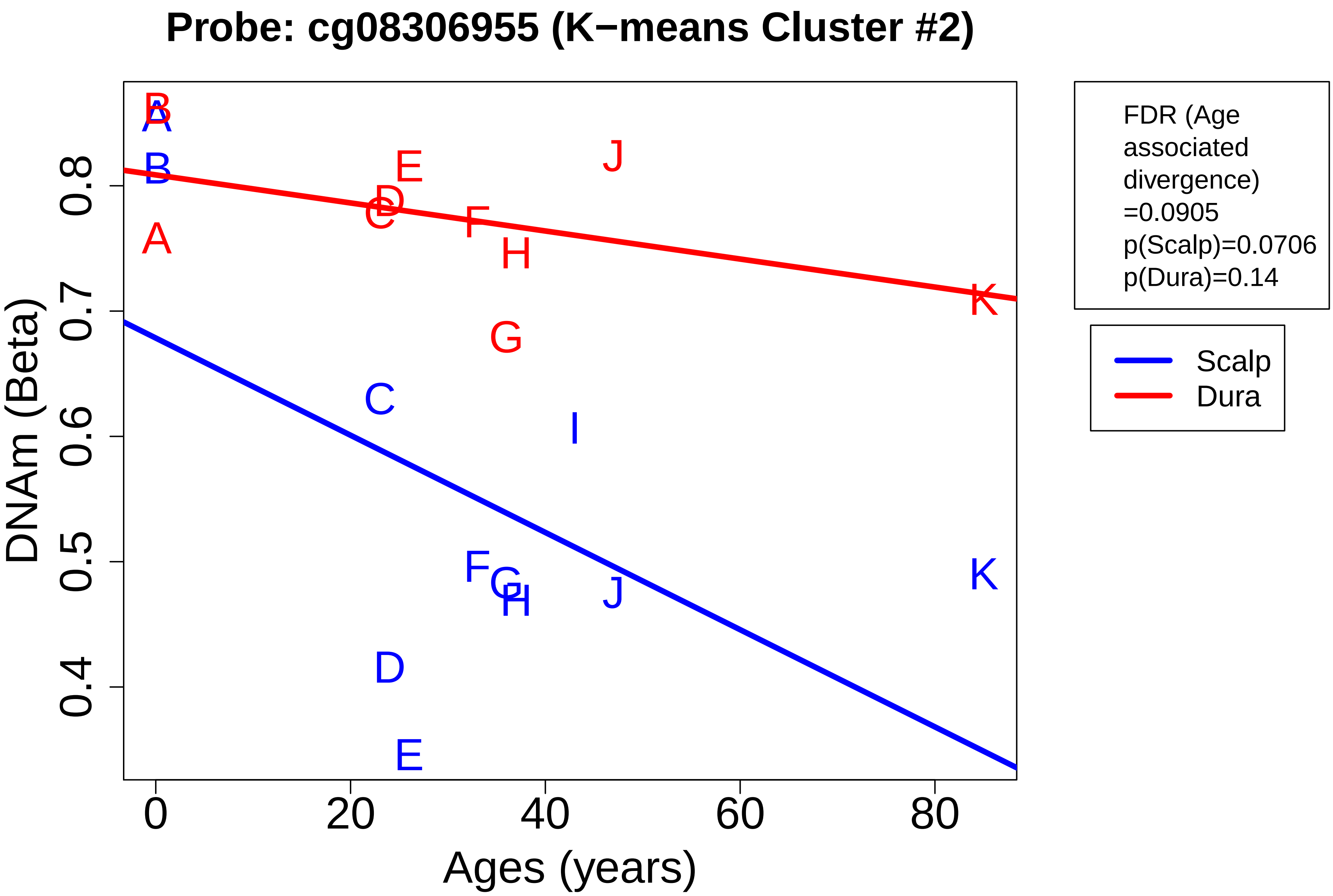

Probe: cg08306955 (K−means Cluster #2)
DNAm (Beta)
0.4
0.5
0.6
0.7
0.8
Ages (years)
0
20
40
60
80
FDR (Age associated divergence) =0.0905
p(Scalp)=0.0706
p(Dura)=0.14
Scalp
Dura

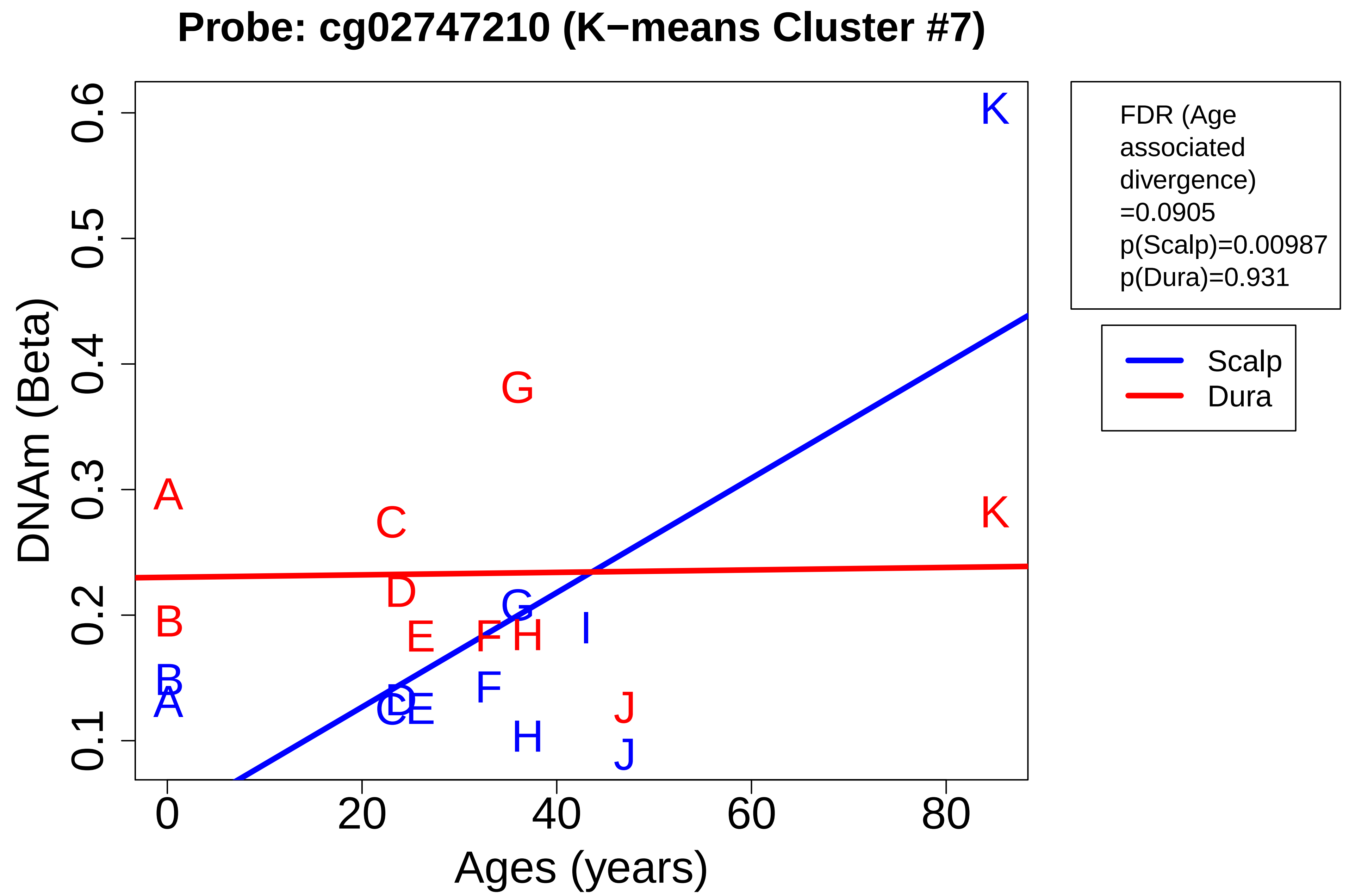

Probe: cg02747210 (K−means Cluster #7)
DNAm (Beta)
0.1
0.2
0.3
0.4
0.5
0.6
0
20
40
60
80
Ages (years)
FDR (Age associated divergence) =0.0905
p(Scalp)=0.00987
p(Dura)=0.931
Scalp
Dura

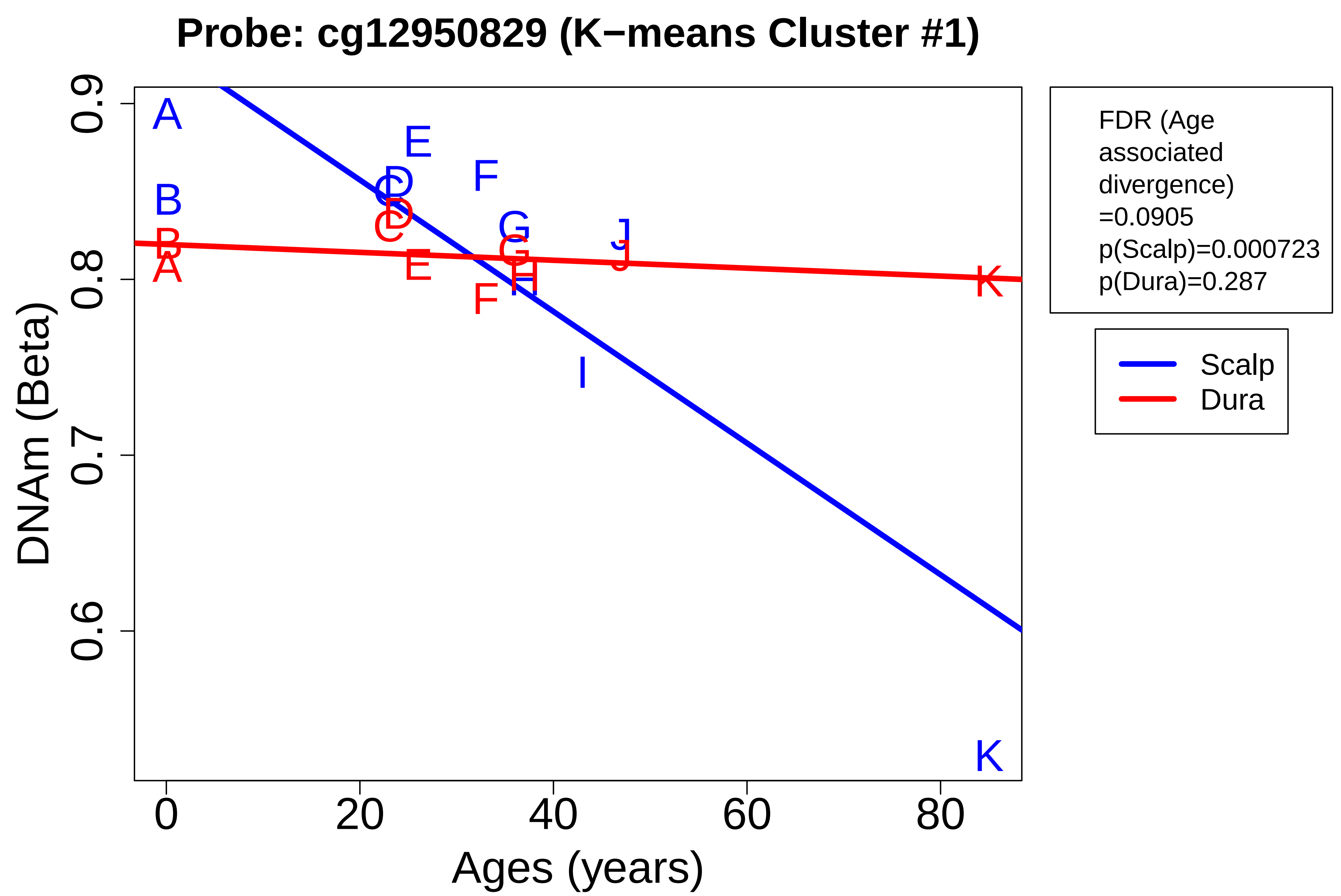

Probe: cg12950829 (K−means Cluster #1)
DNAm (Beta)
0.6
0.7
0.8
0.9
Ages (years)
0
20
40
60
80
A
B
C
D
E
F
G
H
I
J
K
FDR (Age associated divergence) =0.0905
p(Scalp)=0.000723
p(Dura)=0.287
Scalp
Dura

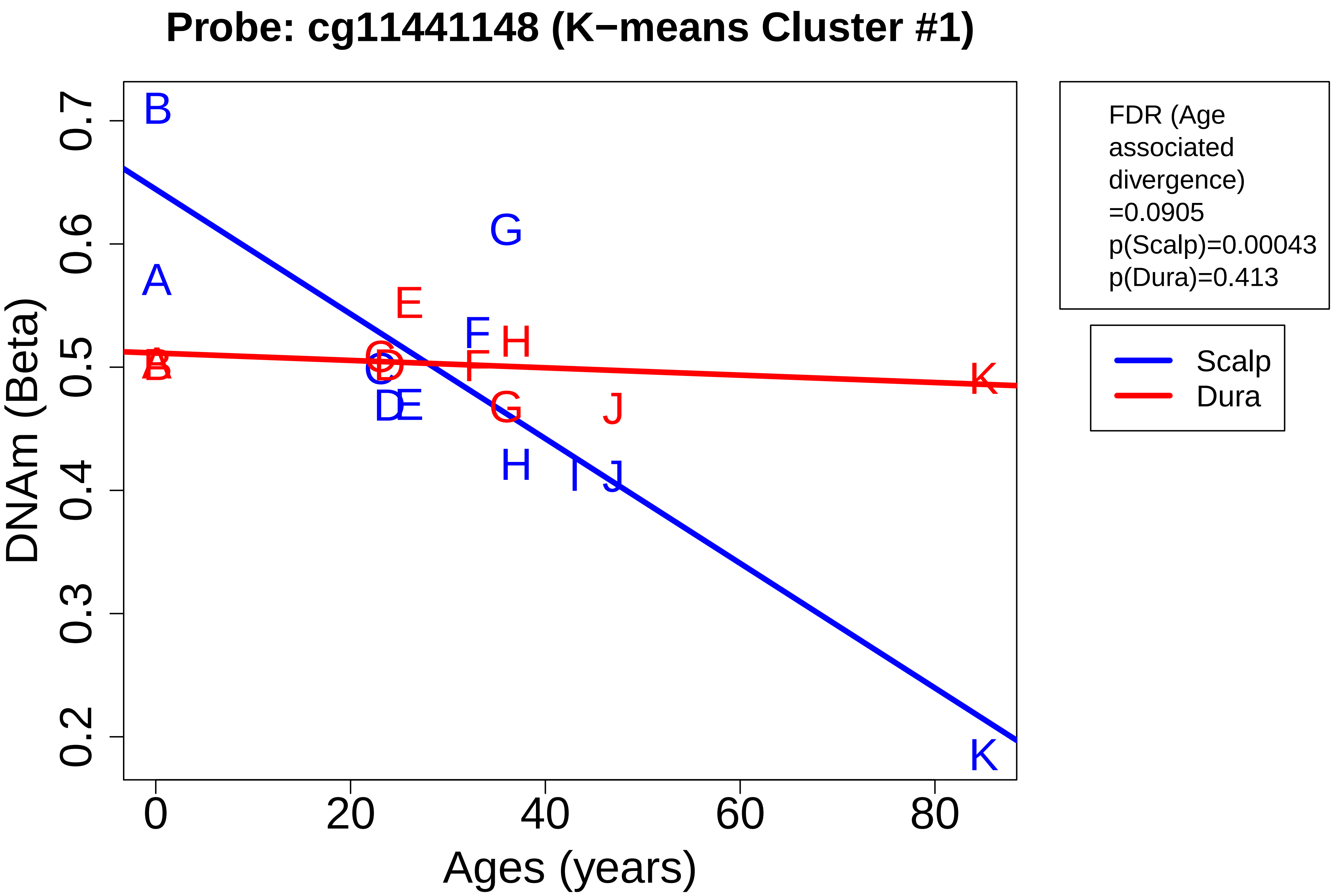
Probe: cg11441148 (K−means Cluster #1)
DNAm (Beta)
0.2
0.3
0.4
0.5
0.6
0.7
0
20
40
60
80
Ages (years)
FDR (Age associated divergence) =0.0905
p(Scalp)=0.00043
p(Dura)=0.413
Scalp
Dura

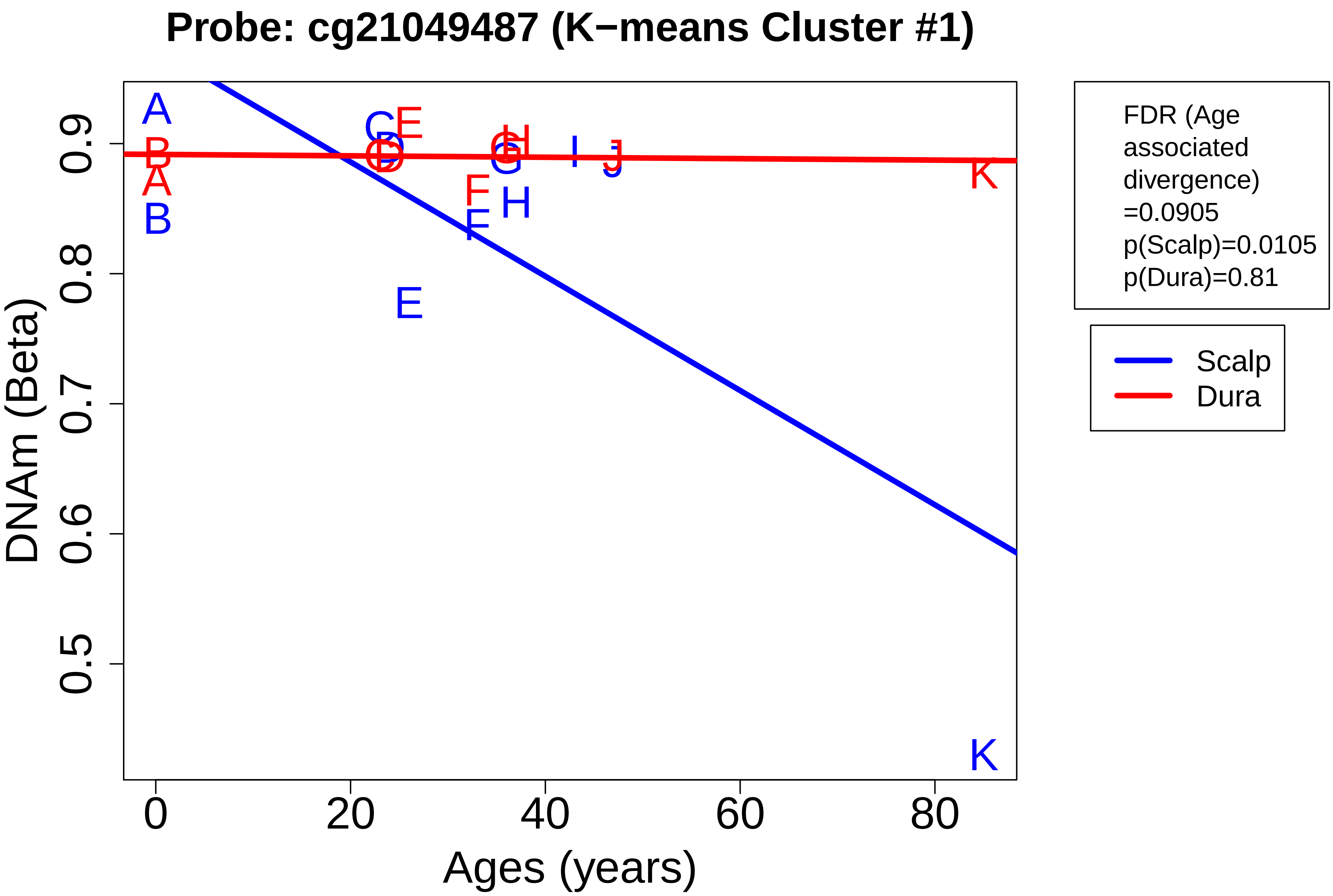

Probe: cg21049487 (K−means Cluster #1)
DNAm (Beta)
0.5
0.6
0.7
0.8
0.9
0
20
40
60
80
Ages (years)
FDR (Age associated divergence) =0.0905
p(Scalp)=0.0105
p(Dura)=0.81
Scalp
Dura

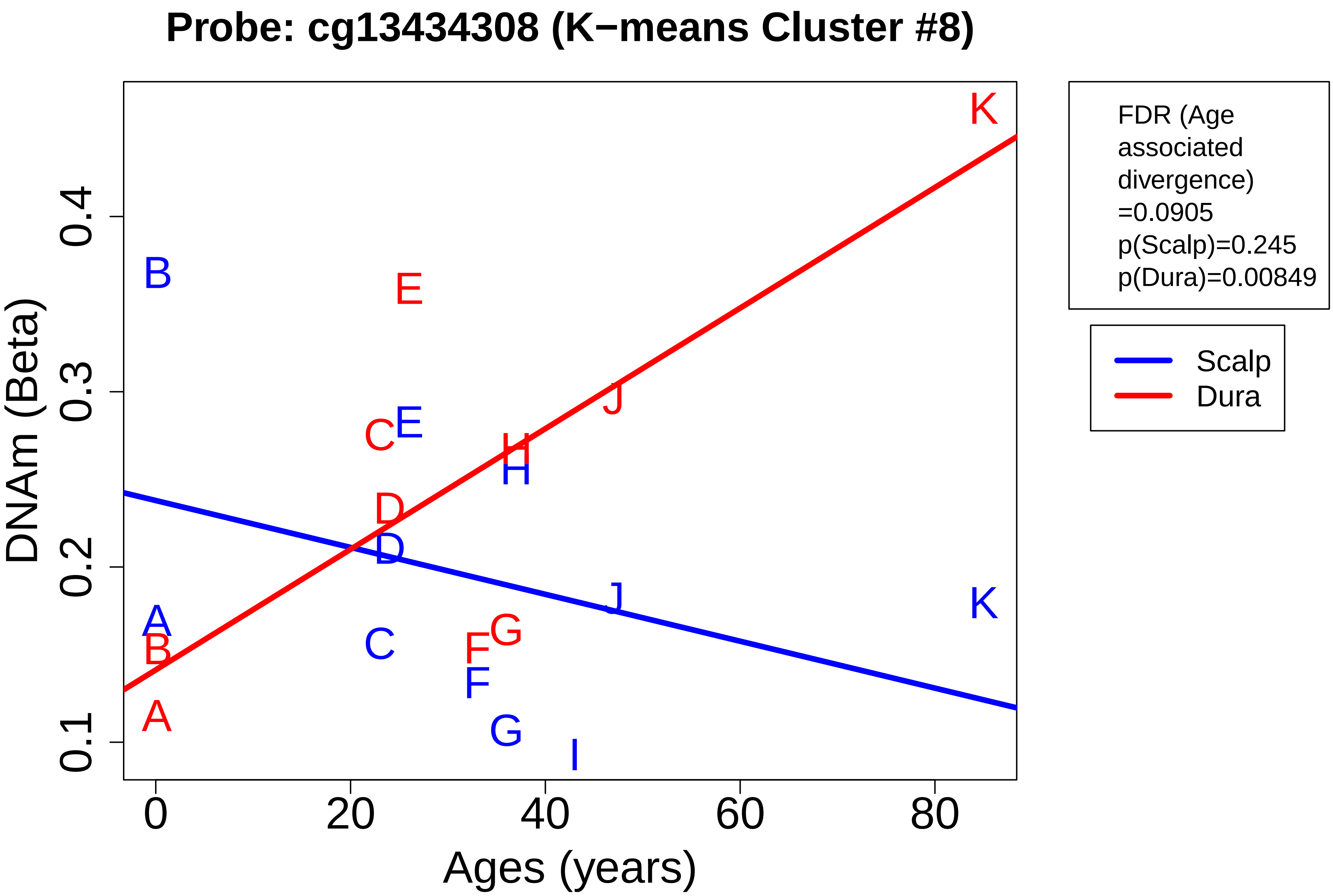

Probe: cg13434308 (K−means Cluster #8)
DNAm (Beta)
0.1
0.2
0.3
0.4
Ages (years)
0
20
40
60
80
FDR (Age associated divergence) =0.0905
p(Scalp)=0.245
p(Dura)=0.00849
Scalp
Dura
A
B
C
D
E
F
G
H
I
J
K

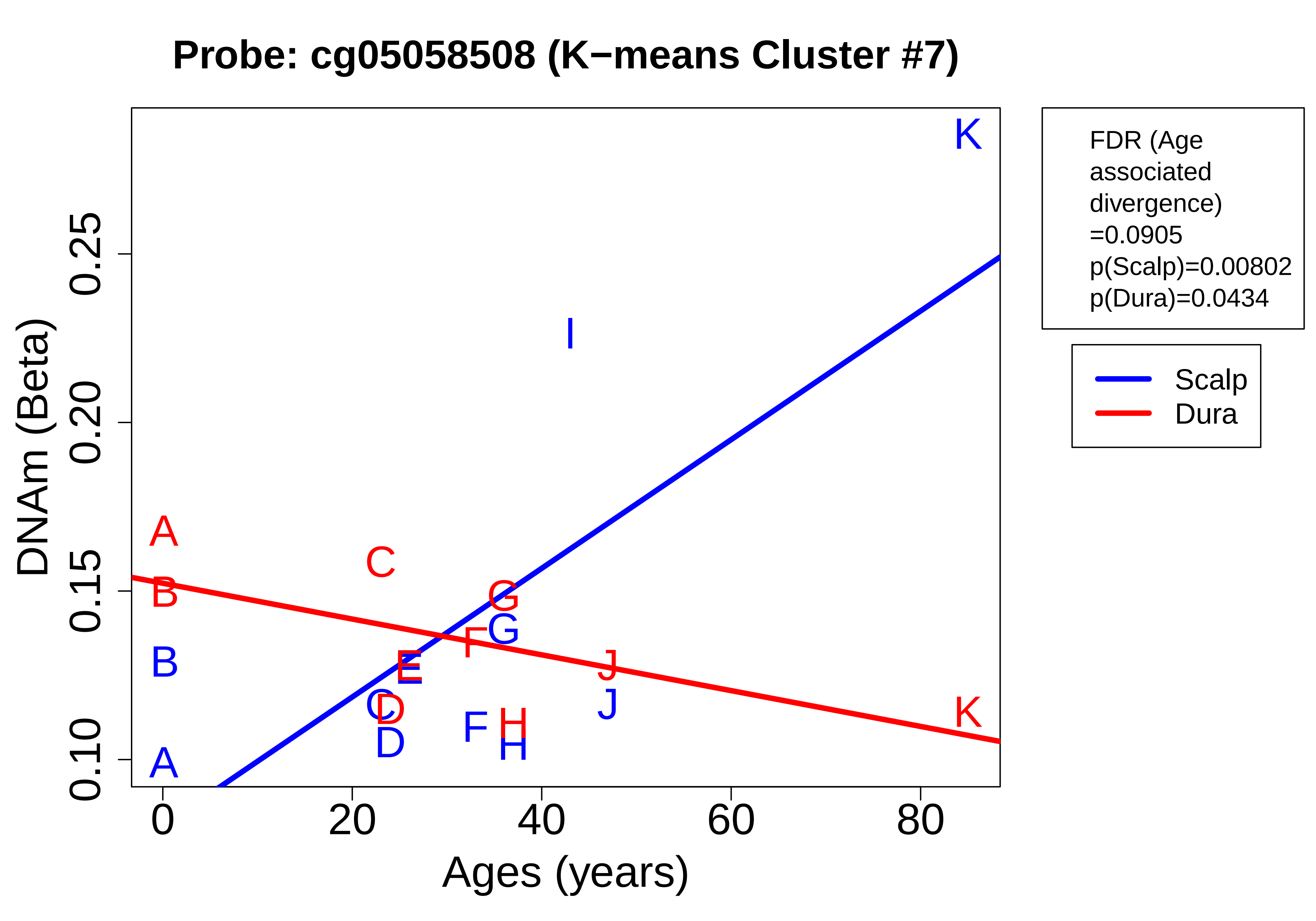
Probe: cg05058508 (K−means Cluster #7)
DNAm (Beta)
0.10
0.15
0.20
0.25
0
20
40
60
80
Ages (years)
FDR (Age associated divergence) =0.0905
p(Scalp)=0.00802
p(Dura)=0.0434
Scalp
Dura
A B C D E F G H I J K

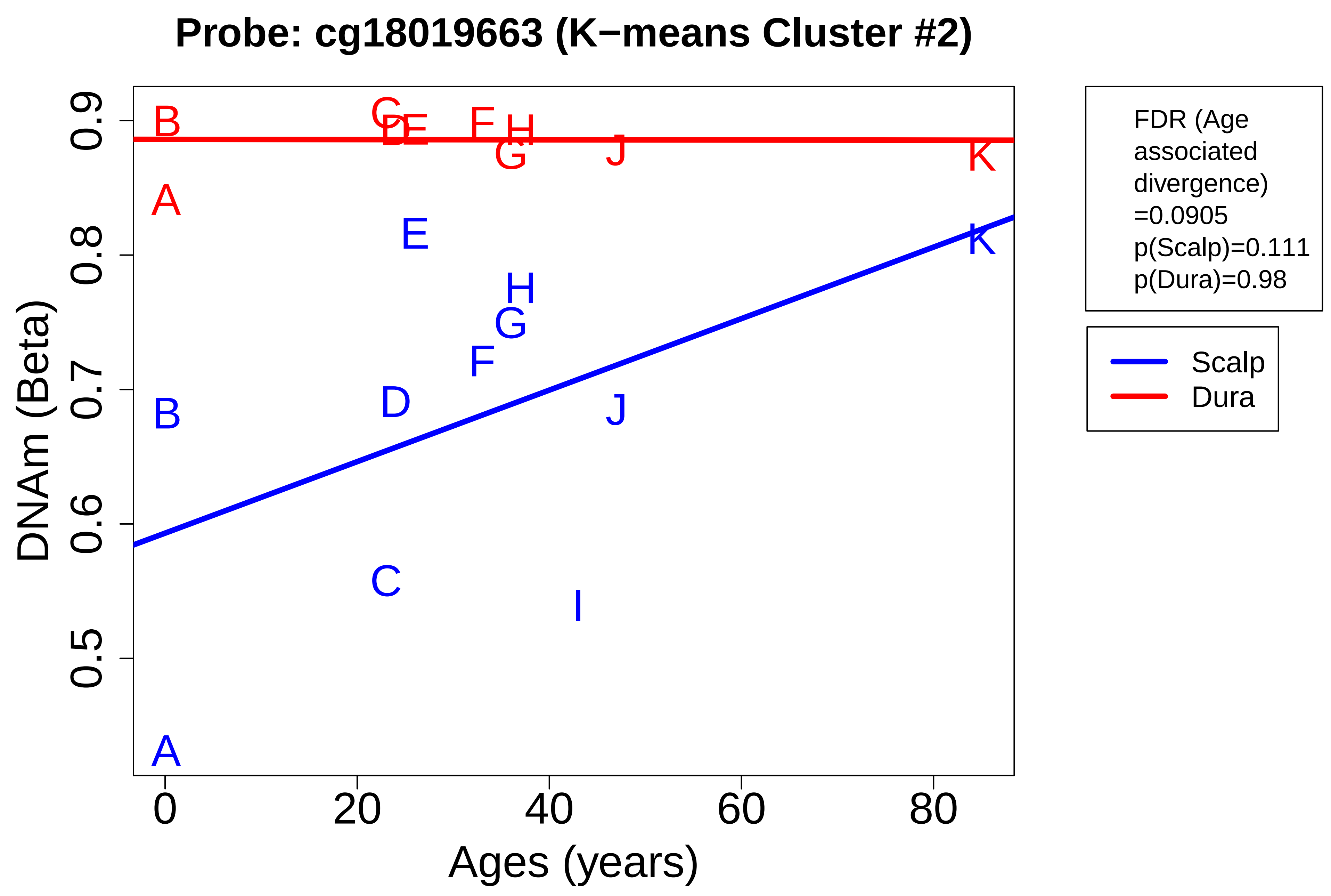

Probe: cg18019663 (K−means Cluster #2)
DNAm (Beta)
0.5
0.6
0.7
0.8
0.9
0
20
40
60
80
Ages (years)
FDR (Age associated divergence) =0.0905
p(Scalp)=0.111
p(Dura)=0.98
Scalp
Dura

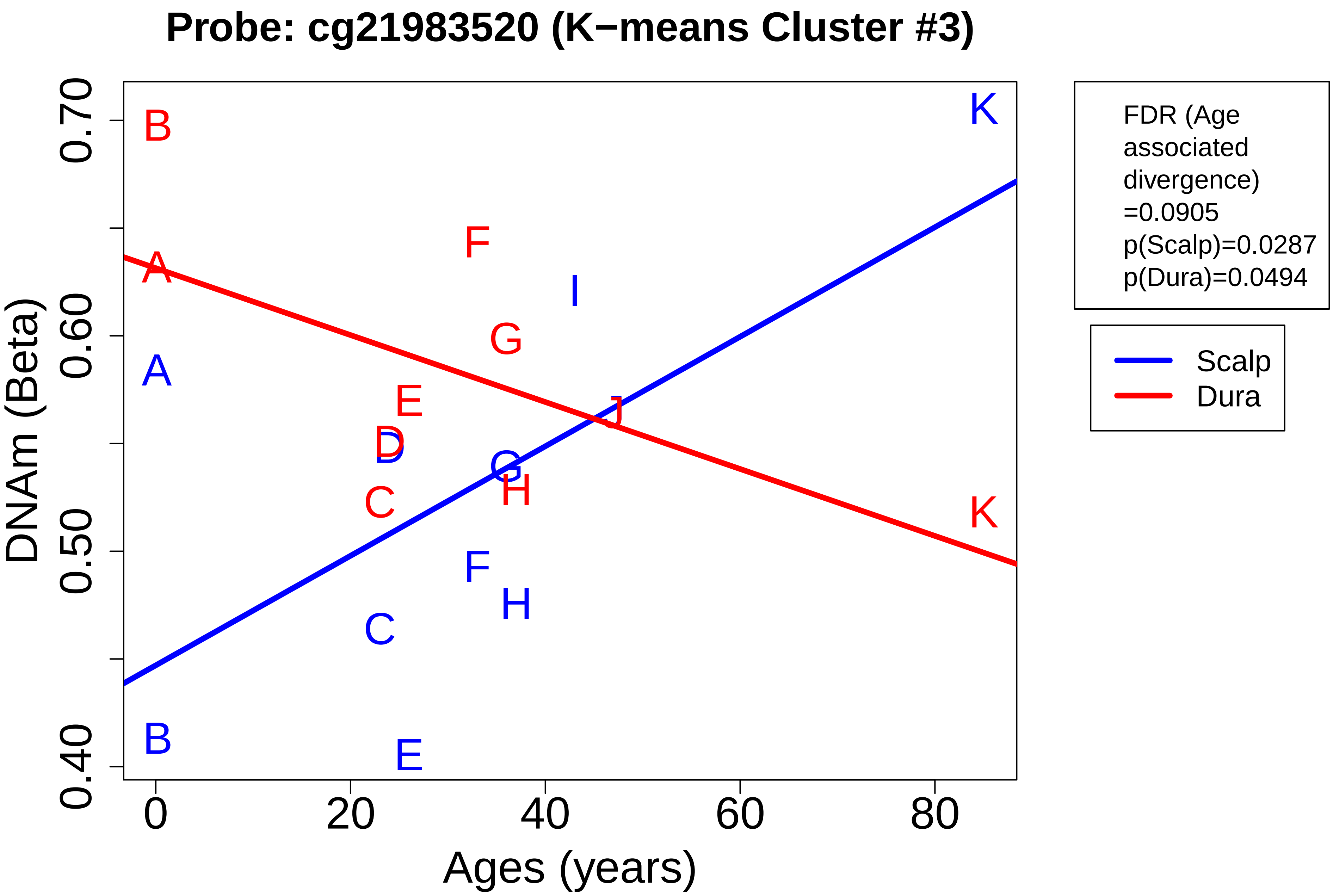
Probe: cg21983520 (K−means Cluster #3)
DNAm (Beta)
0.40
0.50
0.60
0.70
Ages (years)
0
20
40
60
80
FDR (Age associated divergence) =0.0905
p(Scalp)=0.0287
p(Dura)=0.0494
Scalp
Dura

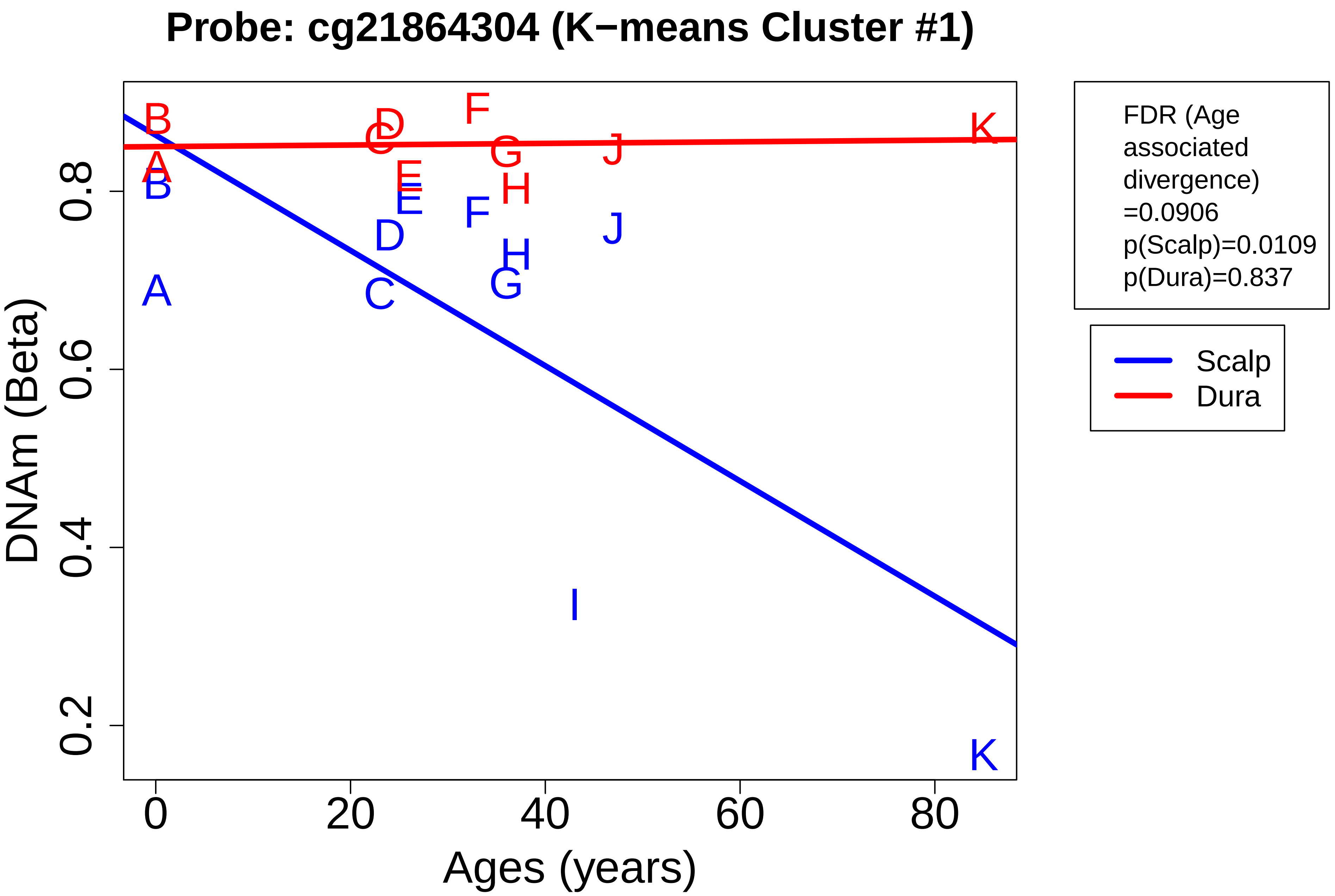

Probe: cg21864304 (K−means Cluster #1)
FDR (Age associated divergence) =0.0906
p(Scalp)=0.0109
p(Dura)=0.837
Scalp
Dura
DNAm (Beta)
Ages (years)
0.2
0.4
0.6
0.8
0
20
40
60
80
A B C D E F G H I J K

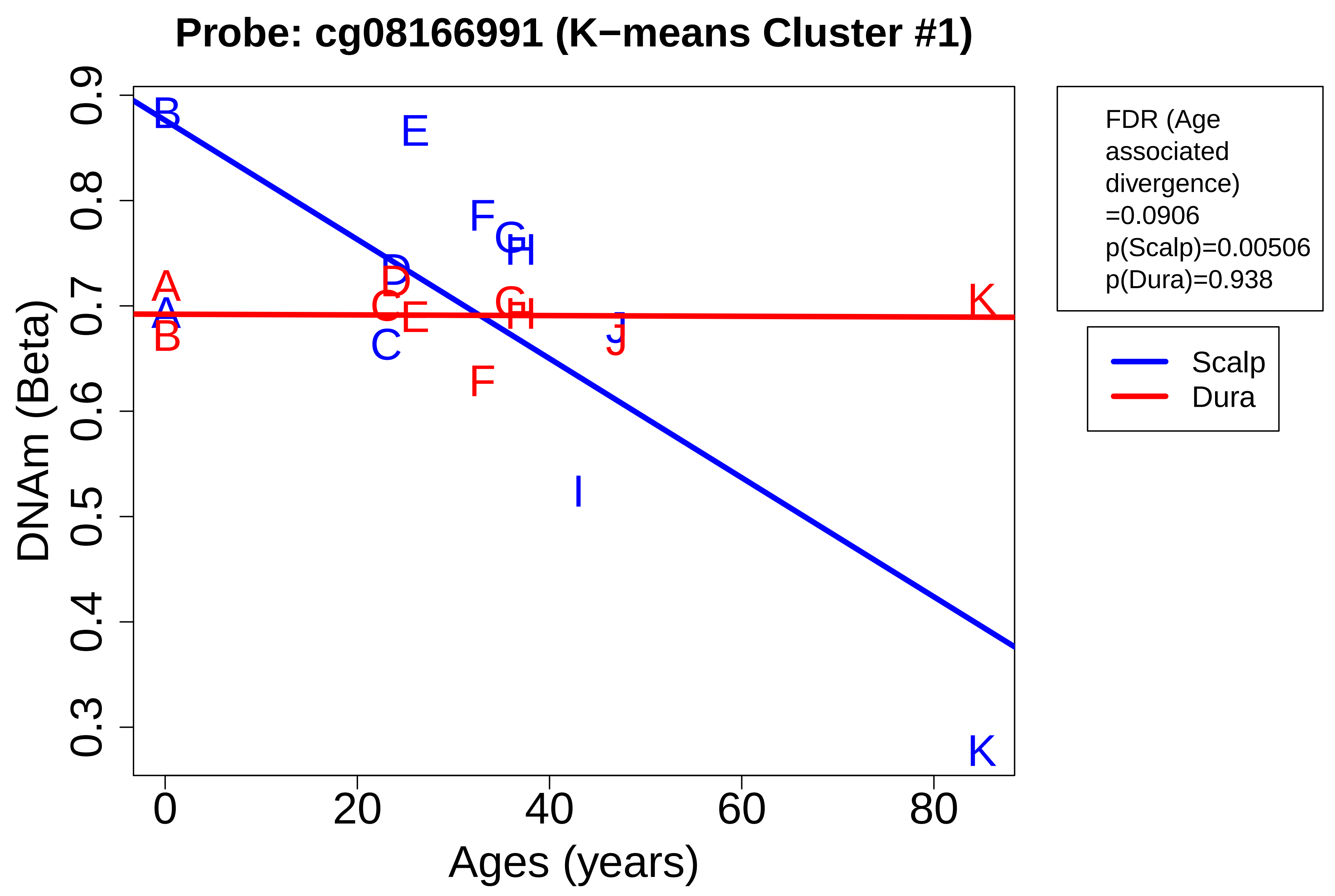

Probe: cg08166991 (K−means Cluster #1)
DNAm (Beta)
Ages (years)
0.3
0.4
0.5
0.6
0.7
0.8
0.9
0
20
40
60
80
FDR (Age associated divergence) =0.0906
p(Scalp)=0.00506
p(Dura)=0.938
Scalp
Dura

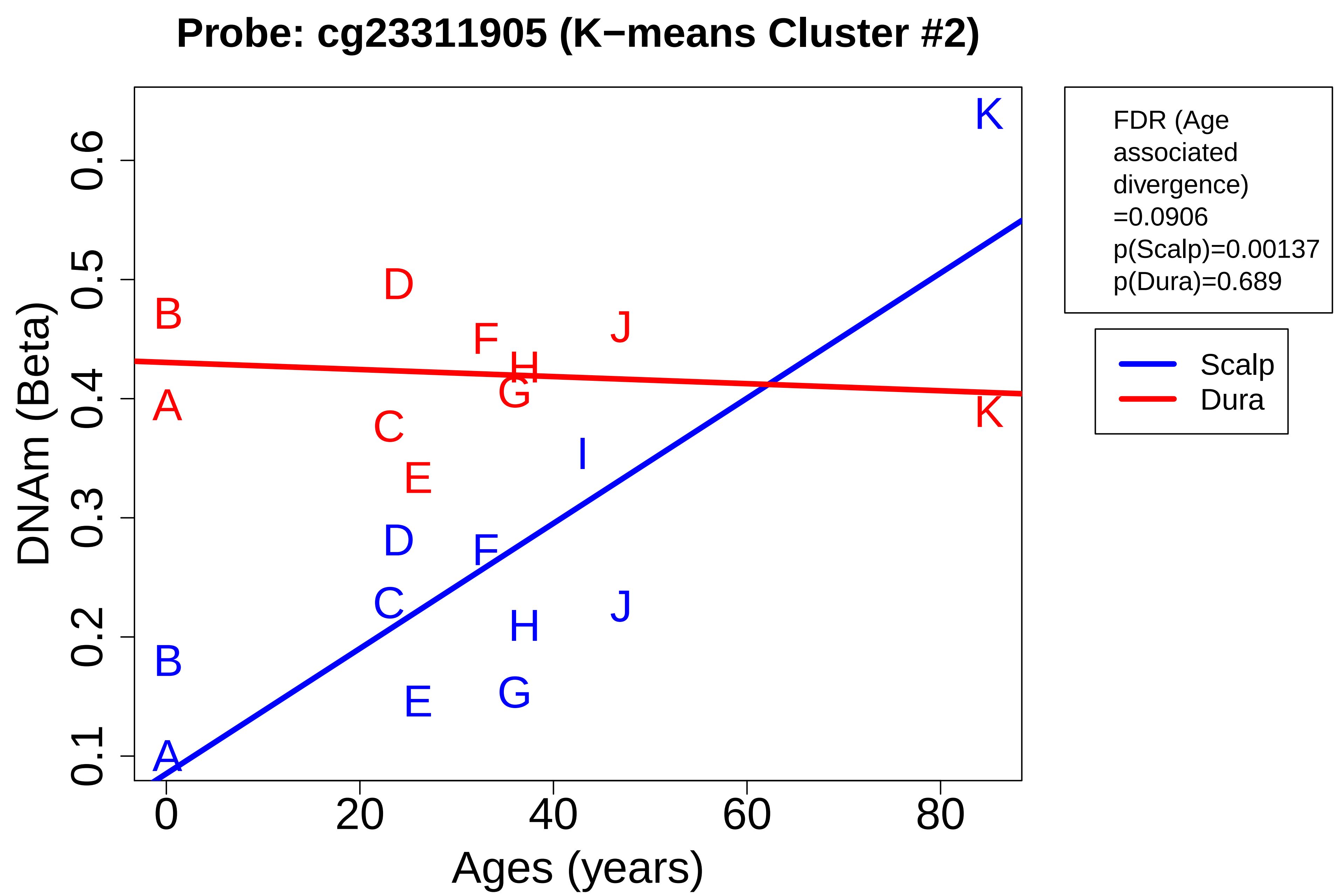
Probe: cg23311905 (K−means Cluster #2)
DNAm (Beta)
0.1
0.2
0.3
0.4
0.5
0.6
Ages (years)
0
20
40
60
80
FDR (Age associated divergence) =0.0906
p(Scalp)=0.00137
p(Dura)=0.689
Scalp
Dura

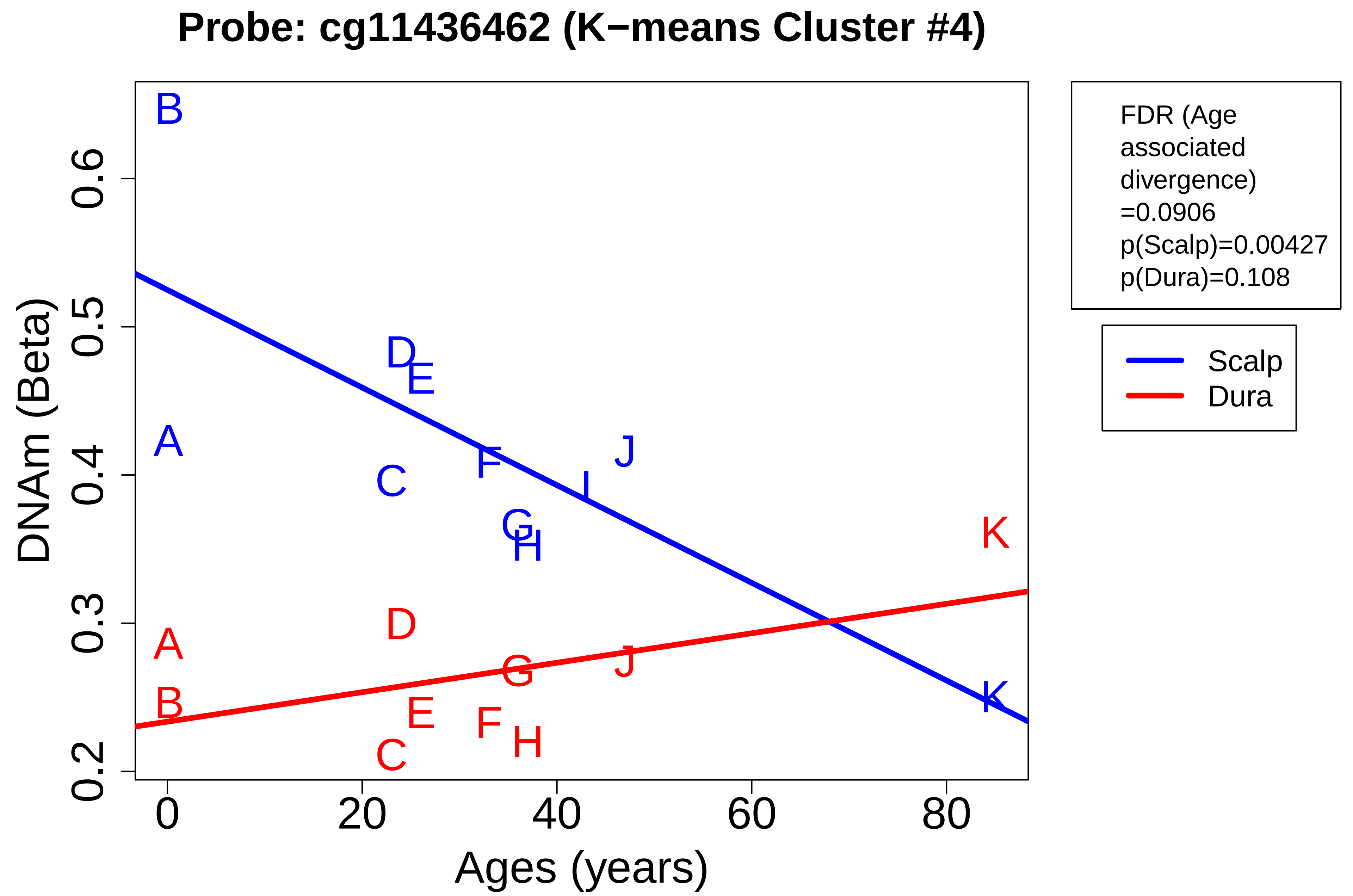

Probe: cg11436462 (K−means Cluster #4)
FDR (Age associated divergence) =0.0906
p(Scalp)=0.00427
p(Dura)=0.108
Scalp
Dura
DNAm (Beta)
0.2
0.3
0.4
0.5
0.6
Ages (years)
0
20
40
60
80
A
B
C
D
E
F
G
H
I
J
K

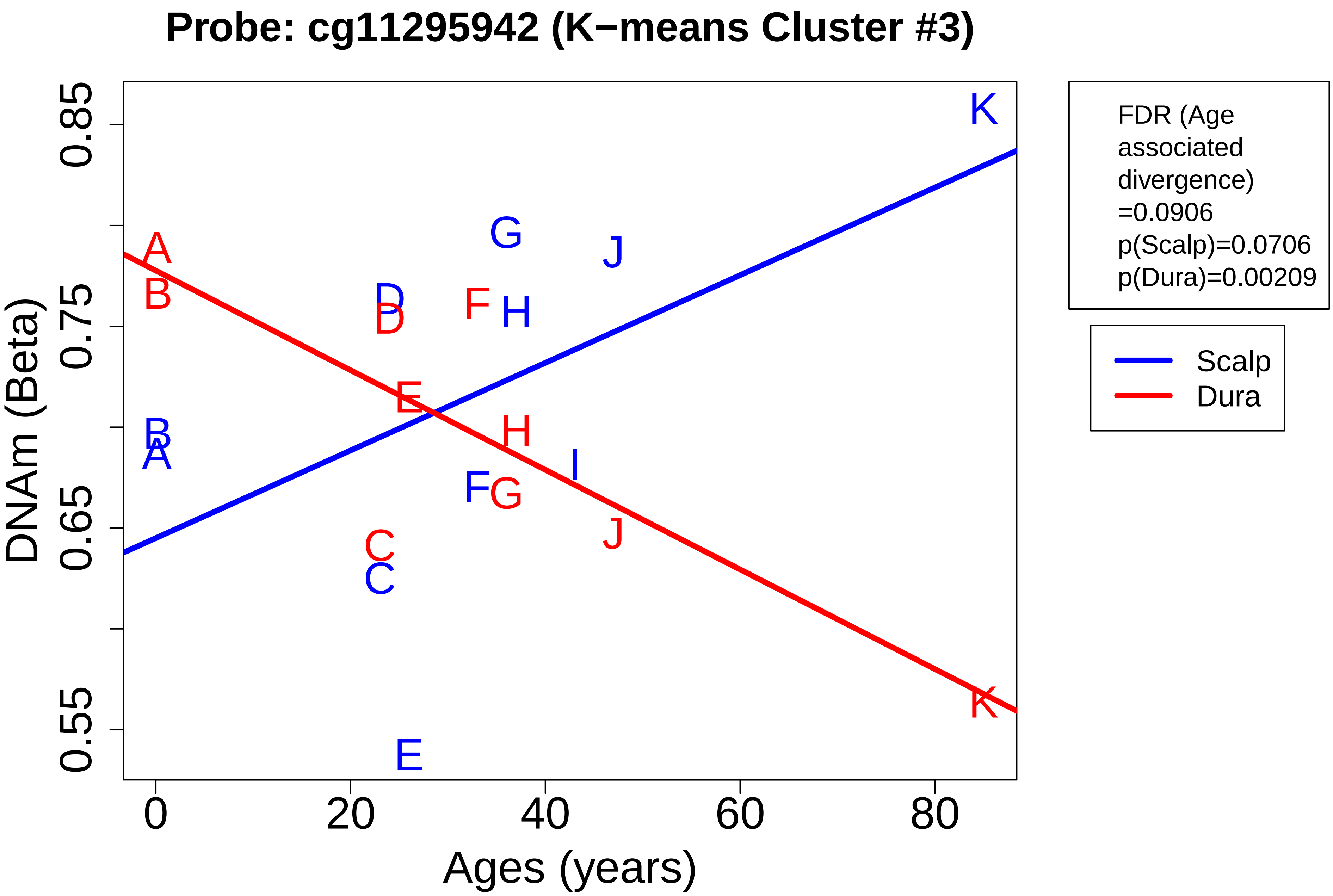

Probe: cg11295942 (K−means Cluster #3)
DNAm (Beta)
0.55
0.65
0.75
0.85
Ages (years)
0
20
40
60
80
FDR (Age associated divergence) =0.0906
p(Scalp)=0.0706
p(Dura)=0.00209
Scalp
Dura

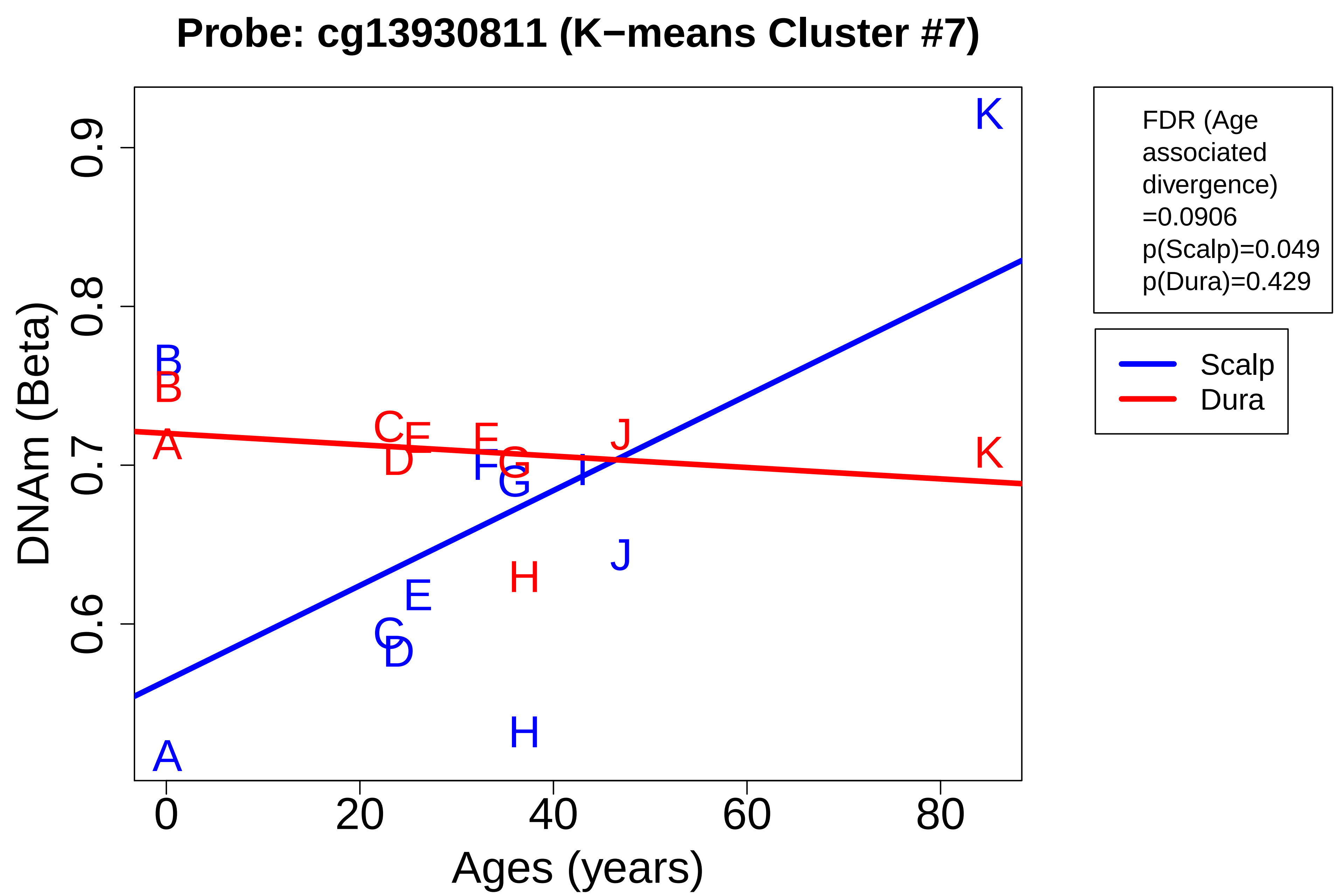

Probe: cg13930811 (K−means Cluster #7)
FDR (Age associated divergence) =0.0906
p(Scalp)=0.049
p(Dura)=0.429
Scalp
Dura
DNAm (Beta)
Ages (years)
0.6
0.7
0.8
0.9
0
20
40
60
80

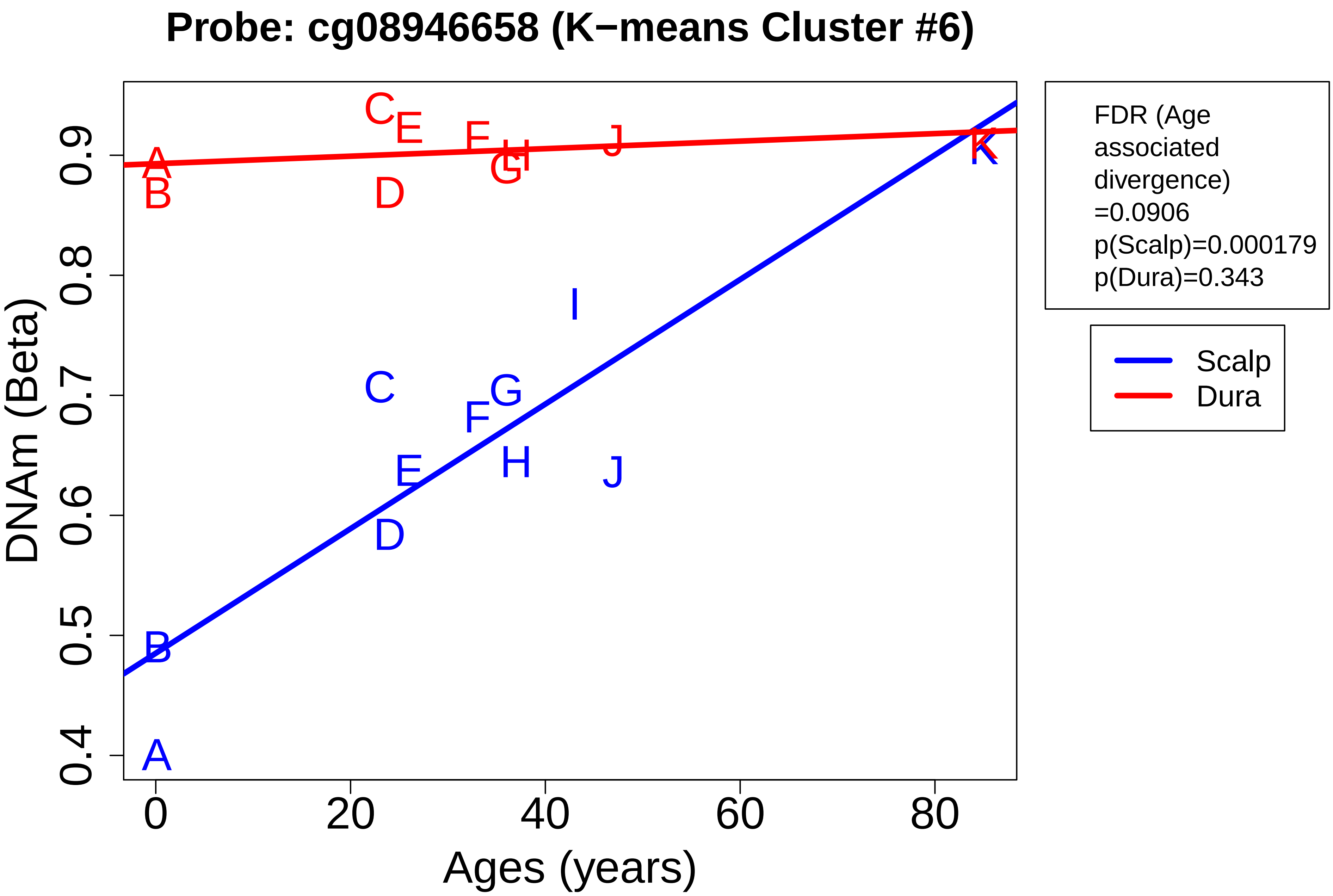

Probe: cg08946658 (K−means Cluster #6)
DNAm (Beta)
0.4
0.5
0.6
0.7
0.8
0.9
0
20
40
60
80
Ages (years)
FDR (Age associated divergence) =0.0906
p(Scalp)=0.000179
p(Dura)=0.343
Scalp
Dura

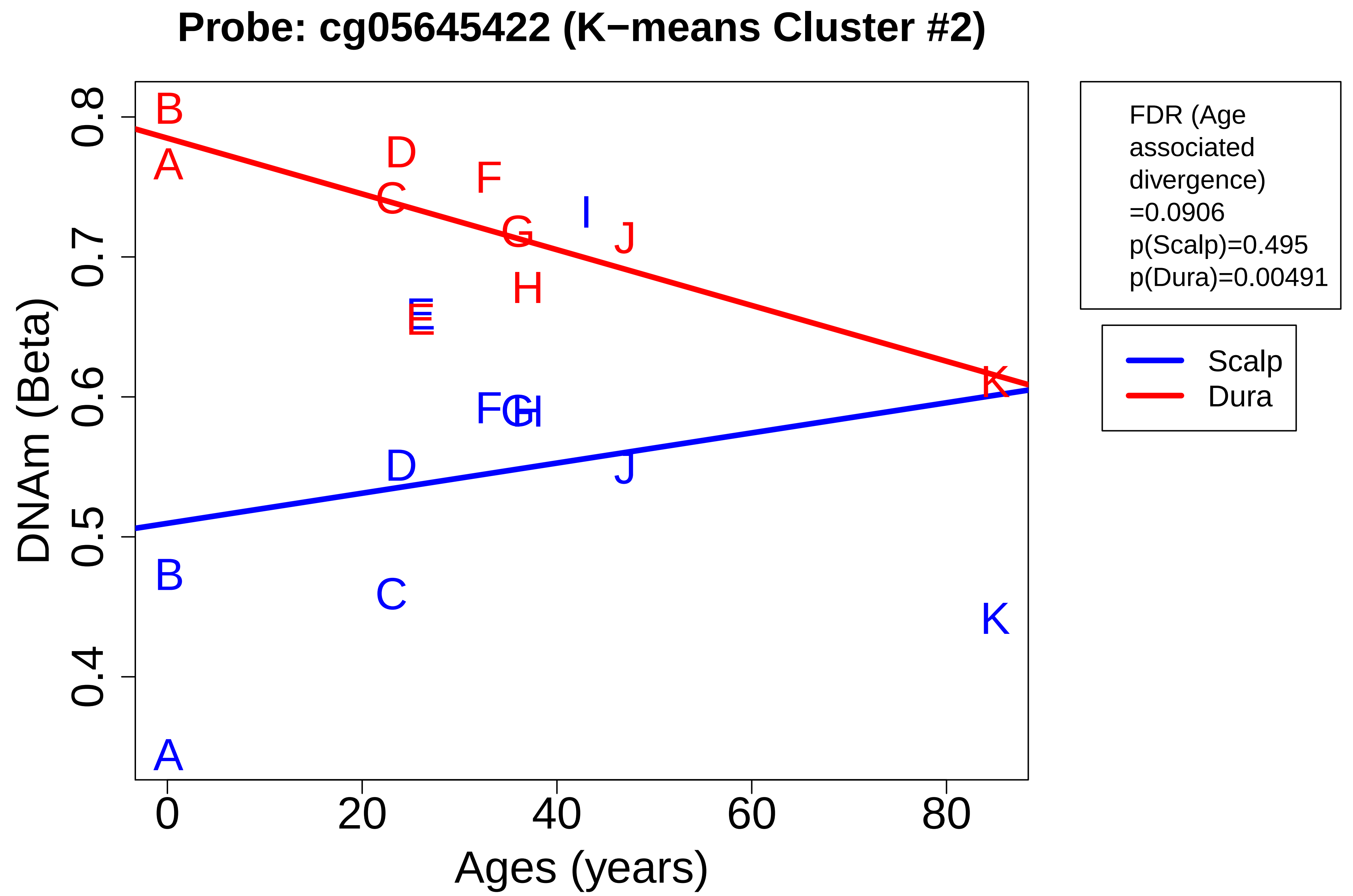

Probe: cg05645422 (K−means Cluster #2)
DNAm (Beta)
0.4
0.5
0.6
0.7
0.8
Ages (years)
0
20
40
60
80
B
A
D
C
F
G
I
J
H
E
E
F
G
H
D
J
K
K
B
C
A
FDR (Age associated divergence) =0.0906
p(Scalp)=0.495
p(Dura)=0.00491
Scalp
Dura

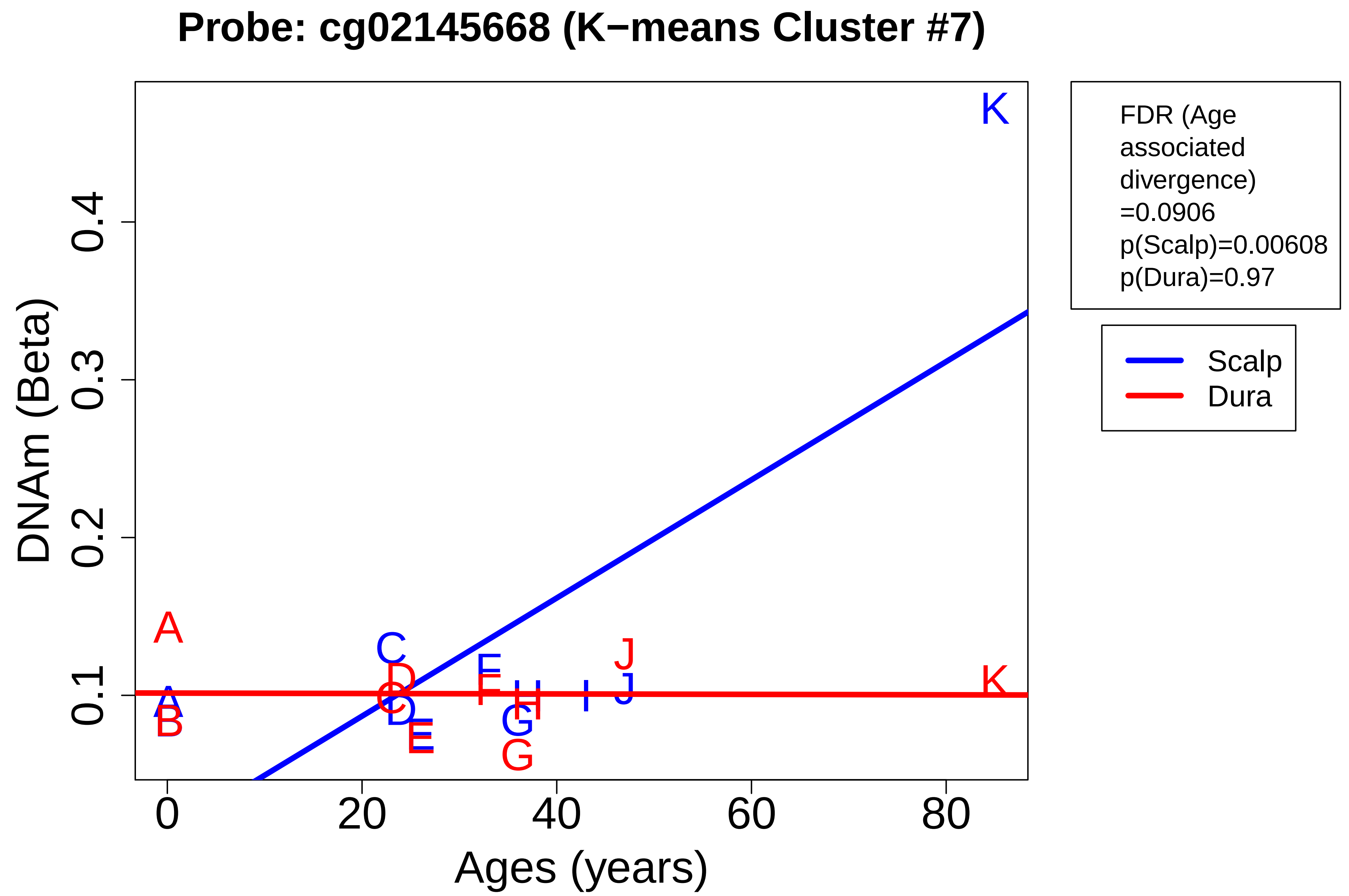

Probe: cg02145668 (K−means Cluster #7)
DNAm (Beta)
Ages (years)
0.1
0.2
0.3
0.4
0
20
40
60
80
FDR (Age associated divergence) =0.0906
p(Scalp)=0.00608
p(Dura)=0.97
Scalp
Dura

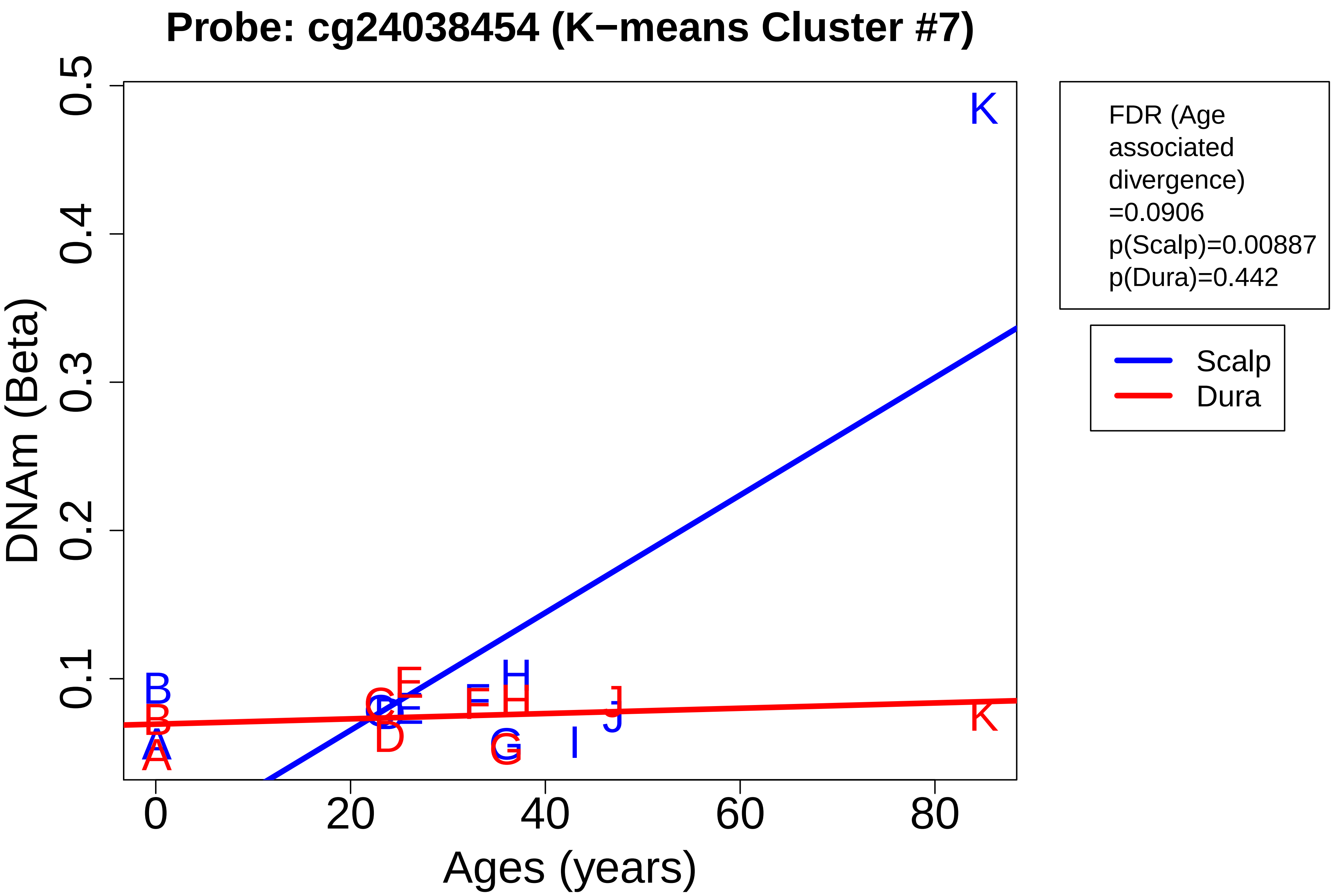

Probe: cg24038454 (K−means Cluster #7)
DNAm (Beta)
0.1
0.2
0.3
0.4
0.5
0
20
40
60
80
Ages (years)
FDR (Age associated divergence) =0.0906
p(Scalp)=0.00887
p(Dura)=0.442
Scalp
Dura
A
B
C
D
E
F
G
H
I
J
K

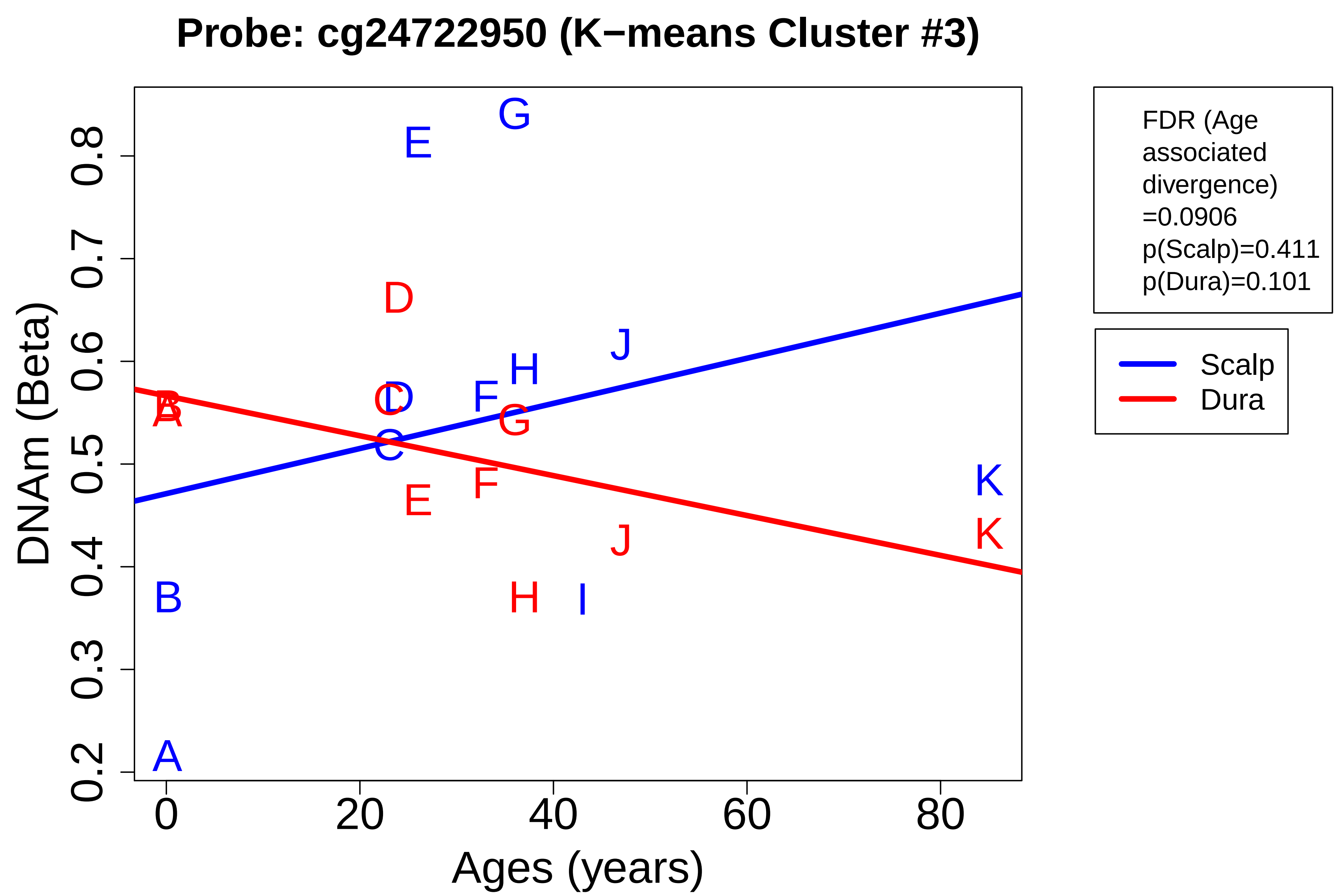

Probe: cg24722950 (K−means Cluster #3)
DNAm (Beta)
0.2
0.3
0.4
0.5
0.6
0.7
0.8
Ages (years)
0
20
40
60
80
FDR (Age associated divergence) =0.0906
p(Scalp)=0.411
p(Dura)=0.101
Scalp
Dura

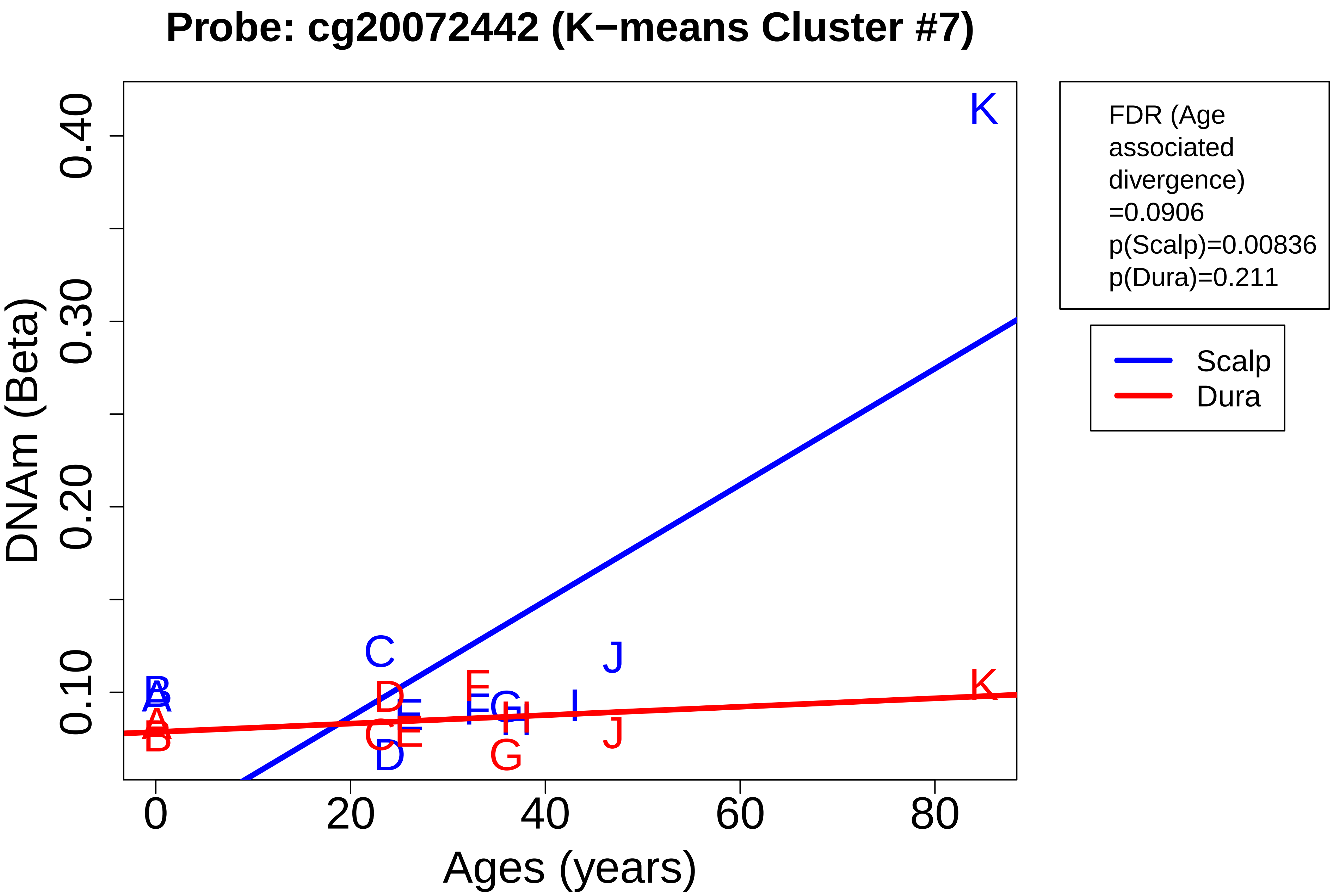

Probe: cg20072442 (K−means Cluster #7)
DNAm (Beta)
0.10
0.20
0.30
0.40
Ages (years)
0
20
40
60
80
FDR (Age associated divergence) =0.0906
p(Scalp)=0.00836
p(Dura)=0.211
Scalp
Dura
A
B
C
D
E
F
G
H
I
J
K

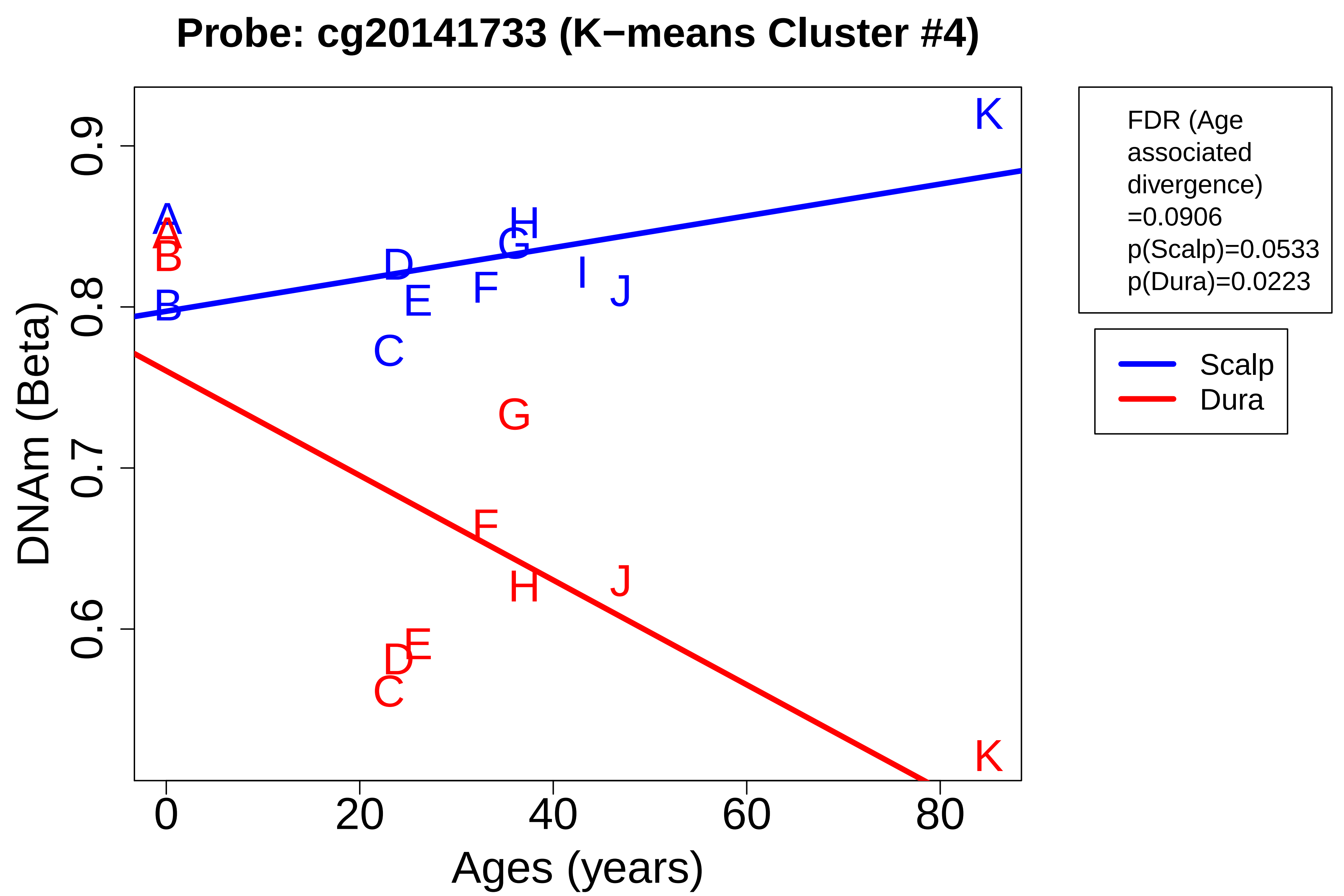

Probe: cg20141733 (K−means Cluster #4)
DNAm (Beta)
0.6
0.7
0.8
0.9
Ages (years)
0
20
40
60
80
FDR (Age associated divergence) =0.0906
p(Scalp)=0.0533
p(Dura)=0.0223
Scalp
Dura
A
B
C
D
E
F
G
H
I
J
K

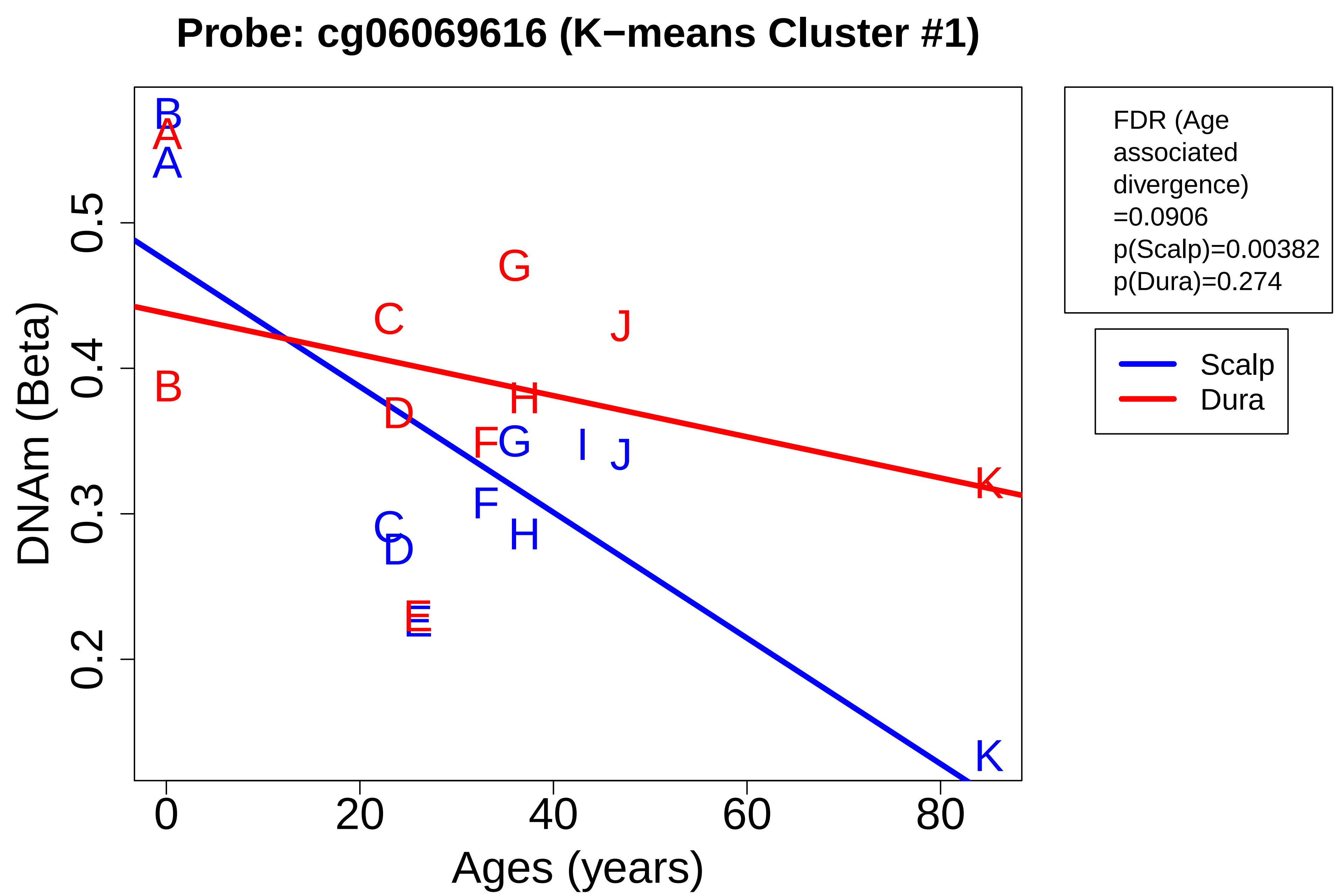

Probe: cg06069616 (K−means Cluster #1)
DNAm (Beta)
0.2
0.3
0.4
0.5
Ages (years)
0
20
40
60
80
FDR (Age associated divergence) =0.0906
p(Scalp)=0.00382
p(Dura)=0.274
Scalp
Dura
A
B
C
D
E
F
G
H
I
J
K

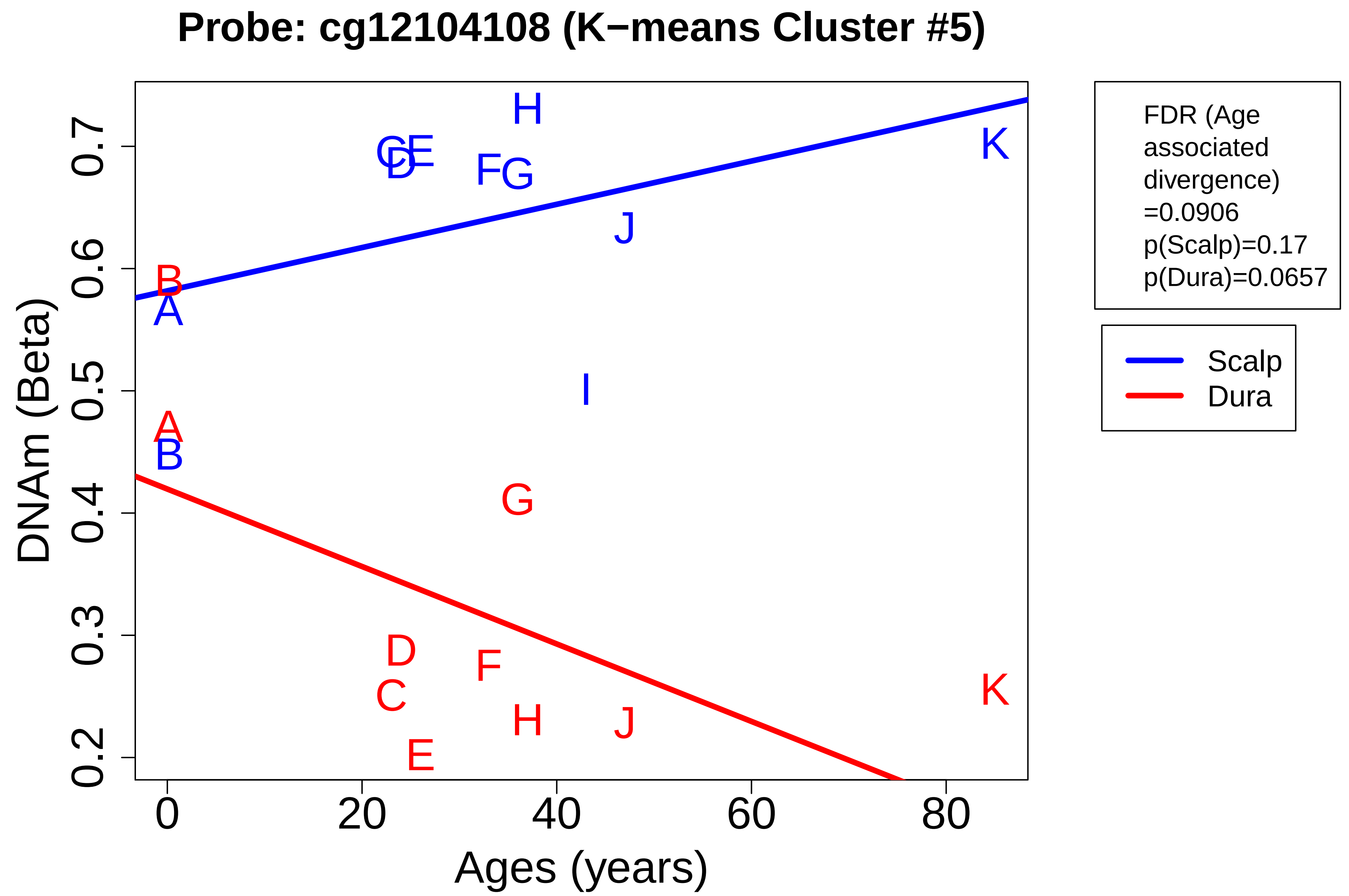

Probe: cg12104108 (K−means Cluster #5)
DNAm (Beta)
Ages (years)
0.2
0.3
0.4
0.5
0.6
0.7
0
20
40
60
80
FDR (Age associated divergence) =0.0906
p(Scalp)=0.17
p(Dura)=0.0657
Scalp
Dura

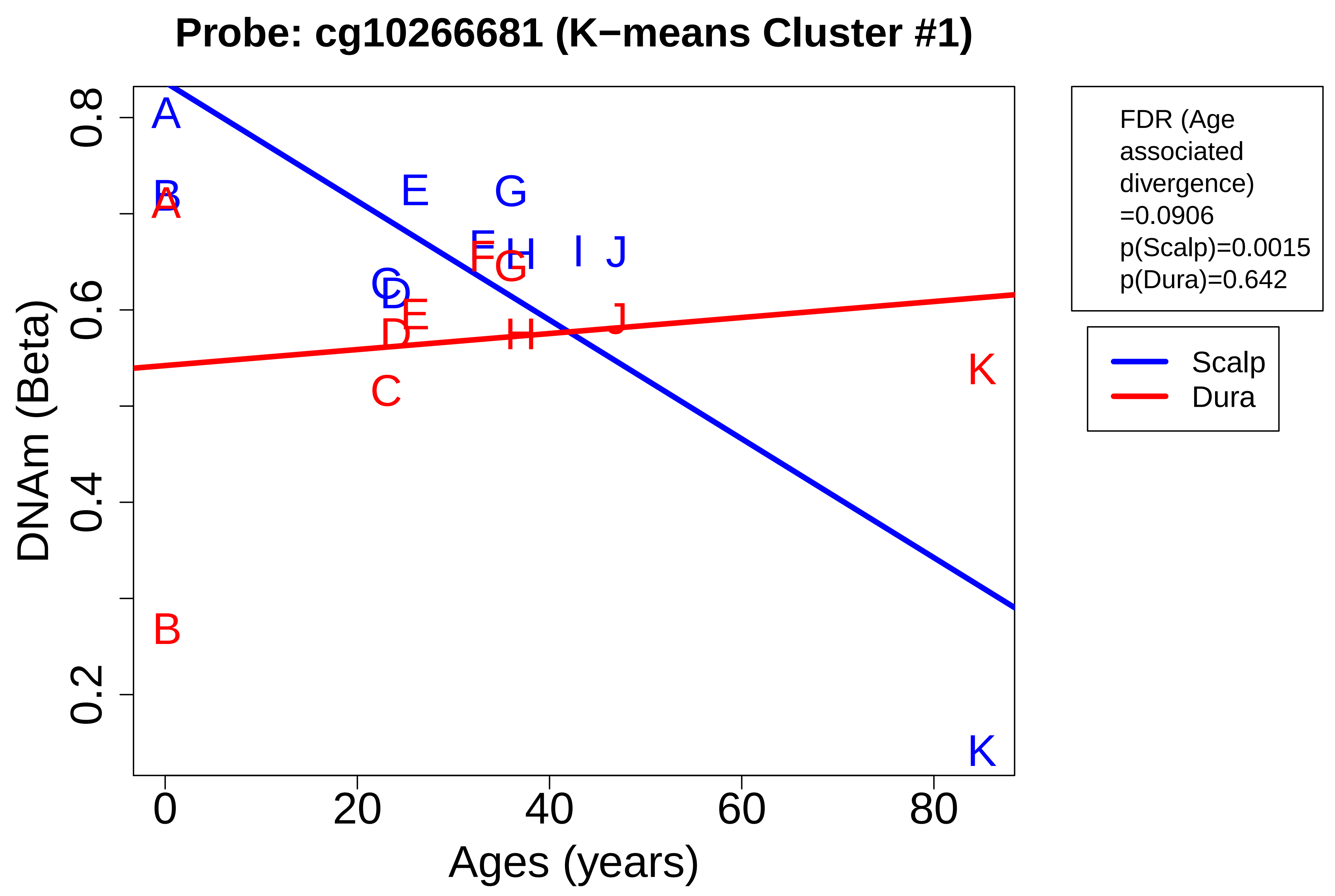

Probe: cg10266681 (K−means Cluster #1)
DNAm (Beta)
0.2
0.4
0.6
0.8
0
20
40
60
80
Ages (years)
FDR (Age associated divergence) =0.0906
p(Scalp)=0.0015
p(Dura)=0.642
Scalp
Dura

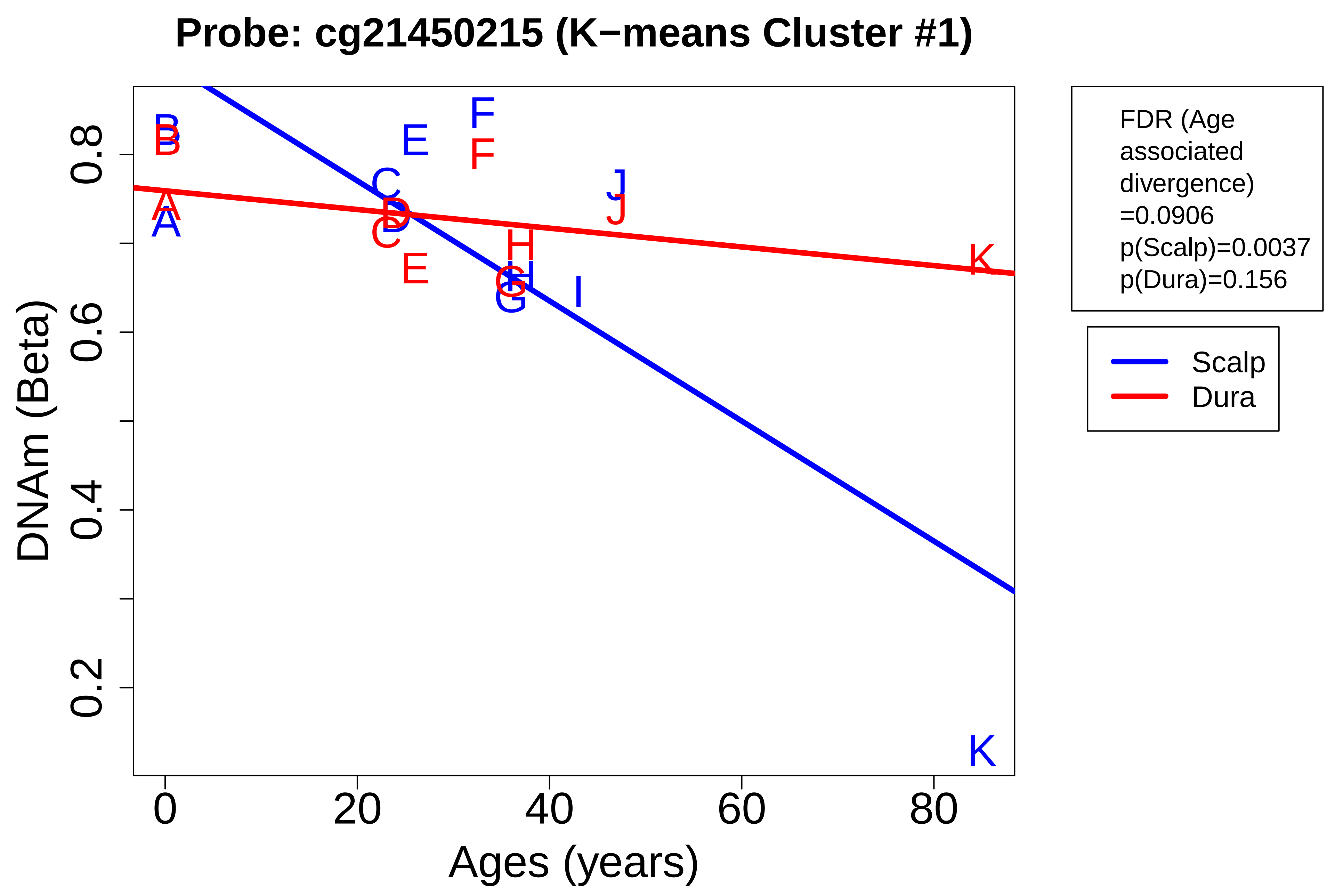
Probe: cg21450215 (K−means Cluster #1)
DNAm (Beta)
0.2
0.4
0.6
0.8
0
20
40
60
80
Ages (years)
A
B
C
D
E
F
G
H
I
J
K
FDR (Age associated divergence) =0.0906
p(Scalp)=0.0037
p(Dura)=0.156
Scalp
Dura

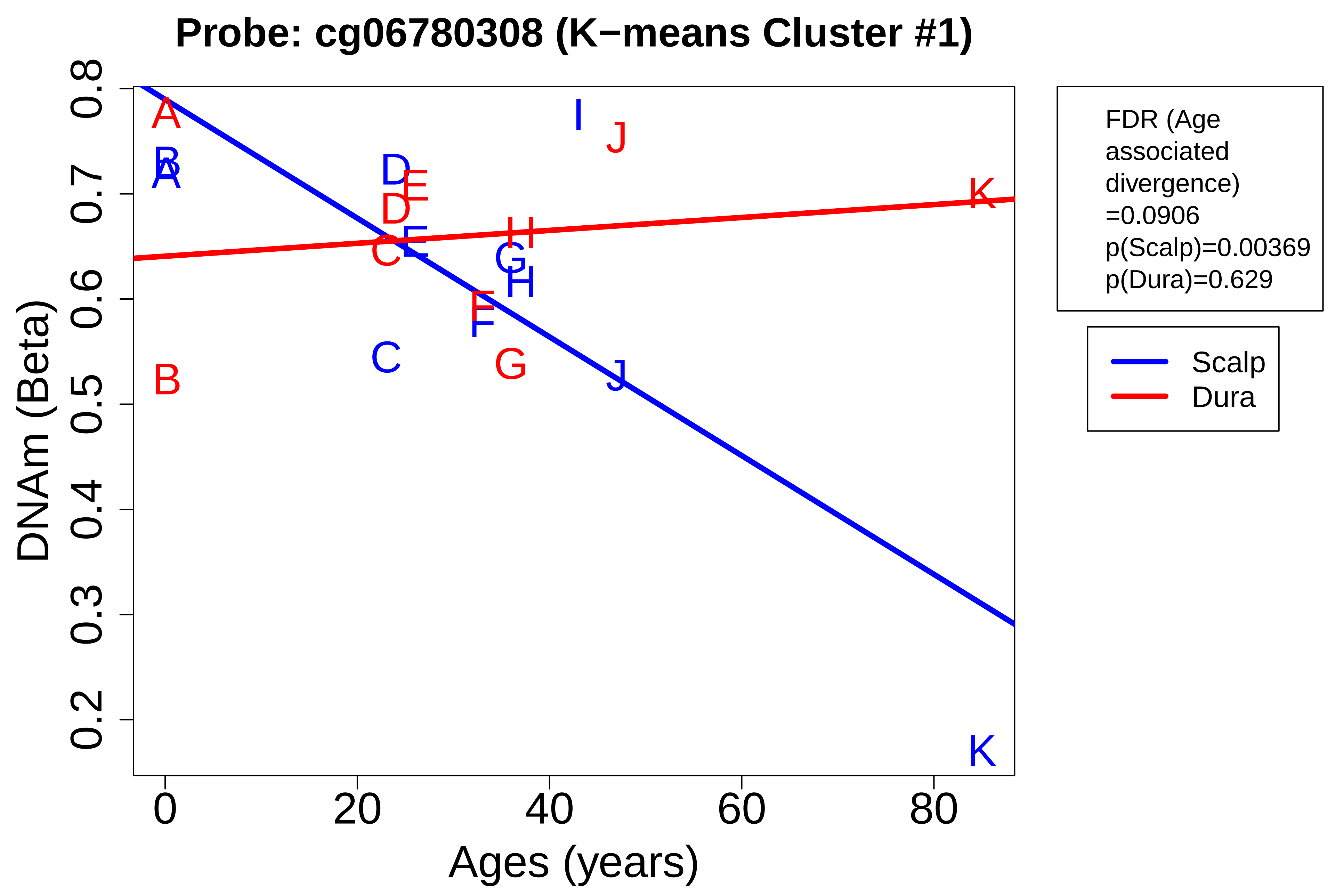

Probe: cg06780308 (K−means Cluster #1)
DNAm (Beta)
0.2
0.3
0.4
0.5
0.6
0.7
0.8
0
20
40
60
80
Ages (years)
FDR (Age associated divergence) =0.0906
p(Scalp)=0.00369
p(Dura)=0.629
Scalp
Dura
A
B
C
D
E
F
G
H
I
J
K

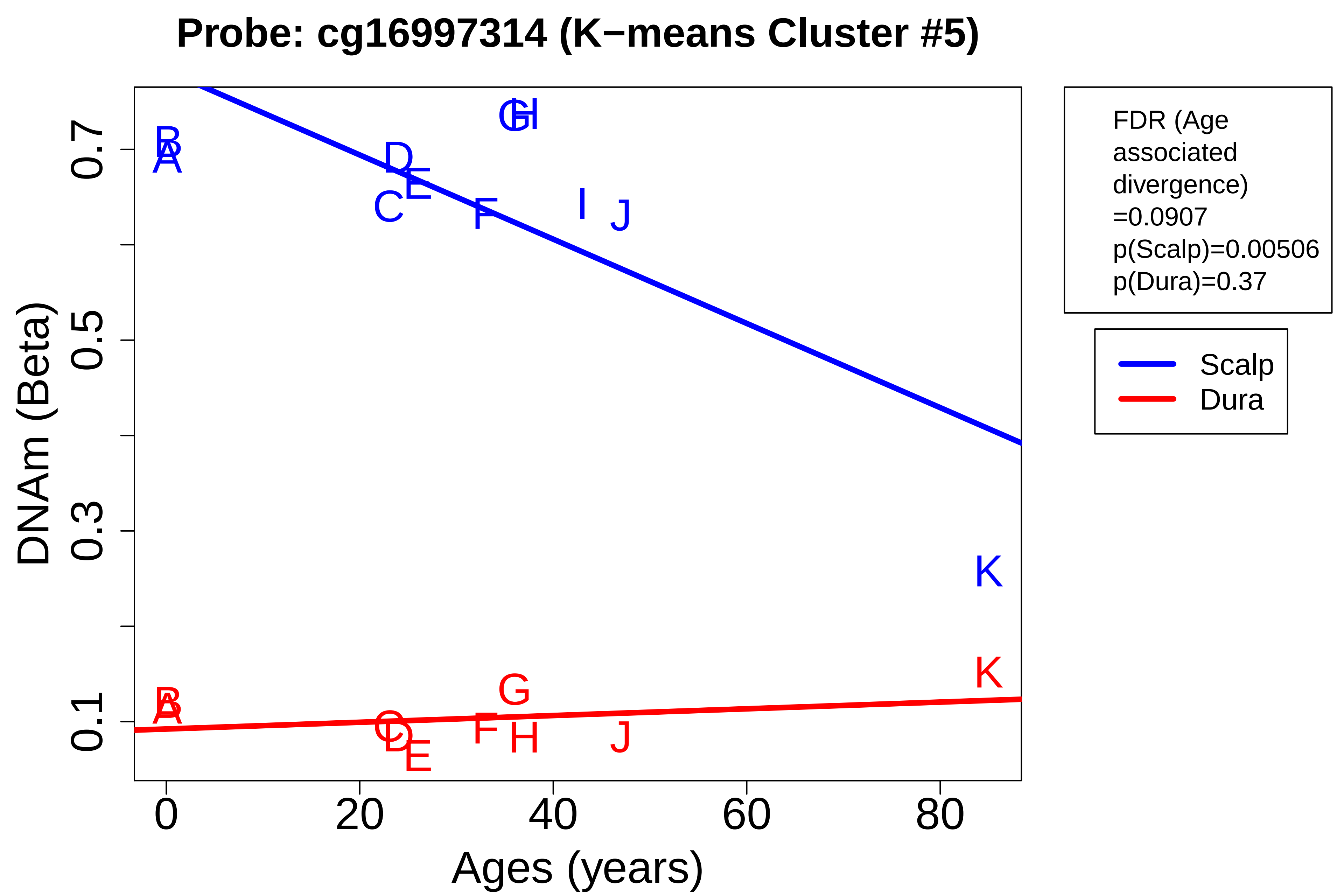
Probe: cg16997314 (K−means Cluster #5)
DNAm (Beta)
0.1
0.3
0.5
0.7
Ages (years)
0
20
40
60
80
FDR (Age associated divergence) =0.0907
p(Scalp)=0.00506
p(Dura)=0.37
Scalp
Dura
A
B
C
D
E
F
G
H
I
J
K

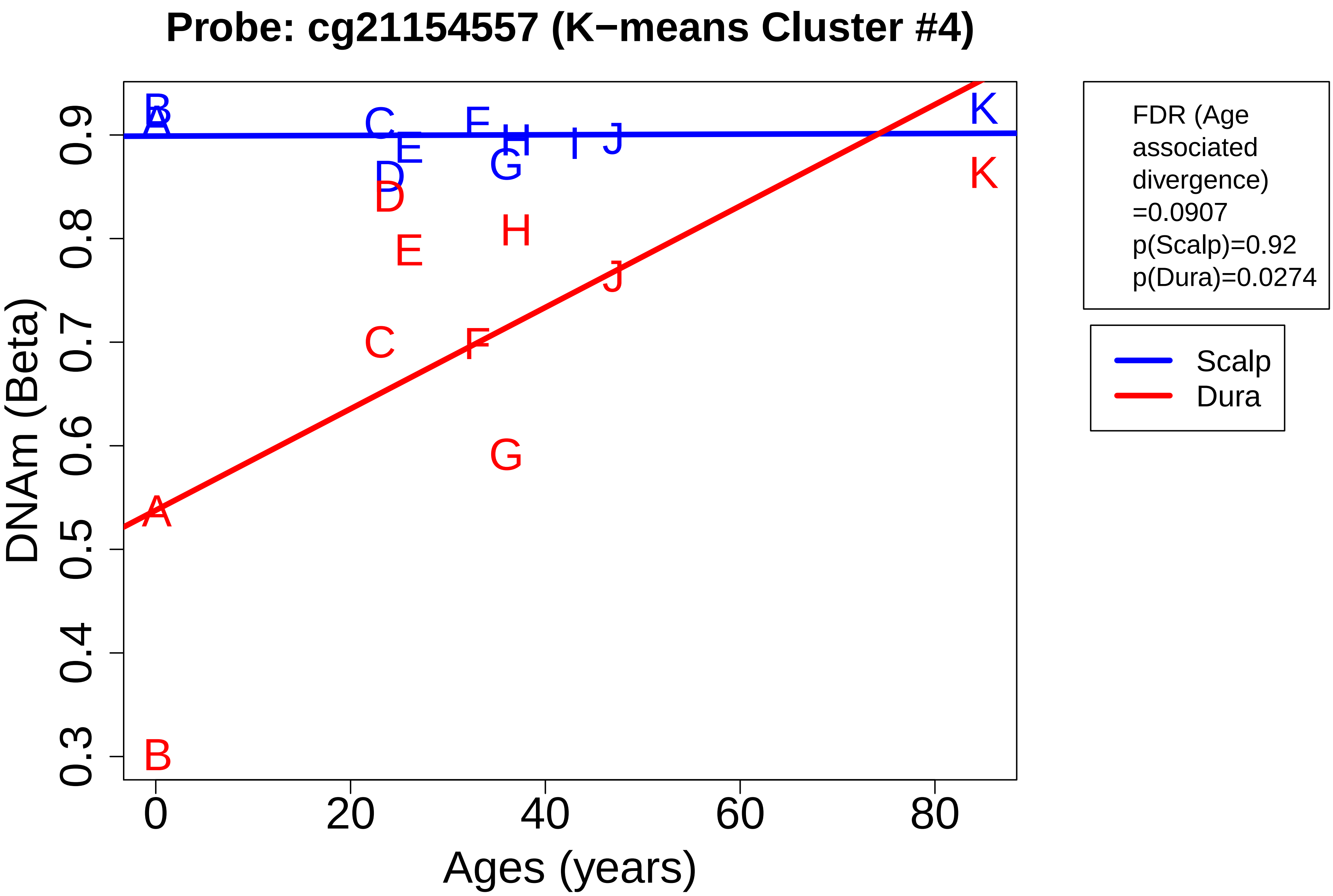

Probe: cg21154557 (K−means Cluster #4)
DNAm (Beta)
Ages (years)
0.3
0.4
0.5
0.6
0.7
0.8
0.9
0
20
40
60
80
FDR (Age associated divergence) =0.0907
p(Scalp)=0.92
p(Dura)=0.0274
Scalp
Dura

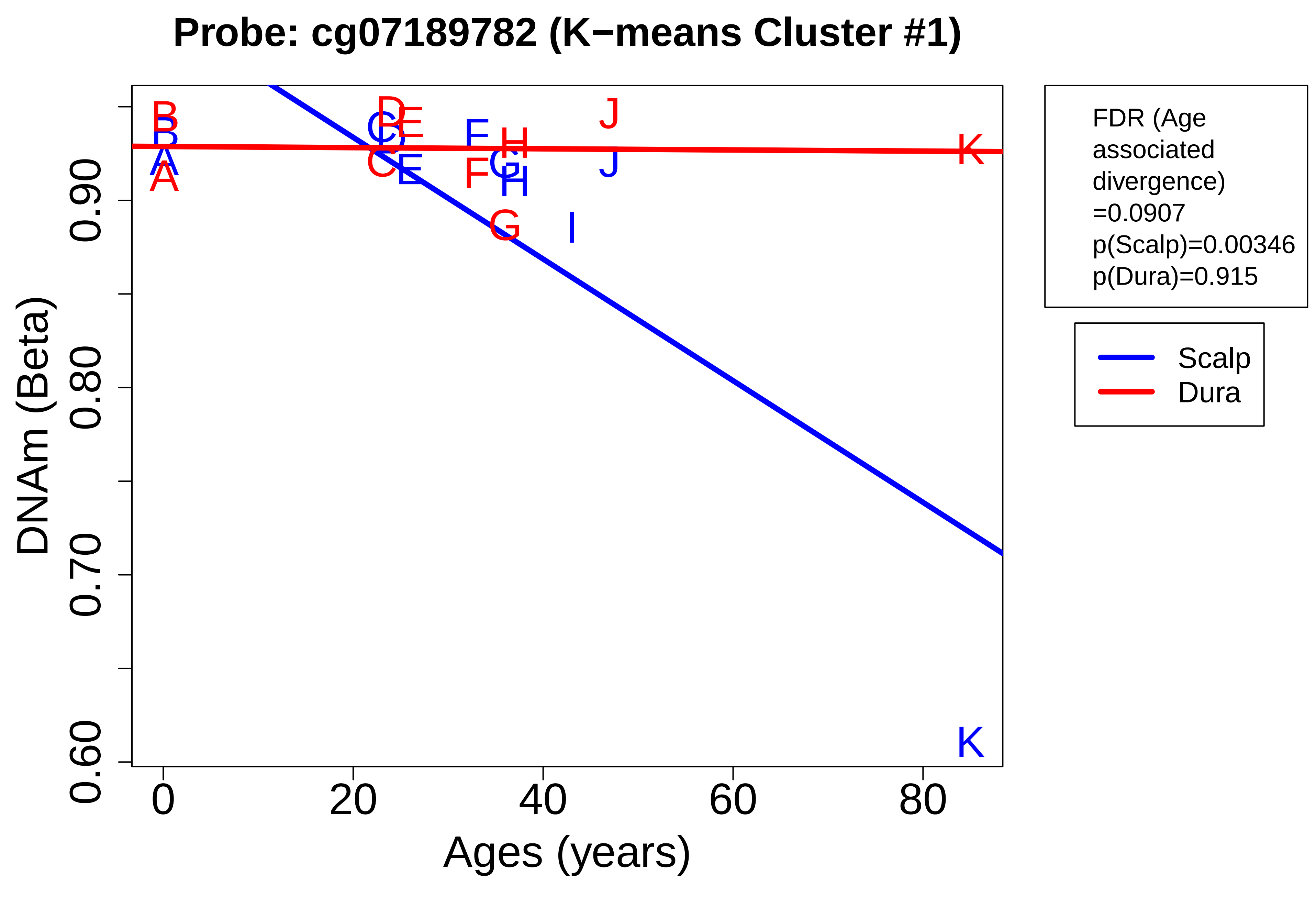

Probe: cg07189782 (K−means Cluster #1)
DNAm (Beta)
0.60
0.70
0.80
0.90
Ages (years)
0
20
40
60
80
A
B
C
D
E
F
G
H
I
J
K
FDR (Age associated divergence) =0.0907
p(Scalp)=0.00346
p(Dura)=0.915
Scalp
Dura

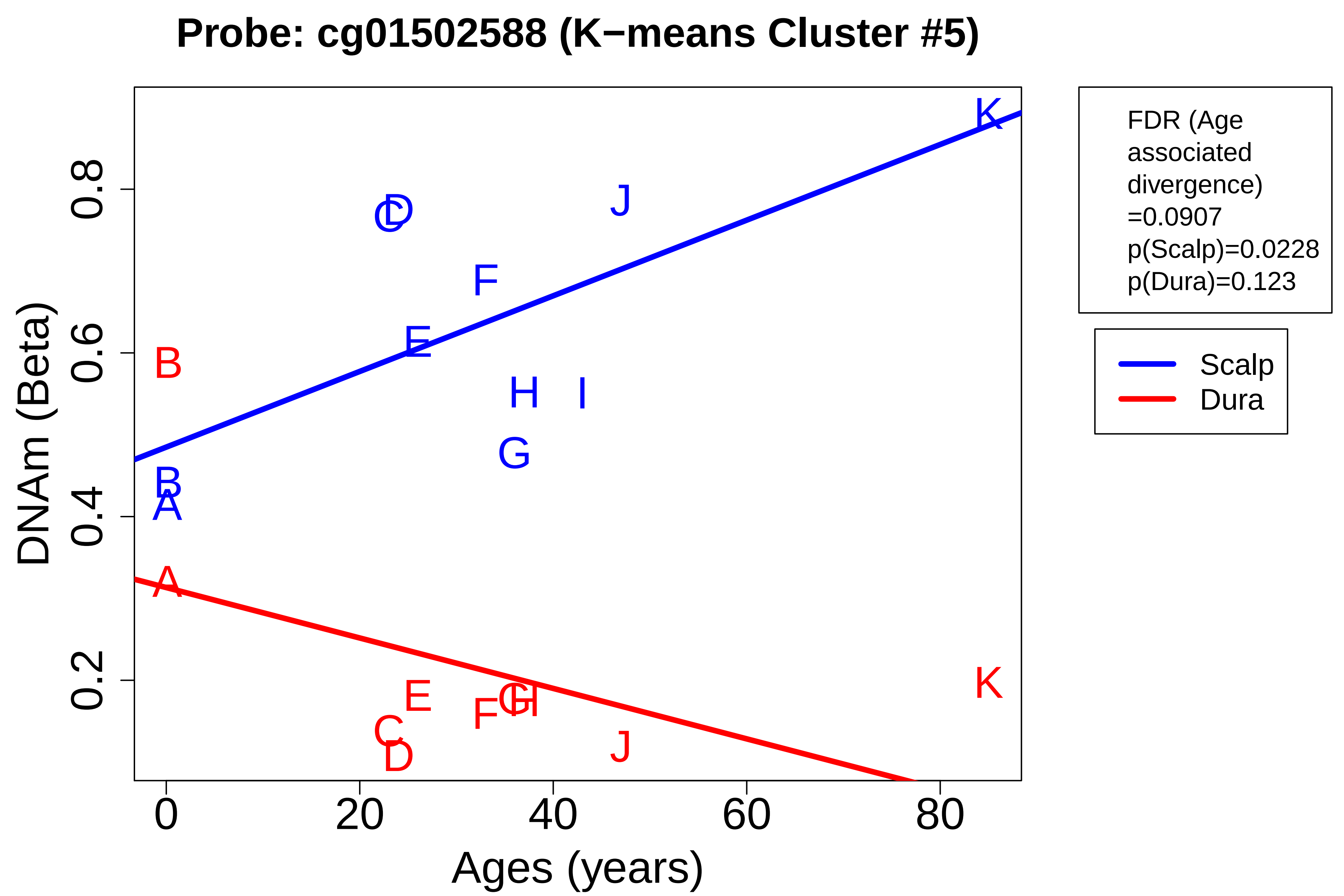

Probe: cg01502588 (K−means Cluster #5)
FDR (Age associated divergence) =0.0907
p(Scalp)=0.0228
p(Dura)=0.123
Scalp
Dura
DNAm (Beta)
0.2
0.4
0.6
0.8
0
20
40
60
80
Ages (years)

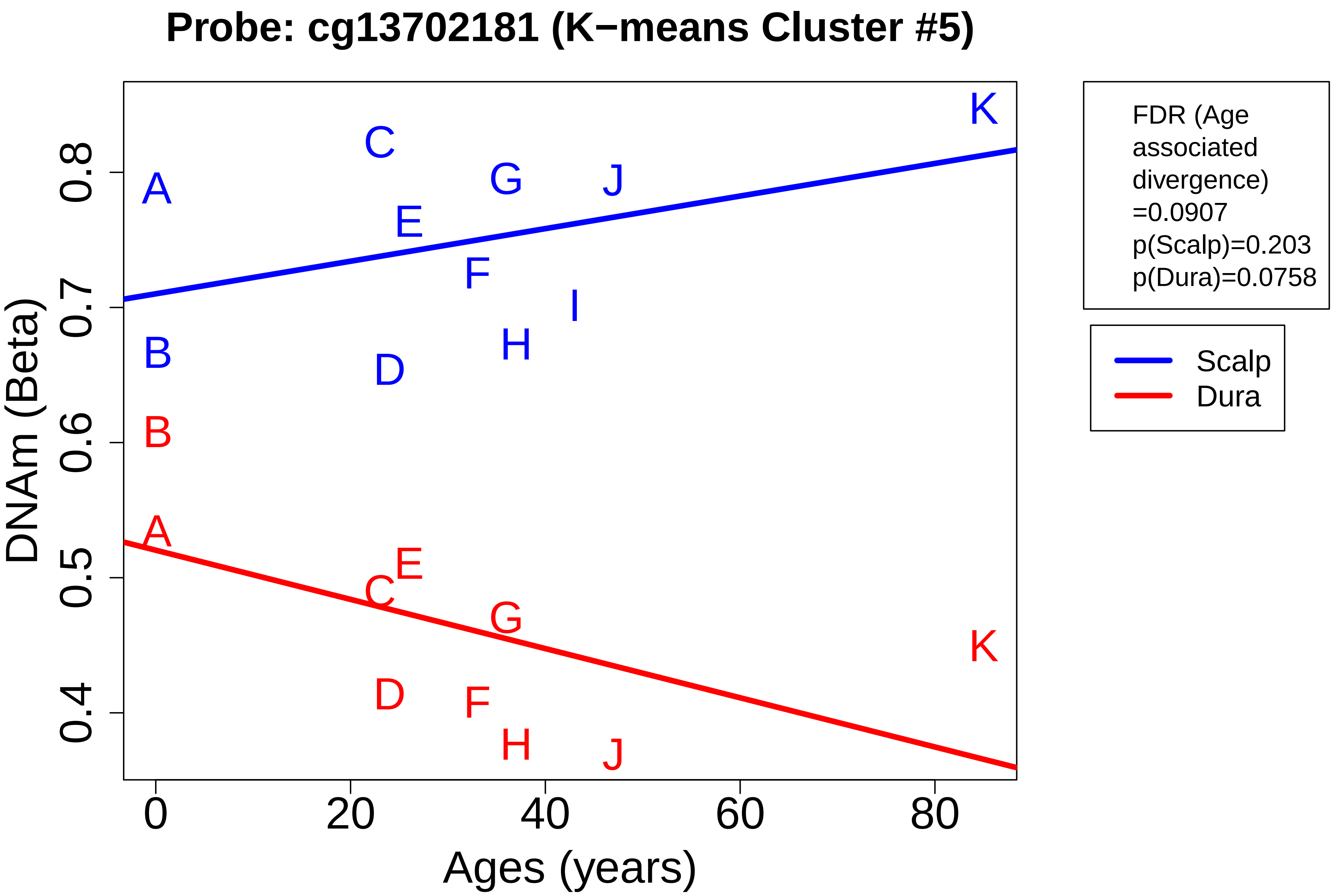

Probe: cg13702181 (K−means Cluster #5)
DNAm (Beta)
0.4
0.5
0.6
0.7
0.8
Ages (years)
0
20
40
60
80
A
B
C
D
E
F
G
H
I
J
K
FDR (Age associated divergence) =0.0907
p(Scalp)=0.203
p(Dura)=0.0758
Scalp
Dura

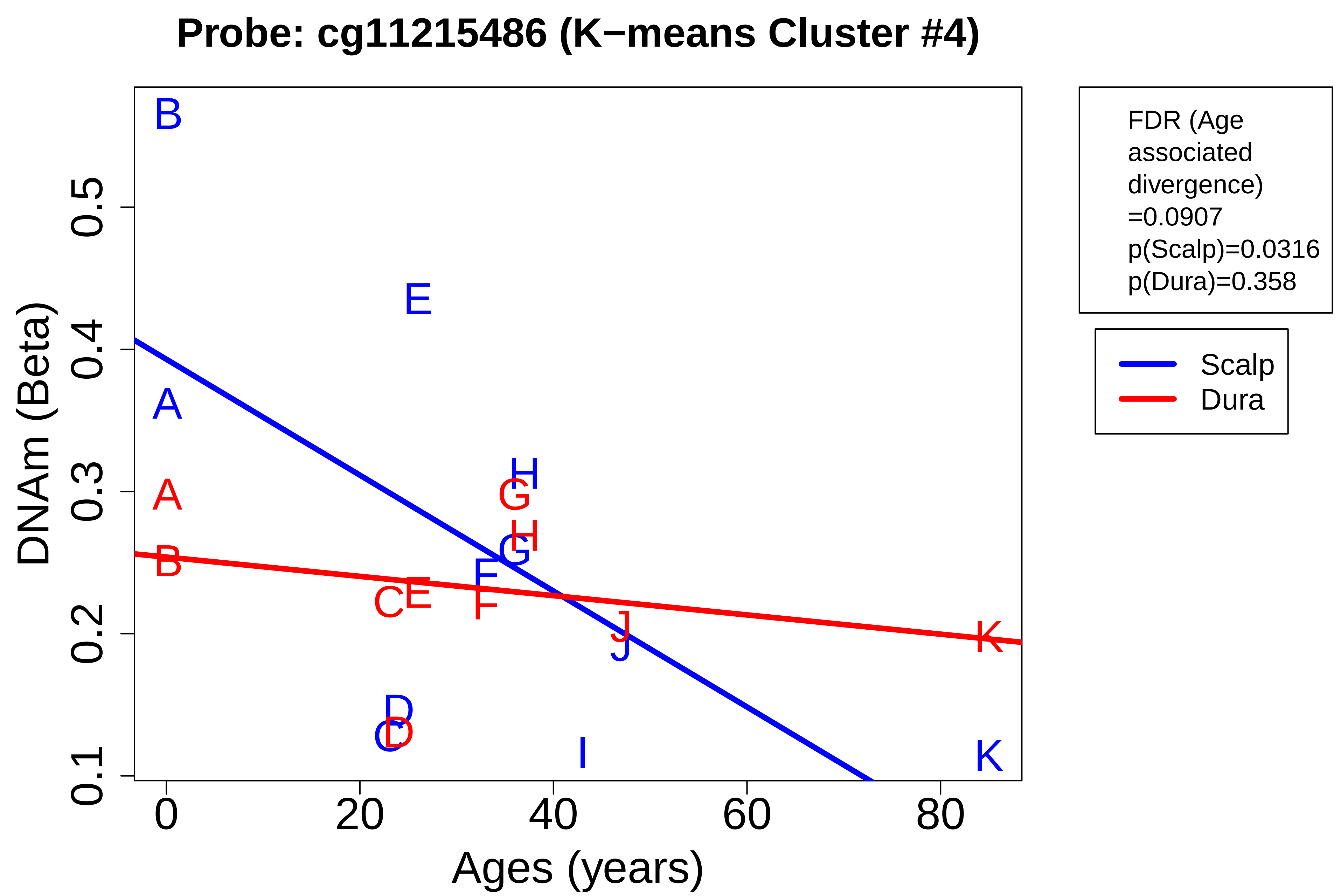

Probe: cg11215486 (K−means Cluster #4)
DNAm (Beta)
Ages (years)
0.1
0.2
0.3
0.4
0.5
0
20
40
60
80
FDR (Age associated divergence) =0.0907
p(Scalp)=0.0316
p(Dura)=0.358
Scalp
Dura
A
B
C
D
E
F
G
H
I
J
K

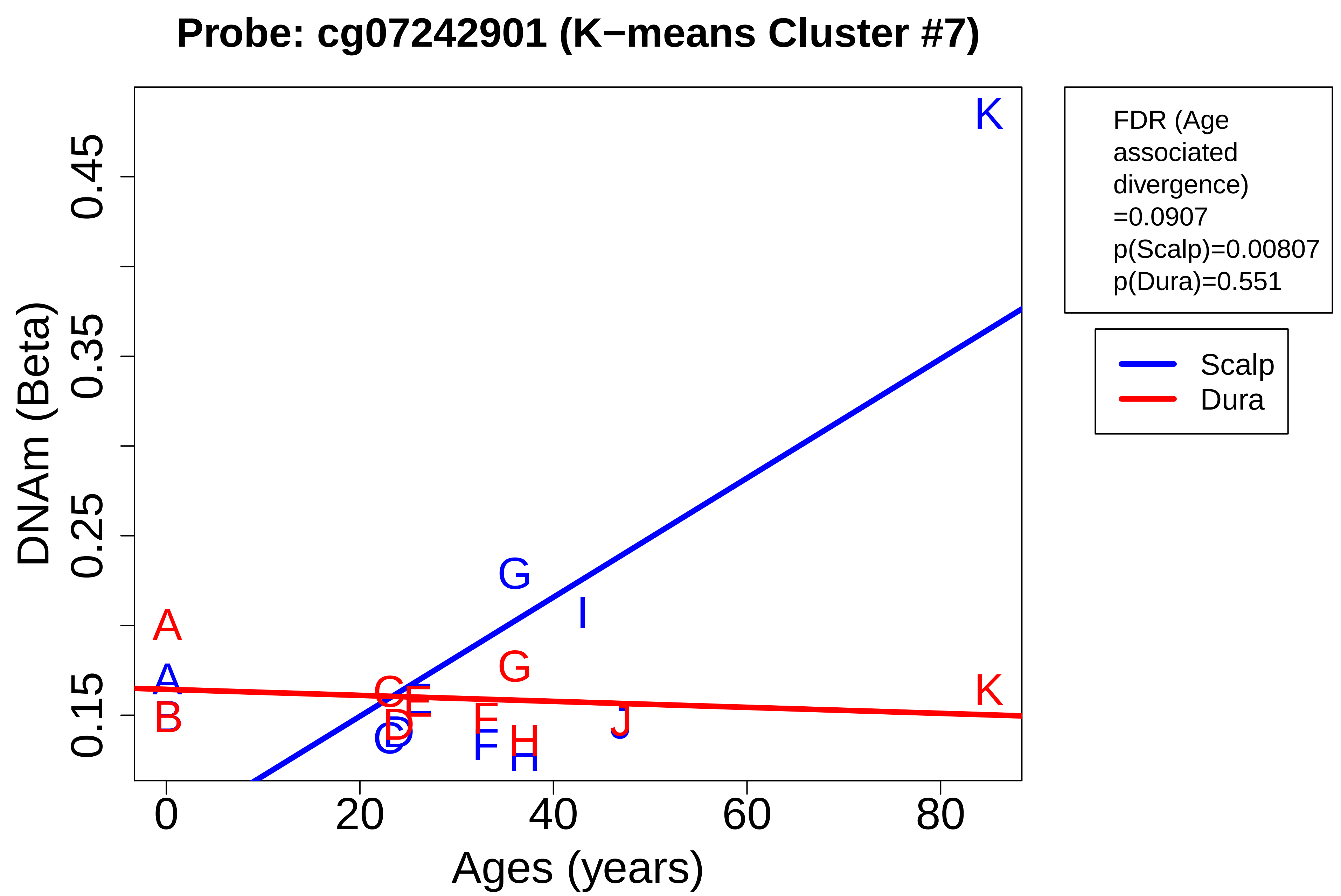

Probe: cg07242901 (K−means Cluster #7)
DNAm (Beta)
0.15
0.25
0.35
0.45
0
20
40
60
80
Ages (years)
A
B
C
D
E
F
G
H
I
J
K
FDR (Age associated divergence) =0.0907
p(Scalp)=0.00807
p(Dura)=0.551
Scalp
Dura

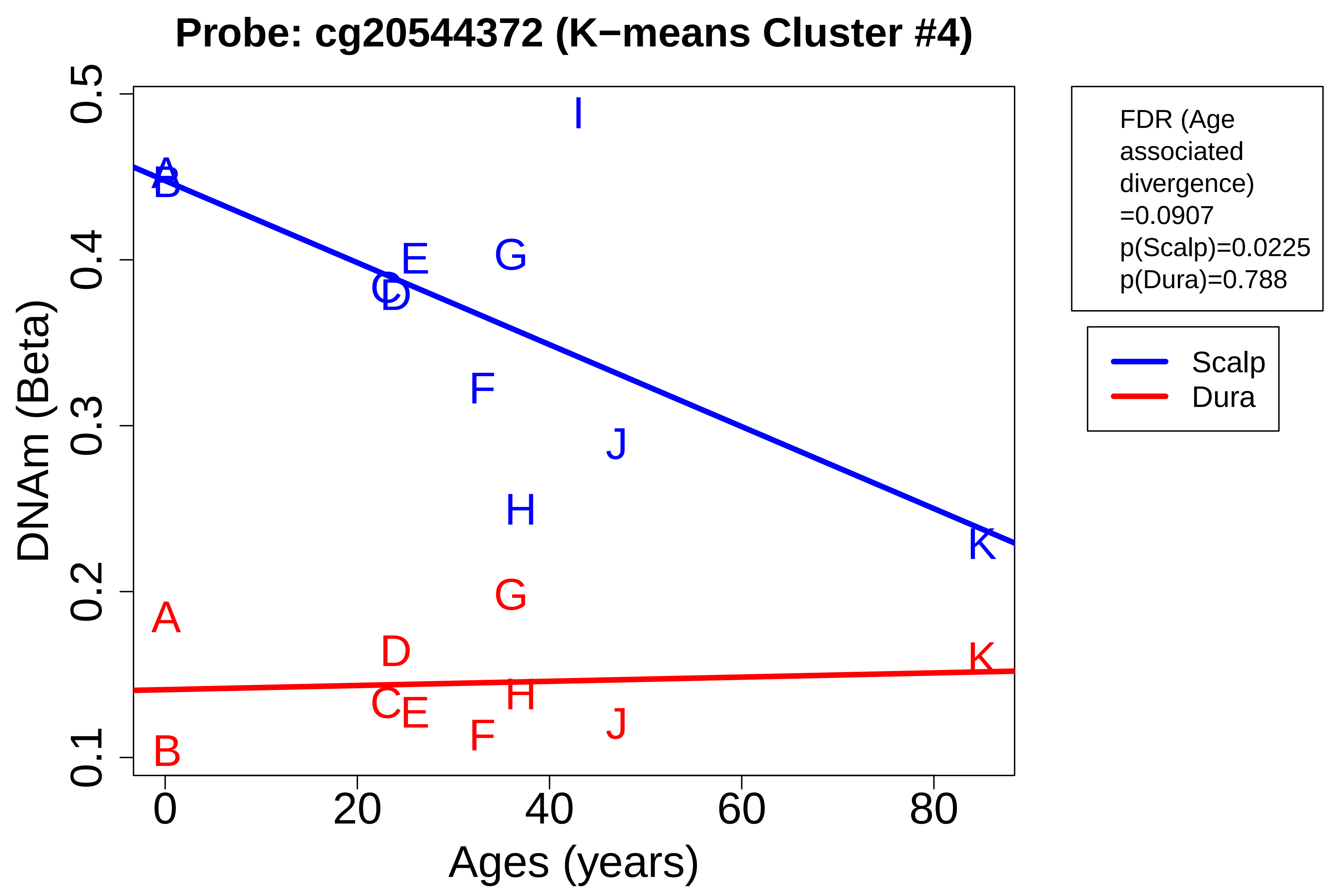
Probe: cg20544372 (K−means Cluster #4)
DNAm (Beta)
0.1
0.2
0.3
0.4
0.5
0
20
40
60
80
Ages (years)
A
B
C
D
E
F
G
H
I
J
K
FDR (Age associated divergence) =0.0907
p(Scalp)=0.0225
p(Dura)=0.788
Scalp
Dura

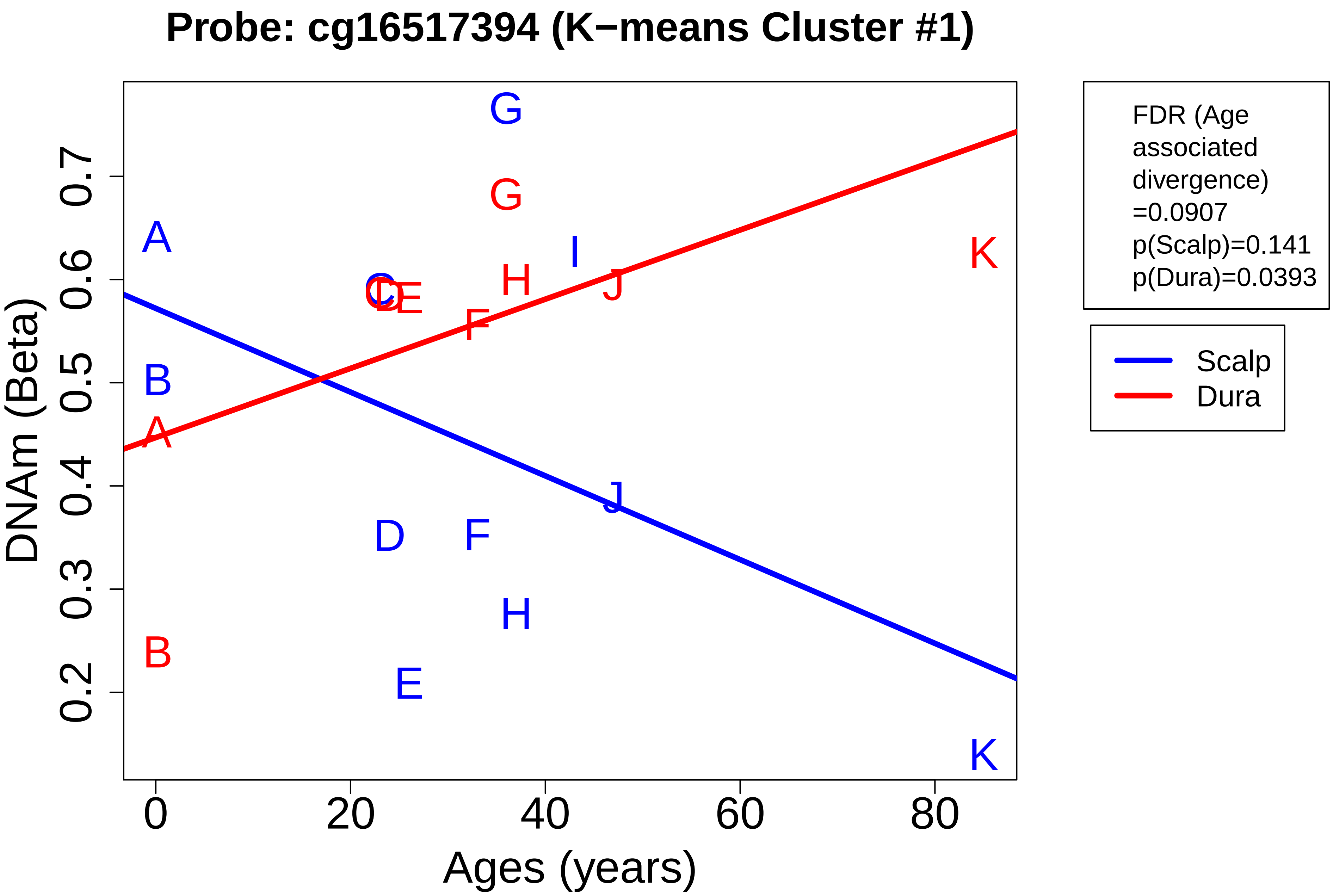

Probe: cg16517394 (K−means Cluster #1)
DNAm (Beta)
0.2
0.3
0.4
0.5
0.6
0.7
0
20
40
60
80
Ages (years)
A
B
C
D
E
F
G
H
I
J
K
FDR (Age associated divergence) =0.0907
p(Scalp)=0.141
p(Dura)=0.0393
Scalp
Dura

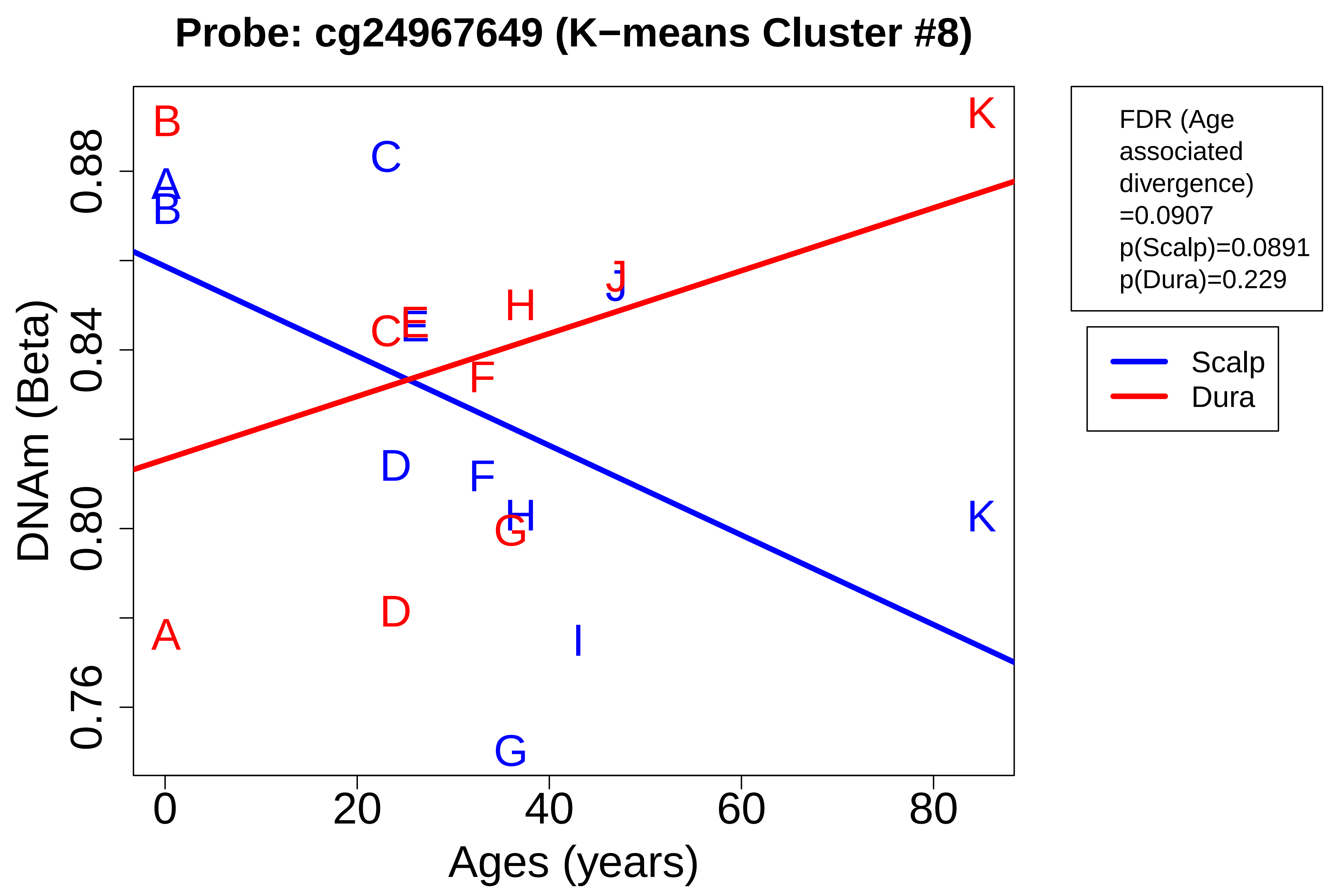

Probe: cg24967649 (K−means Cluster #8)
DNAm (Beta)
0.76
0.80
0.84
0.88
0
20
40
60
80
Ages (years)
FDR (Age associated divergence) =0.0907
p(Scalp)=0.0891
p(Dura)=0.229
Scalp
Dura

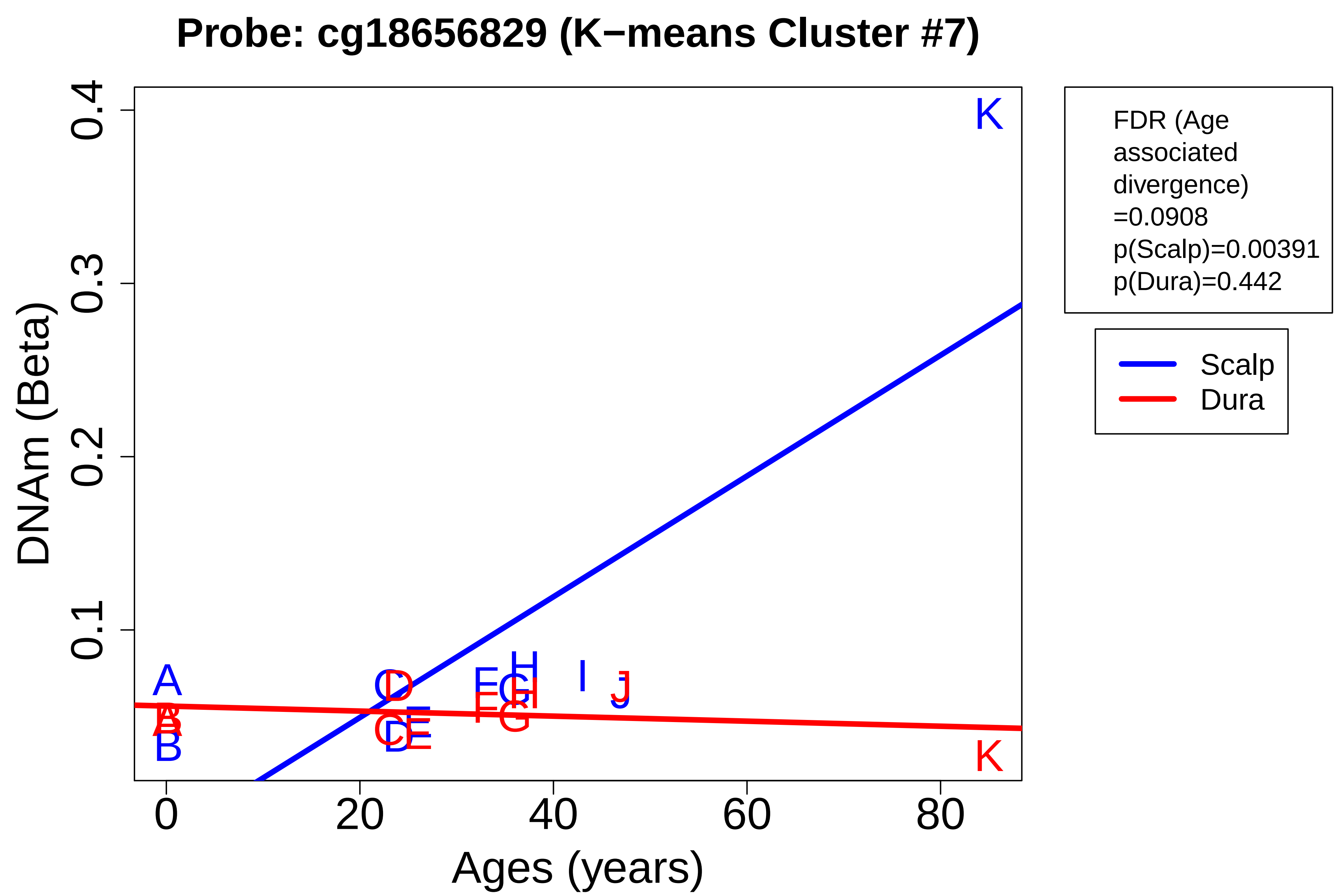

Probe: cg18656829 (K−means Cluster #7)
DNAm (Beta)
0.1
0.2
0.3
0.4
0
20
40
60
80
Ages (years)
FDR (Age associated divergence) =0.0908
p(Scalp)=0.00391
p(Dura)=0.442
Scalp
Dura

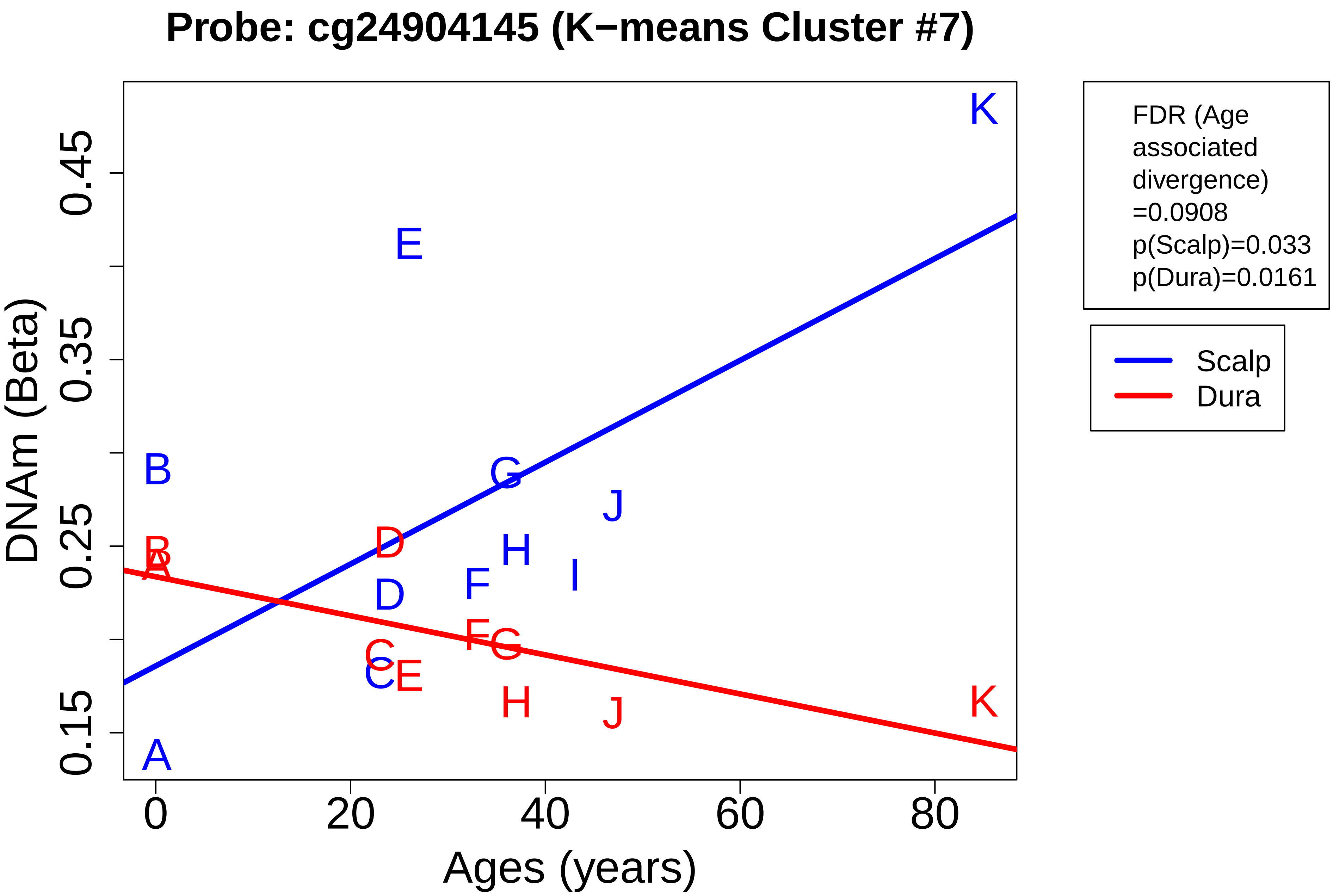

Probe: cg24904145 (K−means Cluster #7)
DNAm (Beta)
Ages (years)
0.15
0.25
0.35
0.45
0
20
40
60
80
FDR (Age associated divergence) =0.0908
p(Scalp)=0.033
p(Dura)=0.0161
Scalp
Dura

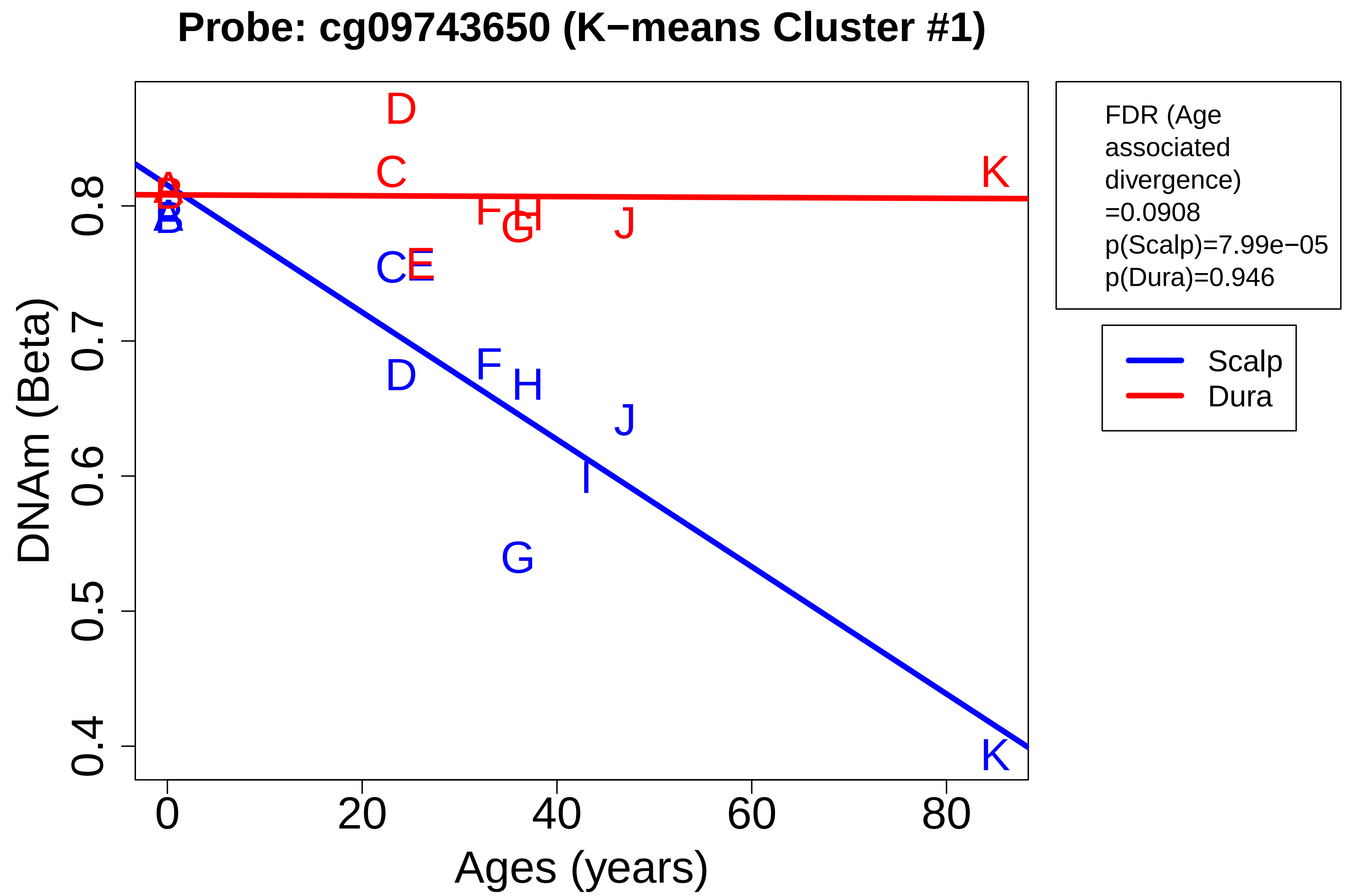

Probe: cg09743650 (K−means Cluster #1)
DNAm (Beta)
0.4
0.5
0.6
0.7
0.8
0
20
40
60
80
Ages (years)
FDR (Age associated divergence) =0.0908
p(Scalp)=7.99e−05
p(Dura)=0.946
Scalp
Dura

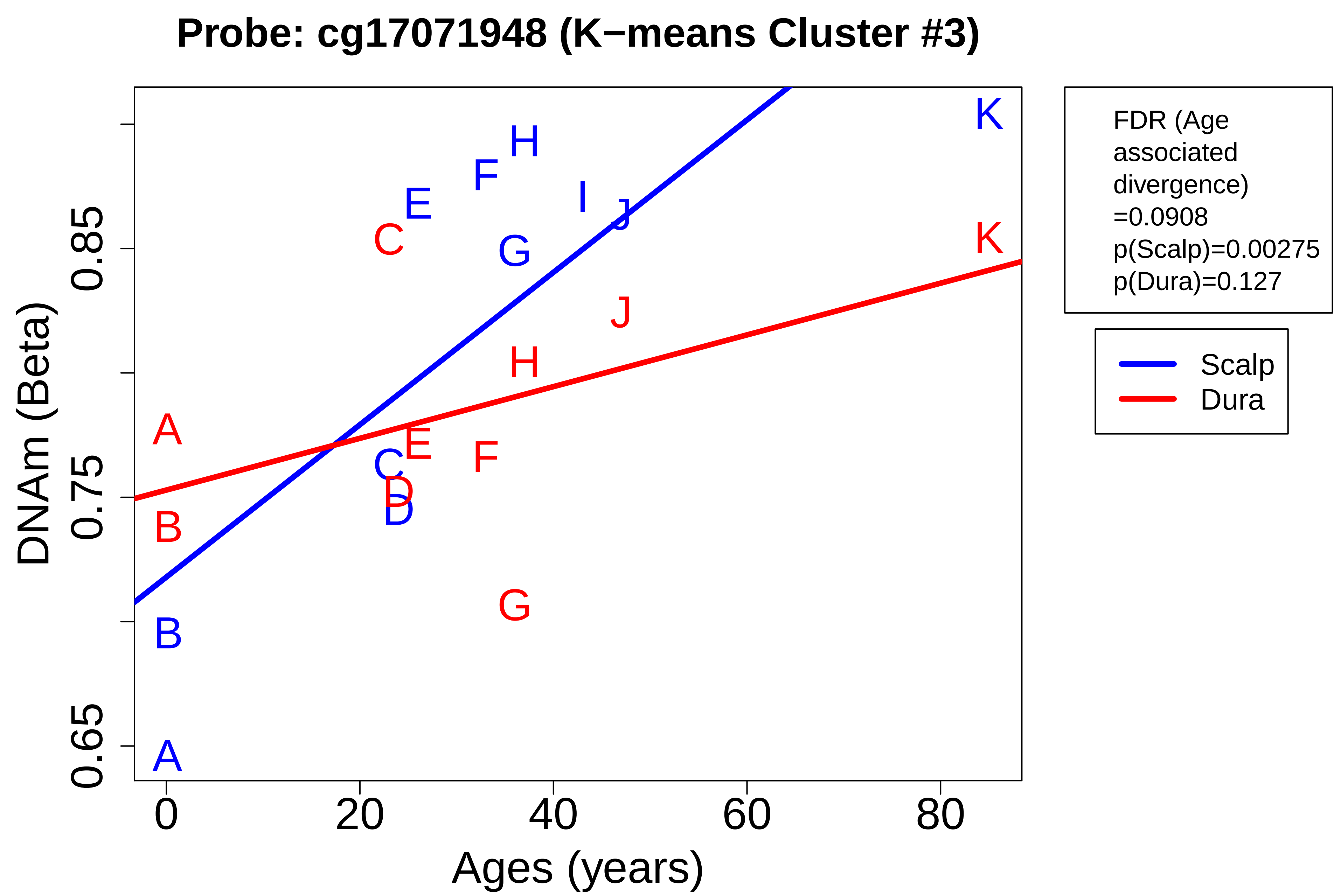

Probe: cg17071948 (K−means Cluster #3)
DNAm (Beta)
0.65
0.75
0.85
Ages (years)
0
20
40
60
80
A
B
C
D
E
F
G
H
I
J
K
A
B
C
D
E
F
G
H
J
K
FDR (Age associated divergence) =0.0908
p(Scalp)=0.00275
p(Dura)=0.127
Scalp
Dura

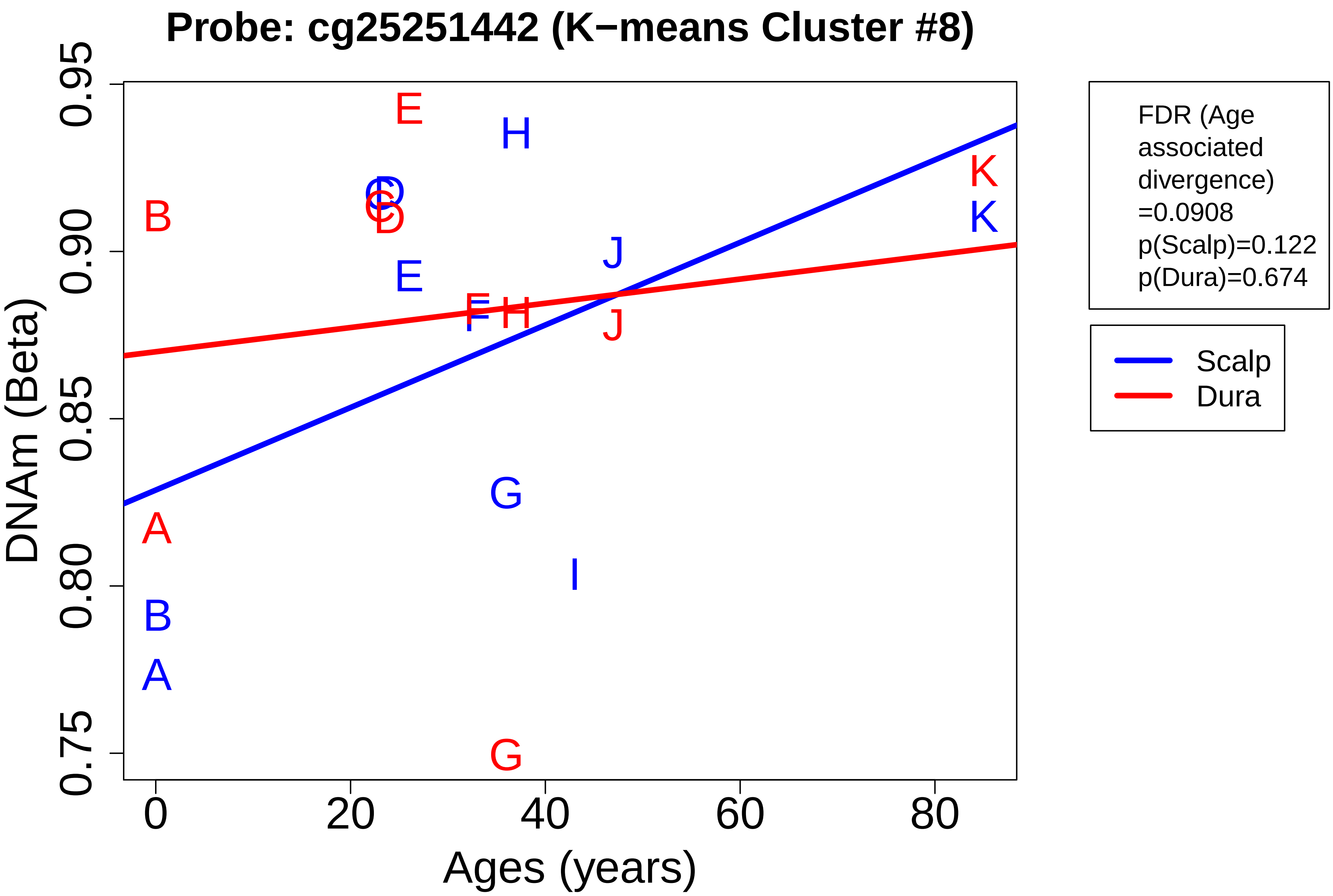

Probe: cg25251442 (K−means Cluster #8)
DNAm (Beta)
0.75
0.80
0.85
0.90
0.95
0
20
40
60
80
Ages (years)
FDR (Age associated divergence) =0.0908
p(Scalp)=0.122
p(Dura)=0.674
Scalp
Dura

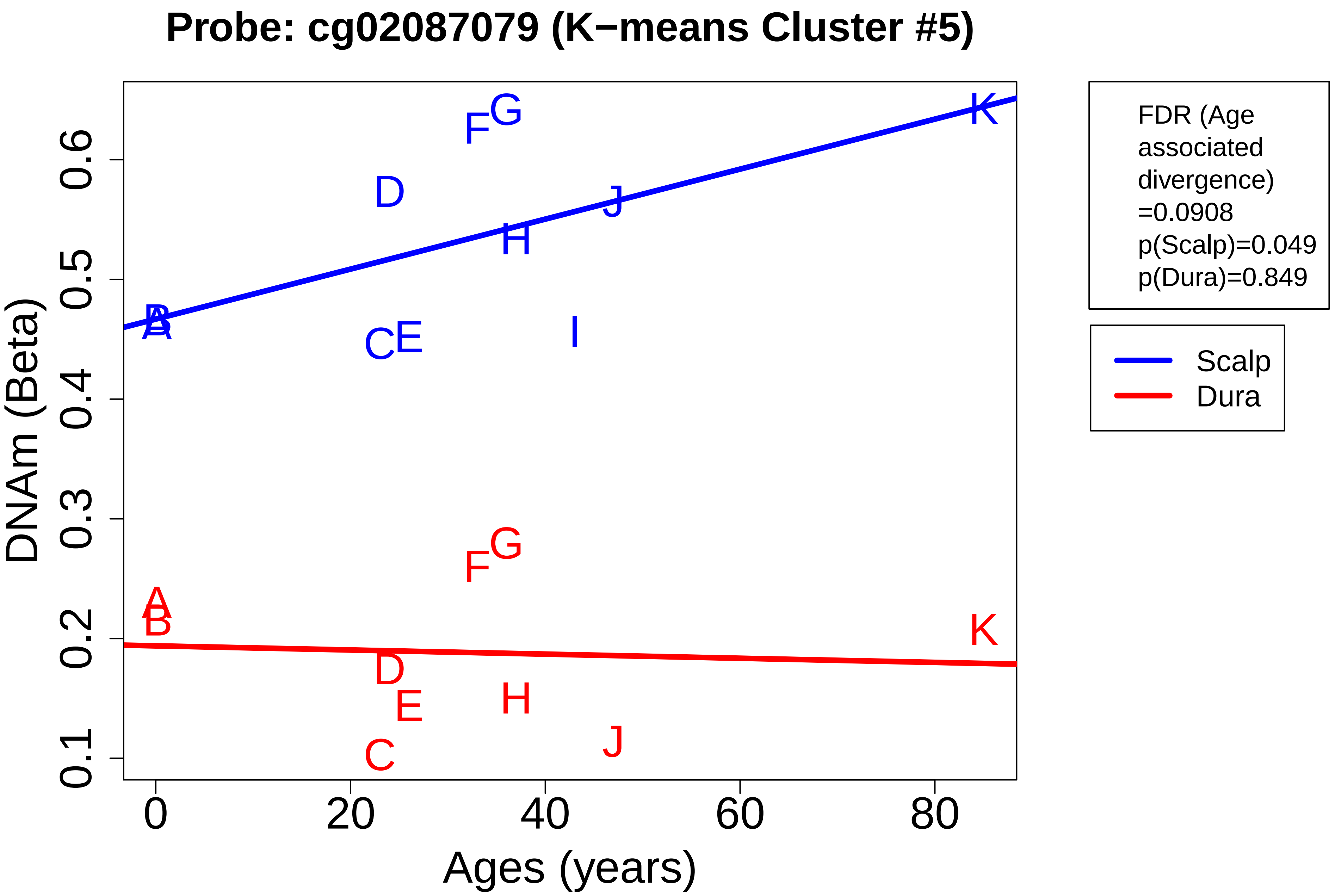

Probe: cg02087079 (K−means Cluster #5)
DNAm (Beta)
0.1
0.2
0.3
0.4
0.5
0.6
Ages (years)
0
20
40
60
80
FDR (Age associated divergence) =0.0908
p(Scalp)=0.049
p(Dura)=0.849
Scalp
Dura
A B C D E F G H I J K
A B C D E F G H J K

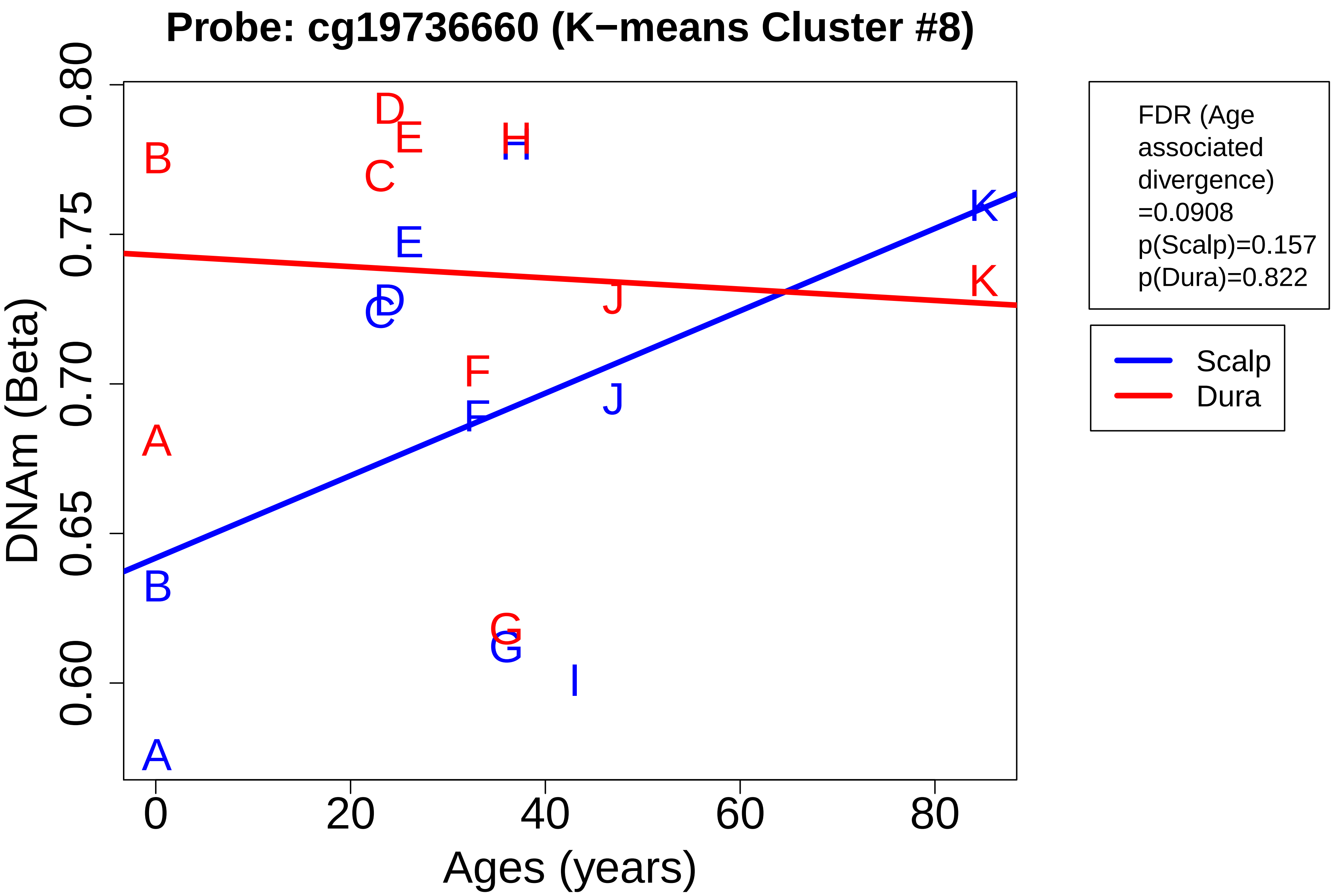

Probe: cg19736660 (K−means Cluster #8)
DNAm (Beta)
0.60
0.65
0.70
0.75
0.80
Ages (years)
0
20
40
60
80
FDR (Age associated divergence) =0.0908
p(Scalp)=0.157
p(Dura)=0.822
Scalp
Dura

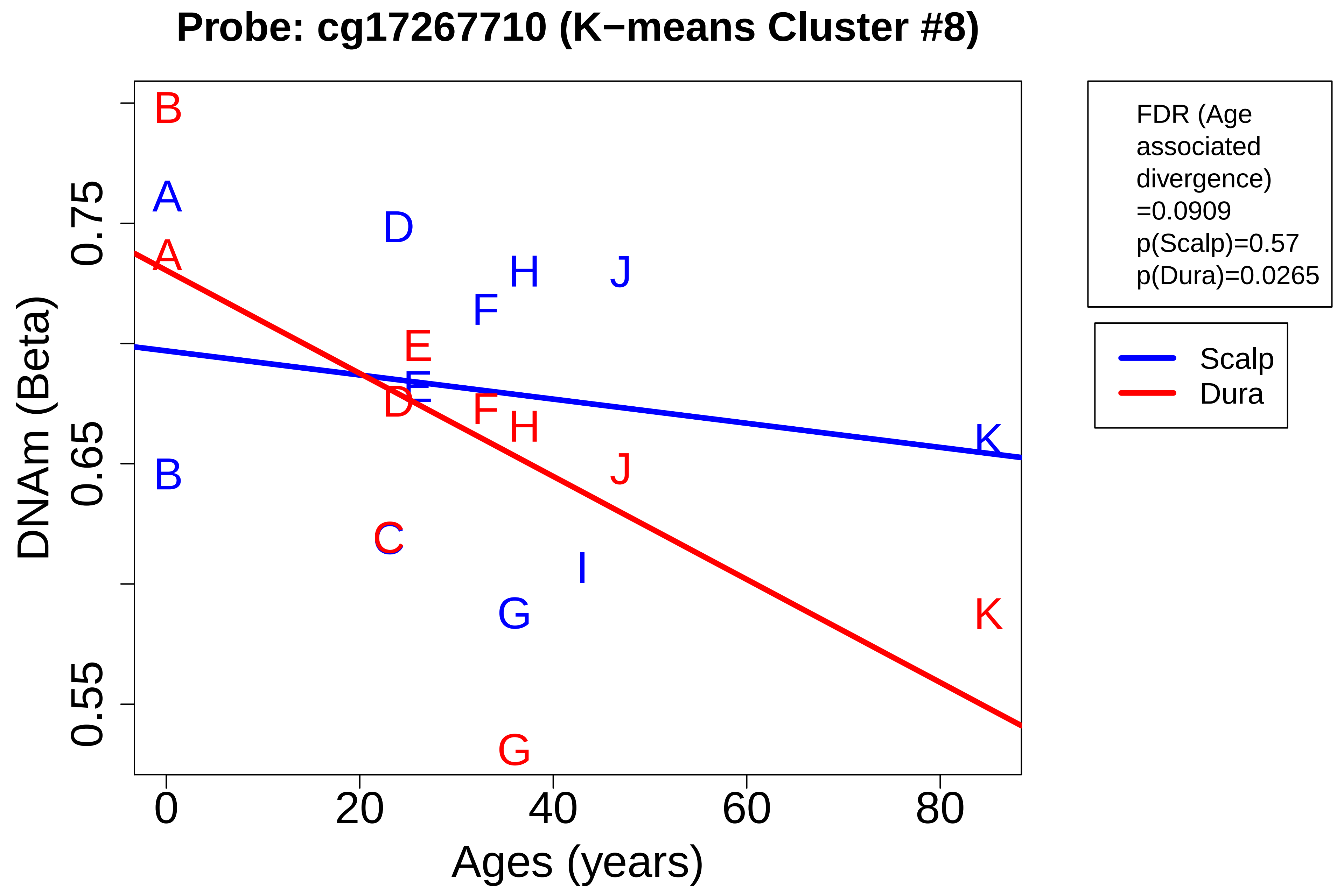

Probe: cg17267710 (K−means Cluster #8)
DNAm (Beta)
0.55
0.65
0.75
Ages (years)
0
20
40
60
80
FDR (Age associated divergence) =0.0909
p(Scalp)=0.57
p(Dura)=0.0265
Scalp
Dura

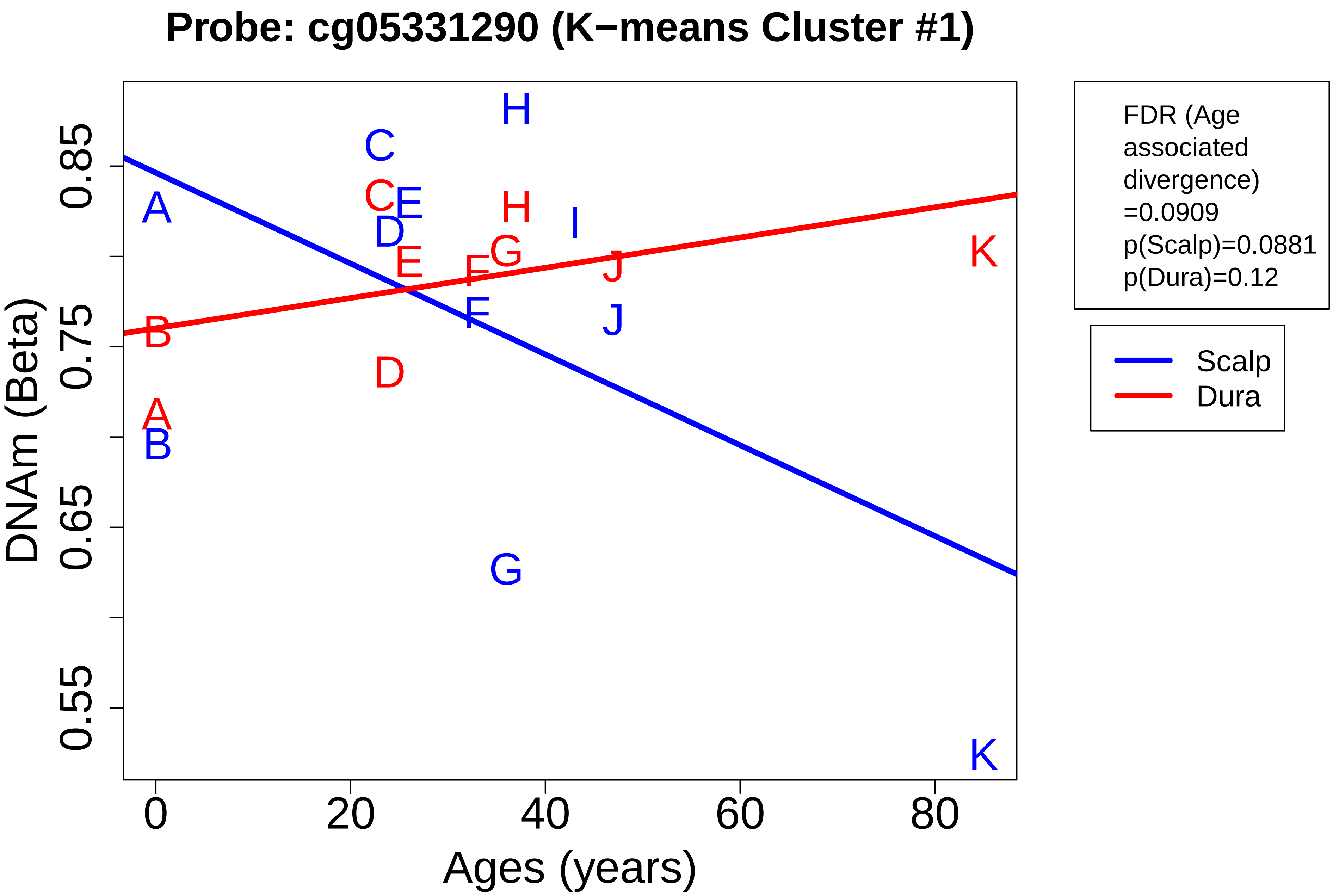

Probe: cg05331290 (K−means Cluster #1)
DNAm (Beta)
0.55
0.65
0.75
0.85
0
20
40
60
80
Ages (years)
A
B
C
D
E
F
G
H
I
J
K
FDR (Age associated divergence) =0.0909
p(Scalp)=0.0881
p(Dura)=0.12
Scalp
Dura

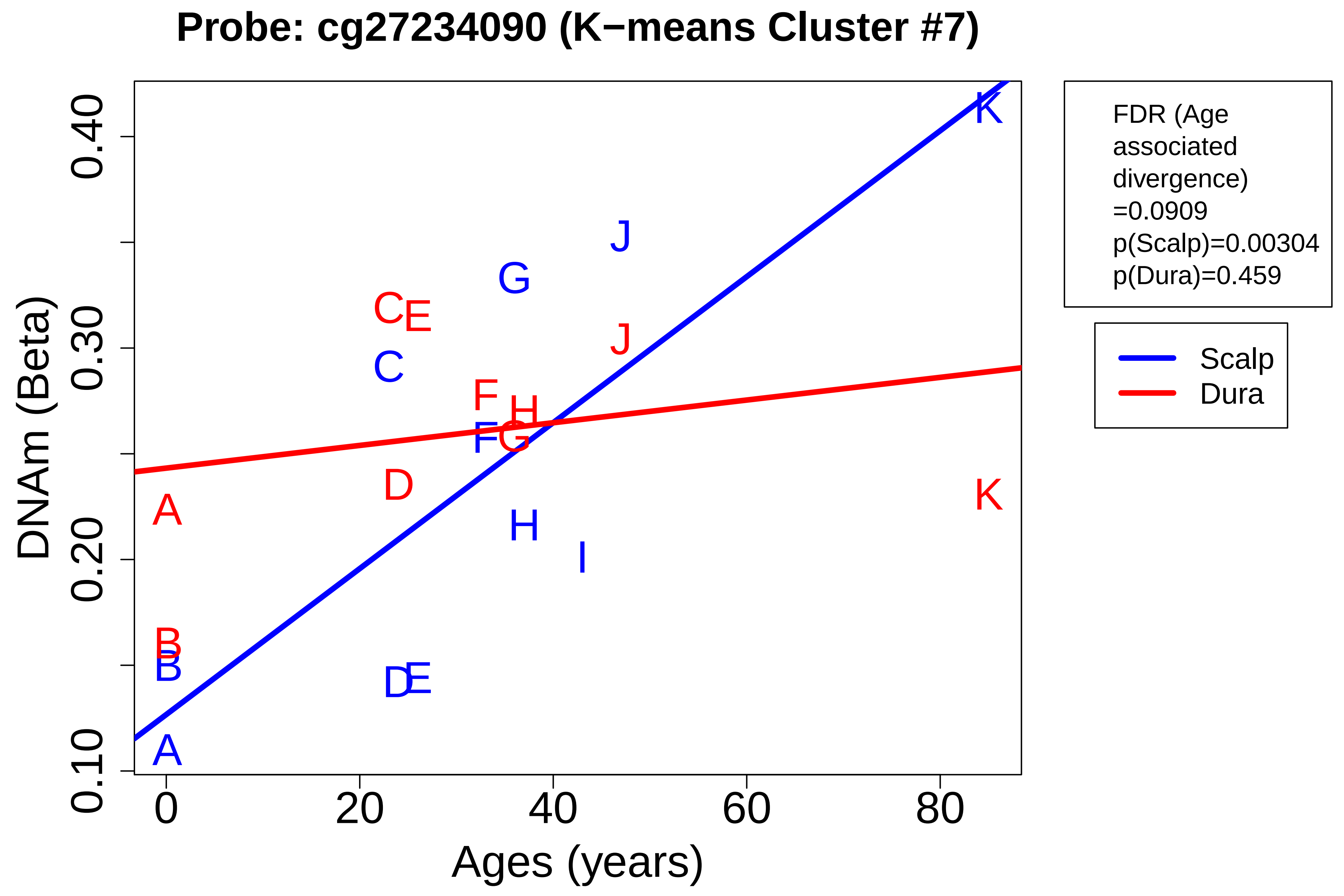
Probe: cg27234090 (K−means Cluster #7)
FDR (Age associated divergence) =0.0909
p(Scalp)=0.00304
p(Dura)=0.459
Scalp
Dura
DNAm (Beta)
0.10
0.20
0.30
0.40
Ages (years)
0
20
40
60
80
A
B
C
D
E
F
G
H
I
J
K

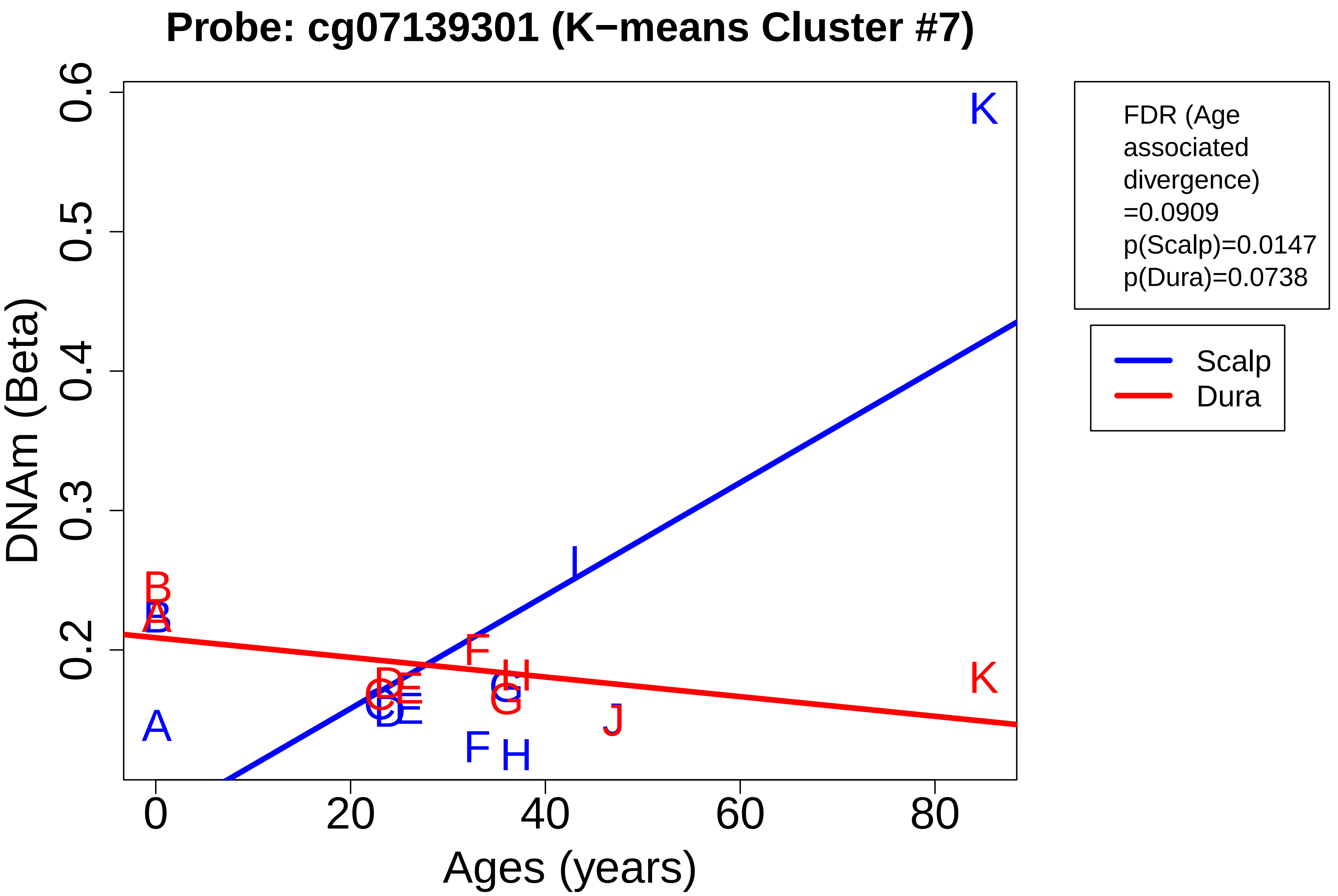

Probe: cg07139301 (K−means Cluster #7)
DNAm (Beta)
0.2
0.3
0.4
0.5
0.6
0
20
40
60
80
Ages (years)
FDR (Age associated divergence) =0.0909
p(Scalp)=0.0147
p(Dura)=0.0738
Scalp
Dura

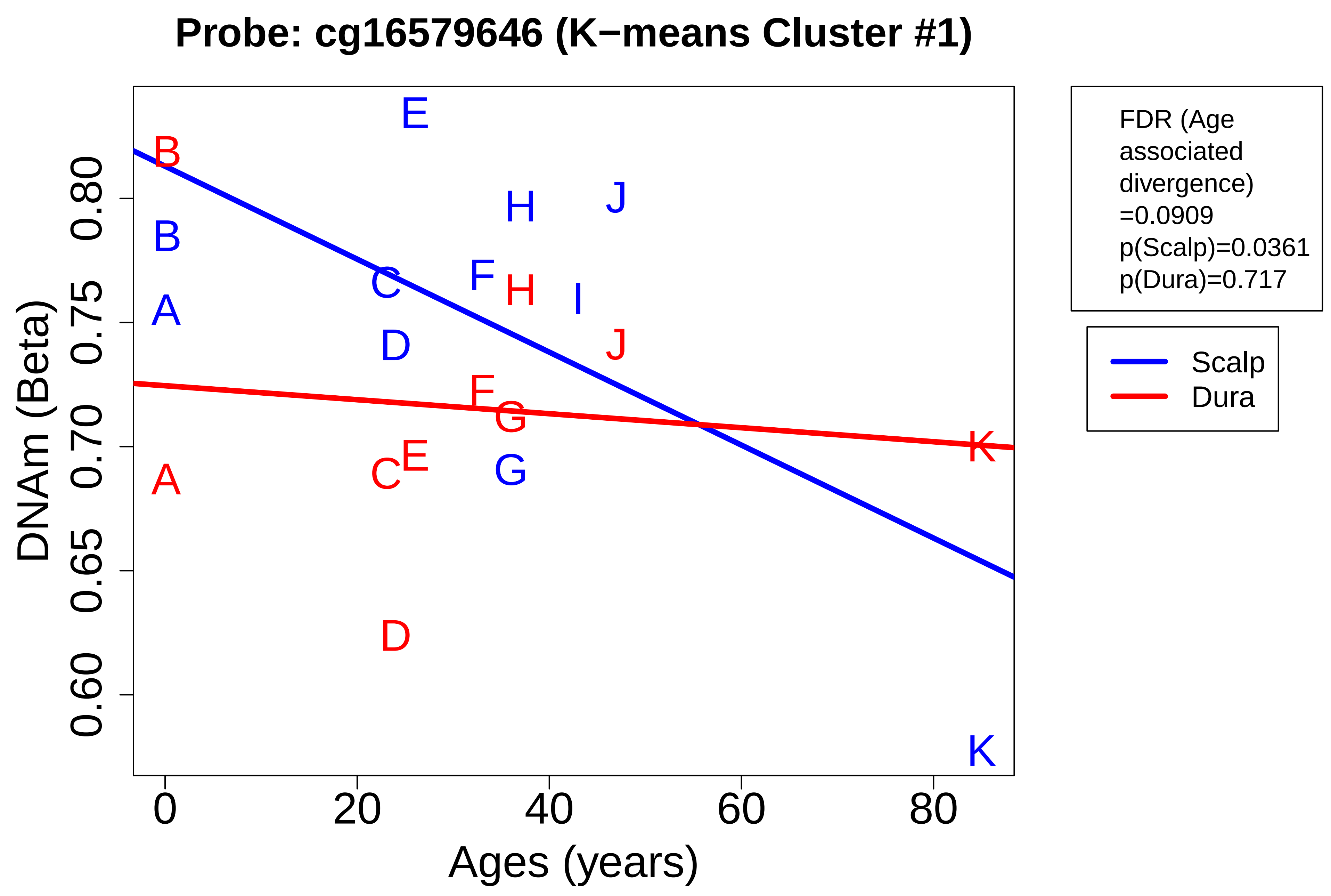
Probe: cg16579646 (K−means Cluster #1)
DNAm (Beta)
0.60
0.65
0.70
0.75
0.80
Ages (years)
0
20
40
60
80
A
B
C
D
E
F
G
H
I
J
K
FDR (Age associated divergence) =0.0909
p(Scalp)=0.0361
p(Dura)=0.717
Scalp
Dura

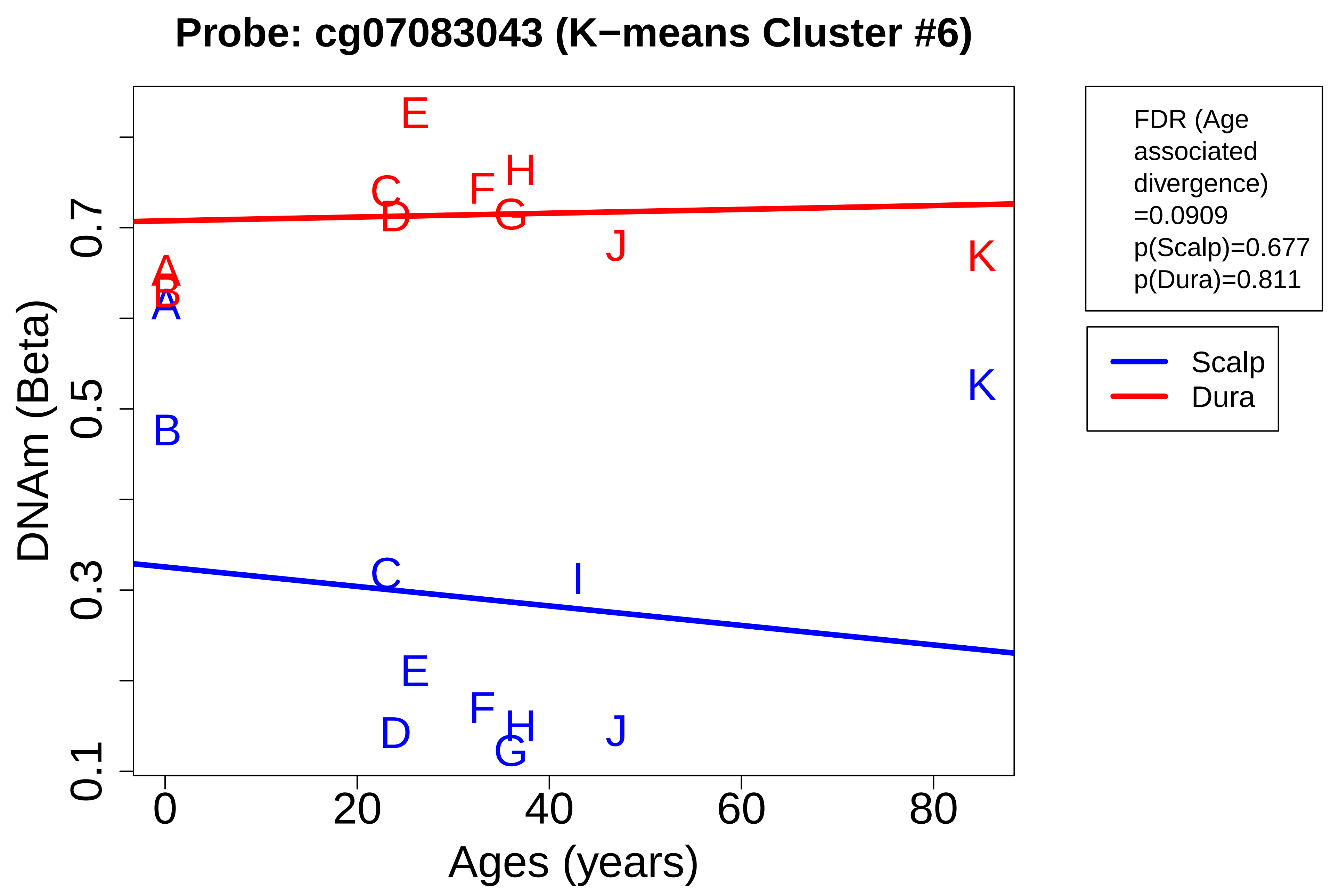

Probe: cg07083043 (K−means Cluster #6)
DNAm (Beta)
0.1
0.3
0.5
0.7
0
20
40
60
80
Ages (years)
FDR (Age associated divergence) =0.0909
p(Scalp)=0.677
p(Dura)=0.811
Scalp
Dura

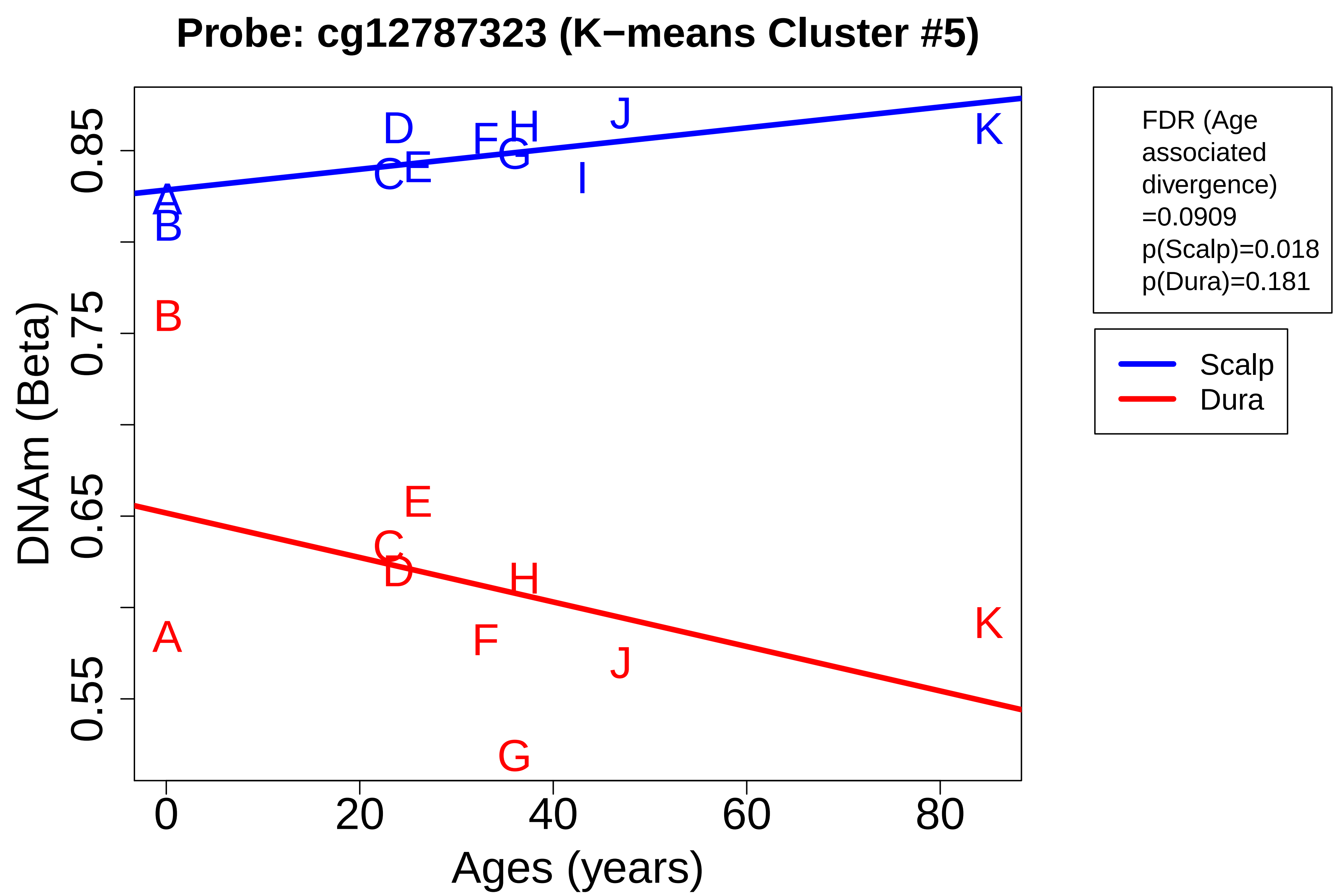

Probe: cg12787323 (K−means Cluster #5)
DNAm (Beta)
0.55
0.65
0.75
0.85
0
20
40
60
80
Ages (years)
FDR (Age associated divergence) =0.0909
p(Scalp)=0.018
p(Dura)=0.181
Scalp
Dura
A
B
C
D
E
F
G
H
I
J
K

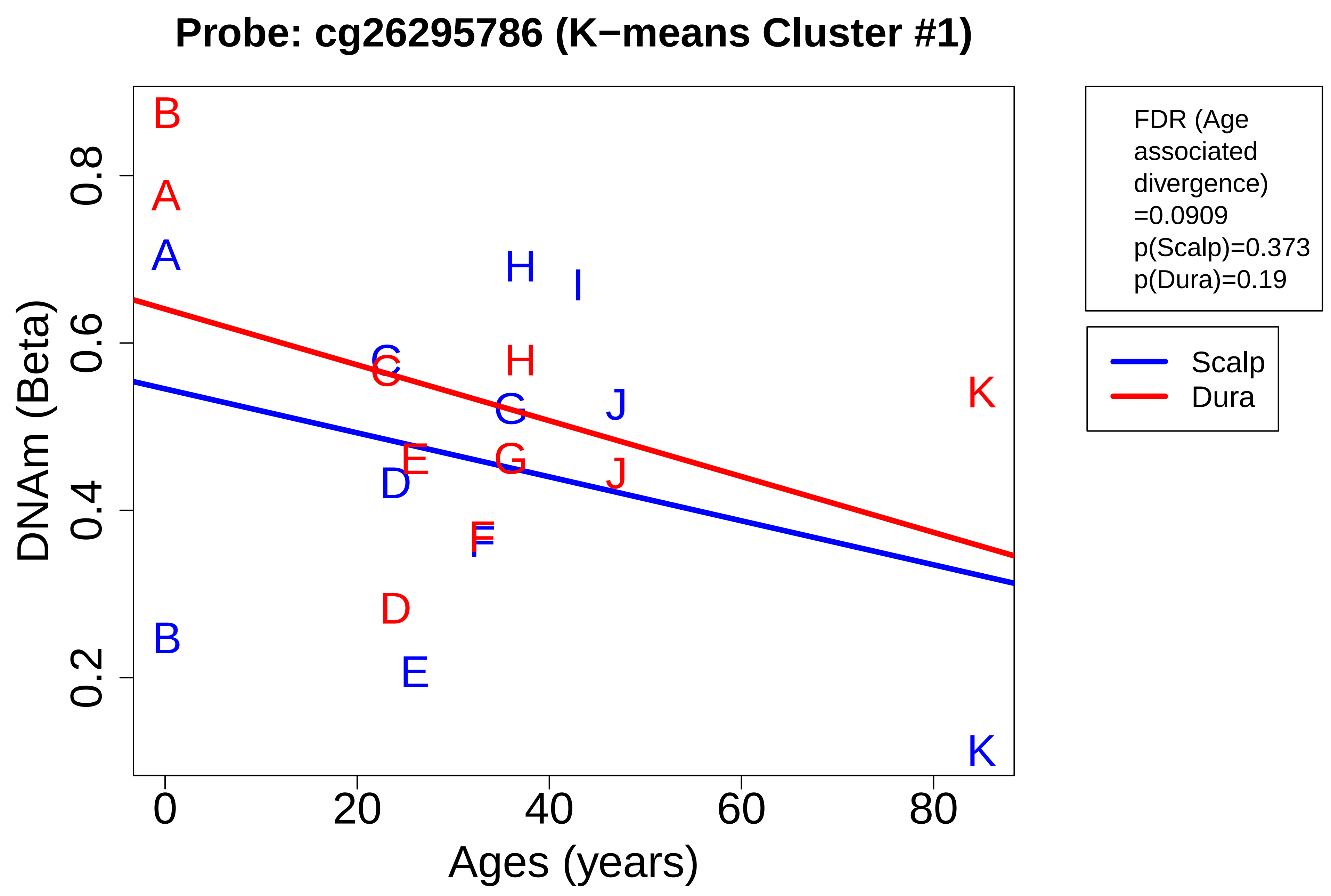

Probe: cg26295786 (K−means Cluster #1)
DNAm (Beta)
0.2
0.4
0.6
0.8
0
20
40
60
80
Ages (years)
A
B
C
D
E
F
G
H
I
J
K
A
B
C
D
E
F
G
H
J
K
FDR (Age associated divergence) =0.0909
p(Scalp)=0.373
p(Dura)=0.19
Scalp
Dura

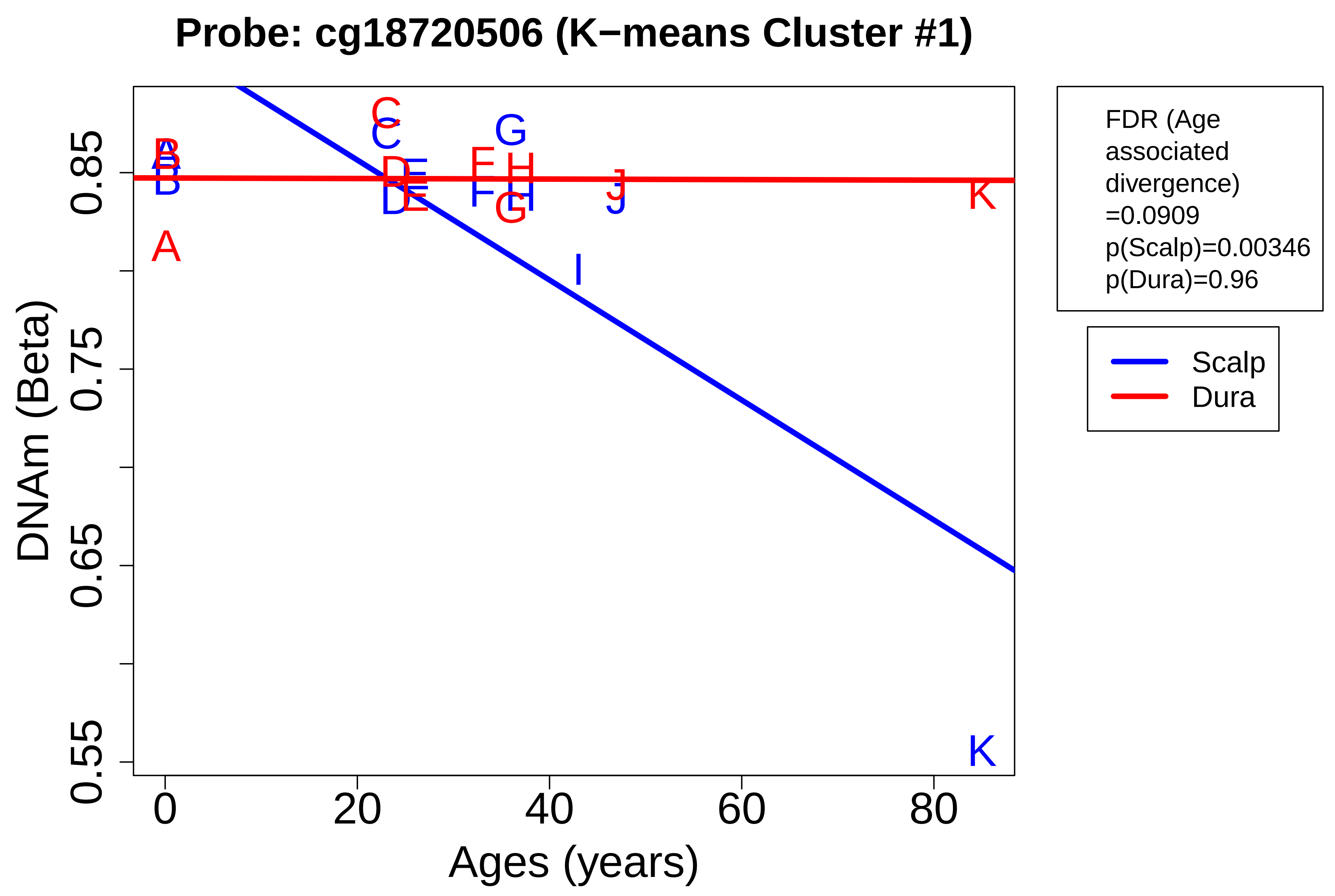

Probe: cg18720506 (K−means Cluster #1)
DNAm (Beta)
0.55
0.65
0.75
0.85
Ages (years)
0
20
40
60
80
A
B
C
D
E
F
G
H
I
J
K
FDR (Age associated divergence) =0.0909
p(Scalp)=0.00346
p(Dura)=0.96
Scalp
Dura

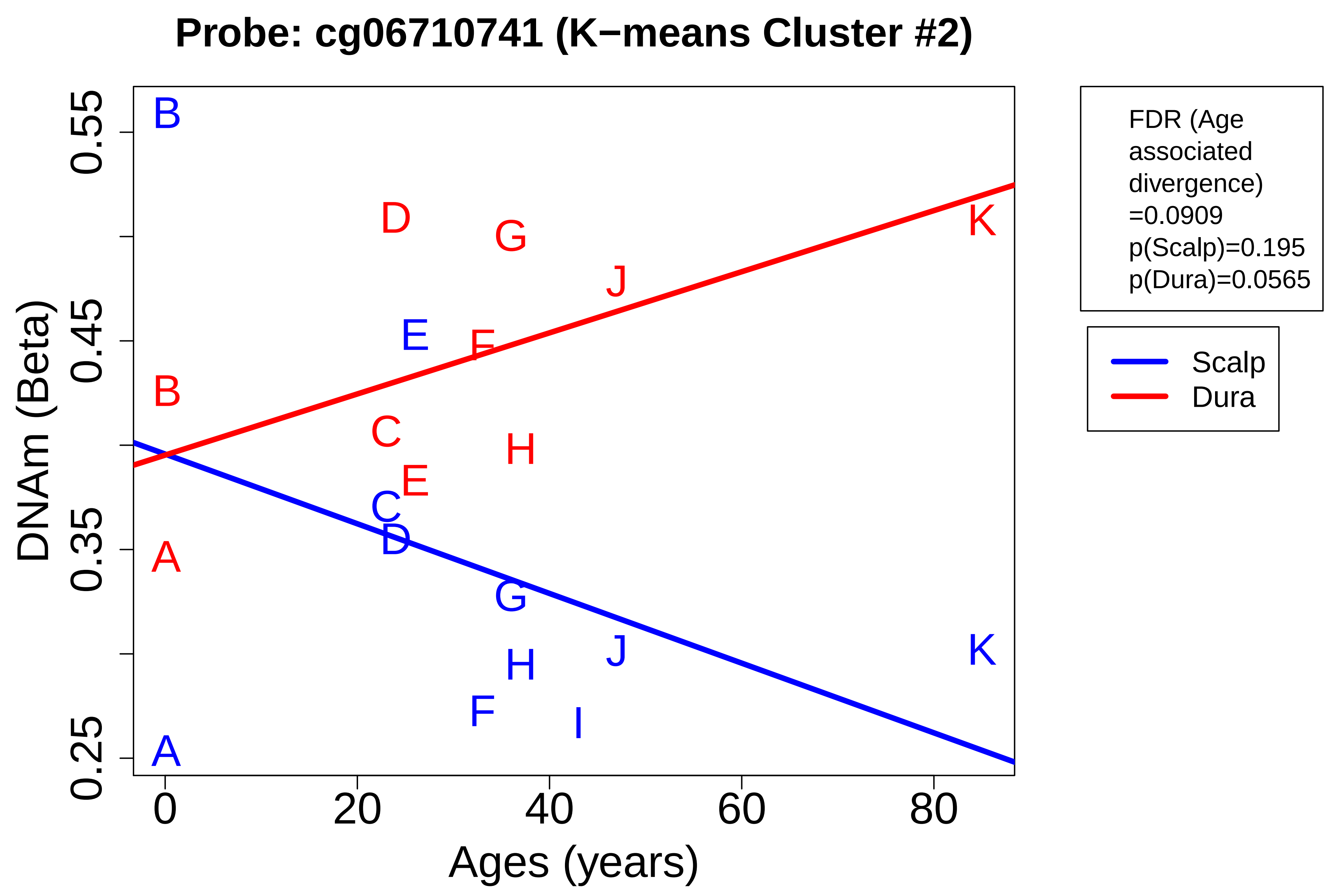

Probe: cg06710741 (K−means Cluster #2)
DNAm (Beta)
0.25
0.35
0.45
0.55
Ages (years)
0
20
40
60
80
FDR (Age associated divergence) =0.0909
p(Scalp)=0.195
p(Dura)=0.0565
Scalp
Dura

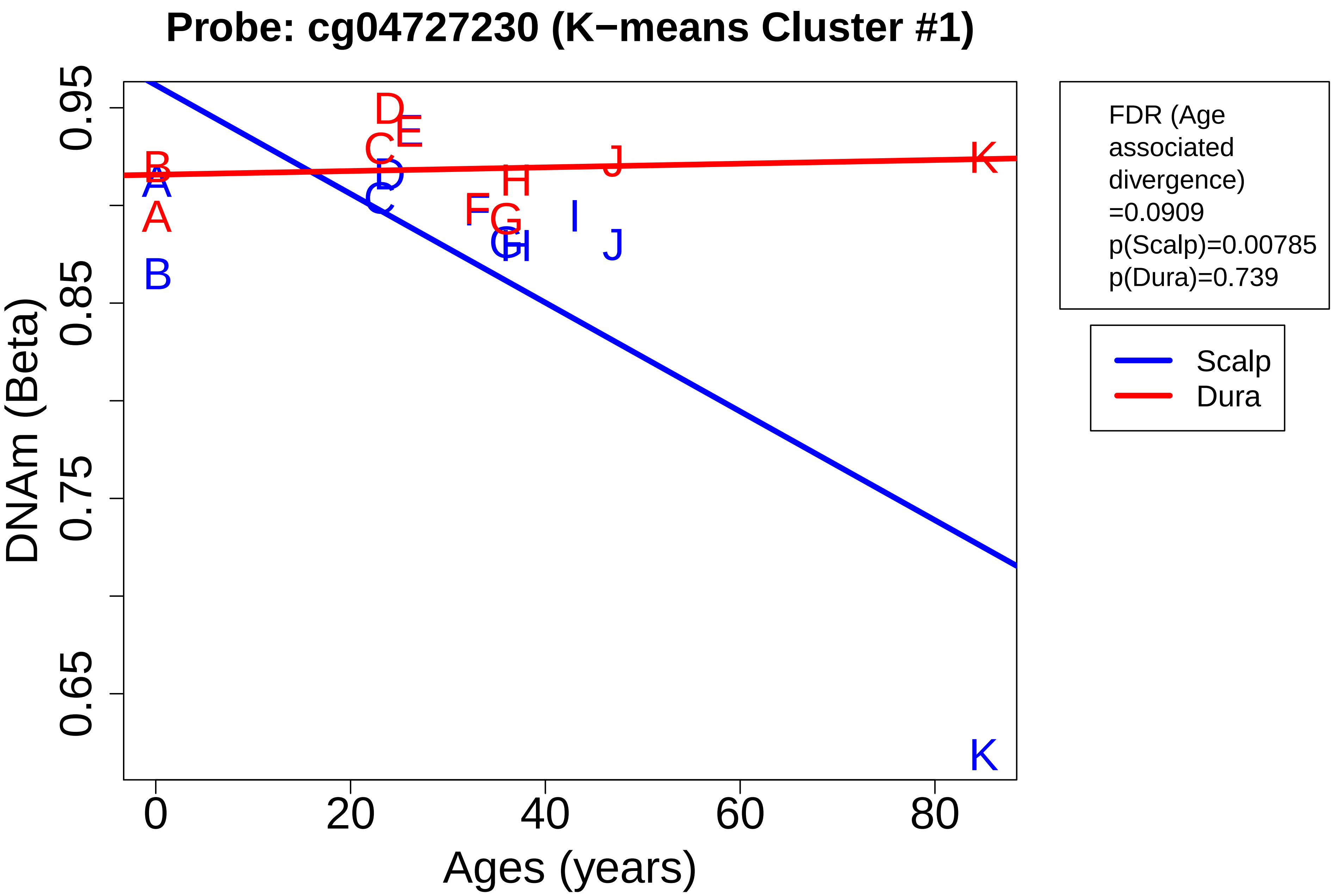

Probe: cg04727230 (K−means Cluster #1)
DNAm (Beta)
0.65
0.75
0.85
0.95
0
20
40
60
80
Ages (years)
FDR (Age associated divergence) =0.0909
p(Scalp)=0.00785
p(Dura)=0.739
Scalp
Dura

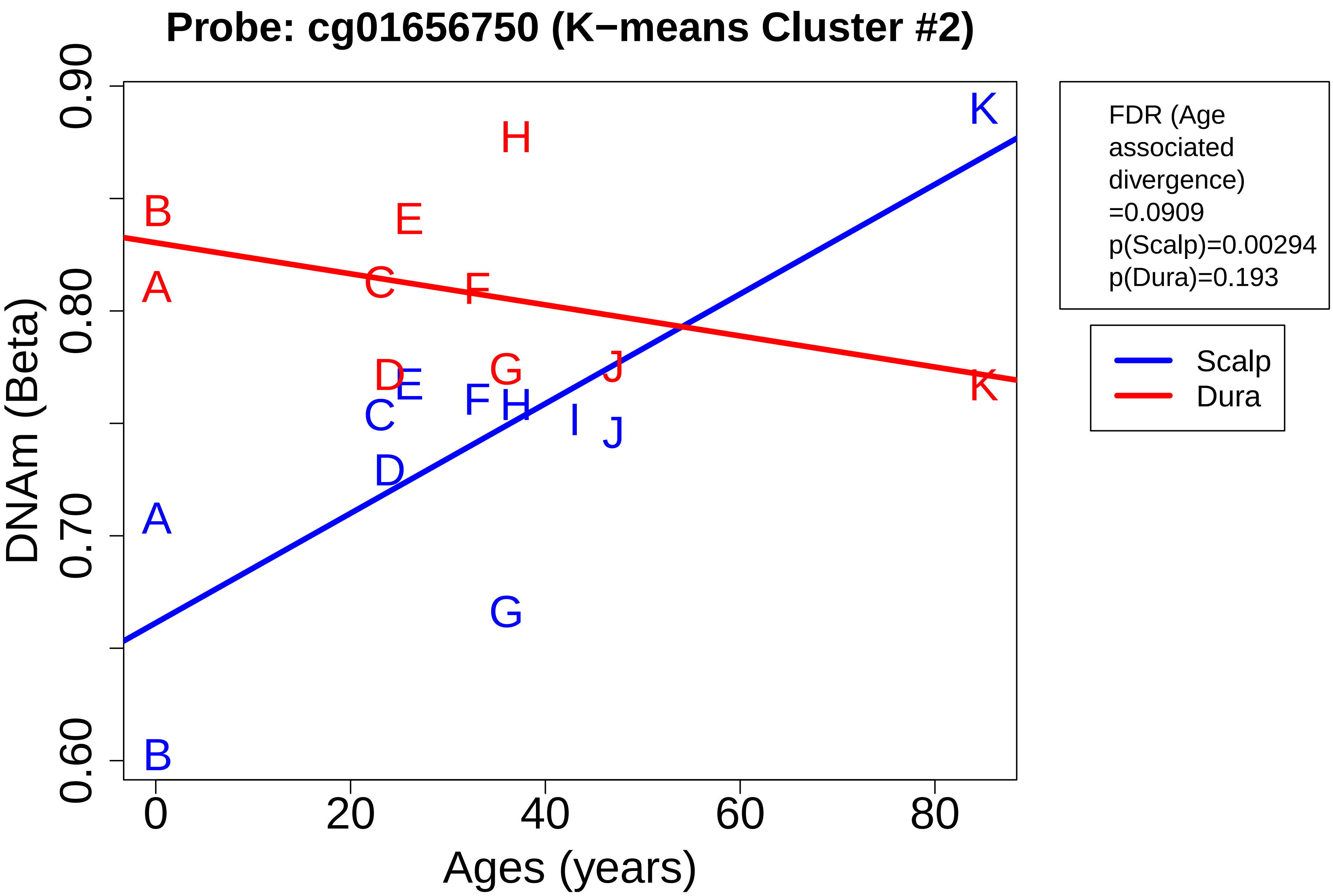

Probe: cg01656750 (K−means Cluster #2)
DNAm (Beta)
0.60
0.70
0.80
0.90
Ages (years)
0
20
40
60
80
FDR (Age associated divergence) =0.0909
p(Scalp)=0.00294
p(Dura)=0.193
Scalp
Dura

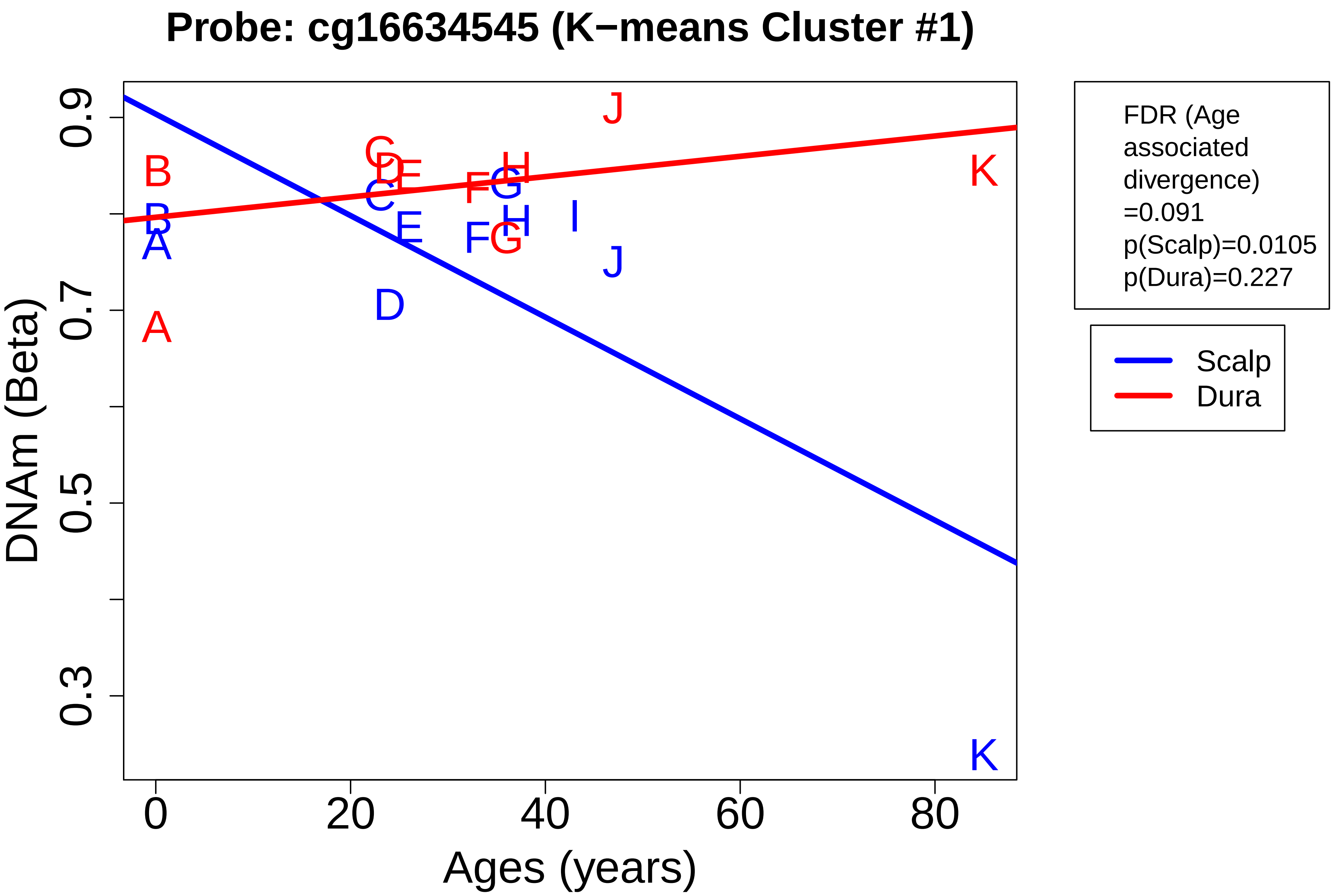
Probe: cg16634545 (K−means Cluster #1)
DNAm (Beta)
0.3
0.5
0.7
0.9
Ages (years)
0
20
40
60
80
FDR (Age associated divergence) =0.091
p(Scalp)=0.0105
p(Dura)=0.227
Scalp
Dura
A B C D E F G H I J K

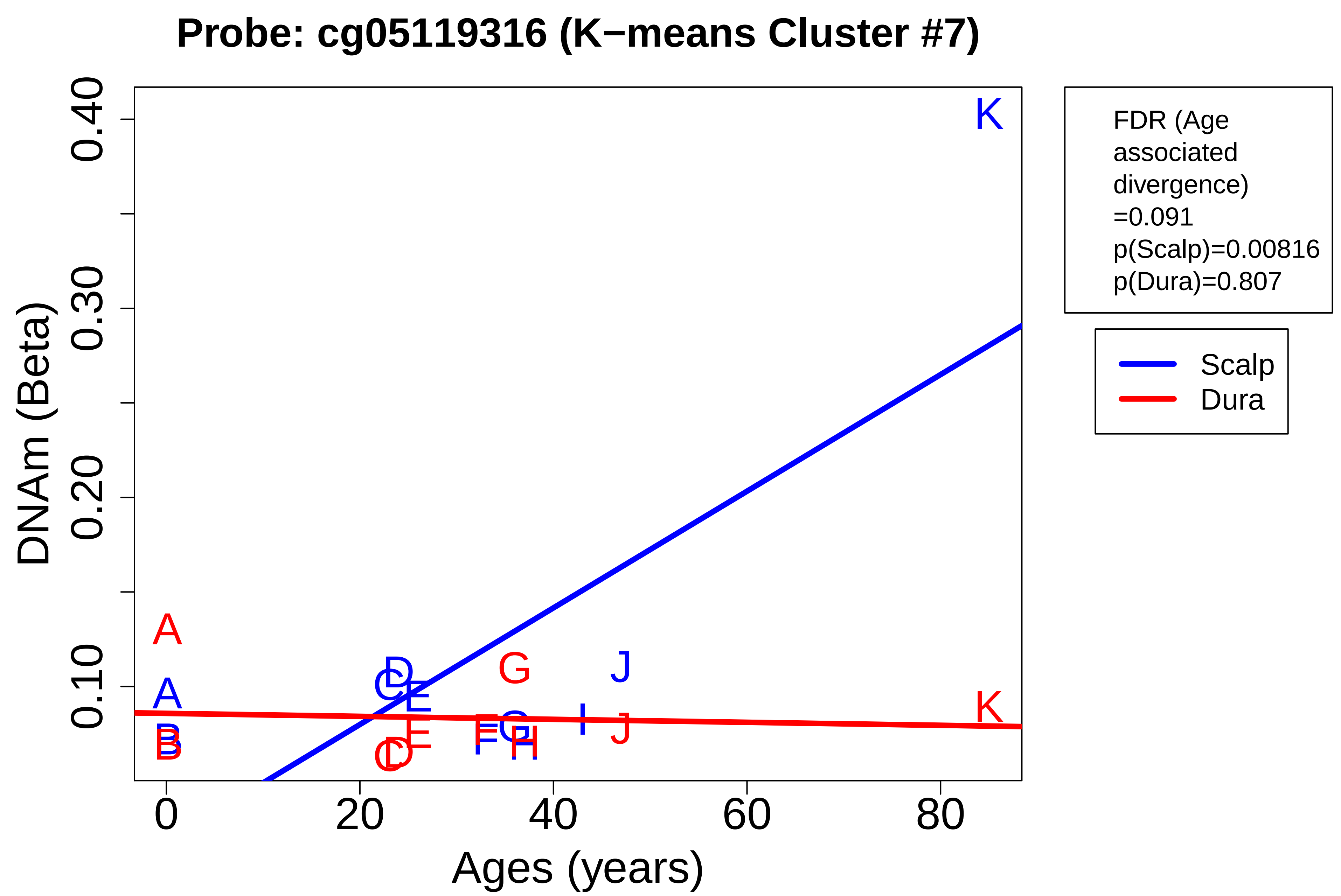

Probe: cg05119316 (K−means Cluster #7)
DNAm (Beta)
0.10
0.20
0.30
0.40
Ages (years)
0
20
40
60
80
FDR (Age associated divergence) =0.091
p(Scalp)=0.00816
p(Dura)=0.807
Scalp
Dura

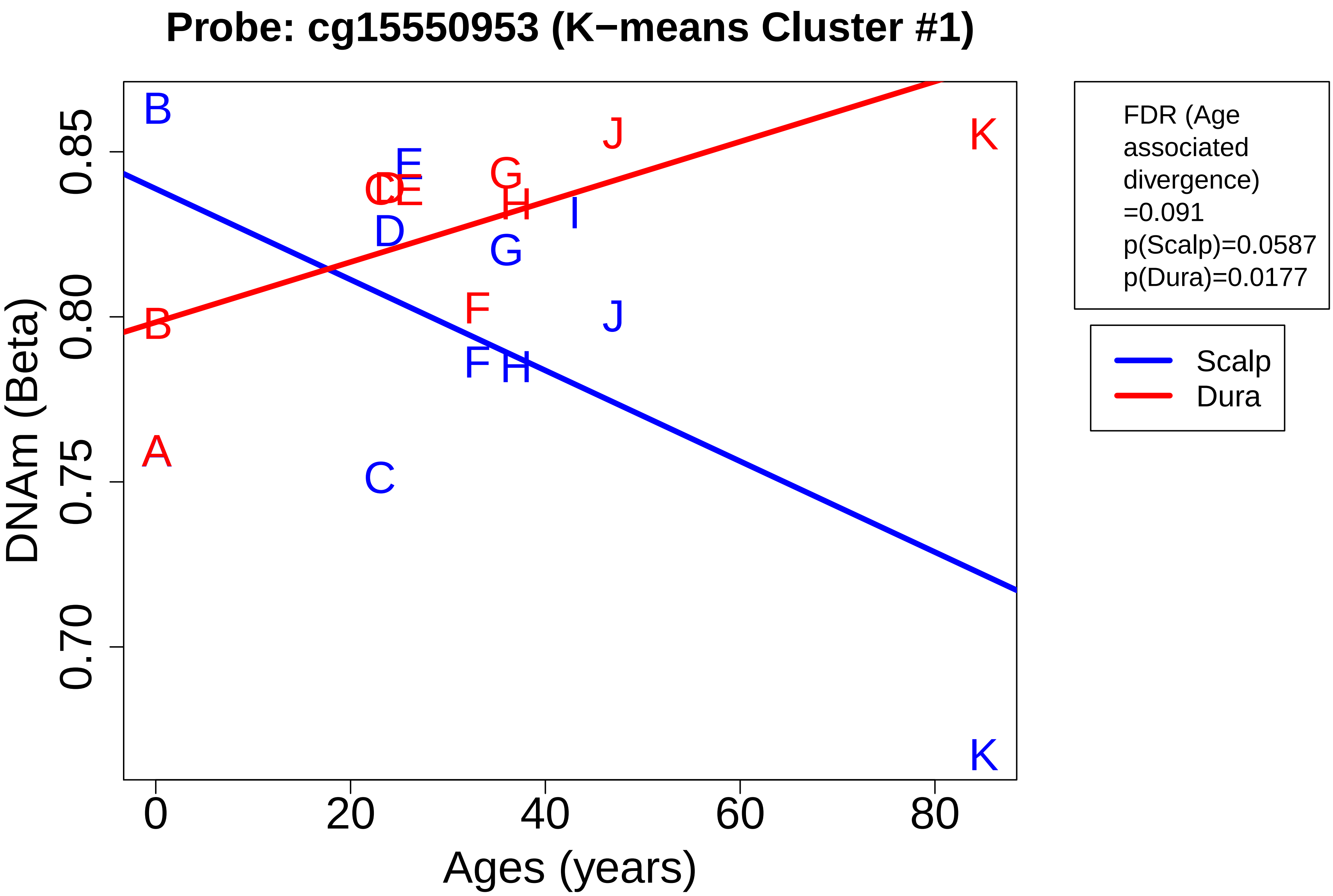
Probe: cg15550953 (K−means Cluster #1)
DNAm (Beta)
Ages (years)
0.70
0.75
0.80
0.85
0
20
40
60
80
FDR (Age associated divergence) =0.091
p(Scalp)=0.0587
p(Dura)=0.0177
Scalp
Dura

Probe: cg25544935 (K−means Cluster #5)

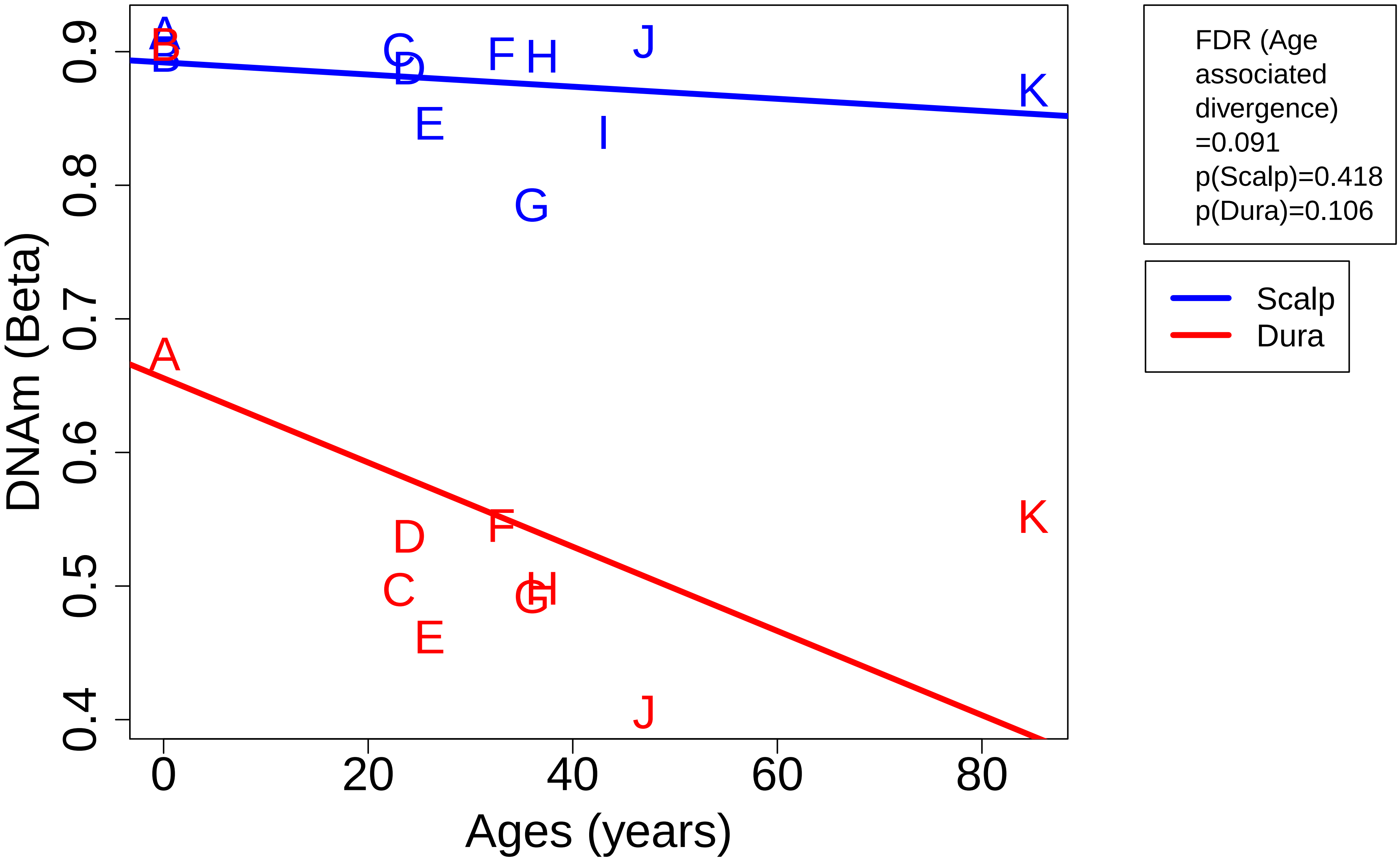

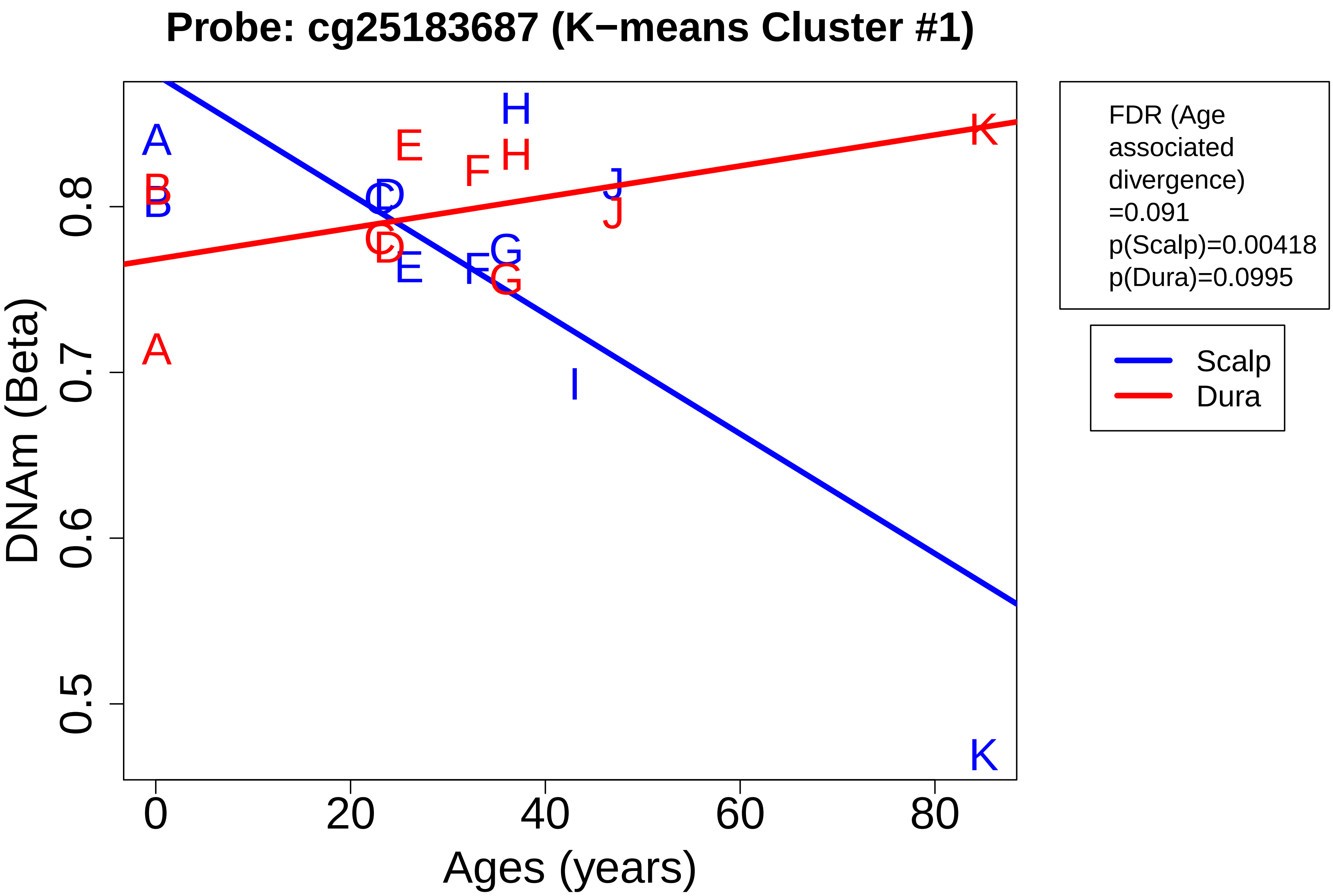

Probe: cg25183687 (K−means Cluster #1)
DNAm (Beta)
0.5
0.6
0.7
0.8
Ages (years)
0
20
40
60
80
FDR (Age associated divergence) =0.091
p(Scalp)=0.00418
p(Dura)=0.0995
Scalp
Dura
A
B
C
D
E
F
G
H
I
J
K

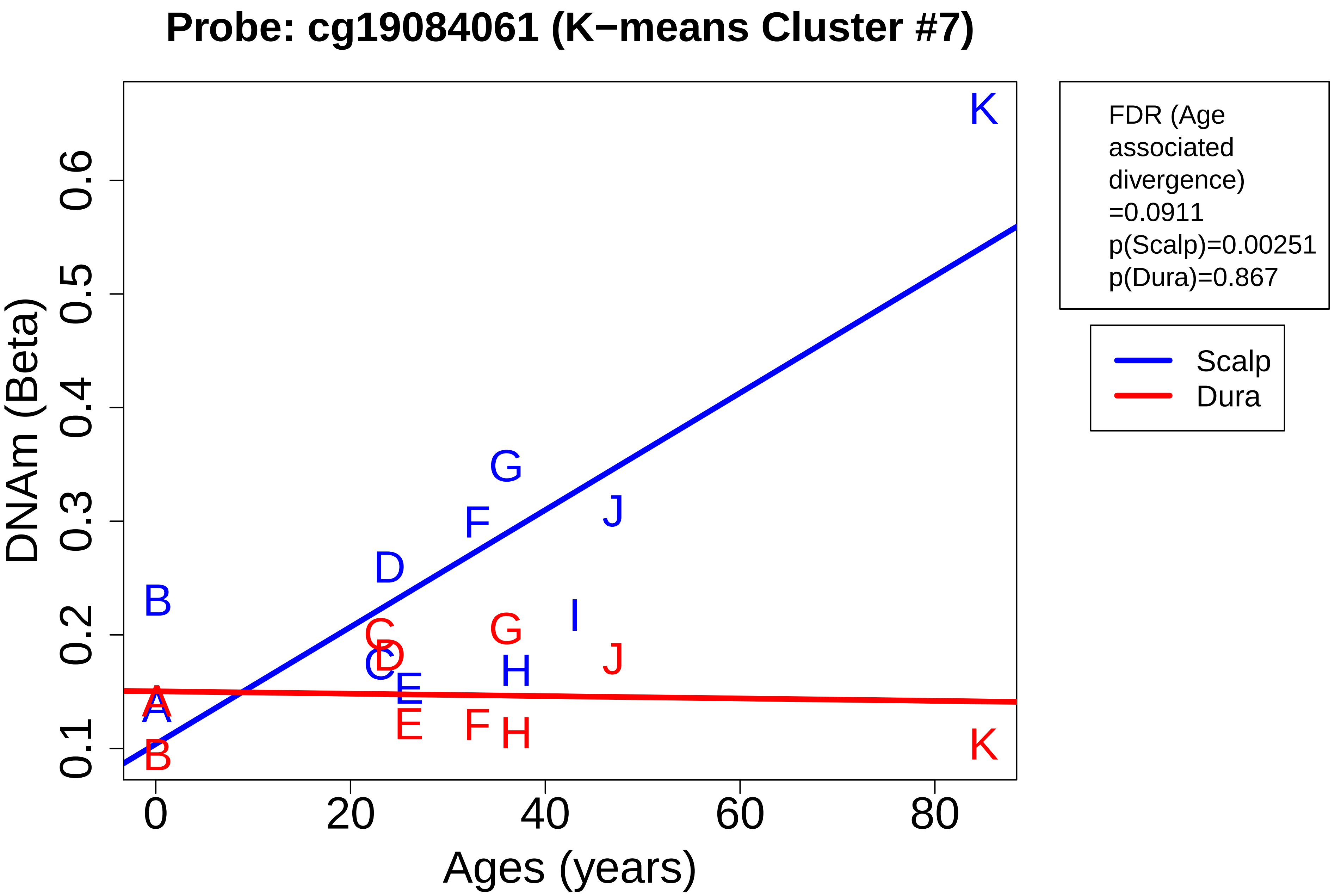

Probe: cg19084061 (K−means Cluster #7)
DNAm (Beta)
0.1
0.2
0.3
0.4
0.5
0.6
0
20
40
60
80
Ages (years)
FDR (Age associated divergence) =0.0911
p(Scalp)=0.00251
p(Dura)=0.867
Scalp
Dura
A
B
C
D
E
F
G
H
I
J
K

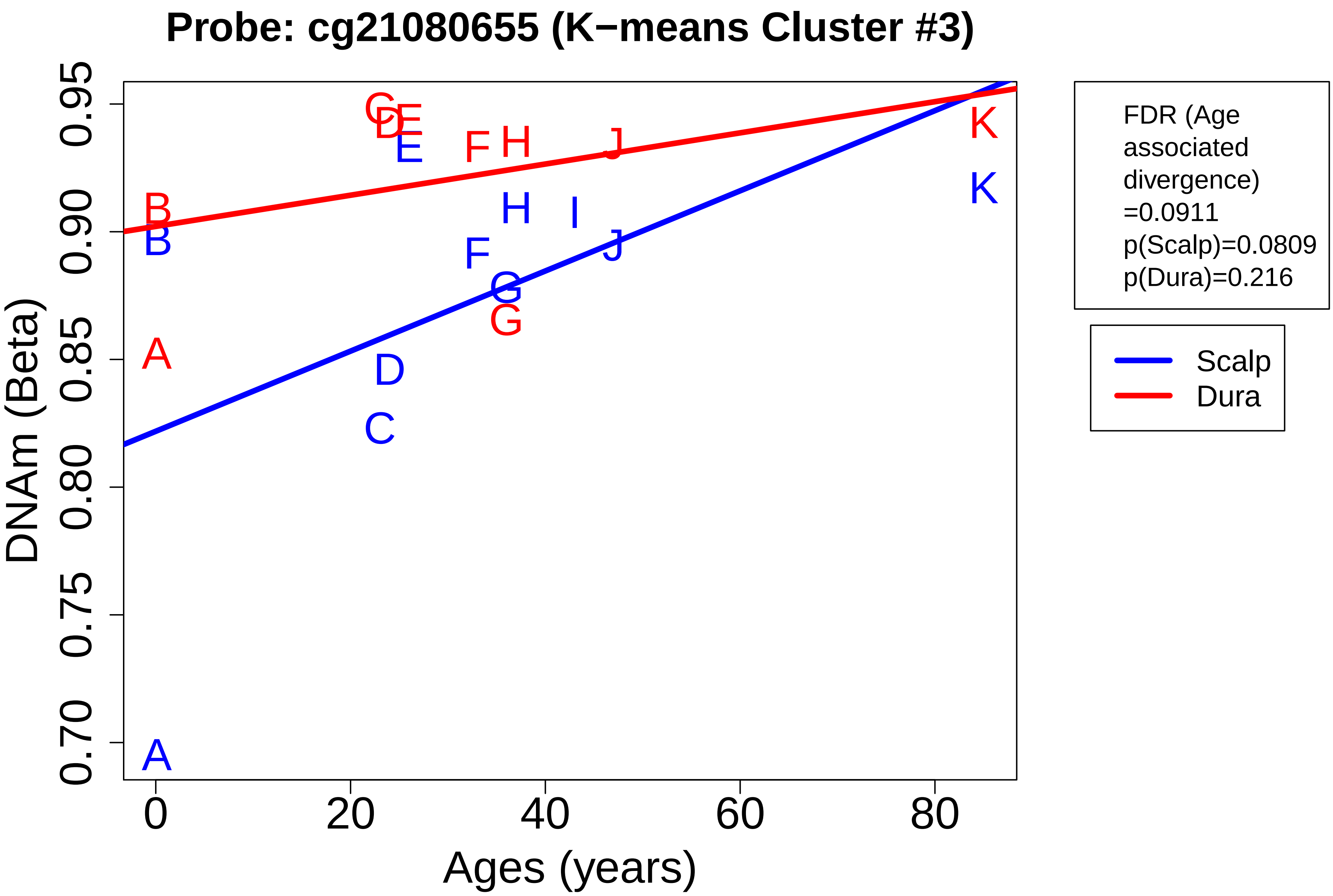

Probe: cg21080655 (K−means Cluster #3)
DNAm (Beta)
0.70
0.75
0.80
0.85
0.90
0.95
0
20
40
60
80
Ages (years)
FDR (Age associated divergence) =0.0911
p(Scalp)=0.0809
p(Dura)=0.216
Scalp
Dura

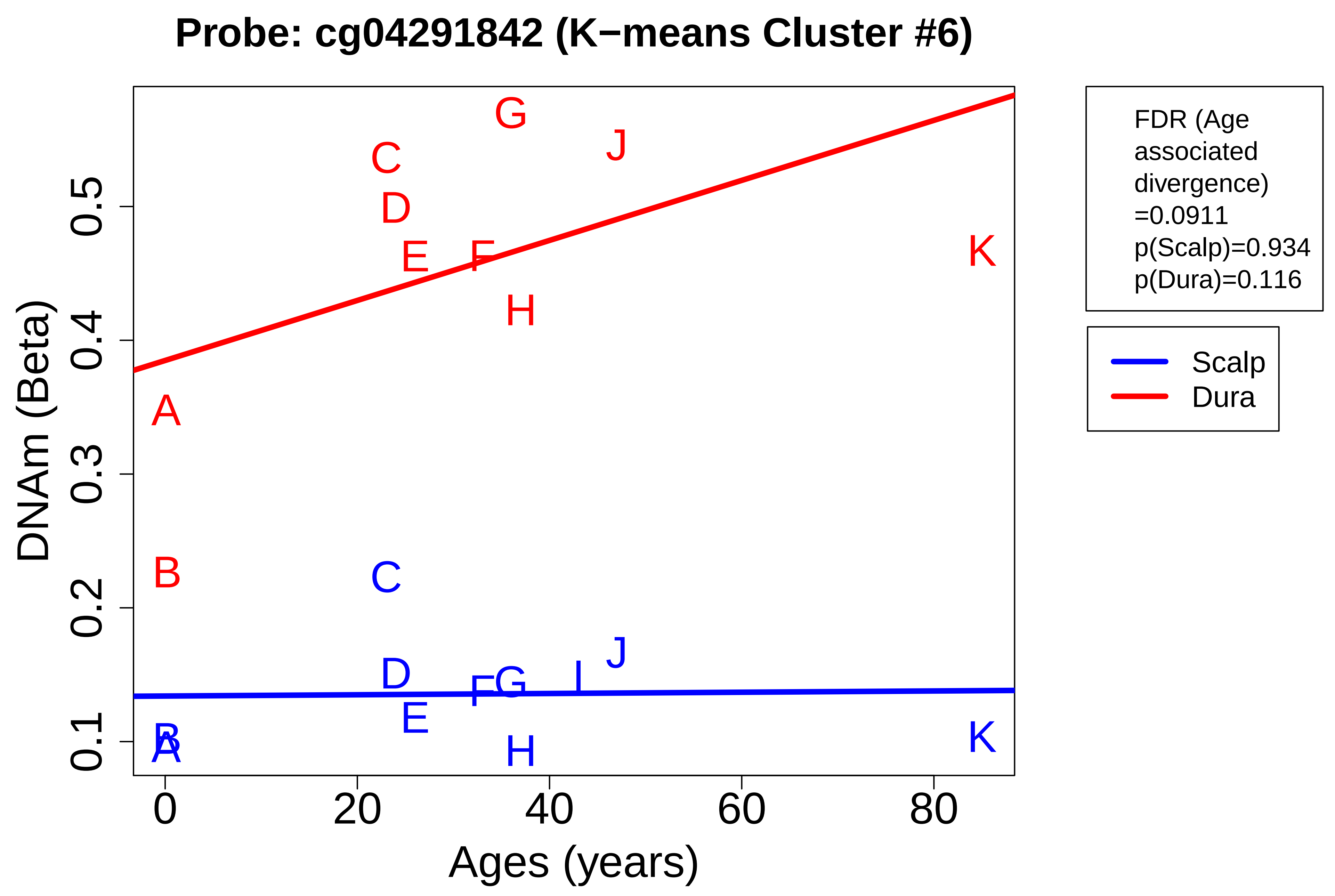

Probe: cg04291842 (K−means Cluster #6)
DNAm (Beta)
0.1
0.2
0.3
0.4
0.5
Ages (years)
0
20
40
60
80
FDR (Age associated divergence) =0.0911
p(Scalp)=0.934
p(Dura)=0.116
Scalp
Dura

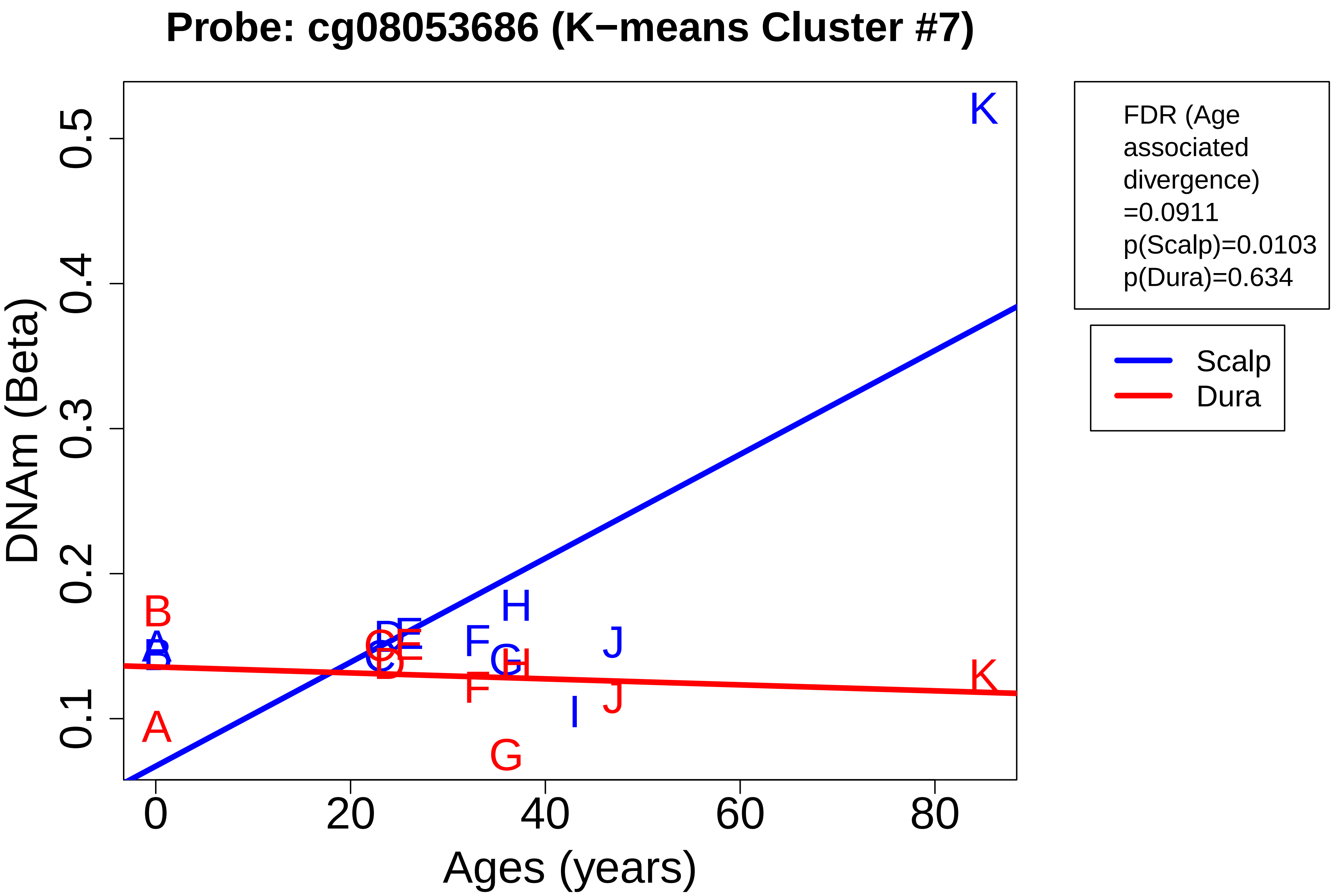

Probe: cg08053686 (K−means Cluster #7)
DNAm (Beta)
0.1
0.2
0.3
0.4
0.5
Ages (years)
0
20
40
60
80
FDR (Age associated divergence) =0.0911
p(Scalp)=0.0103
p(Dura)=0.634
Scalp
Dura

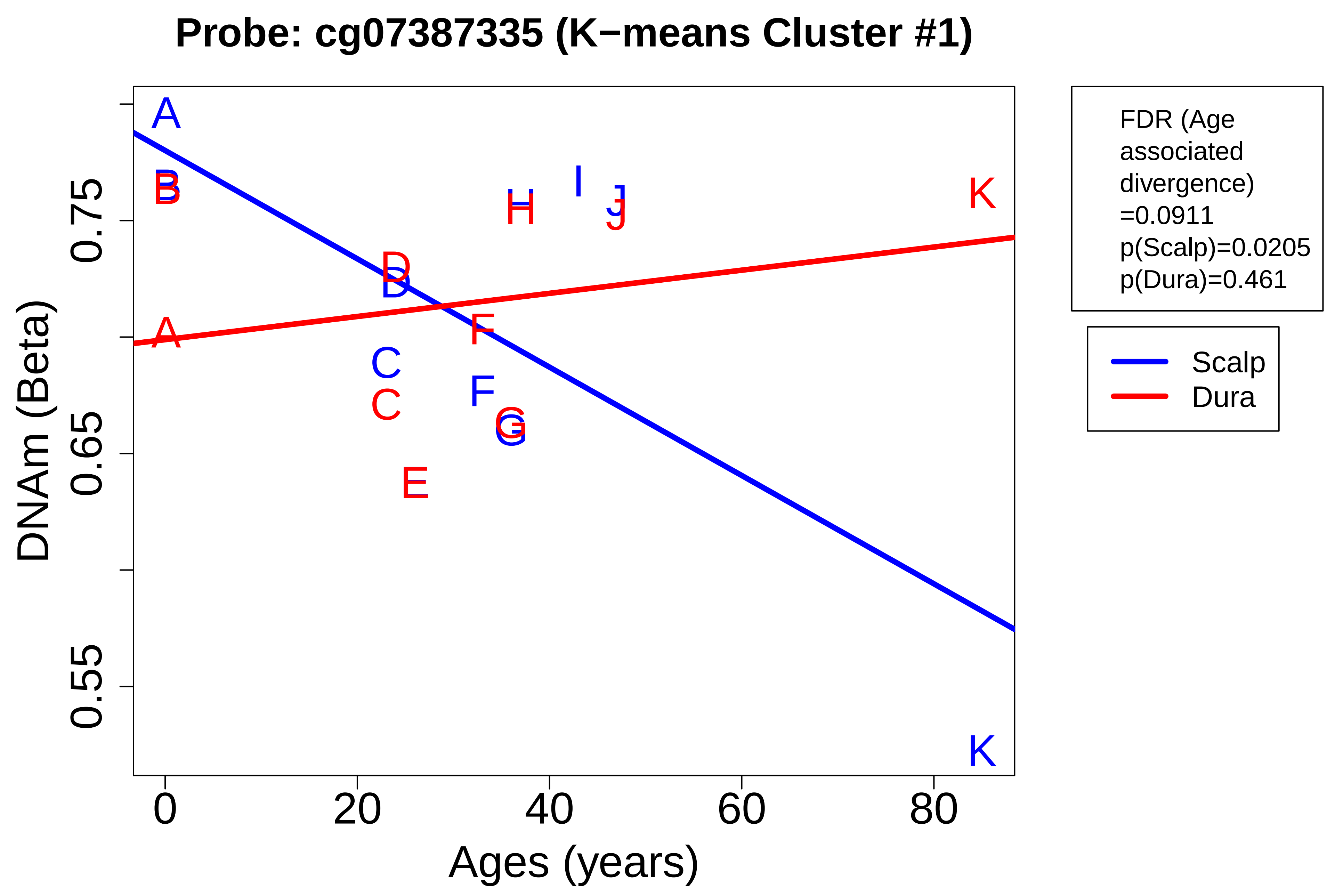

Probe: cg07387335 (K−means Cluster #1)
DNAm (Beta)
0.55
0.65
0.75
Ages (years)
0
20
40
60
80
A
B
C
D
E
F
G
H
I
J
K
FDR (Age associated divergence) =0.0911
p(Scalp)=0.0205
p(Dura)=0.461
Scalp
Dura

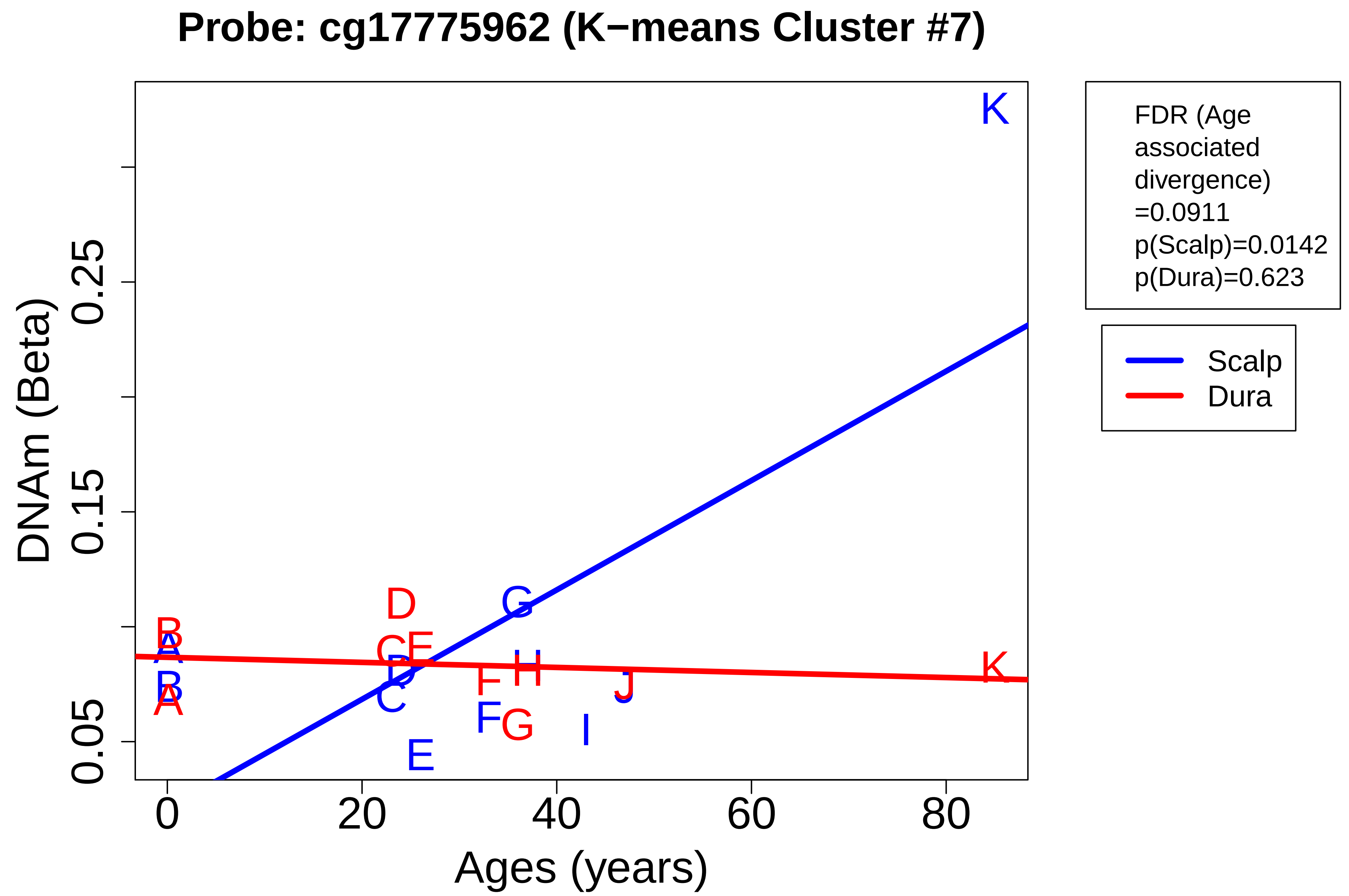
Probe: cg17775962 (K−means Cluster #7)
DNAm (Beta)
0.05
0.15
0.25
0
20
40
60
80
Ages (years)
FDR (Age associated divergence) =0.0911
p(Scalp)=0.0142
p(Dura)=0.623
Scalp
Dura

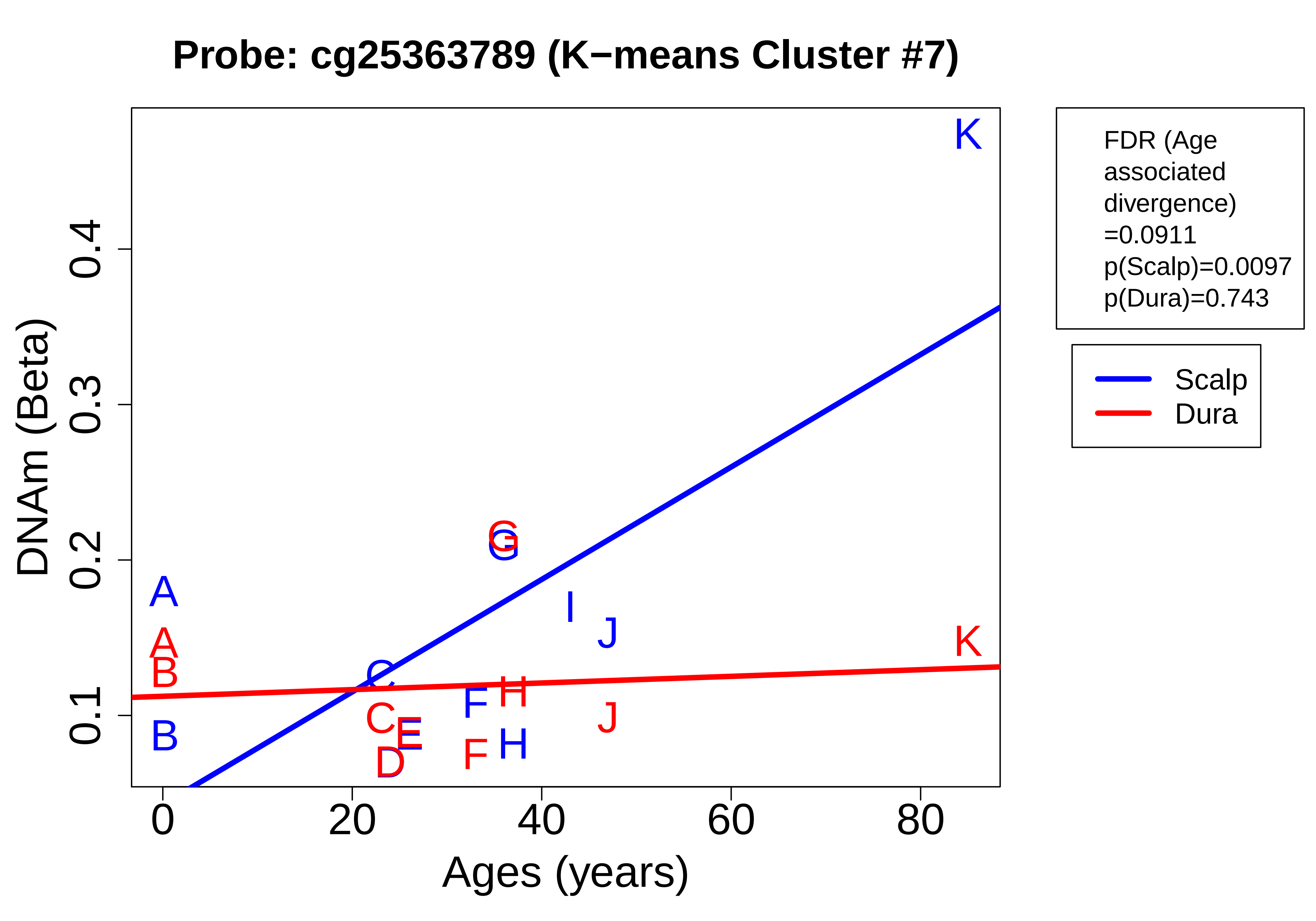

Probe: cg25363789 (K−means Cluster #7)
DNAm (Beta)
0.1
0.2
0.3
0.4
Ages (years)
0
20
40
60
80
FDR (Age associated divergence) =0.0911
p(Scalp)=0.0097
p(Dura)=0.743
Scalp
Dura

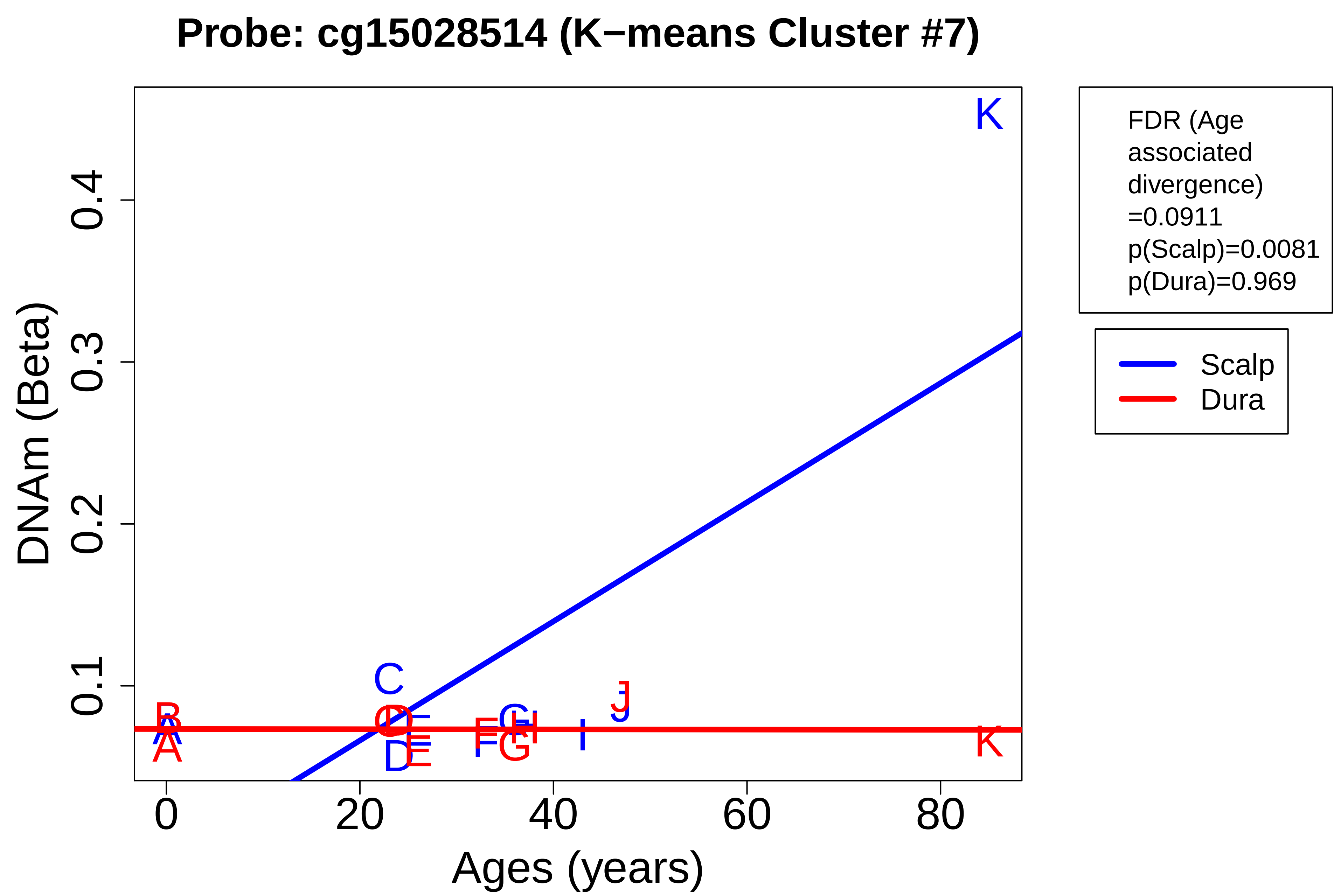

Probe: cg15028514 (K−means Cluster #7)
DNAm (Beta)
0.1
0.2
0.3
0.4
0
20
40
60
80
Ages (years)
A
B
C
D
E
F
G
H
I
J
K
FDR (Age associated divergence) =0.0911
p(Scalp)=0.0081
p(Dura)=0.969
Scalp
Dura

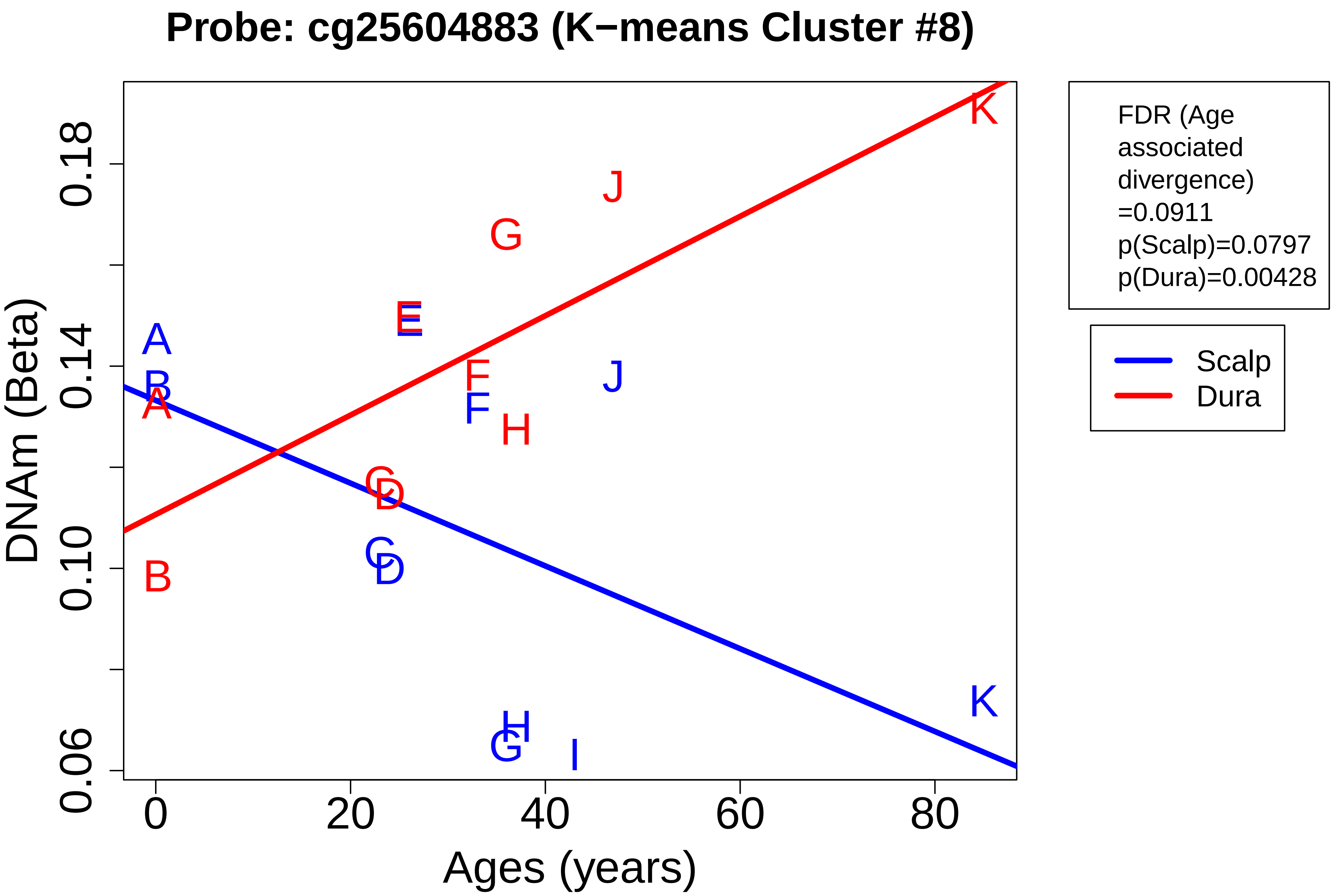
Probe: cg25604883 (K−means Cluster #8)
DNAm (Beta)
0.06
0.10
0.14
0.18
0
20
40
60
80
Ages (years)
FDR (Age associated divergence) =0.0911
p(Scalp)=0.0797
p(Dura)=0.00428
Scalp
Dura
A
B
C
D
E
F
G
H
I
J
K

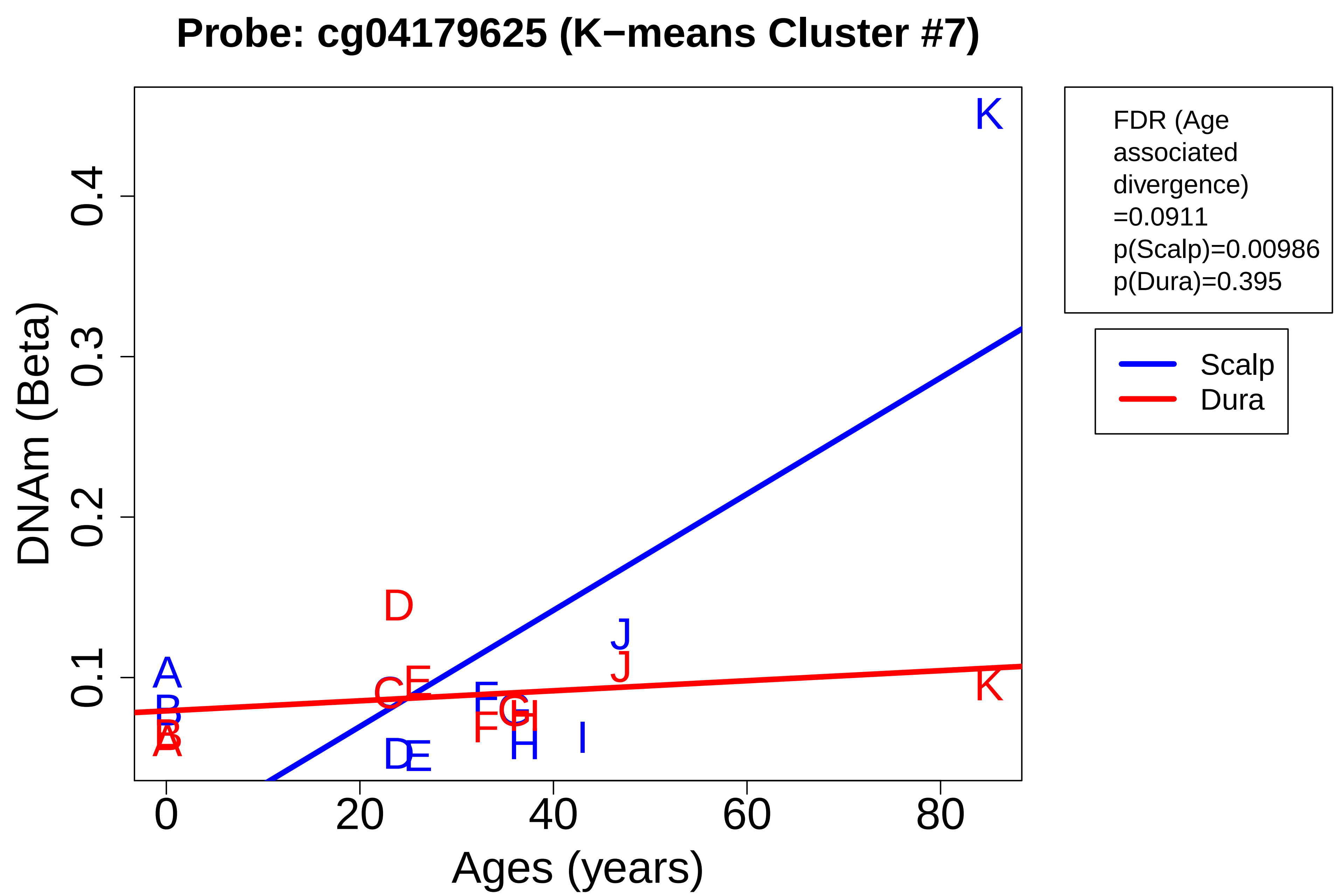

Probe: cg04179625 (K−means Cluster #7)
FDR (Age associated divergence) =0.0911
p(Scalp)=0.00986
p(Dura)=0.395
Scalp
Dura
DNAm (Beta)
Ages (years)
0.1
0.2
0.3
0.4
0
20
40
60
80

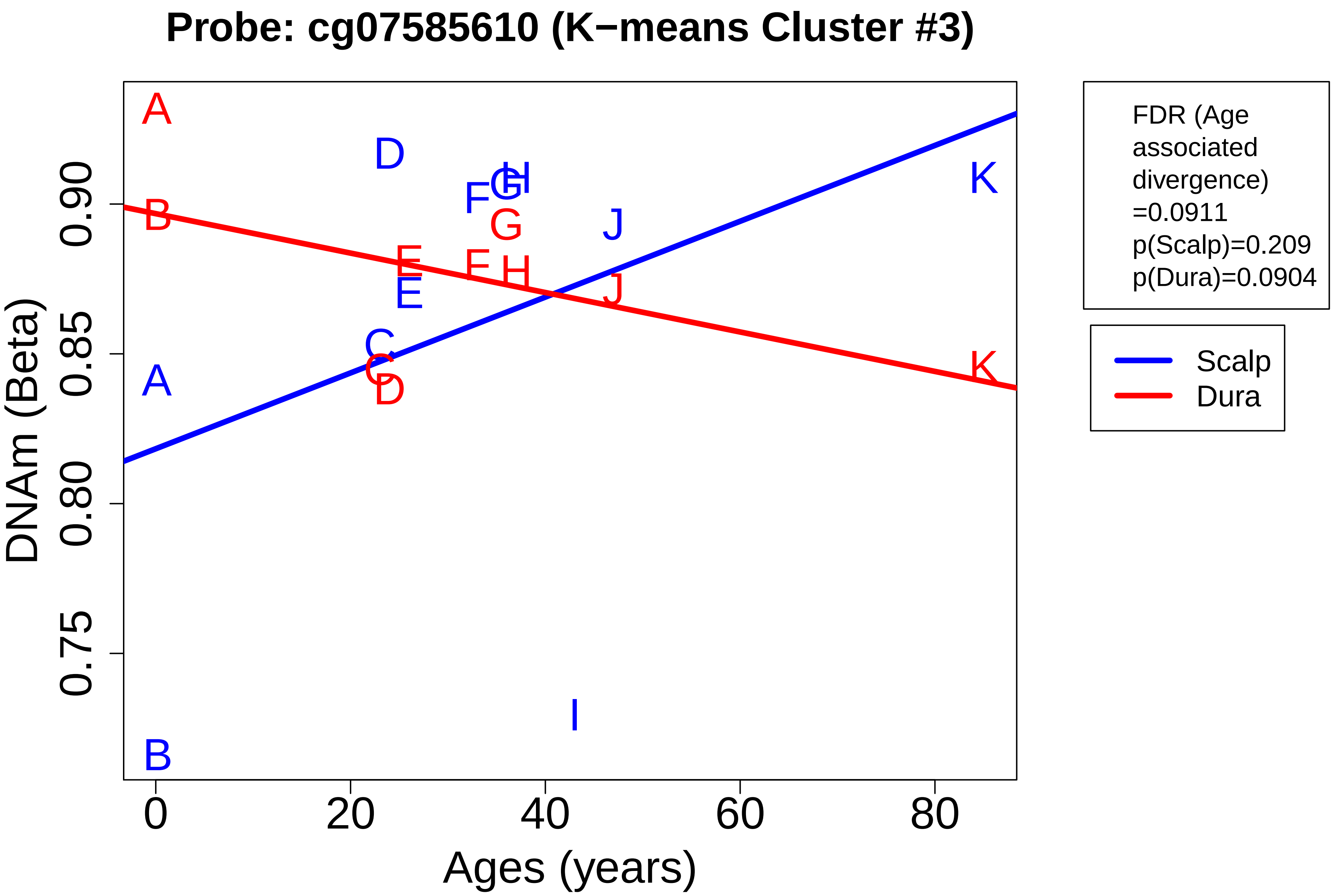

Probe: cg07585610 (K−means Cluster #3)
DNAm (Beta)
0.75
0.80
0.85
0.90
0
20
40
60
80
Ages (years)
FDR (Age associated divergence) =0.0911
p(Scalp)=0.209
p(Dura)=0.0904
Scalp
Dura

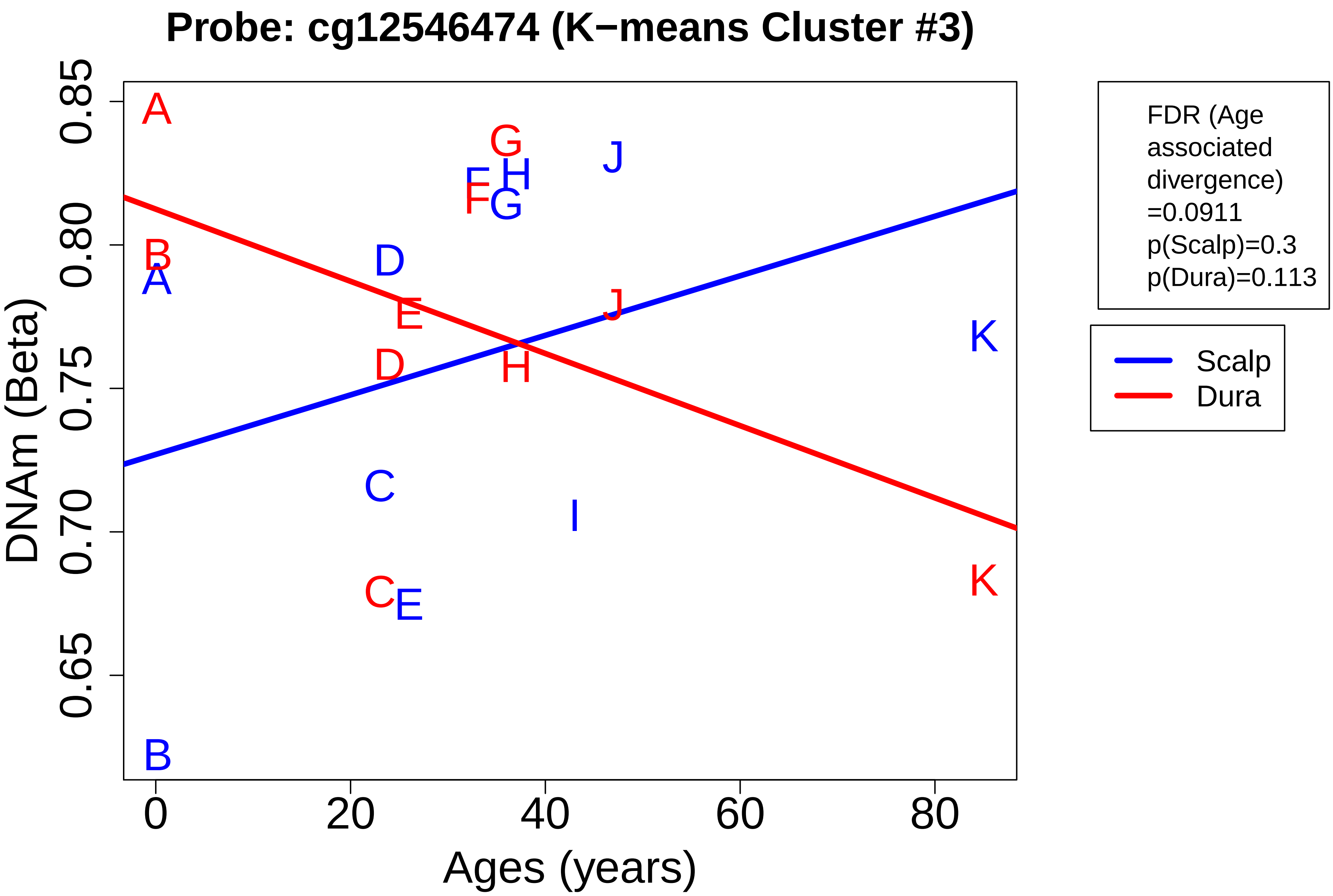

Probe: cg12546474 (K−means Cluster #3)
DNAm (Beta)
0.65
0.70
0.75
0.80
0.85
Ages (years)
0
20
40
60
80
A
B
C
D
E
F
G
H
I
J
K
FDR (Age associated divergence) =0.0911
p(Scalp)=0.3
p(Dura)=0.113
Scalp
Dura

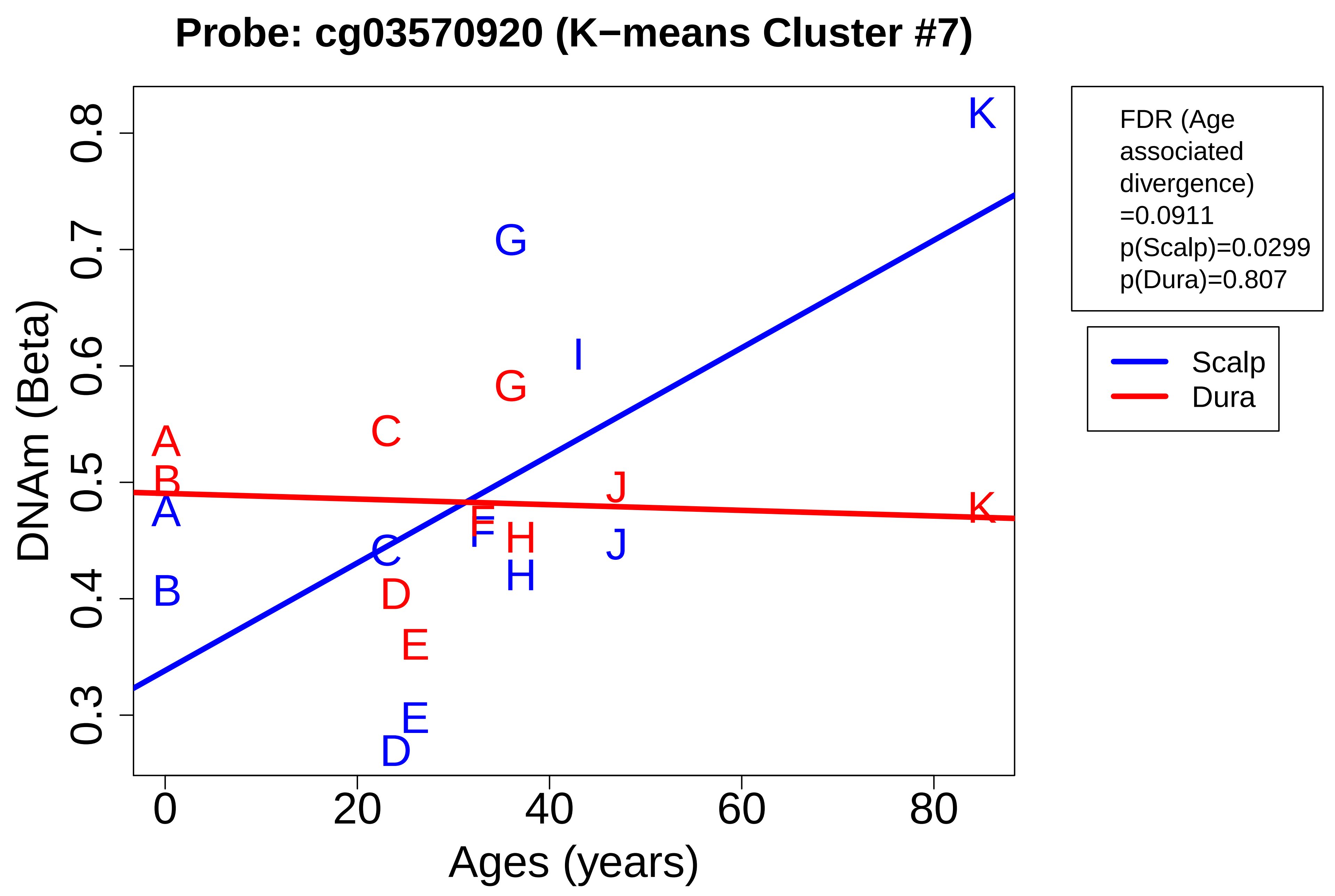
Probe: cg03570920 (K−means Cluster #7)
DNAm (Beta)
0.3
0.4
0.5
0.6
0.7
0.8
Ages (years)
0
20
40
60
80
FDR (Age associated divergence) =0.0911
p(Scalp)=0.0299
p(Dura)=0.807
Scalp
Dura
A
B
C
D
E
F
G
H
I
J
K

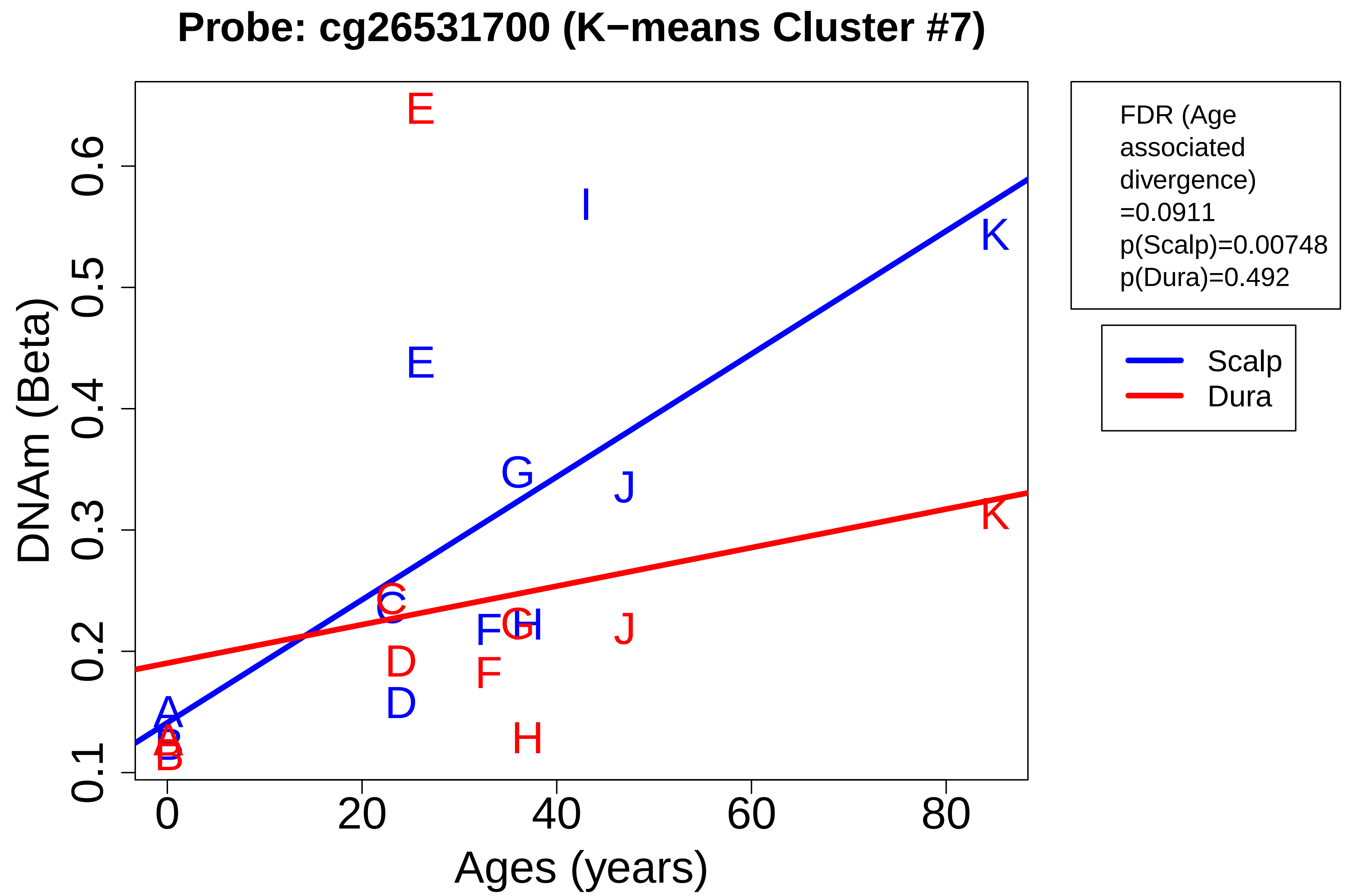

Probe: cg26531700 (K−means Cluster #7)
DNAm (Beta)
0.1
0.2
0.3
0.4
0.5
0.6
0
20
40
60
80
Ages (years)
FDR (Age associated divergence) =0.0911
p(Scalp)=0.00748
p(Dura)=0.492
Scalp
Dura

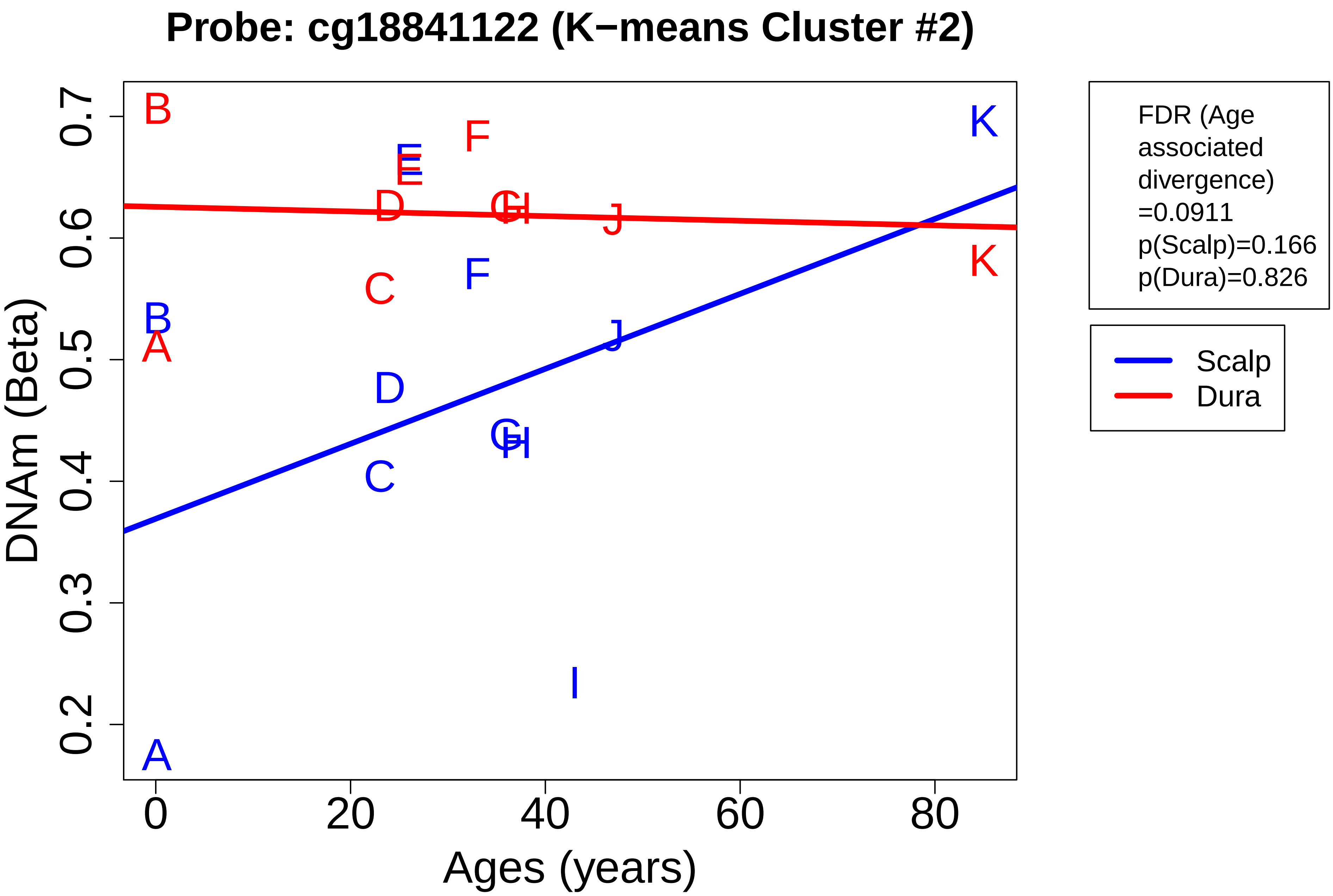

Probe: cg18841122 (K−means Cluster #2)
DNAm (Beta)
0.2
0.3
0.4
0.5
0.6
0.7
0
20
40
60
80
Ages (years)
FDR (Age associated divergence) =0.0911
p(Scalp)=0.166
p(Dura)=0.826
Scalp
Dura

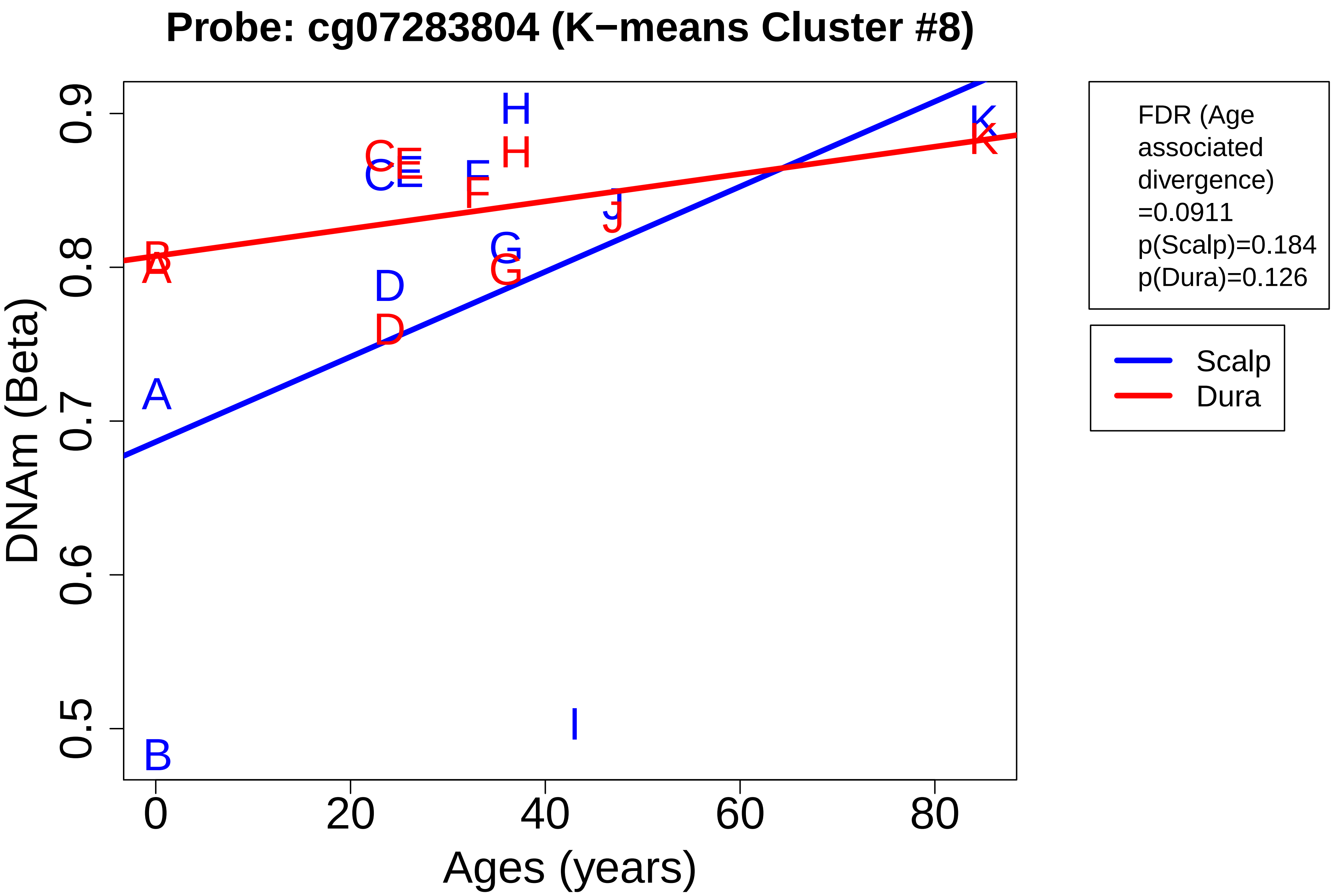

Probe: cg07283804 (K−means Cluster #8)
DNAm (Beta)
0.5
0.6
0.7
0.8
0.9
Ages (years)
0
20
40
60
80
FDR (Age associated divergence) =0.0911
p(Scalp)=0.184
p(Dura)=0.126
Scalp
Dura

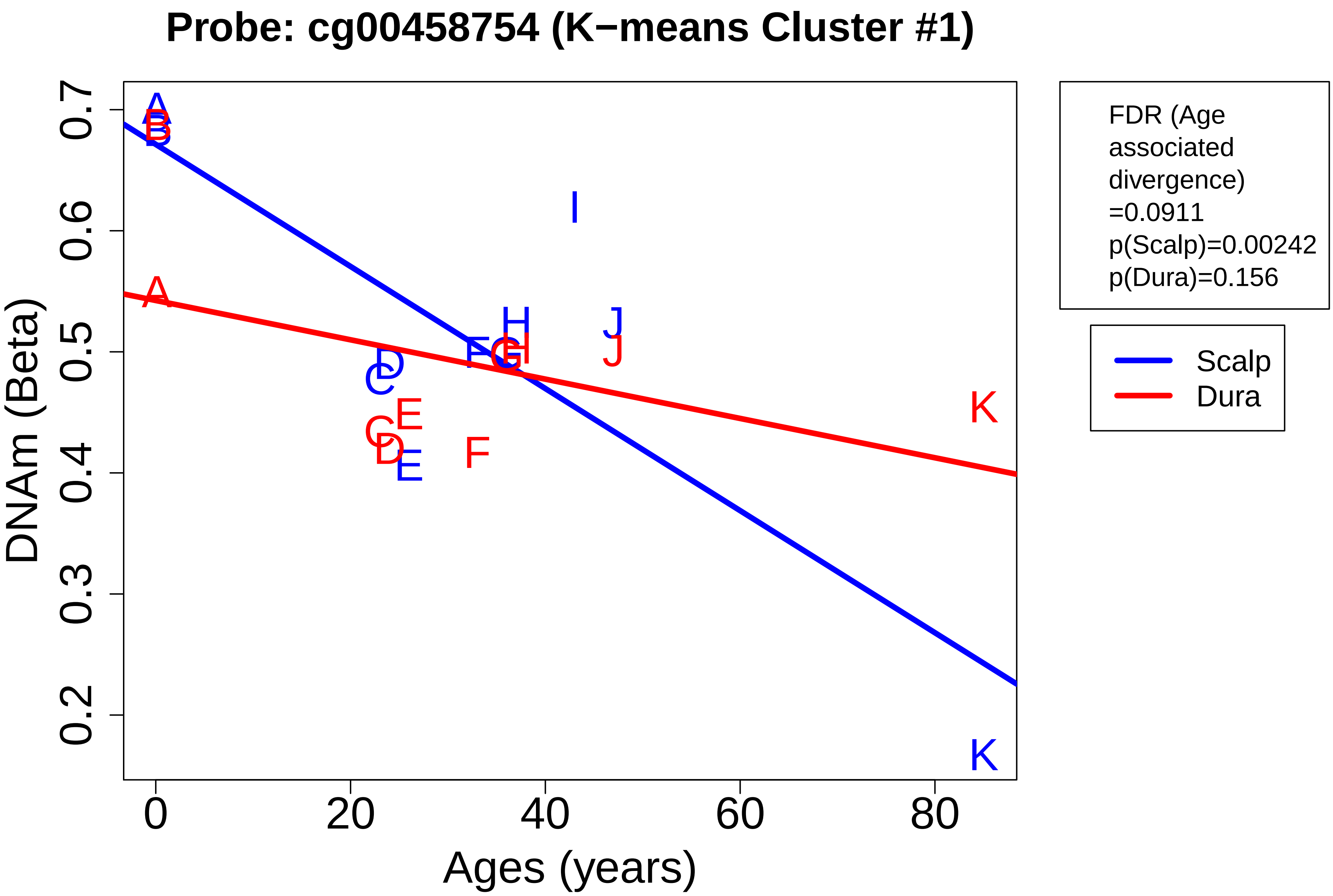

Probe: cg00458754 (K−means Cluster #1)
DNAm (Beta)
0.2
0.3
0.4
0.5
0.6
0.7
Ages (years)
0
20
40
60
80
FDR (Age associated divergence) =0.0911
p(Scalp)=0.00242
p(Dura)=0.156
Scalp
Dura

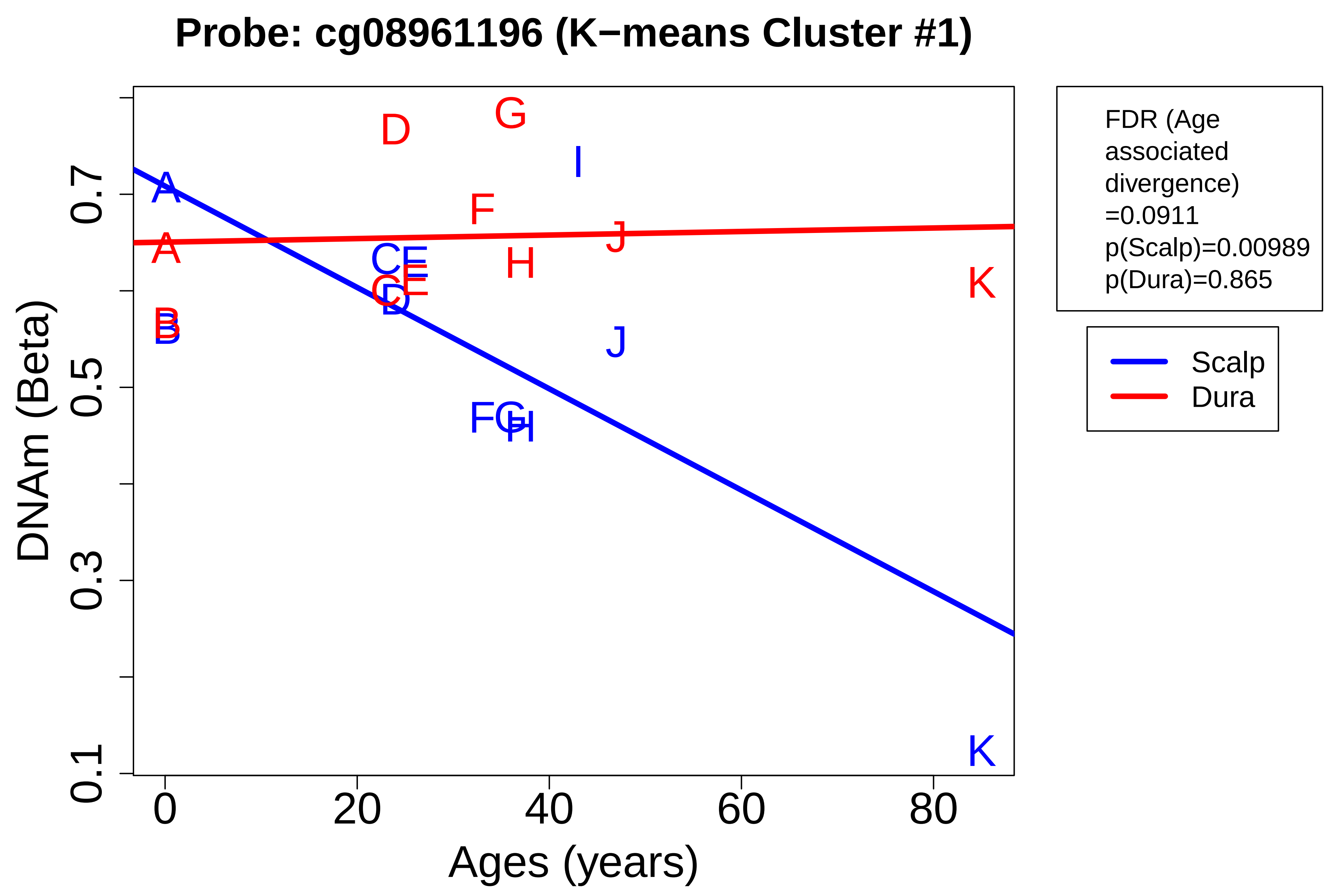

Probe: cg08961196 (K−means Cluster #1)
DNAm (Beta)
0.1
0.3
0.5
0.7
Ages (years)
0
20
40
60
80
FDR (Age associated divergence) =0.0911
p(Scalp)=0.00989
p(Dura)=0.865
Scalp
Dura

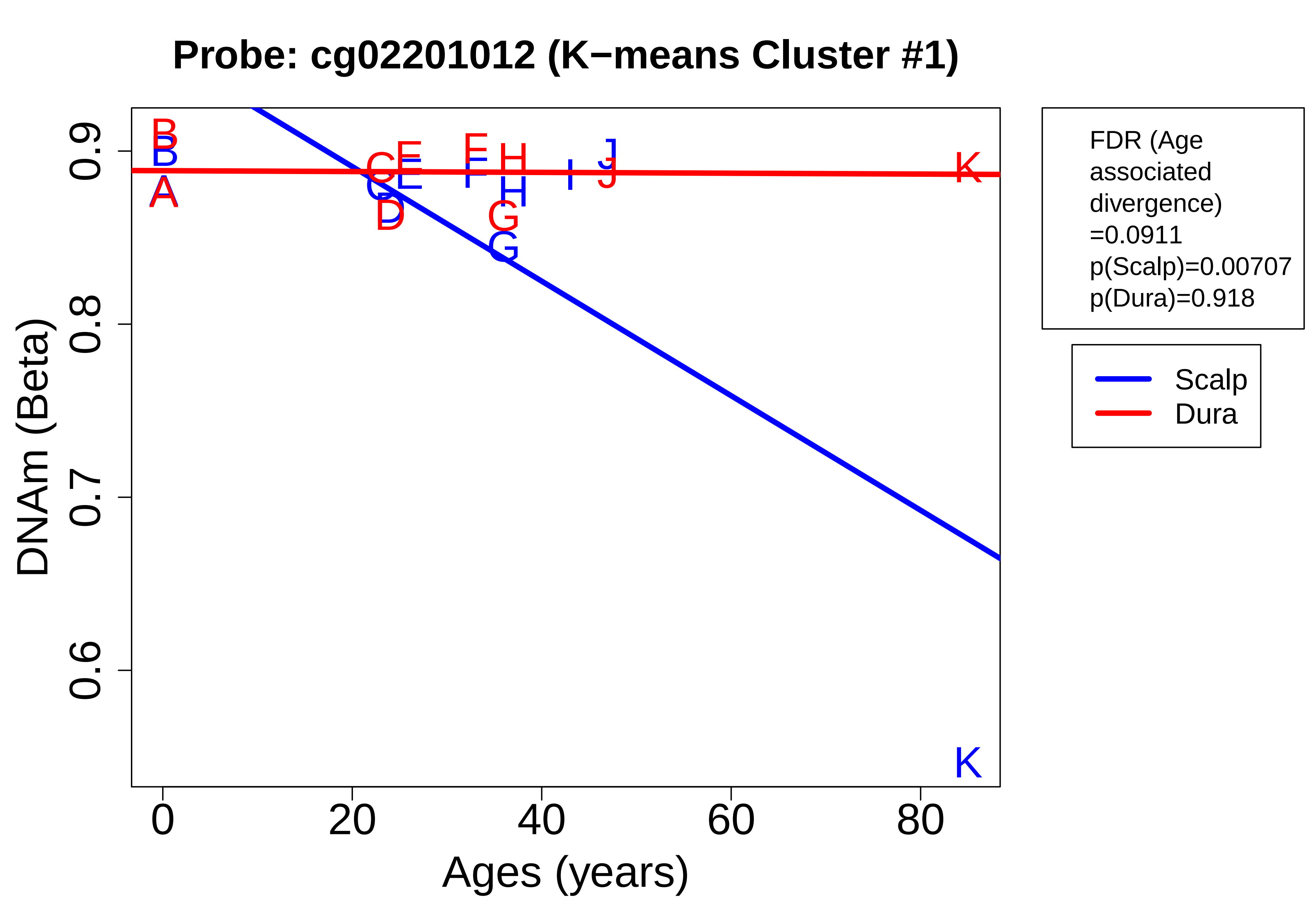

Probe: cg02201012 (K−means Cluster #1)
DNAm (Beta)
0.6
0.7
0.8
0.9
0
20
40
60
80
Ages (years)
A
B
C
D
E
F
G
H
I
J
K
FDR (Age associated divergence) =0.0911
p(Scalp)=0.00707
p(Dura)=0.918
Scalp
Dura

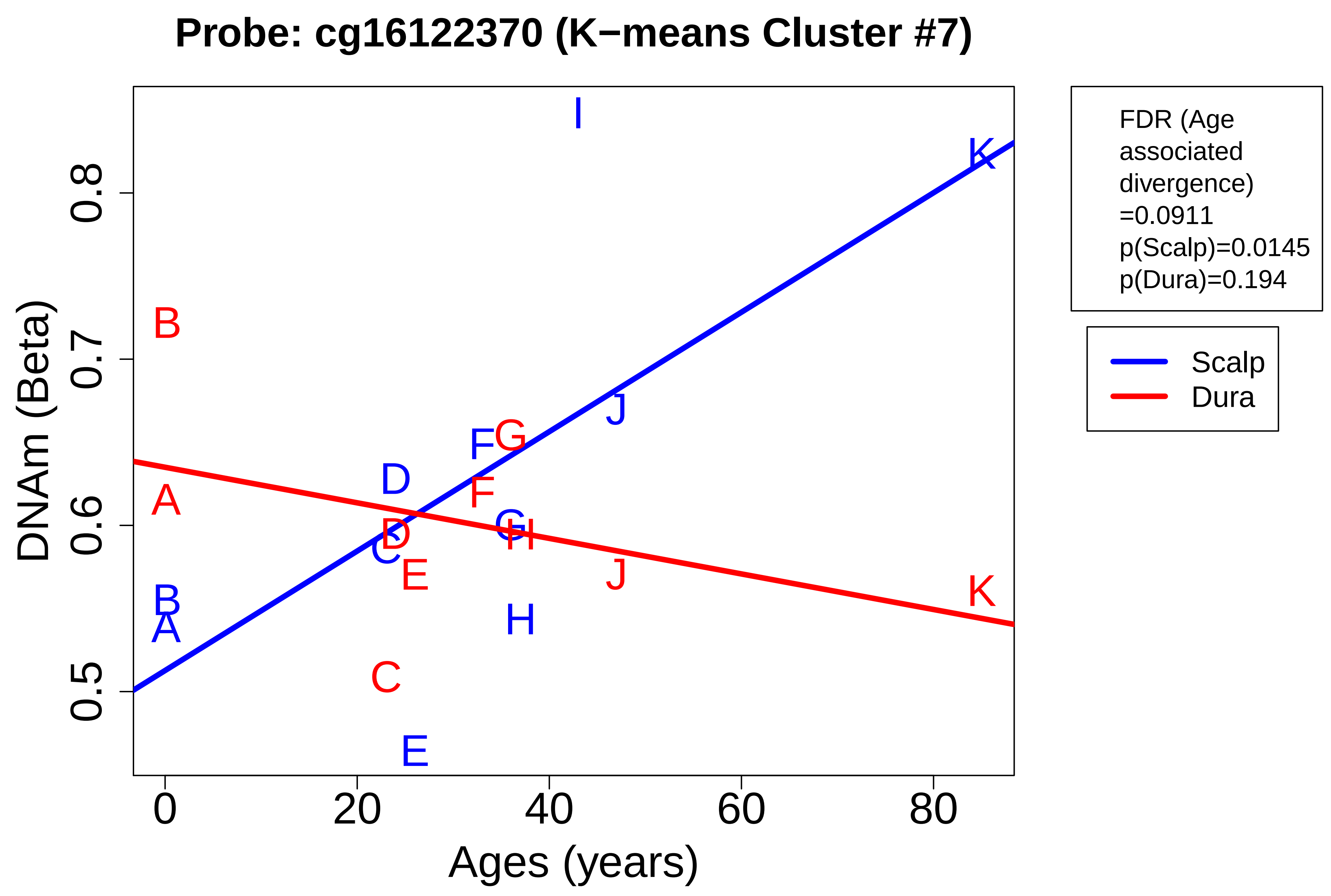
Probe: cg16122370 (K−means Cluster #7)
DNAm (Beta)
Ages (years)
FDR (Age associated divergence) =0.0911
p(Scalp)=0.0145
p(Dura)=0.194
Scalp
Dura

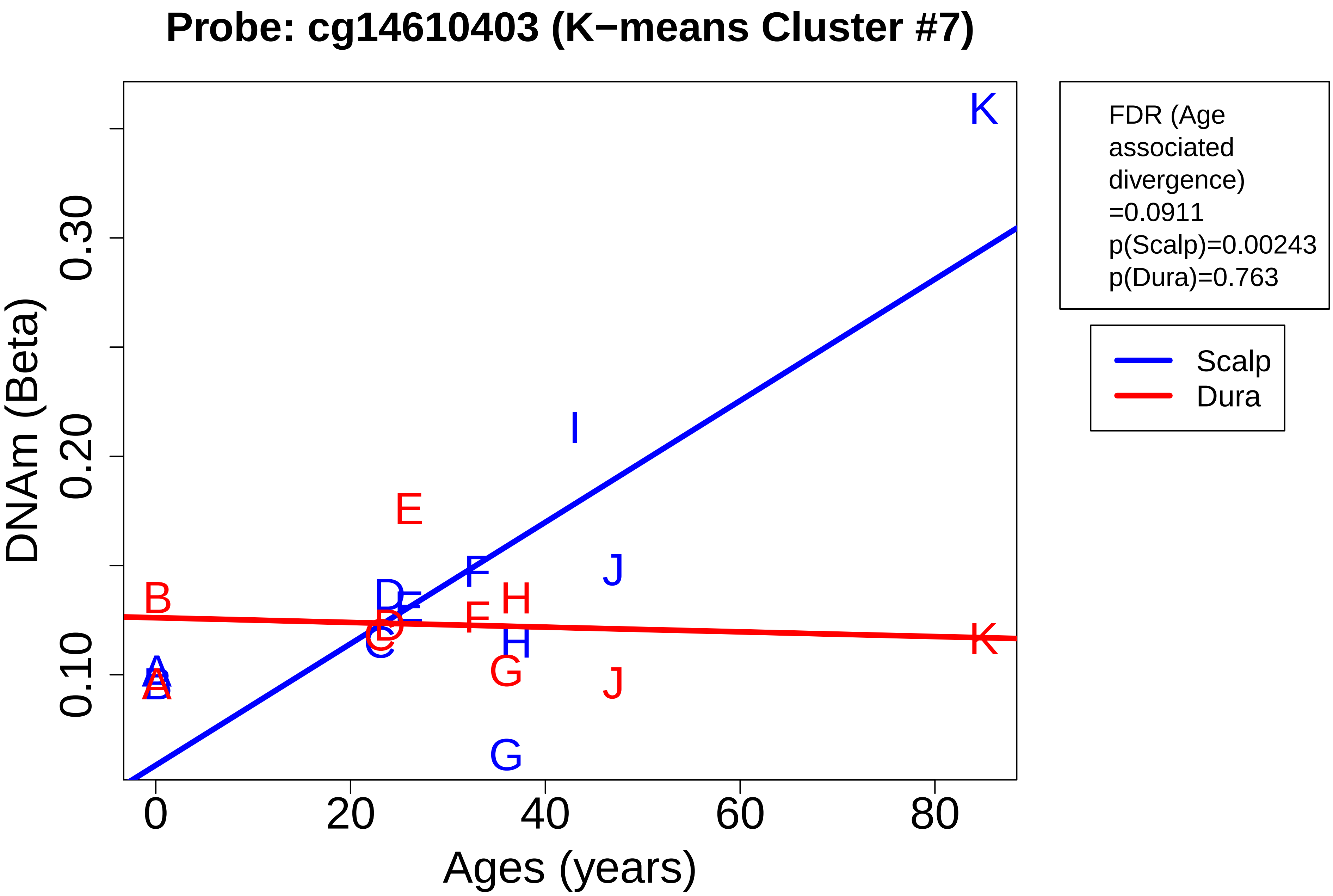

Probe: cg14610403 (K−means Cluster #7)
DNAm (Beta)
0.10
0.20
0.30
0
20
40
60
80
Ages (years)
FDR (Age associated divergence) =0.0911
p(Scalp)=0.00243
p(Dura)=0.763
Scalp
Dura

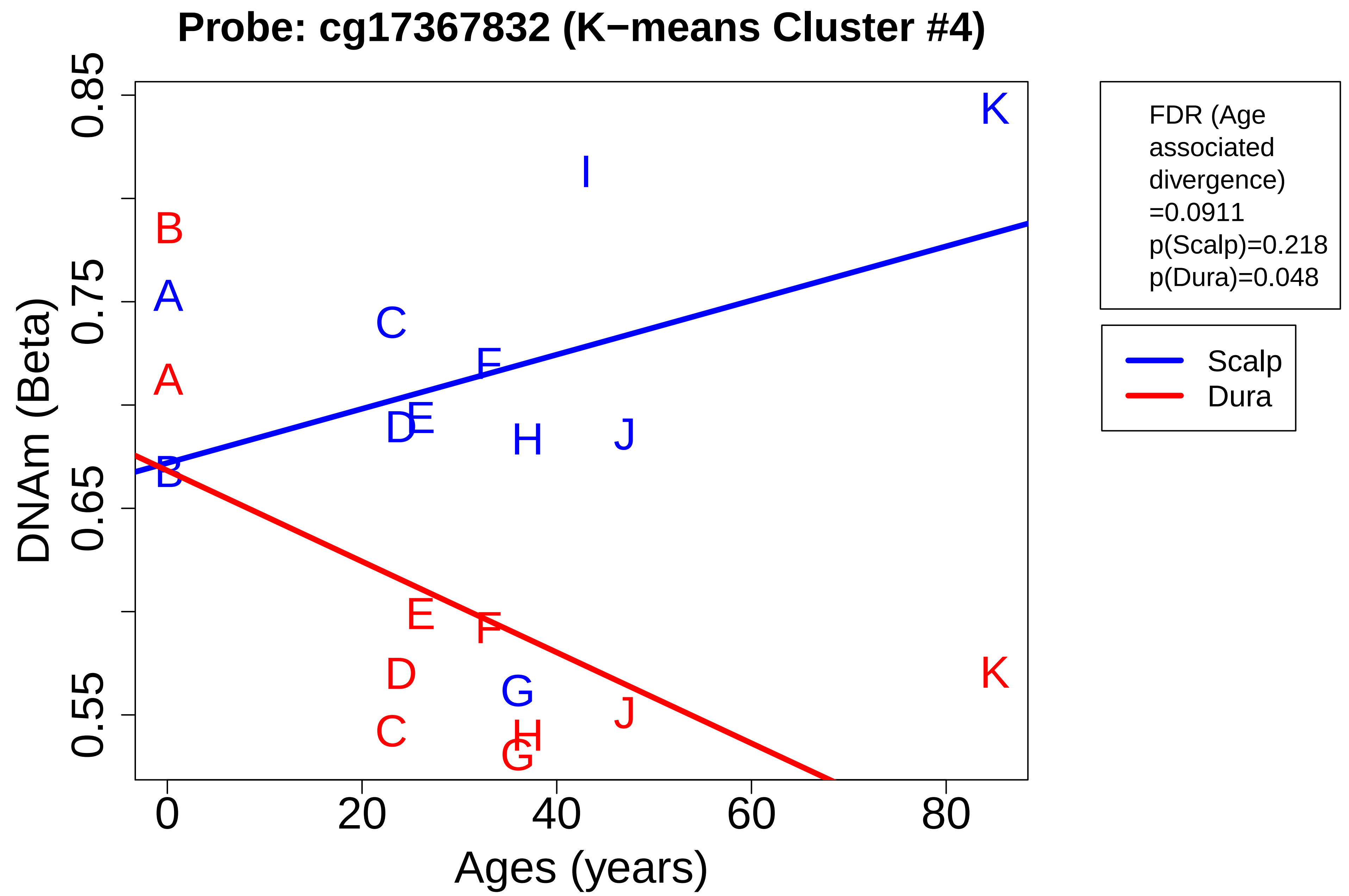

Probe: cg17367832 (K−means Cluster #4)
DNAm (Beta)
0.55
0.65
0.75
0.85
Ages (years)
0
20
40
60
80
FDR (Age associated divergence) =0.0911
p(Scalp)=0.218
p(Dura)=0.048
Scalp
Dura
A
B
C
D
E
F
G
H
I
J
K

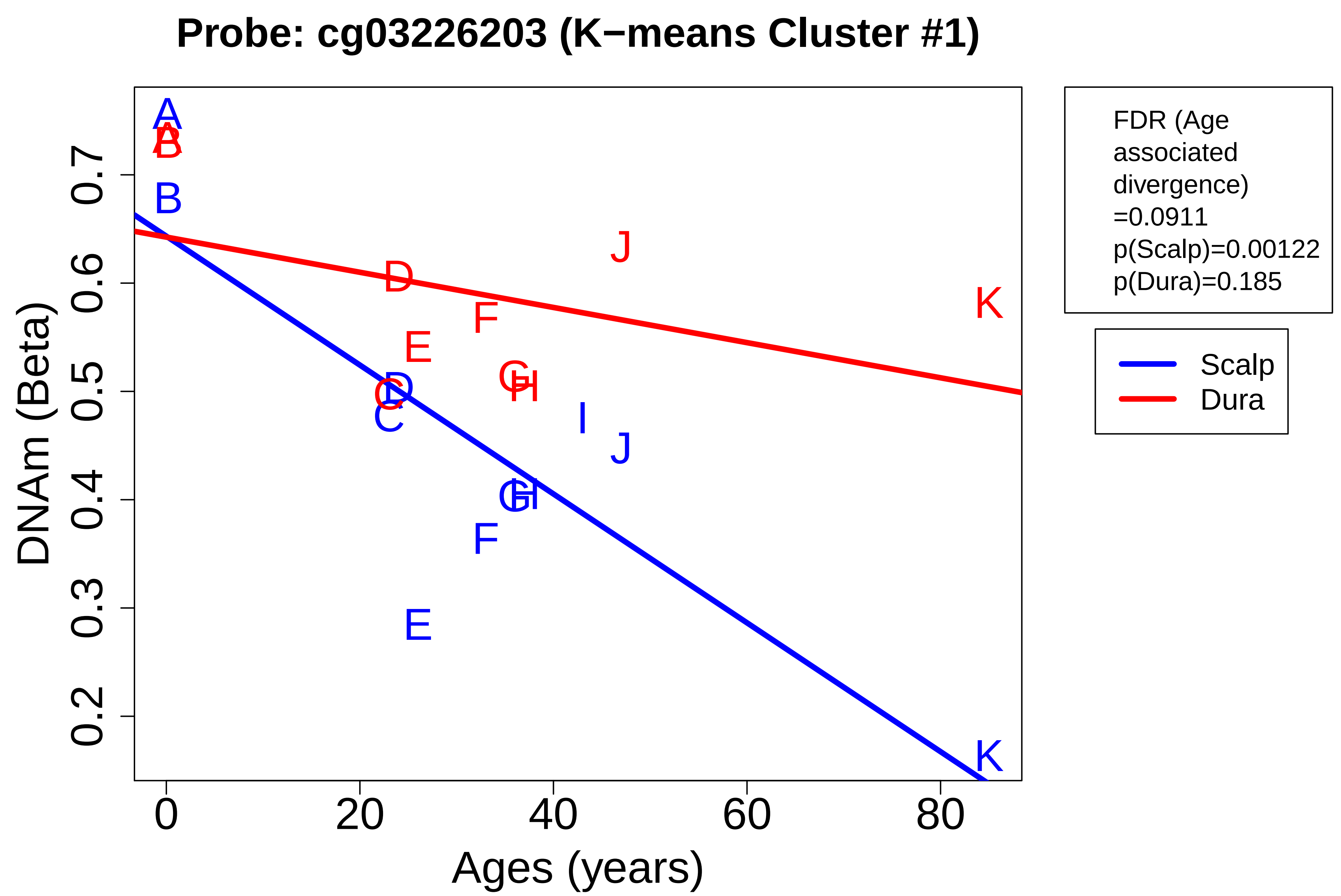

Probe: cg03226203 (K−means Cluster #1)
DNAm (Beta)
0.2
0.3
0.4
0.5
0.6
0.7
Ages (years)
0
20
40
60
80
FDR (Age associated divergence) =0.0911
p(Scalp)=0.00122
p(Dura)=0.185
Scalp
Dura

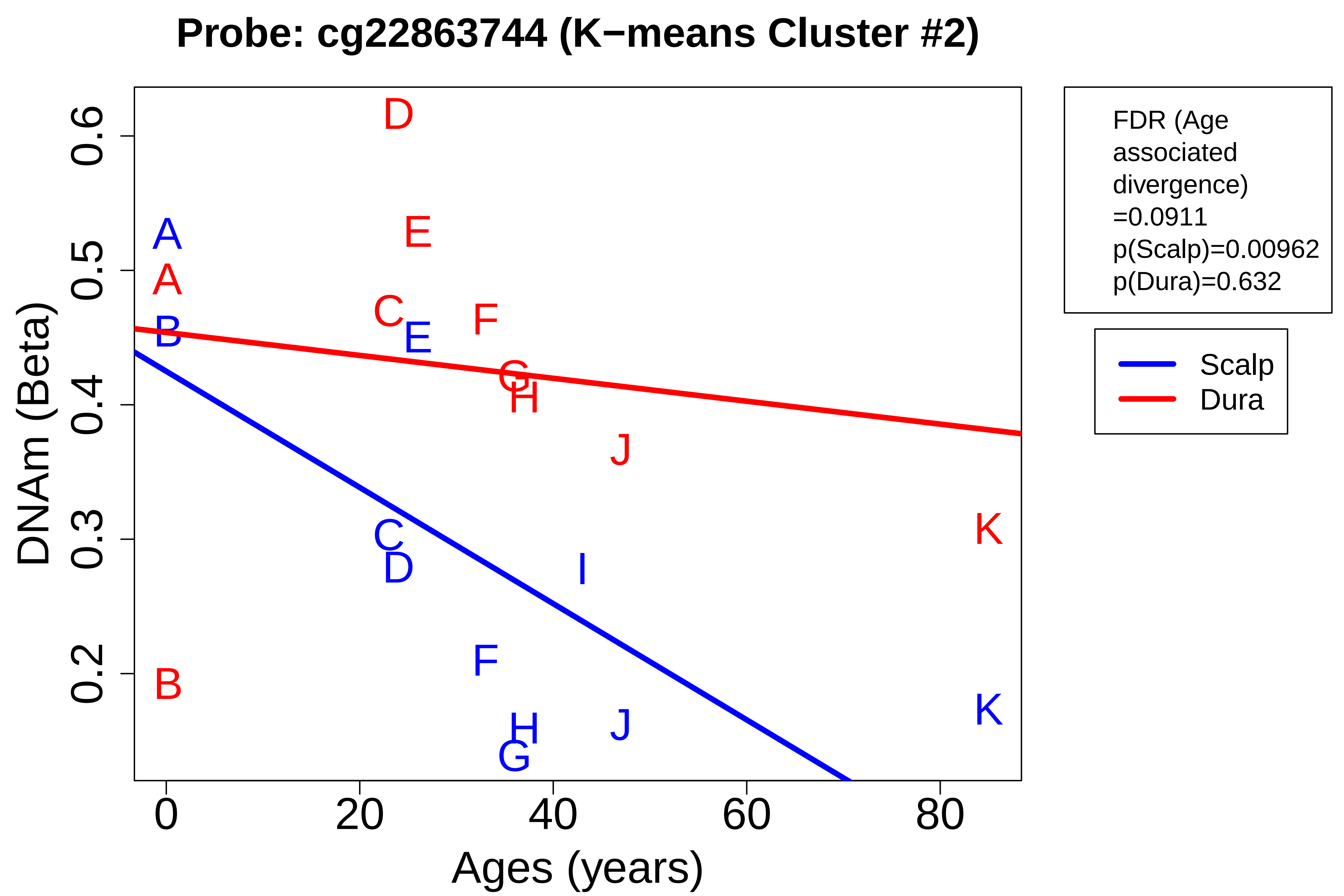

Probe: cg22863744 (K−means Cluster #2)
DNAm (Beta)
Ages (years)
0.2
0.3
0.4
0.5
0.6
0
20
40
60
80
A
B
C
D
E
F
G
H
I
J
K
FDR (Age associated divergence) =0.0911
p(Scalp)=0.00962
p(Dura)=0.632
Scalp
Dura

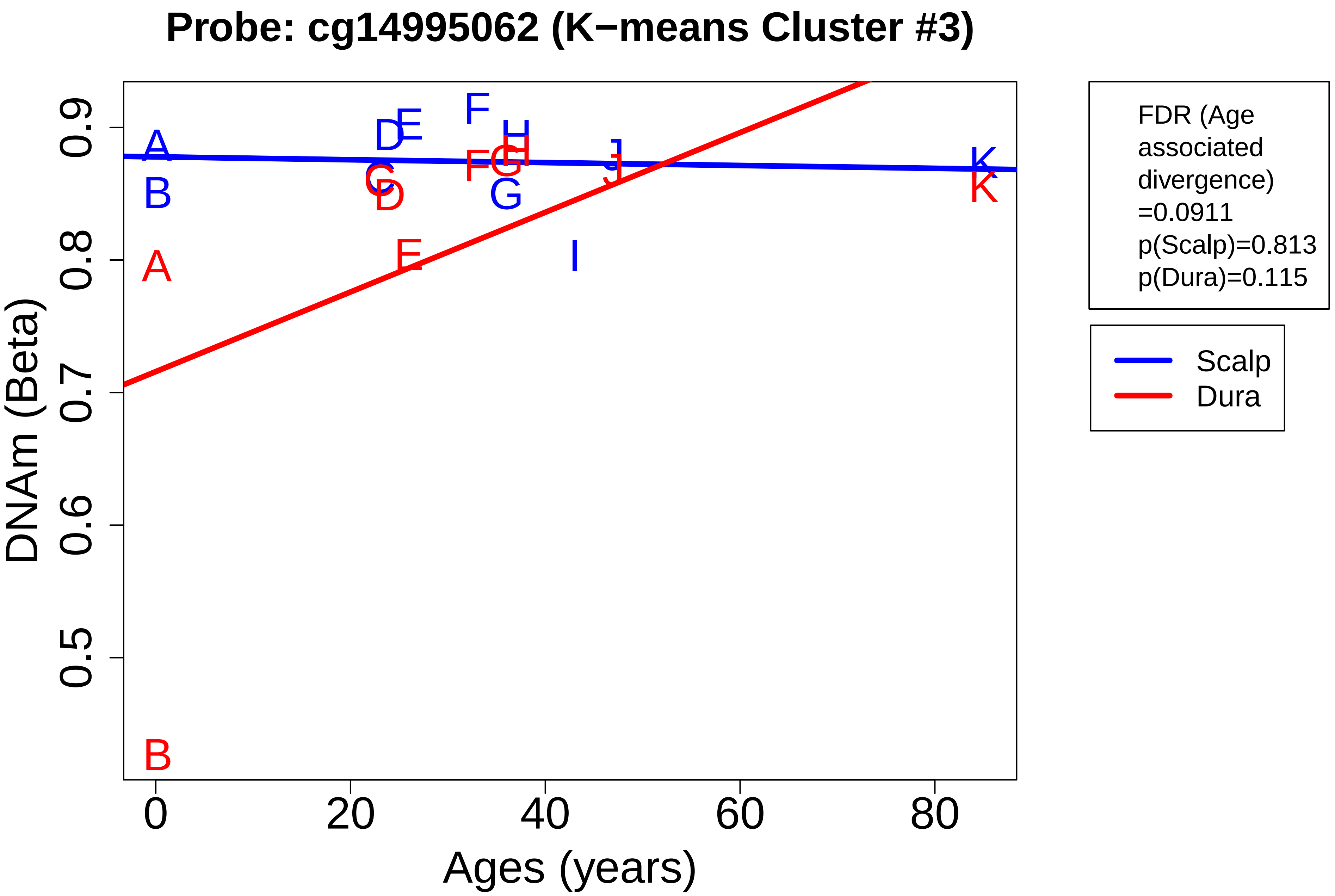

Probe: cg14995062 (K−means Cluster #3)
DNAm (Beta)
0.5
0.6
0.7
0.8
0.9
0
20
40
60
80
Ages (years)
FDR (Age associated divergence) =0.0911
p(Scalp)=0.813
p(Dura)=0.115
Scalp
Dura

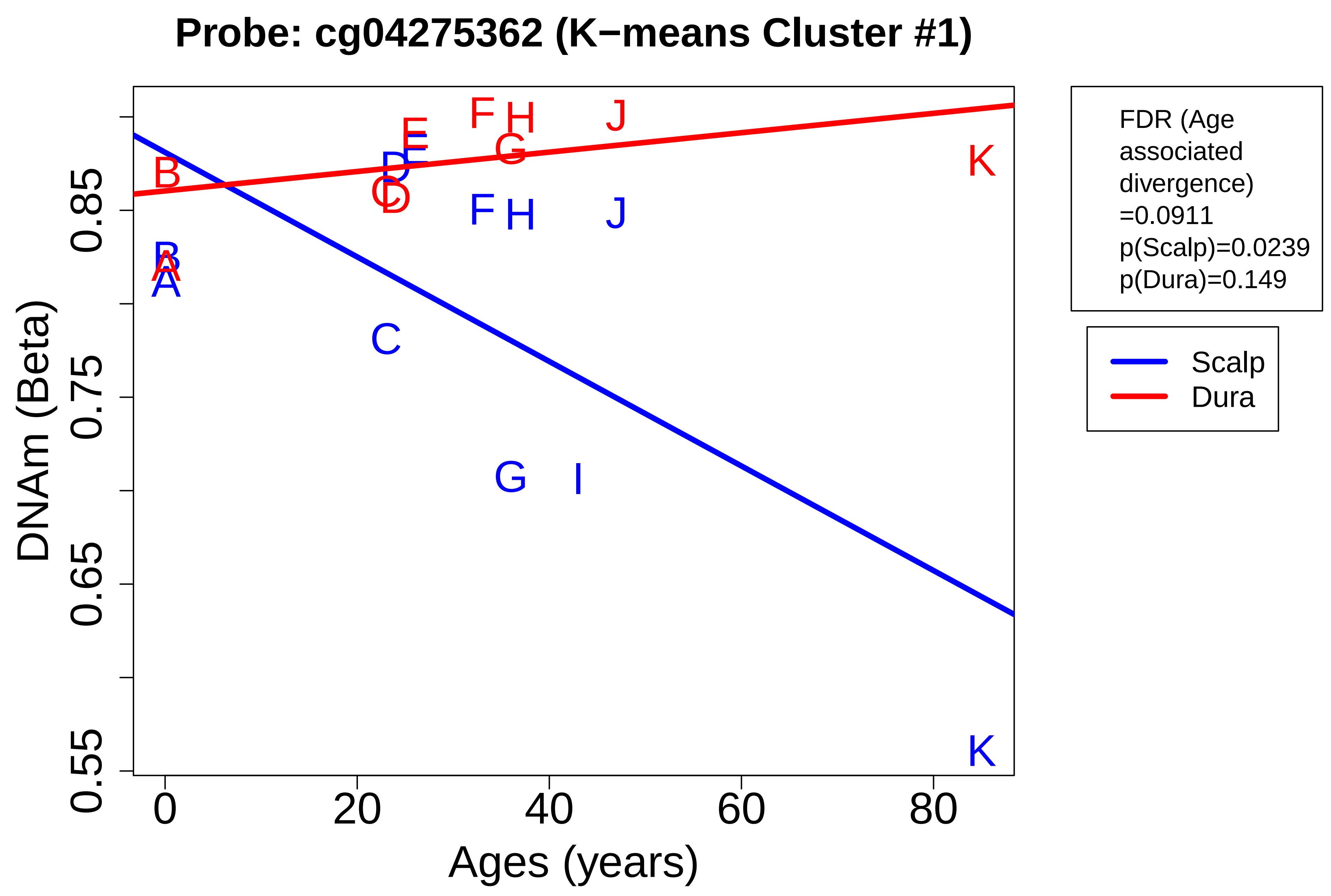

Probe: cg04275362 (K−means Cluster #1)
FDR (Age associated divergence) =0.0911
p(Scalp)=0.0239
p(Dura)=0.149
Scalp
Dura
DNAm (Beta)
0.55
0.65
0.75
0.85
Ages (years)
0
20
40
60
80
A
B
C
D
E
F
G
H
I
J
K

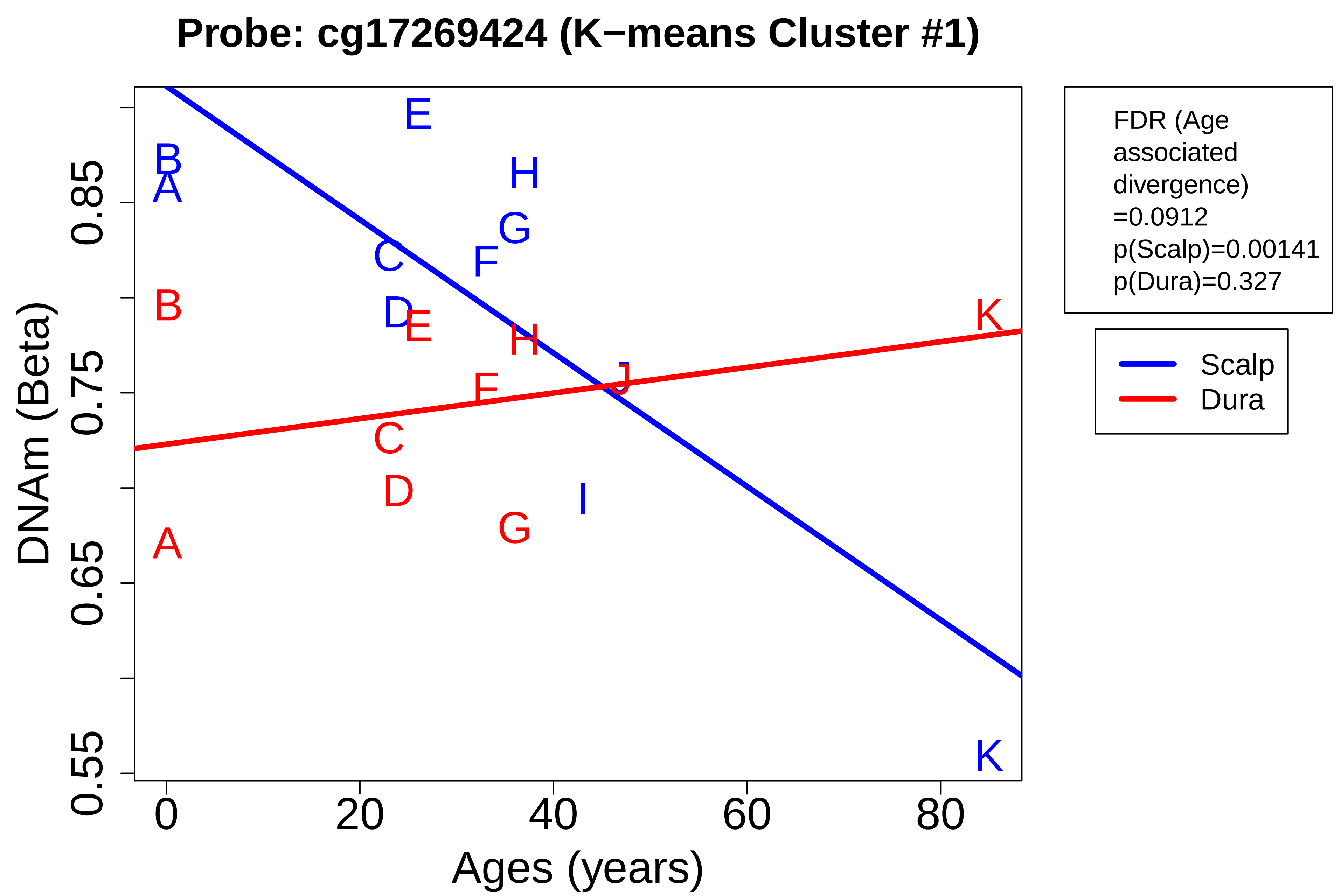

Probe: cg17269424 (K−means Cluster #1)
DNAm (Beta)
0.55
0.65
0.75
0.85
Ages (years)
0
20
40
60
80
B
A
E
H
G
C
F
D
B
E
H
J
F
C
D
G
I
A
K
K
FDR (Age associated divergence) =0.0912
p(Scalp)=0.00141
p(Dura)=0.327
Scalp
Dura

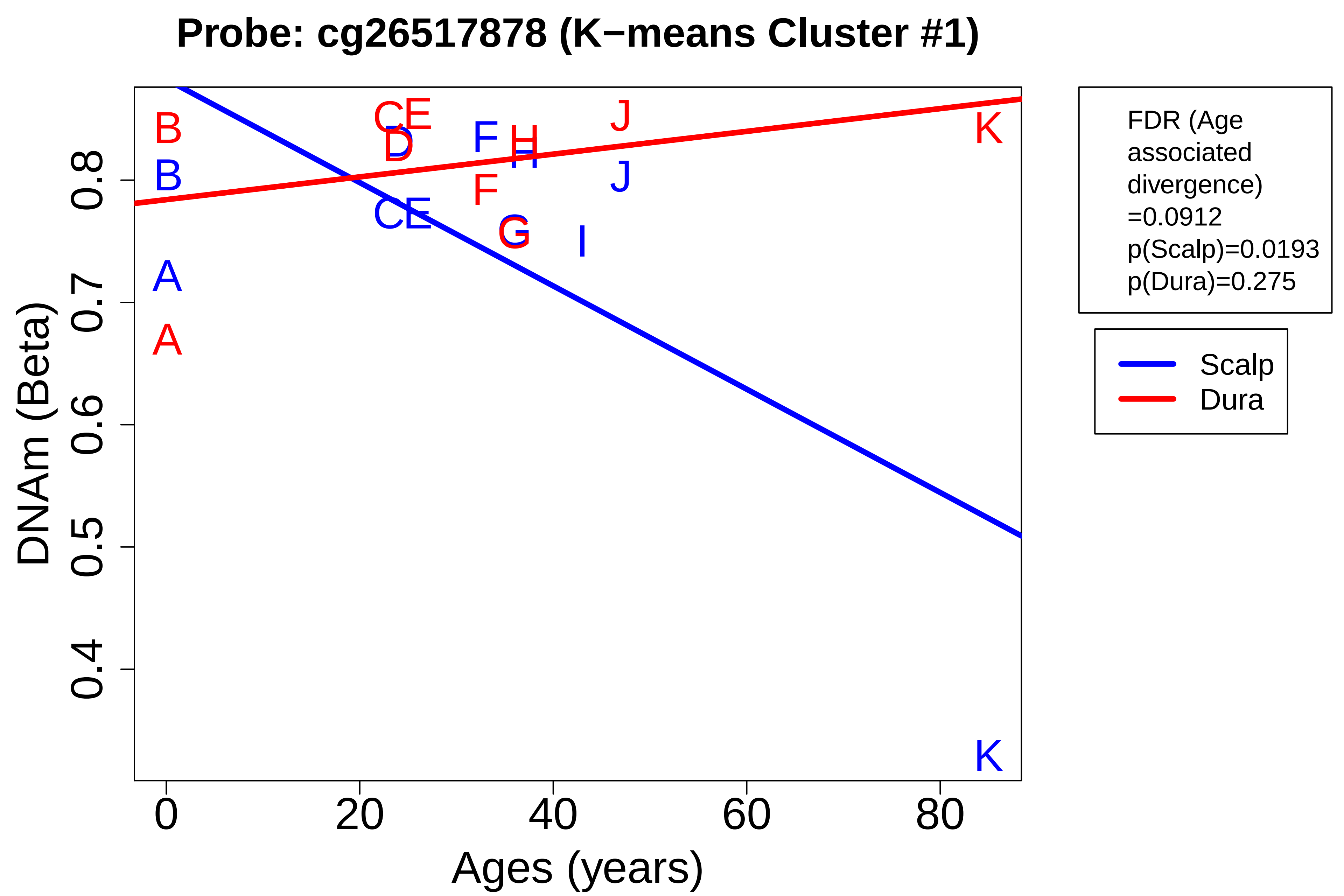

Probe: cg26517878 (K−means Cluster #1)
DNAm (Beta)
0.4
0.5
0.6
0.7
0.8
0
20
40
60
80
Ages (years)
A
B
C
D
E
F
G
H
I
J
K
FDR (Age associated divergence) =0.0912
p(Scalp)=0.0193
p(Dura)=0.275
Scalp
Dura

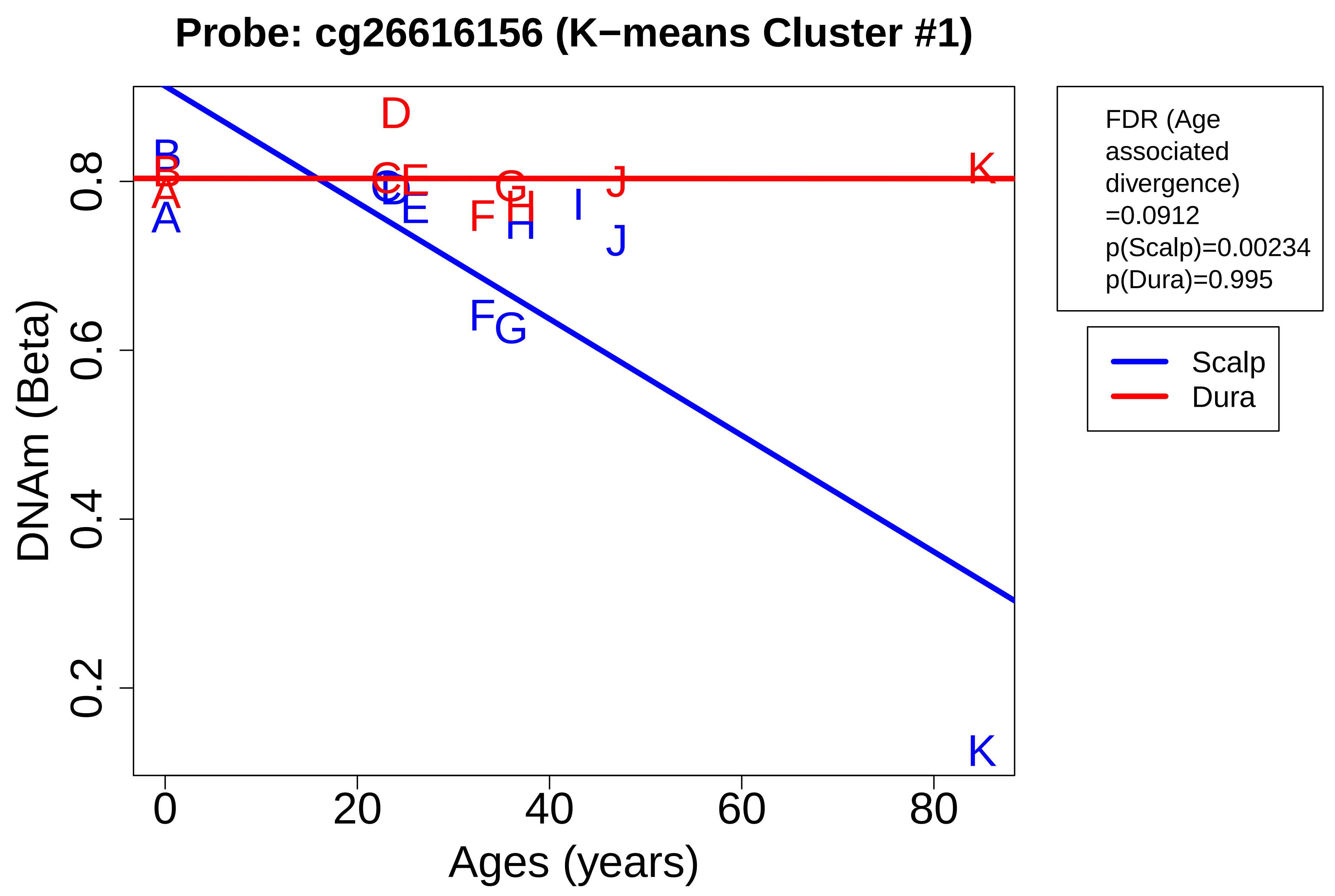
Probe: cg26616156 (K−means Cluster #1)
DNAm (Beta)
0.2
0.4
0.6
0.8
Ages (years)
0
20
40
60
80
FDR (Age associated divergence) =0.0912
p(Scalp)=0.00234
p(Dura)=0.995
Scalp
Dura

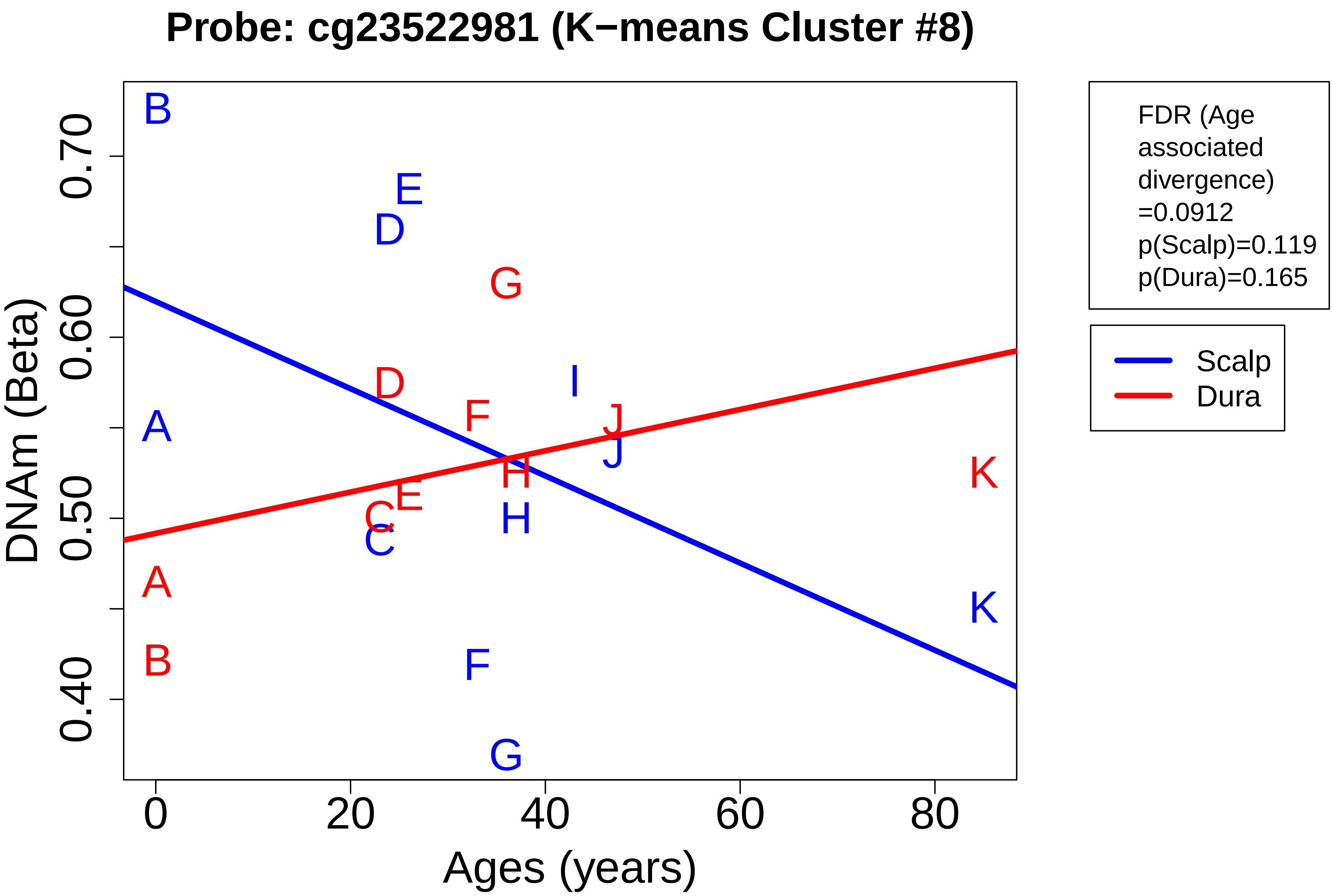

Probe: cg23522981 (K−means Cluster #8)
FDR (Age associated divergence) =0.0912
p(Scalp)=0.119
p(Dura)=0.165
Scalp
Dura
DNAm (Beta)
0.40
0.50
0.60
0.70
Ages (years)
0
20
40
60
80
A B C D E F G H I J K

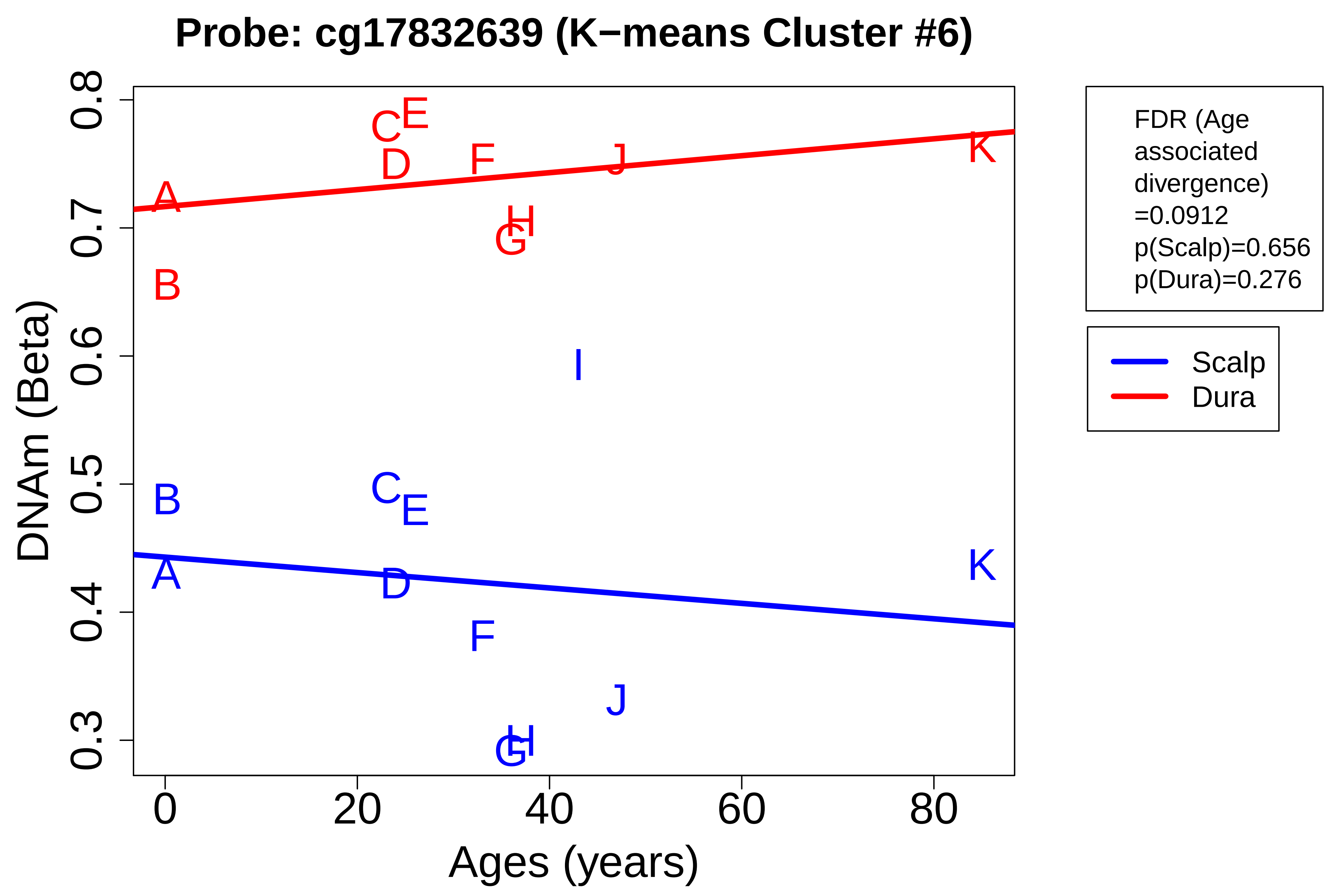

Probe: cg17832639 (K−means Cluster #6)
DNAm (Beta)
Ages (years)
0.3
0.4
0.5
0.6
0.7
0.8
0
20
40
60
80
FDR (Age associated divergence) =0.0912
p(Scalp)=0.656
p(Dura)=0.276
Scalp
Dura
A
B
C
D
E
F
G
H
I
J
K

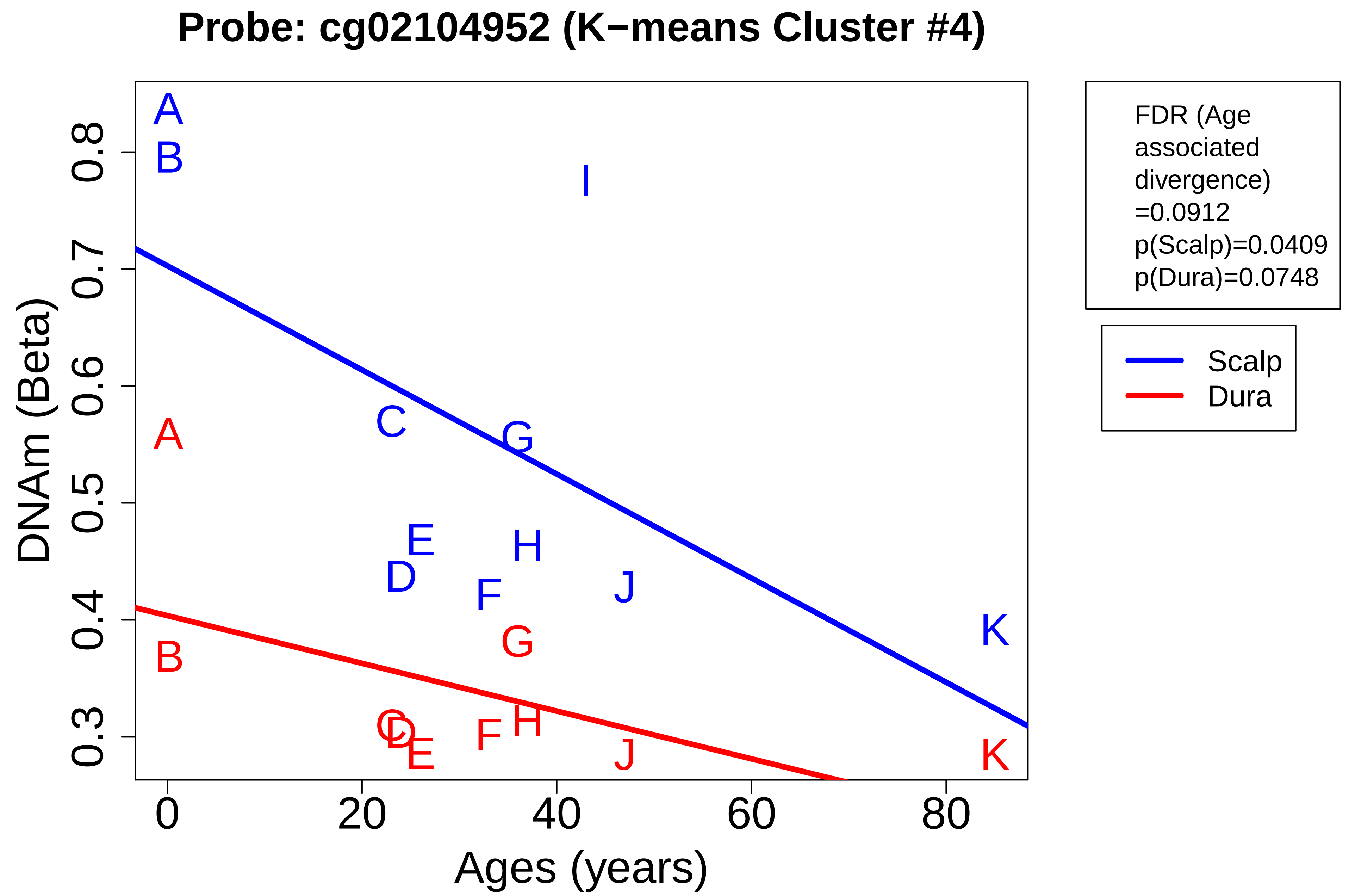

Probe: cg02104952 (K−means Cluster #4)
DNAm (Beta)
0.3
0.4
0.5
0.6
0.7
0.8
0
20
40
60
80
Ages (years)
A
B
I
C
G
E
H
D
F
J
K
A
B
G
C
D
E
F
H
J
K
FDR (Age associated divergence) =0.0912
p(Scalp)=0.0409
p(Dura)=0.0748
Scalp
Dura

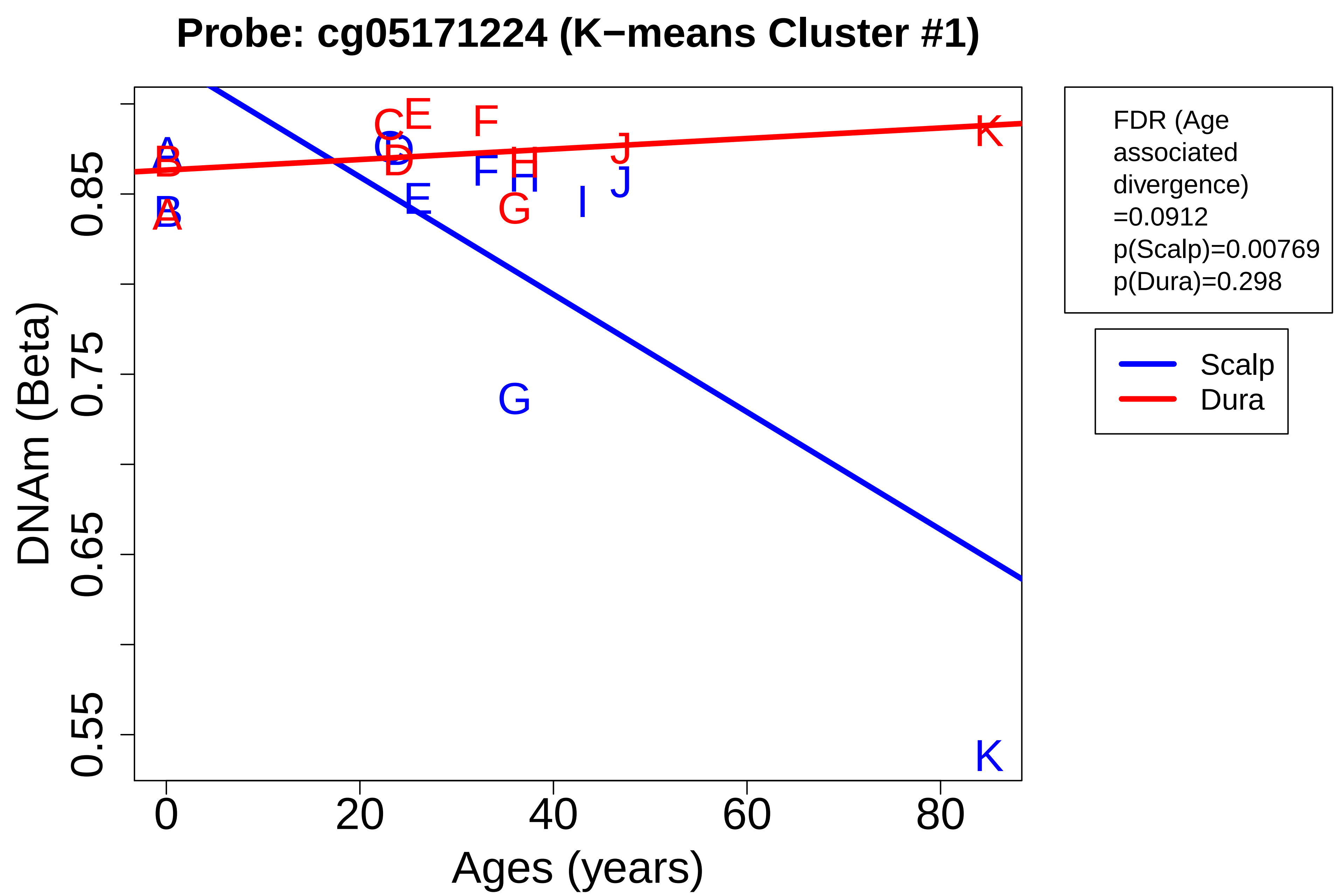

Probe: cg05171224 (K−means Cluster #1)
DNAm (Beta)
0.55
0.65
0.75
0.85
0
20
40
60
80
Ages (years)
A
B
C
D
E
F
G
H
I
J
K
FDR (Age associated divergence) =0.0912
p(Scalp)=0.00769
p(Dura)=0.298
Scalp
Dura

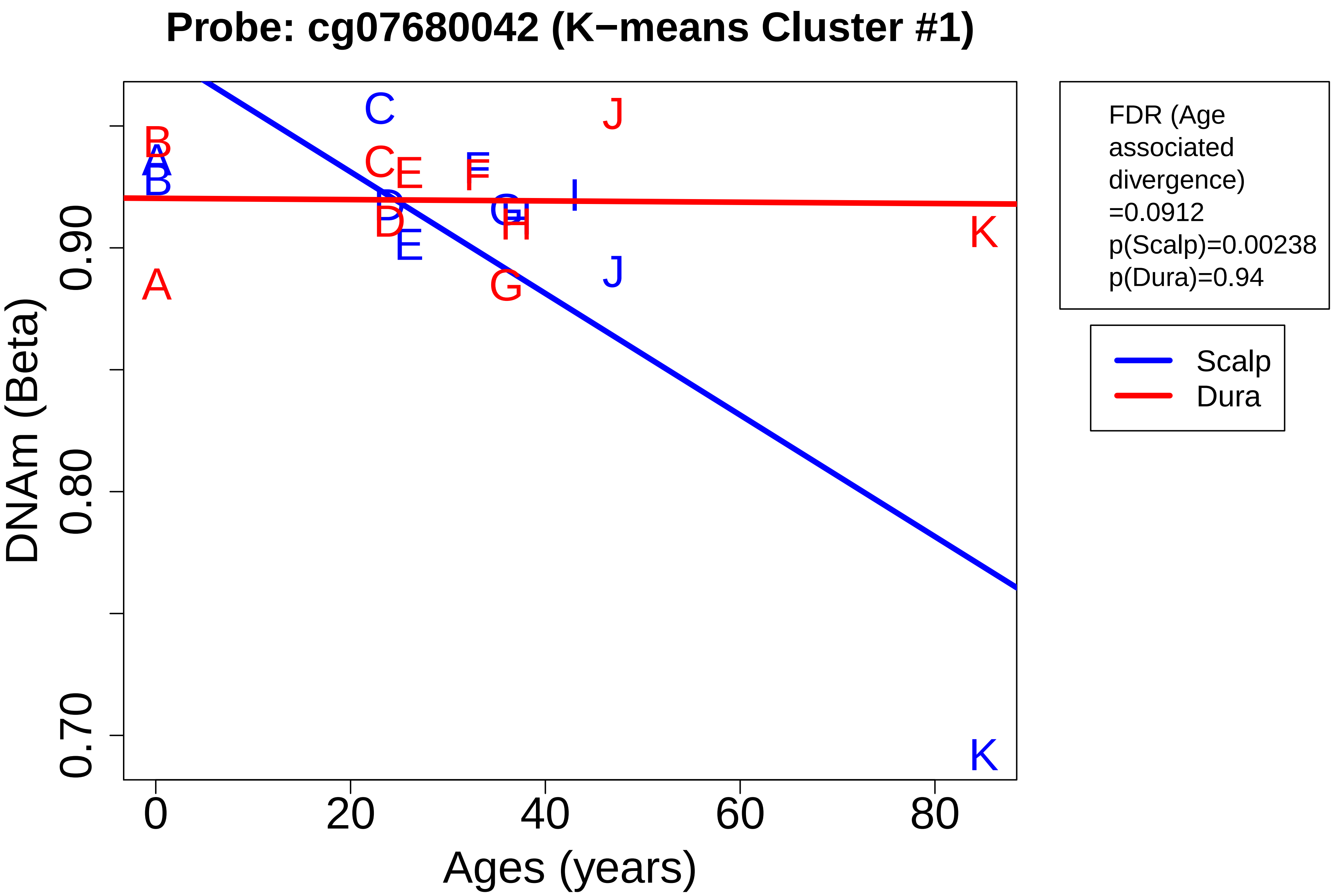

Probe: cg07680042 (K−means Cluster #1)
FDR (Age associated divergence) =0.0912
p(Scalp)=0.00238
p(Dura)=0.94
Scalp
Dura
DNAm (Beta)
0.70
0.80
0.90
0
20
40
60
80
Ages (years)

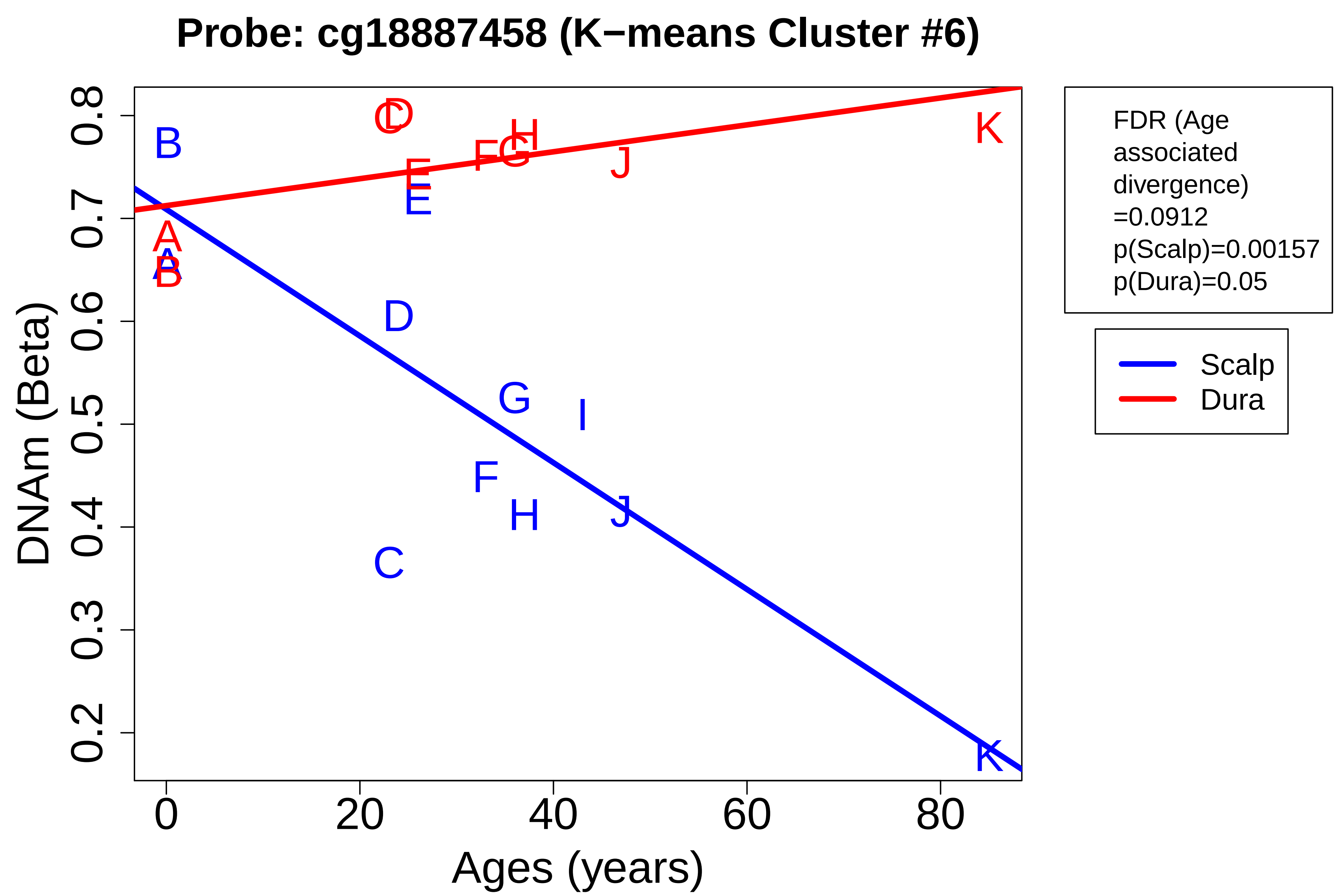
Probe: cg18887458 (K−means Cluster #6)
DNAm (Beta)
Ages (years)
0.2
0.3
0.4
0.5
0.6
0.7
0.8
0
20
40
60
80
FDR (Age associated divergence) =0.0912
p(Scalp)=0.00157
p(Dura)=0.05
Scalp
Dura

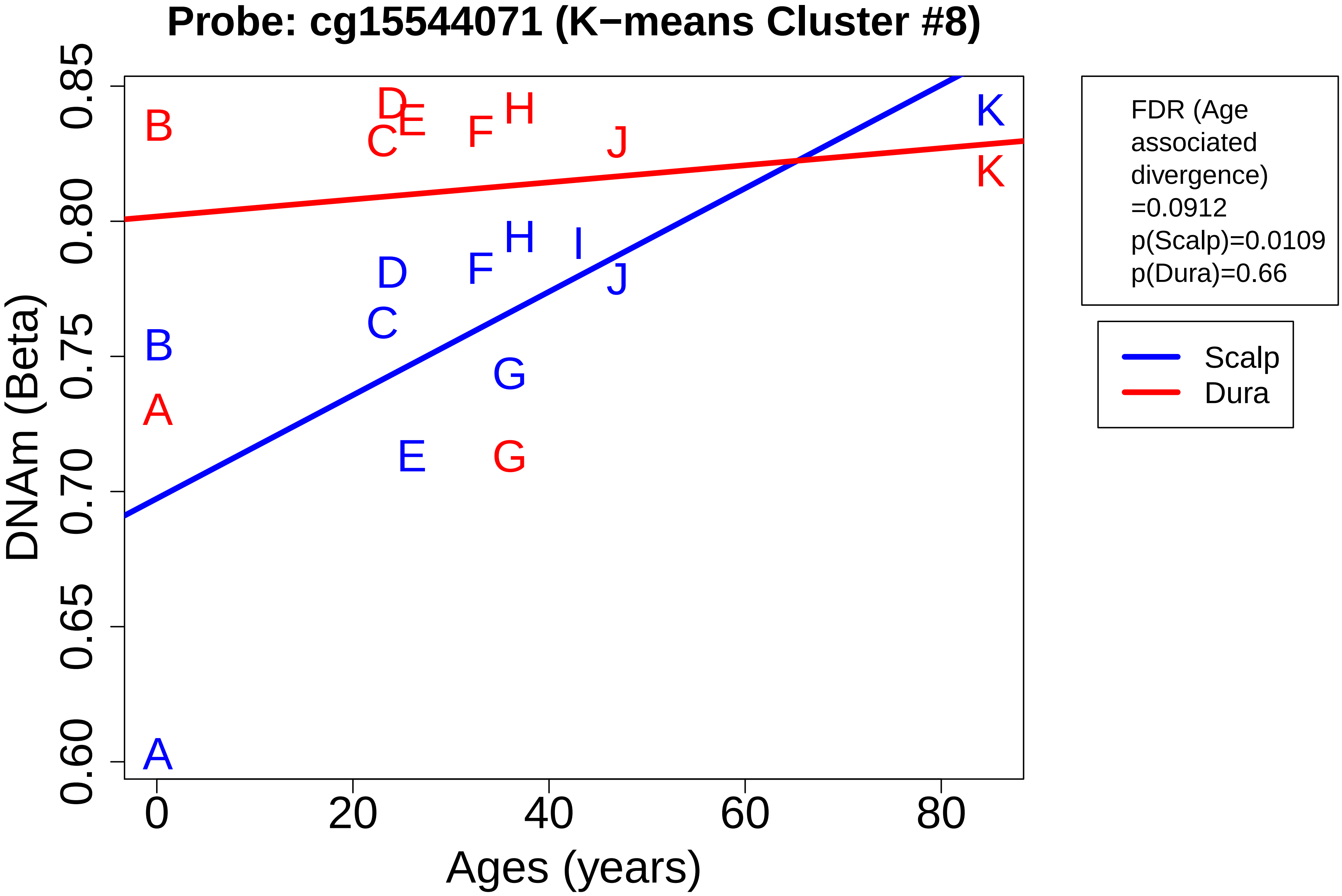

Probe: cg15544071 (K−means Cluster #8)
DNAm (Beta)
0.60
0.65
0.70
0.75
0.80
0.85
0
20
40
60
80
Ages (years)
FDR (Age associated divergence) =0.0912
p(Scalp)=0.0109
p(Dura)=0.66
Scalp
Dura

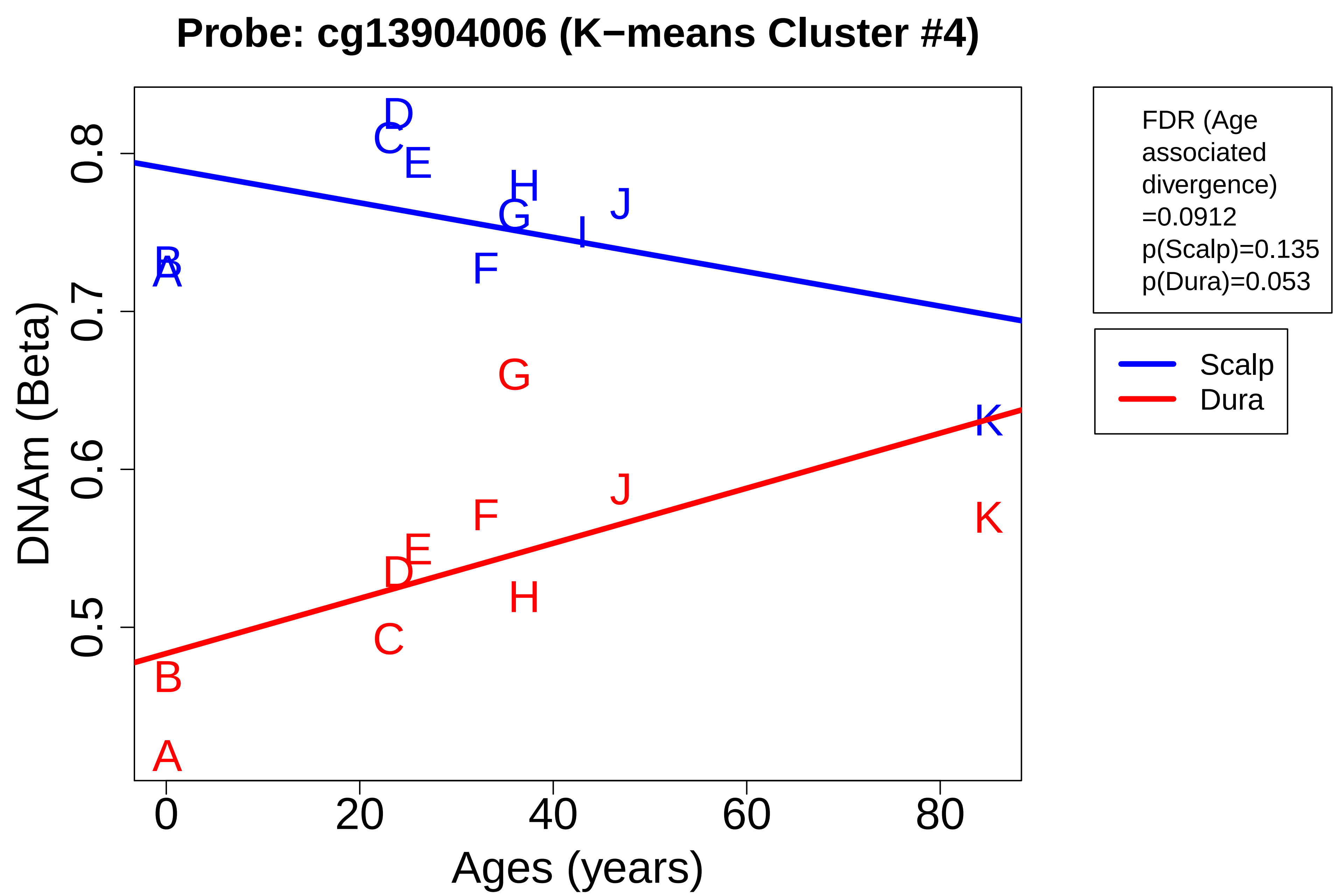

Probe: cg13904006 (K−means Cluster #4)
FDR (Age associated divergence) =0.0912
p(Scalp)=0.135
p(Dura)=0.053
Scalp
Dura
DNAm (Beta)
0.5
0.6
0.7
0.8
Ages (years)
0
20
40
60
80
A
B
C
D
E
F
G
H
I
J
K

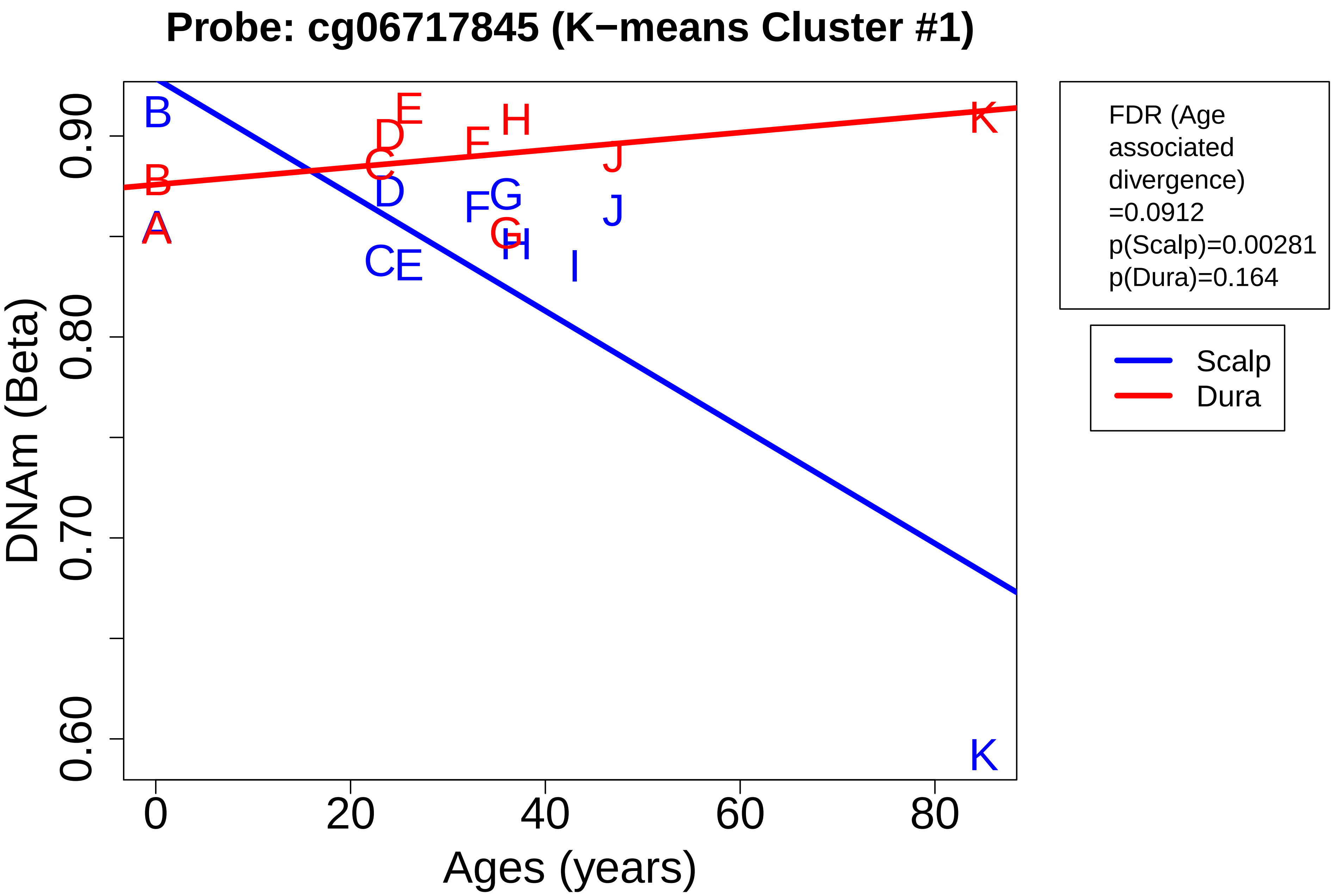

Probe: cg06717845 (K−means Cluster #1)
DNAm (Beta)
0.60
0.70
0.80
0.90
Ages (years)
0
20
40
60
80
A
B
C
D
E
F
G
H
I
J
K
FDR (Age associated divergence) =0.0912
p(Scalp)=0.00281
p(Dura)=0.164
Scalp
Dura

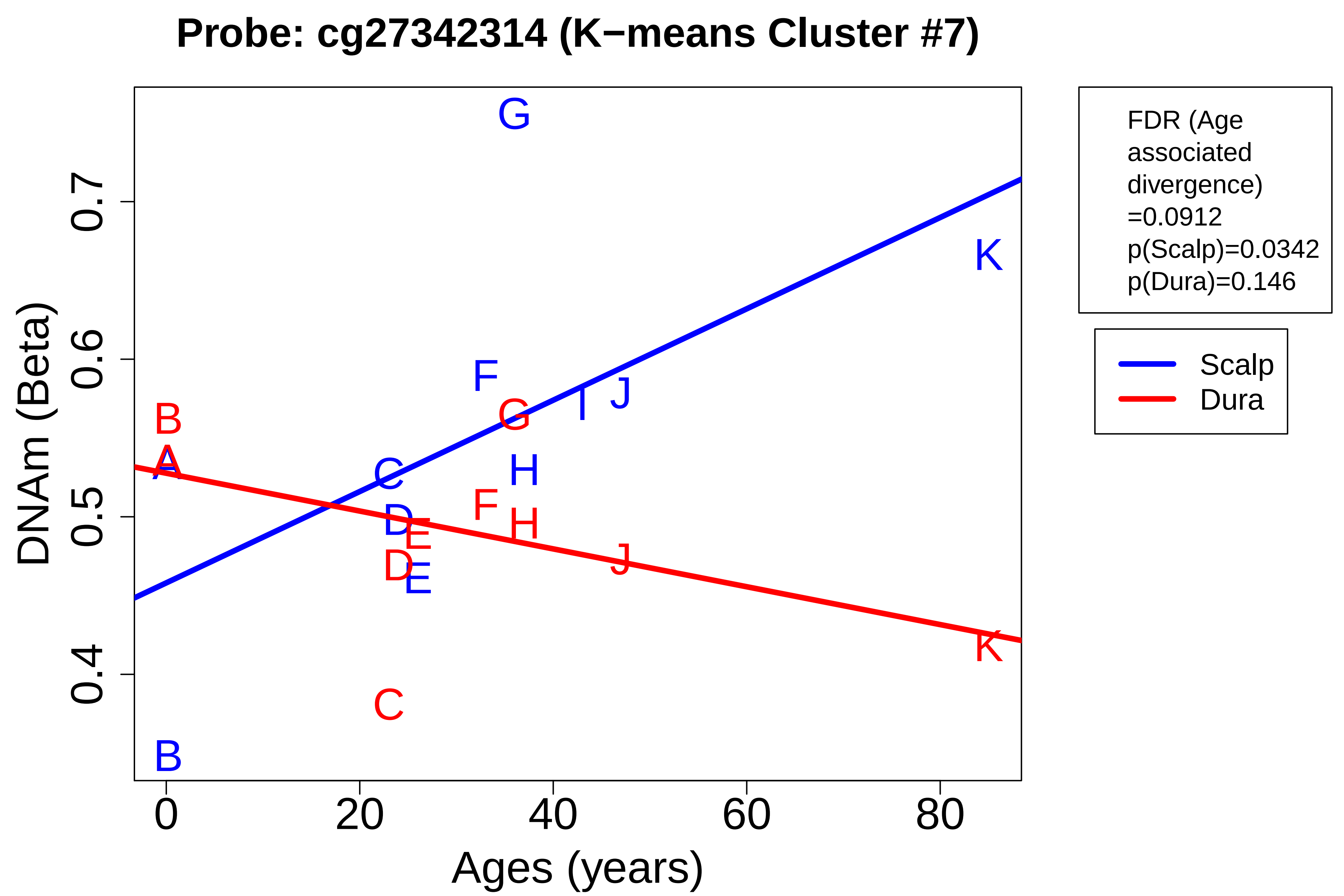
Probe: cg27342314 (K−means Cluster #7)
DNAm (Beta)
0.4
0.5
0.6
0.7
0
20
40
60
80
Ages (years)
FDR (Age associated divergence) =0.0912
p(Scalp)=0.0342
p(Dura)=0.146
Scalp
Dura
A
B
C
D
E
F
G
H
I
J
K

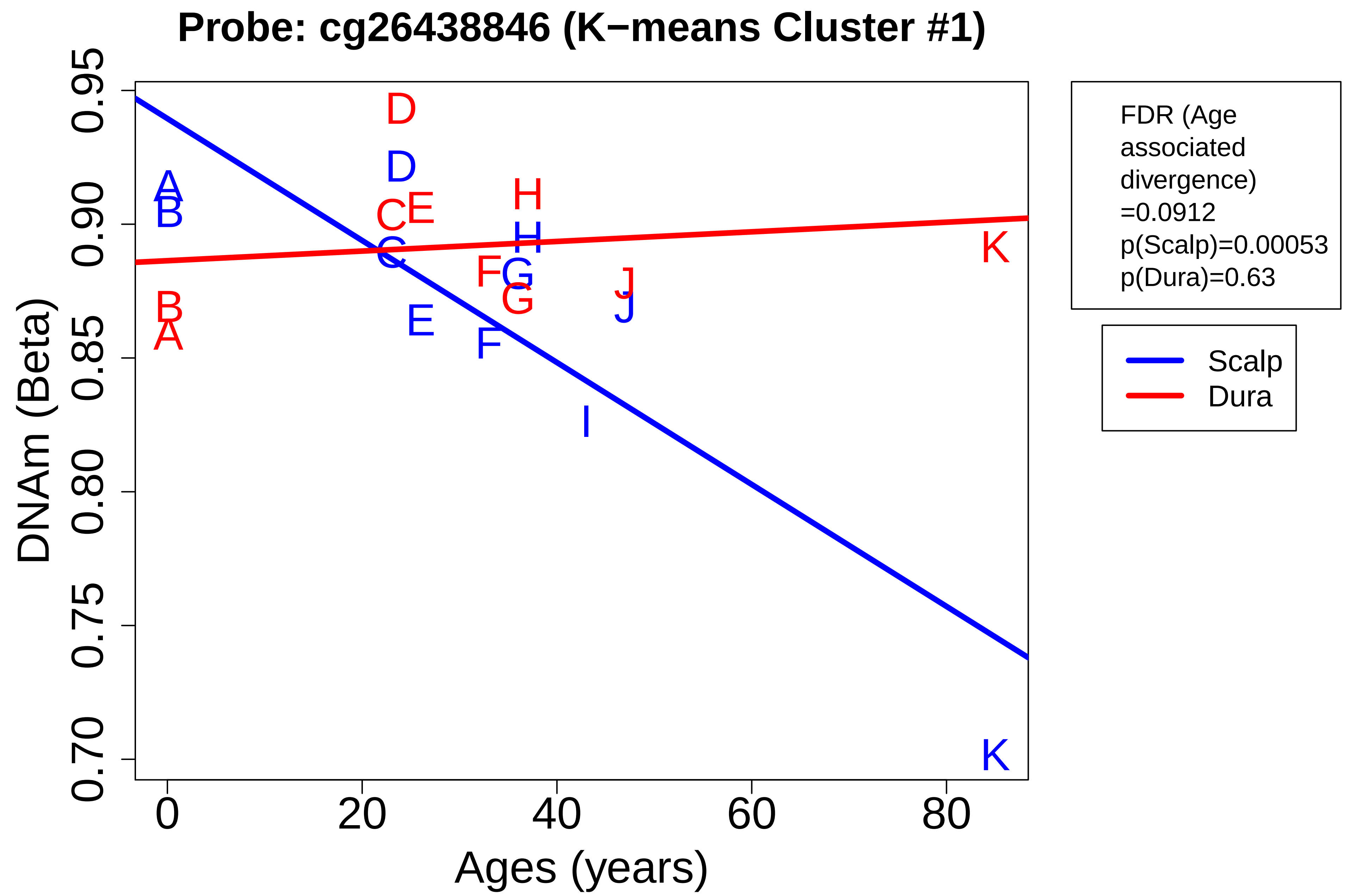

Probe: cg26438846 (K−means Cluster #1)
DNAm (Beta)
0.70
0.75
0.80
0.85
0.90
0.95
0
20
40
60
80
Ages (years)
A
B
C
D
E
F
G
H
I
J
K
FDR (Age associated divergence) =0.0912
p(Scalp)=0.00053
p(Dura)=0.63
Scalp
Dura

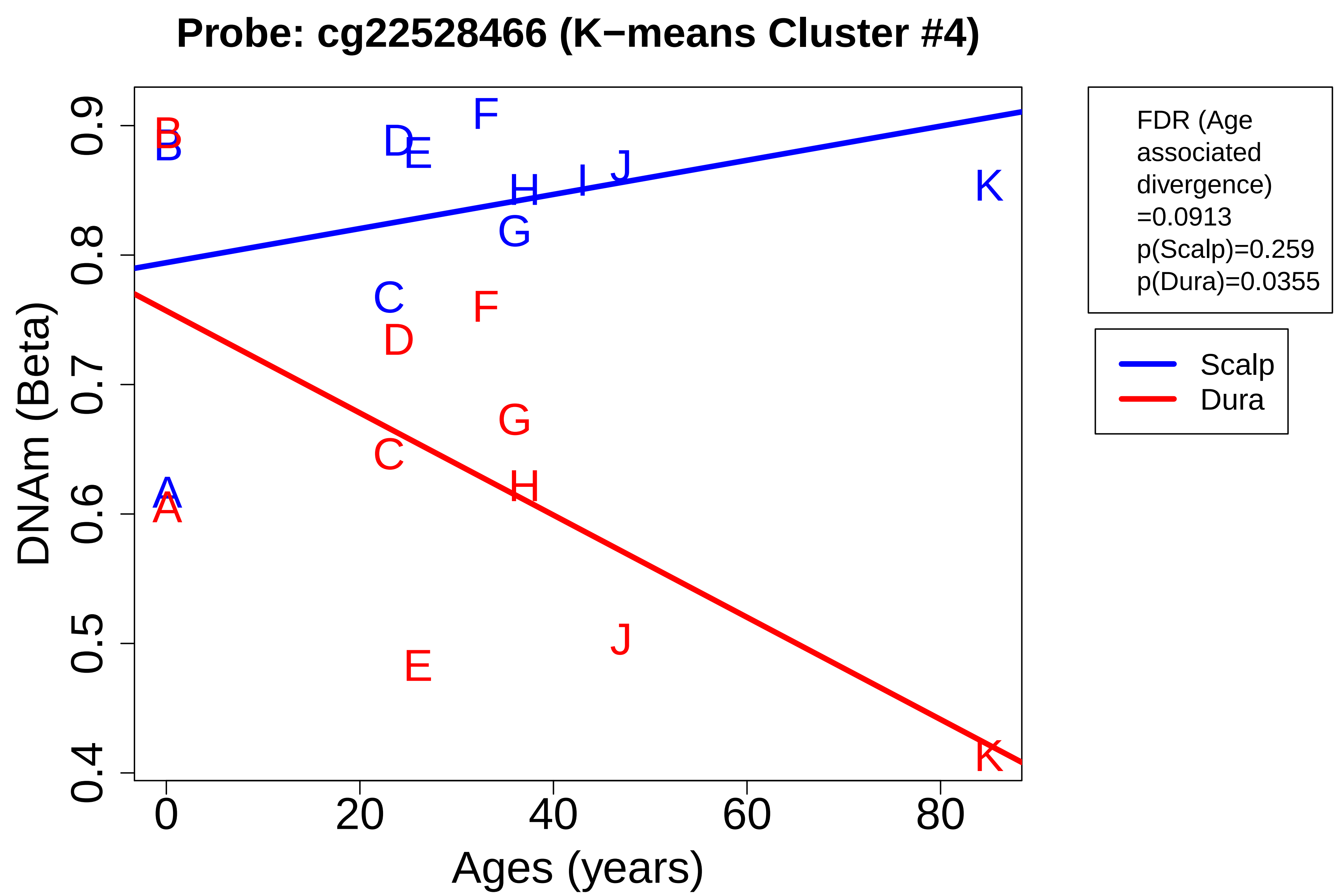
Probe: cg22528466 (K−means Cluster #4)
DNAm (Beta)
Ages (years)
0.4
0.5
0.6
0.7
0.8
0.9
0
20
40
60
80
FDR (Age associated divergence) =0.0913
p(Scalp)=0.259
p(Dura)=0.0355
Scalp
Dura
A
B
C
D
E
F
G
H
I
J
K

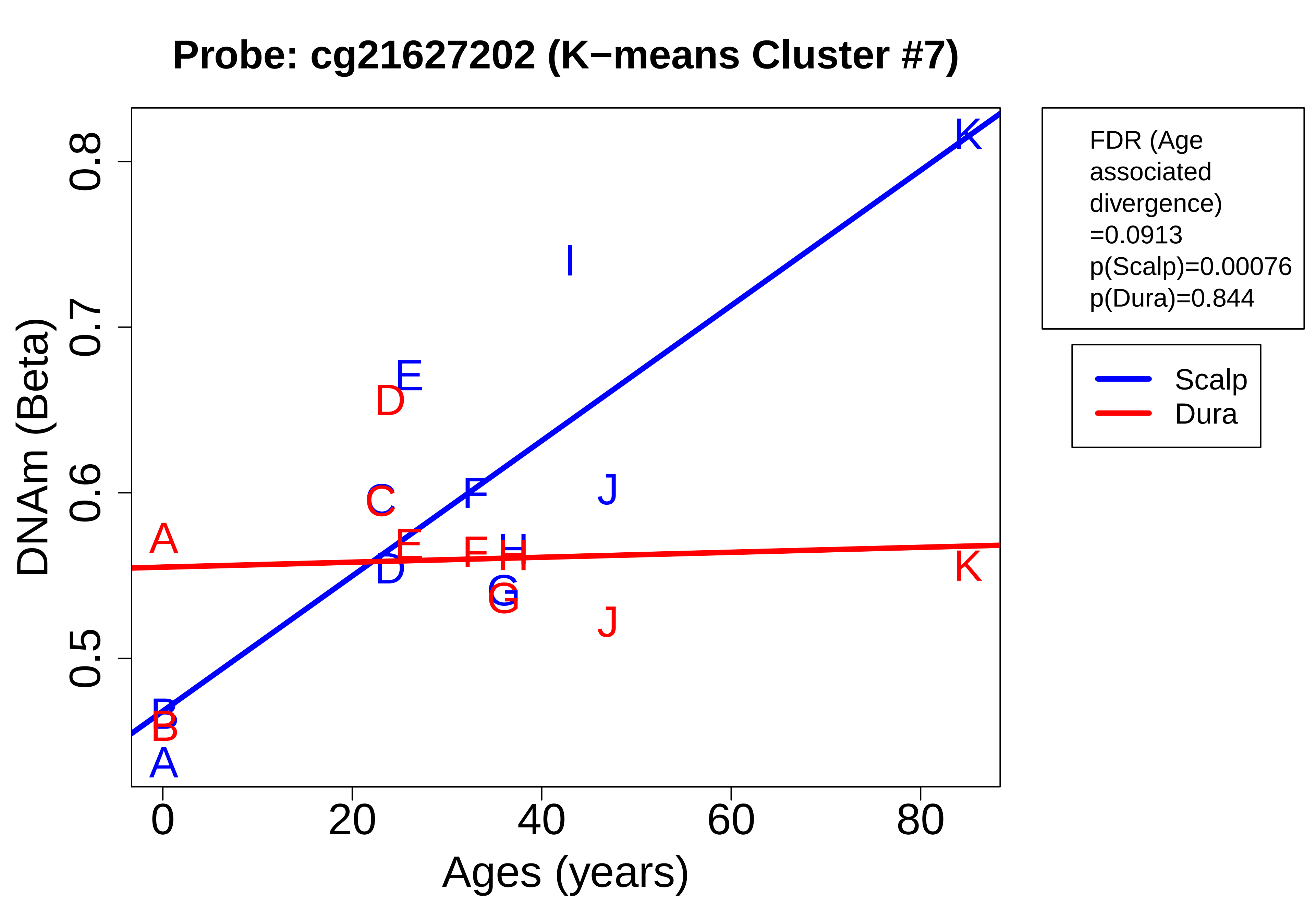
Probe: cg21627202 (K−means Cluster #7)
DNAm (Beta)
Ages (years)
0.5
0.6
0.7
0.8
0
20
40
60
80
FDR (Age associated divergence) =0.0913
p(Scalp)=0.00076
p(Dura)=0.844
Scalp
Dura
A
B
C
D
E
F
G
H
I
J
K

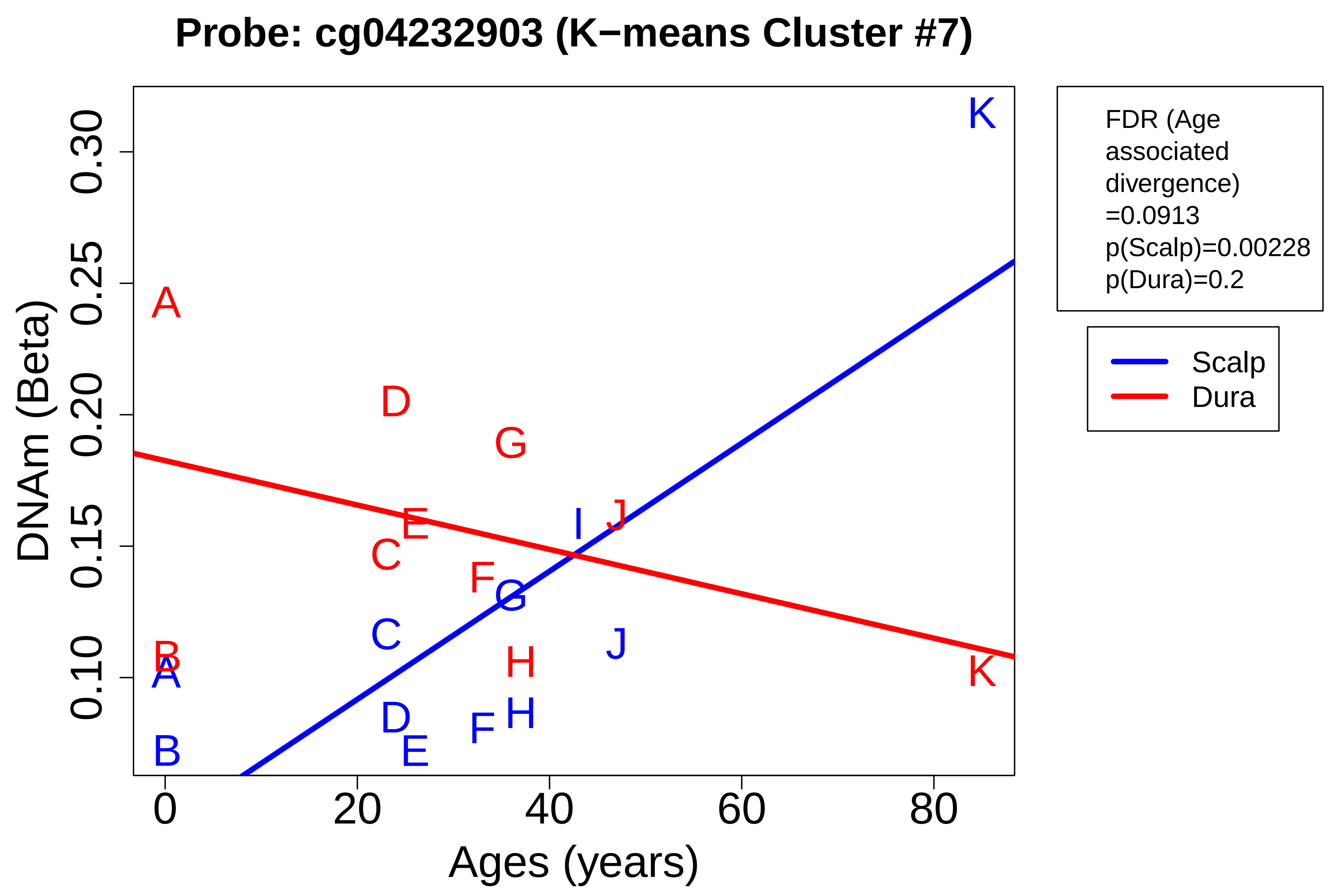

Probe: cg04232903 (K−means Cluster #7)
DNAm (Beta)
0.10
0.15
0.20
0.25
0.30
Ages (years)
0
20
40
60
80
A
B
C
D
E
F
G
H
I
J
K
FDR (Age associated divergence) =0.0913
p(Scalp)=0.00228
p(Dura)=0.2
Scalp
Dura

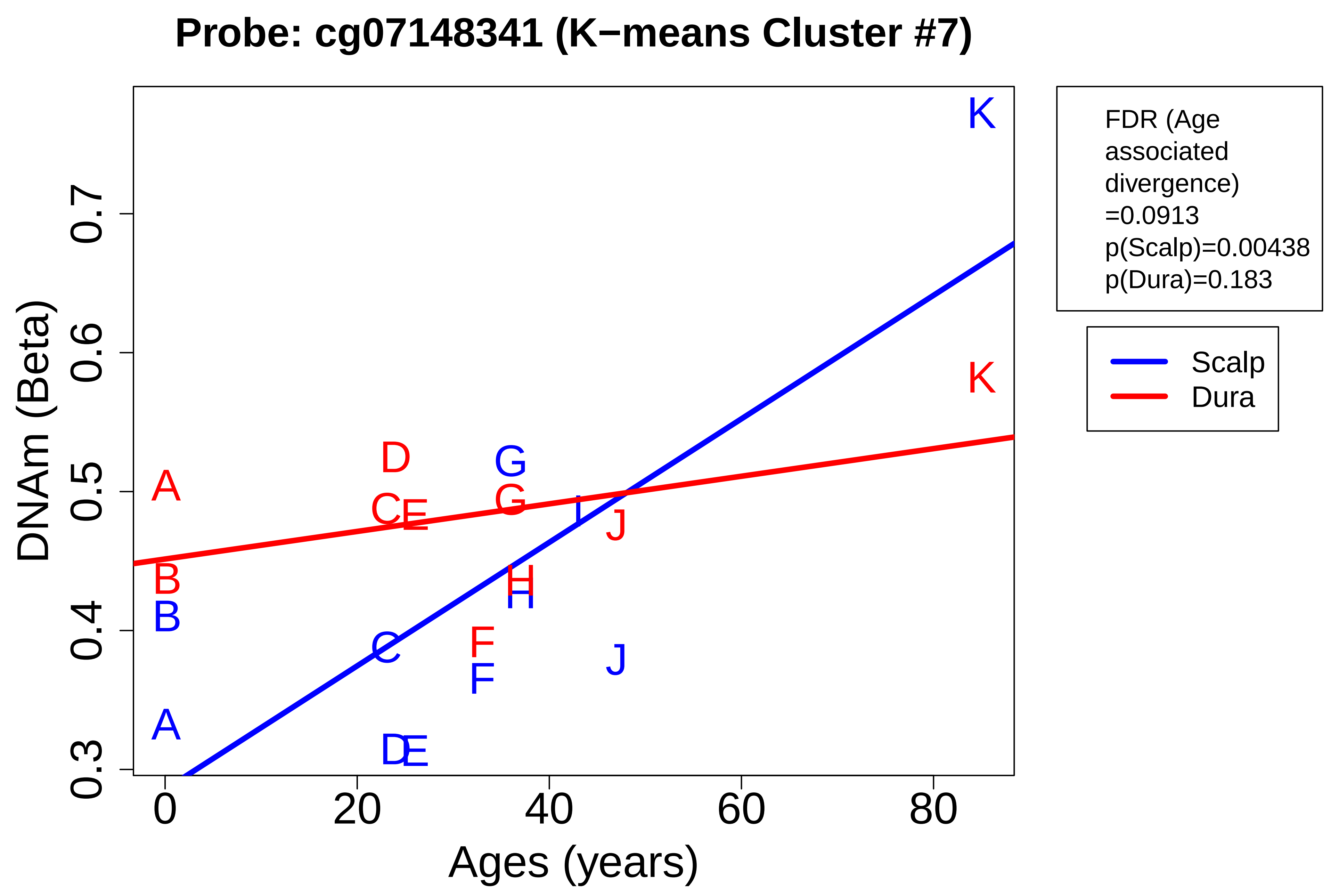

Probe: cg07148341 (K−means Cluster #7)
FDR (Age associated divergence) =0.0913
p(Scalp)=0.00438
p(Dura)=0.183
Scalp
Dura
DNAm (Beta)
0.3
0.4
0.5
0.6
0.7
Ages (years)
0
20
40
60
80
A
B
C
D
E
F
G
H
I
J
K

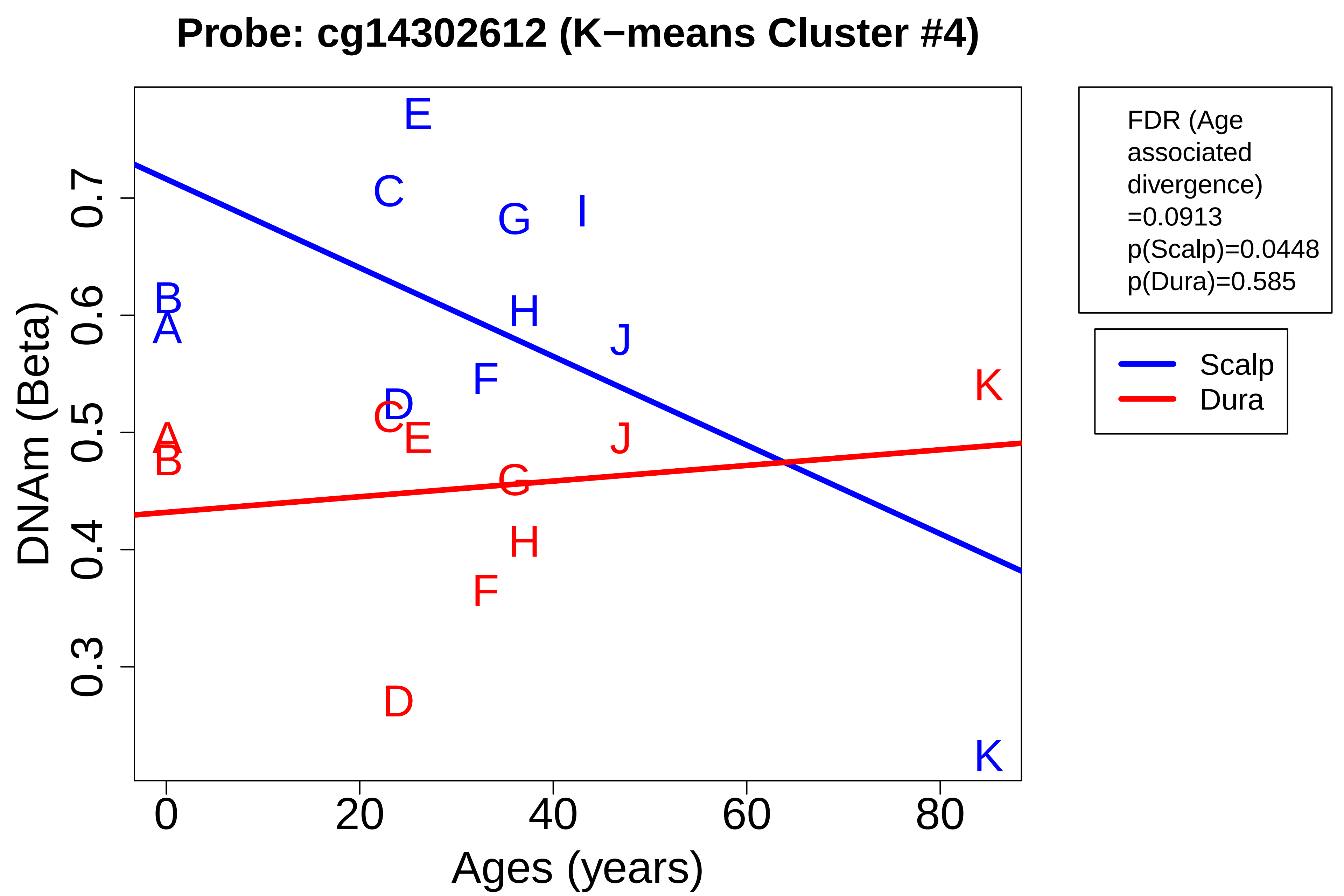

Probe: cg14302612 (K−means Cluster #4)
FDR (Age associated divergence) =0.0913
p(Scalp)=0.0448
p(Dura)=0.585
Scalp
Dura
DNAm (Beta)
0.3
0.4
0.5
0.6
0.7
0
20
40
60
80
Ages (years)
A
B
C
D
E
F
G
H
I
J
K

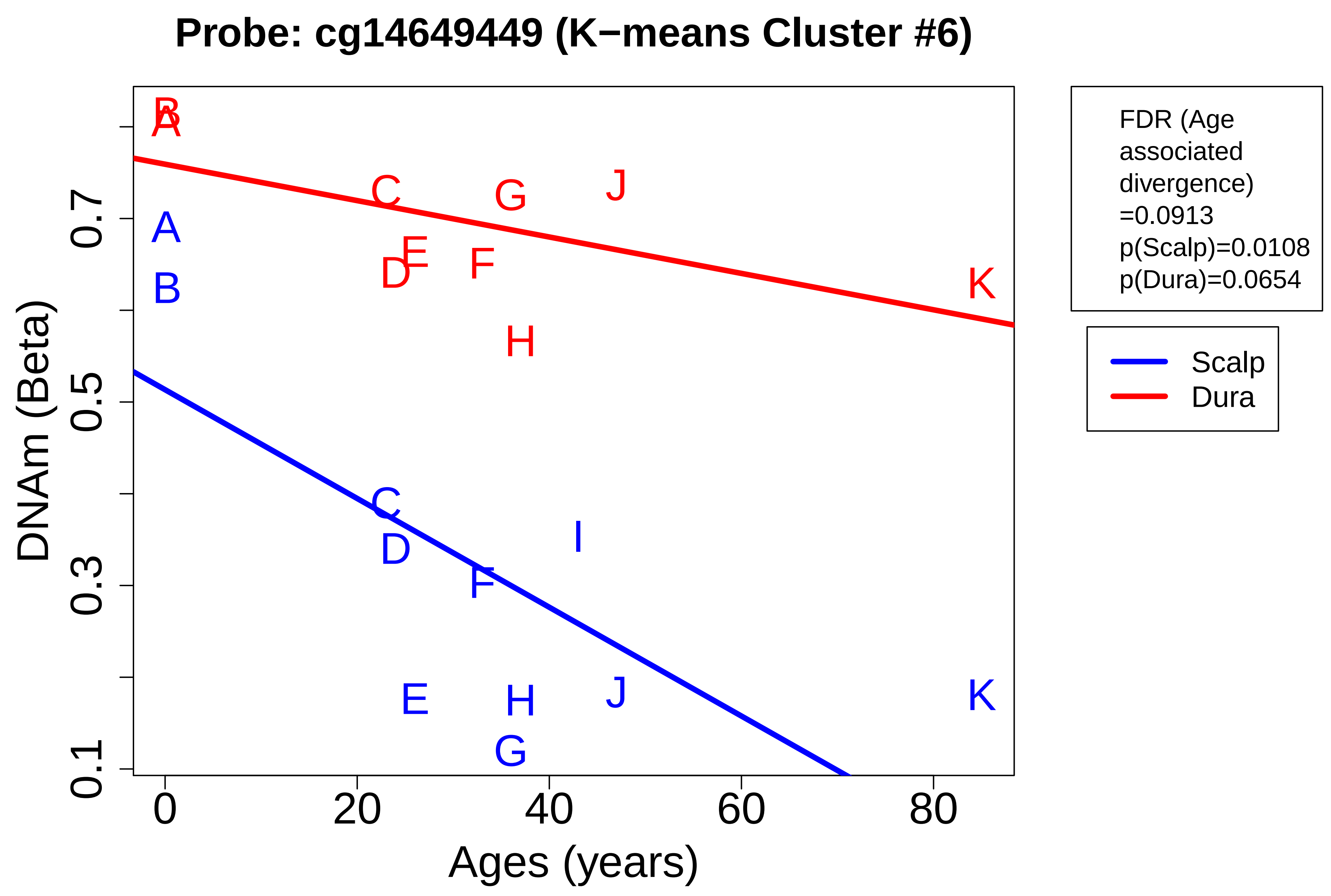
Probe: cg14649449 (K−means Cluster #6)
FDR (Age associated divergence) =0.0913
p(Scalp)=0.0108
p(Dura)=0.0654
Scalp
Dura
DNAm (Beta)
0.1
0.3
0.5
0.7
Ages (years)
0
20
40
60
80
A
B
C
D
E
F
G
H
I
J
K

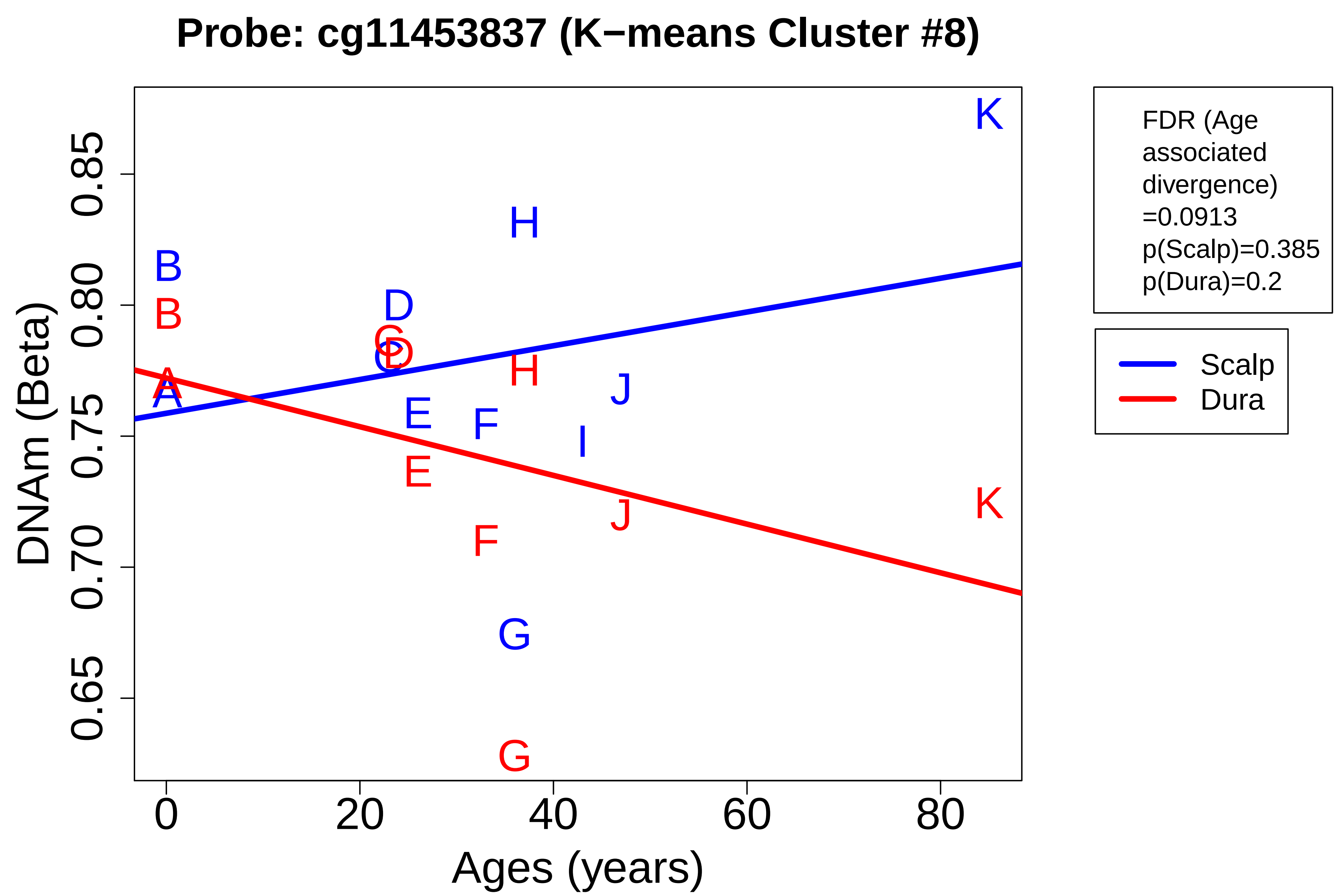

Probe: cg11453837 (K−means Cluster #8)
DNAm (Beta)
0.65
0.70
0.75
0.80
0.85
0
20
40
60
80
Ages (years)
FDR (Age associated divergence) =0.0913
p(Scalp)=0.385
p(Dura)=0.2
Scalp
Dura
A
B
C
D
E
F
G
H
I
J
K

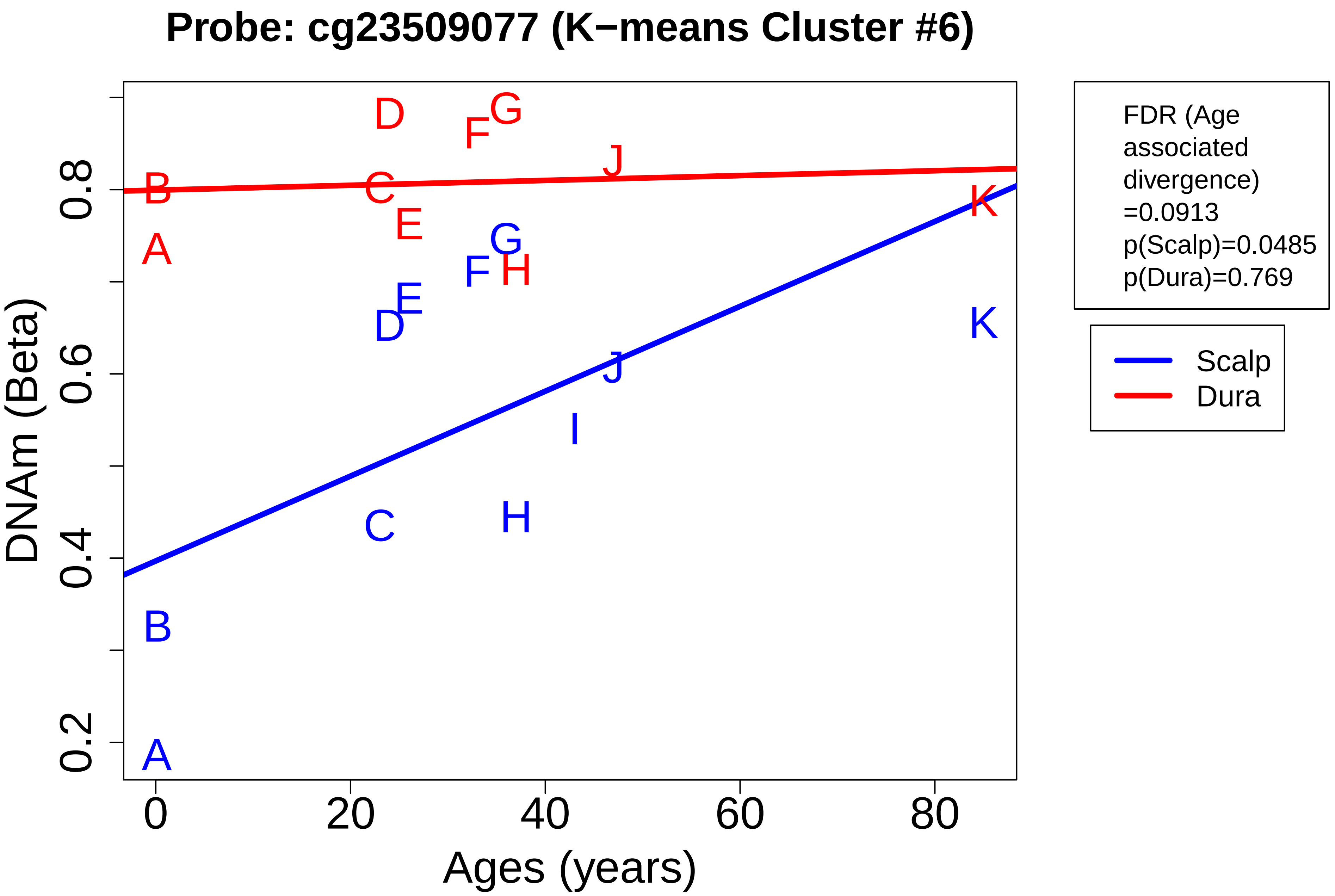
Probe: cg23509077 (K−means Cluster #6)
DNAm (Beta)
0.2
0.4
0.6
0.8
0
20
40
60
80
Ages (years)
A
B
C
D
E
F
G
H
I
J
K
A
B
C
D
E
F
G
H
J
K
FDR (Age associated divergence) =0.0913
p(Scalp)=0.0485
p(Dura)=0.769
Scalp
Dura

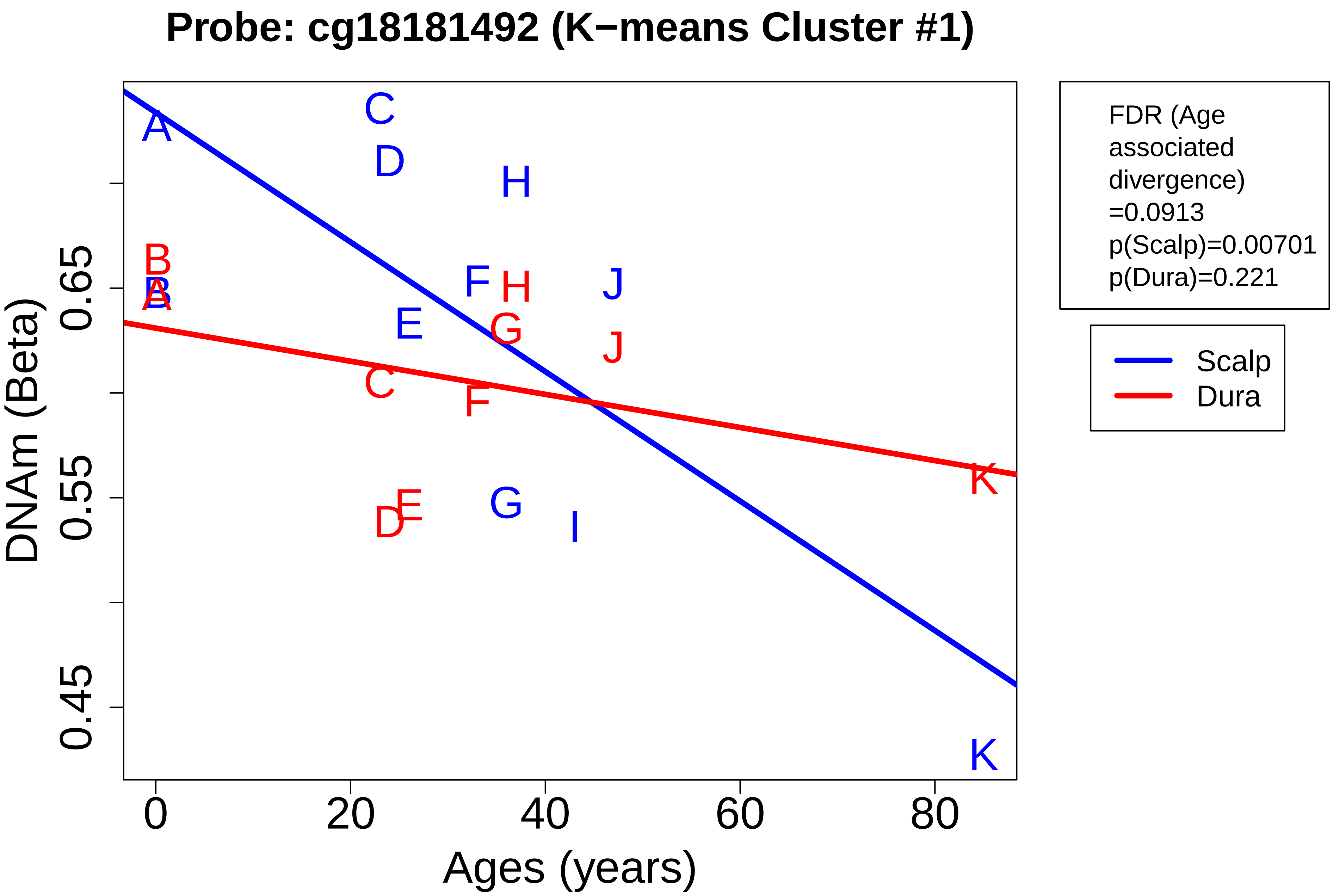

Probe: cg18181492 (K−means Cluster #1)
DNAm (Beta)
0.45
0.55
0.65
Ages (years)
0
20
40
60
80
FDR (Age associated divergence) =0.0913
p(Scalp)=0.00701
p(Dura)=0.221
Scalp
Dura

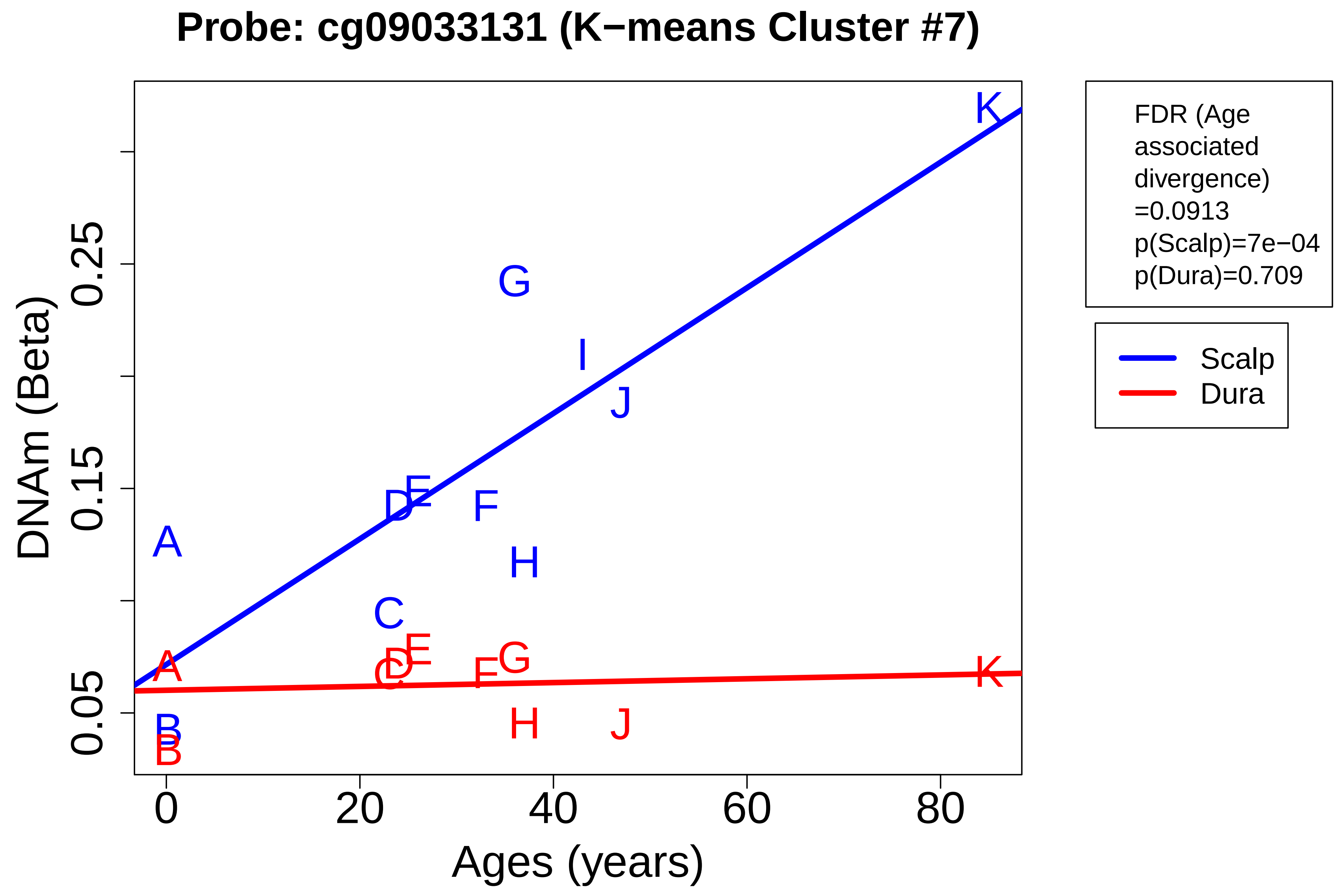

Probe: cg09033131 (K−means Cluster #7)
DNAm (Beta)
0.05
0.15
0.25
0
20
40
60
80
Ages (years)
FDR (Age associated divergence) =0.0913
p(Scalp)=7e−04
p(Dura)=0.709
Scalp
Dura

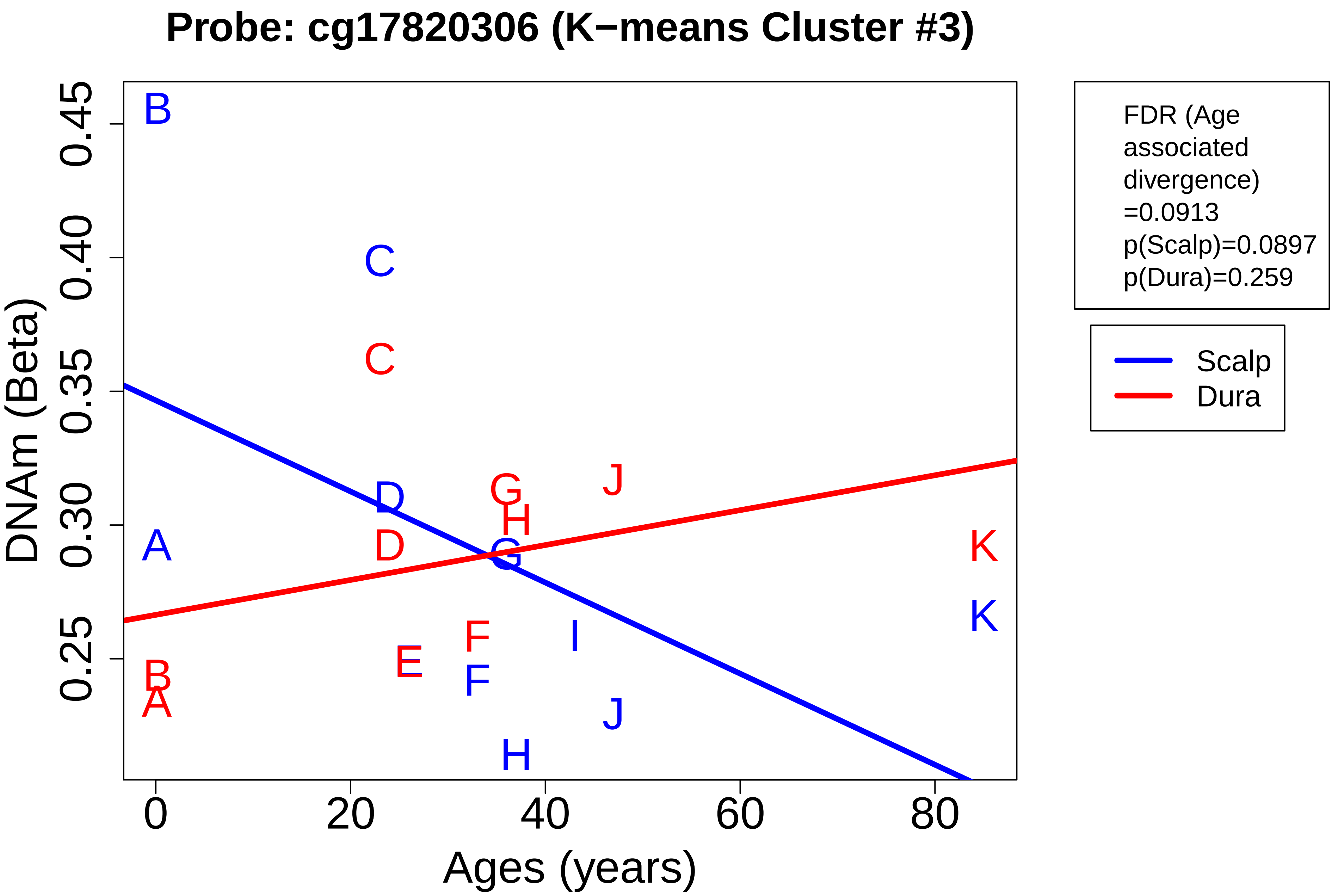

Probe: cg17820306 (K−means Cluster #3)
DNAm (Beta)
0.25
0.30
0.35
0.40
0.45
Ages (years)
0
20
40
60
80
FDR (Age associated divergence) =0.0913
p(Scalp)=0.0897
p(Dura)=0.259
Scalp
Dura
A
B
C
D
E
F
G
H
I
J
K

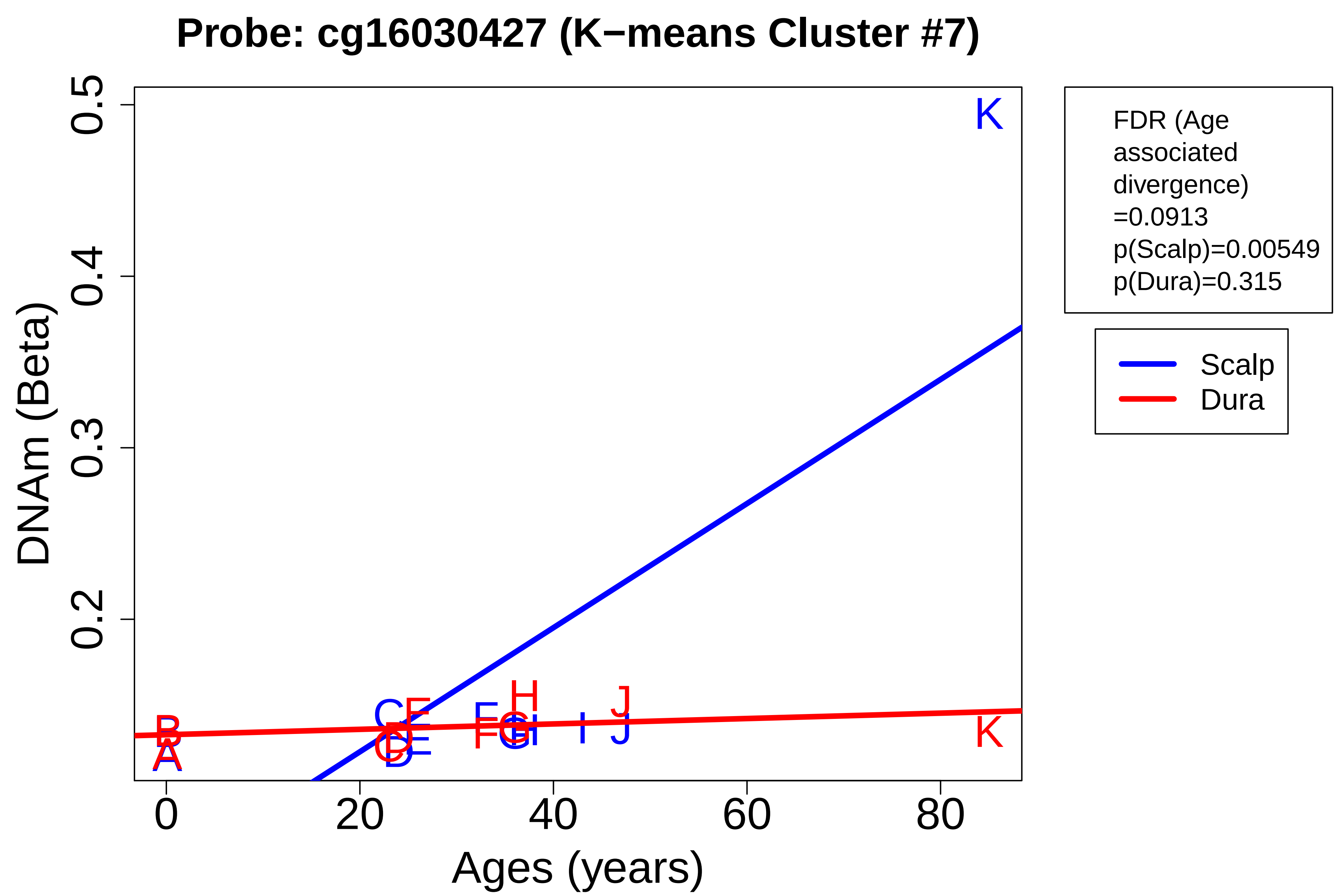

Probe: cg16030427 (K−means Cluster #7)
DNAm (Beta)
0.2
0.3
0.4
0.5
0
20
40
60
80
Ages (years)
FDR (Age associated divergence) =0.0913
p(Scalp)=0.00549
p(Dura)=0.315
Scalp
Dura

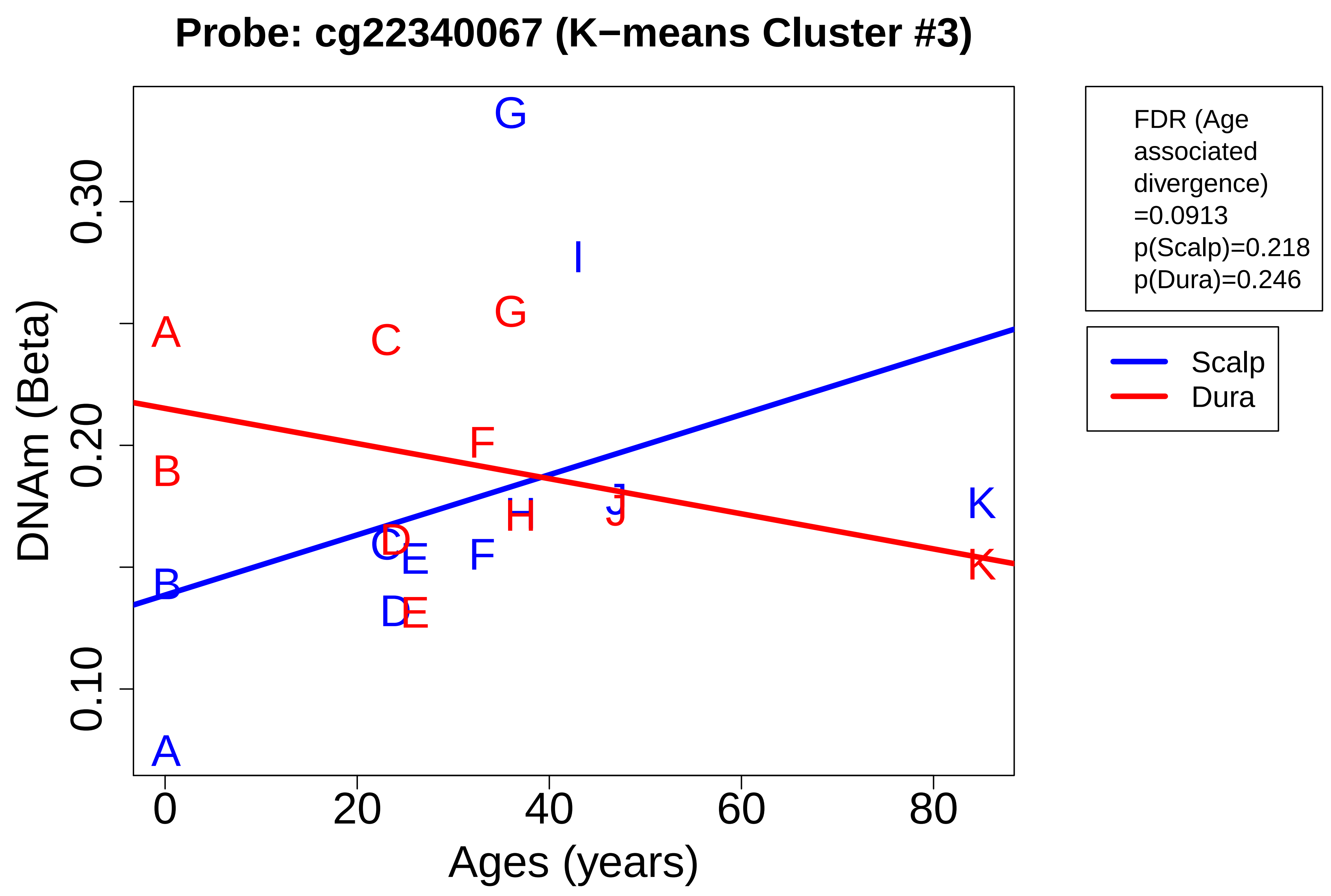

Probe: cg22340067 (K−means Cluster #3)
DNAm (Beta)
0.10
0.20
0.30
Ages (years)
0
20
40
60
80
A
B
C
D
E
F
G
H
I
J
K
FDR (Age associated divergence) =0.0913
p(Scalp)=0.218
p(Dura)=0.246
Scalp
Dura

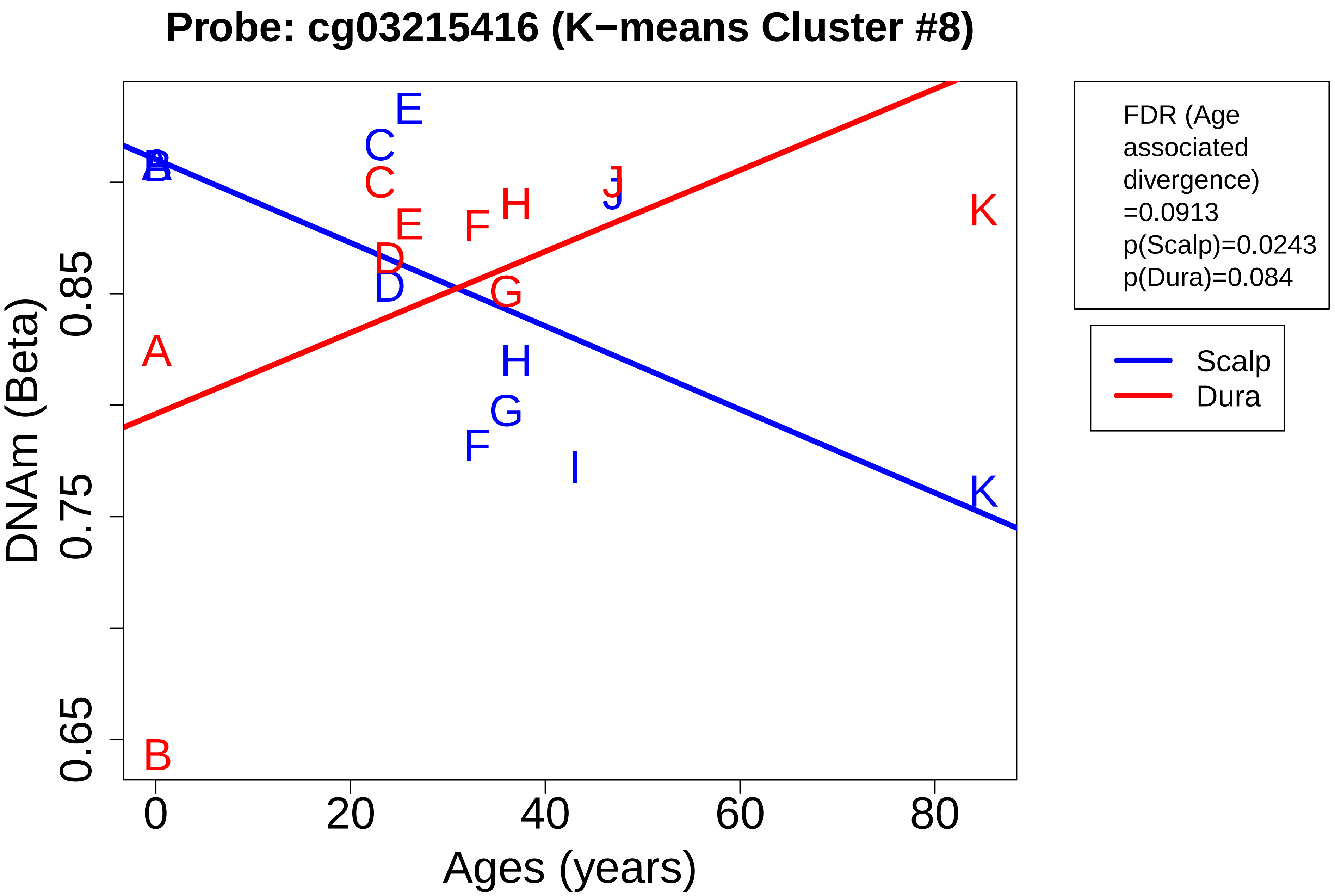

Probe: cg03215416 (K−means Cluster #8)
DNAm (Beta)
0.65
0.75
0.85
Ages (years)
0
20
40
60
80
A
B
C
D
E
F
G
H
I
J
K
FDR (Age associated divergence) =0.0913
p(Scalp)=0.0243
p(Dura)=0.084
Scalp
Dura

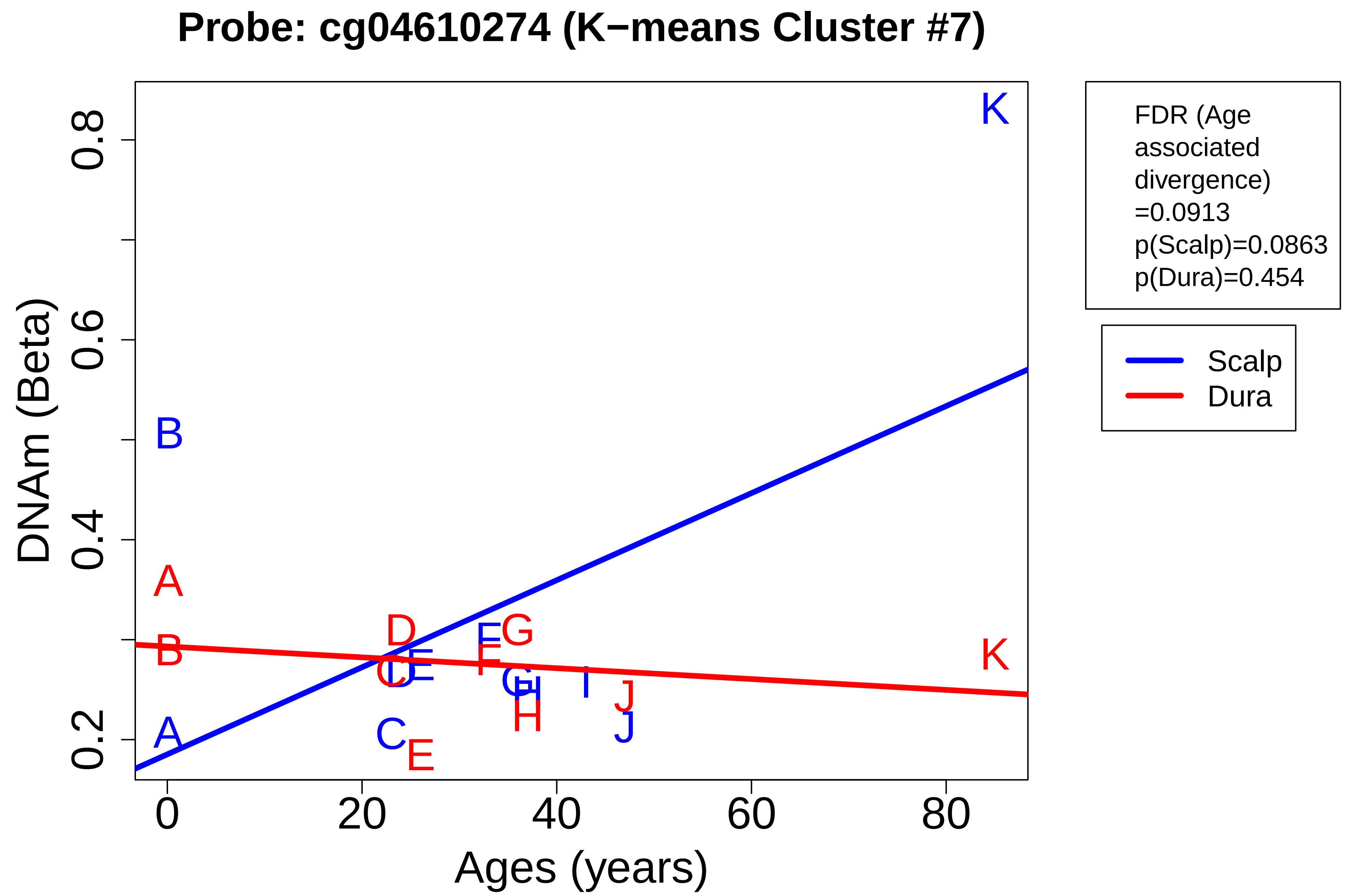

Probe: cg04610274 (K−means Cluster #7)
FDR (Age associated divergence) =0.0913
p(Scalp)=0.0863
p(Dura)=0.454
Scalp
Dura
DNAm (Beta)
0.2
0.4
0.6
0.8
0
20
40
60
80
Ages (years)
A
B
C
D
E
F
G
H
I
J
K

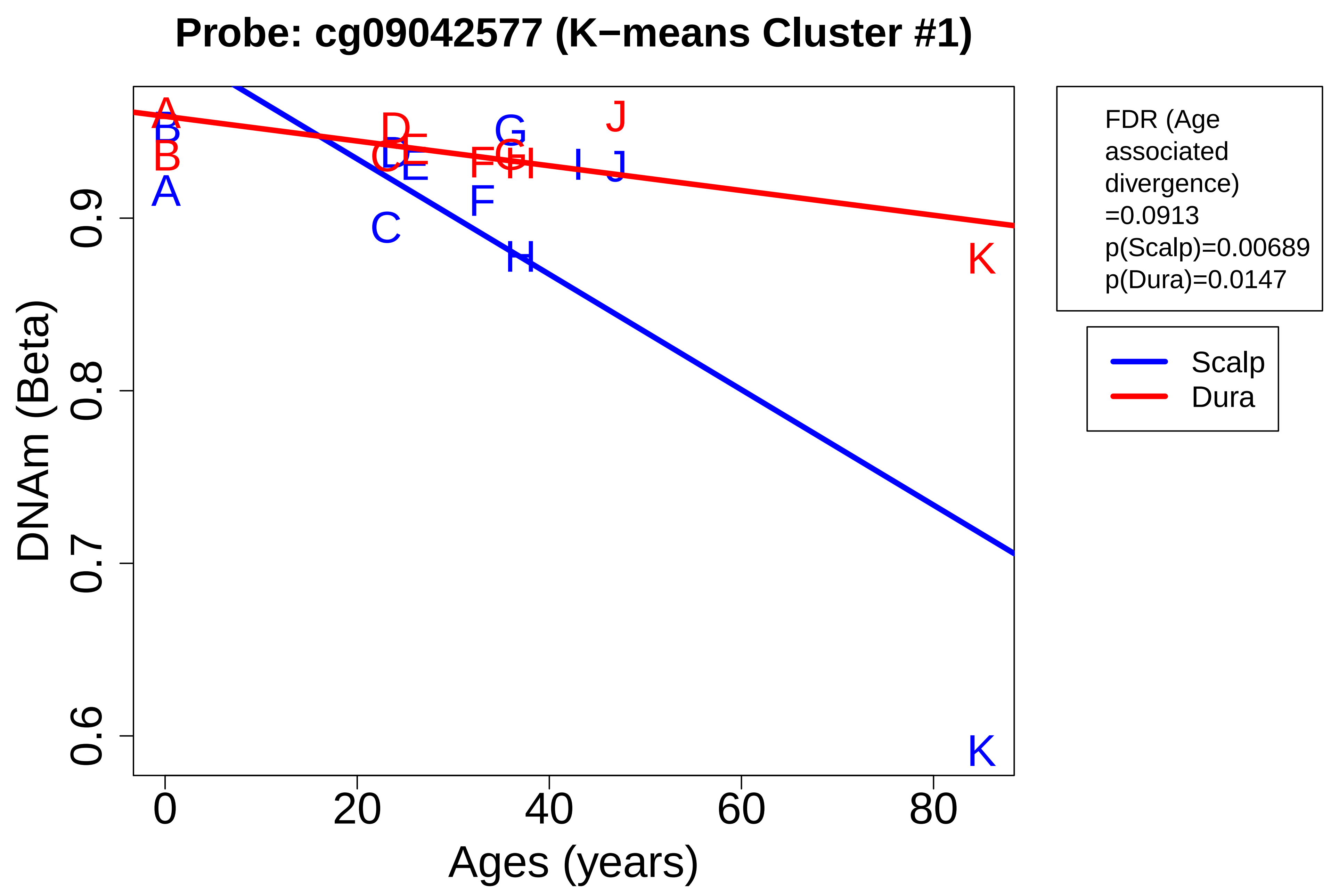

Probe: cg09042577 (K−means Cluster #1)
DNAm (Beta)
0.6
0.7
0.8
0.9
0
20
40
60
80
Ages (years)
A
B
C
D
E
F
G
H
I
J
K
FDR (Age associated divergence) =0.0913
p(Scalp)=0.00689
p(Dura)=0.0147
Scalp
Dura

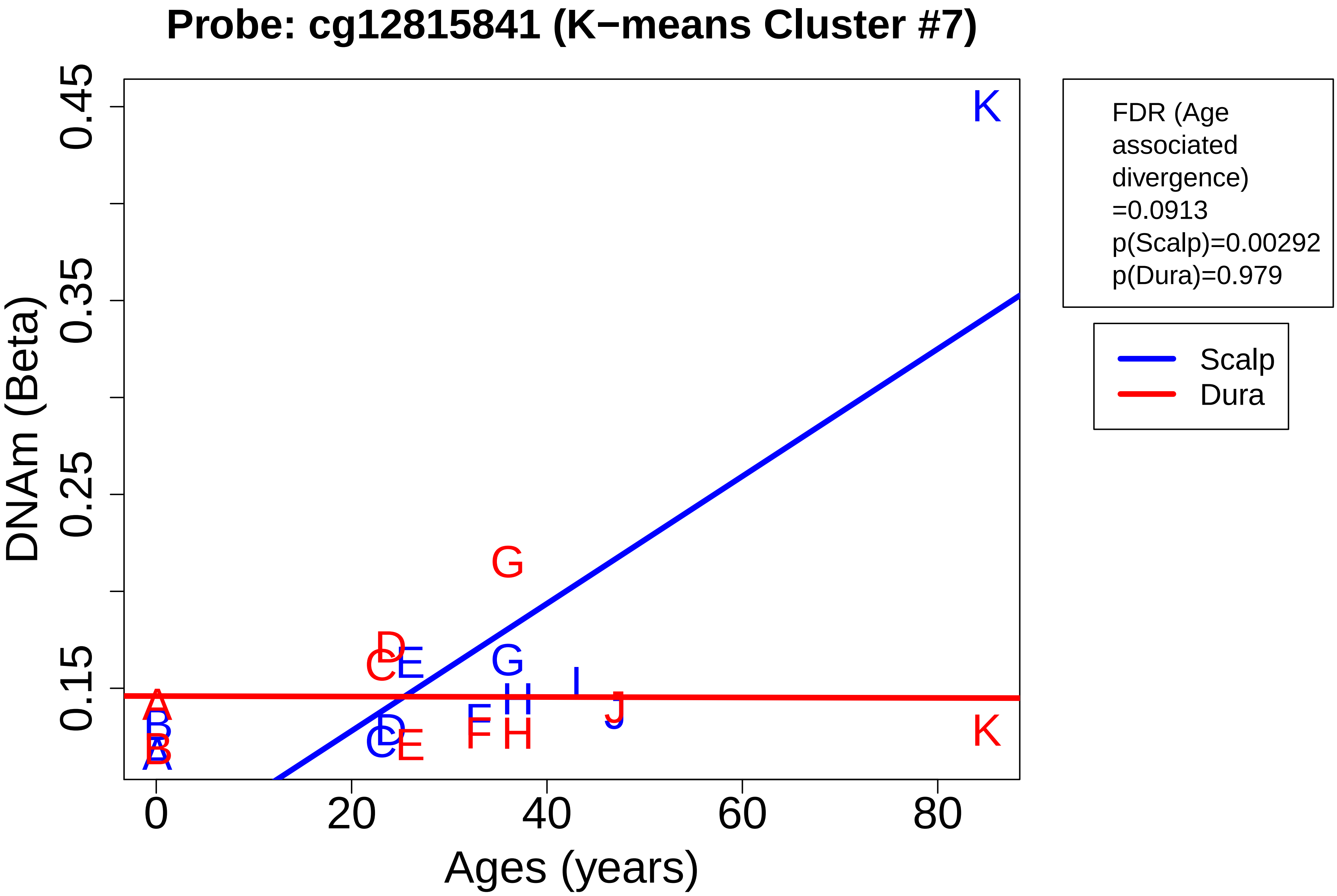

Probe: cg12815841 (K−means Cluster #7)
DNAm (Beta)
0.15
0.25
0.35
0.45
Ages (years)
0
20
40
60
80
FDR (Age associated divergence) =0.0913
p(Scalp)=0.00292
p(Dura)=0.979
Scalp
Dura

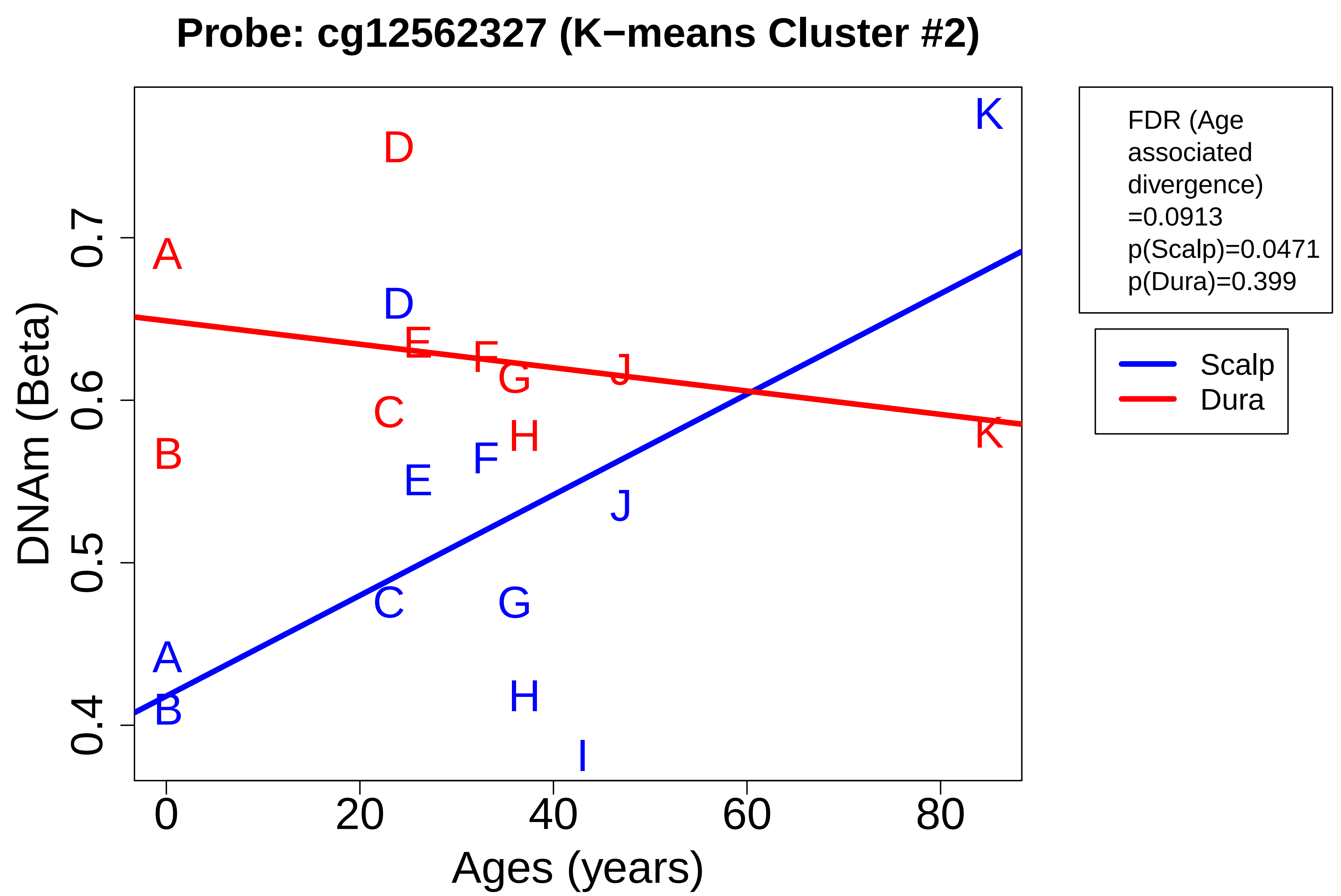

Probe: cg12562327 (K−means Cluster #2)
DNAm (Beta)
0.4
0.5
0.6
0.7
0
20
40
60
80
Ages (years)
A
B
C
D
E
F
G
H
I
J
K
FDR (Age associated divergence) =0.0913
p(Scalp)=0.0471
p(Dura)=0.399
Scalp
Dura

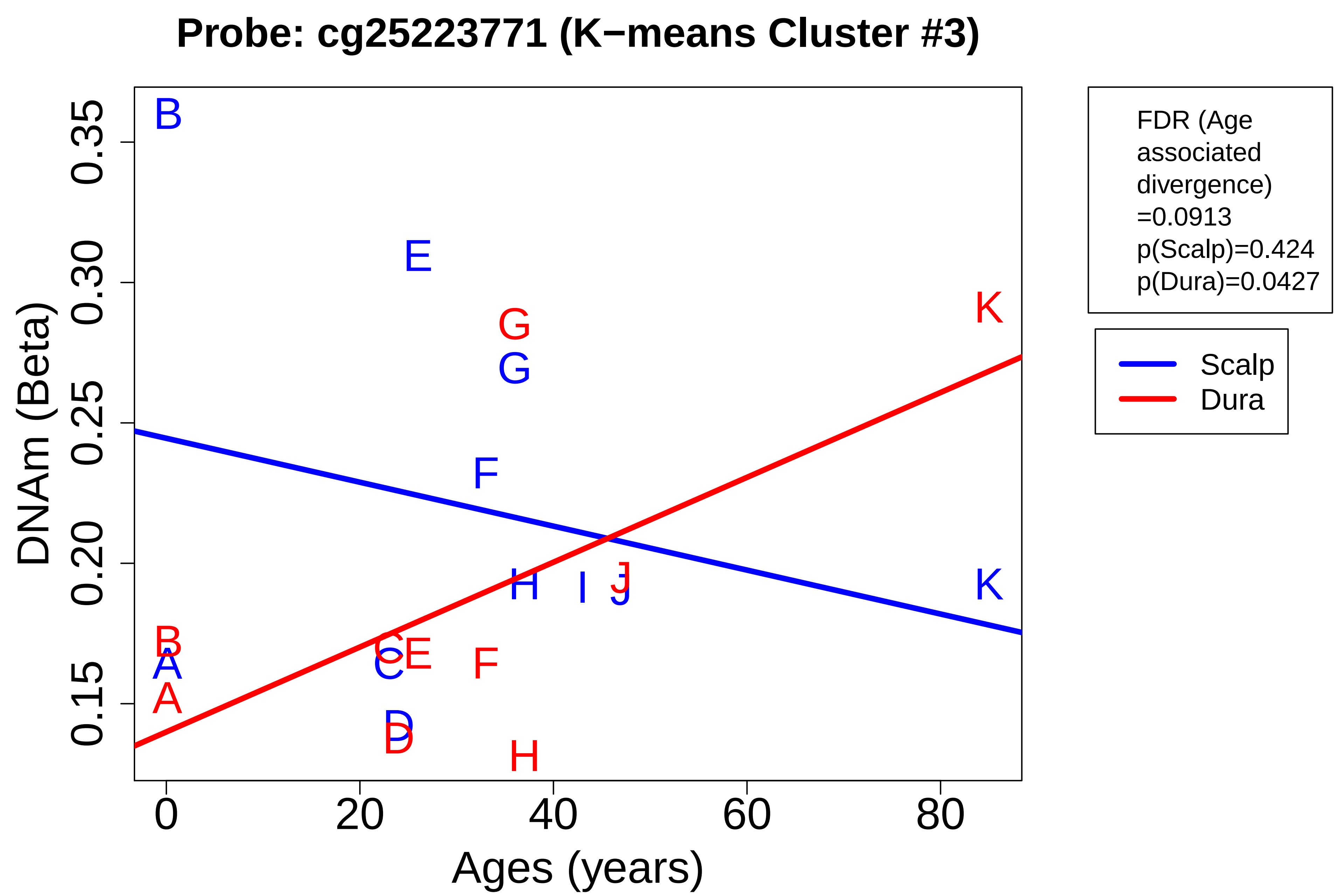
Probe: cg25223771 (K−means Cluster #3)
DNAm (Beta)
0.15
0.20
0.25
0.30
0.35
Ages (years)
0
20
40
60
80
B
E
G
G
F
H
I
J
J
K
K
B
A
A
C
C
E
F
D
D
H
FDR (Age associated divergence) =0.0913
p(Scalp)=0.424
p(Dura)=0.0427
Scalp
Dura

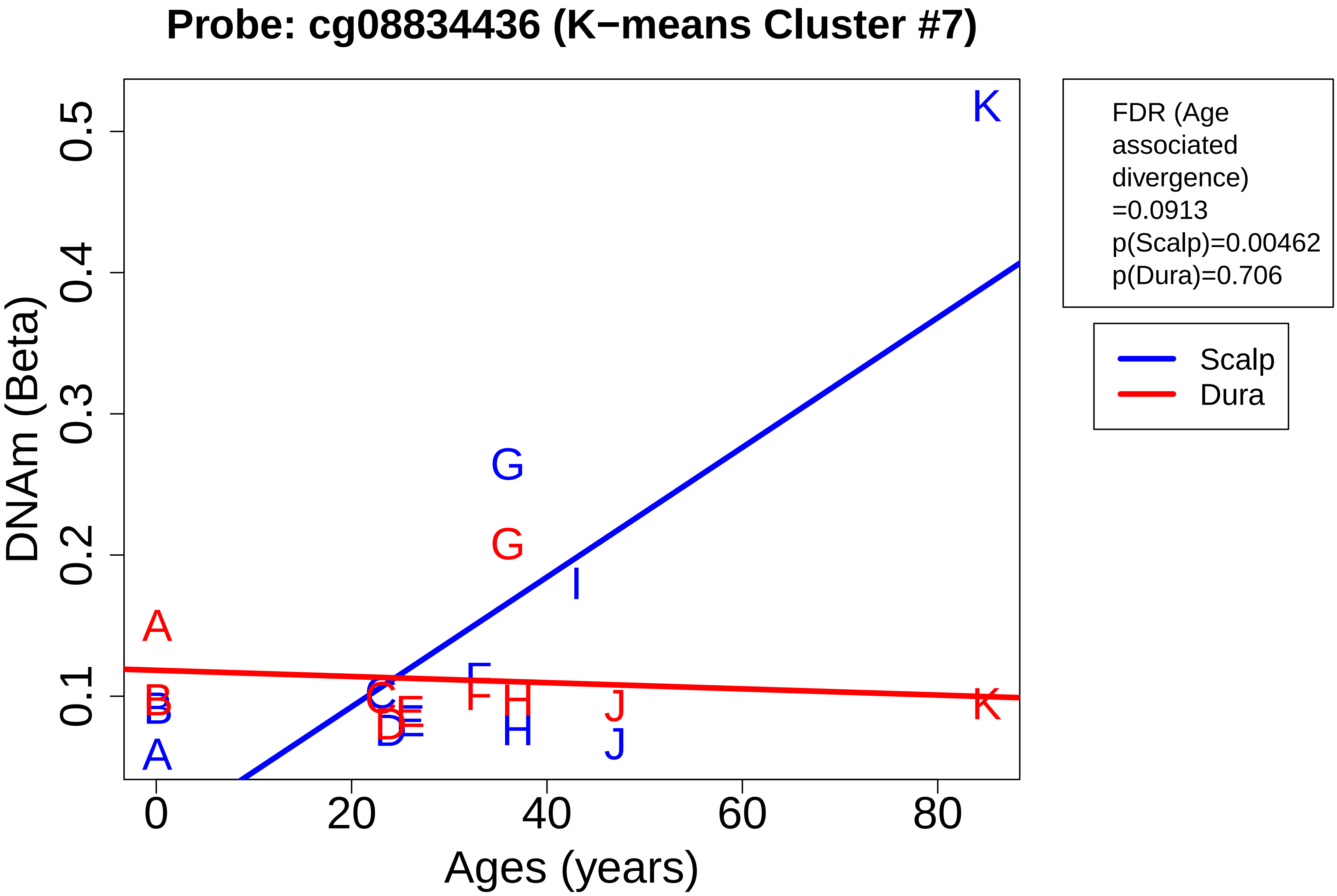

Probe: cg08834436 (K−means Cluster #7)
DNAm (Beta)
0.1
0.2
0.3
0.4
0.5
0
20
40
60
80
Ages (years)
FDR (Age associated divergence) =0.0913
p(Scalp)=0.00462
p(Dura)=0.706
Scalp
Dura

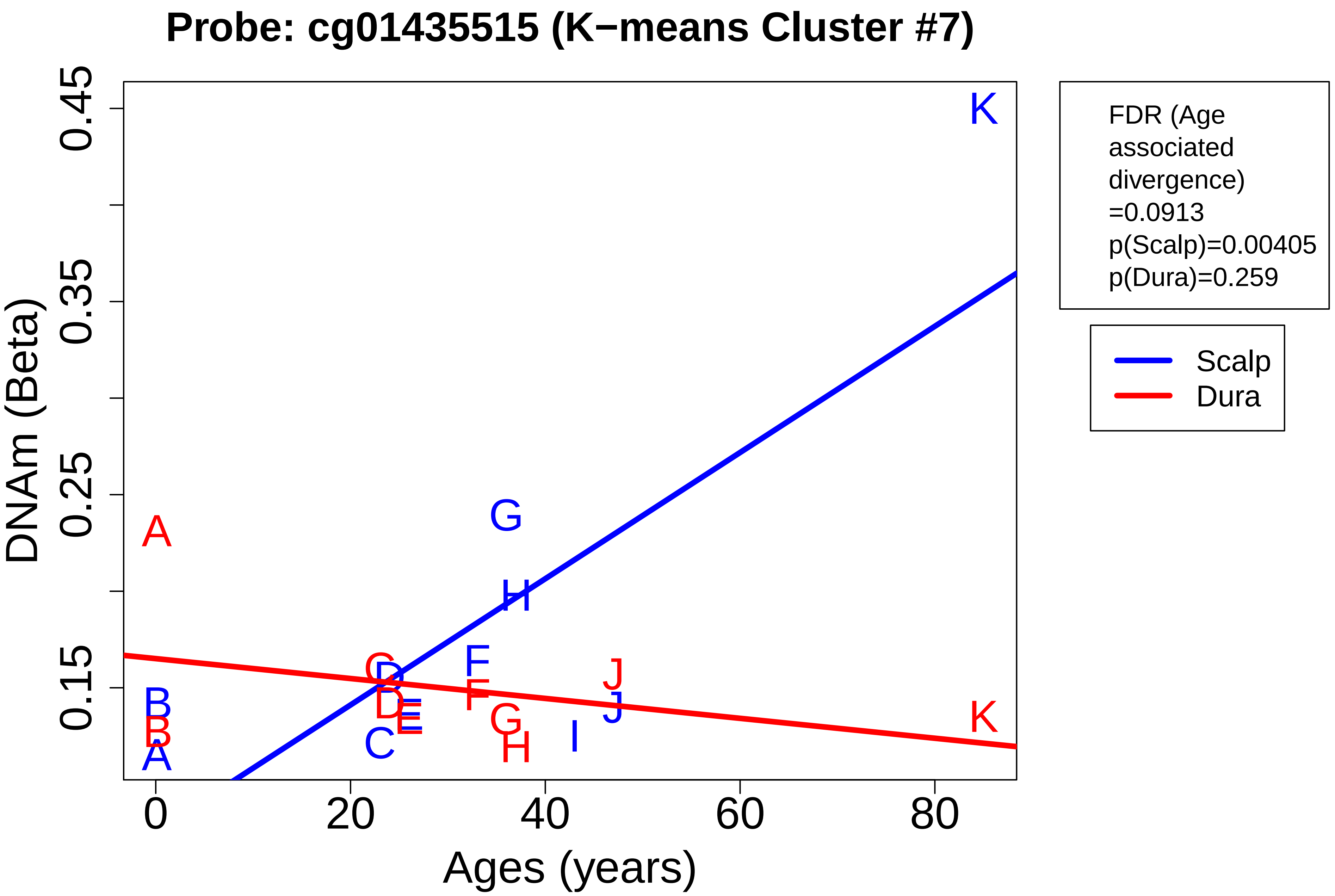

Probe: cg01435515 (K−means Cluster #7)
DNAm (Beta)
0.15
0.25
0.35
0.45
Ages (years)
0
20
40
60
80
FDR (Age associated divergence) =0.0913
p(Scalp)=0.00405
p(Dura)=0.259
Scalp
Dura
A
B
C
D
E
F
G
H
I
J
K

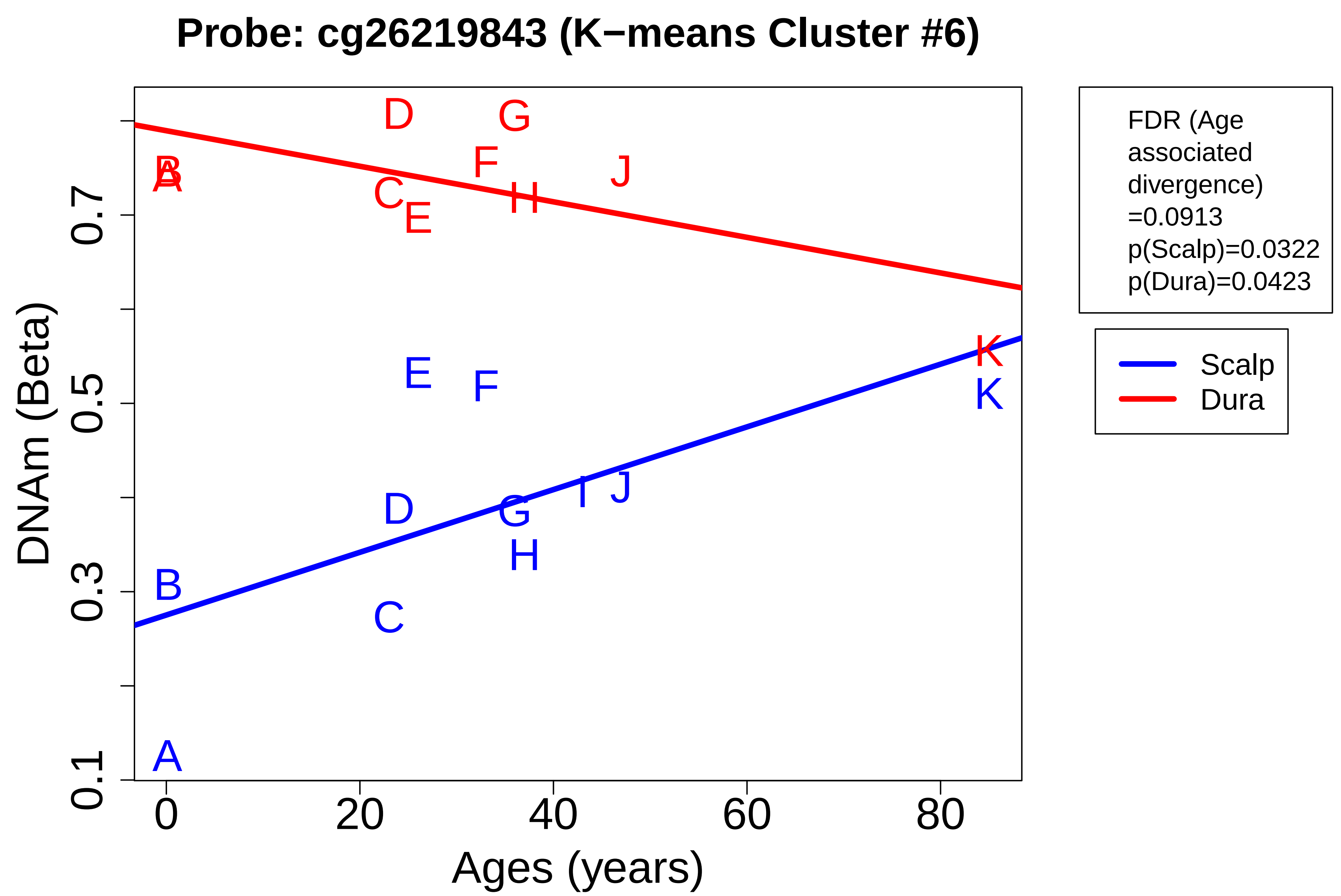

Probe: cg26219843 (K−means Cluster #6)
DNAm (Beta)
0.1
0.3
0.5
0.7
0
20
40
60
80
Ages (years)
A
B
C
D
E
F
G
H
I
J
K
FDR (Age associated divergence) =0.0913
p(Scalp)=0.0322
p(Dura)=0.0423
Scalp
Dura

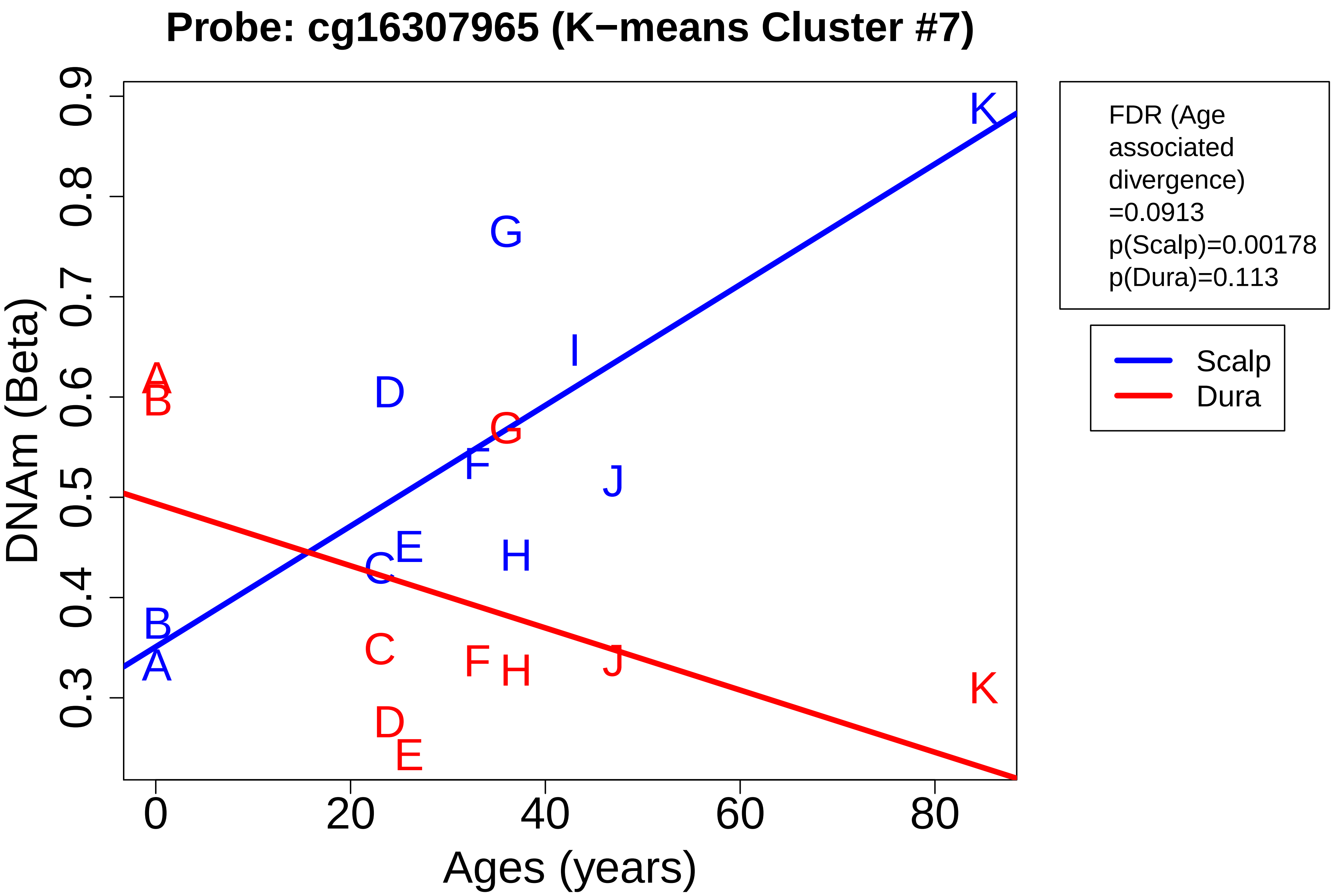
Probe: cg16307965 (K−means Cluster #7)
DNAm (Beta)
Ages (years)
0.3
0.4
0.5
0.6
0.7
0.8
0.9
0
20
40
60
80
FDR (Age associated divergence) =0.0913
p(Scalp)=0.00178
p(Dura)=0.113
Scalp
Dura
A
B
C
D
E
F
G
H
I
J
K

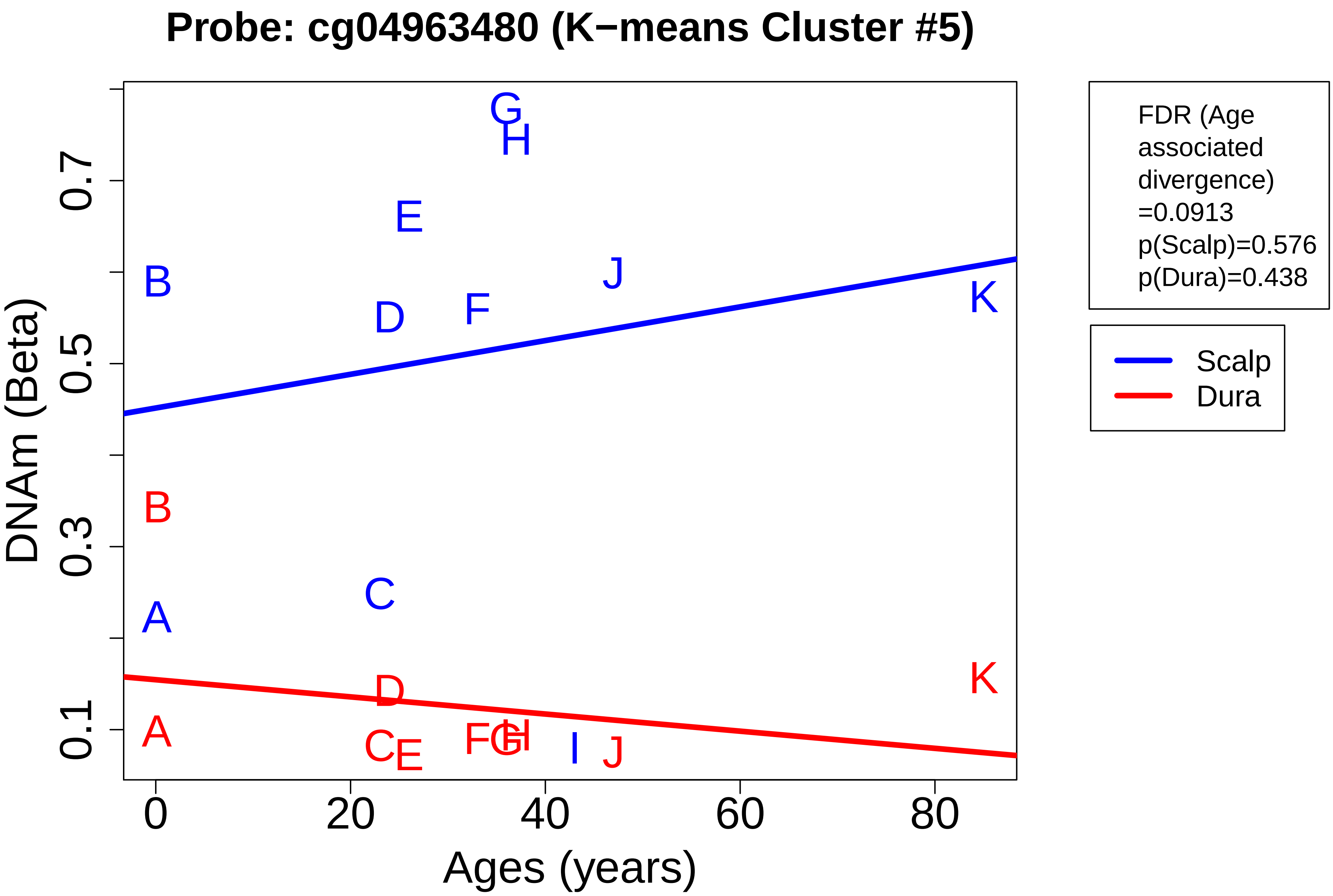

Probe: cg04963480 (K−means Cluster #5)
DNAm (Beta)
0.1
0.3
0.5
0.7
Ages (years)
0
20
40
60
80
A
B
C
D
E
F
G
H
I
J
K
FDR (Age associated divergence) =0.0913
p(Scalp)=0.576
p(Dura)=0.438
Scalp
Dura

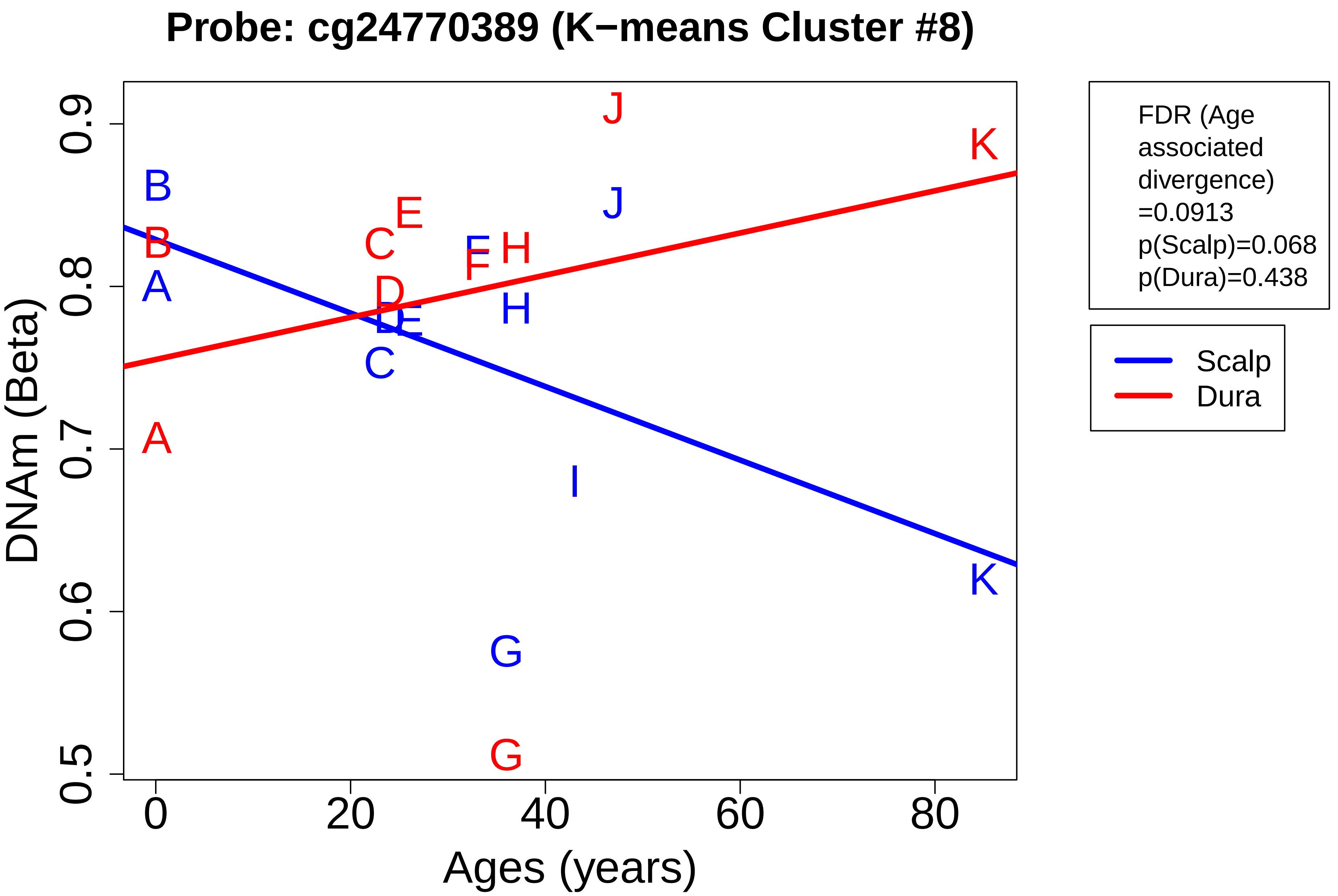
Probe: cg24770389 (K−means Cluster #8)
DNAm (Beta)
0.5
0.6
0.7
0.8
0.9
0
20
40
60
80
Ages (years)
FDR (Age associated divergence) =0.0913
p(Scalp)=0.068
p(Dura)=0.438
Scalp
Dura
A
B
C
D
E
F
G
H
I
J
K

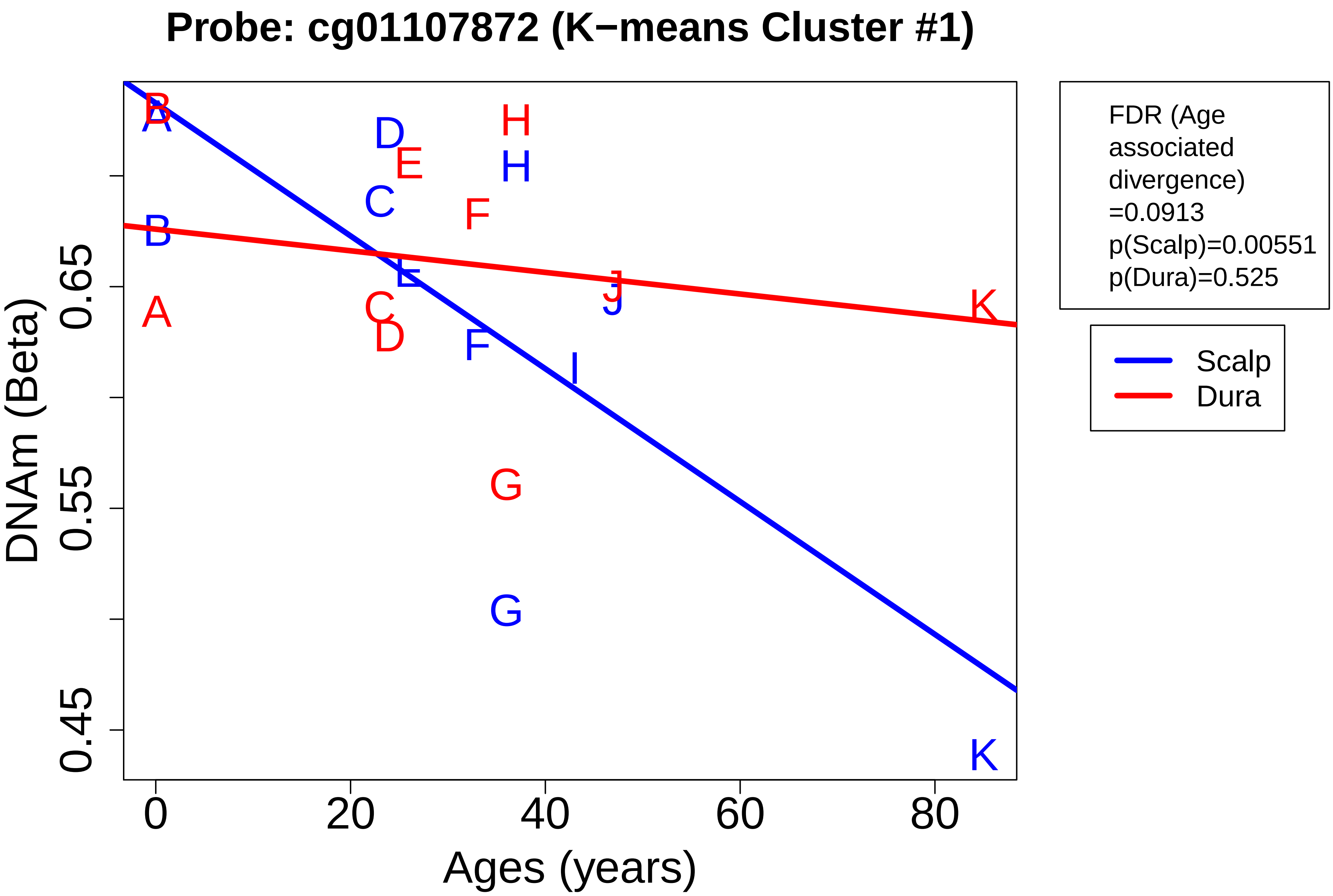

Probe: cg01107872 (K−means Cluster #1)
DNAm (Beta)
0.45
0.55
0.65
0
20
40
60
80
Ages (years)
FDR (Age associated divergence) =0.0913
p(Scalp)=0.00551
p(Dura)=0.525
Scalp
Dura

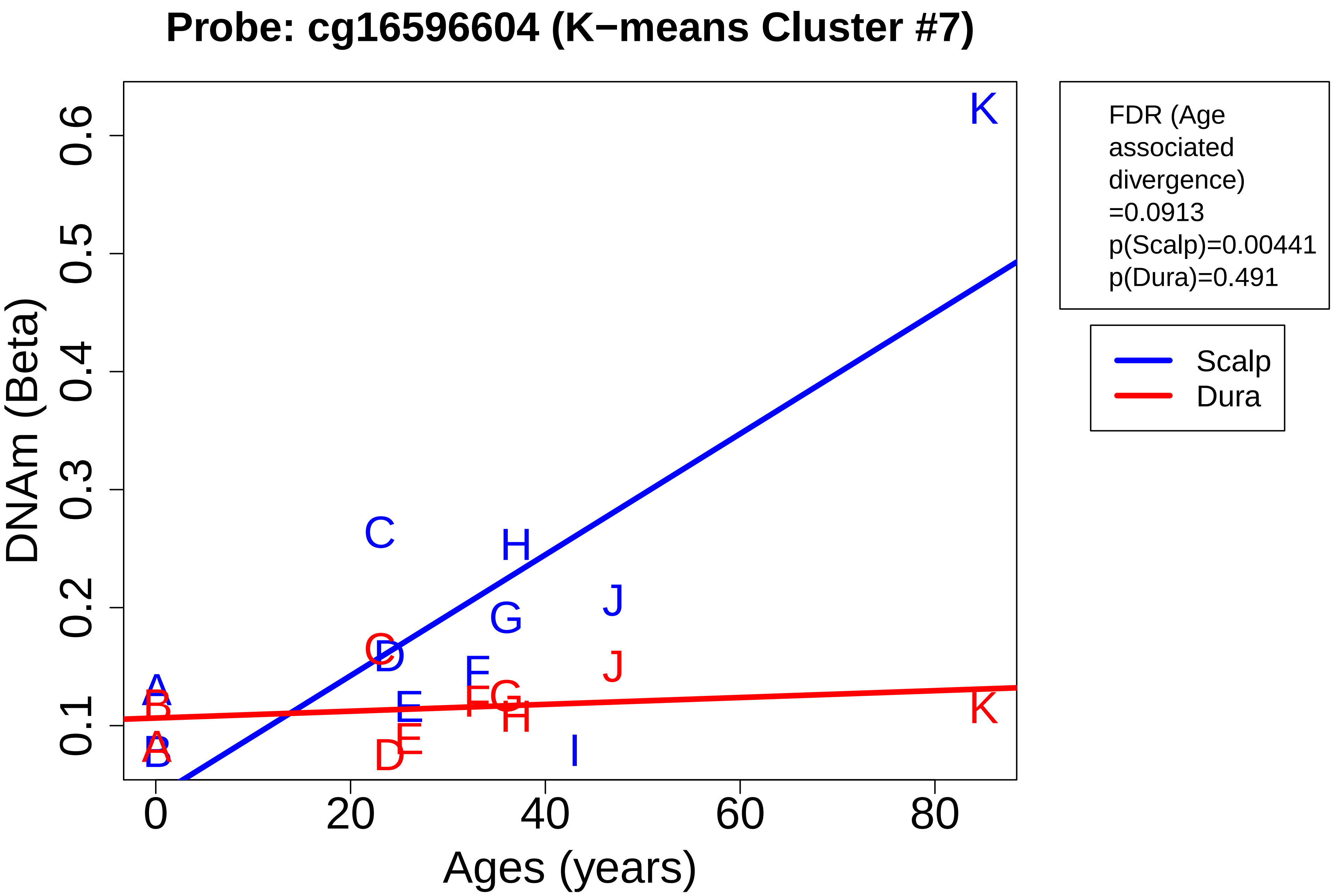

Probe: cg16596604 (K−means Cluster #7)
DNAm (Beta)
0.1
0.2
0.3
0.4
0.5
0.6
0
20
40
60
80
Ages (years)
FDR (Age associated divergence) =0.0913
p(Scalp)=0.00441
p(Dura)=0.491
Scalp
Dura

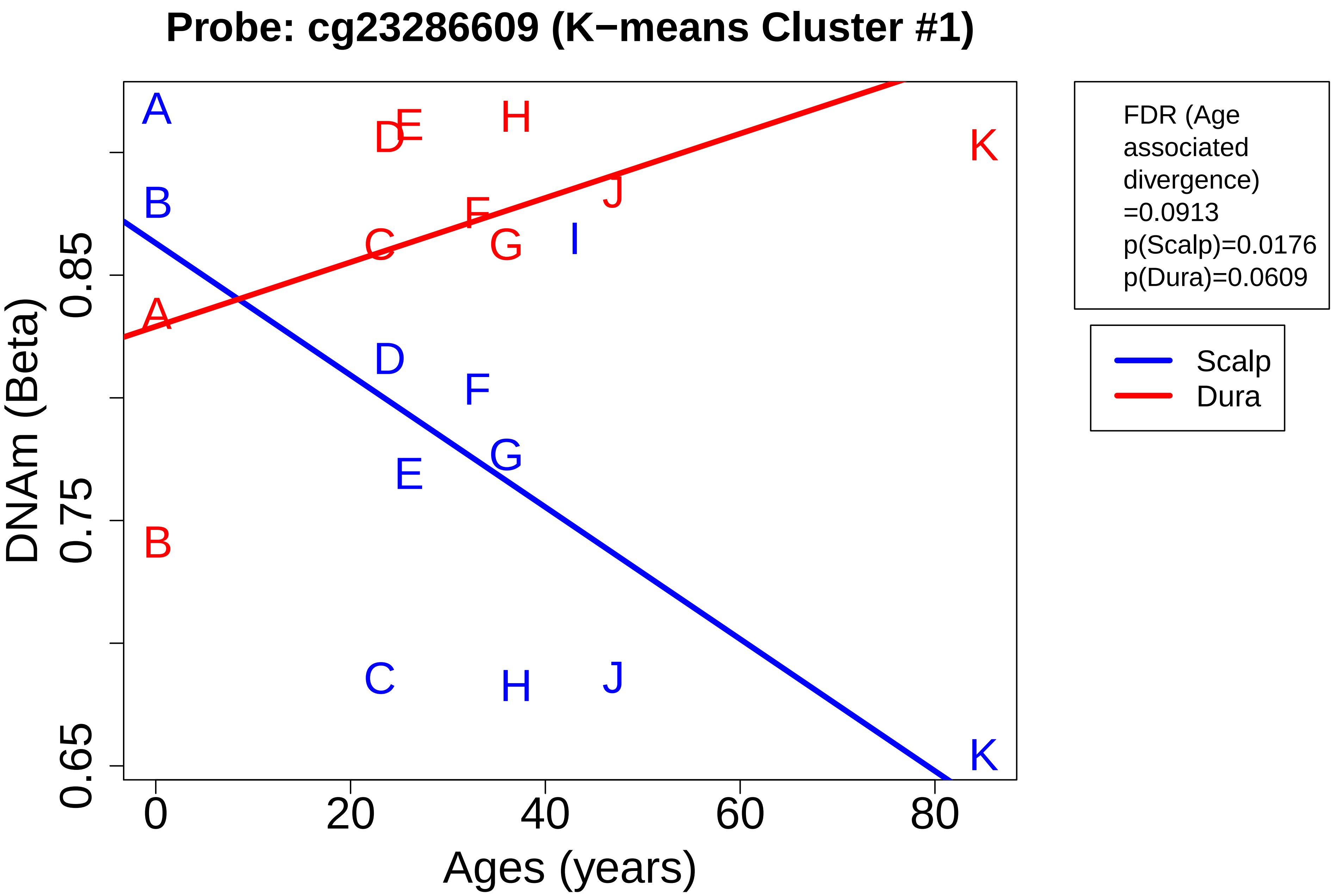

Probe: cg23286609 (K−means Cluster #1)
DNAm (Beta)
0.65
0.75
0.85
Ages (years)
0
20
40
60
80
FDR (Age associated divergence) =0.0913
p(Scalp)=0.0176
p(Dura)=0.0609
Scalp
Dura
A
B
C
D
E
F
G
H
I
J
K

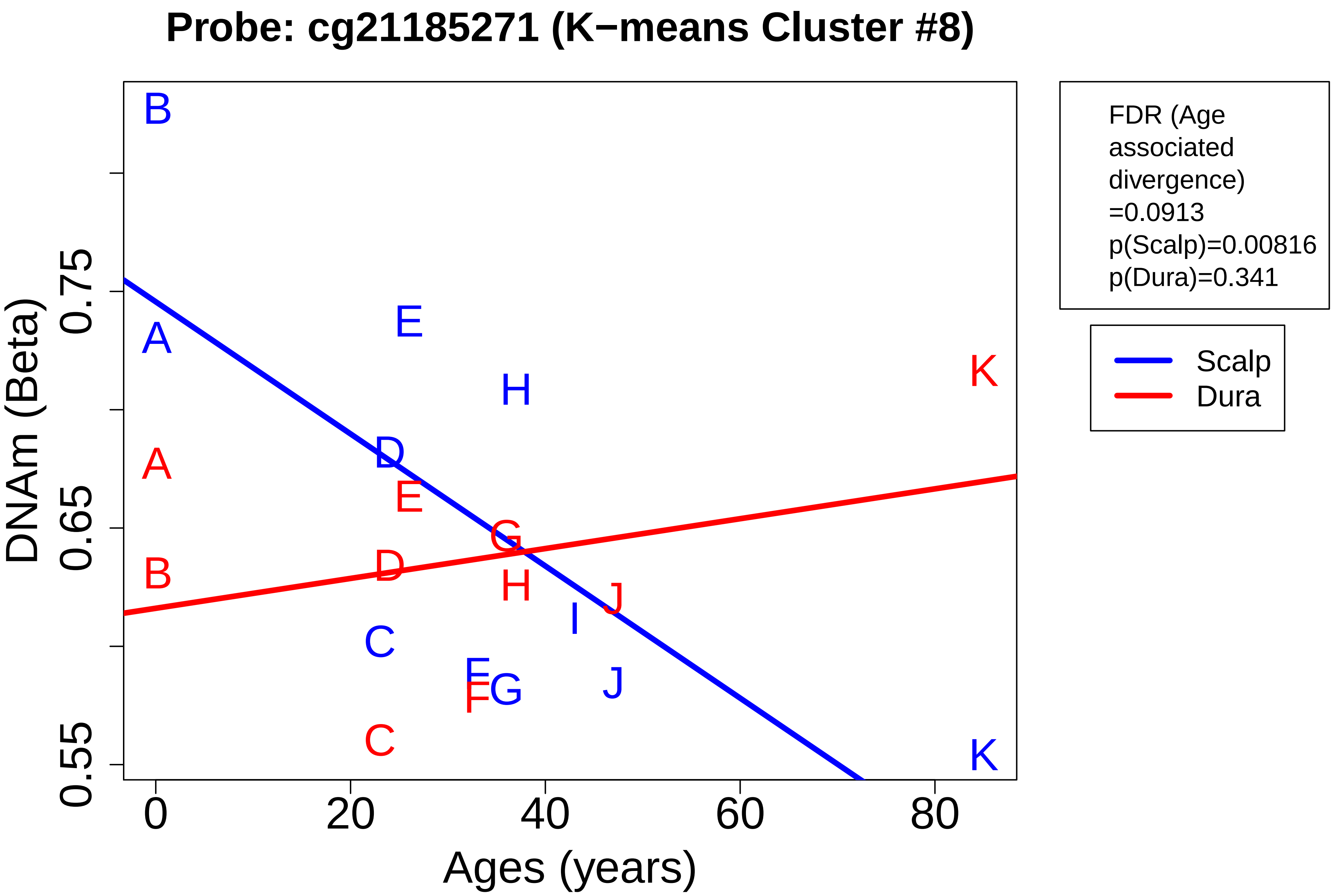
Probe: cg21185271 (K−means Cluster #8)
DNAm (Beta)
0.55
0.65
0.75
0
20
40
60
80
Ages (years)
FDR (Age associated divergence) =0.0913
p(Scalp)=0.00816
p(Dura)=0.341
Scalp
Dura

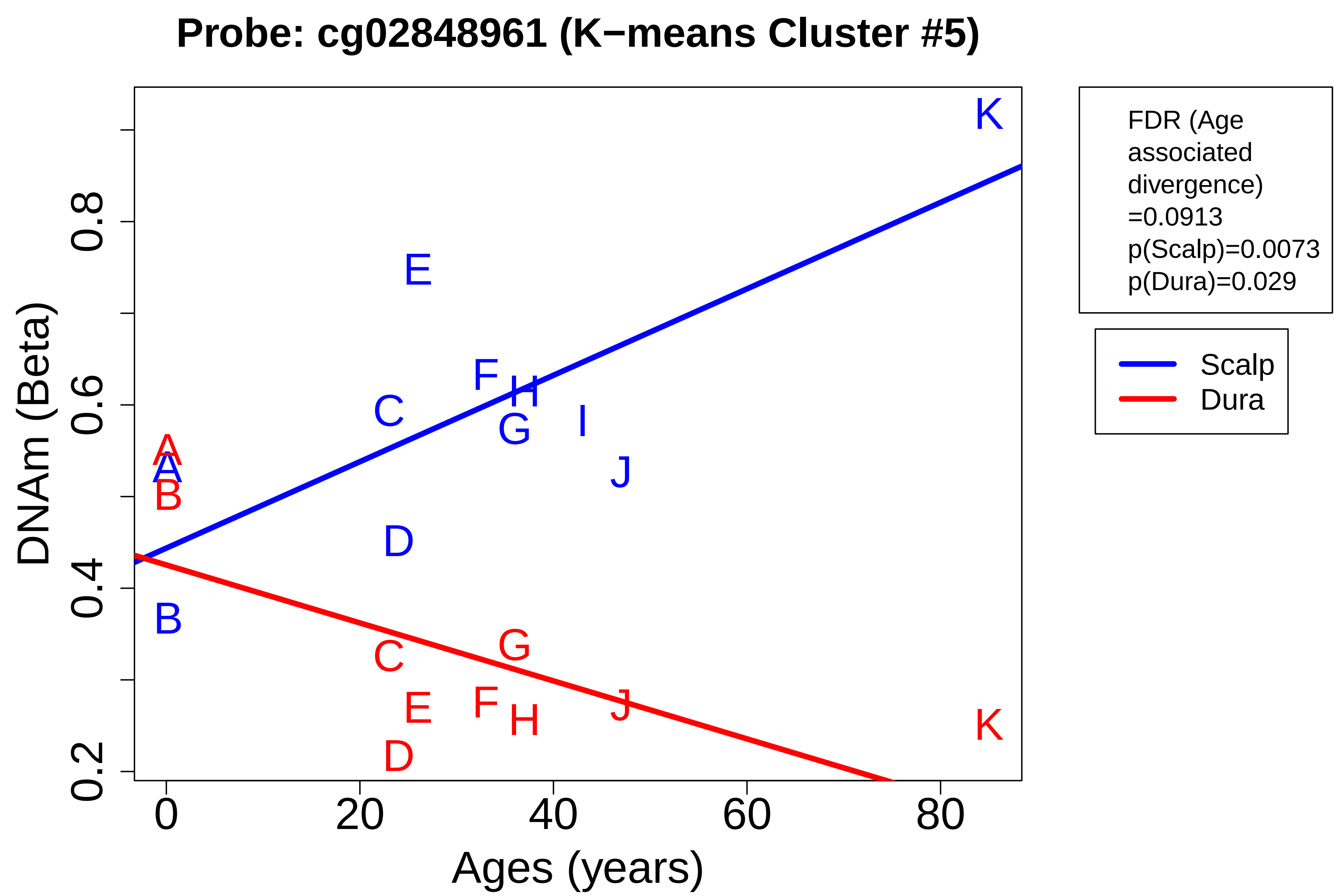

Probe: cg02848961 (K−means Cluster #5)
DNAm (Beta)
0.2
0.4
0.6
0.8
0
20
40
60
80
Ages (years)
A
B
C
D
E
F
G
H
I
J
K
FDR (Age associated divergence) =0.0913
p(Scalp)=0.0073
p(Dura)=0.029
Scalp
Dura

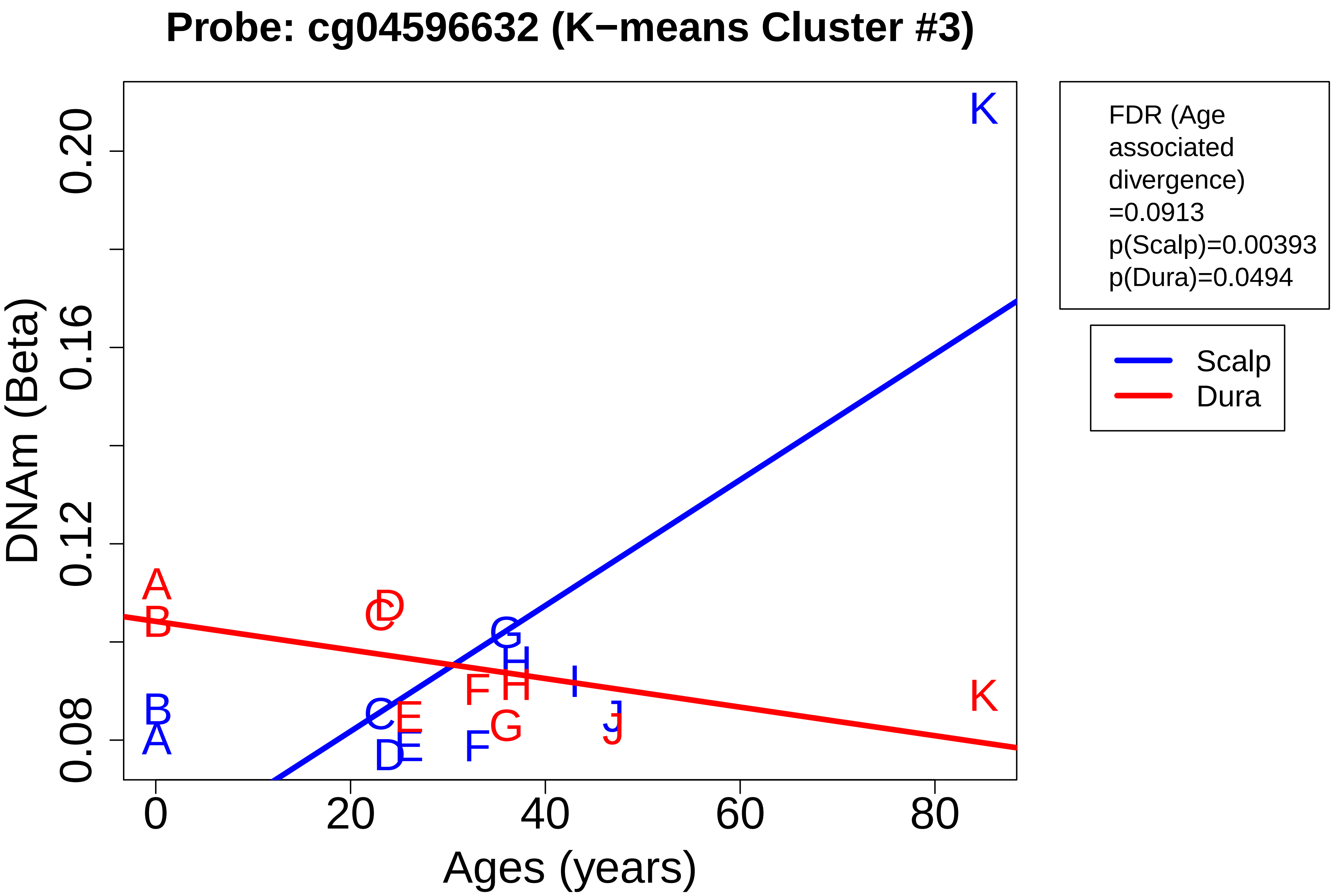

Probe: cg04596632 (K−means Cluster #3)
DNAm (Beta)
0.08
0.12
0.16
0.20
Ages (years)
0
20
40
60
80
FDR (Age associated divergence) =0.0913
p(Scalp)=0.00393
p(Dura)=0.0494
Scalp
Dura

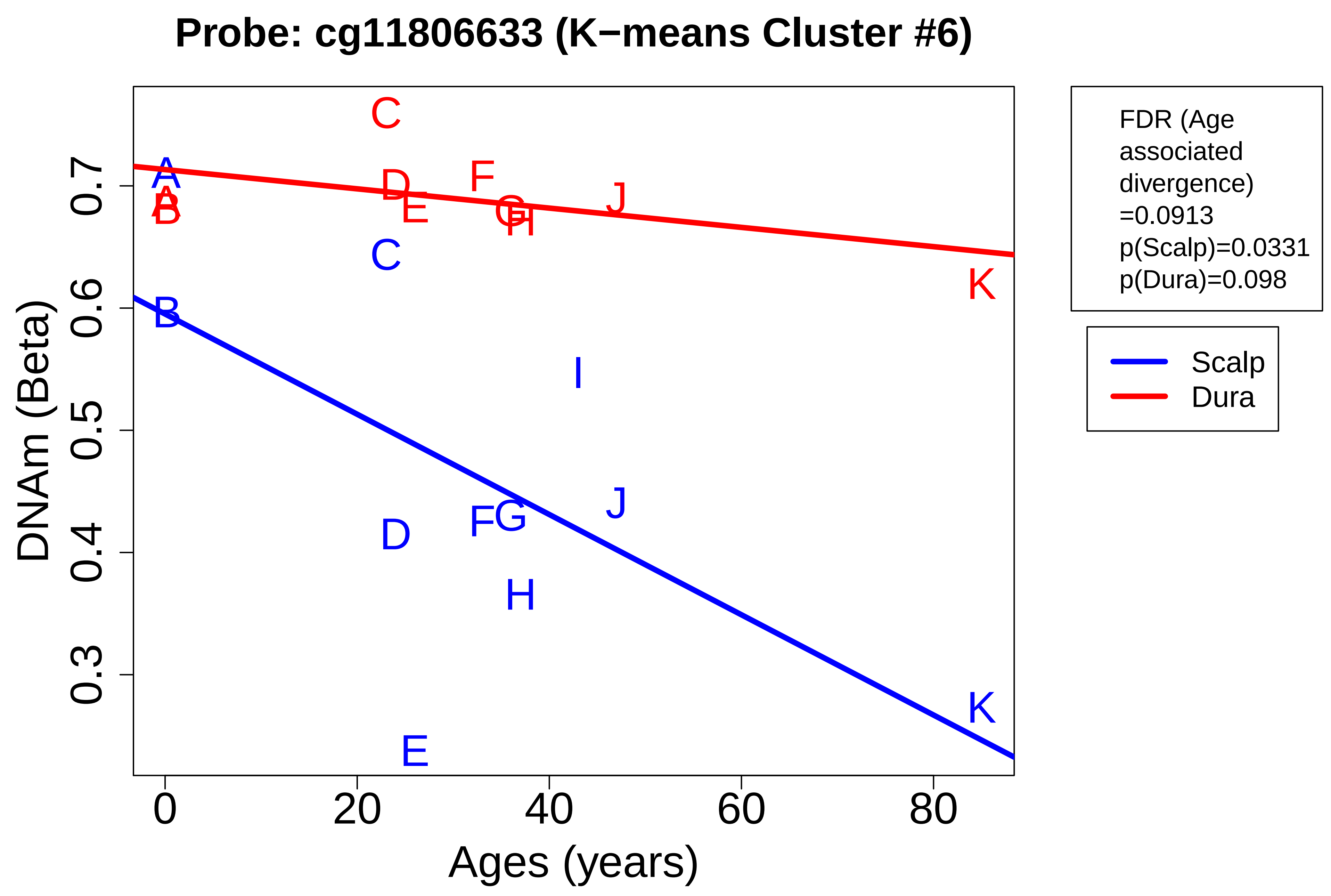
Probe: cg11806633 (K−means Cluster #6)
DNAm (Beta)
0.3
0.4
0.5
0.6
0.7
Ages (years)
0
20
40
60
80
FDR (Age associated divergence) =0.0913
p(Scalp)=0.0331
p(Dura)=0.098
Scalp
Dura

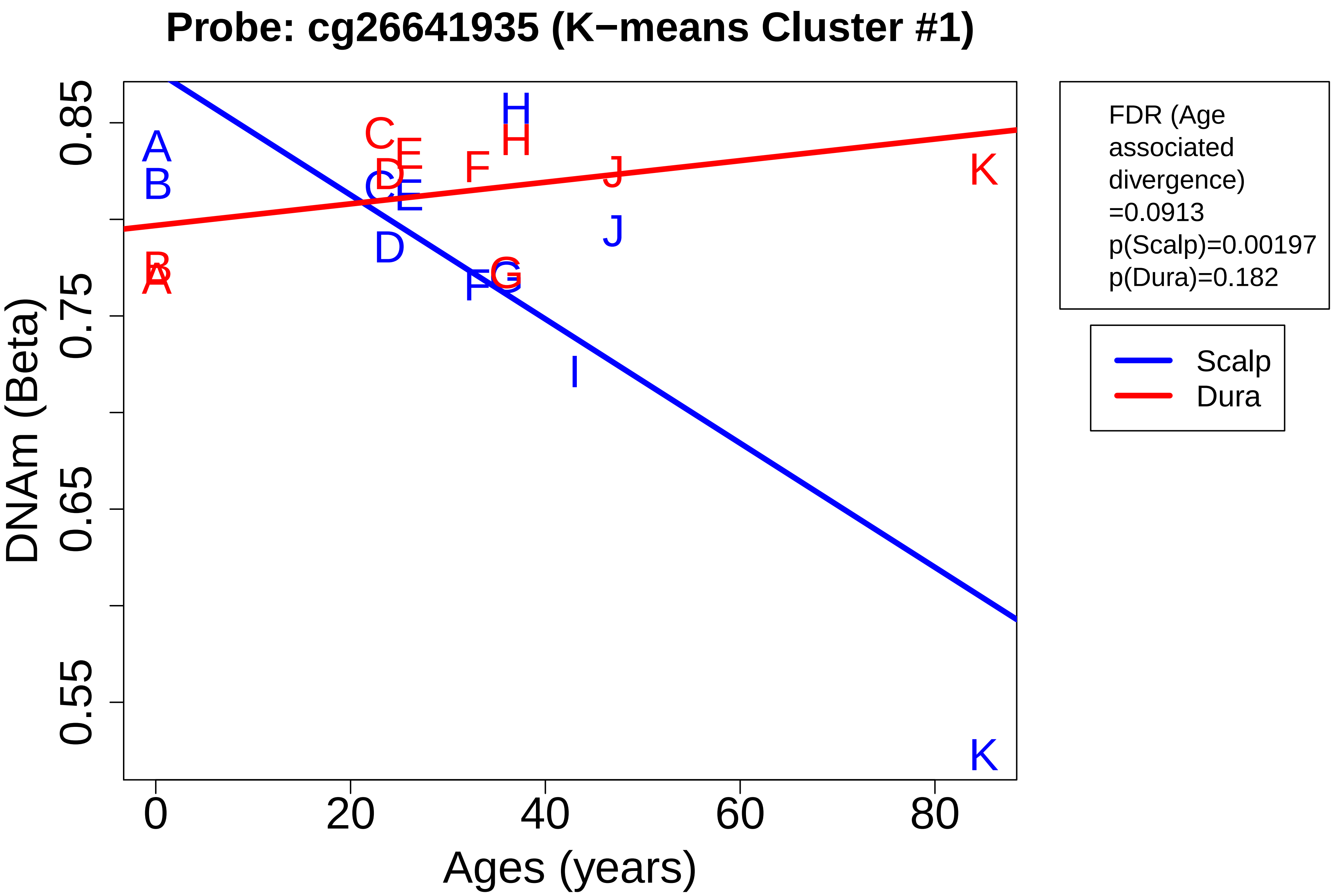

Probe: cg26641935 (K−means Cluster #1)
DNAm (Beta)
0.85
0.75
0.65
0.55
0
20
40
60
80
Ages (years)
FDR (Age associated divergence) =0.0913
p(Scalp)=0.00197
p(Dura)=0.182
Scalp
Dura

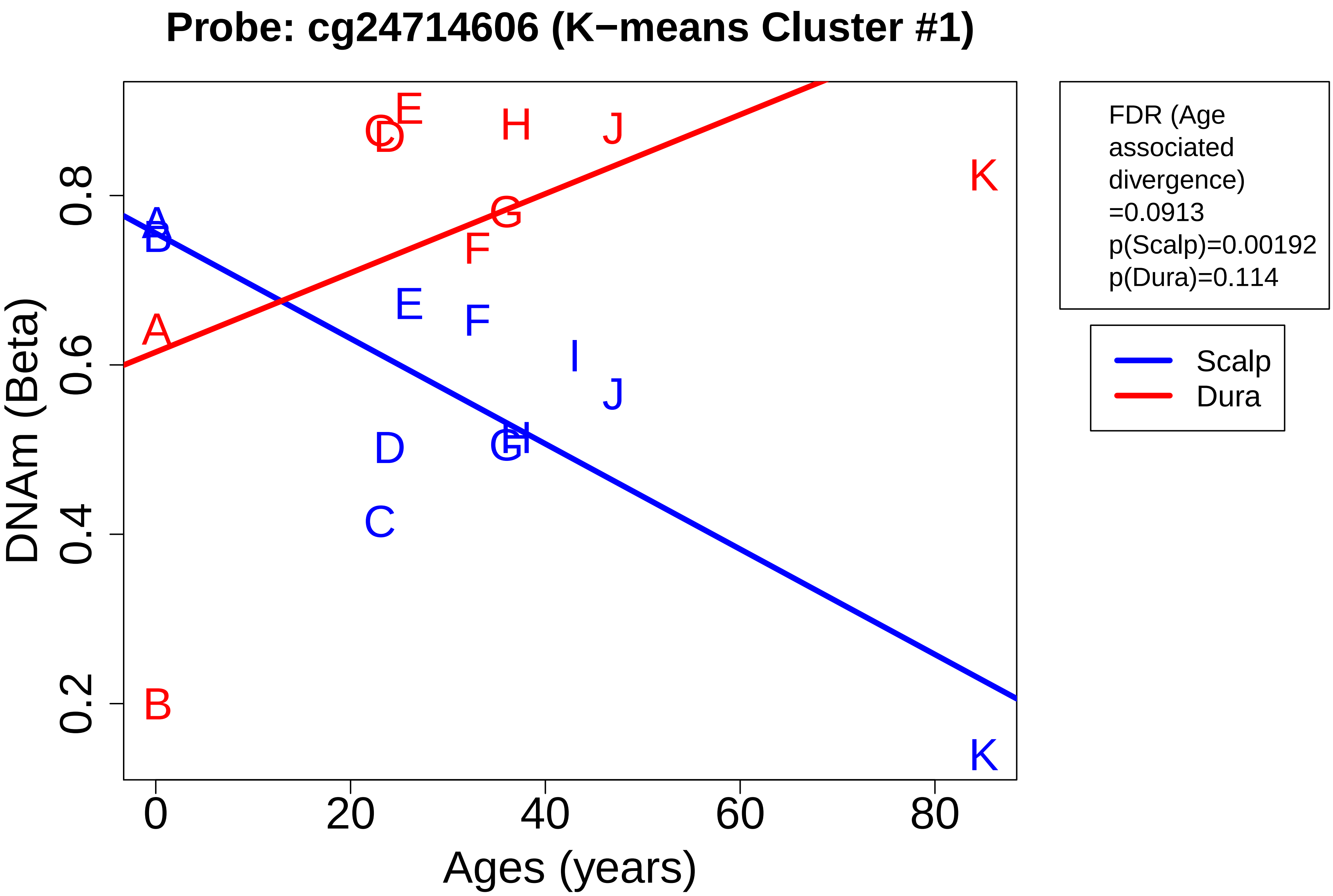

Probe: cg24714606 (K−means Cluster #1)
DNAm (Beta)
0.2
0.4
0.6
0.8
0
20
40
60
80
Ages (years)
A
B
C
D
E
F
G
H
I
J
K
FDR (Age
associated
divergence)
=0.0913
p(Scalp)=0.00192
p(Dura)=0.114
Scalp
Dura

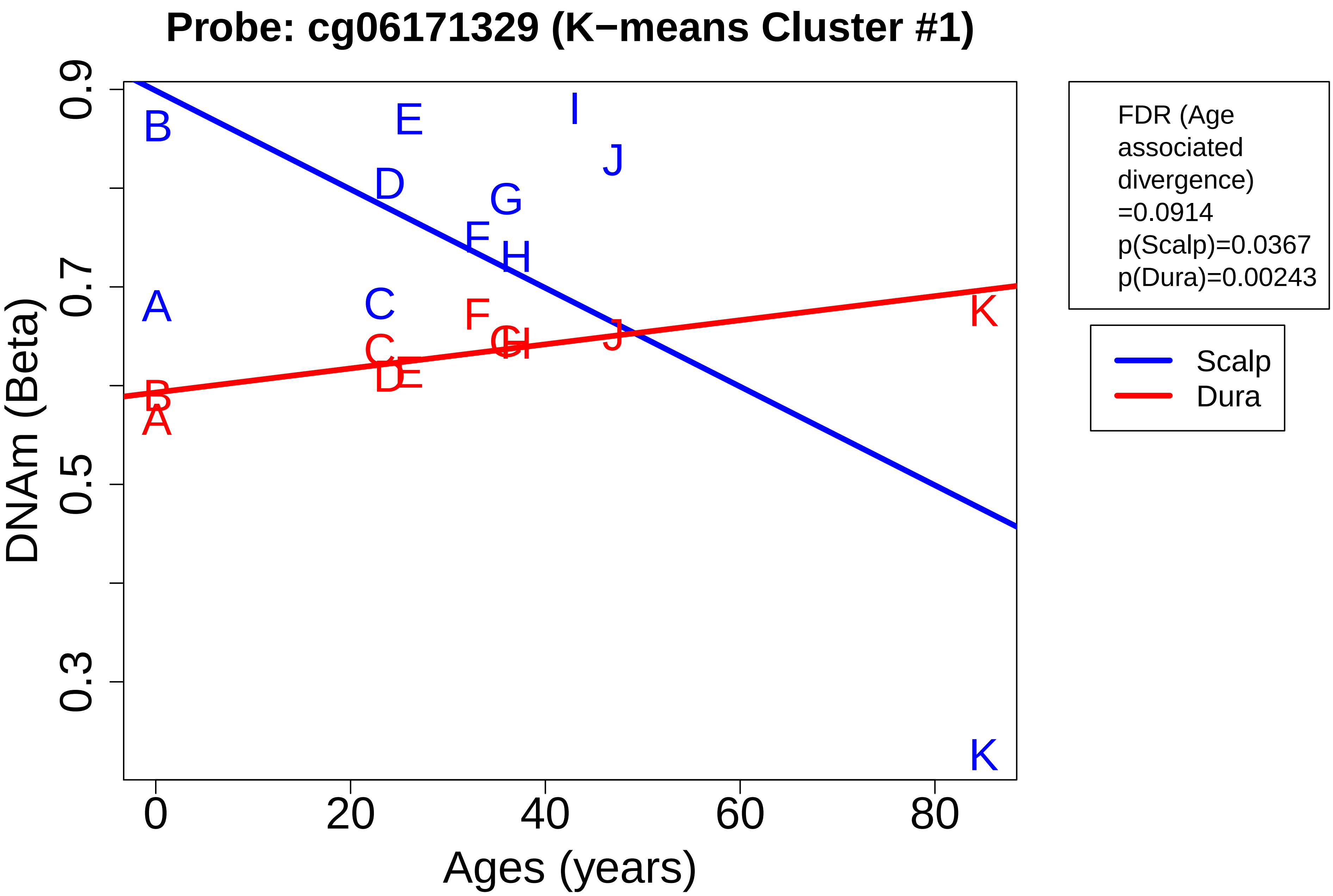

Probe: cg06171329 (K−means Cluster #1)
DNAm (Beta)
0.3
0.5
0.7
0.9
0
20
40
60
80
Ages (years)
FDR (Age associated divergence) =0.0914
p(Scalp)=0.0367
p(Dura)=0.00243
Scalp
Dura

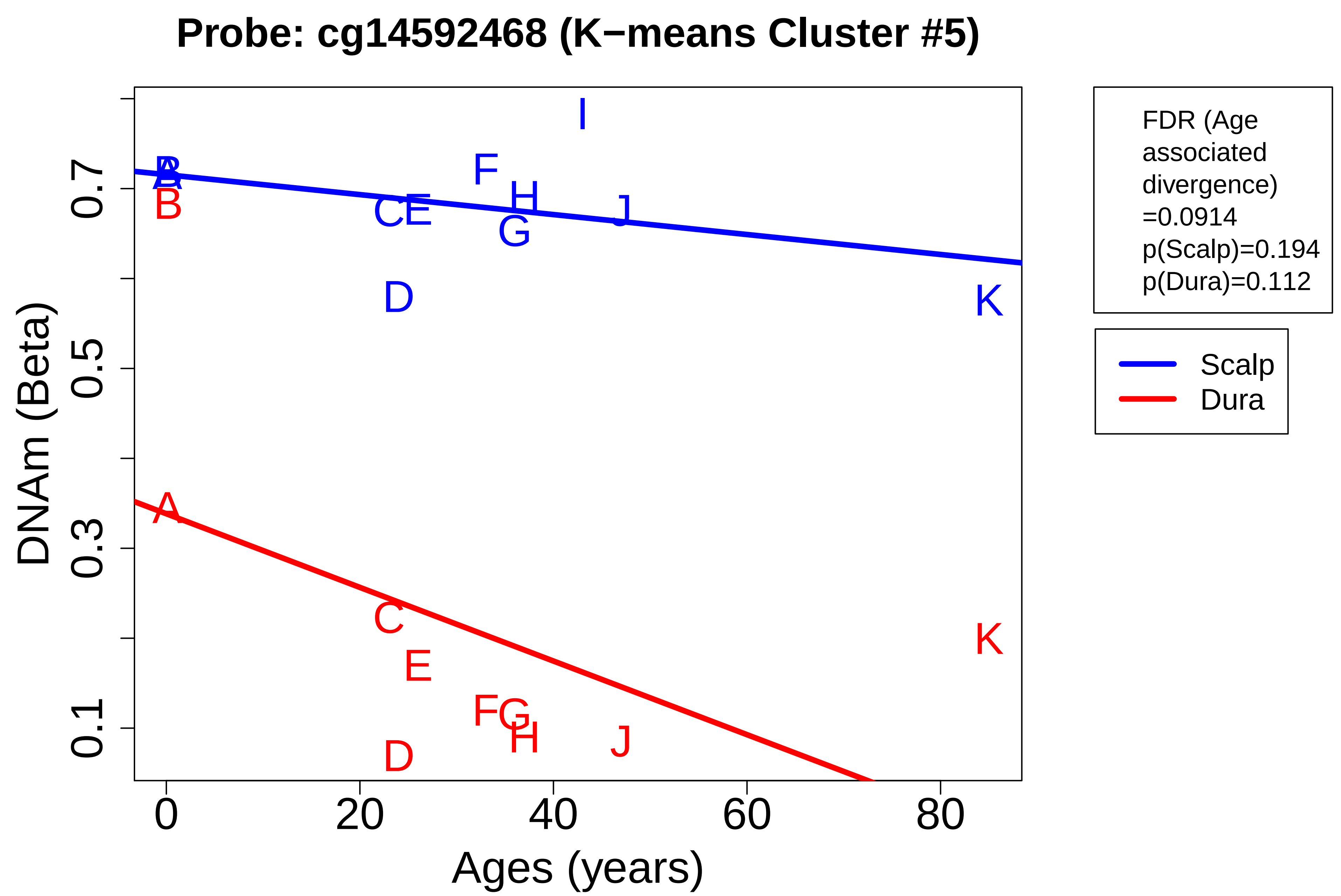

Probe: cg14592468 (K−means Cluster #5)
DNAm (Beta)
0.1
0.3
0.5
0.7
0
20
40
60
80
Ages (years)
FDR (Age associated divergence) =0.0914
p(Scalp)=0.194
p(Dura)=0.112
Scalp
Dura

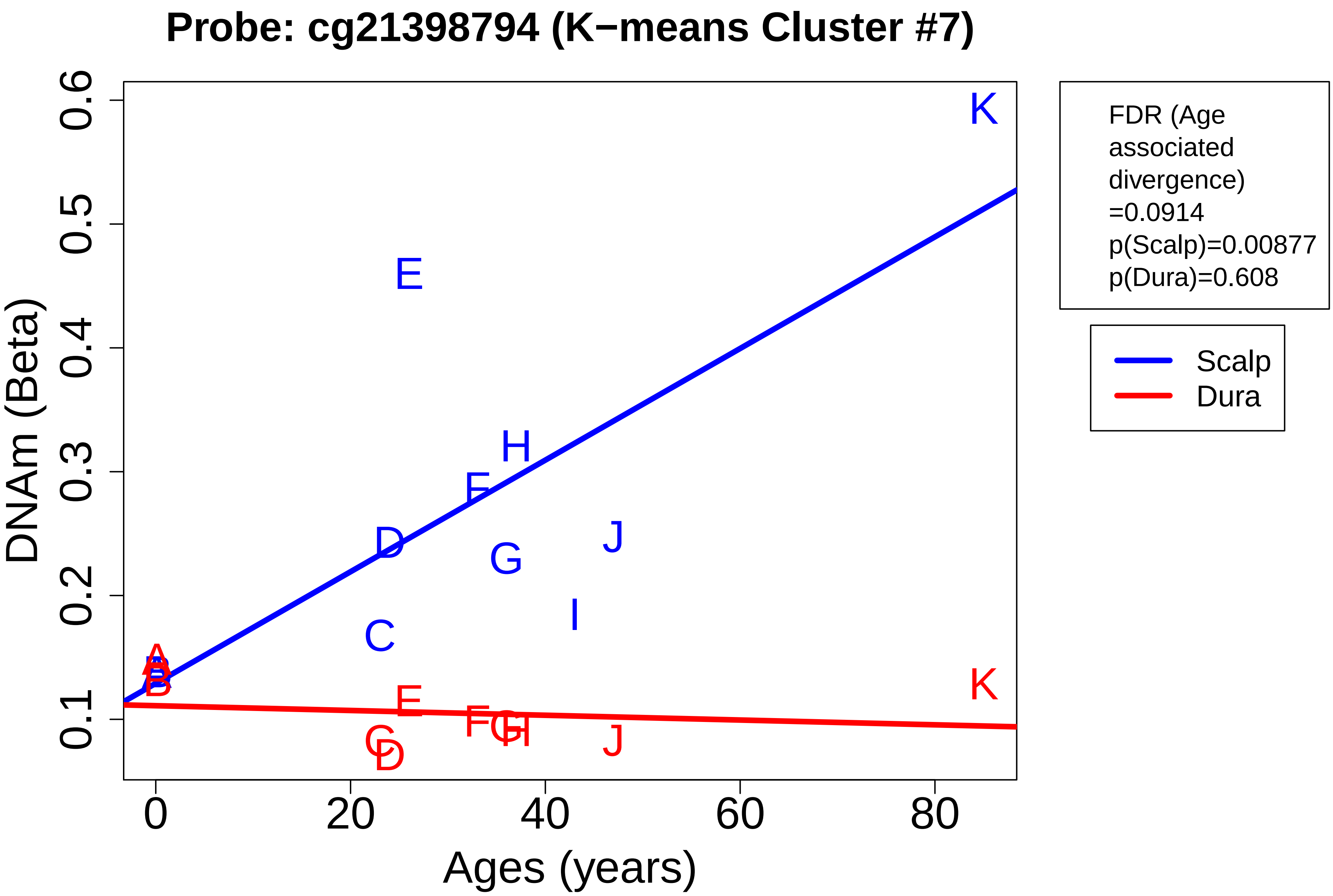
Probe: cg21398794 (K−means Cluster #7)
DNAm (Beta)
0.1
0.2
0.3
0.4
0.5
0.6
0
20
40
60
80
Ages (years)
A
B
C
D
E
F
G
H
I
J
K
FDR (Age associated divergence) =0.0914
p(Scalp)=0.00877
p(Dura)=0.608
Scalp
Dura

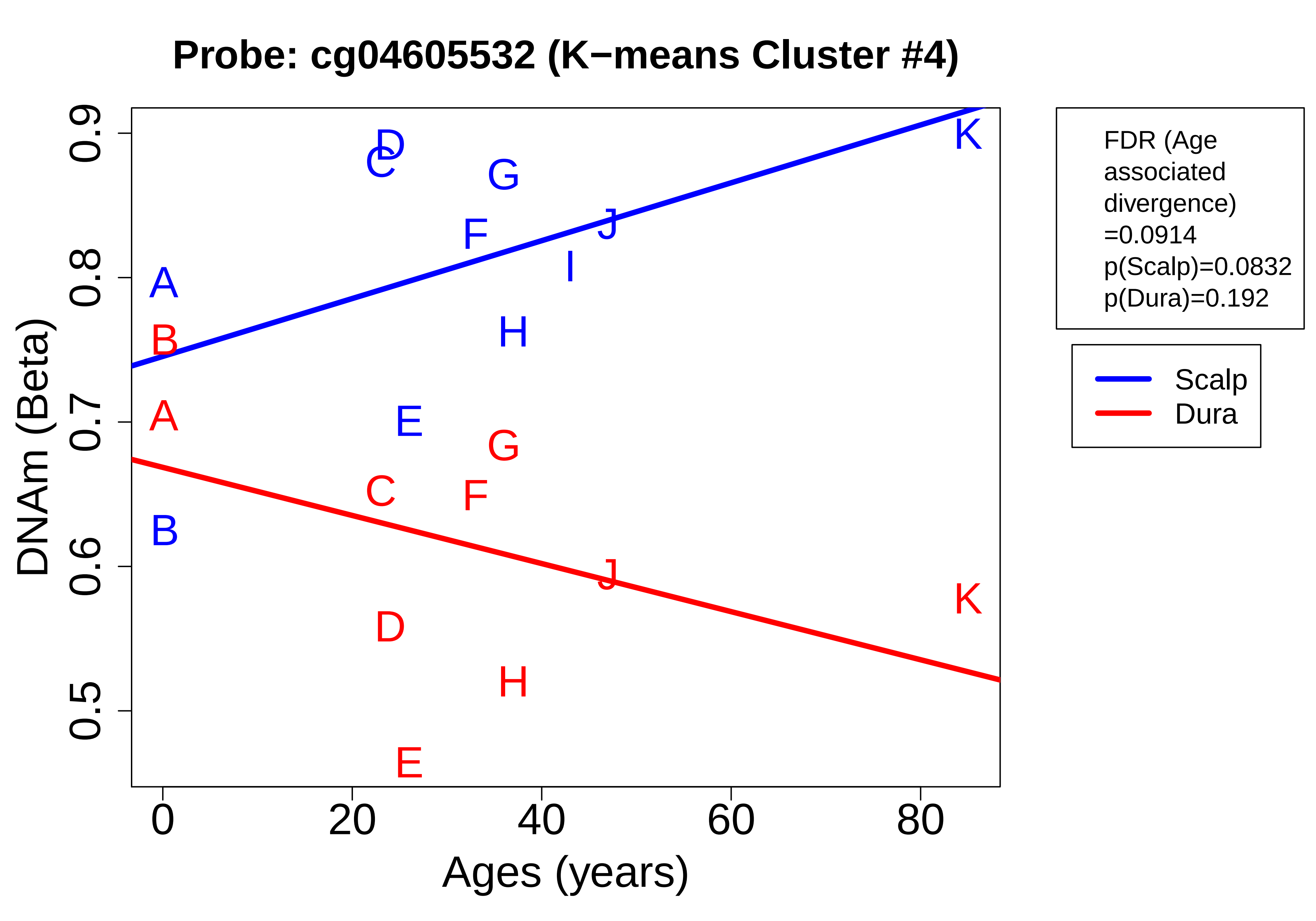
Probe: cg04605532 (K−means Cluster #4)
DNAm (Beta)
0.5
0.6
0.7
0.8
0.9
0
20
40
60
80
Ages (years)
A
B
C
D
E
F
G
H
I
J
K
A
B
C
D
E
F
G
H
J
K
FDR (Age associated divergence) =0.0914
p(Scalp)=0.0832
p(Dura)=0.192
Scalp
Dura

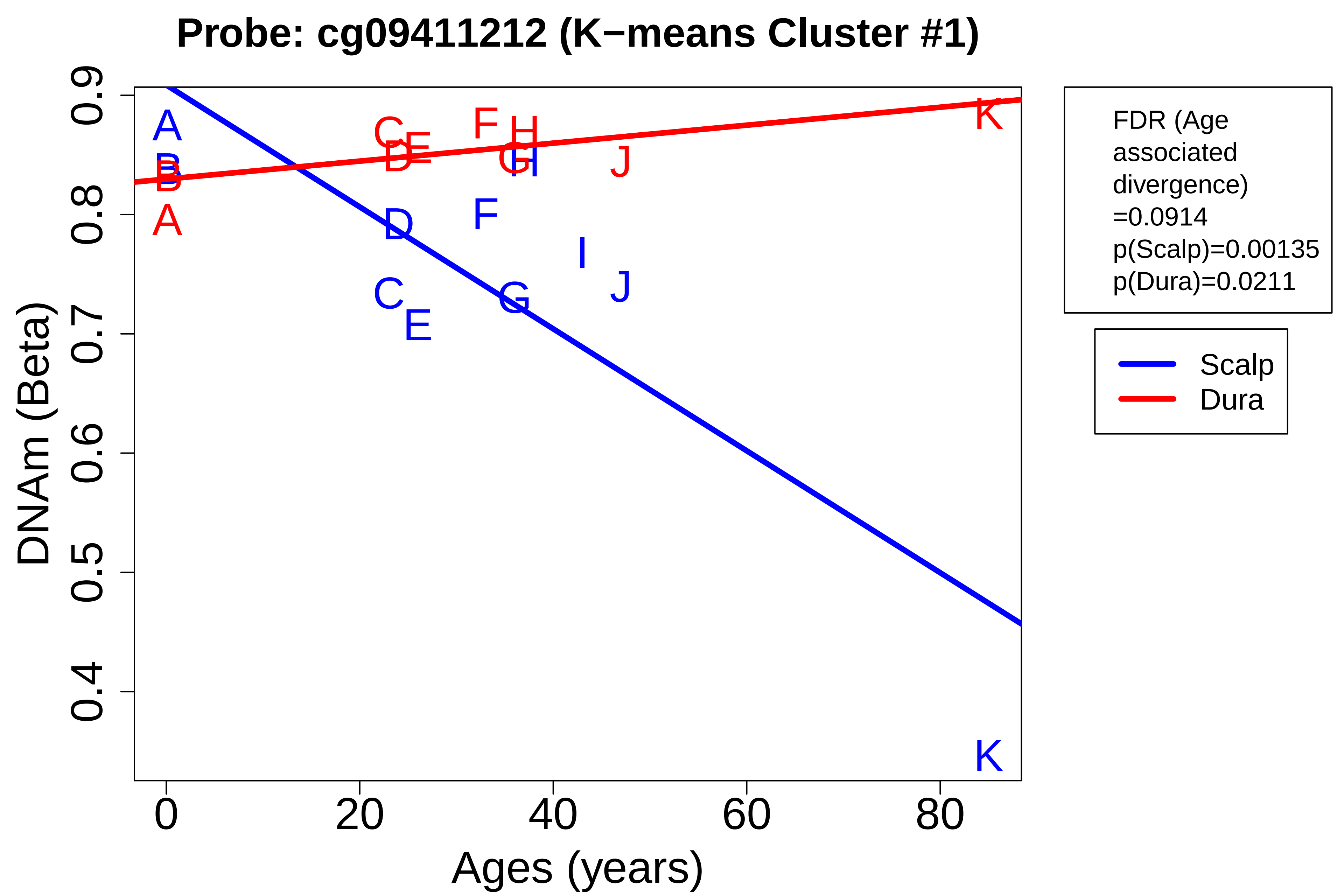

Probe: cg09411212 (K−means Cluster #1)
DNAm (Beta)
Ages (years)
0.4
0.5
0.6
0.7
0.8
0.9
0
20
40
60
80
FDR (Age associated divergence) =0.0914
p(Scalp)=0.00135
p(Dura)=0.0211
Scalp
Dura

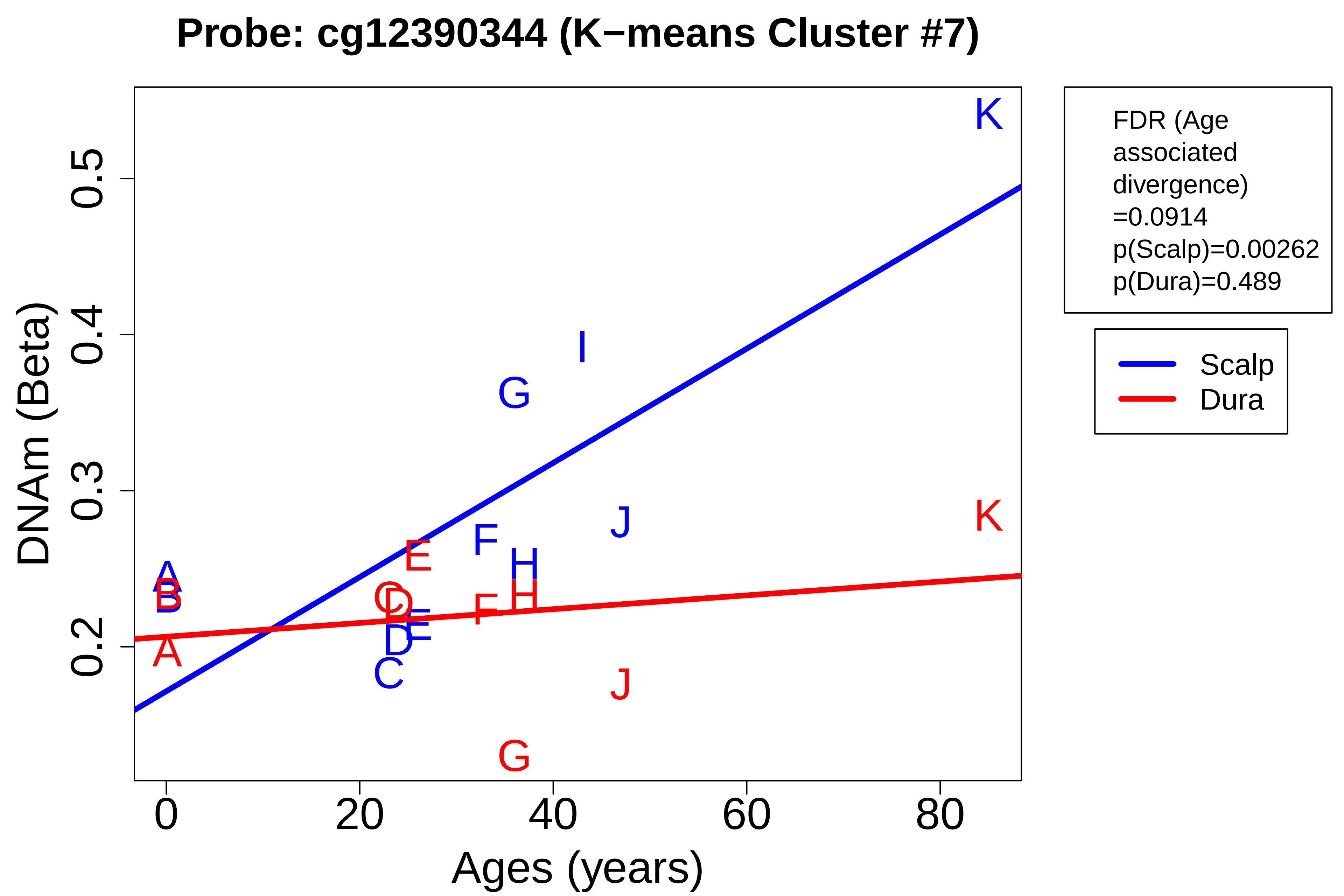

Probe: cg12390344 (K−means Cluster #7)
FDR (Age associated divergence) =0.0914
p(Scalp)=0.00262
p(Dura)=0.489
Scalp
Dura
DNAm (Beta)
0.2
0.3
0.4
0.5
Ages (years)
0
20
40
60
80
A
B
C
D
E
F
G
H
I
J
K

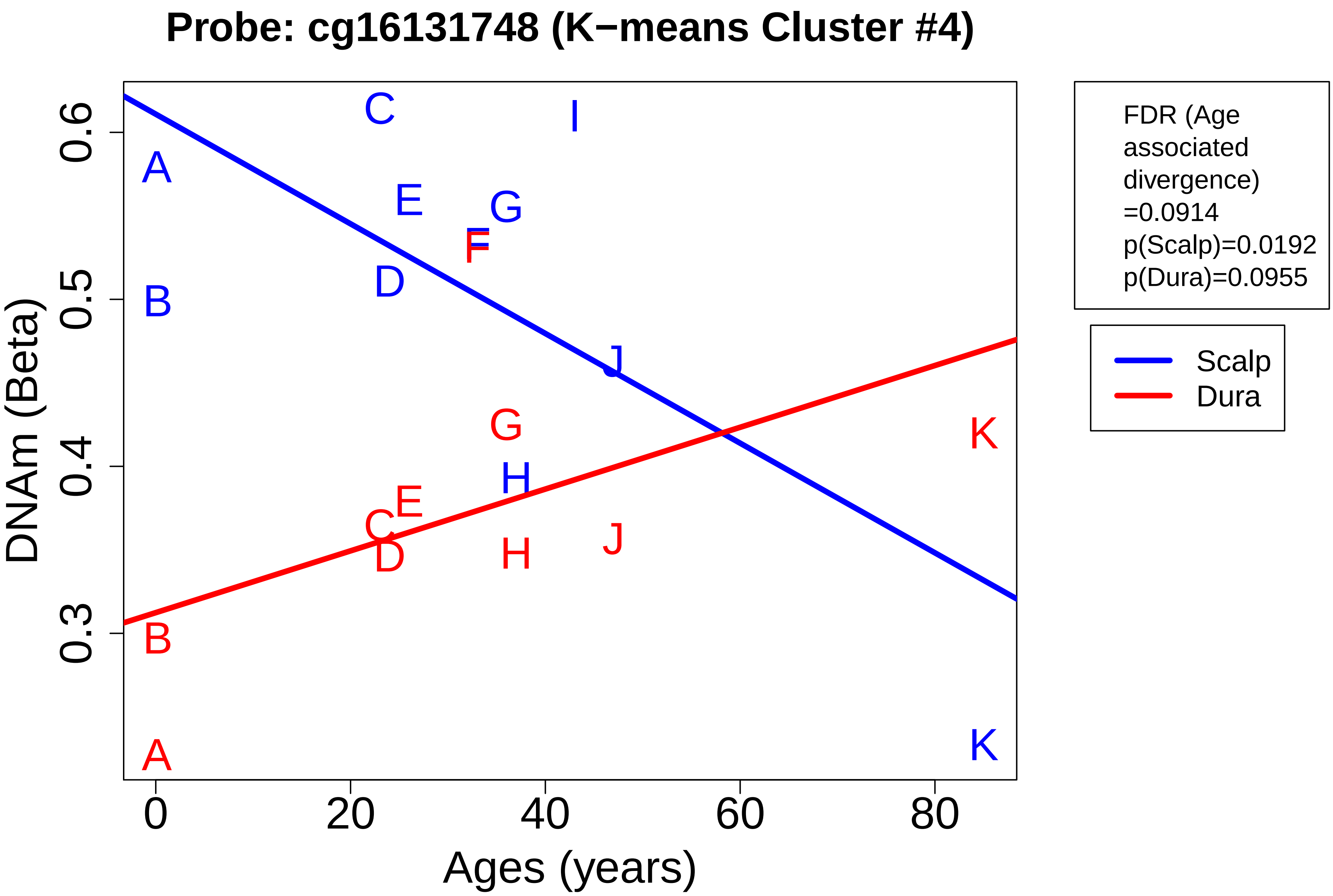

Probe: cg16131748 (K−means Cluster #4)
DNAm (Beta)
0.3
0.4
0.5
0.6
Ages (years)
0
20
40
60
80
A
B
C
D
E
F
G
H
I
J
K
FDR (Age associated divergence) =0.0914
p(Scalp)=0.0192
p(Dura)=0.0955
Scalp
Dura

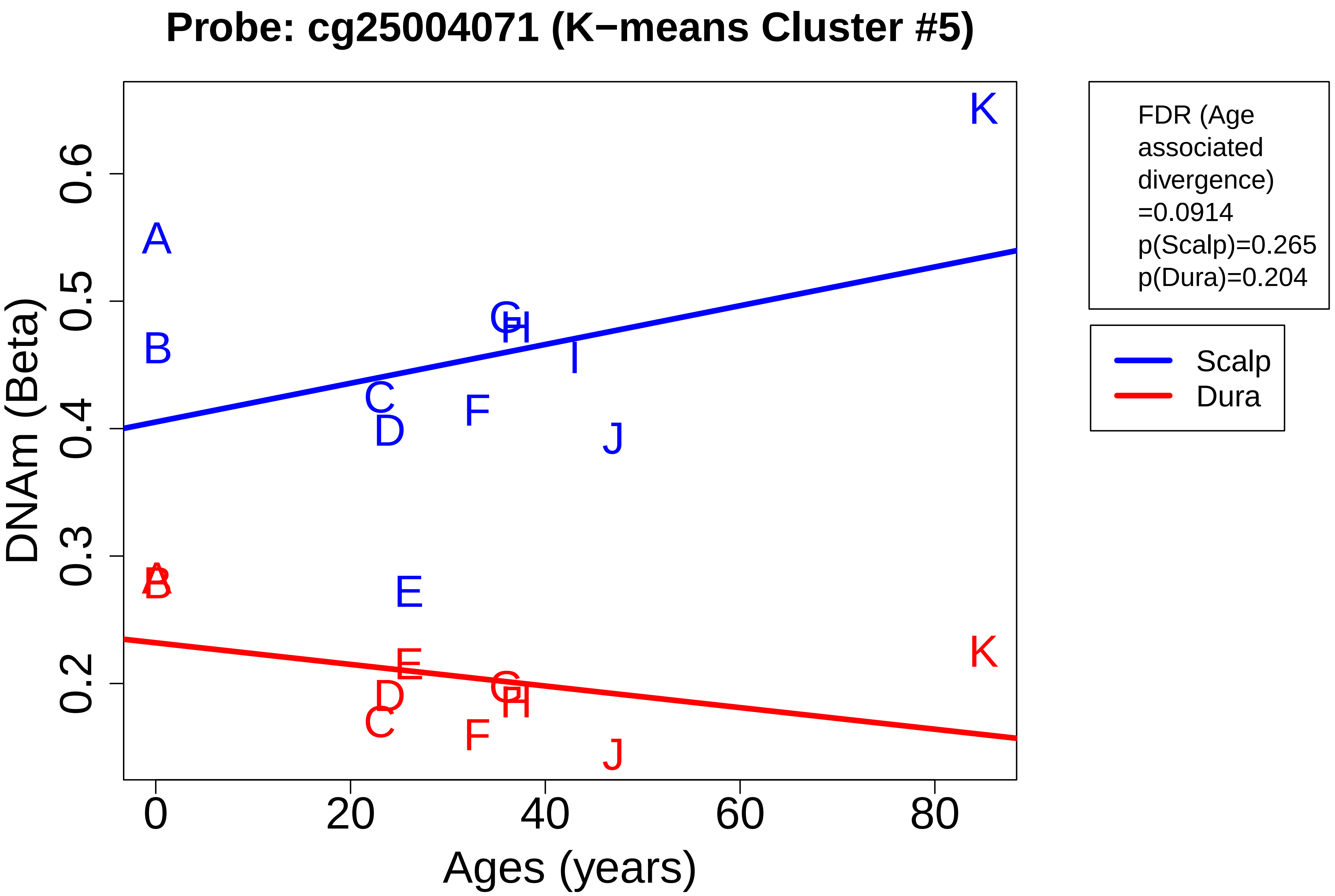

Probe: cg25004071 (K−means Cluster #5)
DNAm (Beta)
0.2
0.3
0.4
0.5
0.6
0
20
40
60
80
Ages (years)
A
B
C
D
E
F
G
H
I
J
K
FDR (Age associated divergence) =0.0914
p(Scalp)=0.265
p(Dura)=0.204
Scalp
Dura

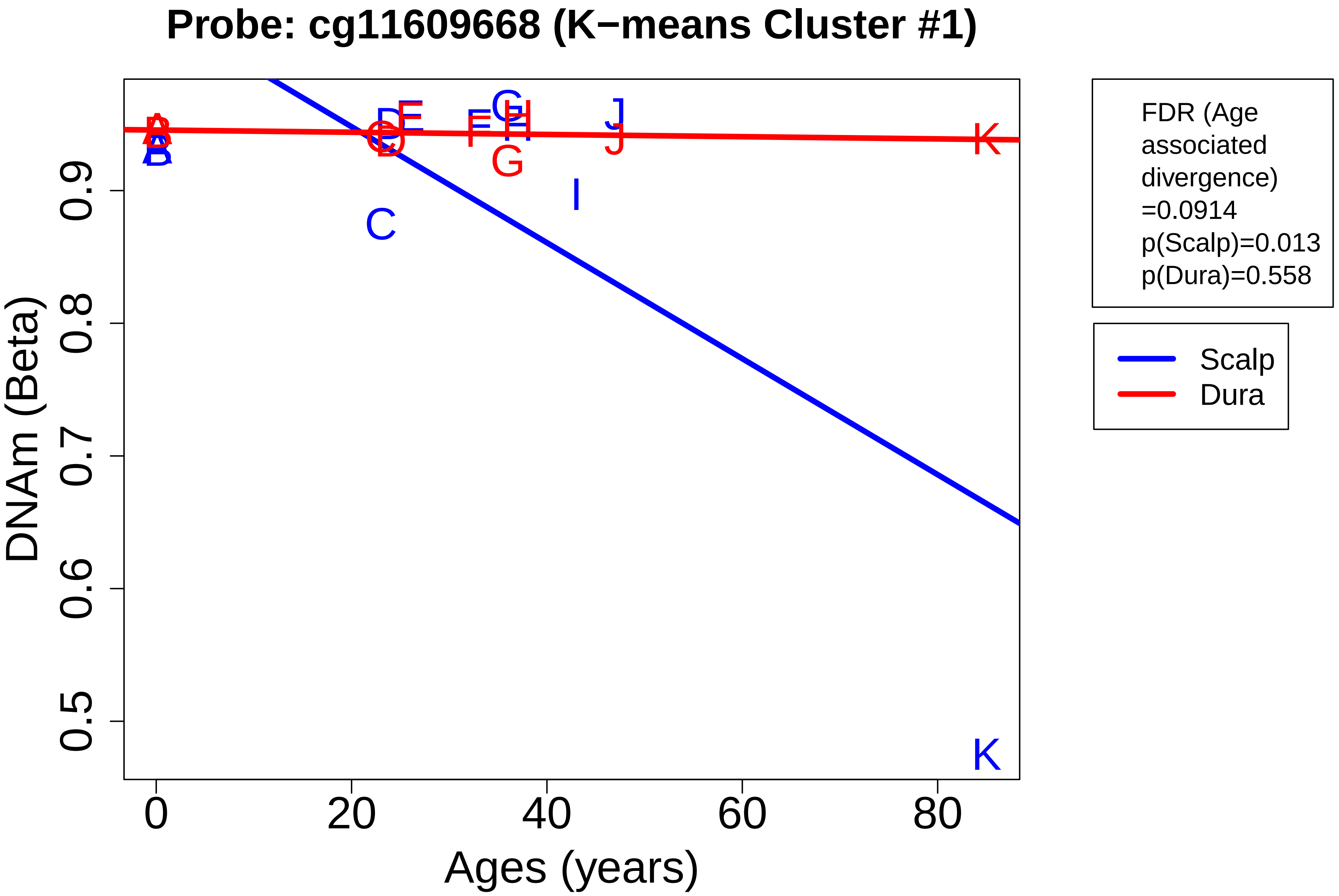

Probe: cg11609668 (K−means Cluster #1)
DNAm (Beta)
Ages (years)
0.5
0.6
0.7
0.8
0.9
0
20
40
60
80
FDR (Age associated divergence) =0.0914
p(Scalp)=0.013
p(Dura)=0.558
Scalp
Dura

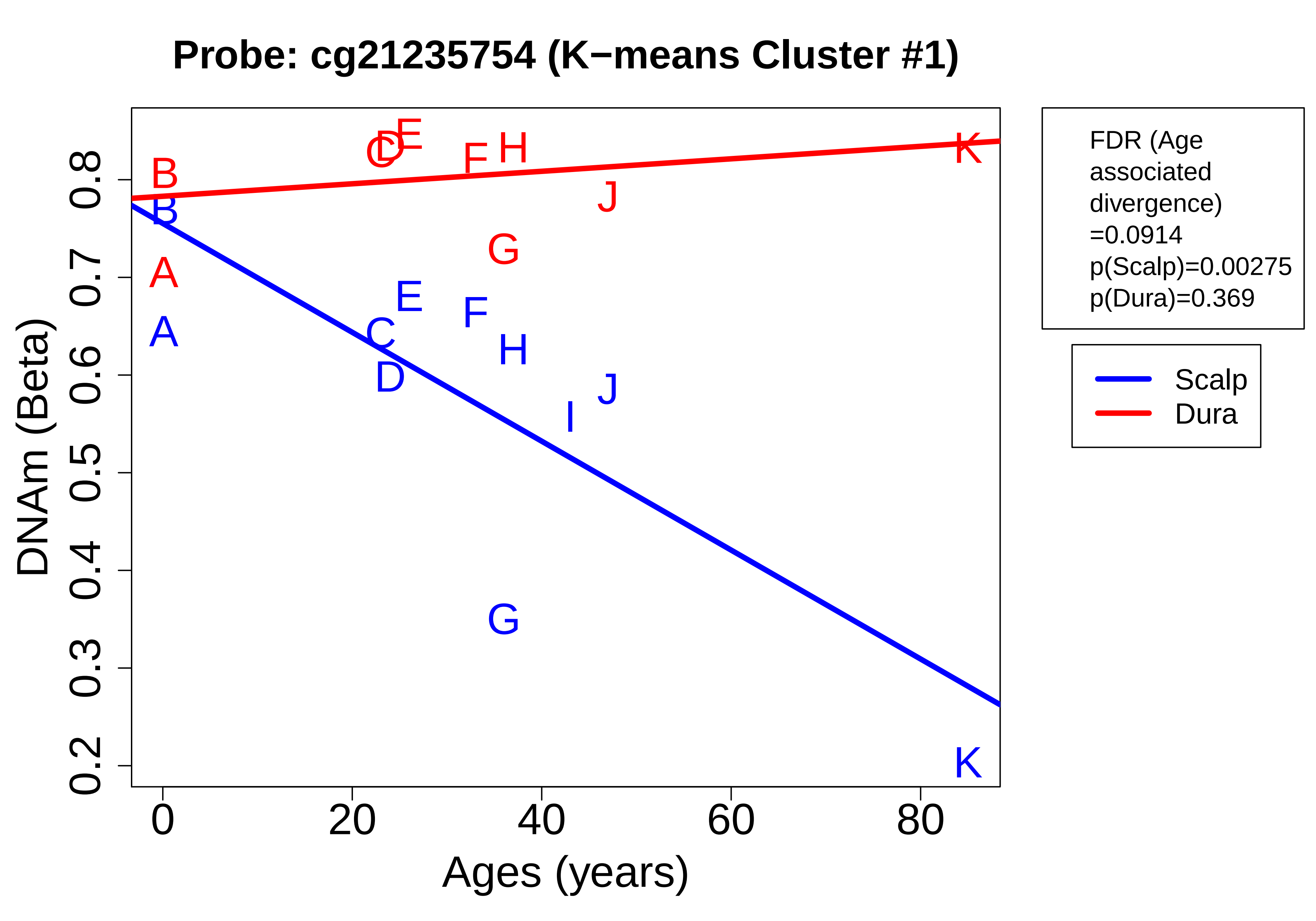
Probe: cg21235754 (K−means Cluster #1)
DNAm (Beta)
Ages (years)
0.2
0.3
0.4
0.5
0.6
0.7
0.8
0
20
40
60
80
A
B
C
D
E
F
G
H
I
J
K
FDR (Age associated divergence) =0.0914
p(Scalp)=0.00275
p(Dura)=0.369
Scalp
Dura

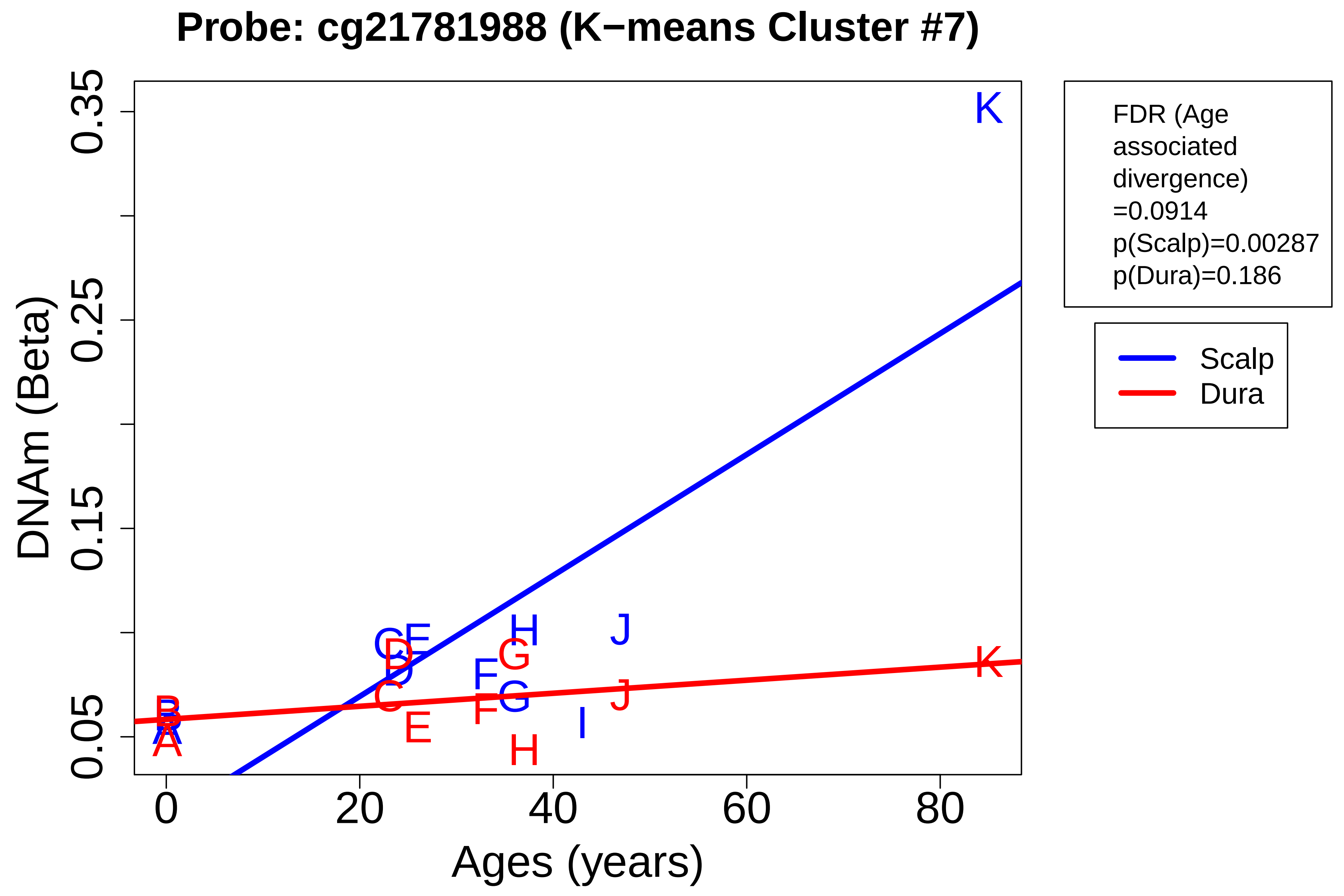
Probe: cg21781988 (K−means Cluster #7)
DNAm (Beta)
0.05
0.15
0.25
0.35
Ages (years)
0
20
40
60
80
FDR (Age associated divergence) =0.0914
p(Scalp)=0.00287
p(Dura)=0.186
Scalp
Dura
A
B
C
D
E
F
G
H
I
J
K

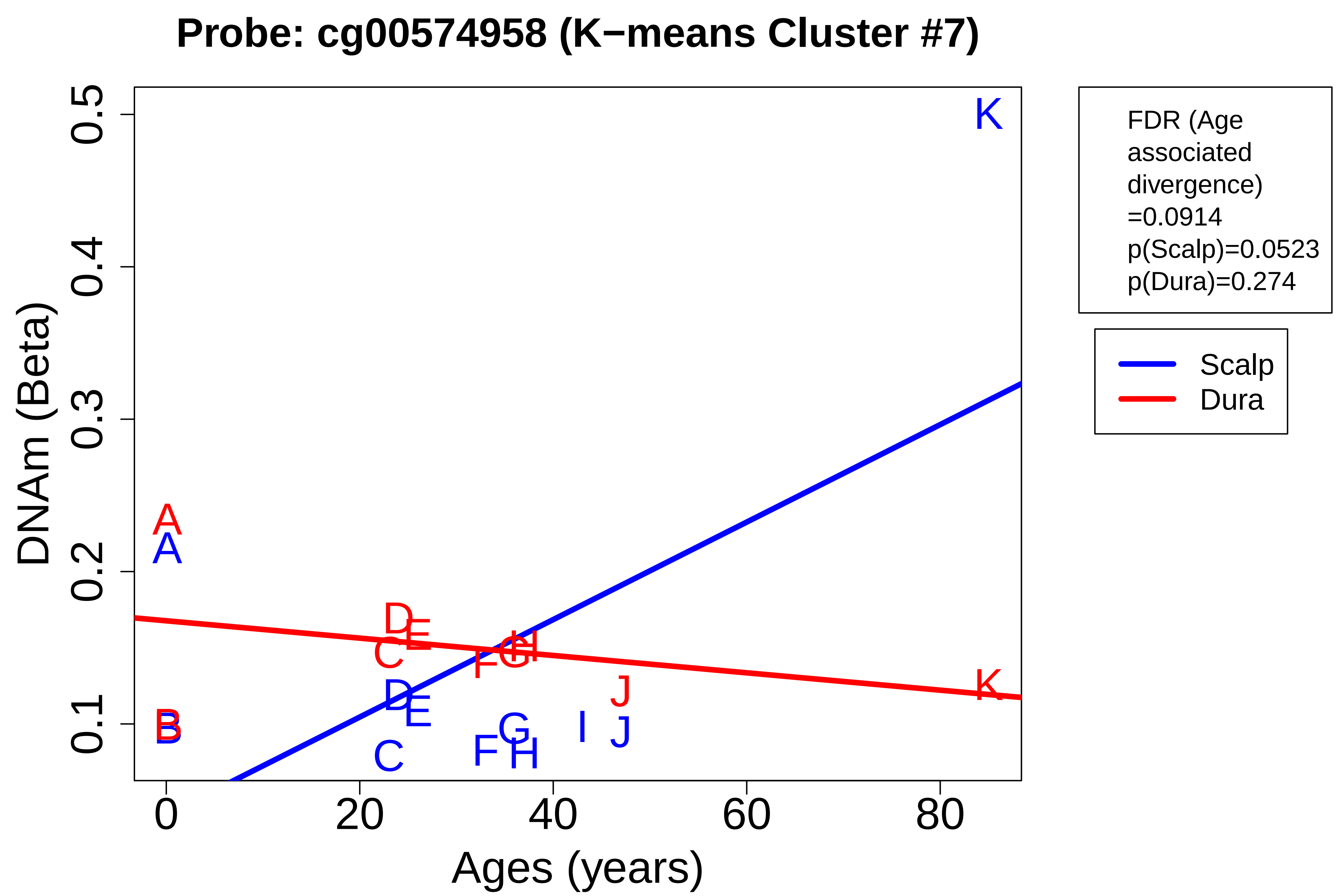

Probe: cg00574958 (K−means Cluster #7)
DNAm (Beta)
0.1
0.2
0.3
0.4
0.5
0
20
40
60
80
Ages (years)
A
B
C
D
E
F
G
H
I
J
K
FDR (Age associated divergence) =0.0914
p(Scalp)=0.0523
p(Dura)=0.274
Scalp
Dura

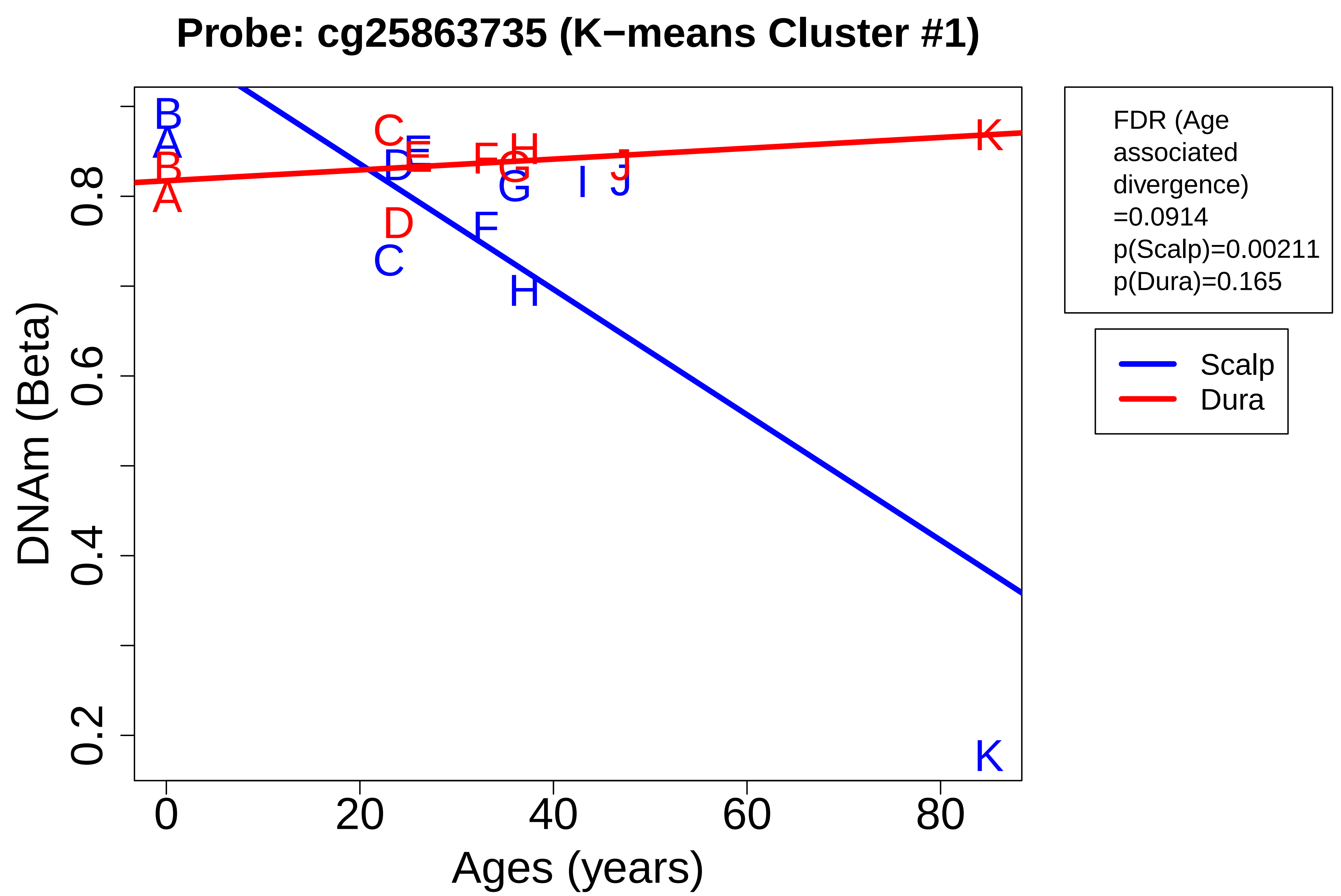

Probe: cg25863735 (K−means Cluster #1)
DNAm (Beta)
0.2
0.4
0.6
0.8
0
20
40
60
80
Ages (years)
B
A
B
A
C
E
D
E
D
C
F
H
F
G
G
I
J
J
H
K
K
FDR (Age associated divergence) =0.0914
p(Scalp)=0.00211
p(Dura)=0.165
Scalp
Dura

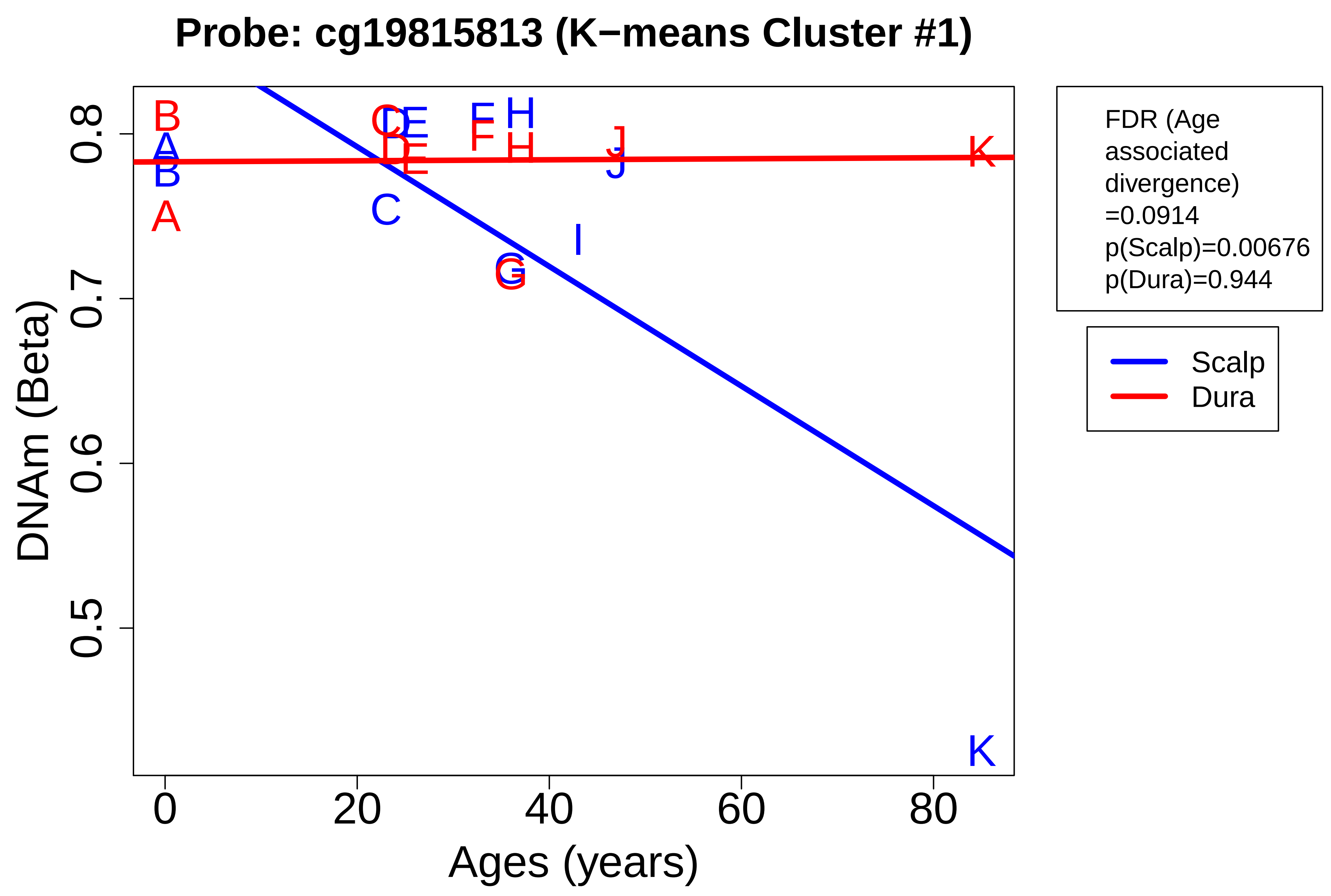

Probe: cg19815813 (K−means Cluster #1)
DNAm (Beta)
0.5
0.6
0.7
0.8
0
20
40
60
80
Ages (years)
A
B
C
D
E
F
G
H
I
J
K
FDR (Age associated divergence) =0.0914
p(Scalp)=0.00676
p(Dura)=0.944
Scalp
Dura

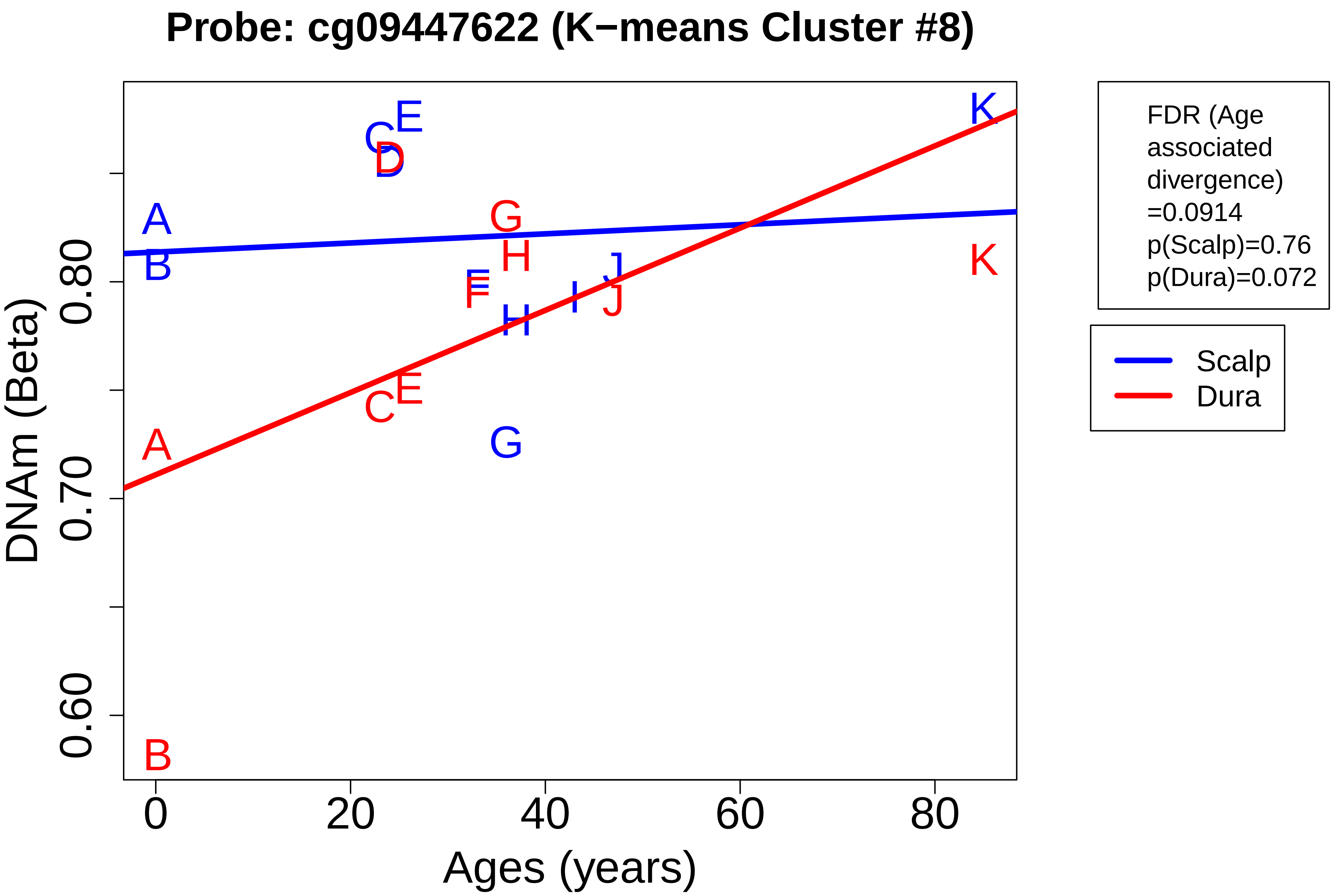

Probe: cg09447622 (K−means Cluster #8)
DNAm (Beta)
0.60
0.70
0.80
Ages (years)
0
20
40
60
80
FDR (Age associated divergence) =0.0914
p(Scalp)=0.76
p(Dura)=0.072
Scalp
Dura

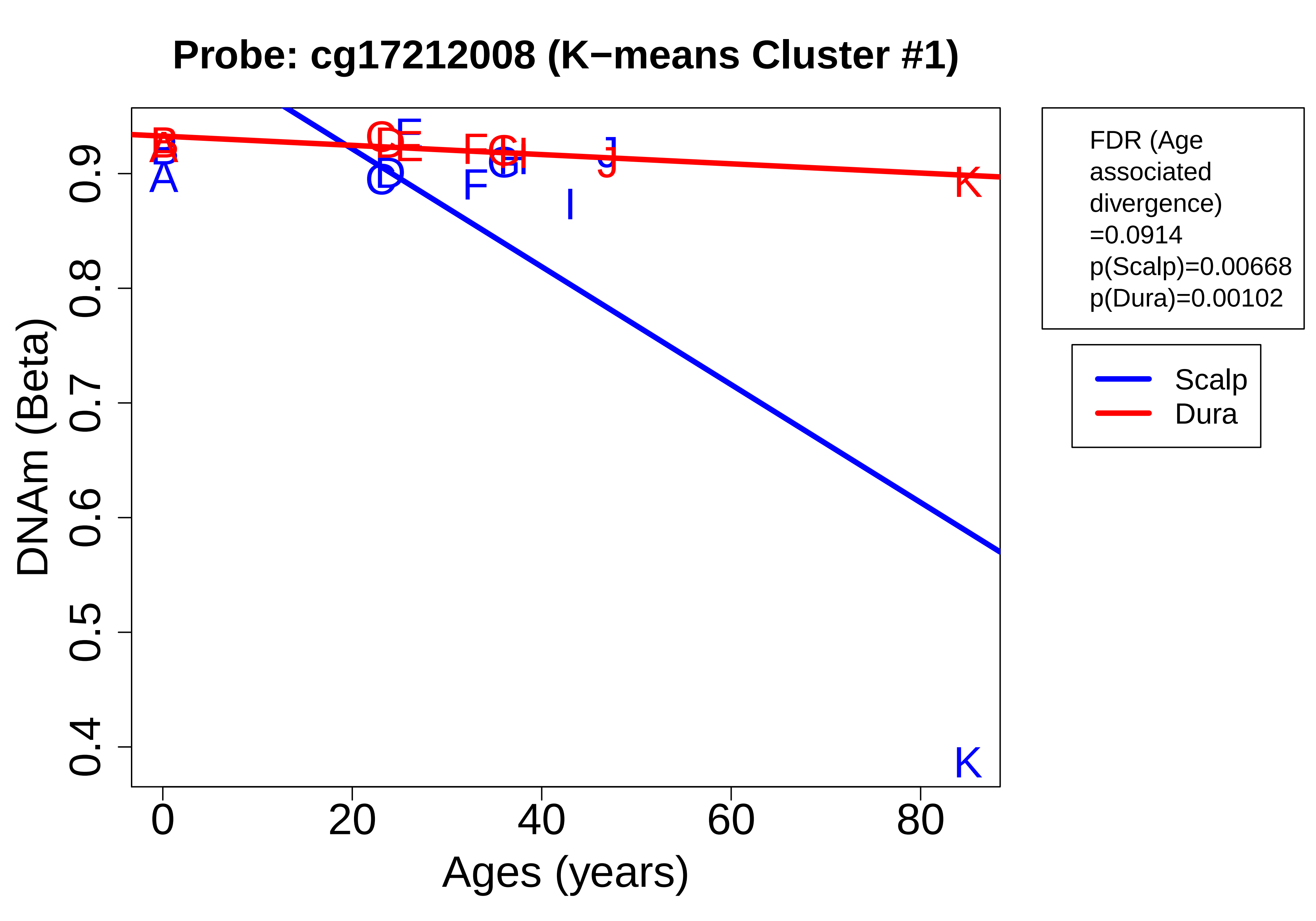

Probe: cg17212008 (K−means Cluster #1)
DNAm (Beta)
0.4
0.5
0.6
0.7
0.8
0.9
Ages (years)
0
20
40
60
80
FDR (Age associated divergence) =0.0914
p(Scalp)=0.00668
p(Dura)=0.00102
Scalp
Dura

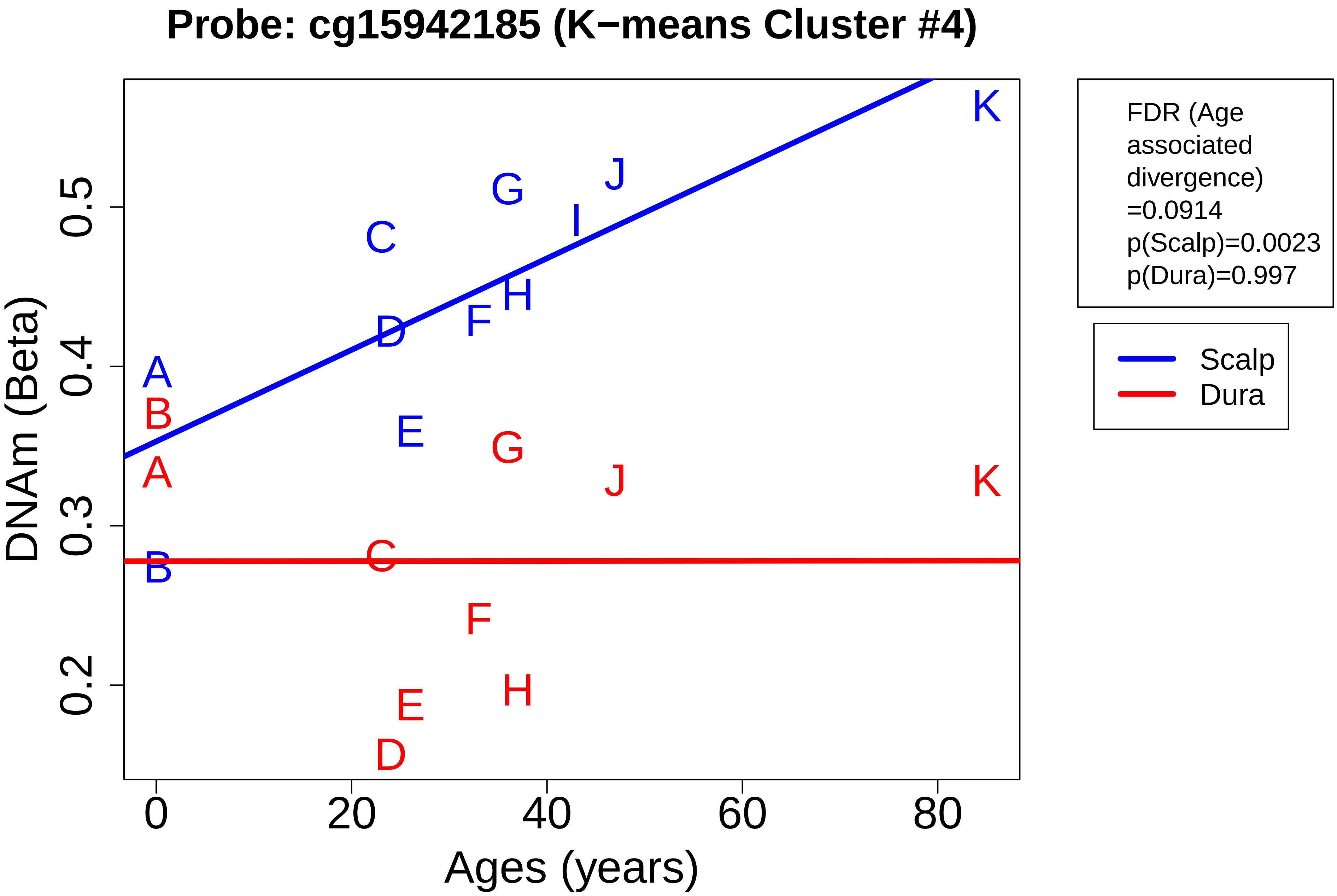

Probe: cg15942185 (K−means Cluster #4)
DNAm (Beta)
0.2
0.3
0.4
0.5
0
20
40
60
80
Ages (years)
A
B
C
D
E
F
G
H
I
J
K
FDR (Age associated divergence) =0.0914
p(Scalp)=0.0023
p(Dura)=0.997
Scalp
Dura

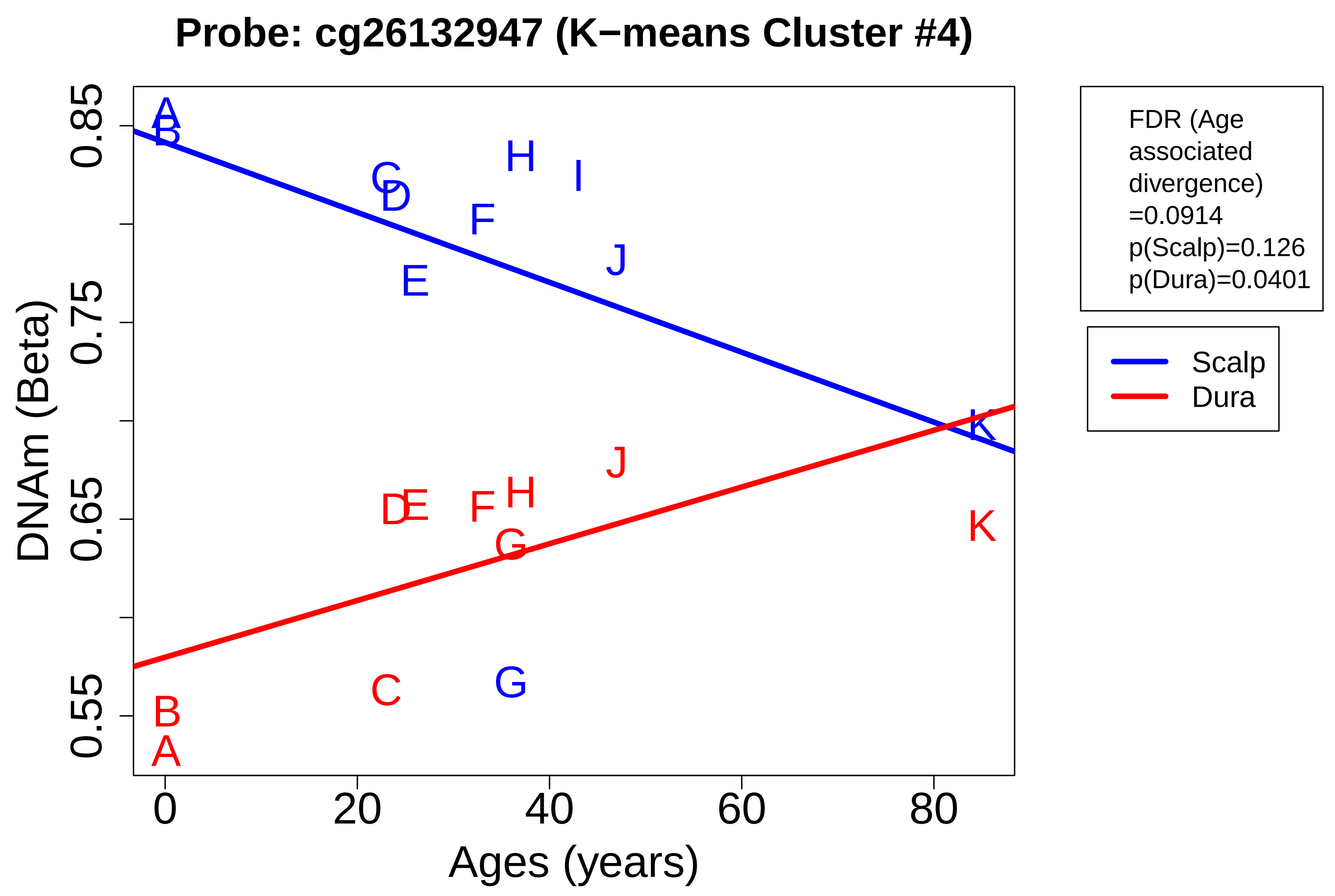

Probe: cg26132947 (K−means Cluster #4)
DNAm (Beta)
0.55
0.65
0.75
0.85
Ages (years)
0
20
40
60
80
FDR (Age associated divergence) =0.0914
p(Scalp)=0.126
p(Dura)=0.0401
Scalp
Dura

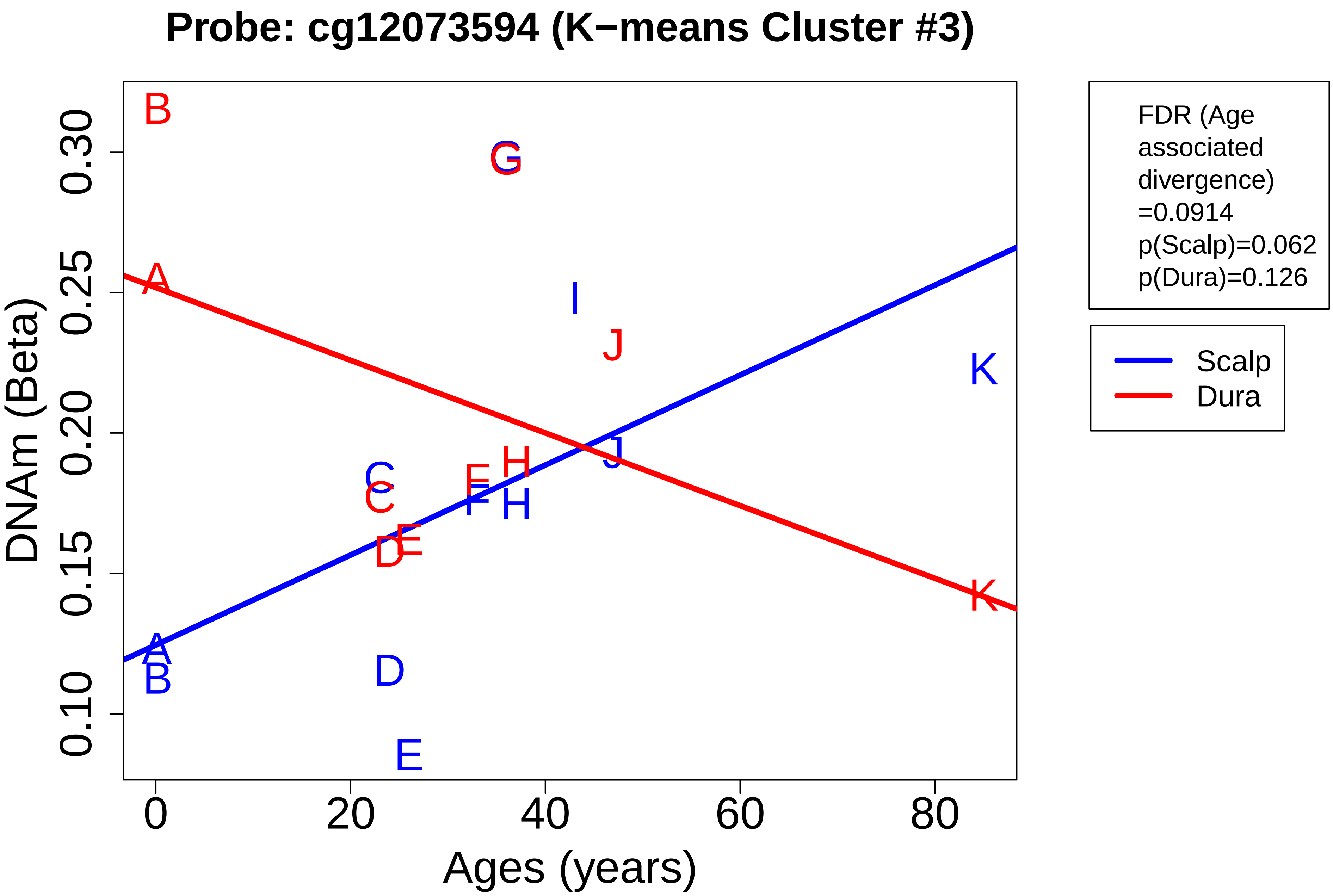

Probe: cg12073594 (K−means Cluster #3)
DNAm (Beta)
0.10
0.15
0.20
0.25
0.30
0
20
40
60
80
Ages (years)
FDR (Age associated divergence) =0.0914
p(Scalp)=0.062
p(Dura)=0.126
Scalp
Dura
A
B
C
D
E
F
G
H
I
J
K

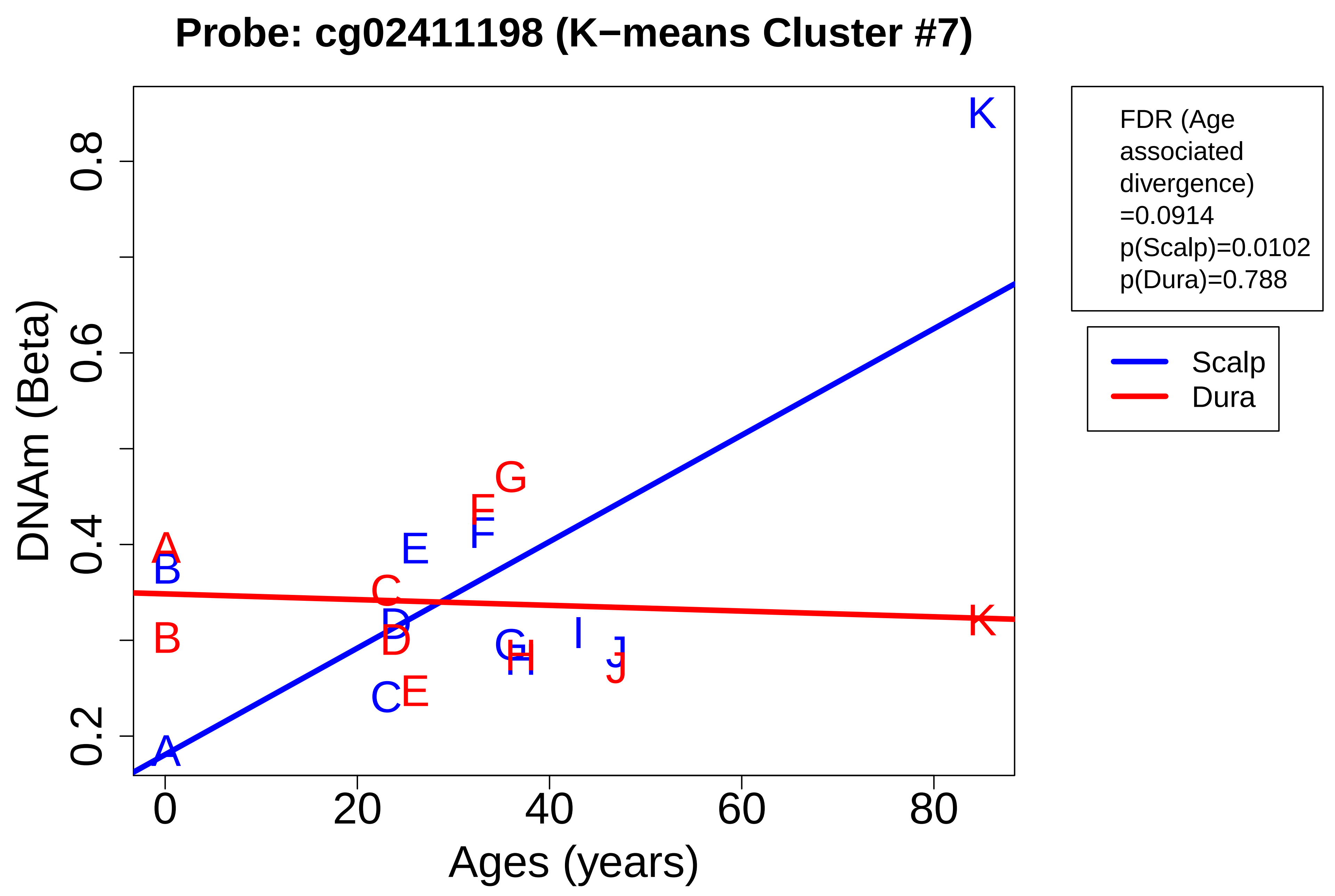
Probe: cg02411198 (K−means Cluster #7)
DNAm (Beta)
0.2
0.4
0.6
0.8
Ages (years)
0
20
40
60
80
FDR (Age associated divergence) =0.0914
p(Scalp)=0.0102
p(Dura)=0.788
Scalp
Dura
A
B
C
D
E
F
G
H
I
J
K

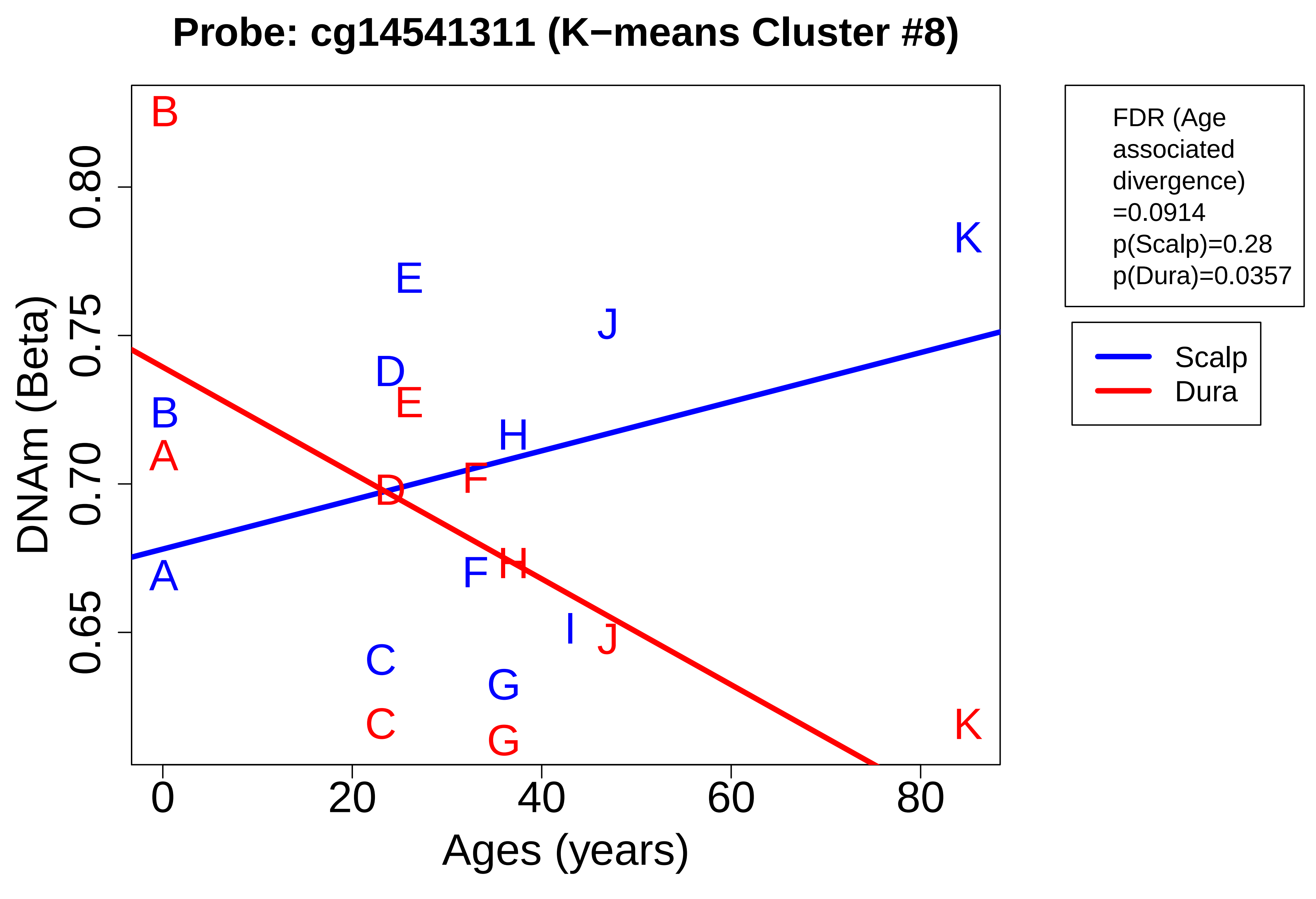

Probe: cg14541311 (K−means Cluster #8)
DNAm (Beta)
0.65
0.70
0.75
0.80
0
20
40
60
80
Ages (years)
FDR (Age associated divergence) =0.0914
p(Scalp)=0.28
p(Dura)=0.0357
Scalp
Dura
A
B
C
D
E
F
G
H
I
J
K

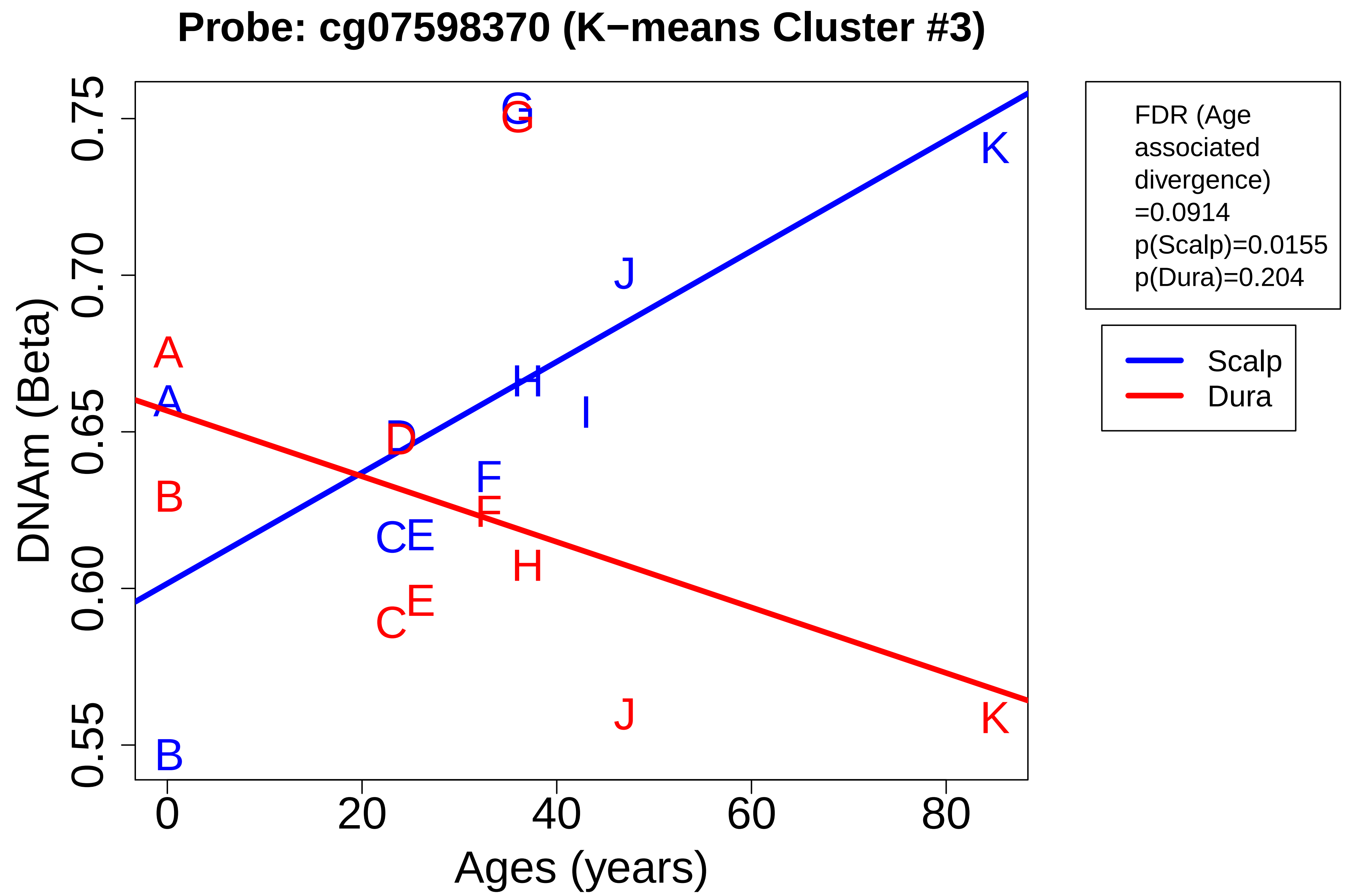

Probe: cg07598370 (K−means Cluster #3)
DNAm (Beta)
0.55
0.60
0.65
0.70
0.75
0
20
40
60
80
Ages (years)
A
B
C
D
E
F
G
H
I
J
K
FDR (Age associated divergence) =0.0914
p(Scalp)=0.0155
p(Dura)=0.204
Scalp
Dura

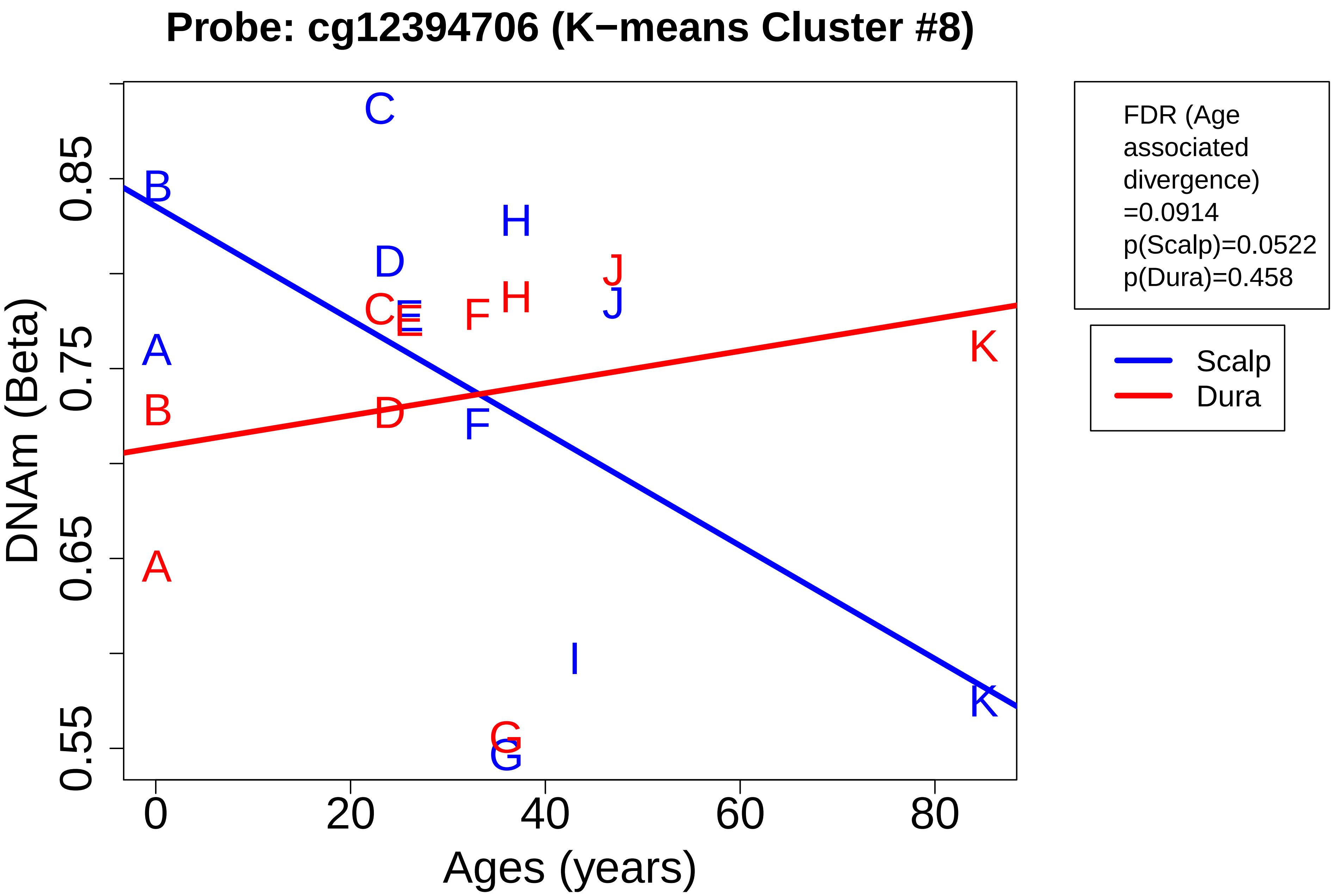

Probe: cg12394706 (K−means Cluster #8)
DNAm (Beta)
0.55
0.65
0.75
0.85
Ages (years)
0
20
40
60
80
FDR (Age associated divergence) =0.0914
p(Scalp)=0.0522
p(Dura)=0.458
Scalp
Dura

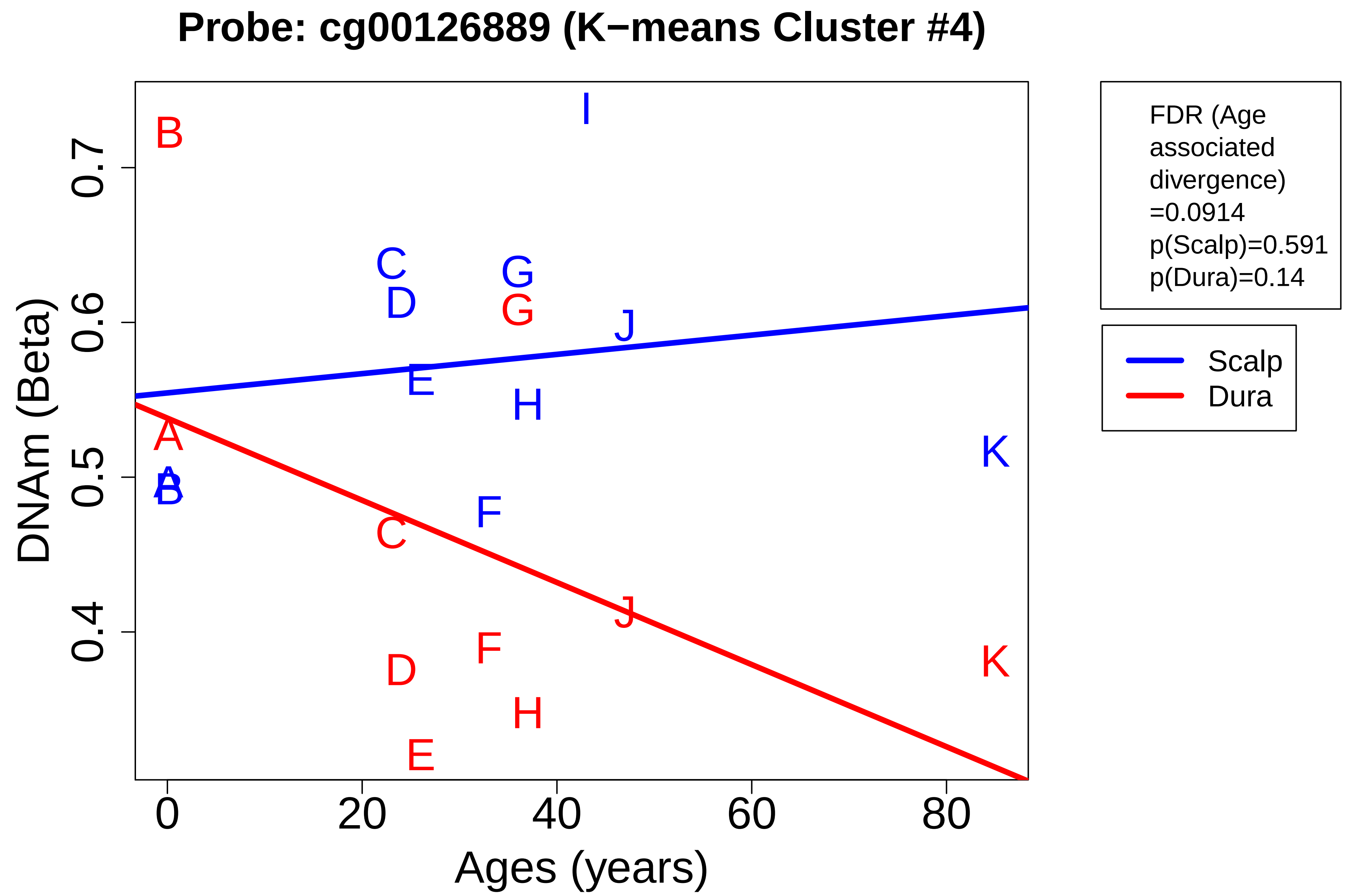

Probe: cg00126889 (K−means Cluster #4)
DNAm (Beta)
0.4
0.5
0.6
0.7
0
20
40
60
80
Ages (years)
FDR (Age associated divergence) =0.0914
p(Scalp)=0.591
p(Dura)=0.14
Scalp
Dura

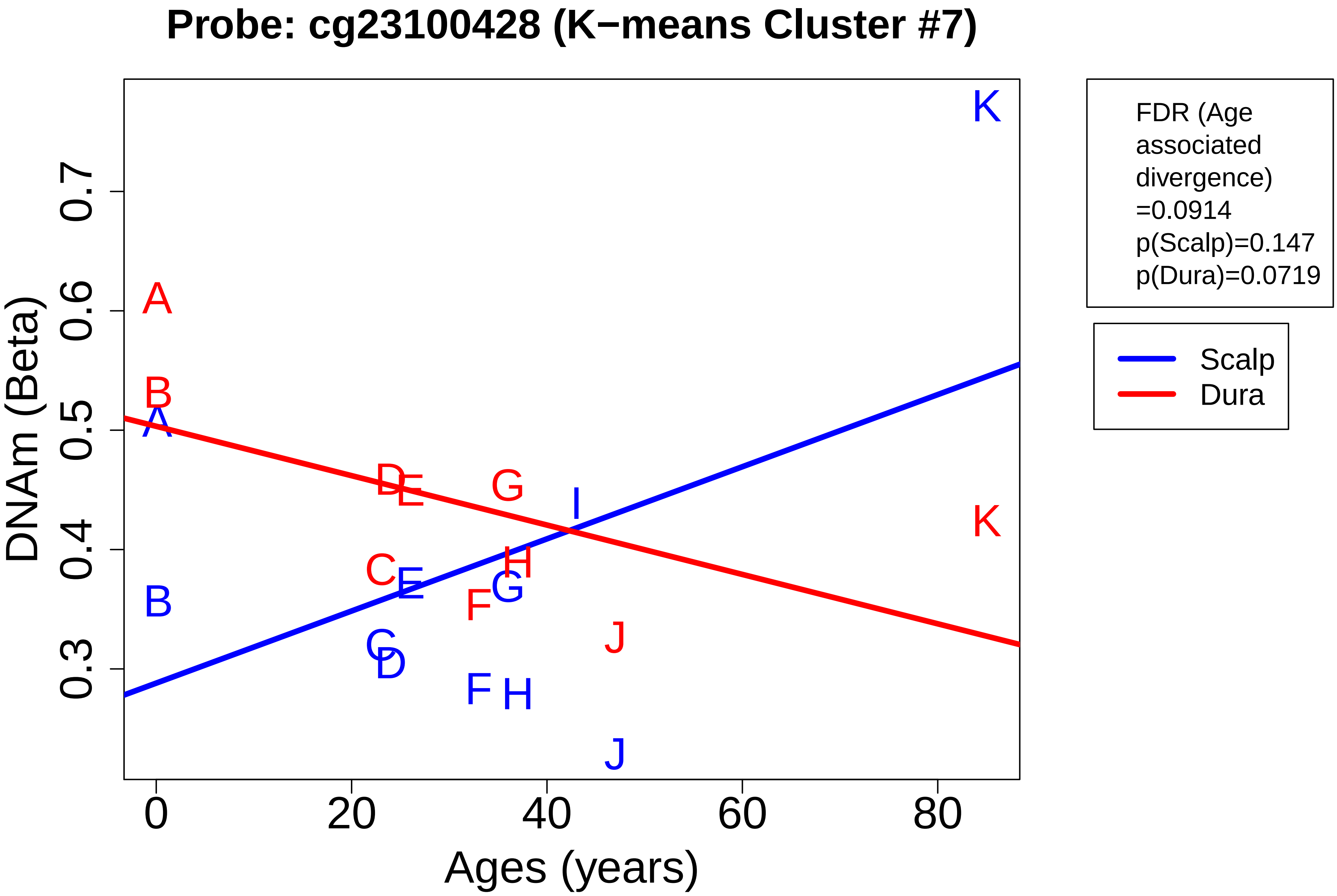

Probe: cg23100428 (K−means Cluster #7)
DNAm (Beta)
0.3
0.4
0.5
0.6
0.7
Ages (years)
0
20
40
60
80
FDR (Age associated divergence) =0.0914
p(Scalp)=0.147
p(Dura)=0.0719
Scalp
Dura
A
B
C
D
E
F
G
H
I
J
K

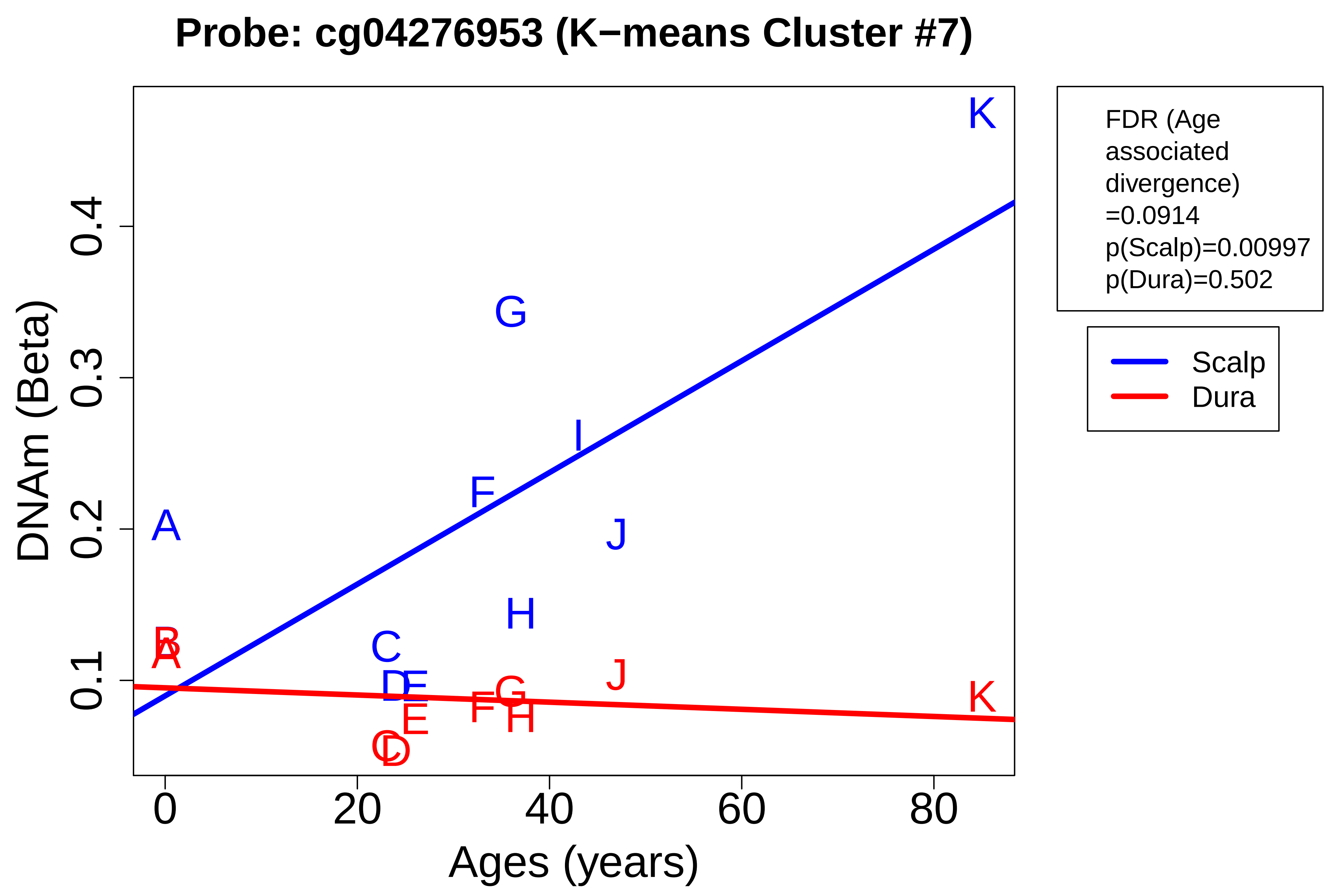

Probe: cg04276953 (K−means Cluster #7)
DNAm (Beta)
0.1
0.2
0.3
0.4
0
20
40
60
80
Ages (years)
FDR (Age associated divergence) =0.0914
p(Scalp)=0.00997
p(Dura)=0.502
Scalp
Dura
A
B
C
D
E
F
G
H
I
J
K

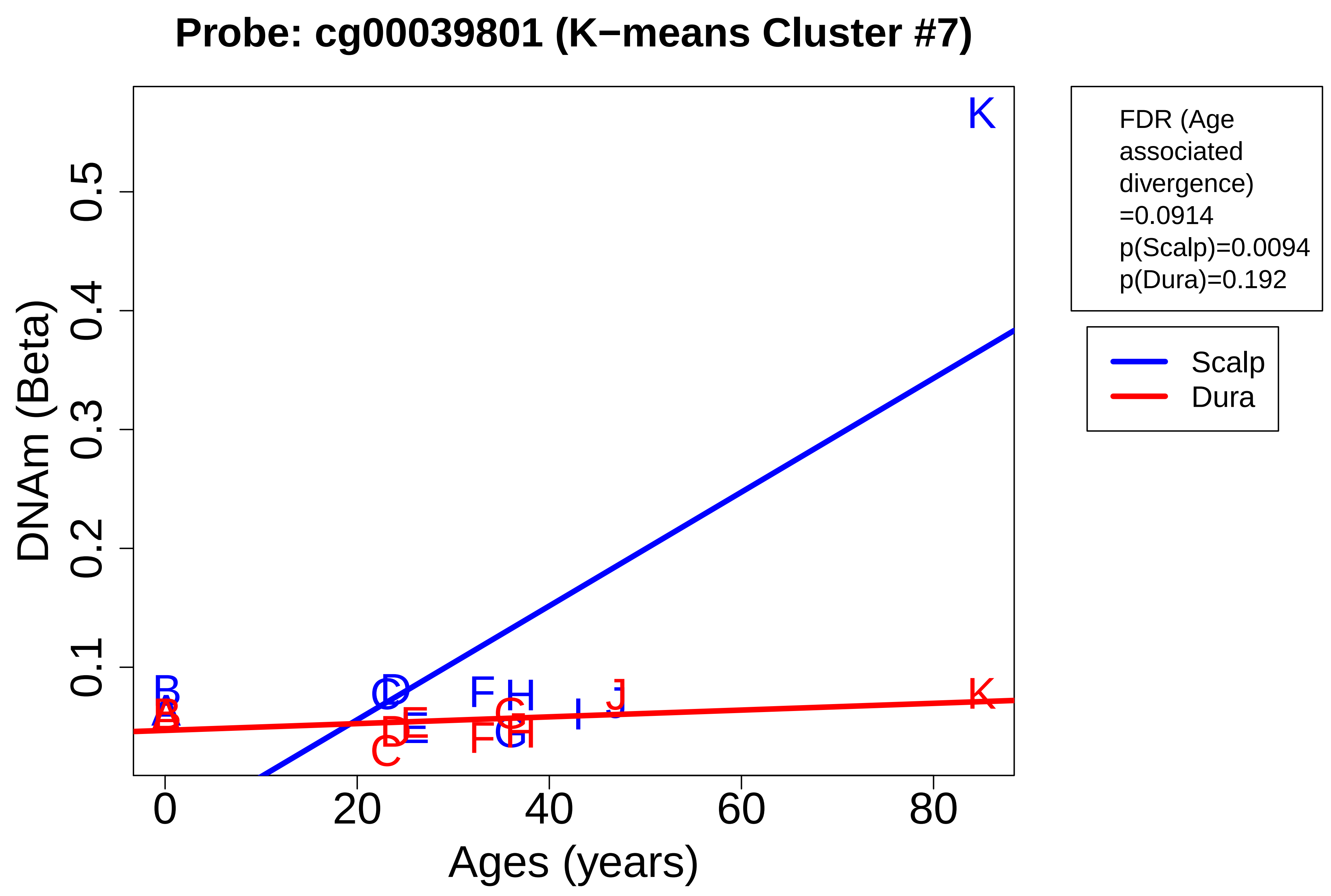

Probe: cg00039801 (K−means Cluster #7)
DNAm (Beta)
0.1
0.2
0.3
0.4
0.5
0
20
40
60
80
Ages (years)
FDR (Age associated divergence) =0.0914
p(Scalp)=0.0094
p(Dura)=0.192
Scalp
Dura
A
B
C
D
E
F
G
H
I
J
K

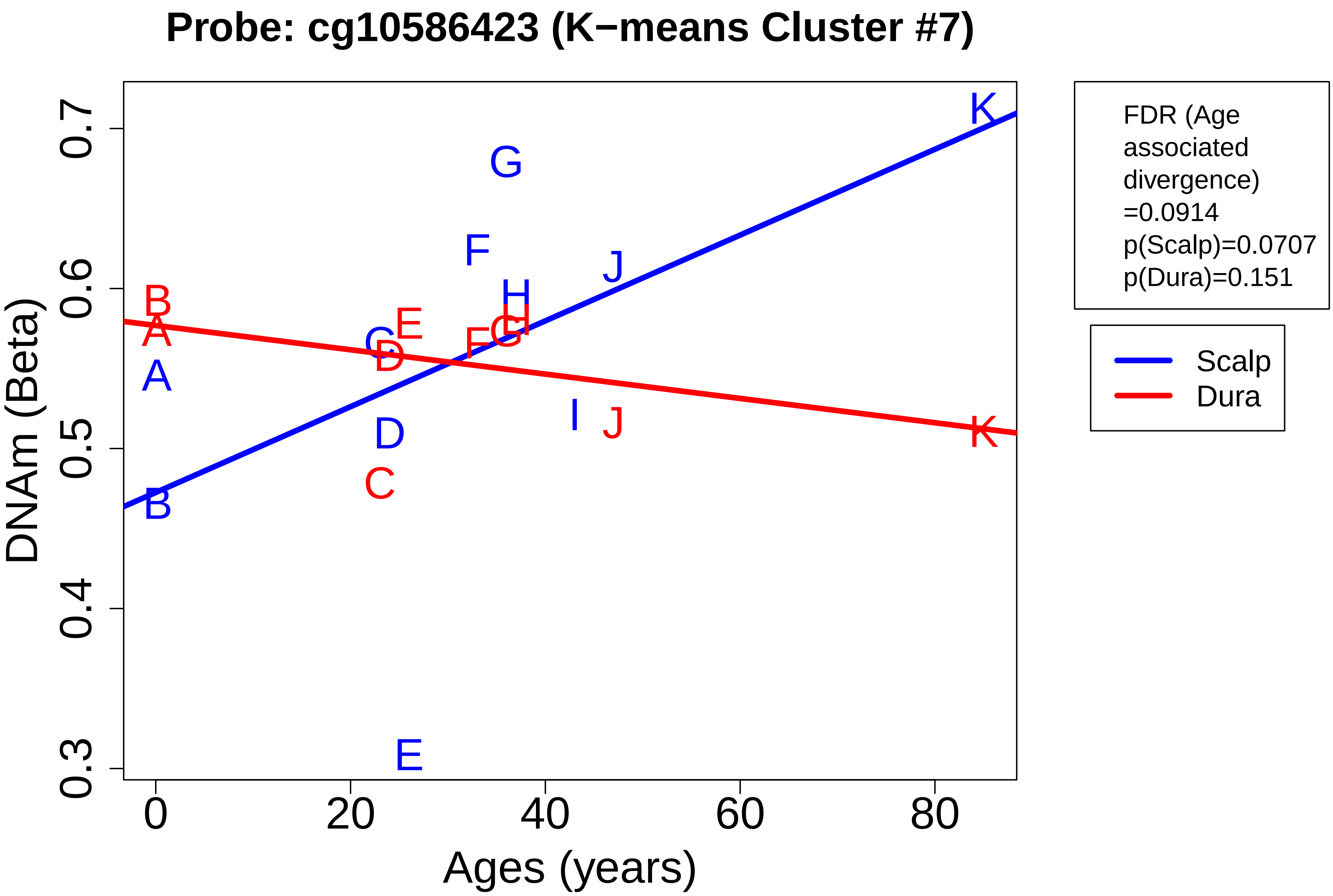

Probe: cg10586423 (K−means Cluster #7)
DNAm (Beta)
0.3
0.4
0.5
0.6
0.7
Ages (years)
0
20
40
60
80
A B C D E F G H I J K
A B C D E F G H J K
FDR (Age associated divergence) =0.0914
p(Scalp)=0.0707
p(Dura)=0.151
Scalp
Dura

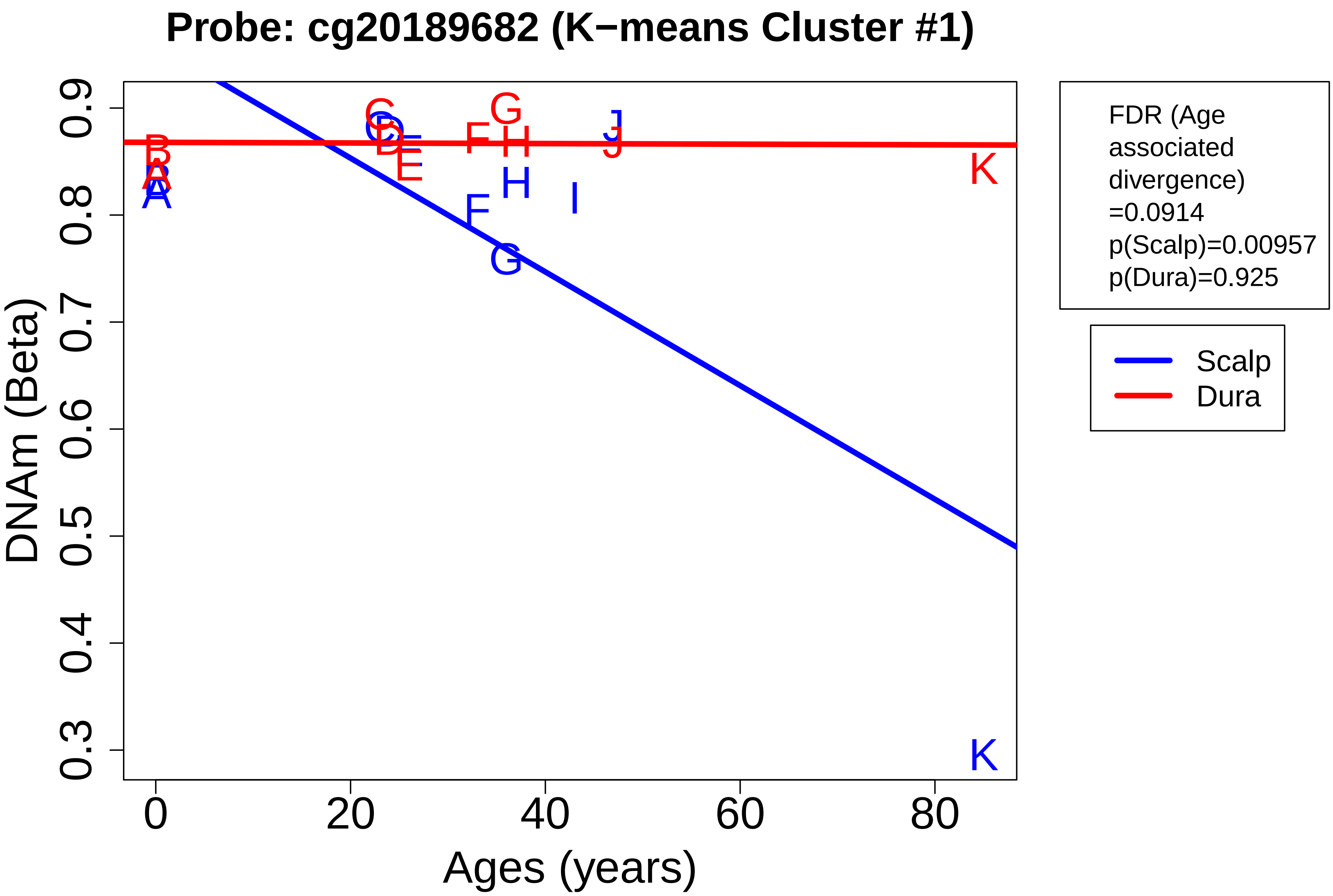
Probe: cg20189682 (K−means Cluster #1)
DNAm (Beta)
Ages (years)
0.3
0.4
0.5
0.6
0.7
0.8
0.9
0
20
40
60
80
FDR (Age associated divergence) =0.0914
p(Scalp)=0.00957
p(Dura)=0.925
Scalp
Dura

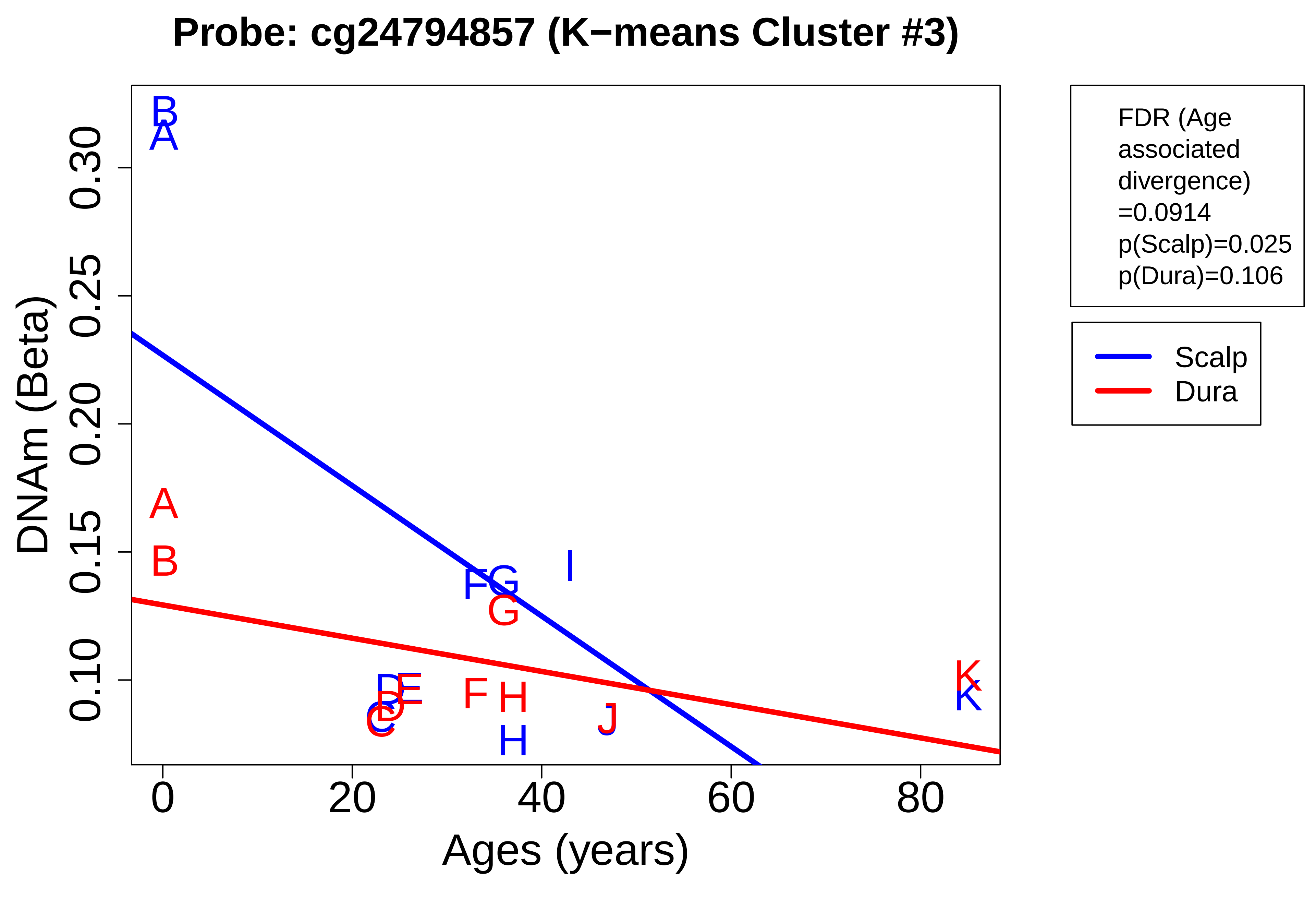

Probe: cg24794857 (K−means Cluster #3)
DNAm (Beta)
0.10
0.15
0.20
0.25
0.30
Ages (years)
0
20
40
60
80
FDR (Age associated divergence) =0.0914
p(Scalp)=0.025
p(Dura)=0.106
Scalp
Dura

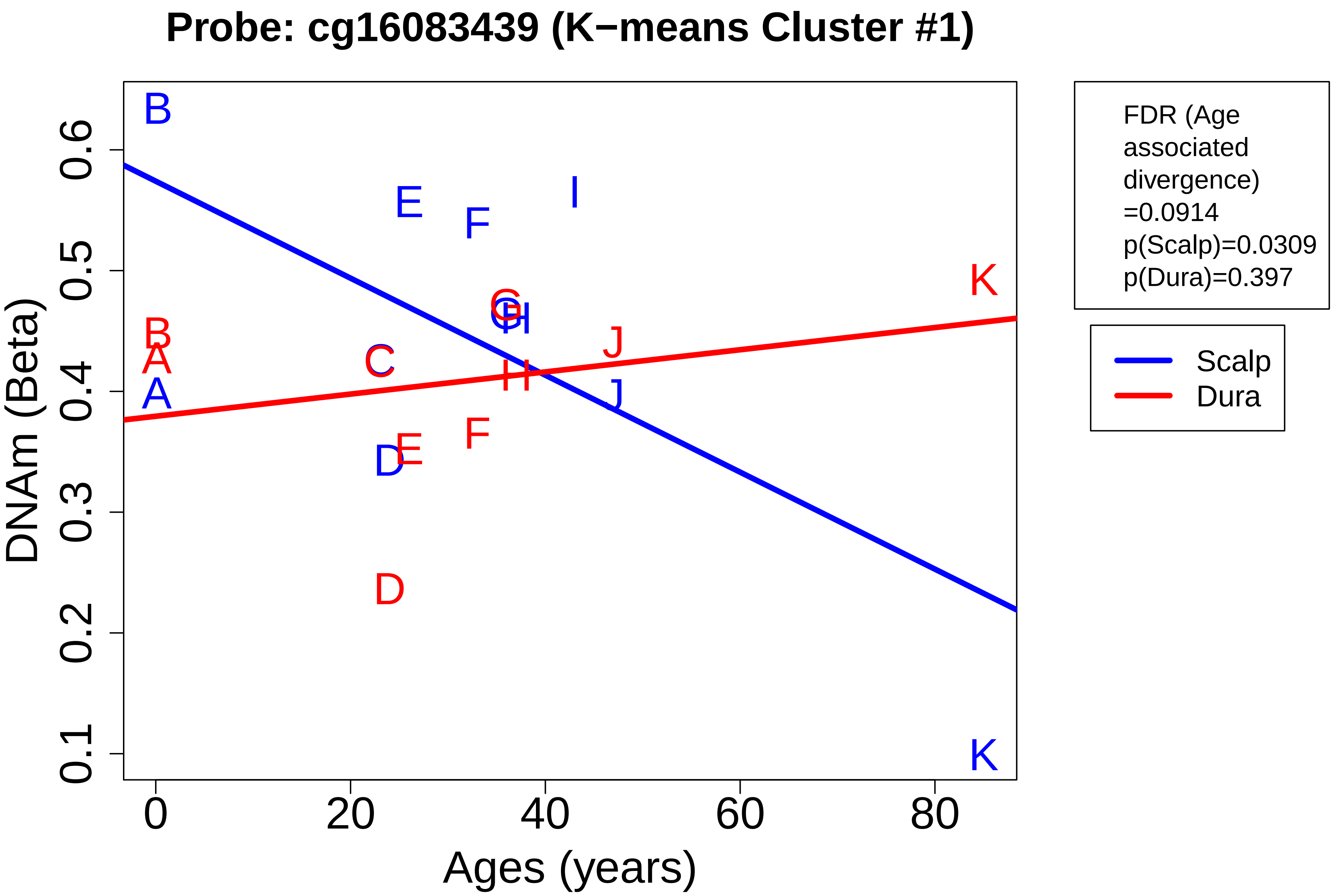

Probe: cg16083439 (K−means Cluster #1)
FDR (Age associated divergence) =0.0914
p(Scalp)=0.0309
p(Dura)=0.397
Scalp
Dura
DNAm (Beta)
Ages (years)
0.1
0.2
0.3
0.4
0.5
0.6
0
20
40
60
80

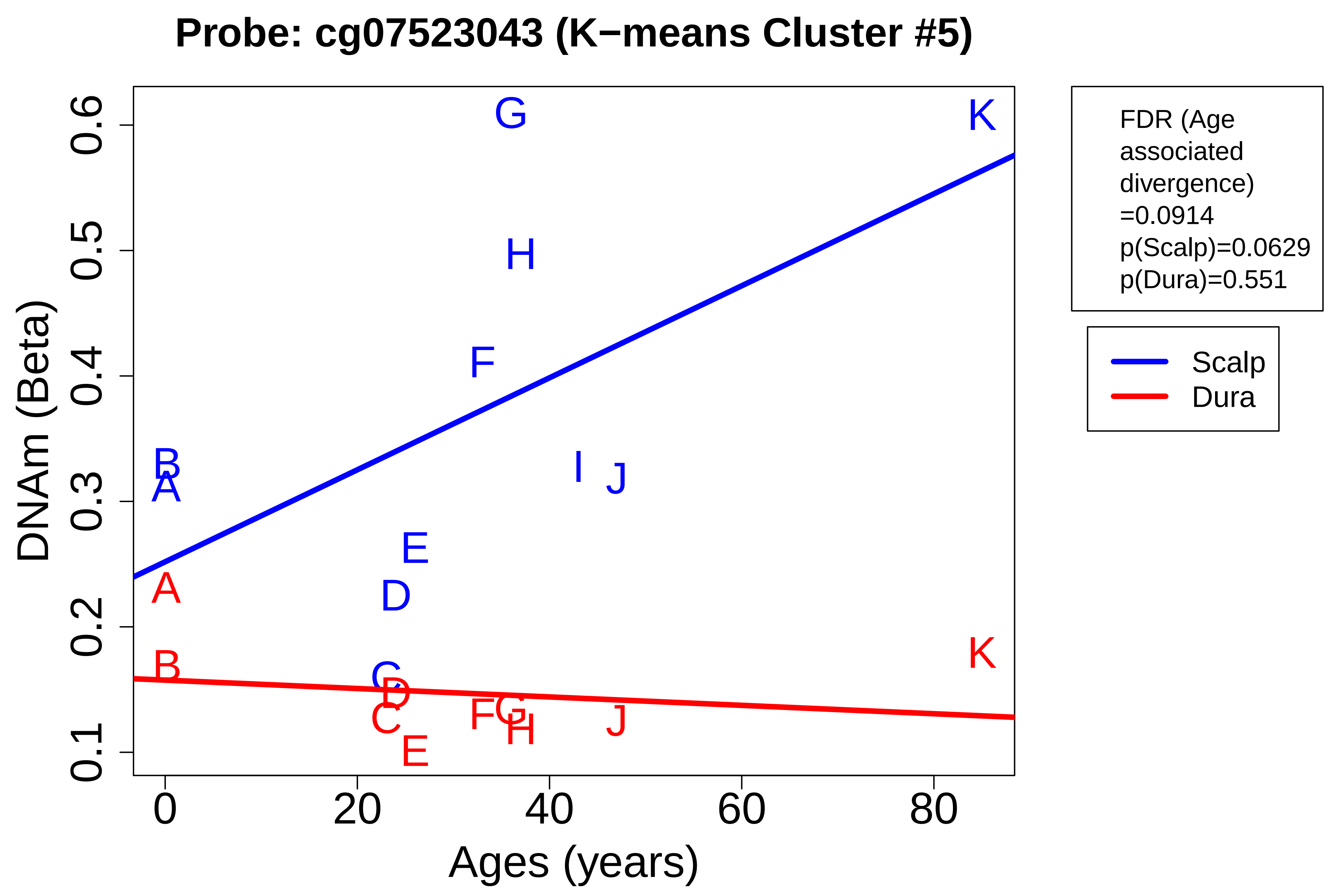

Probe: cg07523043 (K−means Cluster #5)
DNAm (Beta)
0.1
0.2
0.3
0.4
0.5
0.6
0
20
40
60
80
Ages (years)
FDR (Age associated divergence) =0.0914
p(Scalp)=0.0629
p(Dura)=0.551
Scalp
Dura

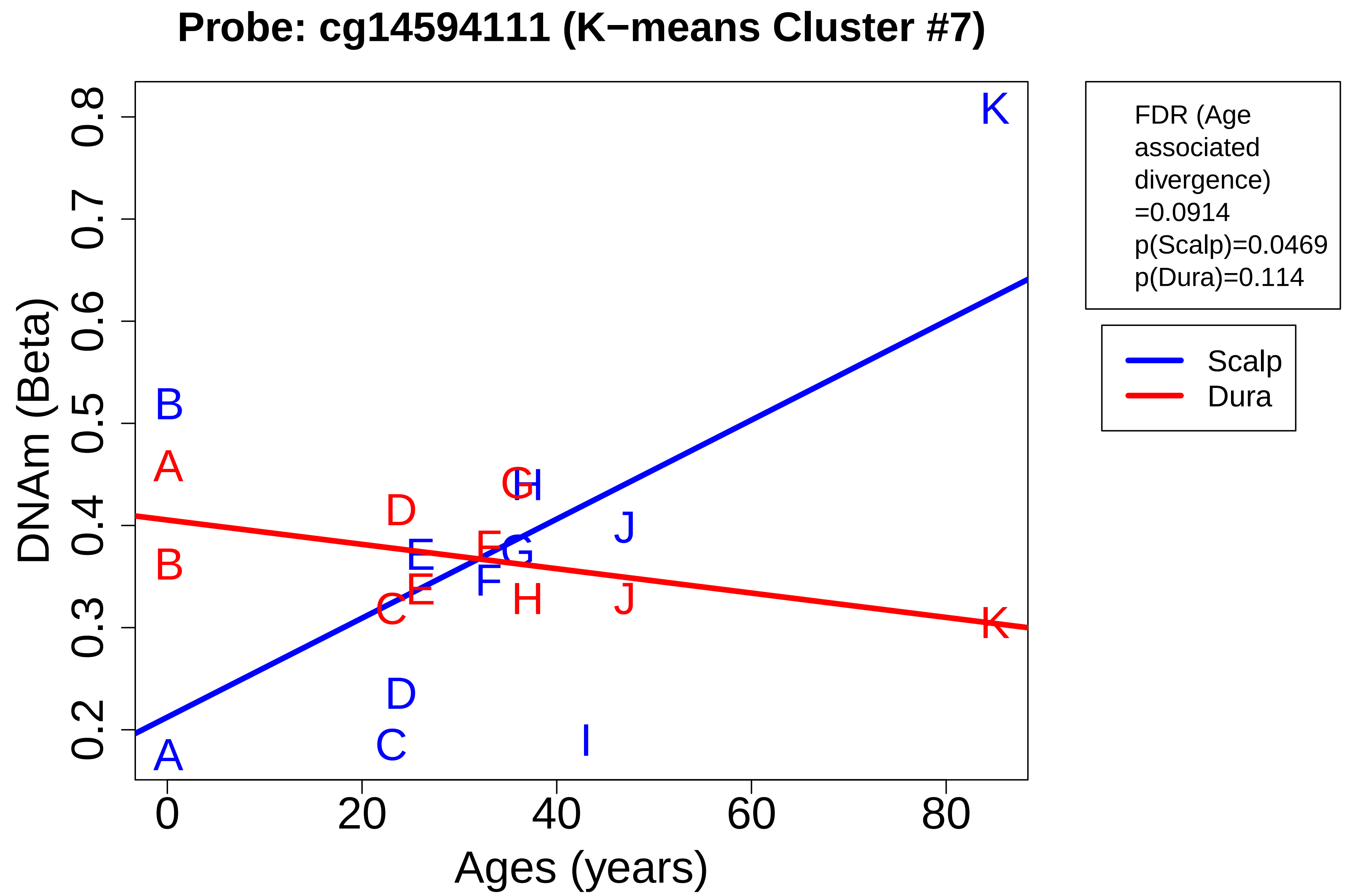

Probe: cg14594111 (K−means Cluster #7)
DNAm (Beta)
0.2
0.3
0.4
0.5
0.6
0.7
0.8
Ages (years)
0
20
40
60
80
FDR (Age associated divergence) =0.0914
p(Scalp)=0.0469
p(Dura)=0.114
Scalp
Dura

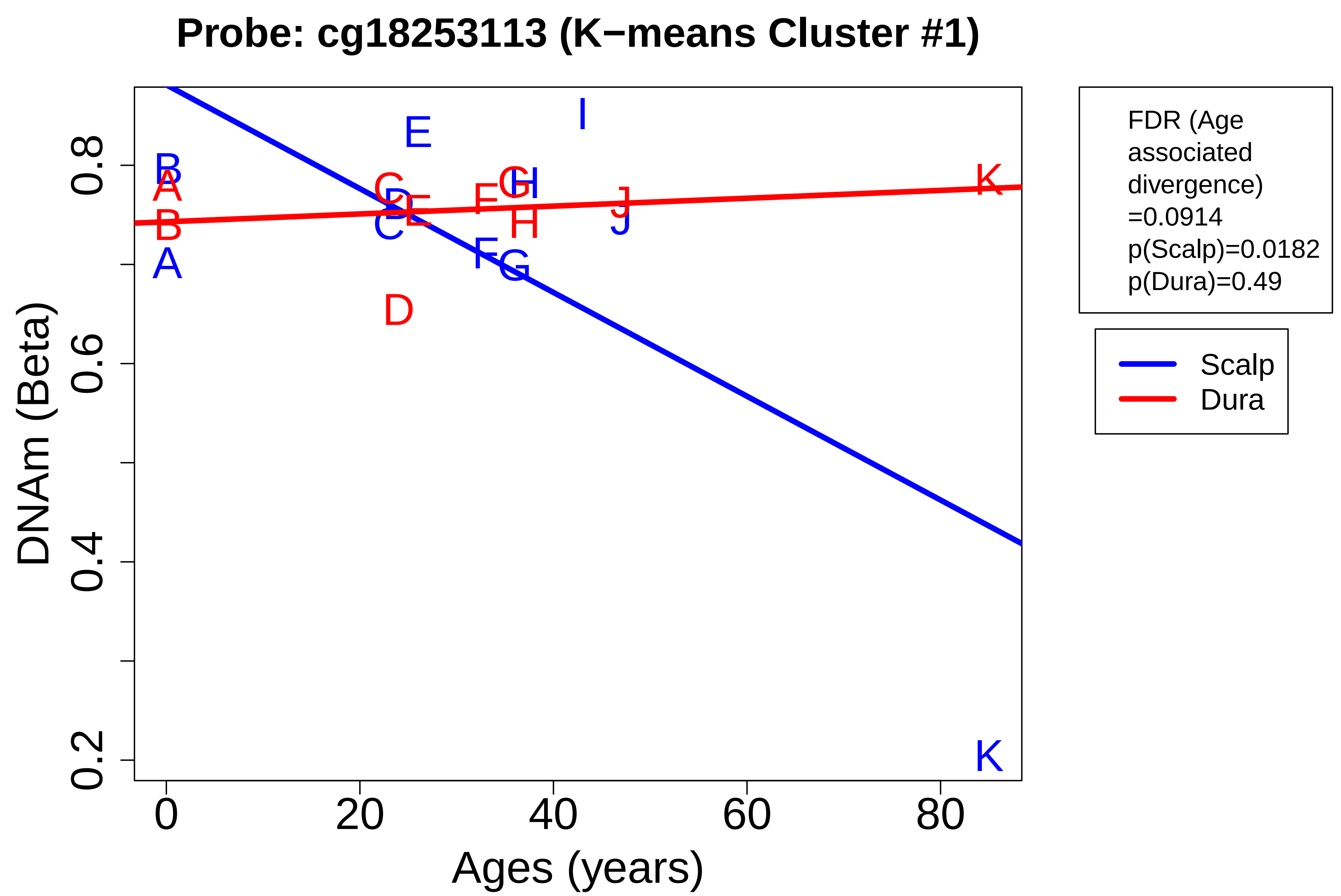

Probe: cg18253113 (K−means Cluster #1)
DNAm (Beta)
0.2
0.4
0.6
0.8
0
20
40
60
80
Ages (years)
A
B
C
D
E
F
G
H
I
J
K
FDR (Age associated divergence) =0.0914
p(Scalp)=0.0182
p(Dura)=0.49
Scalp
Dura

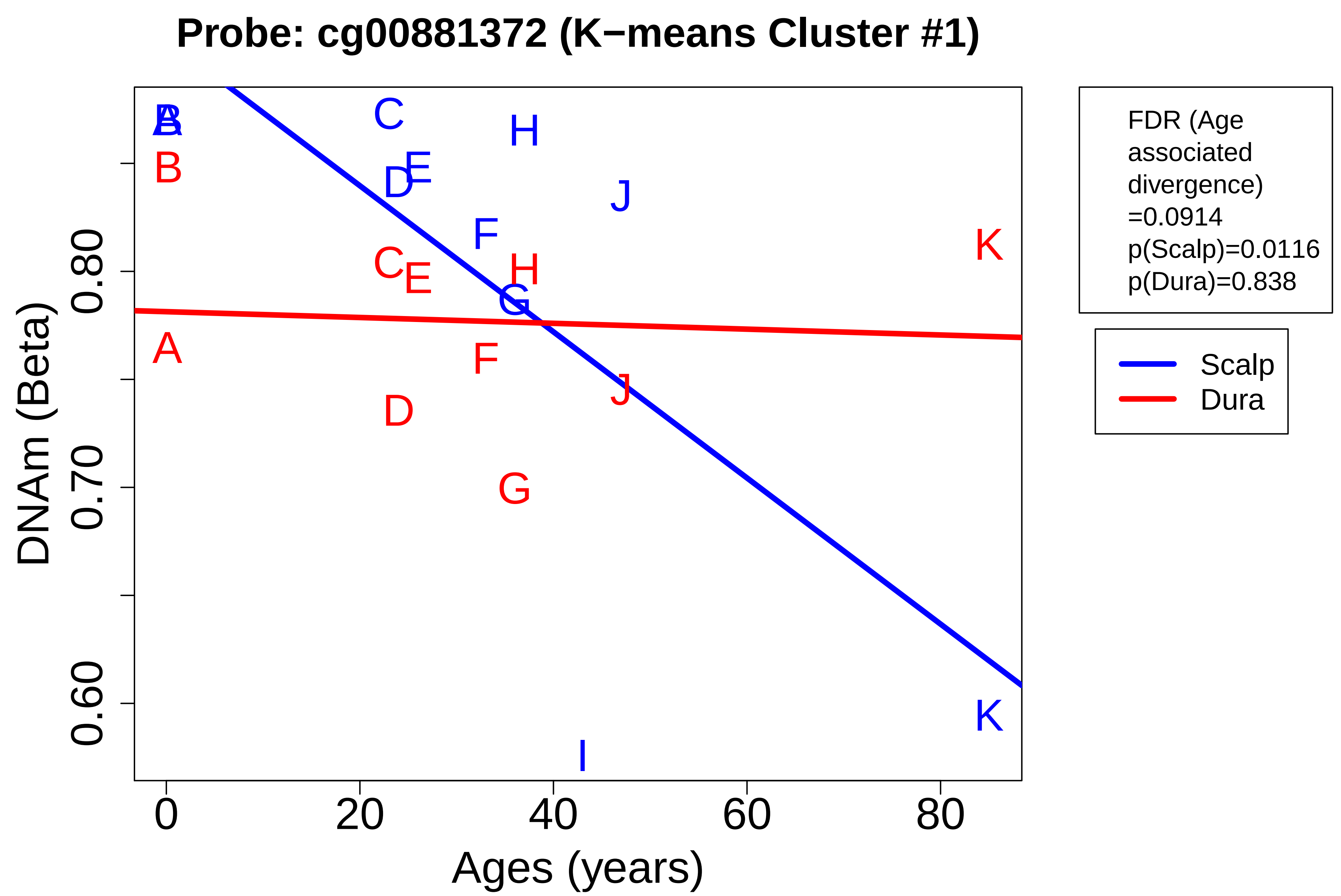

Probe: cg00881372 (K−means Cluster #1)
FDR (Age associated divergence) =0.0914
p(Scalp)=0.0116
p(Dura)=0.838
Scalp
Dura
DNAm (Beta)
0.60
0.70
0.80
Ages (years)
0
20
40
60
80

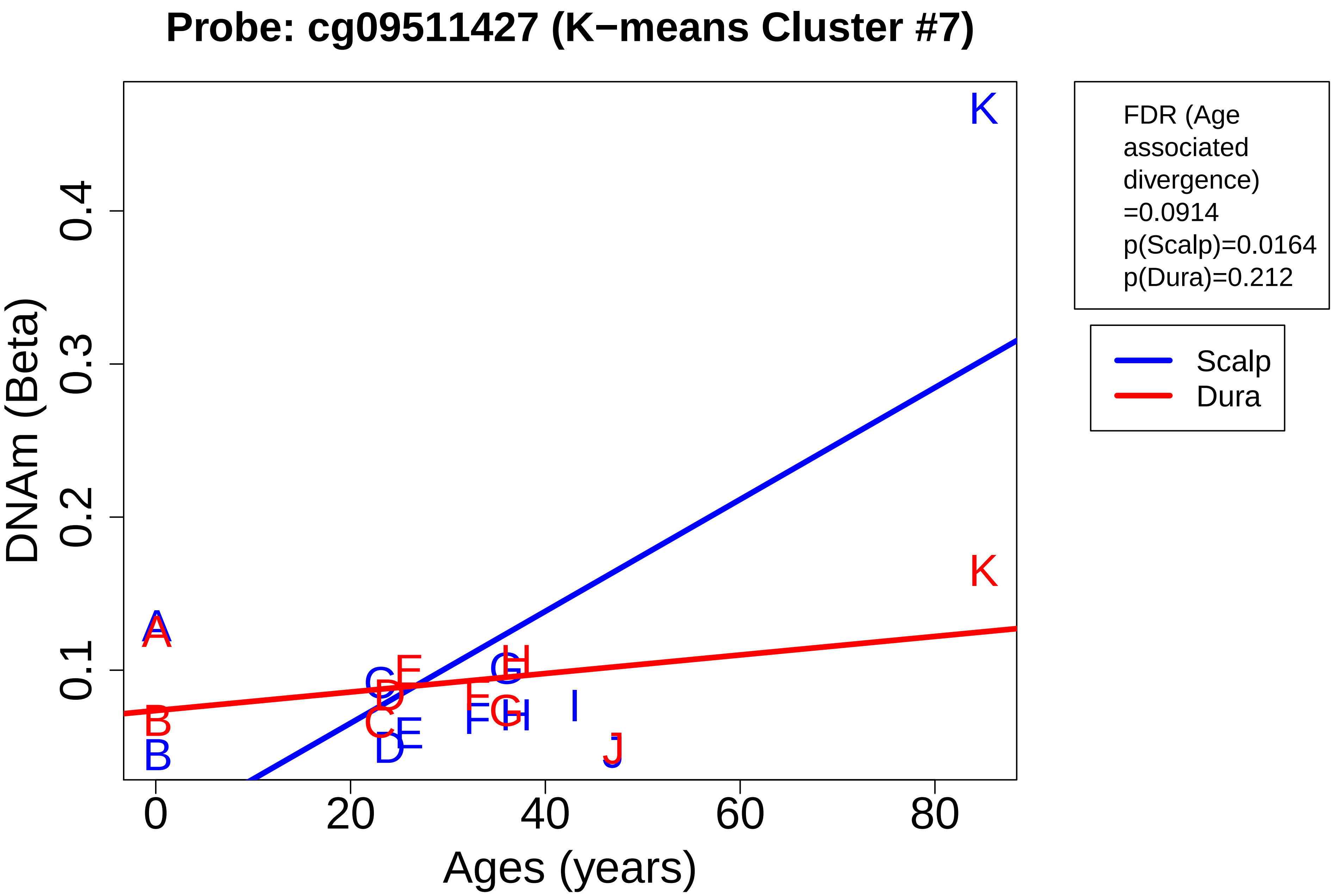

Probe: cg09511427 (K−means Cluster #7)
DNAm (Beta)
0.1
0.2
0.3
0.4
Ages (years)
0
20
40
60
80
FDR (Age
associated
divergence)
=0.0914
p(Scalp)=0.0164
p(Dura)=0.212
Scalp
Dura

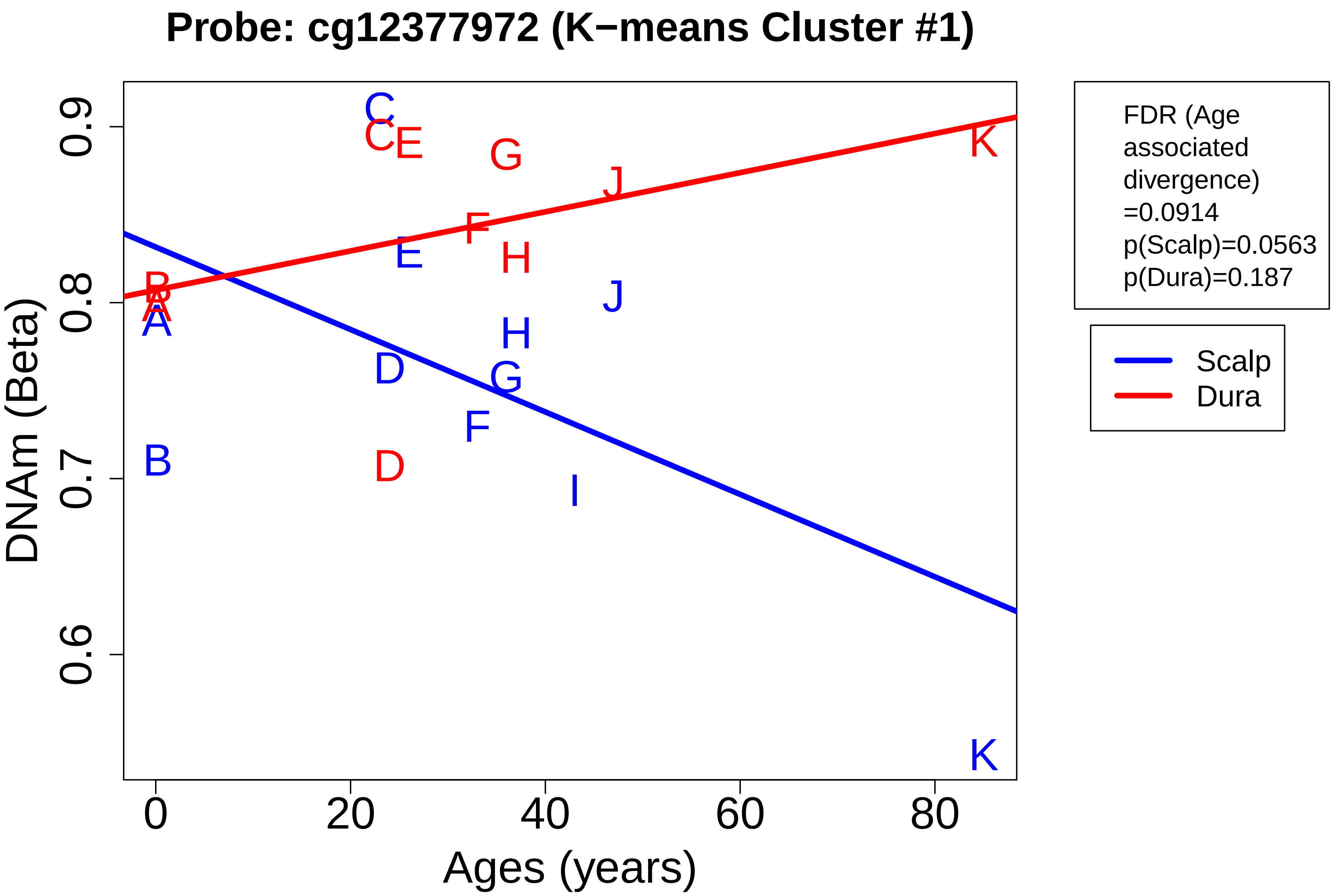

Probe: cg12377972 (K−means Cluster #1)
DNAm (Beta)
Ages (years)
0.6
0.7
0.8
0.9
0
20
40
60
80
FDR (Age associated divergence) =0.0914
p(Scalp)=0.0563
p(Dura)=0.187
Scalp
Dura
A
B
C
D
E
F
G
H
I
J
K

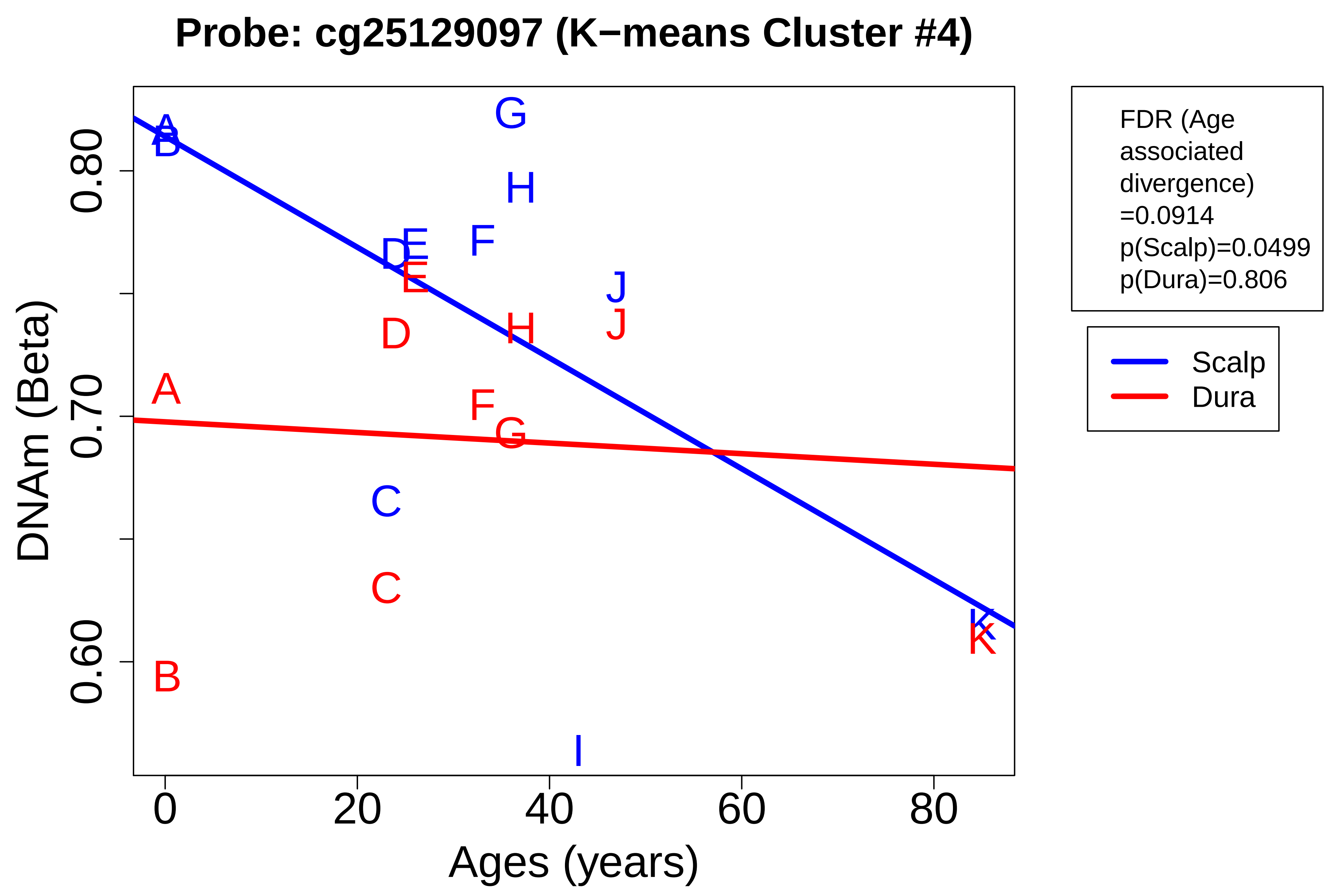

Probe: cg25129097 (K−means Cluster #4)
DNAm (Beta)
0.60
0.70
0.80
Ages (years)
0
20
40
60
80
A
B
C
D
E
F
G
H
I
J
K
FDR (Age associated divergence) =0.0914
p(Scalp)=0.0499
p(Dura)=0.806
Scalp
Dura

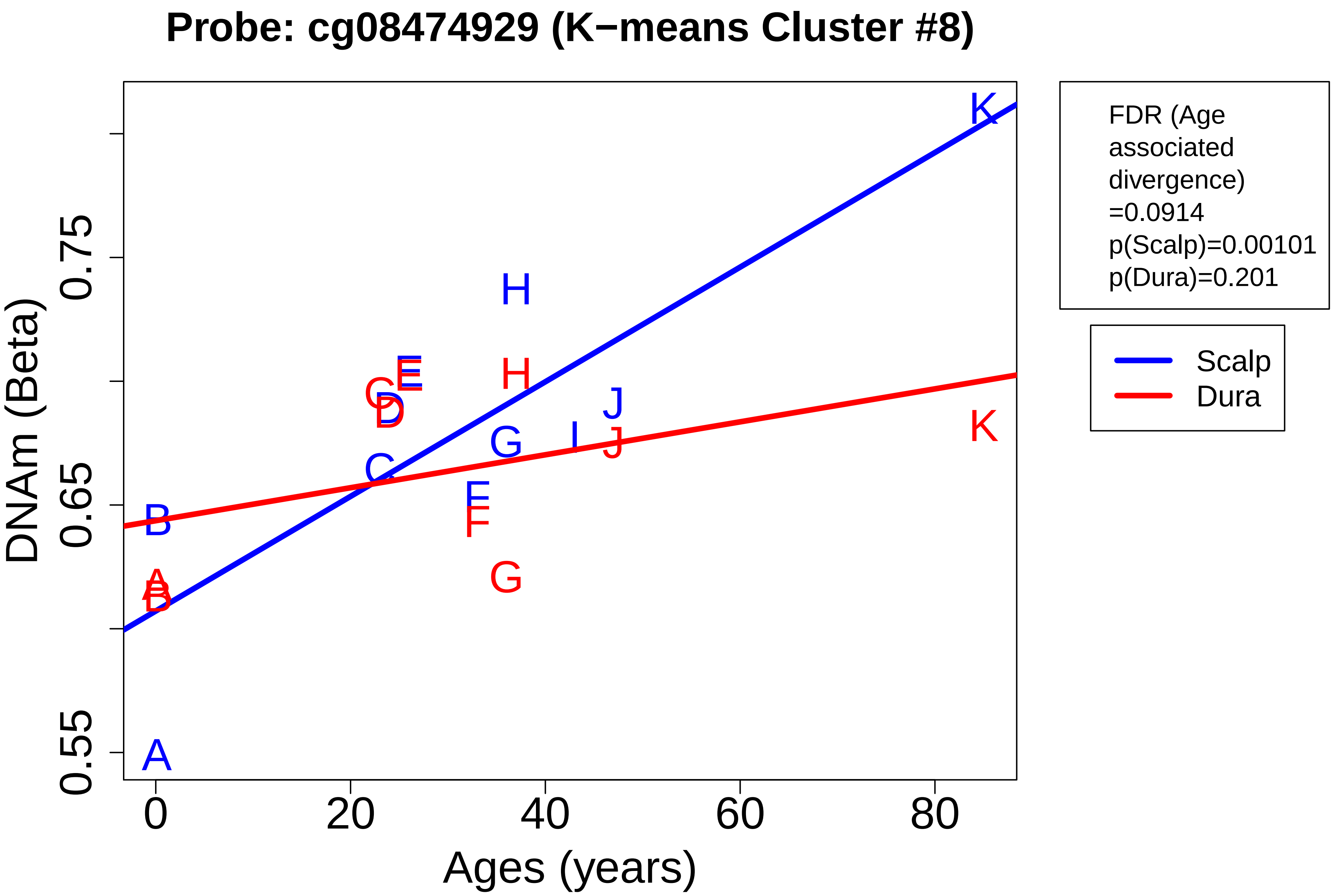

Probe: cg08474929 (K−means Cluster #8)
DNAm (Beta)
0.55
0.65
0.75
0
20
40
60
80
Ages (years)
FDR (Age associated divergence) =0.0914
p(Scalp)=0.00101
p(Dura)=0.201
Scalp
Dura

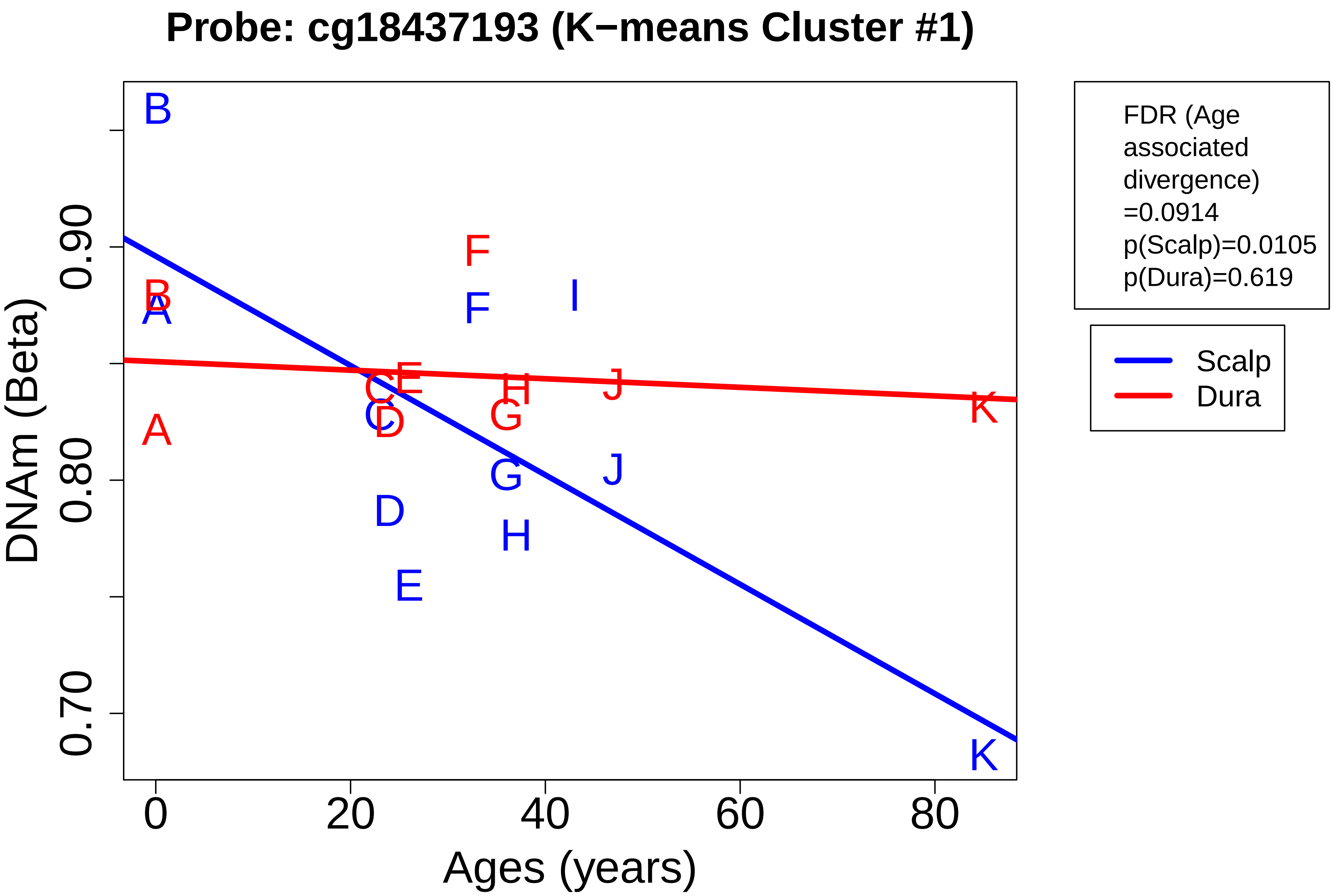

Probe: cg18437193 (K−means Cluster #1)
DNAm (Beta)
0.70
0.80
0.90
Ages (years)
0
20
40
60
80
FDR (Age associated divergence) =0.0914
p(Scalp)=0.0105
p(Dura)=0.619
Scalp
Dura

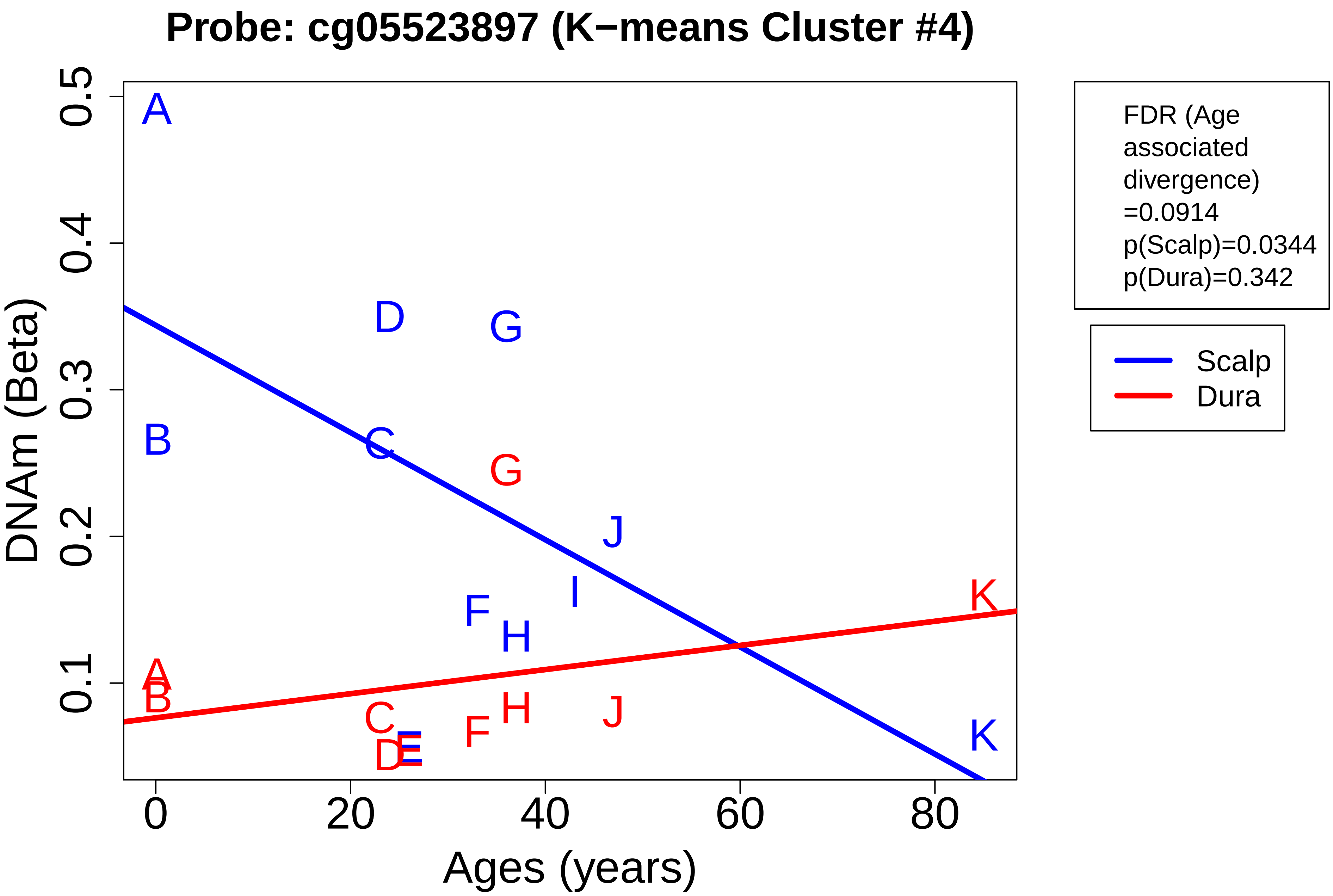

Probe: cg05523897 (K−means Cluster #4)
DNAm (Beta)
0.1
0.2
0.3
0.4
0.5
Ages (years)
0
20
40
60
80
FDR (Age associated divergence) =0.0914
p(Scalp)=0.0344
p(Dura)=0.342
Scalp
Dura

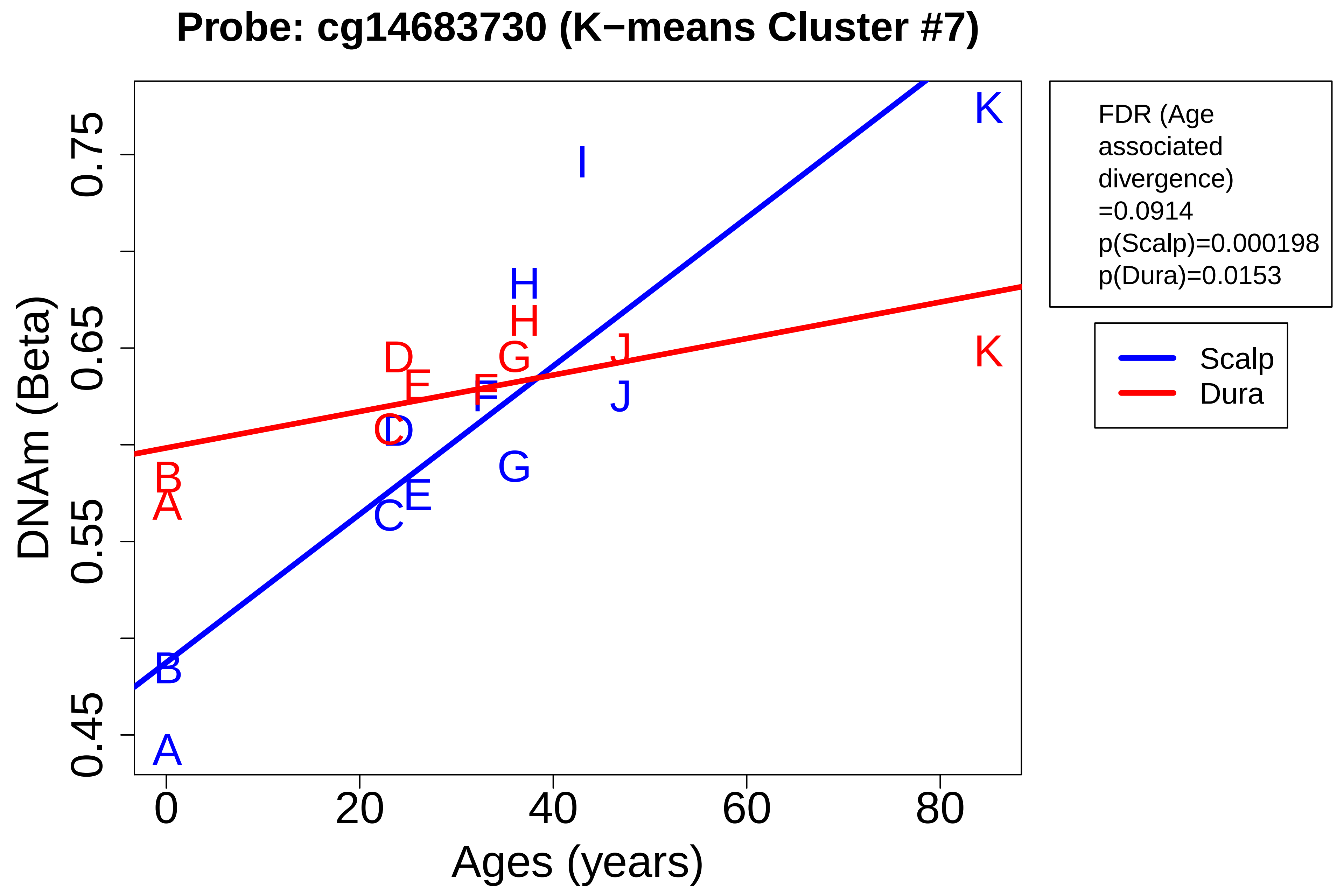

Probe: cg14683730 (K−means Cluster #7)
DNAm (Beta)
0.45
0.55
0.65
0.75
Ages (years)
0
20
40
60
80
FDR (Age associated divergence) =0.0914
p(Scalp)=0.000198
p(Dura)=0.0153
Scalp
Dura

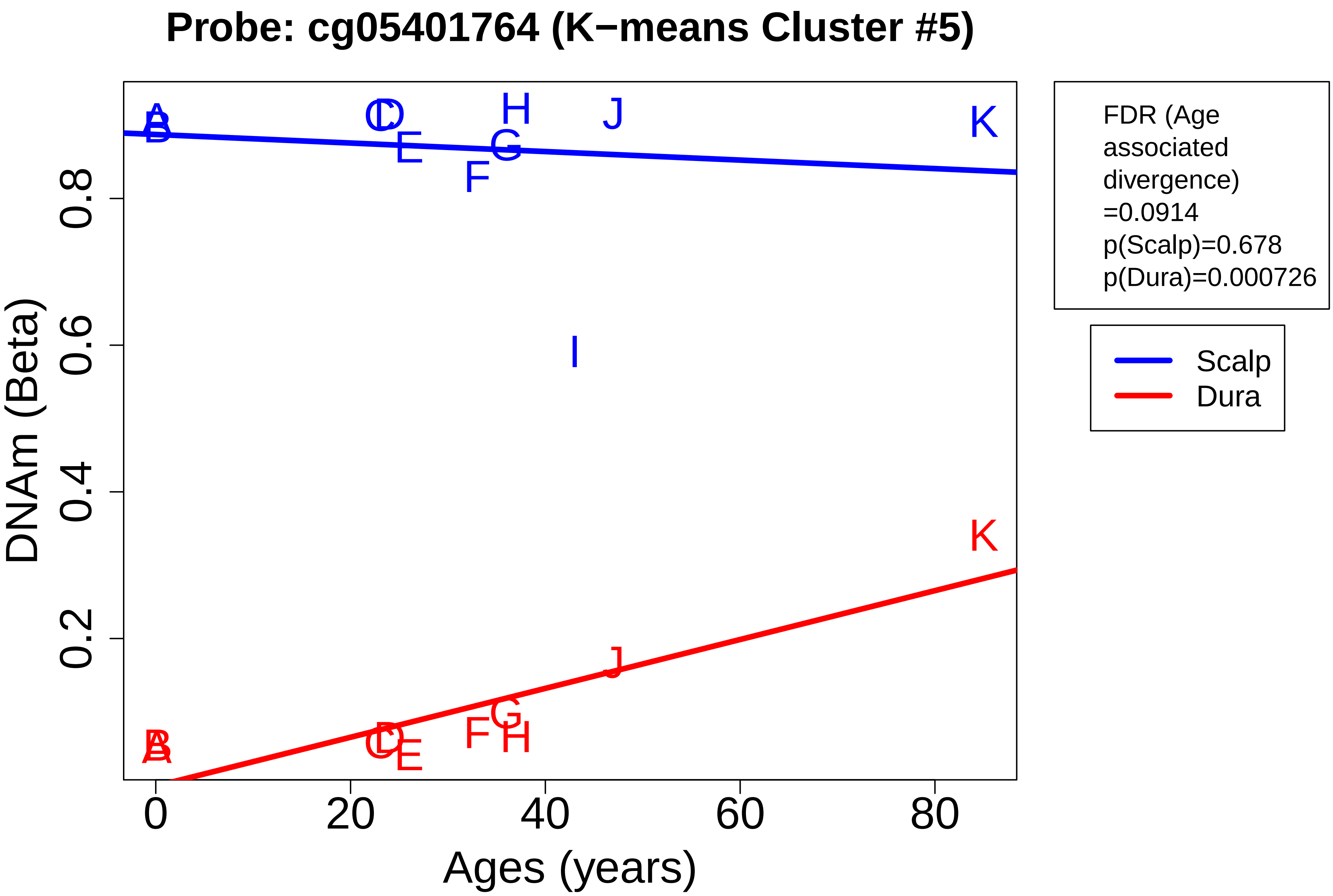

Probe: cg05401764 (K−means Cluster #5)
FDR (Age associated divergence) =0.0914
p(Scalp)=0.678
p(Dura)=0.000726
Scalp
Dura
DNAm (Beta)
0.2
0.4
0.6
0.8
Ages (years)
0
20
40
60
80

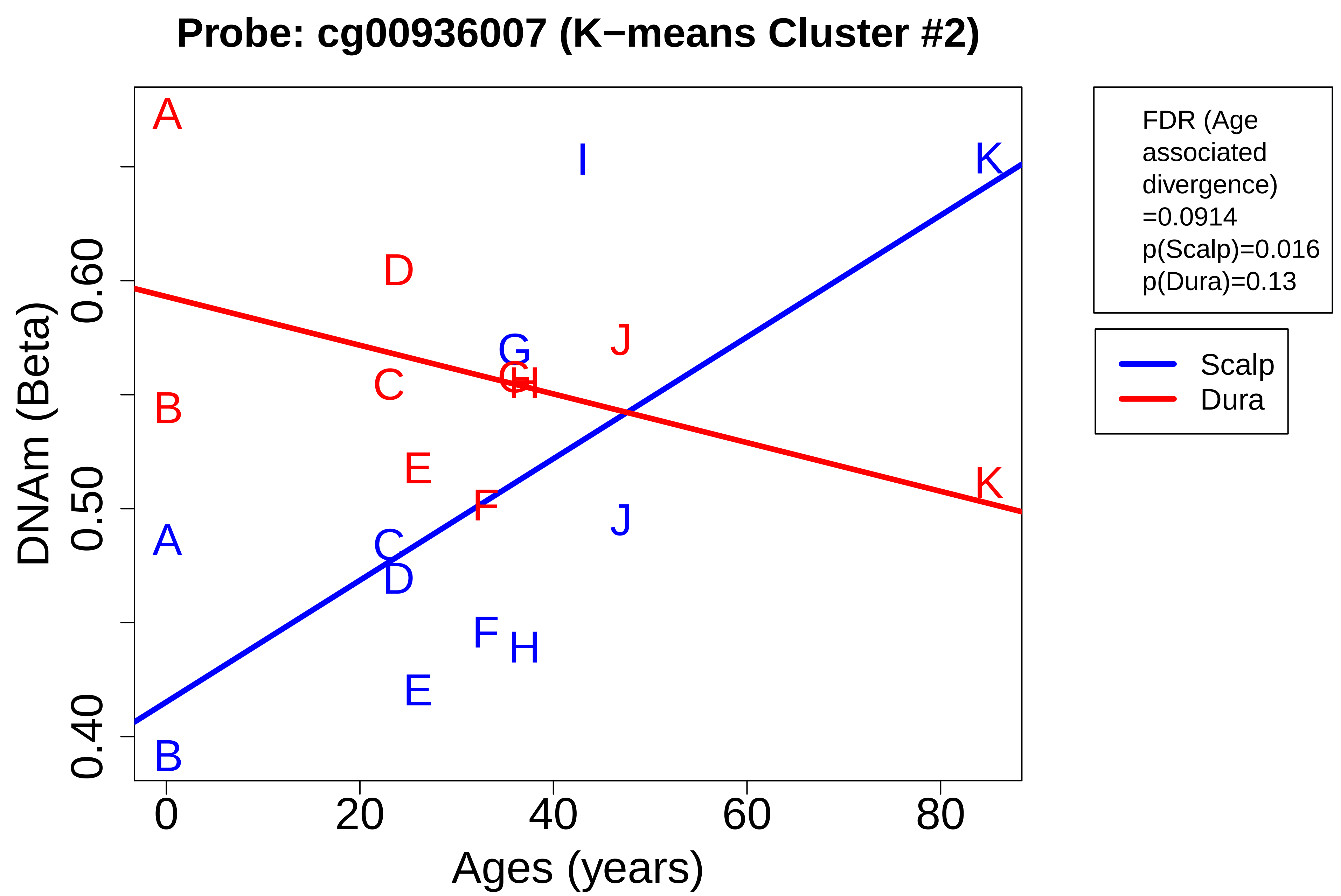

Probe: cg00936007 (K−means Cluster #2)
DNAm (Beta)
0.40
0.50
0.60
Ages (years)
0
20
40
60
80
FDR (Age associated divergence) =0.0914
p(Scalp)=0.016
p(Dura)=0.13
Scalp
Dura

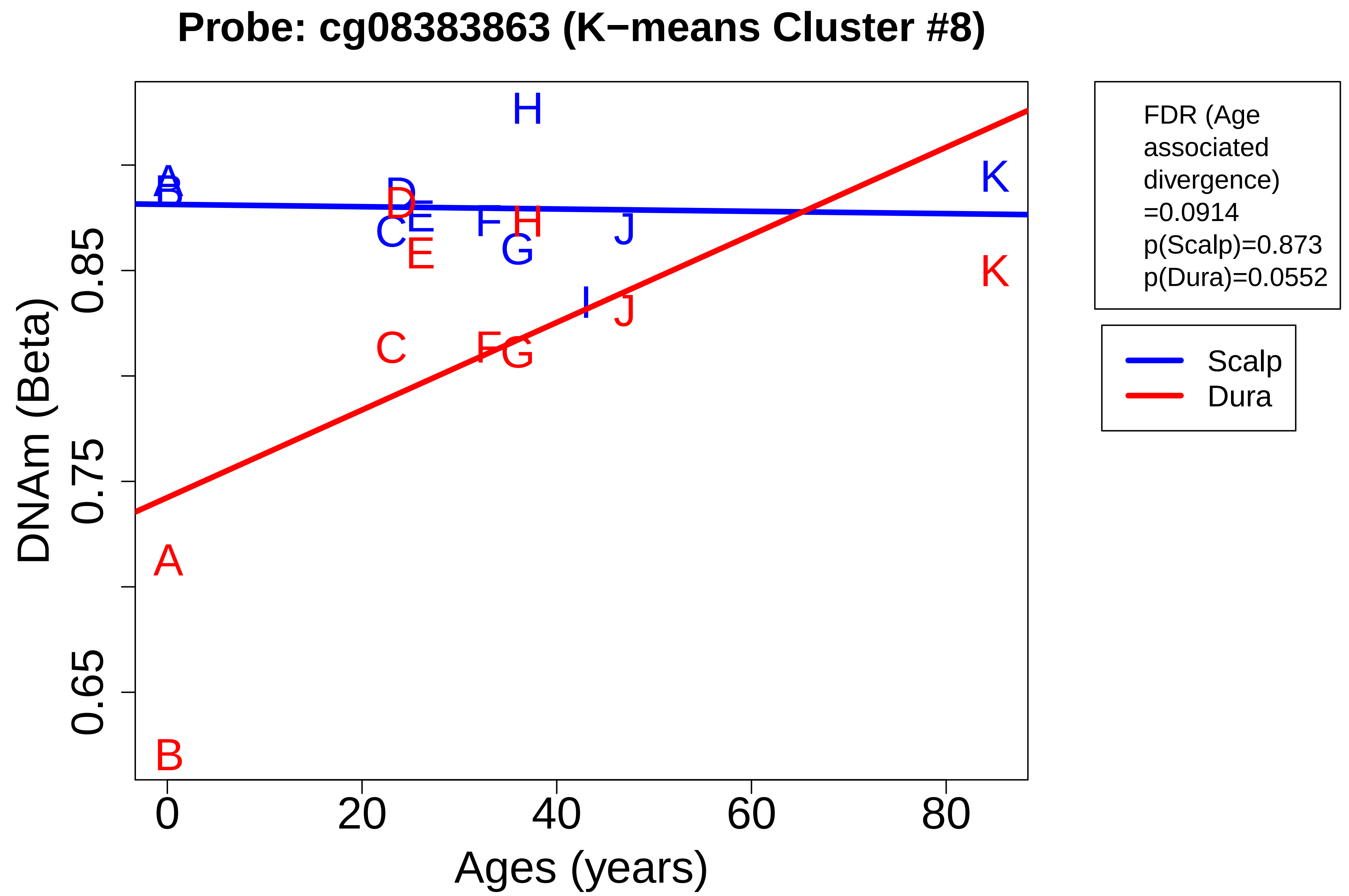

Probe: cg08383863 (K−means Cluster #8)
DNAm (Beta)
0.65
0.75
0.85
Ages (years)
0
20
40
60
80
FDR (Age associated divergence) =0.0914
p(Scalp)=0.873
p(Dura)=0.0552
Scalp
Dura

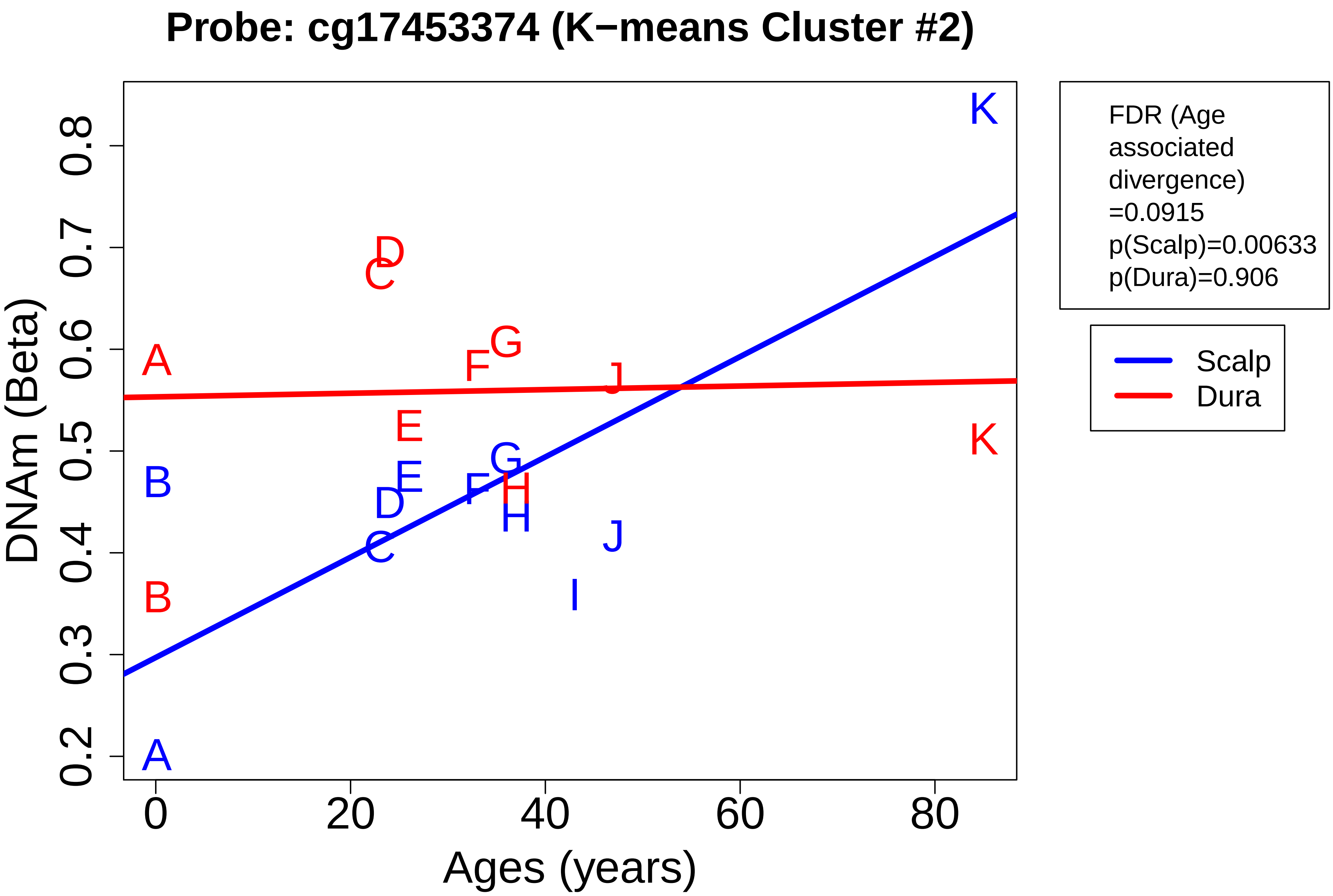

Probe: cg17453374 (K−means Cluster #2)
DNAm (Beta)
0.2
0.3
0.4
0.5
0.6
0.7
0.8
Ages (years)
0
20
40
60
80
FDR (Age associated divergence) =0.0915
p(Scalp)=0.00633
p(Dura)=0.906
Scalp
Dura

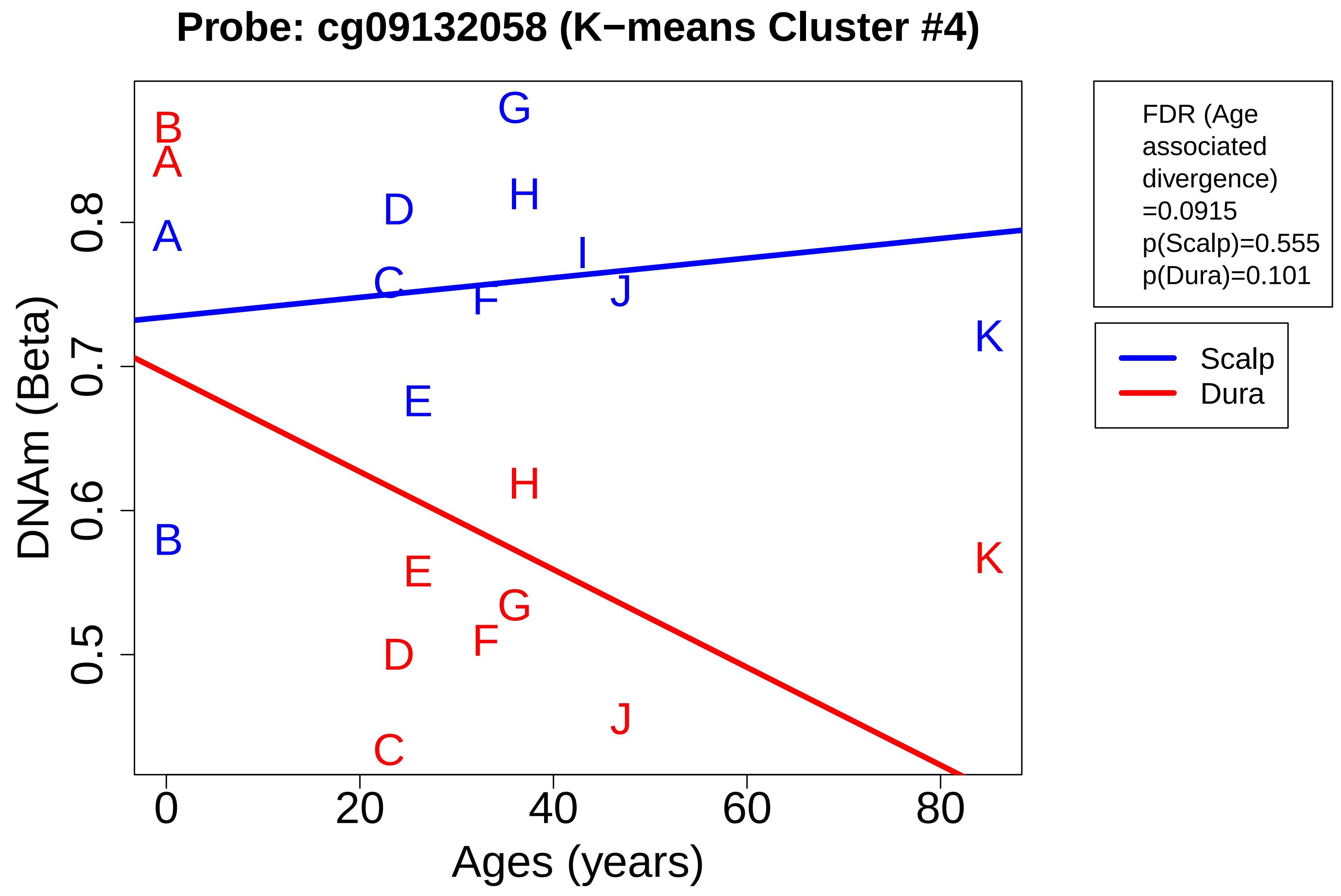

Probe: cg09132058 (K−means Cluster #4)
DNAm (Beta)
0.5
0.6
0.7
0.8
Ages (years)
0
20
40
60
80
FDR (Age associated divergence) =0.0915
p(Scalp)=0.555
p(Dura)=0.101
Scalp
Dura

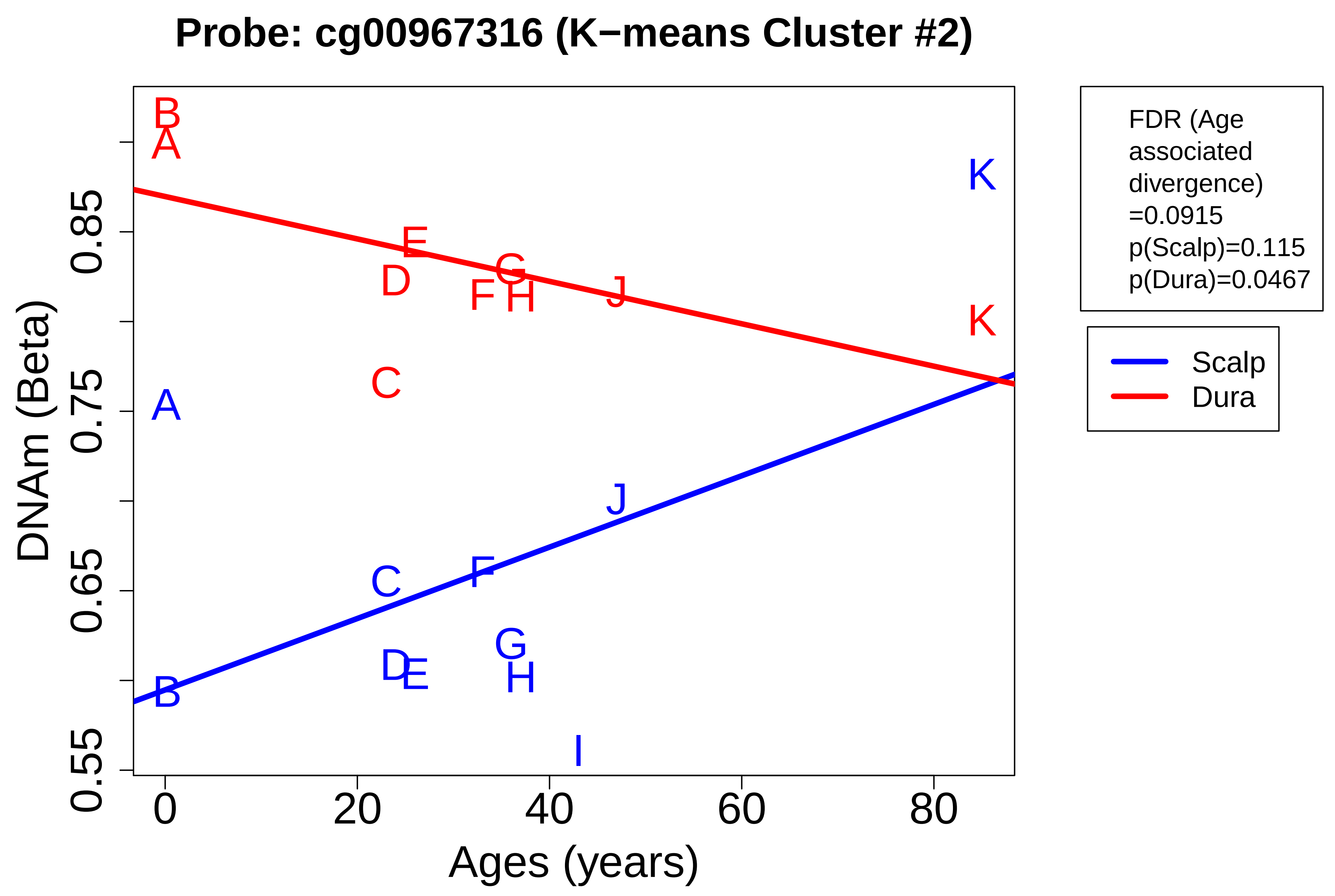
Probe: cg00967316 (K−means Cluster #2)
DNAm (Beta)
0.55
0.65
0.75
0.85
0
20
40
60
80
Ages (years)
FDR (Age associated divergence) =0.0915
p(Scalp)=0.115
p(Dura)=0.0467
Scalp
Dura
A
B
C
D
E
F
G
H
I
J
K

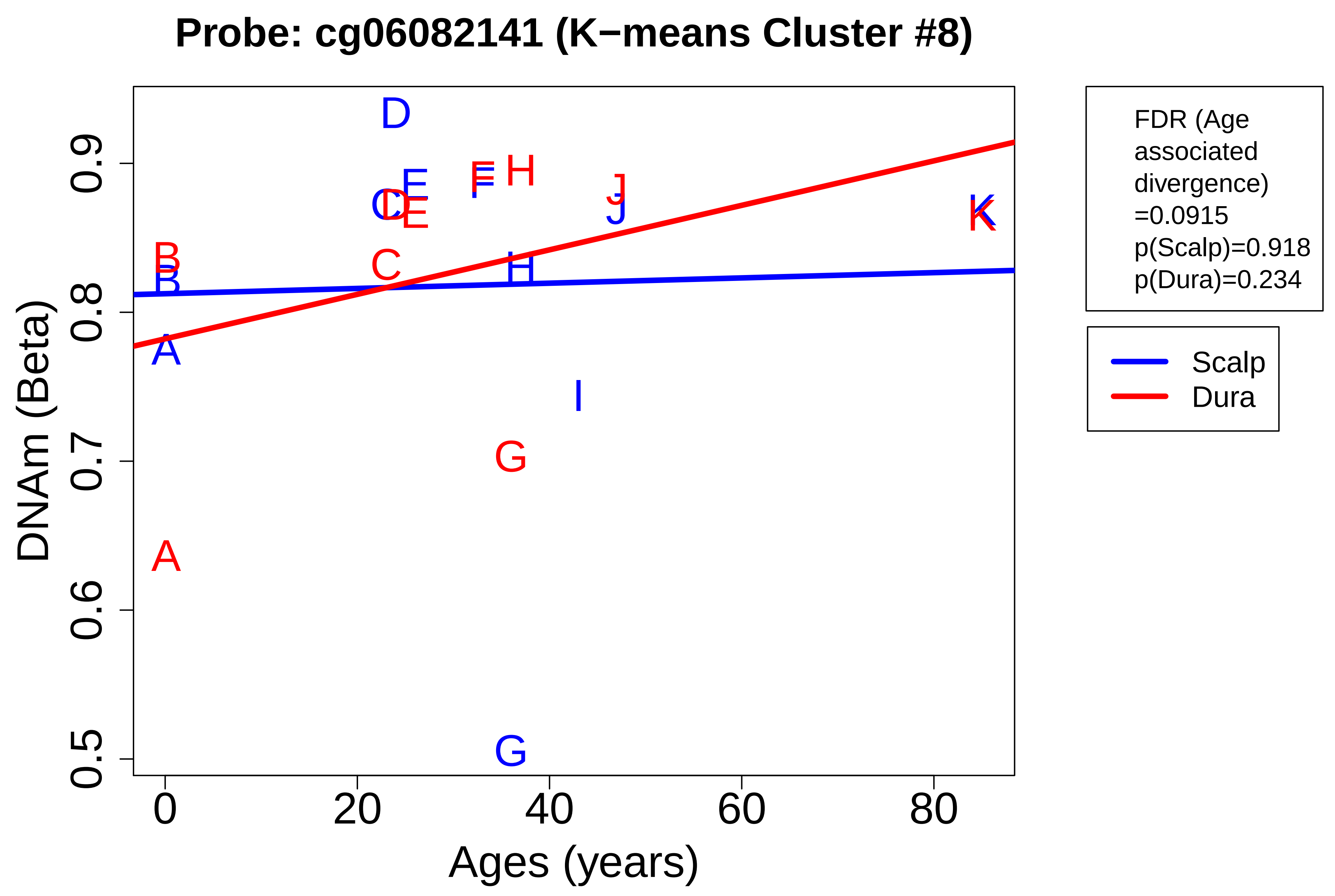

Probe: cg06082141 (K−means Cluster #8)
DNAm (Beta)
0.5
0.6
0.7
0.8
0.9
0
20
40
60
80
Ages (years)
FDR (Age associated divergence) =0.0915
p(Scalp)=0.918
p(Dura)=0.234
Scalp
Dura

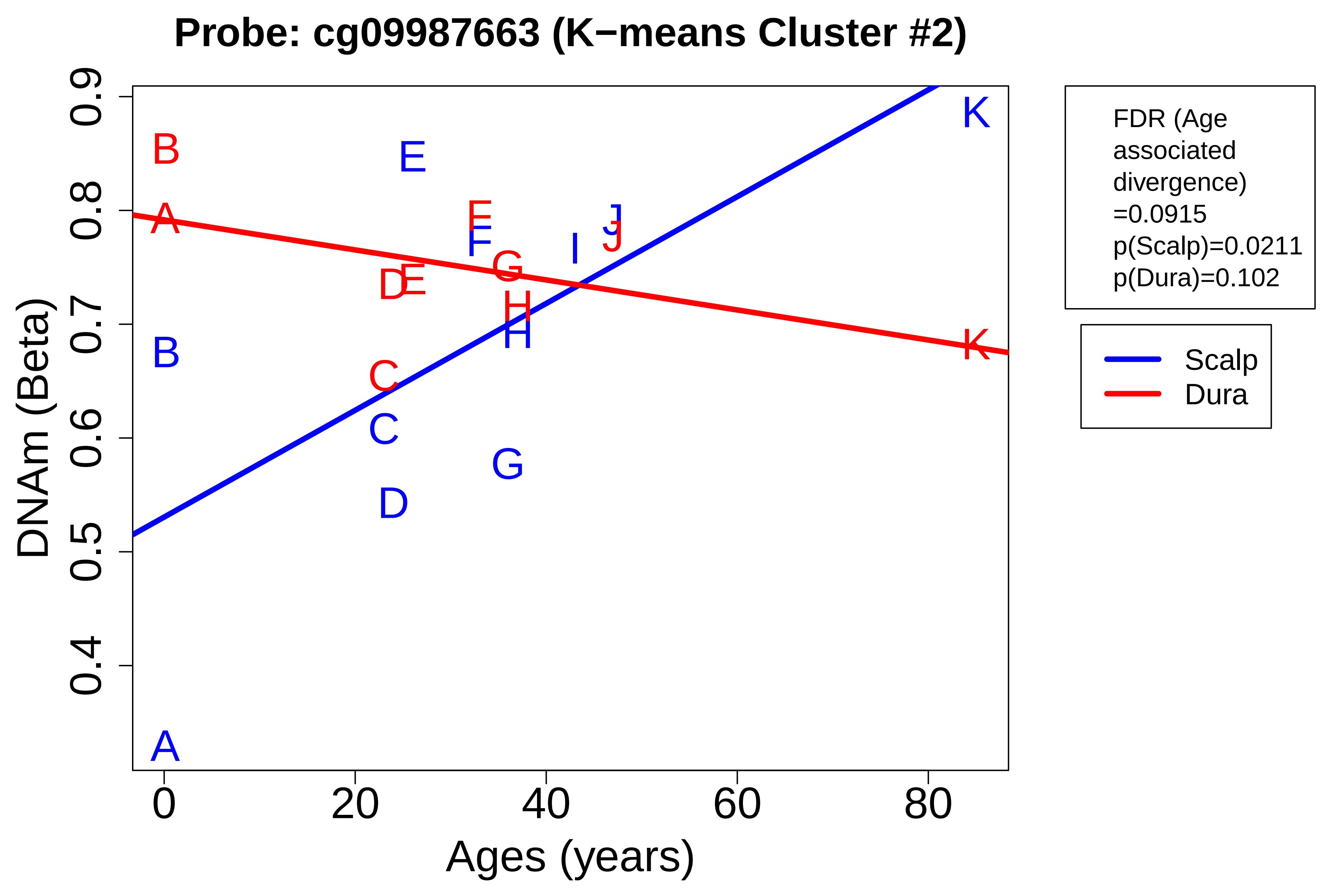

Probe: cg09987663 (K−means Cluster #2)
DNAm (Beta)
0.4
0.5
0.6
0.7
0.8
0.9
0
20
40
60
80
Ages (years)
FDR (Age associated divergence) =0.0915
p(Scalp)=0.0211
p(Dura)=0.102
Scalp
Dura
A
B
C
D
E
F
G
H
I
J
K

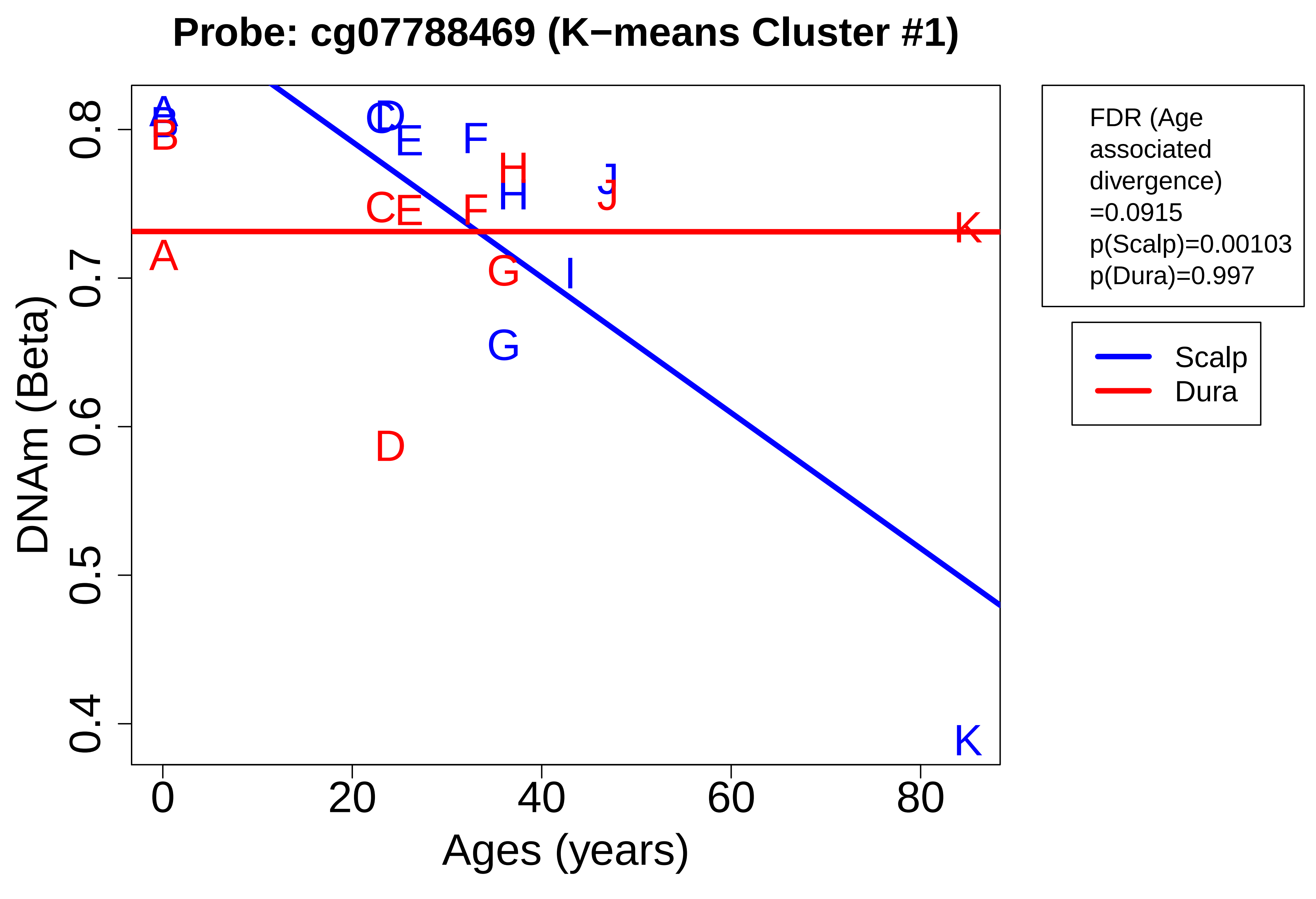
Probe: cg07788469 (K−means Cluster #1)
DNAm (Beta)
Ages (years)
0.4
0.5
0.6
0.7
0.8
0
20
40
60
80
A
B
C
D
E
F
G
H
I
J
K
FDR (Age associated divergence) =0.0915
p(Scalp)=0.00103
p(Dura)=0.997
Scalp
Dura

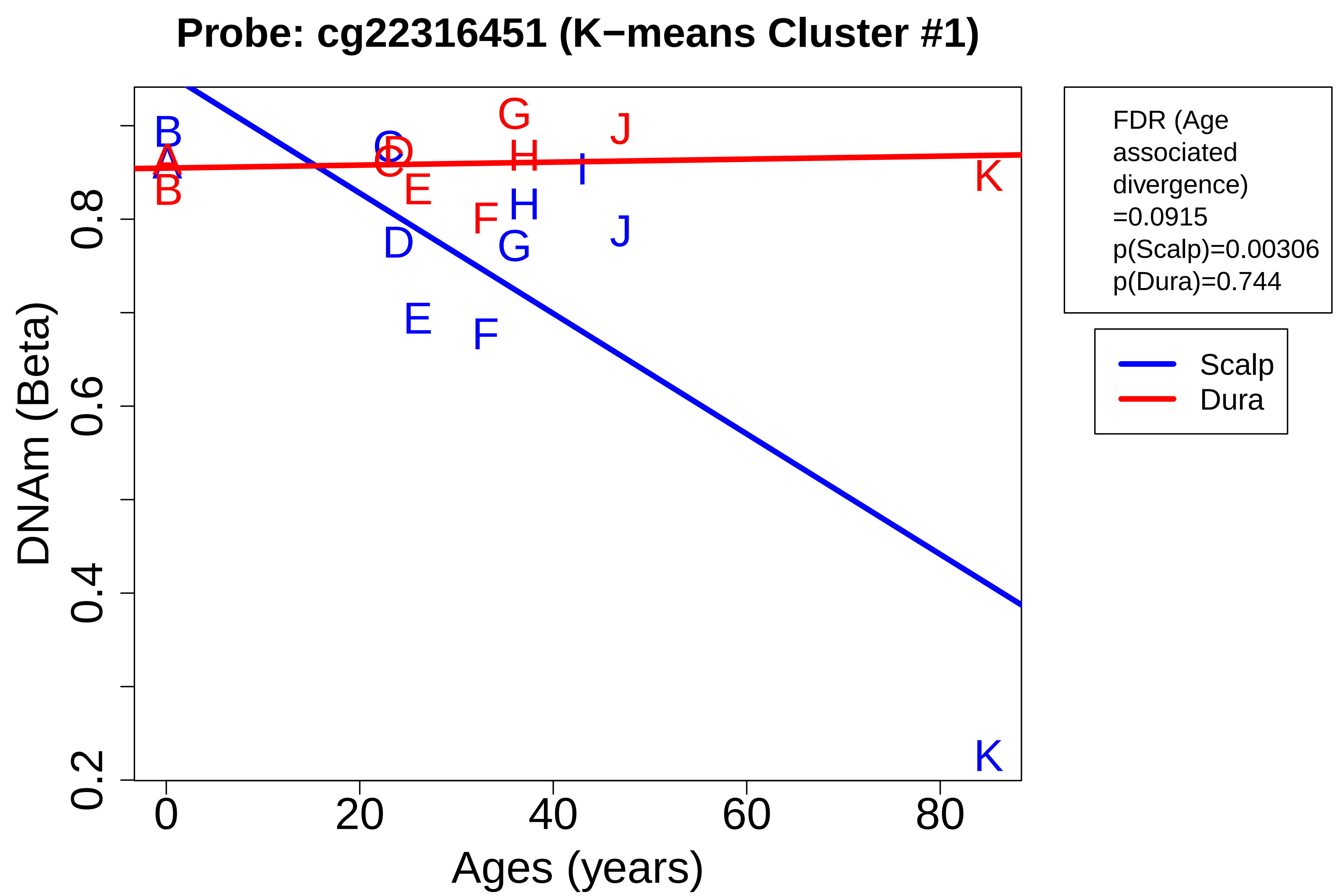

Probe: cg22316451 (K−means Cluster #1)
DNAm (Beta)
0.2
0.4
0.6
0.8
0
20
40
60
80
Ages (years)
FDR (Age associated divergence) =0.0915
p(Scalp)=0.00306
p(Dura)=0.744
Scalp
Dura

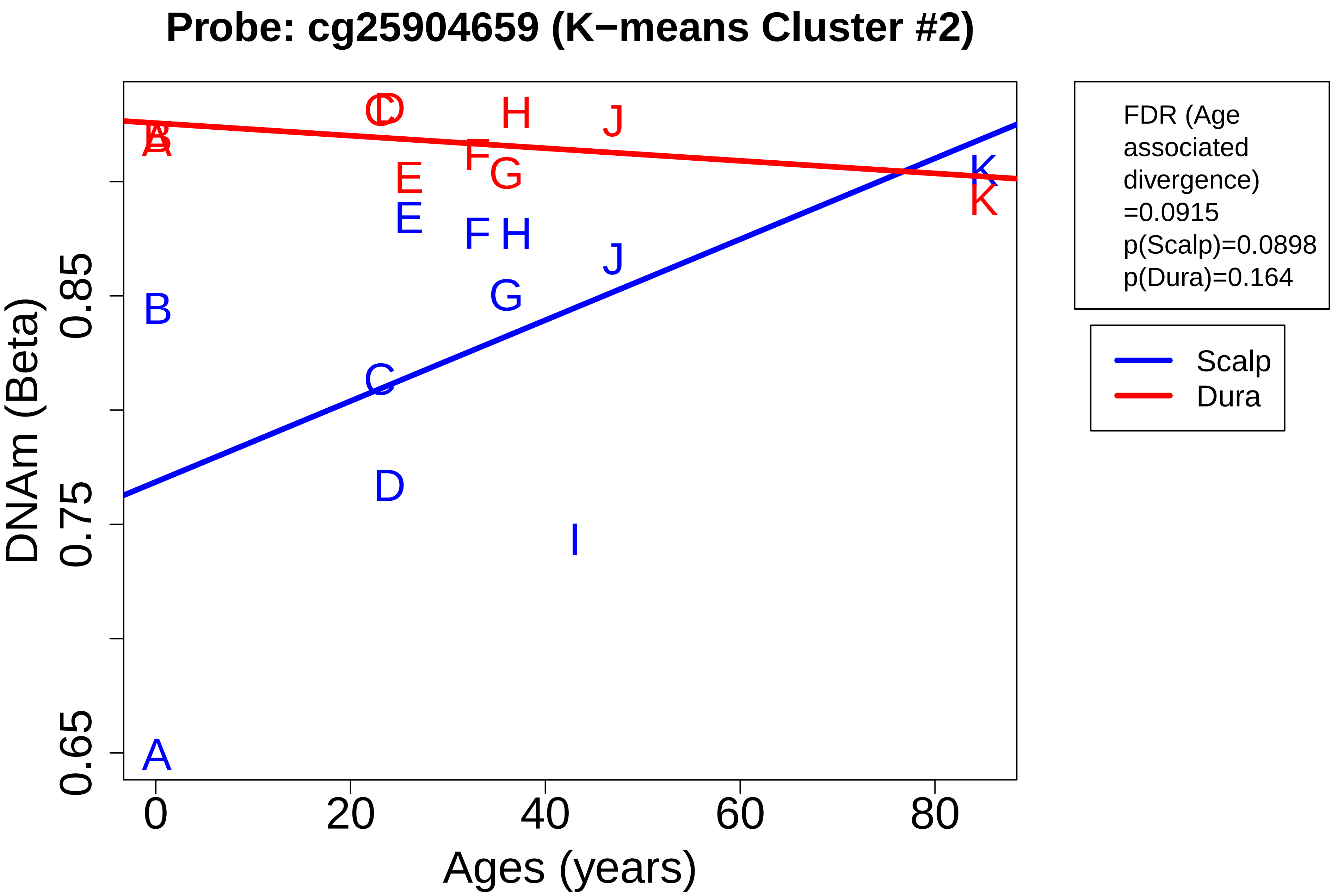

Probe: cg25904659 (K−means Cluster #2)
DNAm (Beta)
0.65
0.75
0.85
Ages (years)
0
20
40
60
80
FDR (Age associated divergence) =0.0915
p(Scalp)=0.0898
p(Dura)=0.164
Scalp
Dura

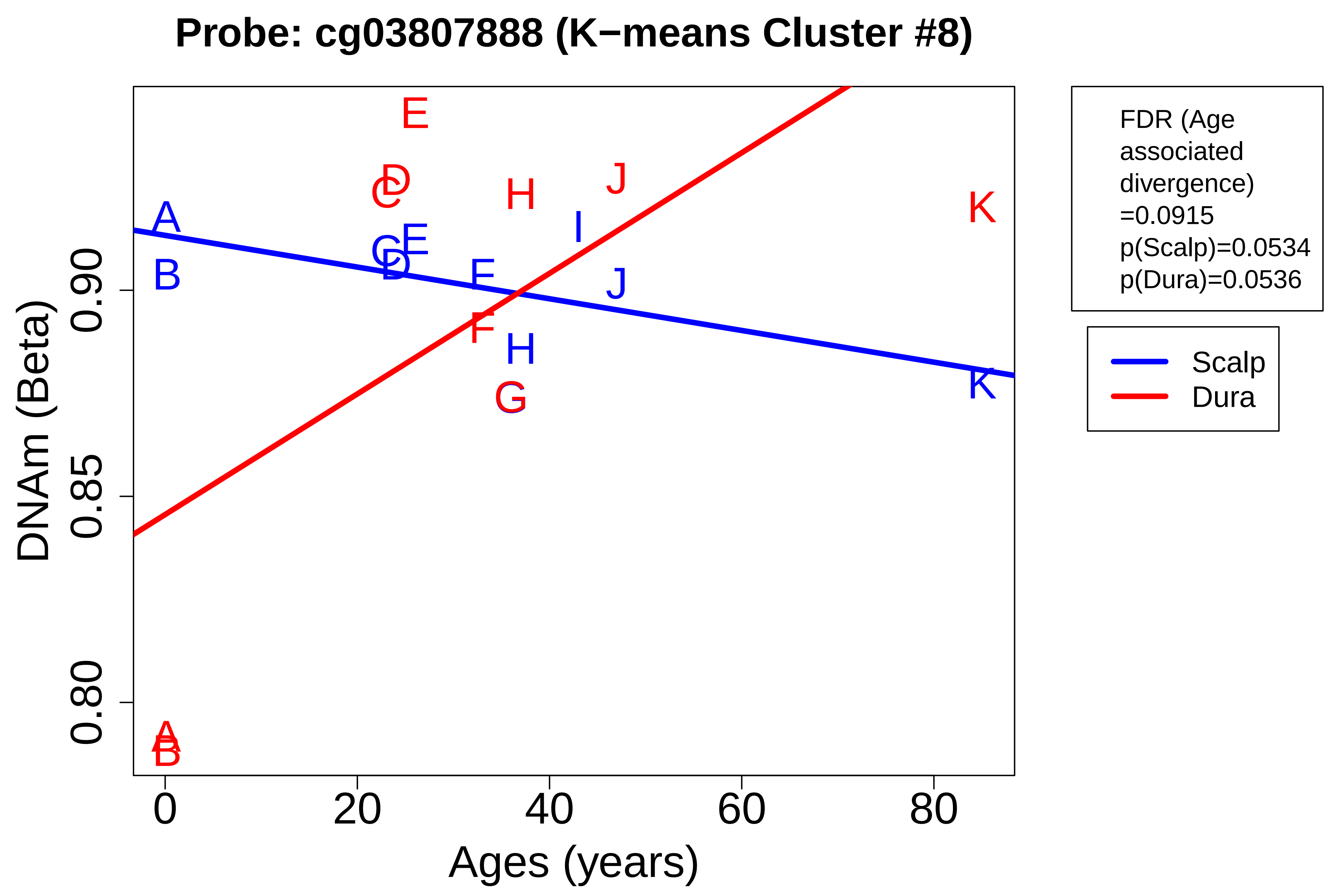

Probe: cg03807888 (K−means Cluster #8)
FDR (Age associated divergence) =0.0915
p(Scalp)=0.0534
p(Dura)=0.0536
Scalp
Dura
DNAm (Beta)
0.80
0.85
0.90
0
20
40
60
80
Ages (years)

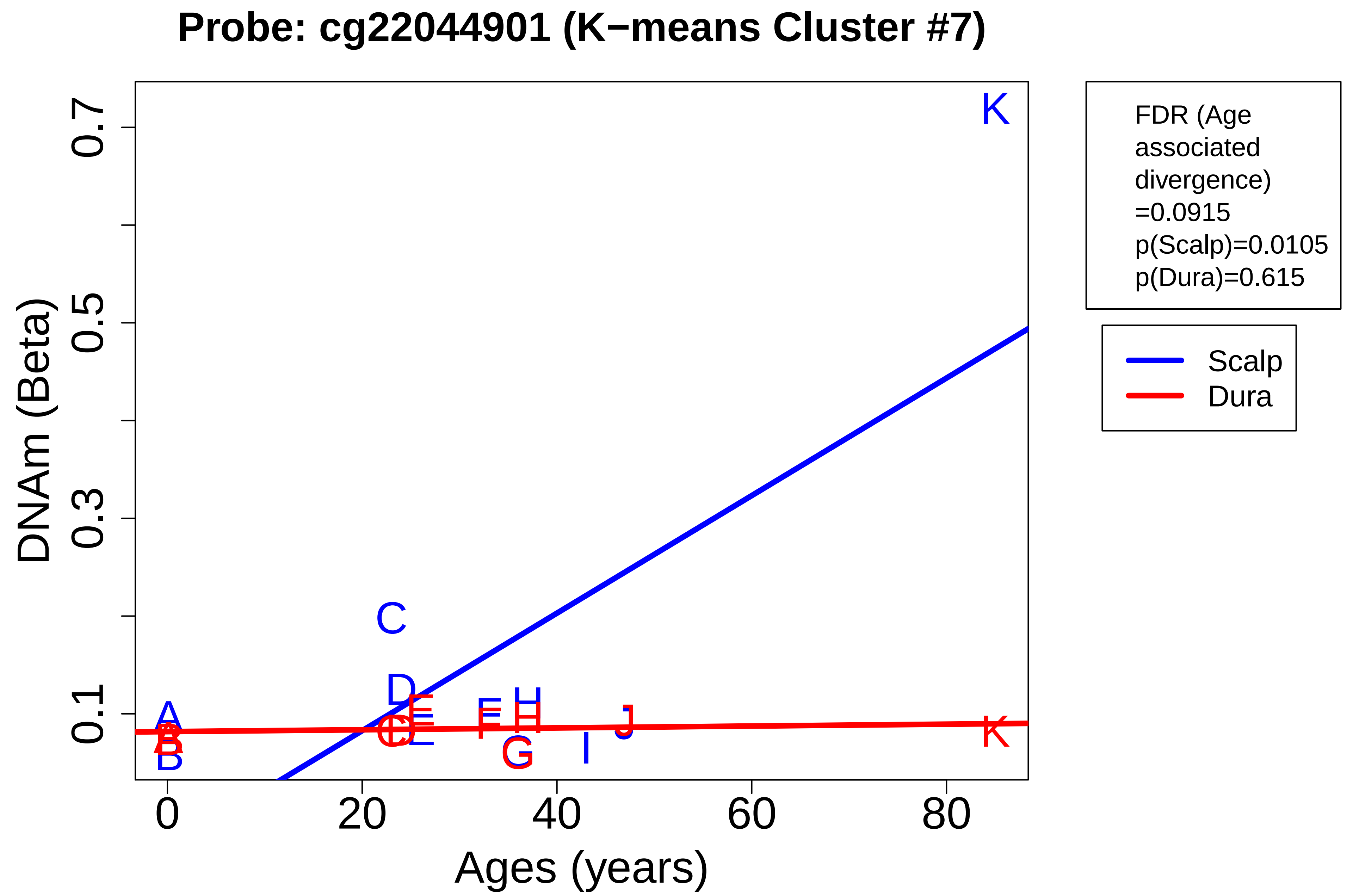

Probe: cg22044901 (K−means Cluster #7)
DNAm (Beta)
0.1
0.3
0.5
0.7
0
20
40
60
80
Ages (years)
A
B
C
D
E
F
G
H
I
J
K
FDR (Age associated divergence) =0.0915
p(Scalp)=0.0105
p(Dura)=0.615
Scalp
Dura

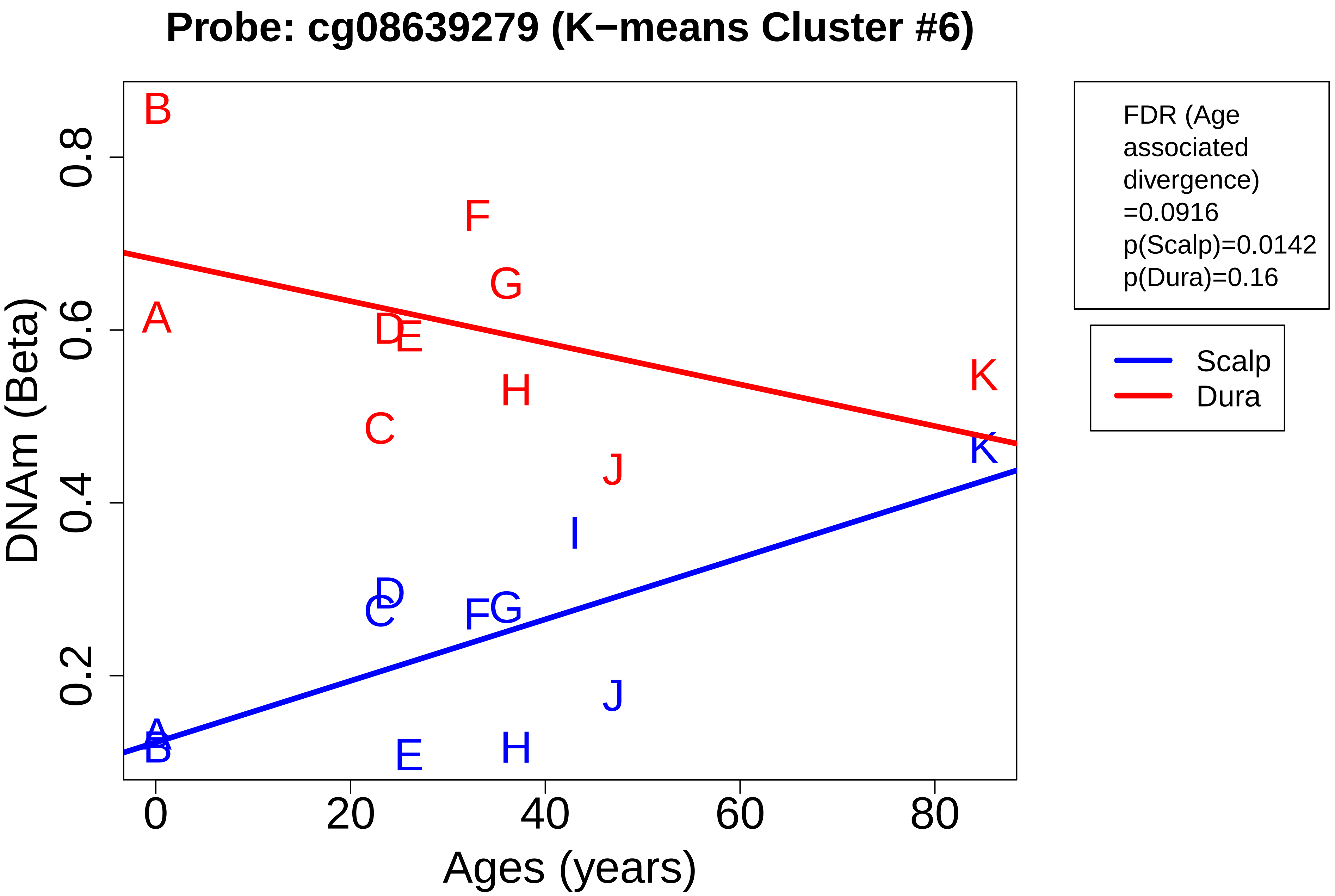
Probe: cg08639279 (K−means Cluster #6)
DNAm (Beta)
0.2
0.4
0.6
0.8
0
20
40
60
80
Ages (years)
FDR (Age associated divergence) =0.0916
p(Scalp)=0.0142
p(Dura)=0.16
Scalp
Dura

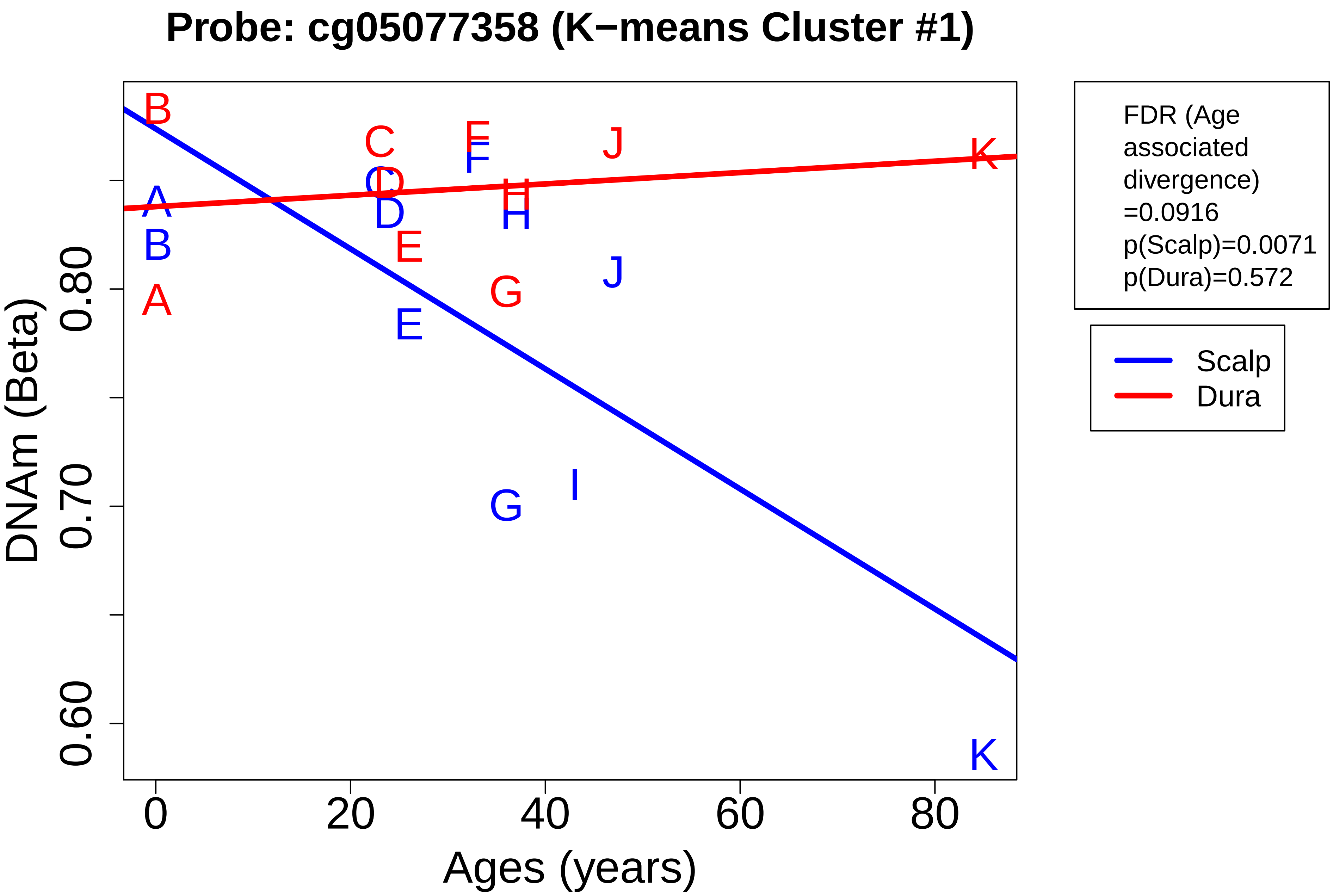

Probe: cg05077358 (K−means Cluster #1)
DNAm (Beta)
0.60
0.70
0.80
0
20
40
60
80
Ages (years)
FDR (Age associated divergence) =0.0916
p(Scalp)=0.0071
p(Dura)=0.572
Scalp
Dura

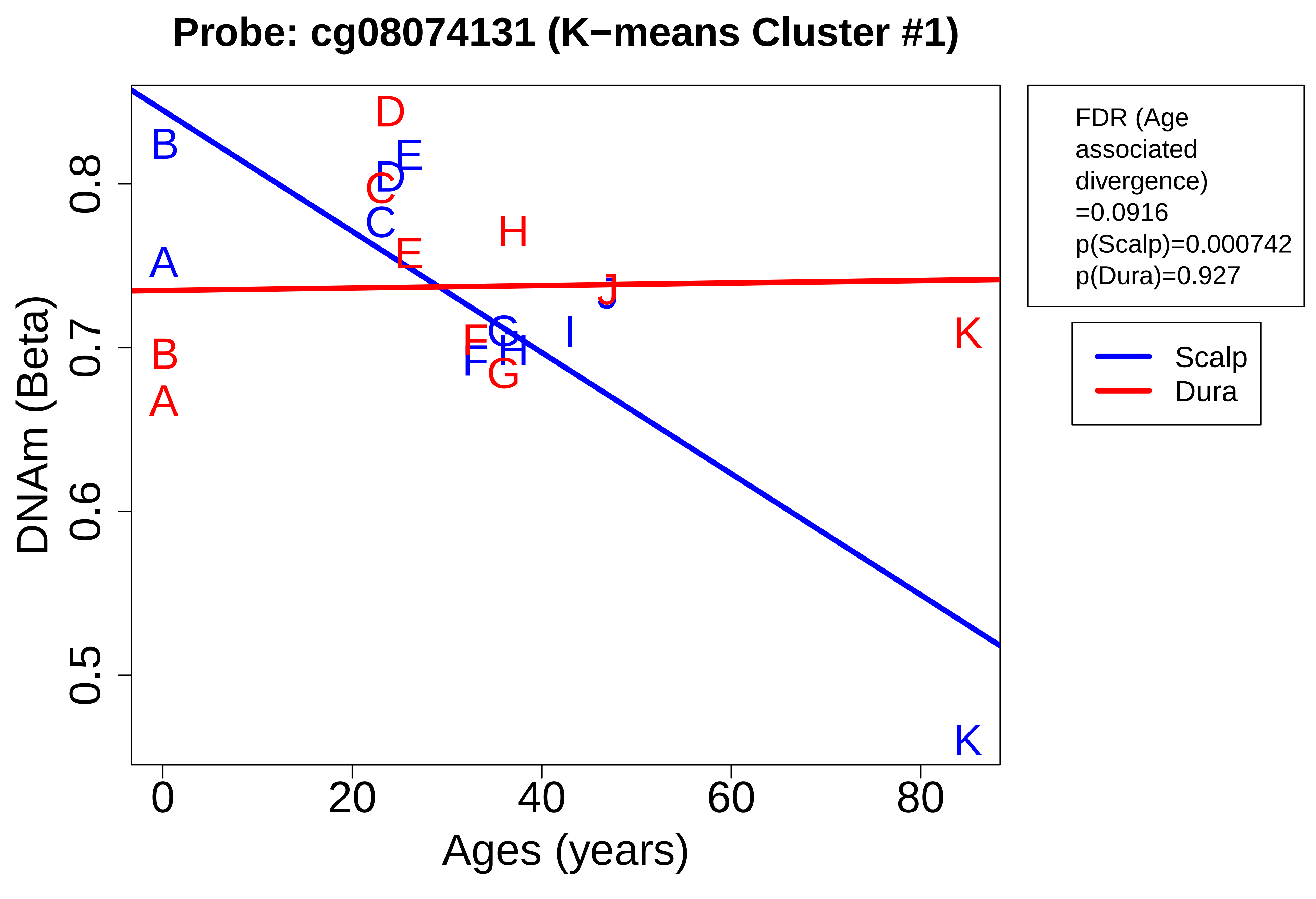

Probe: cg08074131 (K−means Cluster #1)
DNAm (Beta)
0.5
0.6
0.7
0.8
Ages (years)
0
20
40
60
80
FDR (Age associated divergence) =0.0916
p(Scalp)=0.000742
p(Dura)=0.927
Scalp
Dura
A
B
C
D
E
F
G
H
I
J
K

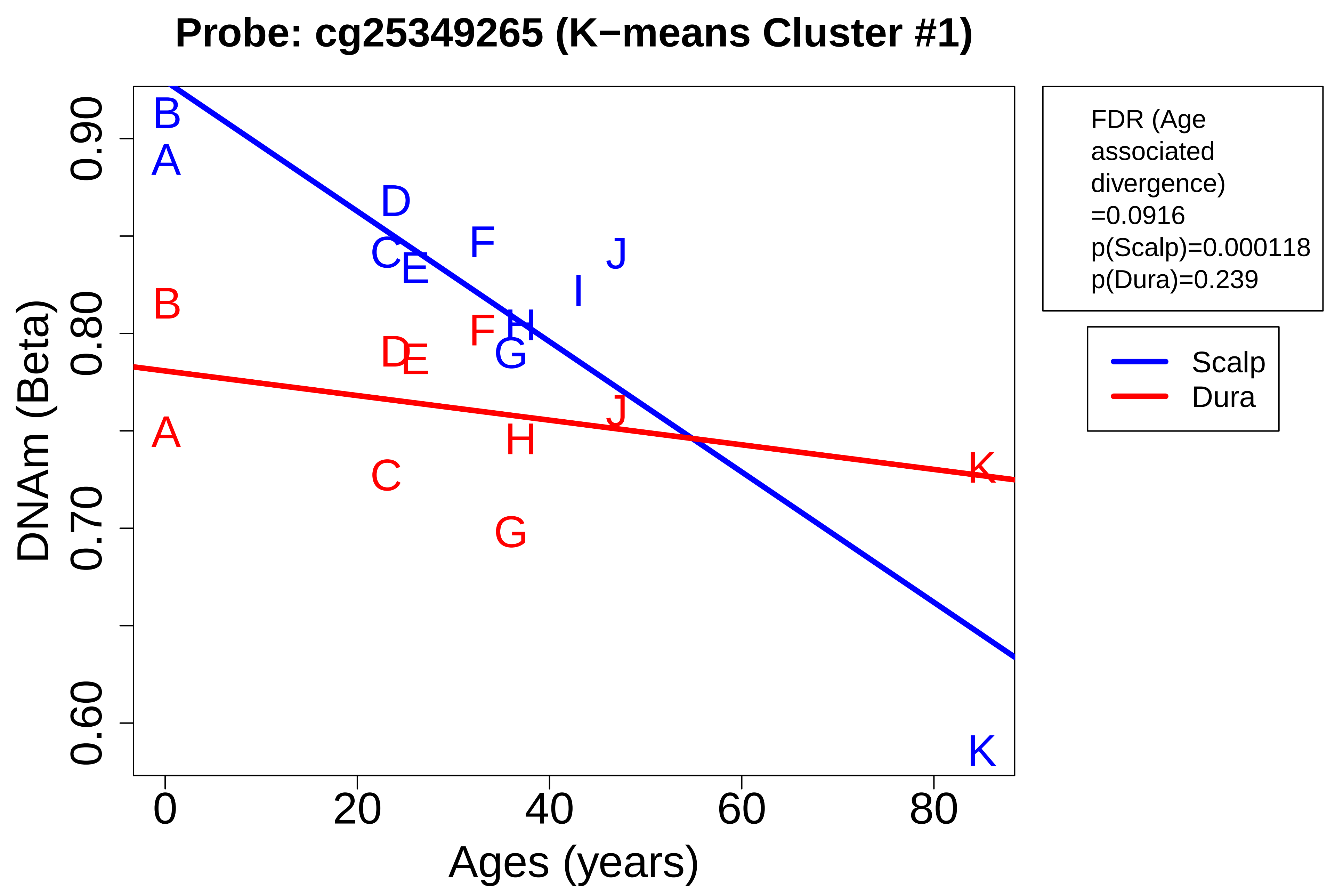

Probe: cg25349265 (K−means Cluster #1)
DNAm (Beta)
0.90
0.80
0.70
0.60
0
20
40
60
80
Ages (years)
FDR (Age associated divergence) =0.0916
p(Scalp)=0.000118
p(Dura)=0.239
Scalp
Dura

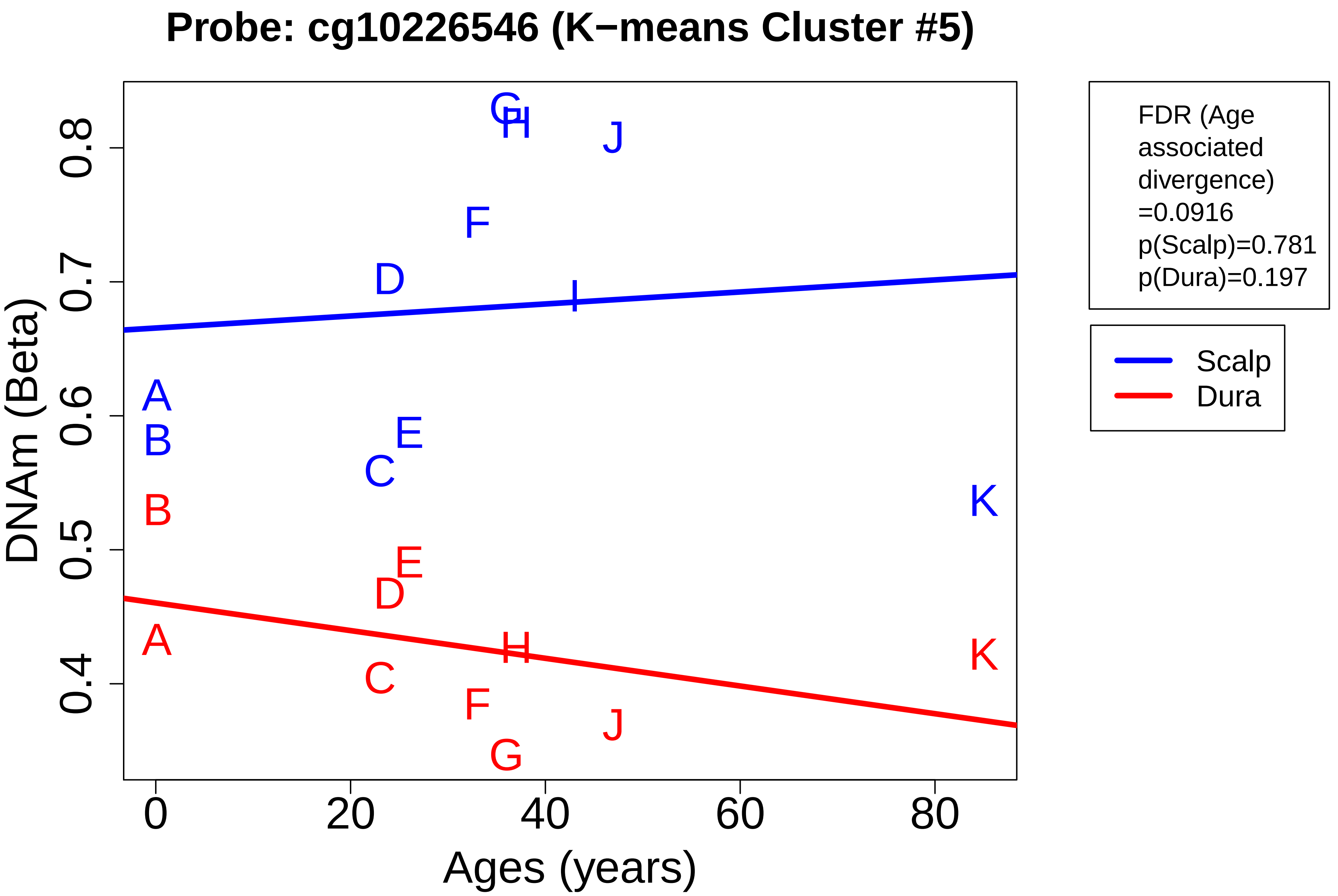
Probe: cg10226546 (K−means Cluster #5)
DNAm (Beta)
0.4
0.5
0.6
0.7
0.8
Ages (years)
0
20
40
60
80
A
B
C
D
E
F
G
H
I
J
K
FDR (Age associated divergence) =0.0916
p(Scalp)=0.781
p(Dura)=0.197
Scalp
Dura

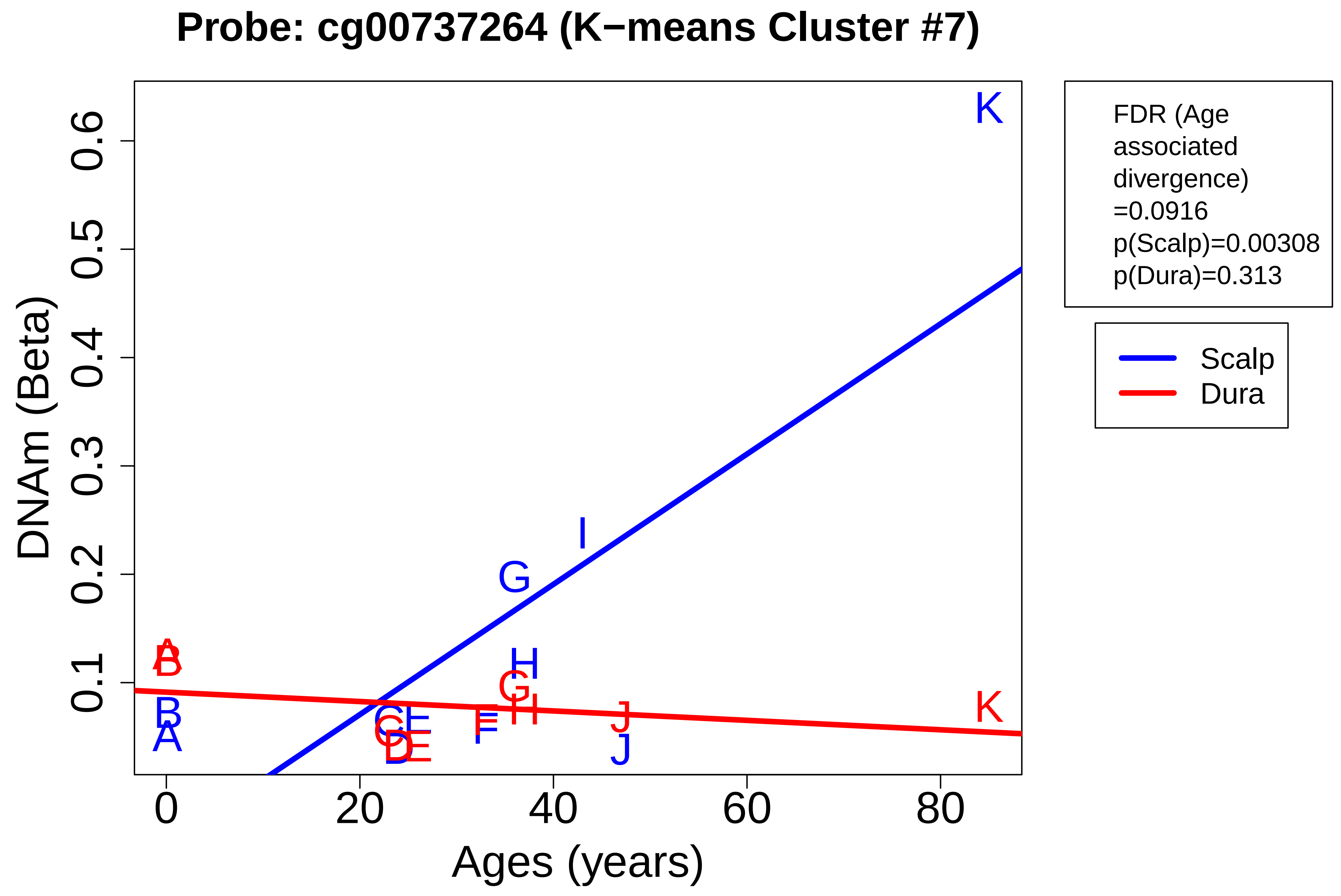
Probe: cg00737264 (K−means Cluster #7)
DNAm (Beta)
0.1
0.2
0.3
0.4
0.5
0.6
Ages (years)
0
20
40
60
80
FDR (Age associated divergence) =0.0916
p(Scalp)=0.00308
p(Dura)=0.313
Scalp
Dura

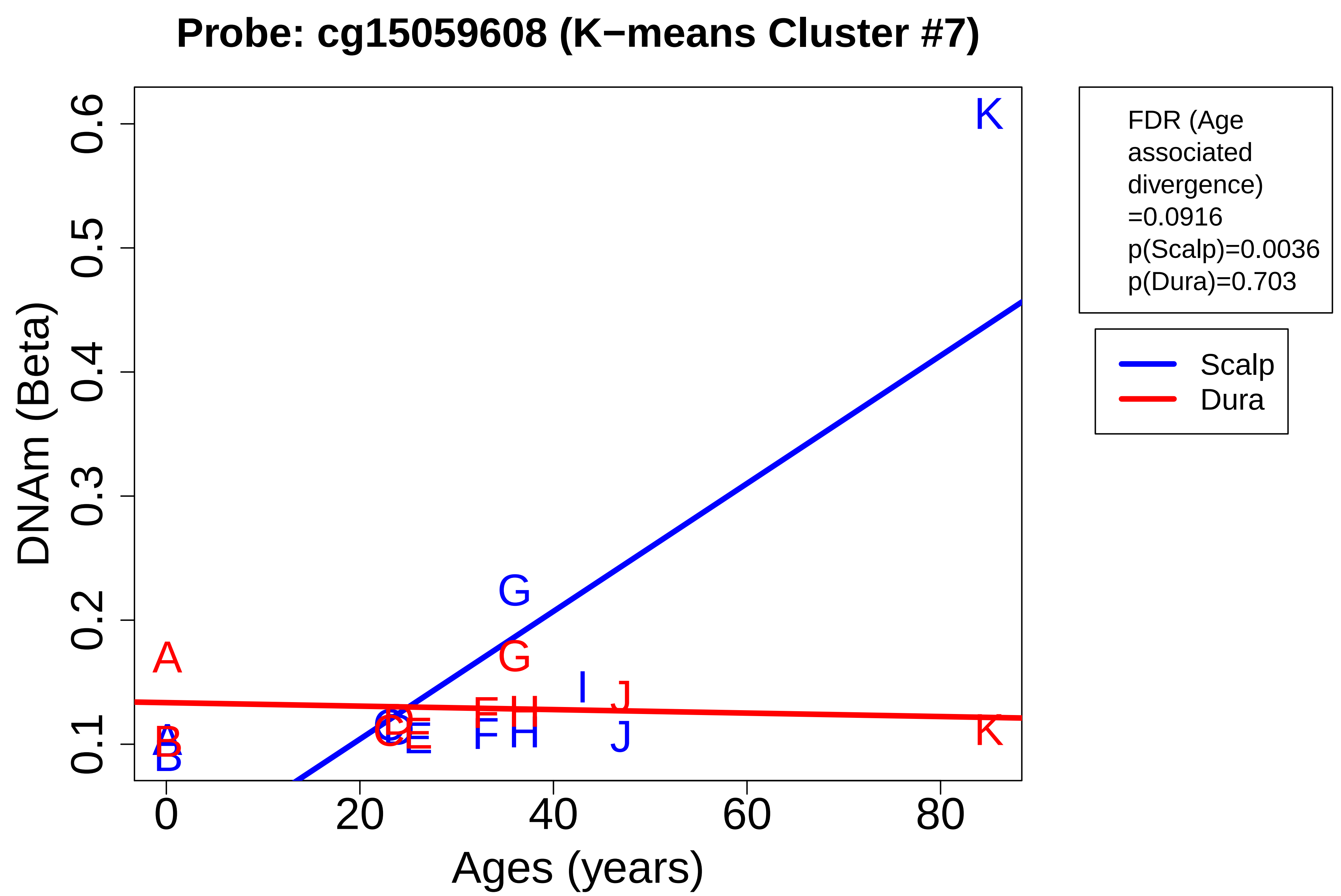

Probe: cg15059608 (K−means Cluster #7)
DNAm (Beta)
Ages (years)
0.1
0.2
0.3
0.4
0.5
0.6
0
20
40
60
80
FDR (Age associated divergence) =0.0916
p(Scalp)=0.0036
p(Dura)=0.703
Scalp
Dura

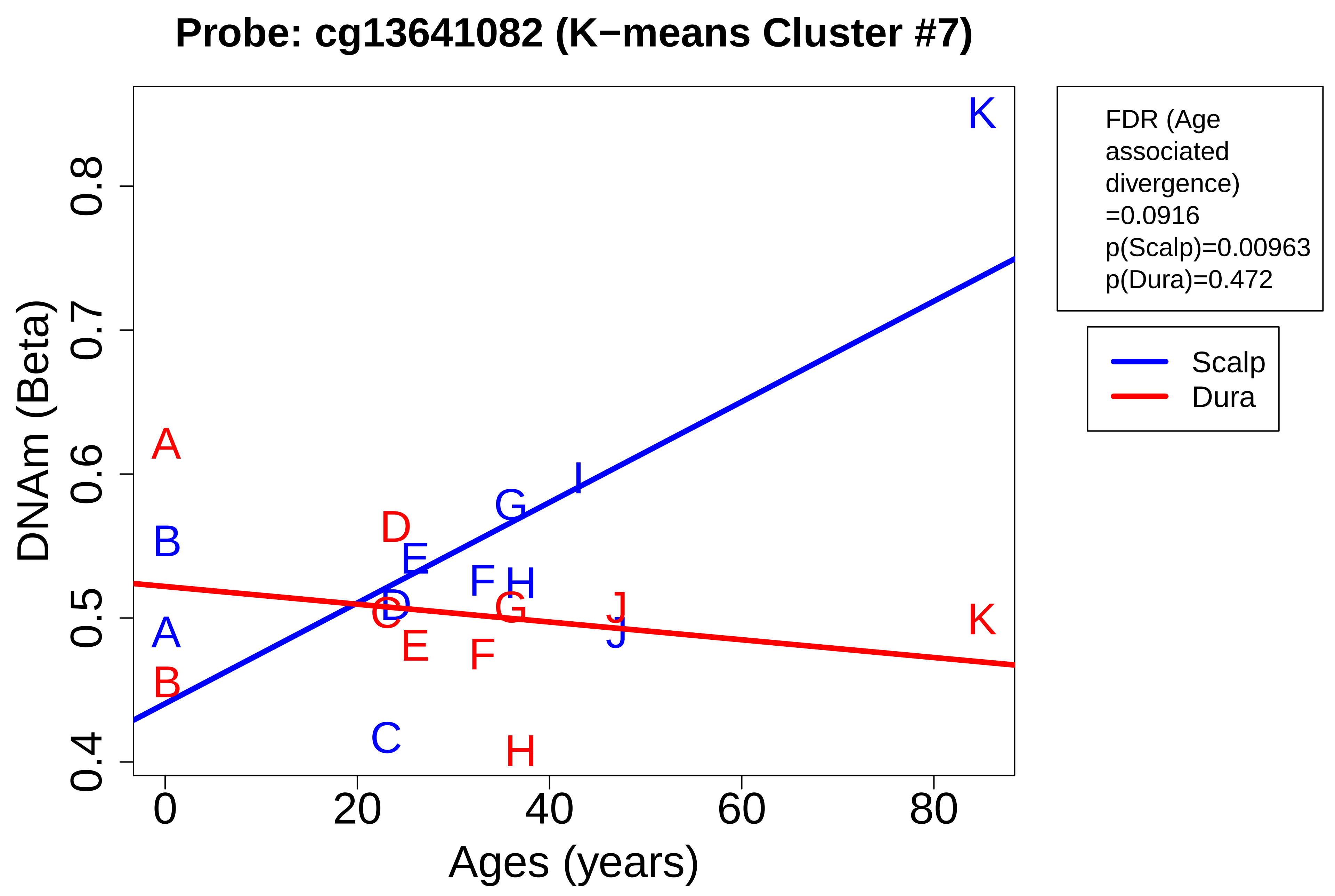

Probe: cg13641082 (K−means Cluster #7)
FDR (Age associated divergence) =0.0916
p(Scalp)=0.00963
p(Dura)=0.472
Scalp
Dura
DNAm (Beta)
0.4
0.5
0.6
0.7
0.8
0
20
40
60
80
Ages (years)
A
B
C
D
E
F
G
H
I
J
K

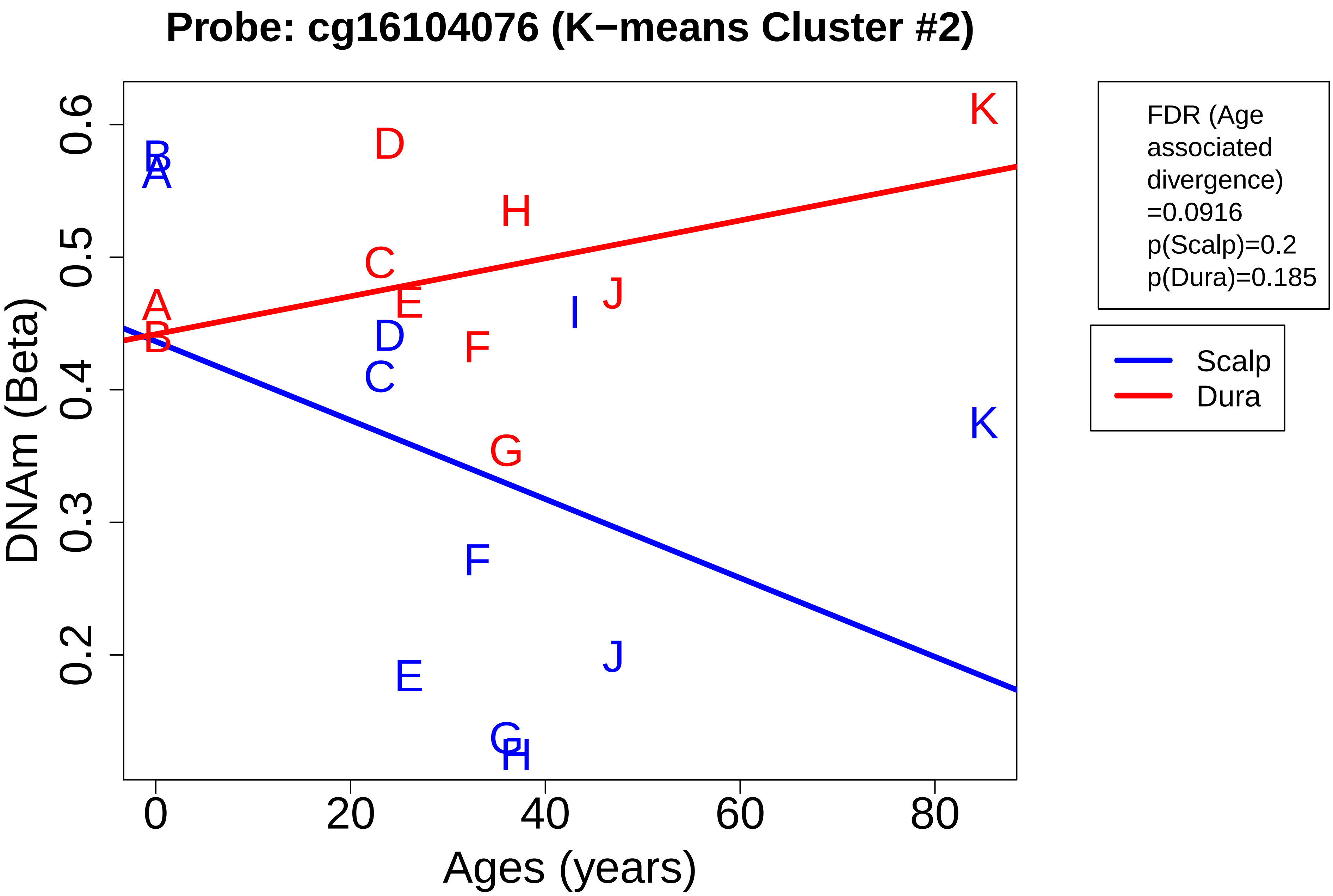

Probe: cg16104076 (K−means Cluster #2)
DNAm (Beta)
0.2
0.3
0.4
0.5
0.6
0
20
40
60
80
Ages (years)
FDR (Age associated divergence) =0.0916
p(Scalp)=0.2
p(Dura)=0.185
Scalp
Dura

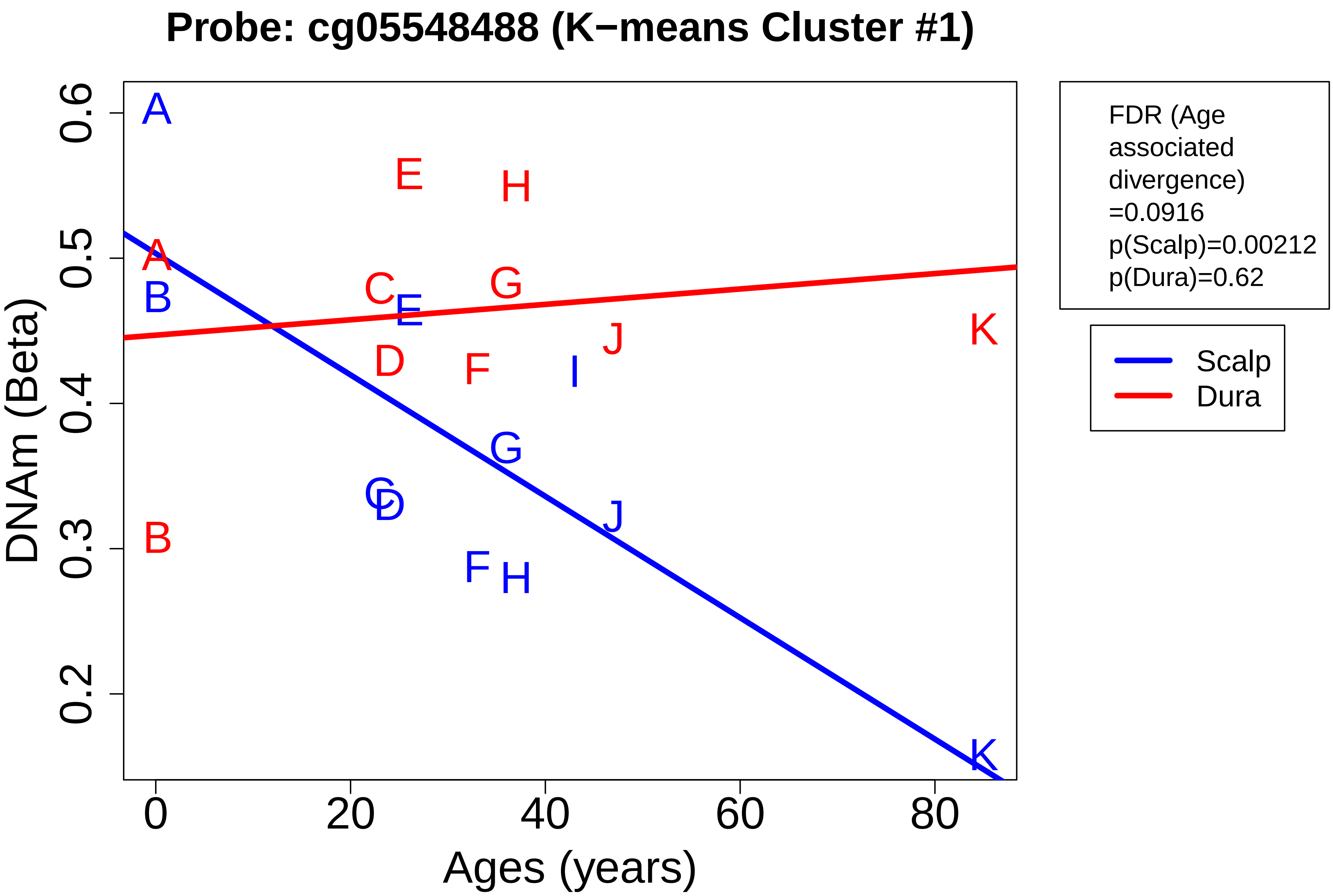

Probe: cg05548488 (K−means Cluster #1)
DNAm (Beta)
Ages (years)
0.2
0.3
0.4
0.5
0.6
0
20
40
60
80
FDR (Age associated divergence) =0.0916
p(Scalp)=0.00212
p(Dura)=0.62
Scalp
Dura

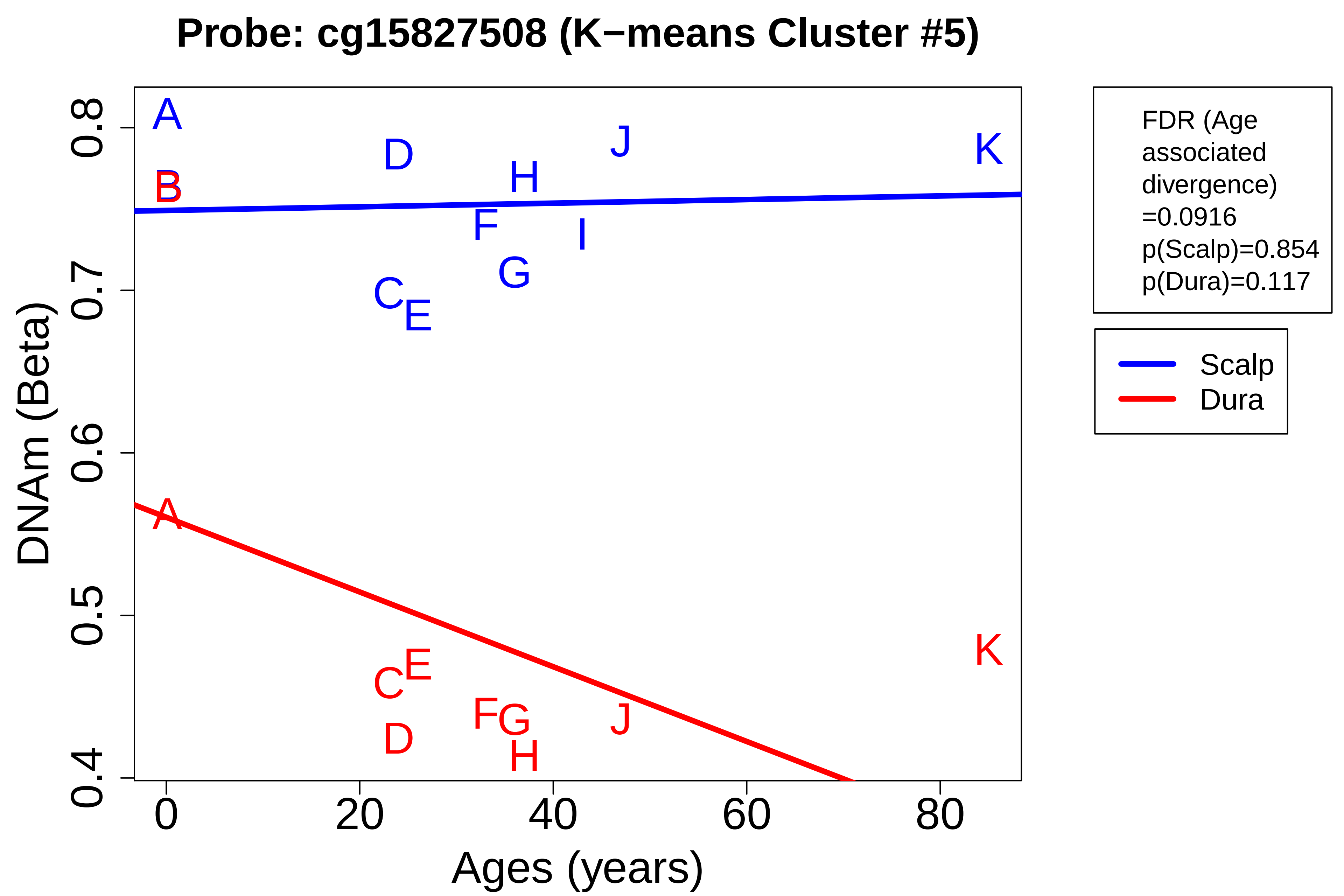

Probe: cg15827508 (K−means Cluster #5)
DNAm (Beta)
0.4
0.5
0.6
0.7
0.8
0
20
40
60
80
Ages (years)
A
B
C
D
E
F
G
H
I
J
K
FDR (Age associated divergence) =0.0916
p(Scalp)=0.854
p(Dura)=0.117
Scalp
Dura

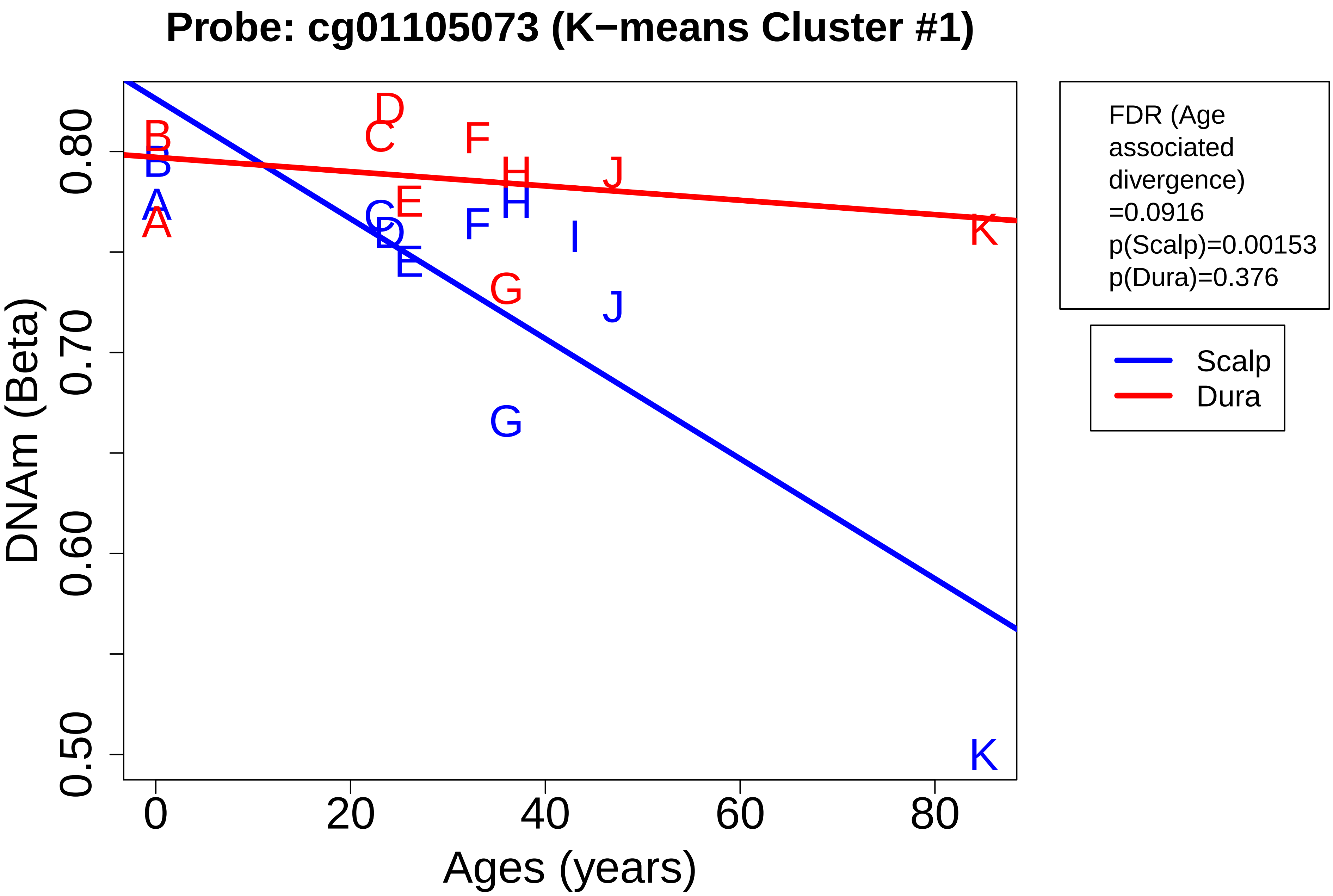

Probe: cg01105073 (K−means Cluster #1)
DNAm (Beta)
0.50
0.60
0.70
0.80
0
20
40
60
80
Ages (years)
A
B
C
D
E
F
G
H
I
J
K
FDR (Age associated divergence) =0.0916
p(Scalp)=0.00153
p(Dura)=0.376
Scalp
Dura

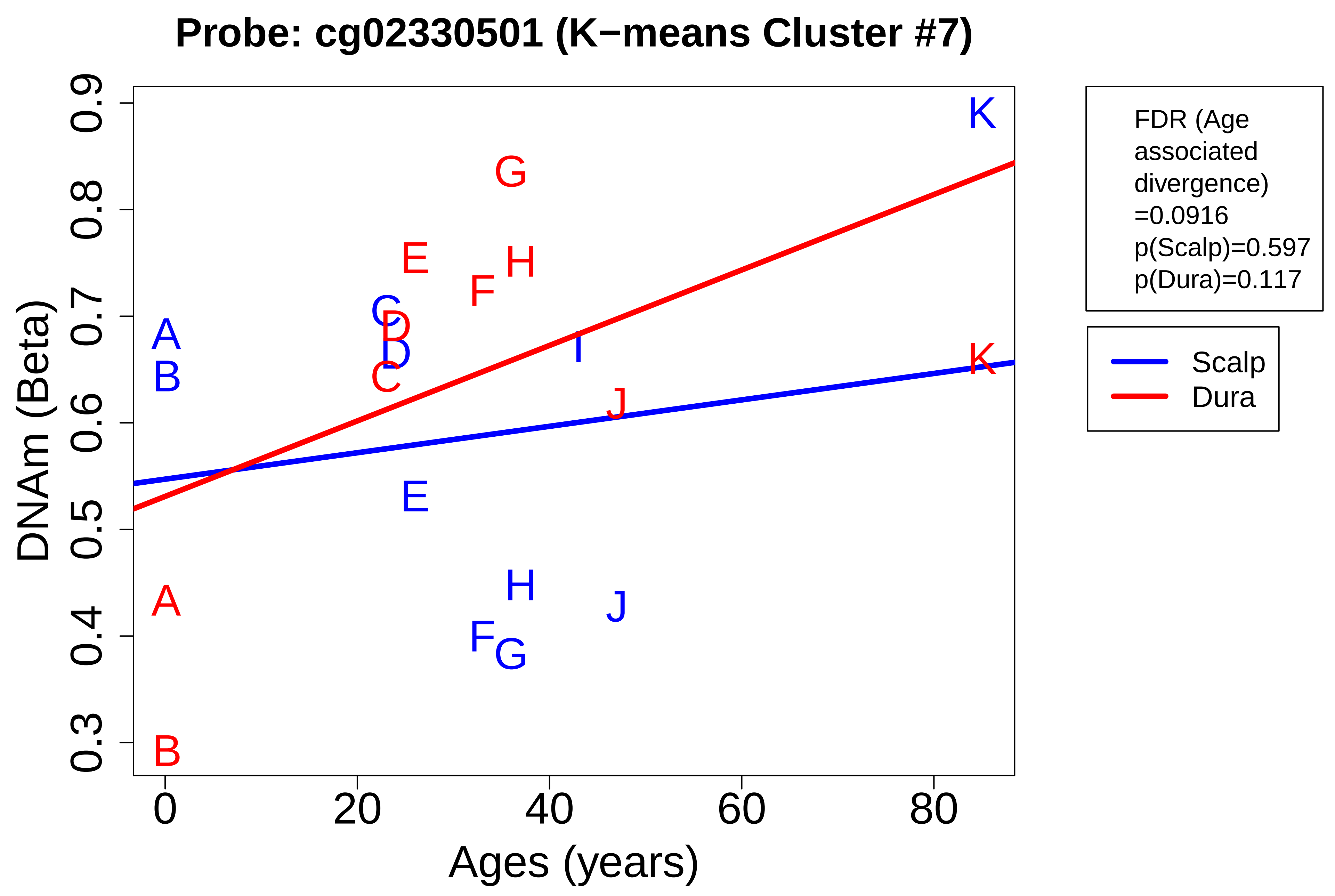

Probe: cg02330501 (K−means Cluster #7)
DNAm (Beta)
0.3
0.4
0.5
0.6
0.7
0.8
0.9
0
20
40
60
80
Ages (years)
FDR (Age associated divergence) =0.0916
p(Scalp)=0.597
p(Dura)=0.117
Scalp
Dura
A
B
C
D
E
F
G
H
I
J
K

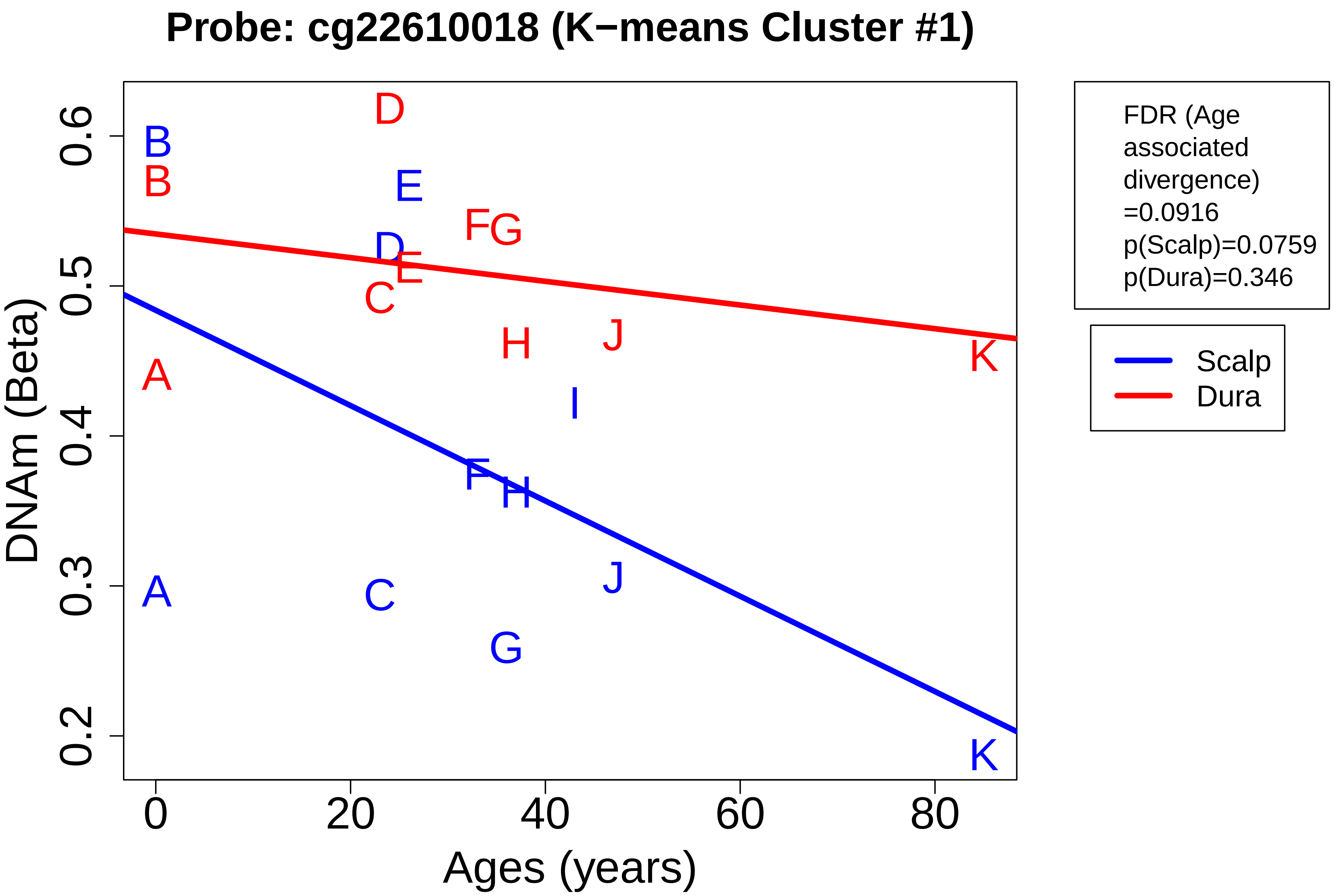

Probe: cg22610018 (K−means Cluster #1)
DNAm (Beta)
0.2
0.3
0.4
0.5
0.6
Ages (years)
0
20
40
60
80
FDR (Age associated divergence) =0.0916
p(Scalp)=0.0759
p(Dura)=0.346
Scalp
Dura
A
B
C
D
E
F
G
H
I
J
K

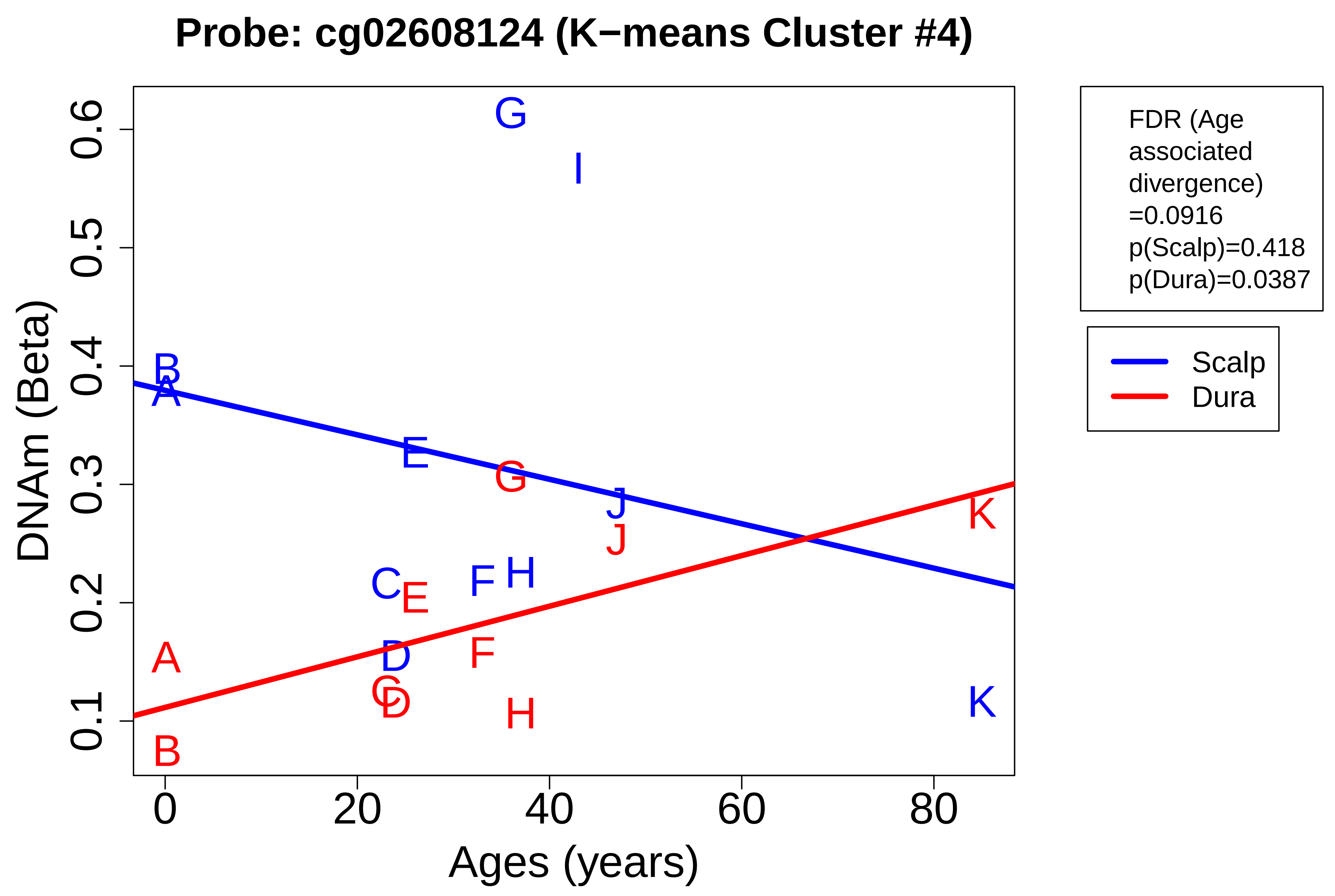

Probe: cg02608124 (K−means Cluster #4)
DNAm (Beta)
Ages (years)
0.1
0.2
0.3
0.4
0.5
0.6
0
20
40
60
80
FDR (Age associated divergence) =0.0916
p(Scalp)=0.418
p(Dura)=0.0387
Scalp
Dura

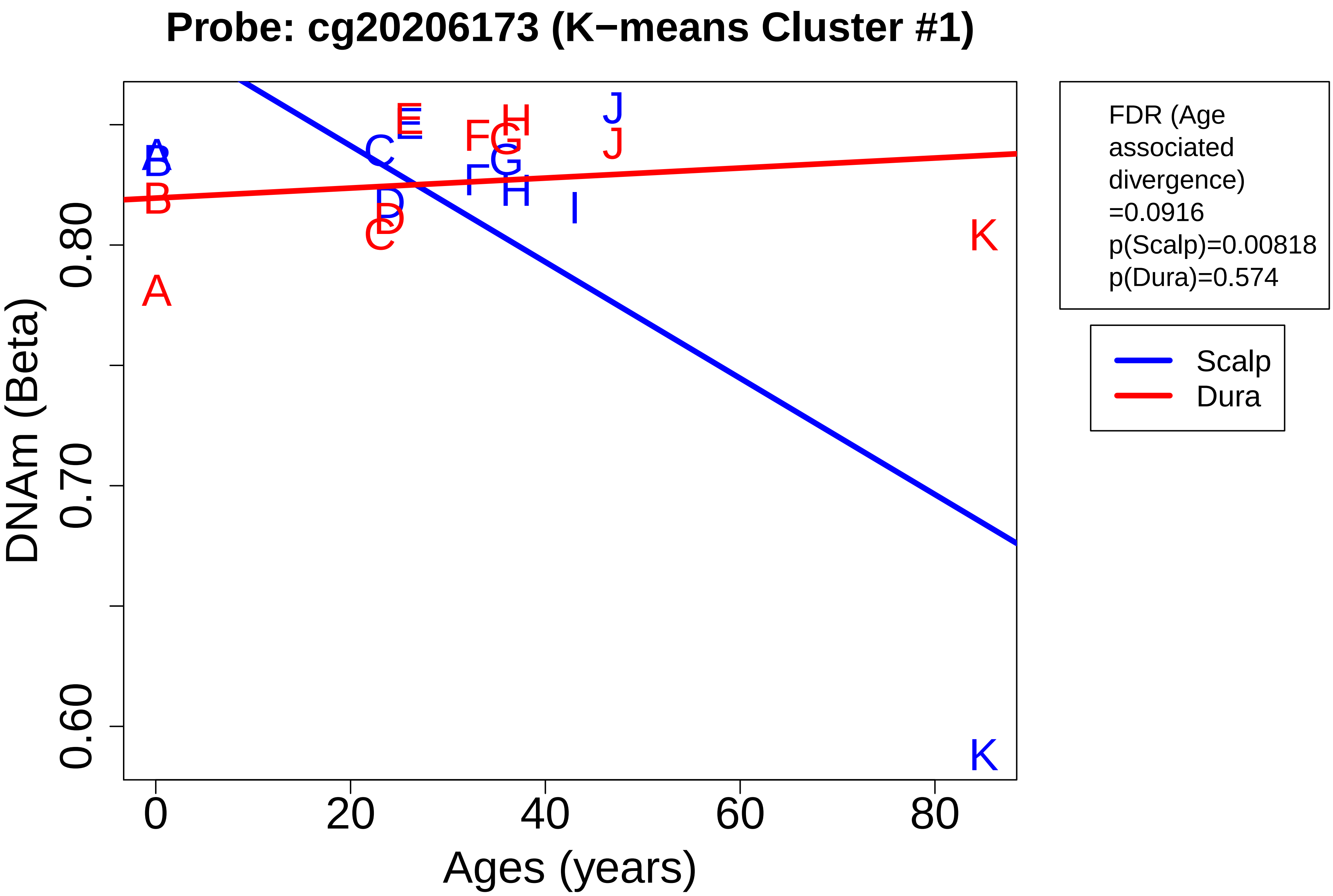

Probe: cg20206173 (K−means Cluster #1)
DNAm (Beta)
0.60
0.70
0.80
Ages (years)
0
20
40
60
80
FDR (Age associated divergence) =0.0916
p(Scalp)=0.00818
p(Dura)=0.574
Scalp
Dura
A
B
C
D
E
F
G
H
I
J
K

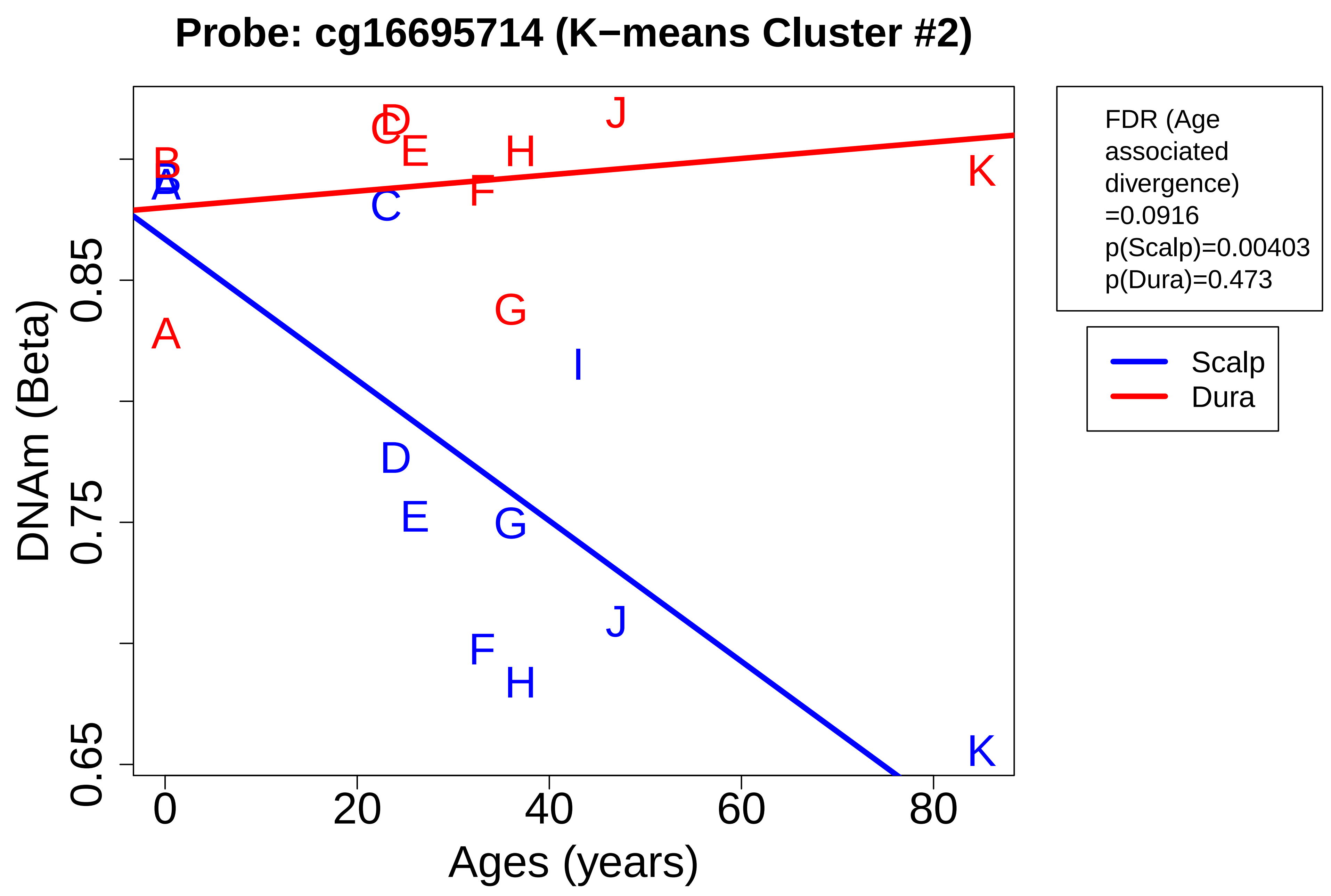

Probe: cg16695714 (K−means Cluster #2)
DNAm (Beta)
0.65
0.75
0.85
0
20
40
60
80
Ages (years)
A
B
C
D
E
F
G
H
I
J
K
FDR (Age associated divergence) =0.0916
p(Scalp)=0.00403
p(Dura)=0.473
Scalp
Dura

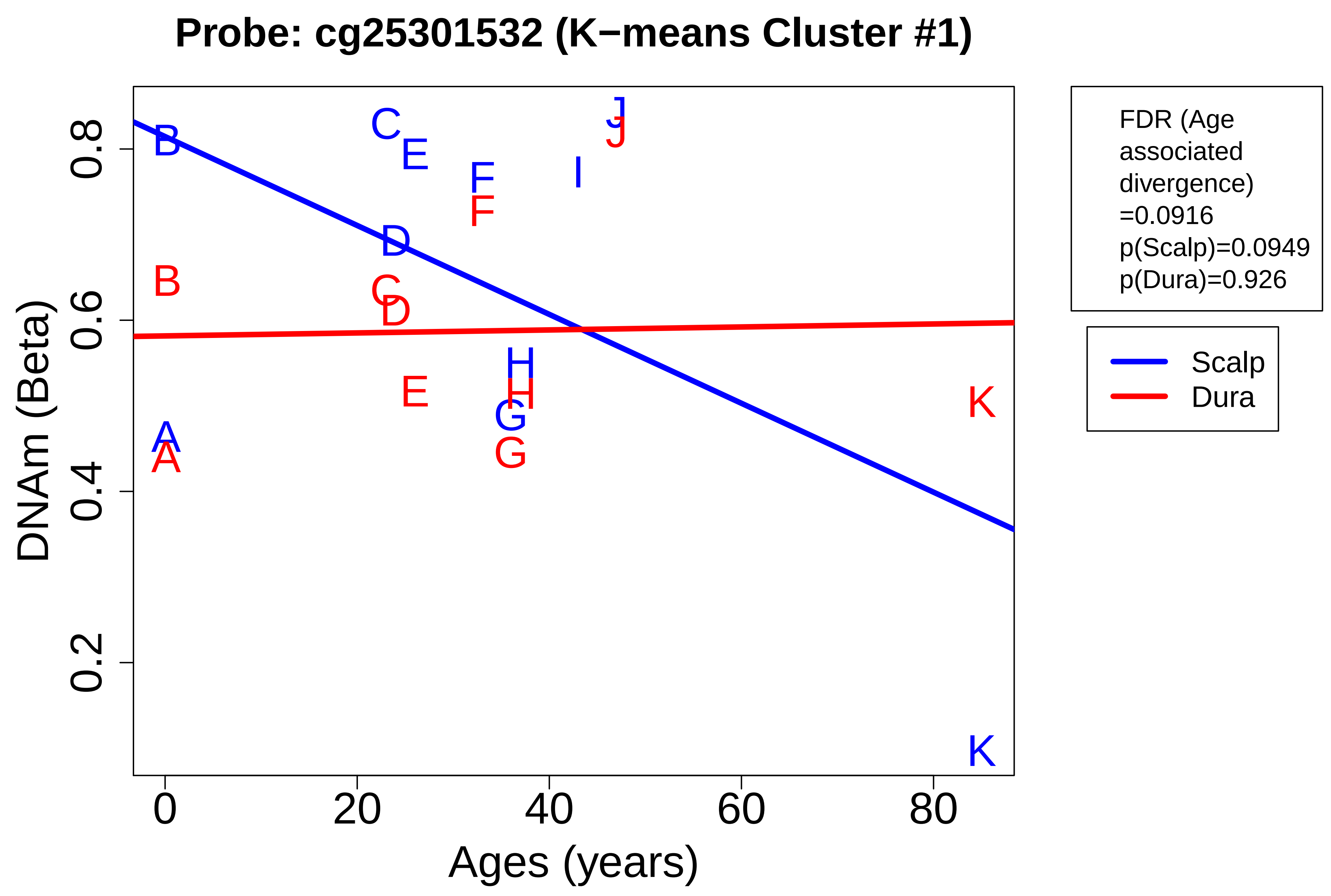

Probe: cg25301532 (K−means Cluster #1)
DNAm (Beta)
0.2
0.4
0.6
0.8
Ages (years)
0
20
40
60
80
A
B
C
D
E
F
G
H
I
J
K
FDR (Age associated divergence) =0.0916
p(Scalp)=0.0949
p(Dura)=0.926
Scalp
Dura

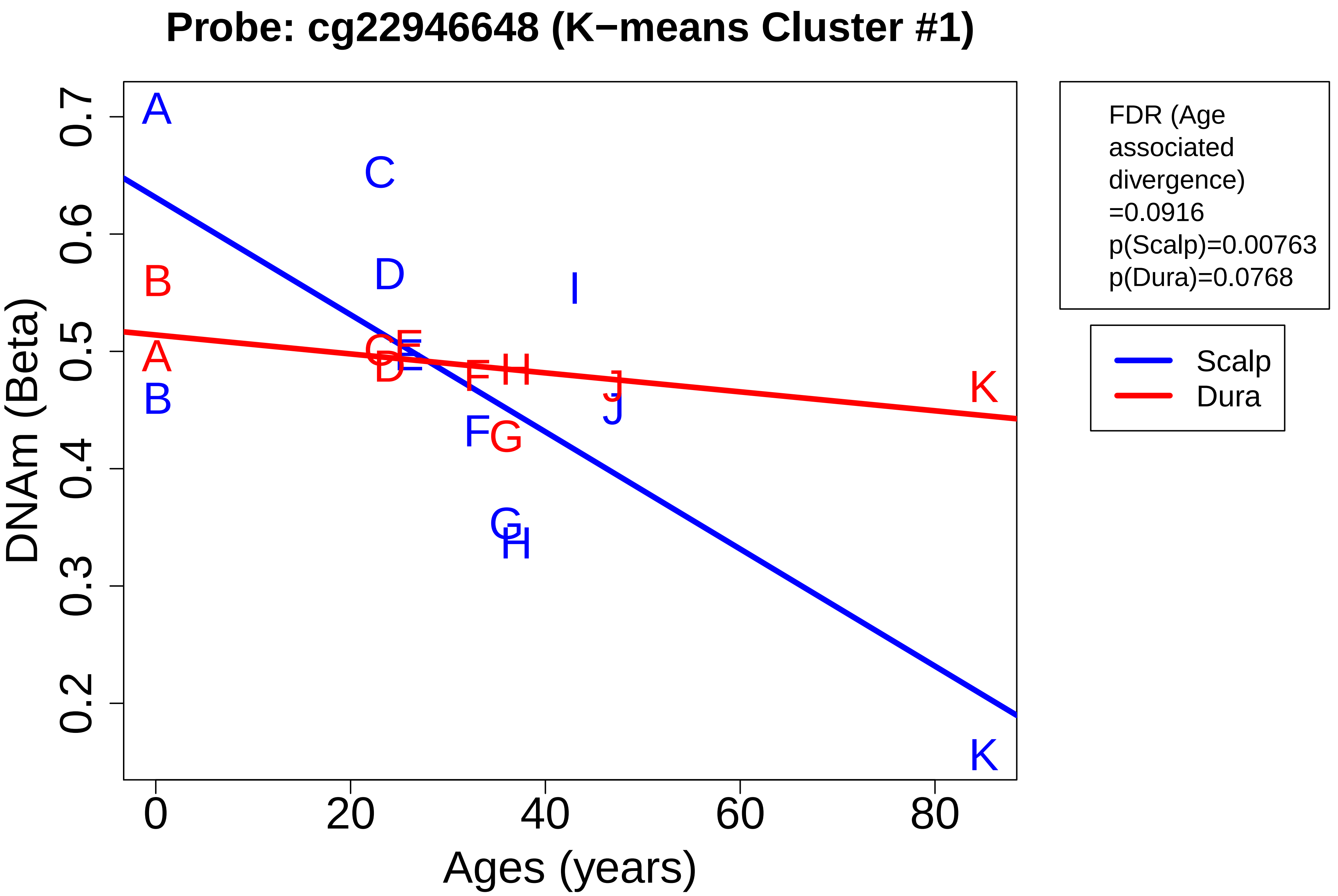

Probe: cg22946648 (K−means Cluster #1)
DNAm (Beta)
0.2
0.3
0.4
0.5
0.6
0.7
0
20
40
60
80
Ages (years)
FDR (Age associated divergence) =0.0916
p(Scalp)=0.00763
p(Dura)=0.0768
Scalp
Dura

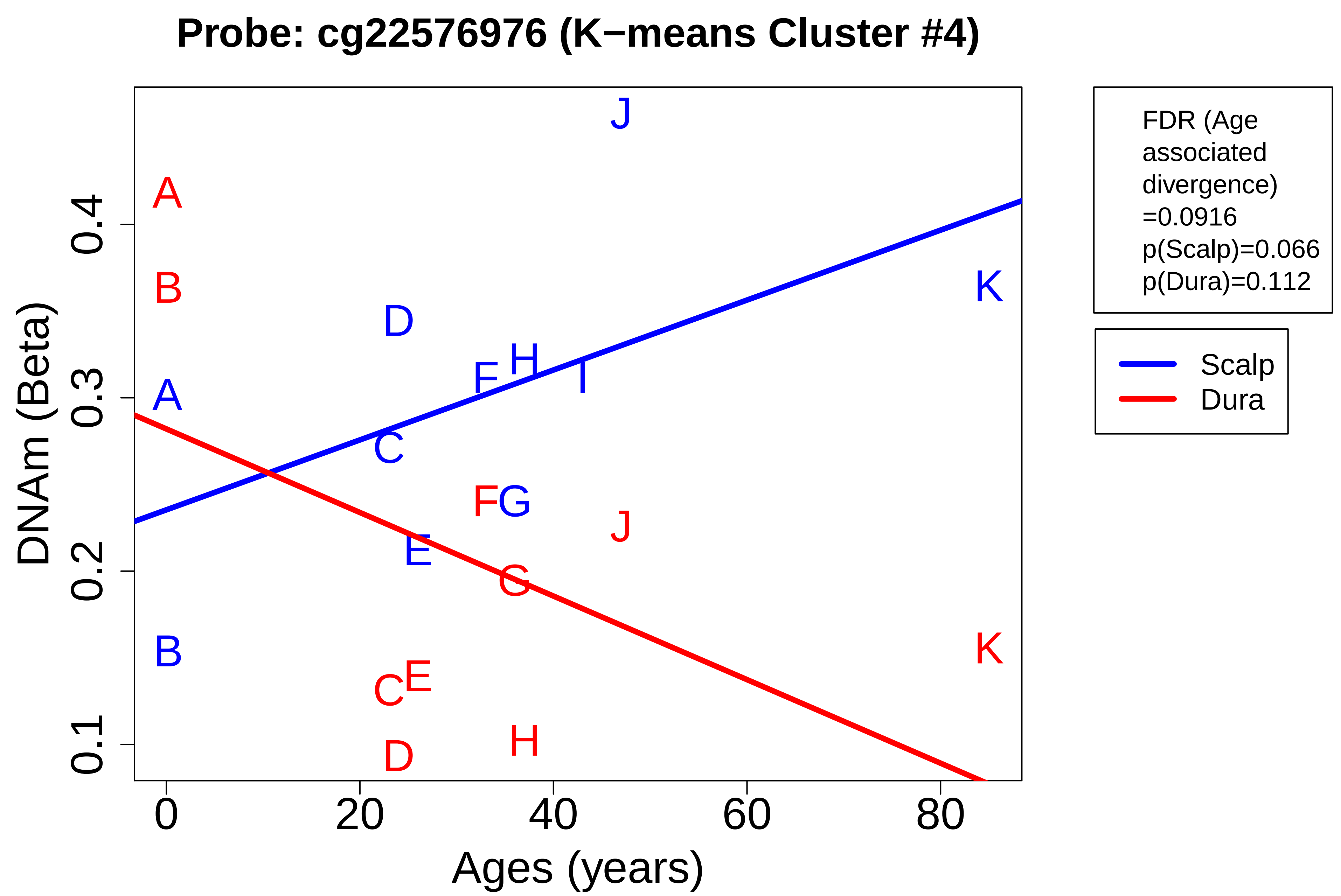

Probe: cg22576976 (K−means Cluster #4)
DNAm (Beta)
0.1
0.2
0.3
0.4
Ages (years)
0
20
40
60
80
A
B
C
D
E
F
G
H
I
J
K
FG
CE
FDR (Age associated divergence) =0.0916
p(Scalp)=0.066
p(Dura)=0.112
Scalp
Dura

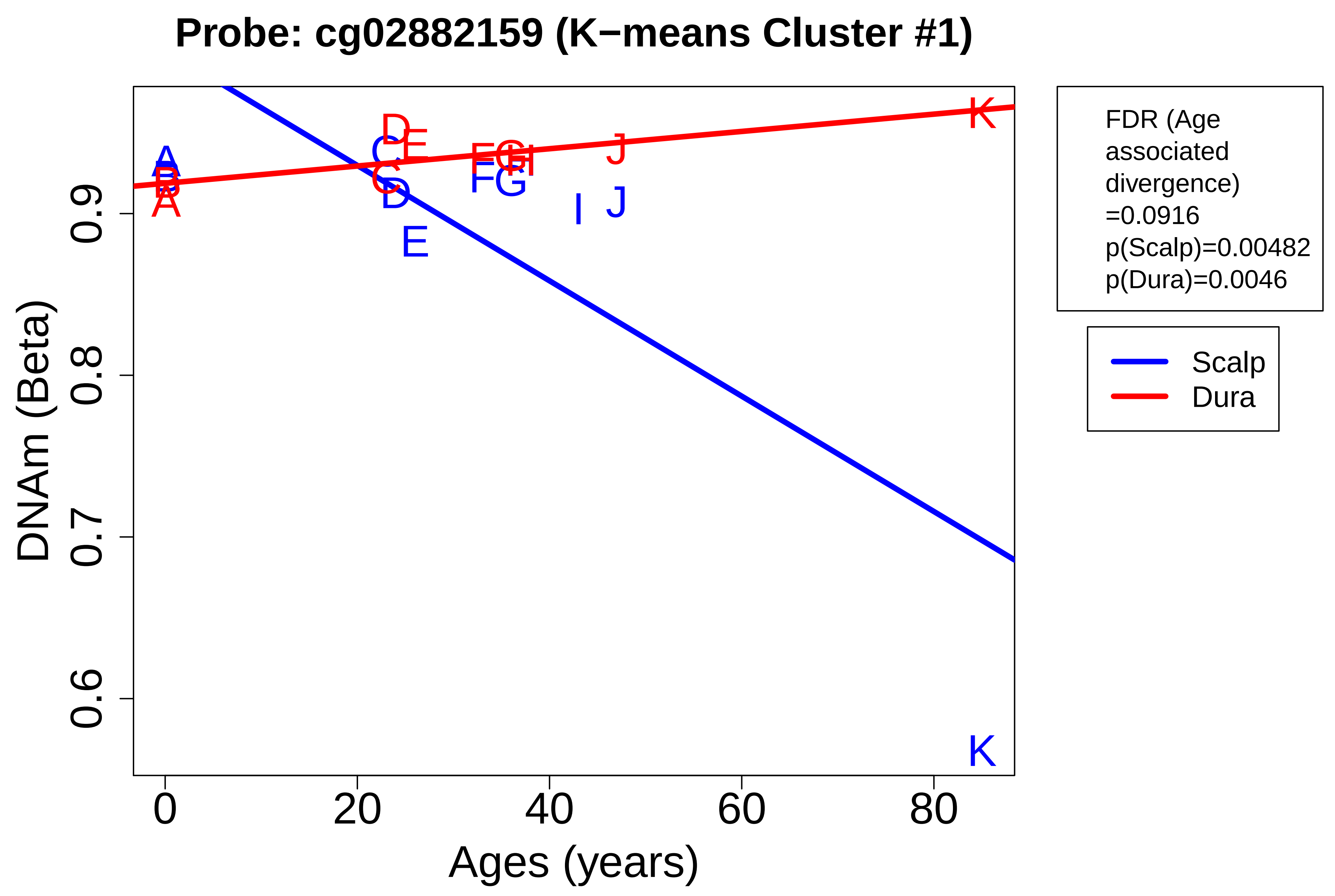

Probe: cg02882159 (K−means Cluster #1)
DNAm (Beta)
0.6
0.7
0.8
0.9
0
20
40
60
80
Ages (years)
A B C D E F G H I J K
FDR (Age associated divergence) =0.0916
p(Scalp)=0.00482
p(Dura)=0.0046
Scalp
Dura

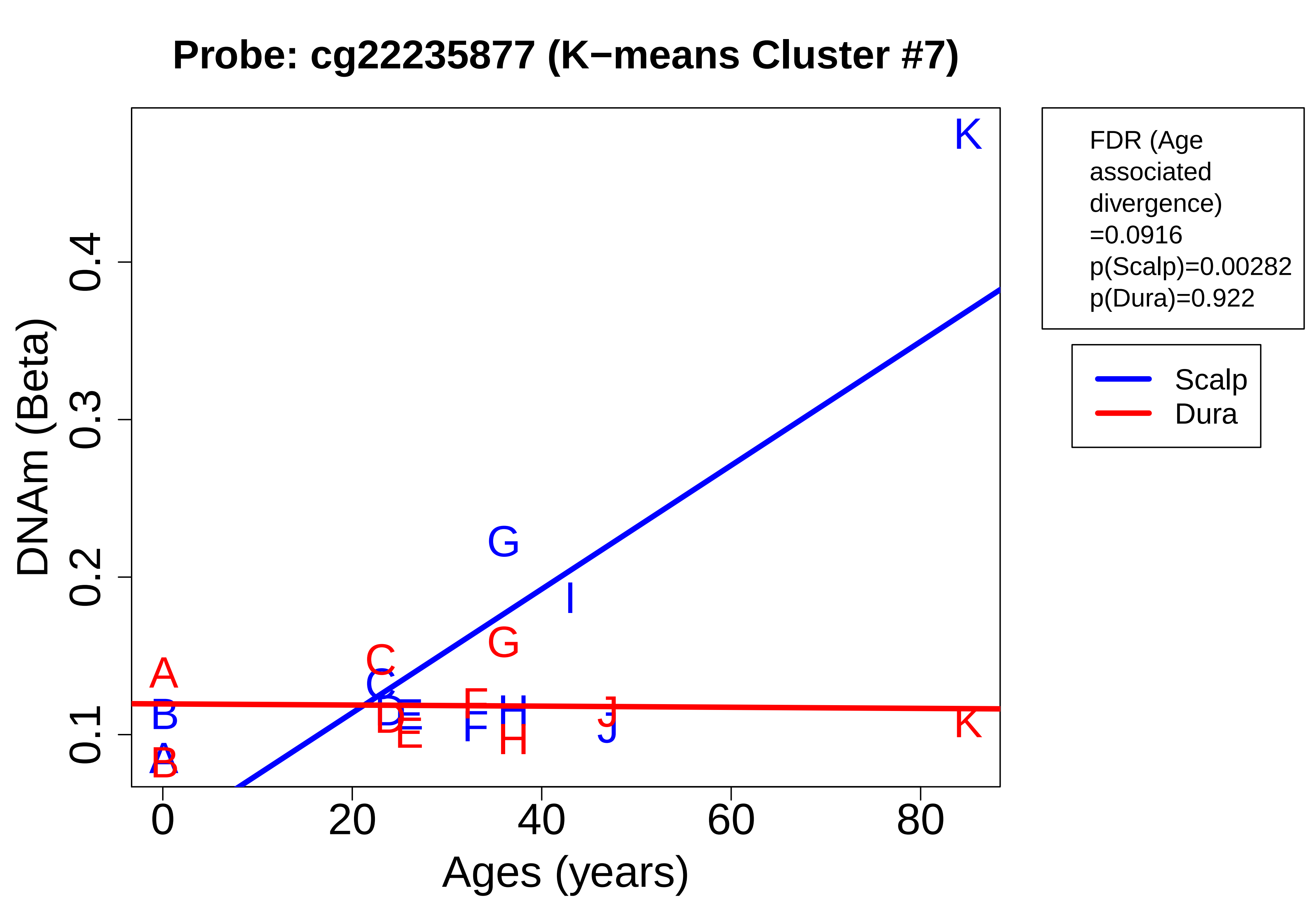

Probe: cg22235877 (K−means Cluster #7)
DNAm (Beta)
0.1
0.2
0.3
0.4
0
20
40
60
80
Ages (years)
FDR (Age associated divergence) =0.0916
p(Scalp)=0.00282
p(Dura)=0.922
Scalp
Dura

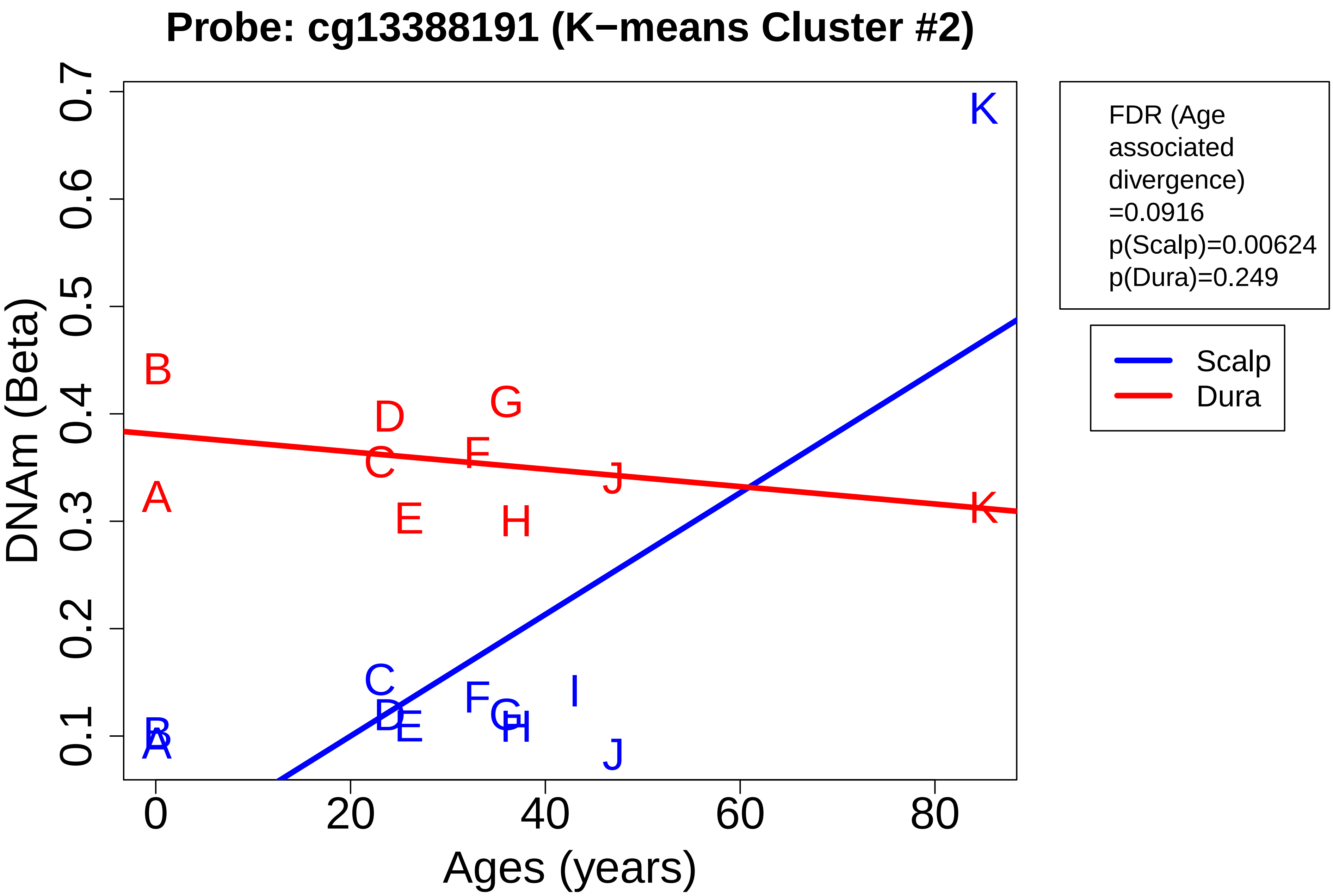

Probe: cg13388191 (K−means Cluster #2)
DNAm (Beta)
0.1
0.2
0.3
0.4
0.5
0.6
0.7
Ages (years)
0
20
40
60
80
A
B
C
D
E
F
G
H
I
J
K
FDR (Age associated divergence) =0.0916
p(Scalp)=0.00624
p(Dura)=0.249
Scalp
Dura

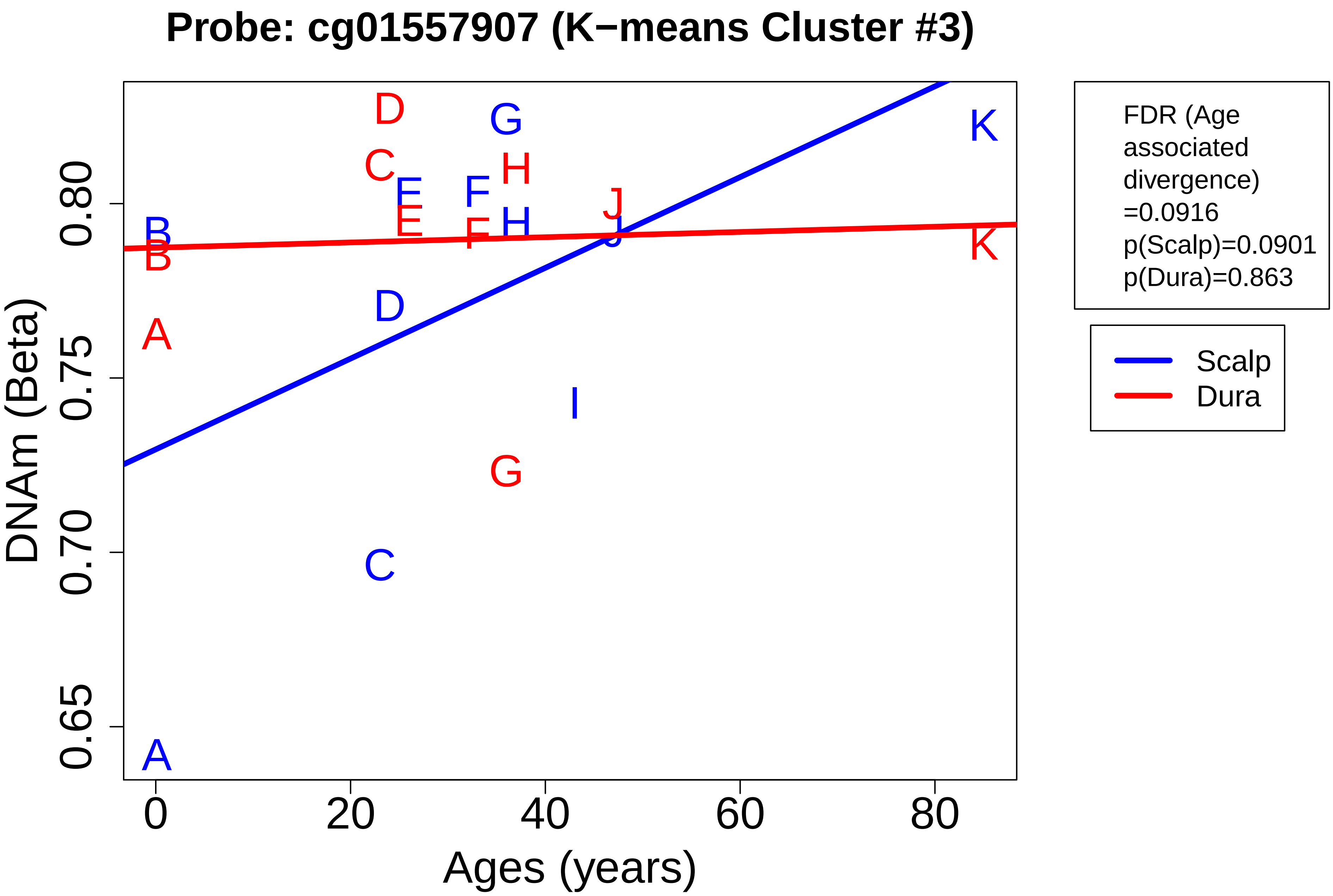
Probe: cg01557907 (K−means Cluster #3)
DNAm (Beta)
0.65
0.70
0.75
0.80
0
20
40
60
80
Ages (years)
FDR (Age associated divergence) =0.0916
p(Scalp)=0.0901
p(Dura)=0.863
Scalp
Dura
A
B
C
D
E
F
G
H
I
J
K

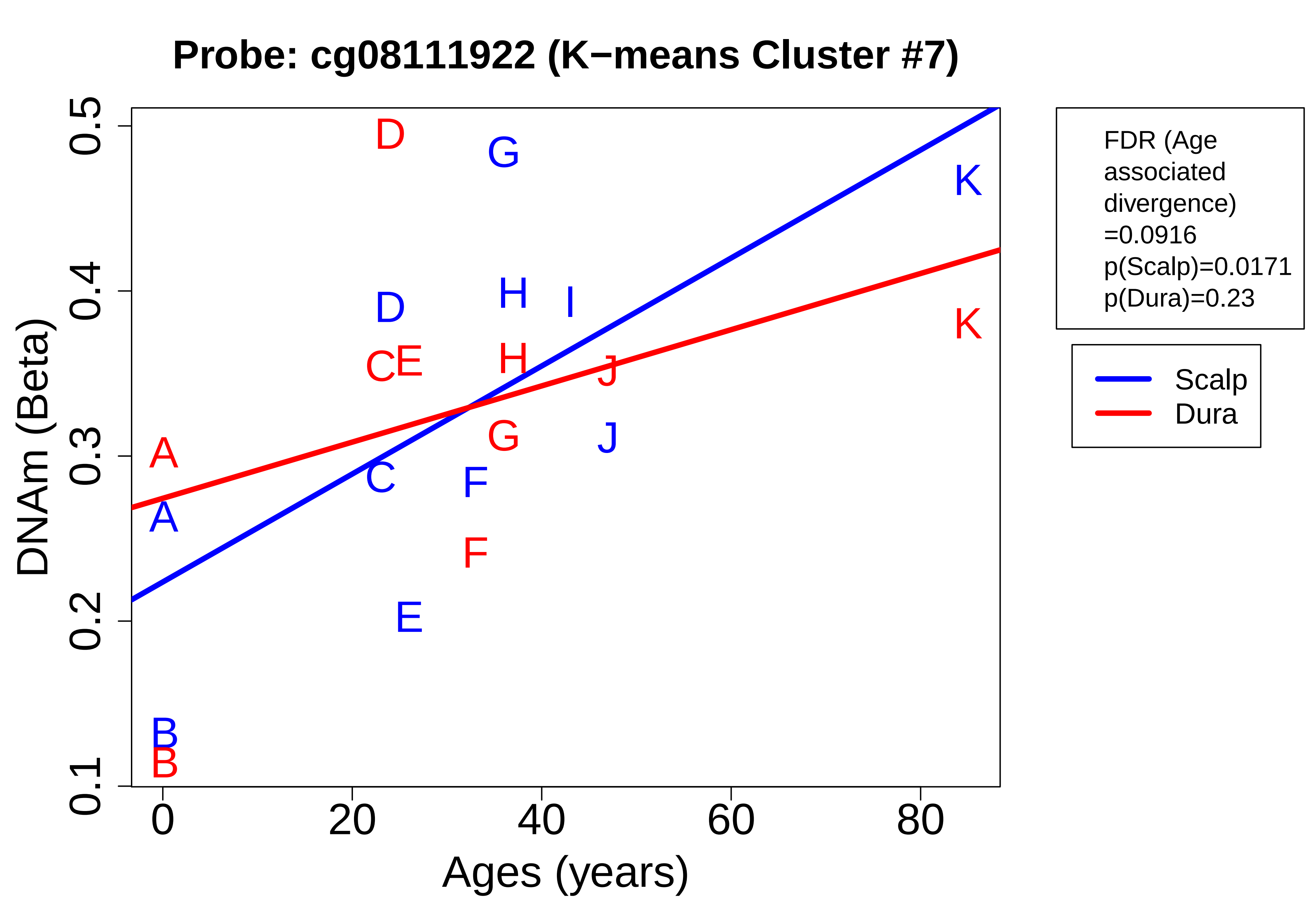

Probe: cg08111922 (K−means Cluster #7)
DNAm (Beta)
Ages (years)
0.1
0.2
0.3
0.4
0.5
0
20
40
60
80
FDR (Age associated divergence) =0.0916
p(Scalp)=0.0171
p(Dura)=0.23
Scalp
Dura

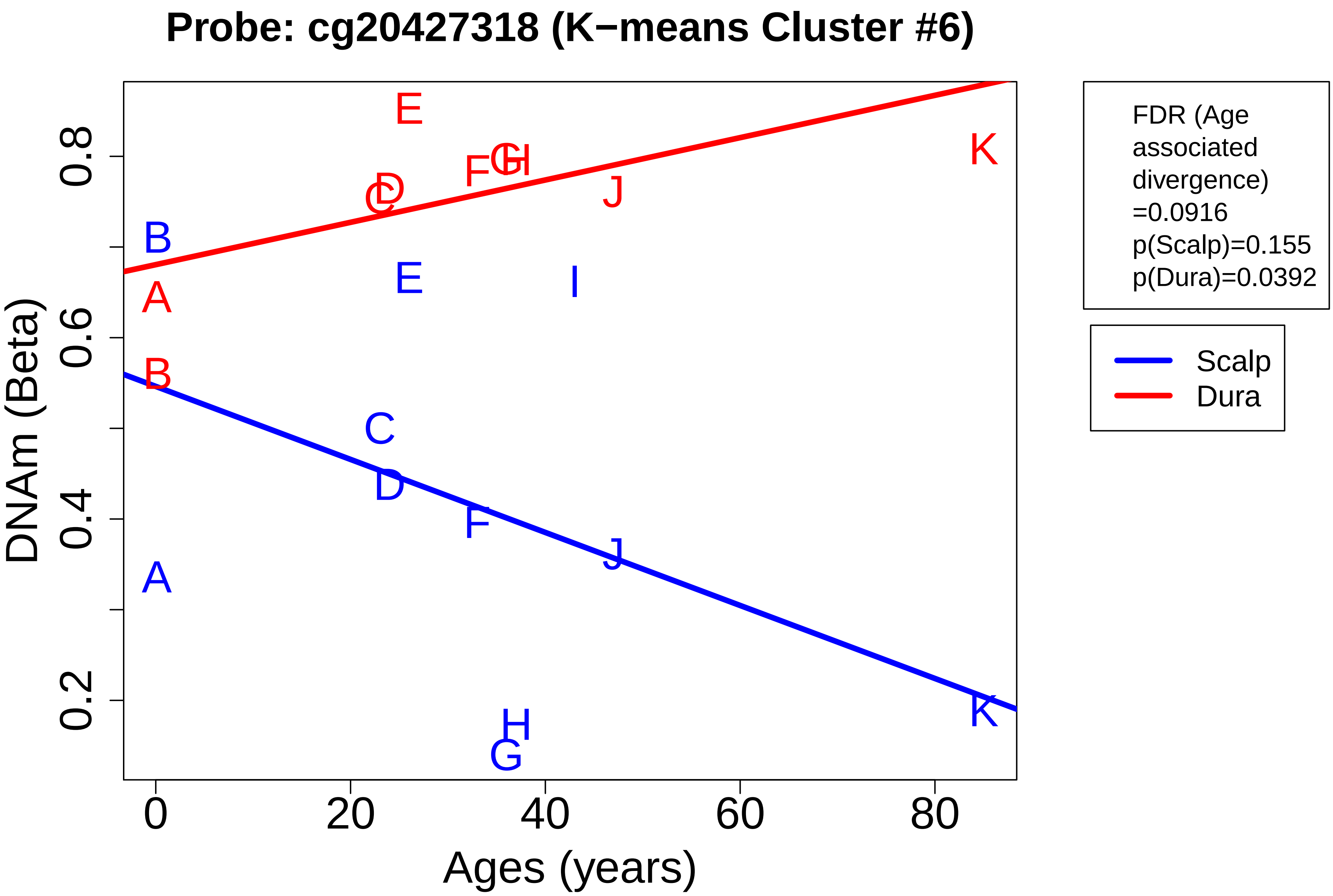

Probe: cg20427318 (K−means Cluster #6)
DNAm (Beta)
0.2
0.4
0.6
0.8
0
20
40
60
80
Ages (years)
FDR (Age associated divergence) =0.0916
p(Scalp)=0.155
p(Dura)=0.0392
Scalp
Dura

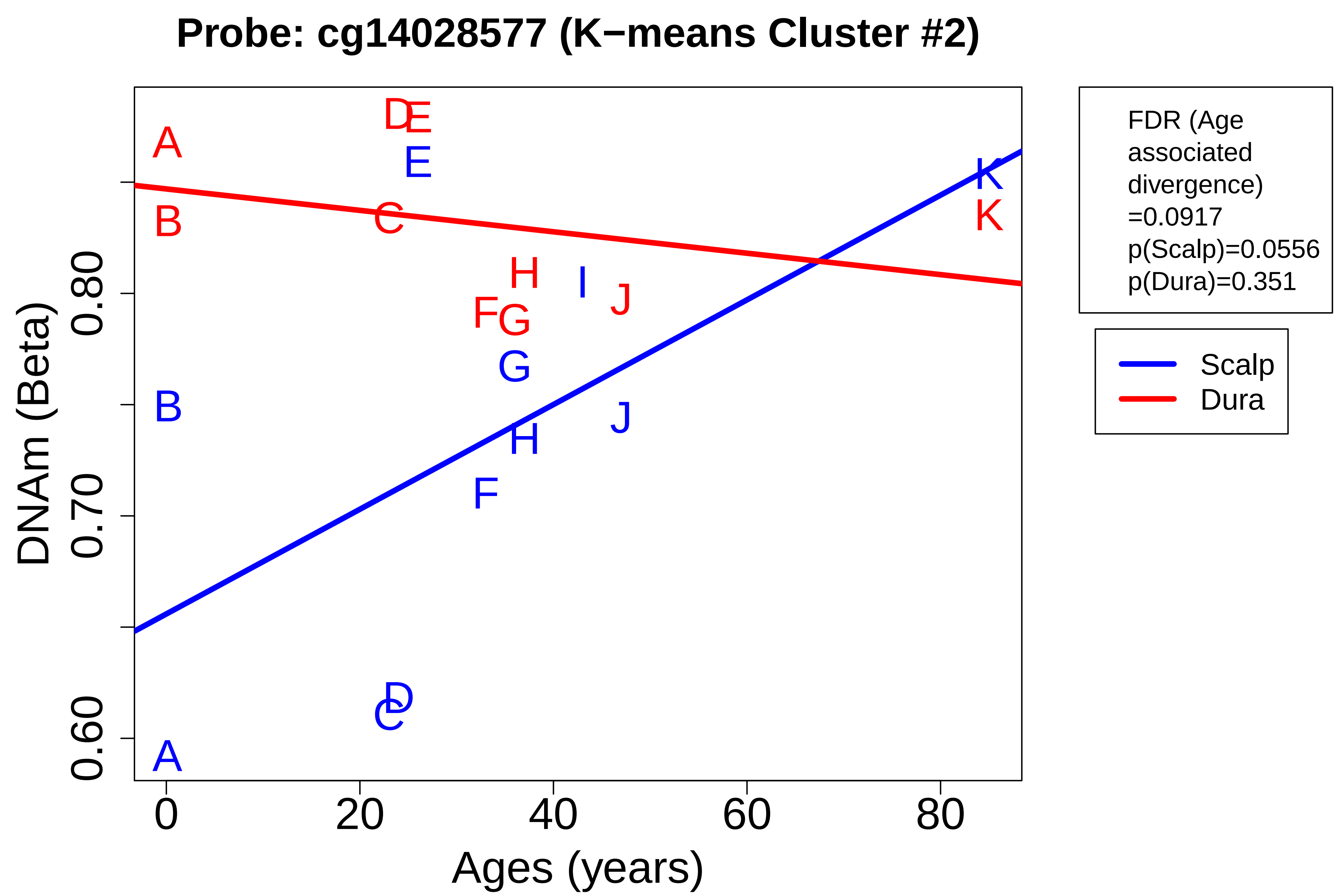

Probe: cg14028577 (K−means Cluster #2)
DNAm (Beta)
0.60
0.70
0.80
Ages (years)
0
20
40
60
80
FDR (Age associated divergence) =0.0917
p(Scalp)=0.0556
p(Dura)=0.351
Scalp
Dura

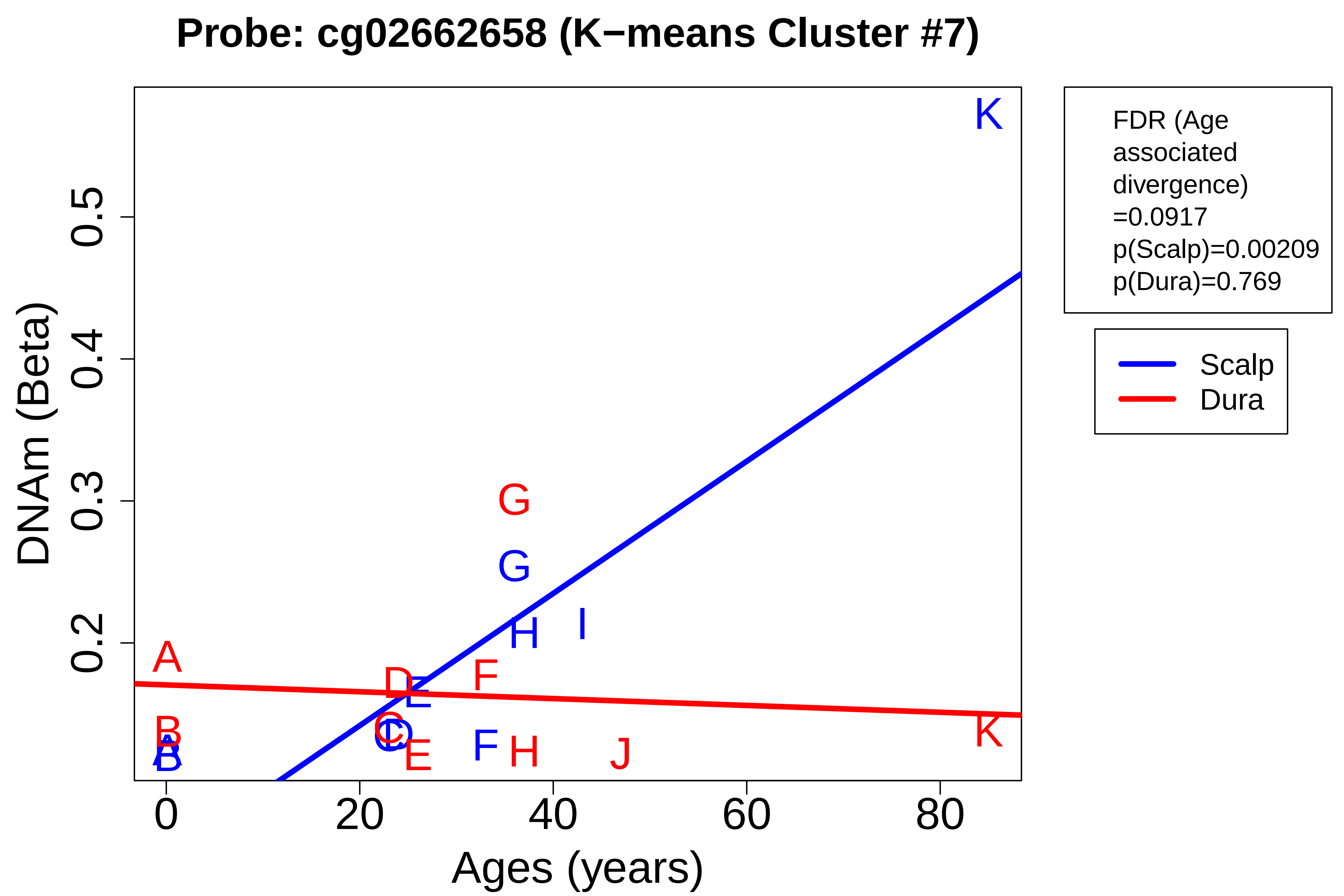
Probe: cg02662658 (K−means Cluster #7)
DNAm (Beta)
0.2
0.3
0.4
0.5
Ages (years)
0
20
40
60
80
FDR (Age associated divergence) =0.0917
p(Scalp)=0.00209
p(Dura)=0.769
Scalp
Dura
A
B
C
D
E
F
G
H
I
J
K

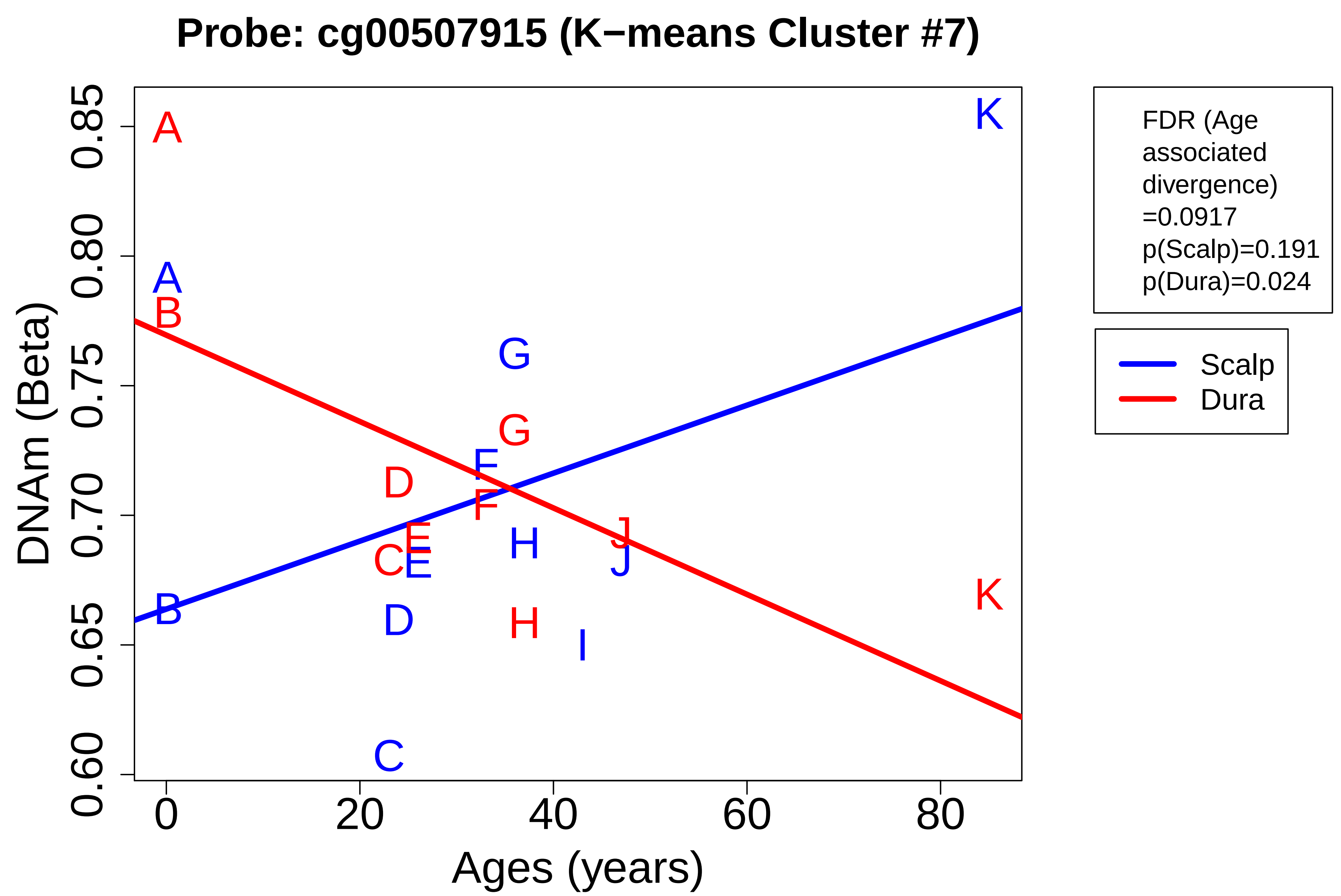

Probe: cg00507915 (K−means Cluster #7)
DNAm (Beta)
0.60
0.65
0.70
0.75
0.80
0.85
0
20
40
60
80
Ages (years)
A
B
C
D
E
F
G
H
I
J
K
FDR (Age associated divergence) =0.0917
p(Scalp)=0.191
p(Dura)=0.024
Scalp
Dura

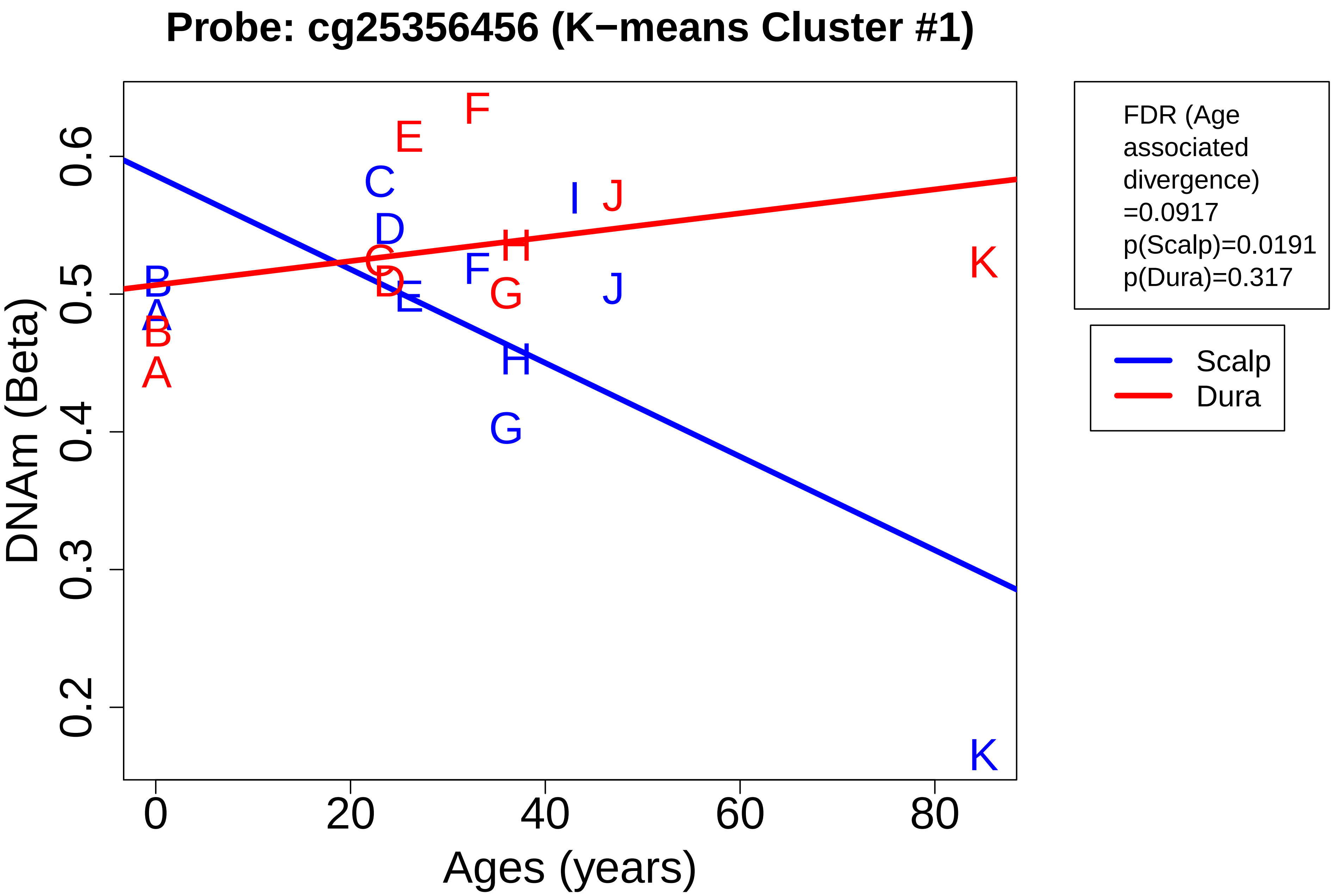
Probe: cg25356456 (K−means Cluster #1)
DNAm (Beta)
0.2
0.3
0.4
0.5
0.6
0
20
40
60
80
Ages (years)
FDR (Age associated divergence) =0.0917
p(Scalp)=0.0191
p(Dura)=0.317
Scalp
Dura

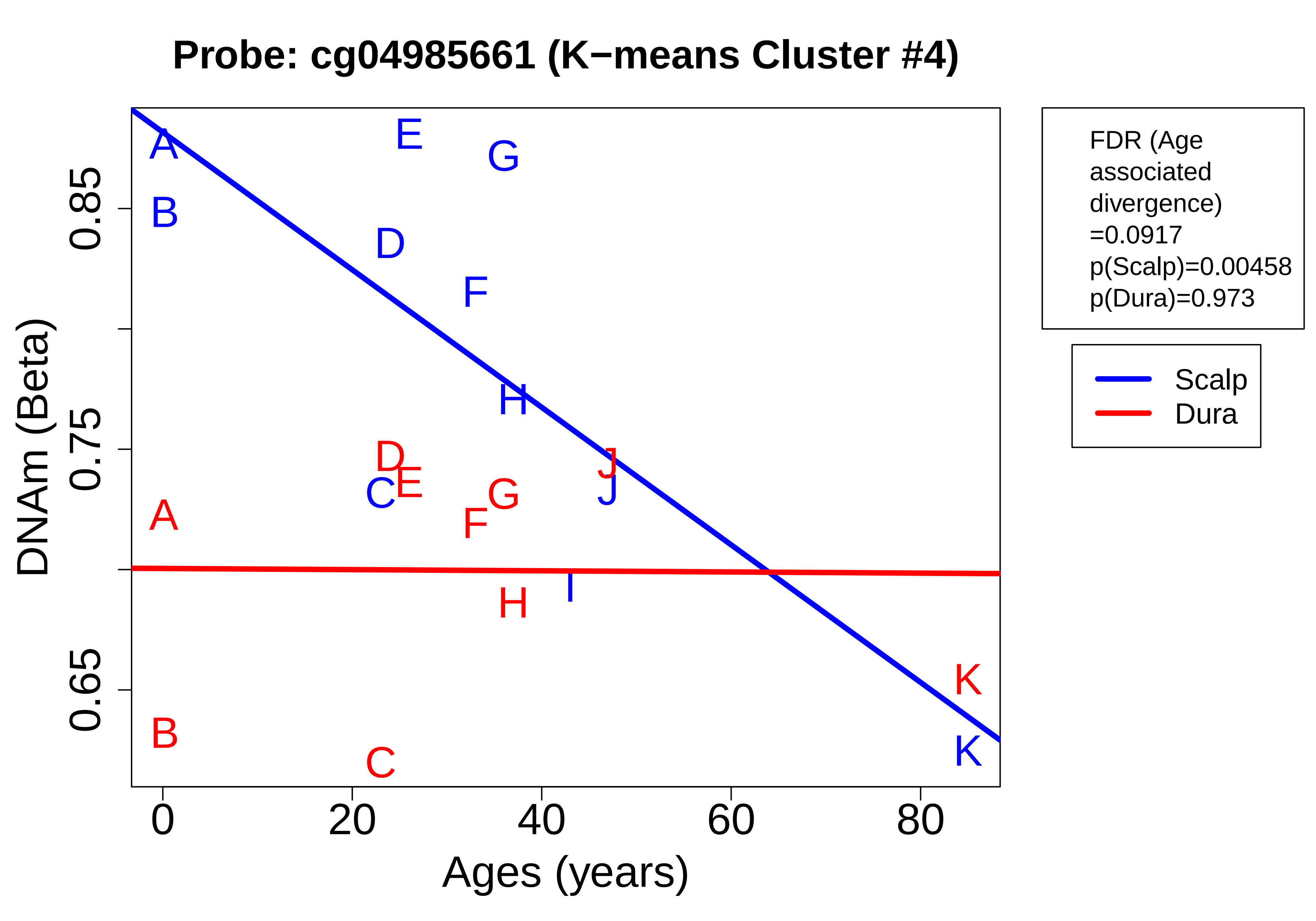

Probe: cg04985661 (K−means Cluster #4)
DNAm (Beta)
Ages (years)
0.65
0.75
0.85
0
20
40
60
80
FDR (Age associated divergence) =0.0917
p(Scalp)=0.00458
p(Dura)=0.973
Scalp
Dura

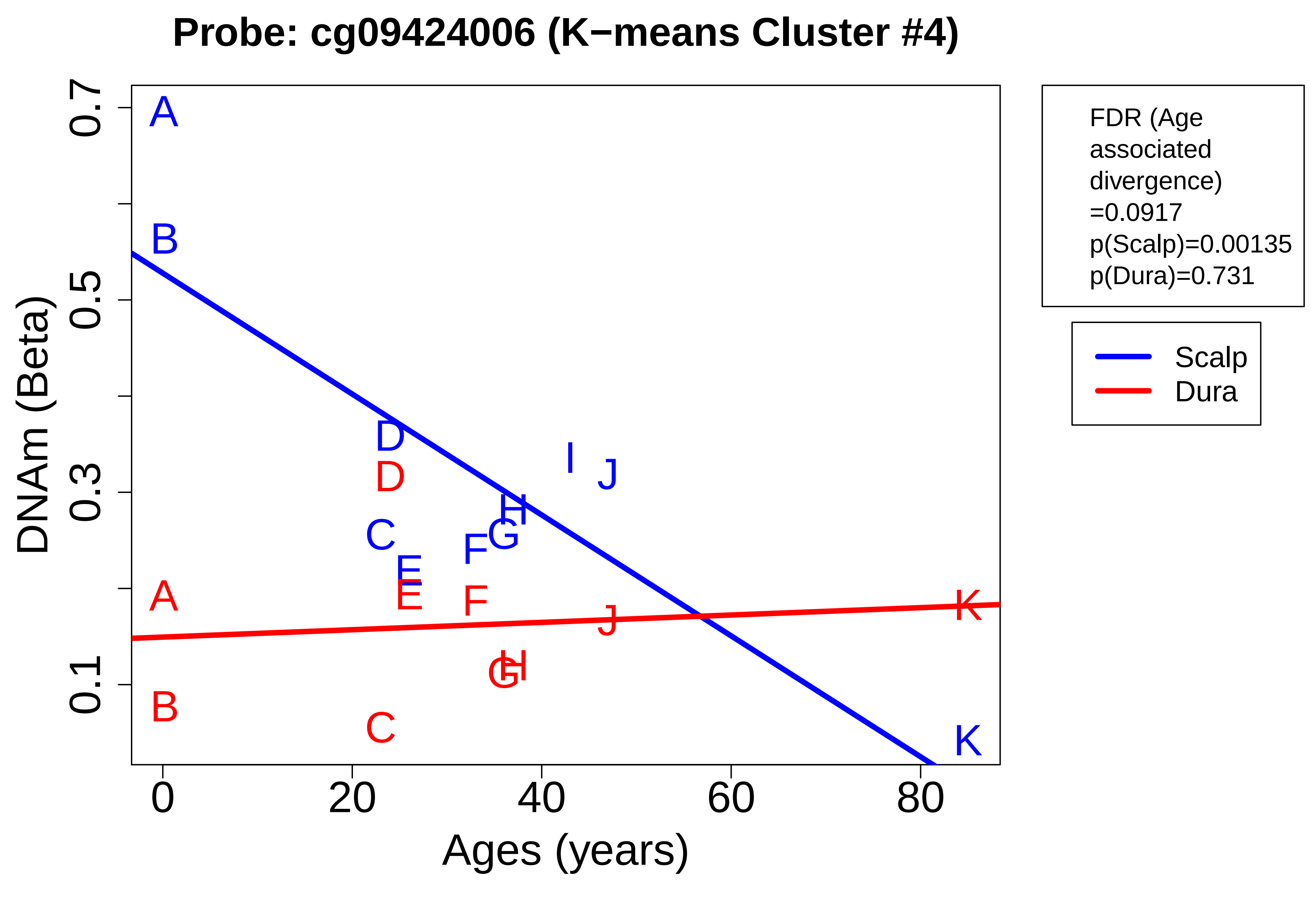

Probe: cg09424006 (K−means Cluster #4)
DNAm (Beta)
Ages (years)
0.1
0.3
0.5
0.7
0
20
40
60
80
A
B
C
D
E
F
G
H
I
J
K
FDR (Age associated divergence) =0.0917
p(Scalp)=0.00135
p(Dura)=0.731
Scalp
Dura

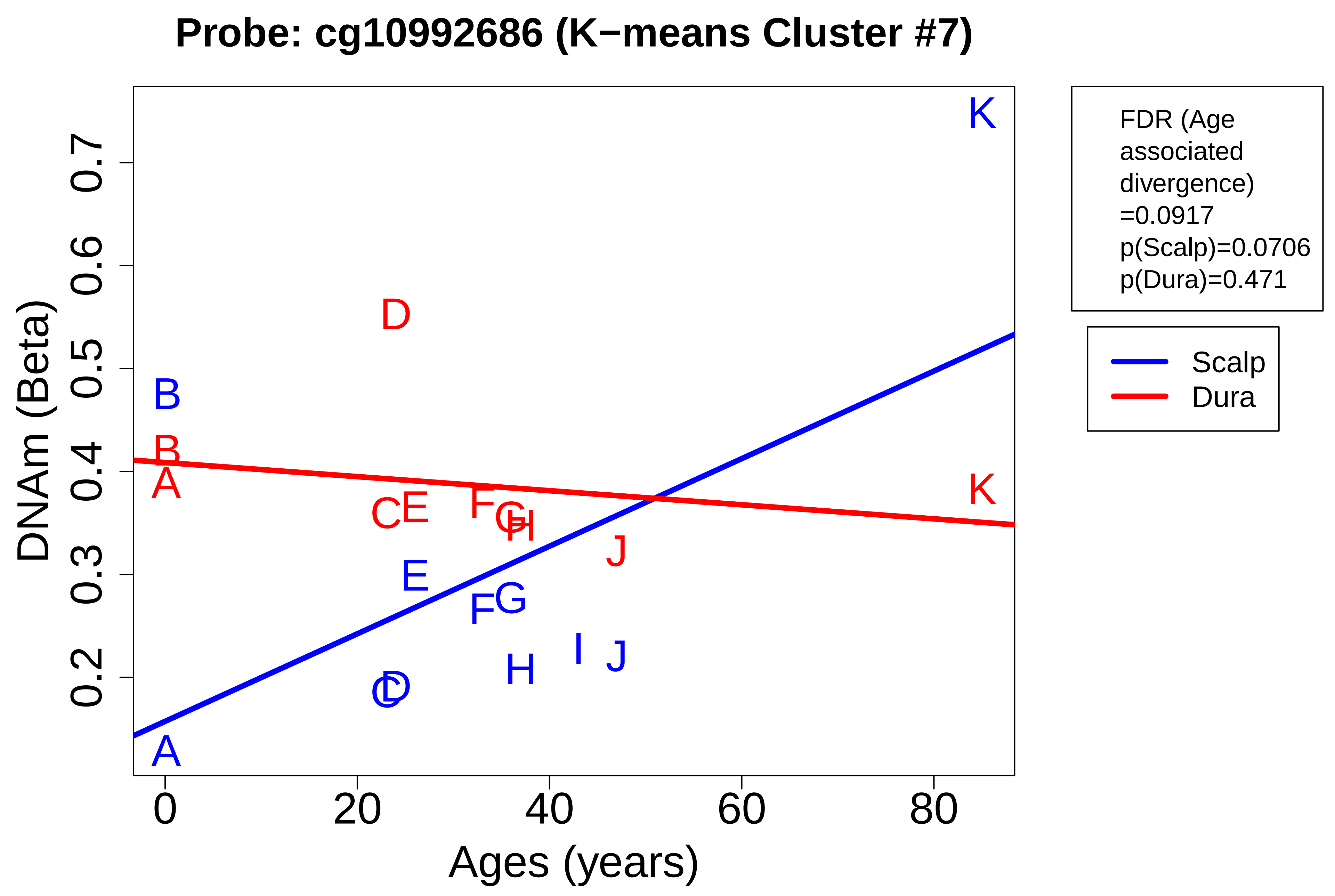

Probe: cg10992686 (K−means Cluster #7)
DNAm (Beta)
0.2
0.3
0.4
0.5
0.6
0.7
0
20
40
60
80
Ages (years)
FDR (Age associated divergence) =0.0917
p(Scalp)=0.0706
p(Dura)=0.471
Scalp
Dura

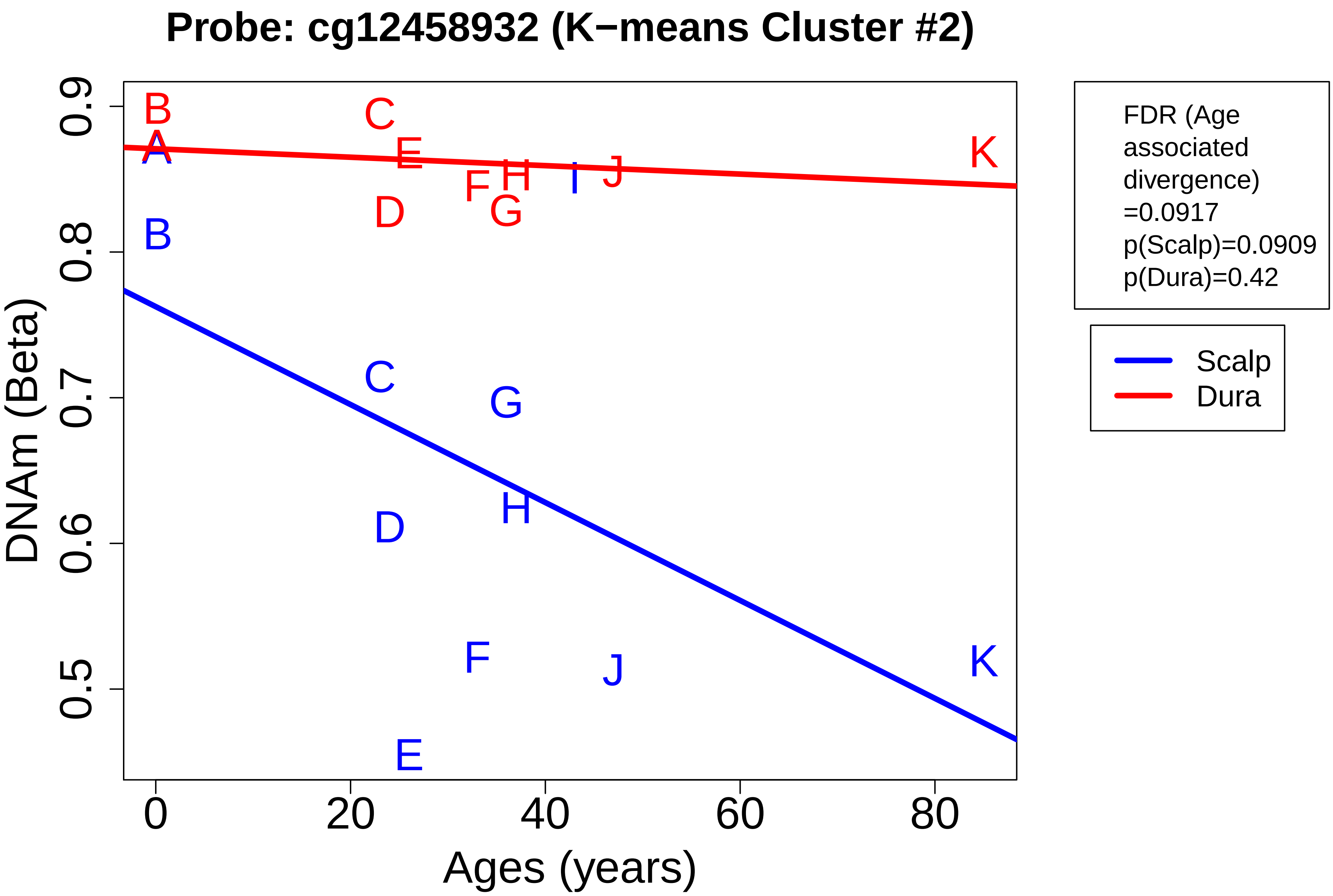
Probe: cg12458932 (K−means Cluster #2)
DNAm (Beta)
0.5
0.6
0.7
0.8
0.9
0
20
40
60
80
Ages (years)
A
B
C
D
E
F
G
H
I
J
K
FDR (Age associated divergence) =0.0917
p(Scalp)=0.0909
p(Dura)=0.42
Scalp
Dura

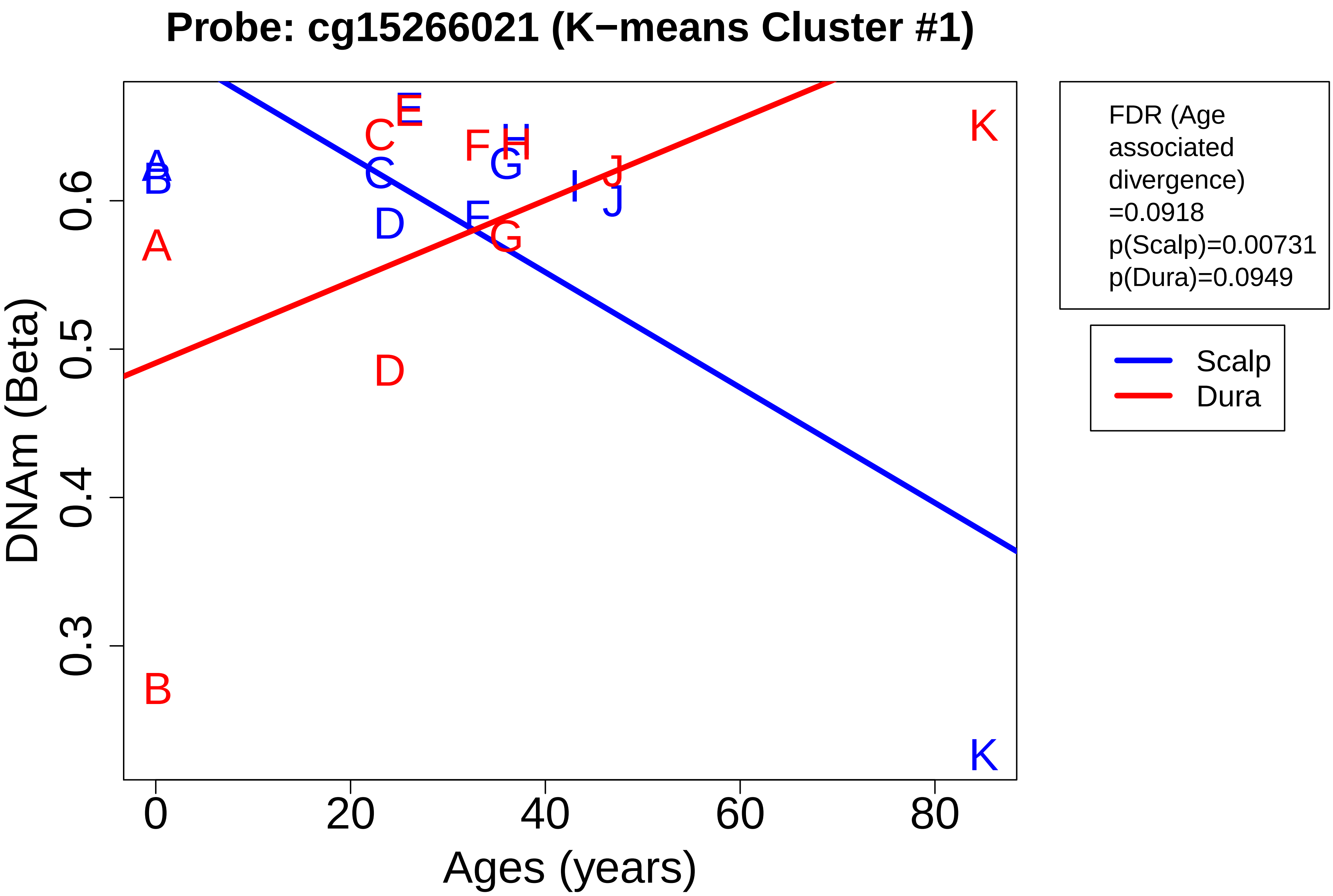

Probe: cg15266021 (K−means Cluster #1)
DNAm (Beta)
0.3
0.4
0.5
0.6
0
20
40
60
80
Ages (years)
FDR (Age associated divergence) =0.0918
p(Scalp)=0.00731
p(Dura)=0.0949
Scalp
Dura

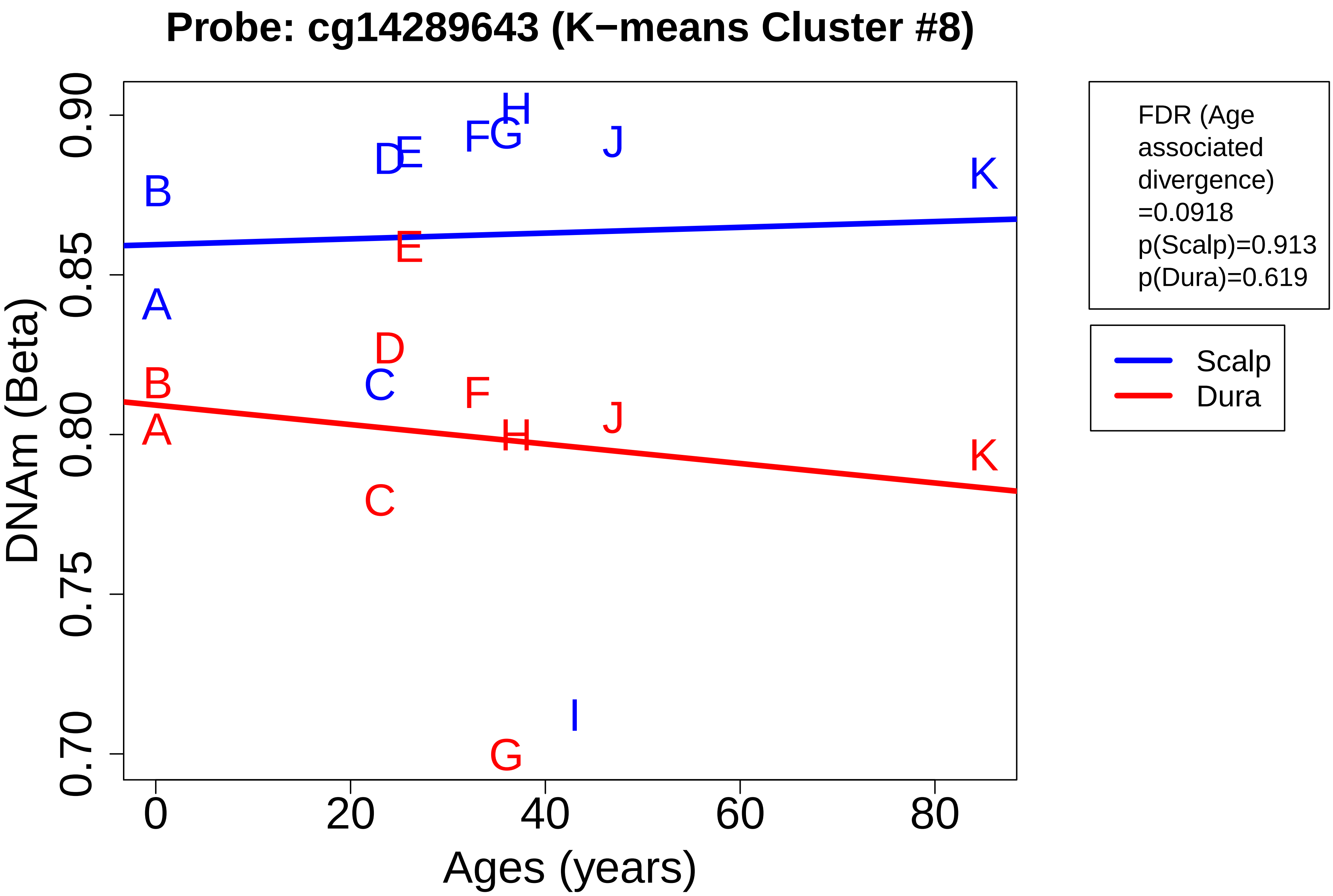
Probe: cg14289643 (K−means Cluster #8)
DNAm (Beta)
0.70
0.75
0.80
0.85
0.90
Ages (years)
0
20
40
60
80
A
B
C
D
E
F
G
H
I
J
K
FDR (Age associated divergence) =0.0918
p(Scalp)=0.913
p(Dura)=0.619
Scalp
Dura

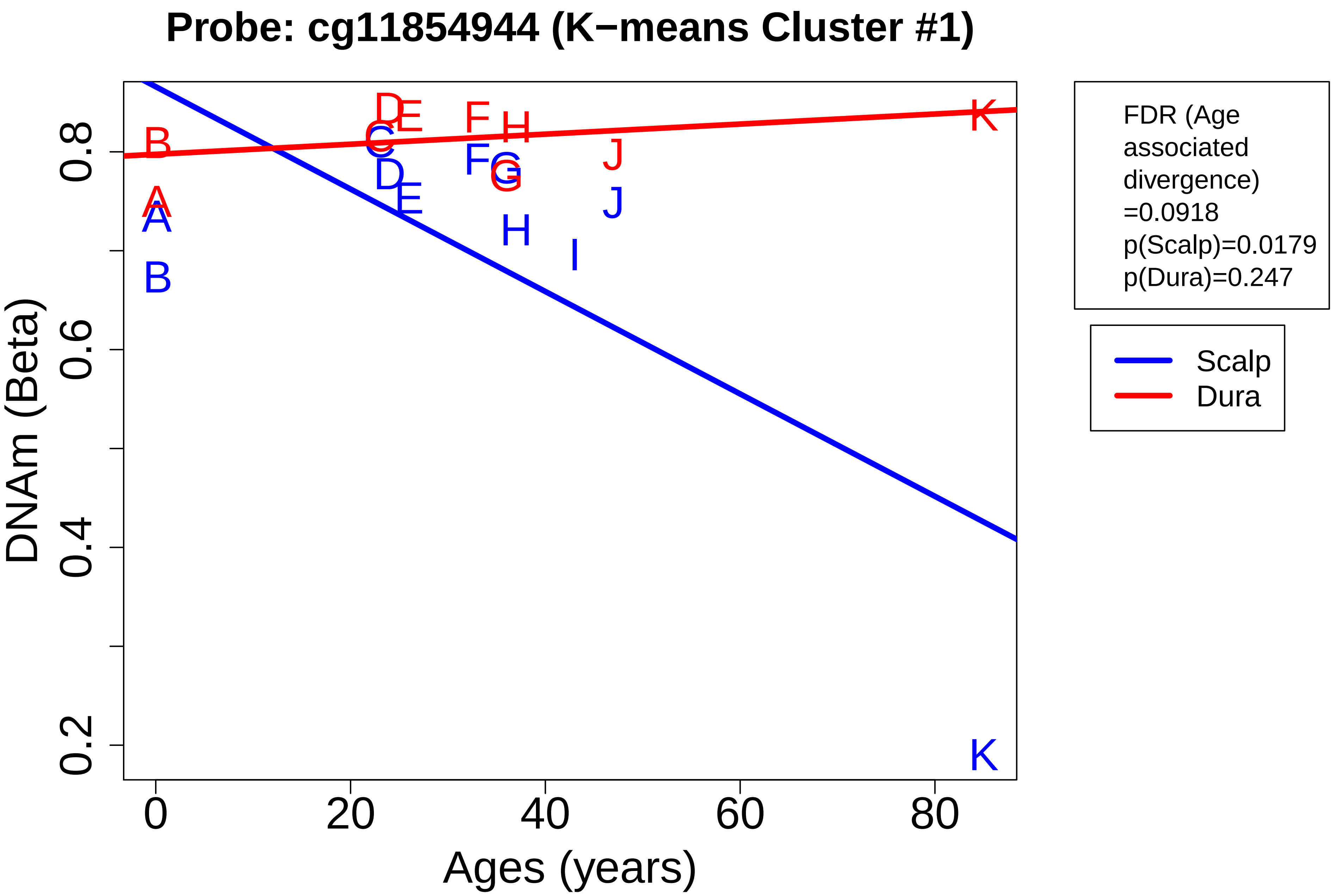

Probe: cg11854944 (K−means Cluster #1)
DNAm (Beta)
0.2
0.4
0.6
0.8
0
20
40
60
80
Ages (years)
FDR (Age associated divergence) =0.0918
p(Scalp)=0.0179
p(Dura)=0.247
Scalp
Dura

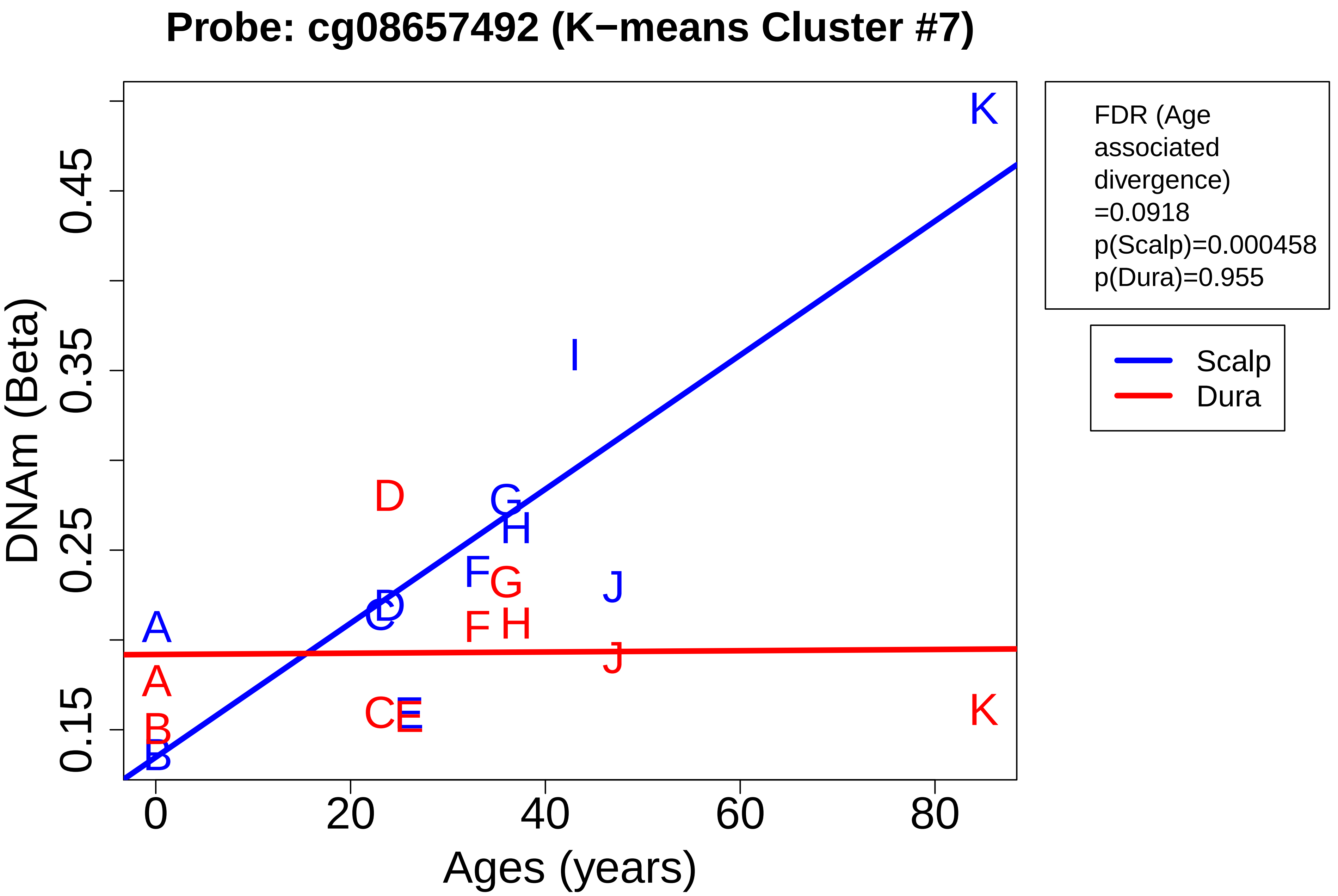

Probe: cg08657492 (K−means Cluster #7)
DNAm (Beta)
0.15
0.25
0.35
0.45
0
20
40
60
80
Ages (years)
FDR (Age associated divergence) =0.0918
p(Scalp)=0.000458
p(Dura)=0.955
Scalp
Dura

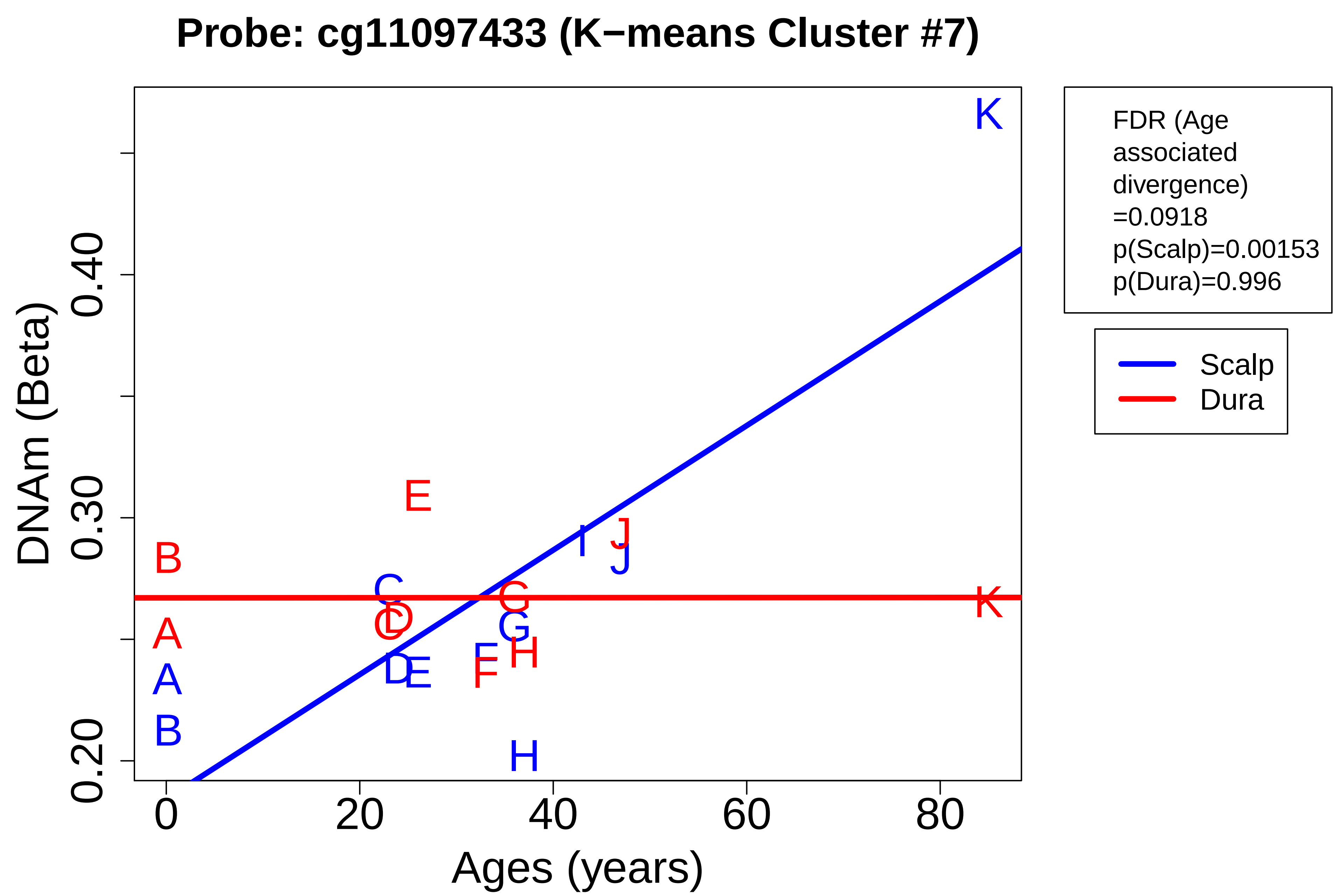

Probe: cg11097433 (K−means Cluster #7)
DNAm (Beta)
0.20
0.30
0.40
0
20
40
60
80
Ages (years)
FDR (Age associated divergence) =0.0918
p(Scalp)=0.00153
p(Dura)=0.996
Scalp
Dura

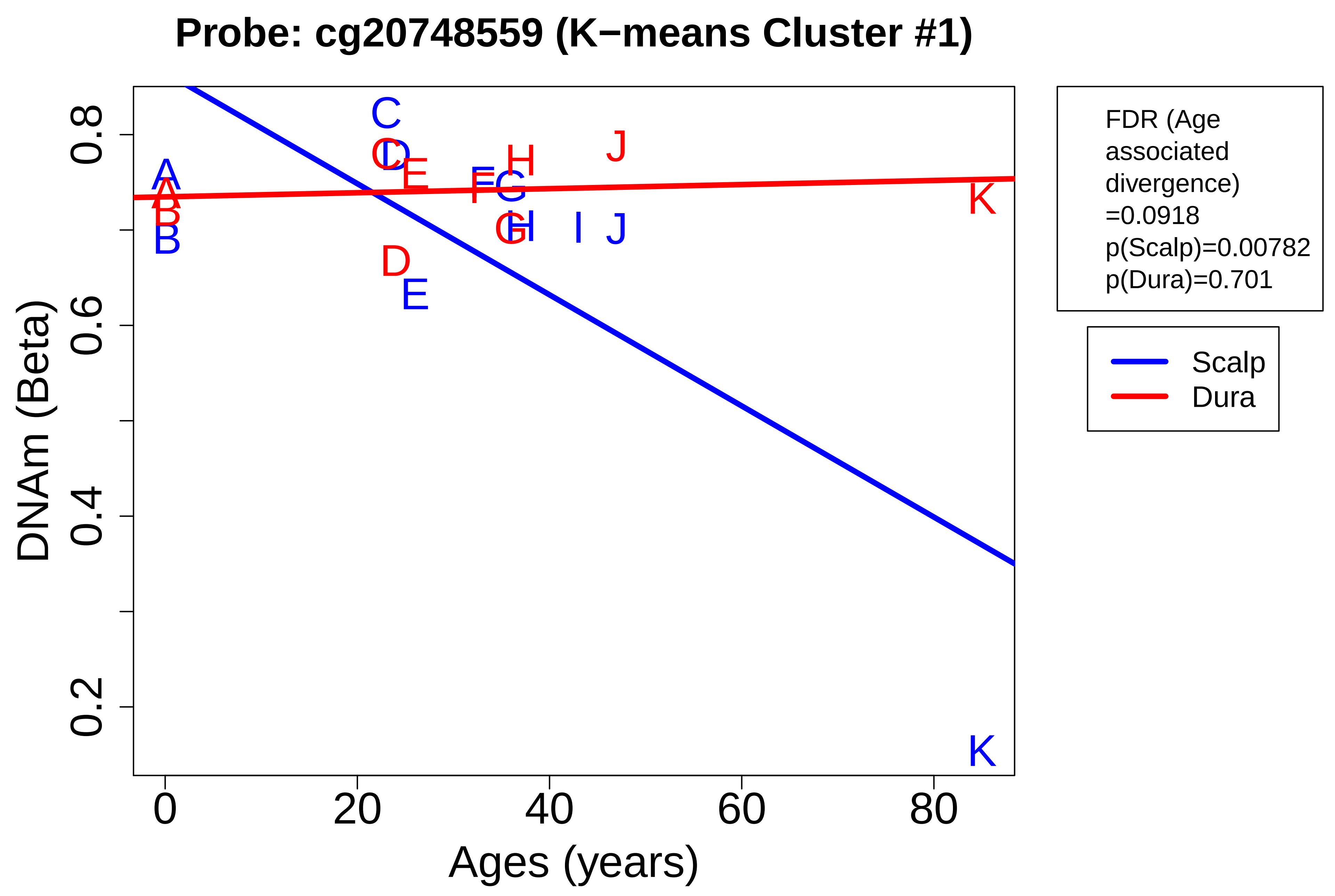

Probe: cg20748559 (K−means Cluster #1)
DNAm (Beta)
0.2
0.4
0.6
0.8
Ages (years)
0
20
40
60
80
FDR (Age associated divergence) =0.0918
p(Scalp)=0.00782
p(Dura)=0.701
Scalp
Dura

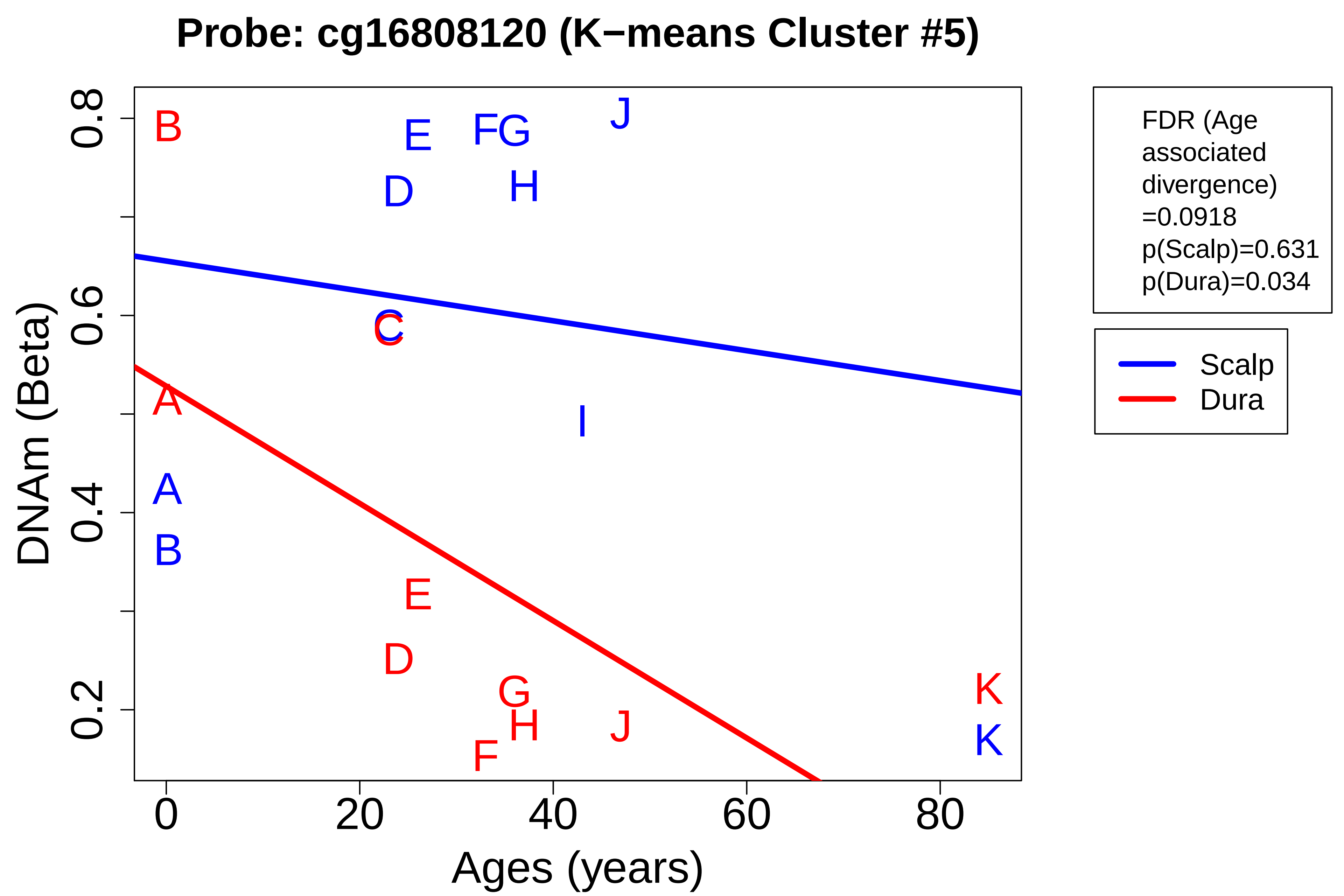
Probe: cg16808120 (K−means Cluster #5)
DNAm (Beta)
0.2
0.4
0.6
0.8
0
20
40
60
80
Ages (years)
FDR (Age associated divergence) =0.0918
p(Scalp)=0.631
p(Dura)=0.034
Scalp
Dura

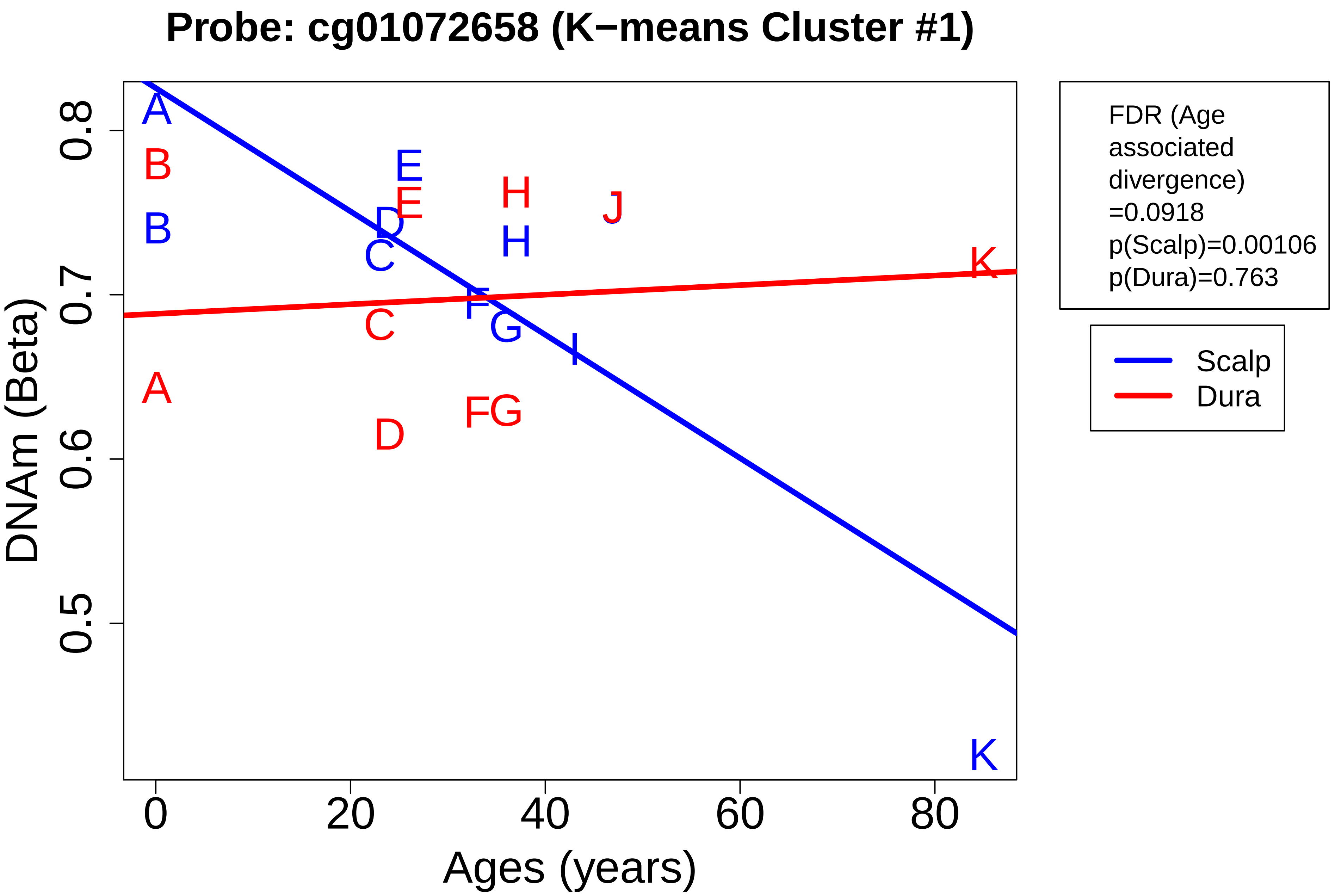
Probe: cg01072658 (K−means Cluster #1)
DNAm (Beta)
Ages (years)
0.5
0.6
0.7
0.8
0
20
40
60
80
FDR (Age associated divergence) =0.0918
p(Scalp)=0.00106
p(Dura)=0.763
Scalp
Dura

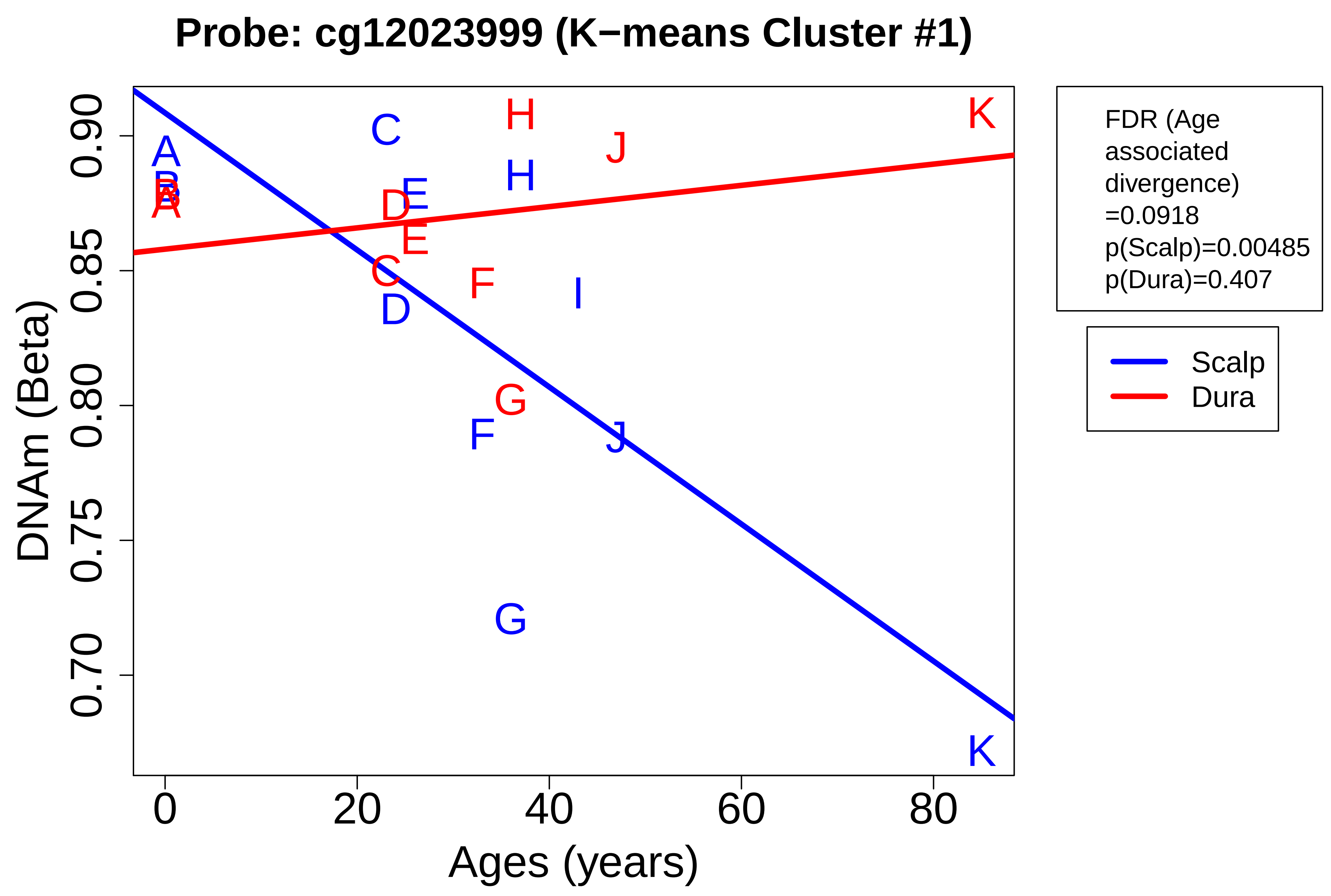

Probe: cg12023999 (K−means Cluster #1)
DNAm (Beta)
0.70
0.75
0.80
0.85
0.90
0
20
40
60
80
Ages (years)
A
B
C
D
E
F
G
H
I
J
K
FDR (Age associated divergence) =0.0918
p(Scalp)=0.00485
p(Dura)=0.407
Scalp
Dura

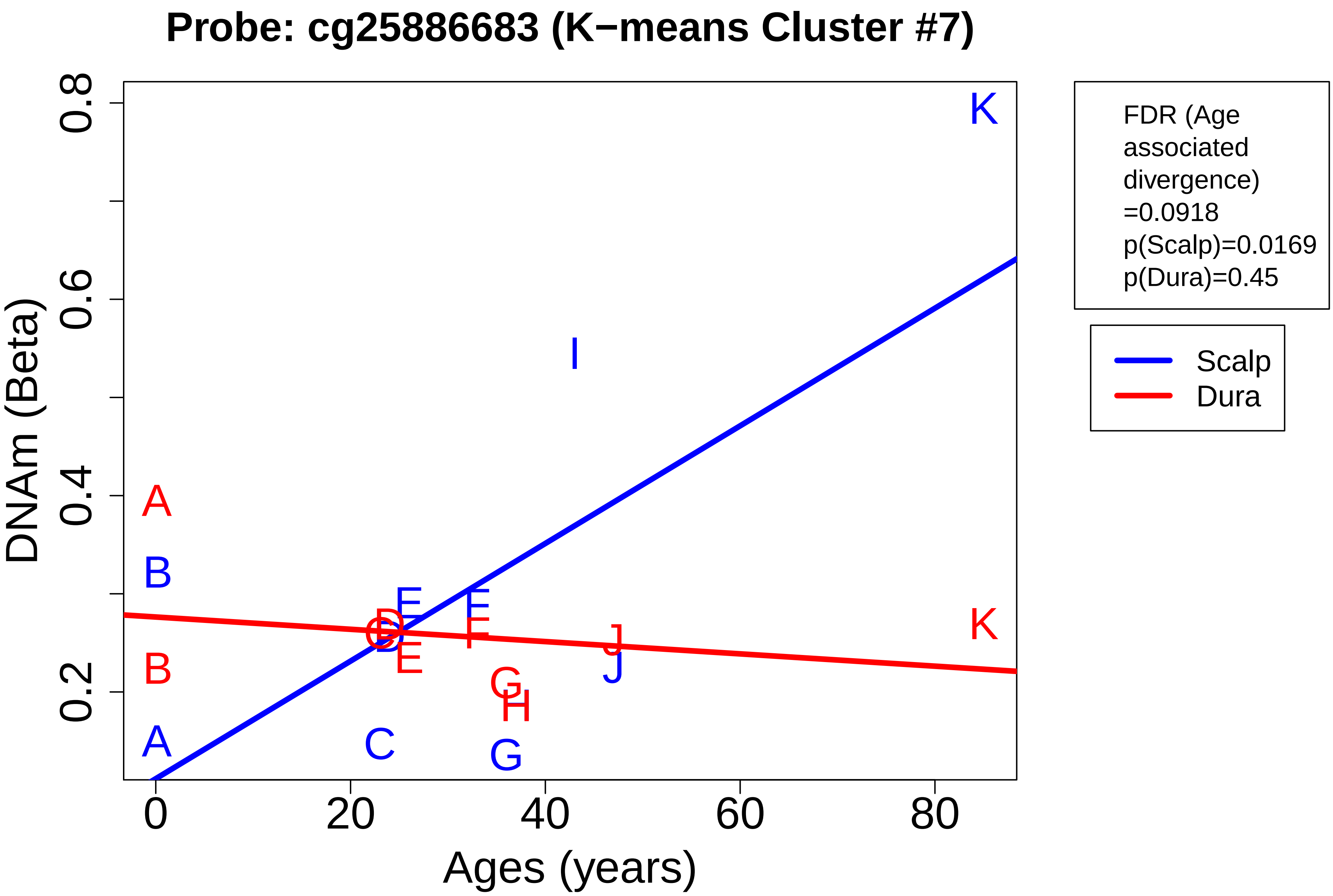

Probe: cg25886683 (K−means Cluster #7)
DNAm (Beta)
0.2
0.4
0.6
0.8
0
20
40
60
80
Ages (years)
FDR (Age associated divergence) =0.0918
p(Scalp)=0.0169
p(Dura)=0.45
Scalp
Dura
A
B
C
D
E
F
G
H
I
J
K

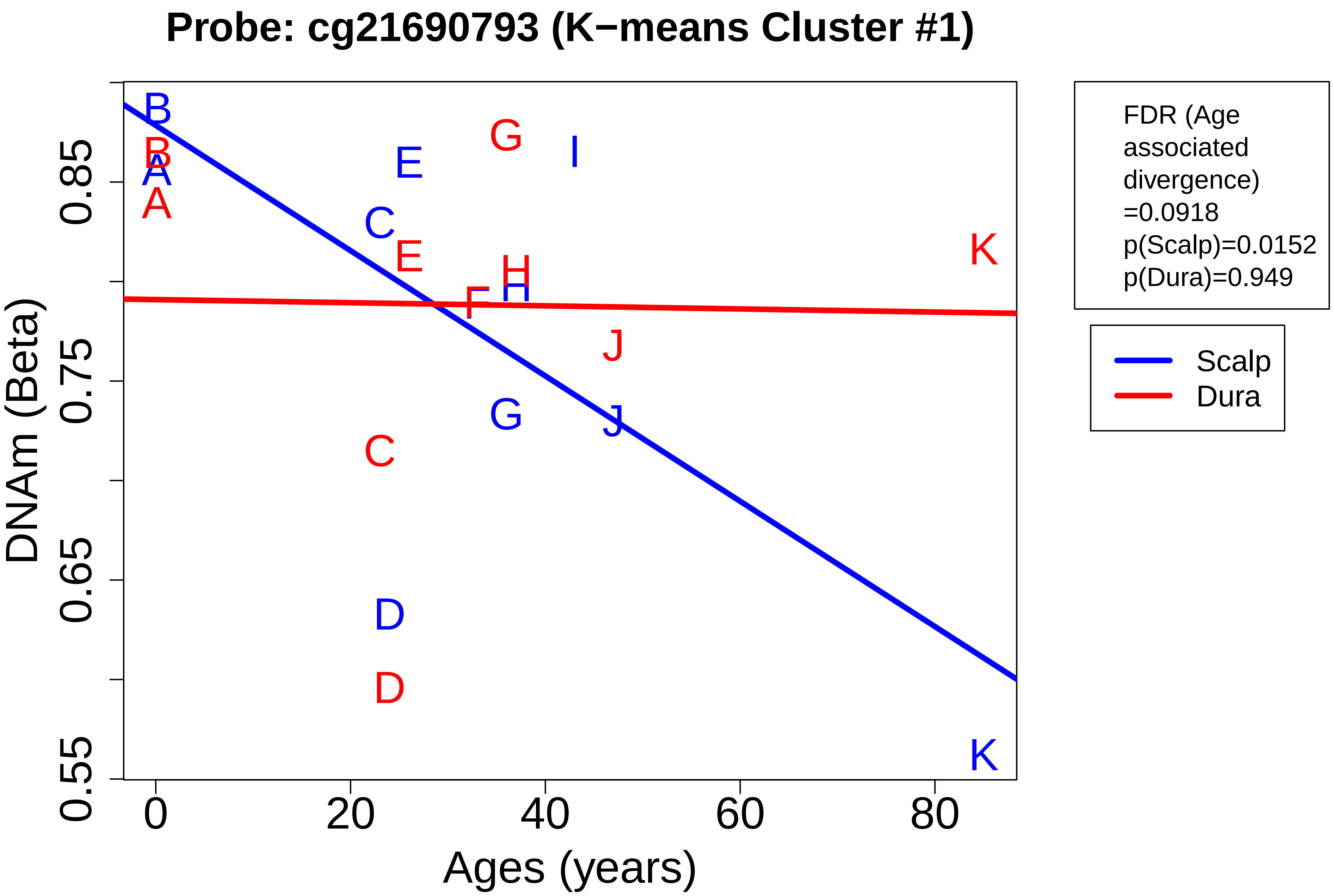

Probe: cg21690793 (K−means Cluster #1)
DNAm (Beta)
0.55
0.65
0.75
0.85
0
20
40
60
80
Ages (years)
FDR (Age associated divergence) =0.0918
p(Scalp)=0.0152
p(Dura)=0.949
Scalp
Dura
A
B
C
D
E
F
G
H
I
J
K

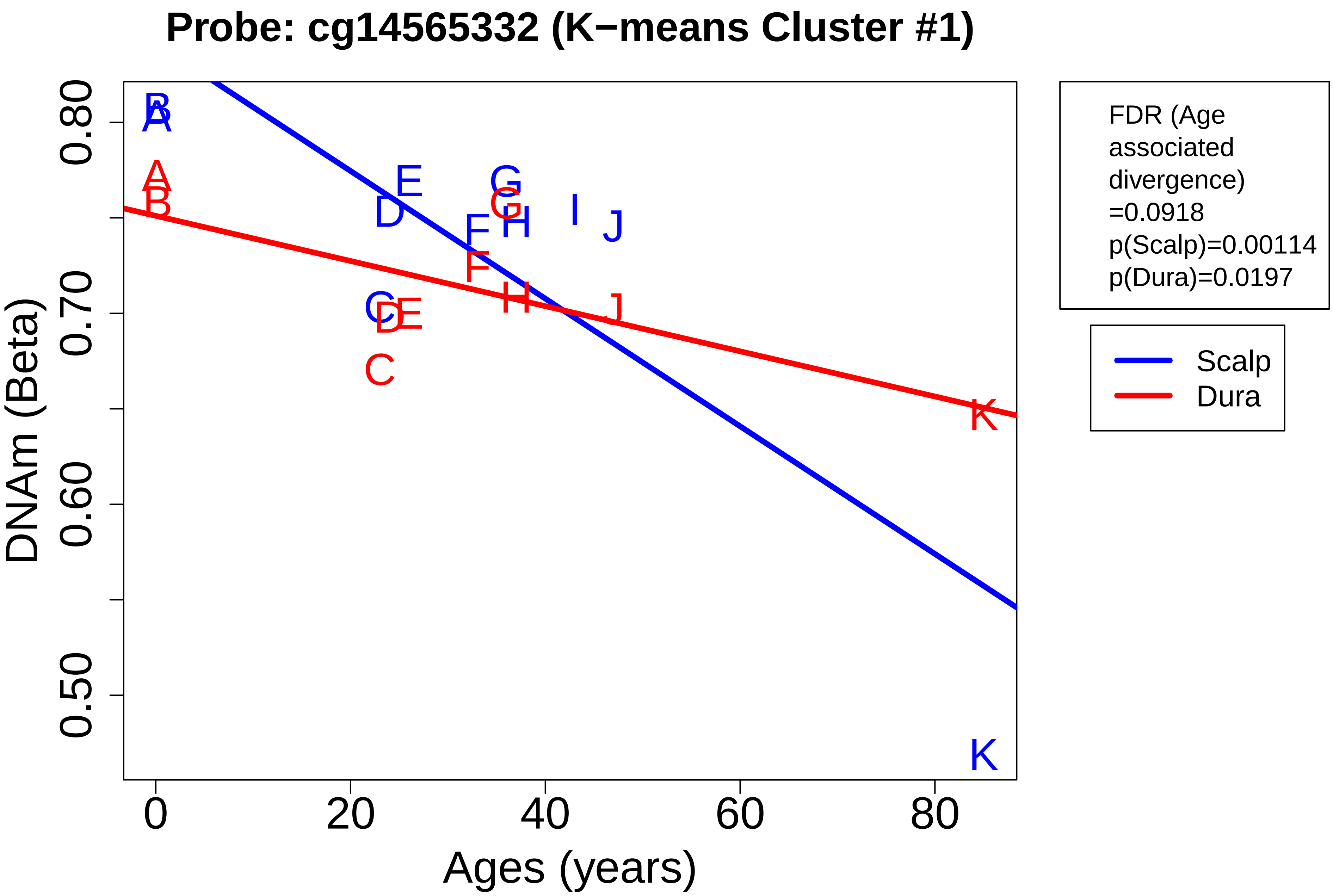

Probe: cg14565332 (K−means Cluster #1)
DNAm (Beta)
0.50
0.60
0.70
0.80
Ages (years)
0
20
40
60
80
FDR (Age associated divergence) =0.0918
p(Scalp)=0.00114
p(Dura)=0.0197
Scalp
Dura

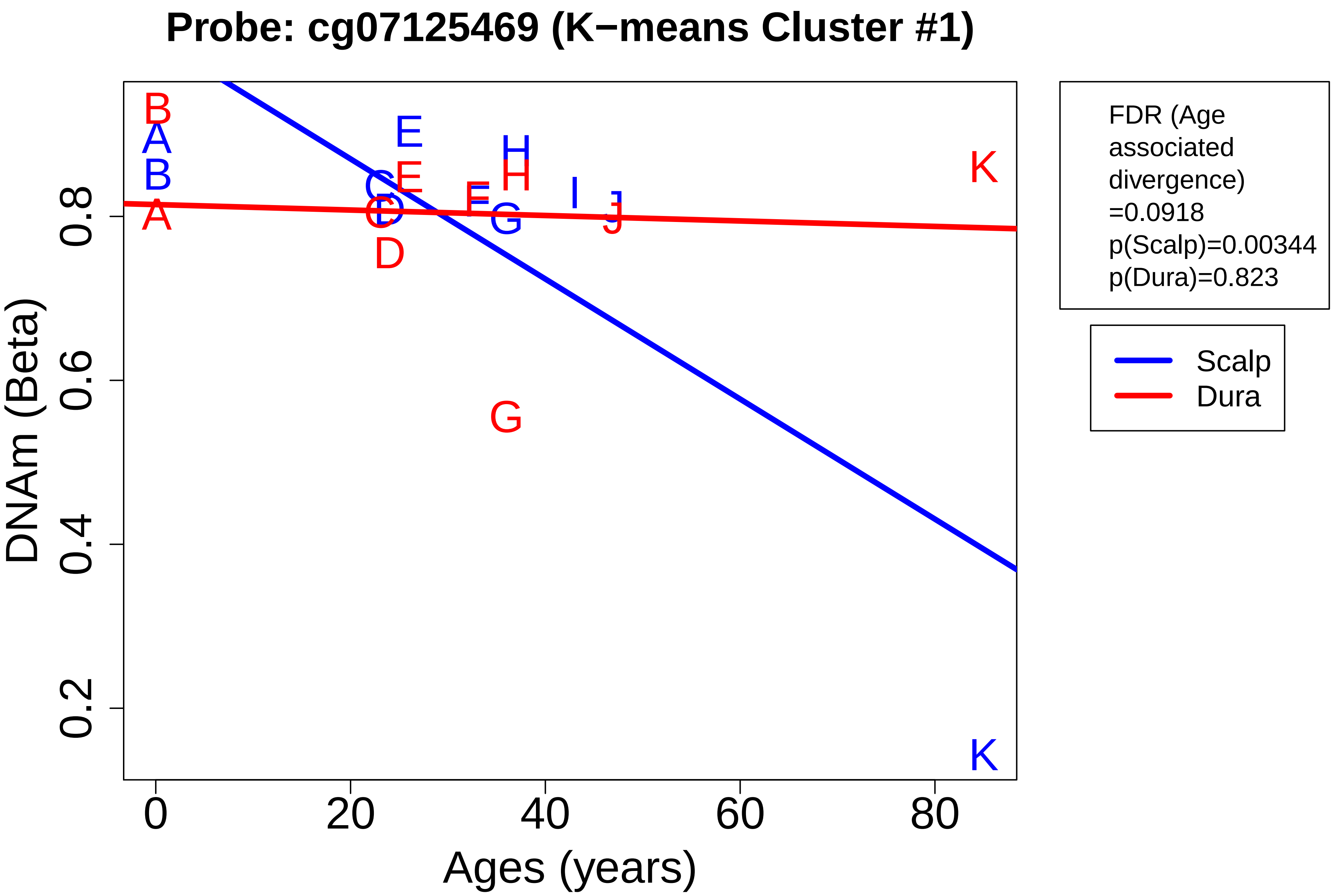

Probe: cg07125469 (K−means Cluster #1)
DNAm (Beta)
0.2
0.4
0.6
0.8
0
20
40
60
80
Ages (years)
A
B
C
D
E
F
G
H
I
J
K
FDR (Age associated divergence) =0.0918
p(Scalp)=0.00344
p(Dura)=0.823
Scalp
Dura

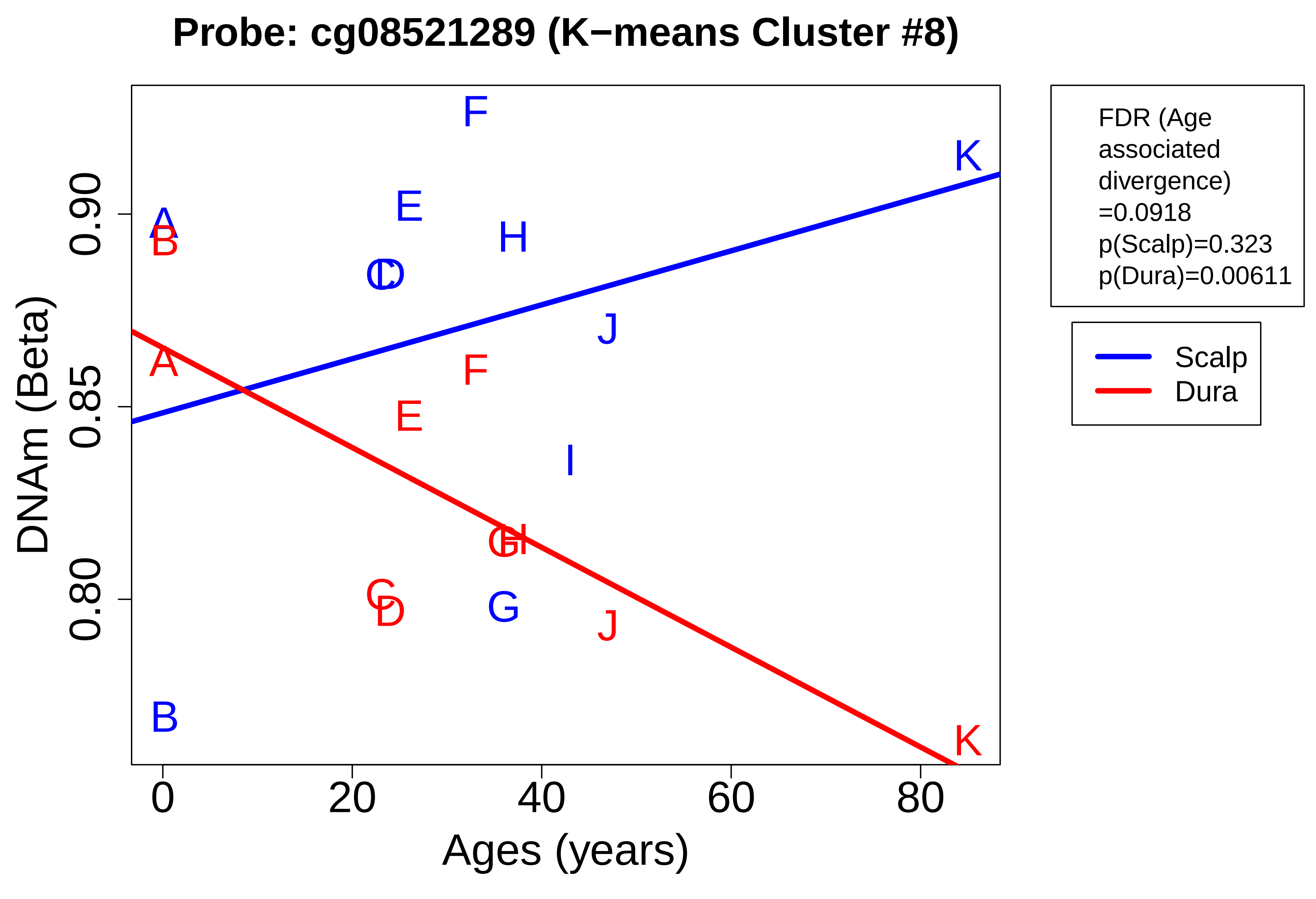
Probe: cg08521289 (K−means Cluster #8)
DNAm (Beta)
0.80
0.85
0.90
Ages (years)
0
20
40
60
80
A
B
C
D
E
F
G
H
I
J
K
FDR (Age associated divergence) =0.0918
p(Scalp)=0.323
p(Dura)=0.00611
Scalp
Dura

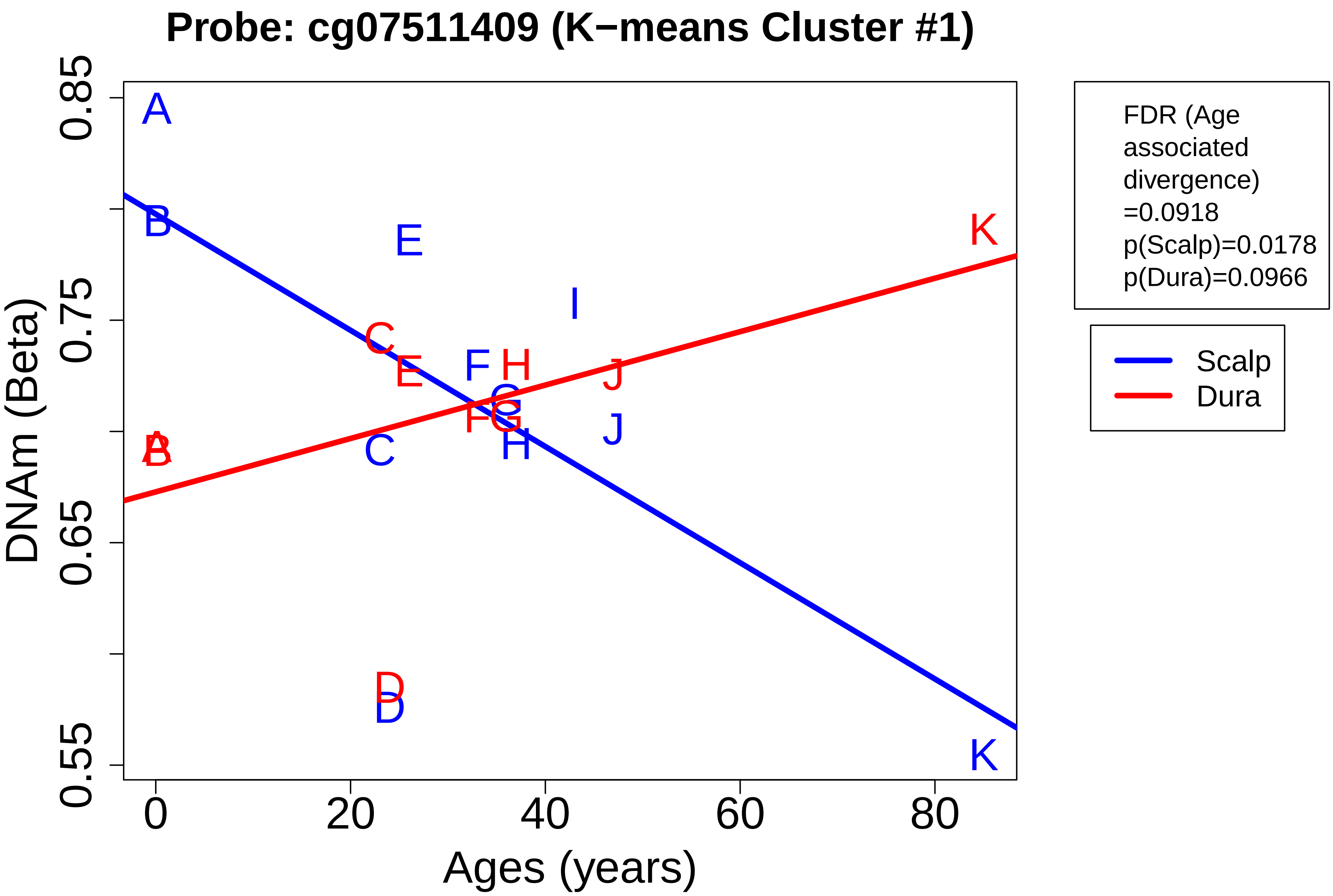

Probe: cg07511409 (K−means Cluster #1)
DNAm (Beta)
0.55
0.65
0.75
0.85
Ages (years)
0
20
40
60
80
A
B
C
D
E
F
G
H
I
J
K
FDR (Age associated divergence) =0.0918
p(Scalp)=0.0178
p(Dura)=0.0966
Scalp
Dura

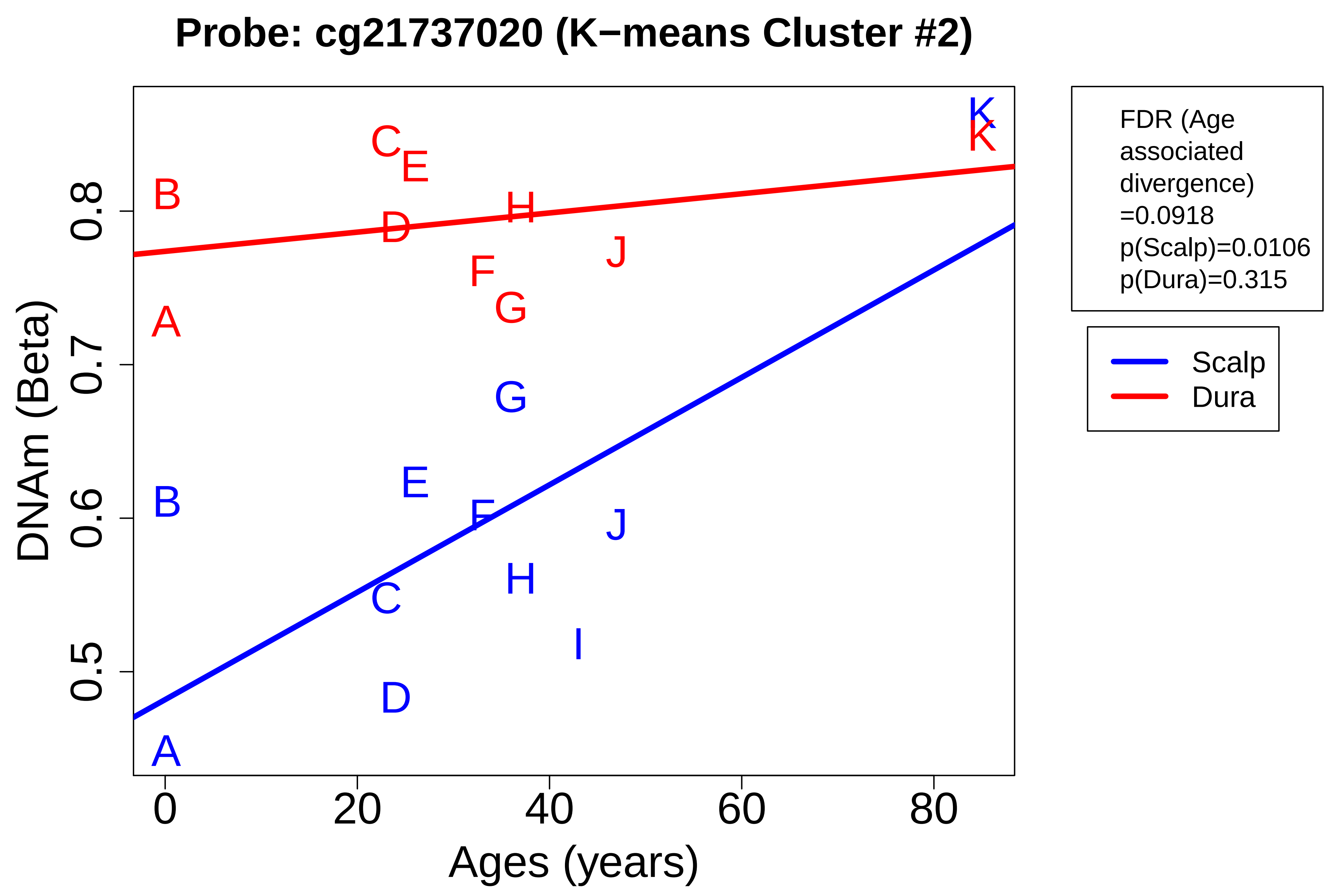

Probe: cg21737020 (K−means Cluster #2)
DNAm (Beta)
0.5
0.6
0.7
0.8
Ages (years)
0
20
40
60
80
A
B
C
D
E
F
G
H
I
J
K
FDR (Age associated divergence) =0.0918
p(Scalp)=0.0106
p(Dura)=0.315
Scalp
Dura

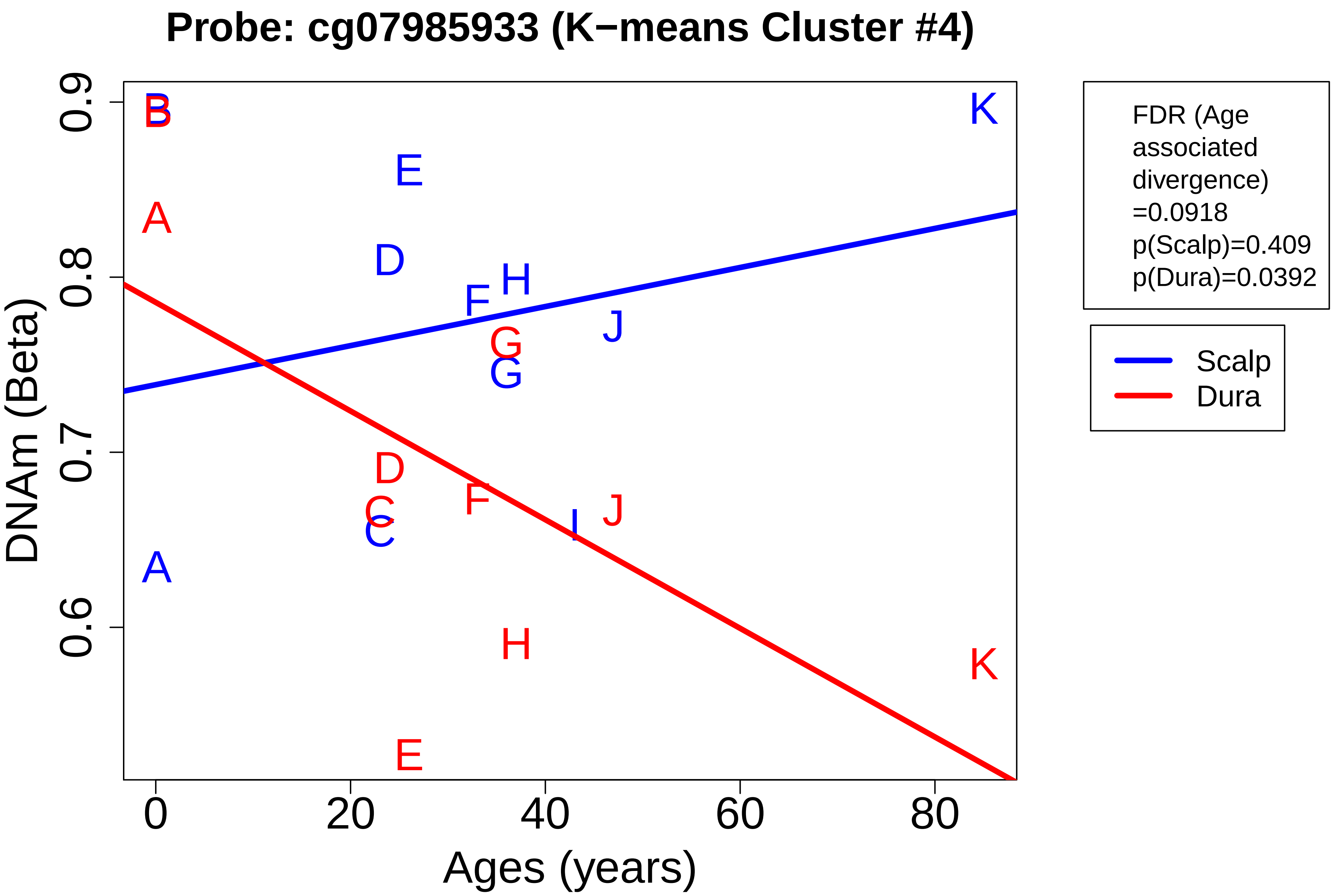

Probe: cg07985933 (K−means Cluster #4)
DNAm (Beta)
0.6
0.7
0.8
0.9
Ages (years)
0
20
40
60
80
FDR (Age associated divergence) =0.0918
p(Scalp)=0.409
p(Dura)=0.0392
Scalp
Dura

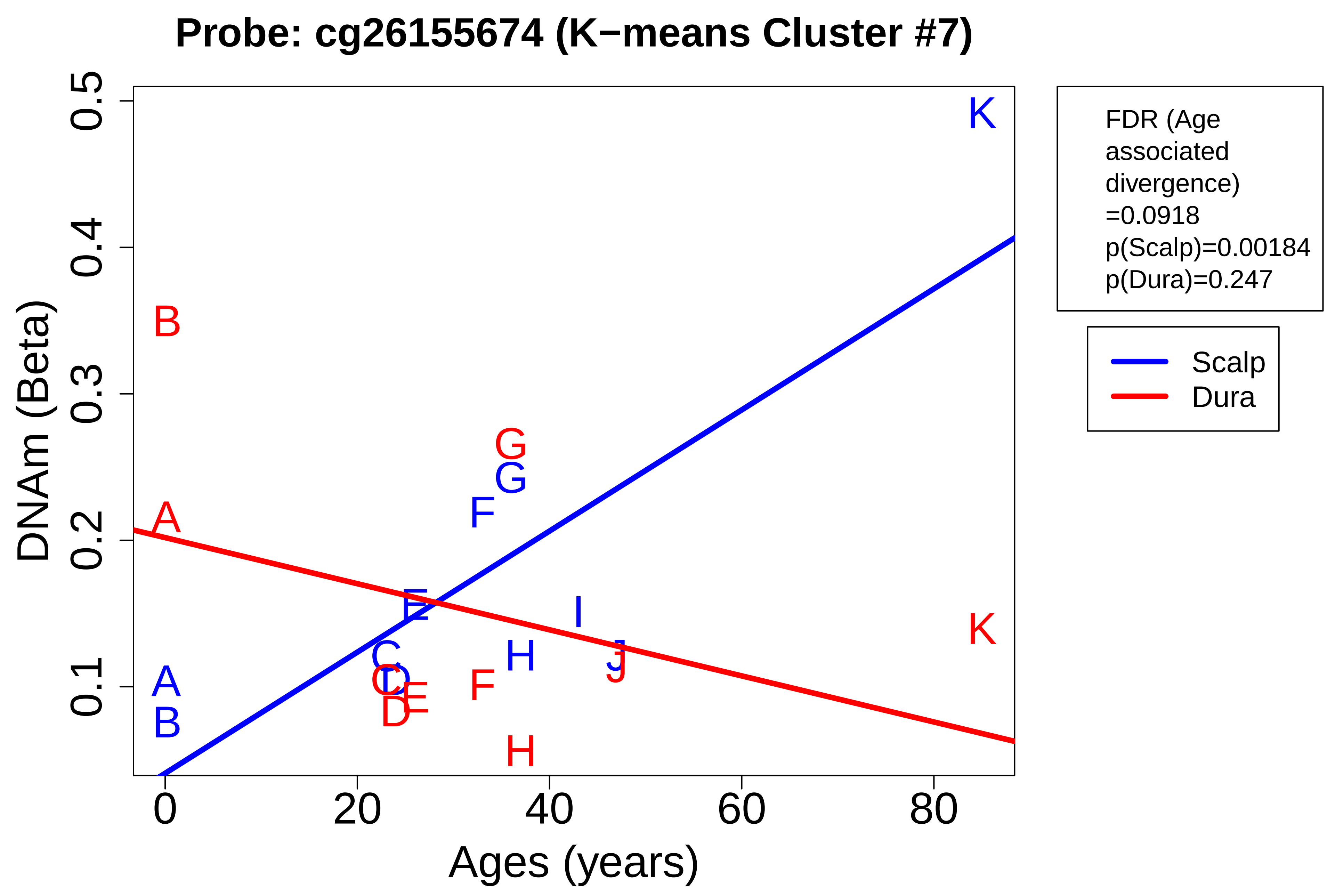

Probe: cg26155674 (K−means Cluster #7)
DNAm (Beta)
0.1
0.2
0.3
0.4
0.5
Ages (years)
0
20
40
60
80
FDR (Age associated divergence) =0.0918
p(Scalp)=0.00184
p(Dura)=0.247
Scalp
Dura

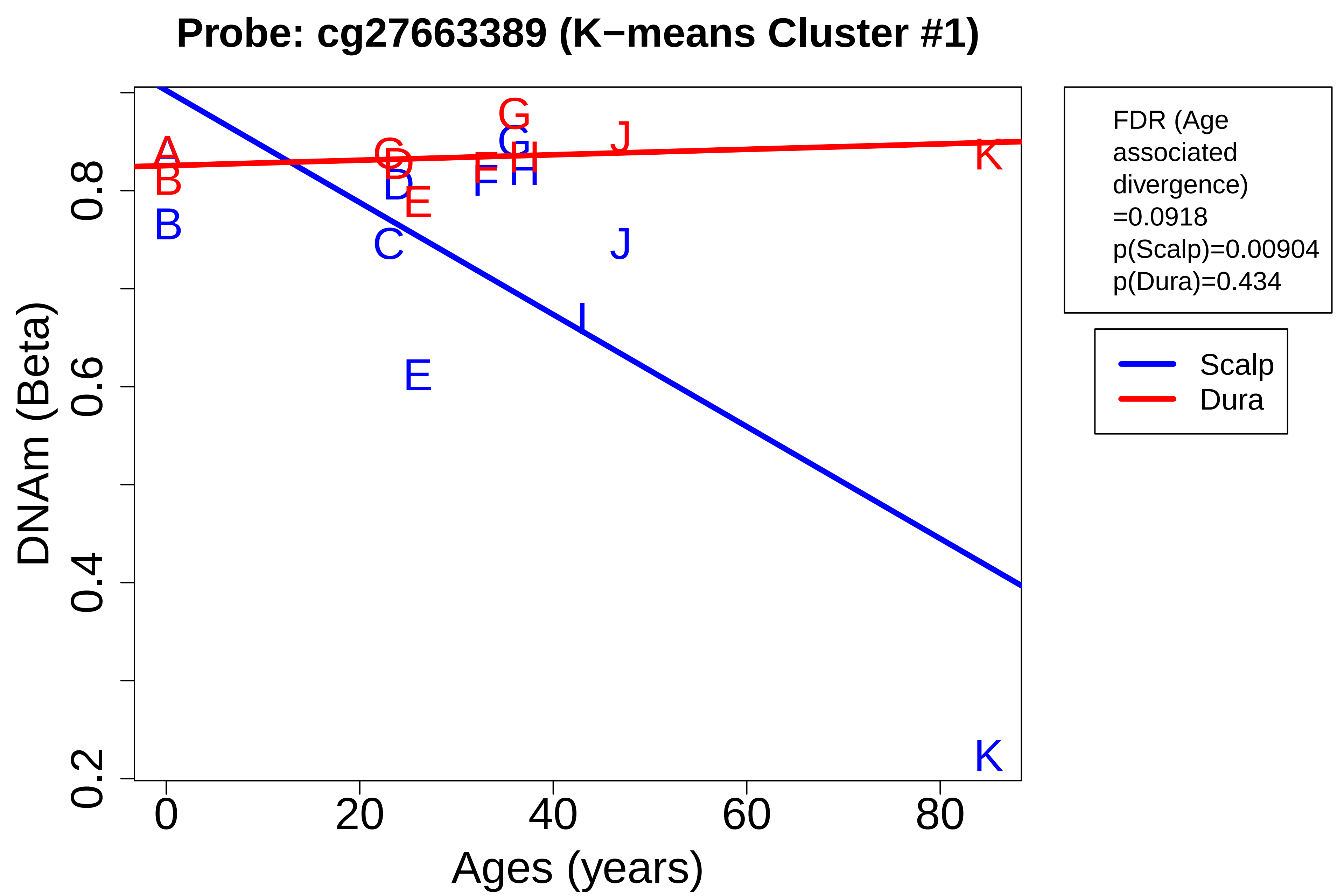

Probe: cg27663389 (K−means Cluster #1)
DNAm (Beta)
0.2
0.4
0.6
0.8
0
20
40
60
80
Ages (years)
A
B
C
D
E
F
G
H
I
J
K
FDR (Age associated divergence) =0.0918
p(Scalp)=0.00904
p(Dura)=0.434
Scalp
Dura

Probe: cg25889998 (K−means Cluster #1)

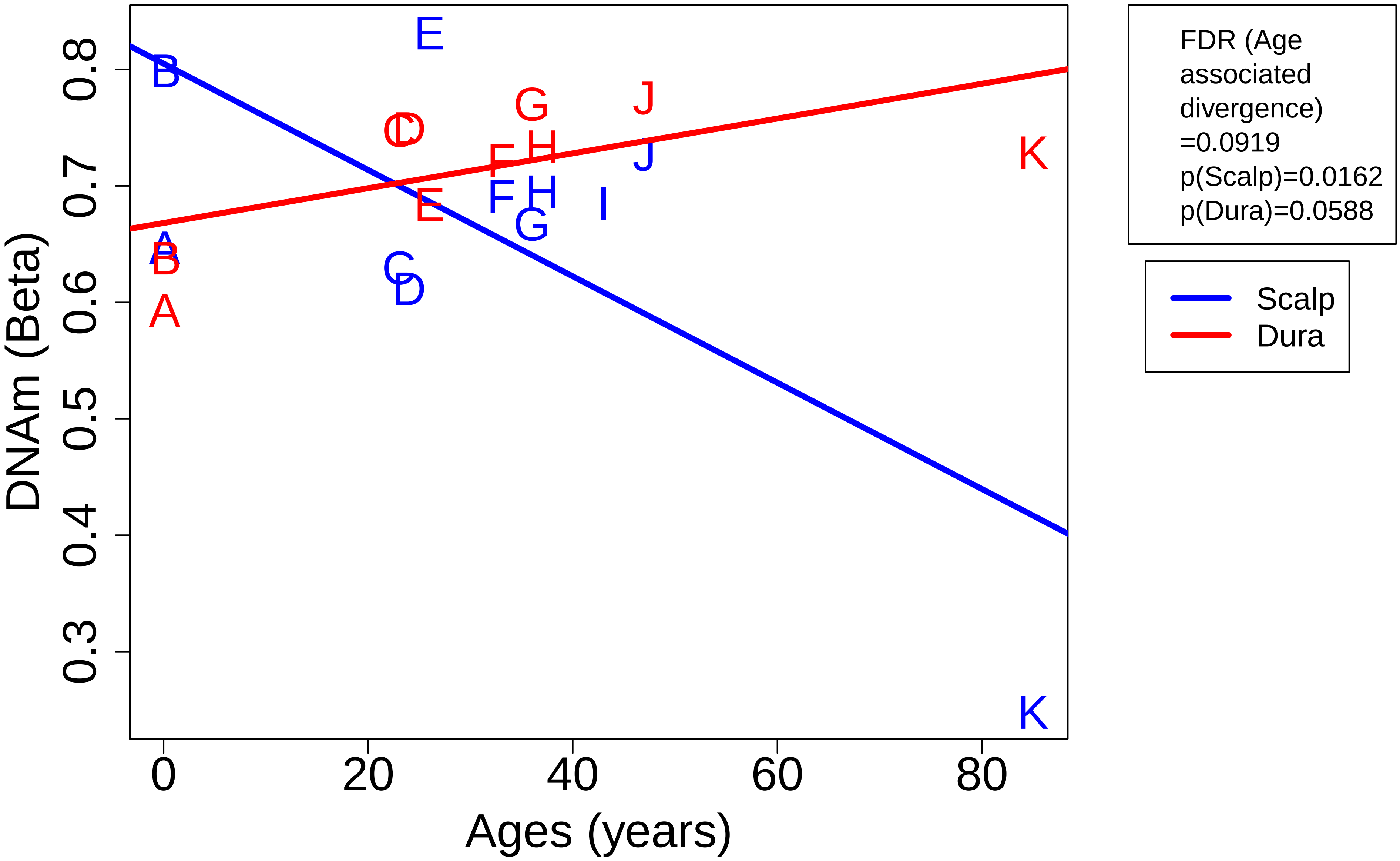

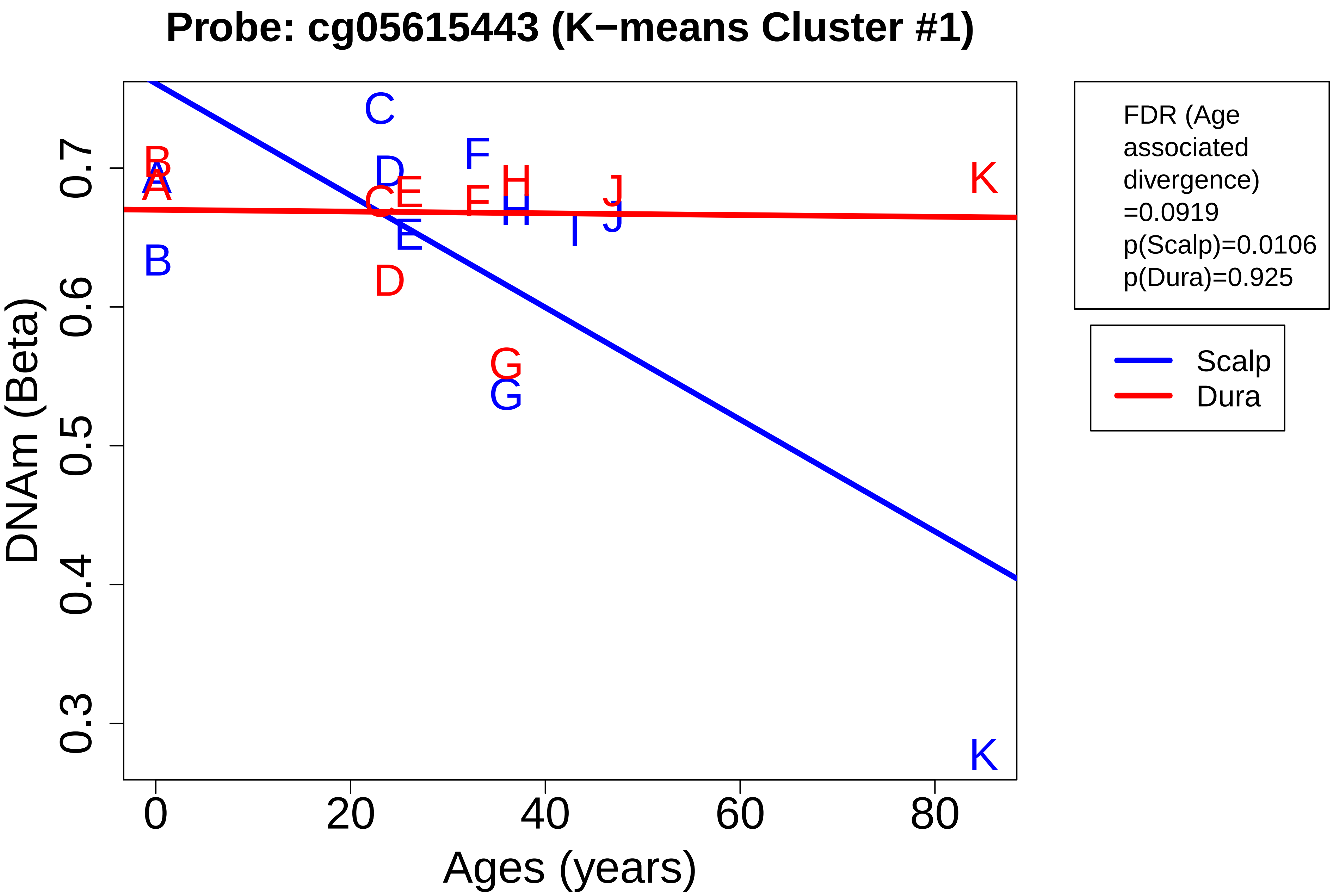
Probe: cg05615443 (K−means Cluster #1)
DNAm (Beta)
0.3
0.4
0.5
0.6
0.7
0
20
40
60
80
Ages (years)
FDR (Age associated divergence) =0.0919
p(Scalp)=0.0106
p(Dura)=0.925
Scalp
Dura

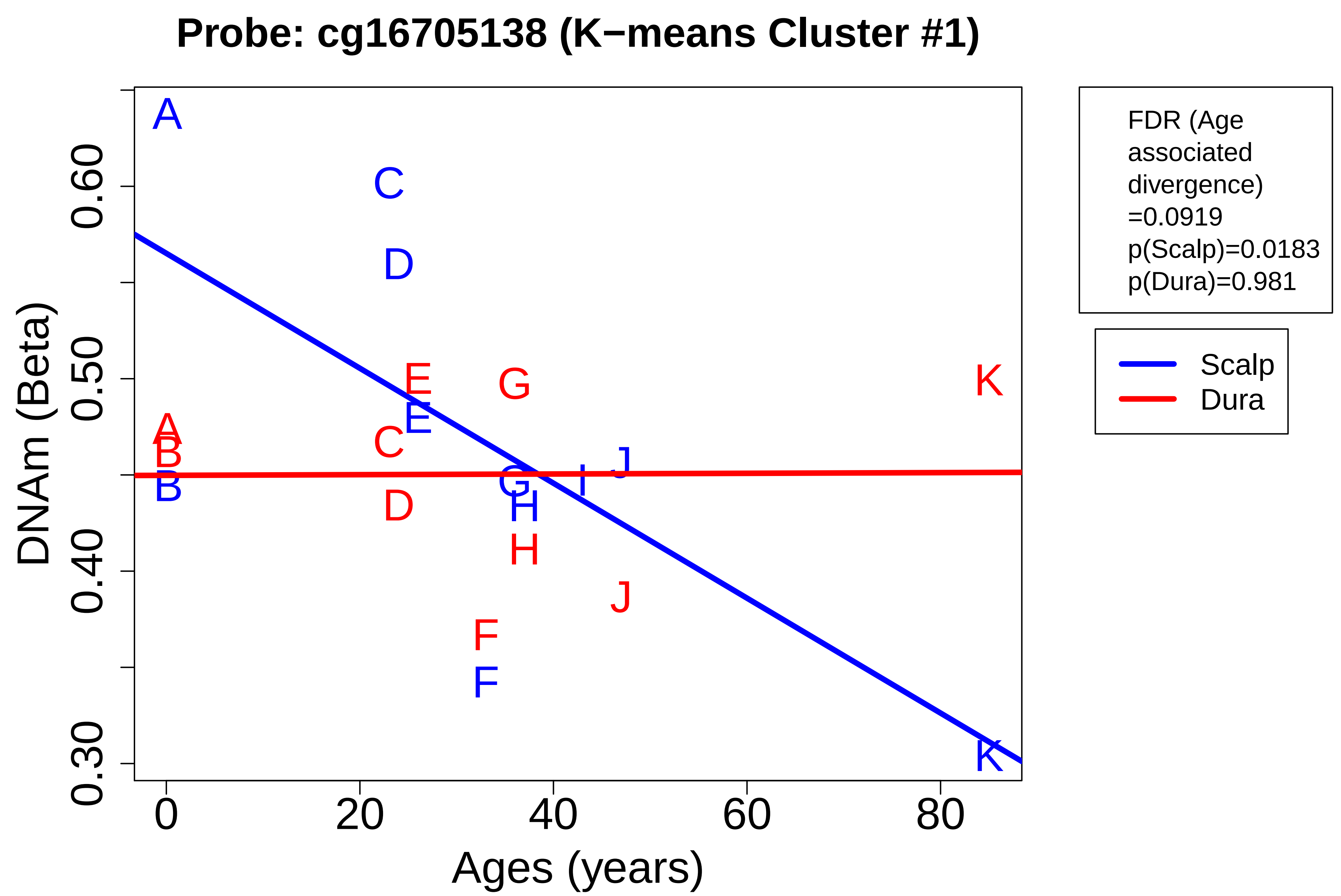

Probe: cg16705138 (K−means Cluster #1)
DNAm (Beta)
0.30
0.40
0.50
0.60
0
20
40
60
80
Ages (years)
A
B
C
D
E
F
G
H
I
J
K
FDR (Age associated divergence) =0.0919
p(Scalp)=0.0183
p(Dura)=0.981
Scalp
Dura

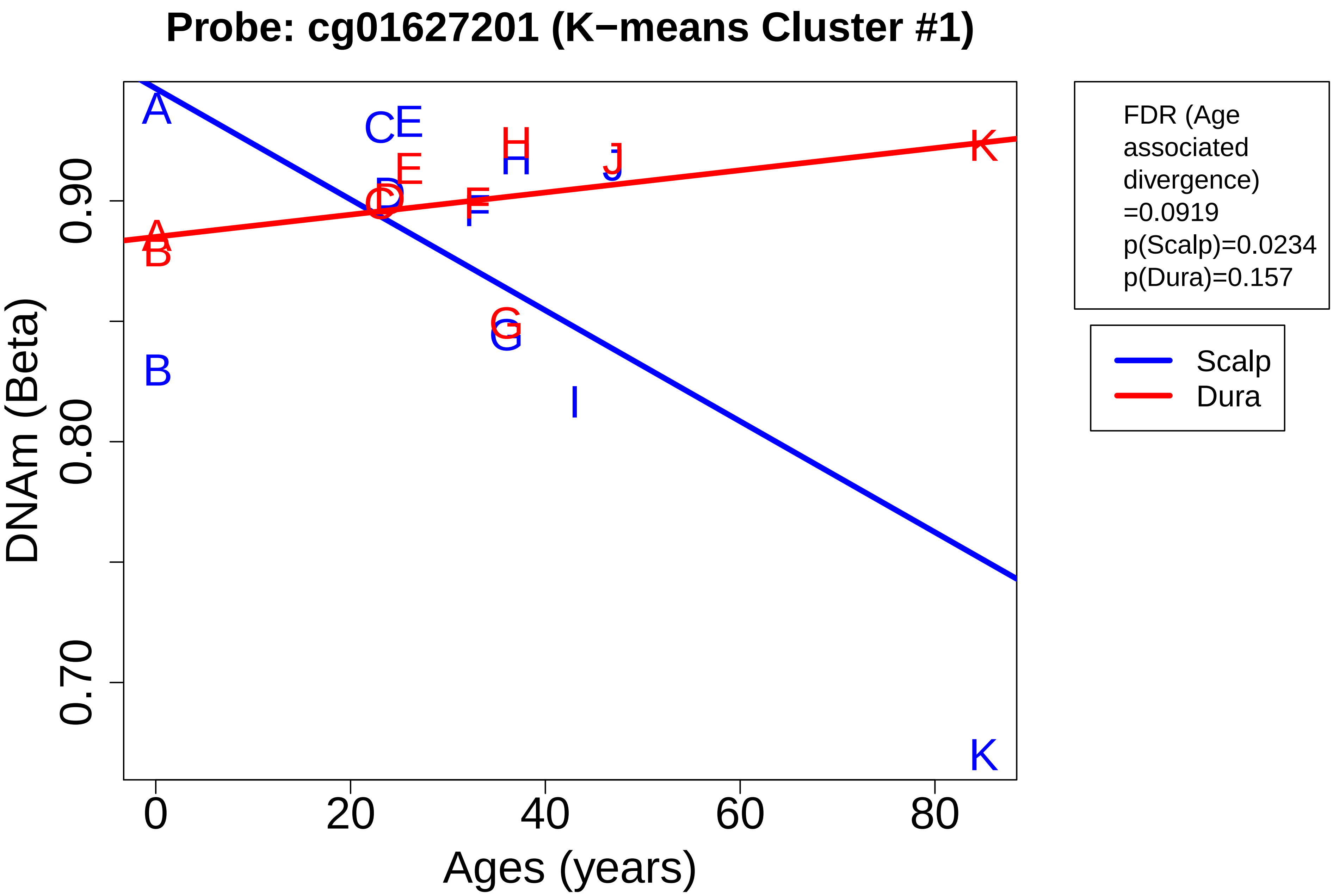

Probe: cg01627201 (K−means Cluster #1)
DNAm (Beta)
0.70
0.80
0.90
0
20
40
60
80
Ages (years)
FDR (Age associated divergence) =0.0919
p(Scalp)=0.0234
p(Dura)=0.157
Scalp
Dura

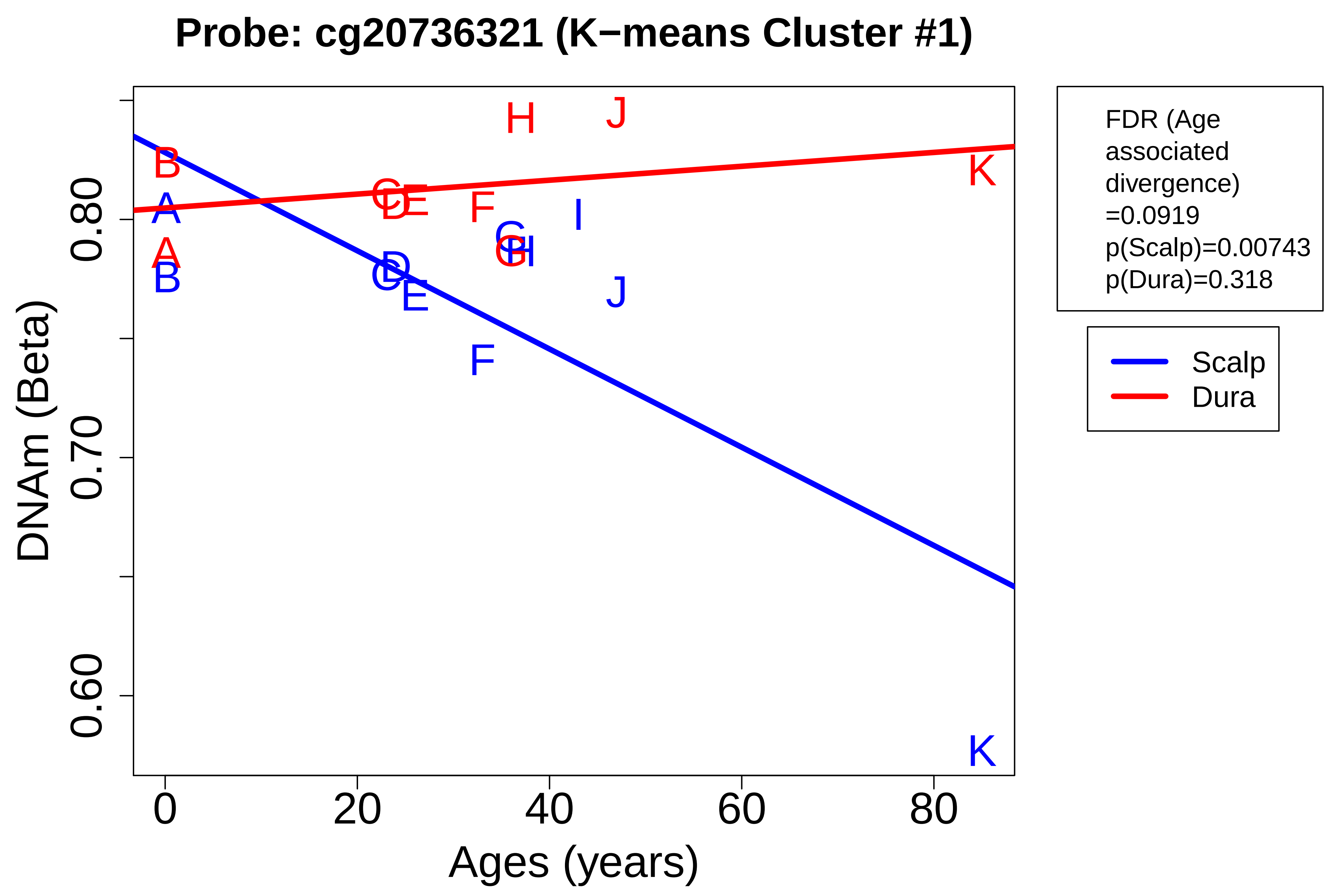
Probe: cg20736321 (K−means Cluster #1)
DNAm (Beta)
0.60
0.70
0.80
Ages (years)
0
20
40
60
80
FDR (Age associated divergence) =0.0919
p(Scalp)=0.00743
p(Dura)=0.318
Scalp
Dura

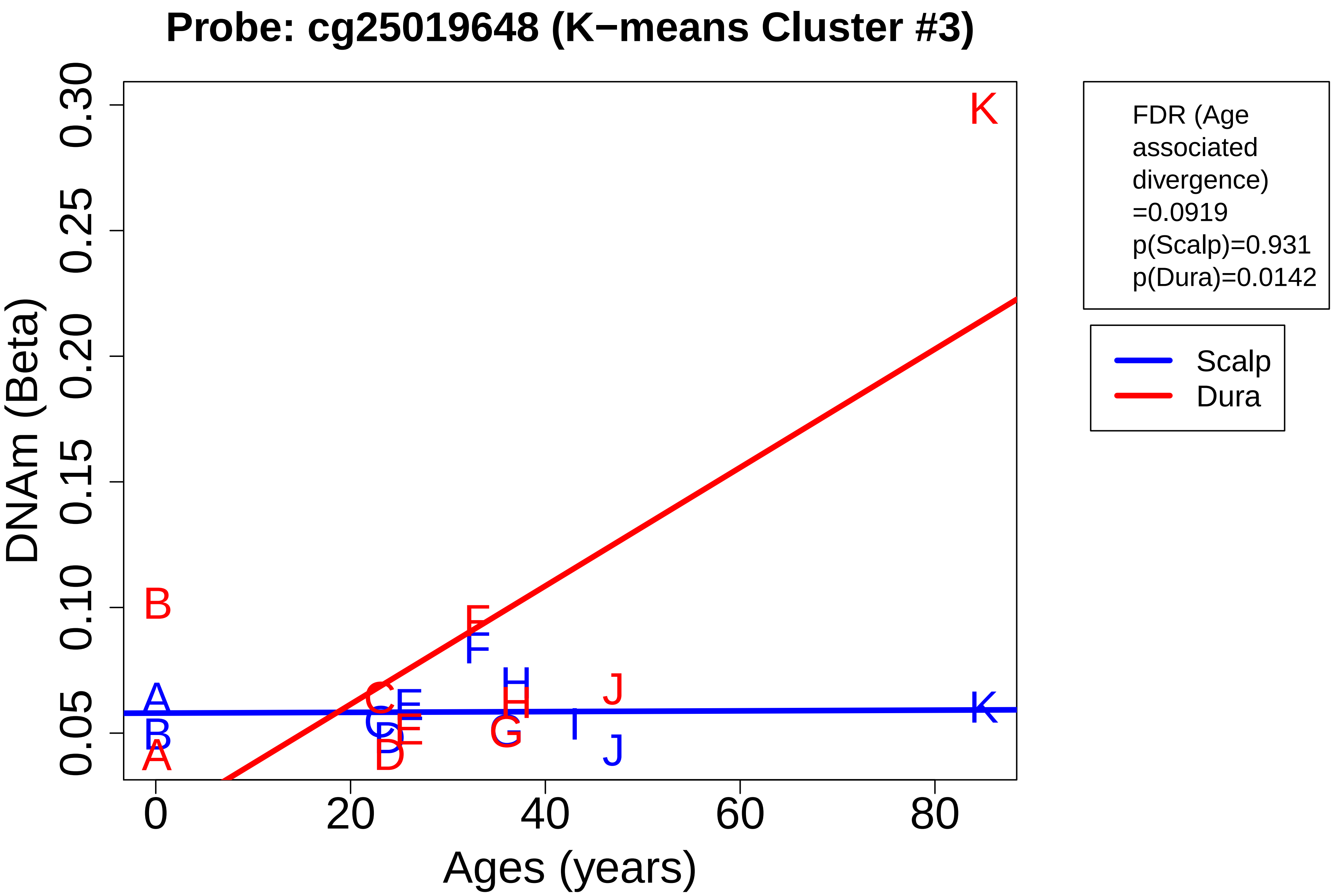

Probe: cg25019648 (K−means Cluster #3)
DNAm (Beta)
0.05
0.10
0.15
0.20
0.25
0.30
Ages (years)
0
20
40
60
80
FDR (Age associated divergence) =0.0919
p(Scalp)=0.931
p(Dura)=0.0142
Scalp
Dura

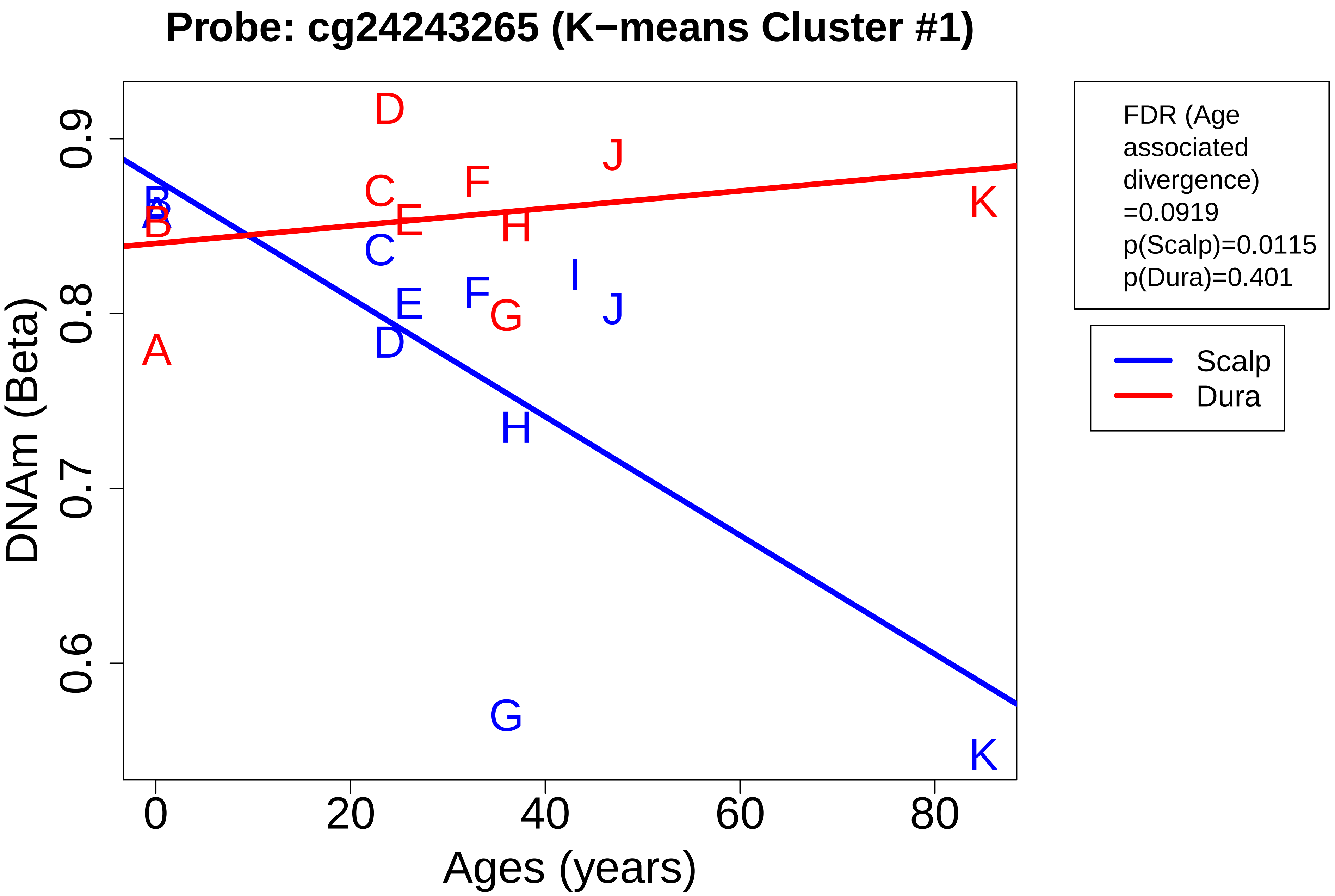

Probe: cg24243265 (K−means Cluster #1)
DNAm (Beta)
0.6
0.7
0.8
0.9
0
20
40
60
80
Ages (years)
FDR (Age associated divergence) =0.0919
p(Scalp)=0.0115
p(Dura)=0.401
Scalp
Dura

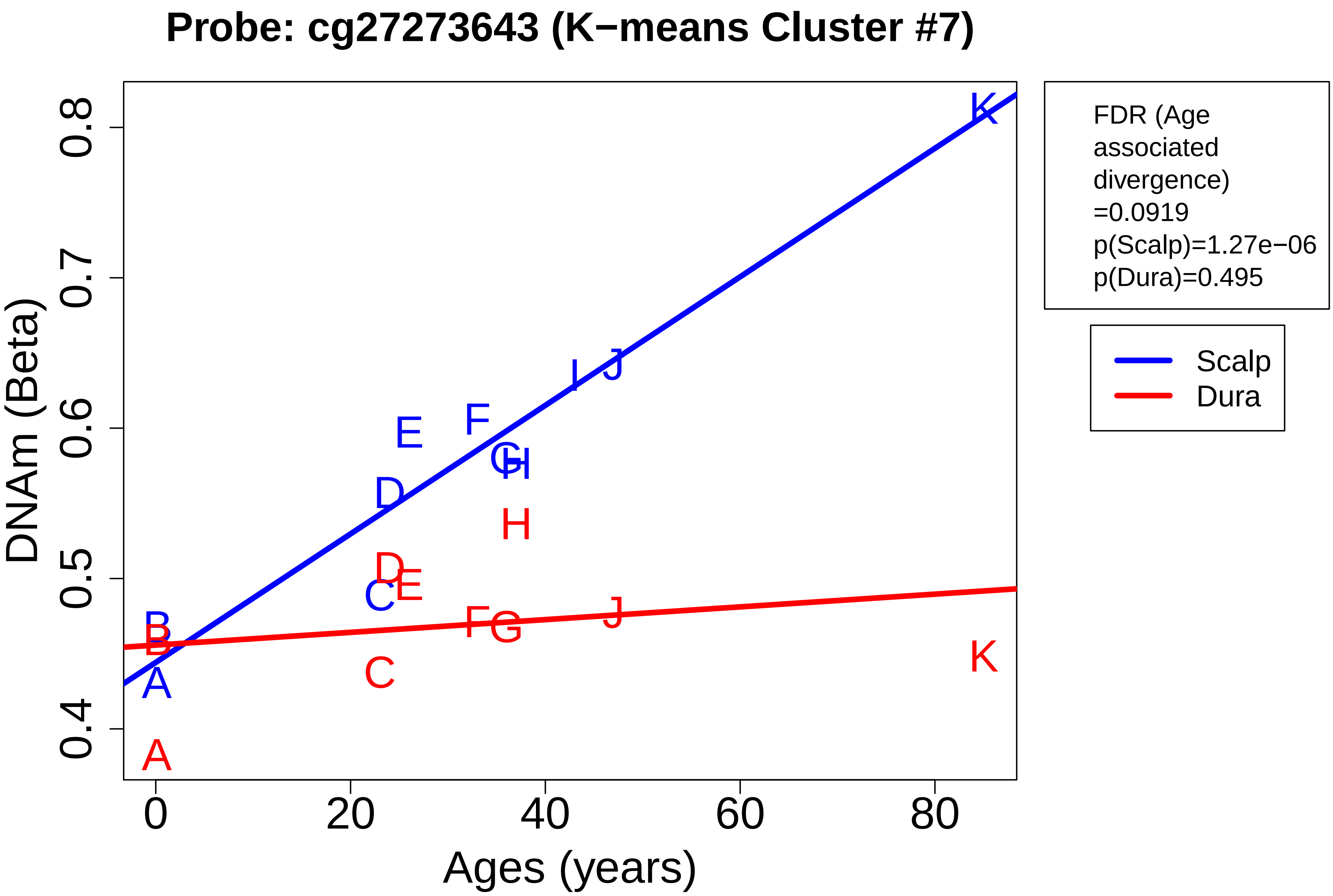
Probe: cg27273643 (K−means Cluster #7)
DNAm (Beta)
0.4
0.5
0.6
0.7
0.8
Ages (years)
0
20
40
60
80
FDR (Age associated divergence) =0.0919
p(Scalp)=1.27e−06
p(Dura)=0.495
Scalp
Dura

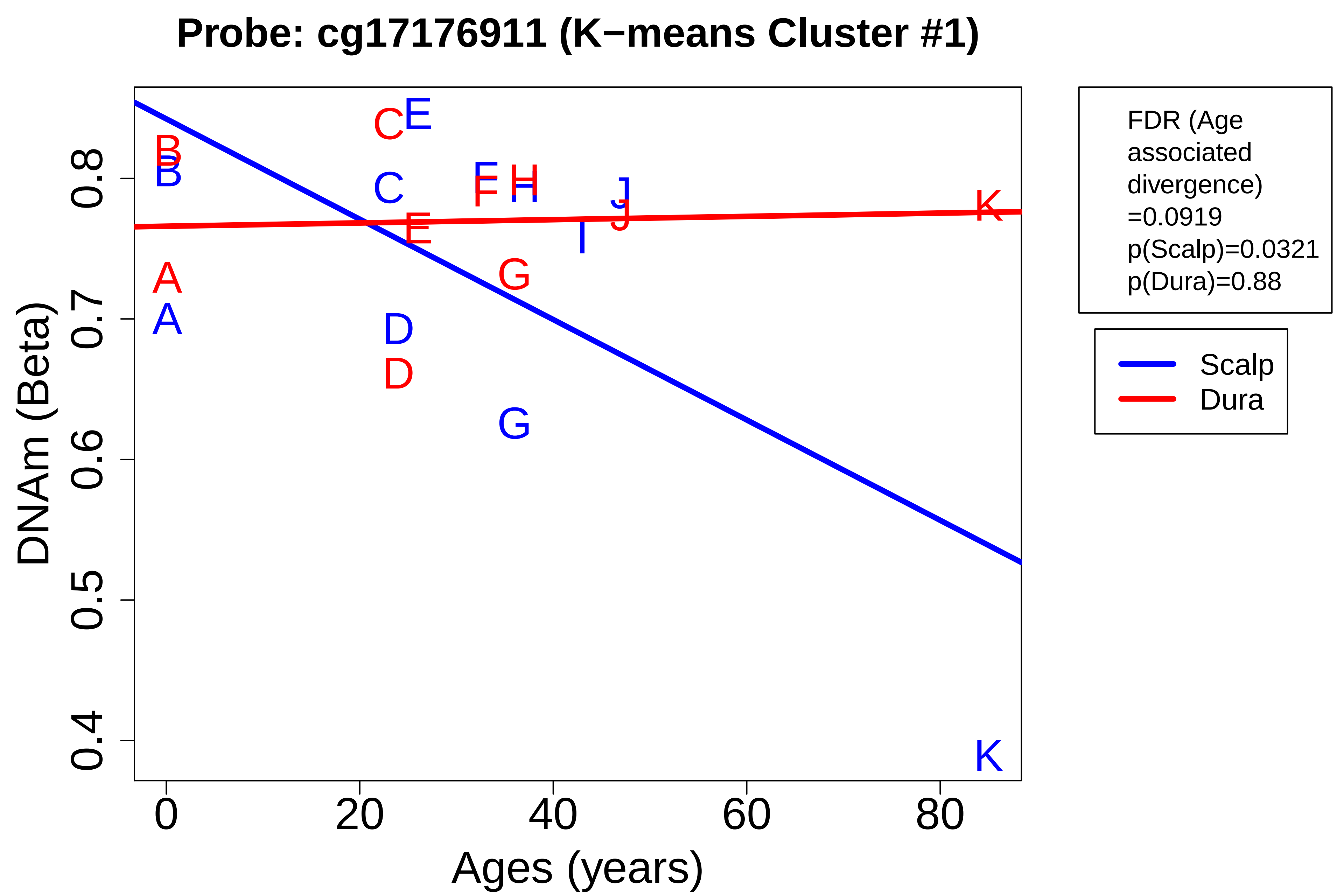

Probe: cg17176911 (K−means Cluster #1)
DNAm (Beta)
0.4
0.5
0.6
0.7
0.8
0
20
40
60
80
Ages (years)
FDR (Age associated divergence) =0.0919
p(Scalp)=0.0321
p(Dura)=0.88
Scalp
Dura
A
B
C
D
E
F
G
H
I
J
K

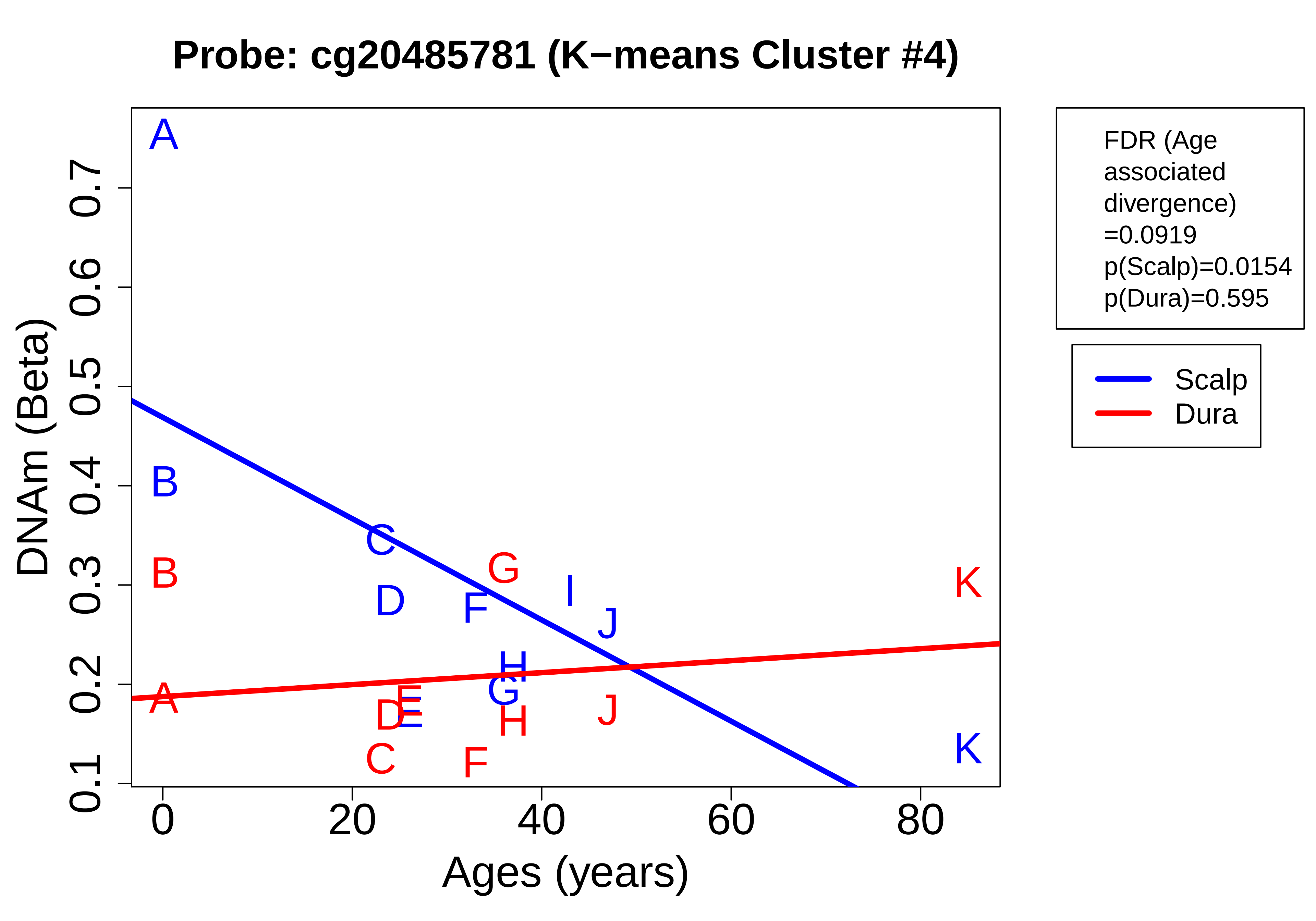

Probe: cg20485781 (K−means Cluster #4)
FDR (Age associated divergence) =0.0919
p(Scalp)=0.0154
p(Dura)=0.595
Scalp
Dura
DNAm (Beta)
Ages (years)
0.1
0.2
0.3
0.4
0.5
0.6
0.7
0
20
40
60
80

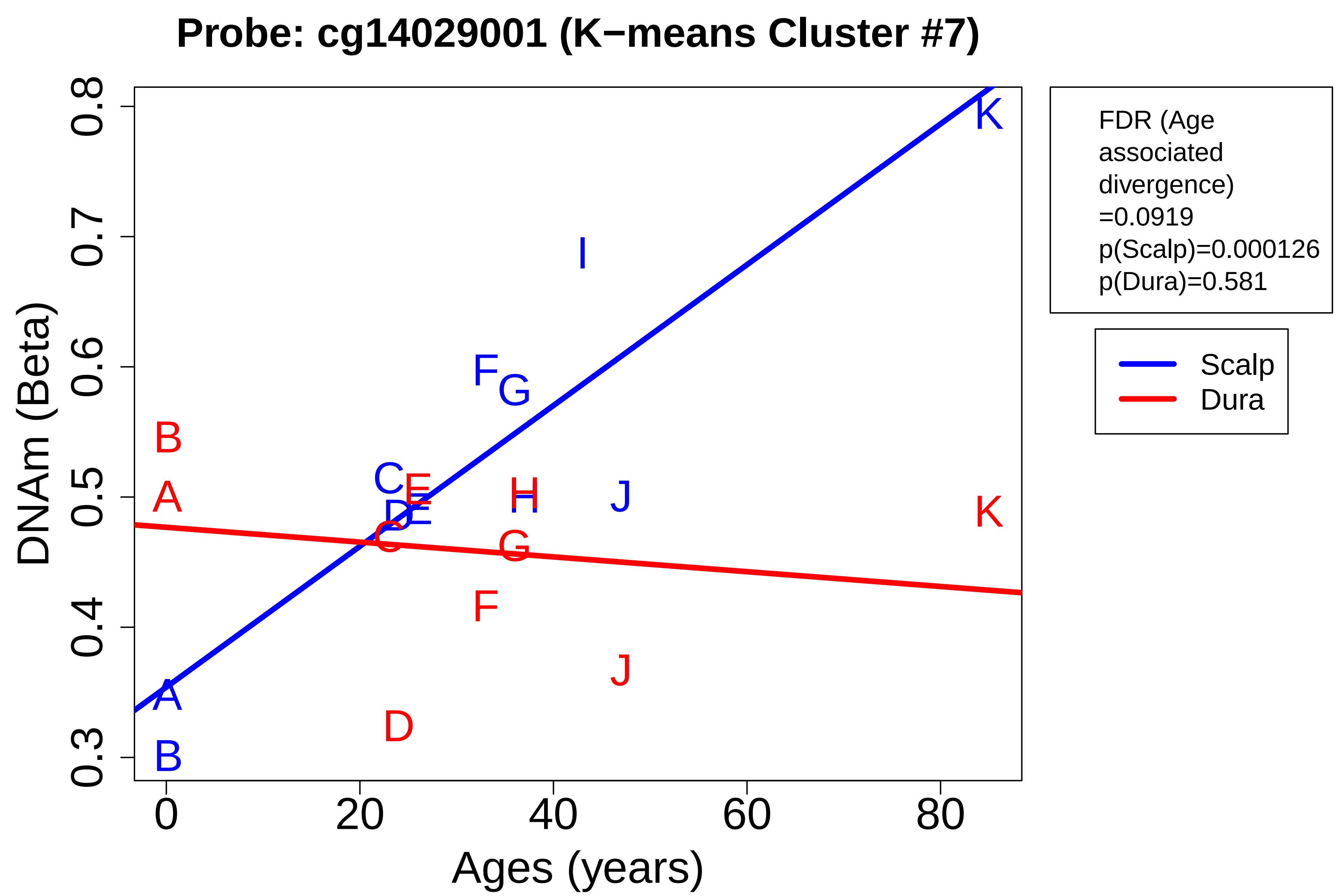

Probe: cg14029001 (K−means Cluster #7)
DNAm (Beta)
Ages (years)
0.3
0.4
0.5
0.6
0.7
0.8
0
20
40
60
80
FDR (Age associated divergence) =0.0919
p(Scalp)=0.000126
p(Dura)=0.581
Scalp
Dura

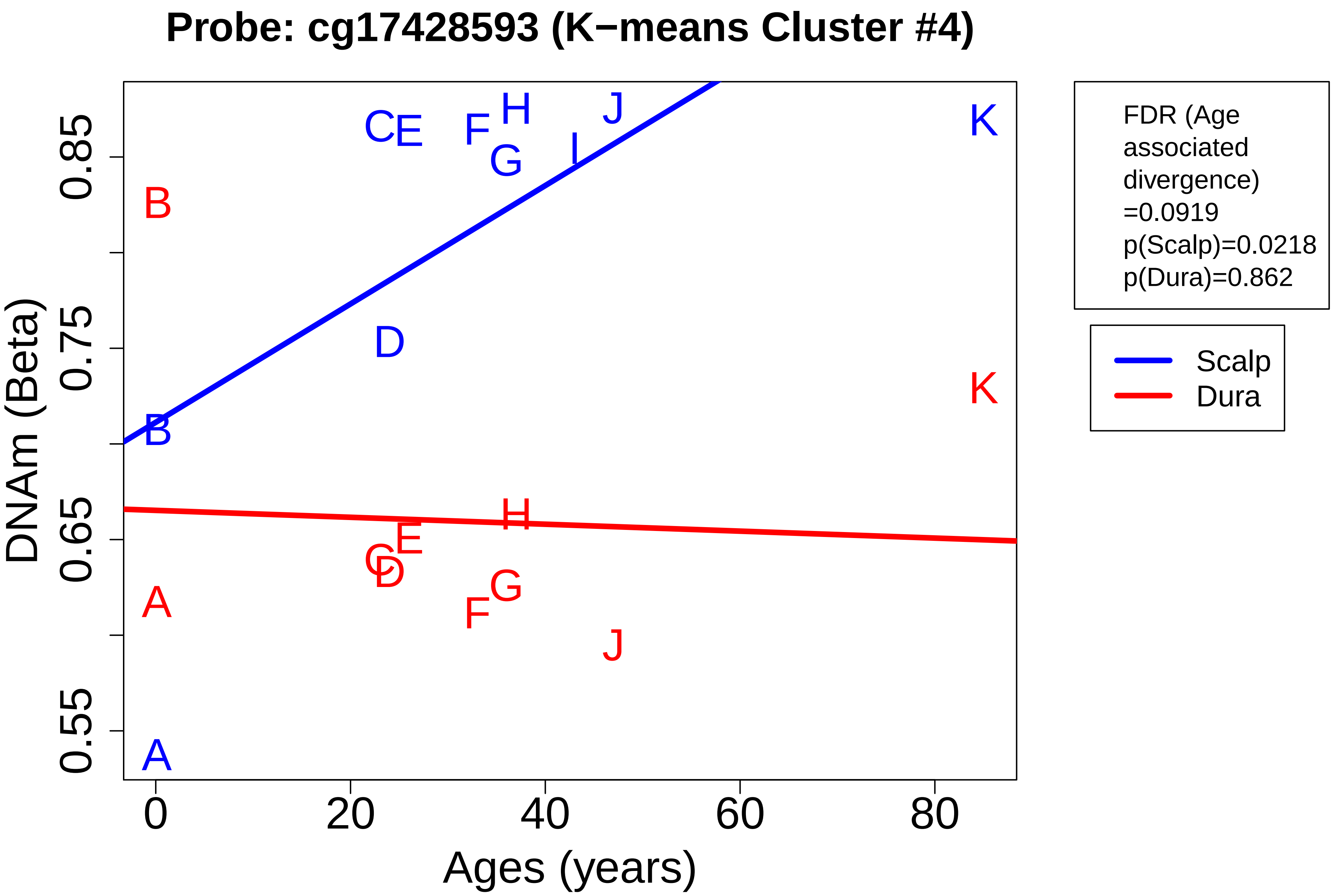

Probe: cg17428593 (K−means Cluster #4)
DNAm (Beta)
0.55
0.65
0.75
0.85
0
20
40
60
80
Ages (years)
FDR (Age associated divergence) =0.0919
p(Scalp)=0.0218
p(Dura)=0.862
Scalp
Dura
A
B
C
D
E
F
G
H
I
J
K

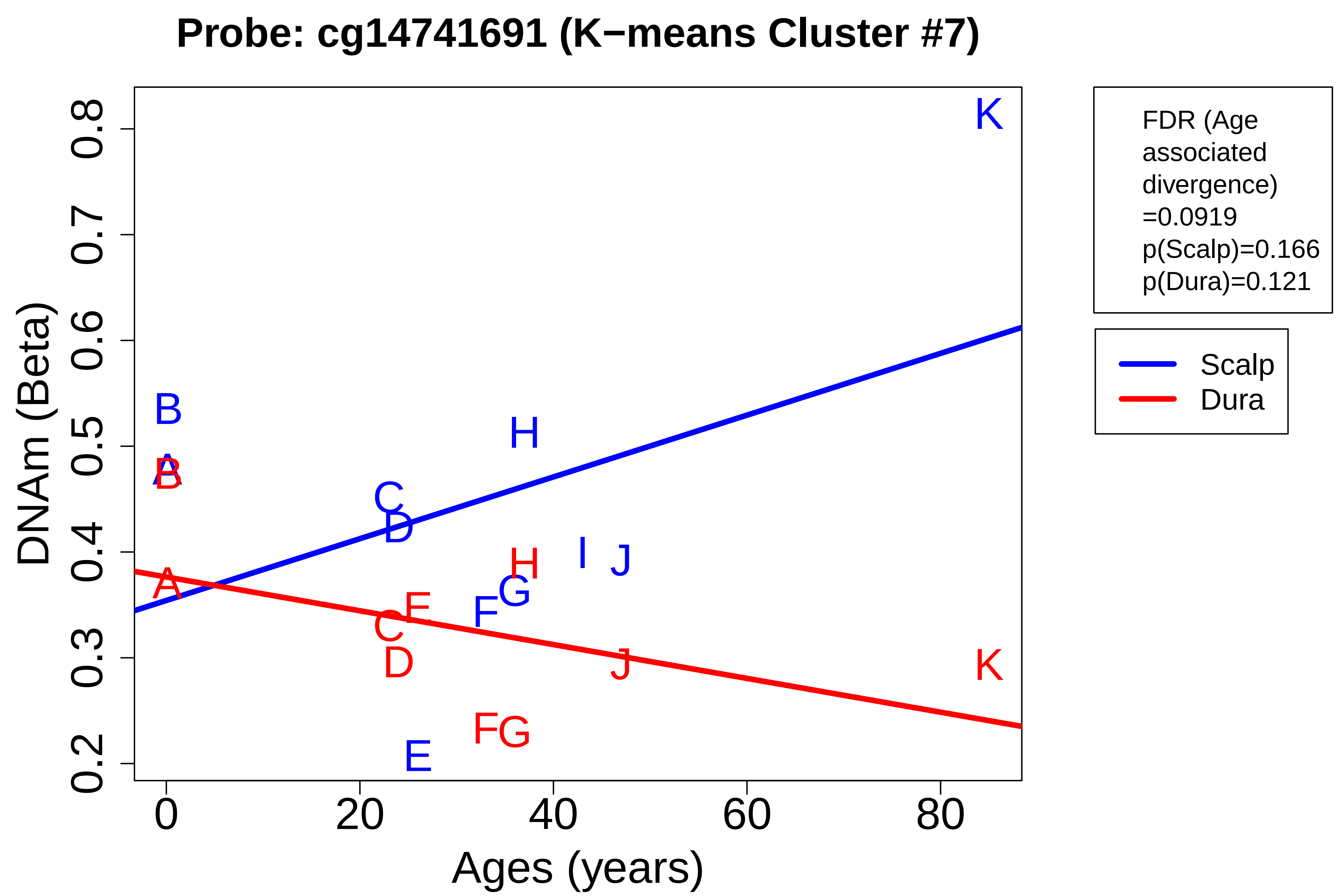

Probe: cg14741691 (K−means Cluster #7)
DNAm (Beta)
0.2
0.3
0.4
0.5
0.6
0.7
0.8
0
20
40
60
80
Ages (years)
A B C D E F G H I J K
A B C D E F G H J K
FDR (Age associated divergence) =0.0919
p(Scalp)=0.166
p(Dura)=0.121
Scalp
Dura

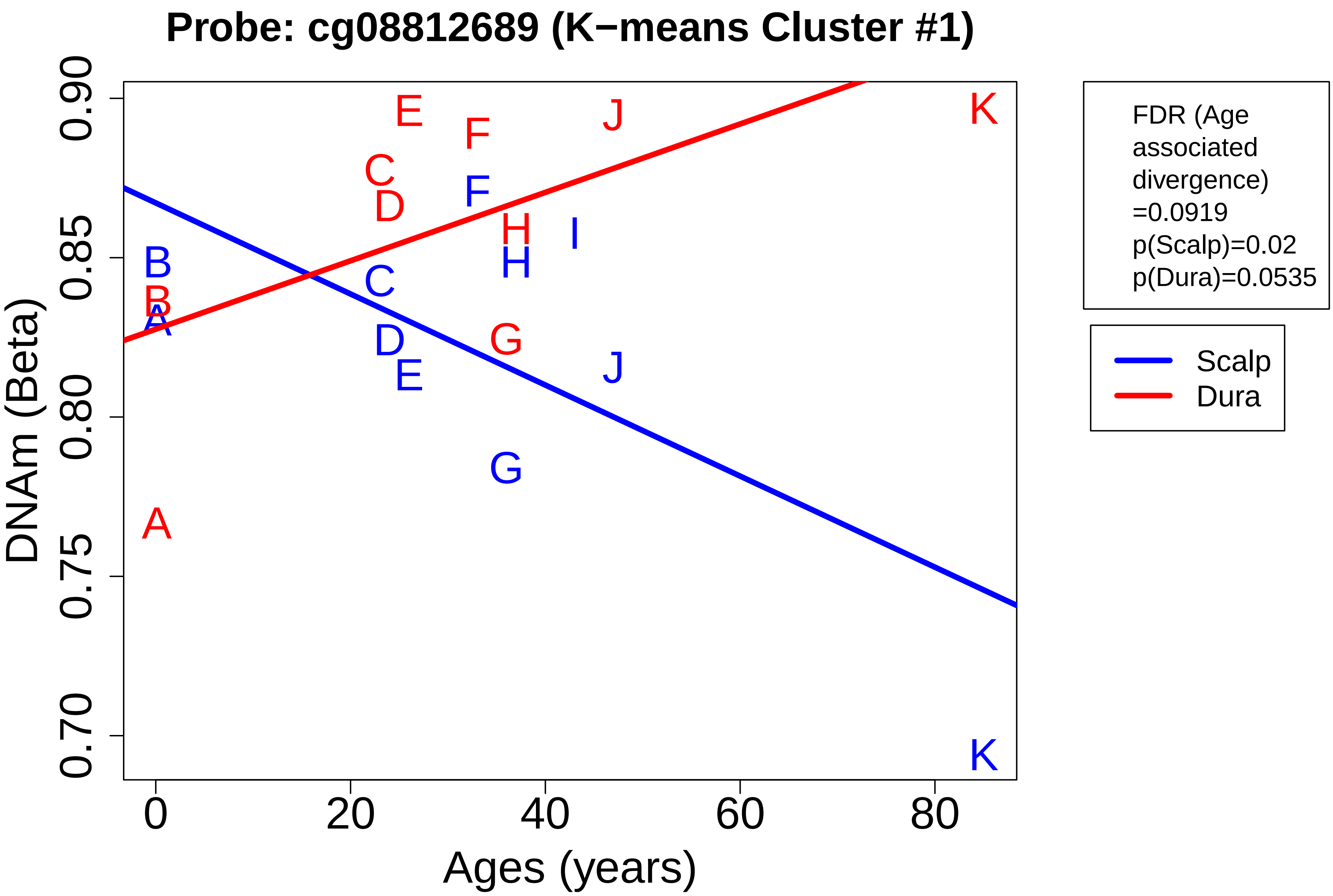
Probe: cg08812689 (K−means Cluster #1)
DNAm (Beta)
0.70
0.75
0.80
0.85
0.90
Ages (years)
0
20
40
60
80
A
B
C
D
E
F
G
H
I
J
K
FDR (Age associated divergence) =0.0919
p(Scalp)=0.02
p(Dura)=0.0535
Scalp
Dura

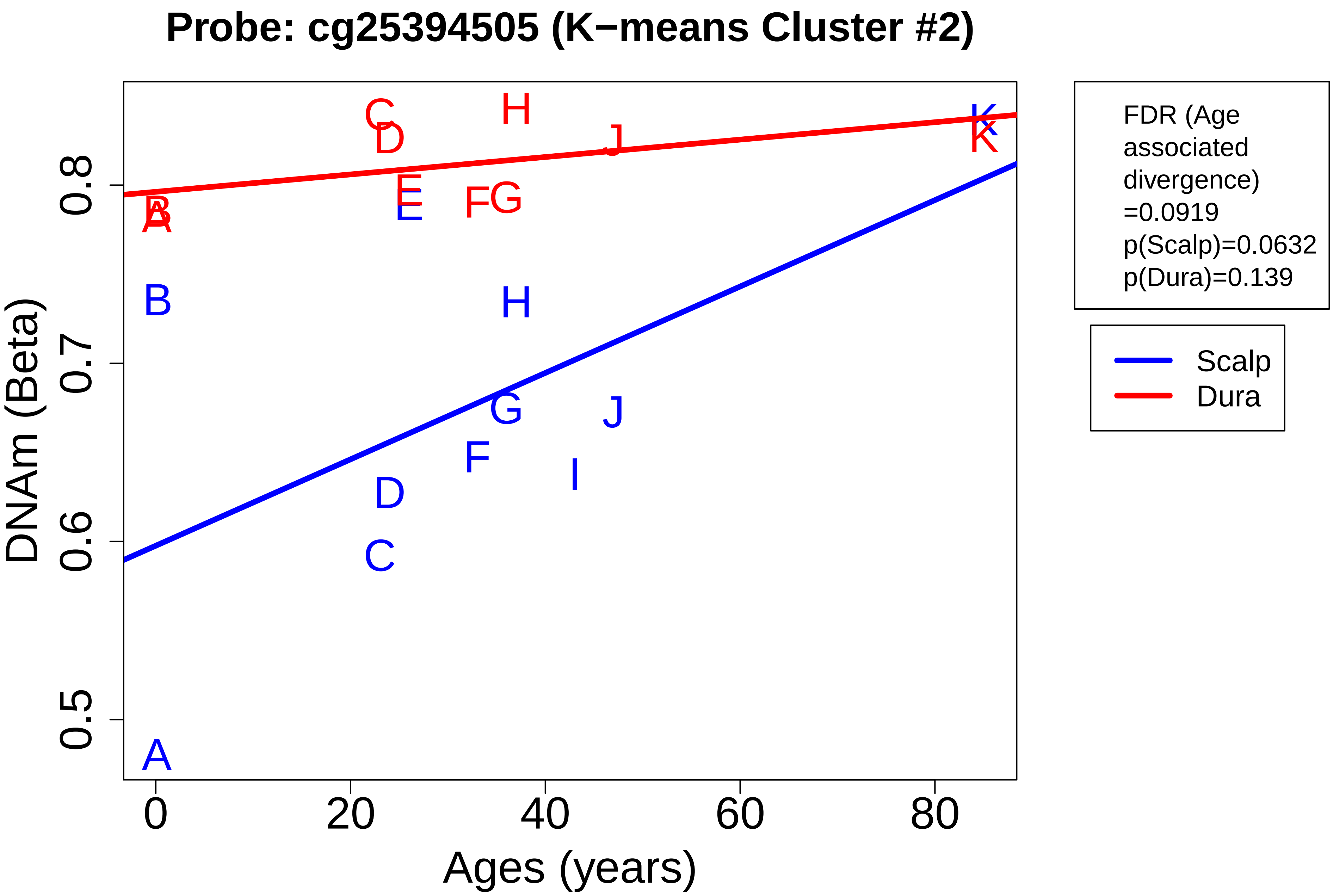

Probe: cg25394505 (K−means Cluster #2)
FDR (Age associated divergence) =0.0919
p(Scalp)=0.0632
p(Dura)=0.139
Scalp
Dura
DNAm (Beta)
0.5
0.6
0.7
0.8
0
20
40
60
80
Ages (years)
A
B
C
D
E
F
G
H
I
J
K

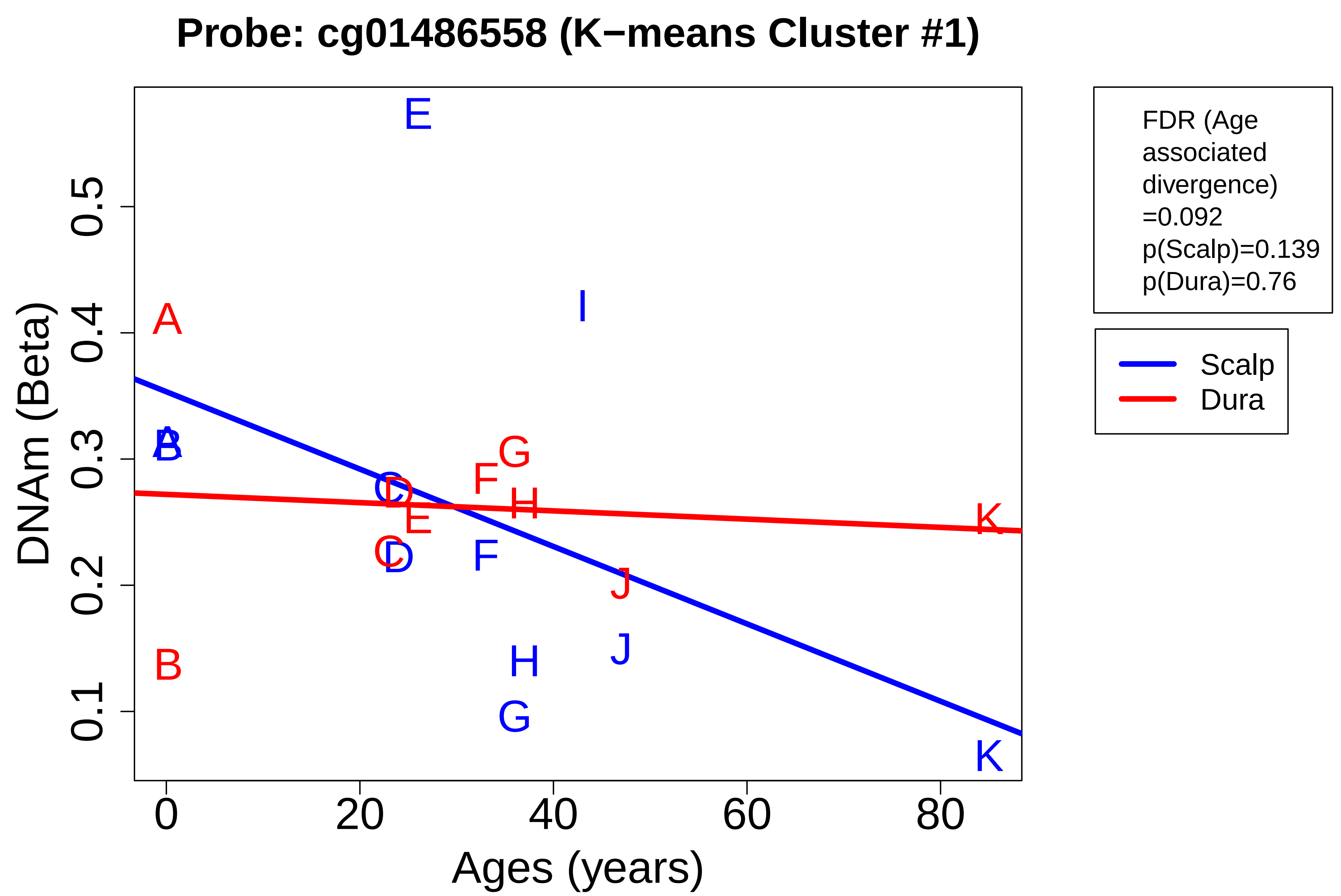

Probe: cg01486558 (K−means Cluster #1)
FDR (Age associated divergence) =0.092
p(Scalp)=0.139
p(Dura)=0.76
Scalp
Dura
DNAm (Beta)
Ages (years)
0.1
0.2
0.3
0.4
0.5
0
20
40
60
80

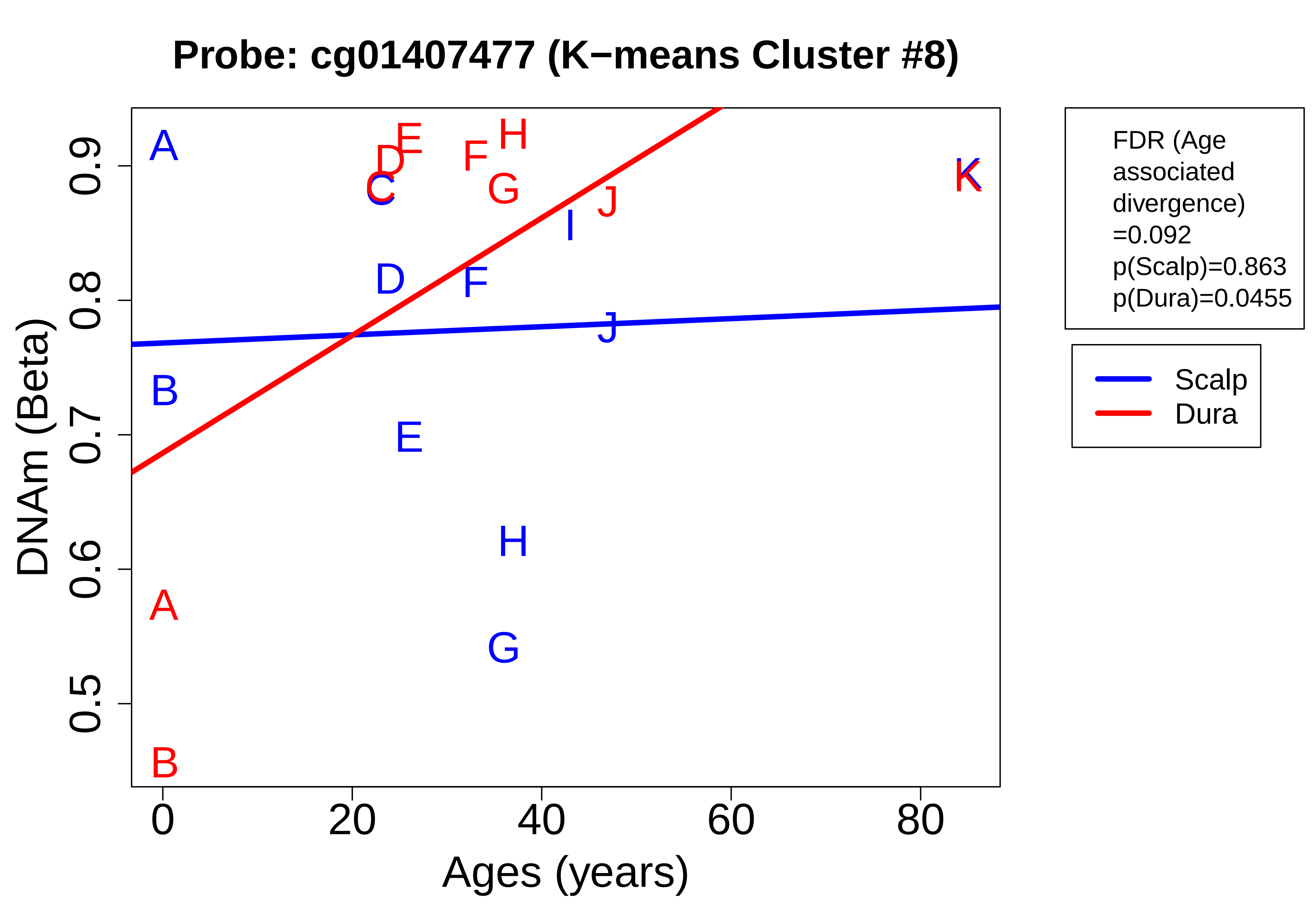

Probe: cg01407477 (K−means Cluster #8)
DNAm (Beta)
0.5
0.6
0.7
0.8
0.9
Ages (years)
0
20
40
60
80
FDR (Age associated divergence) =0.092
p(Scalp)=0.863
p(Dura)=0.0455
Scalp
Dura

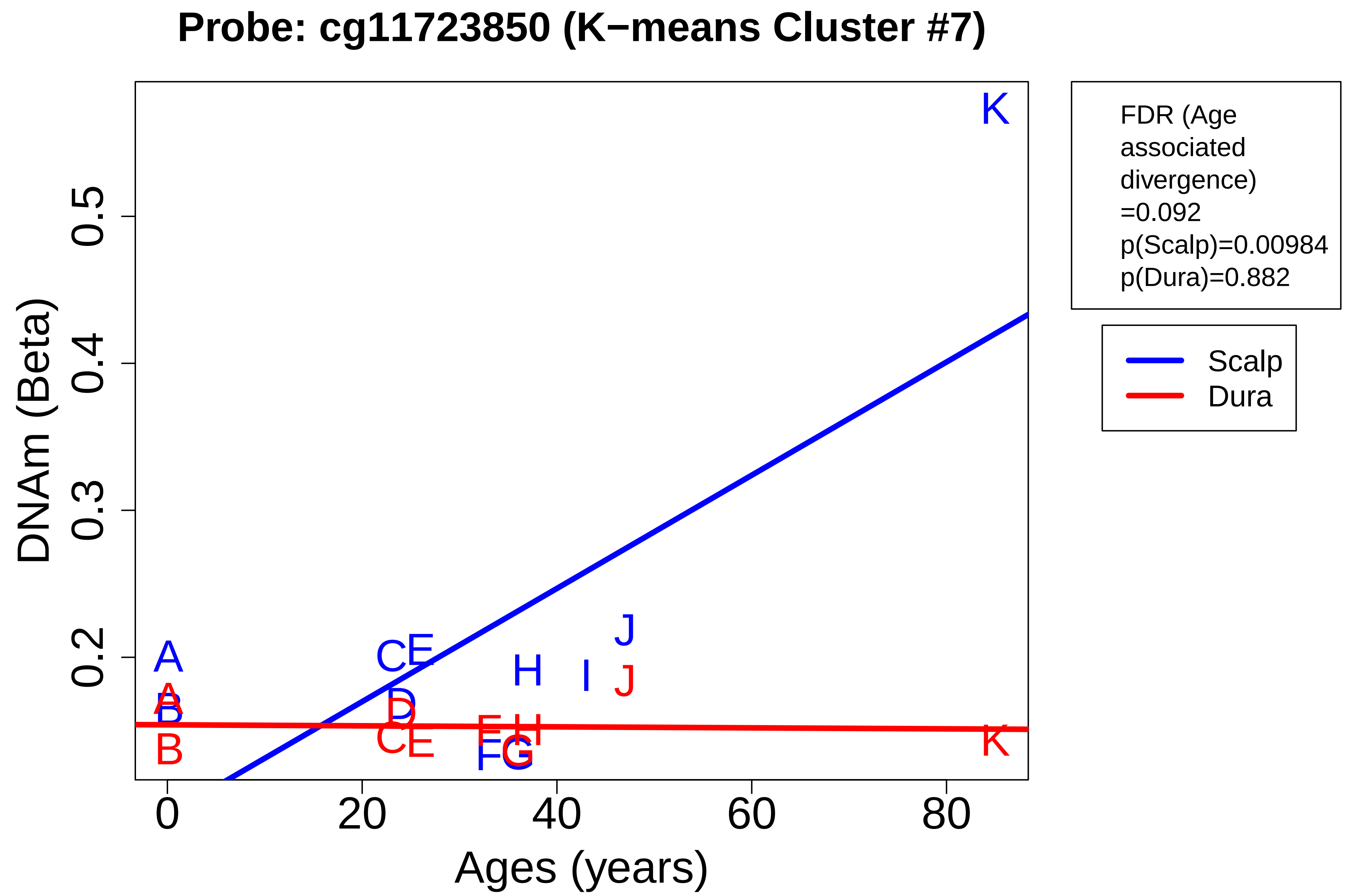

Probe: cg11723850 (K−means Cluster #7)
DNAm (Beta)
0.2
0.3
0.4
0.5
0
20
40
60
80
Ages (years)
A
B
C
D
E
F
G
H
I
J
K
FDR (Age associated divergence) =0.092
p(Scalp)=0.00984
p(Dura)=0.882
Scalp
Dura

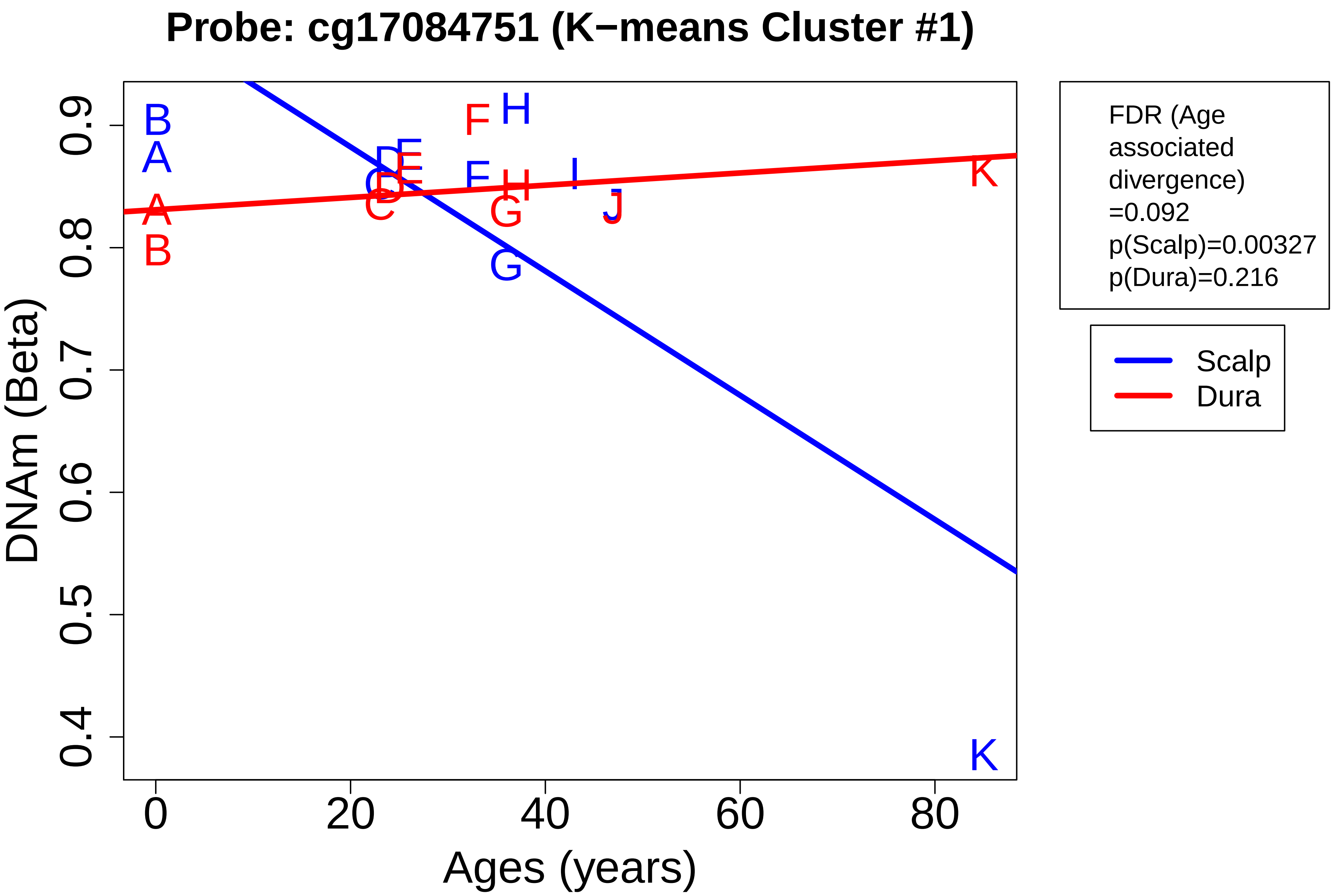
Probe: cg17084751 (K−means Cluster #1)
DNAm (Beta)
Ages (years)
FDR (Age associated divergence) =0.092
p(Scalp)=0.00327
p(Dura)=0.216
Scalp
Dura
0.4
0.5
0.6
0.7
0.8
0.9
0
20
40
60
80

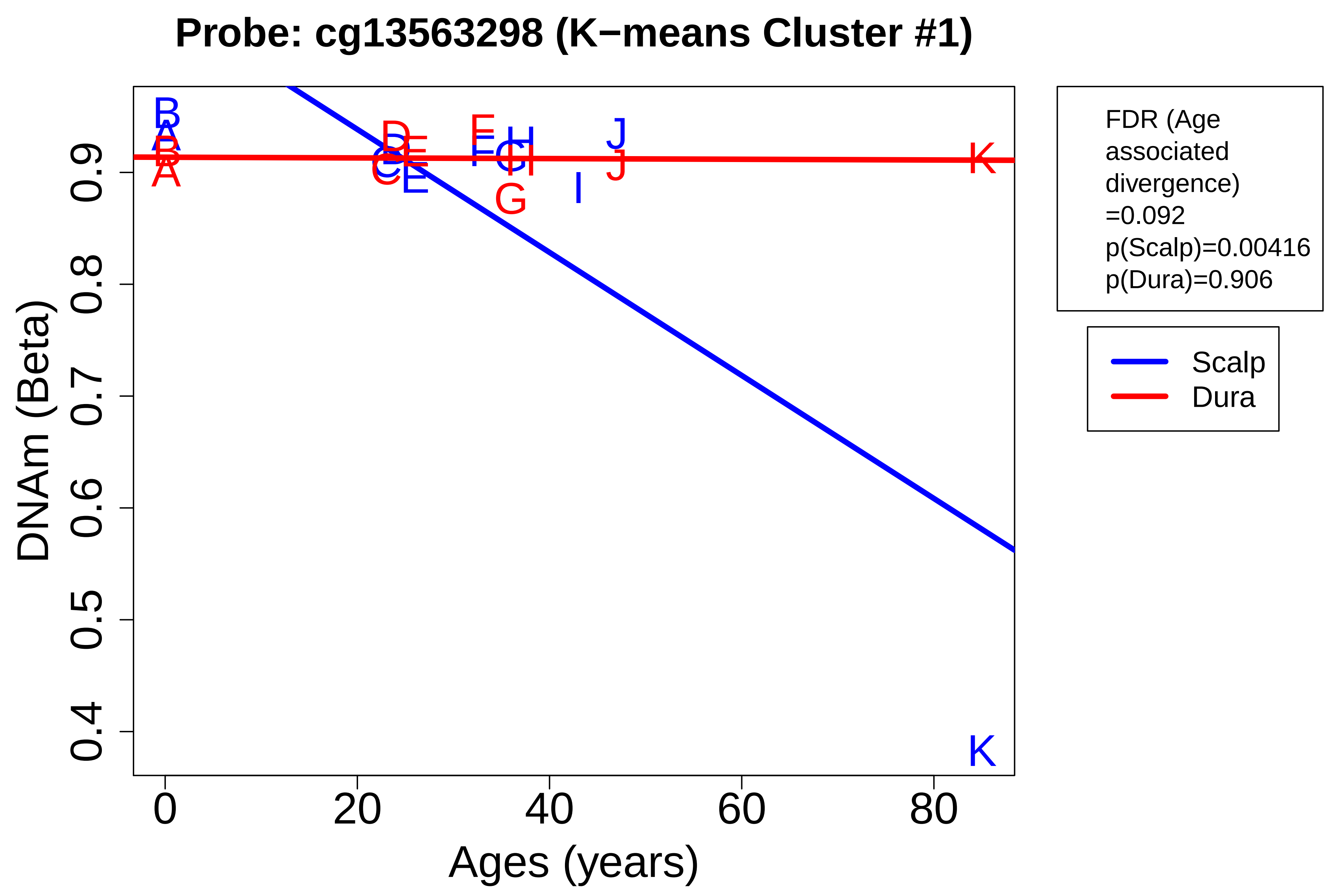

Probe: cg13563298 (K−means Cluster #1)
DNAm (Beta)
0.4
0.5
0.6
0.7
0.8
0.9
0
20
40
60
80
Ages (years)
FDR (Age associated divergence) =0.092
p(Scalp)=0.00416
p(Dura)=0.906
Scalp
Dura

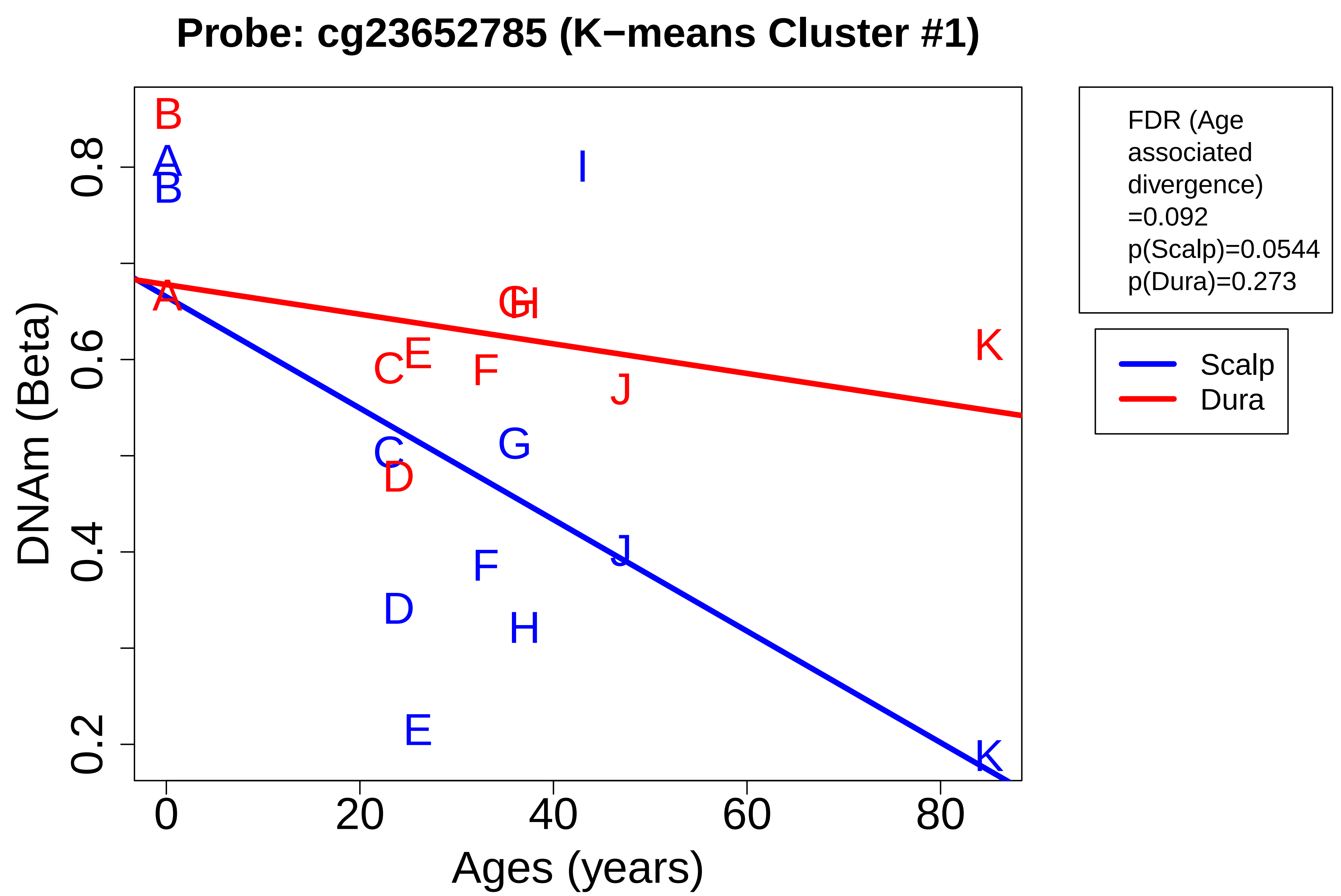
Probe: cg23652785 (K−means Cluster #1)
DNAm (Beta)
0.2
0.4
0.6
0.8
0
20
40
60
80
Ages (years)
FDR (Age associated divergence) =0.092
p(Scalp)=0.0544
p(Dura)=0.273
Scalp
Dura
A
B
C
D
E
F
G
H
I
J
K

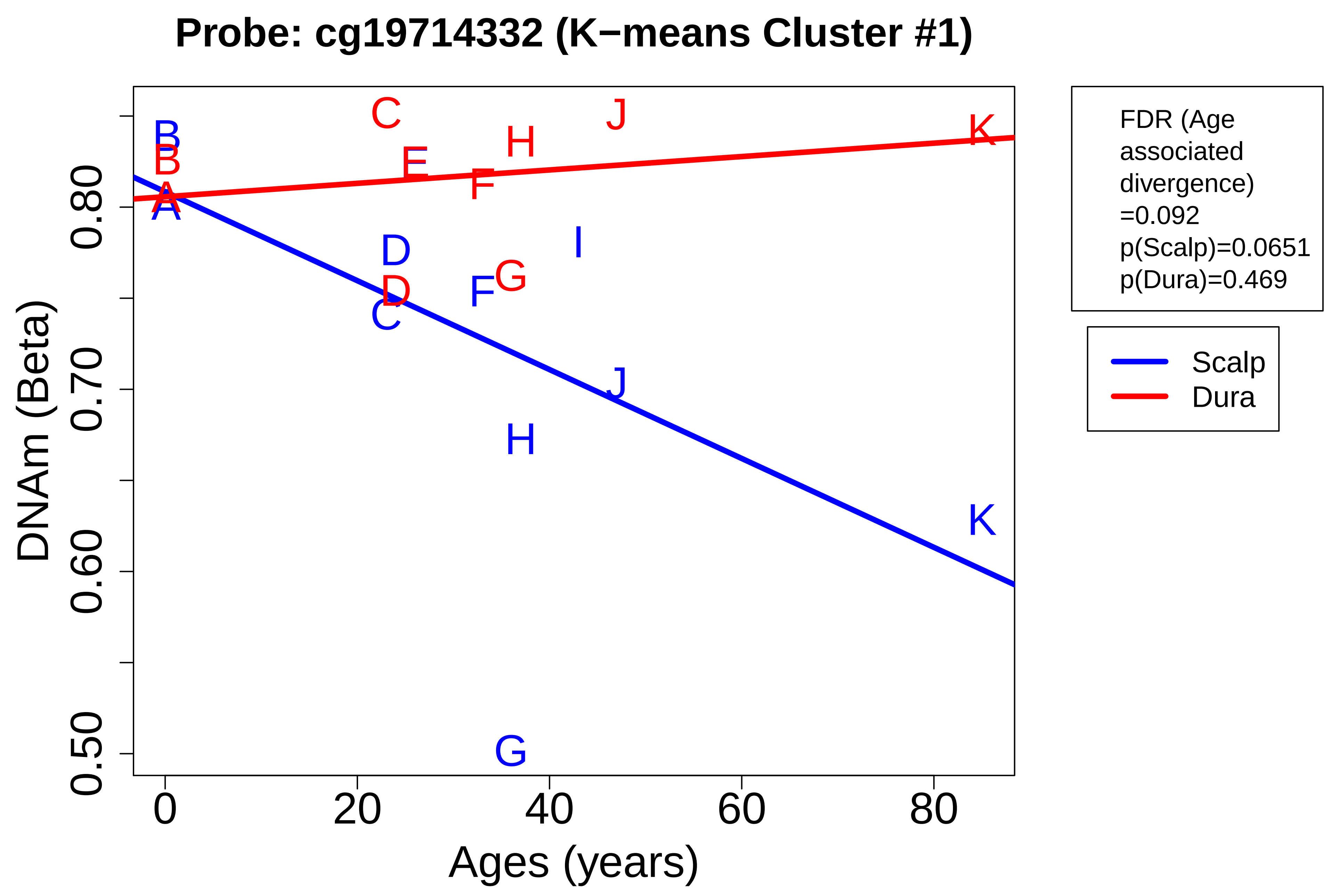

Probe: cg19714332 (K−means Cluster #1)
DNAm (Beta)
0.50
0.60
0.70
0.80
0
20
40
60
80
Ages (years)
FDR (Age associated divergence) =0.092
p(Scalp)=0.0651
p(Dura)=0.469
Scalp
Dura
A
B
C
D
E
F
G
H
I
J
K

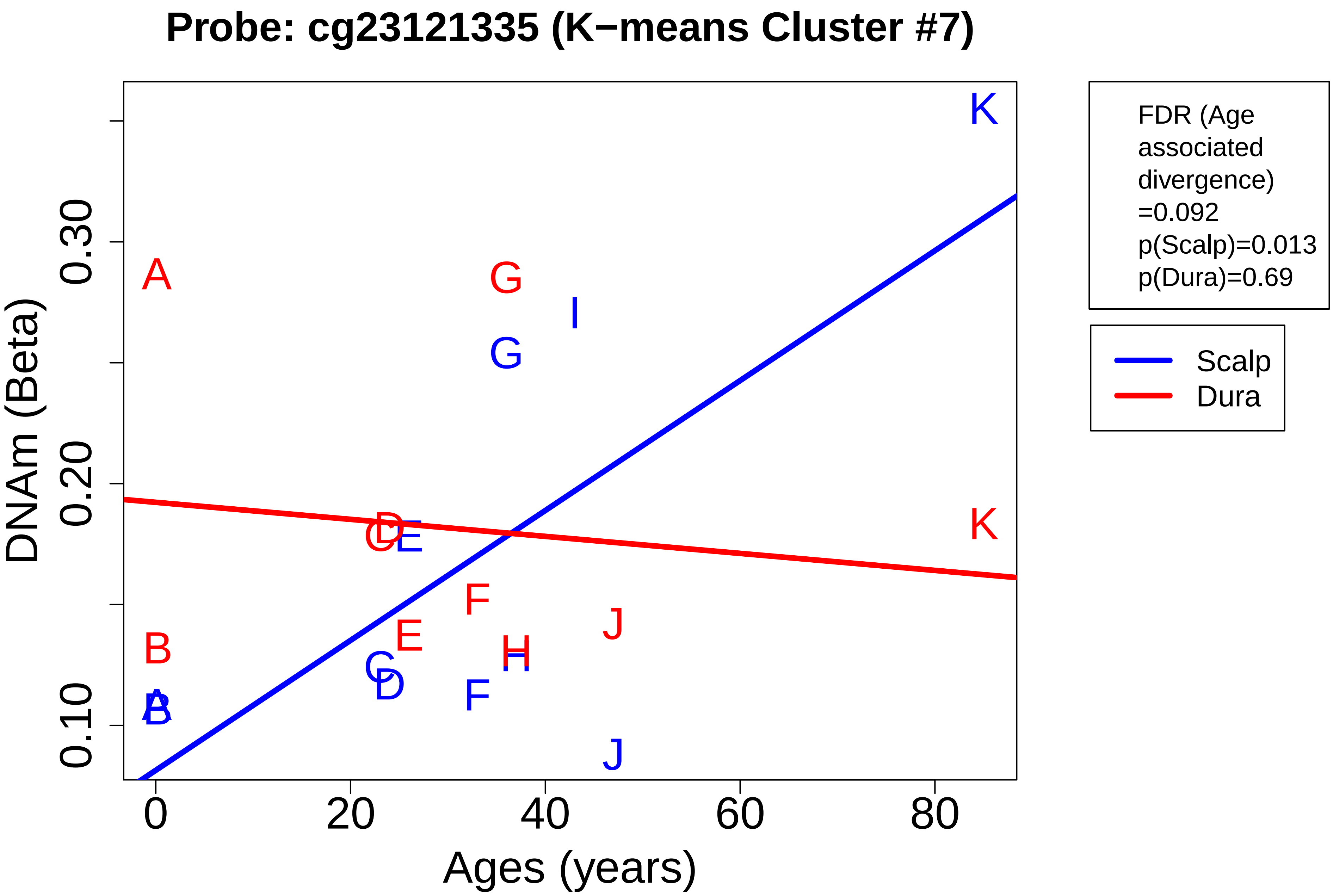

Probe: cg23121335 (K−means Cluster #7)
DNAm (Beta)
0.10
0.20
0.30
0
20
40
60
80
Ages (years)
FDR (Age associated divergence) =0.092
p(Scalp)=0.013
p(Dura)=0.69
Scalp
Dura

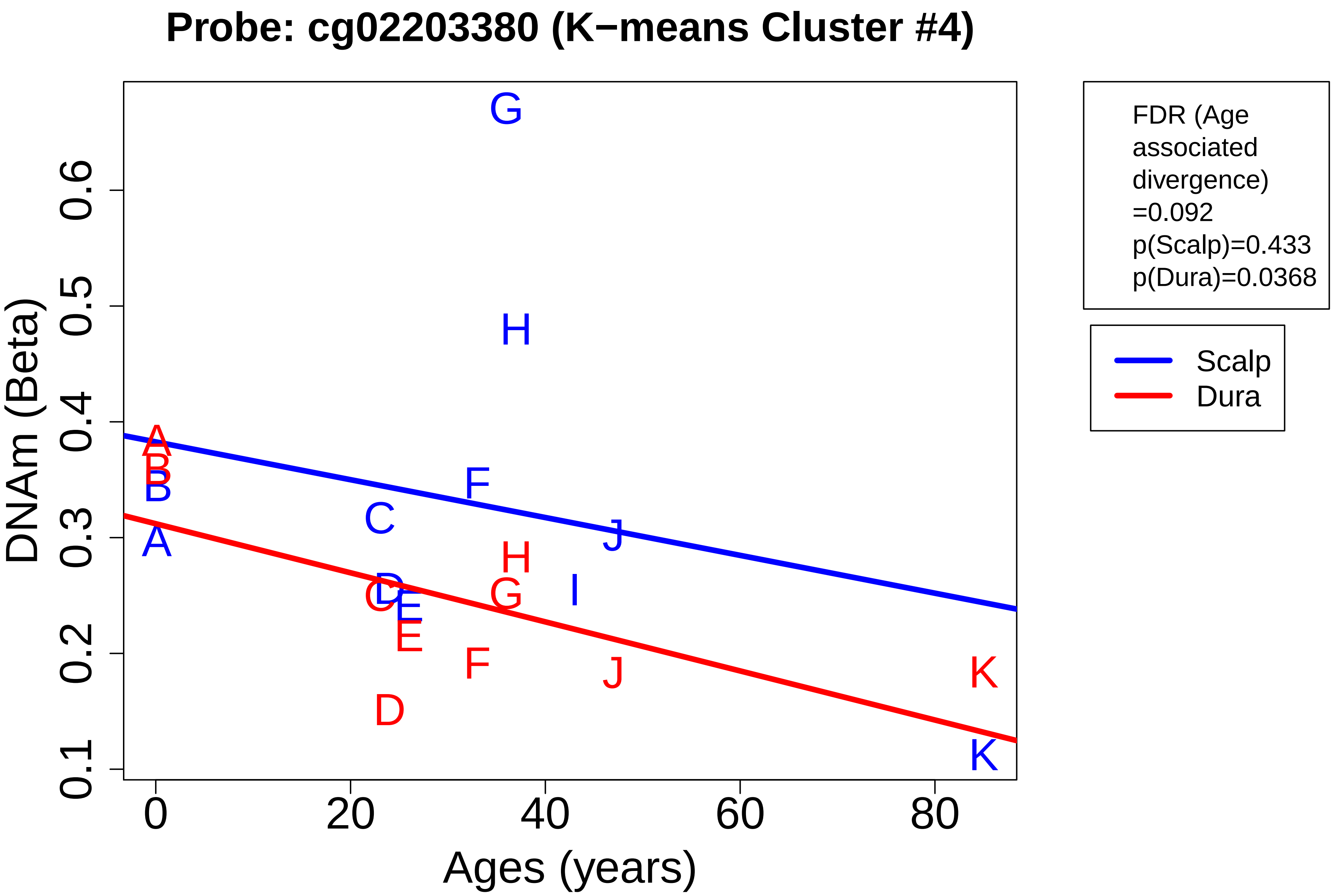

Probe: cg02203380 (K−means Cluster #4)
DNAm (Beta)
0.1
0.2
0.3
0.4
0.5
0.6
0
20
40
60
80
Ages (years)
FDR (Age associated divergence) =0.092
p(Scalp)=0.433
p(Dura)=0.0368
Scalp
Dura

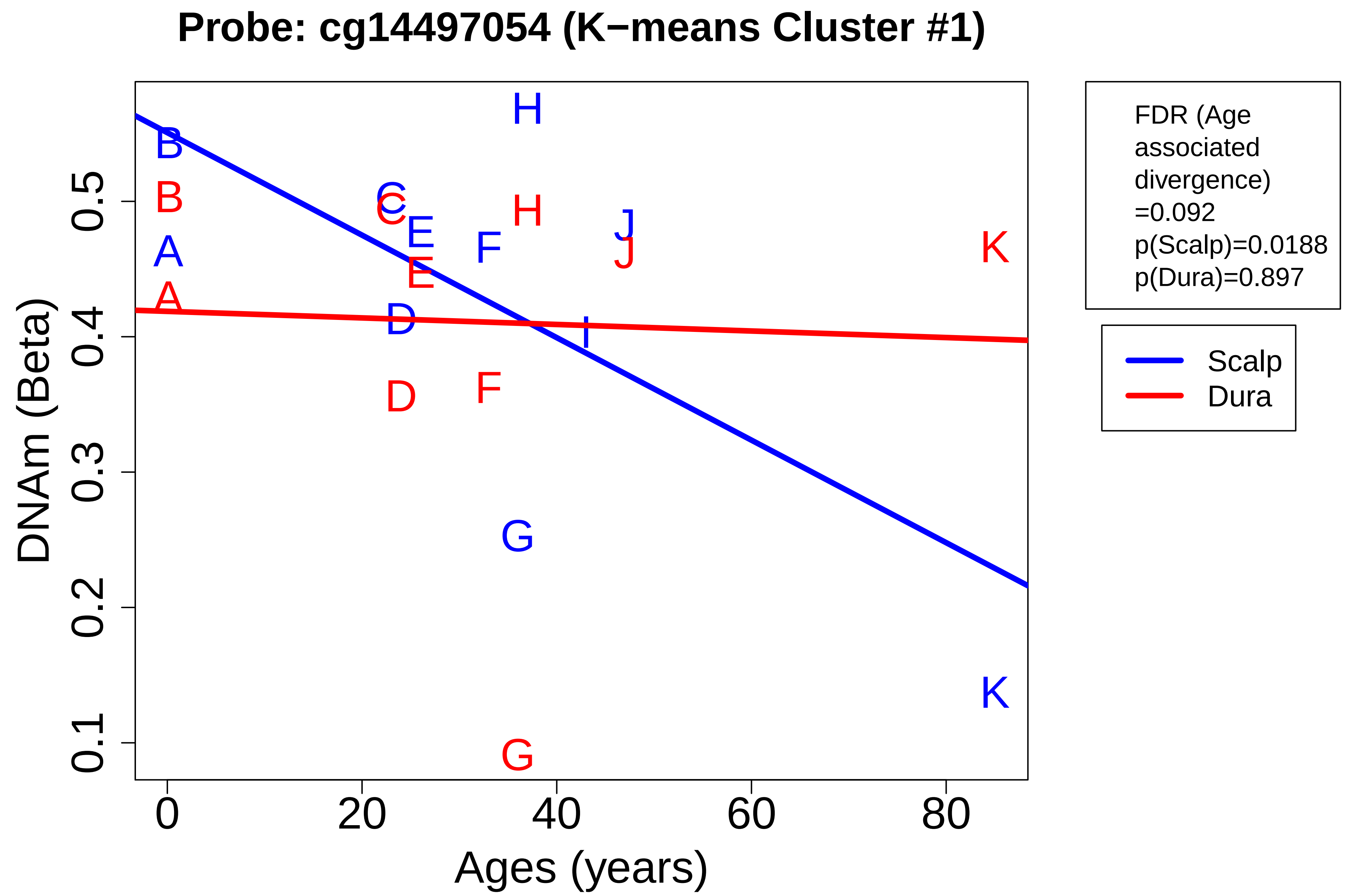

Probe: cg14497054 (K−means Cluster #1)
DNAm (Beta)
Ages (years)
0.1
0.2
0.3
0.4
0.5
0
20
40
60
80
FDR (Age associated divergence) =0.092
p(Scalp)=0.0188
p(Dura)=0.897
Scalp
Dura

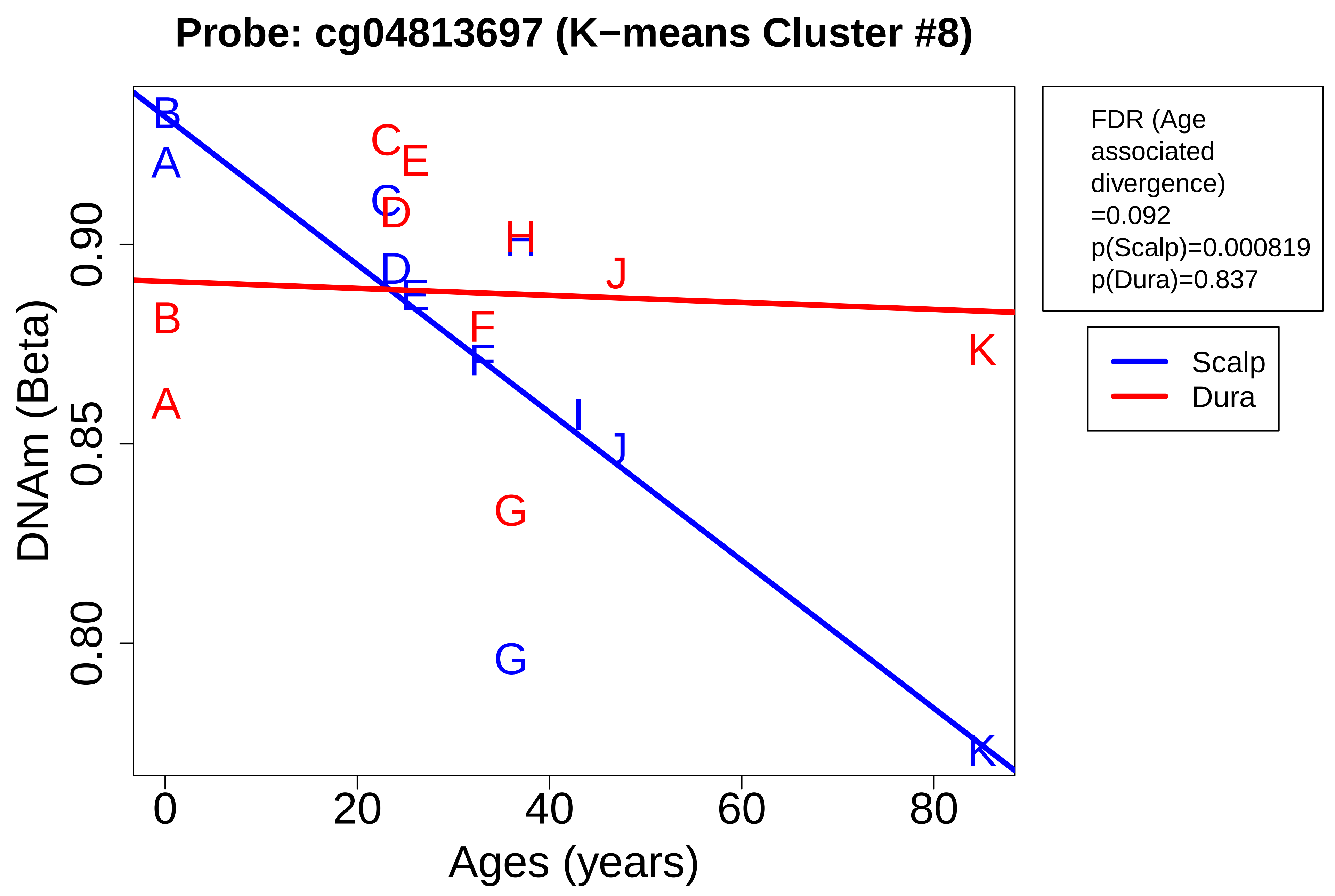

Probe: cg04813697 (K−means Cluster #8)
FDR (Age associated divergence) =0.092
p(Scalp)=0.000819
p(Dura)=0.837
Scalp
Dura
DNAm (Beta)
0.80
0.85
0.90
0
20
40
60
80
Ages (years)
A
B
C
D
E
F
G
H
I
J
K

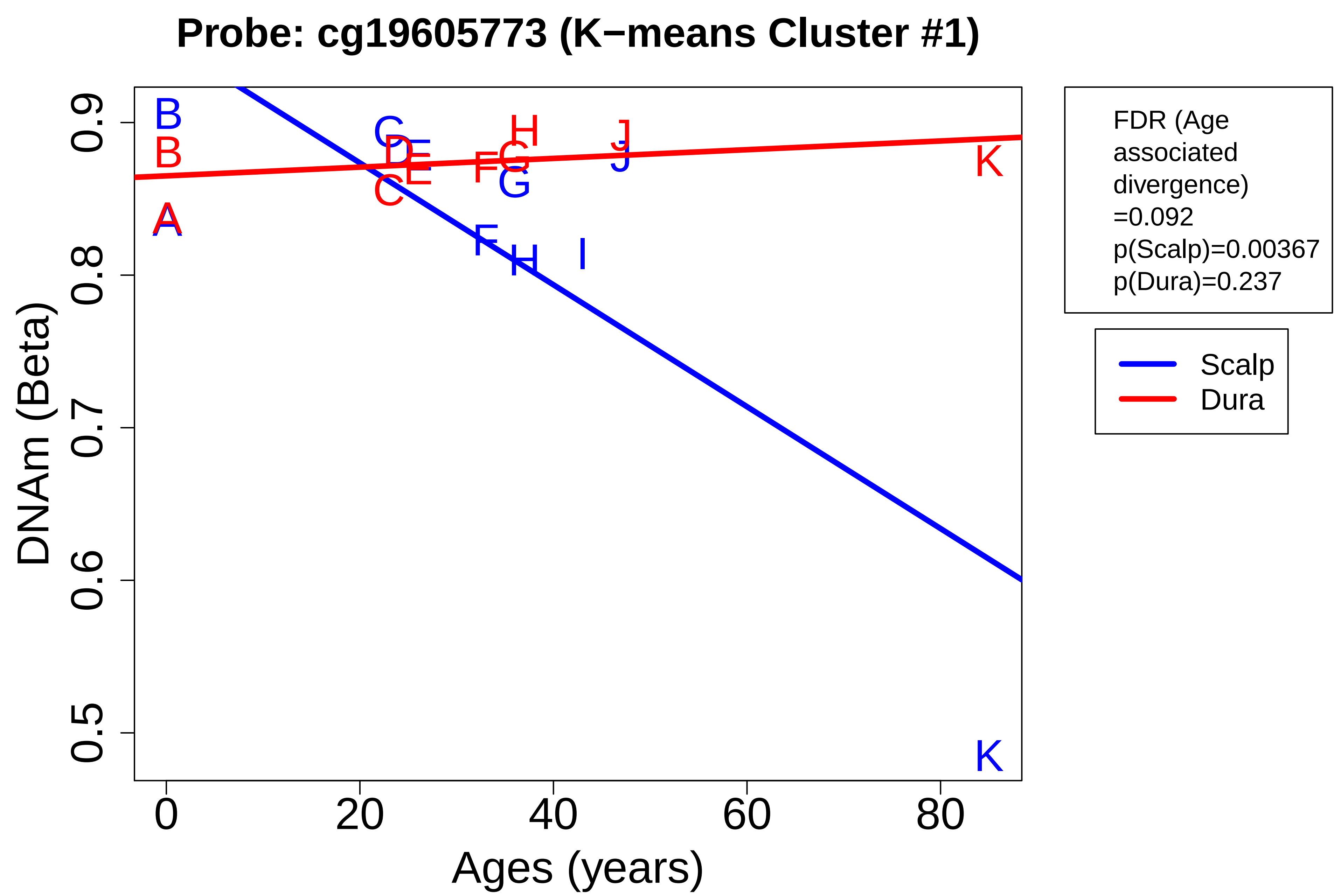

Probe: cg19605773 (K−means Cluster #1)
DNAm (Beta)
Ages (years)
0.5
0.6
0.7
0.8
0.9
0
20
40
60
80
FDR (Age associated divergence) =0.092
p(Scalp)=0.00367
p(Dura)=0.237
Scalp
Dura

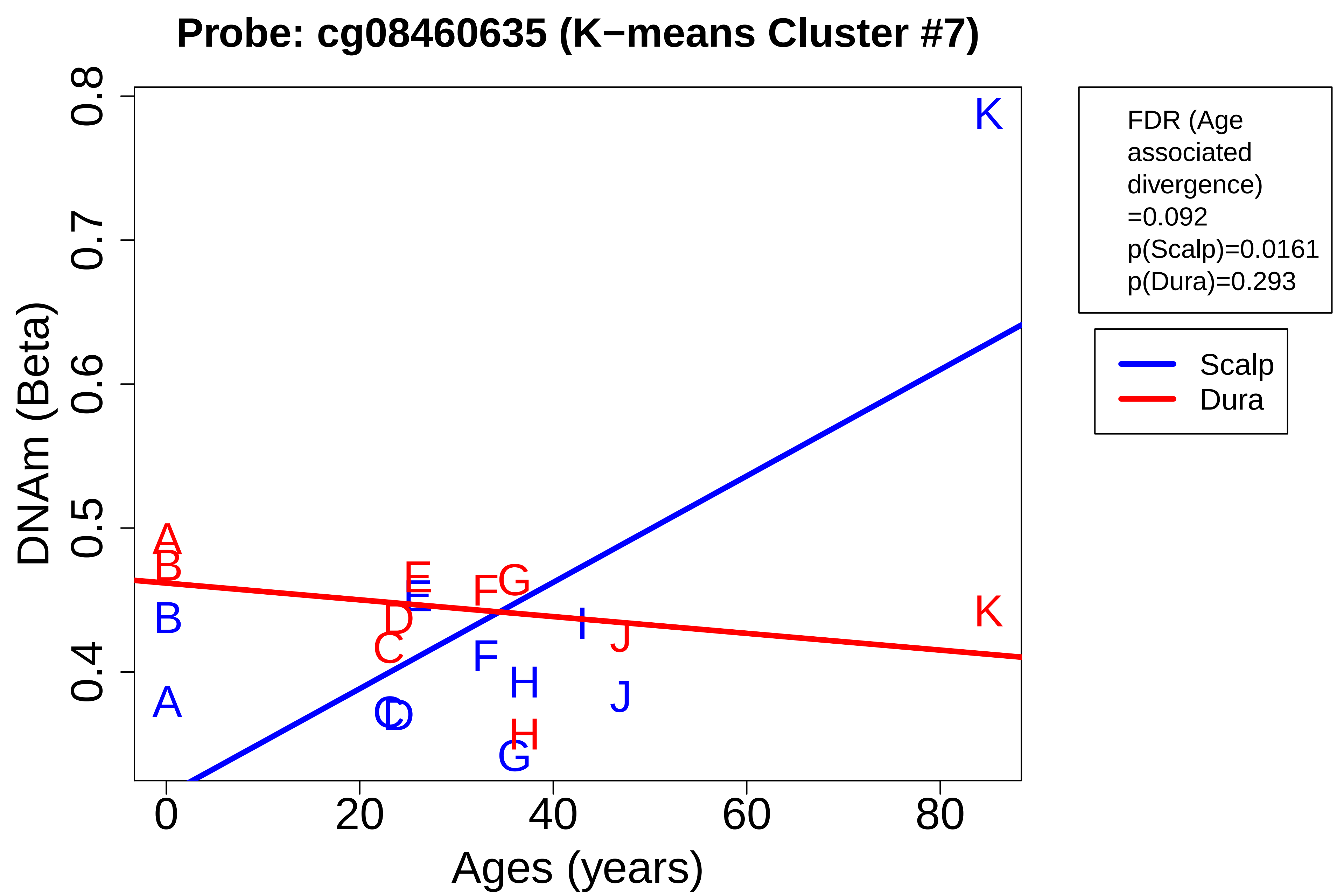

Probe: cg08460635 (K−means Cluster #7)
DNAm (Beta)
0.4
0.5
0.6
0.7
0.8
0
20
40
60
80
Ages (years)
FDR (Age associated divergence) =0.092
p(Scalp)=0.0161
p(Dura)=0.293
Scalp
Dura
A
B
C
D
E
F
G
H
I
J
K

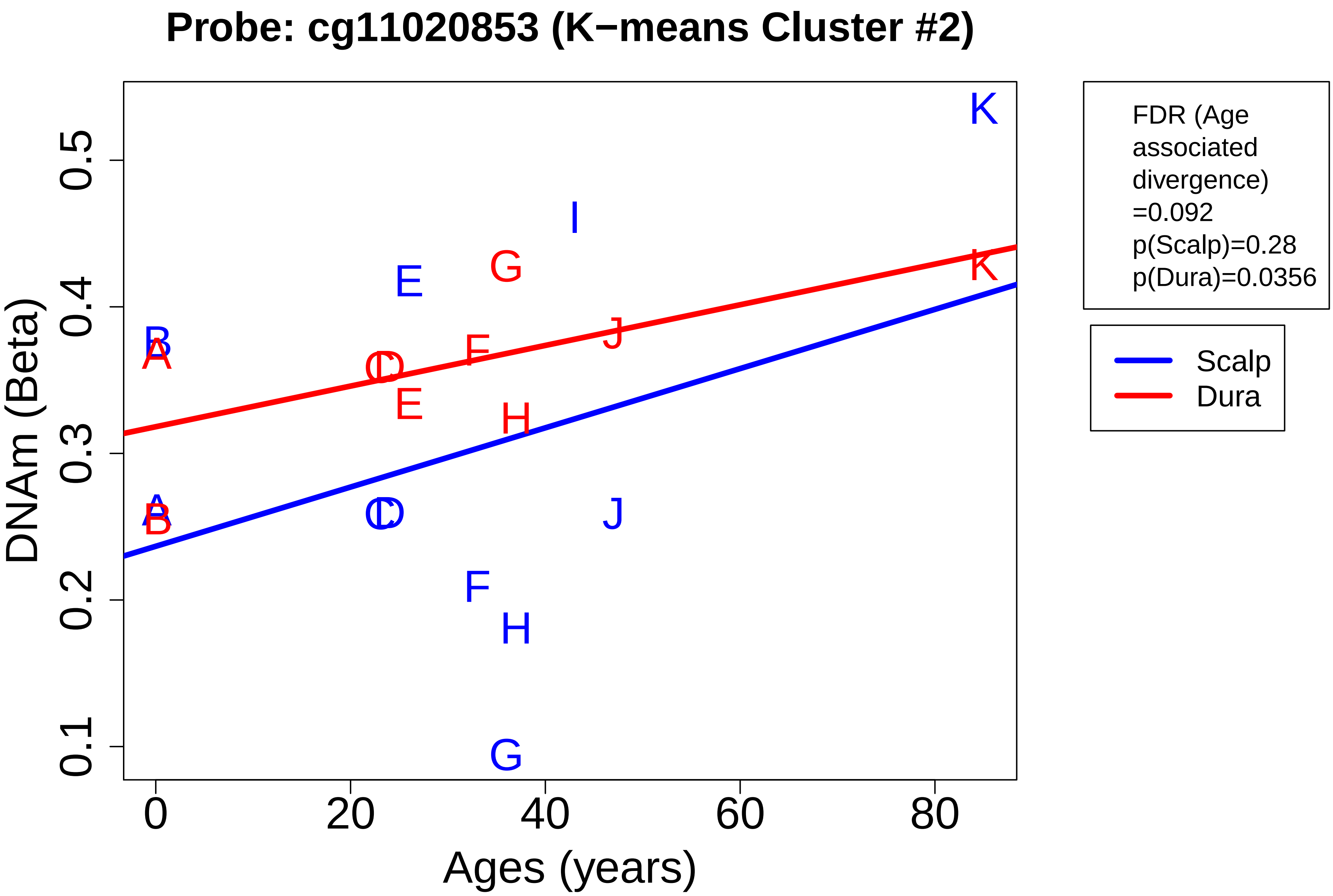

Probe: cg11020853 (K−means Cluster #2)
DNAm (Beta)
0.1
0.2
0.3
0.4
0.5
0
20
40
60
80
Ages (years)
FDR (Age associated divergence) =0.092
p(Scalp)=0.28
p(Dura)=0.0356
Scalp
Dura

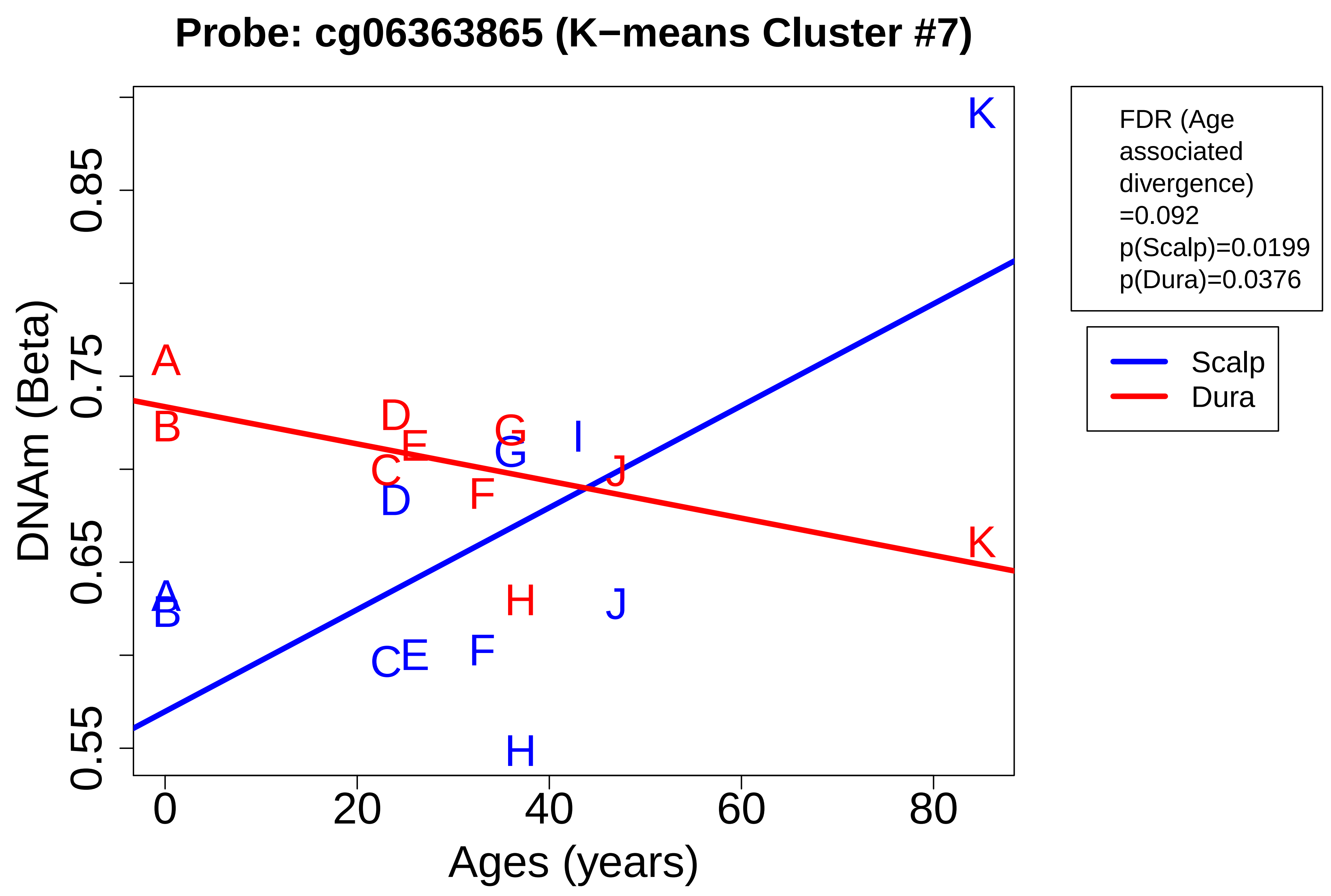

Probe: cg06363865 (K−means Cluster #7)
FDR (Age associated divergence) =0.092
p(Scalp)=0.0199
p(Dura)=0.0376
Scalp
Dura
DNAm (Beta)
0.55
0.65
0.75
0.85
Ages (years)
0
20
40
60
80
A
B
C
D
E
F
G
H
I
J
K

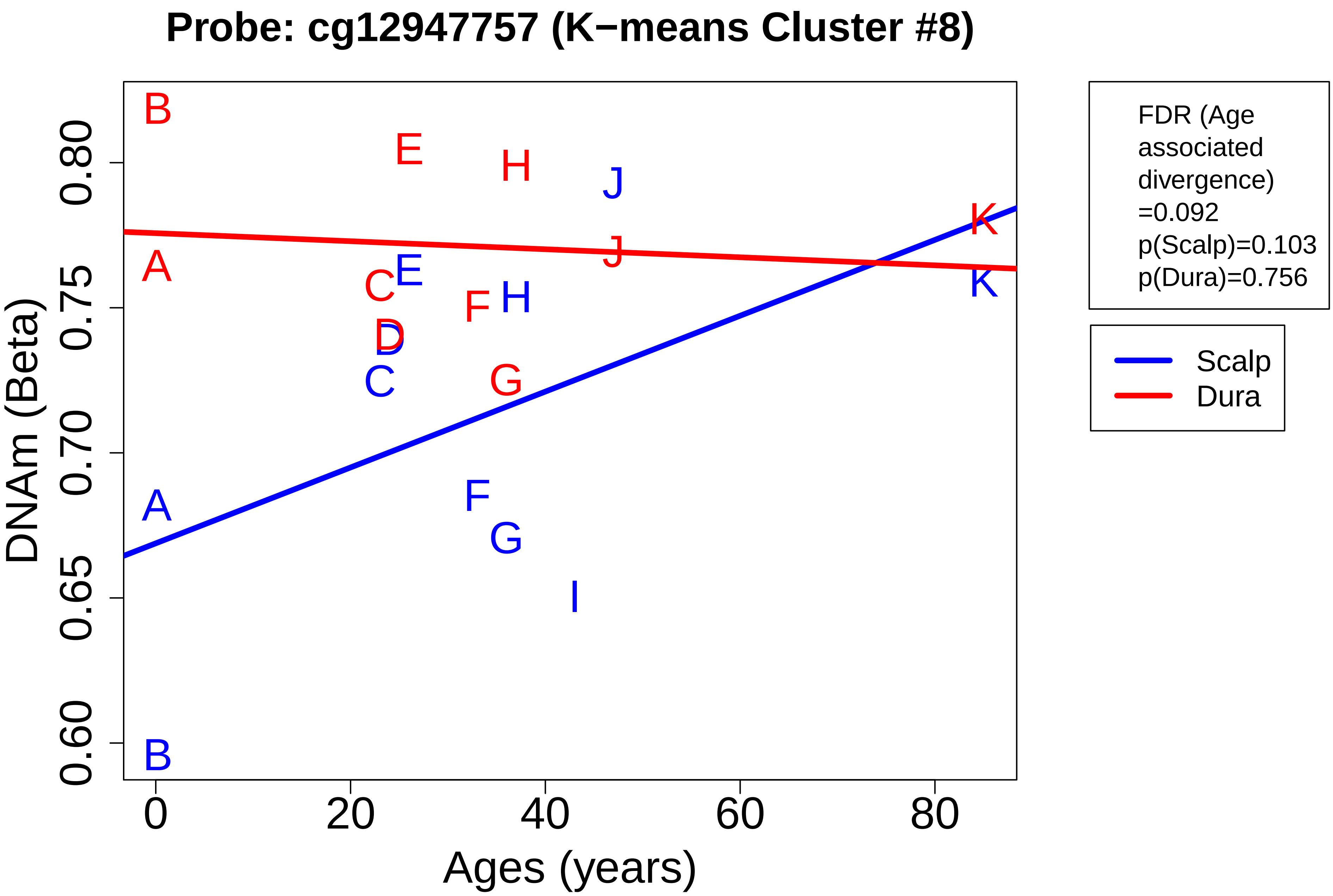

Probe: cg12947757 (K−means Cluster #8)
DNAm (Beta)
0.60
0.65
0.70
0.75
0.80
0
20
40
60
80
Ages (years)
FDR (Age associated divergence) =0.092
p(Scalp)=0.103
p(Dura)=0.756
Scalp
Dura

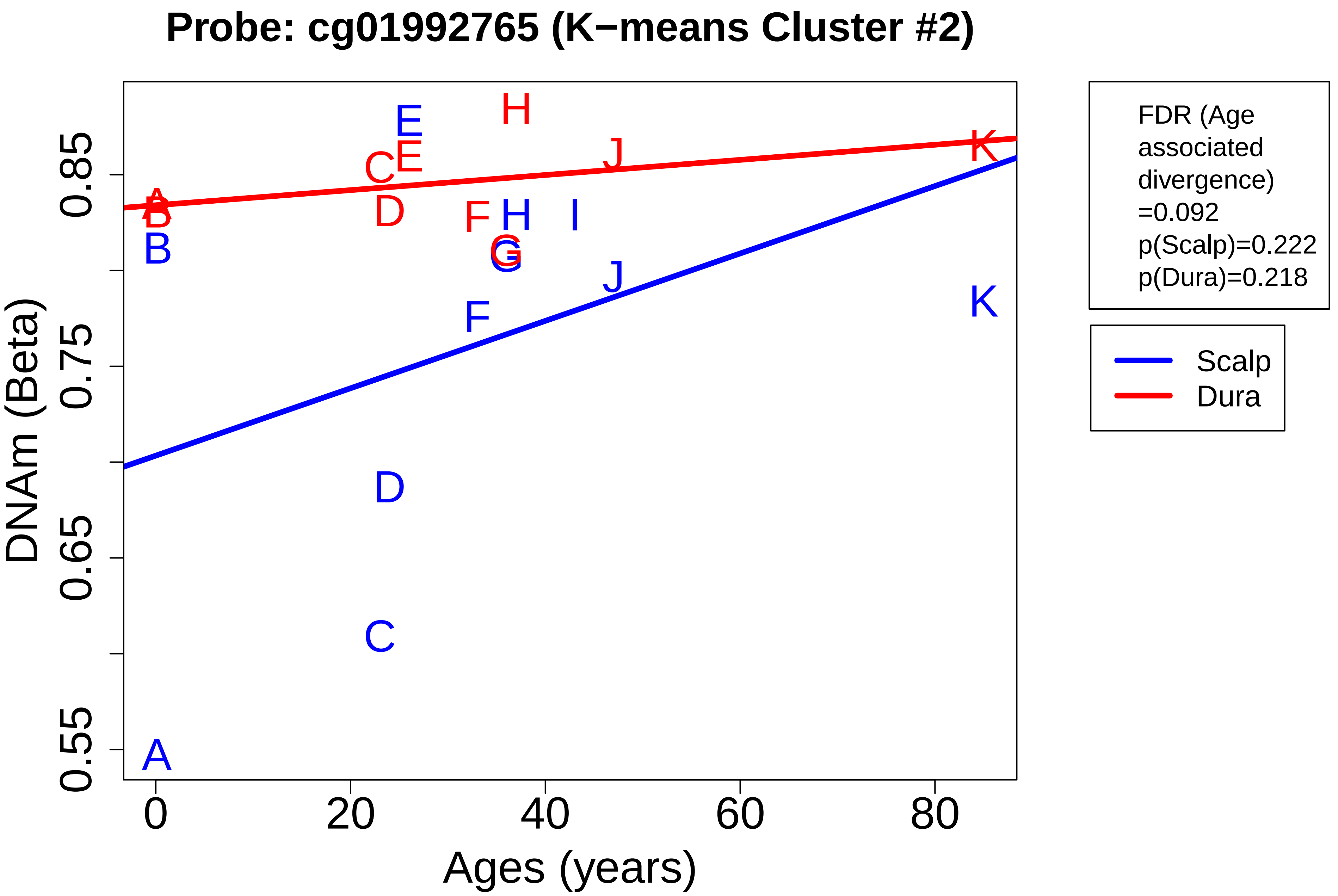

Probe: cg01992765 (K−means Cluster #2)
FDR (Age associated divergence) =0.092
p(Scalp)=0.222
p(Dura)=0.218
Scalp
Dura
DNAm (Beta)
0.55
0.65
0.75
0.85
0
20
40
60
80
Ages (years)

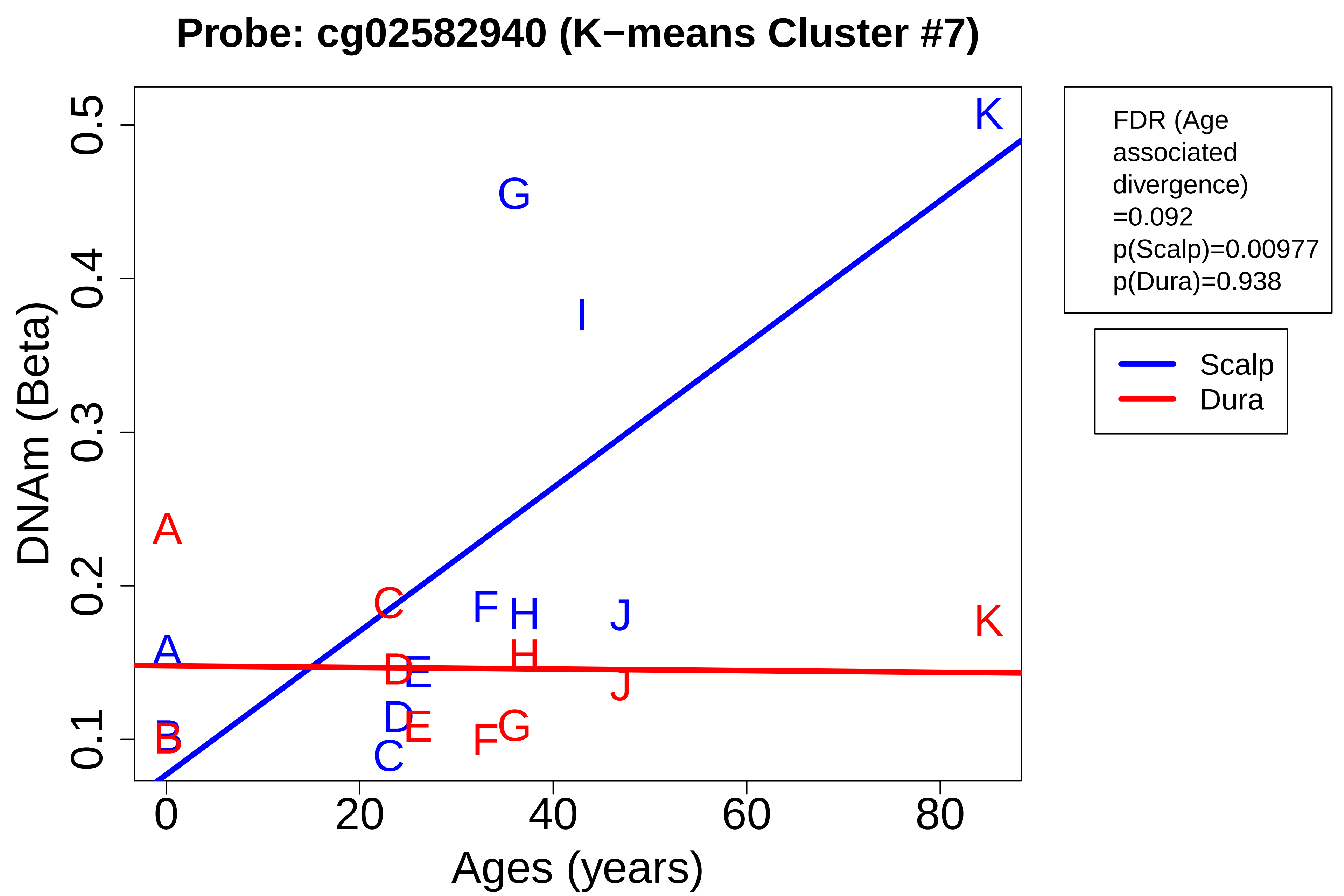

Probe: cg02582940 (K−means Cluster #7)
DNAm (Beta)
0.1
0.2
0.3
0.4
0.5
0
20
40
60
80
Ages (years)
A
B
C
D
E
F
G
H
I
J
K
FDR (Age associated divergence) =0.092
p(Scalp)=0.00977
p(Dura)=0.938
Scalp
Dura

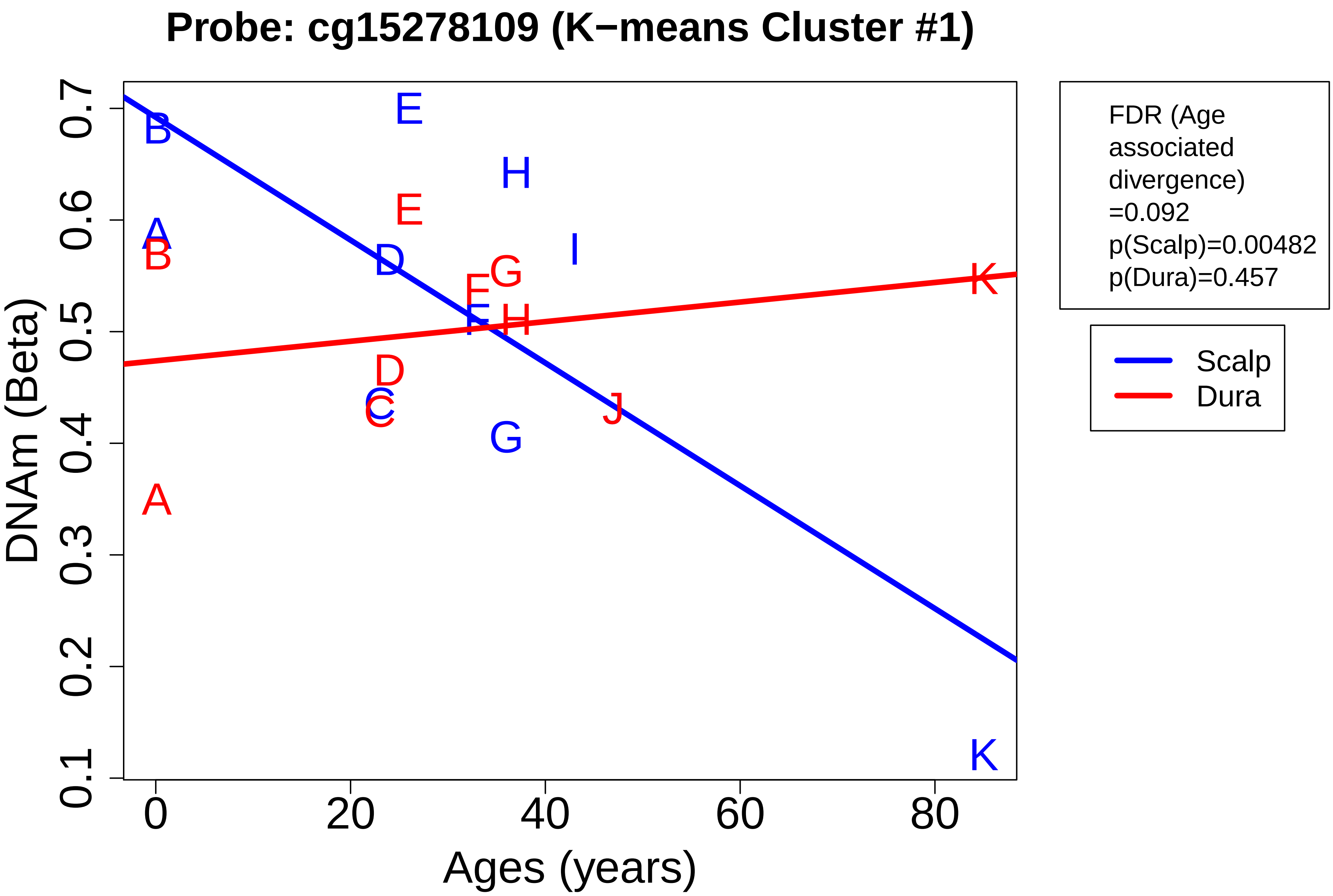

Probe: cg15278109 (K−means Cluster #1)
DNAm (Beta)
0.1
0.2
0.3
0.4
0.5
0.6
0.7
Ages (years)
0
20
40
60
80
FDR (Age associated divergence) =0.092
p(Scalp)=0.00482
p(Dura)=0.457
Scalp
Dura

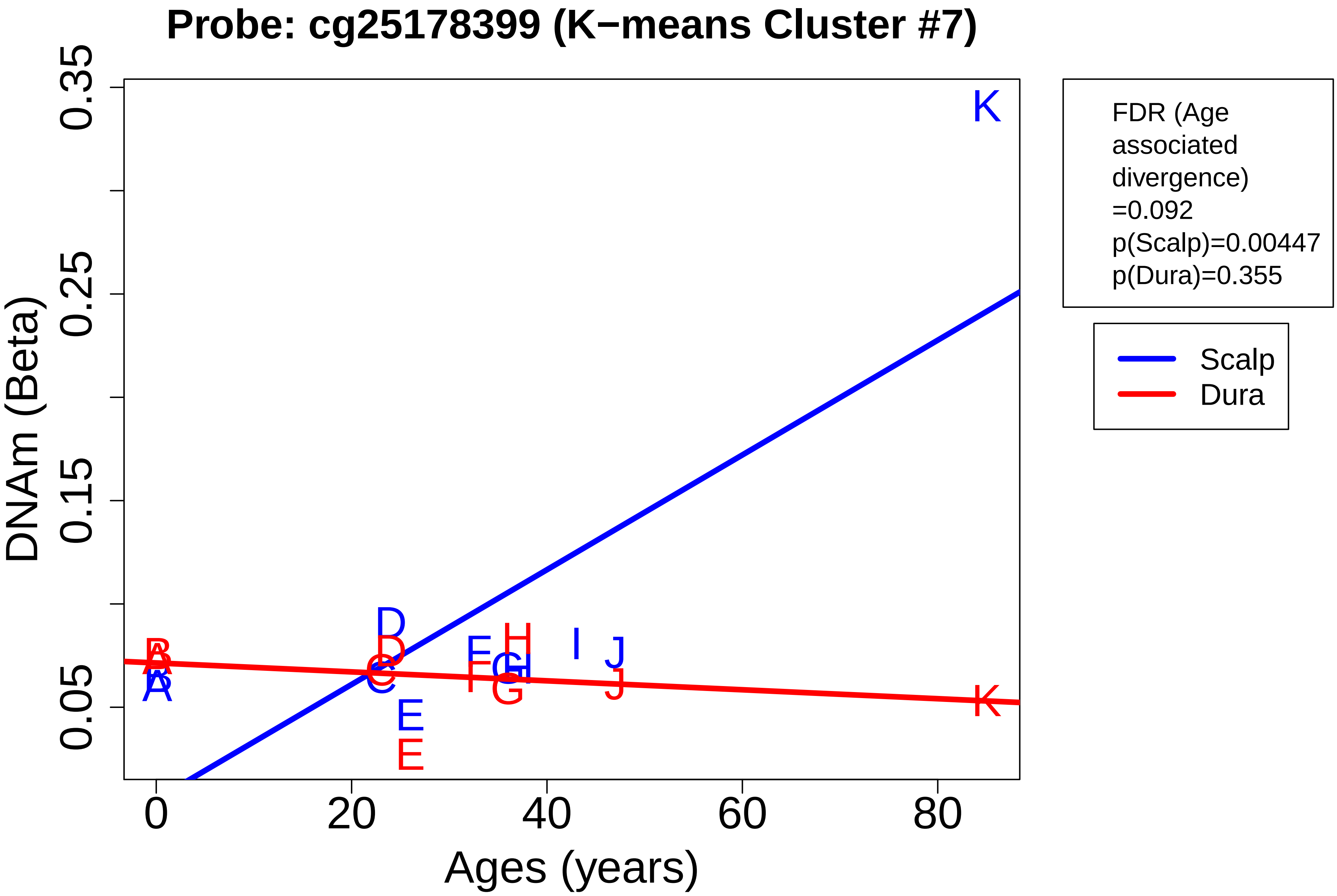

Probe: cg25178399 (K−means Cluster #7)
DNAm (Beta)
0.05
0.15
0.25
0.35
Ages (years)
0
20
40
60
80
FDR (Age associated divergence) =0.092
p(Scalp)=0.00447
p(Dura)=0.355
Scalp
Dura

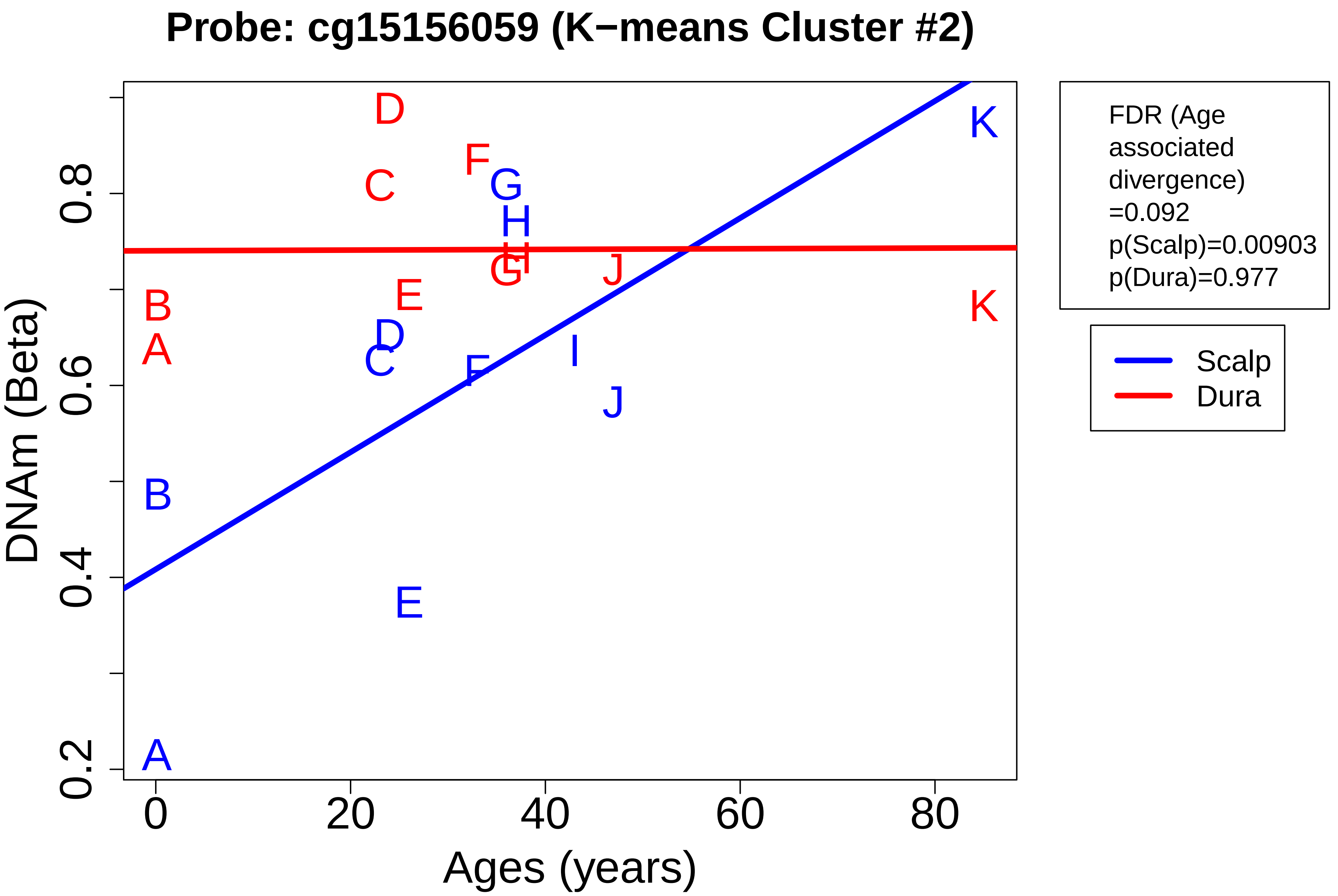

Probe: cg15156059 (K−means Cluster #2)
DNAm (Beta)
0.2
0.4
0.6
0.8
0
20
40
60
80
Ages (years)
FDR (Age associated divergence) =0.092
p(Scalp)=0.00903
p(Dura)=0.977
Scalp
Dura
A
B
C
D
E
F
G
H
I
J
K

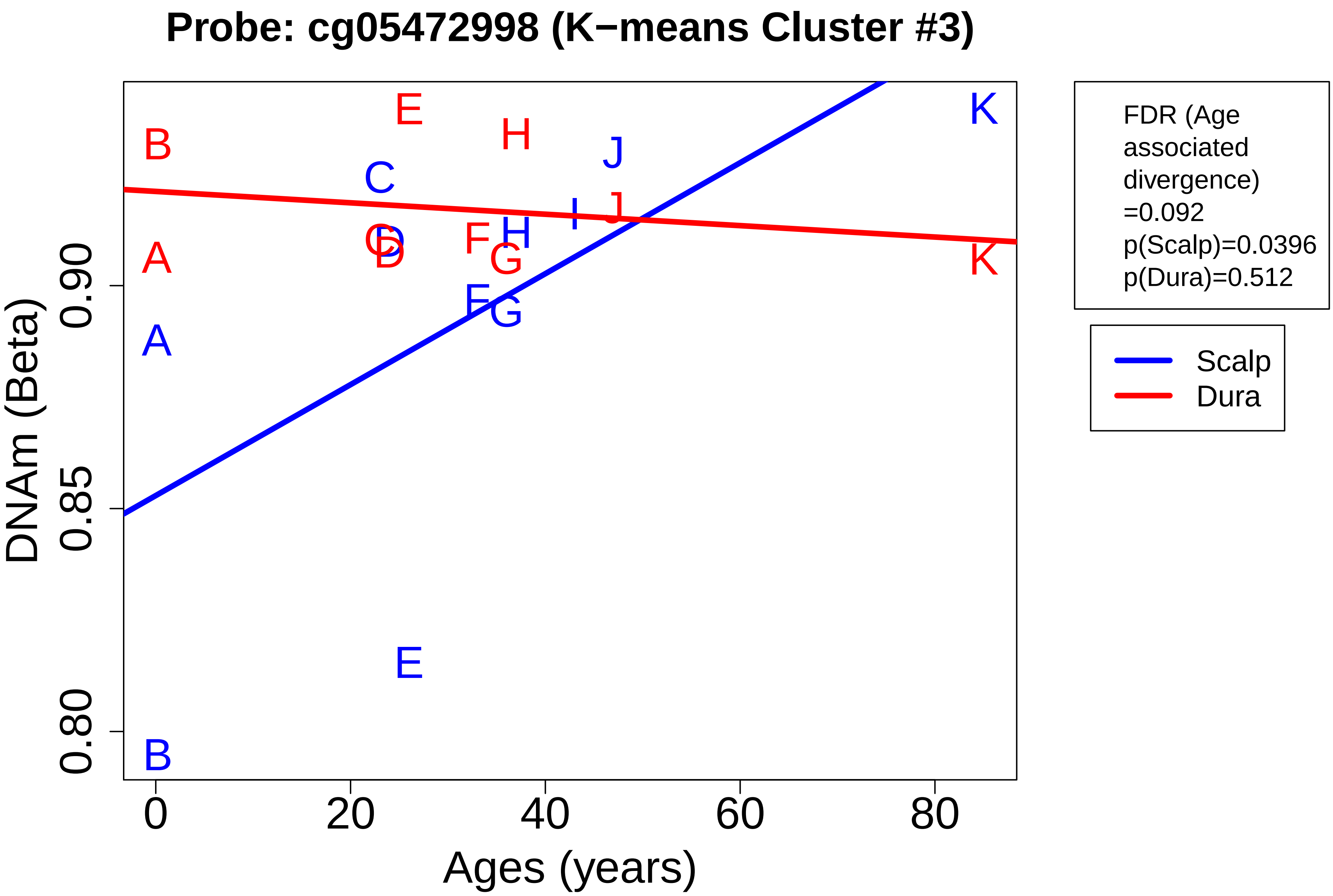

Probe: cg05472998 (K−means Cluster #3)
DNAm (Beta)
0.80
0.85
0.90
0
20
40
60
80
Ages (years)
A
B
C
D
E
F
G
H
I
J
K
FDR (Age associated divergence) =0.092
p(Scalp)=0.0396
p(Dura)=0.512
Scalp
Dura

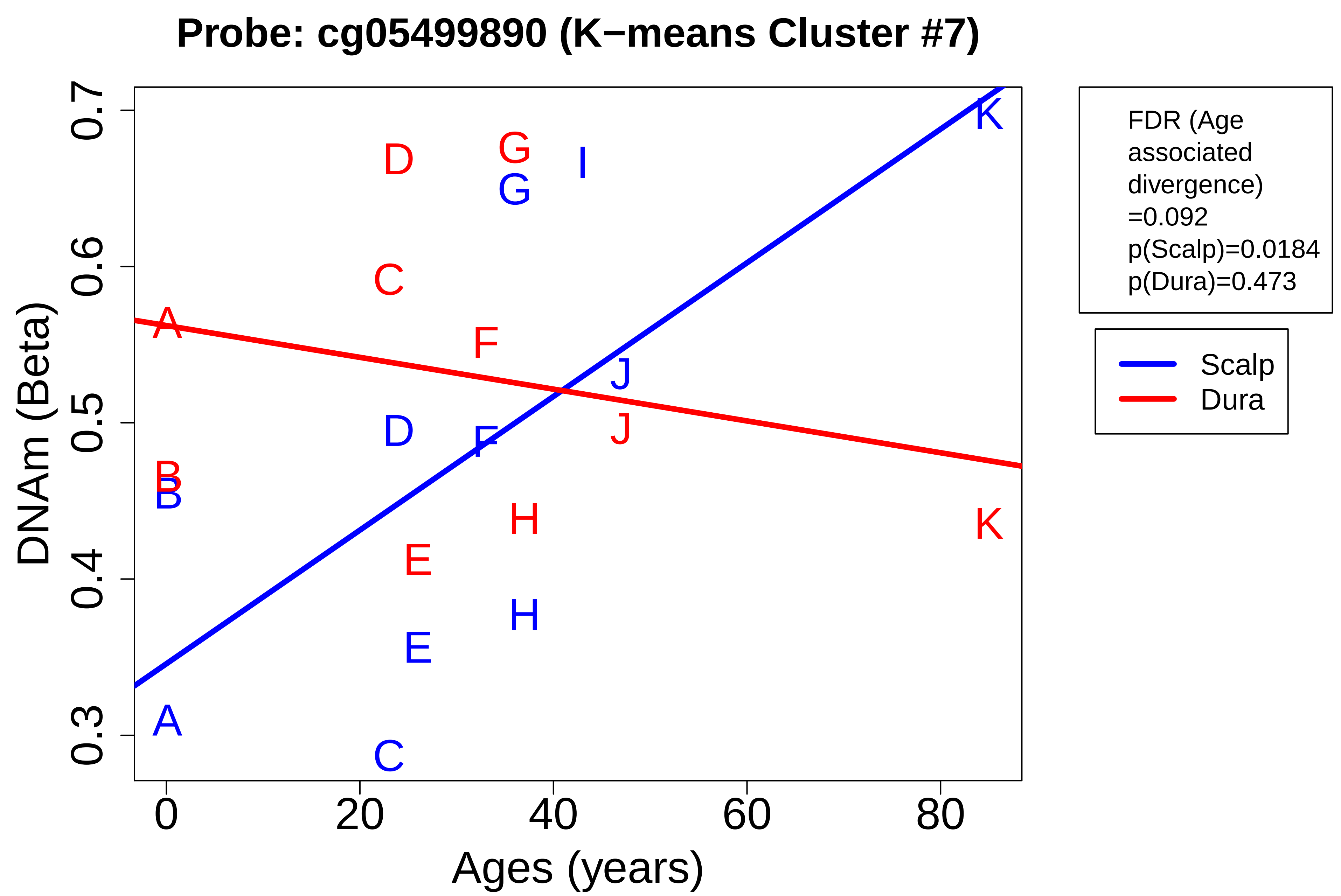
Probe: cg05499890 (K−means Cluster #7)
DNAm (Beta)
0.3
0.4
0.5
0.6
0.7
0
20
40
60
80
Ages (years)
A
B
C
D
E
F
G
H
I
J
K
FDR (Age associated divergence) =0.092
p(Scalp)=0.0184
p(Dura)=0.473
Scalp
Dura

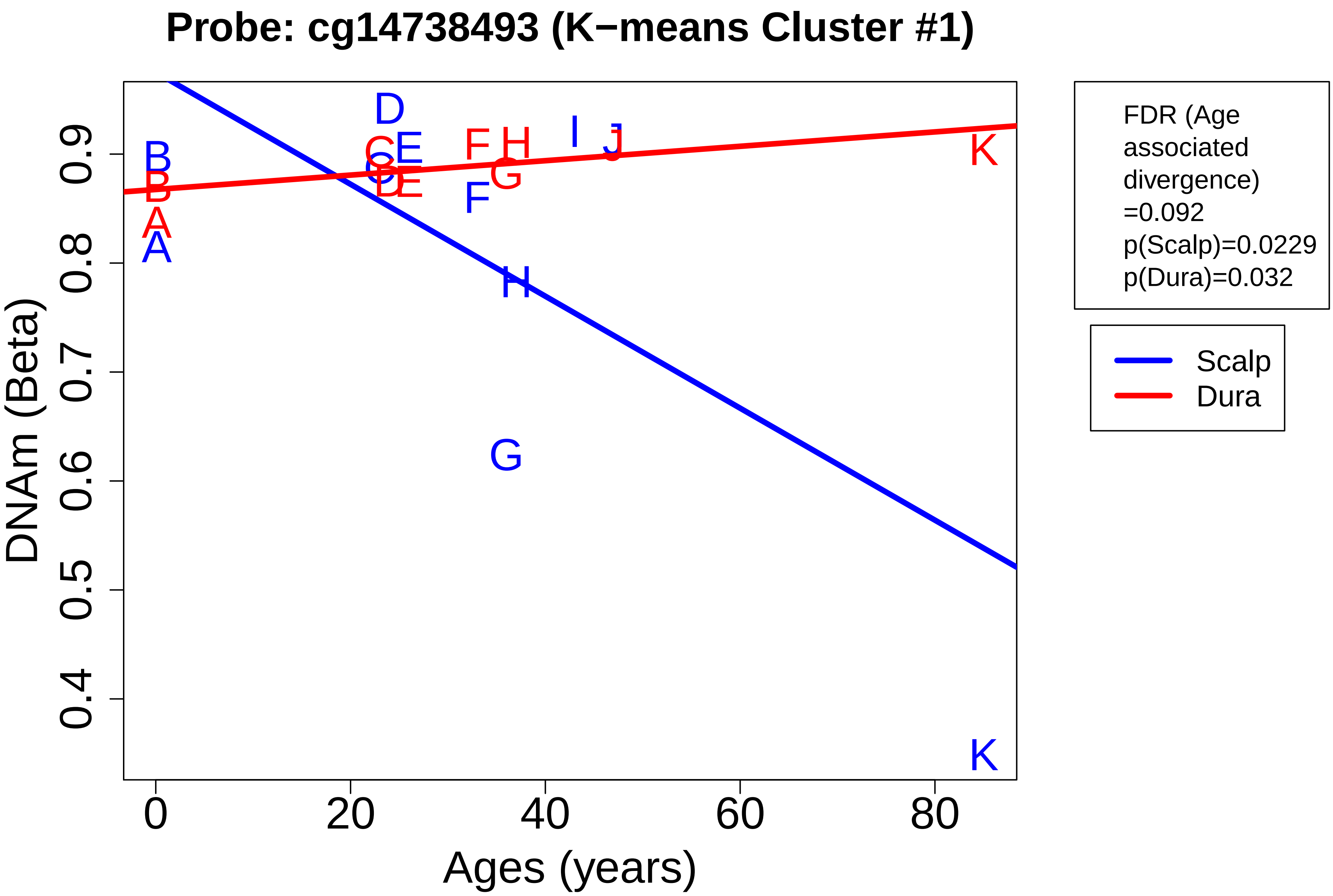

Probe: cg14738493 (K−means Cluster #1)
DNAm (Beta)
0.4
0.5
0.6
0.7
0.8
0.9
0
20
40
60
80
Ages (years)
A
B
C
D
E
F
G
H
I
J
K
FDR (Age associated divergence) =0.092
p(Scalp)=0.0229
p(Dura)=0.032
Scalp
Dura

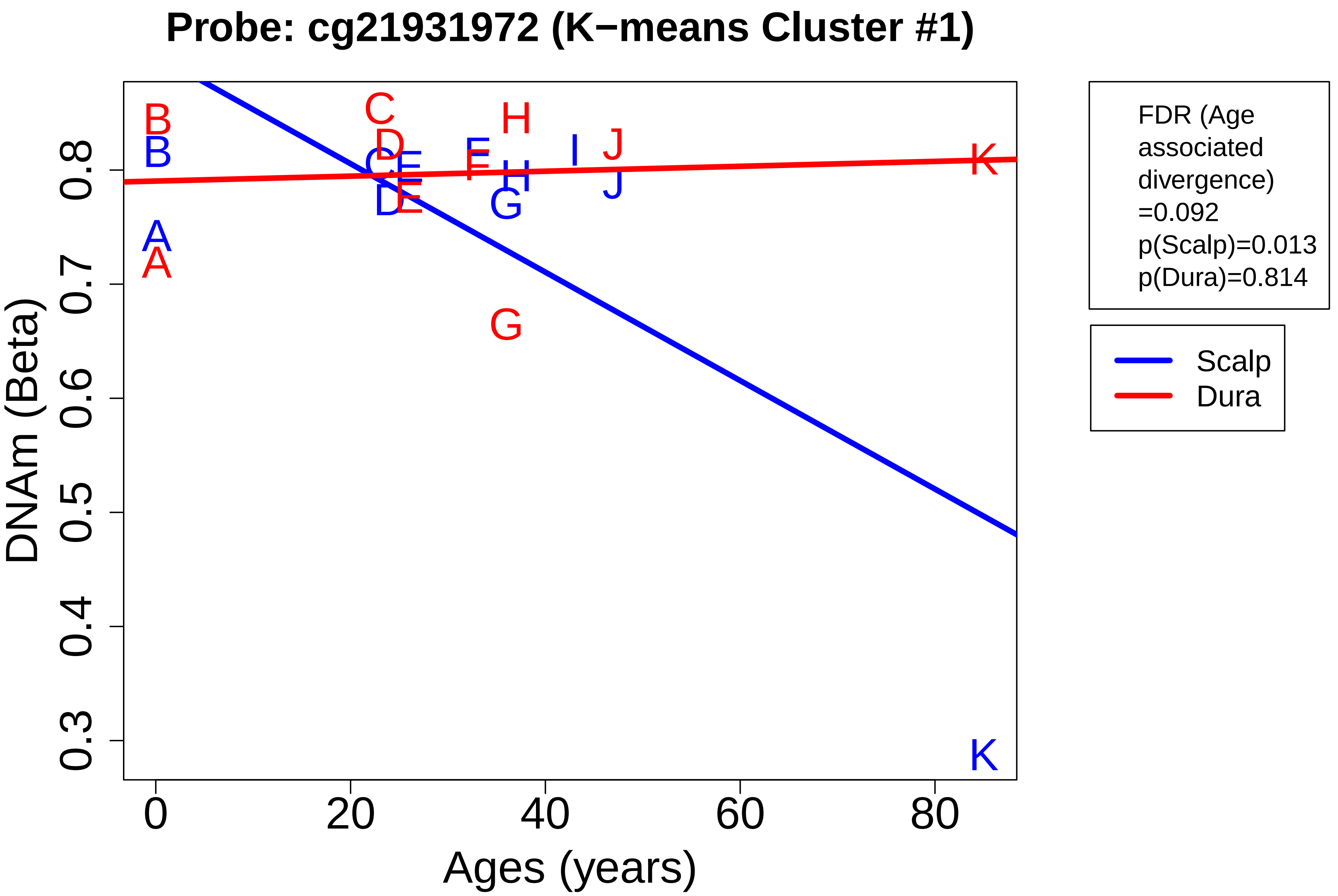
Probe: cg21931972 (K−means Cluster #1)
DNAm (Beta)
0.3
0.4
0.5
0.6
0.7
0.8
0
20
40
60
80
Ages (years)
FDR (Age associated divergence) =0.092
p(Scalp)=0.013
p(Dura)=0.814
Scalp
Dura

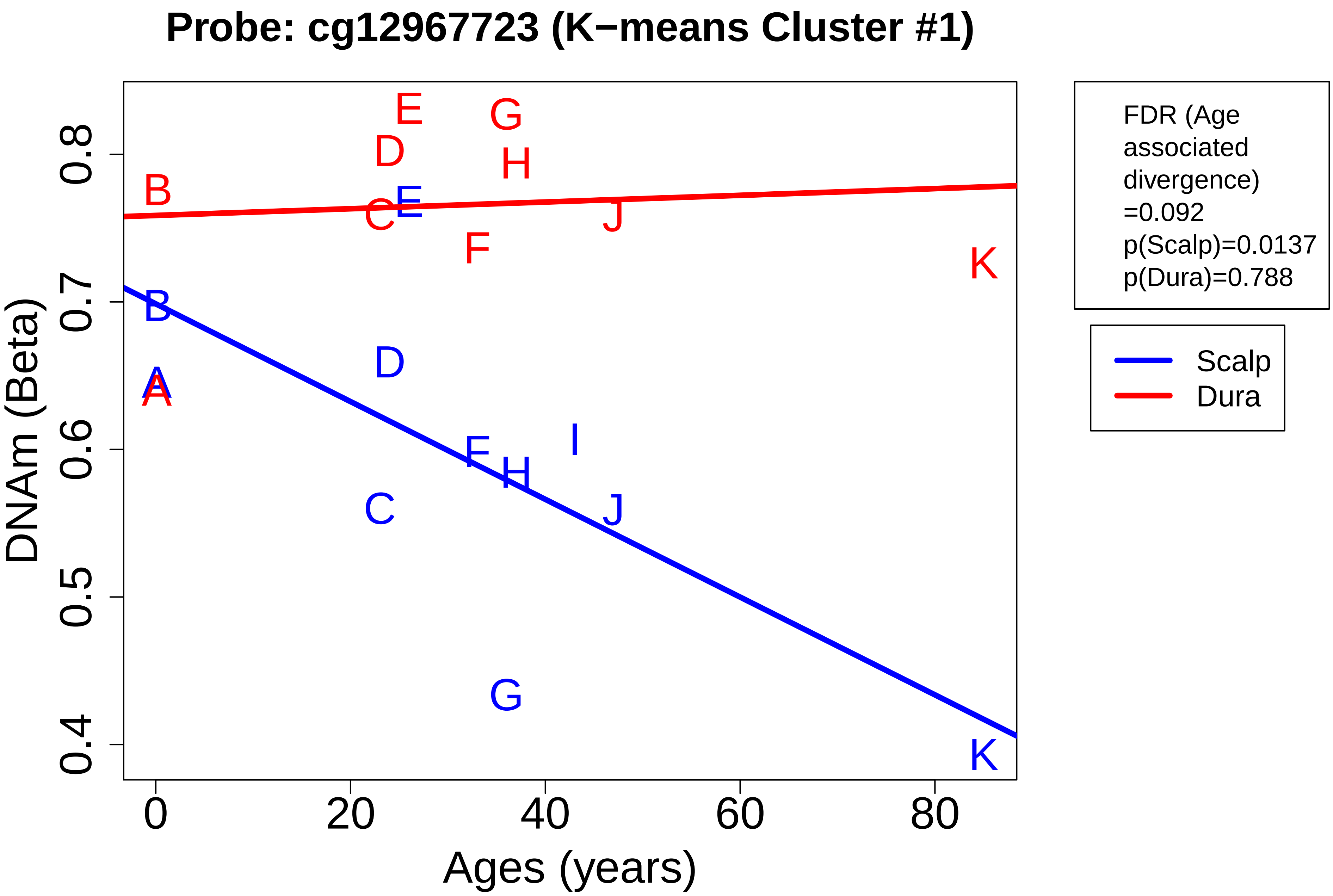

Probe: cg12967723 (K−means Cluster #1)
DNAm (Beta)
0.4
0.5
0.6
0.7
0.8
0
20
40
60
80
Ages (years)
A
B
C
D
E
F
G
H
I
J
K
FDR (Age associated divergence) =0.092
p(Scalp)=0.0137
p(Dura)=0.788
Scalp
Dura

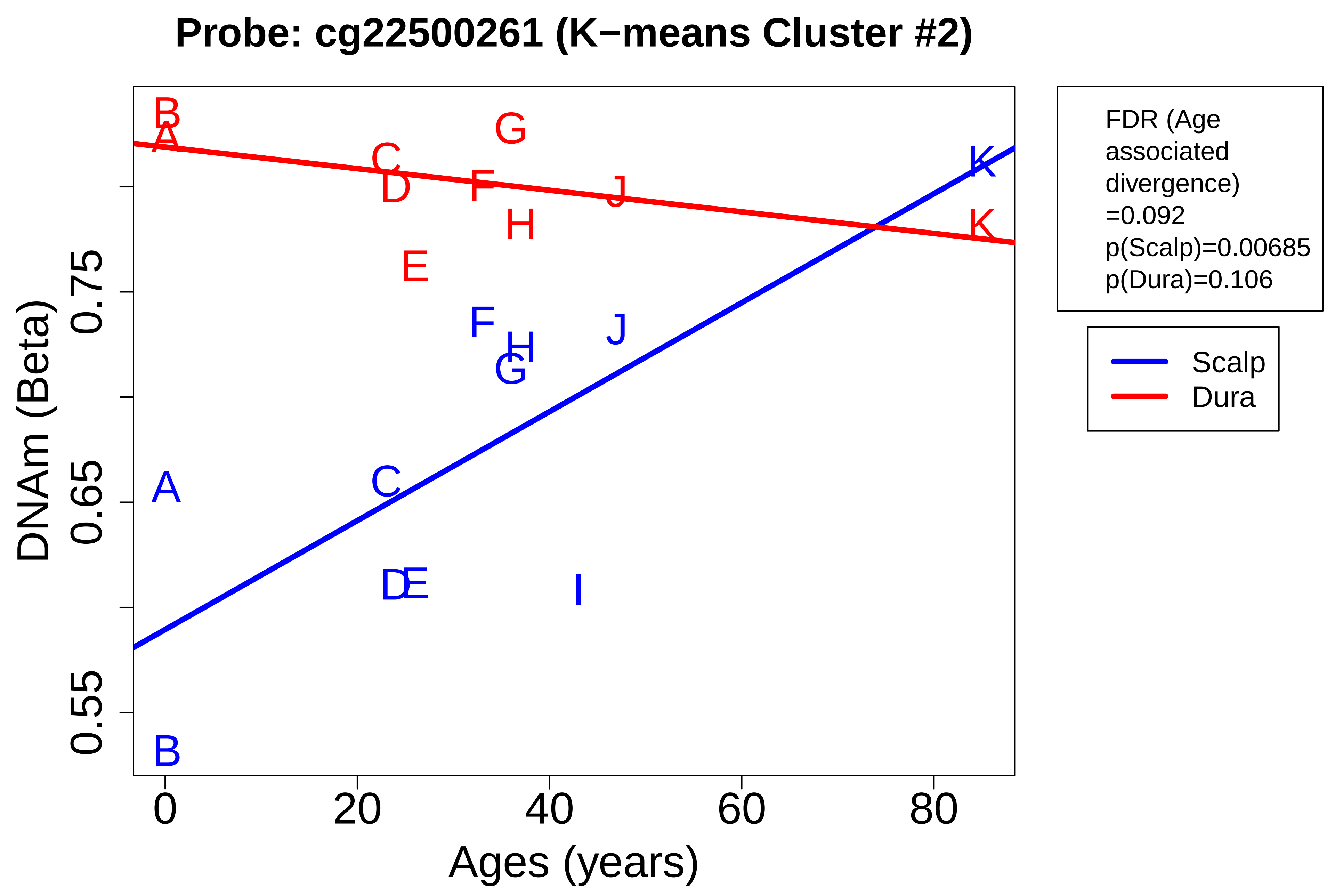

Probe: cg22500261 (K−means Cluster #2)
DNAm (Beta)
0.55
0.65
0.75
Ages (years)
0
20
40
60
80
FDR (Age associated divergence) =0.092
p(Scalp)=0.00685
p(Dura)=0.106
Scalp
Dura
B
A
G
C
D
F
J
H
E
K
K
F
H
G
J
A
C
D
E
I
B

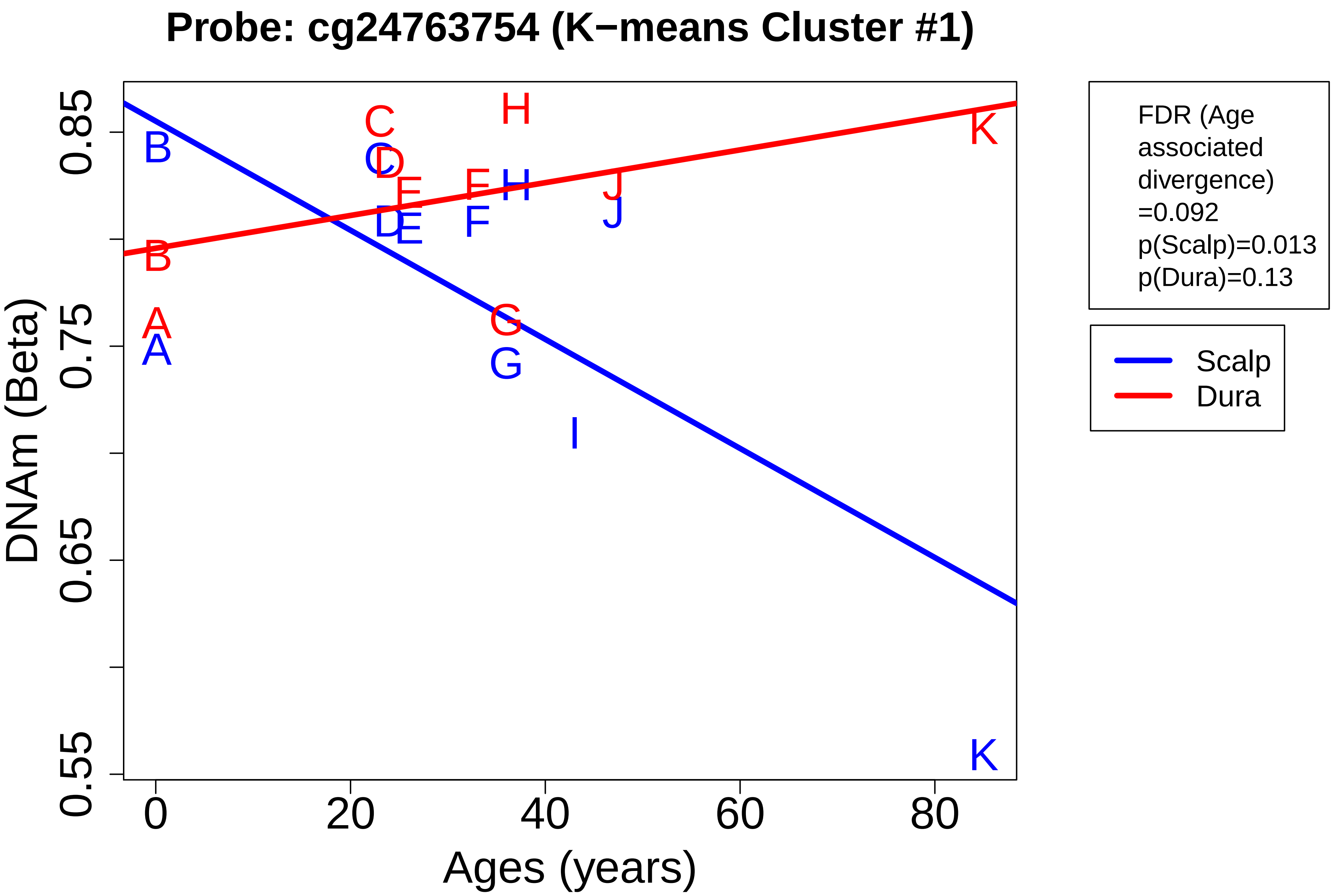

Probe: cg24763754 (K−means Cluster #1)
DNAm (Beta)
0.55
0.65
0.75
0.85
0
20
40
60
80
Ages (years)
A
B
C
D
E
F
G
H
I
J
K
FDR (Age associated divergence) =0.092
p(Scalp)=0.013
p(Dura)=0.13
Scalp
Dura

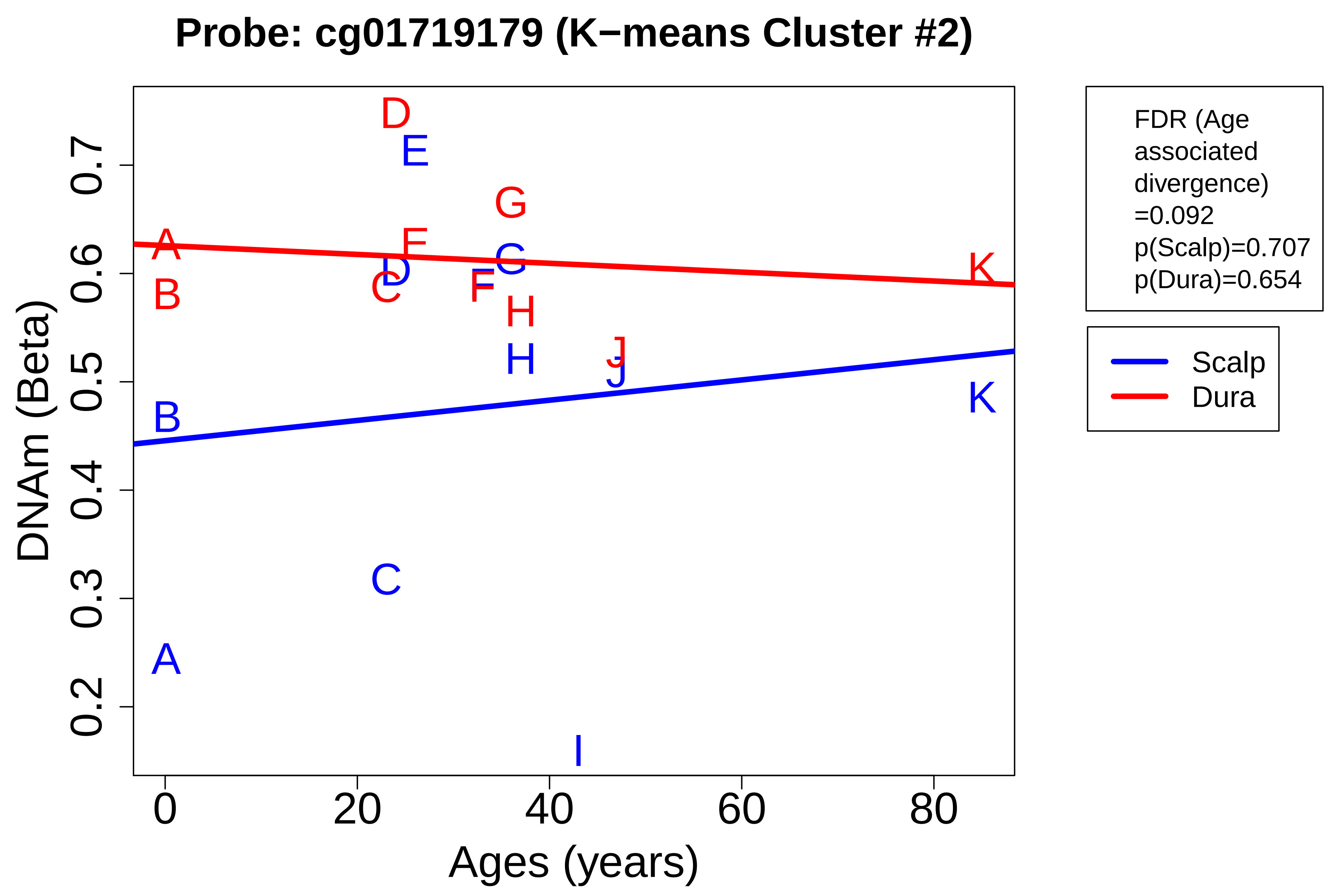
Probe: cg01719179 (K−means Cluster #2)
DNAm (Beta)
0.2
0.3
0.4
0.5
0.6
0.7
0
20
40
60
80
Ages (years)
FDR (Age associated divergence) =0.092
p(Scalp)=0.707
p(Dura)=0.654
Scalp
Dura

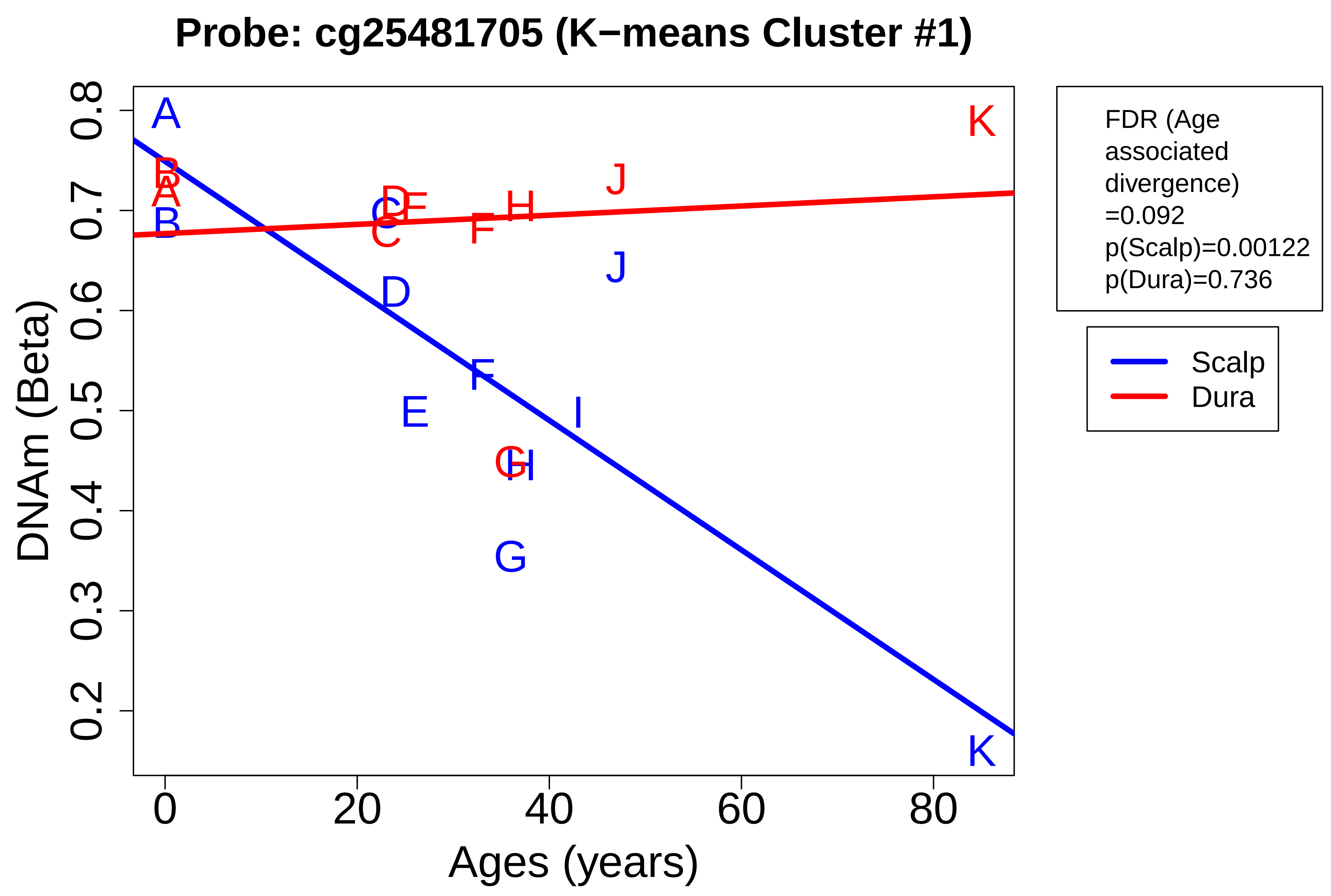

Probe: cg25481705 (K−means Cluster #1)
DNAm (Beta)
0.2
0.3
0.4
0.5
0.6
0.7
0.8
0
20
40
60
80
Ages (years)
A
B
C
D
E
F
G
H
I
J
K
FDR (Age associated divergence) =0.092
p(Scalp)=0.00122
p(Dura)=0.736
Scalp
Dura

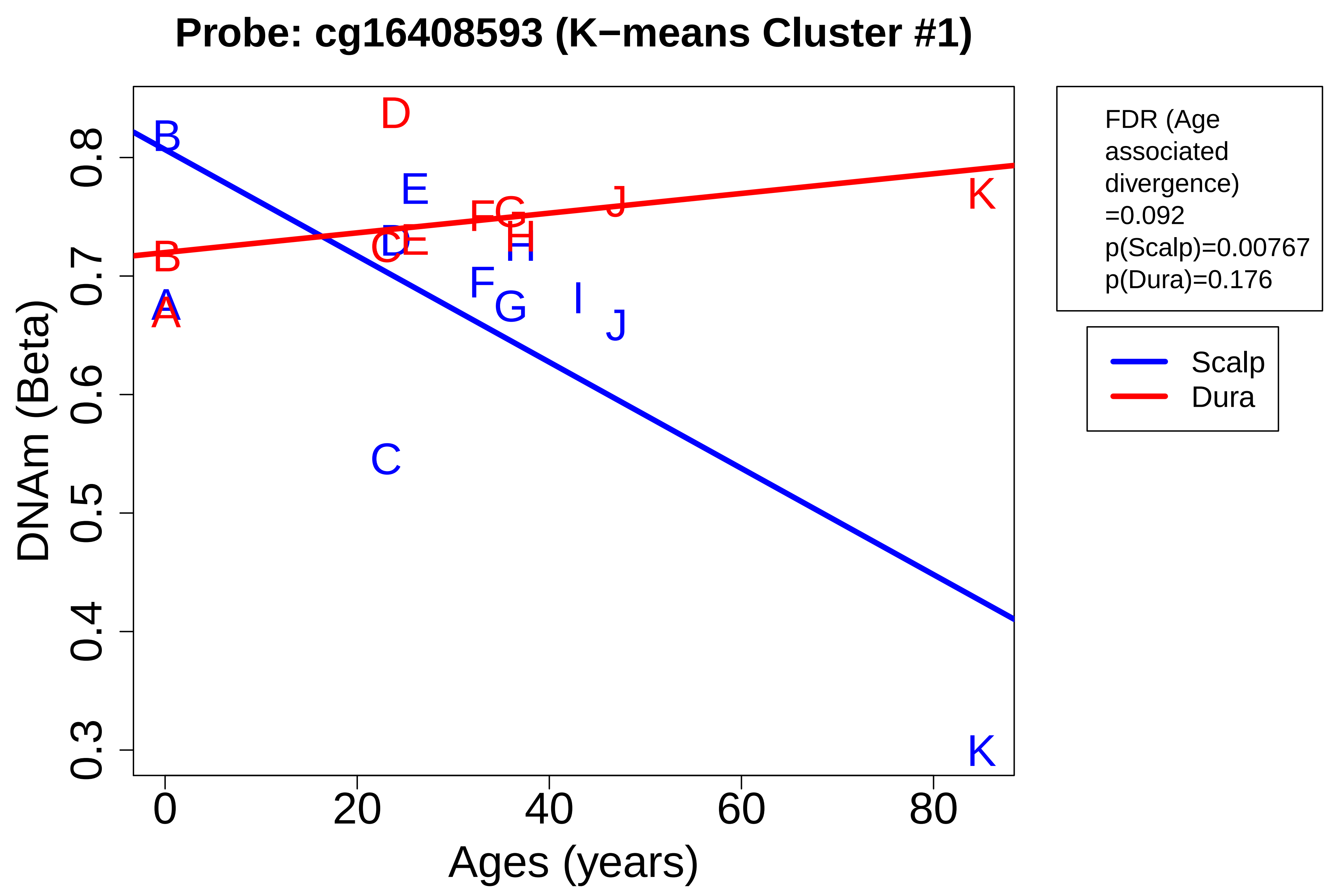

Probe: cg16408593 (K−means Cluster #1)
DNAm (Beta)
0.3
0.4
0.5
0.6
0.7
0.8
0
20
40
60
80
Ages (years)
FDR (Age associated divergence) =0.092
p(Scalp)=0.00767
p(Dura)=0.176
Scalp
Dura
A
B
C
D
E
F
G
H
I
J
K

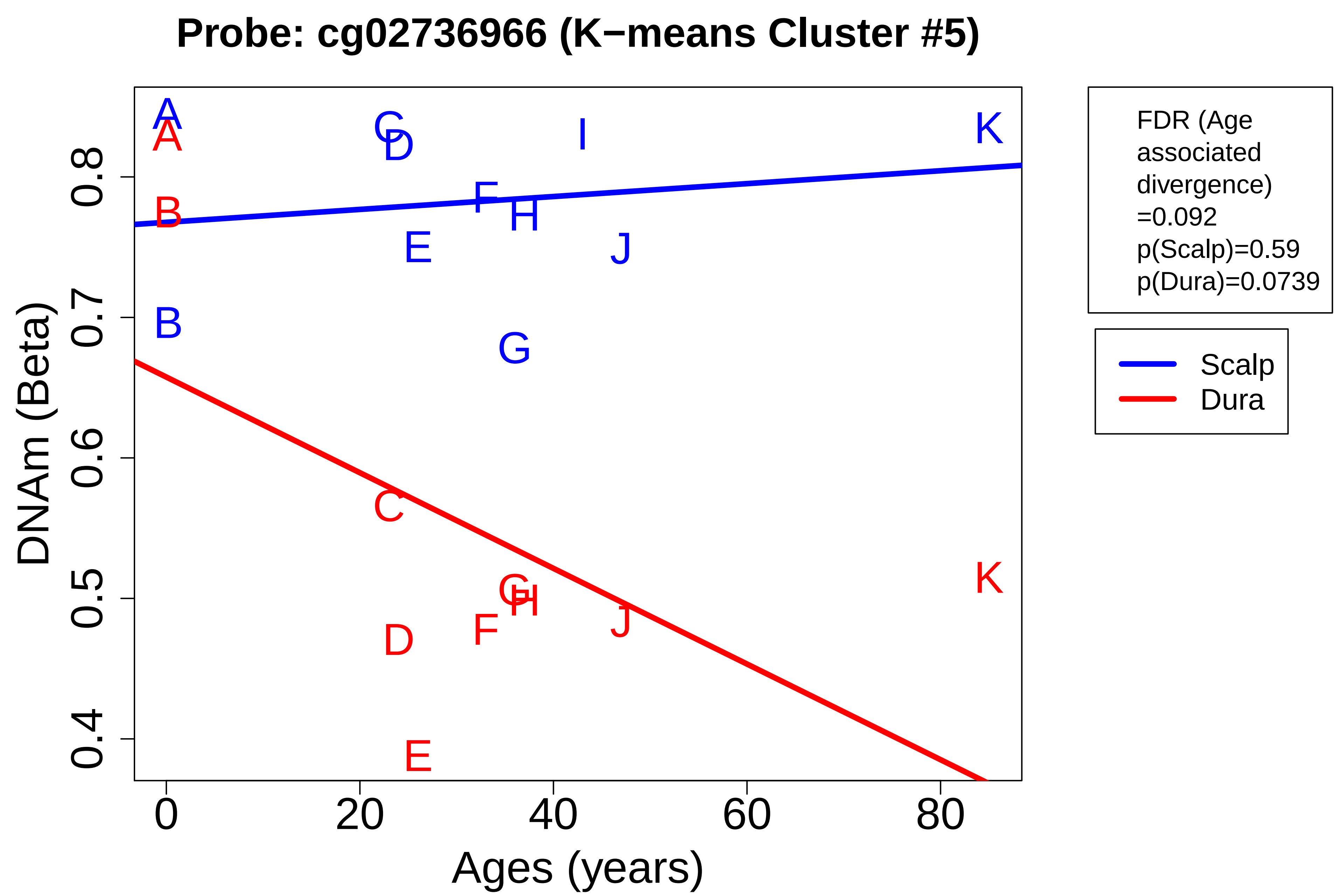
Probe: cg02736966 (K−means Cluster #5)
DNAm (Beta)
0.4
0.5
0.6
0.7
0.8
0
20
40
60
80
Ages (years)
A
A
C
D
I
K
B
F
H
E
J
B
G
C
G
H
K
J
D
F
E
FDR (Age associated divergence) =0.092
p(Scalp)=0.59
p(Dura)=0.0739
Scalp
Dura

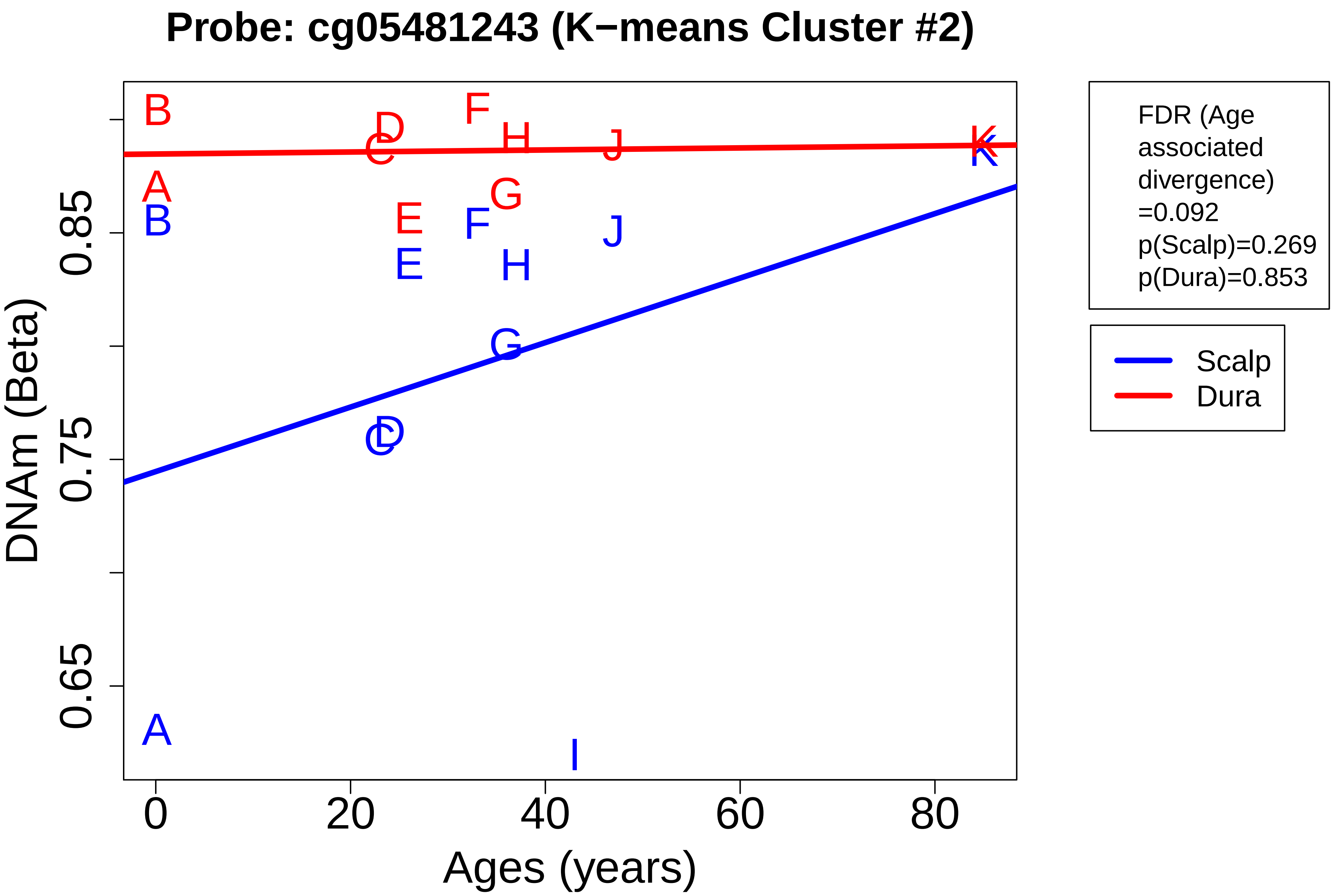

Probe: cg05481243 (K−means Cluster #2)
FDR (Age associated divergence) =0.092
p(Scalp)=0.269
p(Dura)=0.853
Scalp
Dura
DNAm (Beta)
0.65
0.75
0.85
0
20
40
60
80
Ages (years)
A
B
C
D
E
F
G
H
I
J
K

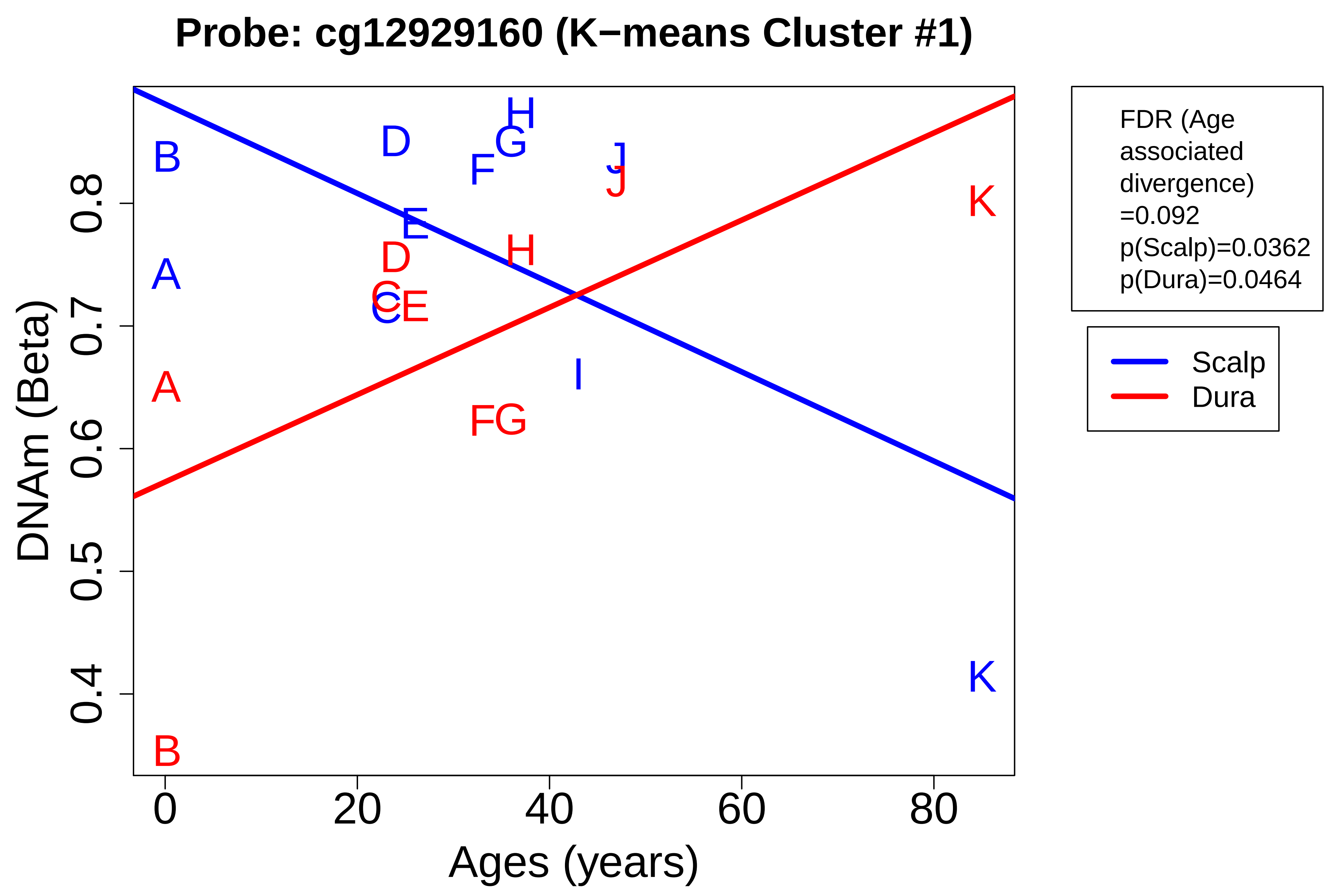

Probe: cg12929160 (K−means Cluster #1)
DNAm (Beta)
0.4
0.5
0.6
0.7
0.8
0
20
40
60
80
Ages (years)
A
B
C
D
E
F
G
H
I
J
K
A
B
C
D
E
FG
H
J
K
FDR (Age associated divergence) =0.092
p(Scalp)=0.0362
p(Dura)=0.0464
Scalp
Dura

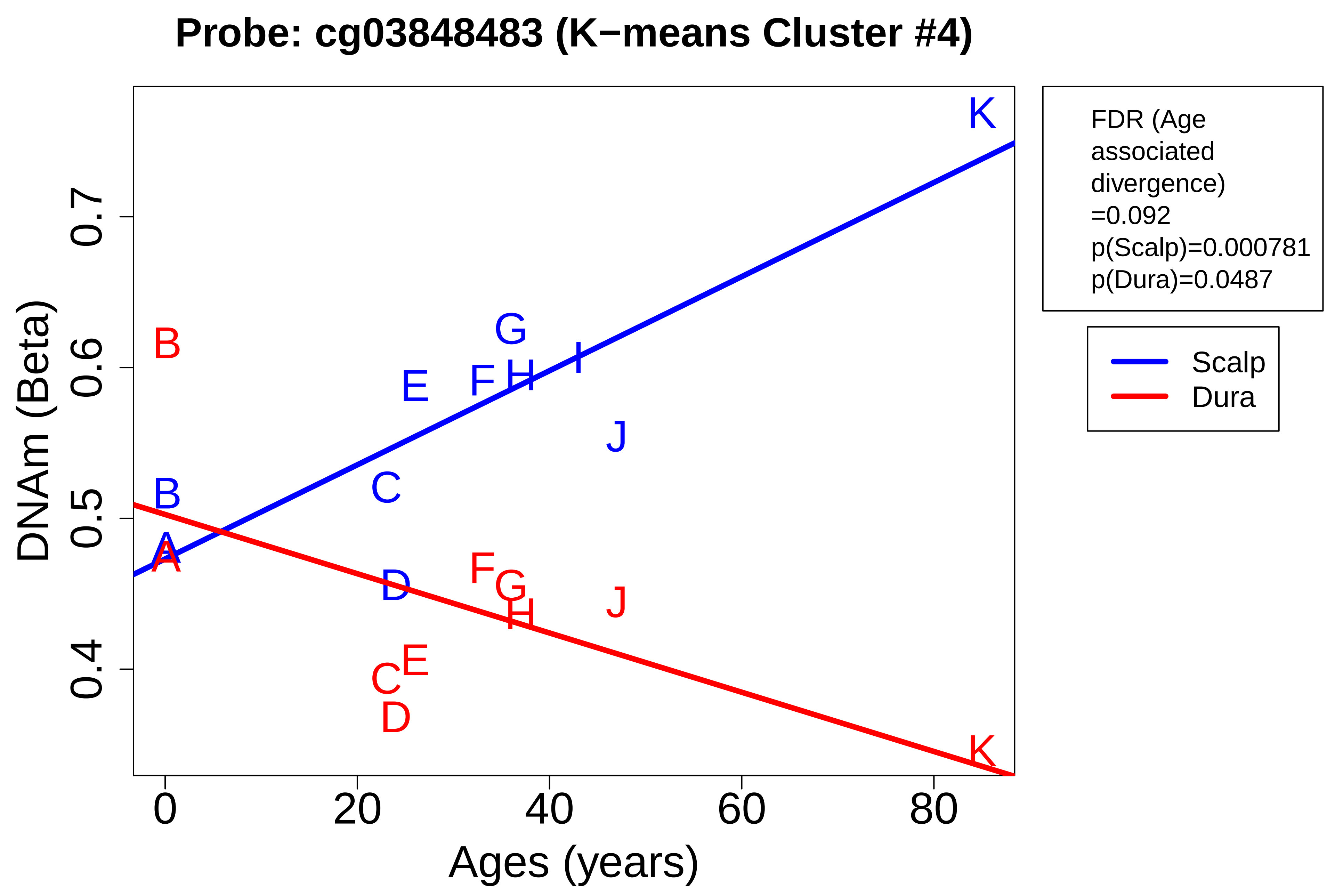

Probe: cg03848483 (K−means Cluster #4)
DNAm (Beta)
0.4
0.5
0.6
0.7
0
20
40
60
80
Ages (years)
FDR (Age associated divergence) =0.092
p(Scalp)=0.000781
p(Dura)=0.0487
Scalp
Dura

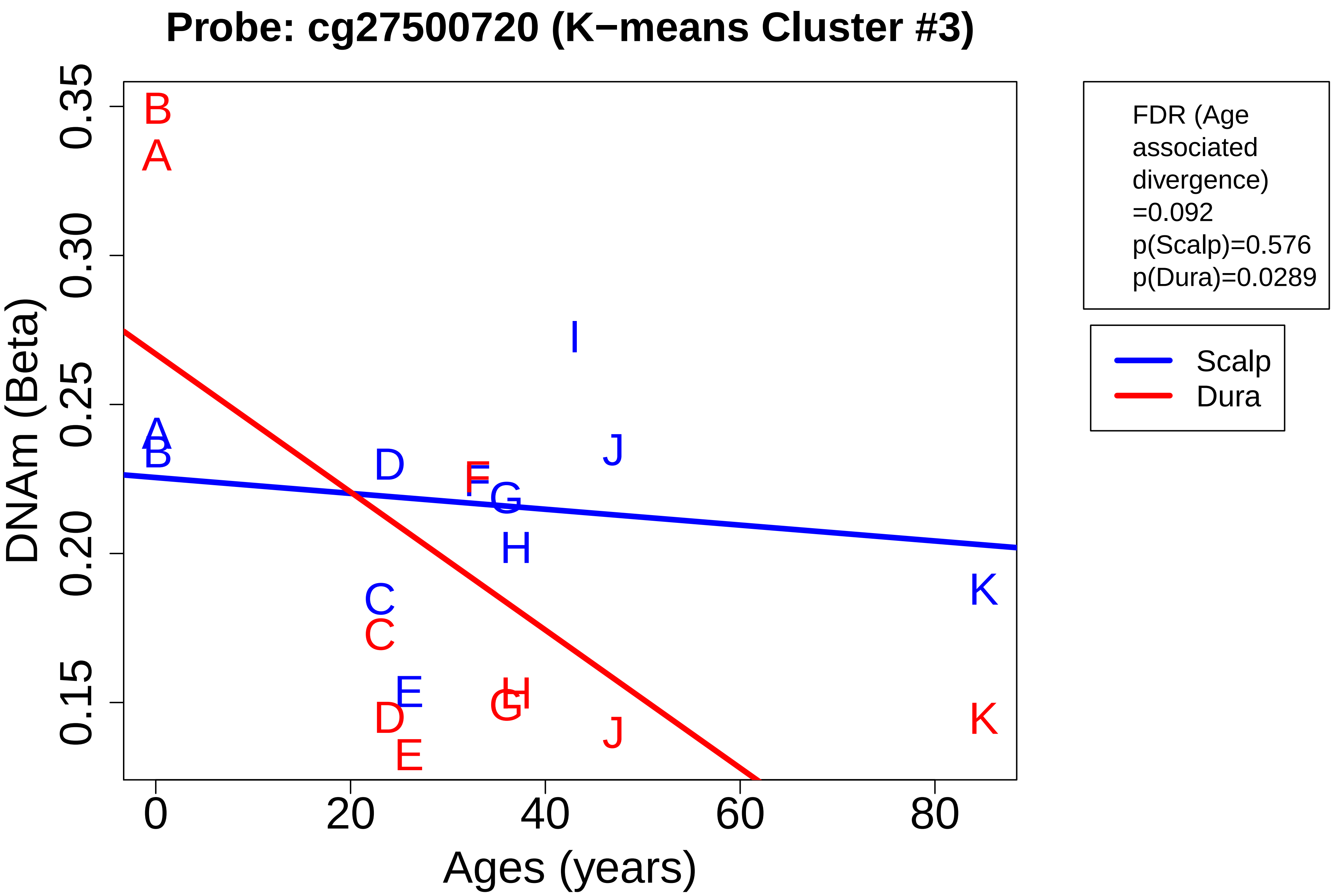

Probe: cg27500720 (K−means Cluster #3)
DNAm (Beta)
0.35
0.30
0.25
0.20
0.15
Ages (years)
0
20
40
60
80
FDR (Age associated divergence) =0.092
p(Scalp)=0.576
p(Dura)=0.0289
Scalp
Dura

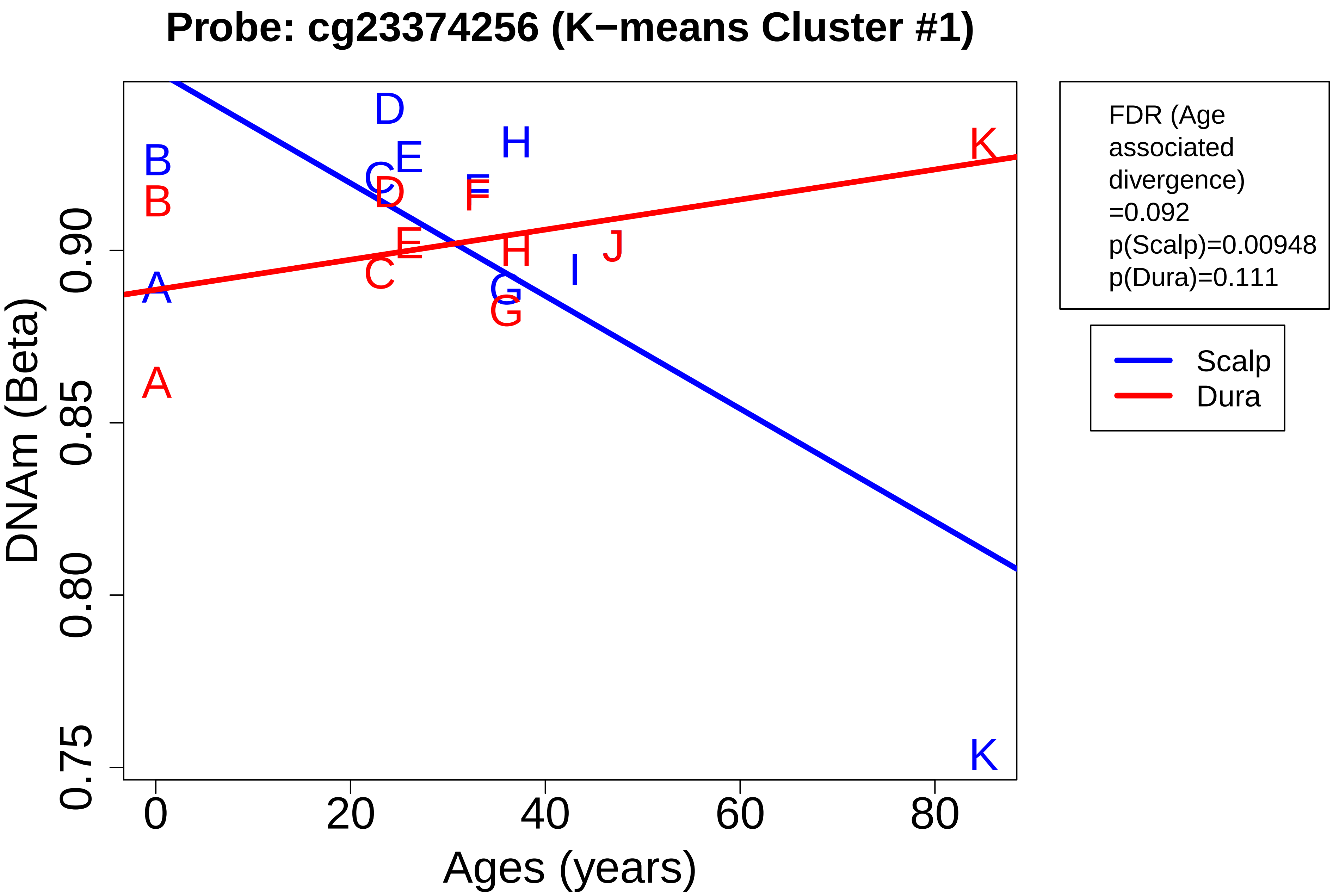
Probe: cg23374256 (K−means Cluster #1)
DNAm (Beta)
0.75
0.80
0.85
0.90
0
20
40
60
80
Ages (years)
A
B
C
D
E
F
G
H
I
J
K
FDR (Age associated divergence) =0.092
p(Scalp)=0.00948
p(Dura)=0.111
Scalp
Dura

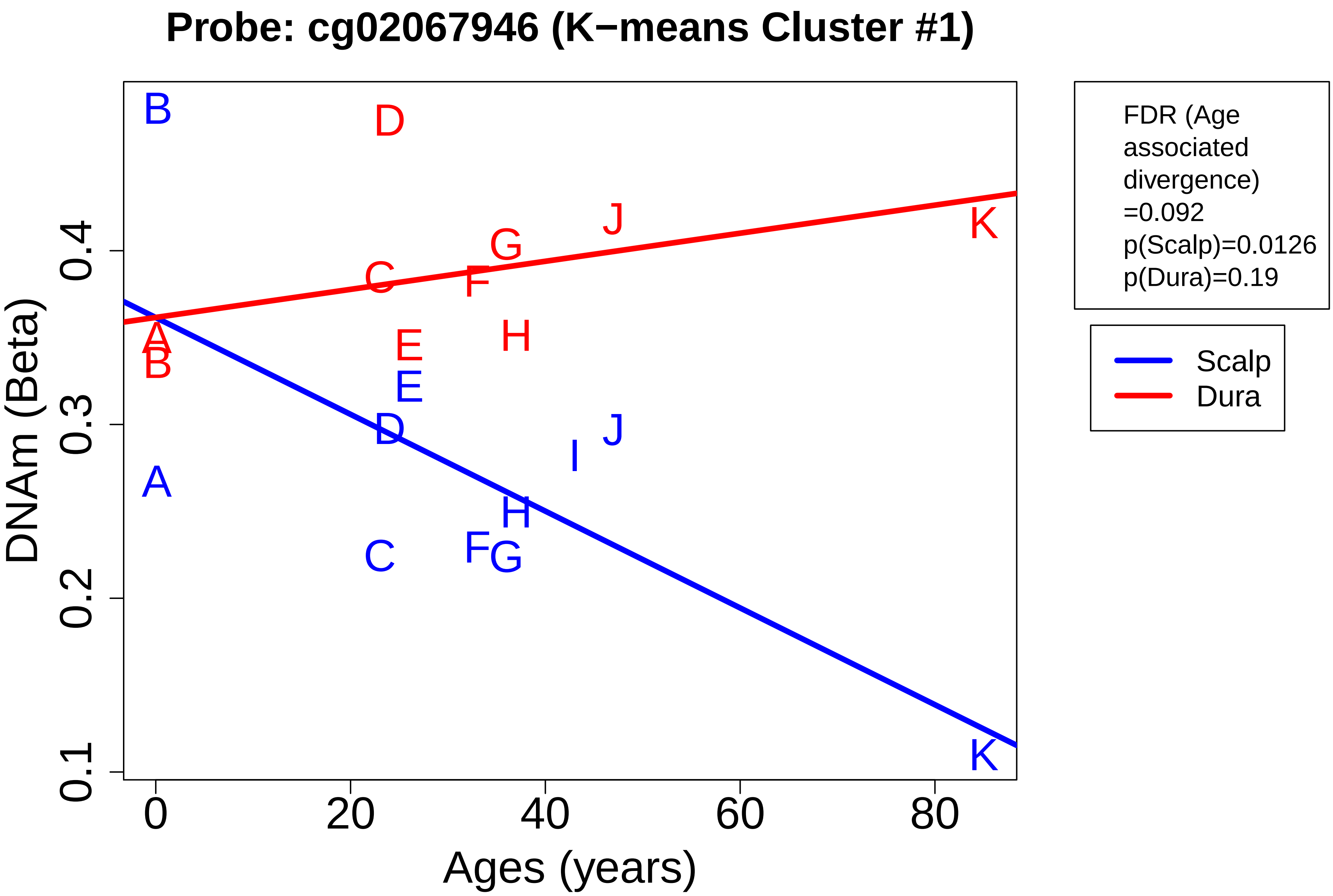
Probe: cg02067946 (K−means Cluster #1)
DNAm (Beta)
0.1
0.2
0.3
0.4
Ages (years)
0
20
40
60
80
FDR (Age associated divergence) =0.092
p(Scalp)=0.0126
p(Dura)=0.19
Scalp
Dura

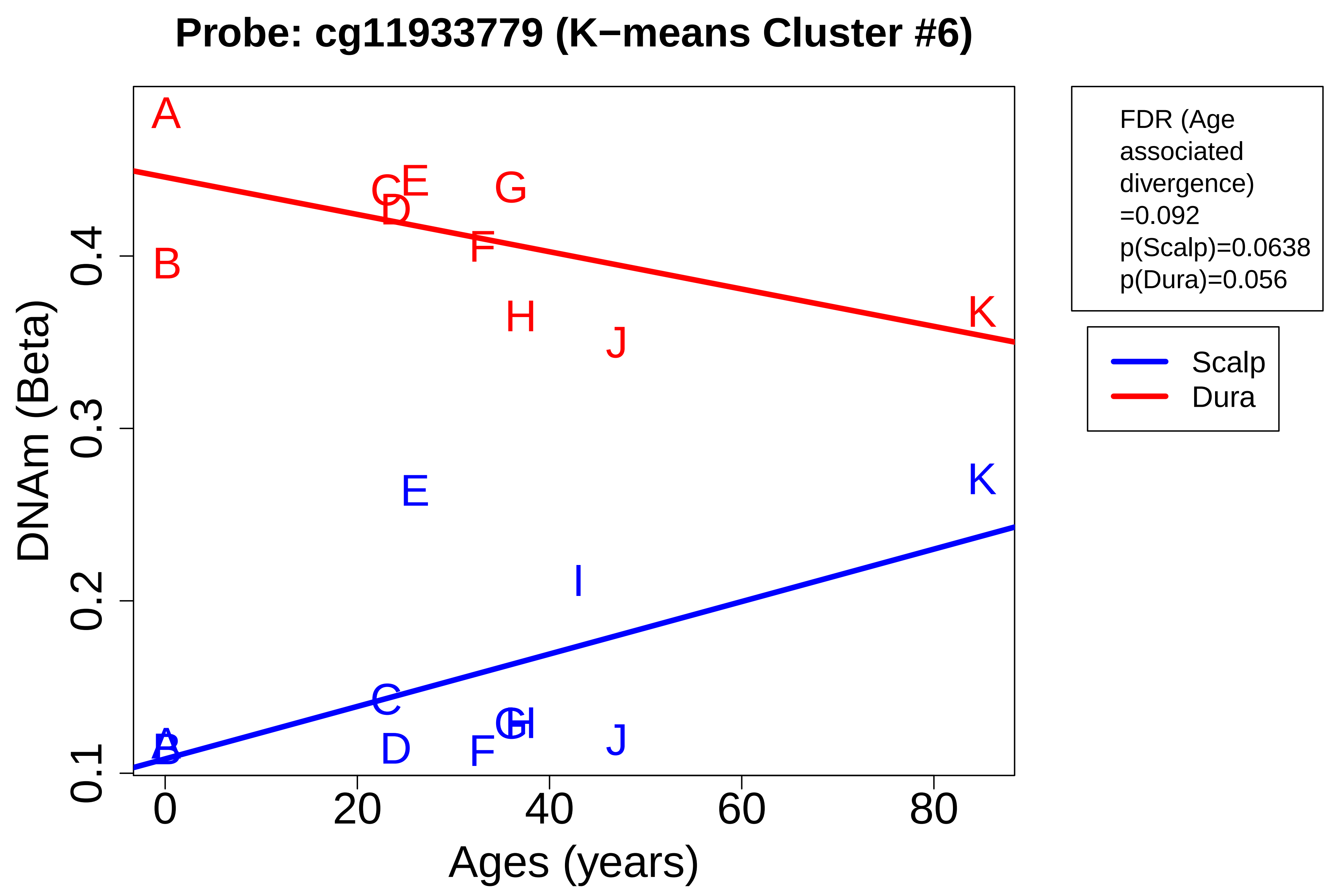
Probe: cg11933779 (K−means Cluster #6)
DNAm (Beta)
0.1
0.2
0.3
0.4
0
20
40
60
80
Ages (years)
A
B
C
D
E
F
G
H
I
J
K
FDR (Age associated divergence) =0.092
p(Scalp)=0.0638
p(Dura)=0.056
Scalp
Dura

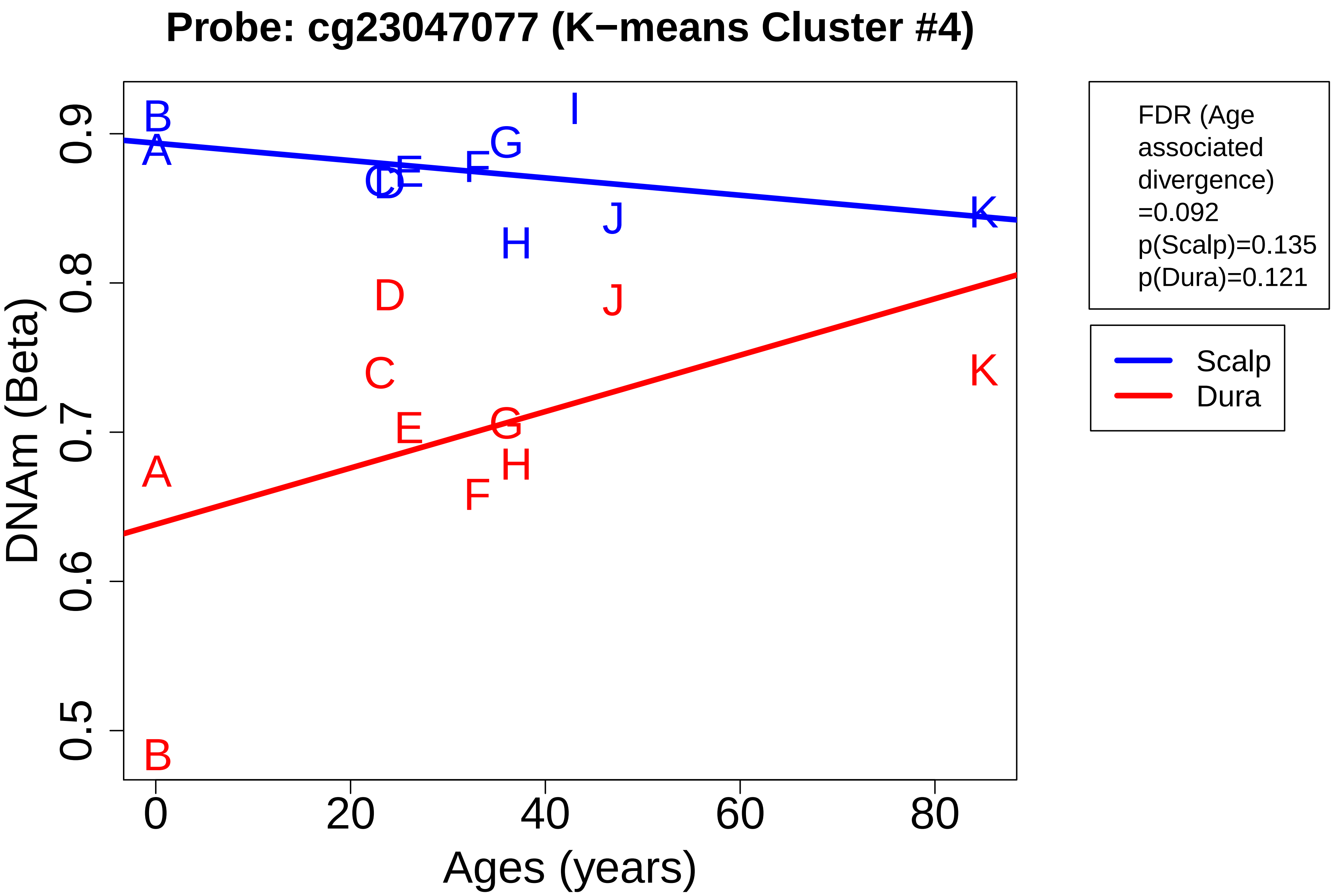

Probe: cg23047077 (K−means Cluster #4)
DNAm (Beta)
0.5
0.6
0.7
0.8
0.9
0
20
40
60
80
Ages (years)
FDR (Age associated divergence) =0.092
p(Scalp)=0.135
p(Dura)=0.121
Scalp
Dura

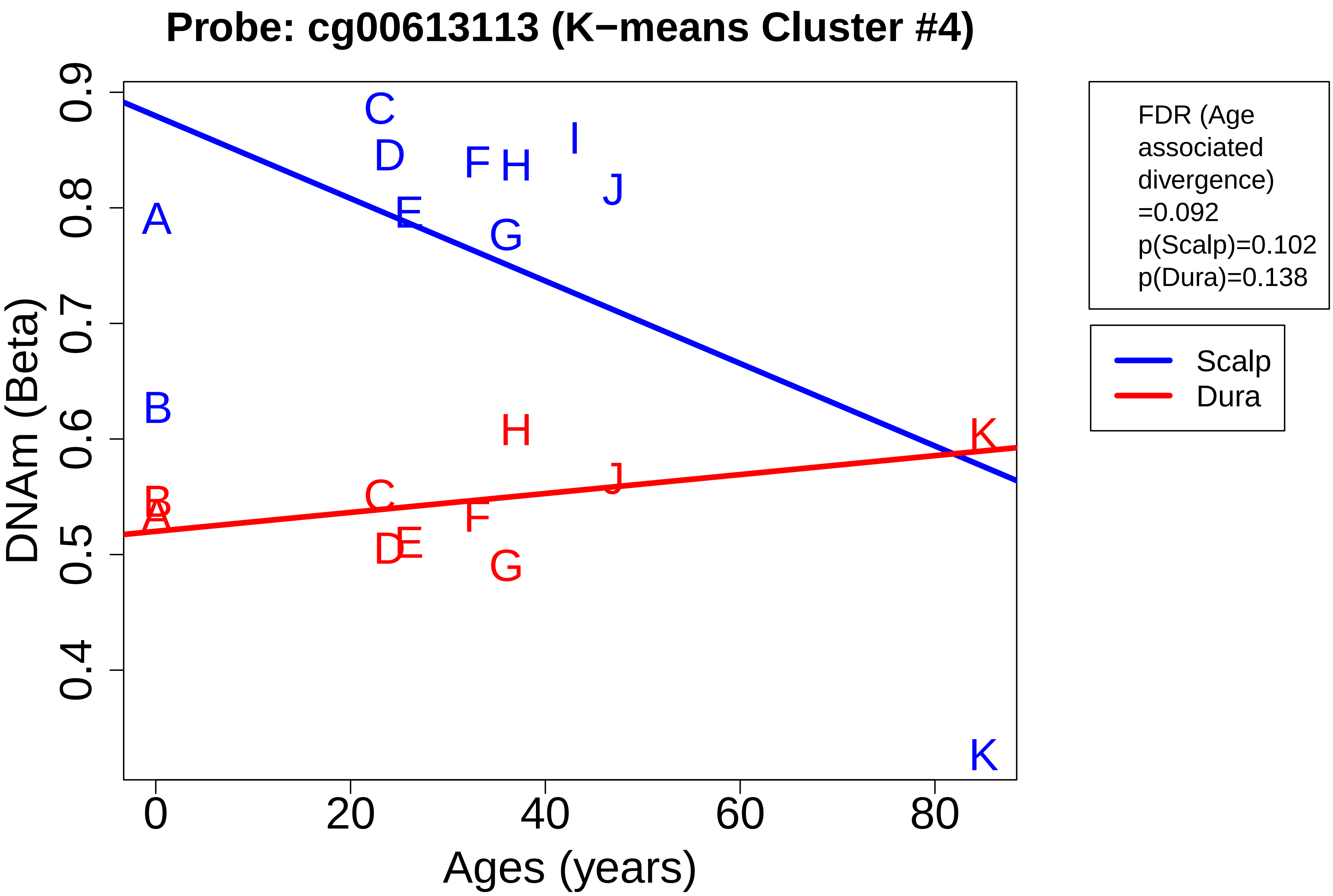

Probe: cg00613113 (K−means Cluster #4)
DNAm (Beta)
0.4
0.5
0.6
0.7
0.8
0.9
0
20
40
60
80
Ages (years)
FDR (Age associated divergence) =0.092
p(Scalp)=0.102
p(Dura)=0.138
Scalp
Dura

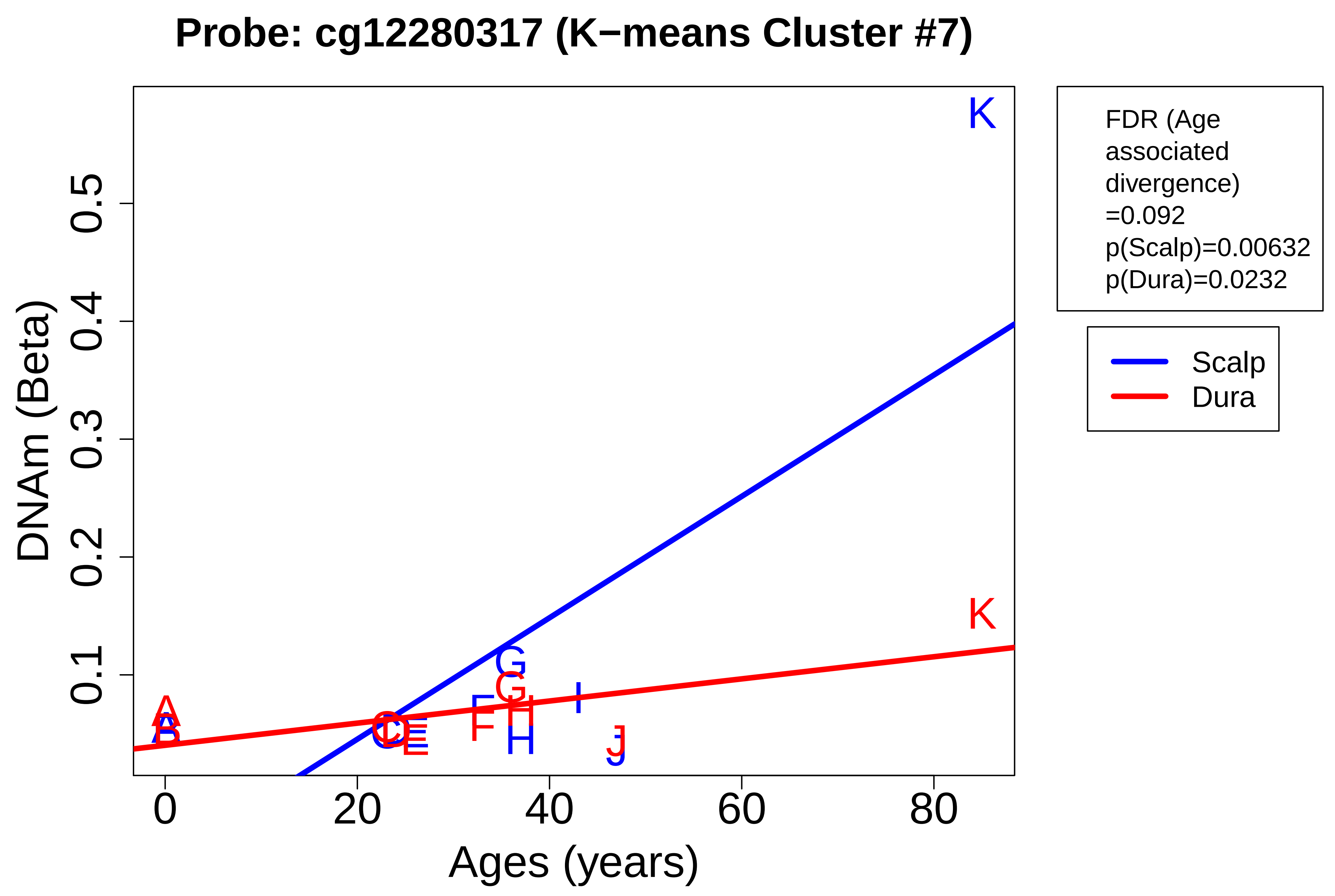

Probe: cg12280317 (K−means Cluster #7)
DNAm (Beta)
0.1
0.2
0.3
0.4
0.5
0
20
40
60
80
Ages (years)
K
K
FDR (Age associated divergence) =0.092
p(Scalp)=0.00632
p(Dura)=0.0232
Scalp
Dura

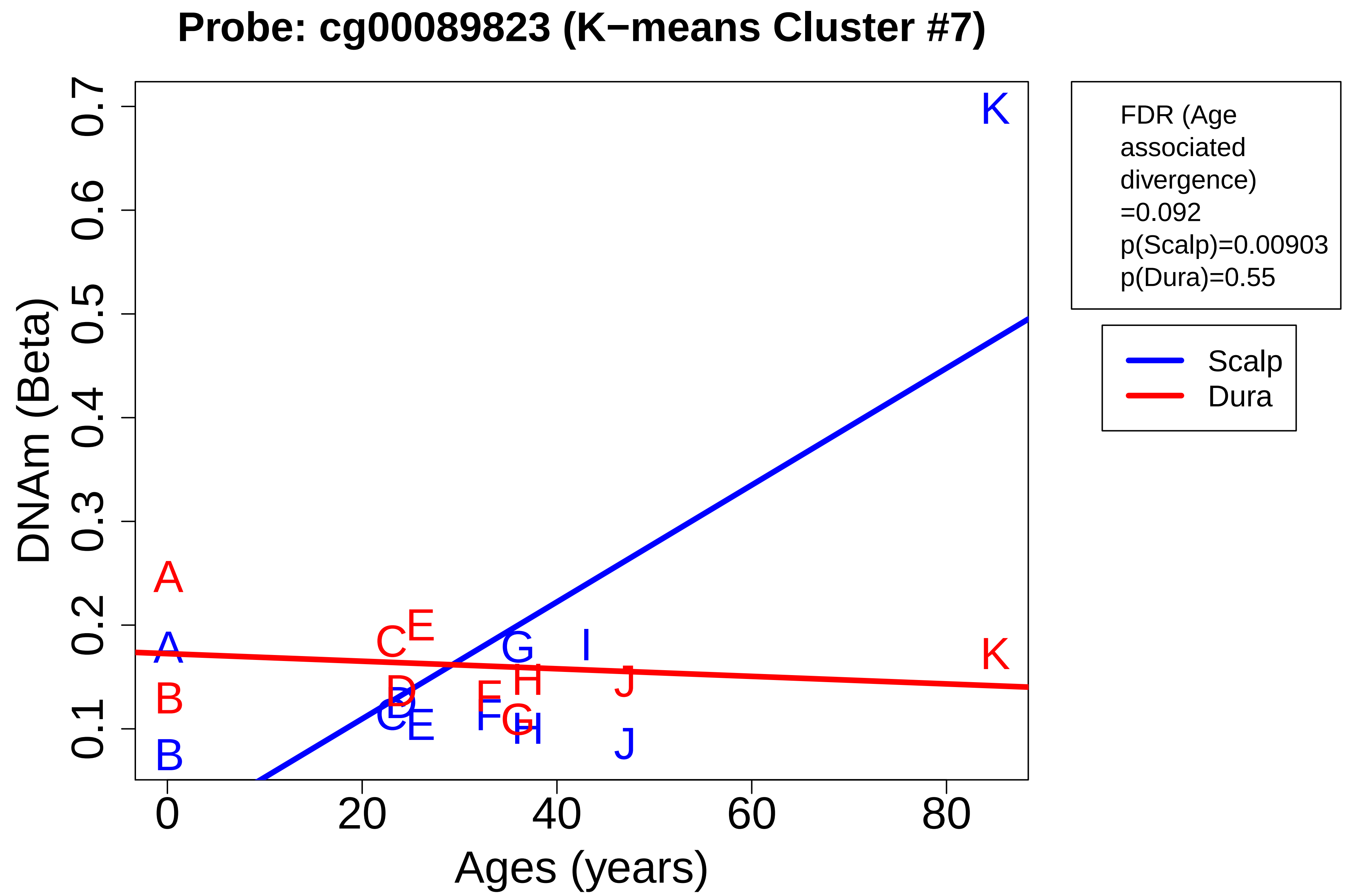

Probe: cg00089823 (K−means Cluster #7)
DNAm (Beta)
0.1
0.2
0.3
0.4
0.5
0.6
0.7
Ages (years)
0
20
40
60
80
FDR (Age associated divergence) =0.092
p(Scalp)=0.00903
p(Dura)=0.55
Scalp
Dura

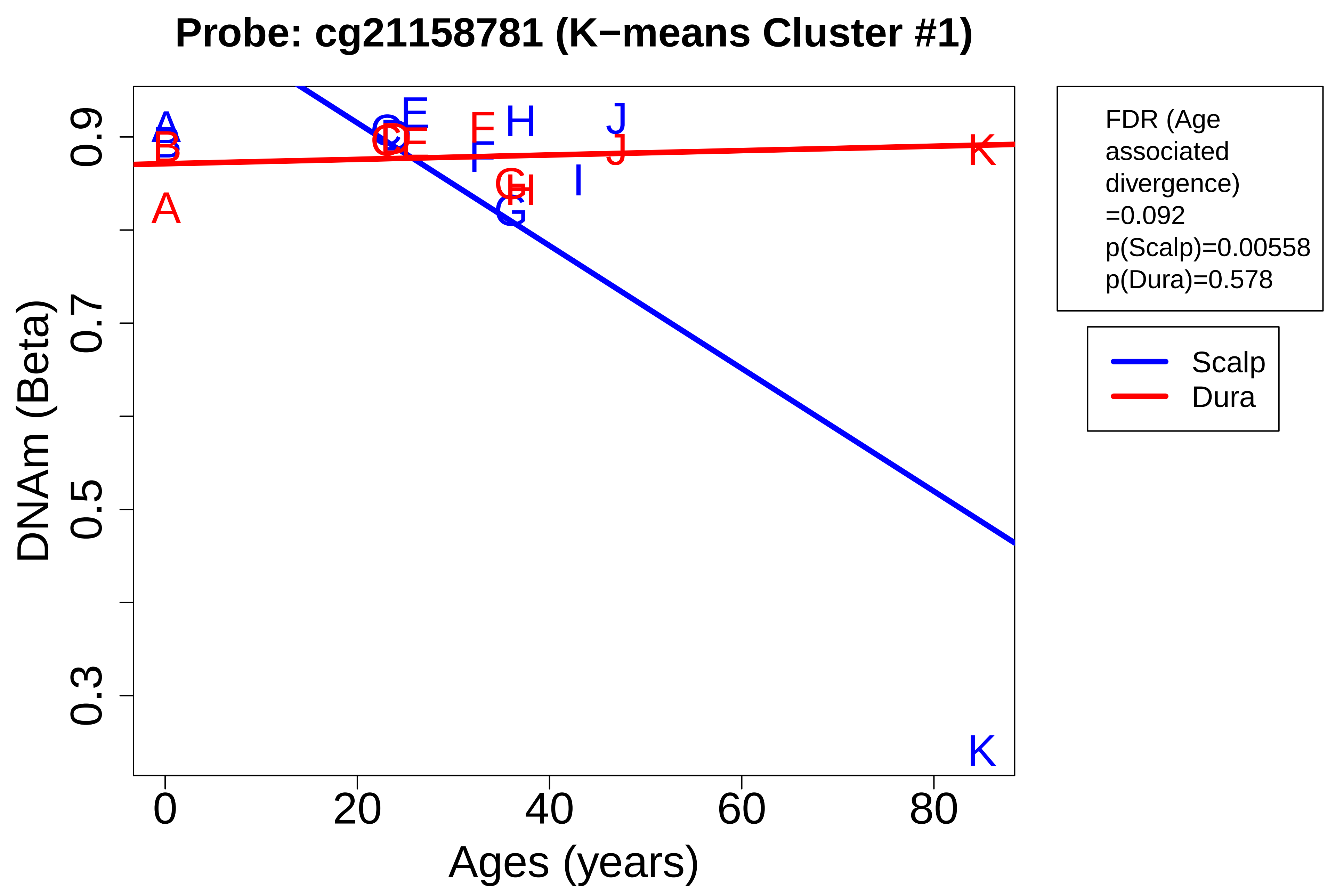

Probe: cg21158781 (K−means Cluster #1)
DNAm (Beta)
0.3
0.5
0.7
0.9
Ages (years)
0
20
40
60
80
A
B
C
D
E
F
G
H
I
J
K
FDR (Age associated divergence) =0.092
p(Scalp)=0.00558
p(Dura)=0.578
Scalp
Dura

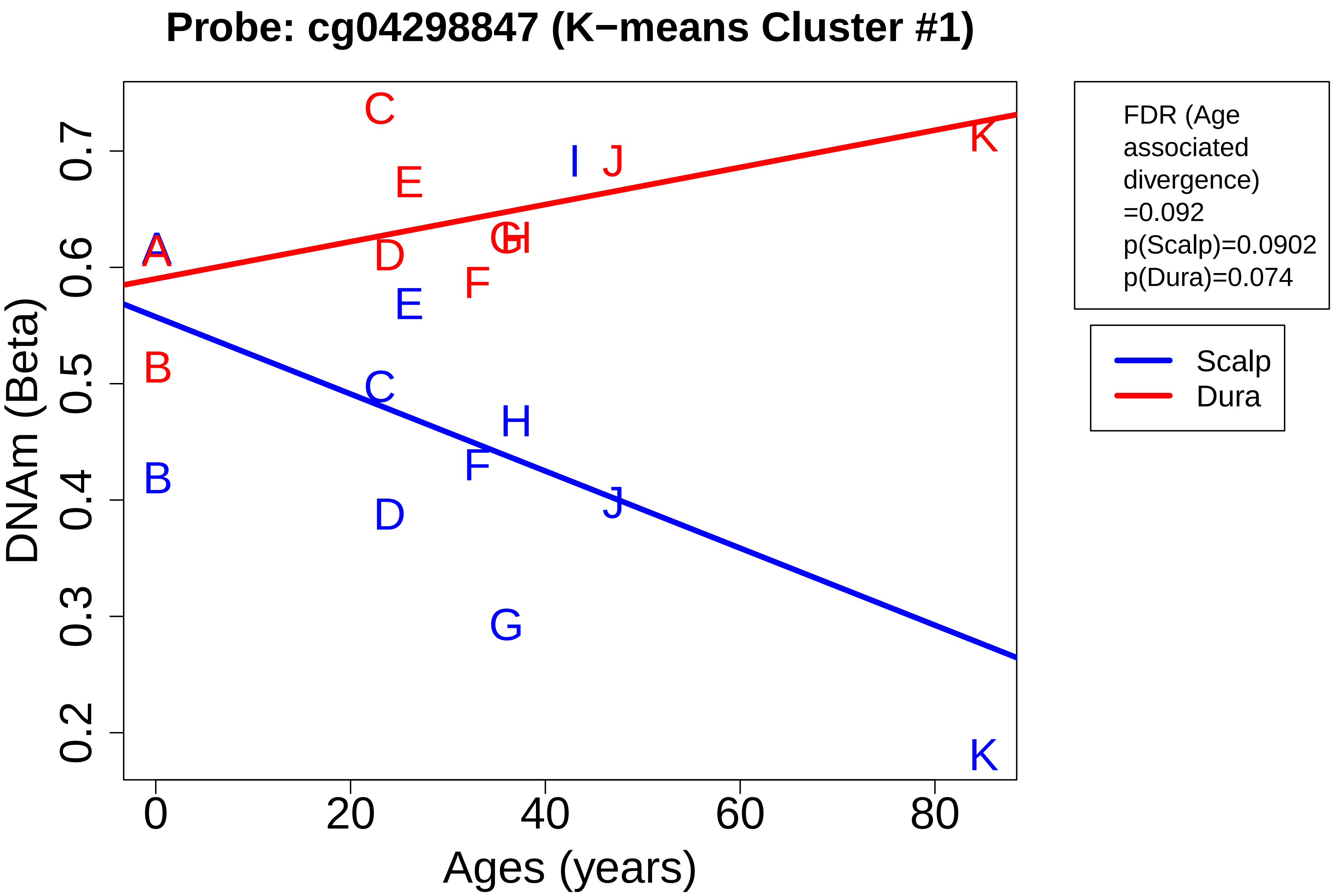
Probe: cg04298847 (K−means Cluster #1)
DNAm (Beta)
0.2
0.3
0.4
0.5
0.6
0.7
0
20
40
60
80
Ages (years)
FDR (Age associated divergence) =0.092
p(Scalp)=0.0902
p(Dura)=0.074
Scalp
Dura

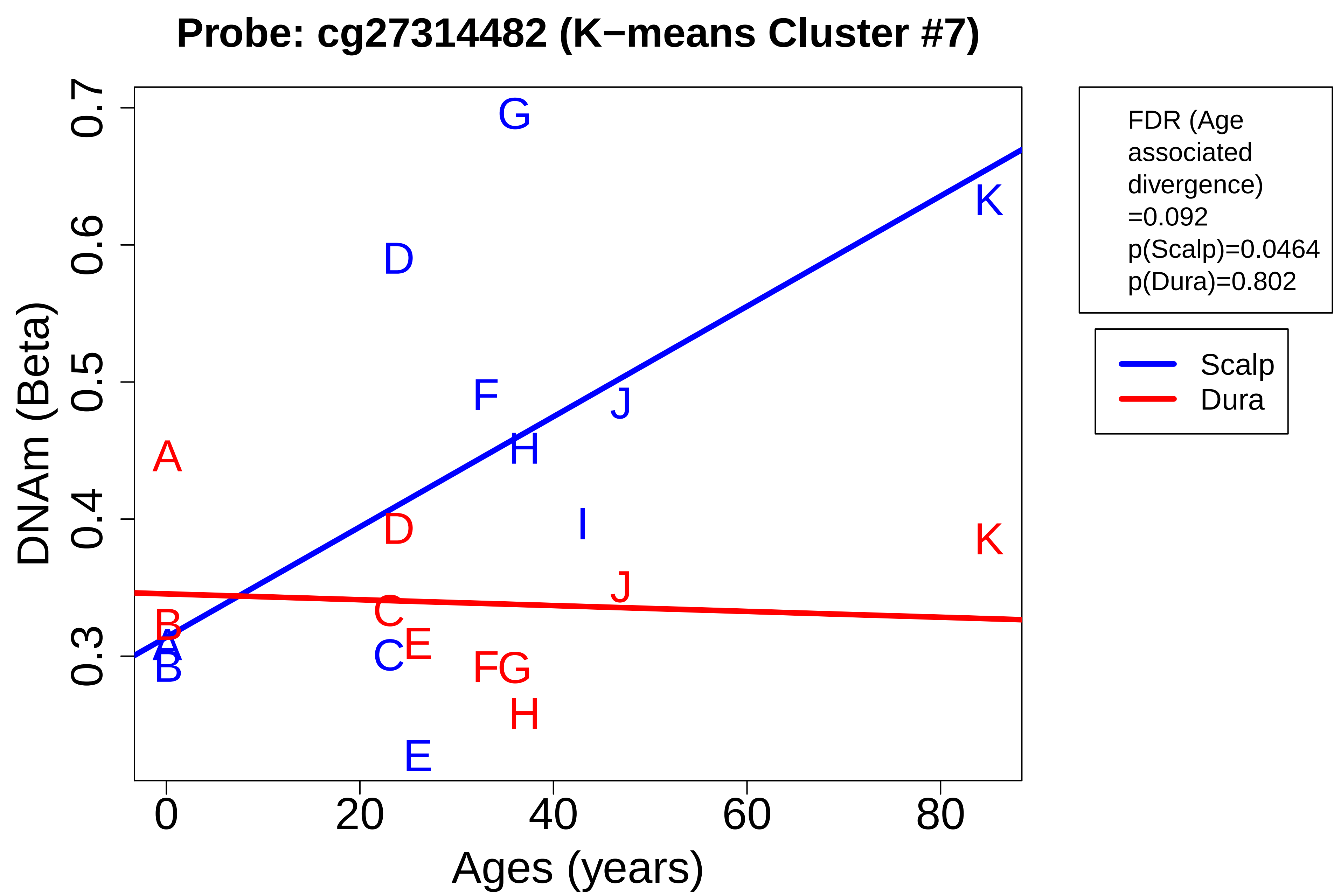
Probe: cg27314482 (K−means Cluster #7)
DNAm (Beta)
0.3
0.4
0.5
0.6
0.7
Ages (years)
0
20
40
60
80
FDR (Age associated divergence) =0.092
p(Scalp)=0.0464
p(Dura)=0.802
Scalp
Dura
A B C D E F G H I J K
A B C D E F G H J K

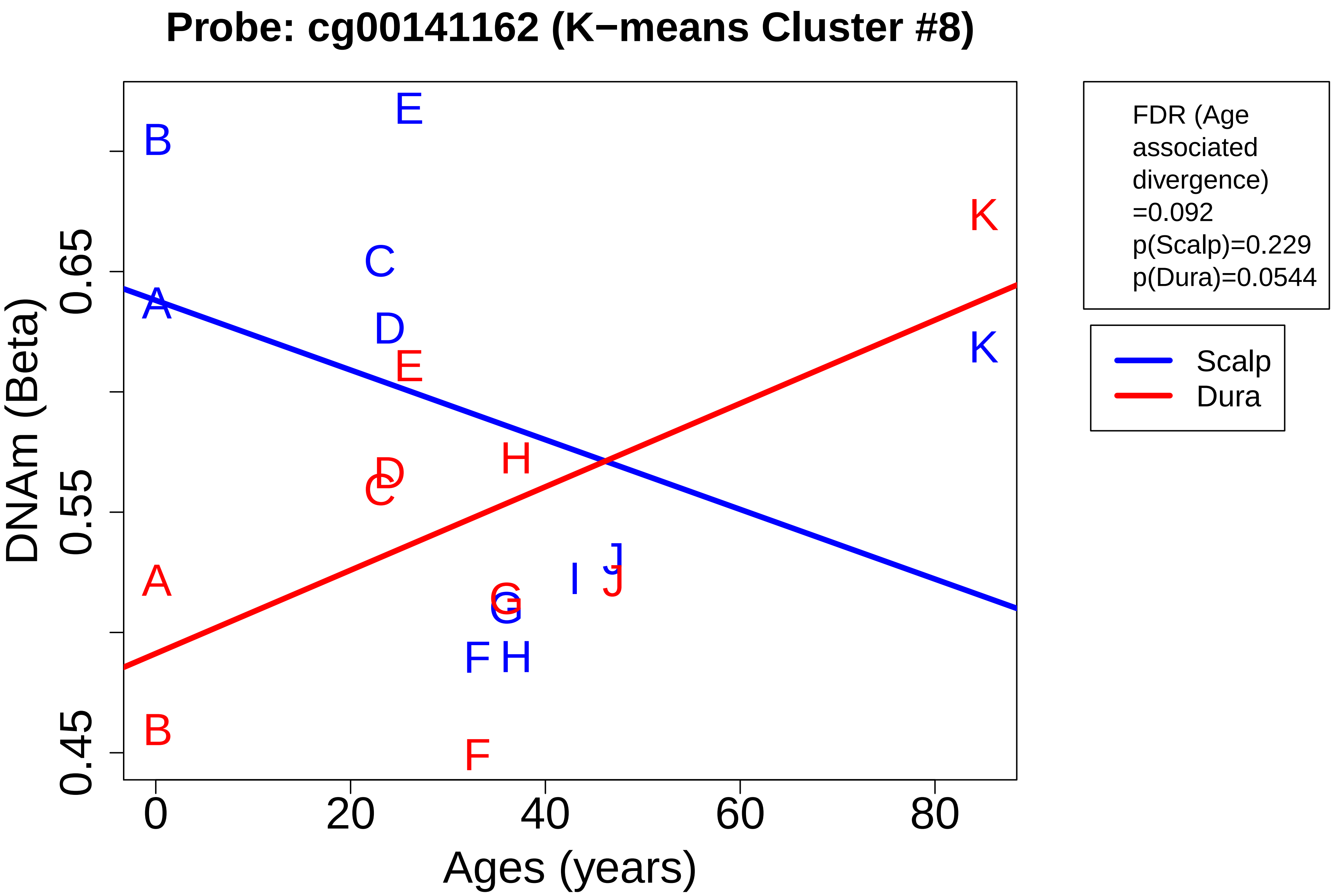

Probe: cg00141162 (K−means Cluster #8)
DNAm (Beta)
0.45
0.55
0.65
0
20
40
60
80
Ages (years)
FDR (Age associated divergence) =0.092
p(Scalp)=0.229
p(Dura)=0.0544
Scalp
Dura

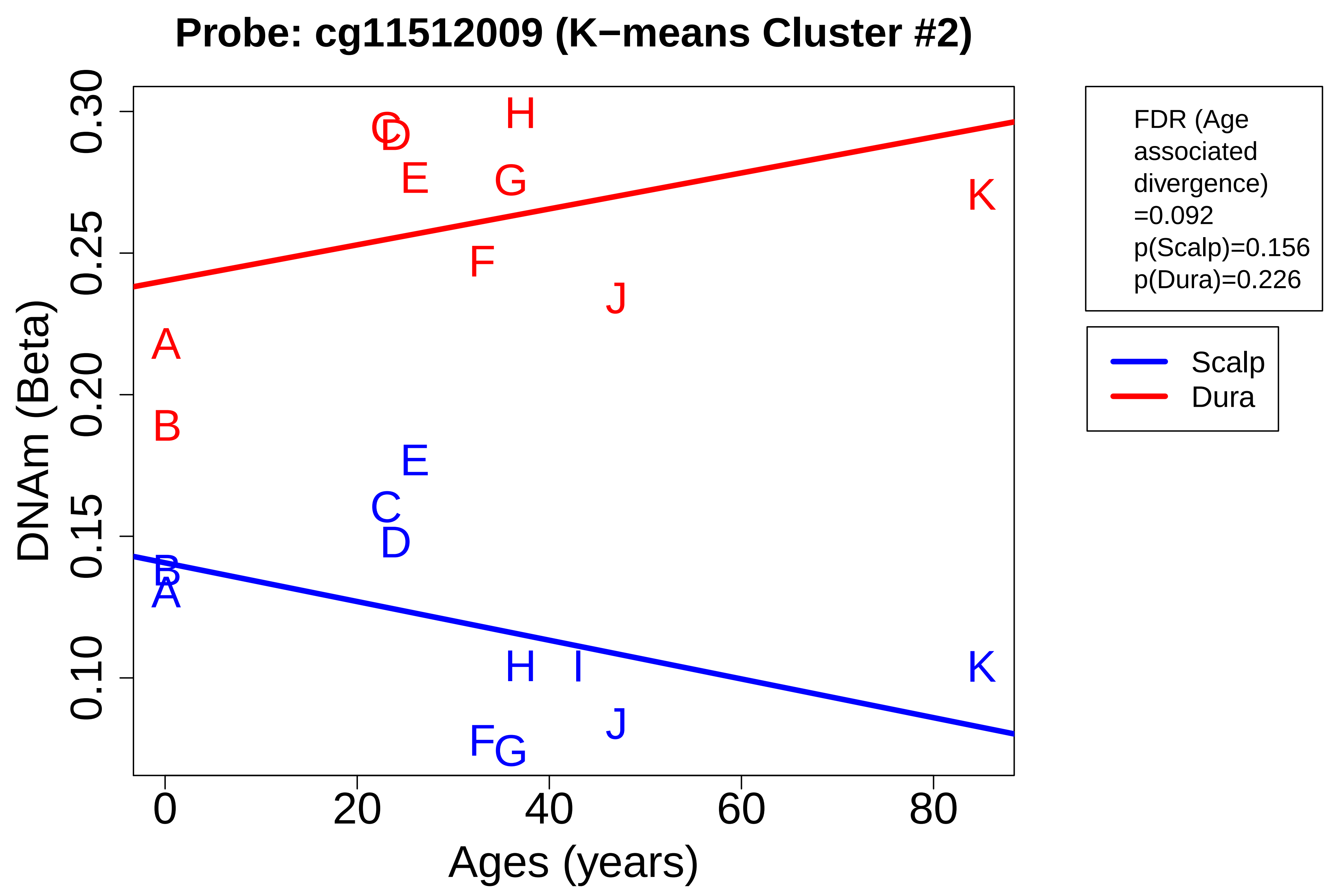
Probe: cg11512009 (K−means Cluster #2)
DNAm (Beta)
0.10
0.15
0.20
0.25
0.30
Ages (years)
0
20
40
60
80
A
B
C
D
E
F
G
H
I
J
K
FDR (Age associated divergence) =0.092
p(Scalp)=0.156
p(Dura)=0.226
Scalp
Dura

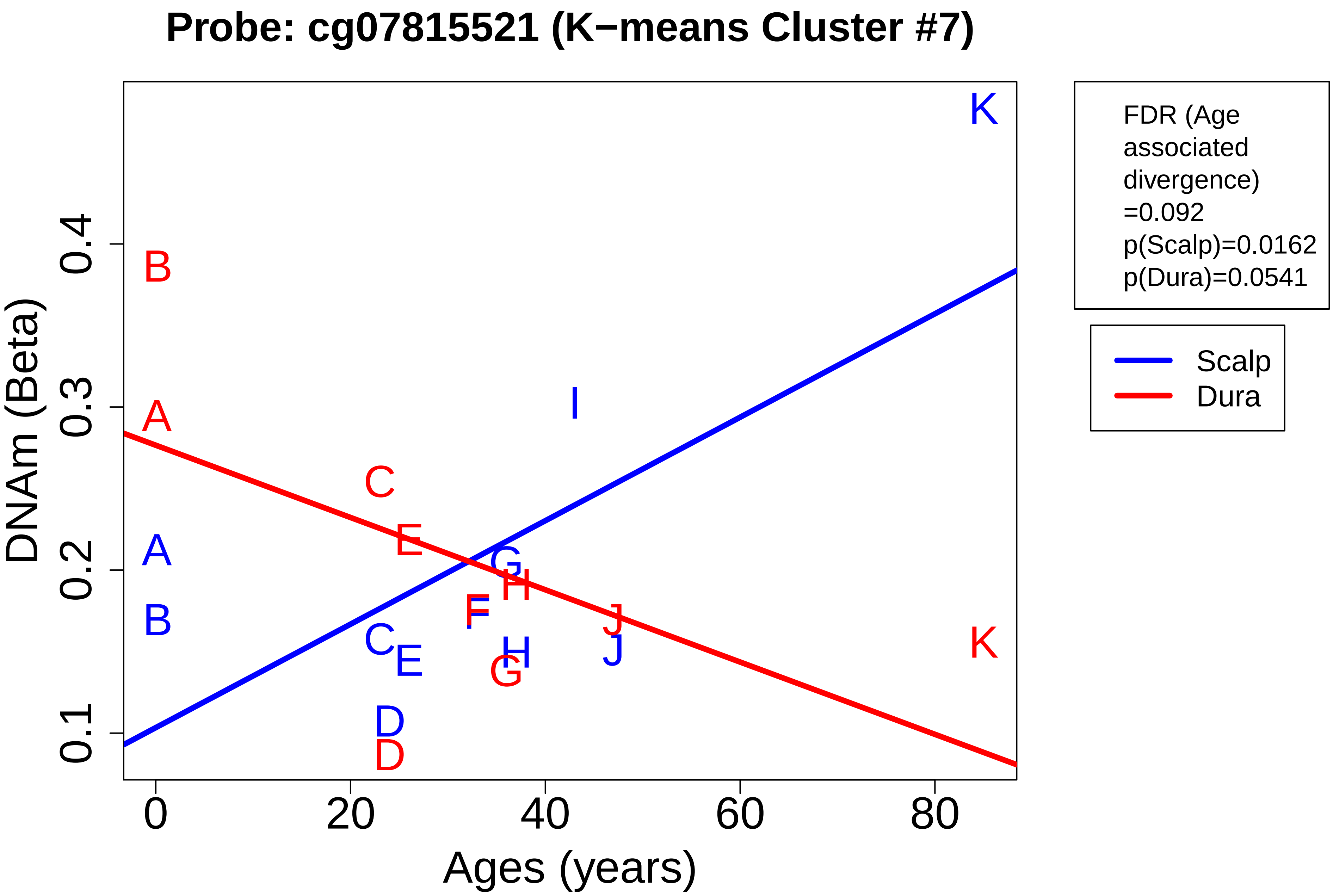

Probe: cg07815521 (K−means Cluster #7)
DNAm (Beta)
0.1
0.2
0.3
0.4
0
20
40
60
80
Ages (years)
FDR (Age associated divergence) =0.092
p(Scalp)=0.0162
p(Dura)=0.0541
Scalp
Dura

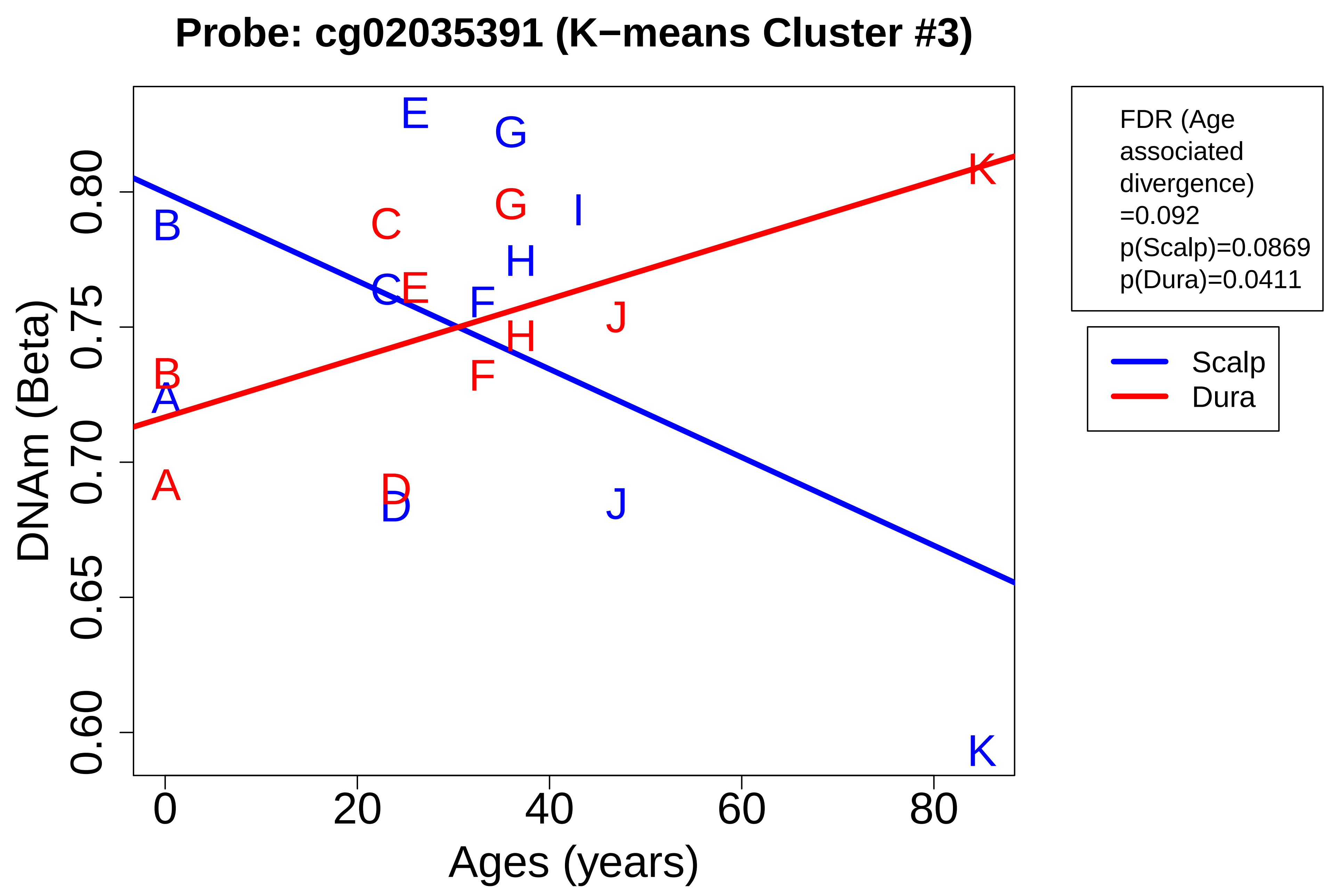

Probe: cg02035391 (K−means Cluster #3)
DNAm (Beta)
0.60
0.65
0.70
0.75
0.80
0
20
40
60
80
Ages (years)
FDR (Age associated divergence) =0.092
p(Scalp)=0.0869
p(Dura)=0.0411
Scalp
Dura

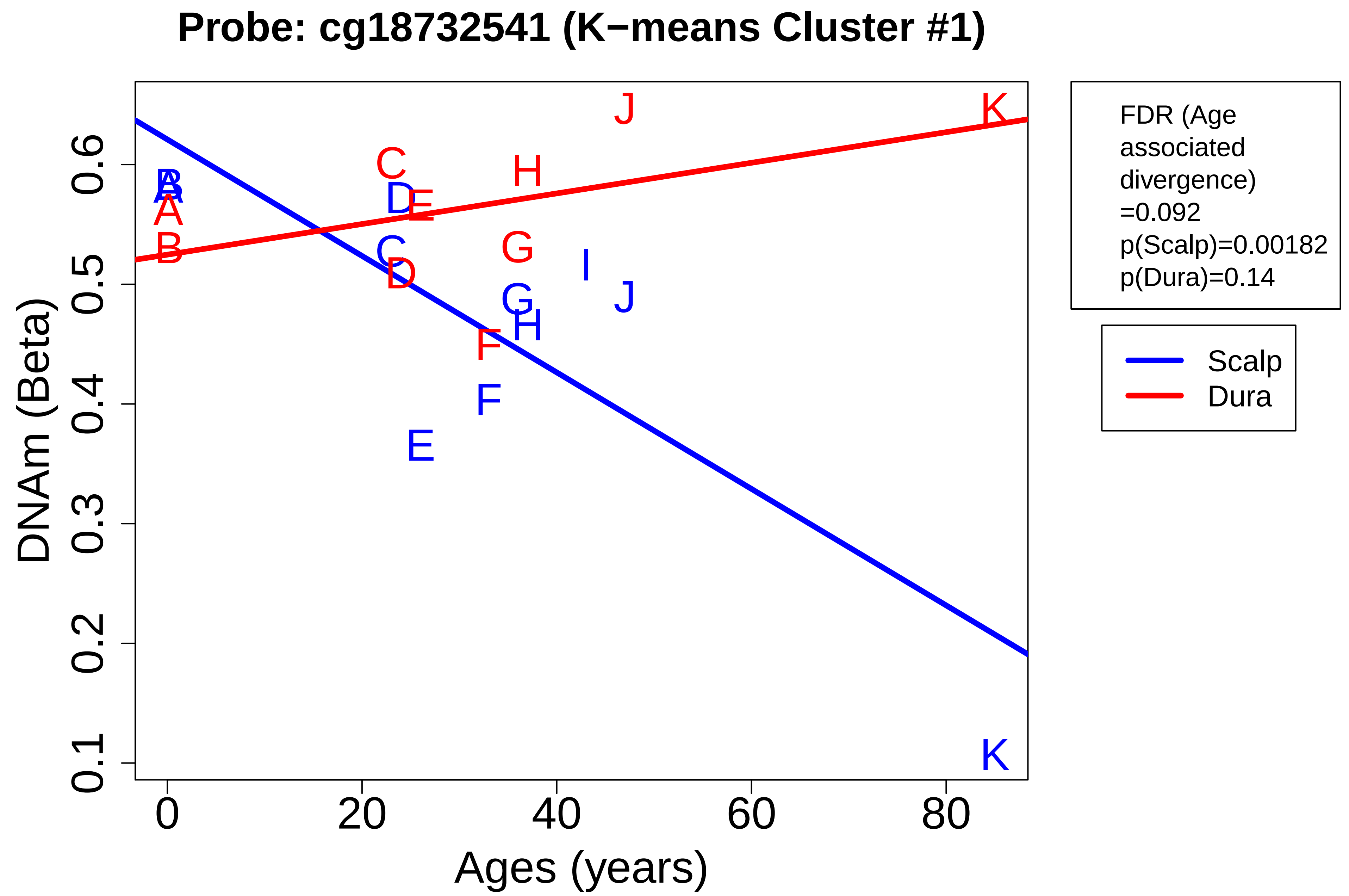

Probe: cg18732541 (K−means Cluster #1)
FDR (Age associated divergence) =0.092
p(Scalp)=0.00182
p(Dura)=0.14
Scalp
Dura
DNAm (Beta)
Ages (years)
0.1
0.2
0.3
0.4
0.5
0.6
0
20
40
60
80

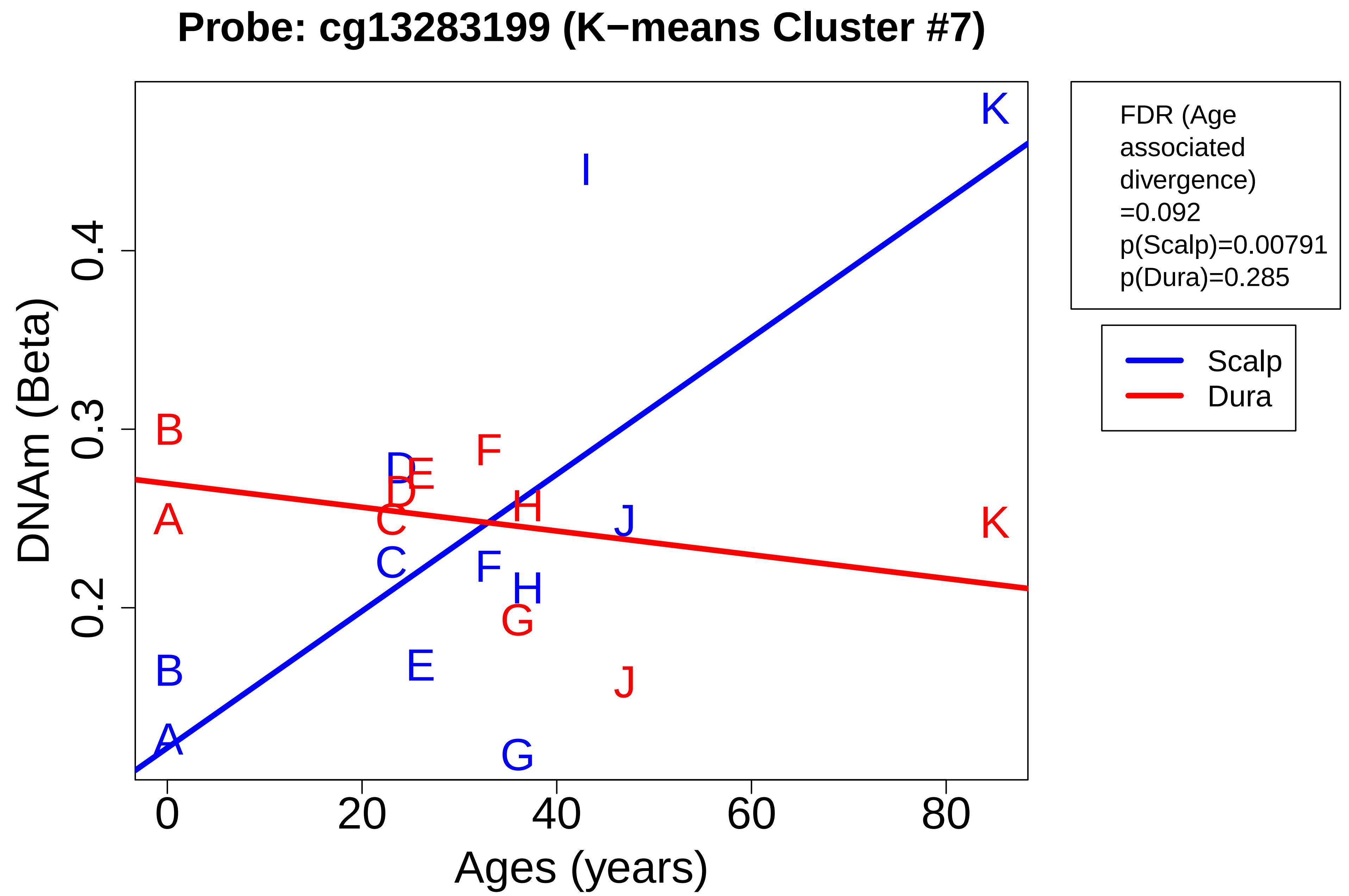

Probe: cg13283199 (K−means Cluster #7)
DNAm (Beta)
0.2
0.3
0.4
0
20
40
60
80
Ages (years)
A
B
C
D
E
F
G
H
I
J
K
A
B
C
D
E
F
G
H
J
K
FDR (Age associated divergence) =0.092
p(Scalp)=0.00791
p(Dura)=0.285
Scalp
Dura

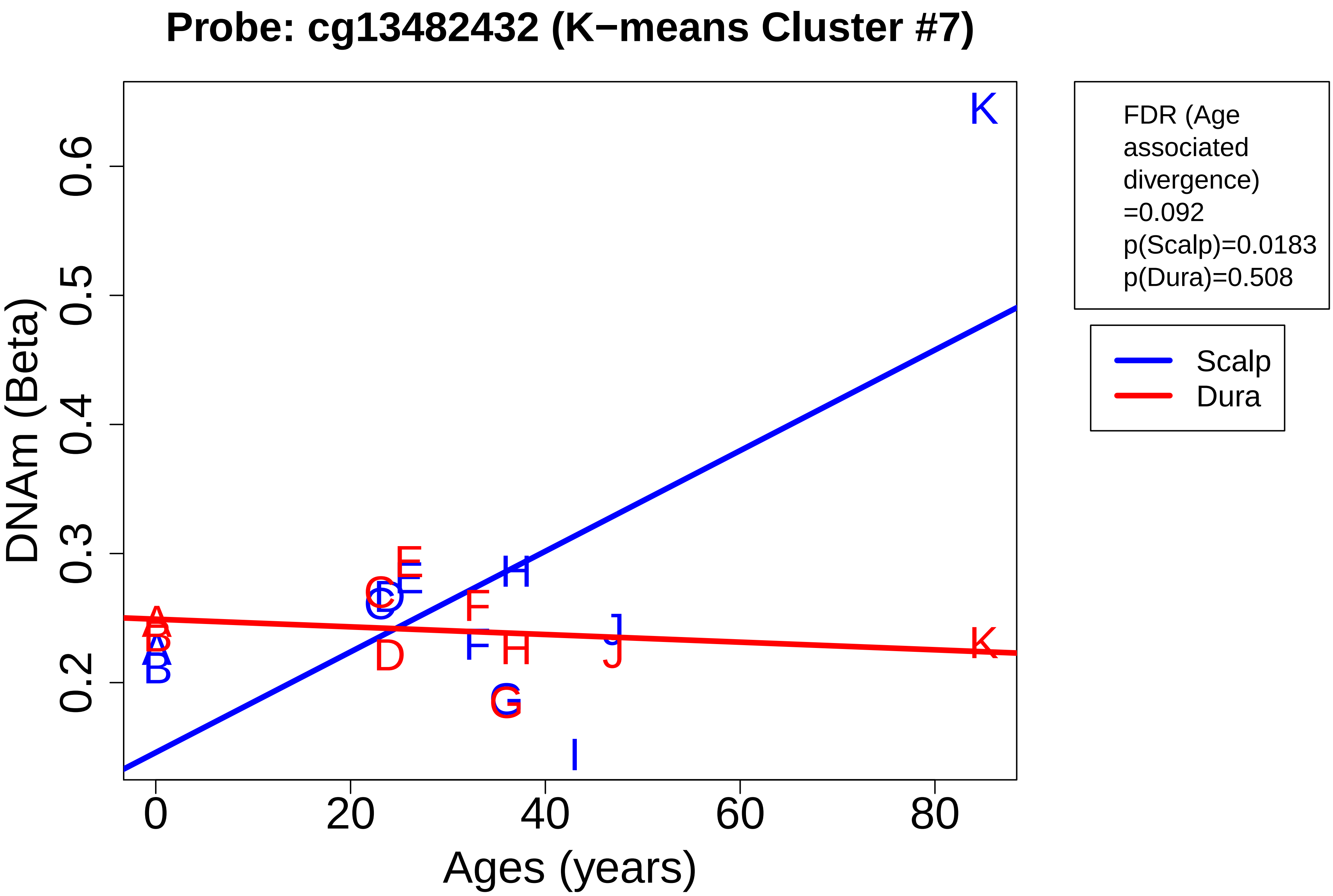

Probe: cg13482432 (K−means Cluster #7)
DNAm (Beta)
Ages (years)
0.2
0.3
0.4
0.5
0.6
0
20
40
60
80
FDR (Age associated divergence) =0.092
p(Scalp)=0.0183
p(Dura)=0.508
Scalp
Dura

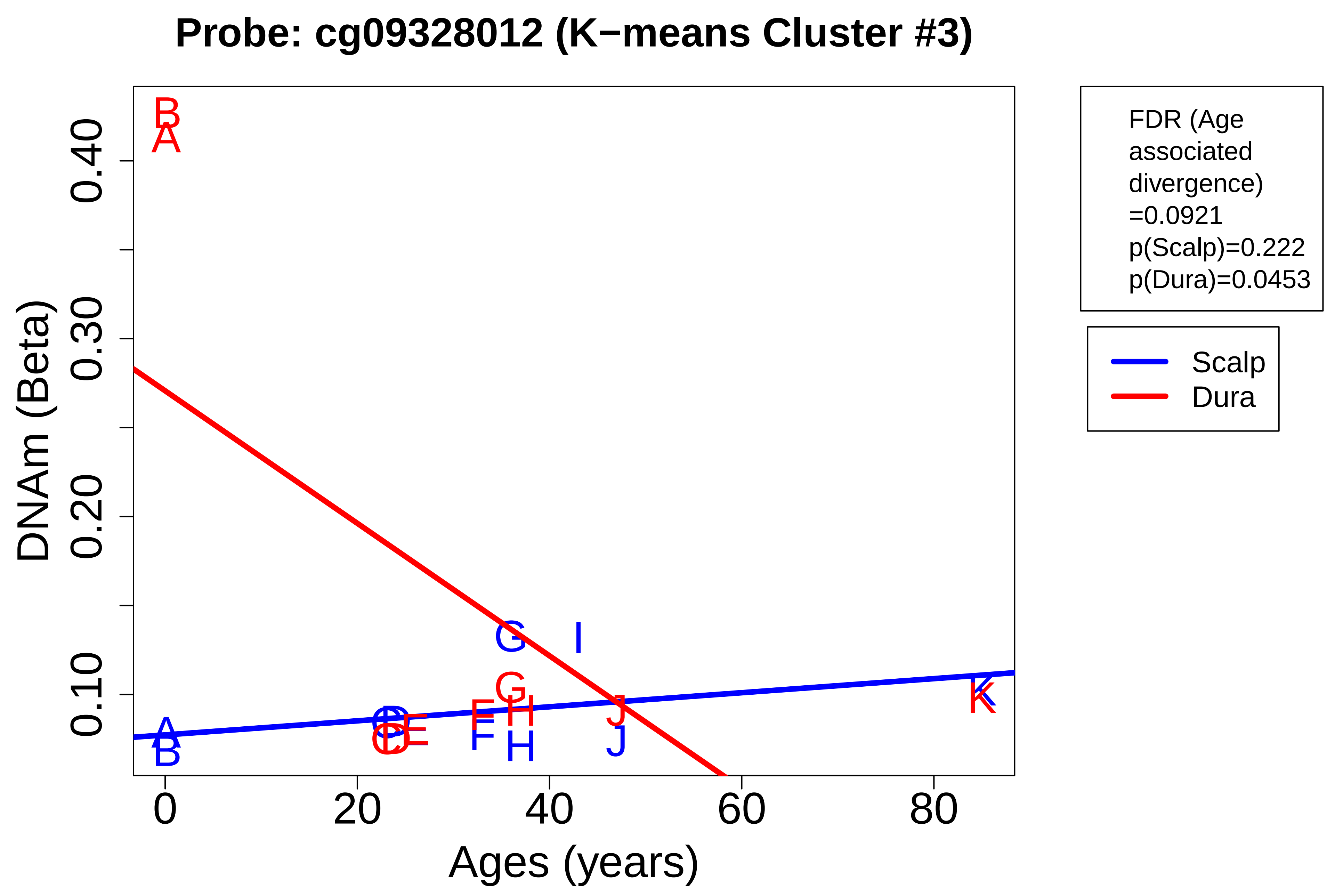

Probe: cg09328012 (K−means Cluster #3)
DNAm (Beta)
0.10
0.20
0.30
0.40
0
20
40
60
80
Ages (years)
FDR (Age associated divergence) =0.0921
p(Scalp)=0.222
p(Dura)=0.0453
Scalp
Dura

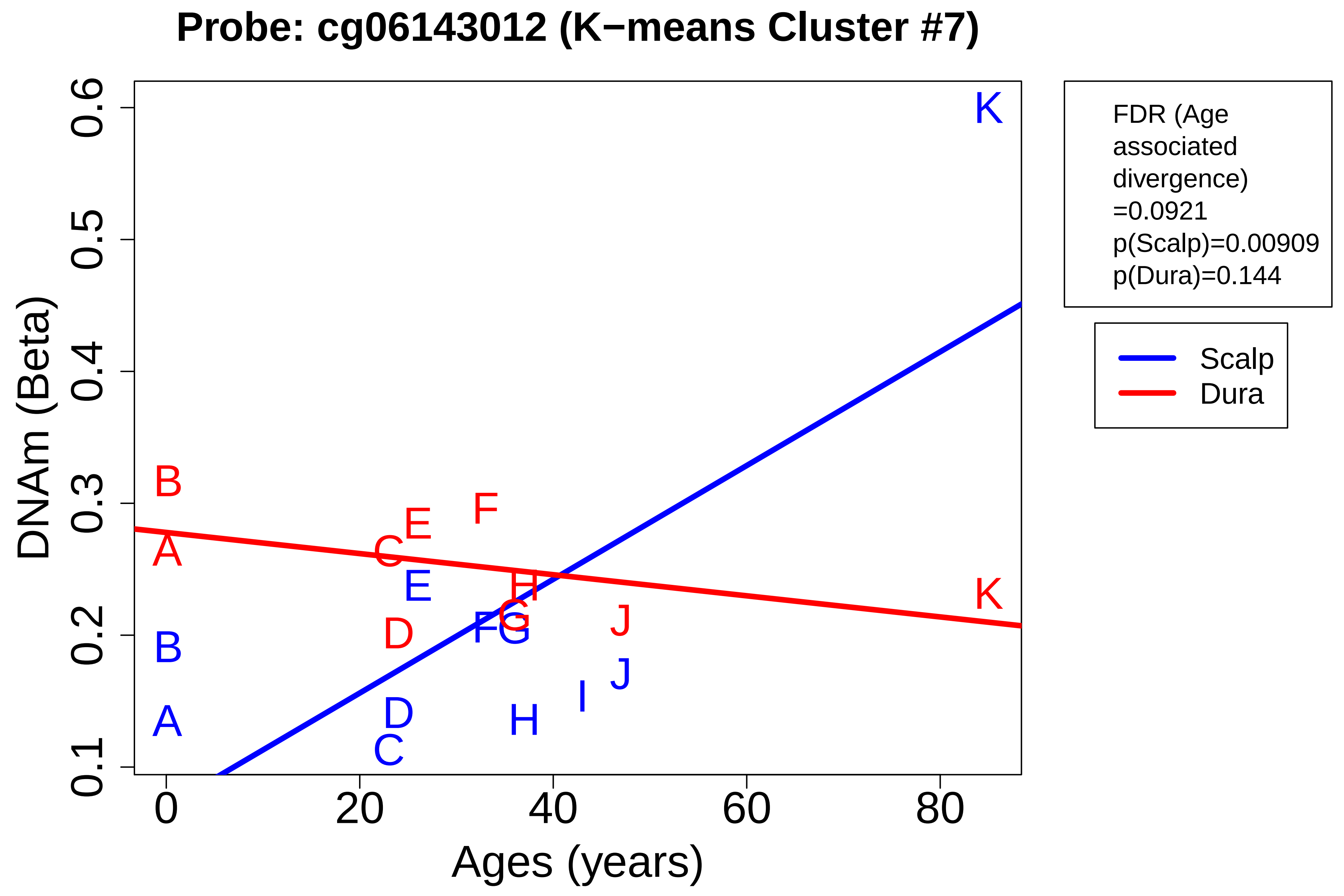

Probe: cg06143012 (K−means Cluster #7)
DNAm (Beta)
0.1
0.2
0.3
0.4
0.5
0.6
Ages (years)
0
20
40
60
80
FDR (Age associated divergence) =0.0921
p(Scalp)=0.00909
p(Dura)=0.144
Scalp
Dura
A
B
C
D
E
F
G
H
I
J
K

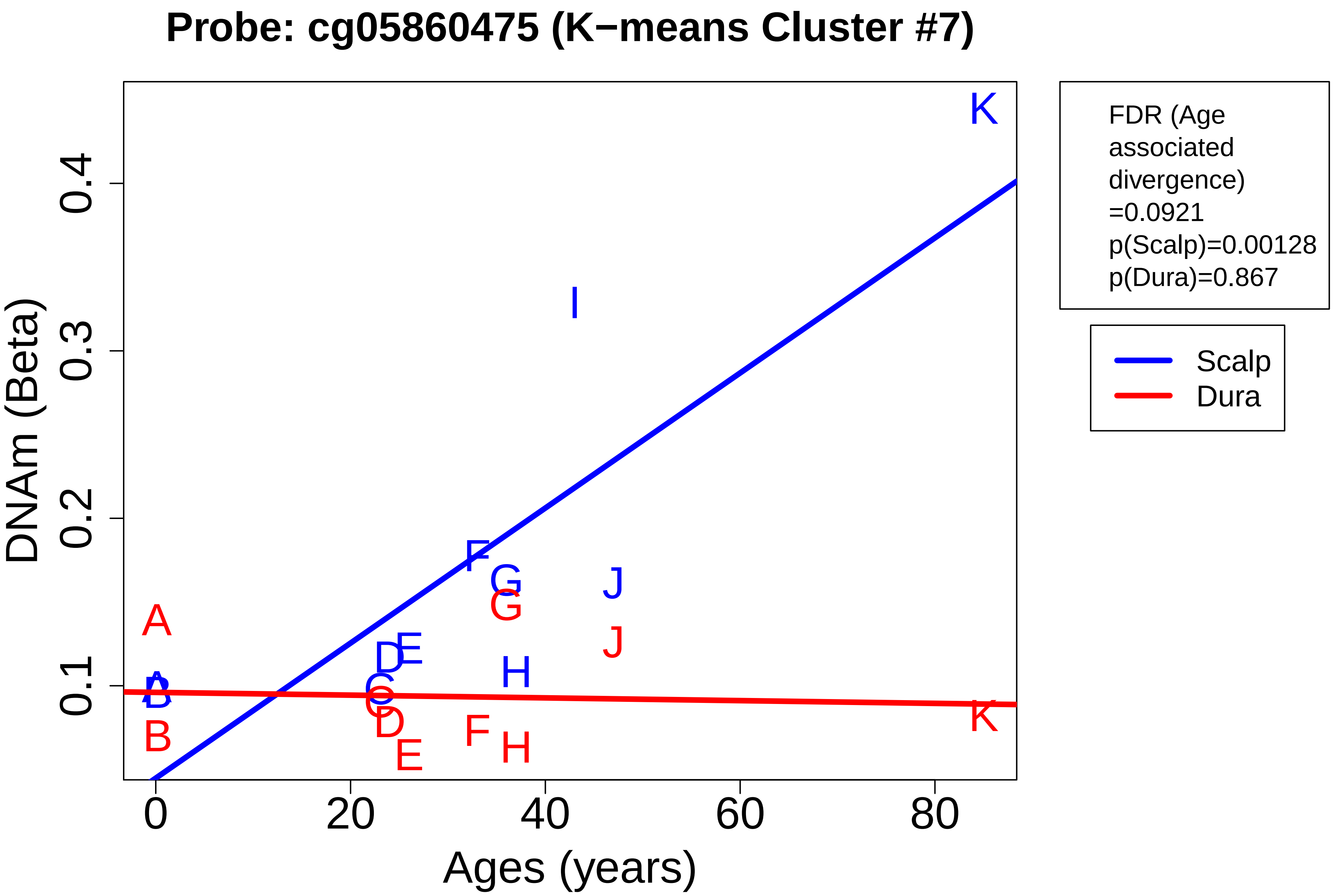

Probe: cg05860475 (K−means Cluster #7)
DNAm (Beta)
0.1
0.2
0.3
0.4
Ages (years)
0
20
40
60
80
A
B
C
D
E
F
G
H
I
J
K
FDR (Age associated divergence) =0.0921
p(Scalp)=0.00128
p(Dura)=0.867
Scalp
Dura

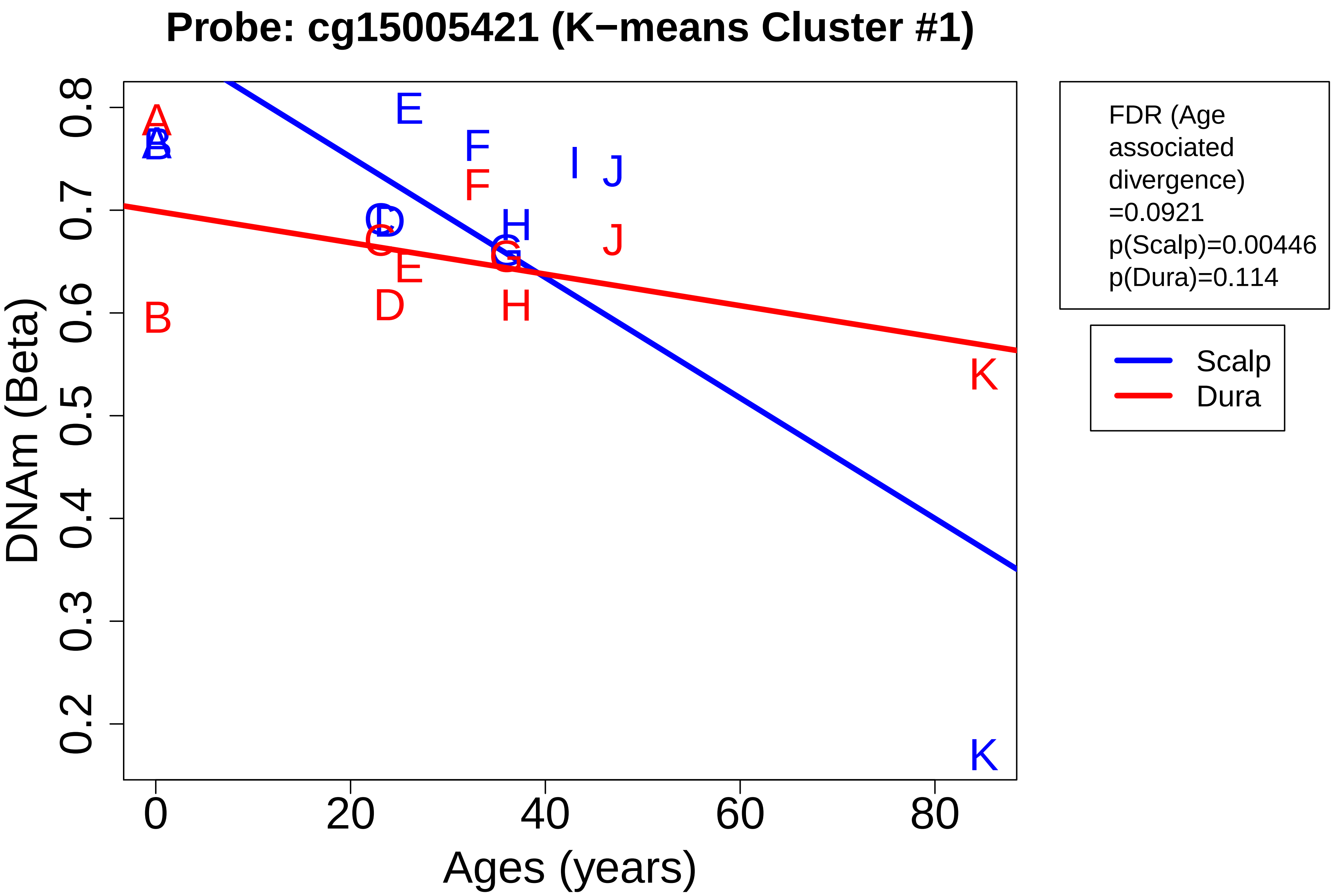
Probe: cg15005421 (K−means Cluster #1)
FDR (Age associated divergence) =0.0921
p(Scalp)=0.00446
p(Dura)=0.114
Scalp
Dura
DNAm (Beta)
Ages (years)
0.2
0.3
0.4
0.5
0.6
0.7
0.8
0
20
40
60
80

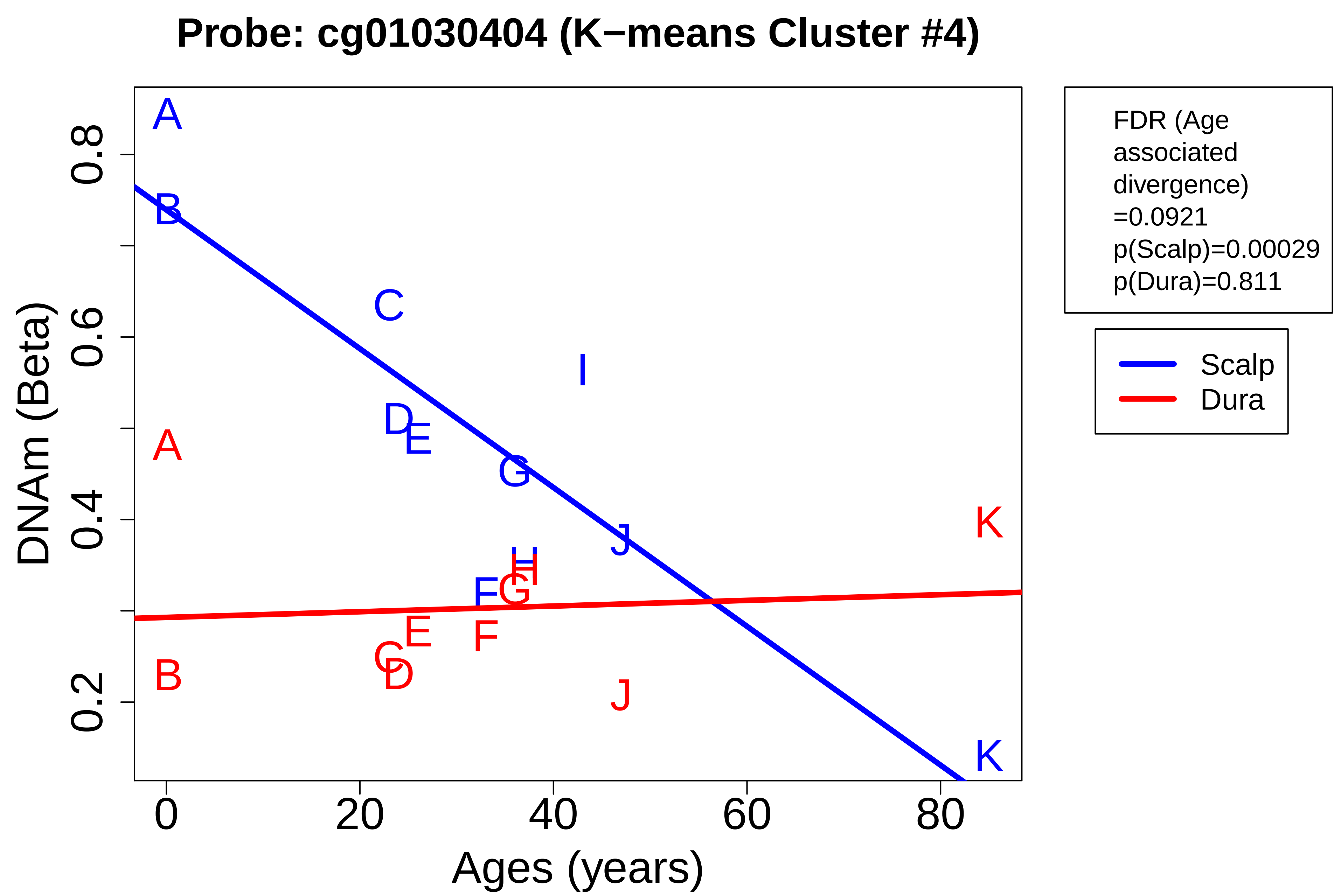

Probe: cg01030404 (K−means Cluster #4)
DNAm (Beta)
0.2
0.4
0.6
0.8
Ages (years)
0
20
40
60
80
A
B
C
D
E
F
G
H
I
J
K
FDR (Age associated divergence) =0.0921
p(Scalp)=0.00029
p(Dura)=0.811
Scalp
Dura

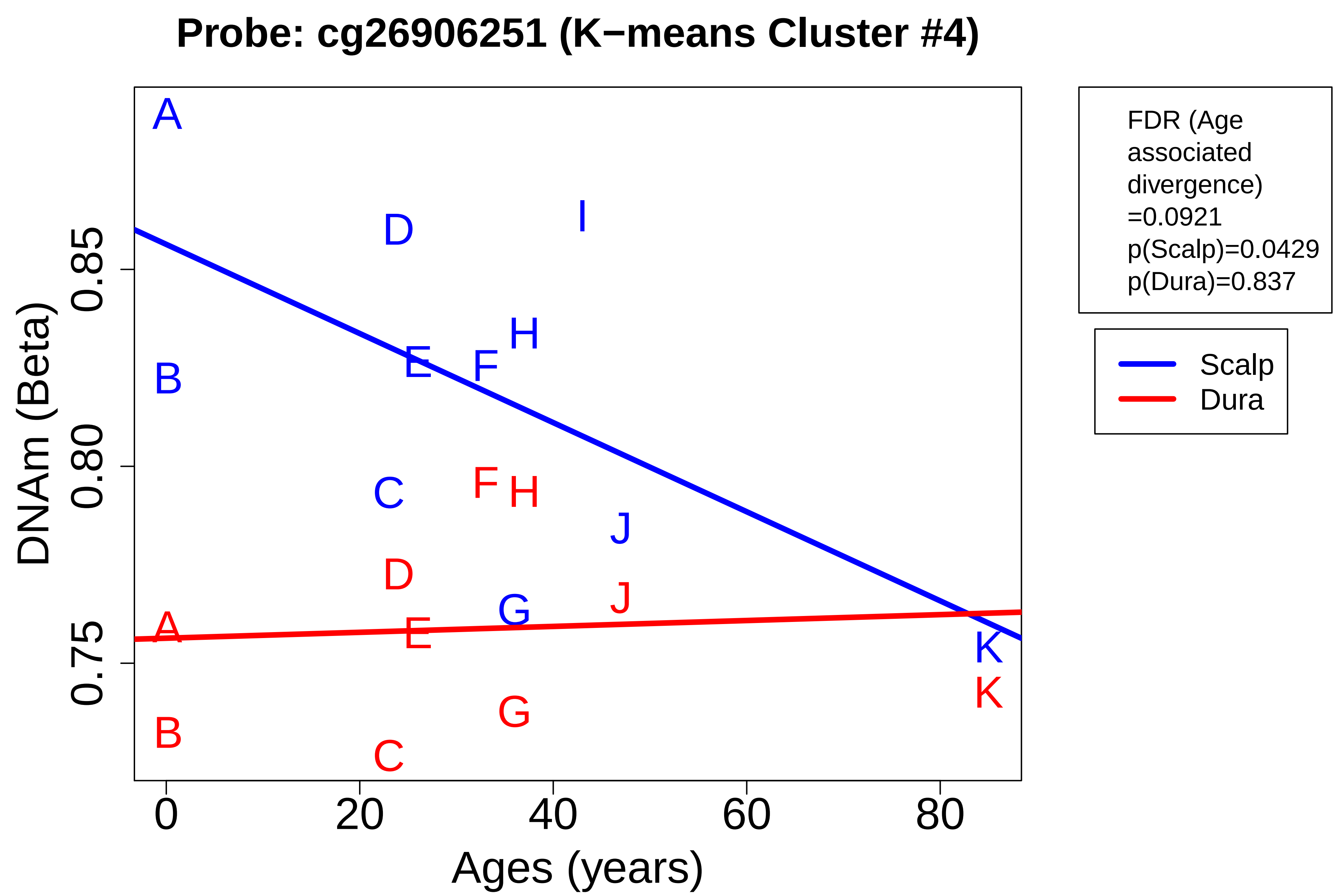

Probe: cg26906251 (K−means Cluster #4)
DNAm (Beta)
0.75
0.80
0.85
0
20
40
60
80
Ages (years)
A
B
C
D
E
F
G
H
I
J
K
FDR (Age associated divergence) =0.0921
p(Scalp)=0.0429
p(Dura)=0.837
Scalp
Dura

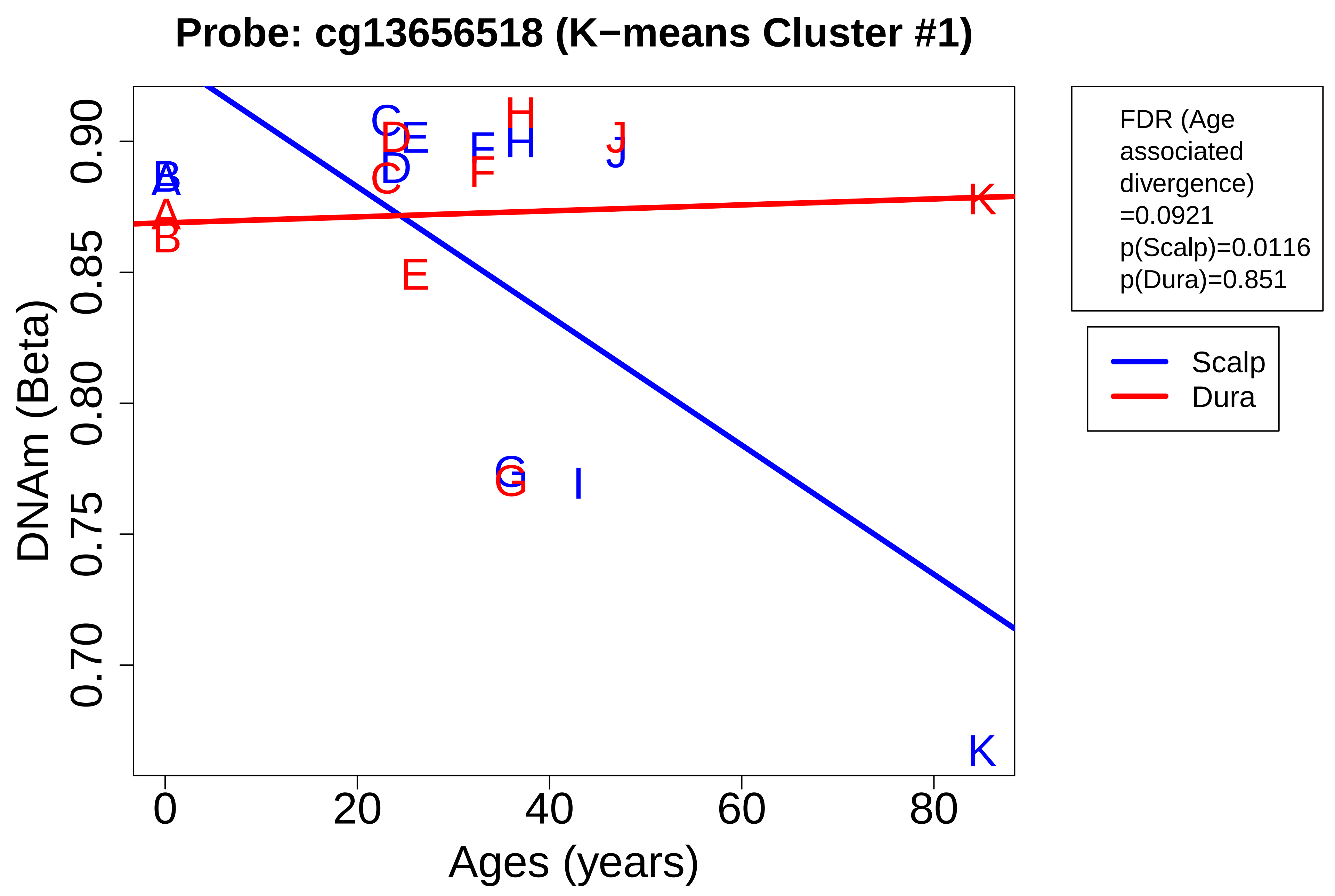

Probe: cg13656518 (K−means Cluster #1)
DNAm (Beta)
0.70
0.75
0.80
0.85
0.90
Ages (years)
0
20
40
60
80
FDR (Age associated divergence) =0.0921
p(Scalp)=0.0116
p(Dura)=0.851
Scalp
Dura

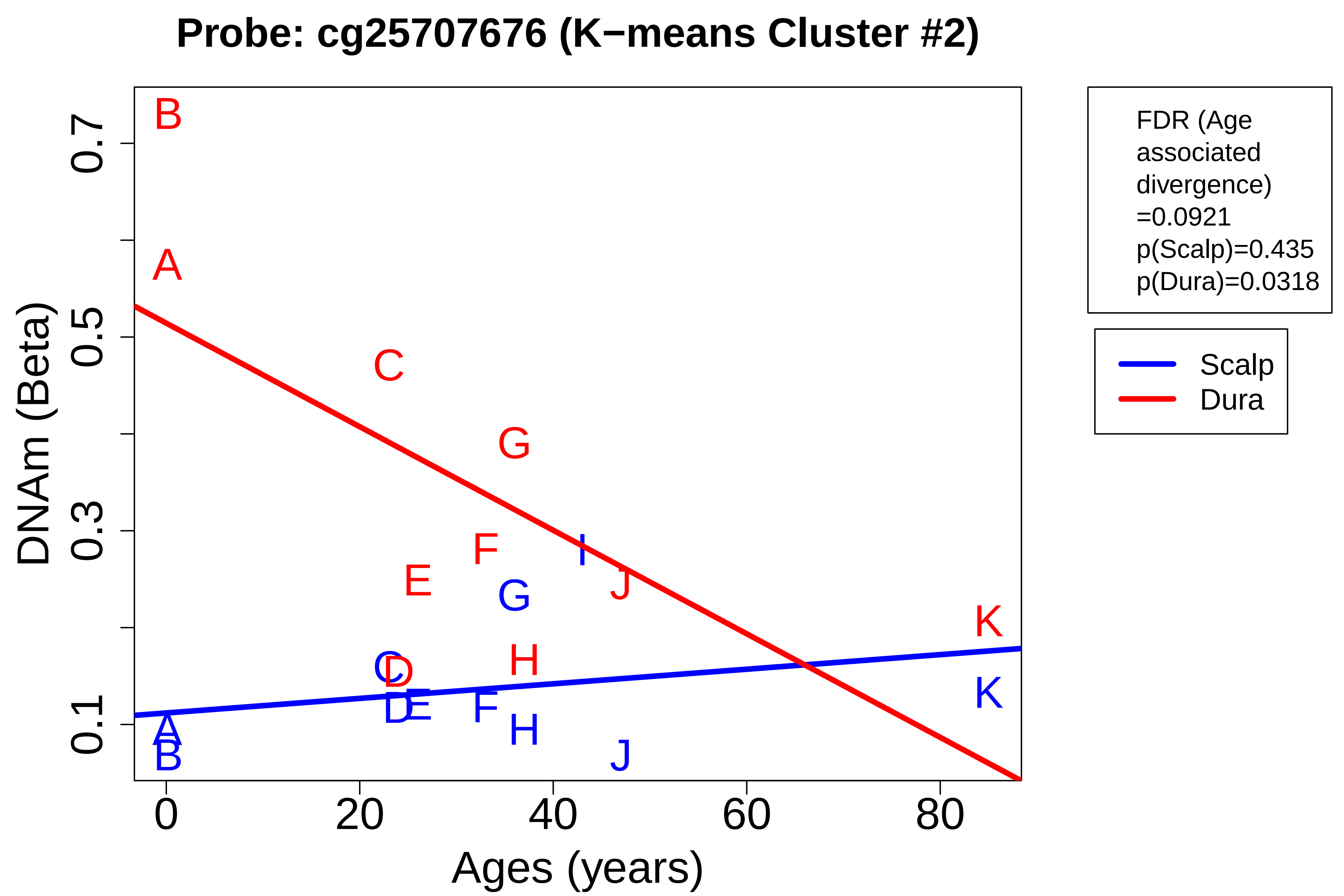

Probe: cg25707676 (K−means Cluster #2)
FDR (Age associated divergence) =0.0921
p(Scalp)=0.435
p(Dura)=0.0318
Scalp
Dura
DNAm (Beta)
0.1
0.3
0.5
0.7
0
20
40
60
80
Ages (years)

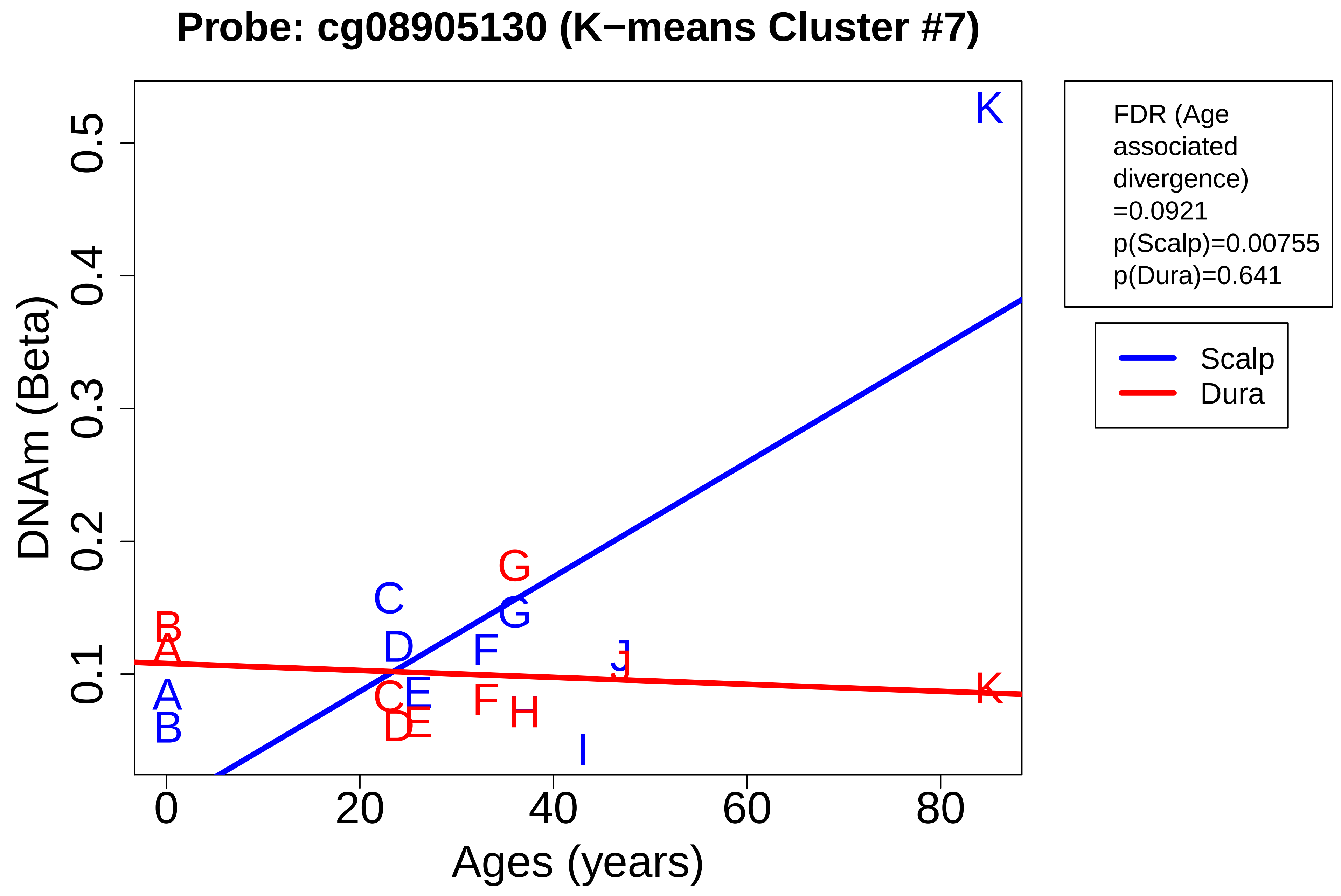

Probe: cg08905130 (K−means Cluster #7)
DNAm (Beta)
0.1
0.2
0.3
0.4
0.5
0
20
40
60
80
Ages (years)
FDR (Age associated divergence) =0.0921
p(Scalp)=0.00755
p(Dura)=0.641
Scalp
Dura

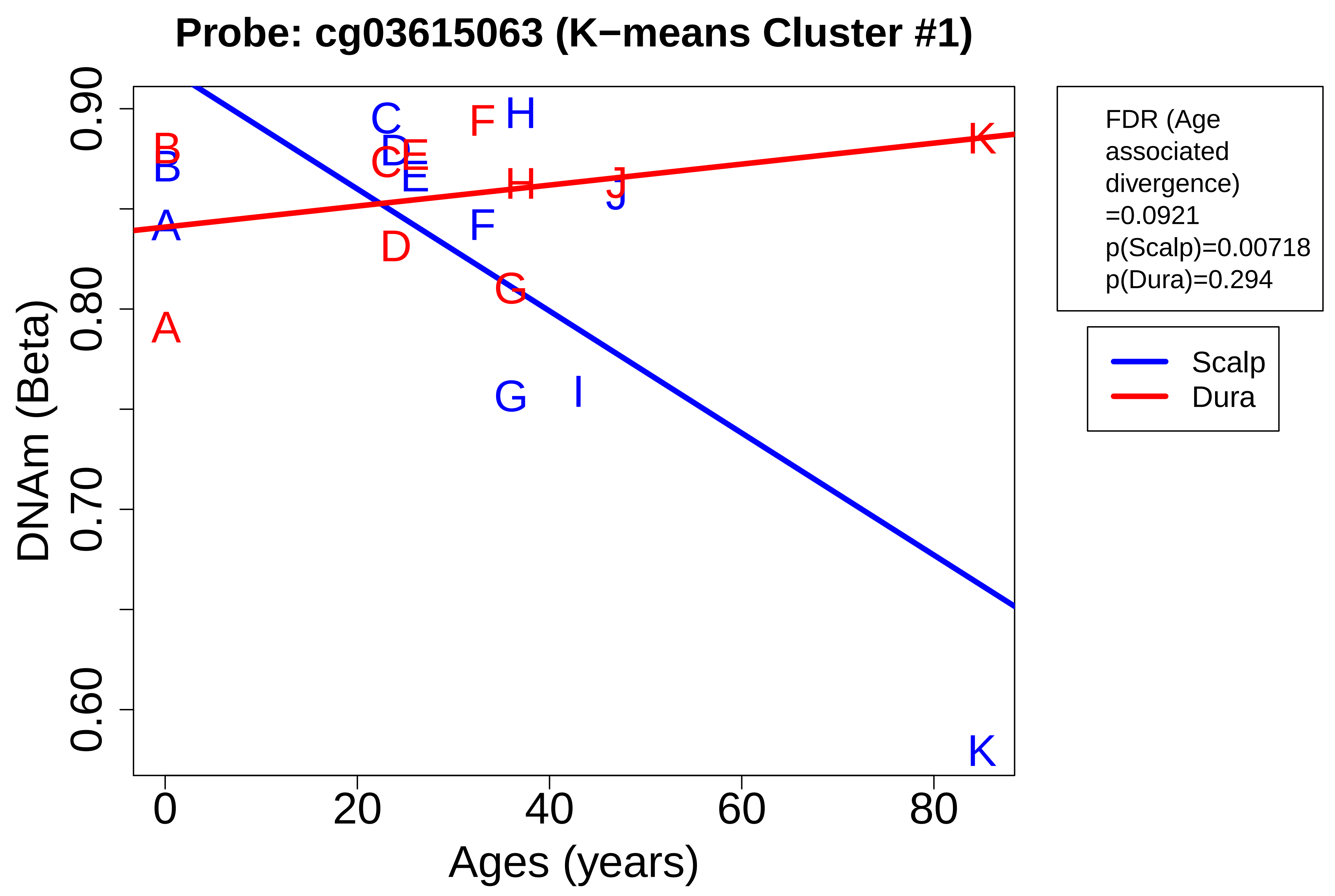
Probe: cg03615063 (K−means Cluster #1)
DNAm (Beta)
0.60
0.70
0.80
0.90
0
20
40
60
80
Ages (years)
A
B
C
D
E
F
G
H
I
J
K
FDR (Age associated divergence) =0.0921
p(Scalp)=0.00718
p(Dura)=0.294
Scalp
Dura

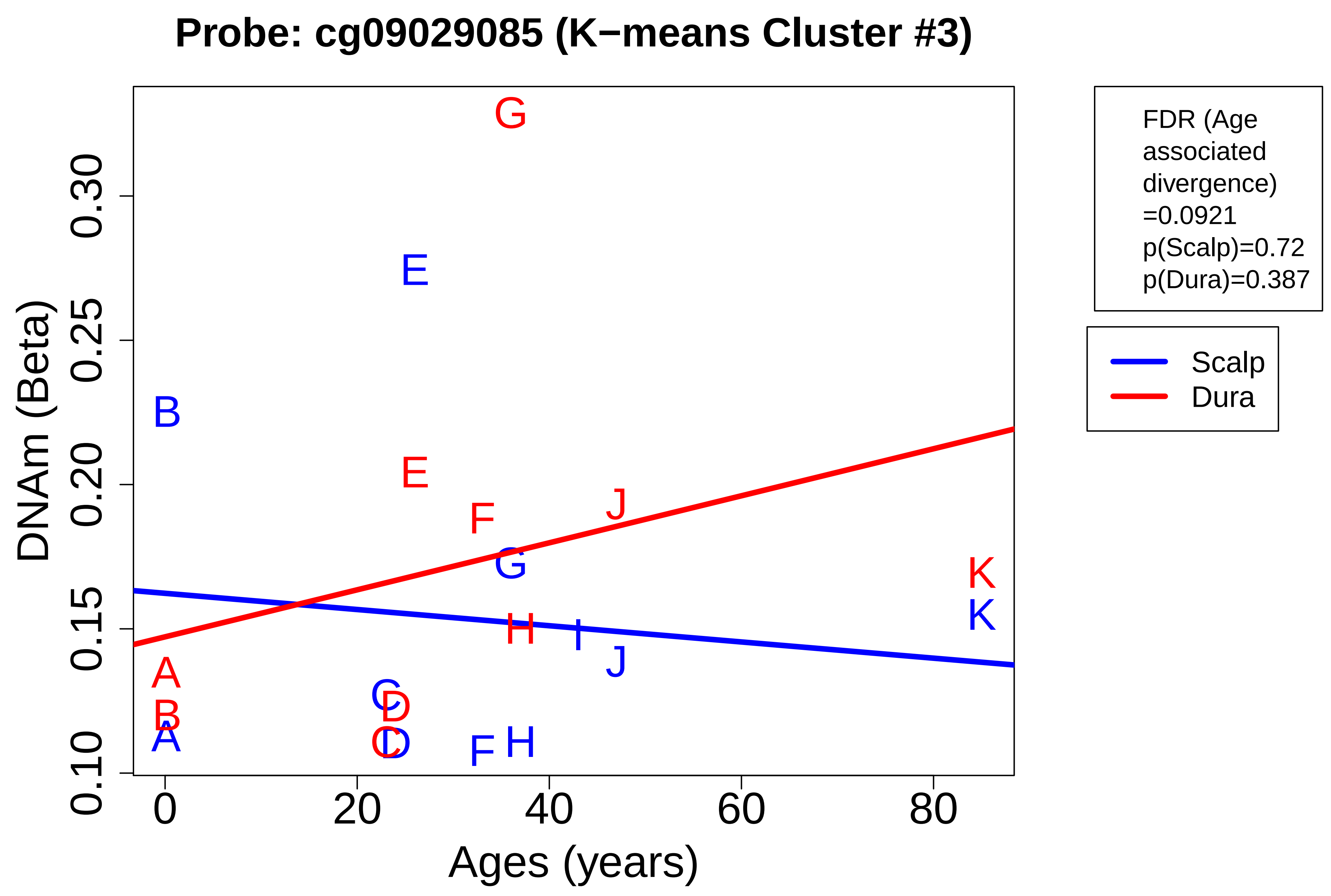
Probe: cg09029085 (K−means Cluster #3)
DNAm (Beta)
0.10
0.15
0.20
0.25
0.30
0
20
40
60
80
Ages (years)
A
B
C
D
E
F
G
H
I
J
K
FDR (Age associated divergence) =0.0921
p(Scalp)=0.72
p(Dura)=0.387
Scalp
Dura

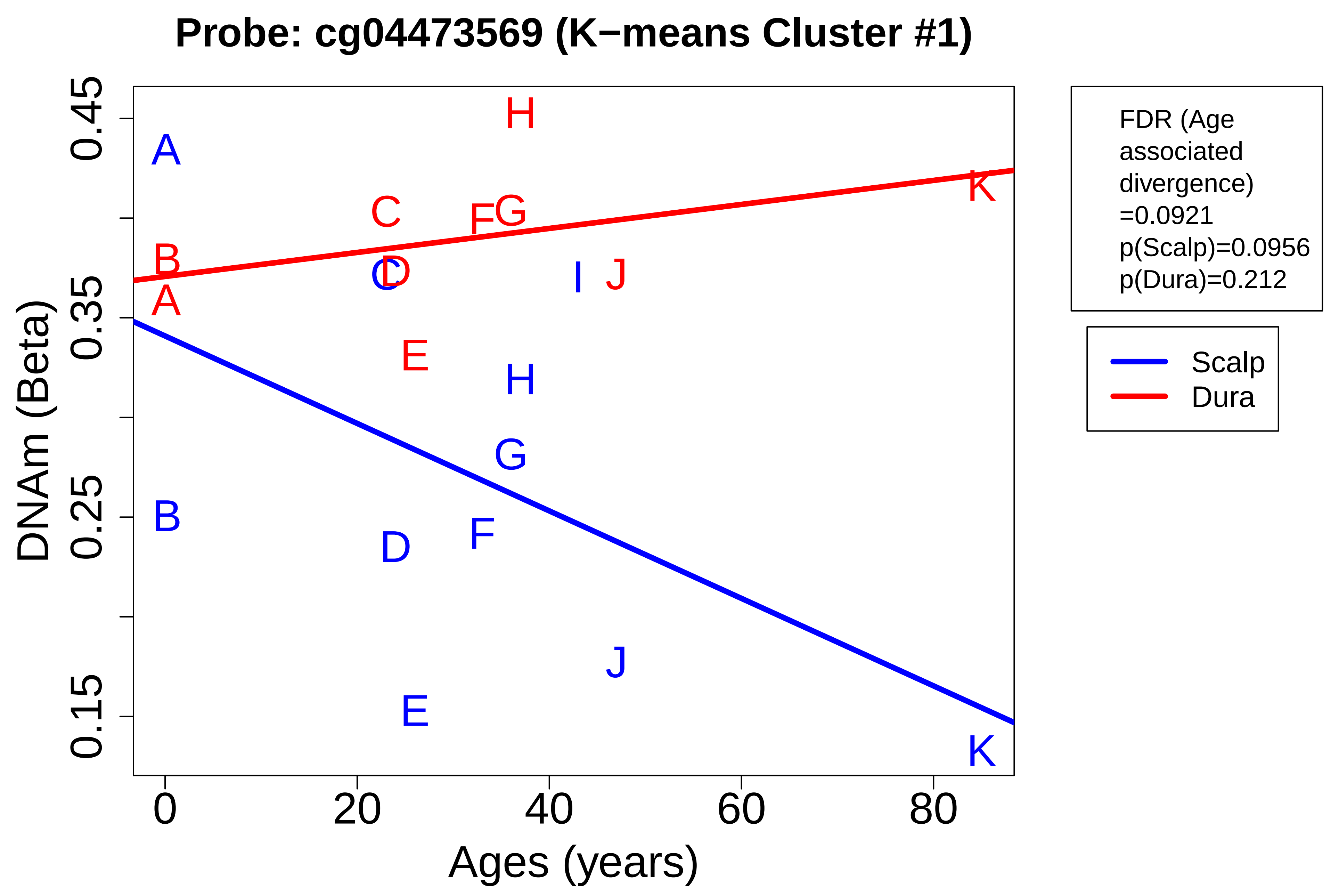

Probe: cg04473569 (K−means Cluster #1)
DNAm (Beta)
0.15
0.25
0.35
0.45
Ages (years)
0
20
40
60
80
FDR (Age associated divergence) =0.0921
p(Scalp)=0.0956
p(Dura)=0.212
Scalp
Dura
A
B
C
D
E
F
G
H
I
J
K

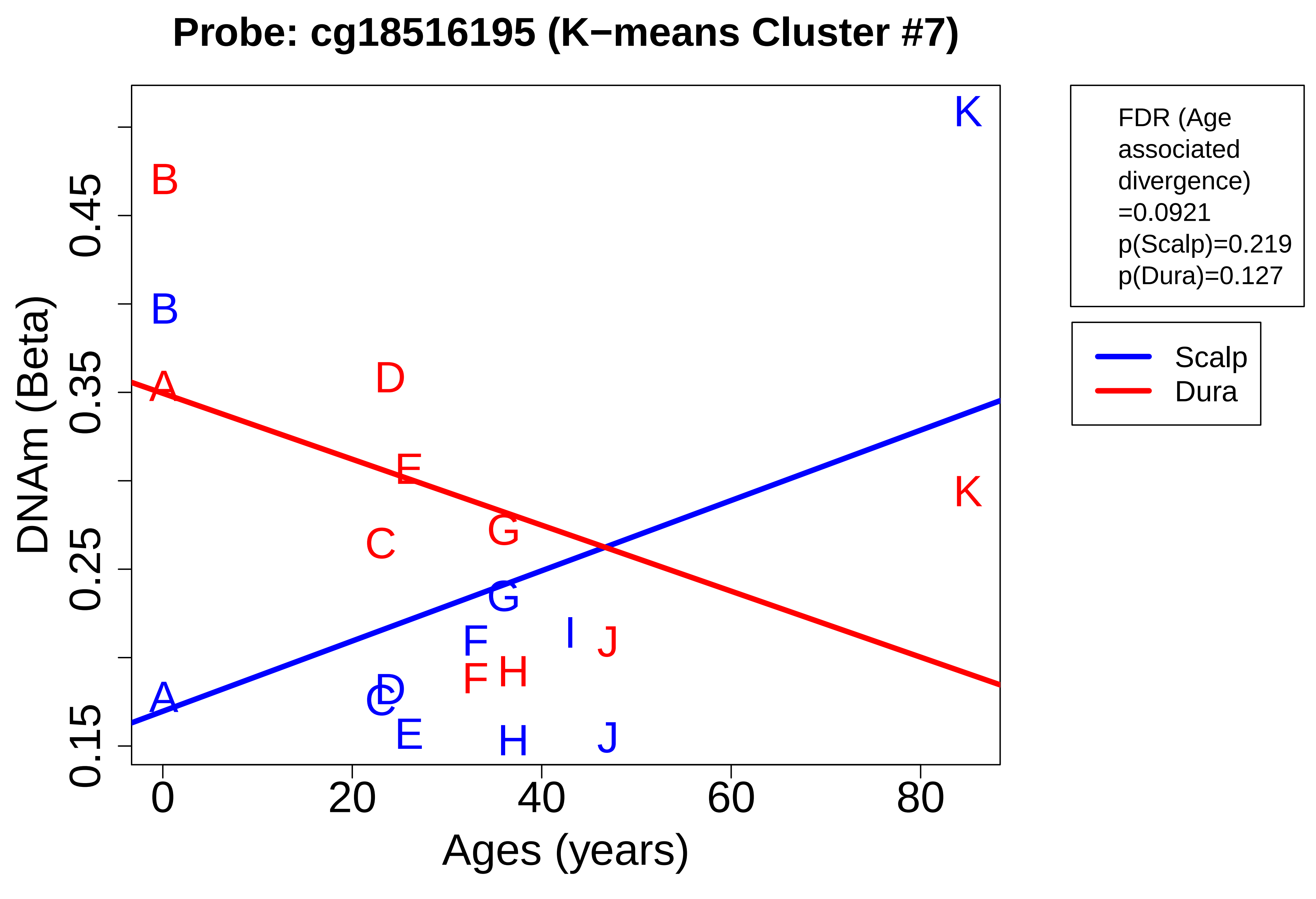
Probe: cg18516195 (K−means Cluster #7)
DNAm (Beta)
Ages (years)
0.15
0.25
0.35
0.45
0
20
40
60
80
FDR (Age associated divergence) =0.0921
p(Scalp)=0.219
p(Dura)=0.127
Scalp
Dura
A
B
C
D
E
F
G
H
I
J
K

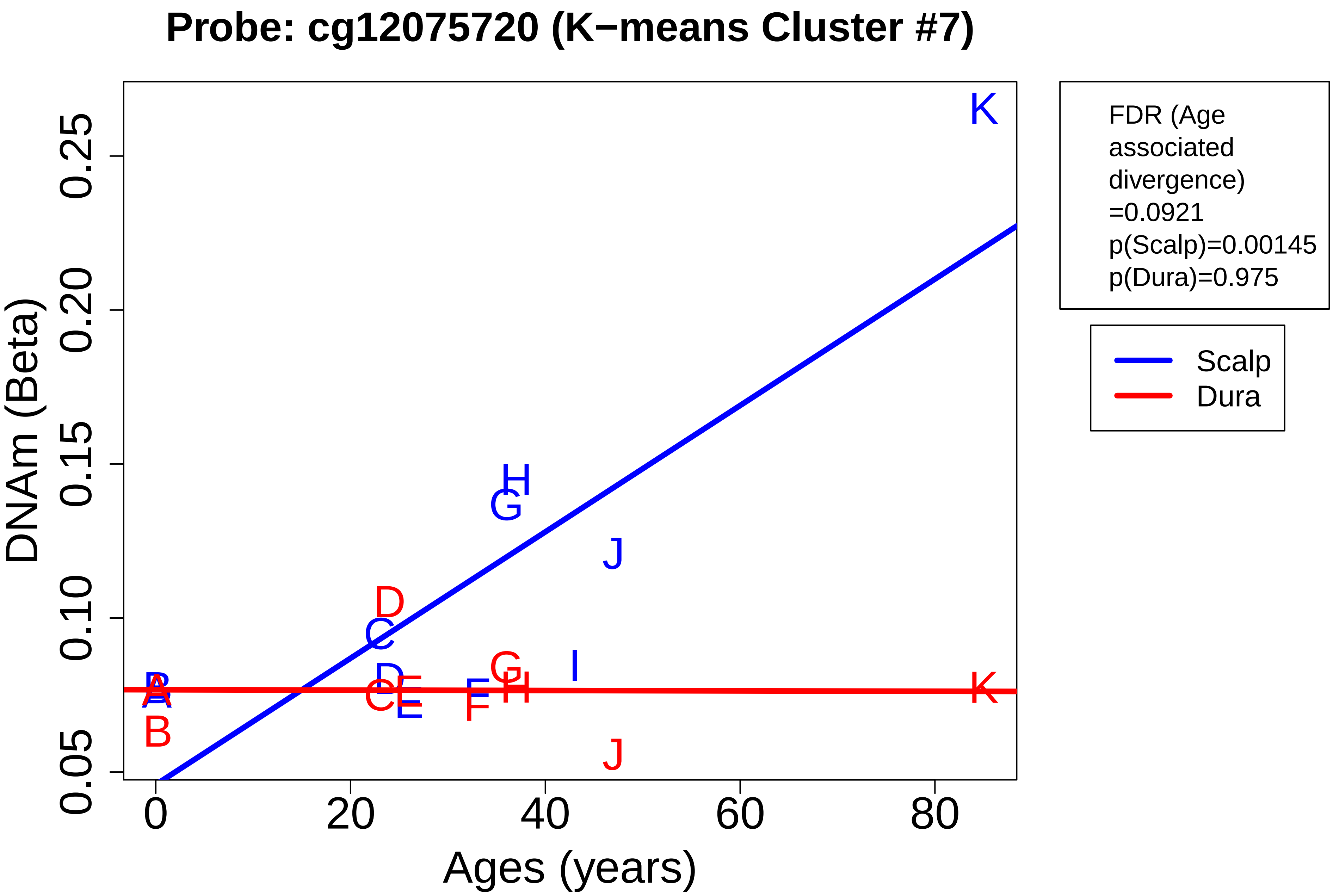

Probe: cg12075720 (K−means Cluster #7)
FDR (Age associated divergence) =0.0921
p(Scalp)=0.00145
p(Dura)=0.975
Scalp
Dura
DNAm (Beta)
0.05
0.10
0.15
0.20
0.25
Ages (years)
0
20
40
60
80

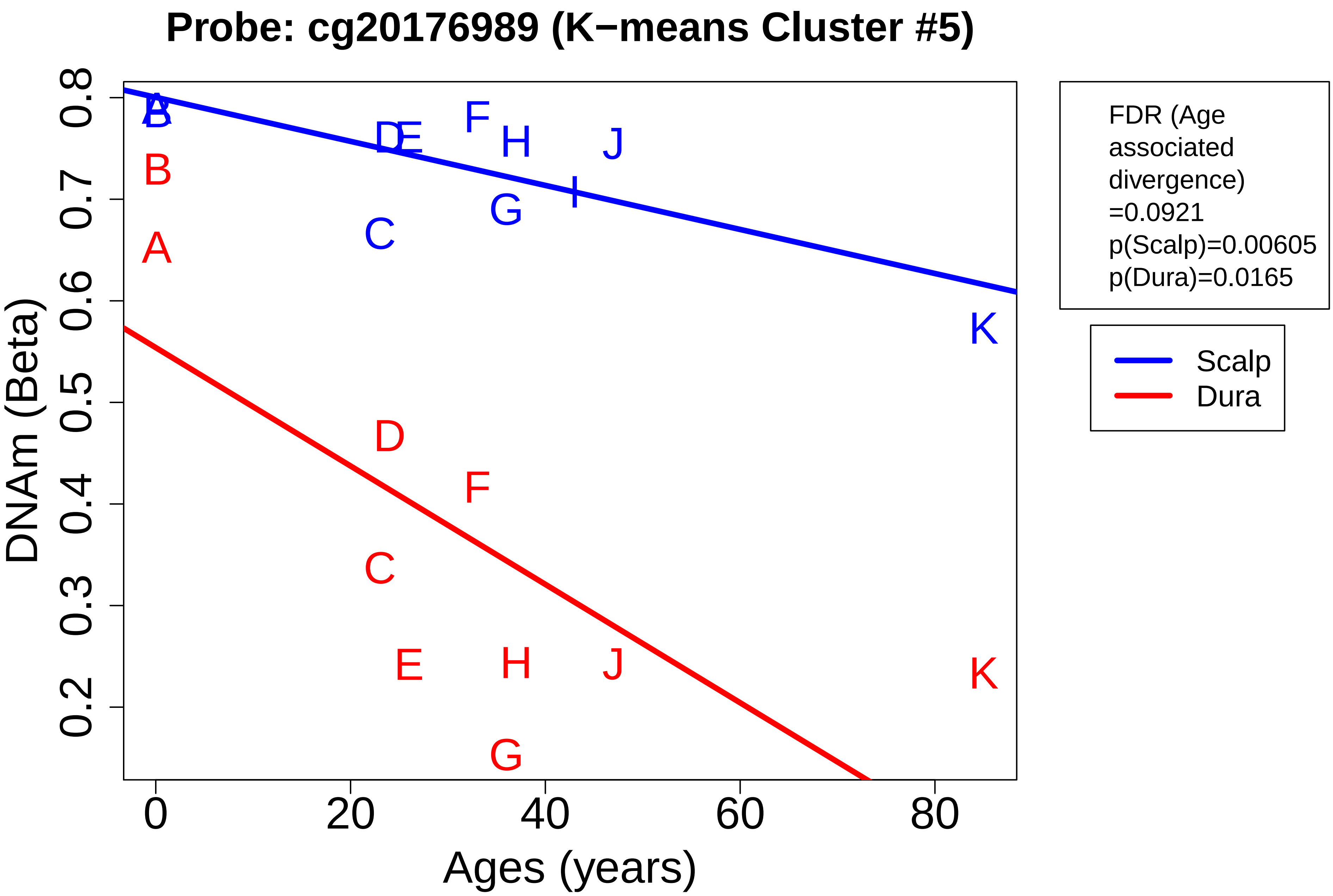

Probe: cg20176989 (K−means Cluster #5)
DNAm (Beta)
Ages (years)
FDR (Age associated divergence) =0.0921
p(Scalp)=0.00605
p(Dura)=0.0165
Scalp
Dura

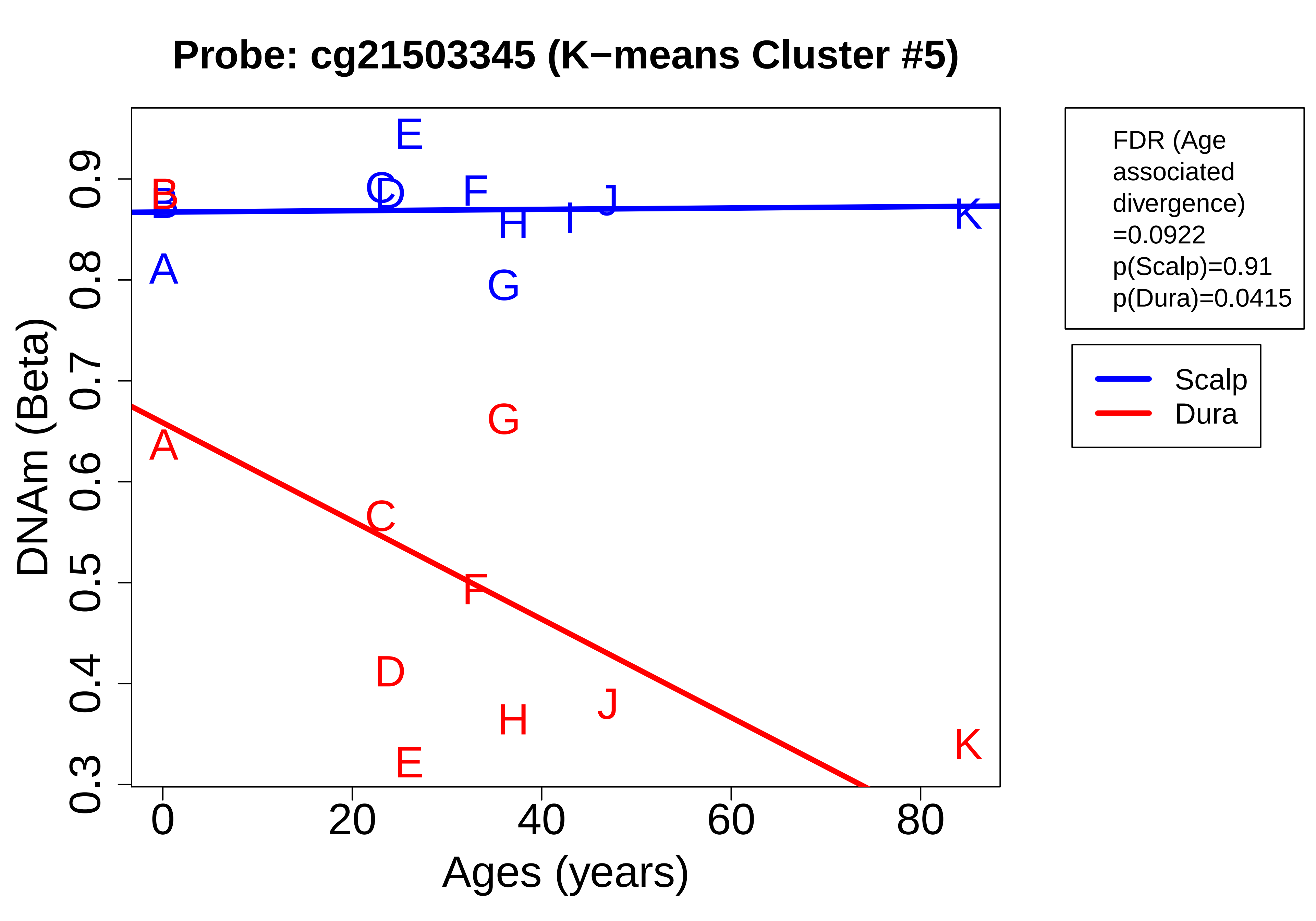

Probe: cg21503345 (K−means Cluster #5)
DNAm (Beta)
Ages (years)
0.3
0.4
0.5
0.6
0.7
0.8
0.9
0
20
40
60
80
FDR (Age associated divergence) =0.0922
p(Scalp)=0.91
p(Dura)=0.0415
Scalp
Dura

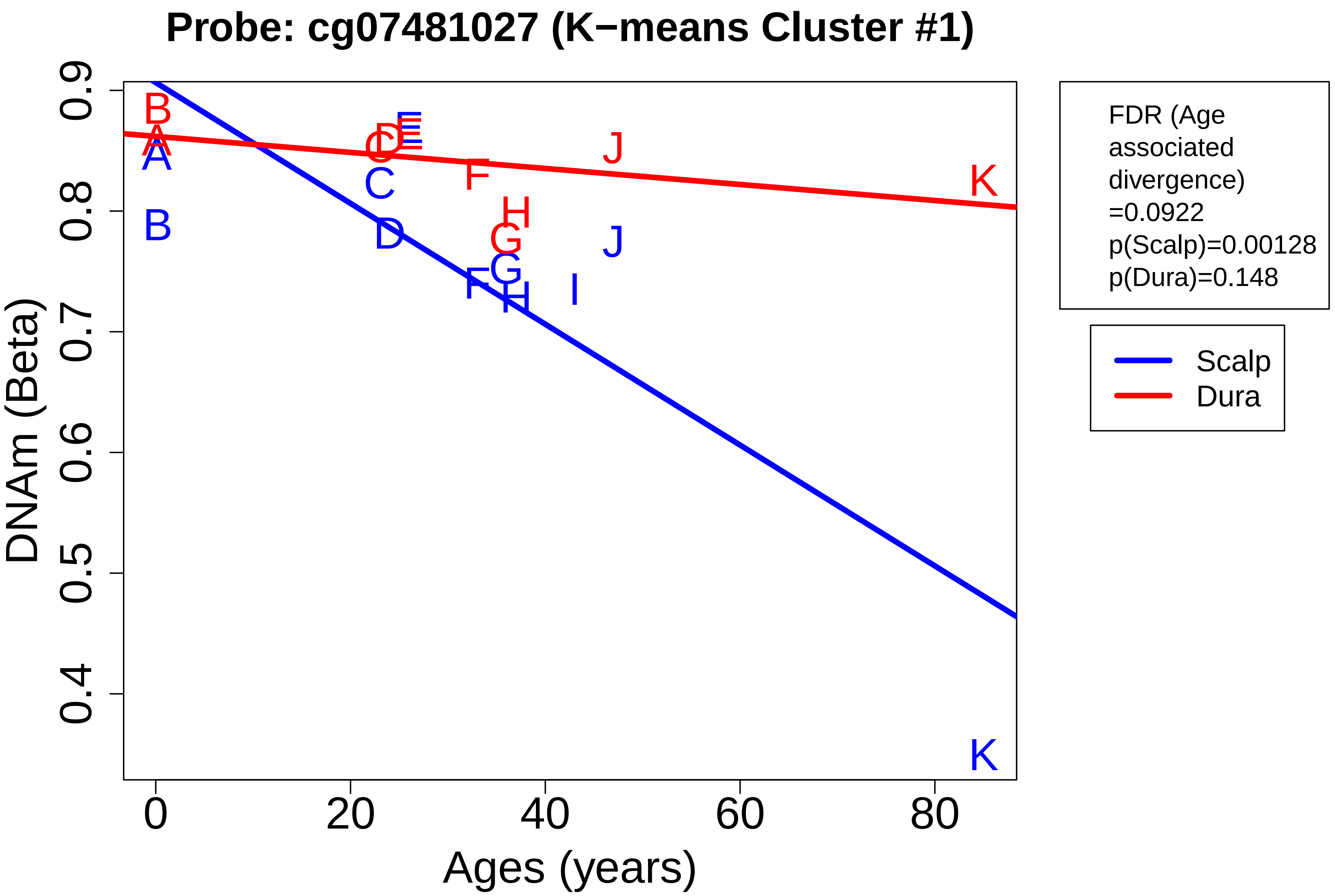
Probe: cg07481027 (K−means Cluster #1)
DNAm (Beta)
0.4
0.5
0.6
0.7
0.8
0.9
Ages (years)
0
20
40
60
80
A
B
C
D
E
F
G
H
I
J
K
FDR (Age associated divergence) =0.0922
p(Scalp)=0.00128
p(Dura)=0.148
Scalp
Dura

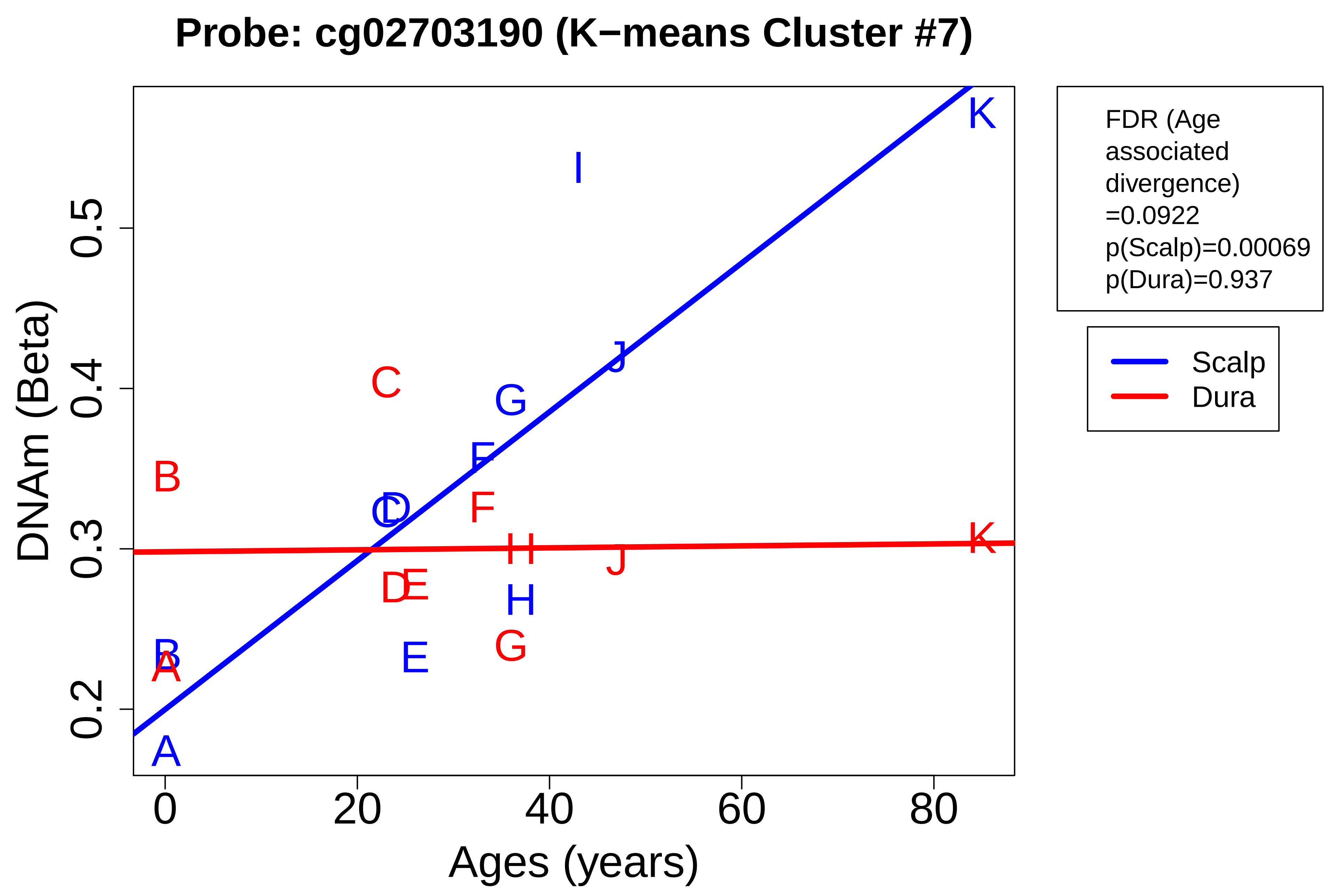

Probe: cg02703190 (K−means Cluster #7)
DNAm (Beta)
0.2
0.3
0.4
0.5
0
20
40
60
80
Ages (years)
FDR (Age associated divergence) =0.0922
p(Scalp)=0.00069
p(Dura)=0.937
Scalp
Dura
A
B
C
D
E
F
G
H
I
J
K

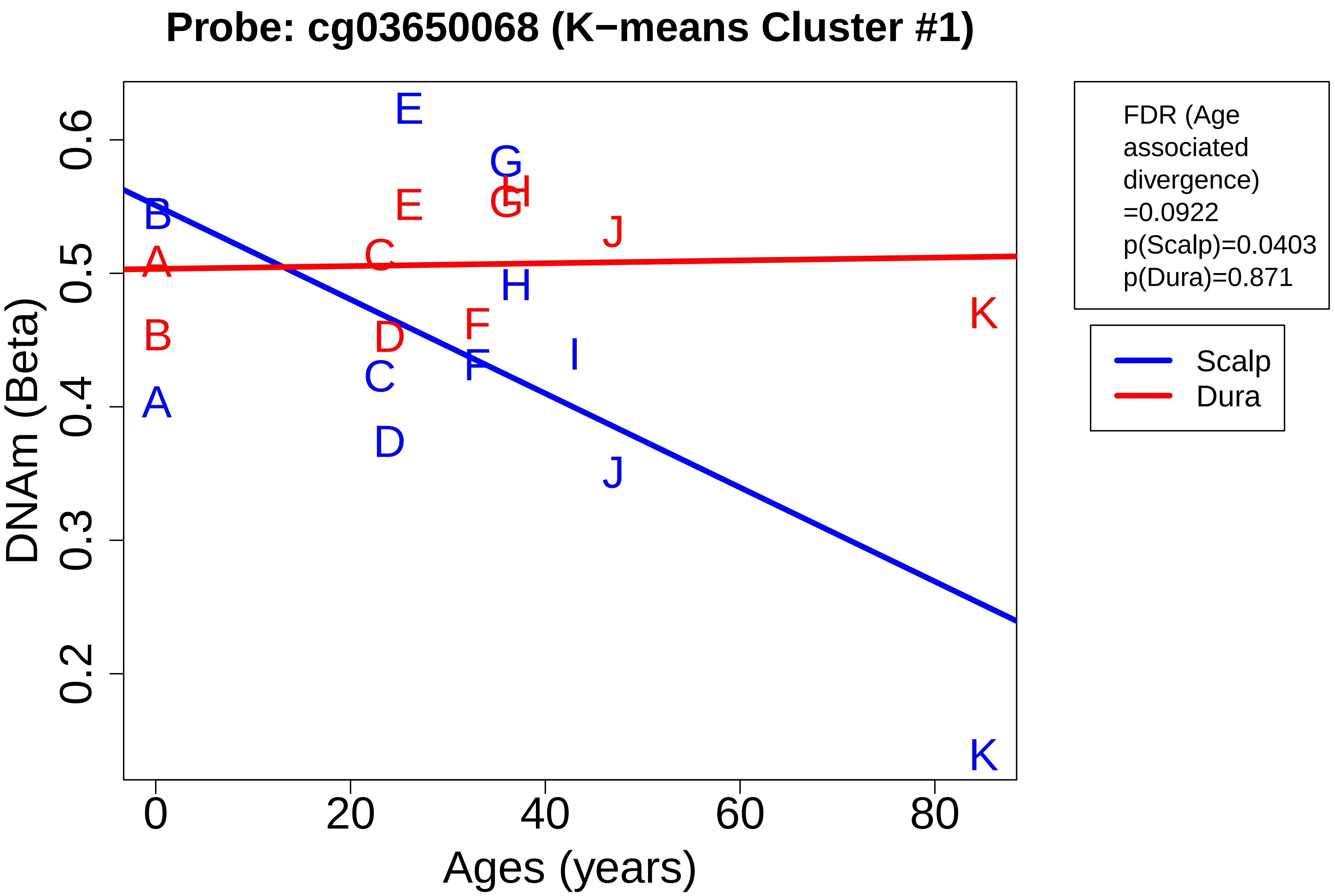

Probe: cg03650068 (K−means Cluster #1)
DNAm (Beta)
0.2
0.3
0.4
0.5
0.6
0
20
40
60
80
Ages (years)
E
G
H
E
G
B
J
A
C
H
K
B
F
D
I
F
C
A
D
J
K
FDR (Age associated divergence) =0.0922
p(Scalp)=0.0403
p(Dura)=0.871
Scalp
Dura

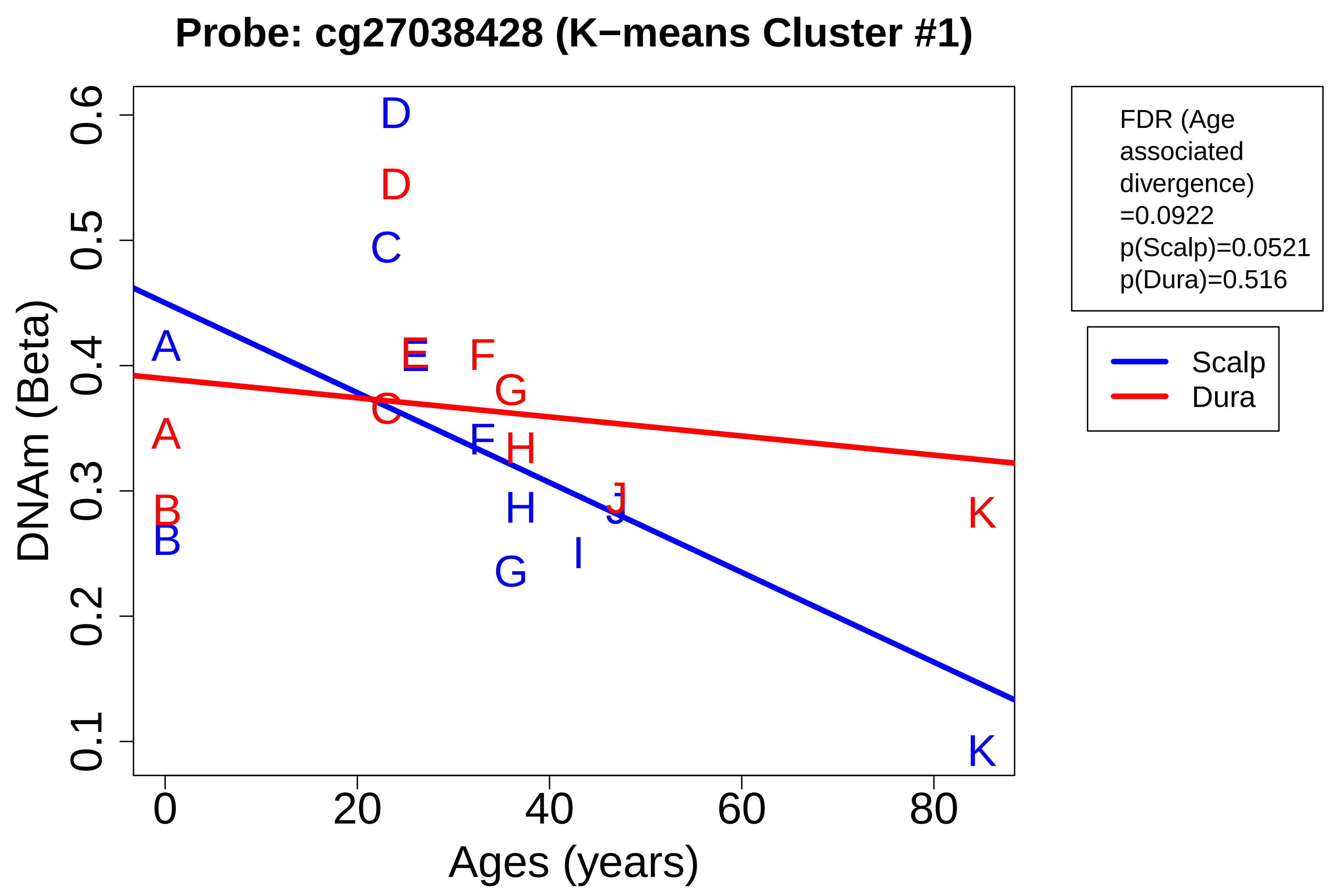

Probe: cg27038428 (K−means Cluster #1)
DNAm (Beta)
0.1
0.2
0.3
0.4
0.5
0.6
0
20
40
60
80
Ages (years)
FDR (Age associated divergence) =0.0922
p(Scalp)=0.0521
p(Dura)=0.516
Scalp
Dura

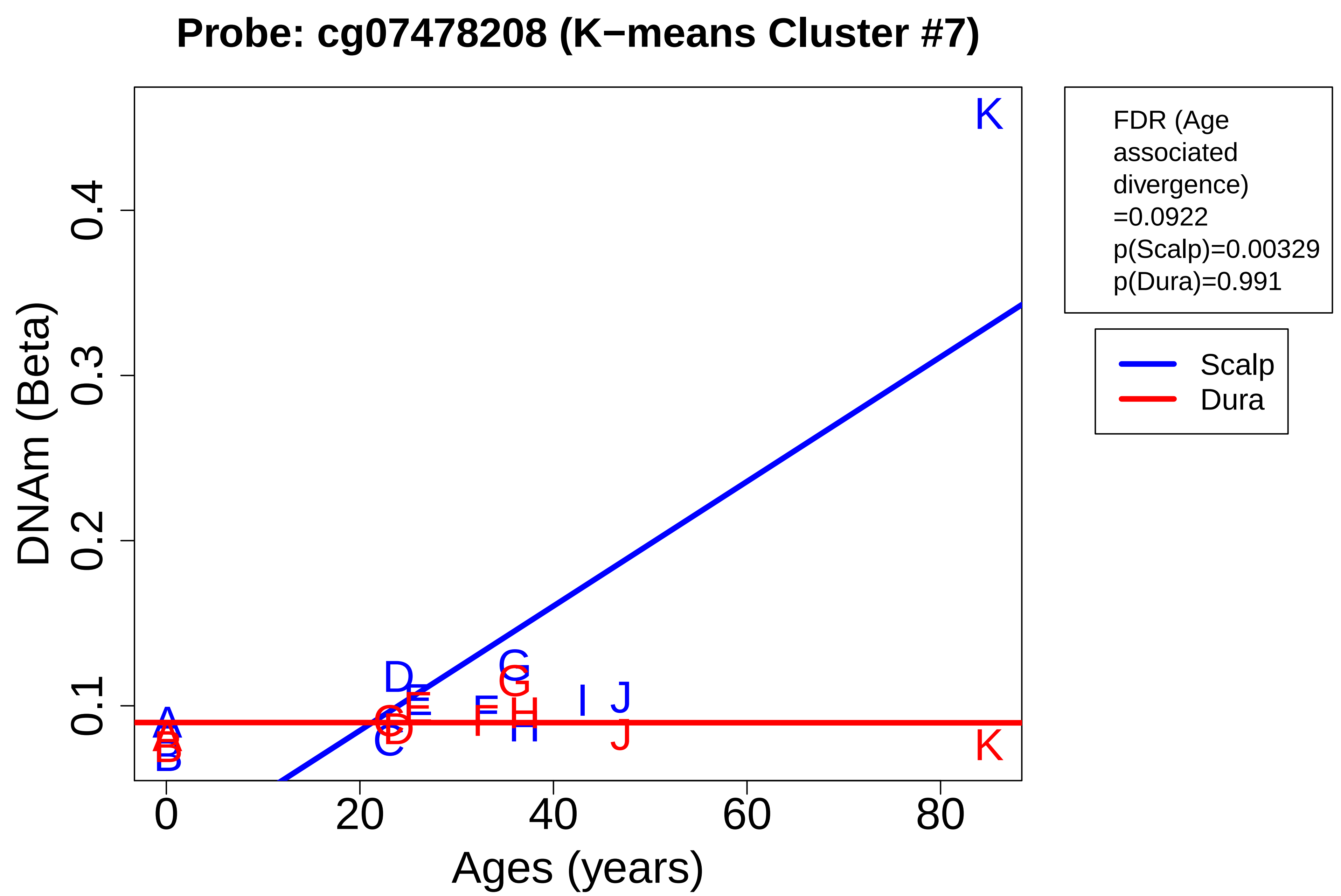
Probe: cg07478208 (K−means Cluster #7)
DNAm (Beta)
0.1
0.2
0.3
0.4
0
20
40
60
80
Ages (years)
FDR (Age associated divergence) =0.0922
p(Scalp)=0.00329
p(Dura)=0.991
Scalp
Dura
A
B
C
D
E
F
G
H
I
J
K

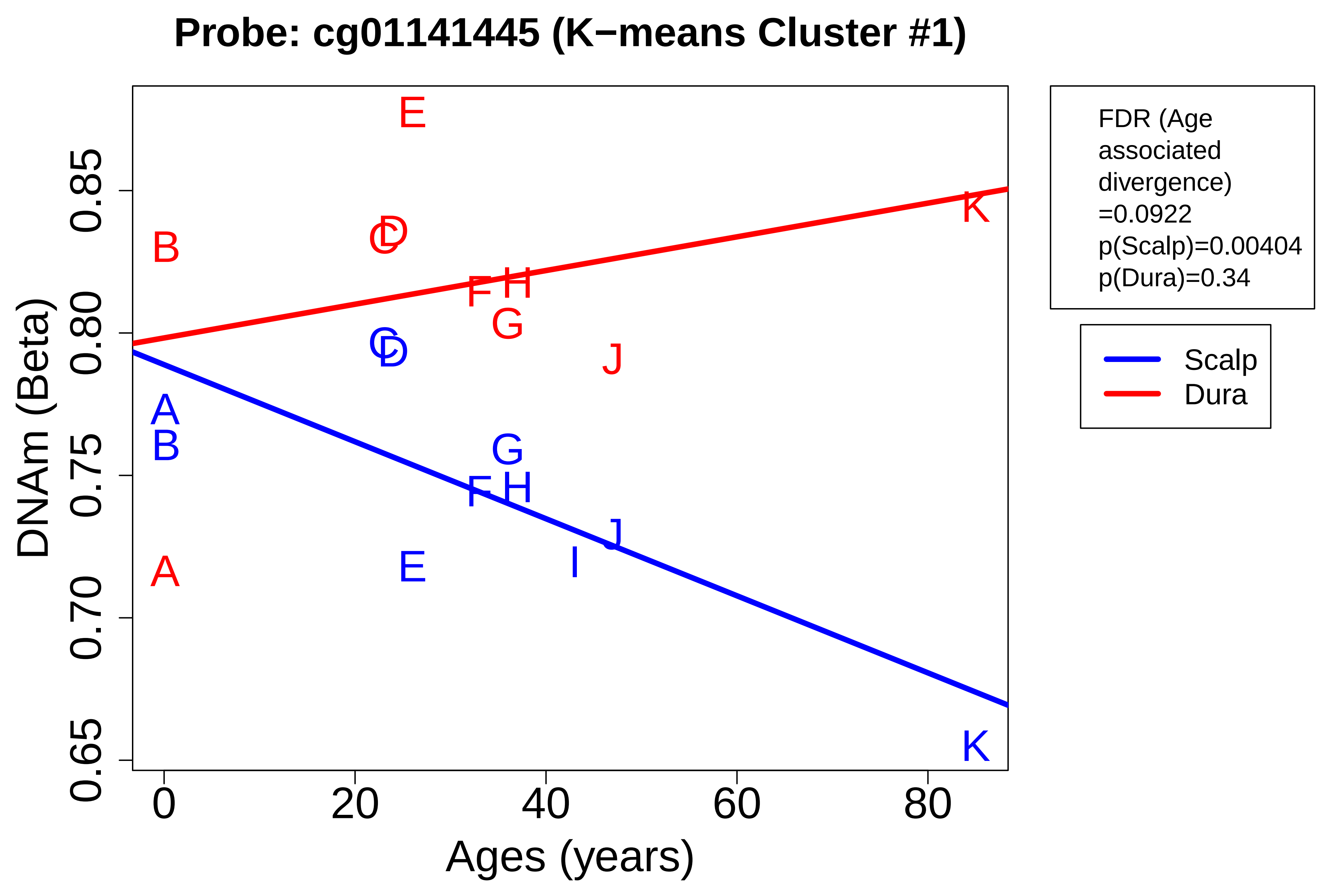

Probe: cg01141445 (K−means Cluster #1)
DNAm (Beta)
0.65
0.70
0.75
0.80
0.85
0
20
40
60
80
Ages (years)
FDR (Age associated divergence) =0.0922
p(Scalp)=0.00404
p(Dura)=0.34
Scalp
Dura
A
B
C
D
E
F
G
H
I
J
K

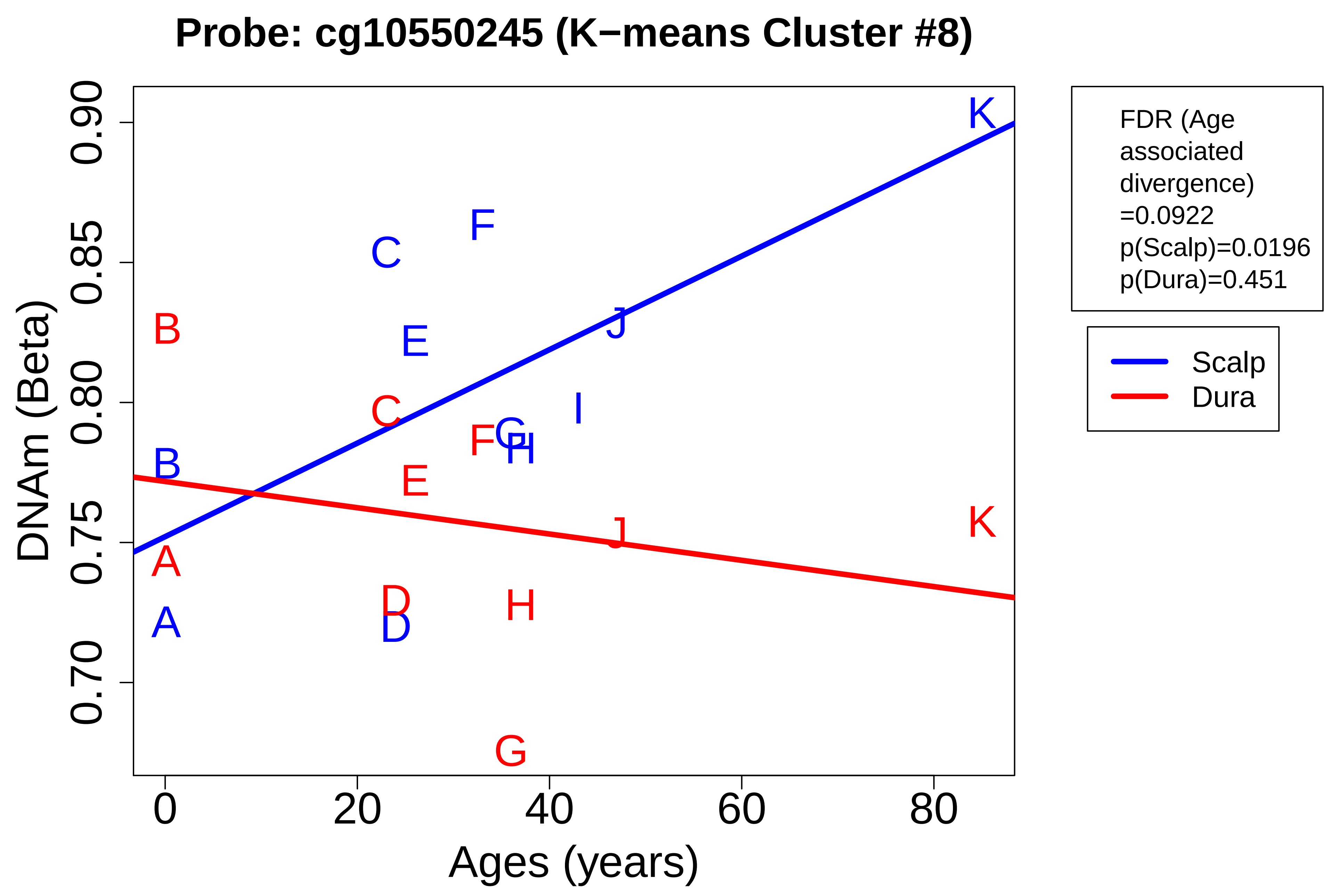

Probe: cg10550245 (K−means Cluster #8)
DNAm (Beta)
Ages (years)
0.70
0.75
0.80
0.85
0.90
0
20
40
60
80
FDR (Age associated divergence) =0.0922
p(Scalp)=0.0196
p(Dura)=0.451
Scalp
Dura

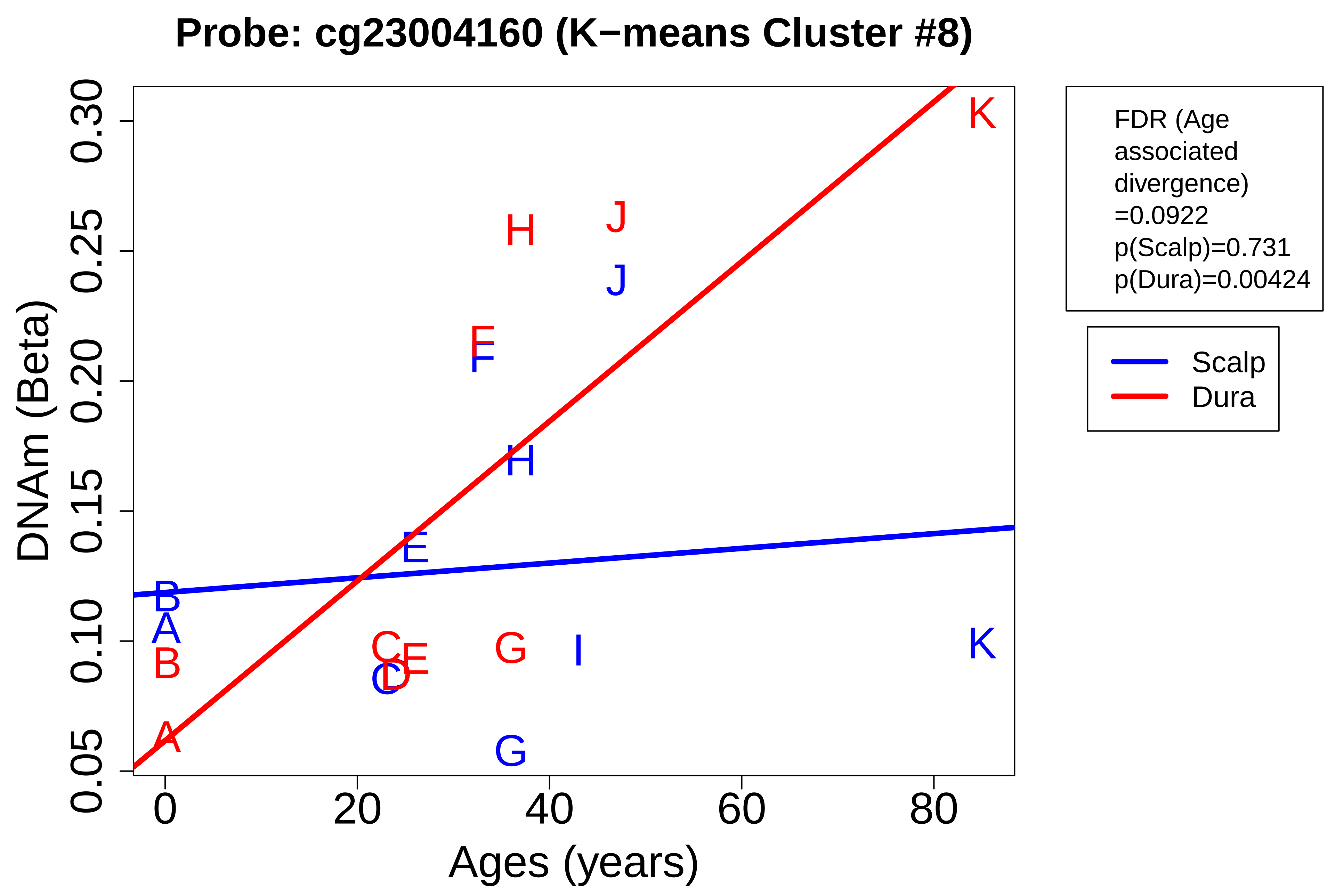

Probe: cg23004160 (K−means Cluster #8)
DNAm (Beta)
0.05
0.10
0.15
0.20
0.25
0.30
0
20
40
60
80
Ages (years)
FDR (Age associated divergence) =0.0922
p(Scalp)=0.731
p(Dura)=0.00424
Scalp
Dura

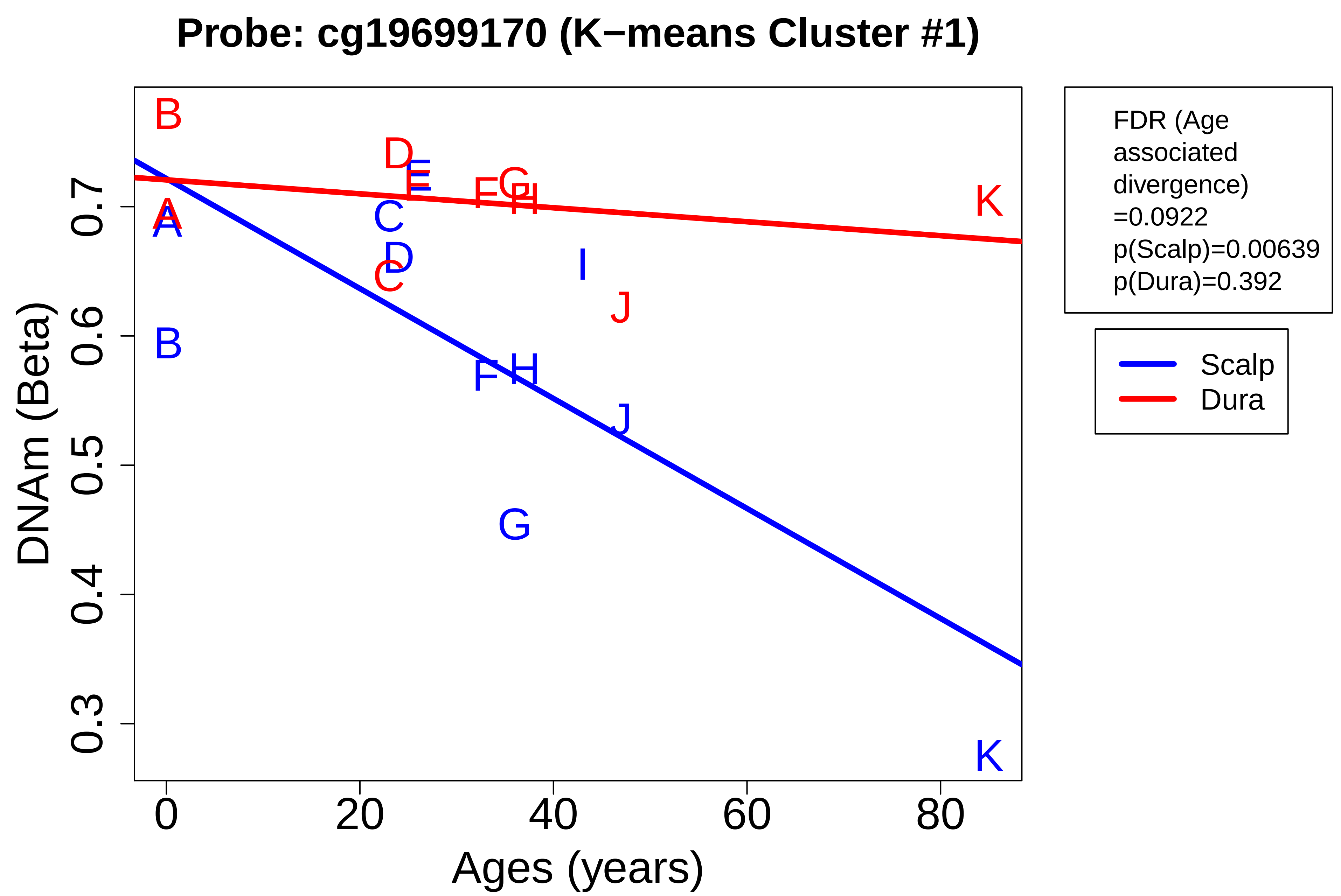
Probe: cg19699170 (K−means Cluster #1)
DNAm (Beta)
Ages (years)
0.3
0.4
0.5
0.6
0.7
0
20
40
60
80
A
B
C
D
E
F
G
H
I
J
K
FDR (Age associated divergence) =0.0922
p(Scalp)=0.00639
p(Dura)=0.392
Scalp
Dura

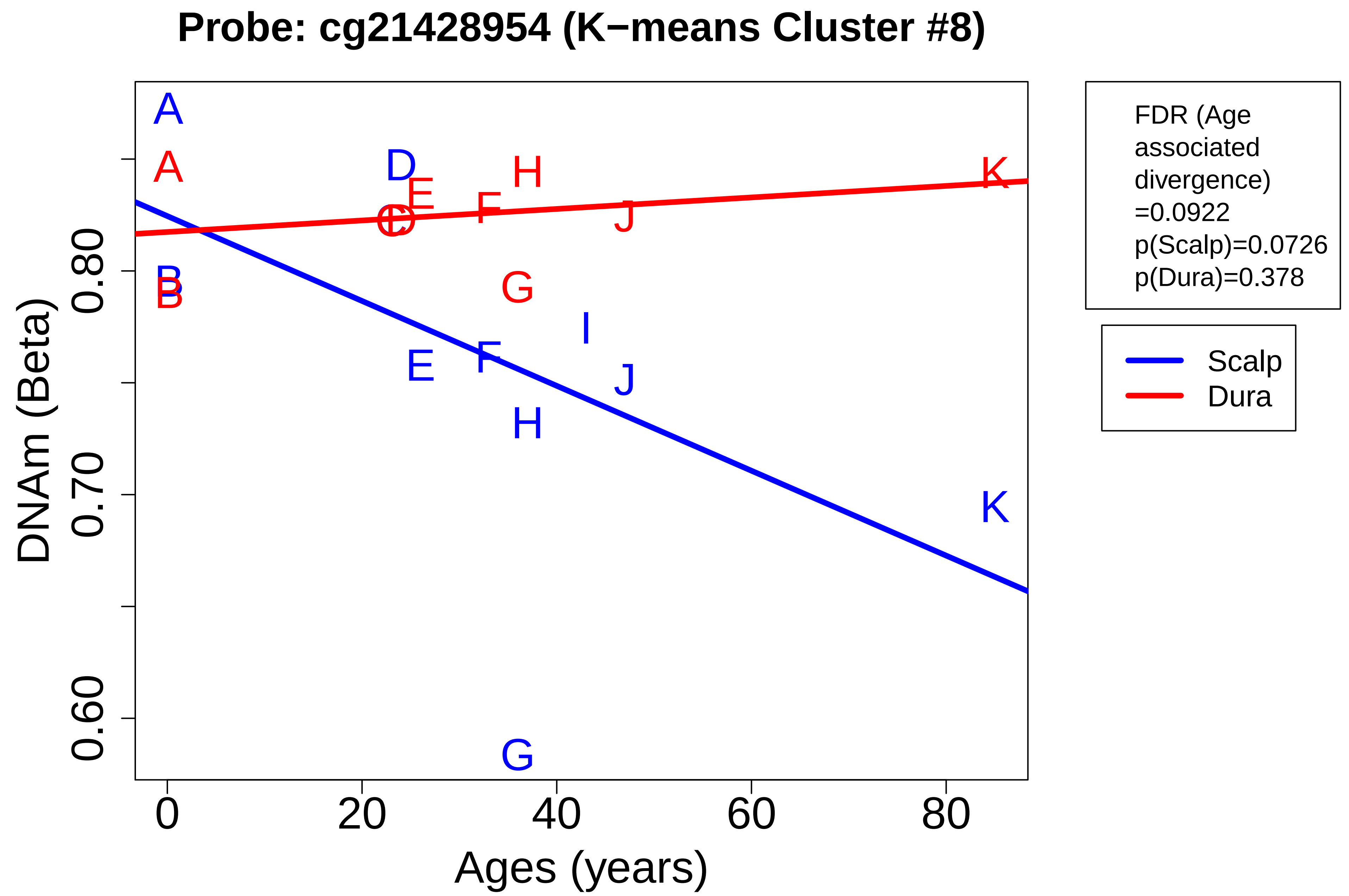
Probe: cg21428954 (K−means Cluster #8)
DNAm (Beta)
0.60
0.70
0.80
Ages (years)
0
20
40
60
80
A
B
D
E
F
G
H
I
J
K
FDR (Age associated divergence) =0.0922
p(Scalp)=0.0726
p(Dura)=0.378
Scalp
Dura

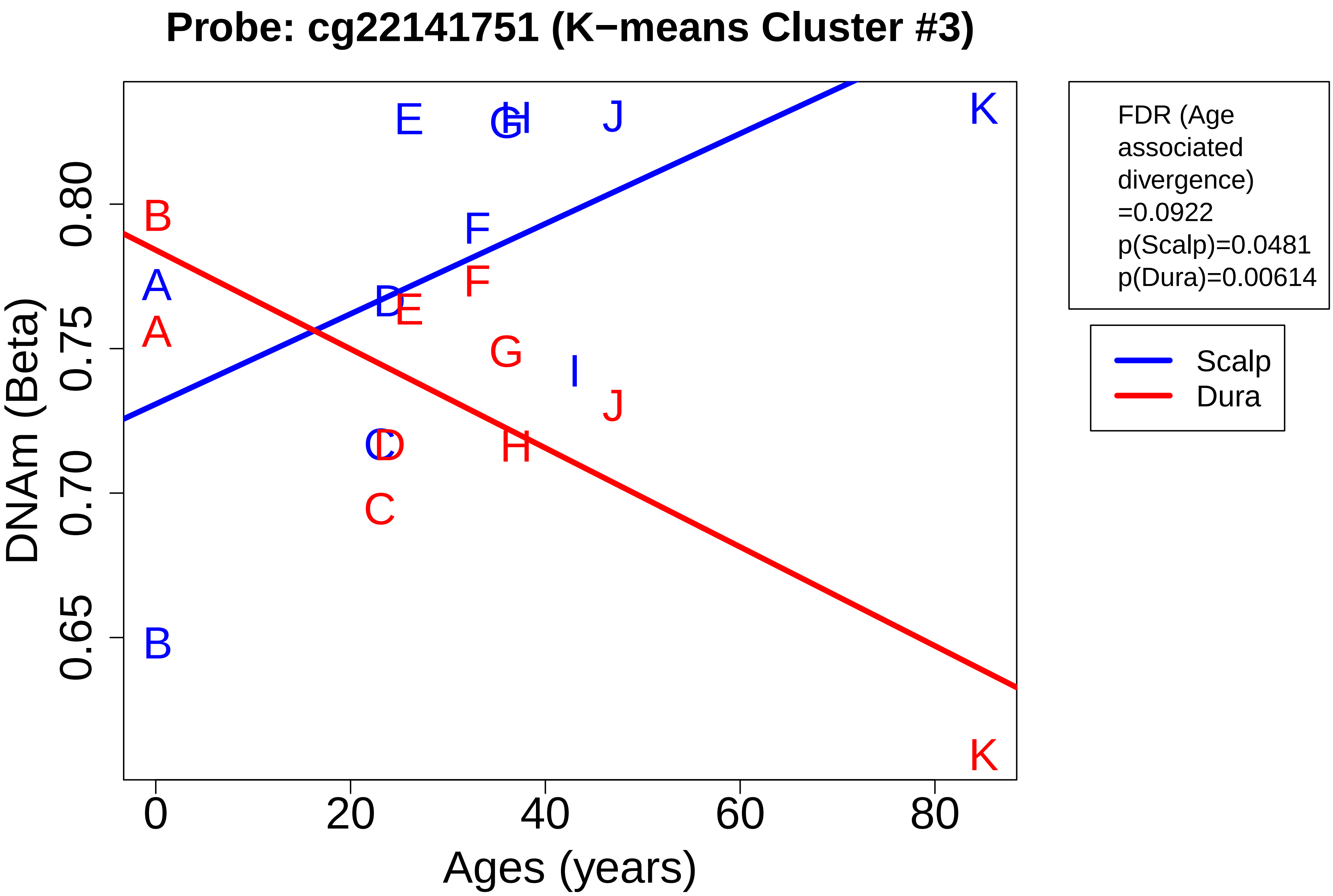
Probe: cg22141751 (K−means Cluster #3)
DNAm (Beta)
0.65
0.70
0.75
0.80
0
20
40
60
80
Ages (years)
A
B
C
D
E
F
G
H
I
J
K
FDR (Age associated divergence) =0.0922
p(Scalp)=0.0481
p(Dura)=0.00614
Scalp
Dura

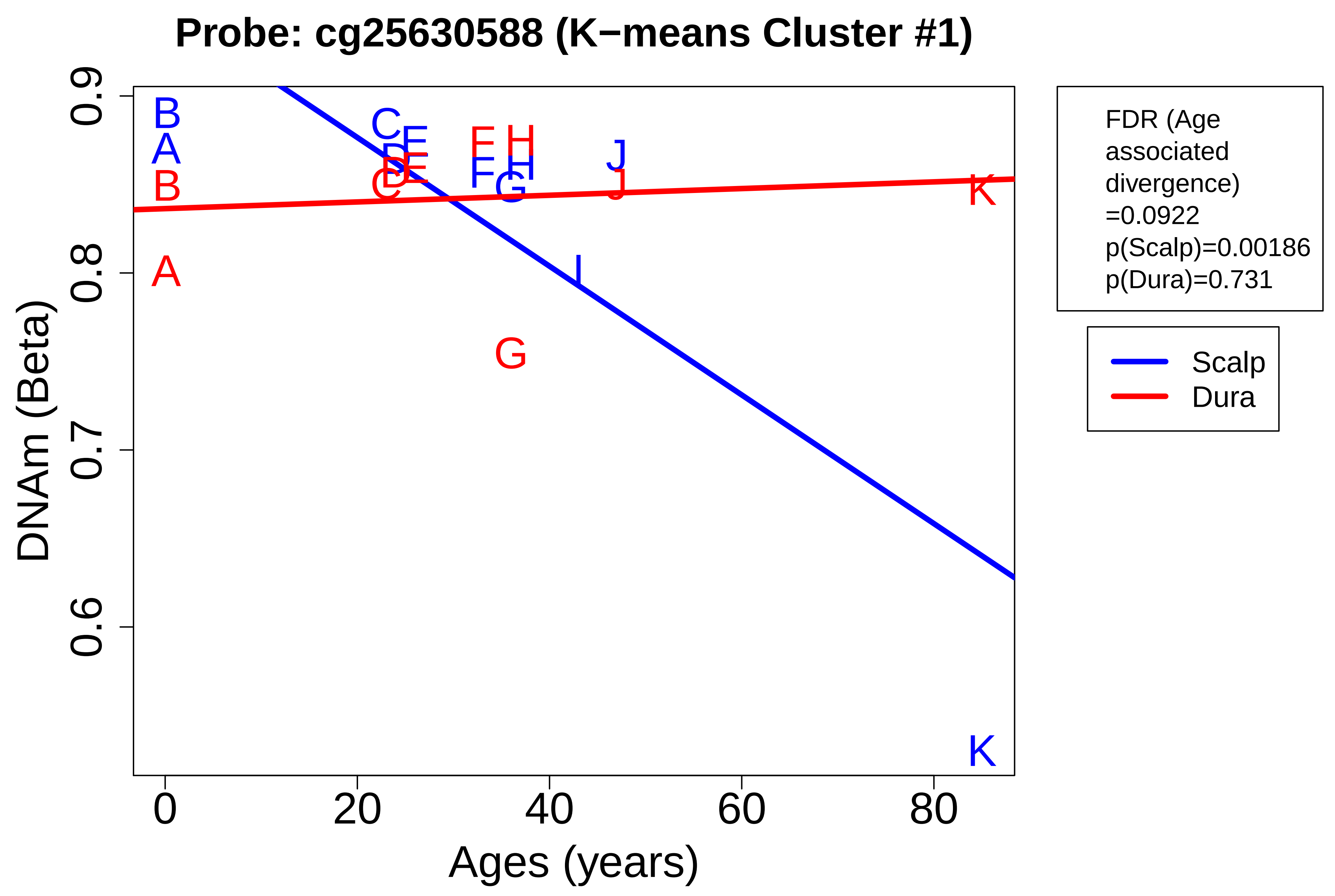

Probe: cg25630588 (K−means Cluster #1)
DNAm (Beta)
0.6
0.7
0.8
0.9
Ages (years)
0
20
40
60
80
FDR (Age associated divergence) =0.0922
p(Scalp)=0.00186
p(Dura)=0.731
Scalp
Dura
A
B
C
D
E
F
G
H
I
J
K

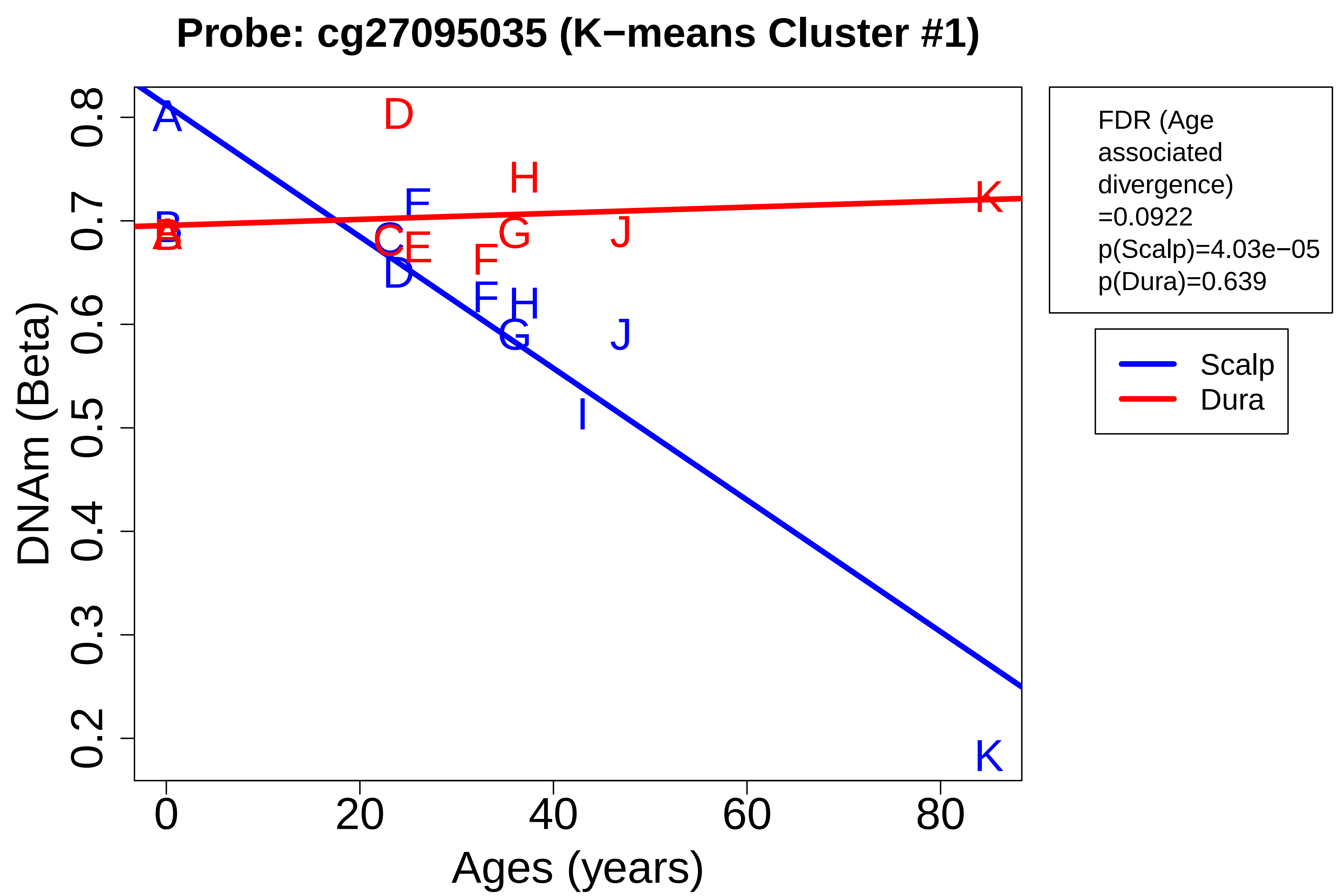

Probe: cg27095035 (K−means Cluster #1)
DNAm (Beta)
Ages (years)
0.2
0.3
0.4
0.5
0.6
0.7
0.8
0
20
40
60
80
FDR (Age associated divergence) =0.0922
p(Scalp)=4.03e−05
p(Dura)=0.639
Scalp
Dura

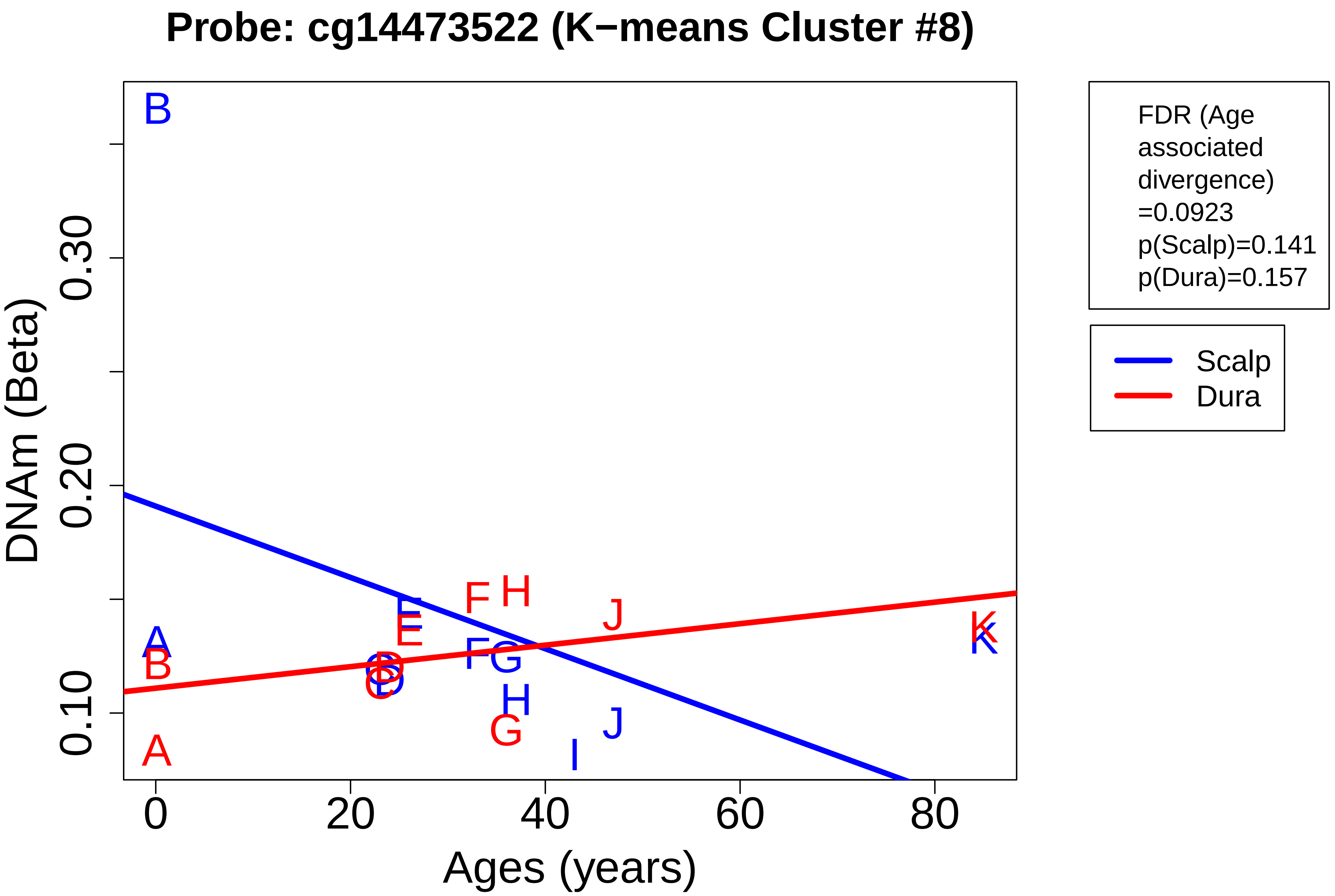

Probe: cg14473522 (K−means Cluster #8)
DNAm (Beta)
0.10
0.20
0.30
Ages (years)
0
20
40
60
80
FDR (Age associated divergence) =0.0923
p(Scalp)=0.141
p(Dura)=0.157
Scalp
Dura
A
B
C
D
E
F
G
H
I
J
K

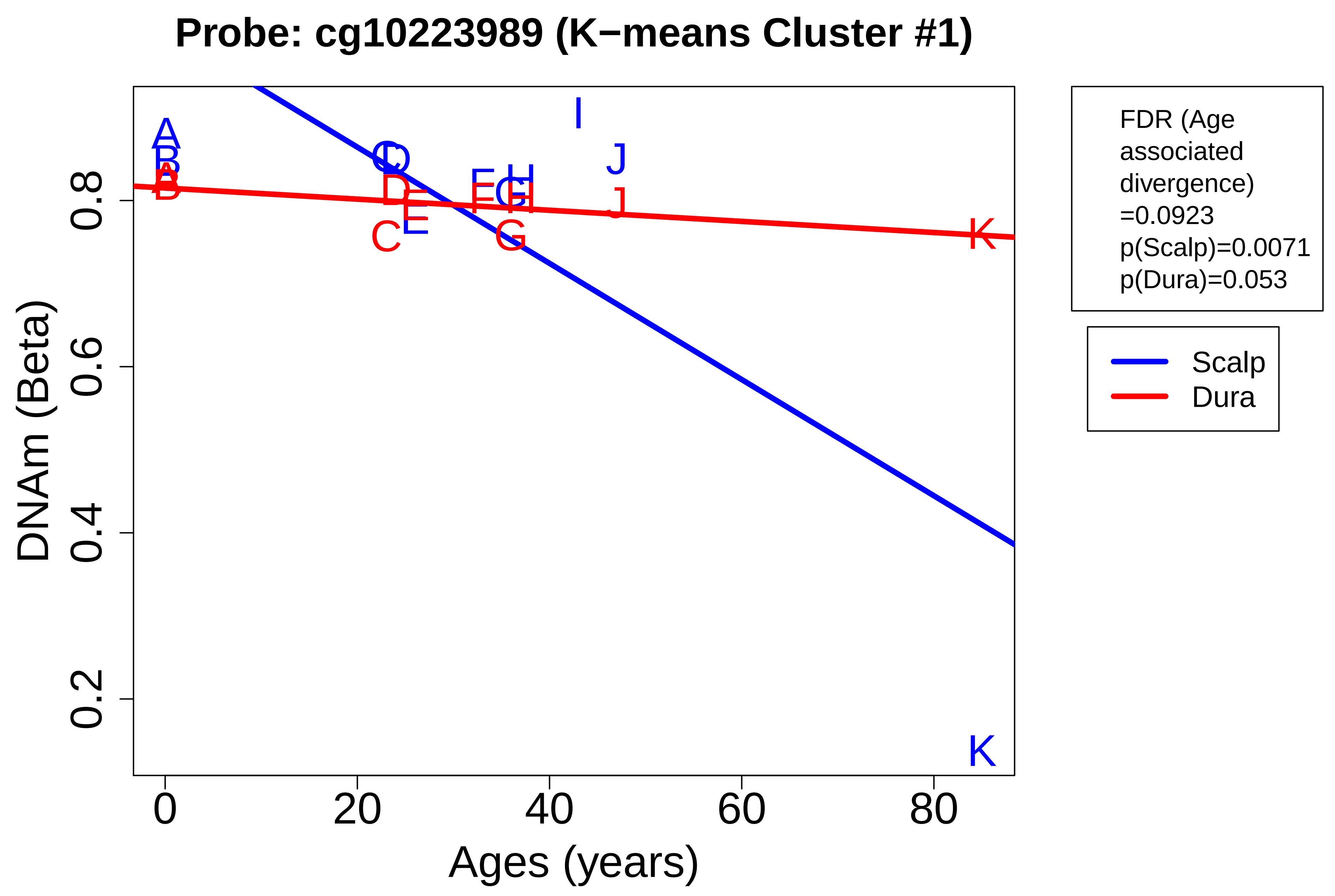

Probe: cg10223989 (K−means Cluster #1)
FDR (Age associated divergence) =0.0923
p(Scalp)=0.0071
p(Dura)=0.053
Scalp
Dura
DNAm (Beta)
0.2
0.4
0.6
0.8
Ages (years)
0
20
40
60
80

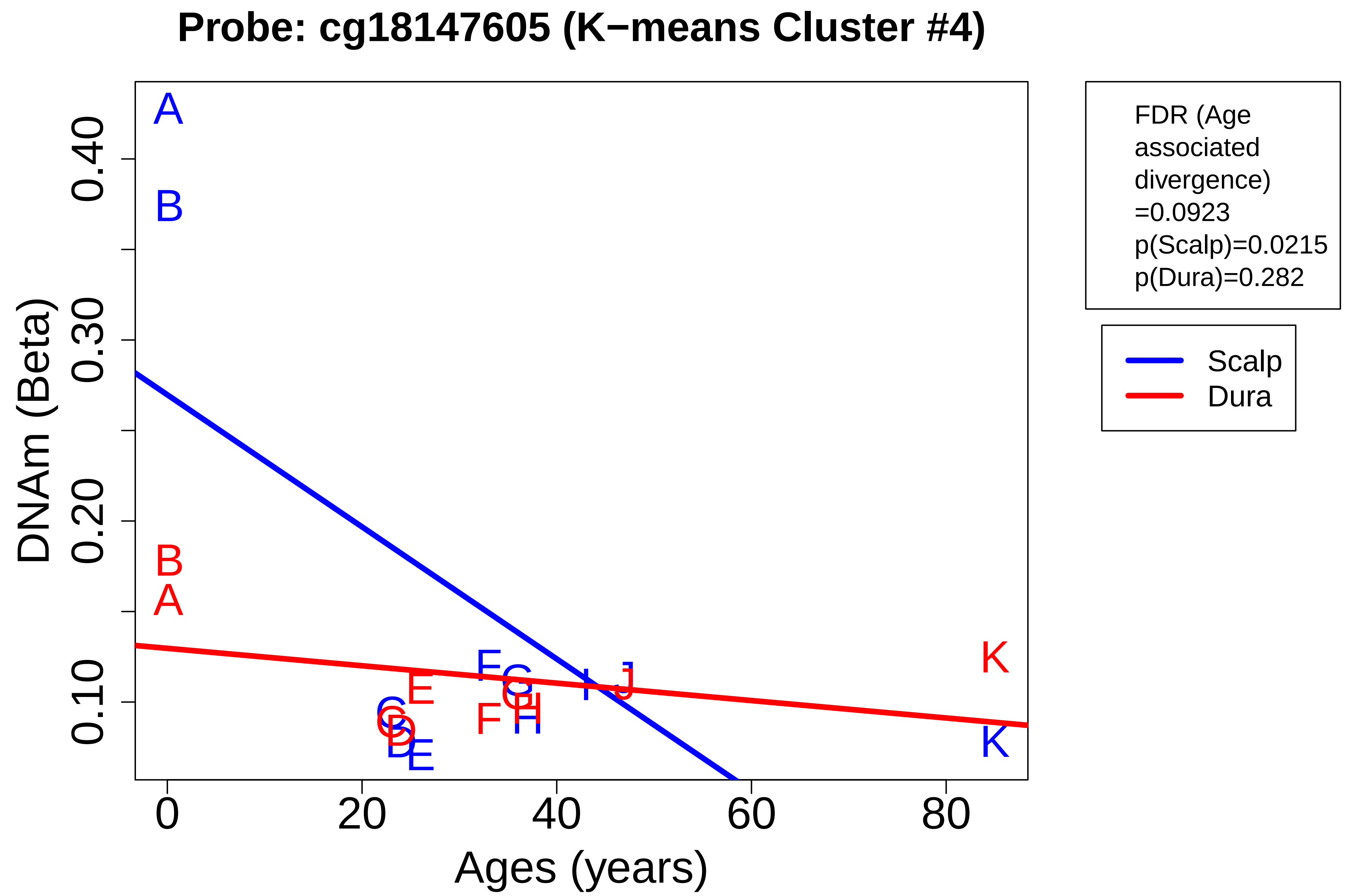
Probe: cg18147605 (K−means Cluster #4)
DNAm (Beta)
0.10
0.20
0.30
0.40
Ages (years)
0
20
40
60
80
FDR (Age associated divergence) =0.0923
p(Scalp)=0.0215
p(Dura)=0.282
Scalp
Dura

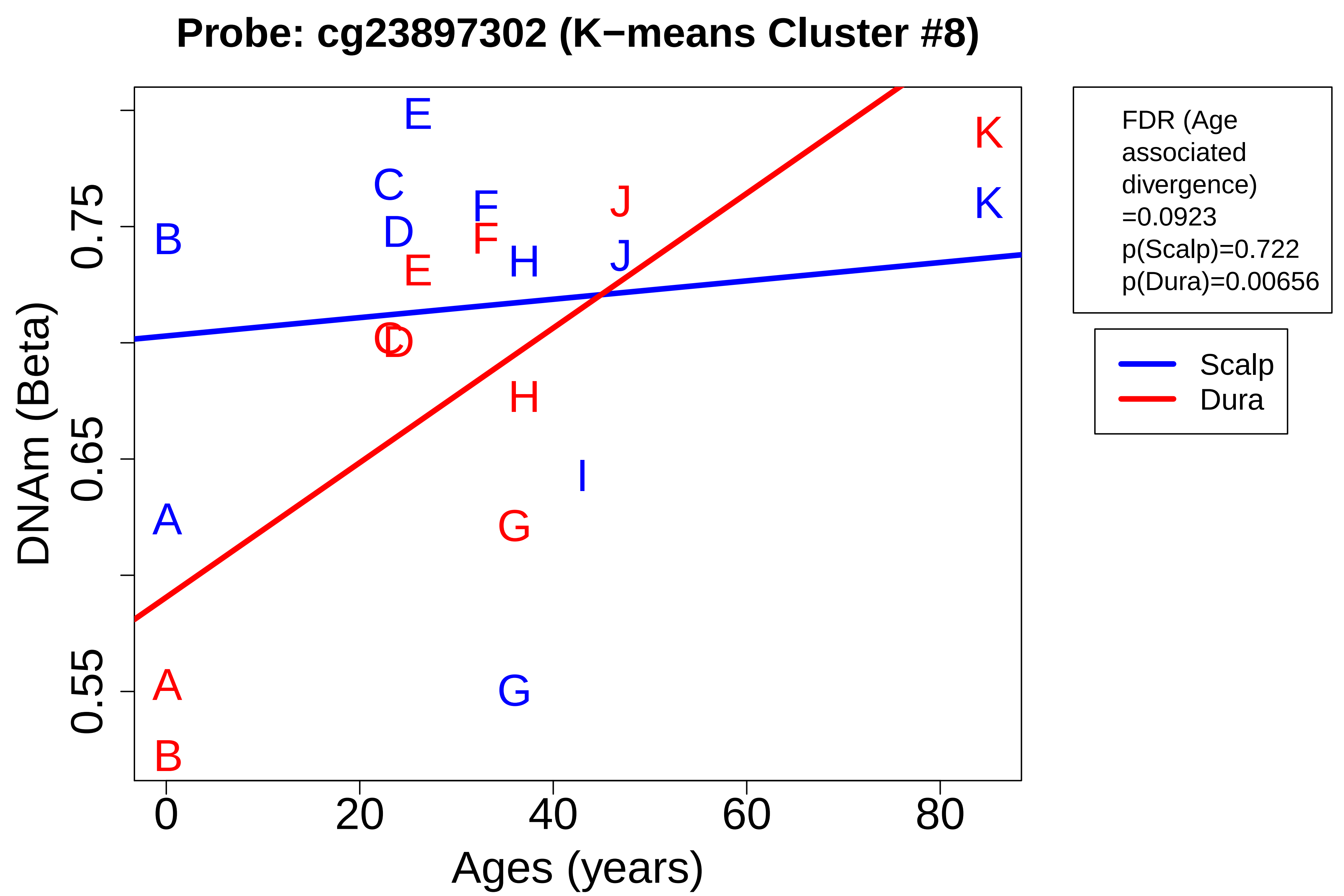

Probe: cg23897302 (K−means Cluster #8)
DNAm (Beta)
0.55
0.65
0.75
Ages (years)
0
20
40
60
80
A
B
C
D
E
F
G
H
I
J
K
FDR (Age associated divergence) =0.0923
p(Scalp)=0.722
p(Dura)=0.00656
Scalp
Dura

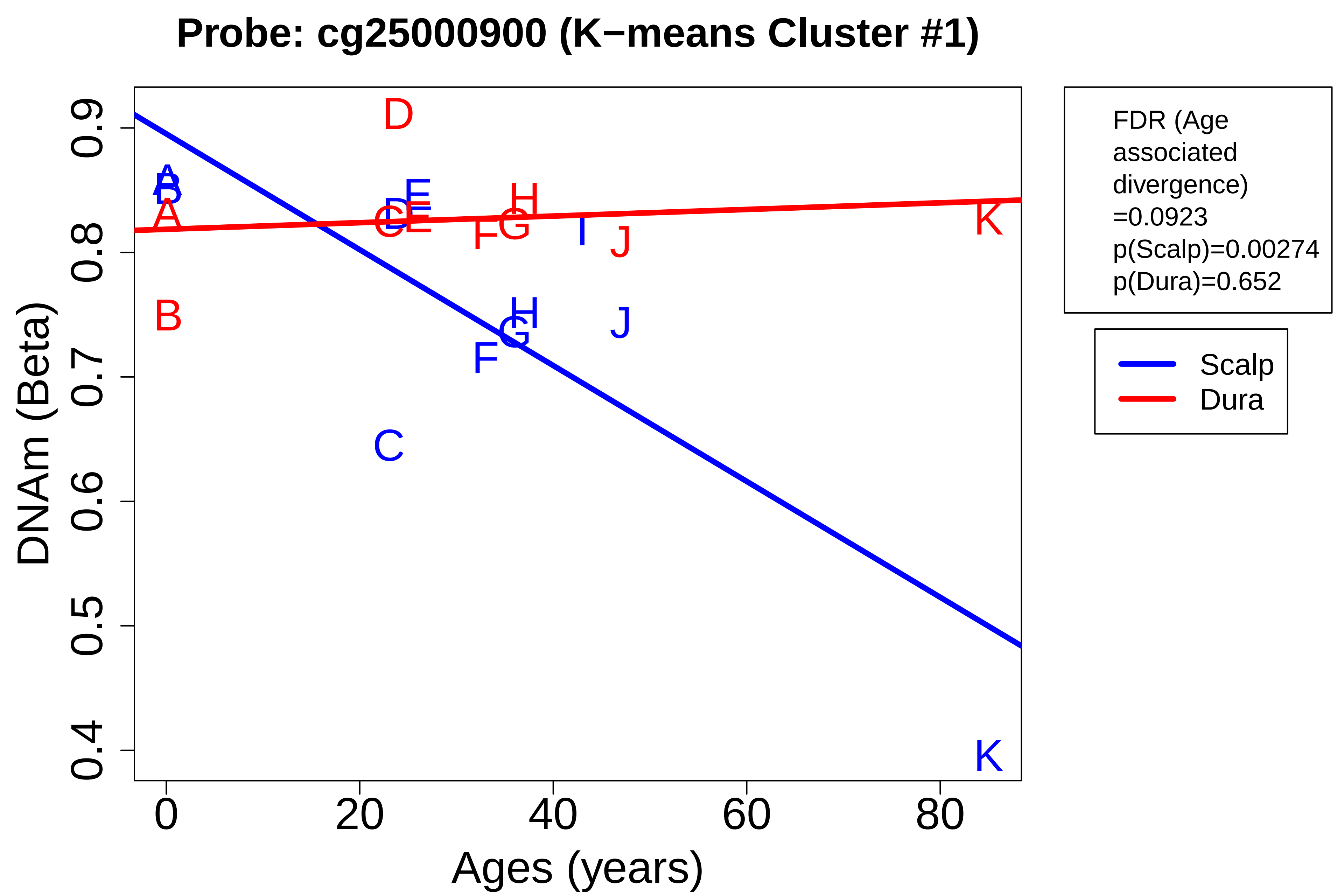

Probe: cg25000900 (K−means Cluster #1)
DNAm (Beta)
0.4
0.5
0.6
0.7
0.8
0.9
Ages (years)
0
20
40
60
80
A B C D E F G H I J K
FDR (Age associated divergence) =0.0923
p(Scalp)=0.00274
p(Dura)=0.652
Scalp
Dura

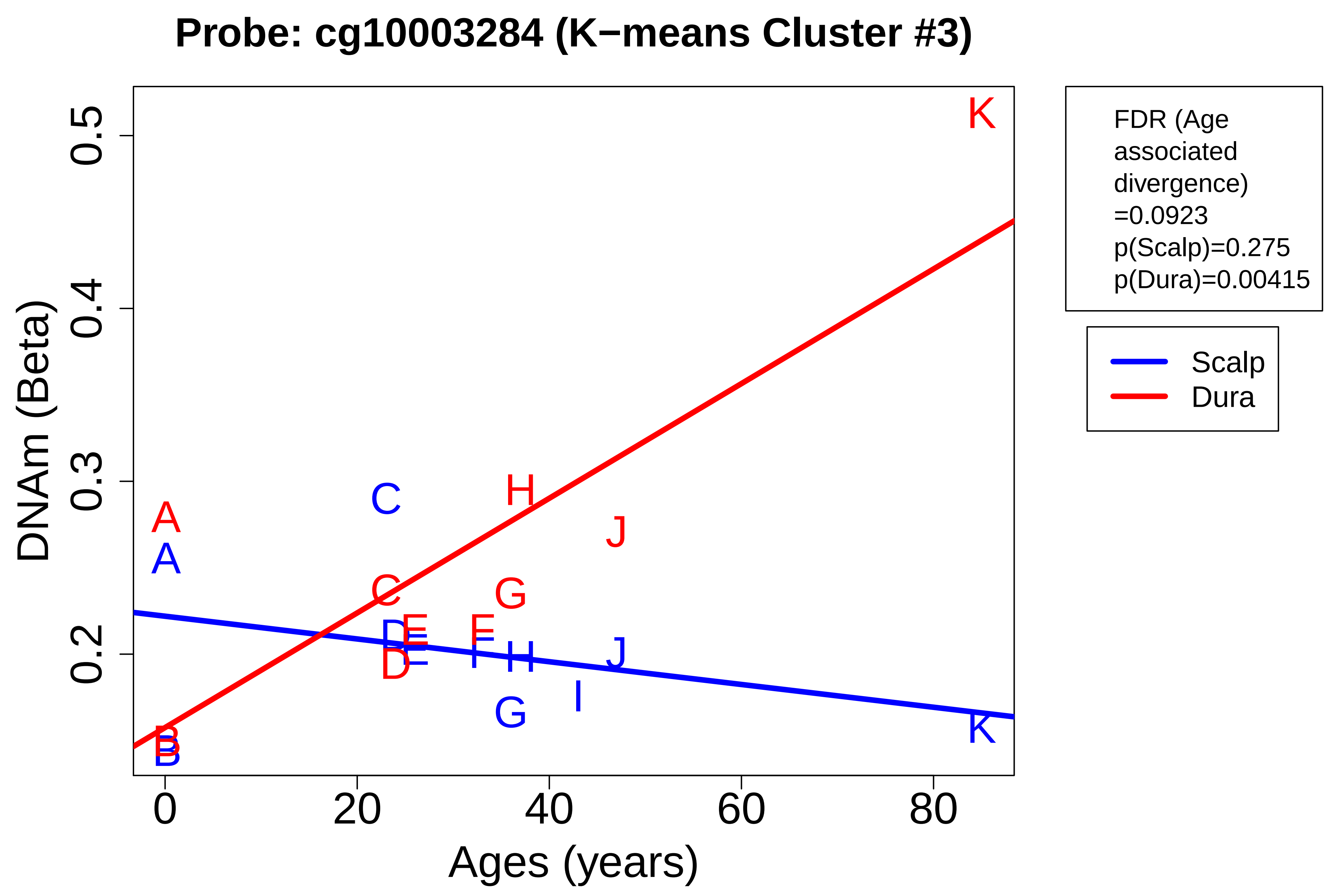
Probe: cg10003284 (K−means Cluster #3)
DNAm (Beta)
0.2
0.3
0.4
0.5
0
20
40
60
80
Ages (years)
FDR (Age associated divergence) =0.0923
p(Scalp)=0.275
p(Dura)=0.00415
Scalp
Dura

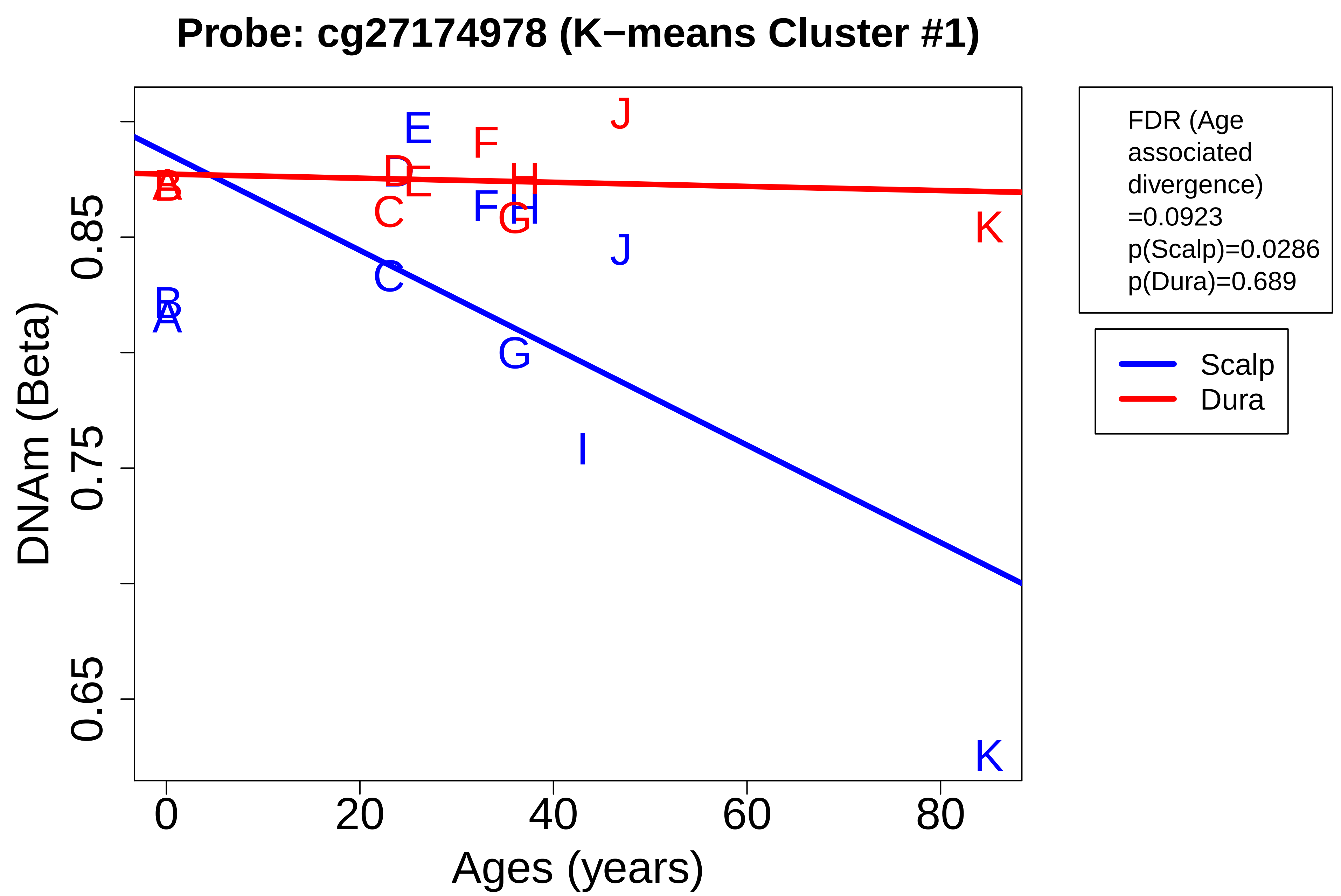

Probe: cg27174978 (K−means Cluster #1)
DNAm (Beta)
0.65
0.75
0.85
0
20
40
60
80
Ages (years)
FDR (Age associated divergence) =0.0923
p(Scalp)=0.0286
p(Dura)=0.689
Scalp
Dura

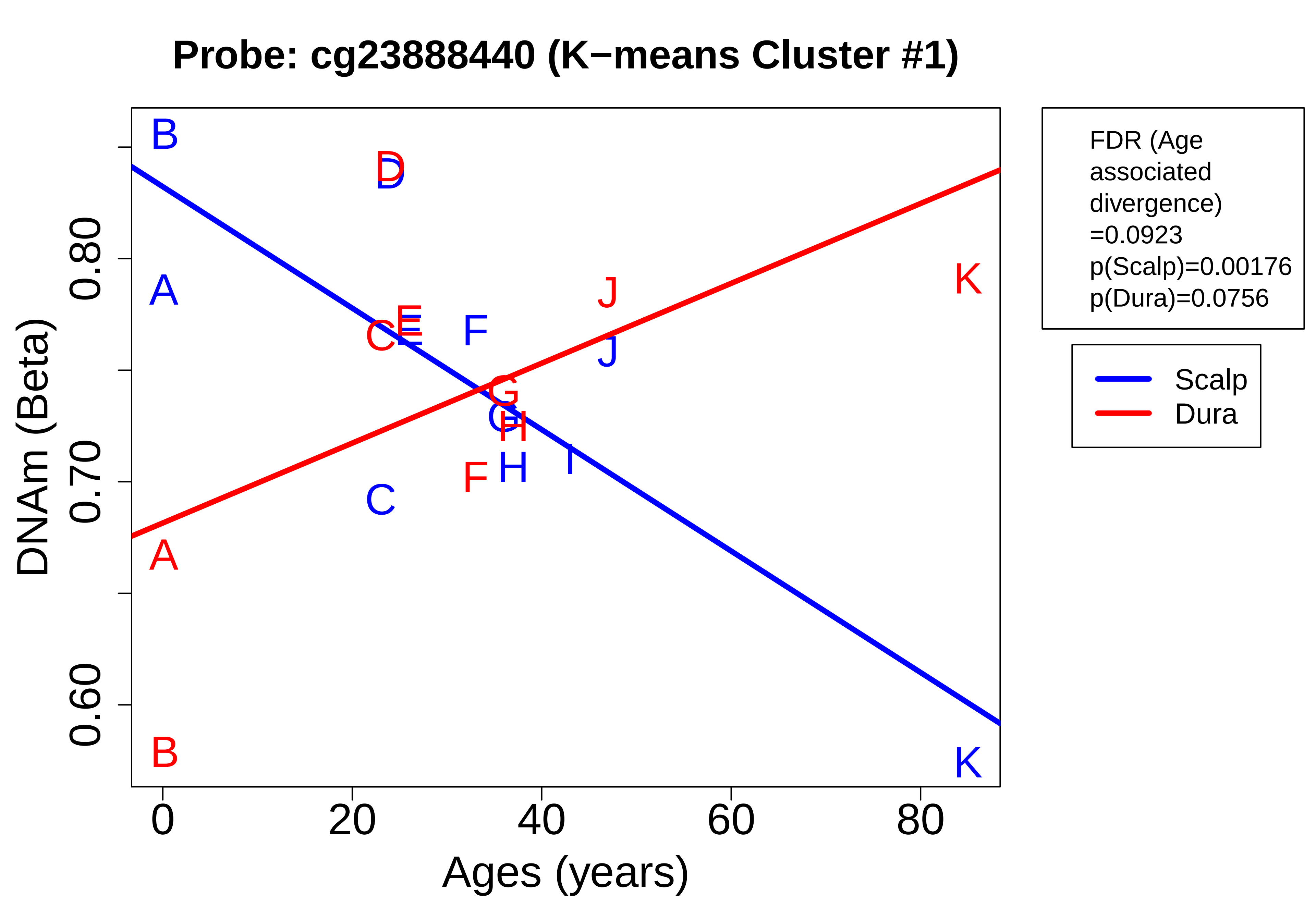

Probe: cg23888440 (K−means Cluster #1)
DNAm (Beta)
0.60
0.70
0.80
Ages (years)
0
20
40
60
80
FDR (Age associated divergence) =0.0923
p(Scalp)=0.00176
p(Dura)=0.0756
Scalp
Dura
A
B
C
D
E
F
G
H
I
J
K

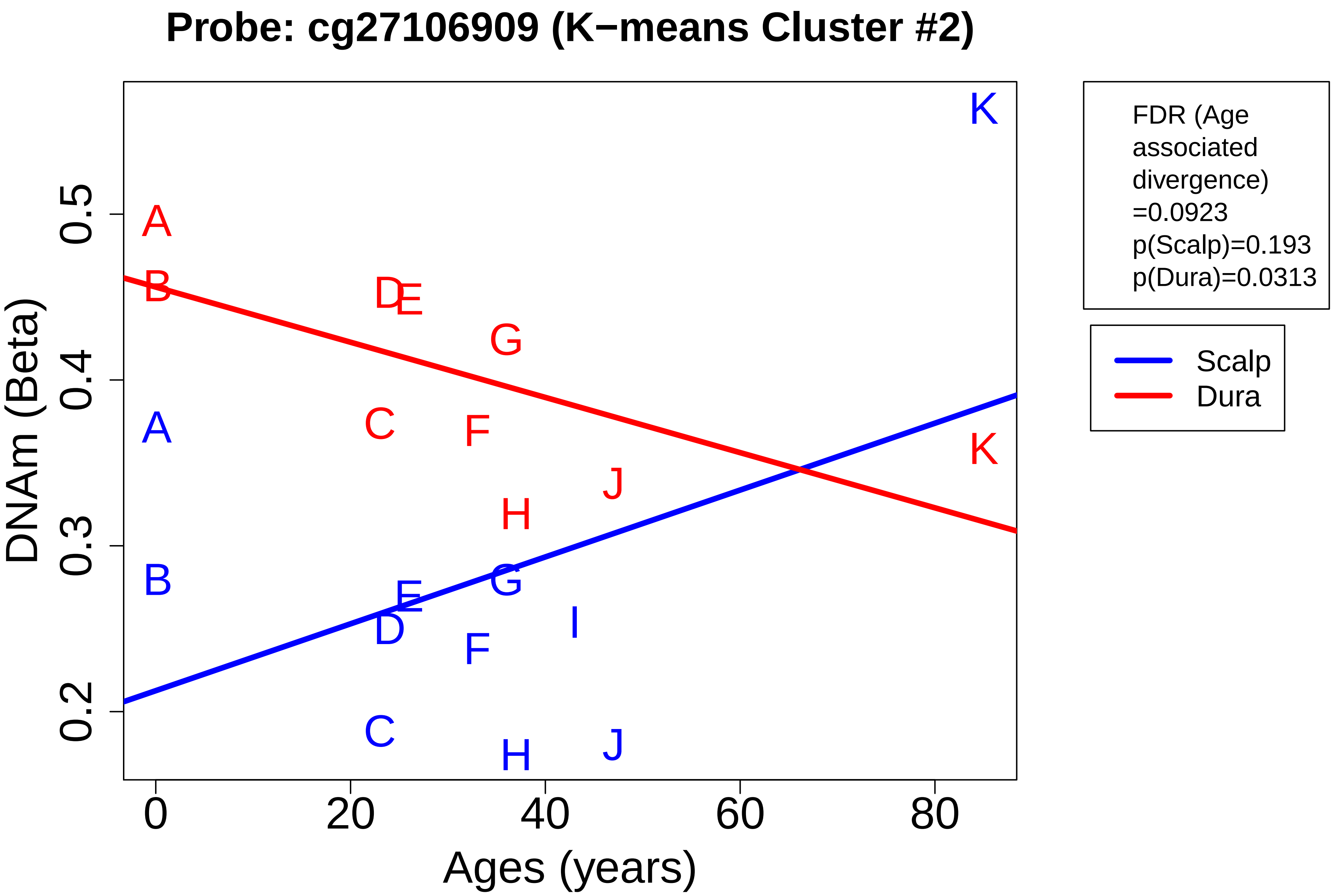

Probe: cg27106909 (K−means Cluster #2)
DNAm (Beta)
0.2
0.3
0.4
0.5
Ages (years)
0
20
40
60
80
A
B
C
D
E
F
G
H
I
J
K
FDR (Age associated divergence) =0.0923
p(Scalp)=0.193
p(Dura)=0.0313
Scalp
Dura

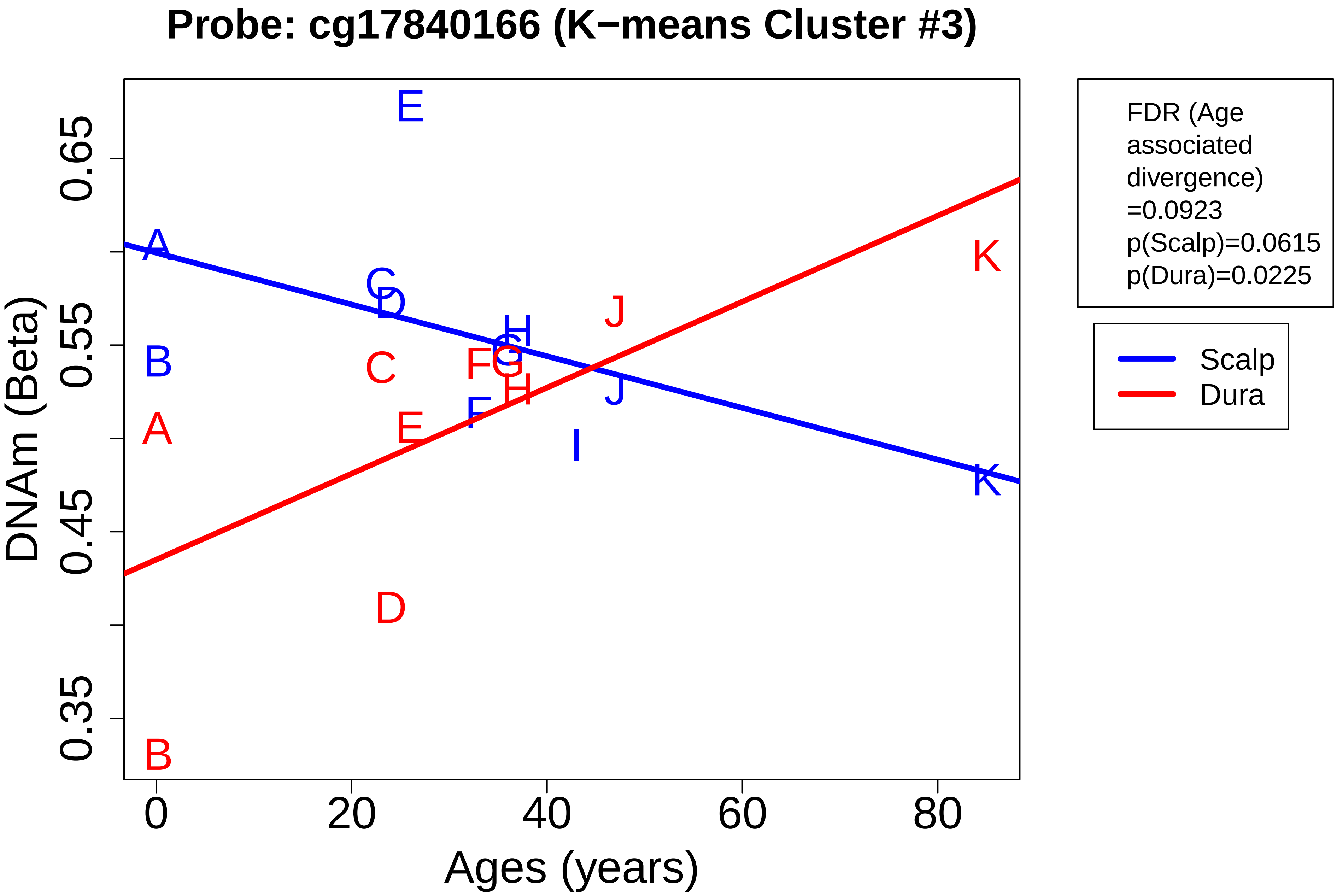

Probe: cg17840166 (K−means Cluster #3)
FDR (Age associated divergence) =0.0923
p(Scalp)=0.0615
p(Dura)=0.0225
Scalp
Dura
DNAm (Beta)
0.35
0.45
0.55
0.65
Ages (years)
0
20
40
60
80
A
B
C
D
E
F
G
H
I
J
K

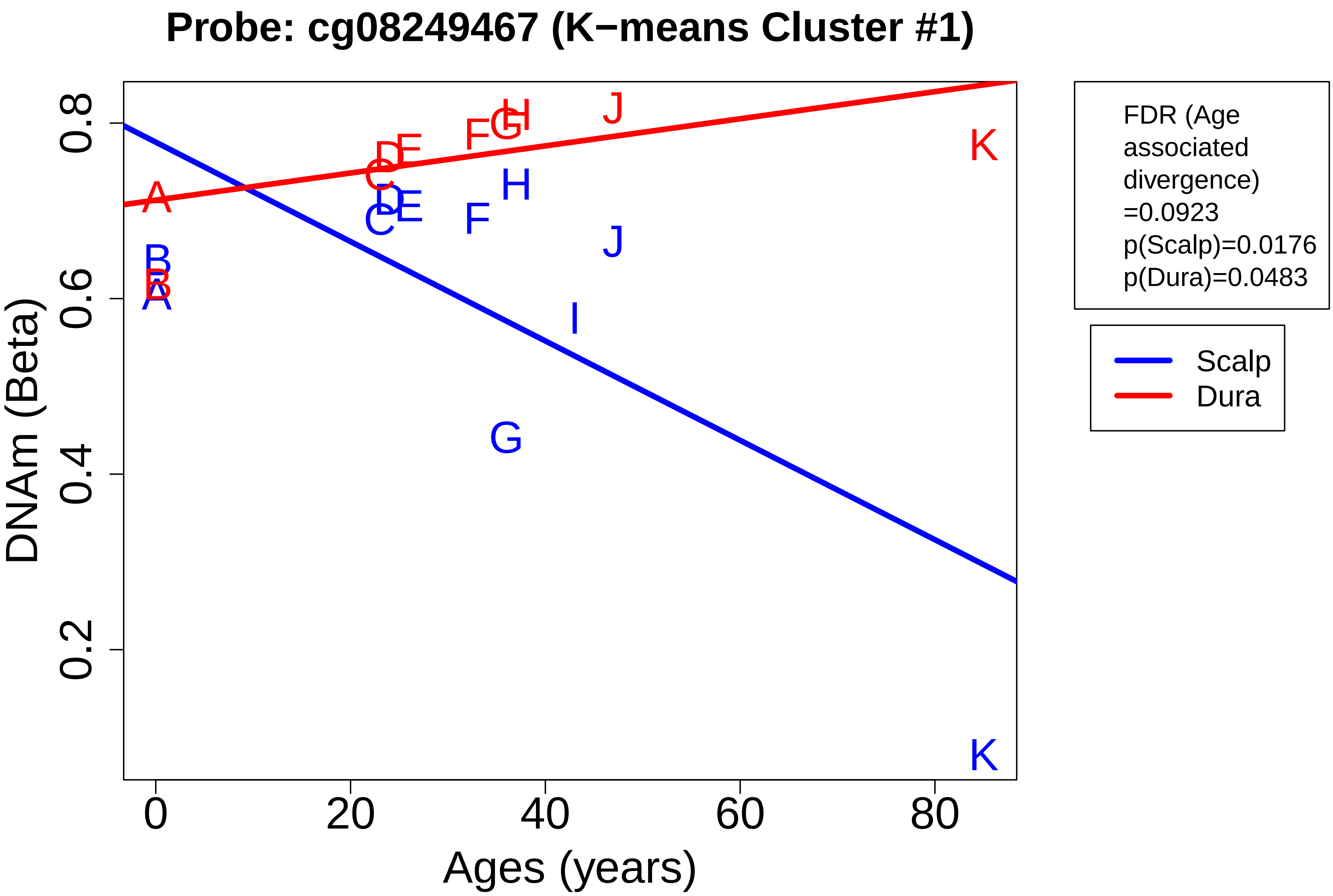
Probe: cg08249467 (K−means Cluster #1)
DNAm (Beta)
0.2
0.4
0.6
0.8
0
20
40
60
80
Ages (years)
FDR (Age associated divergence) =0.0923
p(Scalp)=0.0176
p(Dura)=0.0483
Scalp
Dura

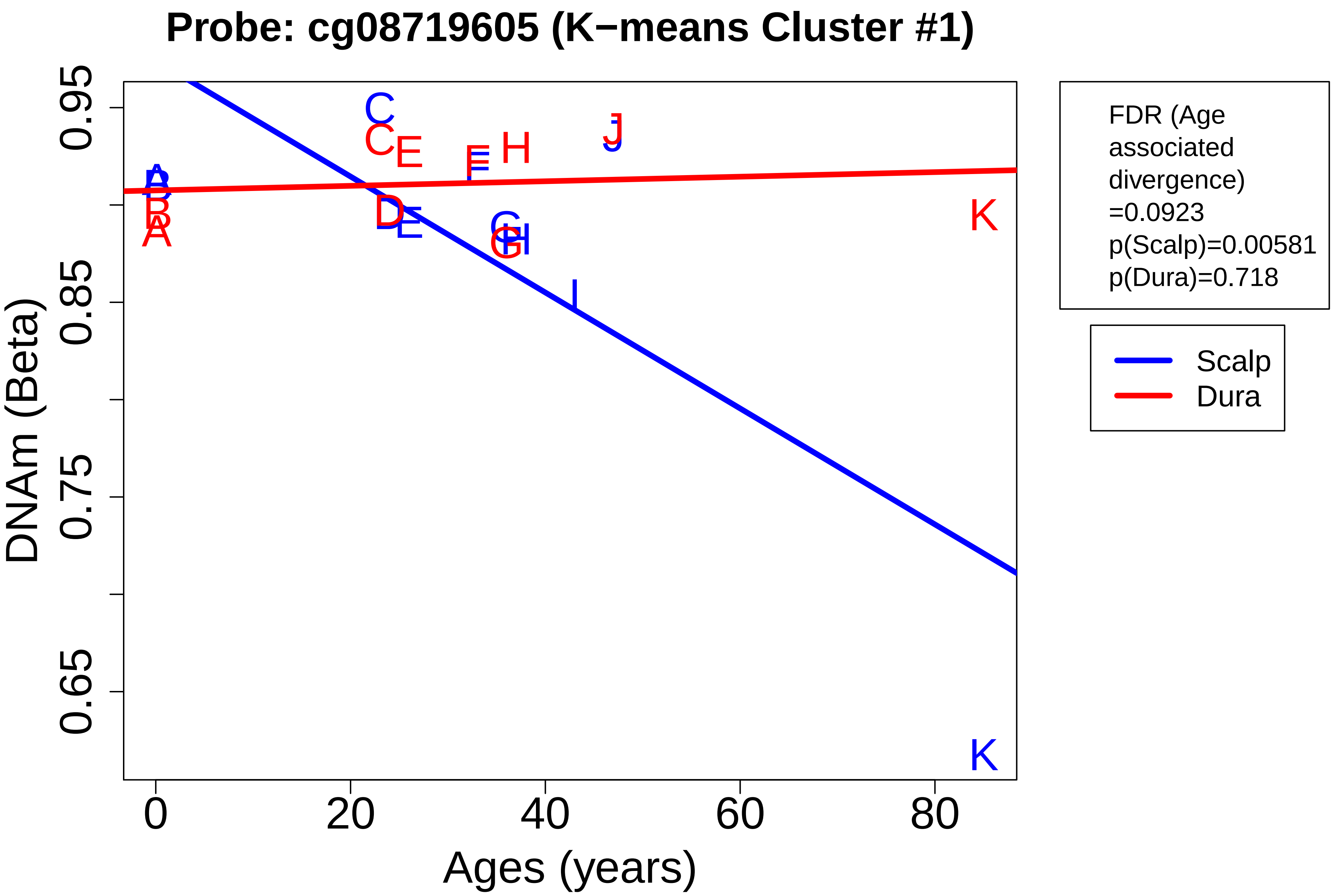

Probe: cg08719605 (K−means Cluster #1)
DNAm (Beta)
0.65
0.75
0.85
0.95
0
20
40
60
80
Ages (years)
FDR (Age associated divergence) =0.0923
p(Scalp)=0.00581
p(Dura)=0.718
Scalp
Dura

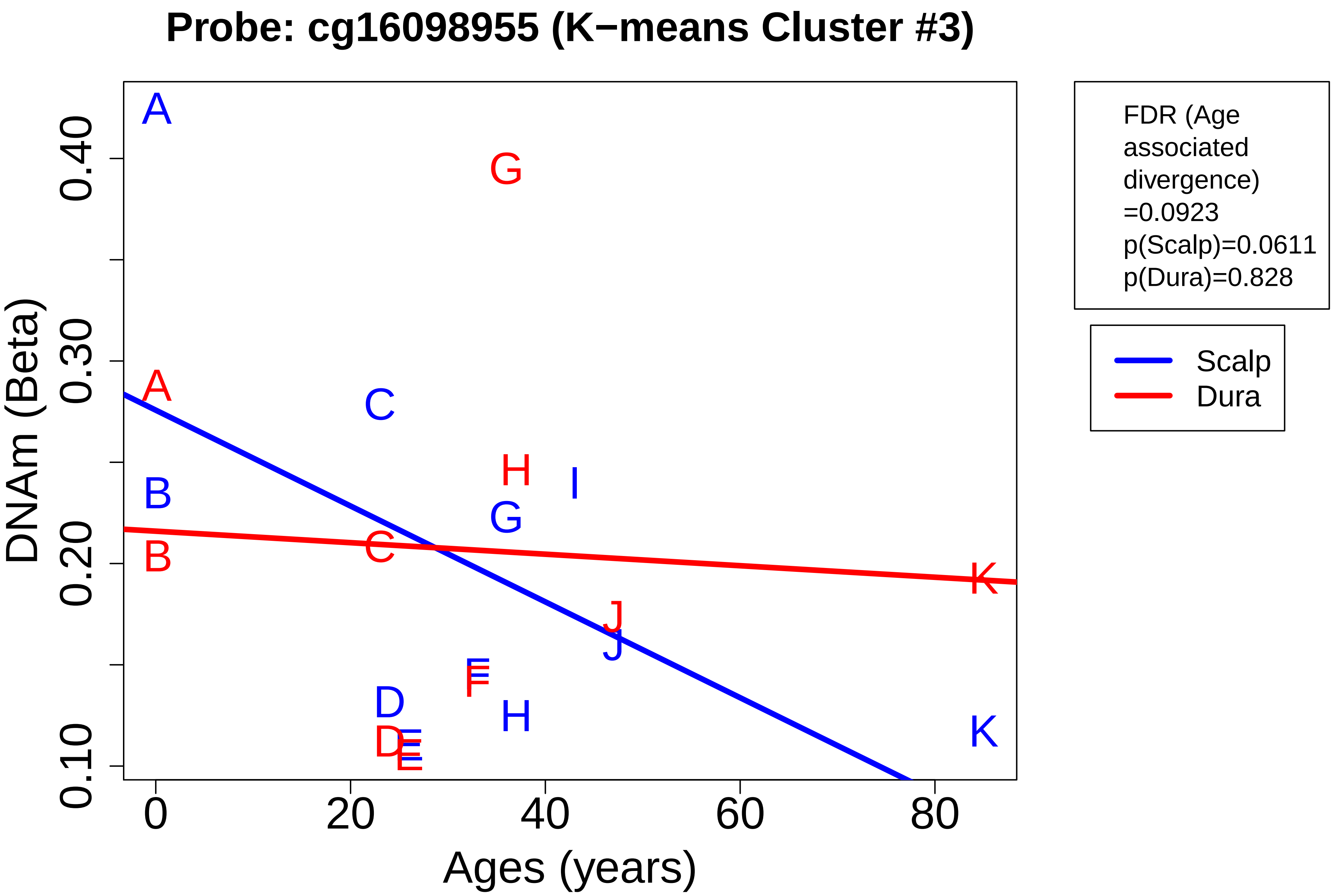

Probe: cg16098955 (K−means Cluster #3)
DNAm (Beta)
0.10
0.20
0.30
0.40
Ages (years)
0
20
40
60
80
A
B
C
D
E
F
G
H
I
J
K
FDR (Age associated divergence) =0.0923
p(Scalp)=0.0611
p(Dura)=0.828
Scalp
Dura

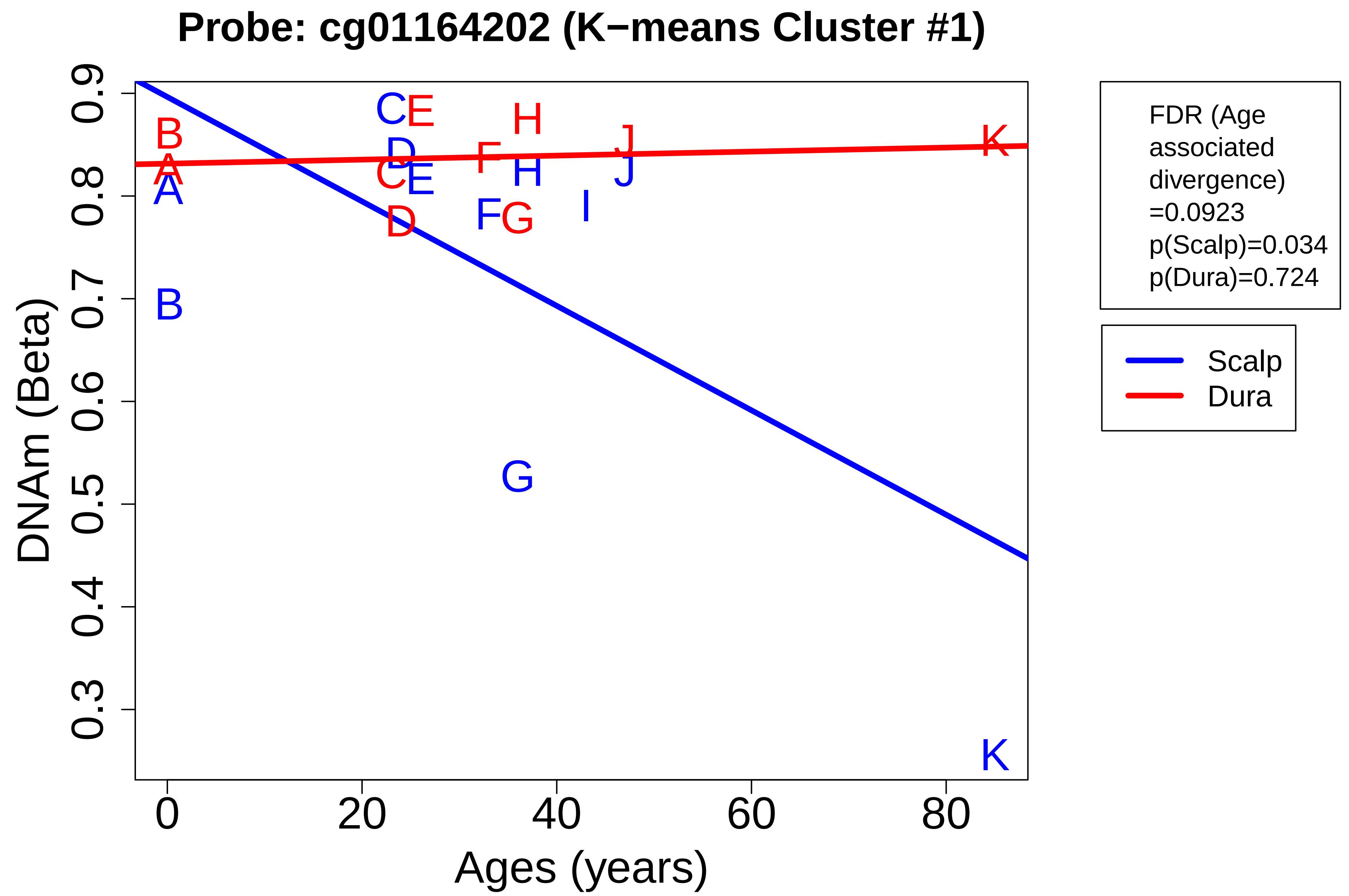

Probe: cg01164202 (K−means Cluster #1)
DNAm (Beta)
Ages (years)
FDR (Age associated divergence) =0.0923
p(Scalp)=0.034
p(Dura)=0.724
Scalp
Dura

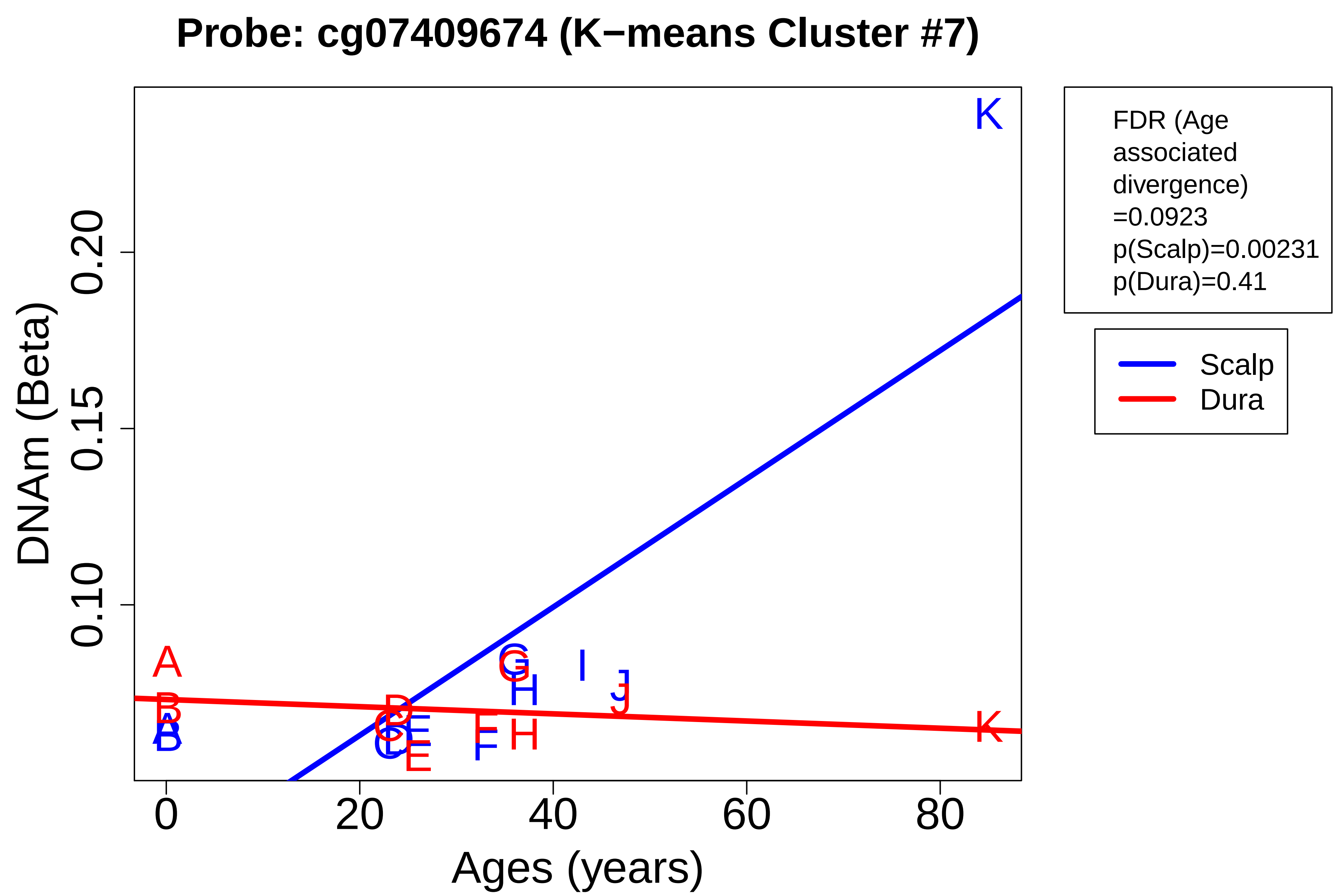

Probe: cg07409674 (K−means Cluster #7)
DNAm (Beta)
0.10
0.15
0.20
Ages (years)
0
20
40
60
80
FDR (Age associated divergence) =0.0923
p(Scalp)=0.00231
p(Dura)=0.41
Scalp
Dura

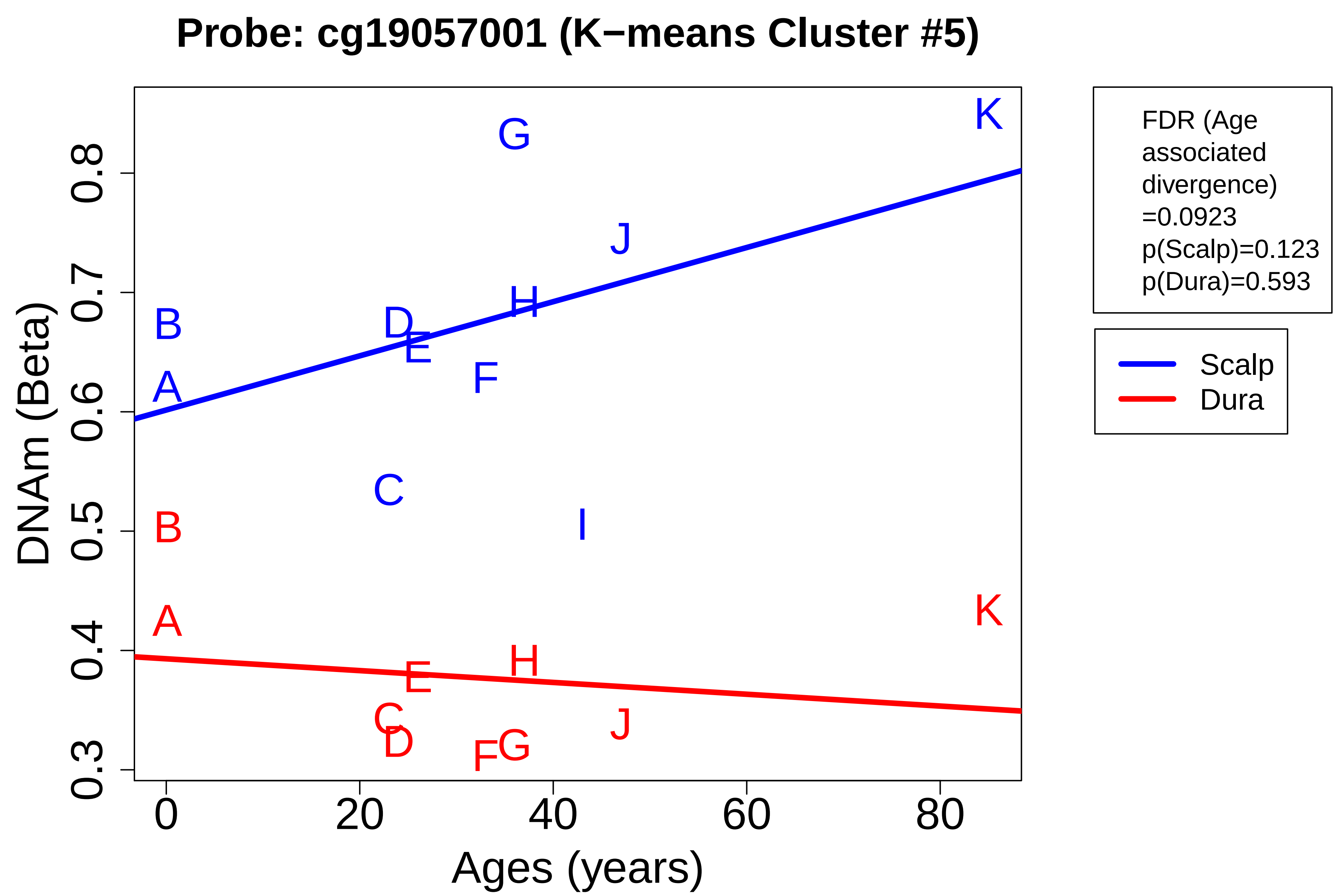

Probe: cg19057001 (K−means Cluster #5)
DNAm (Beta)
Ages (years)
0.3
0.4
0.5
0.6
0.7
0.8
0
20
40
60
80
FDR (Age associated divergence) =0.0923
p(Scalp)=0.123
p(Dura)=0.593
Scalp
Dura
A
B
C
D
E
F
G
H
I
J
K

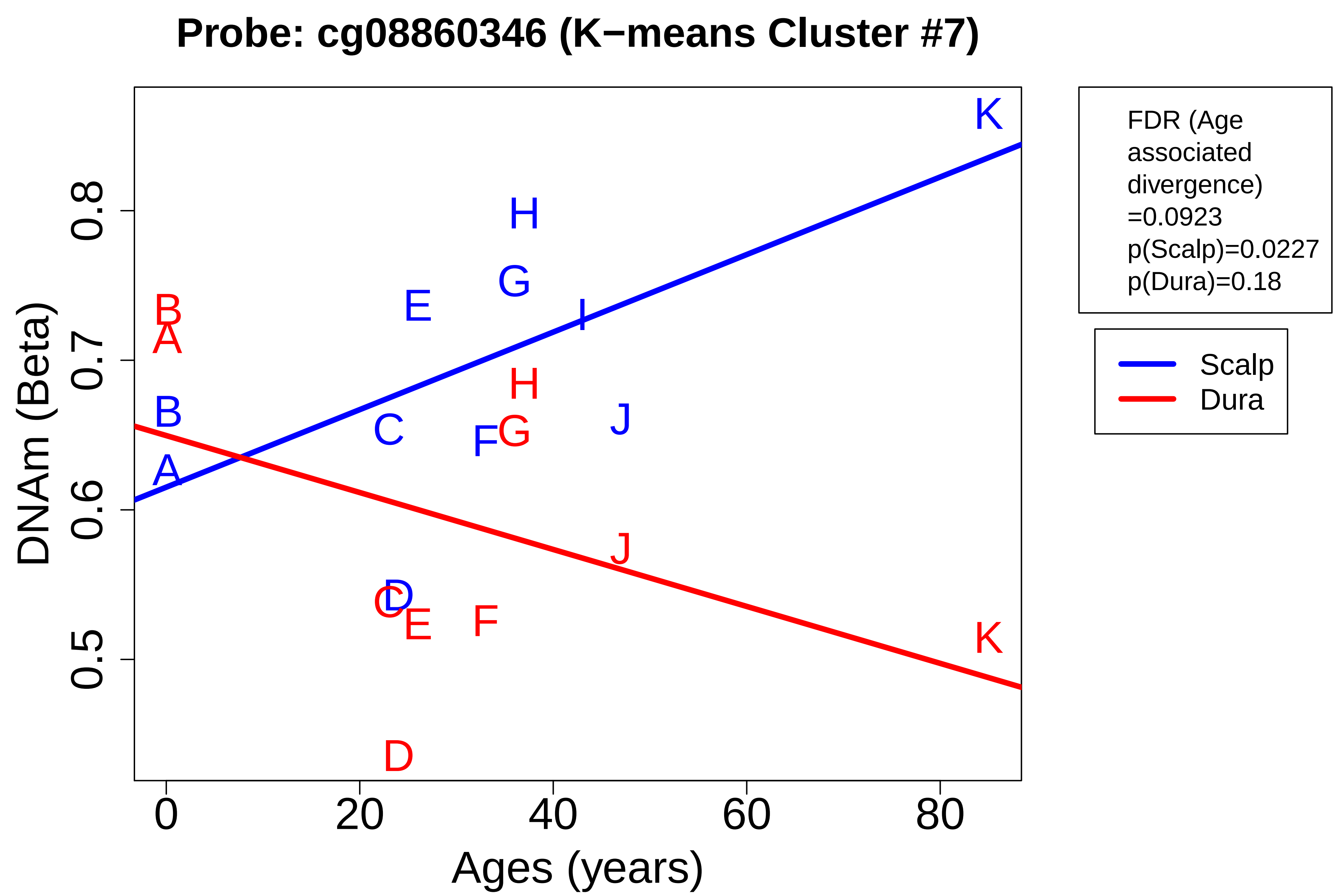
Probe: cg08860346 (K−means Cluster #7)
DNAm (Beta)
0.5
0.6
0.7
0.8
0
20
40
60
80
Ages (years)
A
B
C
D
E
F
G
H
I
J
K
FDR (Age associated divergence) =0.0923
p(Scalp)=0.0227
p(Dura)=0.18
Scalp
Dura

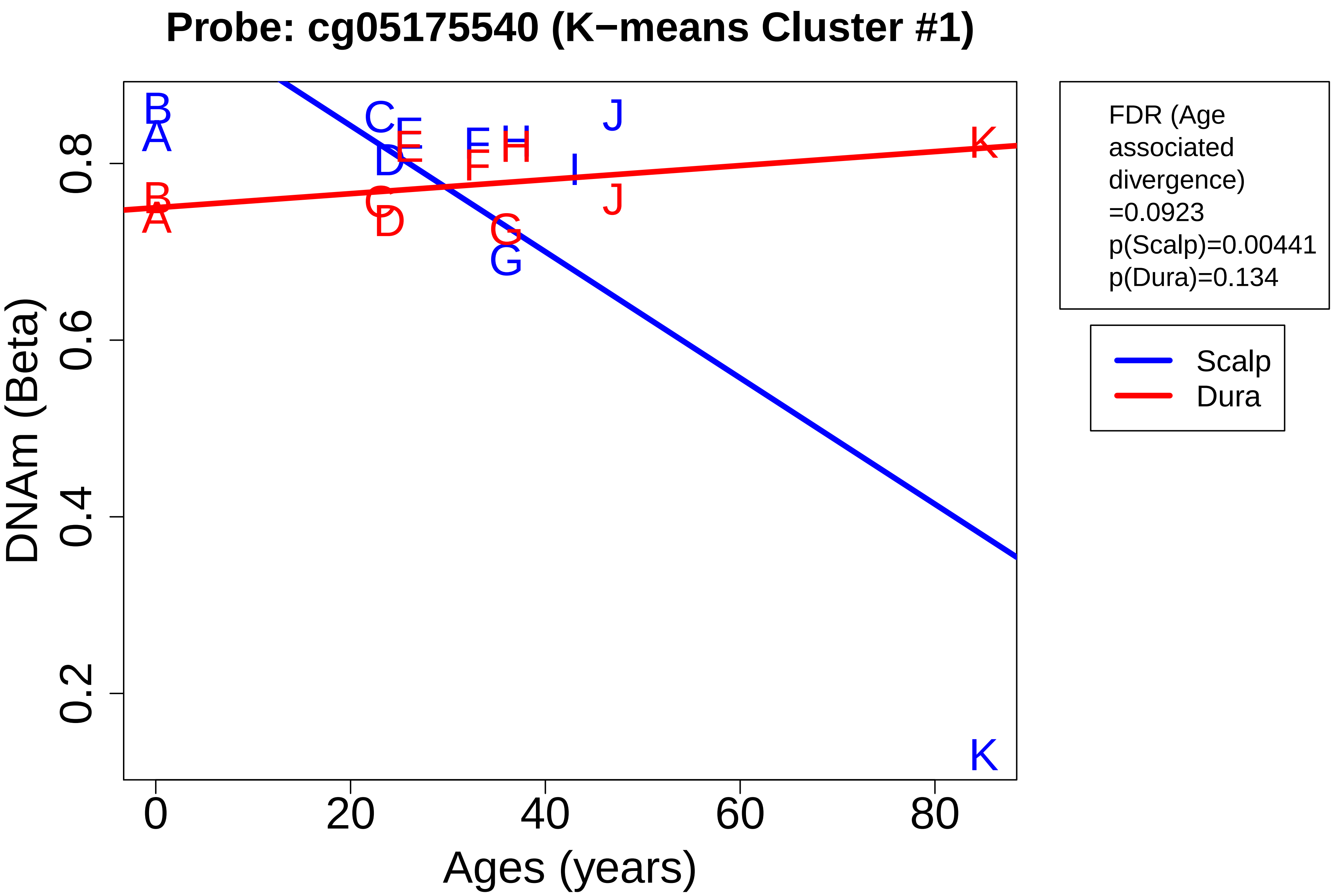

Probe: cg05175540 (K−means Cluster #1)
DNAm (Beta)
0.2
0.4
0.6
0.8
Ages (years)
0
20
40
60
80
A
B
C
D
E
F
G
H
I
J
K
FDR (Age associated divergence) =0.0923
p(Scalp)=0.00441
p(Dura)=0.134
Scalp
Dura

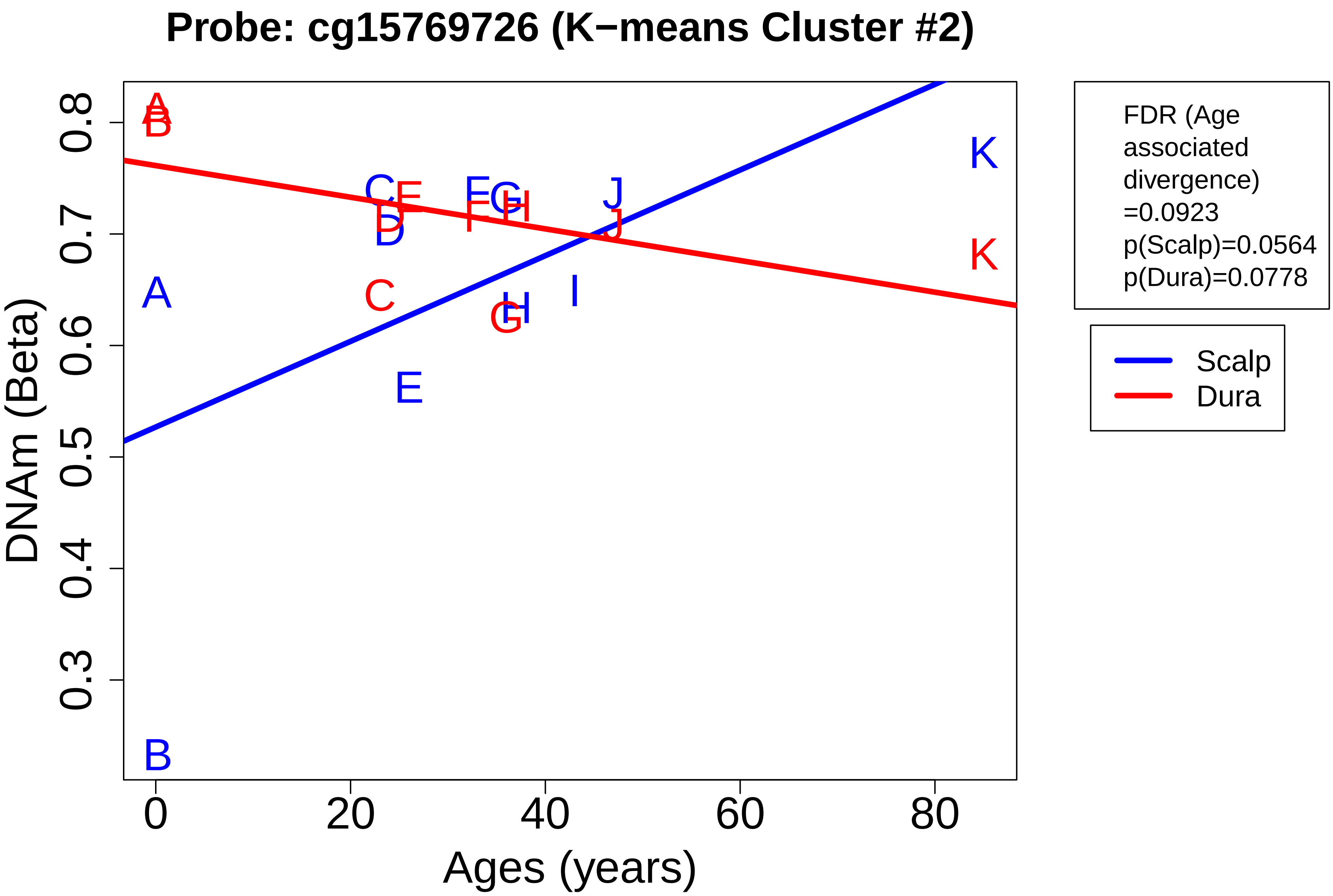

Probe: cg15769726 (K−means Cluster #2)
DNAm (Beta)
Ages (years)
0.3
0.4
0.5
0.6
0.7
0.8
0
20
40
60
80
A
B
C
D
E
F
G
H
I
J
K
FDR (Age associated divergence) =0.0923
p(Scalp)=0.0564
p(Dura)=0.0778
Scalp
Dura

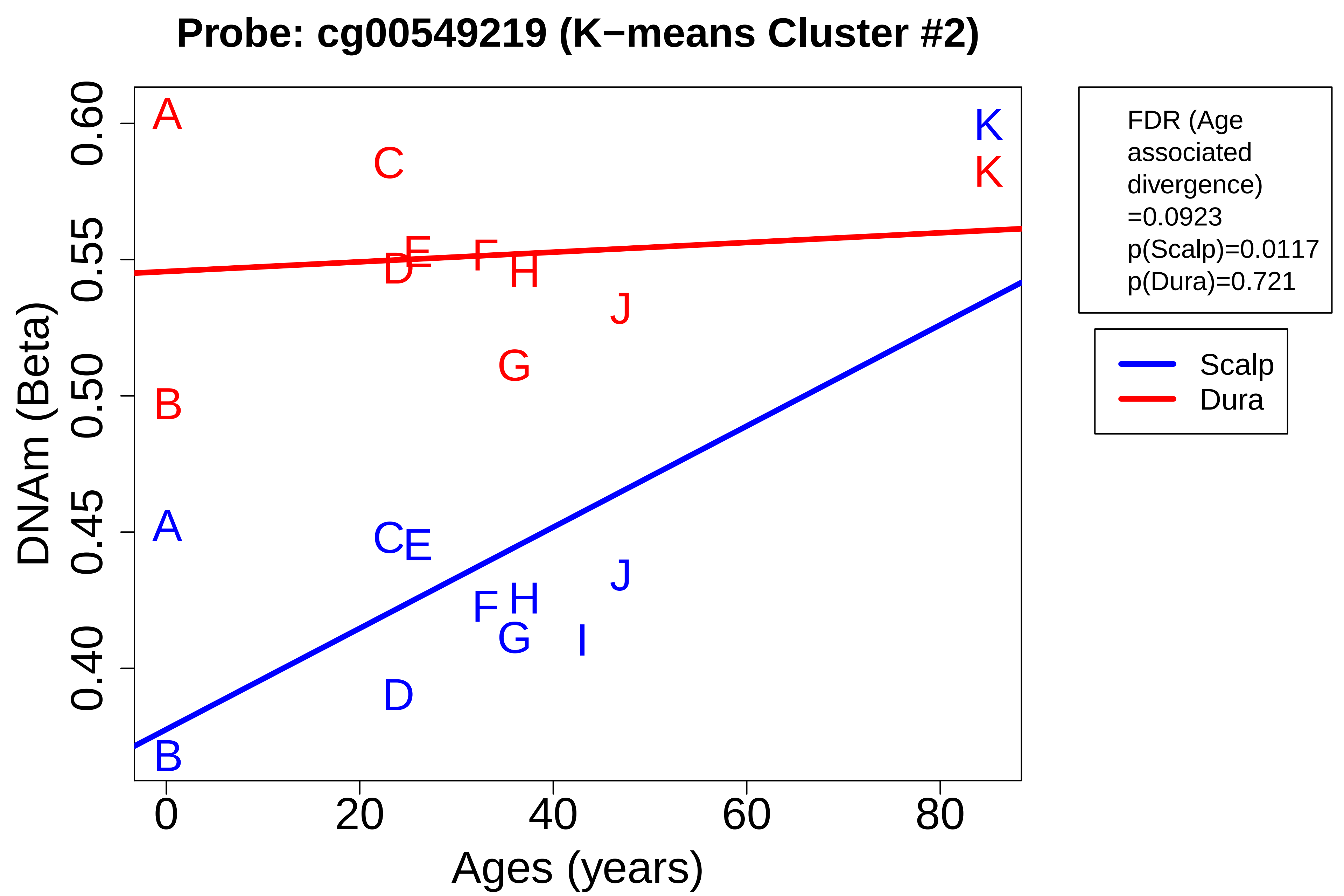

Probe: cg00549219 (K−means Cluster #2)
DNAm (Beta)
0.40
0.45
0.50
0.55
0.60
Ages (years)
0
20
40
60
80
FDR (Age associated divergence) =0.0923
p(Scalp)=0.0117
p(Dura)=0.721
Scalp
Dura
A
B
C
D
E
F
G
H
I
J
K

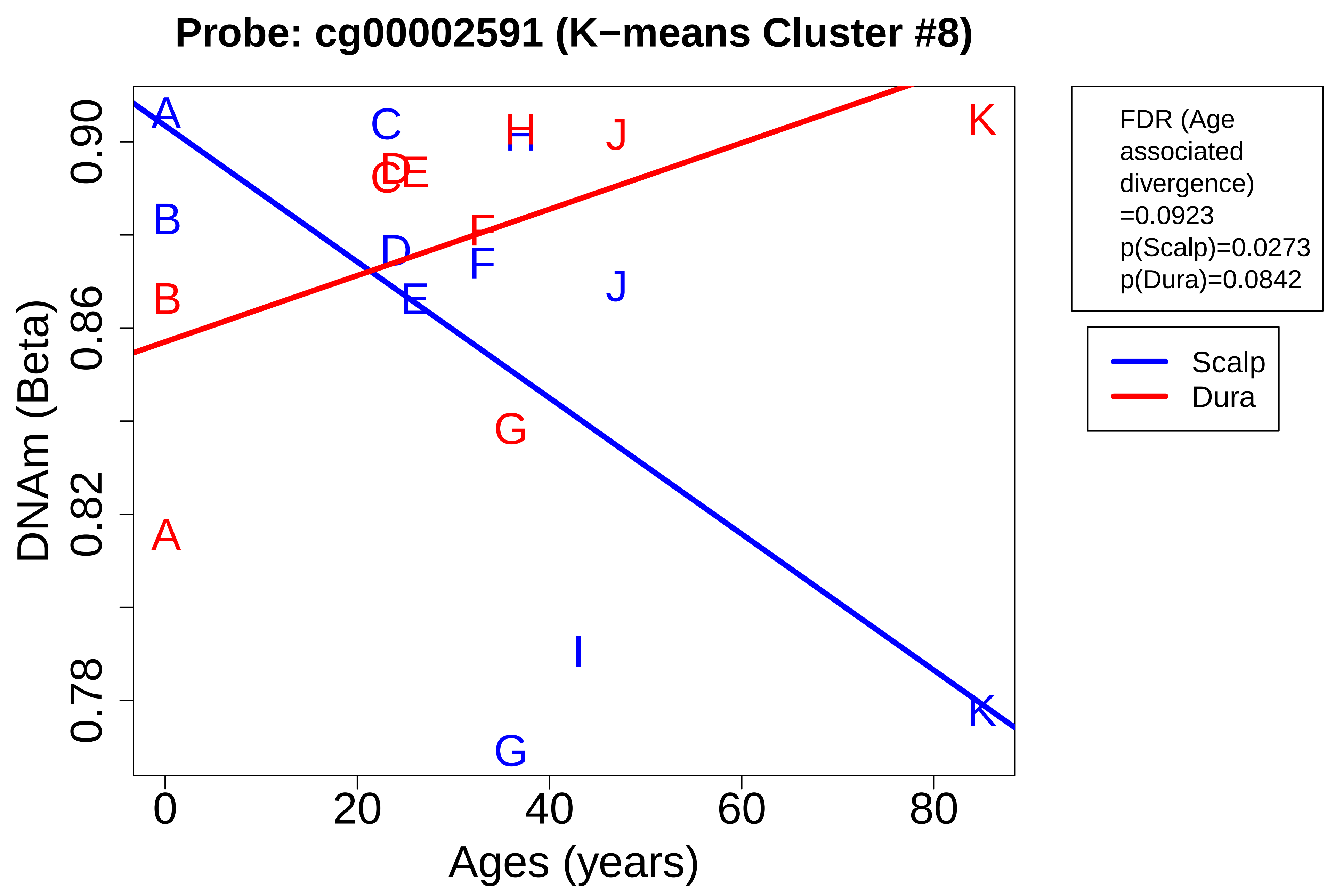

Probe: cg00002591 (K−means Cluster #8)
DNAm (Beta)
0.78
0.82
0.86
0.90
0
20
40
60
80
Ages (years)
A
B
C
D
E
F
G
H
I
J
K
FDR (Age associated divergence) =0.0923
p(Scalp)=0.0273
p(Dura)=0.0842
Scalp
Dura

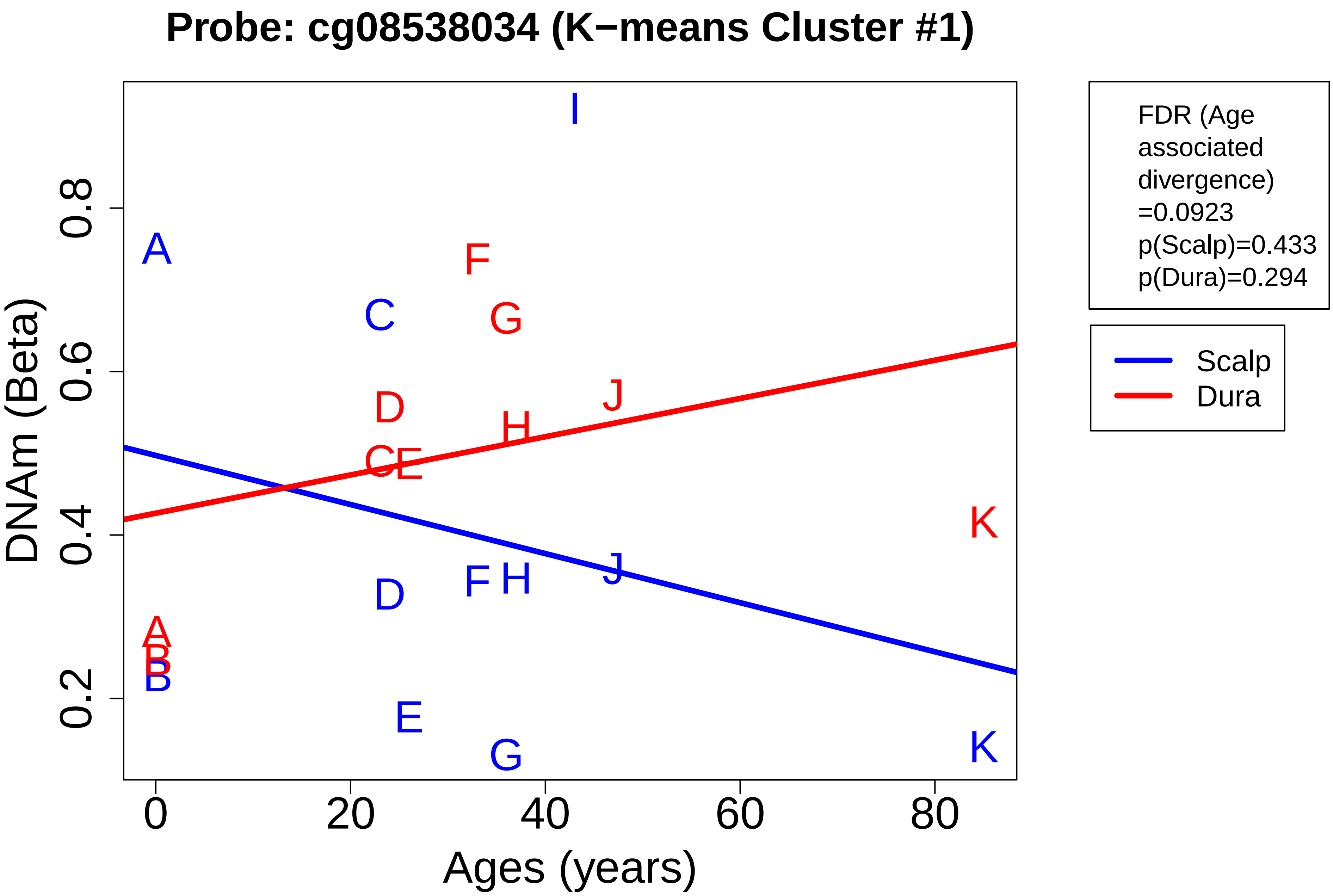
Probe: cg08538034 (K−means Cluster #1)
DNAm (Beta)
0.2
0.4
0.6
0.8
0
20
40
60
80
Ages (years)
FDR (Age associated divergence) =0.0923
p(Scalp)=0.433
p(Dura)=0.294
Scalp
Dura
A
B
C
D
E
F
G
H
I
J
K

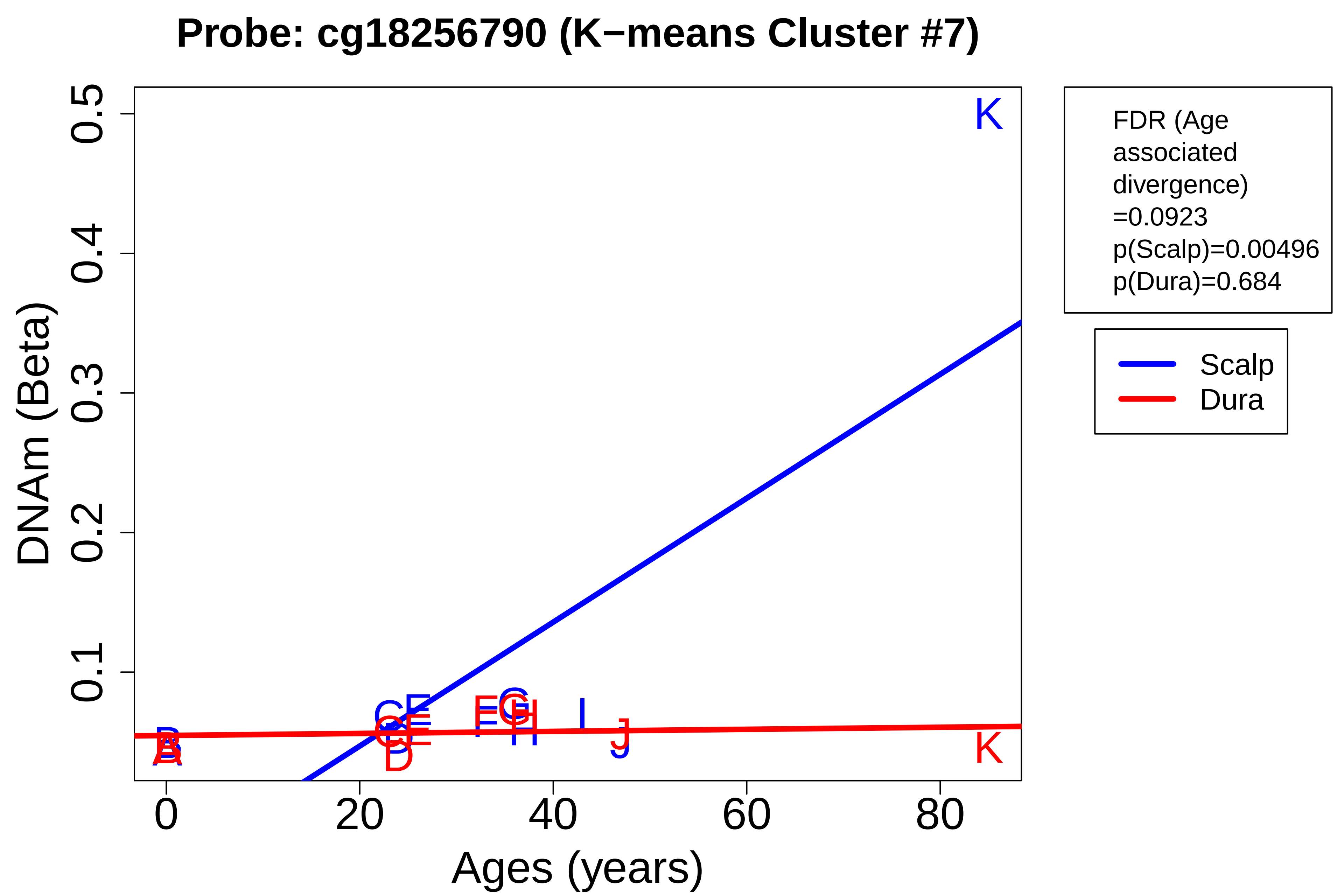

Probe: cg18256790 (K−means Cluster #7)
DNAm (Beta)
0.1
0.2
0.3
0.4
0.5
0
20
40
60
80
Ages (years)
K
FDR (Age associated divergence) =0.0923
p(Scalp)=0.00496
p(Dura)=0.684
Scalp
Dura

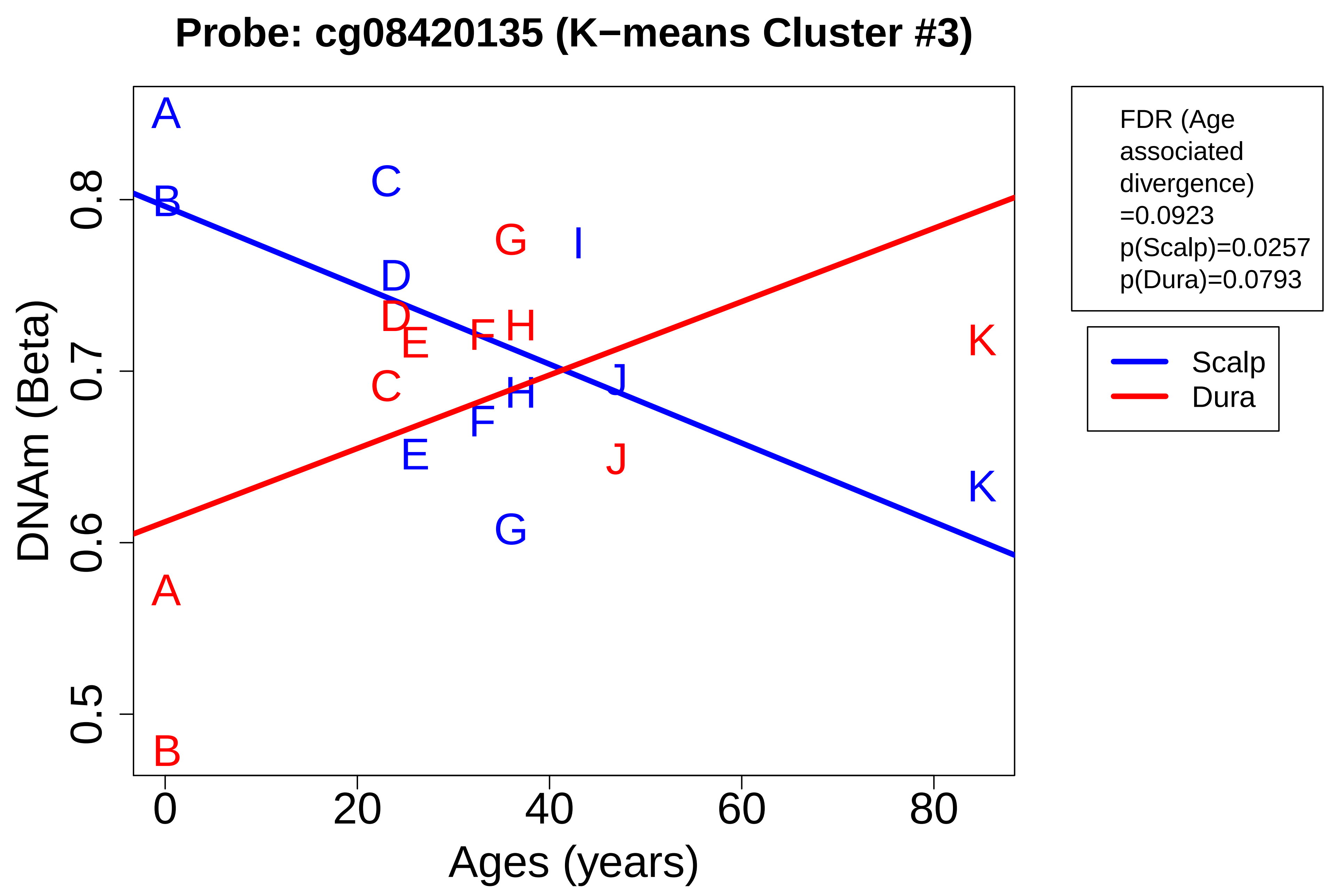

Probe: cg08420135 (K−means Cluster #3)
DNAm (Beta)
0.5
0.6
0.7
0.8
0
20
40
60
80
Ages (years)
FDR (Age associated divergence) =0.0923
p(Scalp)=0.0257
p(Dura)=0.0793
Scalp
Dura
A
B
C
D
E
F
G
H
I
J
K

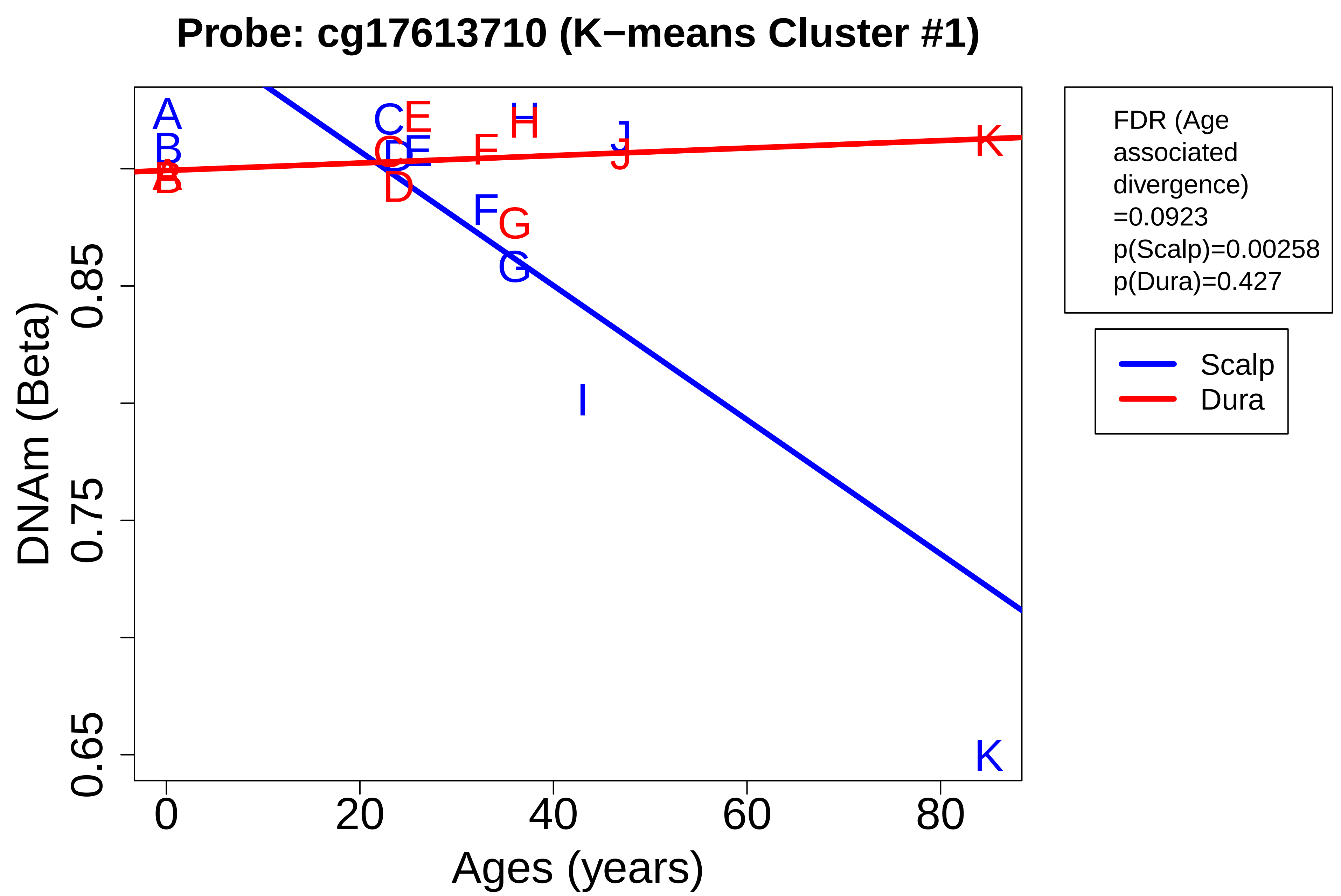
Probe: cg17613710 (K−means Cluster #1)
DNAm (Beta)
0.65
0.75
0.85
0
20
40
60
80
Ages (years)
FDR (Age associated divergence) =0.0923
p(Scalp)=0.00258
p(Dura)=0.427
Scalp
Dura

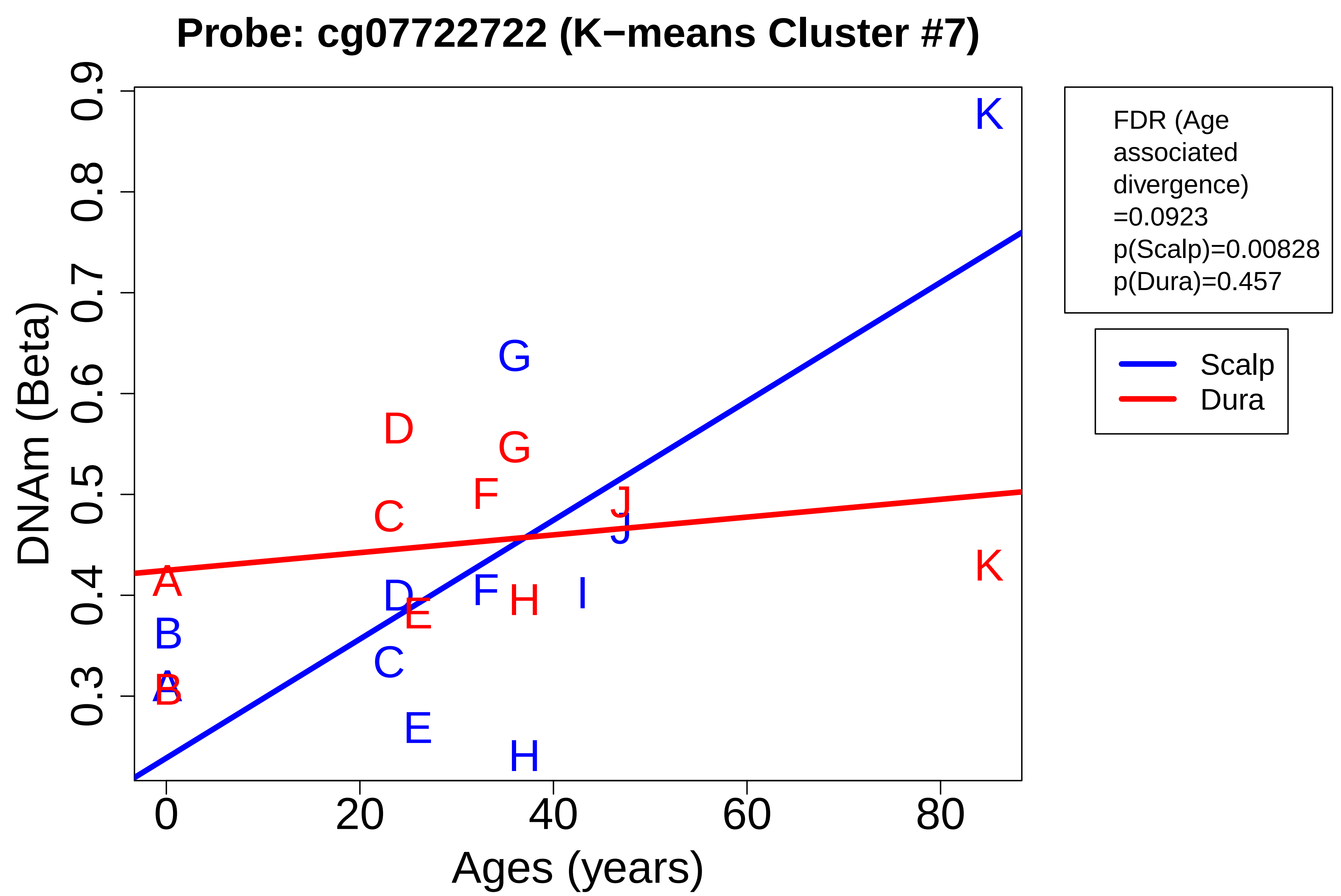

Probe: cg07722722 (K−means Cluster #7)
DNAm (Beta)
0.3
0.4
0.5
0.6
0.7
0.8
0.9
Ages (years)
0
20
40
60
80
FDR (Age associated divergence) =0.0923
p(Scalp)=0.00828
p(Dura)=0.457
Scalp
Dura
A
B
C
D
E
F
G
H
I
J
K

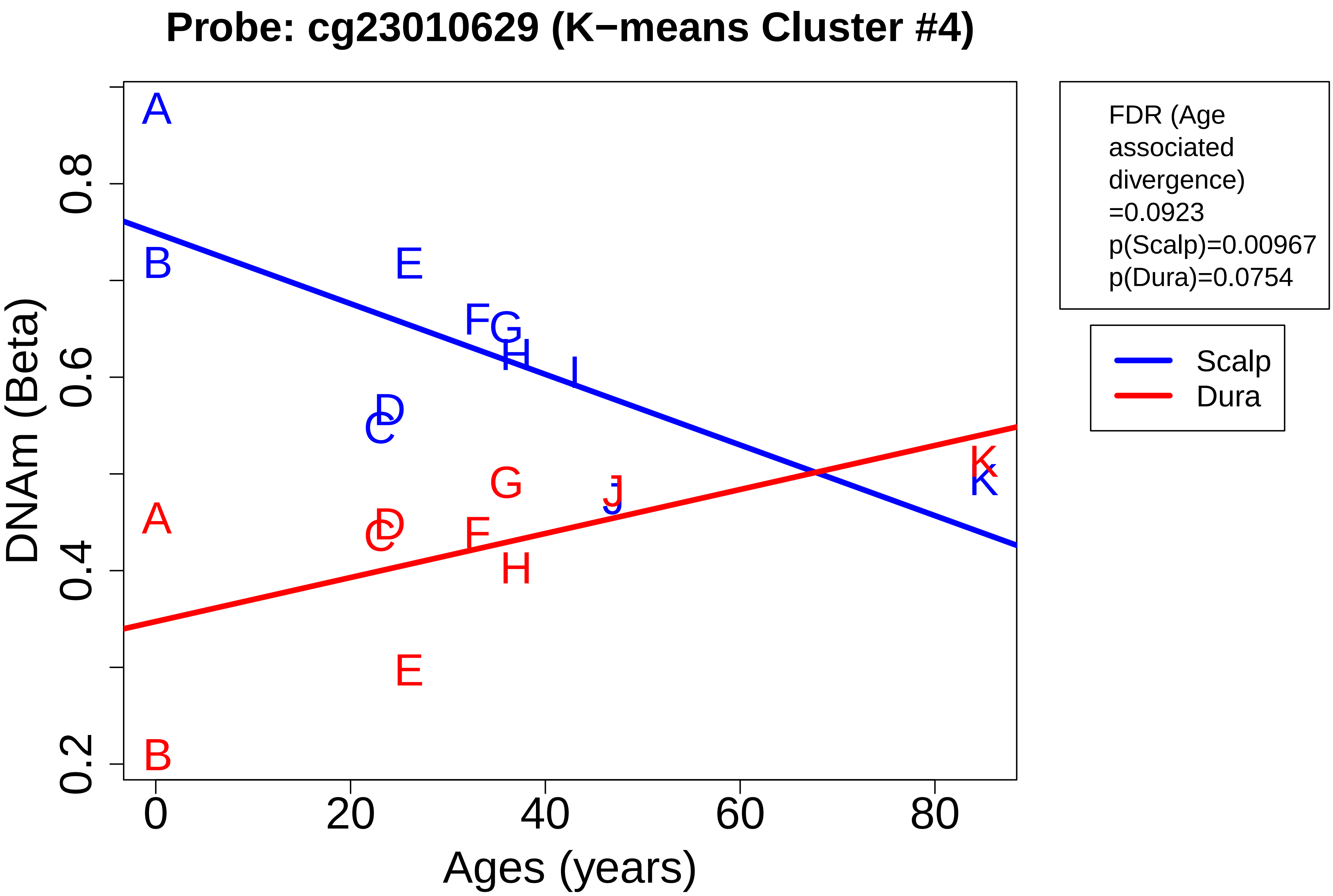

Probe: cg23010629 (K−means Cluster #4)
DNAm (Beta)
0.2
0.4
0.6
0.8
Ages (years)
0
20
40
60
80
A
B
C
D
E
F
G
H
I
J
K
FDR (Age associated divergence) =0.0923
p(Scalp)=0.00967
p(Dura)=0.0754
Scalp
Dura

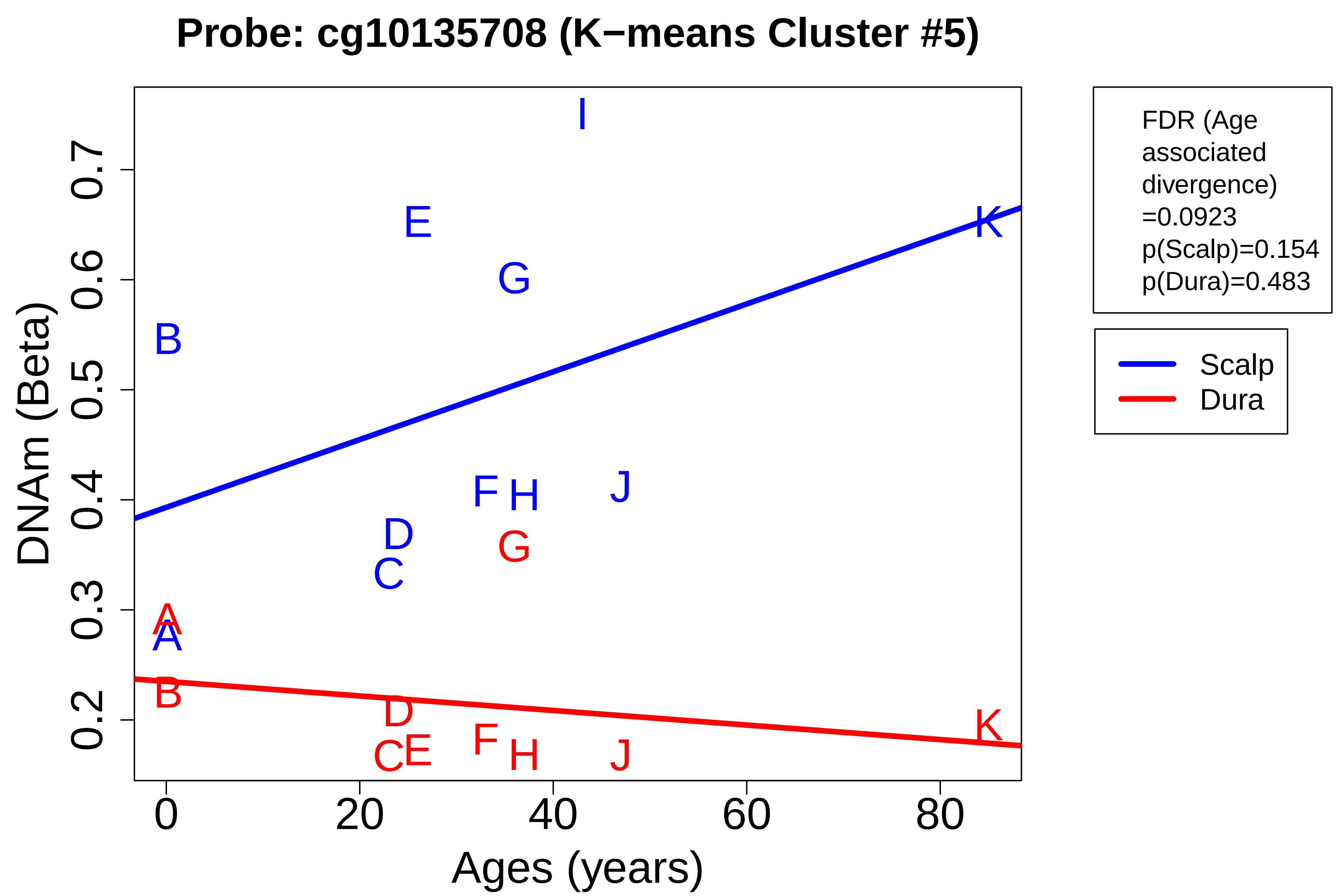
Probe: cg10135708 (K−means Cluster #5)
DNAm (Beta)
0.2
0.3
0.4
0.5
0.6
0.7
0
20
40
60
80
Ages (years)
FDR (Age associated divergence) =0.0923
p(Scalp)=0.154
p(Dura)=0.483
Scalp
Dura
A
B
C
D
E
F
G
H
I
J
K
A
B
C
D
E
F
G
H
J
K

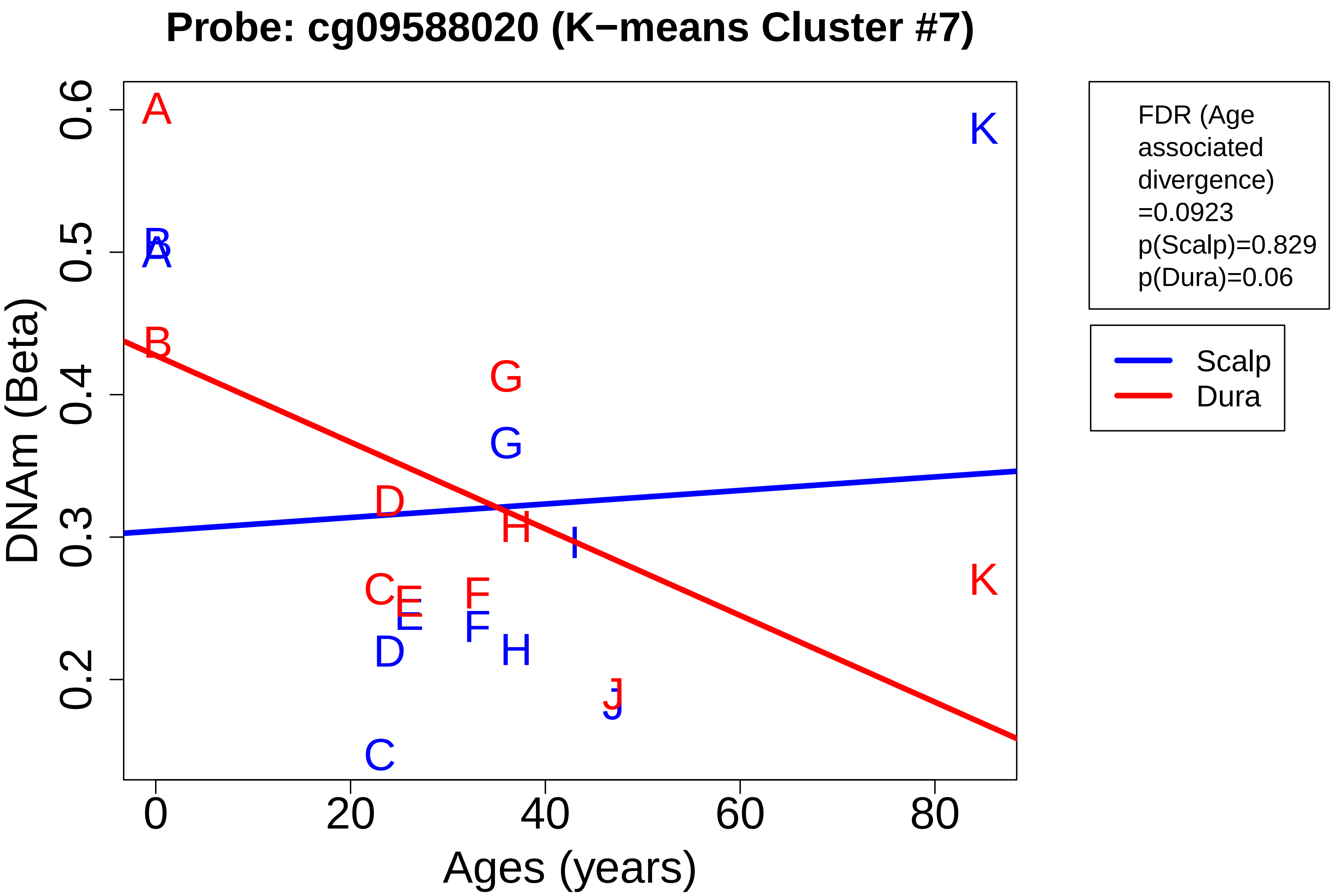

Probe: cg09588020 (K−means Cluster #7)
DNAm (Beta)
0.2
0.3
0.4
0.5
0.6
0
20
40
60
80
Ages (years)
A
B
C
D
E
F
G
H
I
J
K
FDR (Age associated divergence) =0.0923
p(Scalp)=0.829
p(Dura)=0.06
Scalp
Dura

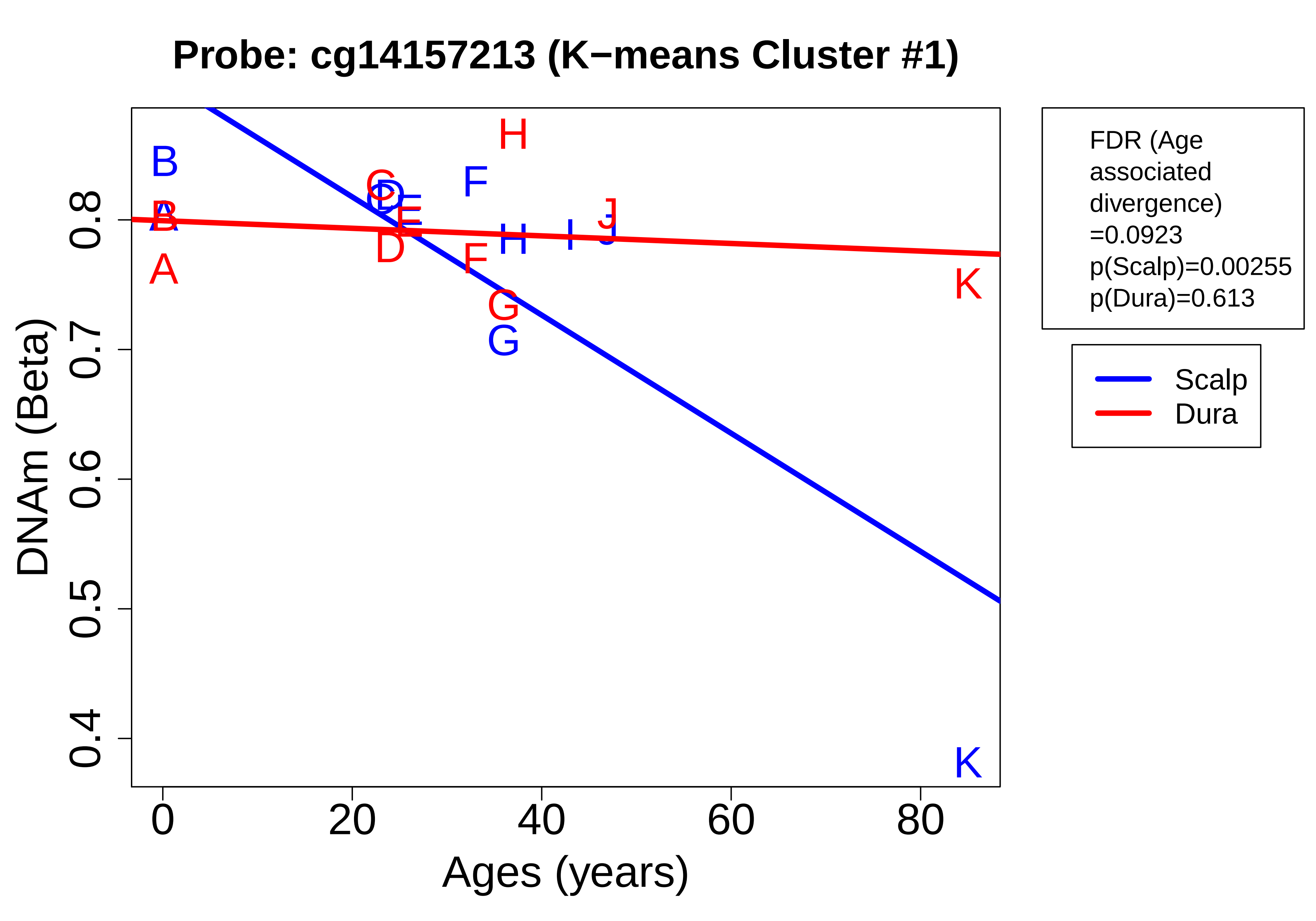
Probe: cg14157213 (K−means Cluster #1)
DNAm (Beta)
0.4
0.5
0.6
0.7
0.8
0
20
40
60
80
Ages (years)
FDR (Age associated divergence) =0.0923
p(Scalp)=0.00255
p(Dura)=0.613
Scalp
Dura

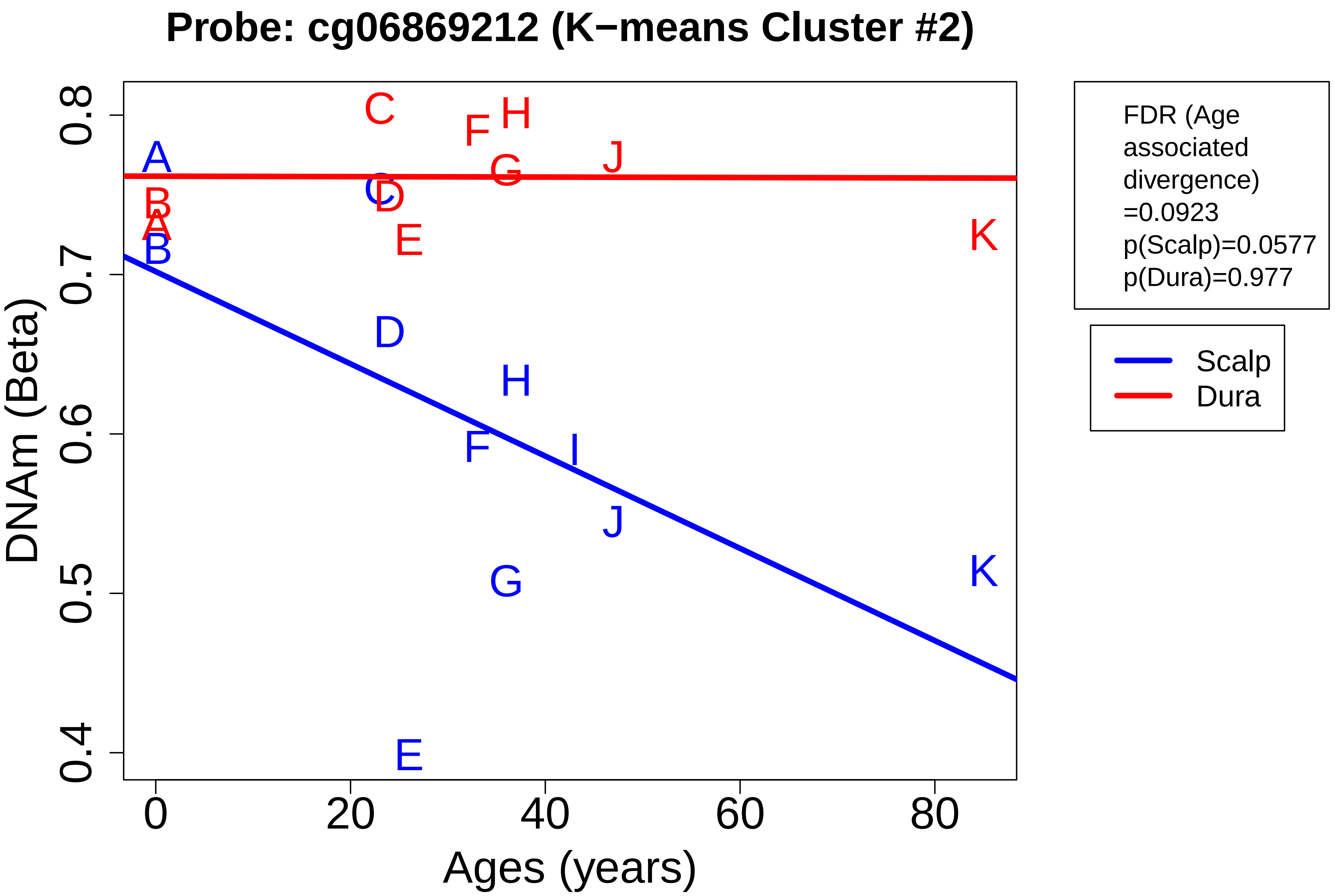

Probe: cg06869212 (K−means Cluster #2)
DNAm (Beta)
0.4
0.5
0.6
0.7
0.8
Ages (years)
0
20
40
60
80
A
B
C
D
E
F
G
H
I
J
K
FDR (Age associated divergence) =0.0923
p(Scalp)=0.0577
p(Dura)=0.977
Scalp
Dura

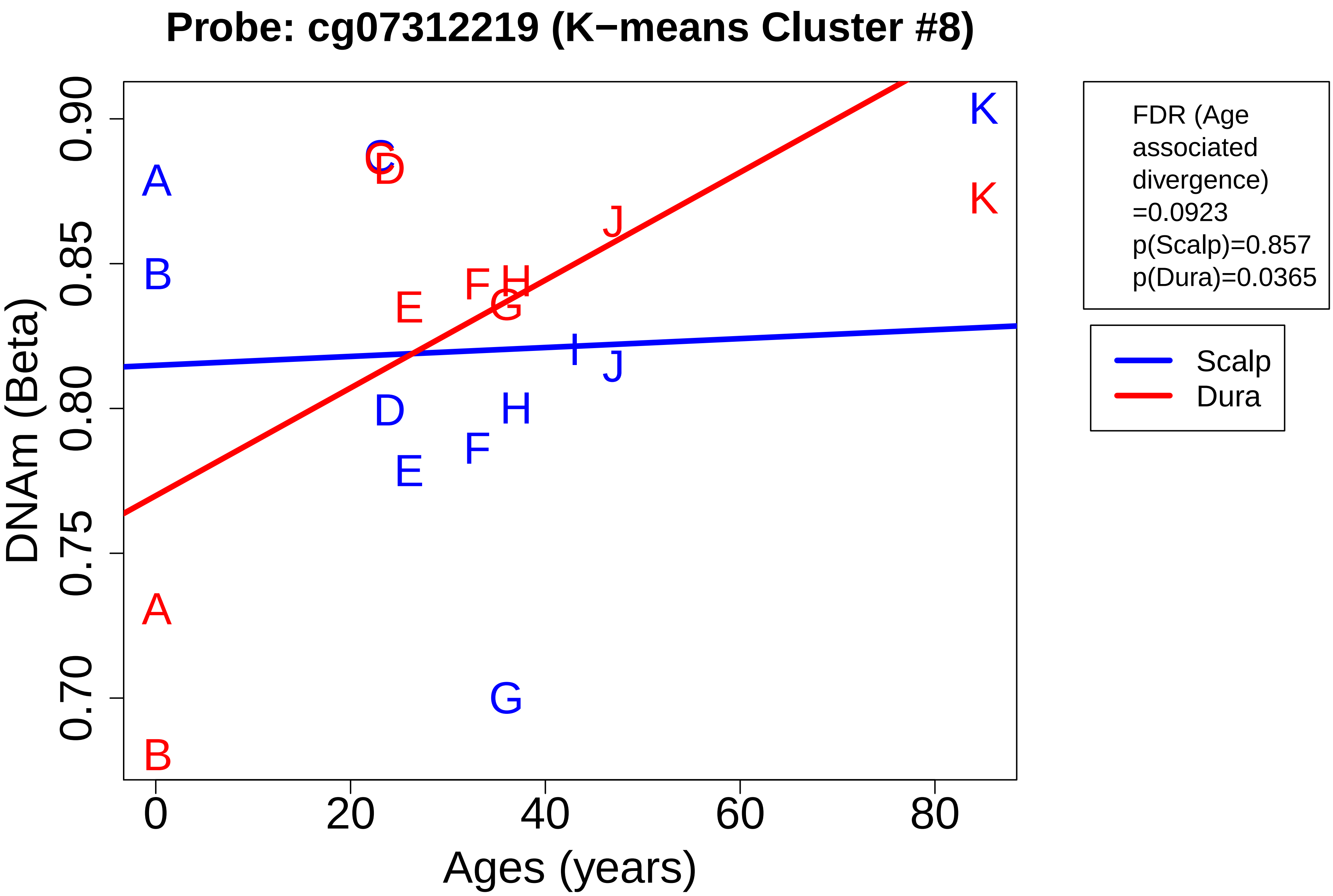

Probe: cg07312219 (K−means Cluster #8)
DNAm (Beta)
0.70
0.75
0.80
0.85
0.90
Ages (years)
0
20
40
60
80
A
B
C
D
E
F
G
H
I
J
K
FDR (Age associated divergence) =0.0923
p(Scalp)=0.857
p(Dura)=0.0365
Scalp
Dura

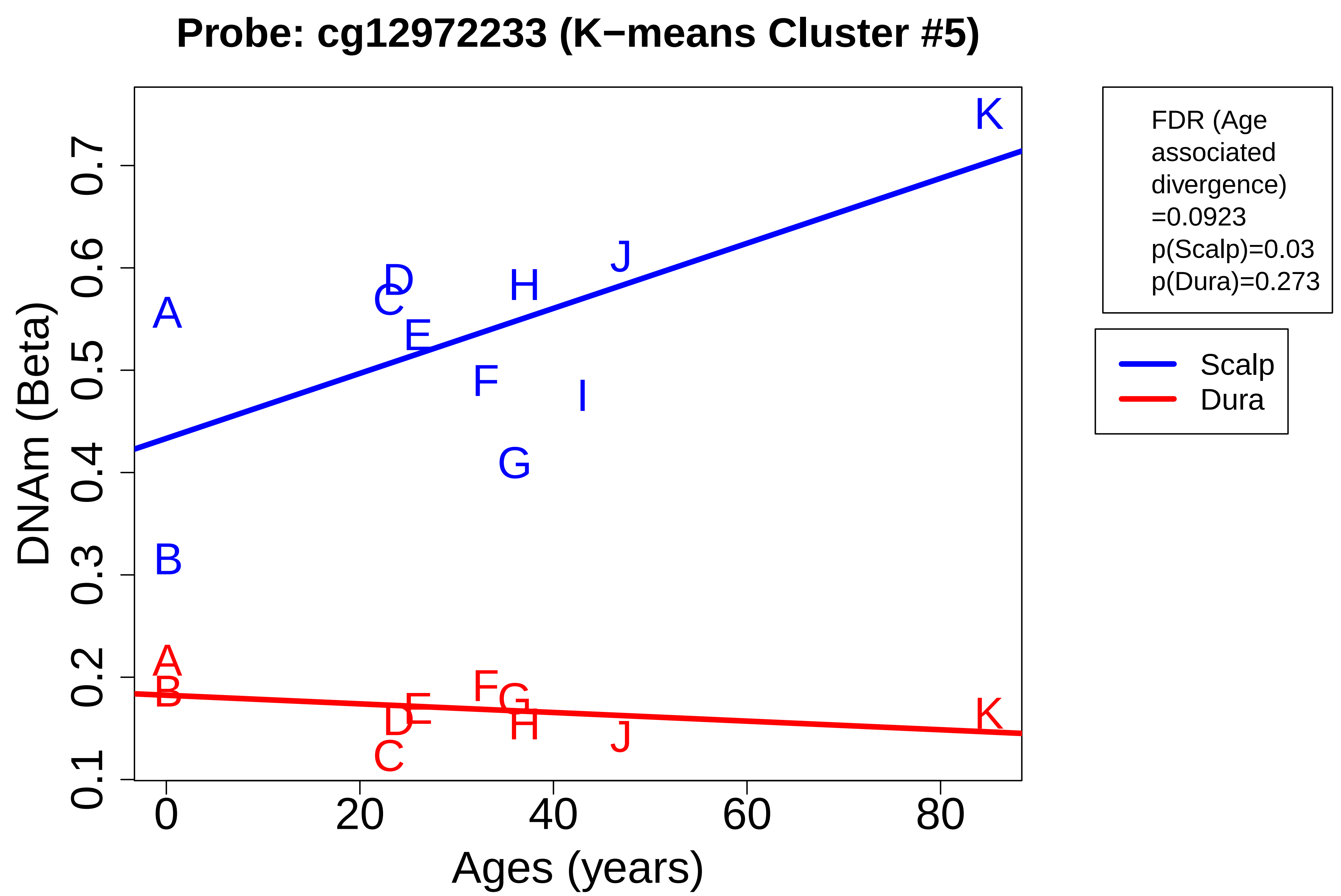

Probe: cg12972233 (K−means Cluster #5)
FDR (Age associated divergence) =0.0923
p(Scalp)=0.03
p(Dura)=0.273
Scalp
Dura
DNAm (Beta)
0.1
0.2
0.3
0.4
0.5
0.6
0.7
0
20
40
60
80
Ages (years)
A
B
C
D
E
F
G
H
I
J
K

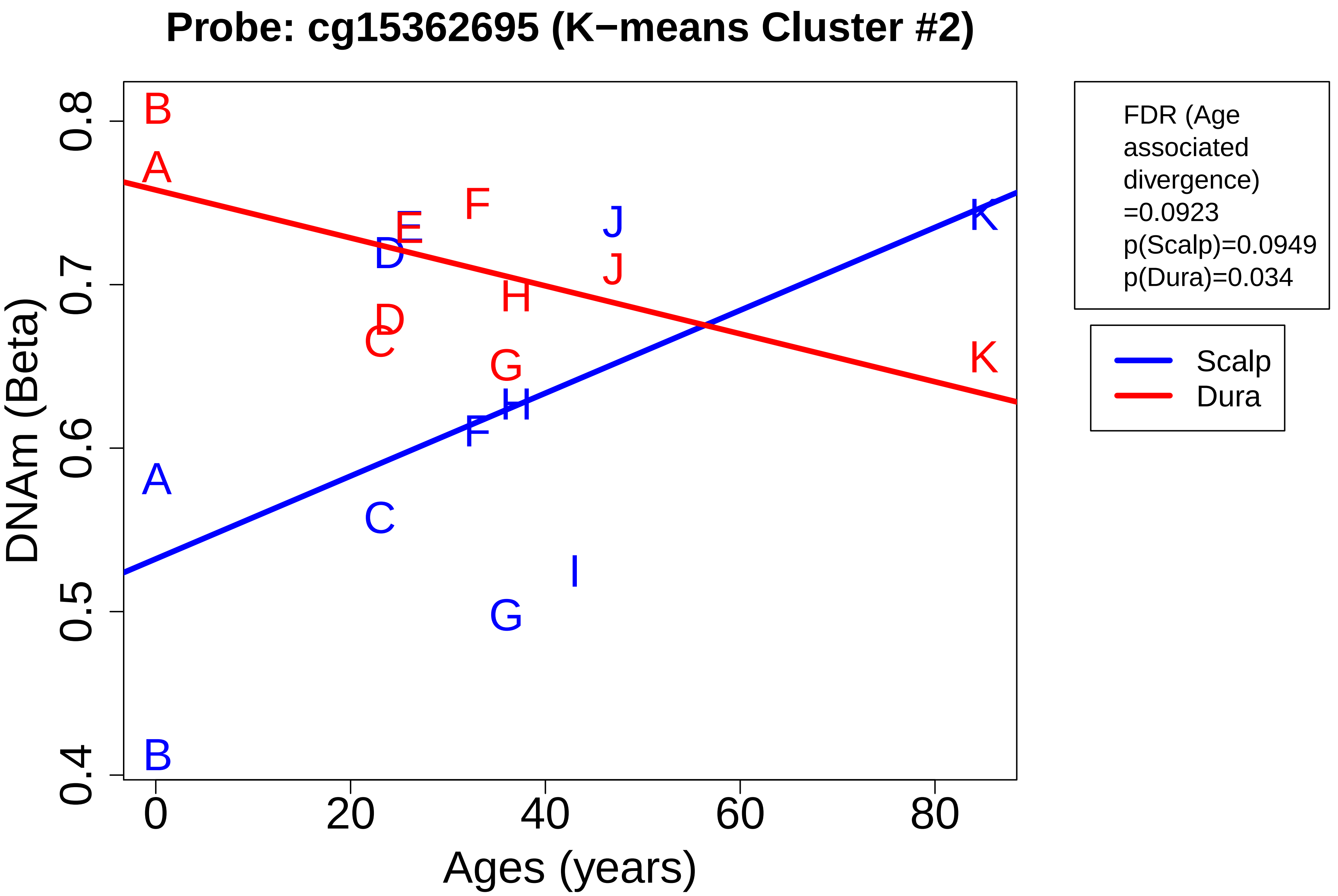

Probe: cg15362695 (K−means Cluster #2)
DNAm (Beta)
Ages (years)
0.4
0.5
0.6
0.7
0.8
0
20
40
60
80
FDR (Age associated divergence) =0.0923
p(Scalp)=0.0949
p(Dura)=0.034
Scalp
Dura

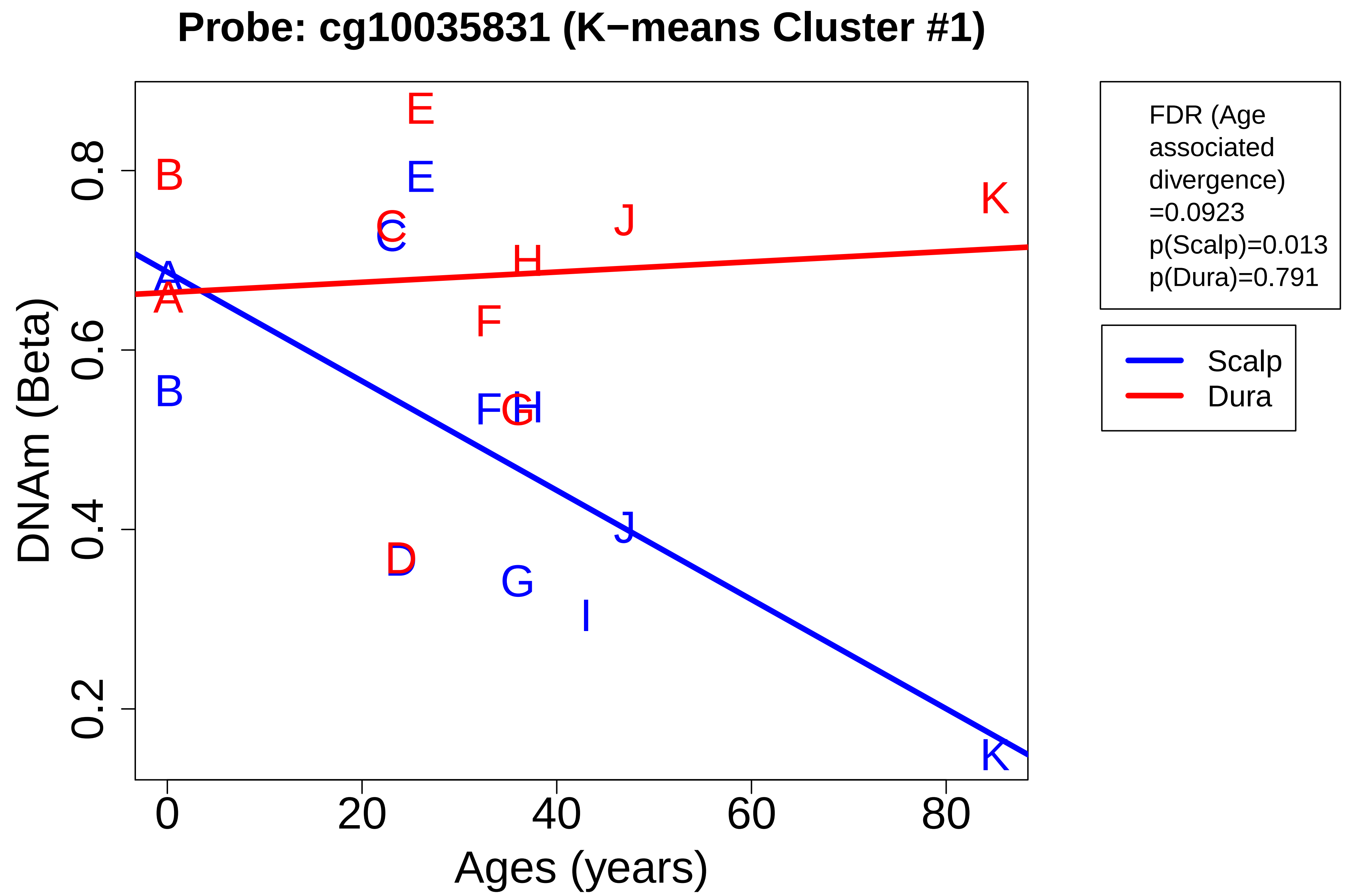

Probe: cg10035831 (K−means Cluster #1)
DNAm (Beta)
0.2
0.4
0.6
0.8
0
20
40
60
80
Ages (years)
FDR (Age associated divergence) =0.0923
p(Scalp)=0.013
p(Dura)=0.791
Scalp
Dura

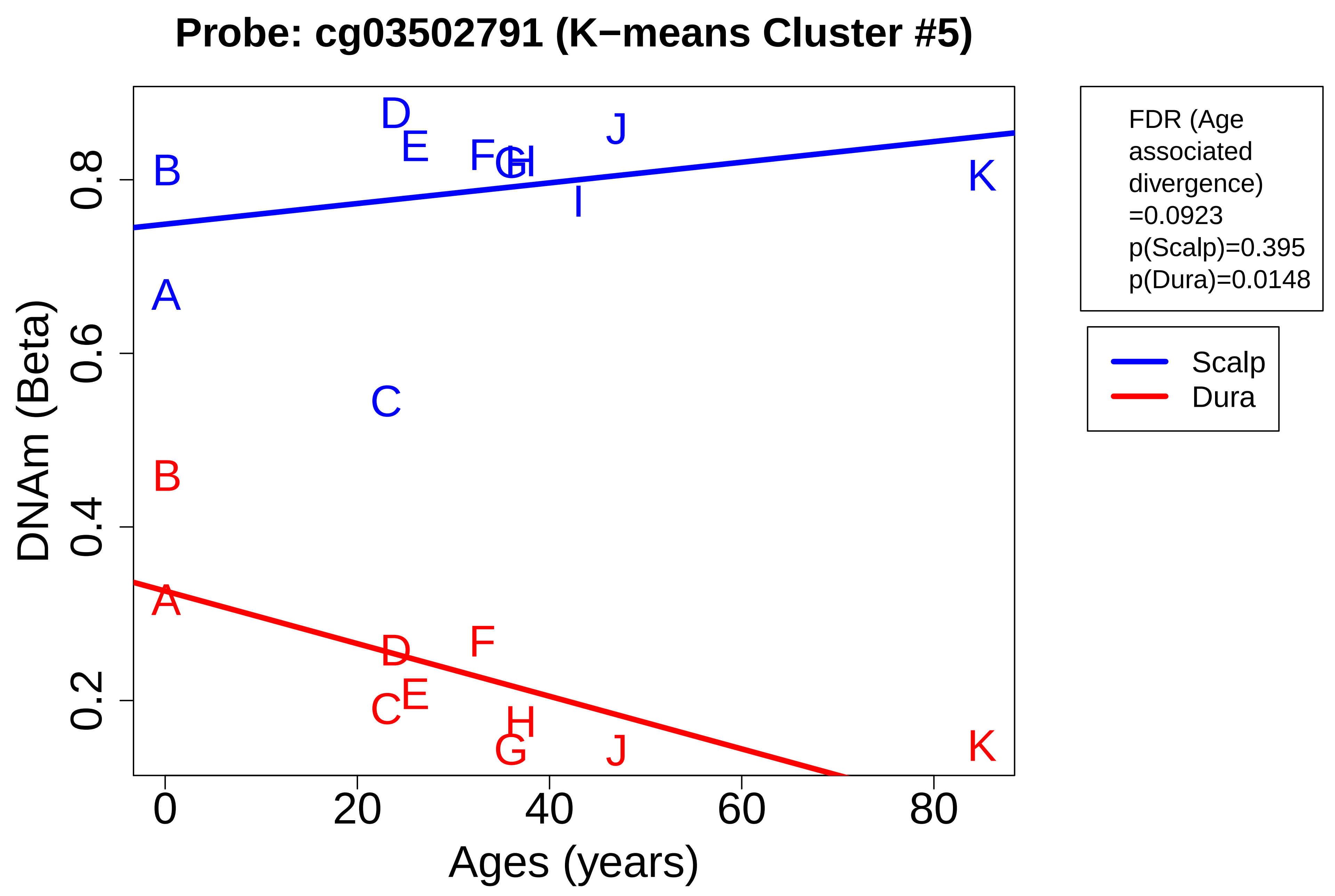
Probe: cg03502791 (K−means Cluster #5)
FDR (Age associated divergence) =0.0923
p(Scalp)=0.395
p(Dura)=0.0148
Scalp
Dura
DNAm (Beta)
0.2
0.4
0.6
0.8
0
20
40
60
80
Ages (years)
A
B
C
D
E
F
G
H
I
J
K

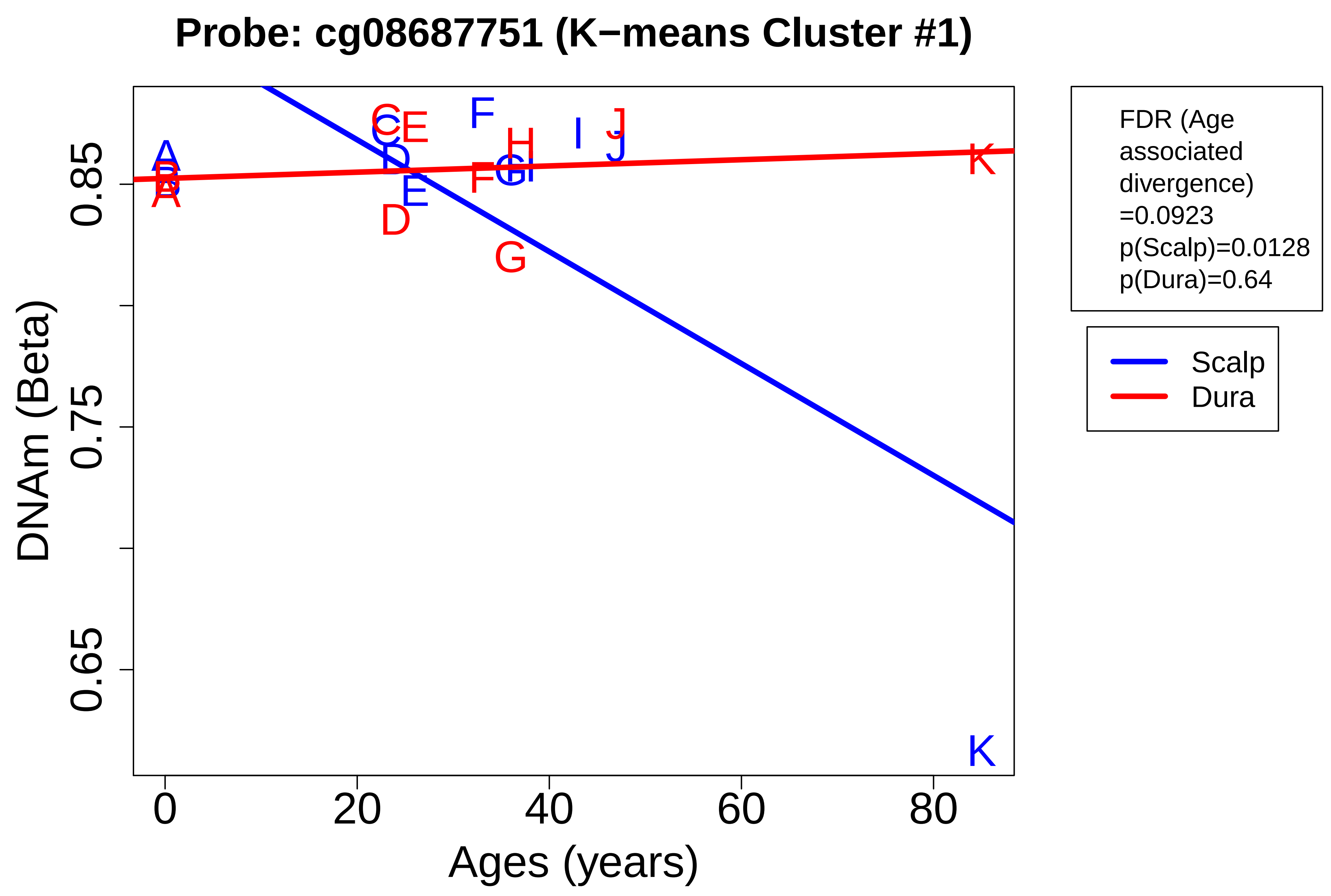

Probe: cg08687751 (K−means Cluster #1)
FDR (Age associated divergence) =0.0923
p(Scalp)=0.0128
p(Dura)=0.64
Scalp
Dura
DNAm (Beta)
0.65
0.75
0.85
0
20
40
60
80
Ages (years)
A
B
C
D
E
F
G
H
I
J
K

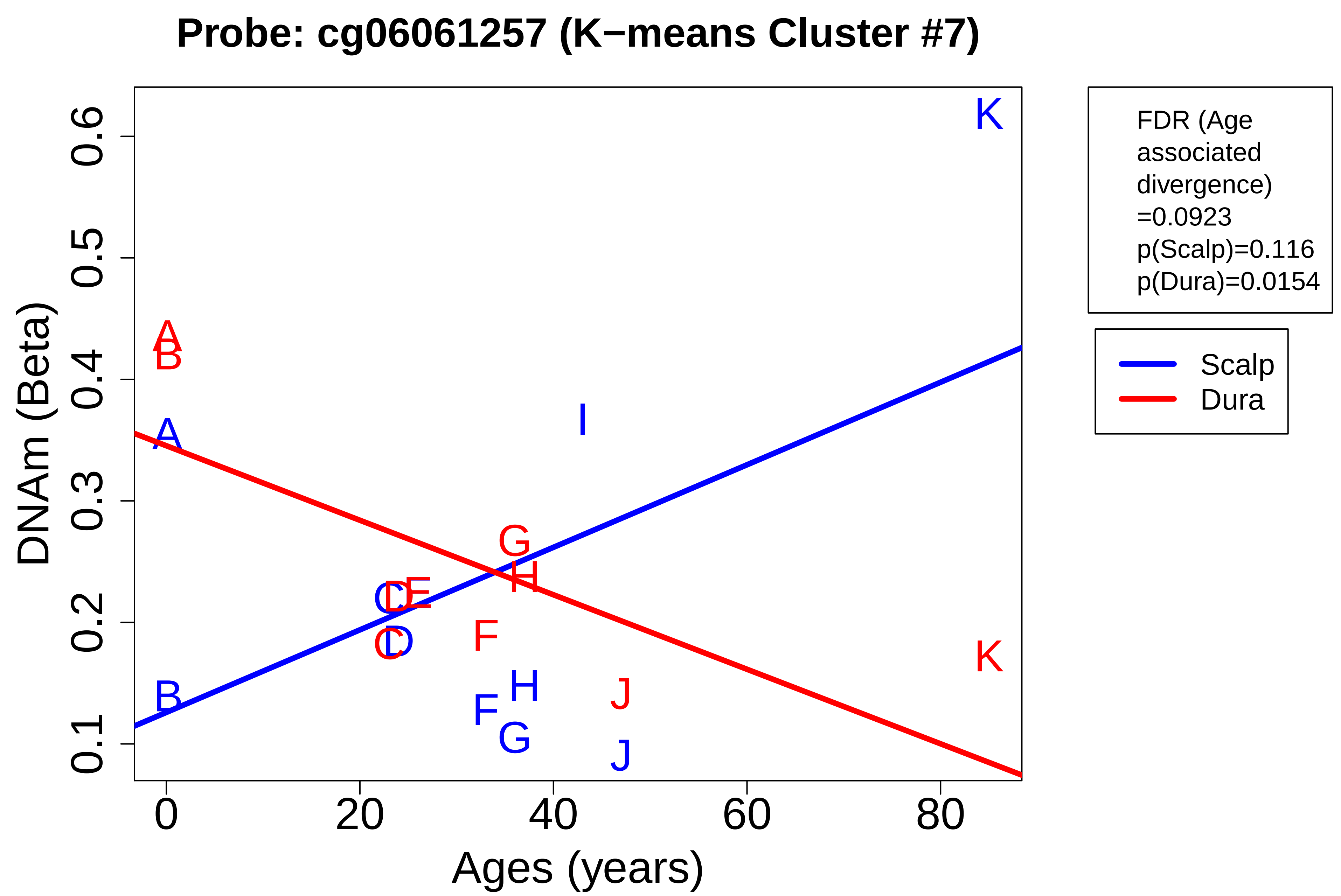

Probe: cg06061257 (K−means Cluster #7)
DNAm (Beta)
0.1
0.2
0.3
0.4
0.5
0.6
0
20
40
60
80
Ages (years)
K
A
B
I
A
G
H
C
D
E
C
D
F
K
B
H
J
F
G
J
FDR (Age associated divergence) =0.0923
p(Scalp)=0.116
p(Dura)=0.0154
Scalp
Dura

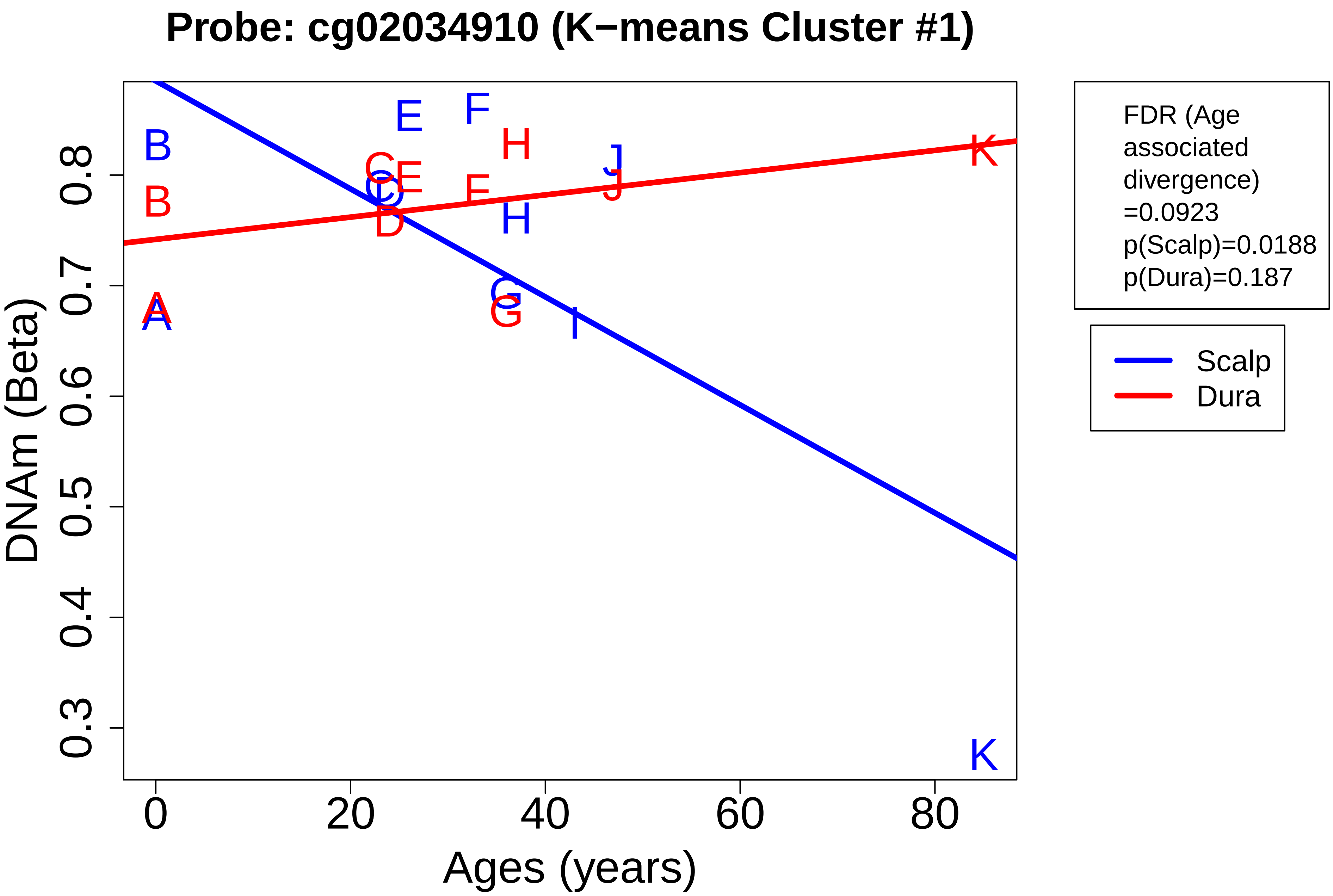

Probe: cg02034910 (K−means Cluster #1)
DNAm (Beta)
0.3
0.4
0.5
0.6
0.7
0.8
0
20
40
60
80
Ages (years)
A
B
C
D
E
F
G
H
I
J
K
FDR (Age associated divergence) =0.0923
p(Scalp)=0.0188
p(Dura)=0.187
Scalp
Dura

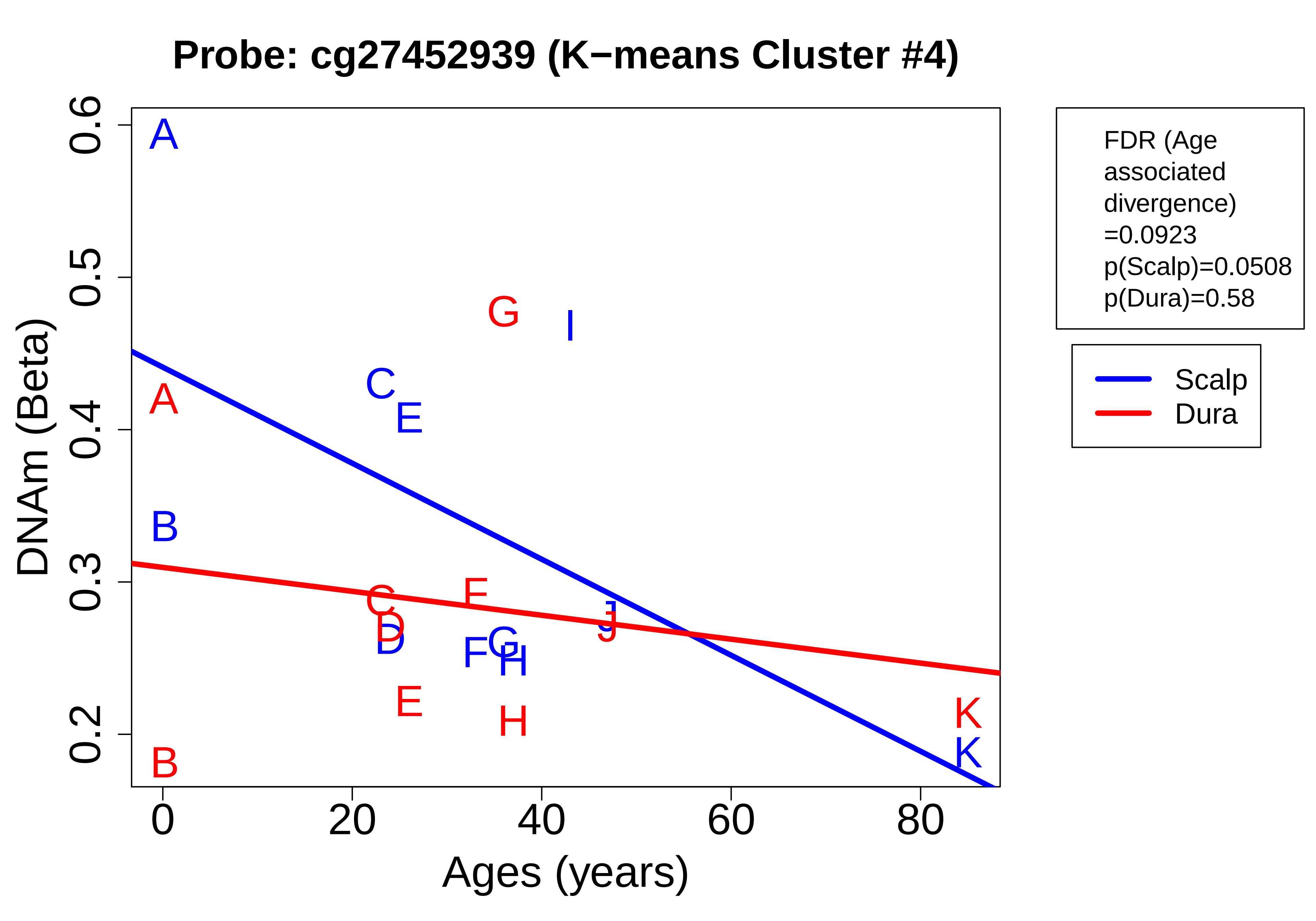
Probe: cg27452939 (K−means Cluster #4)
DNAm (Beta)
0.2
0.3
0.4
0.5
0.6
Ages (years)
0
20
40
60
80
FDR (Age associated divergence) =0.0923
p(Scalp)=0.0508
p(Dura)=0.58
Scalp
Dura
A
B
C
D
E
F
G
H
I
J
K

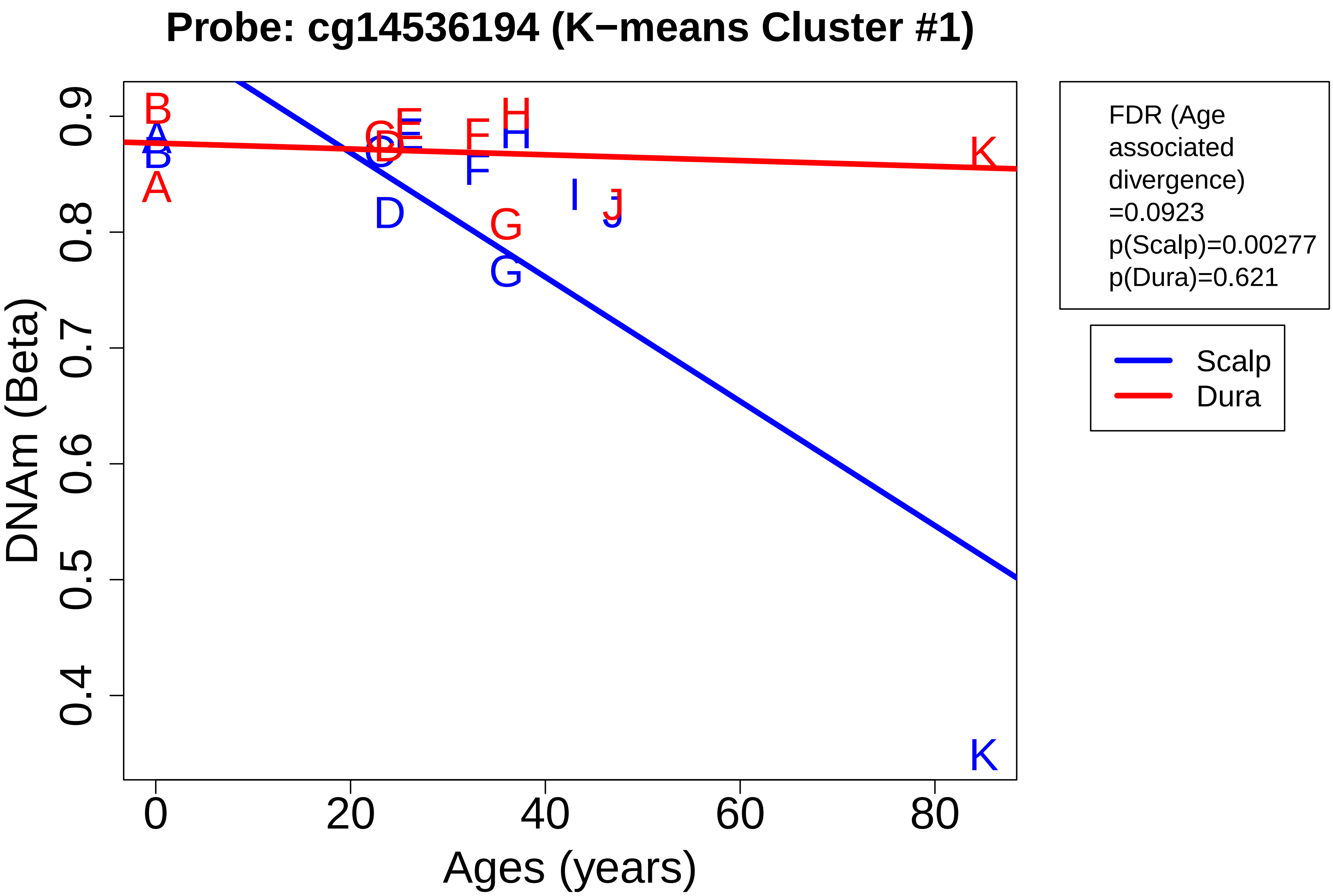
Probe: cg14536194 (K−means Cluster #1)
DNAm (Beta)
0.4
0.5
0.6
0.7
0.8
0.9
0
20
40
60
80
Ages (years)
FDR (Age associated divergence) =0.0923
p(Scalp)=0.00277
p(Dura)=0.621
Scalp
Dura

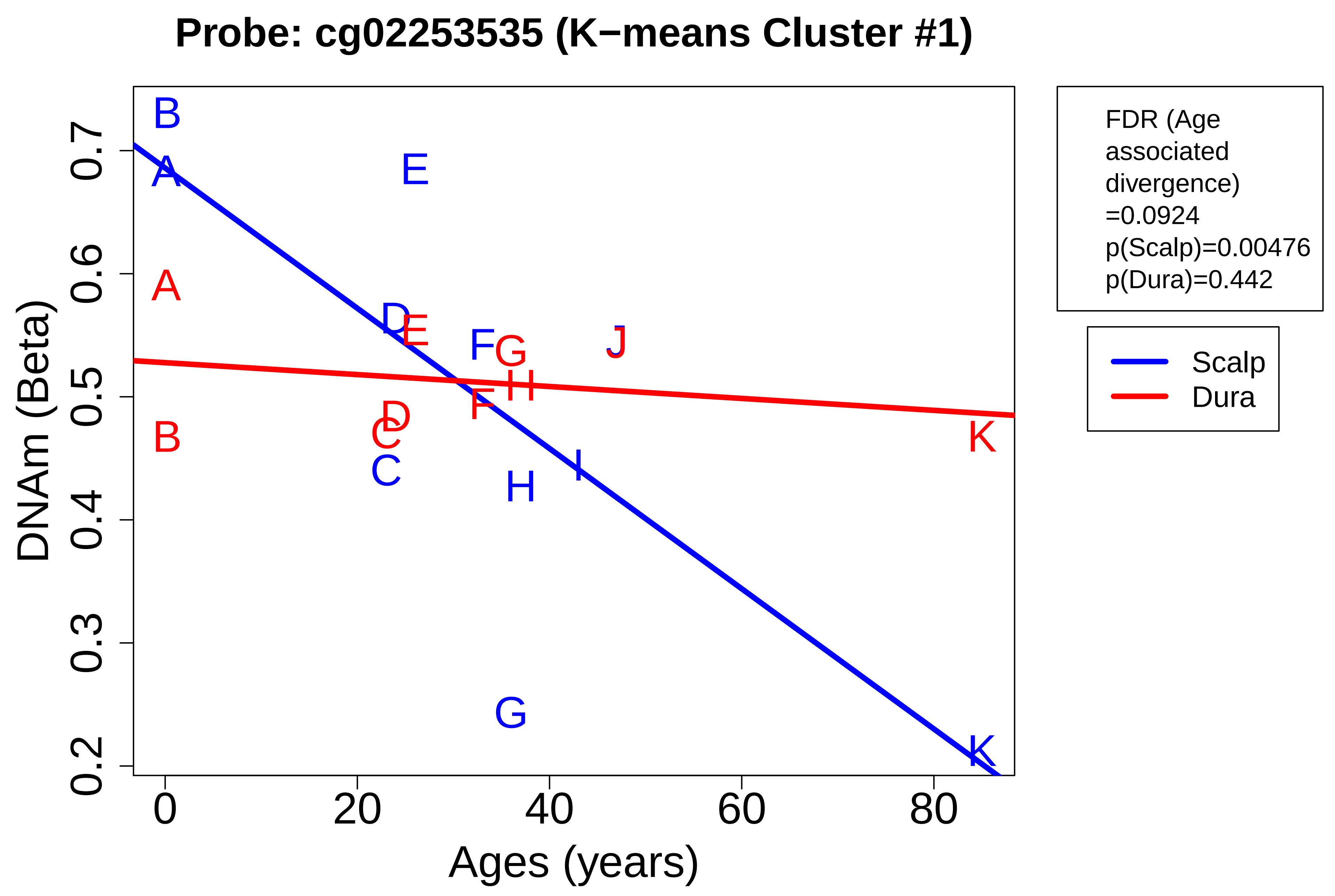
Probe: cg02253535 (K−means Cluster #1)
FDR (Age associated divergence) =0.0924
p(Scalp)=0.00476
p(Dura)=0.442
Scalp
Dura
DNAm (Beta)
Ages (years)
0.2
0.3
0.4
0.5
0.6
0.7
0
20
40
60
80

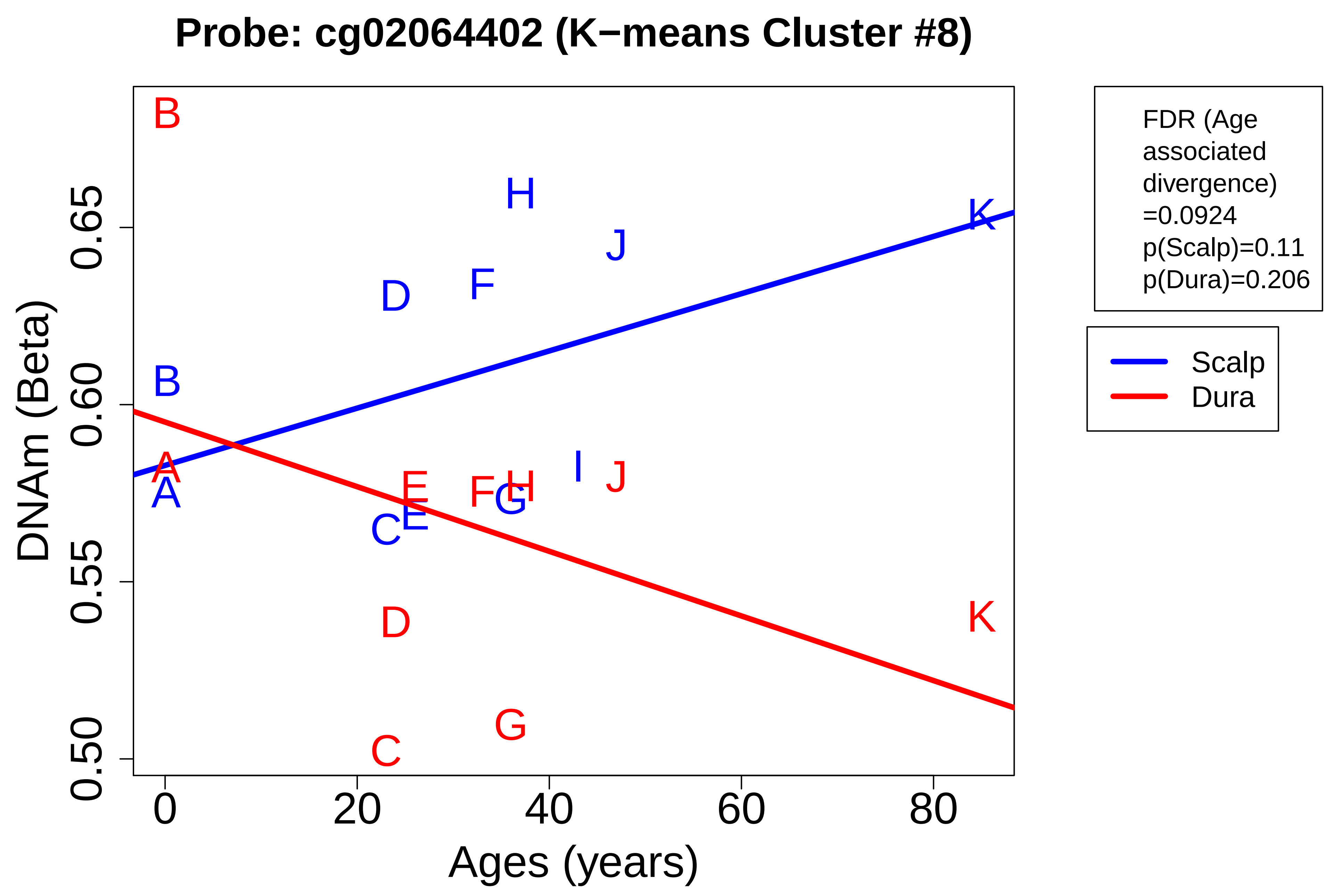

Probe: cg02064402 (K−means Cluster #8)
DNAm (Beta)
0.50
0.55
0.60
0.65
0
20
40
60
80
Ages (years)
FDR (Age associated divergence) =0.0924
p(Scalp)=0.11
p(Dura)=0.206
Scalp
Dura

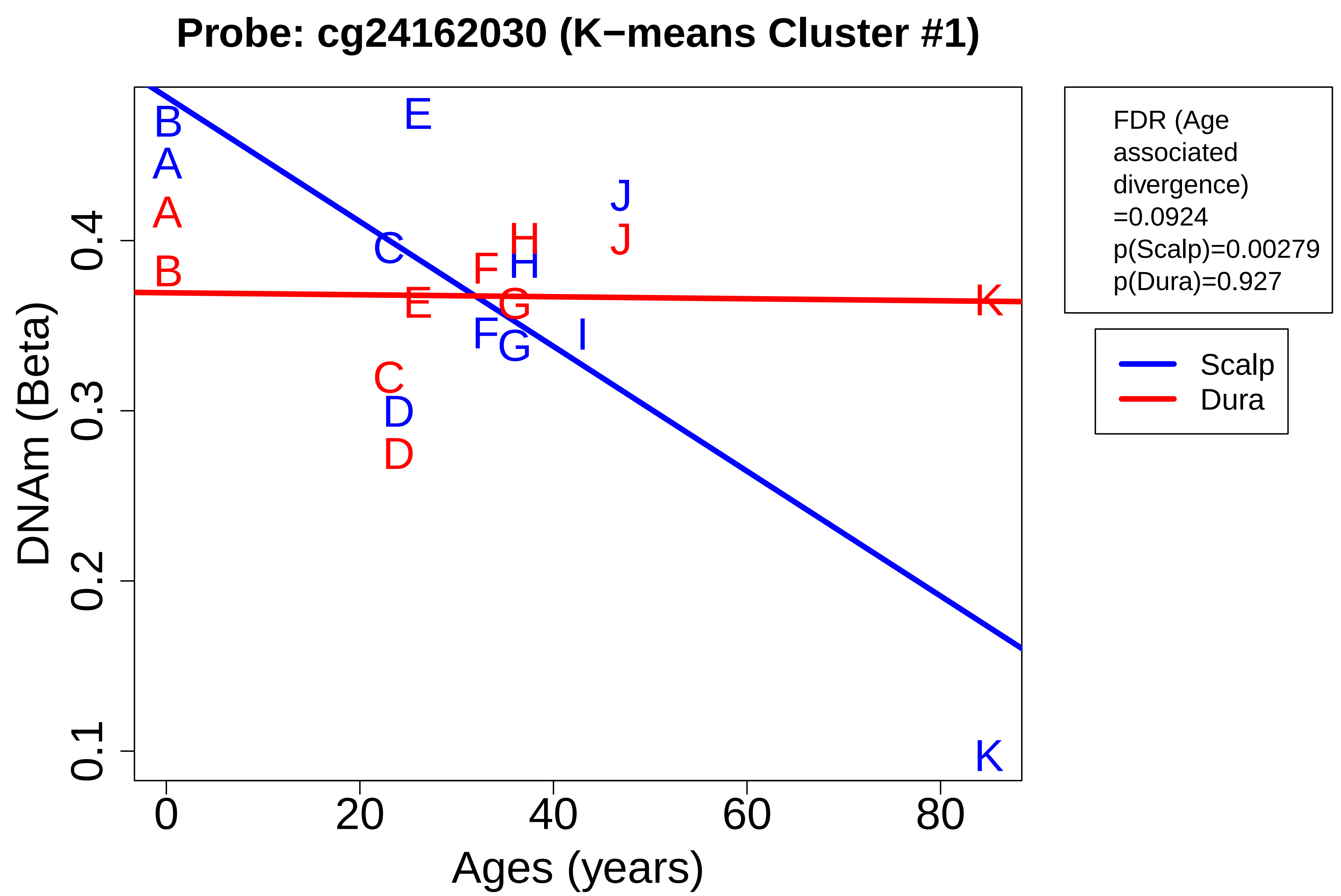
Probe: cg24162030 (K−means Cluster #1)
DNAm (Beta)
0.1
0.2
0.3
0.4
Ages (years)
0
20
40
60
80
FDR (Age associated divergence) =0.0924
p(Scalp)=0.00279
p(Dura)=0.927
Scalp
Dura

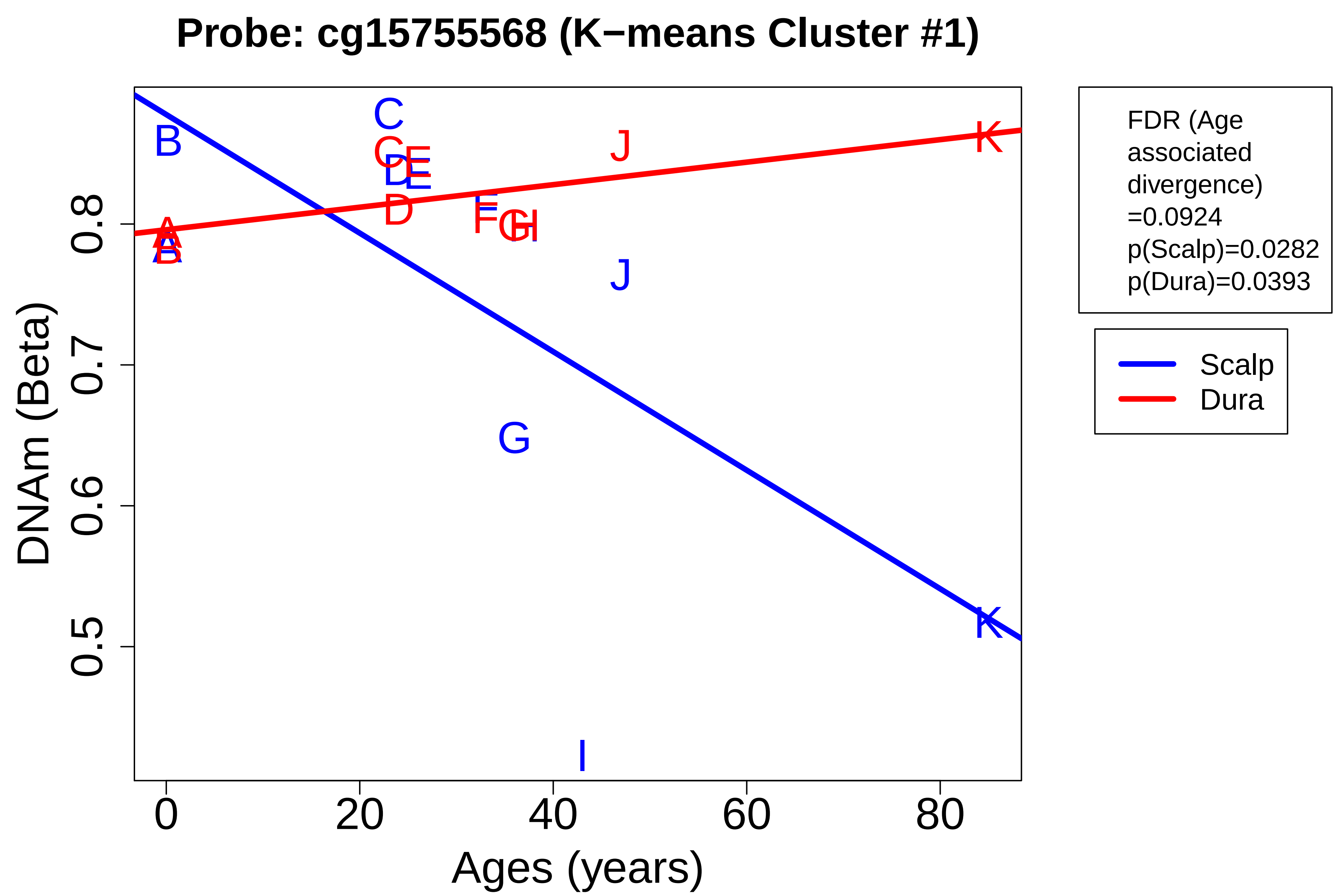

Probe: cg15755568 (K−means Cluster #1)
FDR (Age associated divergence) =0.0924
p(Scalp)=0.0282
p(Dura)=0.0393
Scalp
Dura
DNAm (Beta)
0.5
0.6
0.7
0.8
0
20
40
60
80
Ages (years)

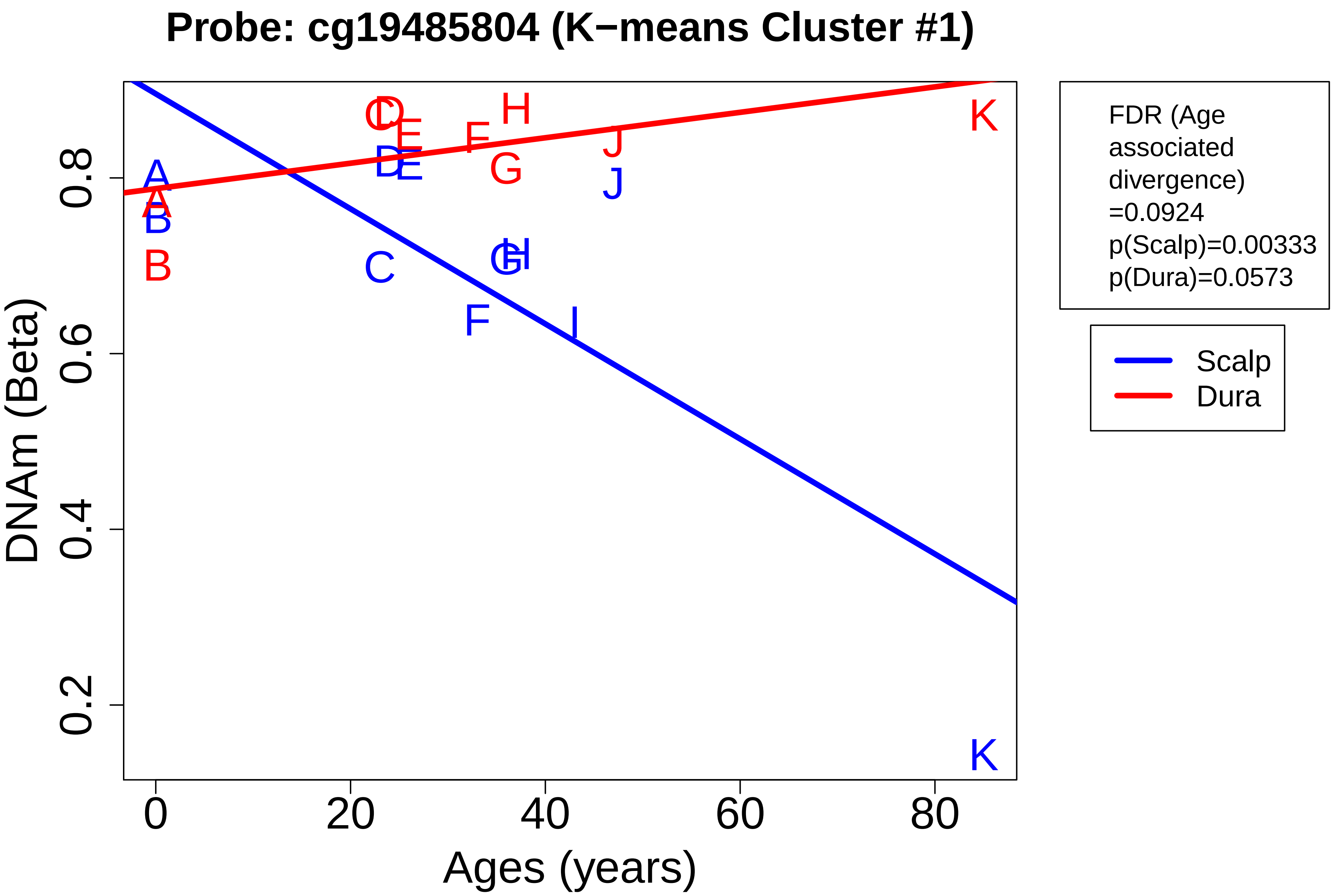
Probe: cg19485804 (K−means Cluster #1)
DNAm (Beta)
0.2
0.4
0.6
0.8
0
20
40
60
80
Ages (years)
FDR (Age associated divergence) =0.0924
p(Scalp)=0.00333
p(Dura)=0.0573
Scalp
Dura

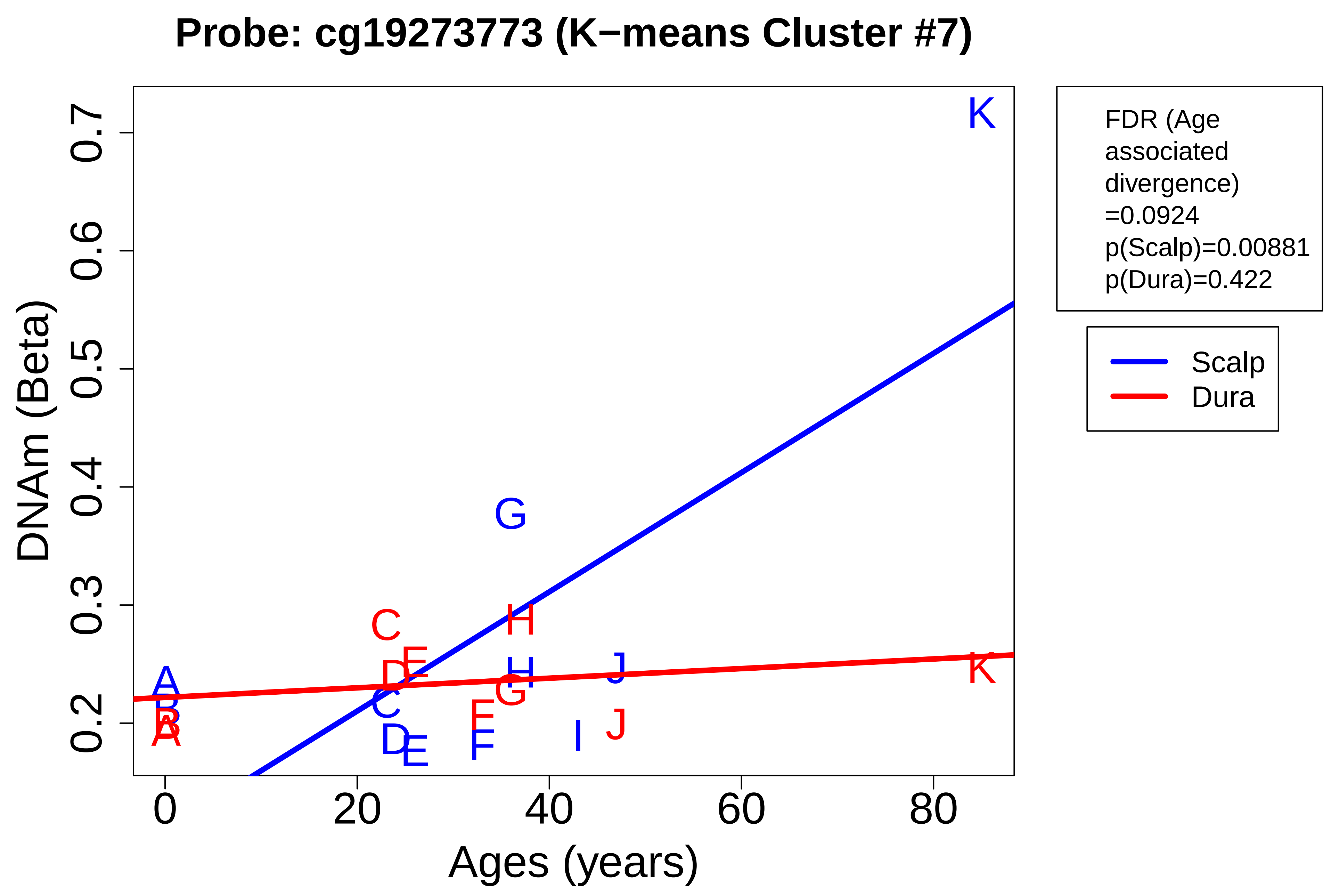

Probe: cg19273773 (K−means Cluster #7)
DNAm (Beta)
0.2
0.3
0.4
0.5
0.6
0.7
Ages (years)
0
20
40
60
80
FDR (Age associated divergence) =0.0924
p(Scalp)=0.00881
p(Dura)=0.422
Scalp
Dura

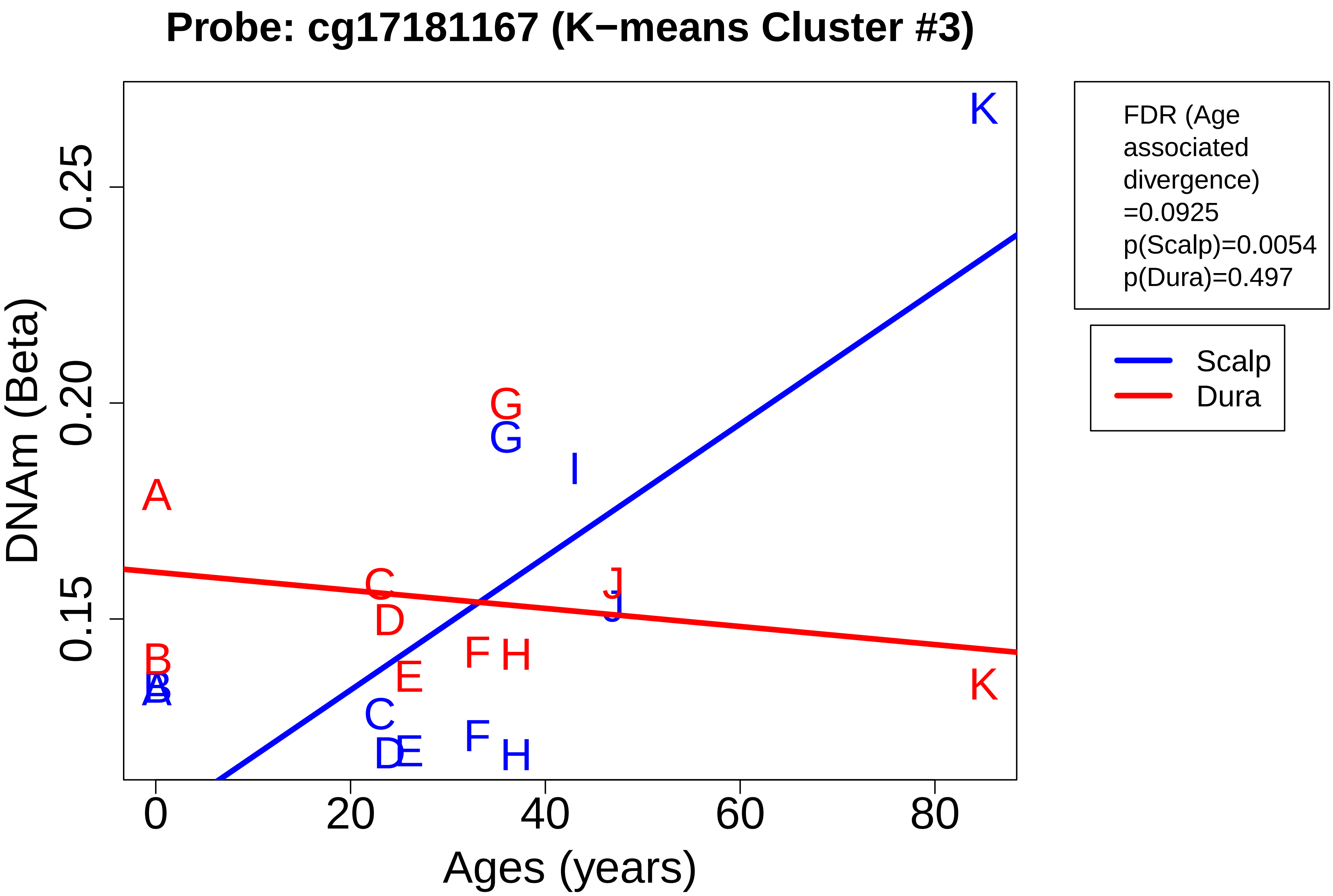

Probe: cg17181167 (K−means Cluster #3)
DNAm (Beta)
0.15
0.20
0.25
Ages (years)
0
20
40
60
80
FDR (Age associated divergence) =0.0925
p(Scalp)=0.0054
p(Dura)=0.497
Scalp
Dura

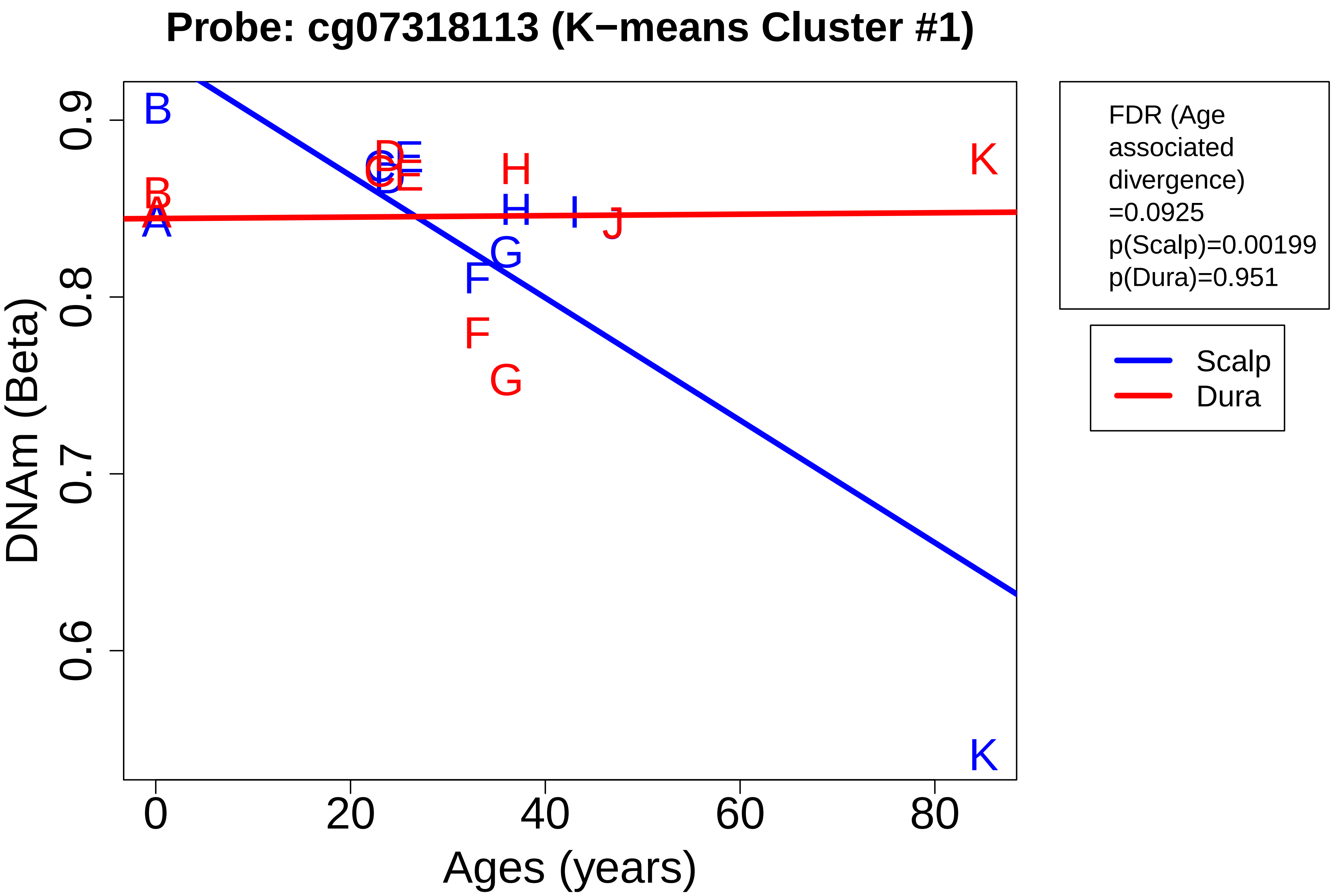

Probe: cg07318113 (K−means Cluster #1)
DNAm (Beta)
0.6
0.7
0.8
0.9
0
20
40
60
80
Ages (years)
FDR (Age associated divergence) =0.0925
p(Scalp)=0.00199
p(Dura)=0.951
Scalp
Dura

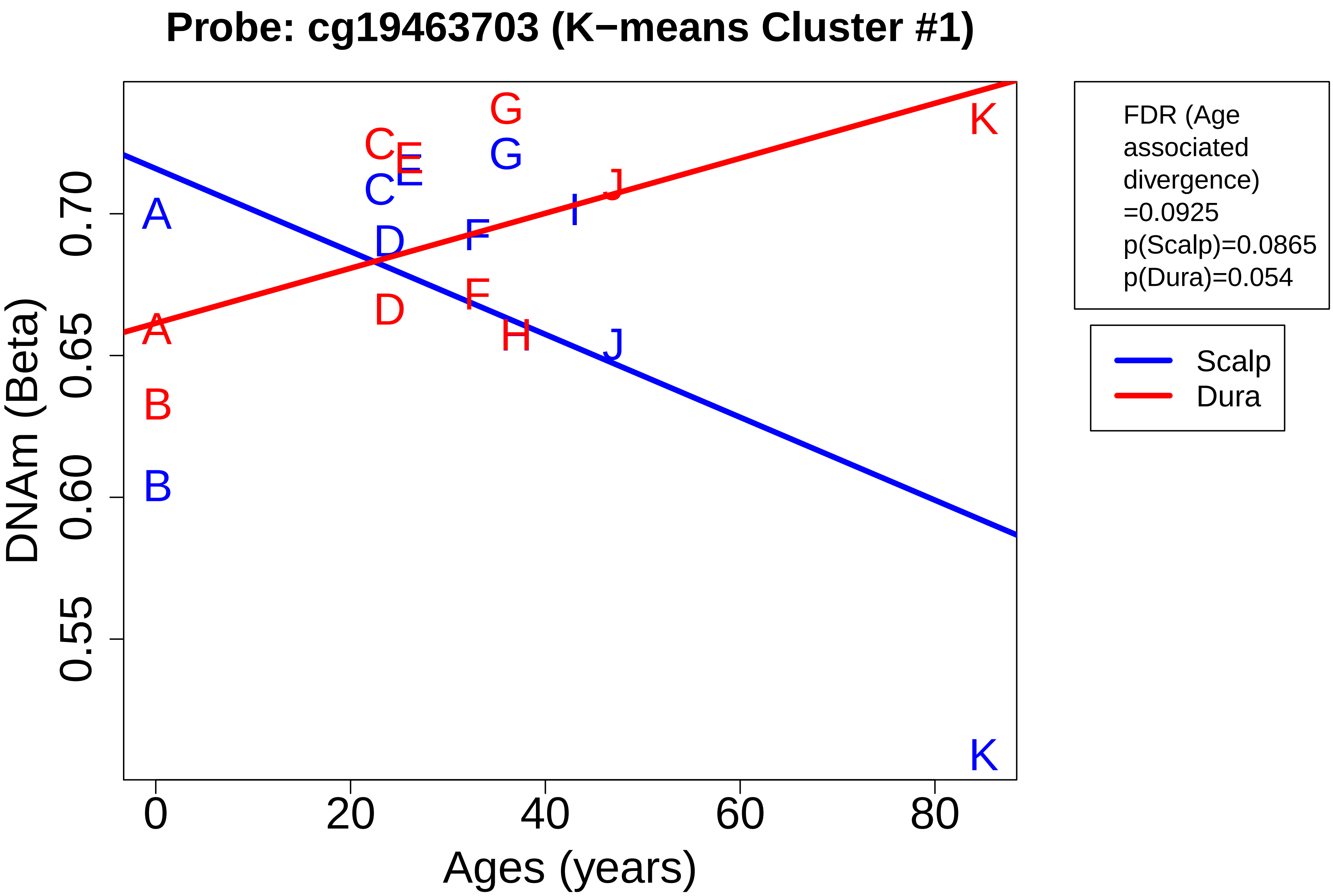

Probe: cg19463703 (K−means Cluster #1)
DNAm (Beta)
0.55
0.60
0.65
0.70
0
20
40
60
80
Ages (years)
A
B
C
D
E
F
G
H
I
J
K
FDR (Age associated divergence) =0.0925
p(Scalp)=0.0865
p(Dura)=0.054
Scalp
Dura

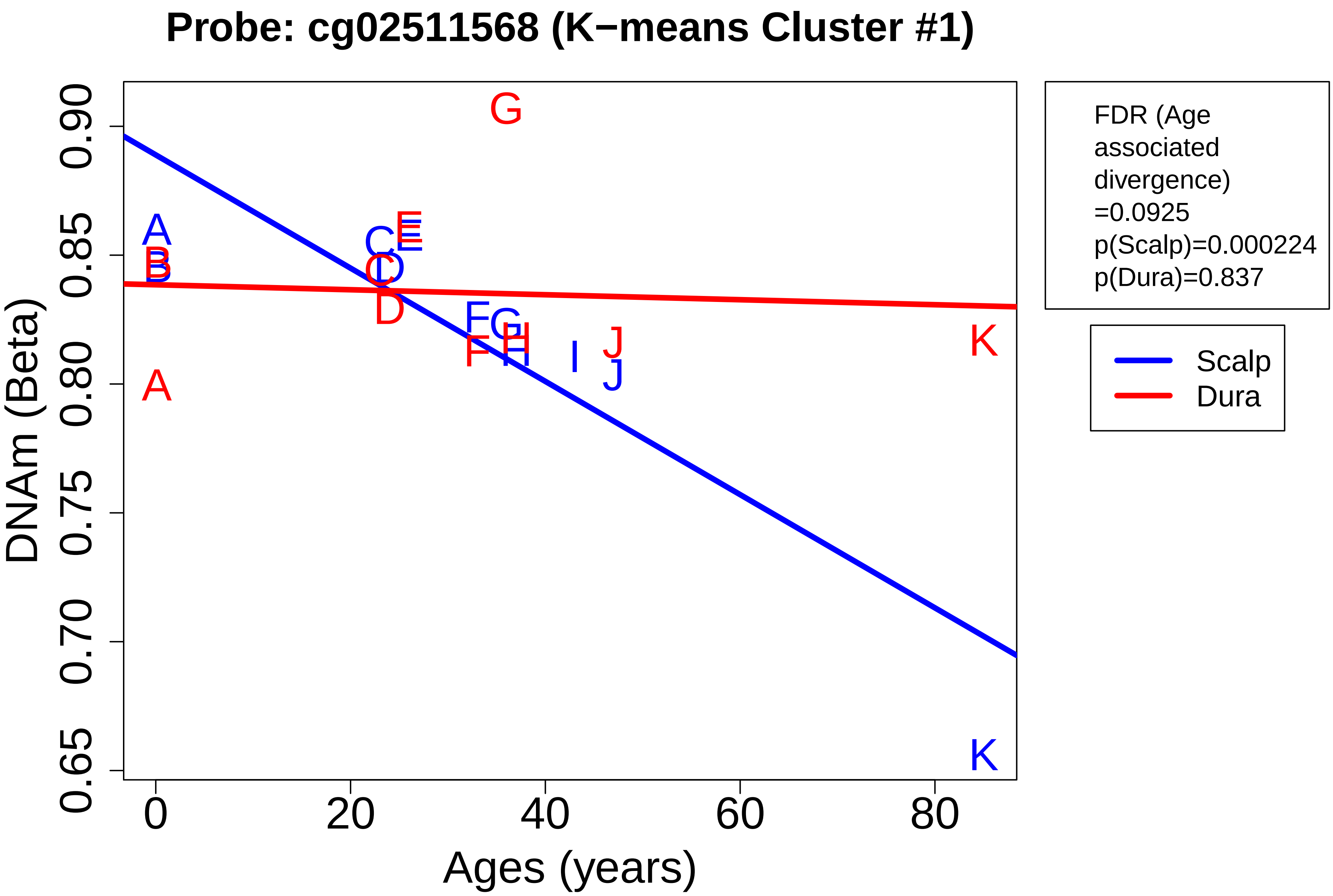

Probe: cg02511568 (K−means Cluster #1)
DNAm (Beta)
0.65
0.70
0.75
0.80
0.85
0.90
0
20
40
60
80
Ages (years)
FDR (Age associated divergence) =0.0925
p(Scalp)=0.000224
p(Dura)=0.837
Scalp
Dura

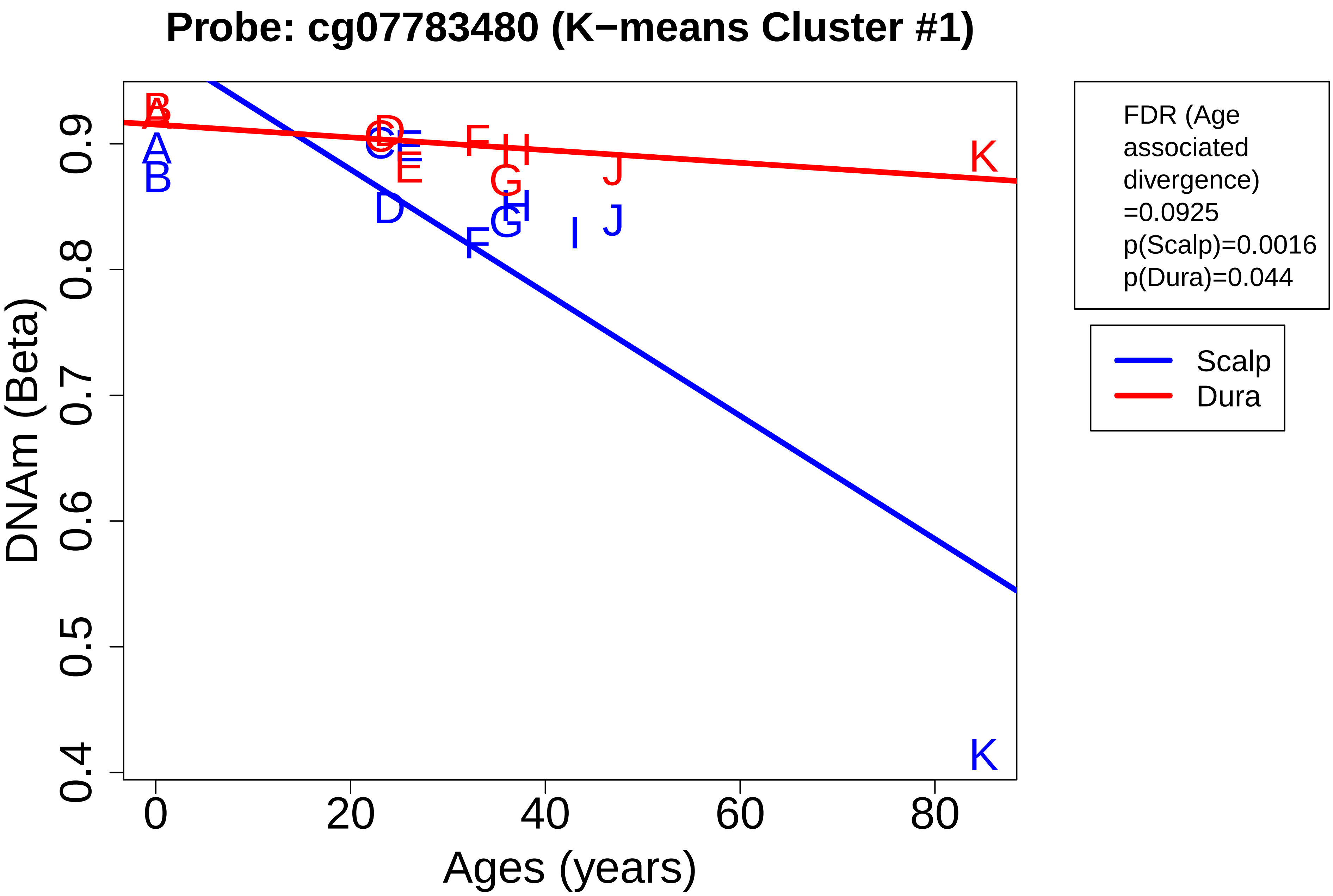
Probe: cg07783480 (K−means Cluster #1)
DNAm (Beta)
0.4
0.5
0.6
0.7
0.8
0.9
0
20
40
60
80
Ages (years)
FDR (Age associated divergence) =0.0925
p(Scalp)=0.0016
p(Dura)=0.044
Scalp
Dura

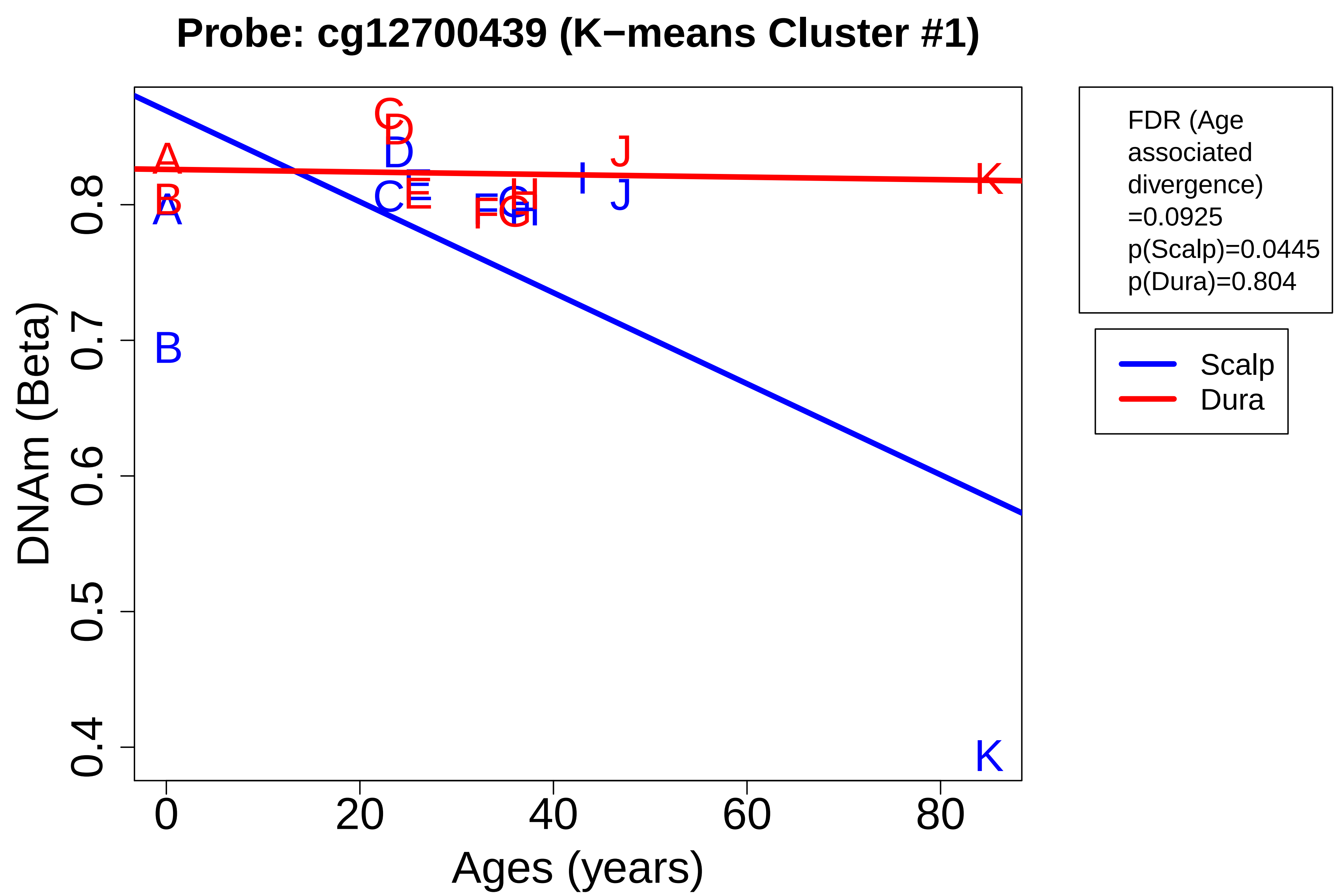

Probe: cg12700439 (K−means Cluster #1)
DNAm (Beta)
0.4
0.5
0.6
0.7
0.8
Ages (years)
0
20
40
60
80
FDR (Age associated divergence) =0.0925
p(Scalp)=0.0445
p(Dura)=0.804
Scalp
Dura

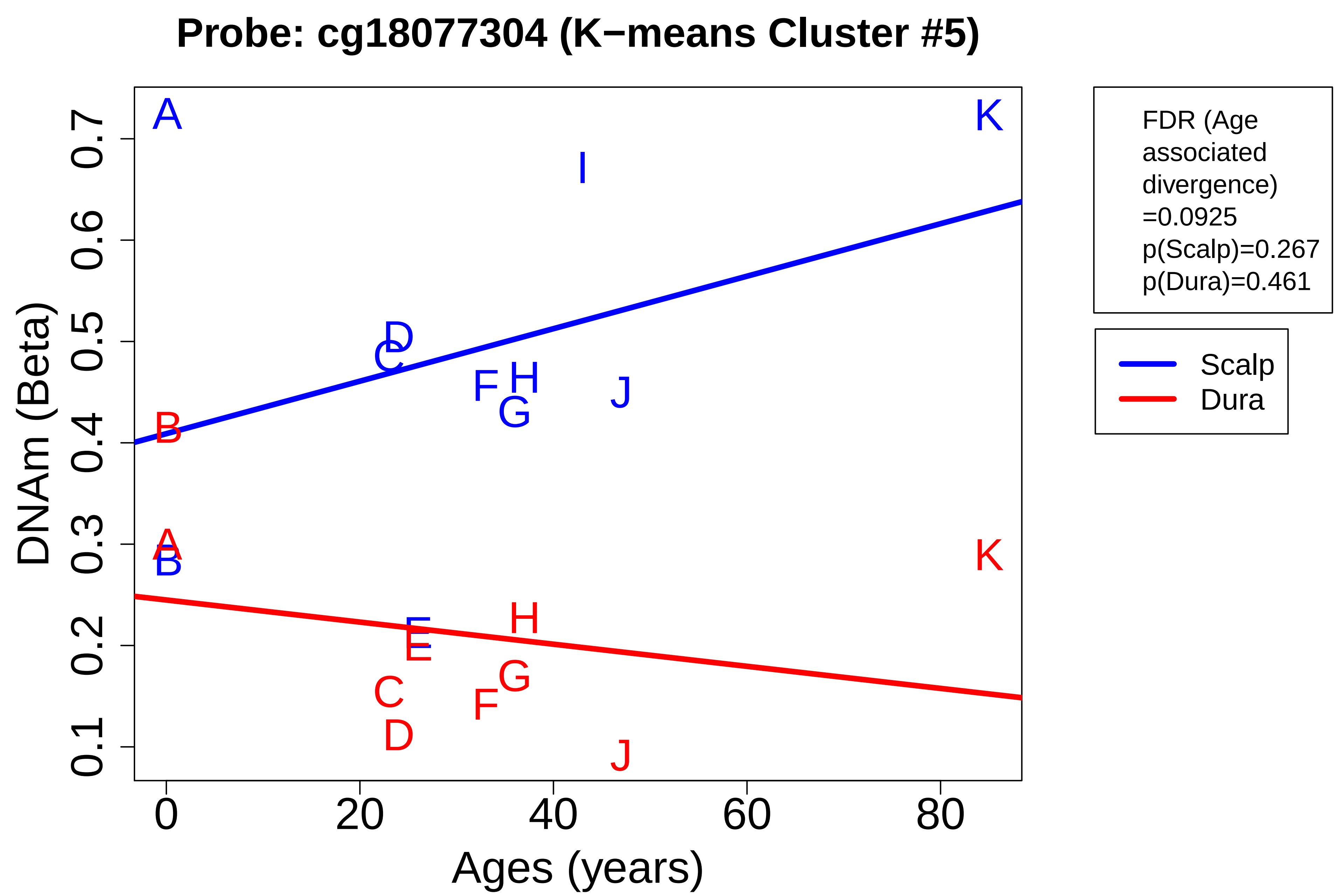
Probe: cg18077304 (K−means Cluster #5)
DNAm (Beta)
0.1
0.2
0.3
0.4
0.5
0.6
0.7
Ages (years)
0
20
40
60
80
A
B
C
D
E
F
G
H
I
J
K
FDR (Age associated divergence) =0.0925
p(Scalp)=0.267
p(Dura)=0.461
Scalp
Dura

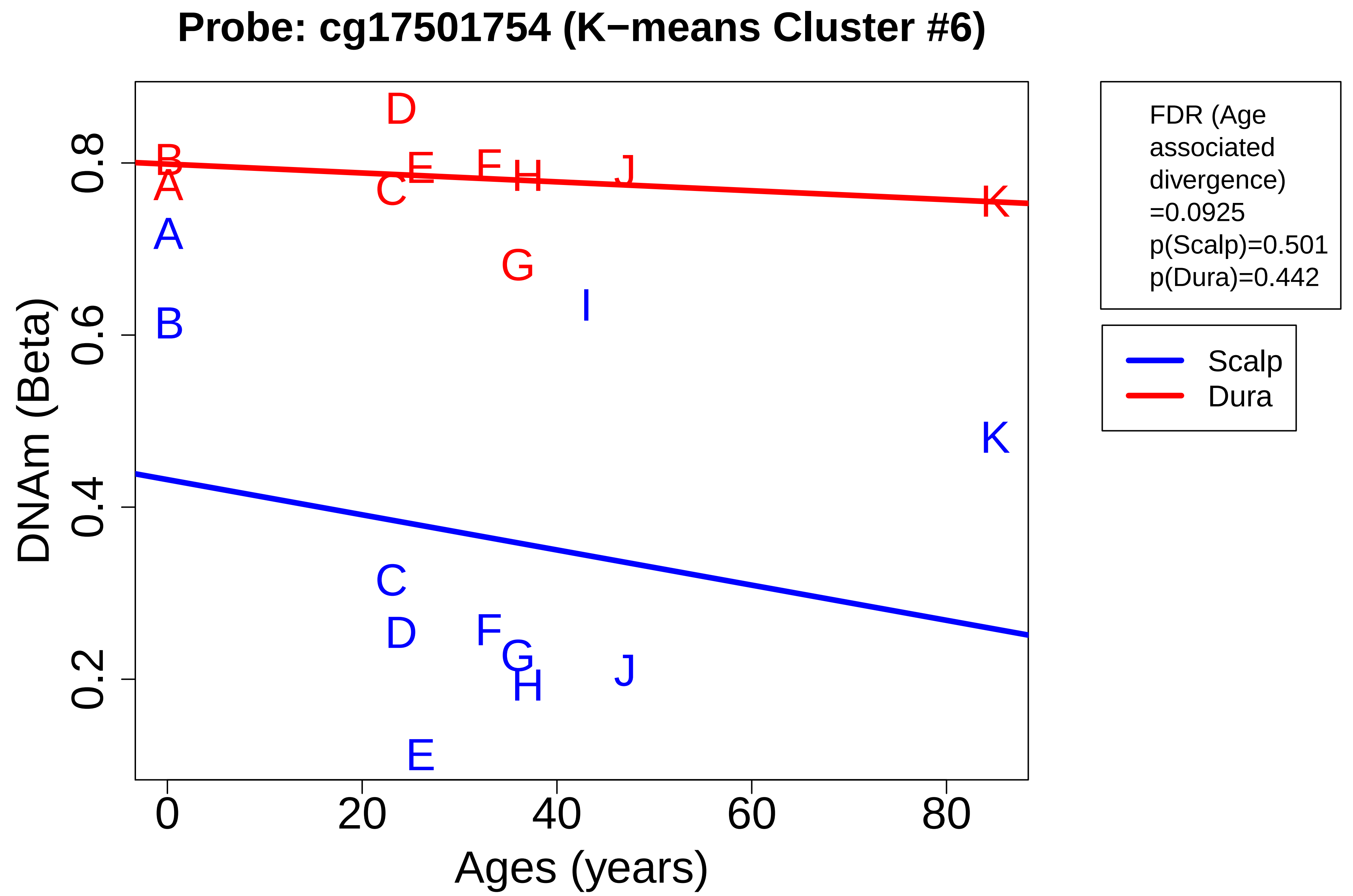

Probe: cg17501754 (K−means Cluster #6)
DNAm (Beta)
0.2
0.4
0.6
0.8
0
20
40
60
80
Ages (years)
FDR (Age associated divergence) =0.0925
p(Scalp)=0.501
p(Dura)=0.442
Scalp
Dura

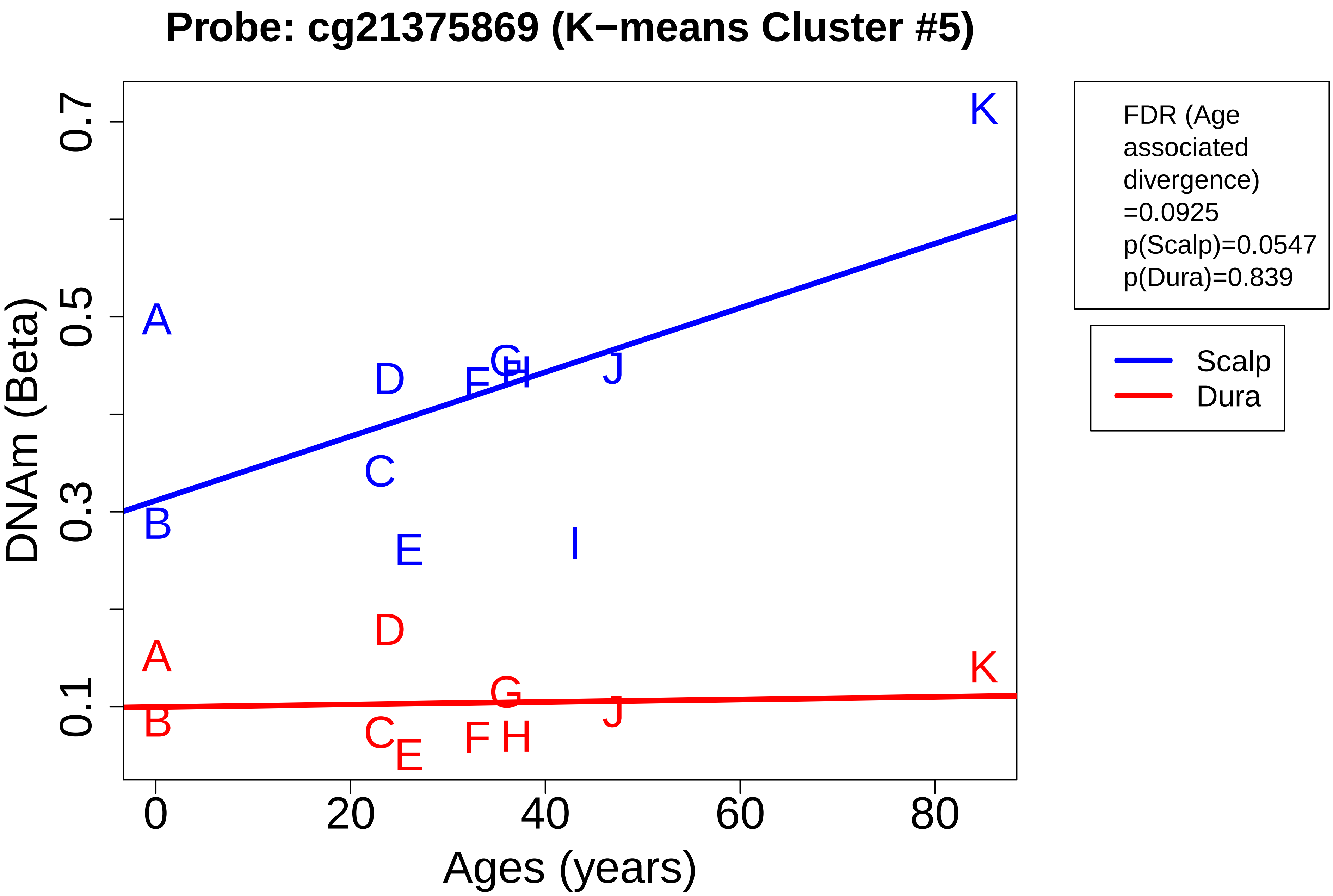

Probe: cg21375869 (K−means Cluster #5)
DNAm (Beta)
0.1
0.3
0.5
0.7
0
20
40
60
80
Ages (years)
A
B
C
D
E
F
G
H
I
J
K
A
B
C
D
E
F
G
H
J
K
FDR (Age associated divergence) =0.0925
p(Scalp)=0.0547
p(Dura)=0.839
Scalp
Dura

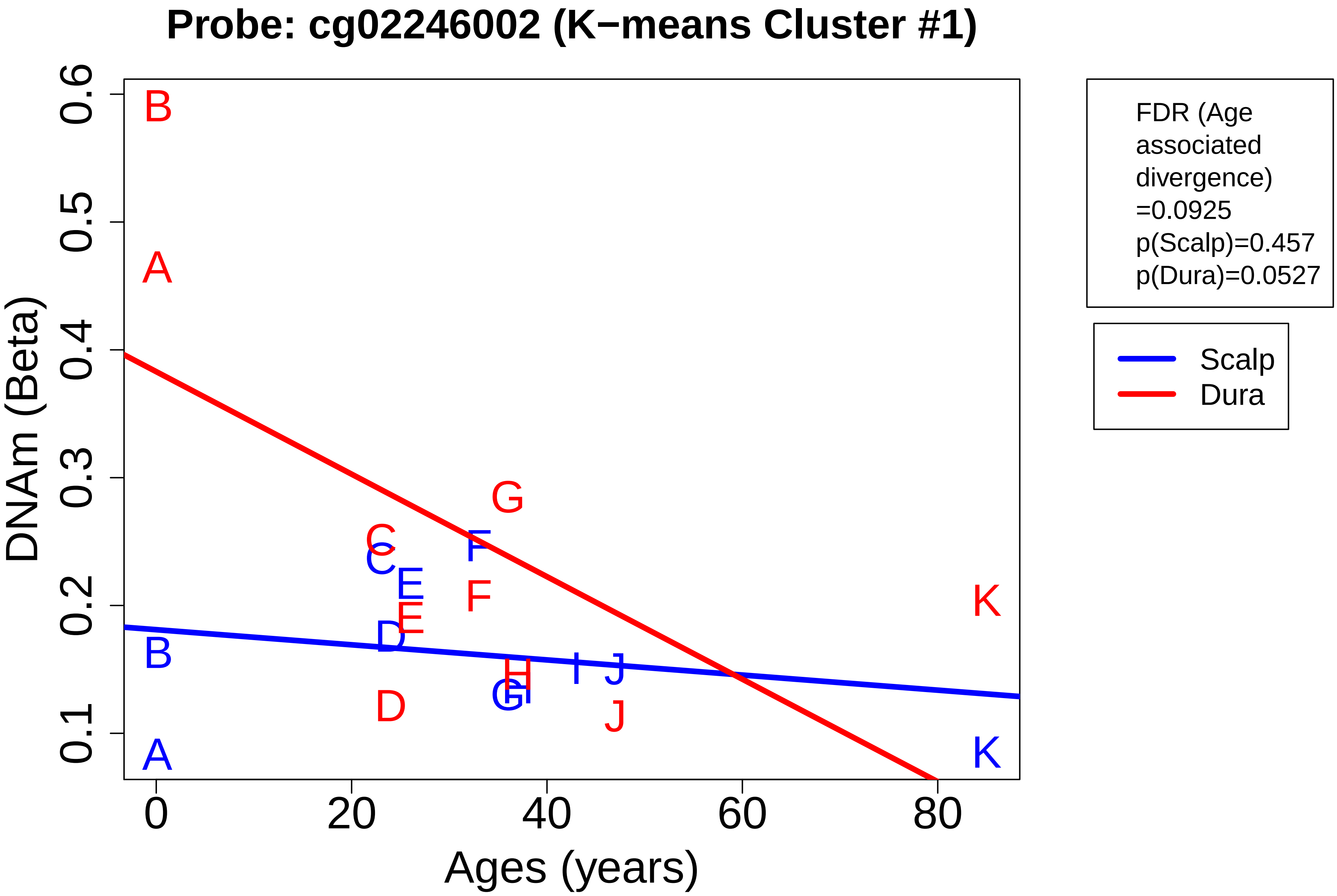
Probe: cg02246002 (K−means Cluster #1)
DNAm (Beta)
0.1
0.2
0.3
0.4
0.5
0.6
Ages (years)
0
20
40
60
80
FDR (Age associated divergence) =0.0925
p(Scalp)=0.457
p(Dura)=0.0527
Scalp
Dura

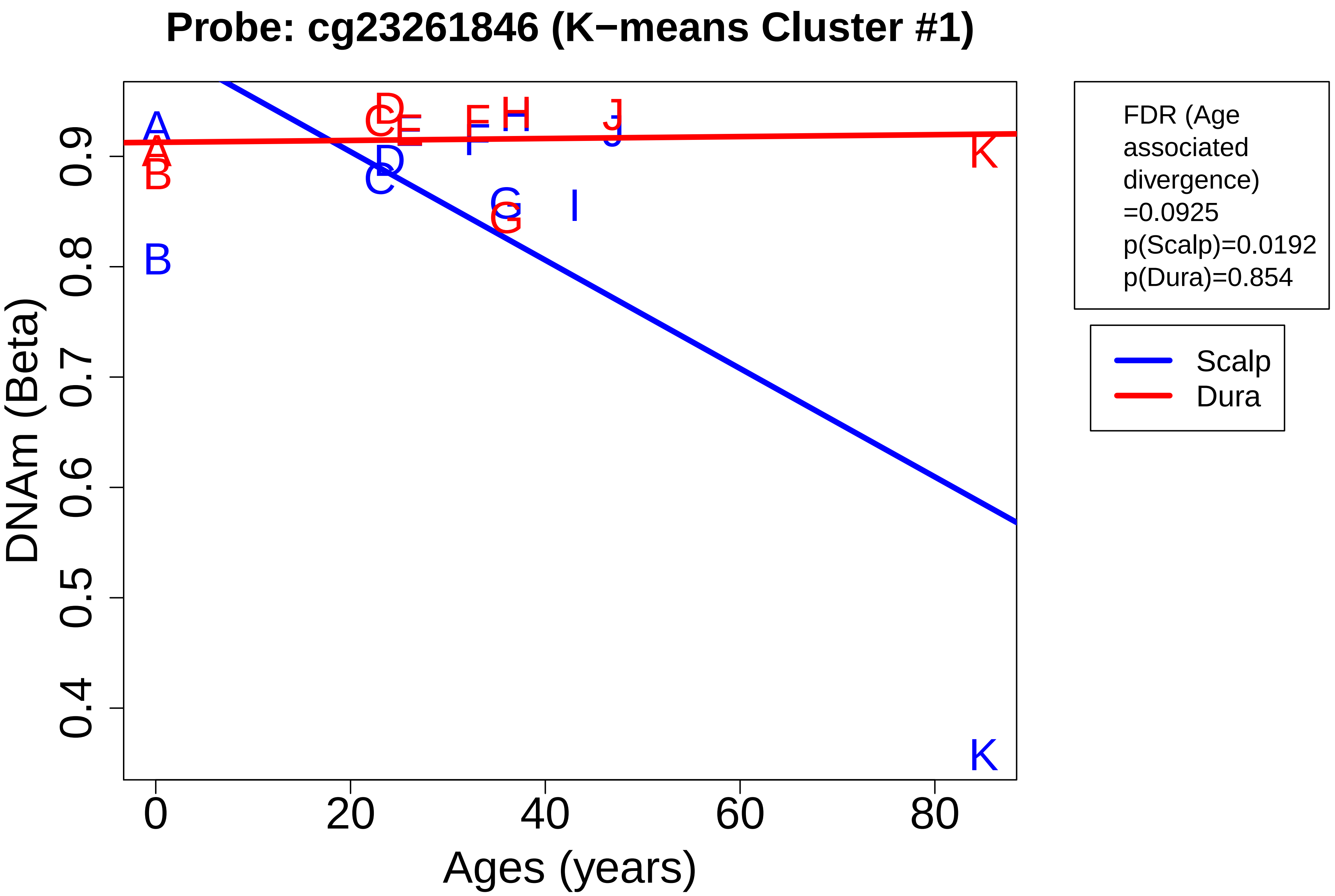
Probe: cg23261846 (K−means Cluster #1)
DNAm (Beta)
0.4
0.5
0.6
0.7
0.8
0.9
Ages (years)
0
20
40
60
80
A
B
C
D
E
F
G
H
I
J
K
FDR (Age associated divergence) =0.0925
p(Scalp)=0.0192
p(Dura)=0.854
Scalp
Dura

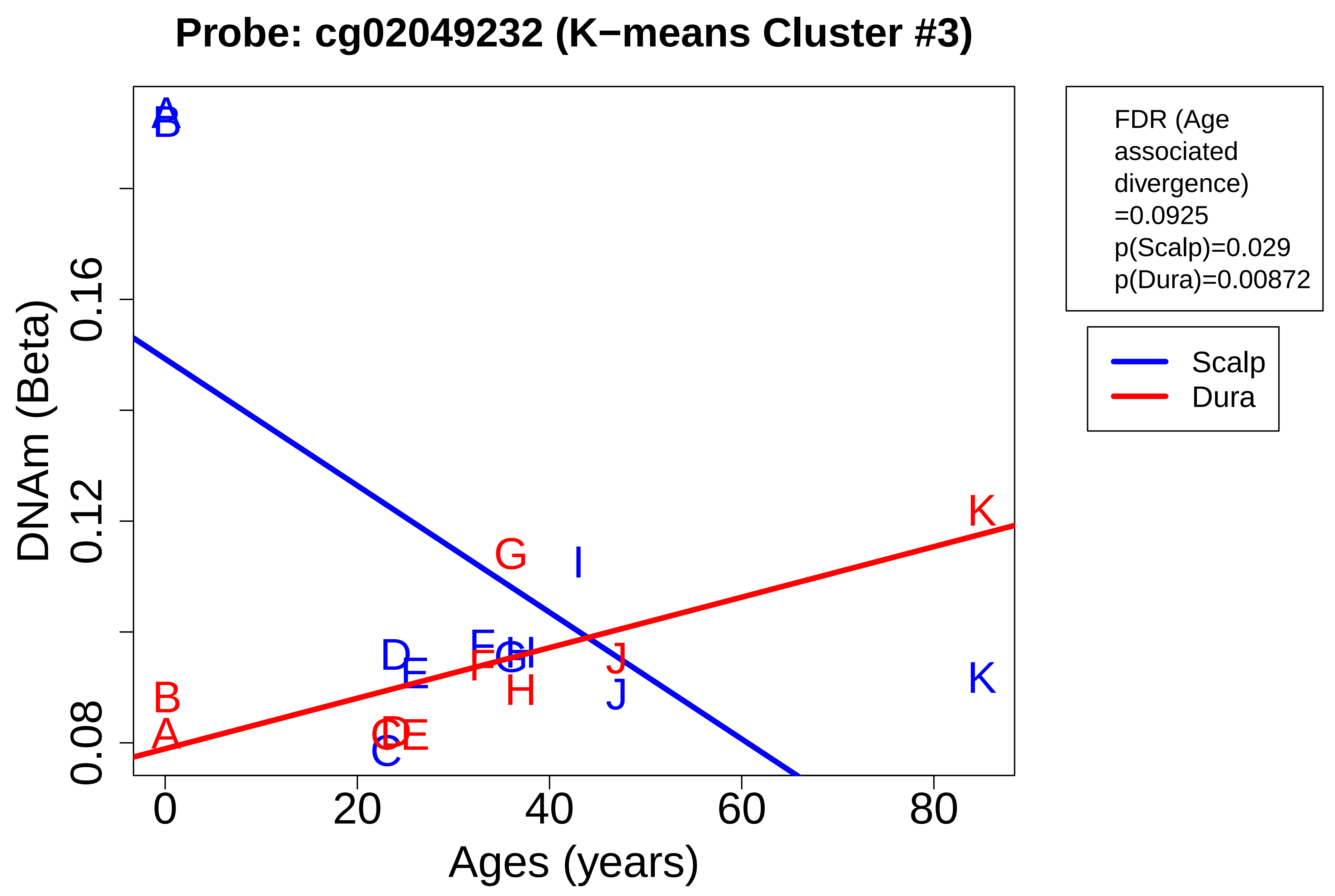

Probe: cg02049232 (K−means Cluster #3)
DNAm (Beta)
0.08
0.12
0.16
Ages (years)
0
20
40
60
80
FDR (Age associated divergence) =0.0925
p(Scalp)=0.029
p(Dura)=0.00872
Scalp
Dura
A
B
C
D
E
F
G
H
I
J
K

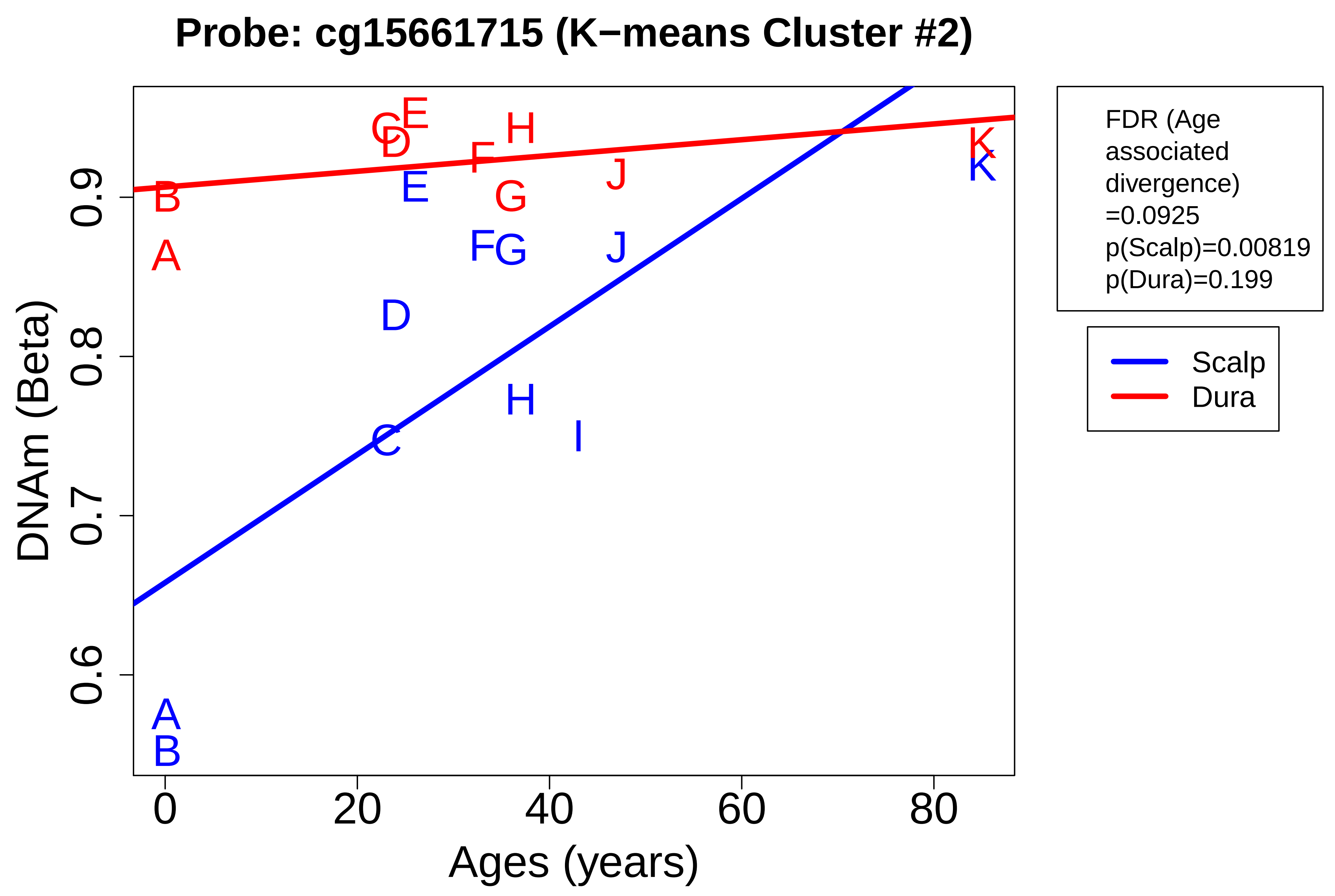

Probe: cg15661715 (K−means Cluster #2)
DNAm (Beta)
0.6
0.7
0.8
0.9
0
20
40
60
80
Ages (years)
A
B
C
D
E
F
G
H
I
J
K
FDR (Age associated divergence) =0.0925
p(Scalp)=0.00819
p(Dura)=0.199
Scalp
Dura

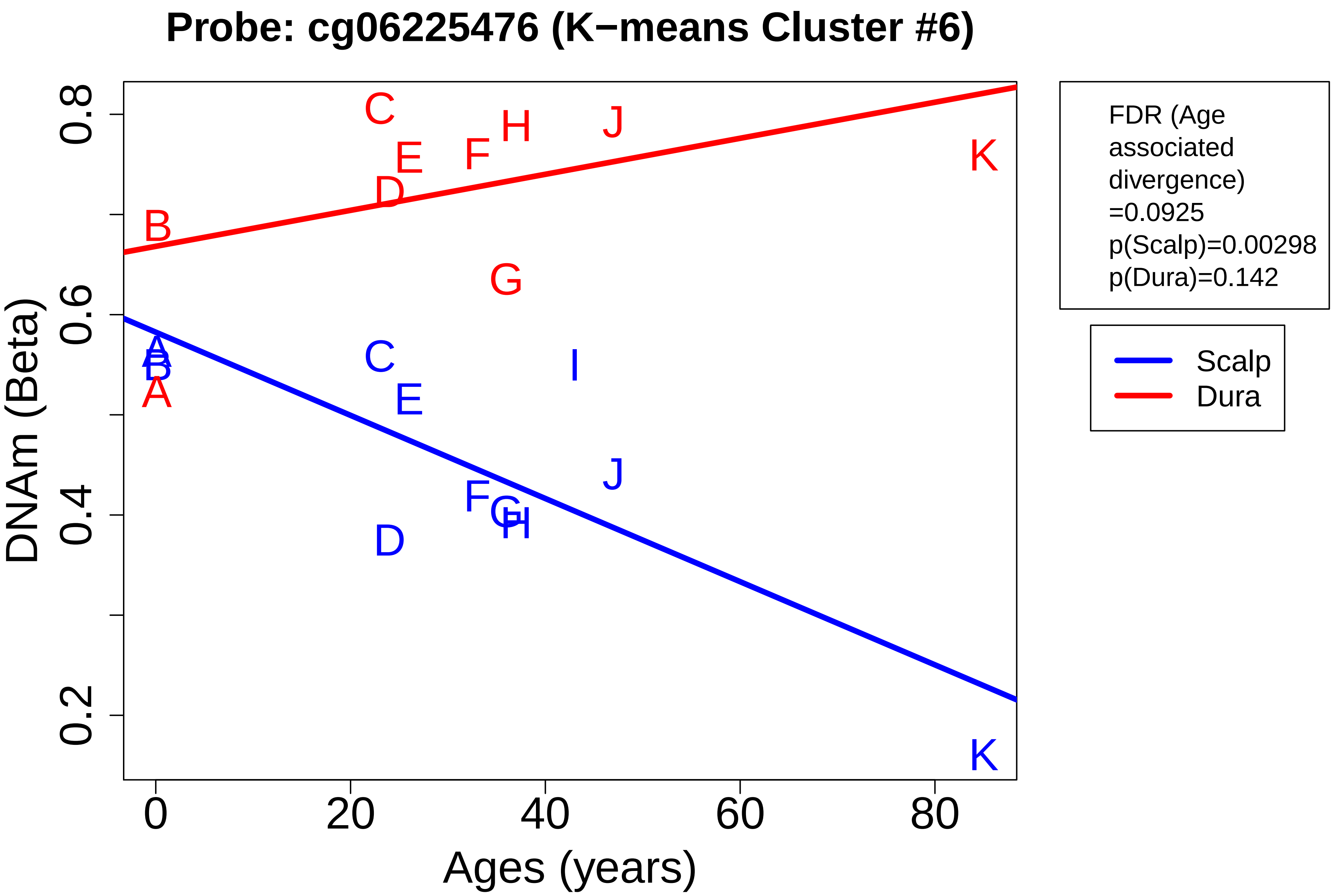

Probe: cg06225476 (K−means Cluster #6)
DNAm (Beta)
0.2
0.4
0.6
0.8
0
20
40
60
80
Ages (years)
FDR (Age associated divergence) =0.0925
p(Scalp)=0.00298
p(Dura)=0.142
Scalp
Dura

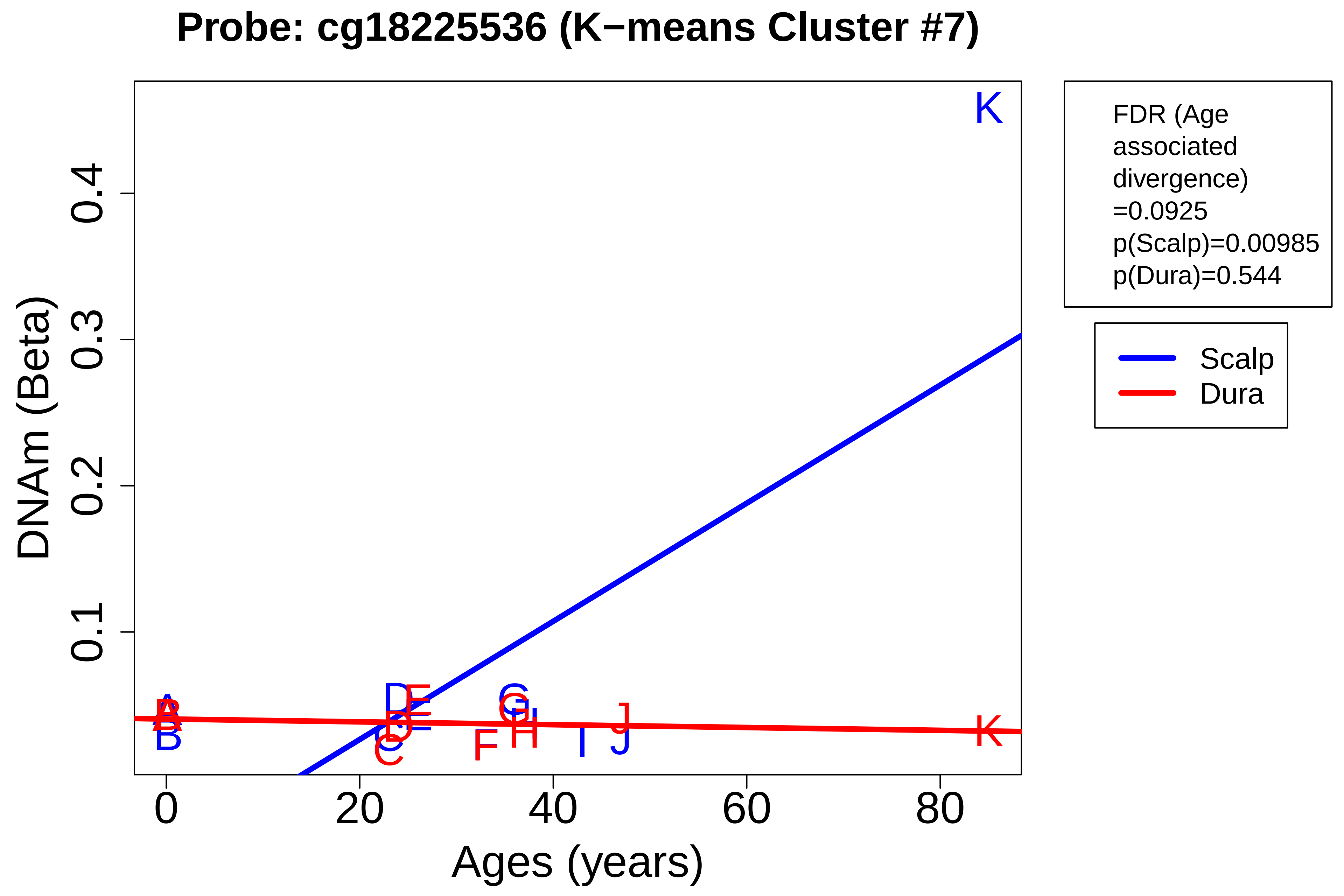

Probe: cg18225536 (K−means Cluster #7)
FDR (Age associated divergence) =0.0925
p(Scalp)=0.00985
p(Dura)=0.544
Scalp
Dura
DNAm (Beta)
0.1
0.2
0.3
0.4
0
20
40
60
80
Ages (years)
A
B
C
D
E
F
G
H
I
J
K

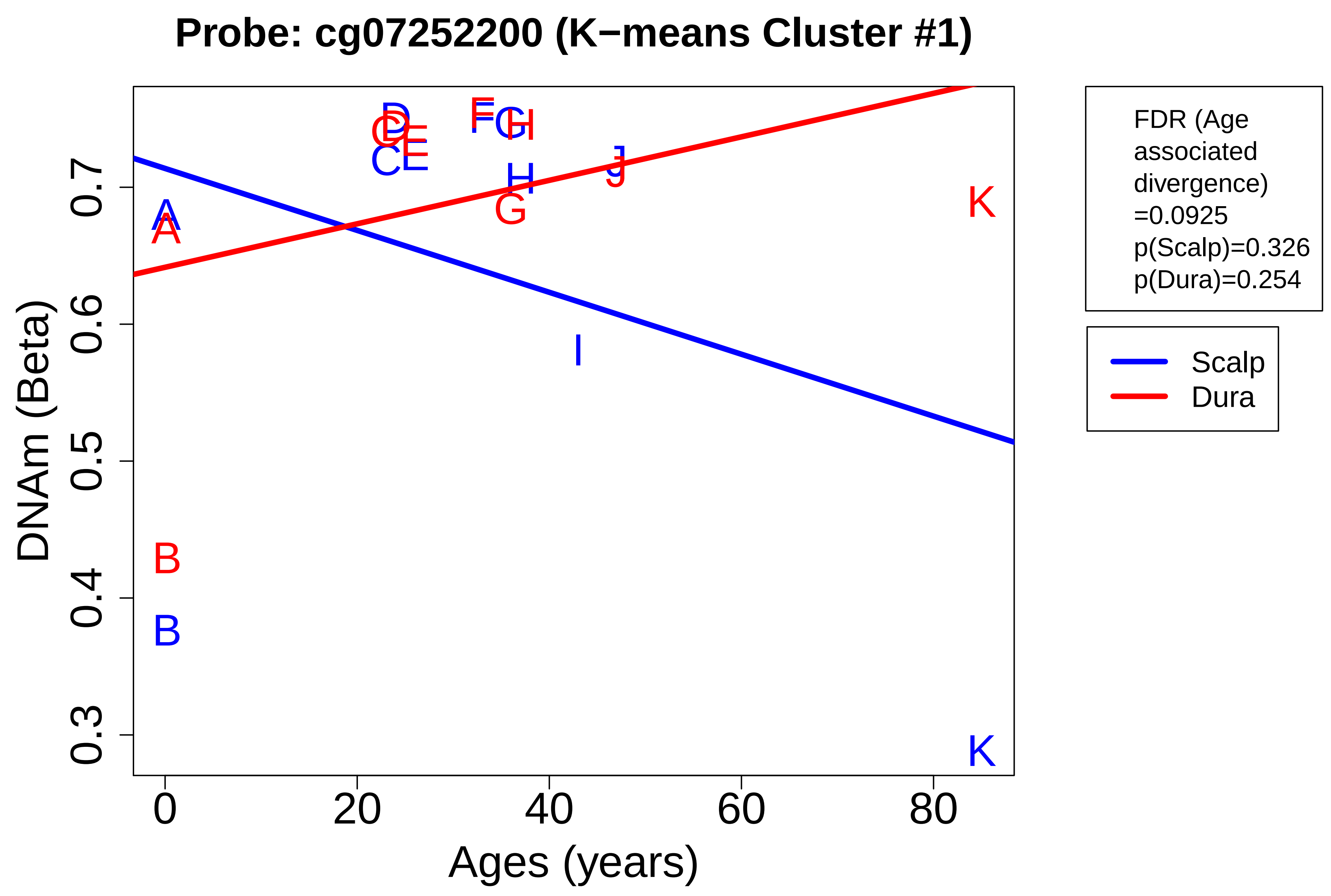

Probe: cg07252200 (K−means Cluster #1)
DNAm (Beta)
0.3
0.4
0.5
0.6
0.7
Ages (years)
0
20
40
60
80
A
A
B
B
C
C
D
D
E
E
F
F
G
G
H
H
I
J
J
K
K
FDR (Age associated divergence) =0.0925
p(Scalp)=0.326
p(Dura)=0.254
Scalp
Dura

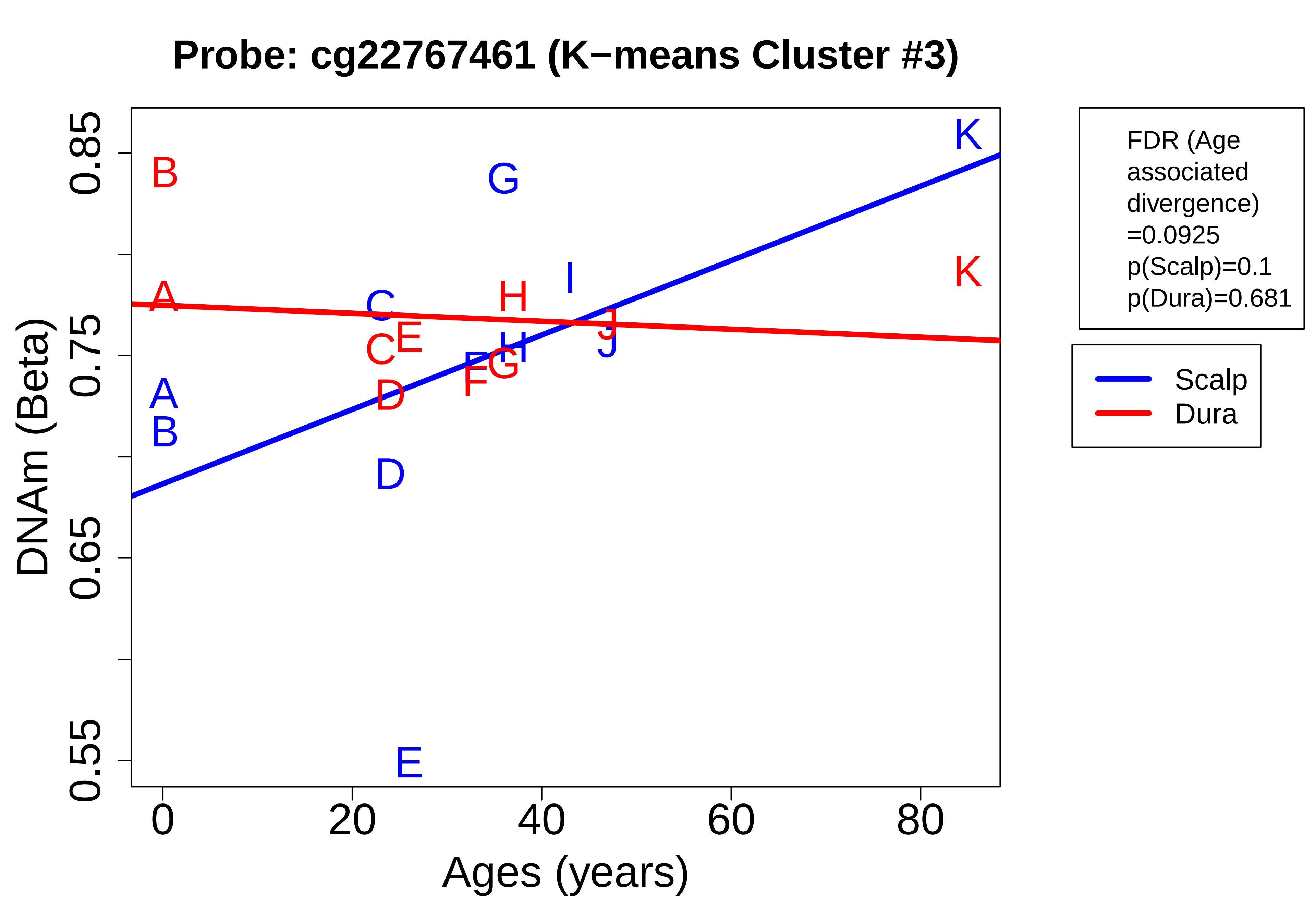

Probe: cg22767461 (K−means Cluster #3)
DNAm (Beta)
0.55
0.65
0.75
0.85
0
20
40
60
80
Ages (years)
A
B
C
D
E
F
G
H
I
J
K
FDR (Age associated divergence) =0.0925
p(Scalp)=0.1
p(Dura)=0.681
Scalp
Dura

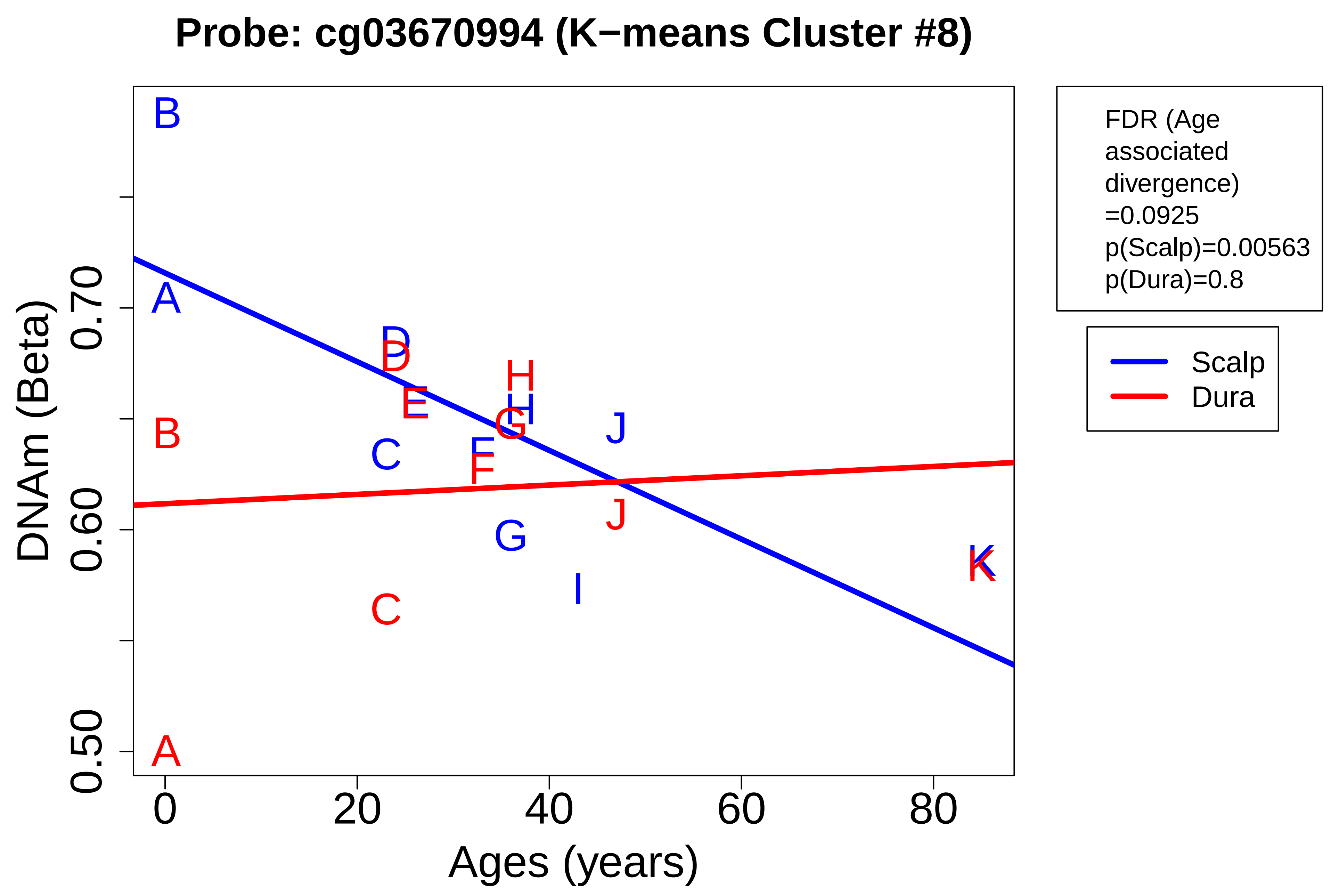

Probe: cg03670994 (K−means Cluster #8)
DNAm (Beta)
0.50
0.60
0.70
Ages (years)
0
20
40
60
80
FDR (Age associated divergence) =0.0925
p(Scalp)=0.00563
p(Dura)=0.8
Scalp
Dura
A
B
C
D
E
F
G
H
I
J
K

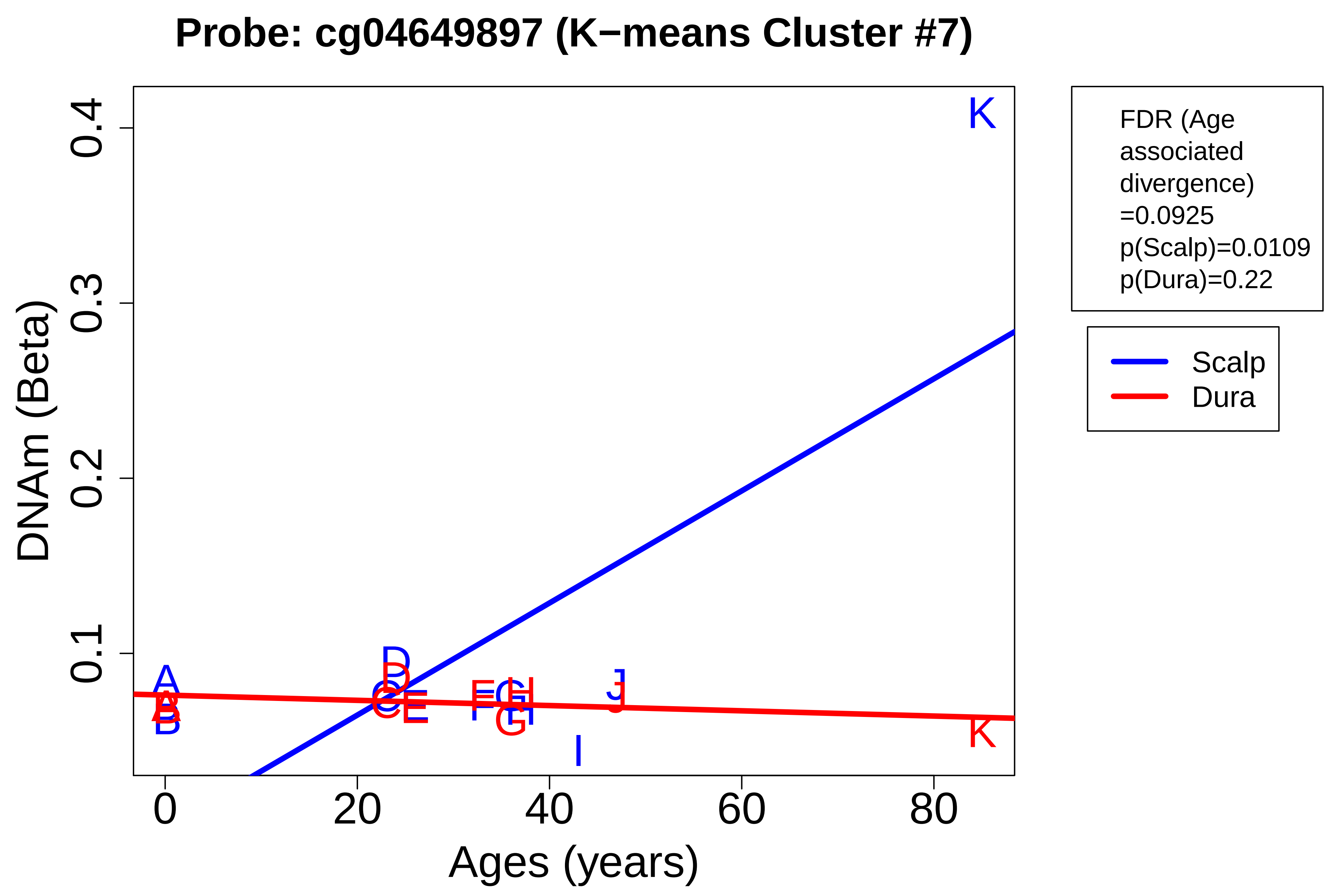

Probe: cg04649897 (K−means Cluster #7)
DNAm (Beta)
0.1
0.2
0.3
0.4
0
20
40
60
80
Ages (years)
FDR (Age associated divergence) =0.0925
p(Scalp)=0.0109
p(Dura)=0.22
Scalp
Dura

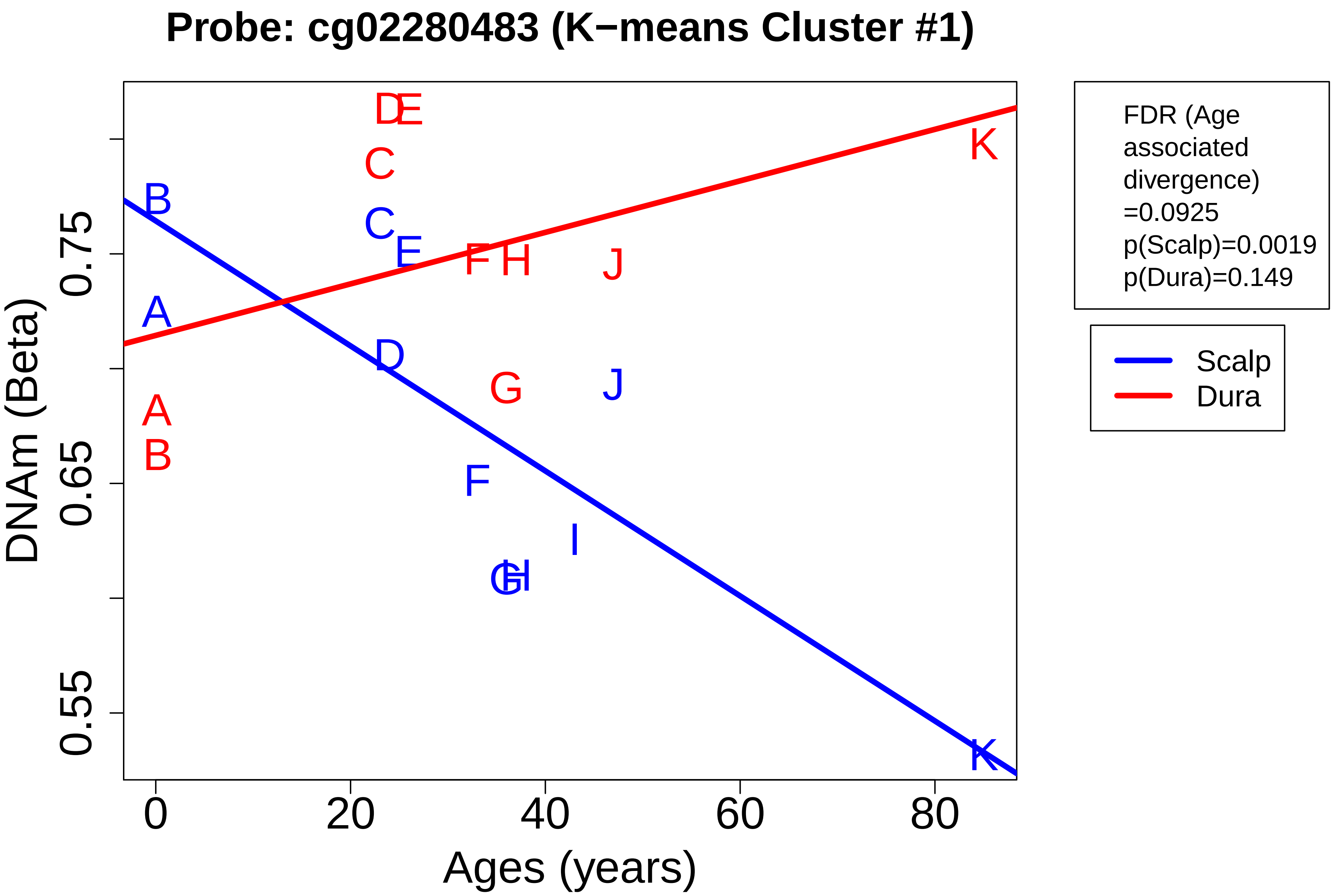

Probe: cg02280483 (K−means Cluster #1)
DNAm (Beta)
0.55
0.65
0.75
Ages (years)
0
20
40
60
80
FDR (Age associated divergence) =0.0925
p(Scalp)=0.0019
p(Dura)=0.149
Scalp
Dura

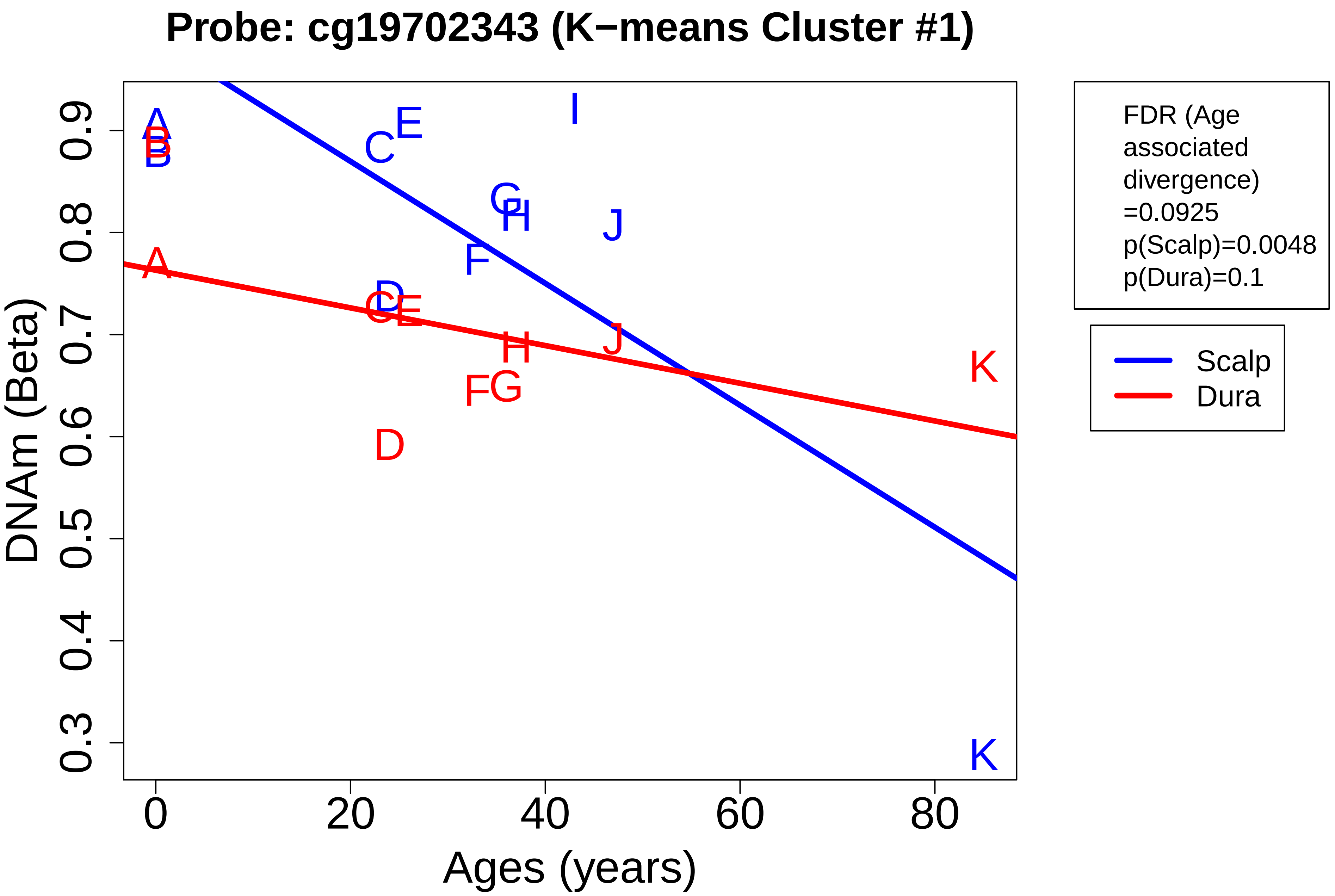
Probe: cg19702343 (K−means Cluster #1)
DNAm (Beta)
0.3
0.4
0.5
0.6
0.7
0.8
0.9
0
20
40
60
80
Ages (years)
FDR (Age associated divergence) =0.0925
p(Scalp)=0.0048
p(Dura)=0.1
Scalp
Dura
A
B
C
D
E
F
G
H
I
J
K

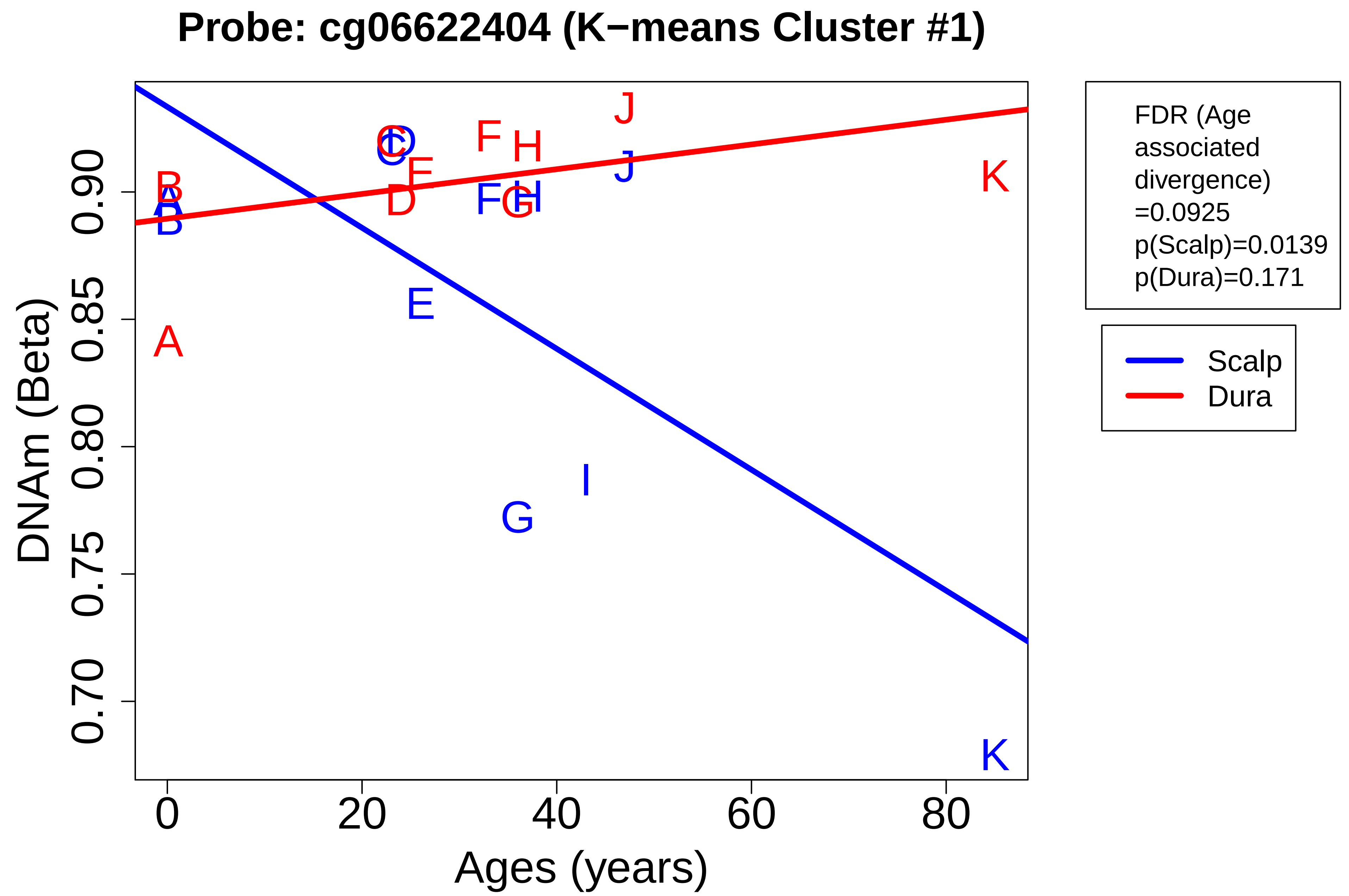

Probe: cg06622404 (K−means Cluster #1)
DNAm (Beta)
0.70
0.75
0.80
0.85
0.90
0
20
40
60
80
Ages (years)
FDR (Age associated divergence) =0.0925
p(Scalp)=0.0139
p(Dura)=0.171
Scalp
Dura

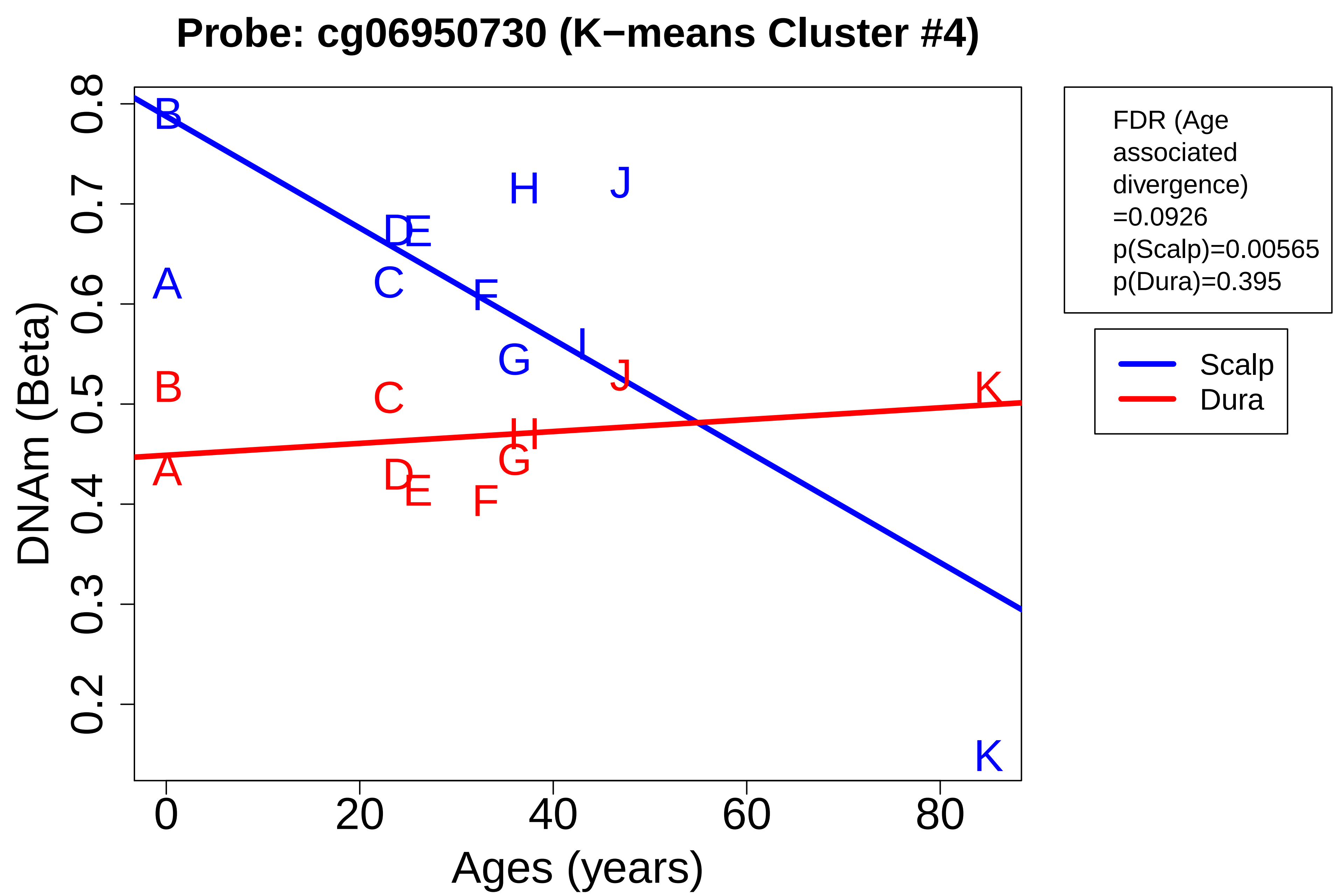

Probe: cg06950730 (K−means Cluster #4)
DNAm (Beta)
0.2
0.3
0.4
0.5
0.6
0.7
0.8
0
20
40
60
80
Ages (years)
FDR (Age associated divergence) =0.0926
p(Scalp)=0.00565
p(Dura)=0.395
Scalp
Dura
A
B
C
D
E
F
G
H
I
J
K

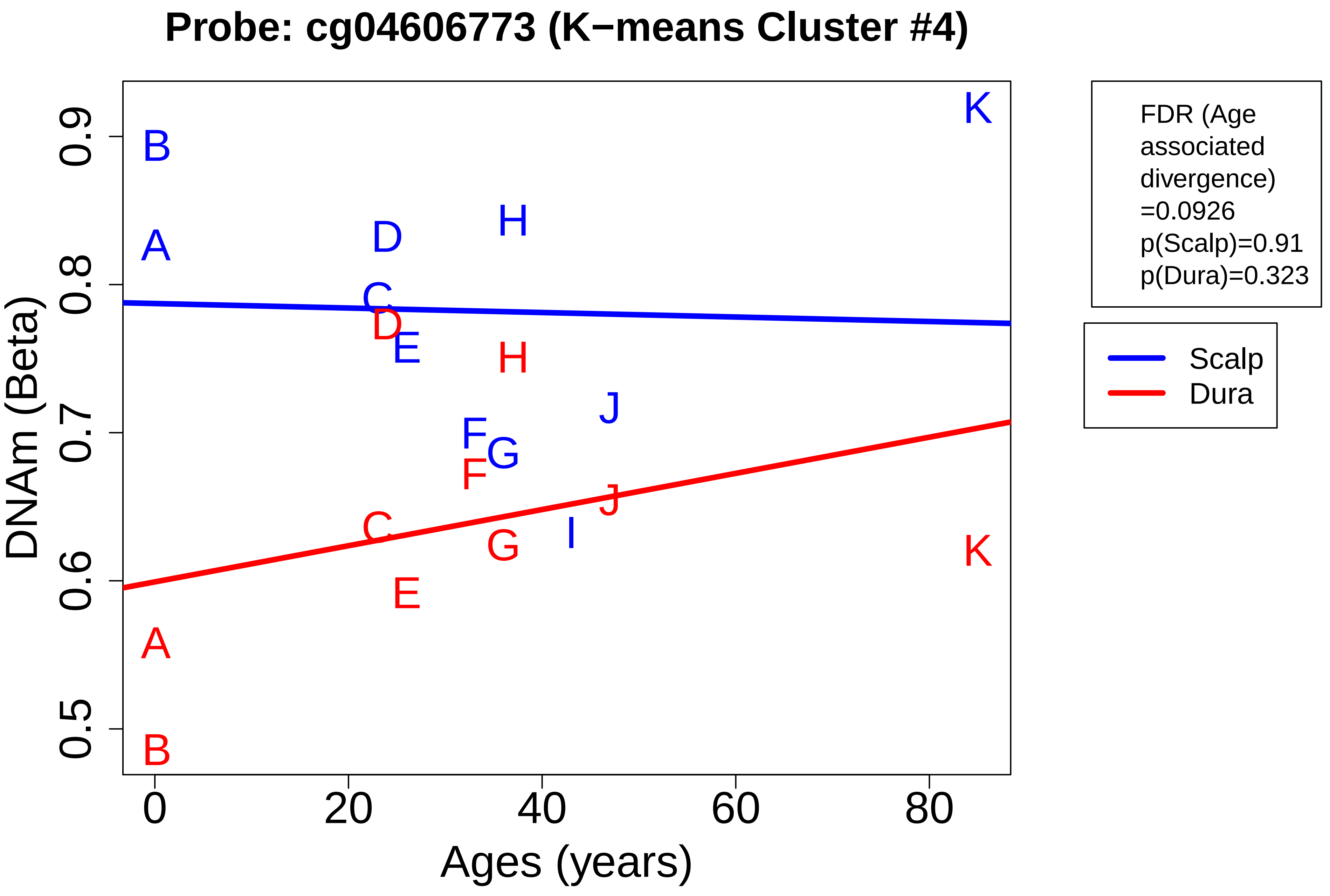

Probe: cg04606773 (K−means Cluster #4)
DNAm (Beta)
Ages (years)
0.5
0.6
0.7
0.8
0.9
0
20
40
60
80
FDR (Age associated divergence) =0.0926
p(Scalp)=0.91
p(Dura)=0.323
Scalp
Dura

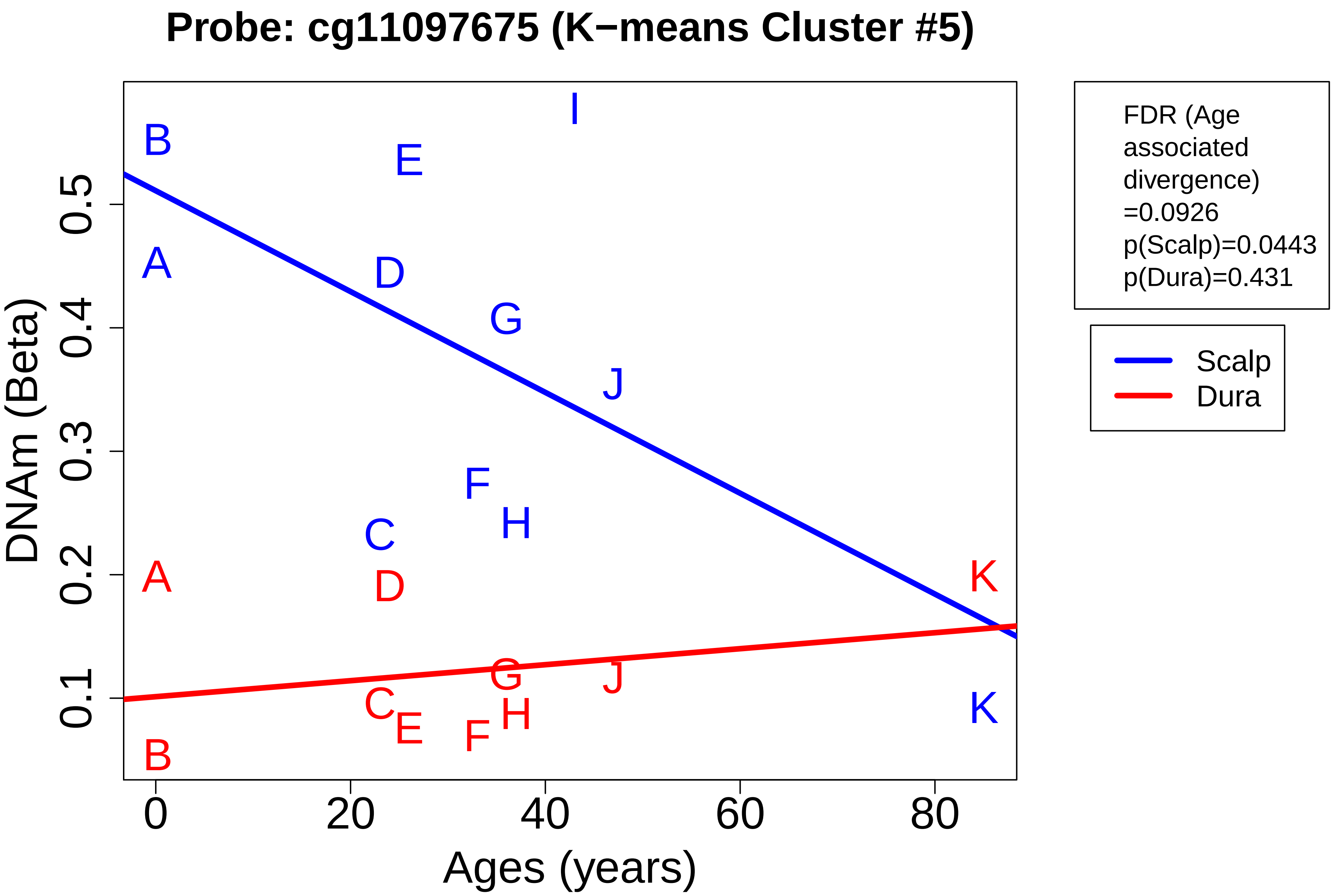
Probe: cg11097675 (K−means Cluster #5)
DNAm (Beta)
0.1
0.2
0.3
0.4
0.5
Ages (years)
0
20
40
60
80
B
E
I
A
D
G
J
F
H
C
A
D
K
G
J
C
E
F
H
K
B
FDR (Age associated divergence) =0.0926
p(Scalp)=0.0443
p(Dura)=0.431
Scalp
Dura

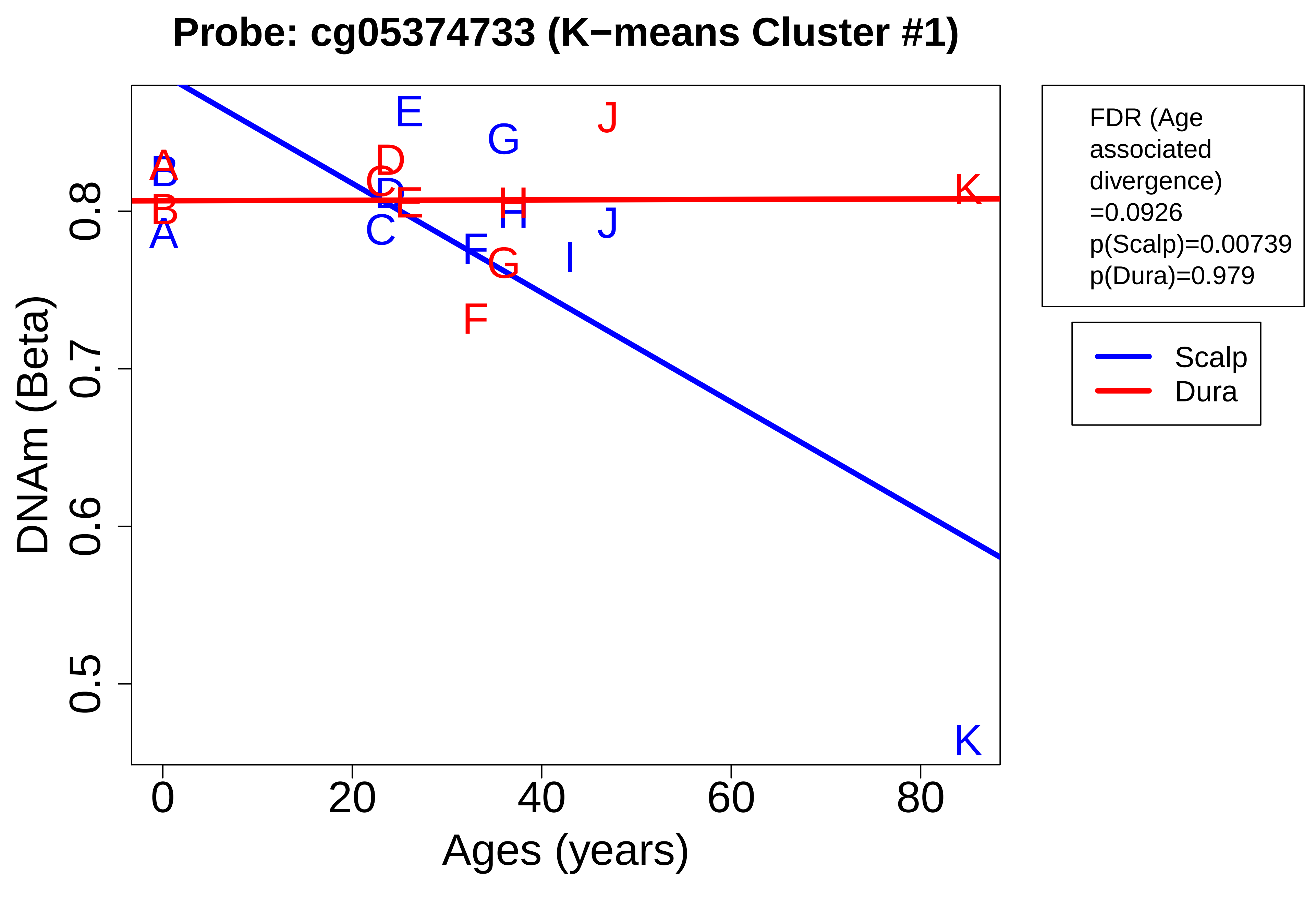

Probe: cg05374733 (K−means Cluster #1)
DNAm (Beta)
0.5
0.6
0.7
0.8
0
20
40
60
80
Ages (years)
FDR (Age associated divergence) =0.0926
p(Scalp)=0.00739
p(Dura)=0.979
Scalp
Dura

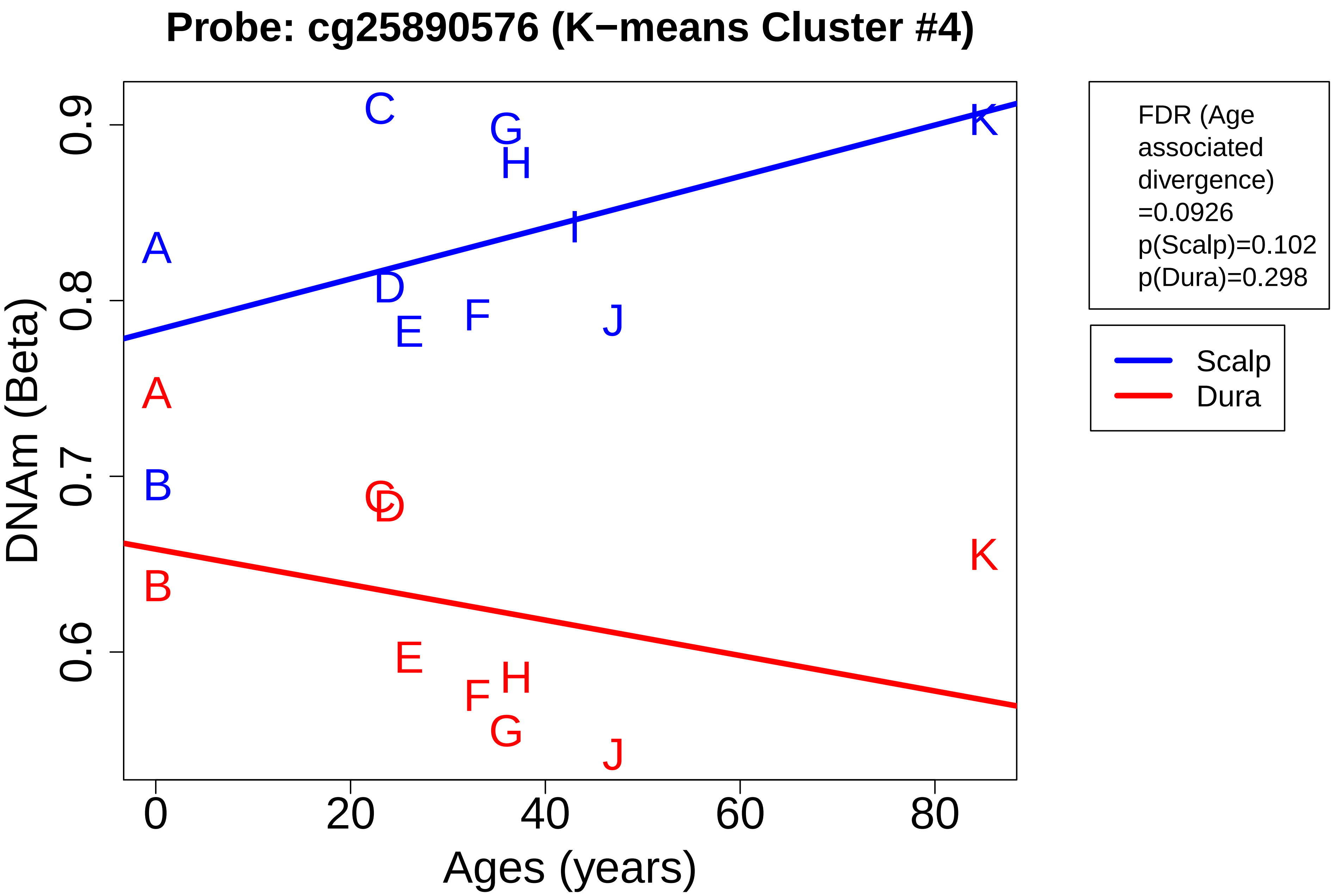

Probe: cg25890576 (K−means Cluster #4)
DNAm (Beta)
0.6
0.7
0.8
0.9
0
20
40
60
80
Ages (years)
A
B
C
D
E
F
G
H
I
J
K
A
B
C
D
E
F
G
H
J
K
FDR (Age associated divergence) =0.0926
p(Scalp)=0.102
p(Dura)=0.298
Scalp
Dura

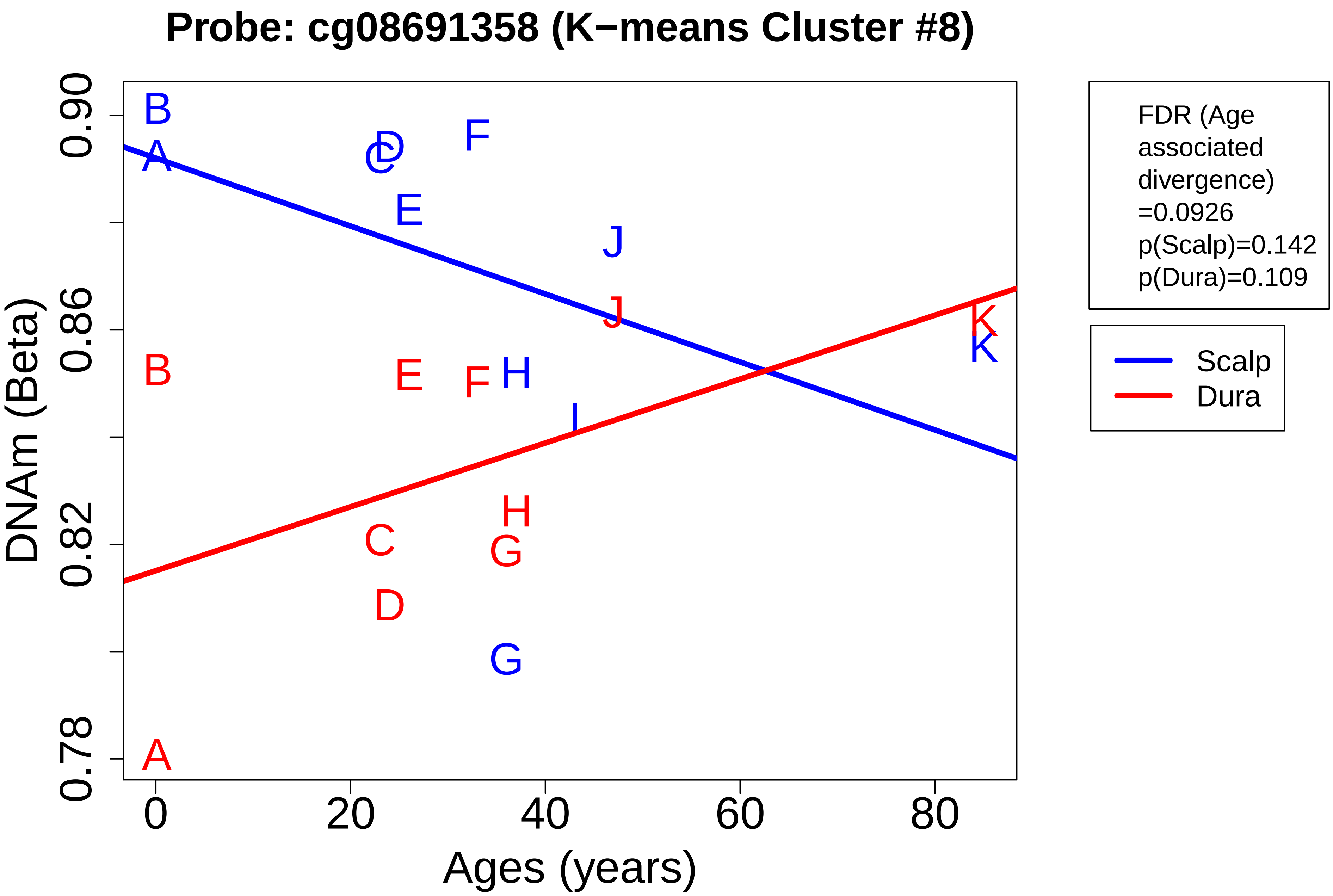

Probe: cg08691358 (K−means Cluster #8)
DNAm (Beta)
0.78
0.82
0.86
0.90
Ages (years)
0
20
40
60
80
A
B
C
D
E
F
G
H
I
J
K
FDR (Age associated divergence) =0.0926
p(Scalp)=0.142
p(Dura)=0.109
Scalp
Dura

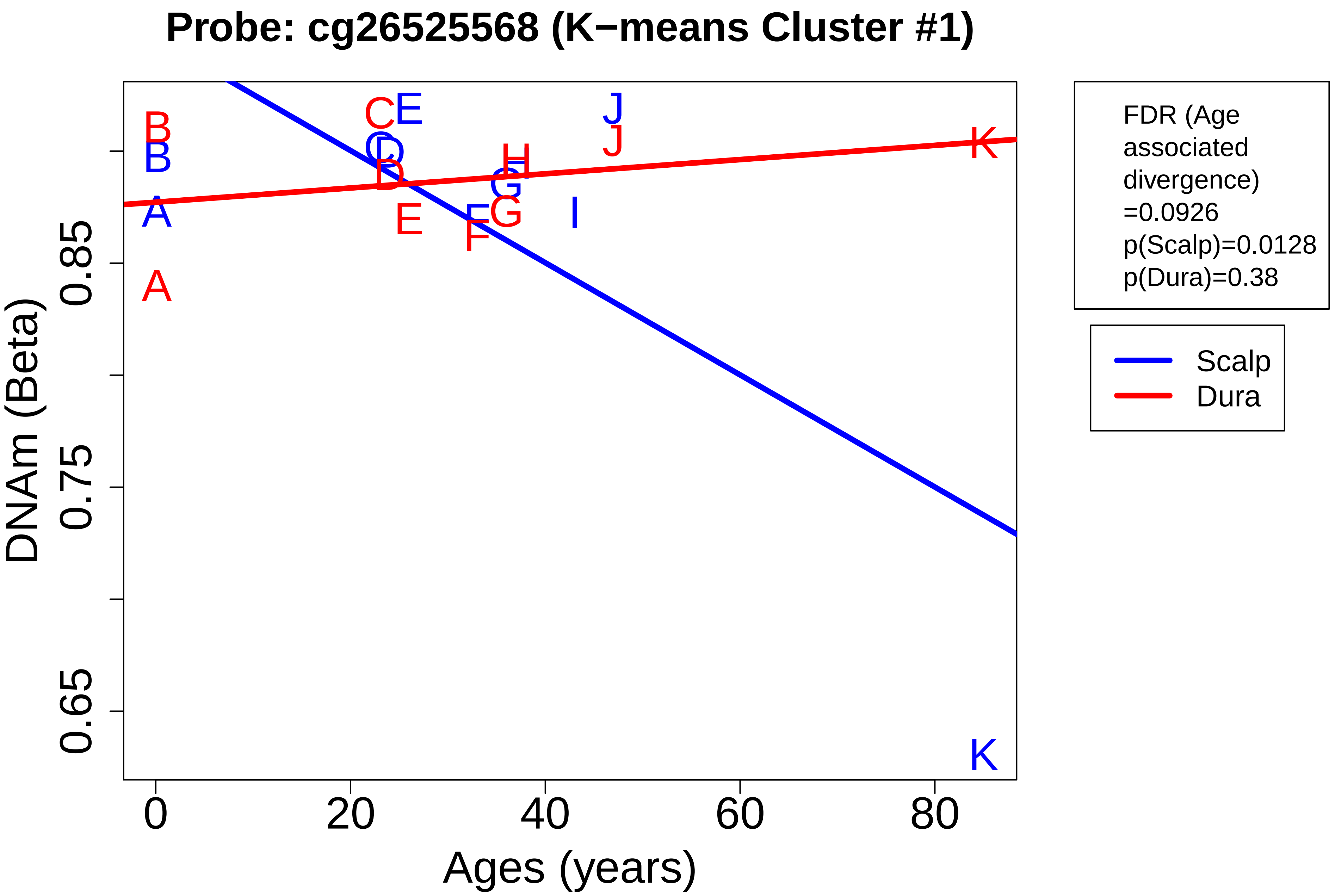

Probe: cg26525568 (K−means Cluster #1)
DNAm (Beta)
0.65
0.75
0.85
Ages (years)
0
20
40
60
80
FDR (Age associated divergence) =0.0926
p(Scalp)=0.0128
p(Dura)=0.38
Scalp
Dura

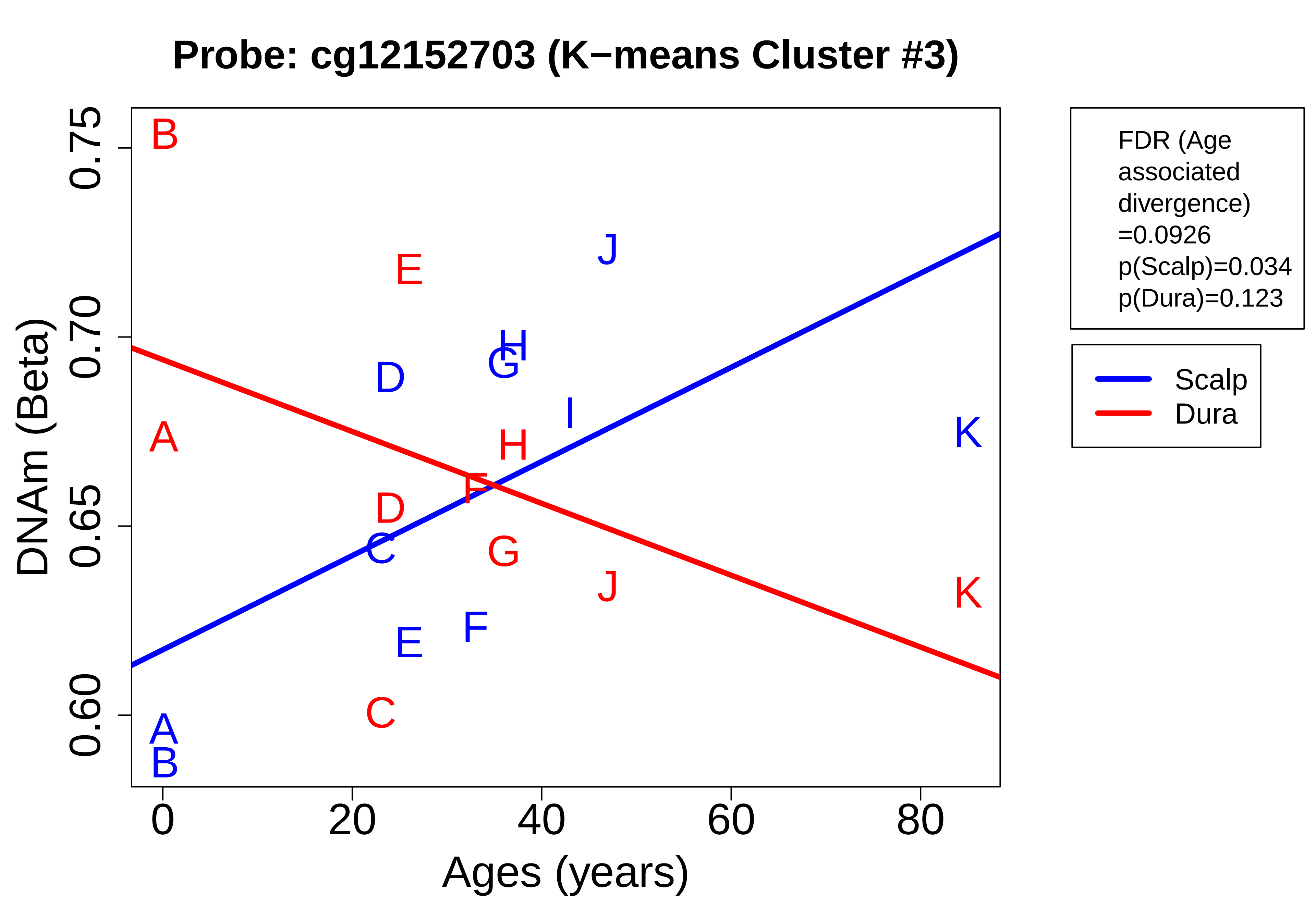

Probe: cg12152703 (K−means Cluster #3)
DNAm (Beta)
0.60
0.65
0.70
0.75
Ages (years)
0
20
40
60
80
FDR (Age associated divergence) =0.0926
p(Scalp)=0.034
p(Dura)=0.123
Scalp
Dura
A
B
C
D
E
F
G
H
I
J
K

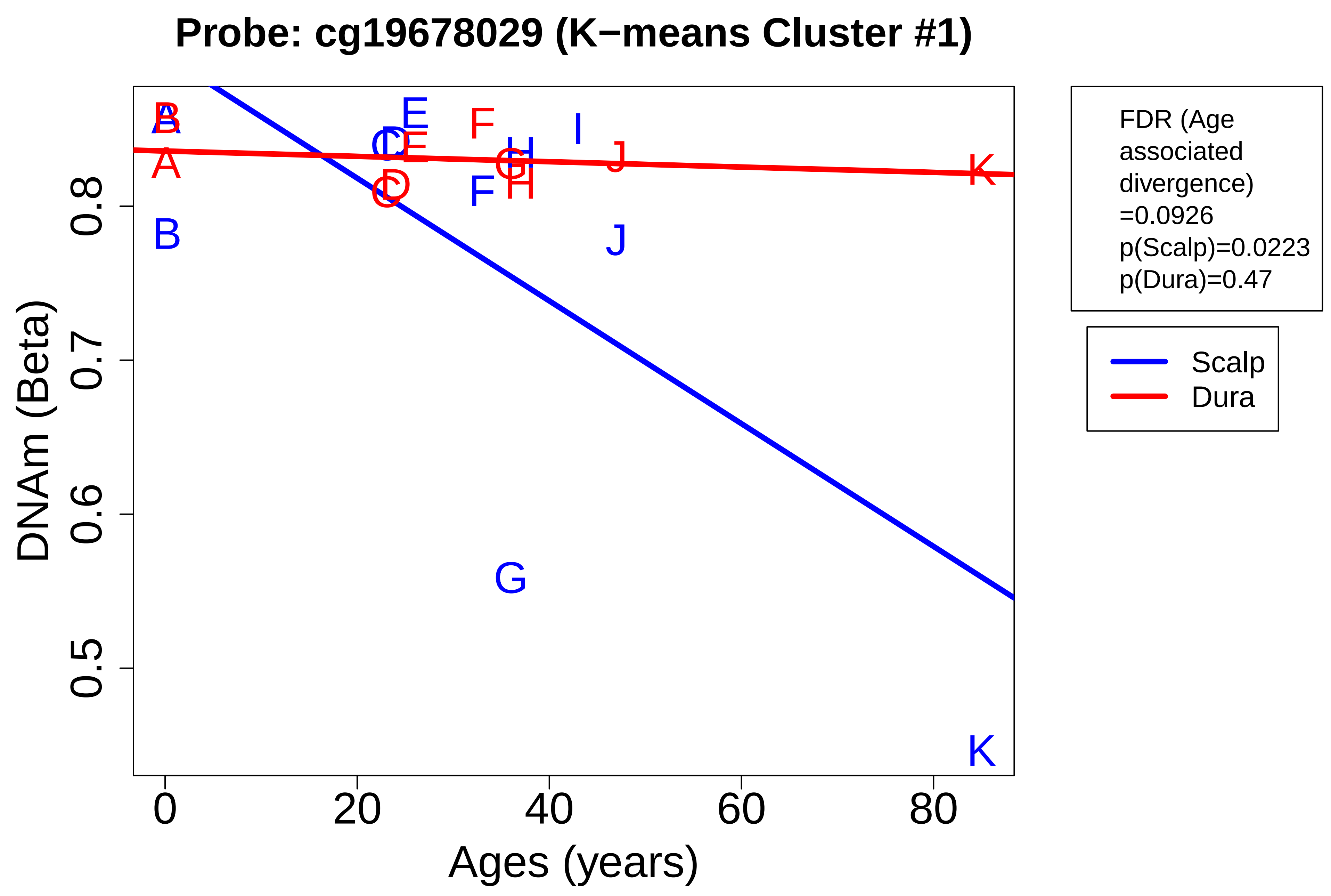

Probe: cg19678029 (K−means Cluster #1)
DNAm (Beta)
0.5
0.6
0.7
0.8
0
20
40
60
80
Ages (years)
A
B
C
D
E
F
G
H
I
J
K
FDR (Age associated divergence) =0.0926
p(Scalp)=0.0223
p(Dura)=0.47
Scalp
Dura

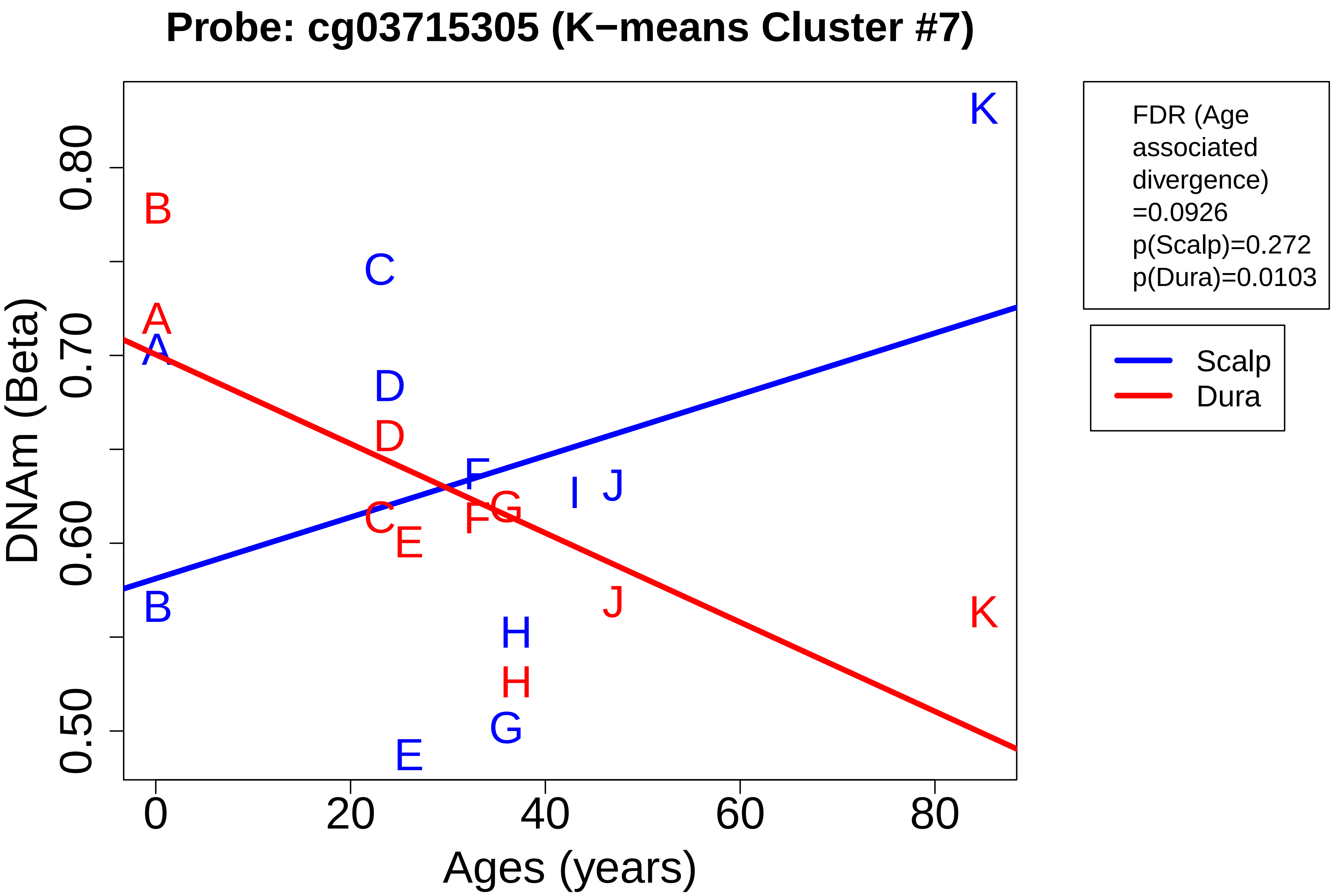

Probe: cg03715305 (K−means Cluster #7)
DNAm (Beta)
0.50
0.60
0.70
0.80
Ages (years)
0
20
40
60
80
B
C
A
A
D
D
F
I
J
C
F
G
E
B
J
K
H
H
G
E
K
FDR (Age associated divergence) =0.0926
p(Scalp)=0.272
p(Dura)=0.0103
Scalp
Dura

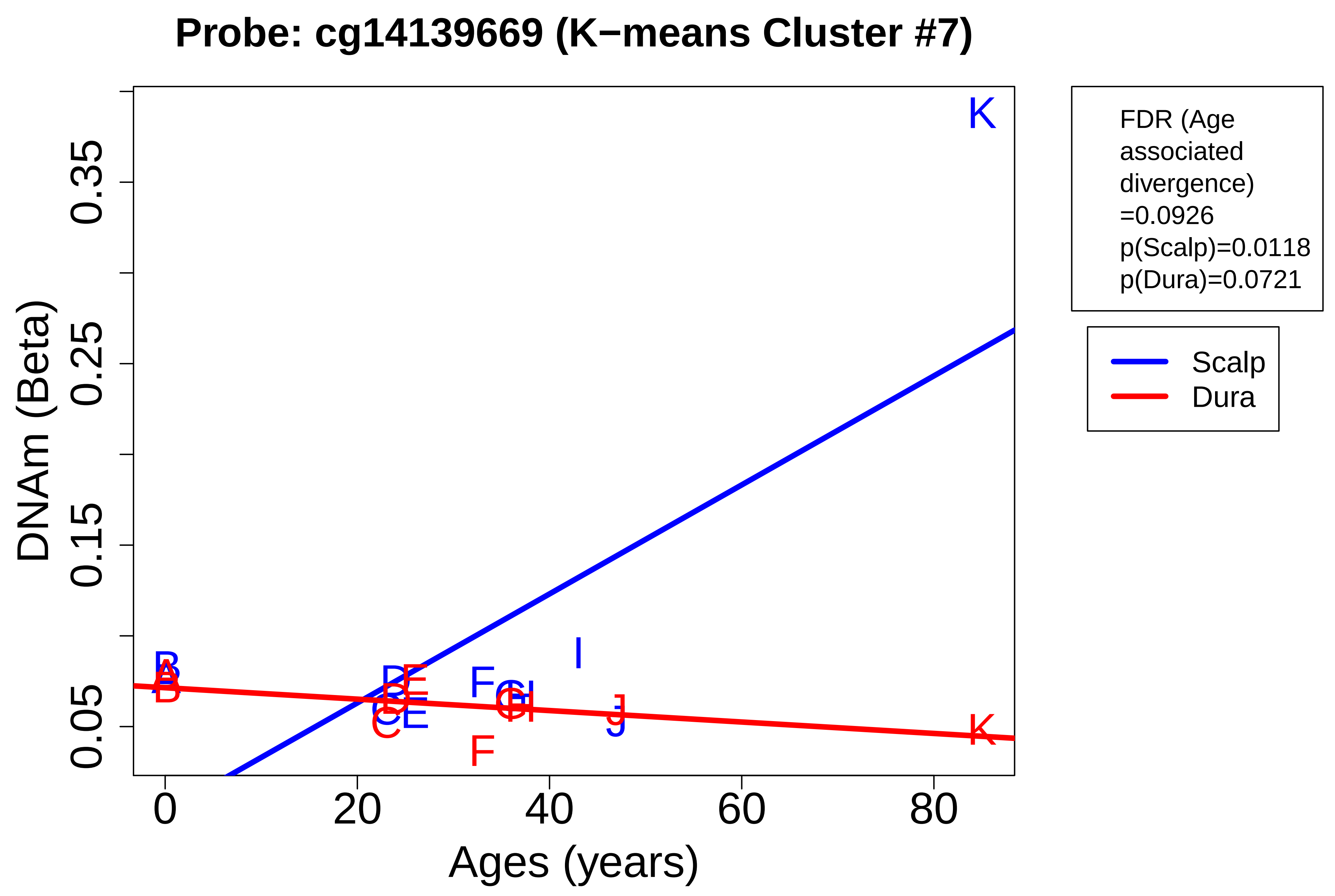

Probe: cg14139669 (K−means Cluster #7)
DNAm (Beta)
0.05
0.15
0.25
0.35
0
20
40
60
80
Ages (years)
FDR (Age associated divergence) =0.0926
p(Scalp)=0.0118
p(Dura)=0.0721
Scalp
Dura

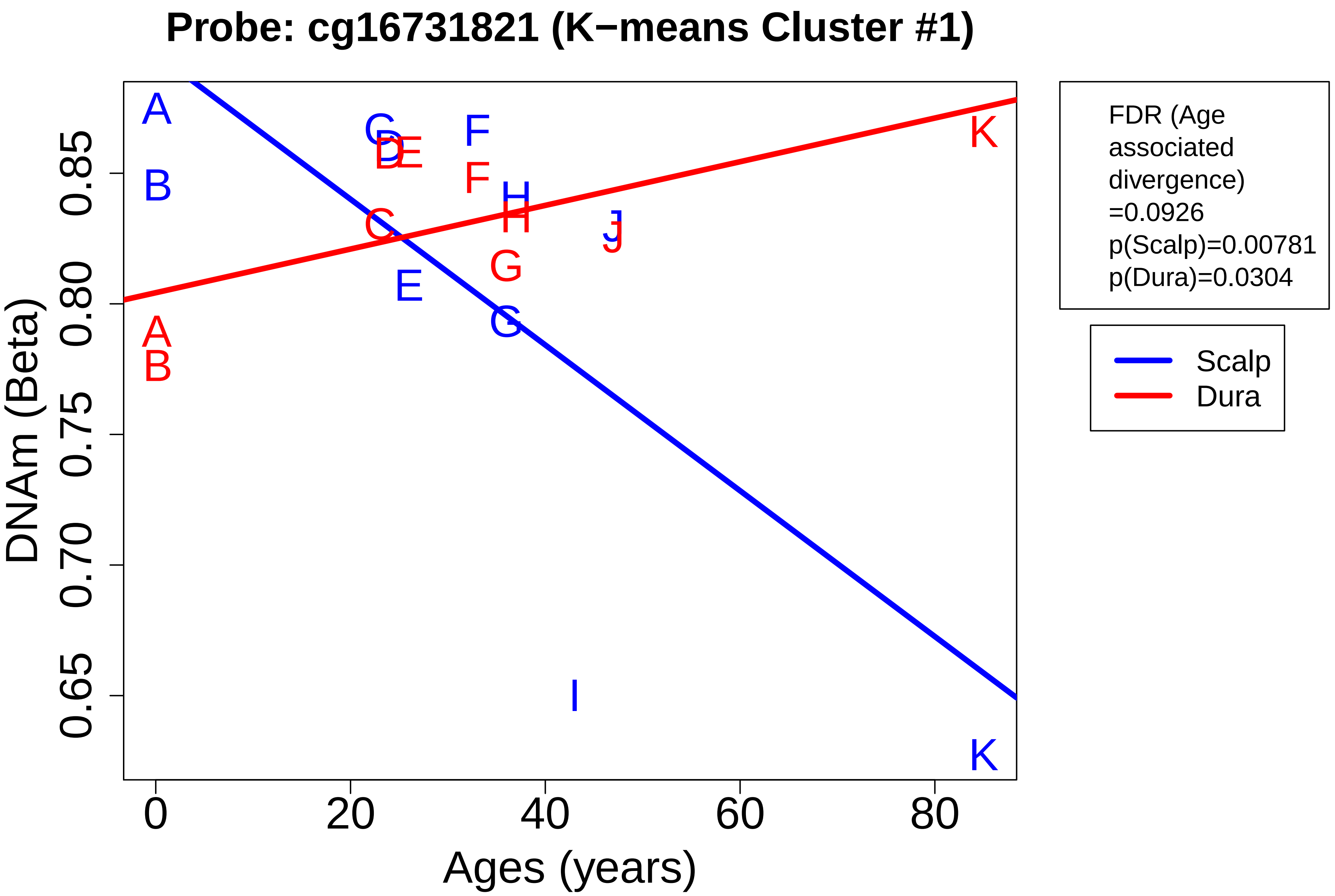

Probe: cg16731821 (K−means Cluster #1)
DNAm (Beta)
0.65
0.70
0.75
0.80
0.85
Ages (years)
0
20
40
60
80
A
B
C
D
E
F
G
H
I
J
K
FDR (Age associated divergence) =0.0926
p(Scalp)=0.00781
p(Dura)=0.0304
Scalp
Dura

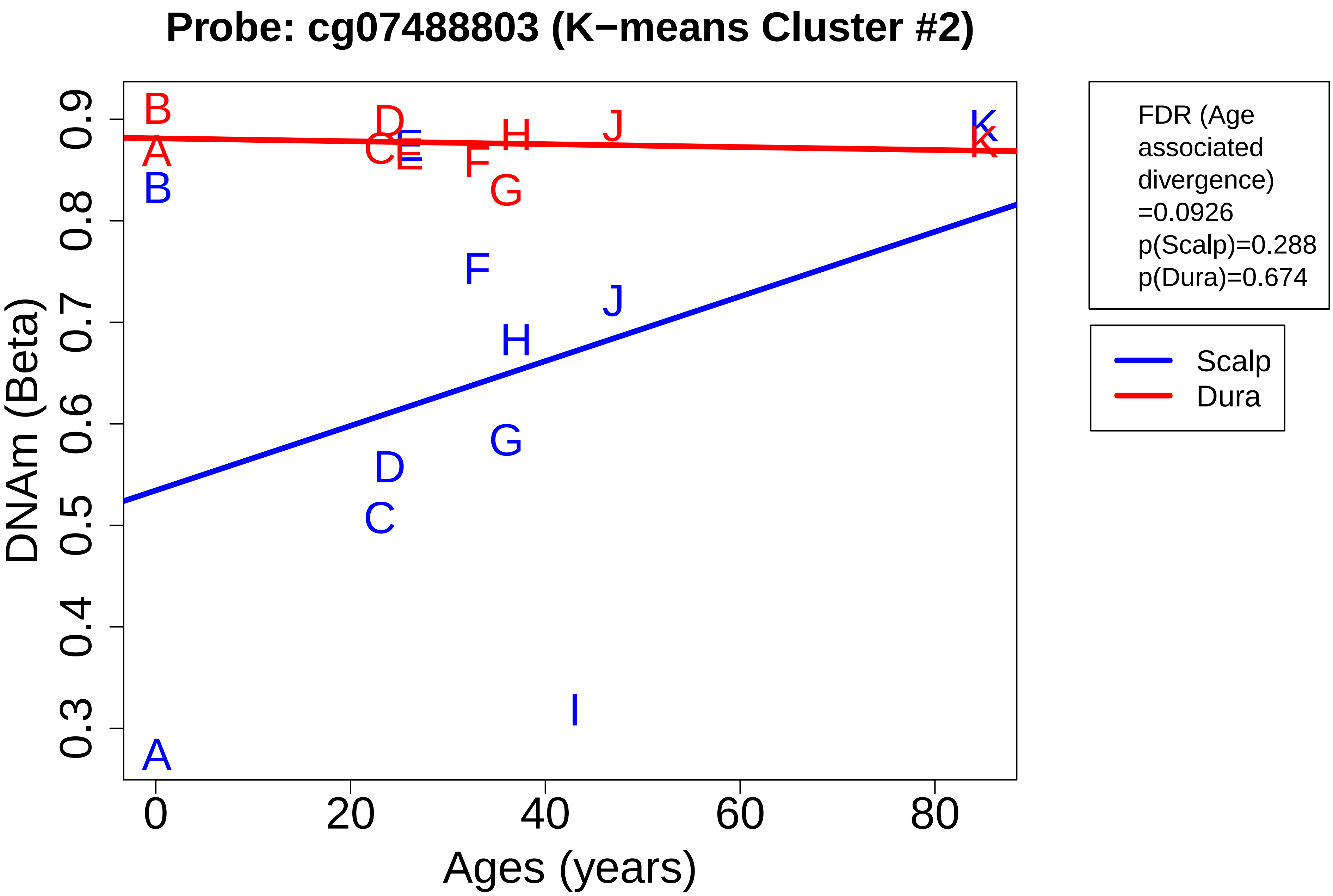
Probe: cg07488803 (K−means Cluster #2)
DNAm (Beta)
0.3
0.4
0.5
0.6
0.7
0.8
0.9
0
20
40
60
80
Ages (years)
FDR (Age associated divergence) =0.0926
p(Scalp)=0.288
p(Dura)=0.674
Scalp
Dura

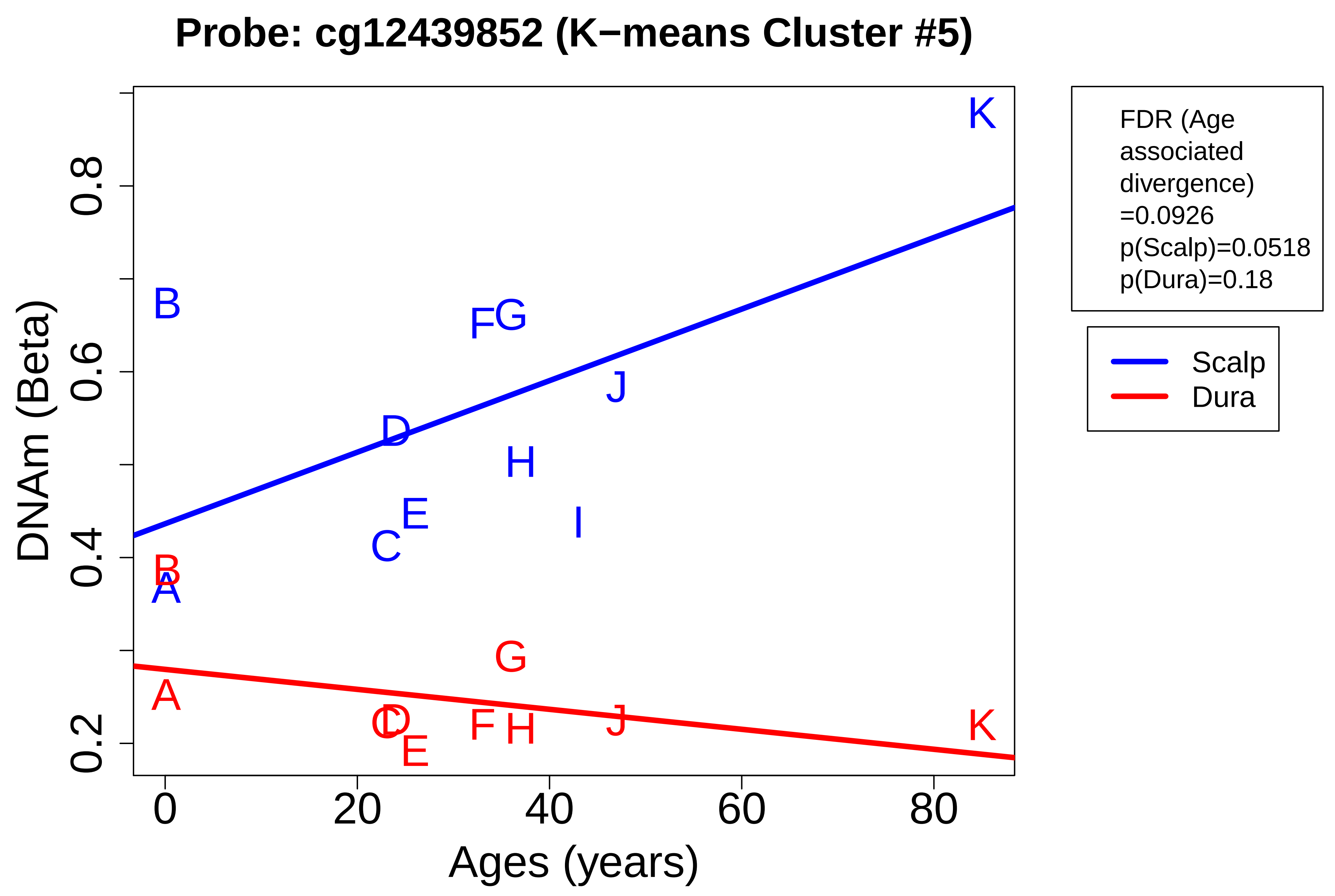

Probe: cg12439852 (K−means Cluster #5)
DNAm (Beta)
0.2
0.4
0.6
0.8
0
20
40
60
80
Ages (years)
FDR (Age associated divergence) =0.0926
p(Scalp)=0.0518
p(Dura)=0.18
Scalp
Dura
A
B
C
D
E
F
G
H
I
J
K
A
B
C
D
E
F
G
H
J
K

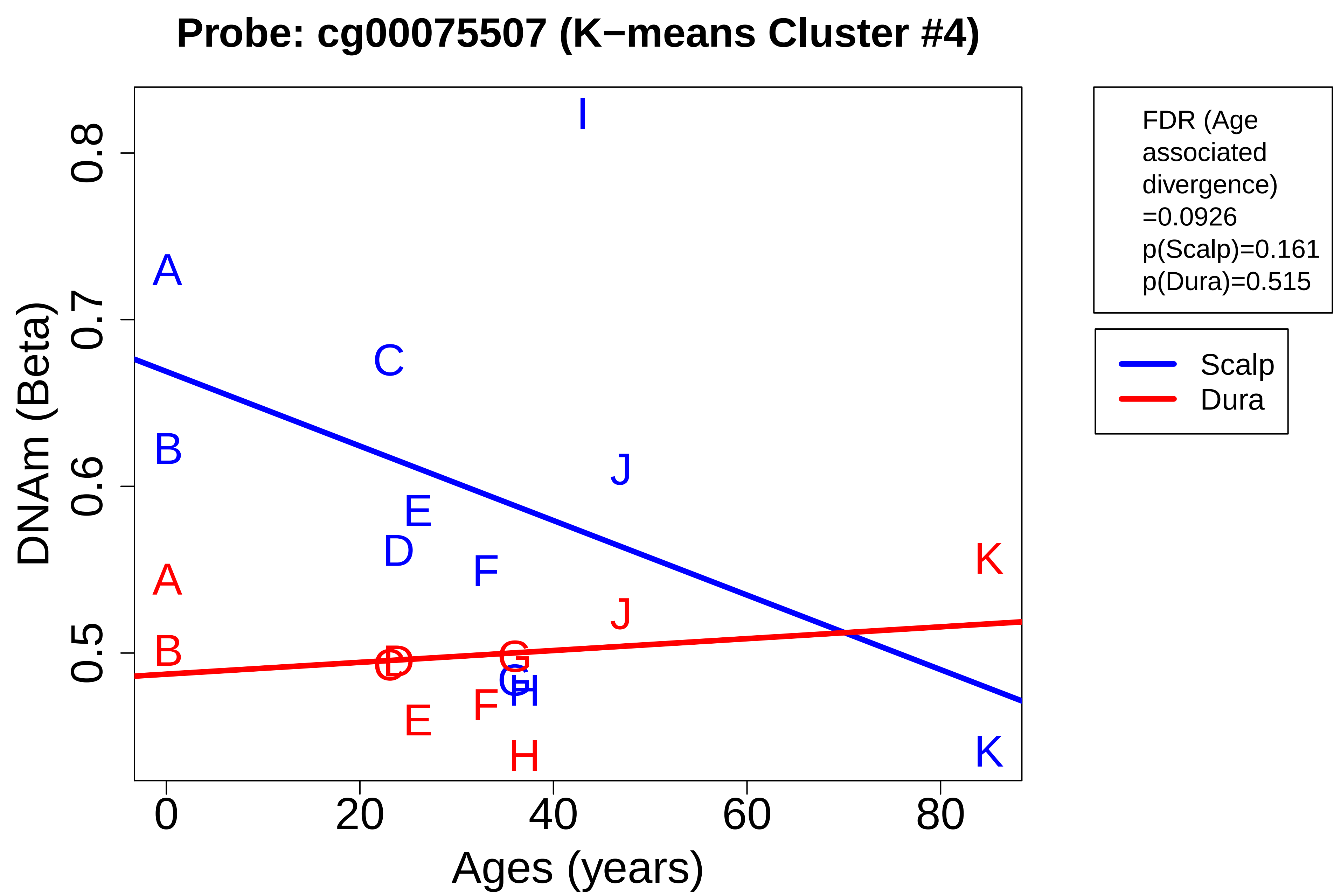

Probe: cg00075507 (K−means Cluster #4)
DNAm (Beta)
0.5
0.6
0.7
0.8
Ages (years)
0
20
40
60
80
A
B
C
D
E
F
G
H
I
J
K
FDR (Age associated divergence) =0.0926
p(Scalp)=0.161
p(Dura)=0.515
Scalp
Dura

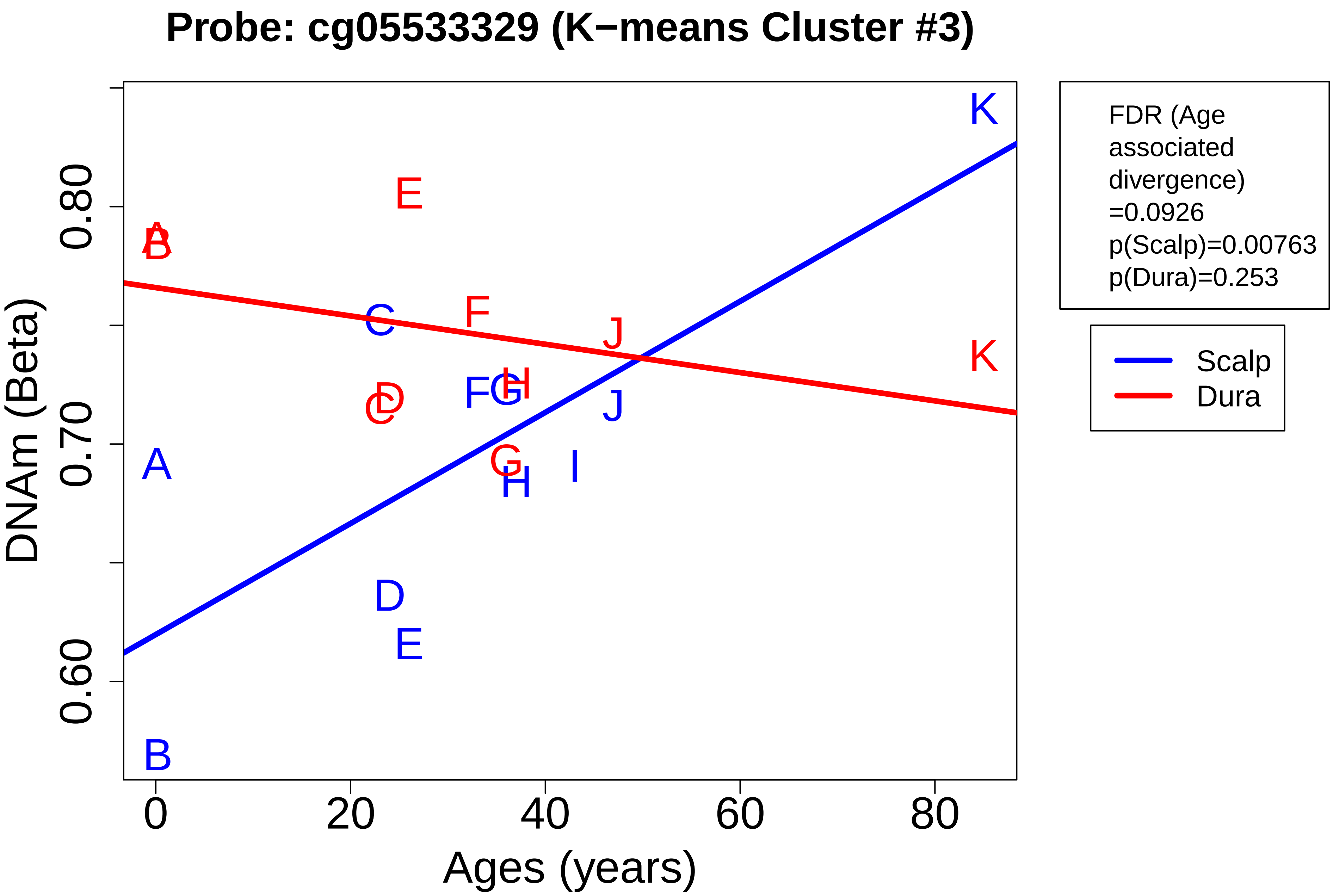

Probe: cg05533329 (K−means Cluster #3)
DNAm (Beta)
0.60
0.70
0.80
Ages (years)
0
20
40
60
80
FDR (Age associated divergence) =0.0926
p(Scalp)=0.00763
p(Dura)=0.253
Scalp
Dura

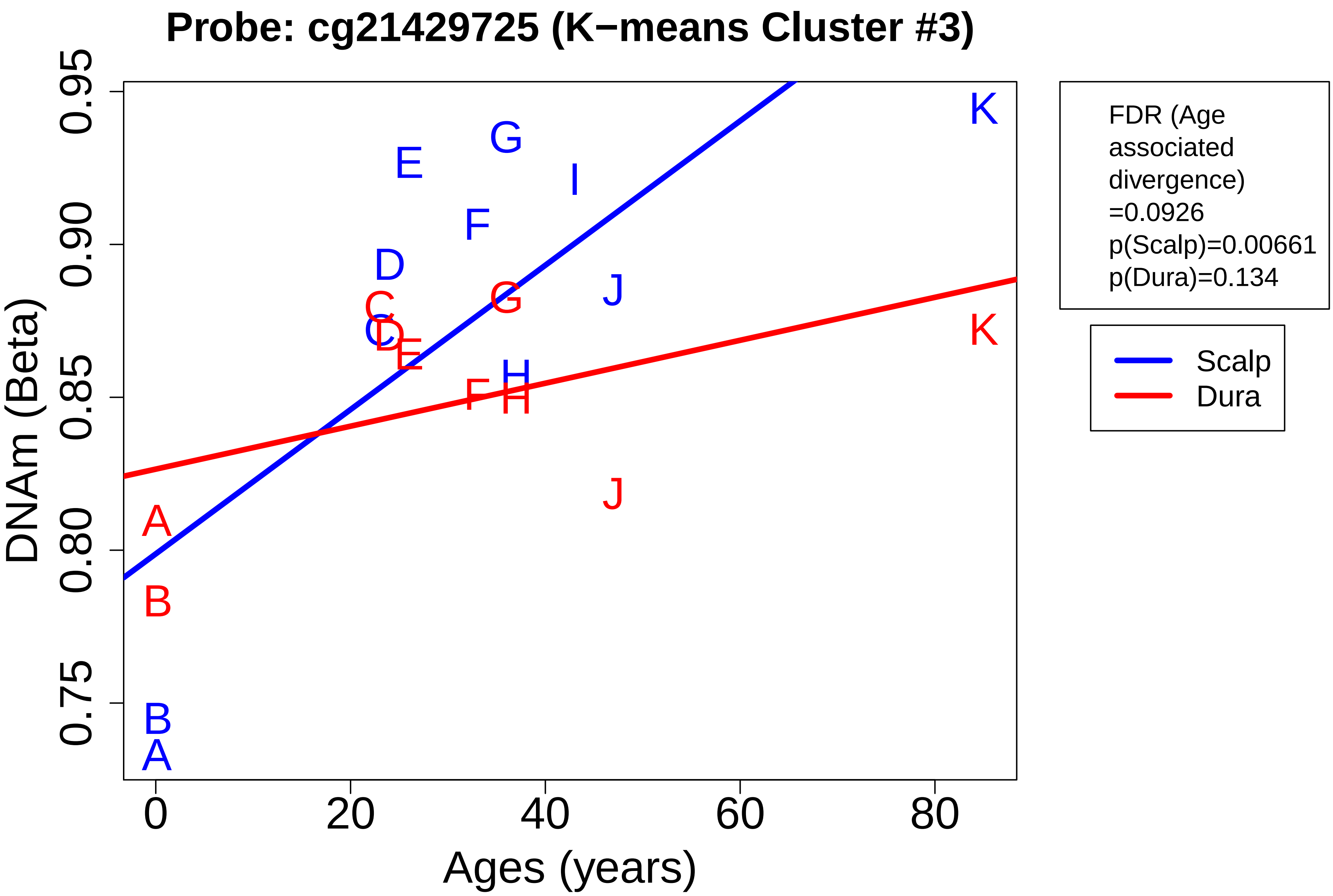
Probe: cg21429725 (K−means Cluster #3)
DNAm (Beta)
0.75
0.80
0.85
0.90
0.95
Ages (years)
0
20
40
60
80
A
B
C
D
E
F
G
H
I
J
K
FDR (Age associated divergence) =0.0926
p(Scalp)=0.00661
p(Dura)=0.134
Scalp
Dura

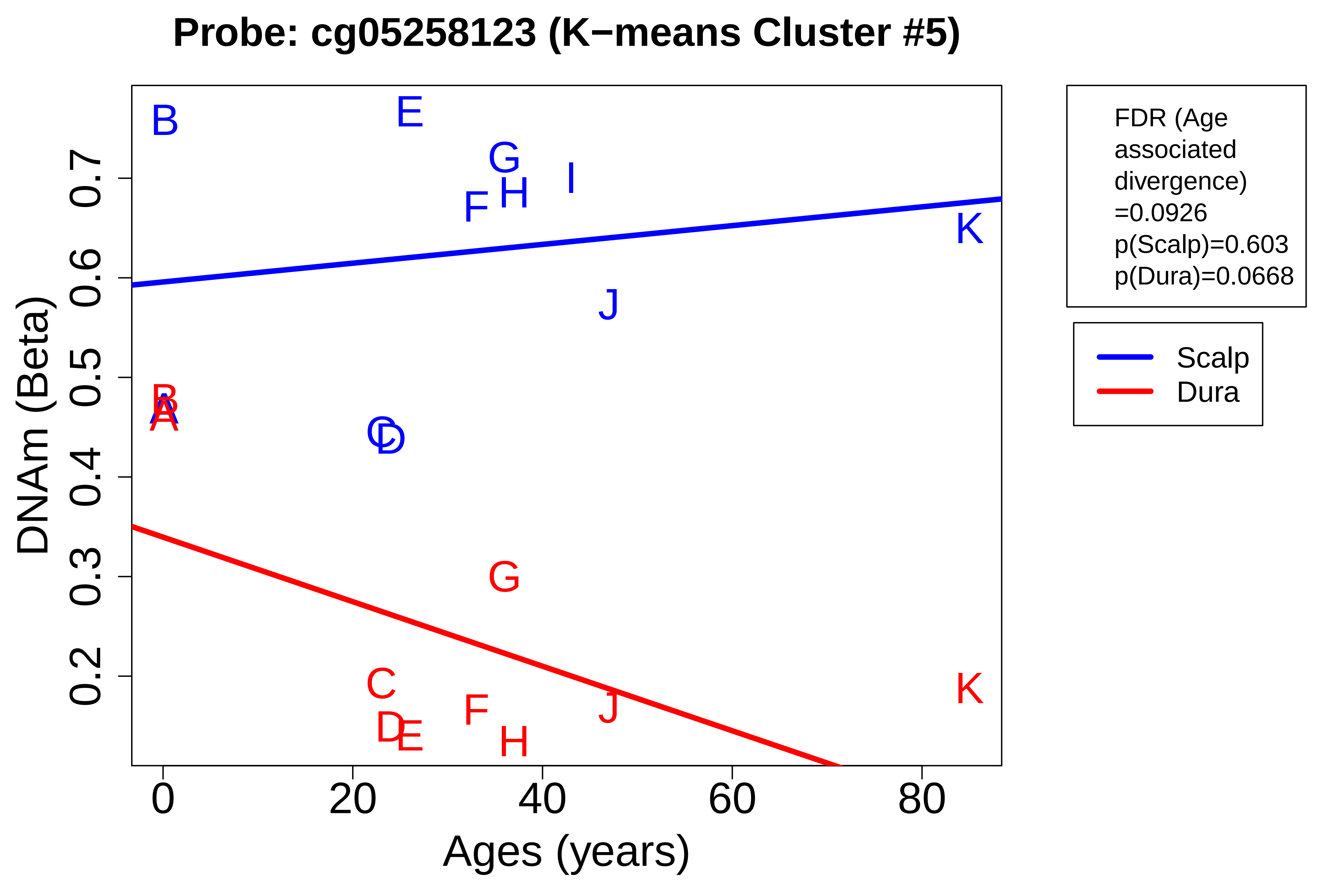

Probe: cg05258123 (K−means Cluster #5)
DNAm (Beta)
0.2
0.3
0.4
0.5
0.6
0.7
Ages (years)
0
20
40
60
80
FDR (Age associated divergence) =0.0926
p(Scalp)=0.603
p(Dura)=0.0668
Scalp
Dura

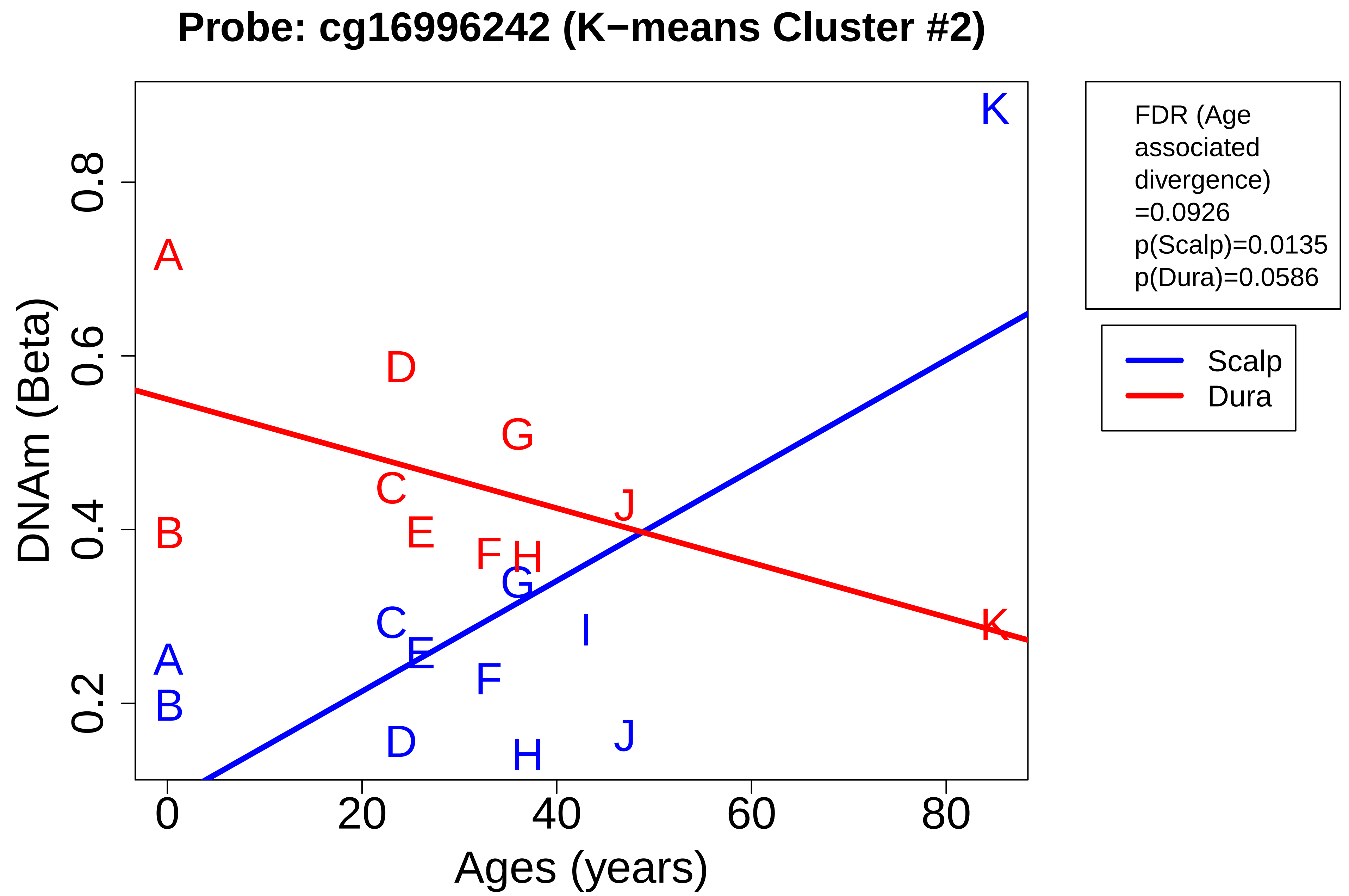
Probe: cg16996242 (K−means Cluster #2)
DNAm (Beta)
0.2
0.4
0.6
0.8
Ages (years)
0
20
40
60
80
A
B
C
D
E
F
G
H
I
J
K
FDR (Age associated divergence) =0.0926
p(Scalp)=0.0135
p(Dura)=0.0586
Scalp
Dura

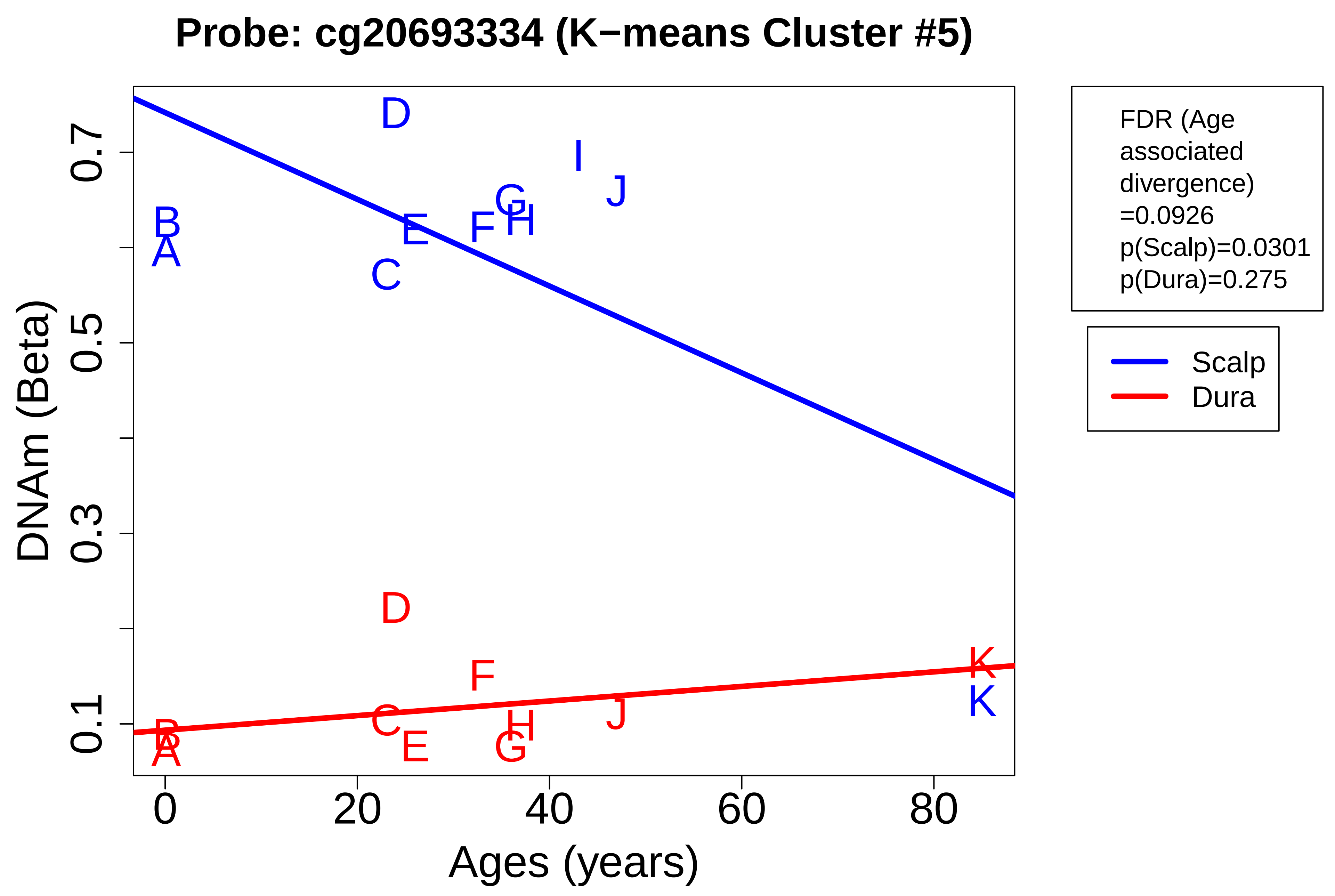
Probe: cg20693334 (K−means Cluster #5)
DNAm (Beta)
Ages (years)
0.1
0.3
0.5
0.7
0
20
40
60
80
FDR (Age associated divergence) =0.0926
p(Scalp)=0.0301
p(Dura)=0.275
Scalp
Dura

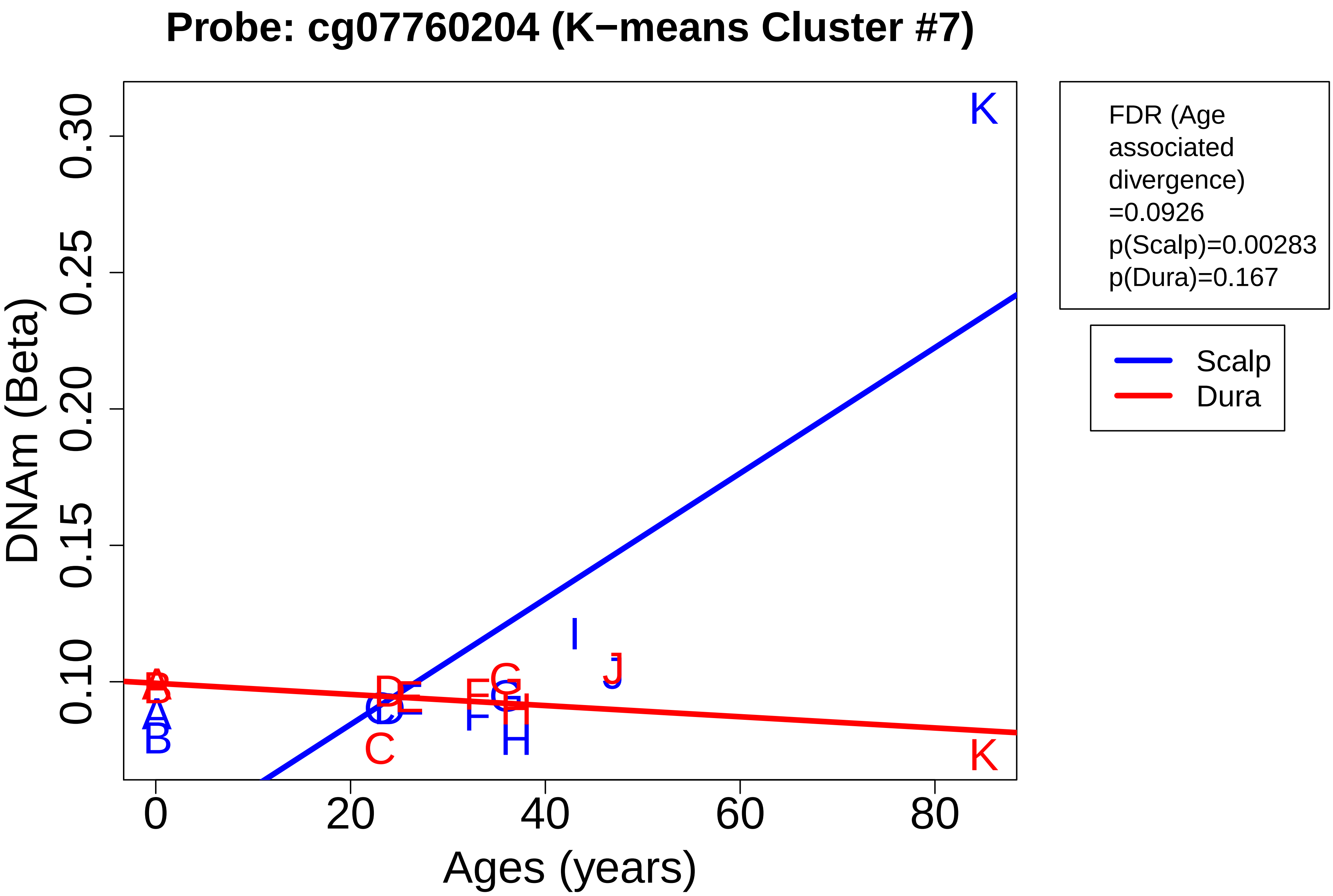

Probe: cg07760204 (K−means Cluster #7)
DNAm (Beta)
0.10
0.15
0.20
0.25
0.30
0
20
40
60
80
Ages (years)
FDR (Age associated divergence) =0.0926
p(Scalp)=0.00283
p(Dura)=0.167
Scalp
Dura

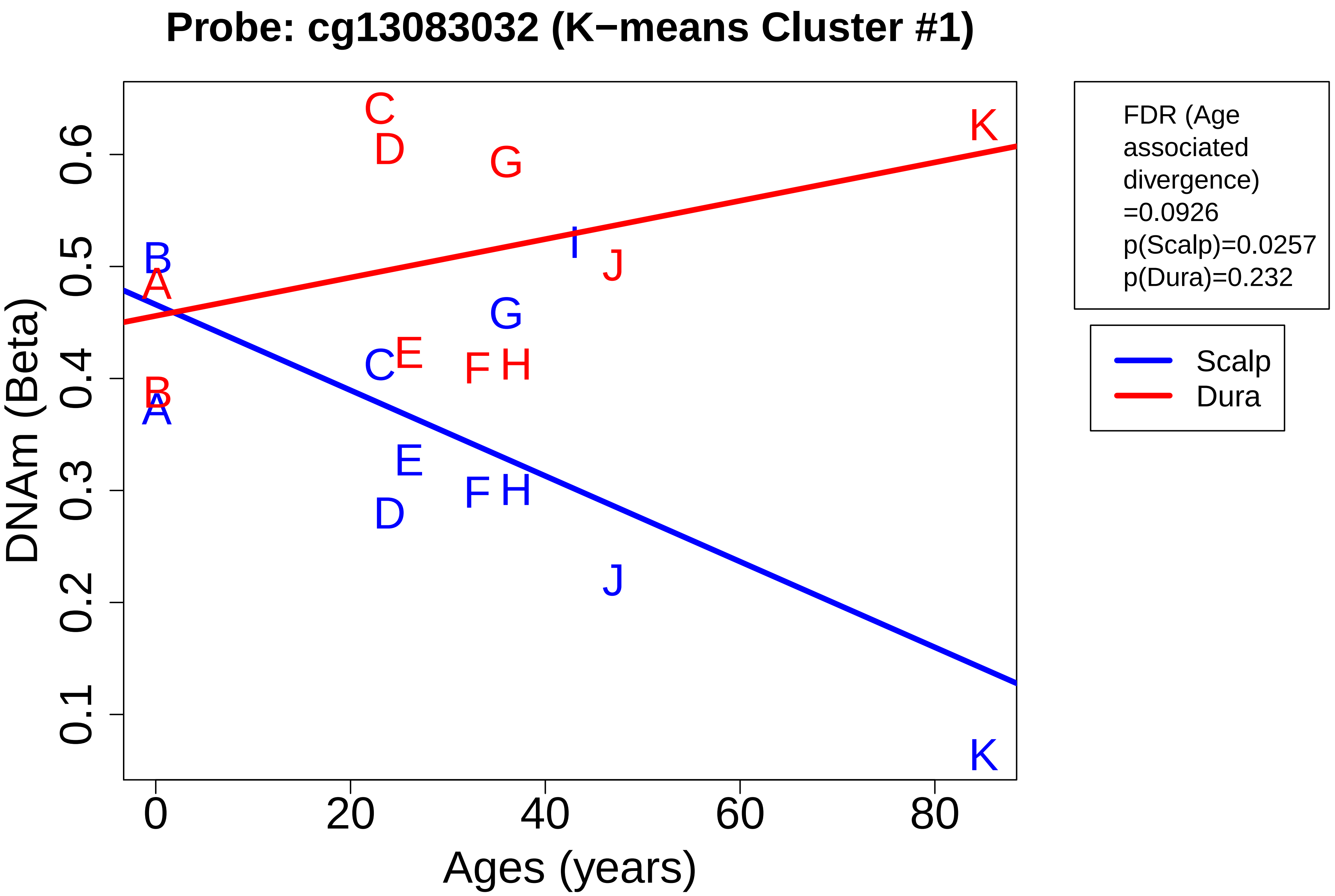

Probe: cg13083032 (K−means Cluster #1)
DNAm (Beta)
0.1
0.2
0.3
0.4
0.5
0.6
Ages (years)
0
20
40
60
80
A
B
C
D
E
F
G
H
I
J
K
FDR (Age associated divergence) =0.0926
p(Scalp)=0.0257
p(Dura)=0.232
Scalp
Dura

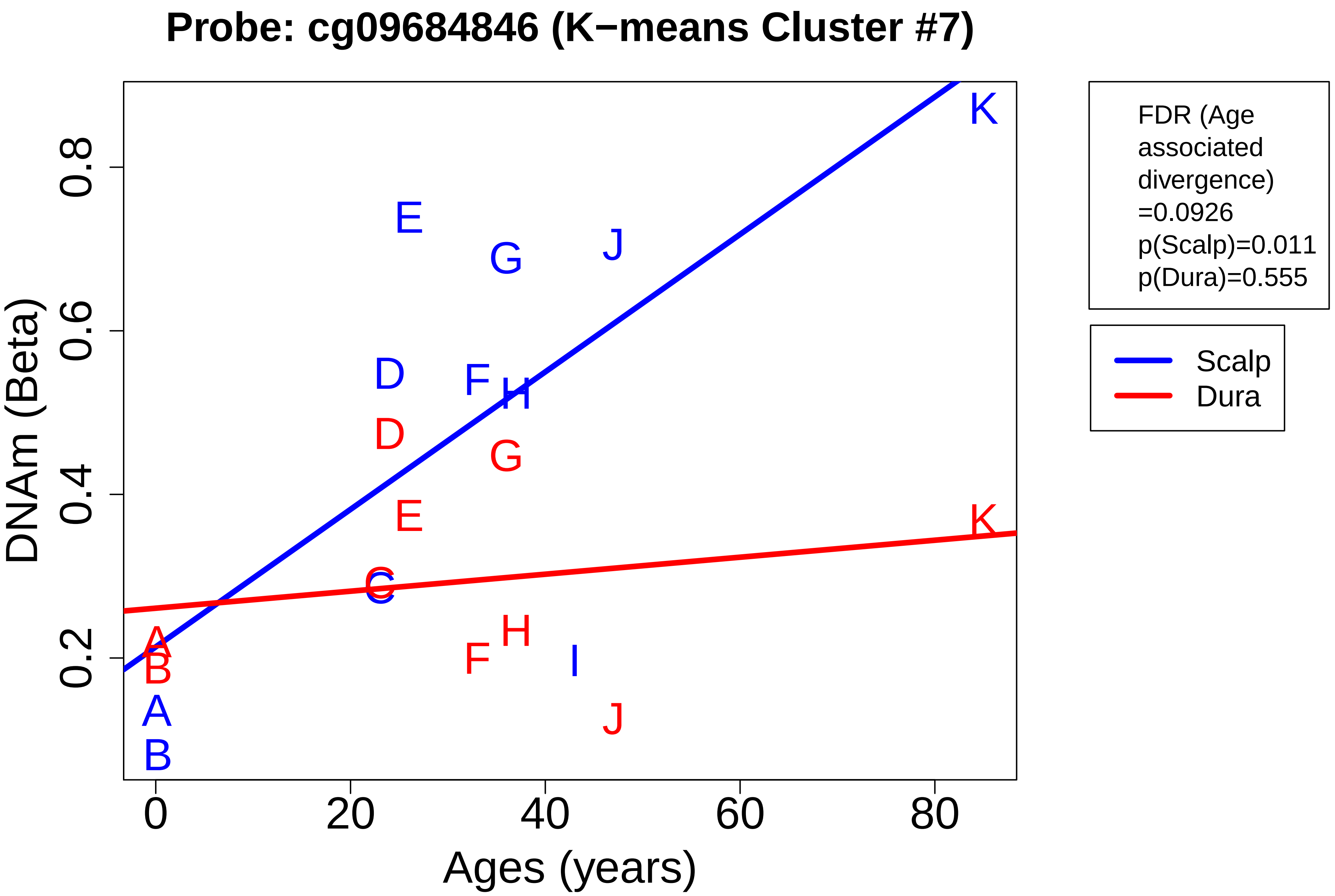

Probe: cg09684846 (K−means Cluster #7)
DNAm (Beta)
0.2
0.4
0.6
0.8
Ages (years)
0
20
40
60
80
A
B
C
D
E
F
G
H
I
J
K
FDR (Age associated divergence) =0.0926
p(Scalp)=0.011
p(Dura)=0.555
Scalp
Dura

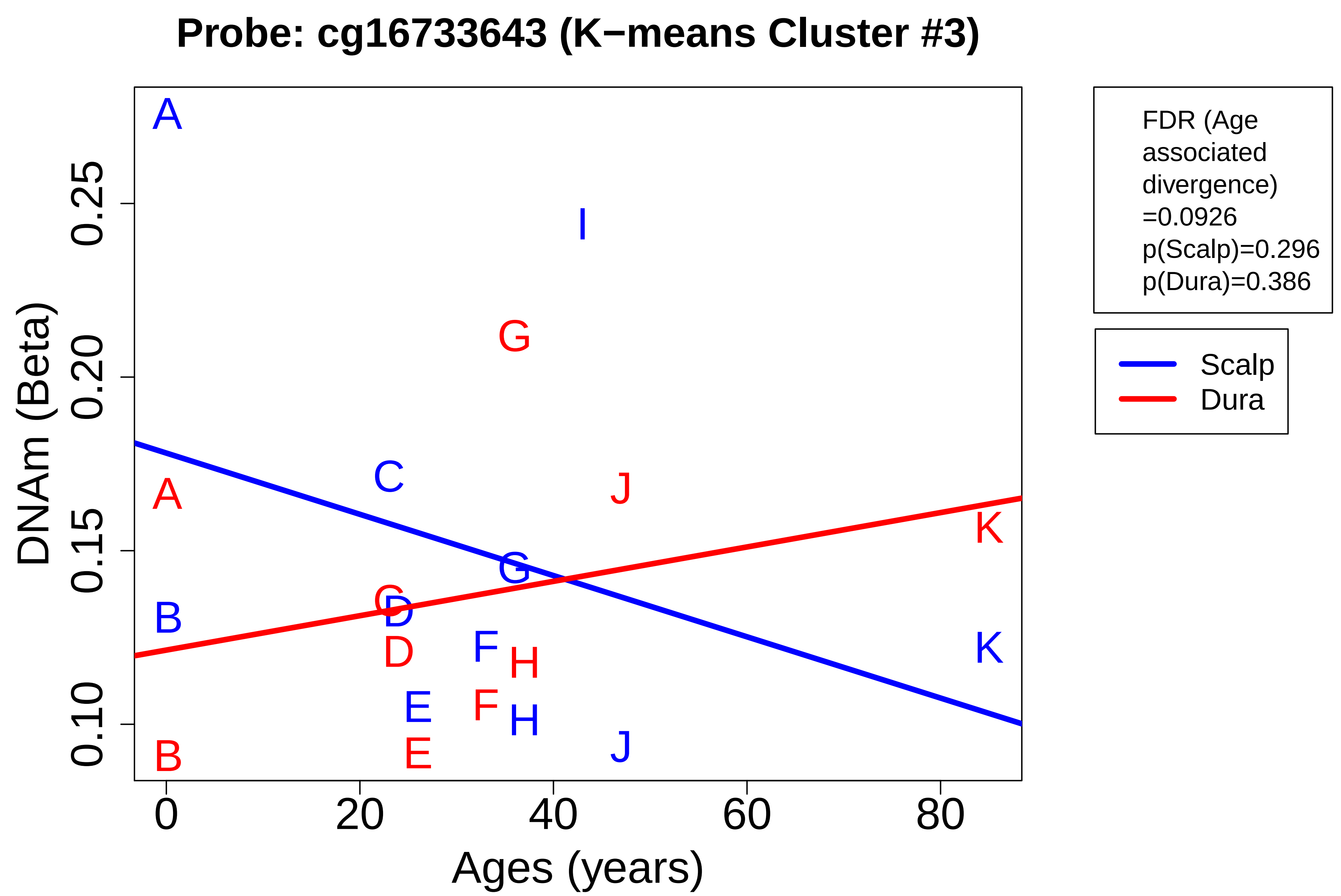

Probe: cg16733643 (K−means Cluster #3)
DNAm (Beta)
0.10
0.15
0.20
0.25
Ages (years)
0
20
40
60
80
A
I
G
C
J
A
K
G
C
D
B
D
F
H
K
E
F
H
B
E
J
FDR (Age associated divergence) =0.0926
p(Scalp)=0.296
p(Dura)=0.386
Scalp
Dura

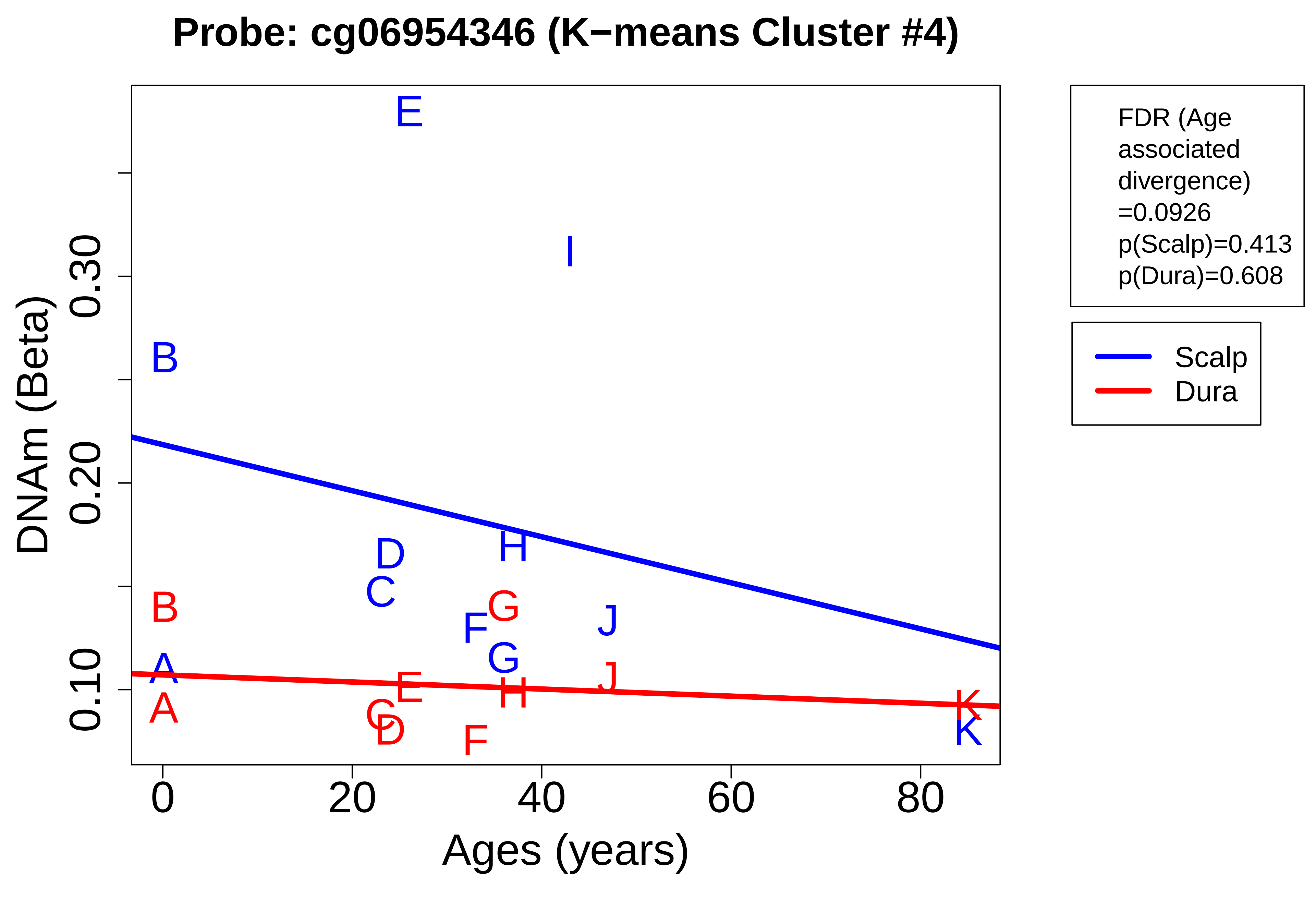

Probe: cg06954346 (K−means Cluster #4)
DNAm (Beta)
0.10
0.20
0.30
0
20
40
60
80
Ages (years)
FDR (Age associated divergence) =0.0926
p(Scalp)=0.413
p(Dura)=0.608
Scalp
Dura
E
I
B
D
H
C
B
G
F
J
G
A
E
J
H
A
G
D
F
K
K

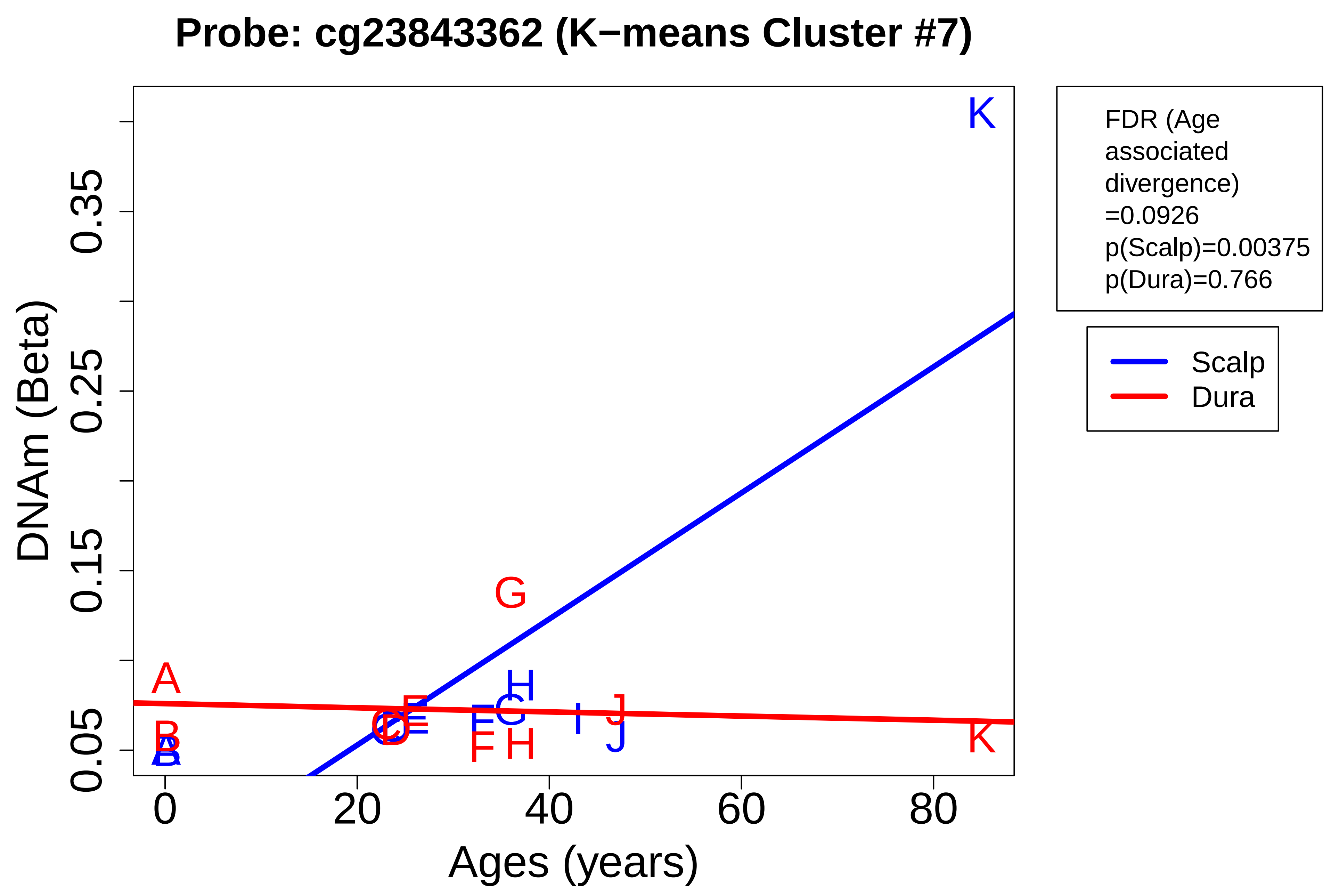

Probe: cg23843362 (K−means Cluster #7)
DNAm (Beta)
0.05
0.15
0.25
0.35
Ages (years)
0
20
40
60
80
FDR (Age associated divergence) =0.0926
p(Scalp)=0.00375
p(Dura)=0.766
Scalp
Dura

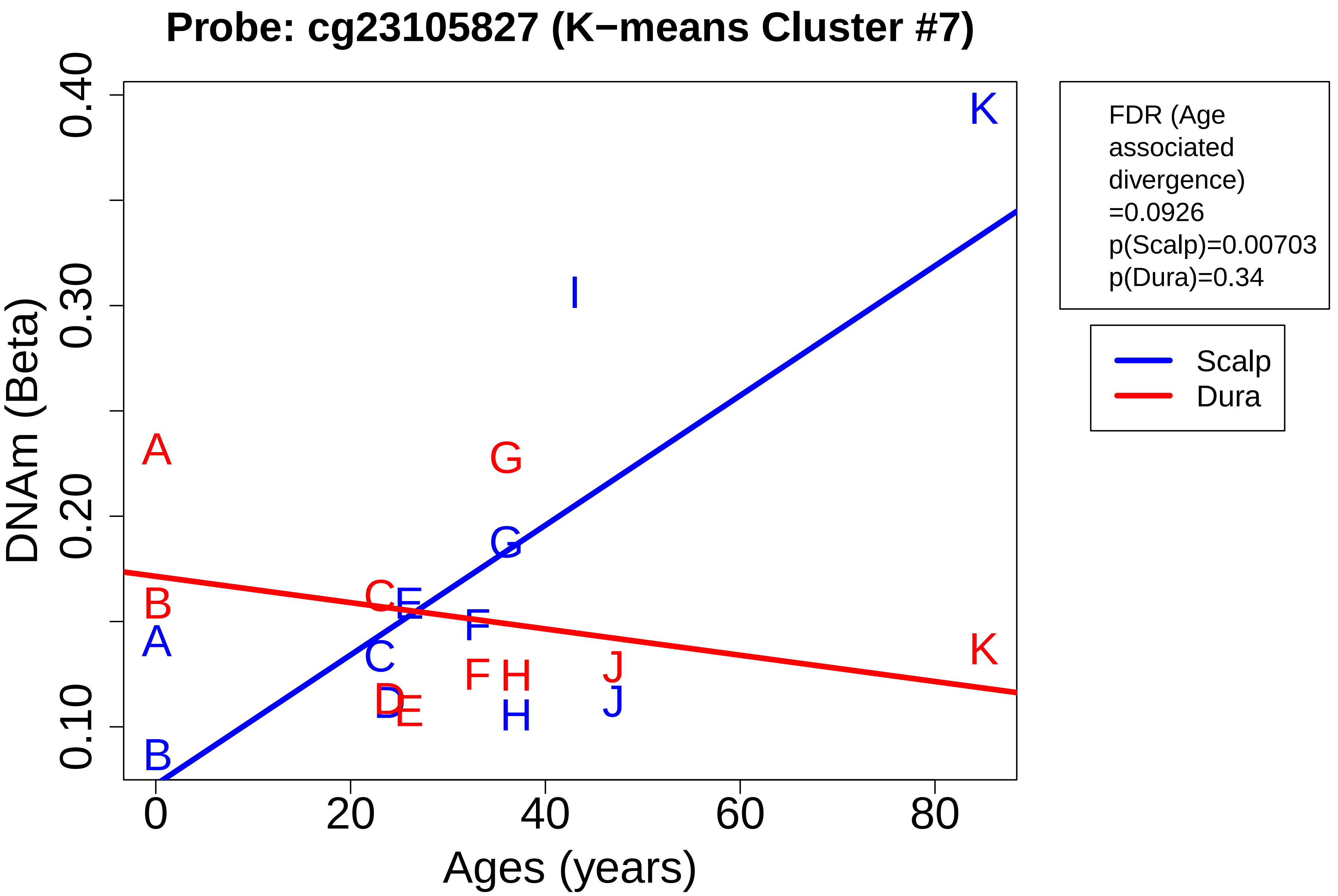

Probe: cg23105827 (K−means Cluster #7)
DNAm (Beta)
0.10
0.20
0.30
0.40
0
20
40
60
80
Ages (years)
FDR (Age associated divergence) =0.0926
p(Scalp)=0.00703
p(Dura)=0.34
Scalp
Dura
A
B
C
D
E
F
G
H
I
J
K

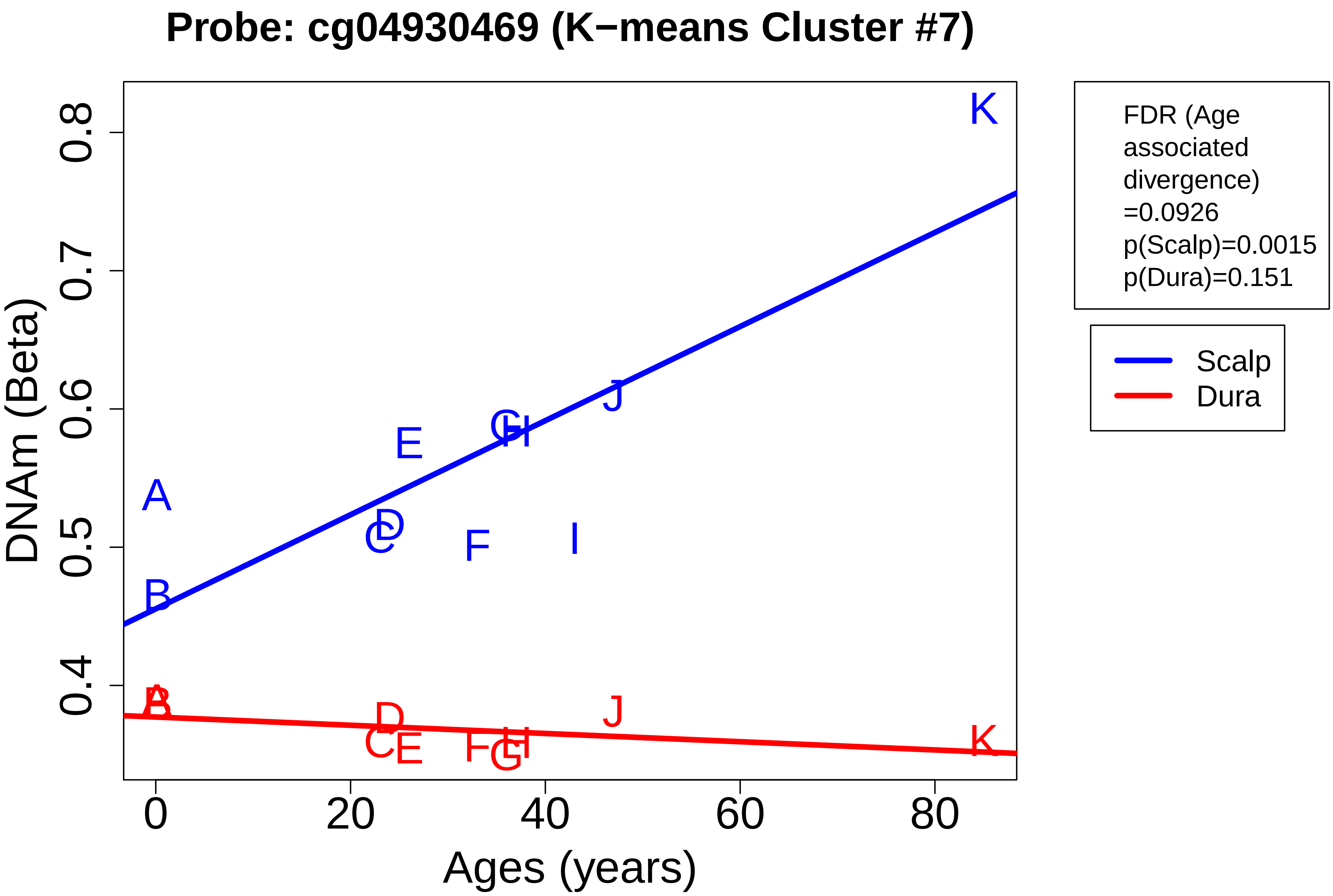

Probe: cg04930469 (K−means Cluster #7)
DNAm (Beta)
0.4
0.5
0.6
0.7
0.8
0
20
40
60
80
Ages (years)
FDR (Age associated divergence) =0.0926
p(Scalp)=0.0015
p(Dura)=0.151
Scalp
Dura

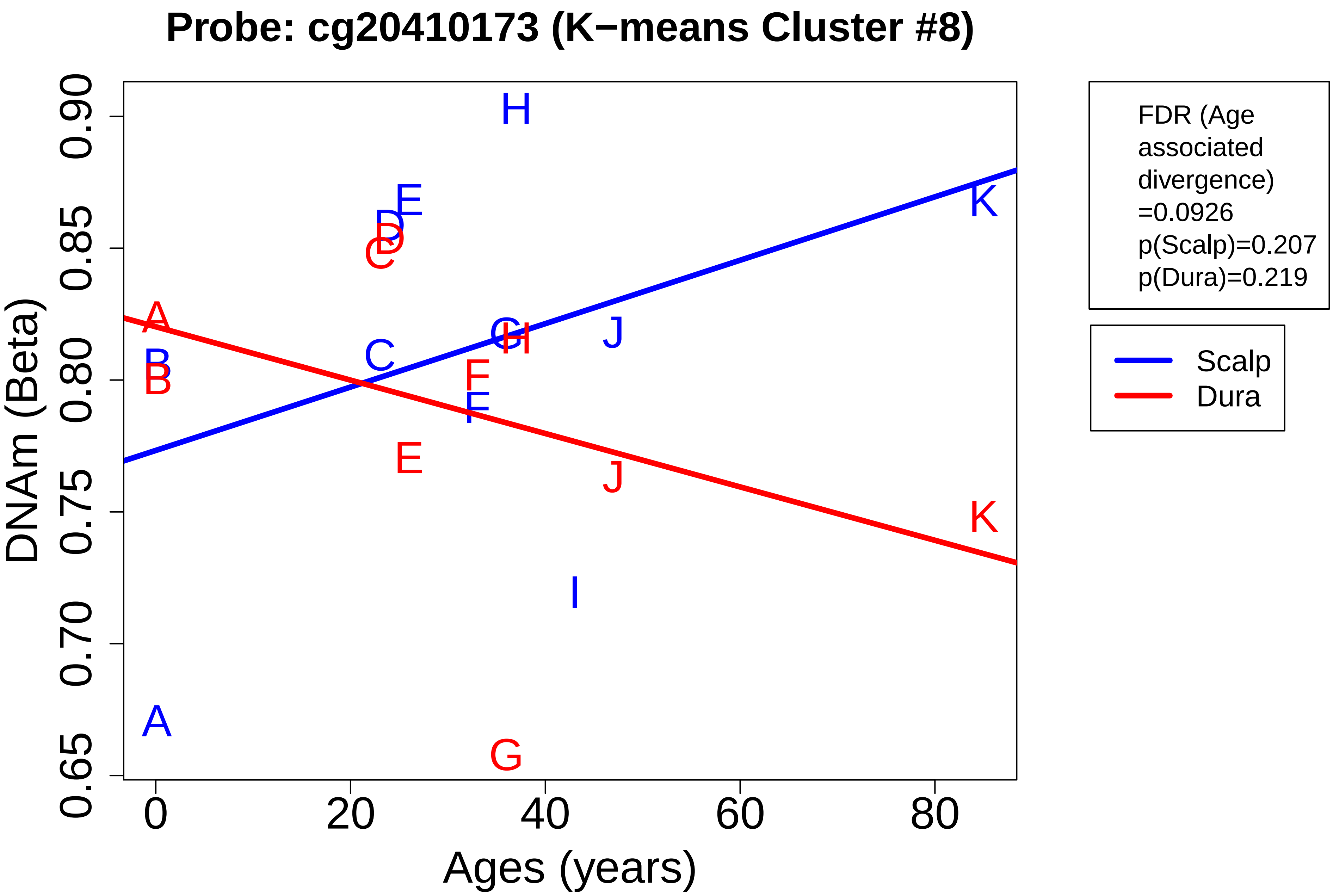
Probe: cg20410173 (K−means Cluster #8)
DNAm (Beta)
Ages (years)
0.65
0.70
0.75
0.80
0.85
0.90
0
20
40
60
80
FDR (Age associated divergence) =0.0926
p(Scalp)=0.207
p(Dura)=0.219
Scalp
Dura

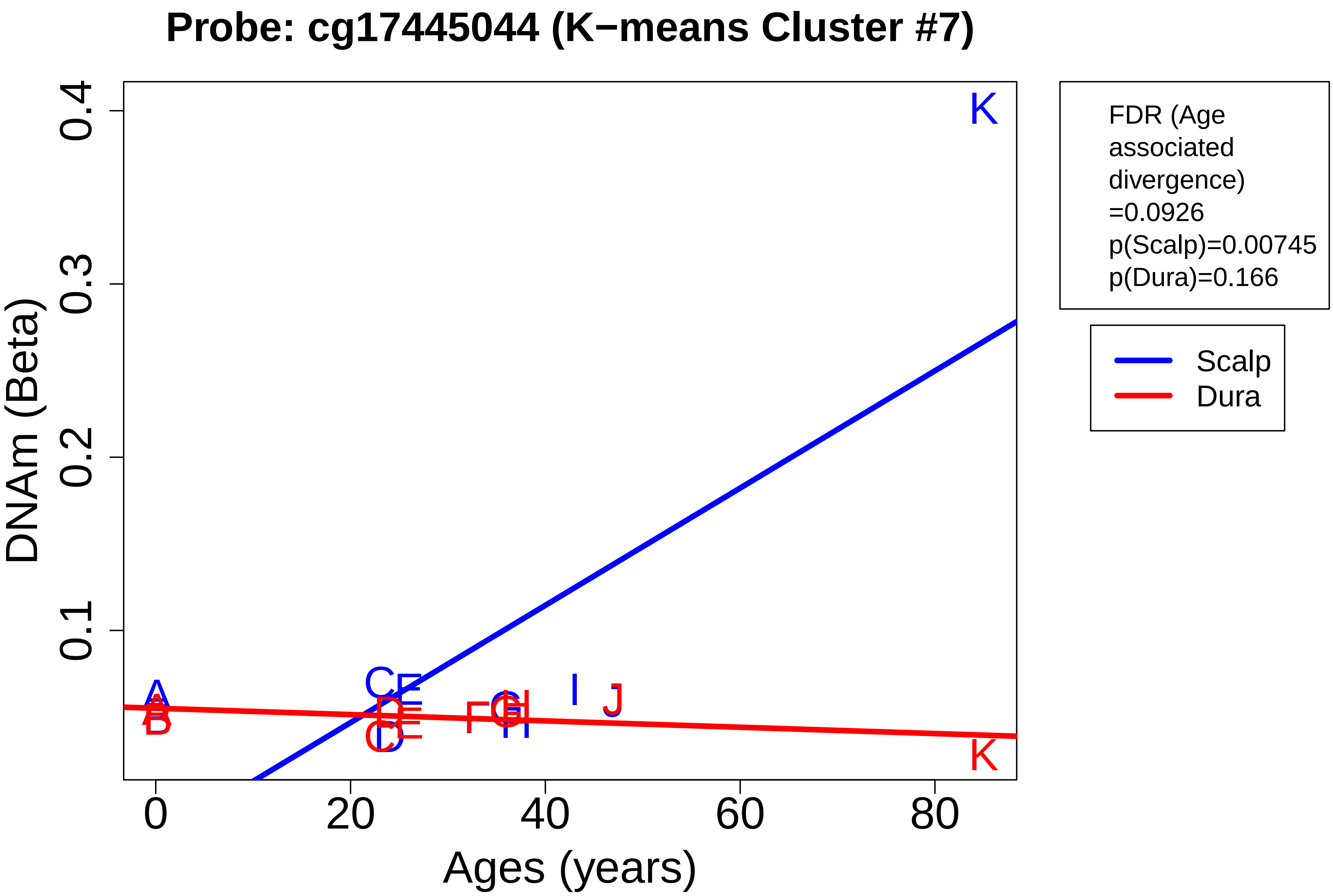

Probe: cg17445044 (K−means Cluster #7)
DNAm (Beta)
0.1
0.2
0.3
0.4
Ages (years)
0
20
40
60
80
FDR (Age associated divergence) =0.0926
p(Scalp)=0.00745
p(Dura)=0.166
Scalp
Dura

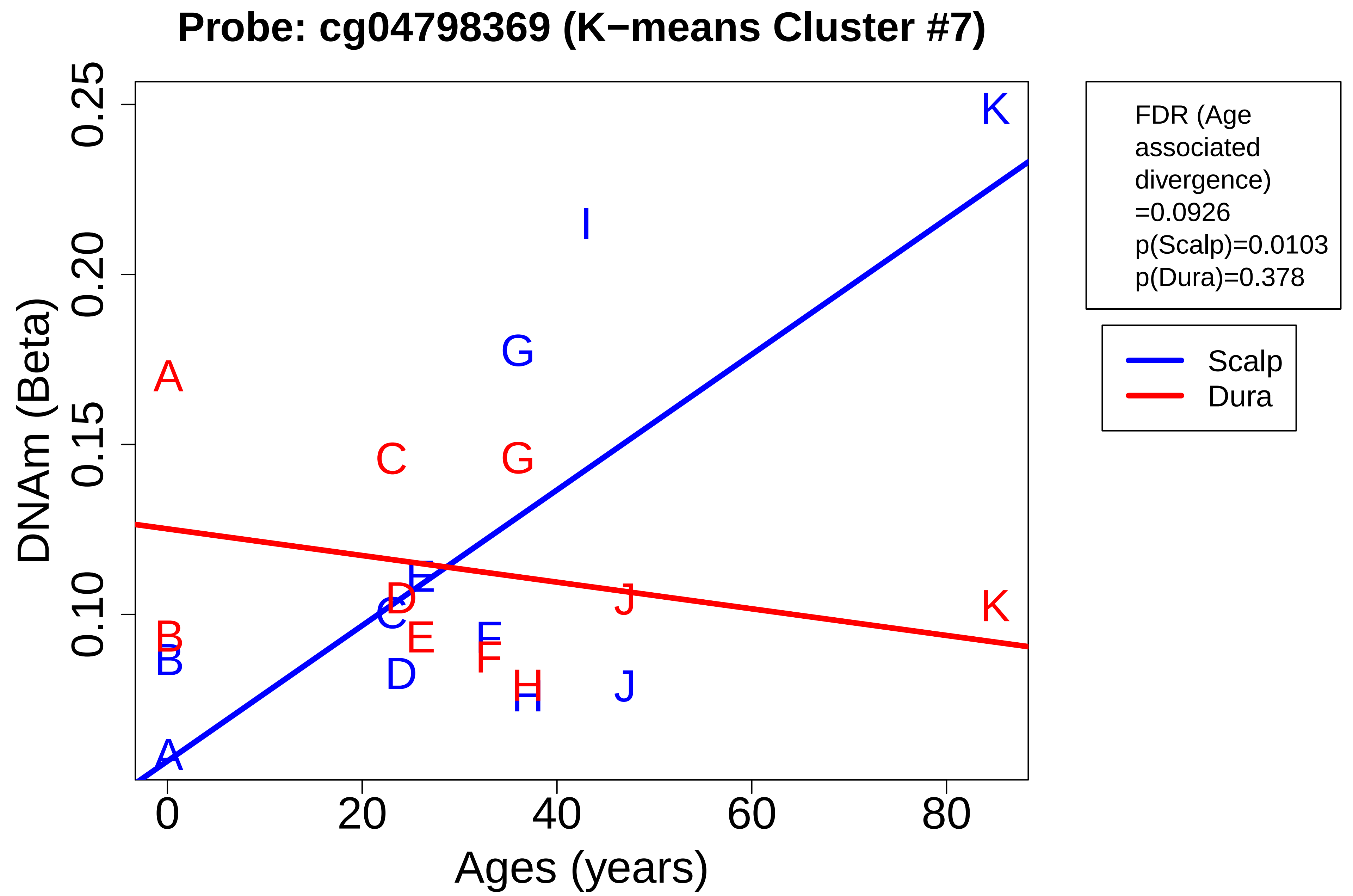

Probe: cg04798369 (K−means Cluster #7)
DNAm (Beta)
0.10
0.15
0.20
0.25
Ages (years)
0
20
40
60
80
FDR (Age associated divergence) =0.0926
p(Scalp)=0.0103
p(Dura)=0.378
Scalp
Dura

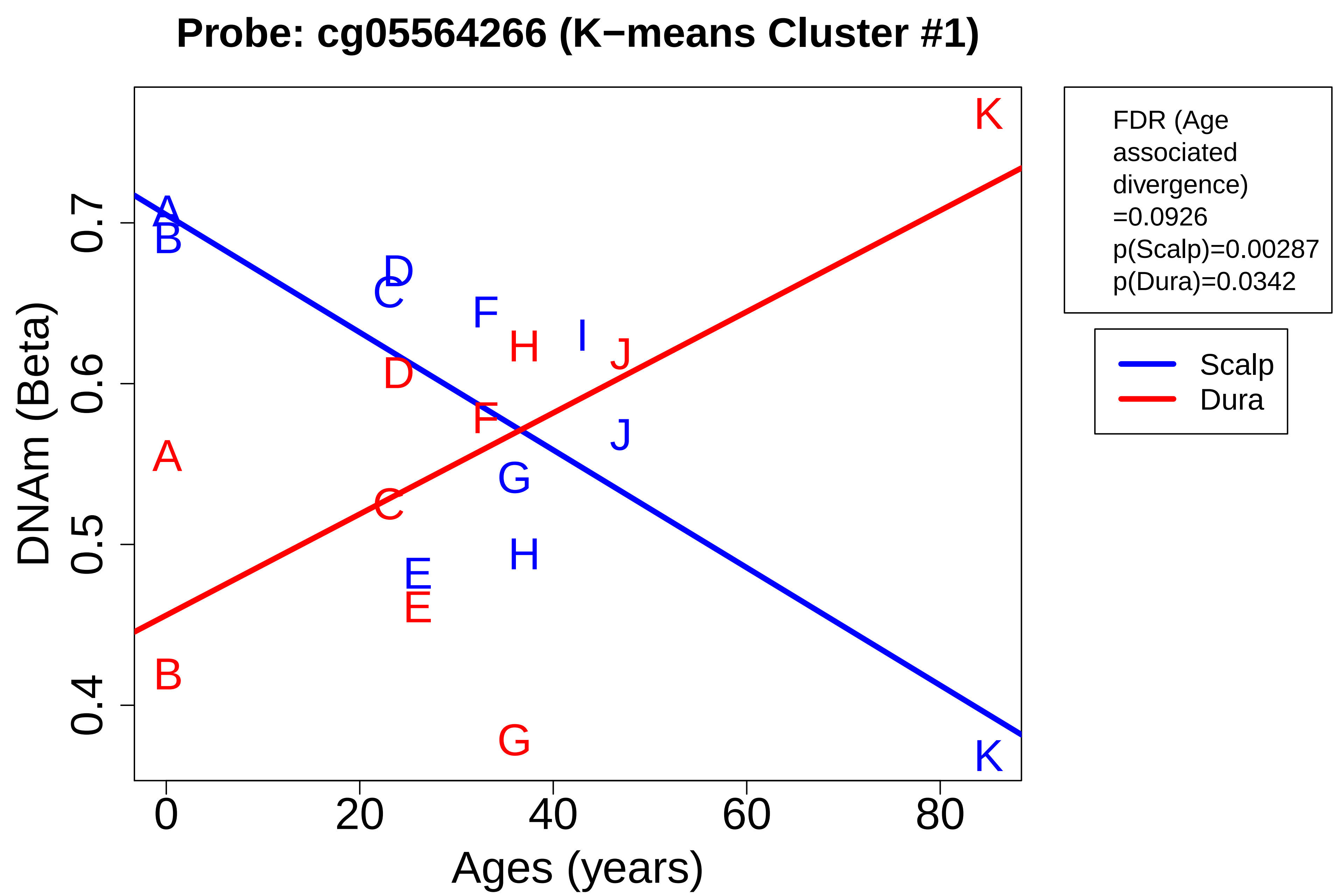
Probe: cg05564266 (K−means Cluster #1)
DNAm (Beta)
0.4
0.5
0.6
0.7
Ages (years)
0
20
40
60
80
A
B
C
D
E
F
G
H
I
J
K
FDR (Age associated divergence) =0.0926
p(Scalp)=0.00287
p(Dura)=0.0342
Scalp
Dura

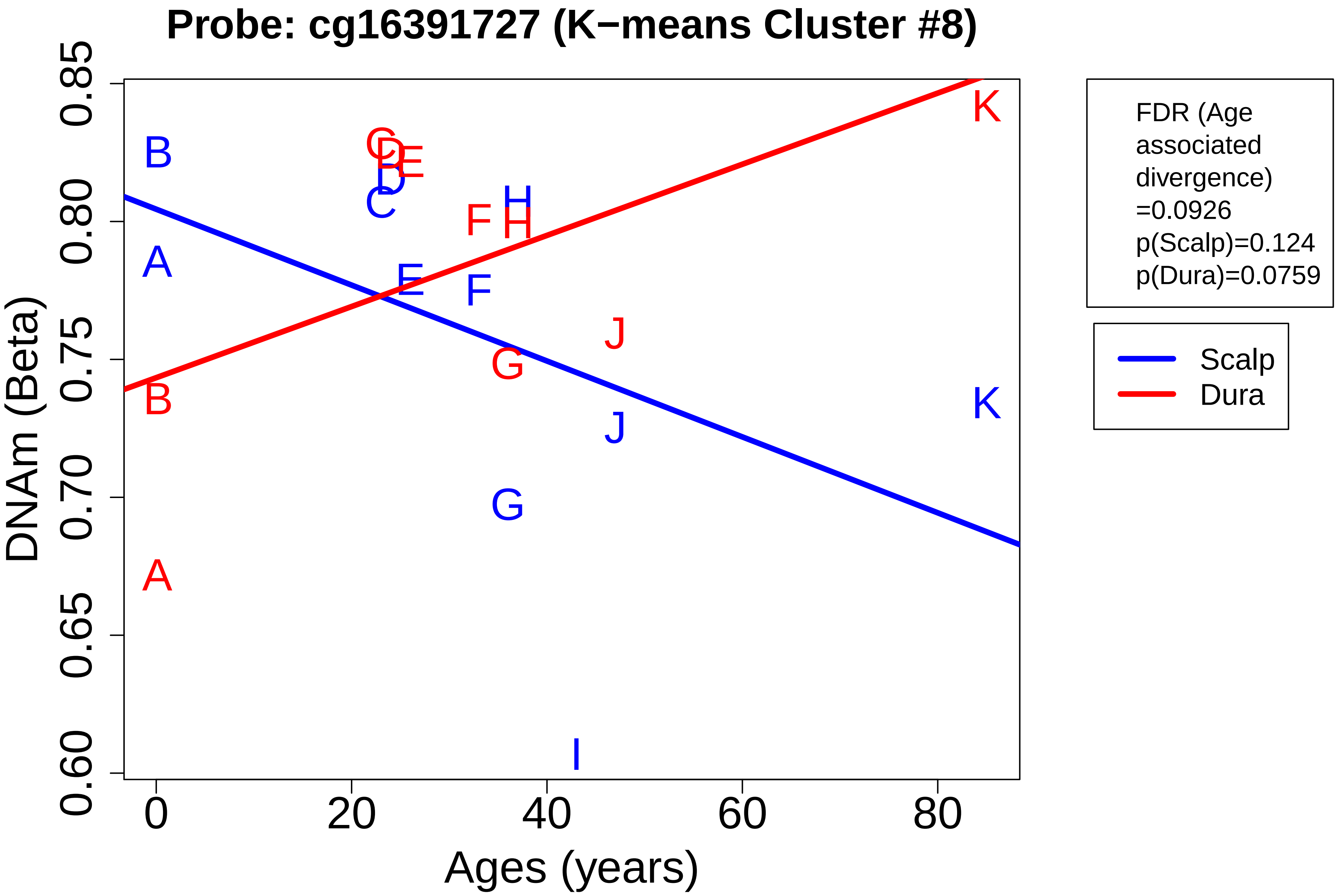

Probe: cg16391727 (K−means Cluster #8)
FDR (Age associated divergence) =0.0926
p(Scalp)=0.124
p(Dura)=0.0759
Scalp
Dura
DNAm (Beta)
Ages (years)
0.60
0.65
0.70
0.75
0.80
0.85
0
20
40
60
80

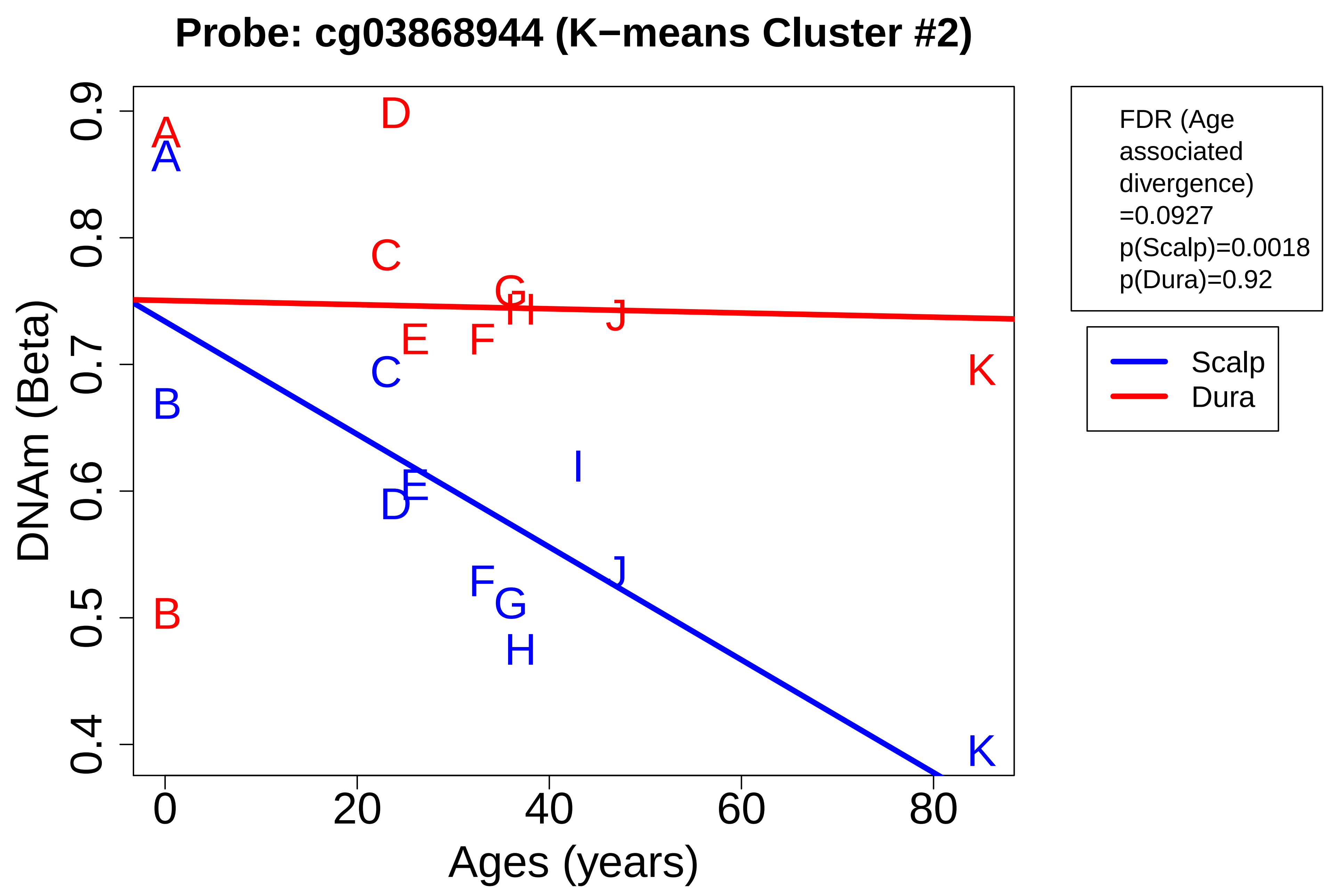

Probe: cg03868944 (K−means Cluster #2)
DNAm (Beta)
0.4
0.5
0.6
0.7
0.8
0.9
0
20
40
60
80
Ages (years)
A
A
B
B
C
C
D
D
E
E
F
F
G
G
H
H
I
J
J
K
K
FDR (Age associated divergence) =0.0927
p(Scalp)=0.0018
p(Dura)=0.92
Scalp
Dura

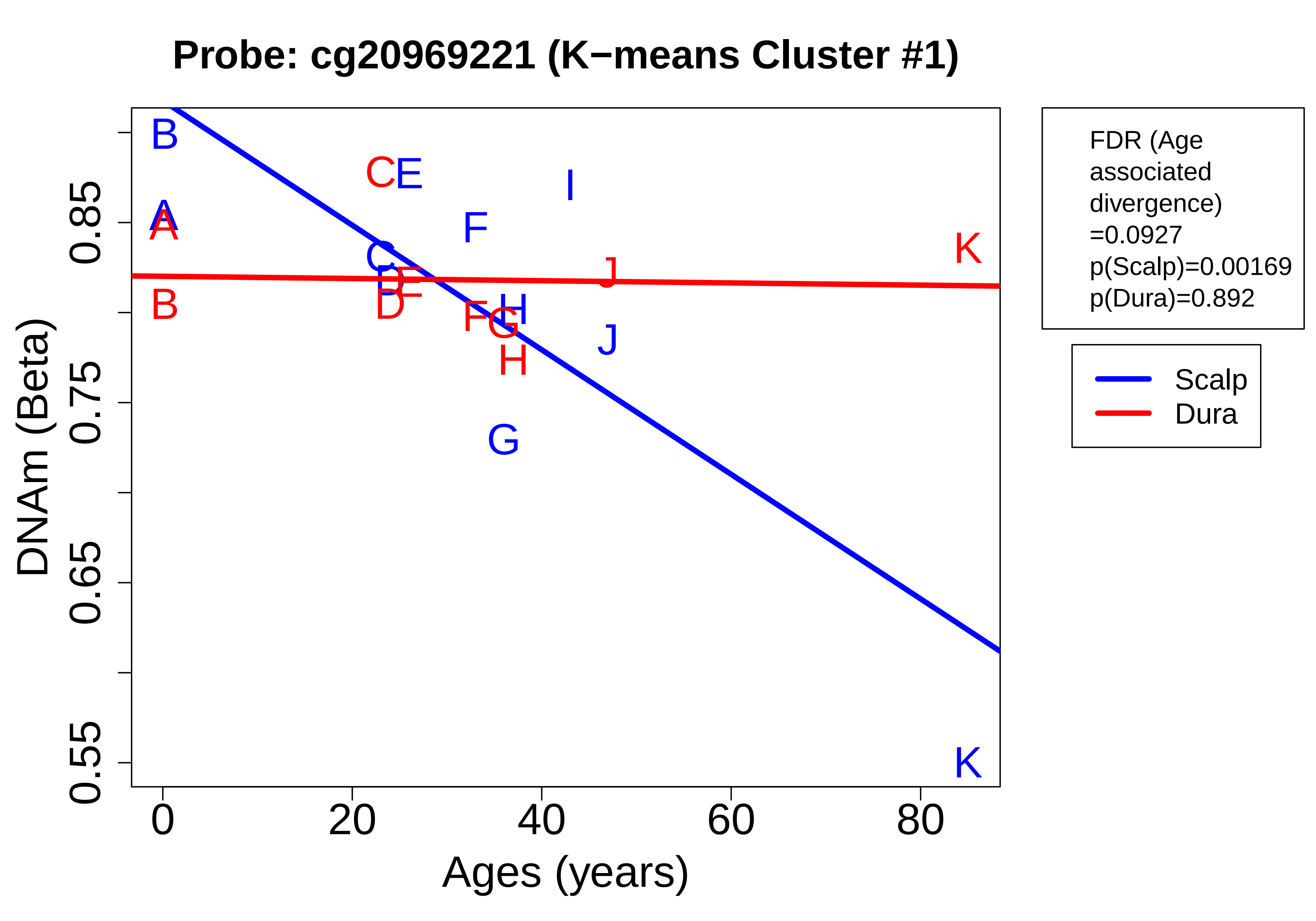

Probe: cg20969221 (K−means Cluster #1)
DNAm (Beta)
0.55
0.65
0.75
0.85
0
20
40
60
80
Ages (years)
FDR (Age associated divergence) =0.0927
p(Scalp)=0.00169
p(Dura)=0.892
Scalp
Dura

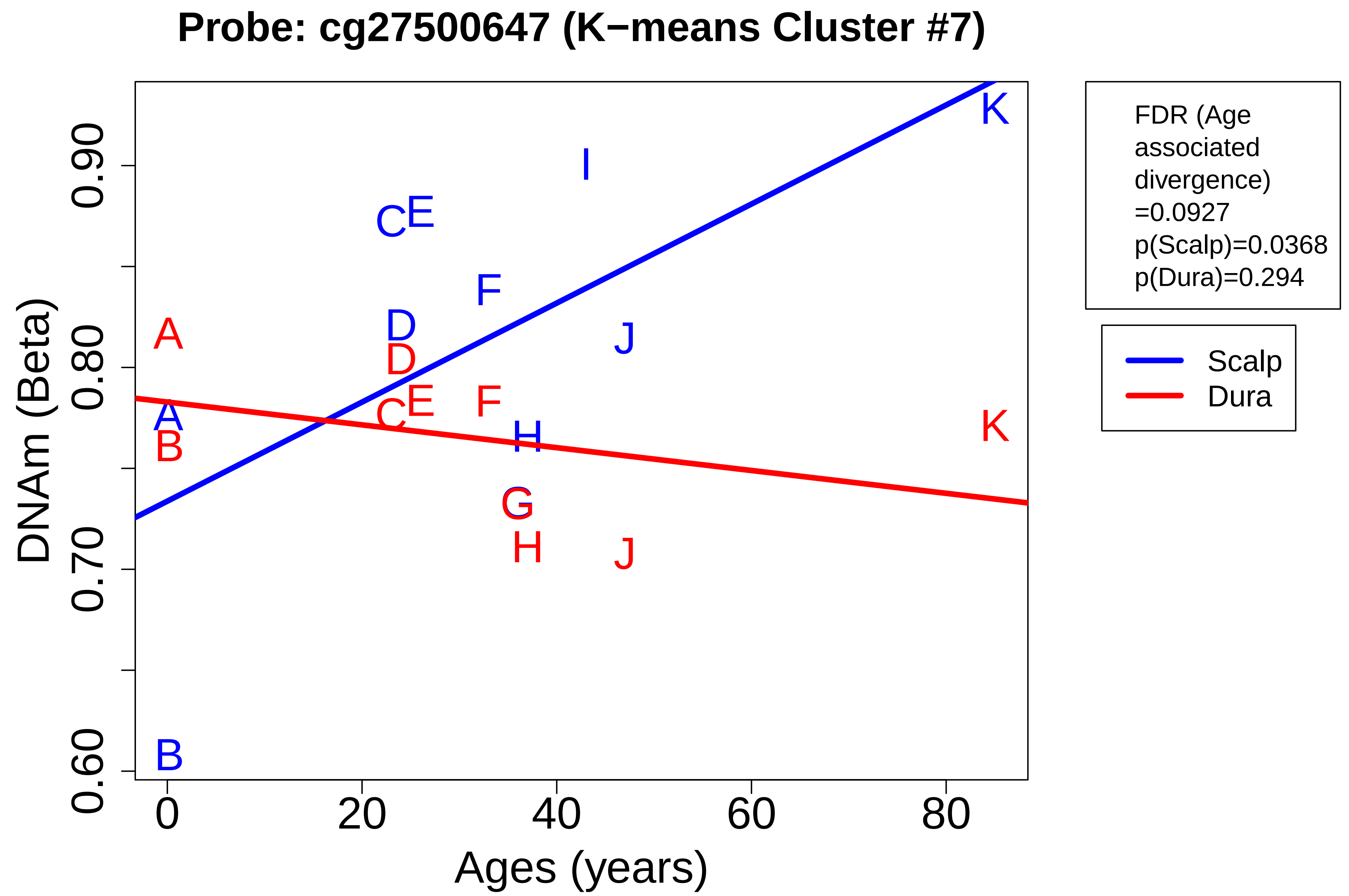

Probe: cg27500647 (K−means Cluster #7)
DNAm (Beta)
0.60
0.70
0.80
0.90
0
20
40
60
80
Ages (years)
A
B
C
D
E
F
G
H
I
J
K
FDR (Age associated divergence) =0.0927
p(Scalp)=0.0368
p(Dura)=0.294
Scalp
Dura

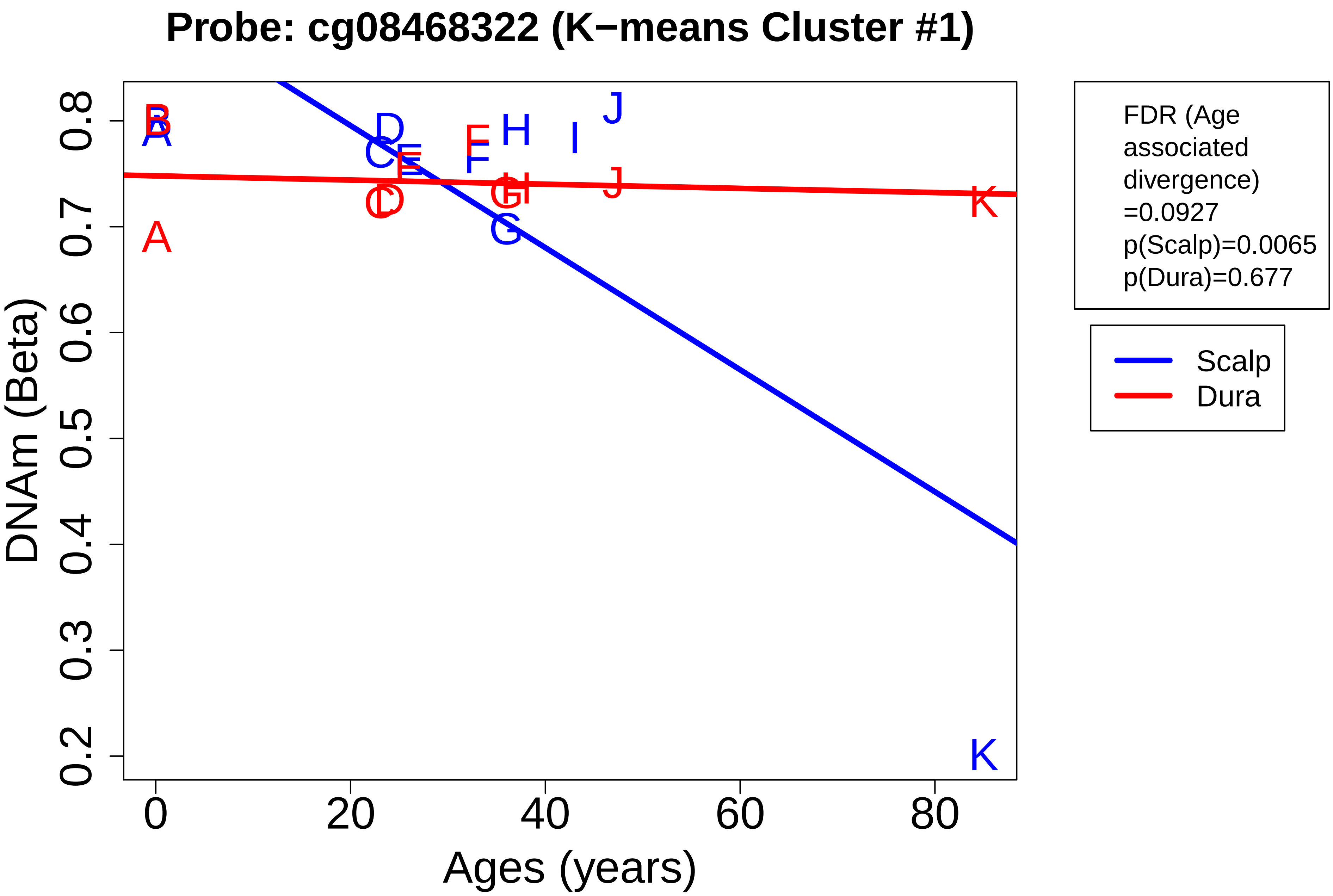

Probe: cg08468322 (K−means Cluster #1)
DNAm (Beta)
0.2
0.3
0.4
0.5
0.6
0.7
0.8
0
20
40
60
80
Ages (years)
FDR (Age associated divergence) =0.0927
p(Scalp)=0.0065
p(Dura)=0.677
Scalp
Dura

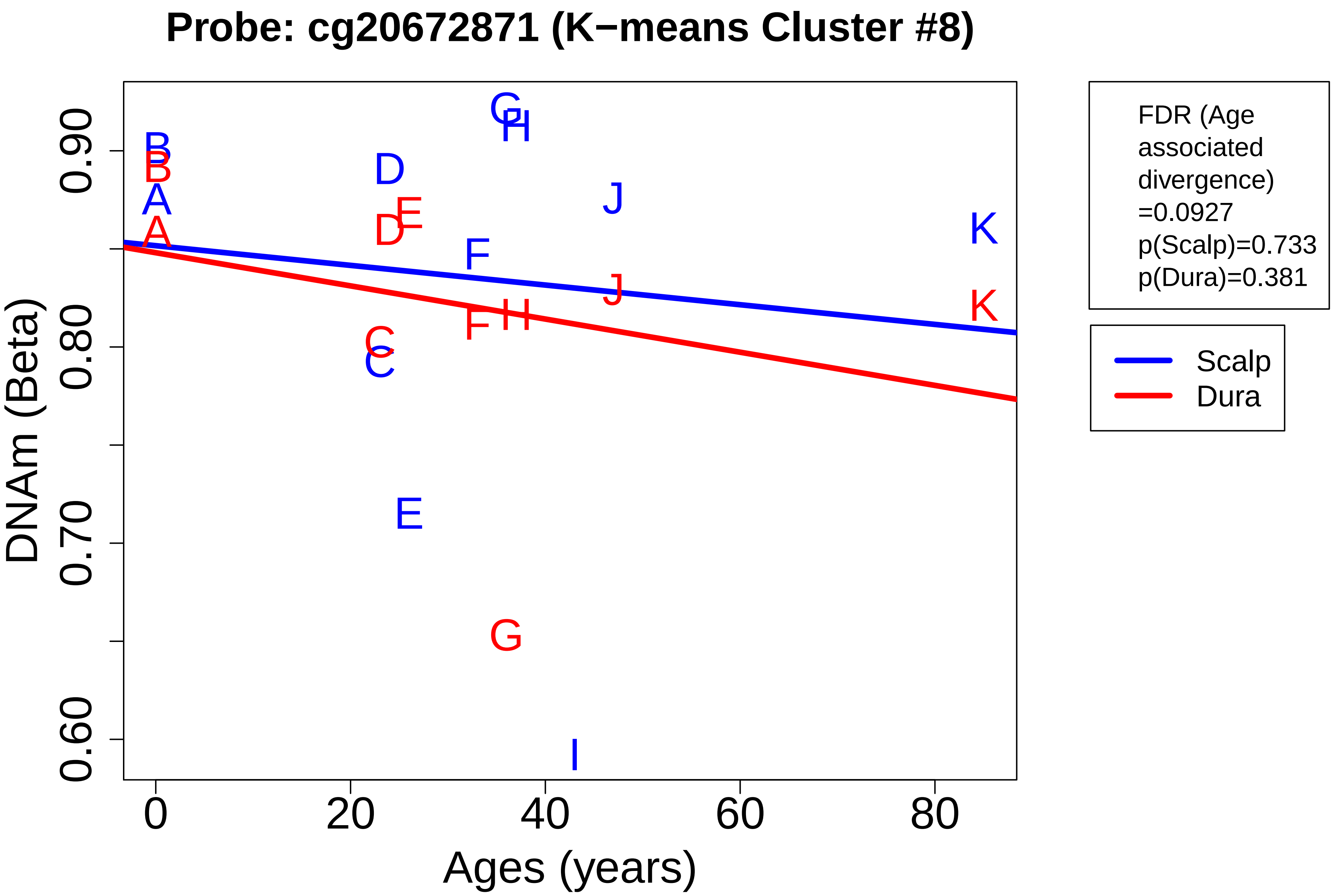

Probe: cg20672871 (K−means Cluster #8)
DNAm (Beta)
0.60
0.70
0.80
0.90
Ages (years)
0
20
40
60
80
FDR (Age associated divergence) =0.0927
p(Scalp)=0.733
p(Dura)=0.381
Scalp
Dura

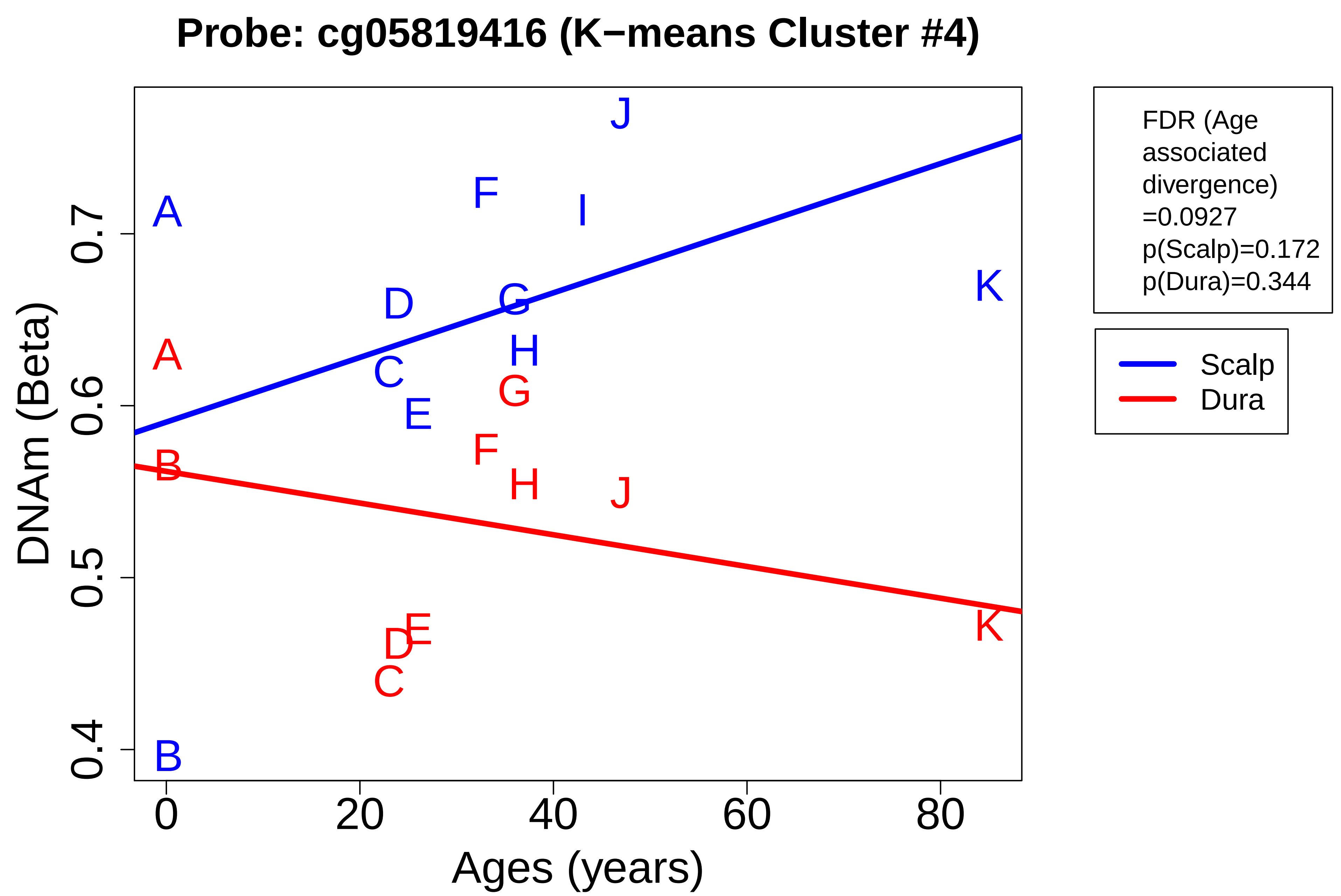

Probe: cg05819416 (K−means Cluster #4)
DNAm (Beta)
0.4
0.5
0.6
0.7
0
20
40
60
80
Ages (years)
A
B
C
D
E
F
G
H
I
J
K
A
B
C
D
E
F
G
H
J
K
FDR (Age associated divergence) =0.0927
p(Scalp)=0.172
p(Dura)=0.344
Scalp
Dura

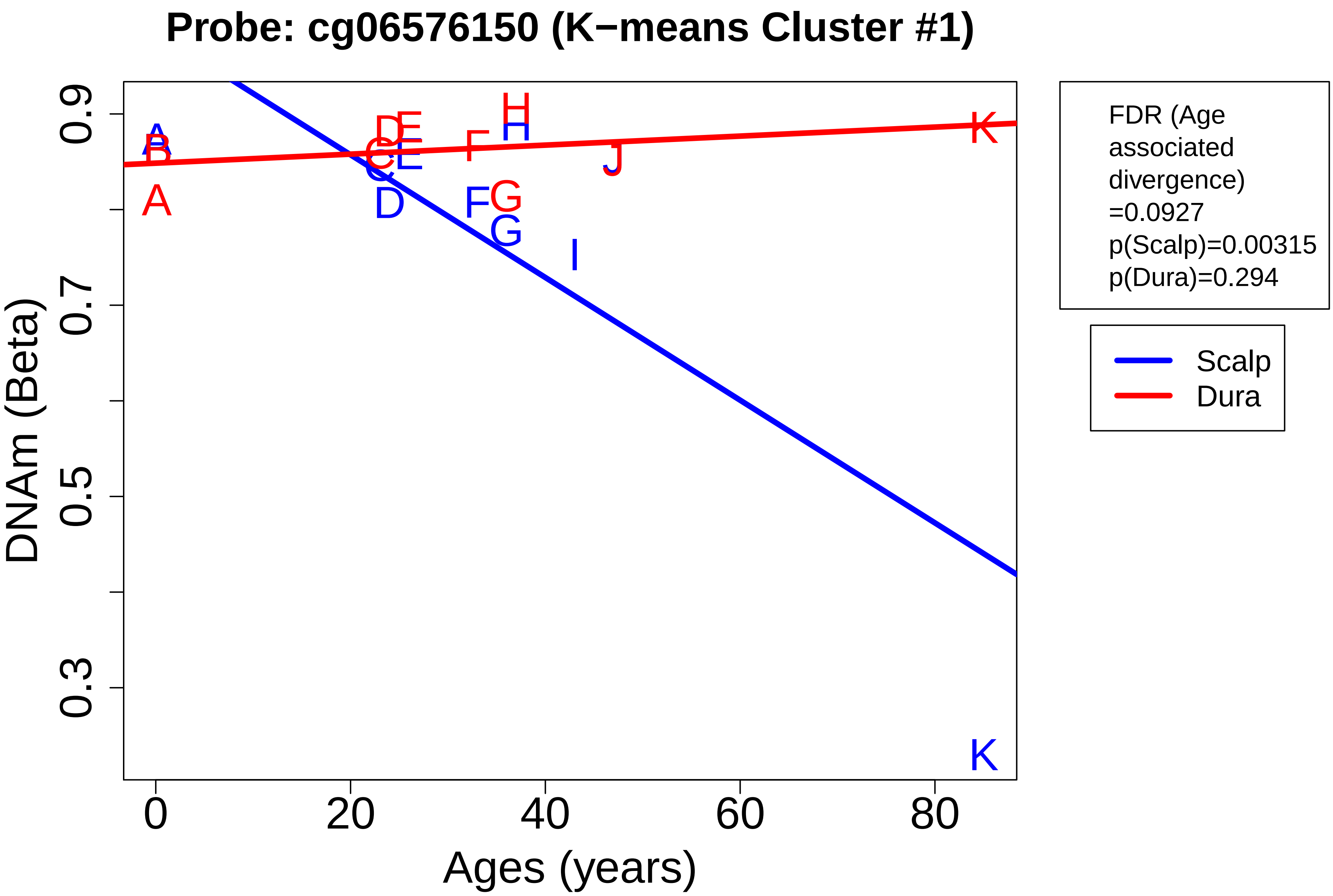

Probe: cg06576150 (K−means Cluster #1)
DNAm (Beta)
0.3
0.5
0.7
0.9
Ages (years)
0
20
40
60
80
FDR (Age associated divergence) =0.0927
p(Scalp)=0.00315
p(Dura)=0.294
Scalp
Dura

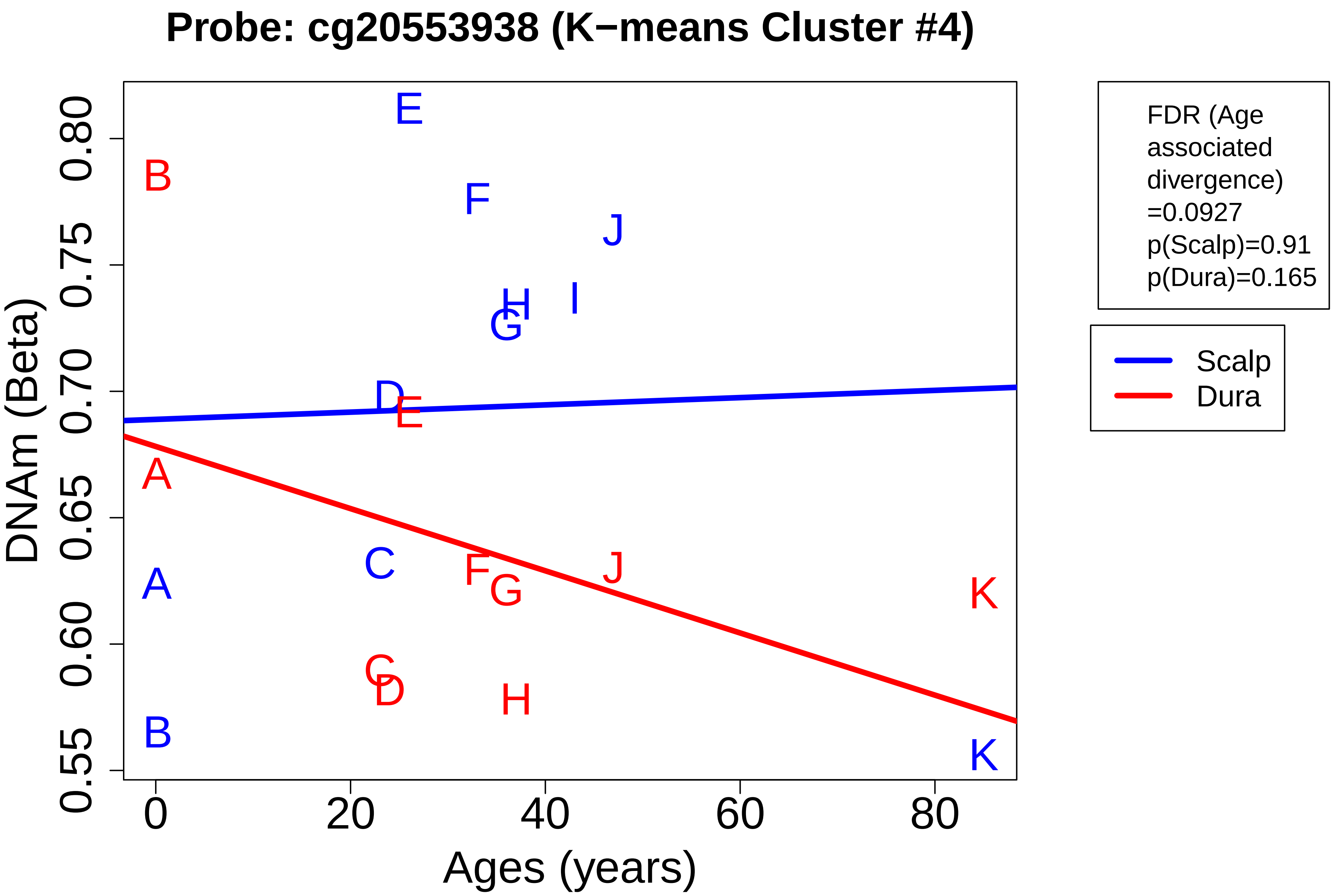
Probe: cg20553938 (K−means Cluster #4)
DNAm (Beta)
0.55
0.60
0.65
0.70
0.75
0.80
0
20
40
60
80
Ages (years)
FDR (Age associated divergence) =0.0927
p(Scalp)=0.91
p(Dura)=0.165
Scalp
Dura

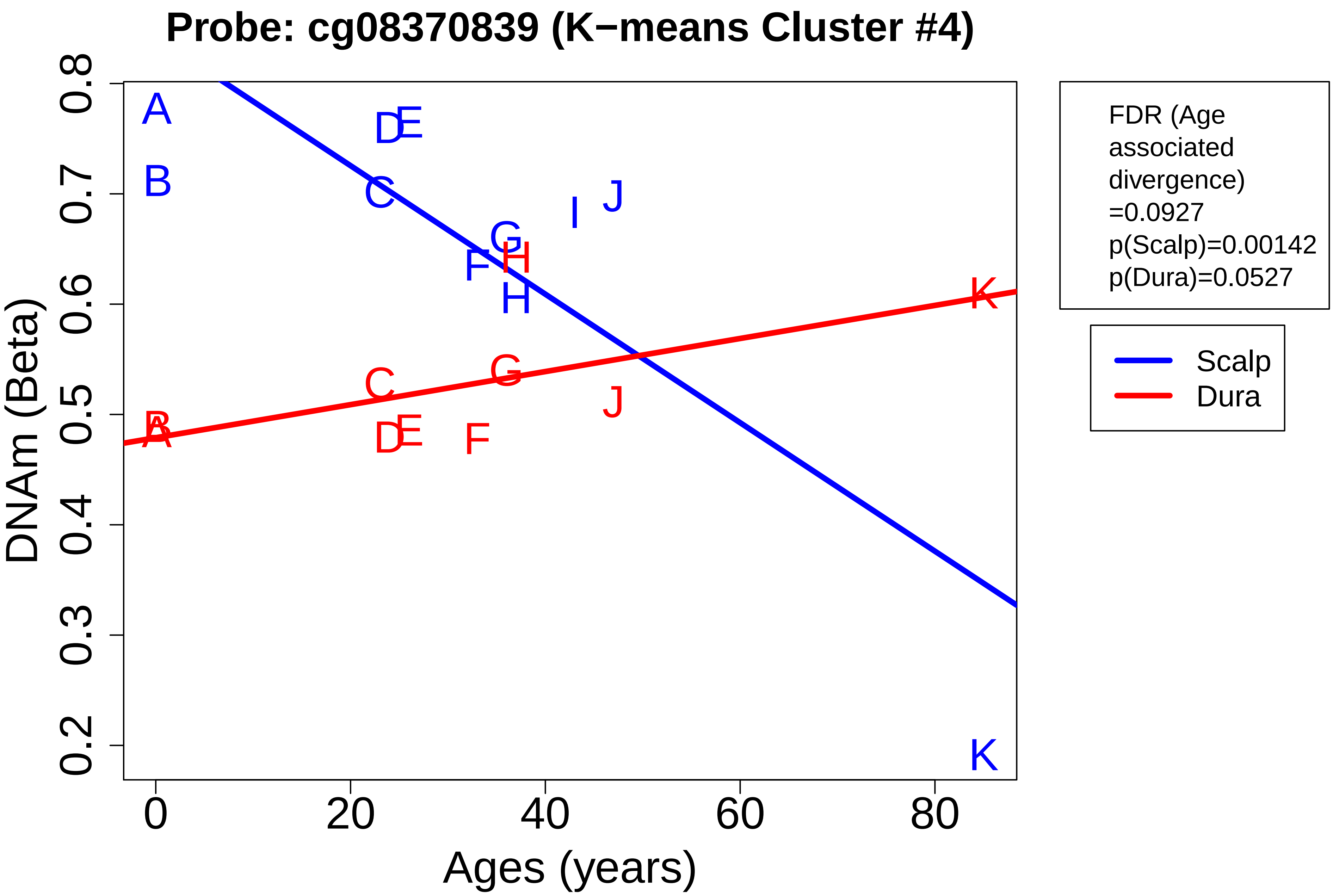
Probe: cg08370839 (K−means Cluster #4)
DNAm (Beta)
Ages (years)
FDR (Age associated divergence) =0.0927
p(Scalp)=0.00142
p(Dura)=0.0527
Scalp
Dura

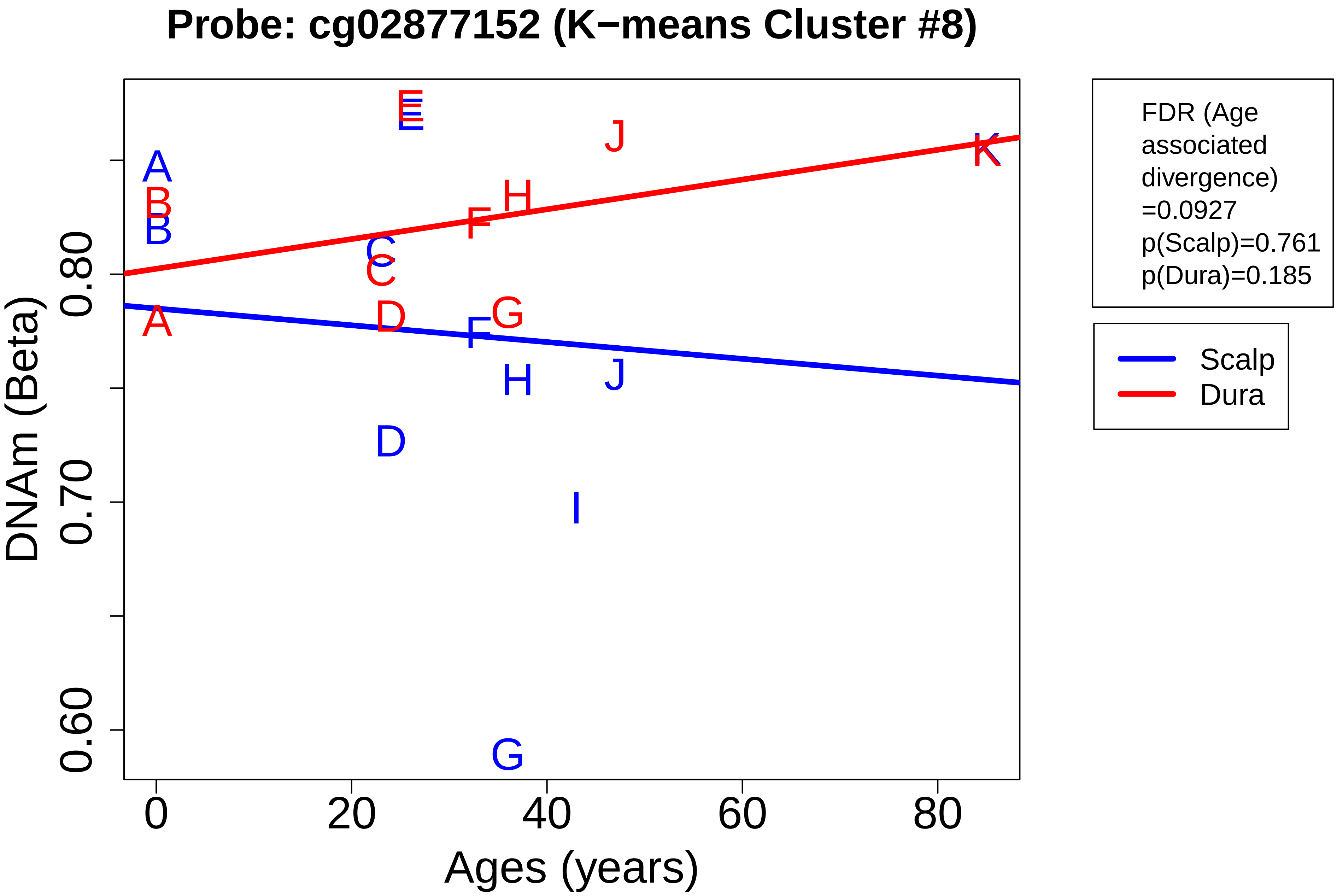
Probe: cg02877152 (K−means Cluster #8)
DNAm (Beta)
0.60
0.70
0.80
0
20
40
60
80
Ages (years)
FDR (Age associated divergence) =0.0927
p(Scalp)=0.761
p(Dura)=0.185
Scalp
Dura
A
B
C
D
E
F
G
H
I
J
K

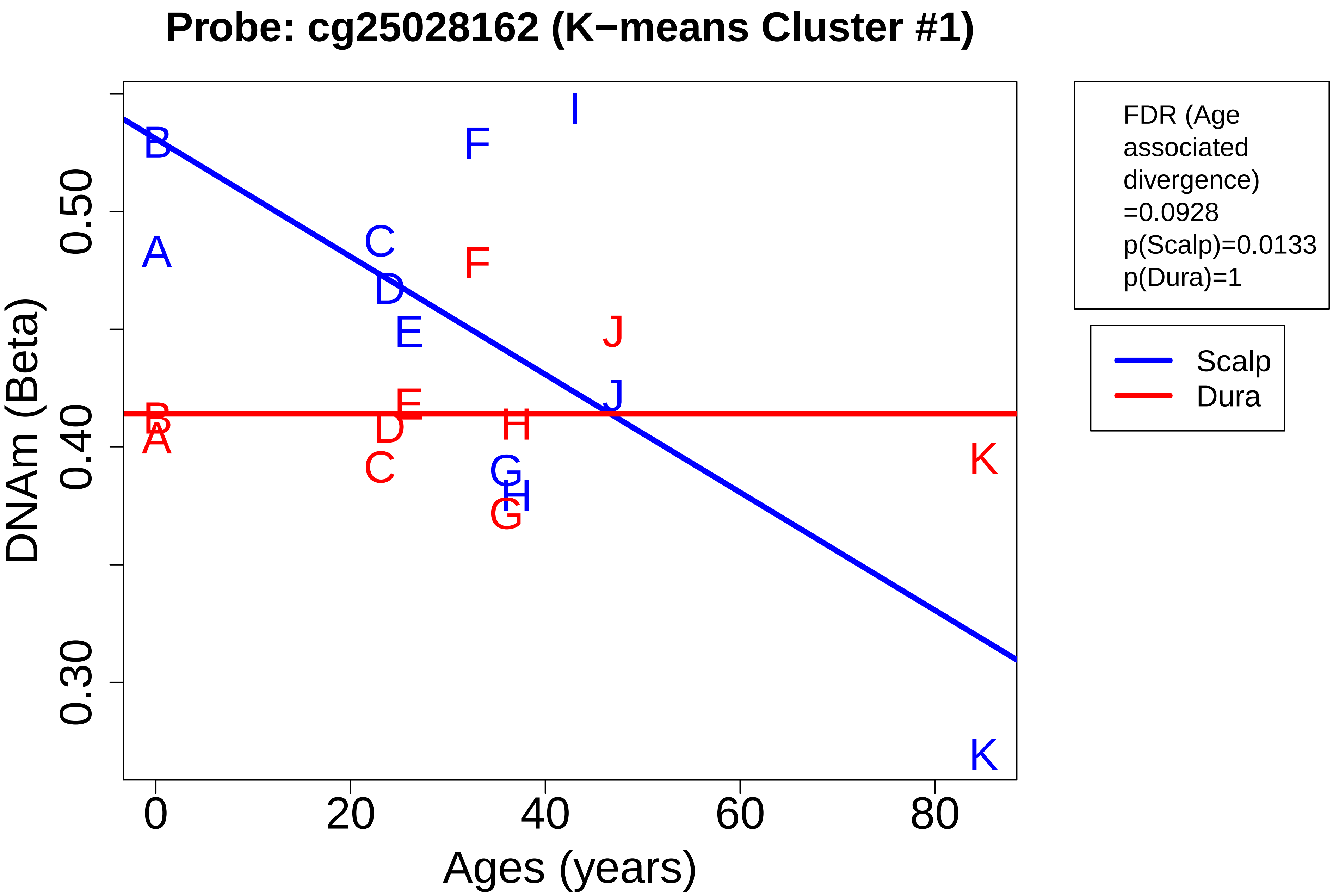

Probe: cg25028162 (K−means Cluster #1)
DNAm (Beta)
0.30
0.40
0.50
0
20
40
60
80
Ages (years)
FDR (Age associated divergence) =0.0928
p(Scalp)=0.0133
p(Dura)=1
Scalp
Dura
A
B
C
D
E
F
G
H
I
J
K

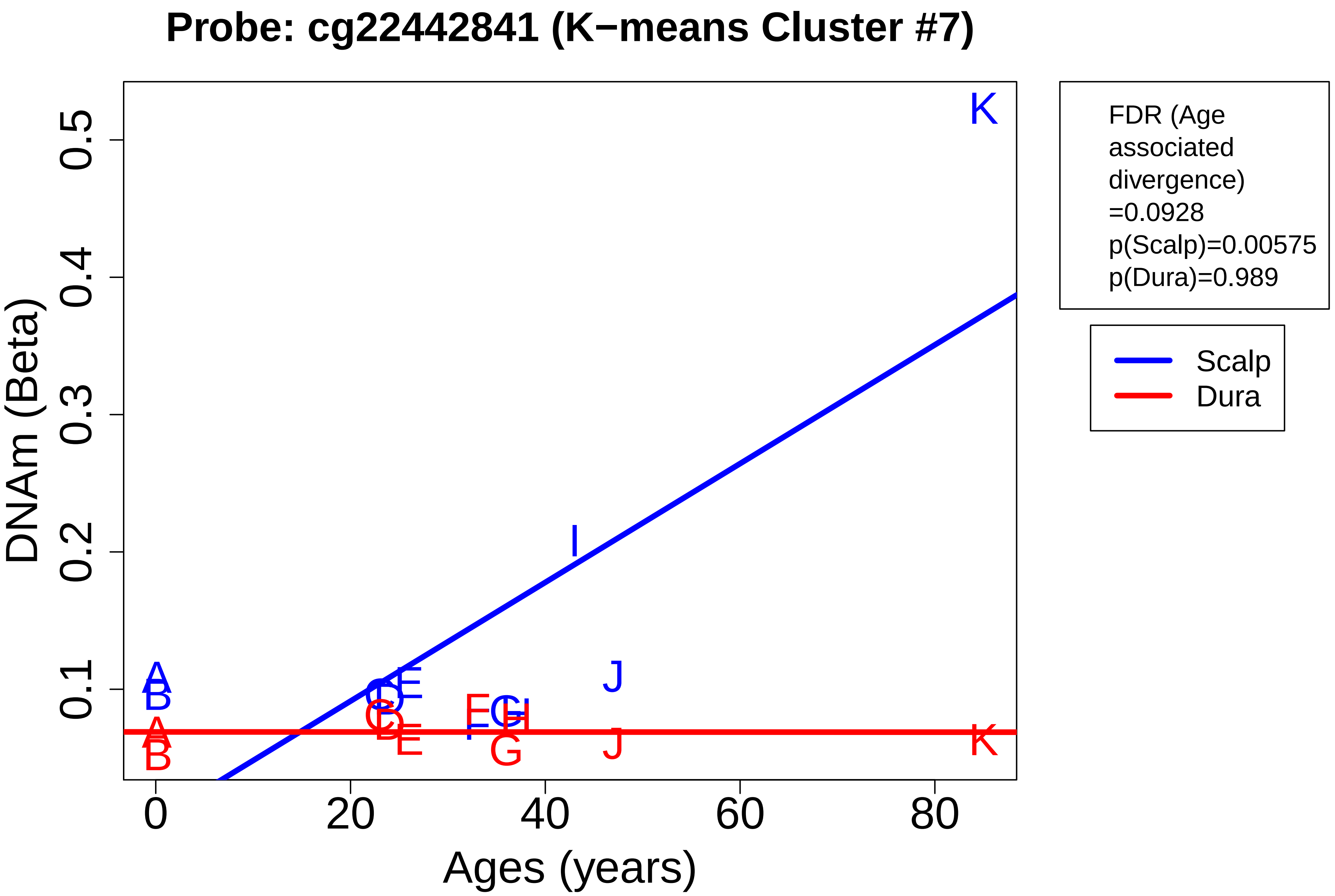

Probe: cg22442841 (K−means Cluster #7)
DNAm (Beta)
0.1
0.2
0.3
0.4
0.5
Ages (years)
0
20
40
60
80
FDR (Age associated divergence) =0.0928
p(Scalp)=0.00575
p(Dura)=0.989
Scalp
Dura

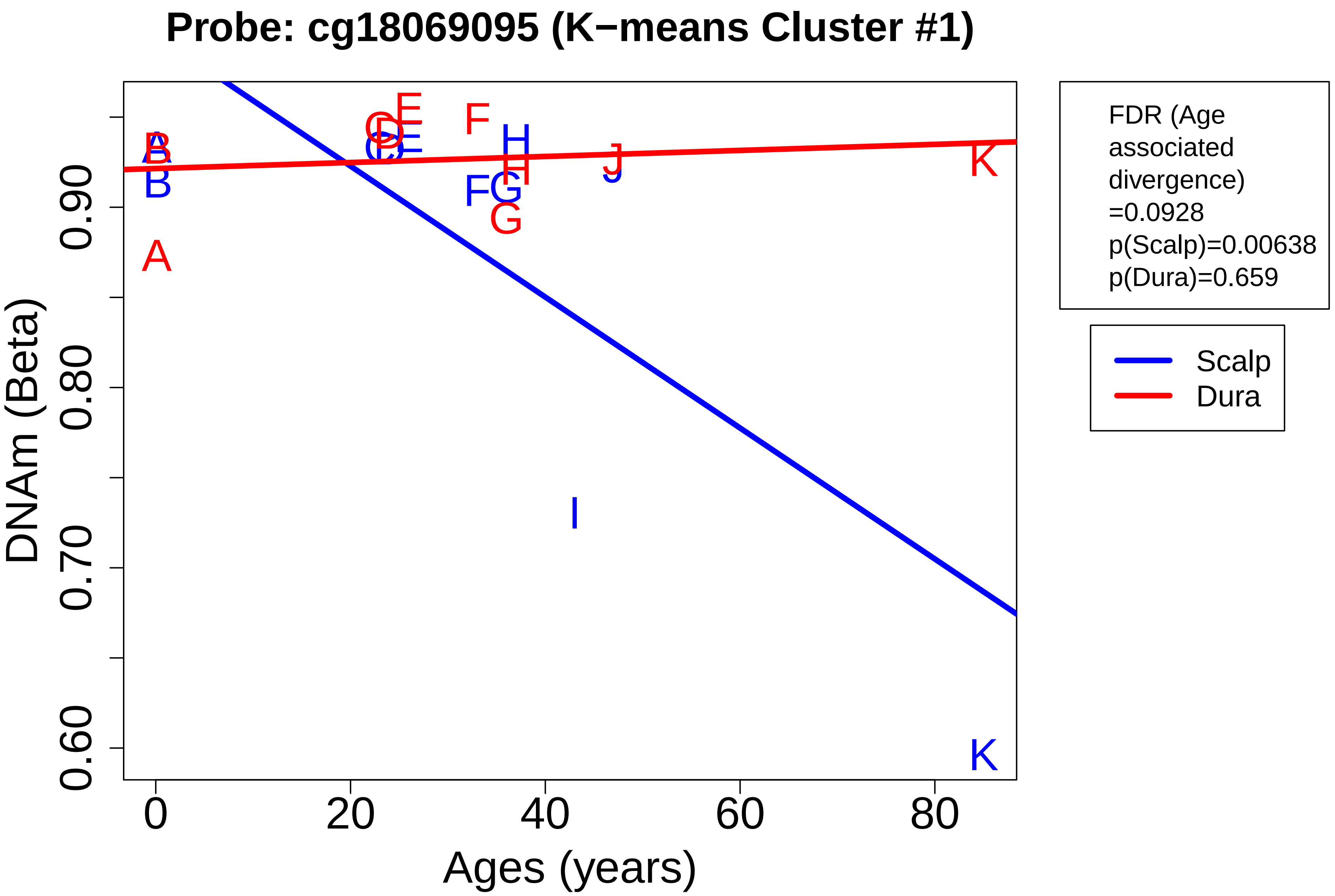

Probe: cg18069095 (K−means Cluster #1)
DNAm (Beta)
0.60
0.70
0.80
0.90
Ages (years)
0
20
40
60
80
FDR (Age associated divergence) =0.0928
p(Scalp)=0.00638
p(Dura)=0.659
Scalp
Dura

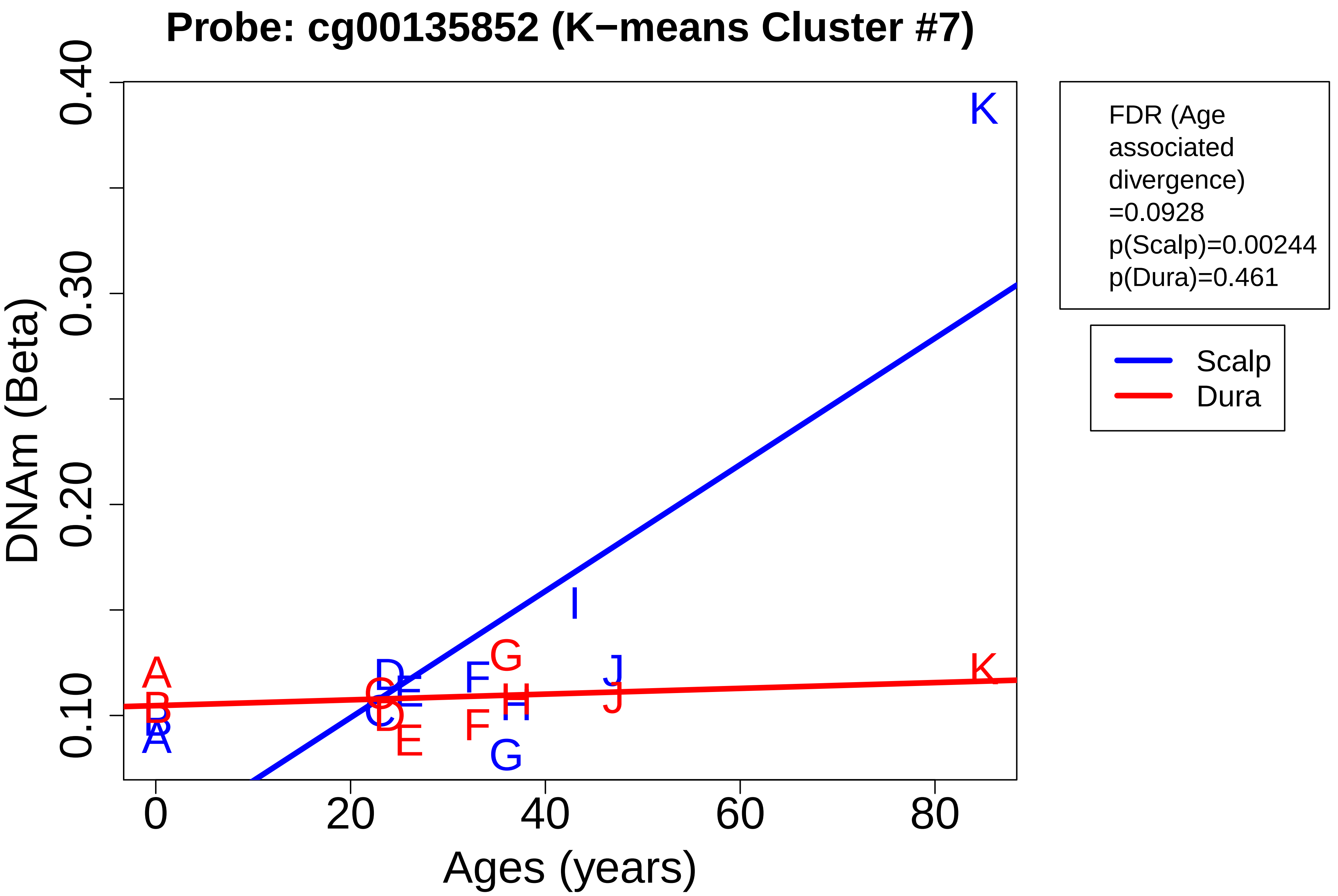

Probe: cg00135852 (K−means Cluster #7)
DNAm (Beta)
0.10
0.20
0.30
0.40
Ages (years)
0
20
40
60
80
FDR (Age associated divergence) =0.0928
p(Scalp)=0.00244
p(Dura)=0.461
Scalp
Dura
A
B
C
D
E
F
G
H
I
J
K

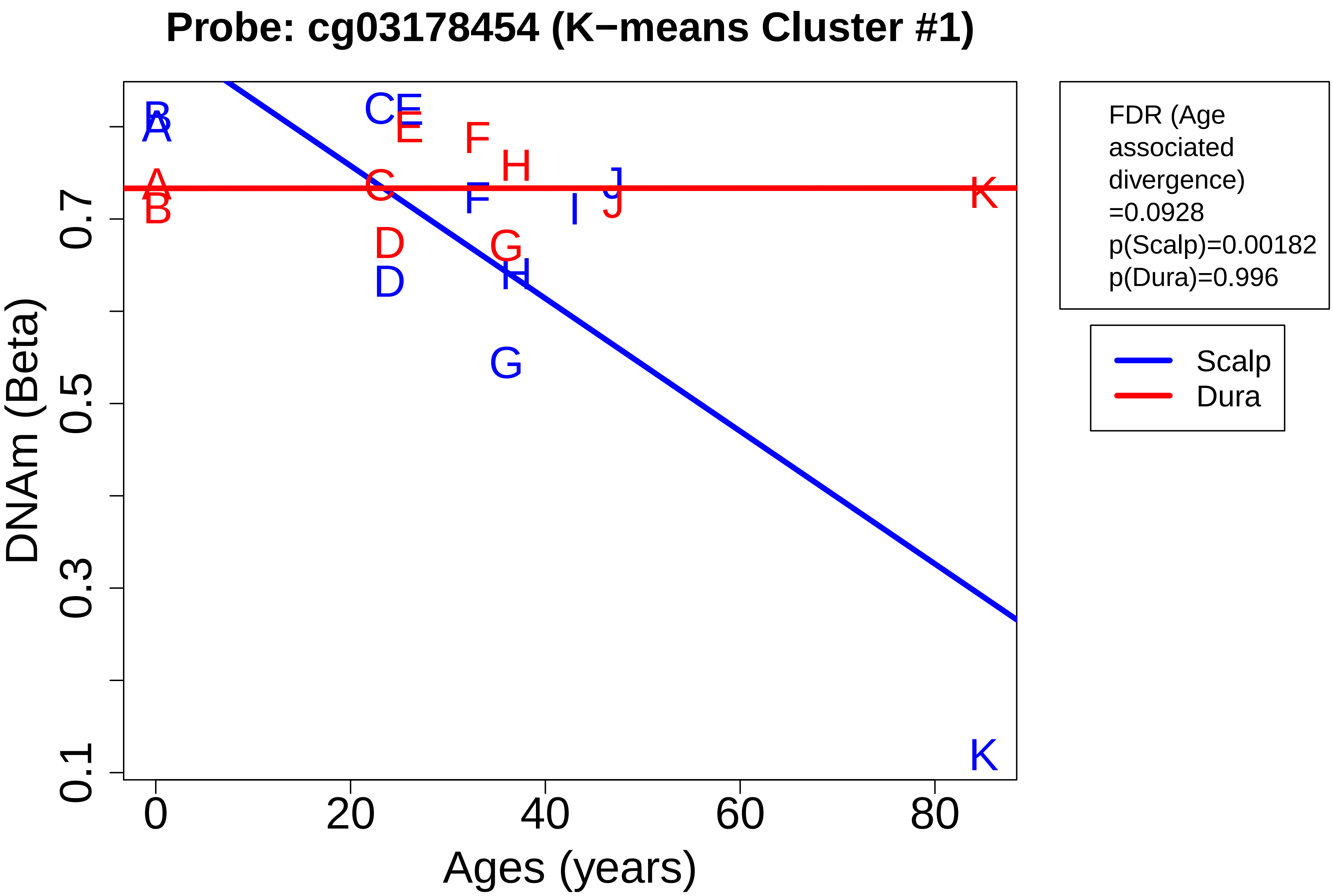
Probe: cg03178454 (K−means Cluster #1)
DNAm (Beta)
0.1
0.3
0.5
0.7
0
20
40
60
80
Ages (years)
A B C D E F G H I J K
FDR (Age associated divergence) =0.0928
p(Scalp)=0.00182
p(Dura)=0.996
Scalp
Dura

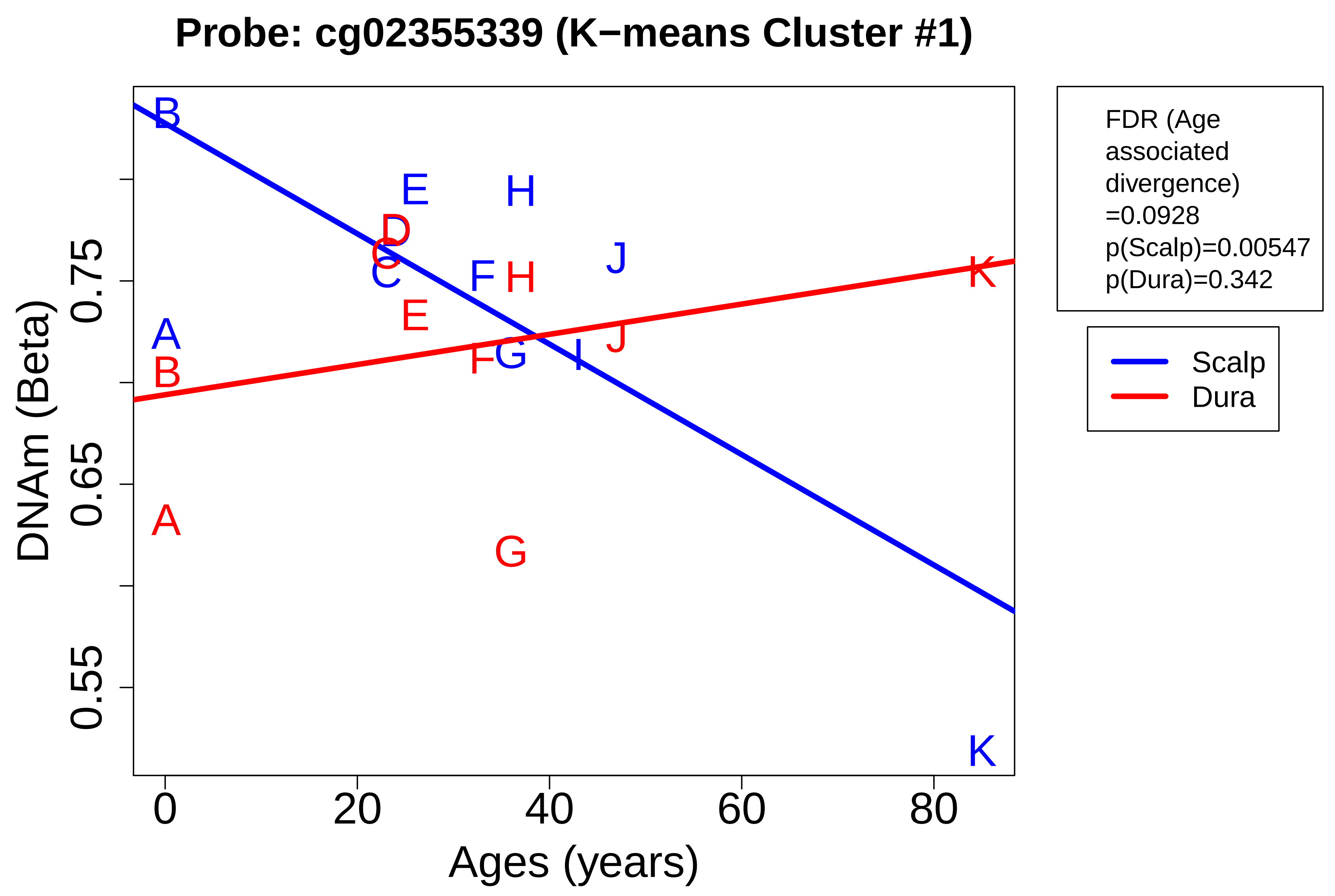

Probe: cg02355339 (K−means Cluster #1)
DNAm (Beta)
0.55
0.65
0.75
Ages (years)
0
20
40
60
80
B
E
H
D
C
C
F
H
J
K
E
A
J
B
F
G
I
A
G
K
FDR (Age associated divergence) =0.0928
p(Scalp)=0.00547
p(Dura)=0.342
Scalp
Dura

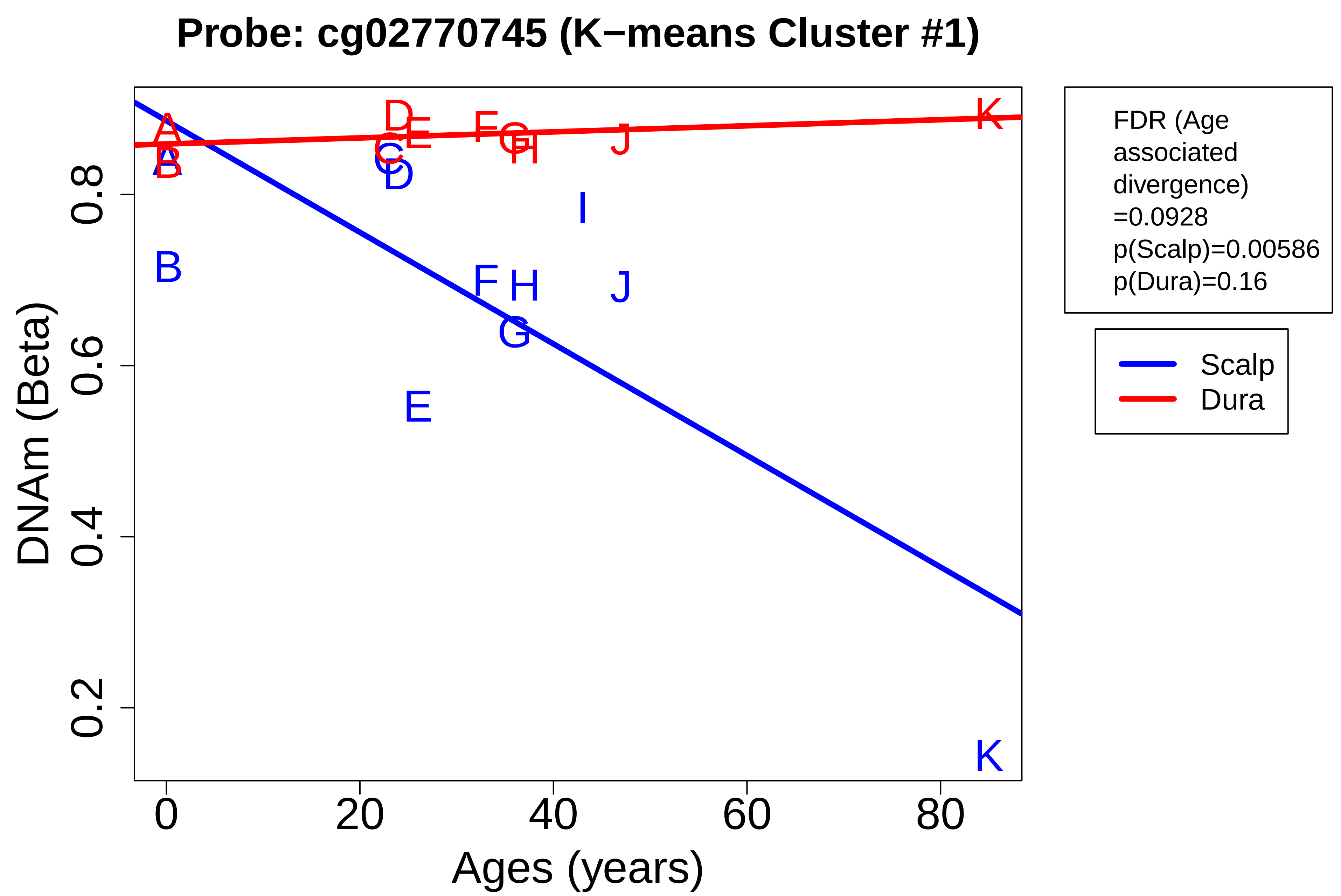

Probe: cg02770745 (K−means Cluster #1)
DNAm (Beta)
0.2
0.4
0.6
0.8
0
20
40
60
80
Ages (years)
A
B
C
D
E
F
G
H
I
J
K
FDR (Age associated divergence) =0.0928
p(Scalp)=0.00586
p(Dura)=0.16
Scalp
Dura

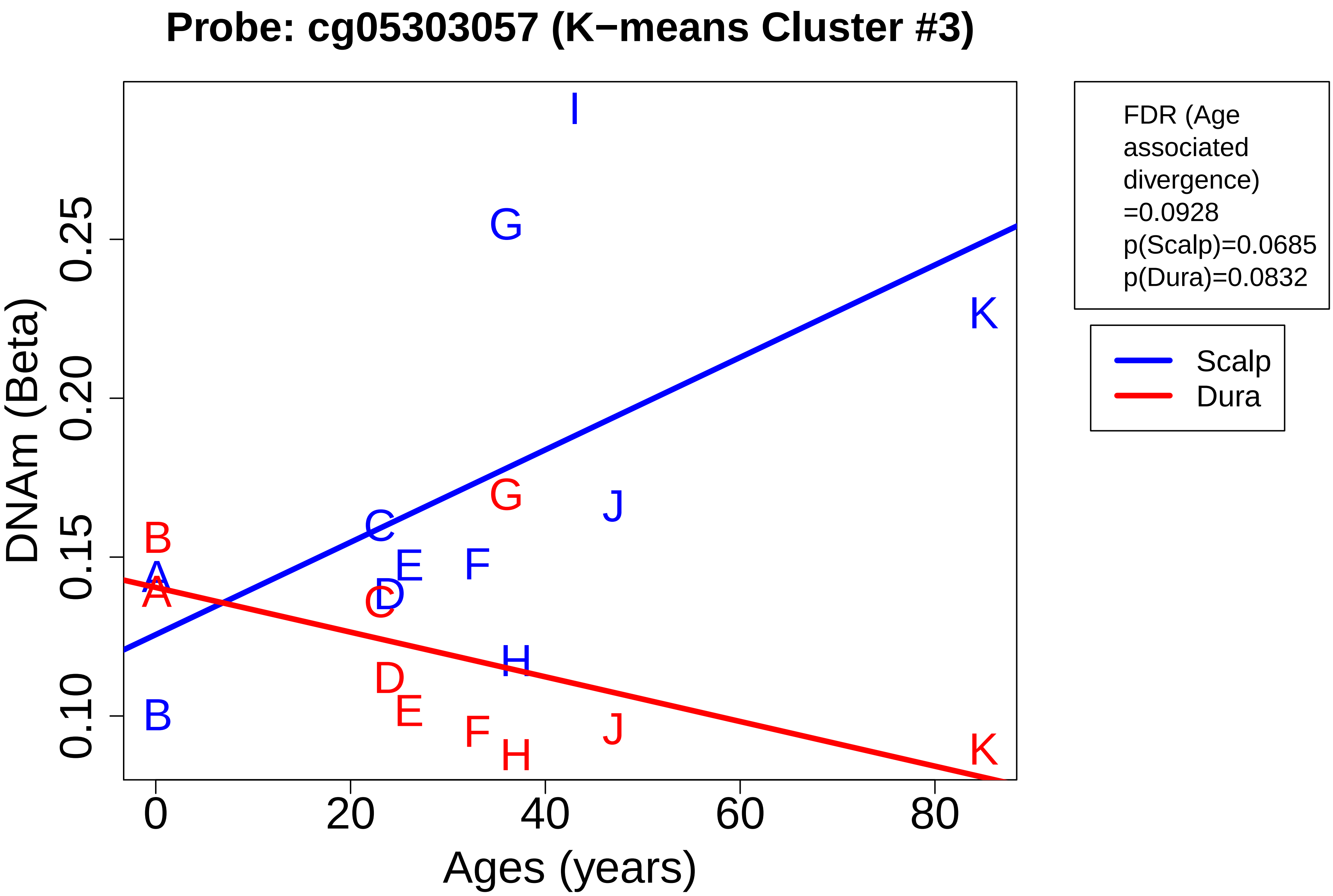

Probe: cg05303057 (K−means Cluster #3)
DNAm (Beta)
0.10
0.15
0.20
0.25
0
20
40
60
80
Ages (years)
FDR (Age associated divergence) =0.0928
p(Scalp)=0.0685
p(Dura)=0.0832
Scalp
Dura

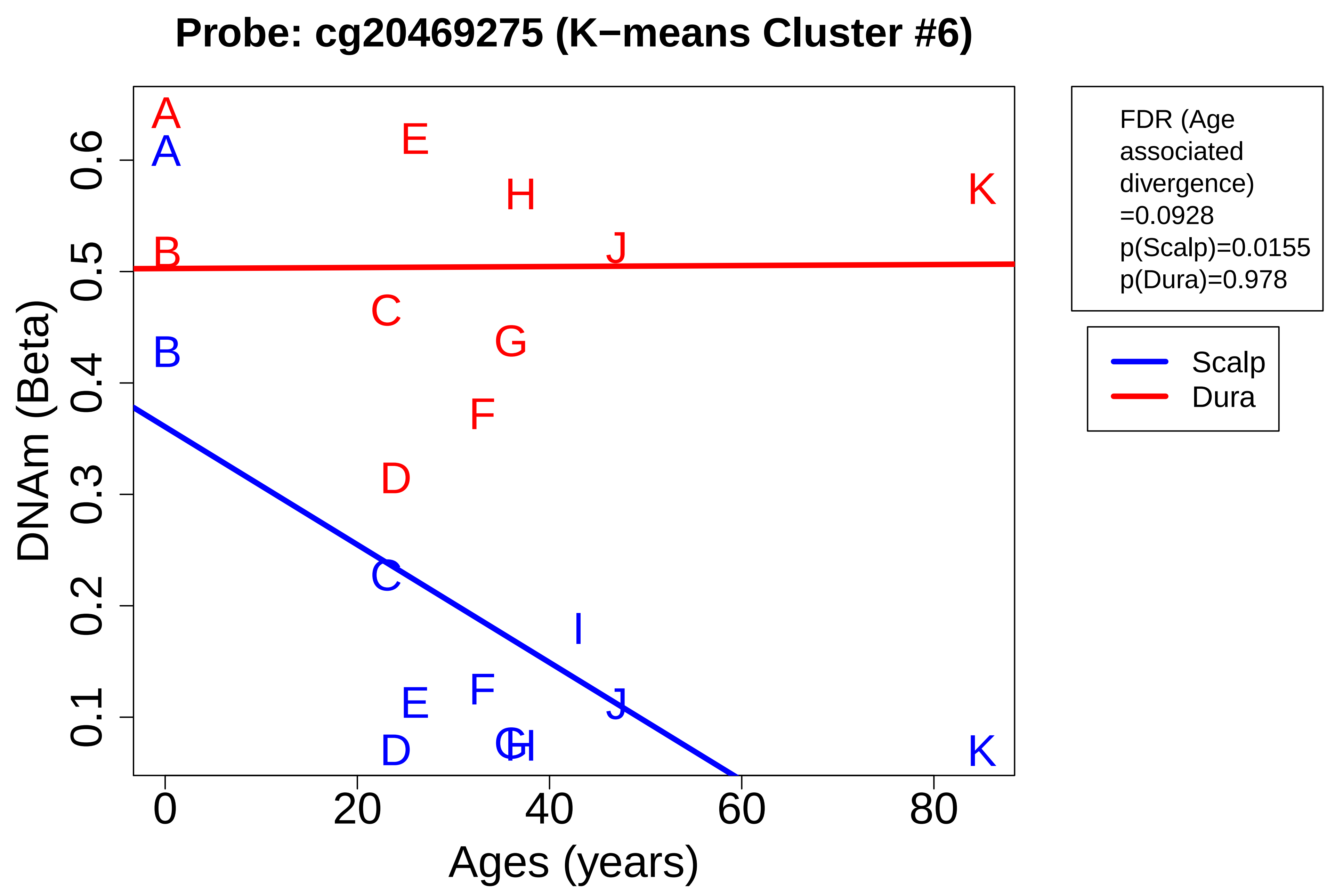

Probe: cg20469275 (K−means Cluster #6)
DNAm (Beta)
Ages (years)
0.1
0.2
0.3
0.4
0.5
0.6
0
20
40
60
80
FDR (Age associated divergence) =0.0928
p(Scalp)=0.0155
p(Dura)=0.978
Scalp
Dura

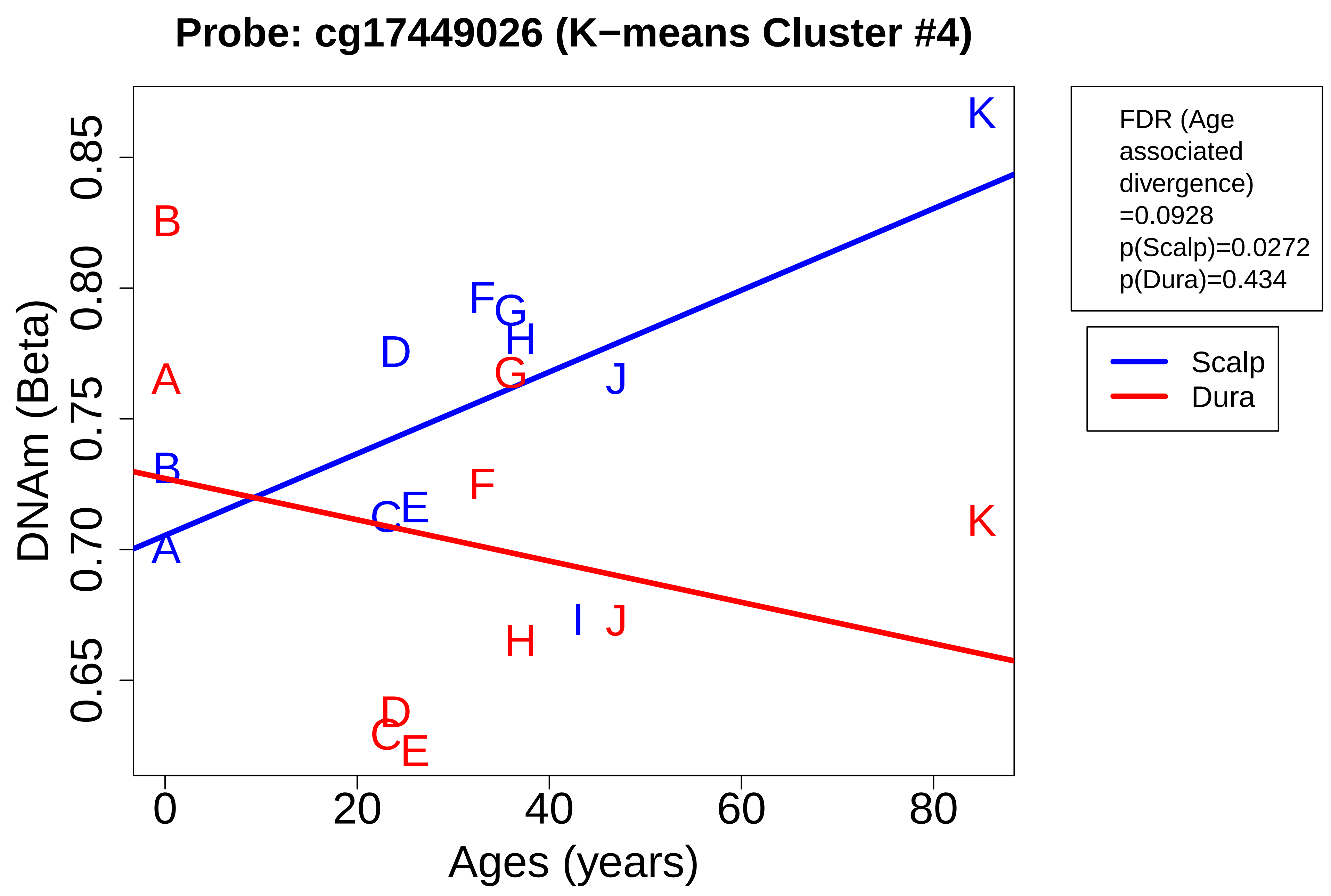

Probe: cg17449026 (K−means Cluster #4)
DNAm (Beta)
0.65
0.70
0.75
0.80
0.85
Ages (years)
0
20
40
60
80
FDR (Age associated divergence) =0.0928
p(Scalp)=0.0272
p(Dura)=0.434
Scalp
Dura
A
B
C
D
E
F
G
H
I
J
K

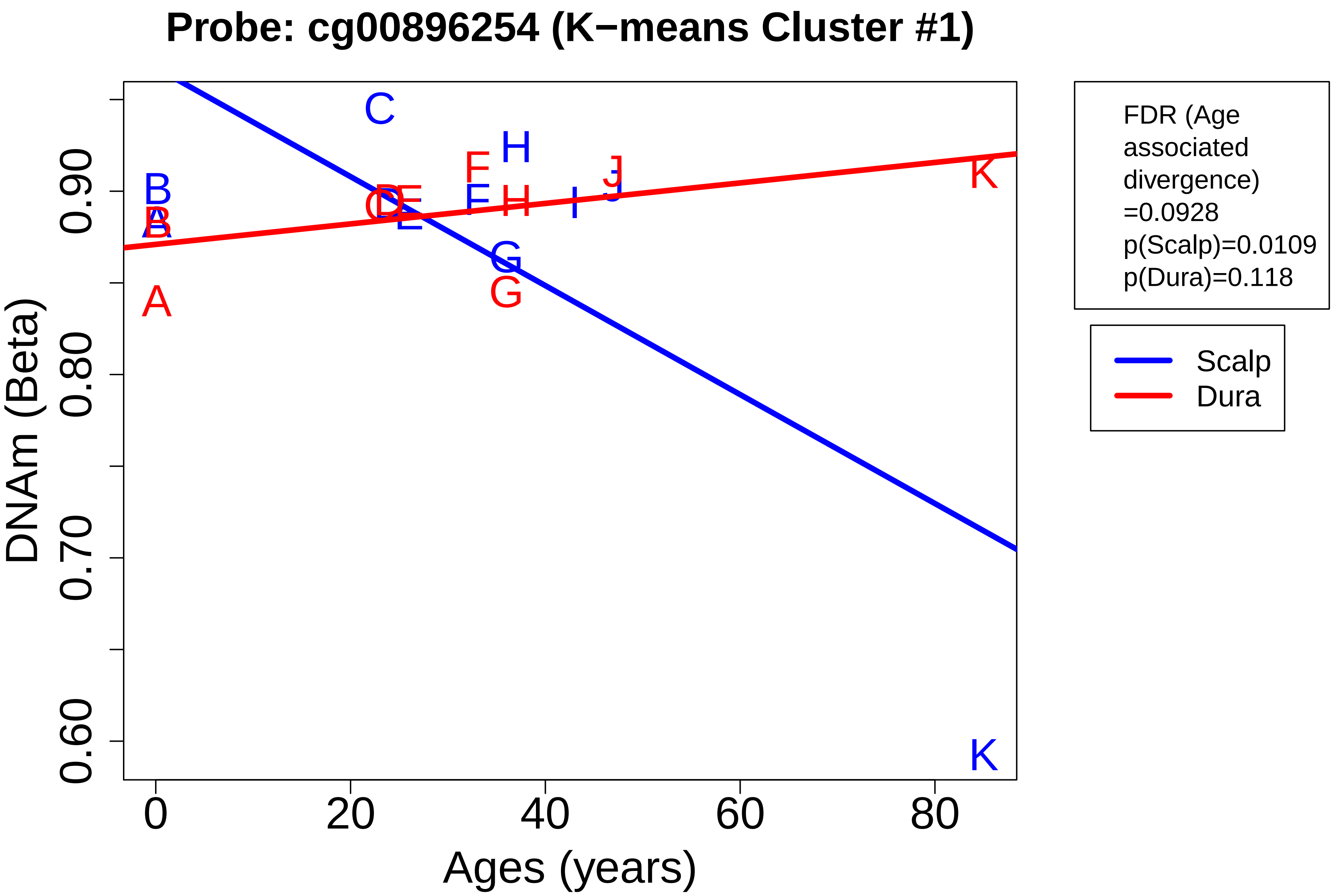

Probe: cg00896254 (K−means Cluster #1)
DNAm (Beta)
0.60
0.70
0.80
0.90
0
20
40
60
80
Ages (years)
A
B
C
D
E
F
G
H
I
J
K
FDR (Age associated divergence) =0.0928
p(Scalp)=0.0109
p(Dura)=0.118
Scalp
Dura

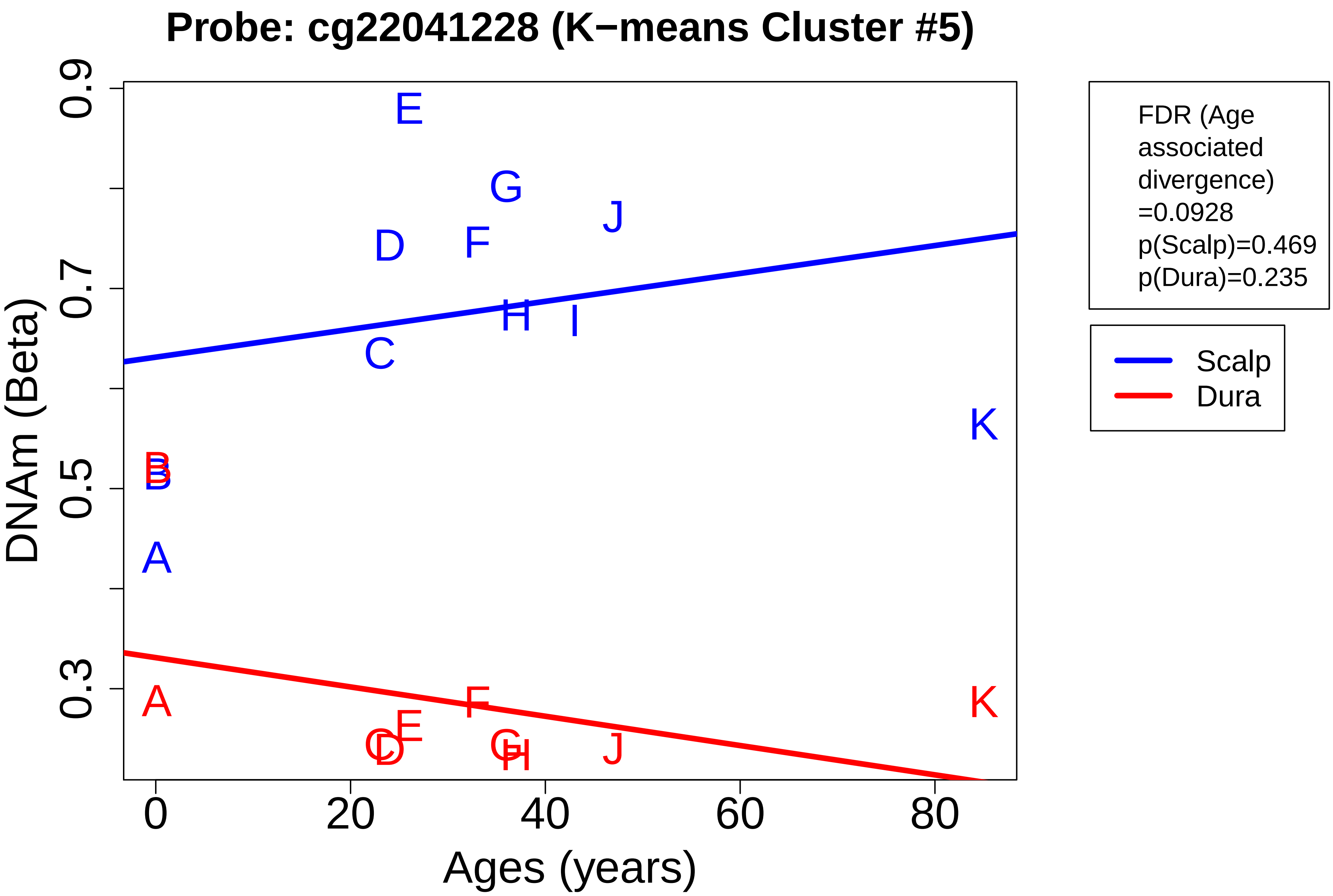
Probe: cg22041228 (K−means Cluster #5)
DNAm (Beta)
0.9
0.7
0.5
0.3
0
20
40
60
80
Ages (years)
FDR (Age associated divergence) =0.0928
p(Scalp)=0.469
p(Dura)=0.235
Scalp
Dura
A
B
C
D
E
F
G
H
I
J
K

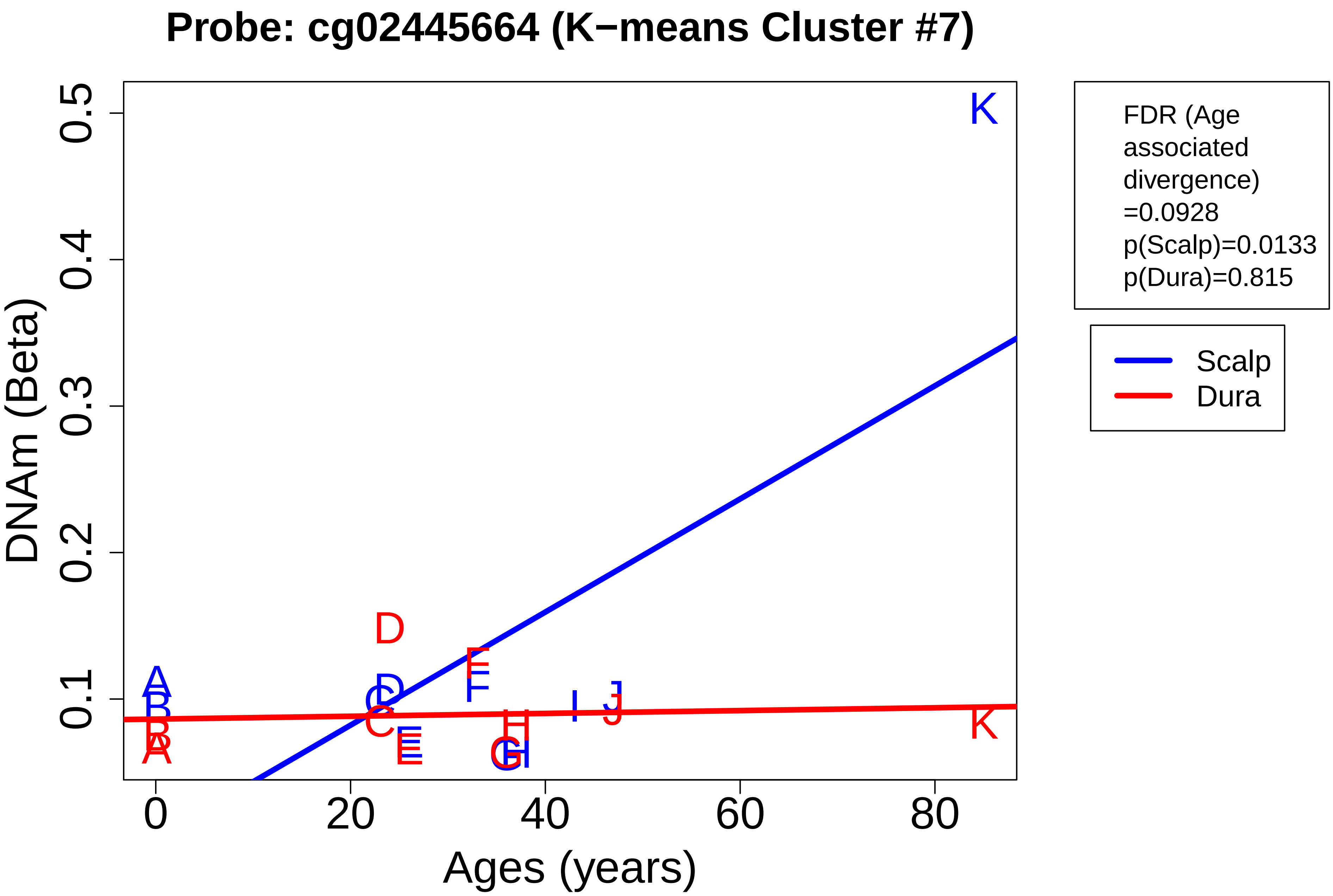

Probe: cg02445664 (K−means Cluster #7)
DNAm (Beta)
0.1
0.2
0.3
0.4
0.5
0
20
40
60
80
Ages (years)
FDR (Age associated divergence) =0.0928
p(Scalp)=0.0133
p(Dura)=0.815
Scalp
Dura

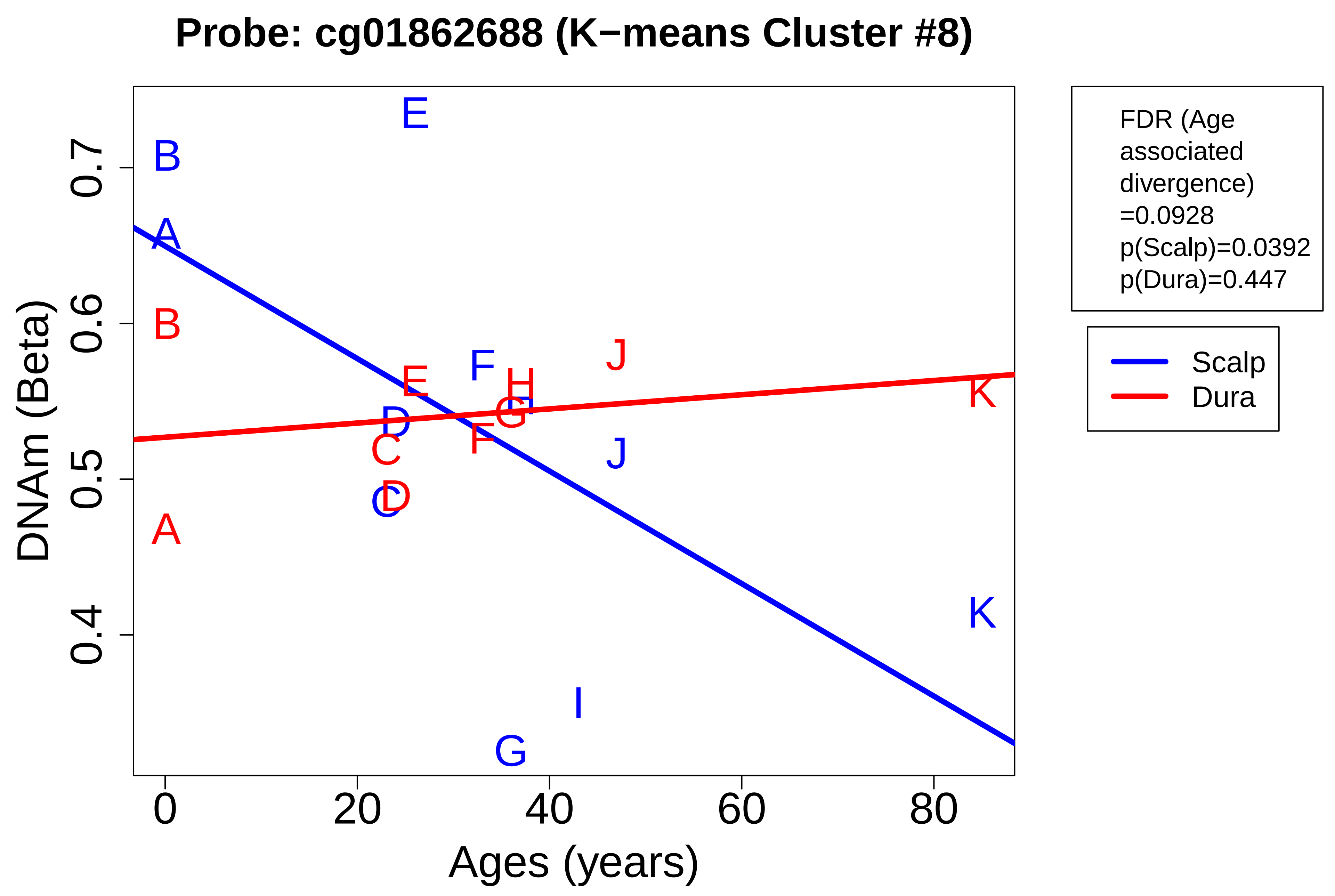
Probe: cg01862688 (K−means Cluster #8)
FDR (Age associated divergence) =0.0928
p(Scalp)=0.0392
p(Dura)=0.447
Scalp
Dura
DNAm (Beta)
0.4
0.5
0.6
0.7
Ages (years)
0
20
40
60
80

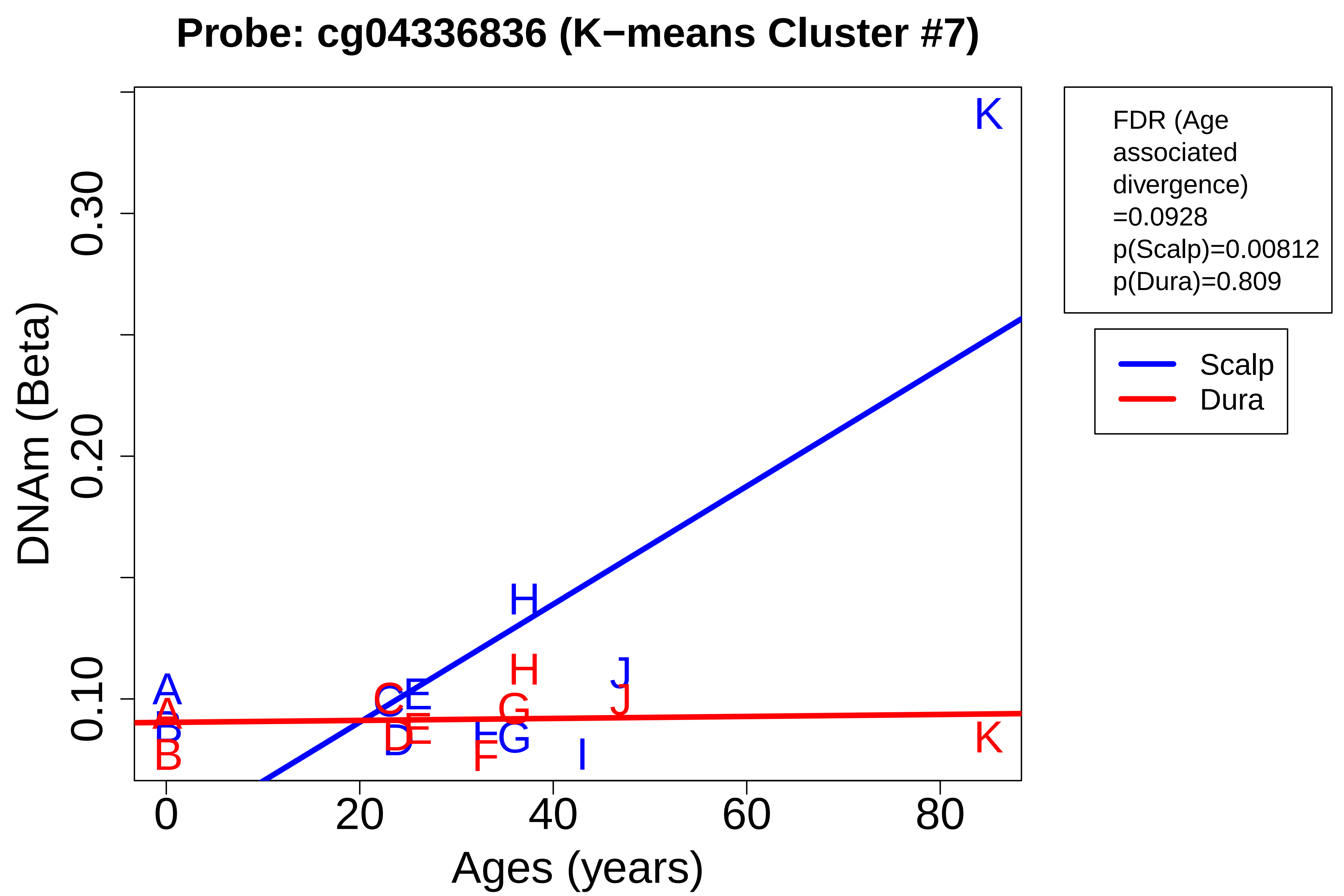

Probe: cg04336836 (K−means Cluster #7)
DNAm (Beta)
0.10
0.20
0.30
Ages (years)
0
20
40
60
80
FDR (Age associated divergence) =0.0928
p(Scalp)=0.00812
p(Dura)=0.809
Scalp
Dura
A B C D E F G H I J K

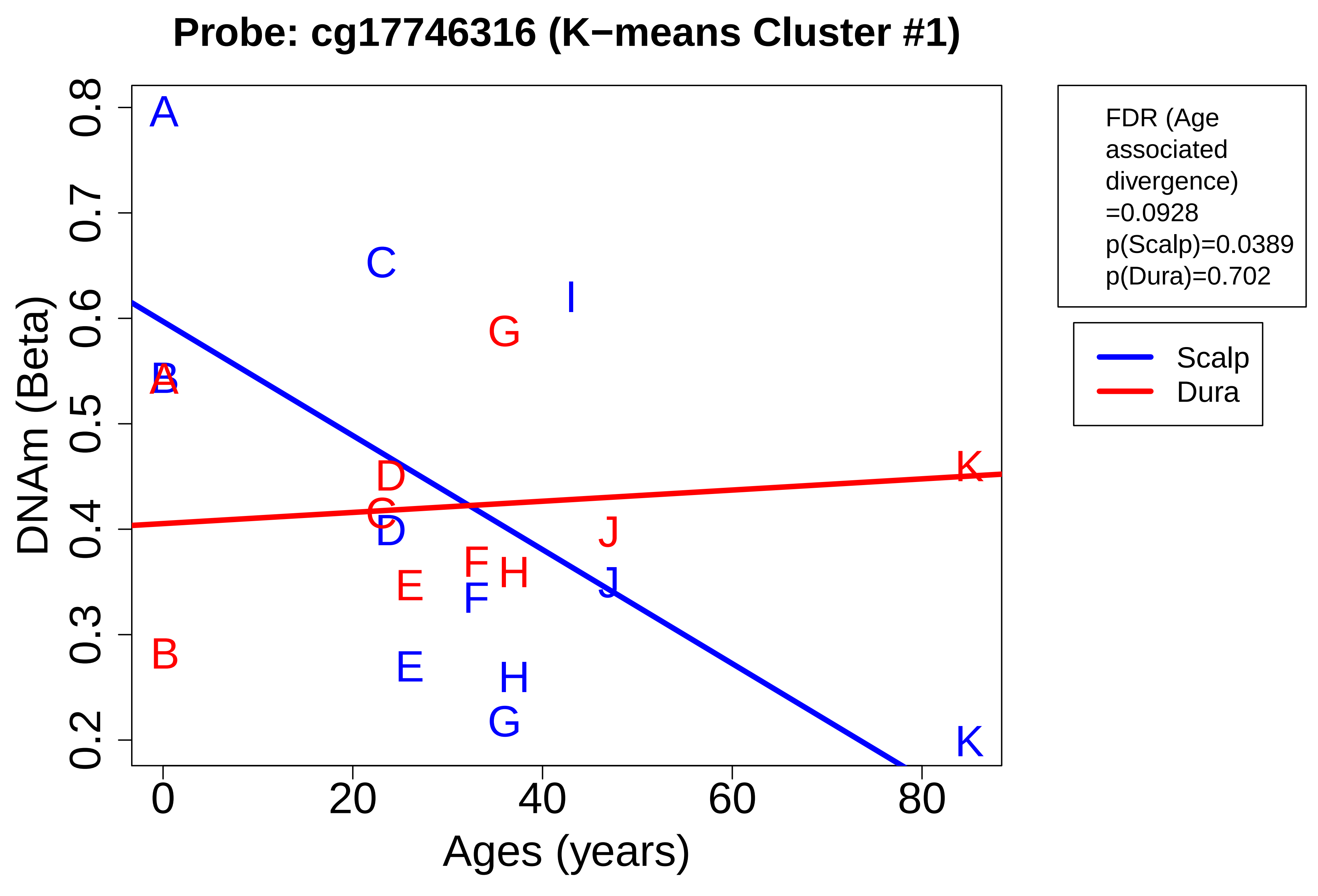

Probe: cg17746316 (K−means Cluster #1)
DNAm (Beta)
0.2
0.3
0.4
0.5
0.6
0.7
0.8
Ages (years)
0
20
40
60
80
A
B
C
D
E
F
G
H
I
J
K
FDR (Age associated divergence) =0.0928
p(Scalp)=0.0389
p(Dura)=0.702
Scalp
Dura

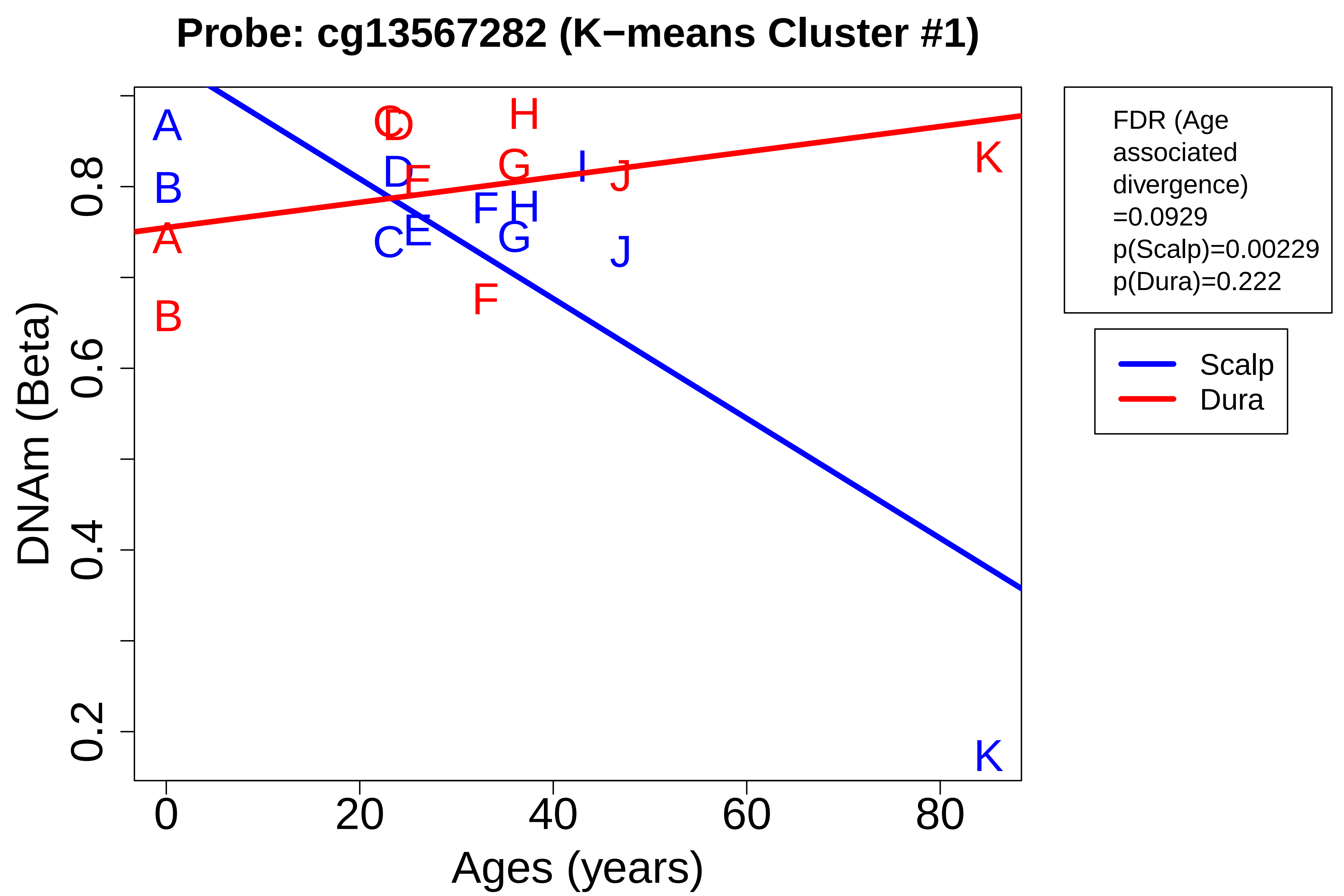

Probe: cg13567282 (K−means Cluster #1)
DNAm (Beta)
0.2
0.4
0.6
0.8
0
20
40
60
80
Ages (years)
A B C D E F G H I J K
A B C D E F G H J K
FDR (Age associated divergence) =0.0929
p(Scalp)=0.00229
p(Dura)=0.222
Scalp
Dura

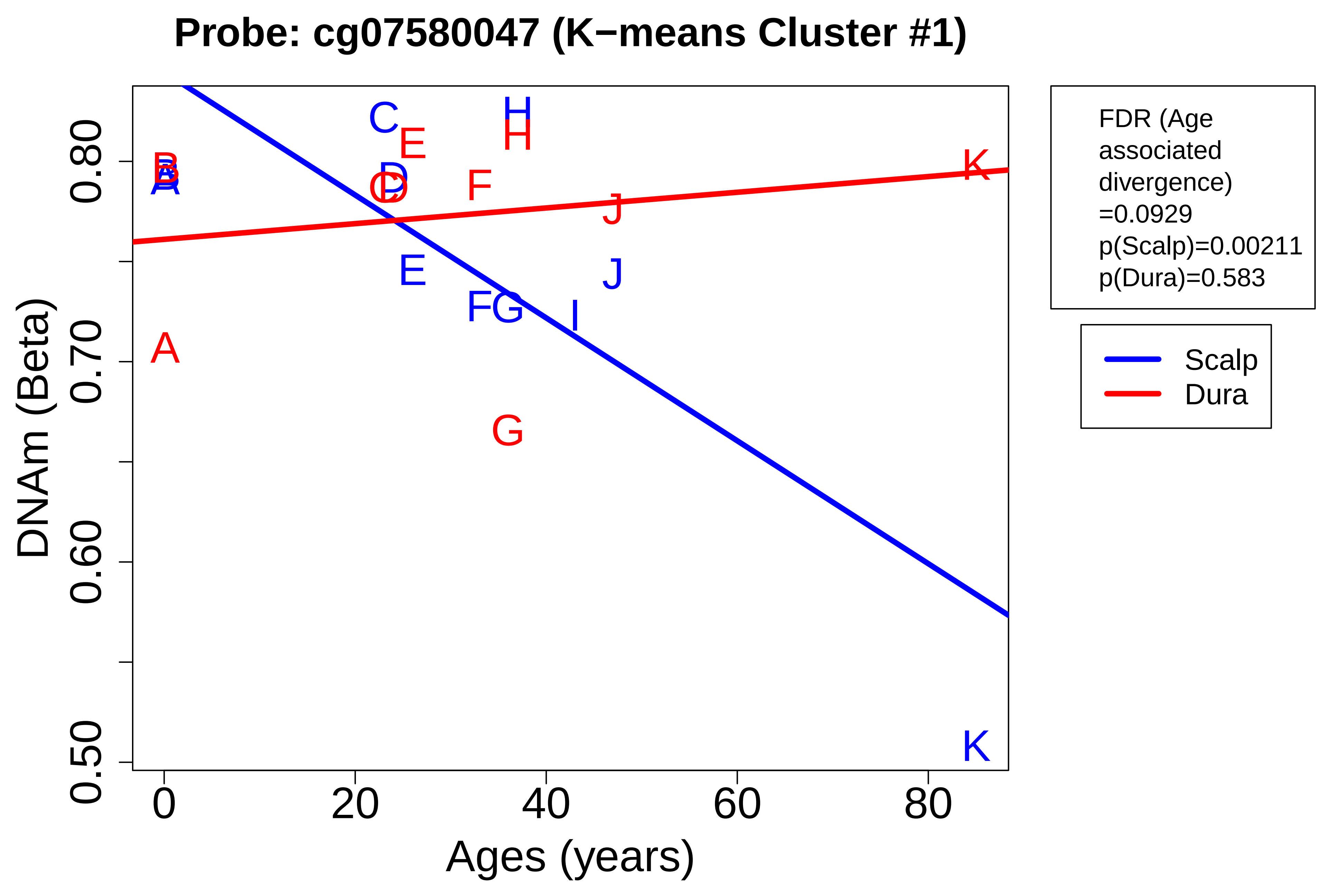

Probe: cg07580047 (K−means Cluster #1)
DNAm (Beta)
0.50
0.60
0.70
0.80
0
20
40
60
80
Ages (years)
FDR (Age associated divergence) =0.0929
p(Scalp)=0.00211
p(Dura)=0.583
Scalp
Dura
A
B
C
D
E
F
G
H
I
J
K

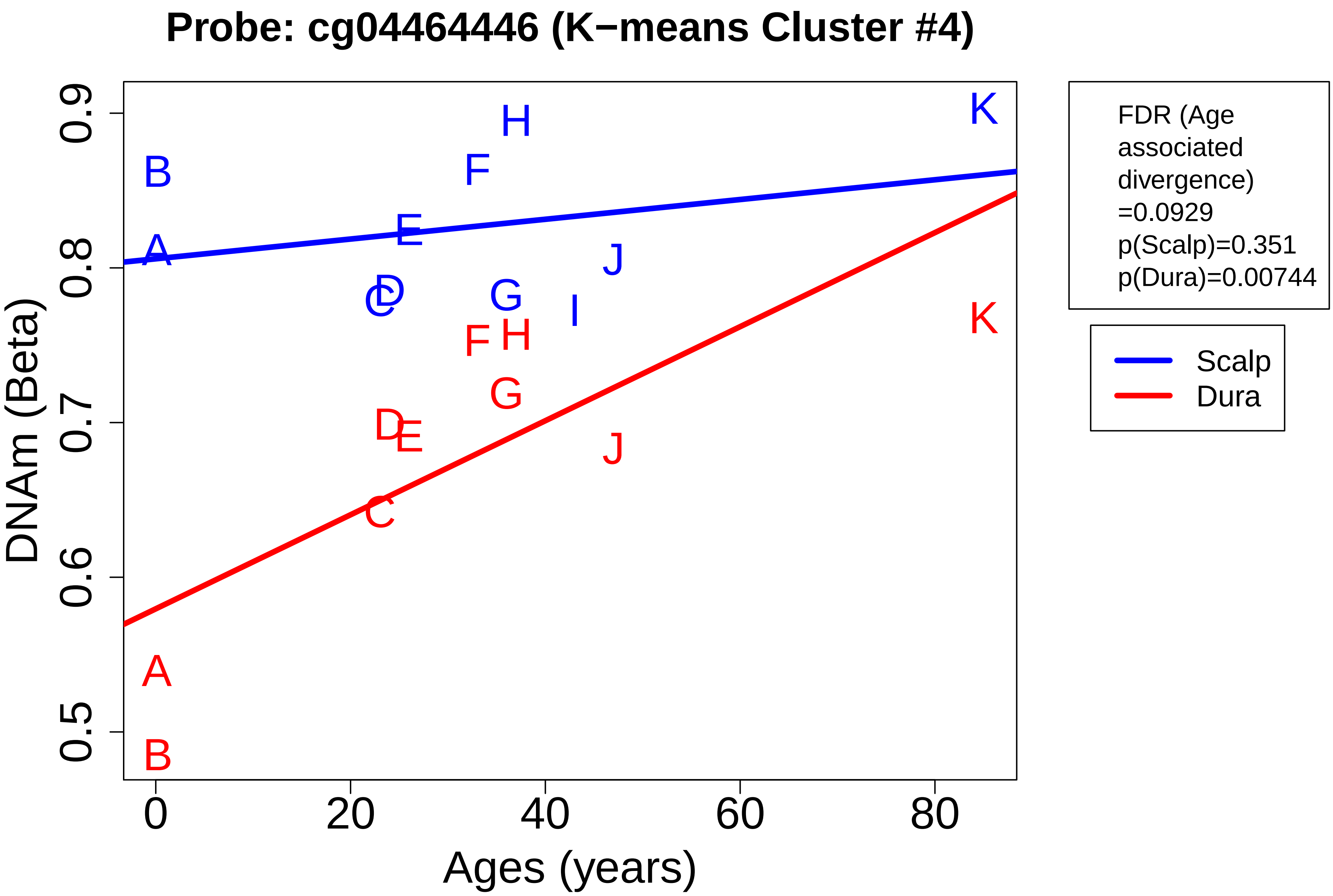

Probe: cg04464446 (K−means Cluster #4)
DNAm (Beta)
0.5
0.6
0.7
0.8
0.9
0
20
40
60
80
Ages (years)
FDR (Age associated divergence) =0.0929
p(Scalp)=0.351
p(Dura)=0.00744
Scalp
Dura

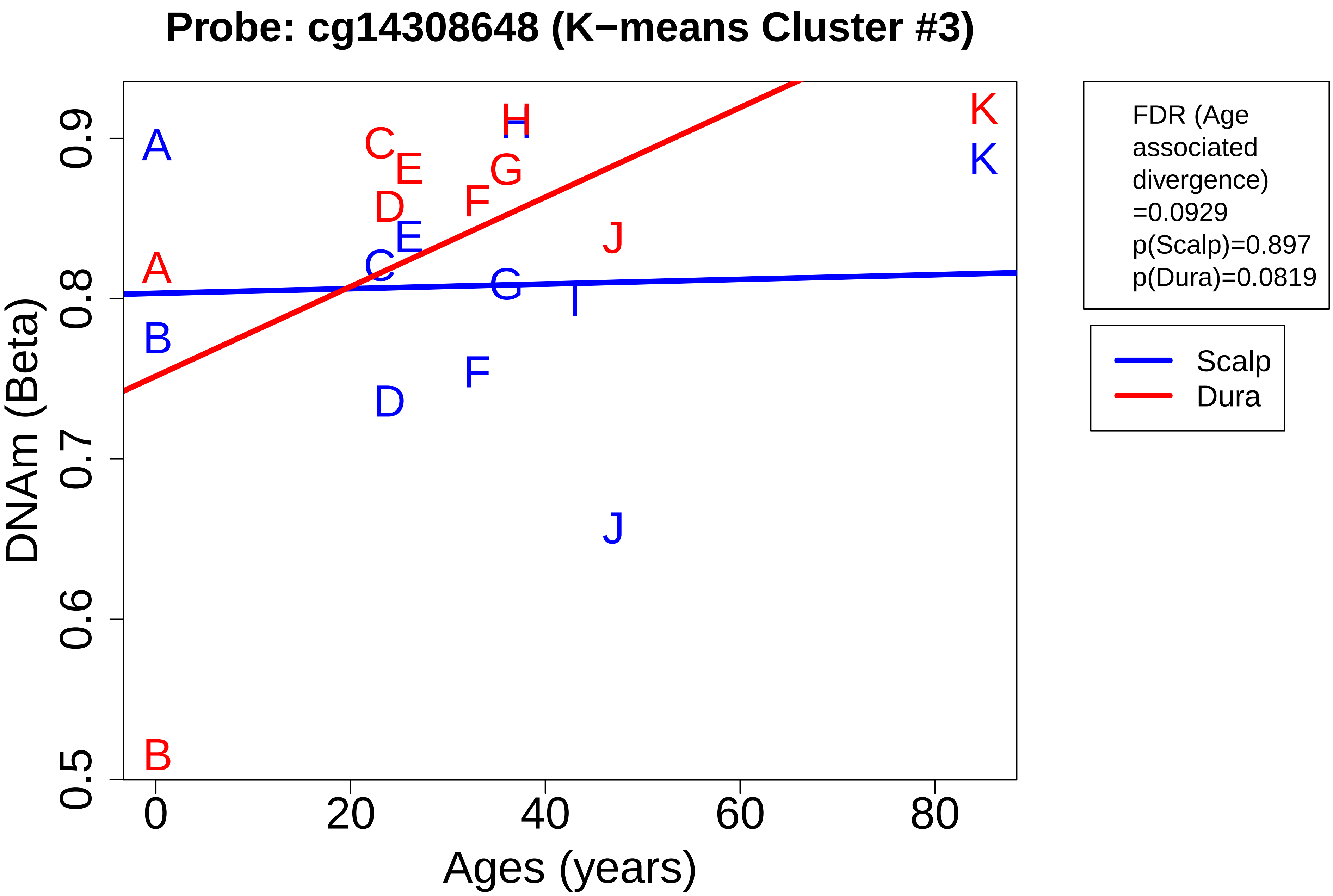

Probe: cg14308648 (K−means Cluster #3)
DNAm (Beta)
Ages (years)
0.5
0.6
0.7
0.8
0.9
0
20
40
60
80
FDR (Age associated divergence) =0.0929
p(Scalp)=0.897
p(Dura)=0.0819
Scalp
Dura
A
B
C
D
E
F
G
H
I
J
K

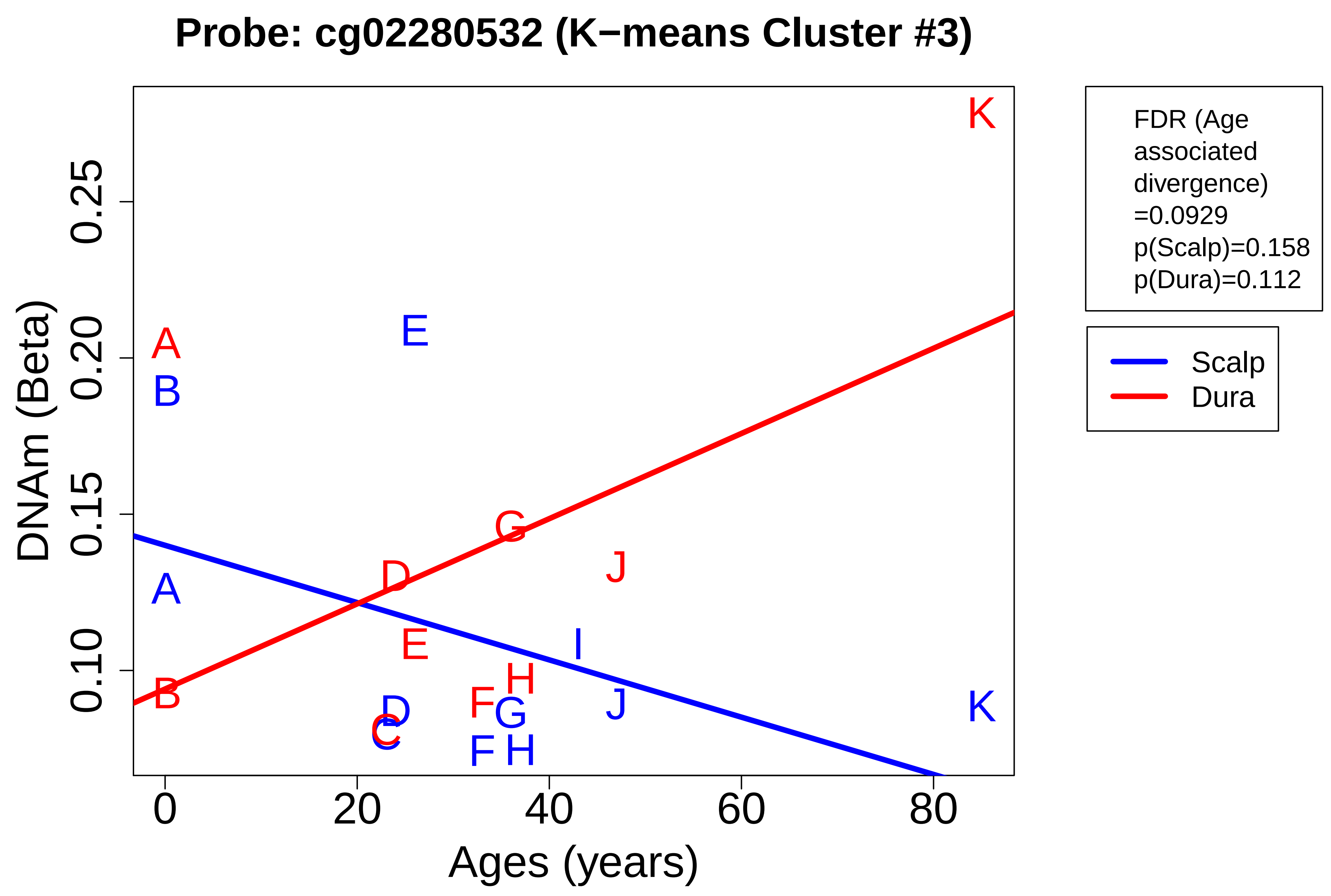

Probe: cg02280532 (K−means Cluster #3)
DNAm (Beta)
0.10
0.15
0.20
0.25
0
20
40
60
80
Ages (years)
A
B
C
D
E
F
G
H
I
J
K
FDR (Age associated divergence) =0.0929
p(Scalp)=0.158
p(Dura)=0.112
Scalp
Dura

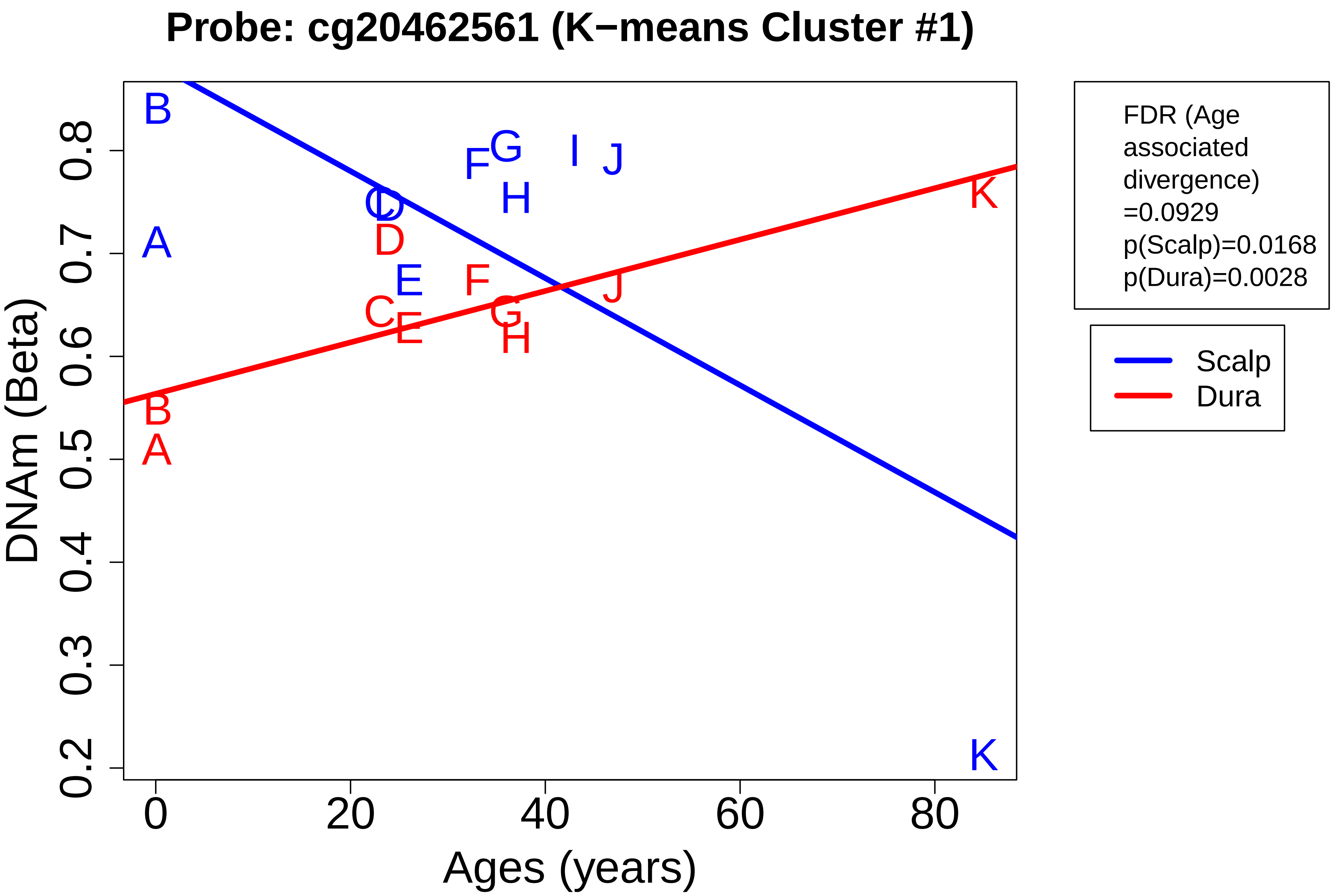

Probe: cg20462561 (K−means Cluster #1)
DNAm (Beta)
0.2
0.3
0.4
0.5
0.6
0.7
0.8
0
20
40
60
80
Ages (years)
B
A
C
D
E
F
G
H
I
J
K
FDR (Age associated divergence) =0.0929
p(Scalp)=0.0168
p(Dura)=0.0028
Scalp
Dura

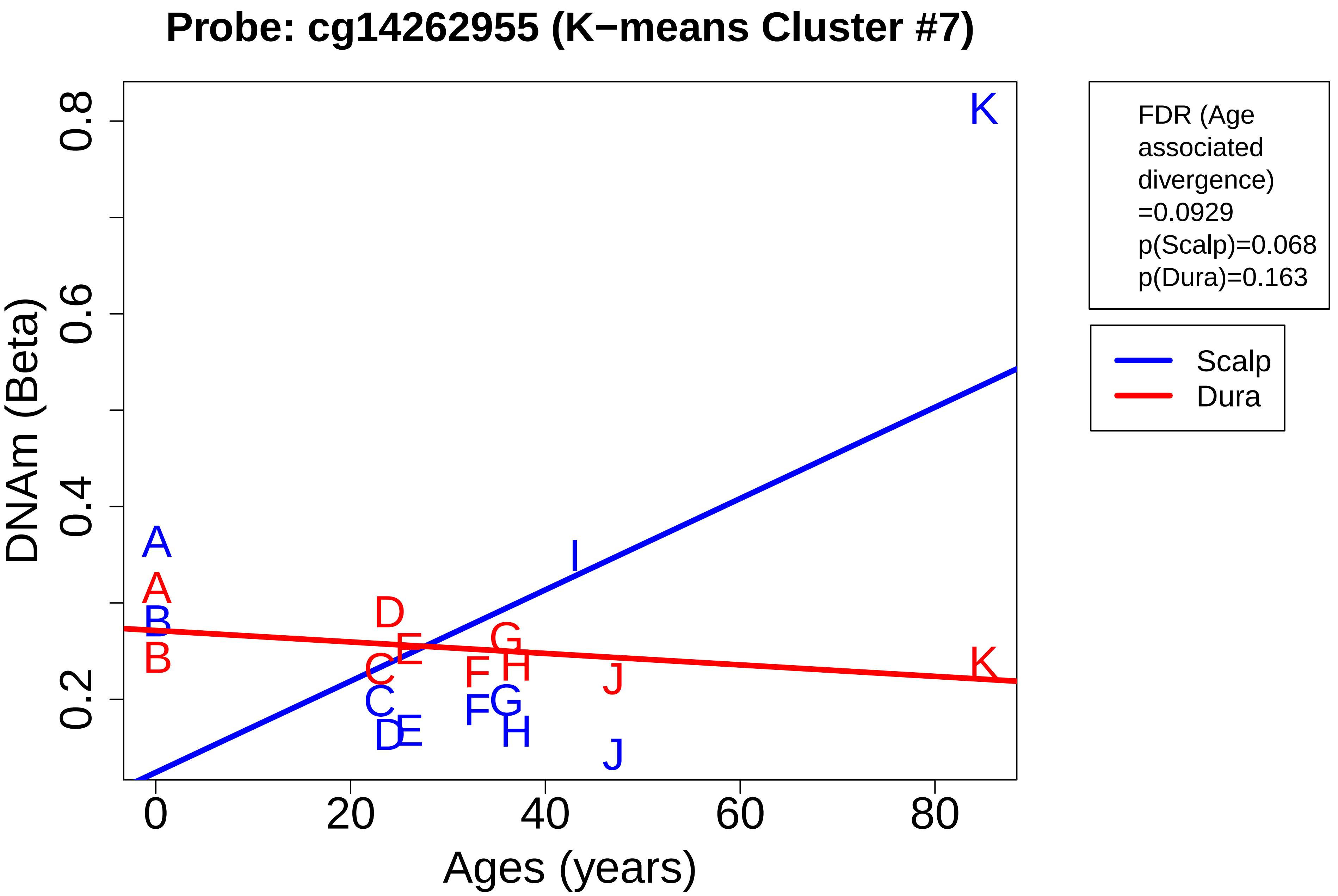

Probe: cg14262955 (K−means Cluster #7)
DNAm (Beta)
0.2
0.4
0.6
0.8
0
20
40
60
80
Ages (years)
FDR (Age associated divergence) =0.0929
p(Scalp)=0.068
p(Dura)=0.163
Scalp
Dura
A
B
C
D
E
F
G
H
I
J
K

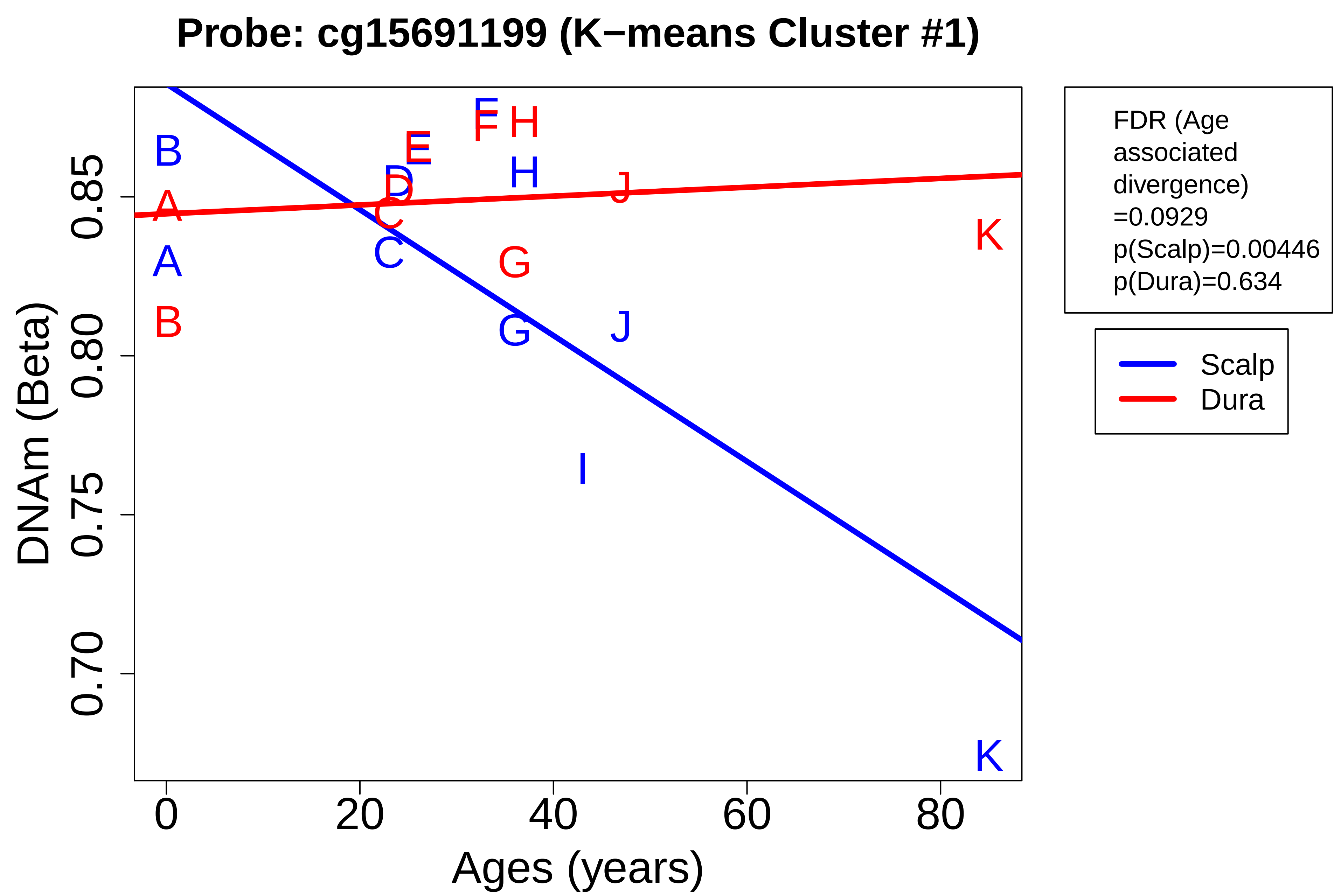

Probe: cg15691199 (K−means Cluster #1)
DNAm (Beta)
0.70
0.75
0.80
0.85
0
20
40
60
80
Ages (years)
A
B
C
D
E
F
G
H
I
J
K
FDR (Age associated divergence) =0.0929
p(Scalp)=0.00446
p(Dura)=0.634
Scalp
Dura

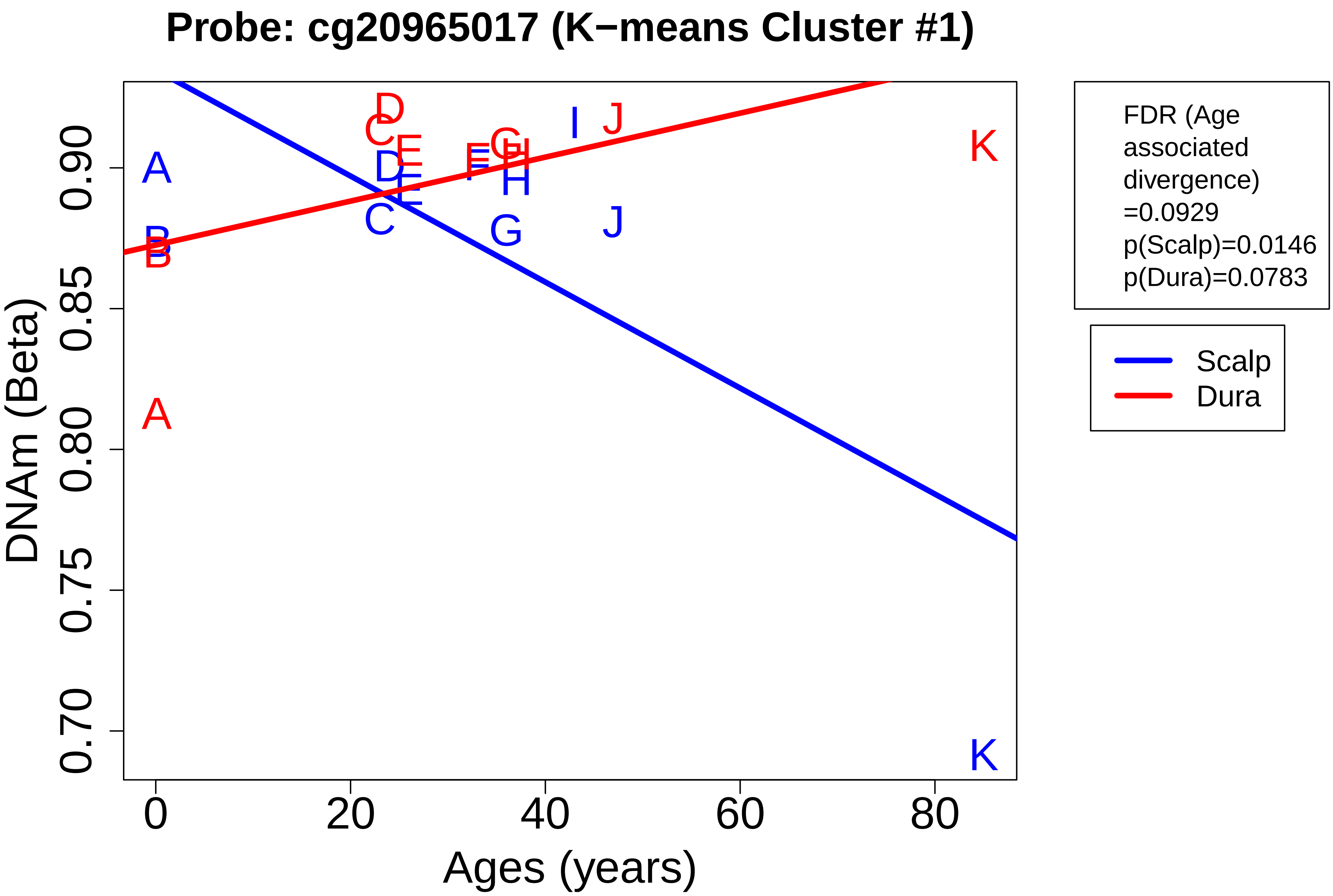
Probe: cg20965017 (K−means Cluster #1)
DNAm (Beta)
Ages (years)
0.70
0.75
0.80
0.85
0.90
0
20
40
60
80
A
B
C
D
E
F
G
H
I
J
K
FDR (Age associated divergence) =0.0929
p(Scalp)=0.0146
p(Dura)=0.0783
Scalp
Dura

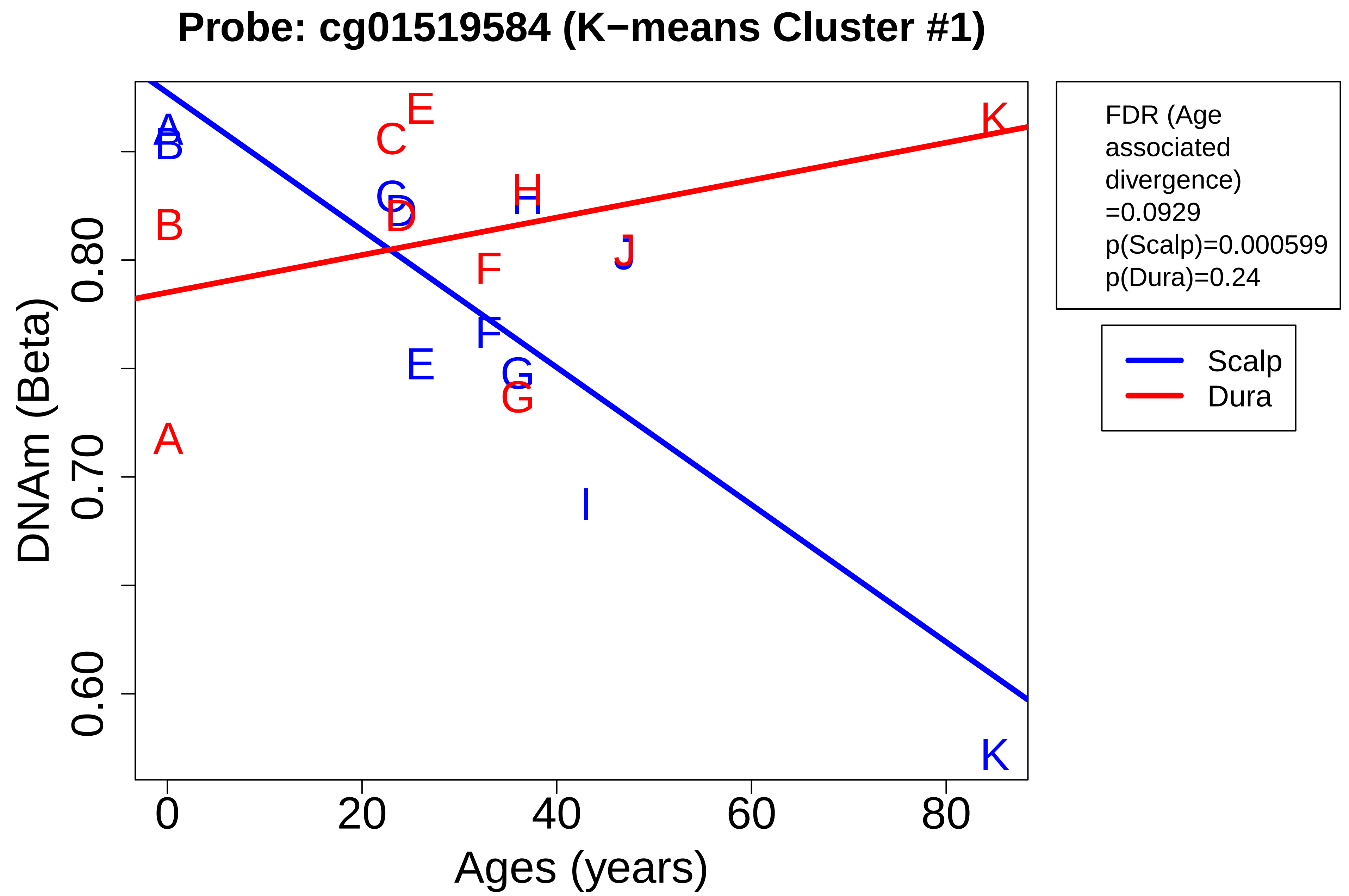

Probe: cg01519584 (K−means Cluster #1)
DNAm (Beta)
0.60
0.70
0.80
Ages (years)
0
20
40
60
80
A
B
C
D
E
F
G
H
I
J
K
FDR (Age associated divergence) =0.0929
p(Scalp)=0.000599
p(Dura)=0.24
Scalp
Dura

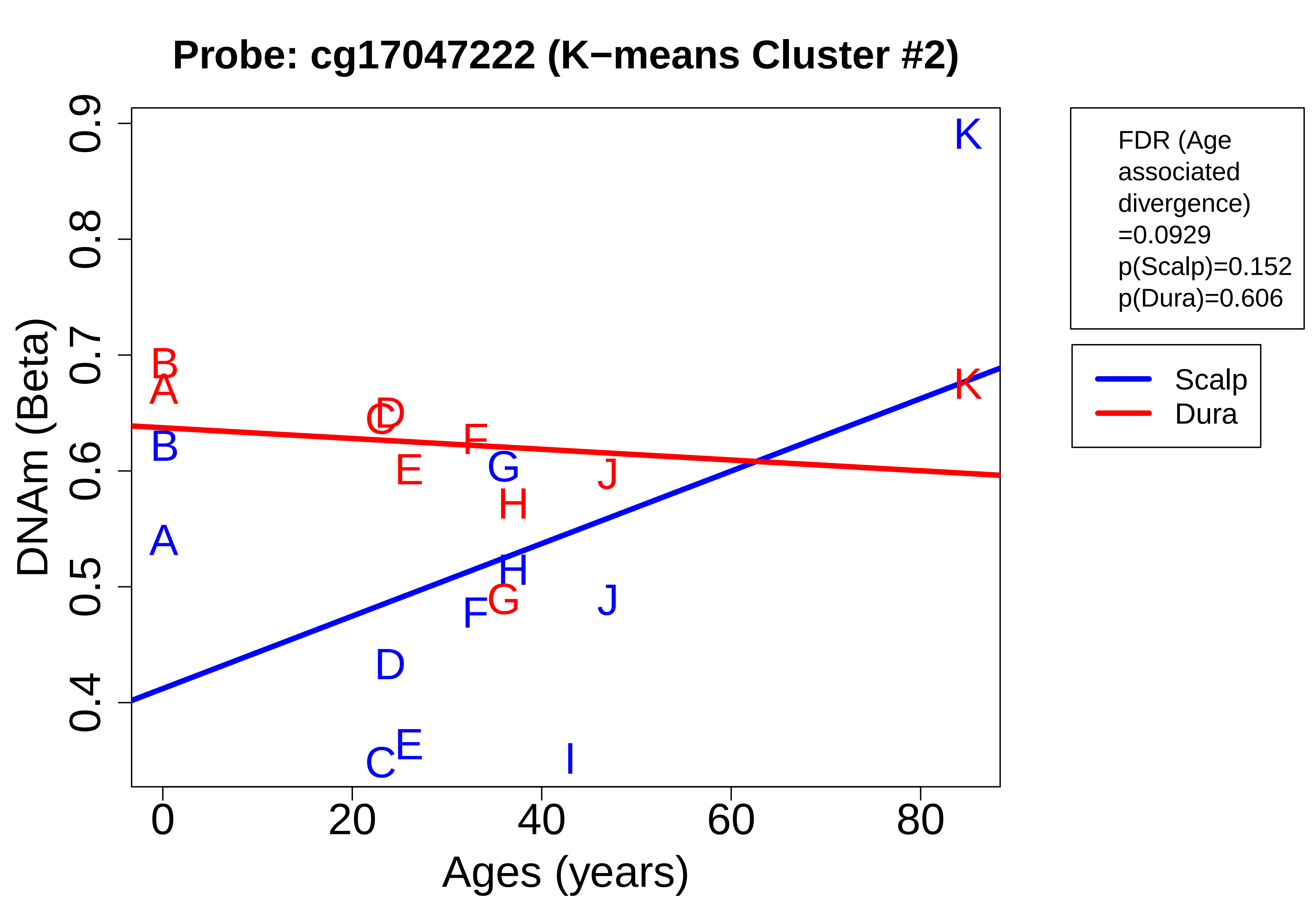

Probe: cg17047222 (K−means Cluster #2)
DNAm (Beta)
Ages (years)
0.4
0.5
0.6
0.7
0.8
0.9
0
20
40
60
80
FDR (Age associated divergence) =0.0929
p(Scalp)=0.152
p(Dura)=0.606
Scalp
Dura

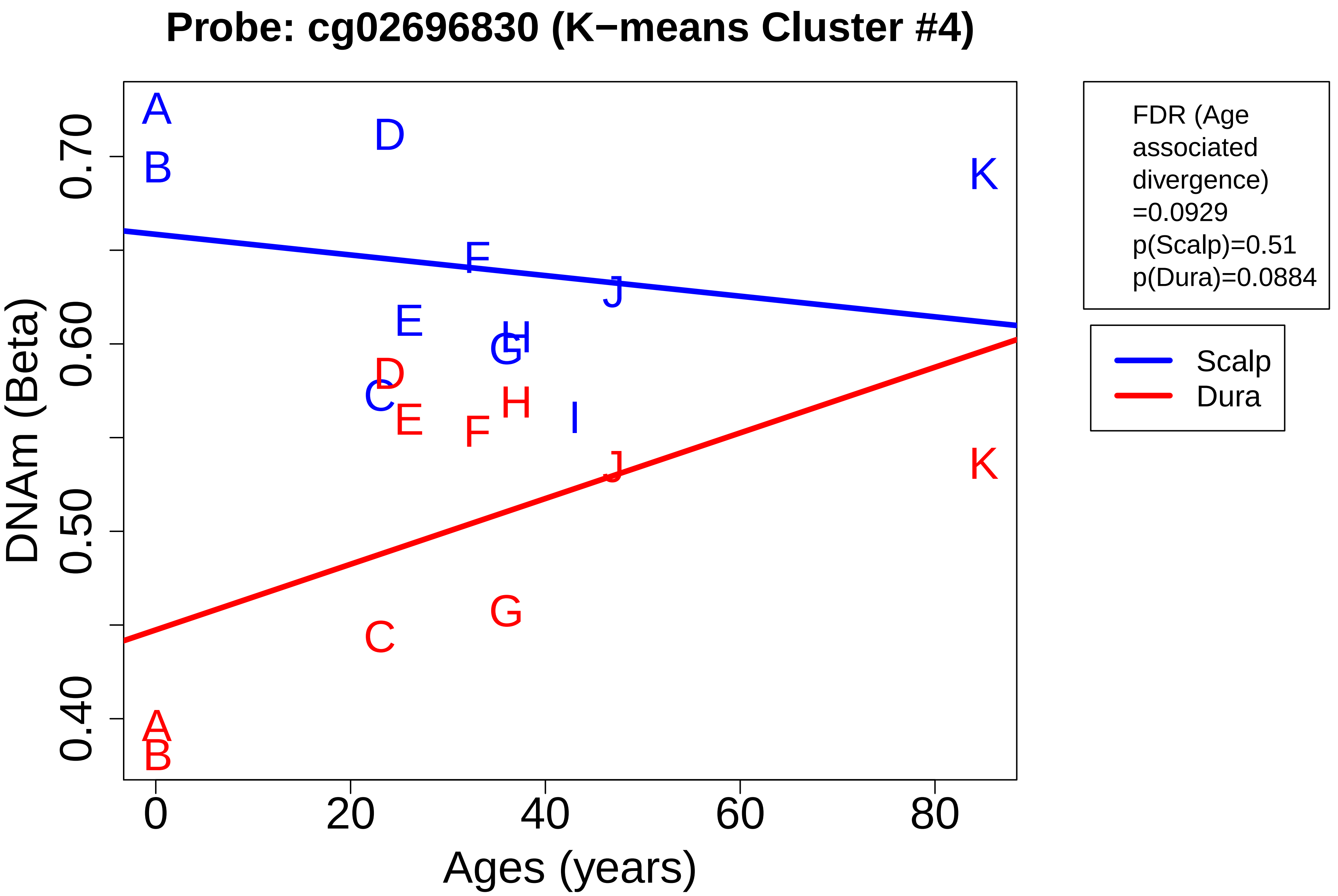
Probe: cg02696830 (K−means Cluster #4)
DNAm (Beta)
0.40
0.50
0.60
0.70
0
20
40
60
80
Ages (years)
FDR (Age associated divergence) =0.0929
p(Scalp)=0.51
p(Dura)=0.0884
Scalp
Dura
A
B
C
D
E
F
G
H
I
J
K

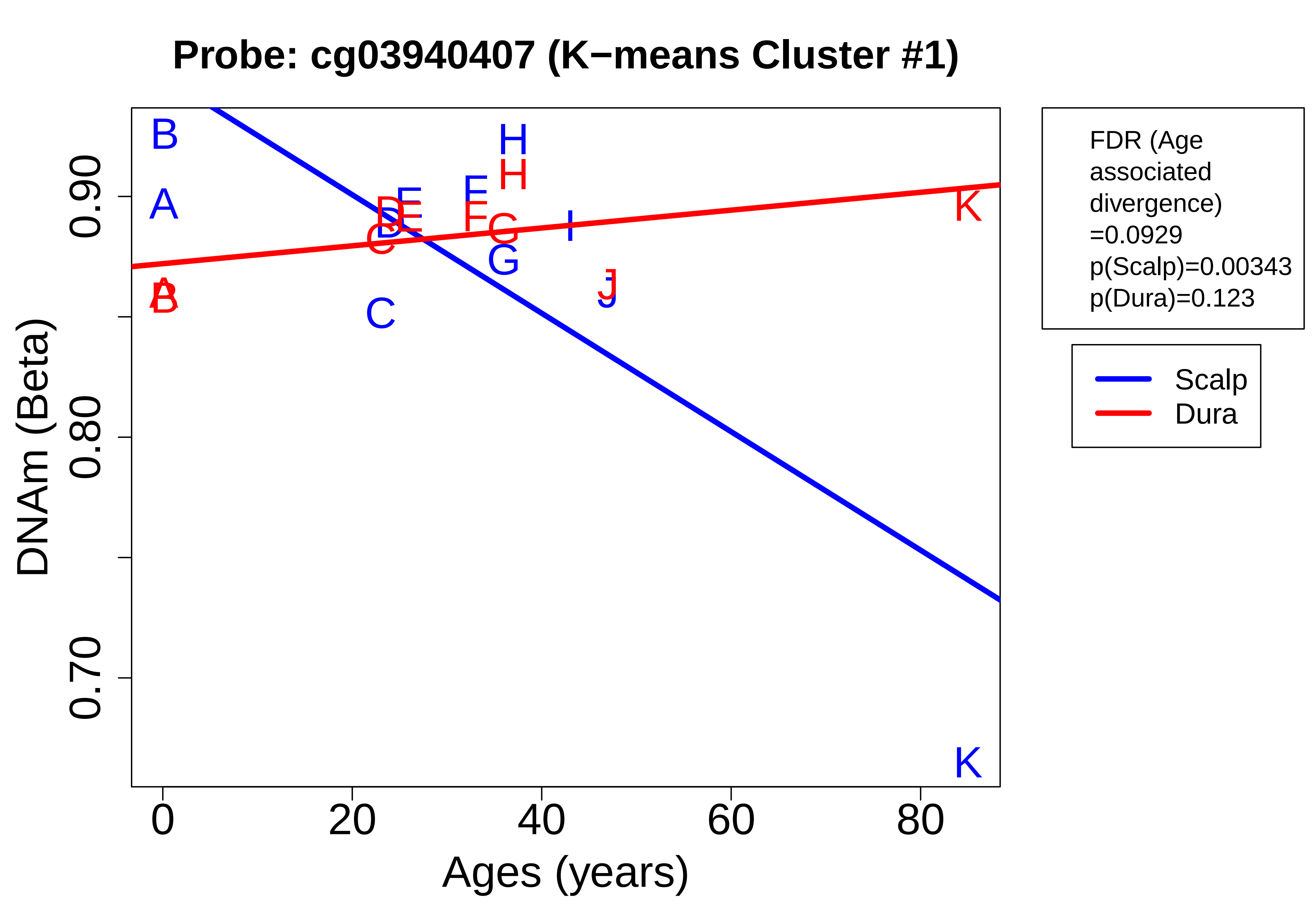

Probe: cg03940407 (K−means Cluster #1)
DNAm (Beta)
0.70
0.80
0.90
0
20
40
60
80
Ages (years)
FDR (Age associated divergence) =0.0929
p(Scalp)=0.00343
p(Dura)=0.123
Scalp
Dura
A
B
C
D
E
F
G
H
I
J
K

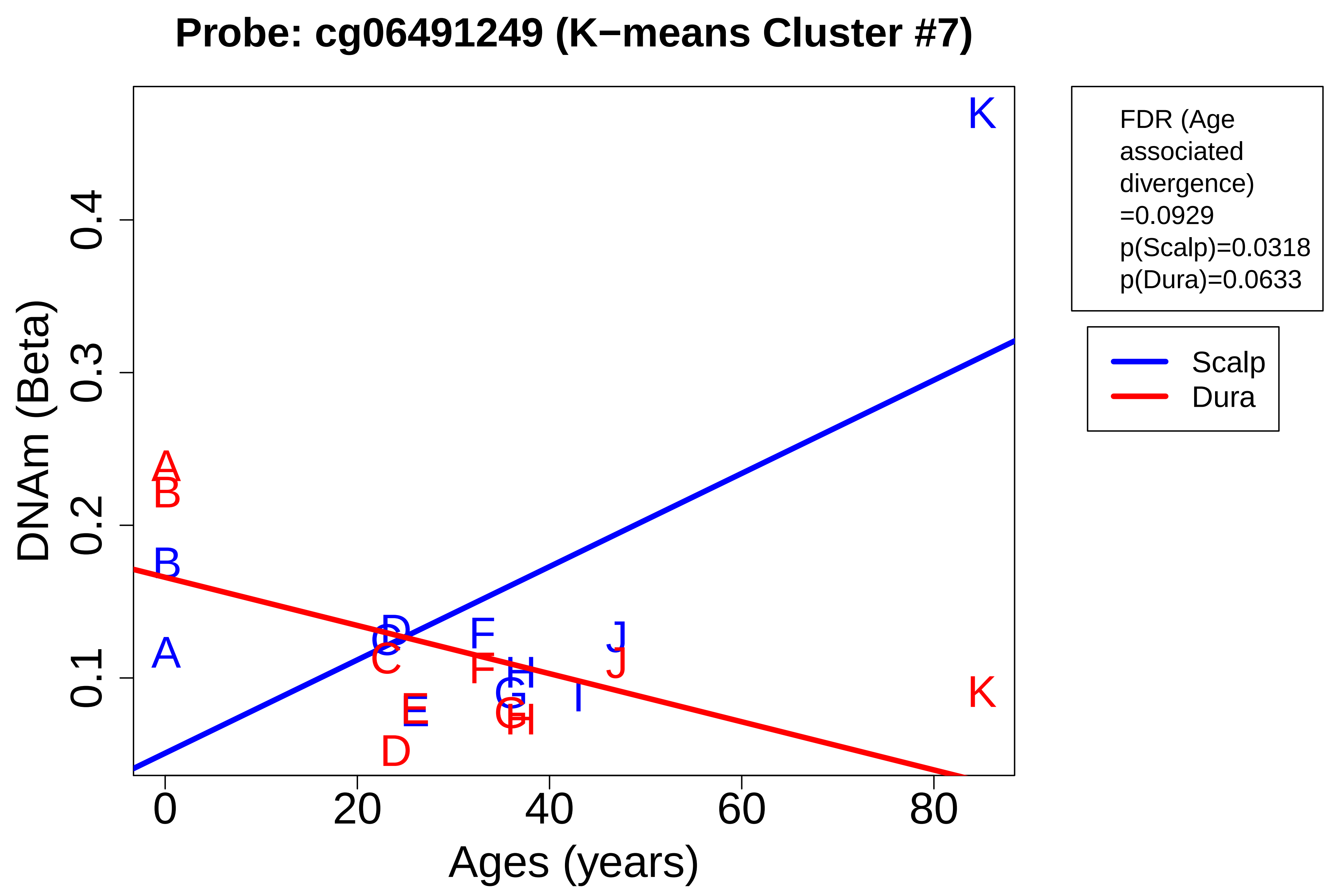

Probe: cg06491249 (K−means Cluster #7)
DNAm (Beta)
0.1
0.2
0.3
0.4
Ages (years)
0
20
40
60
80
FDR (Age associated divergence) =0.0929
p(Scalp)=0.0318
p(Dura)=0.0633
Scalp
Dura
A
B
C
D
E
F
G
H
I
J
K

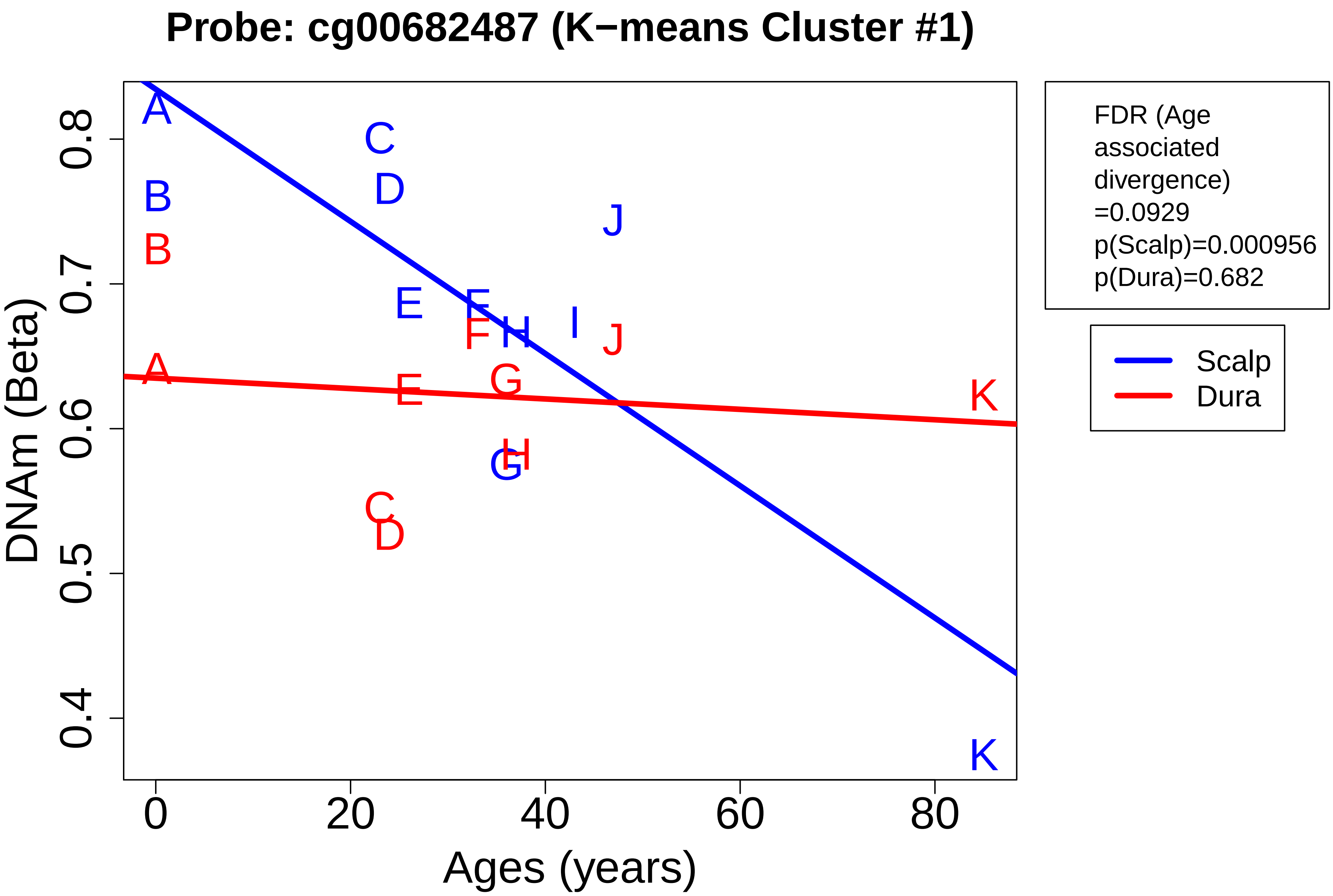
Probe: cg00682487 (K−means Cluster #1)
DNAm (Beta)
0.4
0.5
0.6
0.7
0.8
Ages (years)
0
20
40
60
80
A
B
C
D
E
F
G
H
I
J
K
FDR (Age associated divergence) =0.0929
p(Scalp)=0.000956
p(Dura)=0.682
Scalp
Dura

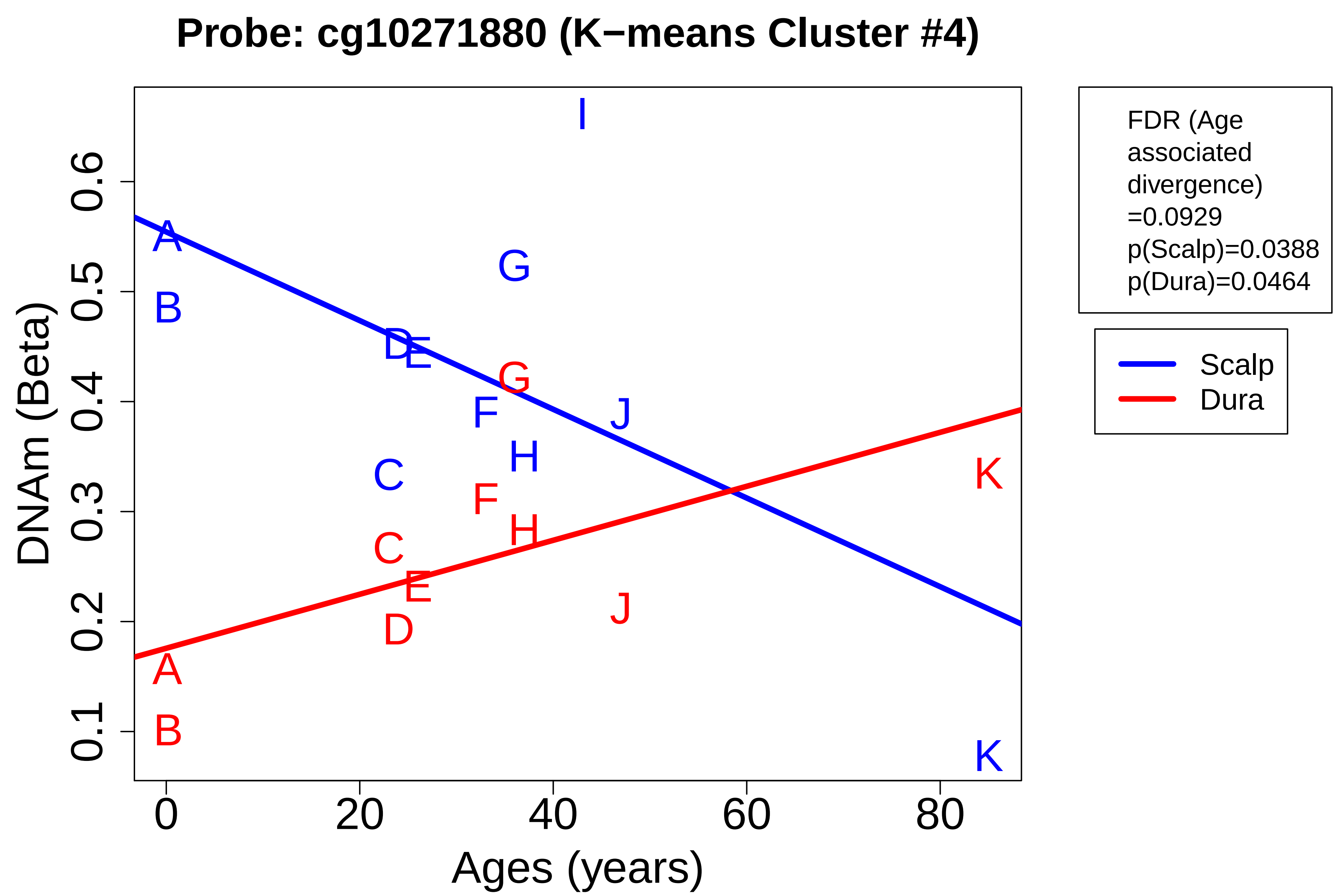
Probe: cg10271880 (K−means Cluster #4)
DNAm (Beta)
0.1
0.2
0.3
0.4
0.5
0.6
Ages (years)
0
20
40
60
80
A
B
C
D
E
F
G
H
I
J
K
FDR (Age associated divergence) =0.0929
p(Scalp)=0.0388
p(Dura)=0.0464
Scalp
Dura

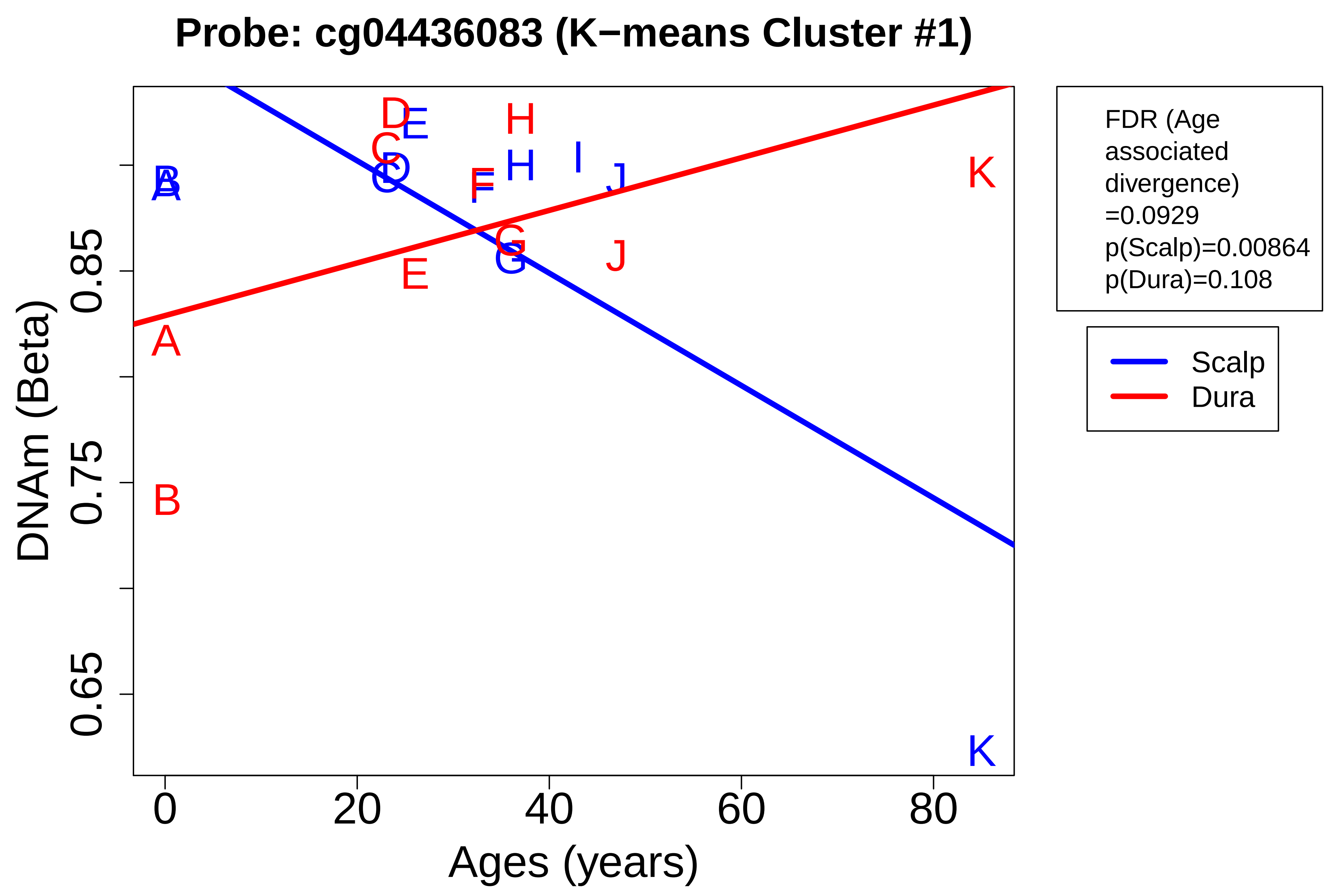

Probe: cg04436083 (K−means Cluster #1)
DNAm (Beta)
0.65
0.75
0.85
0
20
40
60
80
Ages (years)
A
B
C
D
E
F
G
H
I
J
K
FDR (Age associated divergence) =0.0929
p(Scalp)=0.00864
p(Dura)=0.108
Scalp
Dura

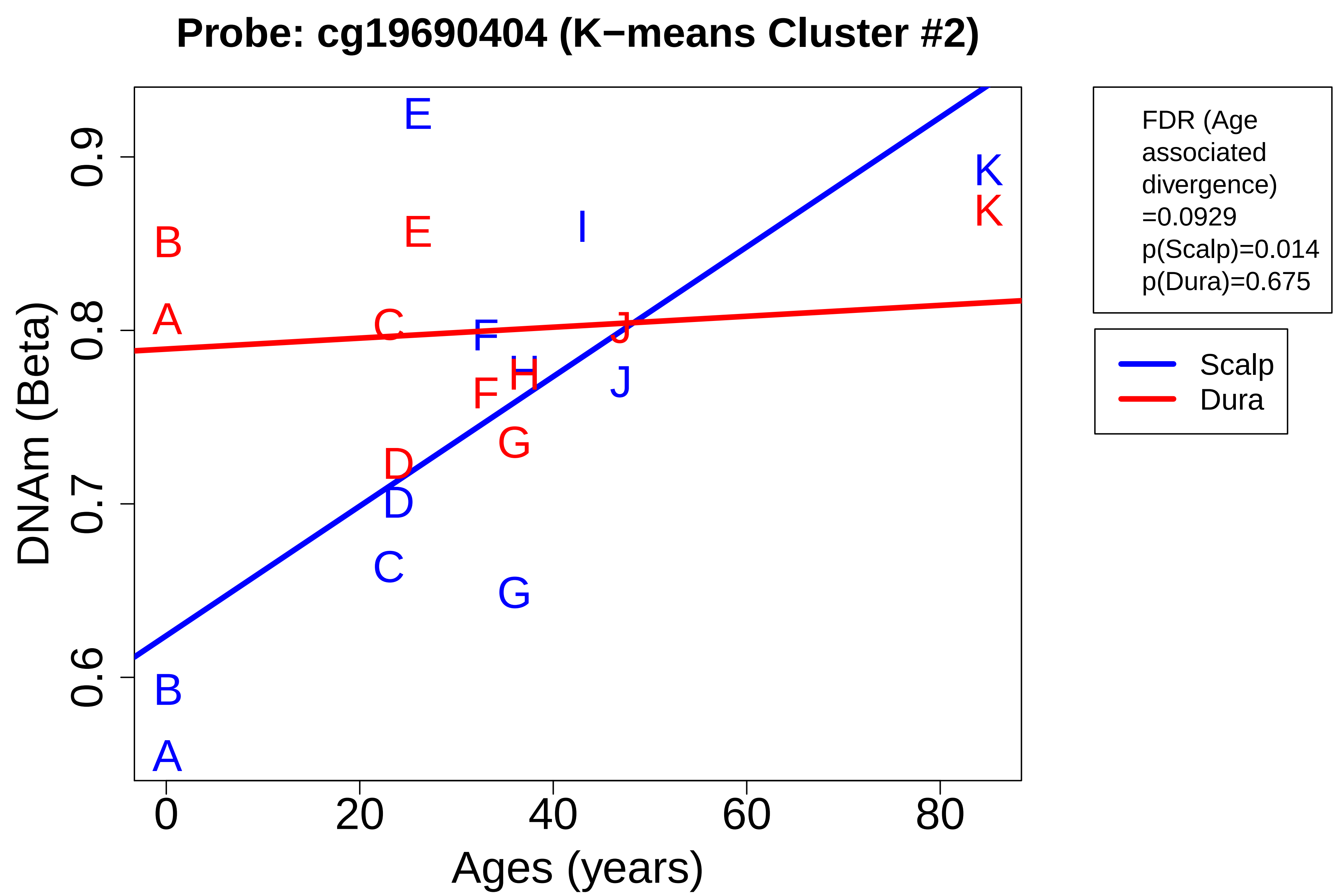

Probe: cg19690404 (K−means Cluster #2)
DNAm (Beta)
0.6
0.7
0.8
0.9
Ages (years)
0
20
40
60
80
FDR (Age associated divergence) =0.0929
p(Scalp)=0.014
p(Dura)=0.675
Scalp
Dura

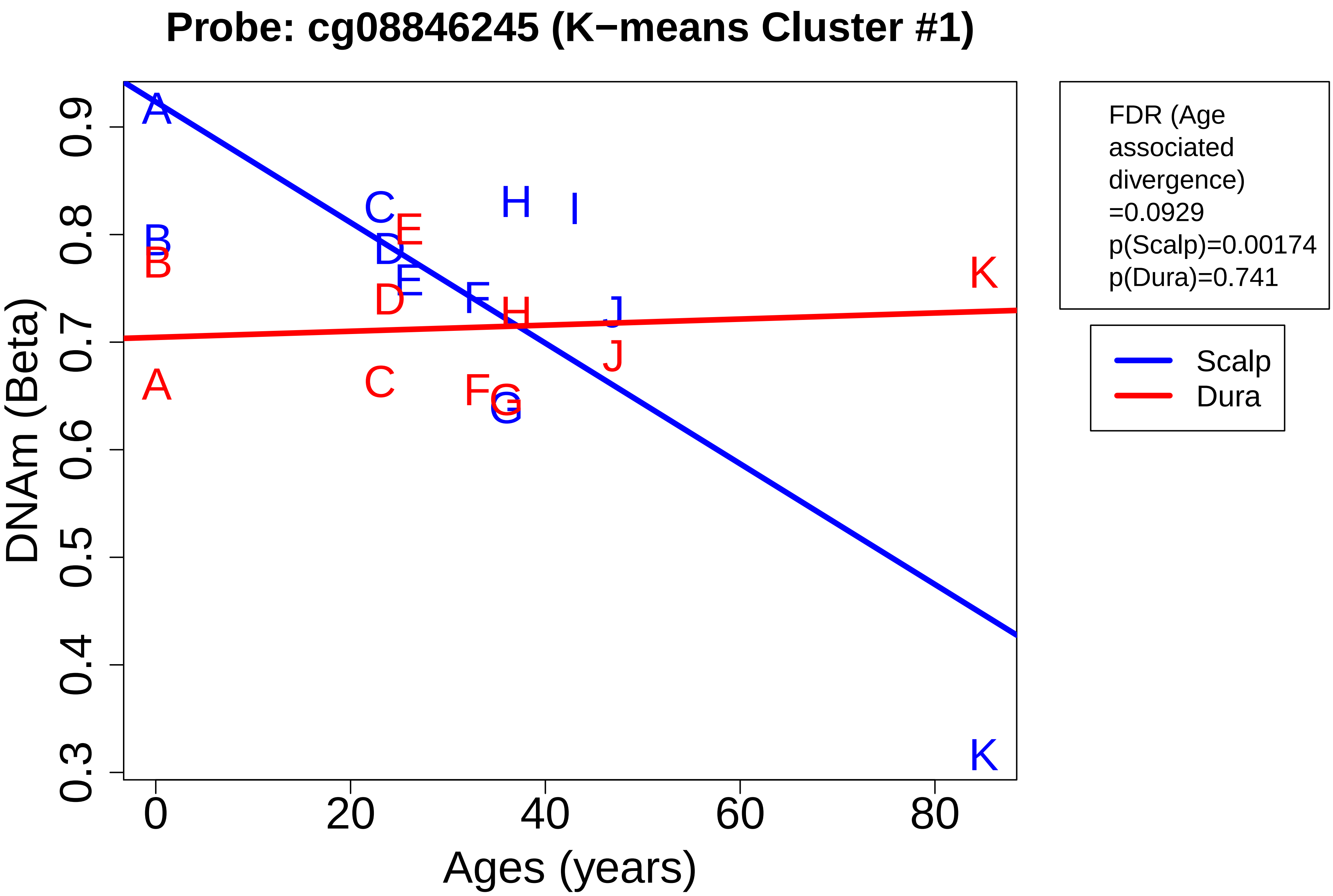

Probe: cg08846245 (K−means Cluster #1)
DNAm (Beta)
0.3
0.4
0.5
0.6
0.7
0.8
0.9
0
20
40
60
80
Ages (years)
A
B
C
D
E
F
G
H
I
J
K
FDR (Age associated divergence) =0.0929
p(Scalp)=0.00174
p(Dura)=0.741
Scalp
Dura

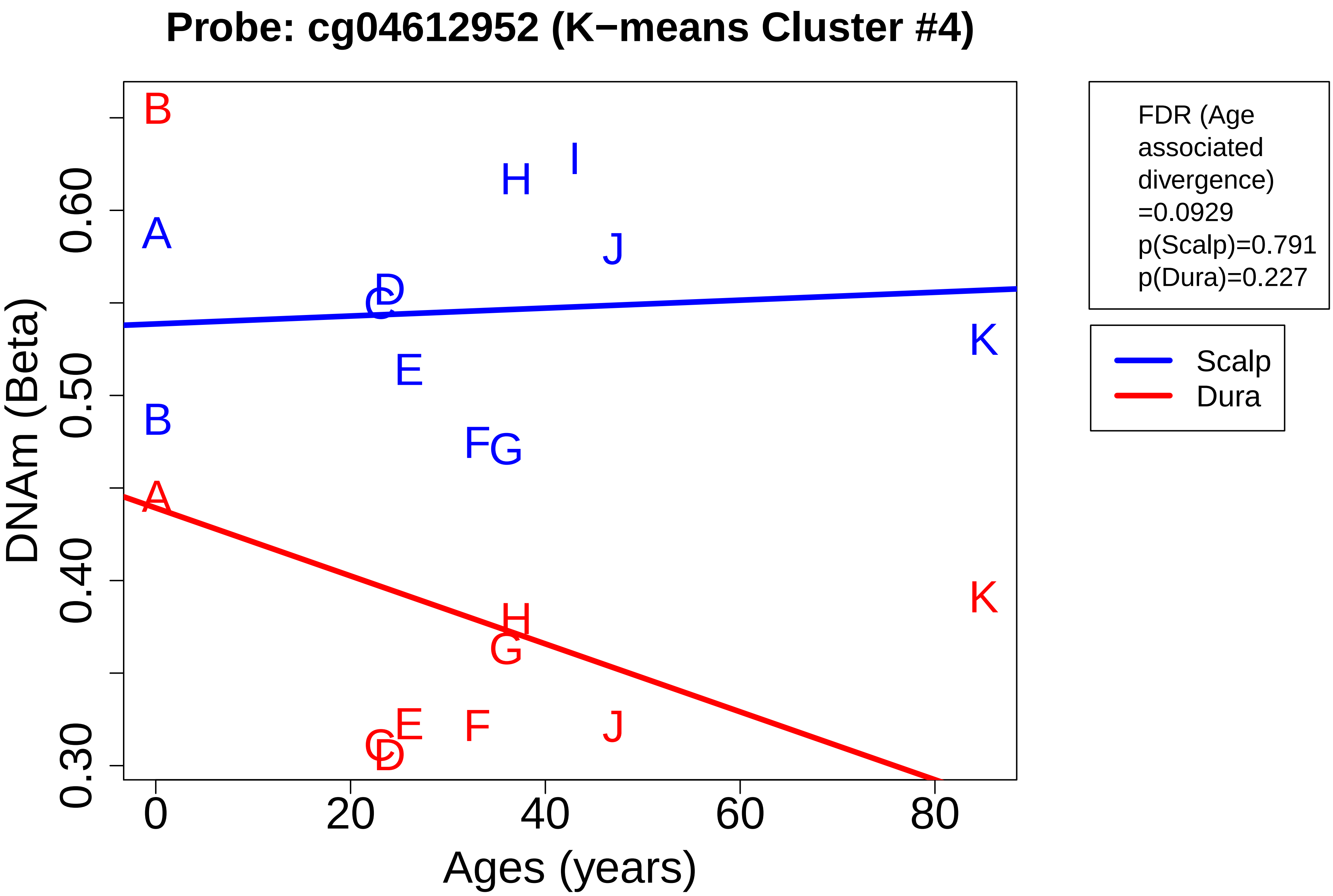

Probe: cg04612952 (K−means Cluster #4)
DNAm (Beta)
0.30
0.40
0.50
0.60
0
20
40
60
80
Ages (years)
B
A
B
A
C
D
E
F
G
H
I
J
K
H
G
E
C
D
F
J
K
FDR (Age associated divergence) =0.0929
p(Scalp)=0.791
p(Dura)=0.227
Scalp
Dura

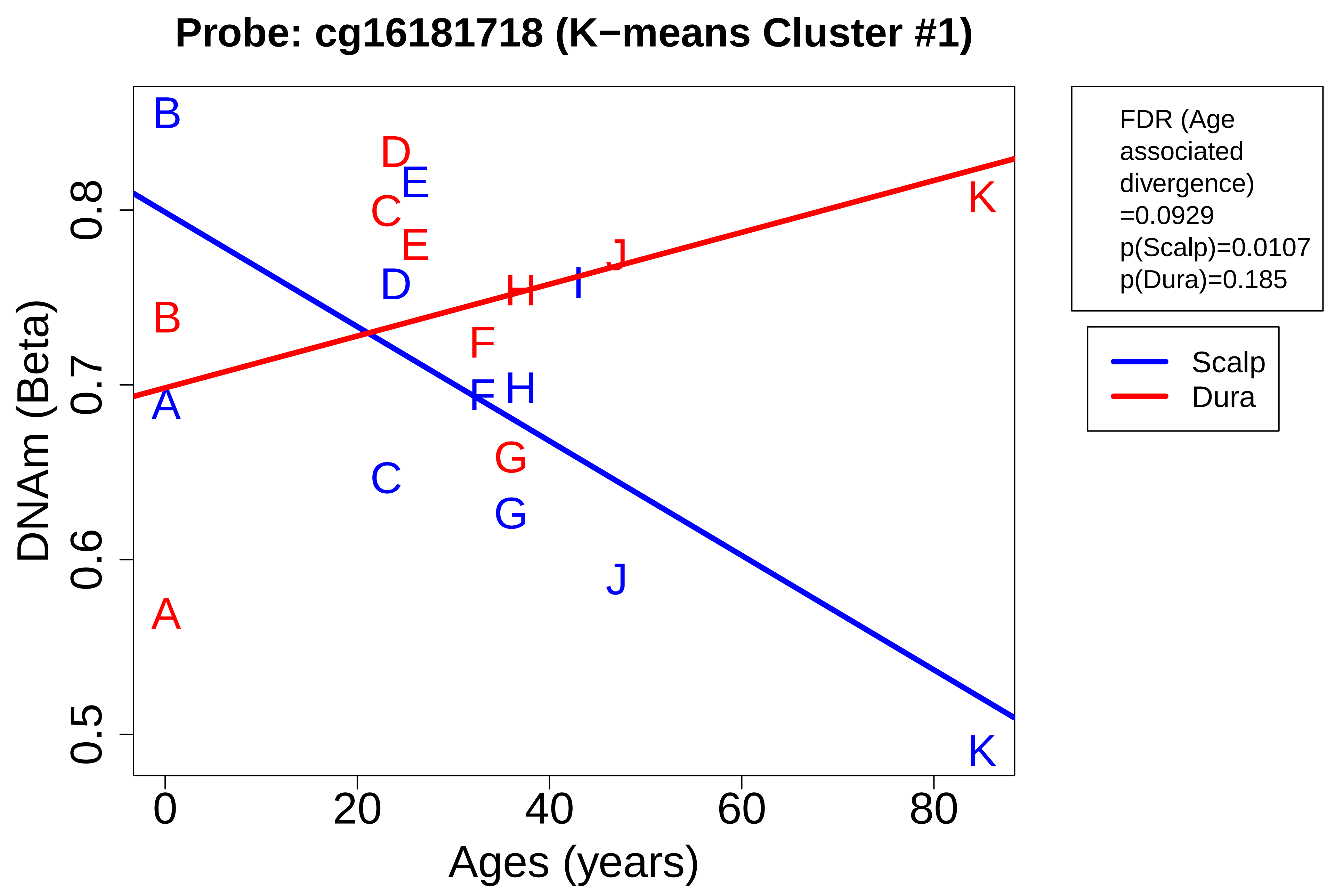

Probe: cg16181718 (K−means Cluster #1)
DNAm (Beta)
0.5
0.6
0.7
0.8
Ages (years)
0
20
40
60
80
B
D
E
C
E
D
B
A
A
C
G
G
F
F
H
H
I
J
J
K
K
FDR (Age associated divergence) =0.0929
p(Scalp)=0.0107
p(Dura)=0.185
Scalp
Dura

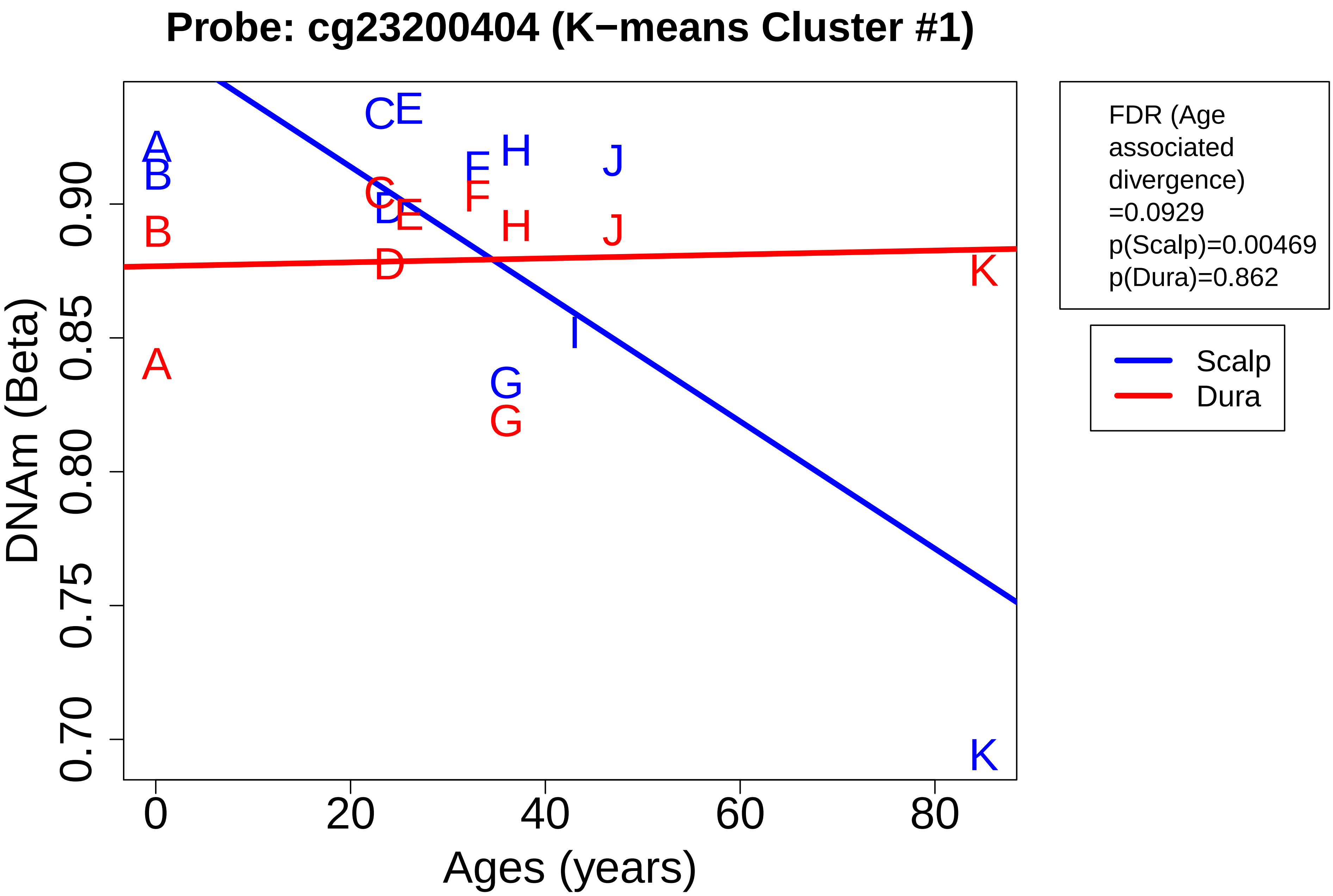

Probe: cg23200404 (K−means Cluster #1)
DNAm (Beta)
0.70
0.75
0.80
0.85
0.90
0
20
40
60
80
Ages (years)
A
B
C
D
E
F
G
H
I
J
K
FDR (Age associated divergence) =0.0929
p(Scalp)=0.00469
p(Dura)=0.862
Scalp
Dura

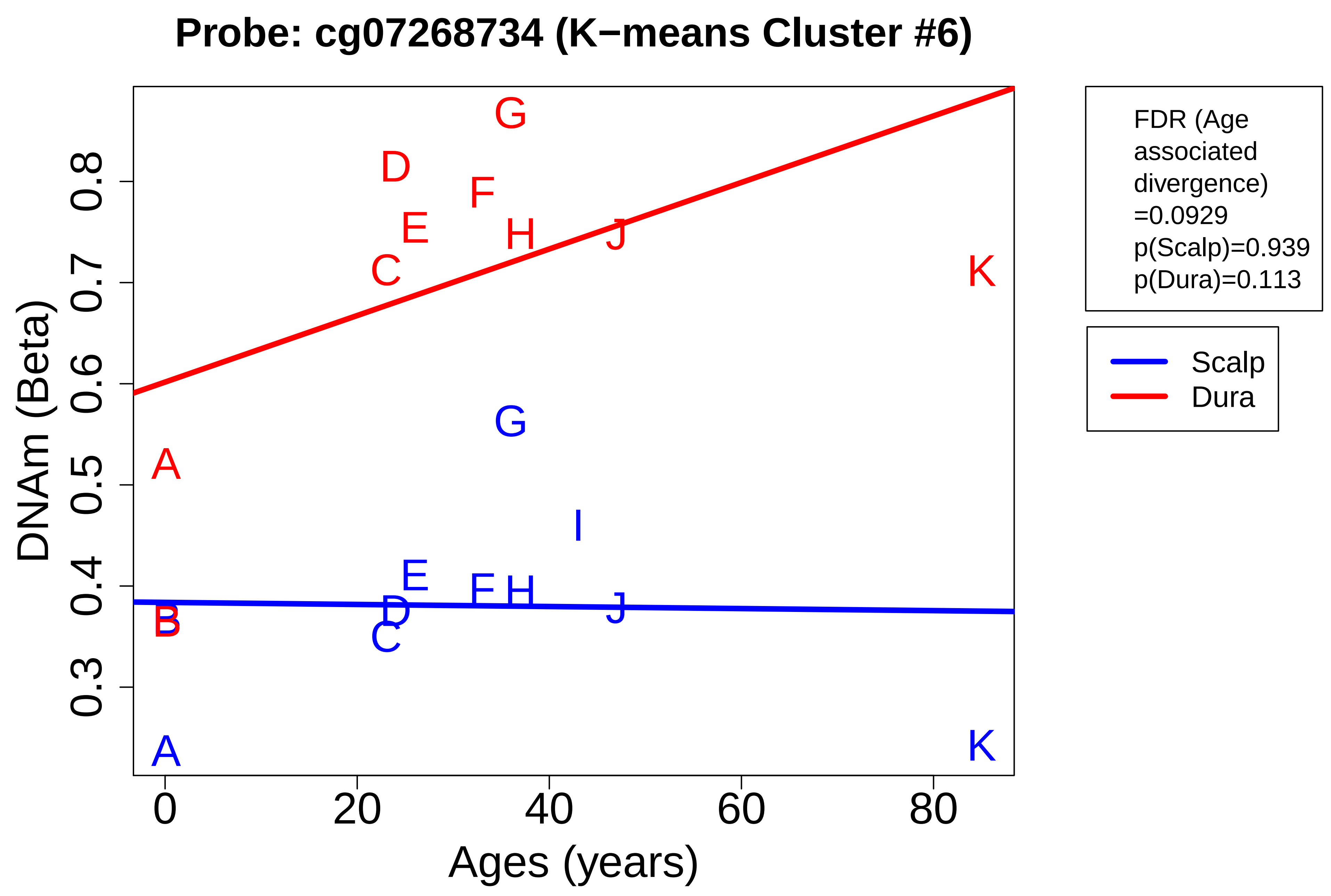
Probe: cg07268734 (K−means Cluster #6)
DNAm (Beta)
0.3
0.4
0.5
0.6
0.7
0.8
Ages (years)
0
20
40
60
80
A
B
C
D
E
F
G
H
I
J
K
FDR (Age associated divergence) =0.0929
p(Scalp)=0.939
p(Dura)=0.113
Scalp
Dura

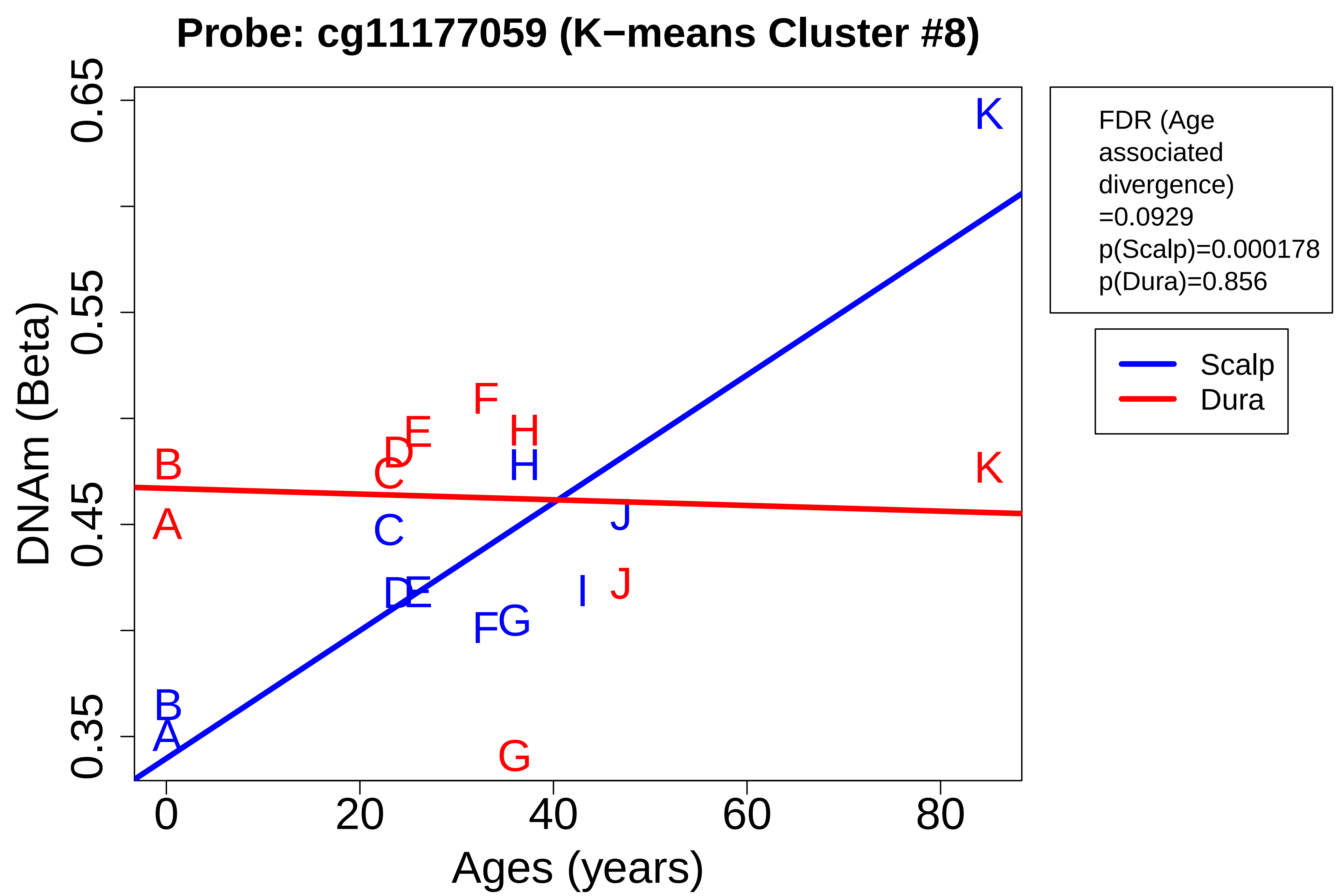
Probe: cg11177059 (K−means Cluster #8)
DNAm (Beta)
0.35
0.45
0.55
0.65
Ages (years)
0
20
40
60
80
FDR (Age associated divergence) =0.0929
p(Scalp)=0.000178
p(Dura)=0.856
Scalp
Dura
A
B
C
D
E
F
G
H
I
J
K

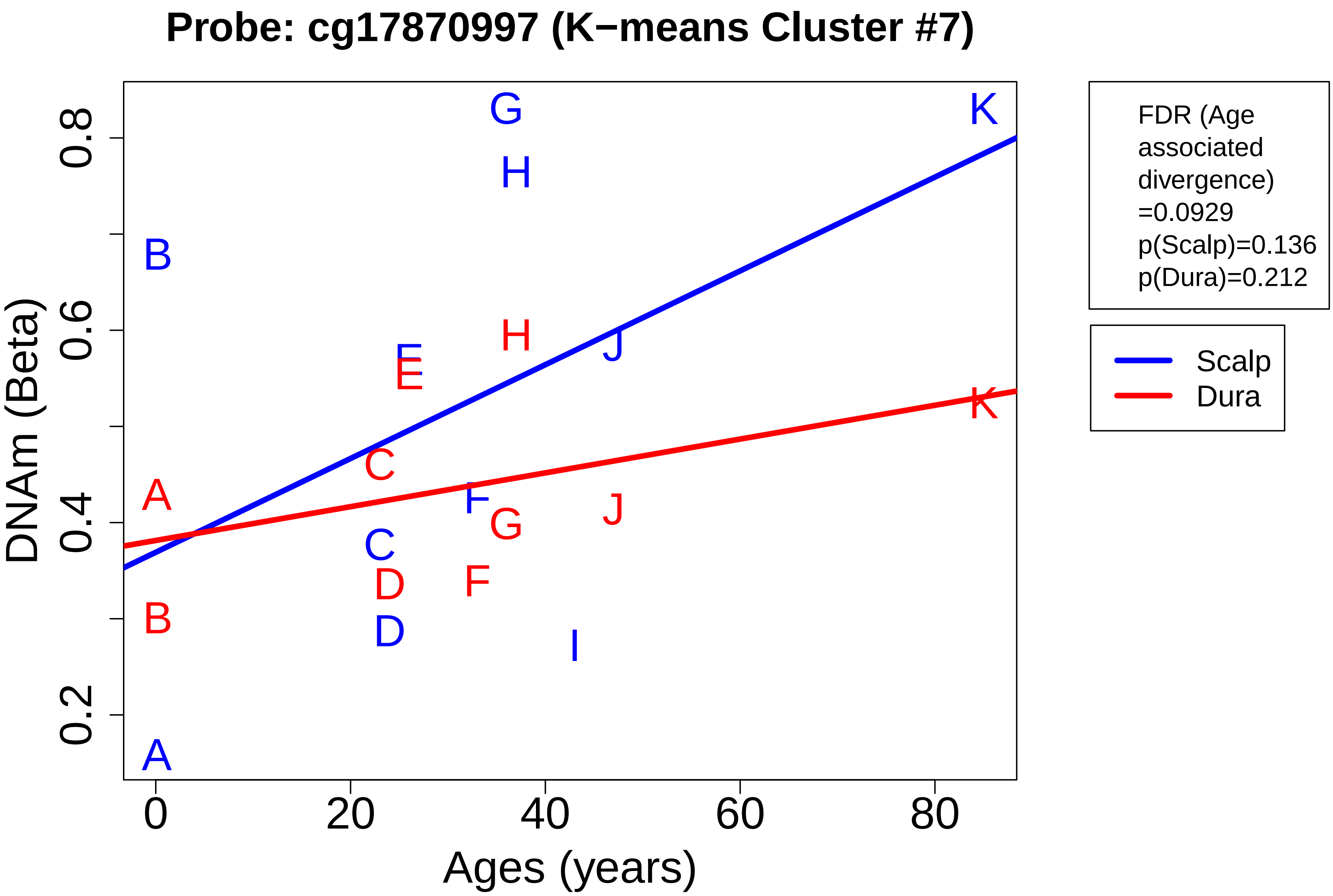

Probe: cg17870997 (K−means Cluster #7)
DNAm (Beta)
0.2
0.4
0.6
0.8
Ages (years)
0
20
40
60
80
FDR (Age associated divergence) =0.0929
p(Scalp)=0.136
p(Dura)=0.212
Scalp
Dura

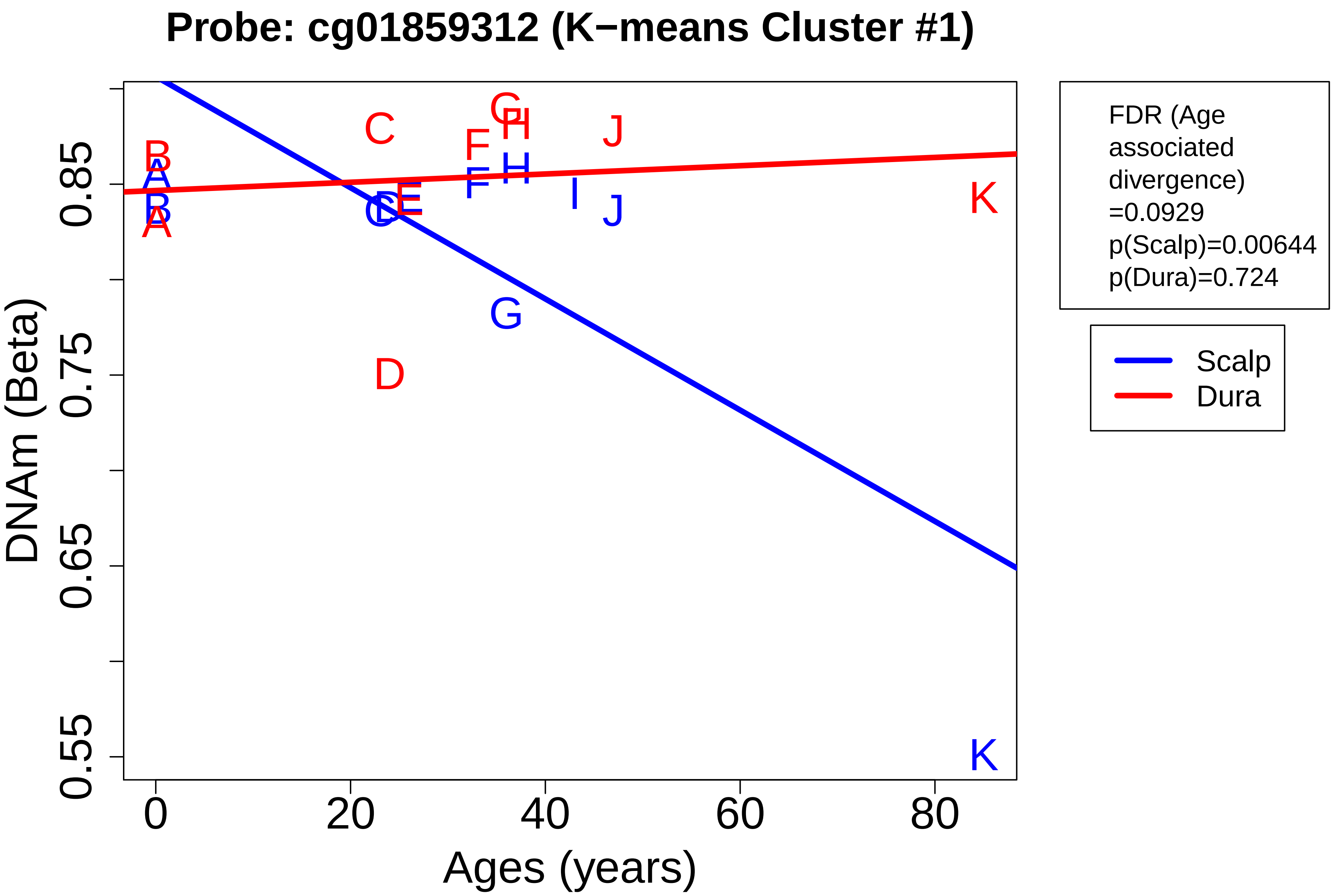

Probe: cg01859312 (K−means Cluster #1)
DNAm (Beta)
0.55
0.65
0.75
0.85
0
20
40
60
80
Ages (years)
A
B
C
D
E
F
G
H
I
J
K
FDR (Age associated divergence) =0.0929
p(Scalp)=0.00644
p(Dura)=0.724
Scalp
Dura

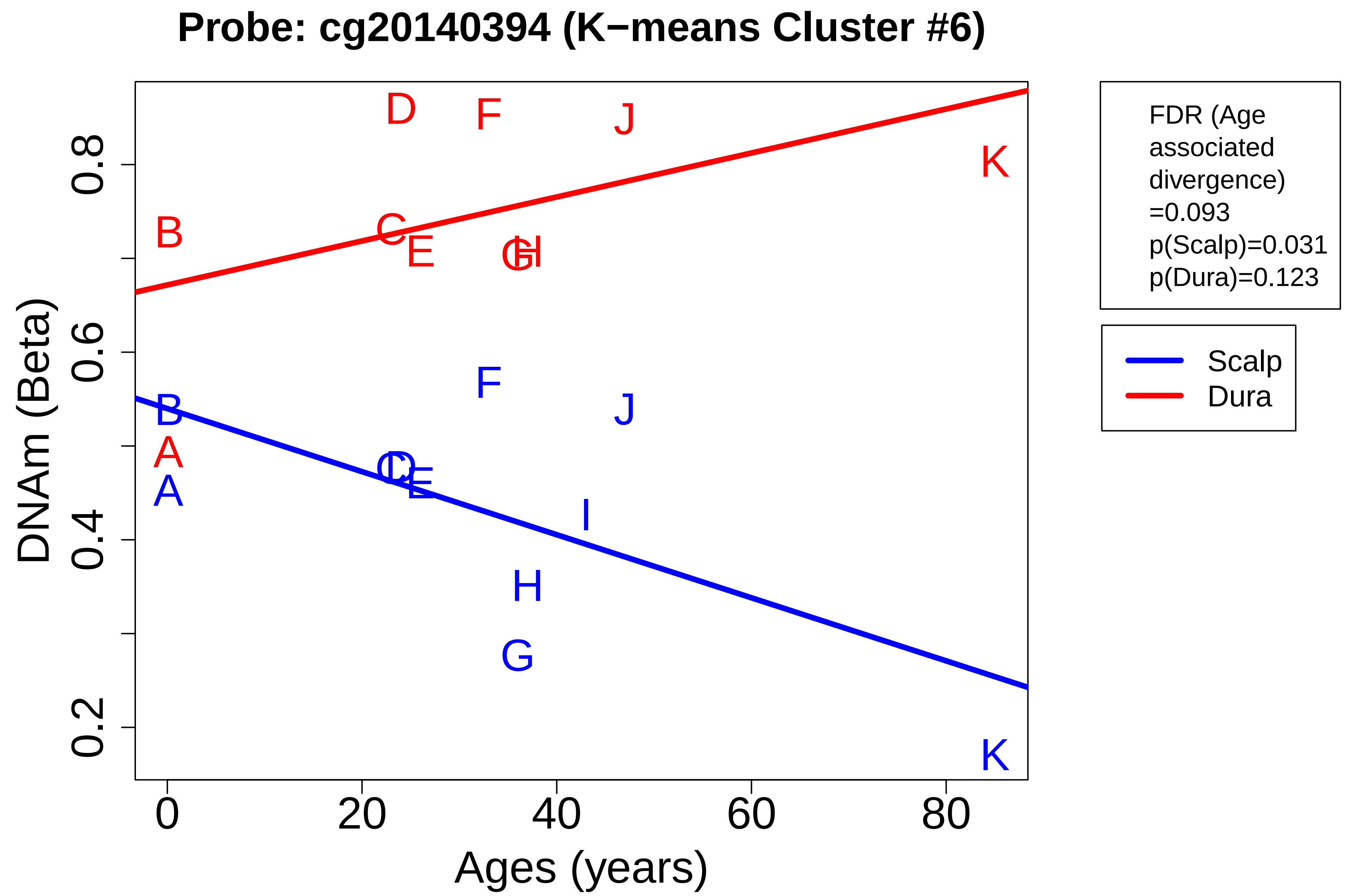
Probe: cg20140394 (K−means Cluster #6)
DNAm (Beta)
0.2
0.4
0.6
0.8
0
20
40
60
80
Ages (years)
FDR (Age associated divergence) =0.093
p(Scalp)=0.031
p(Dura)=0.123
Scalp
Dura
A
B
C
D
E
F
G
H
I
J
K

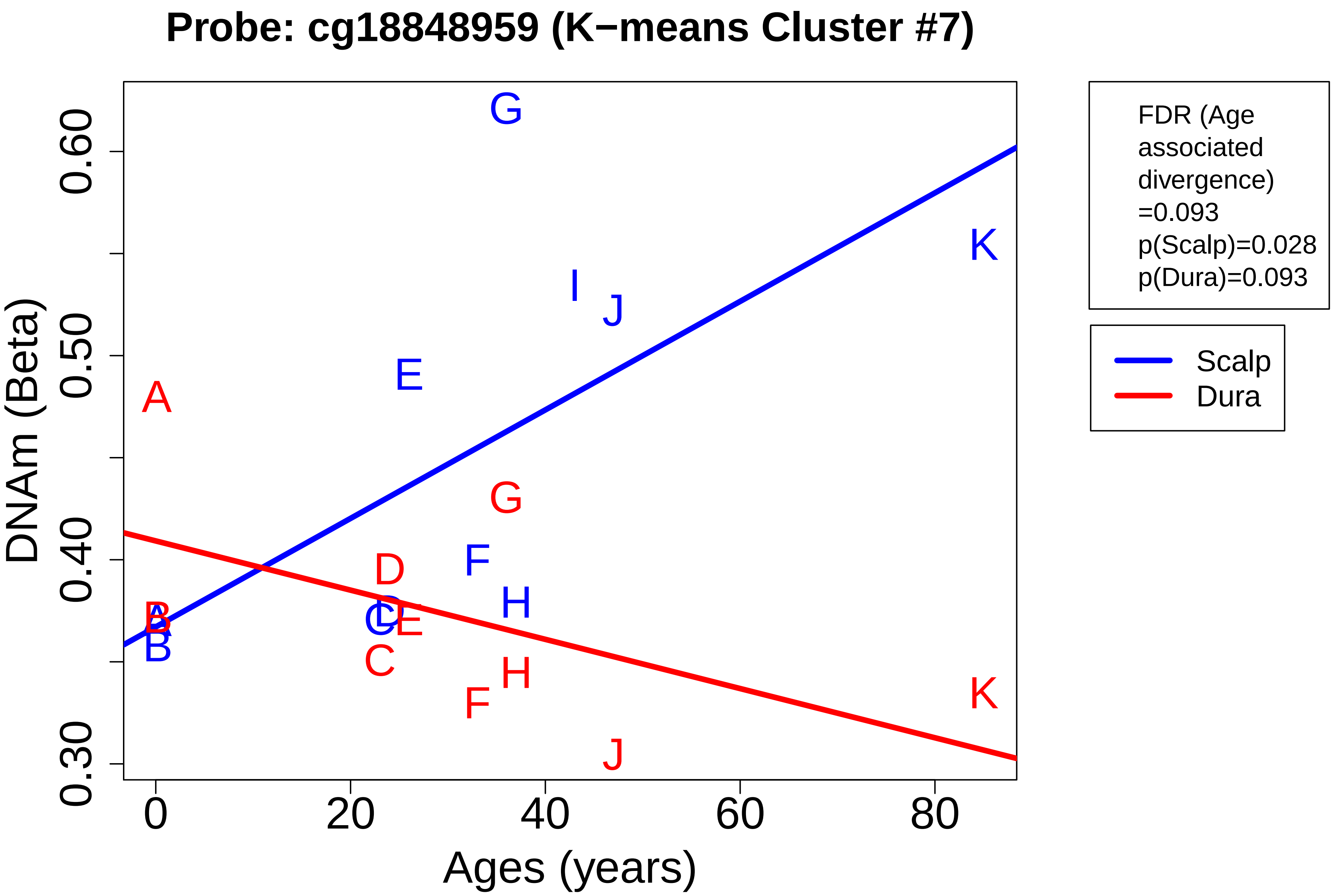
Probe: cg18848959 (K−means Cluster #7)
FDR (Age associated divergence) =0.093
p(Scalp)=0.028
p(Dura)=0.093
Scalp
Dura
DNAm (Beta)
0.30
0.40
0.50
0.60
0
20
40
60
80
Ages (years)
A
B
C
D
E
F
G
H
I
J
K

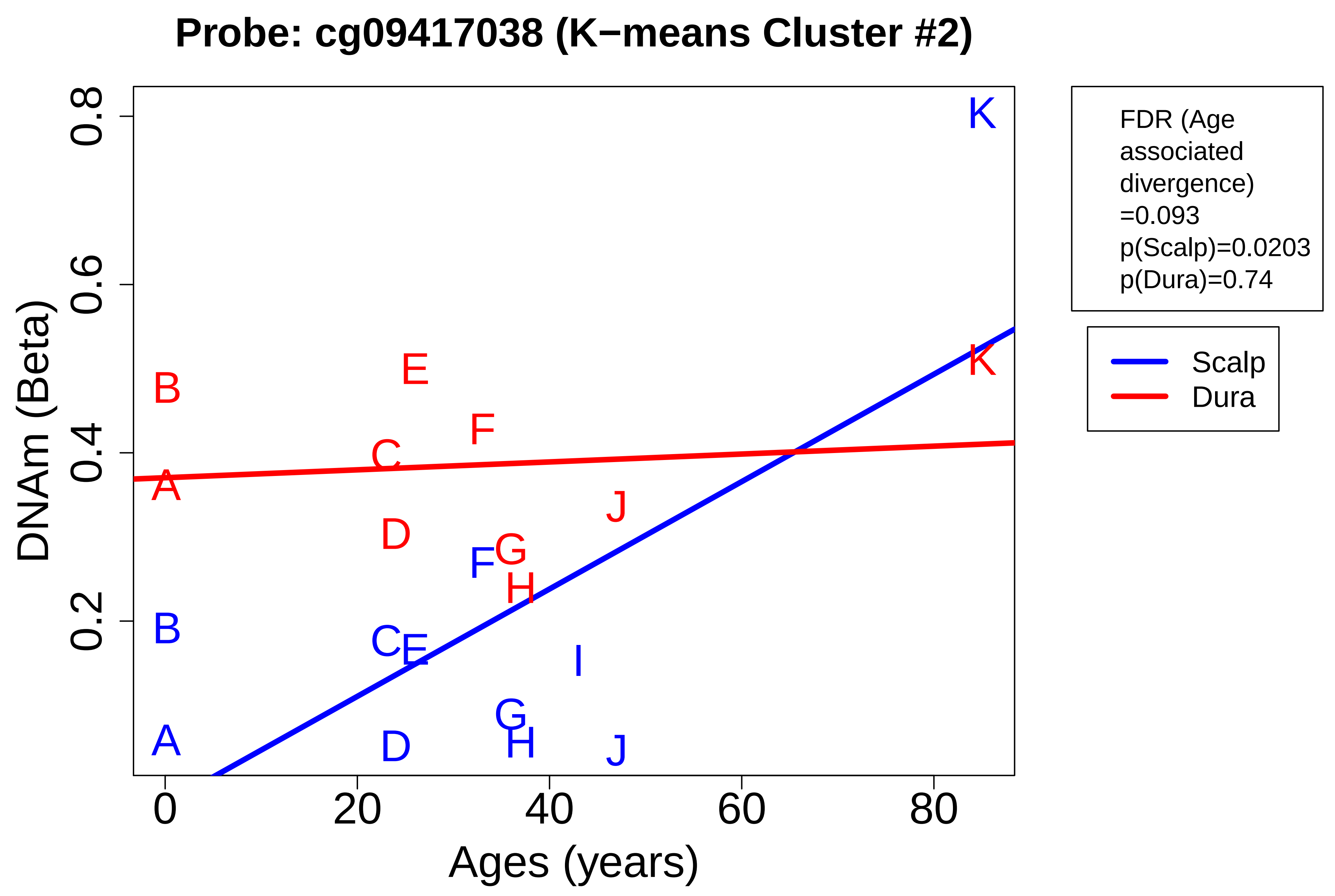

Probe: cg09417038 (K−means Cluster #2)
DNAm (Beta)
0.2
0.4
0.6
0.8
0
20
40
60
80
Ages (years)
FDR (Age associated divergence) =0.093
p(Scalp)=0.0203
p(Dura)=0.74
Scalp
Dura
A B C D E F G H I J K

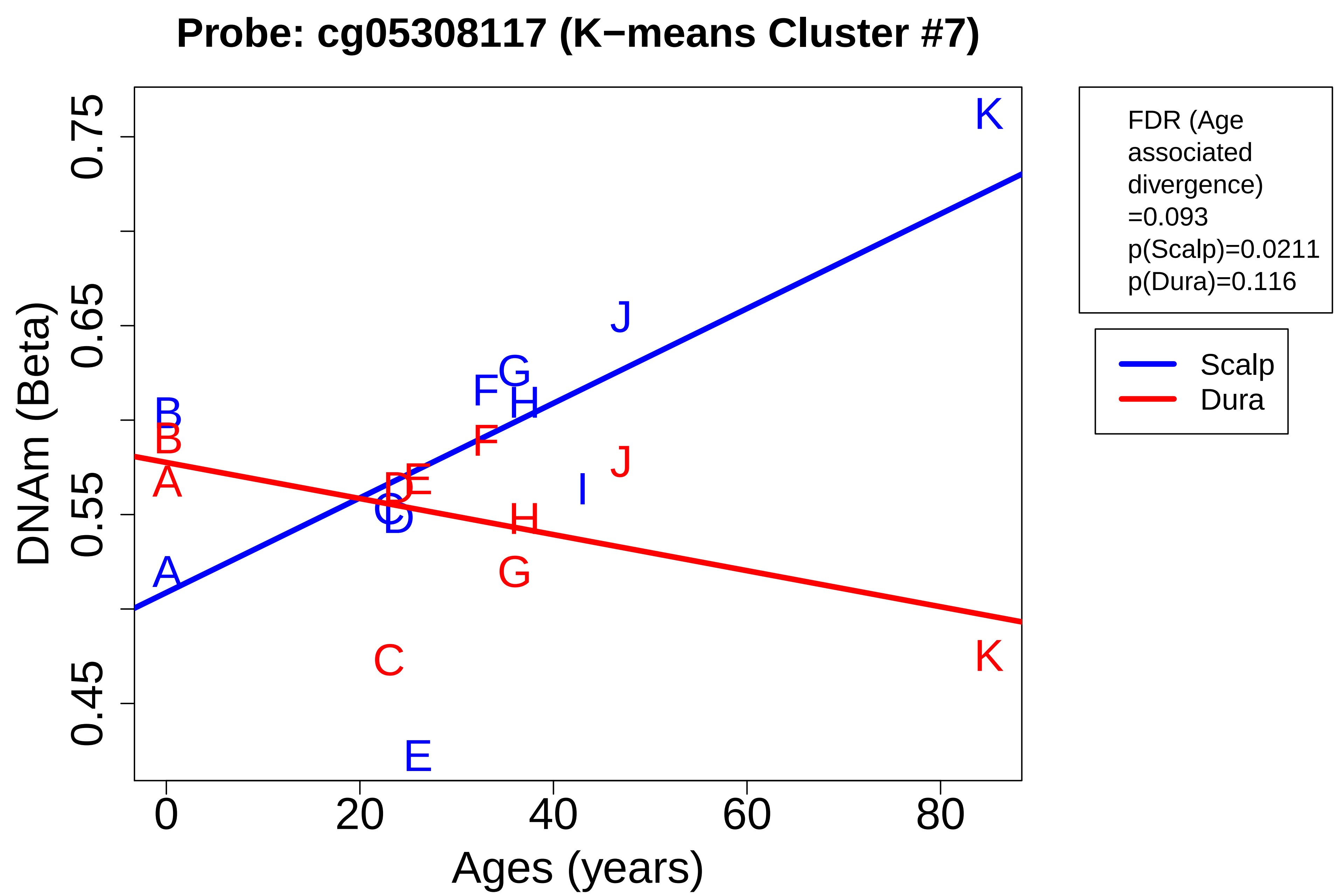

Probe: cg05308117 (K−means Cluster #7)
DNAm (Beta)
0.45
0.55
0.65
0.75
Ages (years)
0
20
40
60
80
FDR (Age associated divergence) =0.093
p(Scalp)=0.0211
p(Dura)=0.116
Scalp
Dura
A
B
C
D
E
F
G
H
I
J
K

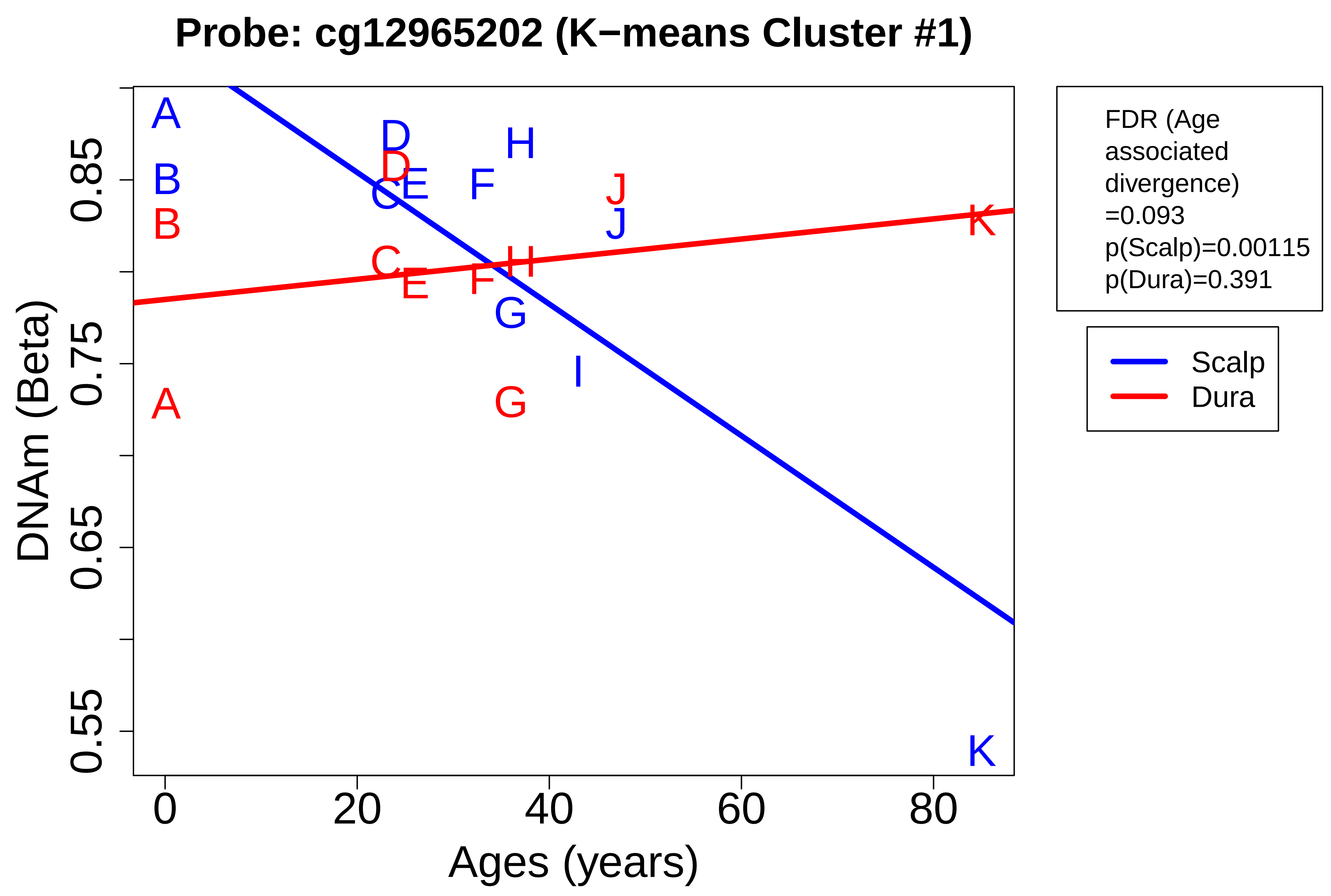

Probe: cg12965202 (K−means Cluster #1)
DNAm (Beta)
0.55
0.65
0.75
0.85
0
20
40
60
80
Ages (years)
A
B
C
D
E
F
G
H
I
J
K
FDR (Age associated divergence) =0.093
p(Scalp)=0.00115
p(Dura)=0.391
Scalp
Dura

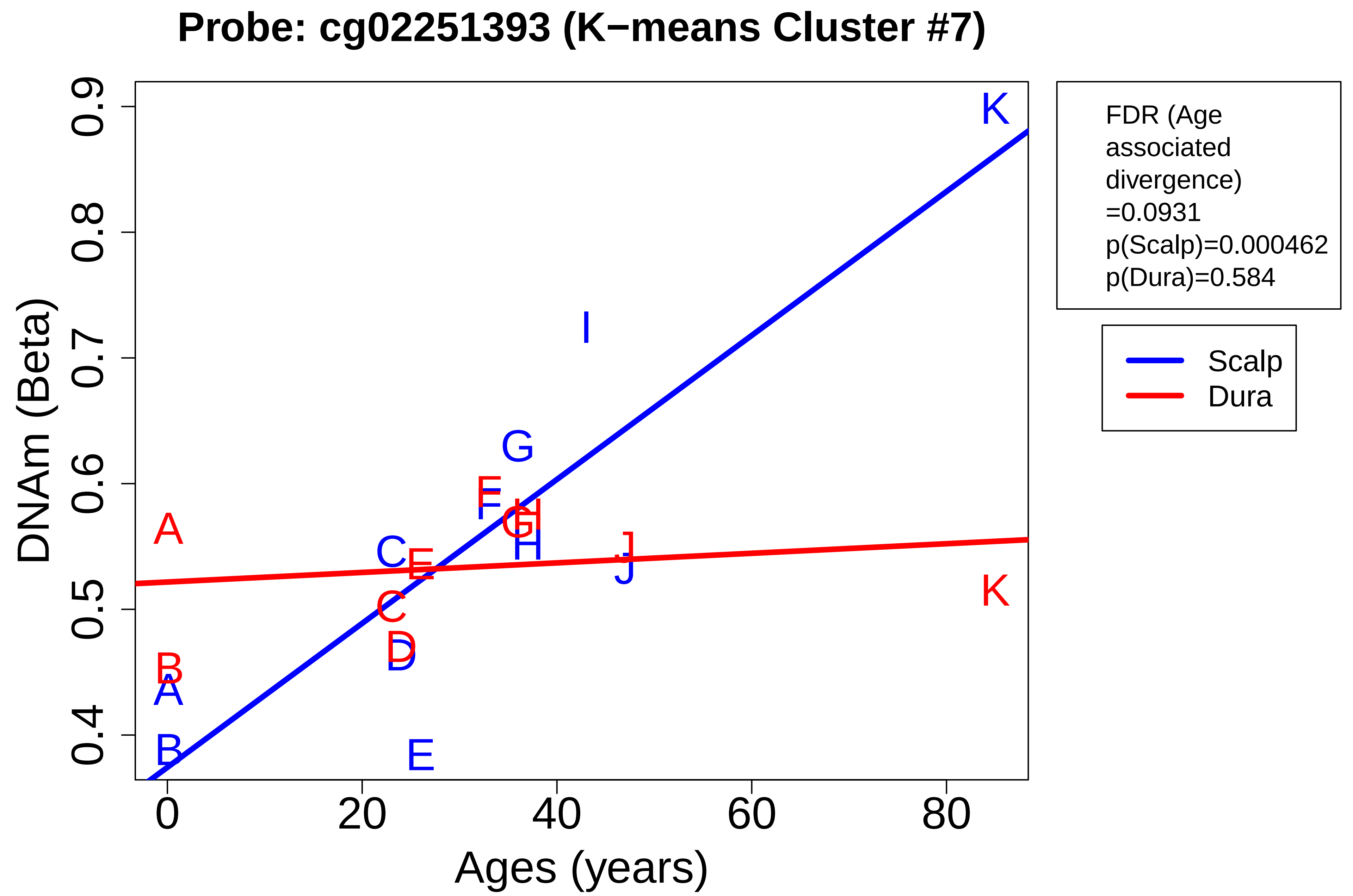

Probe: cg02251393 (K−means Cluster #7)
DNAm (Beta)
Ages (years)
0.4
0.5
0.6
0.7
0.8
0.9
0
20
40
60
80
FDR (Age associated divergence) =0.0931
p(Scalp)=0.000462
p(Dura)=0.584
Scalp
Dura

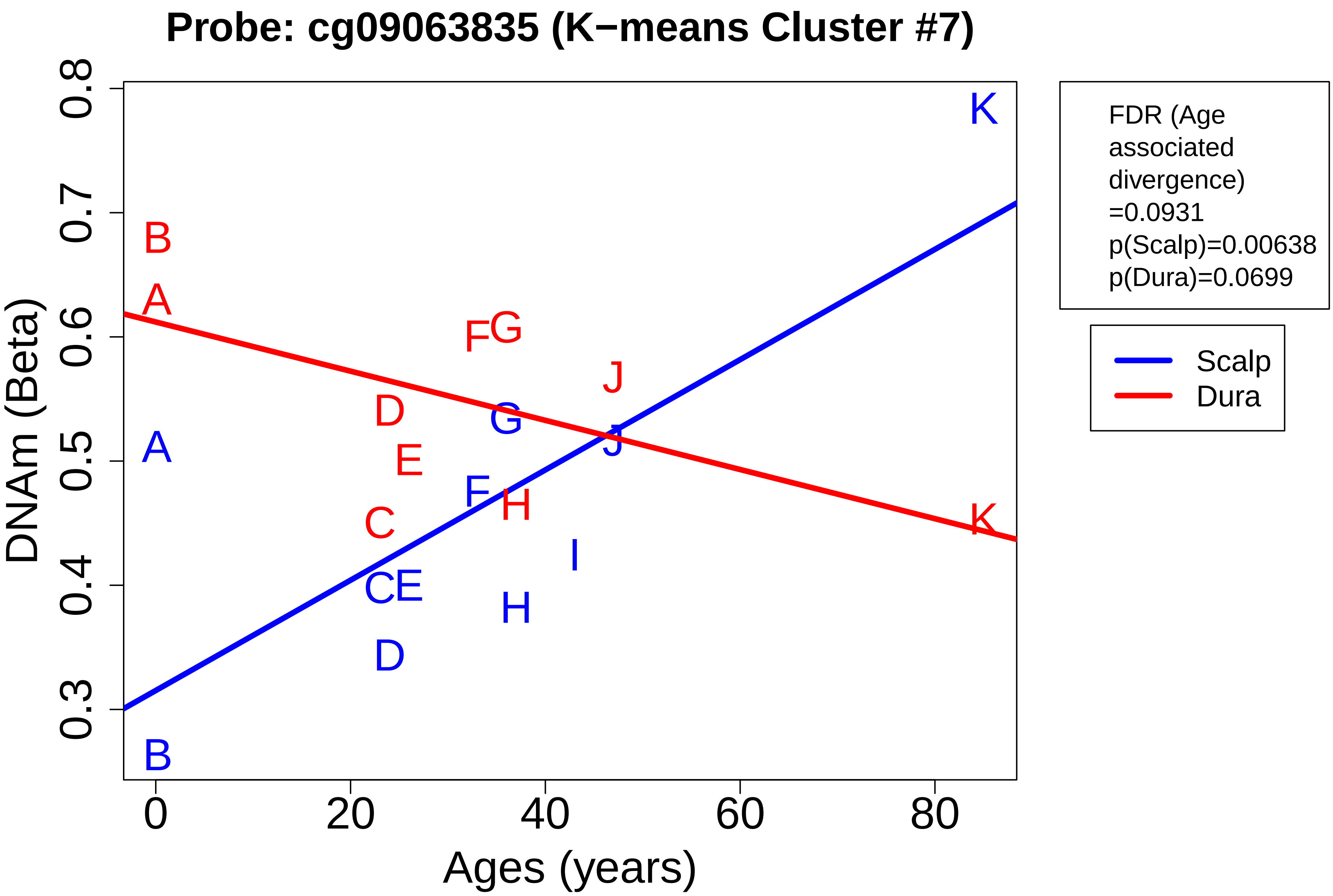

Probe: cg09063835 (K−means Cluster #7)
DNAm (Beta)
0.3
0.4
0.5
0.6
0.7
0.8
Ages (years)
0
20
40
60
80
FDR (Age associated divergence) =0.0931
p(Scalp)=0.00638
p(Dura)=0.0699
Scalp
Dura
A
B
C
D
E
F
G
H
I
J
K

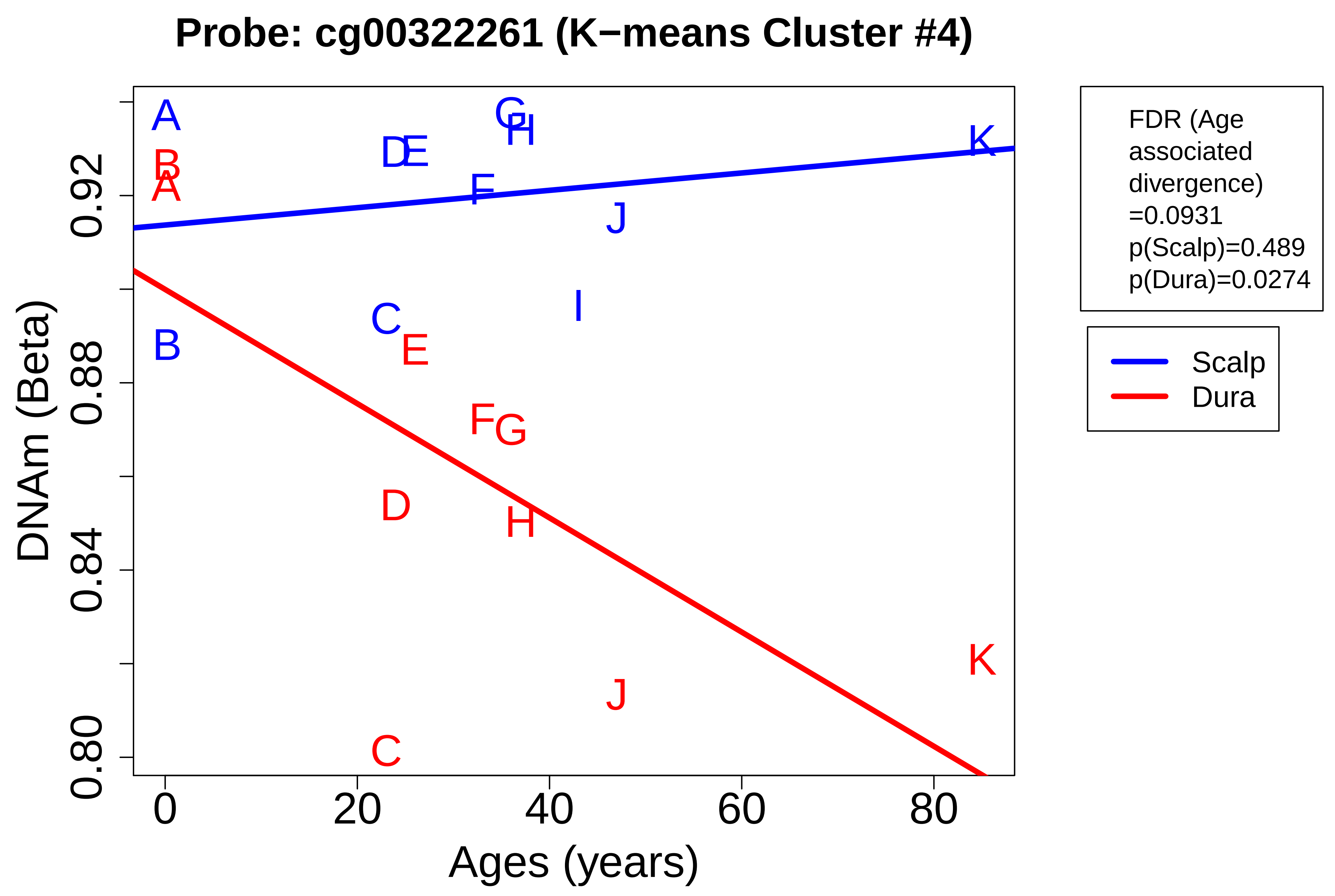

Probe: cg00322261 (K−means Cluster #4)
DNAm (Beta)
0.80
0.84
0.88
0.92
0
20
40
60
80
Ages (years)
FDR (Age associated divergence) =0.0931
p(Scalp)=0.489
p(Dura)=0.0274
Scalp
Dura

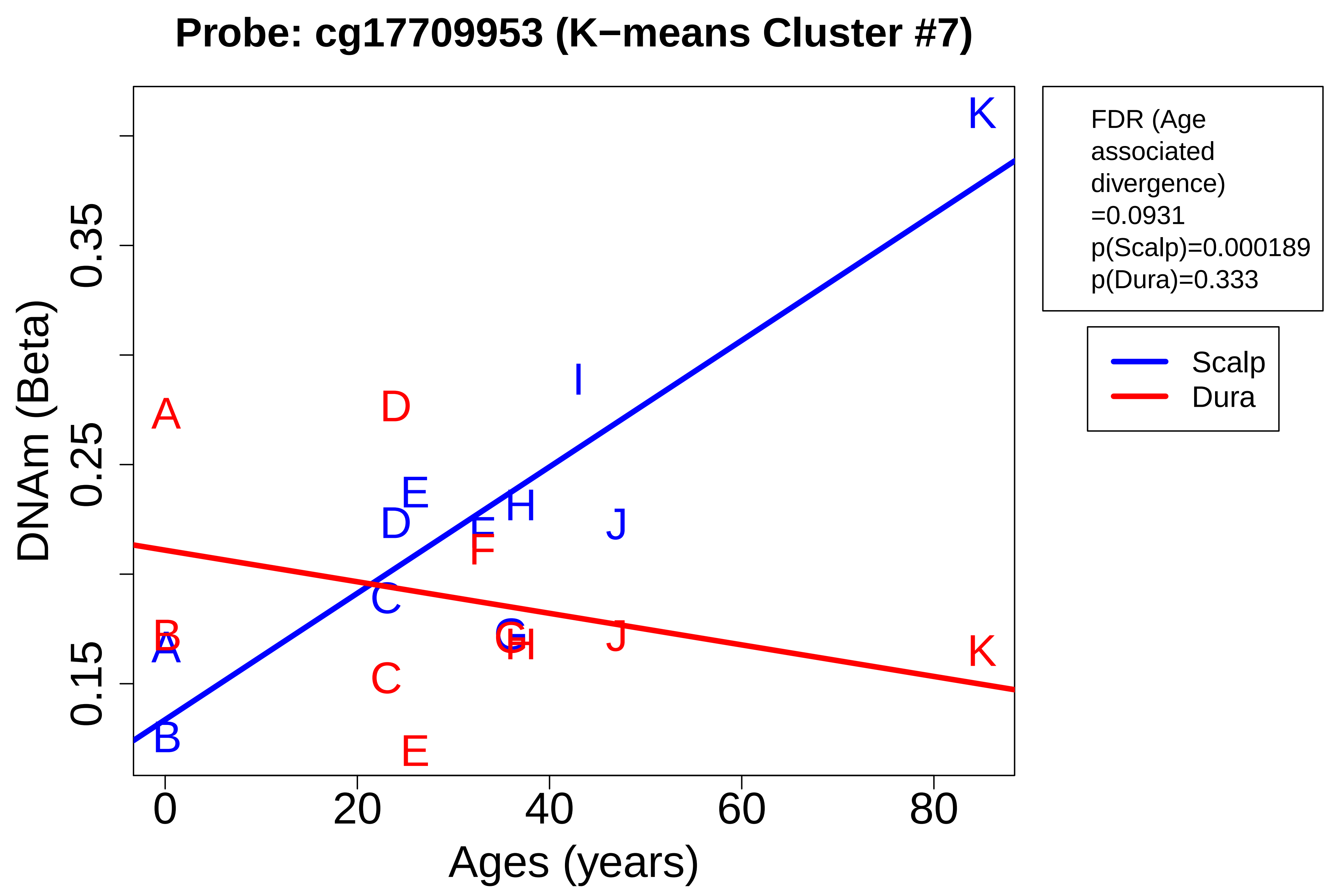

Probe: cg17709953 (K−means Cluster #7)
DNAm (Beta)
0.15
0.25
0.35
Ages (years)
0
20
40
60
80
FDR (Age associated divergence) =0.0931
p(Scalp)=0.000189
p(Dura)=0.333
Scalp
Dura
A
B
C
D
E
F
G
H
I
J
K

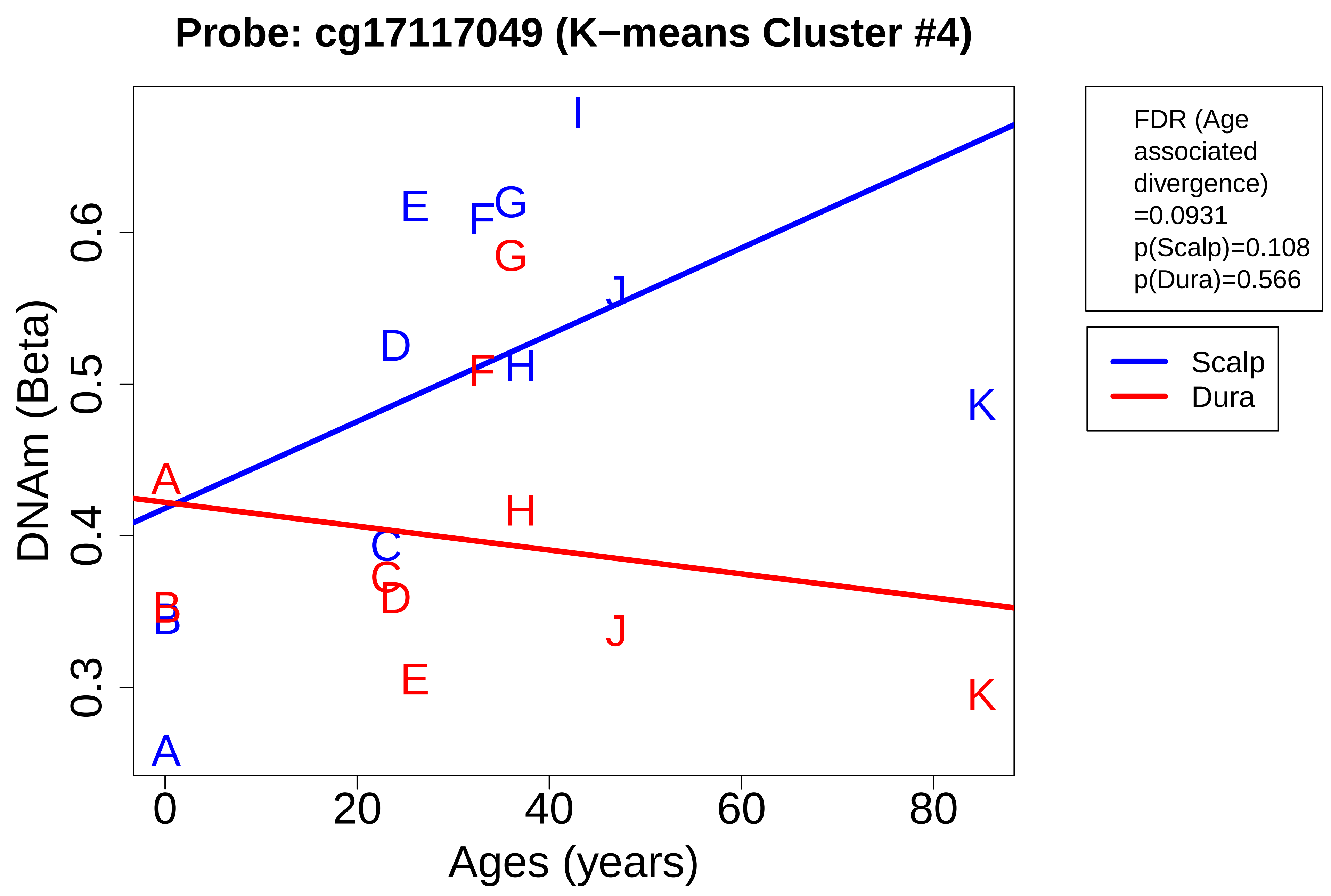

Probe: cg17117049 (K−means Cluster #4)
DNAm (Beta)
0.3
0.4
0.5
0.6
Ages (years)
0
20
40
60
80
FDR (Age associated divergence) =0.0931
p(Scalp)=0.108
p(Dura)=0.566
Scalp
Dura
A
B
C
D
E
F
G
H
I
J
K

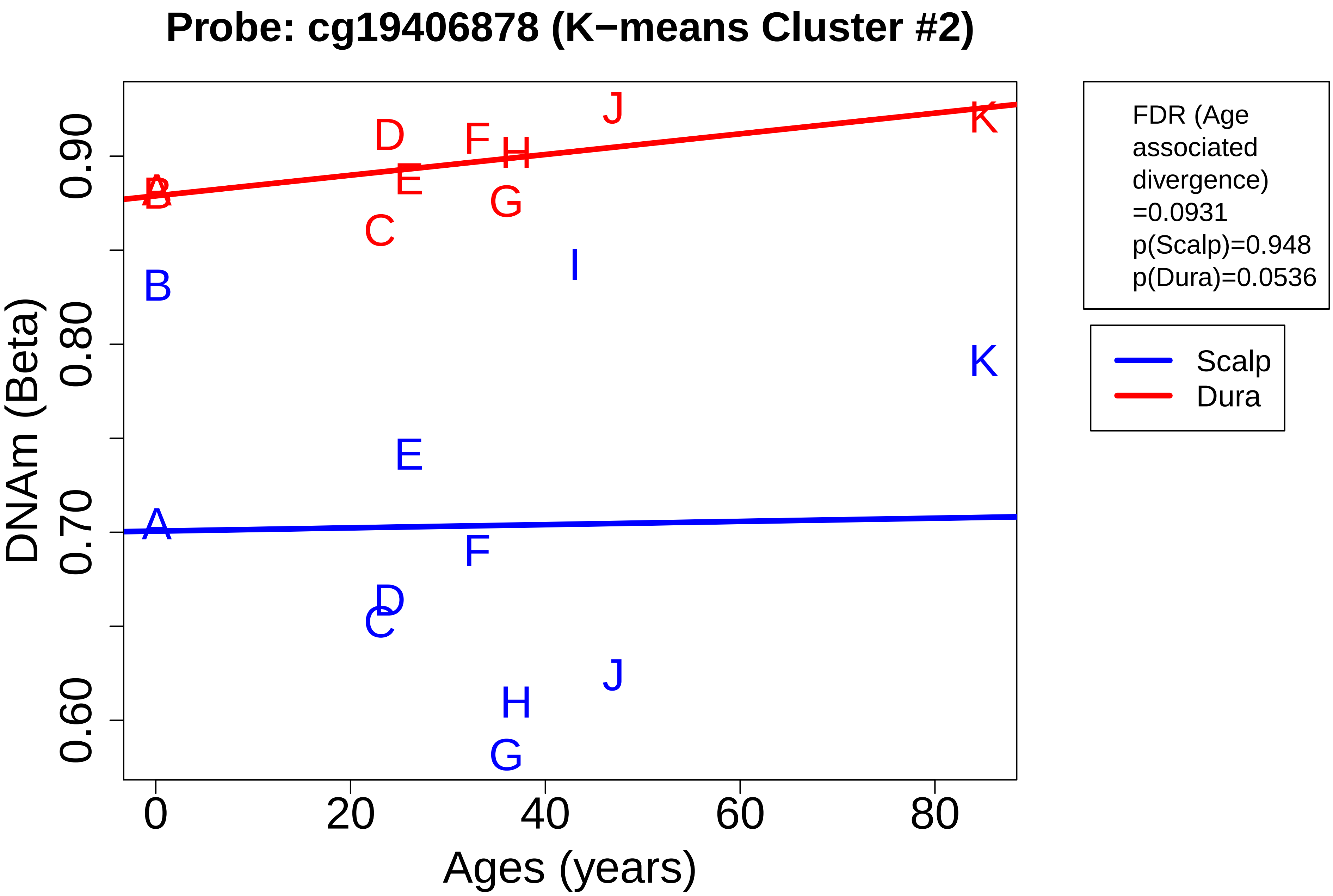

Probe: cg19406878 (K−means Cluster #2)
DNAm (Beta)
0.60
0.70
0.80
0.90
0
20
40
60
80
Ages (years)
FDR (Age associated divergence) =0.0931
p(Scalp)=0.948
p(Dura)=0.0536
Scalp
Dura

Probe: cg16728934 (K−means Cluster #1)

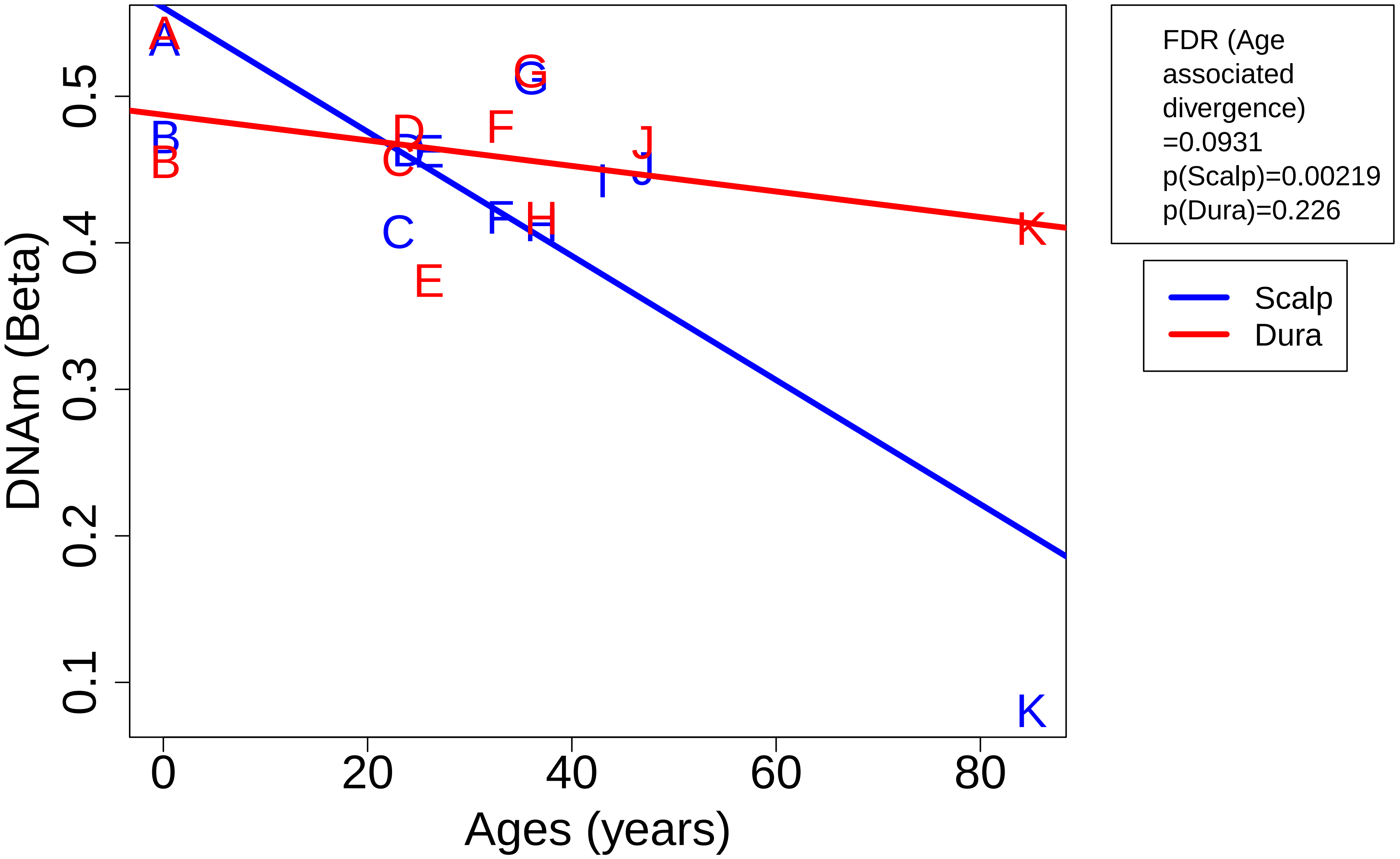

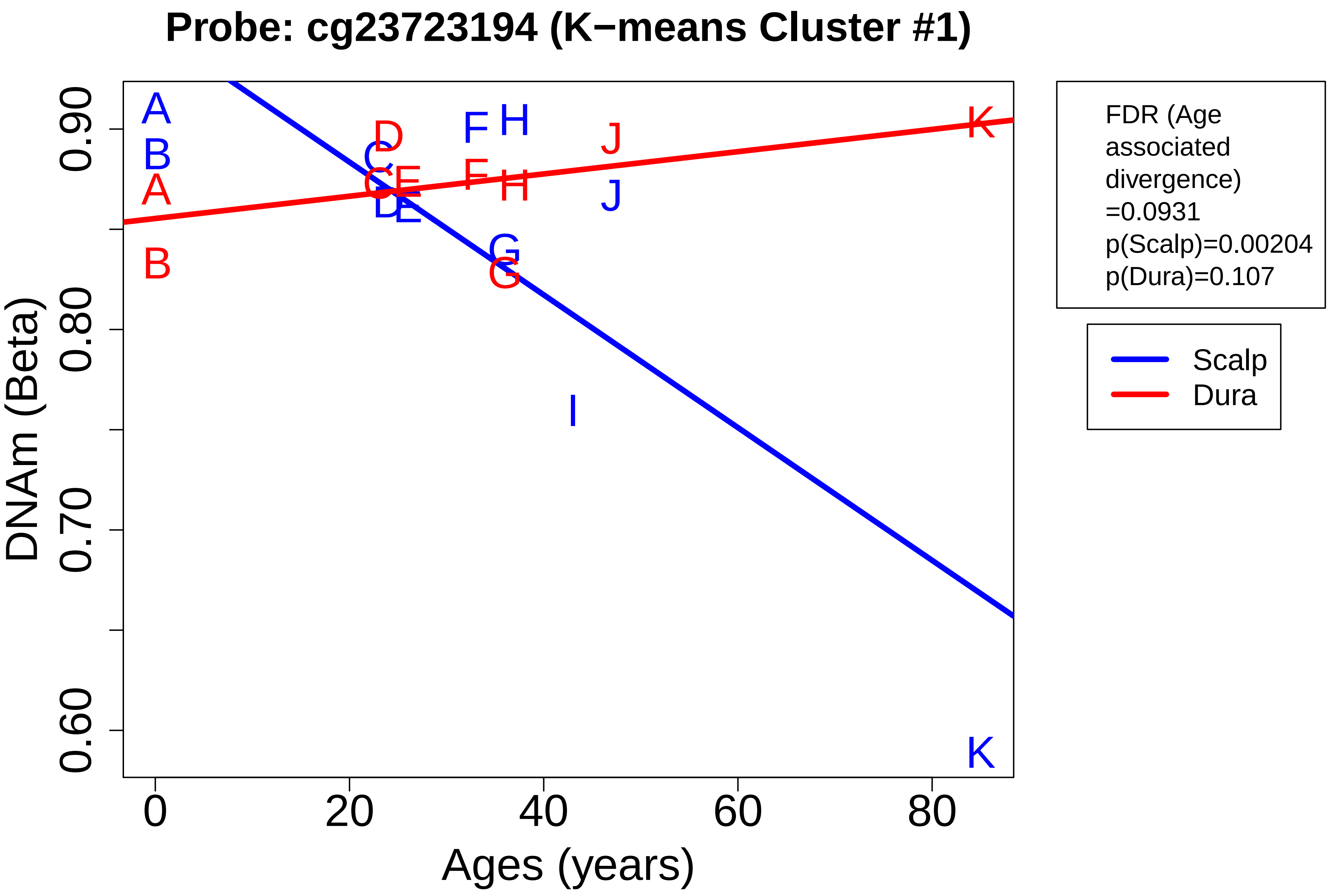
Probe: cg23723194 (K−means Cluster #1)
DNAm (Beta)
0.60
0.70
0.80
0.90
0
20
40
60
80
Ages (years)
FDR (Age associated divergence) =0.0931
p(Scalp)=0.00204
p(Dura)=0.107
Scalp
Dura

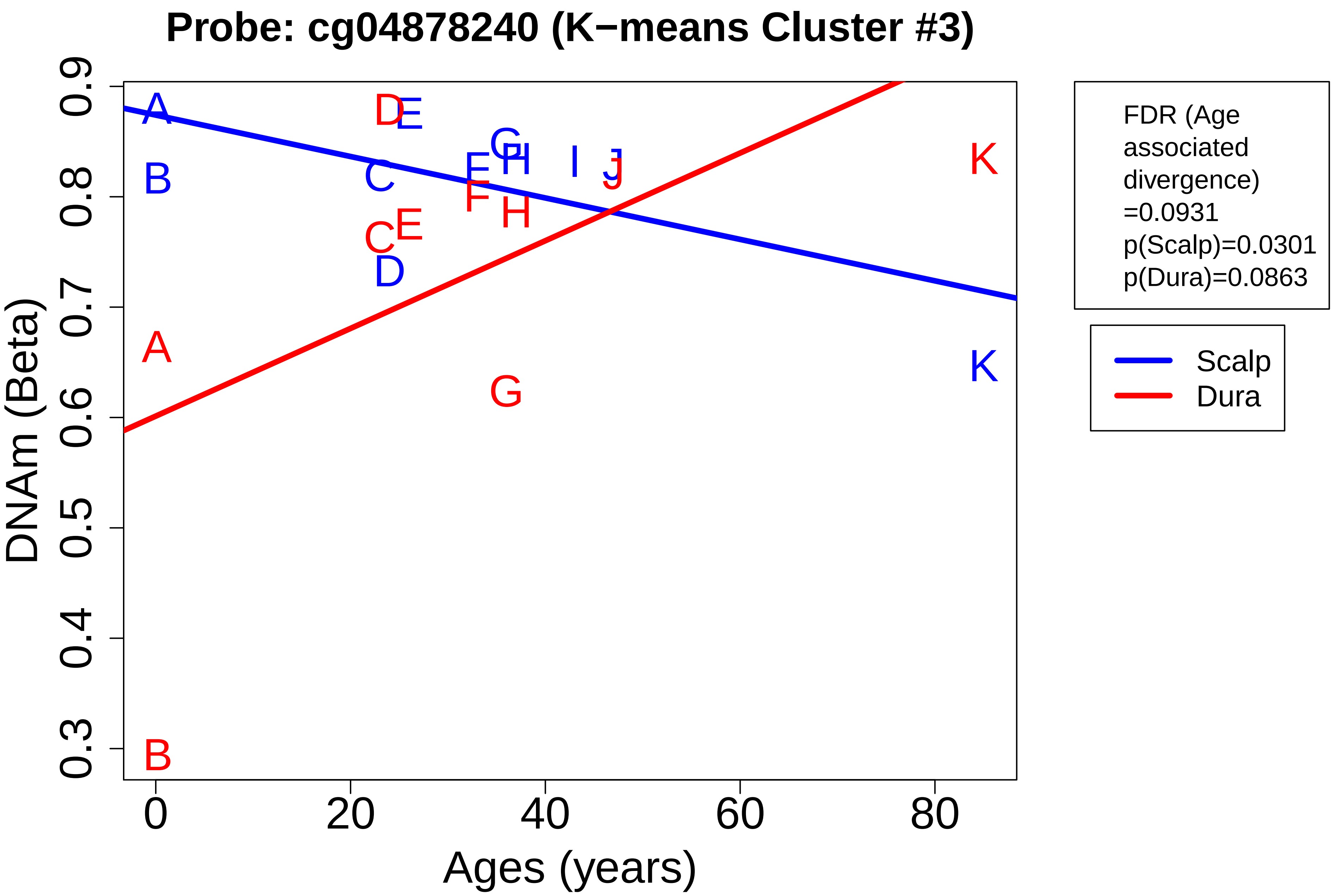
Probe: cg04878240 (K−means Cluster #3)
DNAm (Beta)
0.3
0.4
0.5
0.6
0.7
0.8
0.9
0
20
40
60
80
Ages (years)
A
B
C
D
E
F
G
H
I
J
K
FDR (Age associated divergence) =0.0931
p(Scalp)=0.0301
p(Dura)=0.0863
Scalp
Dura

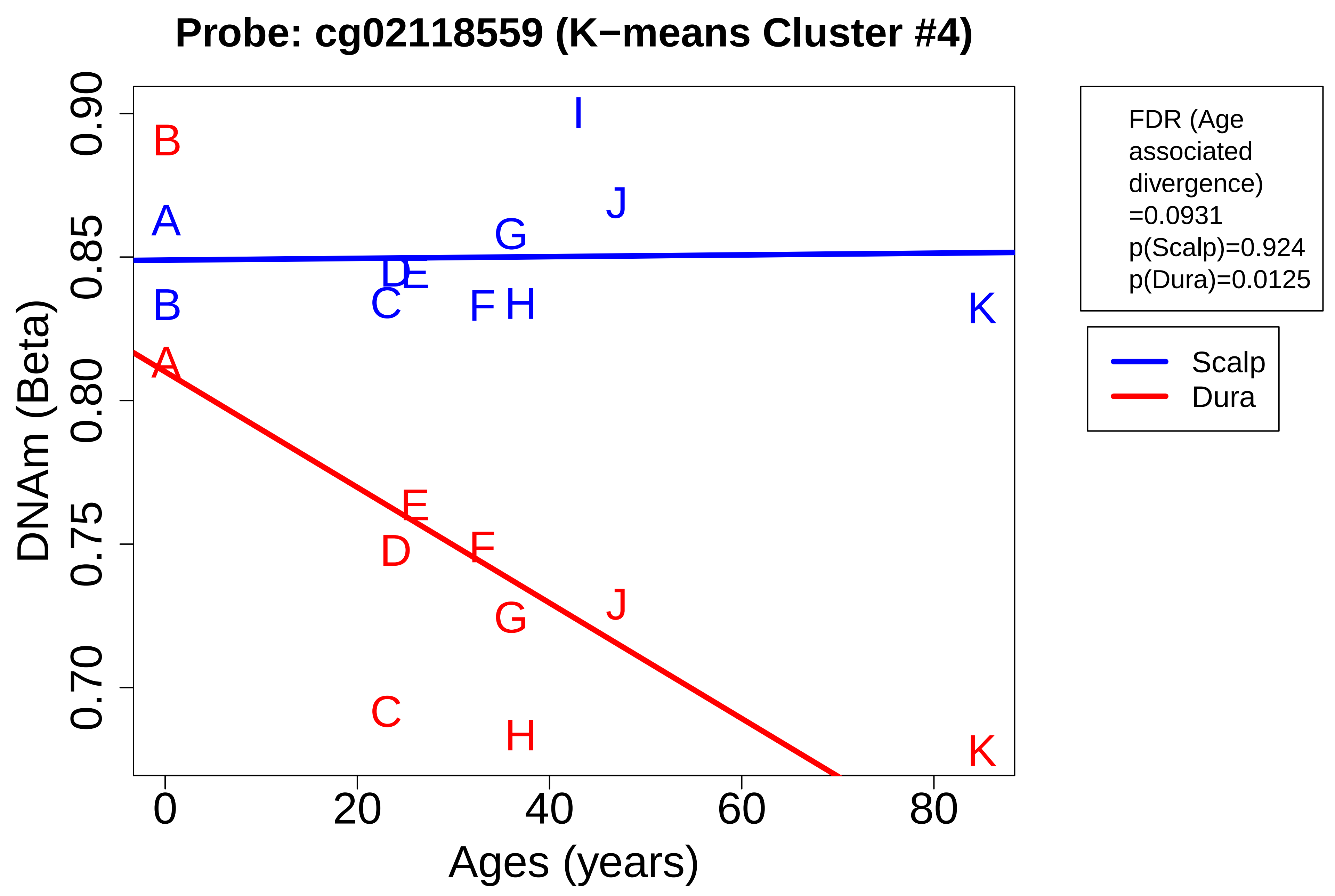
Probe: cg02118559 (K−means Cluster #4)
DNAm (Beta)
0.70
0.75
0.80
0.85
0.90
Ages (years)
0
20
40
60
80
A
B
C
D
E
F
G
H
I
J
K
FDR (Age associated divergence) =0.0931
p(Scalp)=0.924
p(Dura)=0.0125
Scalp
Dura

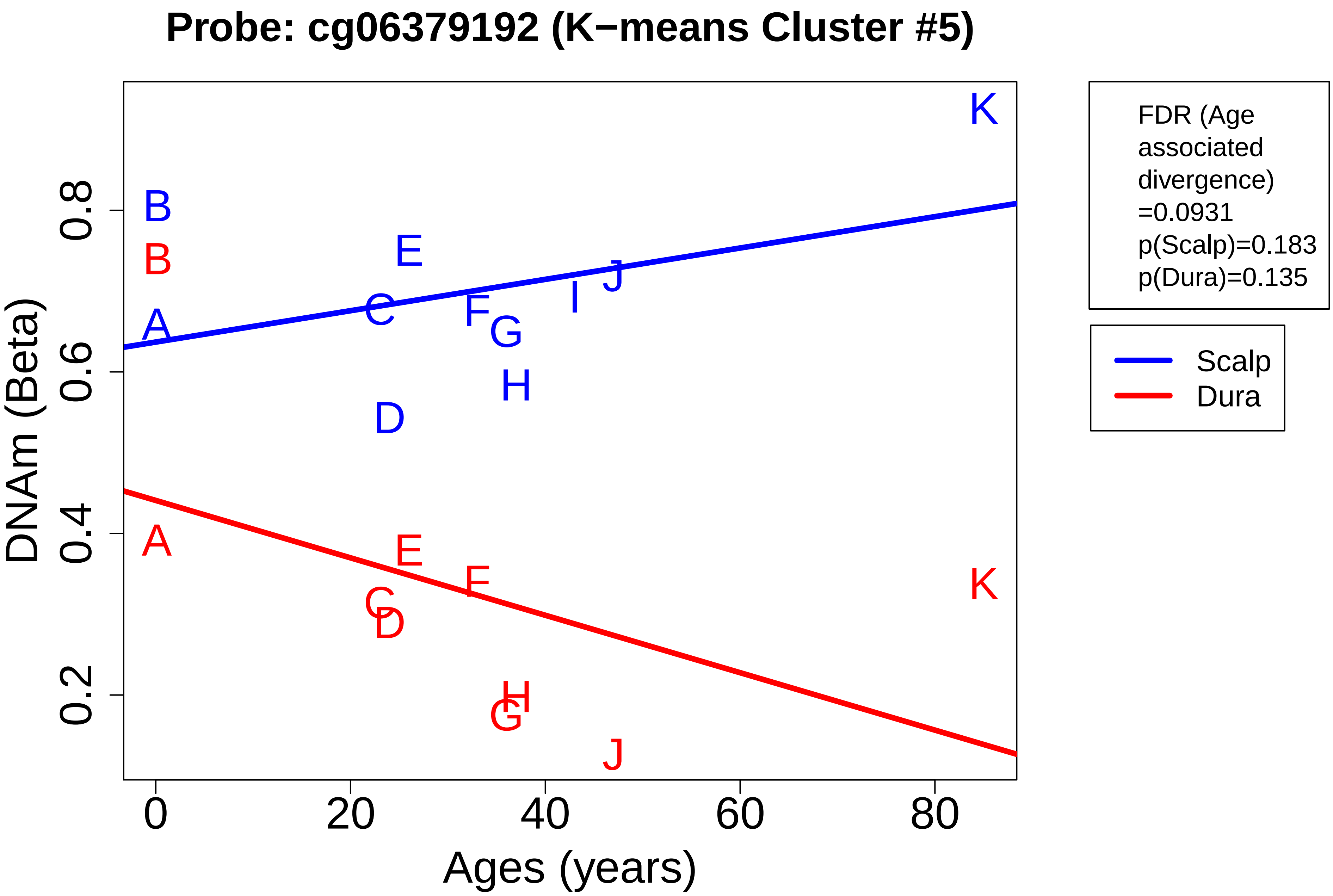

Probe: cg06379192 (K−means Cluster #5)
DNAm (Beta)
0.2
0.4
0.6
0.8
0
20
40
60
80
Ages (years)
A
B
C
D
E
F
G
H
I
J
K
A
B
C
D
E
F
G
H
J
K
FDR (Age associated divergence) =0.0931
p(Scalp)=0.183
p(Dura)=0.135
Scalp
Dura

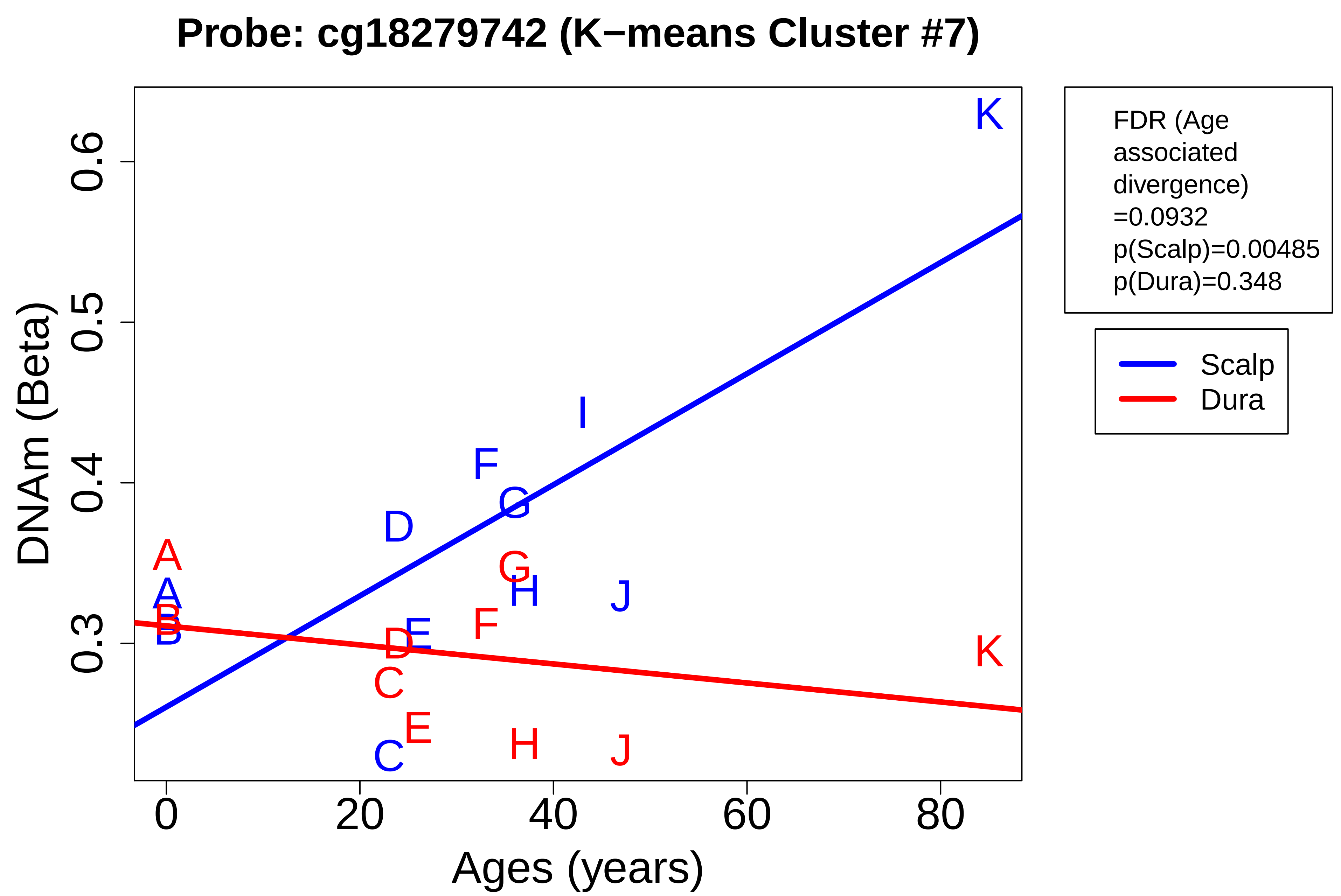

Probe: cg18279742 (K−means Cluster #7)
DNAm (Beta)
0.3
0.4
0.5
0.6
0
20
40
60
80
Ages (years)
A
B
C
D
E
F
G
H
I
J
K
FDR (Age associated divergence) =0.0932
p(Scalp)=0.00485
p(Dura)=0.348
Scalp
Dura

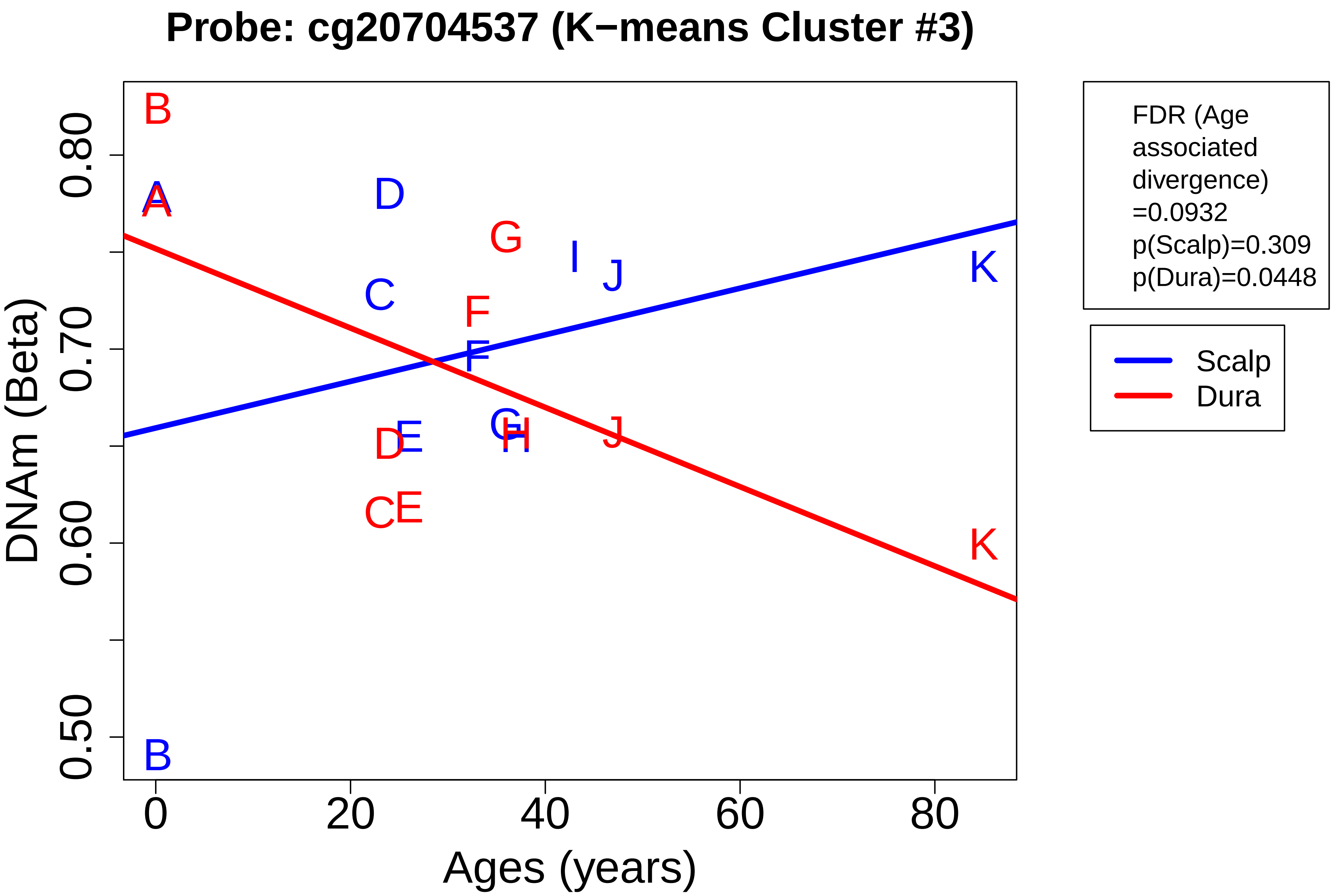

Probe: cg20704537 (K−means Cluster #3)
FDR (Age associated divergence) =0.0932
p(Scalp)=0.309
p(Dura)=0.0448
Scalp
Dura
DNAm (Beta)
0.50
0.60
0.70
0.80
0
20
40
60
80
Ages (years)
A
B
C
D
E
F
G
H
I
J
K

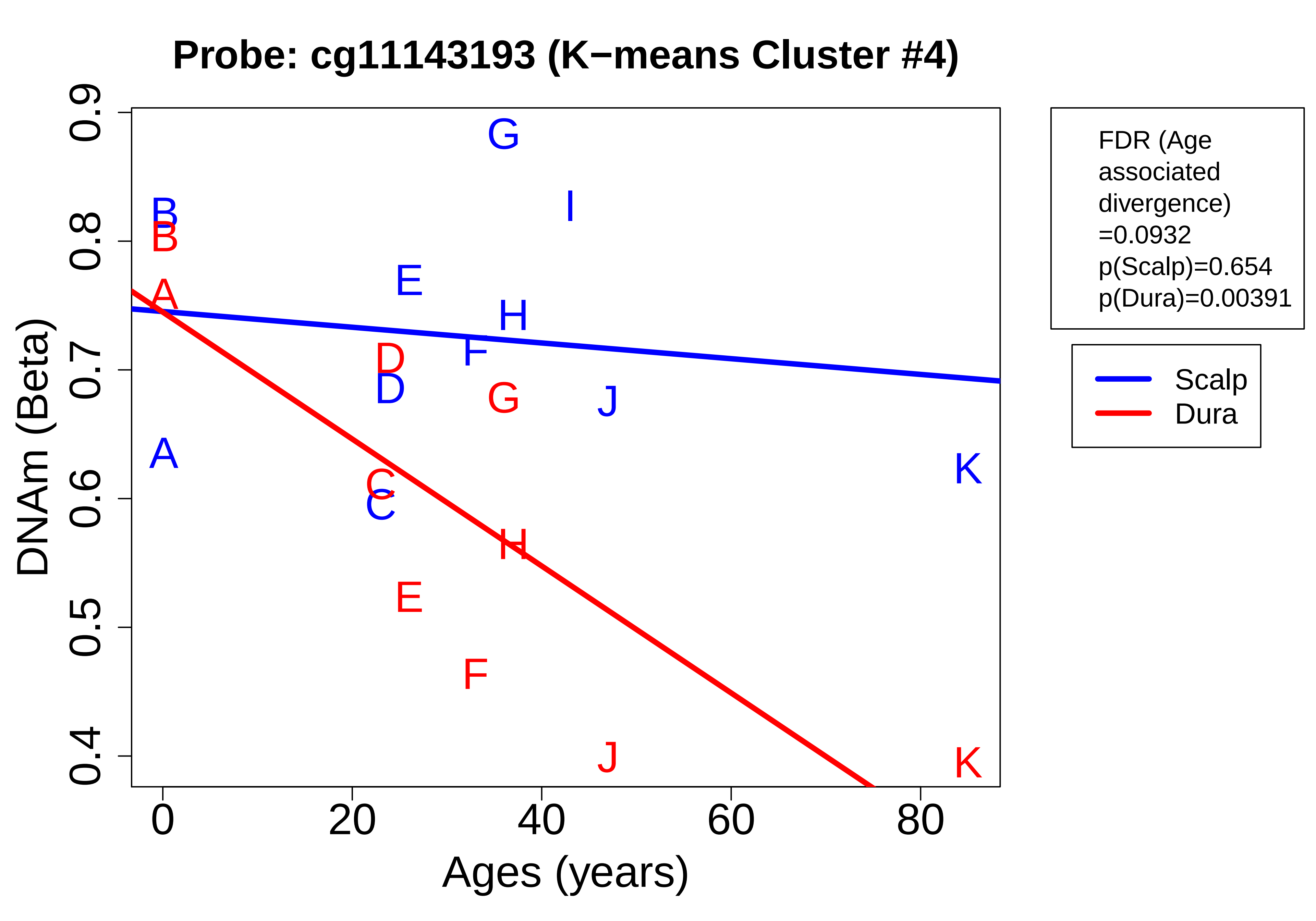

Probe: cg11143193 (K−means Cluster #4)
DNAm (Beta)
0.4
0.5
0.6
0.7
0.8
0.9
0
20
40
60
80
Ages (years)
A
B
C
D
E
F
G
H
I
J
K
FDR (Age associated divergence) =0.0932
p(Scalp)=0.654
p(Dura)=0.00391
Scalp
Dura

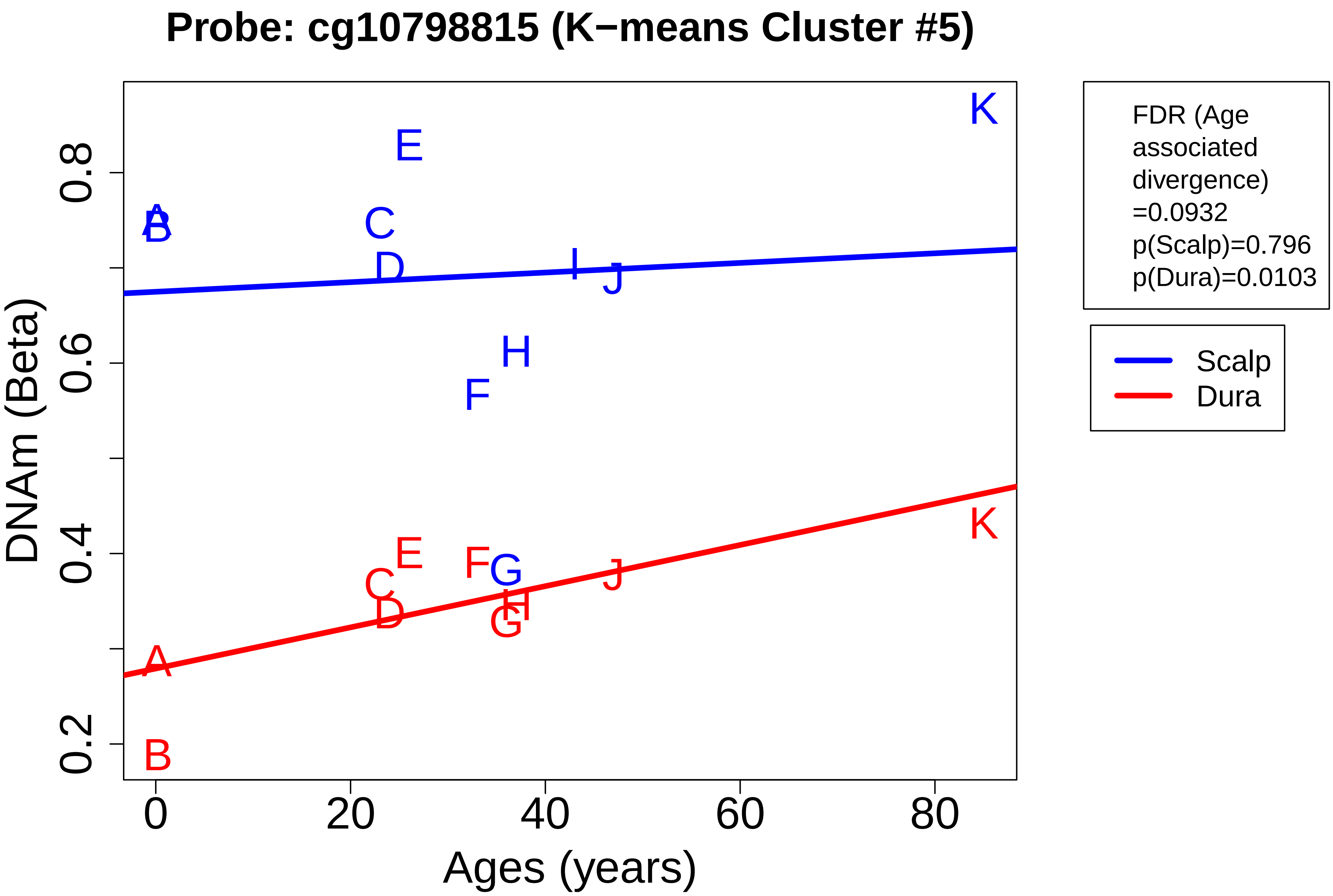

Probe: cg10798815 (K−means Cluster #5)
FDR (Age associated divergence) =0.0932
p(Scalp)=0.796
p(Dura)=0.0103
Scalp
Dura
DNAm (Beta)
0.2
0.4
0.6
0.8
0
20
40
60
80
Ages (years)

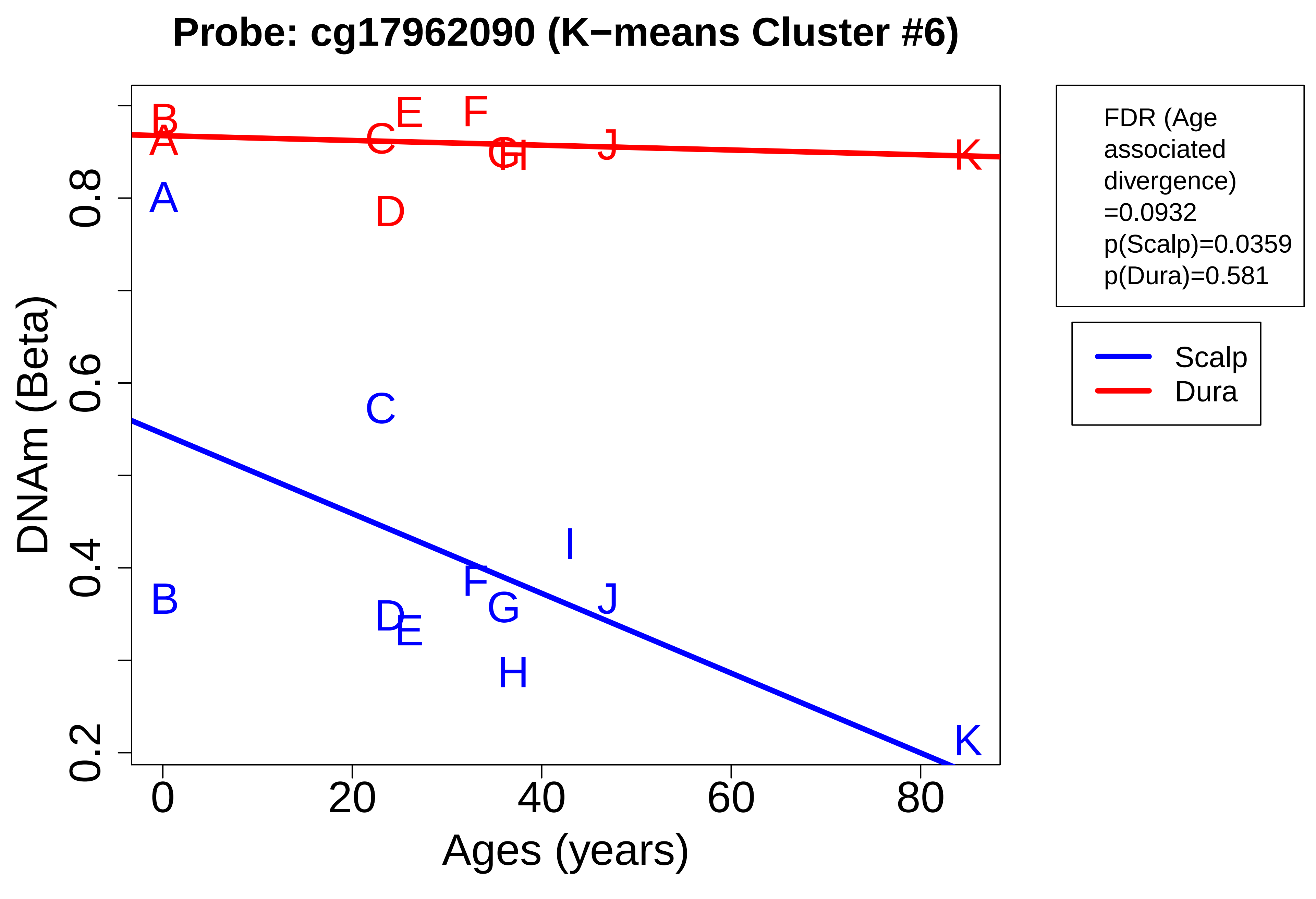

Probe: cg17962090 (K−means Cluster #6)
FDR (Age associated divergence) =0.0932
p(Scalp)=0.0359
p(Dura)=0.581
Scalp
Dura
DNAm (Beta)
Ages (years)
0.2
0.4
0.6
0.8
0
20
40
60
80
A
B
C
D
E
F
G
H
I
J
K

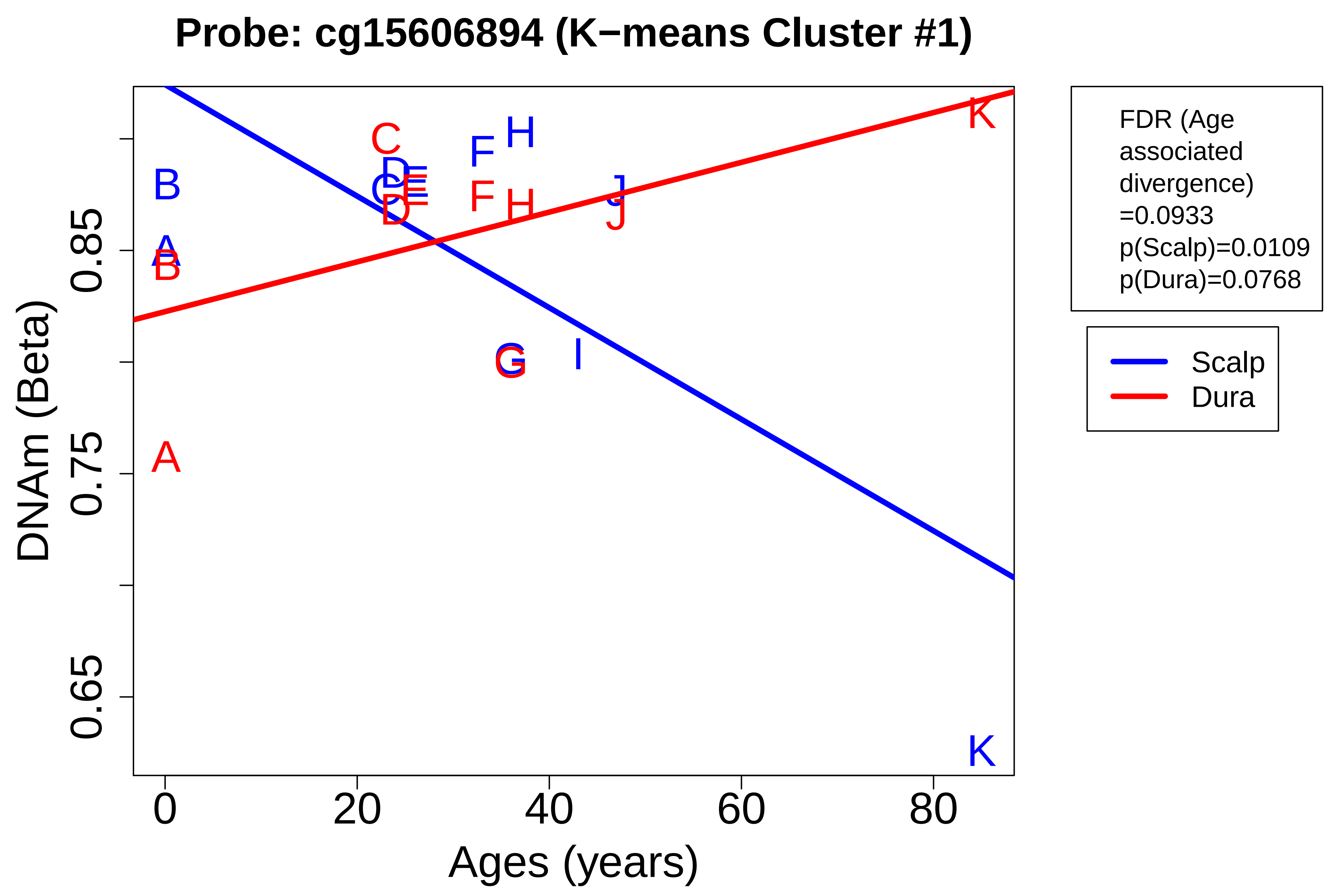

Probe: cg15606894 (K−means Cluster #1)
DNAm (Beta)
0.65
0.75
0.85
0
20
40
60
80
Ages (years)
FDR (Age associated divergence) =0.0933
p(Scalp)=0.0109
p(Dura)=0.0768
Scalp
Dura

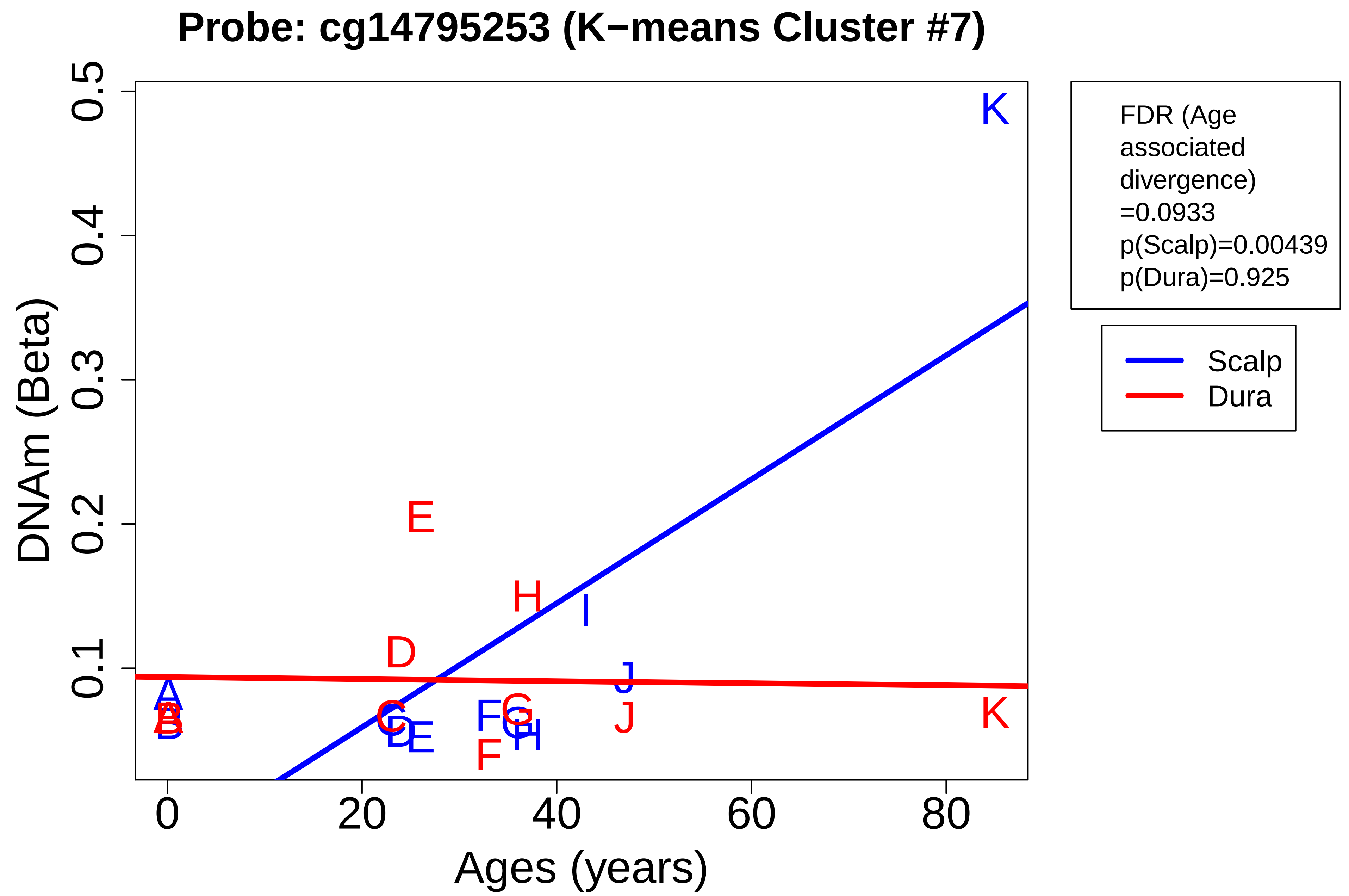
Probe: cg14795253 (K−means Cluster #7)
DNAm (Beta)
0.1
0.2
0.3
0.4
0.5
Ages (years)
0
20
40
60
80
FDR (Age associated divergence) =0.0933
p(Scalp)=0.00439
p(Dura)=0.925
Scalp
Dura

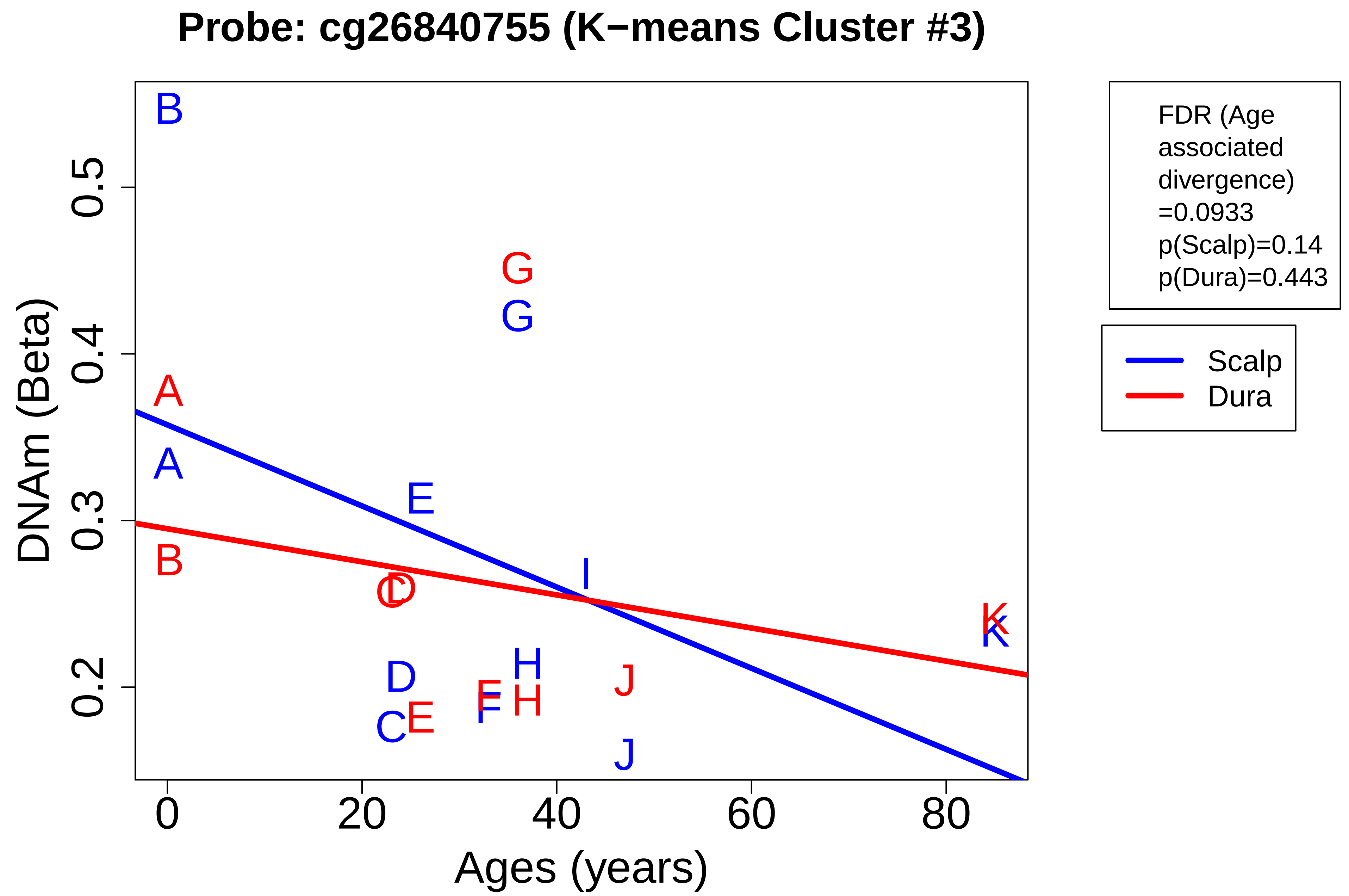
Probe: cg26840755 (K−means Cluster #3)
DNAm (Beta)
0.2
0.3
0.4
0.5
0
20
40
60
80
Ages (years)
B
G
G
A
A
E
B
I
C
D
K
K
H
D
F
H
J
C
E
J
FDR (Age associated divergence) =0.0933
p(Scalp)=0.14
p(Dura)=0.443
Scalp
Dura

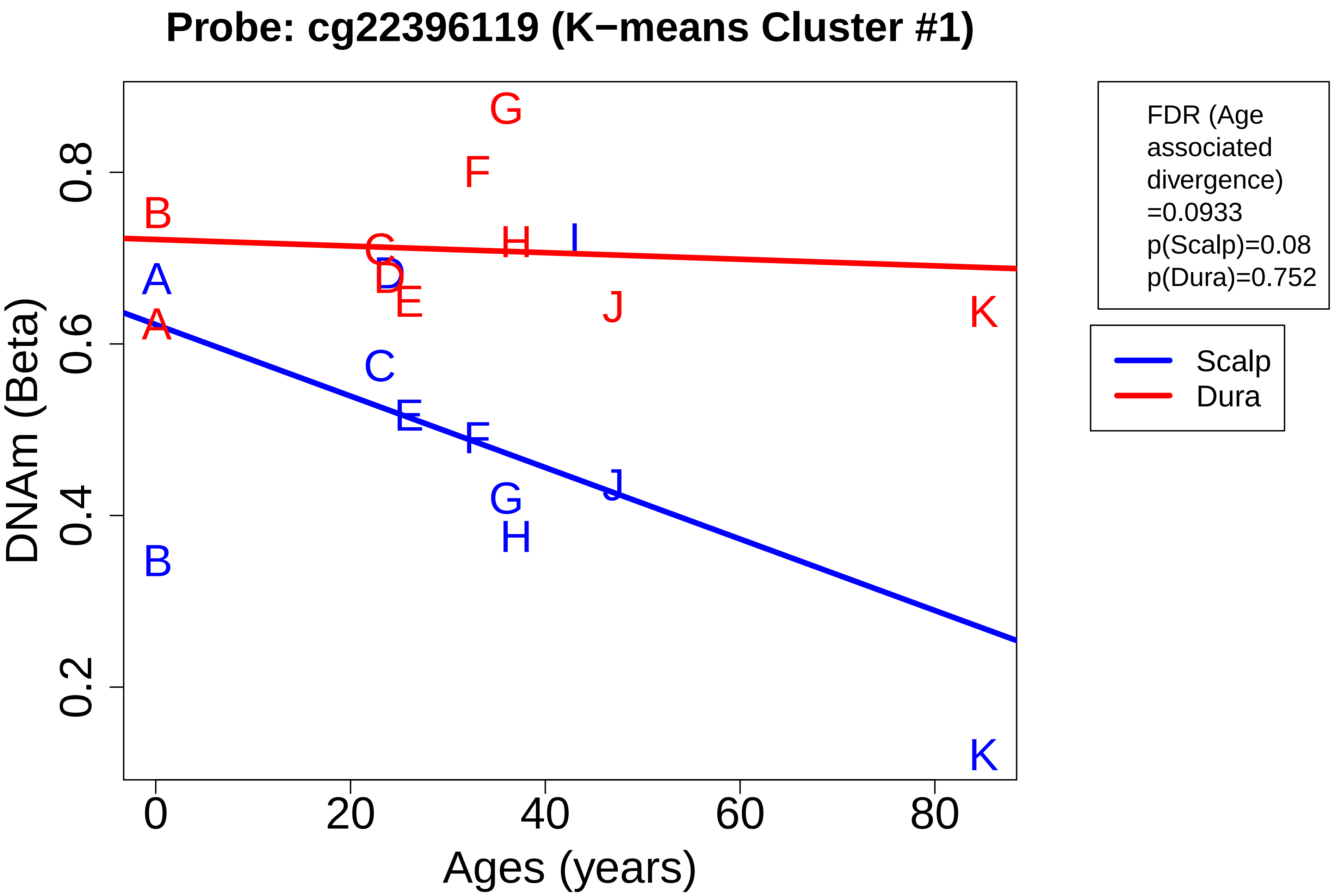
Probe: cg22396119 (K−means Cluster #1)
DNAm (Beta)
0.2
0.4
0.6
0.8
0
20
40
60
80
Ages (years)
FDR (Age associated divergence) =0.0933
p(Scalp)=0.08
p(Dura)=0.752
Scalp
Dura
A
B
C
D
E
F
G
H
I
J
K

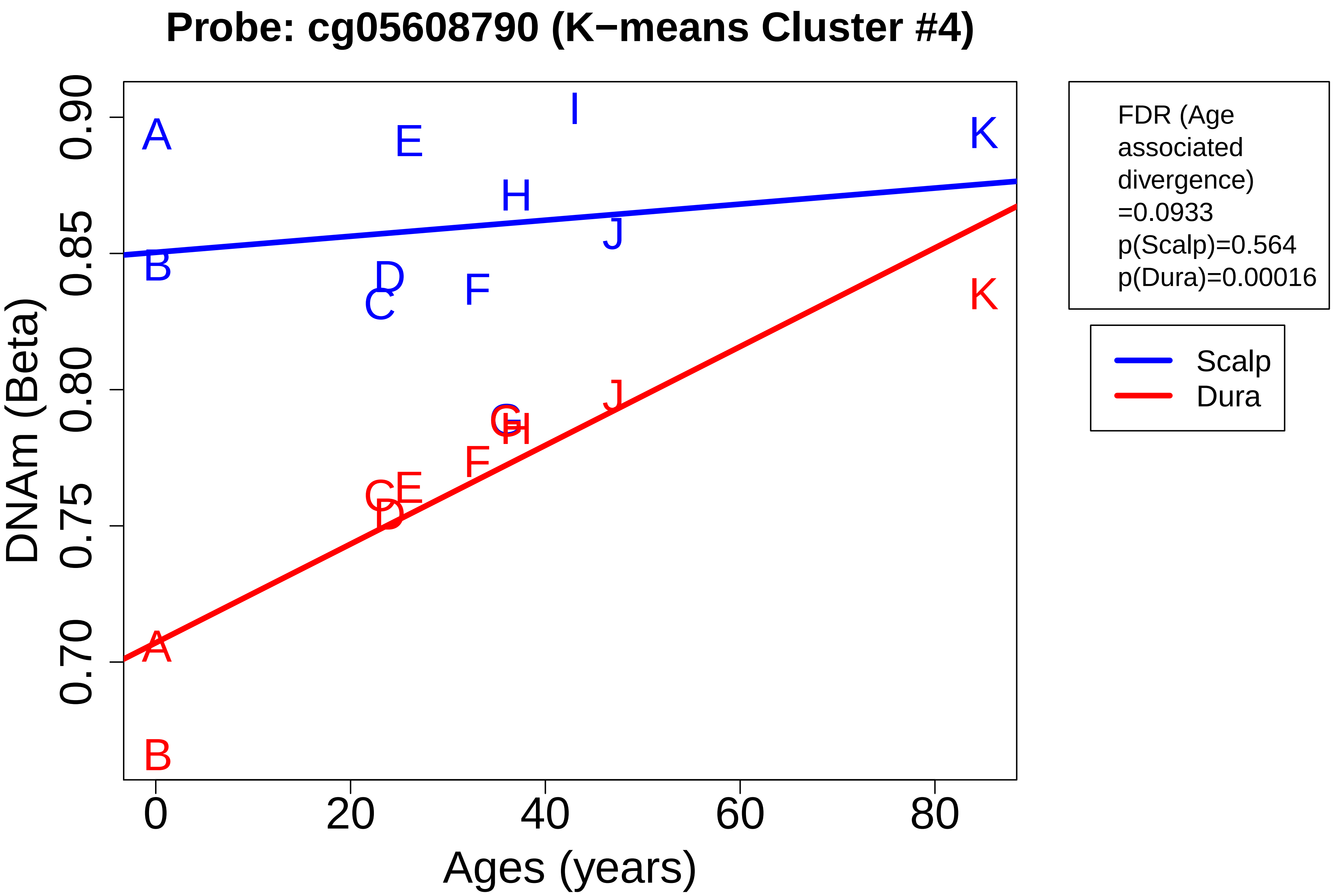

Probe: cg05608790 (K−means Cluster #4)
DNAm (Beta)
0.70
0.75
0.80
0.85
0.90
0
20
40
60
80
Ages (years)
FDR (Age associated divergence) =0.0933
p(Scalp)=0.564
p(Dura)=0.00016
Scalp
Dura

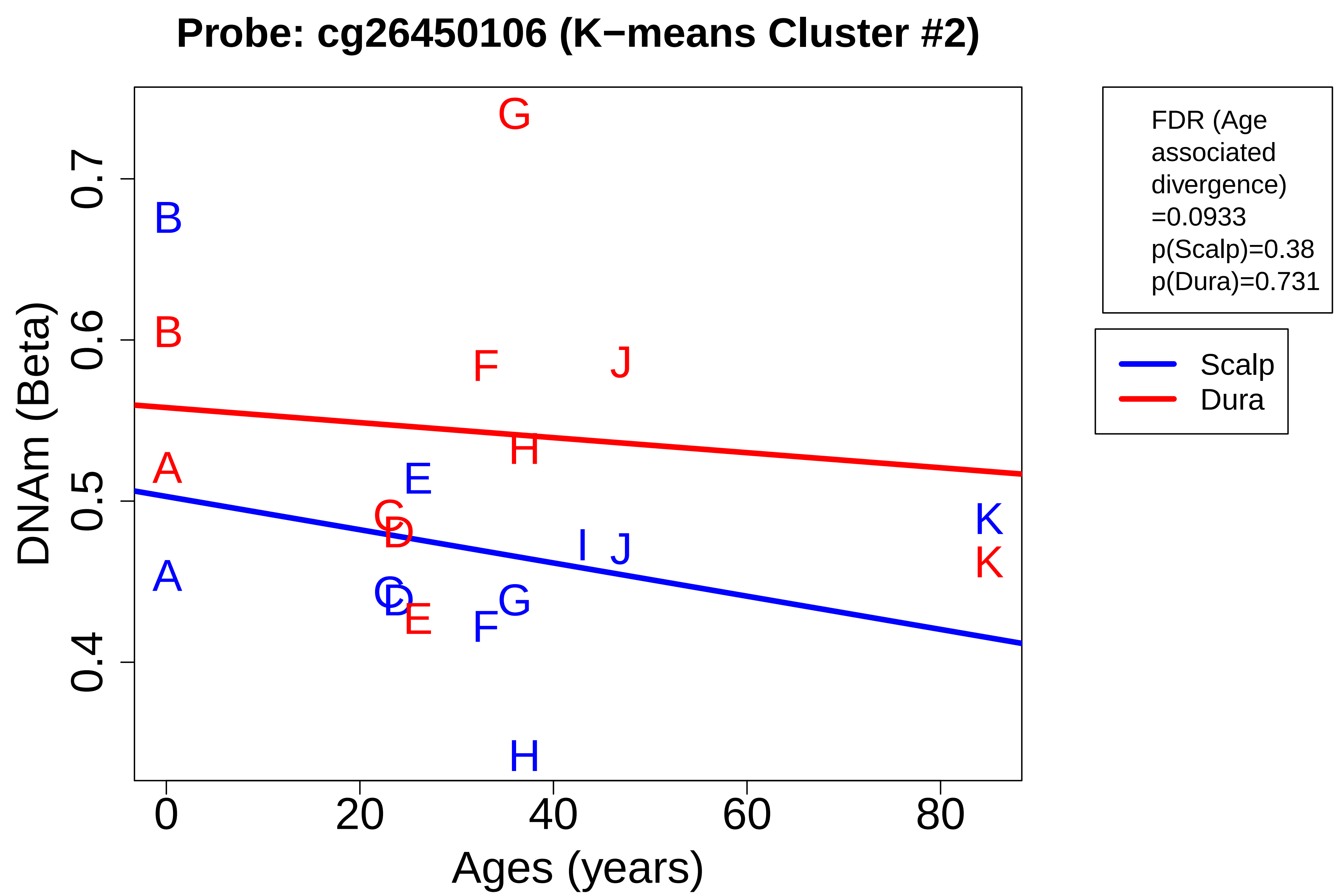

Probe: cg26450106 (K−means Cluster #2)
DNAm (Beta)
Ages (years)
0.4
0.5
0.6
0.7
0
20
40
60
80
FDR (Age associated divergence) =0.0933
p(Scalp)=0.38
p(Dura)=0.731
Scalp
Dura

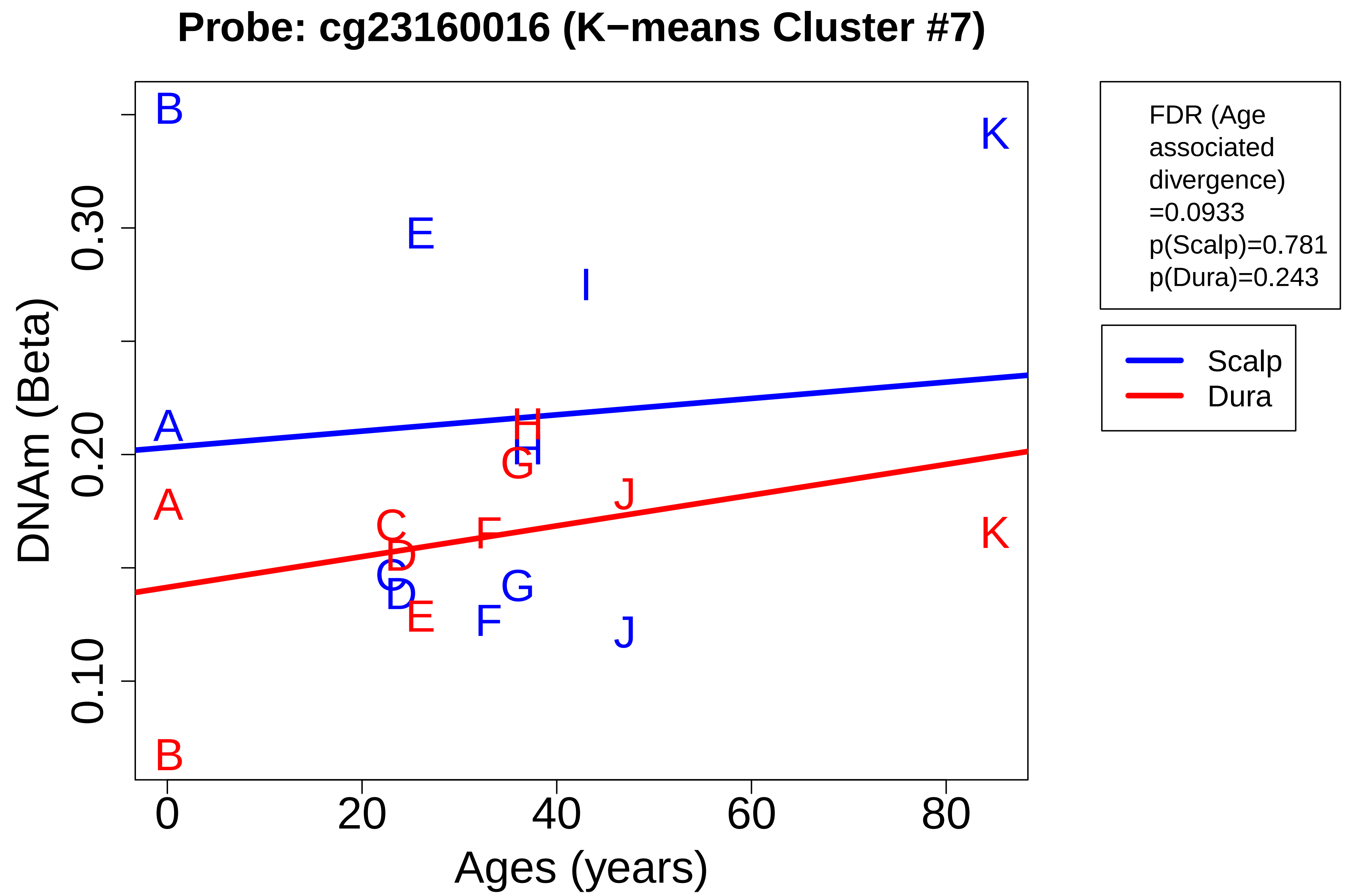

Probe: cg23160016 (K−means Cluster #7)
DNAm (Beta)
0.10
0.20
0.30
0
20
40
60
80
Ages (years)
FDR (Age associated divergence) =0.0933
p(Scalp)=0.781
p(Dura)=0.243
Scalp
Dura
A
B
C
D
E
F
G
H
I
J
K

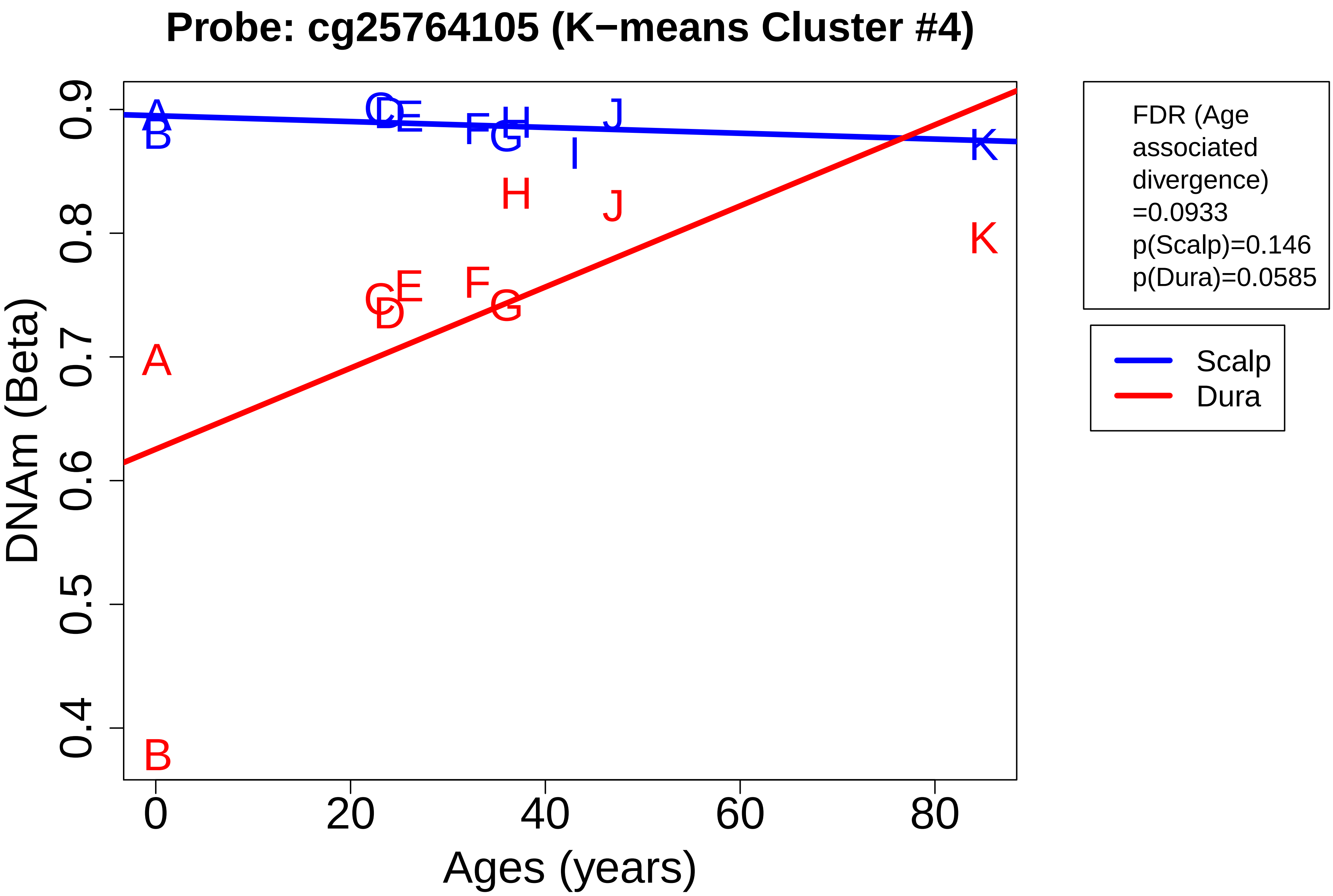

Probe: cg25764105 (K−means Cluster #4)
DNAm (Beta)
0.4
0.5
0.6
0.7
0.8
0.9
0
20
40
60
80
Ages (years)
FDR (Age associated divergence) =0.0933
p(Scalp)=0.146
p(Dura)=0.0585
Scalp
Dura

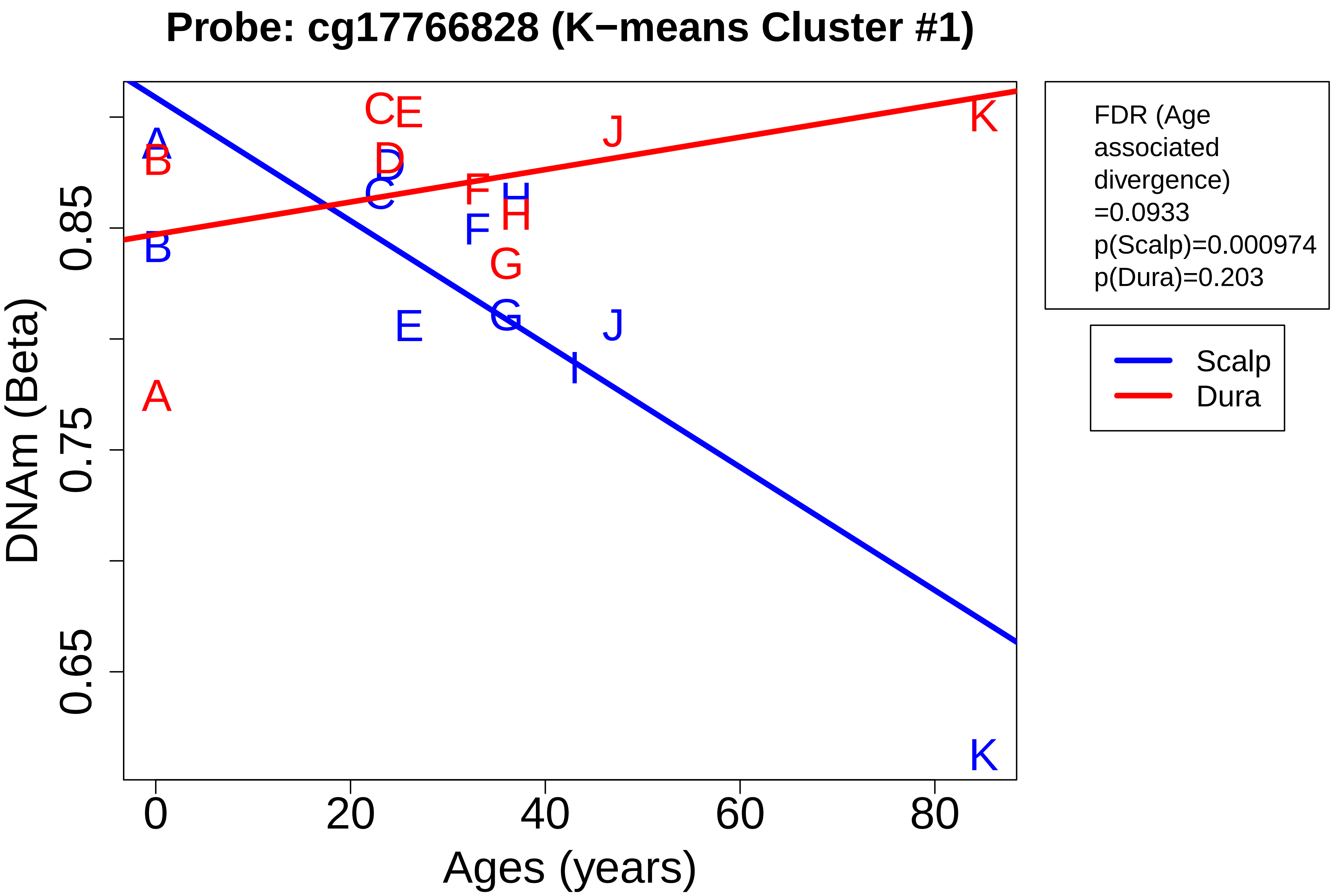

Probe: cg17766828 (K−means Cluster #1)
FDR (Age associated divergence) =0.0933
p(Scalp)=0.000974
p(Dura)=0.203
Scalp
Dura
DNAm (Beta)
0.65
0.75
0.85
Ages (years)
0
20
40
60
80

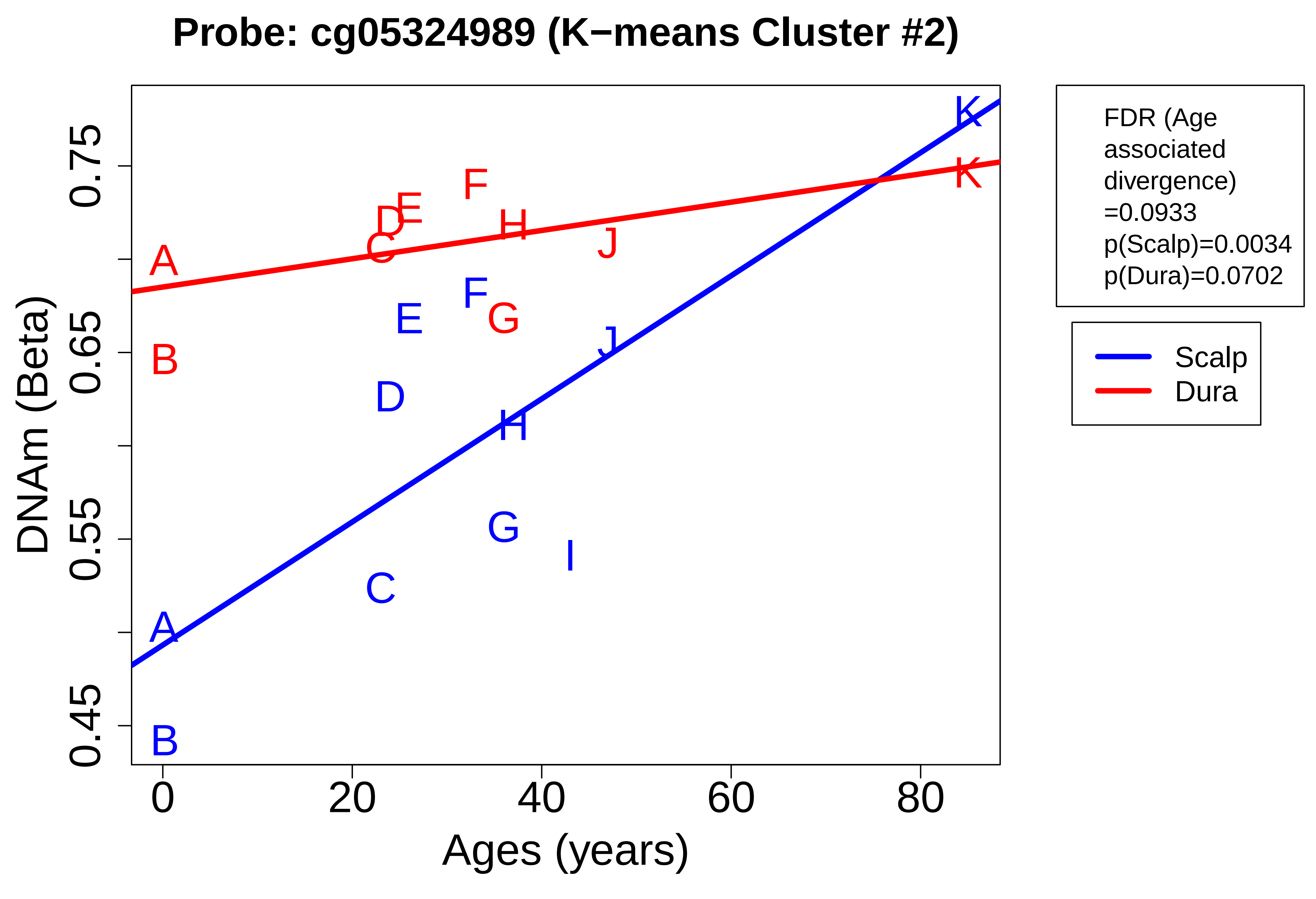

Probe: cg05324989 (K−means Cluster #2)
DNAm (Beta)
0.45
0.55
0.65
0.75
Ages (years)
0
20
40
60
80
A
B
C
D
E
F
G
H
I
J
K
A
B
C
D
E
F
G
H
J
K
FDR (Age associated divergence) =0.0933
p(Scalp)=0.0034
p(Dura)=0.0702
Scalp
Dura

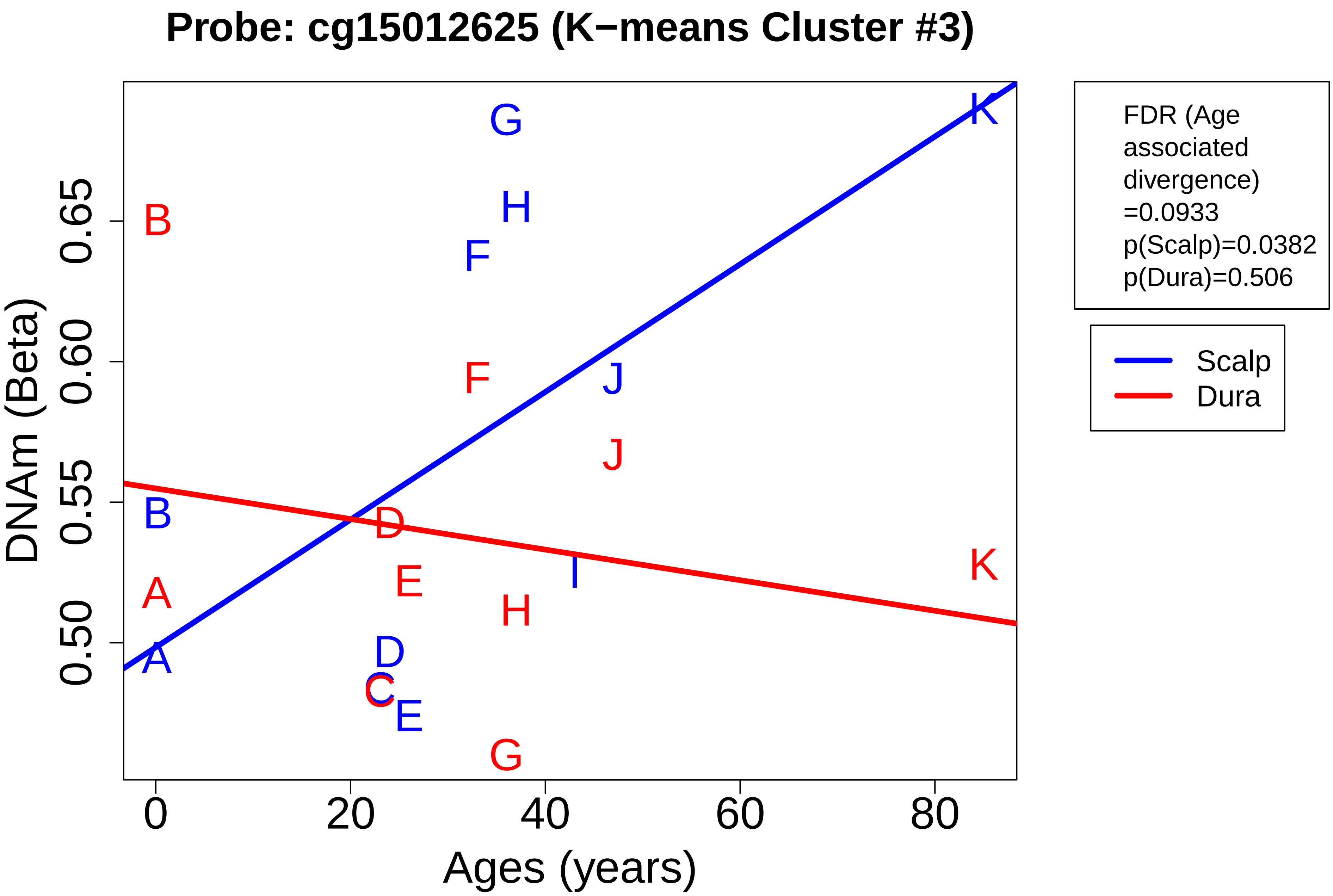

Probe: cg15012625 (K−means Cluster #3)
FDR (Age associated divergence) =0.0933
p(Scalp)=0.0382
p(Dura)=0.506
Scalp
Dura
DNAm (Beta)
0.50
0.55
0.60
0.65
0
20
40
60
80
Ages (years)

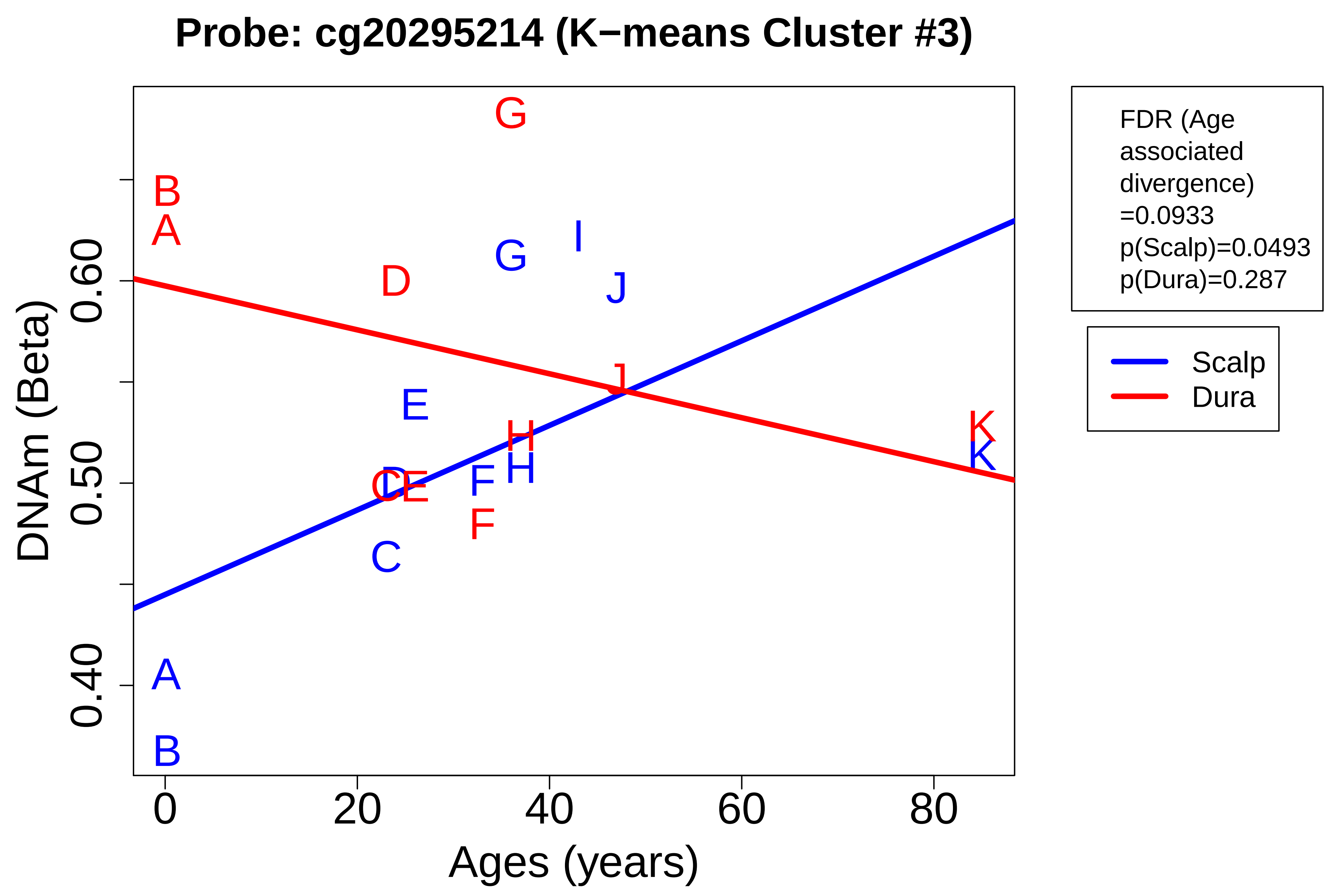
Probe: cg20295214 (K−means Cluster #3)
DNAm (Beta)
0.40
0.50
0.60
0
20
40
60
80
Ages (years)
FDR (Age associated divergence) =0.0933
p(Scalp)=0.0493
p(Dura)=0.287
Scalp
Dura
A
B
C
D
E
F
G
H
I
J
K

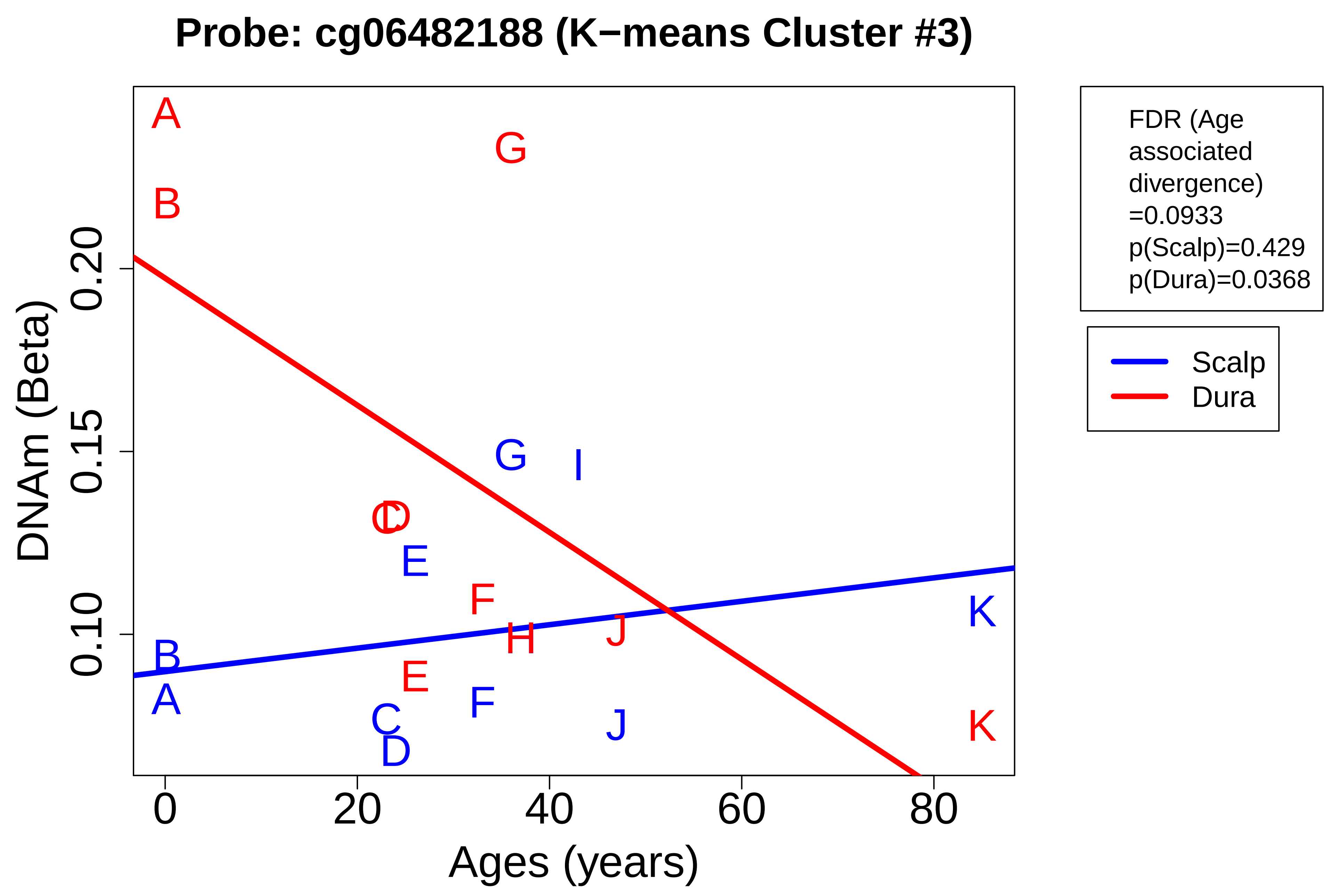

Probe: cg06482188 (K−means Cluster #3)
DNAm (Beta)
0.10
0.15
0.20
Ages (years)
0
20
40
60
80
A
B
C
D
E
F
G
H
I
J
K
FDR (Age associated divergence) =0.0933
p(Scalp)=0.429
p(Dura)=0.0368
Scalp
Dura

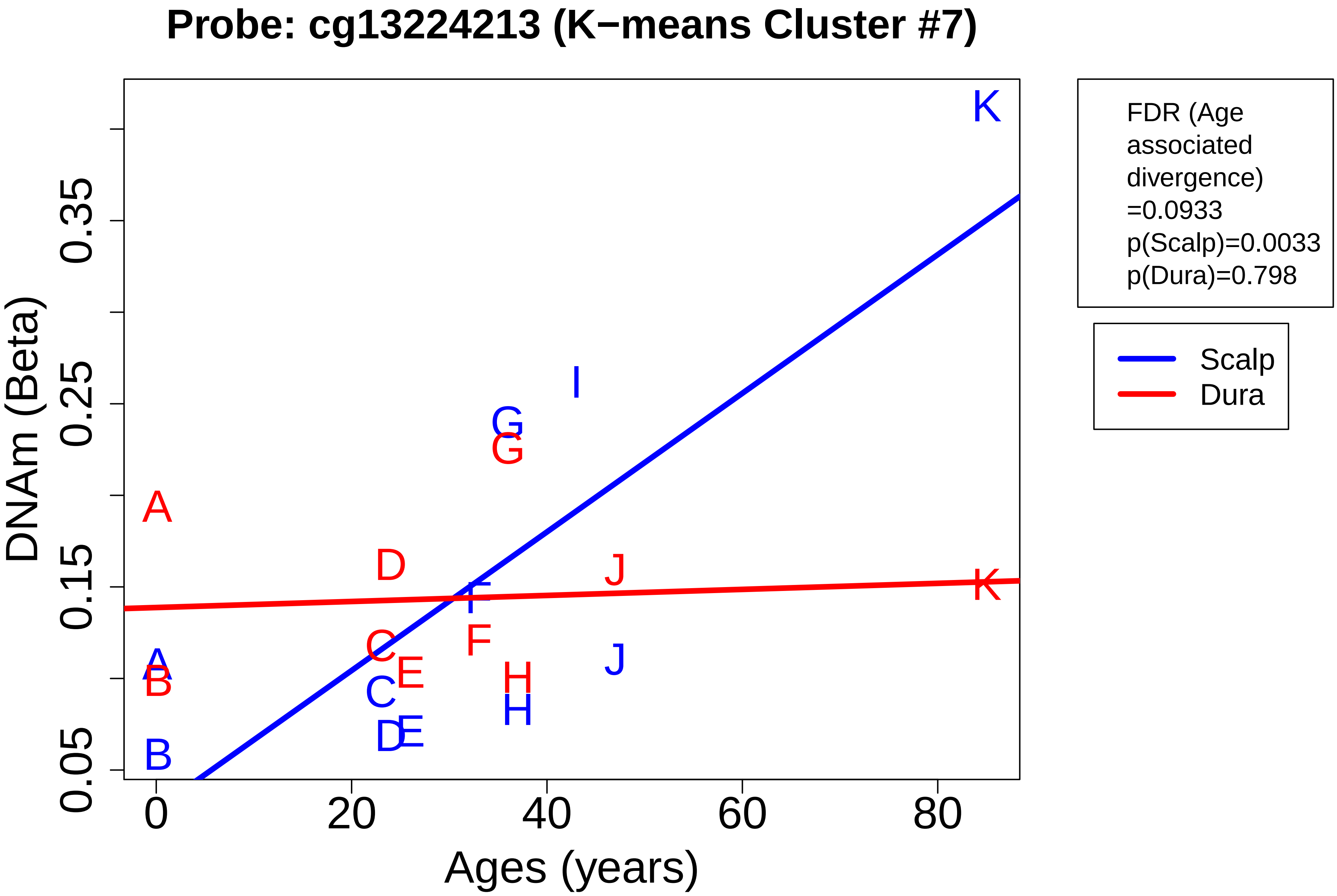
Probe: cg13224213 (K−means Cluster #7)
DNAm (Beta)
0.05
0.15
0.25
0.35
0
20
40
60
80
Ages (years)
FDR (Age associated divergence) =0.0933
p(Scalp)=0.0033
p(Dura)=0.798
Scalp
Dura

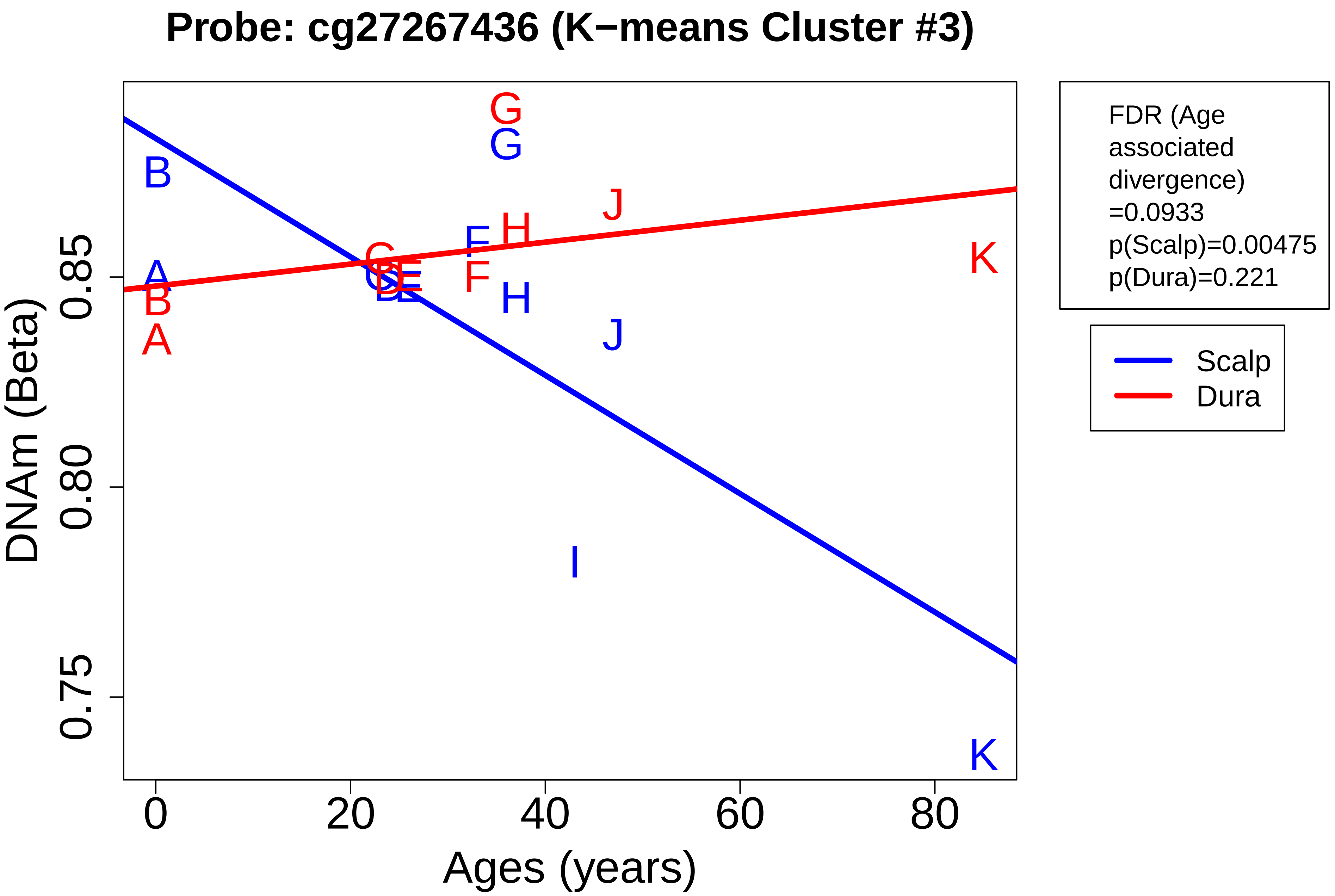
Probe: cg27267436 (K−means Cluster #3)
DNAm (Beta)
0.75
0.80
0.85
Ages (years)
0
20
40
60
80
FDR (Age associated divergence) =0.0933
p(Scalp)=0.00475
p(Dura)=0.221
Scalp
Dura

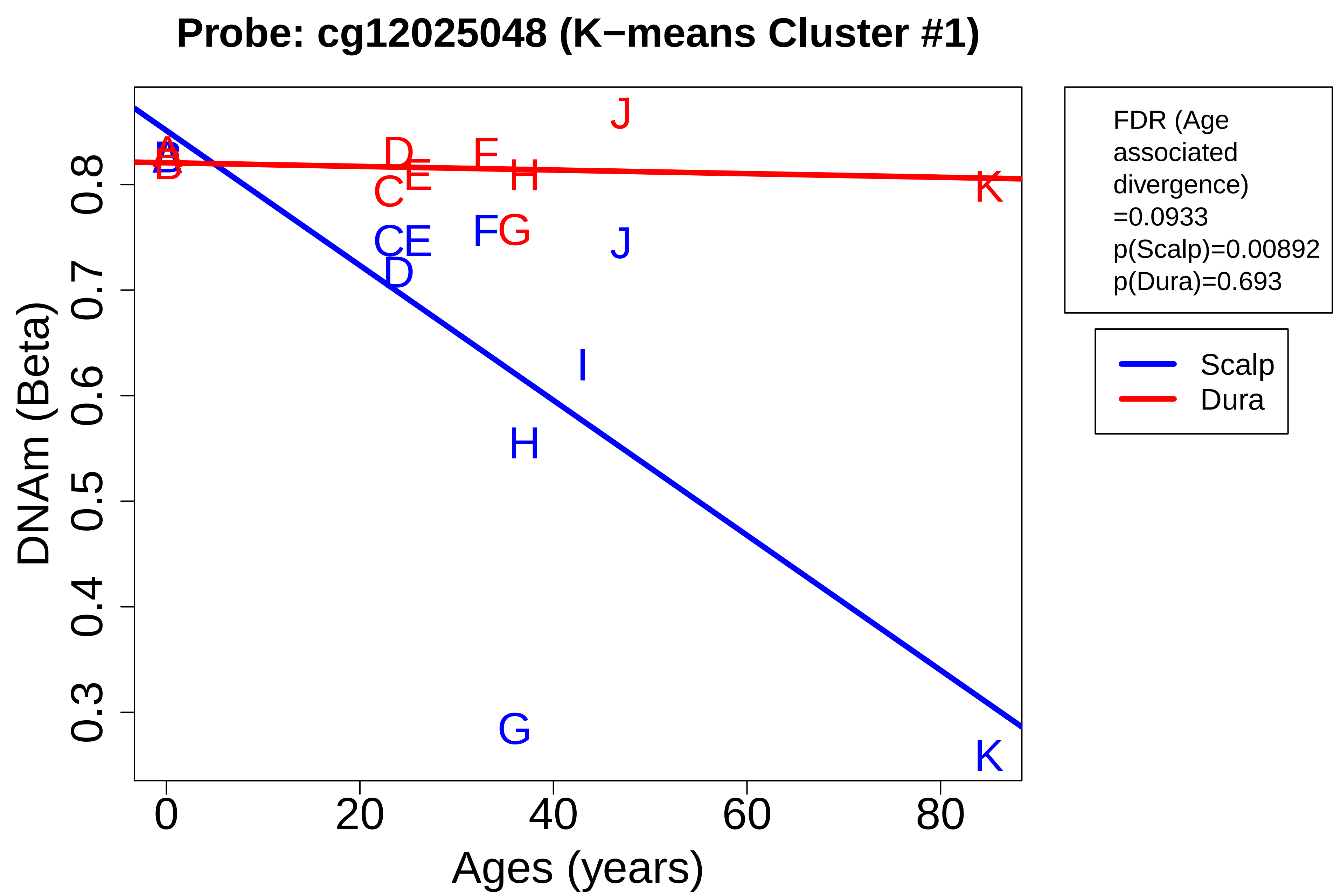
Probe: cg12025048 (K−means Cluster #1)
DNAm (Beta)
Ages (years)
0.3
0.4
0.5
0.6
0.7
0.8
0
20
40
60
80
FDR (Age associated divergence) =0.0933
p(Scalp)=0.00892
p(Dura)=0.693
Scalp
Dura

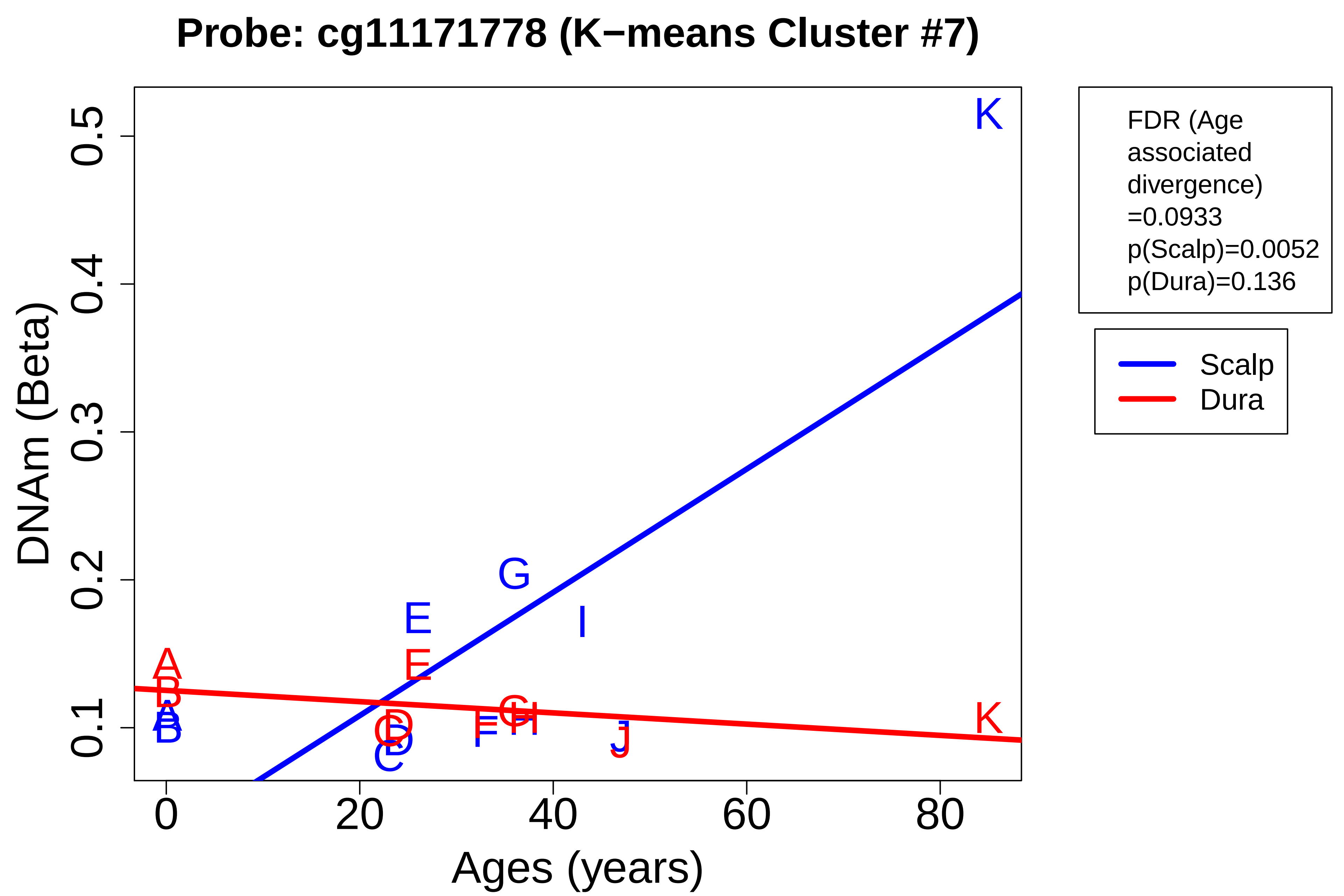

Probe: cg11171778 (K−means Cluster #7)
DNAm (Beta)
0.1
0.2
0.3
0.4
0.5
Ages (years)
0
20
40
60
80
FDR (Age associated divergence) =0.0933
p(Scalp)=0.0052
p(Dura)=0.136
Scalp
Dura

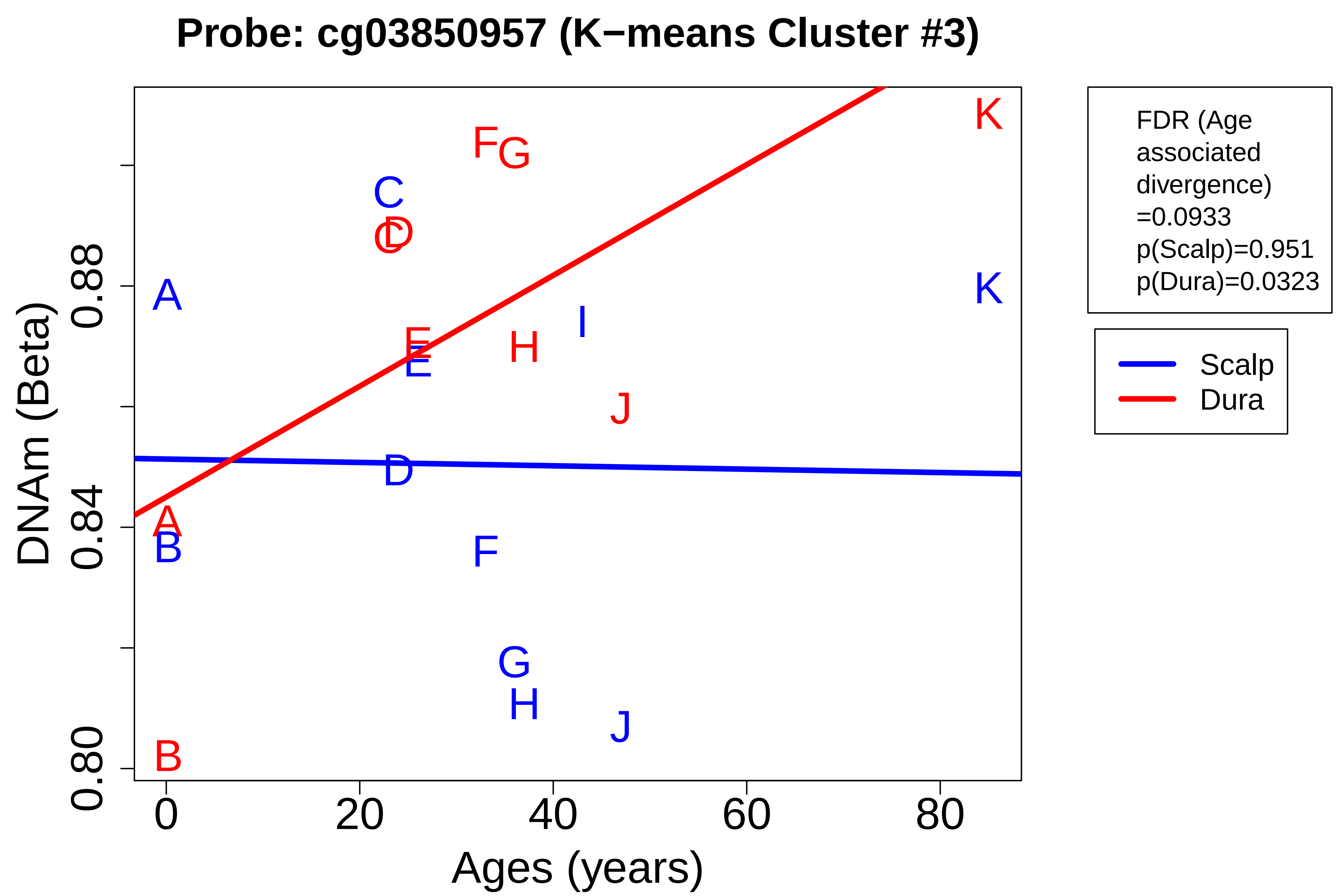

Probe: cg03850957 (K−means Cluster #3)
DNAm (Beta)
0.80
0.84
0.88
0
20
40
60
80
Ages (years)
FDR (Age associated divergence) =0.0933
p(Scalp)=0.951
p(Dura)=0.0323
Scalp
Dura

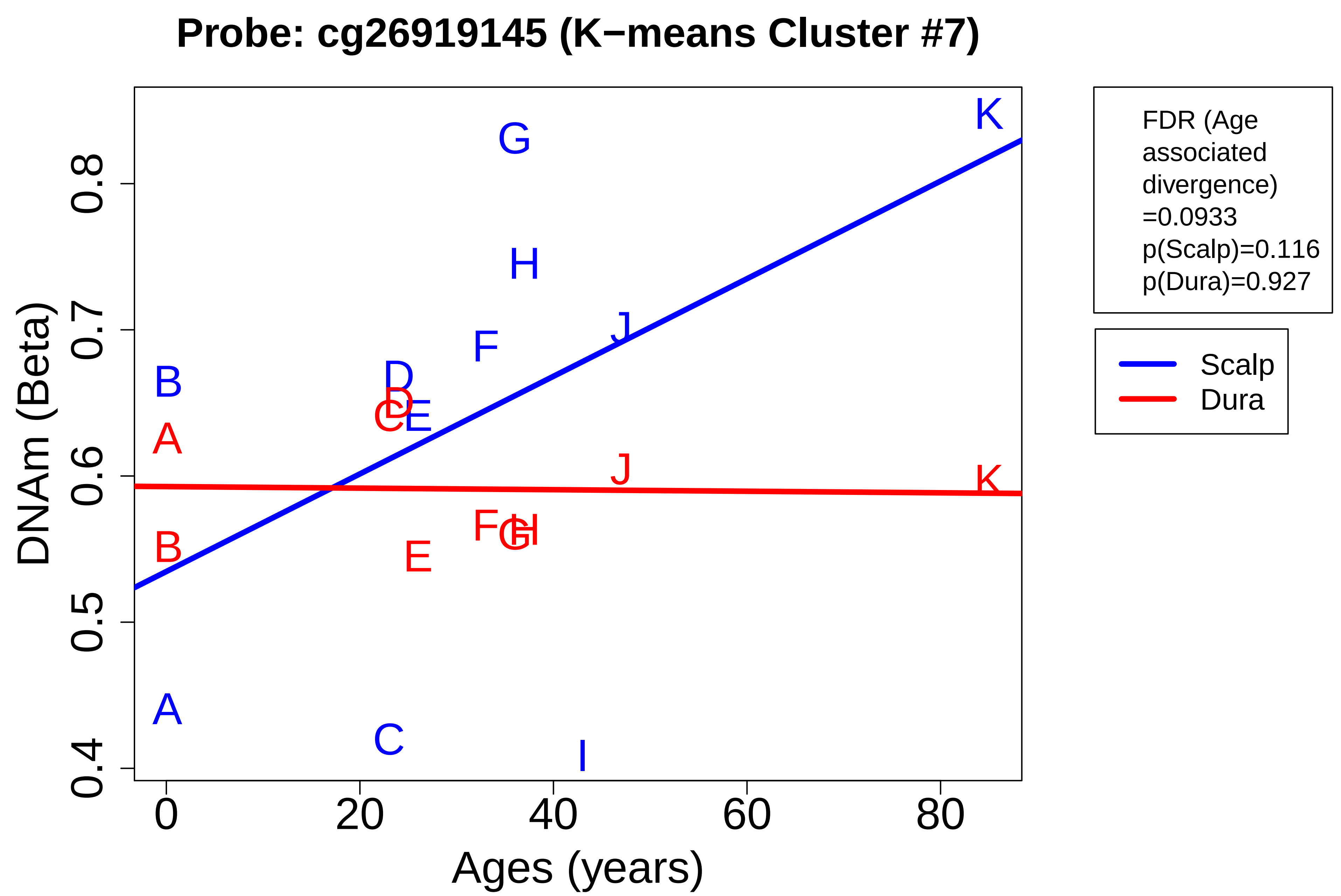

Probe: cg26919145 (K−means Cluster #7)
DNAm (Beta)
0.4
0.5
0.6
0.7
0.8
Ages (years)
0
20
40
60
80
FDR (Age associated divergence) =0.0933
p(Scalp)=0.116
p(Dura)=0.927
Scalp
Dura

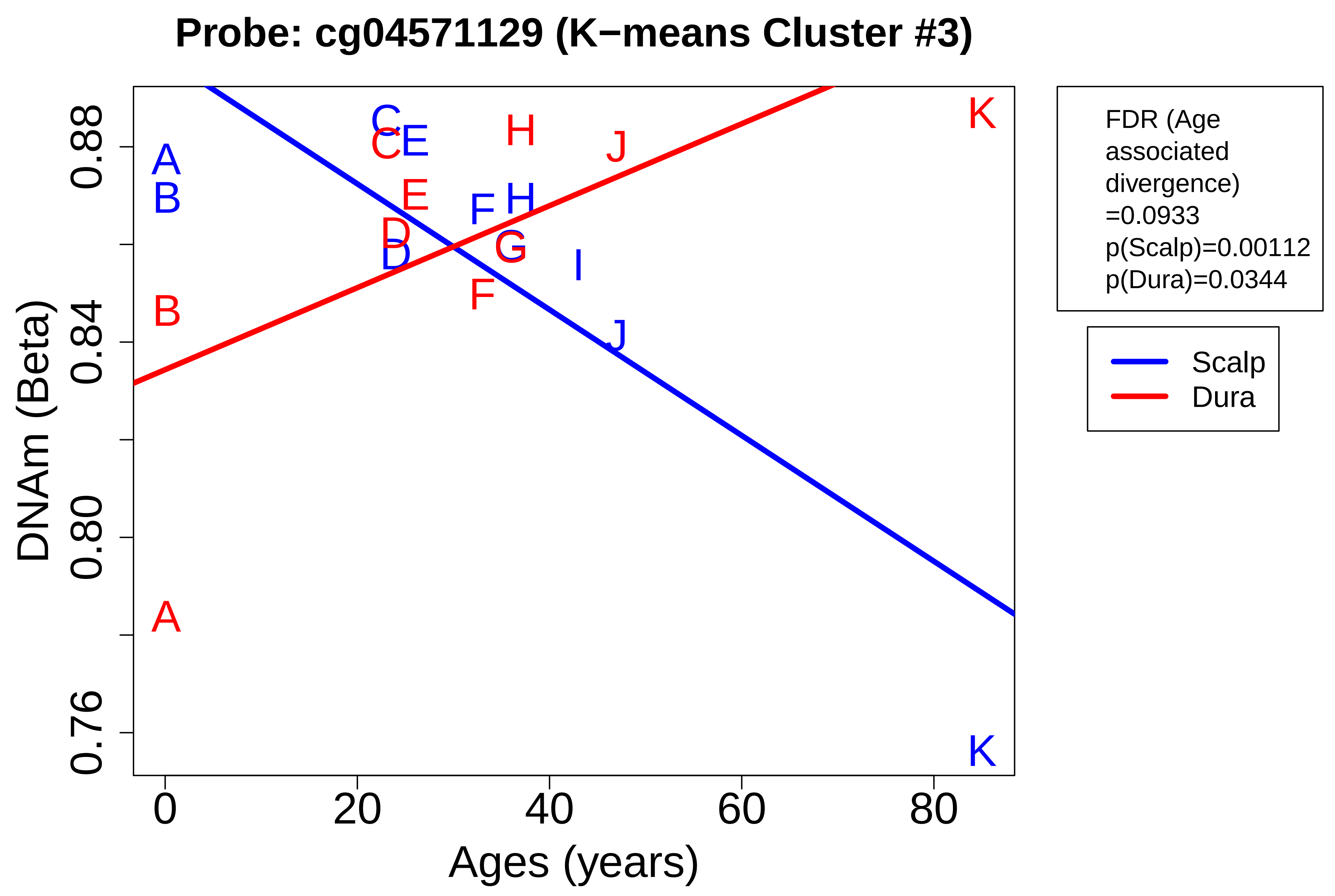

Probe: cg04571129 (K−means Cluster #3)
DNAm (Beta)
0.76
0.80
0.84
0.88
0
20
40
60
80
Ages (years)
A
B
C
D
E
F
G
H
I
J
K
FDR (Age associated divergence) =0.0933
p(Scalp)=0.00112
p(Dura)=0.0344
Scalp
Dura

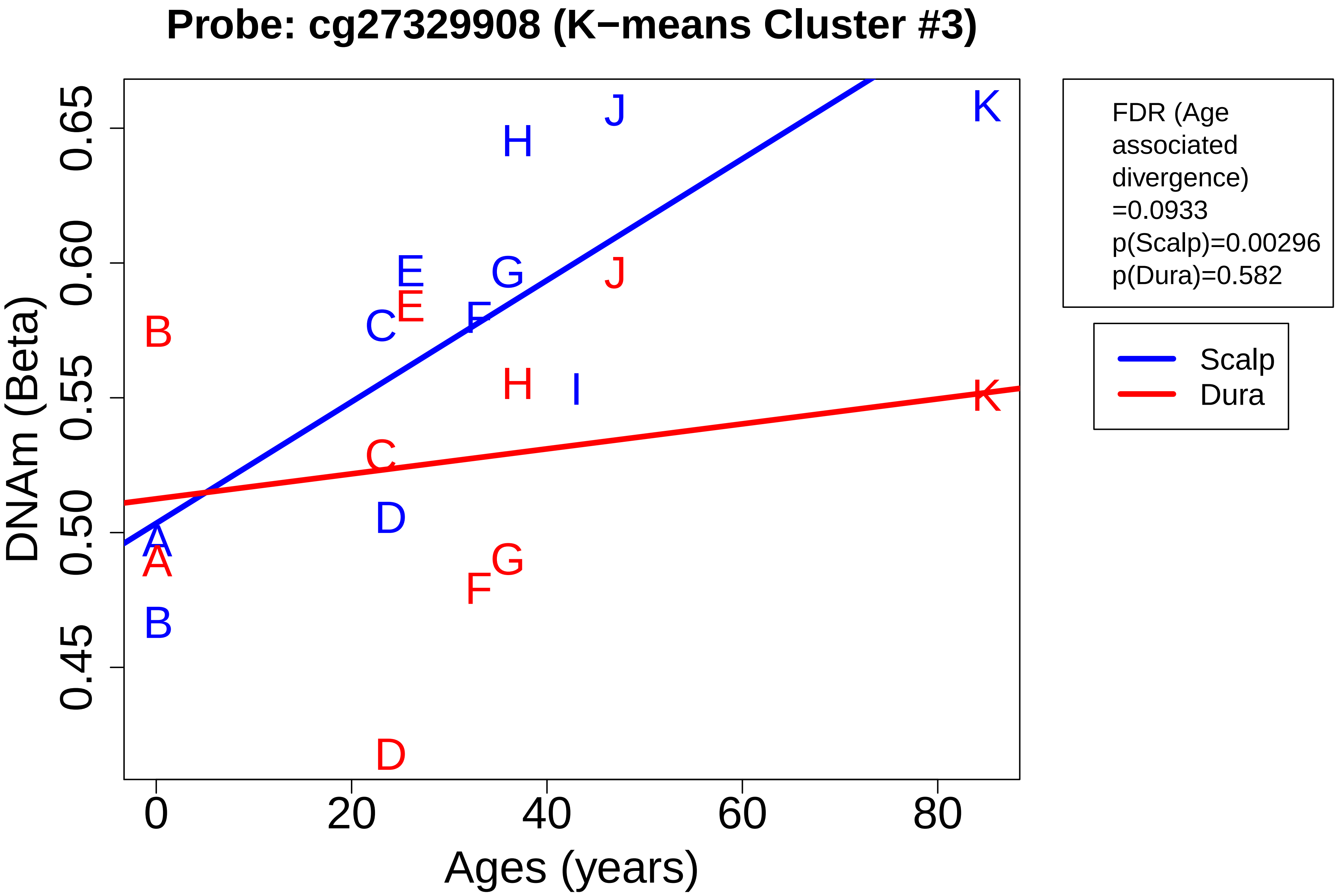

Probe: cg27329908 (K−means Cluster #3)
DNAm (Beta)
0.45
0.50
0.55
0.60
0.65
0
20
40
60
80
Ages (years)
A
B
C
D
E
F
G
H
I
J
K
A
B
C
D
E
F
G
H
J
K
FDR (Age associated divergence) =0.0933
p(Scalp)=0.00296
p(Dura)=0.582
Scalp
Dura

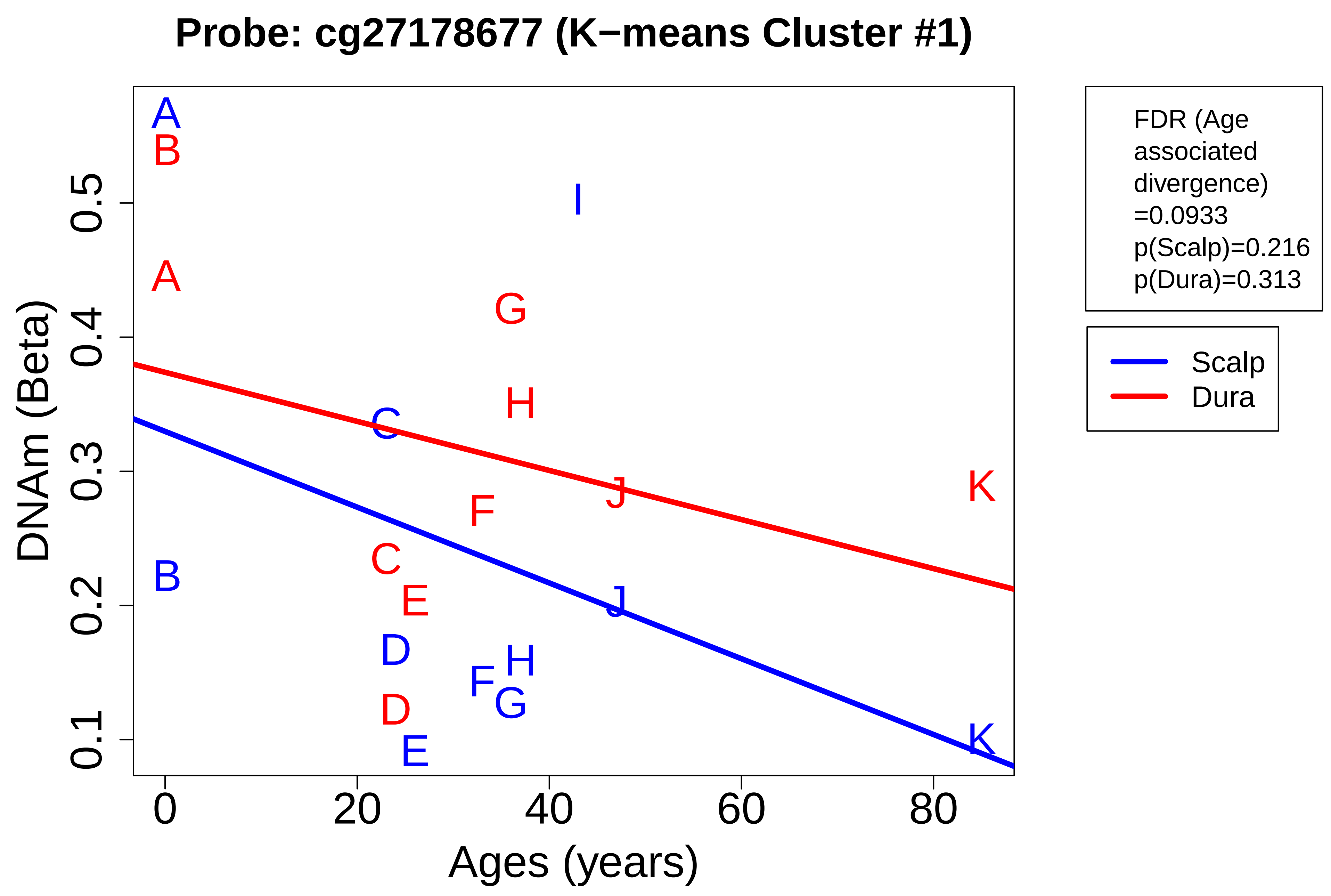

Probe: cg27178677 (K−means Cluster #1)
DNAm (Beta)
0.1
0.2
0.3
0.4
0.5
Ages (years)
0
20
40
60
80
A
B
C
D
E
F
G
H
I
J
K
A
B
C
D
E
F
G
H
J
K
FDR (Age associated divergence) =0.0933
p(Scalp)=0.216
p(Dura)=0.313
Scalp
Dura

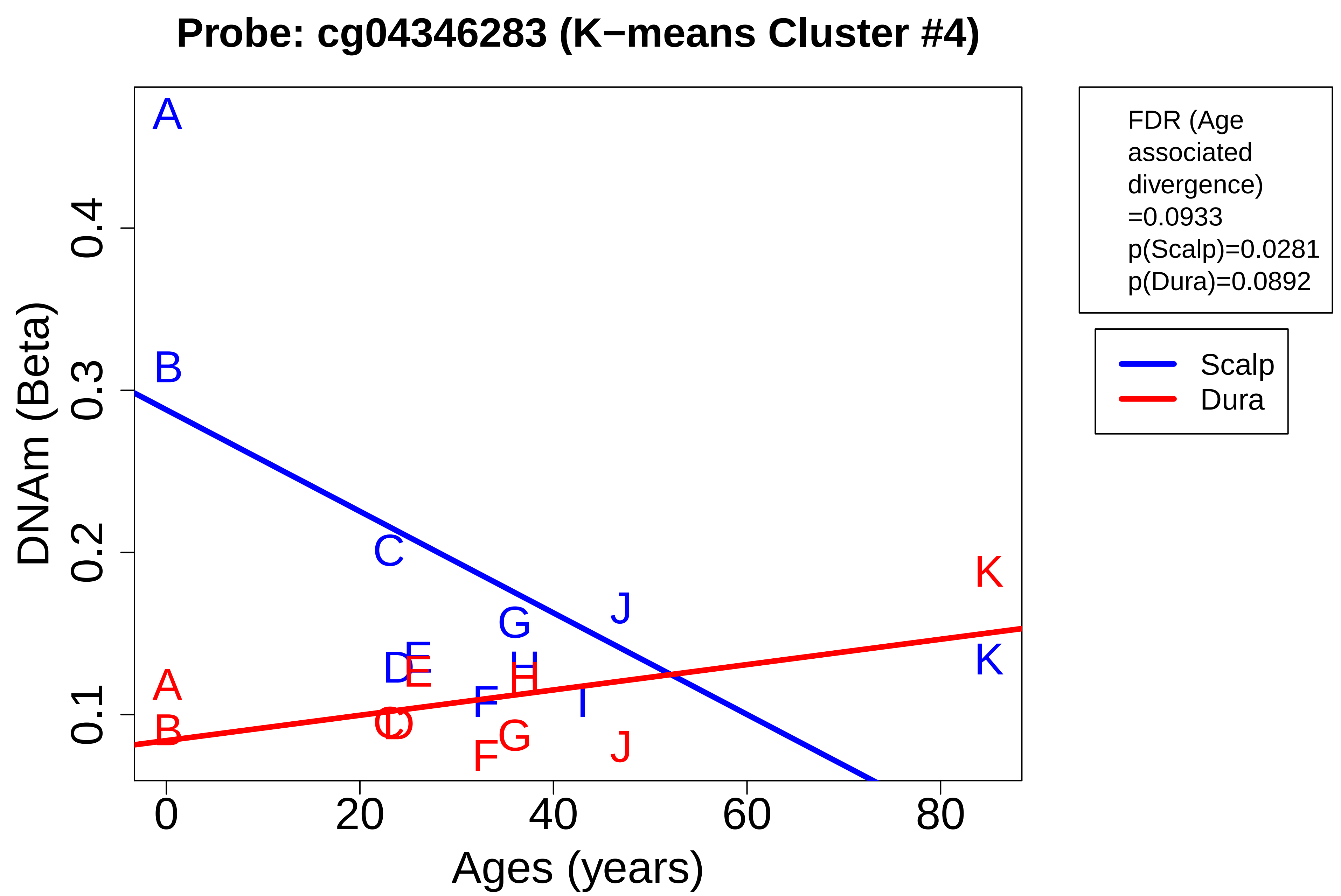

Probe: cg04346283 (K−means Cluster #4)
FDR (Age associated divergence) =0.0933
p(Scalp)=0.0281
p(Dura)=0.0892
Scalp
Dura
DNAm (Beta)
0.1
0.2
0.3
0.4
0
20
40
60
80
Ages (years)
A
B
C
D
E
F
G
H
I
J
K

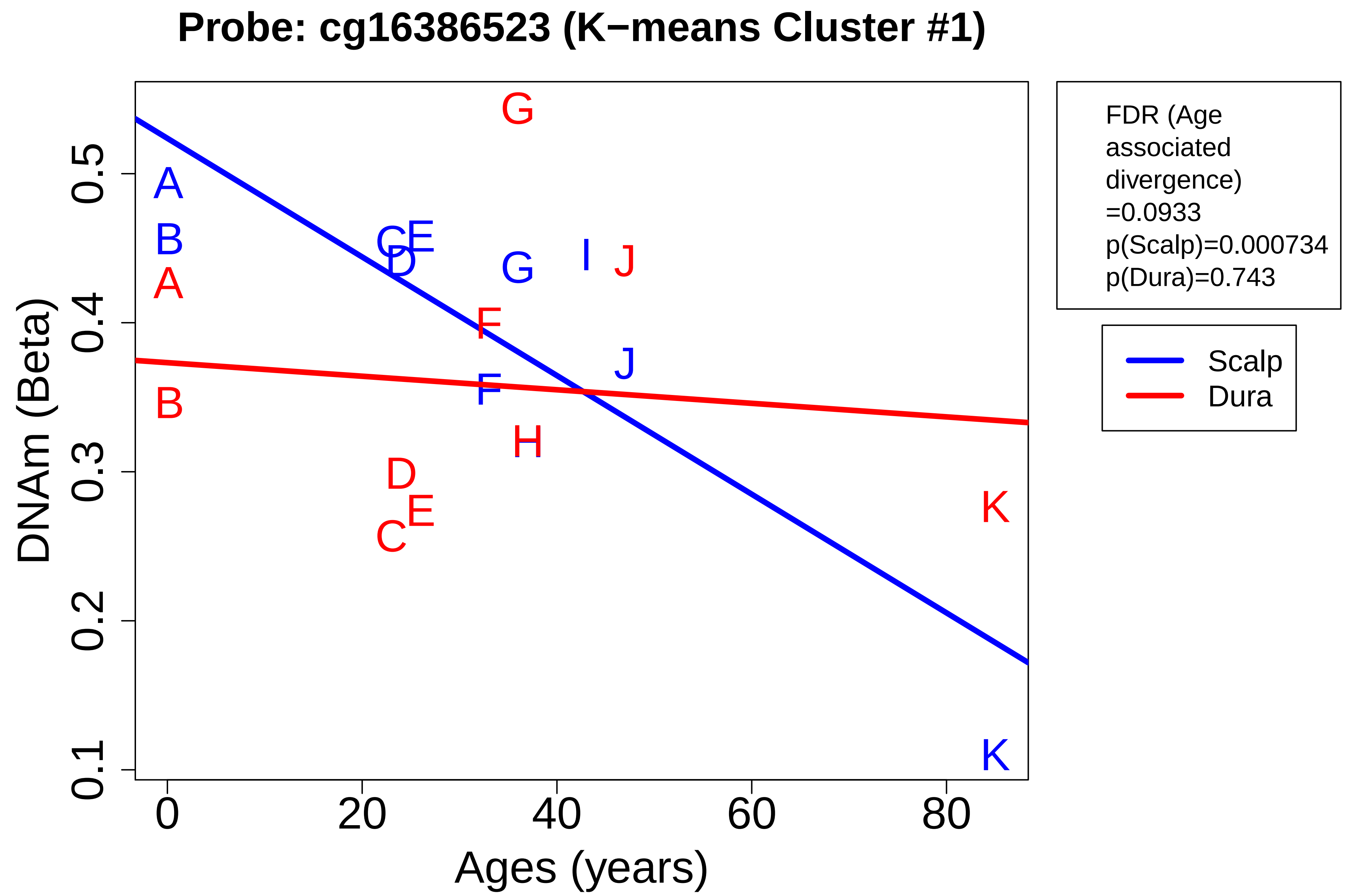
Probe: cg16386523 (K−means Cluster #1)
DNAm (Beta)
0.1
0.2
0.3
0.4
0.5
0
20
40
60
80
Ages (years)
A
B
C
D
E
F
G
H
I
J
K
FDR (Age associated divergence) =0.0933
p(Scalp)=0.000734
p(Dura)=0.743
Scalp
Dura

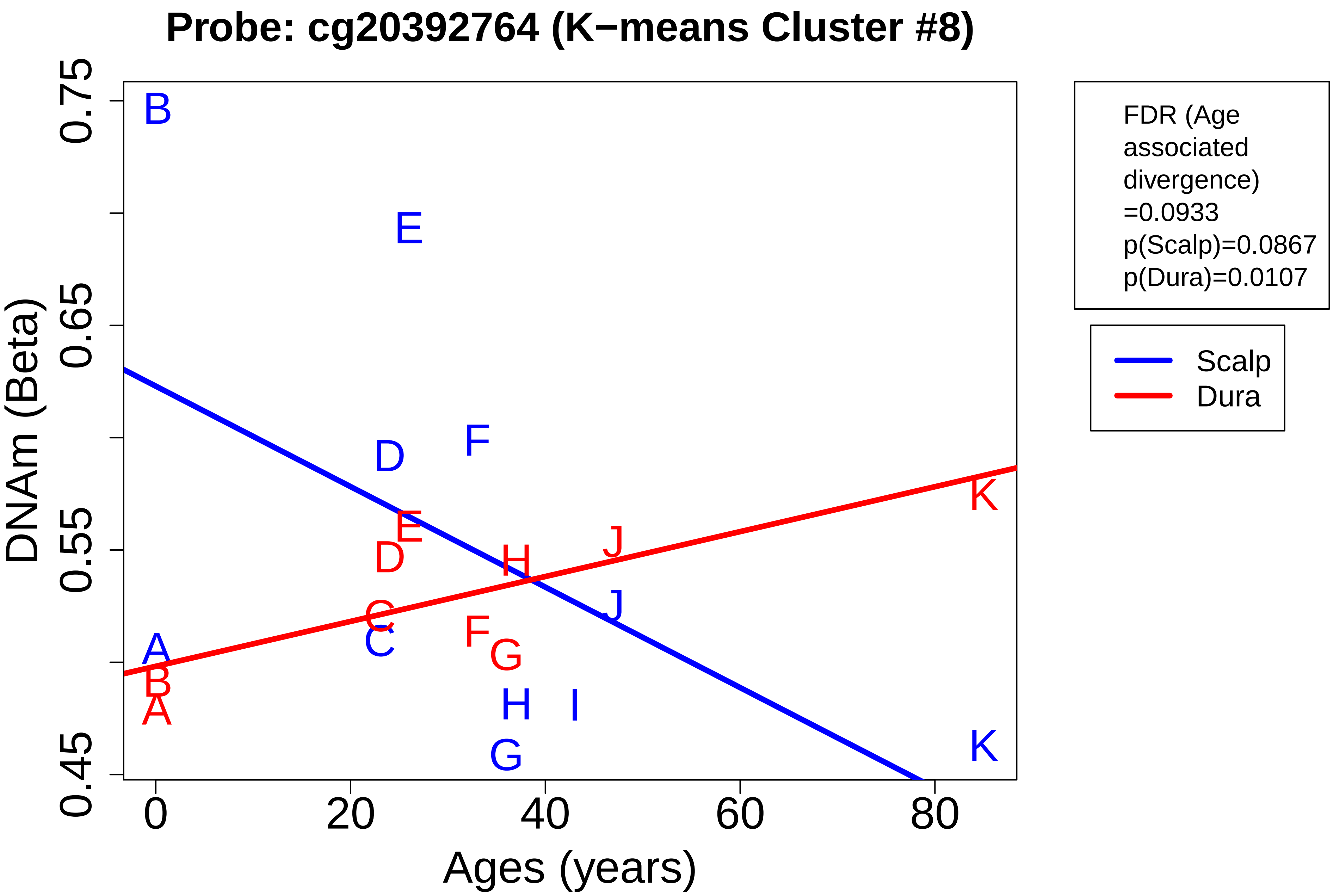

Probe: cg20392764 (K−means Cluster #8)
DNAm (Beta)
0.75
0.65
0.55
0.45
0
20
40
60
80
Ages (years)
FDR (Age associated divergence) =0.0933
p(Scalp)=0.0867
p(Dura)=0.0107
Scalp
Dura

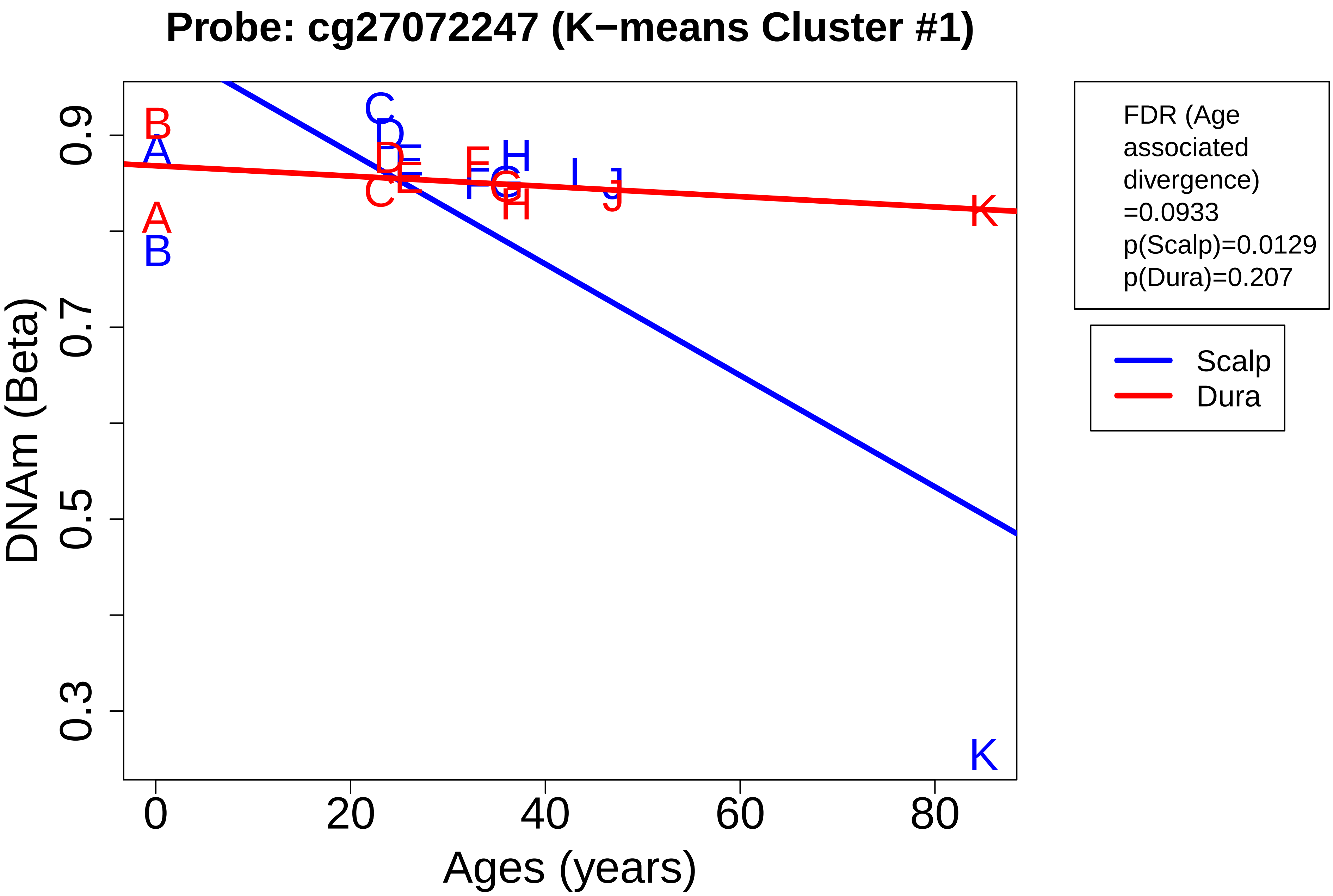

Probe: cg27072247 (K−means Cluster #1)
DNAm (Beta)
0.3
0.5
0.7
0.9
Ages (years)
0
20
40
60
80
A
B
C
D
E
F
G
H
I
J
K
FDR (Age associated divergence) =0.0933
p(Scalp)=0.0129
p(Dura)=0.207
Scalp
Dura

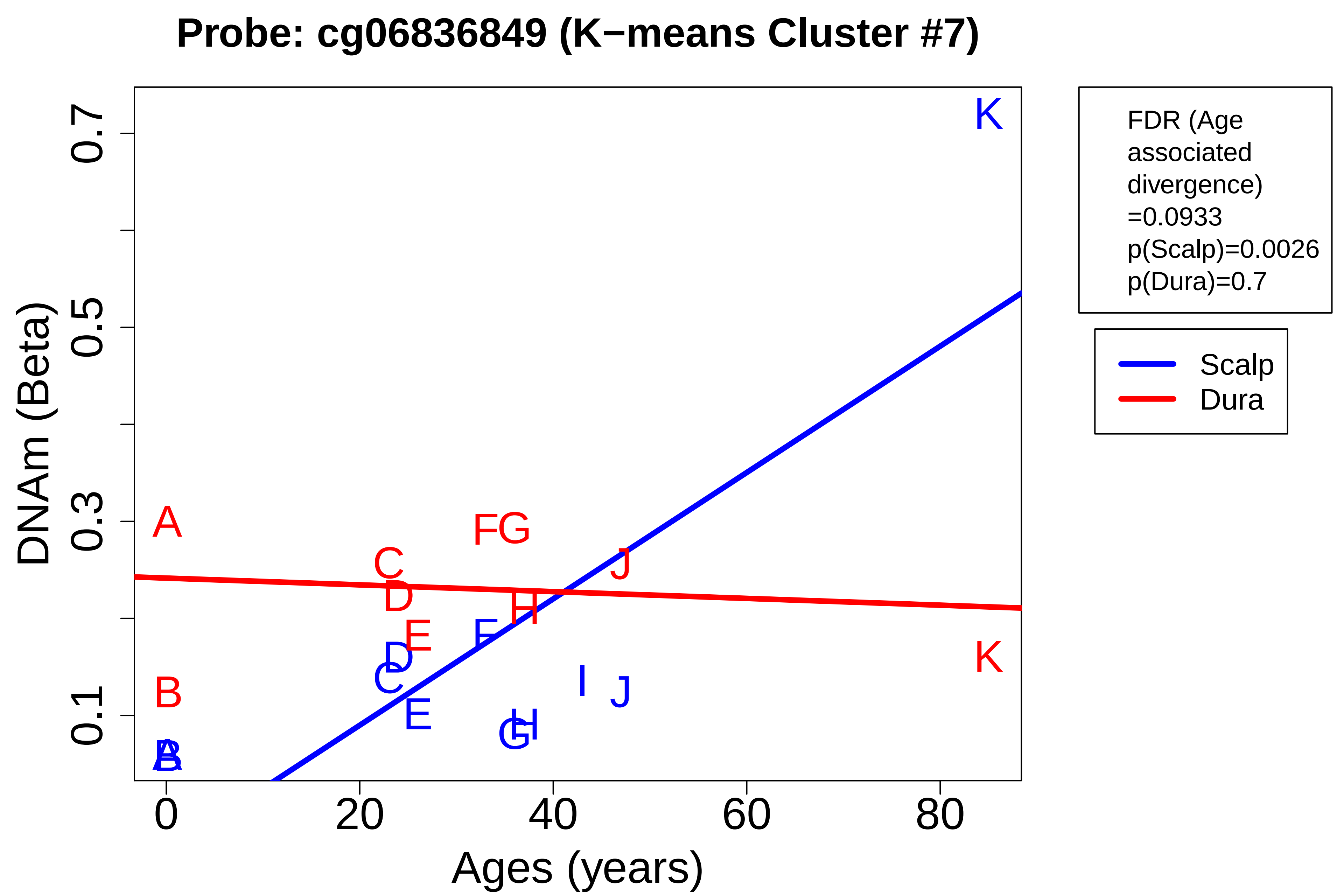

Probe: cg06836849 (K−means Cluster #7)
DNAm (Beta)
0.1
0.3
0.5
0.7
Ages (years)
0
20
40
60
80
A
B
C
D
E
F
G
H
I
J
K
FDR (Age associated divergence) =0.0933
p(Scalp)=0.0026
p(Dura)=0.7
Scalp
Dura

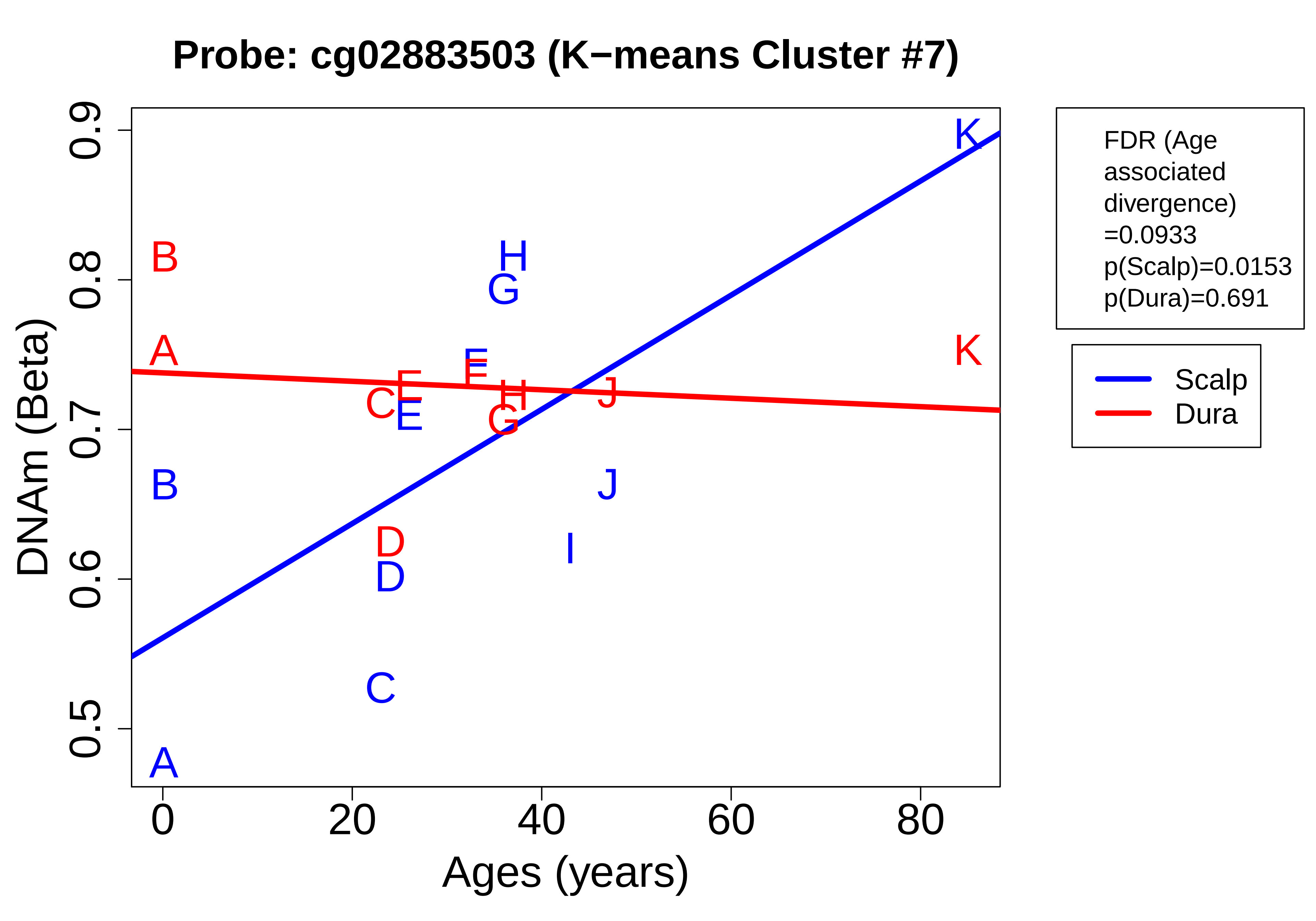

Probe: cg02883503 (K−means Cluster #7)
DNAm (Beta)
0.5
0.6
0.7
0.8
0.9
Ages (years)
0
20
40
60
80
FDR (Age associated divergence) =0.0933
p(Scalp)=0.0153
p(Dura)=0.691
Scalp
Dura

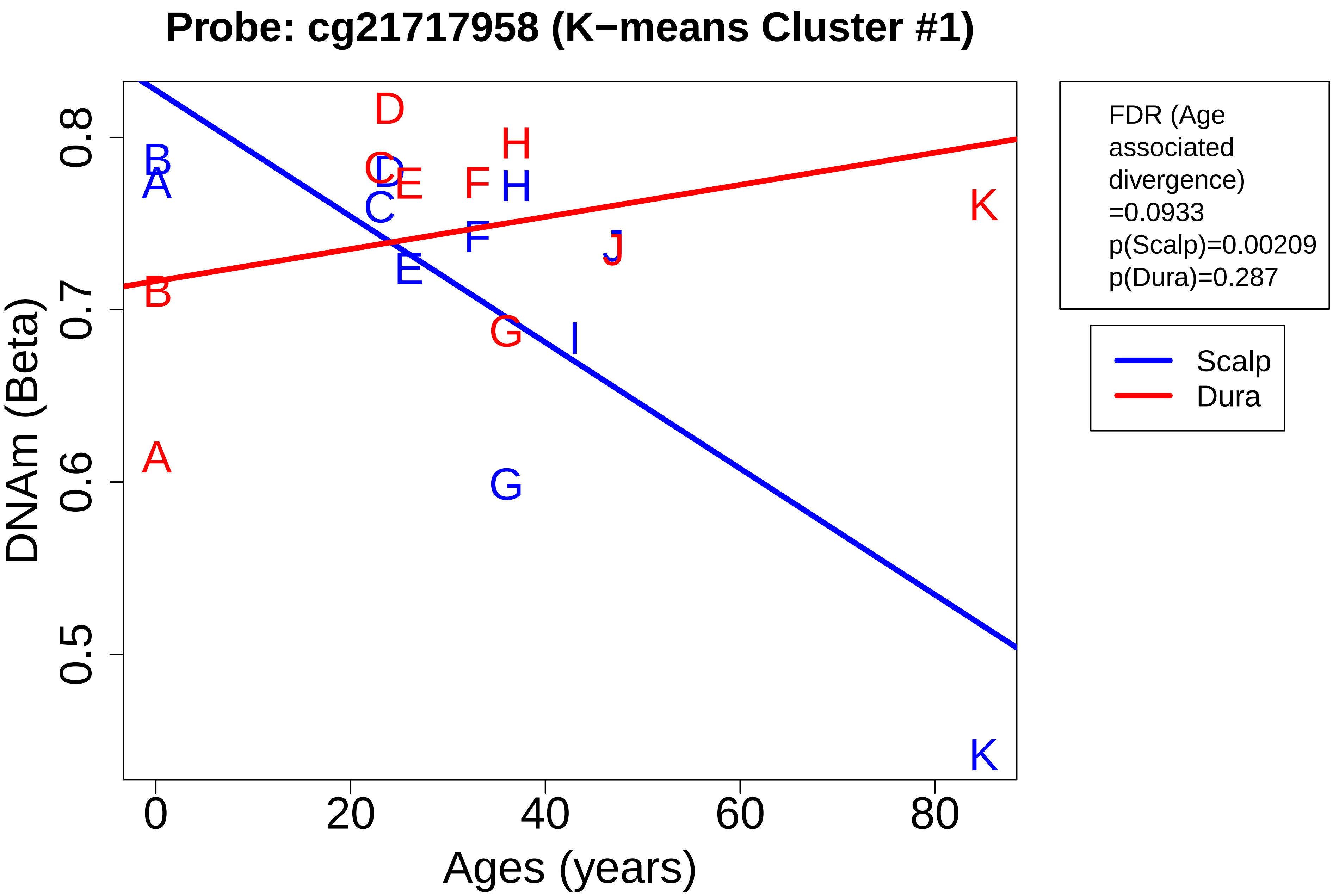

Probe: cg21717958 (K−means Cluster #1)
DNAm (Beta)
0.5
0.6
0.7
0.8
0
20
40
60
80
Ages (years)
FDR (Age associated divergence) =0.0933
p(Scalp)=0.00209
p(Dura)=0.287
Scalp
Dura
A
B
C
D
E
F
G
H
I
J
K

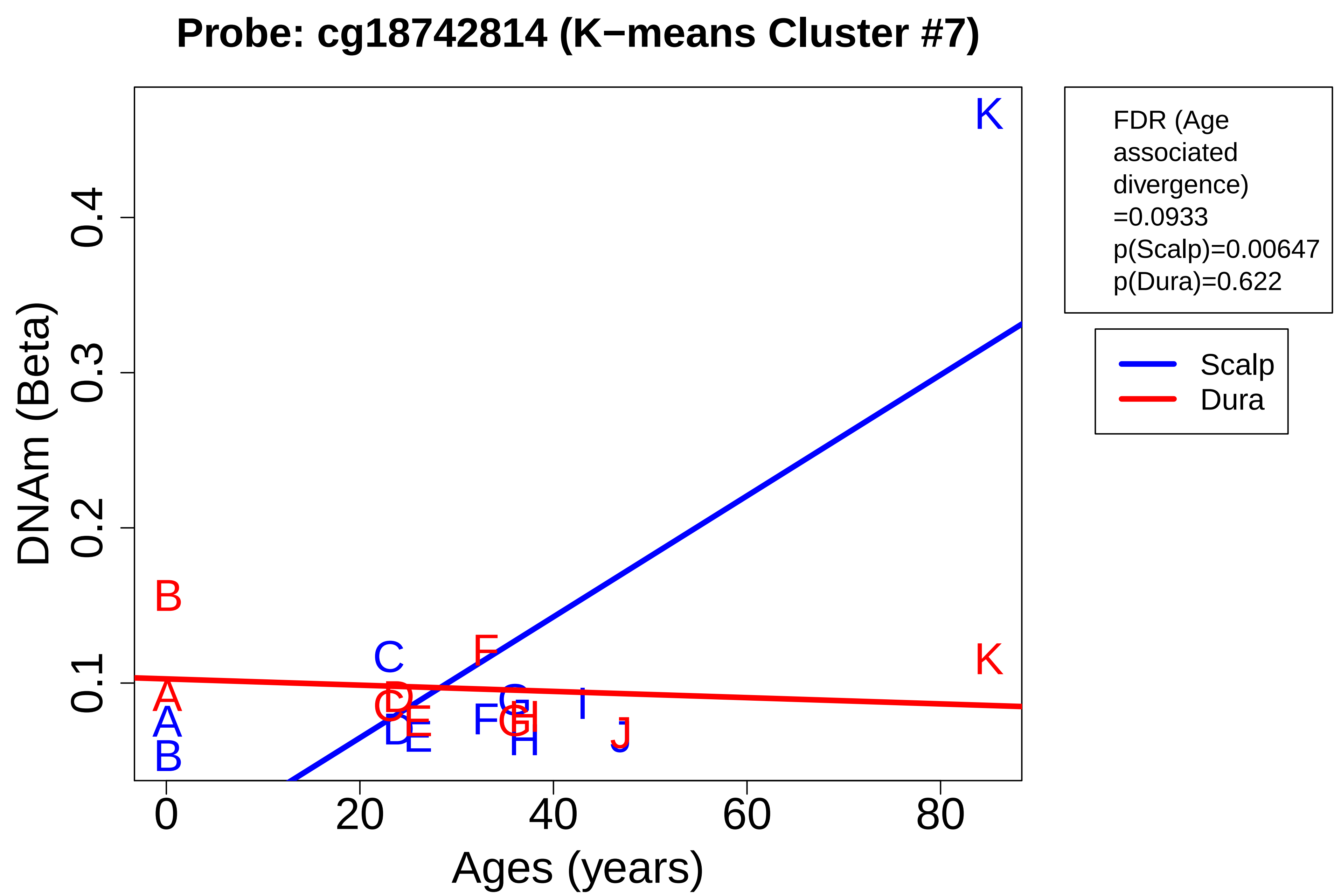

Probe: cg18742814 (K−means Cluster #7)
DNAm (Beta)
0.1
0.2
0.3
0.4
0
20
40
60
80
Ages (years)
FDR (Age associated divergence) =0.0933
p(Scalp)=0.00647
p(Dura)=0.622
Scalp
Dura

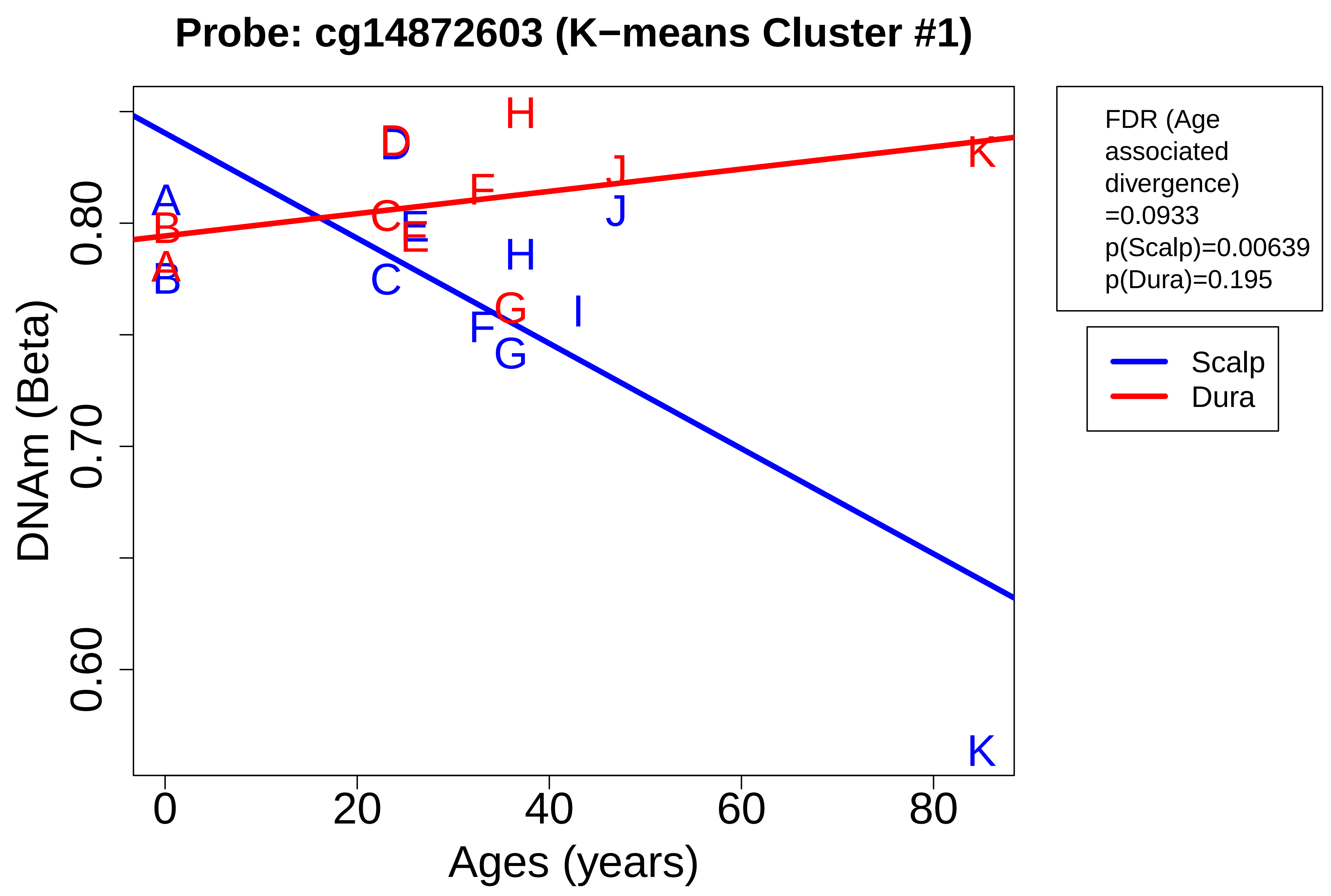

Probe: cg14872603 (K−means Cluster #1)
DNAm (Beta)
0.60
0.70
0.80
0
20
40
60
80
Ages (years)
A
B
C
D
E
F
G
H
I
J
K
FDR (Age associated divergence) =0.0933
p(Scalp)=0.00639
p(Dura)=0.195
Scalp
Dura

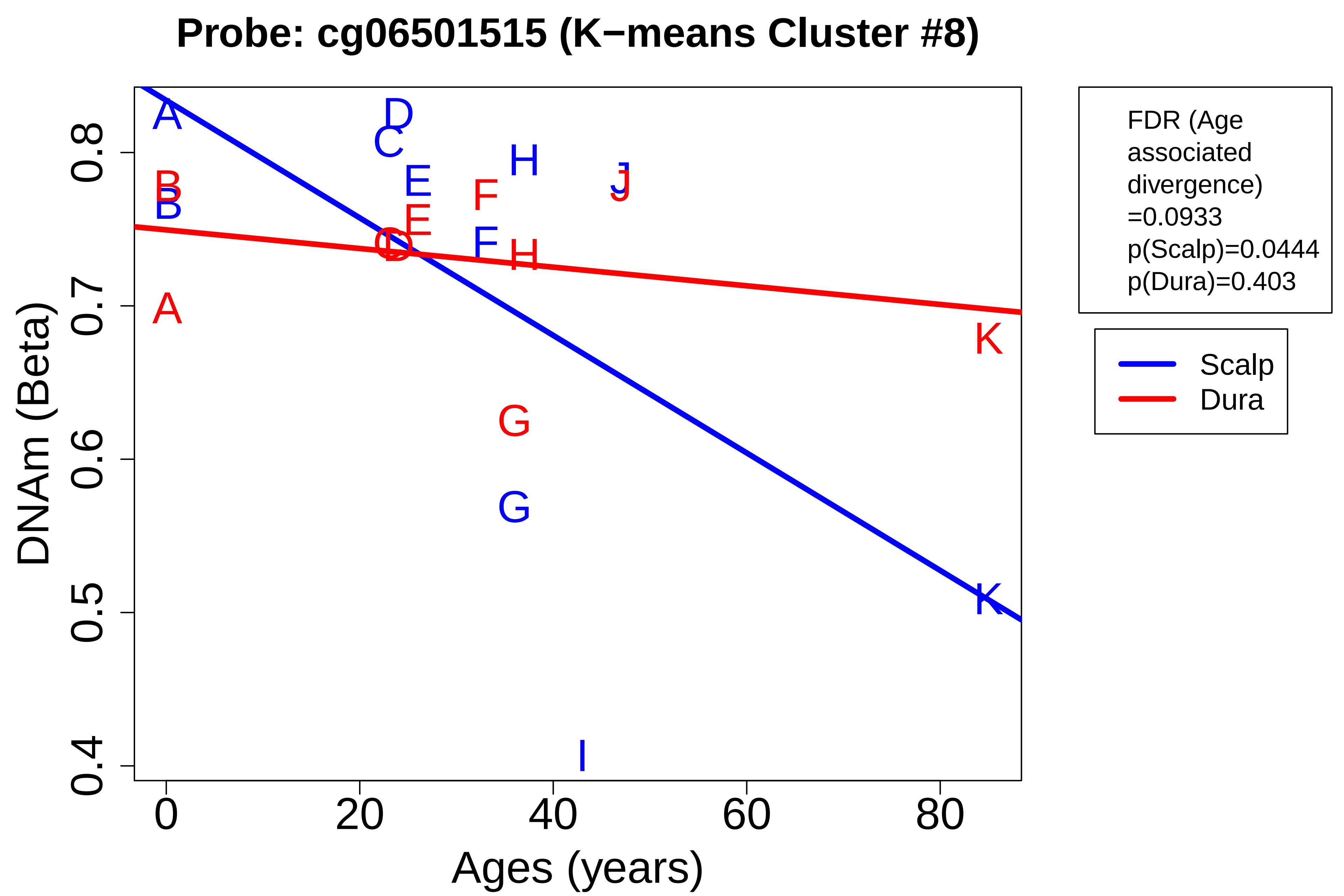

Probe: cg06501515 (K−means Cluster #8)
DNAm (Beta)
0.4
0.5
0.6
0.7
0.8
0
20
40
60
80
Ages (years)
A
B
C
D
E
F
G
H
I
J
K
FDR (Age associated divergence) =0.0933
p(Scalp)=0.0444
p(Dura)=0.403
Scalp
Dura

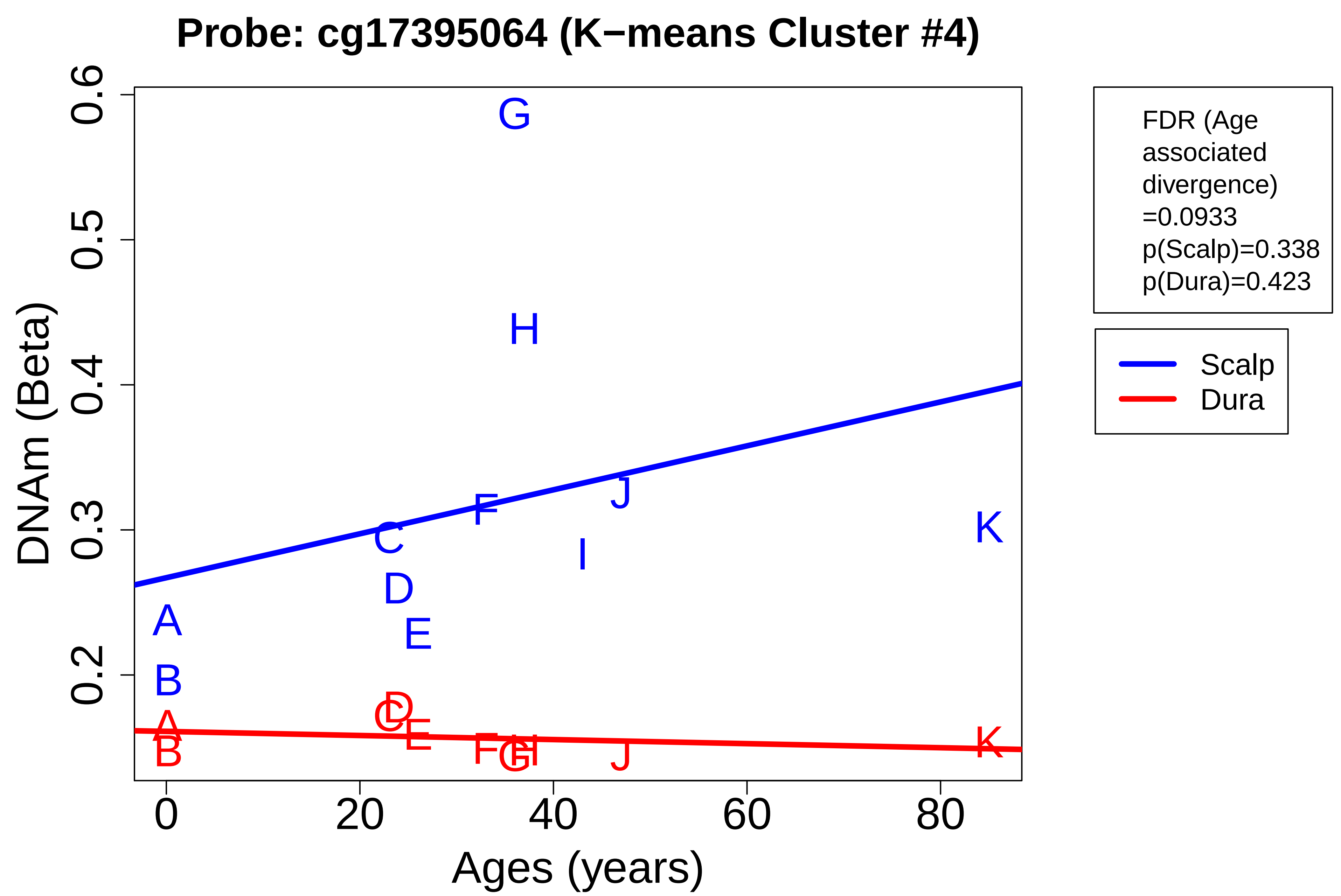

Probe: cg17395064 (K−means Cluster #4)
DNAm (Beta)
0.2
0.3
0.4
0.5
0.6
Ages (years)
0
20
40
60
80
A
B
C
D
E
F
G
H
I
J
K
FDR (Age associated divergence) =0.0933
p(Scalp)=0.338
p(Dura)=0.423
Scalp
Dura

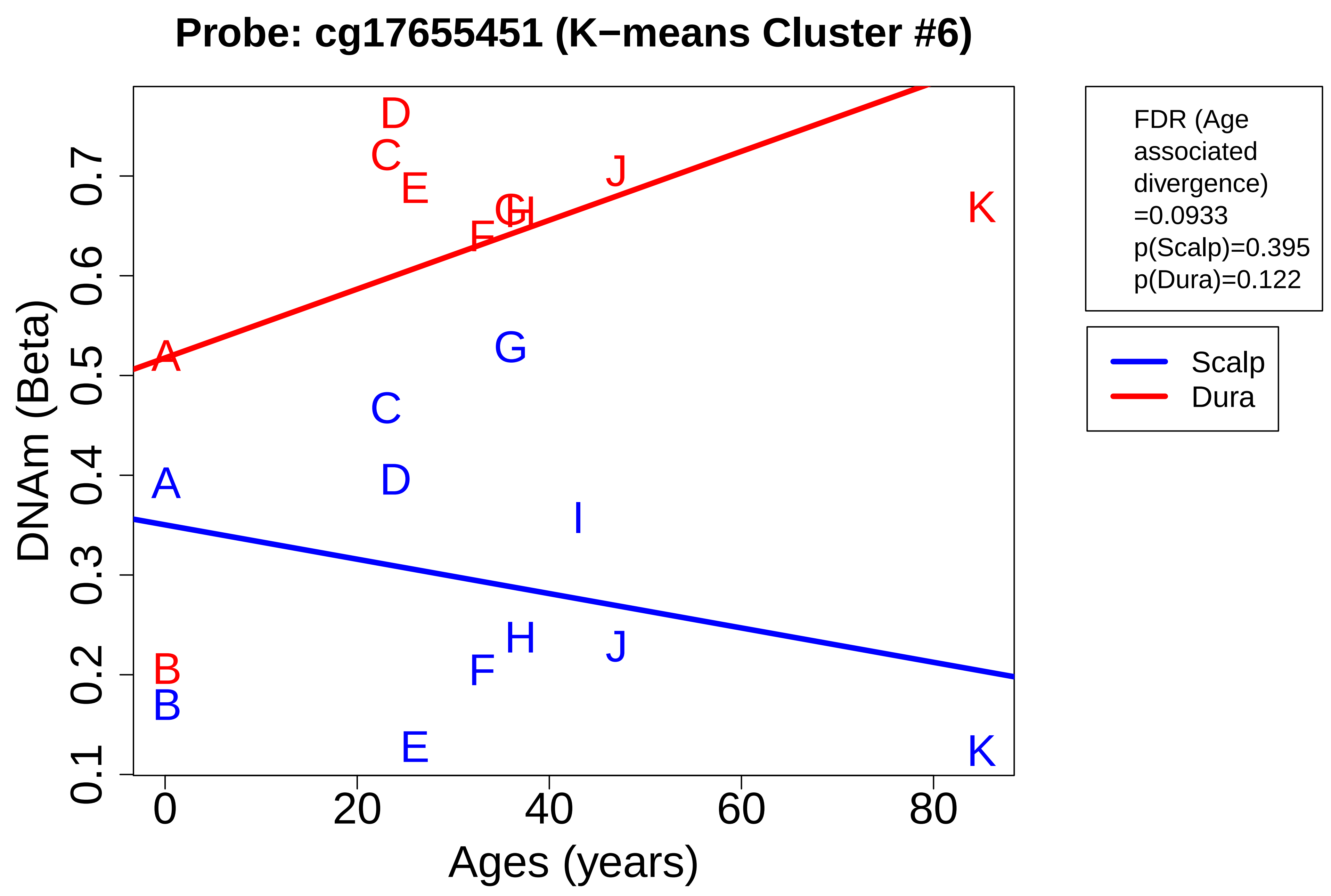

Probe: cg17655451 (K−means Cluster #6)
DNAm (Beta)
0.1
0.2
0.3
0.4
0.5
0.6
0.7
Ages (years)
0
20
40
60
80
FDR (Age associated divergence) =0.0933
p(Scalp)=0.395
p(Dura)=0.122
Scalp
Dura

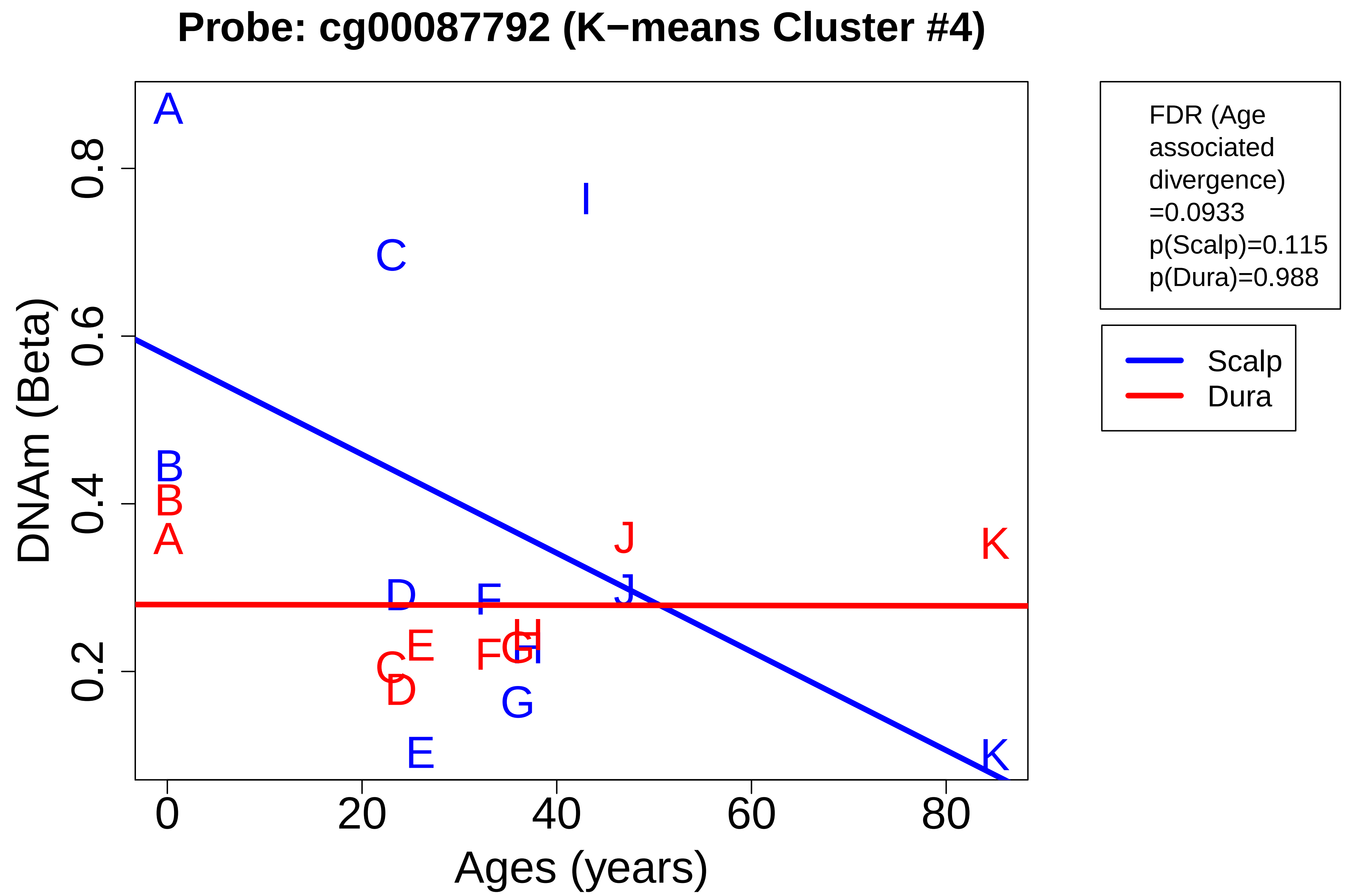

Probe: cg00087792 (K−means Cluster #4)
DNAm (Beta)
0.2
0.4
0.6
0.8
Ages (years)
0
20
40
60
80
FDR (Age associated divergence) =0.0933
p(Scalp)=0.115
p(Dura)=0.988
Scalp
Dura

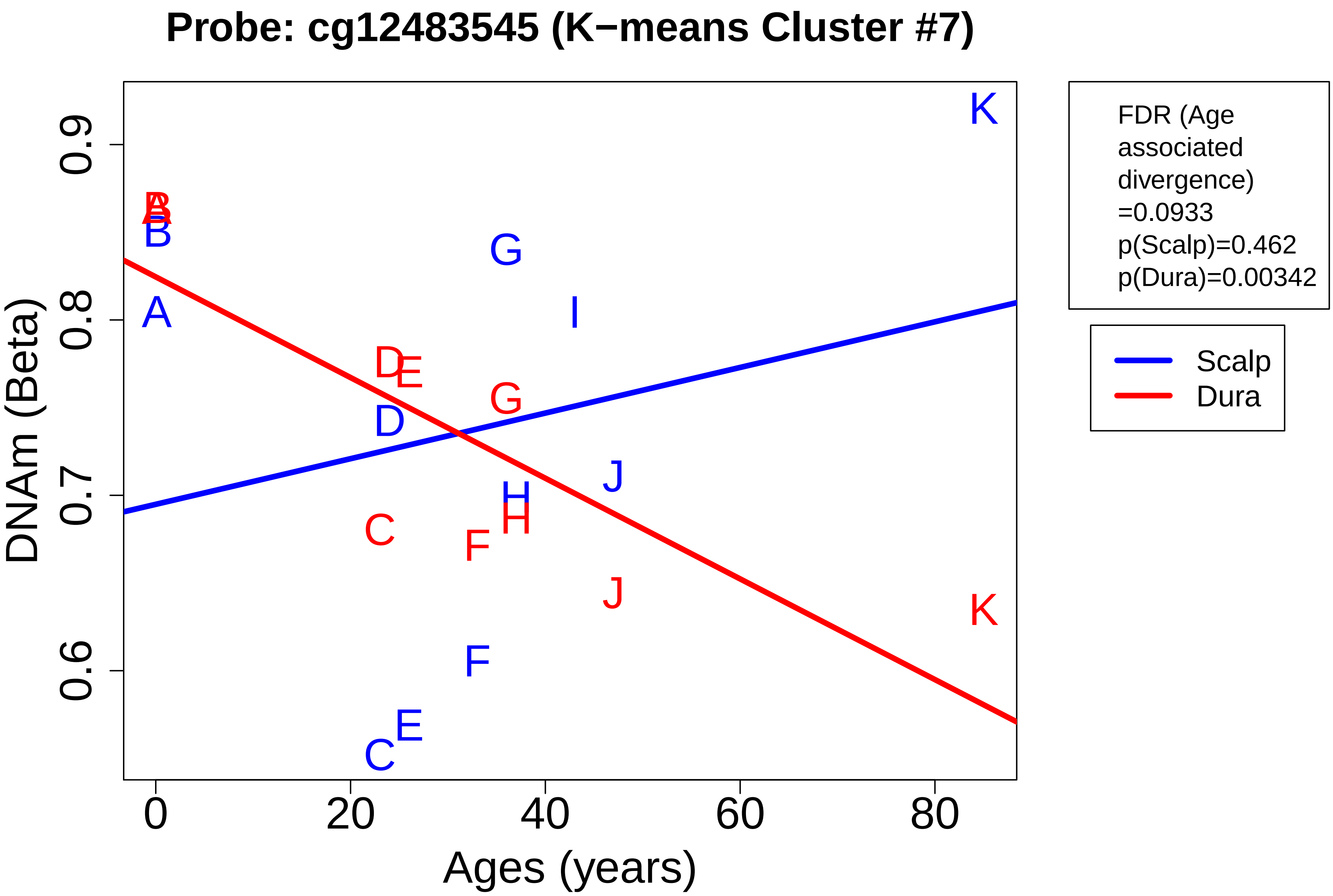

Probe: cg12483545 (K−means Cluster #7)
DNAm (Beta)
0.6
0.7
0.8
0.9
Ages (years)
0
20
40
60
80
A
B
C
D
E
F
G
H
I
J
K
FDR (Age associated divergence) =0.0933
p(Scalp)=0.462
p(Dura)=0.00342
Scalp
Dura

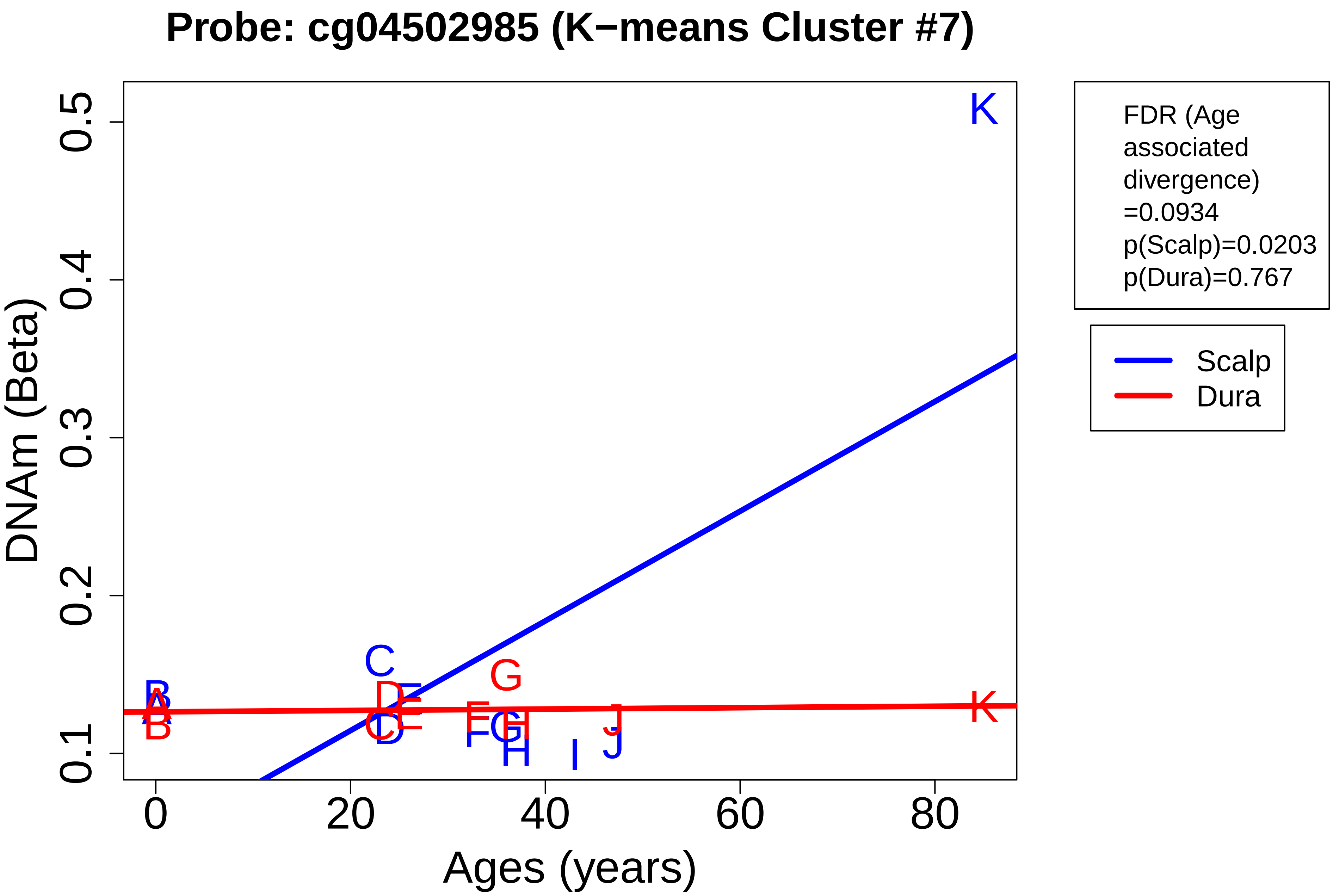

Probe: cg04502985 (K−means Cluster #7)
DNAm (Beta)
Ages (years)
0.1
0.2
0.3
0.4
0.5
0
20
40
60
80
FDR (Age associated divergence) =0.0934
p(Scalp)=0.0203
p(Dura)=0.767
Scalp
Dura

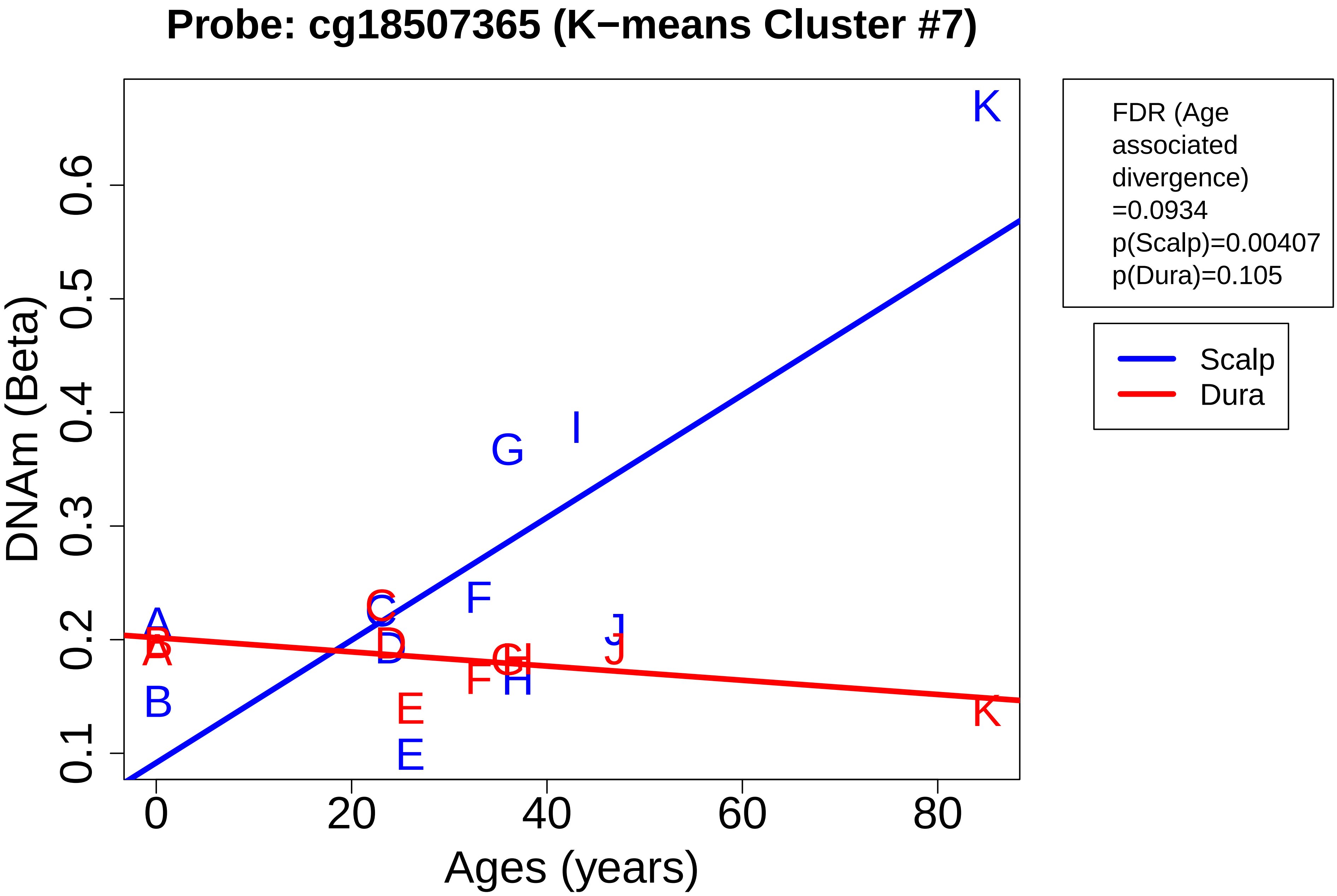

Probe: cg18507365 (K−means Cluster #7)
DNAm (Beta)
Ages (years)
0.1
0.2
0.3
0.4
0.5
0.6
0
20
40
60
80
FDR (Age associated divergence) =0.0934
p(Scalp)=0.00407
p(Dura)=0.105
Scalp
Dura

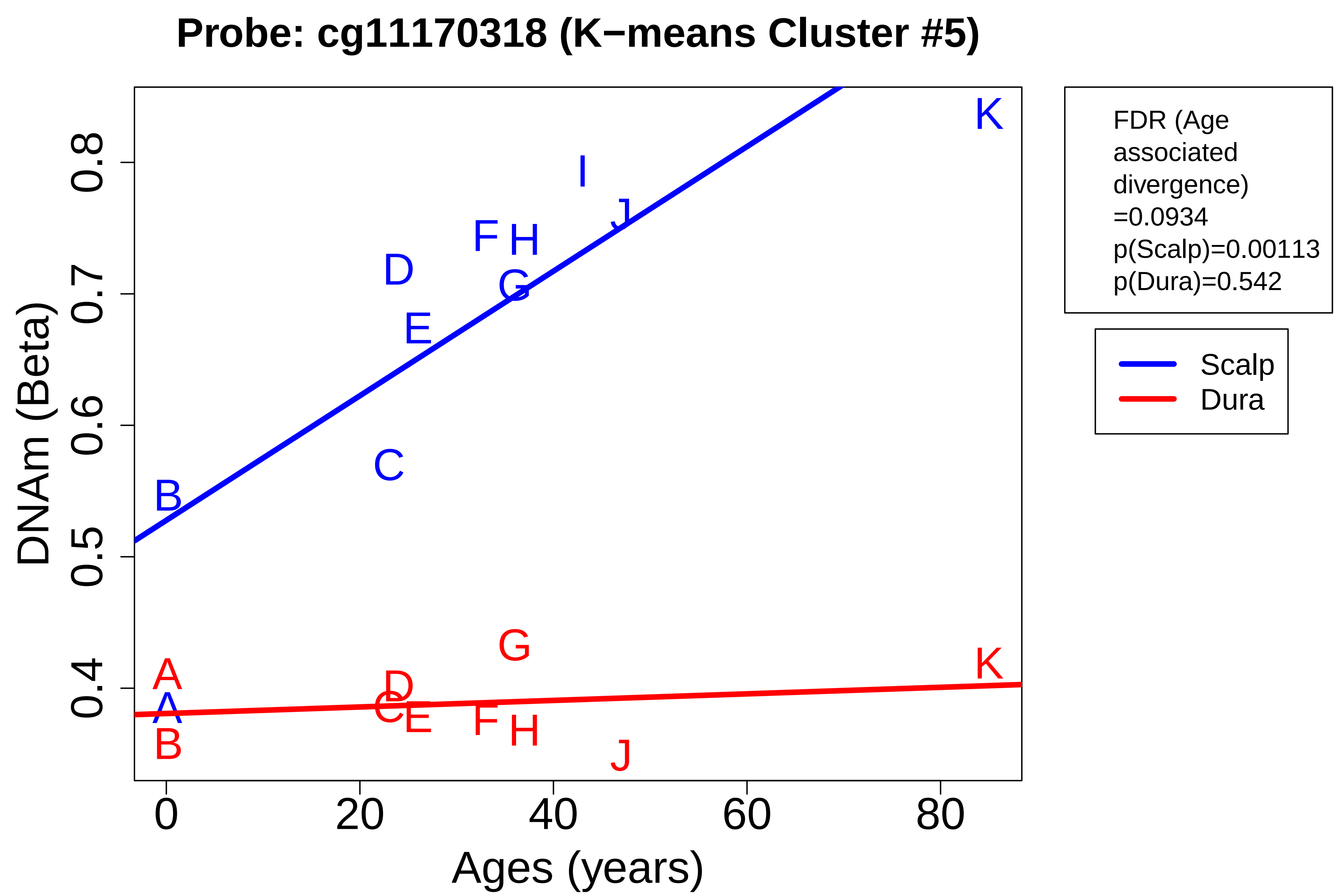

Probe: cg11170318 (K−means Cluster #5)
DNAm (Beta)
0.4
0.5
0.6
0.7
0.8
0
20
40
60
80
Ages (years)
A
B
C
D
E
F
G
H
I
J
K
FDR (Age associated divergence) =0.0934
p(Scalp)=0.00113
p(Dura)=0.542
Scalp
Dura

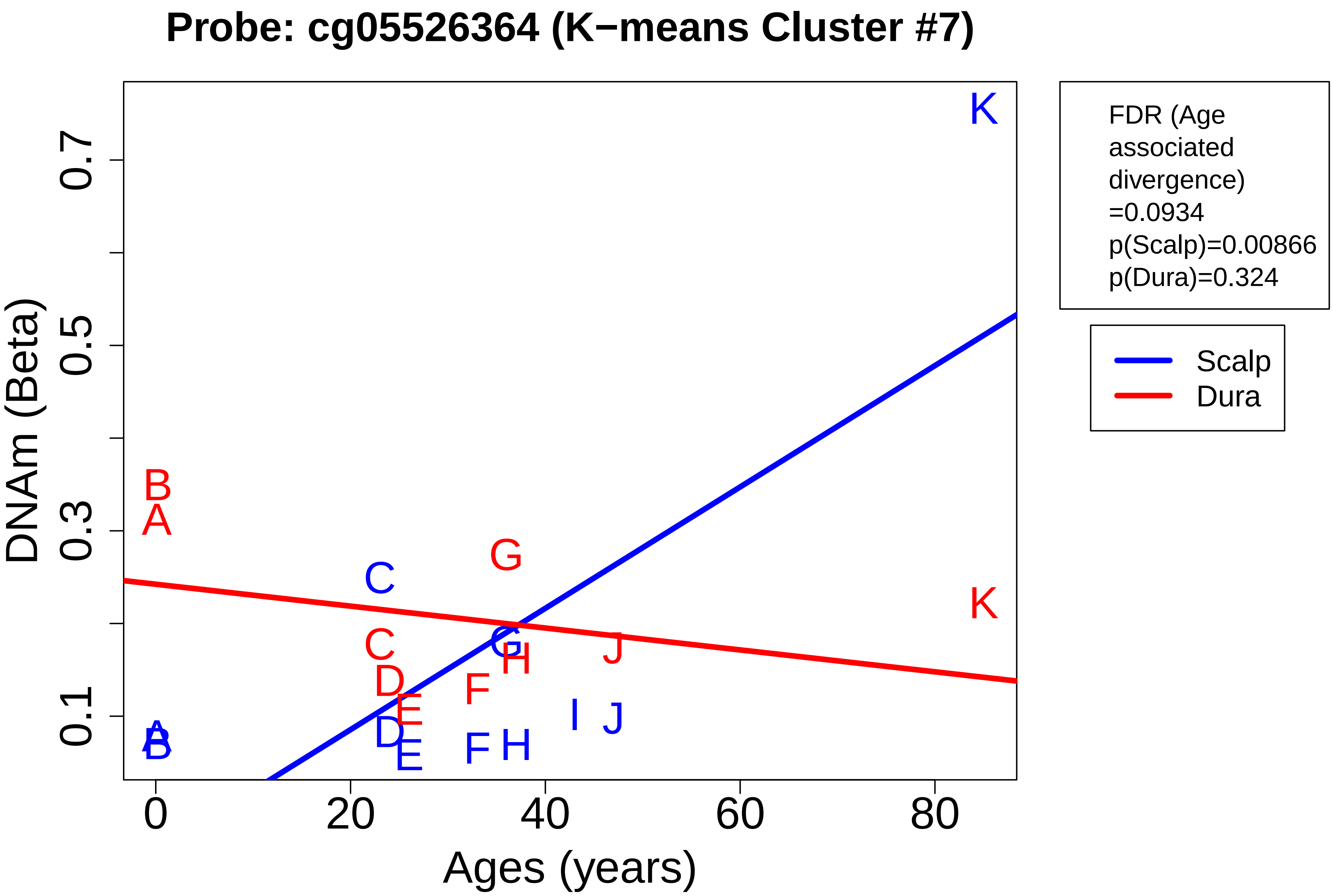
Probe: cg05526364 (K−means Cluster #7)
FDR (Age associated divergence) =0.0934
p(Scalp)=0.00866
p(Dura)=0.324
Scalp
Dura
DNAm (Beta)
Ages (years)
0.1
0.3
0.5
0.7
0
20
40
60
80
A
B
C
D
E
F
G
H
I
J
K

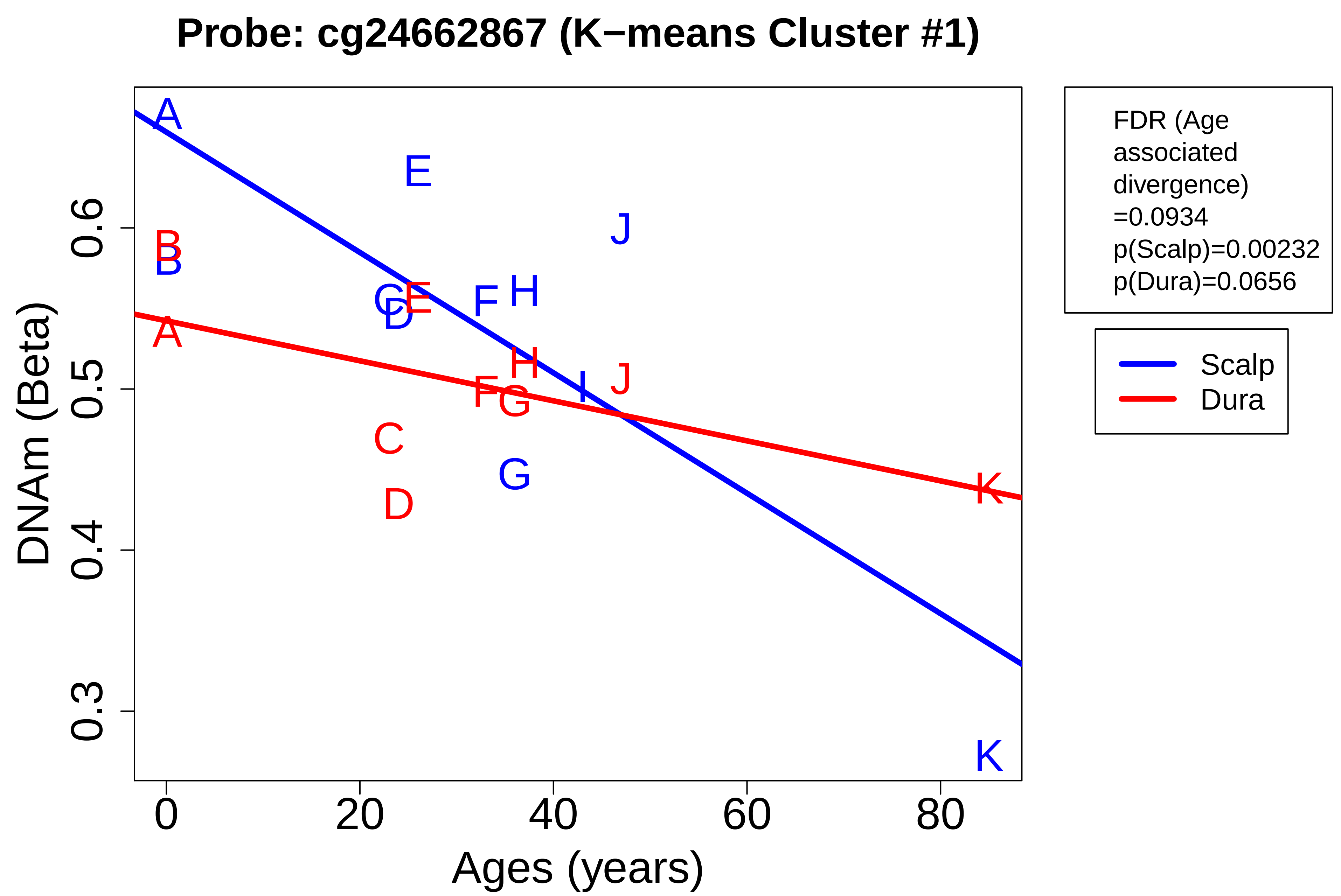

Probe: cg24662867 (K−means Cluster #1)
DNAm (Beta)
0.3
0.4
0.5
0.6
Ages (years)
0
20
40
60
80
A
B
C
D
E
F
G
H
I
J
K
FDR (Age associated divergence) =0.0934
p(Scalp)=0.00232
p(Dura)=0.0656
Scalp
Dura

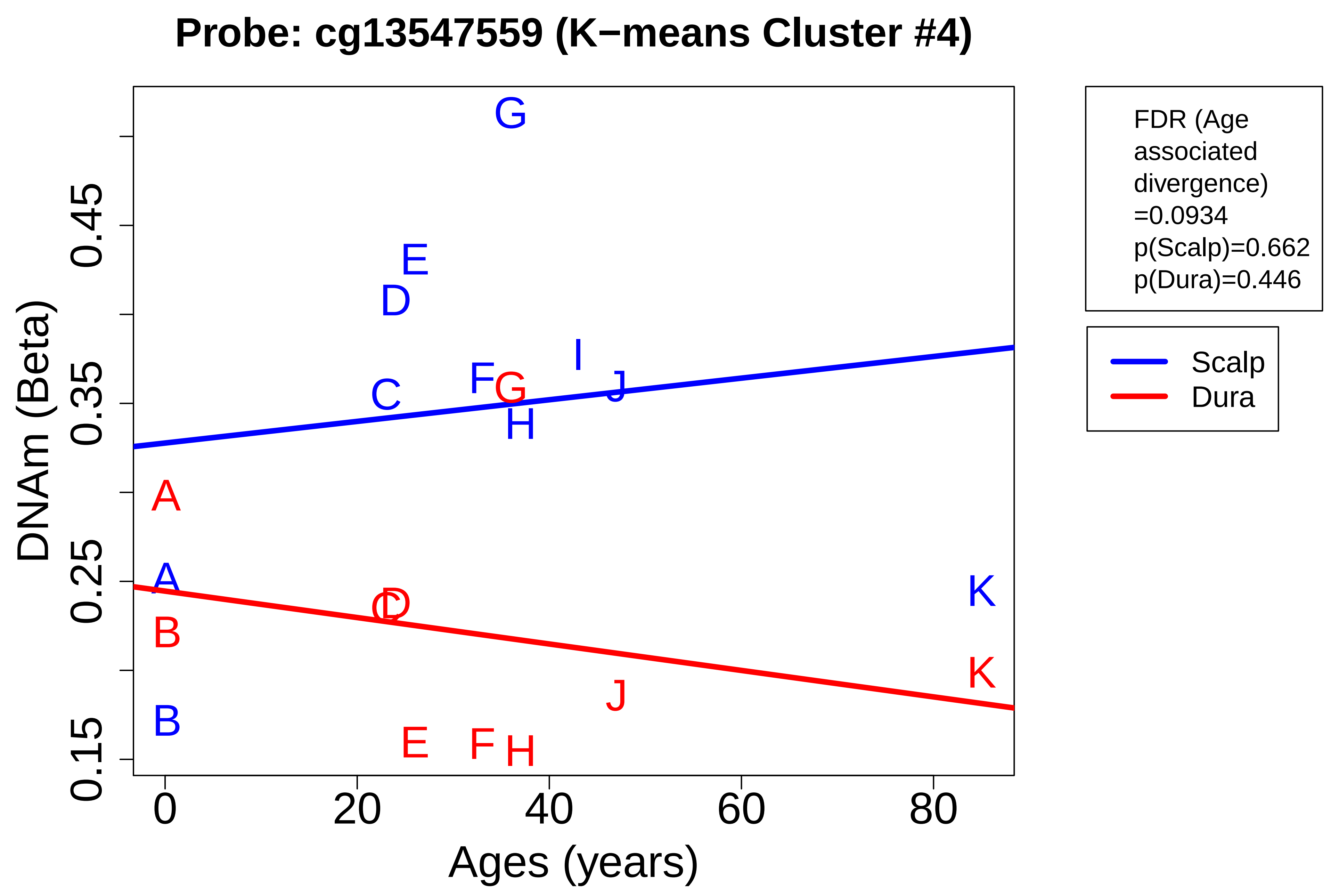

Probe: cg13547559 (K−means Cluster #4)
DNAm (Beta)
0.15
0.25
0.35
0.45
0
20
40
60
80
Ages (years)
FDR (Age associated divergence) =0.0934
p(Scalp)=0.662
p(Dura)=0.446
Scalp
Dura
A
B
C
D
E
F
G
H
I
J
K

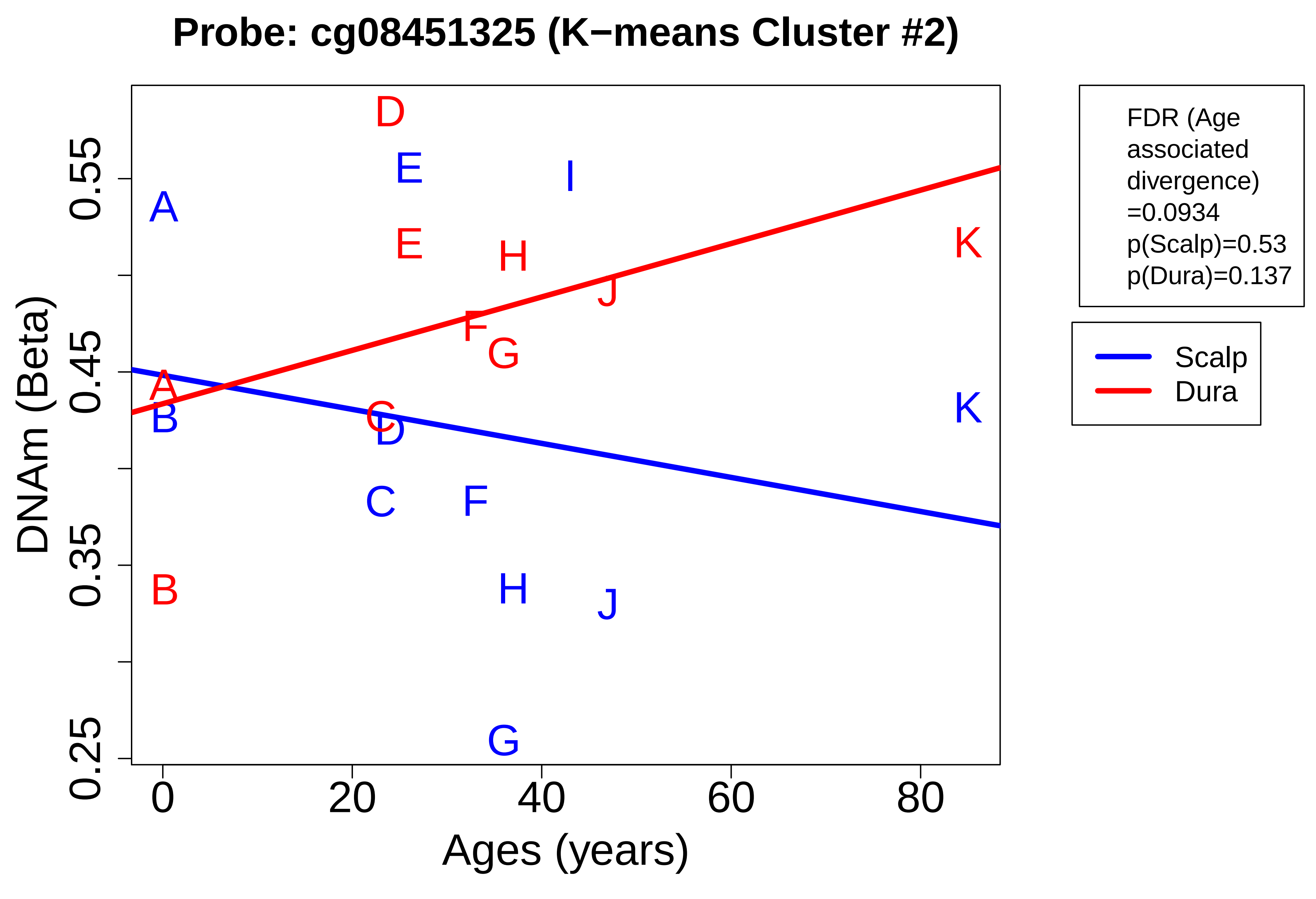

Probe: cg08451325 (K−means Cluster #2)
DNAm (Beta)
0.25
0.35
0.45
0.55
Ages (years)
0
20
40
60
80
A
B
C
D
E
F
G
H
I
J
K
FDR (Age associated divergence) =0.0934
p(Scalp)=0.53
p(Dura)=0.137
Scalp
Dura

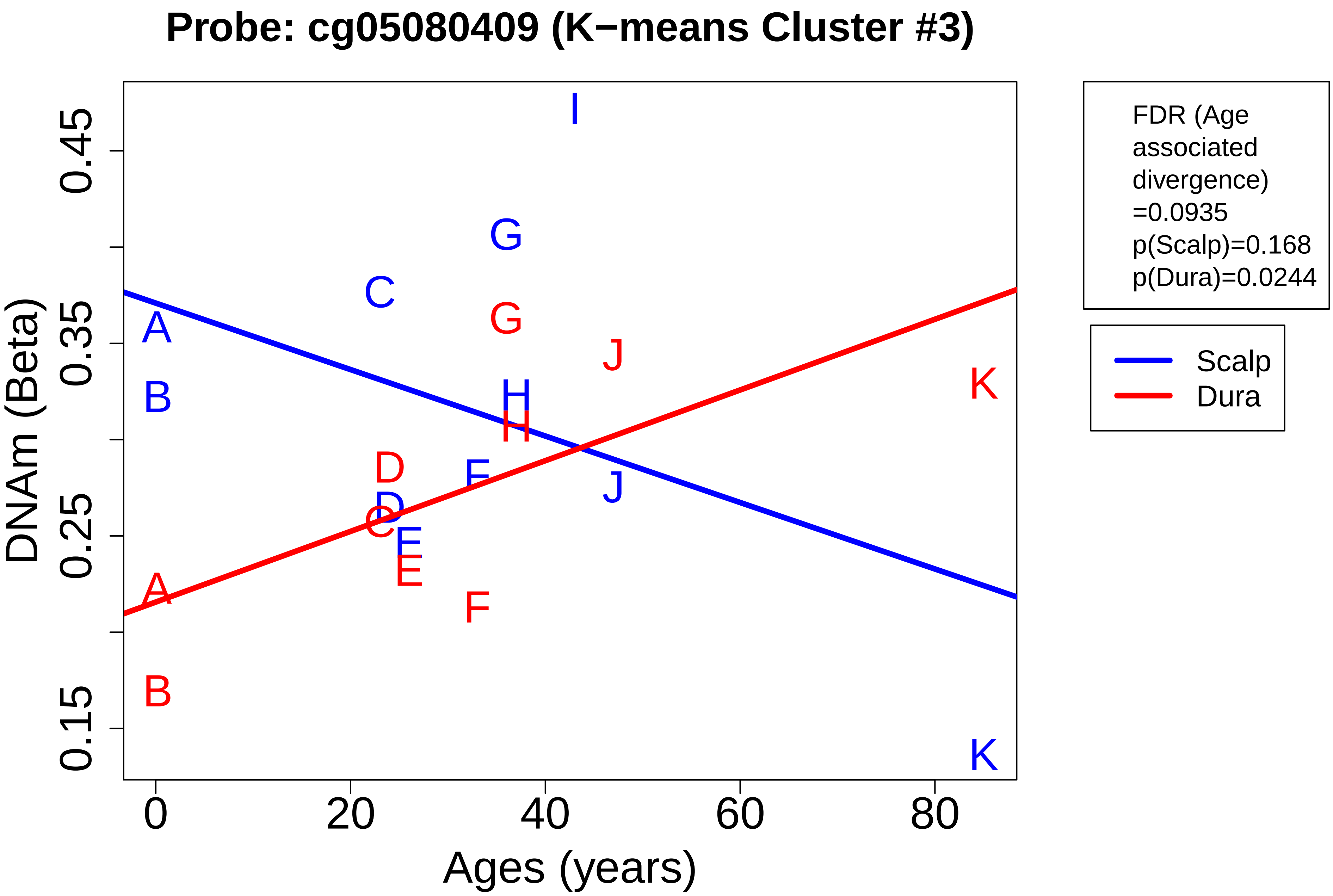

Probe: cg05080409 (K−means Cluster #3)
FDR (Age associated divergence) =0.0935
p(Scalp)=0.168
p(Dura)=0.0244
Scalp
Dura
DNAm (Beta)
0.15
0.25
0.35
0.45
0
20
40
60
80
Ages (years)

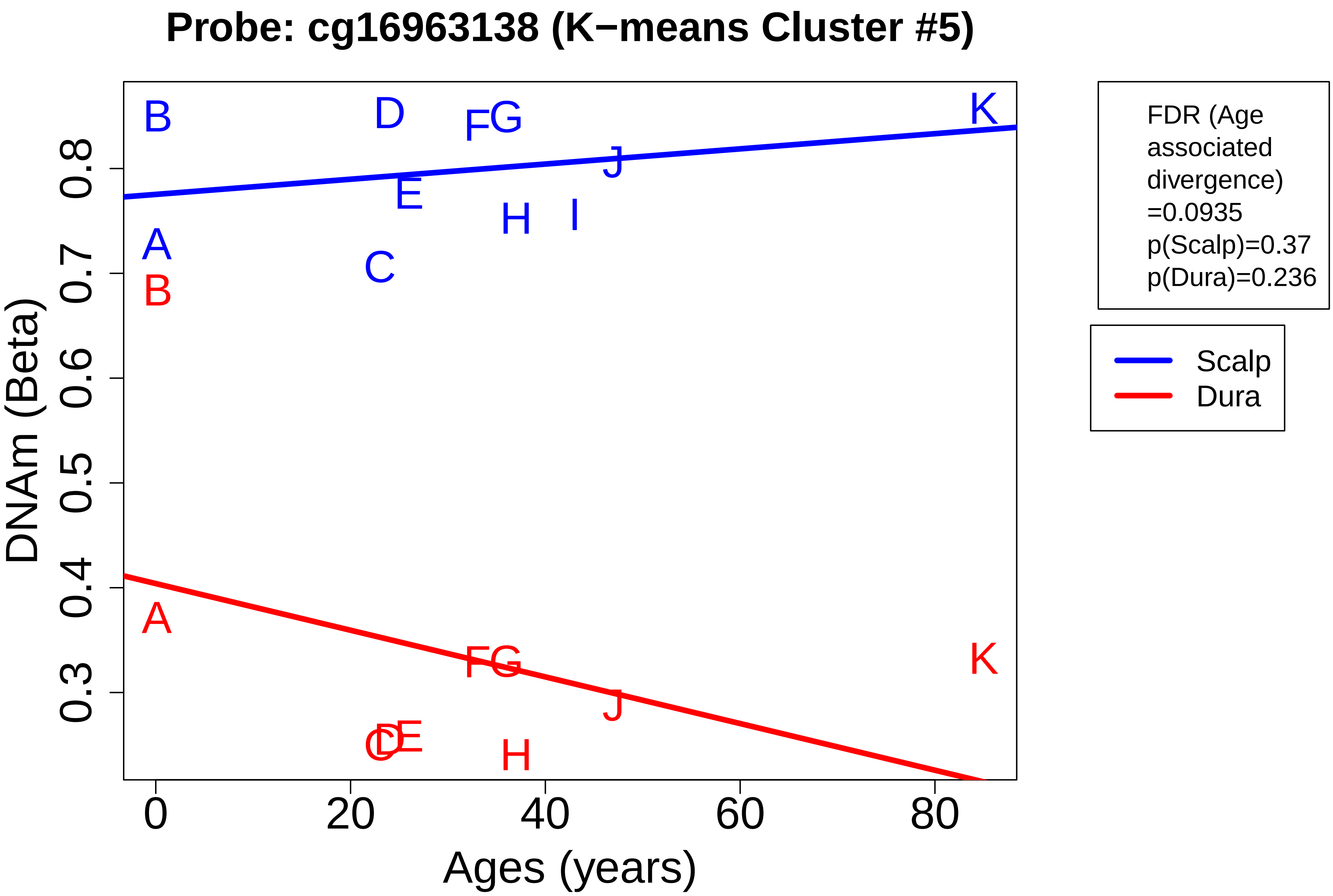

Probe: cg16963138 (K−means Cluster #5)
DNAm (Beta)
0.3
0.4
0.5
0.6
0.7
0.8
Ages (years)
0
20
40
60
80
B
D
FG
K
J
E
H
I
A
C
B
A
FG
K
J
CDE
H
FDR (Age associated divergence) =0.0935
p(Scalp)=0.37
p(Dura)=0.236
Scalp
Dura

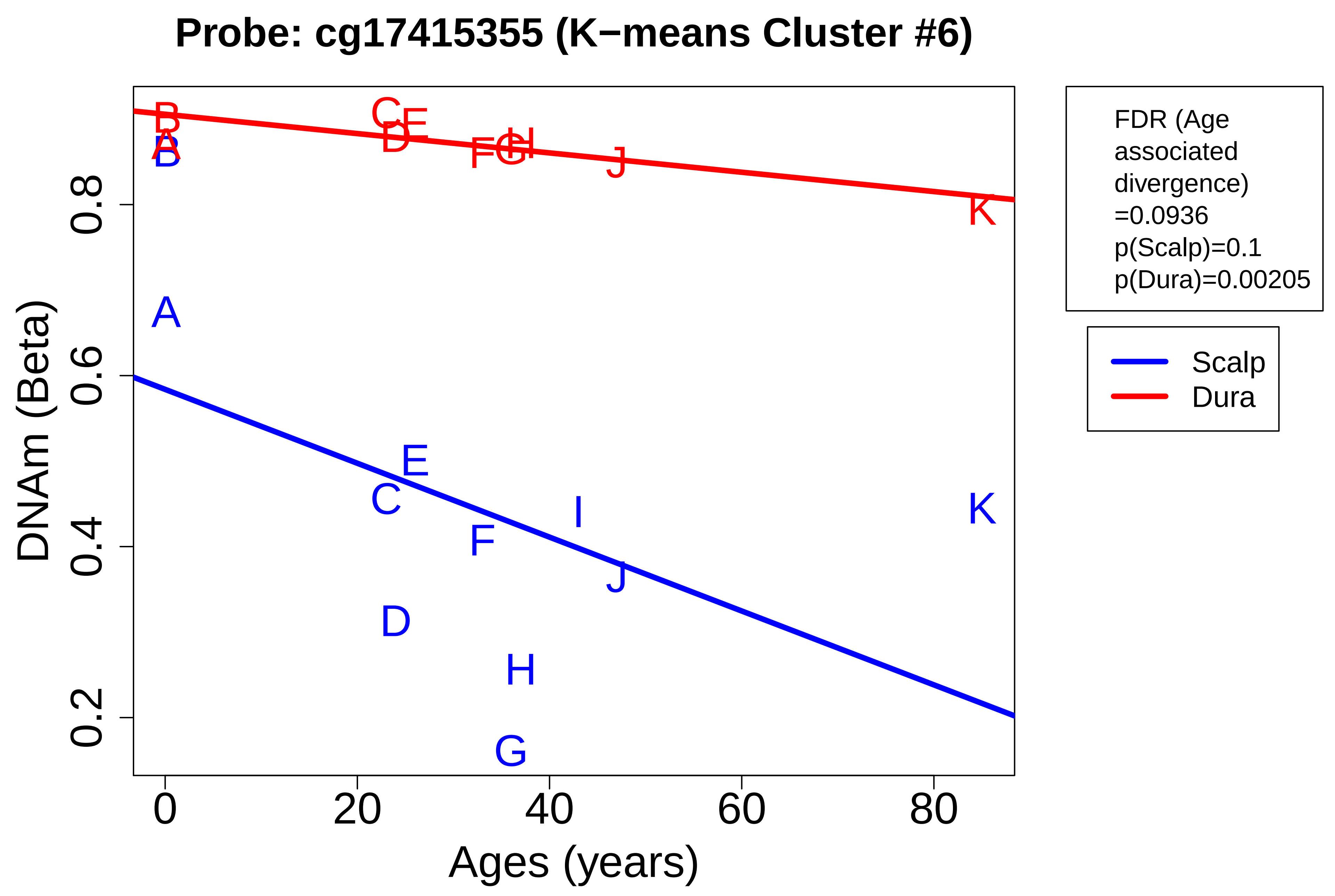

Probe: cg17415355 (K−means Cluster #6)
DNAm (Beta)
0.2
0.4
0.6
0.8
0
20
40
60
80
Ages (years)
FDR (Age associated divergence) =0.0936
p(Scalp)=0.1
p(Dura)=0.00205
Scalp
Dura

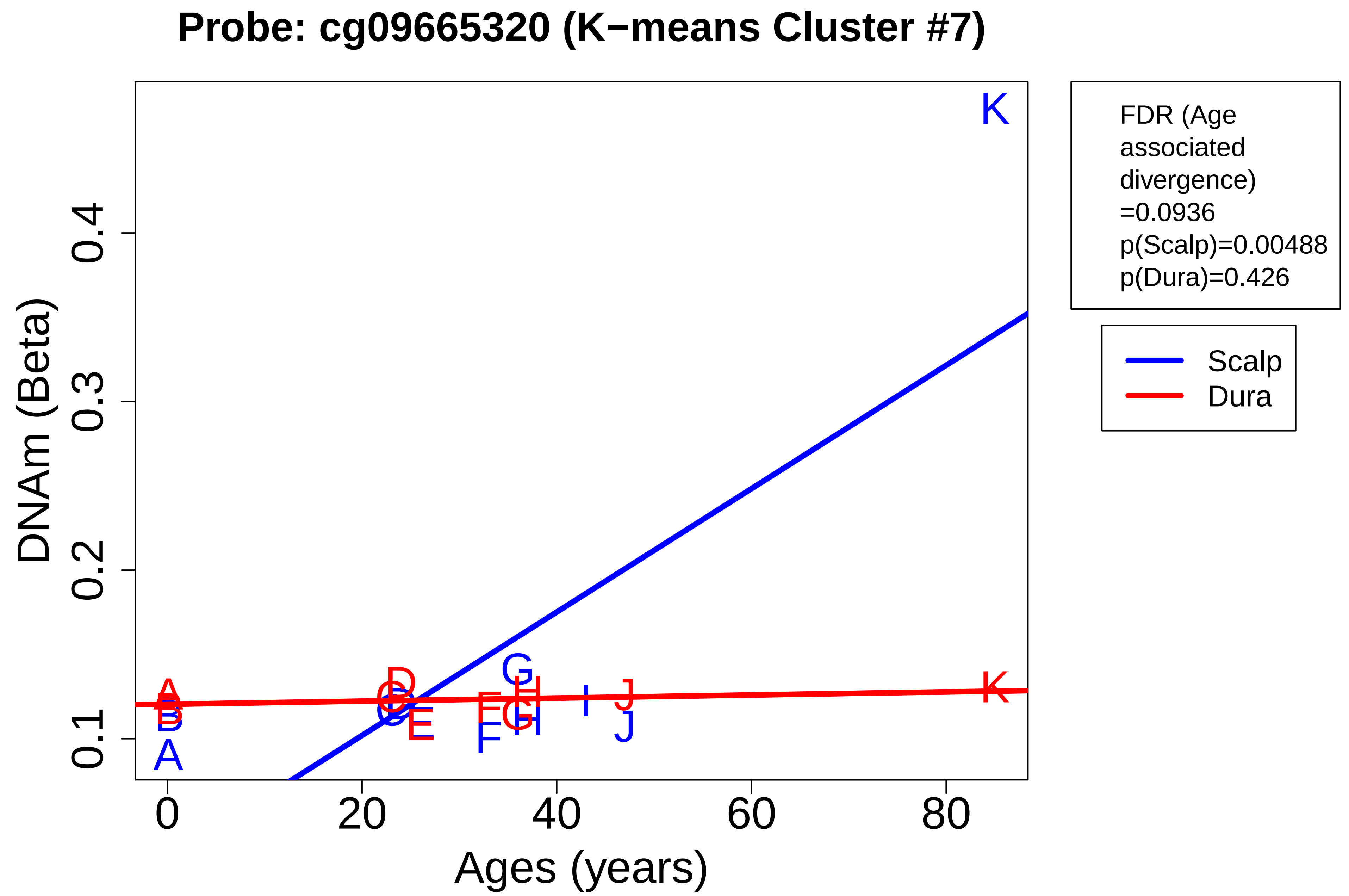

Probe: cg09665320 (K−means Cluster #7)
DNAm (Beta)
0.1
0.2
0.3
0.4
0
20
40
60
80
Ages (years)
FDR (Age associated divergence) =0.0936
p(Scalp)=0.00488
p(Dura)=0.426
Scalp
Dura

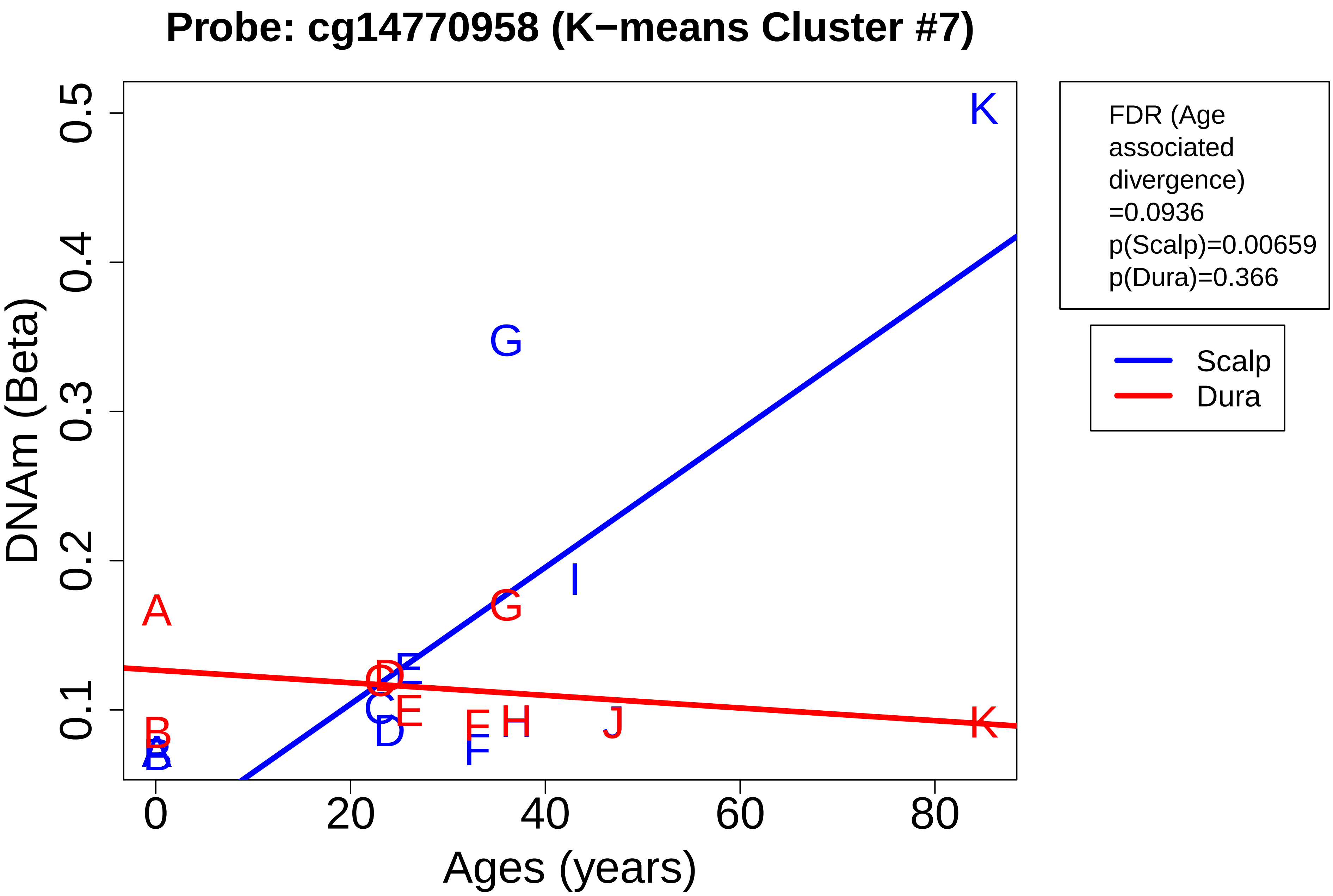
Probe: cg14770958 (K−means Cluster #7)
DNAm (Beta)
Ages (years)
0.1
0.2
0.3
0.4
0.5
0
20
40
60
80
FDR (Age associated divergence) =0.0936
p(Scalp)=0.00659
p(Dura)=0.366
Scalp
Dura
A
B
C
D
E
F
G
H
I
J
K

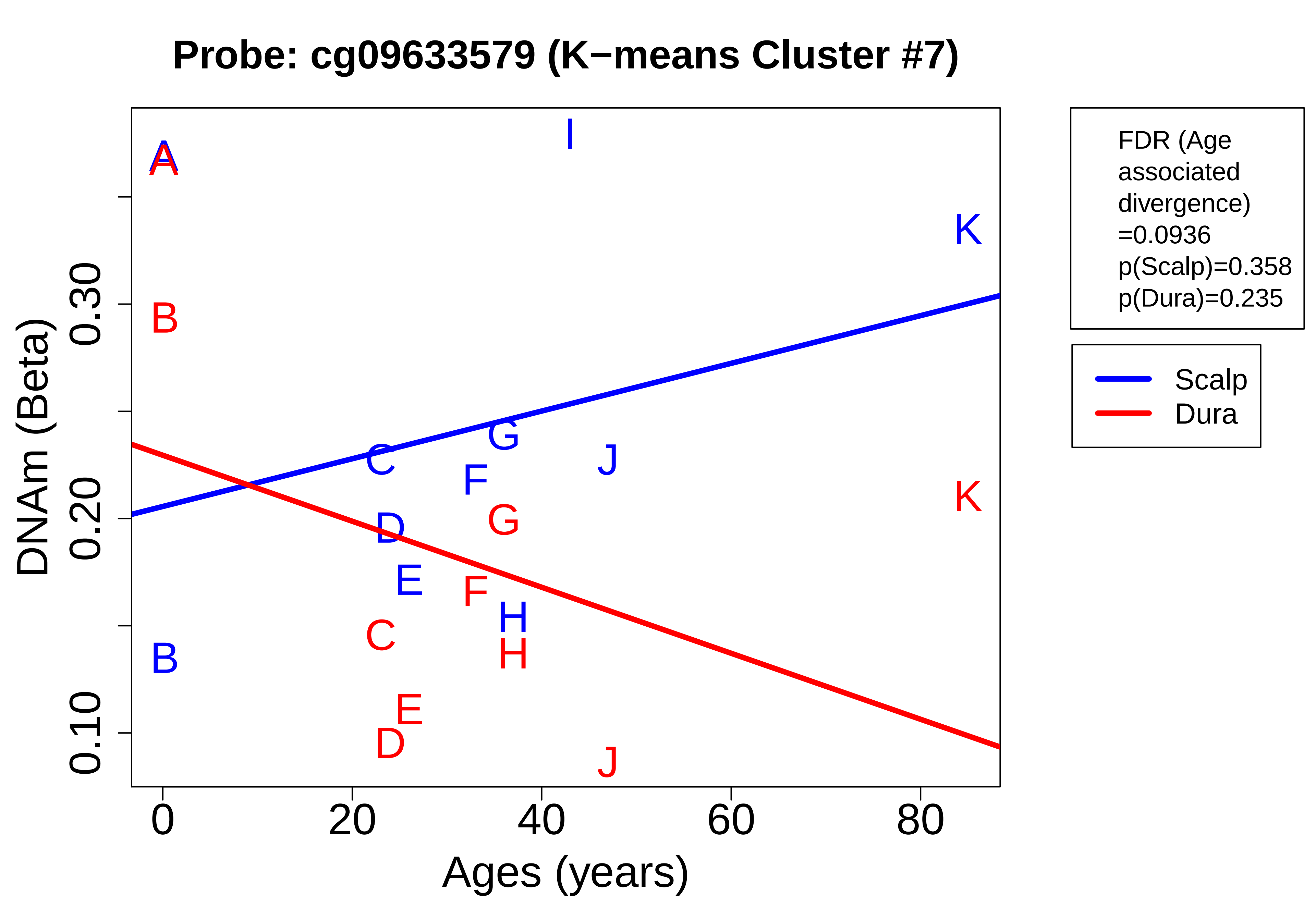

Probe: cg09633579 (K−means Cluster #7)
DNAm (Beta)
0.10
0.20
0.30
0
20
40
60
80
Ages (years)
A
A
B
B
C
C
D
D
E
E
F
F
G
G
H
H
I
J
J
K
K
FDR (Age associated divergence) =0.0936
p(Scalp)=0.358
p(Dura)=0.235
Scalp
Dura

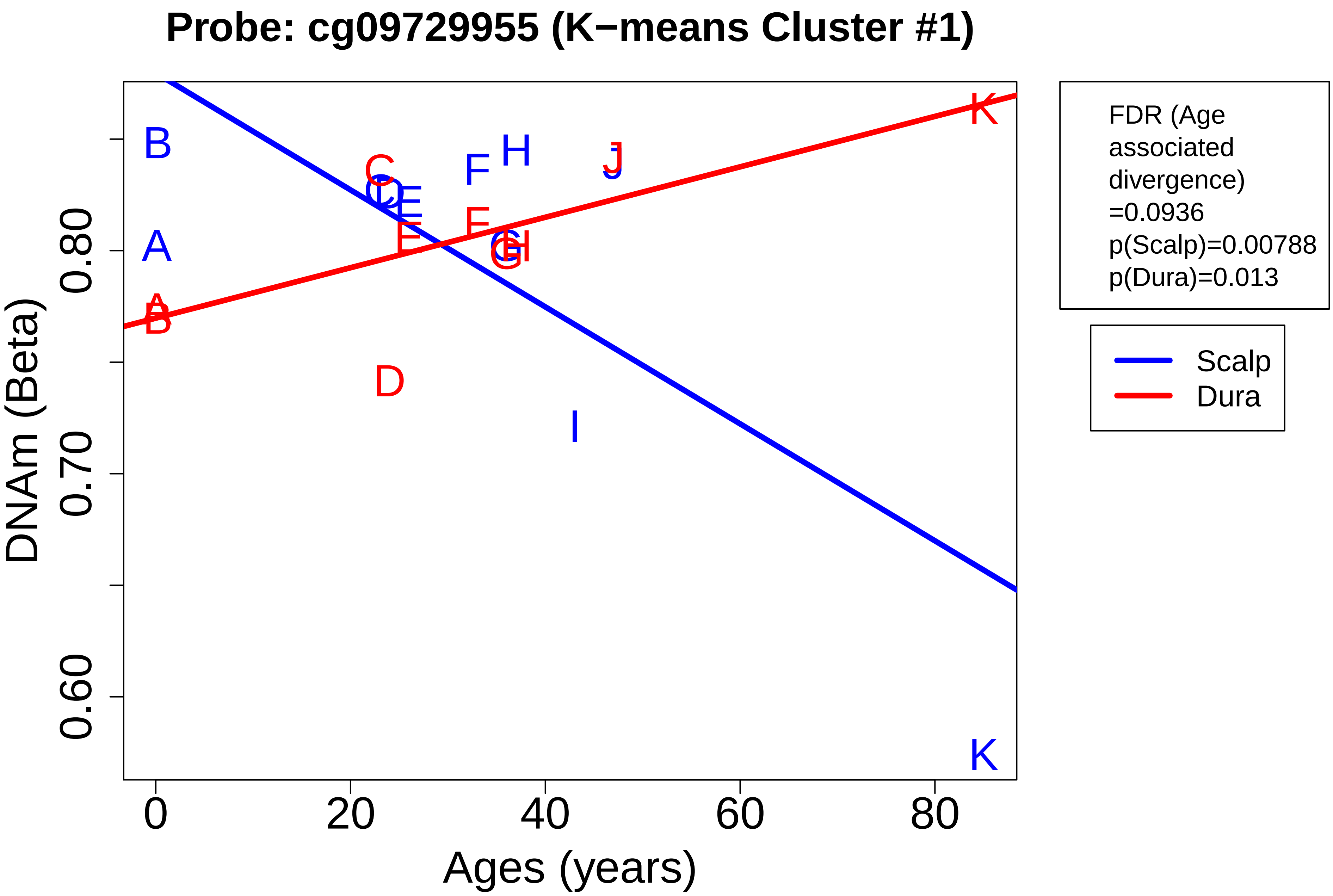
Probe: cg09729955 (K−means Cluster #1)
DNAm (Beta)
0.60
0.70
0.80
0
20
40
60
80
Ages (years)
FDR (Age associated divergence) =0.0936
p(Scalp)=0.00788
p(Dura)=0.013
Scalp
Dura
A
B
C
D
E
F
G
H
I
J
K

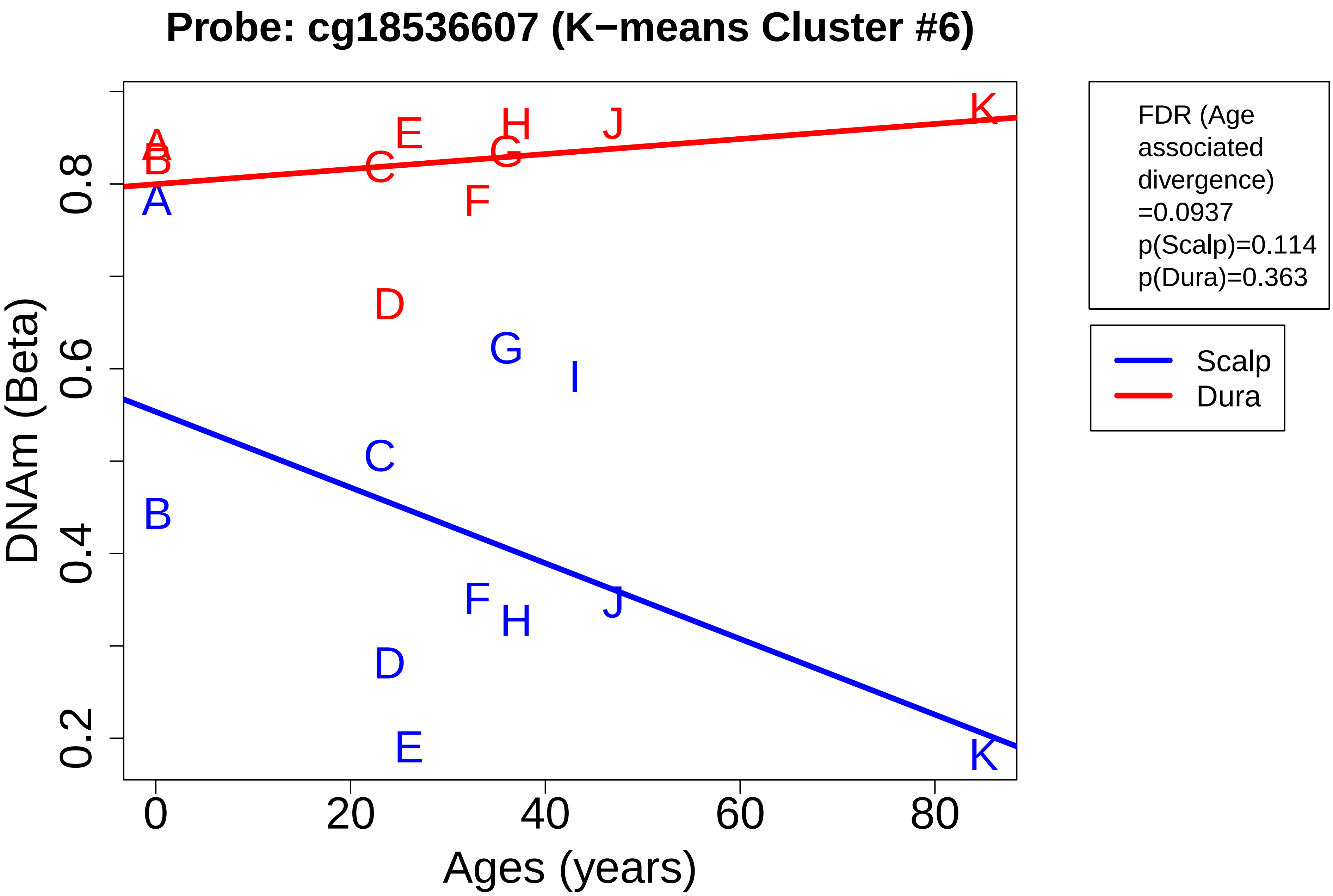

Probe: cg18536607 (K−means Cluster #6)
DNAm (Beta)
0.2
0.4
0.6
0.8
Ages (years)
0
20
40
60
80
A
B
C
D
E
F
G
H
I
J
K
FDR (Age associated divergence) =0.0937
p(Scalp)=0.114
p(Dura)=0.363
Scalp
Dura

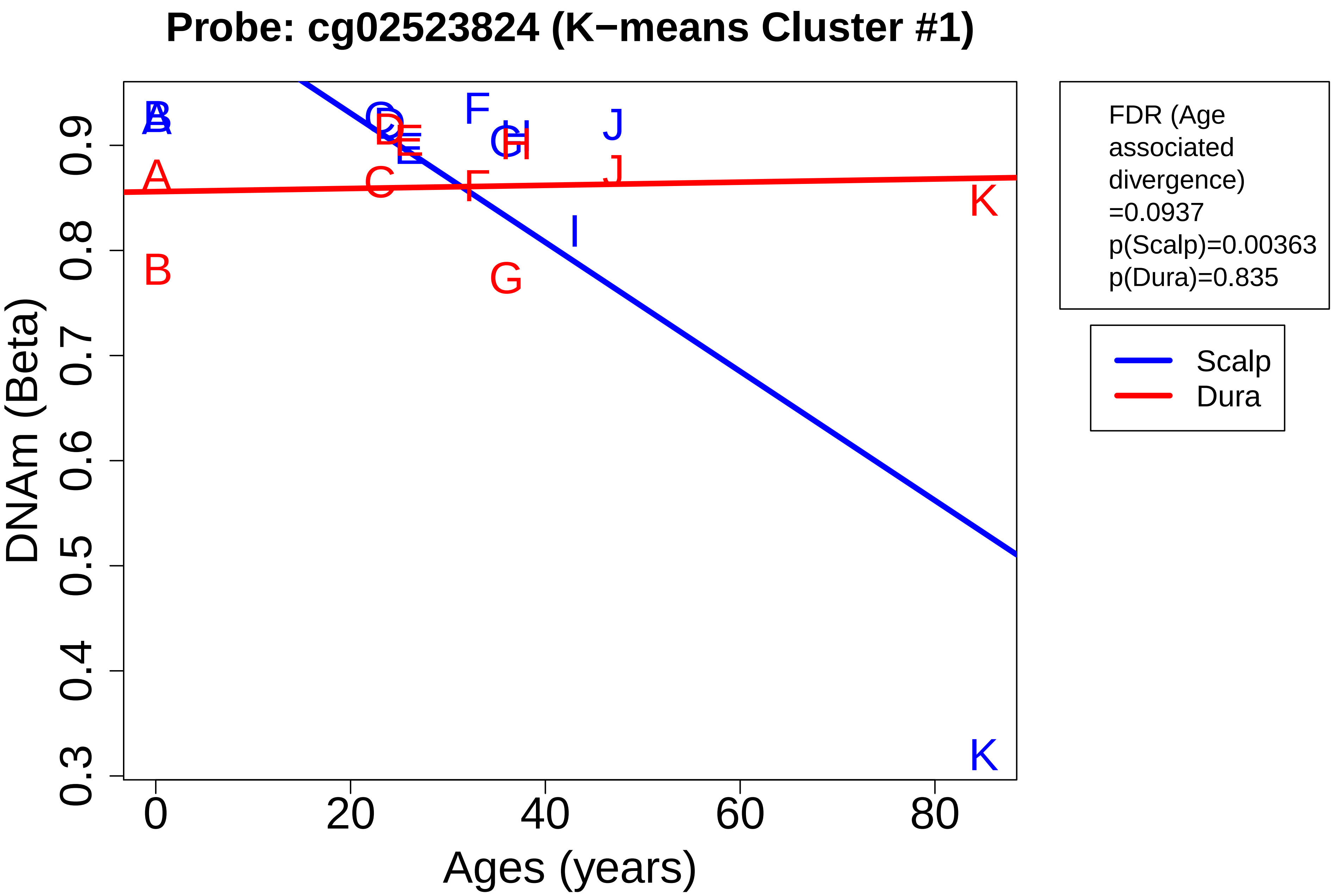

Probe: cg02523824 (K−means Cluster #1)
DNAm (Beta)
Ages (years)
0.3
0.4
0.5
0.6
0.7
0.8
0.9
0
20
40
60
80
FDR (Age associated divergence) =0.0937
p(Scalp)=0.00363
p(Dura)=0.835
Scalp
Dura

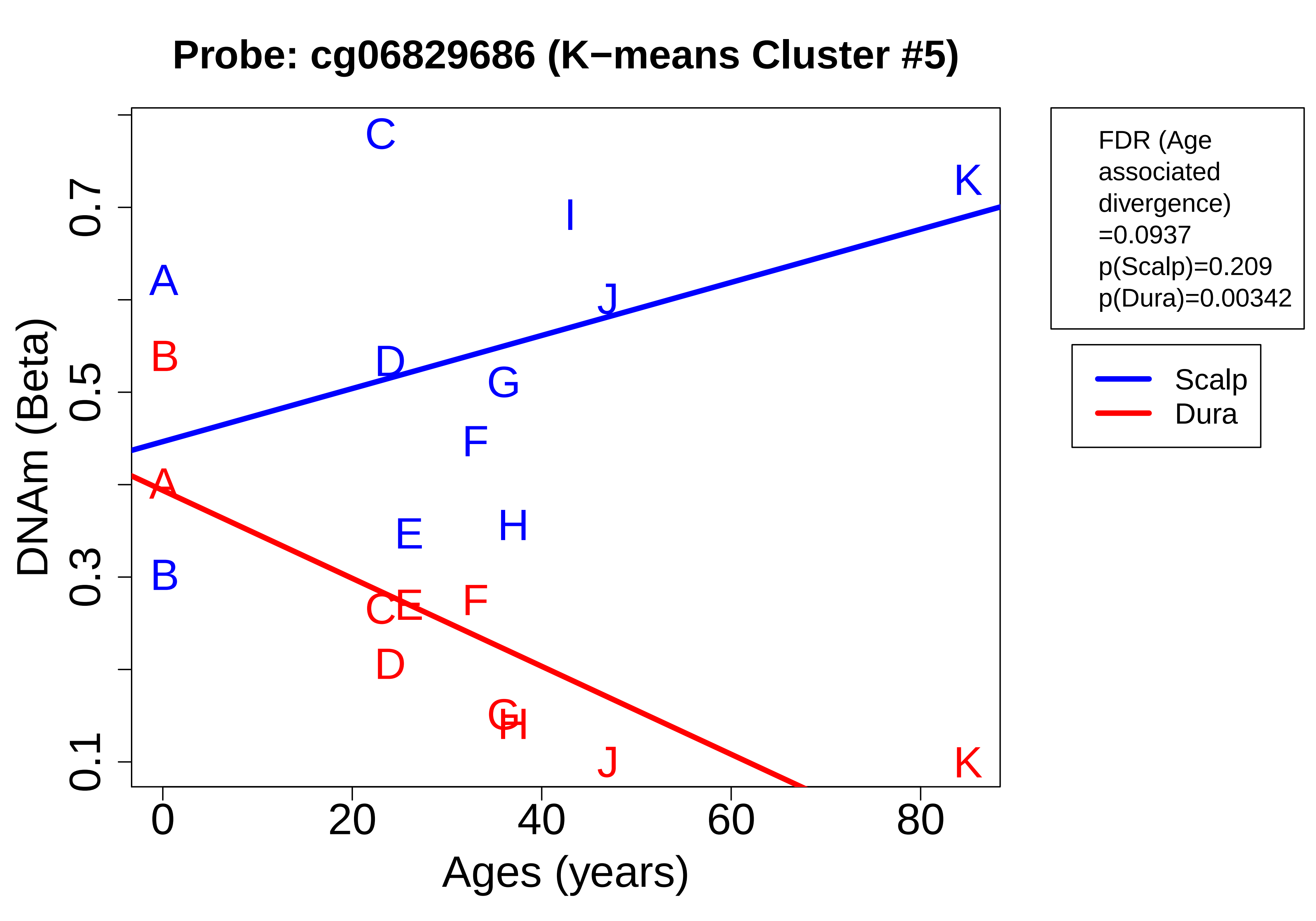
Probe: cg06829686 (K−means Cluster #5)
FDR (Age associated divergence) =0.0937
p(Scalp)=0.209
p(Dura)=0.00342
Scalp
Dura
DNAm (Beta)
0.1
0.3
0.5
0.7
0
20
40
60
80
Ages (years)
A
B
C
D
E
F
G
H
I
J
K

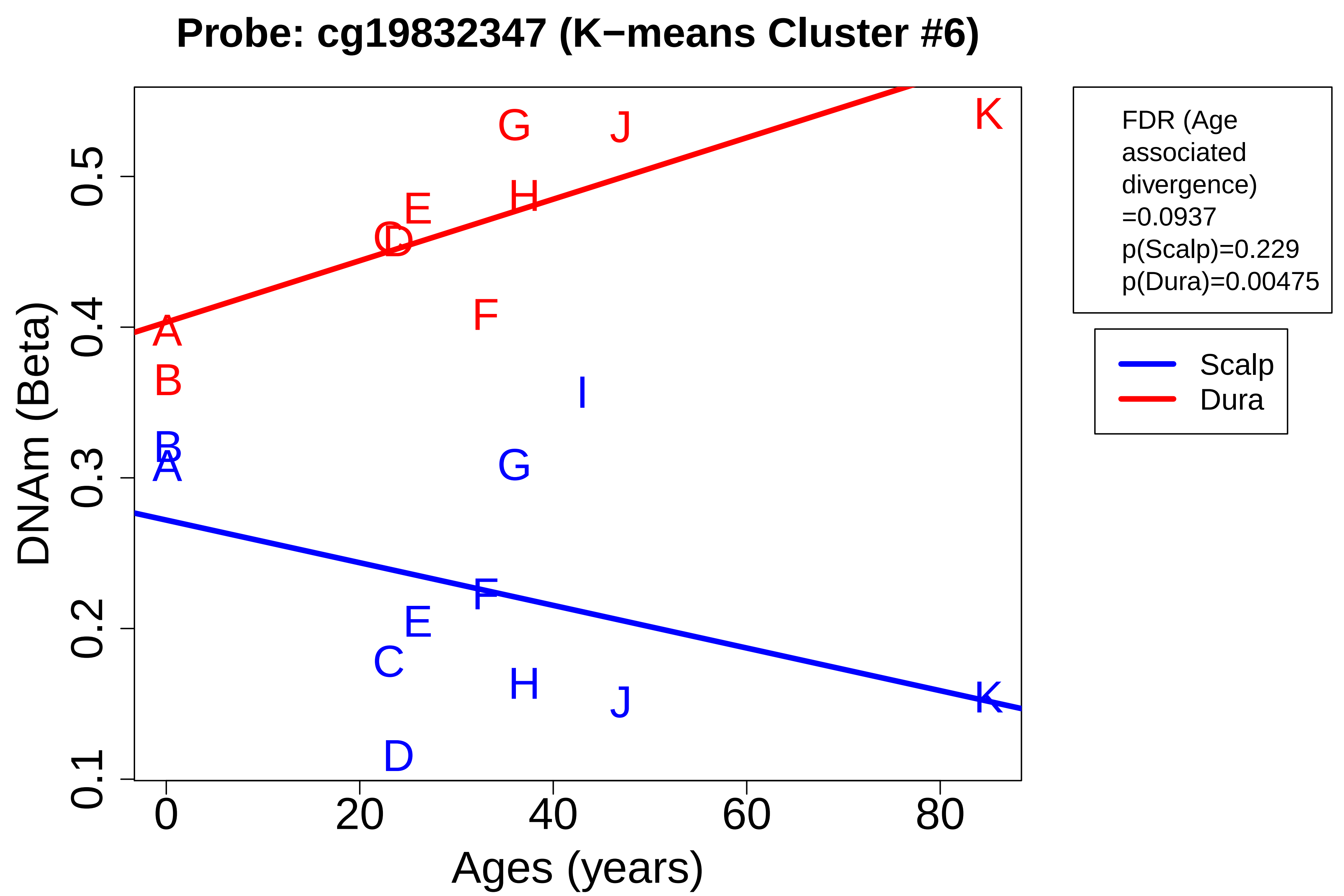

Probe: cg19832347 (K−means Cluster #6)
DNAm (Beta)
0.1
0.2
0.3
0.4
0.5
0
20
40
60
80
Ages (years)
FDR (Age associated divergence) =0.0937
p(Scalp)=0.229
p(Dura)=0.00475
Scalp
Dura

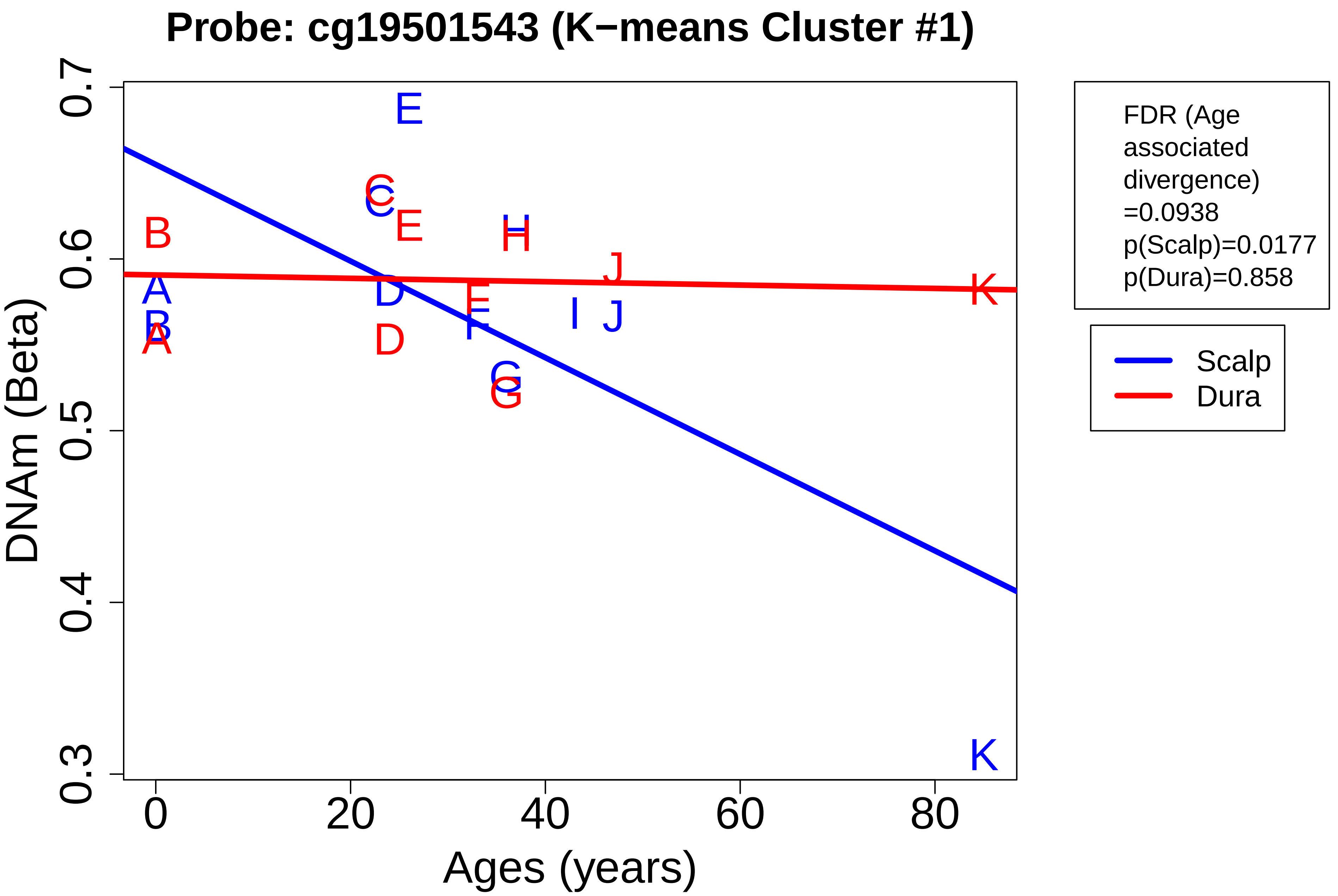

Probe: cg19501543 (K−means Cluster #1)
DNAm (Beta)
0.3
0.4
0.5
0.6
0.7
0
20
40
60
80
Ages (years)
FDR (Age associated divergence) =0.0938
p(Scalp)=0.0177
p(Dura)=0.858
Scalp
Dura
A
B
C
D
E
F
G
H
I
J
K

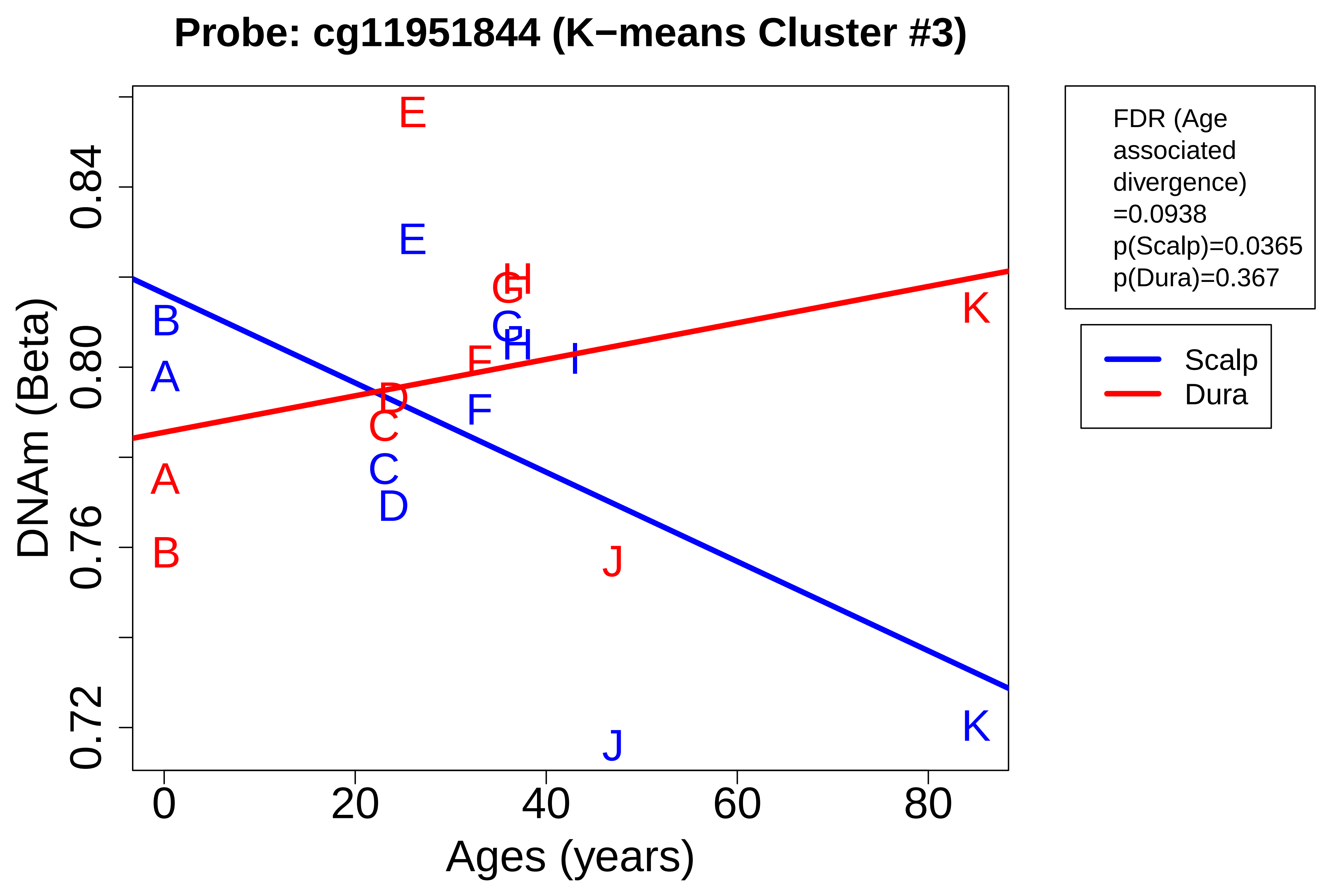

Probe: cg11951844 (K−means Cluster #3)
FDR (Age associated divergence) =0.0938
p(Scalp)=0.0365
p(Dura)=0.367
Scalp
Dura
DNAm (Beta)
0.72
0.76
0.80
0.84
0
20
40
60
80
Ages (years)
A
B
C
D
E
F
G
H
I
J
K

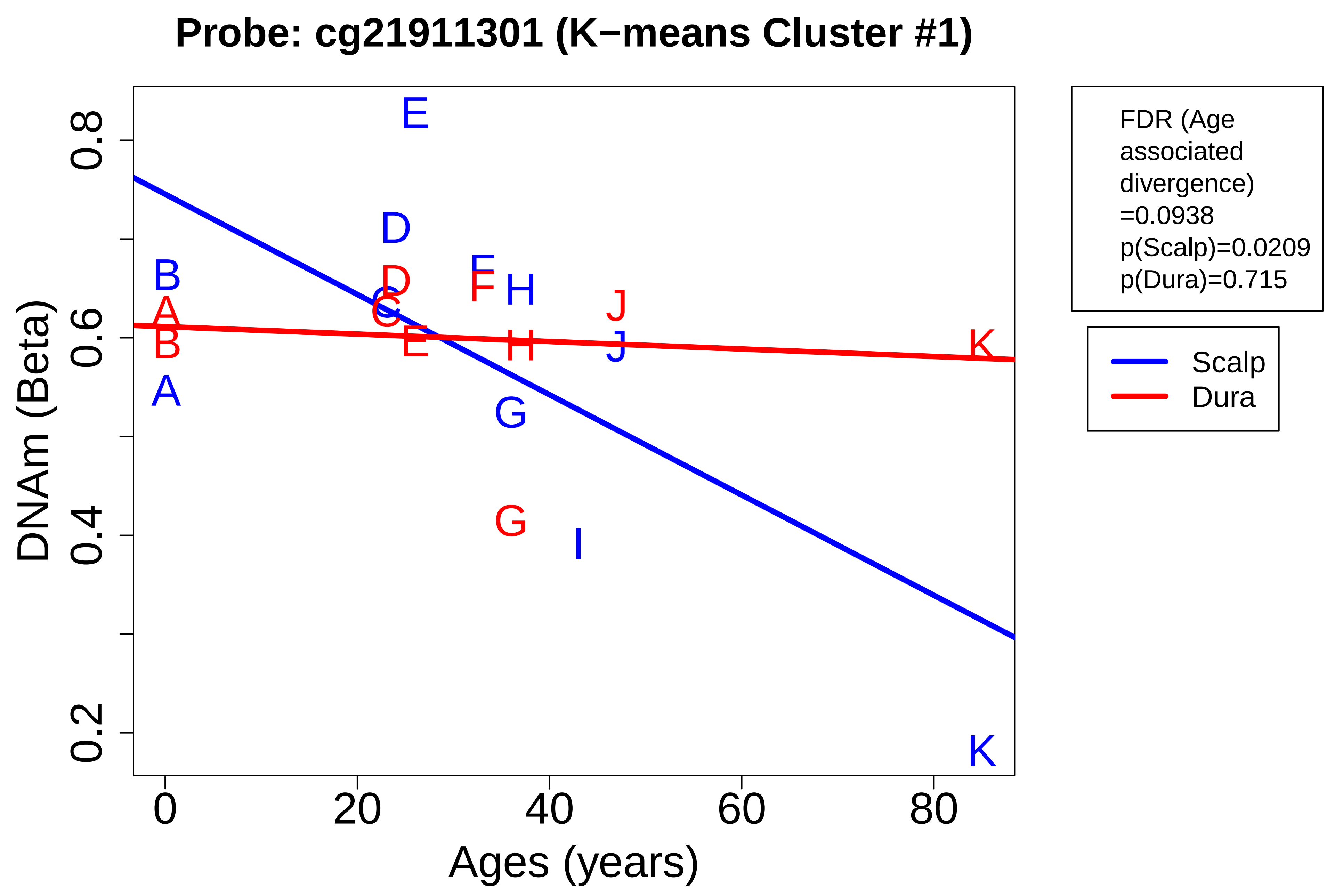

Probe: cg21911301 (K−means Cluster #1)
DNAm (Beta)
Ages (years)
0.2
0.4
0.6
0.8
0
20
40
60
80
FDR (Age associated divergence) =0.0938
p(Scalp)=0.0209
p(Dura)=0.715
Scalp
Dura

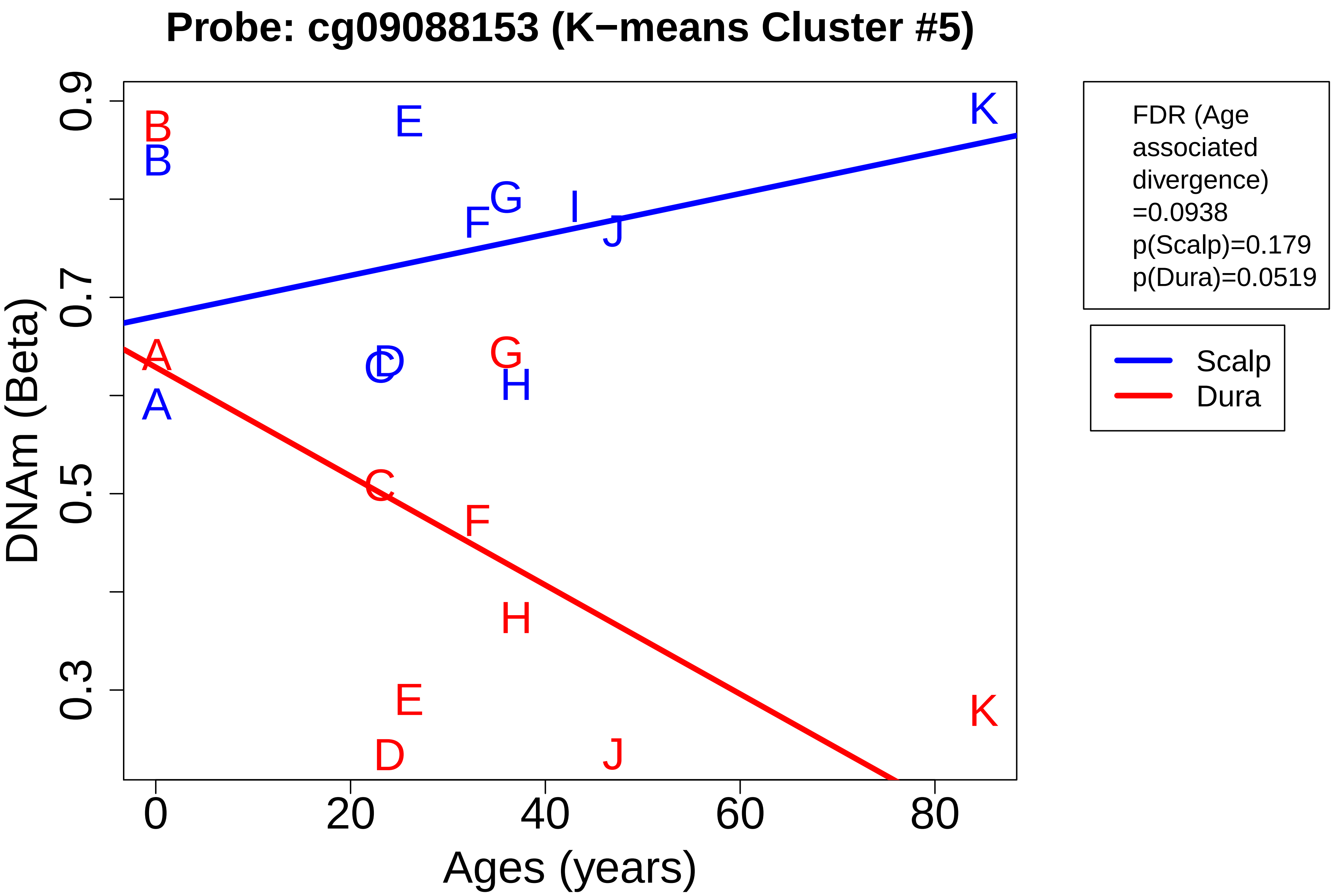

Probe: cg09088153 (K−means Cluster #5)
DNAm (Beta)
0.9
0.7
0.5
0.3
Ages (years)
0
20
40
60
80
A
B
C
D
E
F
G
H
I
J
K
FDR (Age associated divergence) =0.0938
p(Scalp)=0.179
p(Dura)=0.0519
Scalp
Dura

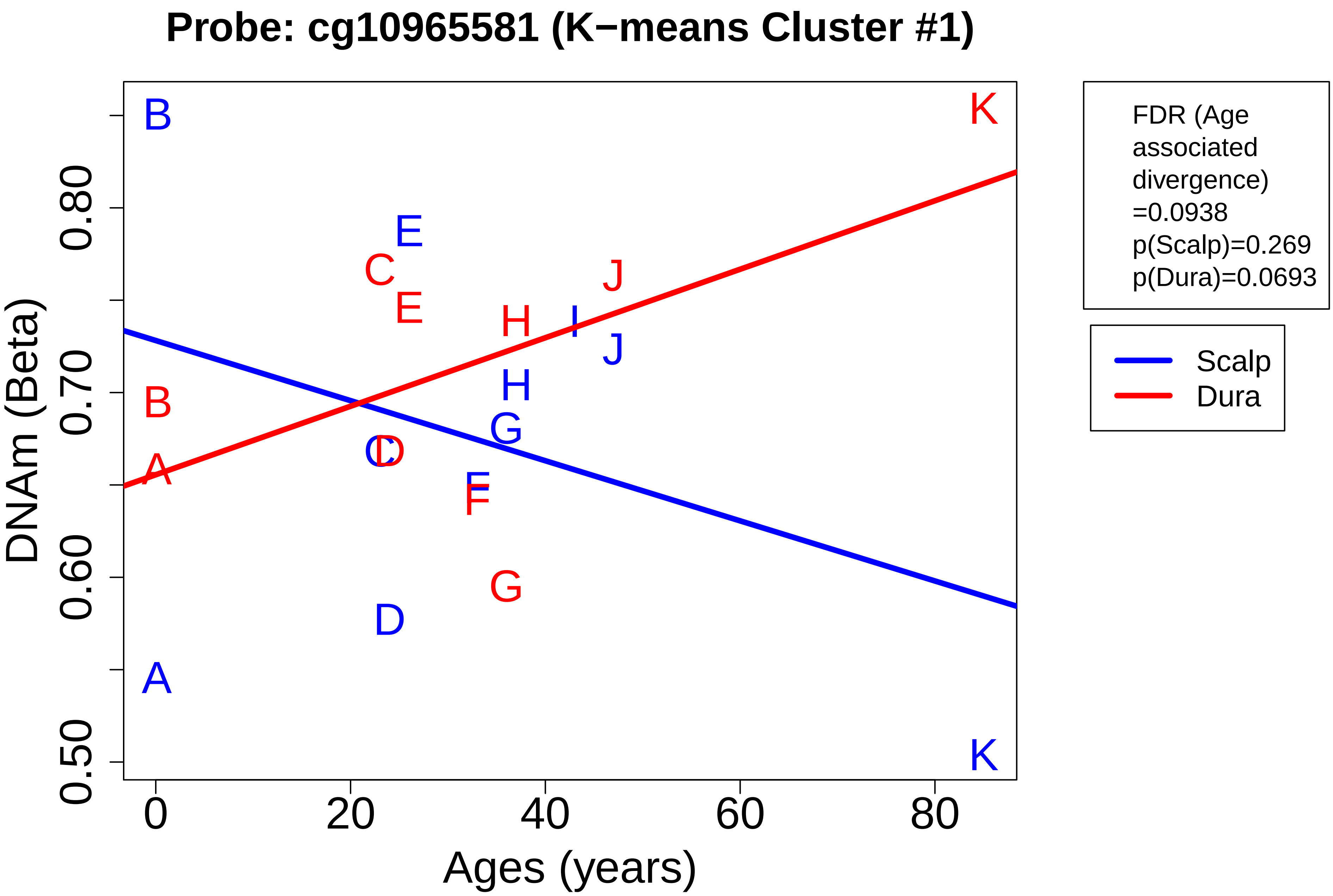
Probe: cg10965581 (K−means Cluster #1)
DNAm (Beta)
0.50
0.60
0.70
0.80
0
20
40
60
80
Ages (years)
B
K
E
C
J
E
H
I
J
H
B
G
C
D
A
F
F
G
D
A
K
FDR (Age associated divergence) =0.0938
p(Scalp)=0.269
p(Dura)=0.0693
Scalp
Dura

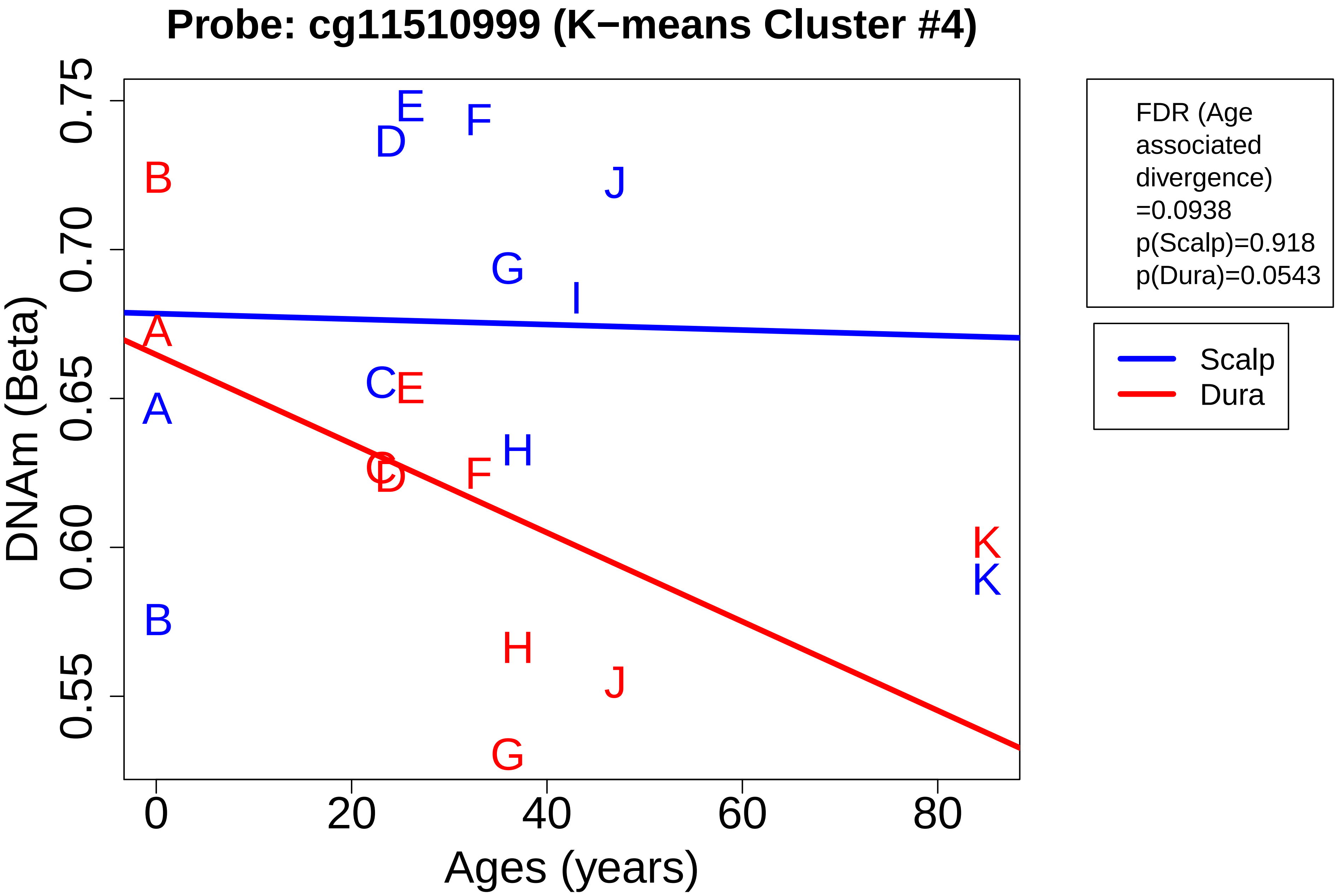

Probe: cg11510999 (K−means Cluster #4)
DNAm (Beta)
0.55
0.60
0.65
0.70
0.75
0
20
40
60
80
Ages (years)
FDR (Age associated divergence) =0.0938
p(Scalp)=0.918
p(Dura)=0.0543
Scalp
Dura

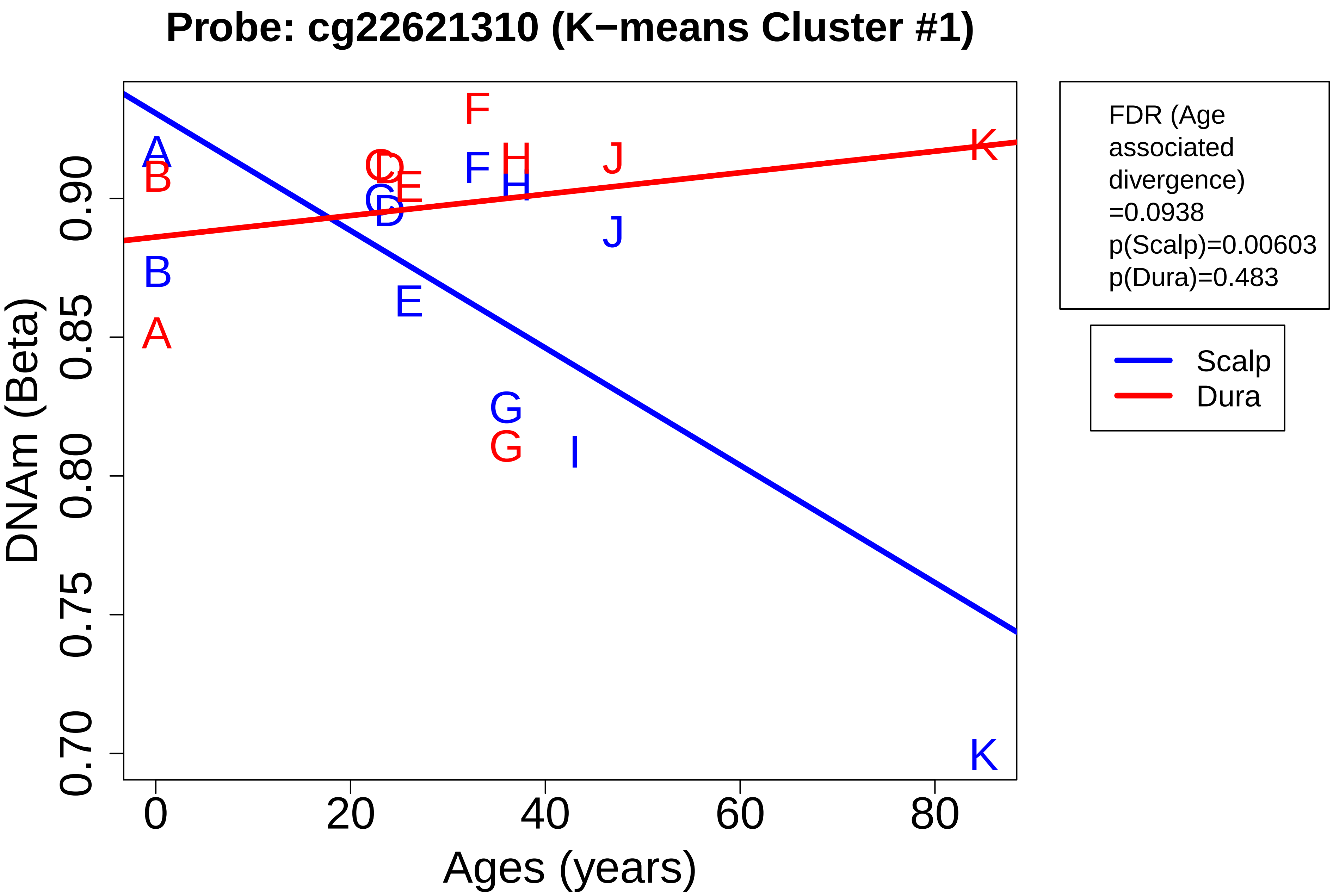

Probe: cg22621310 (K−means Cluster #1)
DNAm (Beta)
0.70
0.75
0.80
0.85
0.90
0
20
40
60
80
Ages (years)
FDR (Age associated divergence) =0.0938
p(Scalp)=0.00603
p(Dura)=0.483
Scalp
Dura

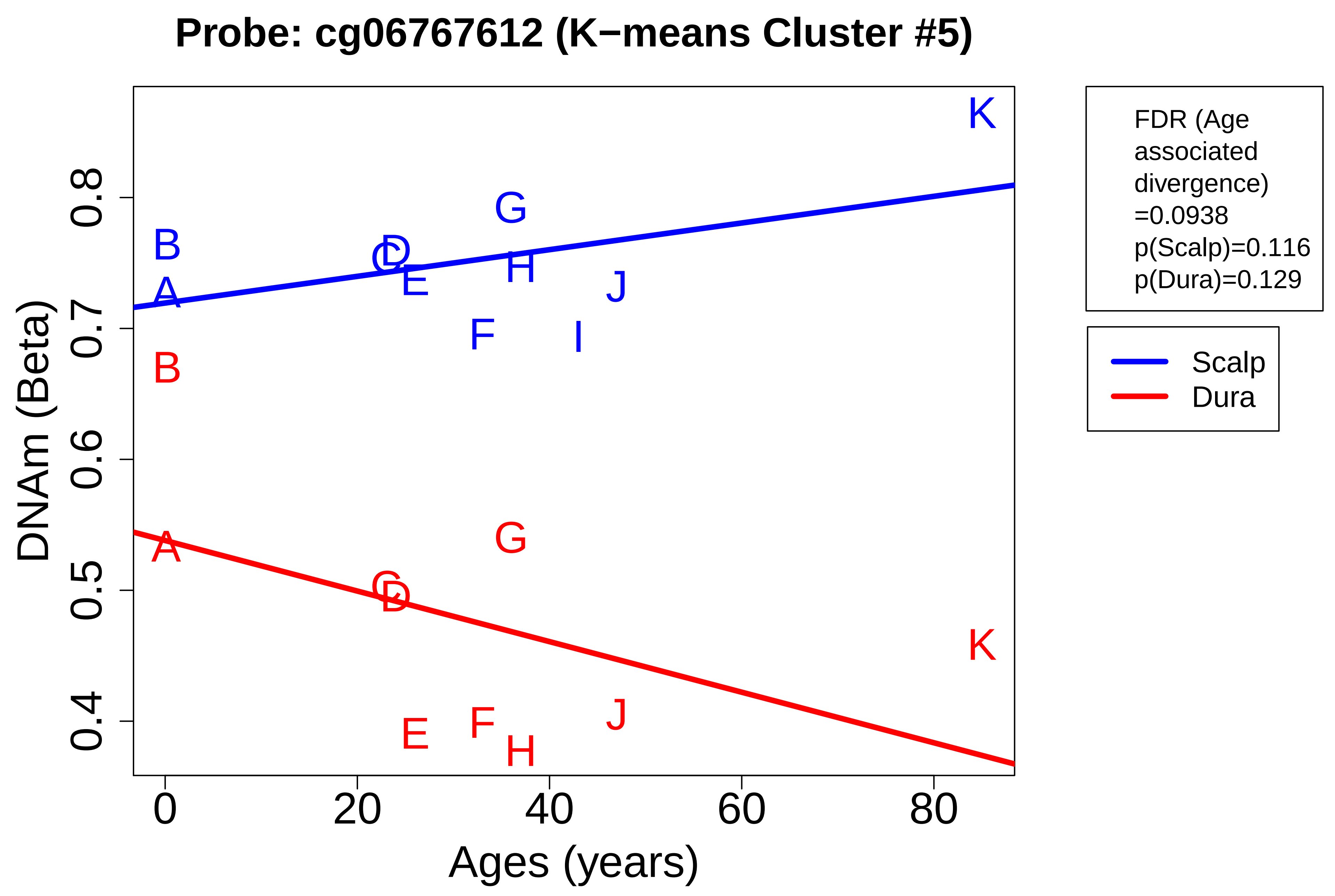

Probe: cg06767612 (K−means Cluster #5)
DNAm (Beta)
0.4
0.5
0.6
0.7
0.8
0
20
40
60
80
Ages (years)
FDR (Age associated divergence) =0.0938
p(Scalp)=0.116
p(Dura)=0.129
Scalp
Dura

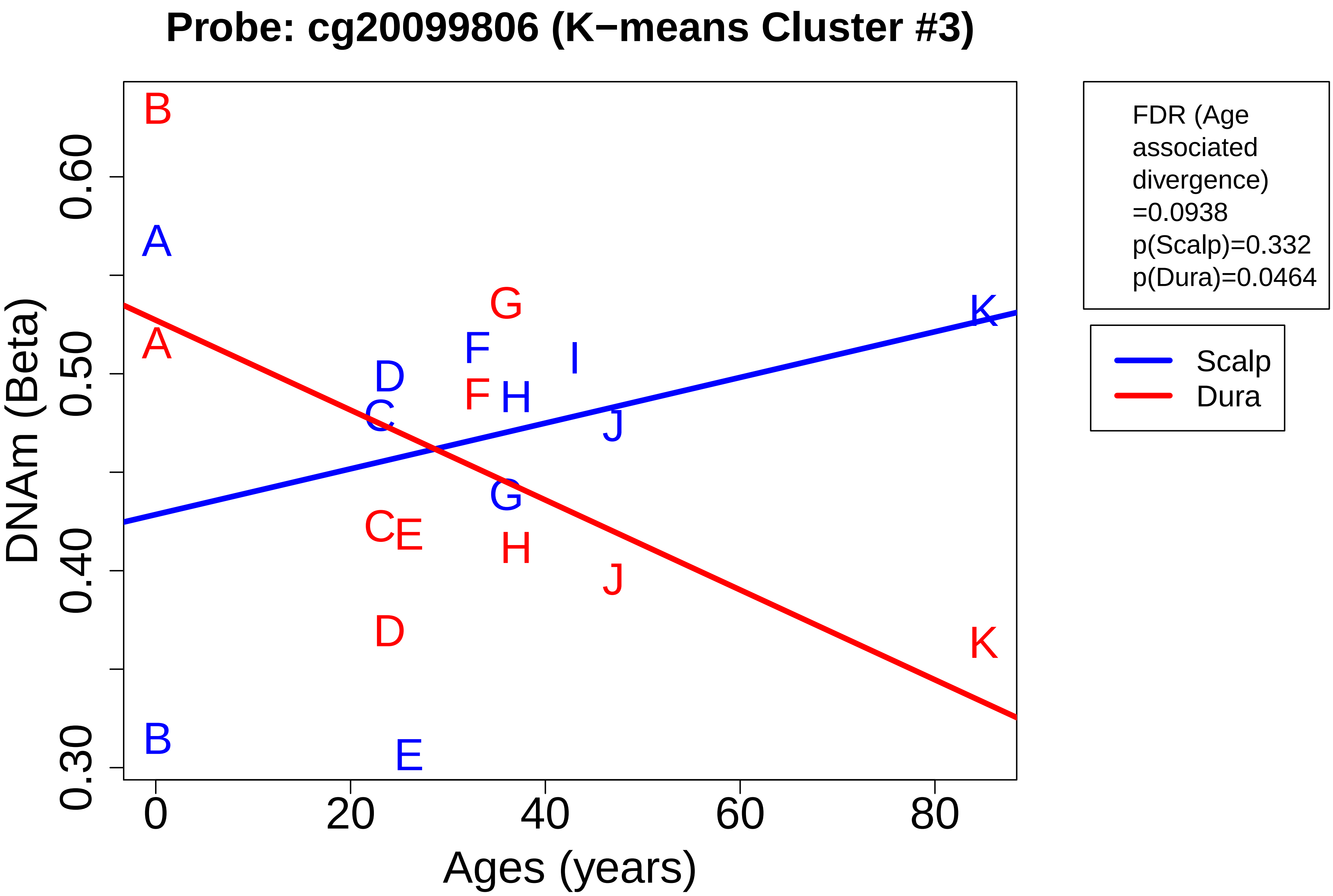

Probe: cg20099806 (K−means Cluster #3)
DNAm (Beta)
0.30
0.40
0.50
0.60
0
20
40
60
80
Ages (years)
FDR (Age associated divergence) =0.0938
p(Scalp)=0.332
p(Dura)=0.0464
Scalp
Dura

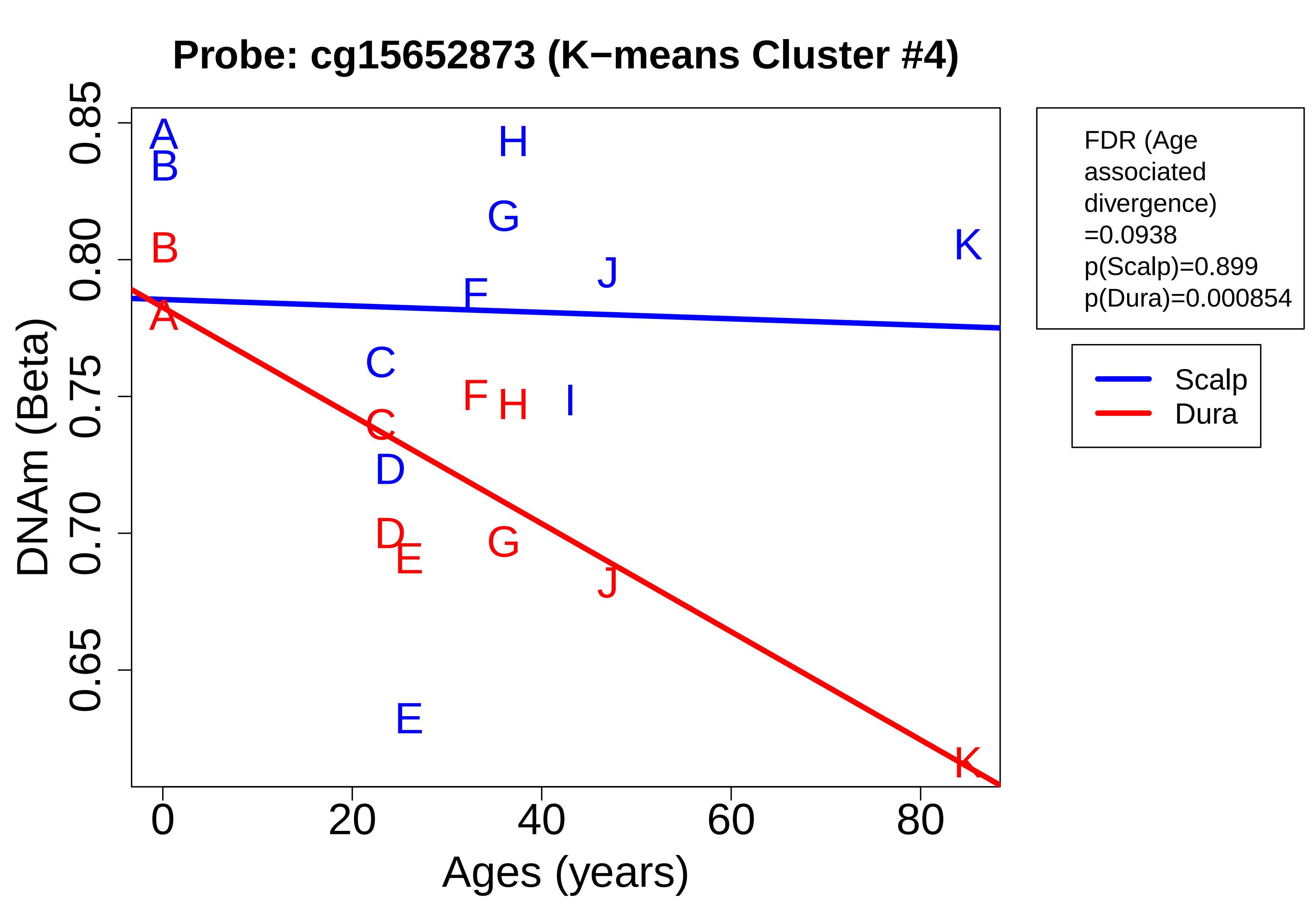

Probe: cg15652873 (K−means Cluster #4)
DNAm (Beta)
0.65
0.70
0.75
0.80
0.85
0
20
40
60
80
Ages (years)
A
B
C
D
E
F
G
H
I
J
K
FDR (Age associated divergence) =0.0938
p(Scalp)=0.899
p(Dura)=0.000854
Scalp
Dura

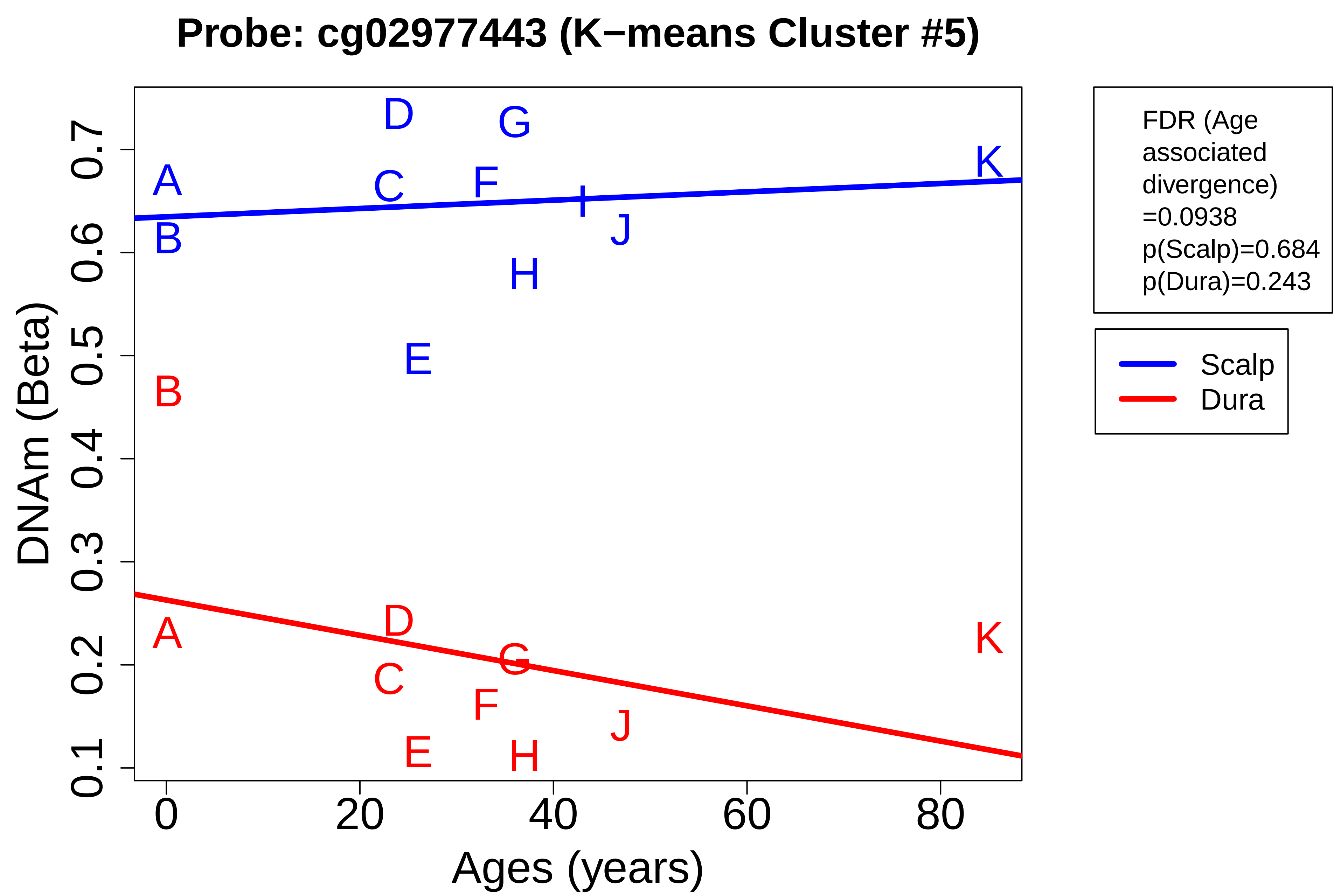

Probe: cg02977443 (K−means Cluster #5)
DNAm (Beta)
0.1
0.2
0.3
0.4
0.5
0.6
0.7
Ages (years)
0
20
40
60
80
FDR (Age associated divergence) =0.0938
p(Scalp)=0.684
p(Dura)=0.243
Scalp
Dura
A
B
C
D
E
F
G
H
J
K

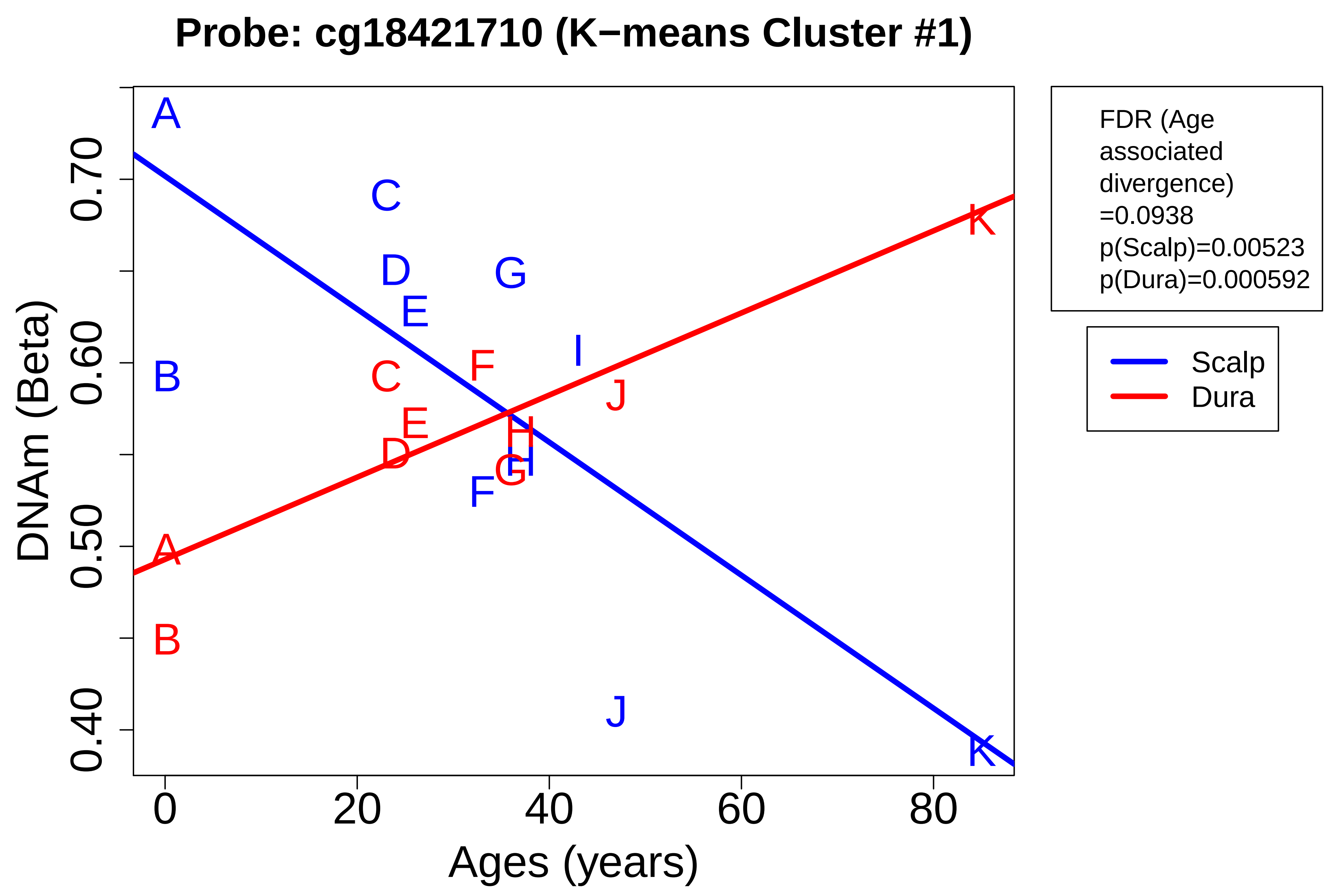

Probe: cg18421710 (K−means Cluster #1)
DNAm (Beta)
0.40
0.50
0.60
0.70
0
20
40
60
80
Ages (years)
A
B
C
D
E
F
G
H
I
J
K
FDR (Age associated divergence) =0.0938
p(Scalp)=0.00523
p(Dura)=0.000592
Scalp
Dura

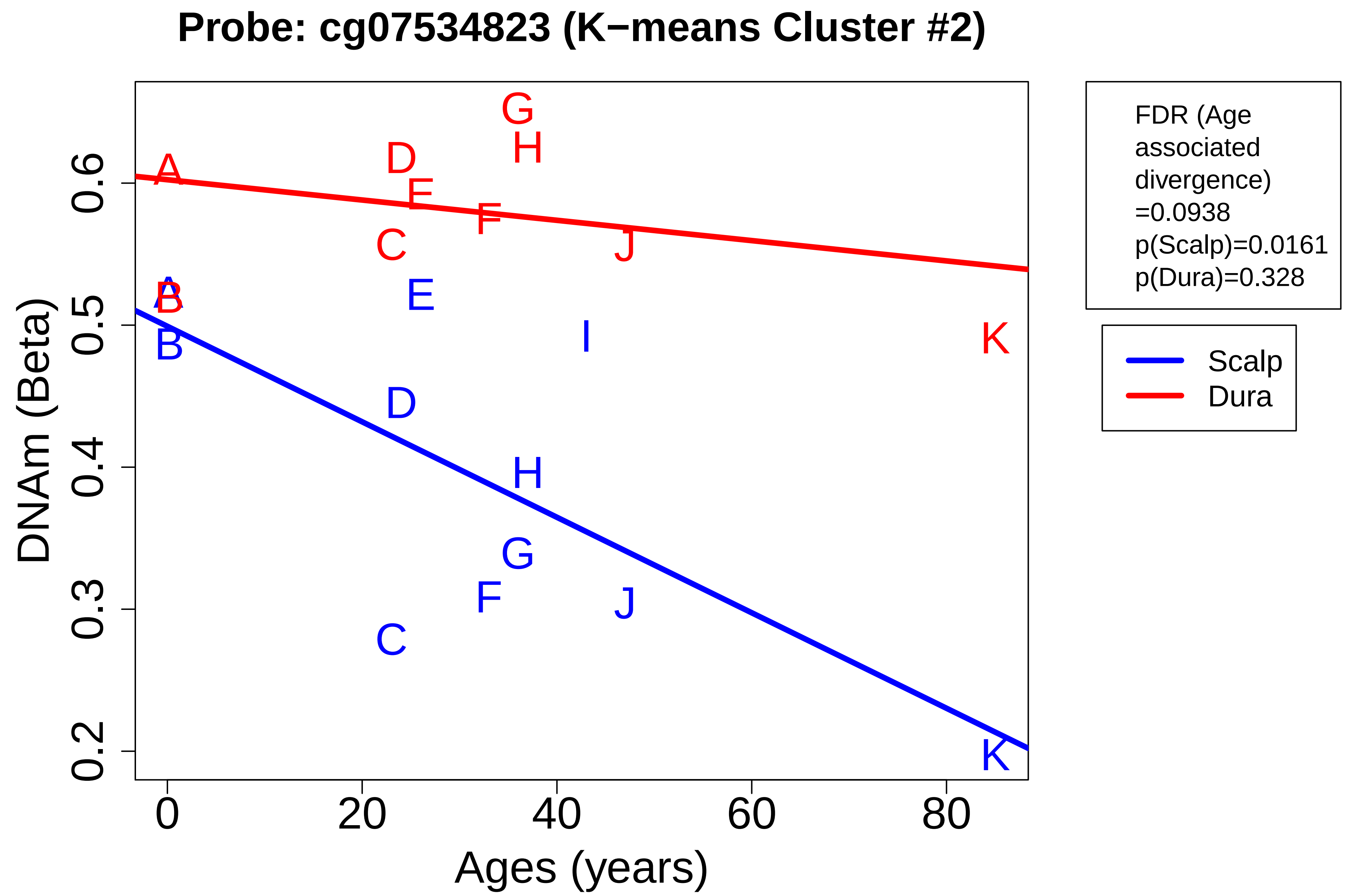

Probe: cg07534823 (K−means Cluster #2)
DNAm (Beta)
Ages (years)
0.2
0.3
0.4
0.5
0.6
0
20
40
60
80
FDR (Age associated divergence) =0.0938
p(Scalp)=0.0161
p(Dura)=0.328
Scalp
Dura

Probe: cg01115569 (K−means Cluster #7)

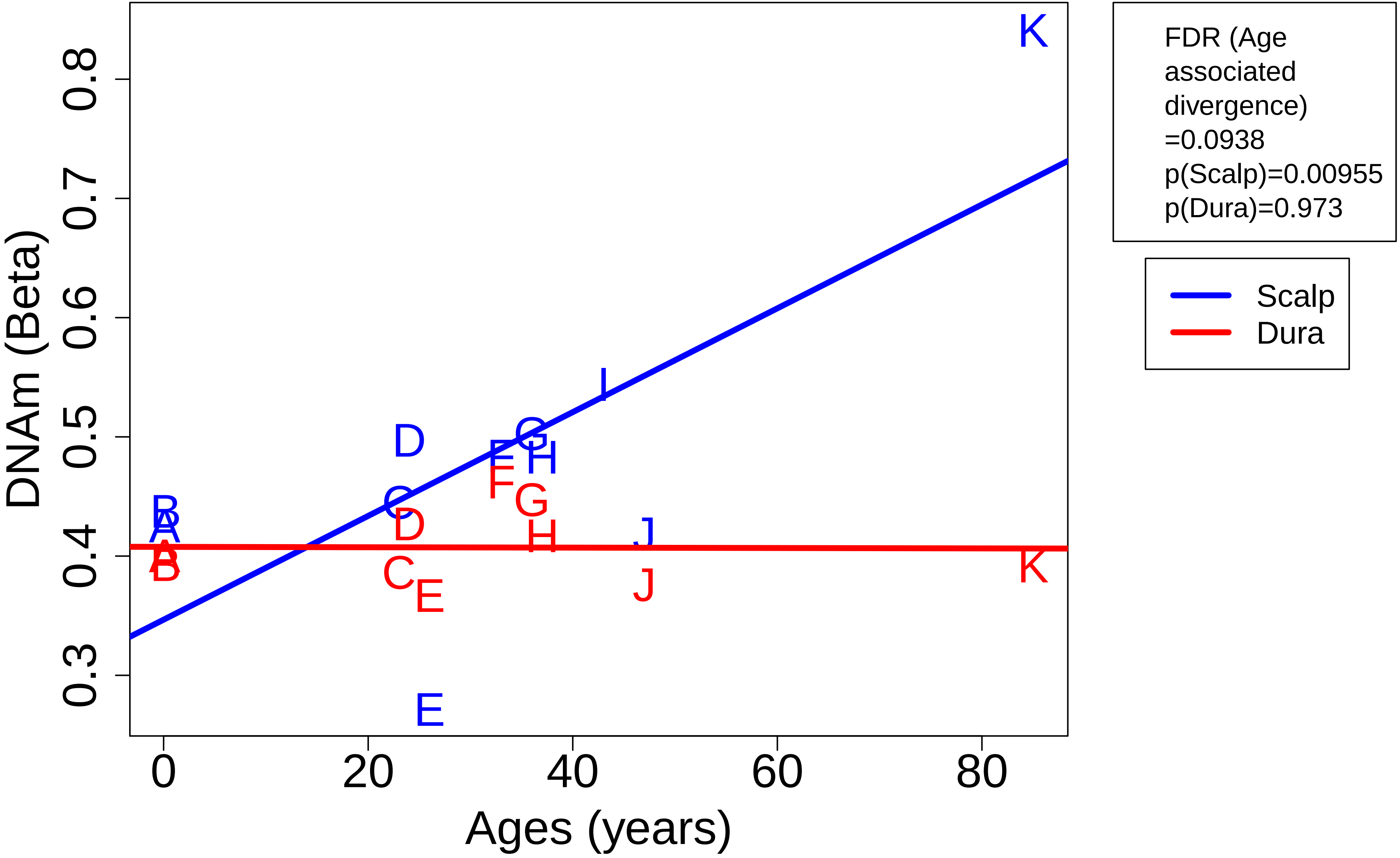

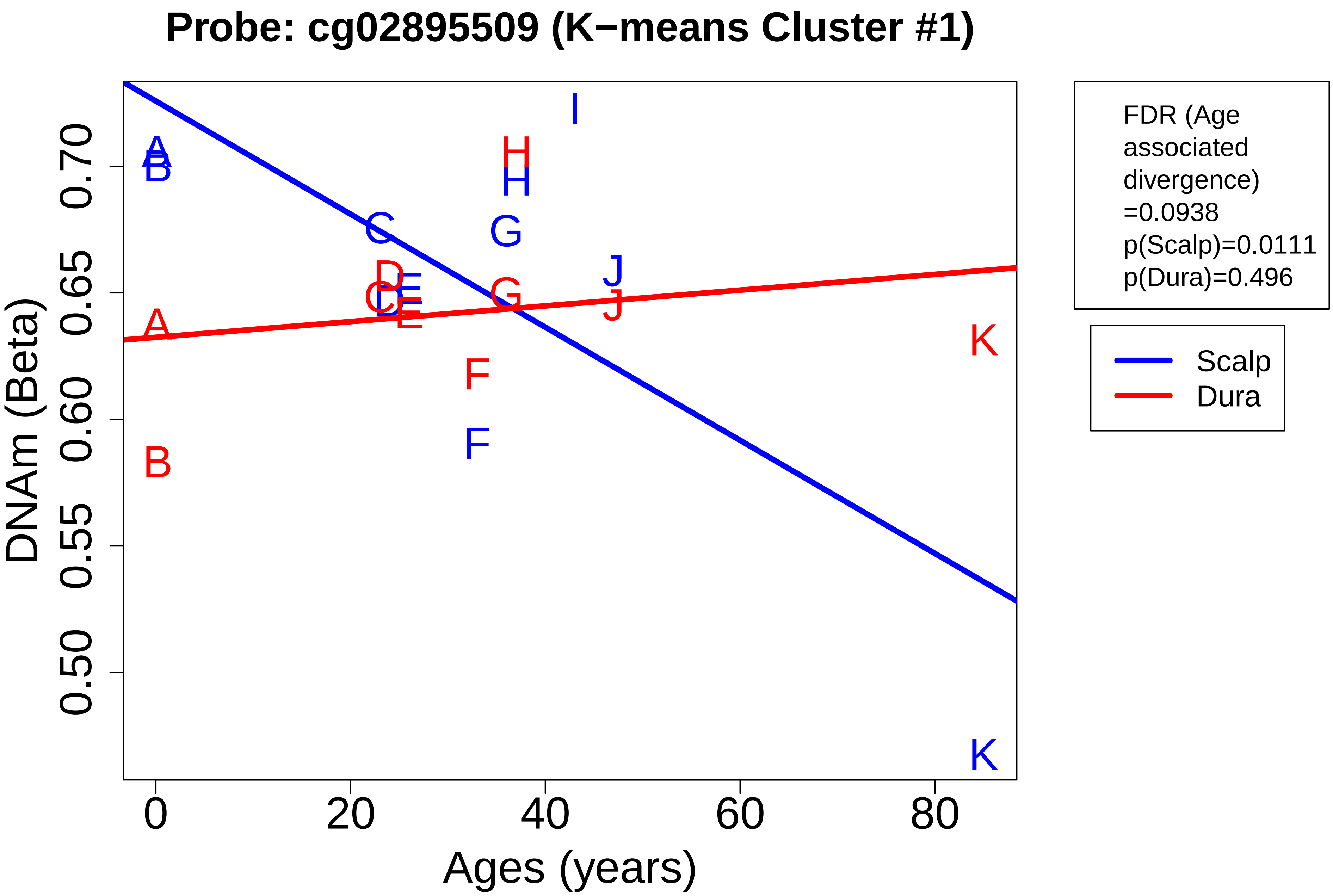

Probe: cg02895509 (K−means Cluster #1)
DNAm (Beta)
0.50
0.55
0.60
0.65
0.70
Ages (years)
0
20
40
60
80
FDR (Age associated divergence) =0.0938
p(Scalp)=0.0111
p(Dura)=0.496
Scalp
Dura
A
B
C
D
E
F
G
H
I
J
K

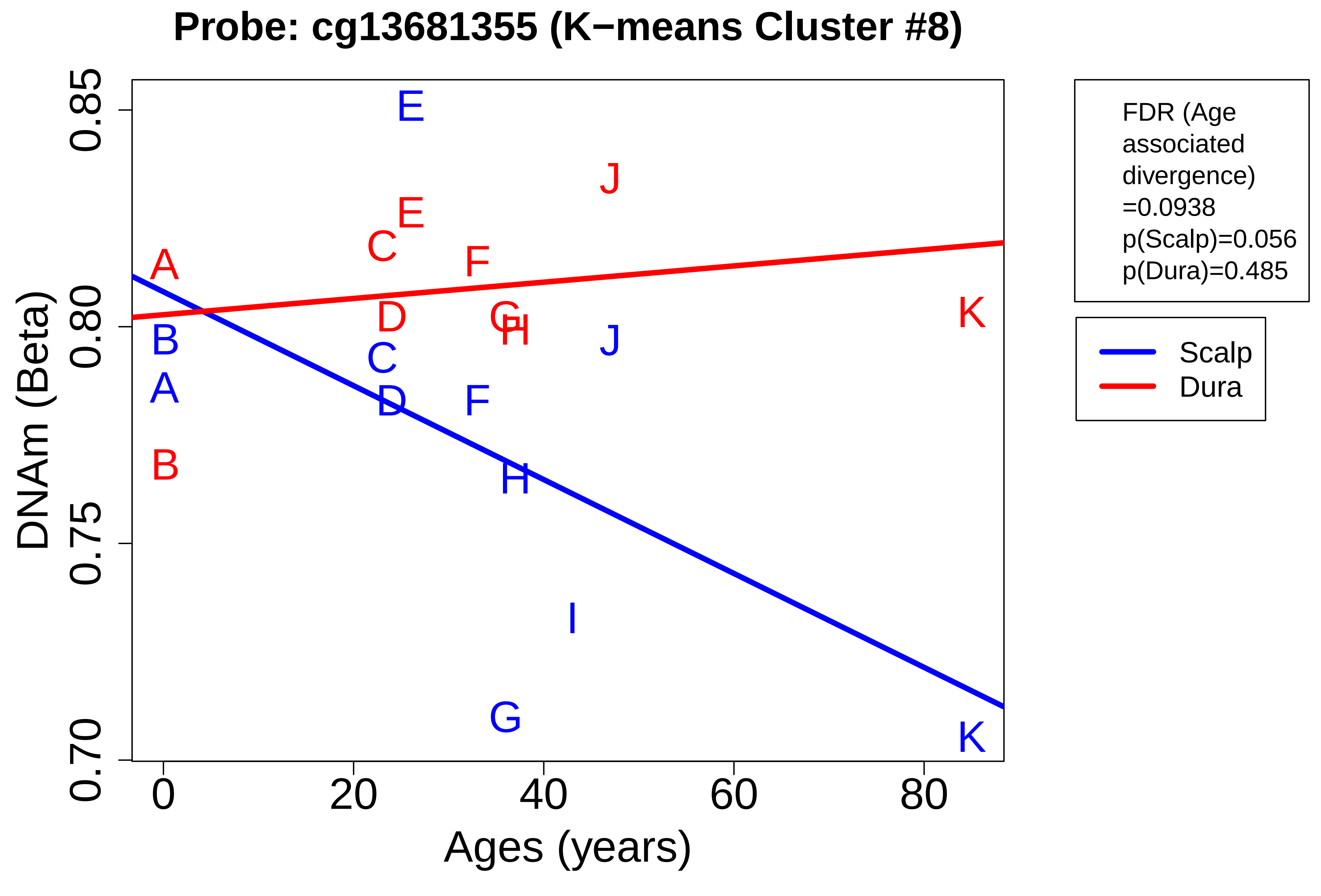

Probe: cg13681355 (K−means Cluster #8)
DNAm (Beta)
0.70
0.75
0.80
0.85
0
20
40
60
80
Ages (years)
FDR (Age associated divergence) =0.0938
p(Scalp)=0.056
p(Dura)=0.485
Scalp
Dura

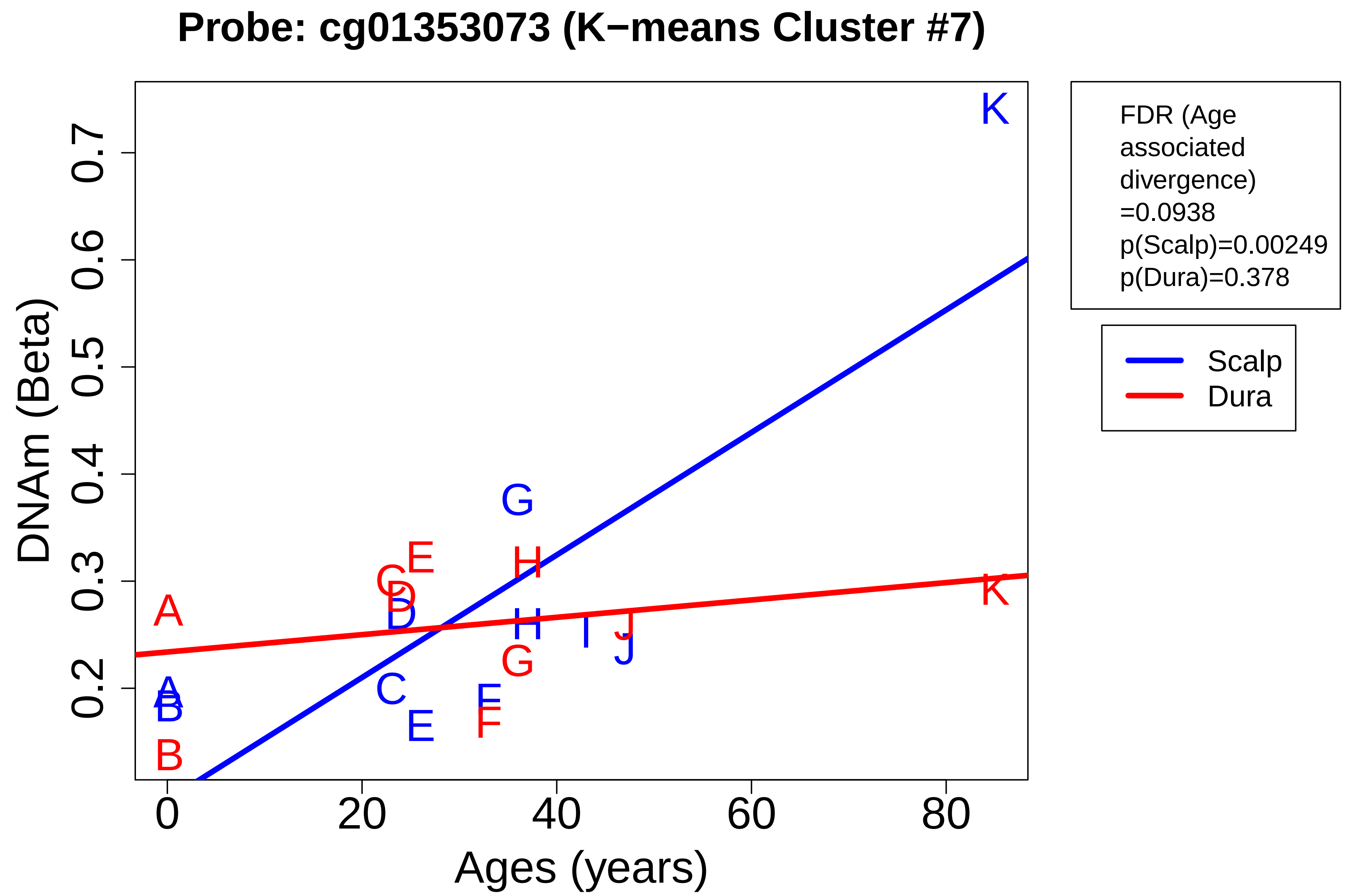

Probe: cg01353073 (K−means Cluster #7)
DNAm (Beta)
0.2
0.3
0.4
0.5
0.6
0.7
0
20
40
60
80
Ages (years)
A
B
C
D
E
F
G
H
I
J
K
FDR (Age associated divergence) =0.0938
p(Scalp)=0.00249
p(Dura)=0.378
Scalp
Dura

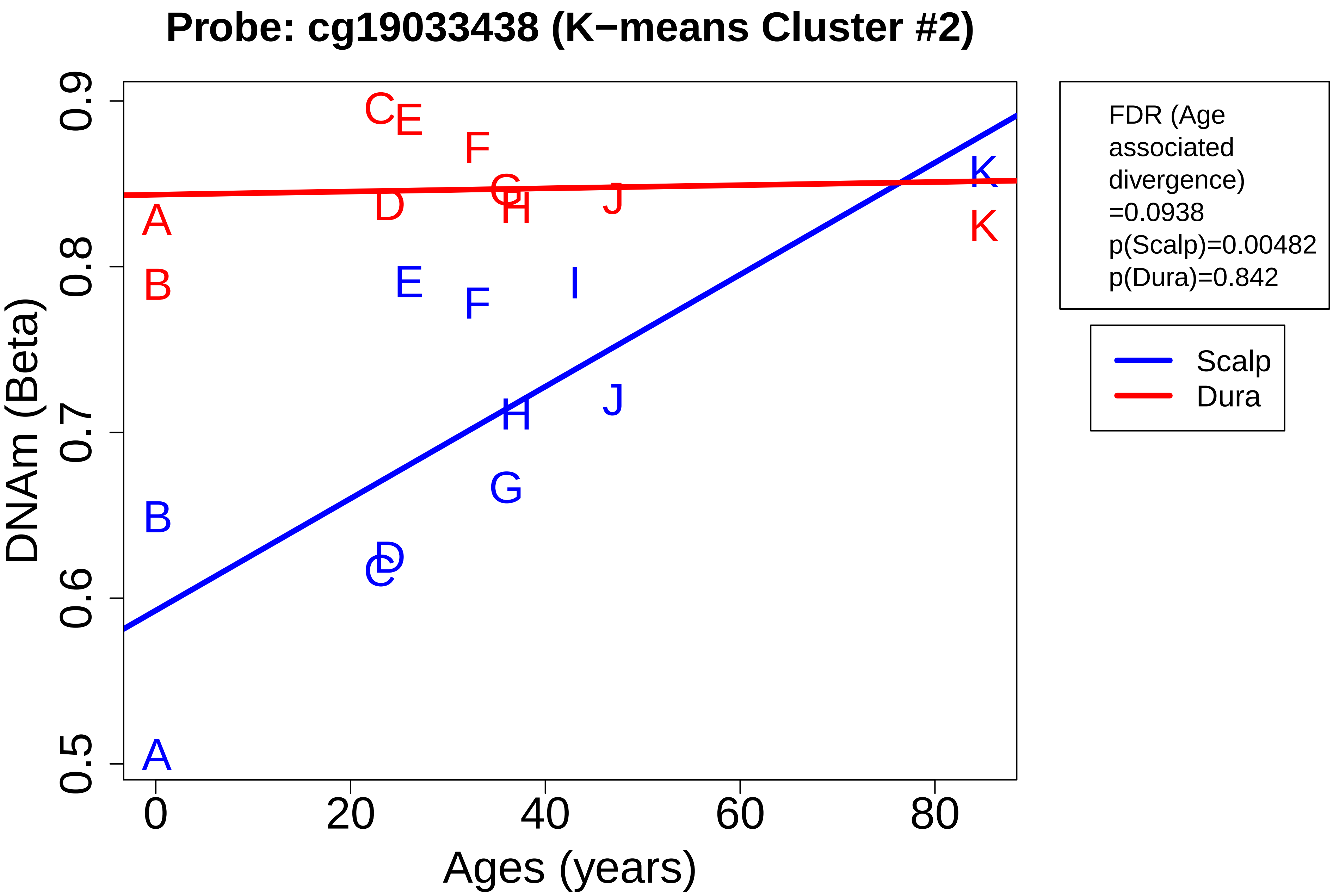
Probe: cg19033438 (K−means Cluster #2)
DNAm (Beta)
0.5
0.6
0.7
0.8
0.9
0
20
40
60
80
Ages (years)
FDR (Age associated divergence) =0.0938
p(Scalp)=0.00482
p(Dura)=0.842
Scalp
Dura

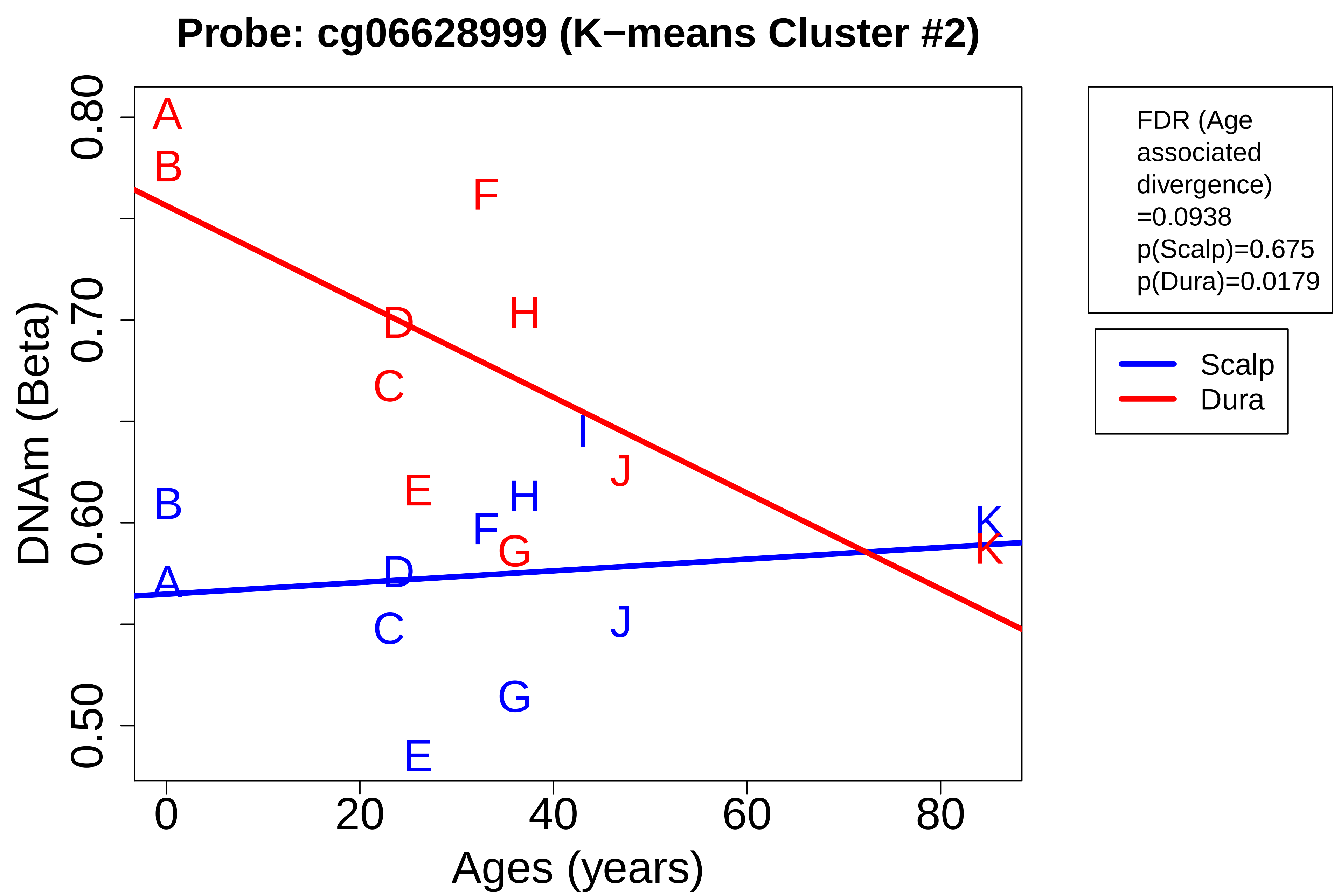

Probe: cg06628999 (K−means Cluster #2)
DNAm (Beta)
0.80
0.70
0.60
0.50
0
20
40
60
80
Ages (years)
FDR (Age associated divergence) =0.0938
p(Scalp)=0.675
p(Dura)=0.0179
Scalp
Dura

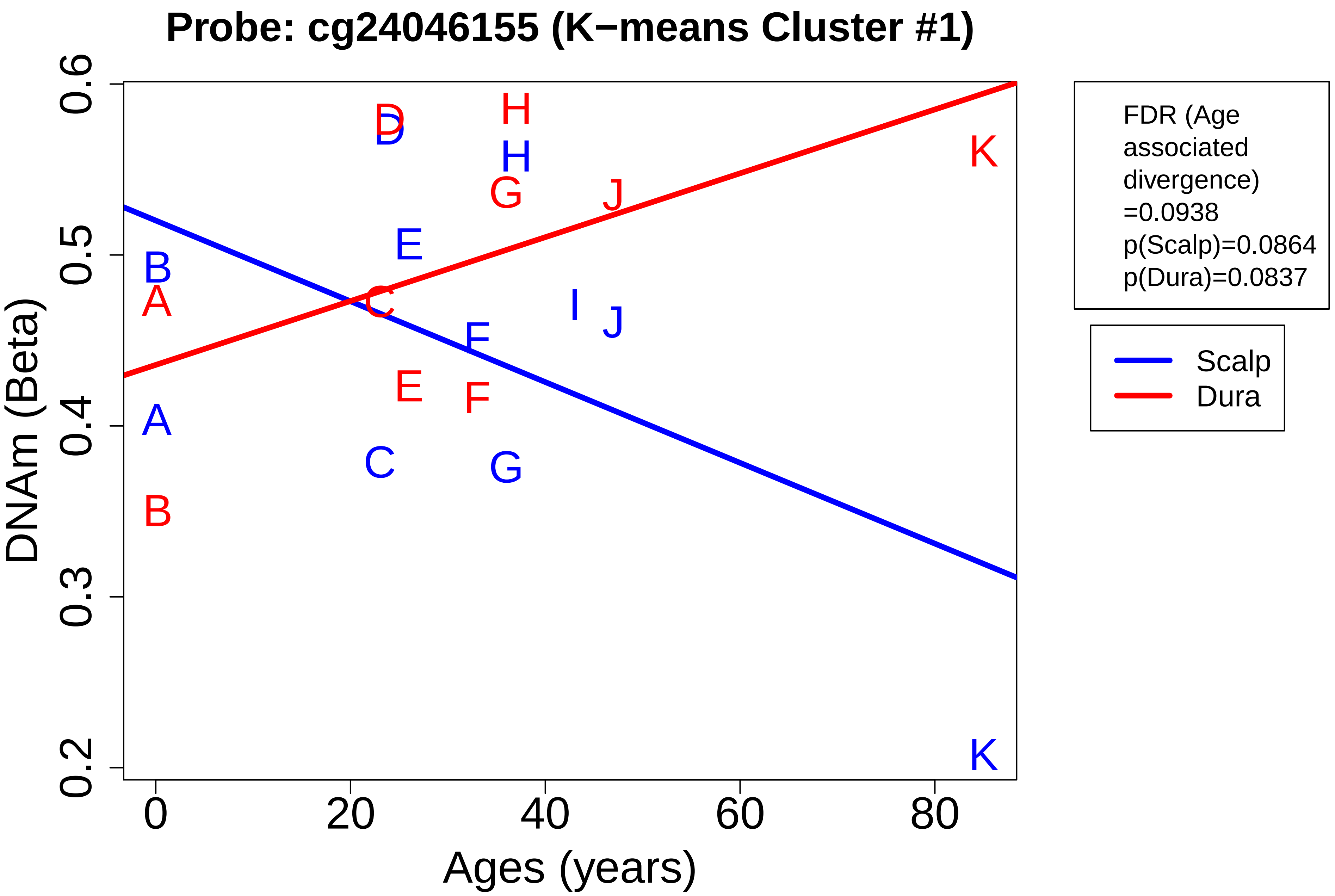

Probe: cg24046155 (K−means Cluster #1)
DNAm (Beta)
0.2
0.3
0.4
0.5
0.6
Ages (years)
0
20
40
60
80
FDR (Age associated divergence) =0.0938
p(Scalp)=0.0864
p(Dura)=0.0837
Scalp
Dura

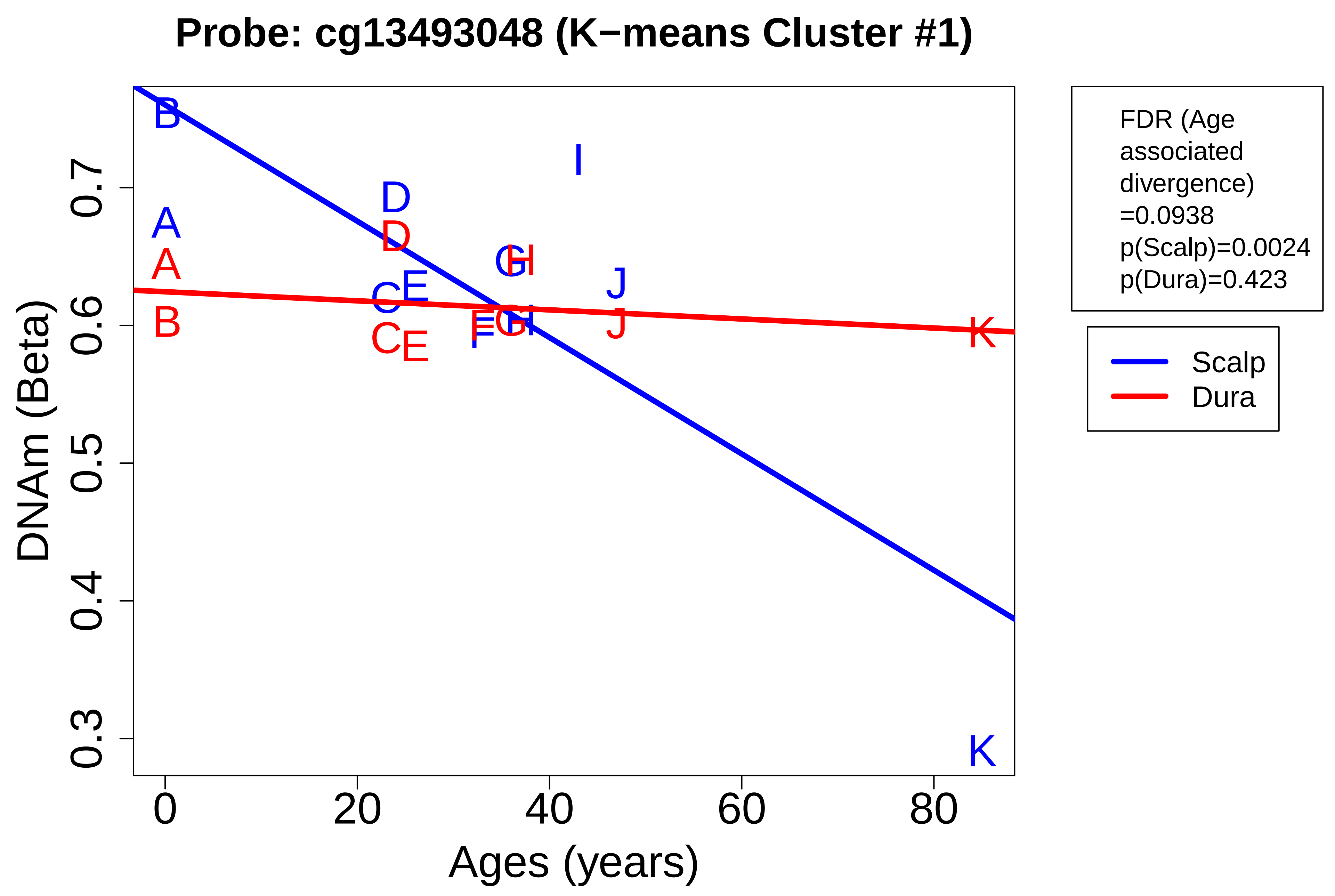

Probe: cg13493048 (K−means Cluster #1)
DNAm (Beta)
0.3
0.4
0.5
0.6
0.7
0
20
40
60
80
Ages (years)
FDR (Age associated divergence) =0.0938
p(Scalp)=0.0024
p(Dura)=0.423
Scalp
Dura

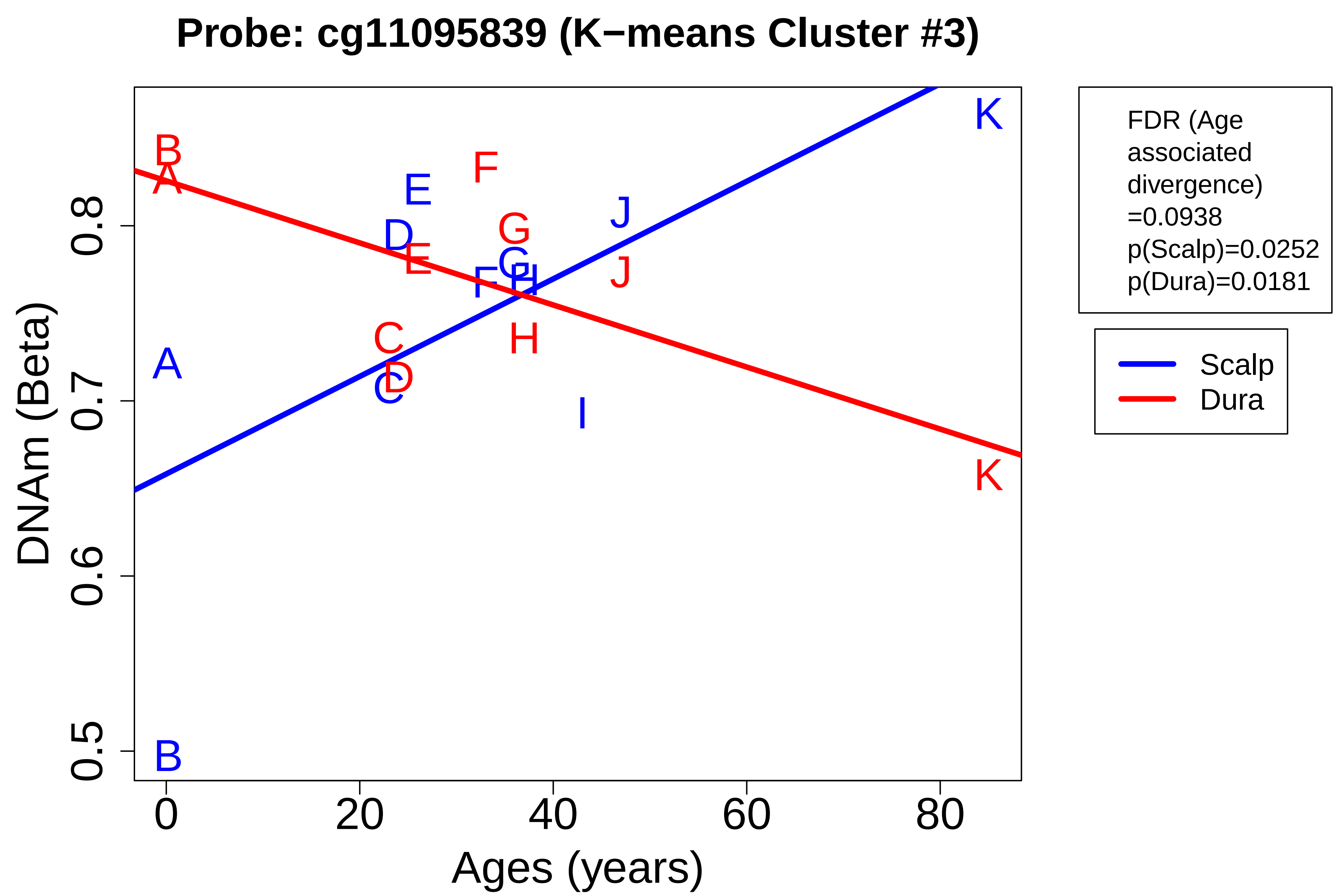

Probe: cg11095839 (K−means Cluster #3)
DNAm (Beta)
0.5
0.6
0.7
0.8
Ages (years)
0
20
40
60
80
FDR (Age associated divergence) =0.0938
p(Scalp)=0.0252
p(Dura)=0.0181
Scalp
Dura
A
B
C
D
E
F
G
H
I
J
K

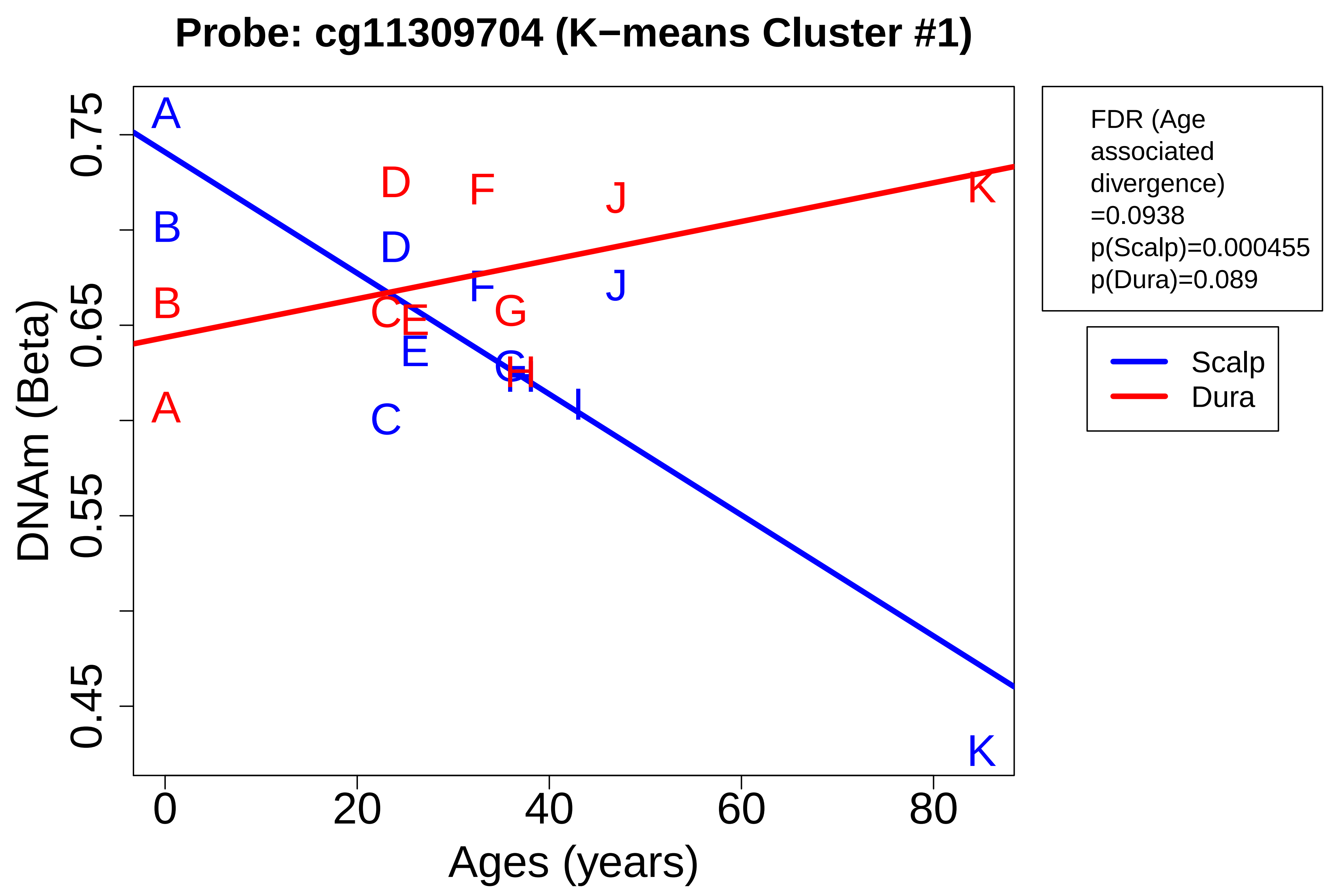
Probe: cg11309704 (K−means Cluster #1)
DNAm (Beta)
0.45
0.55
0.65
0.75
Ages (years)
0
20
40
60
80
A
B
C
D
E
F
G
H
I
J
K
FDR (Age associated divergence) =0.0938
p(Scalp)=0.000455
p(Dura)=0.089
Scalp
Dura

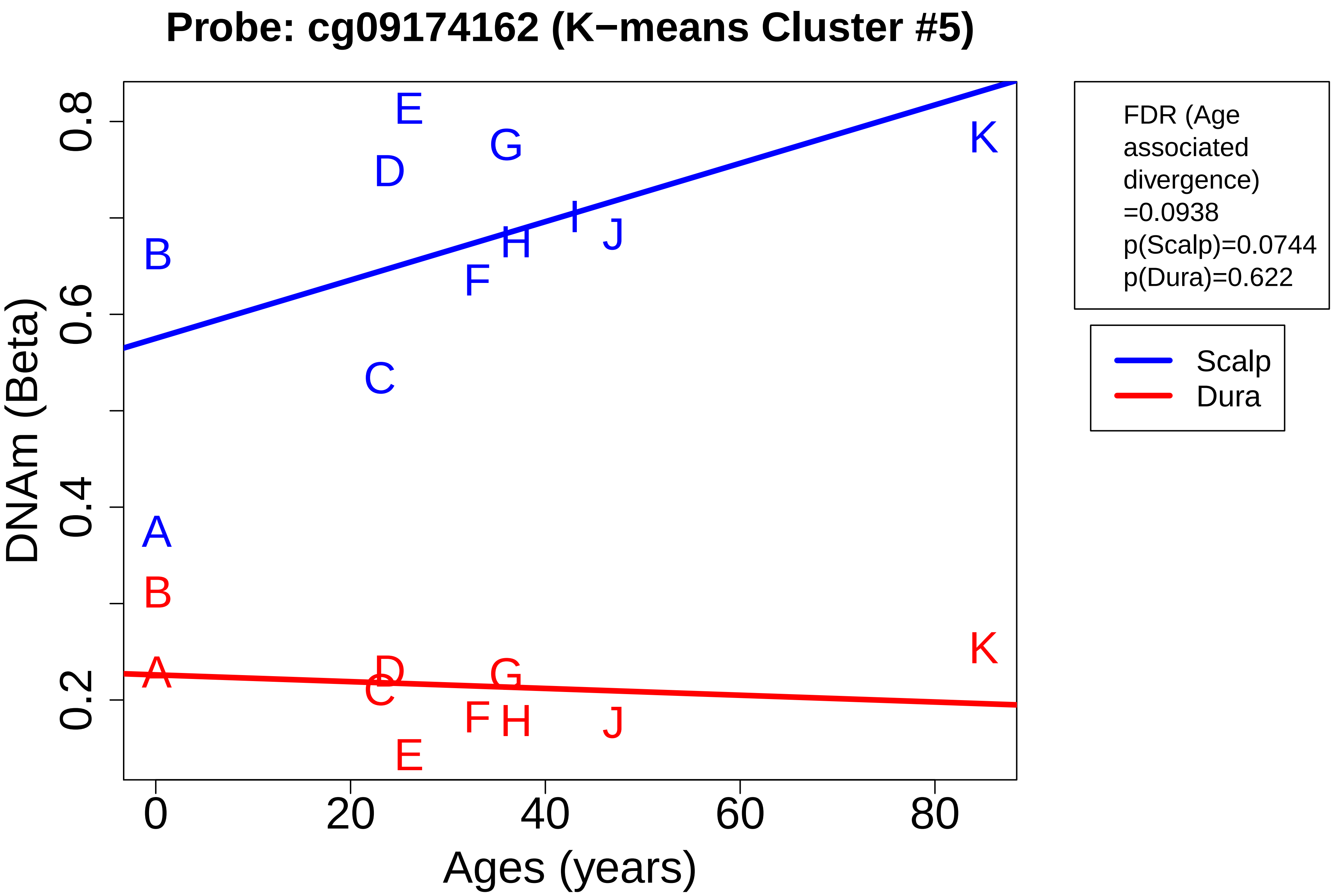

Probe: cg09174162 (K−means Cluster #5)
DNAm (Beta)
0.2
0.4
0.6
0.8
0
20
40
60
80
Ages (years)
FDR (Age associated divergence) =0.0938
p(Scalp)=0.0744
p(Dura)=0.622
Scalp
Dura
A
B
C
D
E
F
G
H
I
J
K

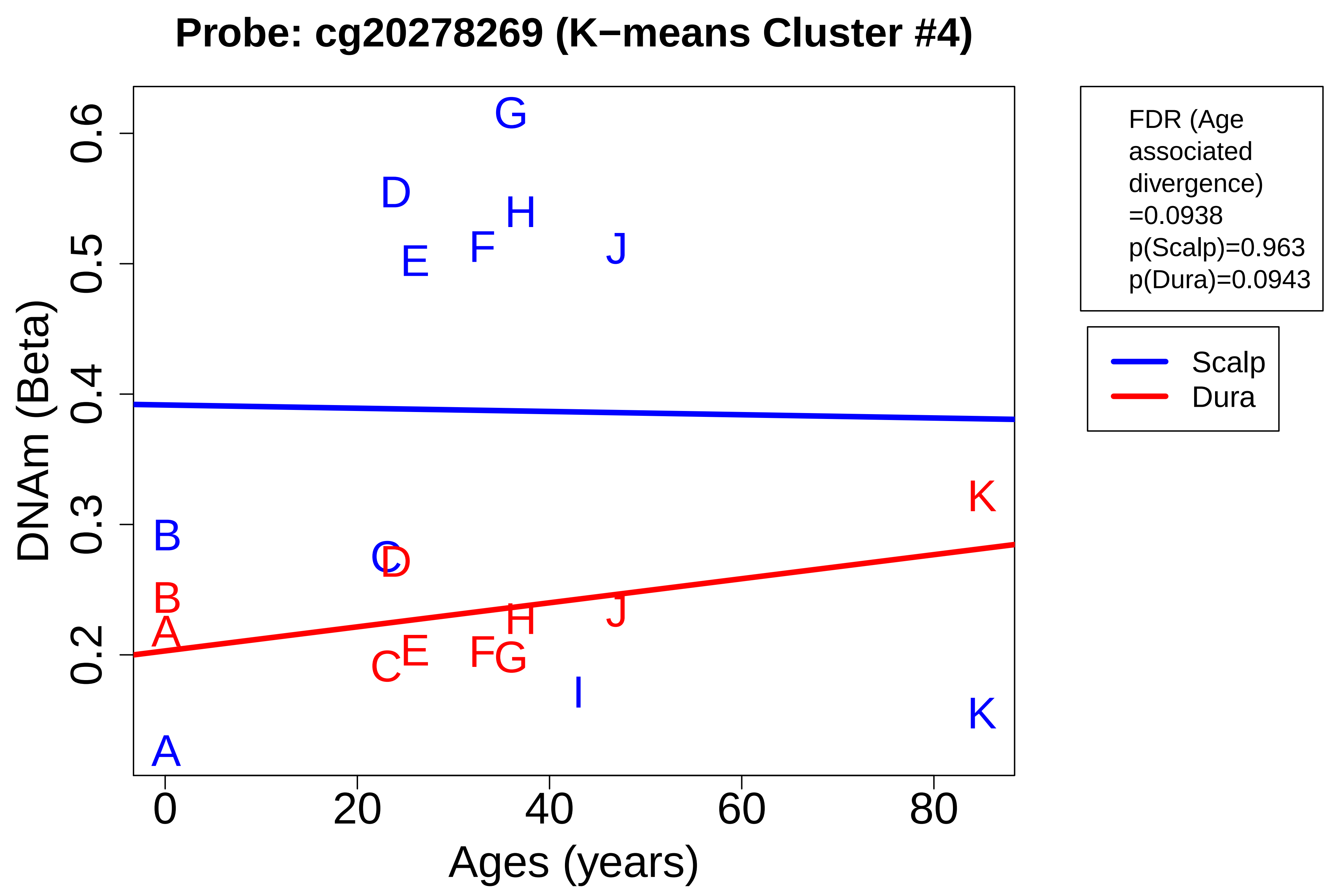

Probe: cg20278269 (K−means Cluster #4)
DNAm (Beta)
0.2
0.3
0.4
0.5
0.6
Ages (years)
0
20
40
60
80
FDR (Age associated divergence) =0.0938
p(Scalp)=0.963
p(Dura)=0.0943
Scalp
Dura
A
B
C
D
E
F
G
H
I
J
K

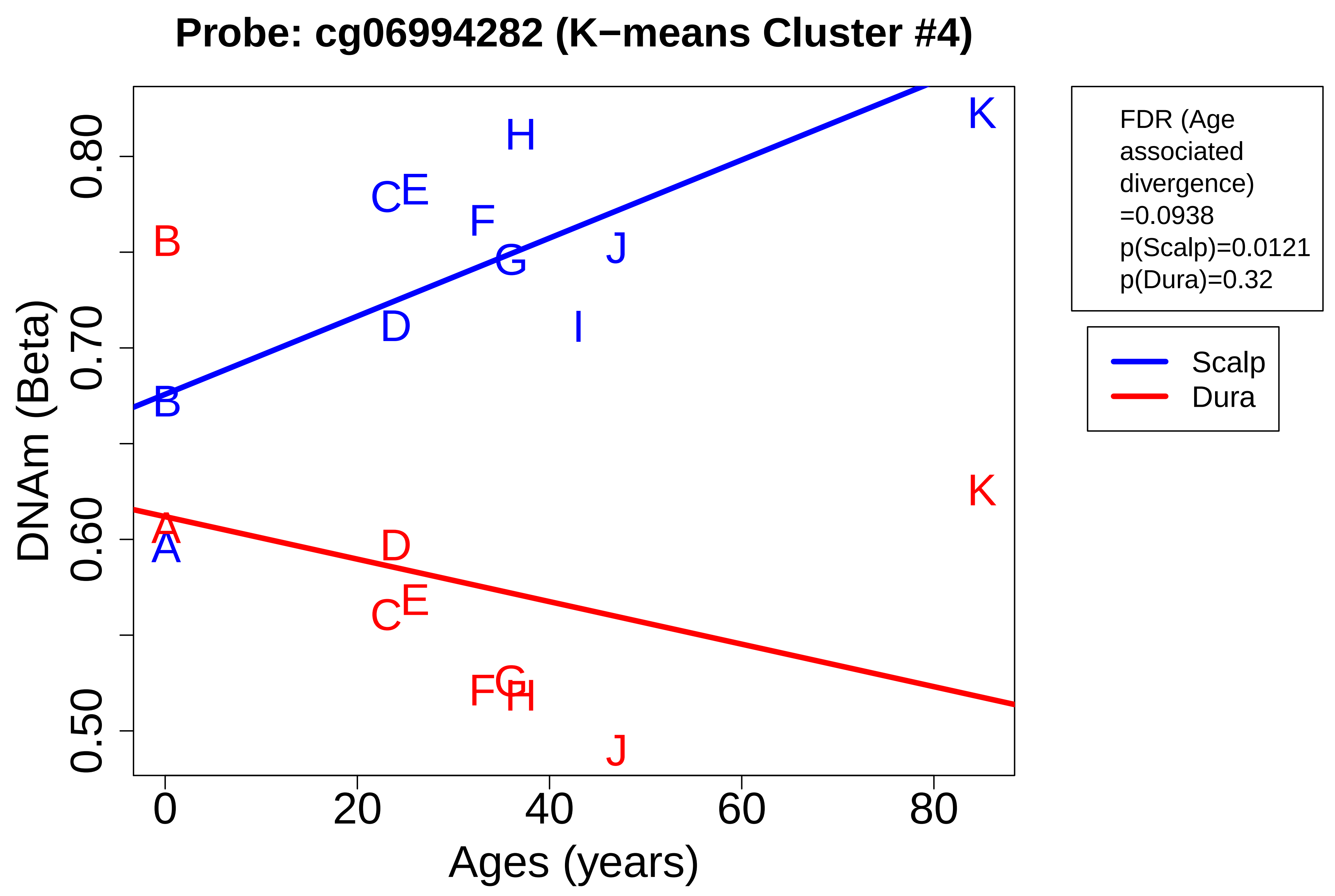

Probe: cg06994282 (K−means Cluster #4)
DNAm (Beta)
0.50
0.60
0.70
0.80
Ages (years)
0
20
40
60
80
FDR (Age associated divergence) =0.0938
p(Scalp)=0.0121
p(Dura)=0.32
Scalp
Dura
A
B
C
D
E
F
G
H
I
J
K

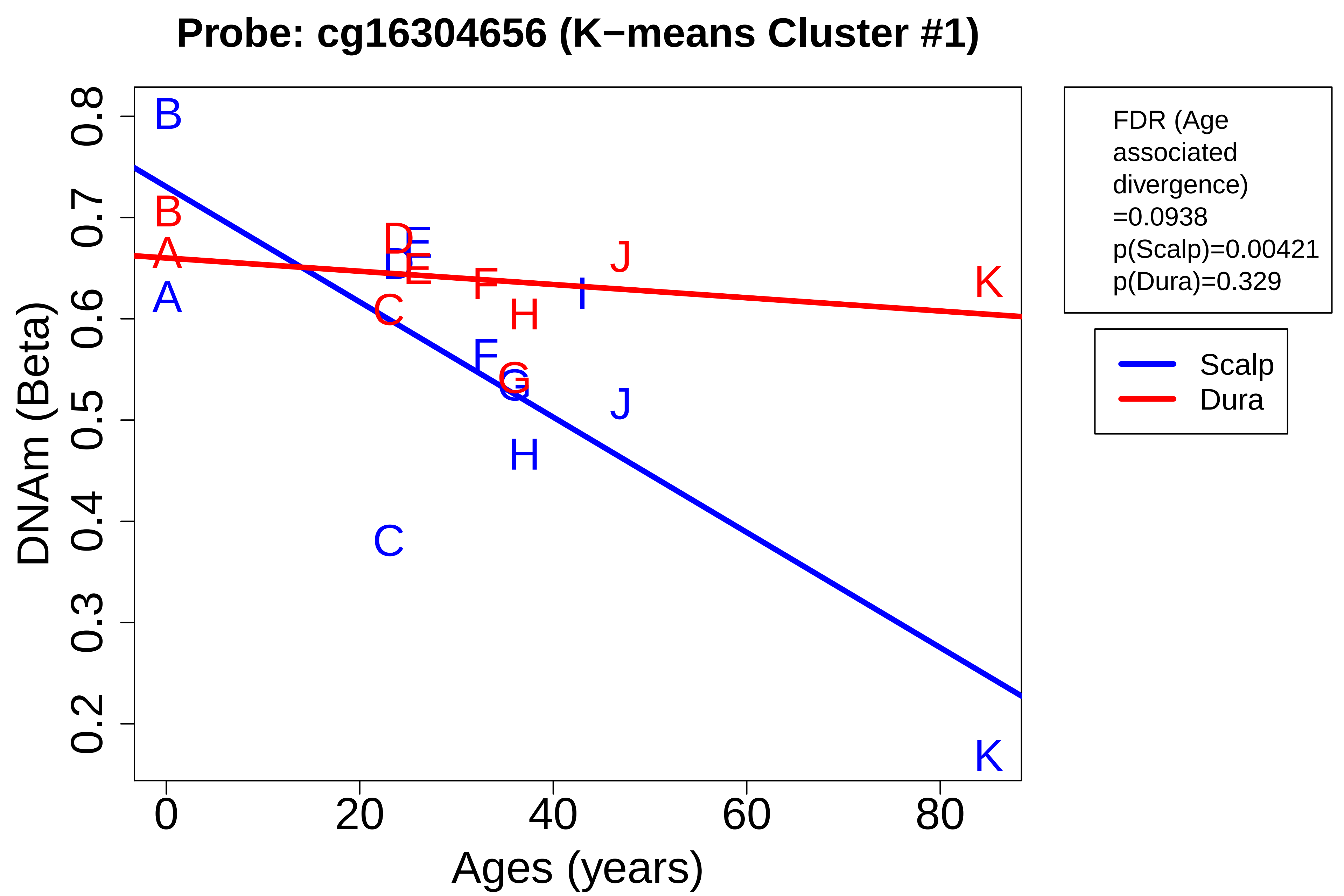

Probe: cg16304656 (K−means Cluster #1)
DNAm (Beta)
0.2
0.3
0.4
0.5
0.6
0.7
0.8
Ages (years)
0
20
40
60
80
A
B
C
D
E
F
G
H
I
J
K
FDR (Age associated divergence) =0.0938
p(Scalp)=0.00421
p(Dura)=0.329
Scalp
Dura

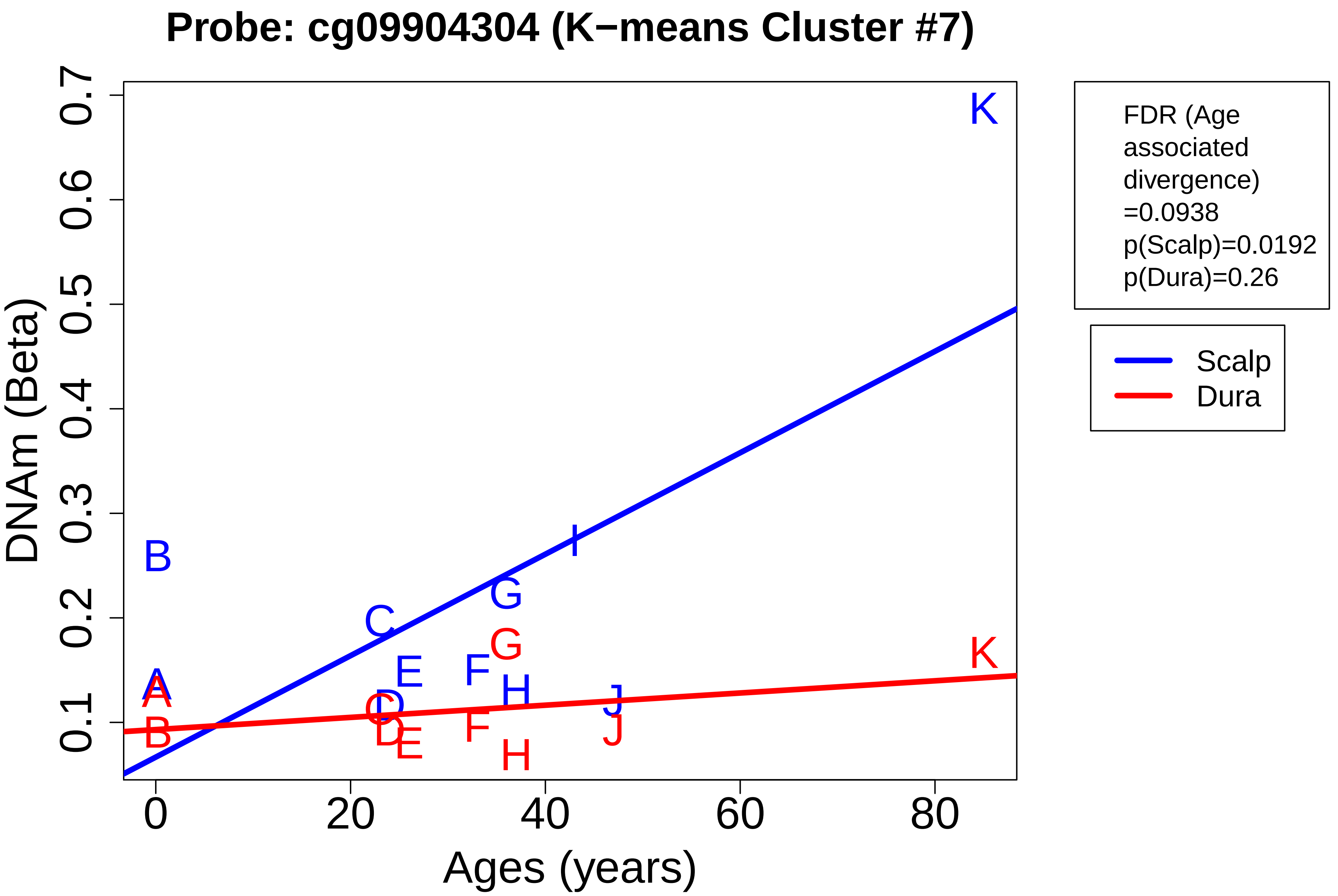

Probe: cg09904304 (K−means Cluster #7)
DNAm (Beta)
0.1
0.2
0.3
0.4
0.5
0.6
0.7
Ages (years)
0
20
40
60
80
FDR (Age associated divergence) =0.0938
p(Scalp)=0.0192
p(Dura)=0.26
Scalp
Dura

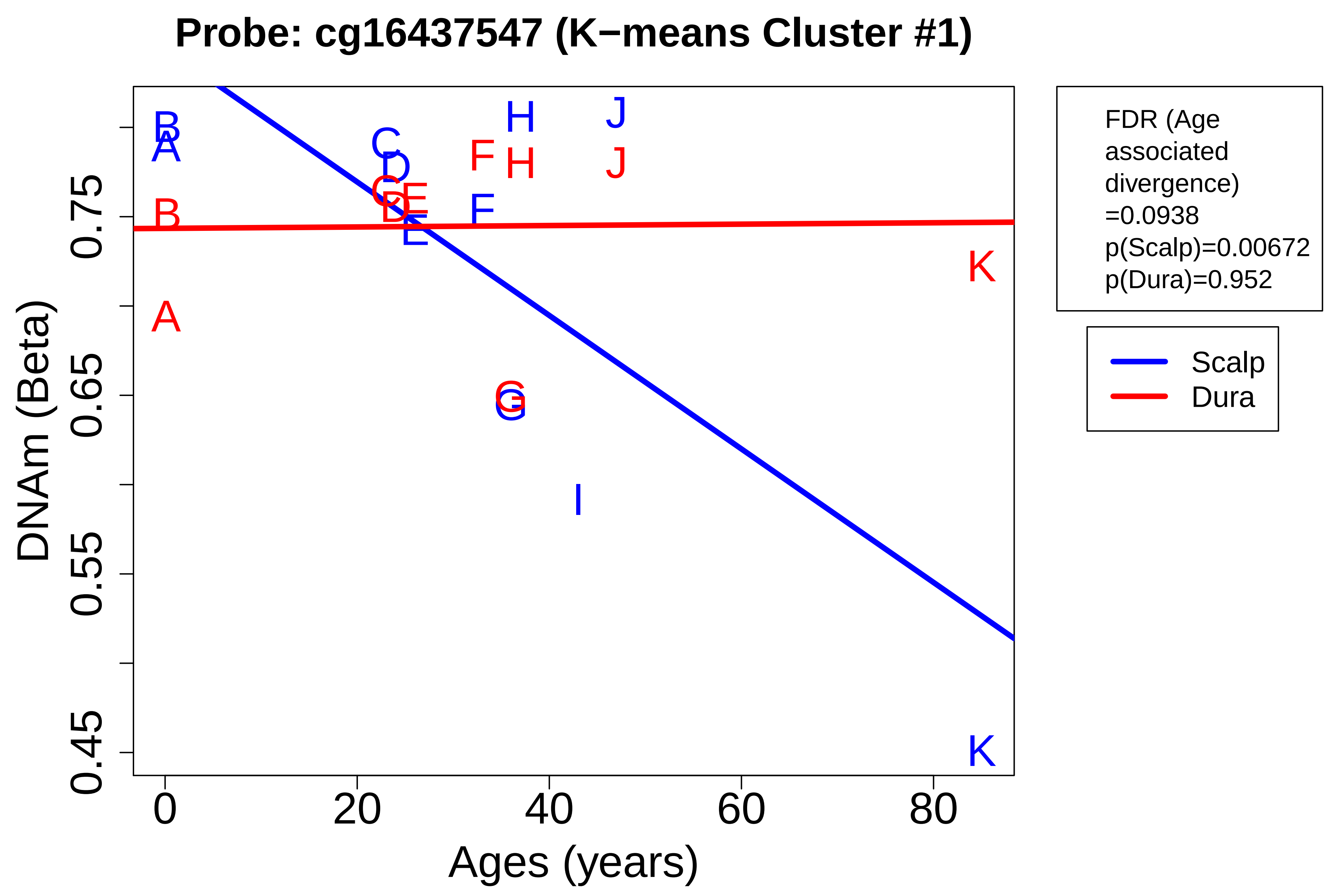

Probe: cg16437547 (K−means Cluster #1)
DNAm (Beta)
0.45
0.55
0.65
0.75
0
20
40
60
80
Ages (years)
FDR (Age associated divergence) =0.0938
p(Scalp)=0.00672
p(Dura)=0.952
Scalp
Dura

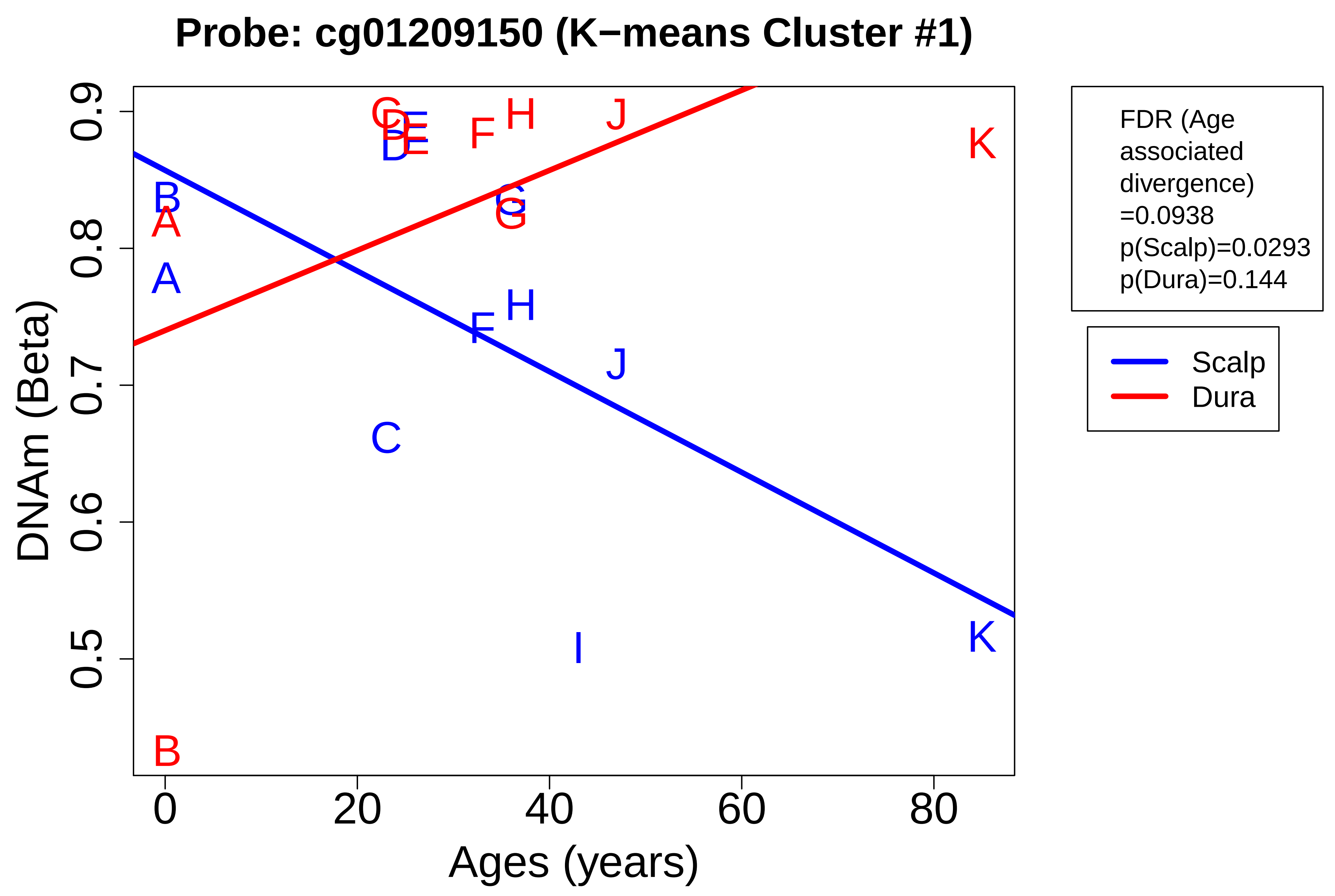

Probe: cg01209150 (K−means Cluster #1)
DNAm (Beta)
0.5
0.6
0.7
0.8
0.9
0
20
40
60
80
Ages (years)
A
B
C
D
E
F
G
H
I
J
K
FDR (Age associated divergence) =0.0938
p(Scalp)=0.0293
p(Dura)=0.144
Scalp
Dura

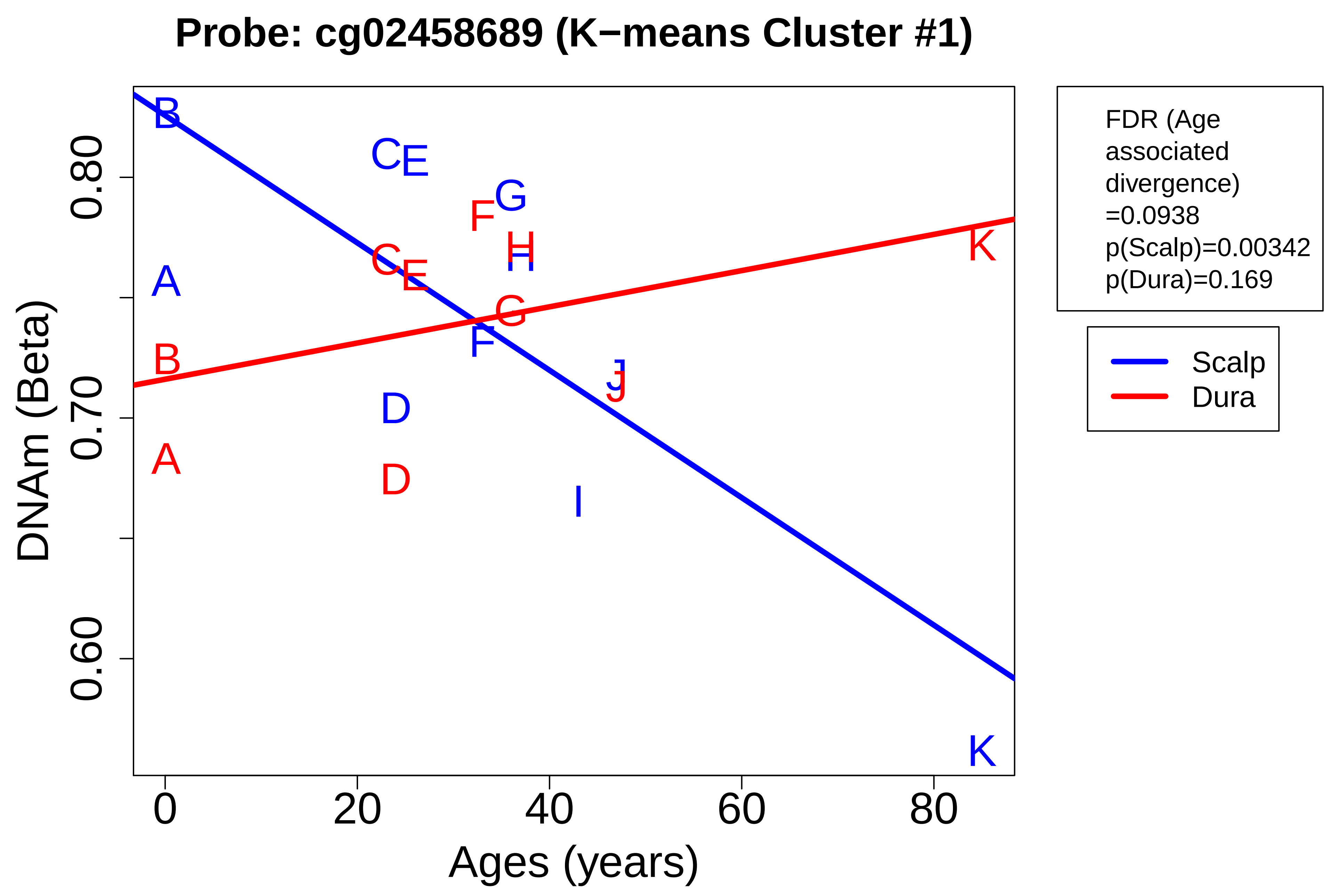
Probe: cg02458689 (K−means Cluster #1)
DNAm (Beta)
0.60
0.70
0.80
0
20
40
60
80
Ages (years)
A
B
C
D
E
F
G
H
I
J
K
FDR (Age associated divergence) =0.0938
p(Scalp)=0.00342
p(Dura)=0.169
Scalp
Dura

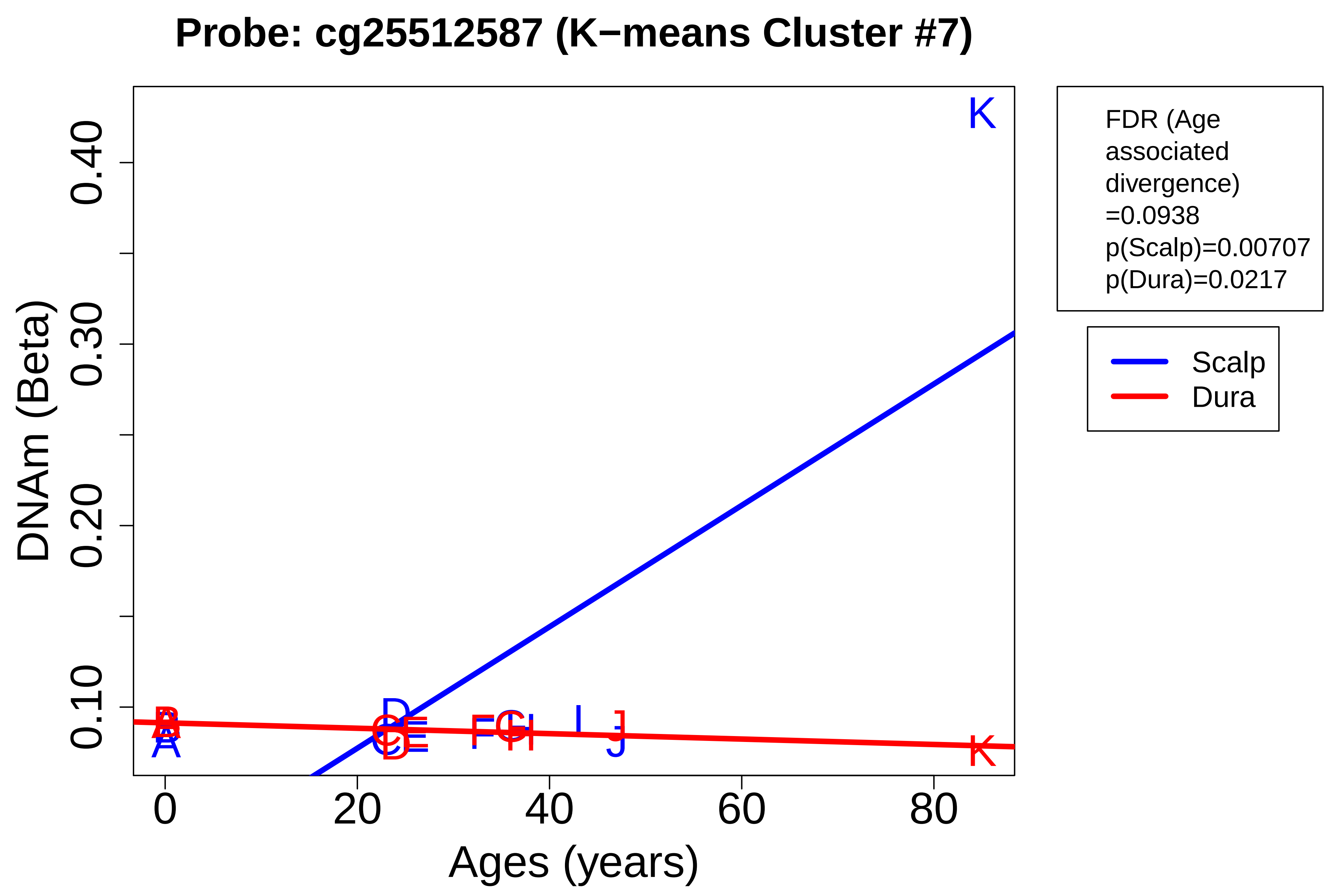

Probe: cg25512587 (K−means Cluster #7)
FDR (Age associated divergence) =0.0938
p(Scalp)=0.00707
p(Dura)=0.0217
Scalp
Dura
DNAm (Beta)
0.10
0.20
0.30
0.40
Ages (years)
0
20
40
60
80

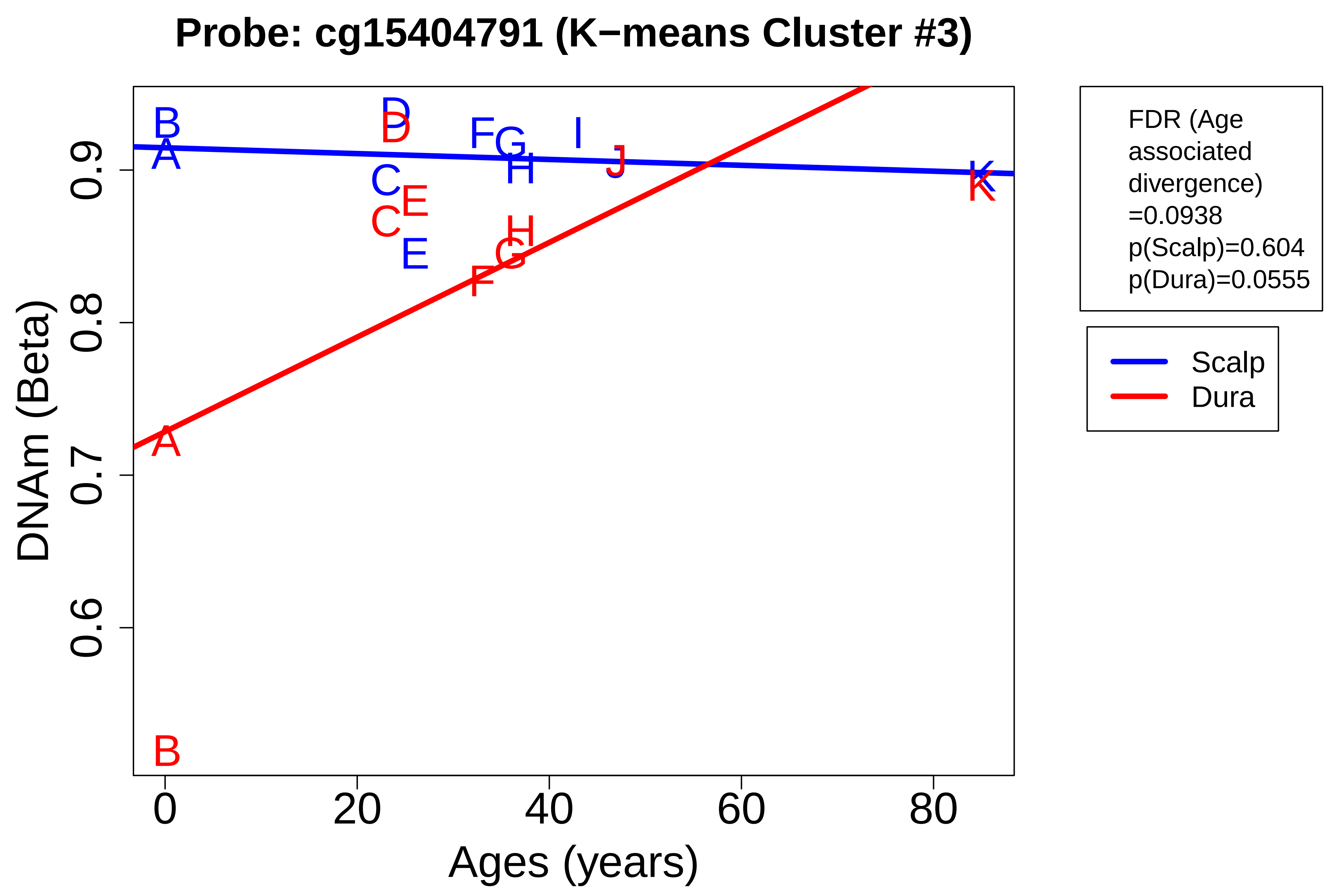
Probe: cg15404791 (K−means Cluster #3)
DNAm (Beta)
0.6
0.7
0.8
0.9
0
20
40
60
80
Ages (years)
A
B
C
D
E
F
G
H
I
J
K
FDR (Age associated divergence) =0.0938
p(Scalp)=0.604
p(Dura)=0.0555
Scalp
Dura

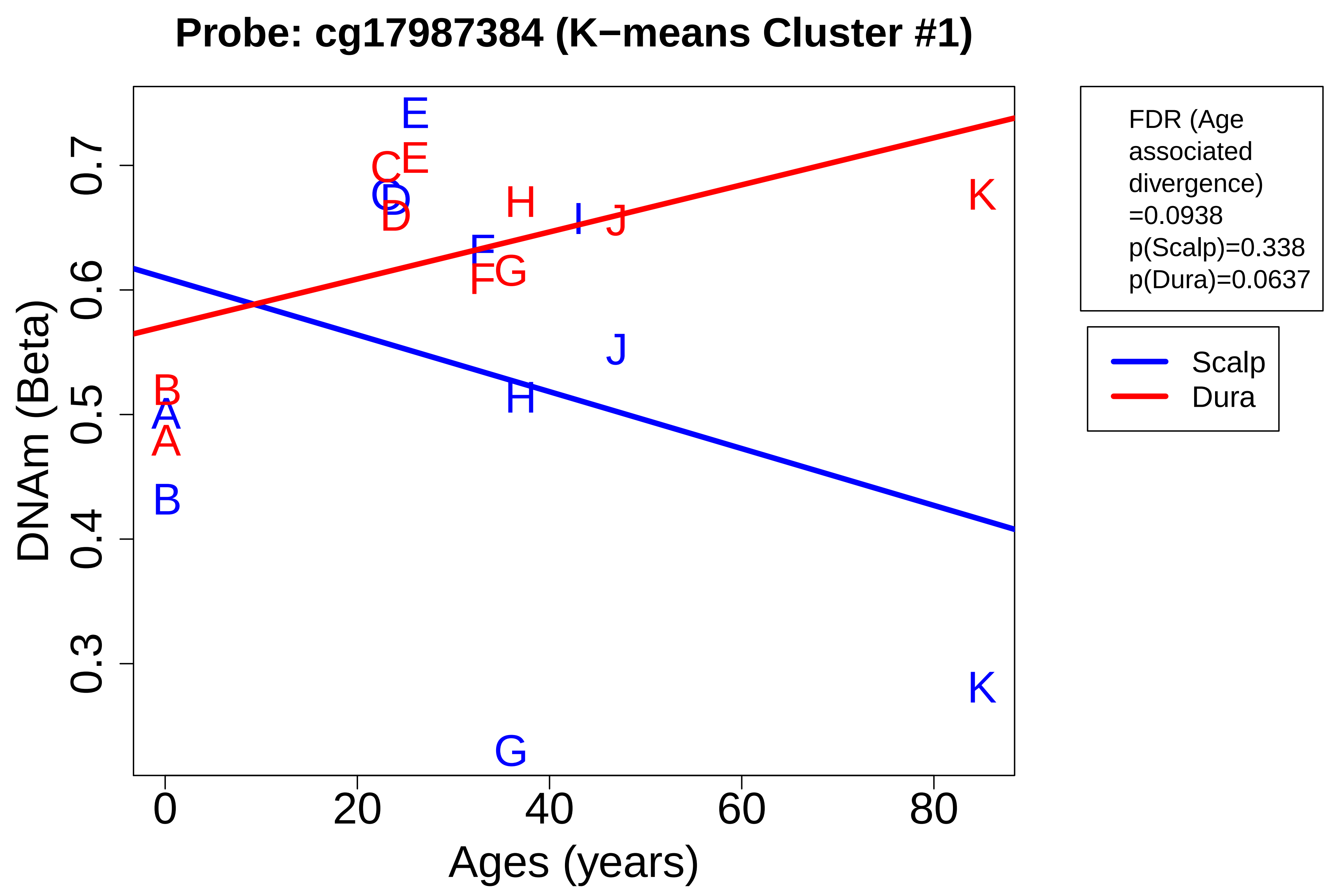

Probe: cg17987384 (K−means Cluster #1)
DNAm (Beta)
0.3
0.4
0.5
0.6
0.7
Ages (years)
0
20
40
60
80
FDR (Age associated divergence) =0.0938
p(Scalp)=0.338
p(Dura)=0.0637
Scalp
Dura

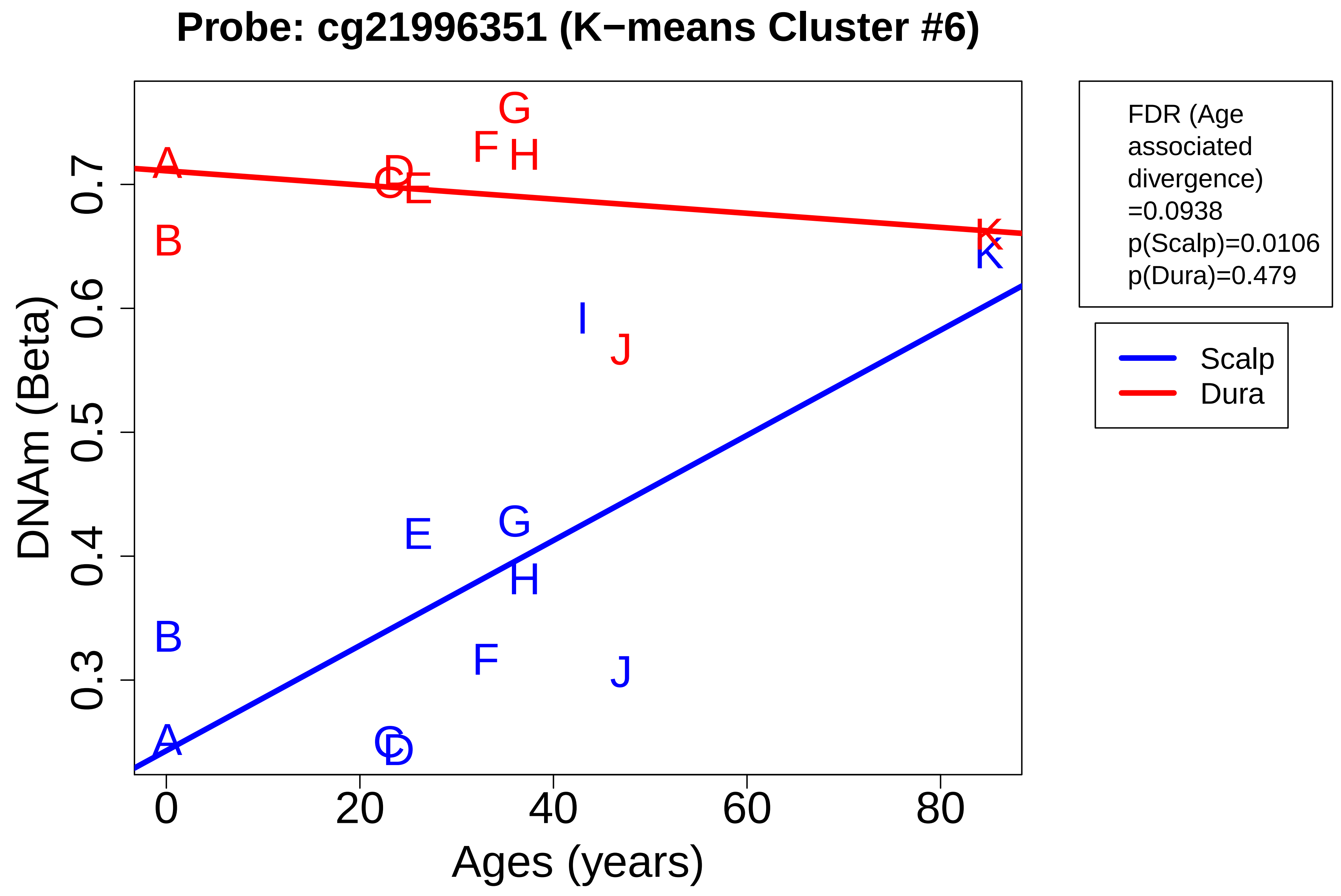
Probe: cg21996351 (K−means Cluster #6)
DNAm (Beta)
0.3
0.4
0.5
0.6
0.7
Ages (years)
0
20
40
60
80
FDR (Age associated divergence) =0.0938
p(Scalp)=0.0106
p(Dura)=0.479
Scalp
Dura
A
B
C
D
E
F
G
H
I
J
K

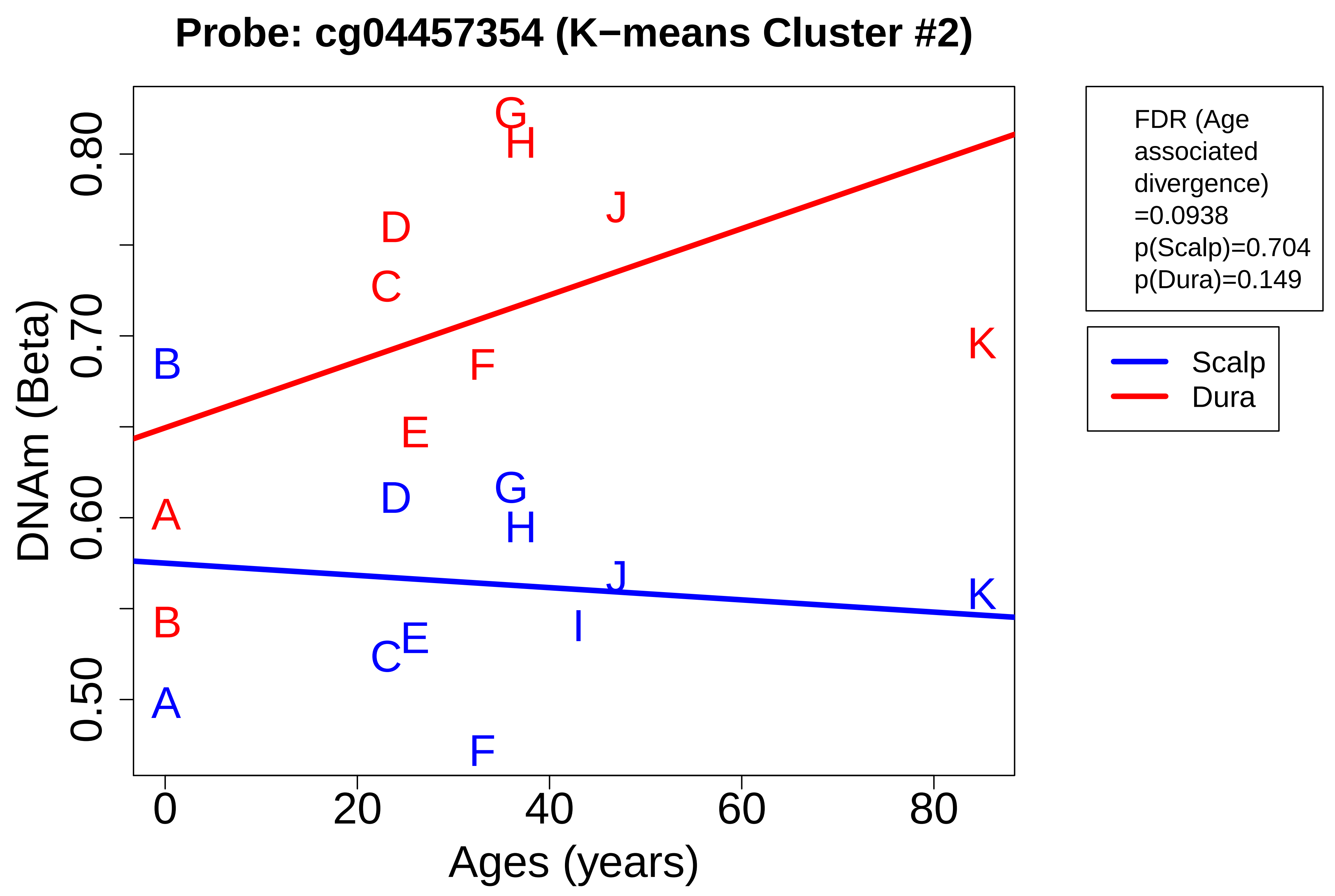
Probe: cg04457354 (K−means Cluster #2)
DNAm (Beta)
0.50
0.60
0.70
0.80
0
20
40
60
80
Ages (years)
FDR (Age associated divergence) =0.0938
p(Scalp)=0.704
p(Dura)=0.149
Scalp
Dura
A
B
C
D
E
F
G
H
I
J
K
A
B
C
D
E
F
G
H
J
K

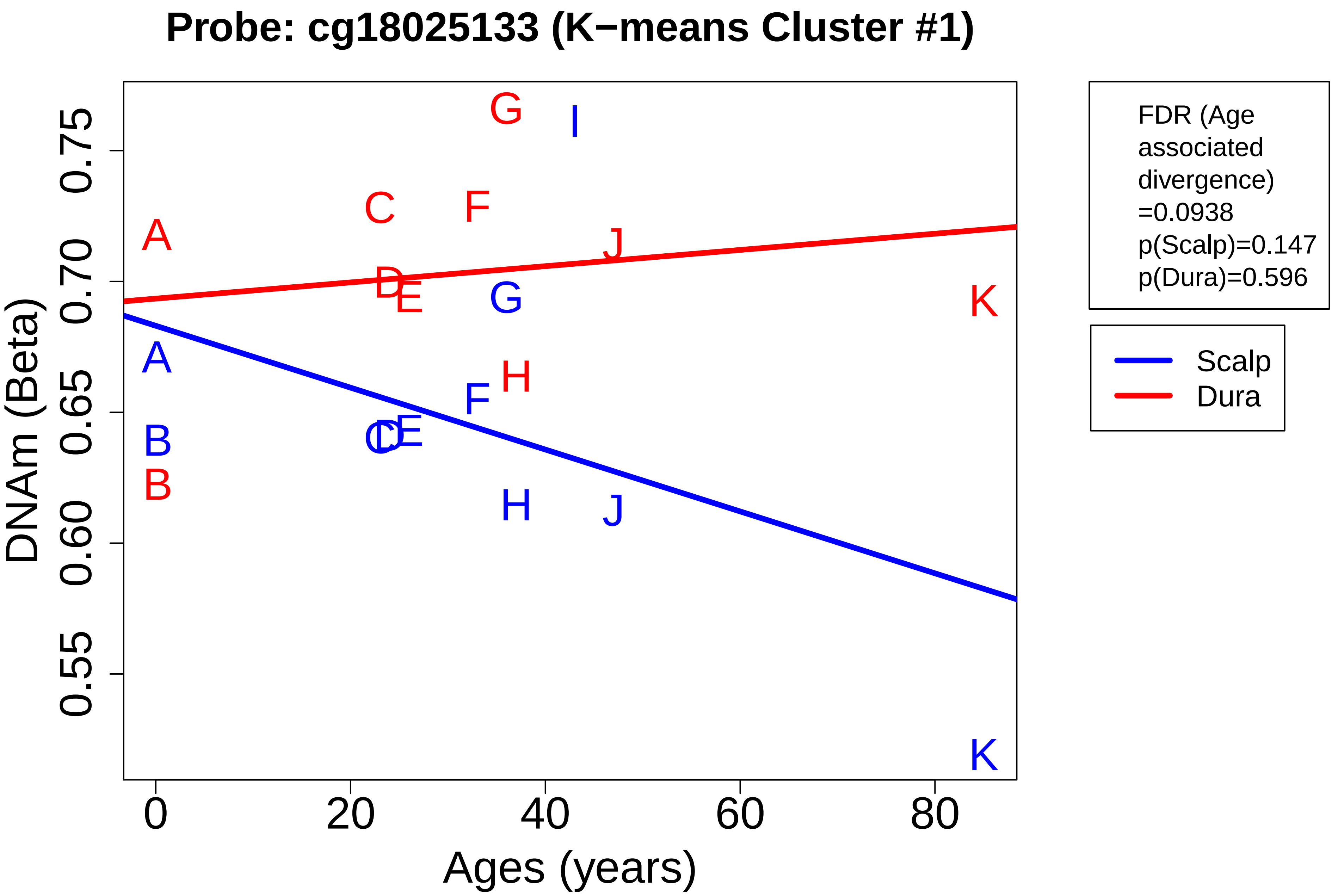

Probe: cg18025133 (K−means Cluster #1)
DNAm (Beta)
0.55
0.60
0.65
0.70
0.75
0
20
40
60
80
Ages (years)
A
B
C
D
E
F
G
H
I
J
K
FDR (Age associated divergence) =0.0938
p(Scalp)=0.147
p(Dura)=0.596
Scalp
Dura

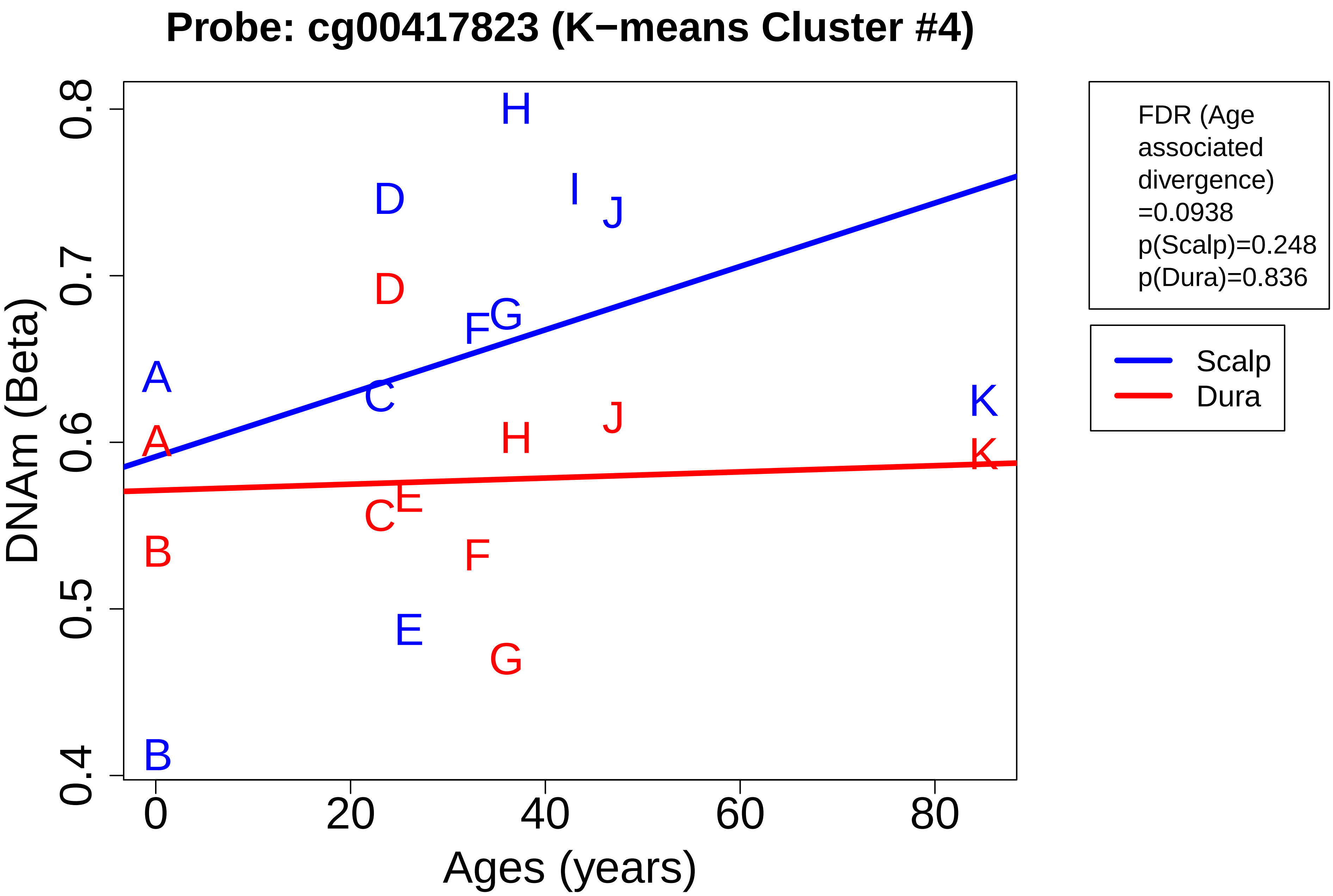
Probe: cg00417823 (K−means Cluster #4)
DNAm (Beta)
0.4
0.5
0.6
0.7
0.8
0
20
40
60
80
Ages (years)
A B C D E F G H I J K
A B C D E F G H J K
FDR (Age associated divergence) =0.0938
p(Scalp)=0.248
p(Dura)=0.836
Scalp
Dura

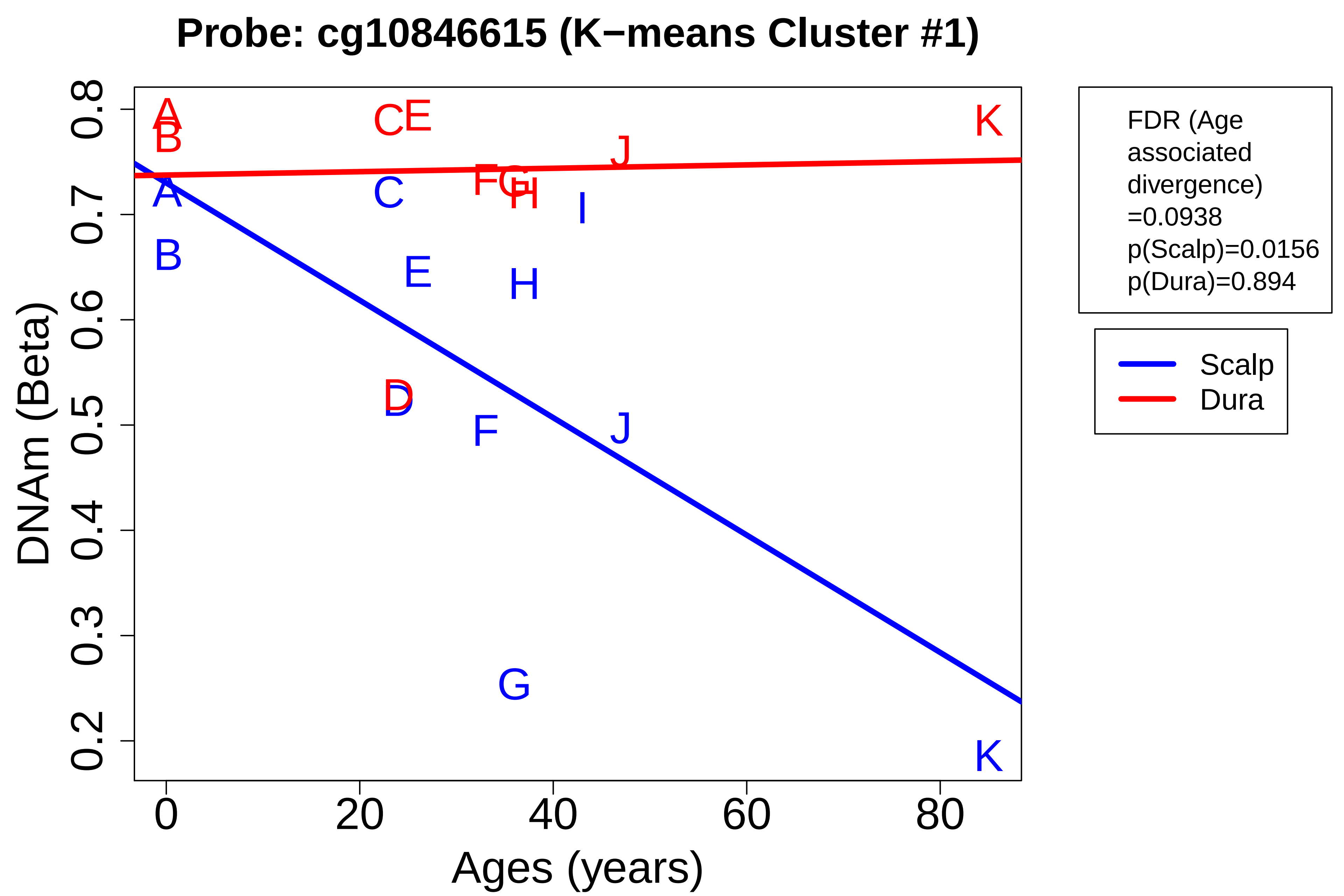
Probe: cg10846615 (K−means Cluster #1)
DNAm (Beta)
0.2
0.3
0.4
0.5
0.6
0.7
0.8
0
20
40
60
80
Ages (years)
A
B
CE
K
J
FG
H
I
A
C
B
E
H
D
D
F
J
G
K
FDR (Age associated divergence) =0.0938
p(Scalp)=0.0156
p(Dura)=0.894
Scalp
Dura

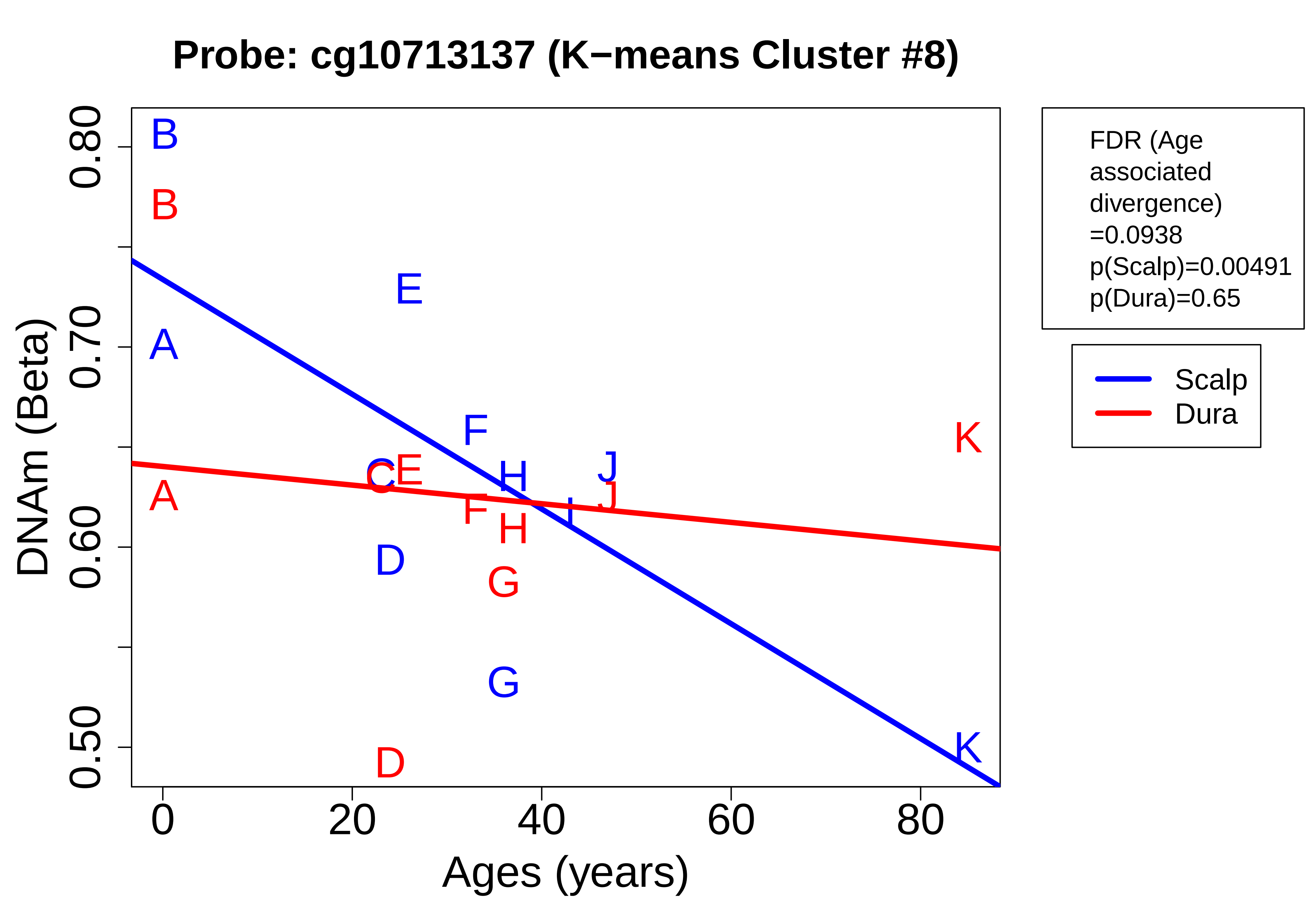

Probe: cg10713137 (K−means Cluster #8)
FDR (Age associated divergence) =0.0938
p(Scalp)=0.00491
p(Dura)=0.65
Scalp
Dura
DNAm (Beta)
0.50
0.60
0.70
0.80
0
20
40
60
80
Ages (years)
A
B
C
D
E
F
G
H
I
J
K

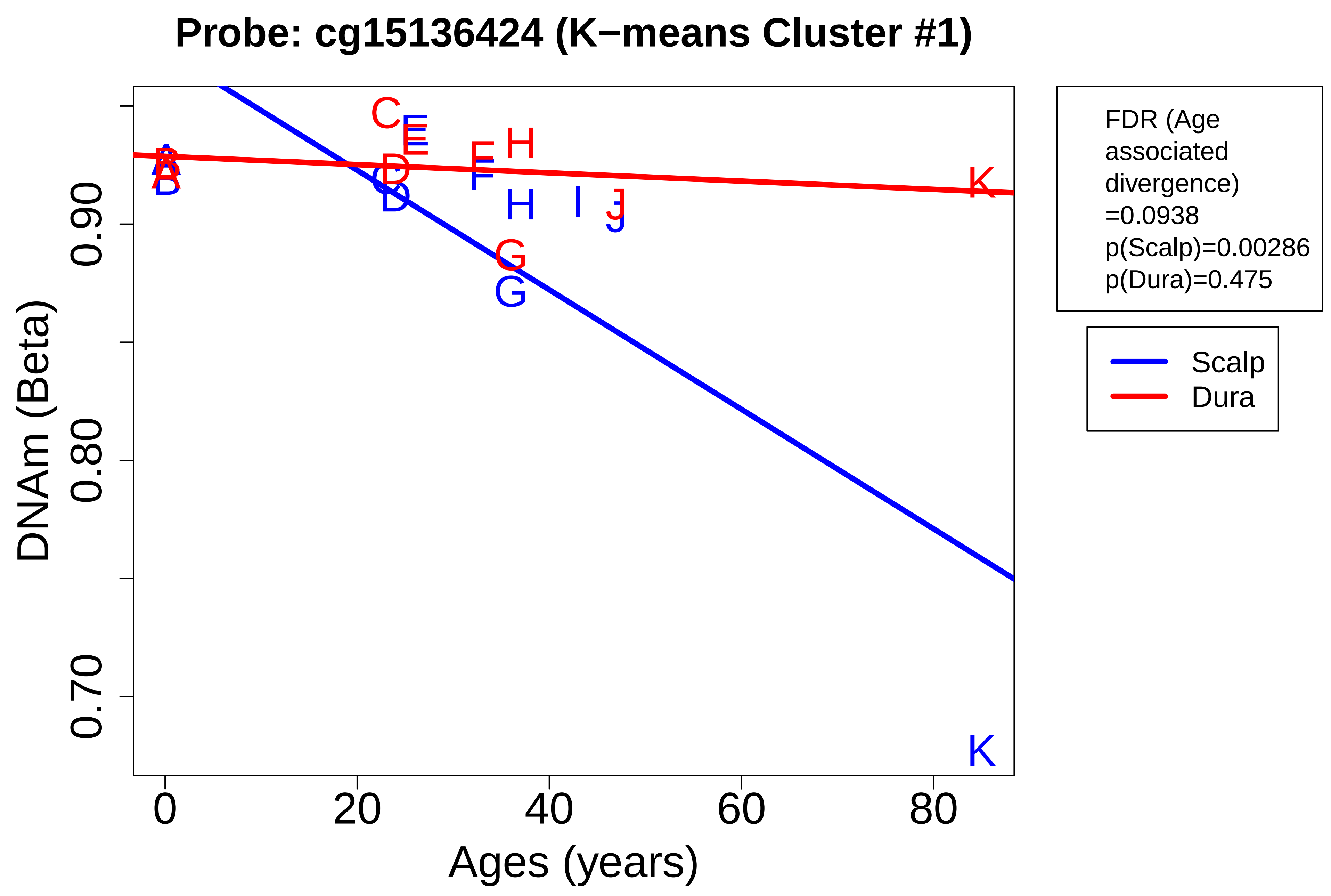

Probe: cg15136424 (K−means Cluster #1)
DNAm (Beta)
0.70
0.80
0.90
0
20
40
60
80
Ages (years)
FDR (Age associated divergence) =0.0938
p(Scalp)=0.00286
p(Dura)=0.475
Scalp
Dura

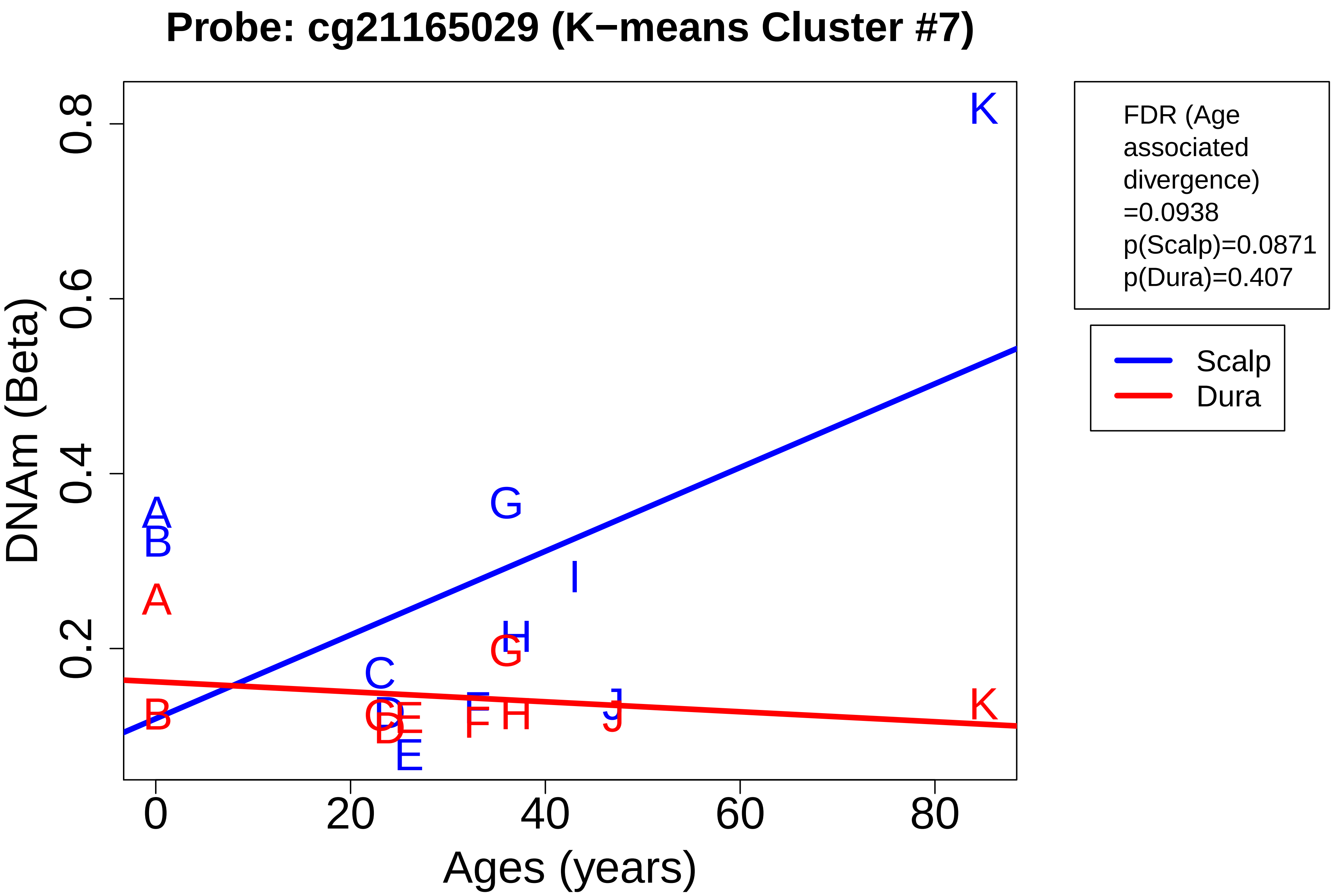

Probe: cg21165029 (K−means Cluster #7)
DNAm (Beta)
Ages (years)
0.2
0.4
0.6
0.8
0
20
40
60
80
FDR (Age associated divergence) =0.0938
p(Scalp)=0.0871
p(Dura)=0.407
Scalp
Dura

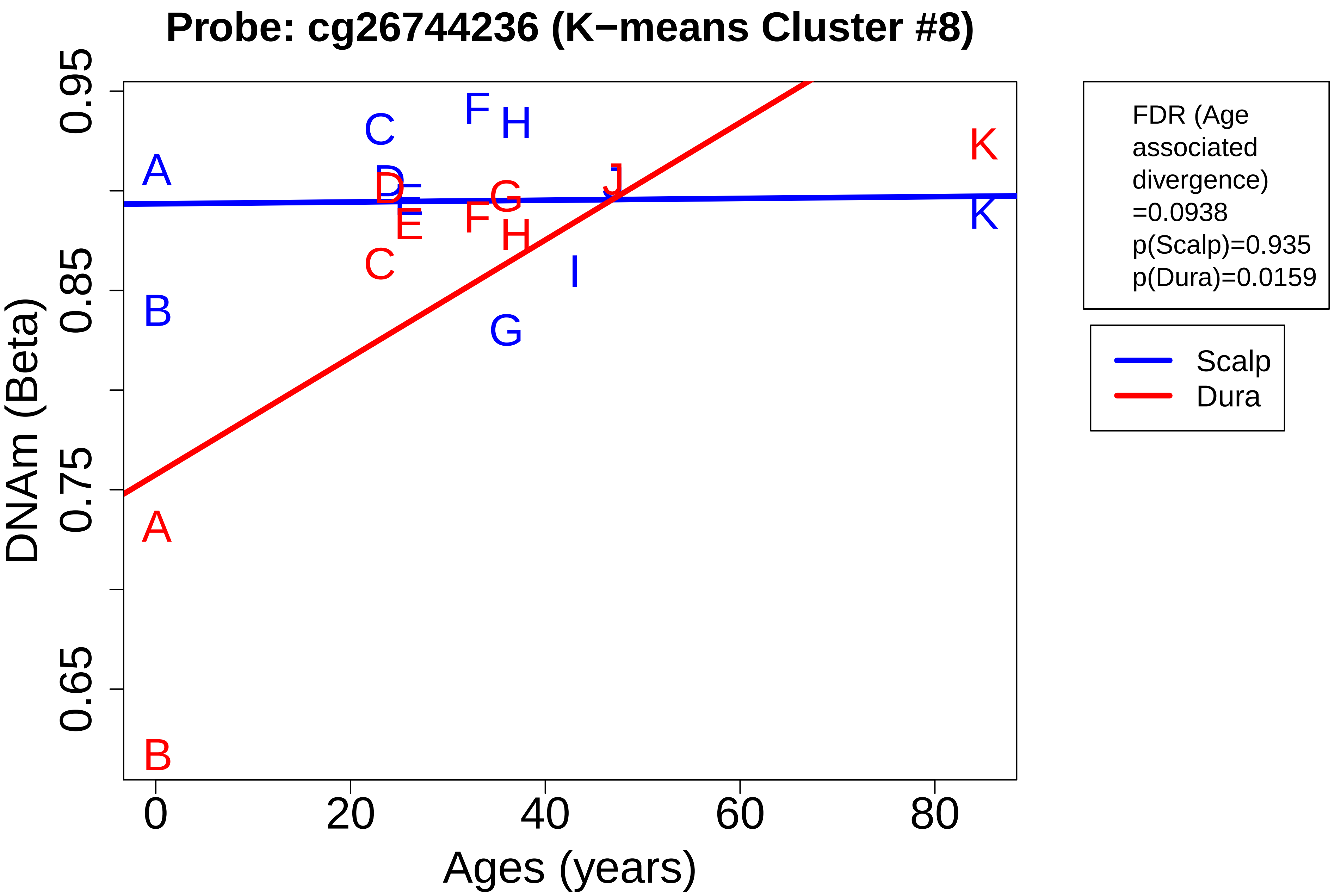

Probe: cg26744236 (K−means Cluster #8)
DNAm (Beta)
0.95
0.85
0.75
0.65
0
20
40
60
80
Ages (years)
FDR (Age associated divergence) =0.0938
p(Scalp)=0.935
p(Dura)=0.0159
Scalp
Dura

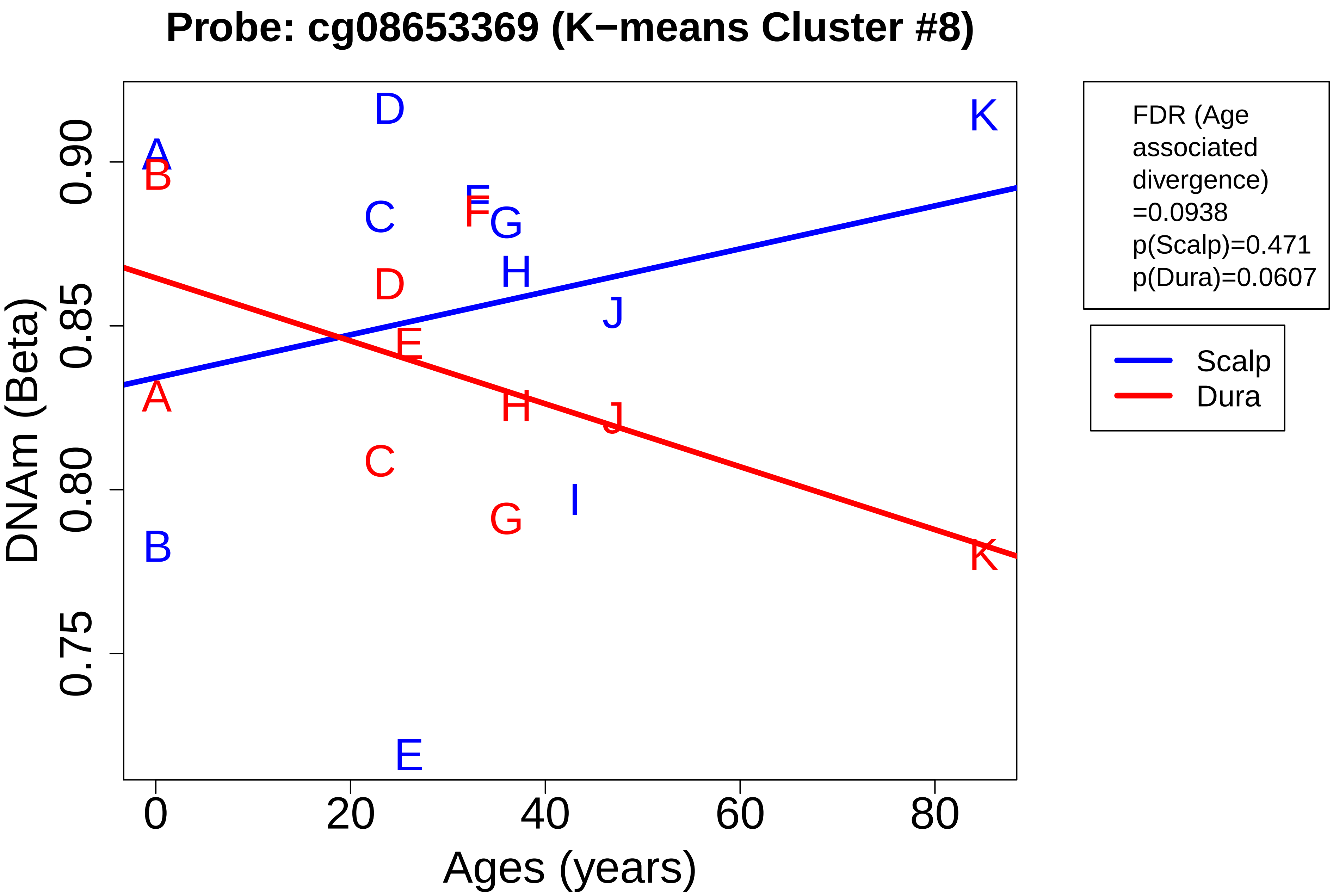
Probe: cg08653369 (K−means Cluster #8)
DNAm (Beta)
0.75
0.80
0.85
0.90
Ages (years)
0
20
40
60
80
A
B
C
D
E
F
G
H
I
J
K
A
B
C
D
E
F
G
H
J
K
FDR (Age associated divergence) =0.0938
p(Scalp)=0.471
p(Dura)=0.0607
Scalp
Dura

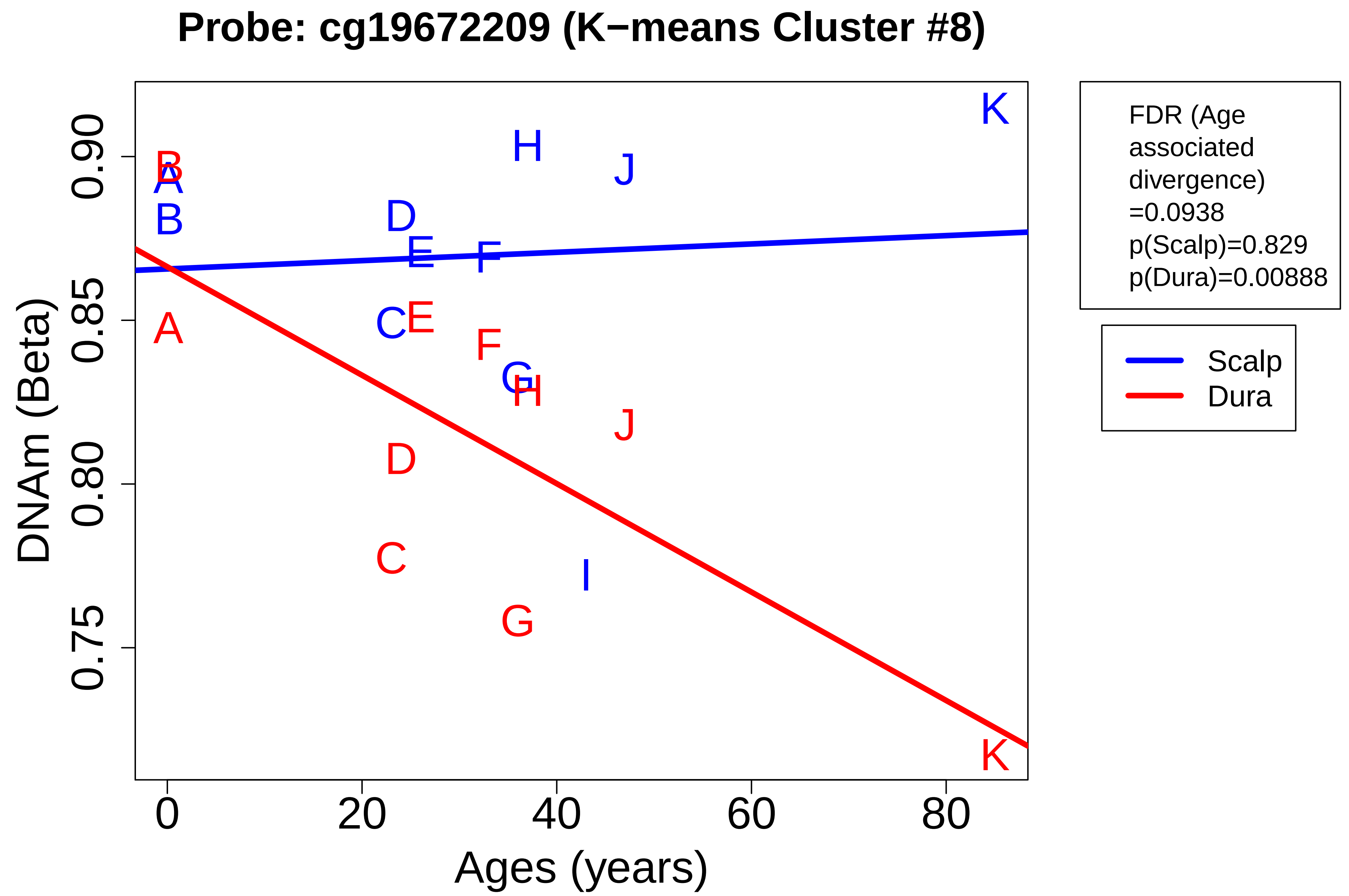

Probe: cg19672209 (K−means Cluster #8)
DNAm (Beta)
0.75
0.80
0.85
0.90
0
20
40
60
80
Ages (years)
FDR (Age associated divergence) =0.0938
p(Scalp)=0.829
p(Dura)=0.00888
Scalp
Dura

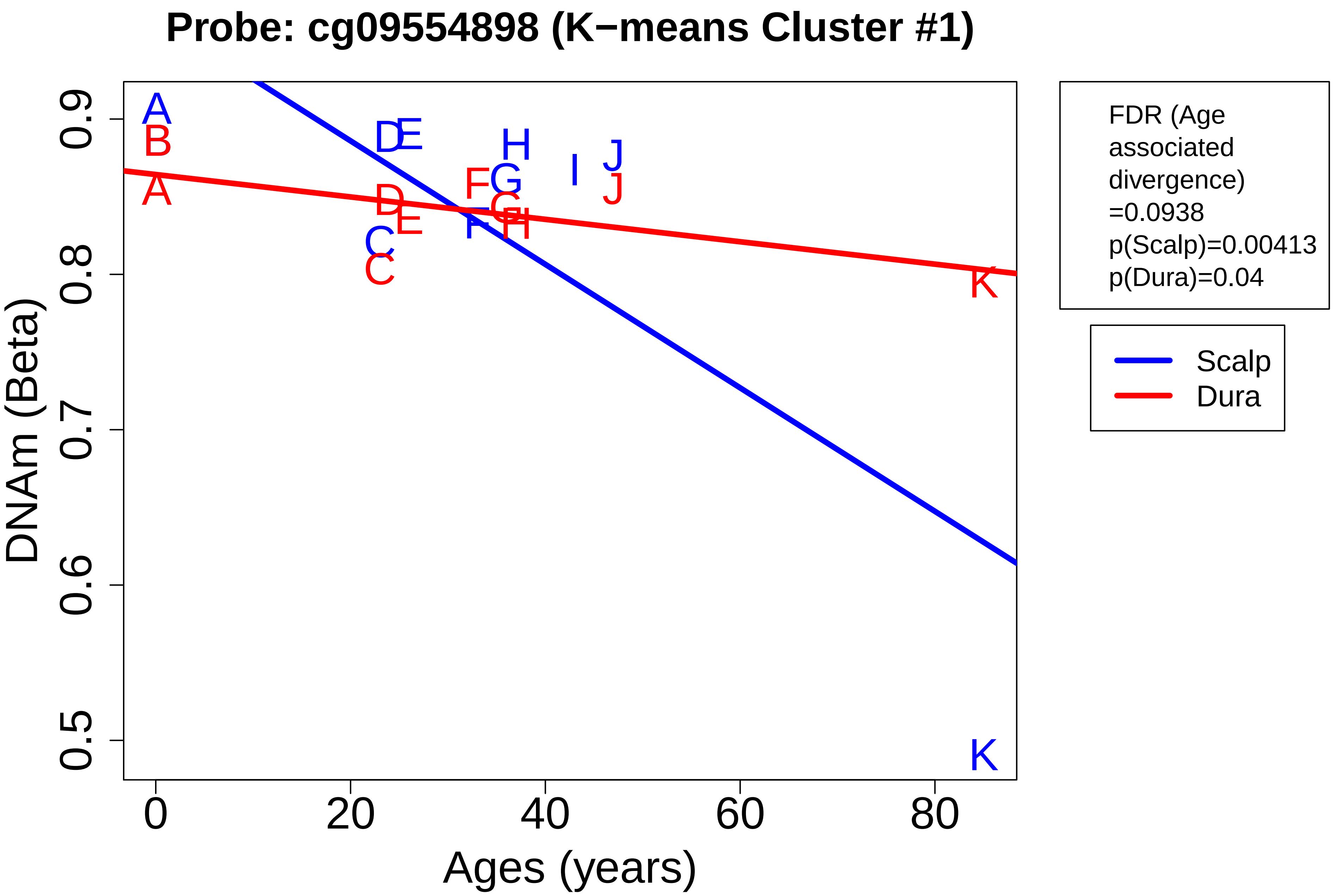

Probe: cg09554898 (K−means Cluster #1)
DNAm (Beta)
0.5
0.6
0.7
0.8
0.9
0
20
40
60
80
Ages (years)
A
B
A
C
C
D
E
D
E
F
G
F
G
H
H
I
J
J
K
K
FDR (Age associated divergence) =0.0938
p(Scalp)=0.00413
p(Dura)=0.04
Scalp
Dura

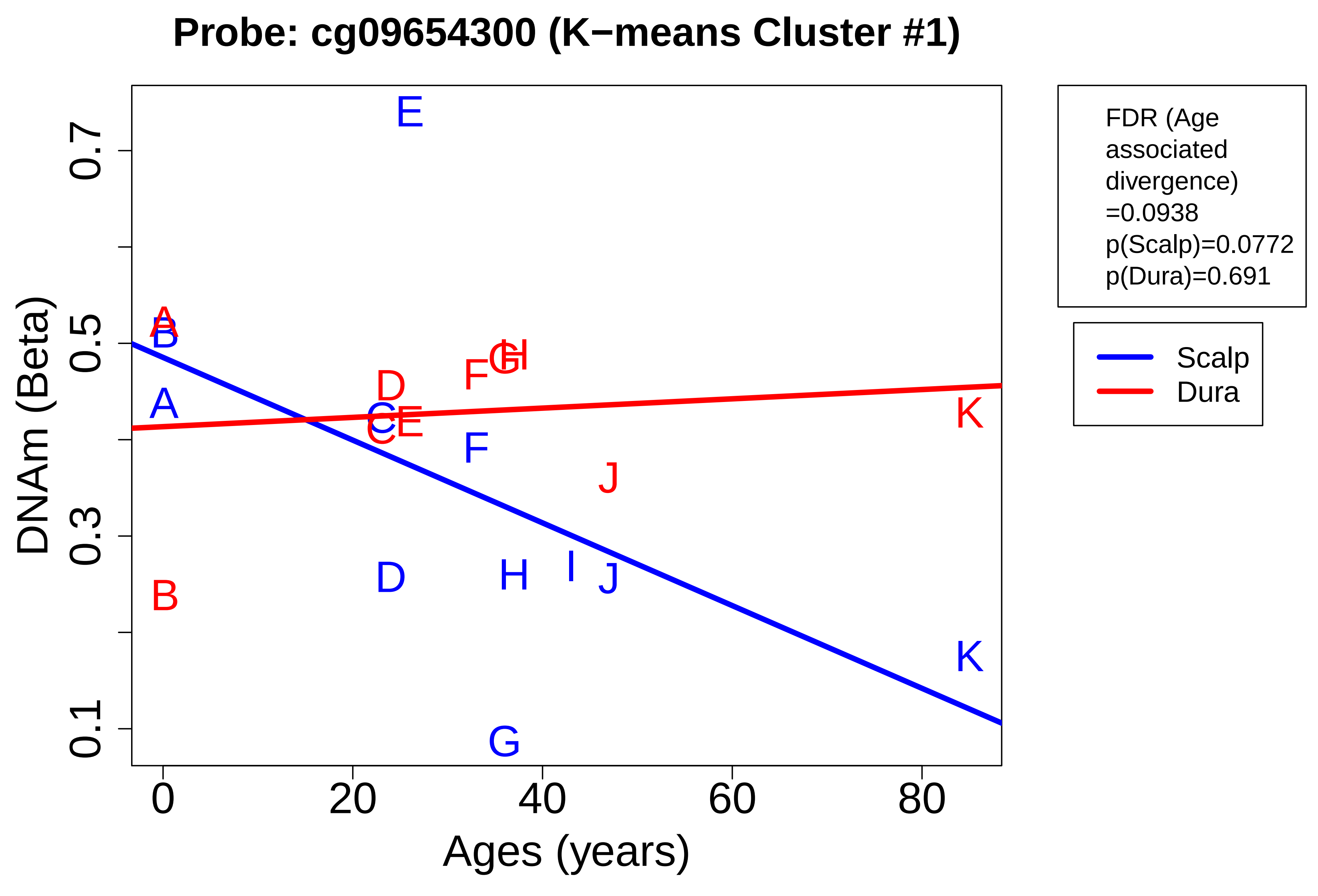
Probe: cg09654300 (K−means Cluster #1)
DNAm (Beta)
0.1
0.3
0.5
0.7
Ages (years)
0
20
40
60
80
FDR (Age associated divergence) =0.0938
p(Scalp)=0.0772
p(Dura)=0.691
Scalp
Dura
A
B
C
D
E
F
G
H
I
J
K

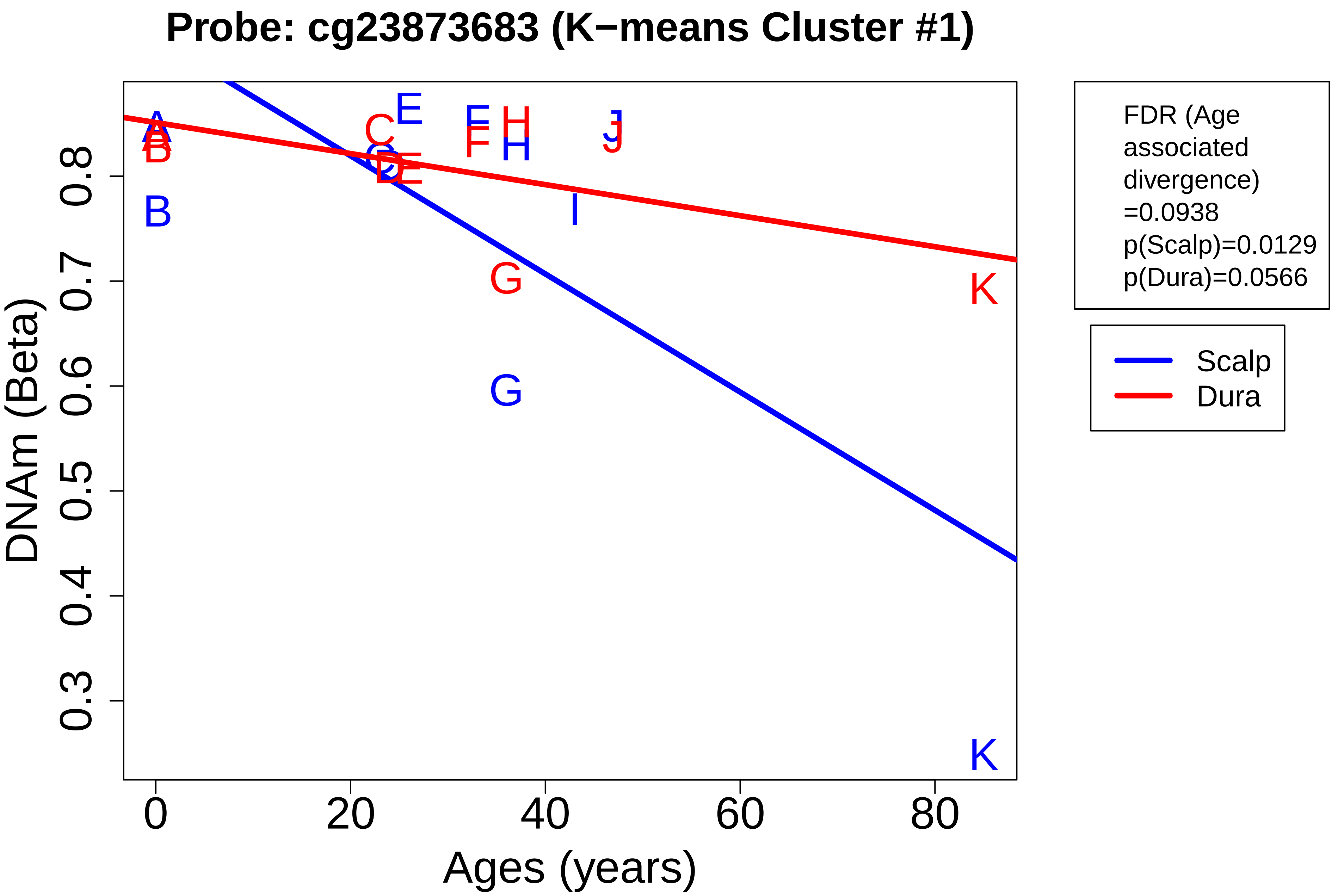
Probe: cg23873683 (K−means Cluster #1)
DNAm (Beta)
0.3
0.4
0.5
0.6
0.7
0.8
0
20
40
60
80
Ages (years)
FDR (Age associated divergence) =0.0938
p(Scalp)=0.0129
p(Dura)=0.0566
Scalp
Dura
A
B
C
D
E
F
G
H
I
J
K

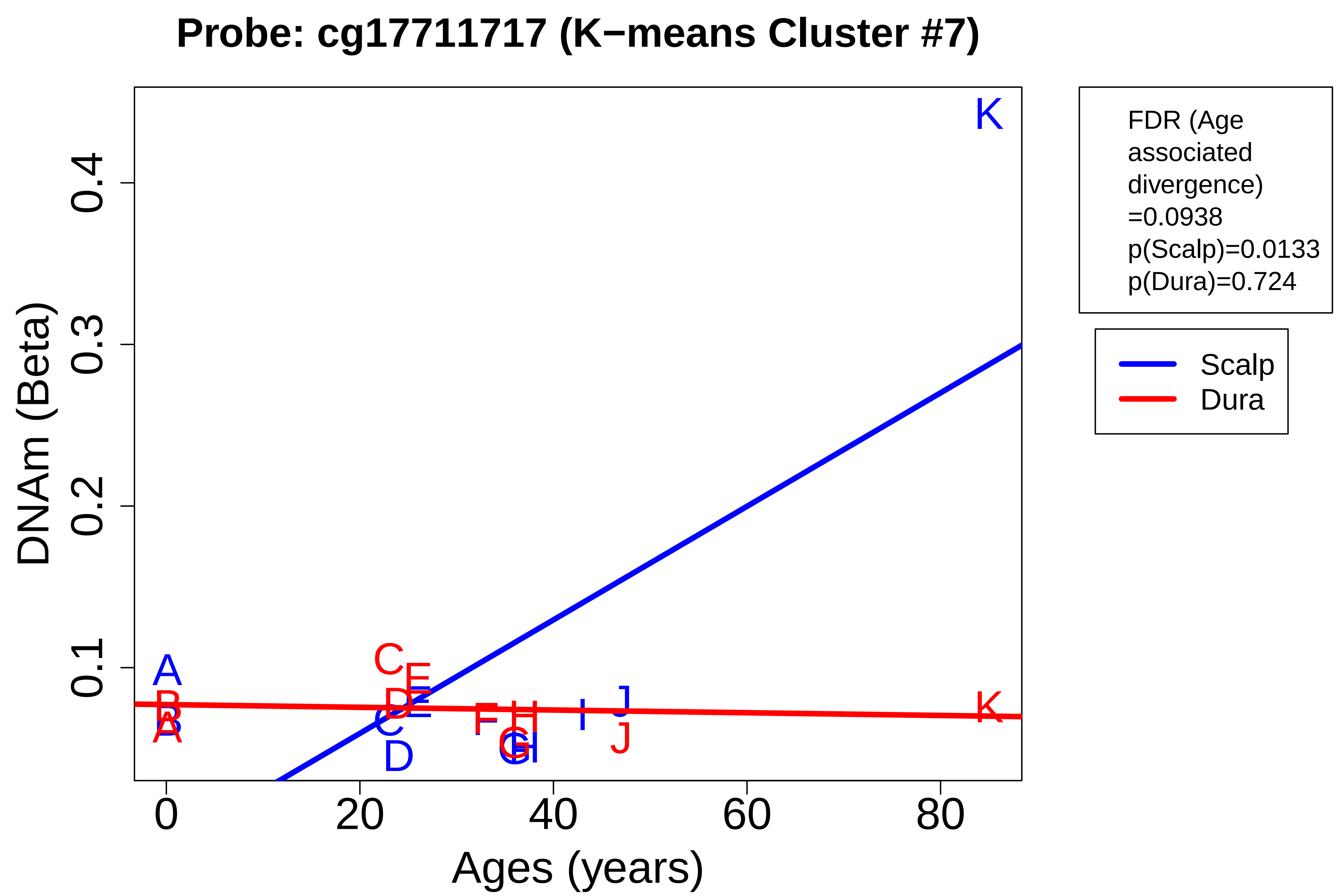

Probe: cg17711717 (K−means Cluster #7)
DNAm (Beta)
0.1
0.2
0.3
0.4
0
20
40
60
80
Ages (years)
FDR (Age associated divergence) =0.0938
p(Scalp)=0.0133
p(Dura)=0.724
Scalp
Dura
A
B
C
D
E
F
G
H
I
J
K

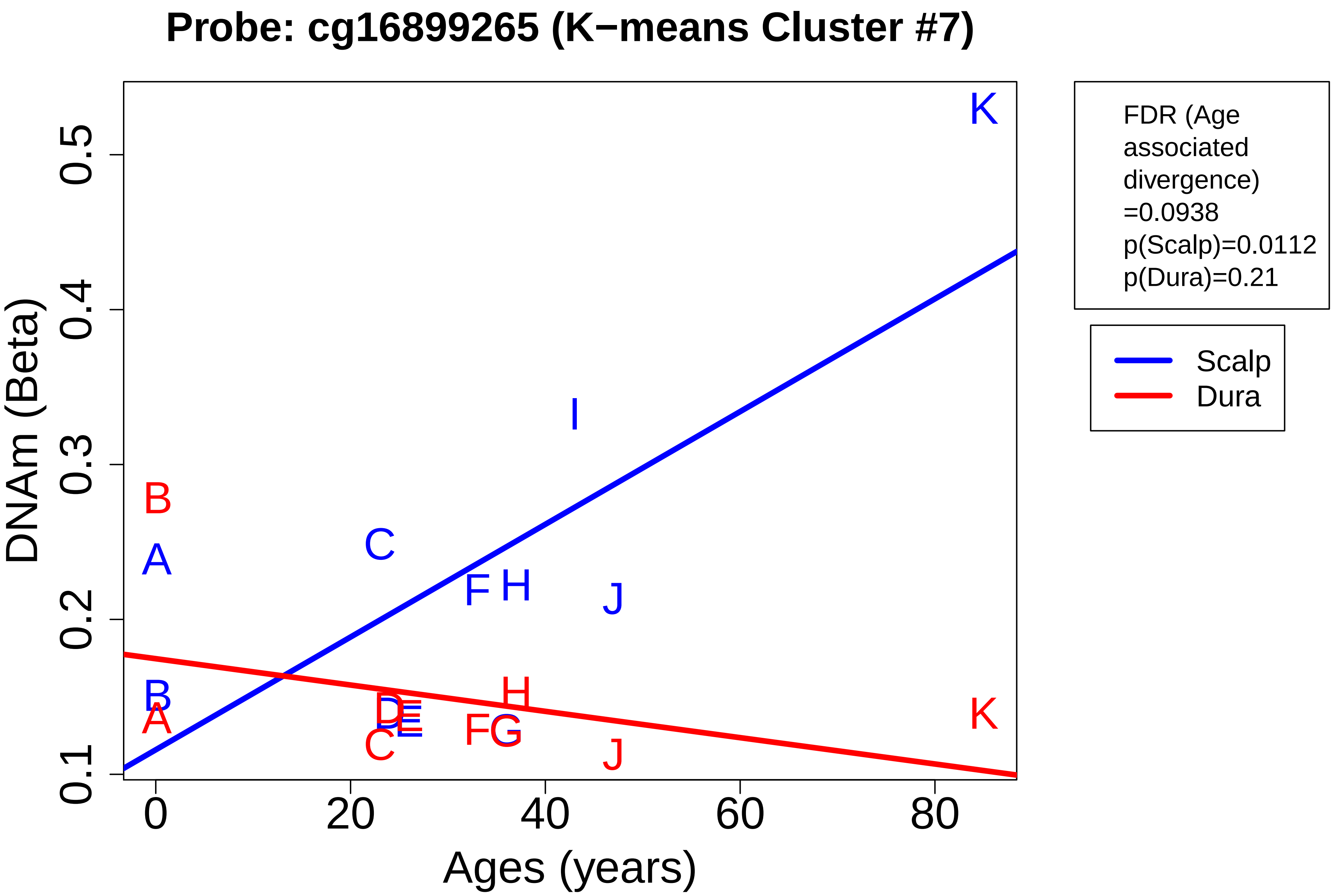
Probe: cg16899265 (K−means Cluster #7)
DNAm (Beta)
0.1
0.2
0.3
0.4
0.5
Ages (years)
0
20
40
60
80
FDR (Age associated divergence) =0.0938
p(Scalp)=0.0112
p(Dura)=0.21
Scalp
Dura
A
B
C
D
E
F
G
H
I
J
K

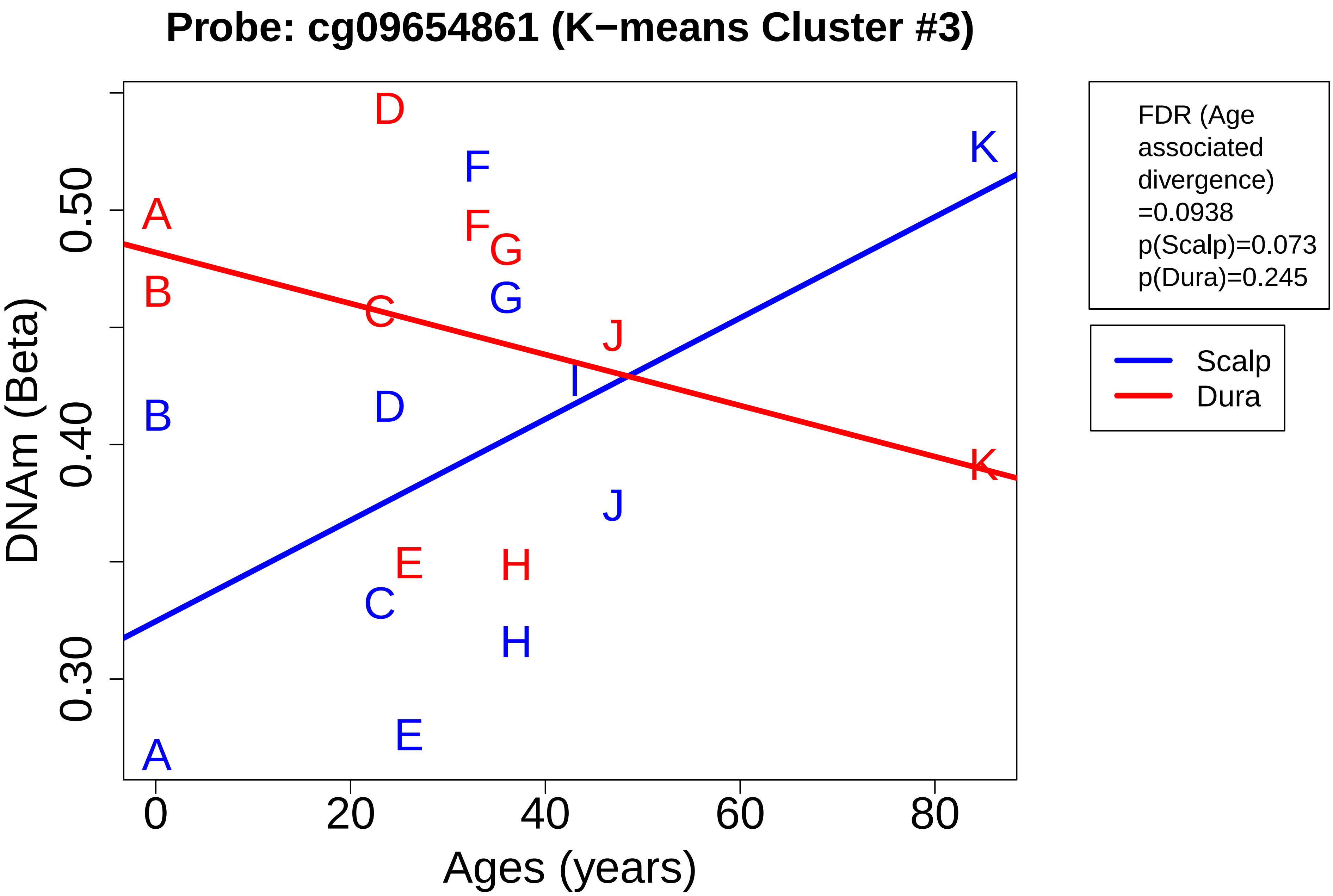

Probe: cg09654861 (K−means Cluster #3)
DNAm (Beta)
Ages (years)
0.30
0.40
0.50
0
20
40
60
80
FDR (Age associated divergence) =0.0938
p(Scalp)=0.073
p(Dura)=0.245
Scalp
Dura

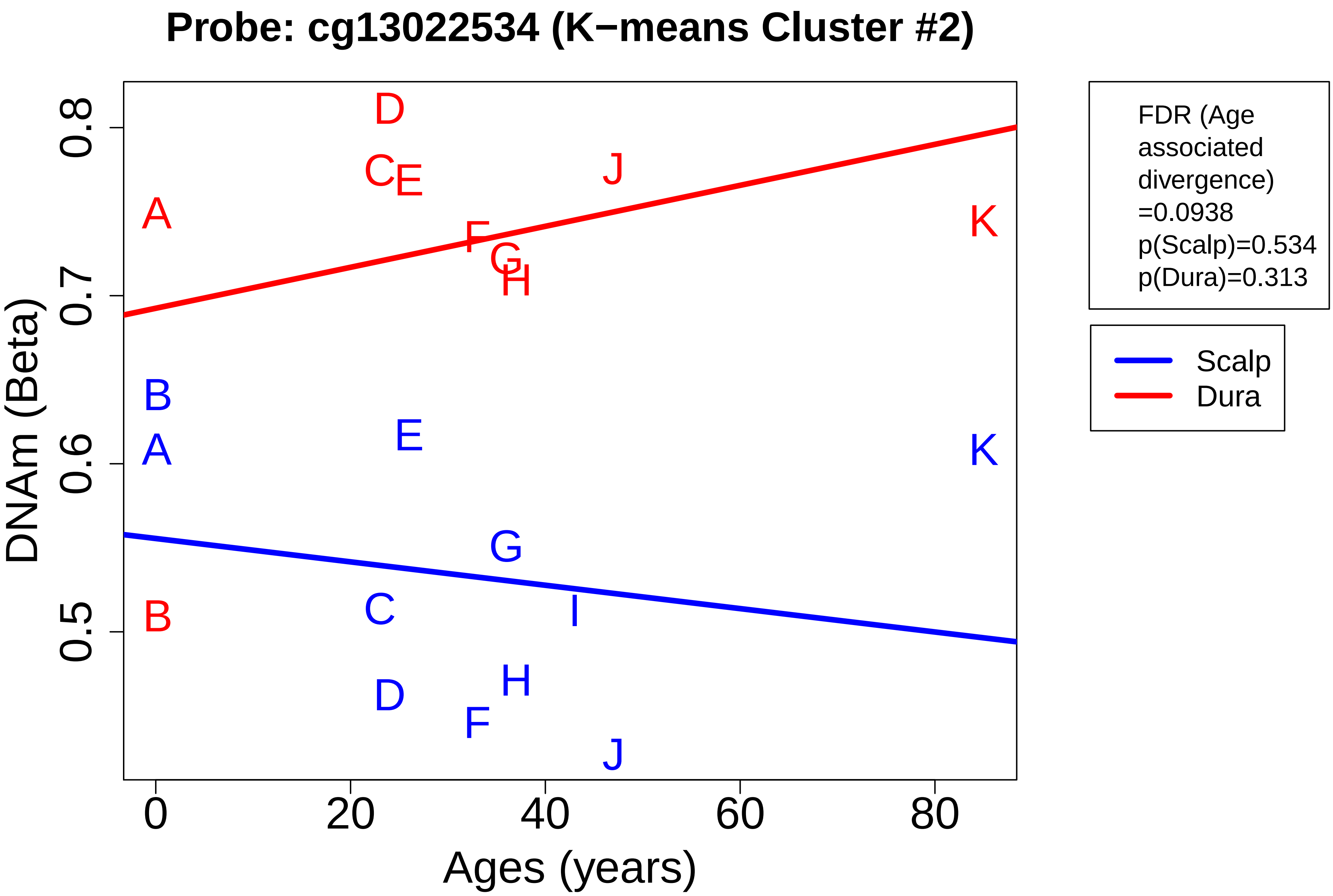

Probe: cg13022534 (K−means Cluster #2)
DNAm (Beta)
0.5
0.6
0.7
0.8
Ages (years)
0
20
40
60
80
A
B
C
D
E
F
G
H
I
J
K
FDR (Age associated divergence) =0.0938
p(Scalp)=0.534
p(Dura)=0.313
Scalp
Dura

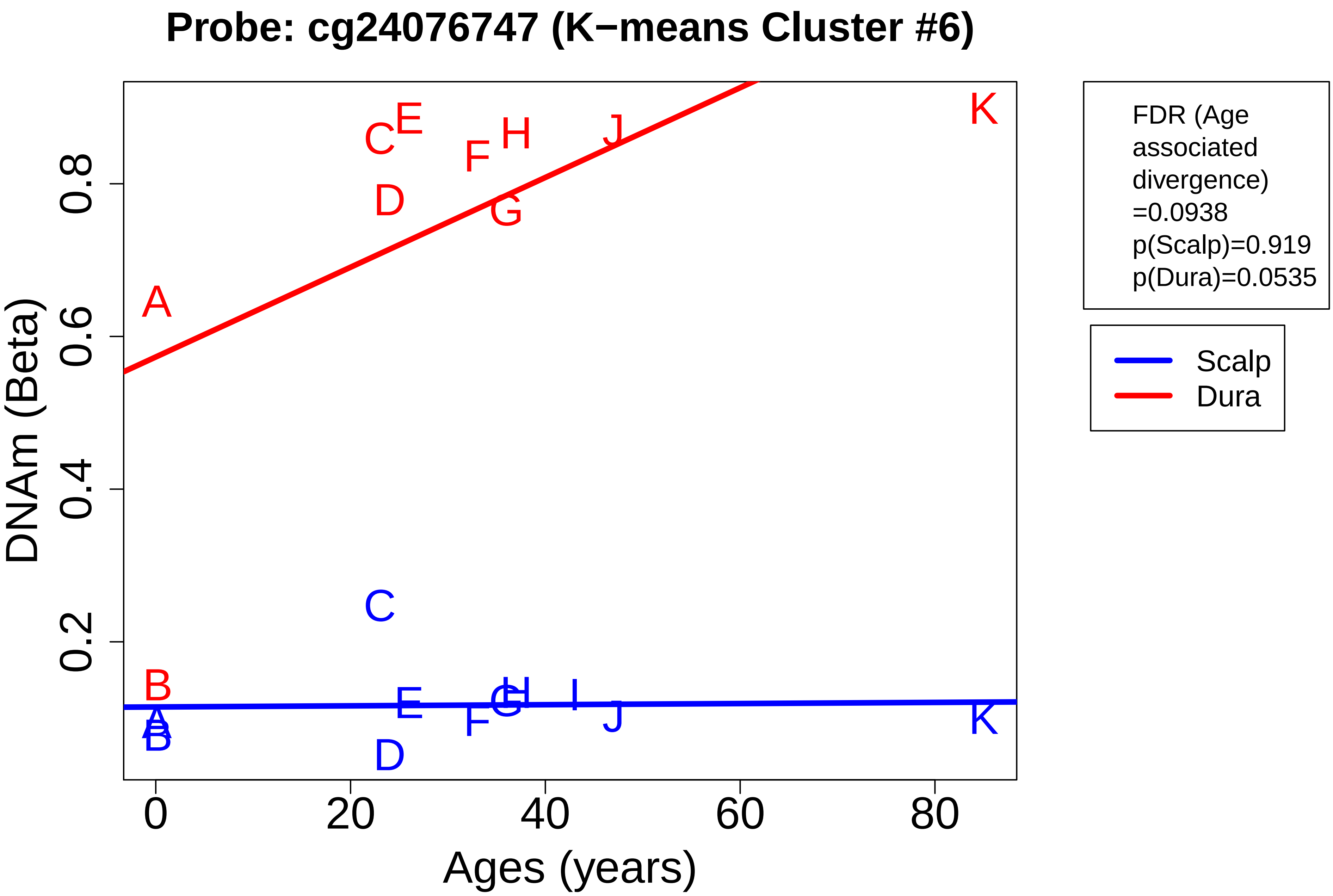
Probe: cg24076747 (K−means Cluster #6)
DNAm (Beta)
0.2
0.4
0.6
0.8
0
20
40
60
80
Ages (years)
FDR (Age associated divergence) =0.0938
p(Scalp)=0.919
p(Dura)=0.0535
Scalp
Dura

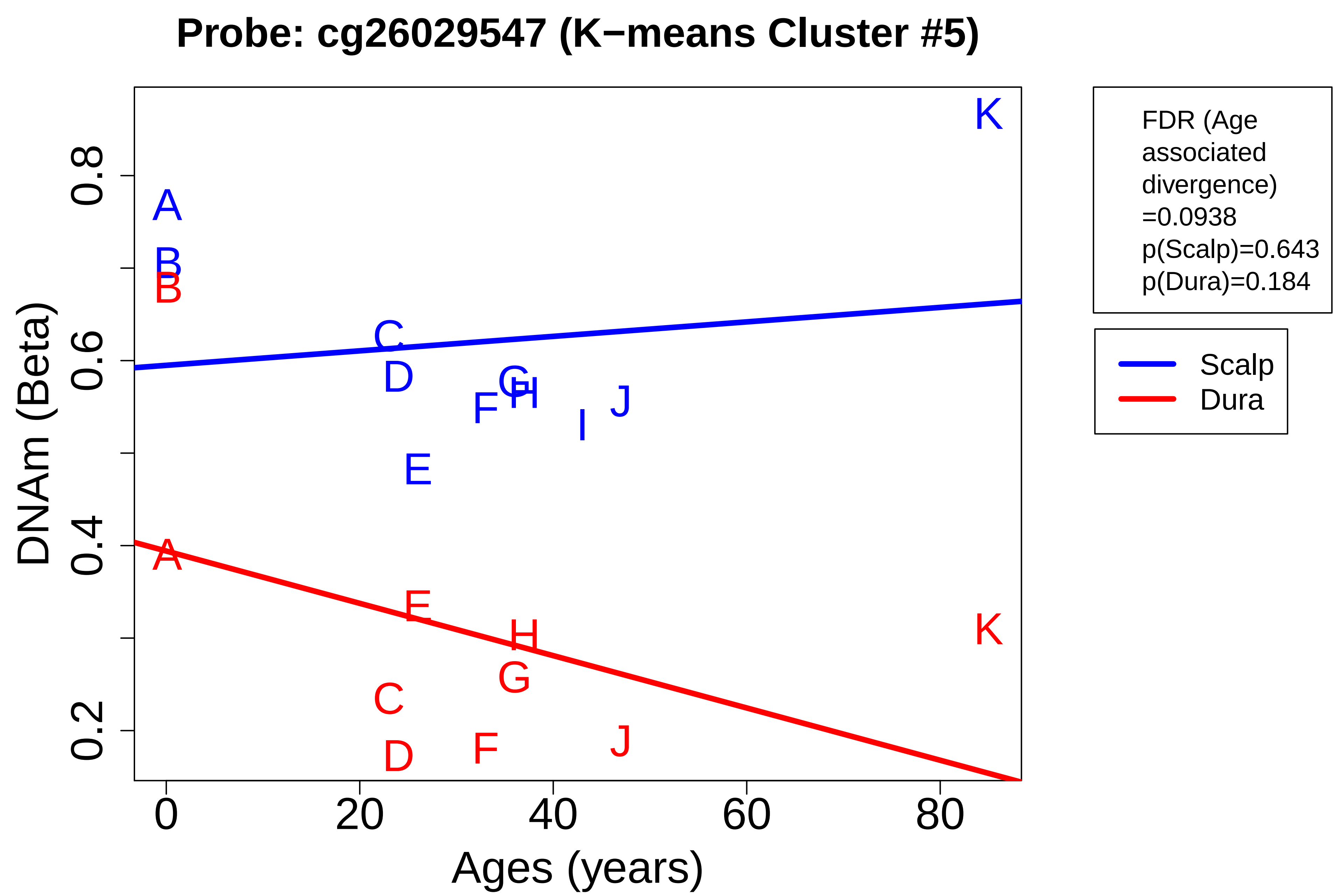

Probe: cg26029547 (K−means Cluster #5)
DNAm (Beta)
0.2
0.4
0.6
0.8
0
20
40
60
80
Ages (years)
A
B
C
D
E
F
G
H
I
J
K
A
B
C
D
E
F
G
H
J
K
FDR (Age associated divergence) =0.0938
p(Scalp)=0.643
p(Dura)=0.184
Scalp
Dura

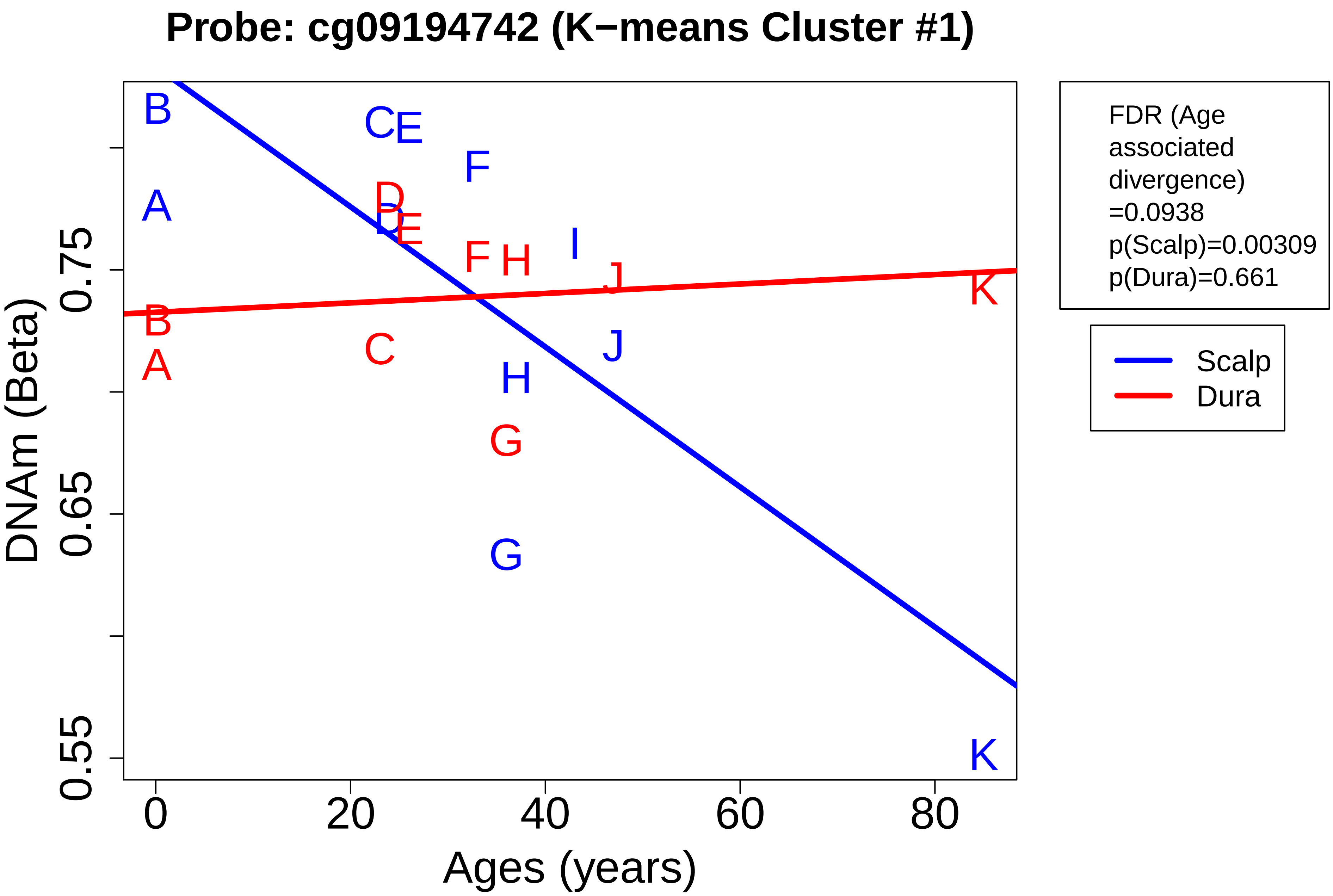

Probe: cg09194742 (K−means Cluster #1)
DNAm (Beta)
0.55
0.65
0.75
Ages (years)
0
20
40
60
80
FDR (Age associated divergence) =0.0938
p(Scalp)=0.00309
p(Dura)=0.661
Scalp
Dura

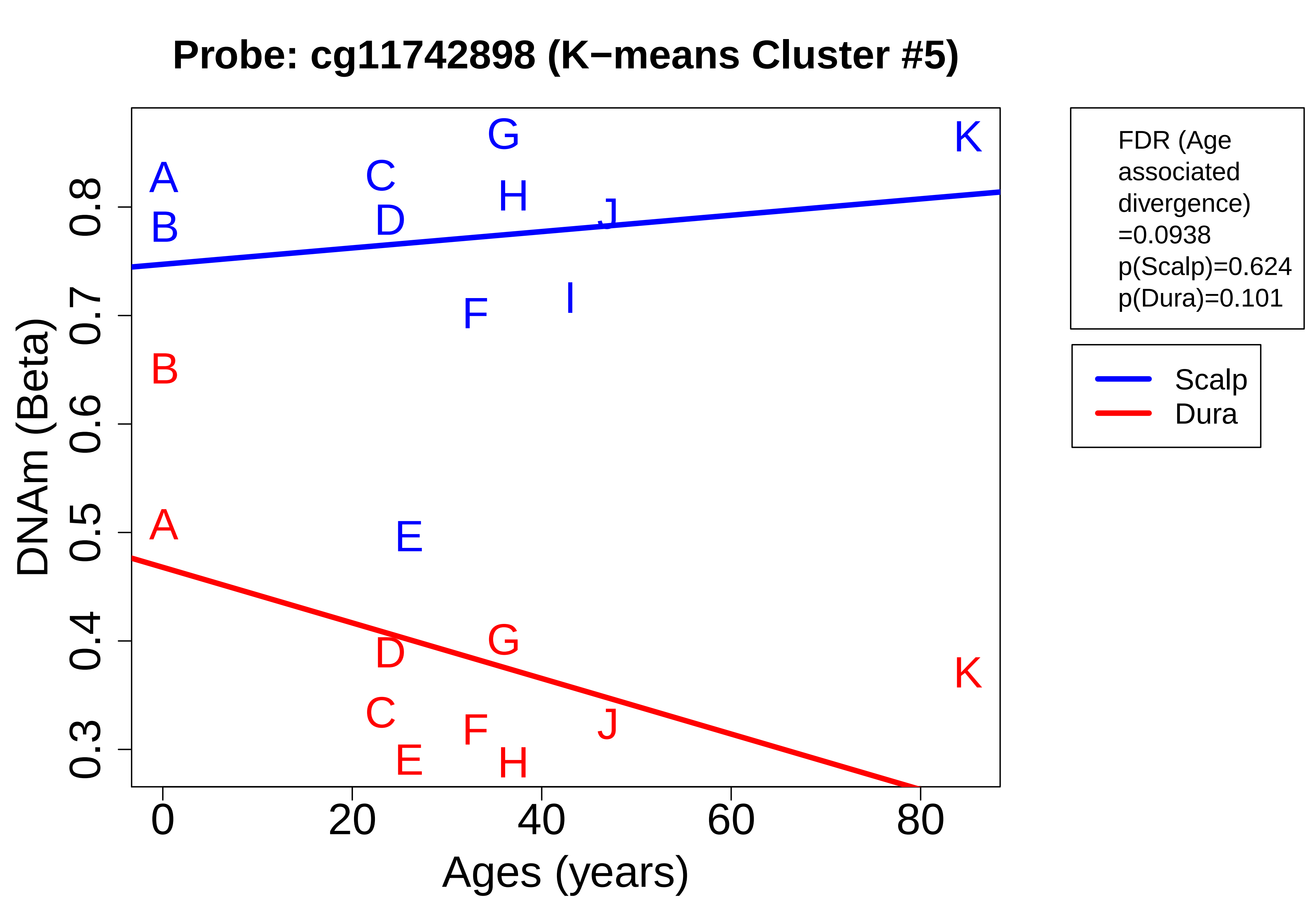
Probe: cg11742898 (K−means Cluster #5)
DNAm (Beta)
Ages (years)
0.3
0.4
0.5
0.6
0.7
0.8
0
20
40
60
80
A
B
C
D
E
F
G
H
I
J
K
FDR (Age associated divergence) =0.0938
p(Scalp)=0.624
p(Dura)=0.101
Scalp
Dura

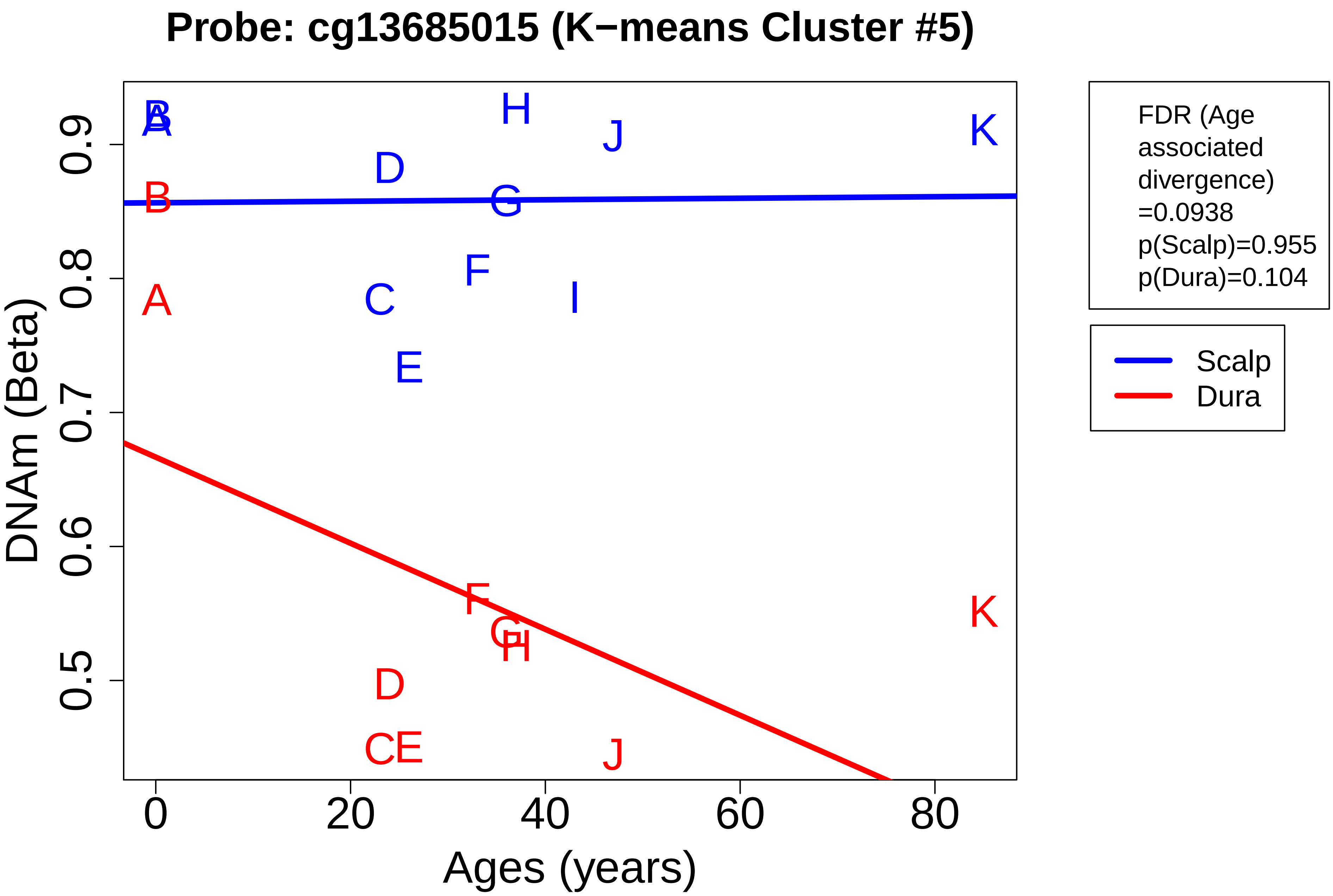

Probe: cg13685015 (K−means Cluster #5)
DNAm (Beta)
0.5
0.6
0.7
0.8
0.9
Ages (years)
0
20
40
60
80
A B C D E F G H I J K
FDR (Age associated divergence) =0.0938
p(Scalp)=0.955
p(Dura)=0.104
Scalp
Dura

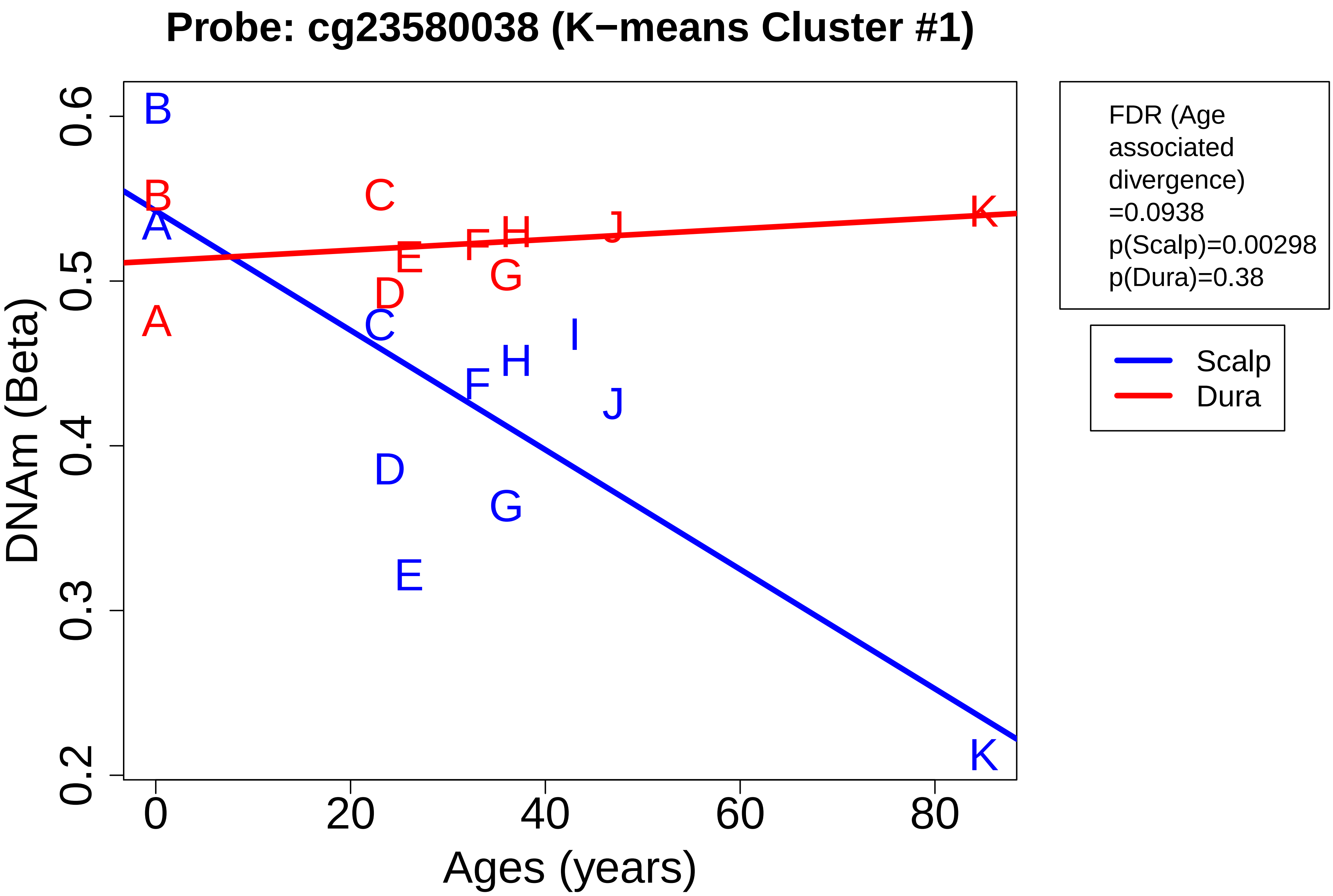

Probe: cg23580038 (K−means Cluster #1)
DNAm (Beta)
0.2
0.3
0.4
0.5
0.6
0
20
40
60
80
Ages (years)
A
B
C
D
E
F
G
H
I
J
K
FDR (Age associated divergence) =0.0938
p(Scalp)=0.00298
p(Dura)=0.38
Scalp
Dura

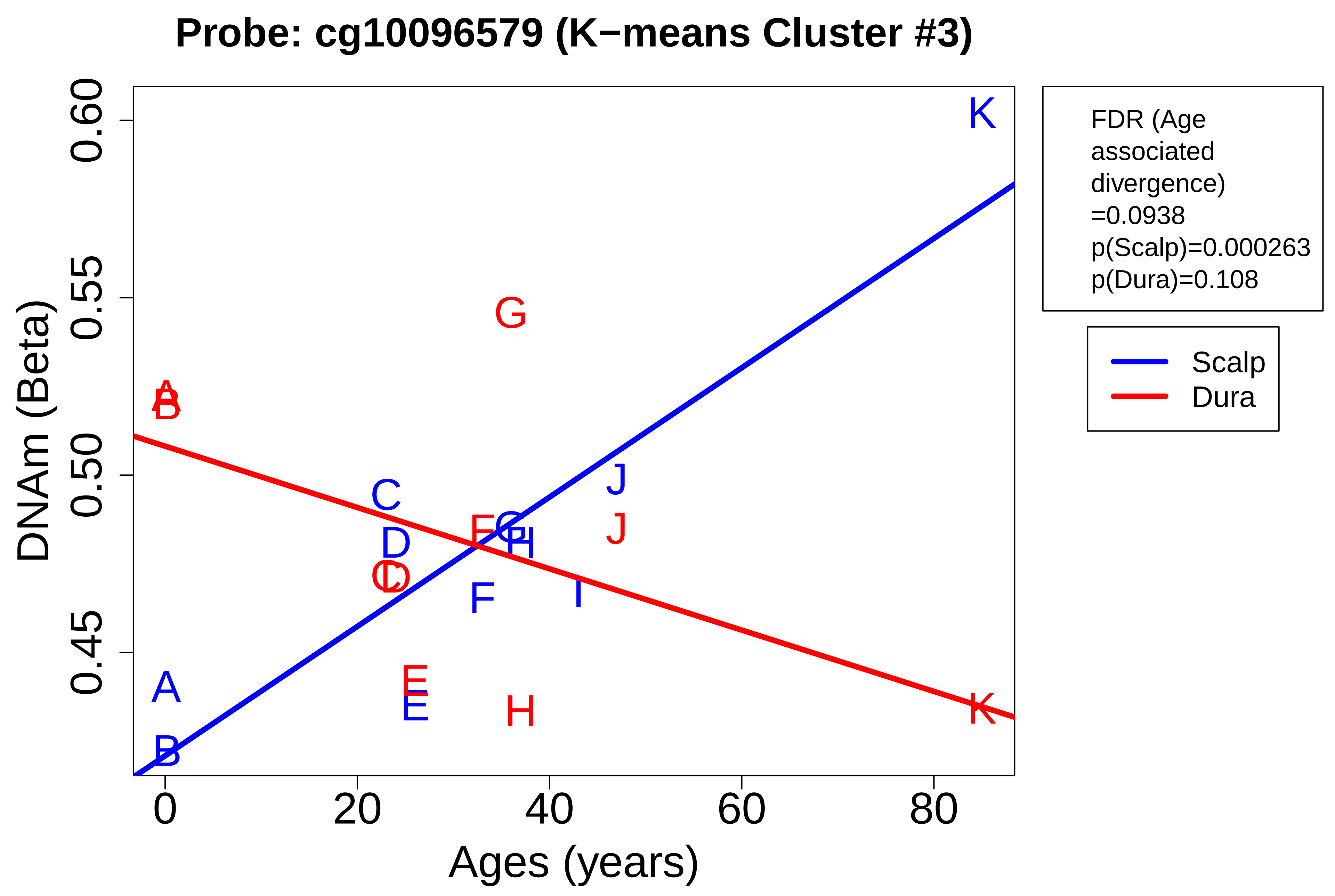

Probe: cg10096579 (K−means Cluster #3)
DNAm (Beta)
0.45
0.50
0.55
0.60
Ages (years)
0
20
40
60
80
FDR (Age associated divergence) =0.0938
p(Scalp)=0.000263
p(Dura)=0.108
Scalp
Dura

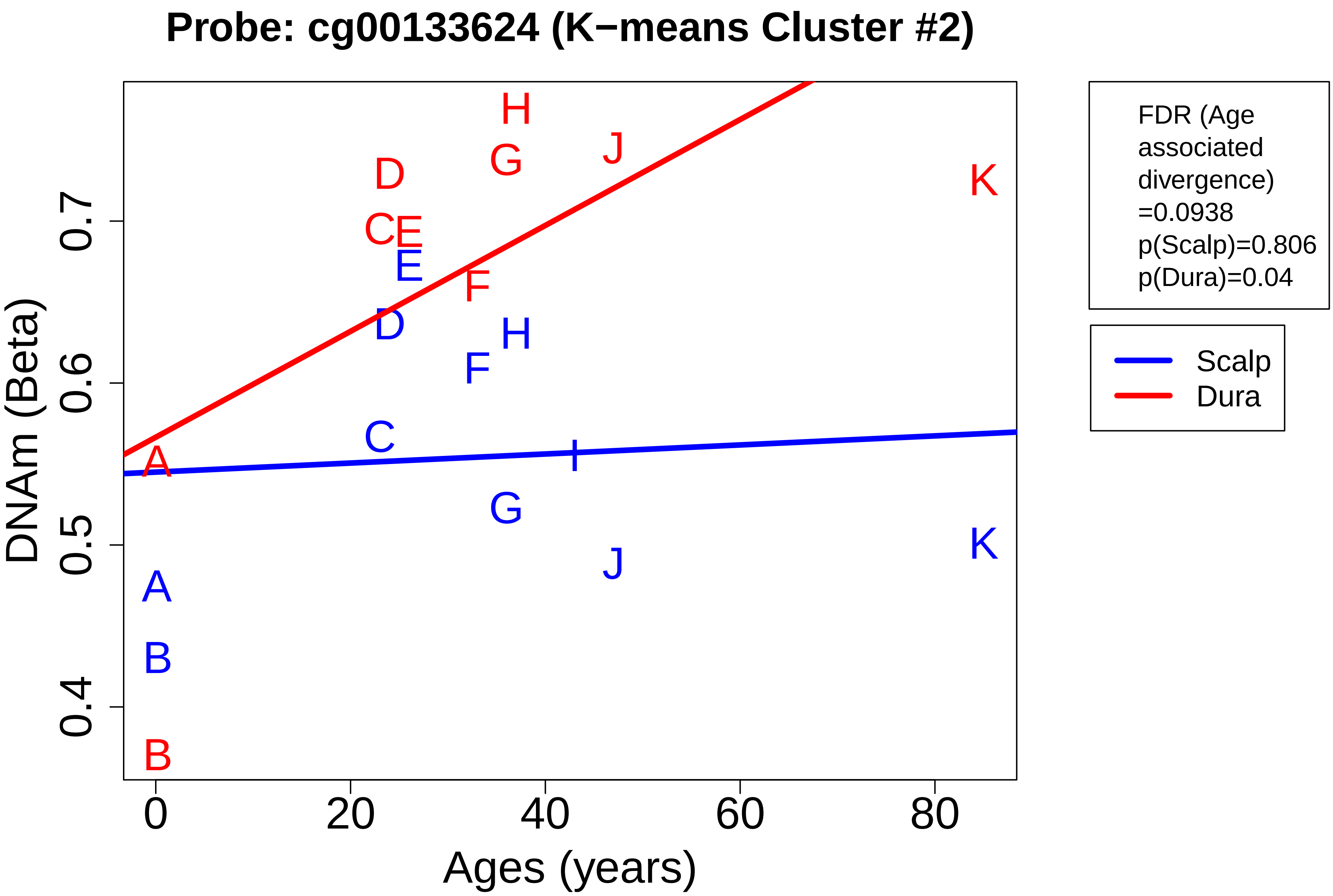
Probe: cg00133624 (K−means Cluster #2)
FDR (Age associated divergence) =0.0938
p(Scalp)=0.806
p(Dura)=0.04
Scalp
Dura
DNAm (Beta)
0.4
0.5
0.6
0.7
Ages (years)
0
20
40
60
80
A
B
C
D
E
F
G
H
I
J
K

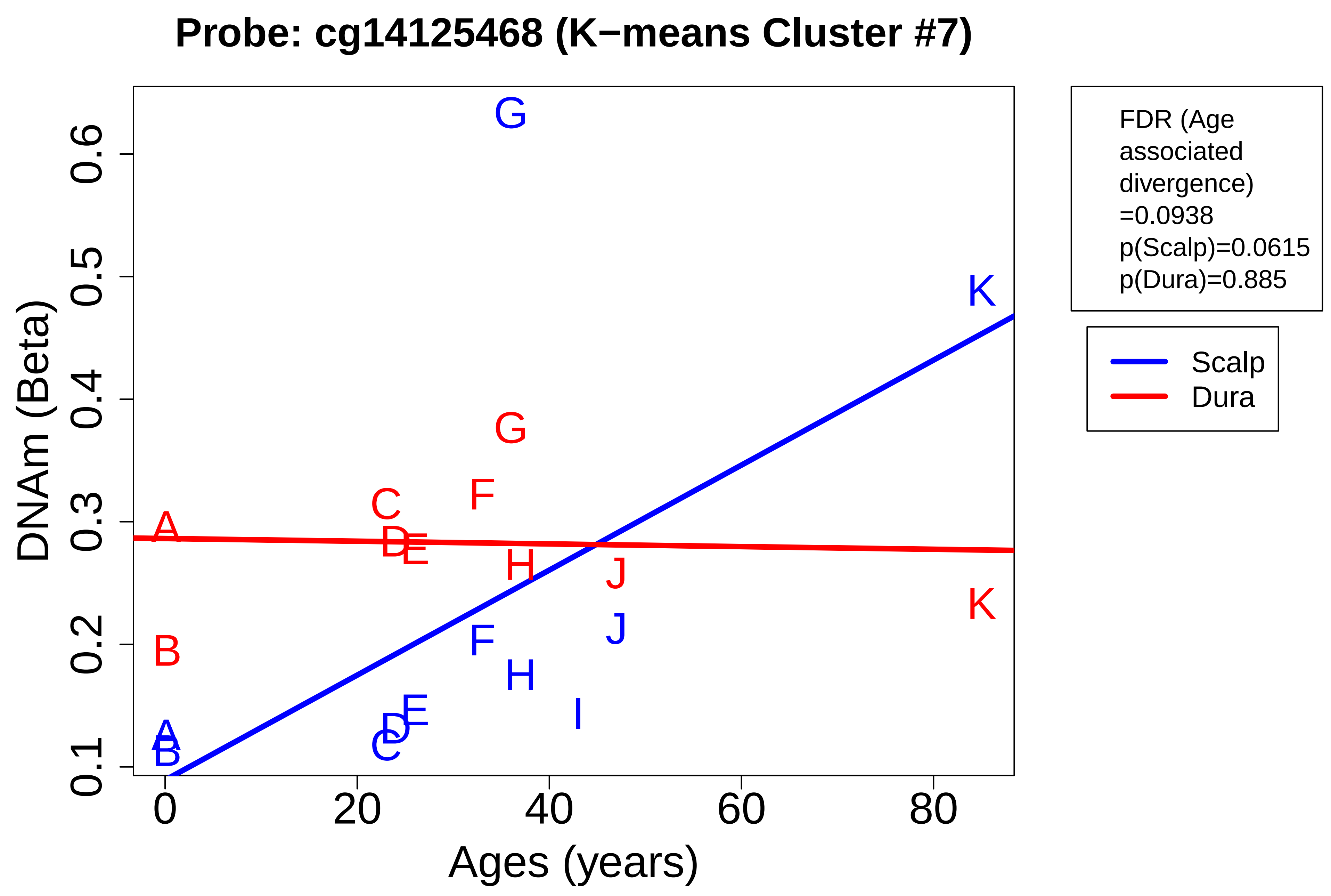

Probe: cg14125468 (K−means Cluster #7)
FDR (Age associated divergence) =0.0938
p(Scalp)=0.0615
p(Dura)=0.885
Scalp
Dura
DNAm (Beta)
0.1
0.2
0.3
0.4
0.5
0.6
0
20
40
60
80
Ages (years)

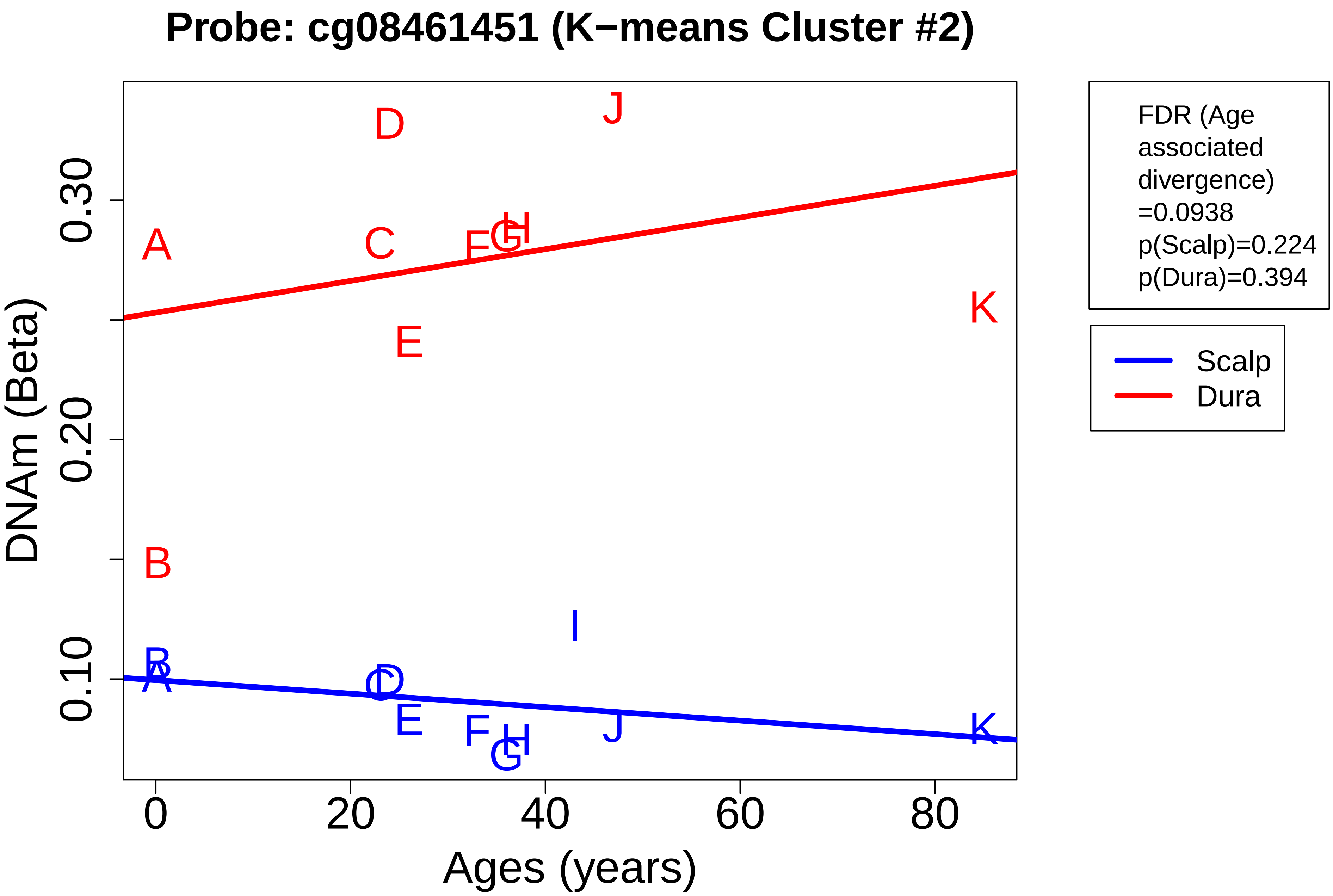

Probe: cg08461451 (K−means Cluster #2)
DNAm (Beta)
0.10
0.20
0.30
Ages (years)
0
20
40
60
80
FDR (Age associated divergence) =0.0938
p(Scalp)=0.224
p(Dura)=0.394
Scalp
Dura

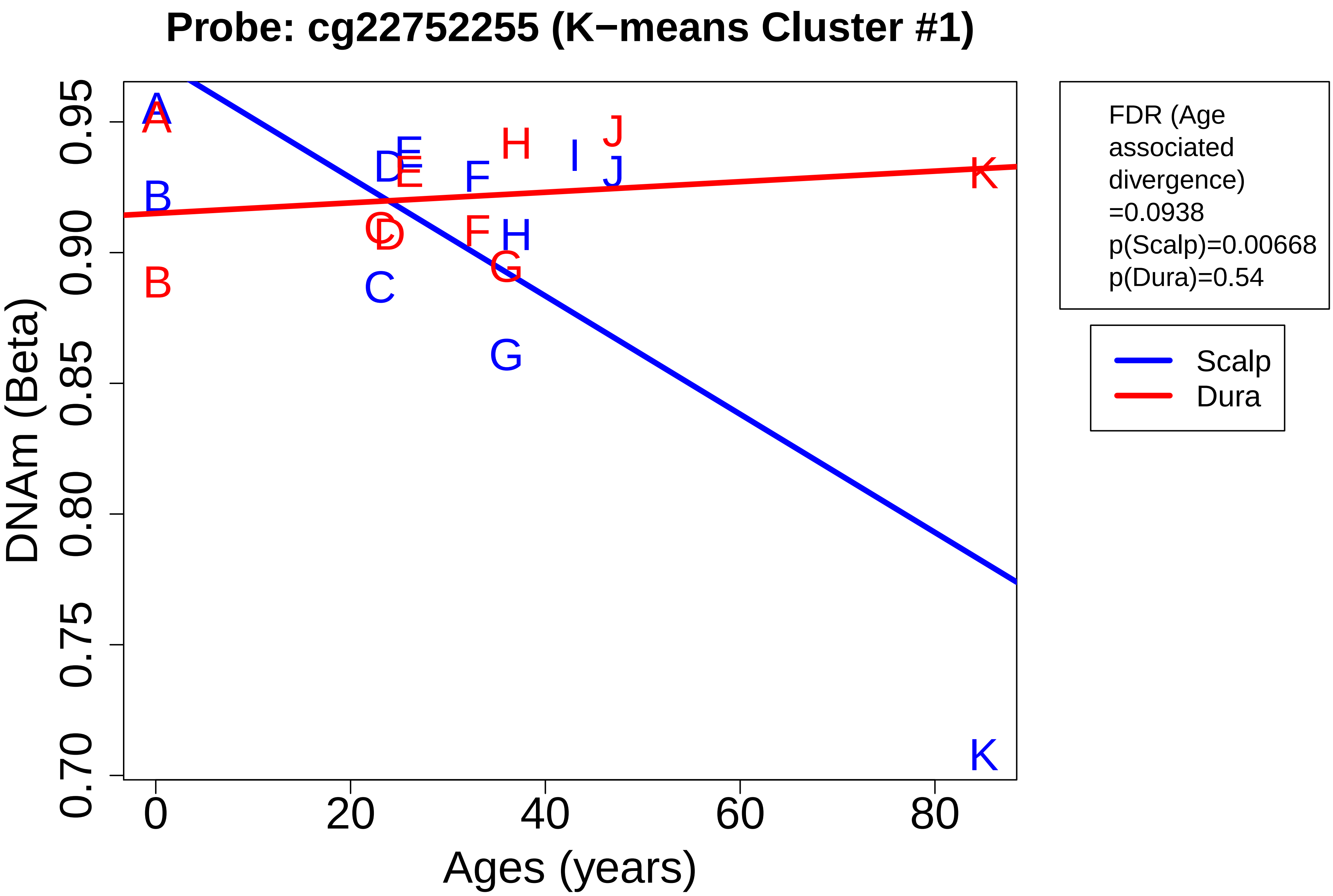

Probe: cg22752255 (K−means Cluster #1)
DNAm (Beta)
0.70
0.75
0.80
0.85
0.90
0.95
0
20
40
60
80
Ages (years)
A
B
C
D
E
F
G
H
I
J
K
FDR (Age associated divergence) =0.0938
p(Scalp)=0.00668
p(Dura)=0.54
Scalp
Dura

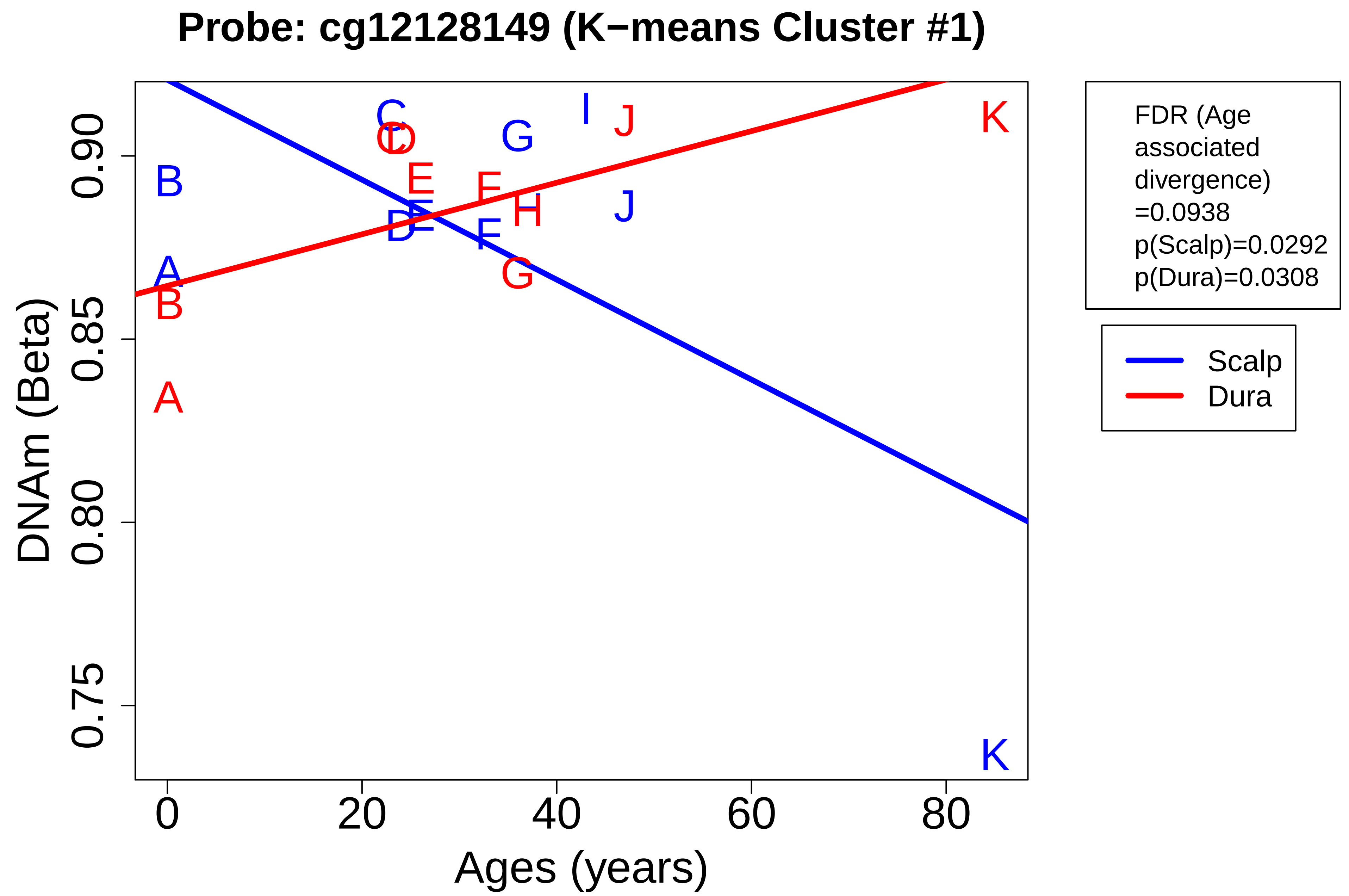

Probe: cg12128149 (K−means Cluster #1)
DNAm (Beta)
0.75
0.80
0.85
0.90
Ages (years)
0
20
40
60
80
FDR (Age associated divergence) =0.0938
p(Scalp)=0.0292
p(Dura)=0.0308
Scalp
Dura

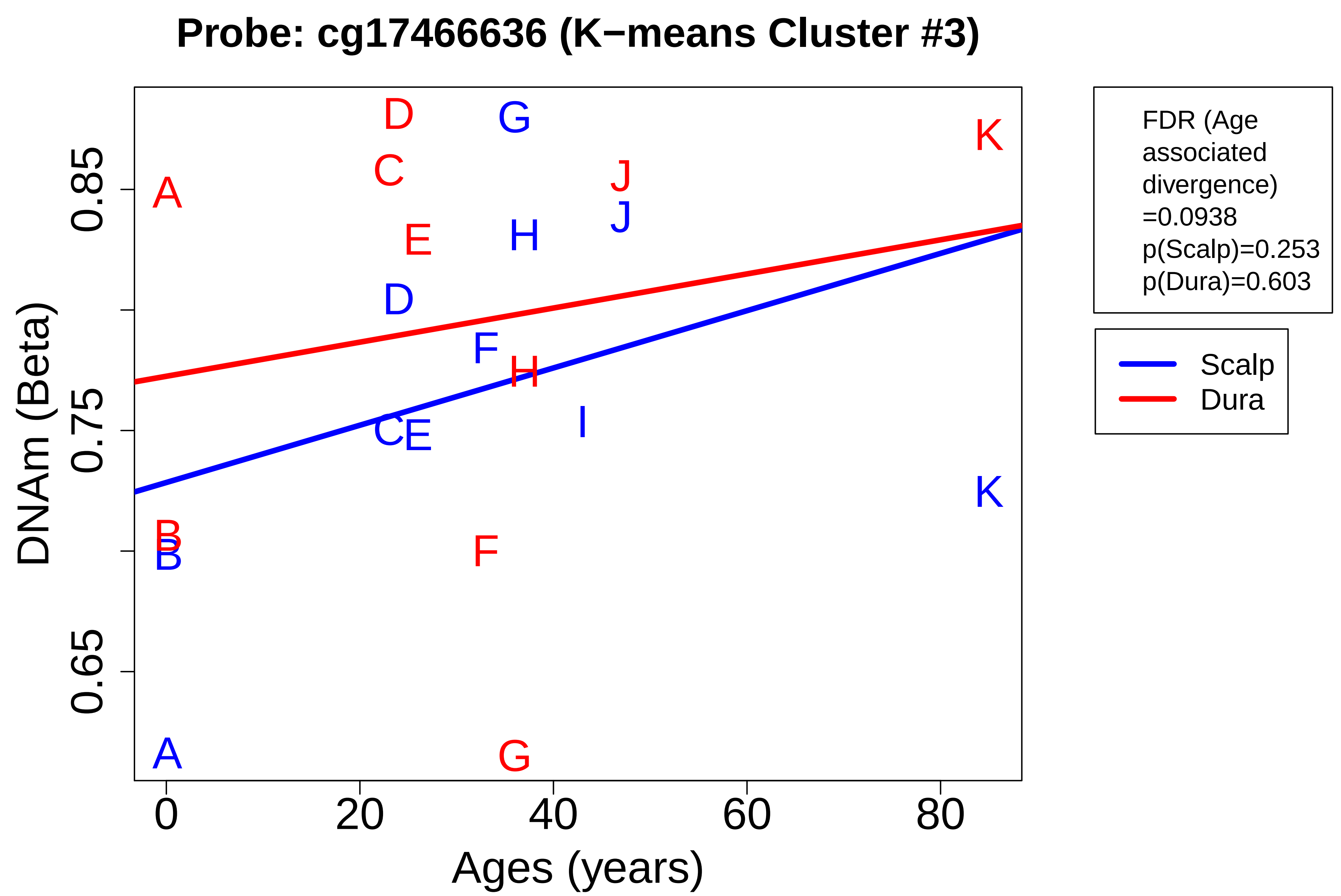

Probe: cg17466636 (K−means Cluster #3)
DNAm (Beta)
0.65
0.75
0.85
Ages (years)
0
20
40
60
80
A
B
C
D
E
F
G
H
I
J
K
A
B
C
D
E
F
G
H
J
K
FDR (Age associated divergence) =0.0938
p(Scalp)=0.253
p(Dura)=0.603
Scalp
Dura

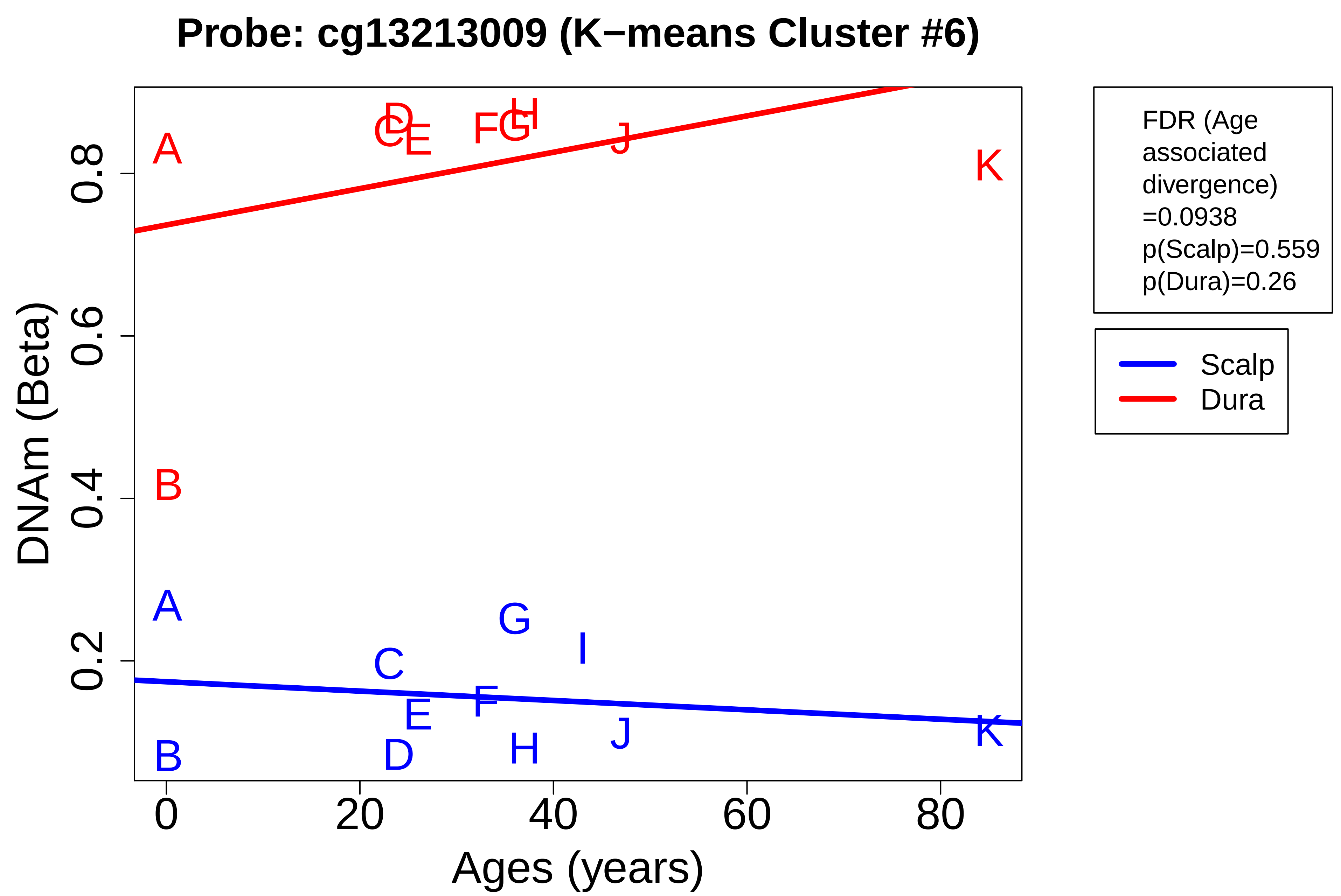

Probe: cg13213009 (K−means Cluster #6)
DNAm (Beta)
0.2
0.4
0.6
0.8
Ages (years)
0
20
40
60
80
FDR (Age associated divergence) =0.0938
p(Scalp)=0.559
p(Dura)=0.26
Scalp
Dura
A
B
C
D
E
F
G
H
I
J
K

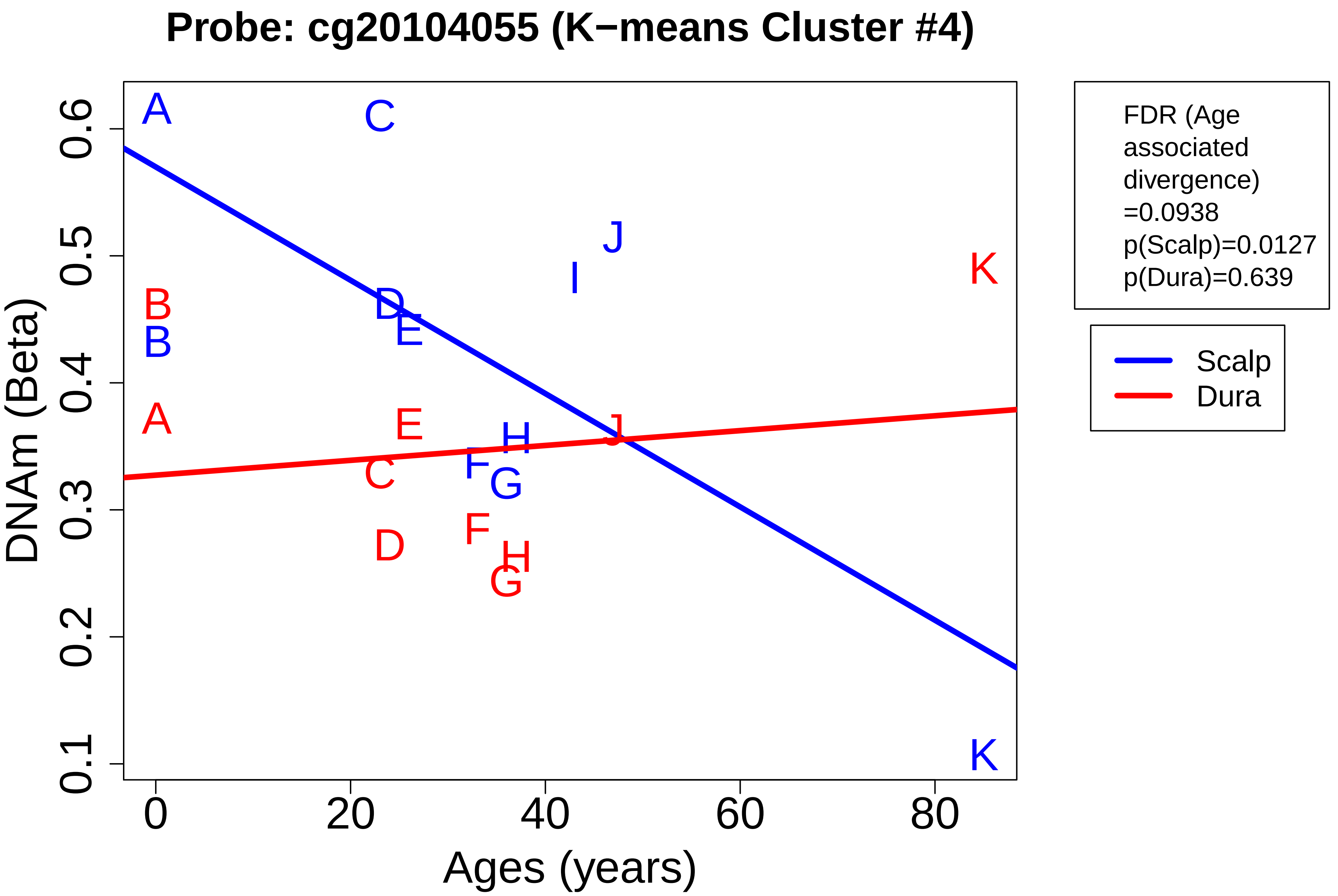
Probe: cg20104055 (K−means Cluster #4)
DNAm (Beta)
Ages (years)
0.1
0.2
0.3
0.4
0.5
0.6
0
20
40
60
80
FDR (Age associated divergence) =0.0938
p(Scalp)=0.0127
p(Dura)=0.639
Scalp
Dura
A
B
C
D
E
F
G
H
I
J
K

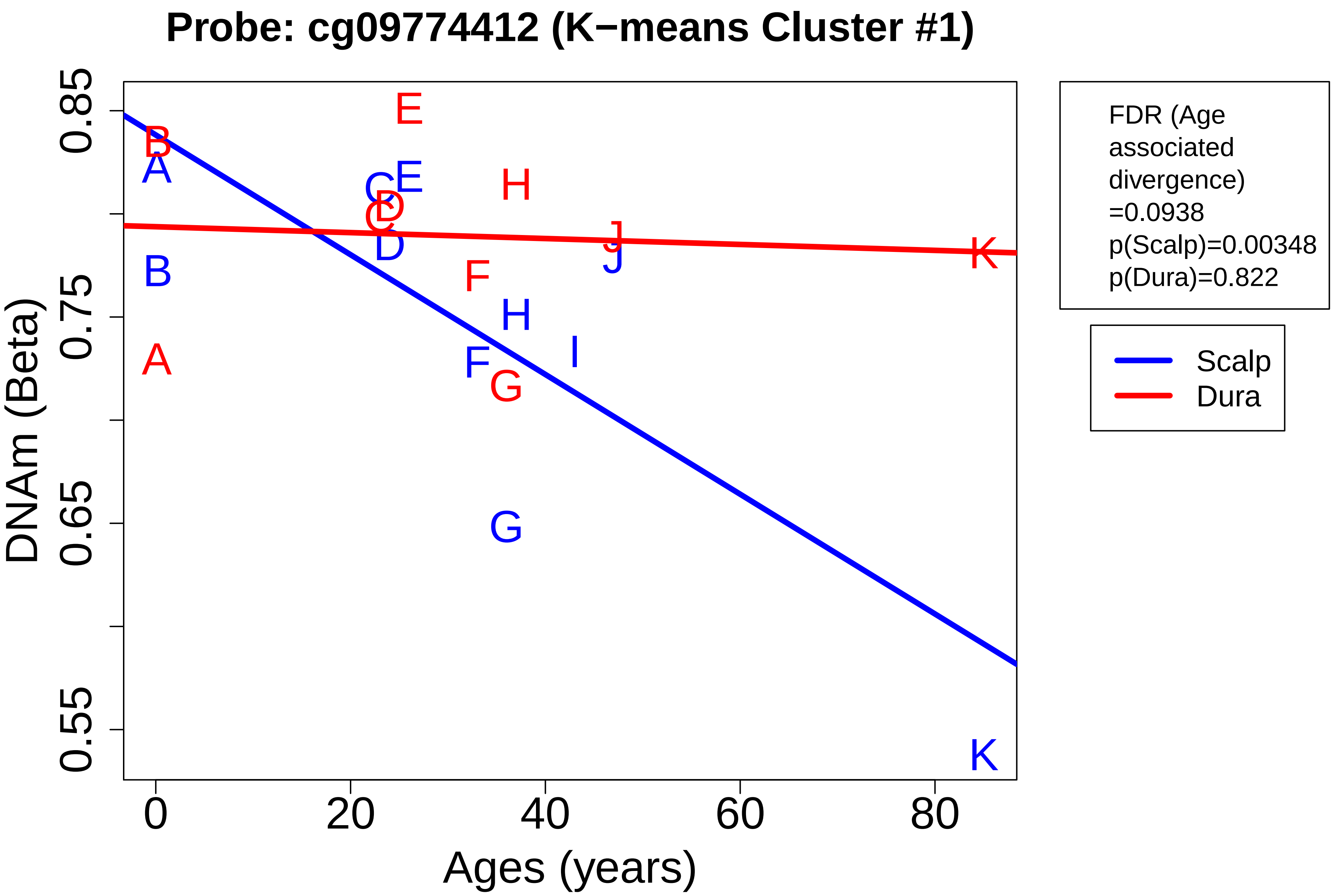

Probe: cg09774412 (K−means Cluster #1)
DNAm (Beta)
0.55
0.65
0.75
0.85
Ages (years)
0
20
40
60
80
FDR (Age associated divergence) =0.0938
p(Scalp)=0.00348
p(Dura)=0.822
Scalp
Dura

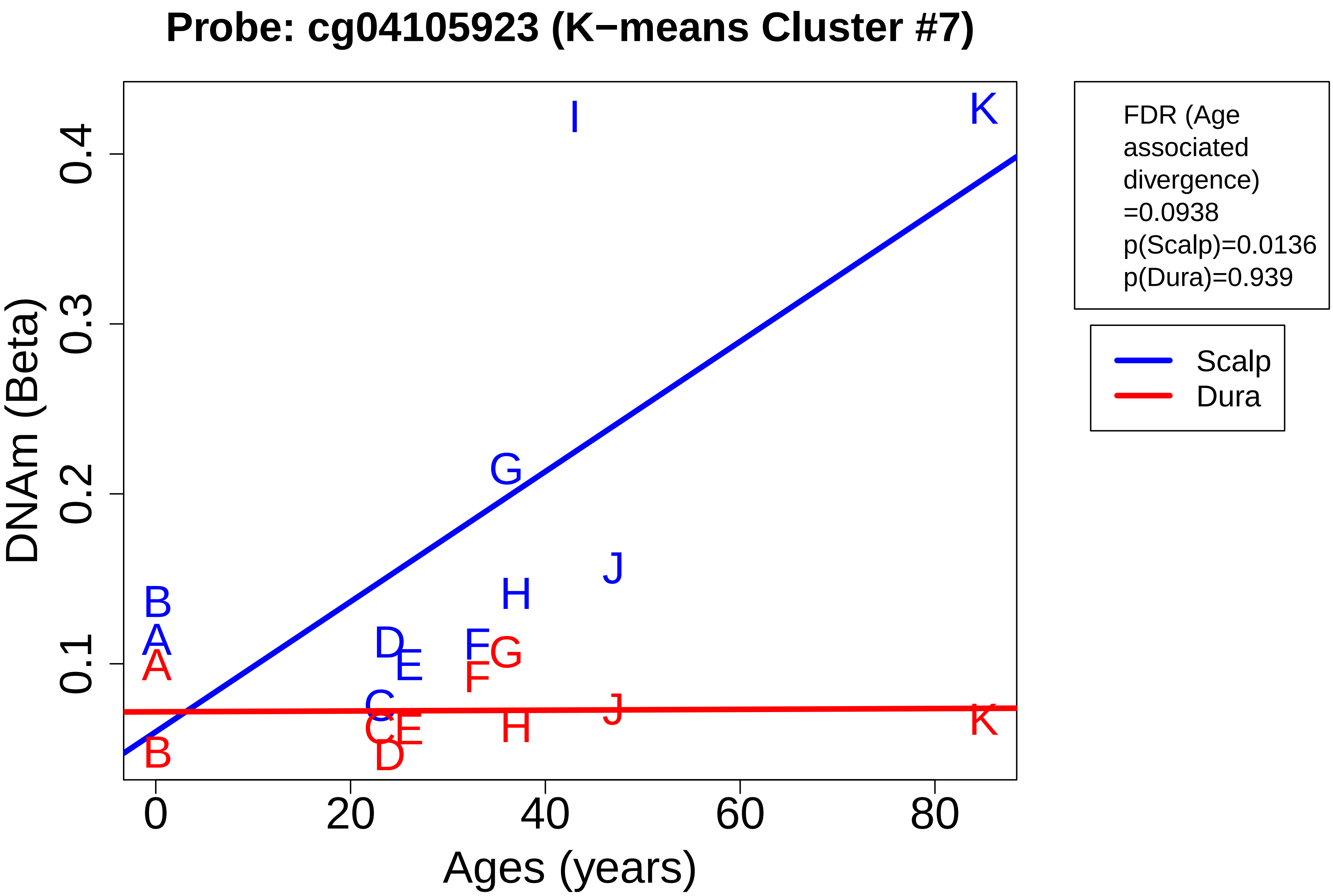
Probe: cg04105923 (K−means Cluster #7)
DNAm (Beta)
0.1
0.2
0.3
0.4
0
20
40
60
80
Ages (years)
FDR (Age associated divergence) =0.0938
p(Scalp)=0.0136
p(Dura)=0.939
Scalp
Dura
A
B
C
D
E
F
G
H
I
J
K

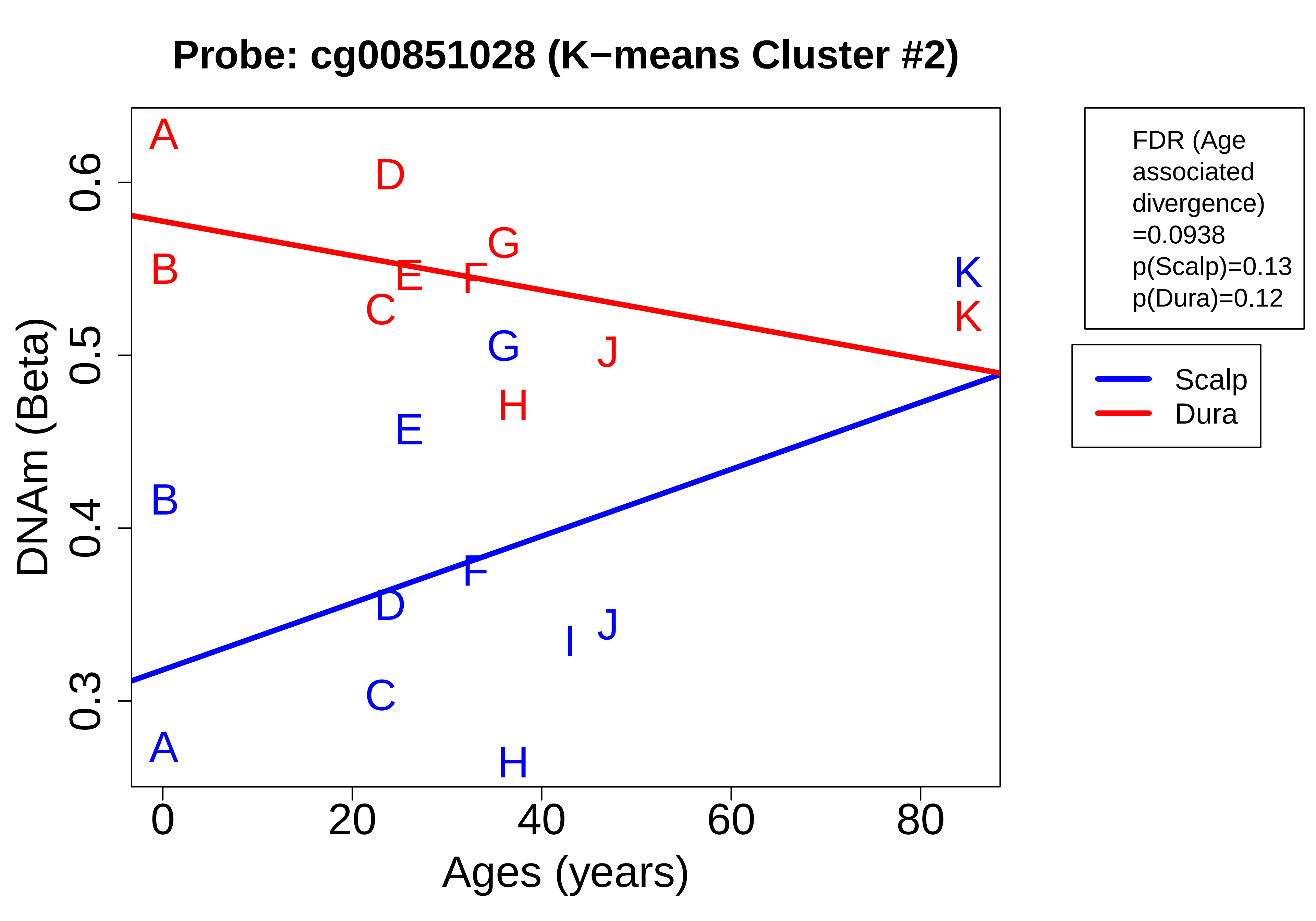
Probe: cg00851028 (K−means Cluster #2)
DNAm (Beta)
0.3
0.4
0.5
0.6
Ages (years)
0
20
40
60
80
A
B
C
D
E
F
G
H
I
J
K
FDR (Age associated divergence) =0.0938
p(Scalp)=0.13
p(Dura)=0.12
Scalp
Dura

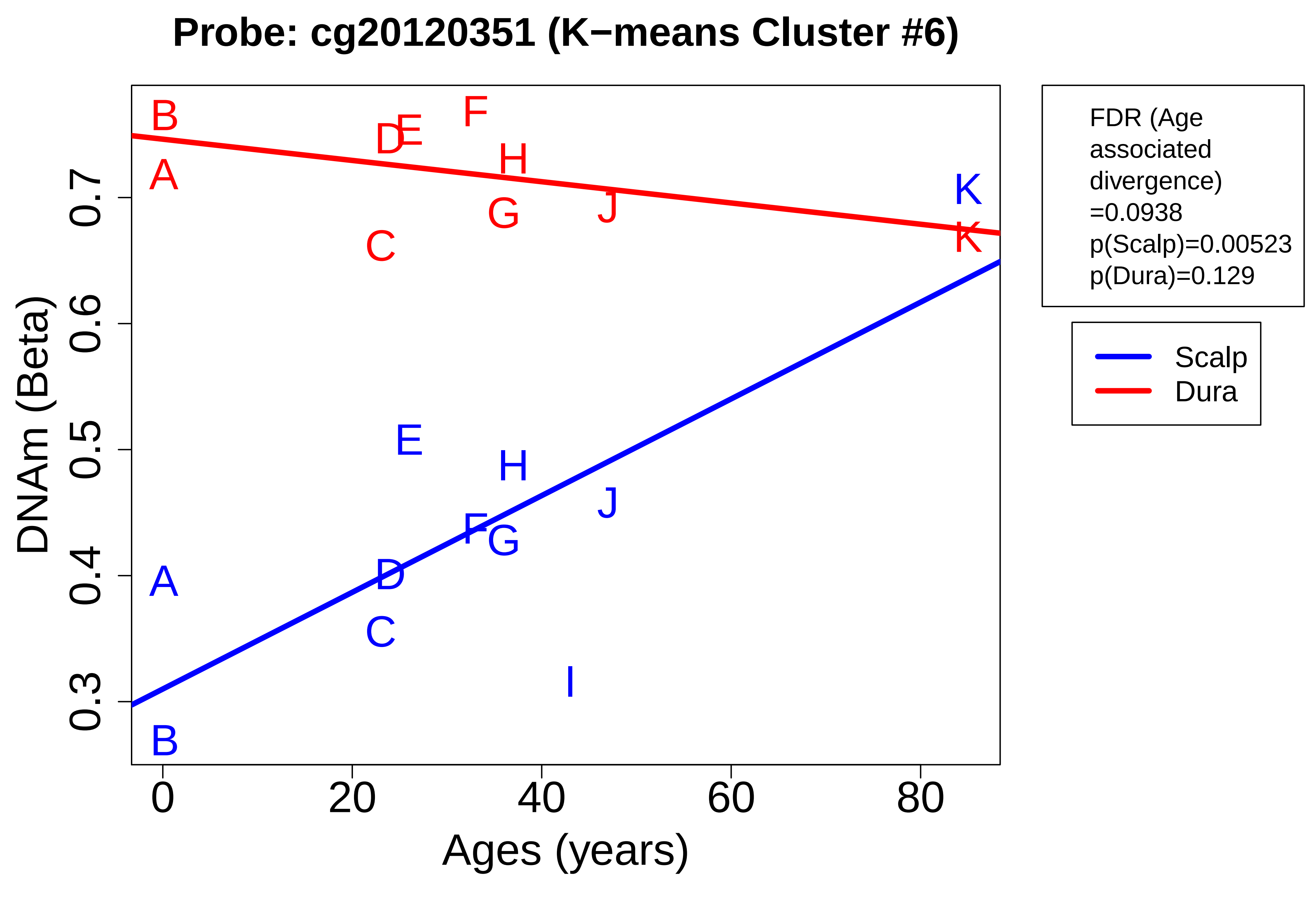

Probe: cg20120351 (K−means Cluster #6)
DNAm (Beta)
0.3
0.4
0.5
0.6
0.7
Ages (years)
0
20
40
60
80
FDR (Age associated divergence) =0.0938
p(Scalp)=0.00523
p(Dura)=0.129
Scalp
Dura

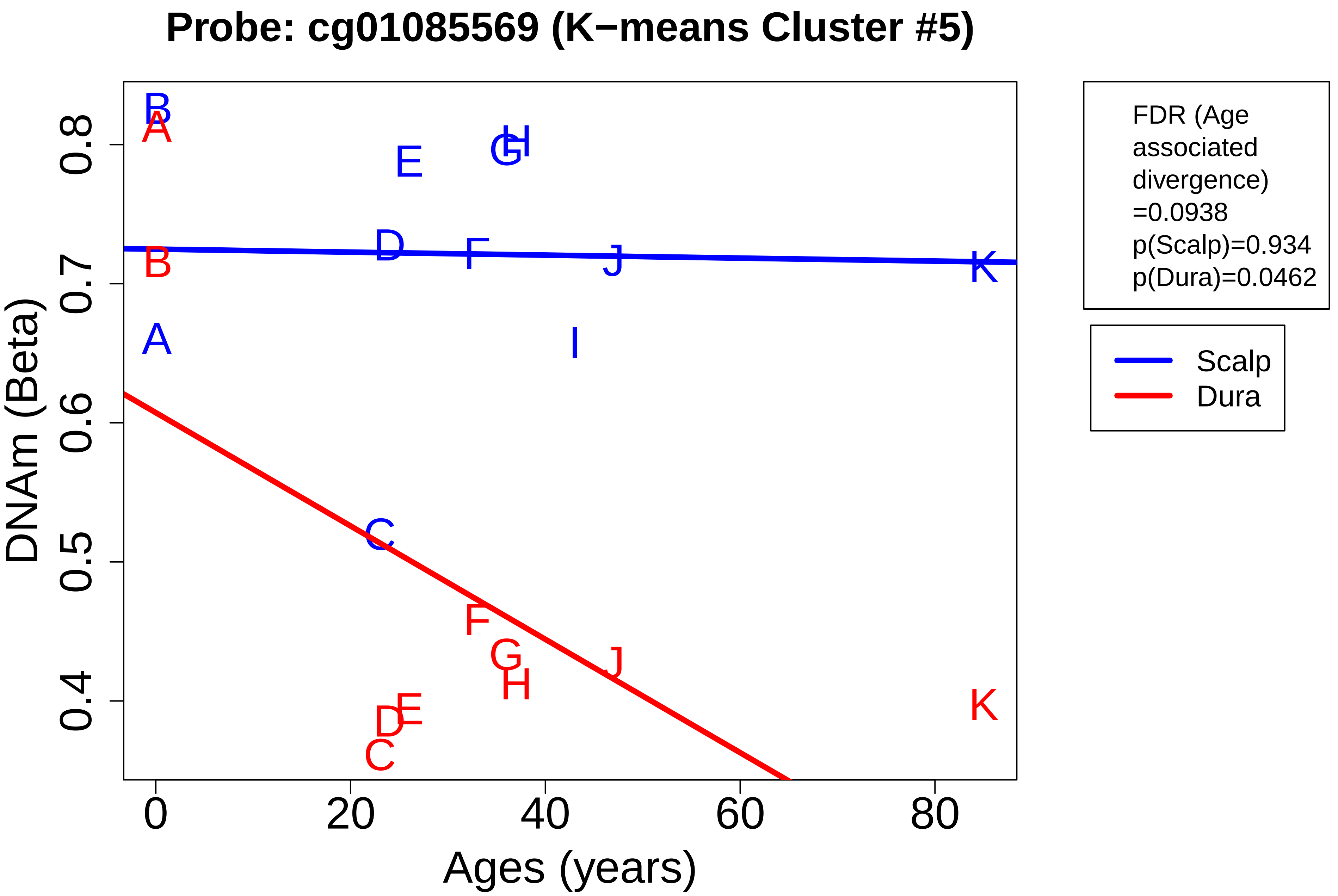

Probe: cg01085569 (K−means Cluster #5)
DNAm (Beta)
Ages (years)
0.4
0.5
0.6
0.7
0.8
0
20
40
60
80
FDR (Age associated divergence) =0.0938
p(Scalp)=0.934
p(Dura)=0.0462
Scalp
Dura

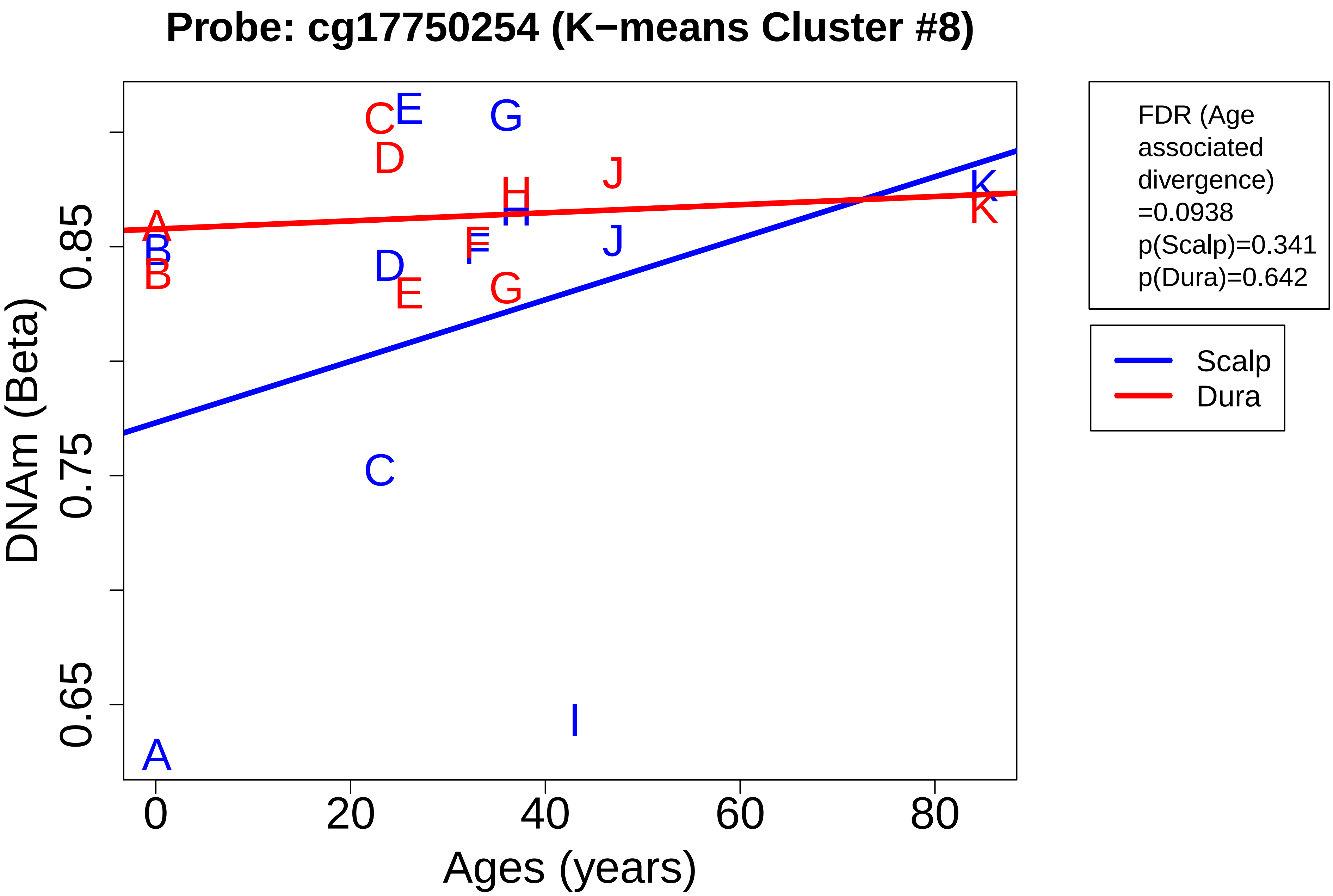

Probe: cg17750254 (K−means Cluster #8)
DNAm (Beta)
0.65
0.75
0.85
Ages (years)
0
20
40
60
80
A
B
C
D
E
F
G
H
I
J
K
FDR (Age associated divergence) =0.0938
p(Scalp)=0.341
p(Dura)=0.642
Scalp
Dura

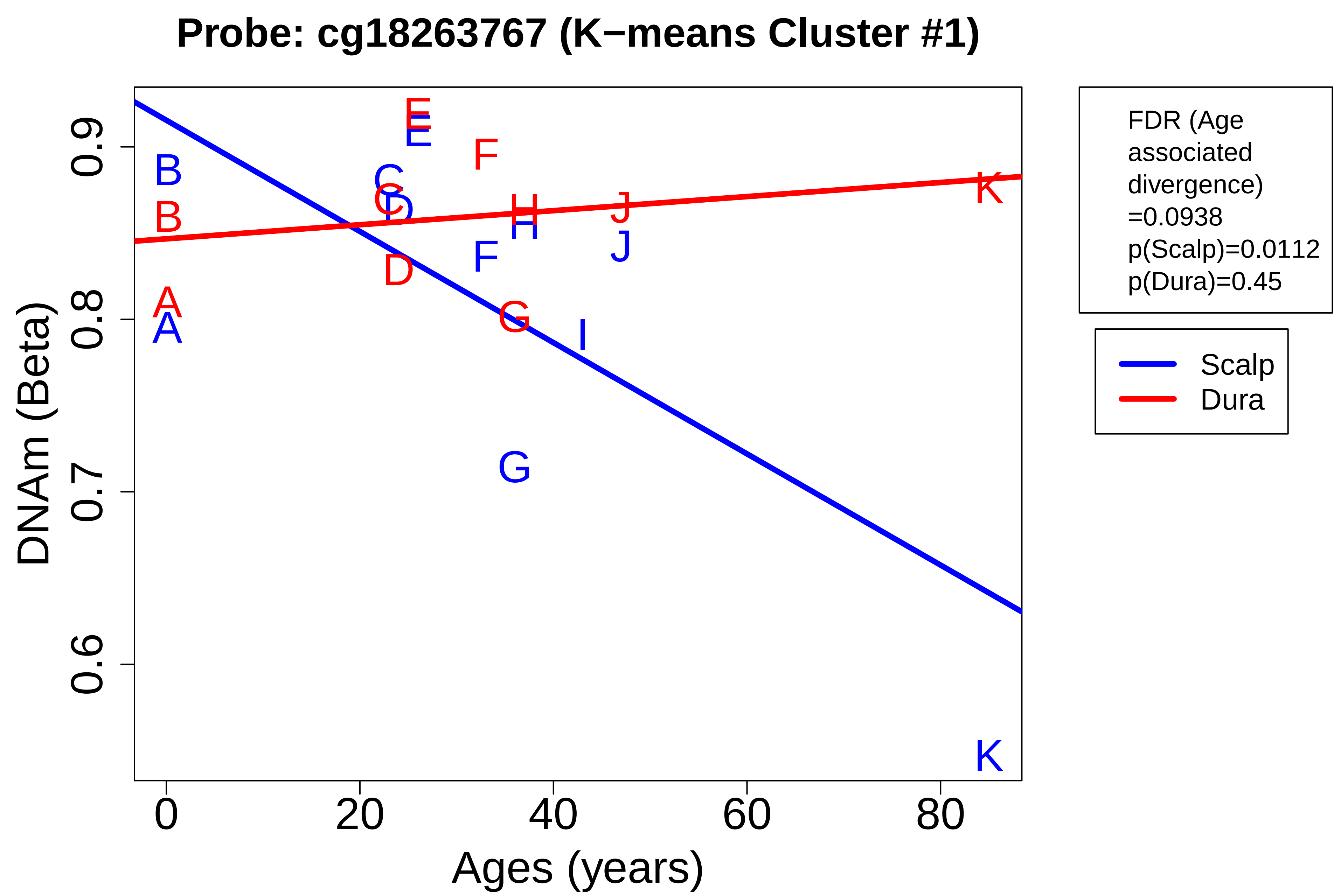
Probe: cg18263767 (K−means Cluster #1)
DNAm (Beta)
0.6
0.7
0.8
0.9
Ages (years)
0
20
40
60
80
A
B
C
D
E
F
G
H
I
J
K
FDR (Age associated divergence) =0.0938
p(Scalp)=0.0112
p(Dura)=0.45
Scalp
Dura

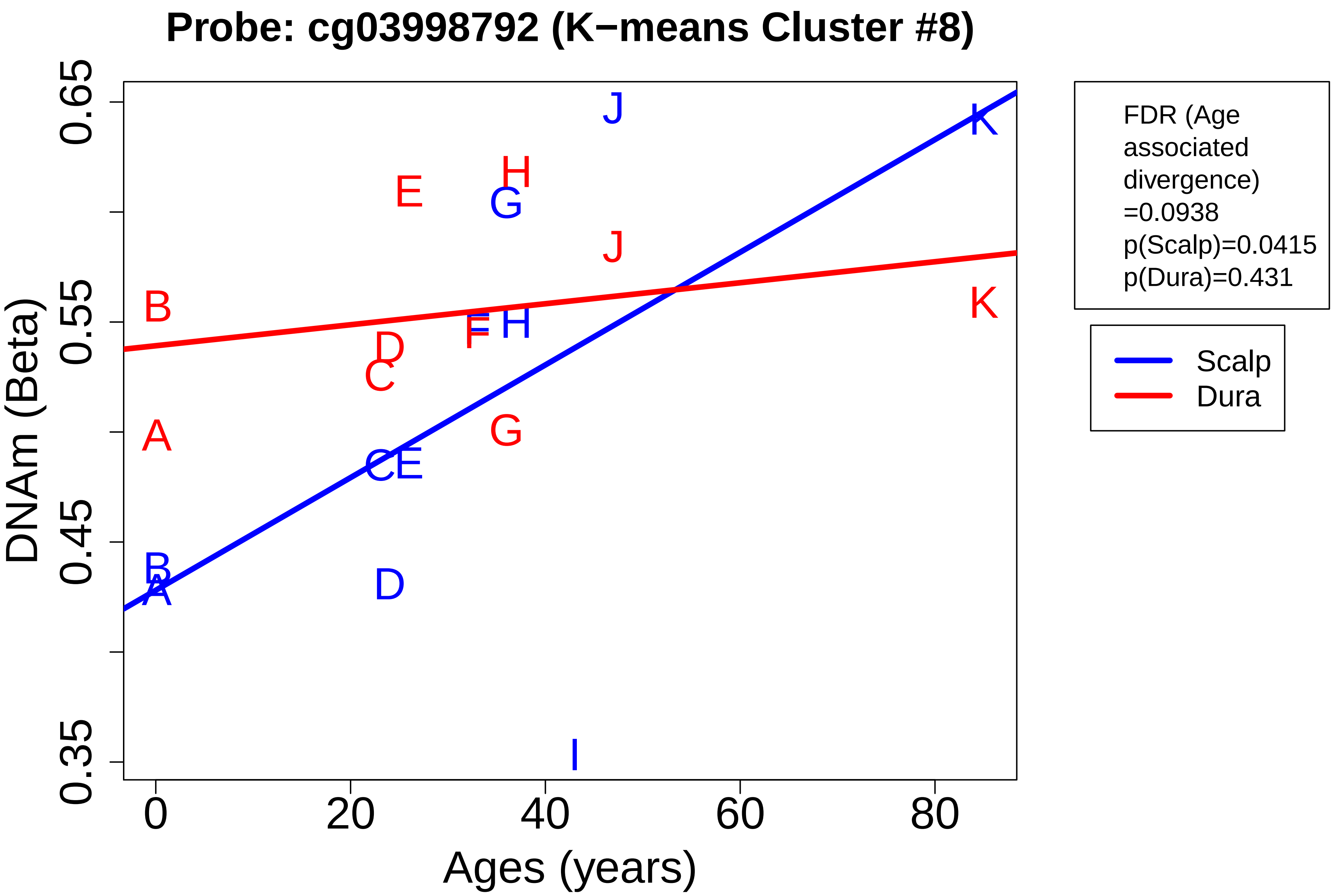

Probe: cg03998792 (K−means Cluster #8)
DNAm (Beta)
0.35
0.45
0.55
0.65
Ages (years)
0
20
40
60
80
FDR (Age associated divergence) =0.0938
p(Scalp)=0.0415
p(Dura)=0.431
Scalp
Dura
A
B
C
D
E
F
G
H
I
J
K

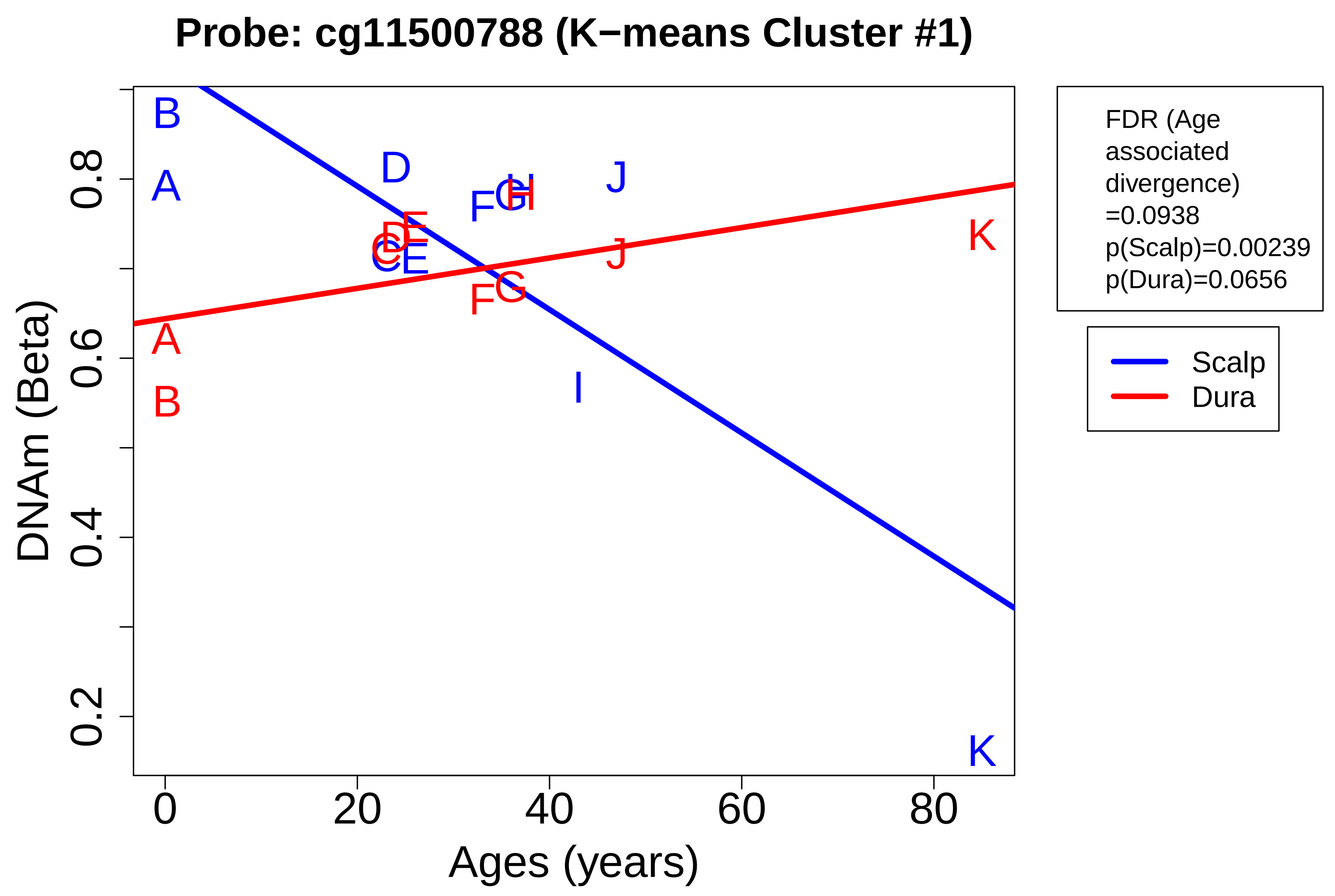

Probe: cg11500788 (K−means Cluster #1)
DNAm (Beta)
0.2
0.4
0.6
0.8
0
20
40
60
80
Ages (years)
A
B
C
D
E
F
G
H
I
J
K
FDR (Age associated divergence) =0.0938
p(Scalp)=0.00239
p(Dura)=0.0656
Scalp
Dura

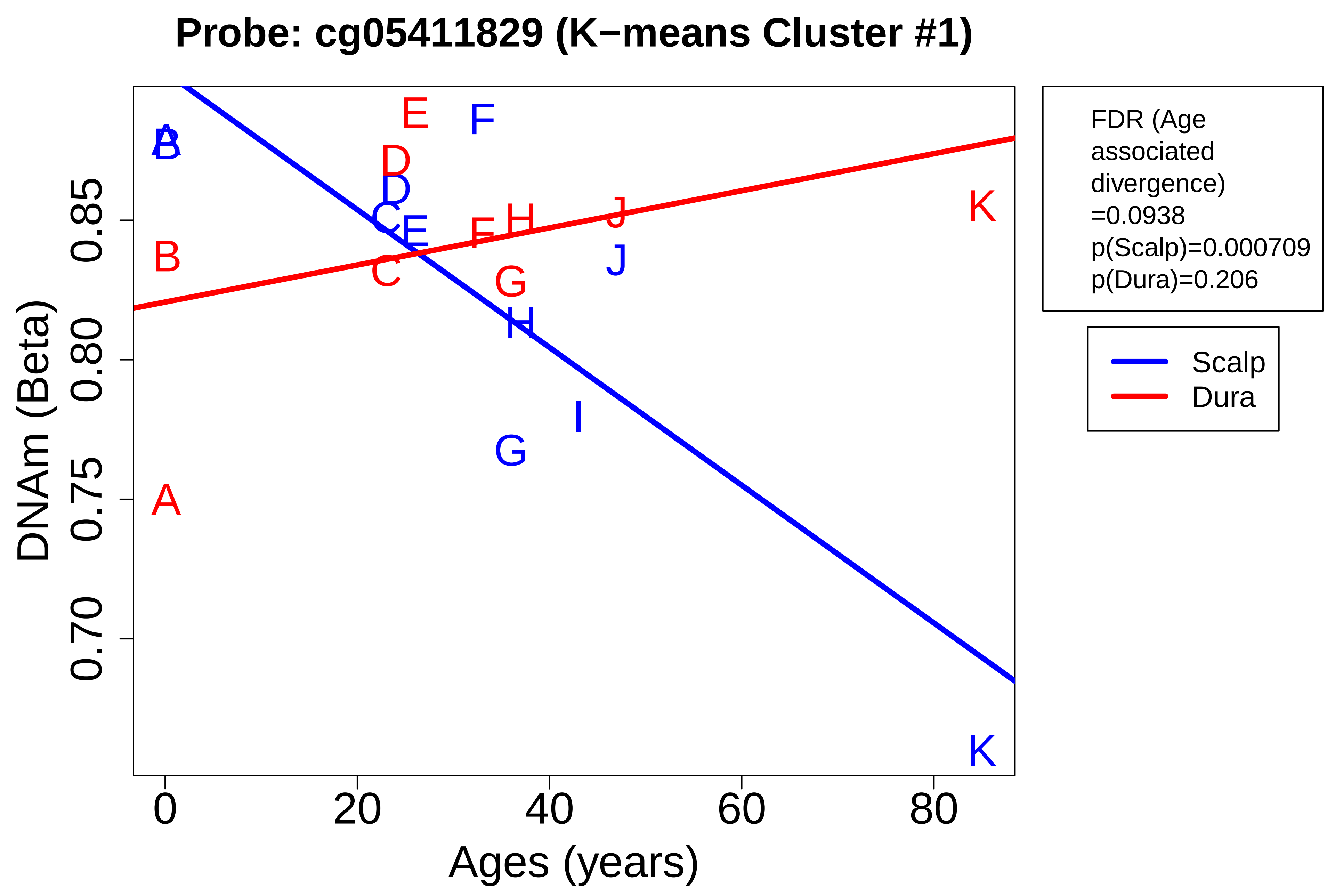

Probe: cg05411829 (K−means Cluster #1)
DNAm (Beta)
0.70
0.75
0.80
0.85
0
20
40
60
80
Ages (years)
A
B
C
D
E
F
G
H
I
J
K
FDR (Age associated divergence) =0.0938
p(Scalp)=0.000709
p(Dura)=0.206
Scalp
Dura

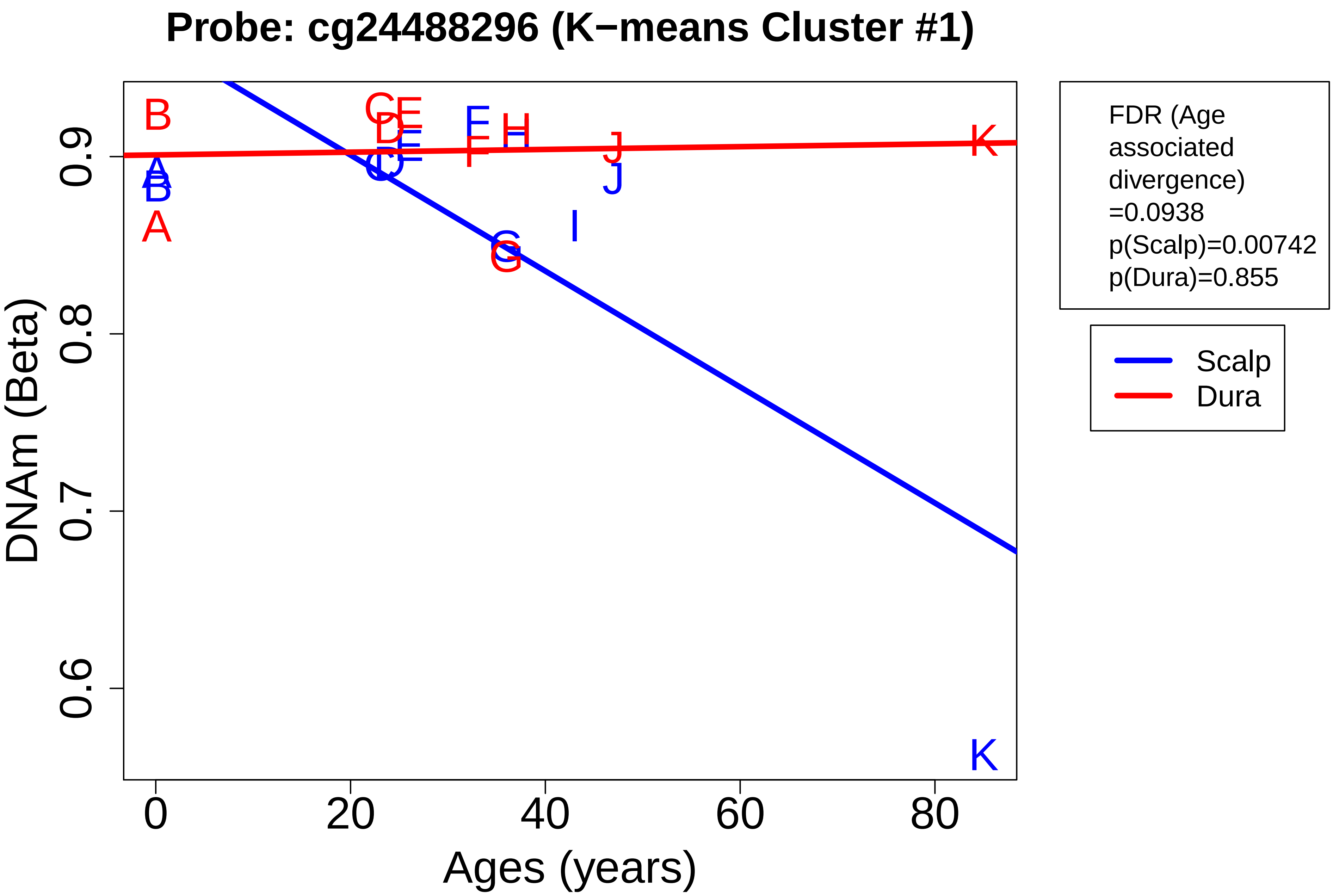
Probe: cg24488296 (K−means Cluster #1)
DNAm (Beta)
0.6
0.7
0.8
0.9
0
20
40
60
80
Ages (years)
FDR (Age associated divergence) =0.0938
p(Scalp)=0.00742
p(Dura)=0.855
Scalp
Dura
A
B
C
D
E
F
G
H
I
J
K

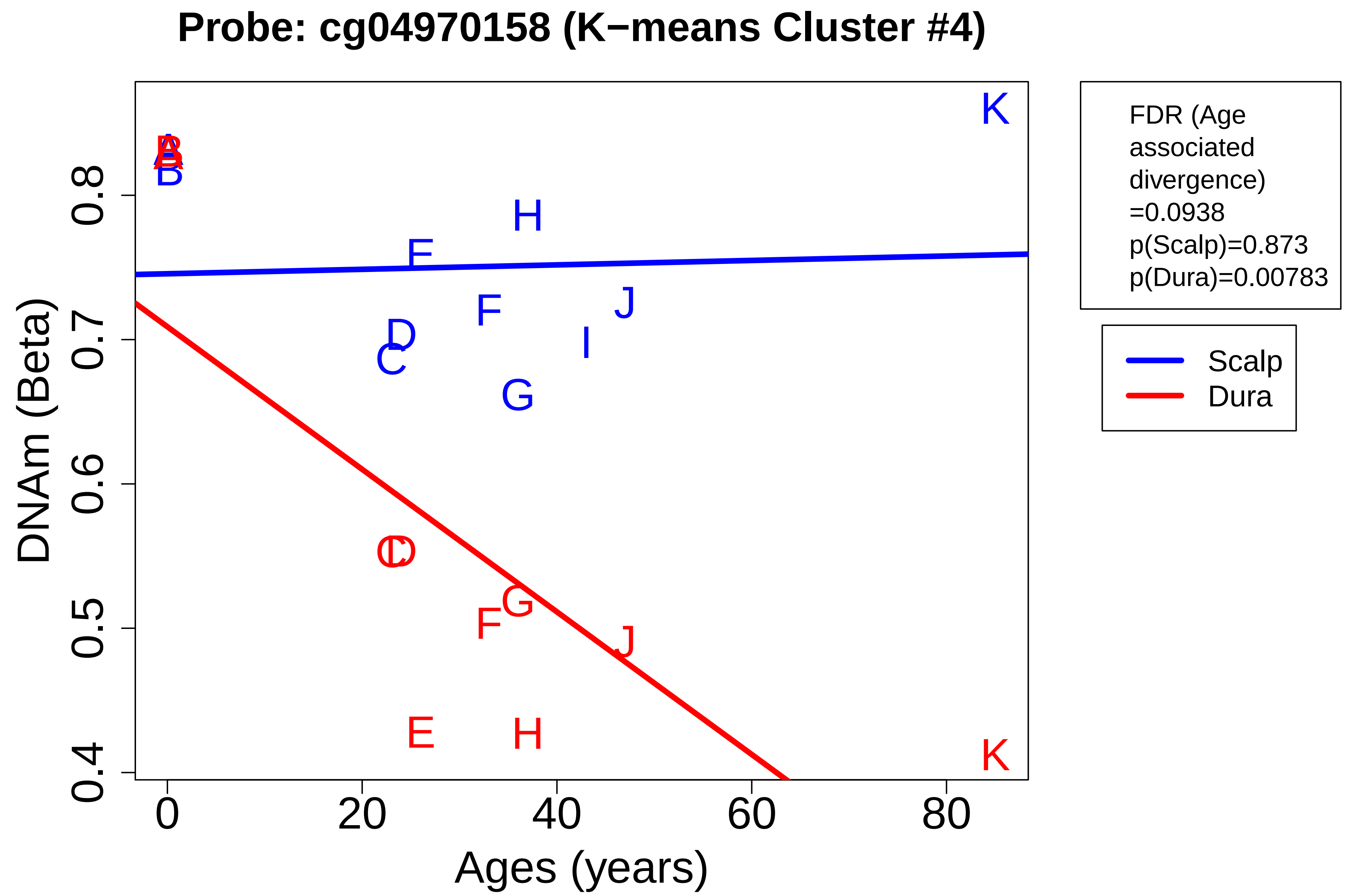

Probe: cg04970158 (K−means Cluster #4)
DNAm (Beta)
Ages (years)
0.4
0.5
0.6
0.7
0.8
0
20
40
60
80
FDR (Age associated divergence) =0.0938
p(Scalp)=0.873
p(Dura)=0.00783
Scalp
Dura

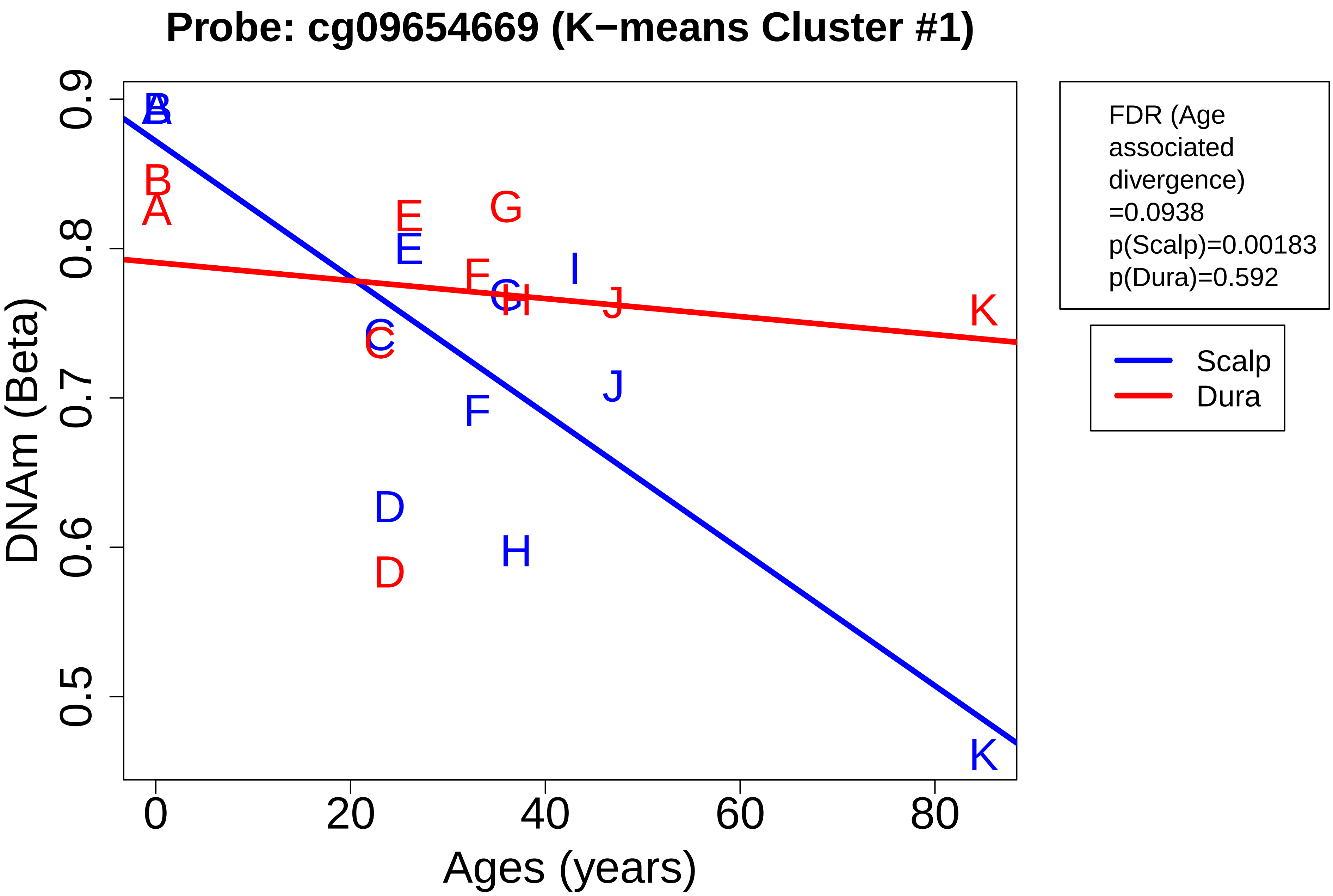

Probe: cg09654669 (K−means Cluster #1)
DNAm (Beta)
0.5
0.6
0.7
0.8
0.9
Ages (years)
0
20
40
60
80
FDR (Age associated divergence) =0.0938
p(Scalp)=0.00183
p(Dura)=0.592
Scalp
Dura

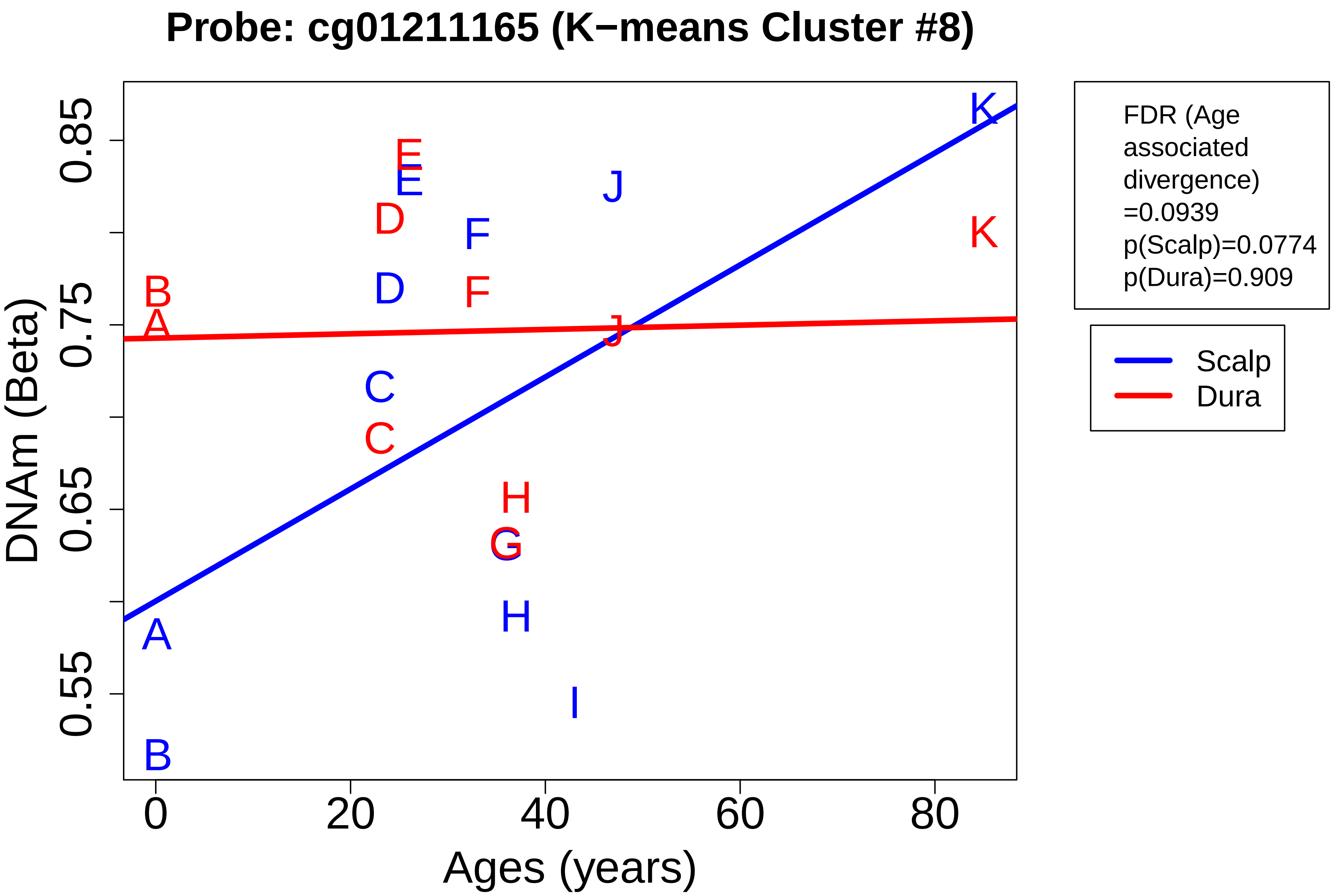

Probe: cg01211165 (K−means Cluster #8)
DNAm (Beta)
0.55
0.65
0.75
0.85
Ages (years)
0
20
40
60
80
A
B
C
D
E
F
G
H
I
J
K
FDR (Age associated divergence) =0.0939
p(Scalp)=0.0774
p(Dura)=0.909
Scalp
Dura

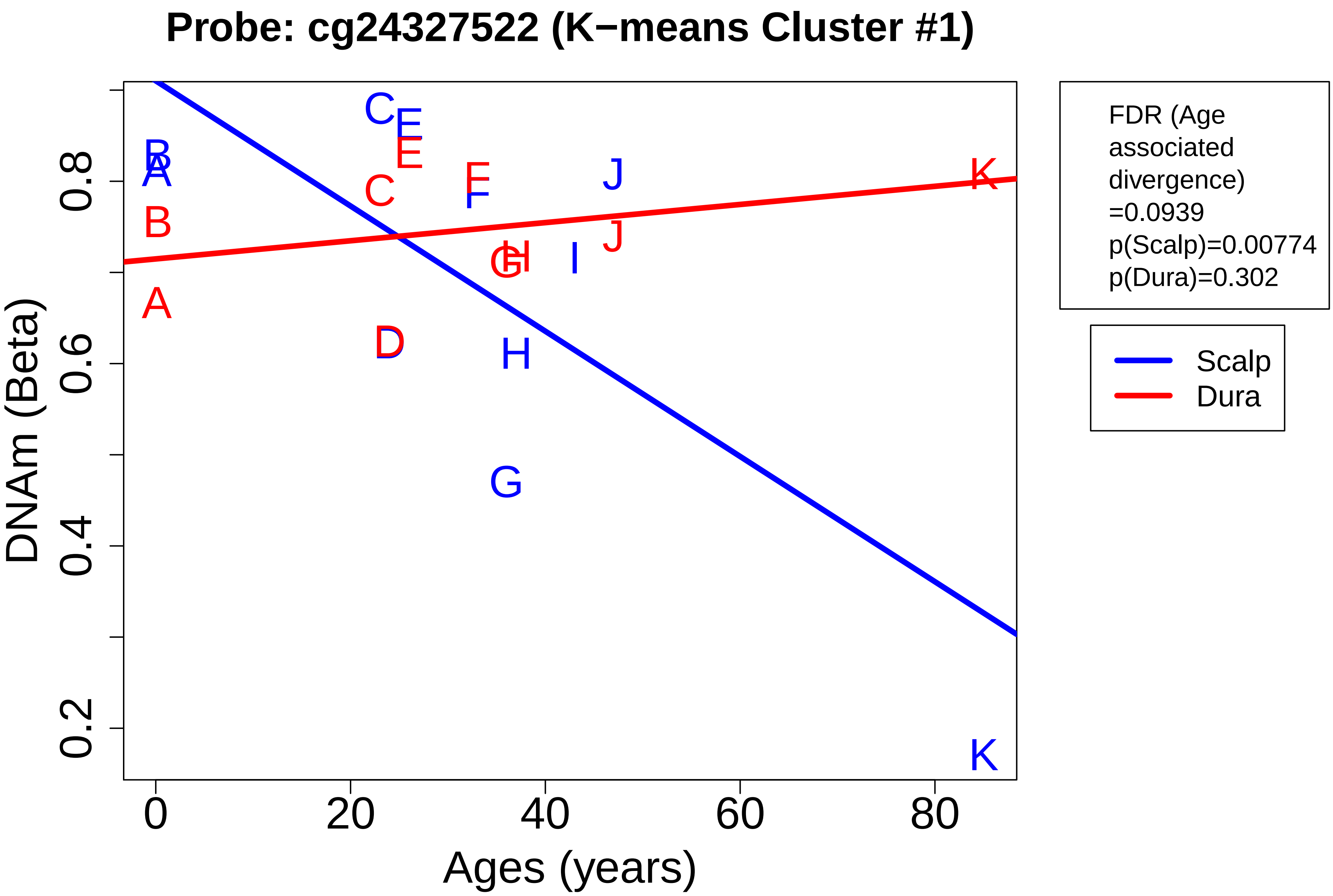

Probe: cg24327522 (K−means Cluster #1)
DNAm (Beta)
0.2
0.4
0.6
0.8
0
20
40
60
80
Ages (years)
FDR (Age associated divergence) =0.0939
p(Scalp)=0.00774
p(Dura)=0.302
Scalp
Dura

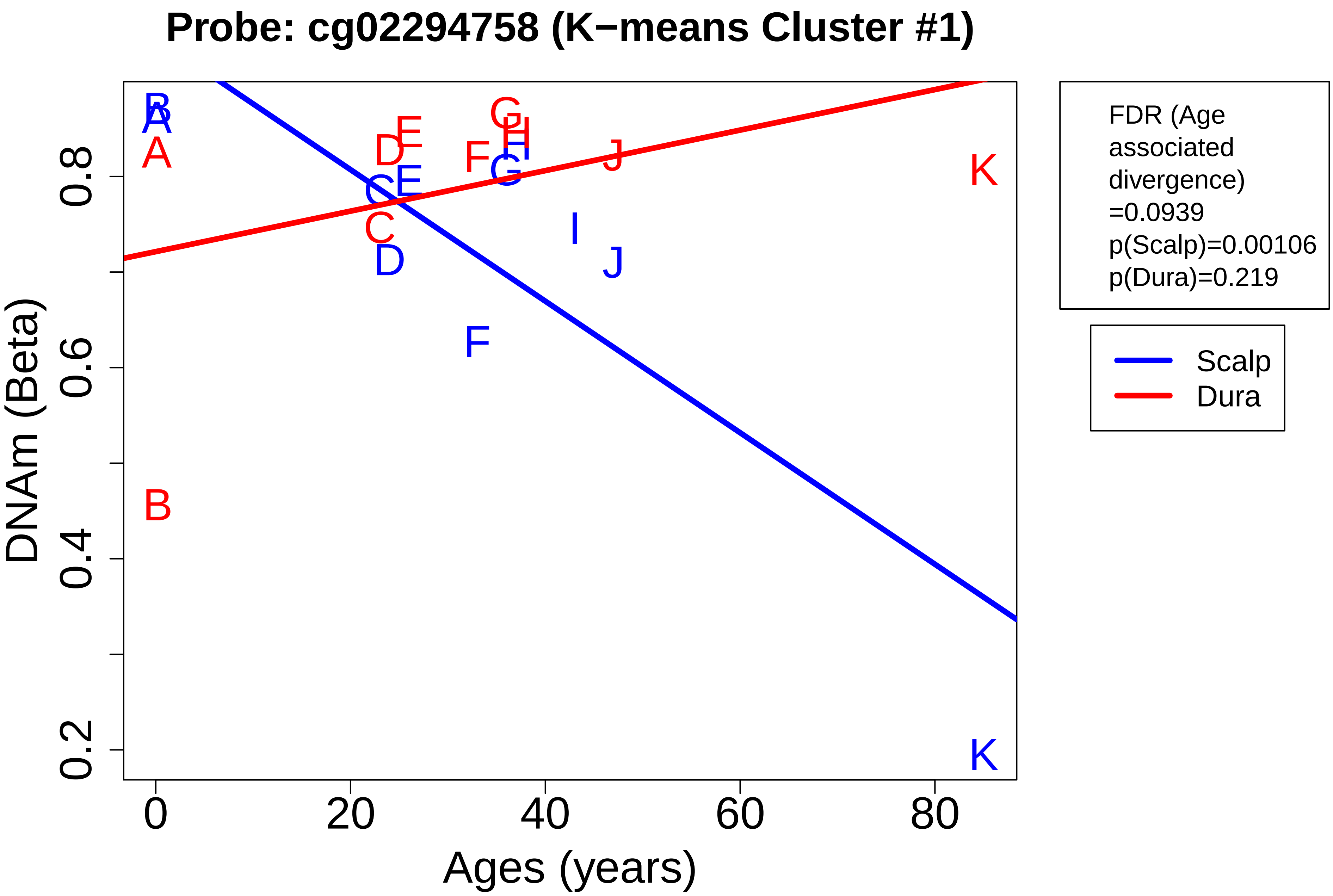
Probe: cg02294758 (K−means Cluster #1)
DNAm (Beta)
0.2
0.4
0.6
0.8
0
20
40
60
80
Ages (years)
FDR (Age associated divergence) =0.0939
p(Scalp)=0.00106
p(Dura)=0.219
Scalp
Dura
A
B
C
D
E
F
G
H
I
J
K

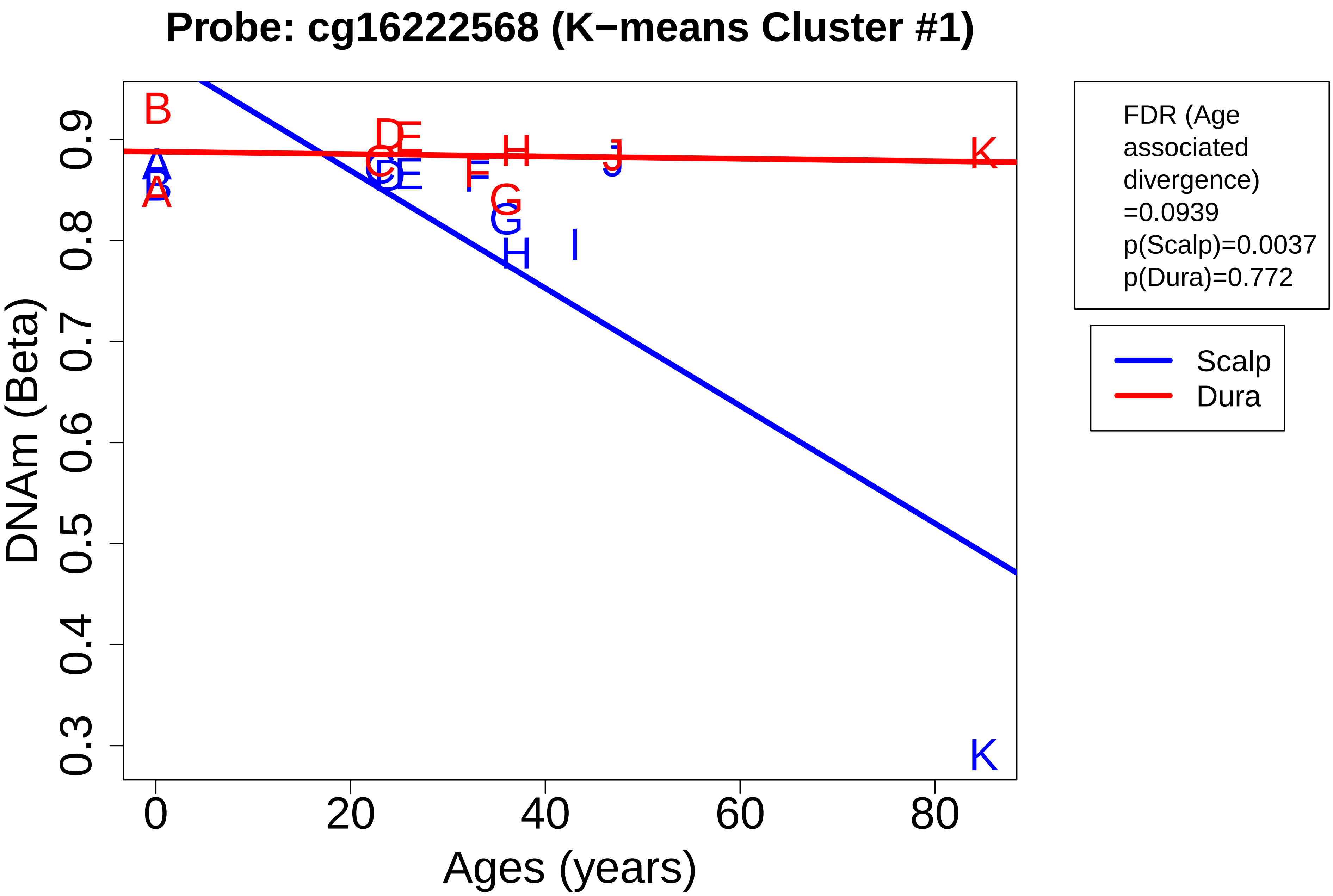

Probe: cg16222568 (K−means Cluster #1)
DNAm (Beta)
0.3
0.4
0.5
0.6
0.7
0.8
0.9
0
20
40
60
80
Ages (years)
FDR (Age associated divergence) =0.0939
p(Scalp)=0.0037
p(Dura)=0.772
Scalp
Dura

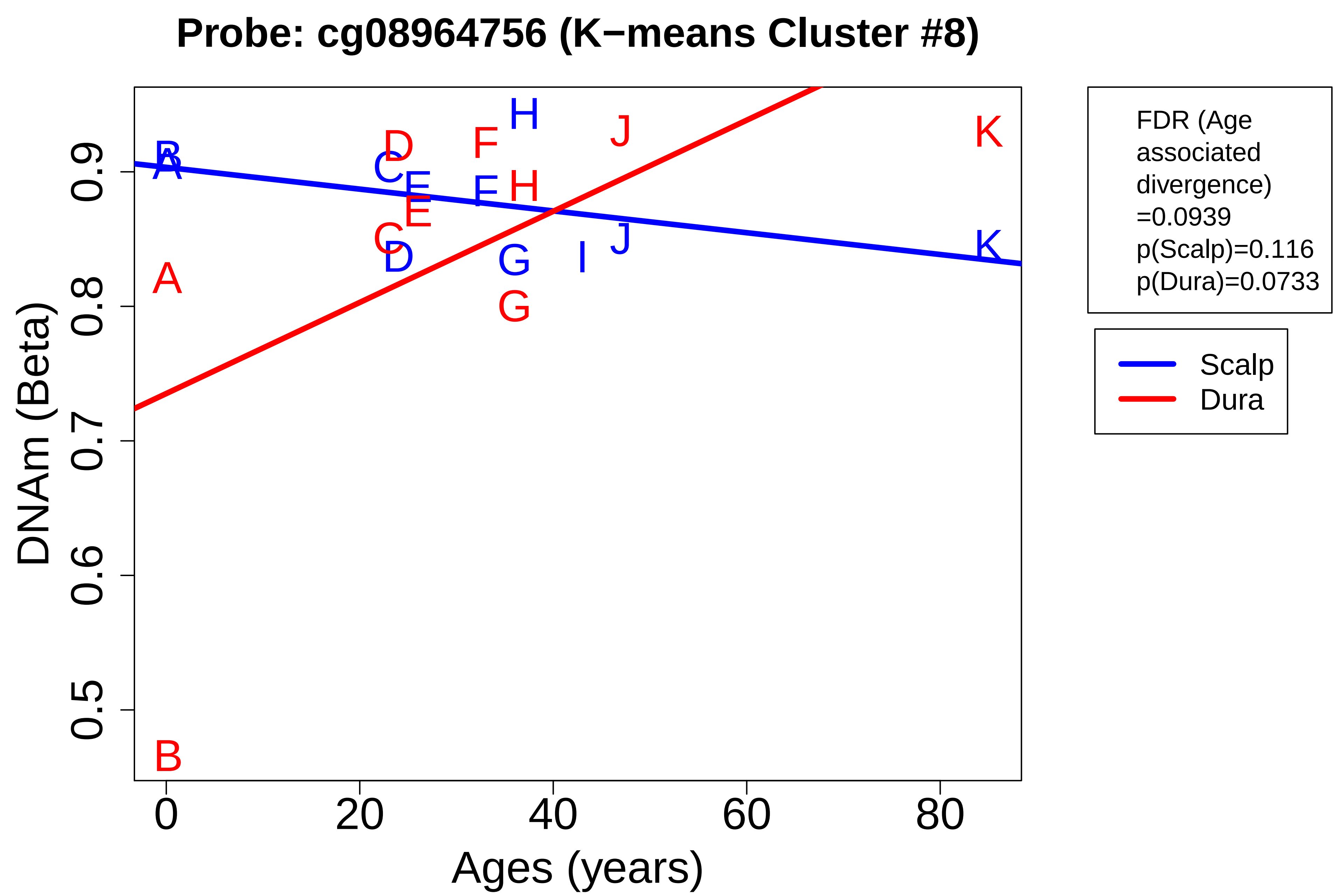

Probe: cg08964756 (K−means Cluster #8)
FDR (Age associated divergence) =0.0939
p(Scalp)=0.116
p(Dura)=0.0733
Scalp
Dura
DNAm (Beta)
0.5
0.6
0.7
0.8
0.9
0
20
40
60
80
Ages (years)

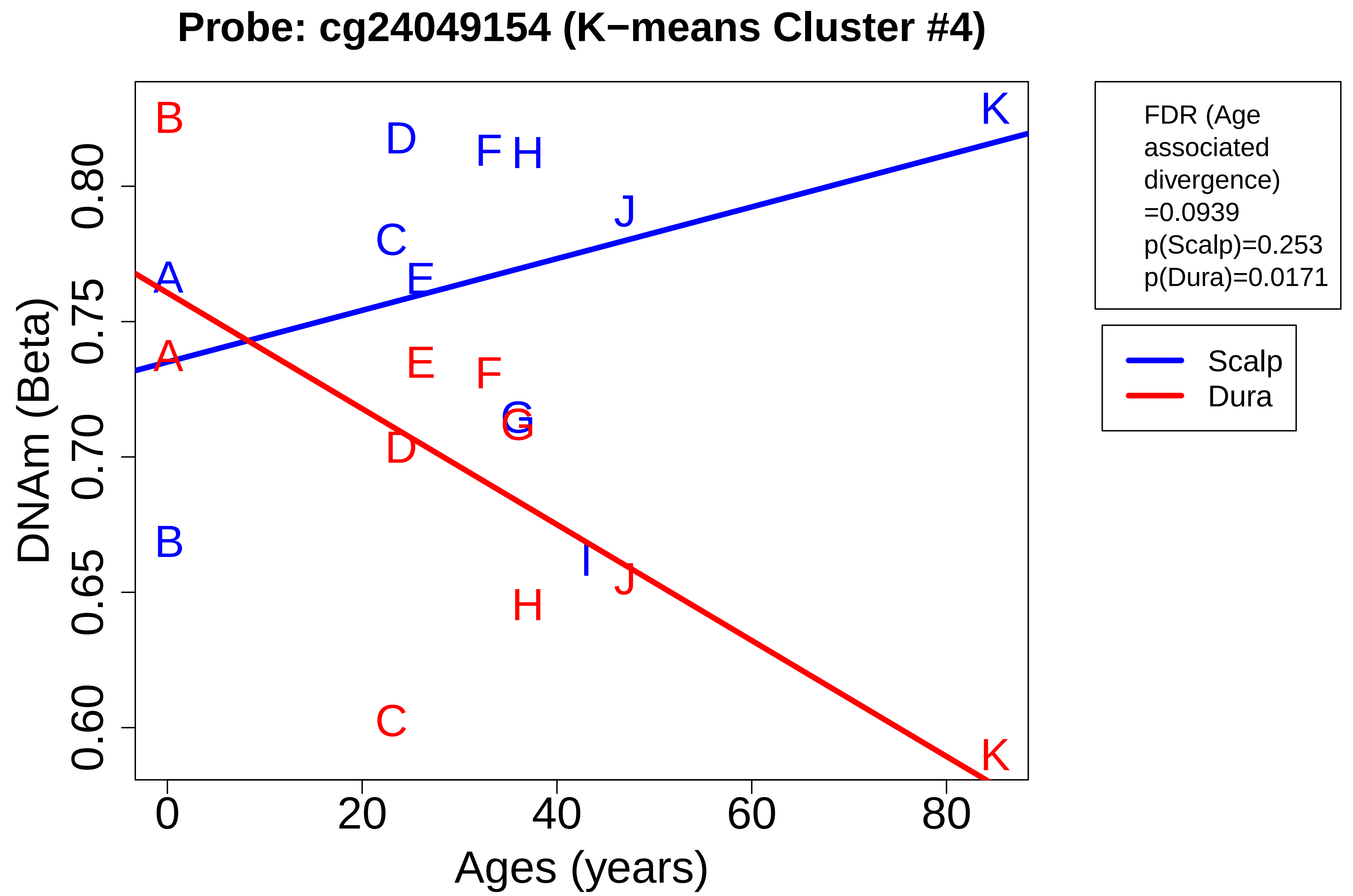

Probe: cg24049154 (K−means Cluster #4)
DNAm (Beta)
0.60
0.65
0.70
0.75
0.80
0
20
40
60
80
Ages (years)
A
B
C
D
E
F
G
H
I
J
K
FDR (Age associated divergence) =0.0939
p(Scalp)=0.253
p(Dura)=0.0171
Scalp
Dura

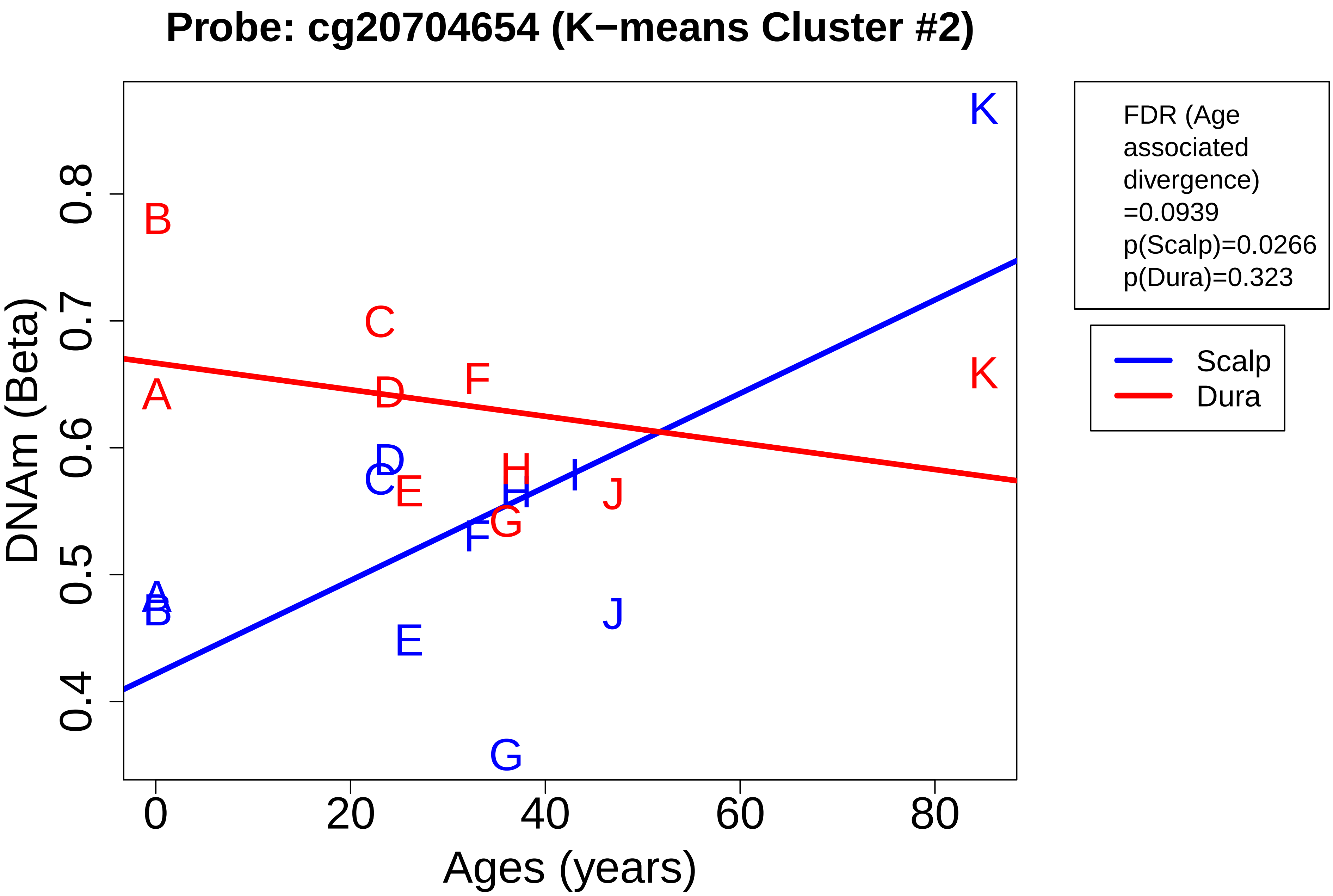
Probe: cg20704654 (K−means Cluster #2)
DNAm (Beta)
0.4
0.5
0.6
0.7
0.8
0
20
40
60
80
Ages (years)
FDR (Age associated divergence) =0.0939
p(Scalp)=0.0266
p(Dura)=0.323
Scalp
Dura
A
B
C
D
E
F
G
H
I
J
K

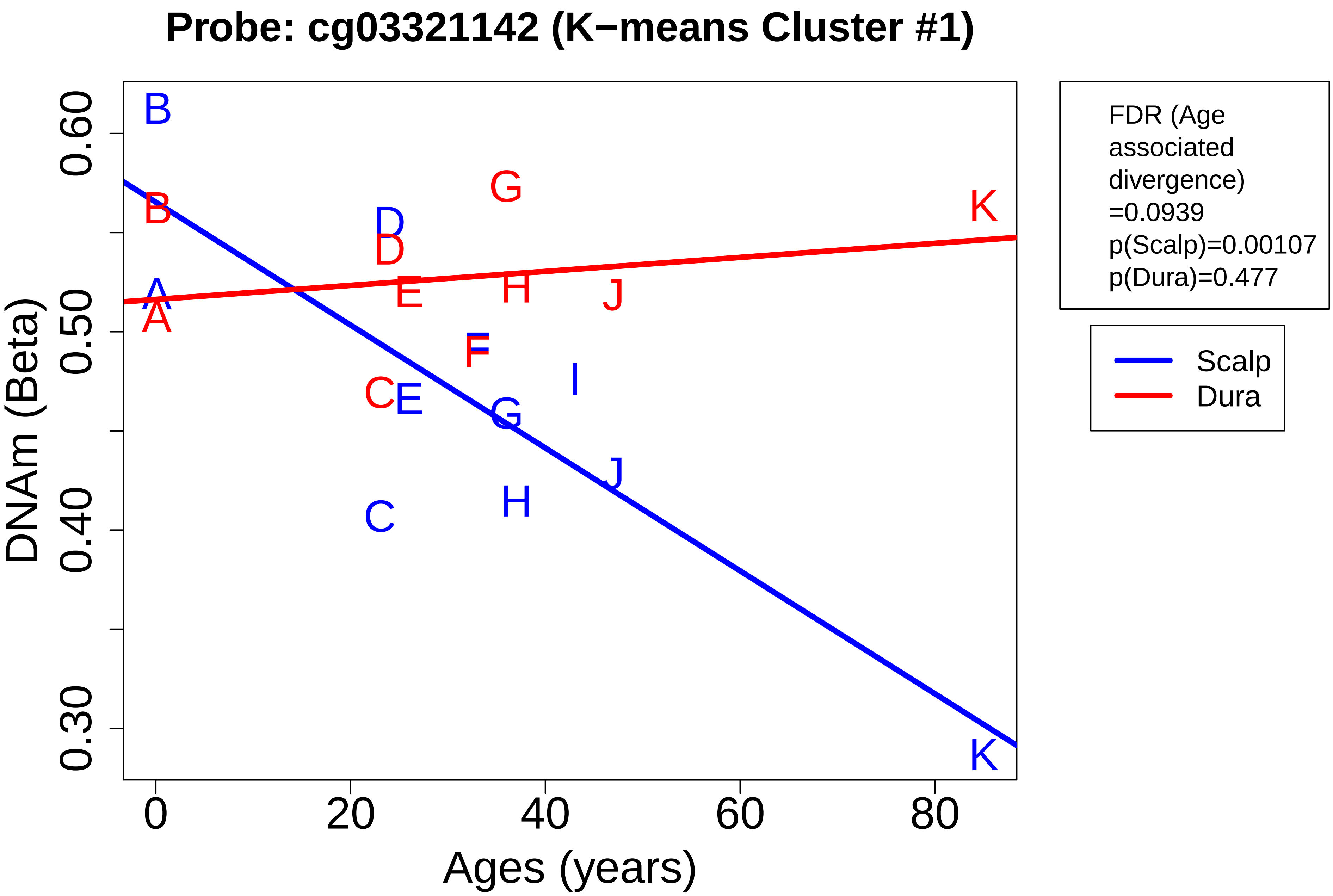

Probe: cg03321142 (K−means Cluster #1)
DNAm (Beta)
0.30
0.40
0.50
0.60
0
20
40
60
80
Ages (years)
FDR (Age associated divergence) =0.0939
p(Scalp)=0.00107
p(Dura)=0.477
Scalp
Dura
A
B
C
D
E
F
G
H
I
J
K

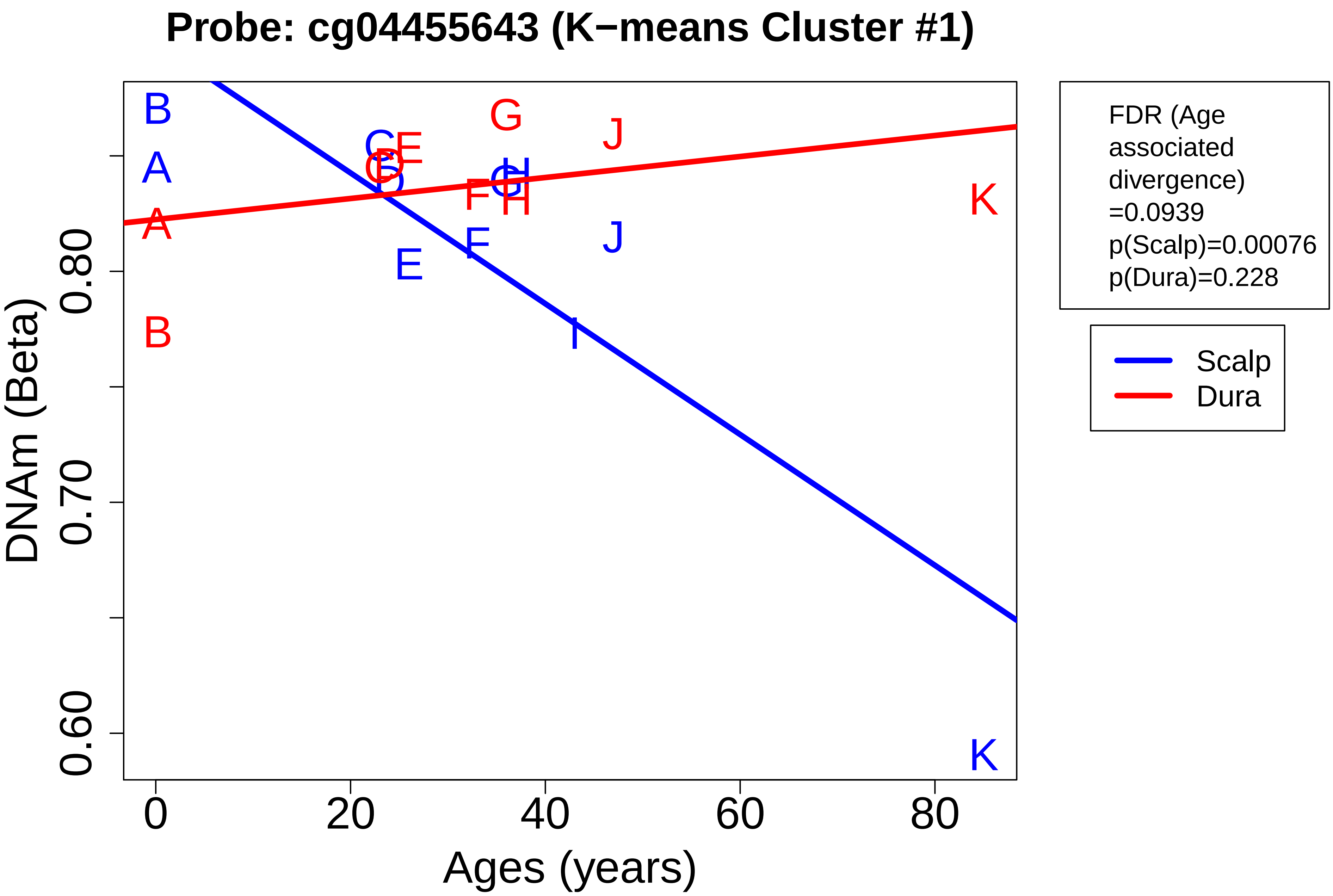

Probe: cg04455643 (K−means Cluster #1)
DNAm (Beta)
0.60
0.70
0.80
0
20
40
60
80
Ages (years)
FDR (Age associated divergence) =0.0939
p(Scalp)=0.00076
p(Dura)=0.228
Scalp
Dura

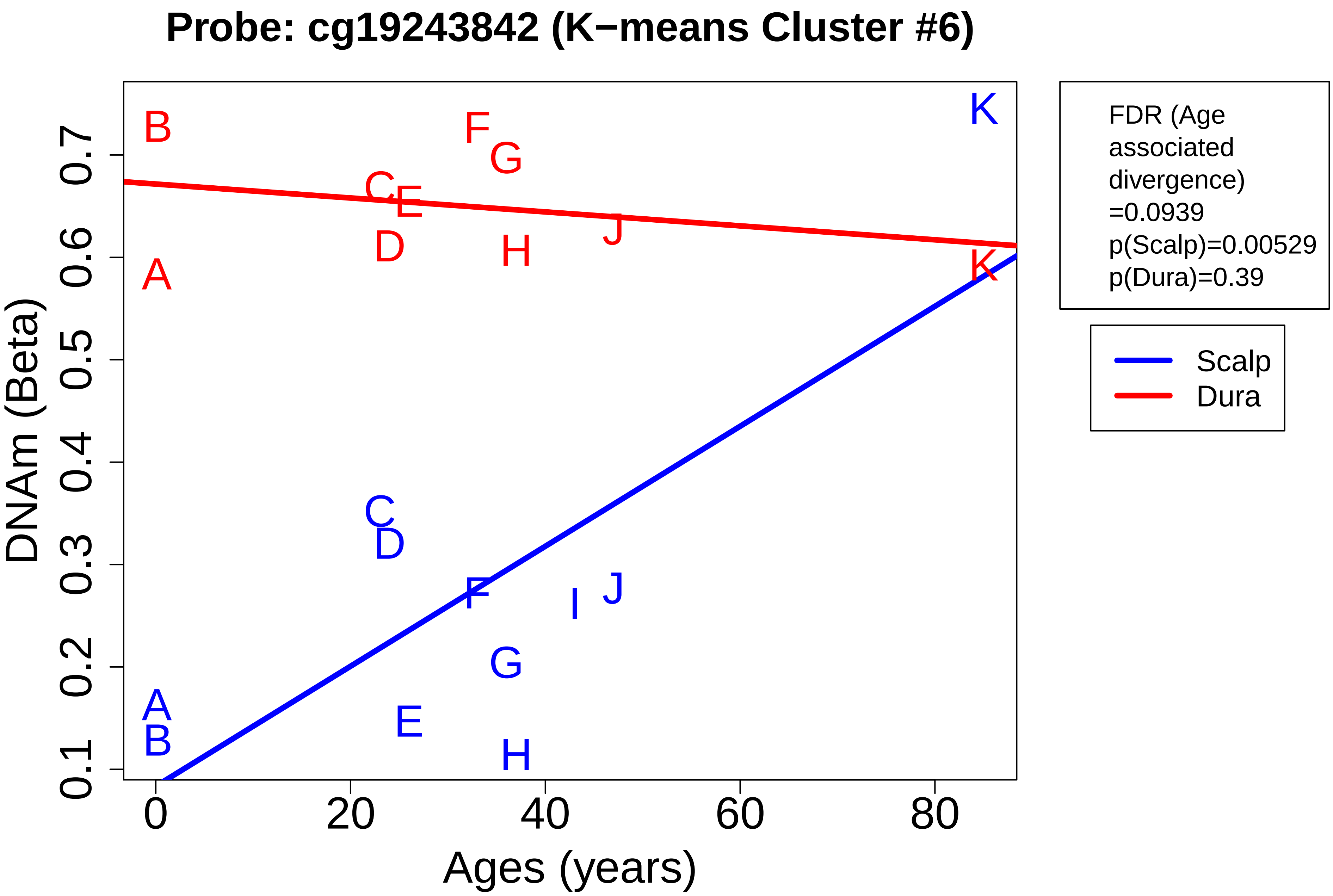

Probe: cg19243842 (K−means Cluster #6)
DNAm (Beta)
0.1
0.2
0.3
0.4
0.5
0.6
0.7
Ages (years)
0
20
40
60
80
B
F
G
C
E
D
H
J
A
K
K
C
D
F
I
J
G
A
B
E
H
FDR (Age associated divergence) =0.0939
p(Scalp)=0.00529
p(Dura)=0.39
Scalp
Dura

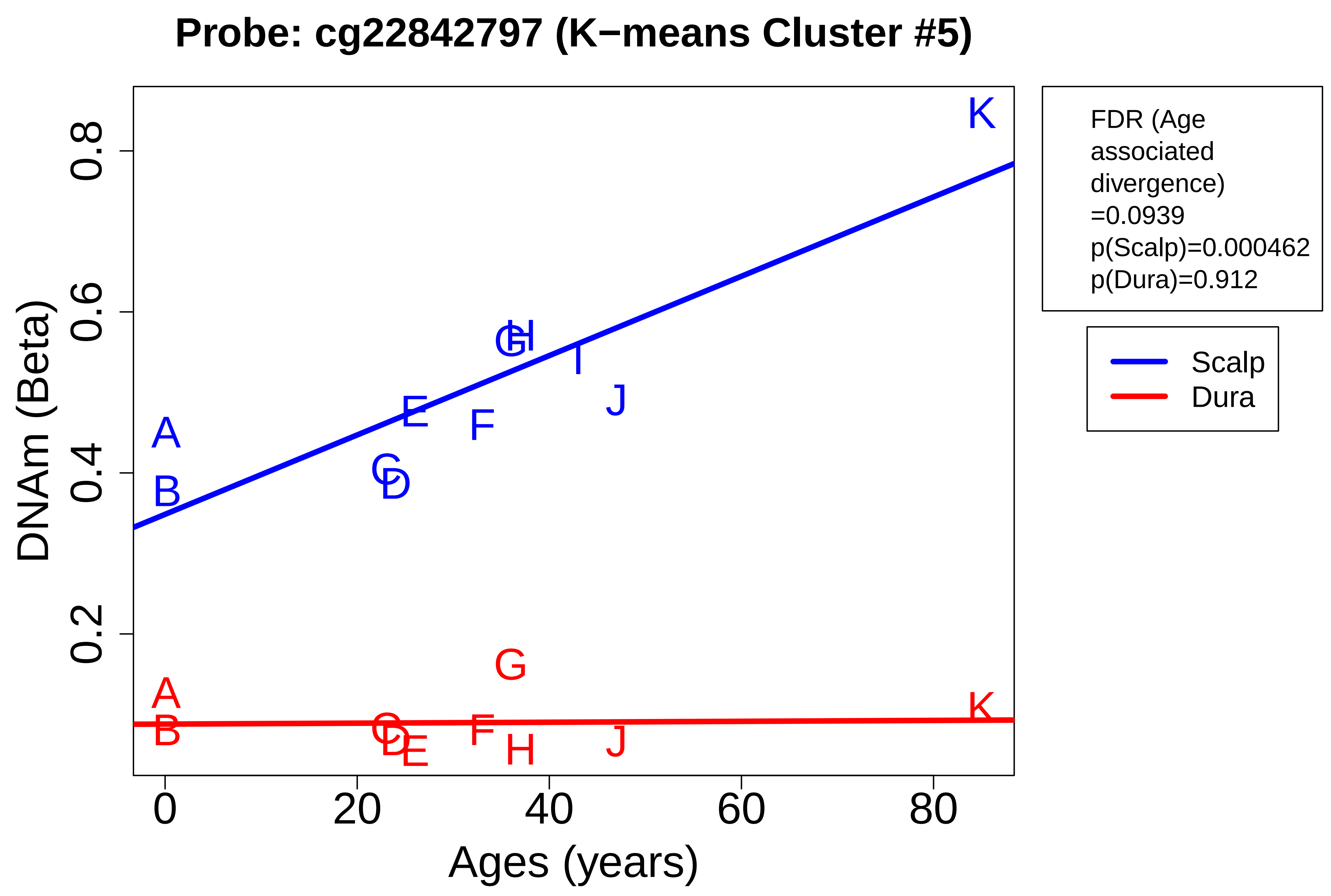

Probe: cg22842797 (K−means Cluster #5)
FDR (Age associated divergence) =0.0939
p(Scalp)=0.000462
p(Dura)=0.912
Scalp
Dura
DNAm (Beta)
0.2
0.4
0.6
0.8
Ages (years)
0
20
40
60
80
A
B
C
D
E
F
G
H
I
J
K

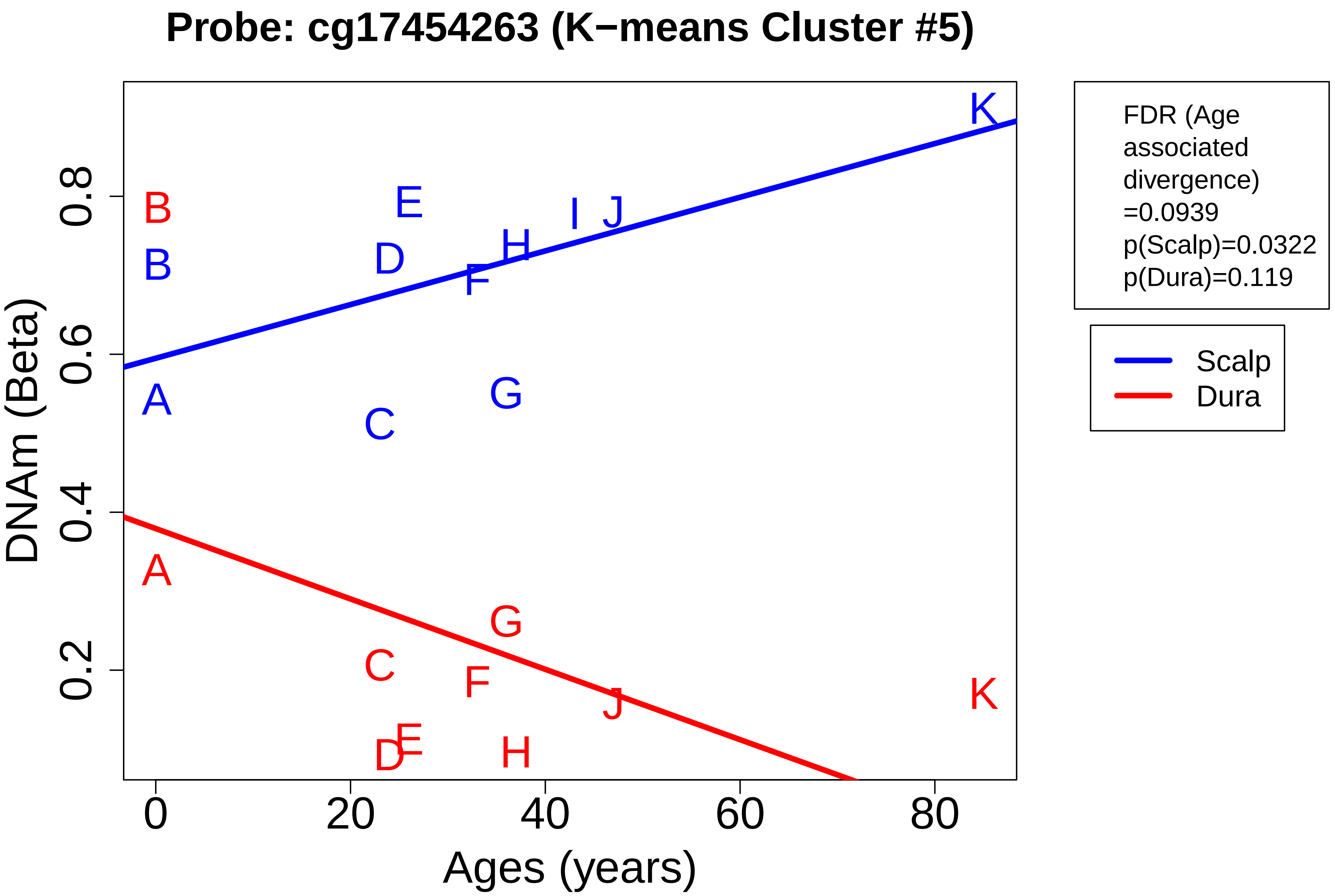

Probe: cg17454263 (K−means Cluster #5)
DNAm (Beta)
0.2
0.4
0.6
0.8
0
20
40
60
80
Ages (years)
A B C D E F G H I J K
A B C D E F G H J K
FDR (Age associated divergence) =0.0939
p(Scalp)=0.0322
p(Dura)=0.119
Scalp
Dura

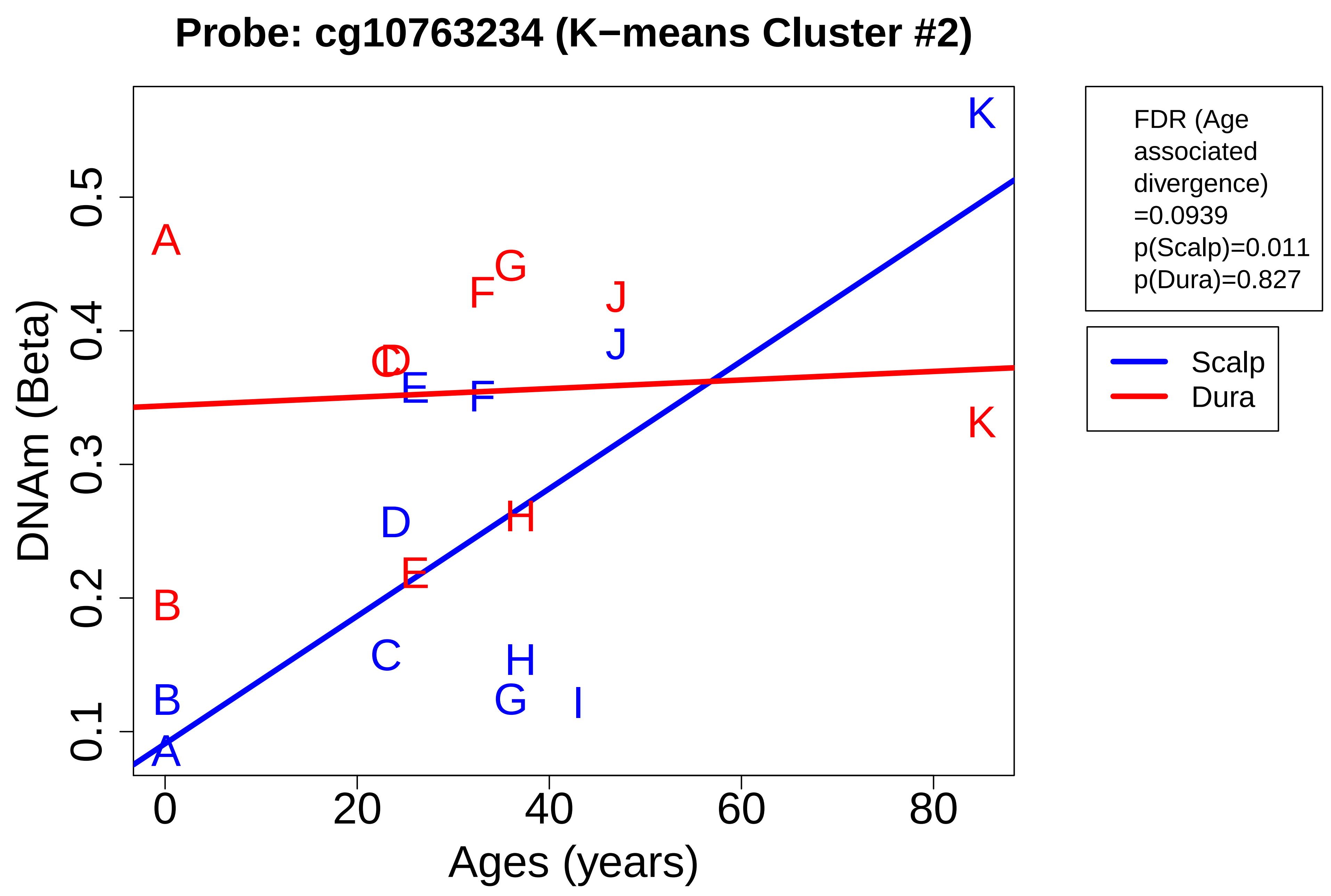

Probe: cg10763234 (K−means Cluster #2)
DNAm (Beta)
0.1
0.2
0.3
0.4
0.5
Ages (years)
0
20
40
60
80
FDR (Age associated divergence) =0.0939
p(Scalp)=0.011
p(Dura)=0.827
Scalp
Dura
A
B
C
D
E
F
G
H
I
J
K

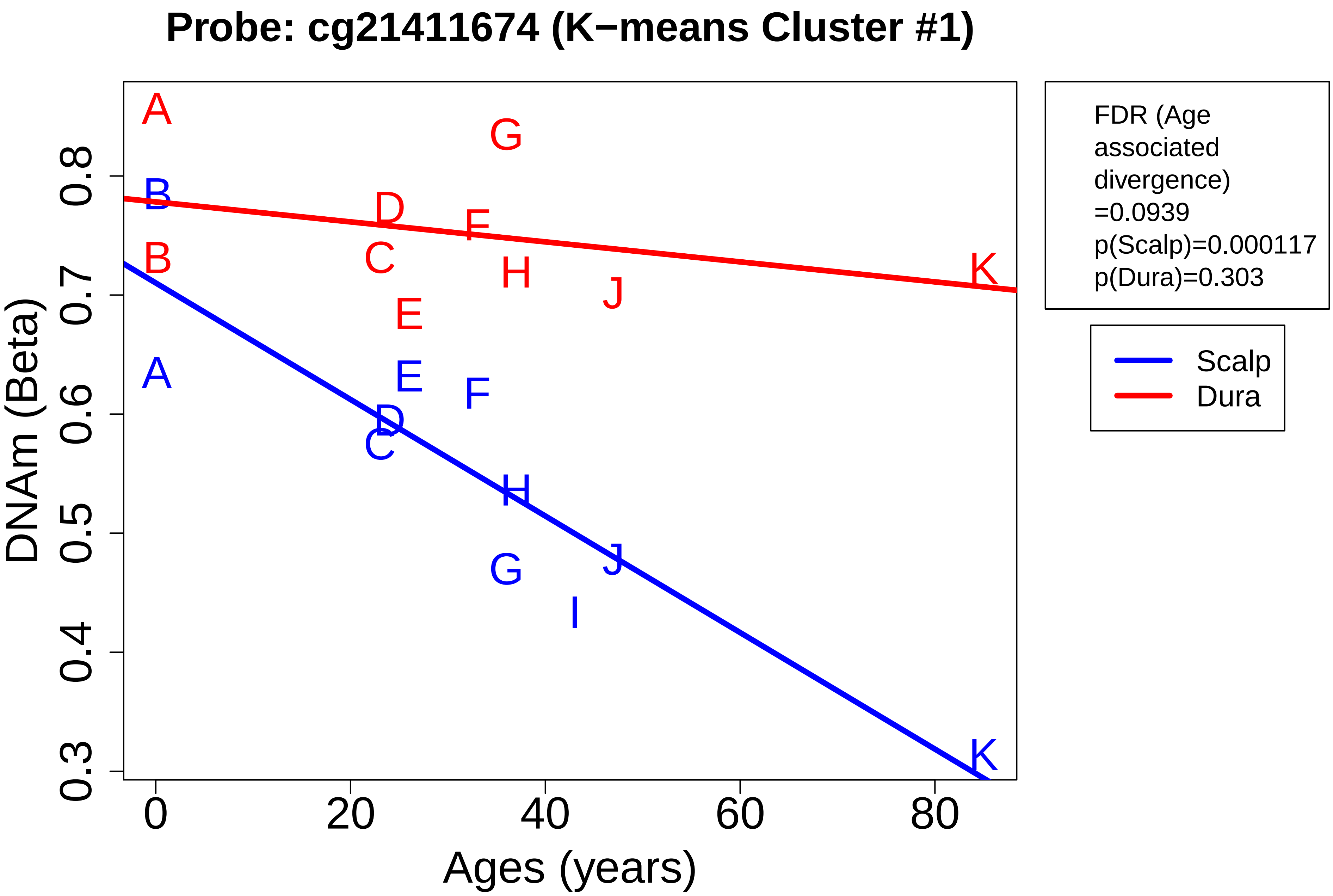

Probe: cg21411674 (K−means Cluster #1)
DNAm (Beta)
0.3
0.4
0.5
0.6
0.7
0.8
0
20
40
60
80
Ages (years)
A
B
C
D
E
F
G
H
I
J
K
FDR (Age associated divergence) =0.0939
p(Scalp)=0.000117
p(Dura)=0.303
Scalp
Dura

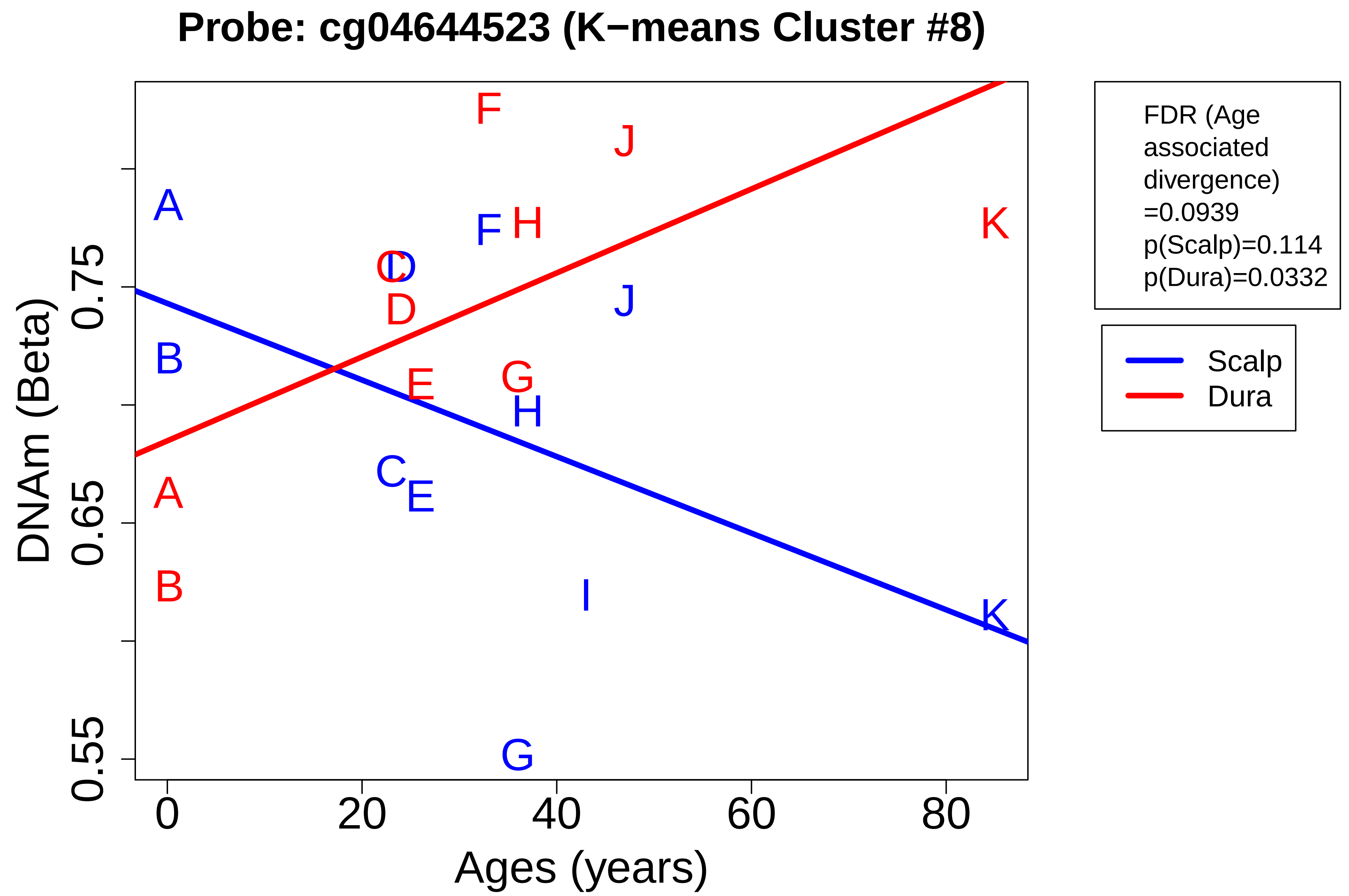

Probe: cg04644523 (K−means Cluster #8)
DNAm (Beta)
0.55
0.65
0.75
0
20
40
60
80
Ages (years)
FDR (Age associated divergence) =0.0939
p(Scalp)=0.114
p(Dura)=0.0332
Scalp
Dura

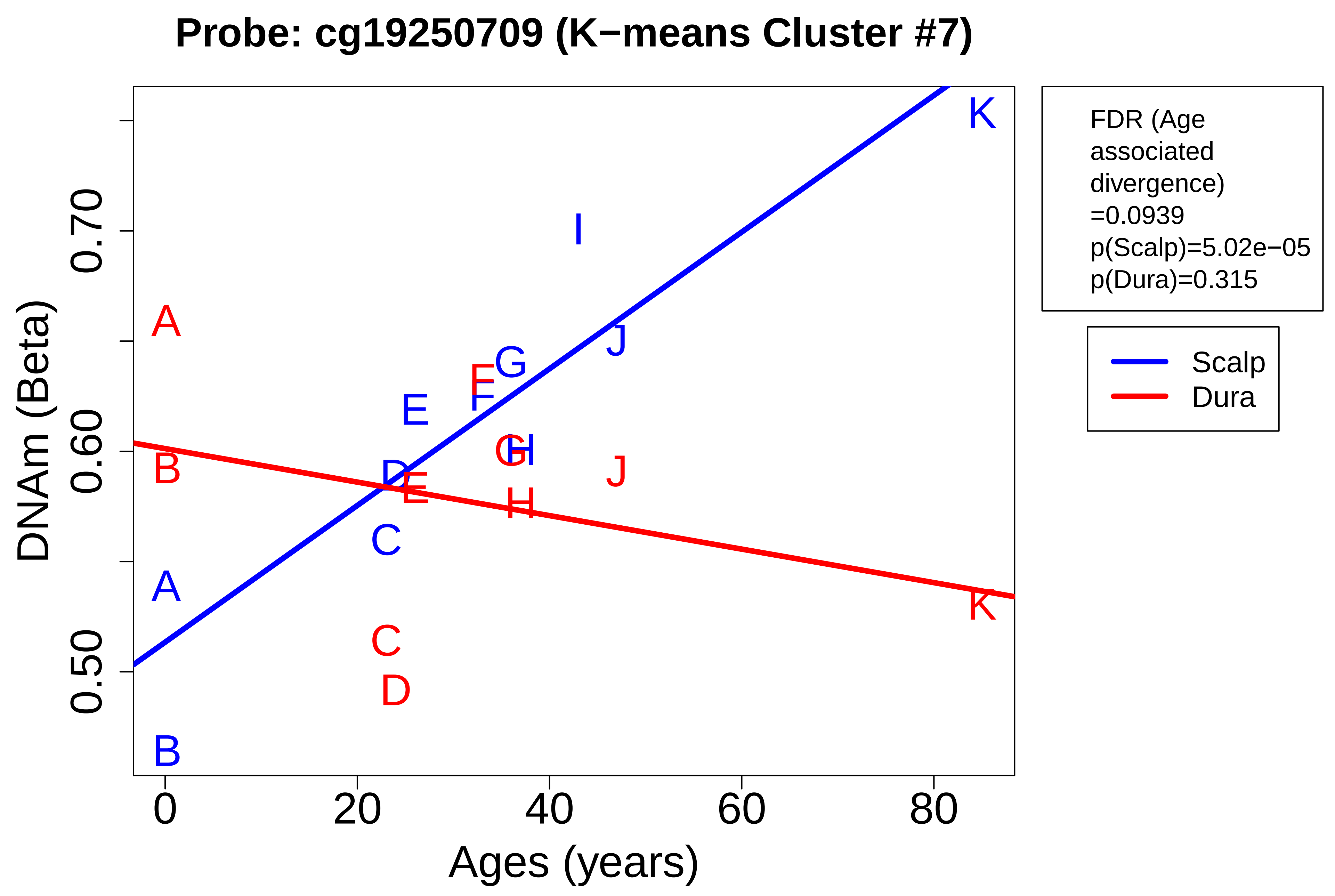

Probe: cg19250709 (K−means Cluster #7)
DNAm (Beta)
0.50
0.60
0.70
0
20
40
60
80
Ages (years)
FDR (Age associated divergence) =0.0939
p(Scalp)=5.02e−05
p(Dura)=0.315
Scalp
Dura
A
B
C
D
E
F
G
H
I
J
K

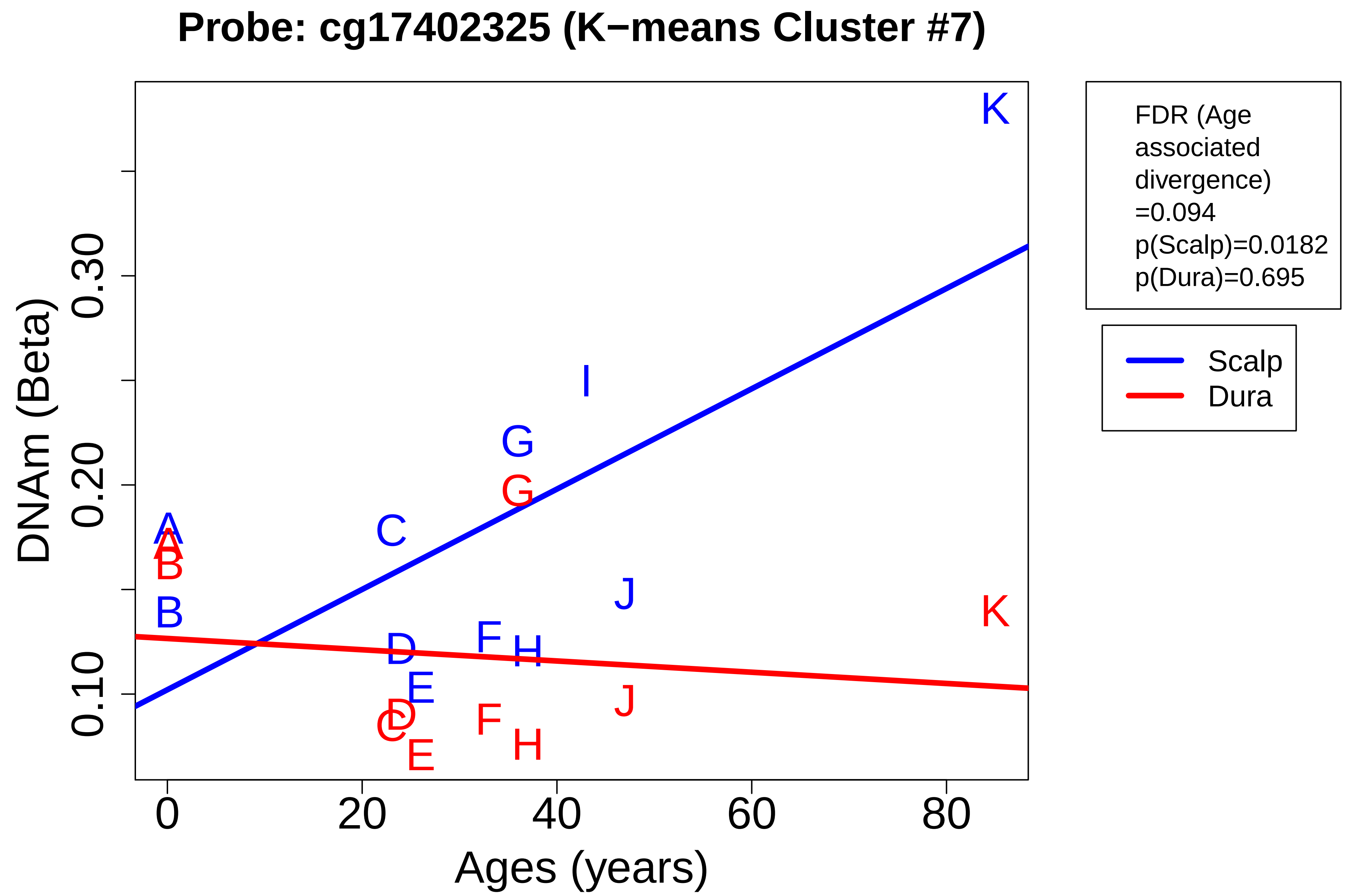

Probe: cg17402325 (K−means Cluster #7)
DNAm (Beta)
0.10
0.20
0.30
0
20
40
60
80
Ages (years)
FDR (Age associated divergence) =0.094
p(Scalp)=0.0182
p(Dura)=0.695
Scalp
Dura
A
B
C
D
E
F
G
H
I
J
K

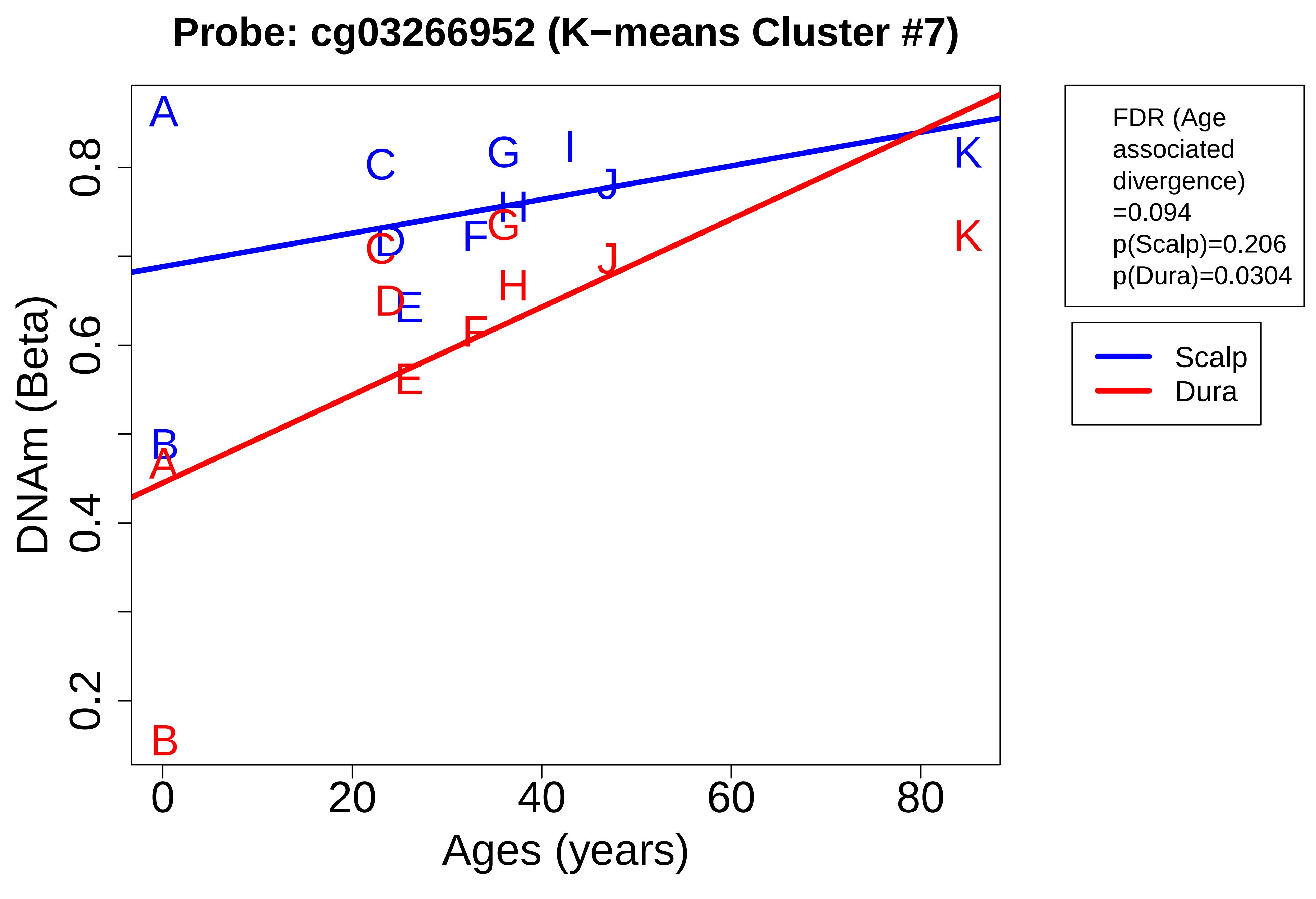
Probe: cg03266952 (K−means Cluster #7)
DNAm (Beta)
0.2
0.4
0.6
0.8
0
20
40
60
80
Ages (years)
A
B
C
D
E
F
G
H
I
J
K
FDR (Age associated divergence) =0.094
p(Scalp)=0.206
p(Dura)=0.0304
Scalp
Dura

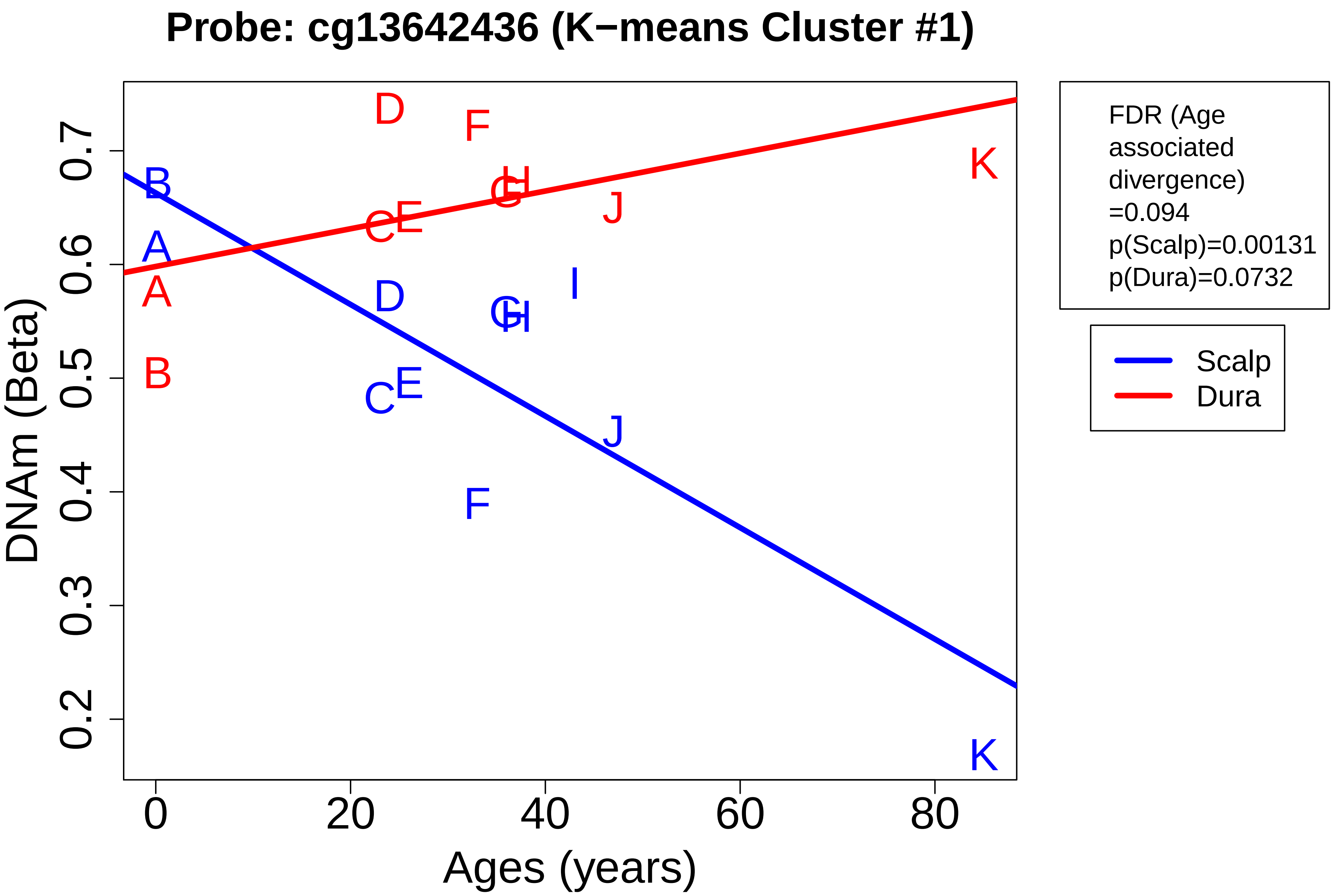

Probe: cg13642436 (K−means Cluster #1)
DNAm (Beta)
0.2
0.3
0.4
0.5
0.6
0.7
0
20
40
60
80
Ages (years)
FDR (Age associated divergence) =0.094
p(Scalp)=0.00131
p(Dura)=0.0732
Scalp
Dura

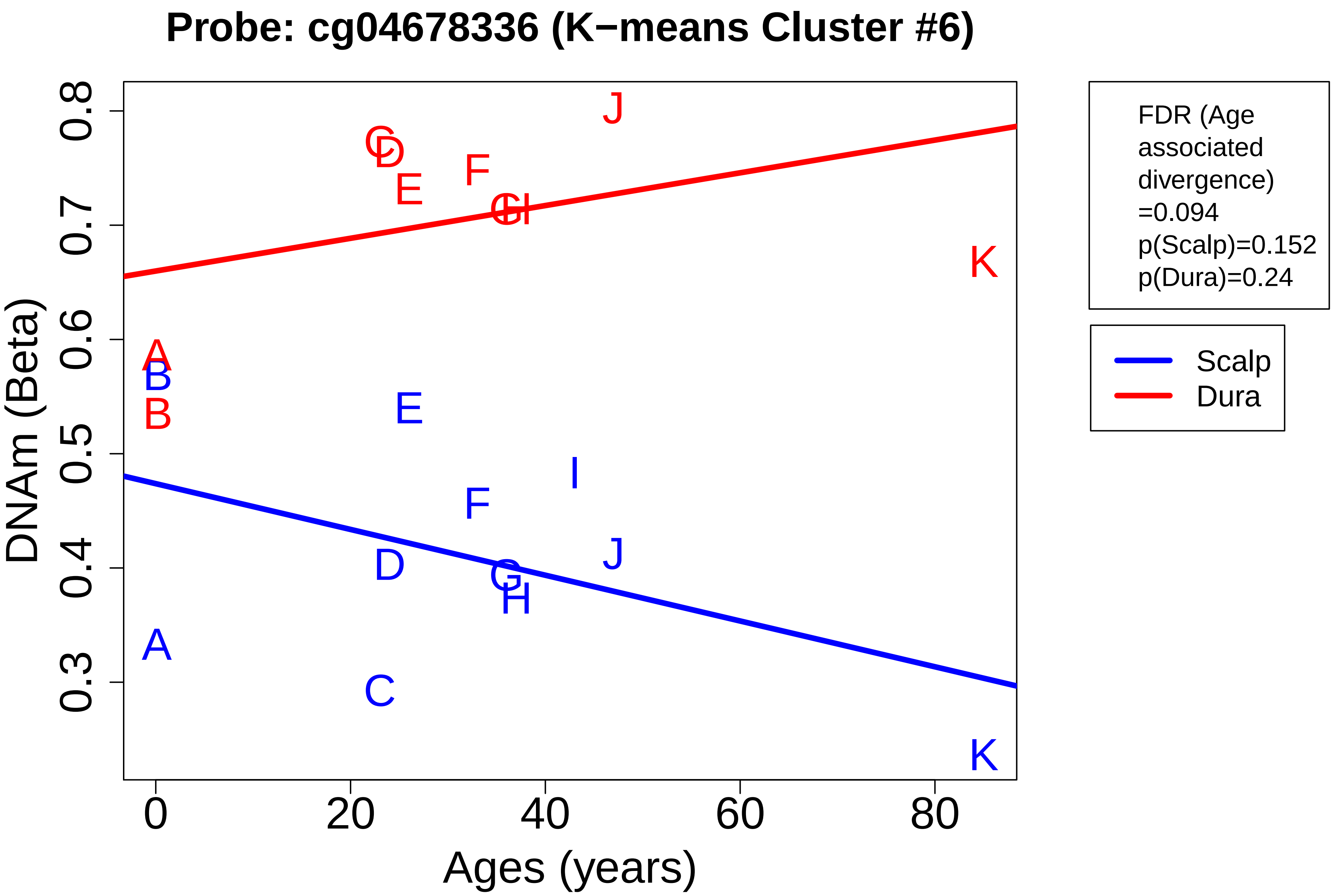

Probe: cg04678336 (K−means Cluster #6)
DNAm (Beta)
0.3
0.4
0.5
0.6
0.7
0.8
0
20
40
60
80
Ages (years)
A
B
B
A
C
D
C
D
E
E
F
F
G
H
G
H
I
J
J
K
K
FDR (Age associated divergence) =0.094
p(Scalp)=0.152
p(Dura)=0.24
Scalp
Dura

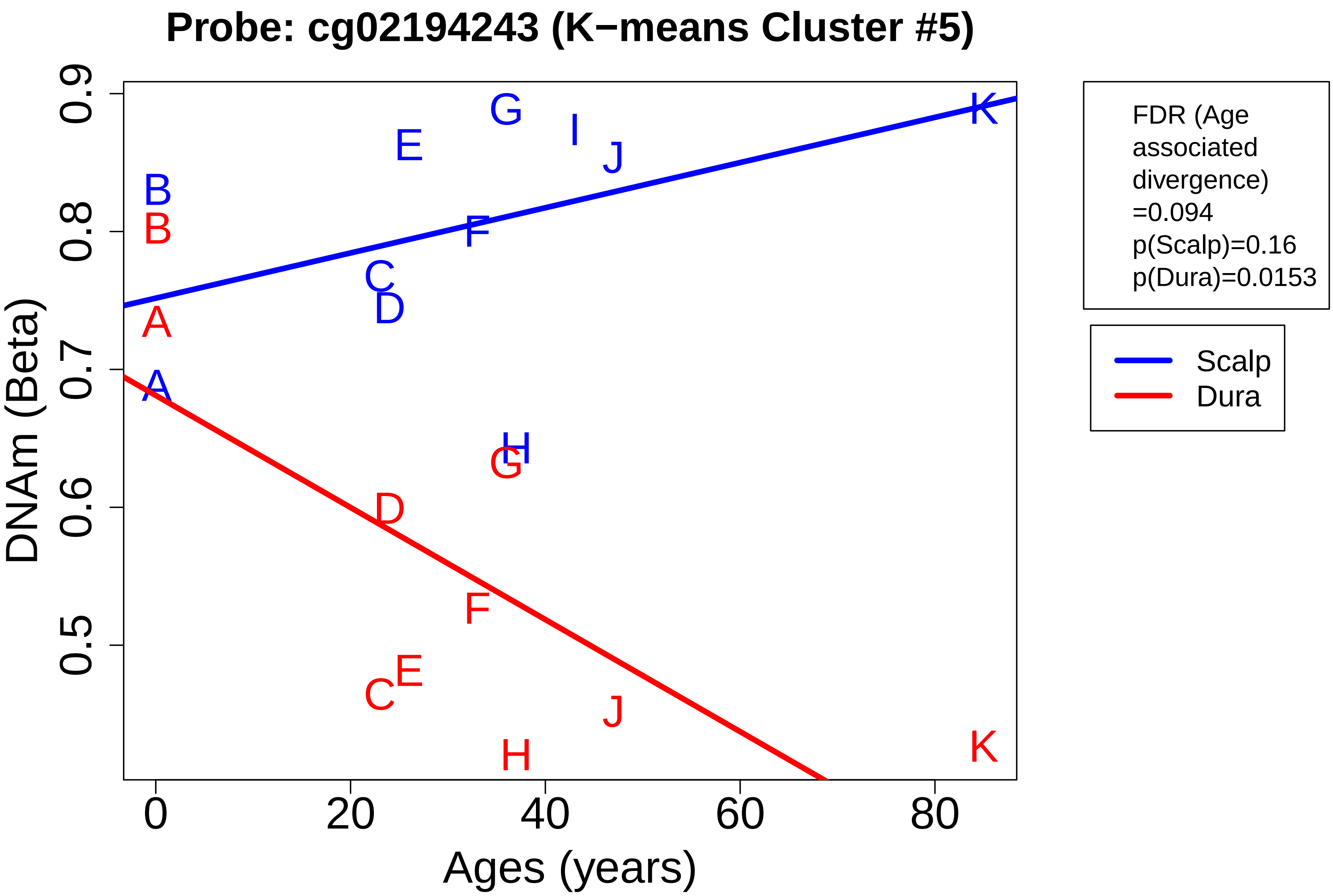

Probe: cg02194243 (K−means Cluster #5)
DNAm (Beta)
0.5
0.6
0.7
0.8
0.9
0
20
40
60
80
Ages (years)
FDR (Age associated divergence) =0.094
p(Scalp)=0.16
p(Dura)=0.0153
Scalp
Dura
A
B
C
D
E
F
G
H
I
J
K

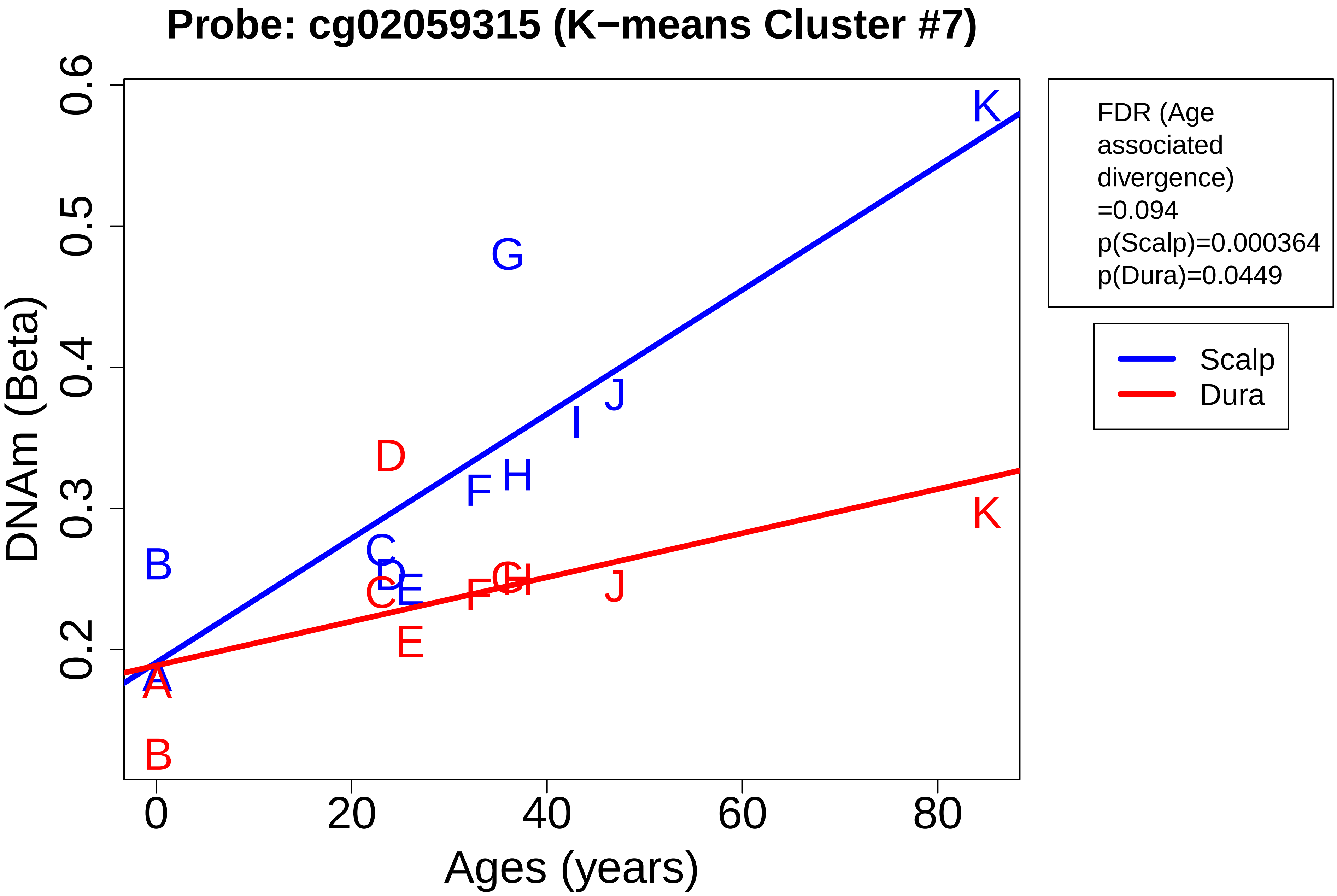
Probe: cg02059315 (K−means Cluster #7)
DNAm (Beta)
0.2
0.3
0.4
0.5
0.6
0
20
40
60
80
Ages (years)
FDR (Age associated divergence) =0.094
p(Scalp)=0.000364
p(Dura)=0.0449
Scalp
Dura
A
B
C
D
E
F
G
H
I
J
K

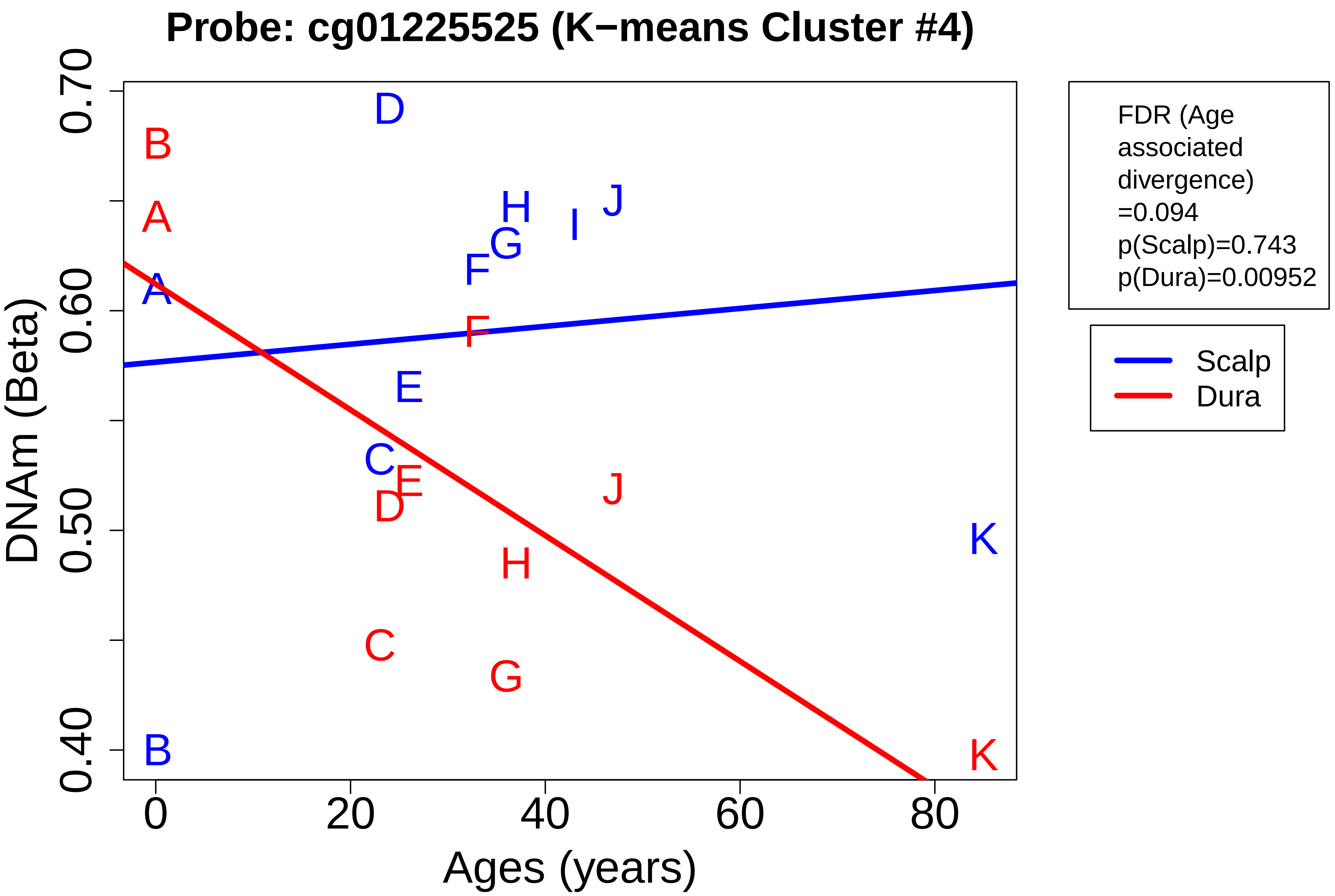

Probe: cg01225525 (K−means Cluster #4)
DNAm (Beta)
0.40
0.50
0.60
0.70
0
20
40
60
80
Ages (years)
FDR (Age associated divergence) =0.094
p(Scalp)=0.743
p(Dura)=0.00952
Scalp
Dura
A
B
C
D
E
F
G
H
I
J
K

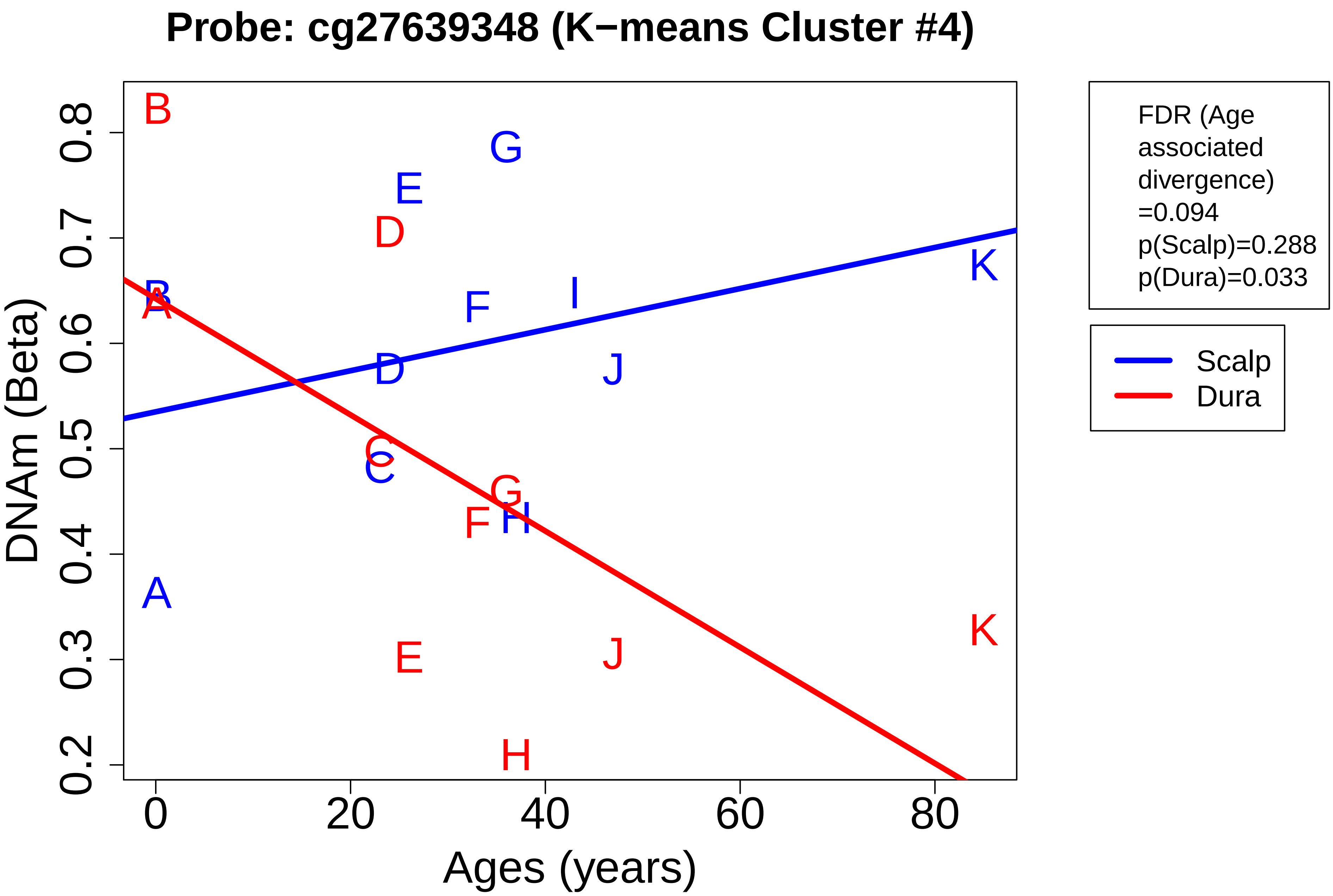

Probe: cg27639348 (K−means Cluster #4)
FDR (Age associated divergence) =0.094
p(Scalp)=0.288
p(Dura)=0.033
Scalp
Dura
DNAm (Beta)
Ages (years)
0.2
0.3
0.4
0.5
0.6
0.7
0.8
0
20
40
60
80

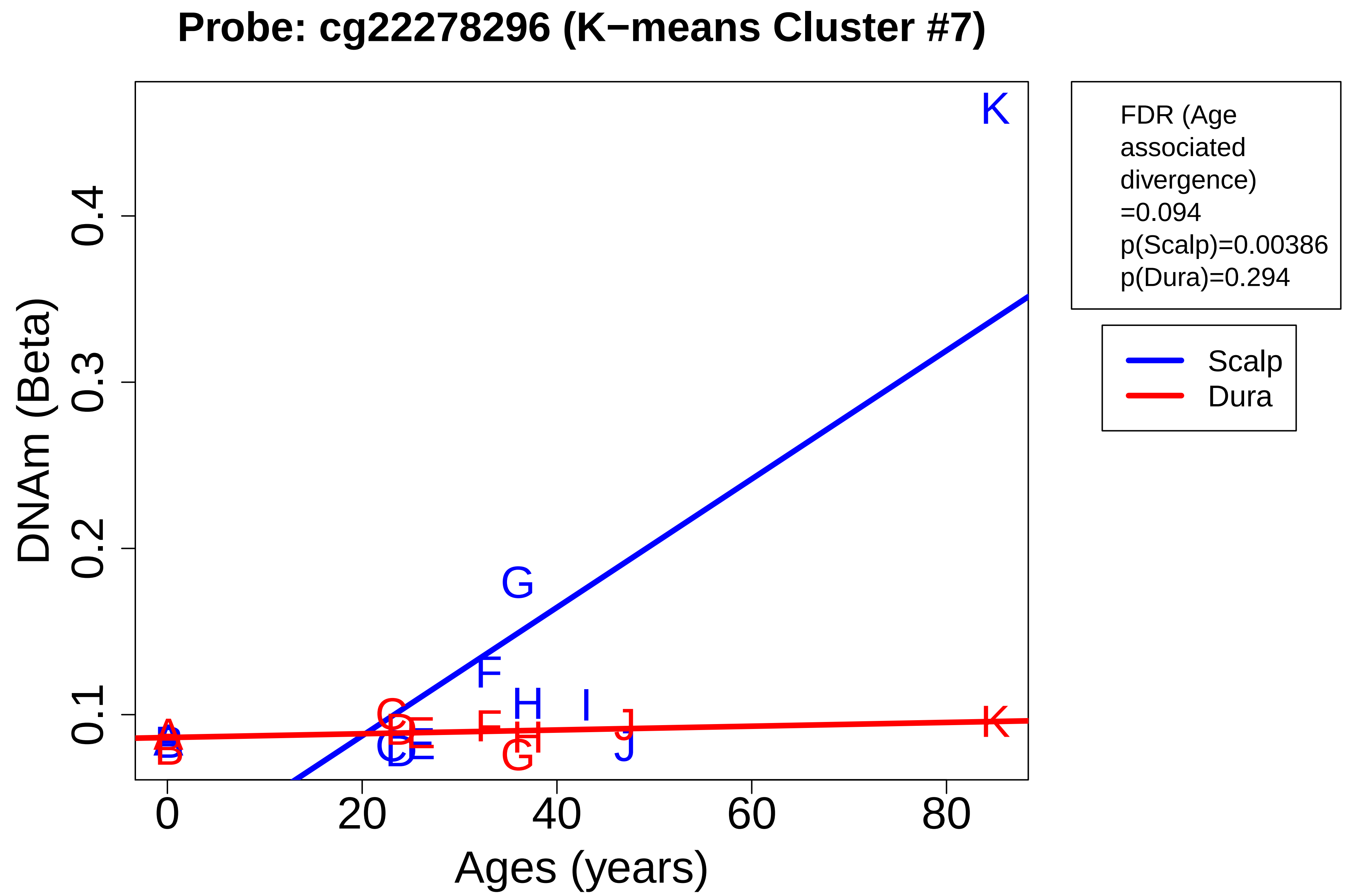
Probe: cg22278296 (K−means Cluster #7)
DNAm (Beta)
0.1
0.2
0.3
0.4
0
20
40
60
80
Ages (years)
FDR (Age associated divergence) =0.094
p(Scalp)=0.00386
p(Dura)=0.294
Scalp
Dura

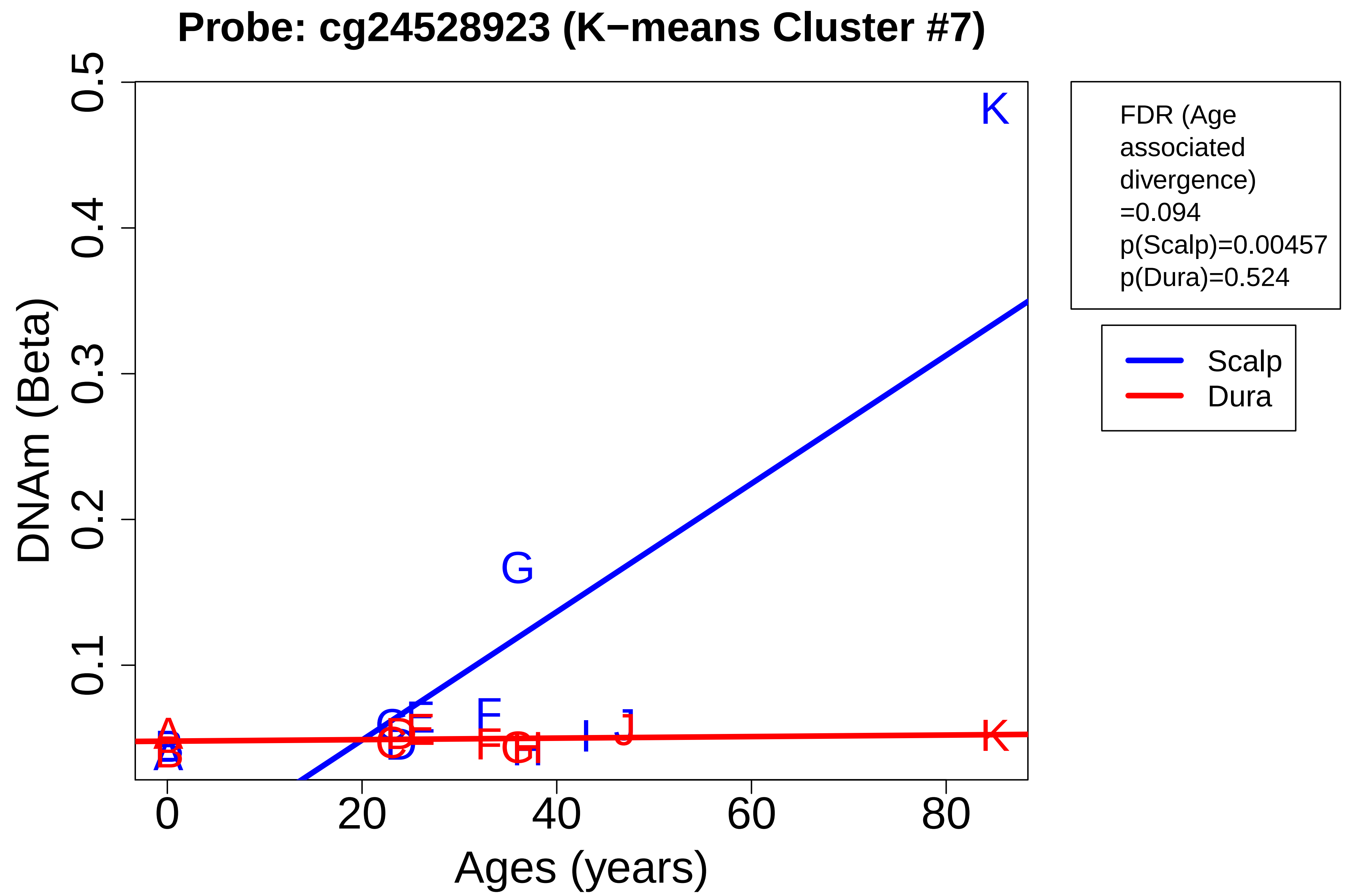

Probe: cg24528923 (K−means Cluster #7)
DNAm (Beta)
0.1
0.2
0.3
0.4
0.5
Ages (years)
0
20
40
60
80
FDR (Age associated divergence) =0.094
p(Scalp)=0.00457
p(Dura)=0.524
Scalp
Dura

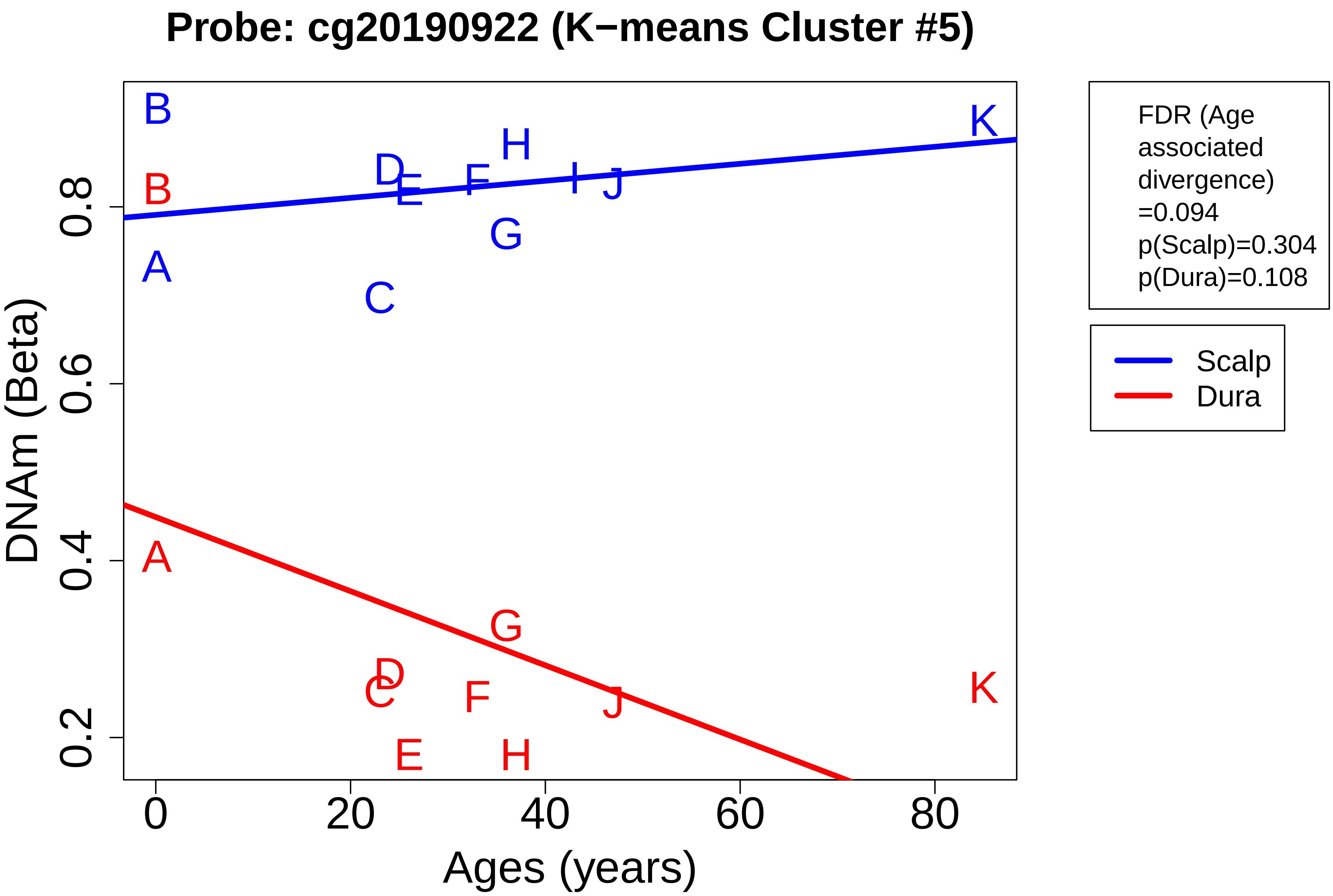

Probe: cg20190922 (K−means Cluster #5)
DNAm (Beta)
0.2
0.4
0.6
0.8
0
20
40
60
80
Ages (years)
FDR (Age associated divergence) =0.094
p(Scalp)=0.304
p(Dura)=0.108
Scalp
Dura

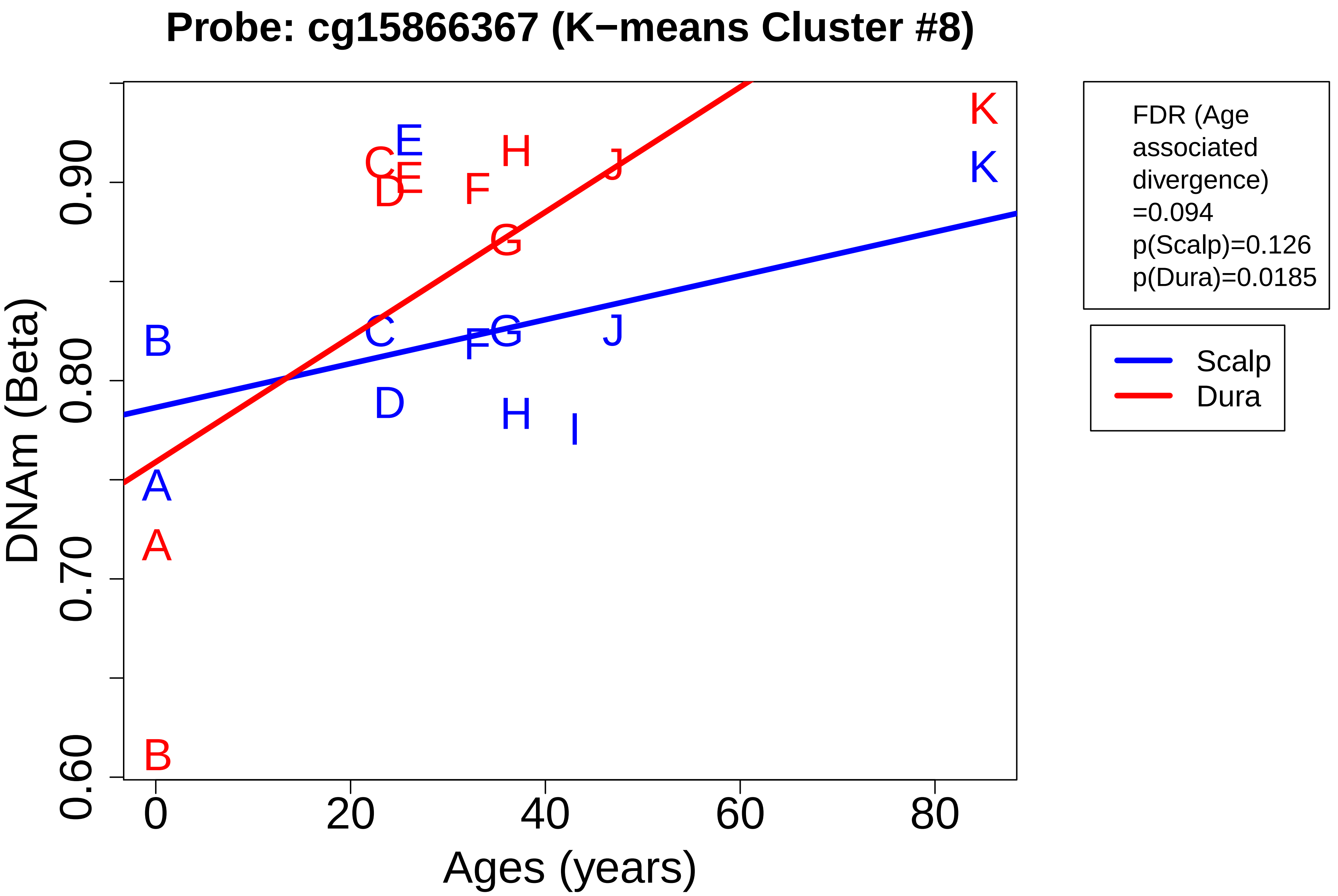
Probe: cg15866367 (K−means Cluster #8)
DNAm (Beta)
0.60
0.70
0.80
0.90
0
20
40
60
80
Ages (years)
FDR (Age associated divergence) =0.094
p(Scalp)=0.126
p(Dura)=0.0185
Scalp
Dura

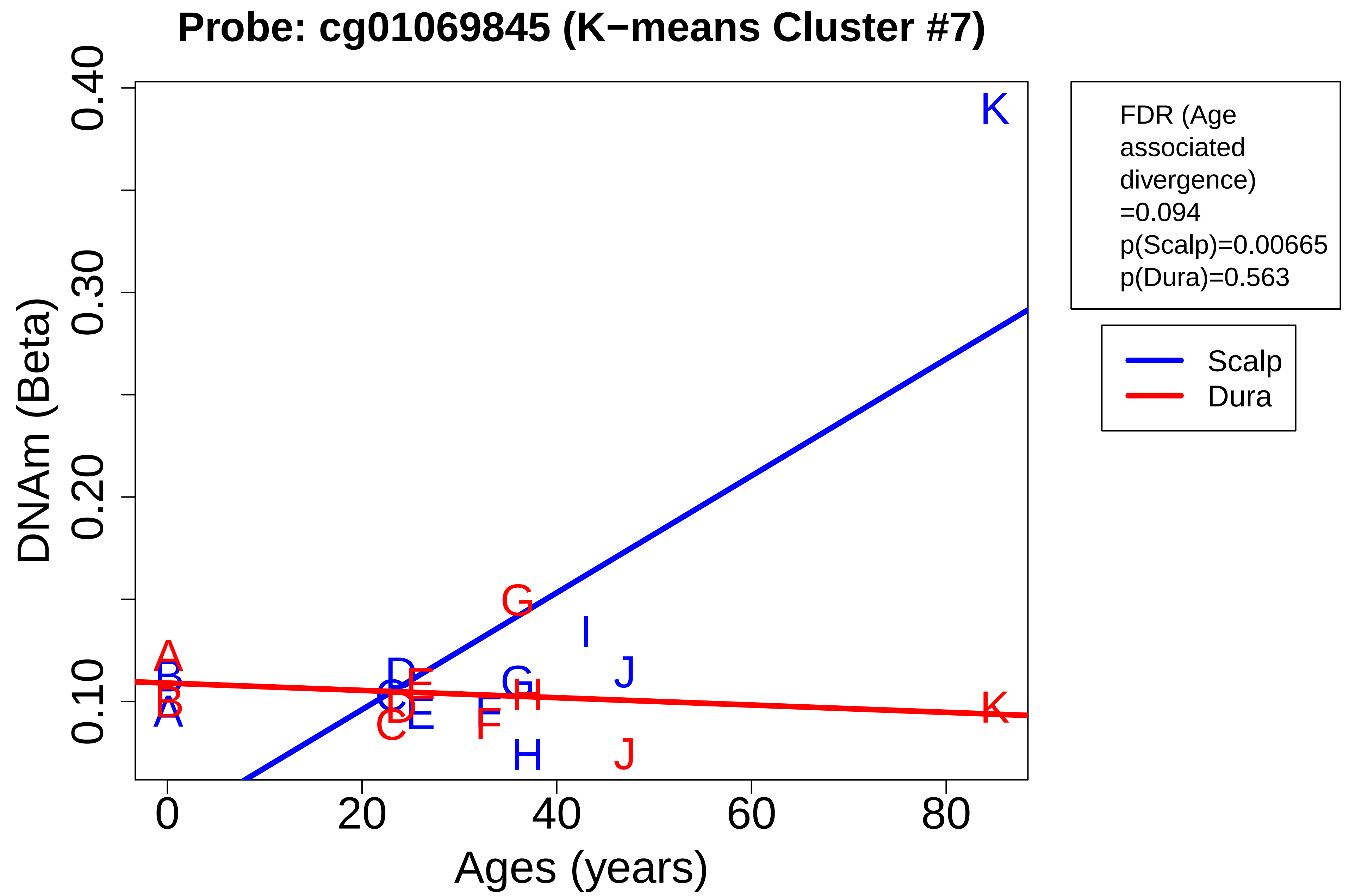

Probe: cg01069845 (K−means Cluster #7)
DNAm (Beta)
0.10
0.20
0.30
0.40
0
20
40
60
80
Ages (years)
FDR (Age associated divergence) =0.094
p(Scalp)=0.00665
p(Dura)=0.563
Scalp
Dura

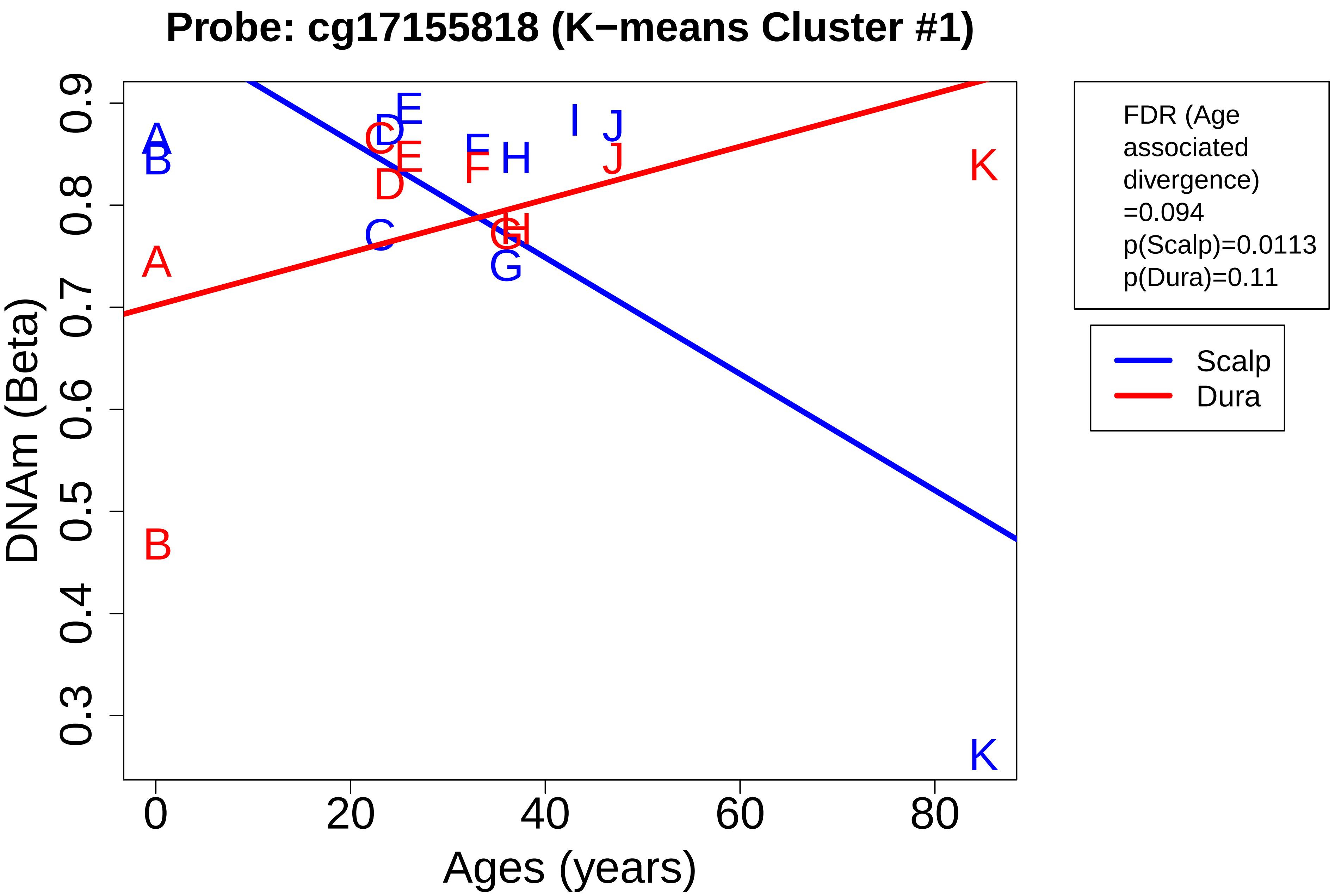

Probe: cg17155818 (K−means Cluster #1)
DNAm (Beta)
0.3
0.4
0.5
0.6
0.7
0.8
0.9
Ages (years)
0
20
40
60
80
A B C D E F G H I J K
FDR (Age associated divergence) =0.094
p(Scalp)=0.0113
p(Dura)=0.11
Scalp
Dura

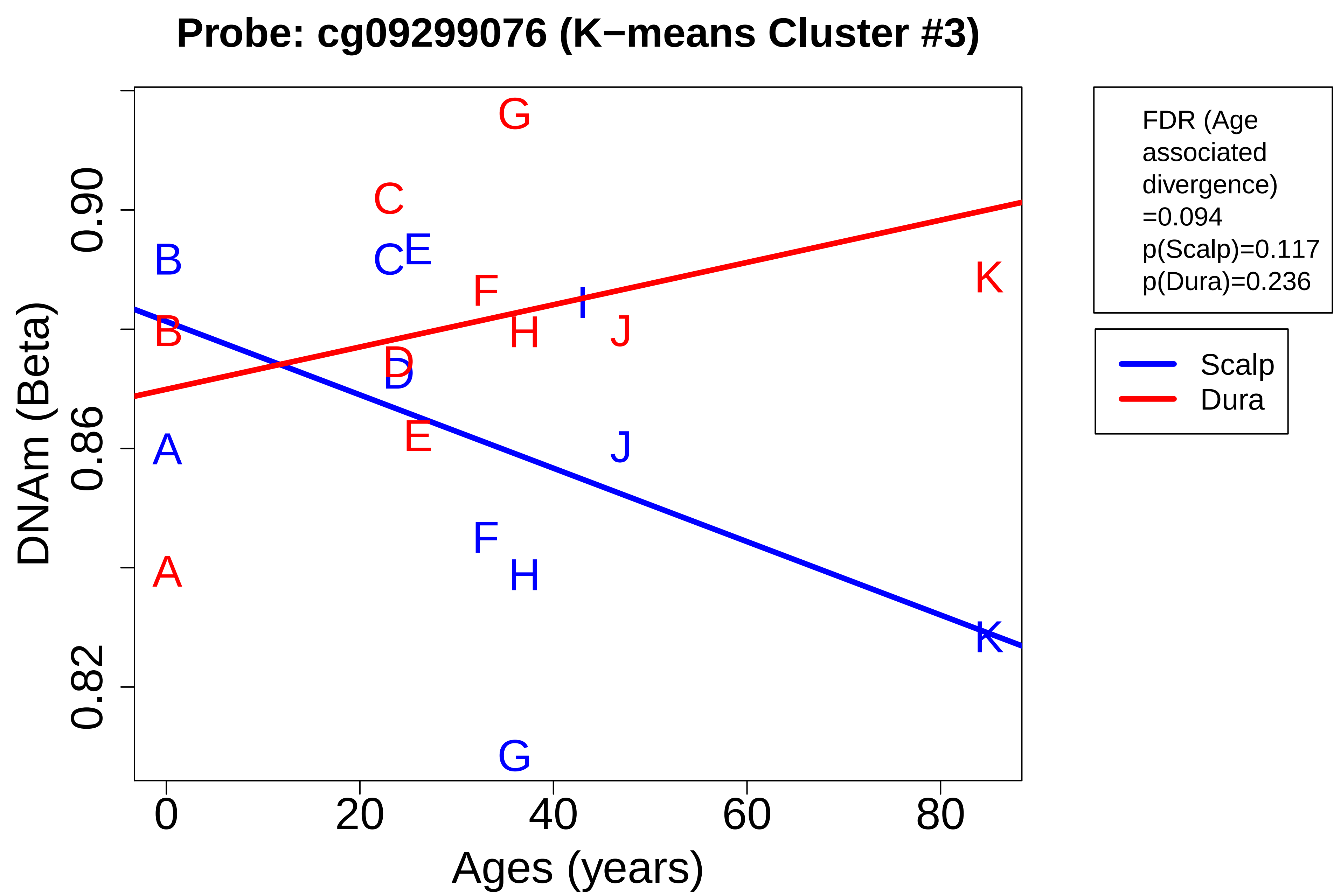

Probe: cg09299076 (K−means Cluster #3)
DNAm (Beta)
0.82
0.86
0.90
Ages (years)
0
20
40
60
80
FDR (Age associated divergence) =0.094
p(Scalp)=0.117
p(Dura)=0.236
Scalp
Dura

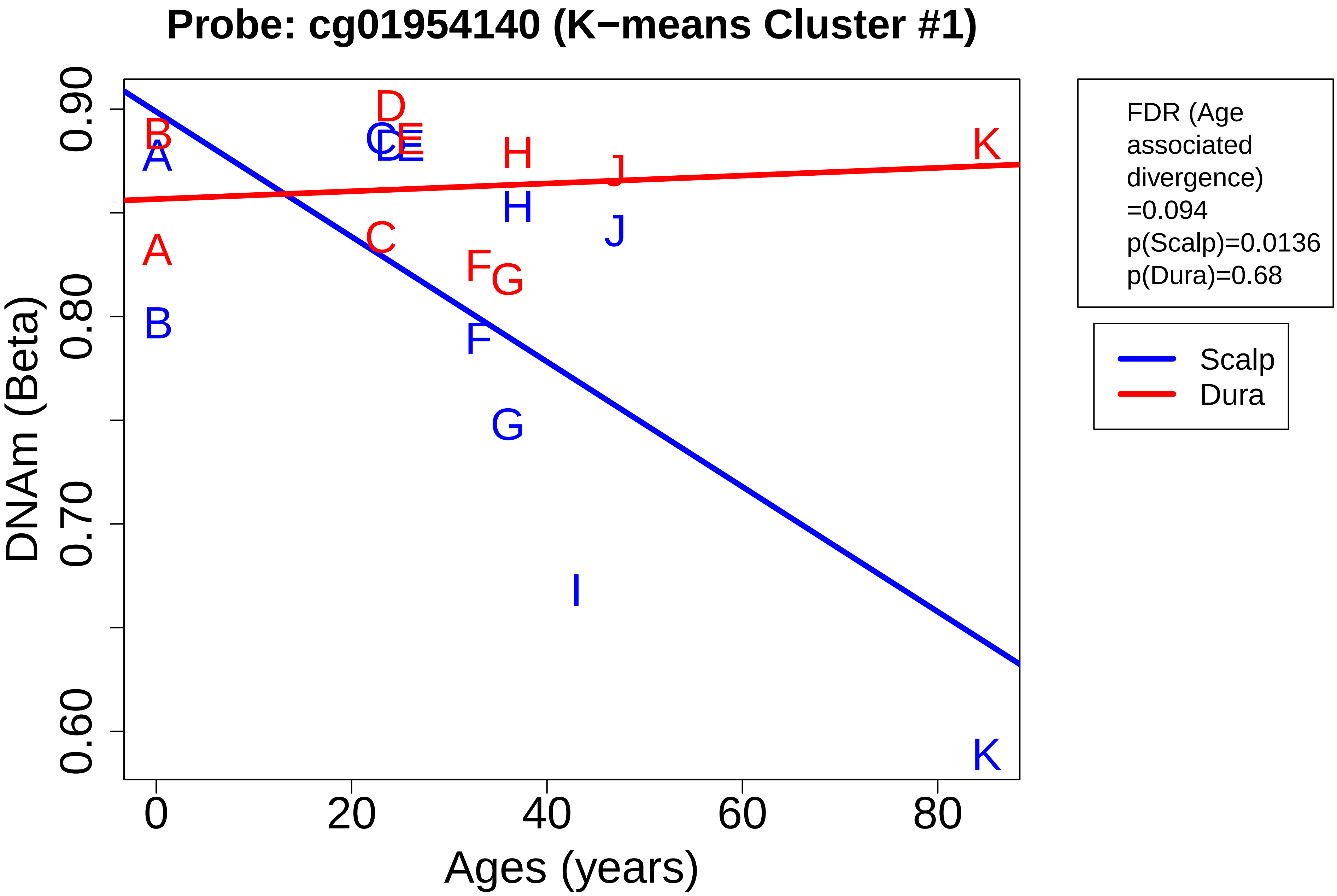
Probe: cg01954140 (K−means Cluster #1)
DNAm (Beta)
0.60
0.70
0.80
0.90
Ages (years)
0
20
40
60
80
FDR (Age associated divergence) =0.094
p(Scalp)=0.0136
p(Dura)=0.68
Scalp
Dura
A
B
C
D
E
F
G
H
I
J
K

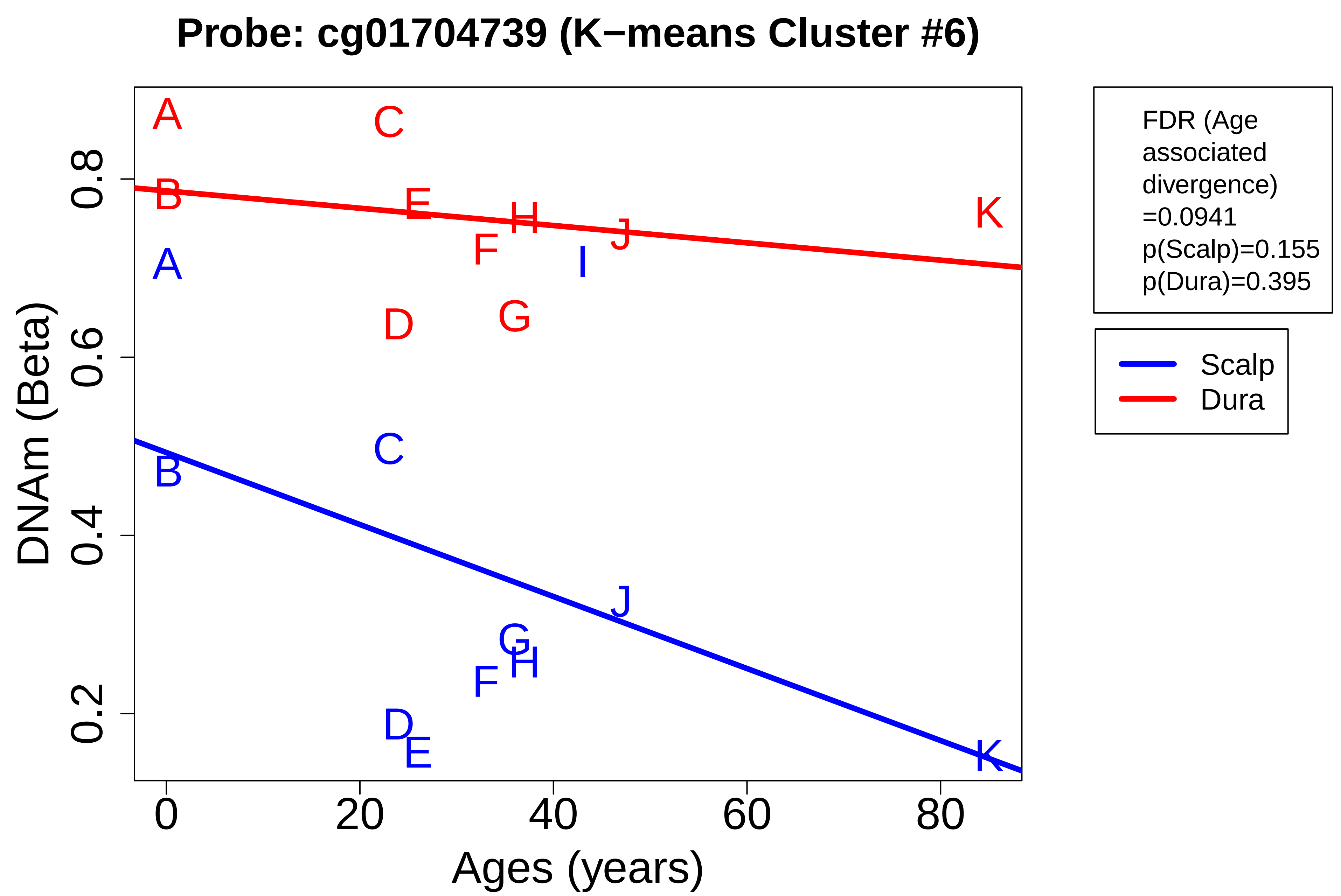

Probe: cg01704739 (K−means Cluster #6)
FDR (Age associated divergence) =0.0941
p(Scalp)=0.155
p(Dura)=0.395
Scalp
Dura
DNAm (Beta)
0.2
0.4
0.6
0.8
0
20
40
60
80
Ages (years)
A
B
C
D
E
F
G
H
I
J
K

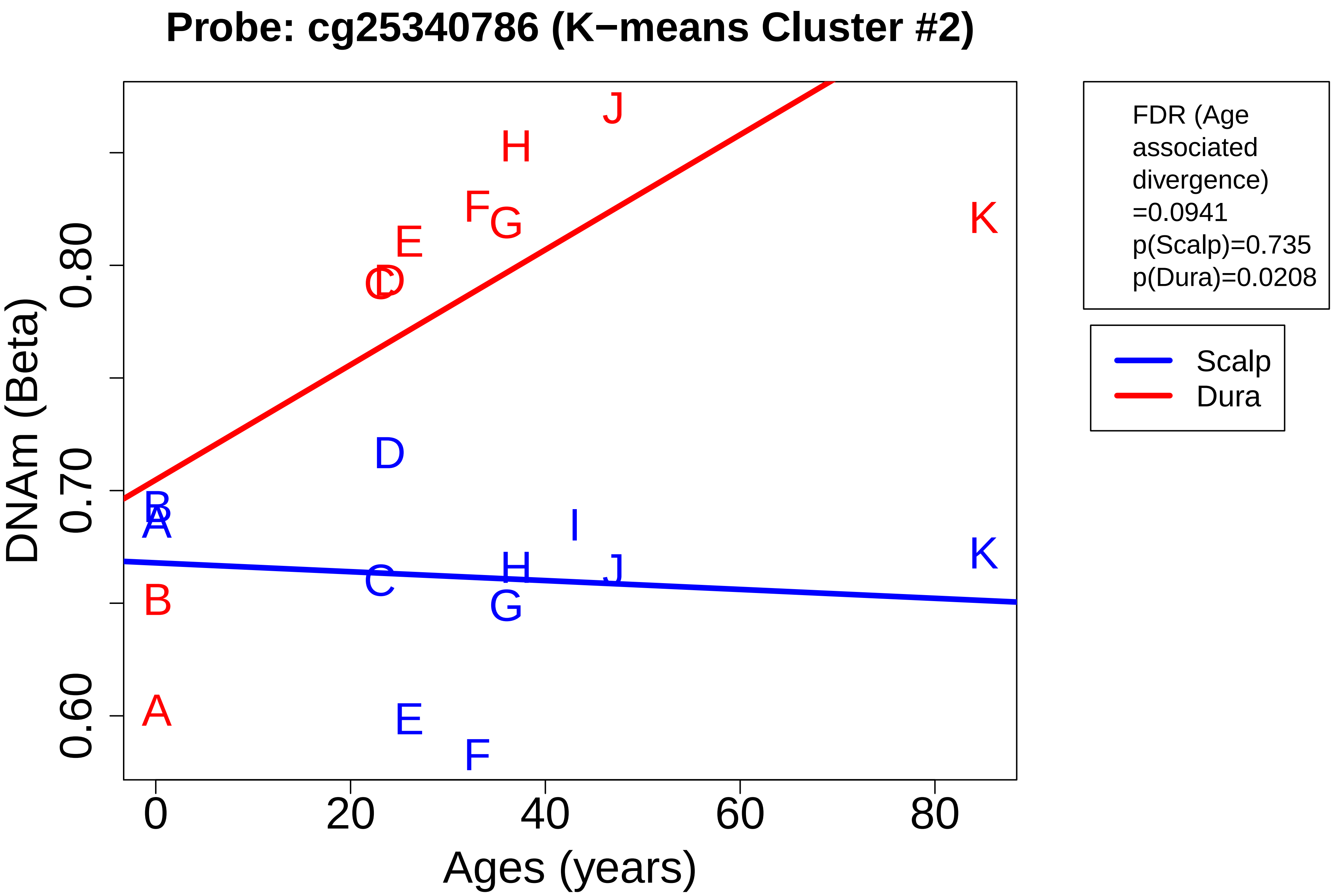
Probe: cg25340786 (K−means Cluster #2)
DNAm (Beta)
0.60
0.70
0.80
Ages (years)
0
20
40
60
80
FDR (Age associated divergence) =0.0941
p(Scalp)=0.735
p(Dura)=0.0208
Scalp
Dura

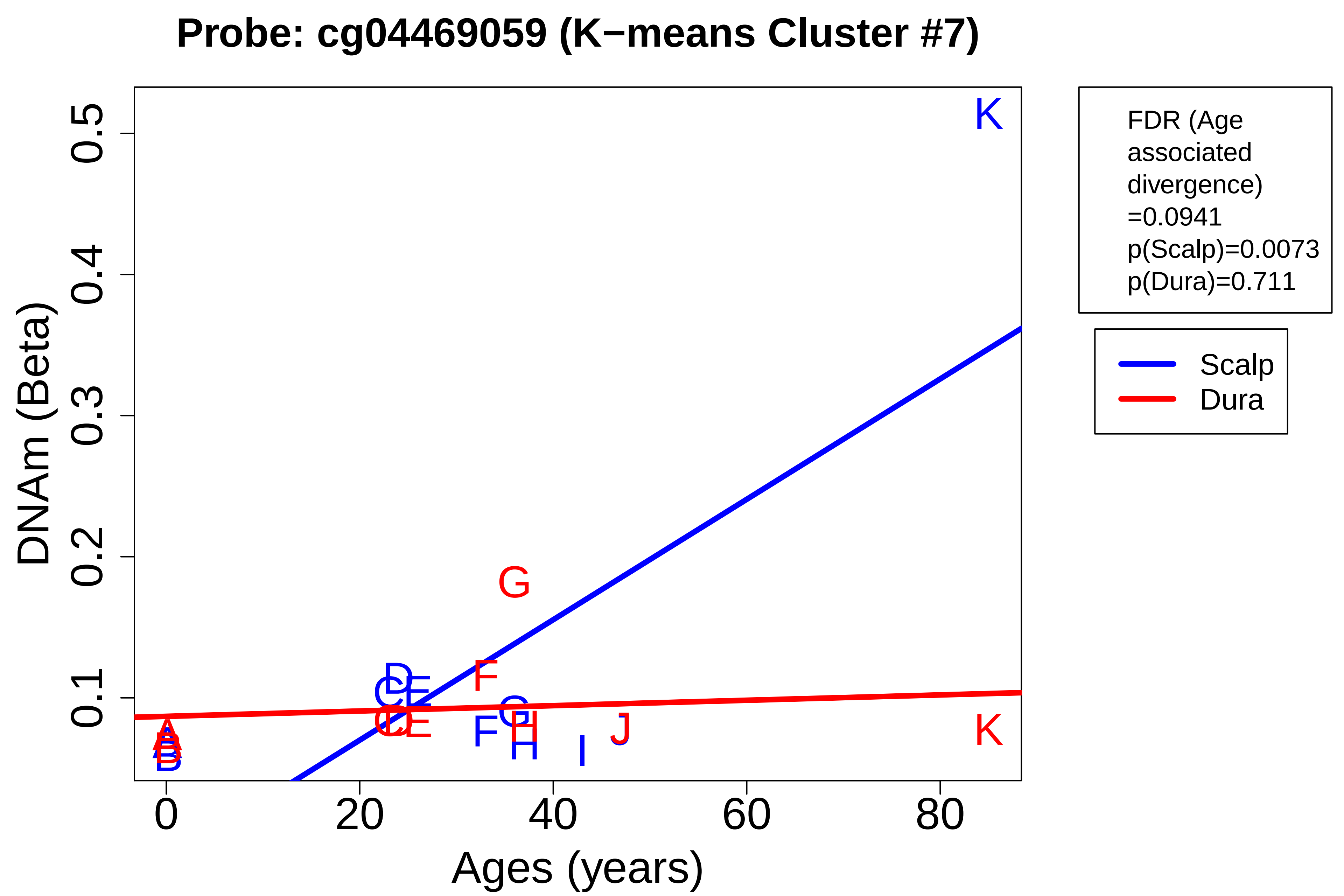

Probe: cg04469059 (K−means Cluster #7)
DNAm (Beta)
0.1
0.2
0.3
0.4
0.5
0
20
40
60
80
Ages (years)
FDR (Age associated divergence) =0.0941
p(Scalp)=0.0073
p(Dura)=0.711
Scalp
Dura

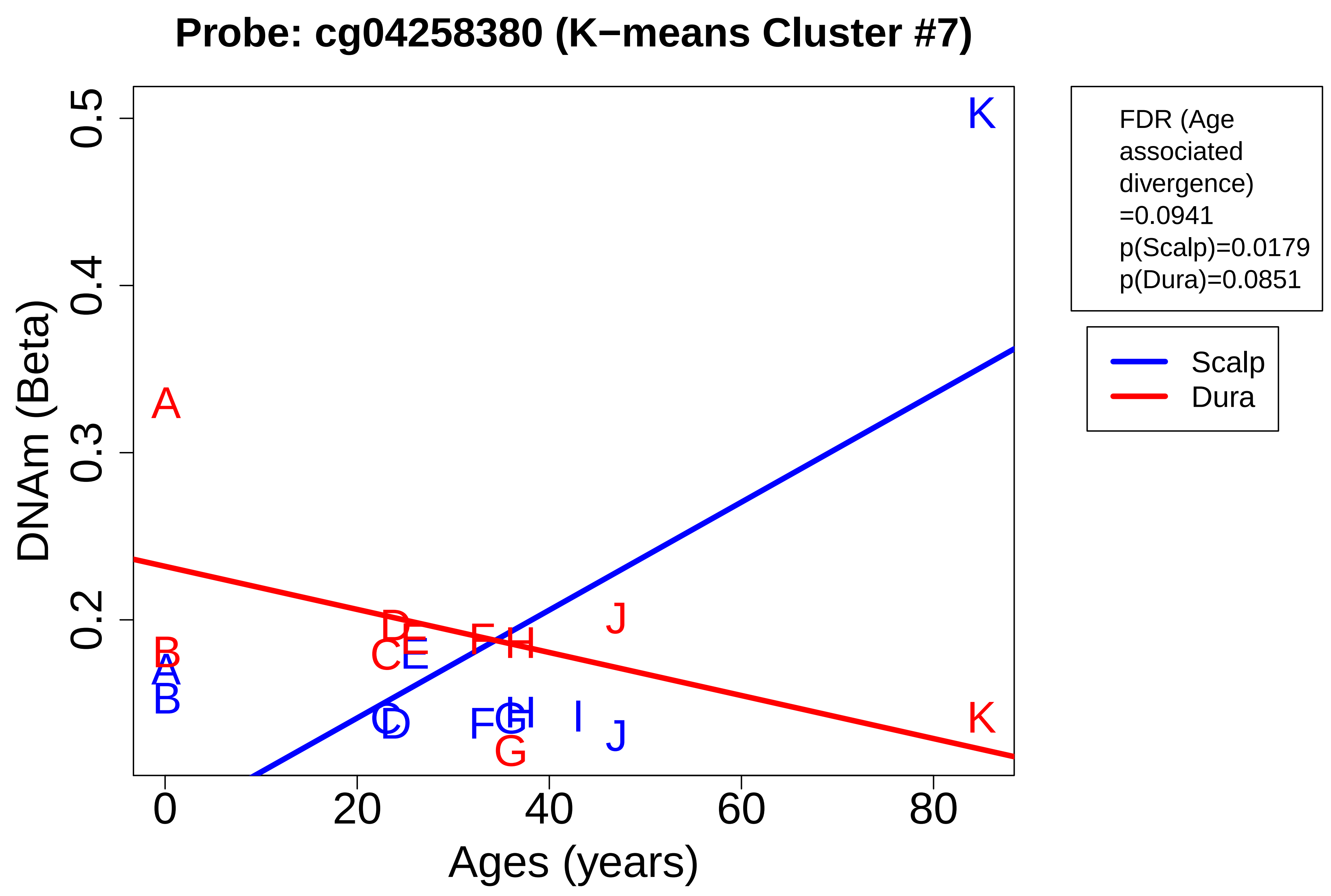

Probe: cg04258380 (K−means Cluster #7)
DNAm (Beta)
0.2
0.3
0.4
0.5
0
20
40
60
80
Ages (years)
A
B
C
D
E
F
G
H
I
J
K
FDR (Age associated divergence) =0.0941
p(Scalp)=0.0179
p(Dura)=0.0851
Scalp
Dura

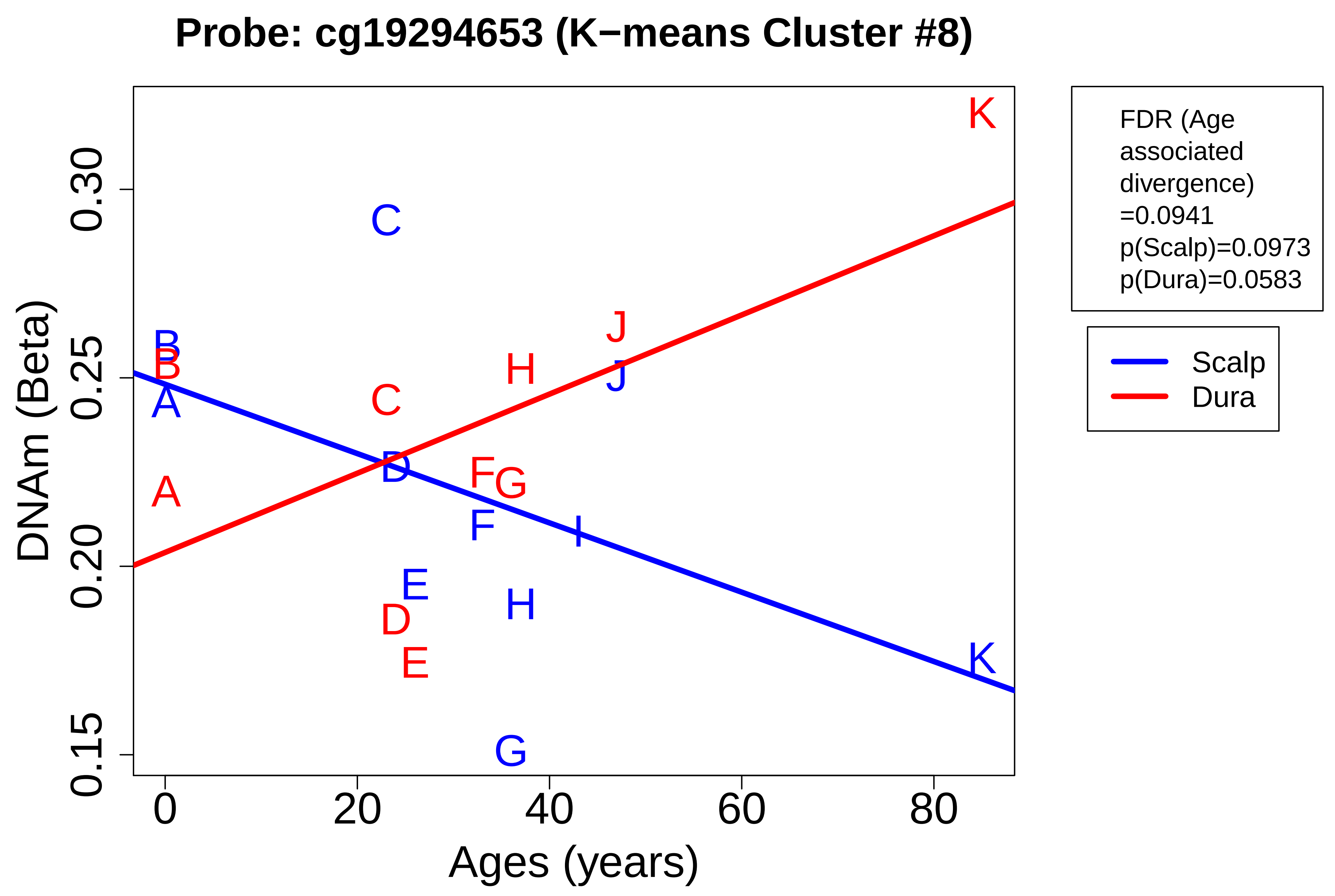

Probe: cg19294653 (K−means Cluster #8)
DNAm (Beta)
0.15
0.20
0.25
0.30
Ages (years)
0
20
40
60
80
FDR (Age associated divergence) =0.0941
p(Scalp)=0.0973
p(Dura)=0.0583
Scalp
Dura

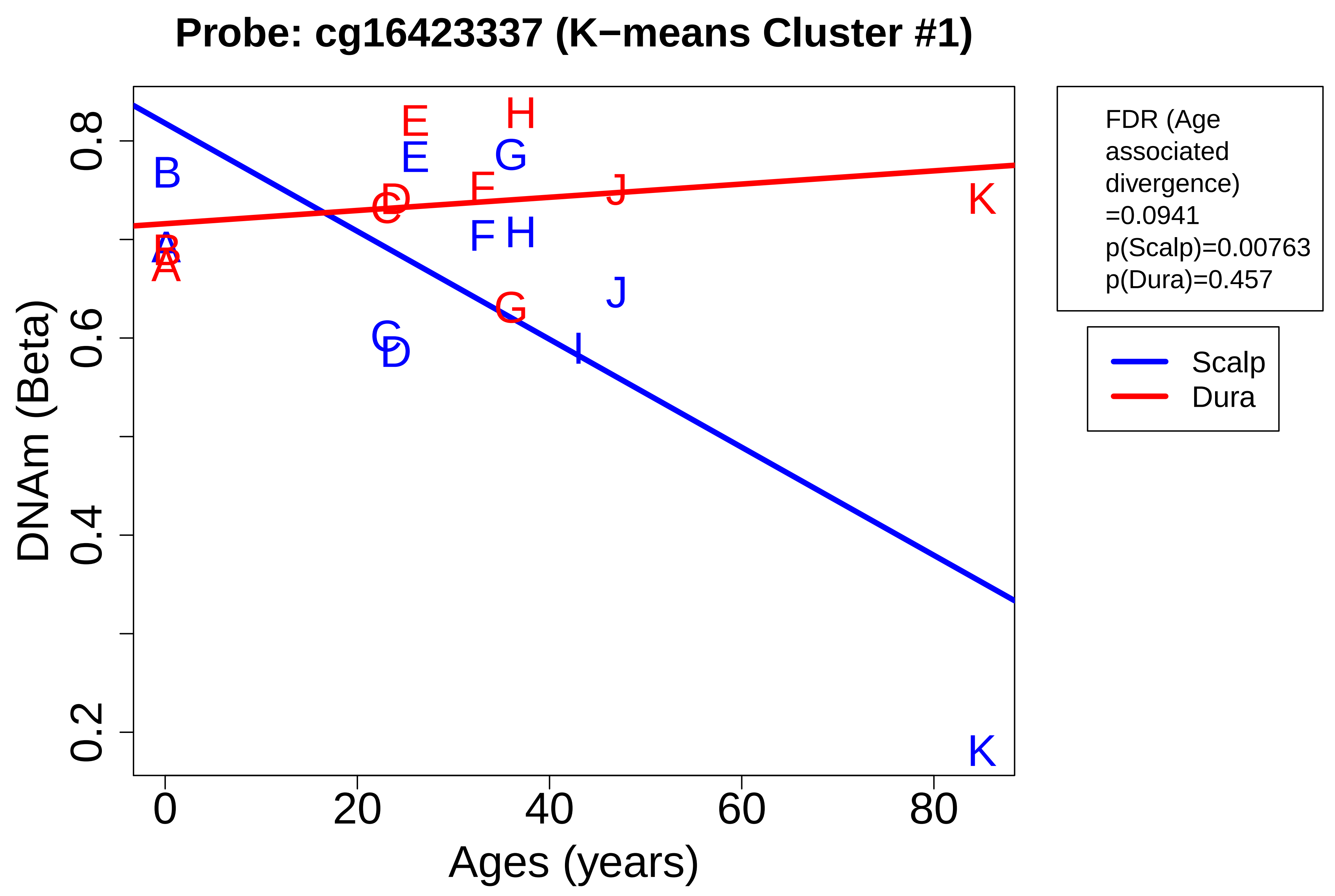

Probe: cg16423337 (K−means Cluster #1)
DNAm (Beta)
0.2
0.4
0.6
0.8
Ages (years)
0
20
40
60
80
A B C D E F G H I J K
FDR (Age associated divergence) =0.0941
p(Scalp)=0.00763
p(Dura)=0.457
Scalp
Dura

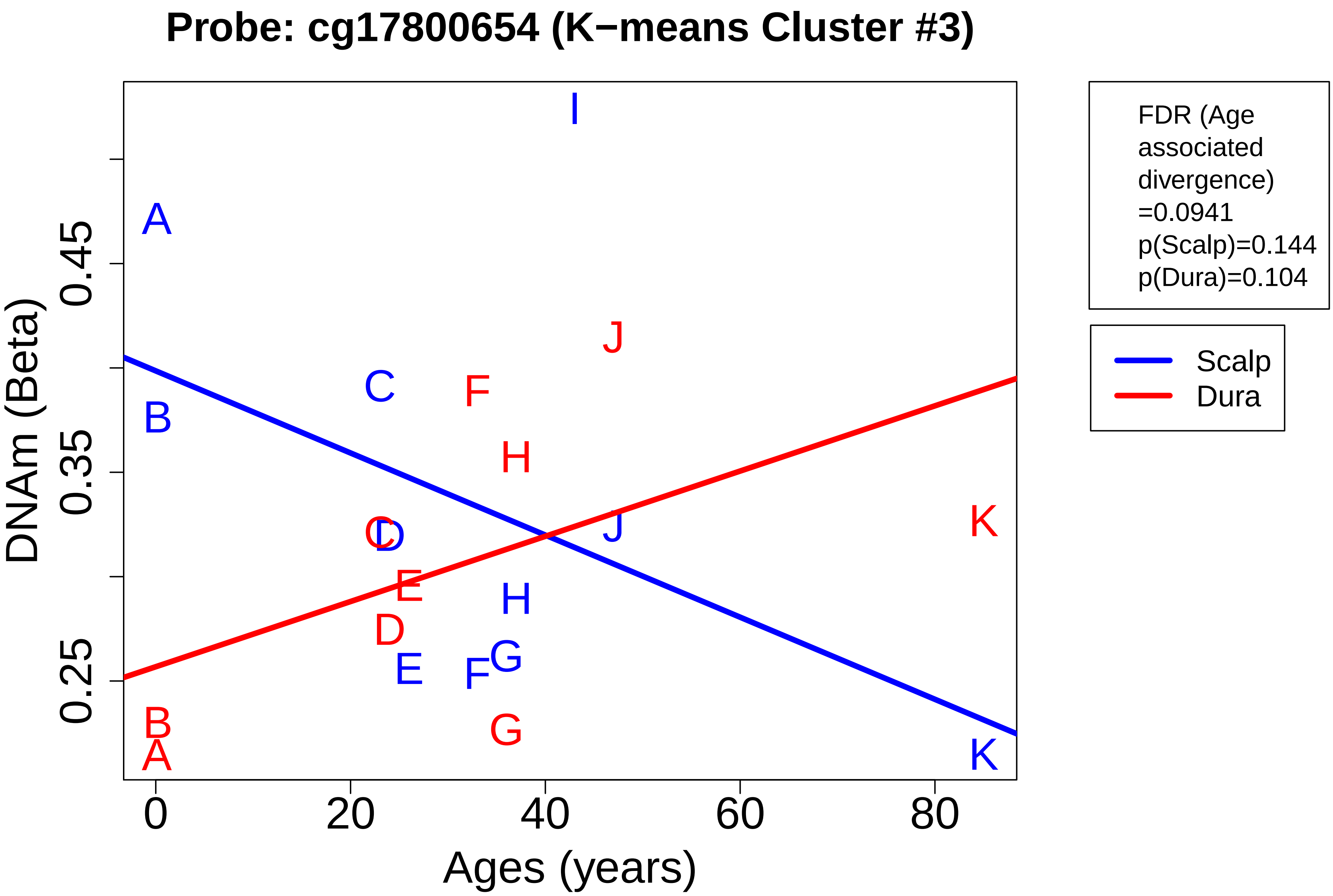

Probe: cg17800654 (K−means Cluster #3)
DNAm (Beta)
0.25
0.35
0.45
Ages (years)
0
20
40
60
80
A
B
C
D
E
F
G
H
I
J
K
FDR (Age associated divergence) =0.0941
p(Scalp)=0.144
p(Dura)=0.104
Scalp
Dura

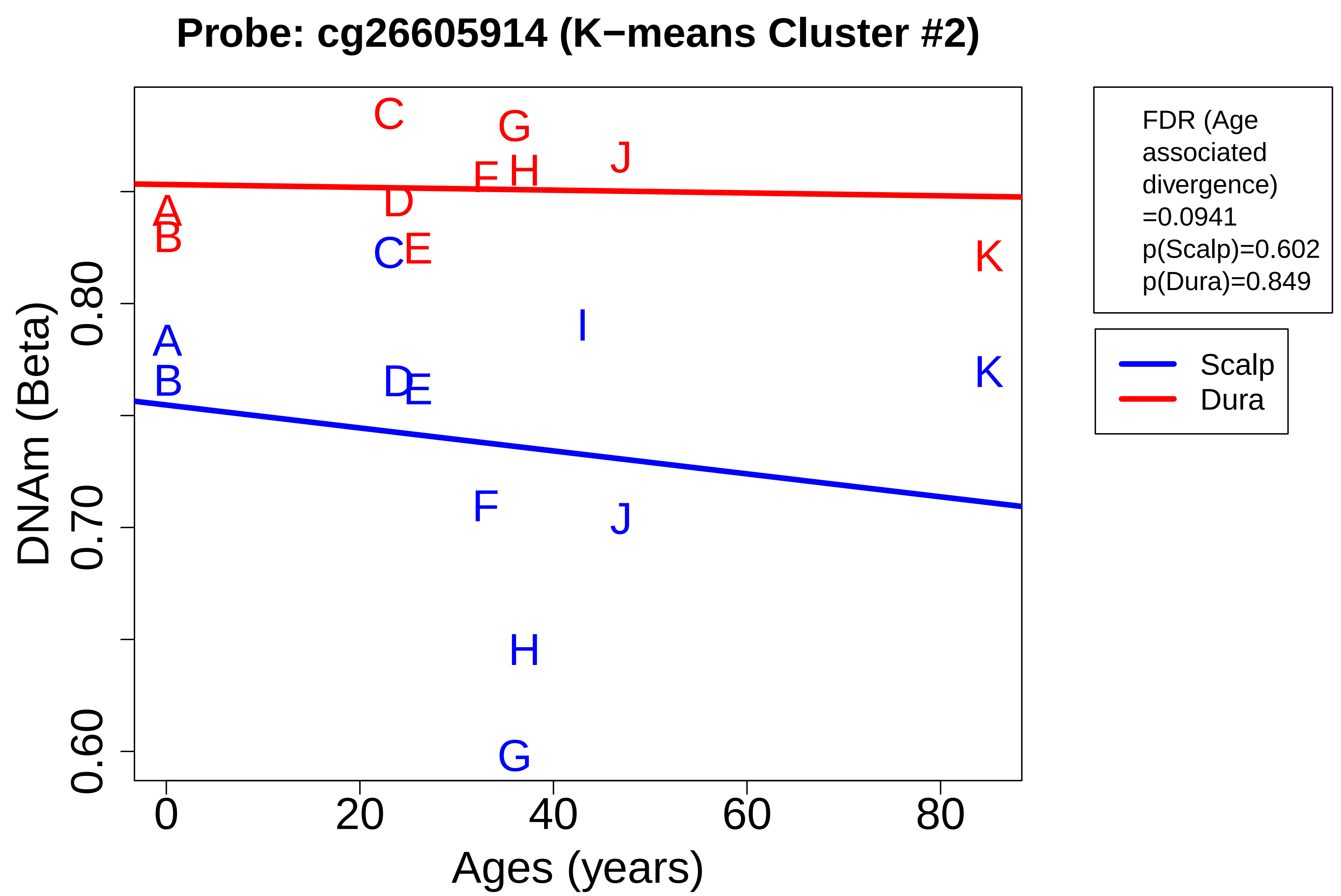

Probe: cg26605914 (K−means Cluster #2)
DNAm (Beta)
0.60
0.70
0.80
0
20
40
60
80
Ages (years)
FDR (Age associated divergence) =0.0941
p(Scalp)=0.602
p(Dura)=0.849
Scalp
Dura

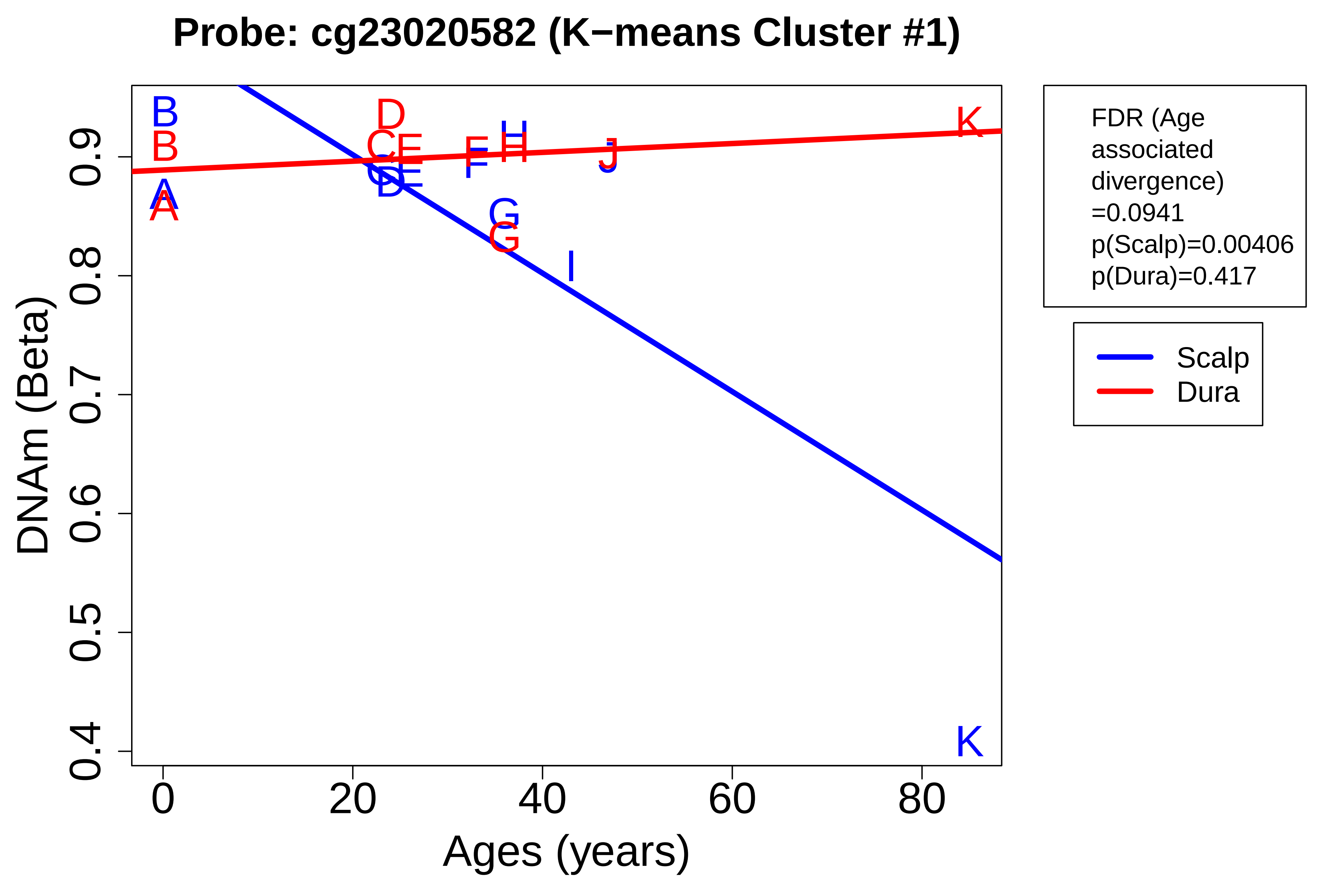

Probe: cg23020582 (K−means Cluster #1)
DNAm (Beta)
0.4
0.5
0.6
0.7
0.8
0.9
0
20
40
60
80
Ages (years)
FDR (Age associated divergence) =0.0941
p(Scalp)=0.00406
p(Dura)=0.417
Scalp
Dura

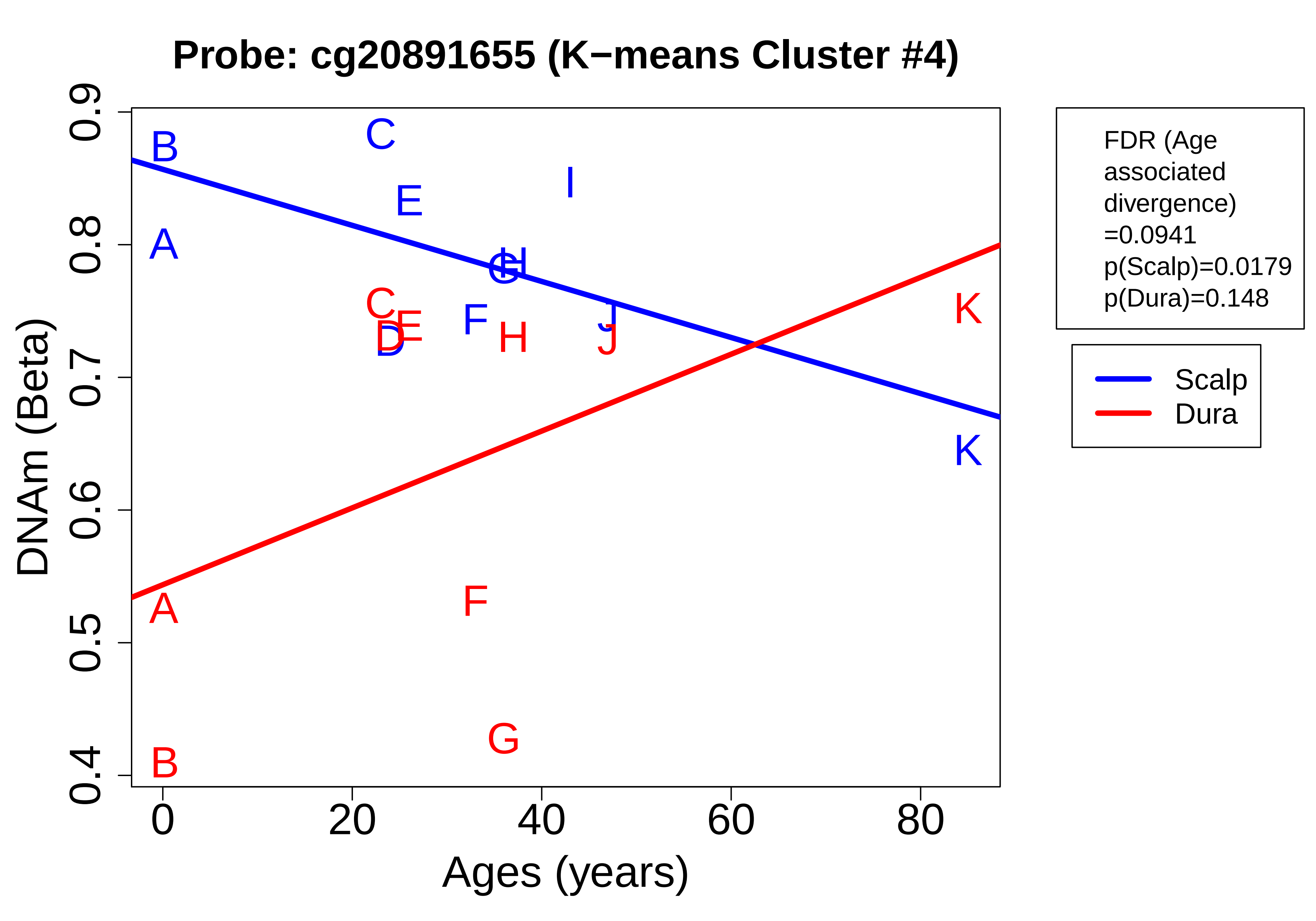

Probe: cg20891655 (K−means Cluster #4)
DNAm (Beta)
0.4
0.5
0.6
0.7
0.8
0.9
0
20
40
60
80
Ages (years)
A
B
C
D
E
F
G
H
I
J
K
FDR (Age associated divergence) =0.0941
p(Scalp)=0.0179
p(Dura)=0.148
Scalp
Dura

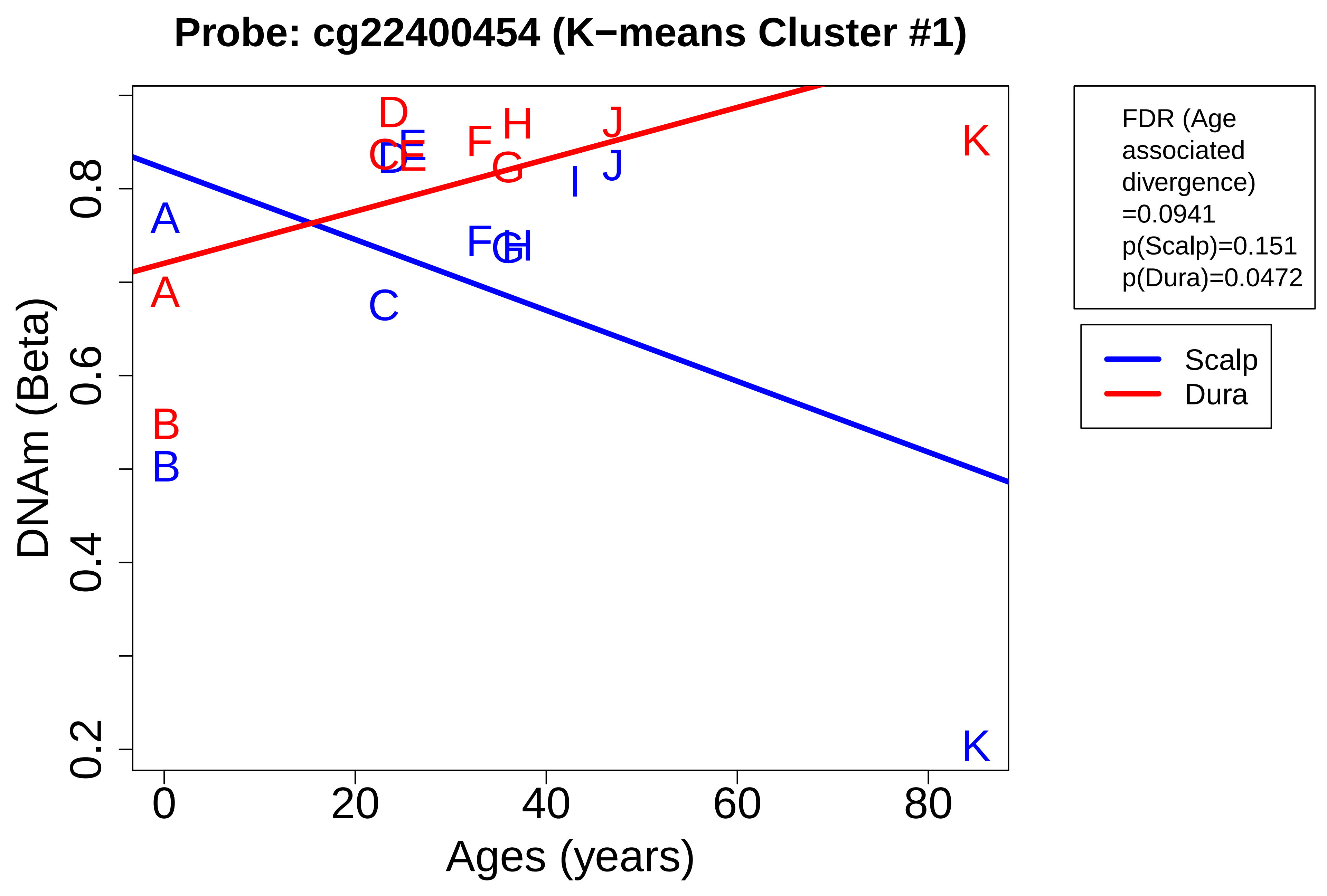

Probe: cg22400454 (K−means Cluster #1)
DNAm (Beta)
0.2
0.4
0.6
0.8
0
20
40
60
80
Ages (years)
A
B
C
D
E
F
G
H
I
J
K
FDR (Age associated divergence) =0.0941
p(Scalp)=0.151
p(Dura)=0.0472
Scalp
Dura

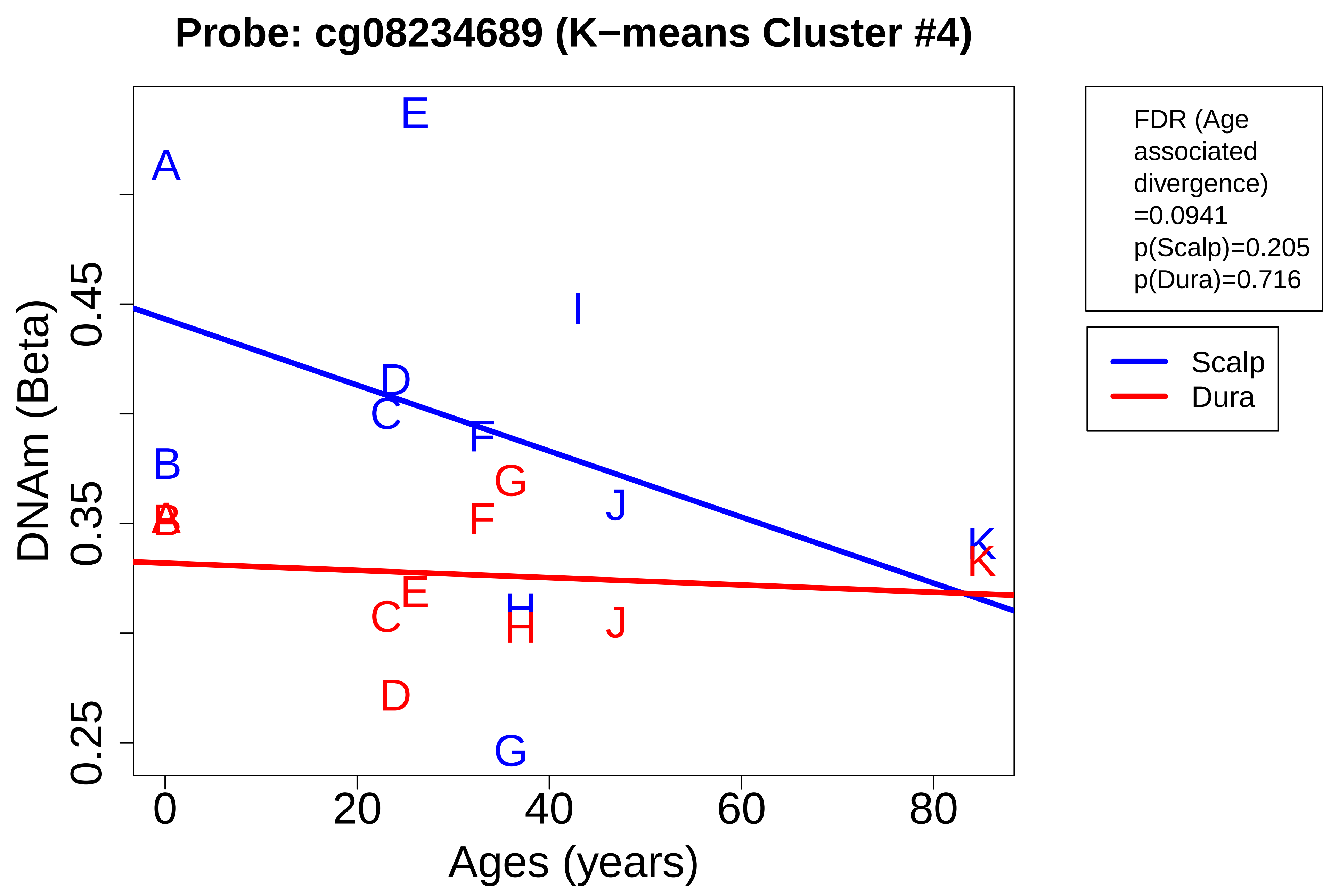
Probe: cg08234689 (K−means Cluster #4)
FDR (Age associated divergence) =0.0941
p(Scalp)=0.205
p(Dura)=0.716
Scalp
Dura
DNAm (Beta)
0.25
0.35
0.45
Ages (years)
0
20
40
60
80
A
B
C
D
E
F
G
H
I
J
K

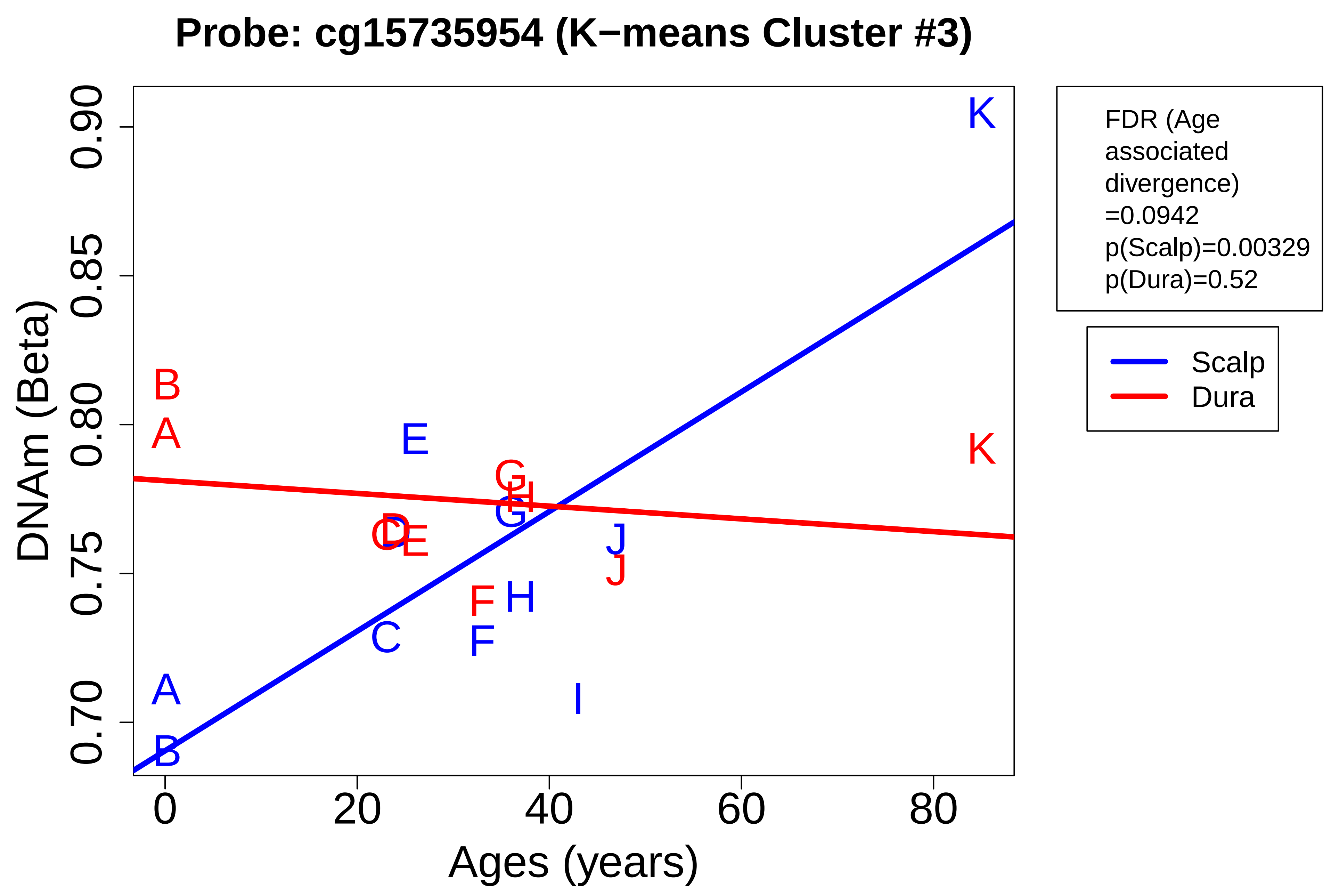

Probe: cg15735954 (K−means Cluster #3)
DNAm (Beta)
0.70
0.75
0.80
0.85
0.90
Ages (years)
0
20
40
60
80
FDR (Age associated divergence) =0.0942
p(Scalp)=0.00329
p(Dura)=0.52
Scalp
Dura
A
B
C
D
E
F
G
H
I
J
K

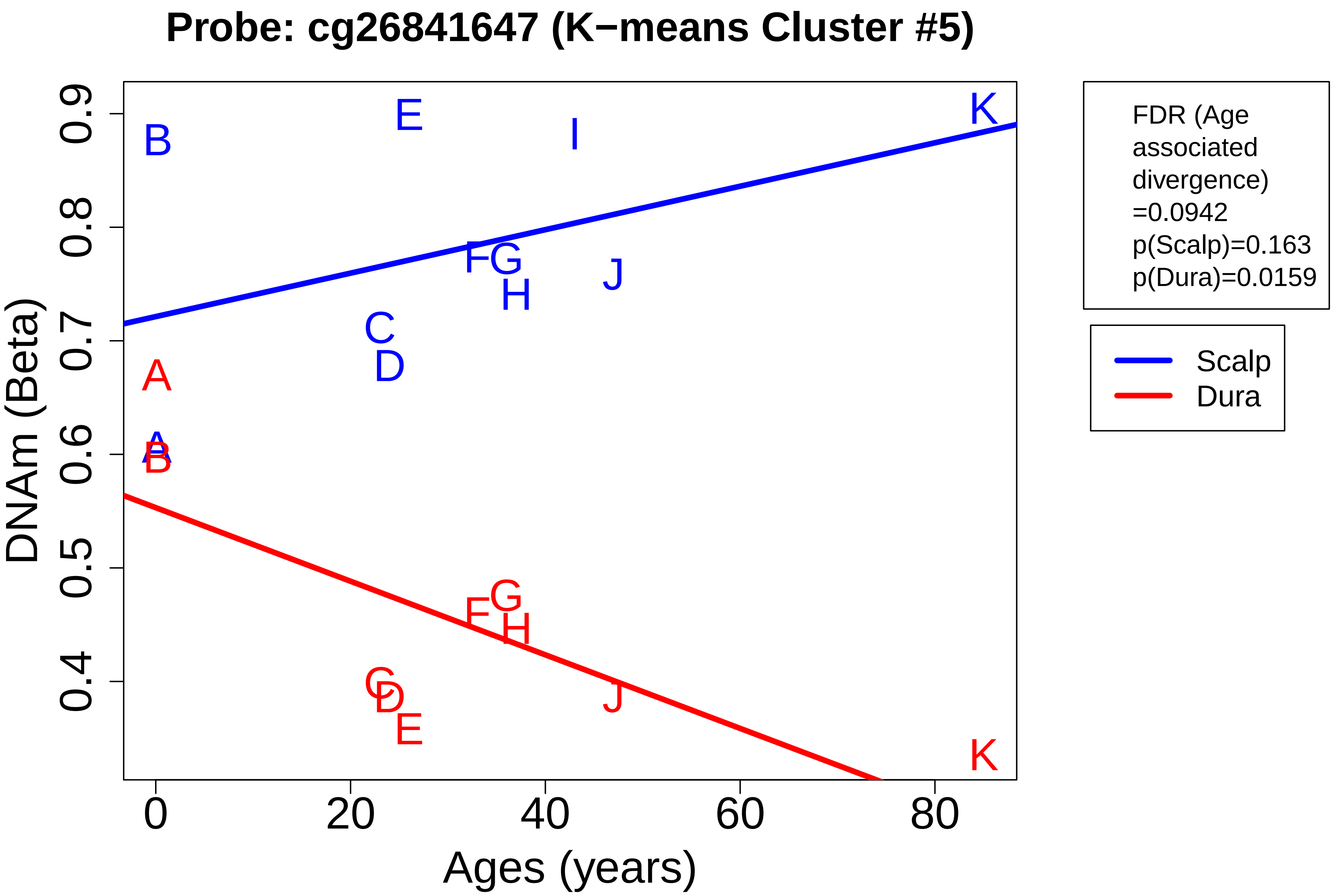
Probe: cg26841647 (K−means Cluster #5)
DNAm (Beta)
Ages (years)
0.4
0.5
0.6
0.7
0.8
0.9
0
20
40
60
80
A
B
C
D
E
F
G
H
I
J
K
FDR (Age associated divergence) =0.0942
p(Scalp)=0.163
p(Dura)=0.0159
Scalp
Dura

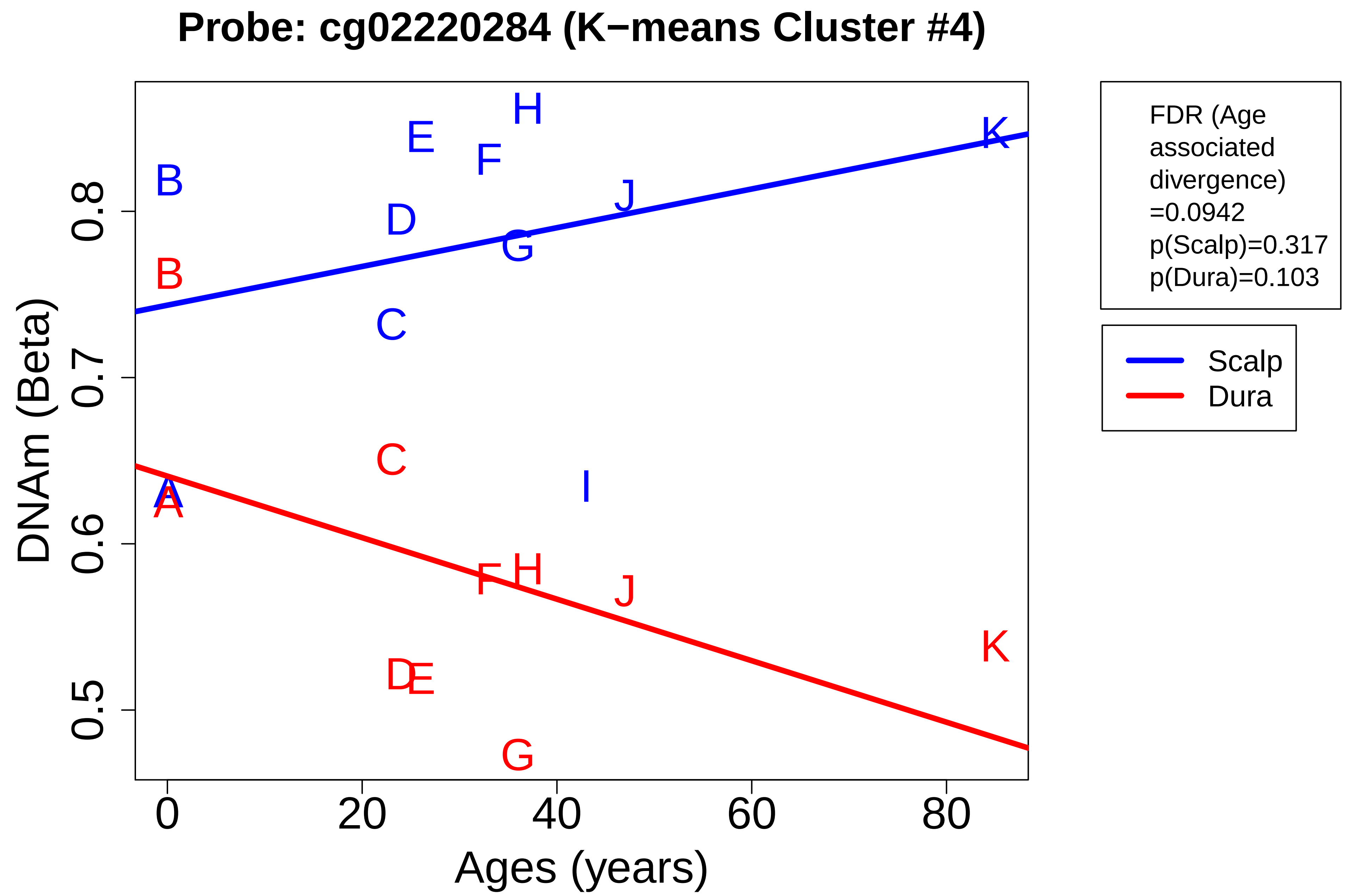

Probe: cg02220284 (K−means Cluster #4)
DNAm (Beta)
0.5
0.6
0.7
0.8
0
20
40
60
80
Ages (years)
FDR (Age associated divergence) =0.0942
p(Scalp)=0.317
p(Dura)=0.103
Scalp
Dura

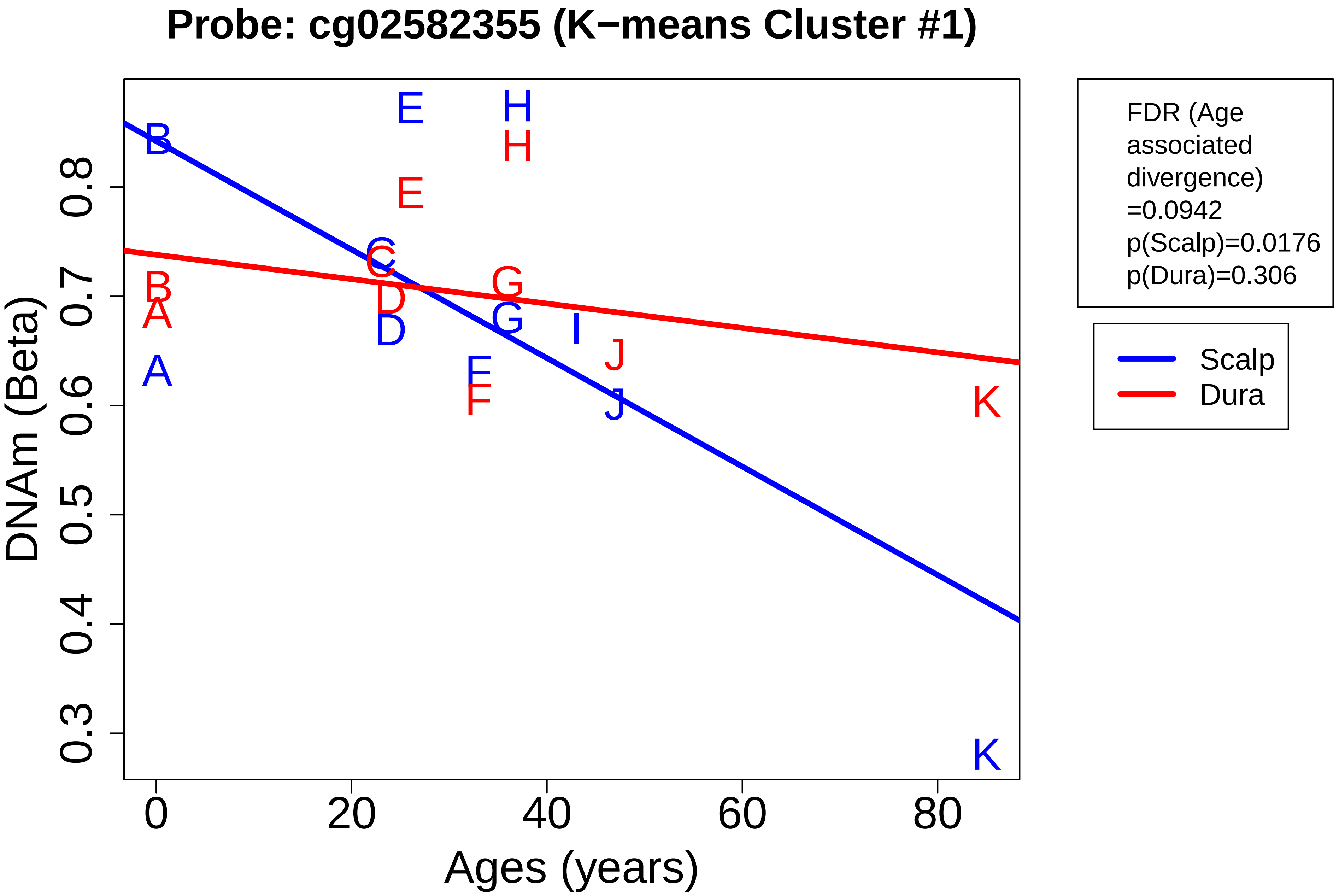

Probe: cg02582355 (K−means Cluster #1)
DNAm (Beta)
0.3
0.4
0.5
0.6
0.7
0.8
Ages (years)
0
20
40
60
80
FDR (Age associated divergence) =0.0942
p(Scalp)=0.0176
p(Dura)=0.306
Scalp
Dura

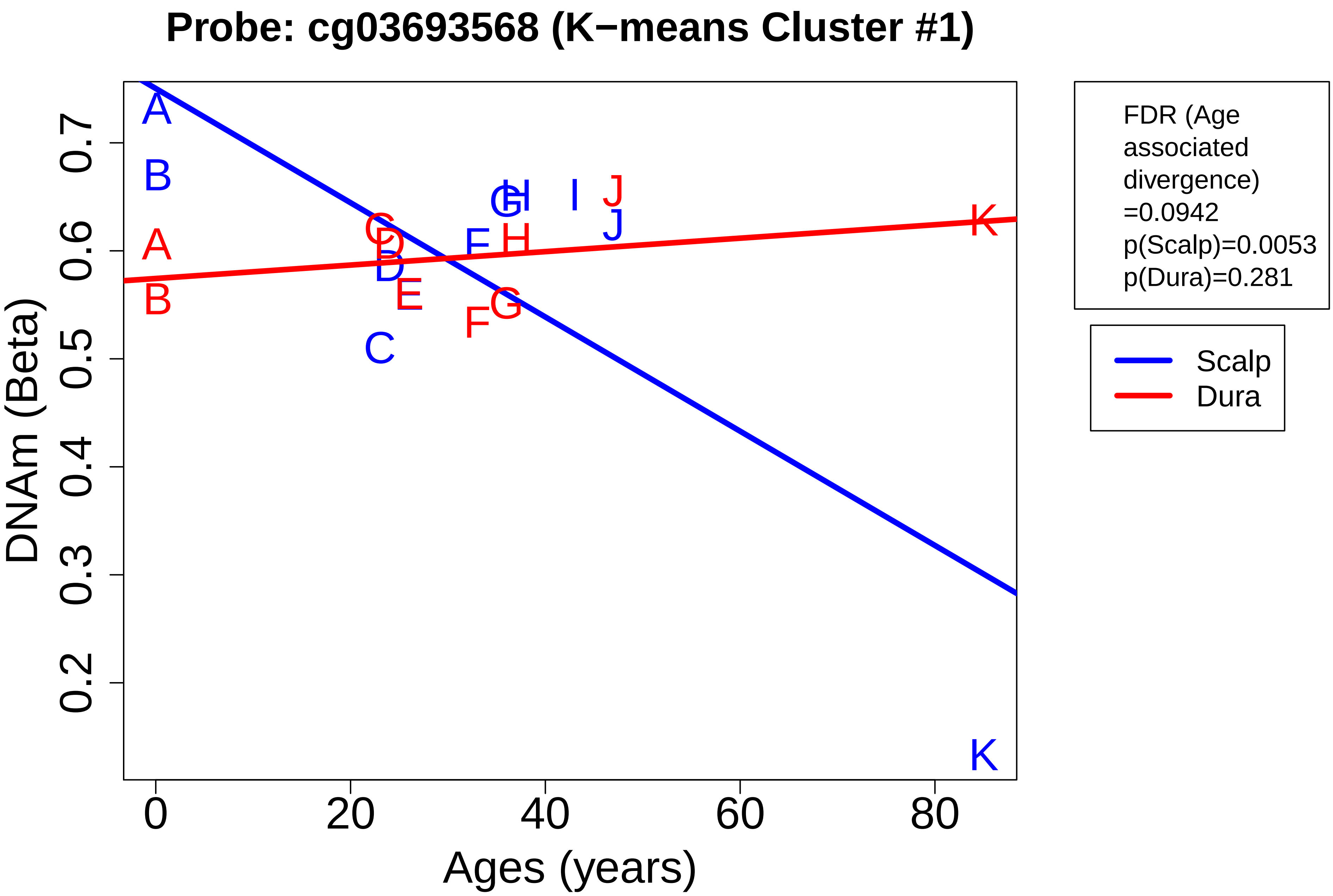
Probe: cg03693568 (K−means Cluster #1)
DNAm (Beta)
0.2
0.3
0.4
0.5
0.6
0.7
Ages (years)
0
20
40
60
80
FDR (Age associated divergence) =0.0942
p(Scalp)=0.0053
p(Dura)=0.281
Scalp
Dura

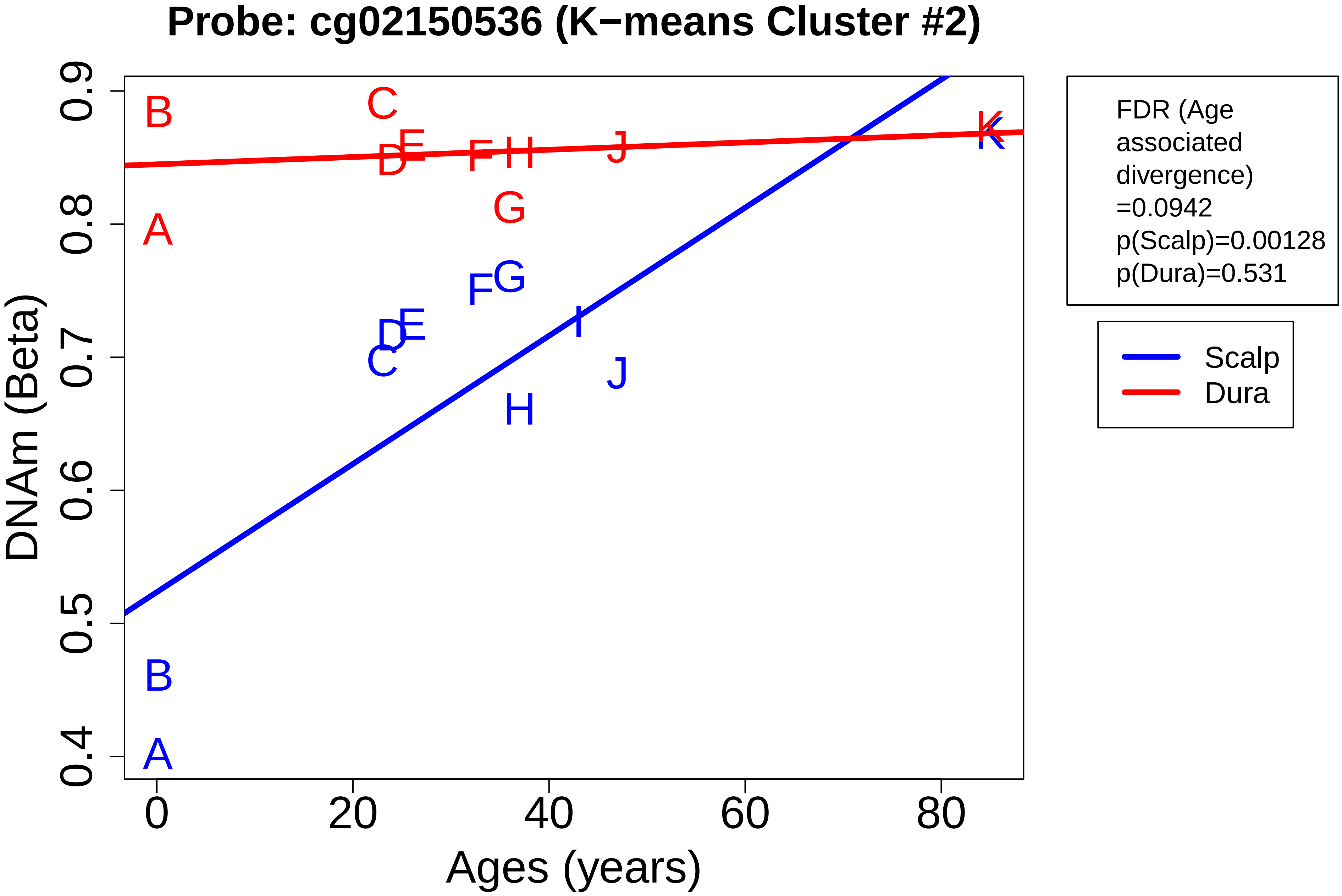

Probe: cg02150536 (K−means Cluster #2)
DNAm (Beta)
0.4
0.5
0.6
0.7
0.8
0.9
0
20
40
60
80
Ages (years)
FDR (Age associated divergence) =0.0942
p(Scalp)=0.00128
p(Dura)=0.531
Scalp
Dura

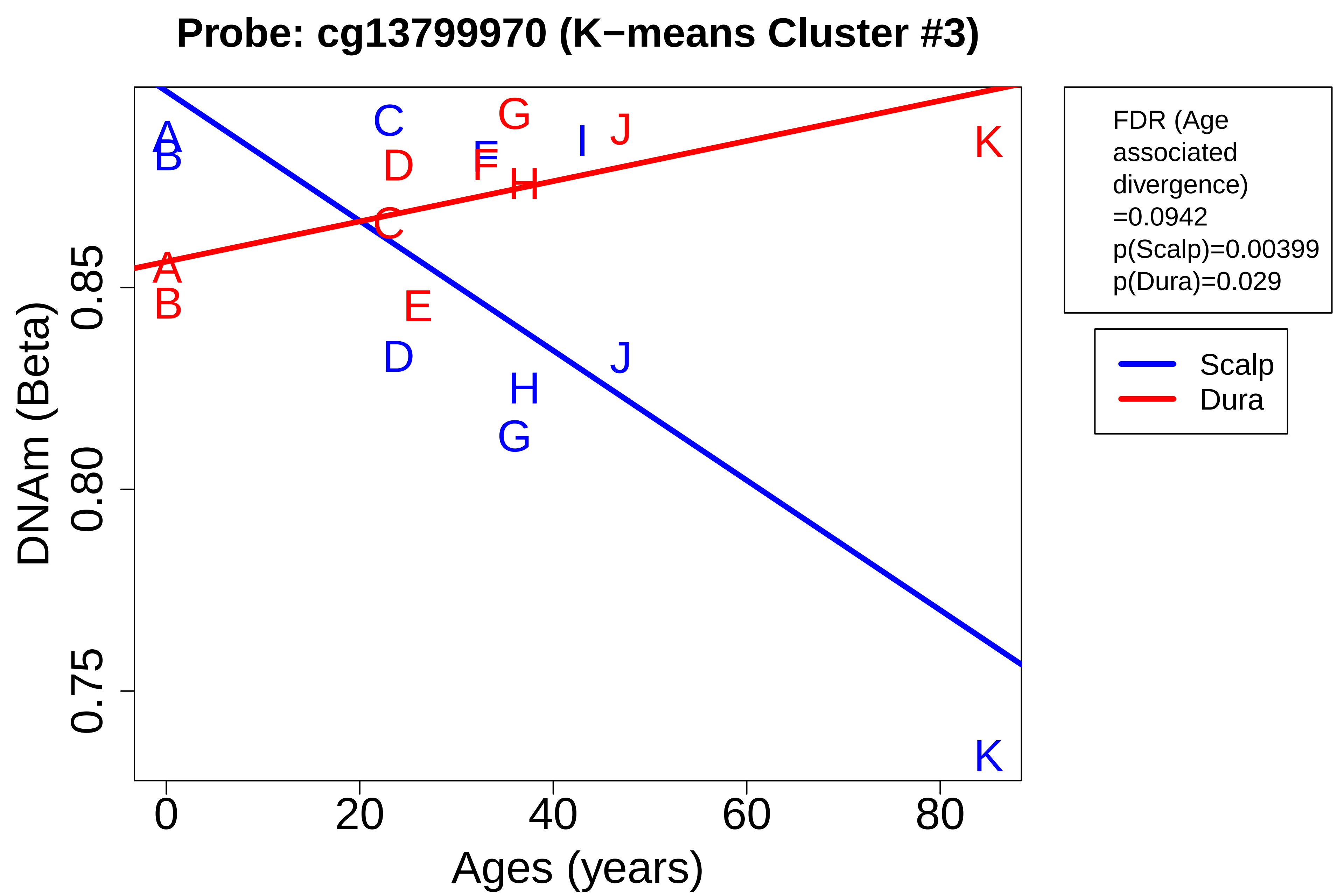

Probe: cg13799970 (K−means Cluster #3)
DNAm (Beta)
0.75
0.80
0.85
Ages (years)
0
20
40
60
80
A
B
C
D
E
F
G
H
I
J
K
FDR (Age associated divergence) =0.0942
p(Scalp)=0.00399
p(Dura)=0.029
Scalp
Dura

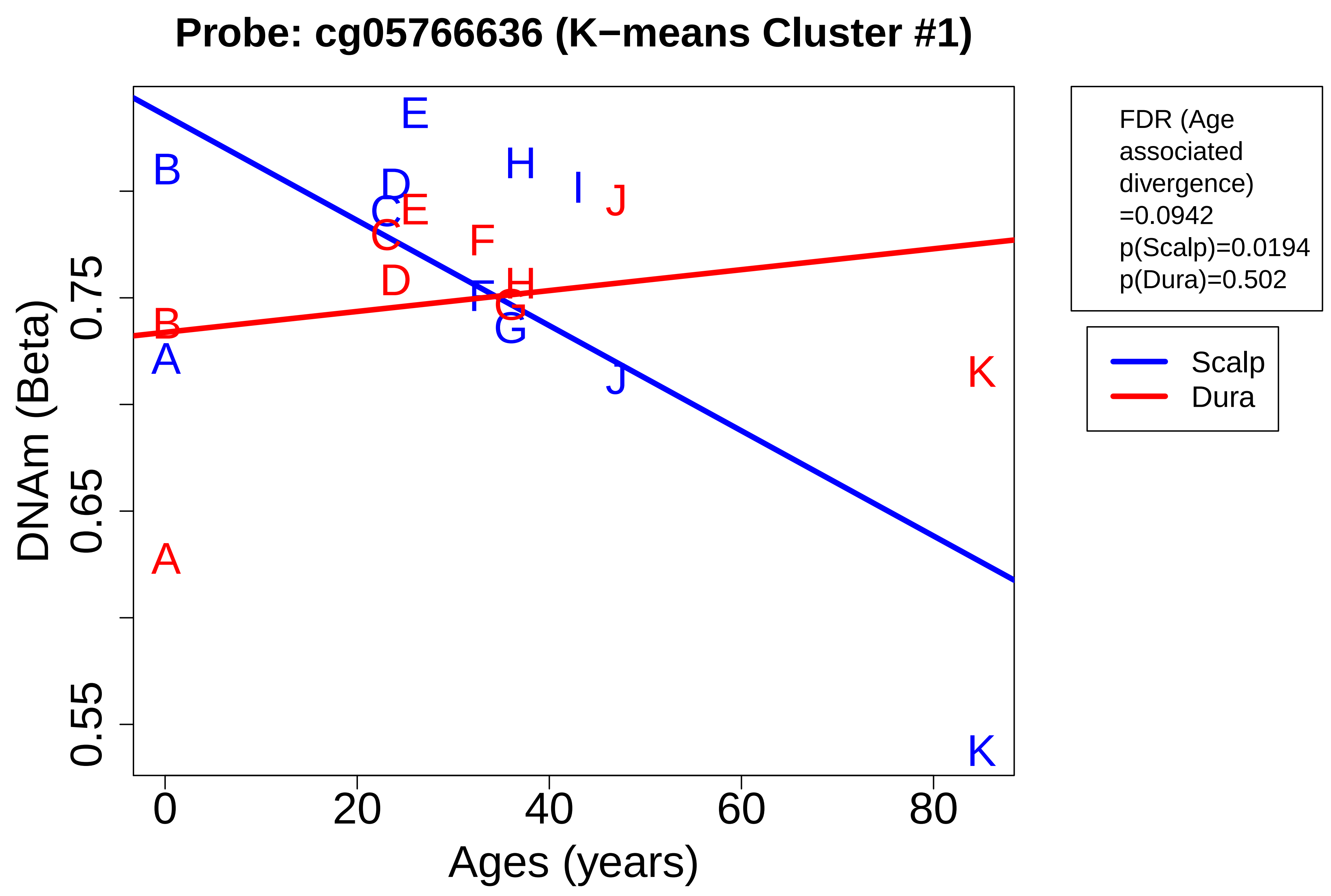

Probe: cg05766636 (K−means Cluster #1)
DNAm (Beta)
0.55
0.65
0.75
Ages (years)
0
20
40
60
80
FDR (Age associated divergence) =0.0942
p(Scalp)=0.0194
p(Dura)=0.502
Scalp
Dura

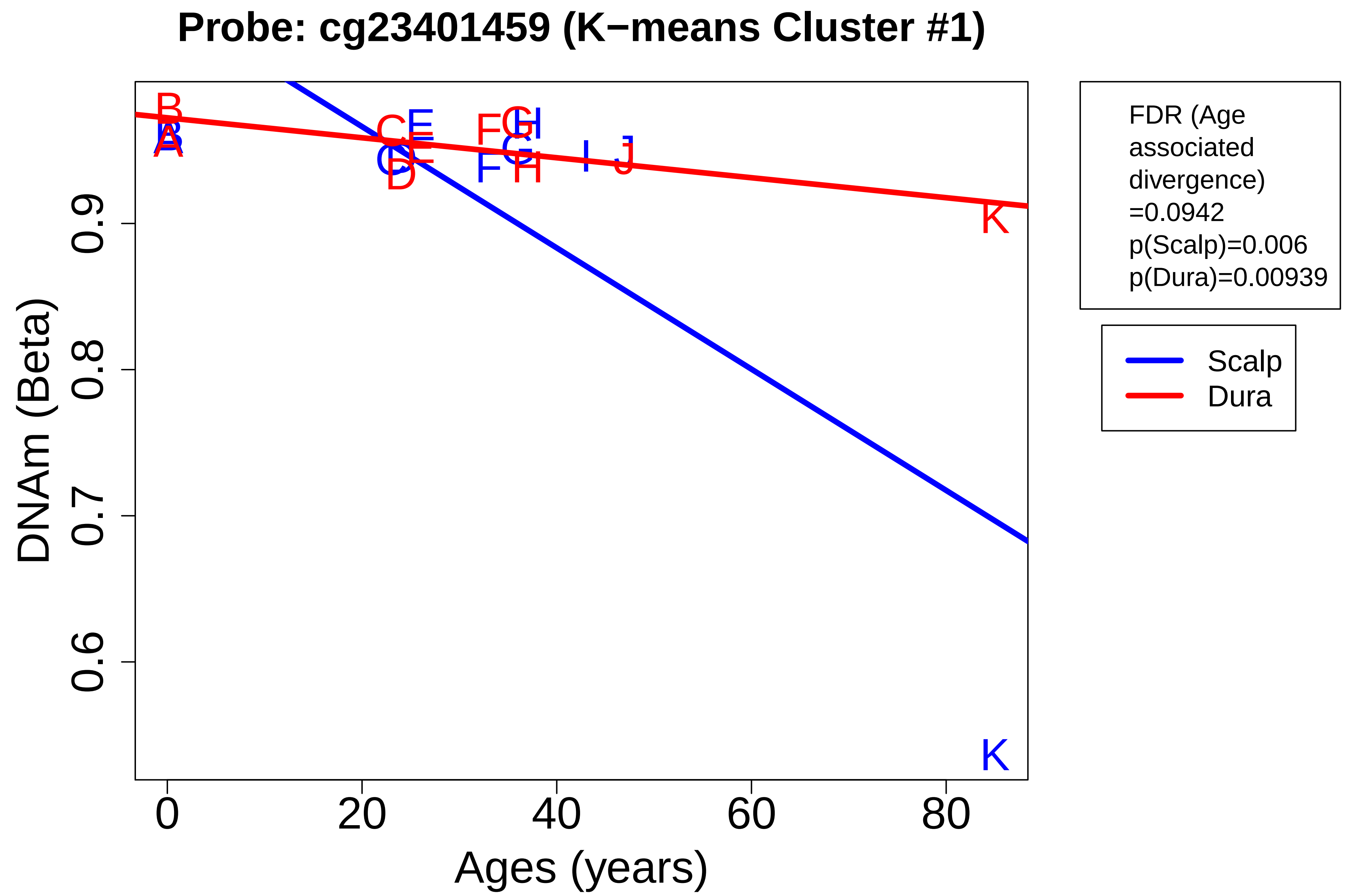

Probe: cg23401459 (K−means Cluster #1)
DNAm (Beta)
0.6
0.7
0.8
0.9
0
20
40
60
80
Ages (years)
A
B
C
D
E
F
G
H
I
J
K
FDR (Age associated divergence) =0.0942
p(Scalp)=0.006
p(Dura)=0.00939
Scalp
Dura

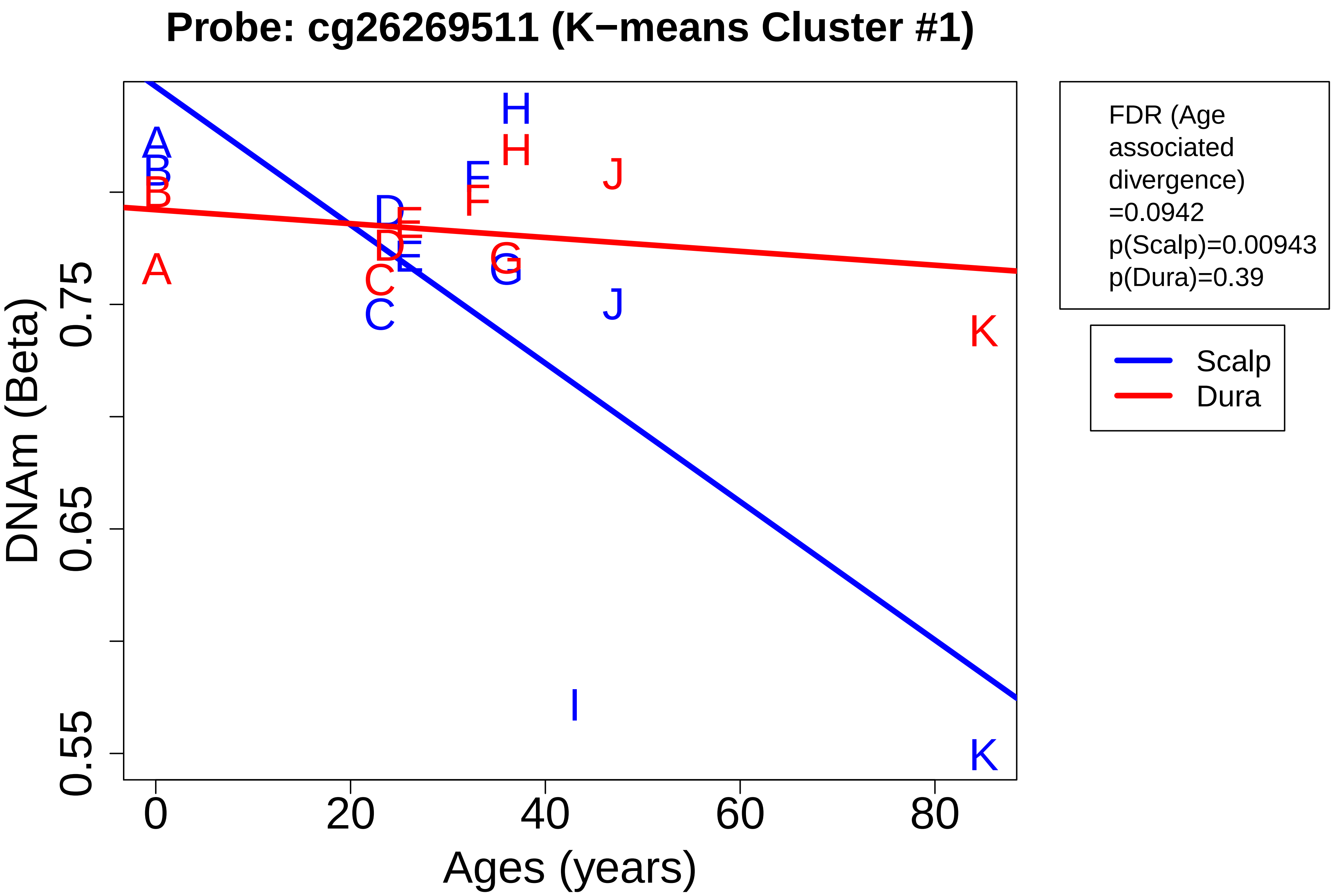

Probe: cg26269511 (K−means Cluster #1)
DNAm (Beta)
0.55
0.65
0.75
0
20
40
60
80
Ages (years)
FDR (Age associated divergence) =0.0942
p(Scalp)=0.00943
p(Dura)=0.39
Scalp
Dura

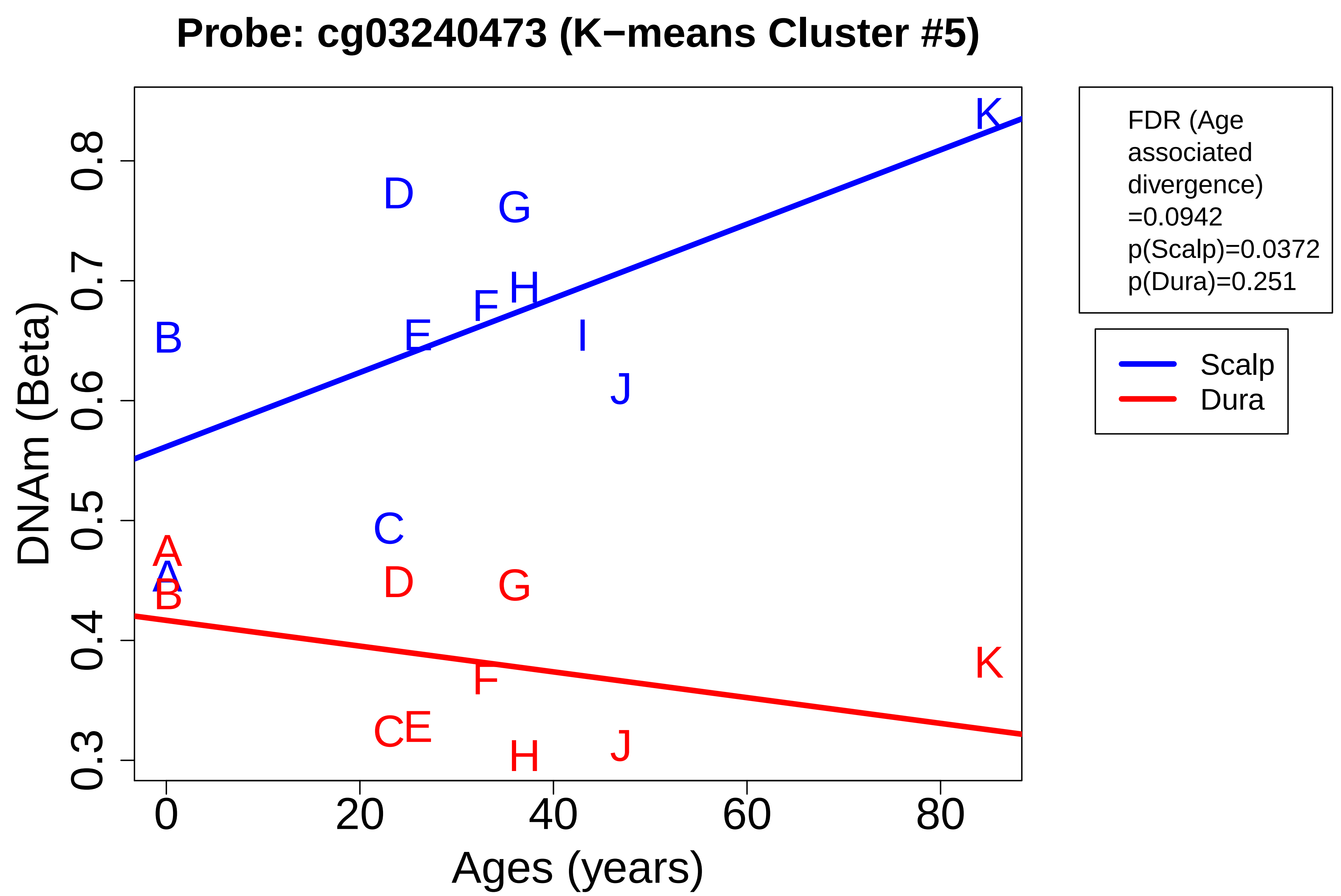

Probe: cg03240473 (K−means Cluster #5)
DNAm (Beta)
0.3
0.4
0.5
0.6
0.7
0.8
0
20
40
60
80
Ages (years)
FDR (Age associated divergence) =0.0942
p(Scalp)=0.0372
p(Dura)=0.251
Scalp
Dura
A
B
C
D
E
F
G
H
I
J
K

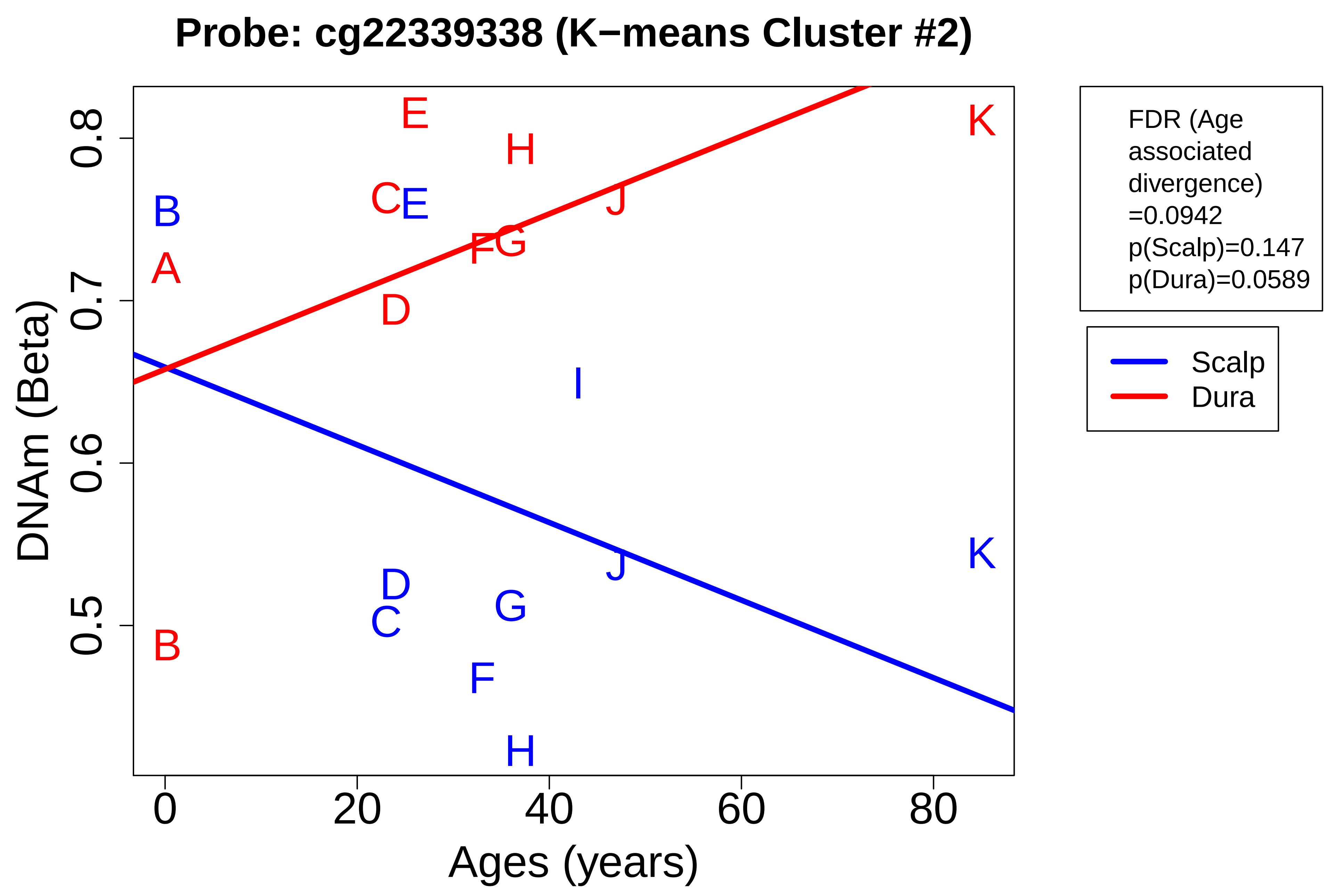

Probe: cg22339338 (K−means Cluster #2)
DNAm (Beta)
0.5
0.6
0.7
0.8
0
20
40
60
80
Ages (years)
E
H
K
C
E
J
B
F
G
A
D
I
K
J
D
G
C
B
F
H
FDR (Age associated divergence) =0.0942
p(Scalp)=0.147
p(Dura)=0.0589
Scalp
Dura

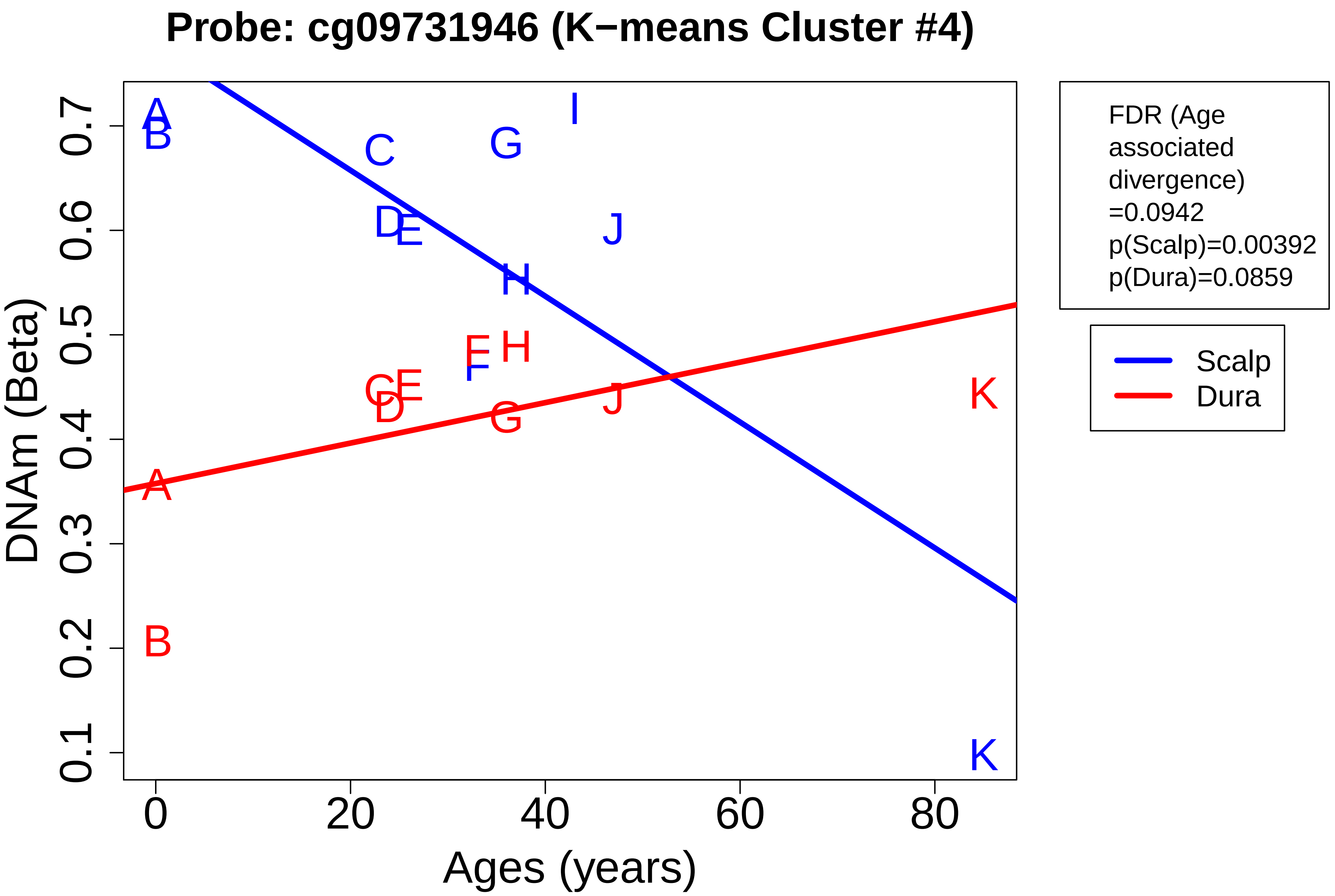

Probe: cg09731946 (K−means Cluster #4)
DNAm (Beta)
Ages (years)
0.1
0.2
0.3
0.4
0.5
0.6
0.7
0
20
40
60
80
A
B
C
D
E
F
G
H
I
J
K
FDR (Age associated divergence) =0.0942
p(Scalp)=0.00392
p(Dura)=0.0859
Scalp
Dura

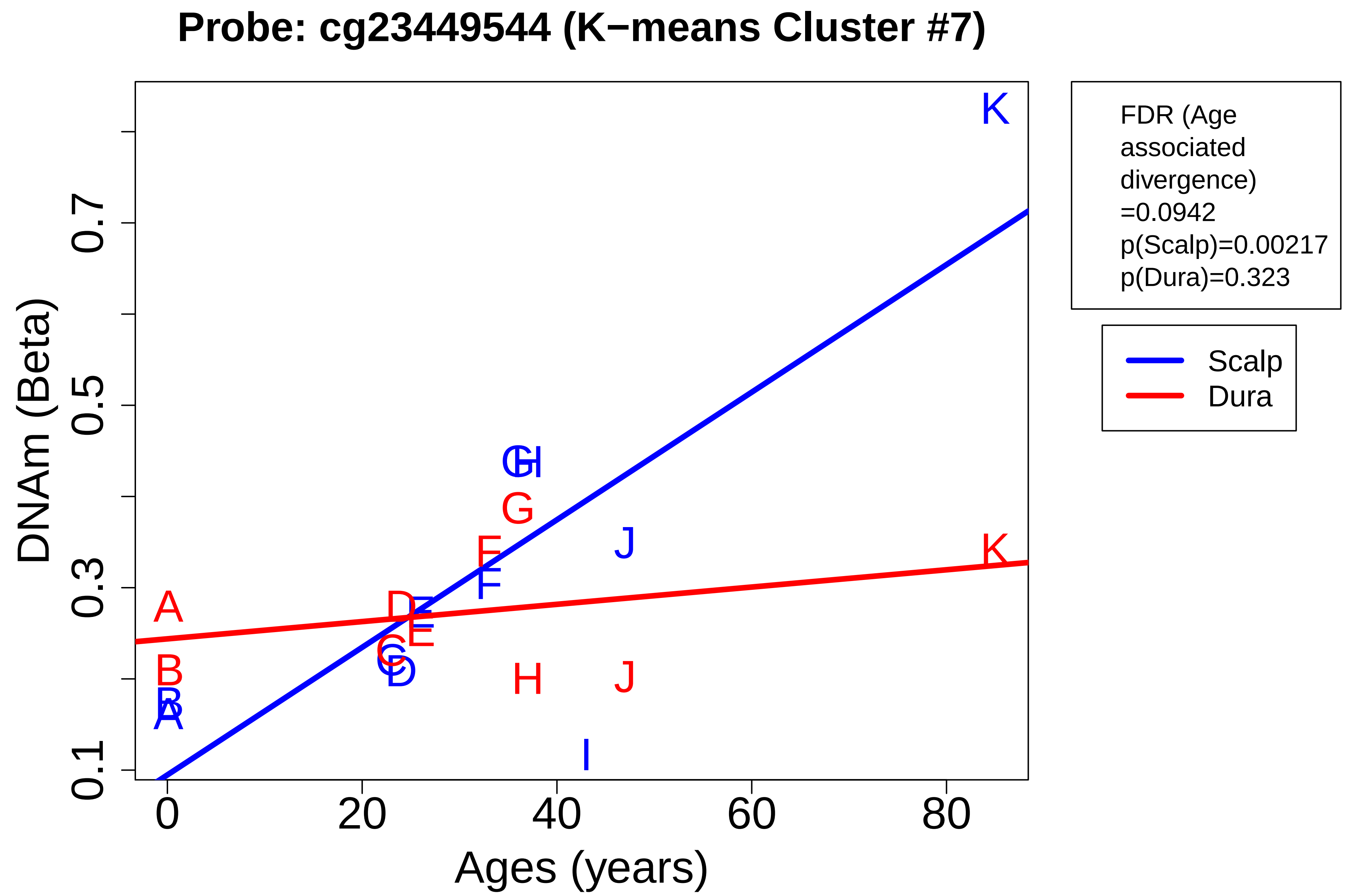

Probe: cg23449544 (K−means Cluster #7)
DNAm (Beta)
0.1
0.3
0.5
0.7
0
20
40
60
80
Ages (years)
K
GH
G
J
F
F
K
A
D
E
E
C
C
D
H
J
B
B
A
I
FDR (Age associated divergence) =0.0942
p(Scalp)=0.00217
p(Dura)=0.323
Scalp
Dura

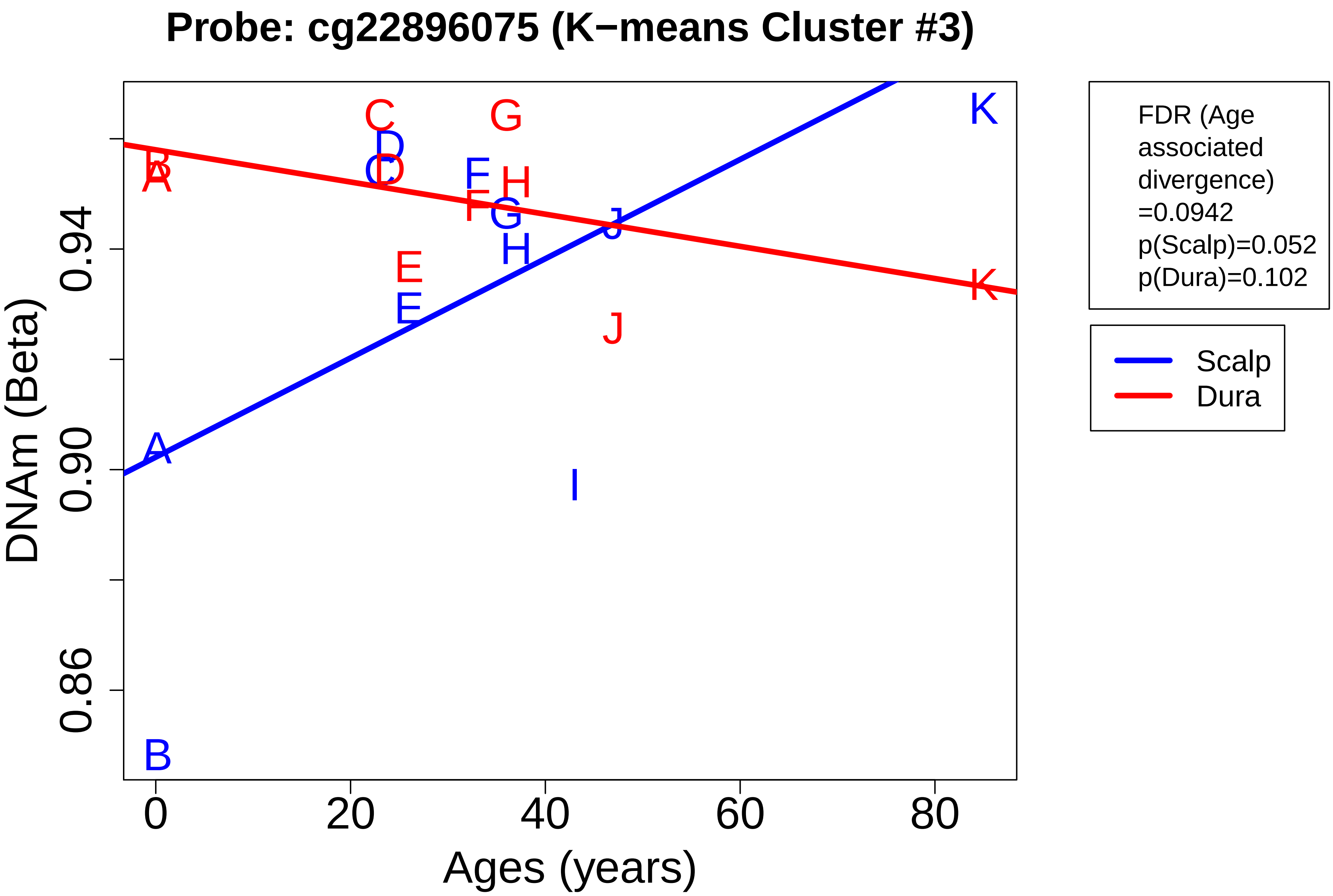

Probe: cg22896075 (K−means Cluster #3)
DNAm (Beta)
0.86
0.90
0.94
0
20
40
60
80
Ages (years)
FDR (Age associated divergence) =0.0942
p(Scalp)=0.052
p(Dura)=0.102
Scalp
Dura
A
B
C
D
E
F
G
H
I
J
K

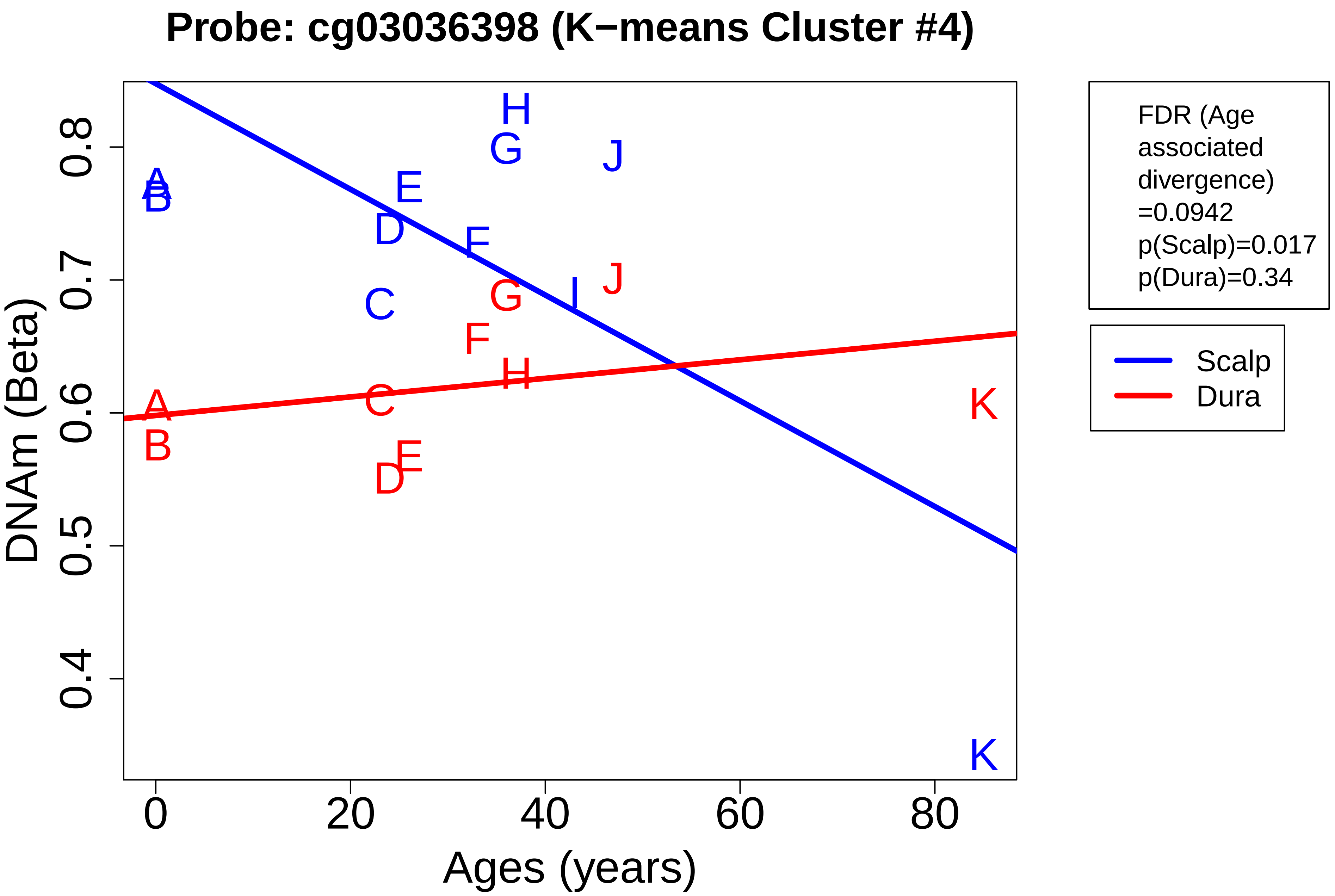

Probe: cg03036398 (K−means Cluster #4)
DNAm (Beta)
0.4
0.5
0.6
0.7
0.8
0
20
40
60
80
Ages (years)
A
B
C
D
E
F
G
H
I
J
K
FDR (Age associated divergence) =0.0942
p(Scalp)=0.017
p(Dura)=0.34
Scalp
Dura

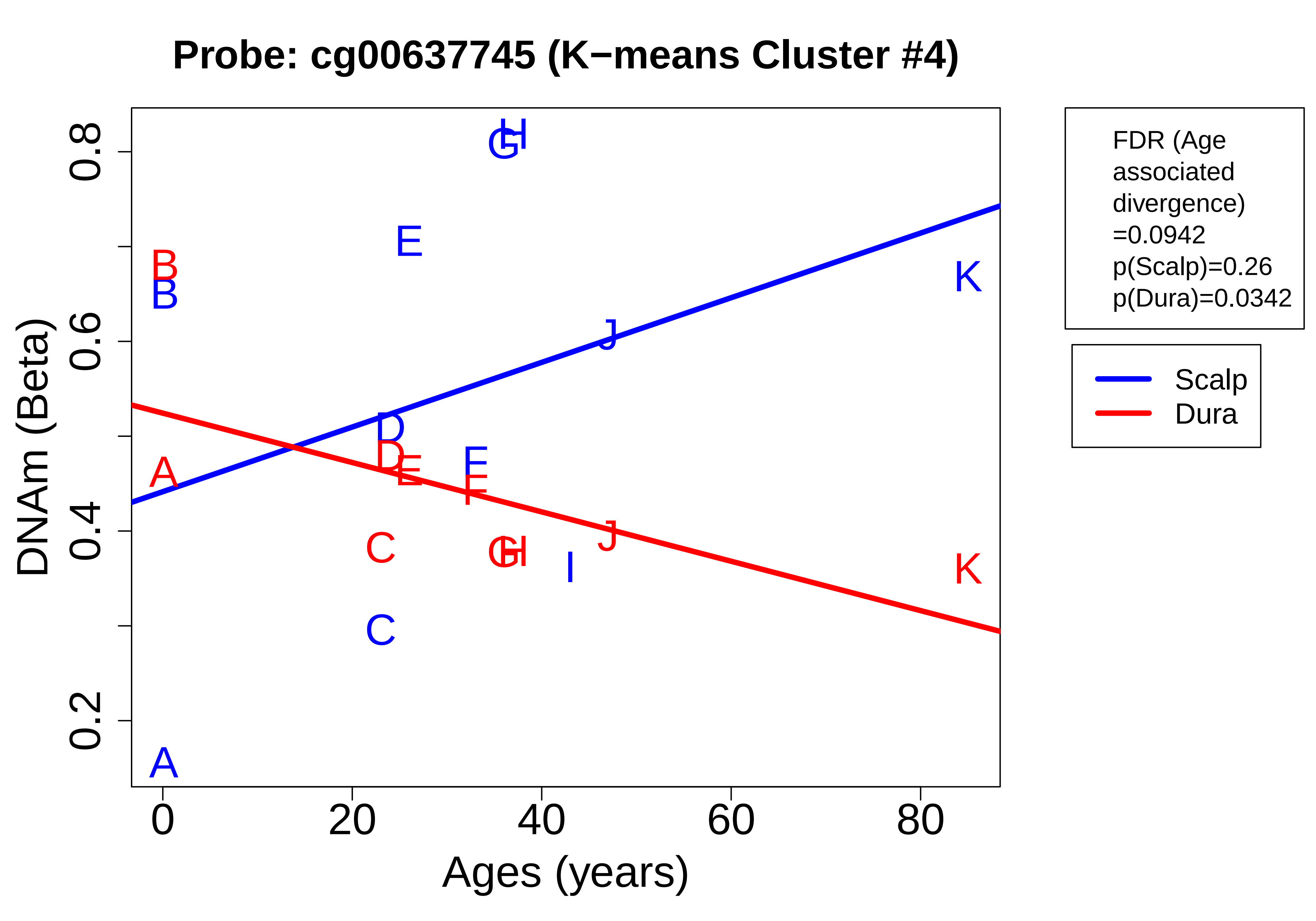

Probe: cg00637745 (K−means Cluster #4)
DNAm (Beta)
0.2
0.4
0.6
0.8
Ages (years)
0
20
40
60
80
A
B
C
D
E
F
G
H
I
J
K
FDR (Age associated divergence) =0.0942
p(Scalp)=0.26
p(Dura)=0.0342
Scalp
Dura

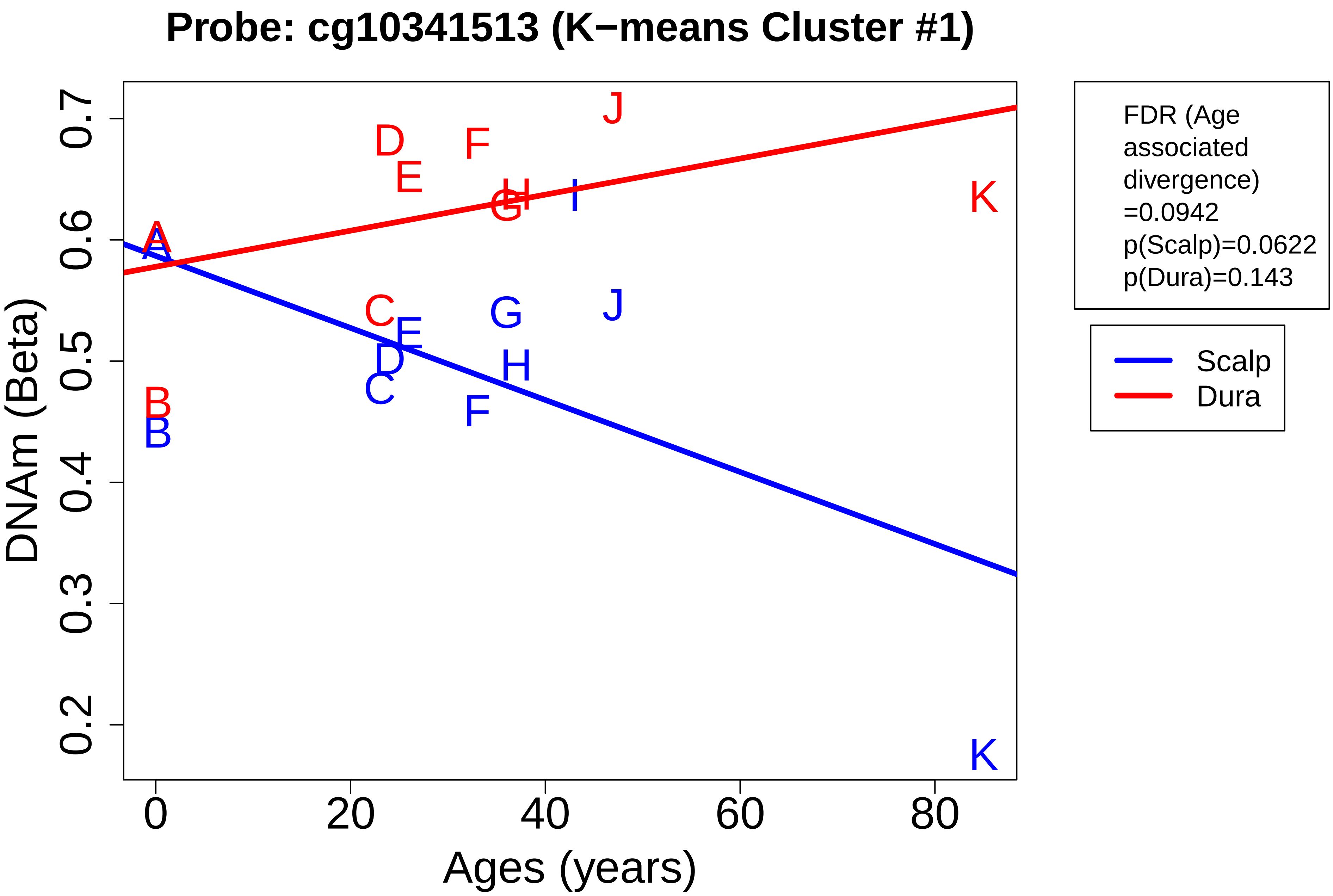

Probe: cg10341513 (K−means Cluster #1)
DNAm (Beta)
0.2
0.3
0.4
0.5
0.6
0.7
Ages (years)
0
20
40
60
80
A
B
C
D
E
F
G
H
I
J
K
FDR (Age associated divergence) =0.0942
p(Scalp)=0.0622
p(Dura)=0.143
Scalp
Dura

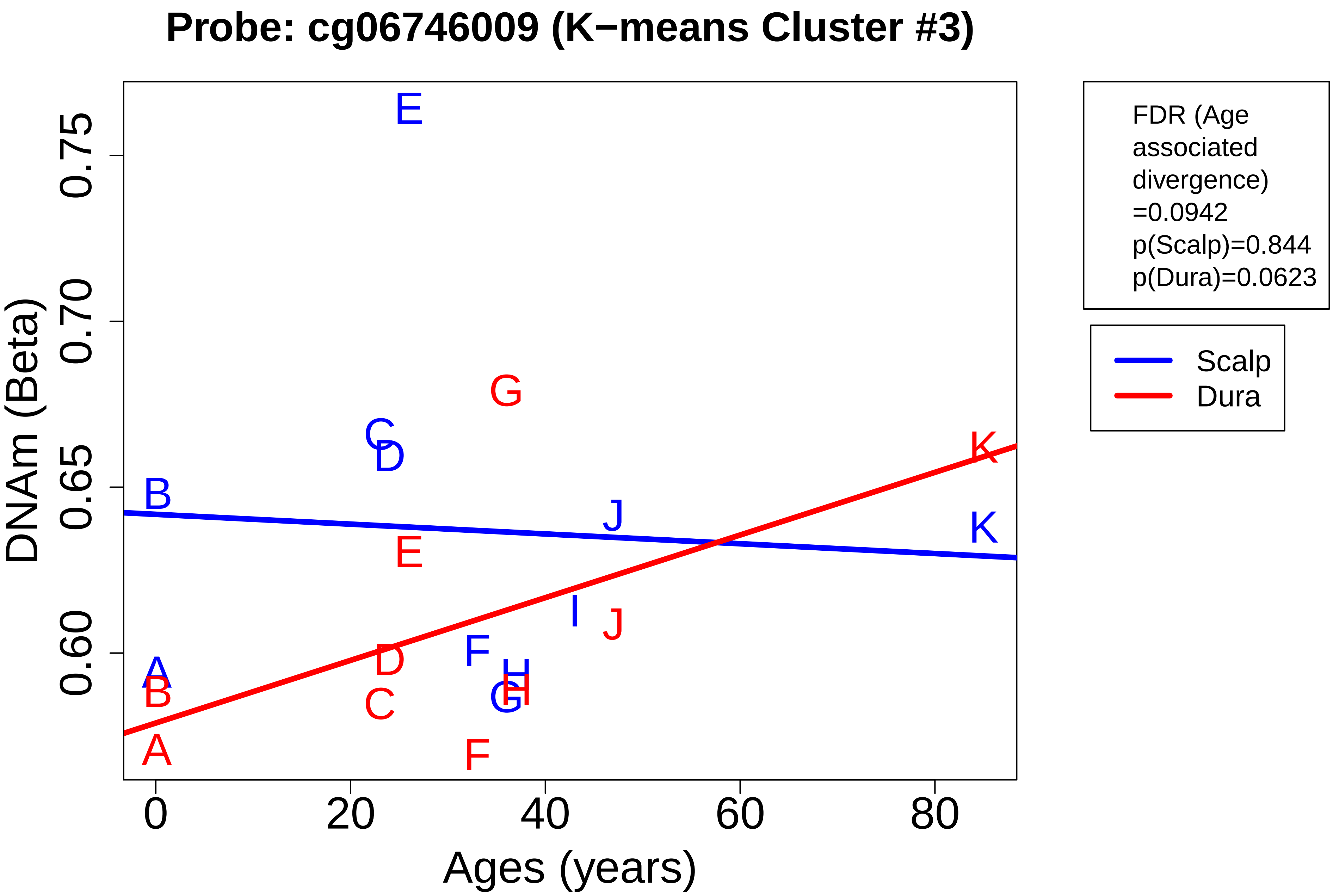

Probe: cg06746009 (K−means Cluster #3)
DNAm (Beta)
0.60
0.65
0.70
0.75
Ages (years)
0
20
40
60
80
FDR (Age associated divergence) =0.0942
p(Scalp)=0.844
p(Dura)=0.0623
Scalp
Dura

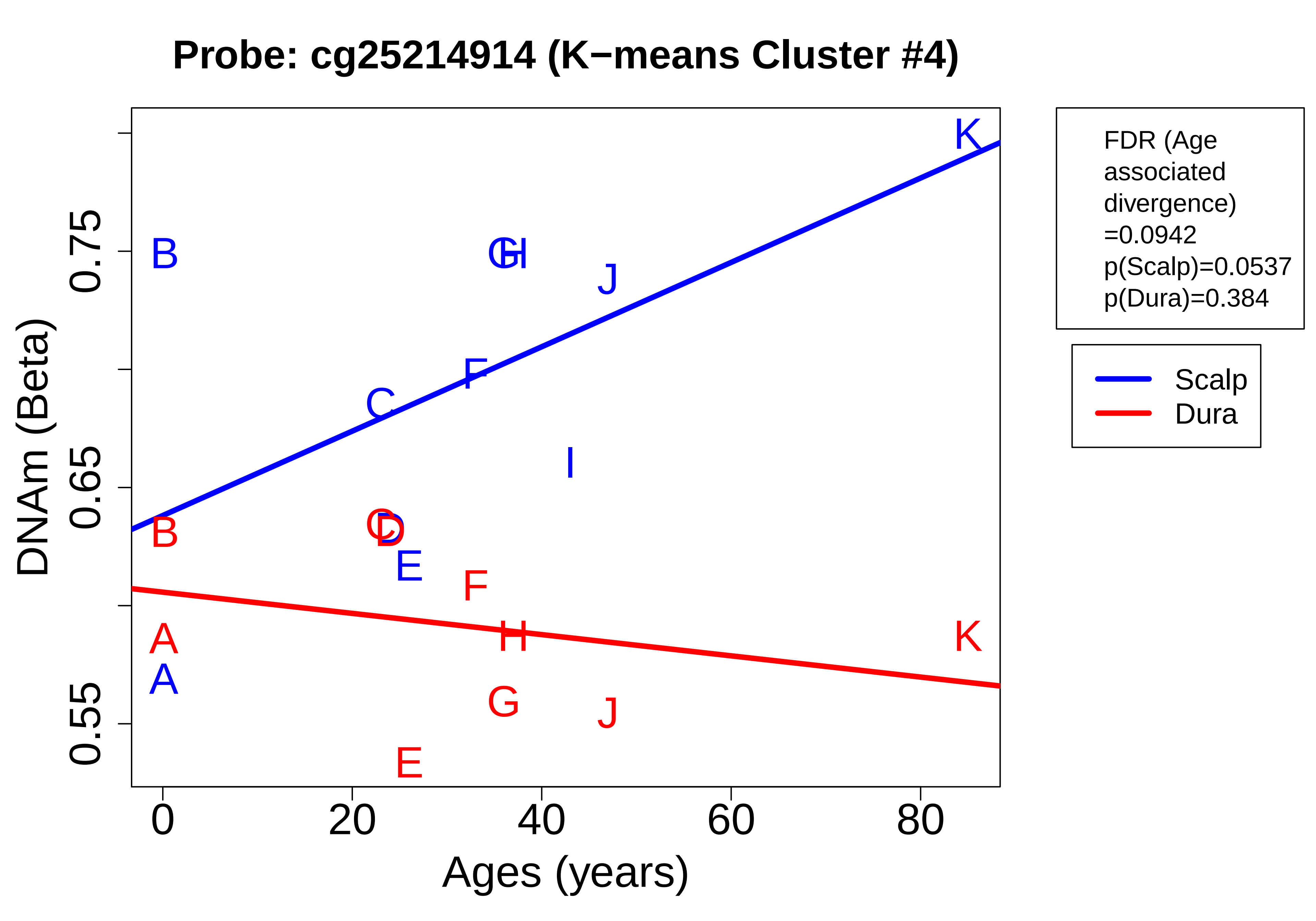
Probe: cg25214914 (K−means Cluster #4)
FDR (Age associated divergence) =0.0942
p(Scalp)=0.0537
p(Dura)=0.384
Scalp
Dura
DNAm (Beta)
0.55
0.65
0.75
Ages (years)
0
20
40
60
80

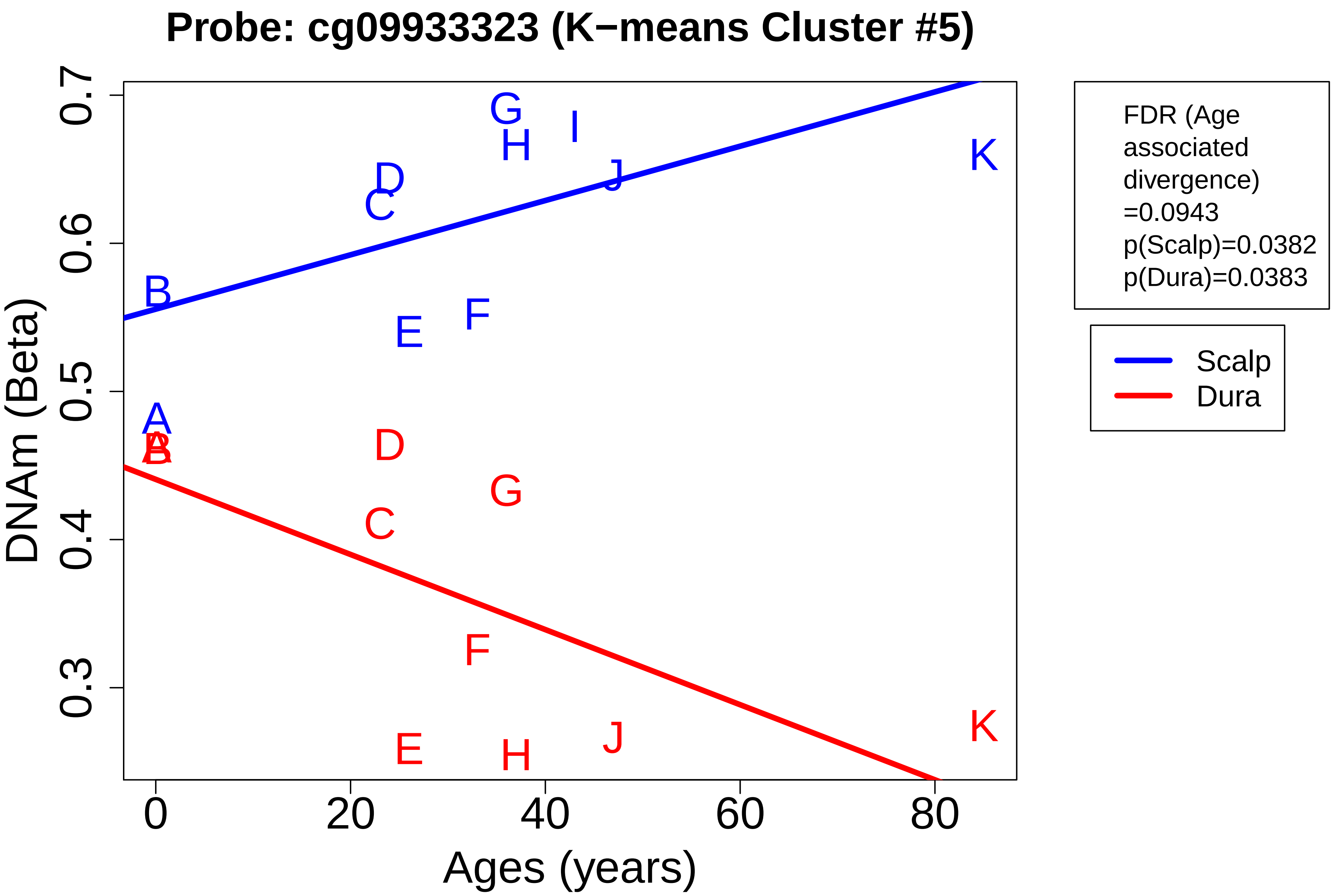

Probe: cg09933323 (K−means Cluster #5)
DNAm (Beta)
0.3
0.4
0.5
0.6
0.7
Ages (years)
0
20
40
60
80
FDR (Age associated divergence) =0.0943
p(Scalp)=0.0382
p(Dura)=0.0383
Scalp
Dura

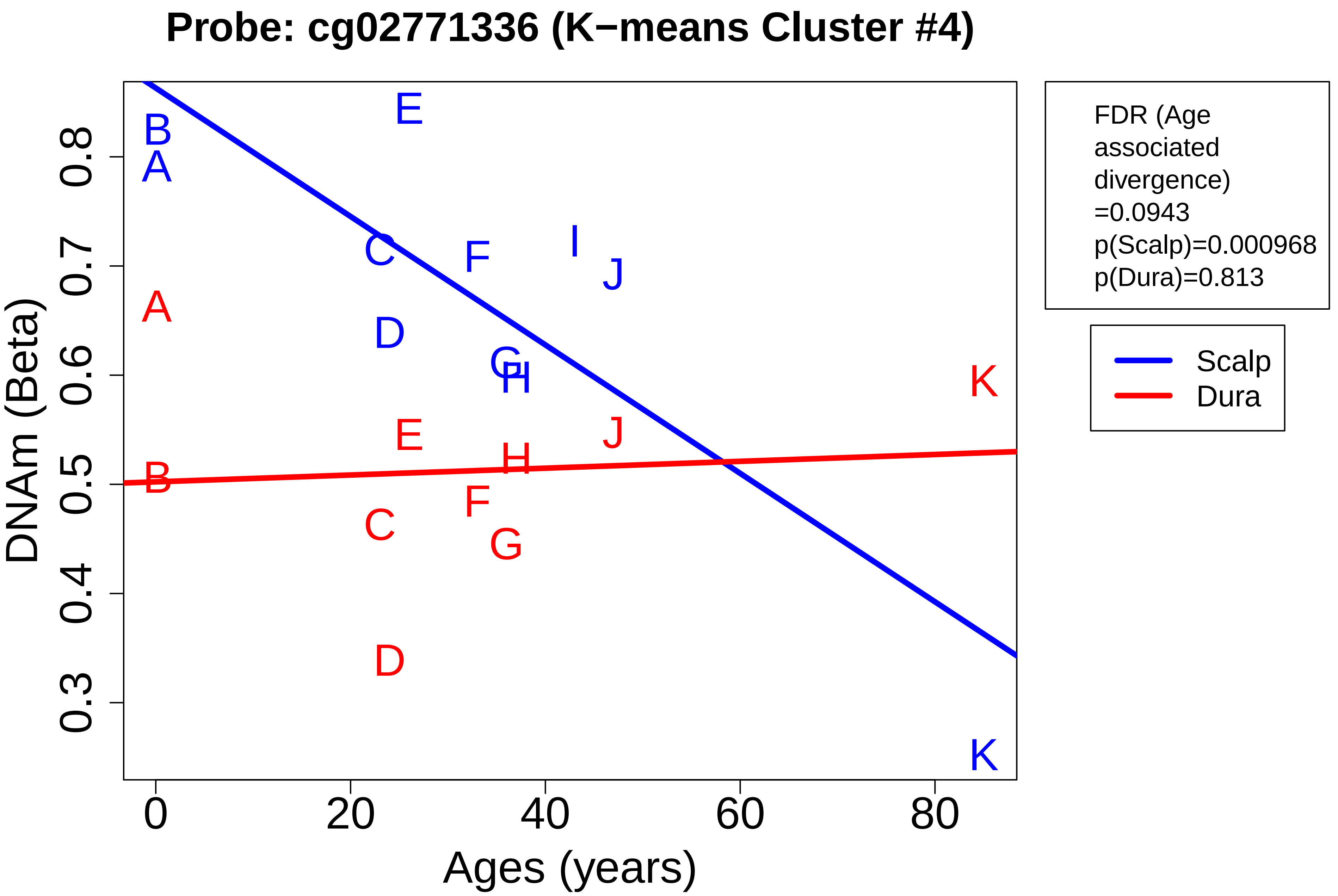
Probe: cg02771336 (K−means Cluster #4)
DNAm (Beta)
Ages (years)
FDR (Age associated divergence) =0.0943
p(Scalp)=0.000968
p(Dura)=0.813
Scalp
Dura

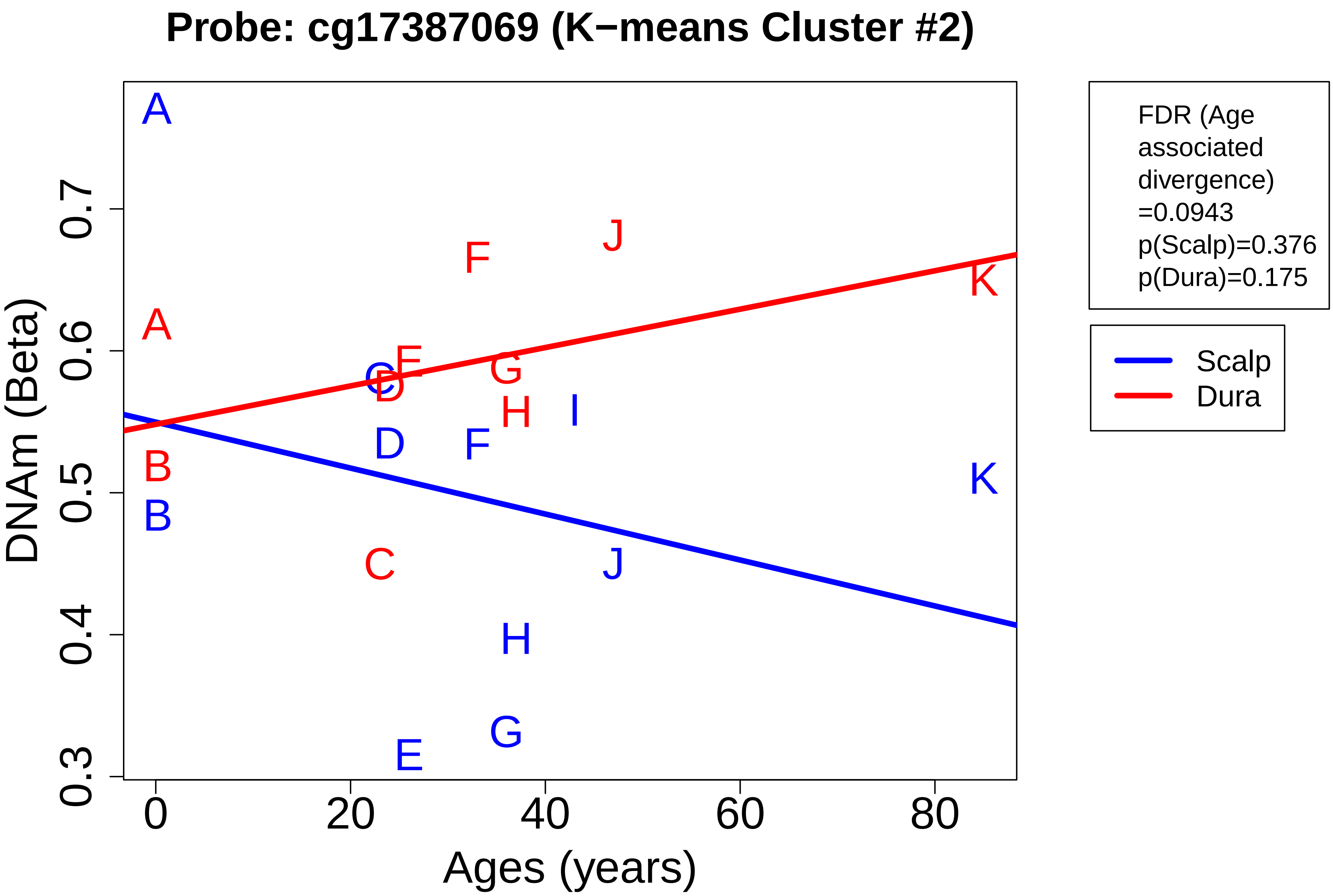

Probe: cg17387069 (K−means Cluster #2)
DNAm (Beta)
0.3
0.4
0.5
0.6
0.7
0
20
40
60
80
Ages (years)
A
B
C
D
E
F
G
H
I
J
K
FDR (Age associated divergence) =0.0943
p(Scalp)=0.376
p(Dura)=0.175
Scalp
Dura

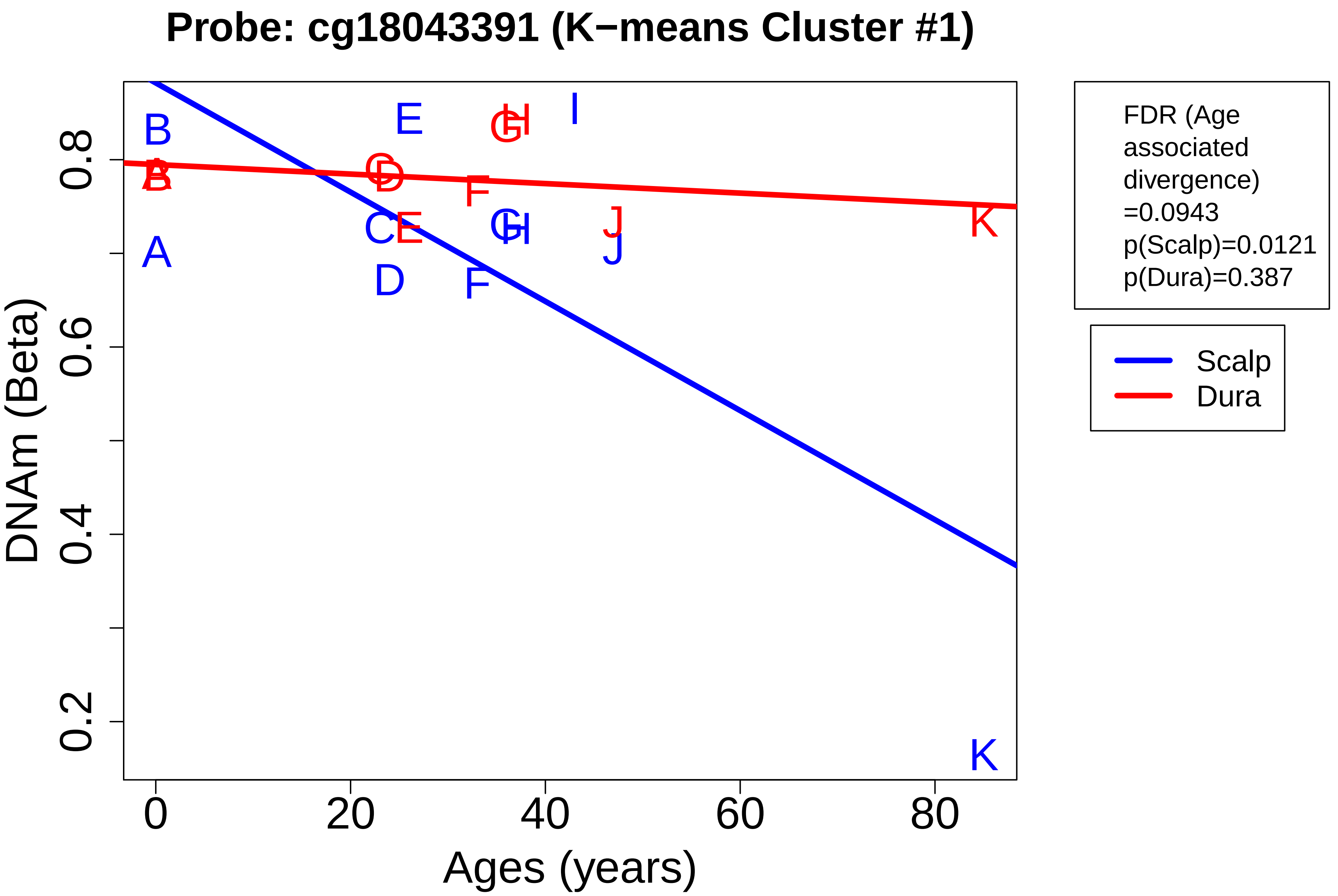

Probe: cg18043391 (K−means Cluster #1)
DNAm (Beta)
0.2
0.4
0.6
0.8
Ages (years)
0
20
40
60
80
FDR (Age associated divergence) =0.0943
p(Scalp)=0.0121
p(Dura)=0.387
Scalp
Dura

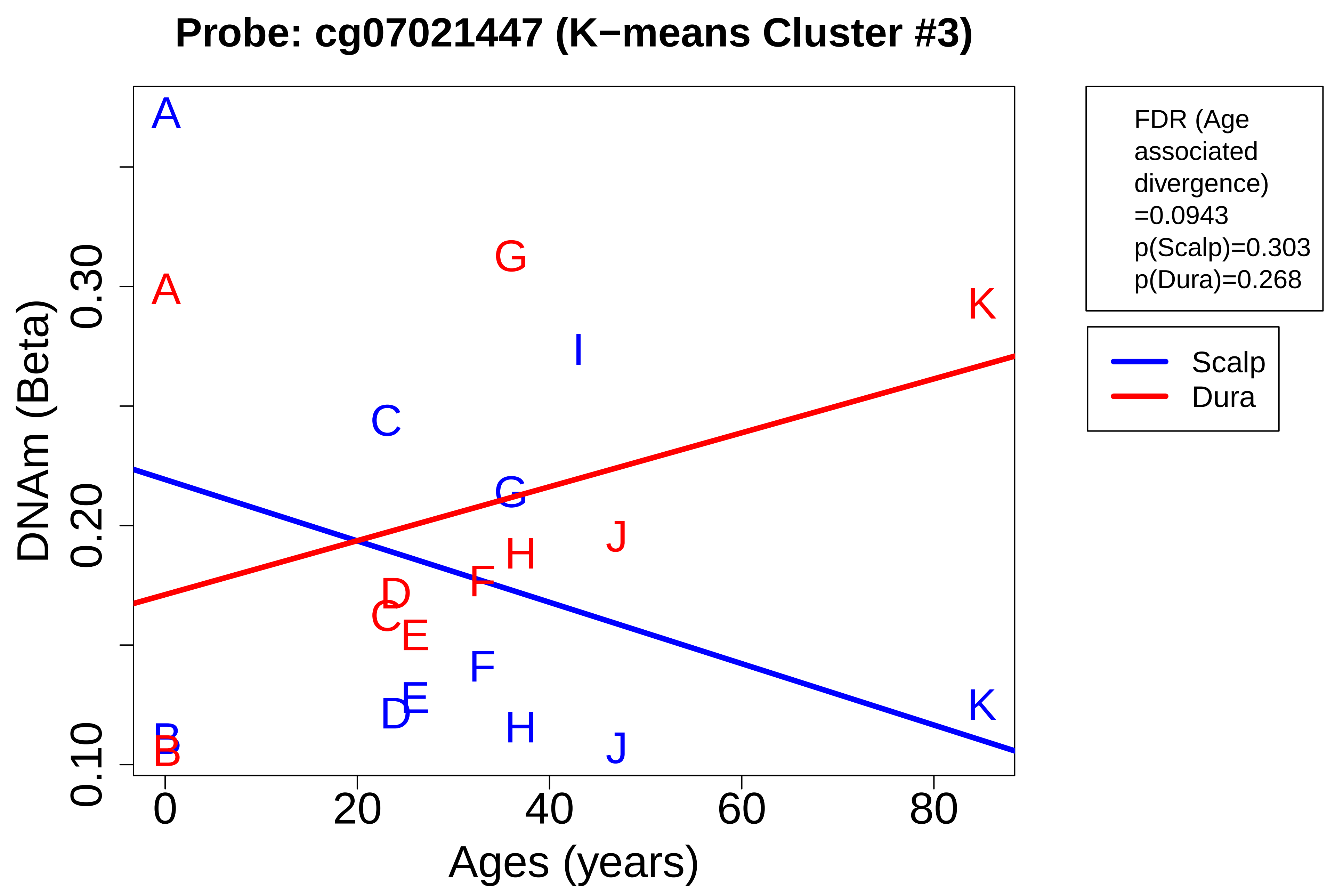
Probe: cg07021447 (K−means Cluster #3)
FDR (Age associated divergence) =0.0943
p(Scalp)=0.303
p(Dura)=0.268
Scalp
Dura
DNAm (Beta)
0.10
0.20
0.30
0
20
40
60
80
Ages (years)
A
B
C
D
E
F
G
H
I
J
K

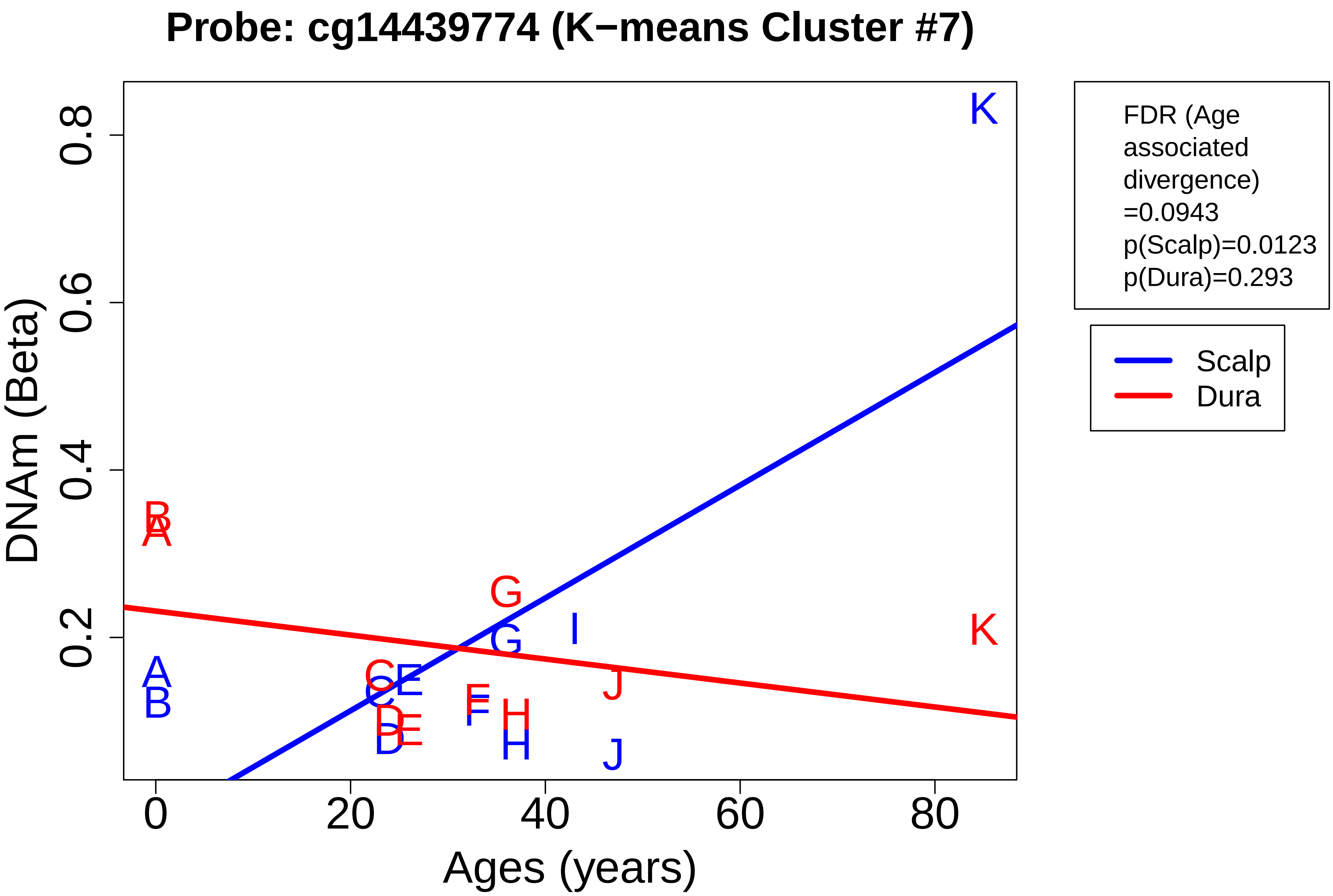

Probe: cg14439774 (K−means Cluster #7)
DNAm (Beta)
0.2
0.4
0.6
0.8
0
20
40
60
80
Ages (years)
FDR (Age associated divergence) =0.0943
p(Scalp)=0.0123
p(Dura)=0.293
Scalp
Dura

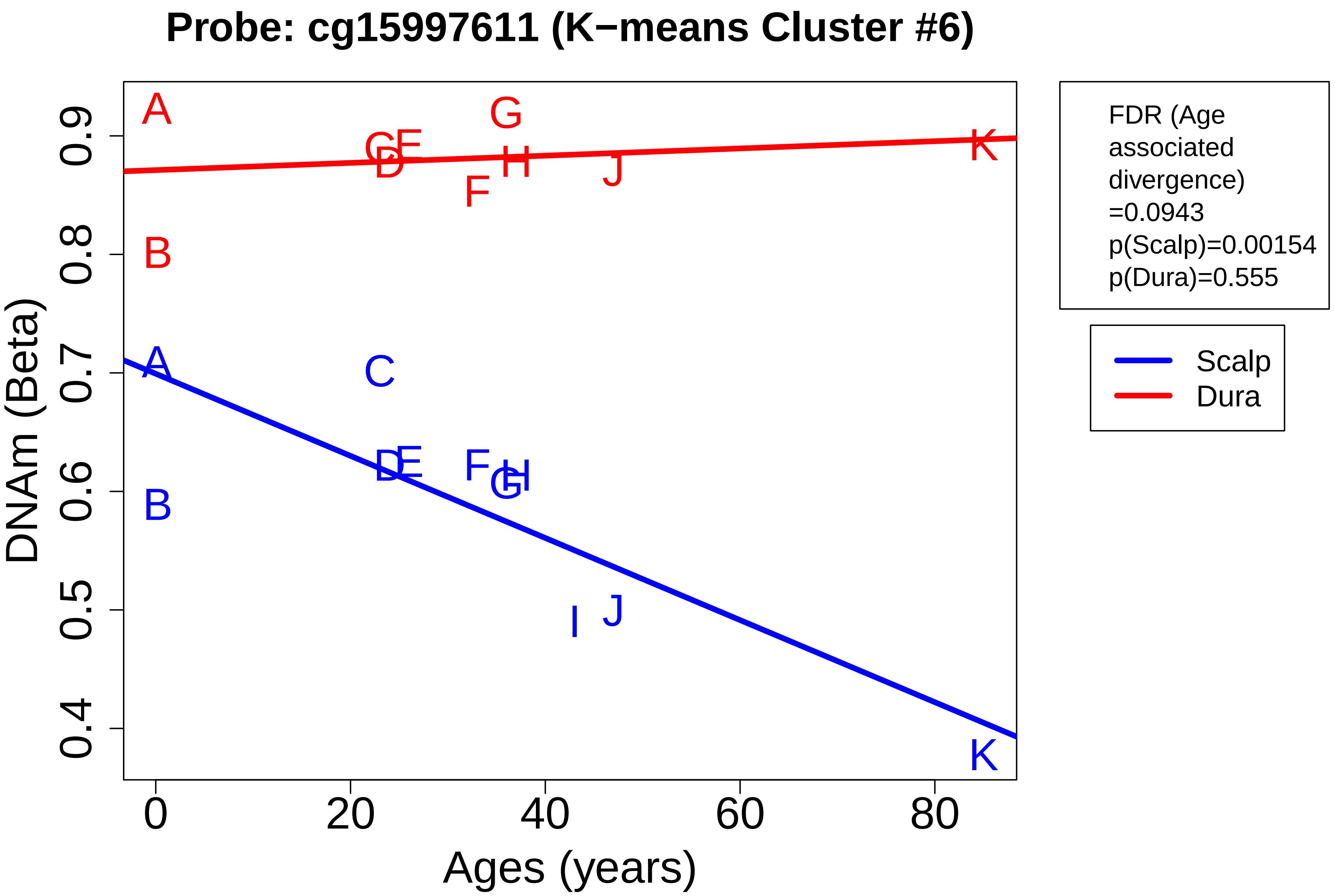

Probe: cg15997611 (K−means Cluster #6)
DNAm (Beta)
Ages (years)
0.4
0.5
0.6
0.7
0.8
0.9
0
20
40
60
80
FDR (Age associated divergence) =0.0943
p(Scalp)=0.00154
p(Dura)=0.555
Scalp
Dura

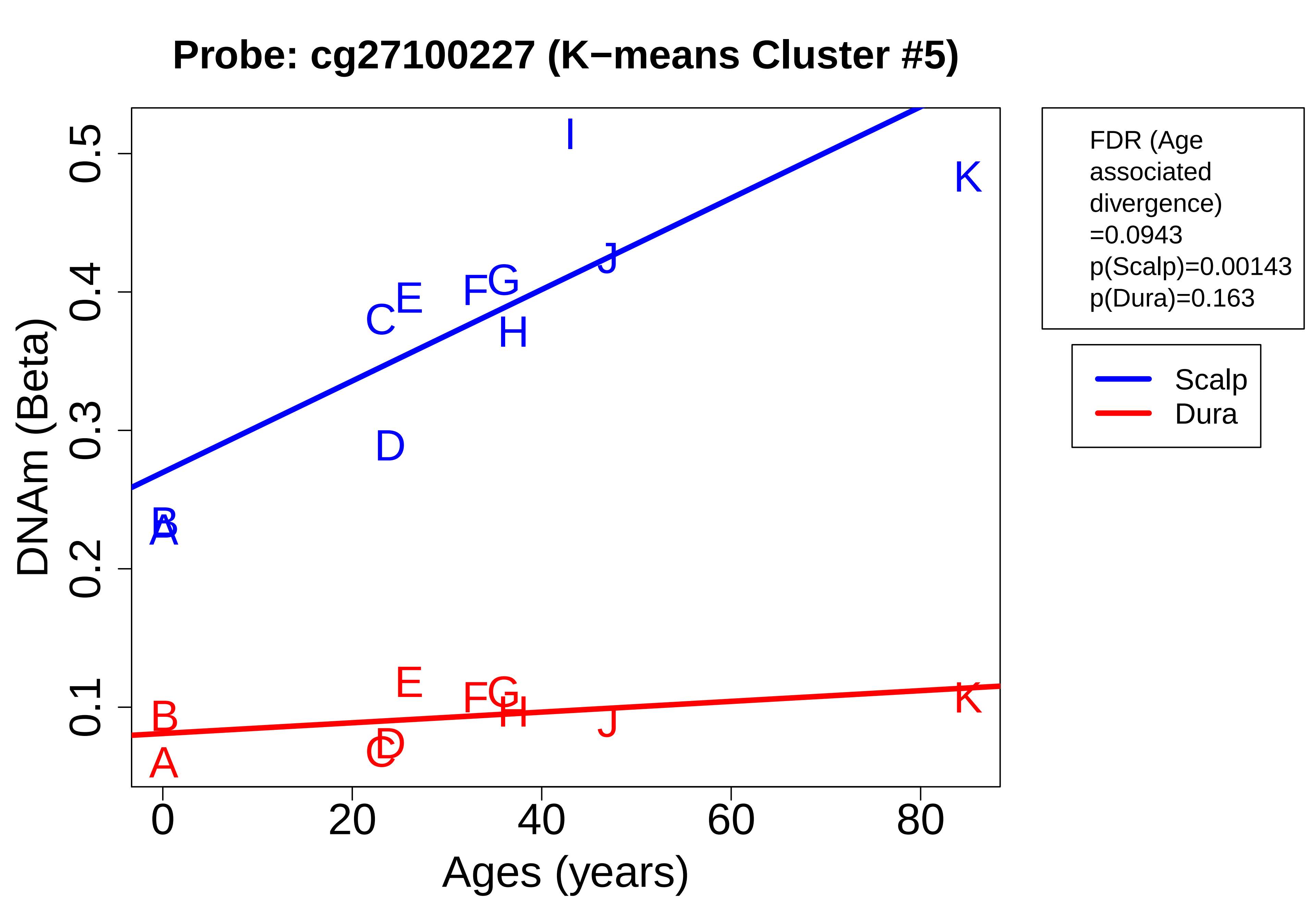

Probe: cg27100227 (K−means Cluster #5)
DNAm (Beta)
0.1
0.2
0.3
0.4
0.5
Ages (years)
0
20
40
60
80
FDR (Age associated divergence) =0.0943
p(Scalp)=0.00143
p(Dura)=0.163
Scalp
Dura

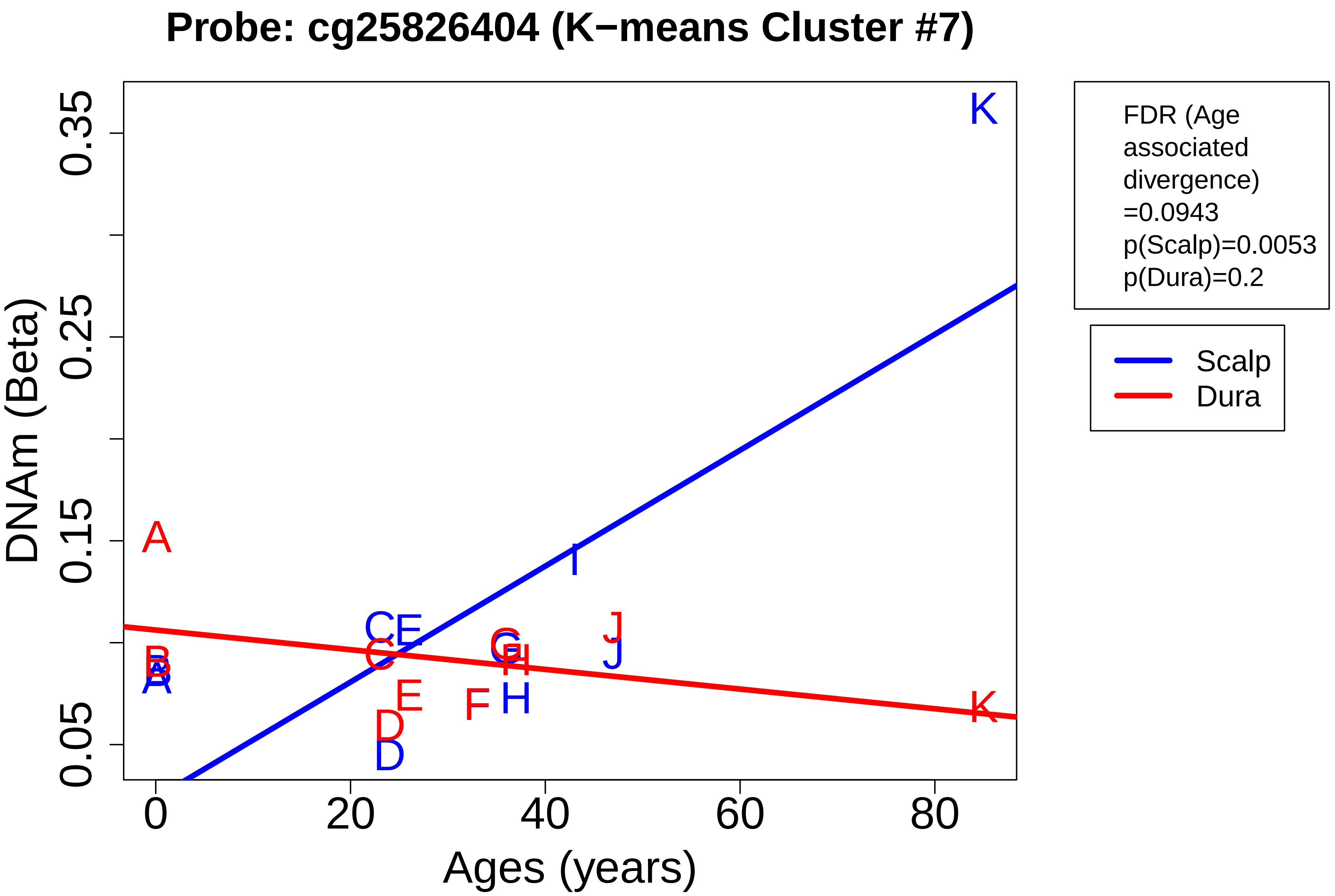

Probe: cg25826404 (K−means Cluster #7)
DNAm (Beta)
0.05
0.15
0.25
0.35
Ages (years)
0
20
40
60
80
FDR (Age associated divergence) =0.0943
p(Scalp)=0.0053
p(Dura)=0.2
Scalp
Dura

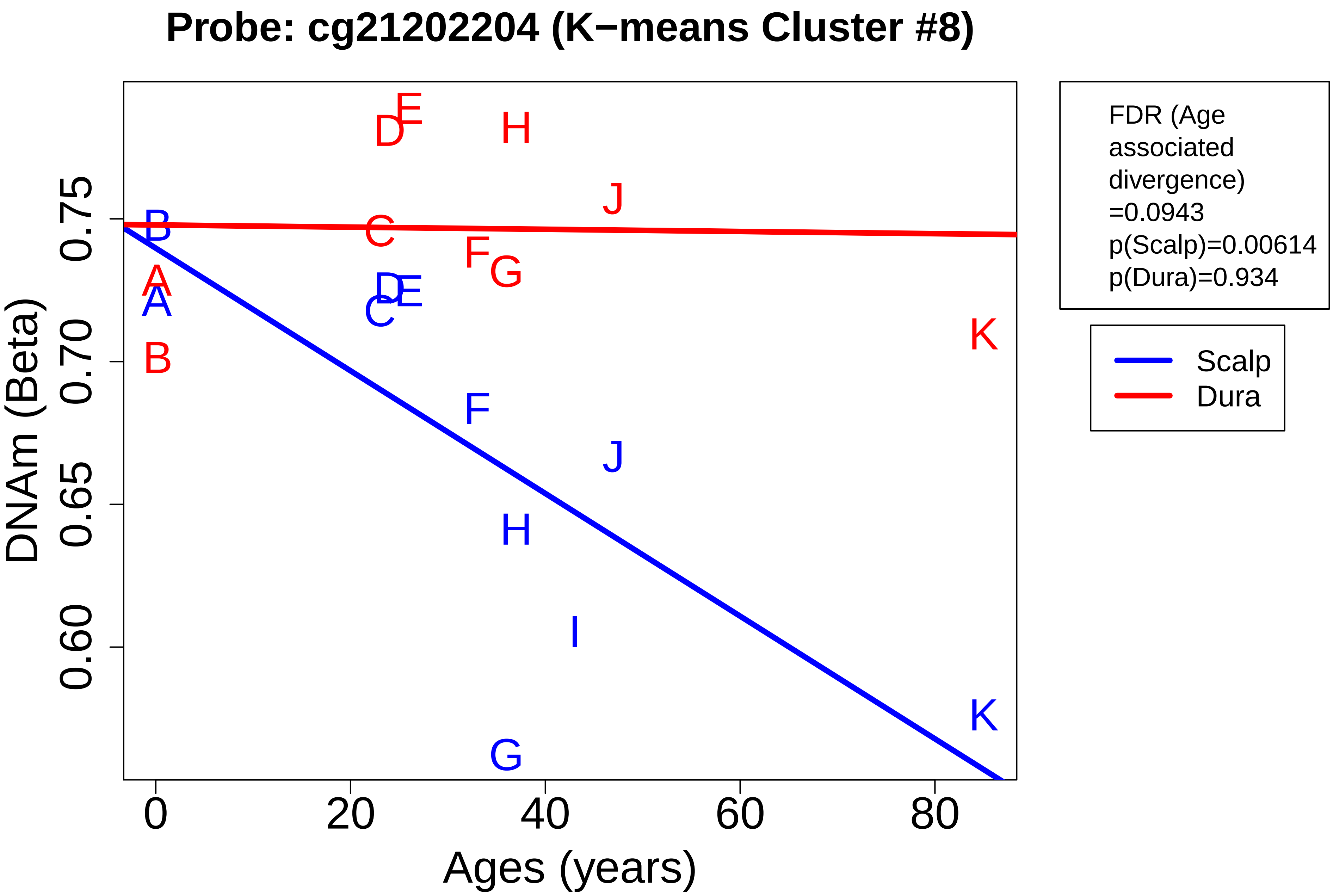

Probe: cg21202204 (K−means Cluster #8)
DNAm (Beta)
0.60
0.65
0.70
0.75
0
20
40
60
80
Ages (years)
FDR (Age associated divergence) =0.0943
p(Scalp)=0.00614
p(Dura)=0.934
Scalp
Dura

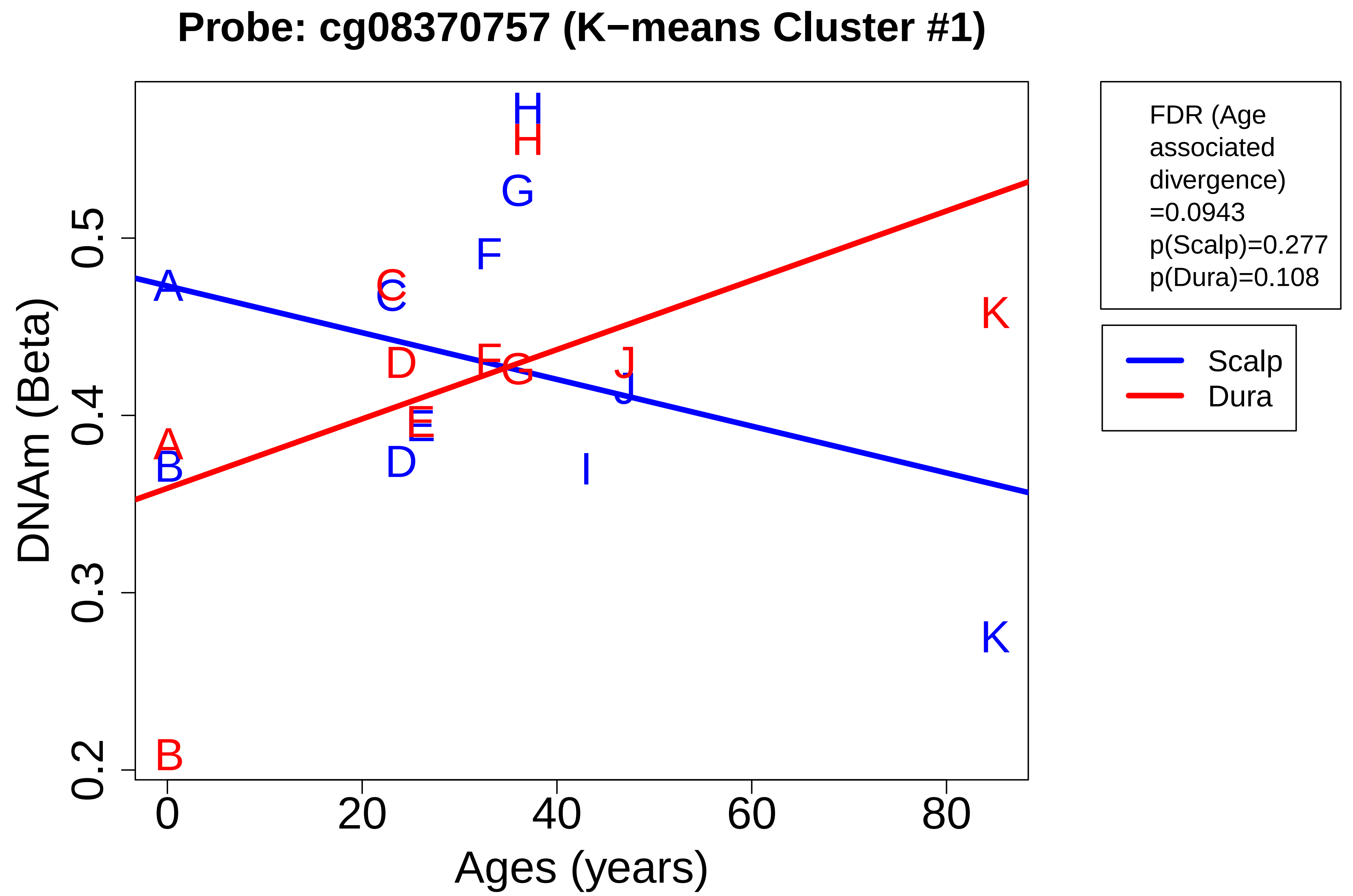

Probe: cg08370757 (K−means Cluster #1)
DNAm (Beta)
0.2
0.3
0.4
0.5
0
20
40
60
80
Ages (years)
A
B
C
D
E
F
G
H
I
J
K
FDR (Age associated divergence) =0.0943
p(Scalp)=0.277
p(Dura)=0.108
Scalp
Dura

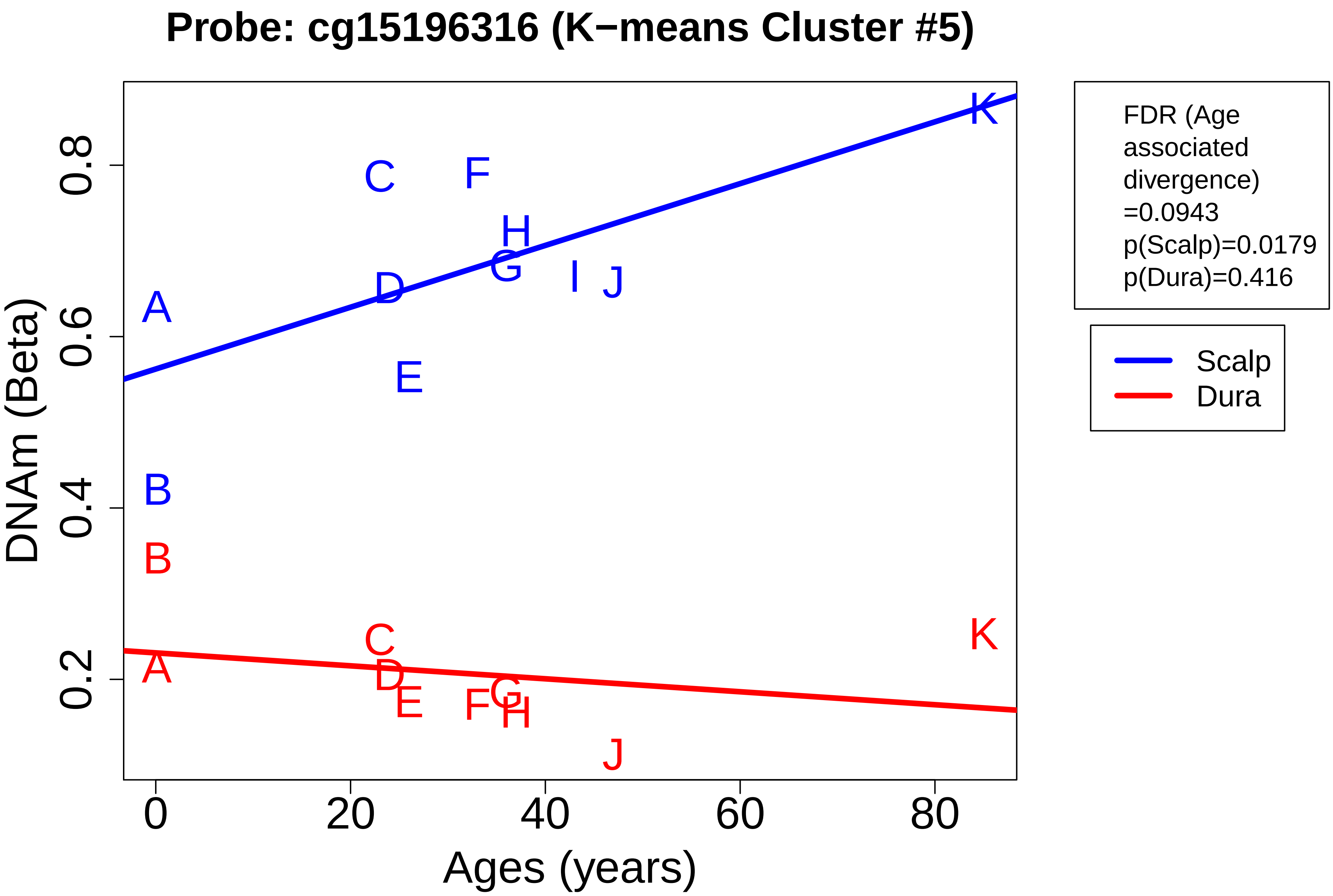

Probe: cg15196316 (K−means Cluster #5)
DNAm (Beta)
0.2
0.4
0.6
0.8
0
20
40
60
80
Ages (years)
A
B
C
D
E
F
G
H
I
J
K
FDR (Age associated divergence) =0.0943
p(Scalp)=0.0179
p(Dura)=0.416
Scalp
Dura

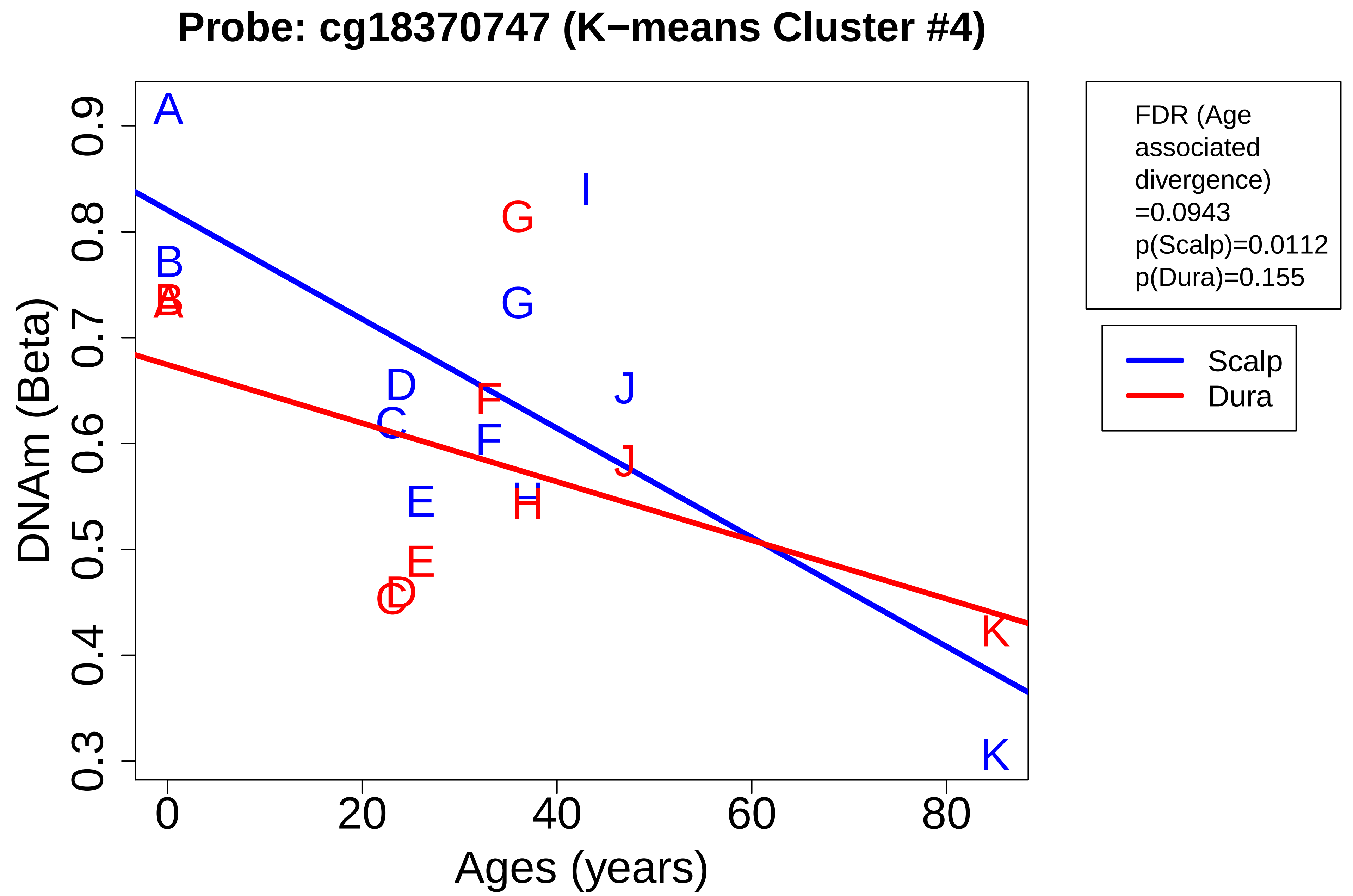

Probe: cg18370747 (K−means Cluster #4)
DNAm (Beta)
0.3
0.4
0.5
0.6
0.7
0.8
0.9
0
20
40
60
80
Ages (years)
A
B
C
D
E
F
G
H
I
J
K
FDR (Age associated divergence) =0.0943
p(Scalp)=0.0112
p(Dura)=0.155
Scalp
Dura

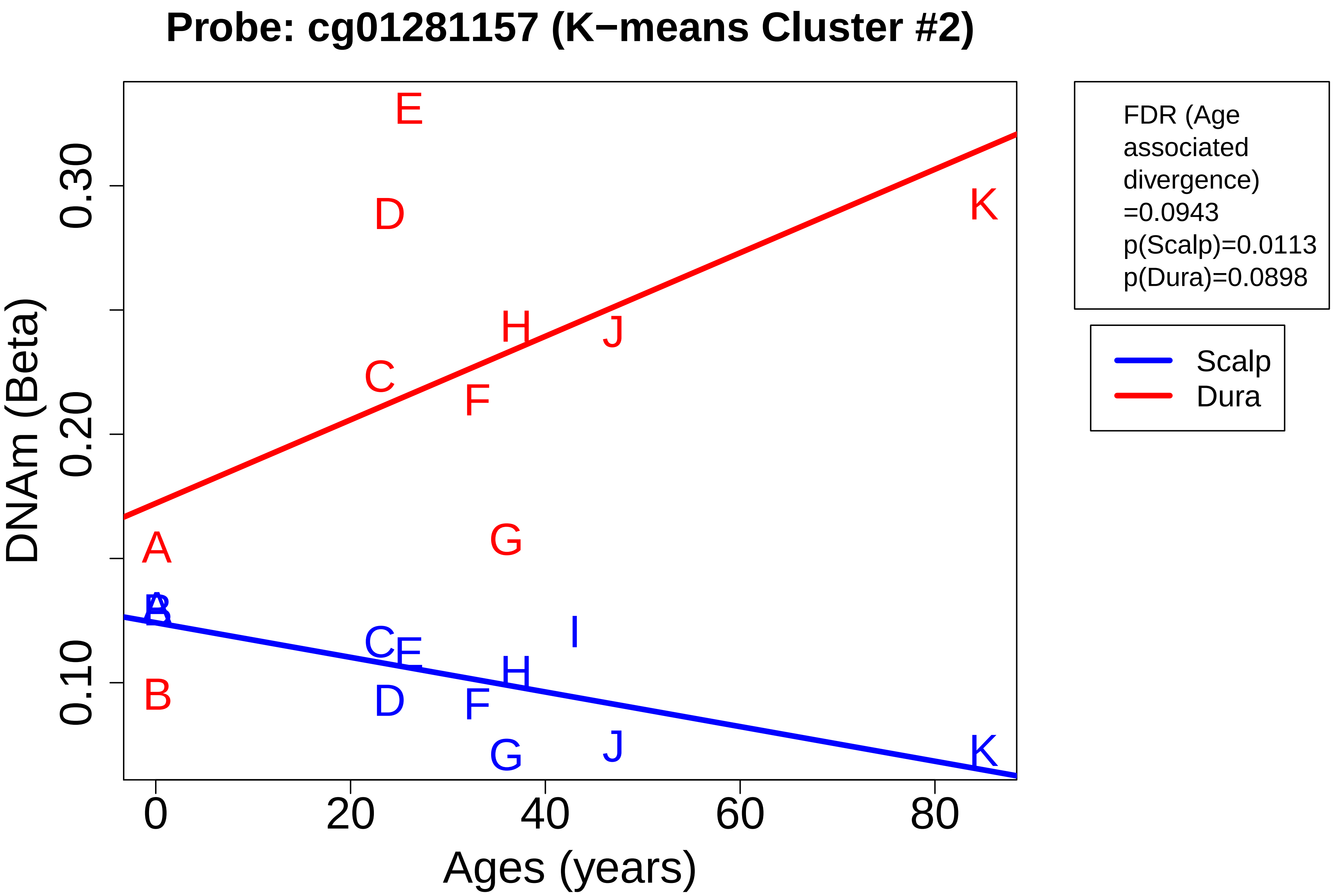

Probe: cg01281157 (K−means Cluster #2)
FDR (Age associated divergence) =0.0943
p(Scalp)=0.0113
p(Dura)=0.0898
Scalp
Dura
DNAm (Beta)
0.10
0.20
0.30
0
20
40
60
80
Ages (years)

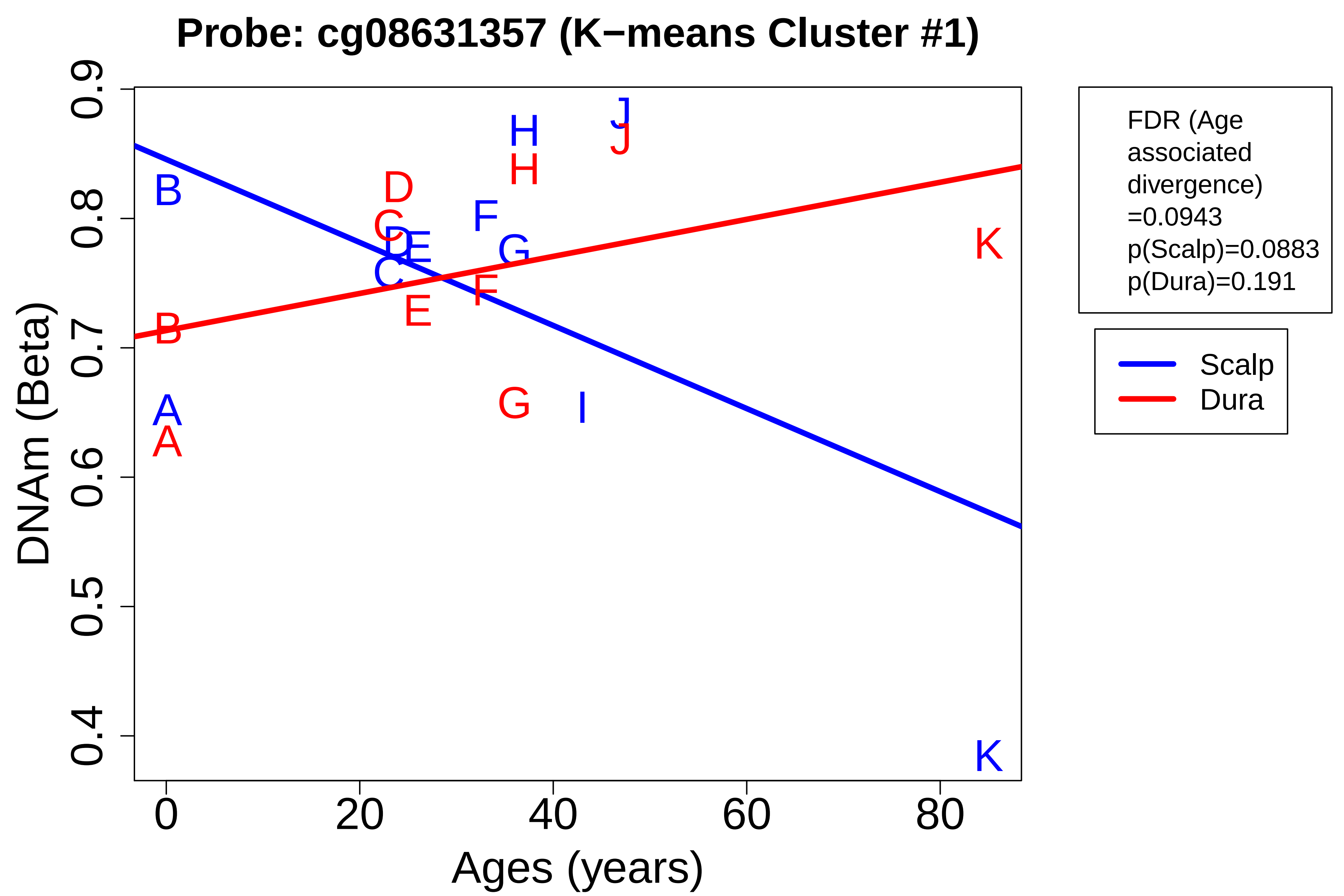

Probe: cg08631357 (K−means Cluster #1)
DNAm (Beta)
0.4
0.5
0.6
0.7
0.8
0.9
Ages (years)
0
20
40
60
80
A
B
C
D
E
F
G
H
I
J
K
FDR (Age associated divergence) =0.0943
p(Scalp)=0.0883
p(Dura)=0.191
Scalp
Dura

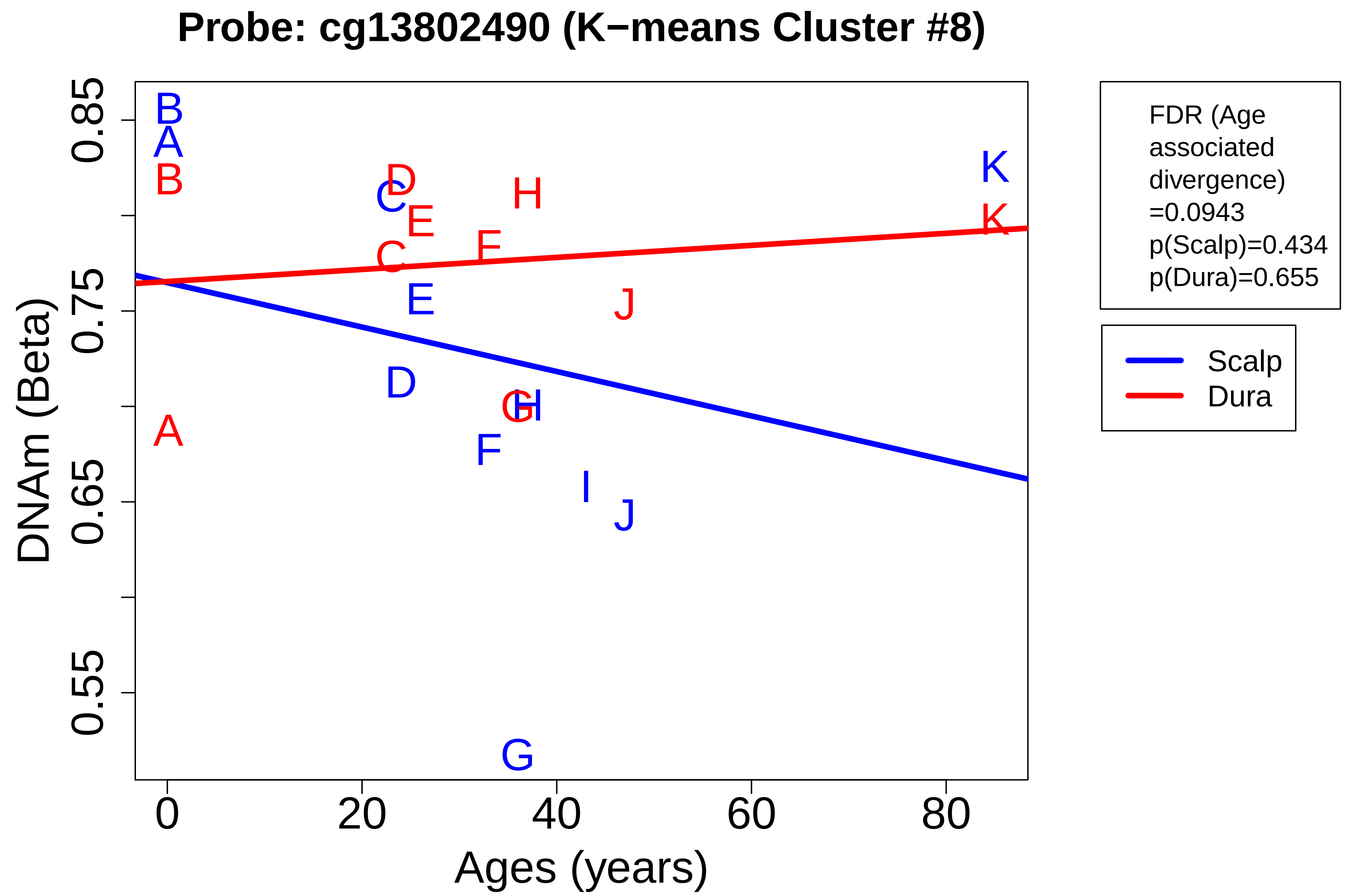

Probe: cg13802490 (K−means Cluster #8)
DNAm (Beta)
0.55
0.65
0.75
0.85
0
20
40
60
80
Ages (years)
A
B
C
D
E
F
G
H
I
J
K
FDR (Age associated divergence) =0.0943
p(Scalp)=0.434
p(Dura)=0.655
Scalp
Dura

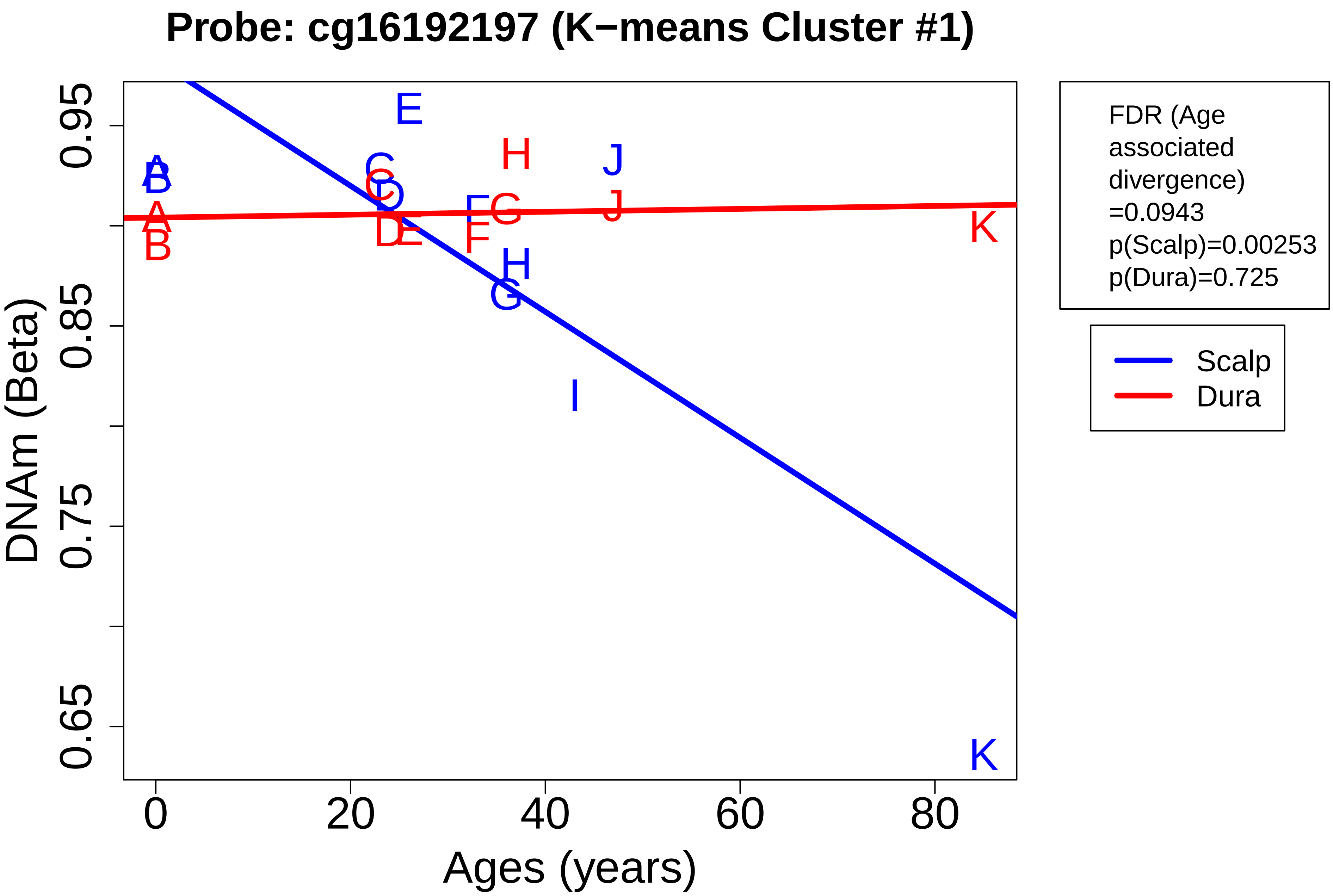

Probe: cg16192197 (K−means Cluster #1)
DNAm (Beta)
0.95
0.85
0.75
0.65
Ages (years)
0
20
40
60
80
FDR (Age associated divergence) =0.0943
p(Scalp)=0.00253
p(Dura)=0.725
Scalp
Dura
A
B
C
D
E
F
G
H
I
J
K

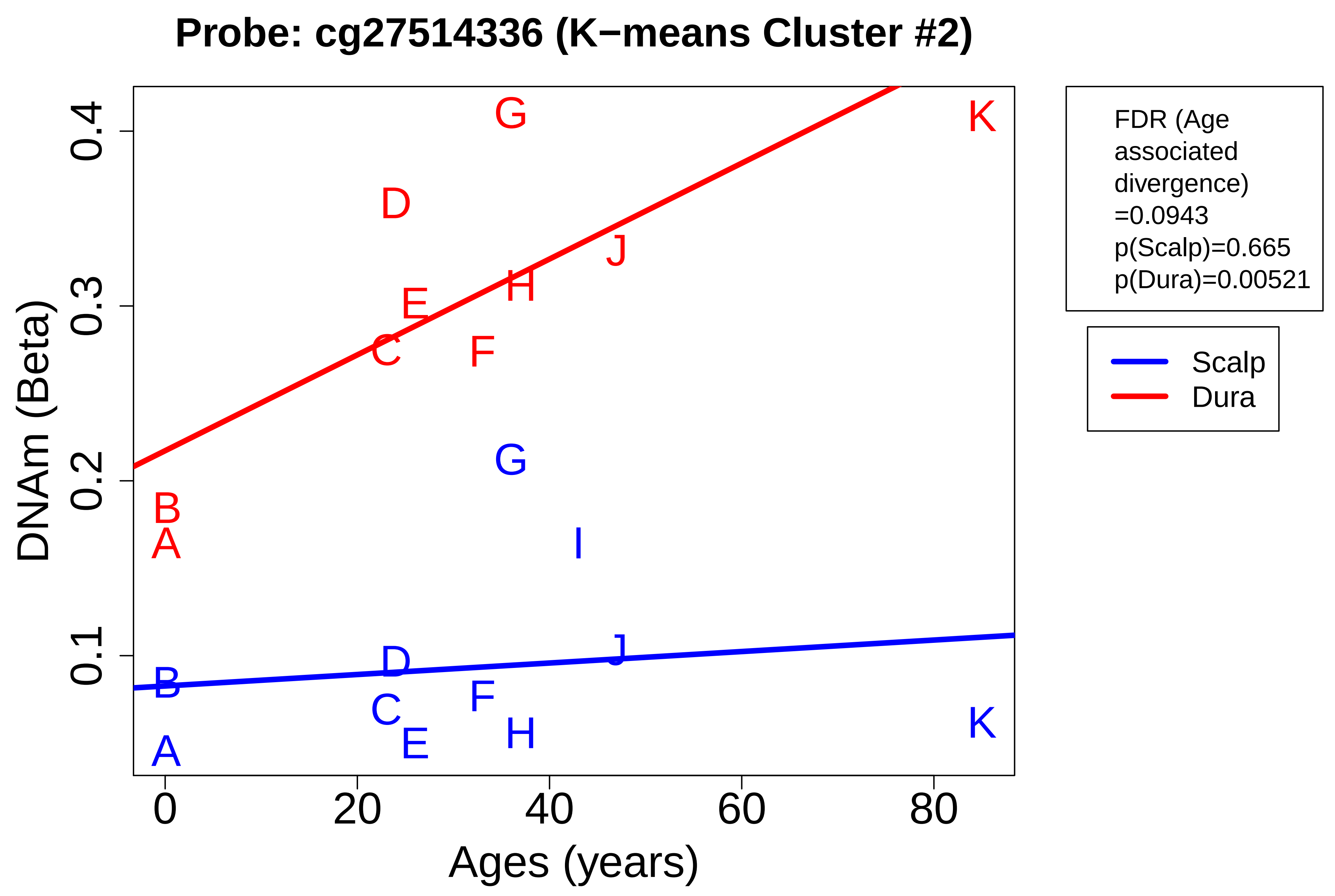
Probe: cg27514336 (K−means Cluster #2)
FDR (Age associated divergence) =0.0943
p(Scalp)=0.665
p(Dura)=0.00521
Scalp
Dura
DNAm (Beta)
0.1
0.2
0.3
0.4
Ages (years)
0
20
40
60
80
A
B
C
D
E
F
G
H
I
J
K

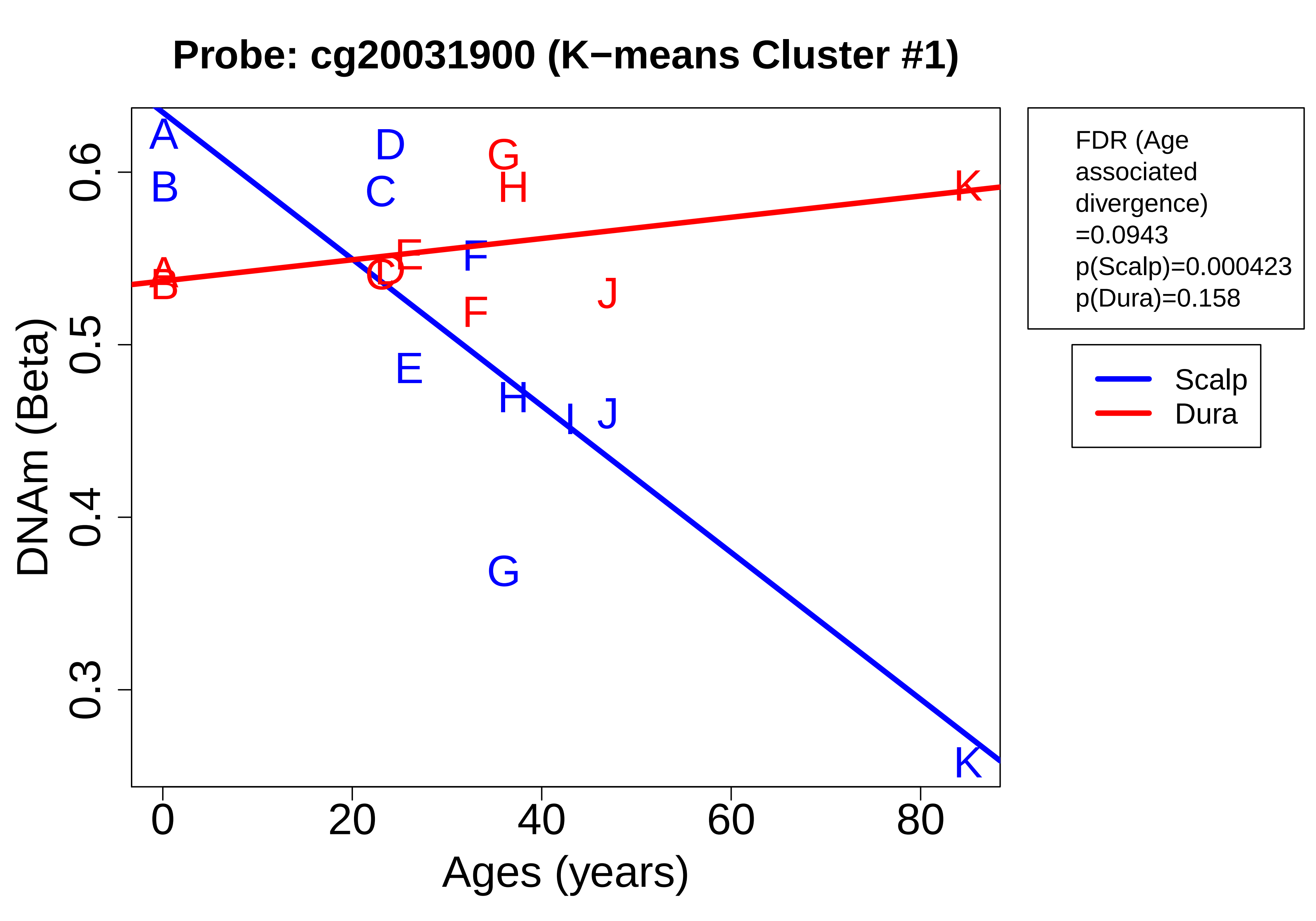

Probe: cg20031900 (K−means Cluster #1)
DNAm (Beta)
Ages (years)
0.3
0.4
0.5
0.6
0
20
40
60
80
FDR (Age associated divergence) =0.0943
p(Scalp)=0.000423
p(Dura)=0.158
Scalp
Dura

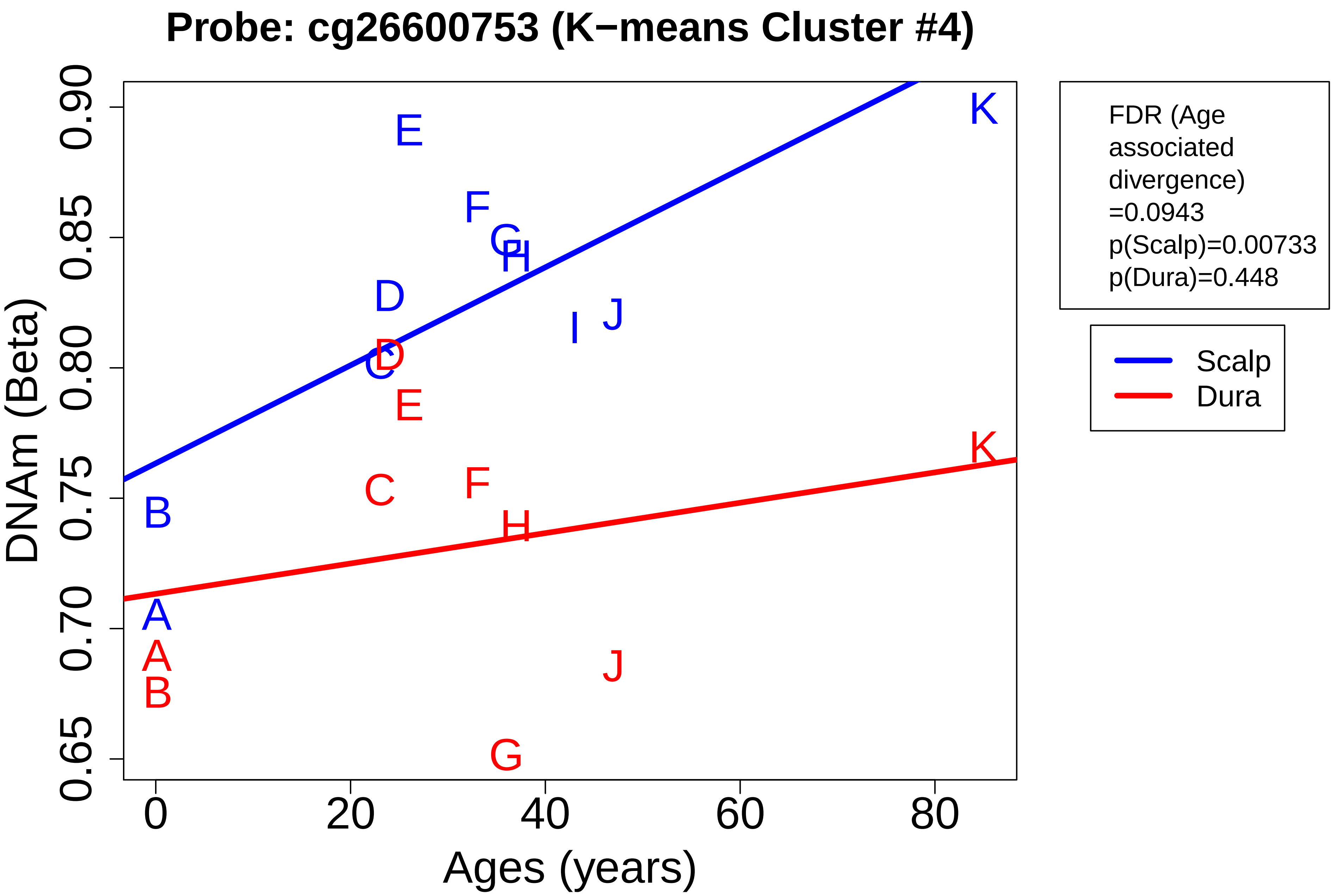

Probe: cg26600753 (K−means Cluster #4)
DNAm (Beta)
0.65
0.70
0.75
0.80
0.85
0.90
0
20
40
60
80
Ages (years)
FDR (Age associated divergence) =0.0943
p(Scalp)=0.00733
p(Dura)=0.448
Scalp
Dura

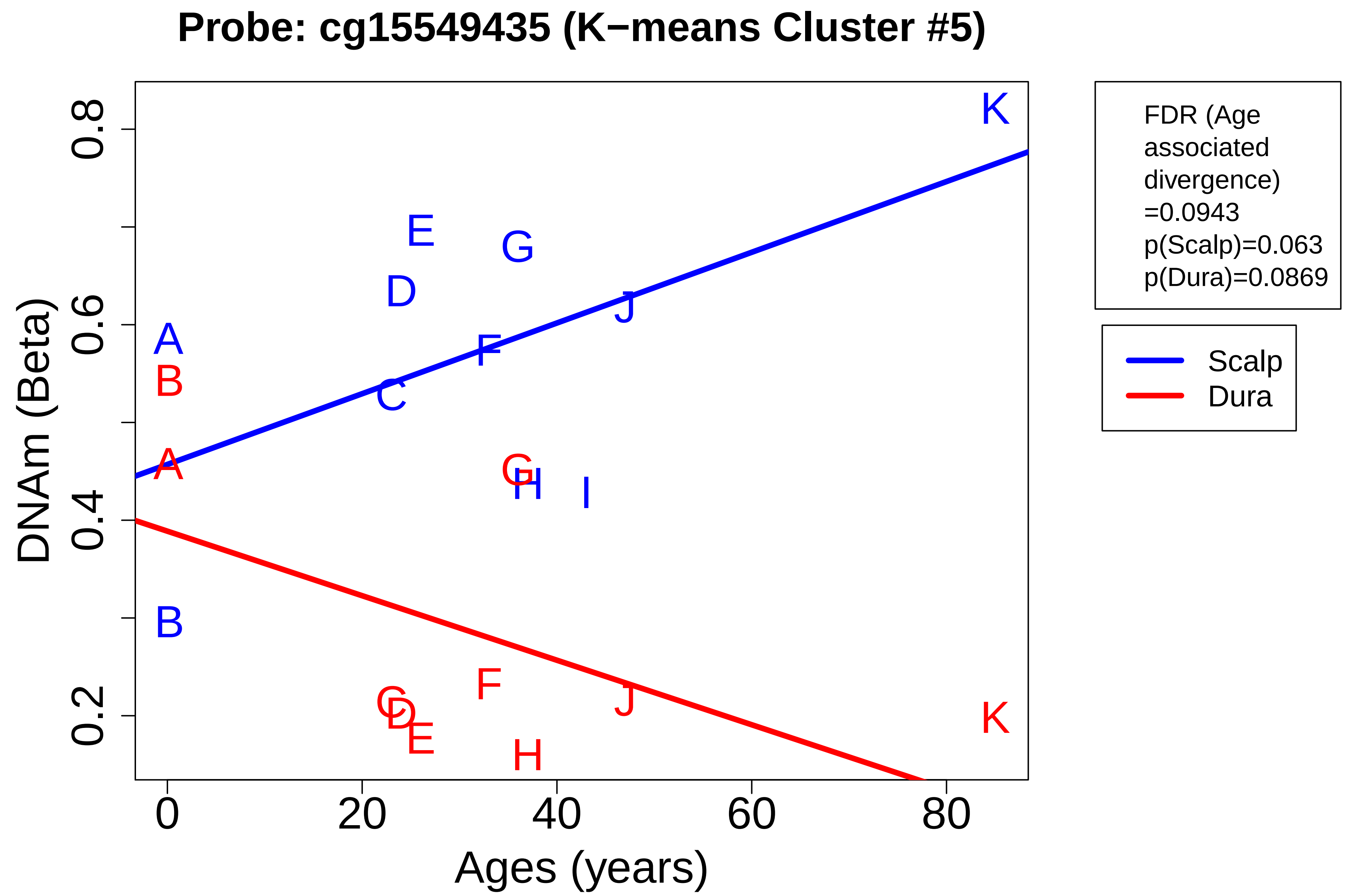

Probe: cg15549435 (K−means Cluster #5)
DNAm (Beta)
0.2
0.4
0.6
0.8
0
20
40
60
80
Ages (years)
A
B
C
D
E
F
G
H
I
J
K
A
B
C
D
E
F
G
H
J
K
FDR (Age associated divergence) =0.0943
p(Scalp)=0.063
p(Dura)=0.0869
Scalp
Dura

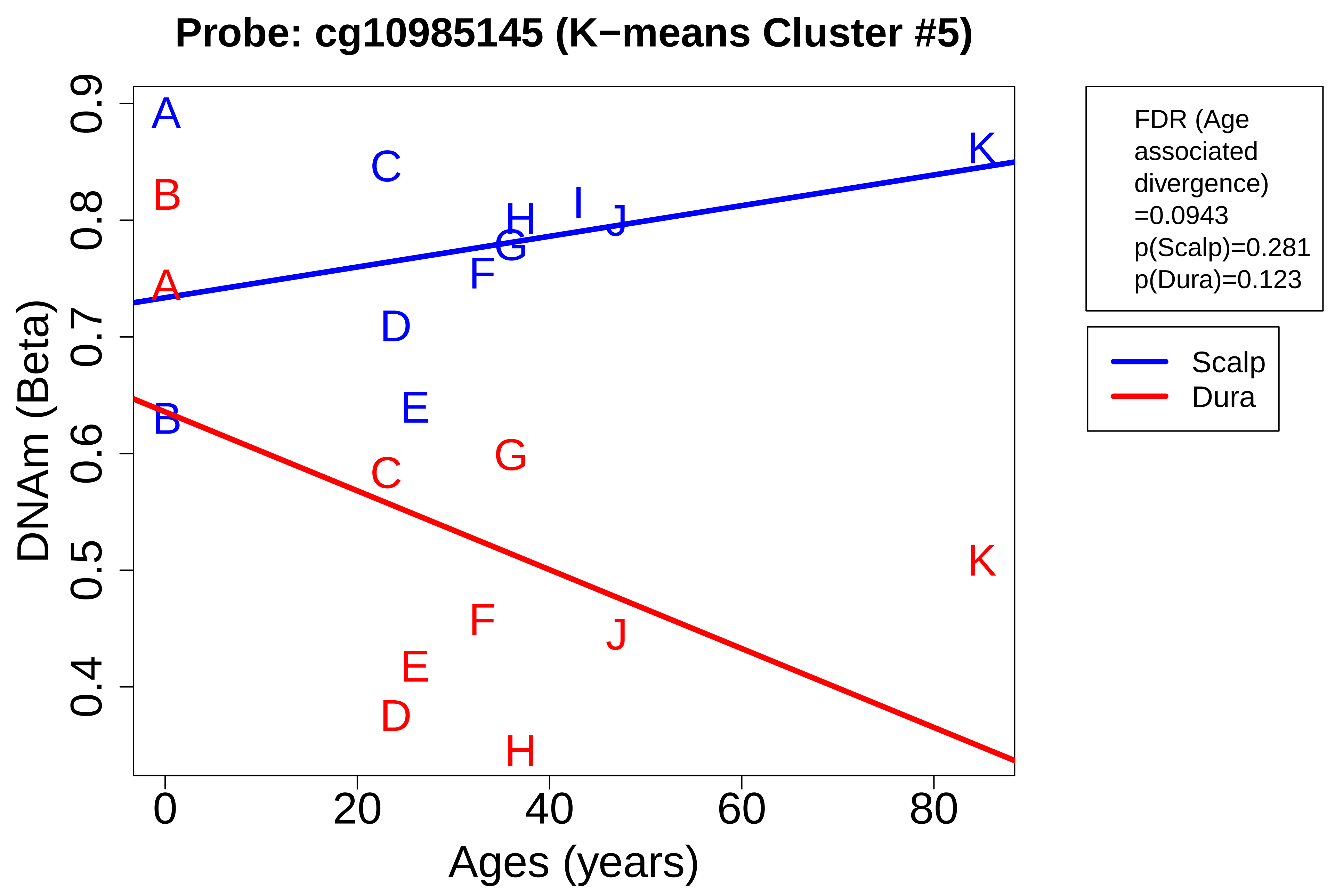

Probe: cg10985145 (K−means Cluster #5)
DNAm (Beta)
0.4
0.5
0.6
0.7
0.8
0.9
Ages (years)
0
20
40
60
80
FDR (Age associated divergence) =0.0943
p(Scalp)=0.281
p(Dura)=0.123
Scalp
Dura

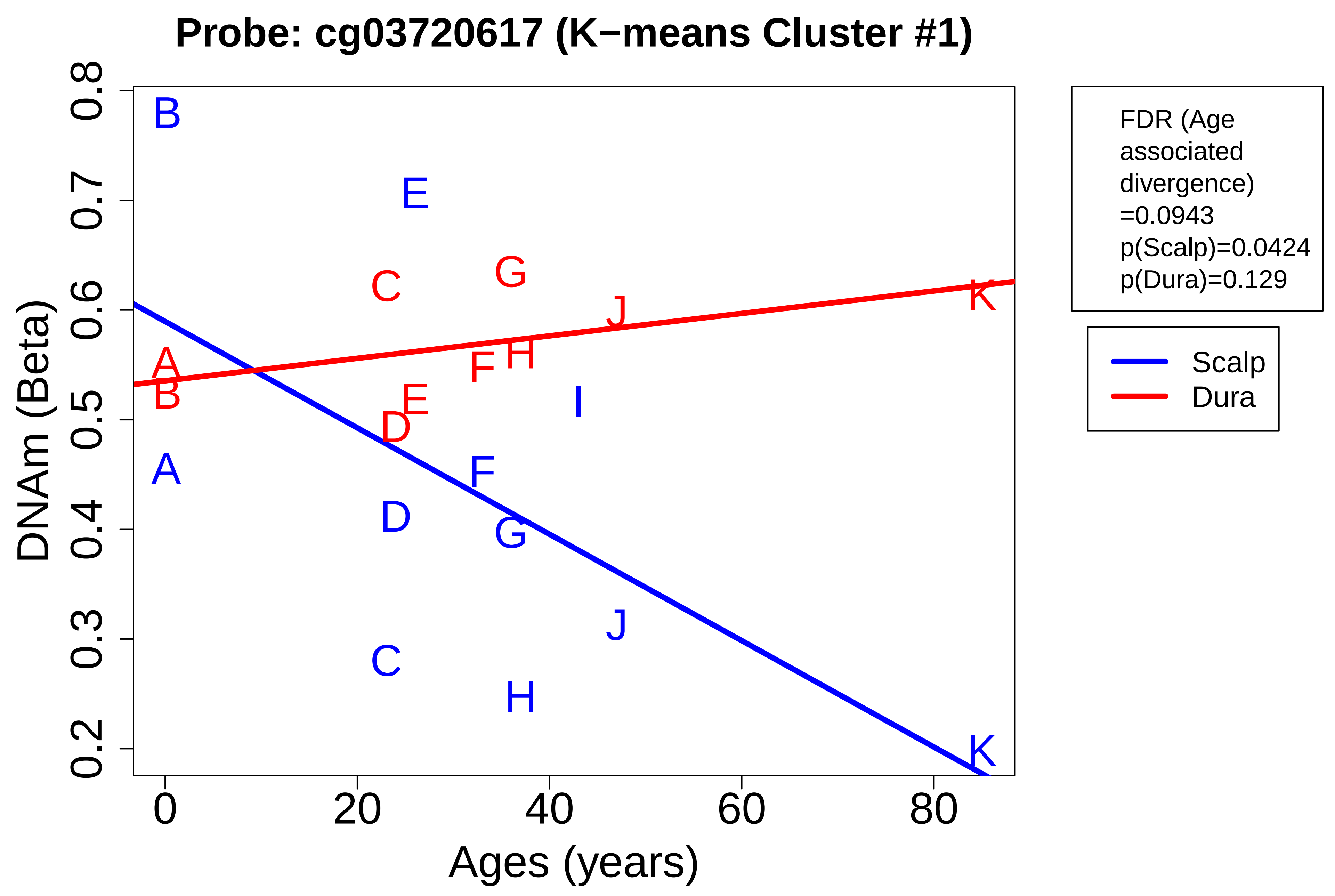

Probe: cg03720617 (K−means Cluster #1)
DNAm (Beta)
0.2
0.3
0.4
0.5
0.6
0.7
0.8
Ages (years)
0
20
40
60
80
FDR (Age associated divergence) =0.0943
p(Scalp)=0.0424
p(Dura)=0.129
Scalp
Dura

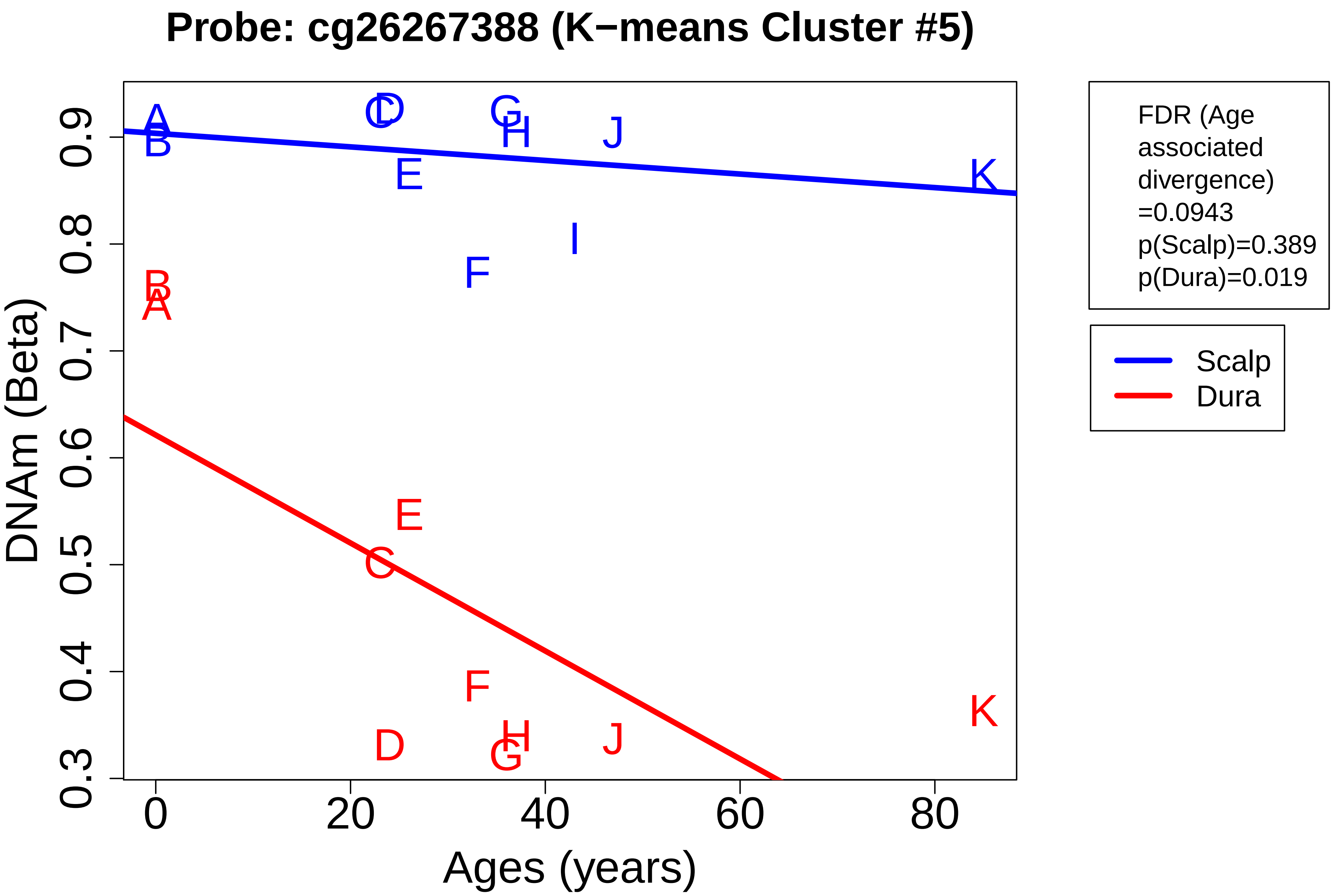

Probe: cg26267388 (K−means Cluster #5)
DNAm (Beta)
0.3
0.4
0.5
0.6
0.7
0.8
0.9
Ages (years)
0
20
40
60
80
A
B
C
D
E
F
G
H
I
J
K
FDR (Age associated divergence) =0.0943
p(Scalp)=0.389
p(Dura)=0.019
Scalp
Dura

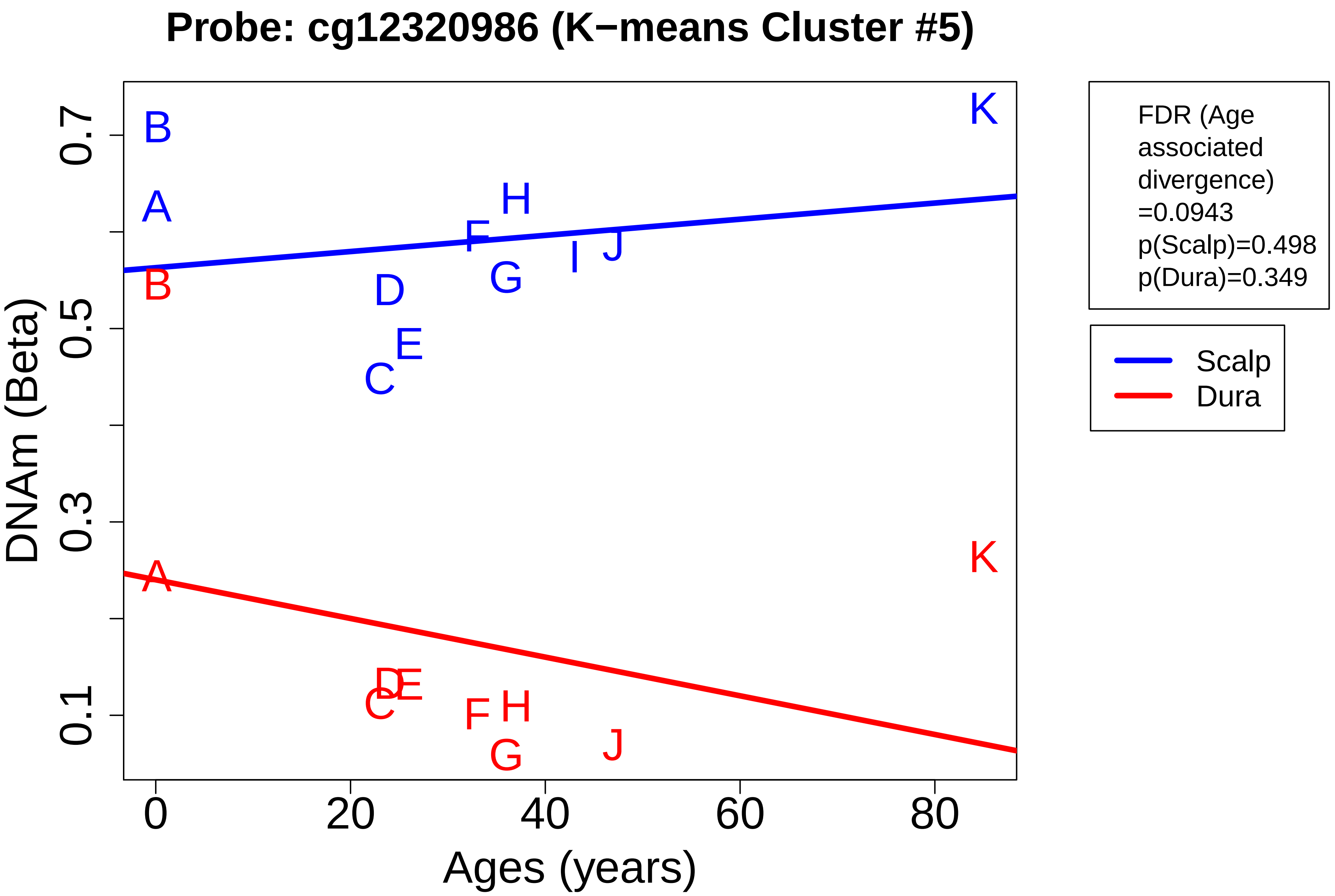
Probe: cg12320986 (K−means Cluster #5)
DNAm (Beta)
0.1
0.3
0.5
0.7
Ages (years)
0
20
40
60
80
B
A
B
D
E
C
F
H
G
I
J
K
A
K
D
E
C
F
H
G
J
FDR (Age associated divergence) =0.0943
p(Scalp)=0.498
p(Dura)=0.349
Scalp
Dura

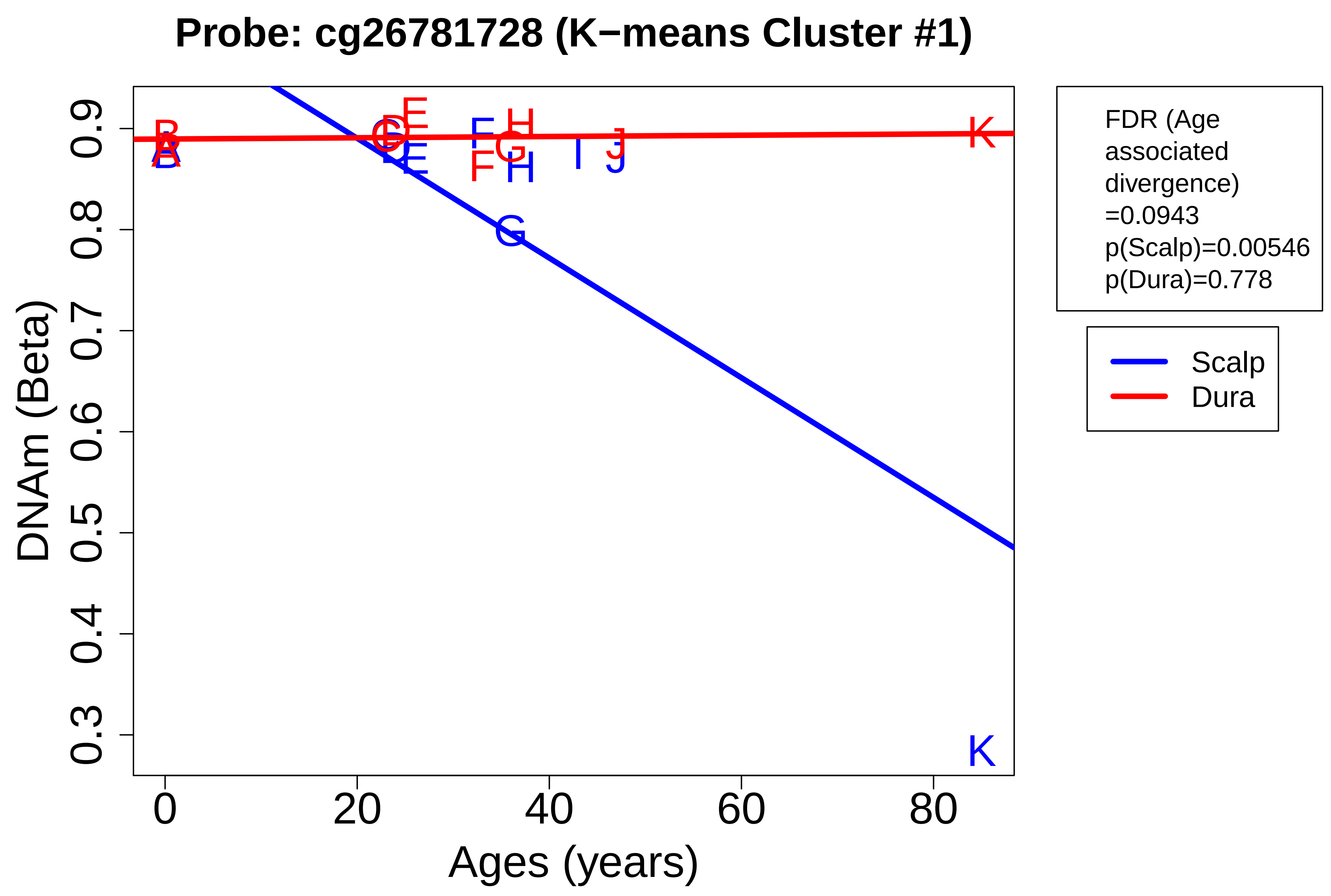

Probe: cg26781728 (K−means Cluster #1)
DNAm (Beta)
Ages (years)
0.3
0.4
0.5
0.6
0.7
0.8
0.9
0
20
40
60
80
FDR (Age associated divergence) =0.0943
p(Scalp)=0.00546
p(Dura)=0.778
Scalp
Dura

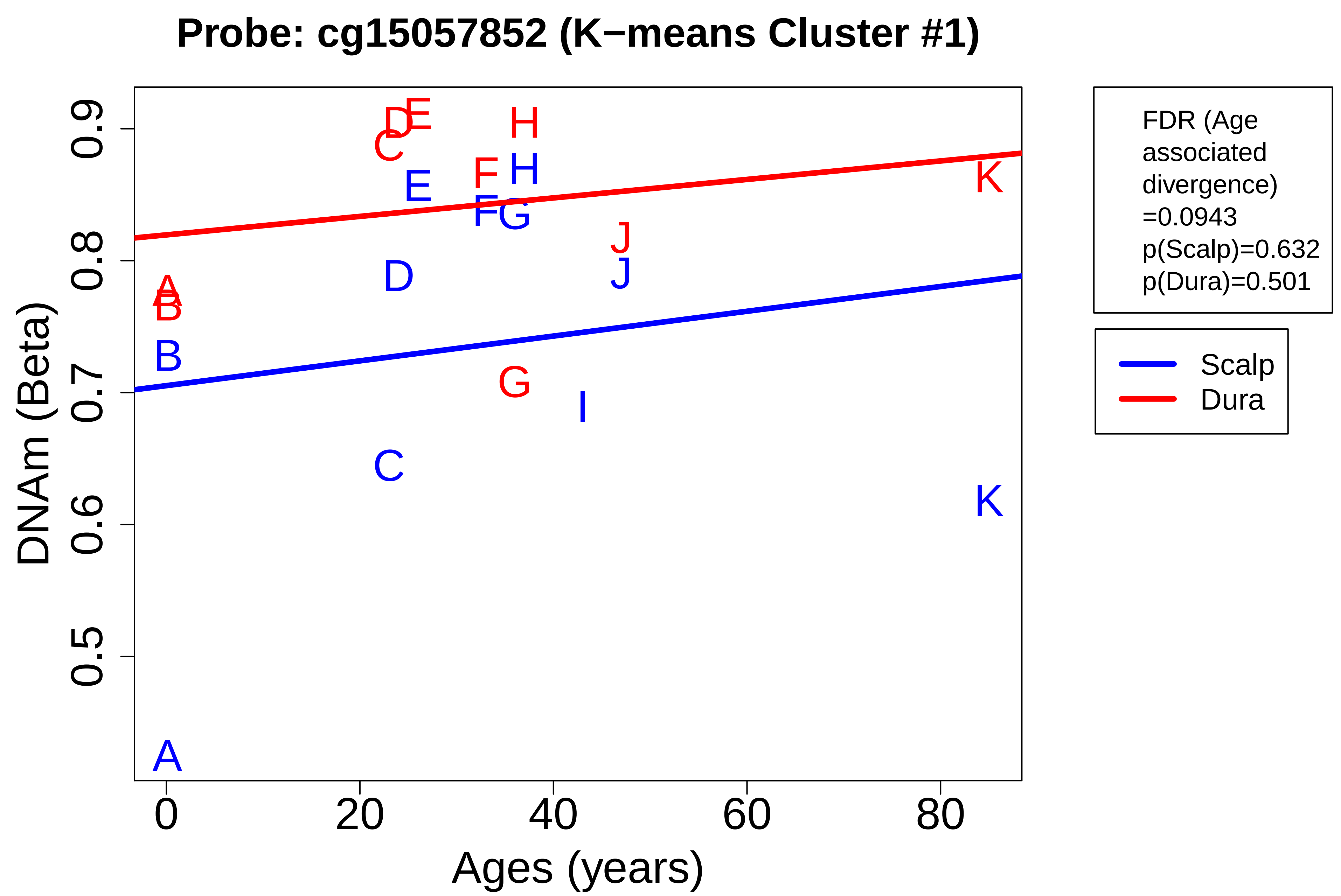

Probe: cg15057852 (K−means Cluster #1)
FDR (Age associated divergence) =0.0943
p(Scalp)=0.632
p(Dura)=0.501
Scalp
Dura
DNAm (Beta)
0.5
0.6
0.7
0.8
0.9
0
20
40
60
80
Ages (years)
A
B
C
D
E
F
G
H
I
J
K

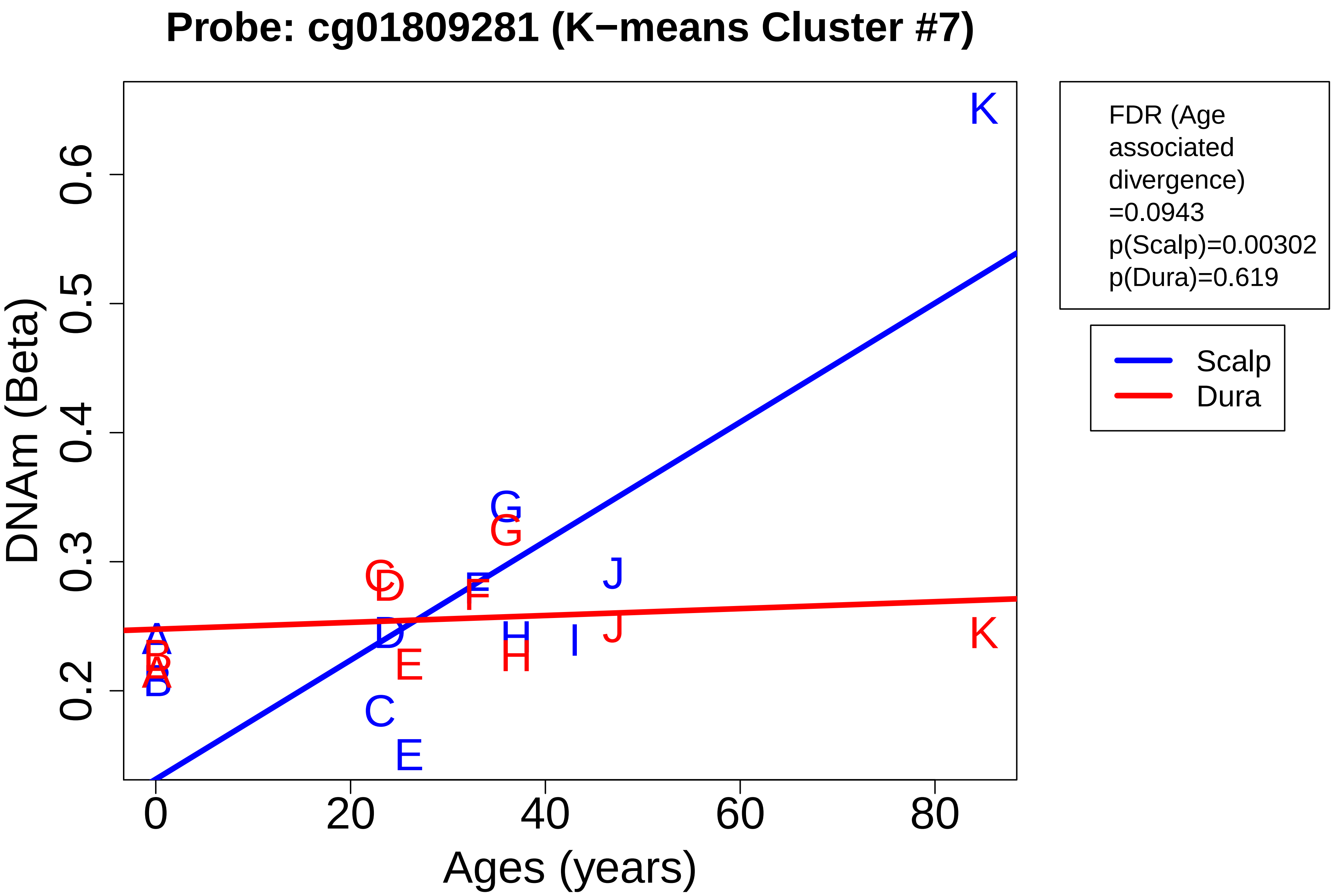

Probe: cg01809281 (K−means Cluster #7)
DNAm (Beta)
0.2
0.3
0.4
0.5
0.6
Ages (years)
0
20
40
60
80
FDR (Age associated divergence) =0.0943
p(Scalp)=0.00302
p(Dura)=0.619
Scalp
Dura

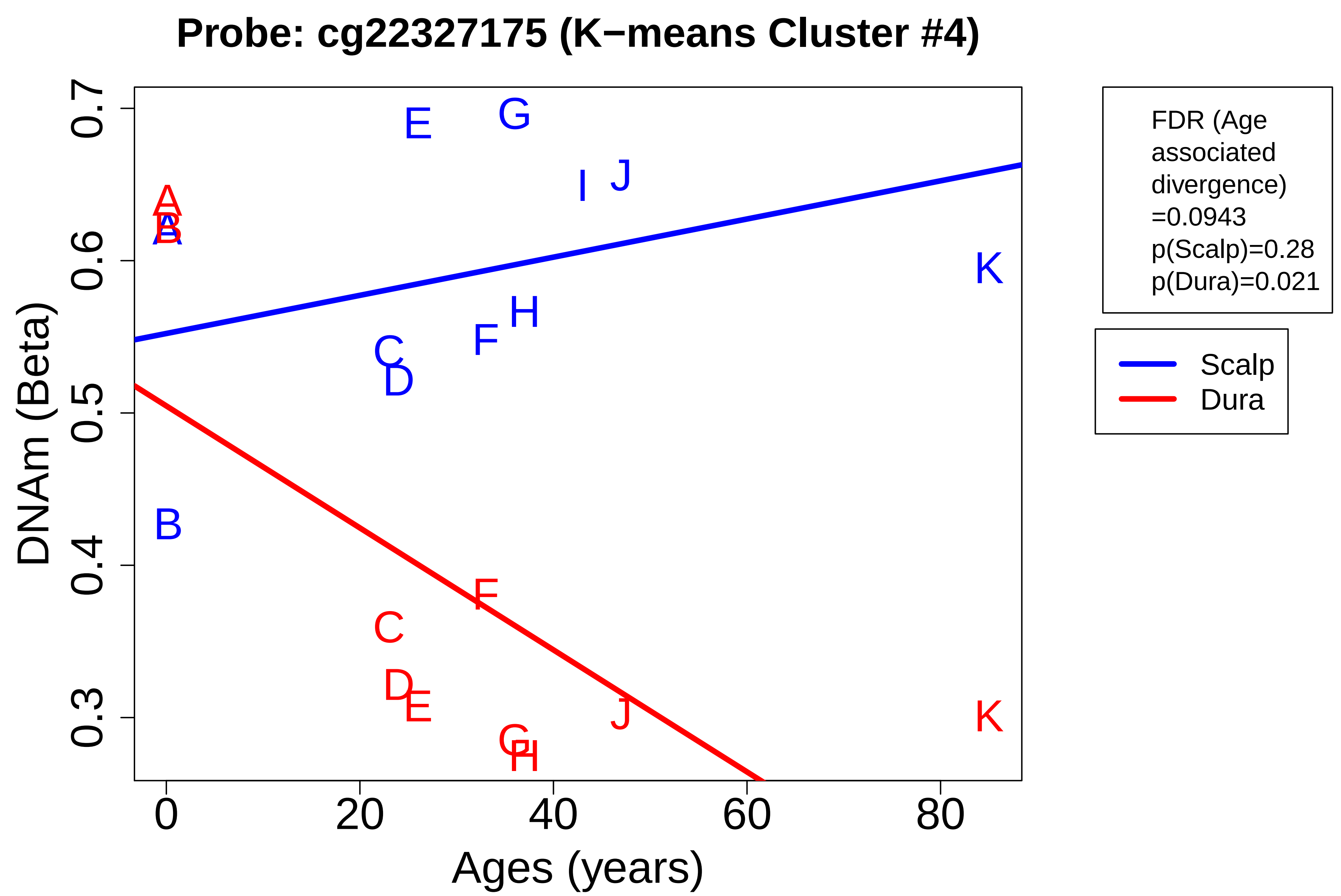

Probe: cg22327175 (K−means Cluster #4)
DNAm (Beta)
0.3
0.4
0.5
0.6
0.7
0
20
40
60
80
Ages (years)
A
B
C
D
E
F
G
H
I
J
K
FDR (Age associated divergence) =0.0943
p(Scalp)=0.28
p(Dura)=0.021
Scalp
Dura

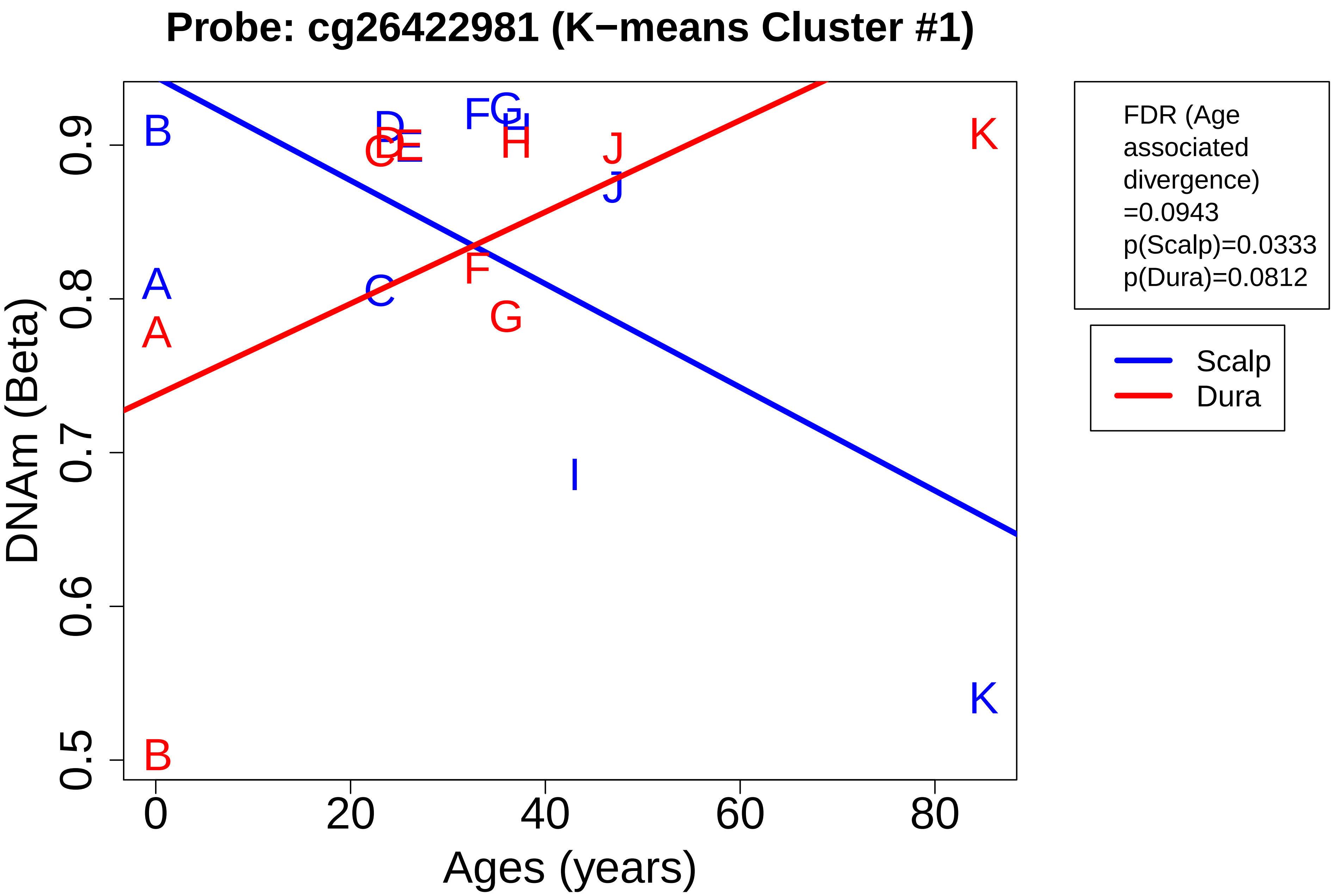

Probe: cg26422981 (K−means Cluster #1)
DNAm (Beta)
0.5
0.6
0.7
0.8
0.9
0
20
40
60
80
Ages (years)
FDR (Age associated divergence) =0.0943
p(Scalp)=0.0333
p(Dura)=0.0812
Scalp
Dura

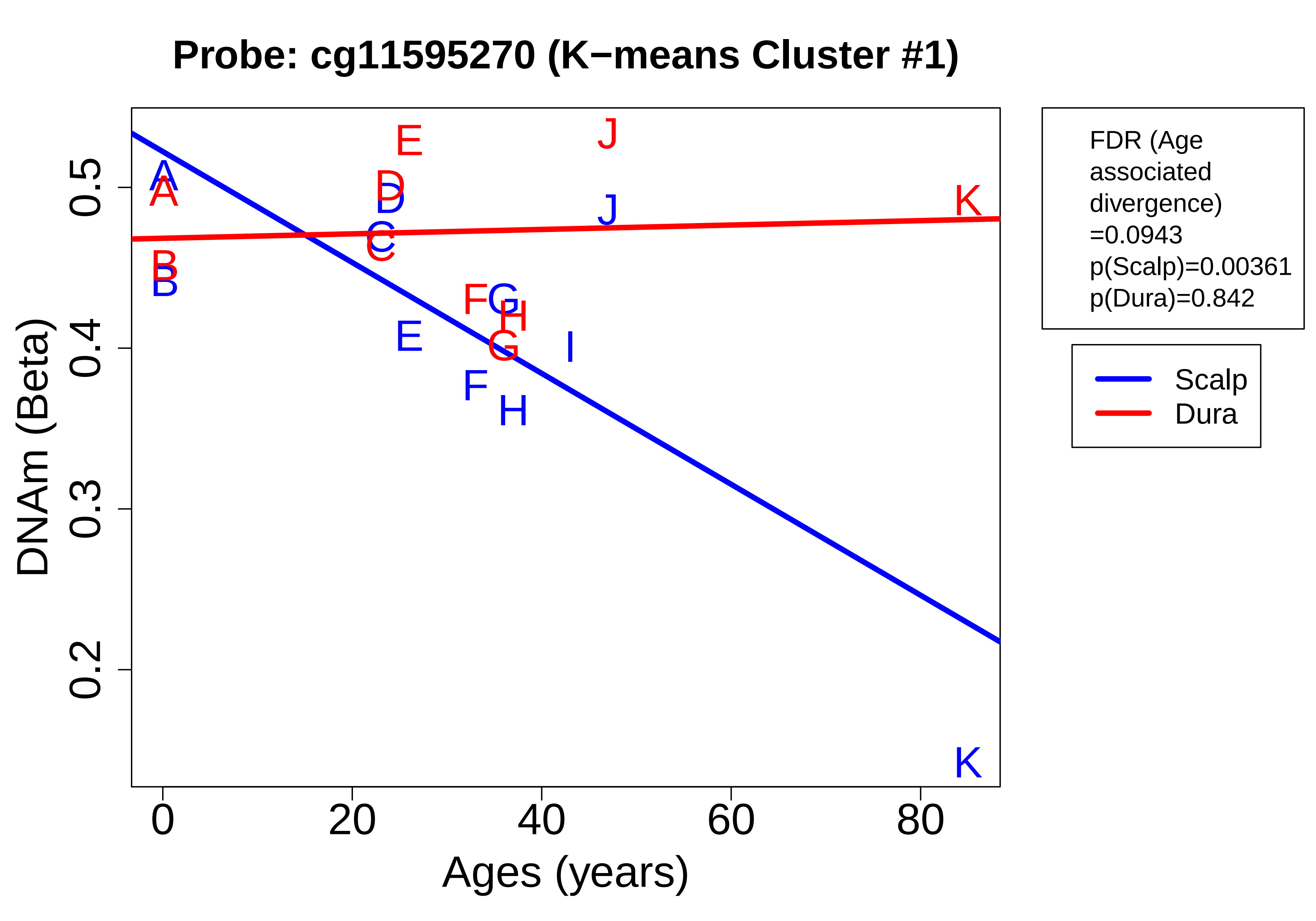

Probe: cg11595270 (K−means Cluster #1)
DNAm (Beta)
0.2
0.3
0.4
0.5
Ages (years)
0
20
40
60
80
A
B
C
D
E
F
G
H
I
J
K
FDR (Age associated divergence) =0.0943
p(Scalp)=0.00361
p(Dura)=0.842
Scalp
Dura

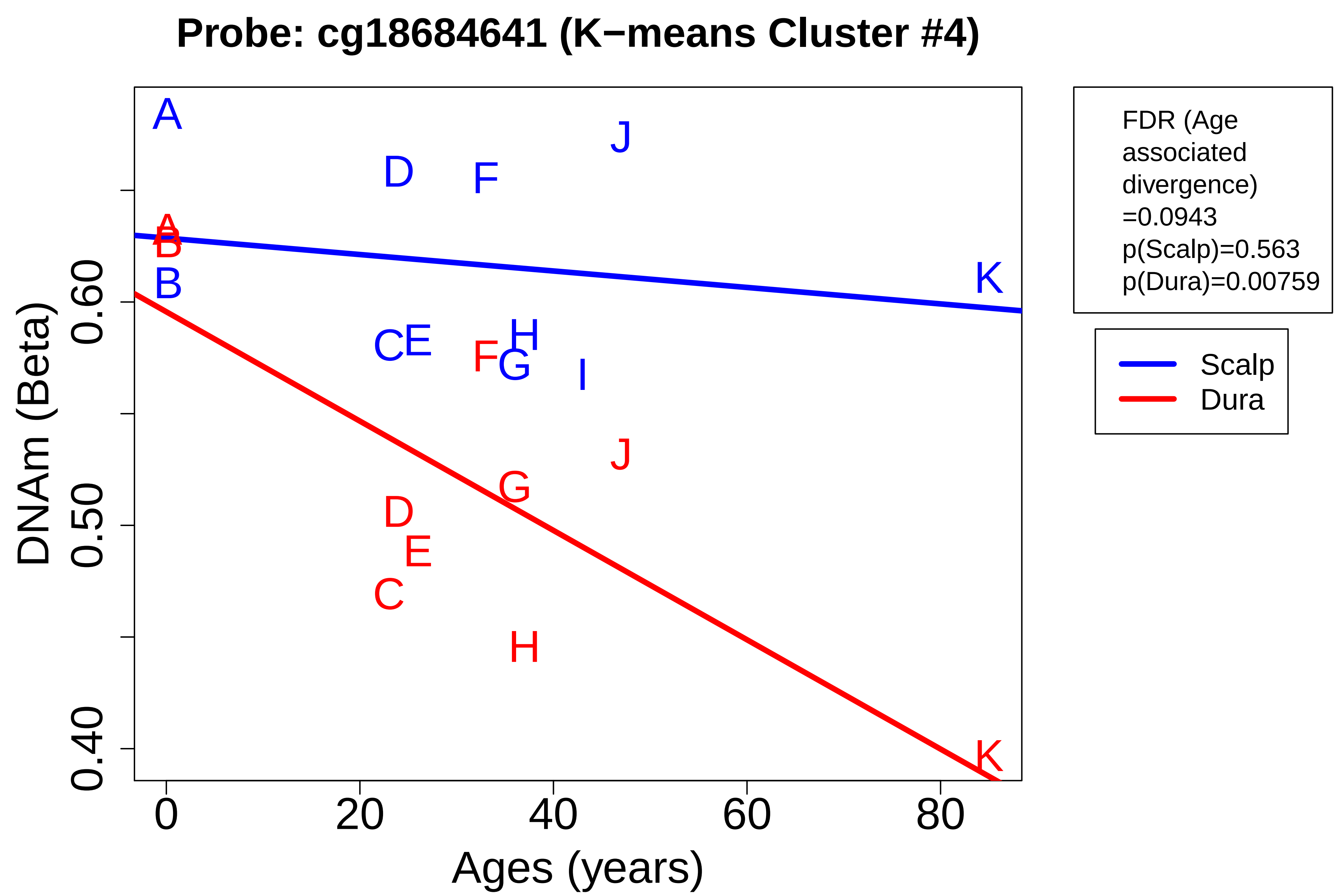

Probe: cg18684641 (K−means Cluster #4)
DNAm (Beta)
0.40
0.50
0.60
0
20
40
60
80
Ages (years)
FDR (Age associated divergence) =0.0943
p(Scalp)=0.563
p(Dura)=0.00759
Scalp
Dura

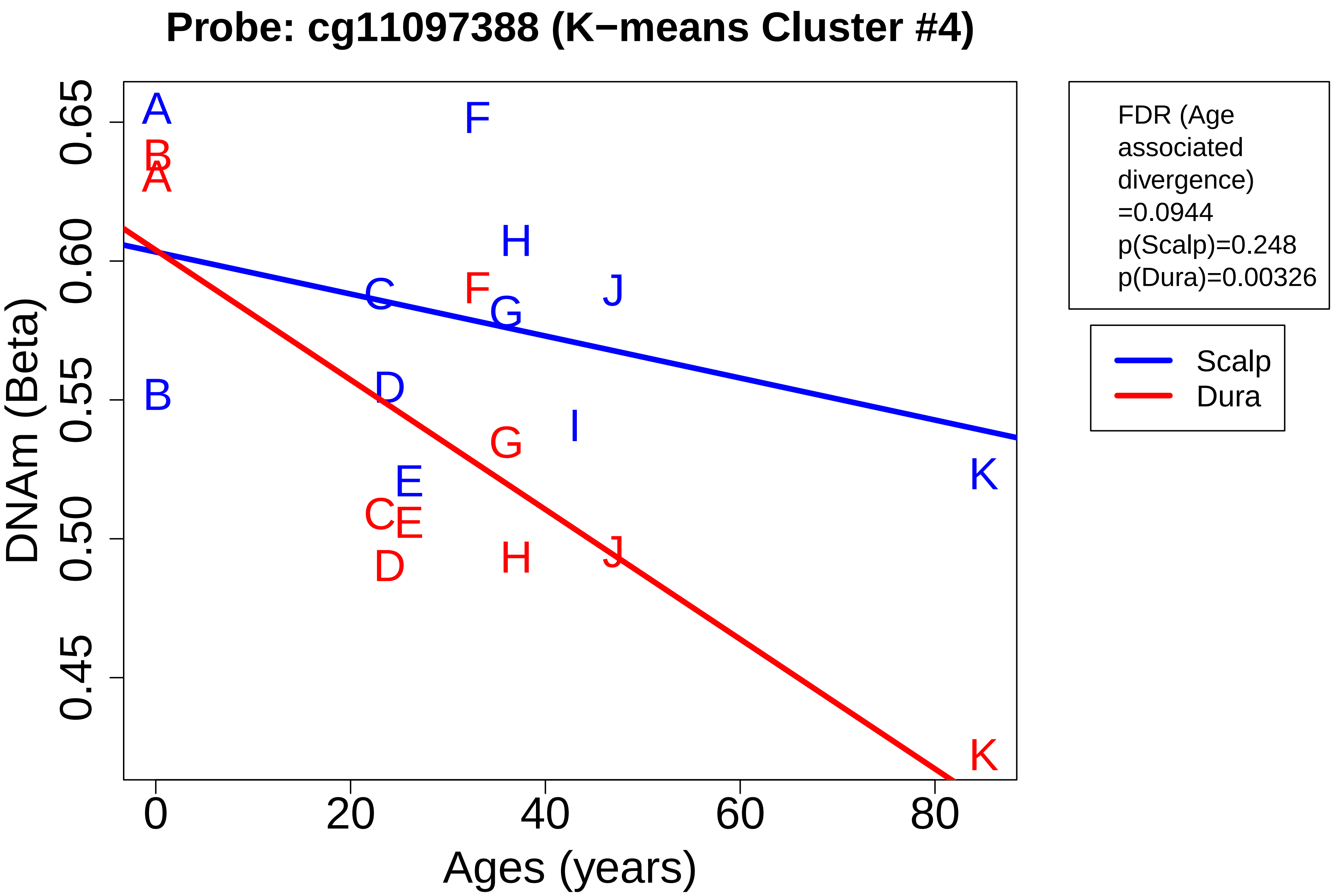

Probe: cg11097388 (K−means Cluster #4)
DNAm (Beta)
0.45
0.50
0.55
0.60
0.65
Ages (years)
0
20
40
60
80
A
B
C
D
E
F
G
H
I
J
K
FDR (Age associated divergence) =0.0944
p(Scalp)=0.248
p(Dura)=0.00326
Scalp
Dura

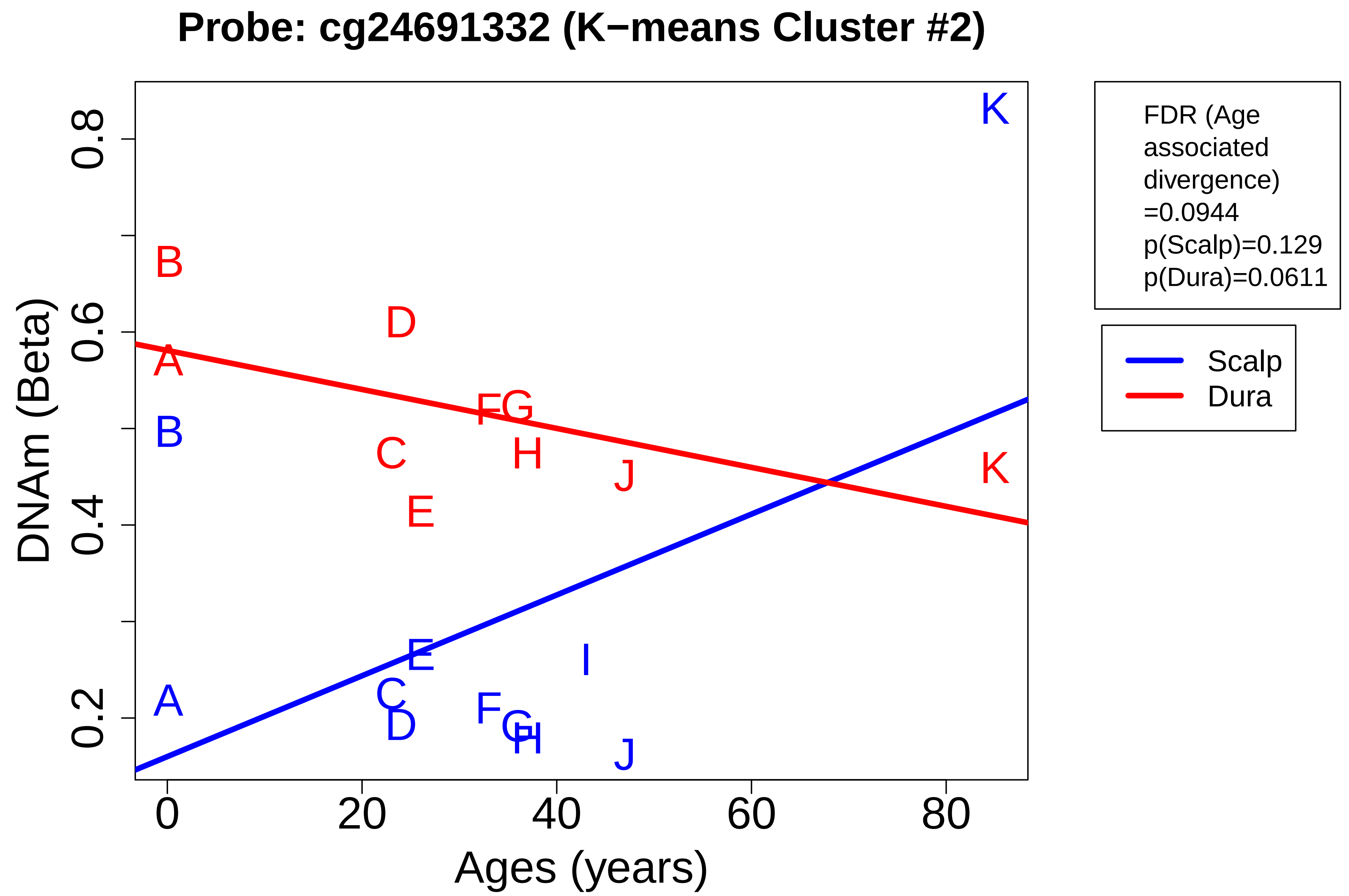
Probe: cg24691332 (K−means Cluster #2)
DNAm (Beta)
0.2
0.4
0.6
0.8
0
20
40
60
80
Ages (years)
FDR (Age associated divergence) =0.0944
p(Scalp)=0.129
p(Dura)=0.0611
Scalp
Dura
A
B
C
D
E
F
G
H
I
J
K

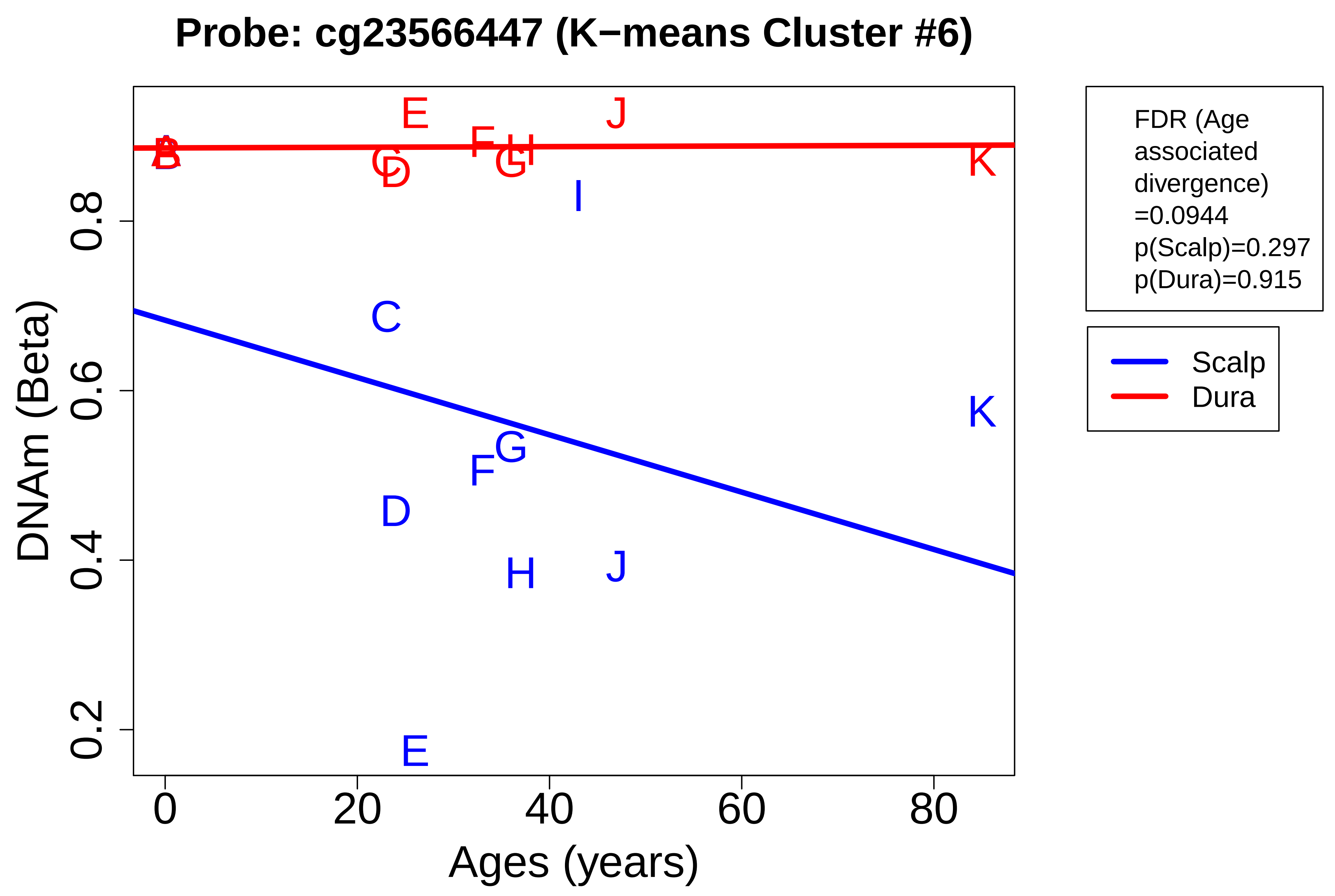

Probe: cg23566447 (K−means Cluster #6)
DNAm (Beta)
0.2
0.4
0.6
0.8
Ages (years)
0
20
40
60
80
FDR (Age associated divergence) =0.0944
p(Scalp)=0.297
p(Dura)=0.915
Scalp
Dura

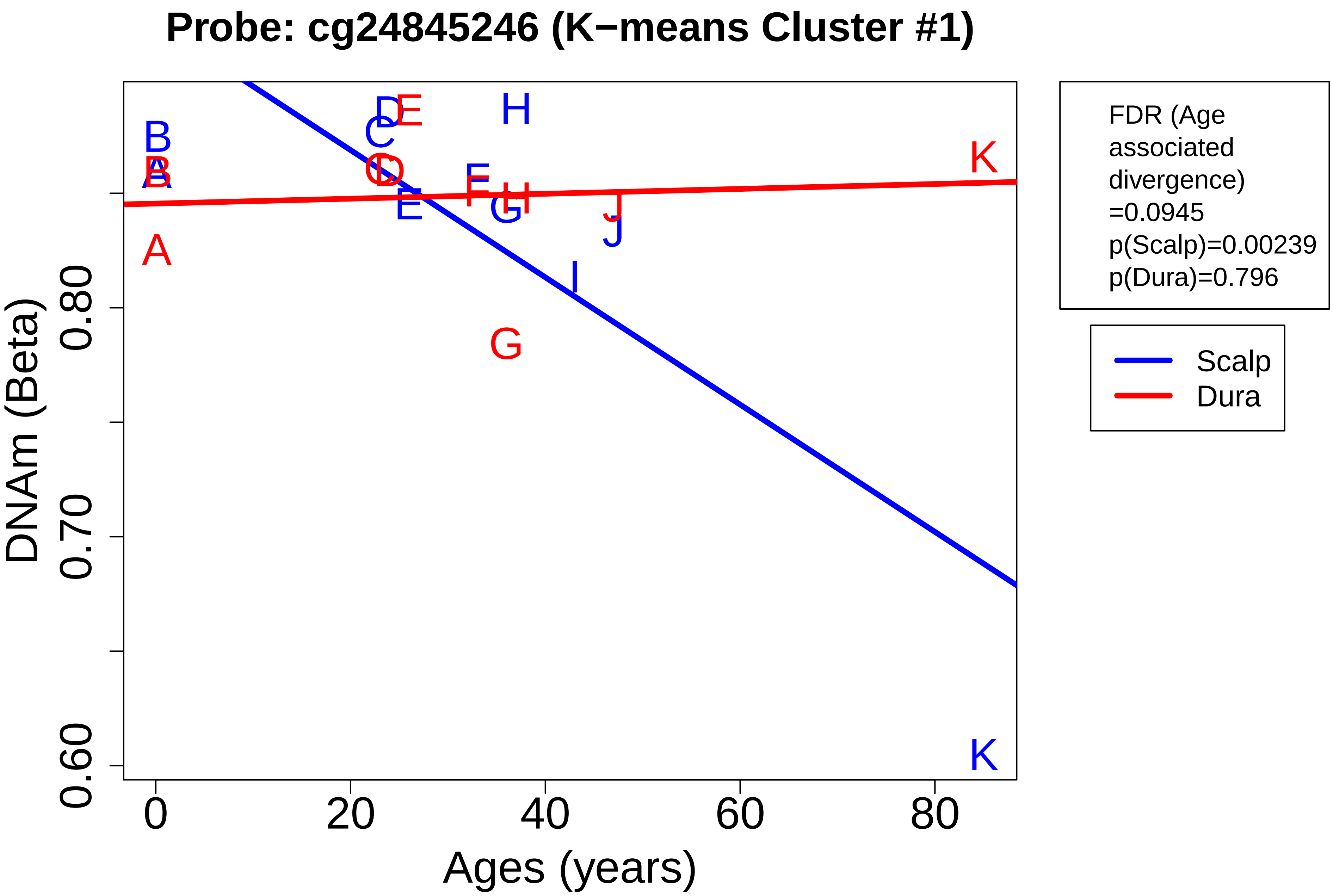

Probe: cg24845246 (K−means Cluster #1)
DNAm (Beta)
0.60
0.70
0.80
0
20
40
60
80
Ages (years)
FDR (Age associated divergence) =0.0945
p(Scalp)=0.00239
p(Dura)=0.796
Scalp
Dura

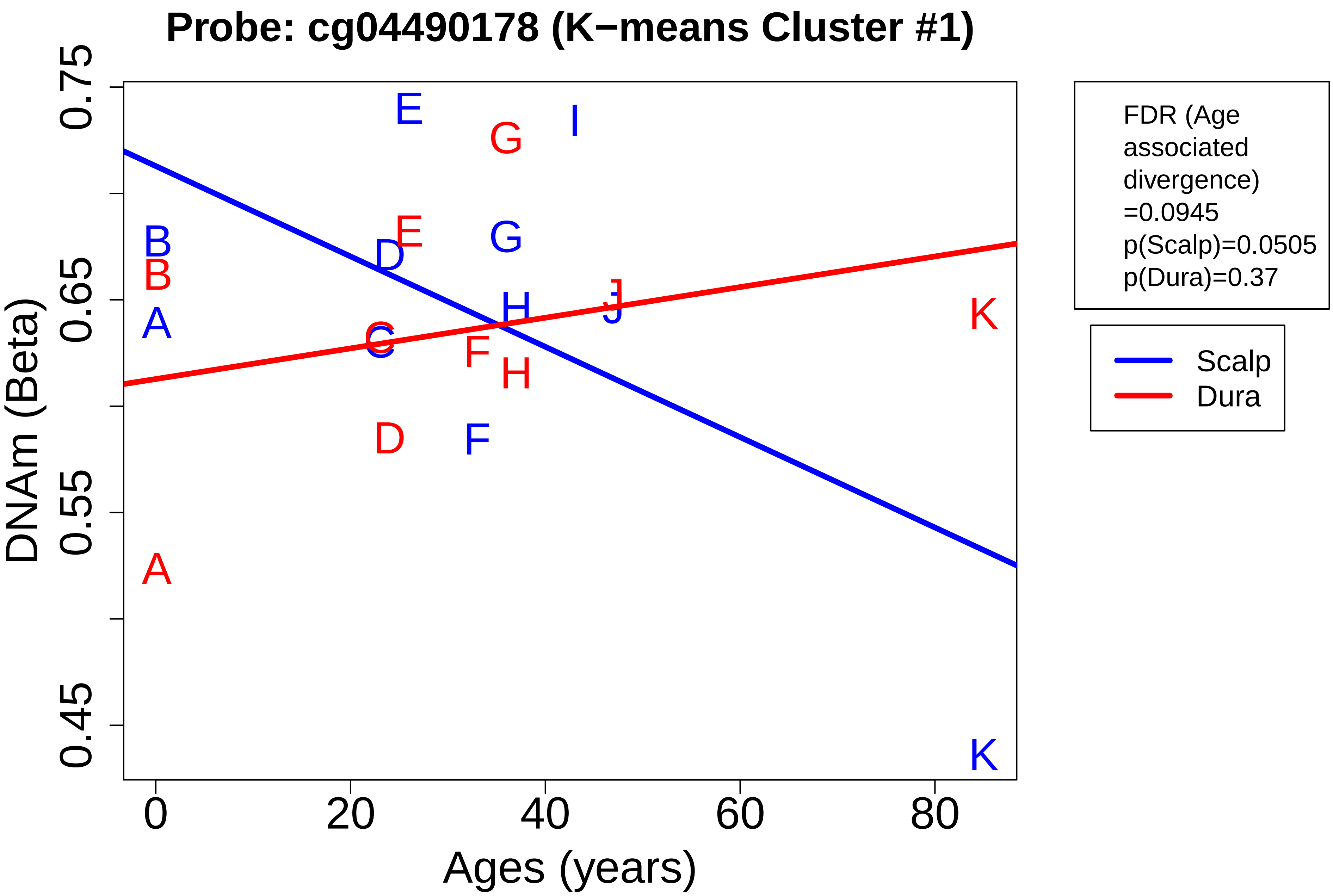

Probe: cg04490178 (K−means Cluster #1)
DNAm (Beta)
0.45
0.55
0.65
0.75
Ages (years)
0
20
40
60
80
FDR (Age associated divergence) =0.0945
p(Scalp)=0.0505
p(Dura)=0.37
Scalp
Dura
A
B
C
D
E
F
G
H
I
J
K

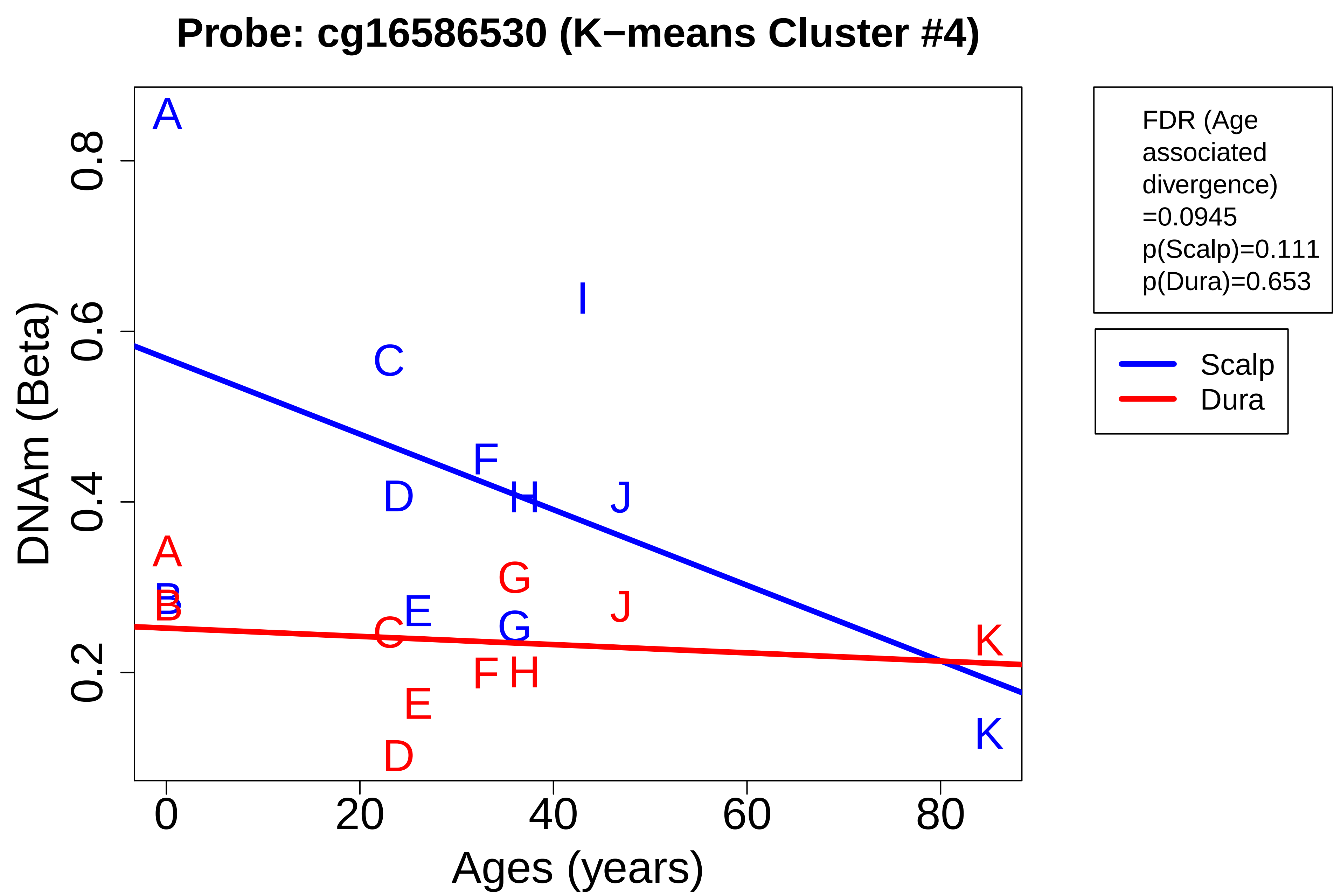
Probe: cg16586530 (K−means Cluster #4)
DNAm (Beta)
0.2
0.4
0.6
0.8
0
20
40
60
80
Ages (years)
A
B
C
D
E
F
G
H
I
J
K
FDR (Age associated divergence) =0.0945
p(Scalp)=0.111
p(Dura)=0.653
Scalp
Dura

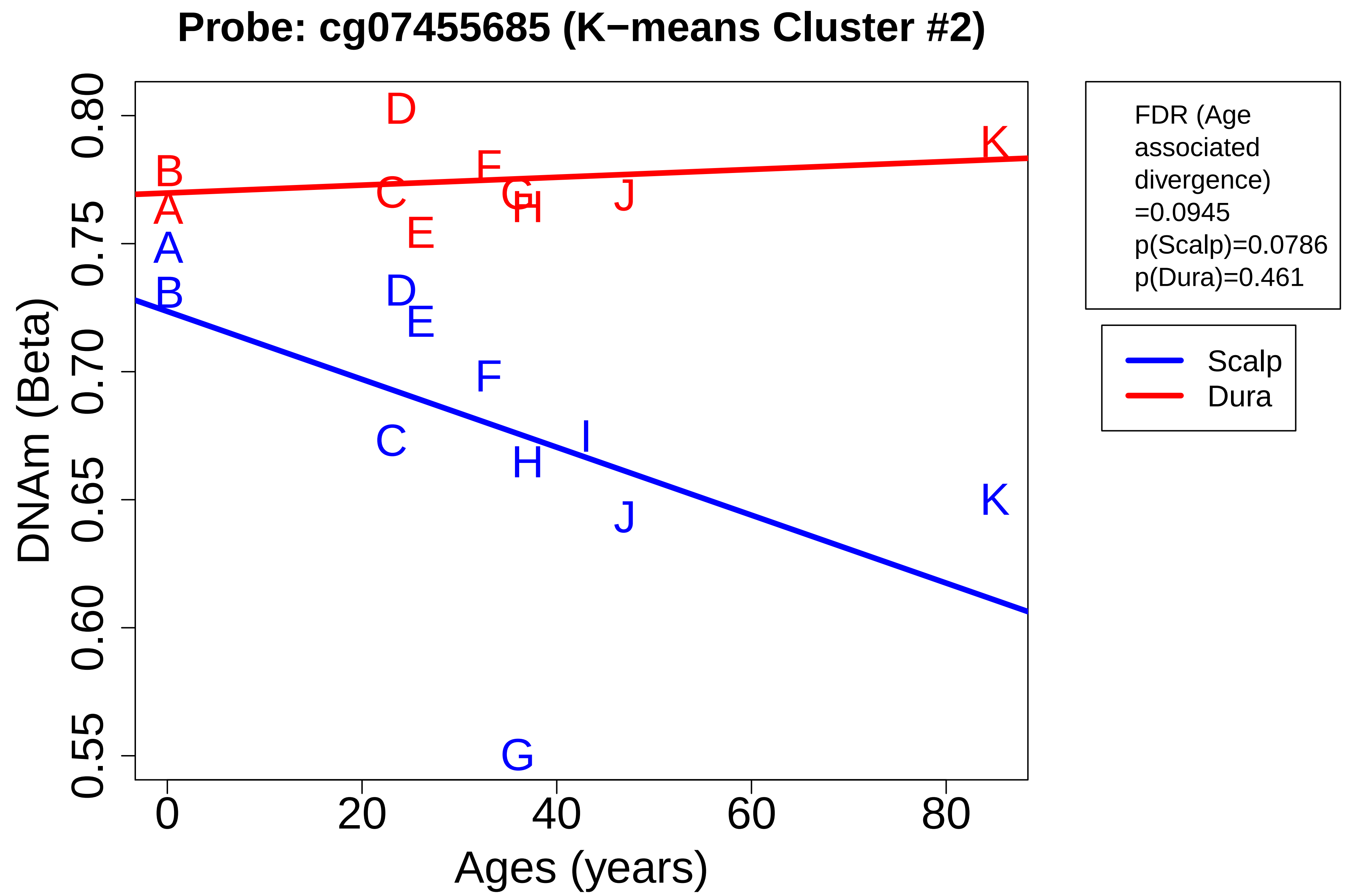

Probe: cg07455685 (K−means Cluster #2)
DNAm (Beta)
0.55
0.60
0.65
0.70
0.75
0.80
0
20
40
60
80
Ages (years)
FDR (Age associated divergence) =0.0945
p(Scalp)=0.0786
p(Dura)=0.461
Scalp
Dura

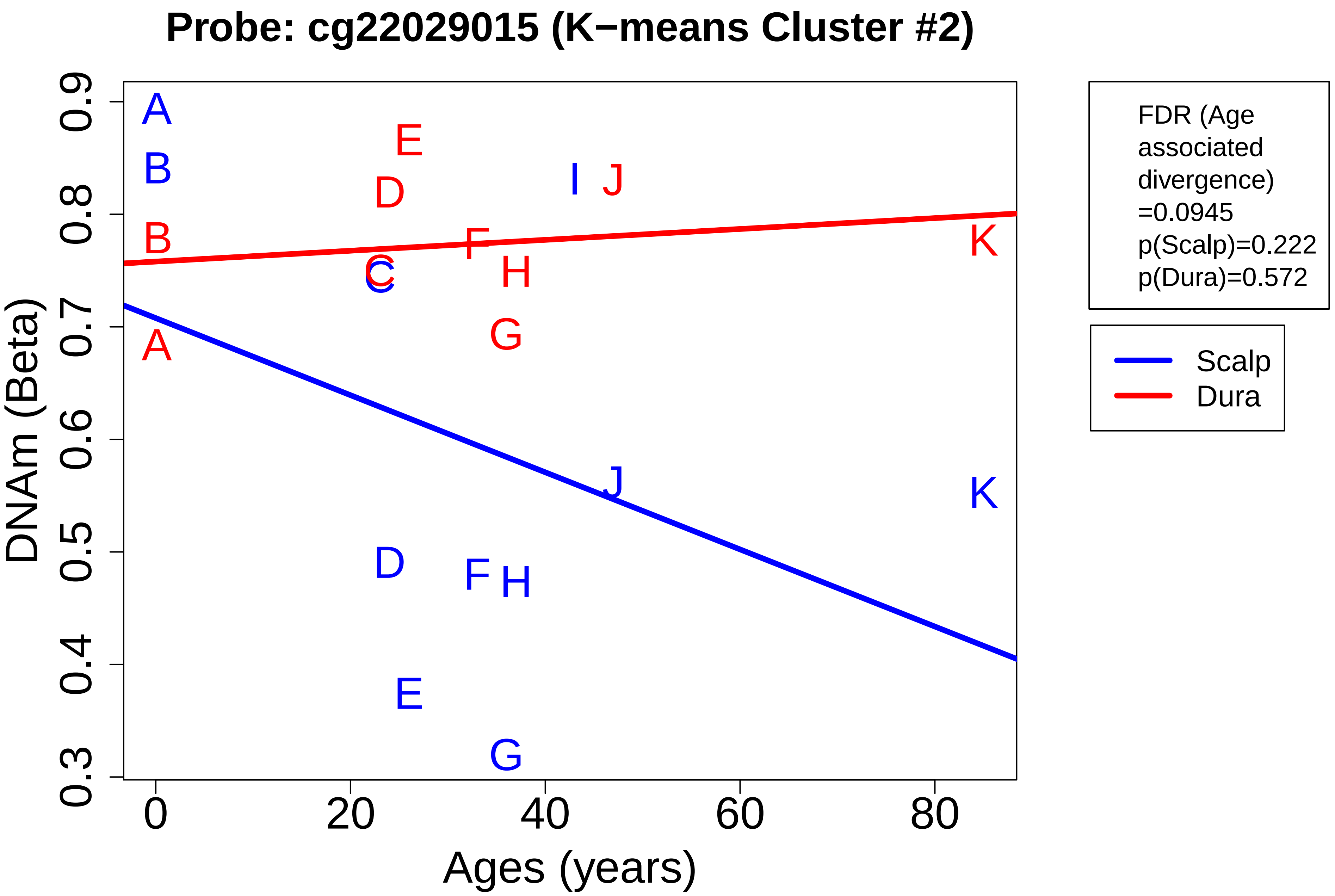

Probe: cg22029015 (K−means Cluster #2)
DNAm (Beta)
Ages (years)
0.3
0.4
0.5
0.6
0.7
0.8
0.9
0
20
40
60
80
A
B
B
C
C
A
D
E
E
D
F
F
H
H
G
G
I
J
J
K
K
FDR (Age associated divergence) =0.0945
p(Scalp)=0.222
p(Dura)=0.572
Scalp
Dura

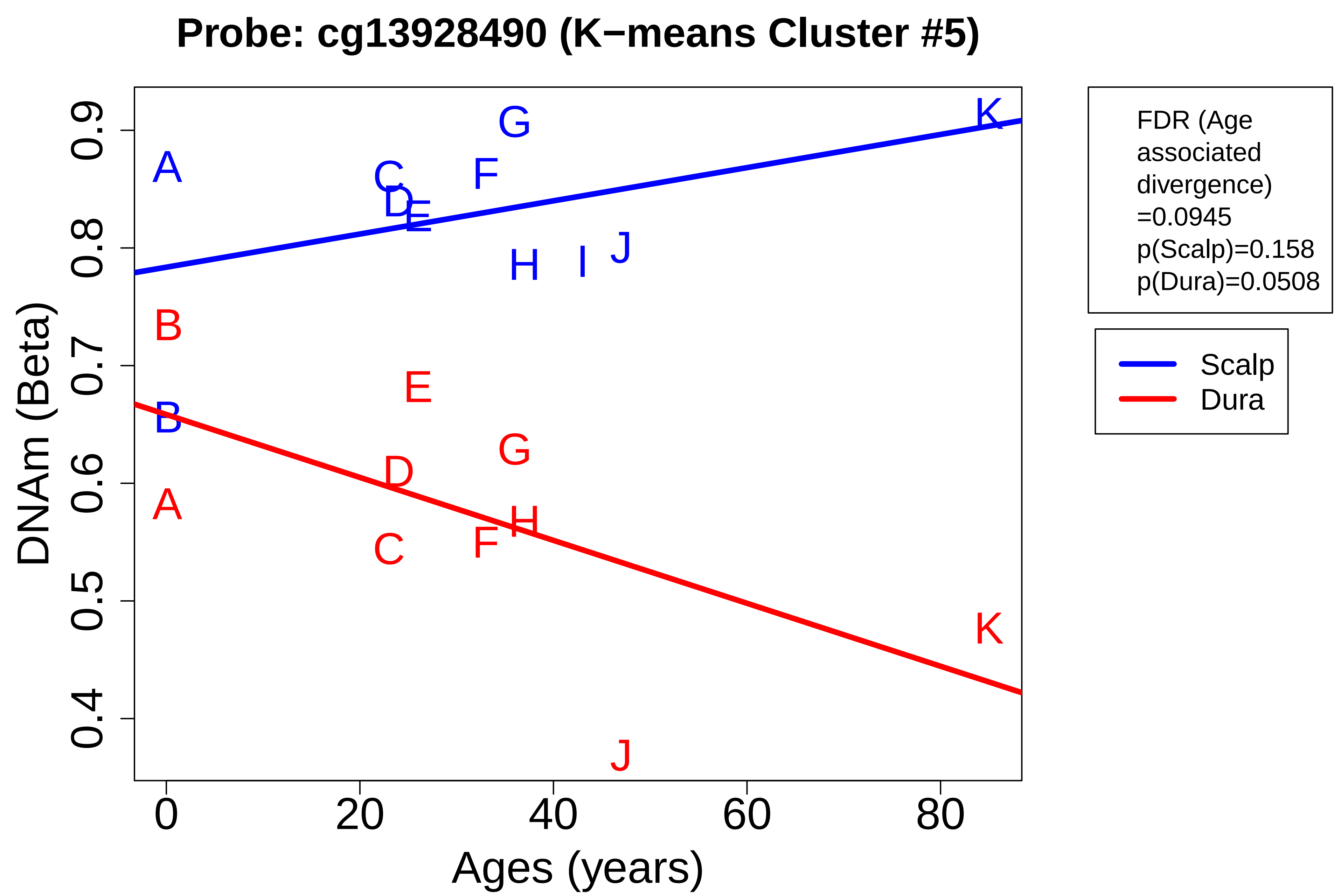

Probe: cg13928490 (K−means Cluster #5)
DNAm (Beta)
0.4
0.5
0.6
0.7
0.8
0.9
0
20
40
60
80
Ages (years)
A
B
C
D
E
F
G
H
I
J
K
A
B
C
D
E
F
G
H
J
K
FDR (Age associated divergence) =0.0945
p(Scalp)=0.158
p(Dura)=0.0508
Scalp
Dura

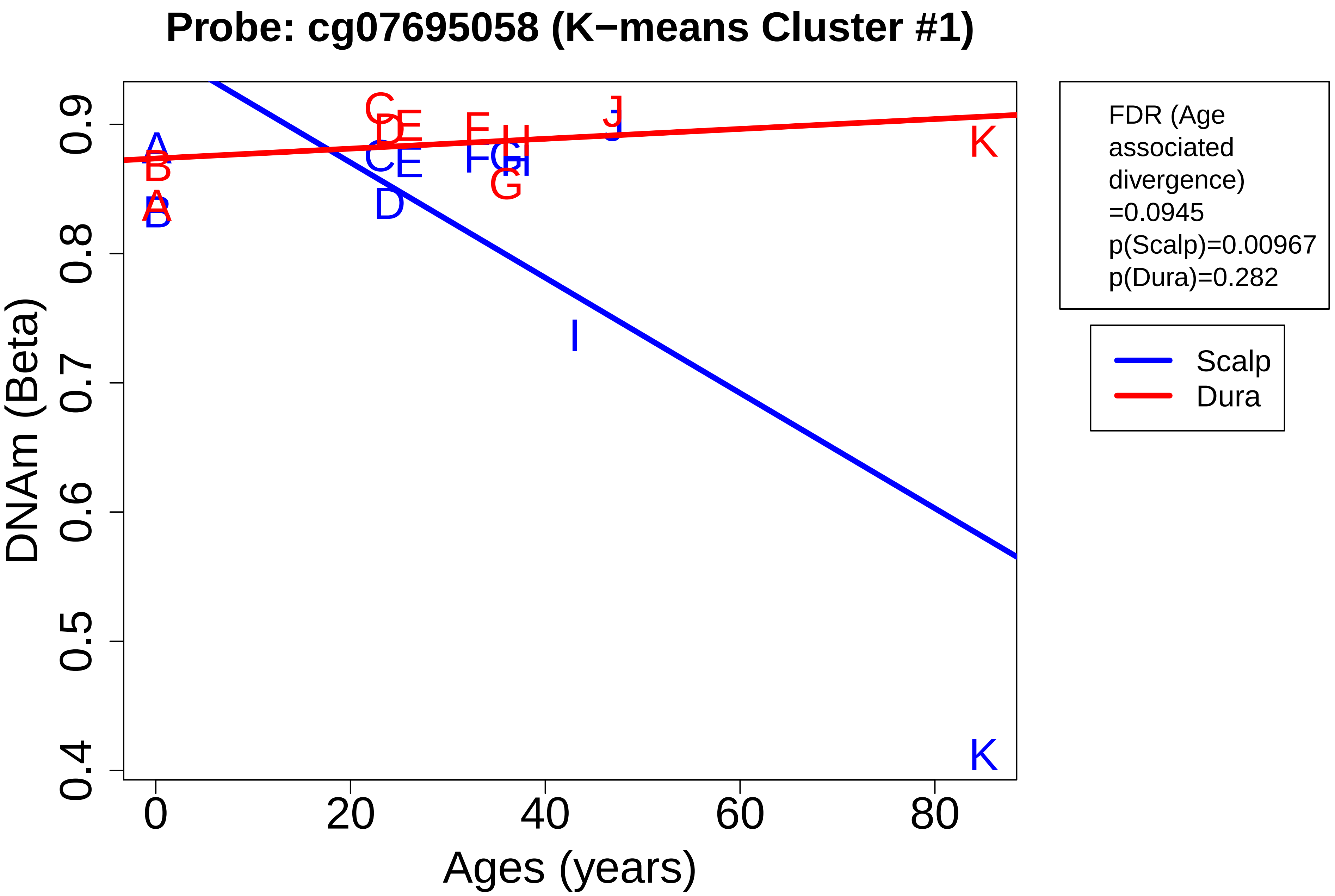

Probe: cg07695058 (K−means Cluster #1)
DNAm (Beta)
0.4
0.5
0.6
0.7
0.8
0.9
0
20
40
60
80
Ages (years)
A
B
C
D
E
F
G
H
I
J
K
FDR (Age associated divergence) =0.0945
p(Scalp)=0.00967
p(Dura)=0.282
Scalp
Dura

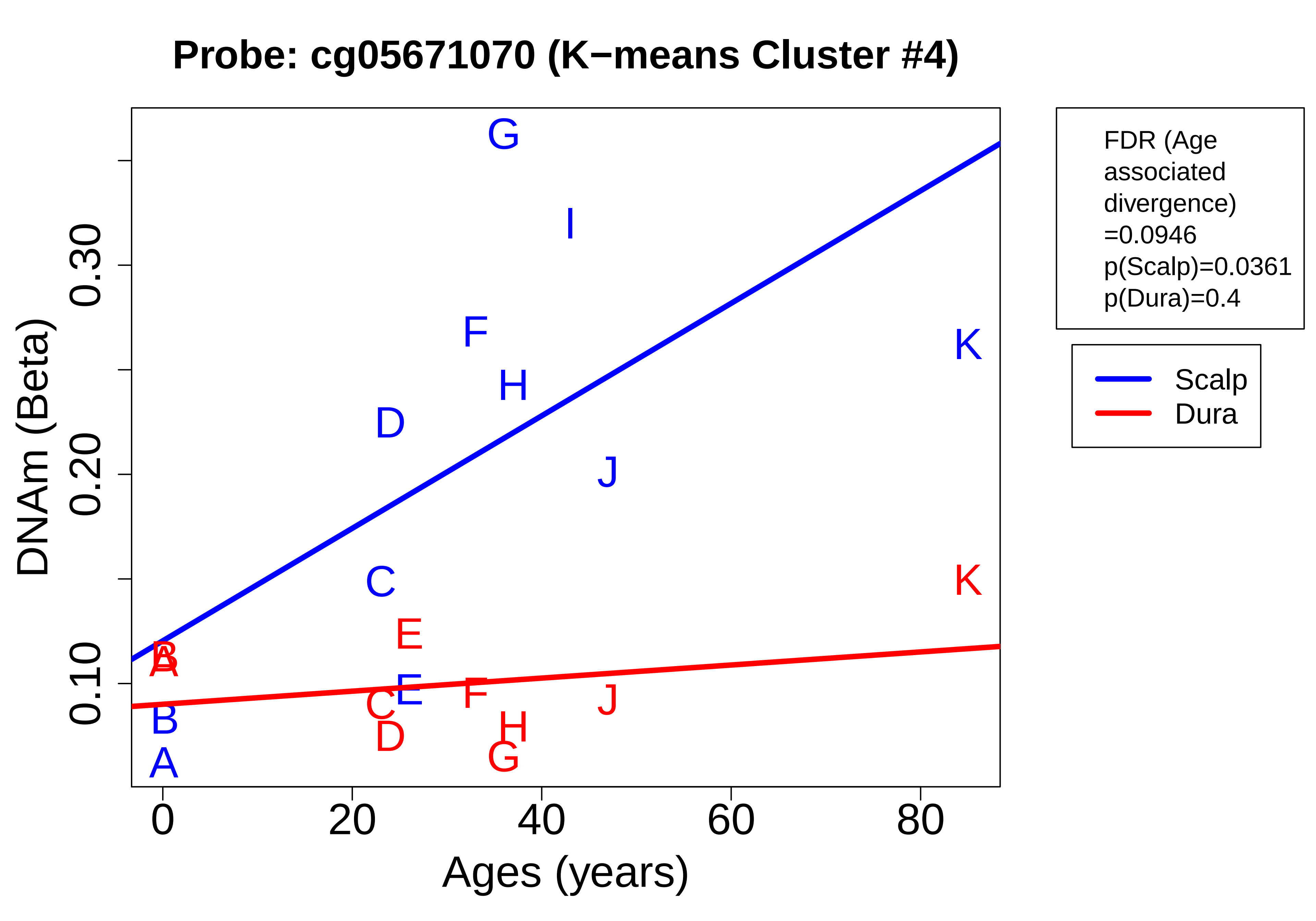

Probe: cg05671070 (K−means Cluster #4)
DNAm (Beta)
0.10
0.20
0.30
0
20
40
60
80
Ages (years)
FDR (Age associated divergence) =0.0946
p(Scalp)=0.0361
p(Dura)=0.4
Scalp
Dura
A
B
C
D
E
F
G
H
I
J
K

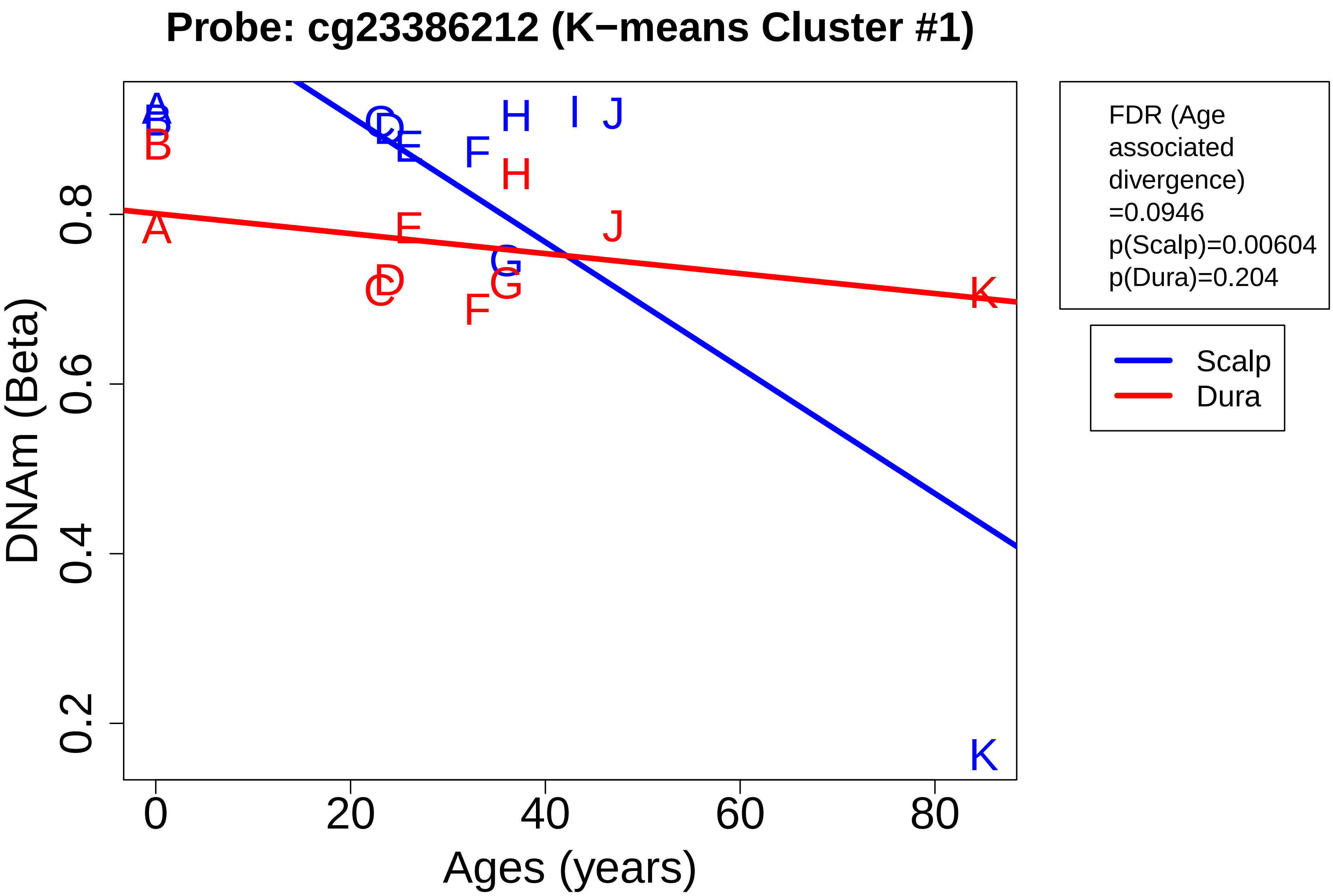

Probe: cg23386212 (K−means Cluster #1)
DNAm (Beta)
0.2
0.4
0.6
0.8
0
20
40
60
80
Ages (years)
FDR (Age associated divergence) =0.0946
p(Scalp)=0.00604
p(Dura)=0.204
Scalp
Dura

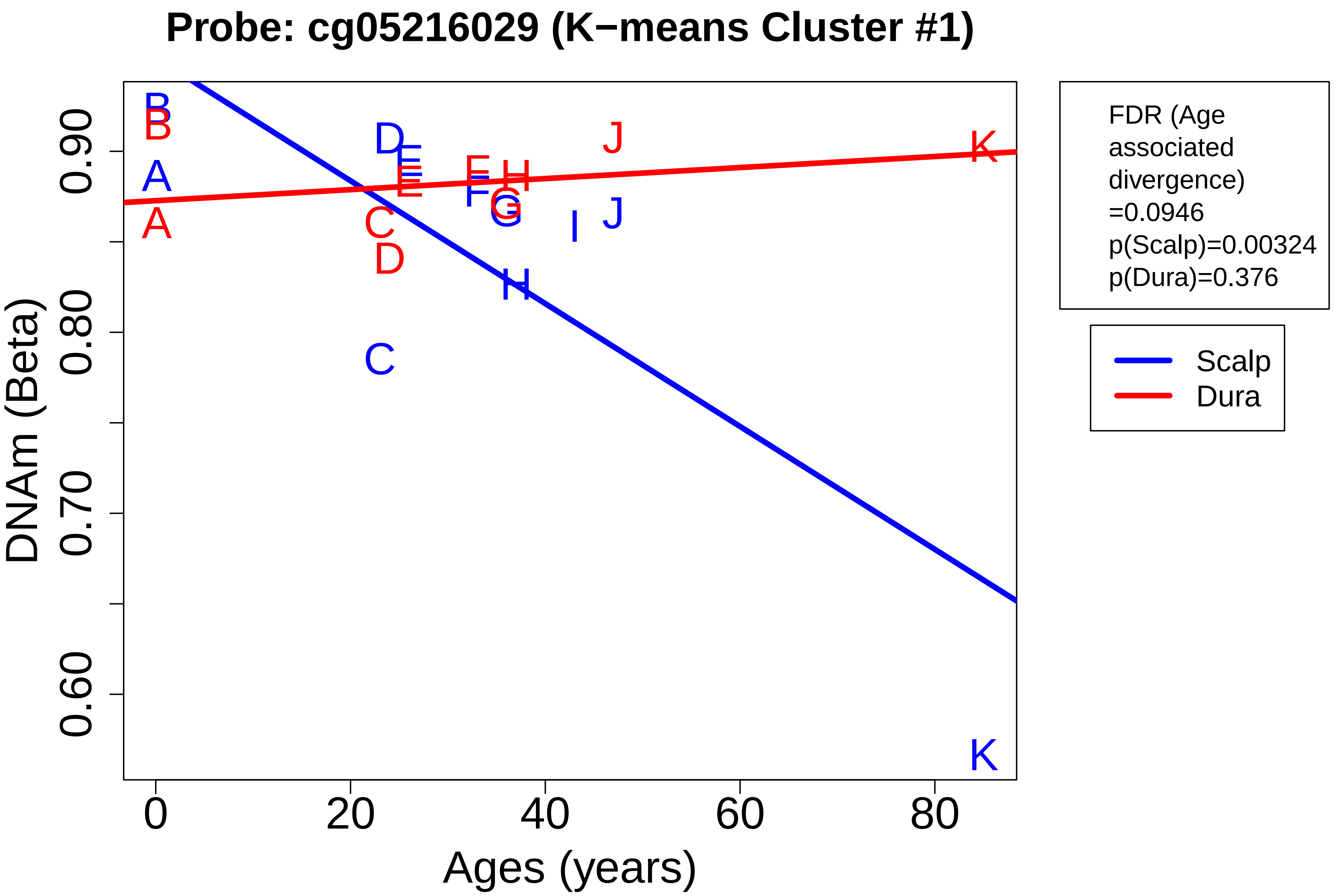
Probe: cg05216029 (K−means Cluster #1)
DNAm (Beta)
0.60
0.70
0.80
0.90
Ages (years)
0
20
40
60
80
A
B
C
D
E
F
G
H
I
J
K
FDR (Age associated divergence) =0.0946
p(Scalp)=0.00324
p(Dura)=0.376
Scalp
Dura

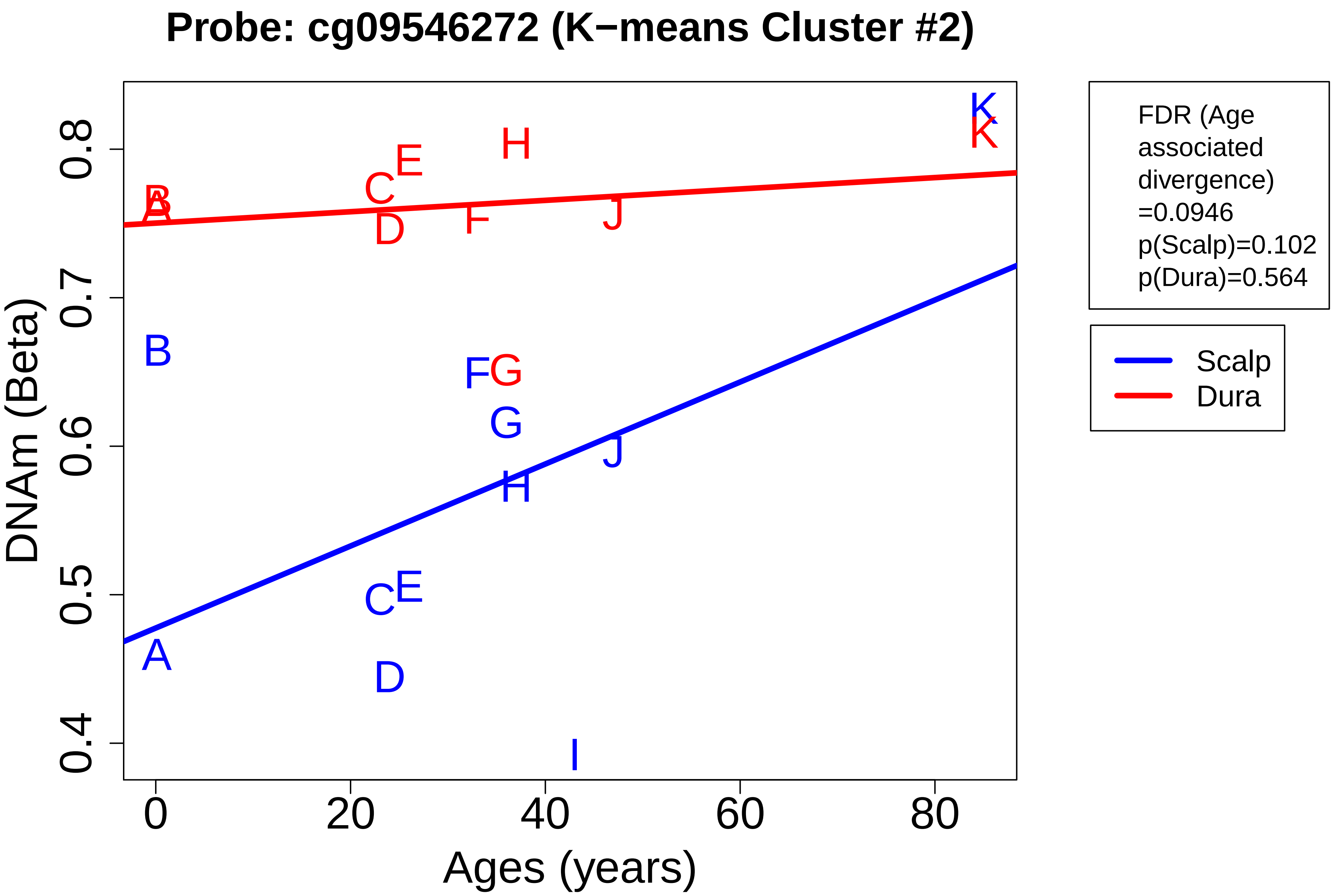

Probe: cg09546272 (K−means Cluster #2)
DNAm (Beta)
0.4
0.5
0.6
0.7
0.8
0
20
40
60
80
Ages (years)
FDR (Age associated divergence) =0.0946
p(Scalp)=0.102
p(Dura)=0.564
Scalp
Dura

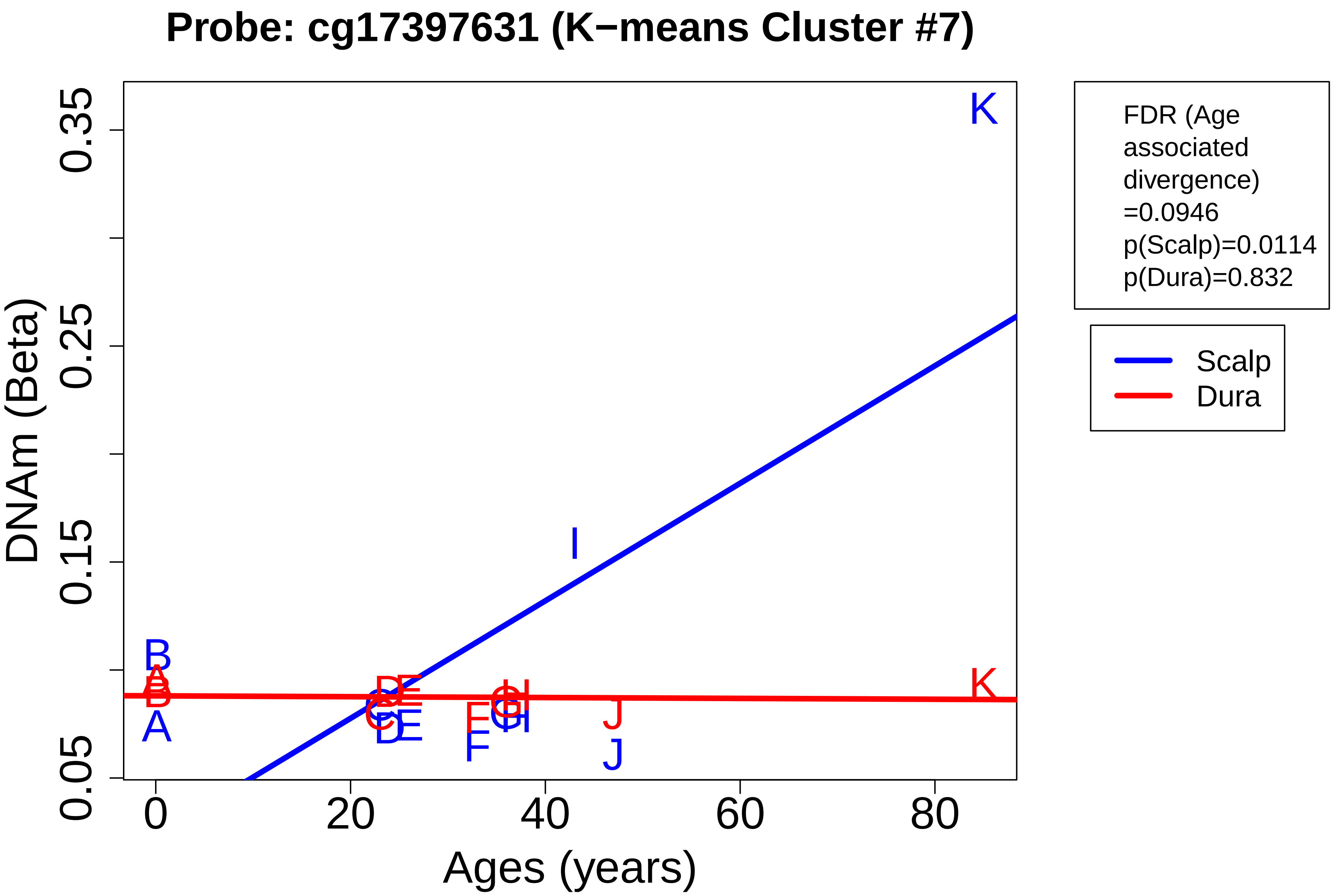

Probe: cg17397631 (K−means Cluster #7)
DNAm (Beta)
0.05
0.15
0.25
0.35
0
20
40
60
80
Ages (years)
FDR (Age associated divergence) =0.0946
p(Scalp)=0.0114
p(Dura)=0.832
Scalp
Dura

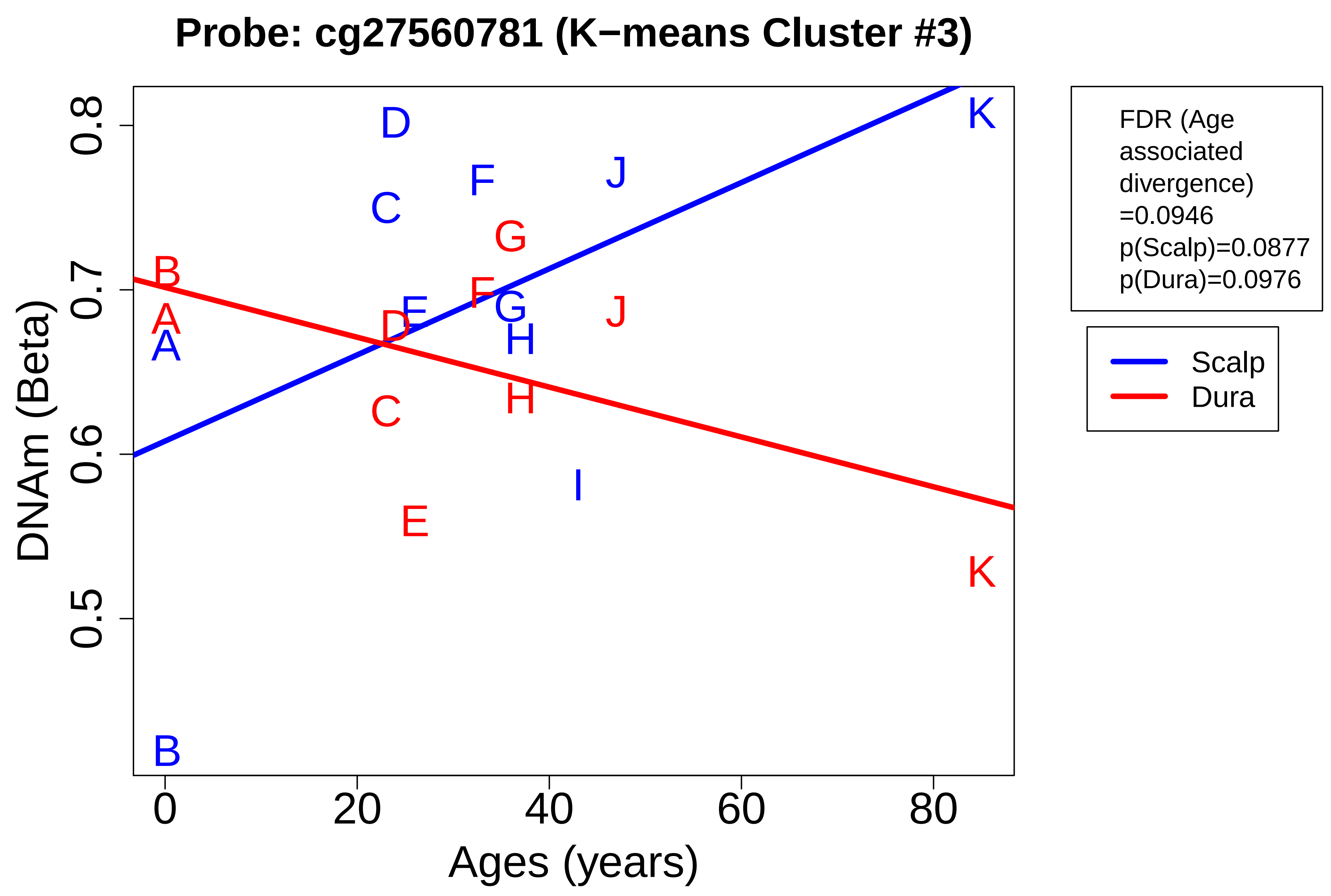

Probe: cg27560781 (K−means Cluster #3)
DNAm (Beta)
Ages (years)
0.5
0.6
0.7
0.8
0
20
40
60
80
FDR (Age associated divergence) =0.0946
p(Scalp)=0.0877
p(Dura)=0.0976
Scalp
Dura
A
B
C
D
E
F
G
H
I
J
K

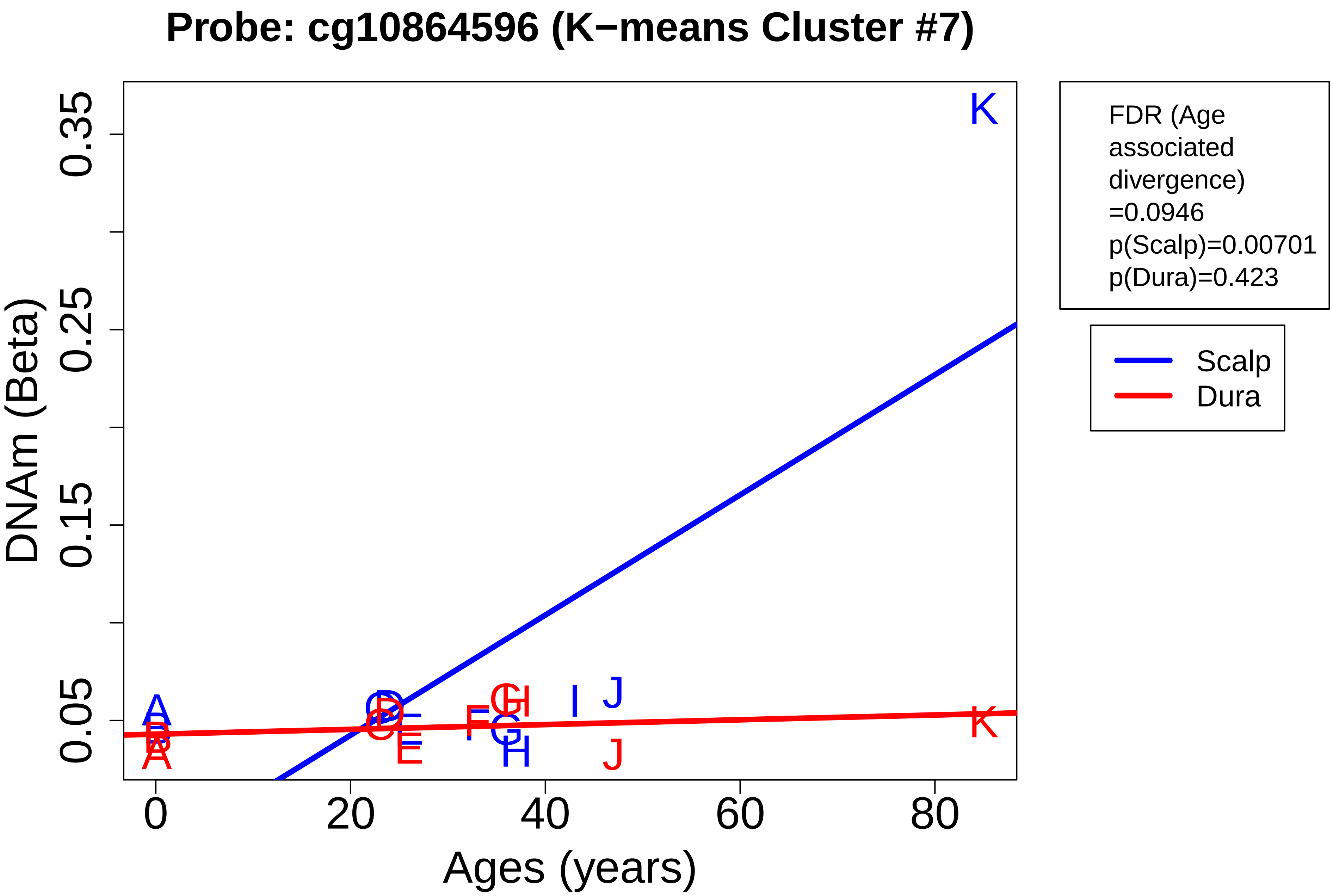

Probe: cg10864596 (K−means Cluster #7)
DNAm (Beta)
0.05
0.15
0.25
0.35
0
20
40
60
80
Ages (years)
FDR (Age associated divergence) =0.0946
p(Scalp)=0.00701
p(Dura)=0.423
Scalp
Dura

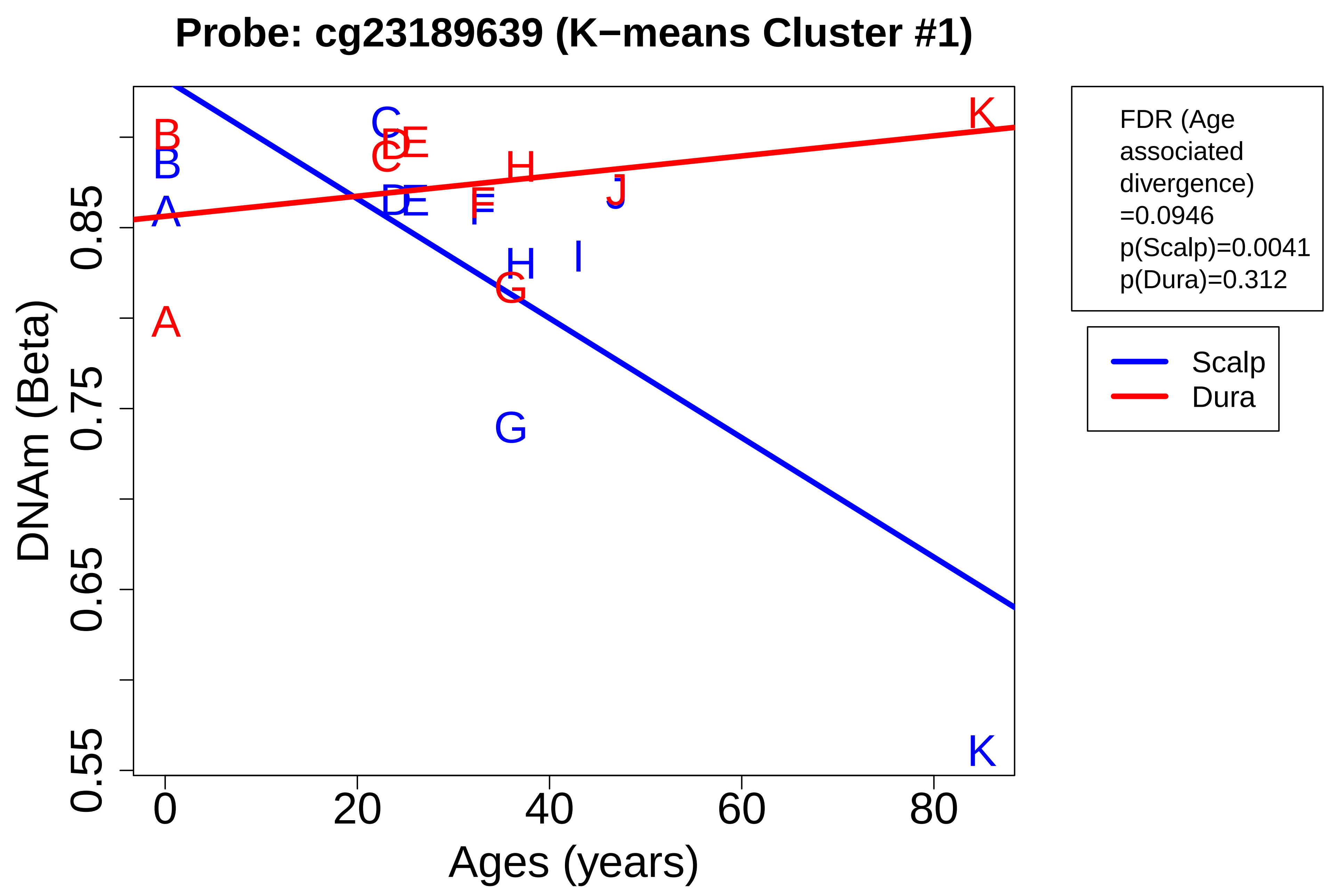

Probe: cg23189639 (K−means Cluster #1)
DNAm (Beta)
0.55
0.65
0.75
0.85
0
20
40
60
80
Ages (years)
FDR (Age associated divergence) =0.0946
p(Scalp)=0.0041
p(Dura)=0.312
Scalp
Dura

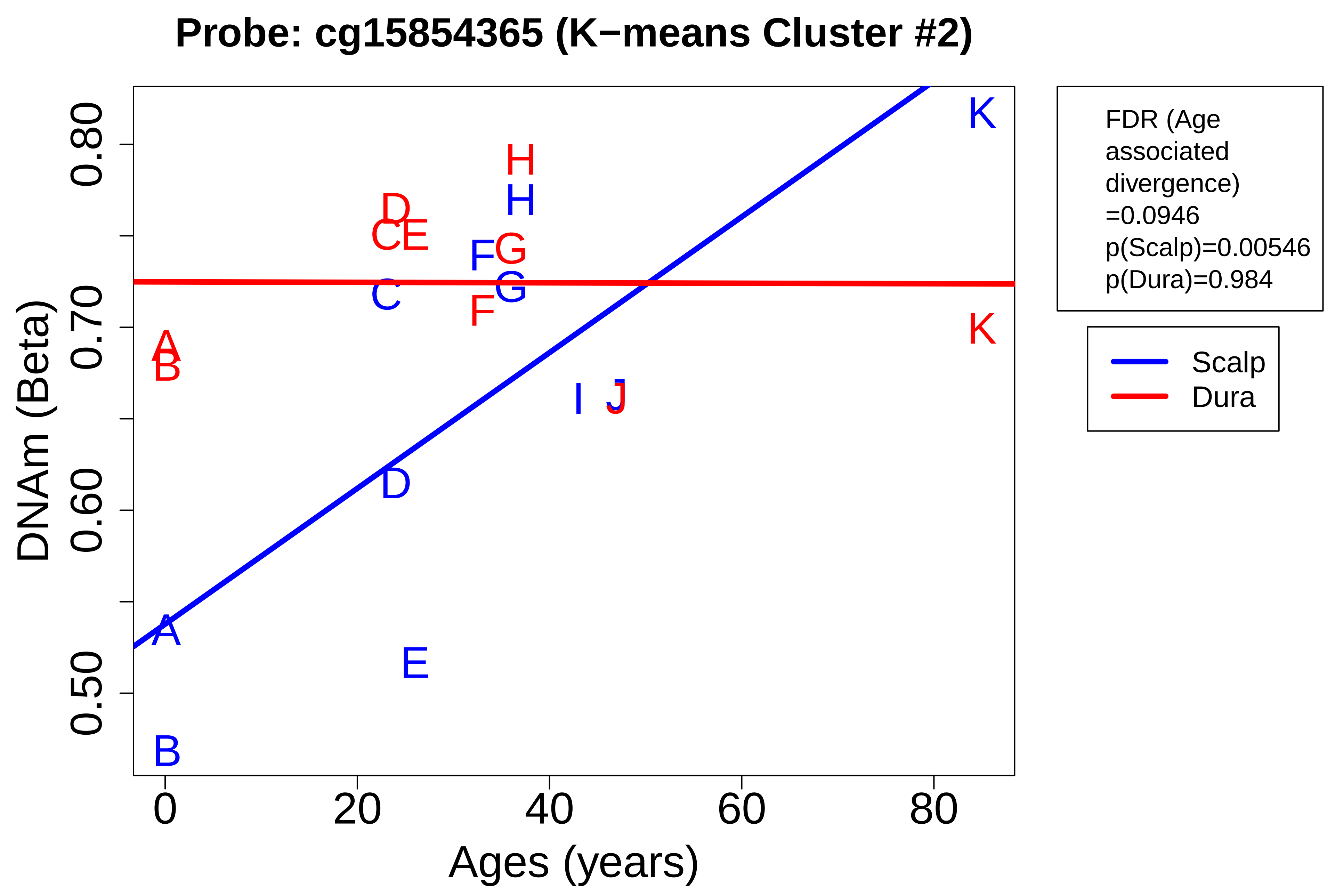

Probe: cg15854365 (K−means Cluster #2)
DNAm (Beta)
0.50
0.60
0.70
0.80
0
20
40
60
80
Ages (years)
FDR (Age associated divergence) =0.0946
p(Scalp)=0.00546
p(Dura)=0.984
Scalp
Dura
A
B
C
D
E
F
G
H
I
J
K

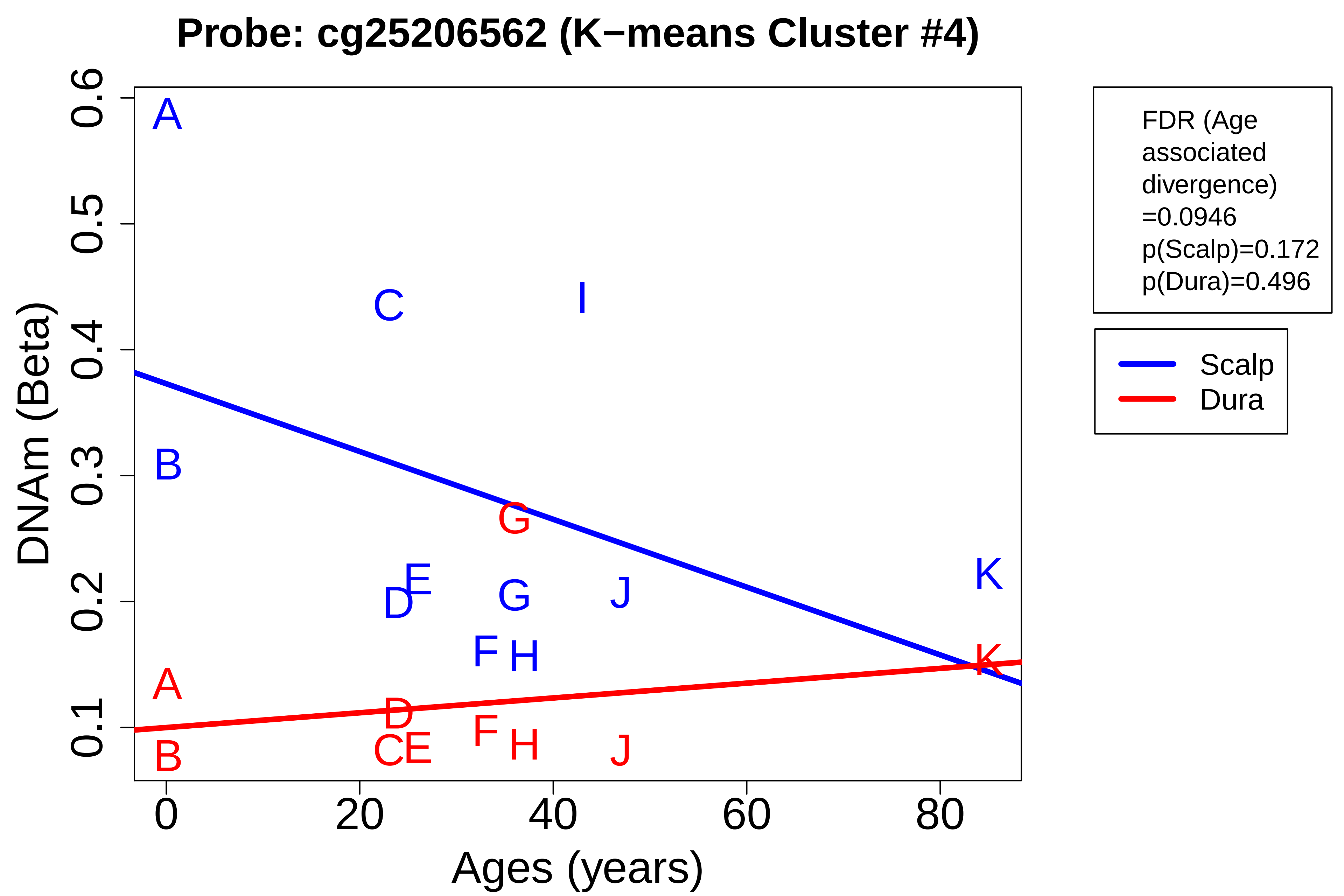
Probe: cg25206562 (K−means Cluster #4)
DNAm (Beta)
Ages (years)
0.1
0.2
0.3
0.4
0.5
0.6
0
20
40
60
80
FDR (Age associated divergence) =0.0946
p(Scalp)=0.172
p(Dura)=0.496
Scalp
Dura
A
B
C
D
E
F
G
H
I
J
K

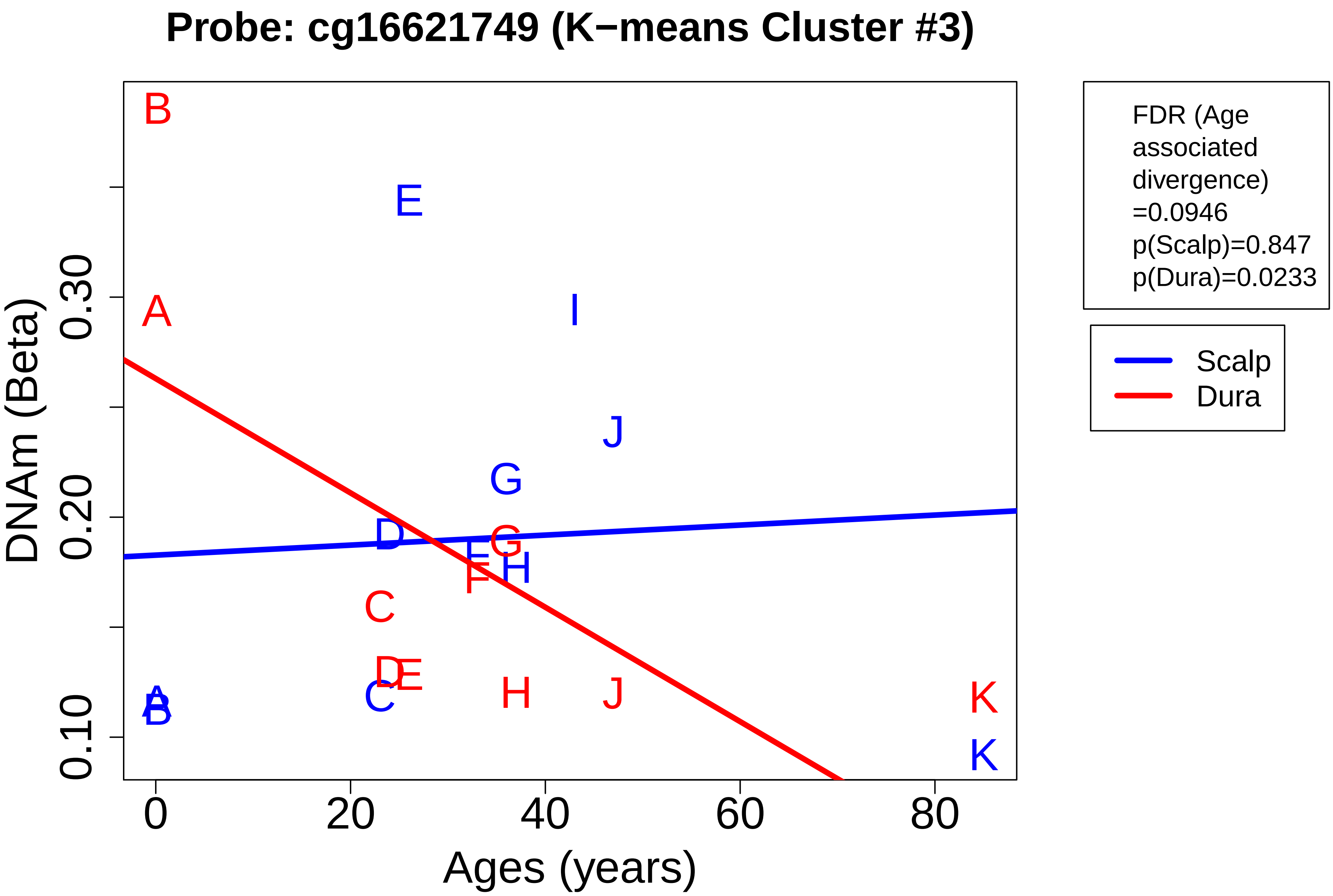

Probe: cg16621749 (K−means Cluster #3)
DNAm (Beta)
0.10
0.20
0.30
0
20
40
60
80
Ages (years)
FDR (Age associated divergence) =0.0946
p(Scalp)=0.847
p(Dura)=0.0233
Scalp
Dura
A
B
C
D
E
F
G
H
I
J
K

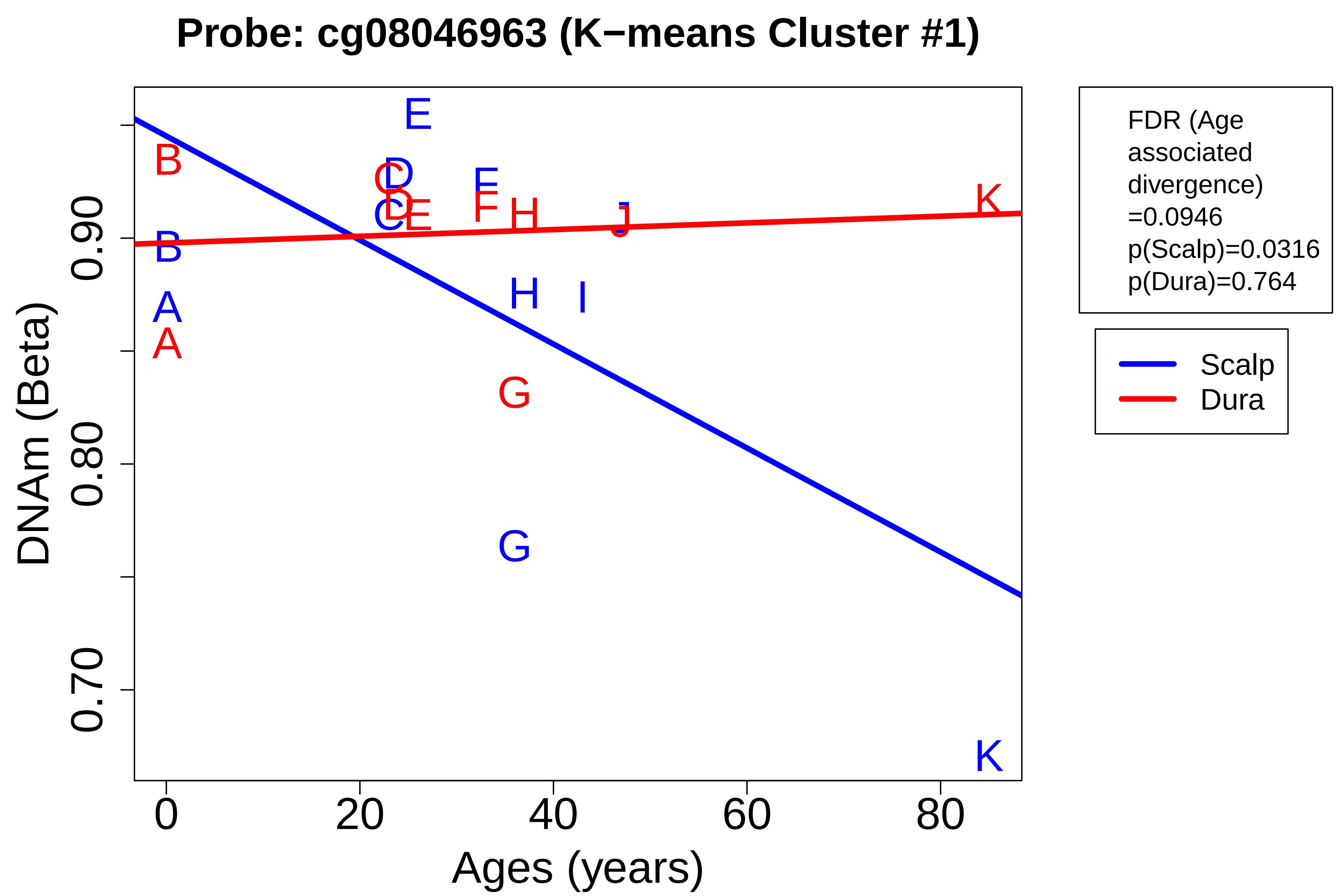

Probe: cg08046963 (K−means Cluster #1)
DNAm (Beta)
0.70
0.80
0.90
0
20
40
60
80
Ages (years)
FDR (Age associated divergence) =0.0946
p(Scalp)=0.0316
p(Dura)=0.764
Scalp
Dura

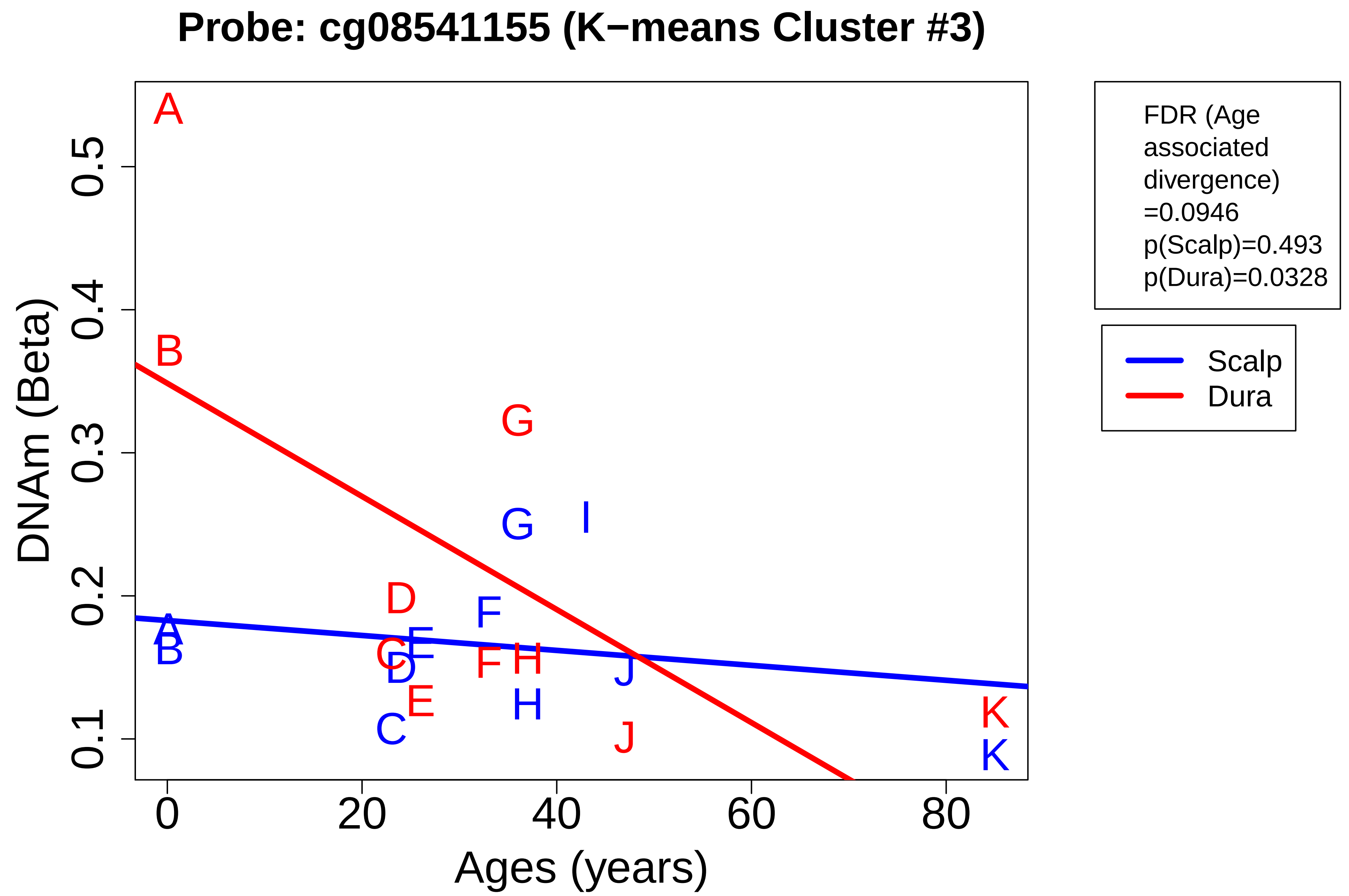

Probe: cg08541155 (K−means Cluster #3)
DNAm (Beta)
0.1
0.2
0.3
0.4
0.5
0
20
40
60
80
Ages (years)
FDR (Age associated divergence) =0.0946
p(Scalp)=0.493
p(Dura)=0.0328
Scalp
Dura

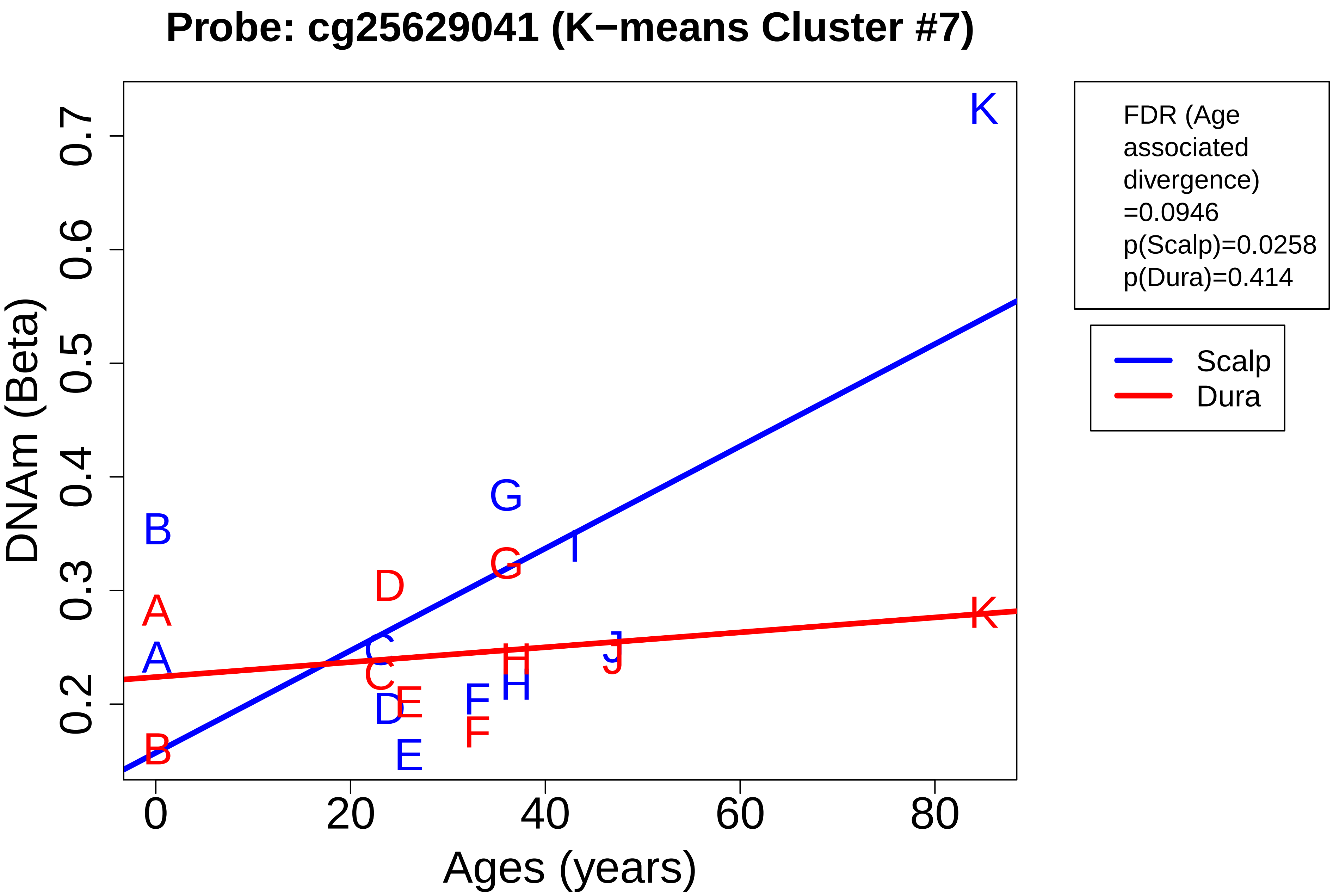

Probe: cg25629041 (K−means Cluster #7)
DNAm (Beta)
0.2
0.3
0.4
0.5
0.6
0.7
0
20
40
60
80
Ages (years)
FDR (Age associated divergence) =0.0946
p(Scalp)=0.0258
p(Dura)=0.414
Scalp
Dura

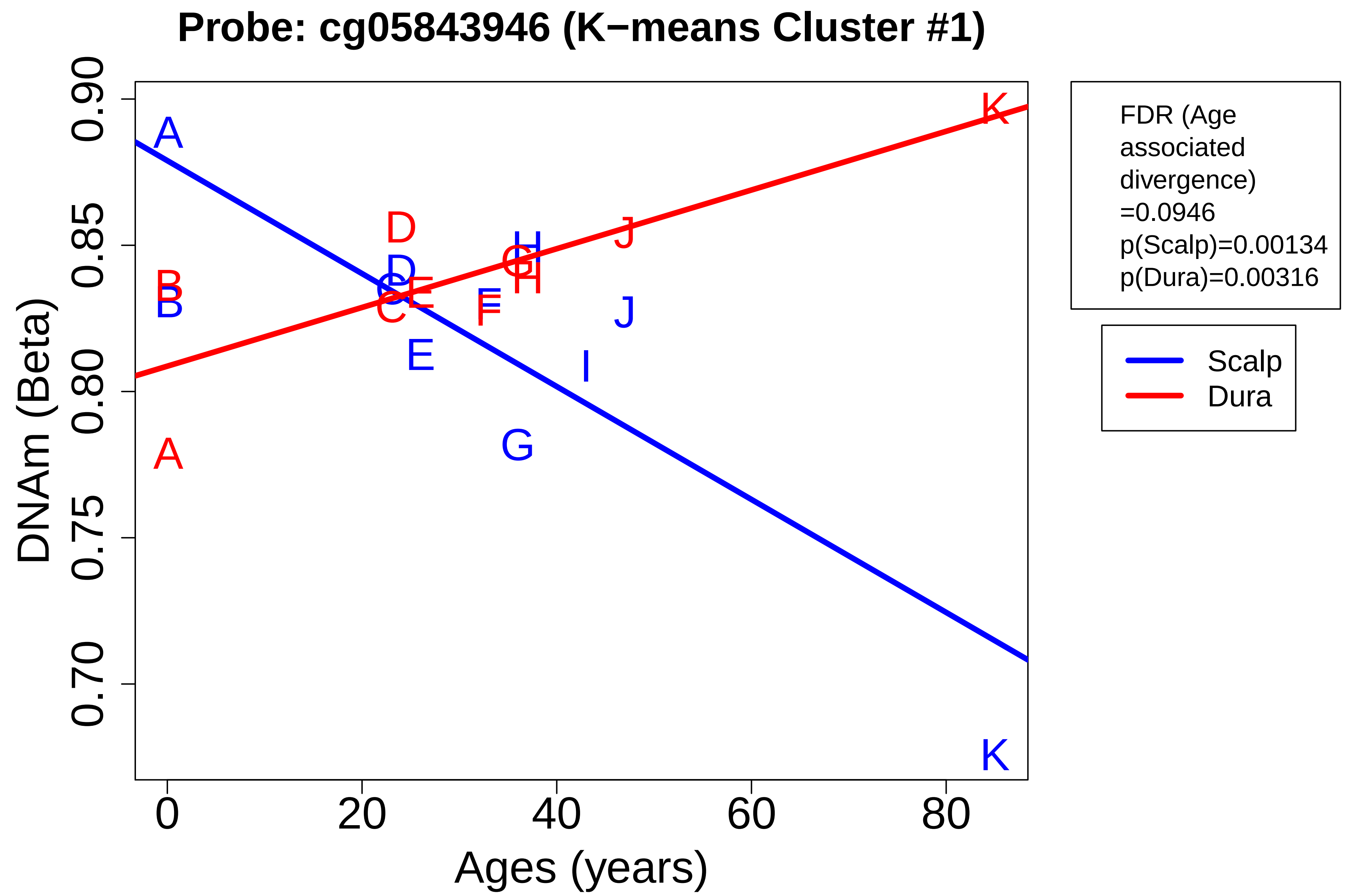

Probe: cg05843946 (K−means Cluster #1)
DNAm (Beta)
0.70
0.75
0.80
0.85
0.90
Ages (years)
0
20
40
60
80
A
B
C
D
E
F
G
H
I
J
K
FDR (Age associated divergence) =0.0946
p(Scalp)=0.00134
p(Dura)=0.00316
Scalp
Dura

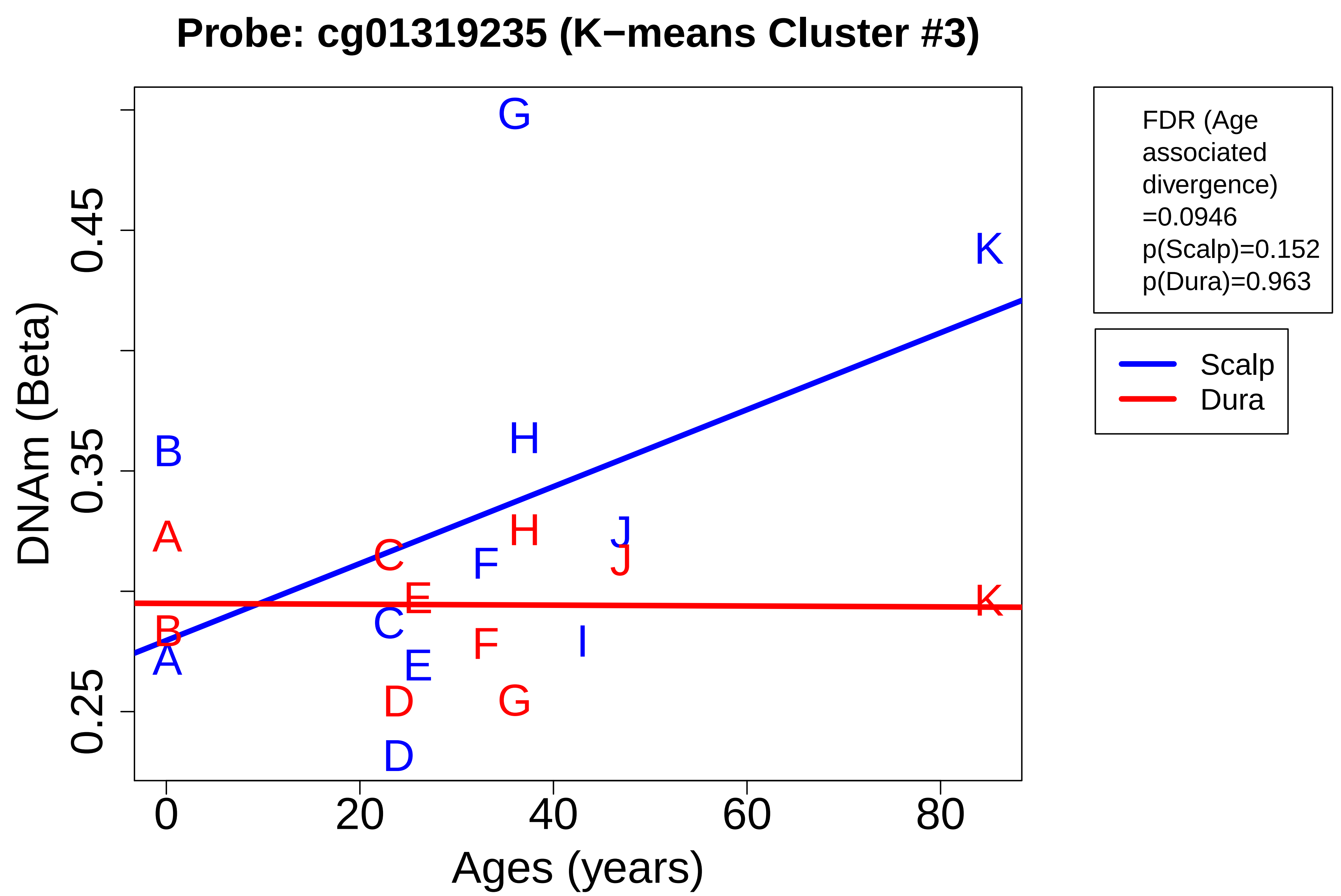

Probe: cg01319235 (K−means Cluster #3)
DNAm (Beta)
0.25
0.35
0.45
Ages (years)
0
20
40
60
80
A
B
C
D
E
F
G
H
I
J
K
FDR (Age associated divergence) =0.0946
p(Scalp)=0.152
p(Dura)=0.963
Scalp
Dura

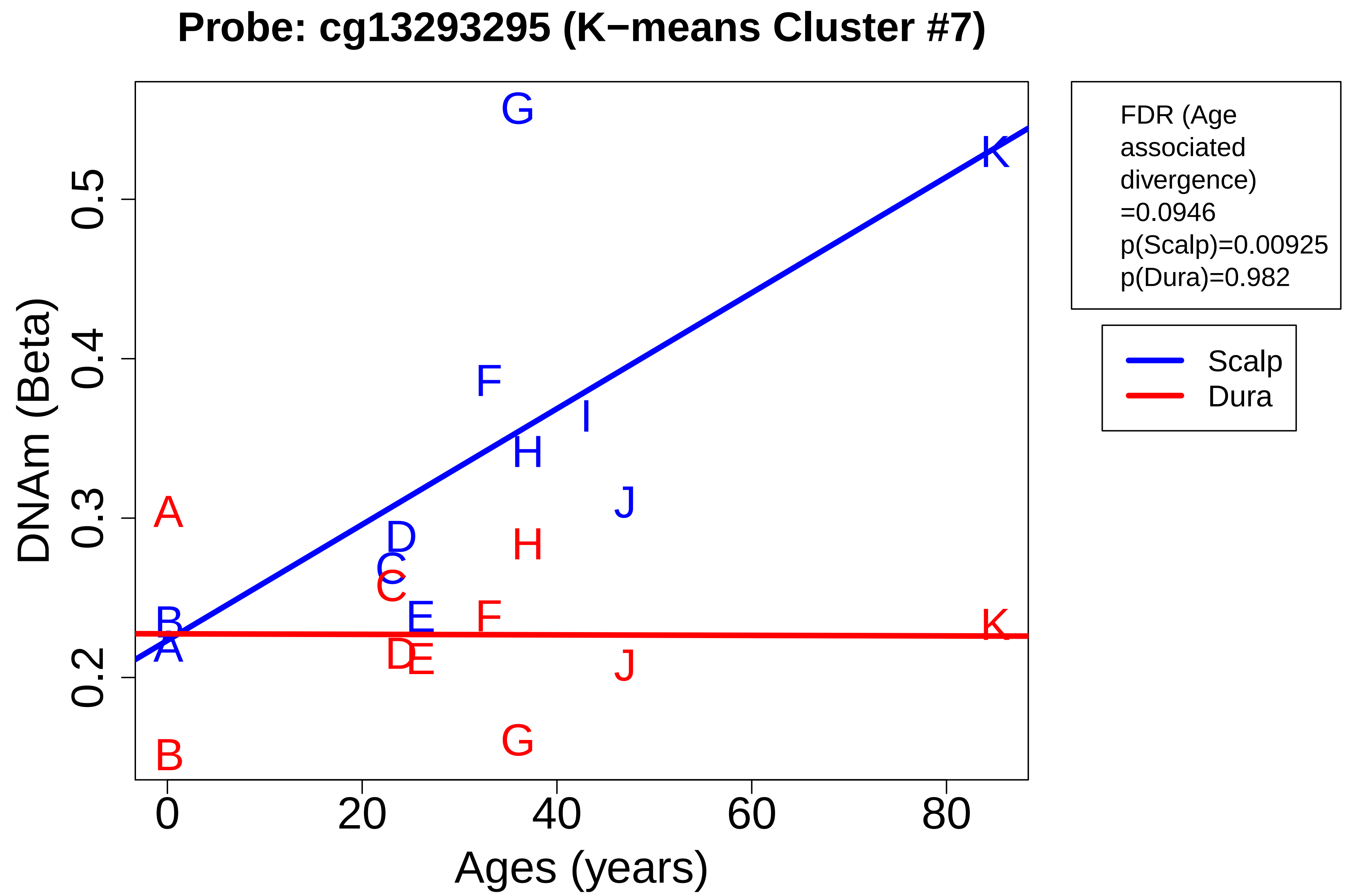
Probe: cg13293295 (K−means Cluster #7)
DNAm (Beta)
0.2
0.3
0.4
0.5
0
20
40
60
80
Ages (years)
FDR (Age associated divergence) =0.0946
p(Scalp)=0.00925
p(Dura)=0.982
Scalp
Dura

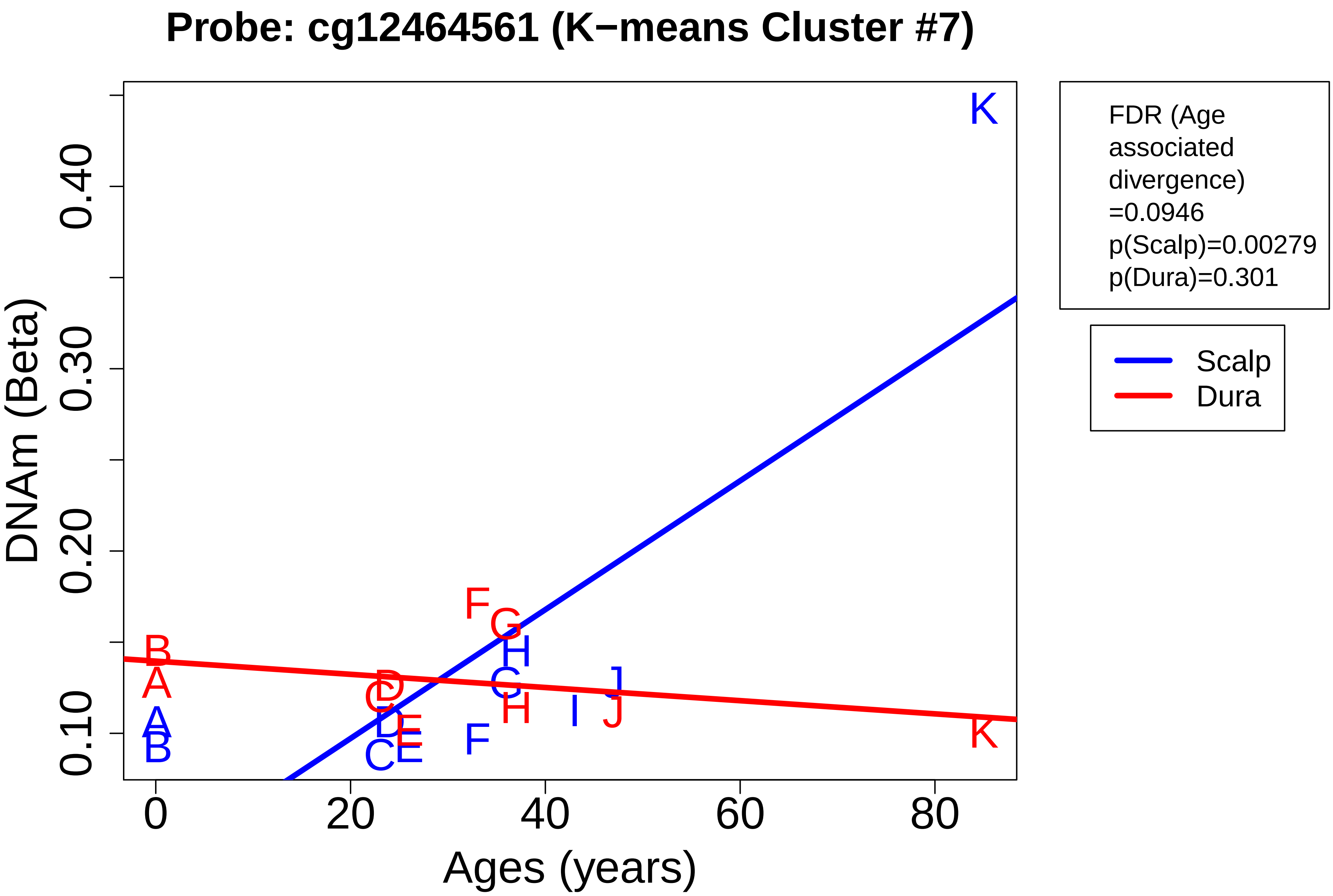

Probe: cg12464561 (K−means Cluster #7)
DNAm (Beta)
0.10
0.20
0.30
0.40
0
20
40
60
80
Ages (years)
FDR (Age associated divergence) =0.0946
p(Scalp)=0.00279
p(Dura)=0.301
Scalp
Dura

Probe: cg01950074 (K−means Cluster #7)

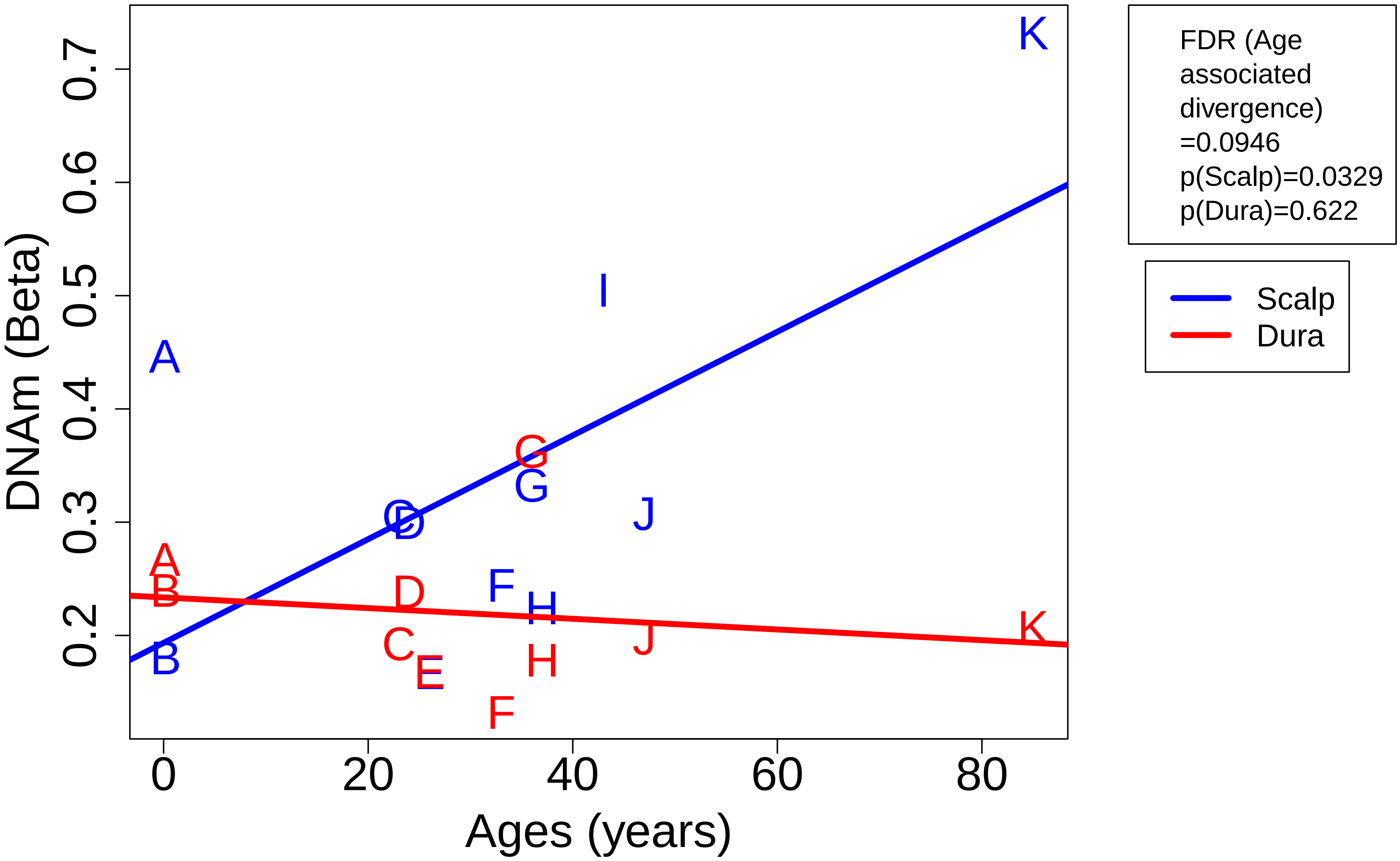

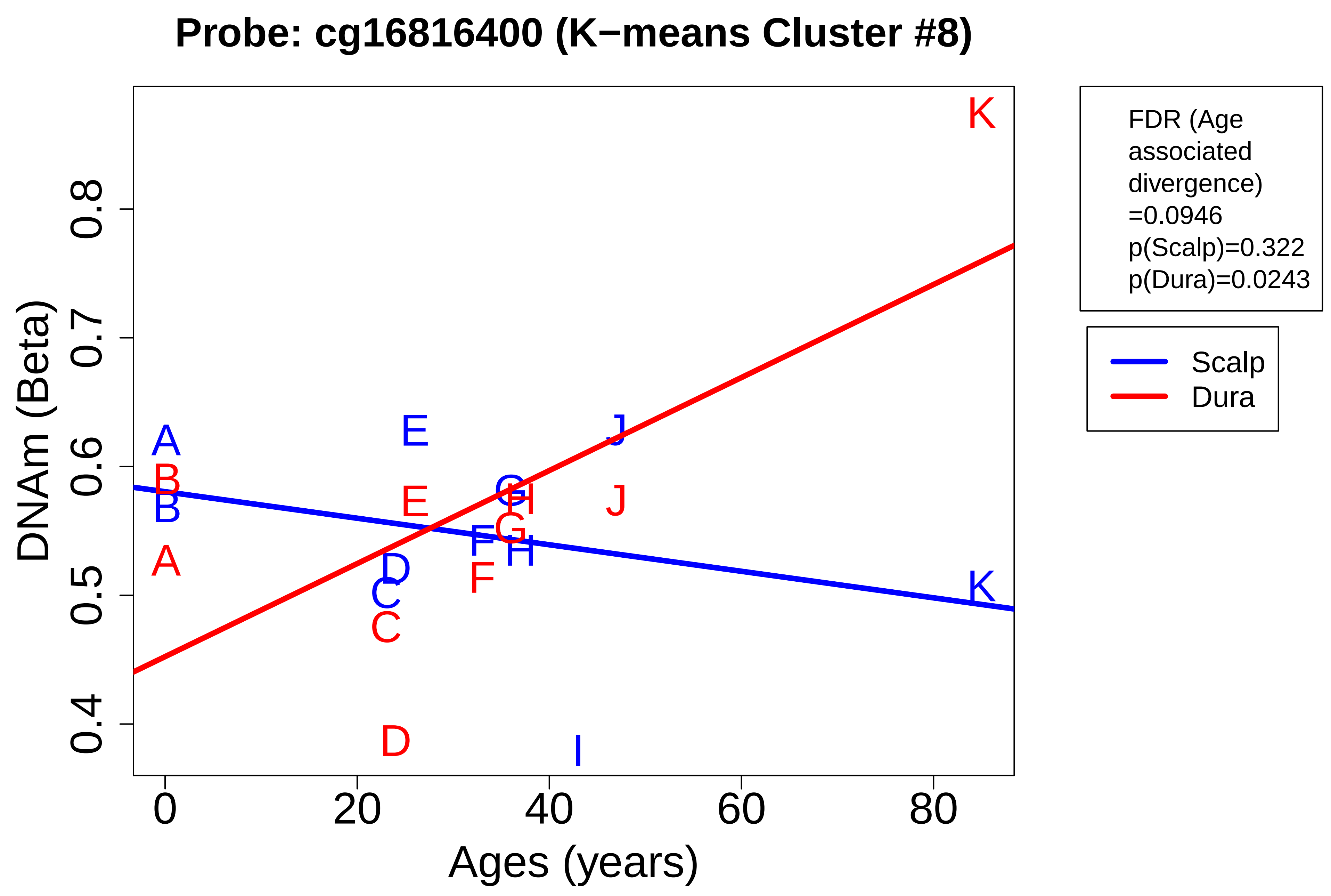

Probe: cg16816400 (K−means Cluster #8)
FDR (Age associated divergence) =0.0946
p(Scalp)=0.322
p(Dura)=0.0243
Scalp
Dura
DNAm (Beta)
0.4
0.5
0.6
0.7
0.8
Ages (years)
0
20
40
60
80
A
B
C
D
E
F
G
H
I
J
K

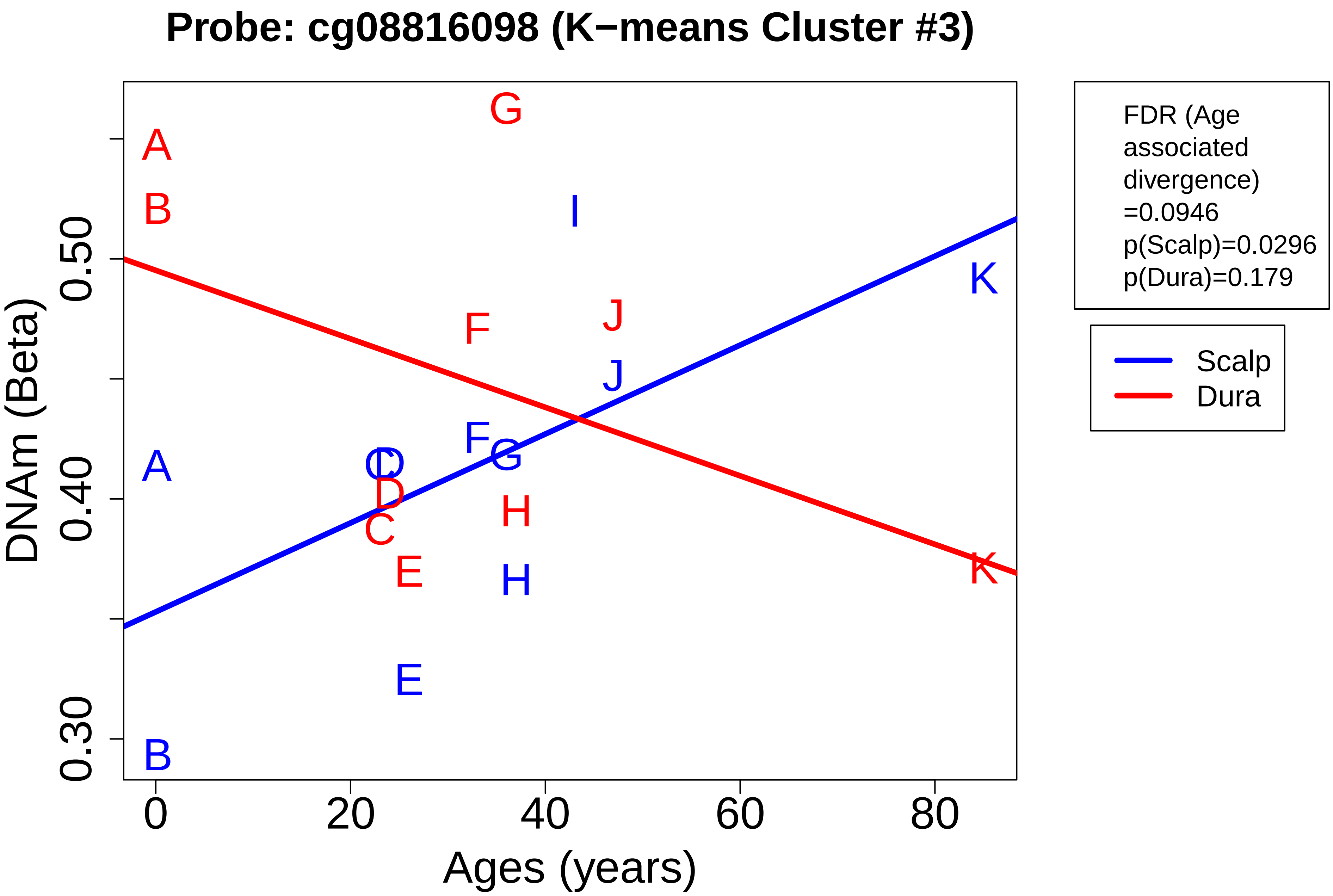

Probe: cg08816098 (K−means Cluster #3)
DNAm (Beta)
0.30
0.40
0.50
Ages (years)
0
20
40
60
80
FDR (Age associated divergence) =0.0946
p(Scalp)=0.0296
p(Dura)=0.179
Scalp
Dura

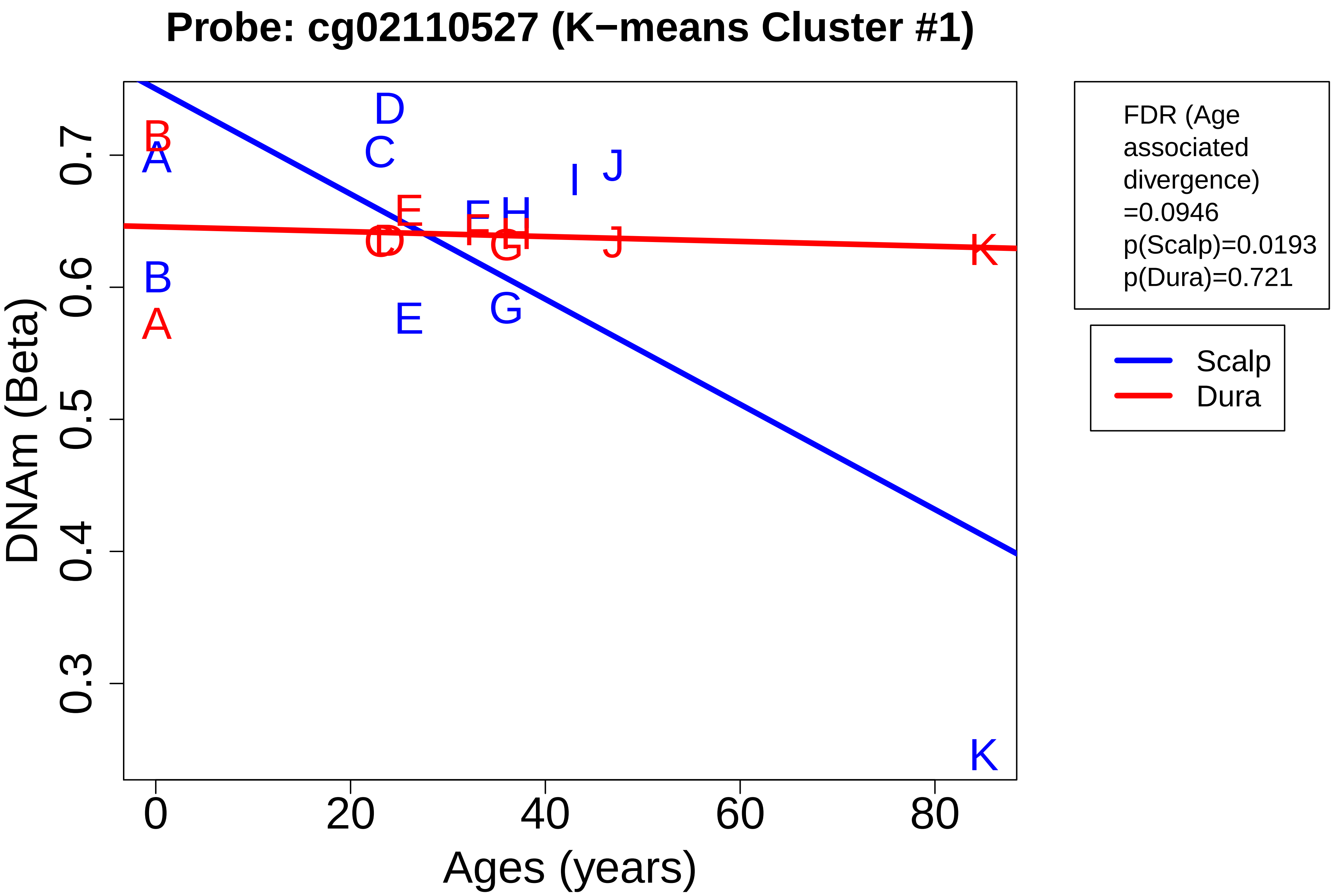

Probe: cg02110527 (K−means Cluster #1)
DNAm (Beta)
Ages (years)
0.3
0.4
0.5
0.6
0.7
0
20
40
60
80
FDR (Age associated divergence) =0.0946
p(Scalp)=0.0193
p(Dura)=0.721
Scalp
Dura

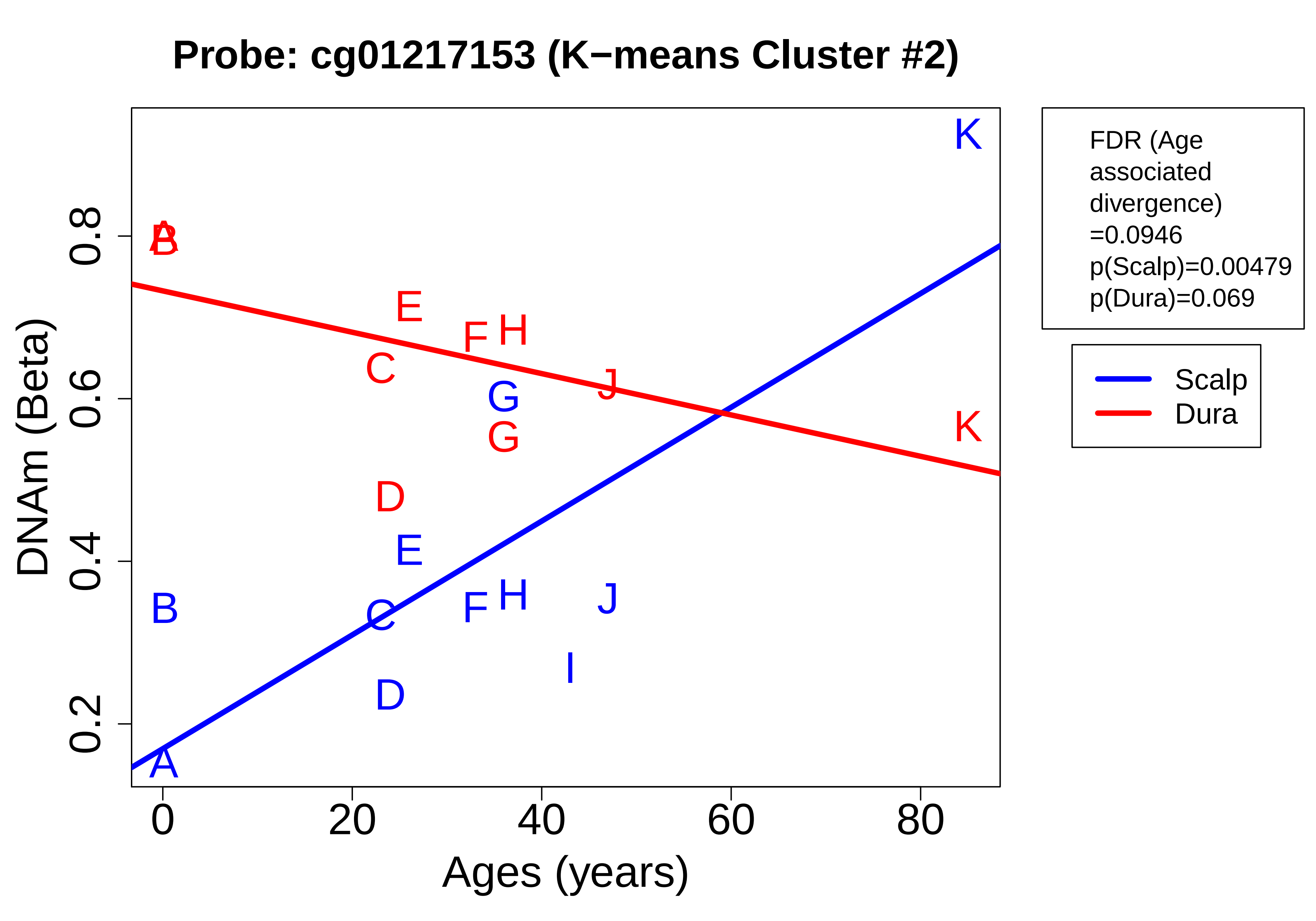

Probe: cg01217153 (K−means Cluster #2)
DNAm (Beta)
0.2
0.4
0.6
0.8
Ages (years)
0
20
40
60
80
FDR (Age associated divergence) =0.0946
p(Scalp)=0.00479
p(Dura)=0.069
Scalp
Dura
A
B
C
D
E
F
G
H
I
J
K

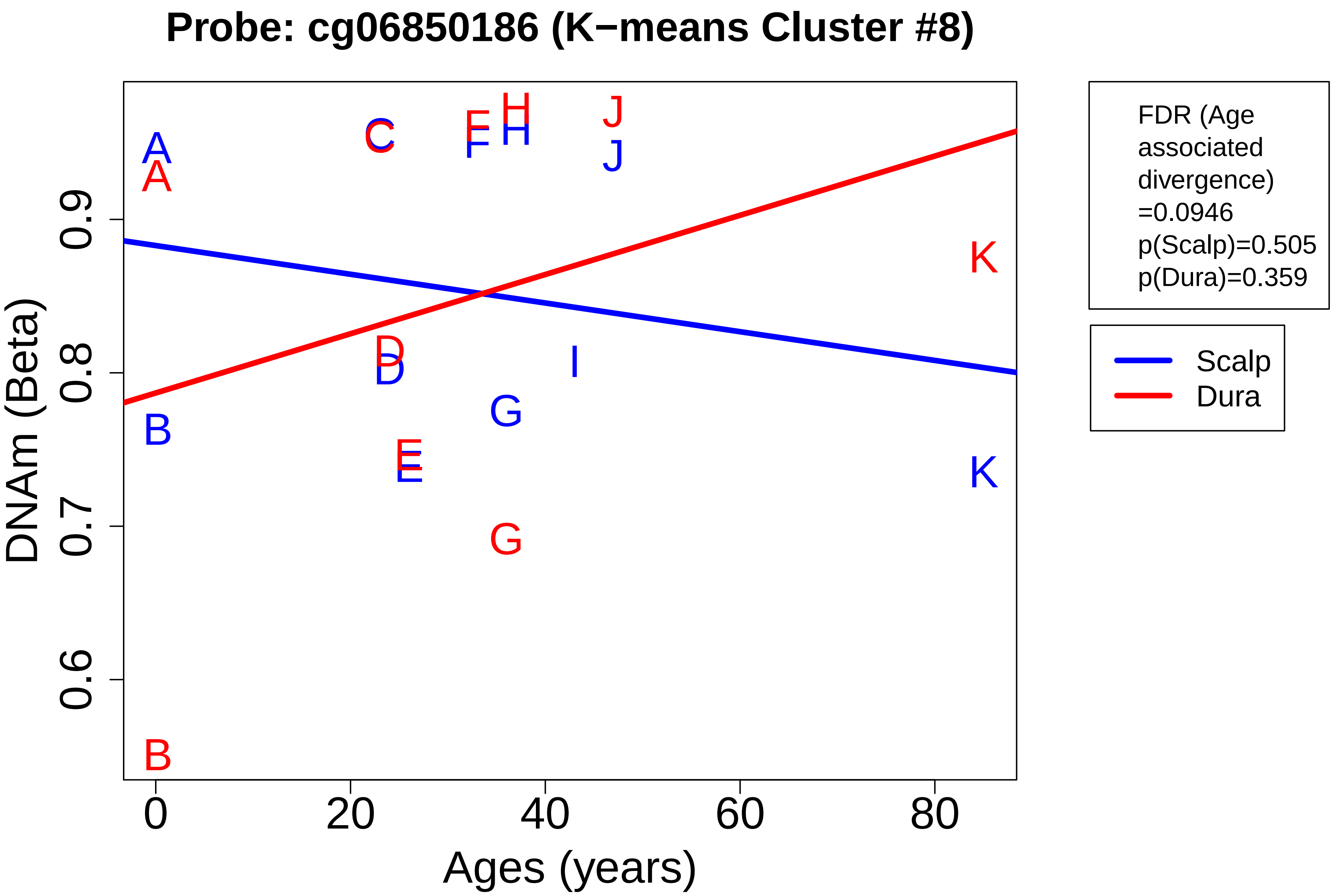

Probe: cg06850186 (K−means Cluster #8)
DNAm (Beta)
Ages (years)
0.6
0.7
0.8
0.9
0
20
40
60
80
FDR (Age associated divergence) =0.0946
p(Scalp)=0.505
p(Dura)=0.359
Scalp
Dura
A
B
C
D
E
F
G
H
I
J
K

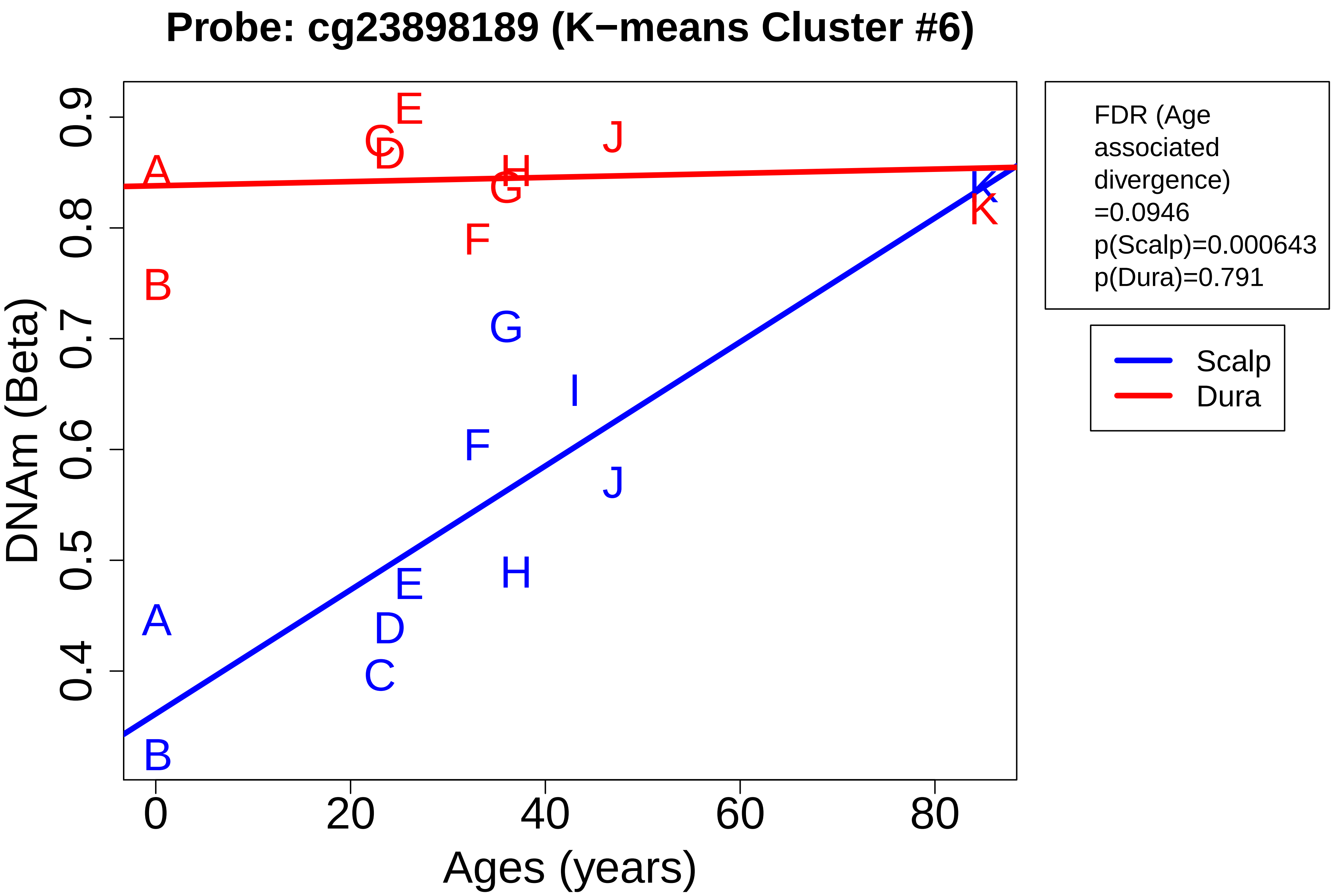
Probe: cg23898189 (K−means Cluster #6)
DNAm (Beta)
Ages (years)
0.4
0.5
0.6
0.7
0.8
0.9
0
20
40
60
80
FDR (Age associated divergence) =0.0946
p(Scalp)=0.000643
p(Dura)=0.791
Scalp
Dura
A
B
C
D
E
F
G
H
I
J
K

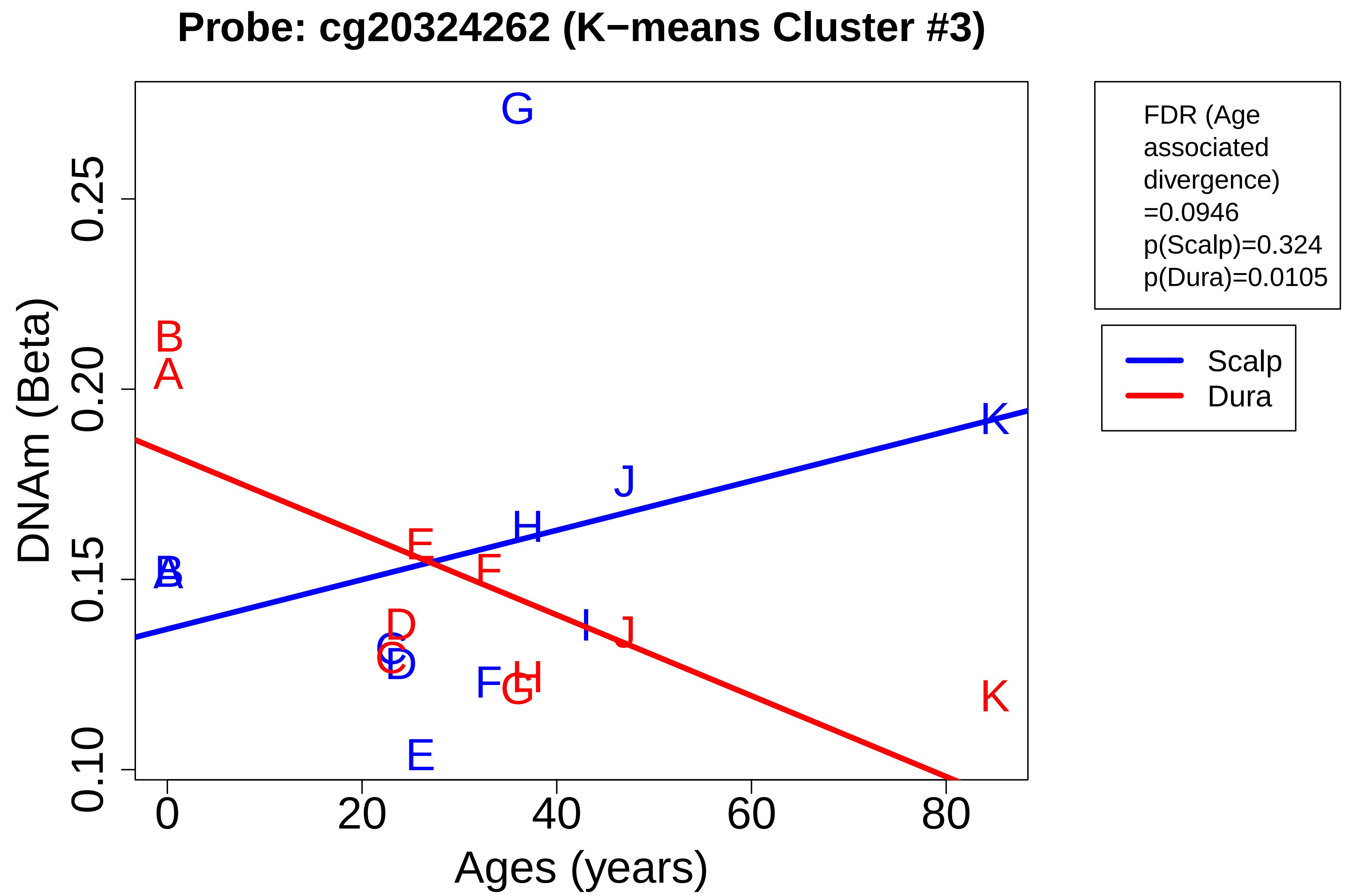

Probe: cg20324262 (K−means Cluster #3)
FDR (Age associated divergence) =0.0946
p(Scalp)=0.324
p(Dura)=0.0105
Scalp
Dura
DNAm (Beta)
0.10
0.15
0.20
0.25
0
20
40
60
80
Ages (years)

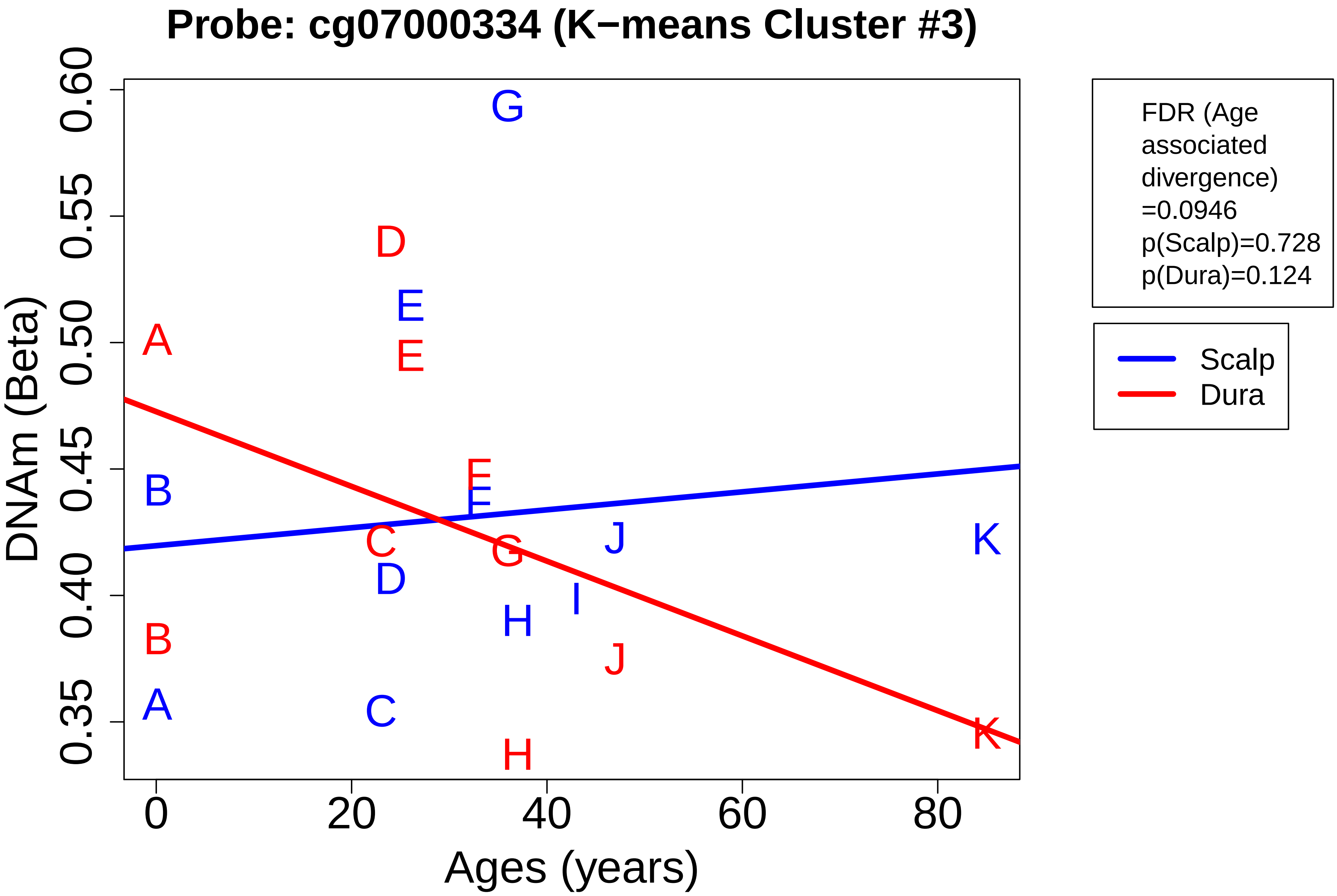

Probe: cg07000334 (K−means Cluster #3)
DNAm (Beta)
0.60
0.55
0.50
0.45
0.40
0.35
0
20
40
60
80
Ages (years)
A
B
C
D
E
F
G
H
I
J
K
FDR (Age associated divergence) =0.0946
p(Scalp)=0.728
p(Dura)=0.124
Scalp
Dura

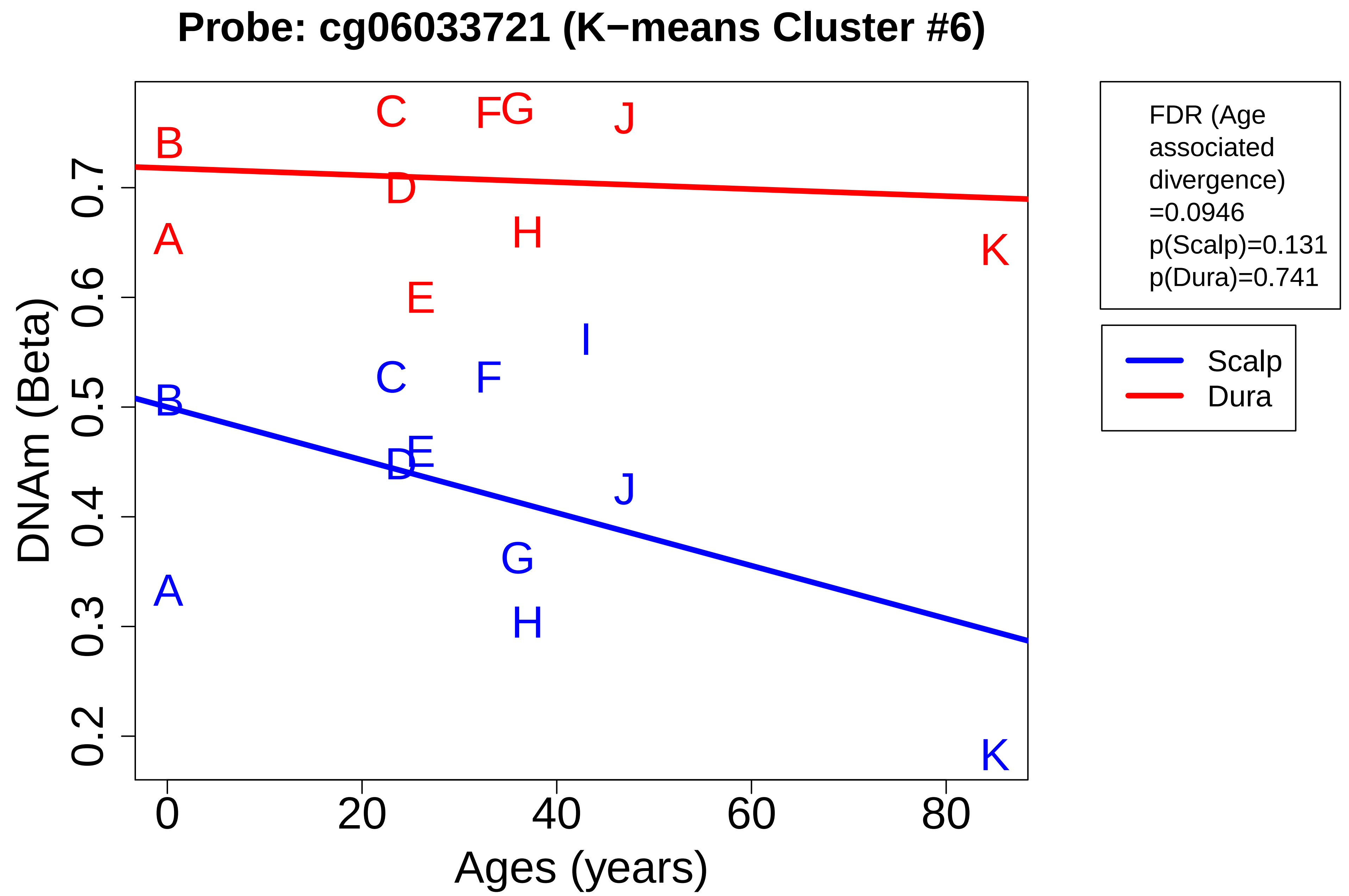
Probe: cg06033721 (K−means Cluster #6)
DNAm (Beta)
0.2
0.3
0.4
0.5
0.6
0.7
0
20
40
60
80
Ages (years)
FDR (Age associated divergence) =0.0946
p(Scalp)=0.131
p(Dura)=0.741
Scalp
Dura
A
B
C
D
E
F
G
H
I
J
K

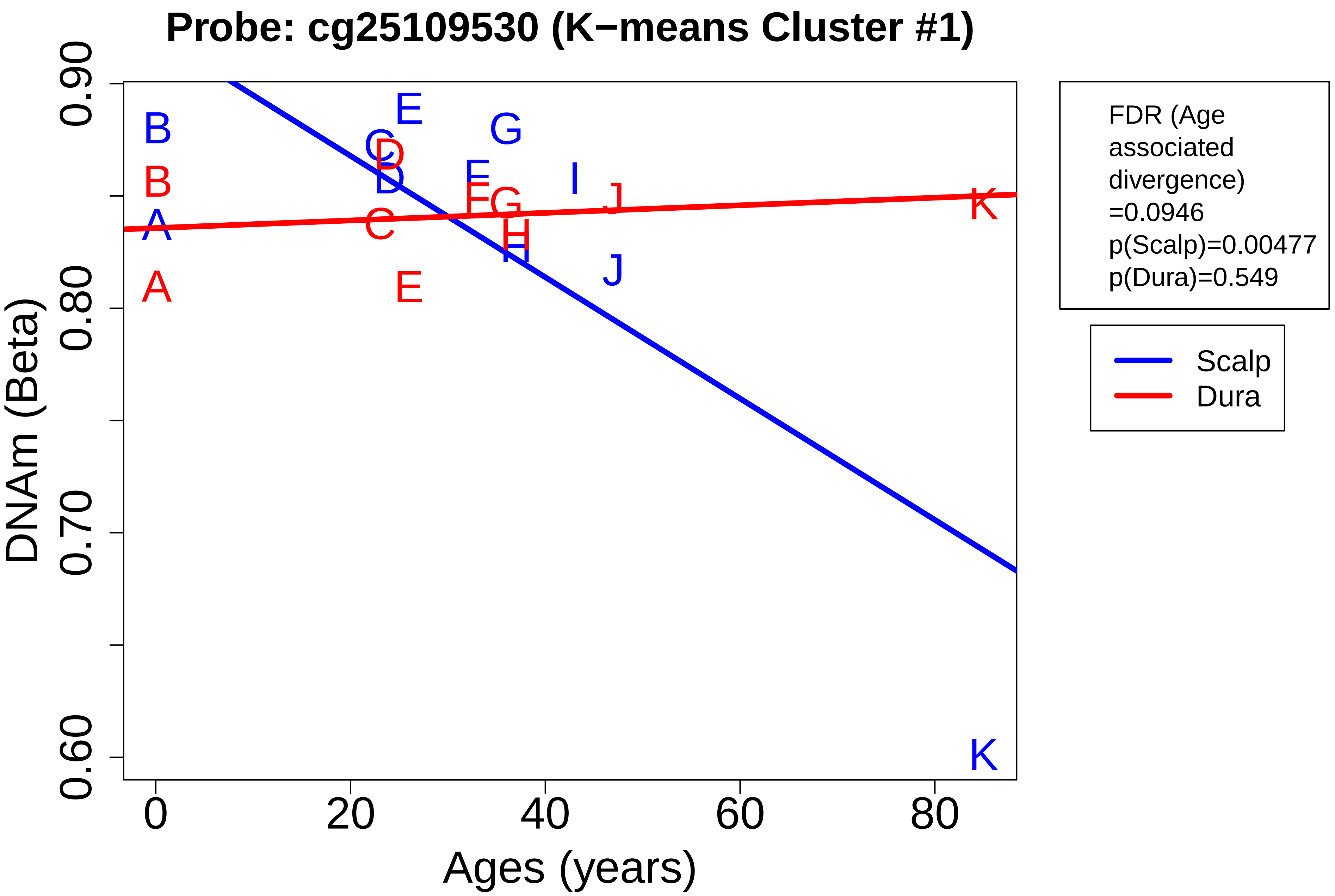

Probe: cg25109530 (K−means Cluster #1)
DNAm (Beta)
0.60
0.70
0.80
0.90
0
20
40
60
80
Ages (years)
B
B
A
A
C
D
D
C
E
E
E
F
F
G
G
H
H
I
J
J
K
K
FDR (Age associated divergence) =0.0946
p(Scalp)=0.00477
p(Dura)=0.549
Scalp
Dura

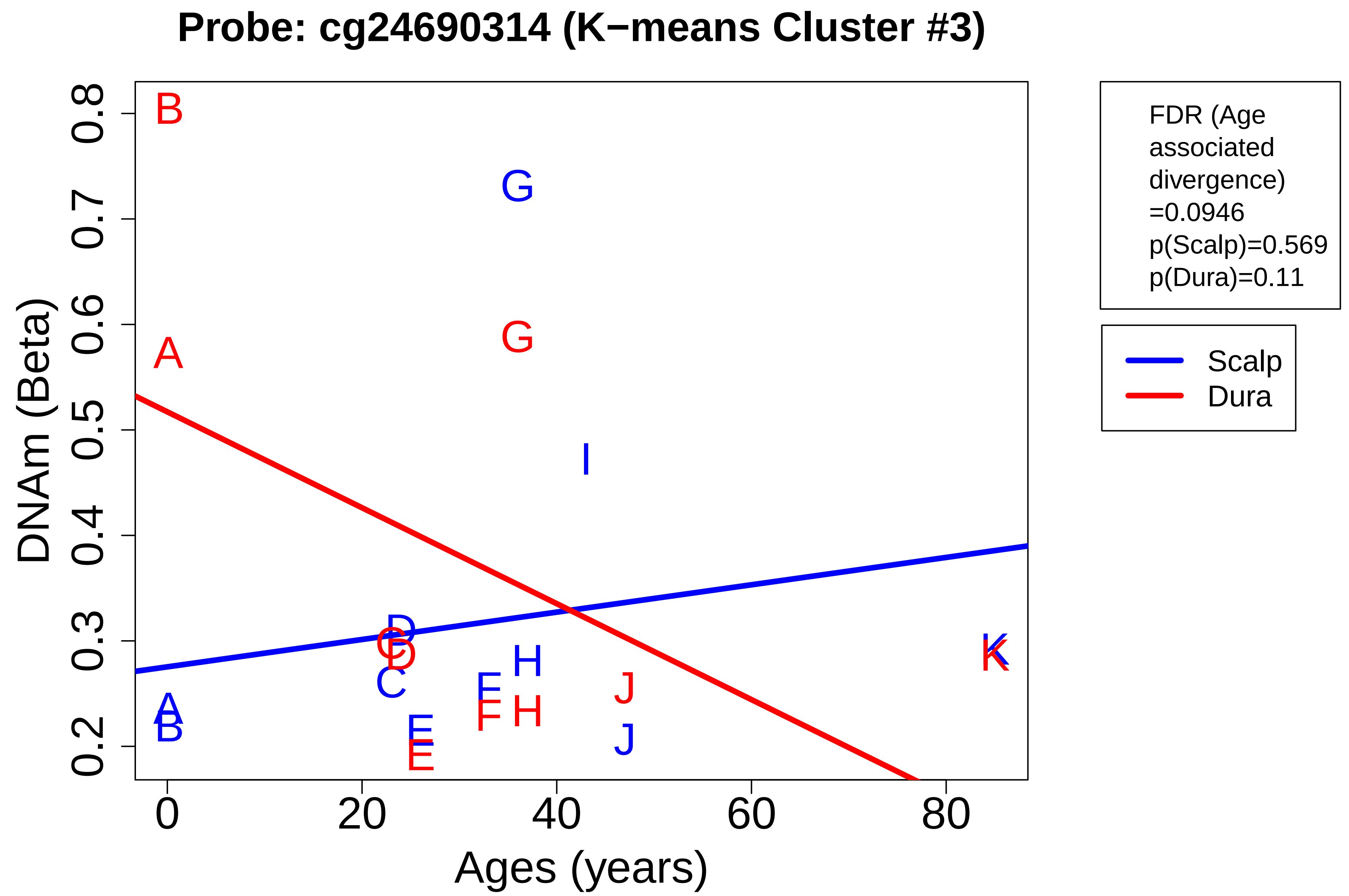

Probe: cg24690314 (K−means Cluster #3)
DNAm (Beta)
0.2
0.3
0.4
0.5
0.6
0.7
0.8
Ages (years)
0
20
40
60
80
FDR (Age associated divergence) =0.0946
p(Scalp)=0.569
p(Dura)=0.11
Scalp
Dura

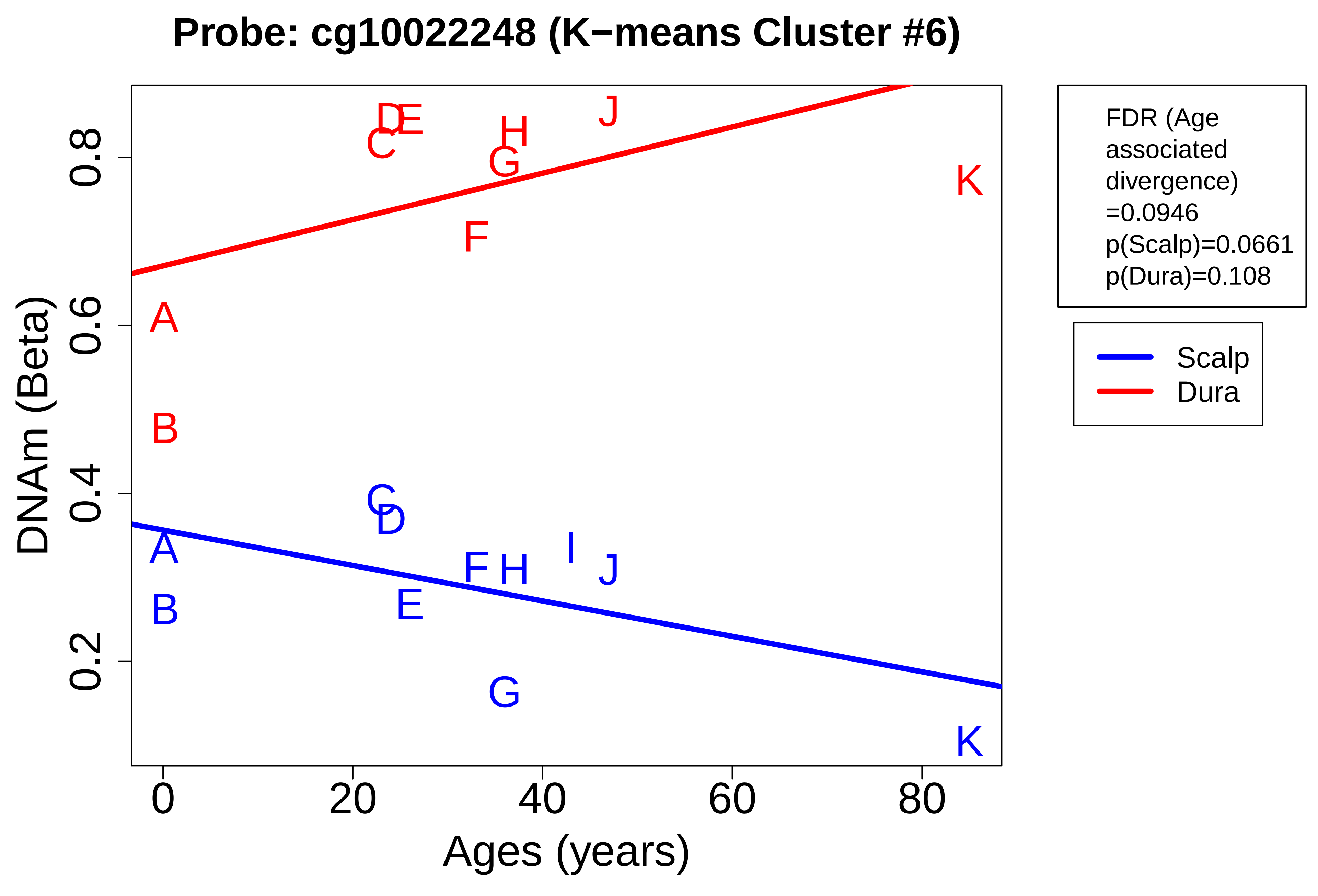

Probe: cg10022248 (K−means Cluster #6)
DNAm (Beta)
0.2
0.4
0.6
0.8
0
20
40
60
80
Ages (years)
FDR (Age associated divergence) =0.0946
p(Scalp)=0.0661
p(Dura)=0.108
Scalp
Dura
A
B
C
D
E
F
G
H
I
J
K

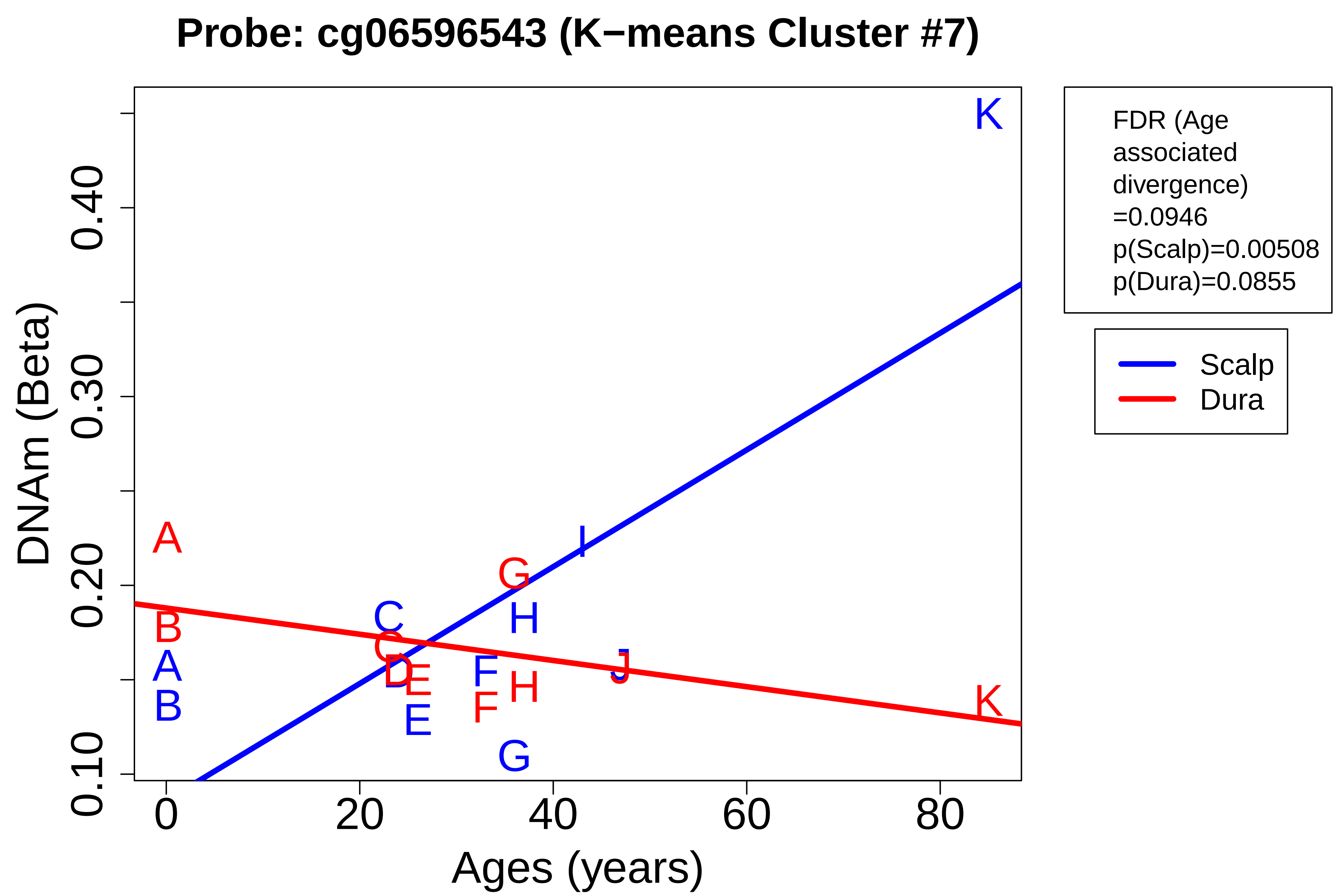

Probe: cg06596543 (K−means Cluster #7)
DNAm (Beta)
0.10
0.20
0.30
0.40
Ages (years)
0
20
40
60
80
FDR (Age associated divergence) =0.0946
p(Scalp)=0.00508
p(Dura)=0.0855
Scalp
Dura
A
B
C
D
E
F
G
H
I
J
K

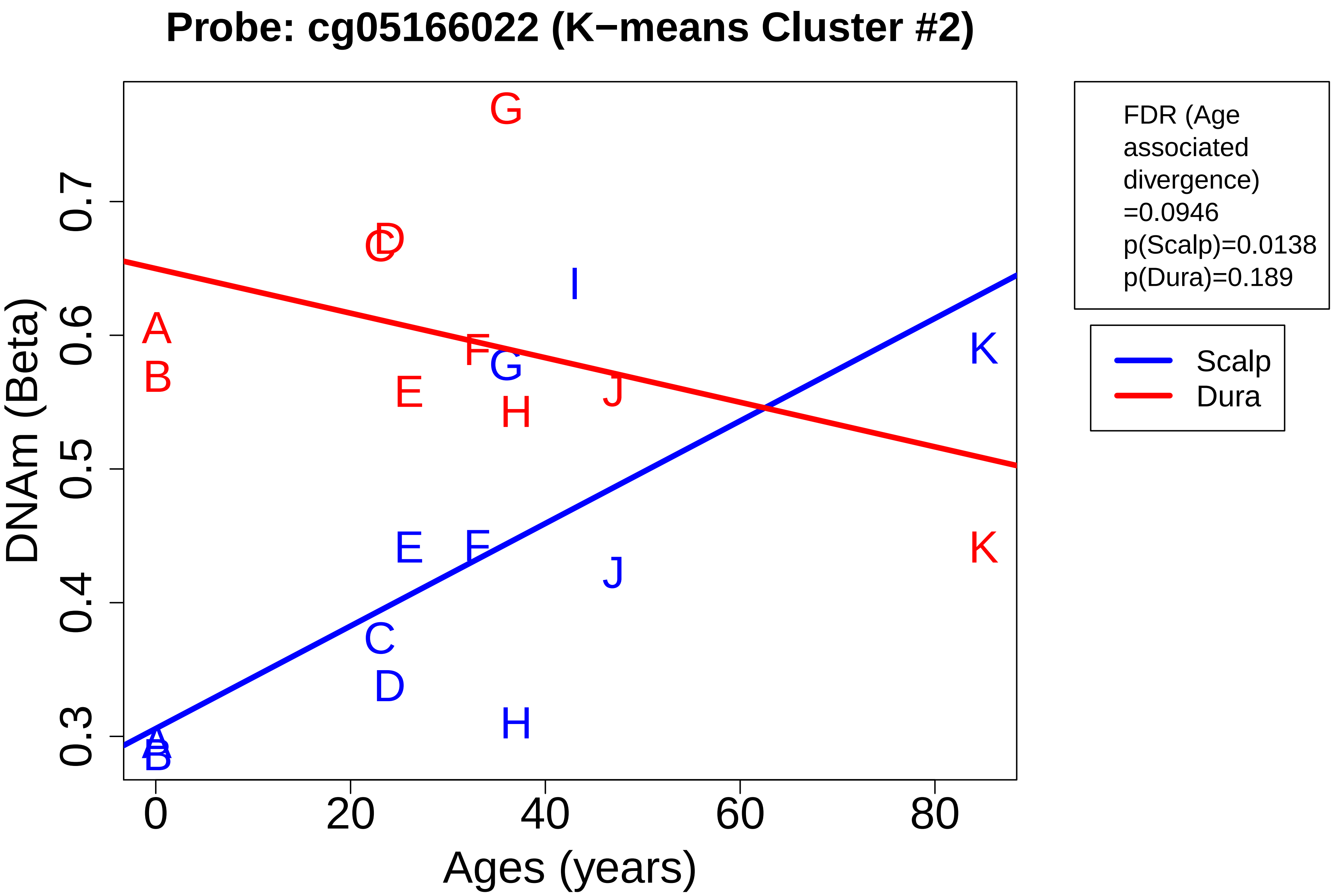
Probe: cg05166022 (K−means Cluster #2)
DNAm (Beta)
0.3
0.4
0.5
0.6
0.7
Ages (years)
0
20
40
60
80
A
B
C
D
E
F
G
H
I
J
K
FDR (Age associated divergence) =0.0946
p(Scalp)=0.0138
p(Dura)=0.189
Scalp
Dura

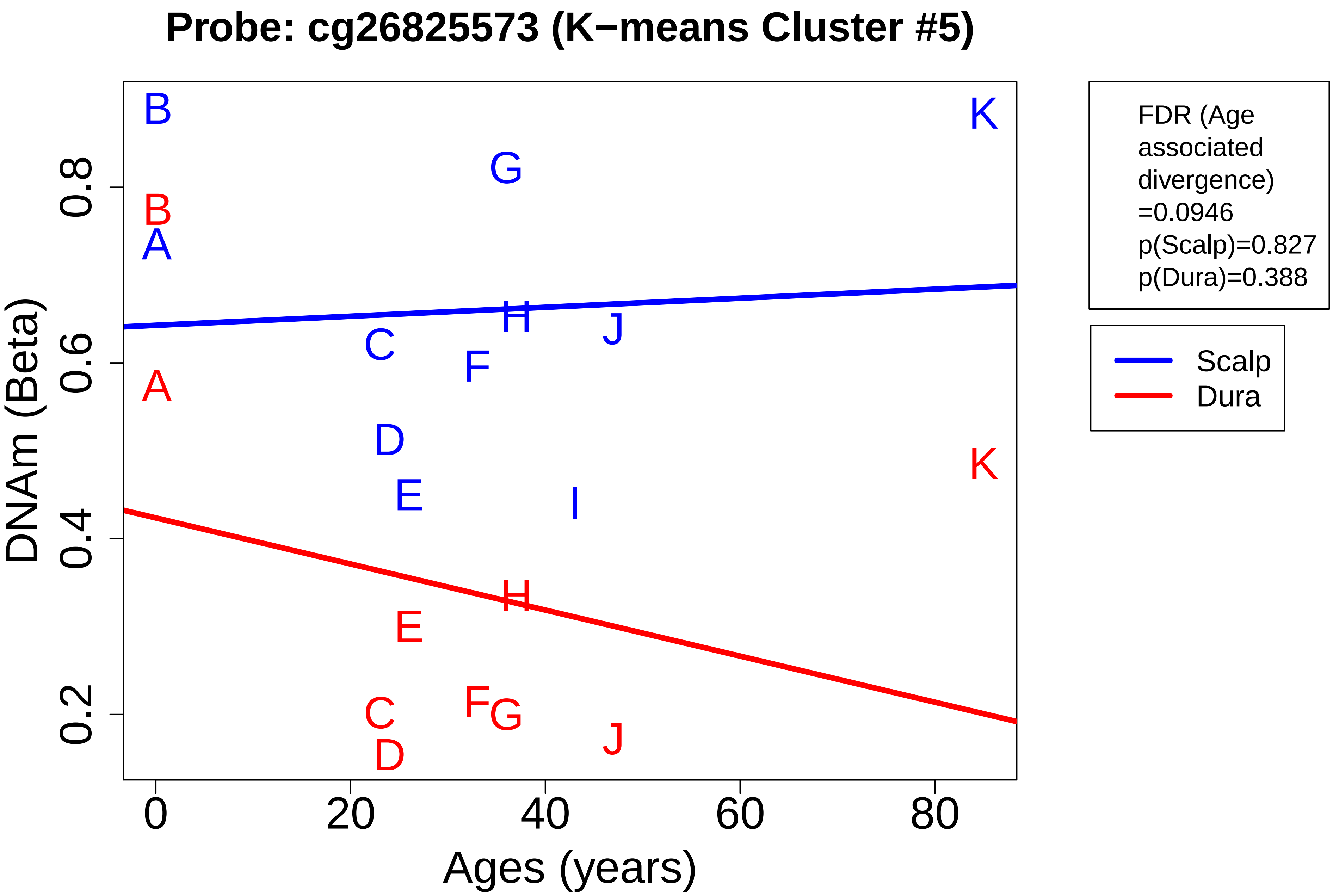

Probe: cg26825573 (K−means Cluster #5)
DNAm (Beta)
0.2
0.4
0.6
0.8
Ages (years)
0
20
40
60
80
A
B
C
D
E
F
G
H
I
J
K
FDR (Age associated divergence) =0.0946
p(Scalp)=0.827
p(Dura)=0.388
Scalp
Dura

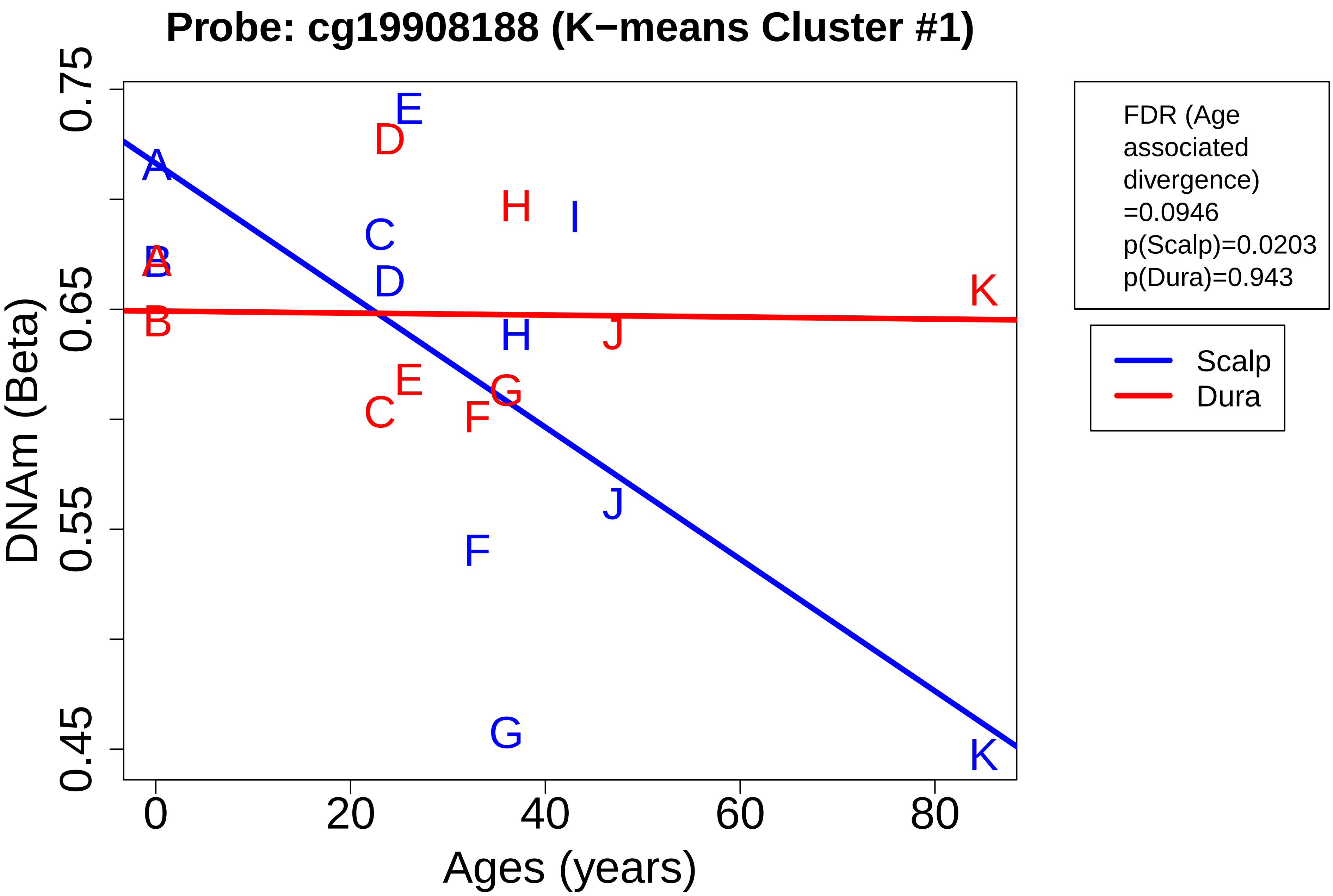

Probe: cg19908188 (K−means Cluster #1)
DNAm (Beta)
0.45
0.55
0.65
0.75
0
20
40
60
80
Ages (years)
FDR (Age associated divergence) =0.0946
p(Scalp)=0.0203
p(Dura)=0.943
Scalp
Dura
A
B
C
D
E
F
G
H
I
J
K

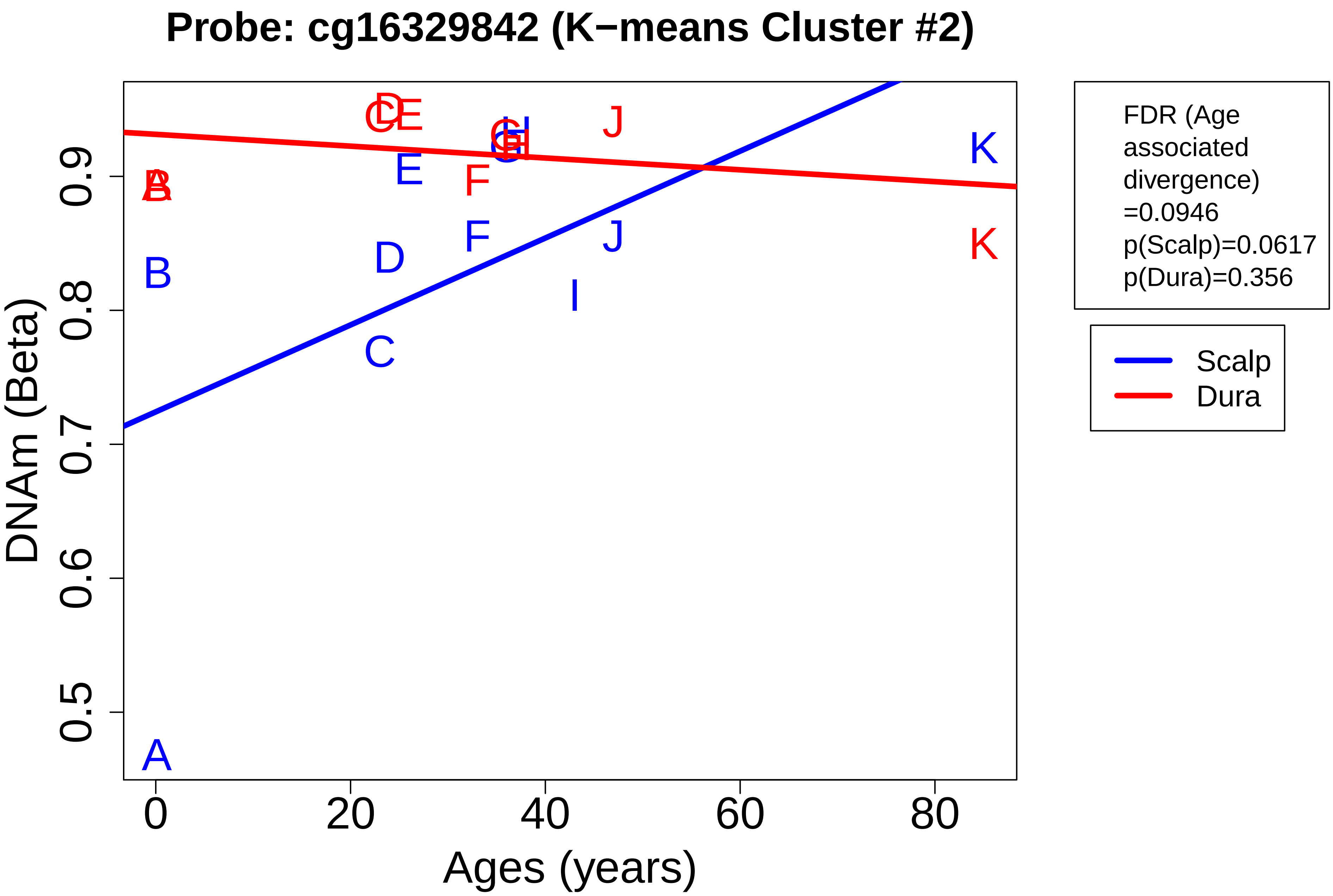

Probe: cg16329842 (K−means Cluster #2)
DNAm (Beta)
0.5
0.6
0.7
0.8
0.9
0
20
40
60
80
Ages (years)
FDR (Age associated divergence) =0.0946
p(Scalp)=0.0617
p(Dura)=0.356
Scalp
Dura

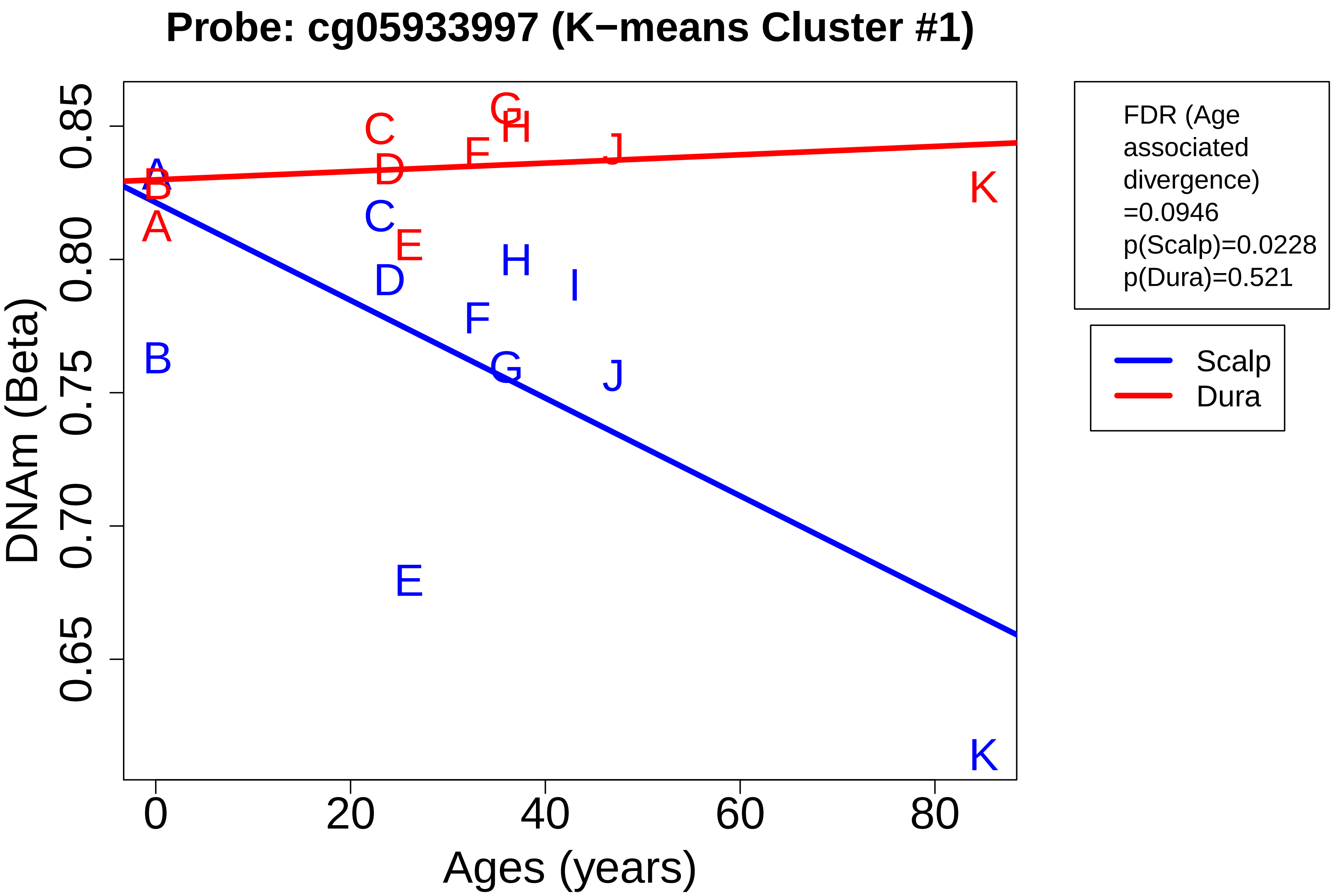

Probe: cg05933997 (K−means Cluster #1)
DNAm (Beta)
0.65
0.70
0.75
0.80
0.85
0
20
40
60
80
Ages (years)
A
B
C
D
E
F
G
H
I
J
K
FDR (Age associated divergence) =0.0946
p(Scalp)=0.0228
p(Dura)=0.521
Scalp
Dura

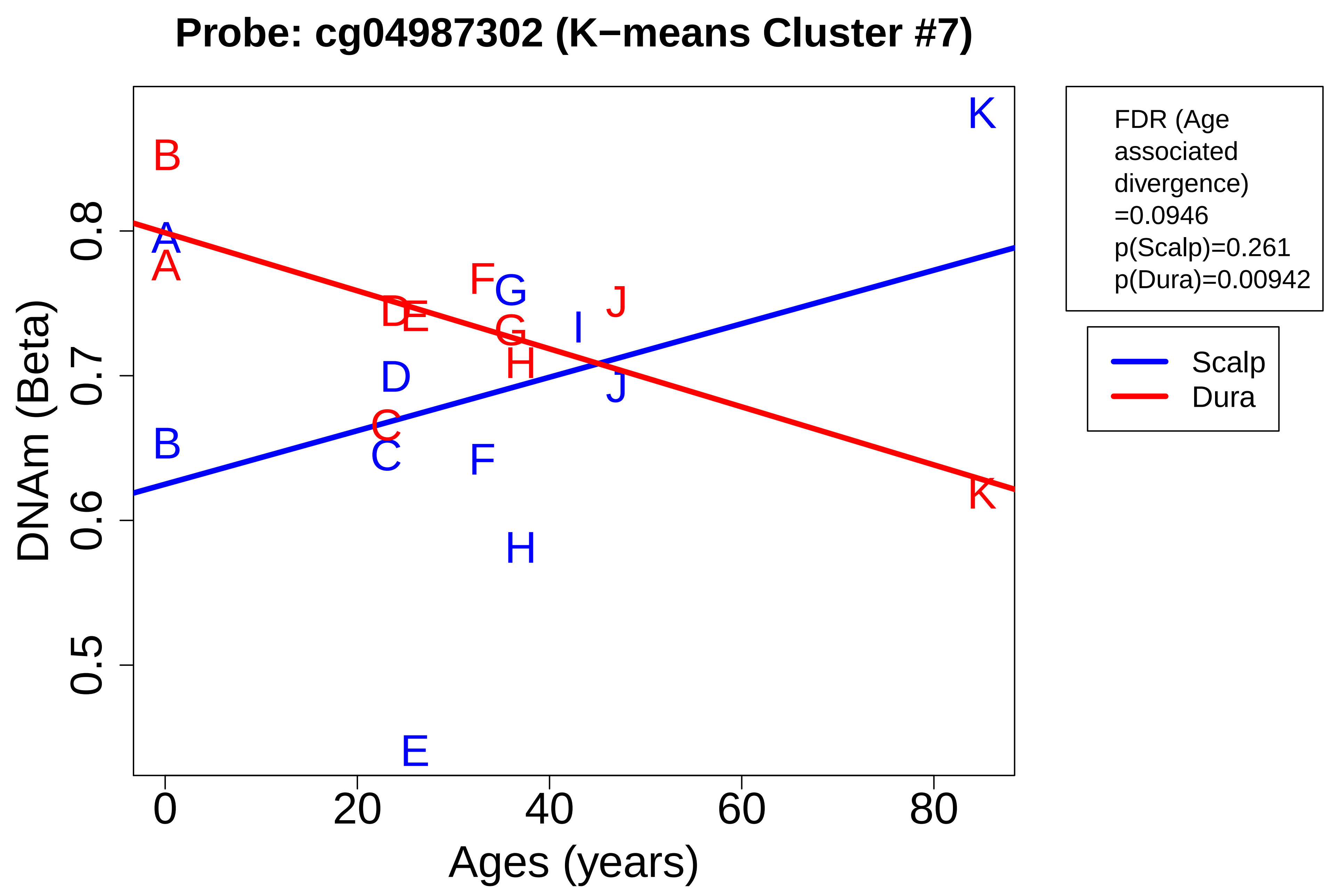

Probe: cg04987302 (K−means Cluster #7)
DNAm (Beta)
0.5
0.6
0.7
0.8
Ages (years)
0
20
40
60
80
A
B
C
D
E
F
G
H
I
J
K
FDR (Age associated divergence) =0.0946
p(Scalp)=0.261
p(Dura)=0.00942
Scalp
Dura

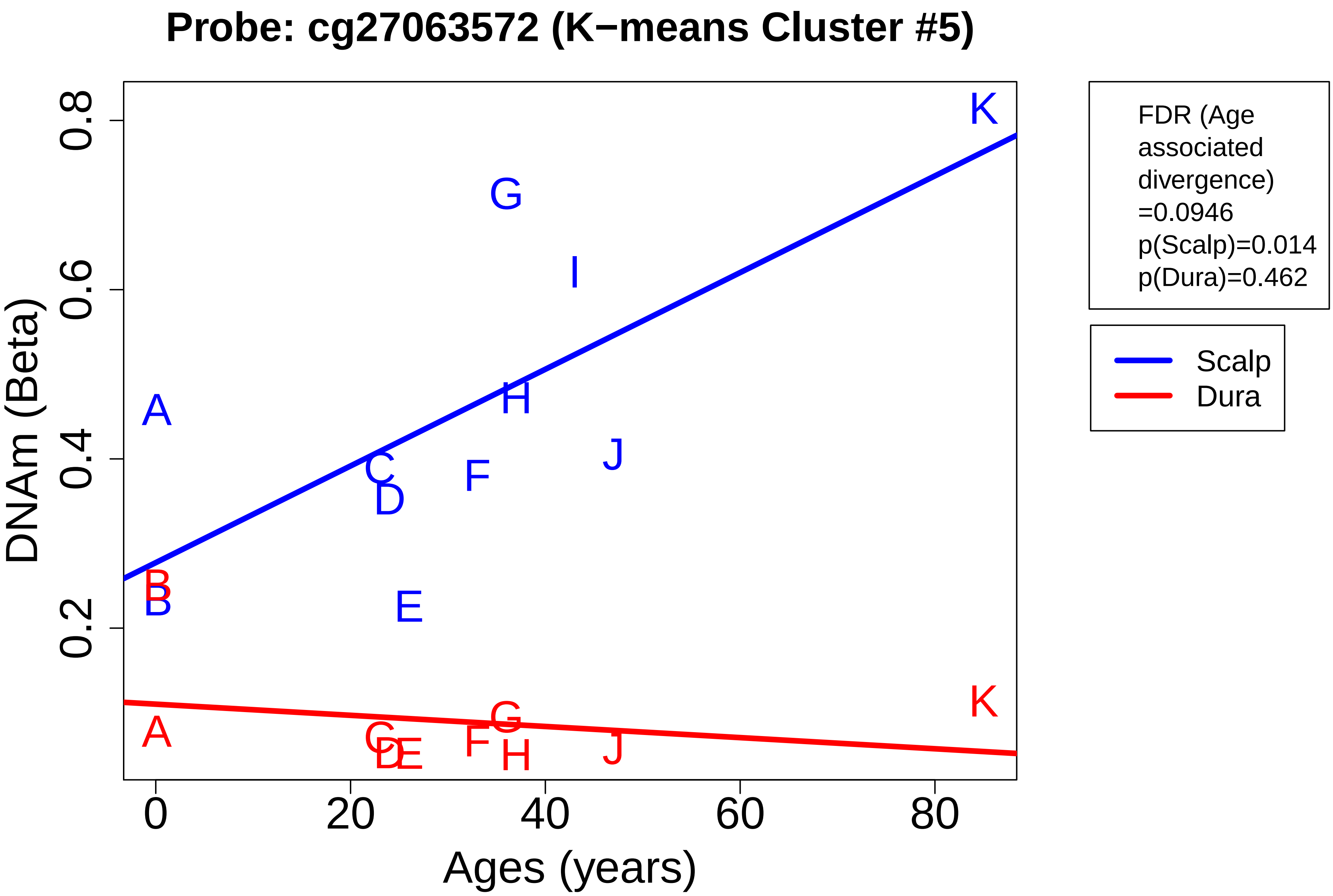

Probe: cg27063572 (K−means Cluster #5)
DNAm (Beta)
0.2
0.4
0.6
0.8
0
20
40
60
80
Ages (years)
FDR (Age associated divergence) =0.0946
p(Scalp)=0.014
p(Dura)=0.462
Scalp
Dura
A
B
C
D
E
F
G
H
I
J
K

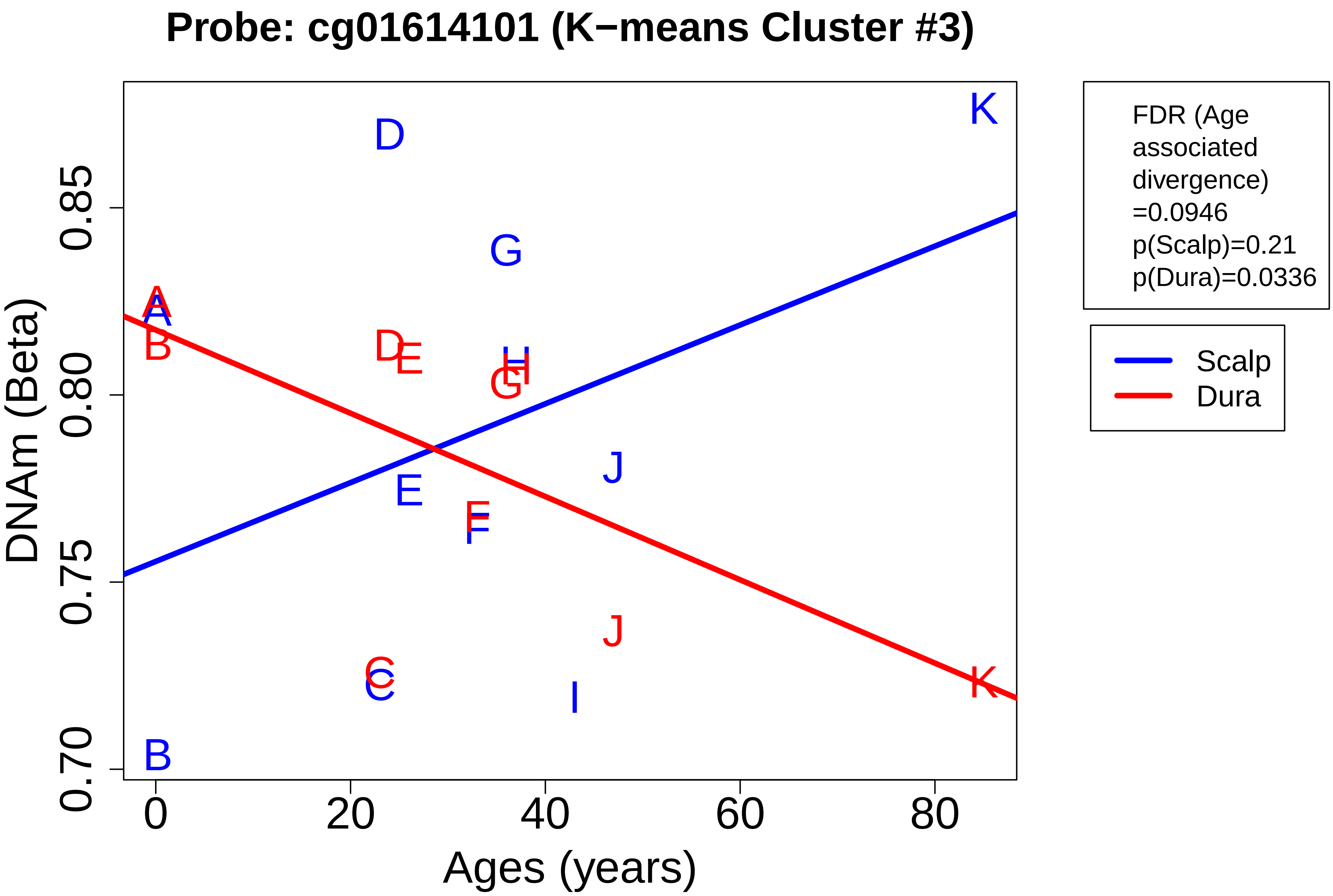

Probe: cg01614101 (K−means Cluster #3)
DNAm (Beta)
0.70
0.75
0.80
0.85
Ages (years)
0
20
40
60
80
FDR (Age associated divergence) =0.0946
p(Scalp)=0.21
p(Dura)=0.0336
Scalp
Dura

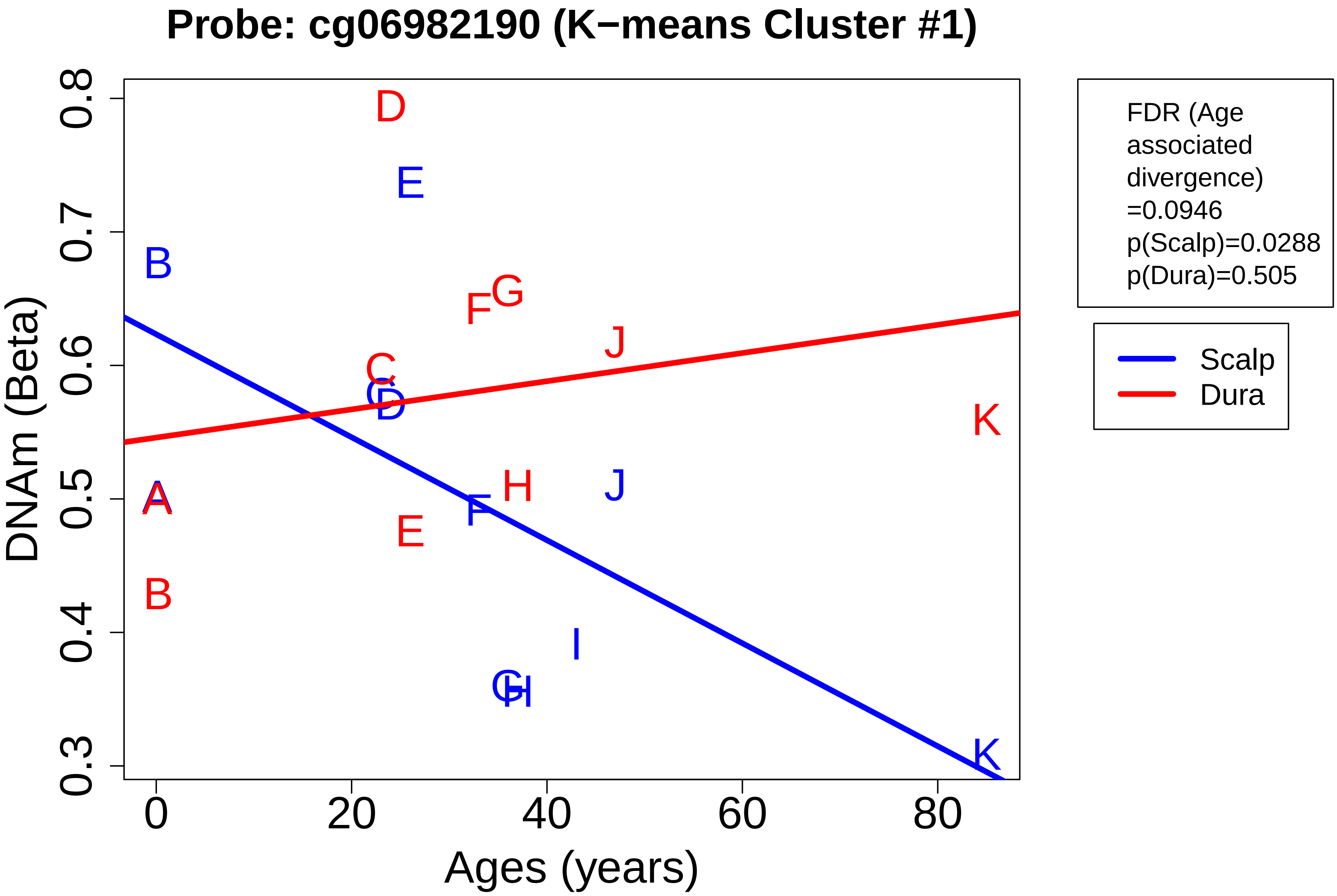

Probe: cg06982190 (K−means Cluster #1)
DNAm (Beta)
0.3
0.4
0.5
0.6
0.7
0.8
0
20
40
60
80
Ages (years)
FDR (Age associated divergence) =0.0946
p(Scalp)=0.0288
p(Dura)=0.505
Scalp
Dura

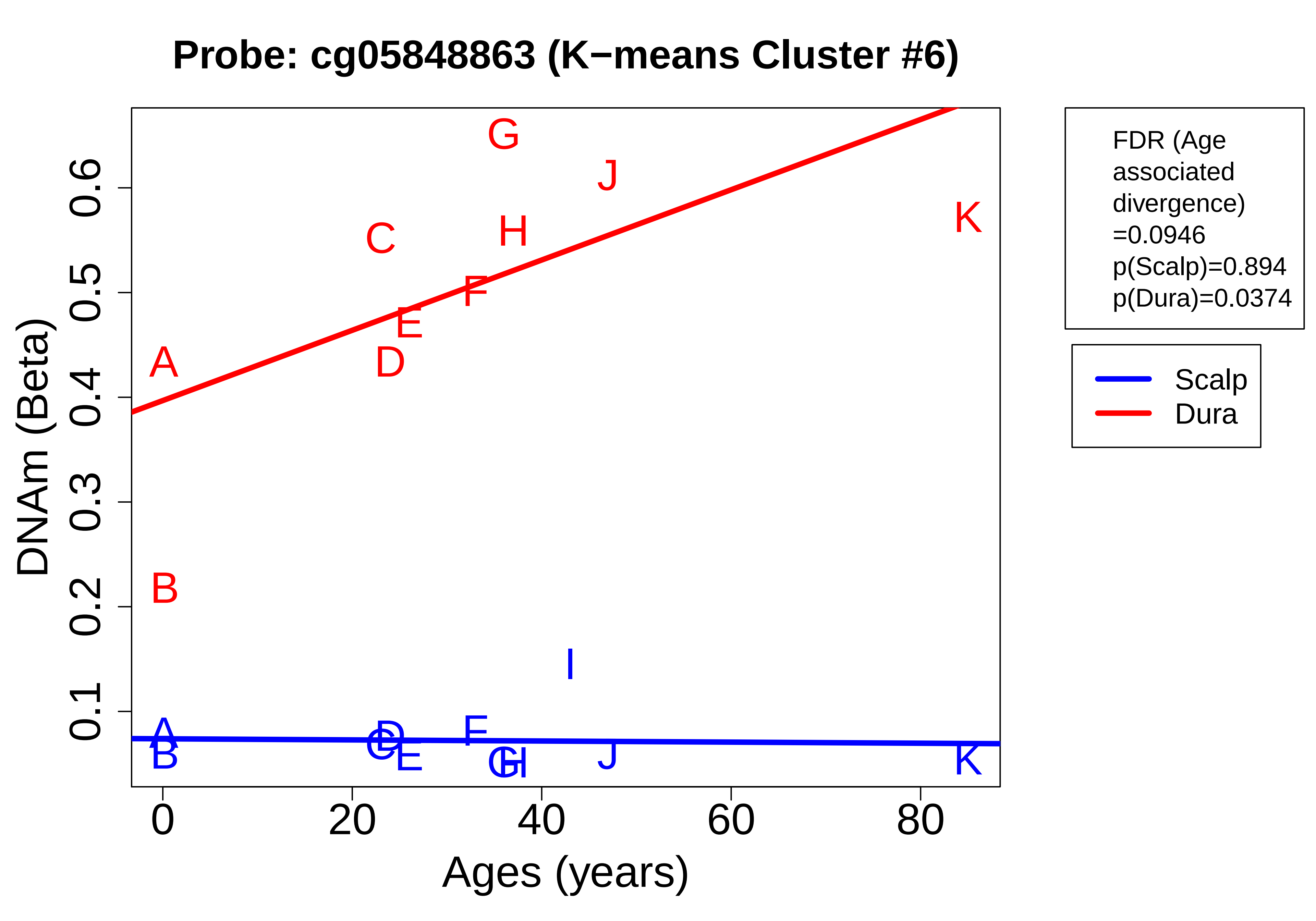

Probe: cg05848863 (K−means Cluster #6)
DNAm (Beta)
0.1
0.2
0.3
0.4
0.5
0.6
Ages (years)
0
20
40
60
80
FDR (Age associated divergence) =0.0946
p(Scalp)=0.894
p(Dura)=0.0374
Scalp
Dura
A
B
C
D
E
F
G
H
I
J
K

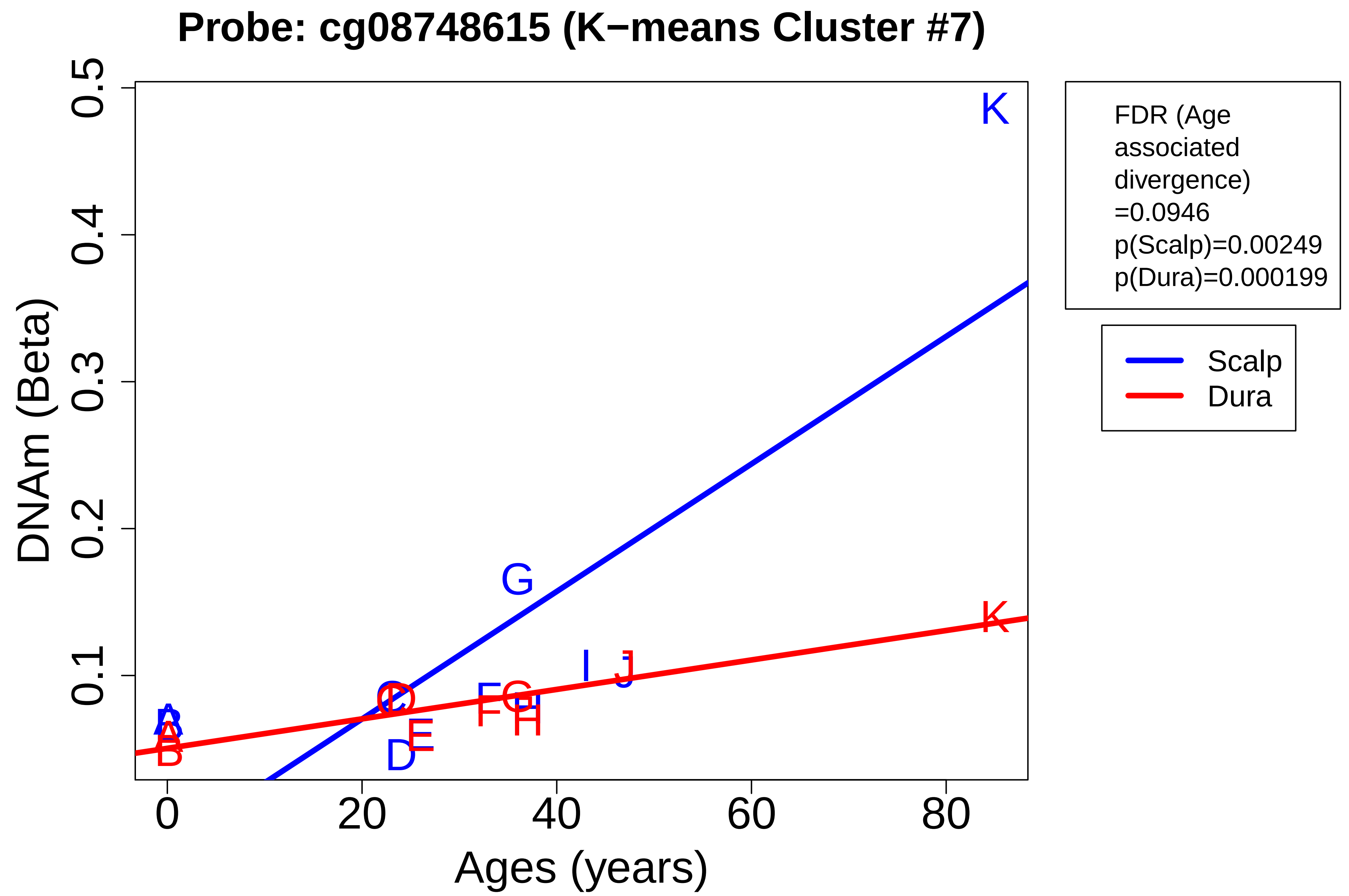
Probe: cg08748615 (K−means Cluster #7)
DNAm (Beta)
0.1
0.2
0.3
0.4
0.5
0
20
40
60
80
Ages (years)
FDR (Age associated divergence) =0.0946
p(Scalp)=0.00249
p(Dura)=0.000199
Scalp
Dura

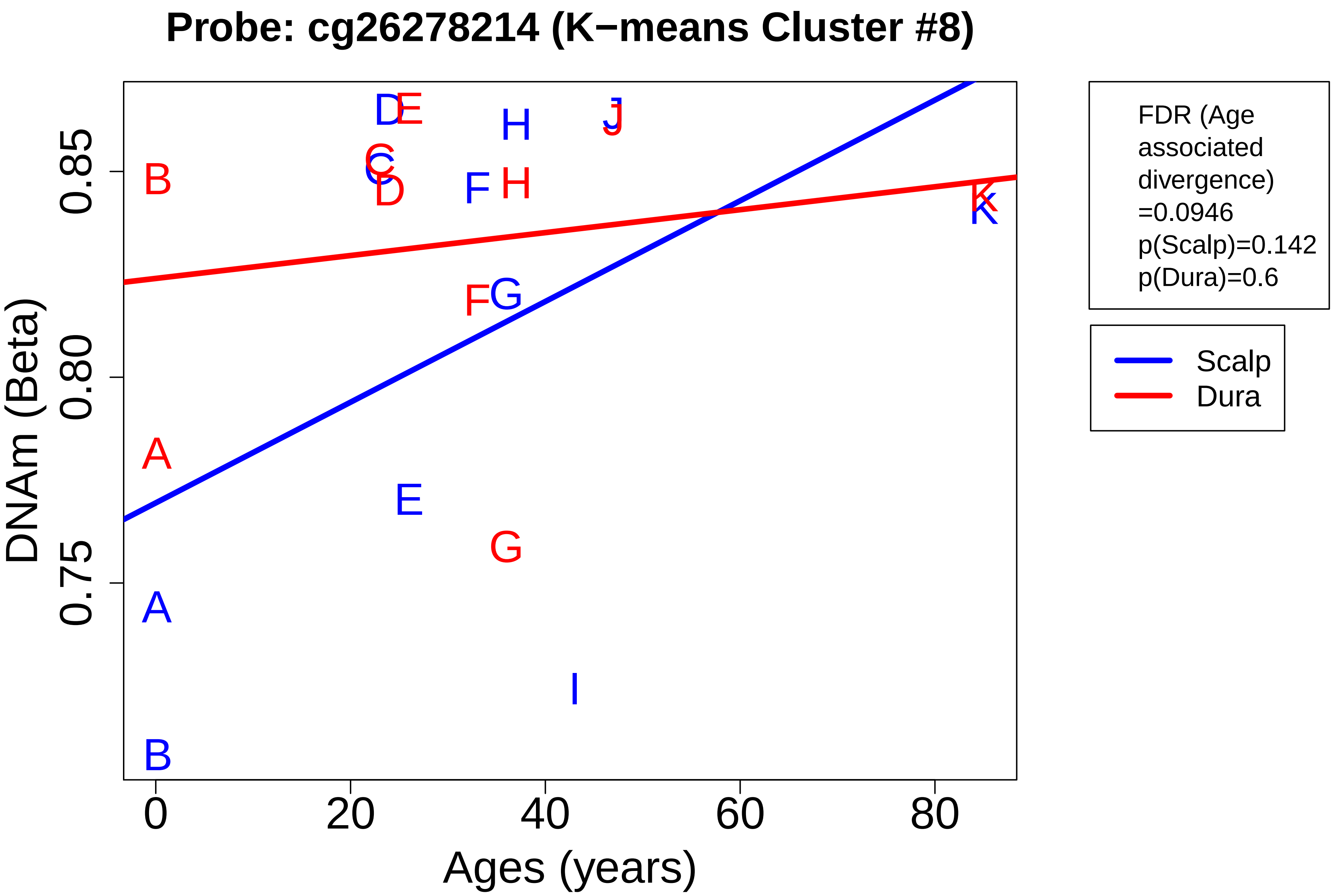
Probe: cg26278214 (K−means Cluster #8)
DNAm (Beta)
0.75
0.80
0.85
Ages (years)
0
20
40
60
80
FDR (Age associated divergence) =0.0946
p(Scalp)=0.142
p(Dura)=0.6
Scalp
Dura

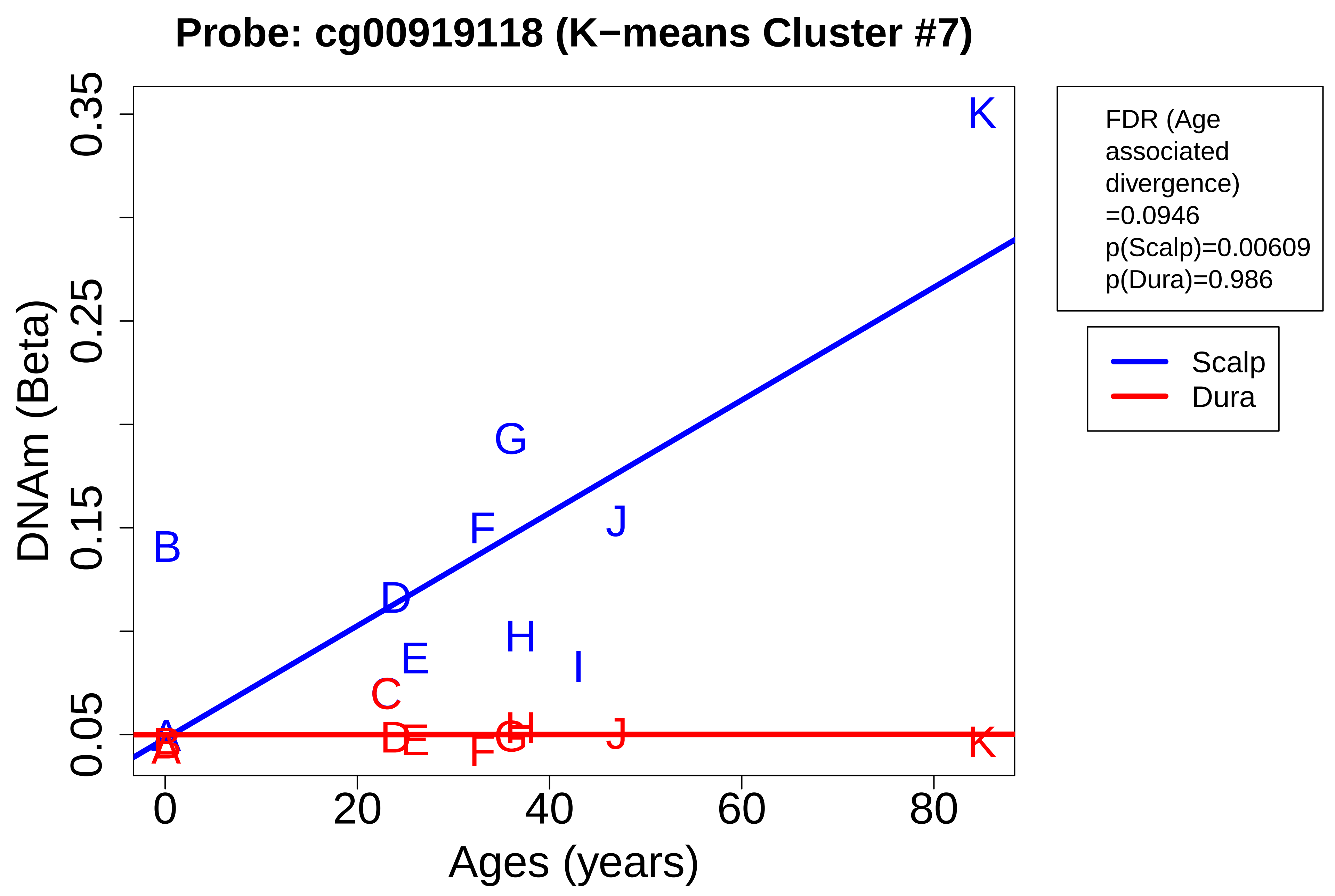

Probe: cg00919118 (K−means Cluster #7)
DNAm (Beta)
0.05
0.15
0.25
0.35
0
20
40
60
80
Ages (years)
FDR (Age associated divergence) =0.0946
p(Scalp)=0.00609
p(Dura)=0.986
Scalp
Dura
A
B
C
D
E
F
G
H
I
J
K

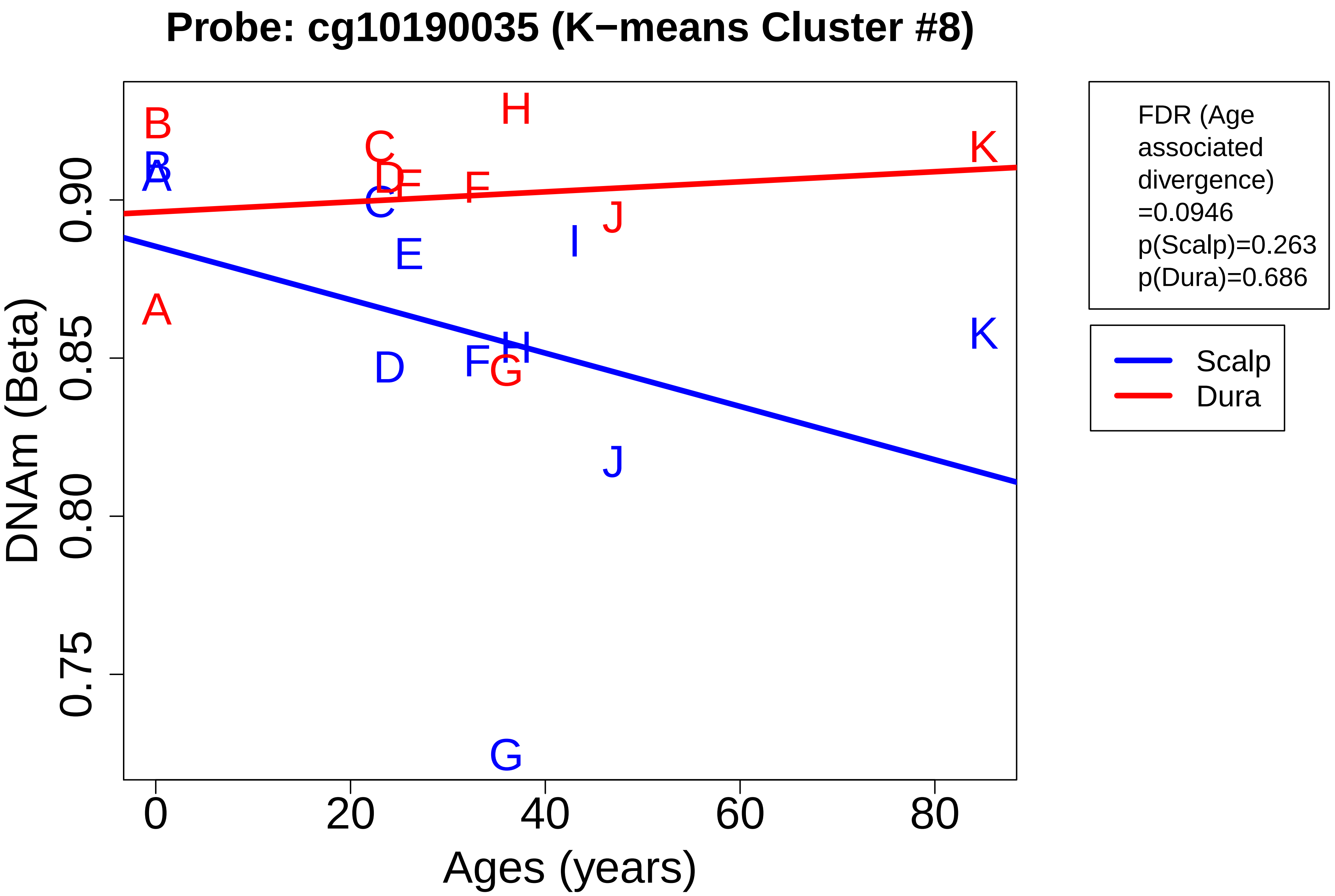

Probe: cg10190035 (K−means Cluster #8)
FDR (Age associated divergence) =0.0946
p(Scalp)=0.263
p(Dura)=0.686
Scalp
Dura
DNAm (Beta)
0.75
0.80
0.85
0.90
0
20
40
60
80
Ages (years)

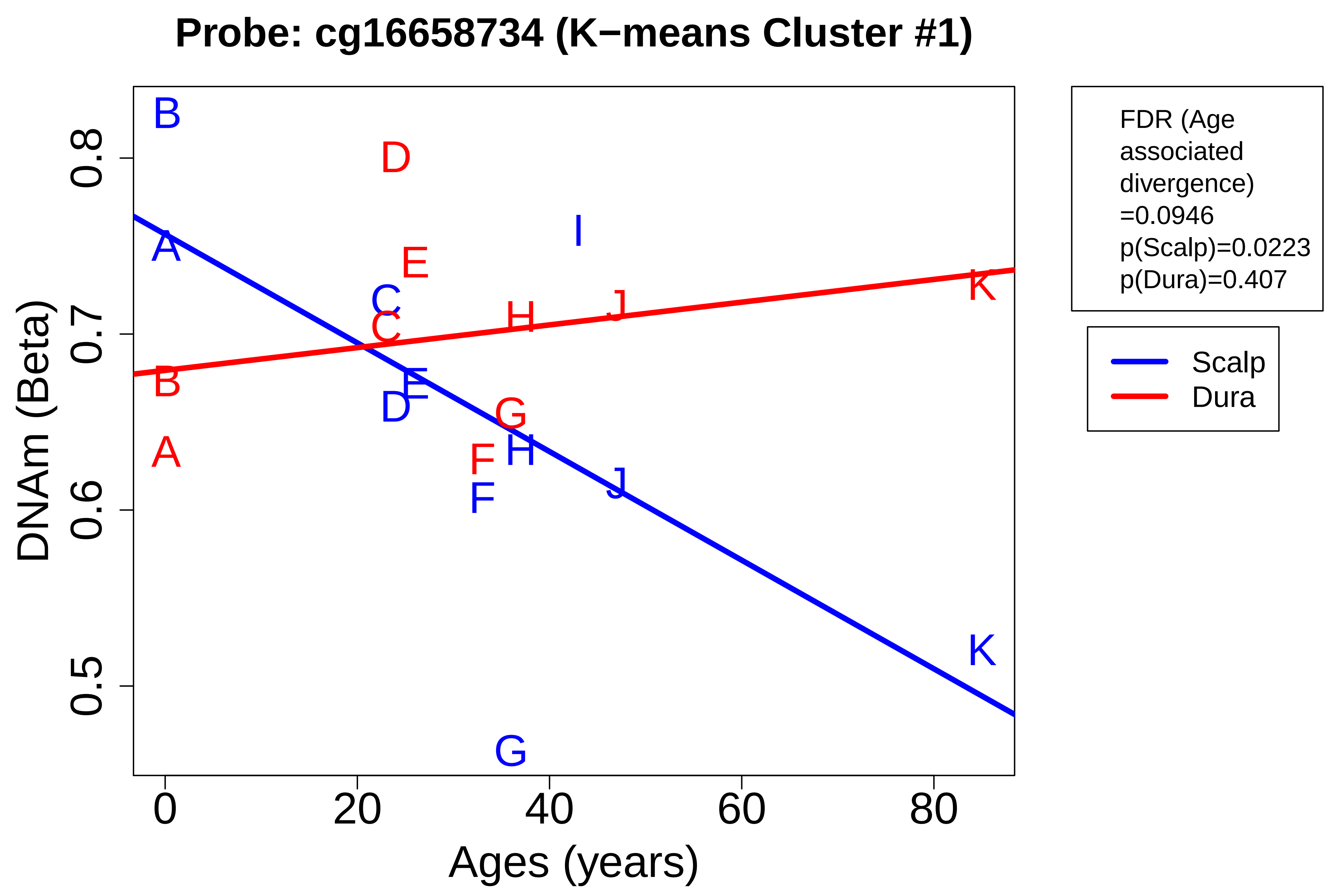
Probe: cg16658734 (K−means Cluster #1)
DNAm (Beta)
0.5
0.6
0.7
0.8
Ages (years)
0
20
40
60
80
B
D
I
A
E
C
C
K
H
J
B
E
D
G
A
F
H
F
J
K
G
FDR (Age associated divergence) =0.0946
p(Scalp)=0.0223
p(Dura)=0.407
Scalp
Dura

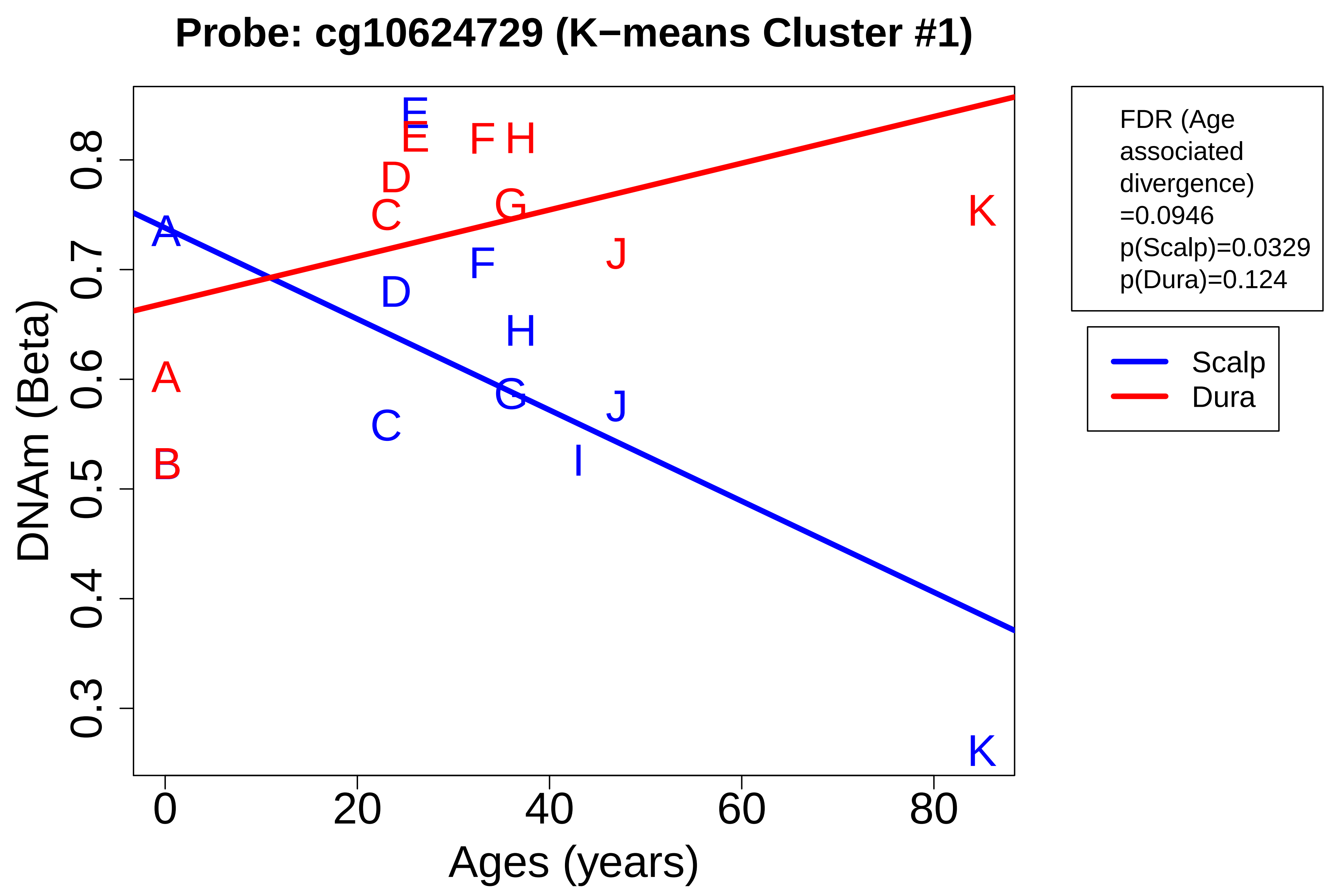
Probe: cg10624729 (K−means Cluster #1)
DNAm (Beta)
0.3
0.4
0.5
0.6
0.7
0.8
0
20
40
60
80
Ages (years)
FDR (Age associated divergence) =0.0946
p(Scalp)=0.0329
p(Dura)=0.124
Scalp
Dura
A
B
C
D
E
F
G
H
I
J
K

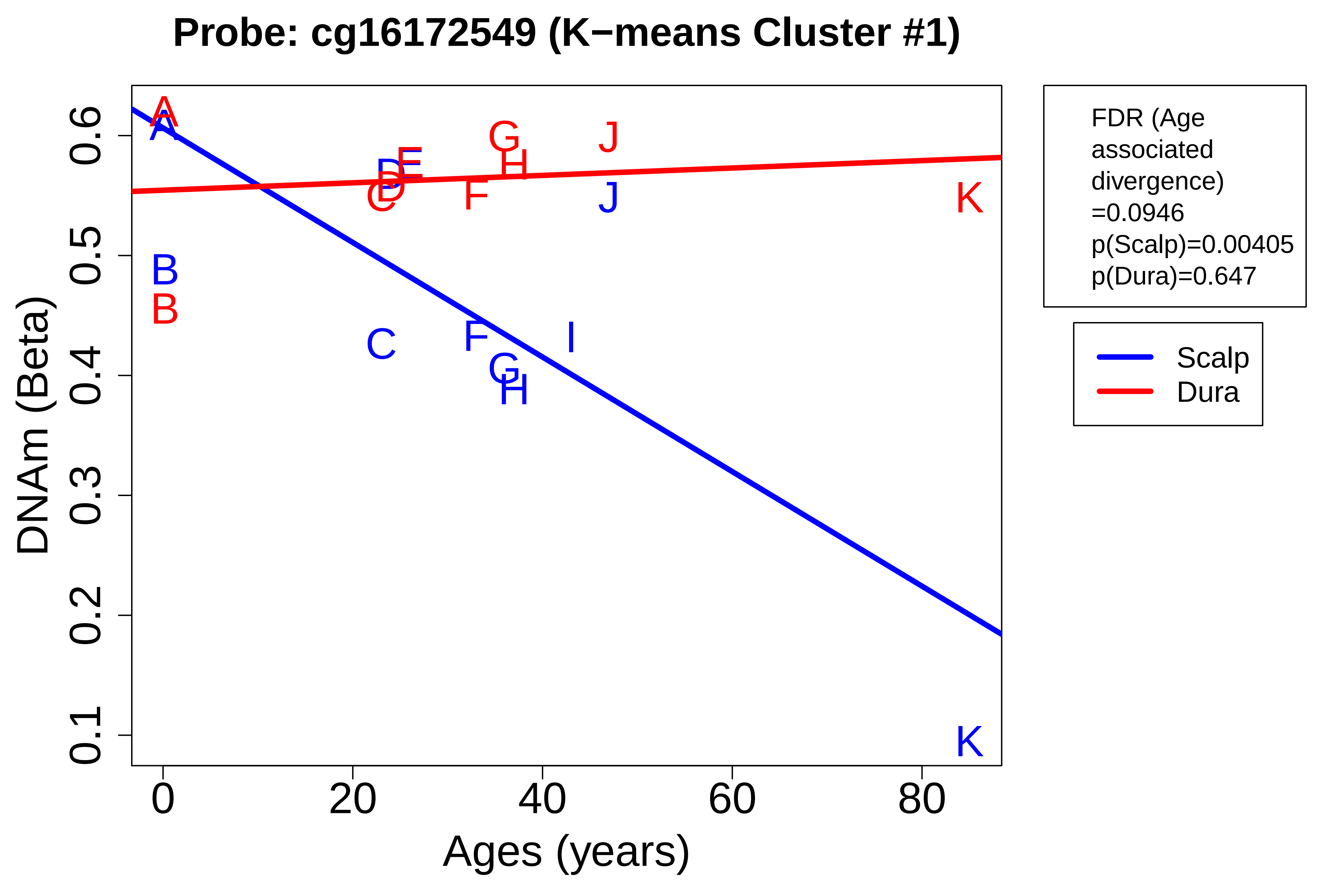

Probe: cg16172549 (K−means Cluster #1)
DNAm (Beta)
Ages (years)
0.1
0.2
0.3
0.4
0.5
0.6
0
20
40
60
80
A
B
C
D
E
F
G
H
I
J
K
FDR (Age associated divergence) =0.0946
p(Scalp)=0.00405
p(Dura)=0.647
Scalp
Dura

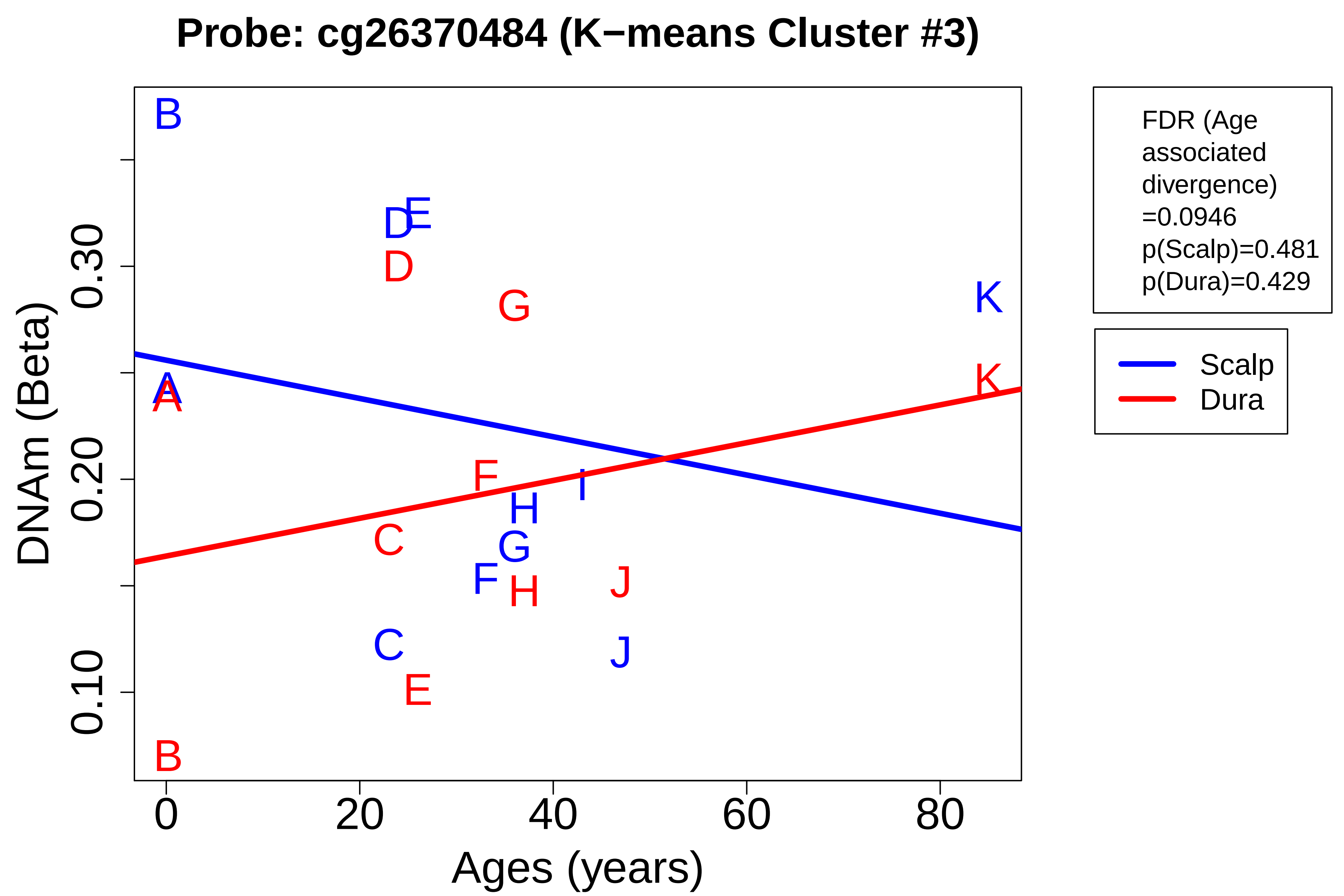
Probe: cg26370484 (K−means Cluster #3)
DNAm (Beta)
0.10
0.20
0.30
Ages (years)
0
20
40
60
80
FDR (Age associated divergence) =0.0946
p(Scalp)=0.481
p(Dura)=0.429
Scalp
Dura
A
B
C
D
E
F
G
H
I
J
K

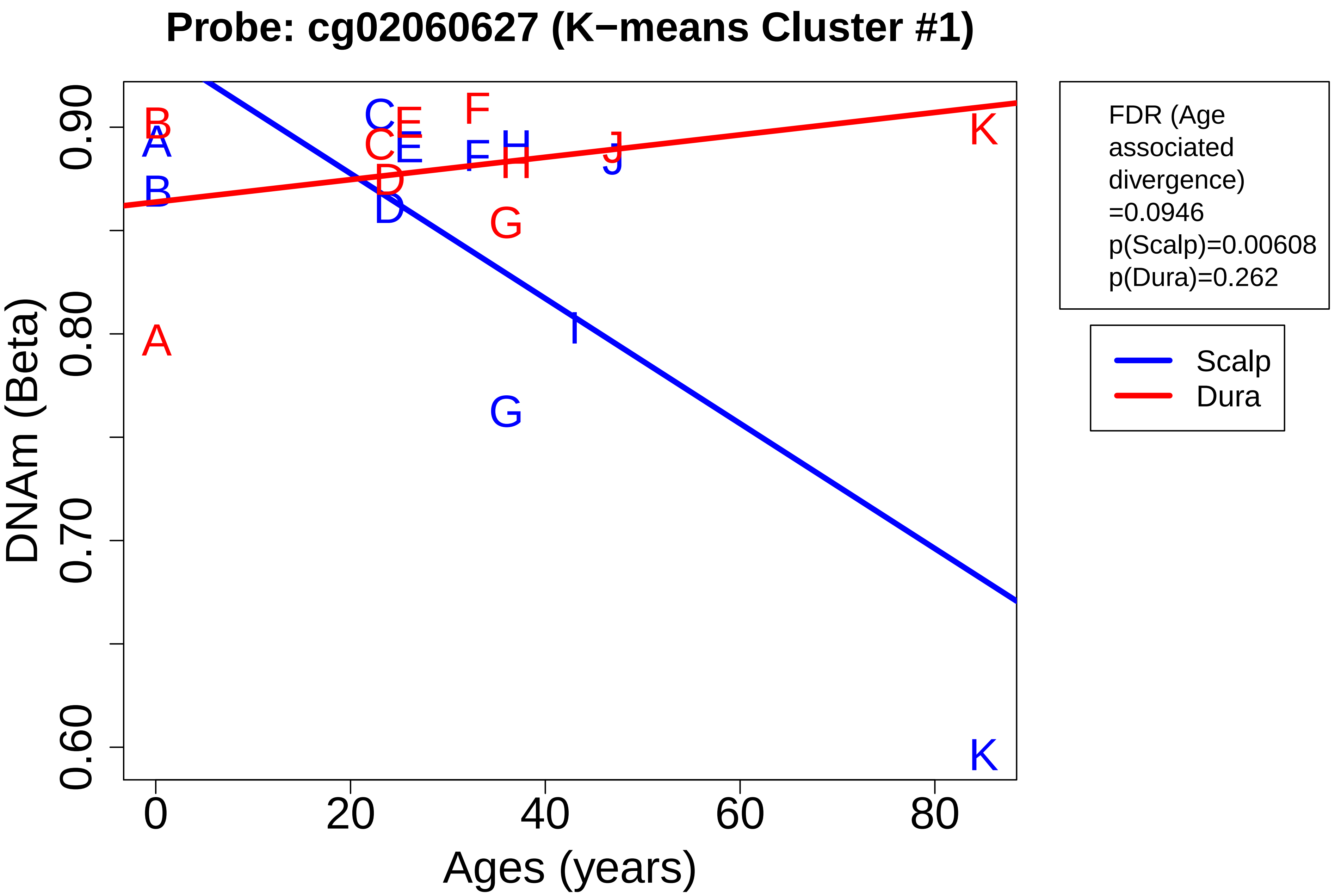

Probe: cg02060627 (K−means Cluster #1)
DNAm (Beta)
0.60
0.70
0.80
0.90
Ages (years)
0
20
40
60
80
FDR (Age associated divergence) =0.0946
p(Scalp)=0.00608
p(Dura)=0.262
Scalp
Dura

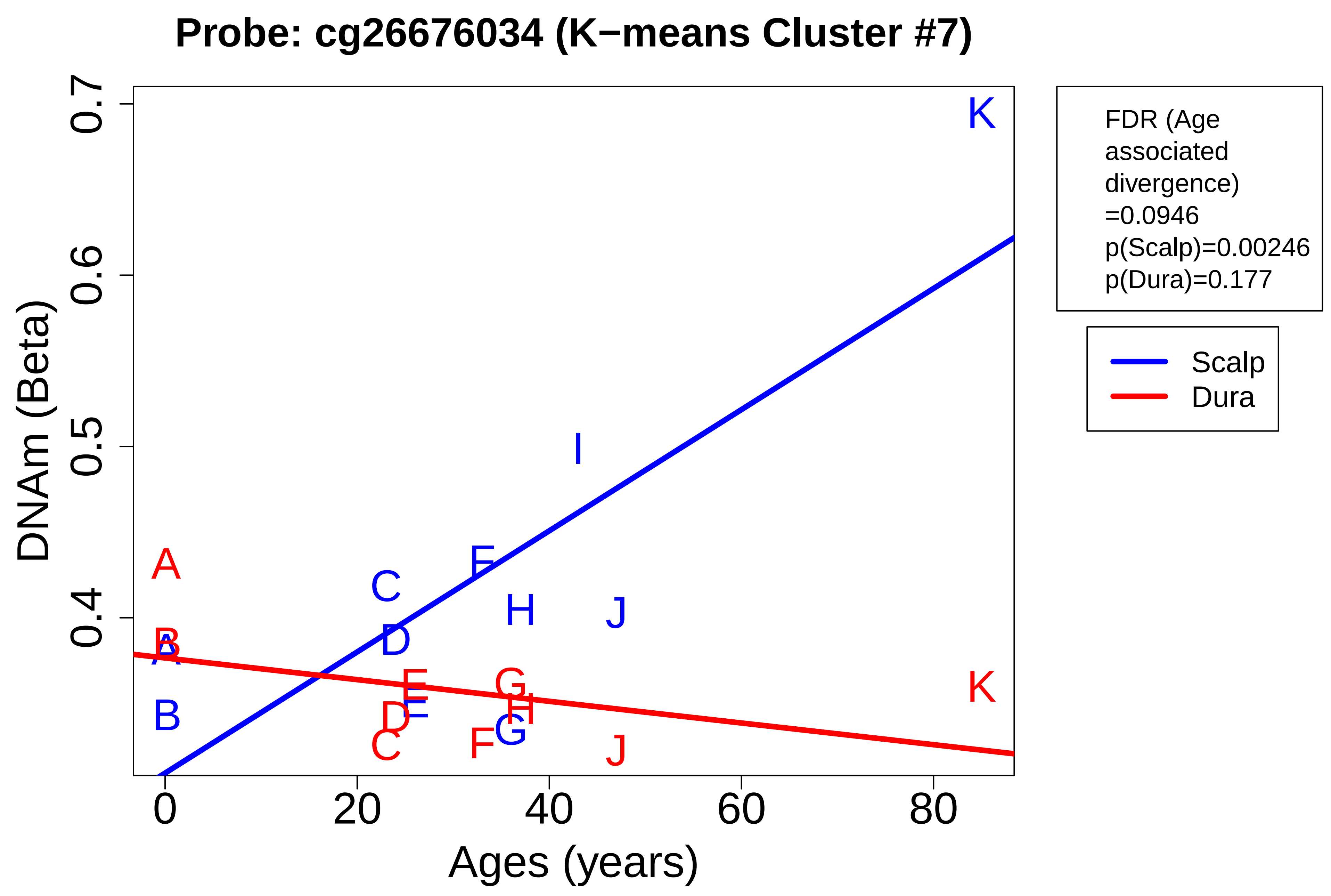

Probe: cg26676034 (K−means Cluster #7)
DNAm (Beta)
0.4
0.5
0.6
0.7
0
20
40
60
80
Ages (years)
FDR (Age associated divergence) =0.0946
p(Scalp)=0.00246
p(Dura)=0.177
Scalp
Dura

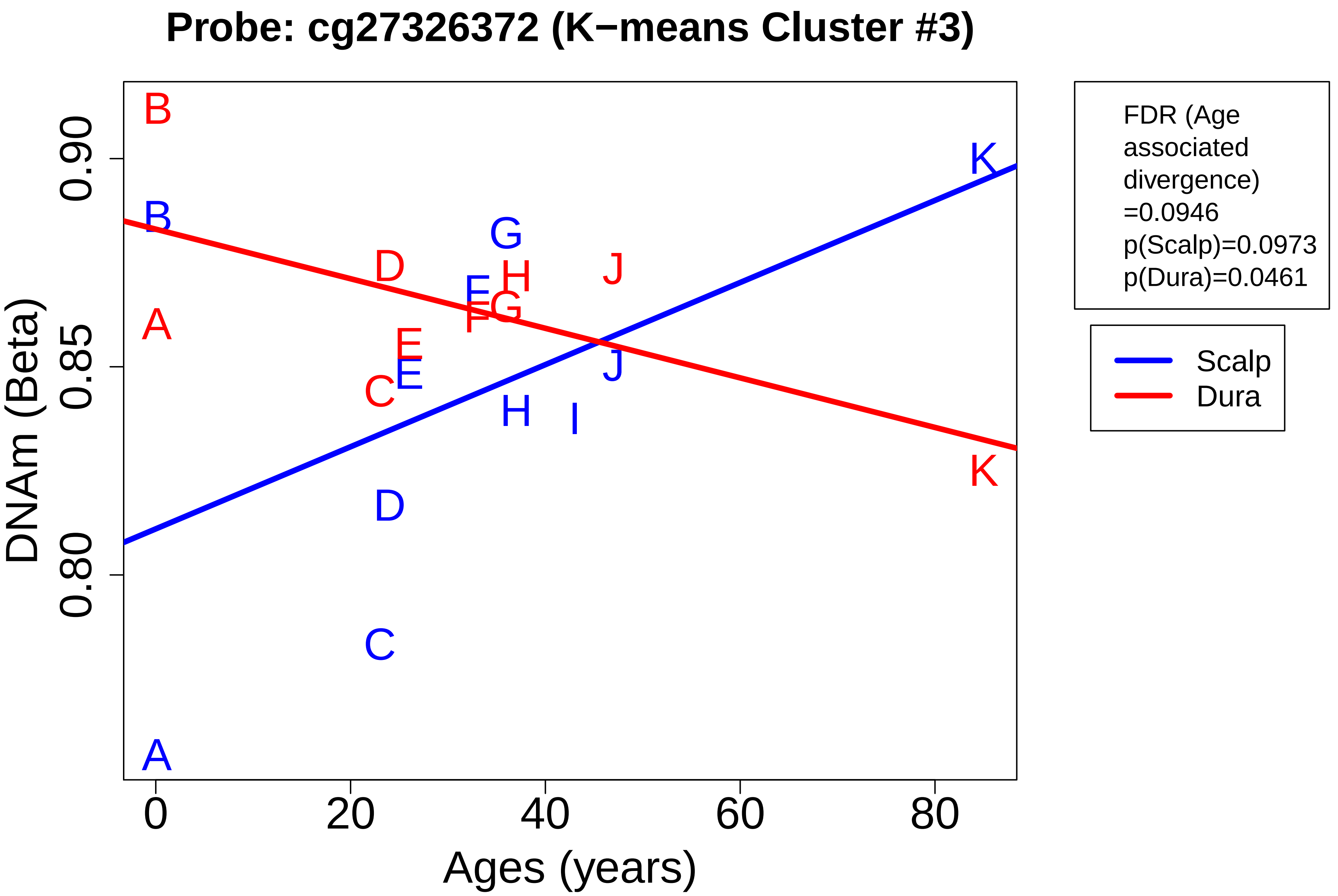
Probe: cg27326372 (K−means Cluster #3)
DNAm (Beta)
0.80
0.85
0.90
Ages (years)
0
20
40
60
80
A
B
C
D
E
F
G
H
I
J
K
FDR (Age associated divergence) =0.0946
p(Scalp)=0.0973
p(Dura)=0.0461
Scalp
Dura

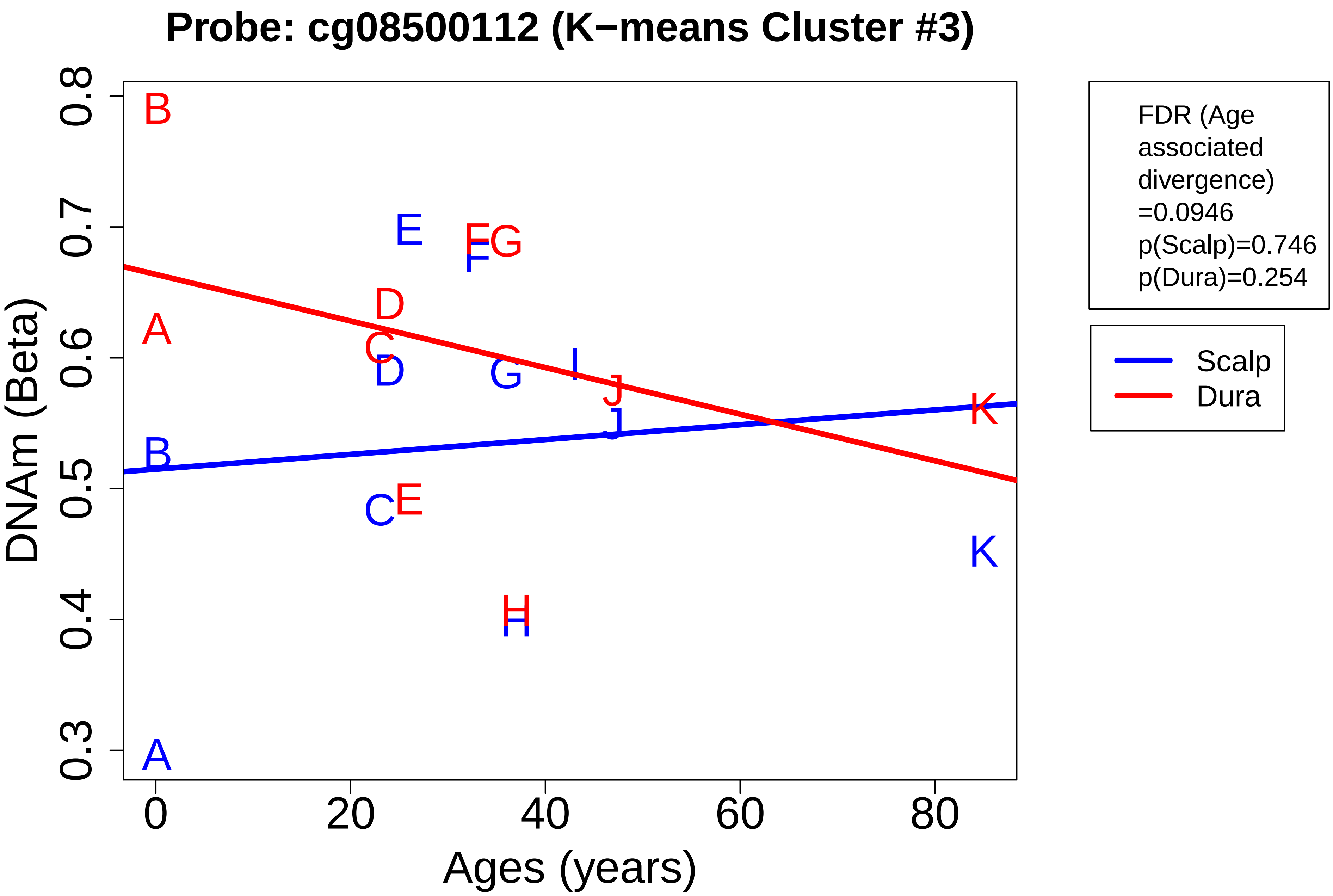

Probe: cg08500112 (K−means Cluster #3)
DNAm (Beta)
0.3
0.4
0.5
0.6
0.7
0.8
0
20
40
60
80
Ages (years)
FDR (Age associated divergence) =0.0946
p(Scalp)=0.746
p(Dura)=0.254
Scalp
Dura

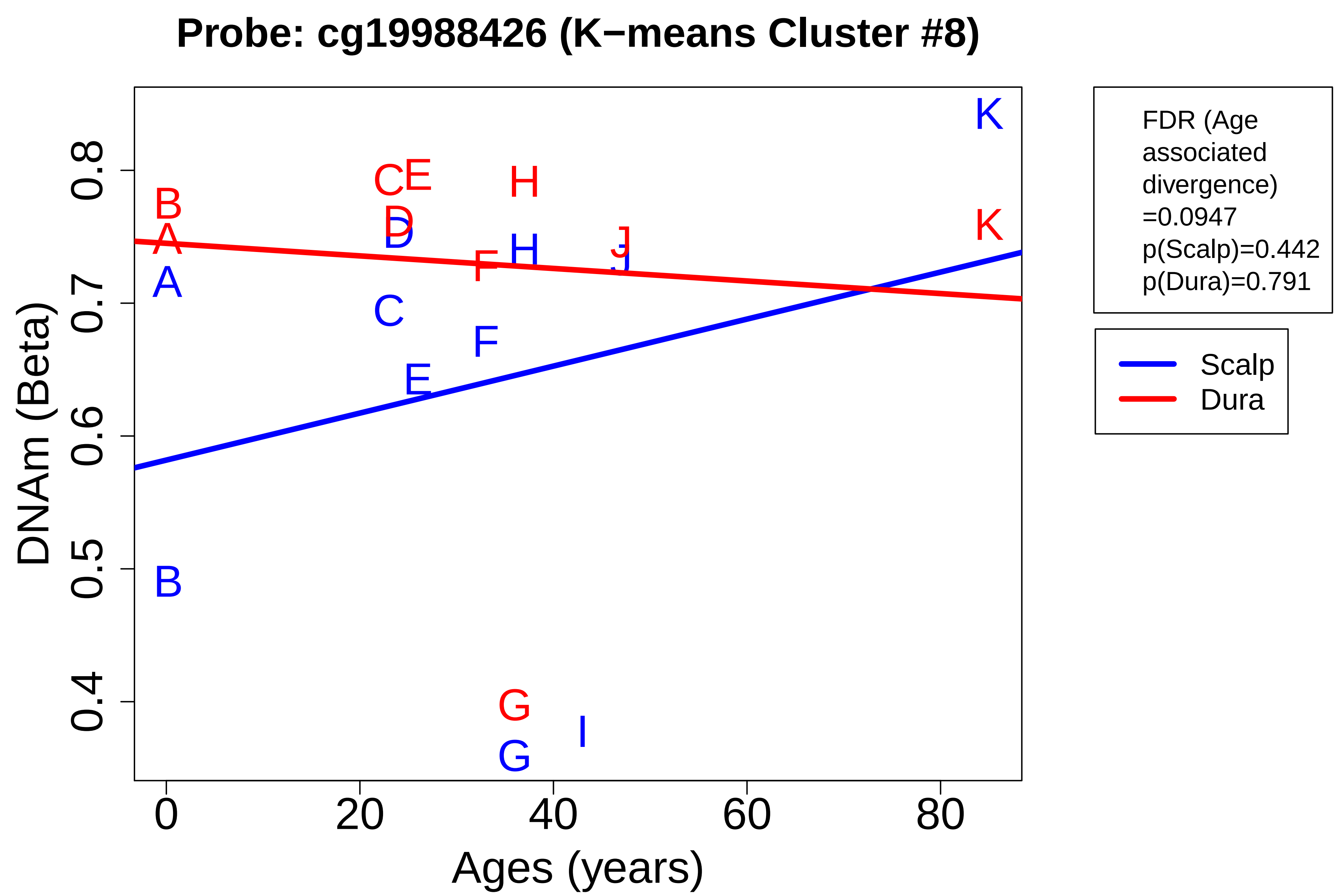

Probe: cg19988426 (K−means Cluster #8)
DNAm (Beta)
0.4
0.5
0.6
0.7
0.8
0
20
40
60
80
Ages (years)
A
B
C
D
E
F
G
H
I
J
K
A
B
CE
D
F
G
H
J
K
FDR (Age associated divergence) =0.0947
p(Scalp)=0.442
p(Dura)=0.791
Scalp
Dura

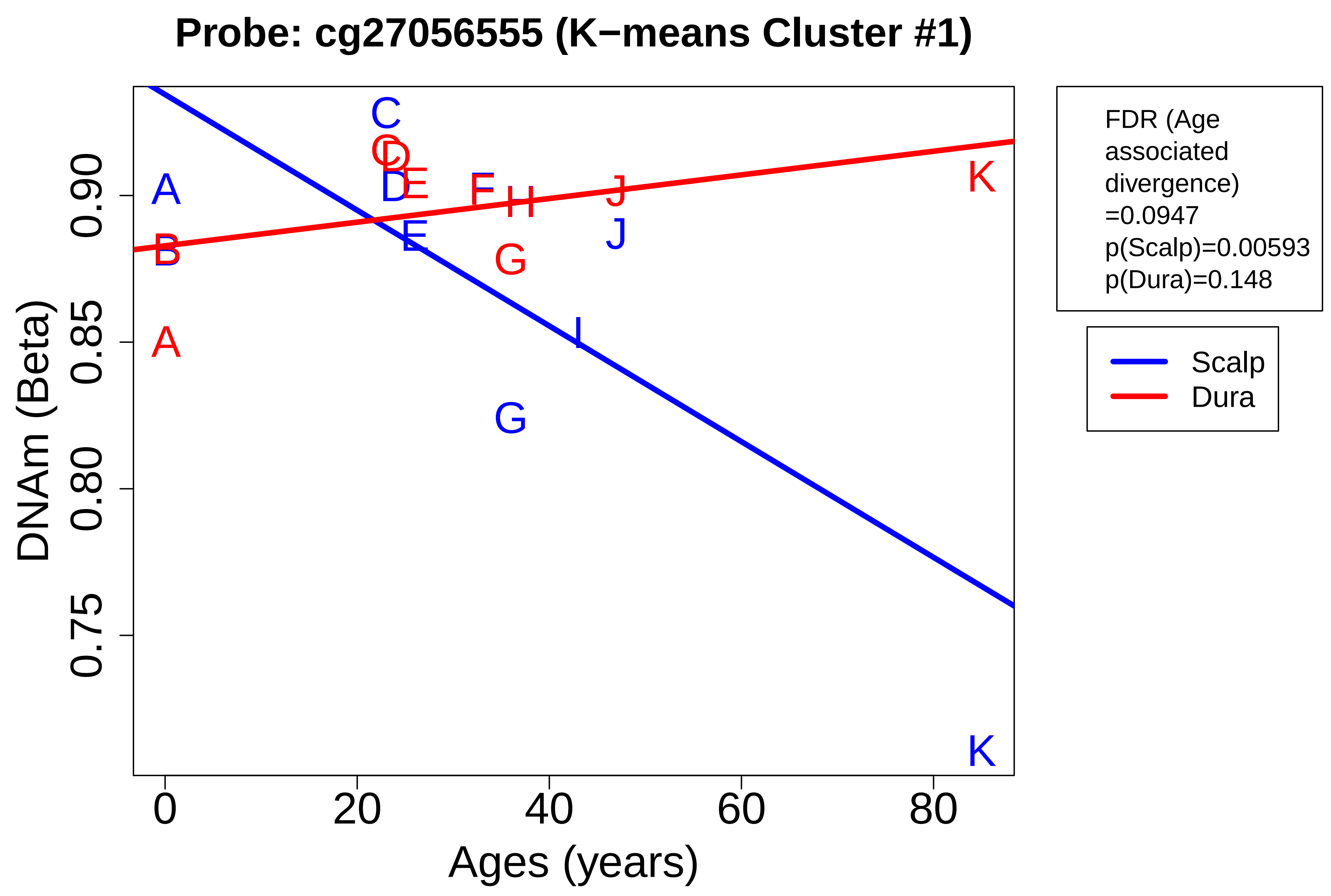
Probe: cg27056555 (K−means Cluster #1)
DNAm (Beta)
0.75
0.80
0.85
0.90
Ages (years)
0
20
40
60
80
A
B
C
D
E
F
G
H
I
J
K
FDR (Age associated divergence) =0.0947
p(Scalp)=0.00593
p(Dura)=0.148
Scalp
Dura

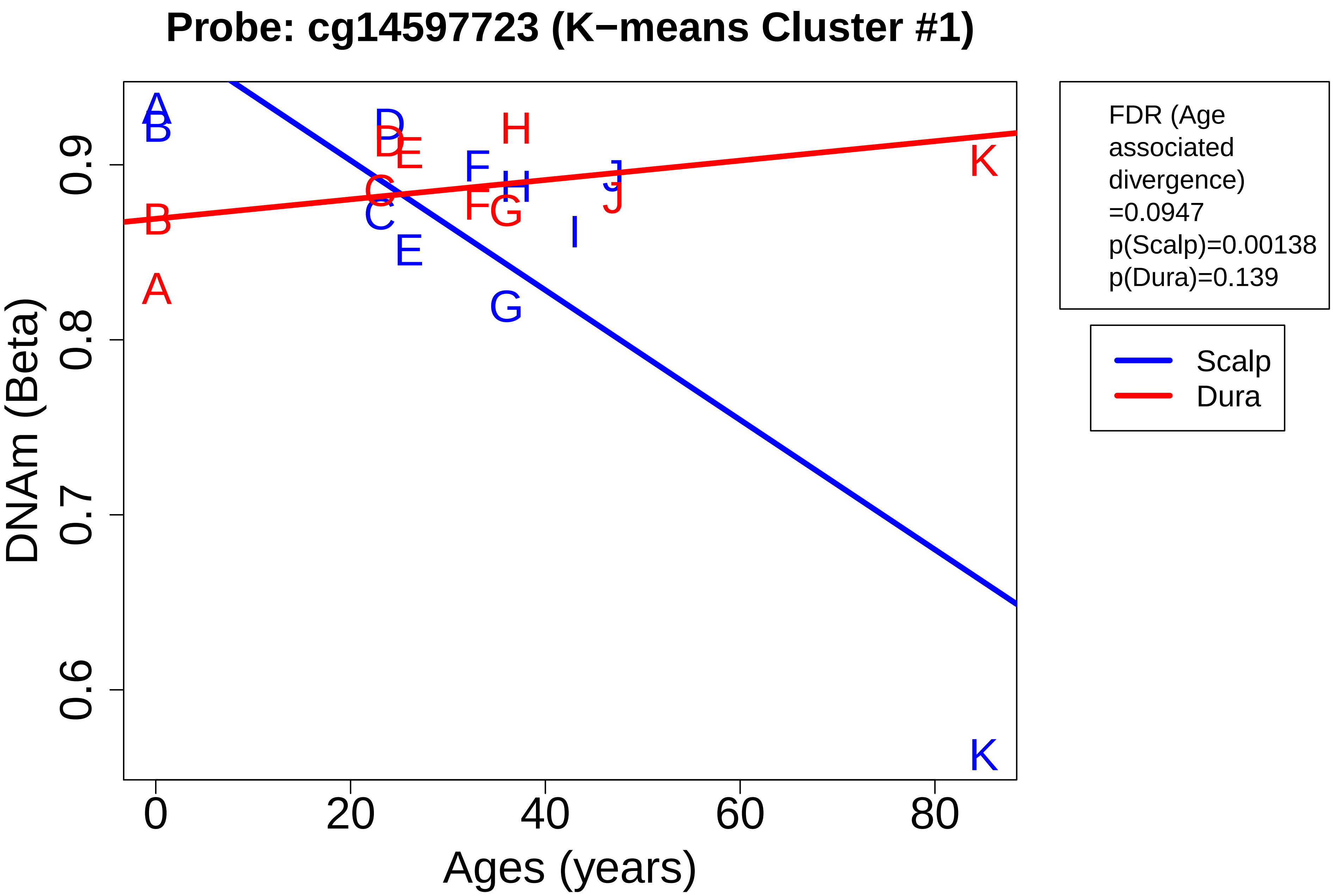

Probe: cg14597723 (K−means Cluster #1)
DNAm (Beta)
0.6
0.7
0.8
0.9
Ages (years)
0
20
40
60
80
FDR (Age associated divergence) =0.0947
p(Scalp)=0.00138
p(Dura)=0.139
Scalp
Dura

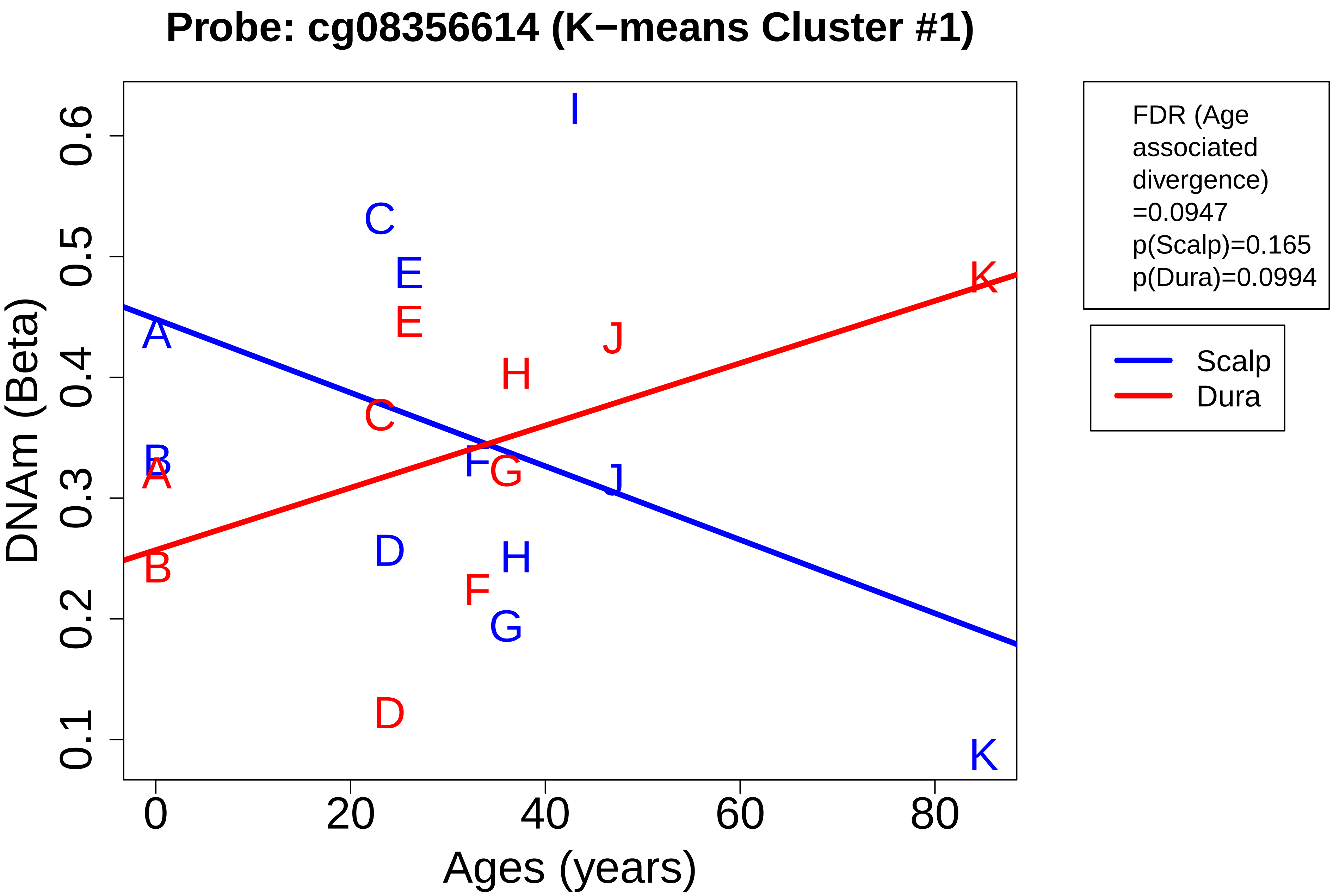
Probe: cg08356614 (K−means Cluster #1)
DNAm (Beta)
0.1
0.2
0.3
0.4
0.5
0.6
0
20
40
60
80
Ages (years)
FDR (Age associated divergence) =0.0947
p(Scalp)=0.165
p(Dura)=0.0994
Scalp
Dura

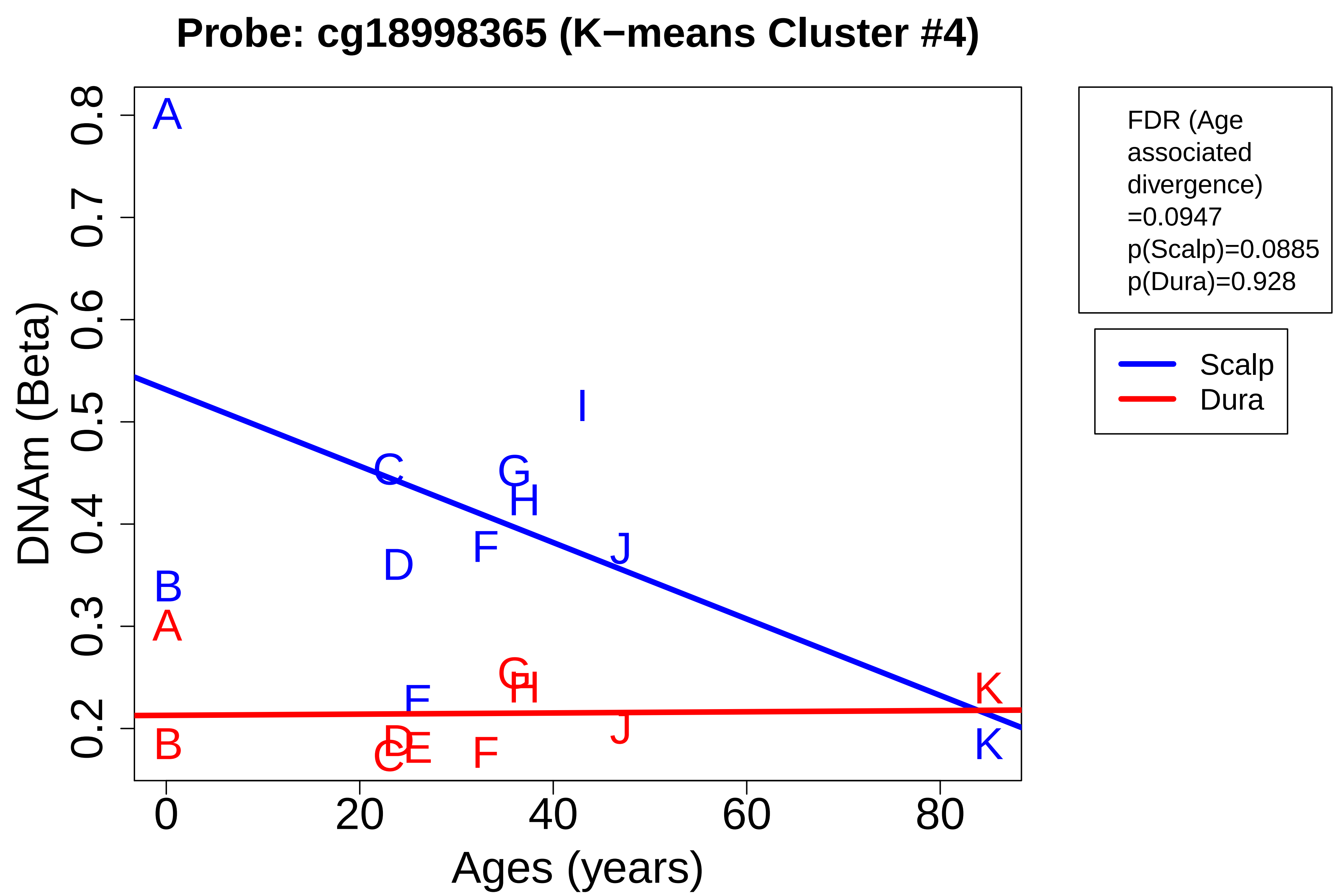

Probe: cg18998365 (K−means Cluster #4)
DNAm (Beta)
0.2
0.3
0.4
0.5
0.6
0.7
0.8
0
20
40
60
80
Ages (years)
A
B
C
D
E
F
G
H
I
J
K
A
B
C
D
E
F
G
H
J
K
FDR (Age associated divergence) =0.0947
p(Scalp)=0.0885
p(Dura)=0.928
Scalp
Dura

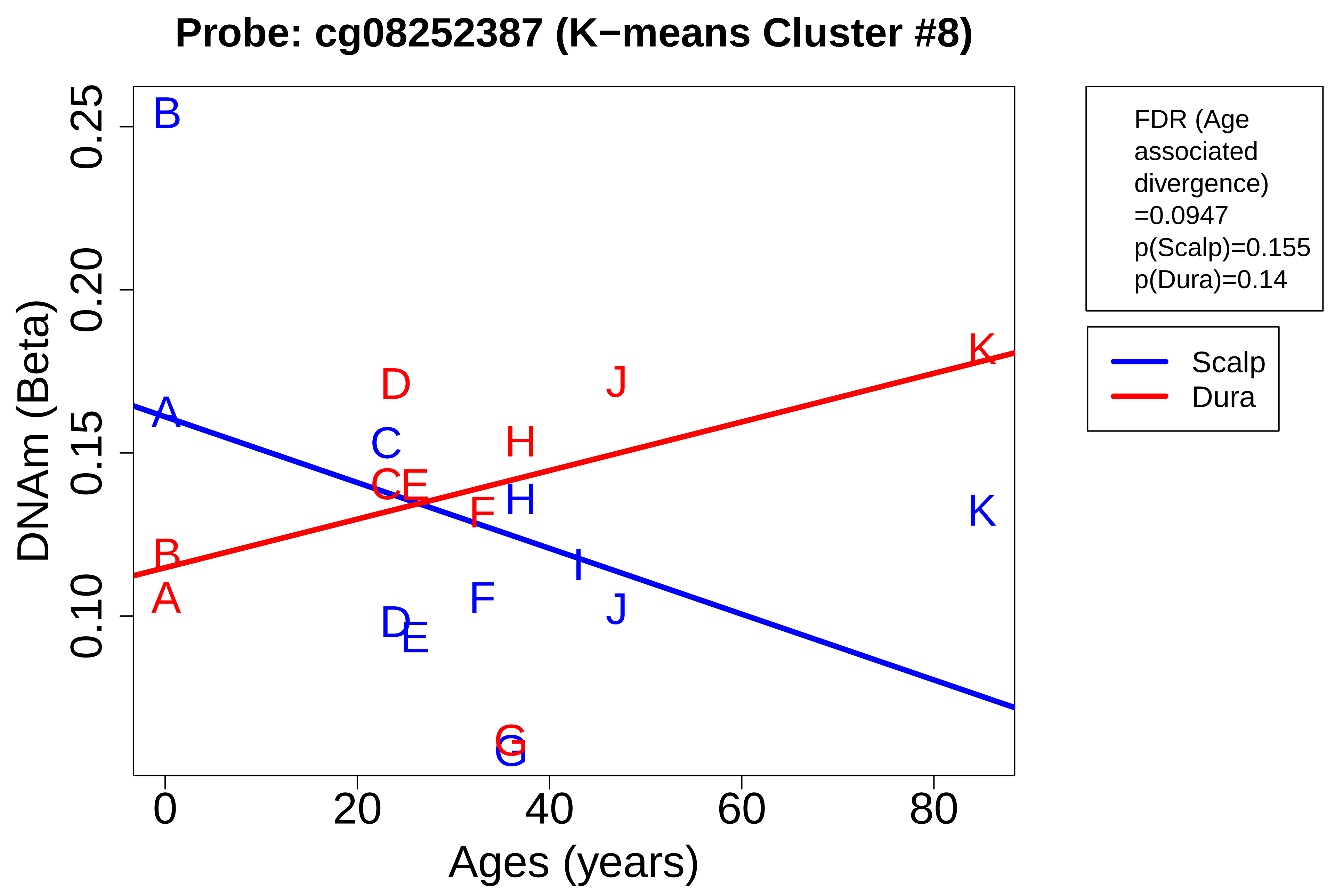

Probe: cg08252387 (K−means Cluster #8)
DNAm (Beta)
0.10
0.15
0.20
0.25
Ages (years)
0
20
40
60
80
FDR (Age associated divergence) =0.0947
p(Scalp)=0.155
p(Dura)=0.14
Scalp
Dura
A
B
C
D
E
F
G
H
I
J
K

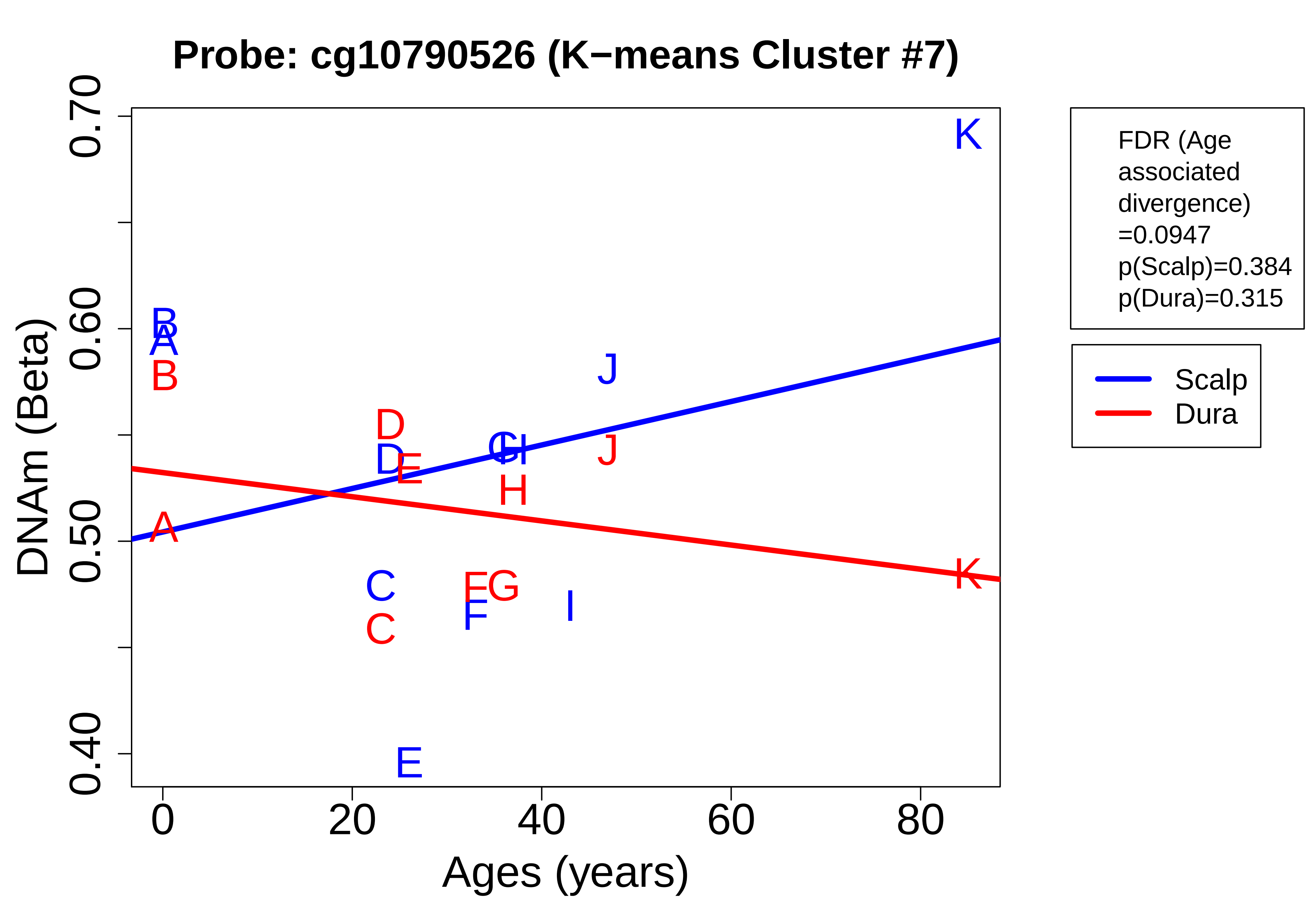

Probe: cg10790526 (K−means Cluster #7)
DNAm (Beta)
0.40
0.50
0.60
0.70
0
20
40
60
80
Ages (years)
FDR (Age associated divergence) =0.0947
p(Scalp)=0.384
p(Dura)=0.315
Scalp
Dura

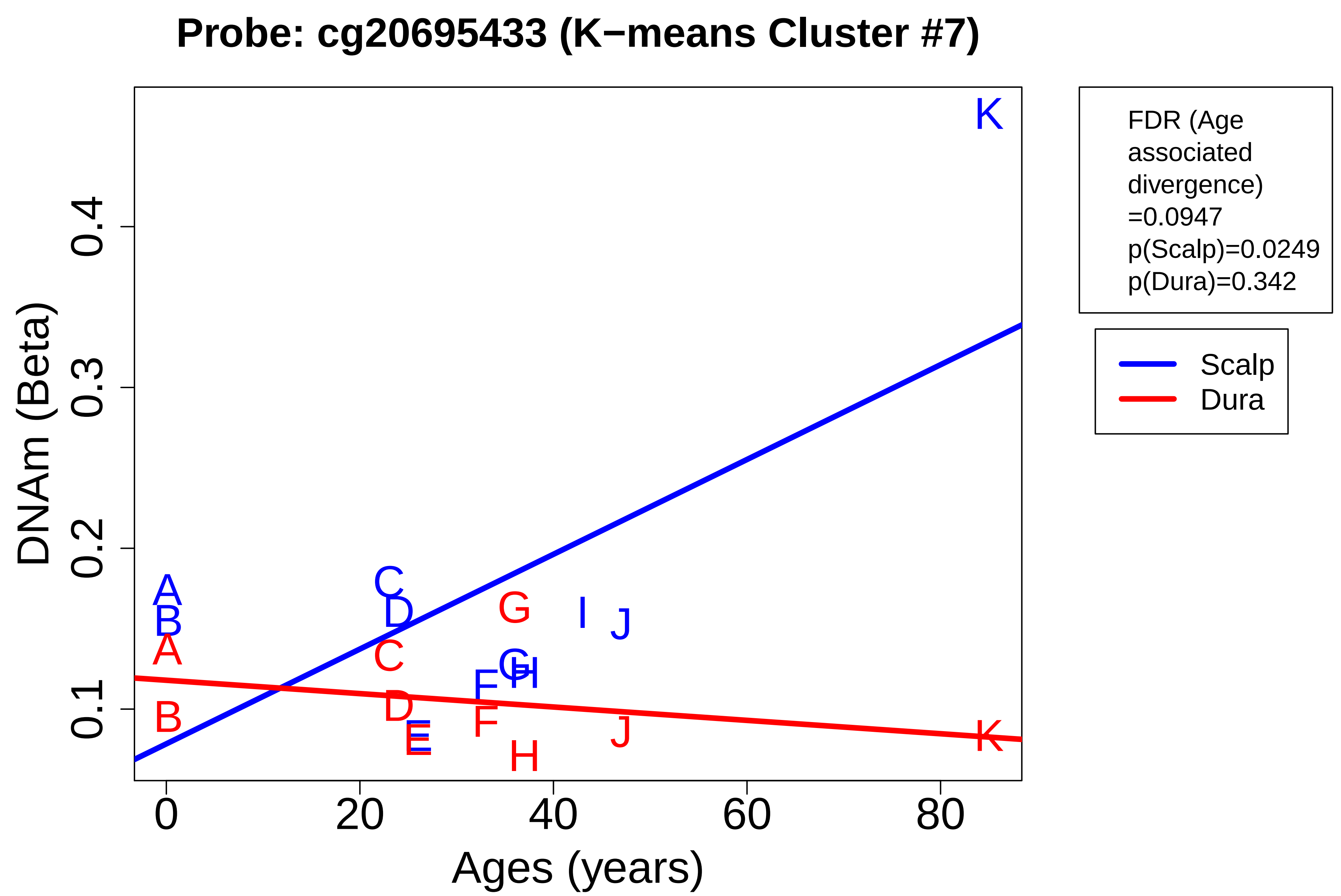

Probe: cg20695433 (K−means Cluster #7)
FDR (Age associated divergence) =0.0947
p(Scalp)=0.0249
p(Dura)=0.342
Scalp
Dura
DNAm (Beta)
0.1
0.2
0.3
0.4
0
20
40
60
80
Ages (years)
A
B
C
D
E
F
G
H
I
J
K

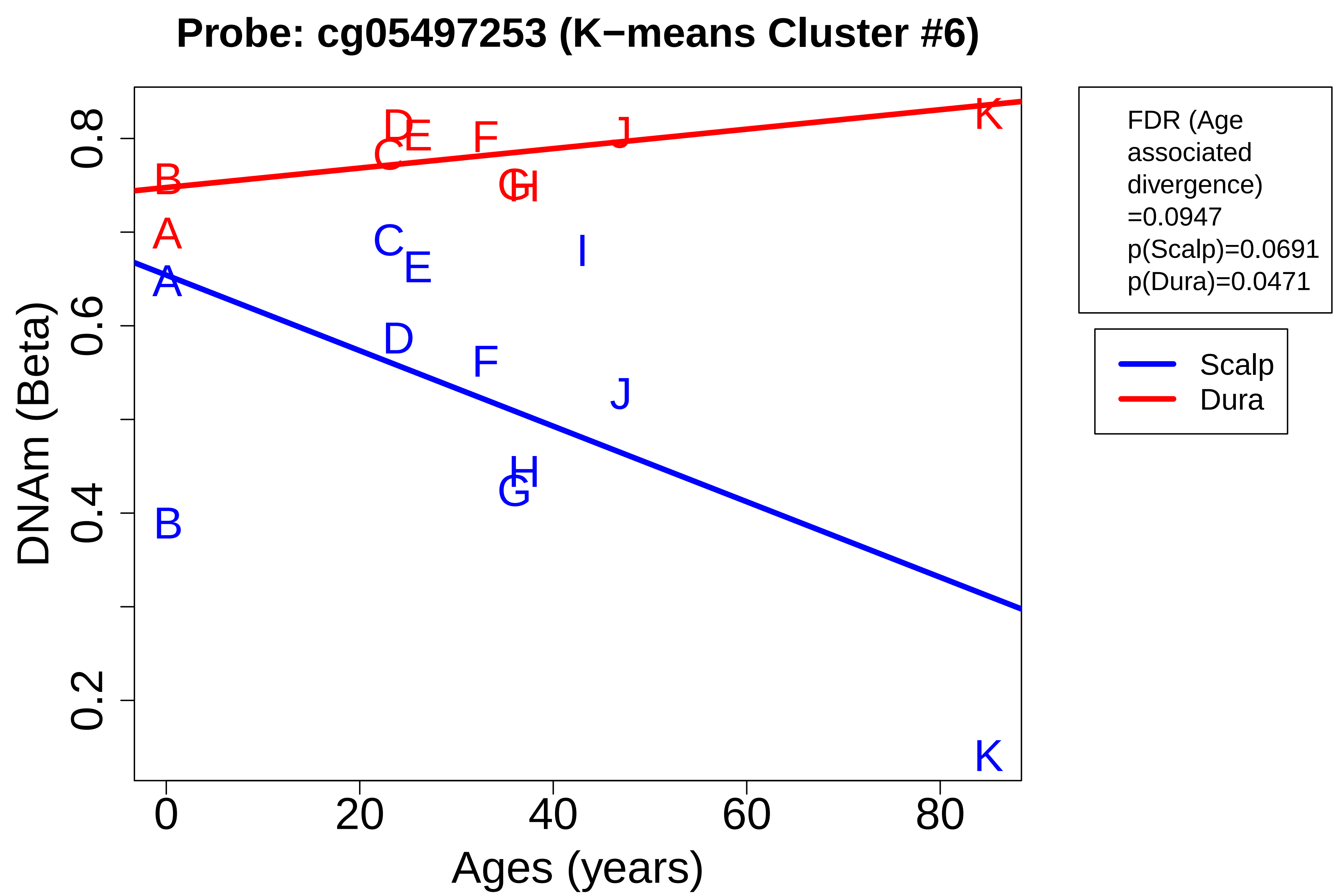
Probe: cg05497253 (K−means Cluster #6)
DNAm (Beta)
0.2
0.4
0.6
0.8
0
20
40
60
80
Ages (years)
FDR (Age associated divergence) =0.0947
p(Scalp)=0.0691
p(Dura)=0.0471
Scalp
Dura
A
B
C
D
E
F
G
H
I
J
K

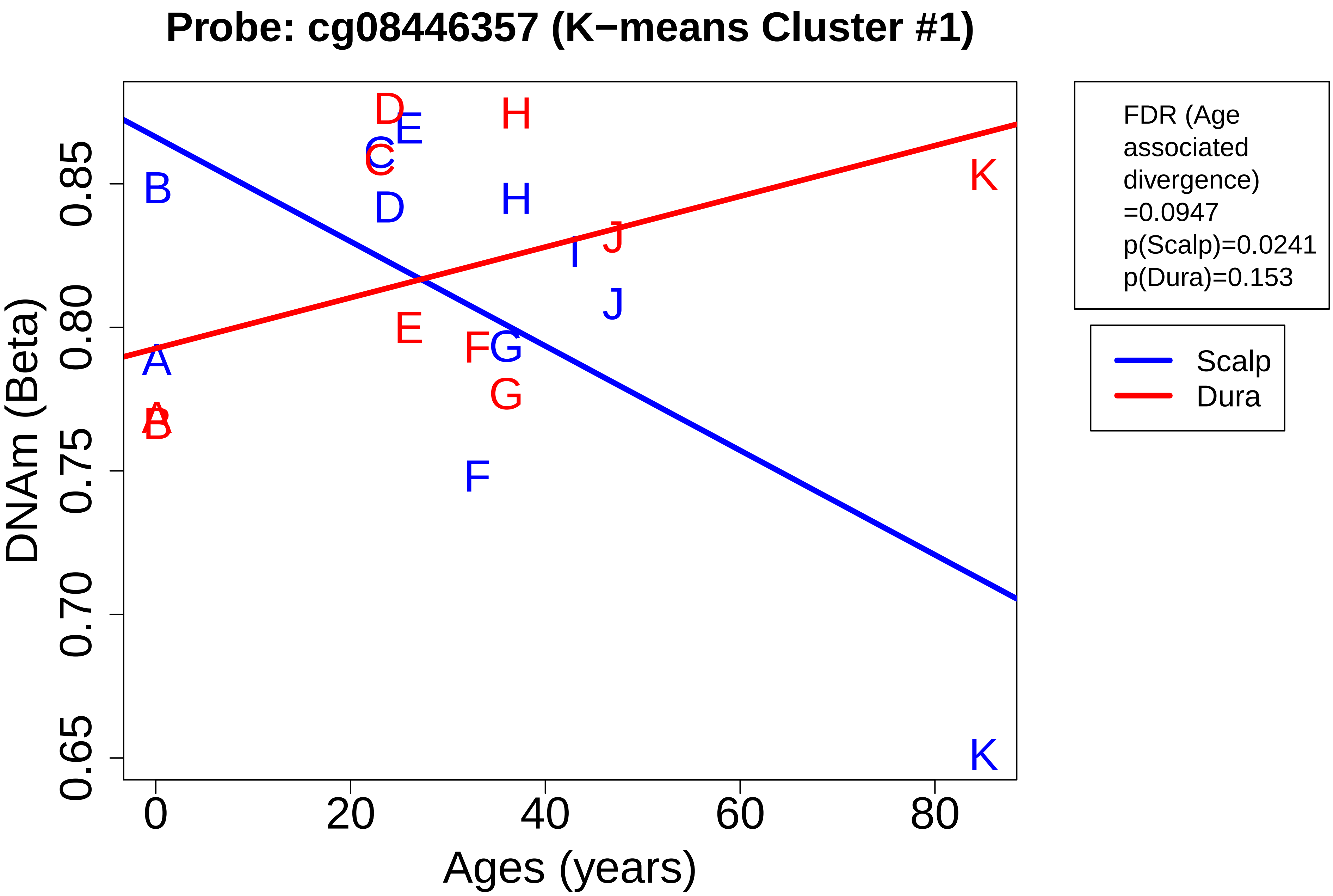
Probe: cg08446357 (K−means Cluster #1)
DNAm (Beta)
0.65
0.70
0.75
0.80
0.85
0
20
40
60
80
Ages (years)
FDR (Age associated divergence) =0.0947
p(Scalp)=0.0241
p(Dura)=0.153
Scalp
Dura

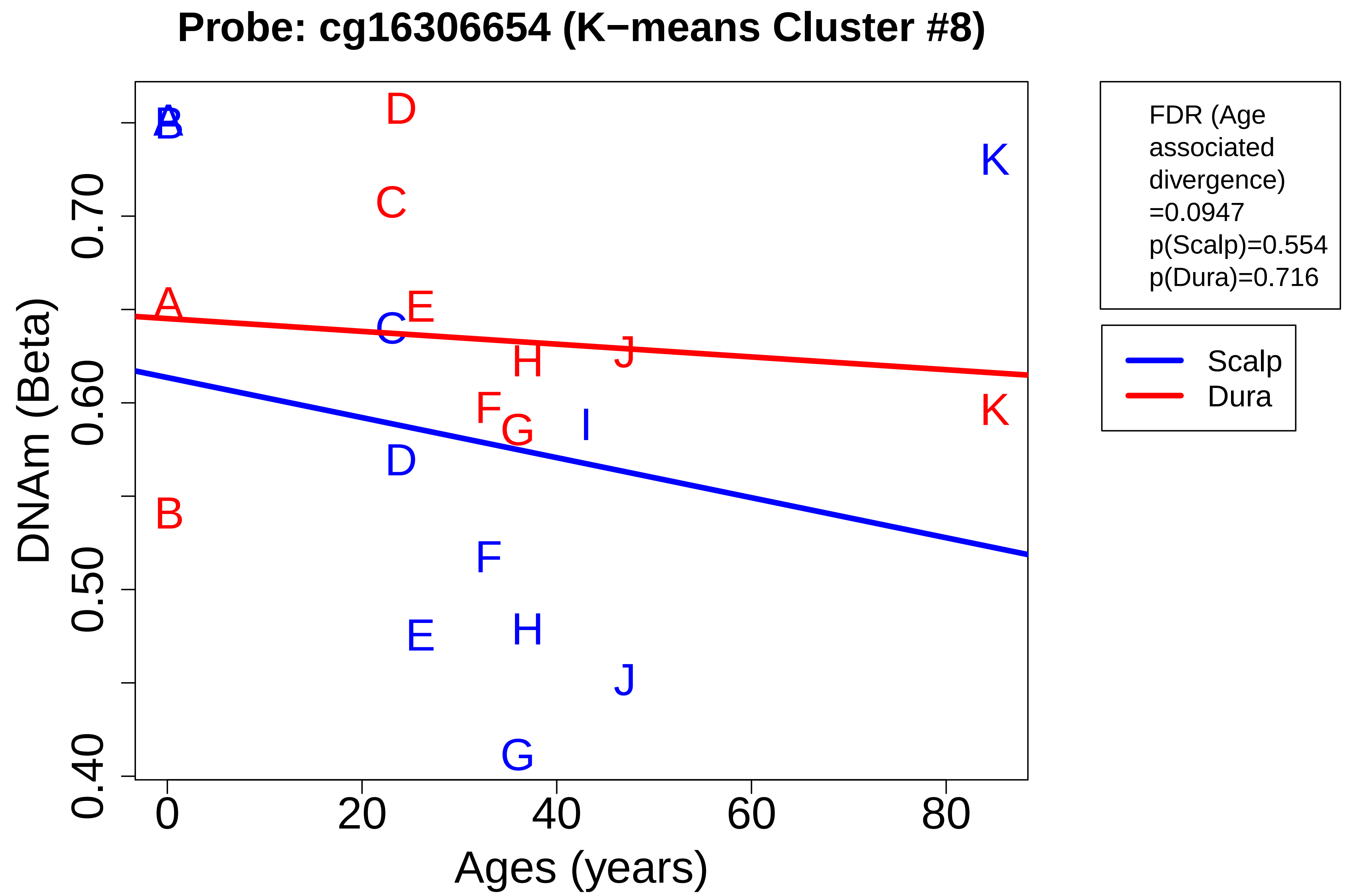

Probe: cg16306654 (K−means Cluster #8)
DNAm (Beta)
0.40
0.50
0.60
0.70
0
20
40
60
80
Ages (years)
FDR (Age associated divergence) =0.0947
p(Scalp)=0.554
p(Dura)=0.716
Scalp
Dura

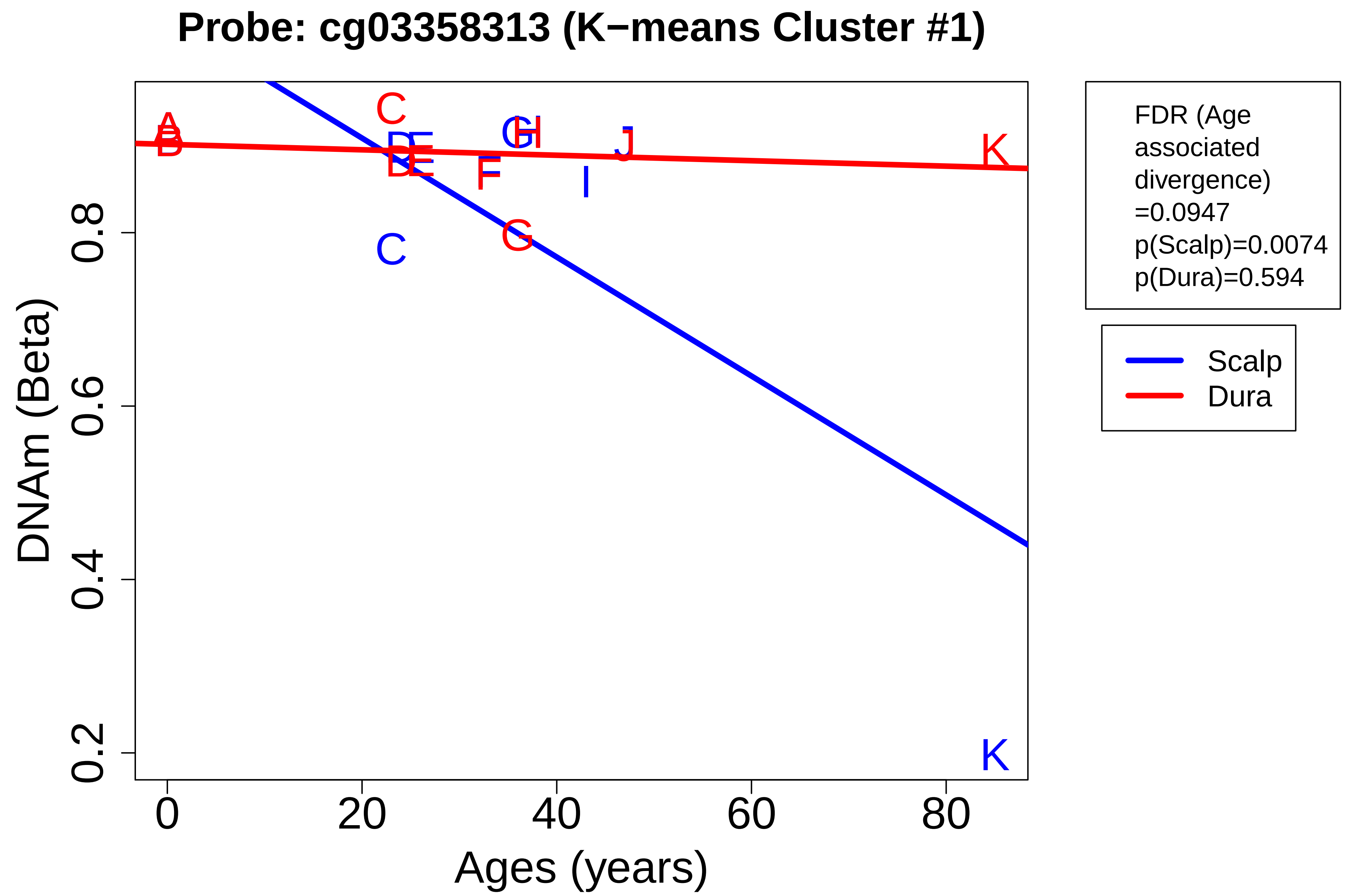

Probe: cg03358313 (K−means Cluster #1)
FDR (Age associated divergence) =0.0947
p(Scalp)=0.0074
p(Dura)=0.594
Scalp
Dura
DNAm (Beta)
0.2
0.4
0.6
0.8
0
20
40
60
80
Ages (years)
A
B
C
D
E
F
G
H
I
J
K

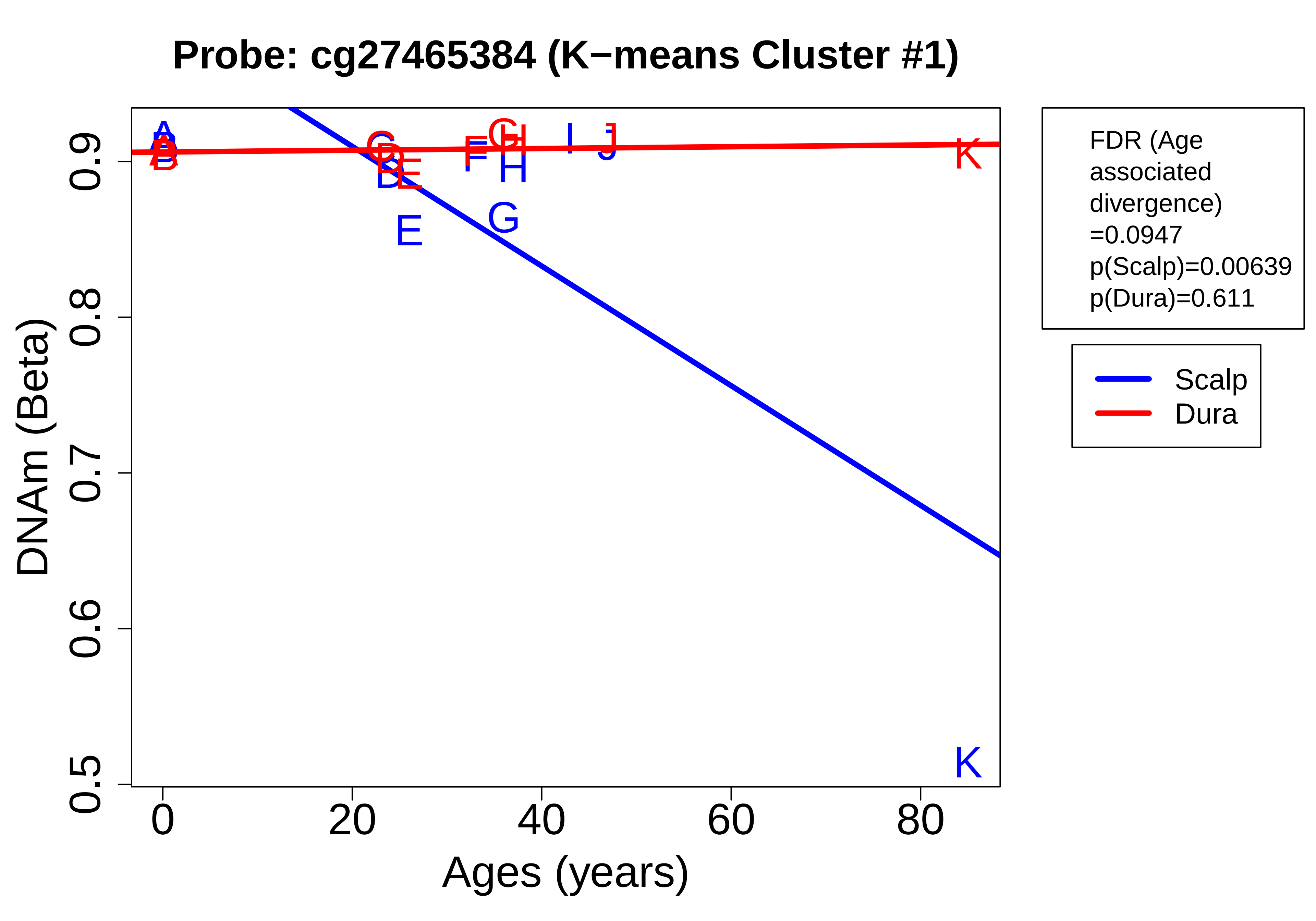

Probe: cg27465384 (K−means Cluster #1)
DNAm (Beta)
Ages (years)
0.5
0.6
0.7
0.8
0.9
0
20
40
60
80
FDR (Age associated divergence) =0.0947
p(Scalp)=0.00639
p(Dura)=0.611
Scalp
Dura

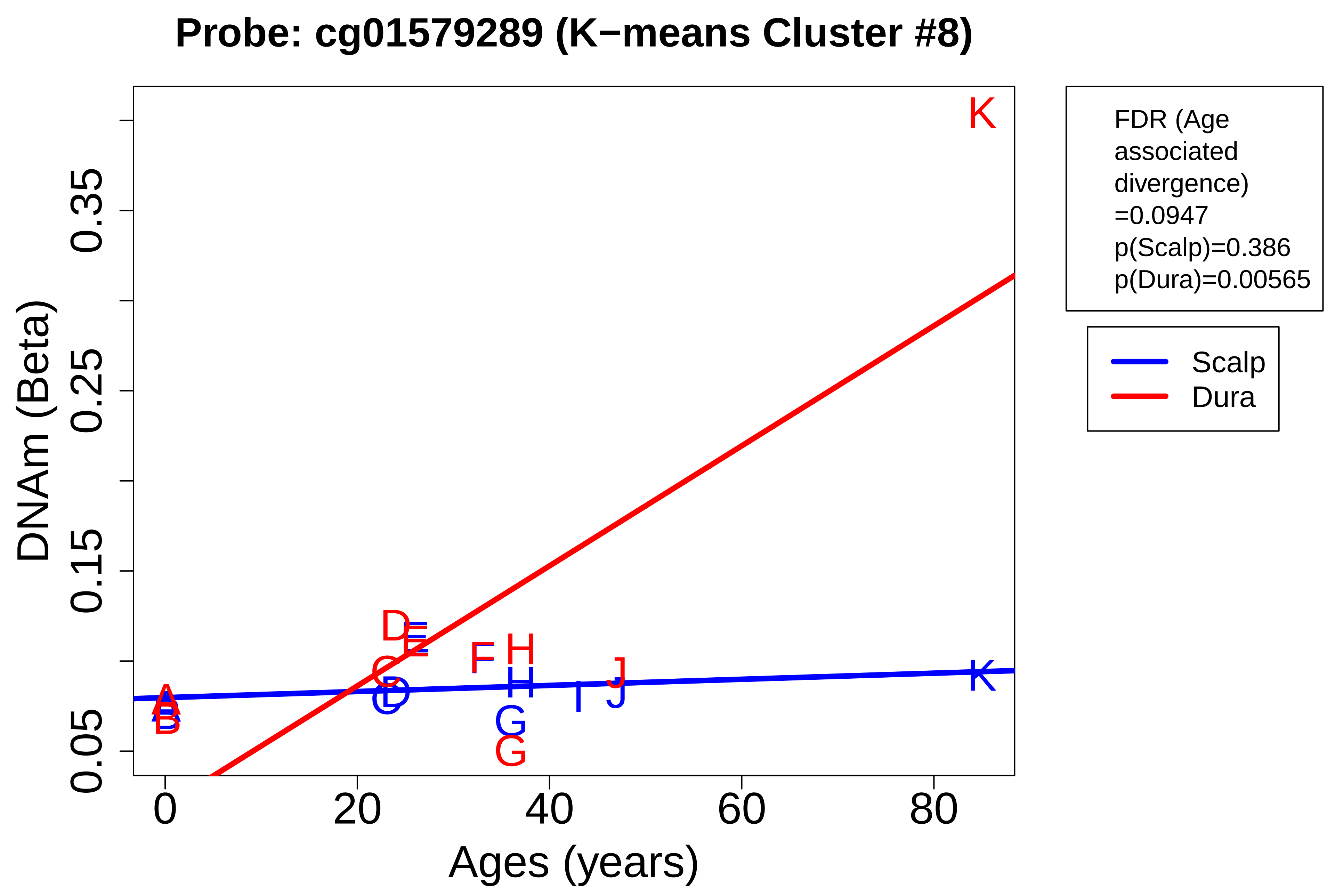
Probe: cg01579289 (K−means Cluster #8)
DNAm (Beta)
0.05
0.15
0.25
0.35
0
20
40
60
80
Ages (years)
FDR (Age associated divergence) =0.0947
p(Scalp)=0.386
p(Dura)=0.00565
Scalp
Dura
A
B
C
D
E
F
G
H
I
J
K

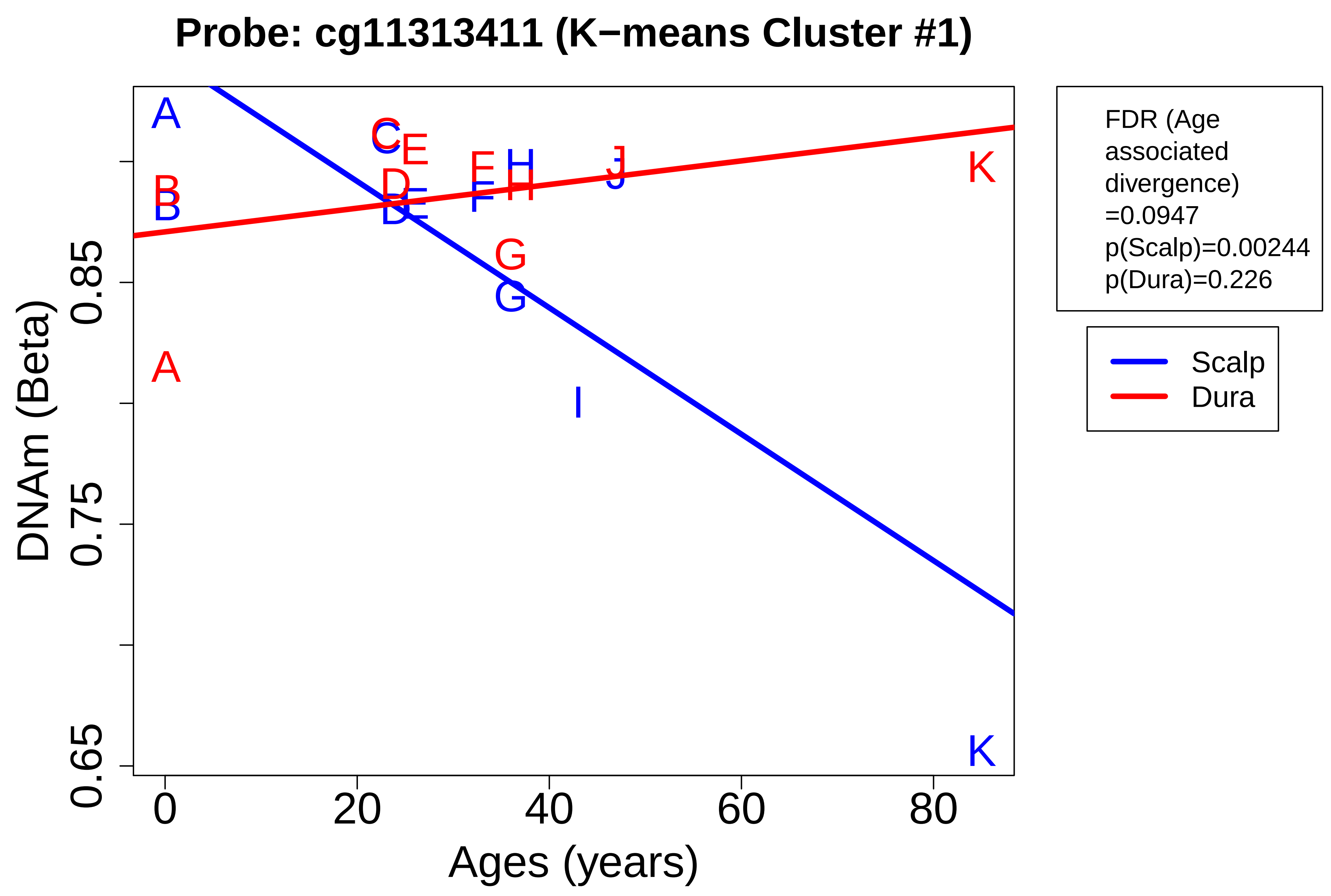
Probe: cg11313411 (K−means Cluster #1)
DNAm (Beta)
0.65
0.75
0.85
0
20
40
60
80
Ages (years)
A
B
C
D
E
F
G
H
I
J
K
FDR (Age associated divergence) =0.0947
p(Scalp)=0.00244
p(Dura)=0.226
Scalp
Dura

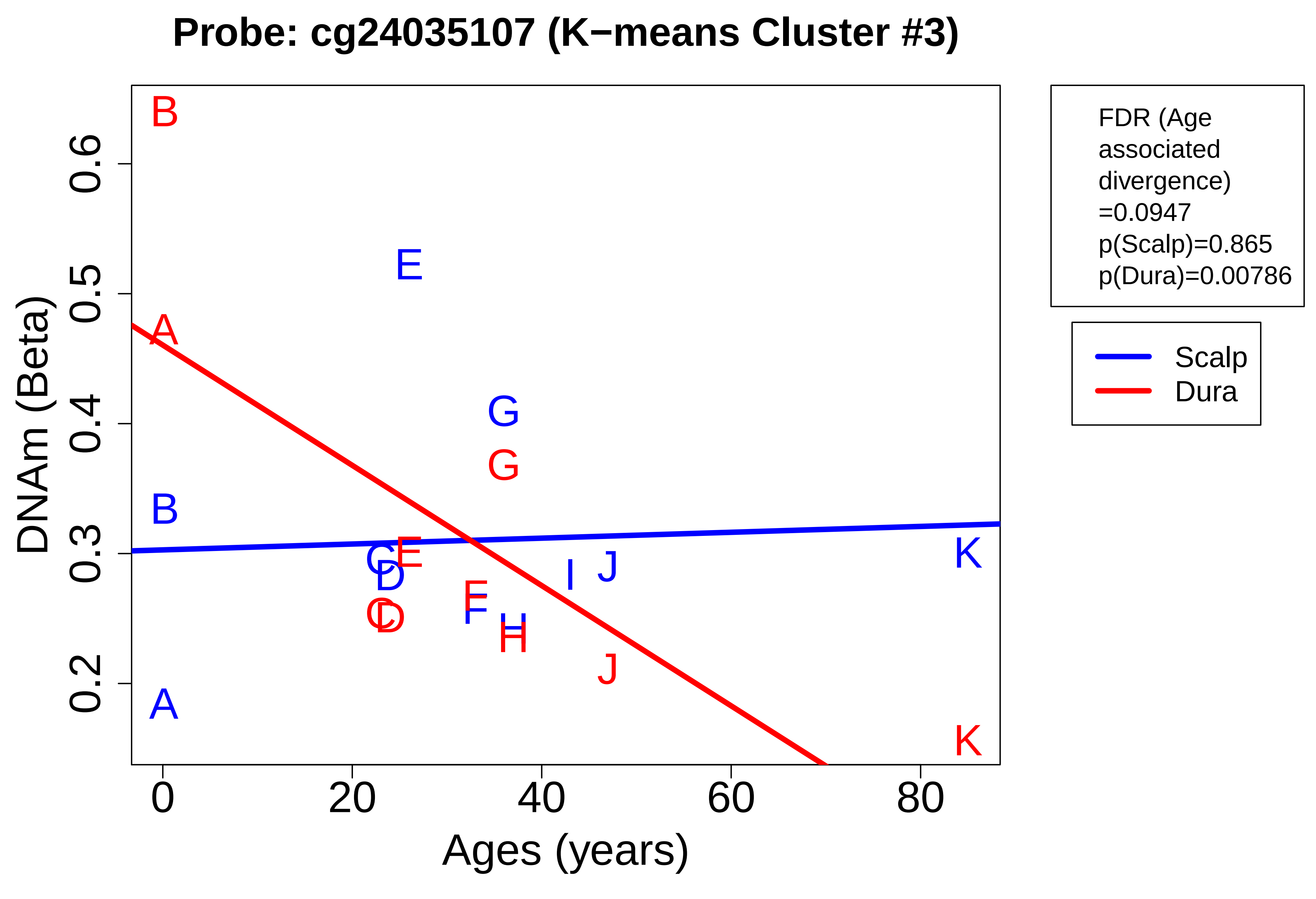

Probe: cg24035107 (K−means Cluster #3)
DNAm (Beta)
0.2
0.3
0.4
0.5
0.6
0
20
40
60
80
Ages (years)
B
A
E
G
G
B
C
E
D
I
J
K
F
F
C
D
H
H
J
A
K
FDR (Age
associated
divergence)
=0.0947
p(Scalp)=0.865
p(Dura)=0.00786
Scalp
Dura

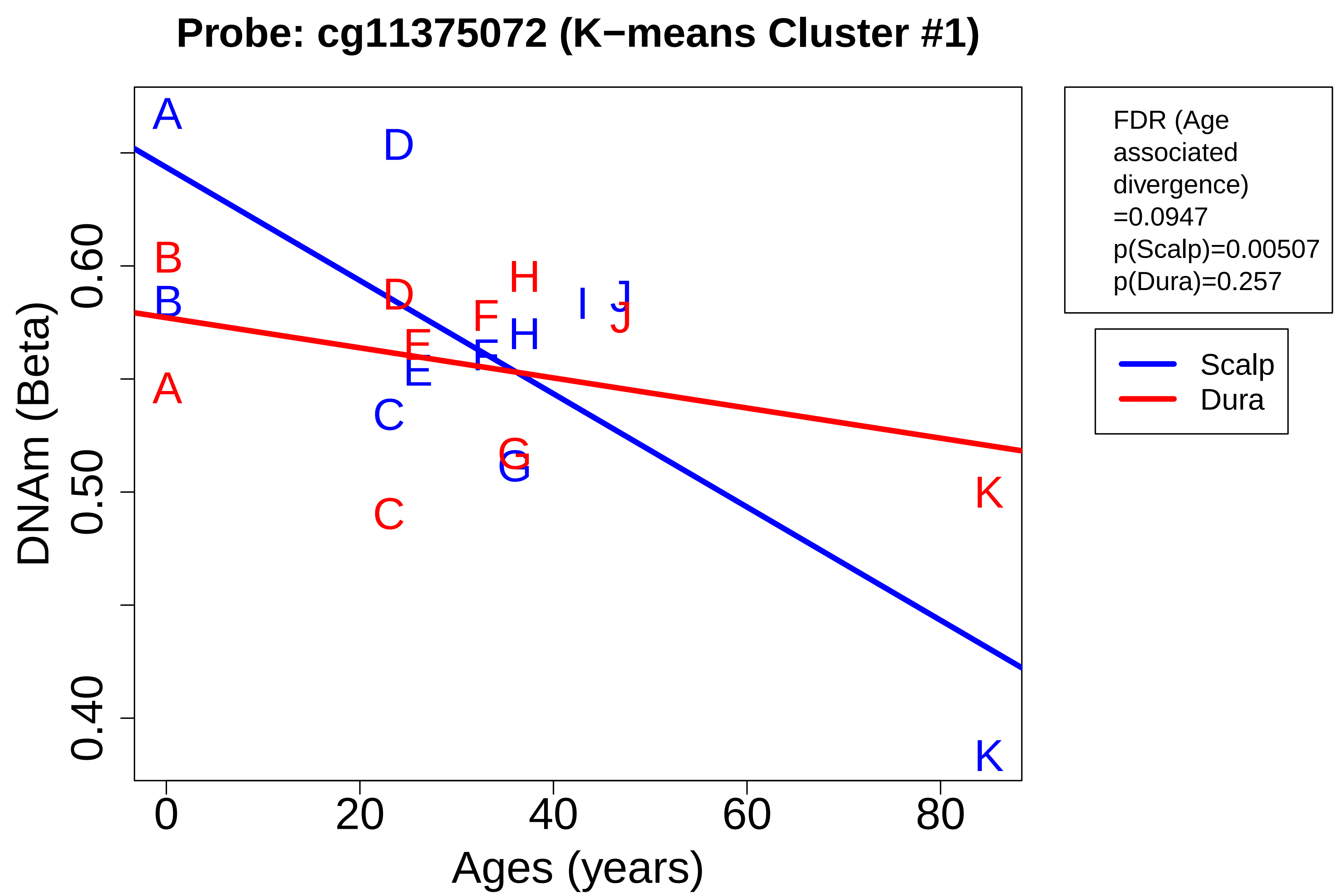

Probe: cg11375072 (K−means Cluster #1)
FDR (Age associated divergence) =0.0947
p(Scalp)=0.00507
p(Dura)=0.257
Scalp
Dura
DNAm (Beta)
0.40
0.50
0.60
Ages (years)
0
20
40
60
80
A
B
C
D
E
F
G
H
I
J
K

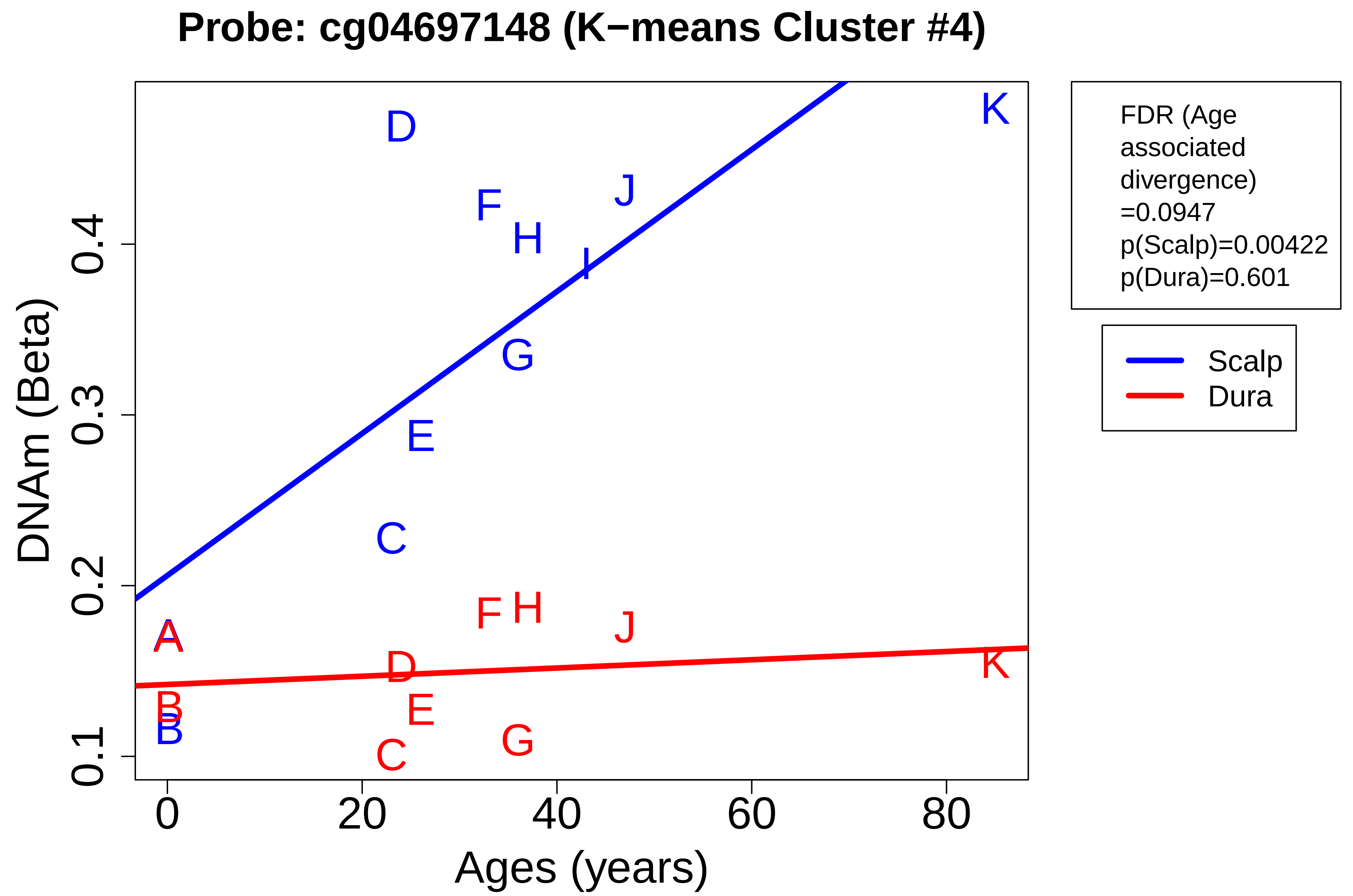

Probe: cg04697148 (K−means Cluster #4)
DNAm (Beta)
Ages (years)
0.1
0.2
0.3
0.4
0
20
40
60
80
FDR (Age associated divergence) =0.0947
p(Scalp)=0.00422
p(Dura)=0.601
Scalp
Dura
A
B
C
D
E
F
G
H
I
J
K

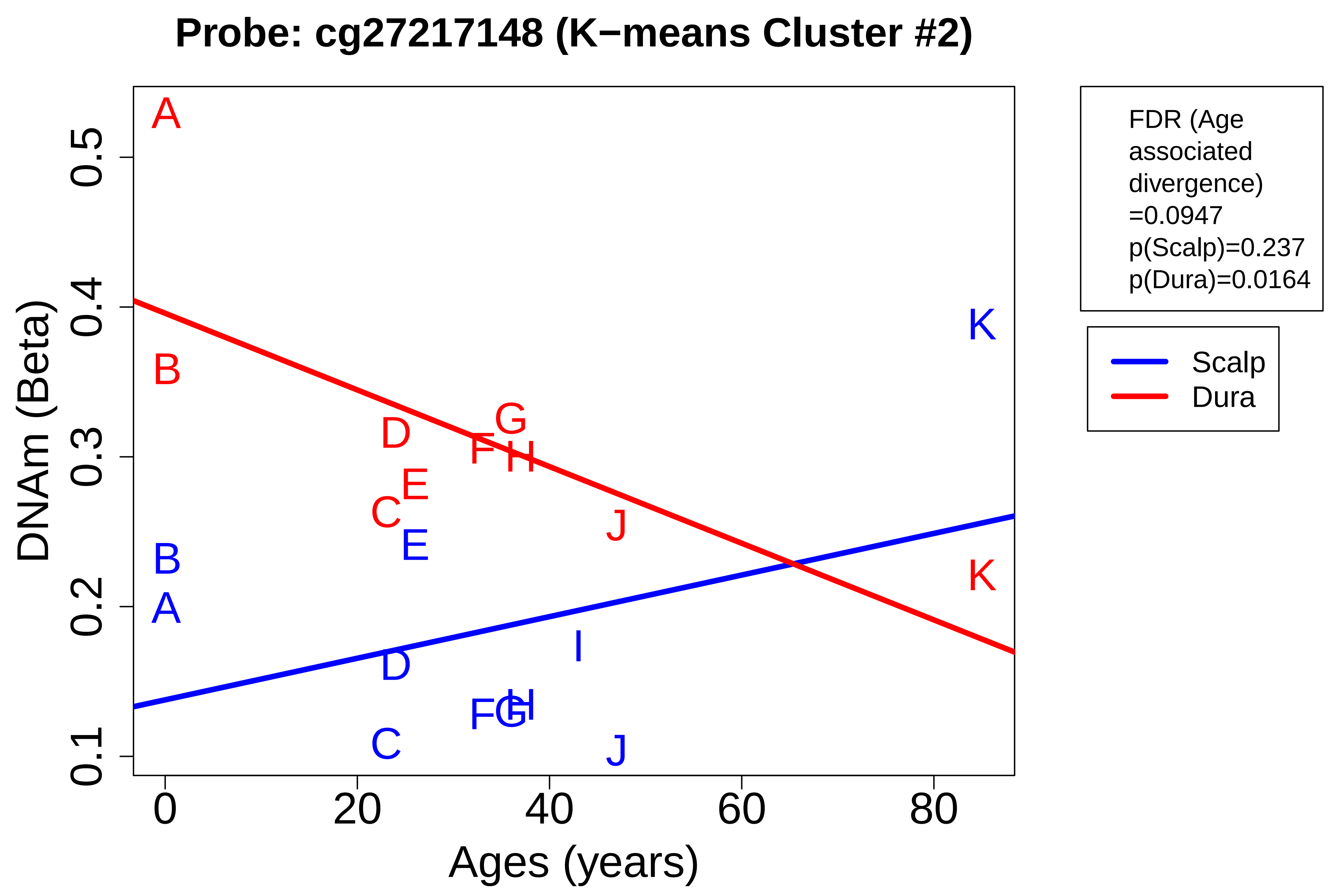

Probe: cg27217148 (K−means Cluster #2)
DNAm (Beta)
0.1
0.2
0.3
0.4
0.5
0
20
40
60
80
Ages (years)
A
B
C
D
E
F
G
H
I
J
K
FDR (Age associated divergence) =0.0947
p(Scalp)=0.237
p(Dura)=0.0164
Scalp
Dura

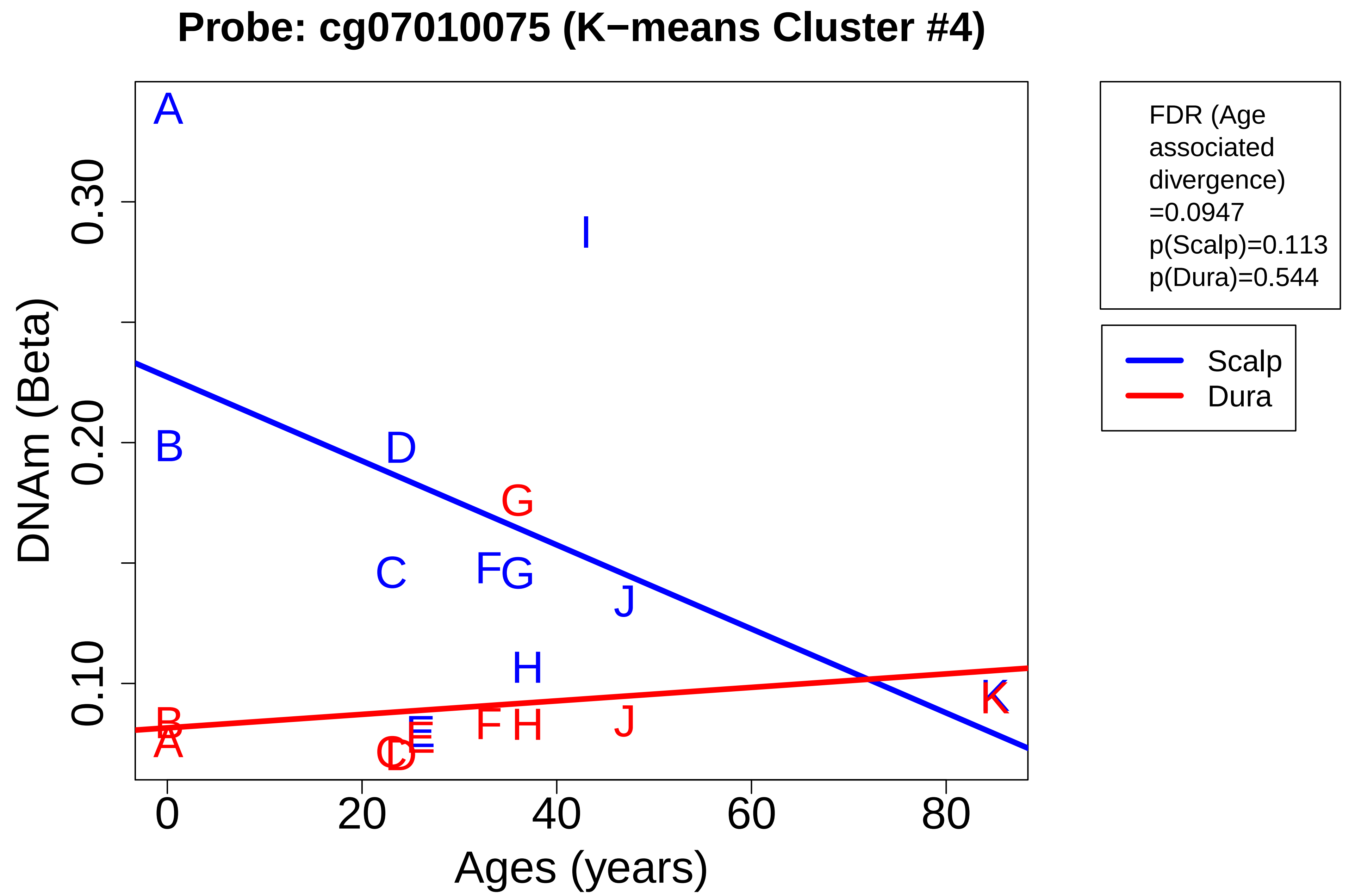

Probe: cg07010075 (K−means Cluster #4)
DNAm (Beta)
0.10
0.20
0.30
0
20
40
60
80
Ages (years)
FDR (Age associated divergence) =0.0947
p(Scalp)=0.113
p(Dura)=0.544
Scalp
Dura
A
B
C
D
E
F
G
H
I
J
K

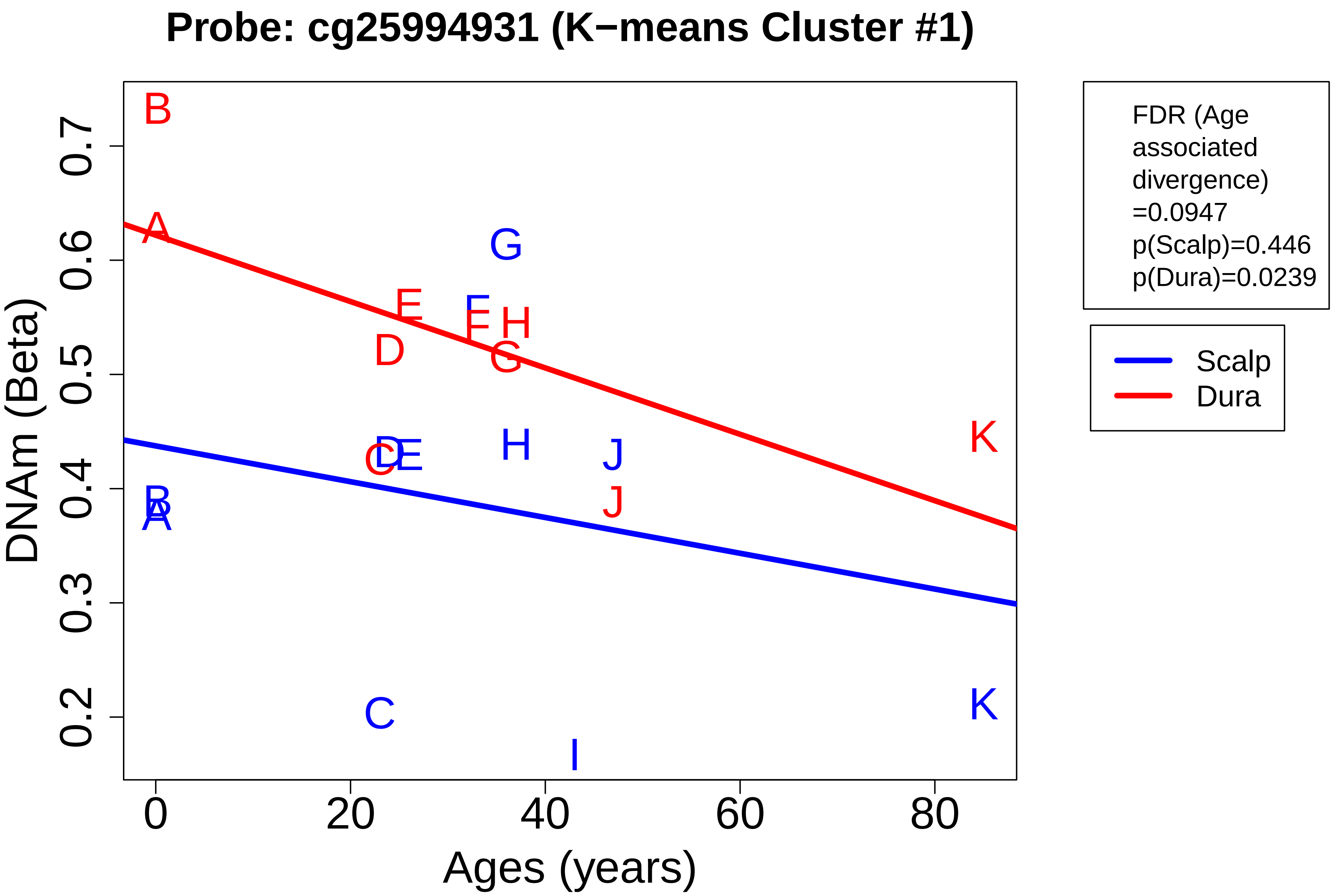

Probe: cg25994931 (K−means Cluster #1)
FDR (Age associated divergence) =0.0947
p(Scalp)=0.446
p(Dura)=0.0239
Scalp
Dura
DNAm (Beta)
0.2
0.3
0.4
0.5
0.6
0.7
0
20
40
60
80
Ages (years)

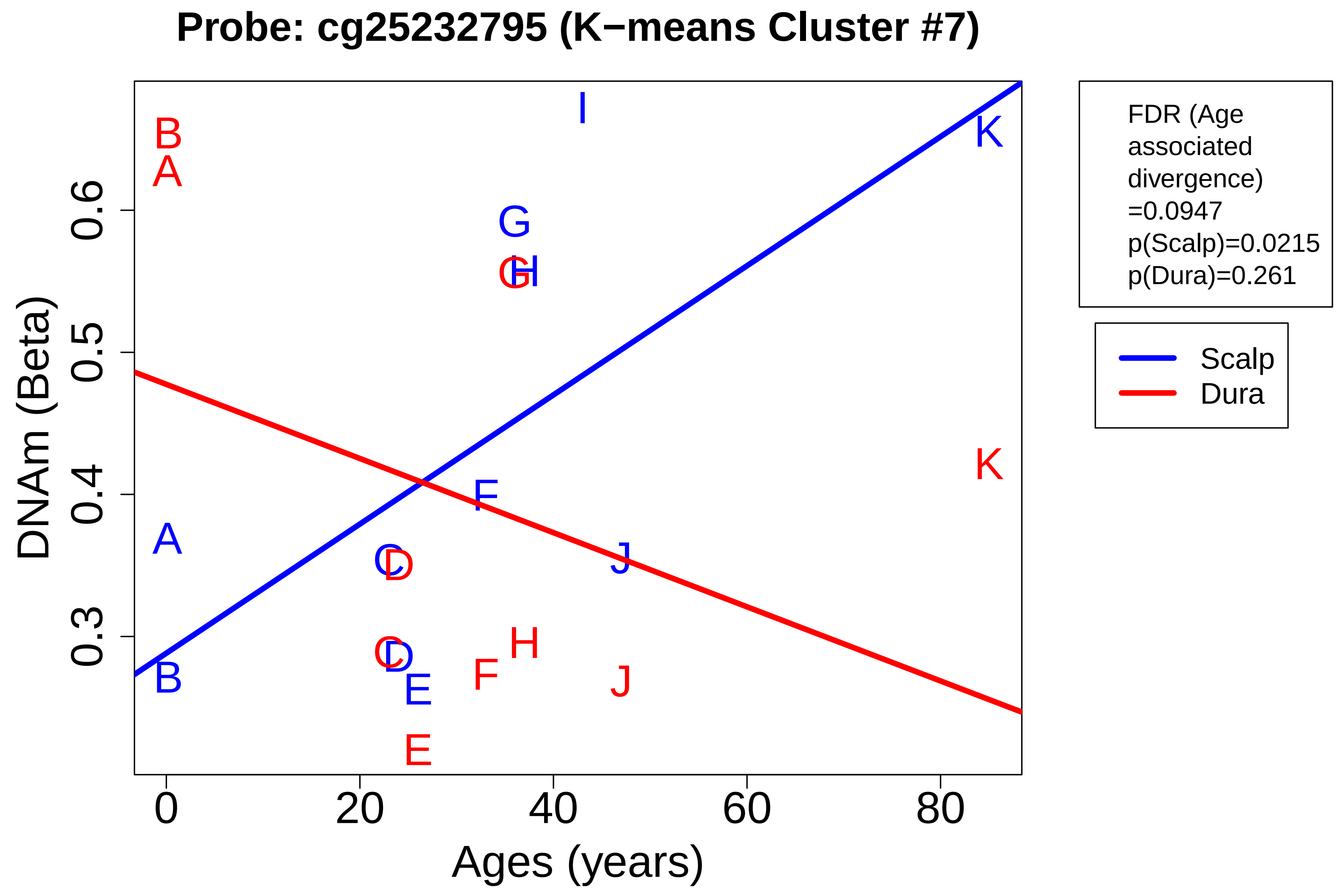

Probe: cg25232795 (K−means Cluster #7)
DNAm (Beta)
0.3
0.4
0.5
0.6
0
20
40
60
80
Ages (years)
A
B
C
D
E
F
G
H
I
J
K
FDR (Age associated divergence) =0.0947
p(Scalp)=0.0215
p(Dura)=0.261
Scalp
Dura

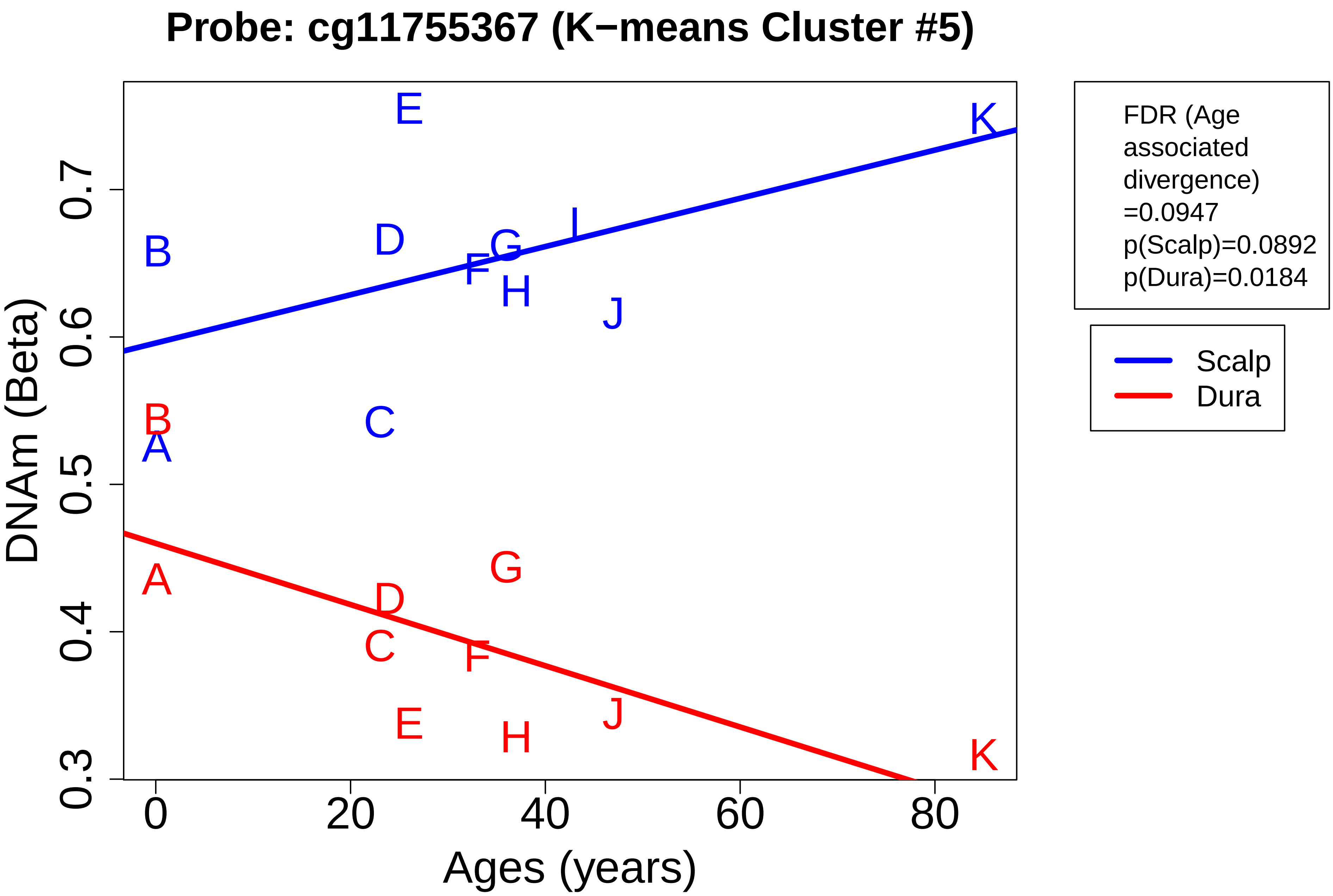

Probe: cg11755367 (K−means Cluster #5)
DNAm (Beta)
0.3
0.4
0.5
0.6
0.7
Ages (years)
0
20
40
60
80
A B C D E F G H I J K
A B C D E F G H J K
FDR (Age associated divergence) =0.0947
p(Scalp)=0.0892
p(Dura)=0.0184
Scalp
Dura

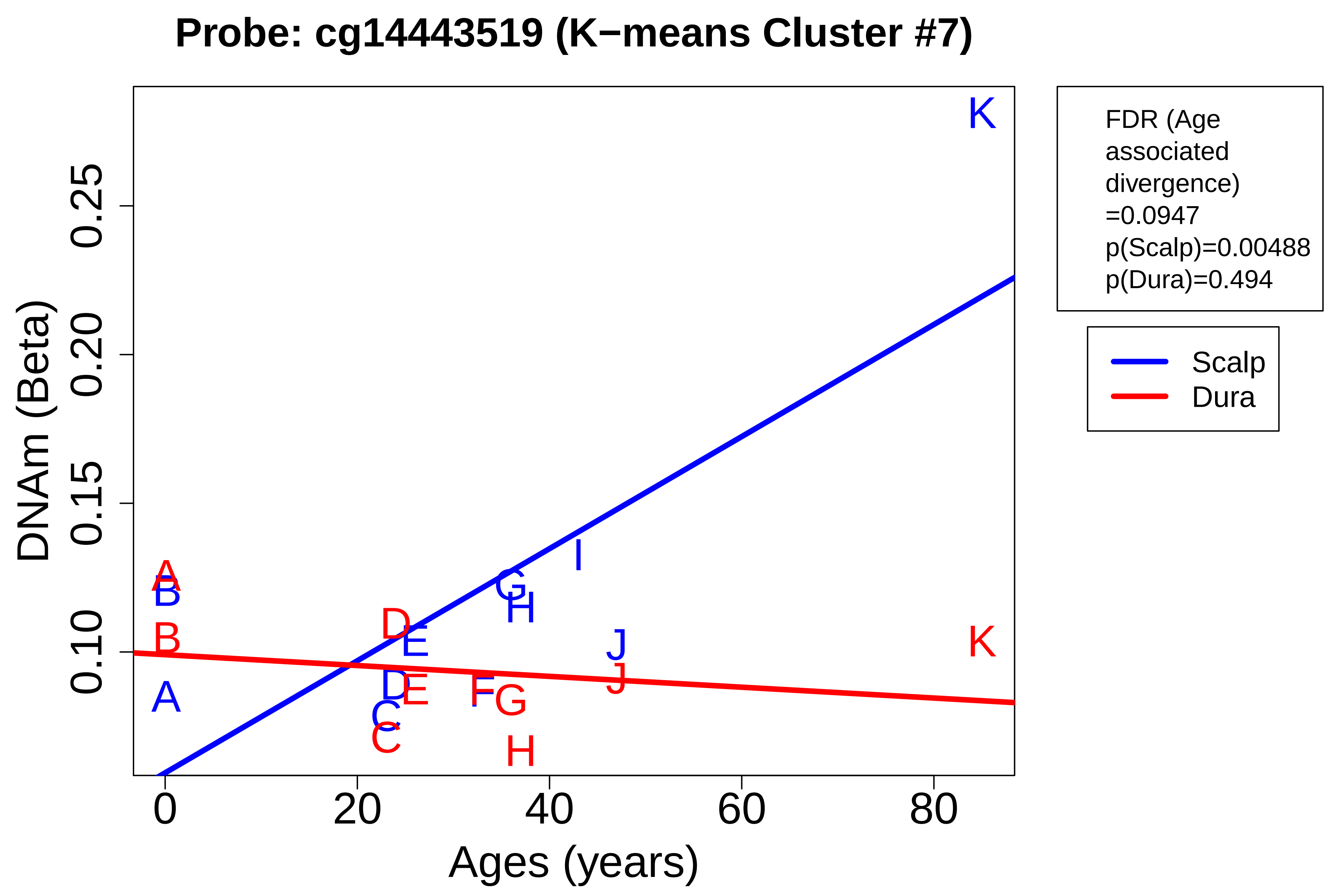

Probe: cg14443519 (K−means Cluster #7)
DNAm (Beta)
0.10
0.15
0.20
0.25
Ages (years)
0
20
40
60
80
A
B
C
D
E
F
G
H
I
J
K
FDR (Age associated divergence) =0.0947
p(Scalp)=0.00488
p(Dura)=0.494
Scalp
Dura

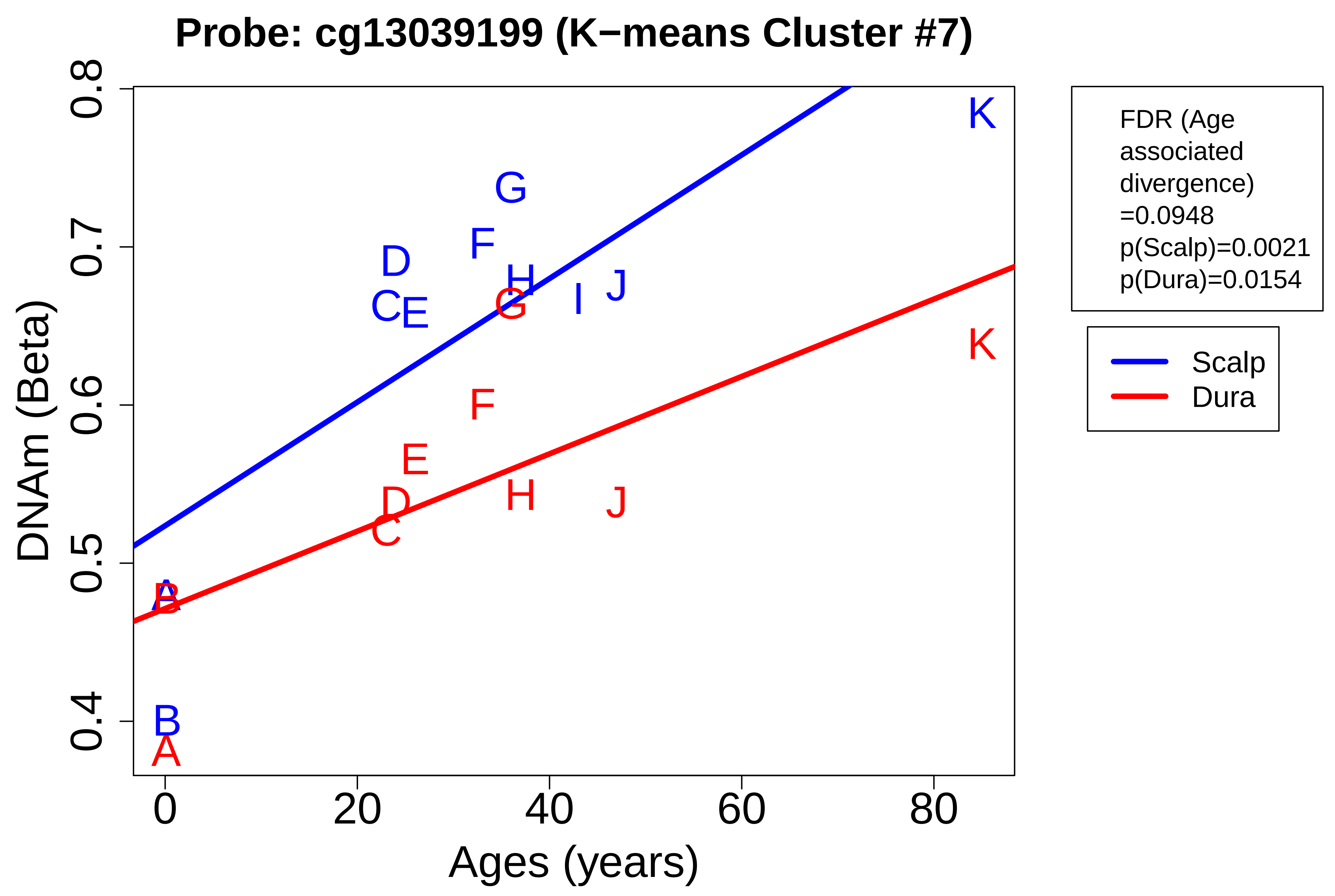

Probe: cg13039199 (K−means Cluster #7)
DNAm (Beta)
0.4
0.5
0.6
0.7
0.8
Ages (years)
0
20
40
60
80
A
B
C
D
E
F
G
H
I
J
K
FDR (Age associated divergence) =0.0948
p(Scalp)=0.0021
p(Dura)=0.0154
Scalp
Dura

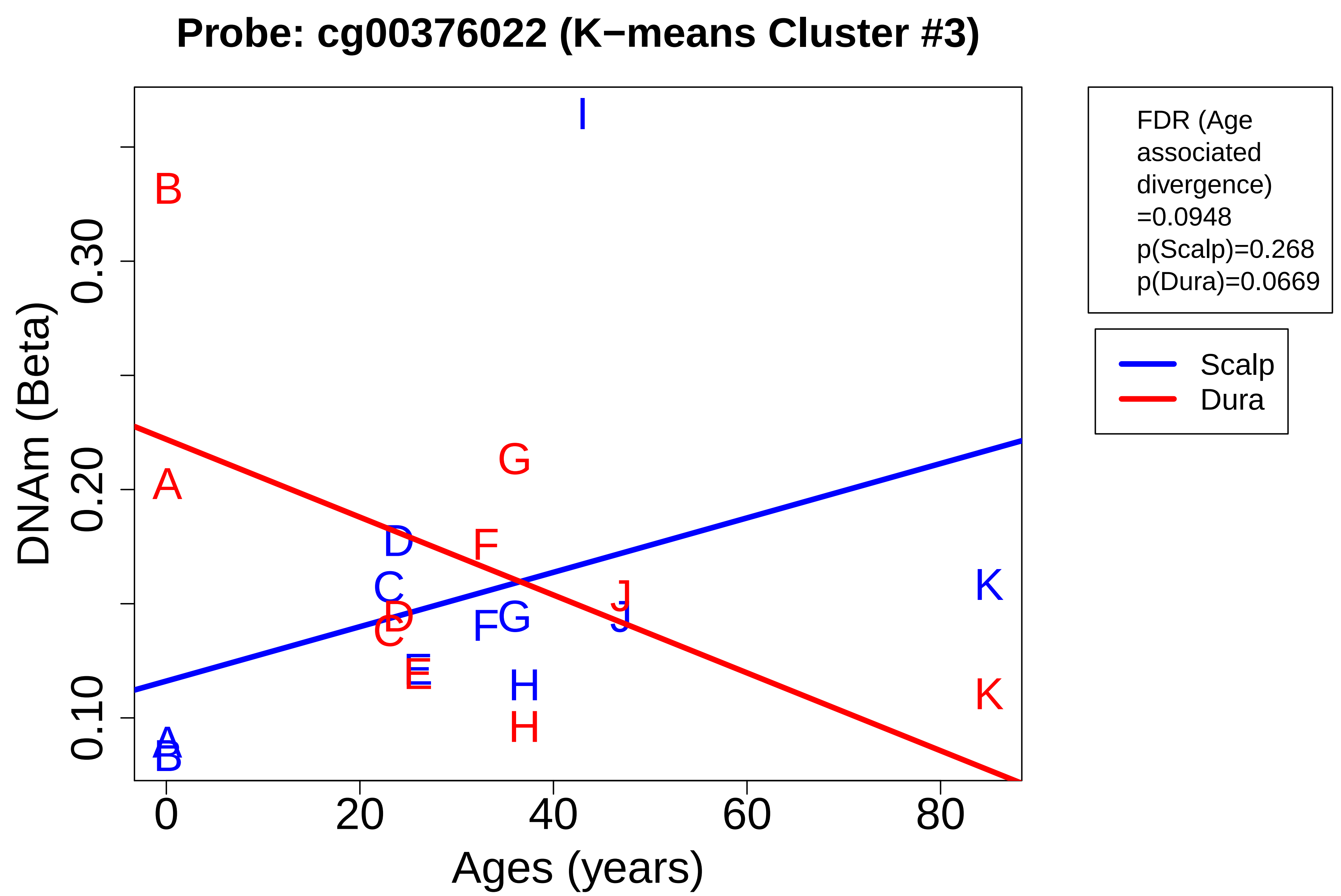

Probe: cg00376022 (K−means Cluster #3)
DNAm (Beta)
0.10
0.20
0.30
0
20
40
60
80
Ages (years)
FDR (Age associated divergence) =0.0948
p(Scalp)=0.268
p(Dura)=0.0669
Scalp
Dura

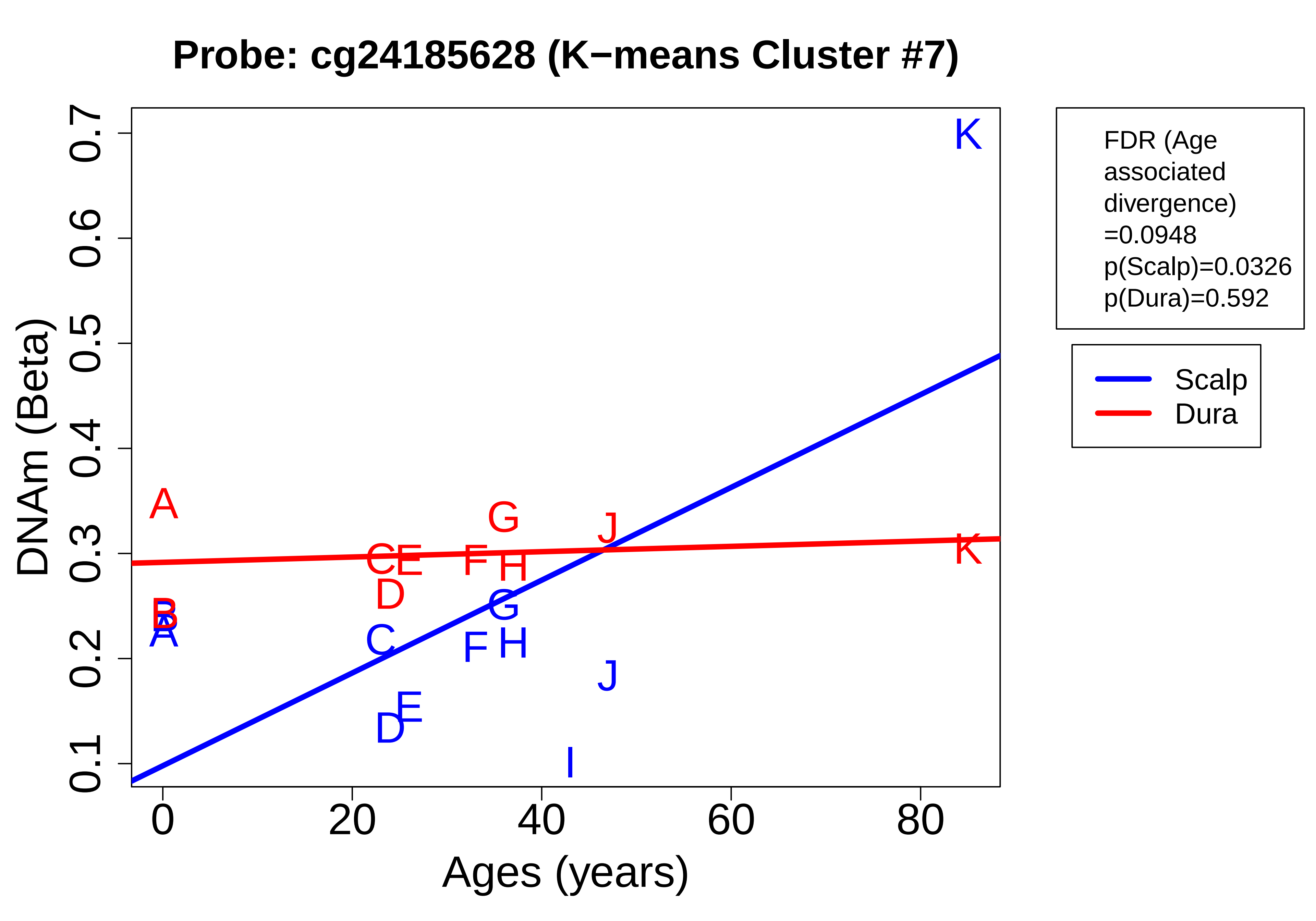
Probe: cg24185628 (K−means Cluster #7)
DNAm (Beta)
Ages (years)
0.1
0.2
0.3
0.4
0.5
0.6
0.7
0
20
40
60
80
FDR (Age associated divergence) =0.0948
p(Scalp)=0.0326
p(Dura)=0.592
Scalp
Dura
A
B
C
D
E
F
G
H
I
J
K

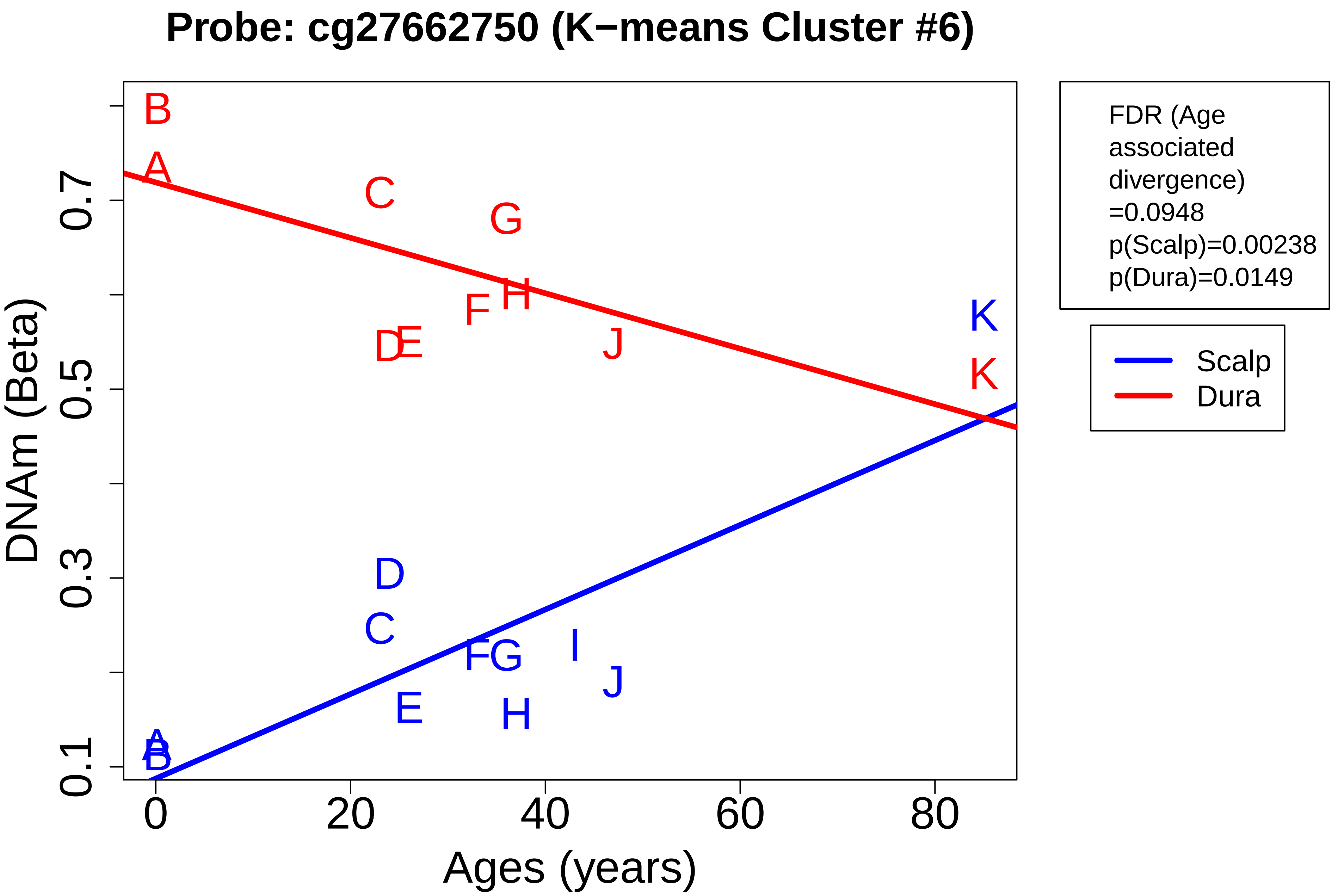

Probe: cg27662750 (K−means Cluster #6)
DNAm (Beta)
0.1
0.3
0.5
0.7
Ages (years)
0
20
40
60
80
FDR (Age associated divergence) =0.0948
p(Scalp)=0.00238
p(Dura)=0.0149
Scalp
Dura

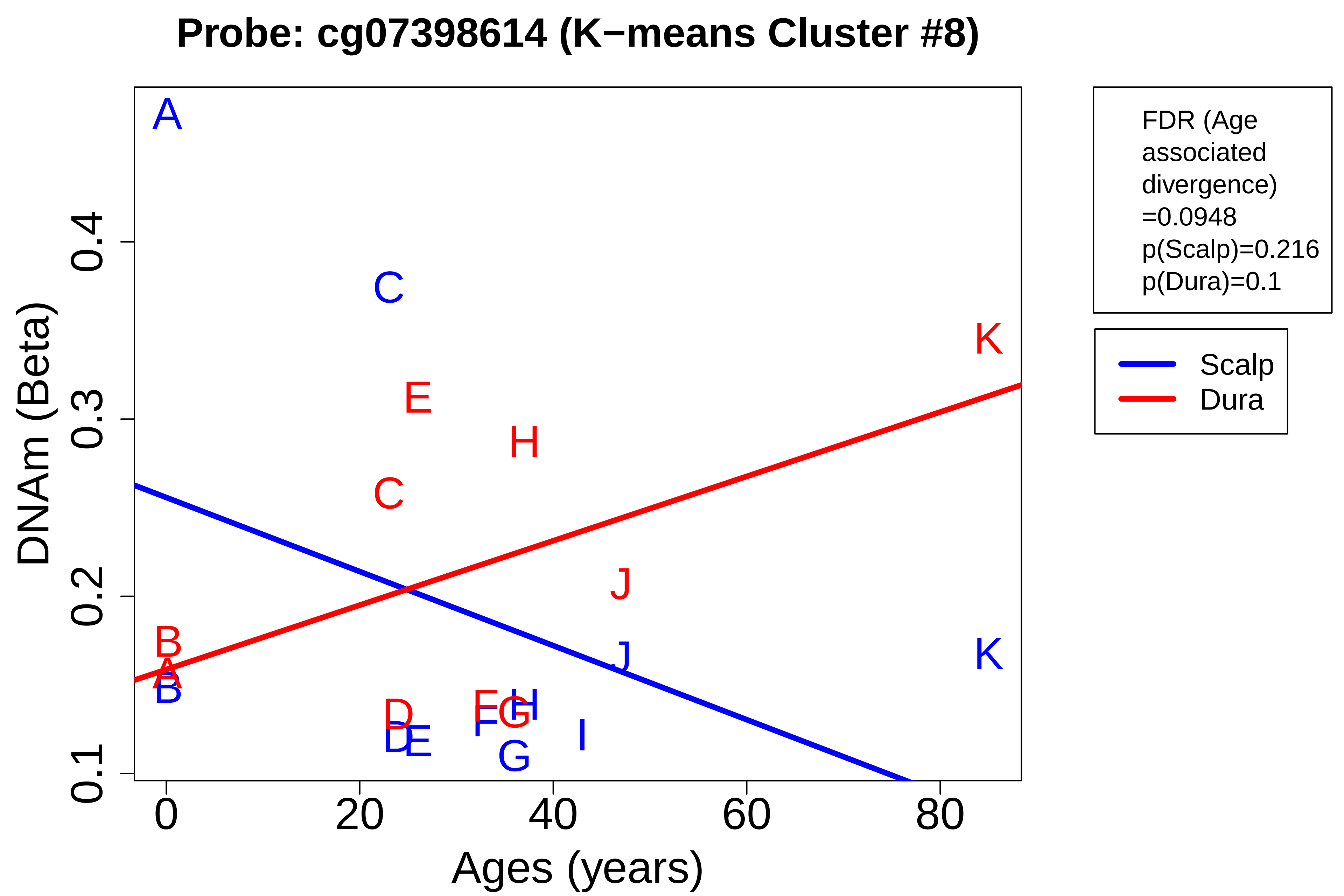

Probe: cg07398614 (K−means Cluster #8)
FDR (Age associated divergence) =0.0948
p(Scalp)=0.216
p(Dura)=0.1
Scalp
Dura
DNAm (Beta)
0.1
0.2
0.3
0.4
0
20
40
60
80
Ages (years)
A
B
C
D
E
F
G
H
I
J
K

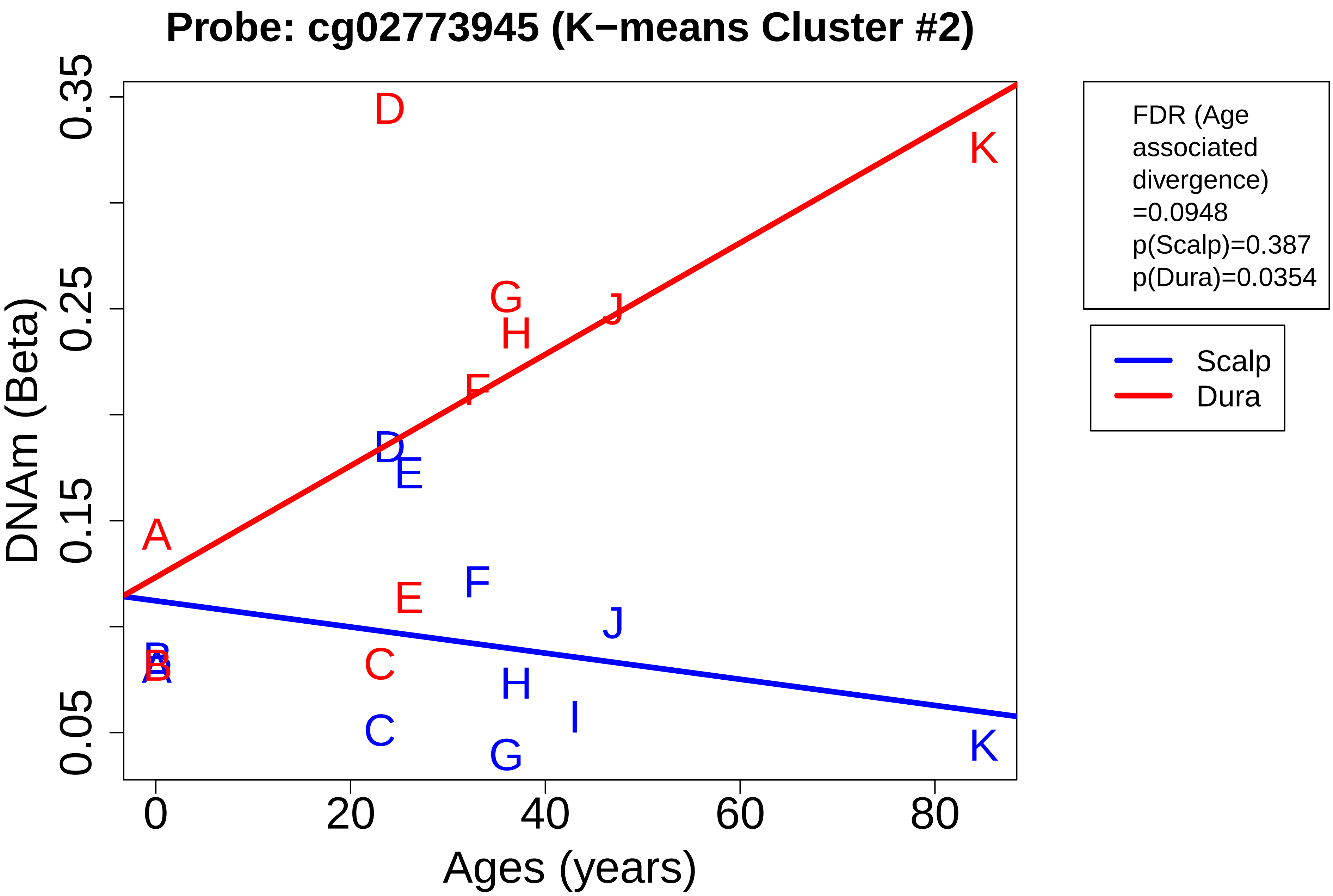

Probe: cg02773945 (K−means Cluster #2)
FDR (Age associated divergence) =0.0948
p(Scalp)=0.387
p(Dura)=0.0354
Scalp
Dura
DNAm (Beta)
0.05
0.15
0.25
0.35
0
20
40
60
80
Ages (years)

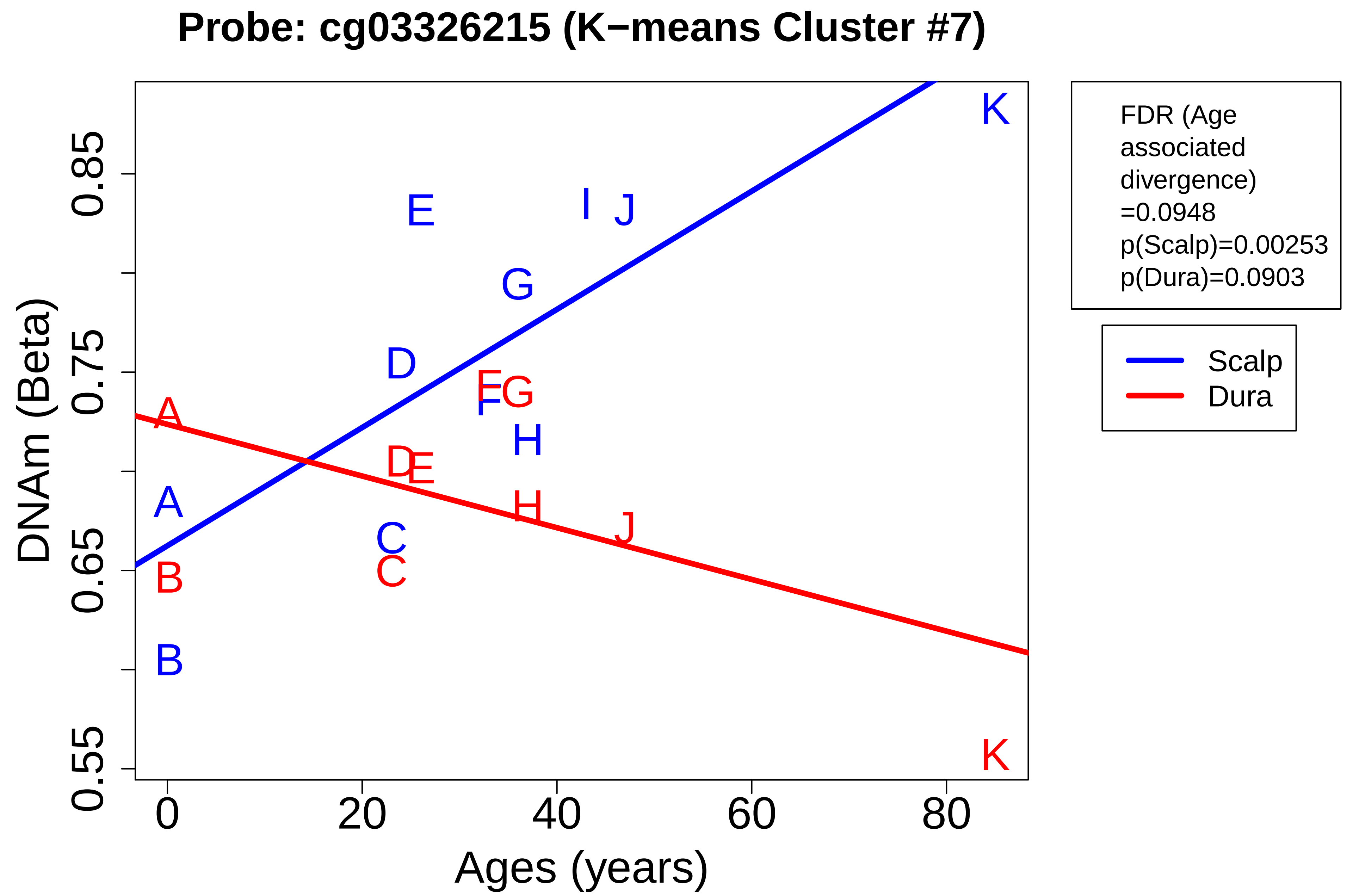

Probe: cg03326215 (K−means Cluster #7)
DNAm (Beta)
0.55
0.65
0.75
0.85
0
20
40
60
80
Ages (years)
FDR (Age associated divergence) =0.0948
p(Scalp)=0.00253
p(Dura)=0.0903
Scalp
Dura
A
B
C
D
E
F
G
H
I
J
K

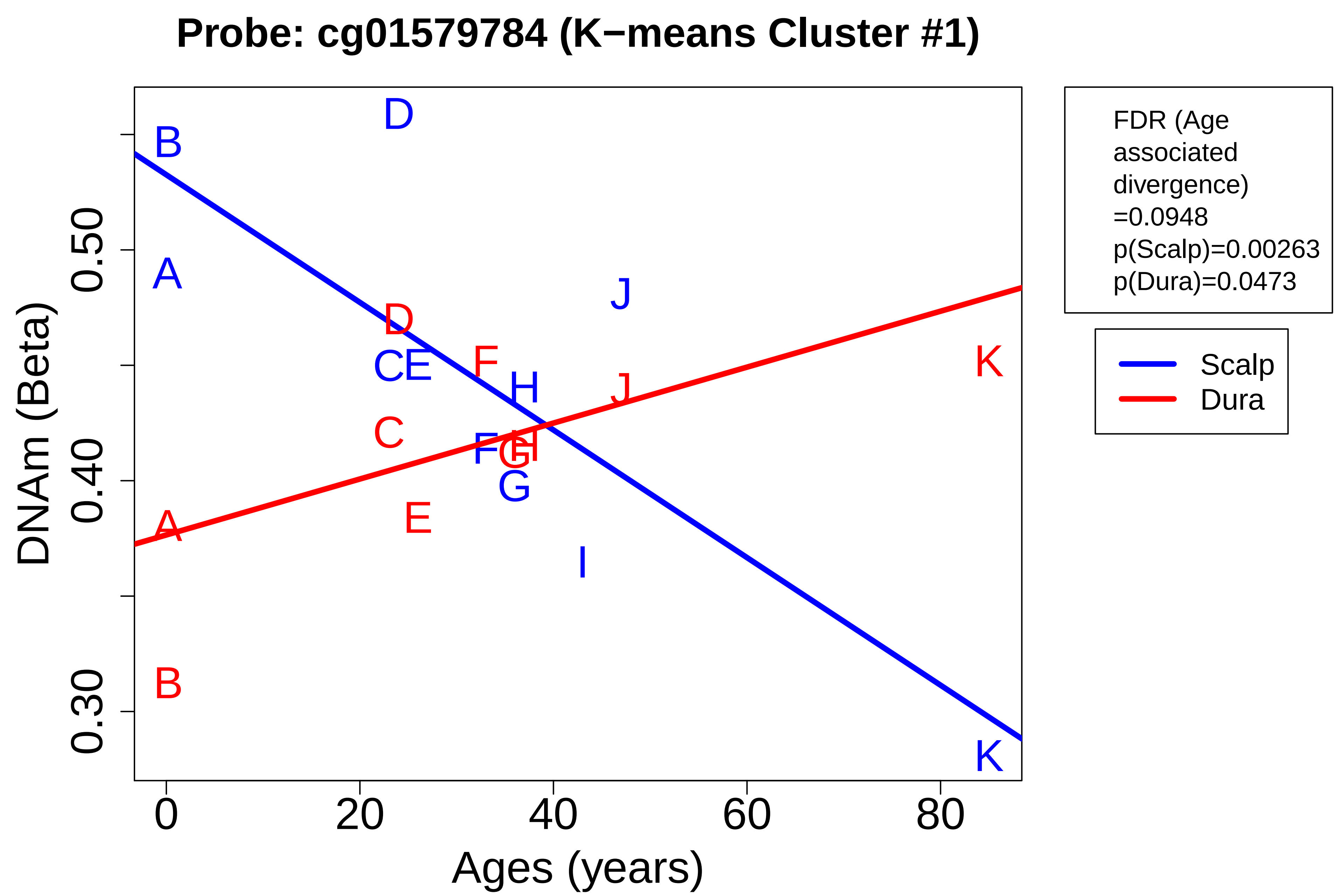

Probe: cg01579784 (K−means Cluster #1)
DNAm (Beta)
0.30
0.40
0.50
0
20
40
60
80
Ages (years)
FDR (Age associated divergence) =0.0948
p(Scalp)=0.00263
p(Dura)=0.0473
Scalp
Dura

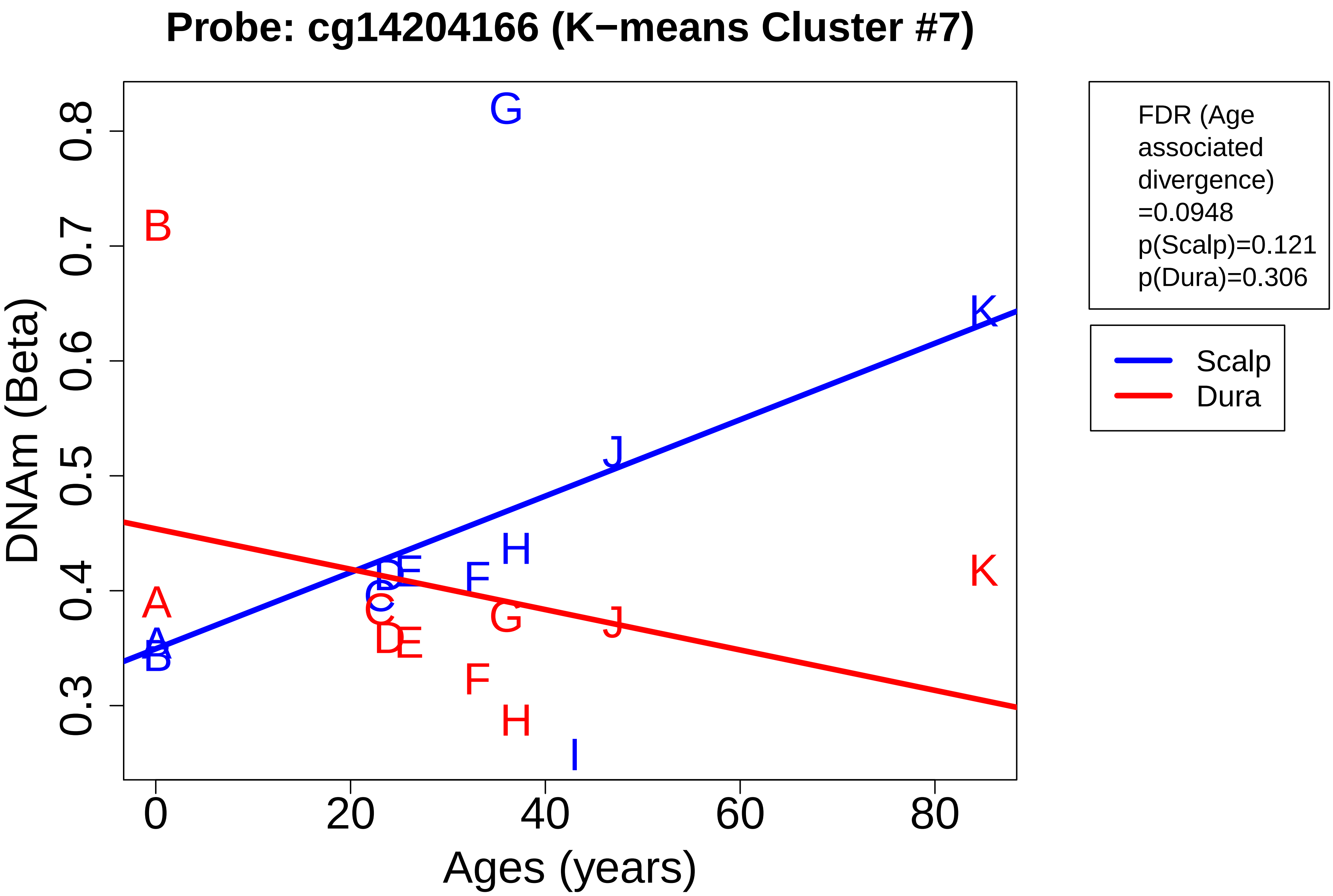

Probe: cg14204166 (K−means Cluster #7)
DNAm (Beta)
0.3
0.4
0.5
0.6
0.7
0.8
Ages (years)
0
20
40
60
80
FDR (Age associated divergence) =0.0948
p(Scalp)=0.121
p(Dura)=0.306
Scalp
Dura

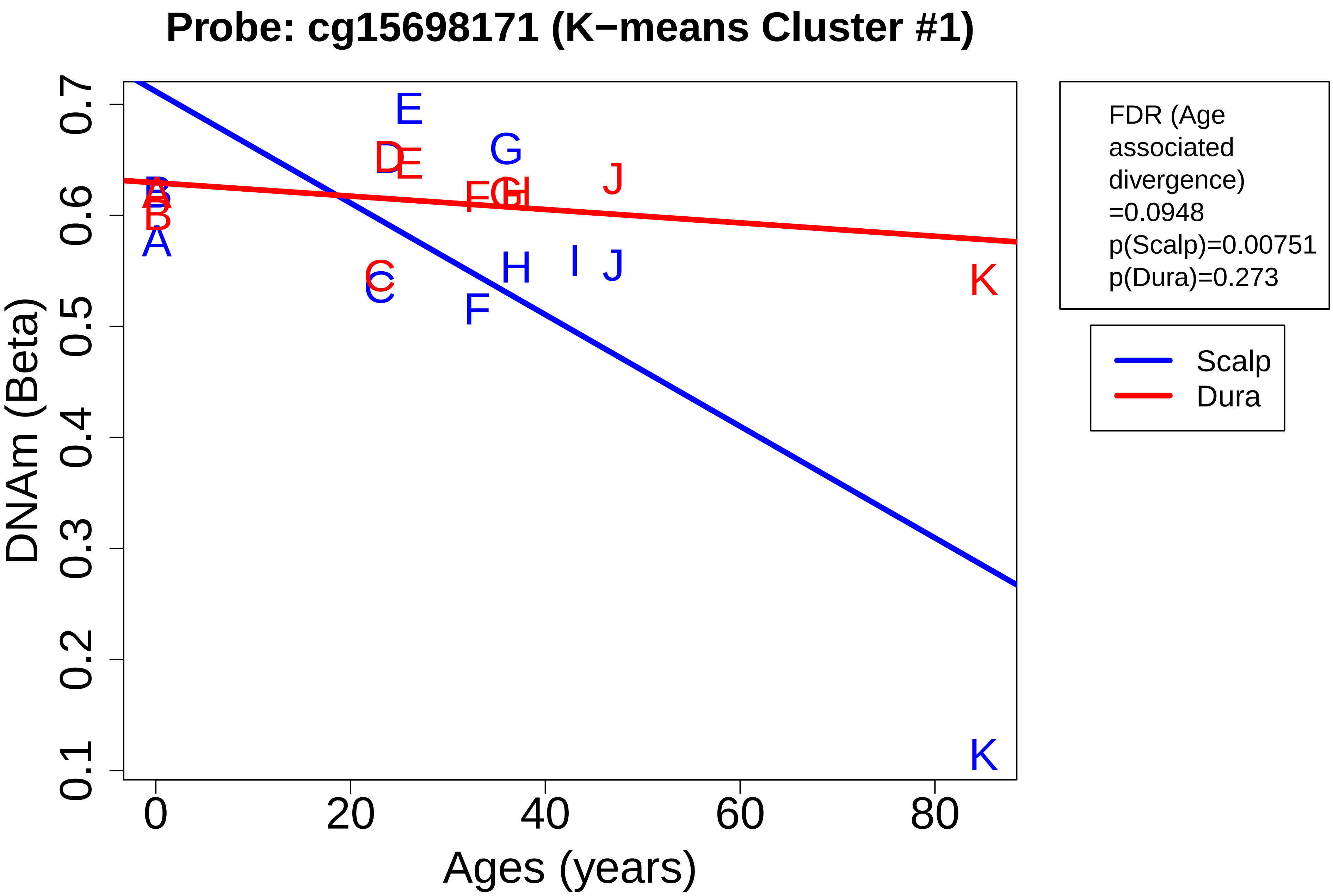

Probe: cg15698171 (K−means Cluster #1)
DNAm (Beta)
0.1
0.2
0.3
0.4
0.5
0.6
0.7
Ages (years)
0
20
40
60
80
A
B
C
D
E
F
G
H
I
J
K
FDR (Age associated divergence) =0.0948
p(Scalp)=0.00751
p(Dura)=0.273
Scalp
Dura

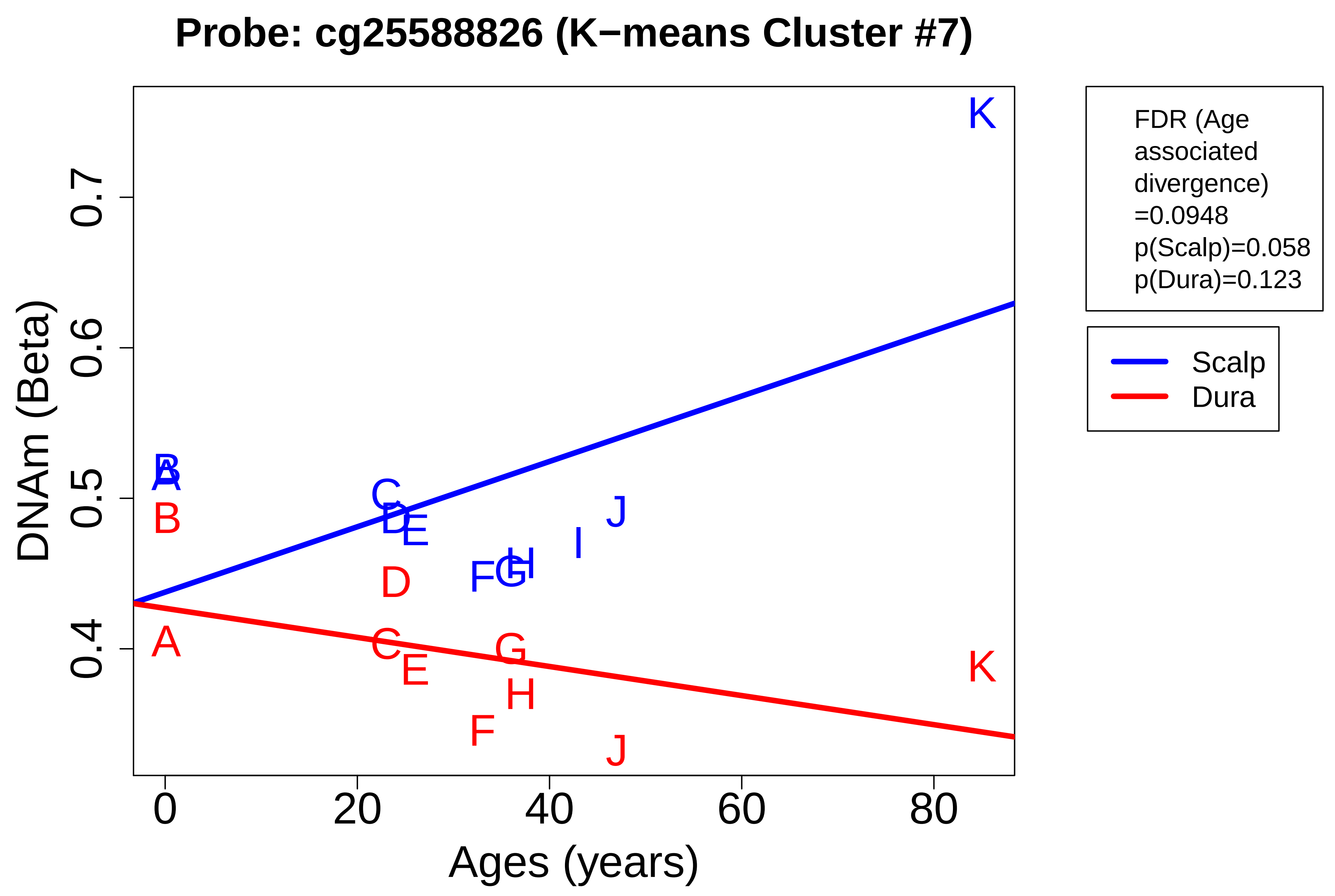

Probe: cg25588826 (K−means Cluster #7)
DNAm (Beta)
0.4
0.5
0.6
0.7
Ages (years)
0
20
40
60
80
FDR (Age associated divergence) =0.0948
p(Scalp)=0.058
p(Dura)=0.123
Scalp
Dura
A
B
C
D
E
F
G
H
I
J
K

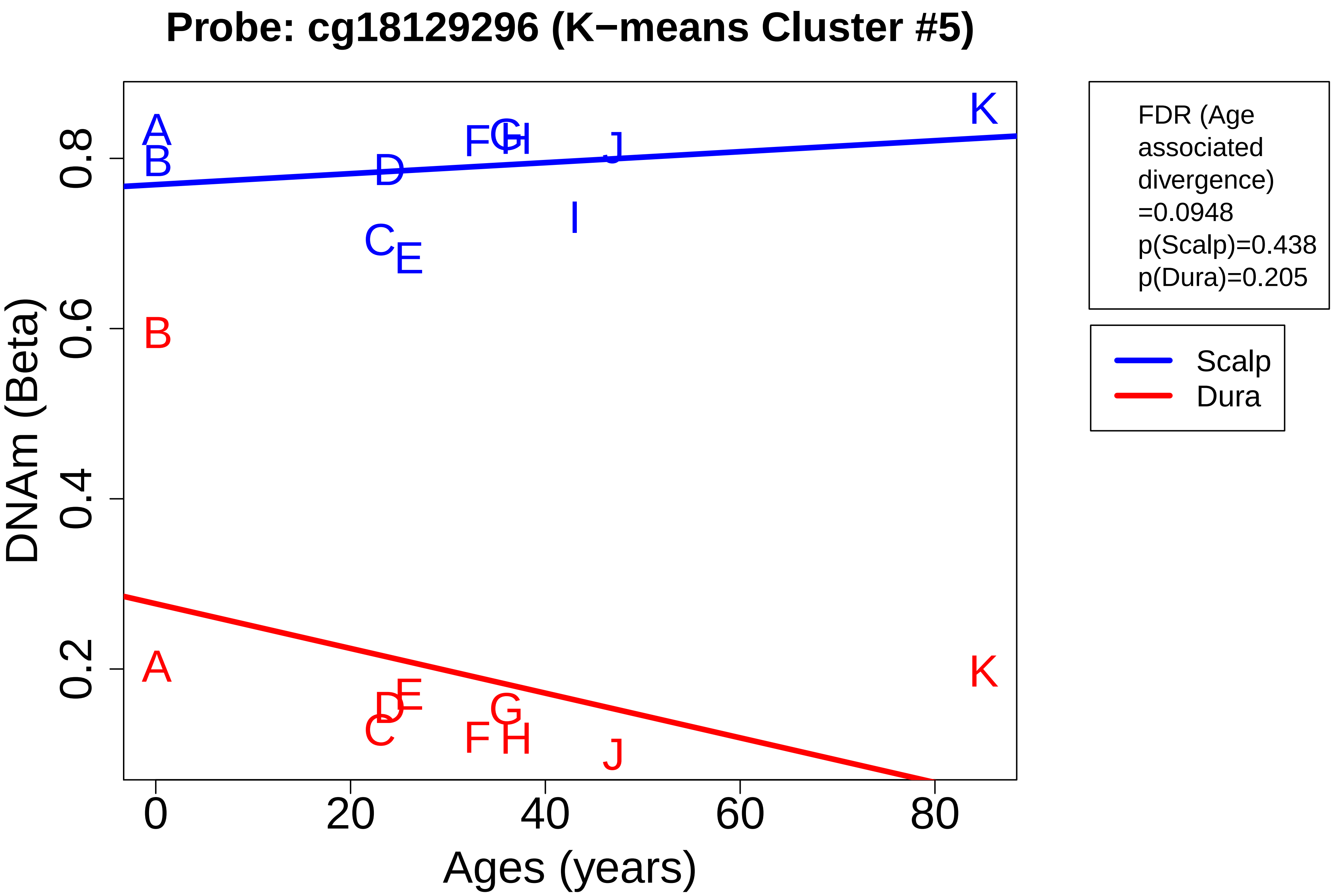

Probe: cg18129296 (K−means Cluster #5)
FDR (Age associated divergence) =0.0948
p(Scalp)=0.438
p(Dura)=0.205
Scalp
Dura
DNAm (Beta)
0.2
0.4
0.6
0.8
0
20
40
60
80
Ages (years)
A
B
C
D
E
F
G
H
I
J
K

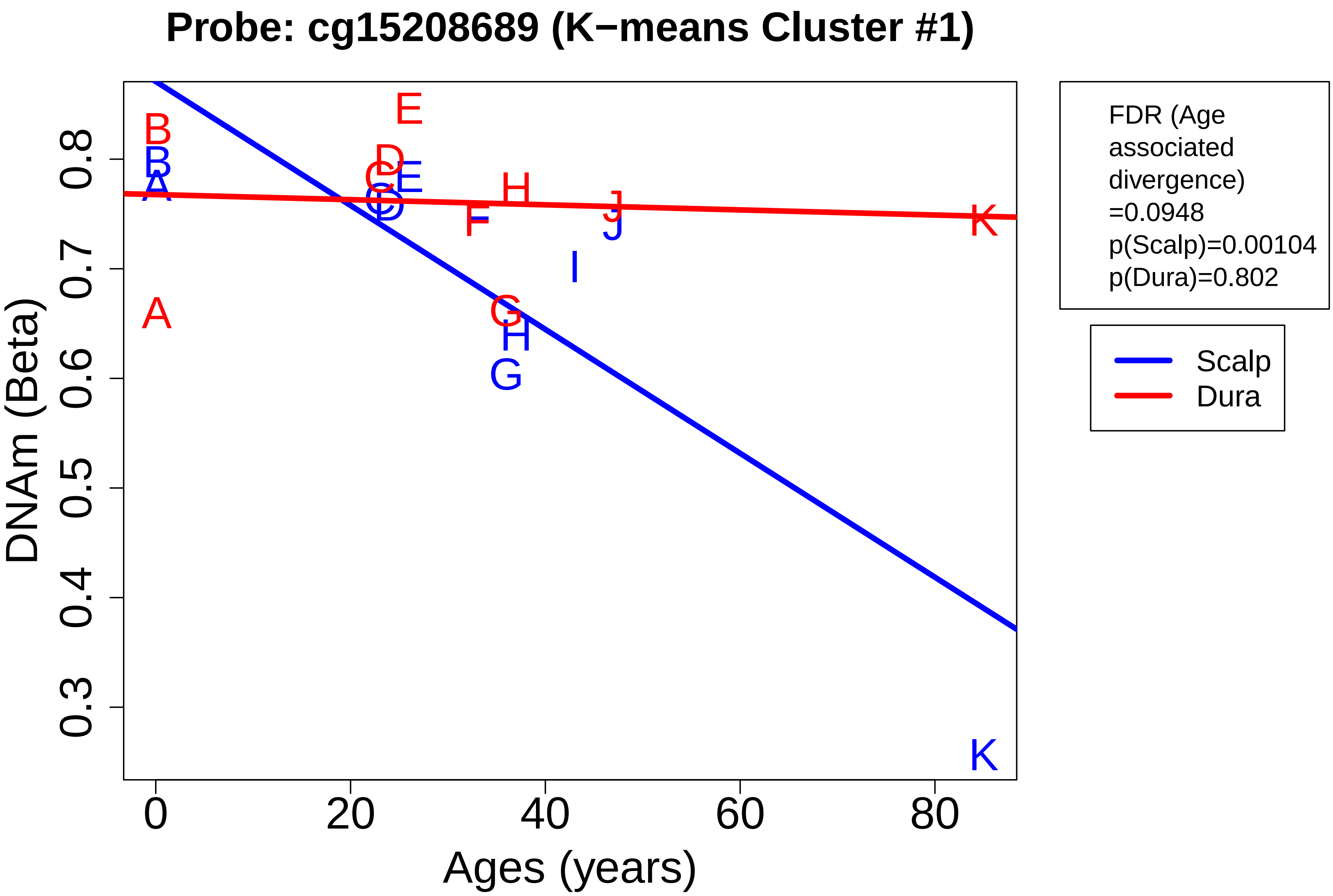

Probe: cg15208689 (K−means Cluster #1)
DNAm (Beta)
0.3
0.4
0.5
0.6
0.7
0.8
0
20
40
60
80
Ages (years)
FDR (Age associated divergence) =0.0948
p(Scalp)=0.00104
p(Dura)=0.802
Scalp
Dura

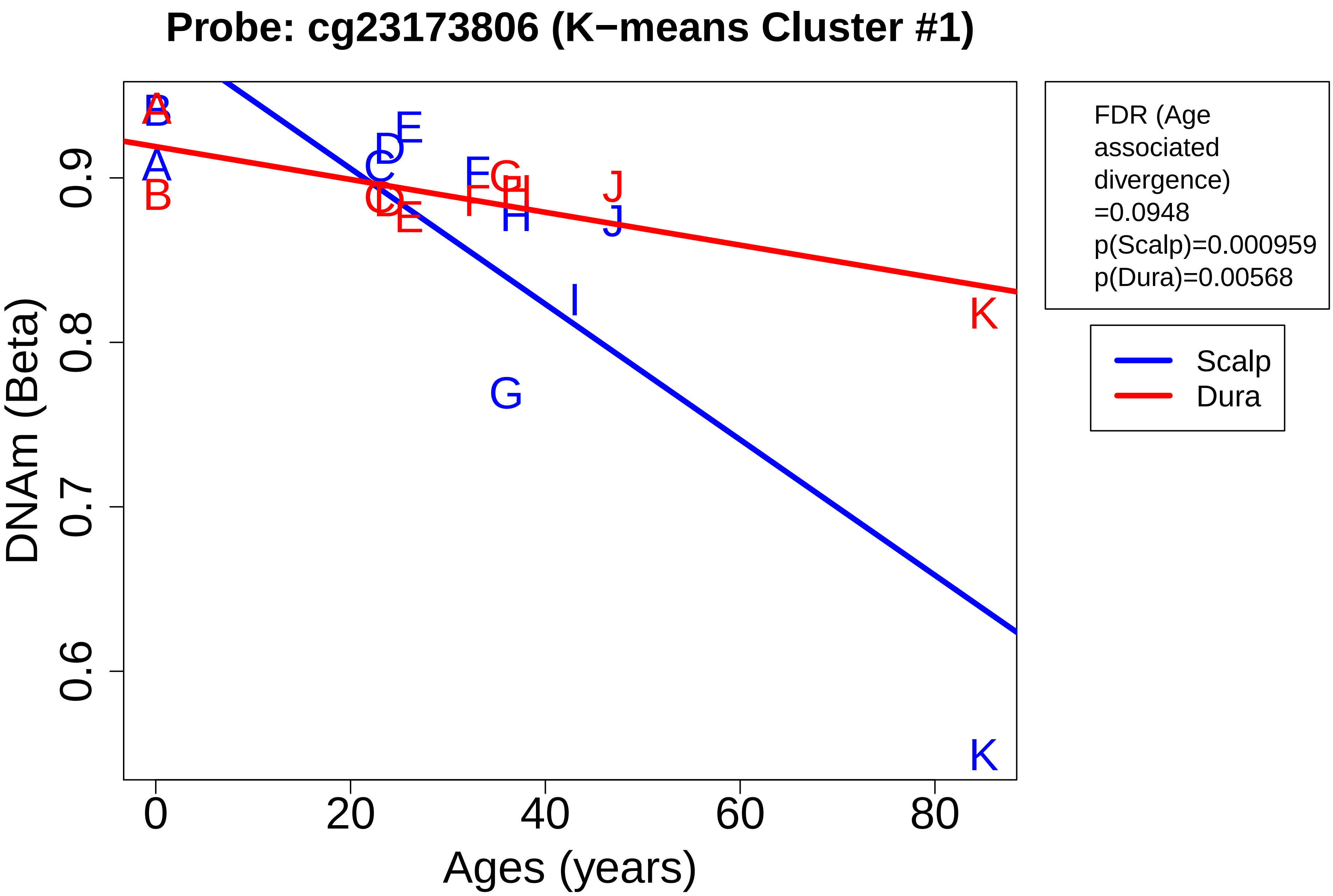

Probe: cg23173806 (K−means Cluster #1)
FDR (Age associated divergence) =0.0948
p(Scalp)=0.000959
p(Dura)=0.00568
Scalp
Dura
DNAm (Beta)
0.6
0.7
0.8
0.9
0
20
40
60
80
Ages (years)

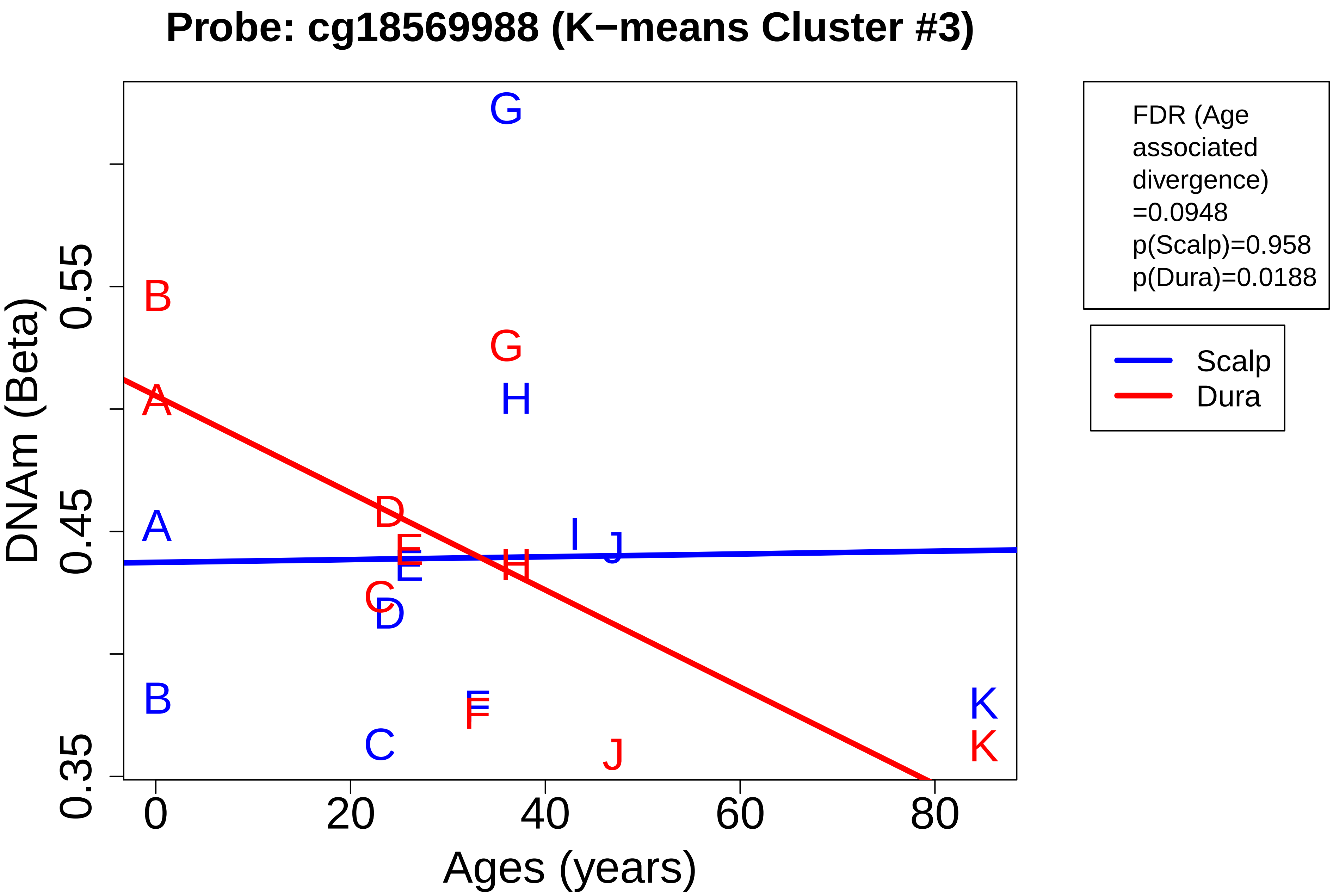

Probe: cg18569988 (K−means Cluster #3)
DNAm (Beta)
0.35
0.45
0.55
Ages (years)
0
20
40
60
80
G
B
G
A
H
D
A
I
J
E
E
H
C
D
B
F
F
K
C
J
K
FDR (Age associated divergence) =0.0948
p(Scalp)=0.958
p(Dura)=0.0188
Scalp
Dura

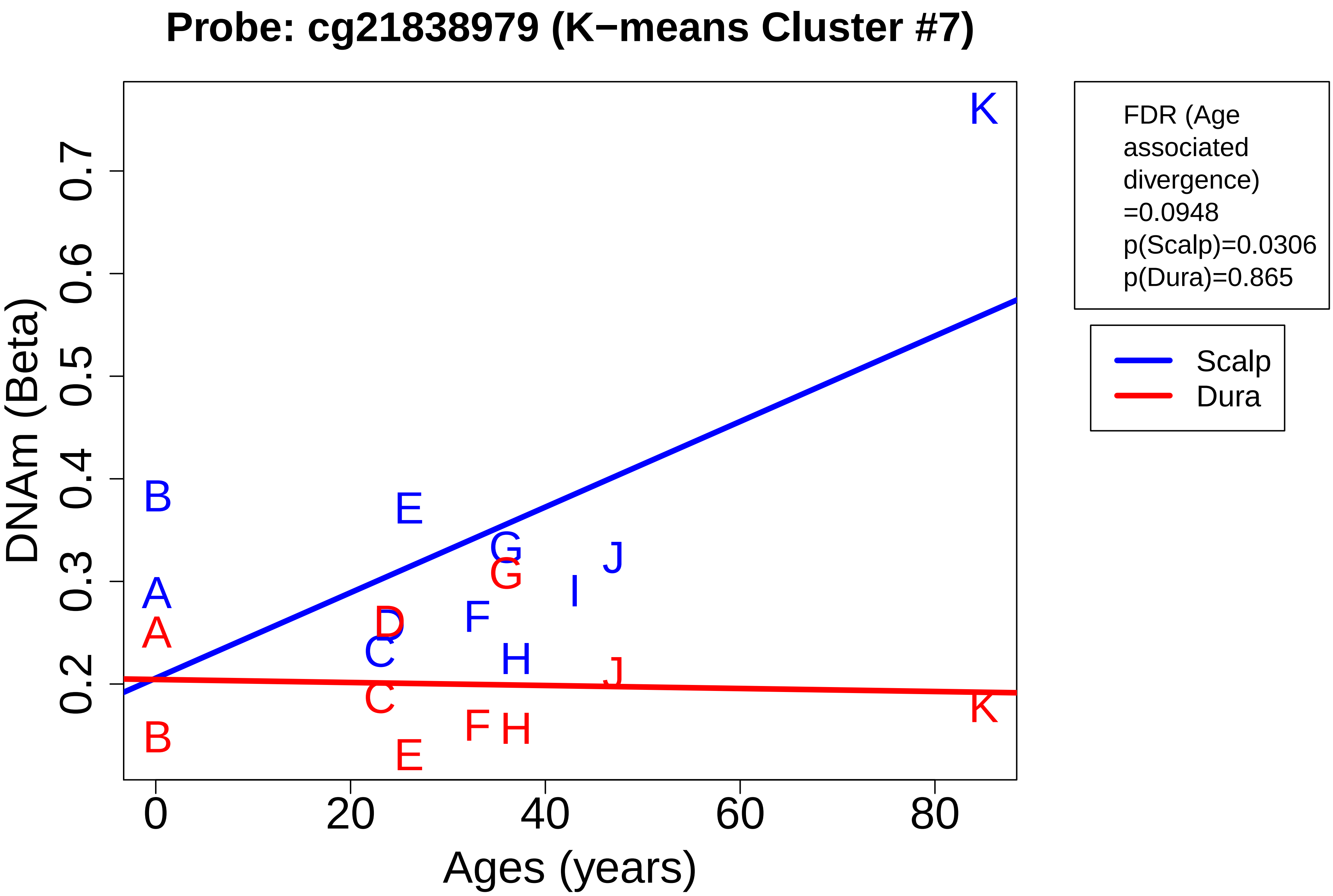
Probe: cg21838979 (K−means Cluster #7)
DNAm (Beta)
0.2
0.3
0.4
0.5
0.6
0.7
Ages (years)
0
20
40
60
80
FDR (Age associated divergence) =0.0948
p(Scalp)=0.0306
p(Dura)=0.865
Scalp
Dura
A
B
C
D
E
F
G
H
I
J
K

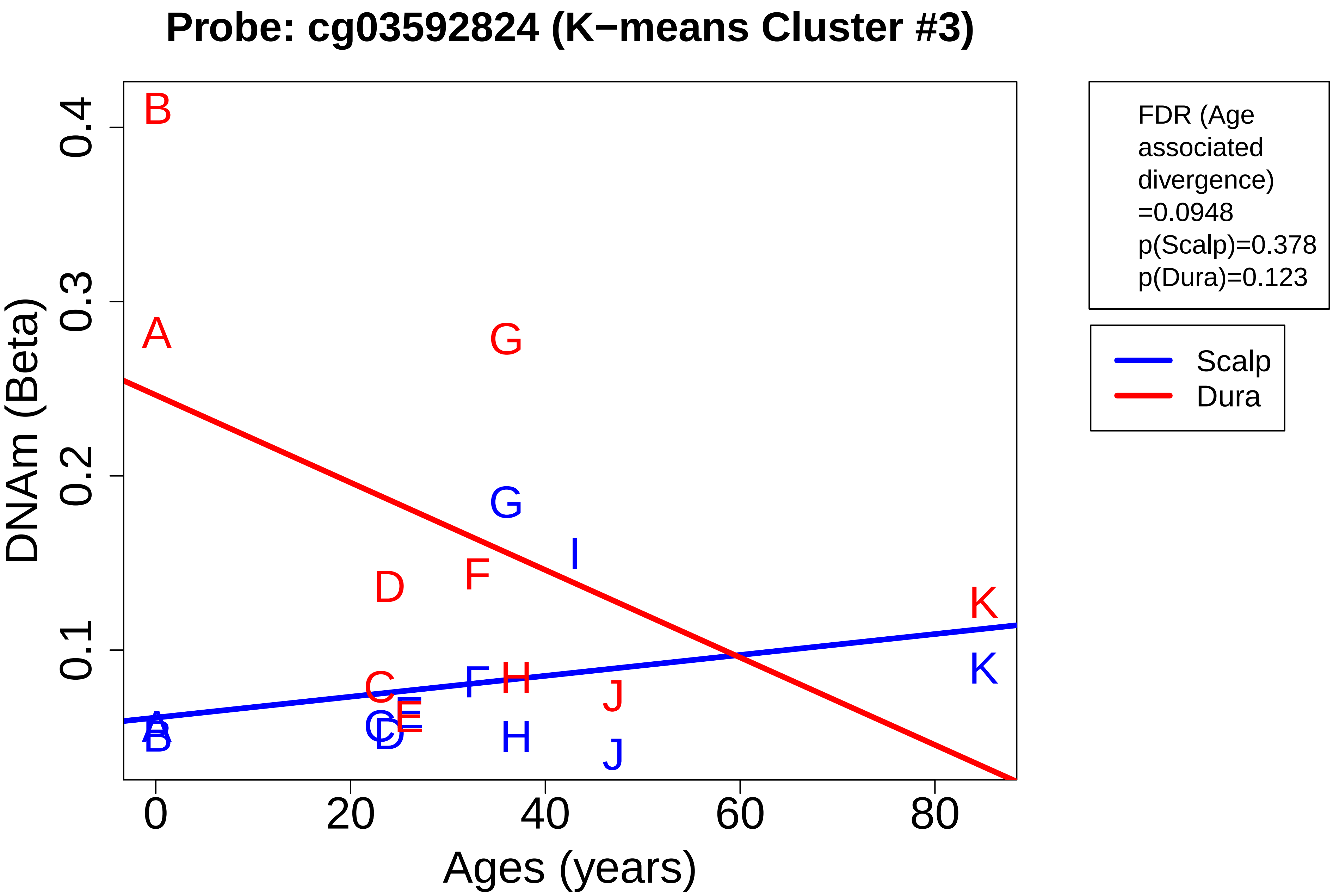
Probe: cg03592824 (K−means Cluster #3)
DNAm (Beta)
0.1
0.2
0.3
0.4
Ages (years)
0
20
40
60
80
FDR (Age associated divergence) =0.0948
p(Scalp)=0.378
p(Dura)=0.123
Scalp
Dura

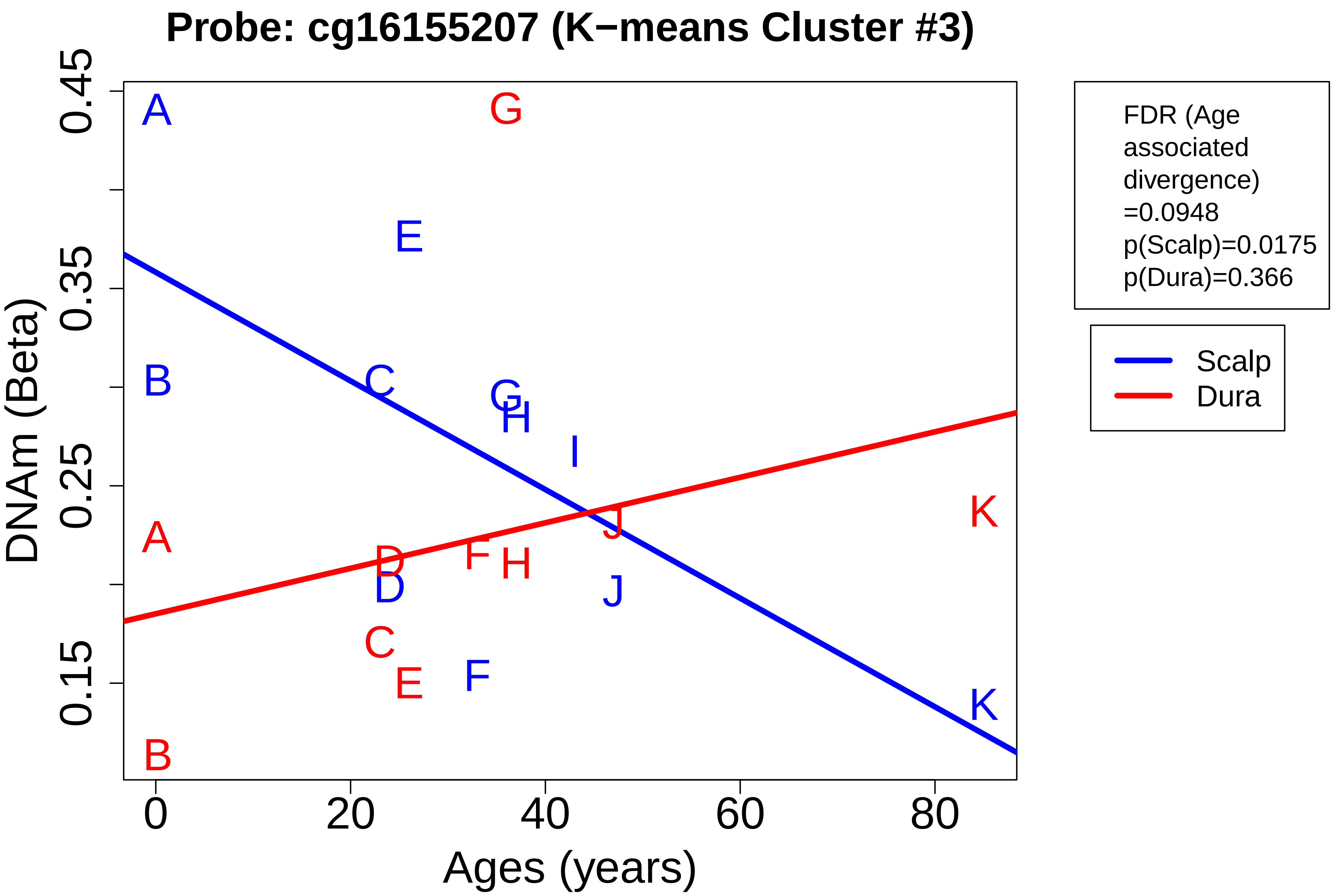

Probe: cg16155207 (K−means Cluster #3)
FDR (Age associated divergence) =0.0948
p(Scalp)=0.0175
p(Dura)=0.366
Scalp
Dura
DNAm (Beta)
0.15
0.25
0.35
0.45
0
20
40
60
80
Ages (years)

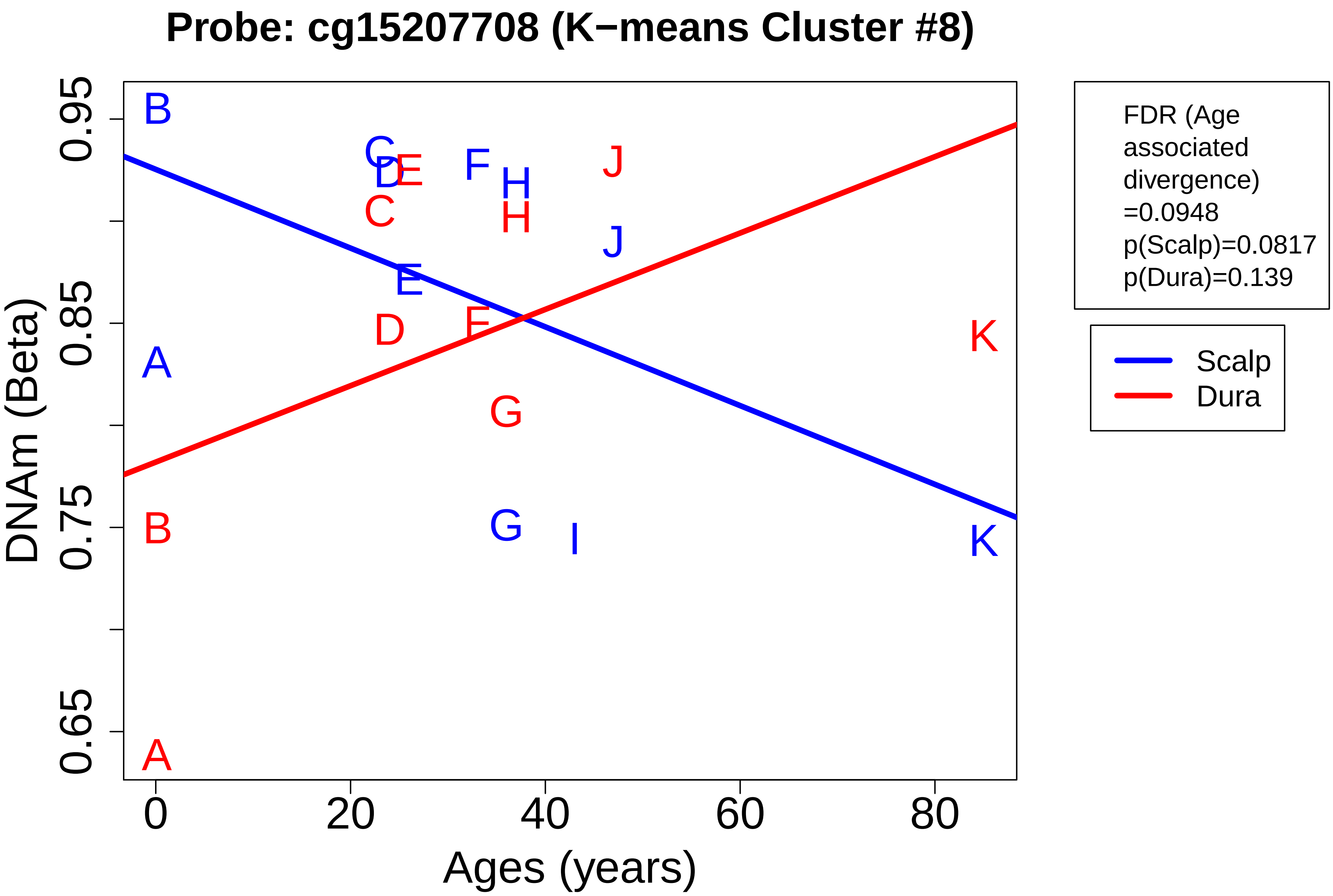

Probe: cg15207708 (K−means Cluster #8)
DNAm (Beta)
0.65
0.75
0.85
0.95
Ages (years)
0
20
40
60
80
FDR (Age associated divergence) =0.0948
p(Scalp)=0.0817
p(Dura)=0.139
Scalp
Dura

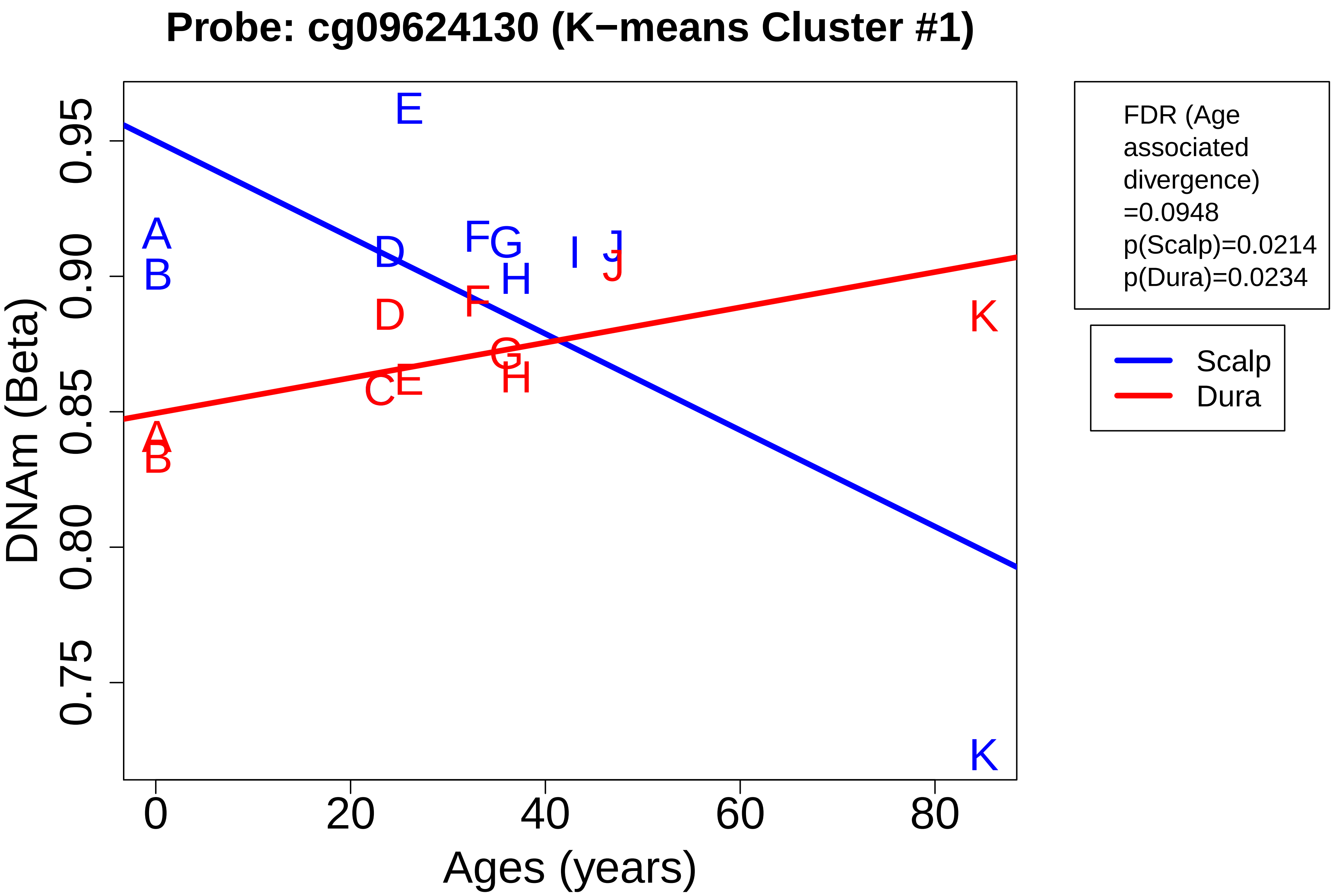
Probe: cg09624130 (K−means Cluster #1)
DNAm (Beta)
0.75
0.80
0.85
0.90
0.95
0
20
40
60
80
Ages (years)
A
B
C
D
E
F
G
H
I
J
K
FDR (Age associated divergence) =0.0948
p(Scalp)=0.0214
p(Dura)=0.0234
Scalp
Dura

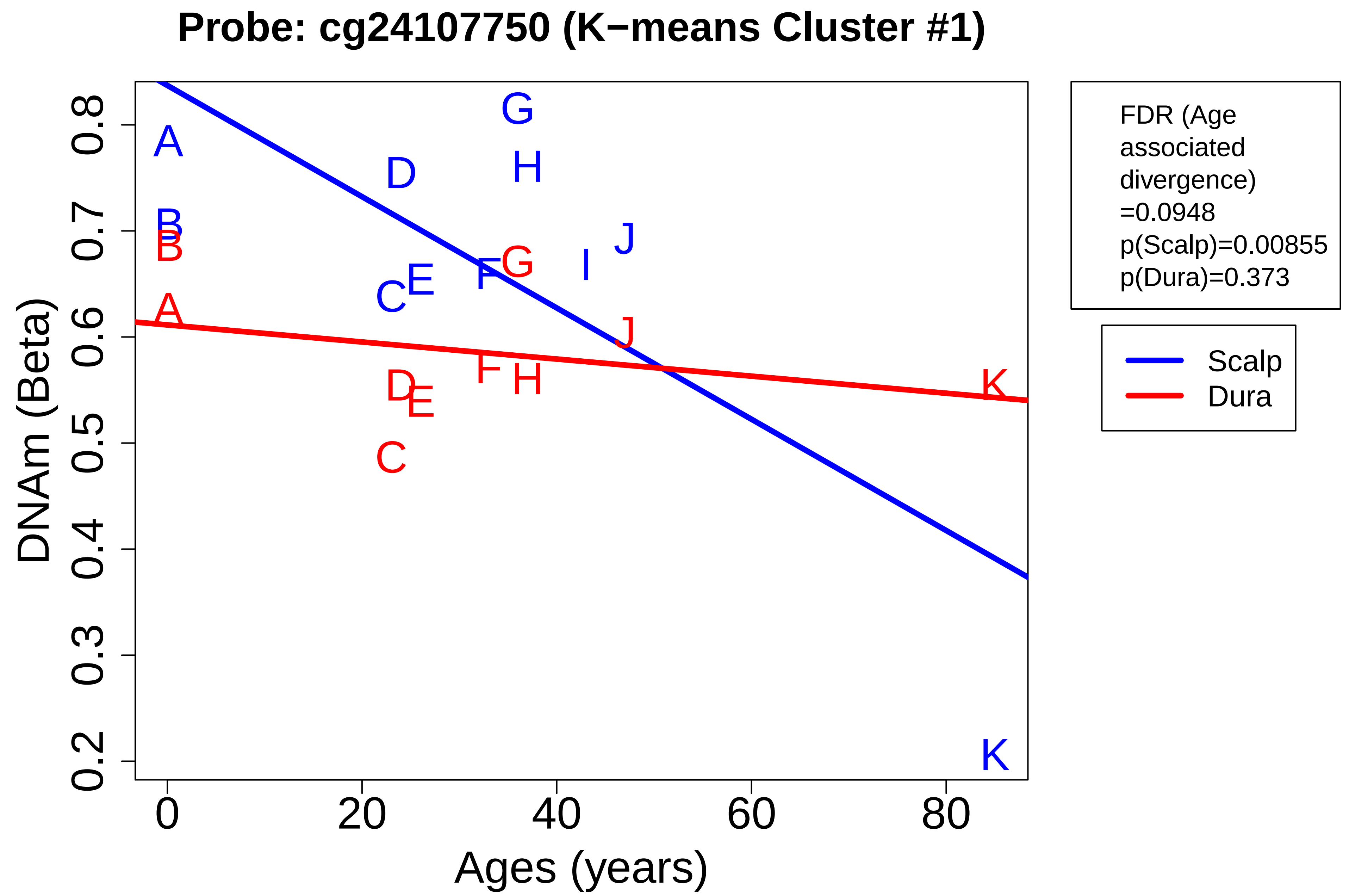

Probe: cg24107750 (K−means Cluster #1)
DNAm (Beta)
0.2
0.3
0.4
0.5
0.6
0.7
0.8
0
20
40
60
80
Ages (years)
A
B
C
D
E
F
G
H
I
J
K
FDR (Age associated divergence) =0.0948
p(Scalp)=0.00855
p(Dura)=0.373
Scalp
Dura

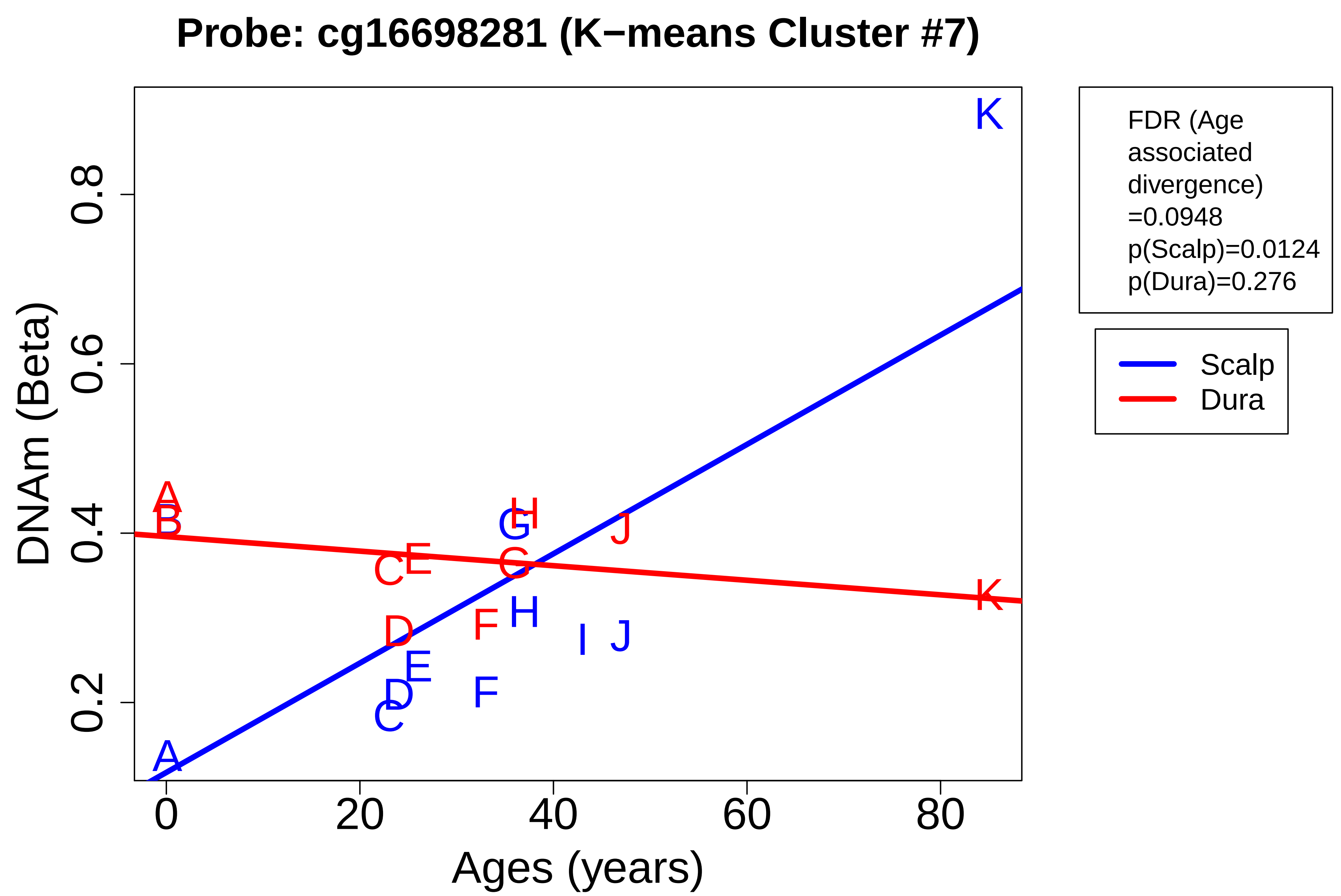
Probe: cg16698281 (K−means Cluster #7)
FDR (Age associated divergence) =0.0948
p(Scalp)=0.0124
p(Dura)=0.276
Scalp
Dura
DNAm (Beta)
0.2
0.4
0.6
0.8
0
20
40
60
80
Ages (years)
A
B
C
D
E
F
G
H
I
J
K

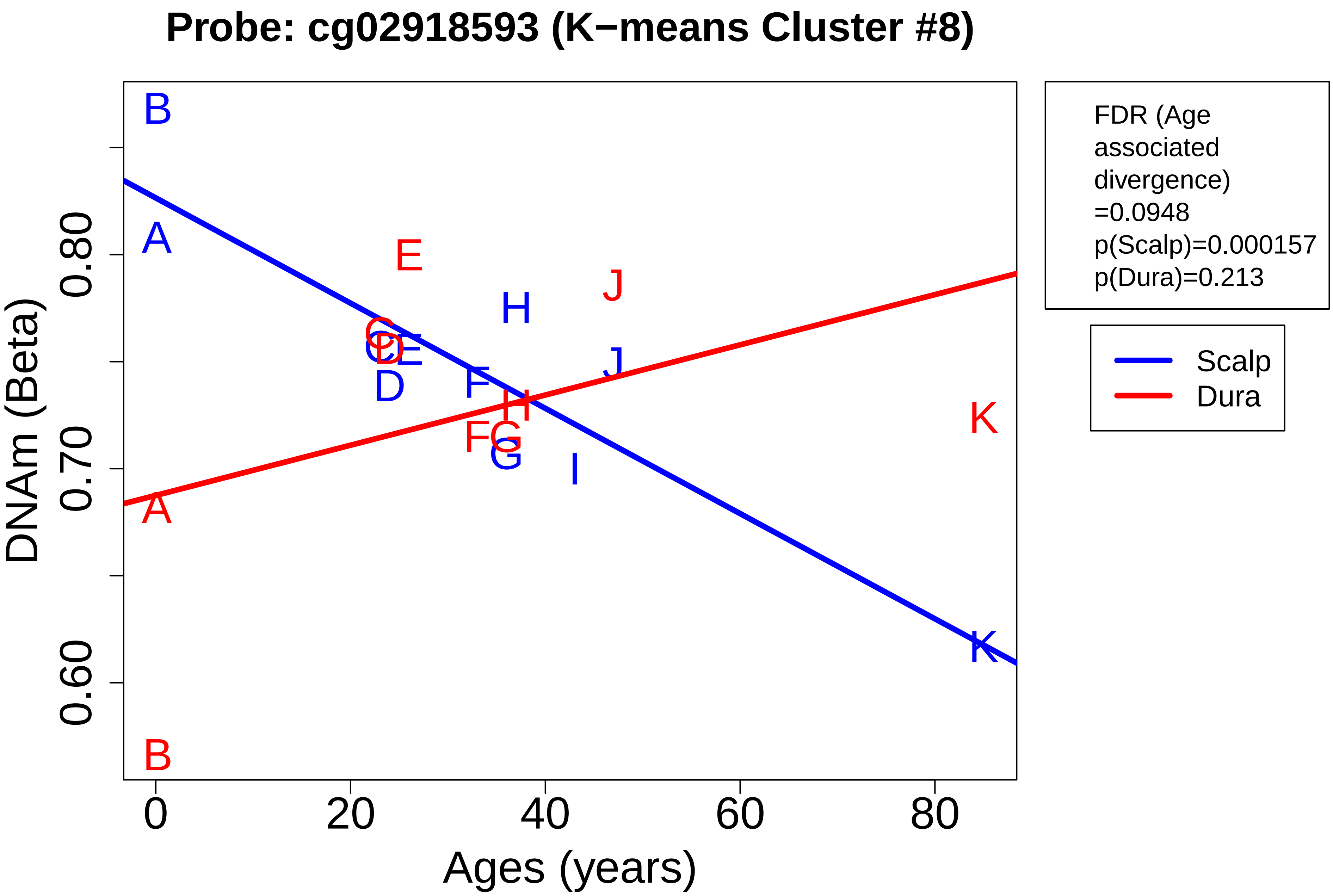

Probe: cg02918593 (K−means Cluster #8)
DNAm (Beta)
0.60
0.70
0.80
Ages (years)
0
20
40
60
80
FDR (Age associated divergence) =0.0948
p(Scalp)=0.000157
p(Dura)=0.213
Scalp
Dura

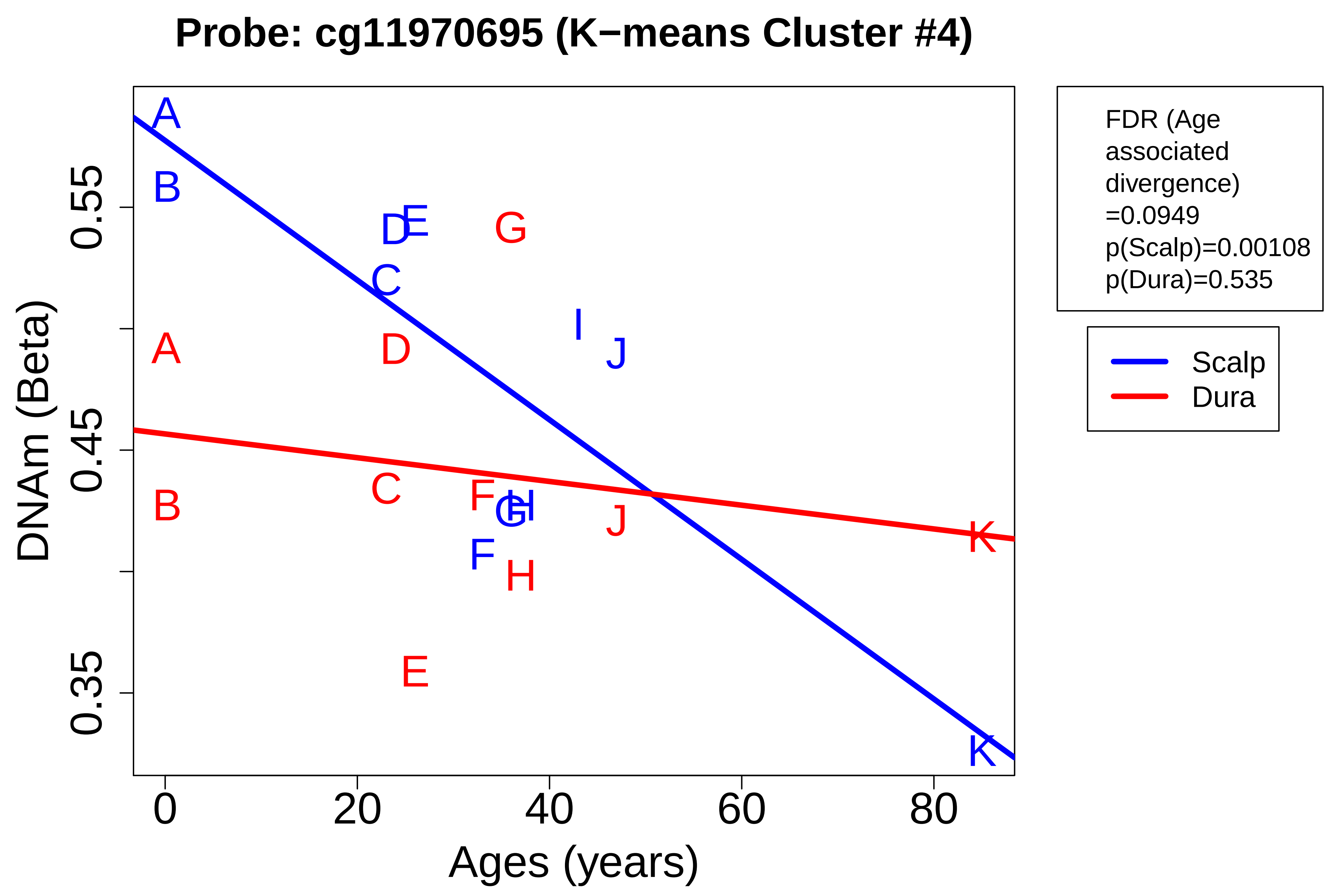
Probe: cg11970695 (K−means Cluster #4)
DNAm (Beta)
0.35
0.45
0.55
Ages (years)
0
20
40
60
80
A
B
C
D
E
F
G
H
I
J
K
FDR (Age associated divergence) =0.0949
p(Scalp)=0.00108
p(Dura)=0.535
Scalp
Dura

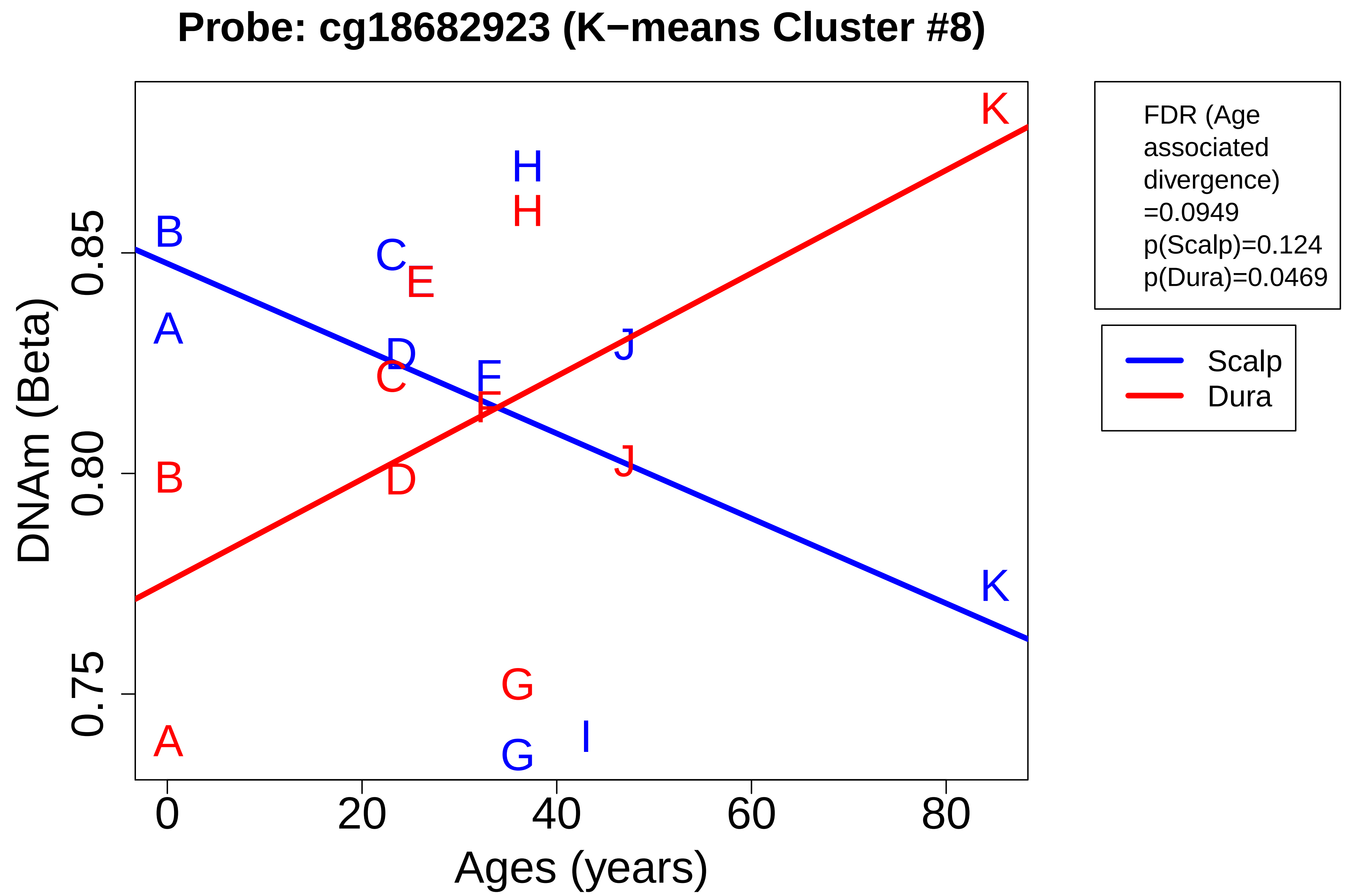
Probe: cg18682923 (K−means Cluster #8)
FDR (Age associated divergence) =0.0949
p(Scalp)=0.124
p(Dura)=0.0469
Scalp
Dura
DNAm (Beta)
0.75
0.80
0.85
0
20
40
60
80
Ages (years)
A
B
C
D
E
F
G
H
I
J
K

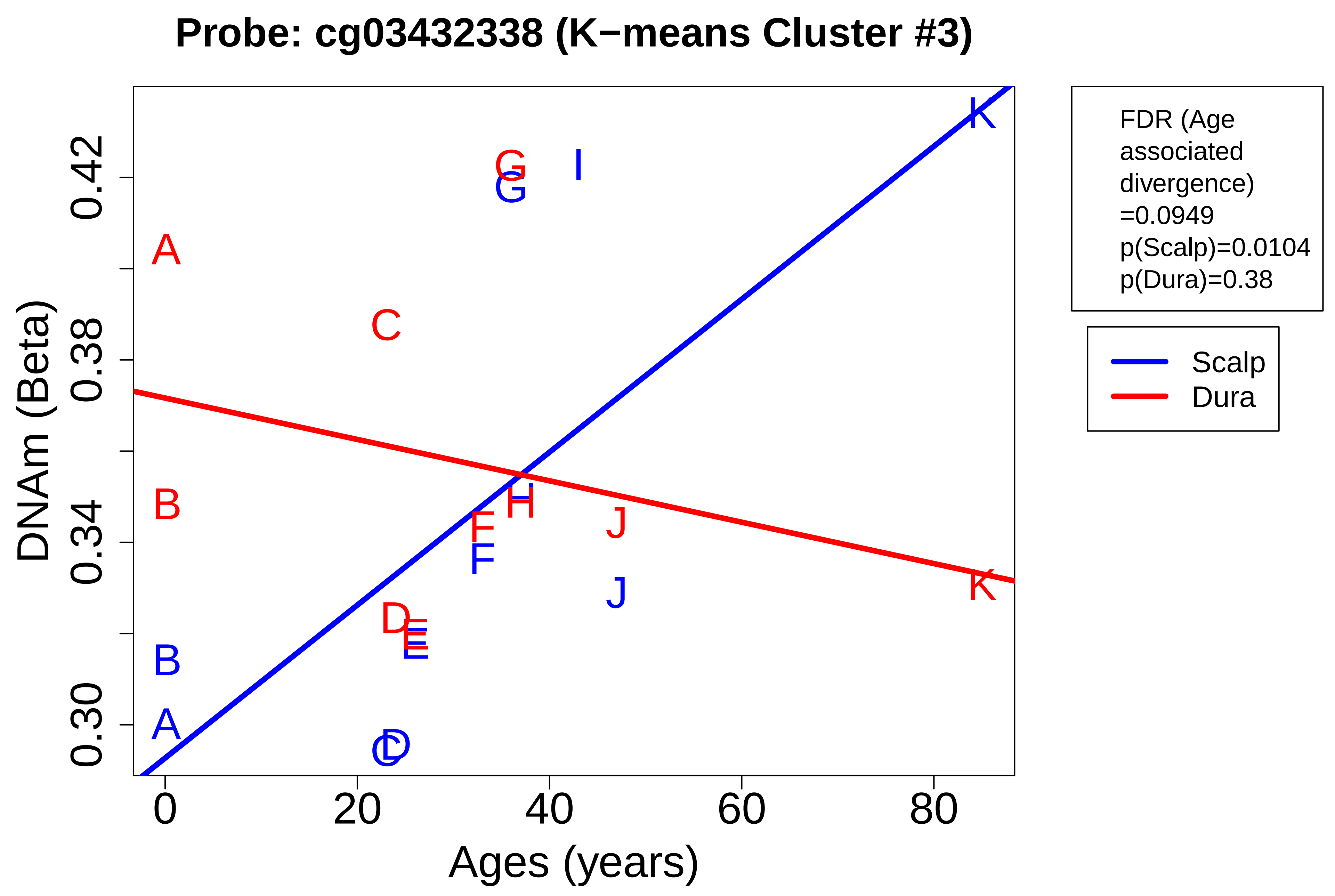

Probe: cg03432338 (K−means Cluster #3)
DNAm (Beta)
0.30
0.34
0.38
0.42
0
20
40
60
80
Ages (years)
A
B
C
D
E
F
G
H
I
J
K
FDR (Age associated divergence) =0.0949
p(Scalp)=0.0104
p(Dura)=0.38
Scalp
Dura

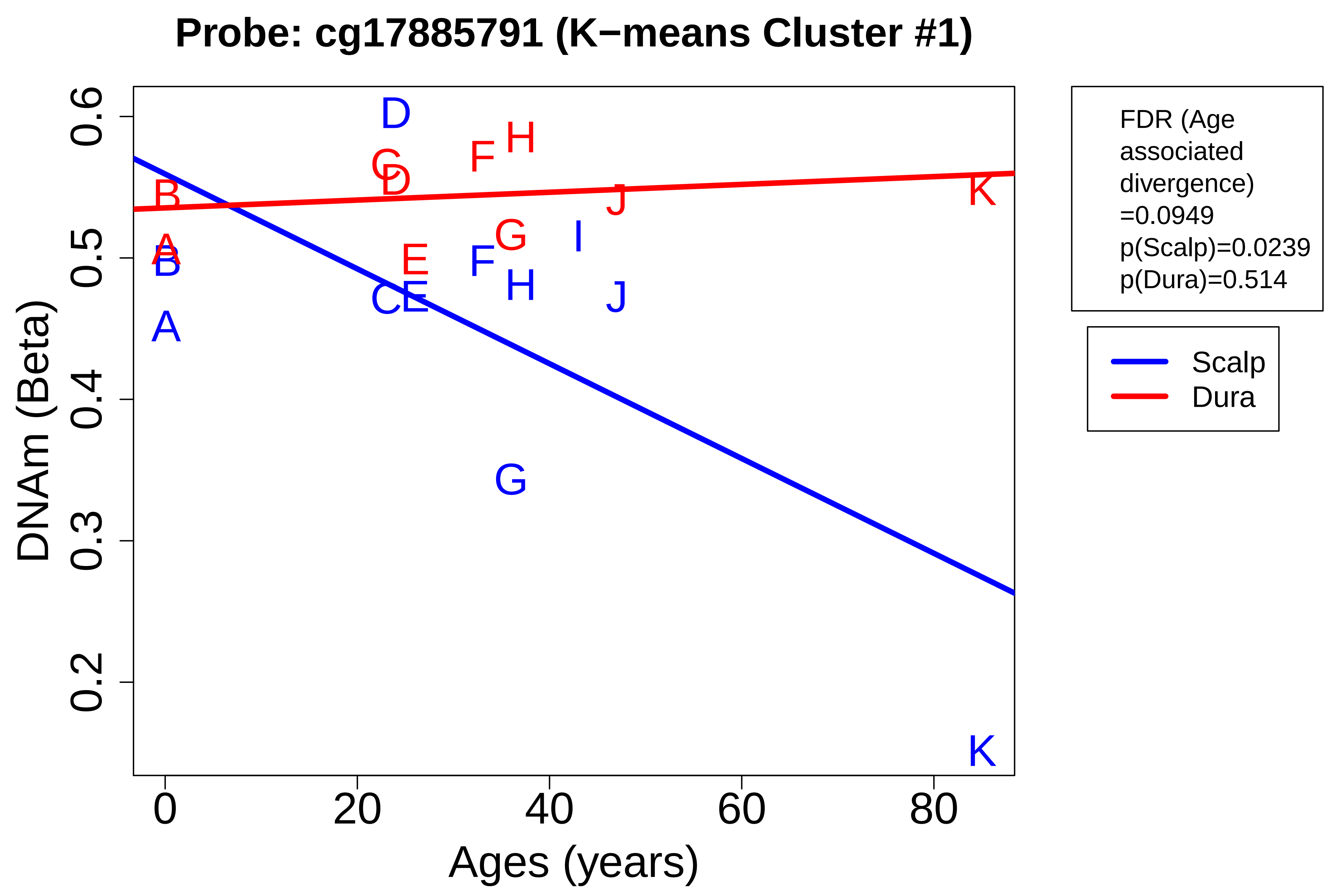

Probe: cg17885791 (K−means Cluster #1)
DNAm (Beta)
0.2
0.3
0.4
0.5
0.6
0
20
40
60
80
Ages (years)
A
B
C
D
E
F
G
H
I
J
K
FDR (Age associated divergence) =0.0949
p(Scalp)=0.0239
p(Dura)=0.514
Scalp
Dura

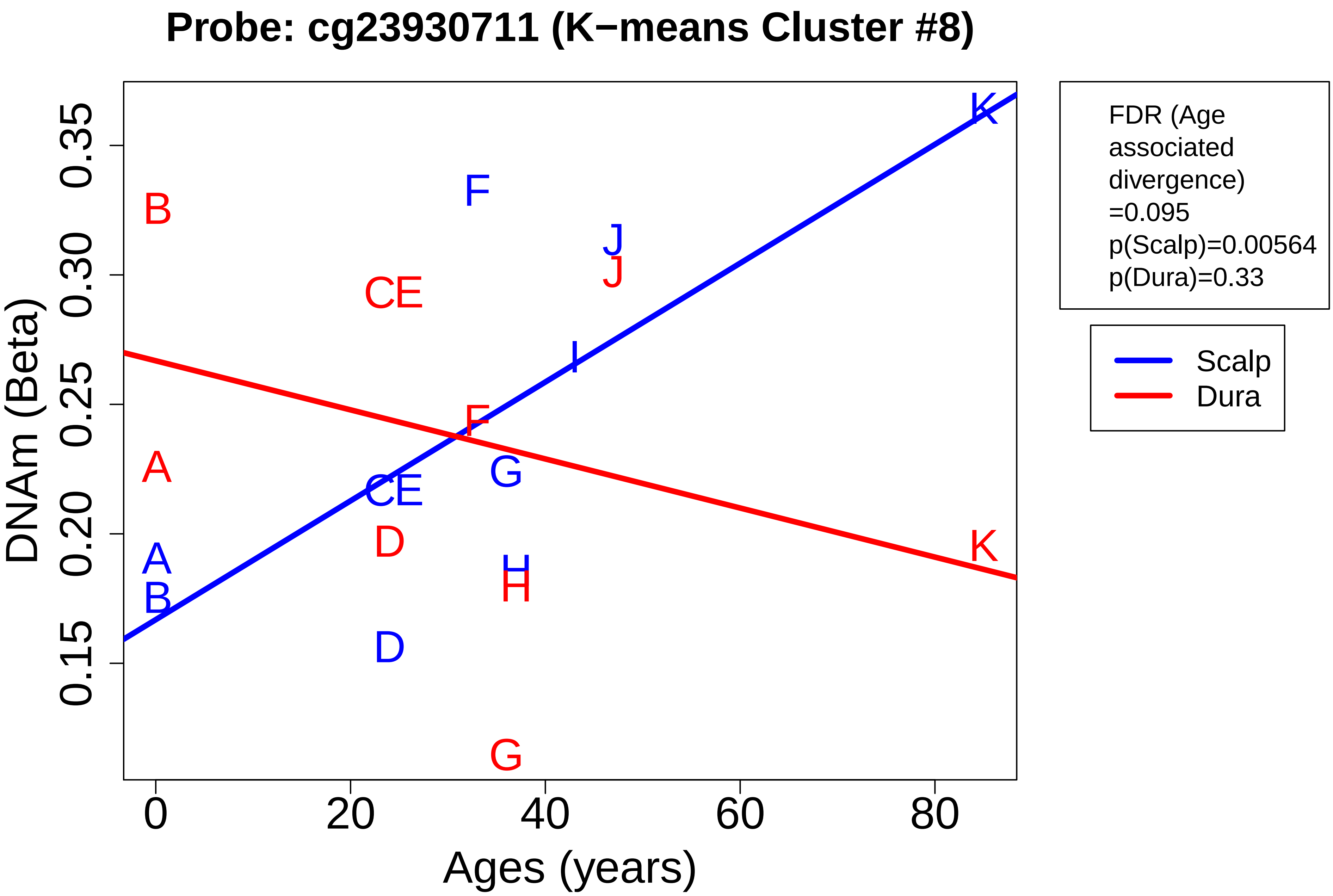

Probe: cg23930711 (K−means Cluster #8)
DNAm (Beta)
0.15
0.20
0.25
0.30
0.35
Ages (years)
0
20
40
60
80
FDR (Age associated divergence) =0.095
p(Scalp)=0.00564
p(Dura)=0.33
Scalp
Dura
A
B
CE
D
F
G
H
I
J
K

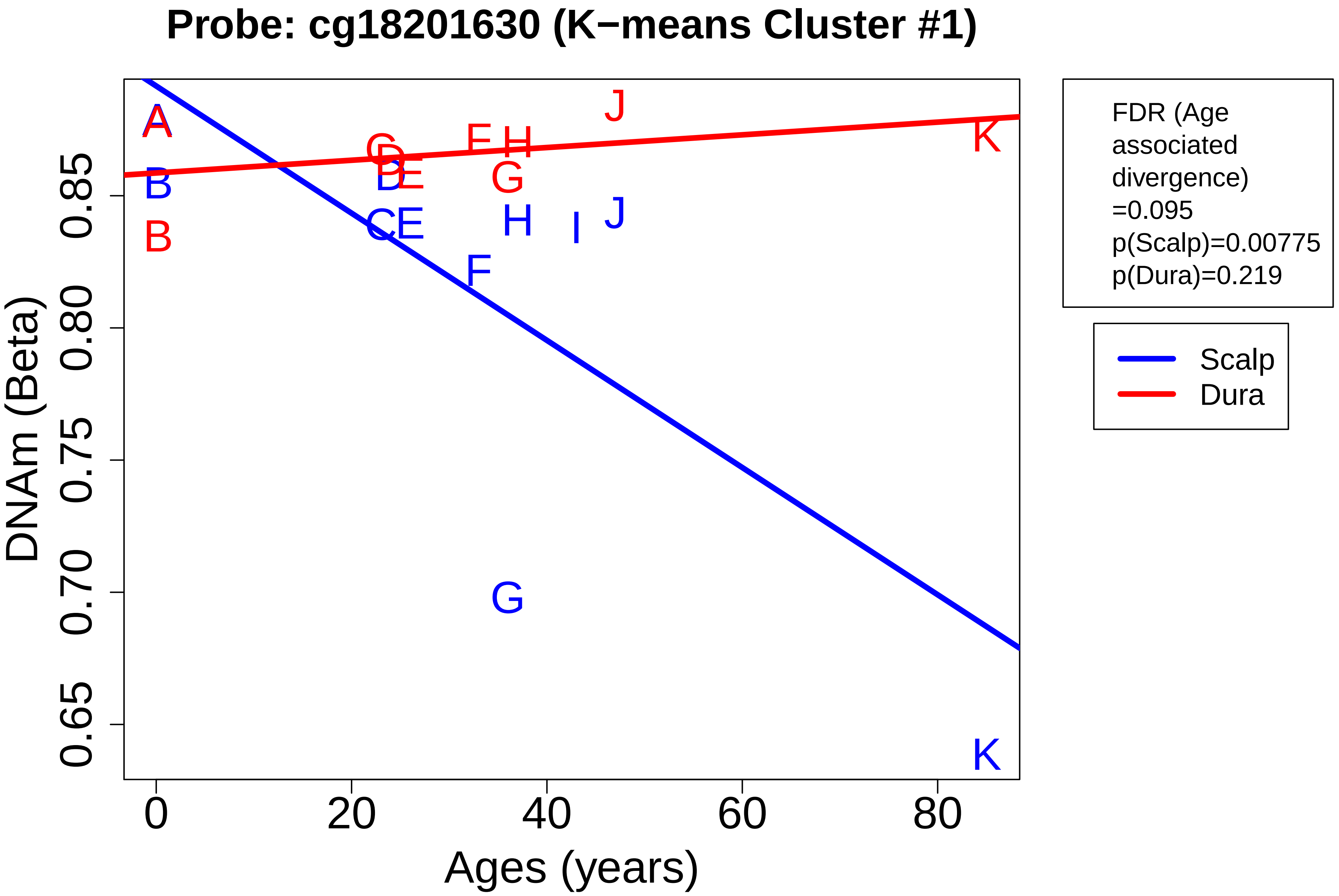
Probe: cg18201630 (K−means Cluster #1)
FDR (Age associated divergence) =0.095
p(Scalp)=0.00775
p(Dura)=0.219
Scalp
Dura
DNAm (Beta)
0.65
0.70
0.75
0.80
0.85
Ages (years)
0
20
40
60
80
A B C D E F G H I J K

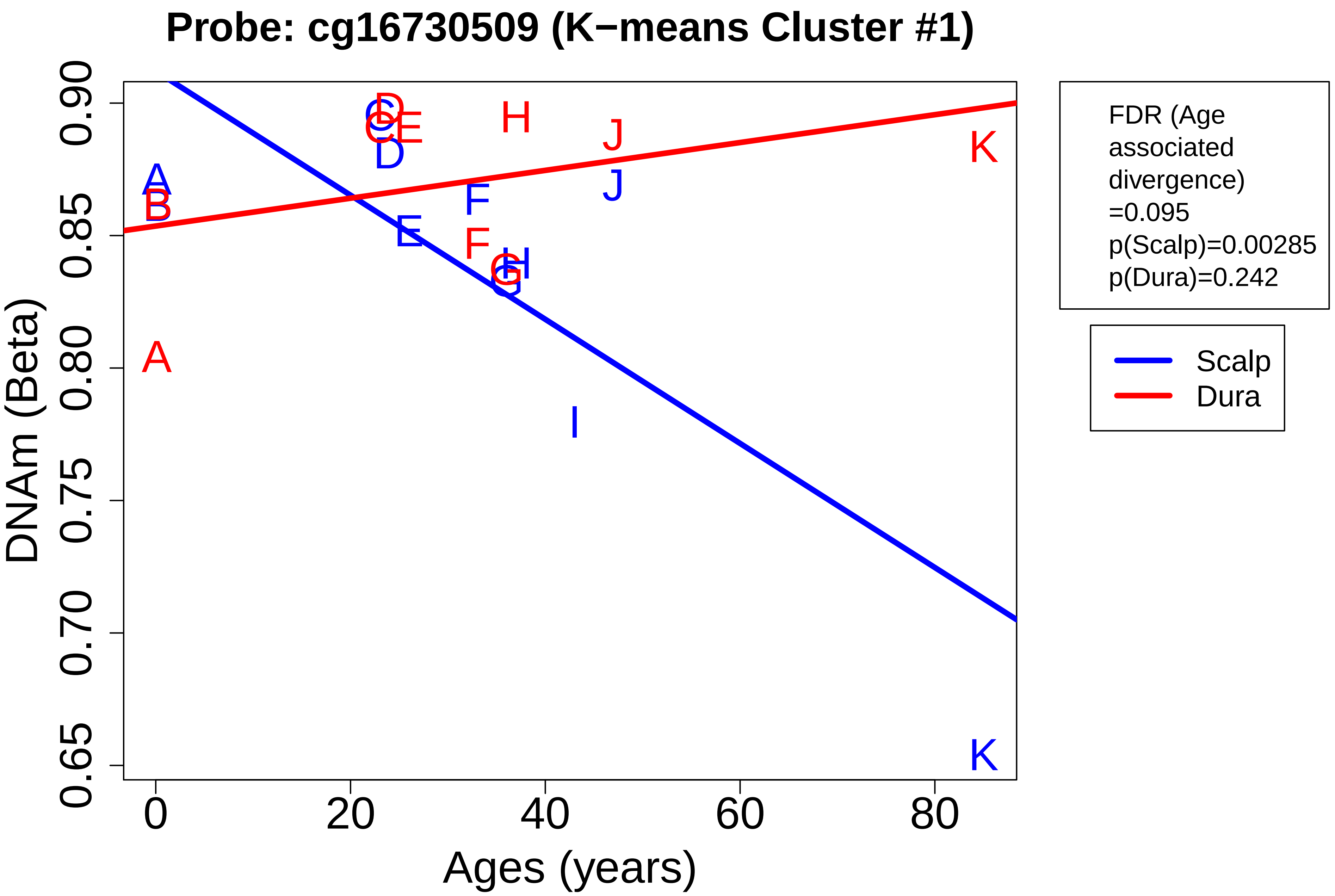

Probe: cg16730509 (K−means Cluster #1)
DNAm (Beta)
0.65
0.70
0.75
0.80
0.85
0.90
0
20
40
60
80
Ages (years)
A
B
C
D
E
F
G
H
I
J
K
FDR (Age associated divergence) =0.095
p(Scalp)=0.00285
p(Dura)=0.242
Scalp
Dura

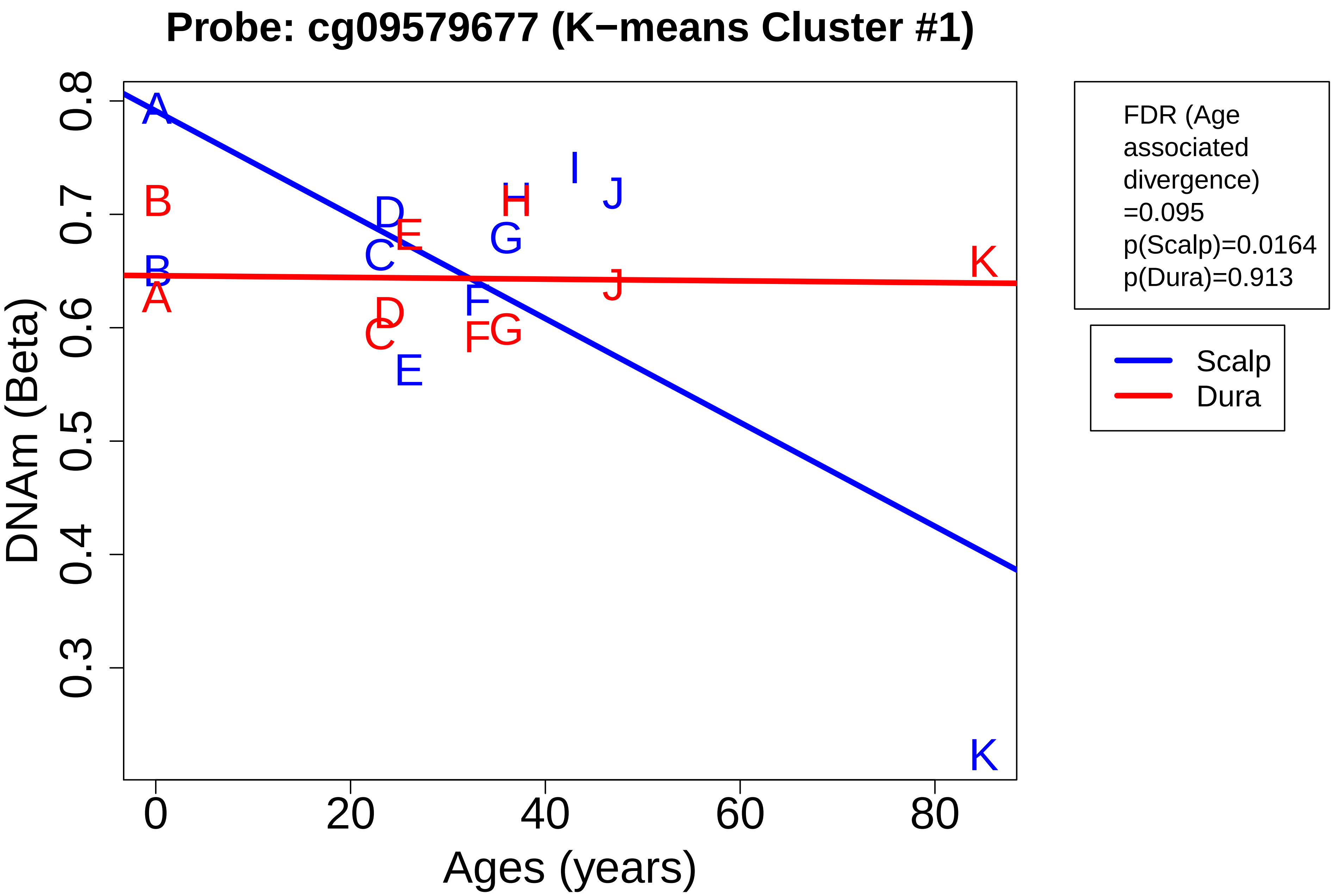

Probe: cg09579677 (K−means Cluster #1)
DNAm (Beta)
0.3
0.4
0.5
0.6
0.7
0.8
Ages (years)
0
20
40
60
80
A
B
C
D
E
F
G
H
I
J
K
FDR (Age associated divergence) =0.095
p(Scalp)=0.0164
p(Dura)=0.913
Scalp
Dura

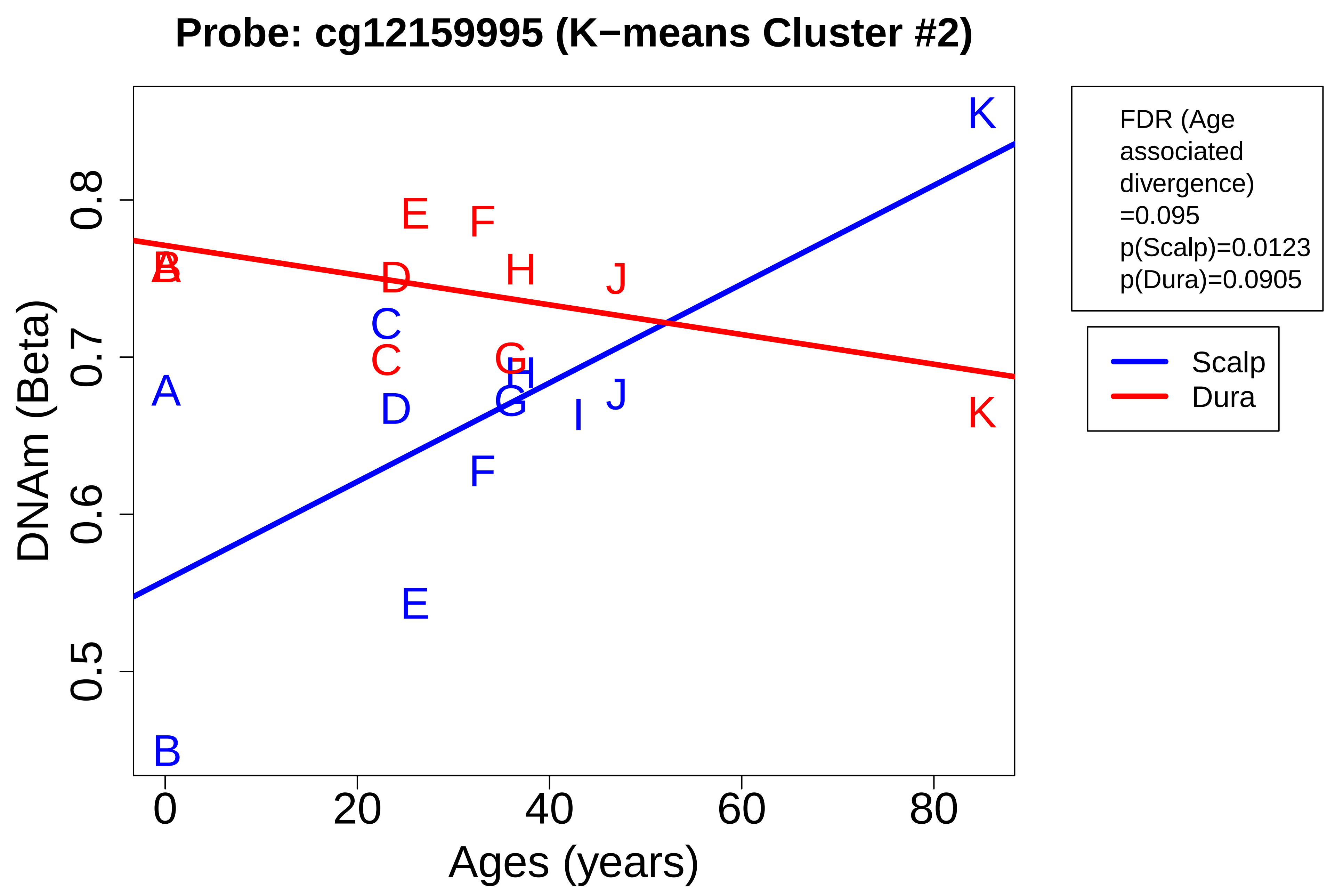

Probe: cg12159995 (K−means Cluster #2)
DNAm (Beta)
0.5
0.6
0.7
0.8
0
20
40
60
80
Ages (years)
FDR (Age associated divergence) =0.095
p(Scalp)=0.0123
p(Dura)=0.0905
Scalp
Dura
A
B
C
D
E
F
G
H
I
J
K

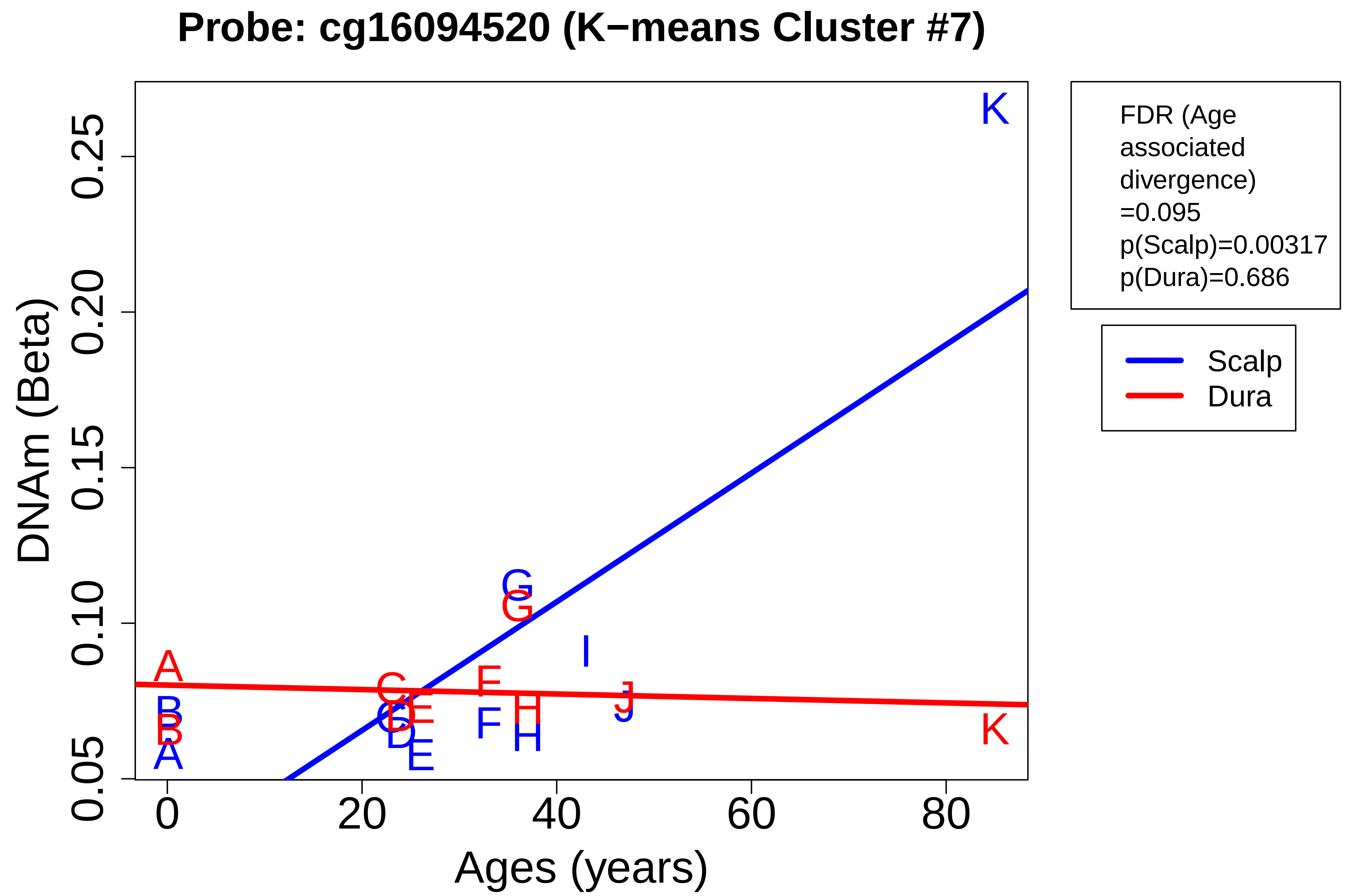
Probe: cg16094520 (K−means Cluster #7)
DNAm (Beta)
0.05
0.10
0.15
0.20
0.25
0
20
40
60
80
Ages (years)
FDR (Age associated divergence) =0.095
p(Scalp)=0.00317
p(Dura)=0.686
Scalp
Dura
A
B
C
D
E
F
G
H
I
J
K

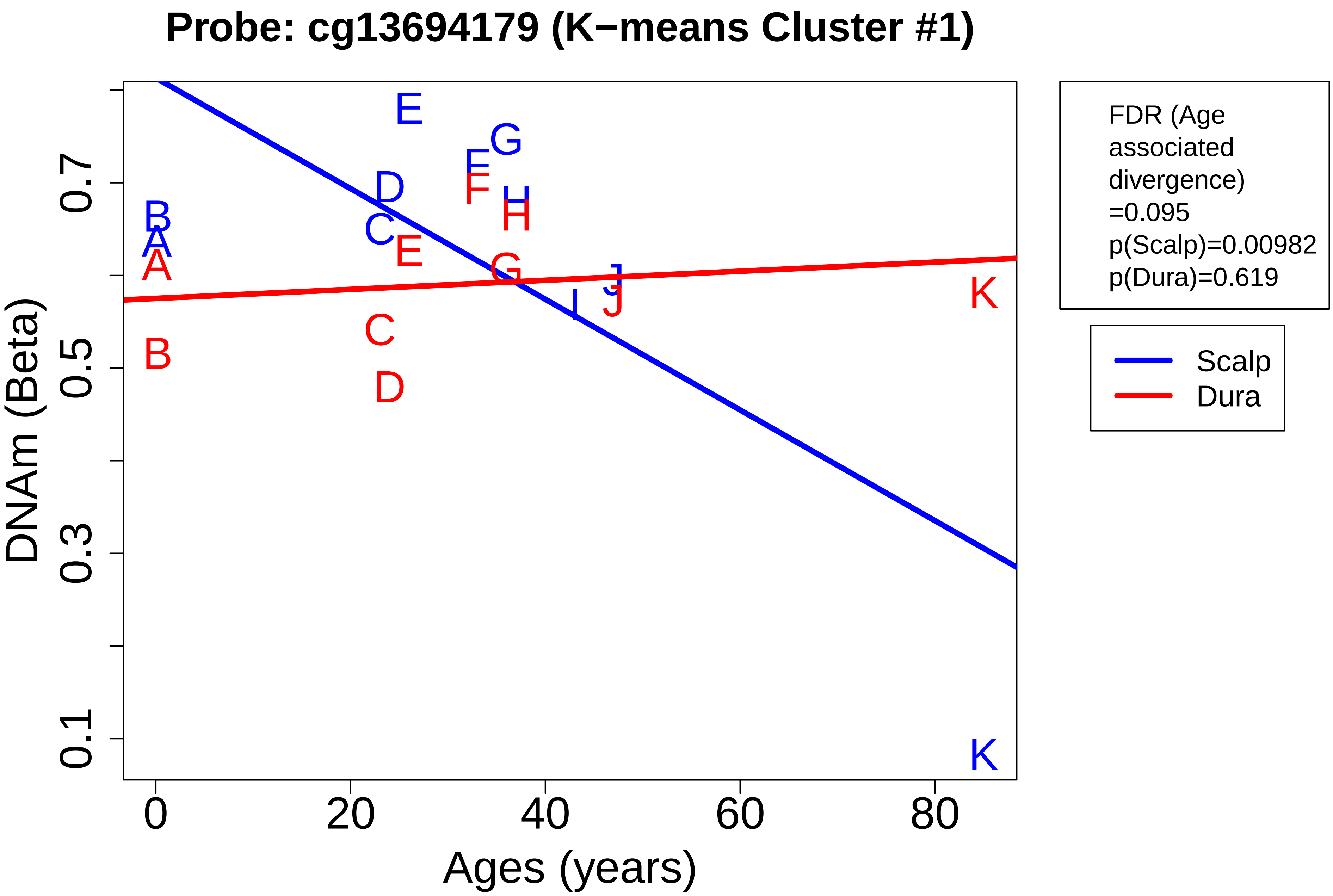

Probe: cg13694179 (K−means Cluster #1)
DNAm (Beta)
Ages (years)
0.1
0.3
0.5
0.7
0
20
40
60
80
FDR (Age associated divergence) =0.095
p(Scalp)=0.00982
p(Dura)=0.619
Scalp
Dura

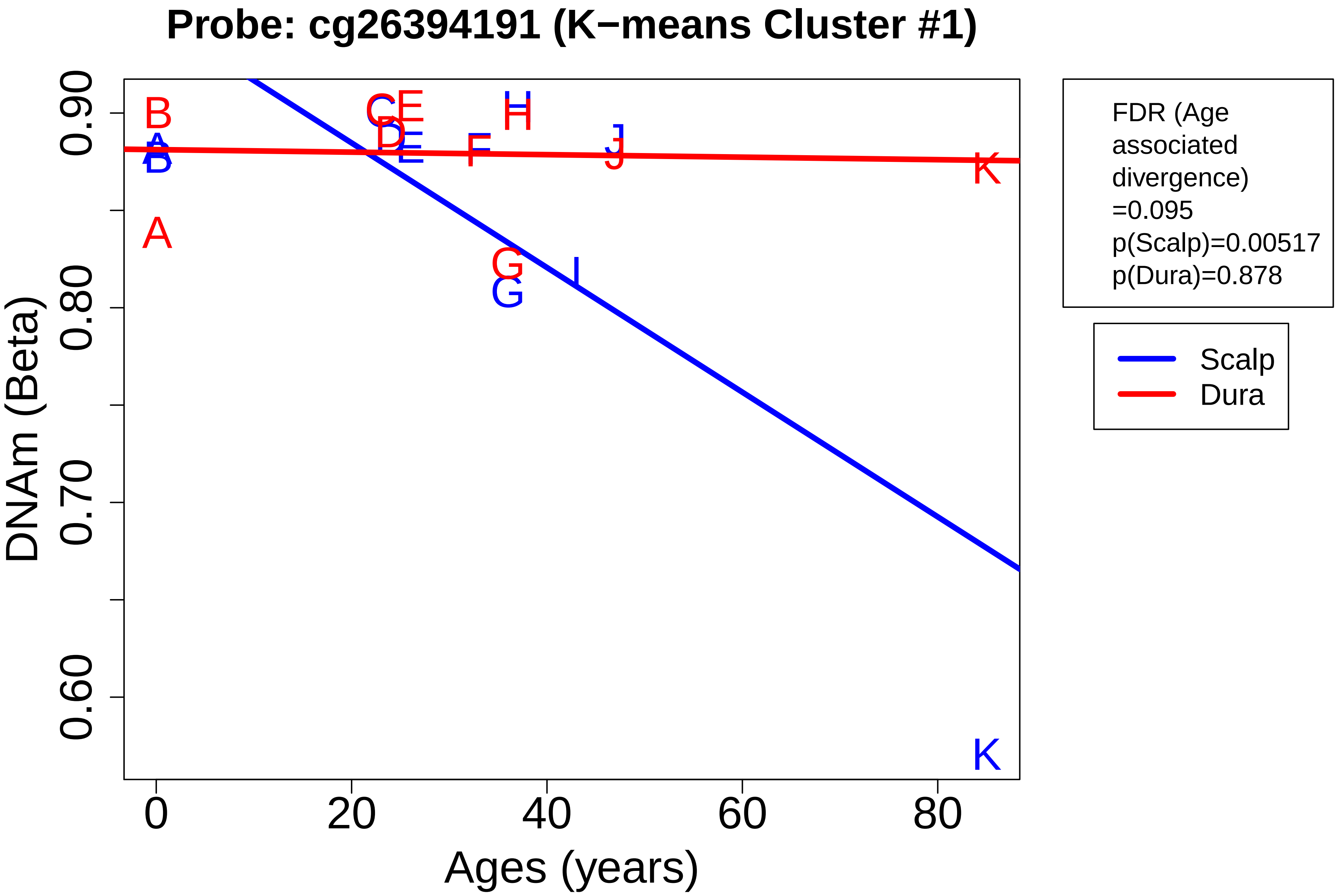
Probe: cg26394191 (K−means Cluster #1)
DNAm (Beta)
0.60
0.70
0.80
0.90
0
20
40
60
80
Ages (years)
FDR (Age associated divergence) =0.095
p(Scalp)=0.00517
p(Dura)=0.878
Scalp
Dura
A
B
C
D
E
F
G
H
I
J
K

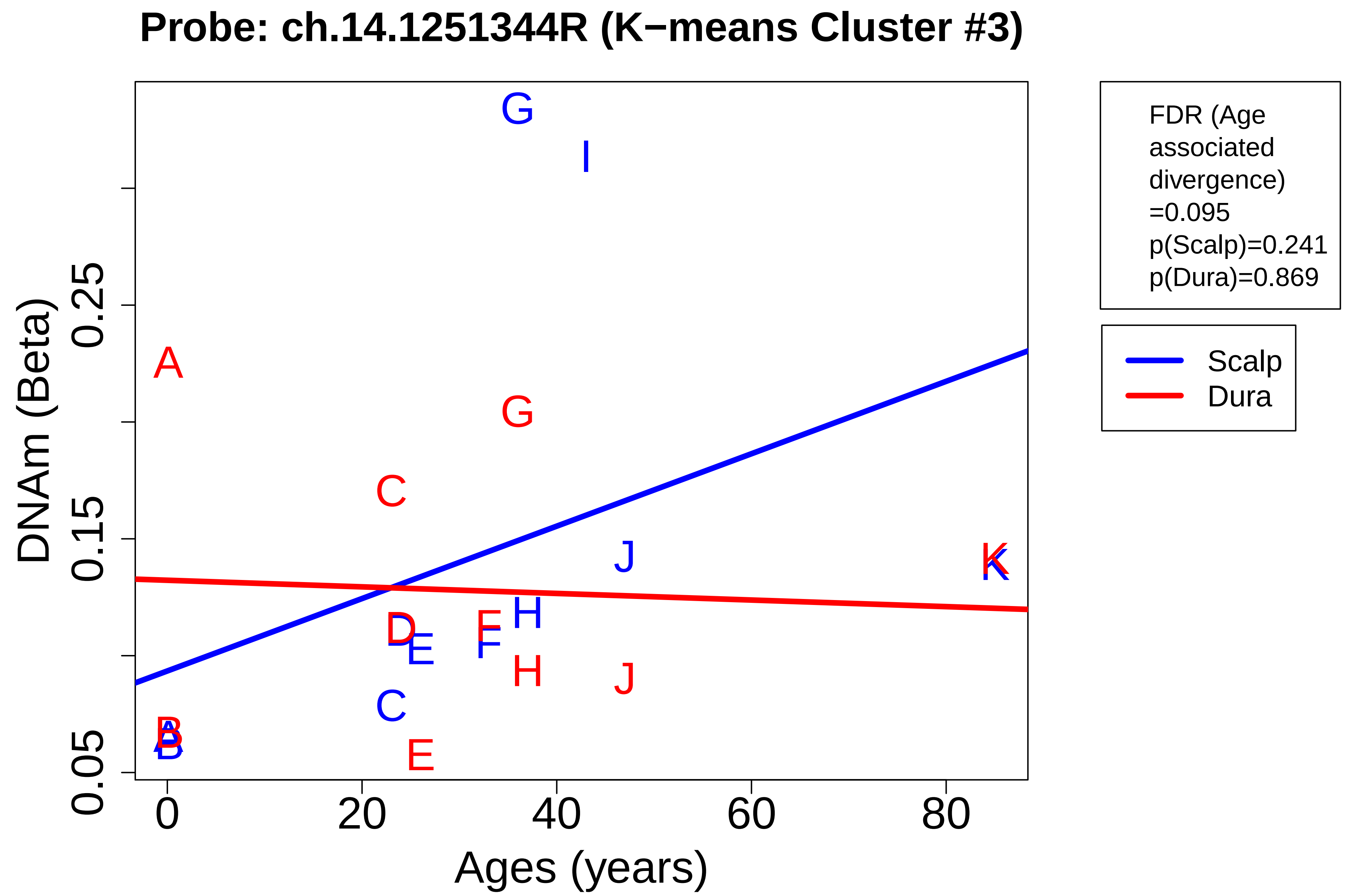

Probe: ch.14.1251344R (K−means Cluster #3)
FDR (Age associated divergence) =0.095
p(Scalp)=0.241
p(Dura)=0.869
Scalp
Dura
DNAm (Beta)
0.05
0.15
0.25
0
20
40
60
80
Ages (years)

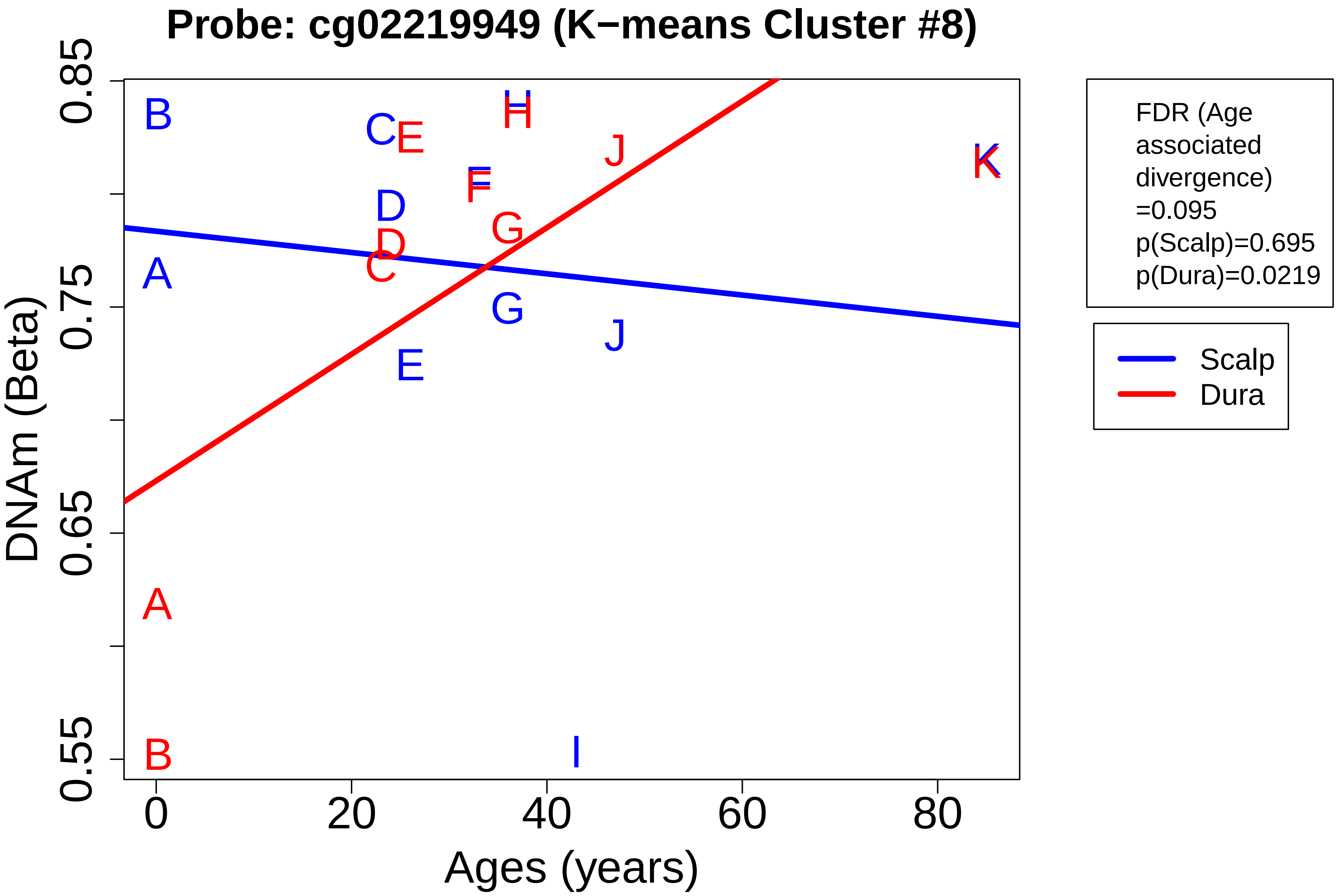

Probe: cg02219949 (K−means Cluster #8)
DNAm (Beta)
0.55
0.65
0.75
0.85
0
20
40
60
80
Ages (years)
FDR (Age associated divergence) =0.095
p(Scalp)=0.695
p(Dura)=0.0219
Scalp
Dura

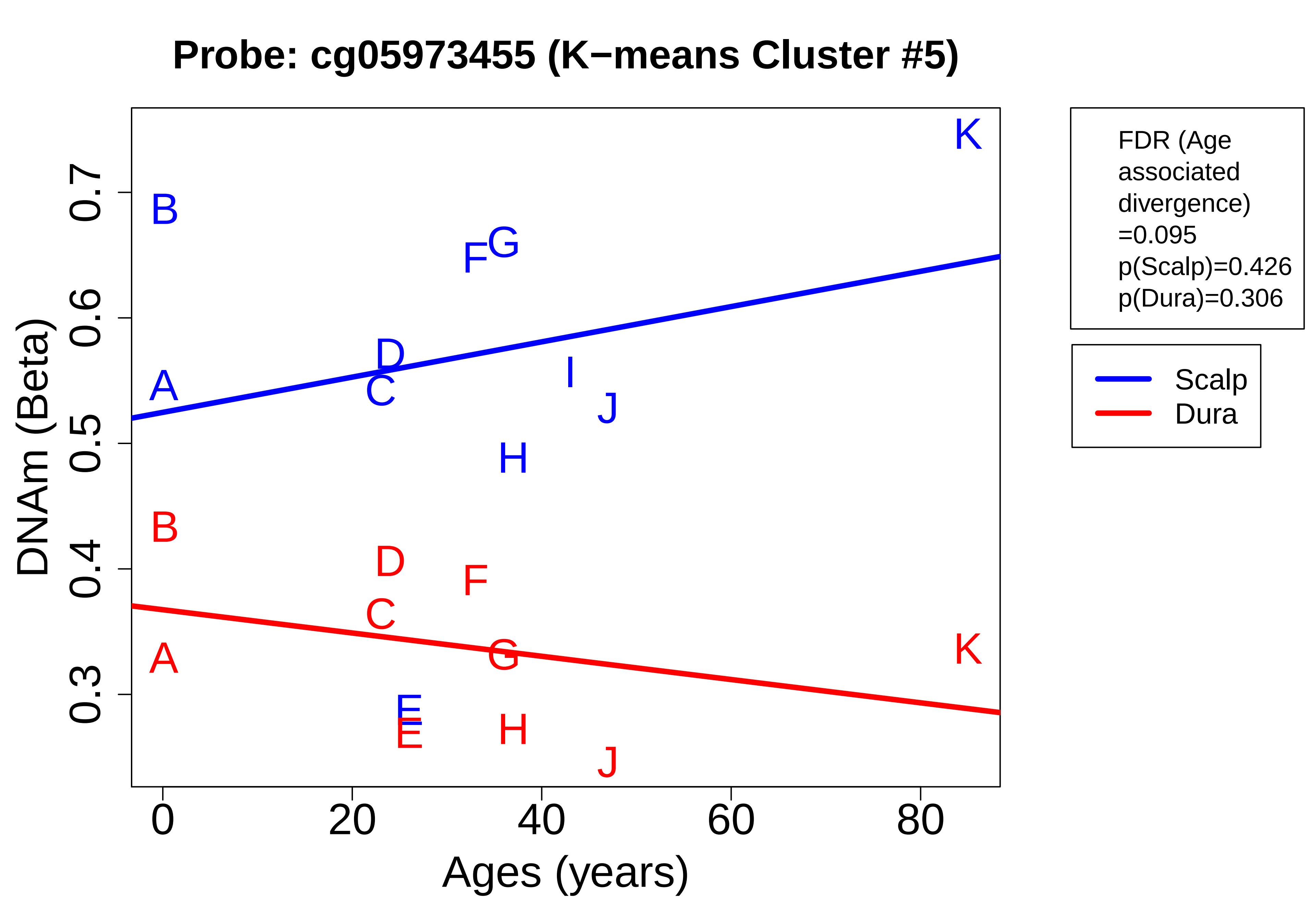

Probe: cg05973455 (K−means Cluster #5)
DNAm (Beta)
0.3
0.4
0.5
0.6
0.7
Ages (years)
0
20
40
60
80
FDR (Age associated divergence) =0.095
p(Scalp)=0.426
p(Dura)=0.306
Scalp
Dura
A
B
C
D
E
F
G
H
I
J
K

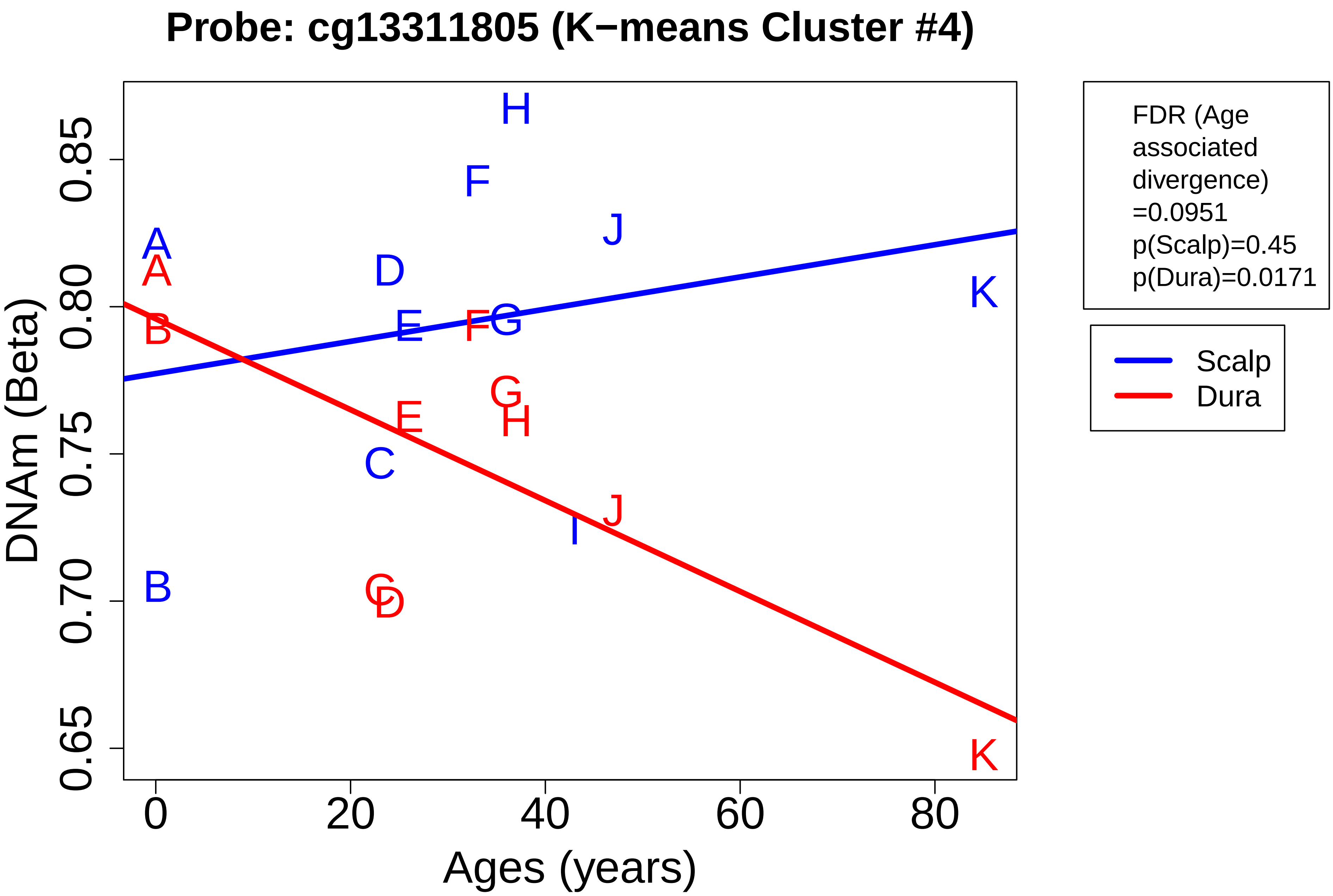

Probe: cg13311805 (K−means Cluster #4)
DNAm (Beta)
0.65
0.70
0.75
0.80
0.85
Ages (years)
0
20
40
60
80
FDR (Age associated divergence) =0.0951
p(Scalp)=0.45
p(Dura)=0.0171
Scalp
Dura
A
B
C
D
E
F
G
H
I
J
K

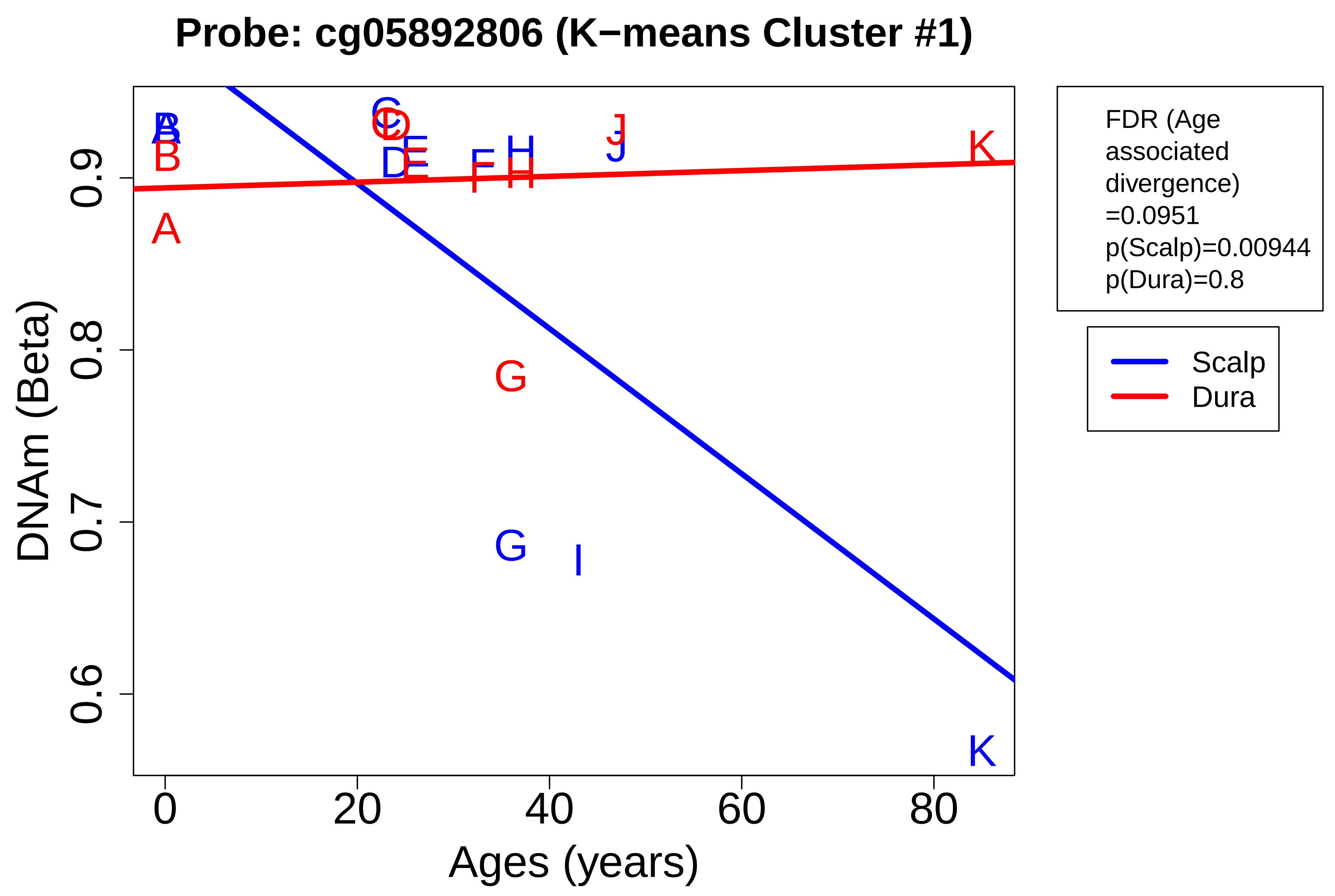

Probe: cg05892806 (K−means Cluster #1)
DNAm (Beta)
0.6
0.7
0.8
0.9
0
20
40
60
80
Ages (years)
A
B
C
D
E
F
G
H
I
J
K
FDR (Age associated divergence) =0.0951
p(Scalp)=0.00944
p(Dura)=0.8
Scalp
Dura

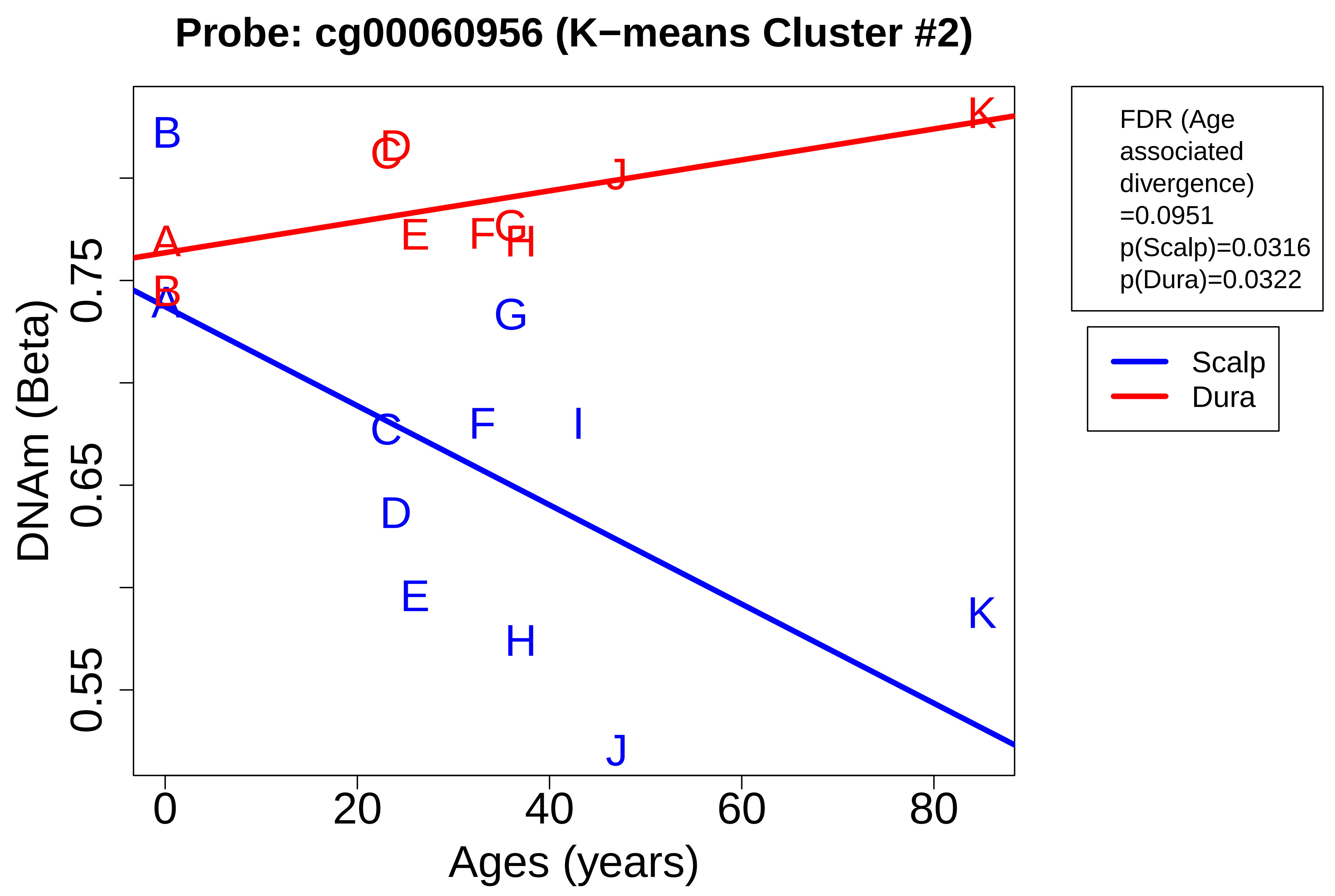

Probe: cg00060956 (K−means Cluster #2)
DNAm (Beta)
0.55
0.65
0.75
Ages (years)
0
20
40
60
80
FDR (Age associated divergence) =0.0951
p(Scalp)=0.0316
p(Dura)=0.0322
Scalp
Dura
A
B
C
D
E
F
G
H
I
J
K

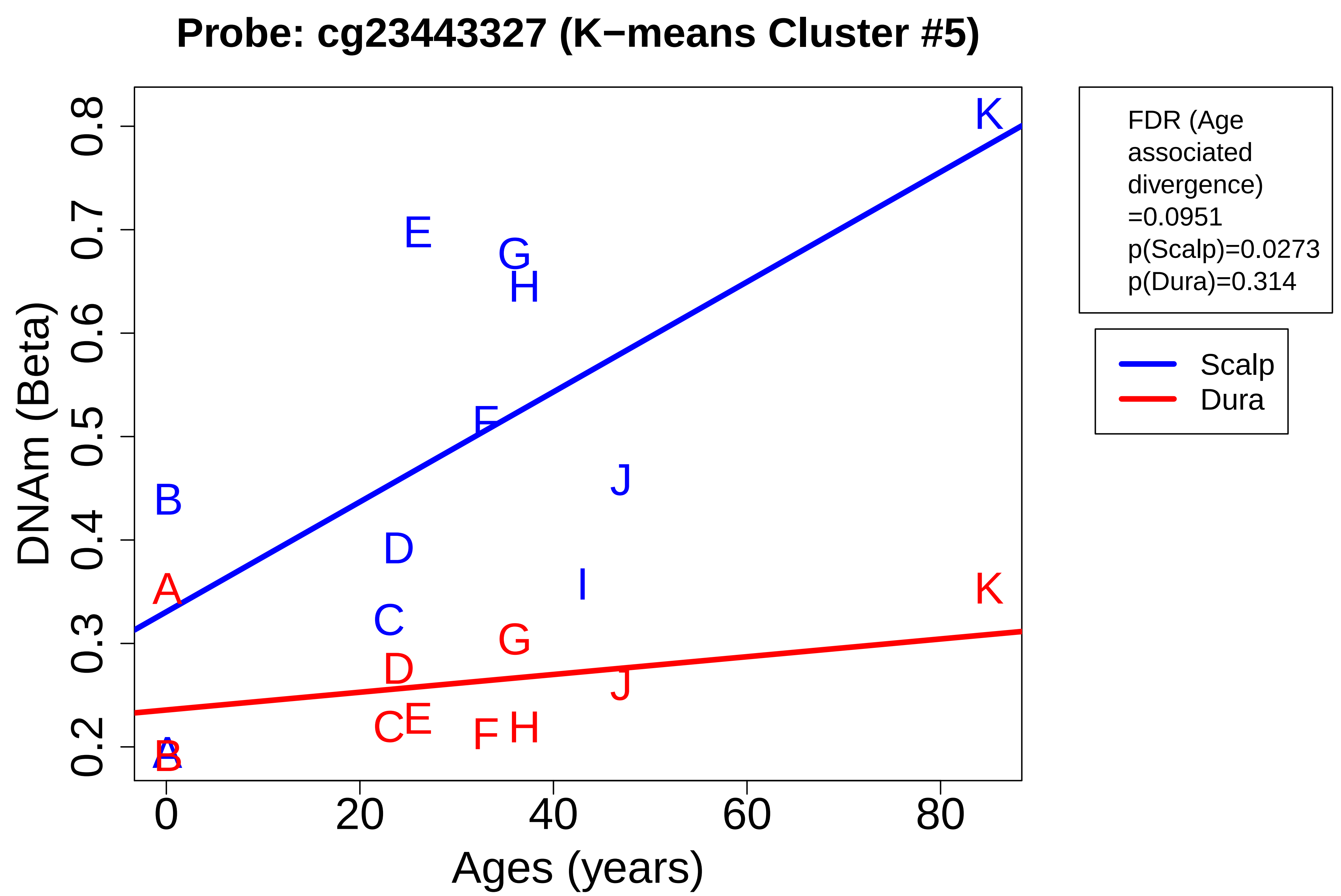

Probe: cg23443327 (K−means Cluster #5)
DNAm (Beta)
Ages (years)
0.2
0.3
0.4
0.5
0.6
0.7
0.8
0
20
40
60
80
FDR (Age associated divergence) =0.0951
p(Scalp)=0.0273
p(Dura)=0.314
Scalp
Dura
A
B
C
D
E
F
G
H
I
J
K

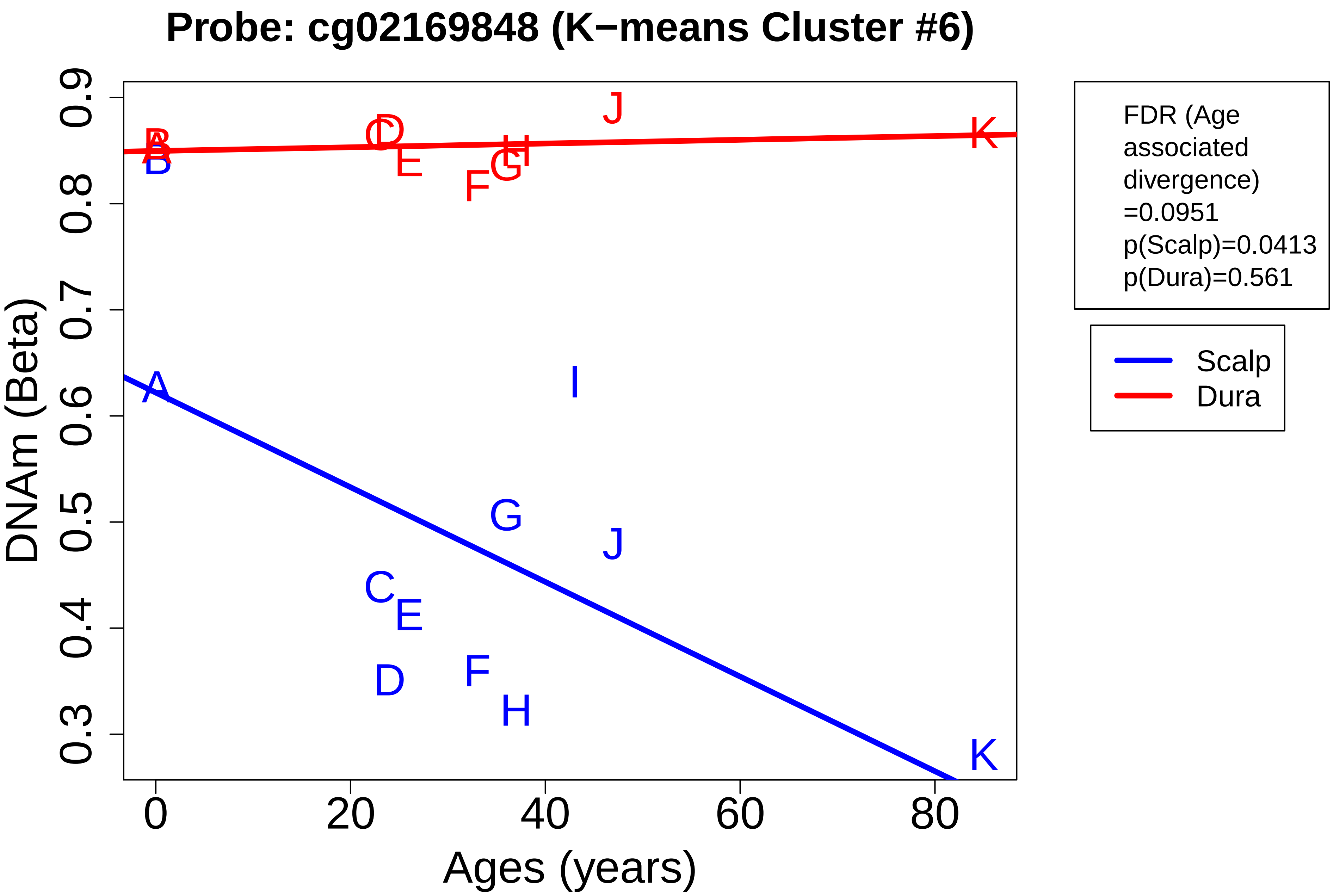
Probe: cg02169848 (K−means Cluster #6)
DNAm (Beta)
0.3
0.4
0.5
0.6
0.7
0.8
0.9
Ages (years)
0
20
40
60
80
FDR (Age associated divergence) =0.0951
p(Scalp)=0.0413
p(Dura)=0.561
Scalp
Dura

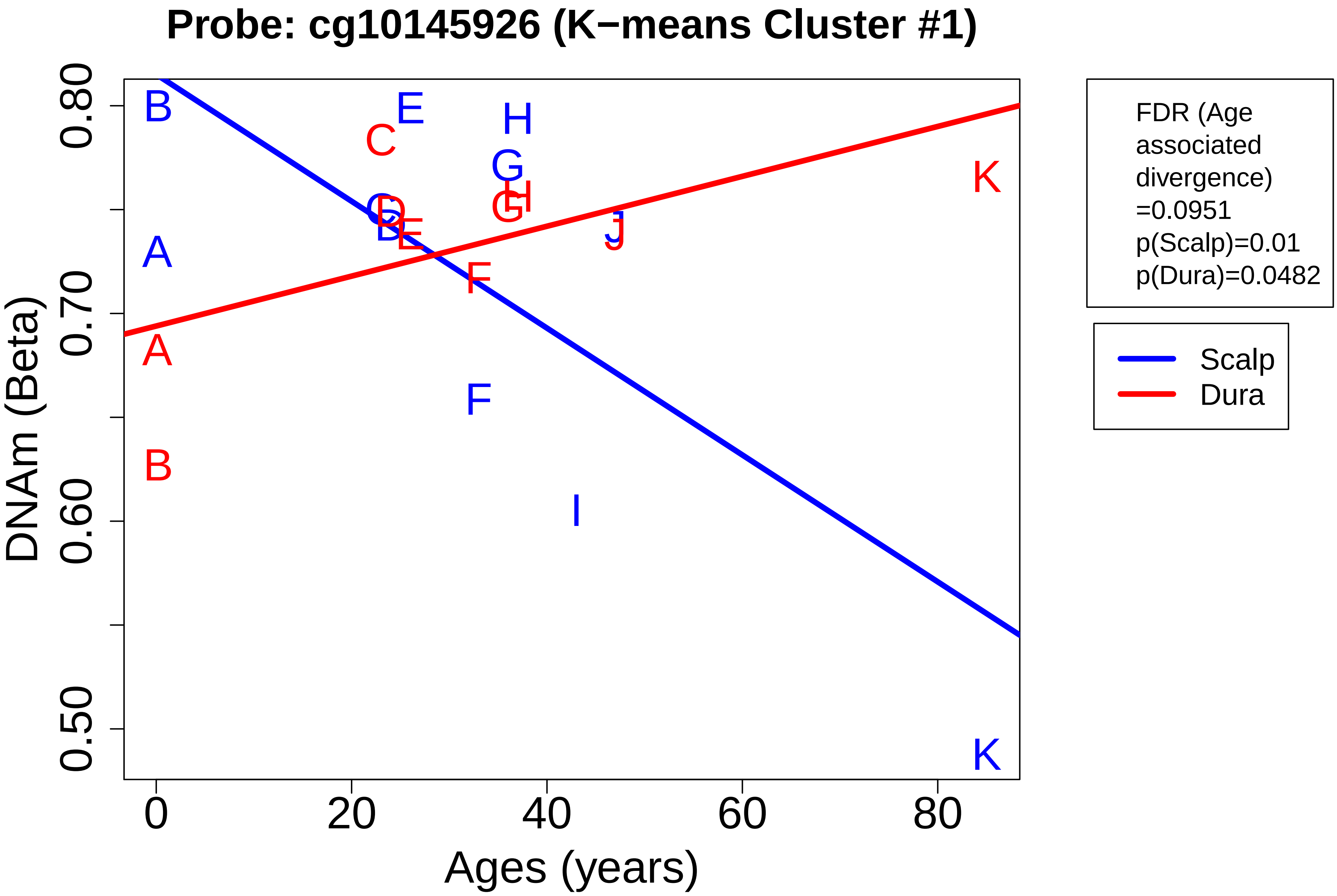
Probe: cg10145926 (K−means Cluster #1)
DNAm (Beta)
0.50
0.60
0.70
0.80
0
20
40
60
80
Ages (years)
FDR (Age associated divergence) =0.0951
p(Scalp)=0.01
p(Dura)=0.0482
Scalp
Dura

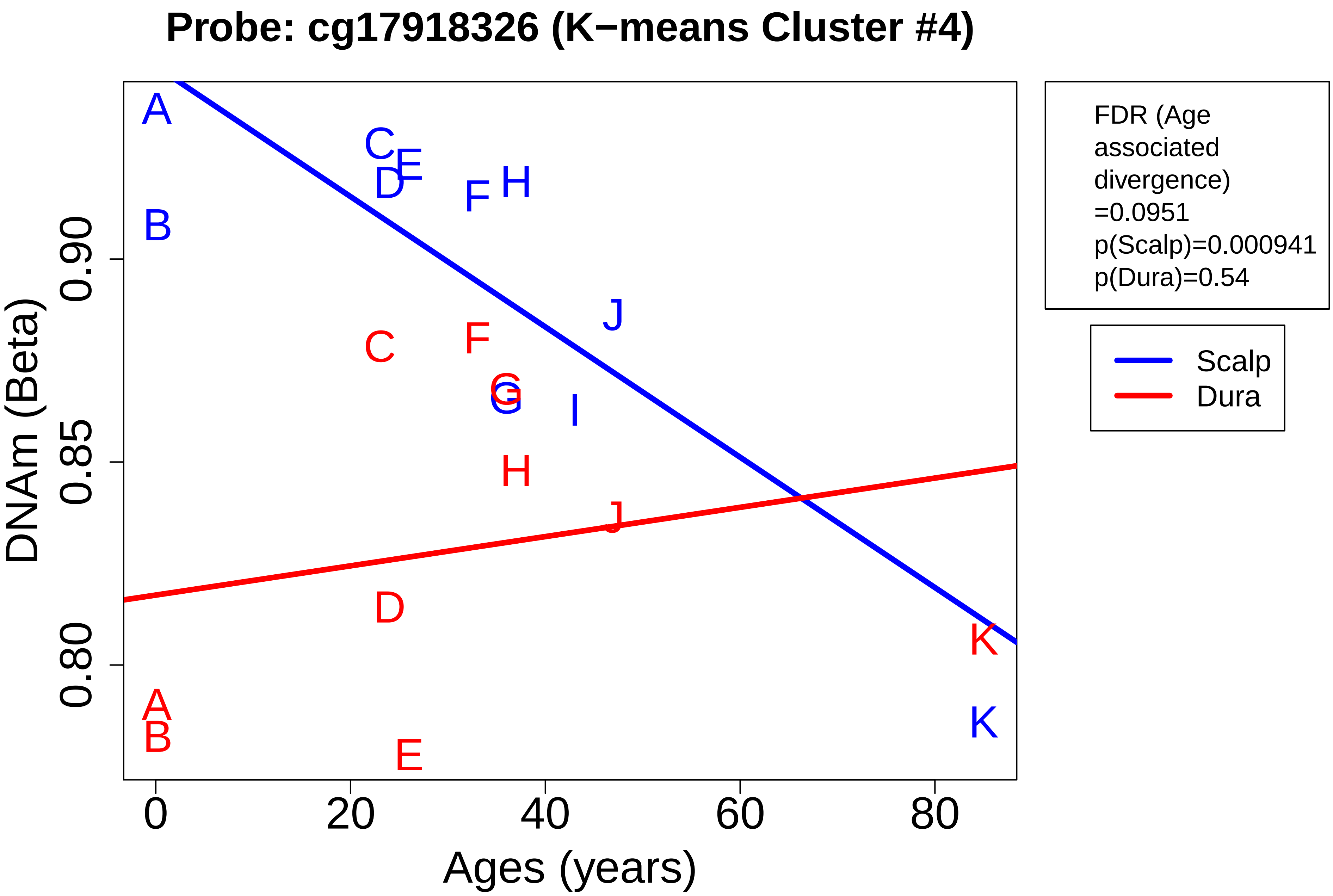

Probe: cg17918326 (K−means Cluster #4)
DNAm (Beta)
0.80
0.85
0.90
Ages (years)
0
20
40
60
80
FDR (Age associated divergence) =0.0951
p(Scalp)=0.000941
p(Dura)=0.54
Scalp
Dura

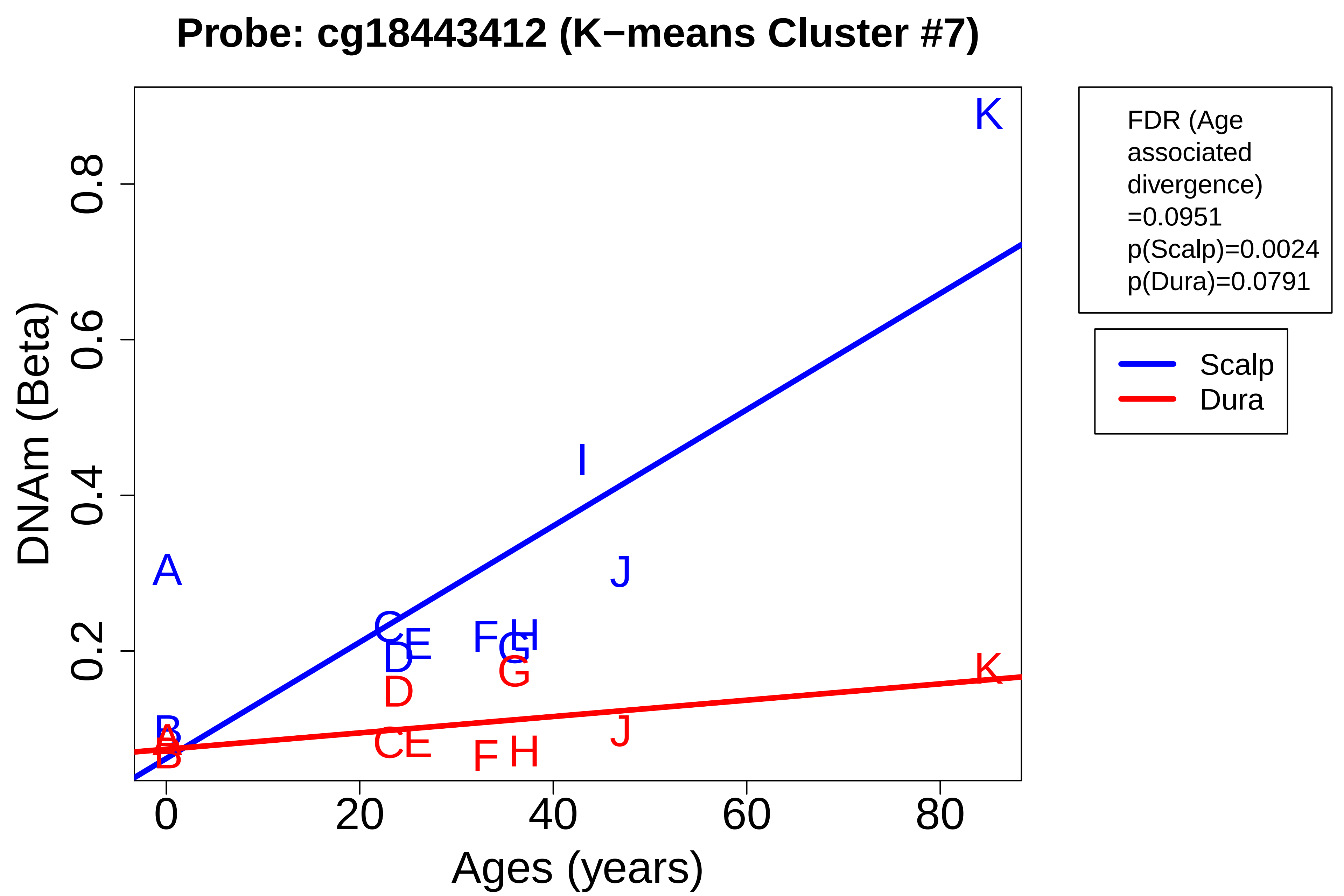

Probe: cg18443412 (K−means Cluster #7)
DNAm (Beta)
0.2
0.4
0.6
0.8
0
20
40
60
80
Ages (years)
FDR (Age associated divergence) =0.0951
p(Scalp)=0.0024
p(Dura)=0.0791
Scalp
Dura

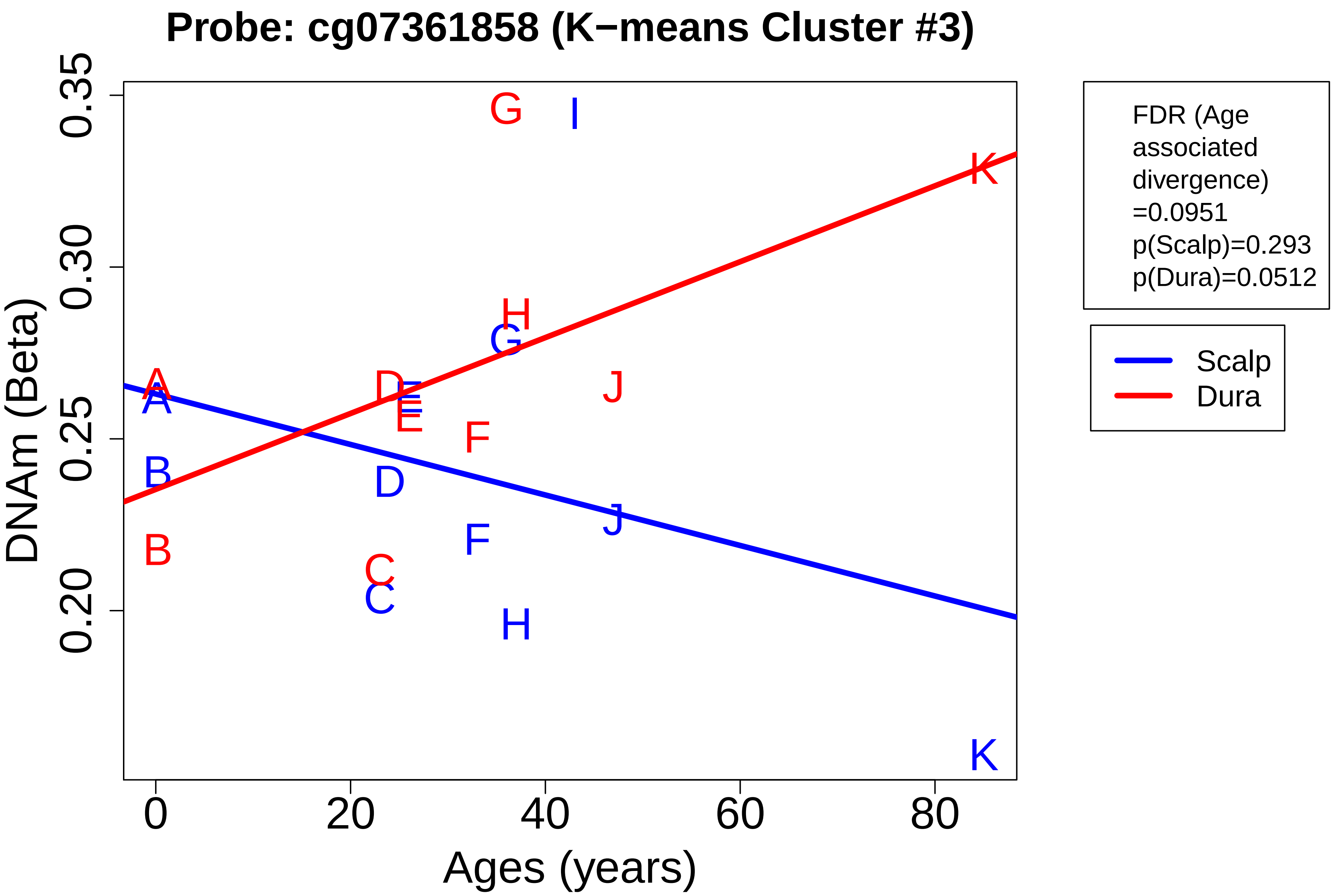
Probe: cg07361858 (K−means Cluster #3)
DNAm (Beta)
0.20
0.25
0.30
0.35
0
20
40
60
80
Ages (years)
A
B
C
D
E
F
G
H
I
J
K
FDR (Age associated divergence) =0.0951
p(Scalp)=0.293
p(Dura)=0.0512
Scalp
Dura

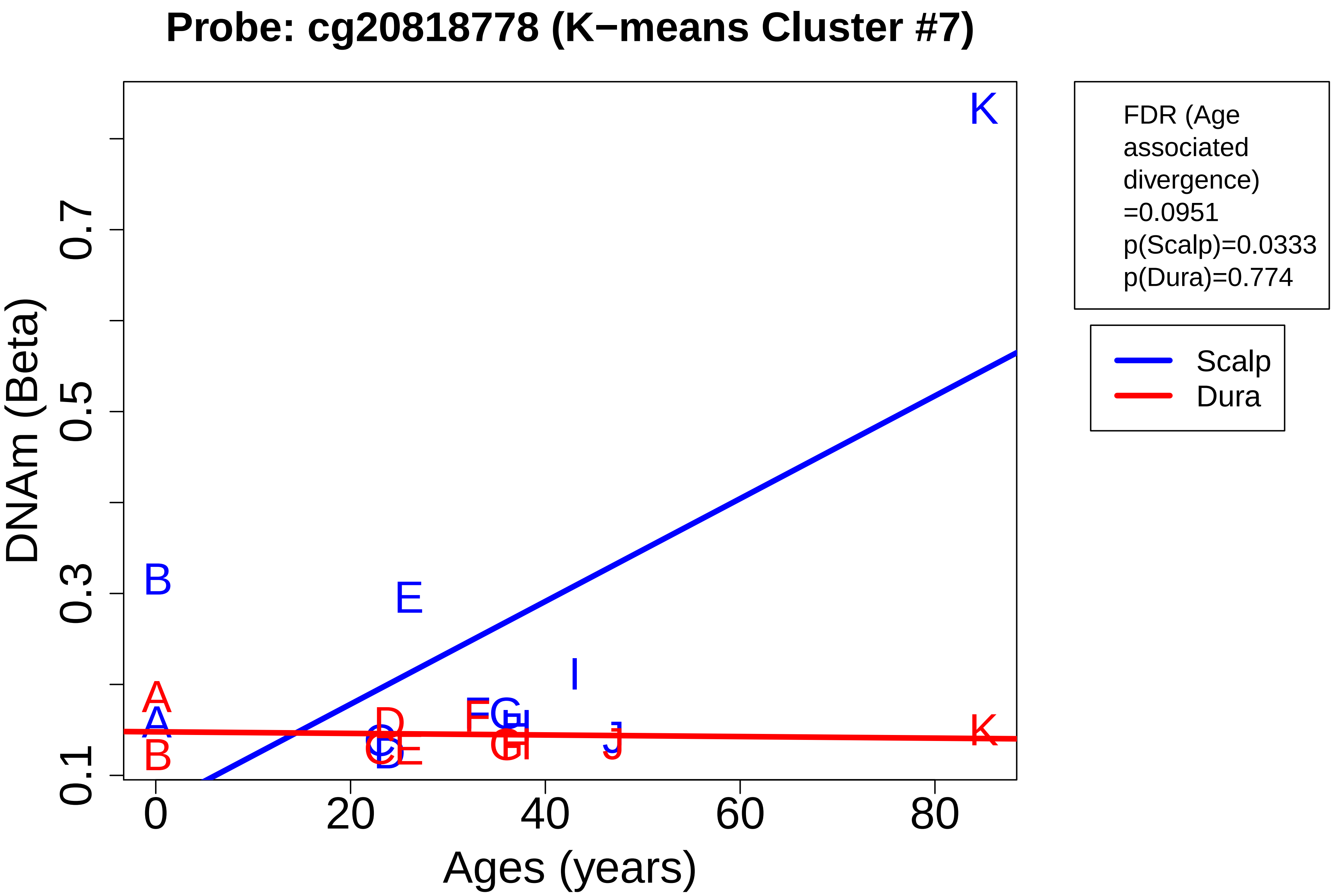

Probe: cg20818778 (K−means Cluster #7)
DNAm (Beta)
0.1
0.3
0.5
0.7
0
20
40
60
80
Ages (years)
FDR (Age associated divergence) =0.0951
p(Scalp)=0.0333
p(Dura)=0.774
Scalp
Dura

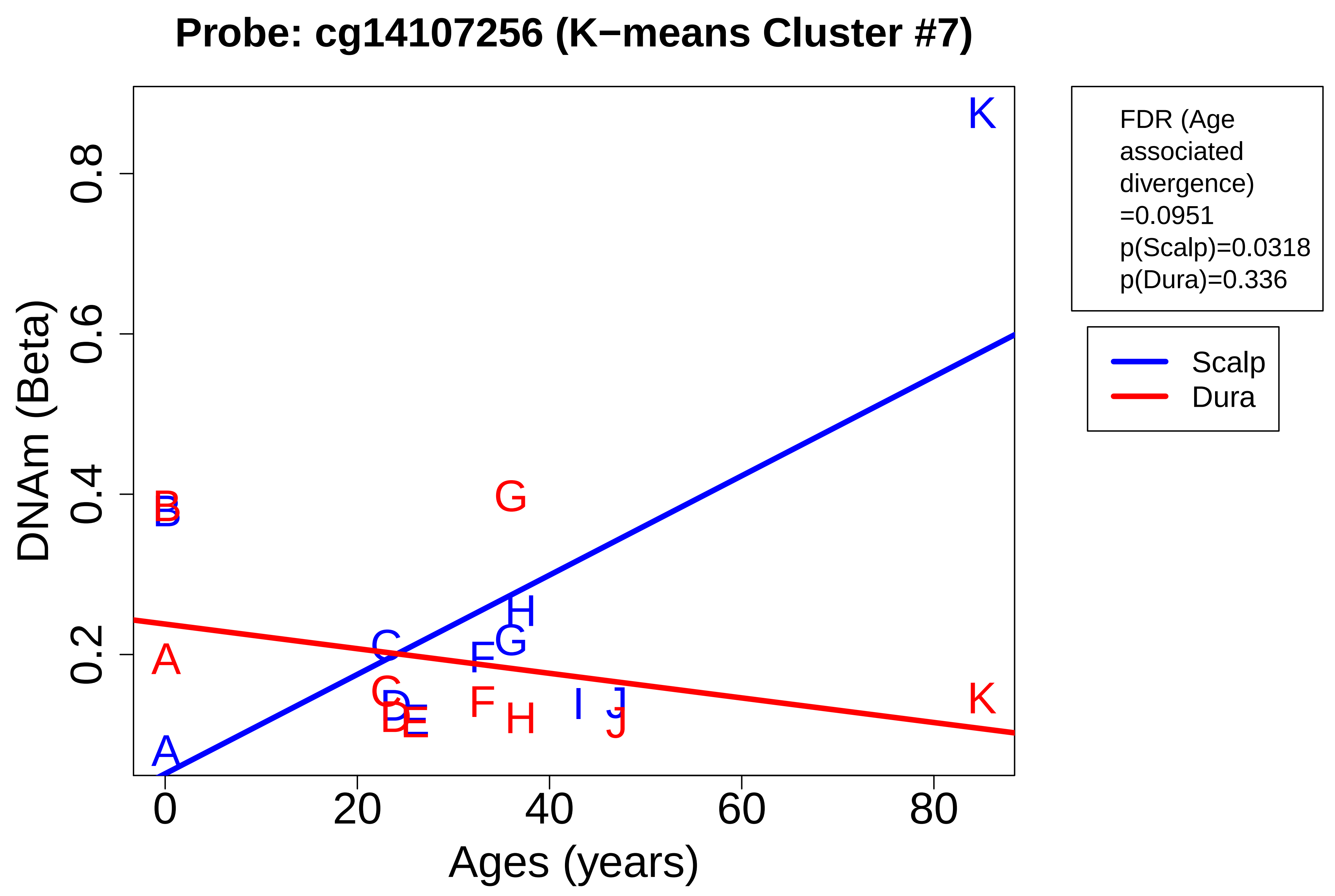
Probe: cg14107256 (K−means Cluster #7)
DNAm (Beta)
0.2
0.4
0.6
0.8
0
20
40
60
80
Ages (years)
FDR (Age associated divergence) =0.0951
p(Scalp)=0.0318
p(Dura)=0.336
Scalp
Dura
A
B
C
D
E
F
G
H
I
J
K

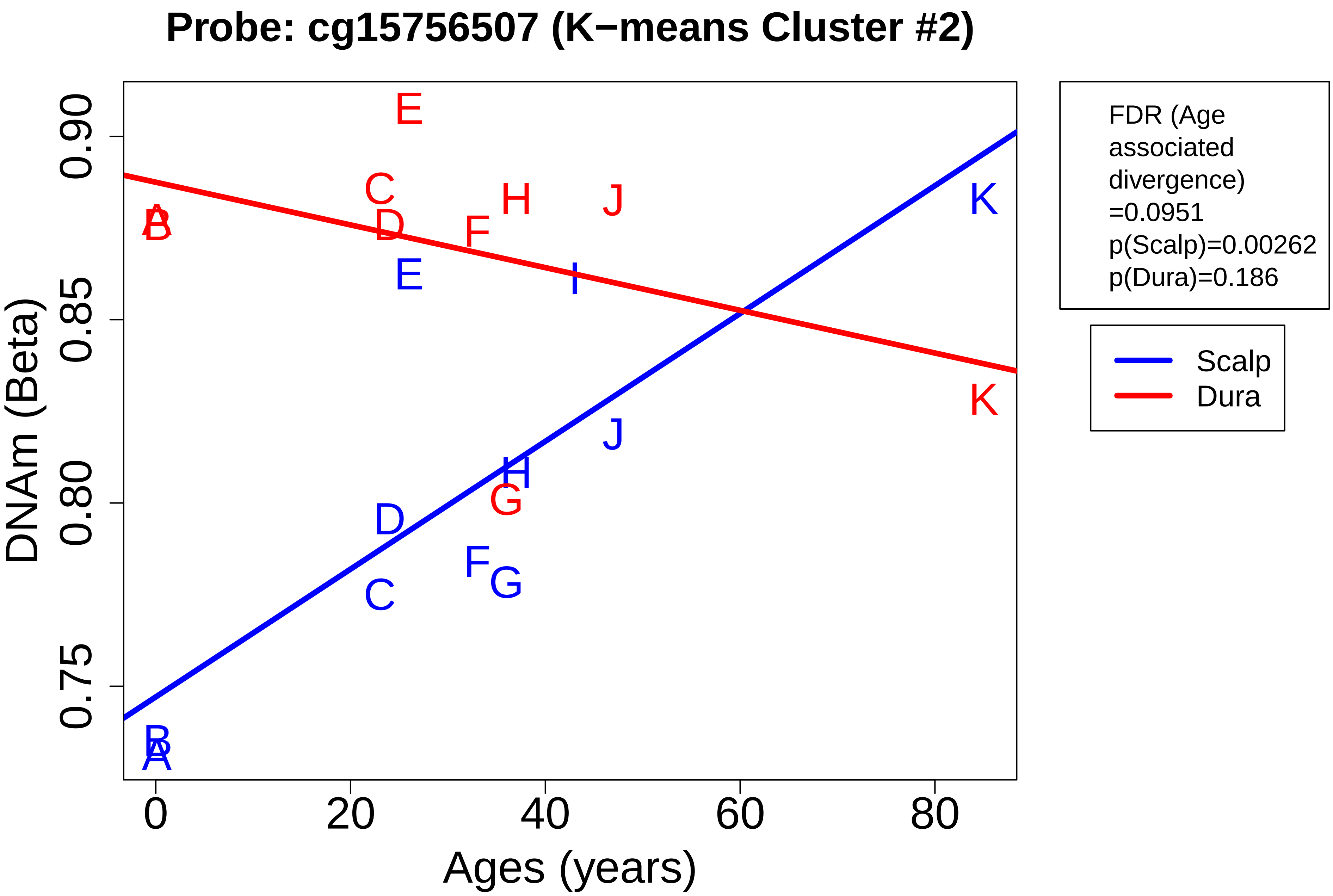

Probe: cg15756507 (K−means Cluster #2)
DNAm (Beta)
0.75
0.80
0.85
0.90
0
20
40
60
80
Ages (years)
A
B
C
D
E
F
G
H
I
J
K
FDR (Age associated divergence) =0.0951
p(Scalp)=0.00262
p(Dura)=0.186
Scalp
Dura

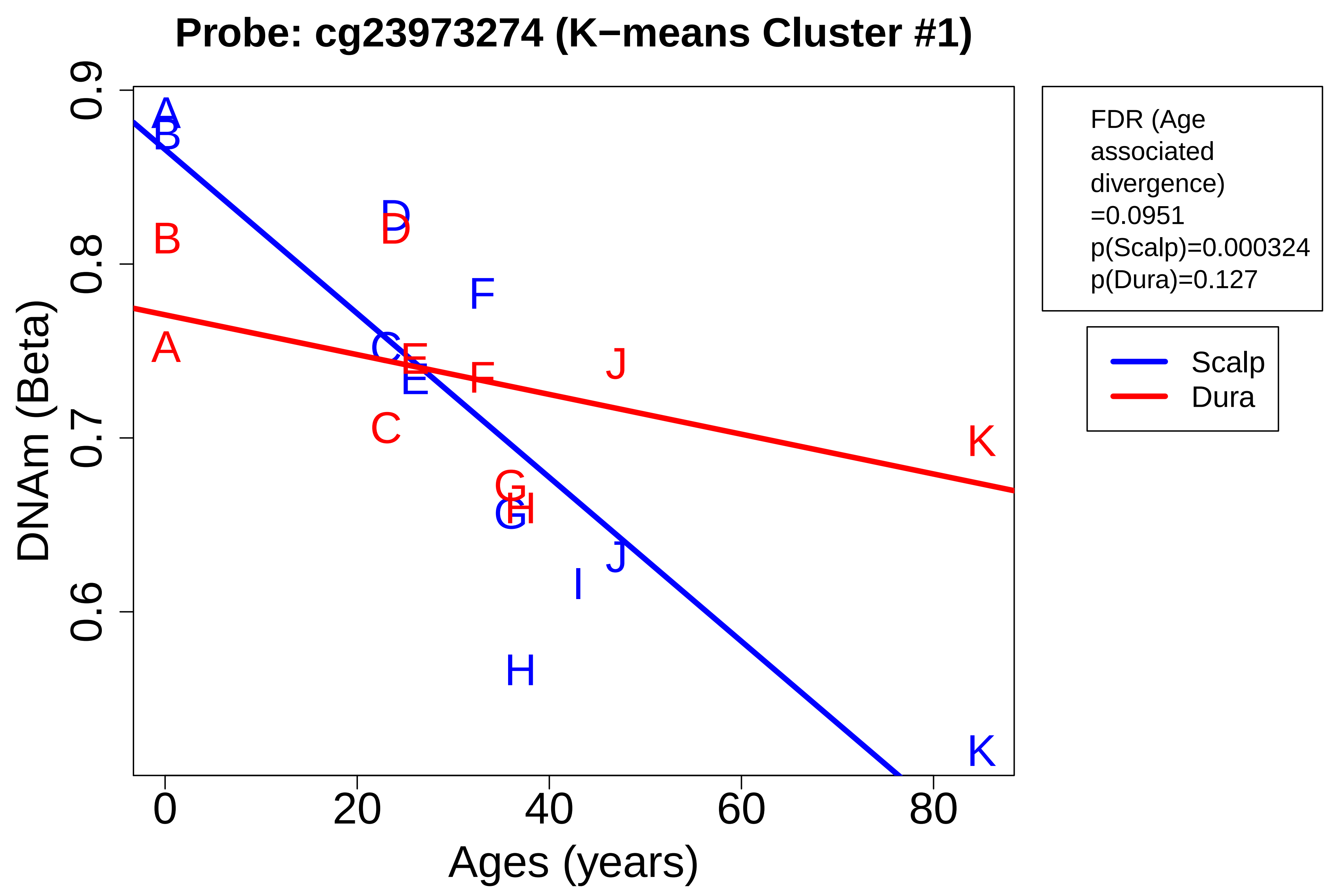

Probe: cg23973274 (K−means Cluster #1)
DNAm (Beta)
0.9
0.8
0.7
0.6
0
20
40
60
80
Ages (years)
A
B
C
D
E
F
G
H
I
J
K
FDR (Age associated divergence) =0.0951
p(Scalp)=0.000324
p(Dura)=0.127
Scalp
Dura

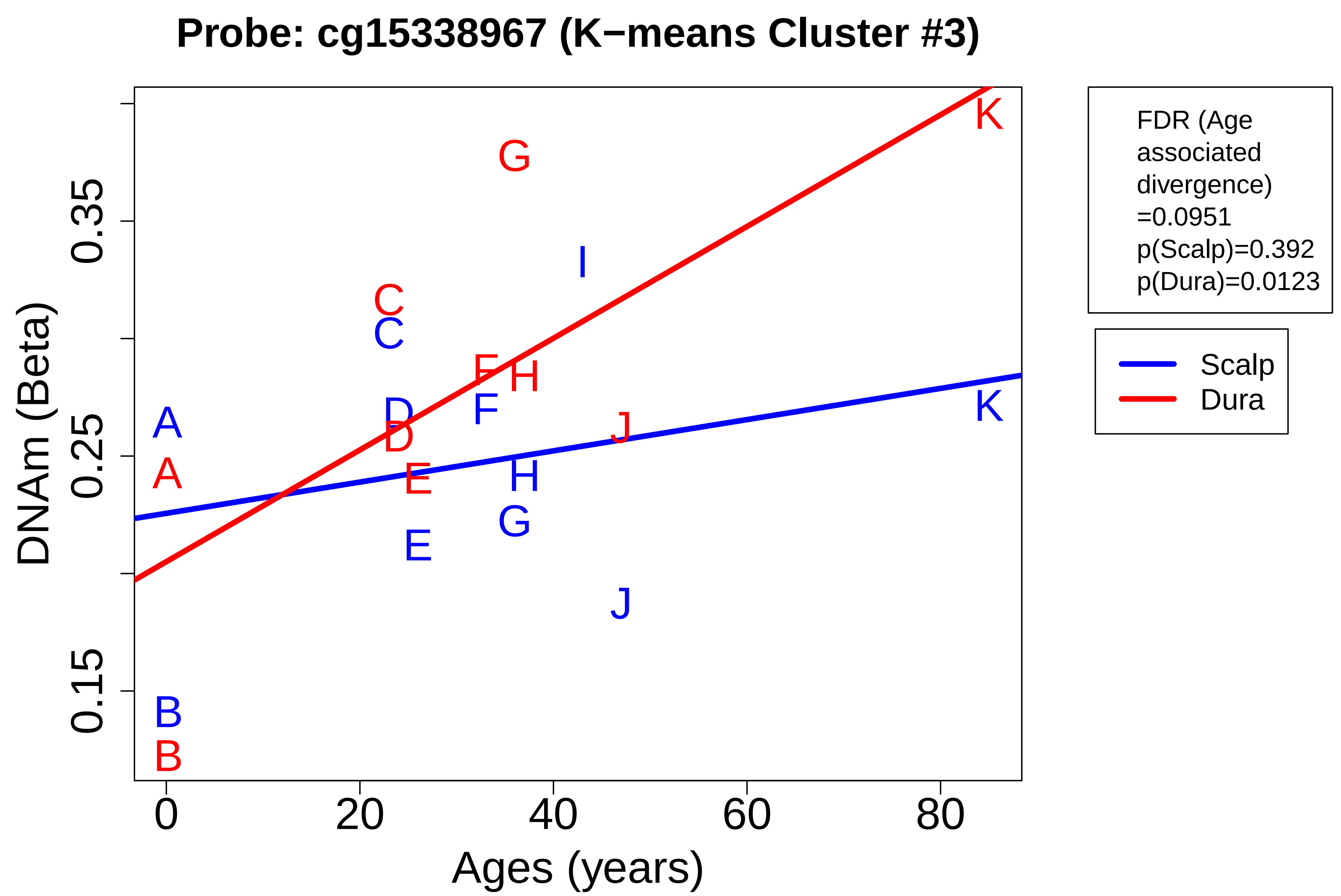

Probe: cg15338967 (K−means Cluster #3)
DNAm (Beta)
0.15
0.25
0.35
Ages (years)
0
20
40
60
80
A
B
C
D
E
F
G
H
I
J
K
FDR (Age associated divergence) =0.0951
p(Scalp)=0.392
p(Dura)=0.0123
Scalp
Dura

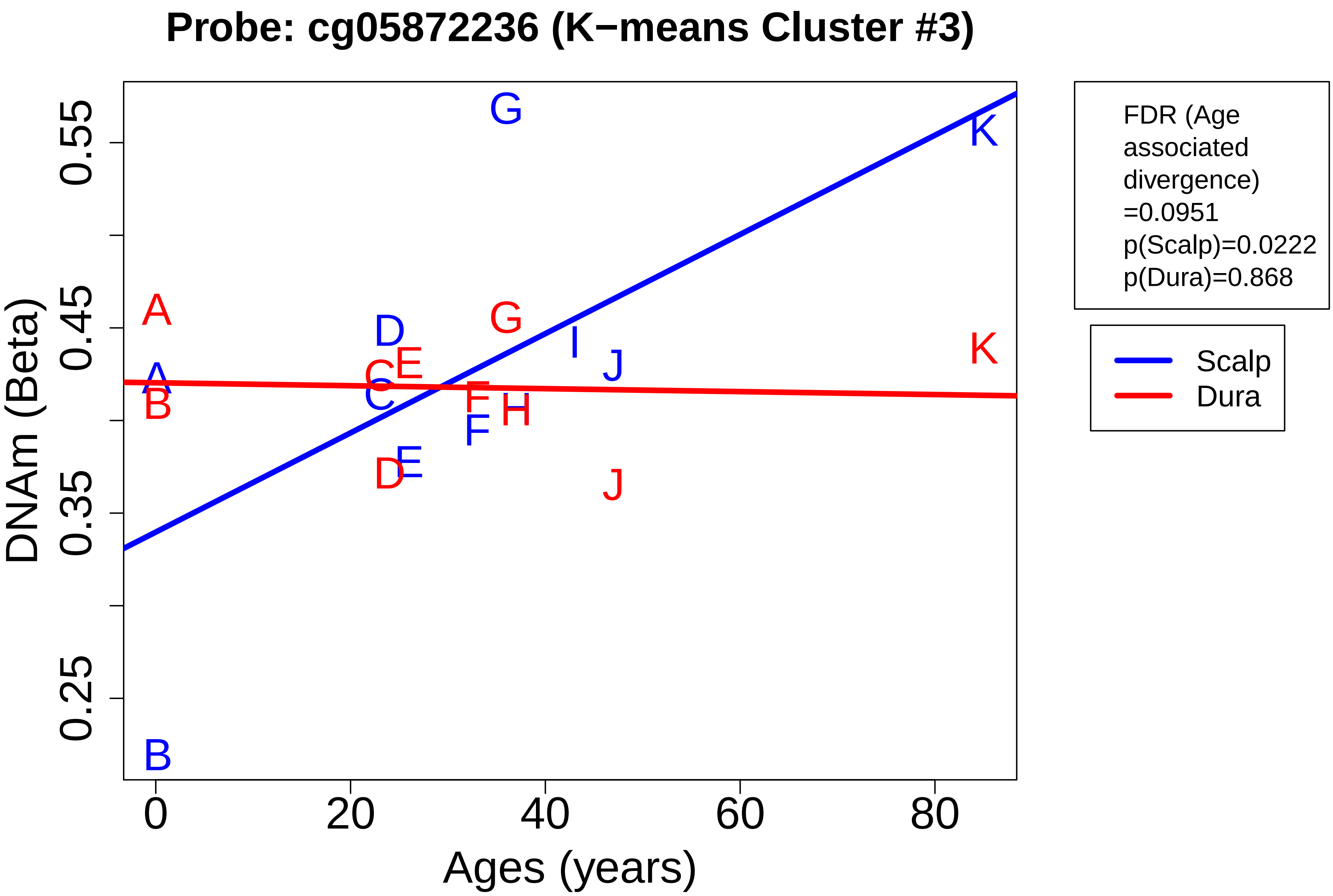

Probe: cg05872236 (K−means Cluster #3)
DNAm (Beta)
0.25
0.35
0.45
0.55
Ages (years)
0
20
40
60
80
A
B
C
D
E
F
G
H
I
J
K
FDR (Age associated divergence) =0.0951
p(Scalp)=0.0222
p(Dura)=0.868
Scalp
Dura

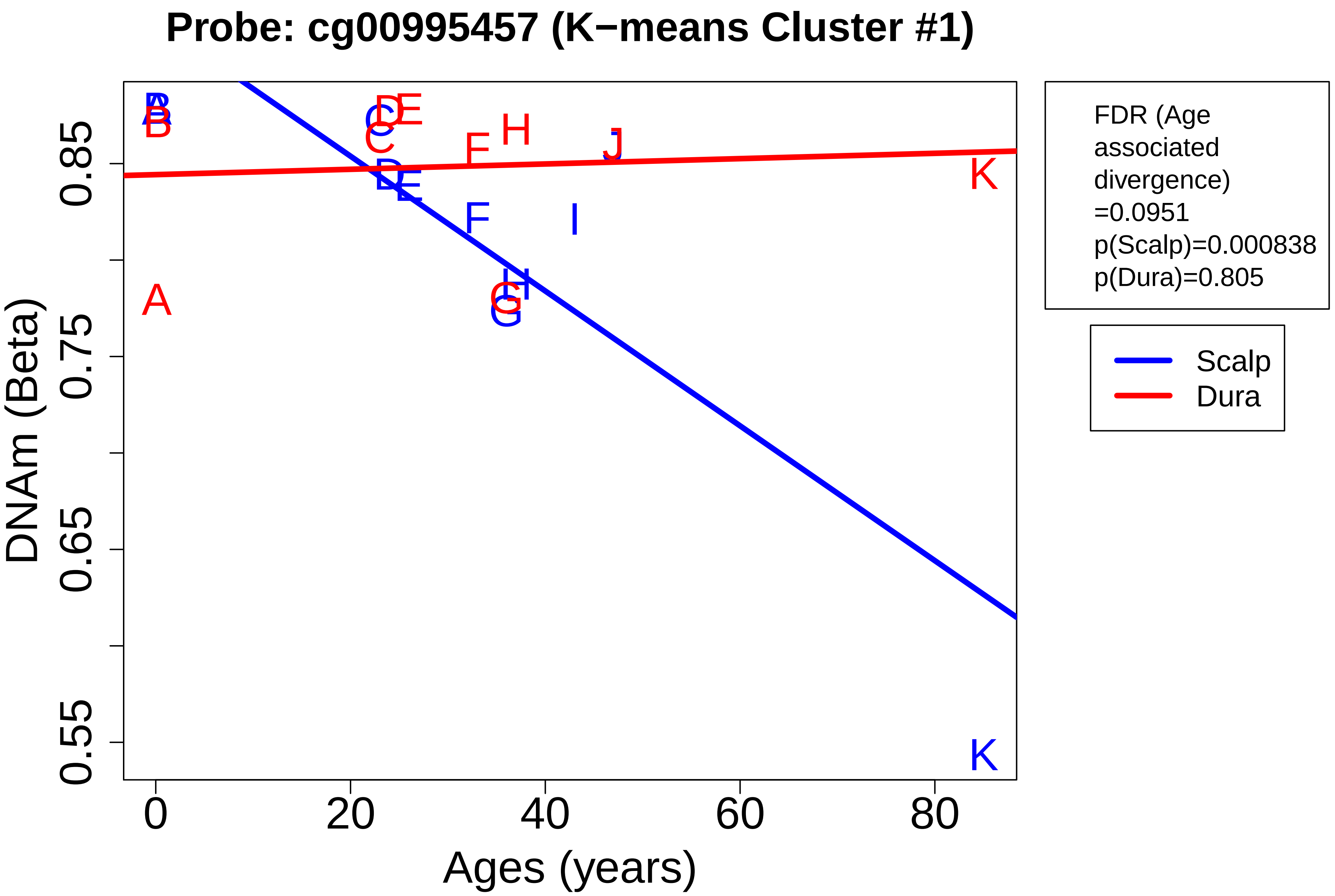
Probe: cg00995457 (K−means Cluster #1)
FDR (Age associated divergence) =0.0951
p(Scalp)=0.000838
p(Dura)=0.805
Scalp
Dura
DNAm (Beta)
0.55
0.65
0.75
0.85
0
20
40
60
80
Ages (years)

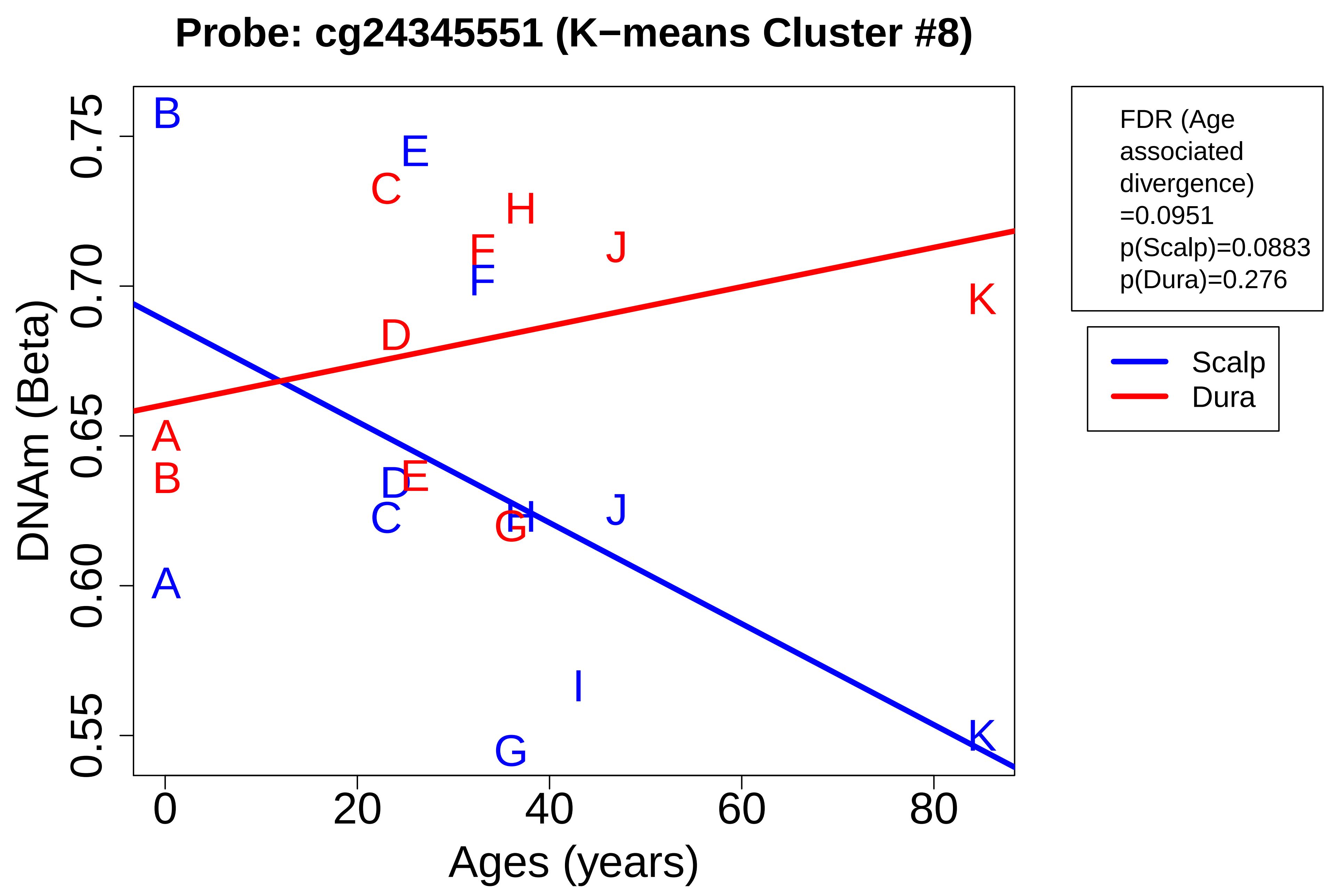

Probe: cg24345551 (K−means Cluster #8)
DNAm (Beta)
0.55
0.60
0.65
0.70
0.75
0
20
40
60
80
Ages (years)
B
E
C
H
F
J
F
K
D
A
B
D
E
C
H
G
J
A
I
K
G
FDR (Age associated divergence) =0.0951
p(Scalp)=0.0883
p(Dura)=0.276
Scalp
Dura

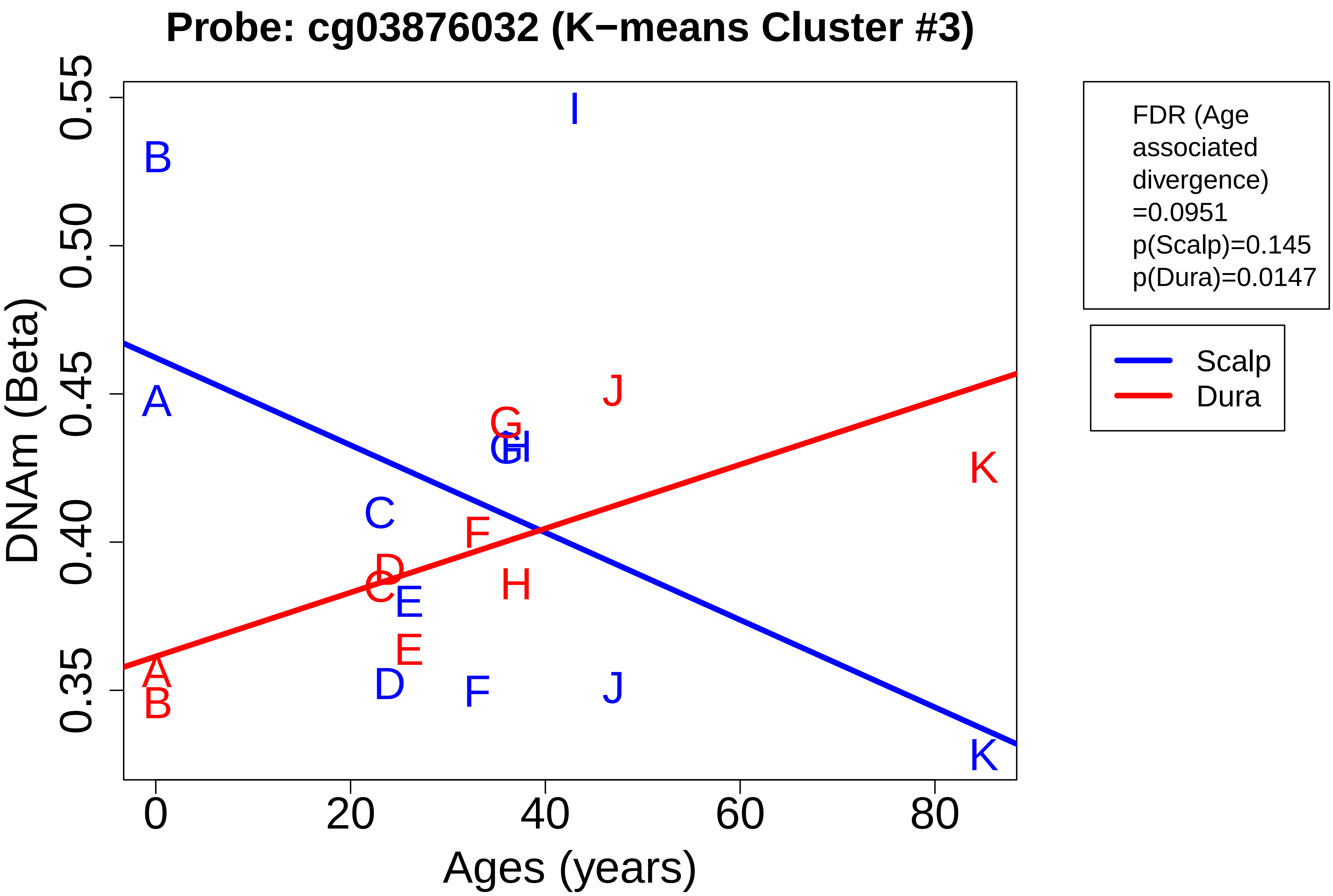

Probe: cg03876032 (K−means Cluster #3)
DNAm (Beta)
0.35
0.40
0.45
0.50
0.55
0
20
40
60
80
Ages (years)
FDR (Age associated divergence) =0.0951
p(Scalp)=0.145
p(Dura)=0.0147
Scalp
Dura

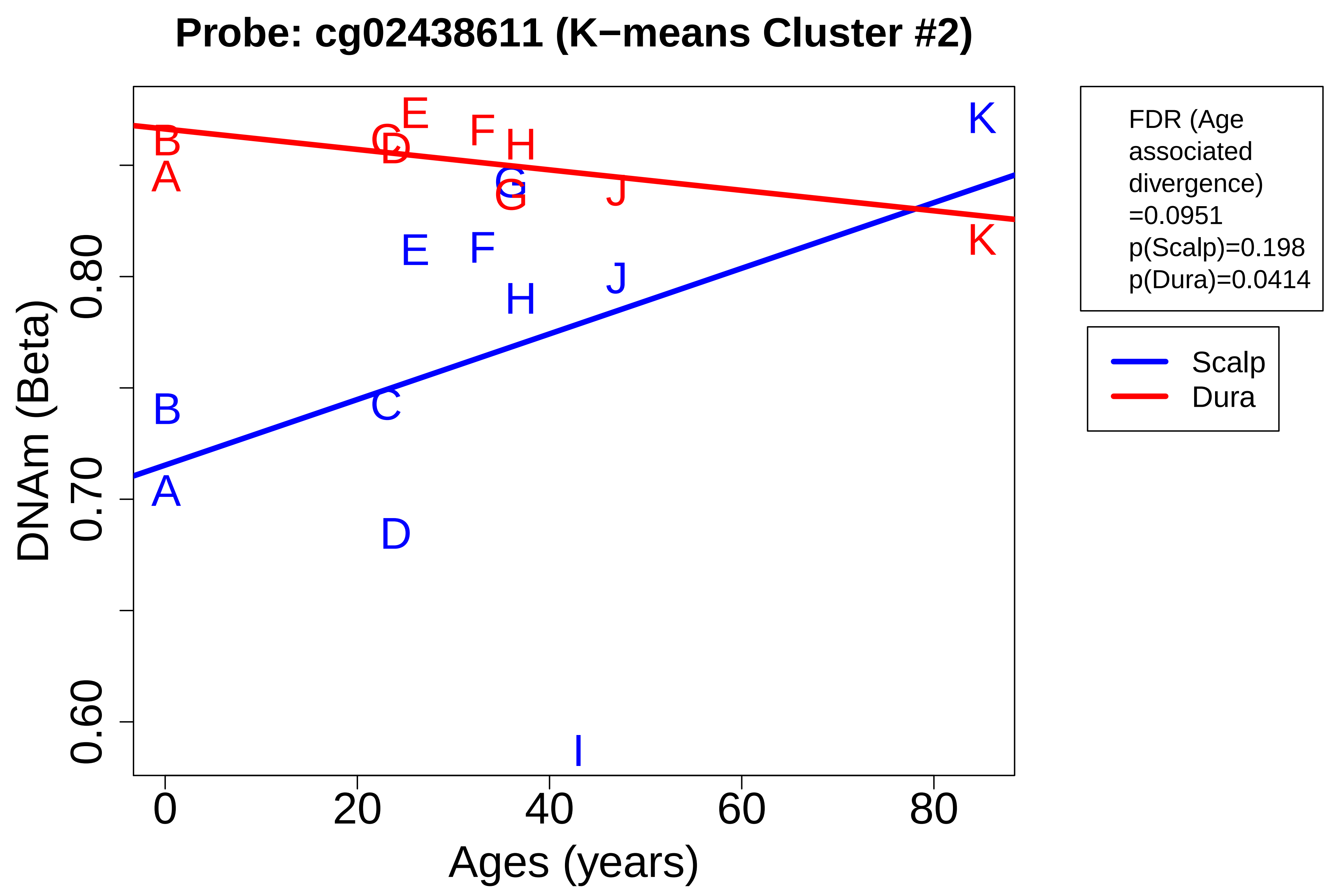

Probe: cg02438611 (K−means Cluster #2)
DNAm (Beta)
0.60
0.70
0.80
0
20
40
60
80
Ages (years)
A
B
C
D
E
F
G
H
I
J
K
FDR (Age associated divergence) =0.0951
p(Scalp)=0.198
p(Dura)=0.0414
Scalp
Dura

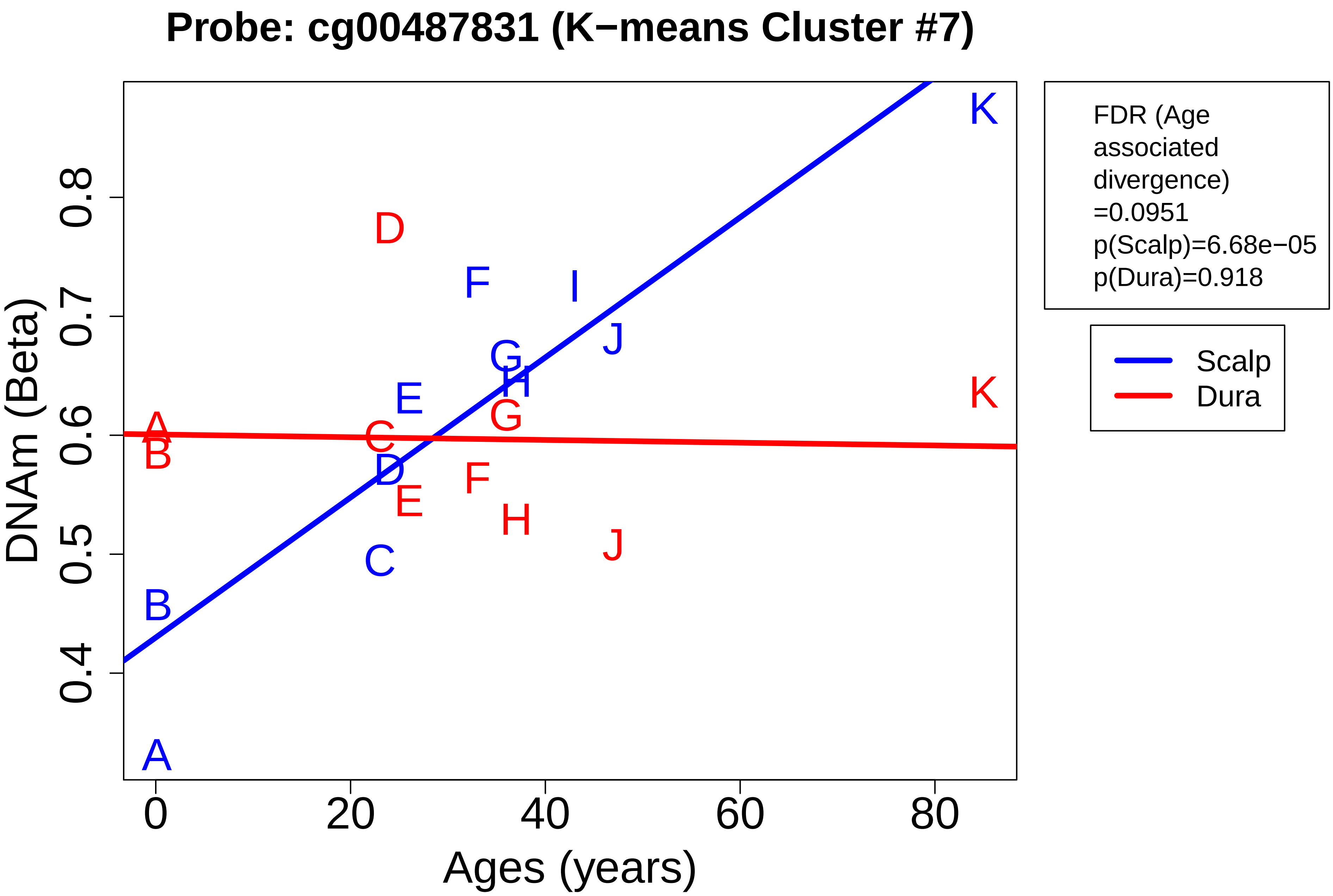

Probe: cg00487831 (K−means Cluster #7)
FDR (Age associated divergence) =0.0951
p(Scalp)=6.68e−05
p(Dura)=0.918
Scalp
Dura
DNAm (Beta)
0.4
0.5
0.6
0.7
0.8
0
20
40
60
80
Ages (years)
A
B
C
D
E
F
G
H
I
J
K

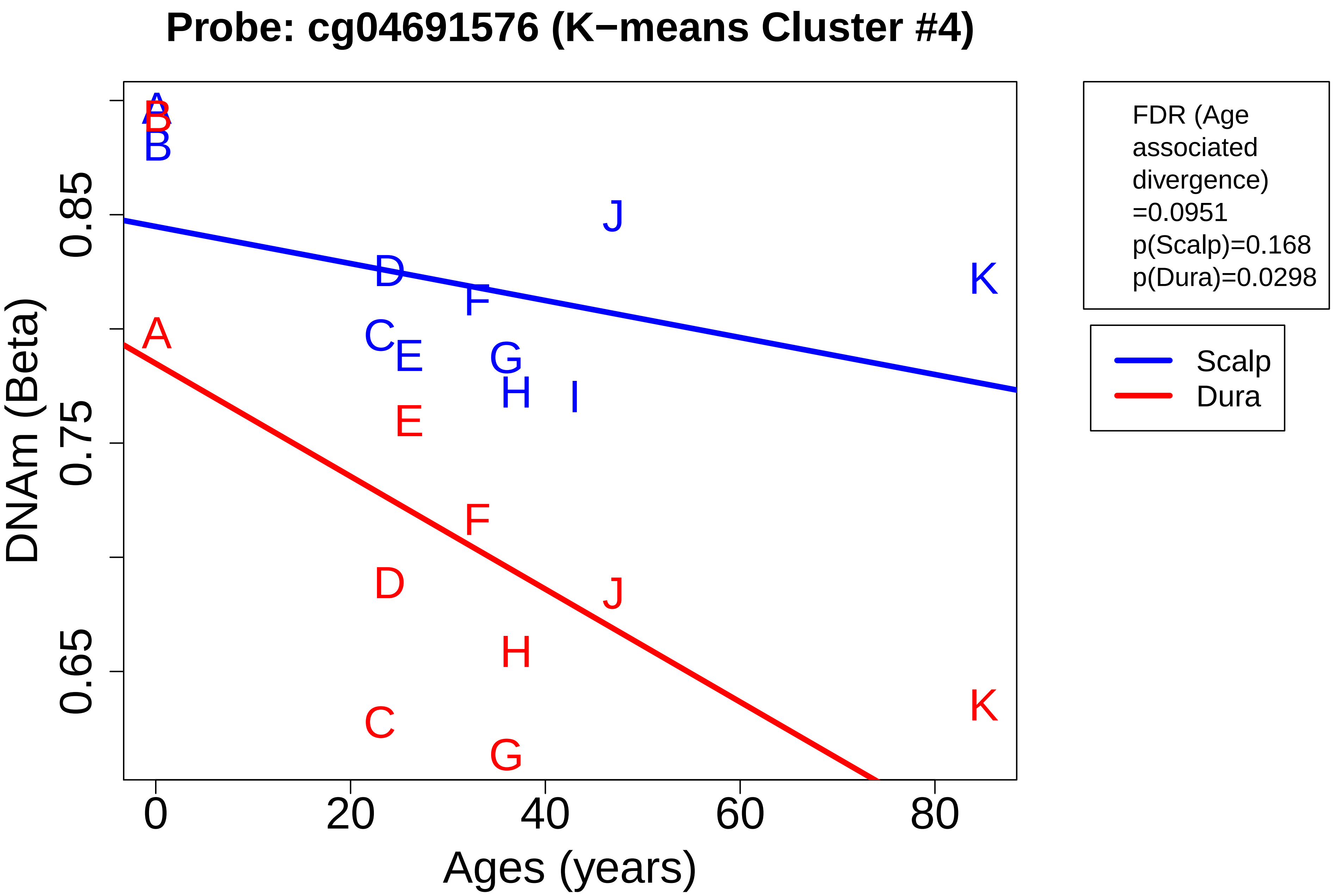

Probe: cg04691576 (K−means Cluster #4)
DNAm (Beta)
0.65
0.75
0.85
Ages (years)
0
20
40
60
80
A
B
C
D
E
F
G
H
I
J
K
FDR (Age associated divergence) =0.0951
p(Scalp)=0.168
p(Dura)=0.0298
Scalp
Dura

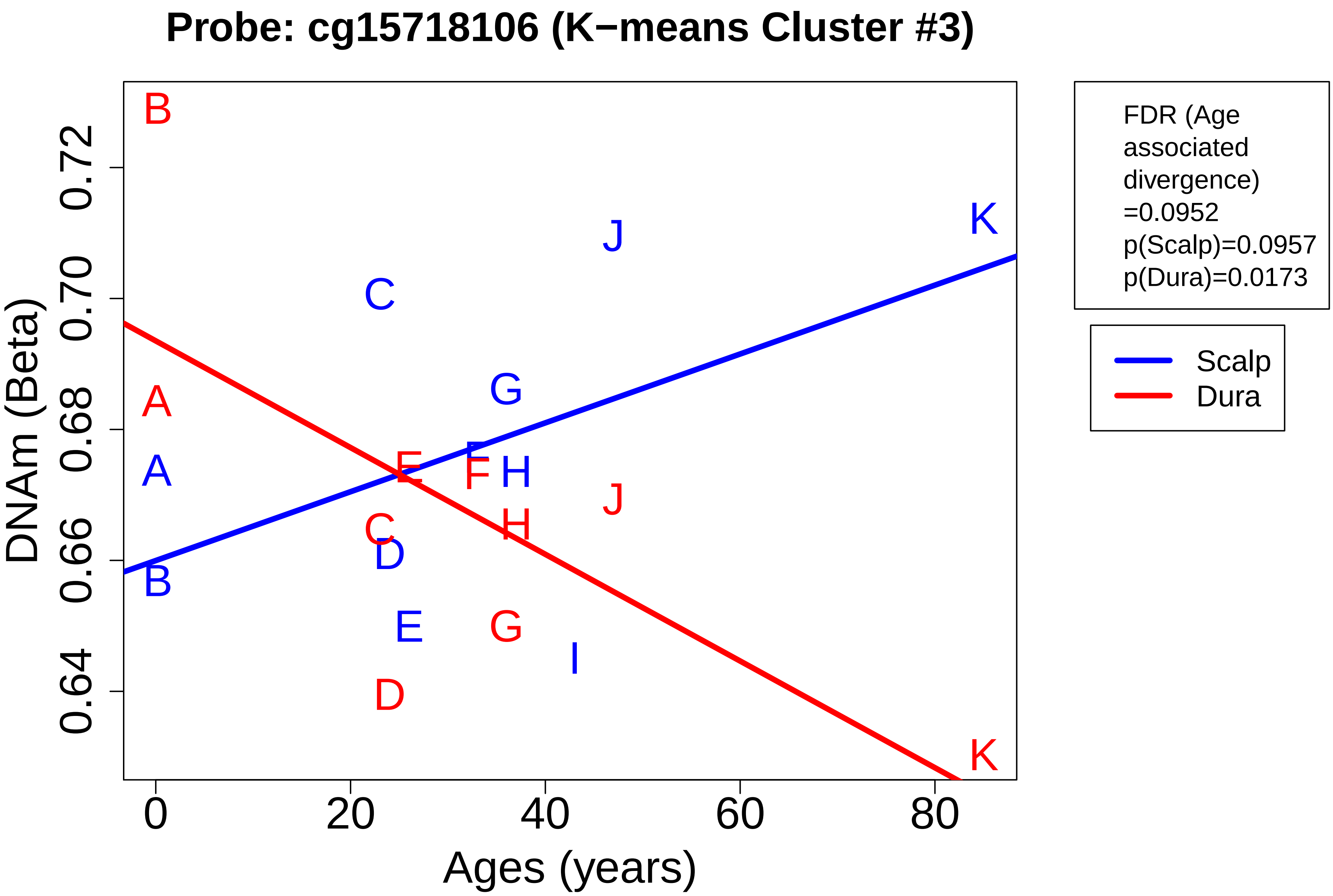
Probe: cg15718106 (K−means Cluster #3)
DNAm (Beta)
0.64
0.66
0.68
0.70
0.72
Ages (years)
0
20
40
60
80
B
A
A
B
C
C
D
D
E
E
E
F
F
G
G
H
H
I
J
J
K
K
FDR (Age associated divergence) =0.0952
p(Scalp)=0.0957
p(Dura)=0.0173
Scalp
Dura

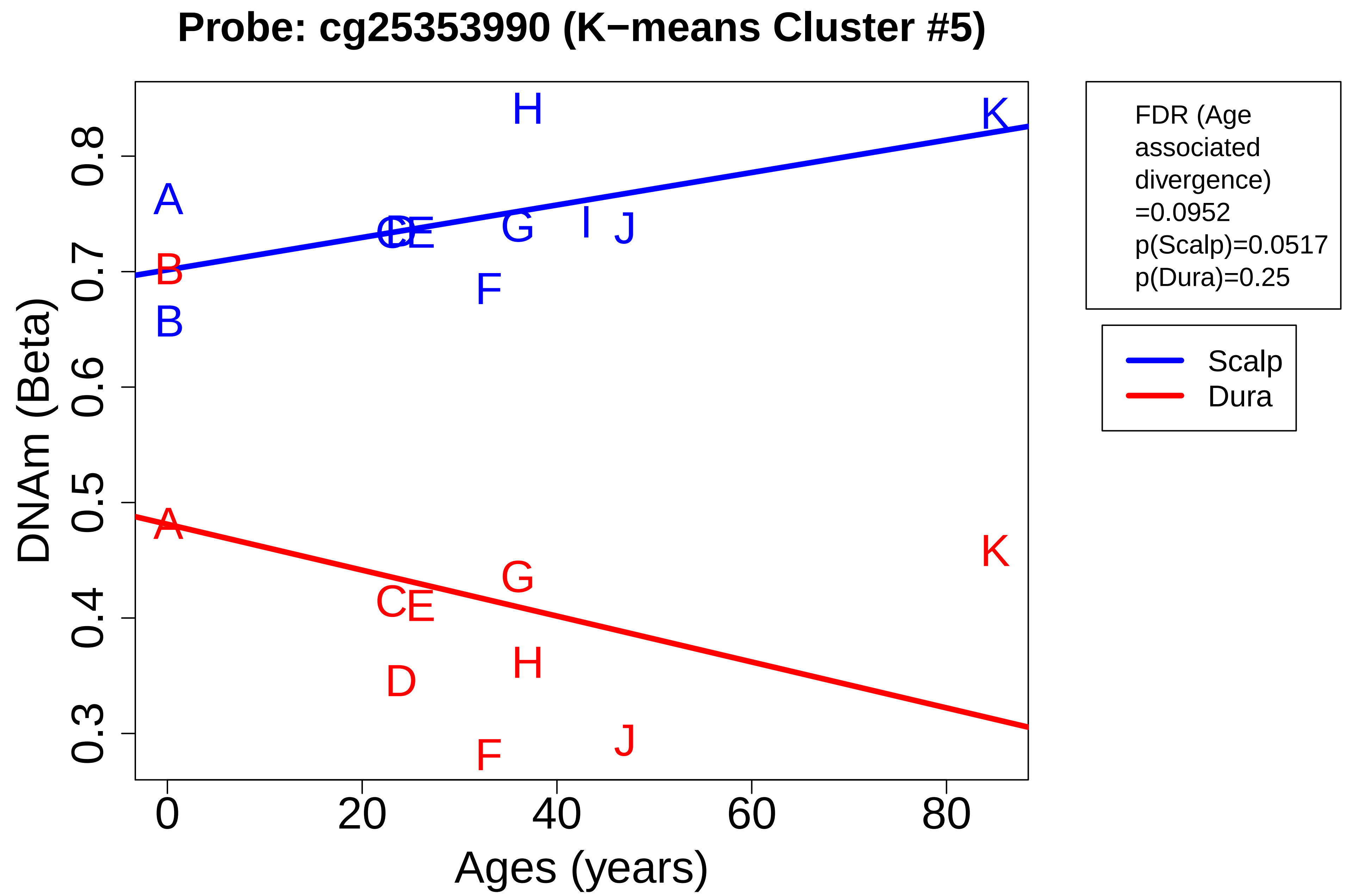

Probe: cg25353990 (K−means Cluster #5)
DNAm (Beta)
0.3
0.4
0.5
0.6
0.7
0.8
0
20
40
60
80
Ages (years)
FDR (Age associated divergence) =0.0952
p(Scalp)=0.0517
p(Dura)=0.25
Scalp
Dura

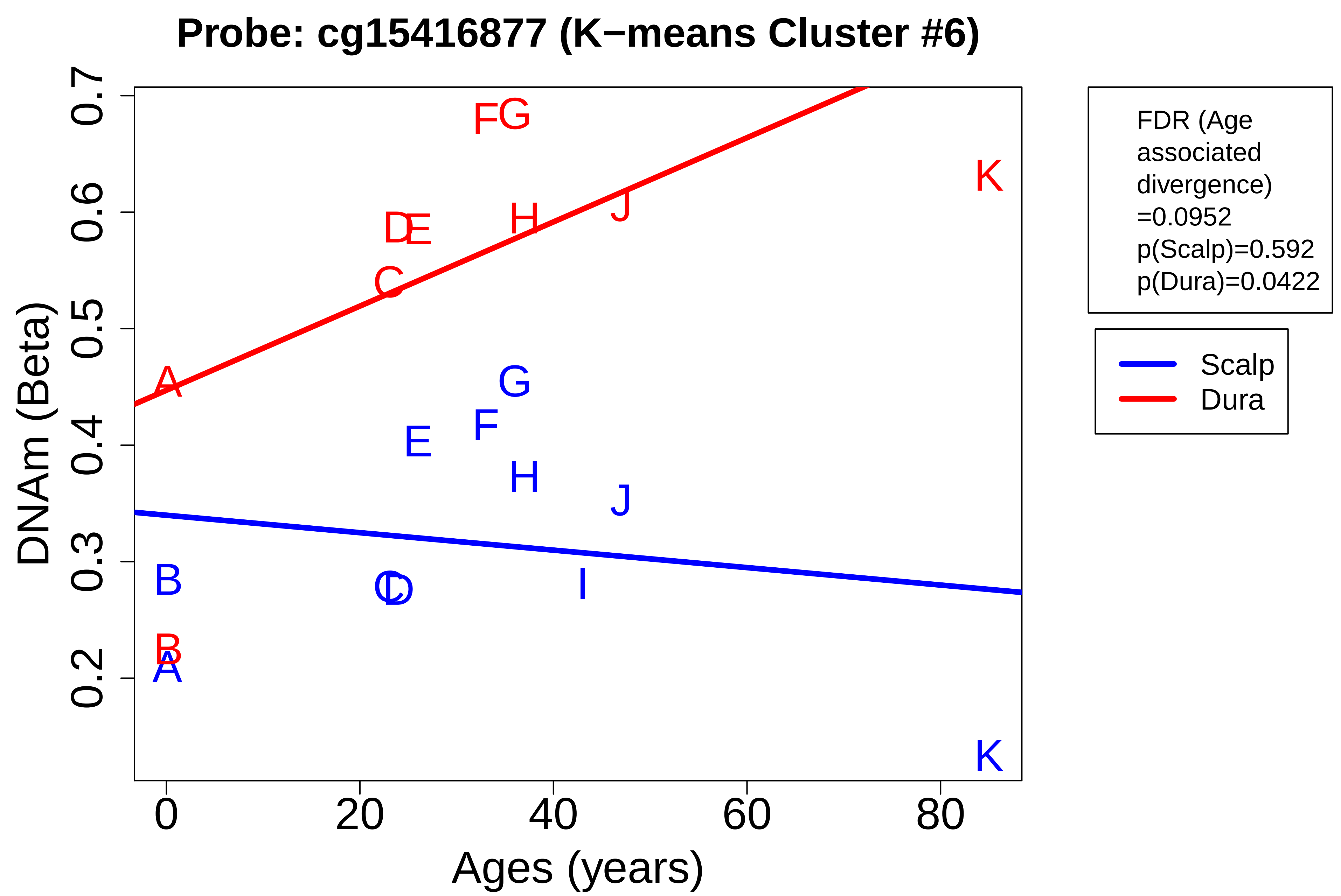

Probe: cg15416877 (K−means Cluster #6)
DNAm (Beta)
0.2
0.3
0.4
0.5
0.6
0.7
Ages (years)
0
20
40
60
80
FDR (Age associated divergence) =0.0952
p(Scalp)=0.592
p(Dura)=0.0422
Scalp
Dura
A
B
C
D
E
F
G
H
I
J
K

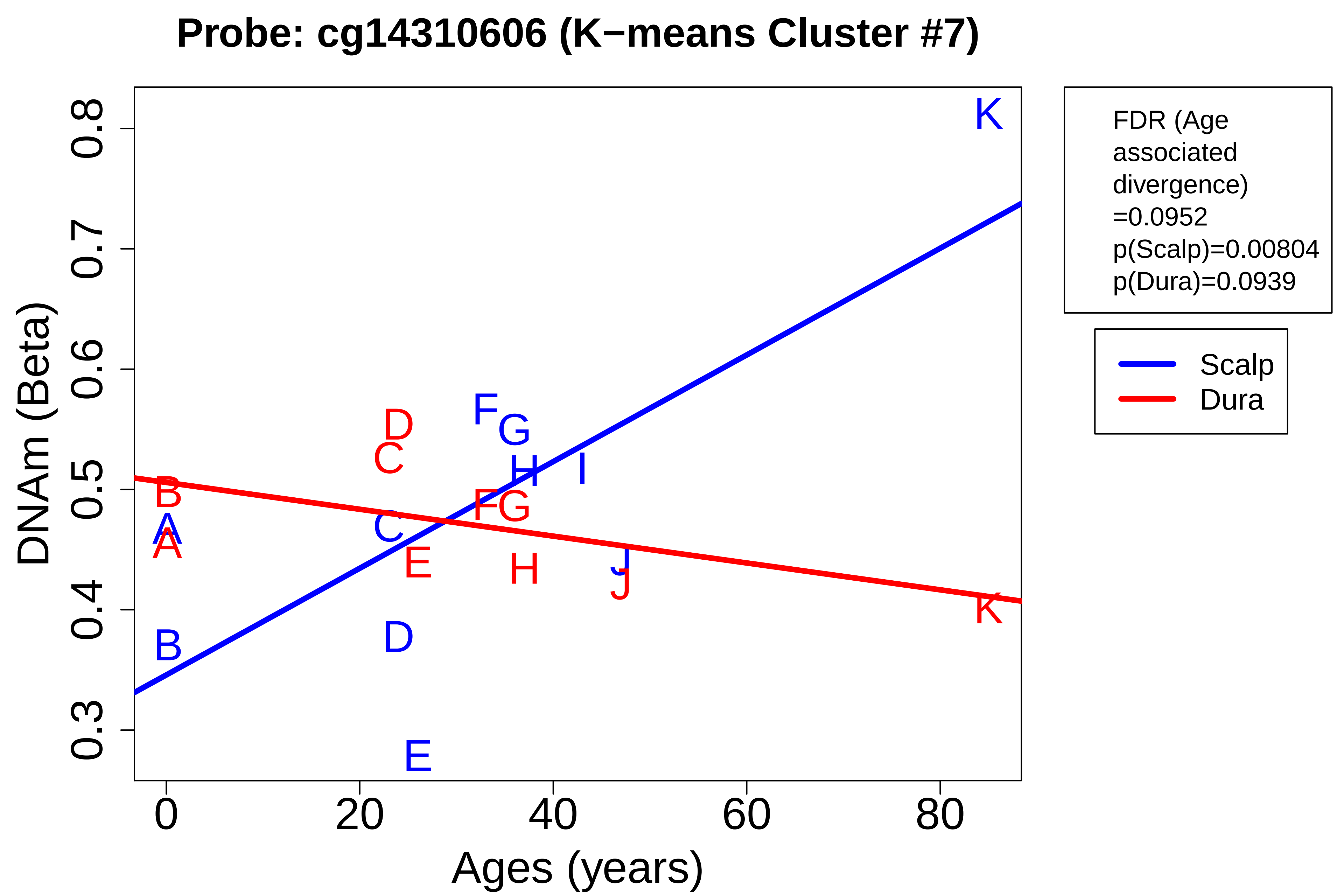

Probe: cg14310606 (K−means Cluster #7)
DNAm (Beta)
0.3
0.4
0.5
0.6
0.7
0.8
0
20
40
60
80
Ages (years)
FDR (Age associated divergence) =0.0952
p(Scalp)=0.00804
p(Dura)=0.0939
Scalp
Dura

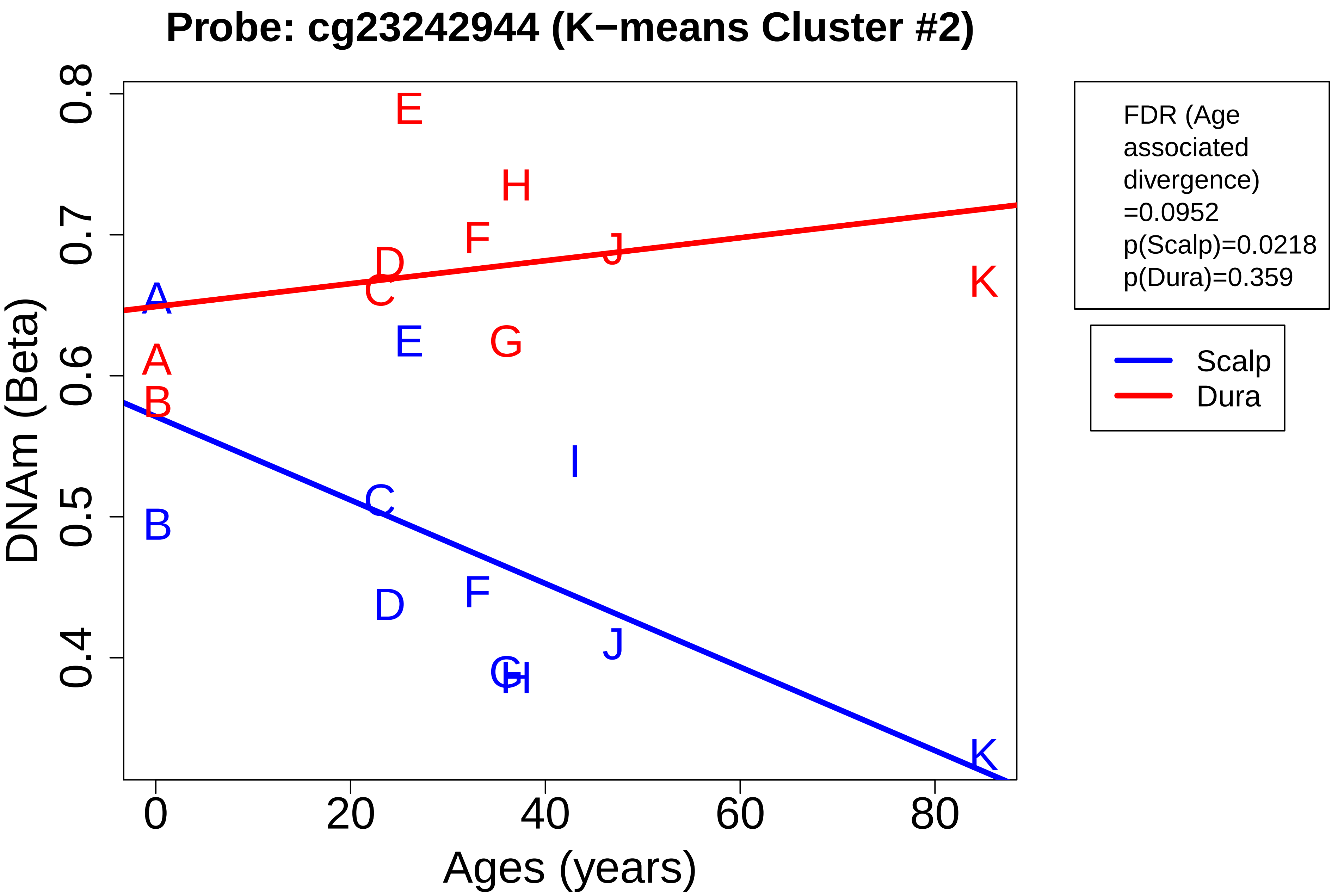

Probe: cg23242944 (K−means Cluster #2)
DNAm (Beta)
Ages (years)
0.4
0.5
0.6
0.7
0.8
0
20
40
60
80
FDR (Age associated divergence) =0.0952
p(Scalp)=0.0218
p(Dura)=0.359
Scalp
Dura

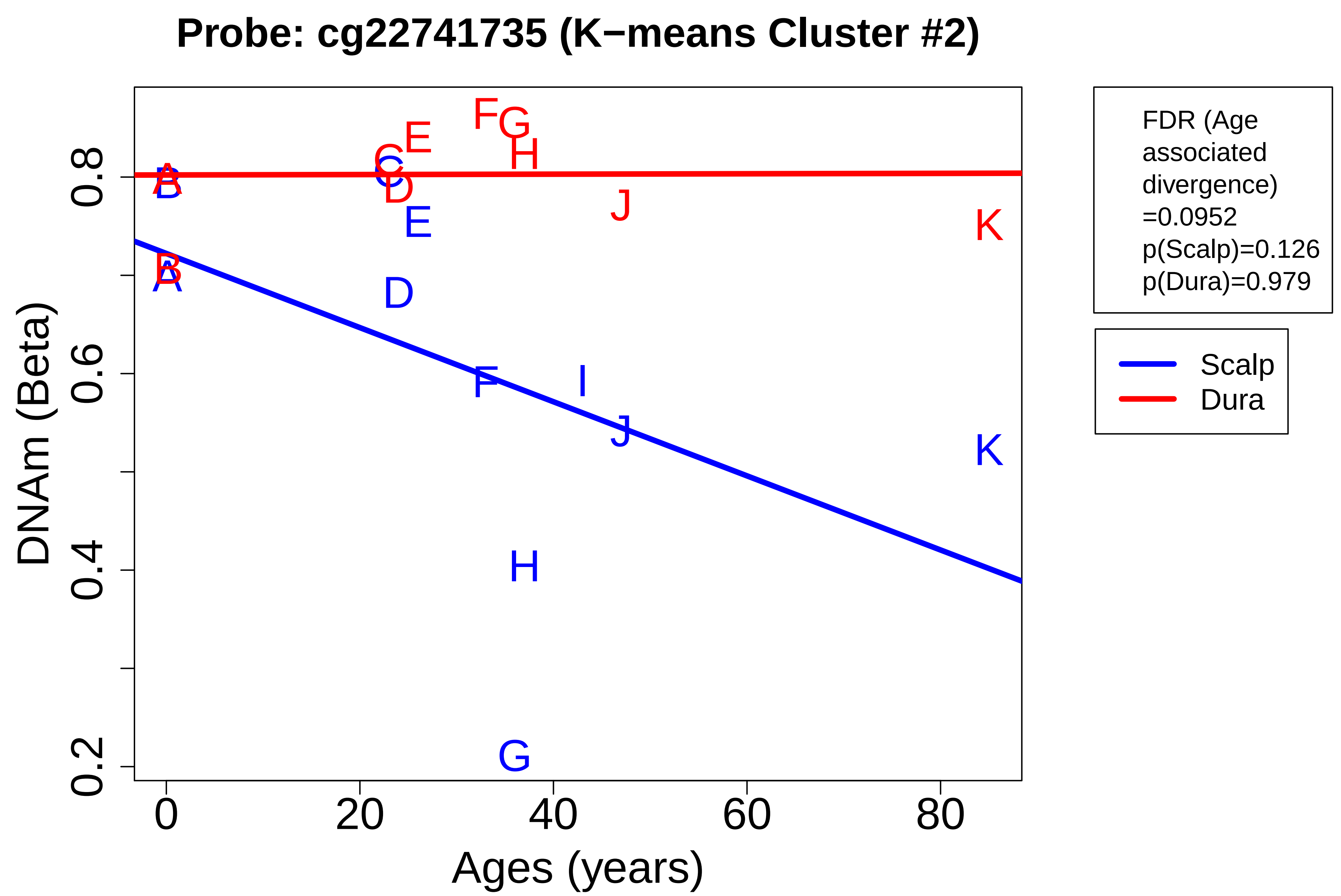

Probe: cg22741735 (K−means Cluster #2)
DNAm (Beta)
0.2
0.4
0.6
0.8
0
20
40
60
80
Ages (years)
FDR (Age associated divergence) =0.0952
p(Scalp)=0.126
p(Dura)=0.979
Scalp
Dura

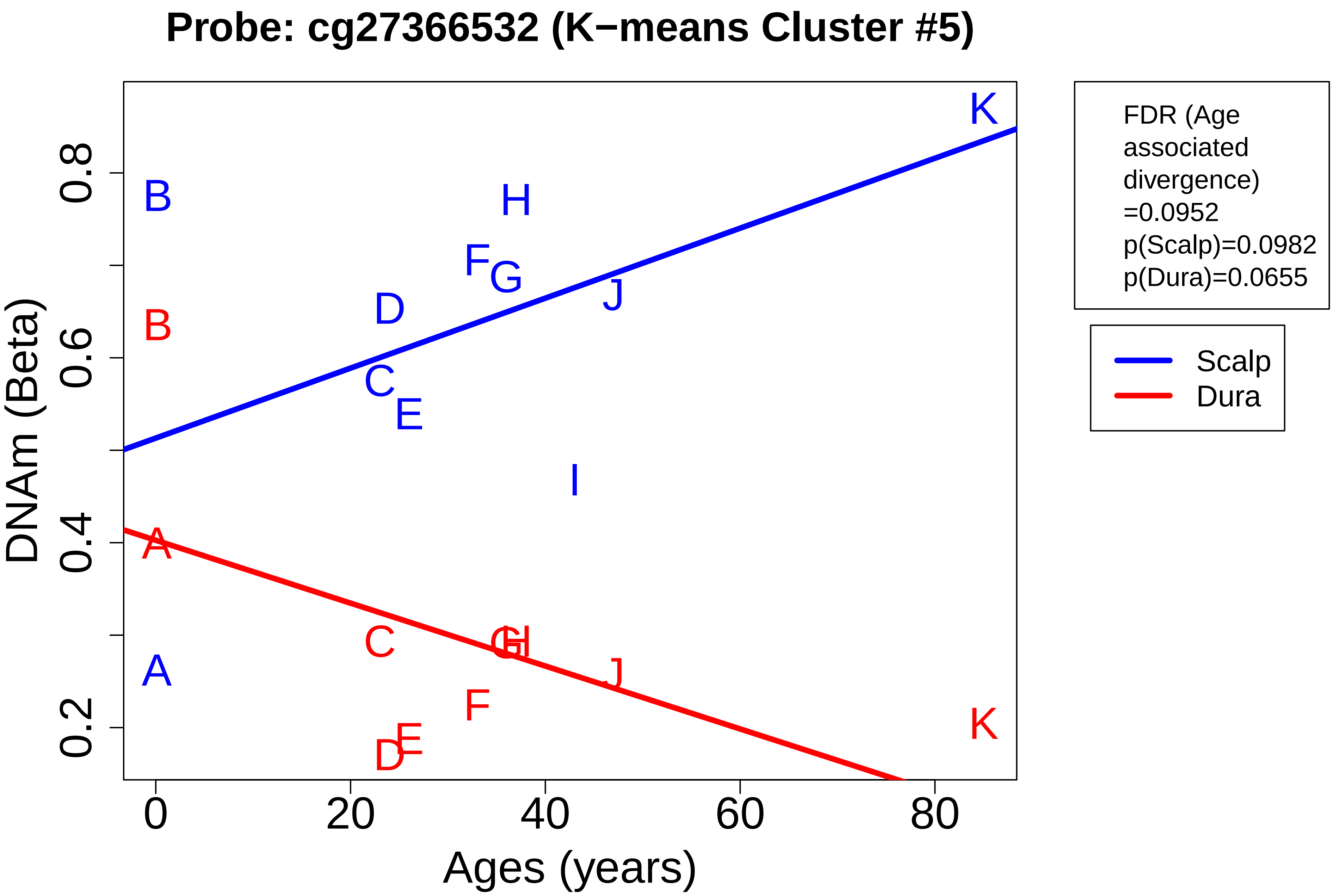

Probe: cg27366532 (K−means Cluster #5)
DNAm (Beta)
0.2
0.4
0.6
0.8
0
20
40
60
80
Ages (years)
FDR (Age associated divergence) =0.0952
p(Scalp)=0.0982
p(Dura)=0.0655
Scalp
Dura
A
B
C
D
E
F
G
H
I
J
K

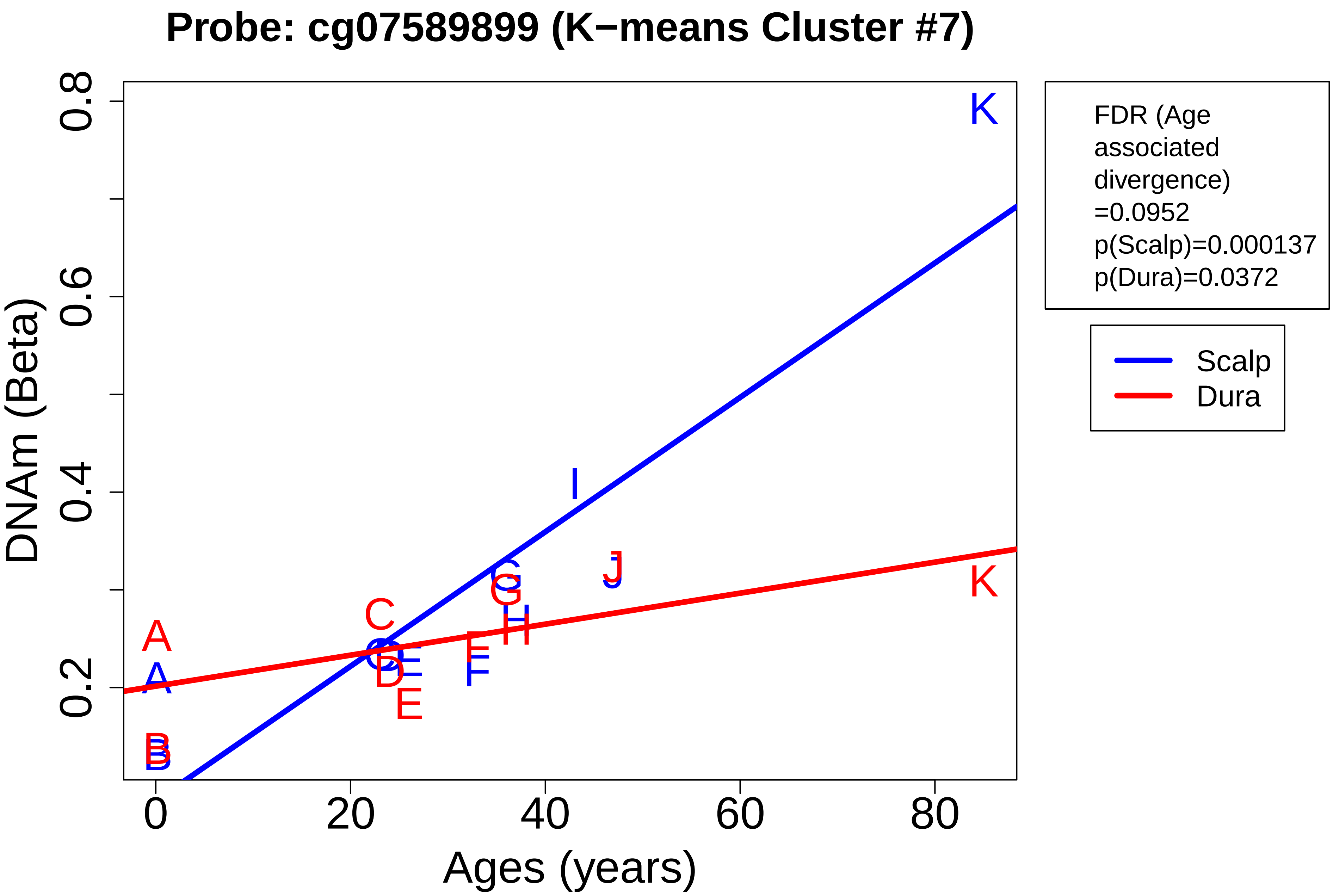

Probe: cg07589899 (K−means Cluster #7)
DNAm (Beta)
0.2
0.4
0.6
0.8
0
20
40
60
80
Ages (years)
FDR (Age associated divergence) =0.0952
p(Scalp)=0.000137
p(Dura)=0.0372
Scalp
Dura
A
B
C
D
E
F
G
H
I
J
K

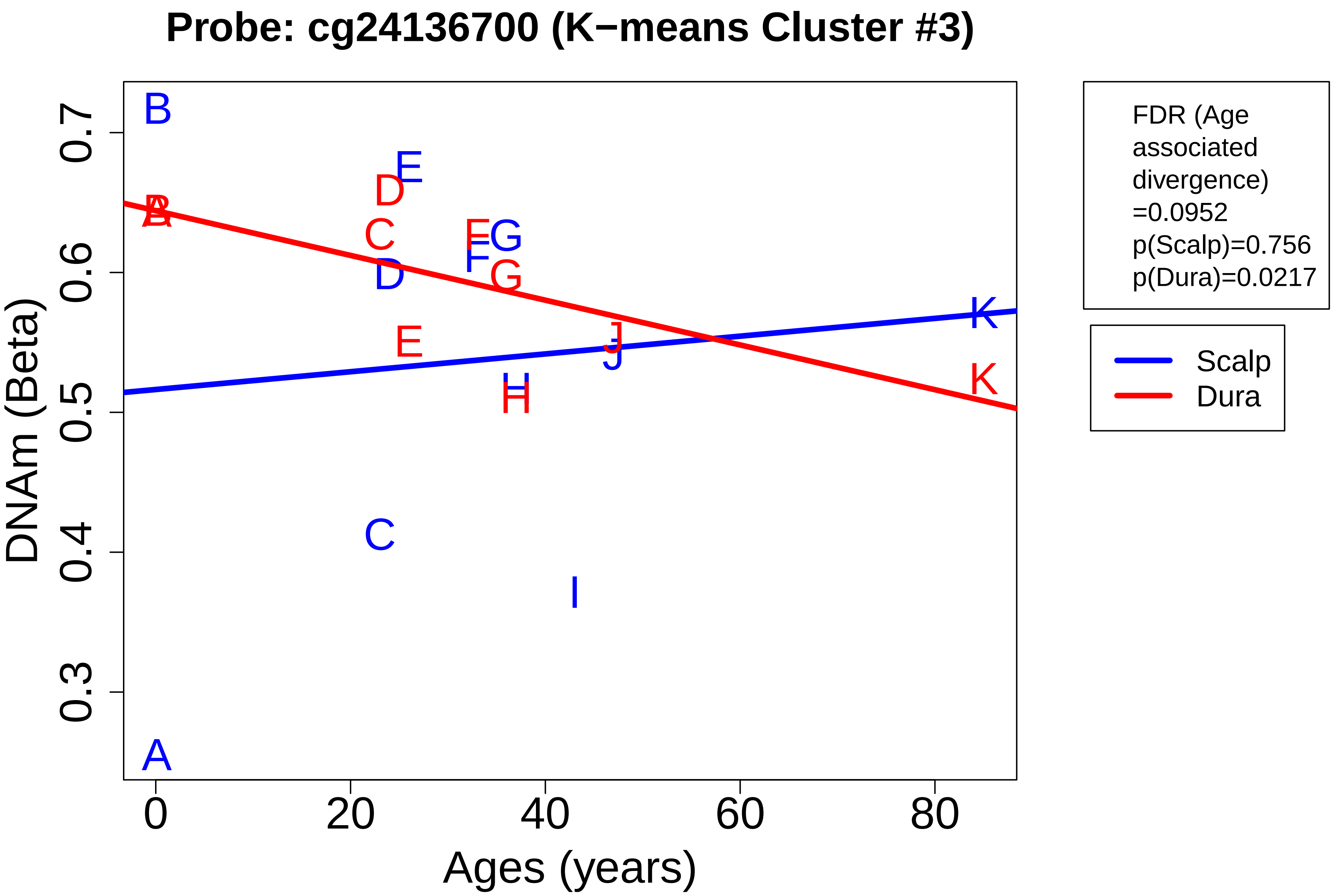

Probe: cg24136700 (K−means Cluster #3)
FDR (Age associated divergence) =0.0952
p(Scalp)=0.756
p(Dura)=0.0217
Scalp
Dura
DNAm (Beta)
0.3
0.4
0.5
0.6
0.7
Ages (years)
0
20
40
60
80

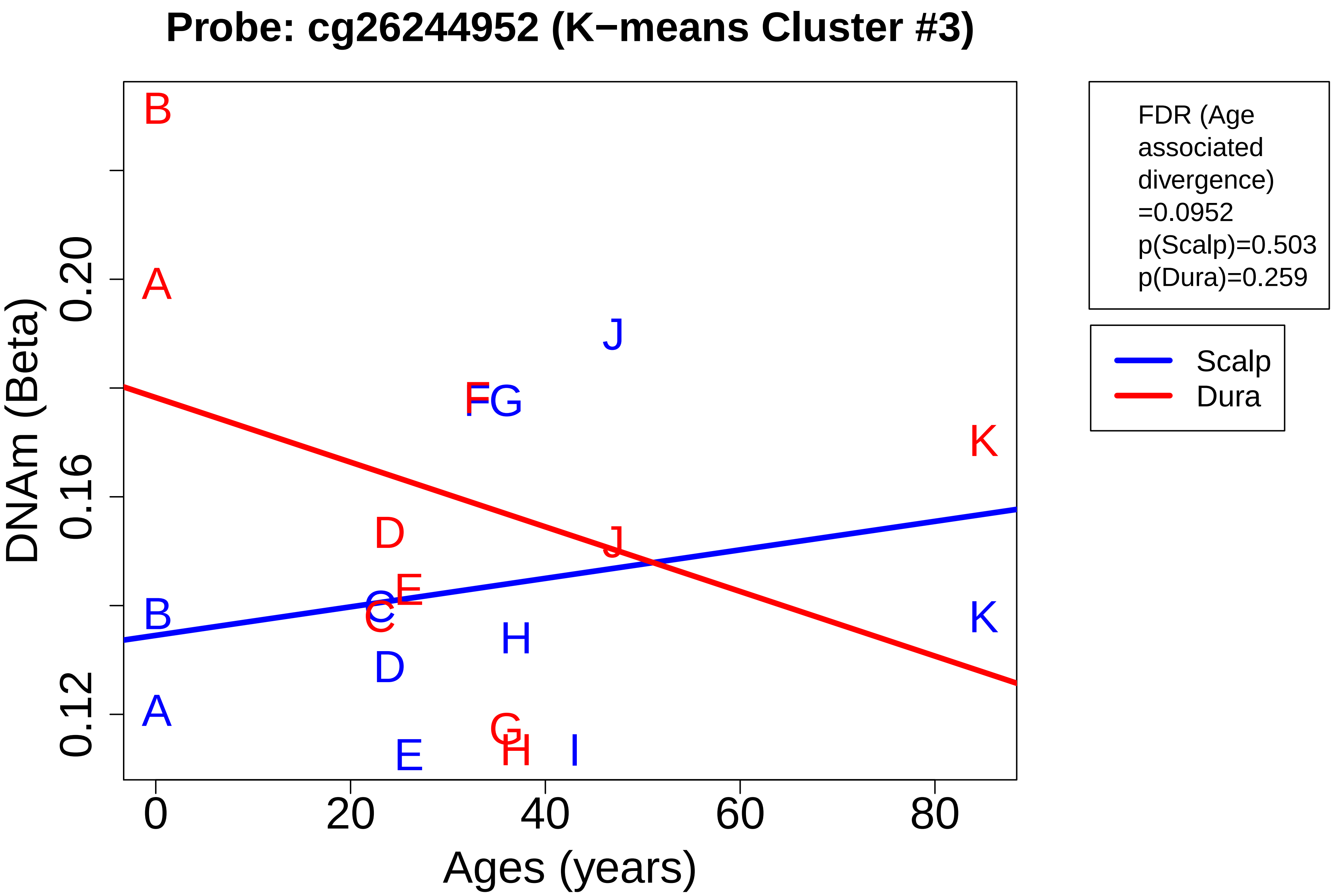

Probe: cg26244952 (K−means Cluster #3)
FDR (Age associated divergence) =0.0952
p(Scalp)=0.503
p(Dura)=0.259
Scalp
Dura
DNAm (Beta)
0.12
0.16
0.20
Ages (years)
0
20
40
60
80

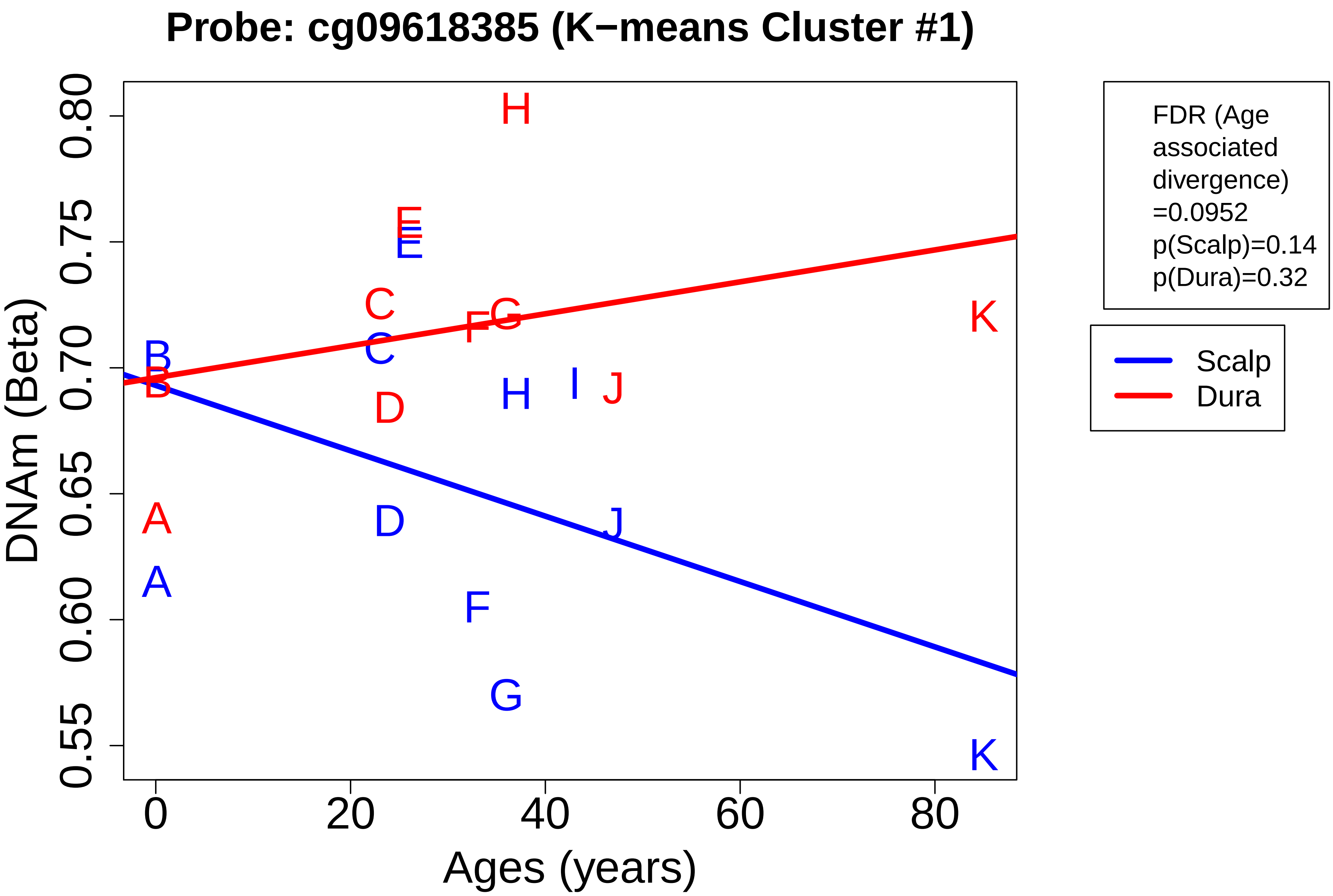

Probe: cg09618385 (K−means Cluster #1)
DNAm (Beta)
0.55
0.60
0.65
0.70
0.75
0.80
Ages (years)
0
20
40
60
80
FDR (Age associated divergence) =0.0952
p(Scalp)=0.14
p(Dura)=0.32
Scalp
Dura

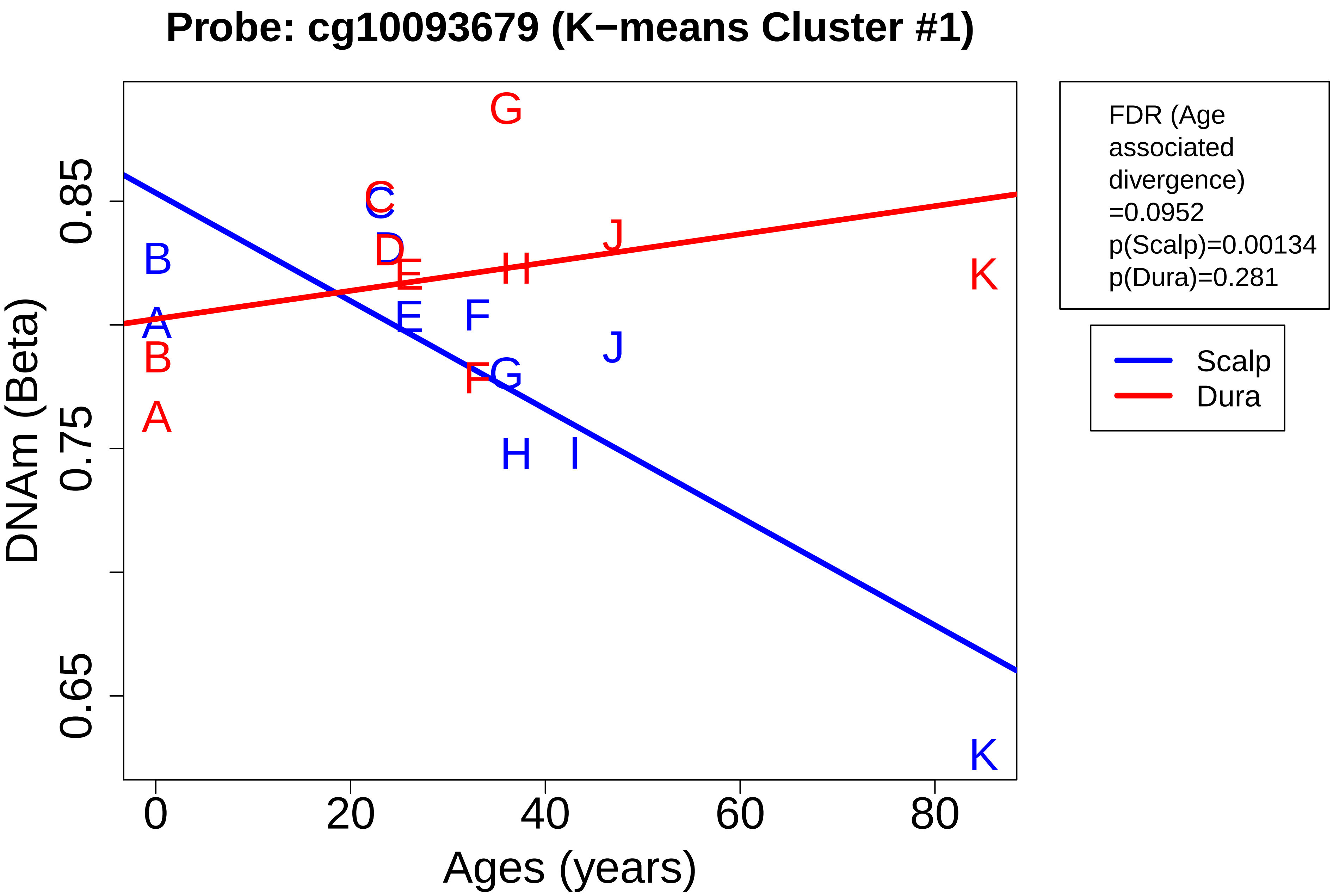

Probe: cg10093679 (K−means Cluster #1)
DNAm (Beta)
0.65
0.75
0.85
Ages (years)
0
20
40
60
80
FDR (Age associated divergence) =0.0952
p(Scalp)=0.00134
p(Dura)=0.281
Scalp
Dura
A
B
C
D
E
F
G
H
I
J
K

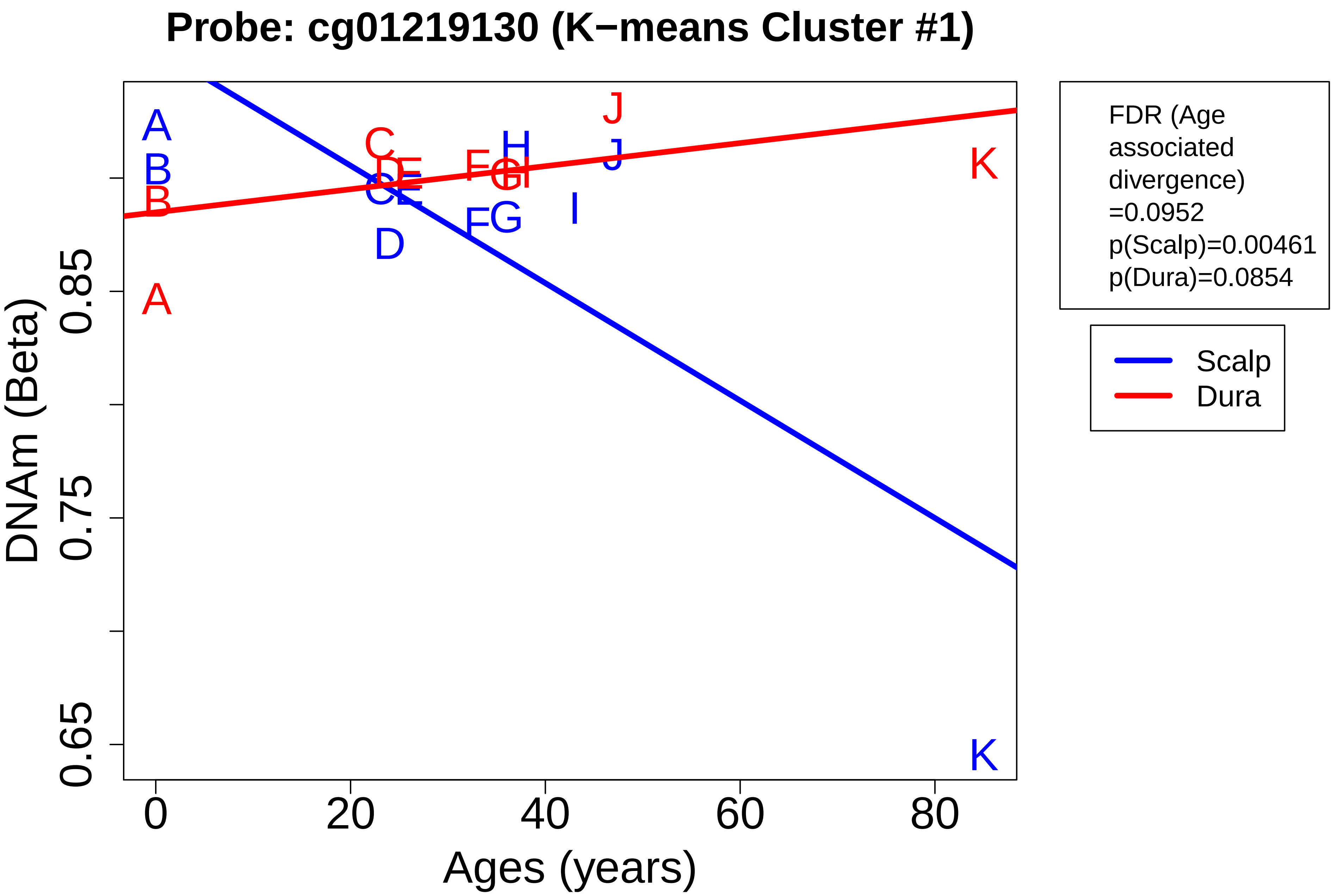

Probe: cg01219130 (K−means Cluster #1)
FDR (Age associated divergence) =0.0952
p(Scalp)=0.00461
p(Dura)=0.0854
Scalp
Dura
DNAm (Beta)
0.65
0.75
0.85
Ages (years)
0
20
40
60
80
A
B
C
D
E
F
G
H
I
J
K

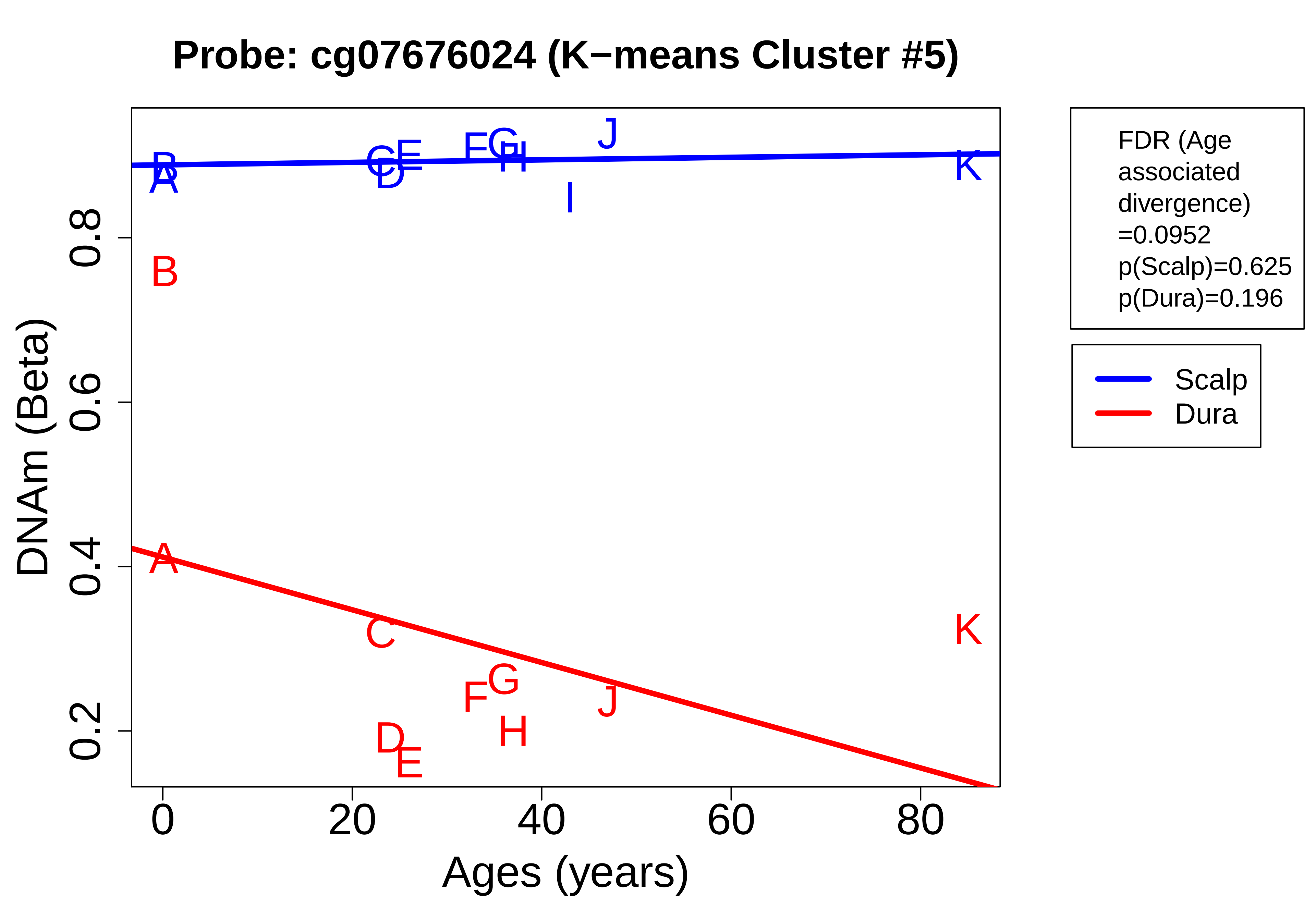

Probe: cg07676024 (K−means Cluster #5)
DNAm (Beta)
0.2
0.4
0.6
0.8
0
20
40
60
80
Ages (years)
FDR (Age associated divergence) =0.0952
p(Scalp)=0.625
p(Dura)=0.196
Scalp
Dura

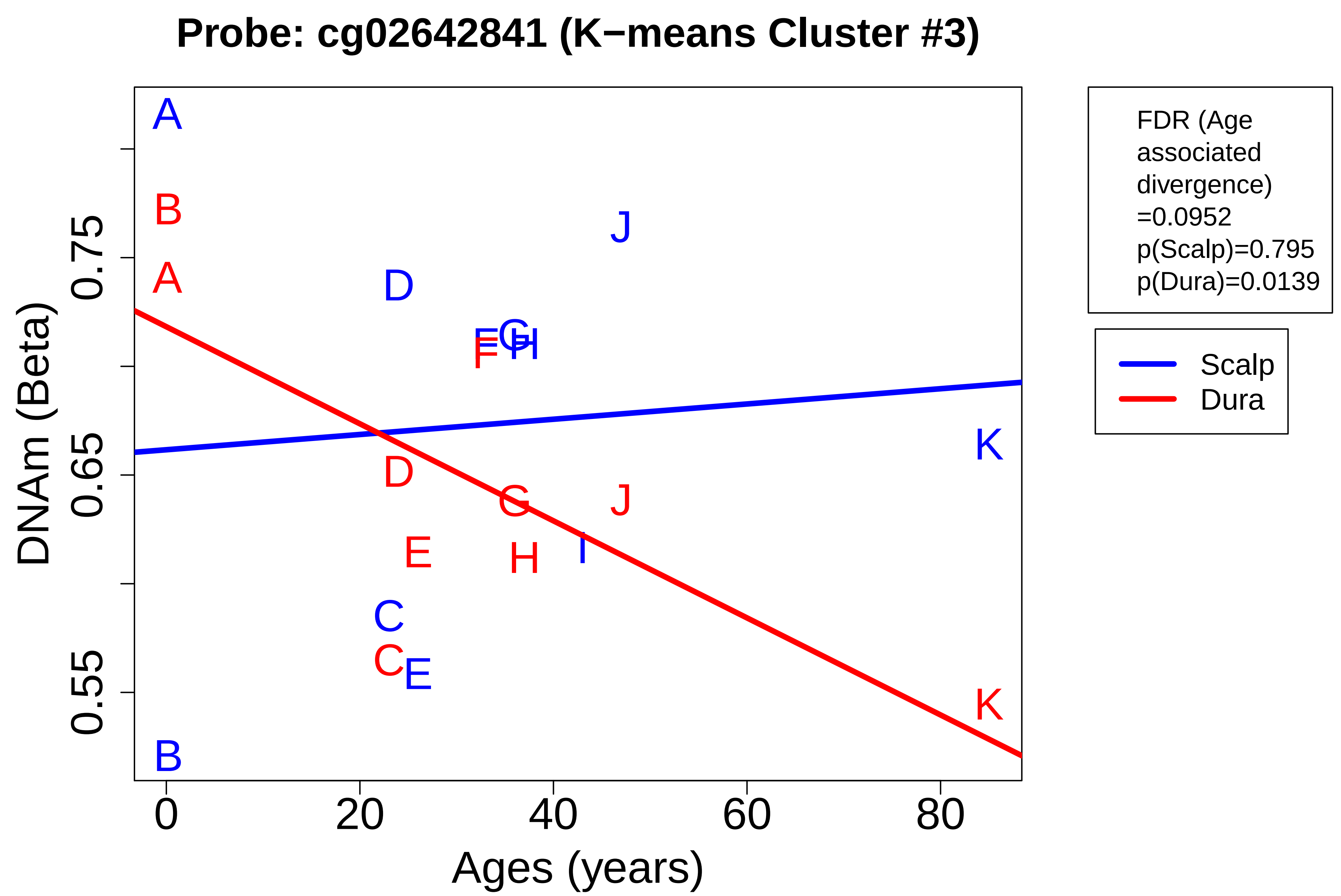

Probe: cg02642841 (K−means Cluster #3)
DNAm (Beta)
0.55
0.65
0.75
Ages (years)
0
20
40
60
80
A
B
A
B
C
C
D
D
E
E
F
F
G
G
H
H
I
J
J
K
K
FDR (Age associated divergence) =0.0952
p(Scalp)=0.795
p(Dura)=0.0139
Scalp
Dura

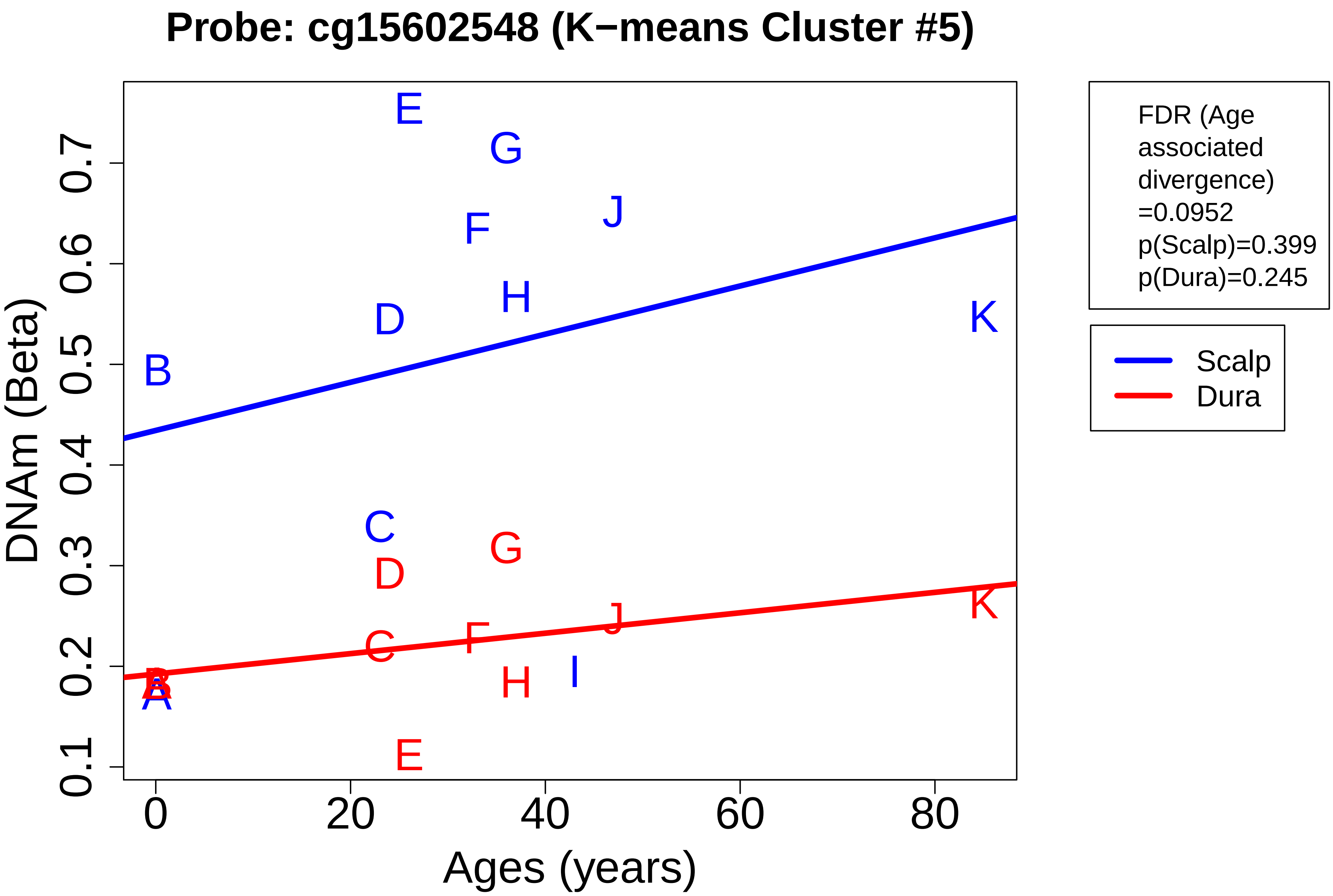

Probe: cg15602548 (K−means Cluster #5)
DNAm (Beta)
0.1
0.2
0.3
0.4
0.5
0.6
0.7
Ages (years)
0
20
40
60
80
FDR (Age associated divergence) =0.0952
p(Scalp)=0.399
p(Dura)=0.245
Scalp
Dura

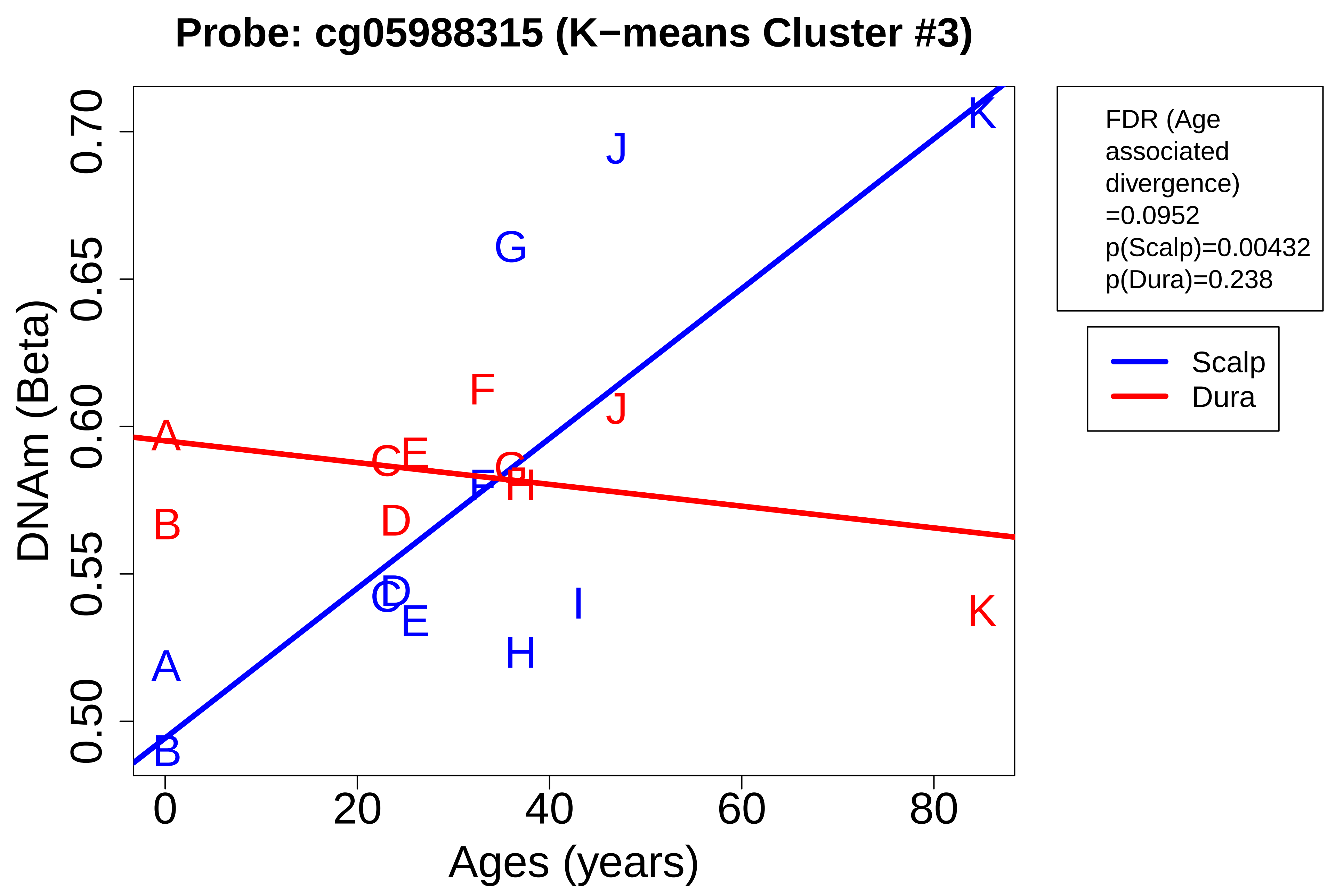
Probe: cg05988315 (K−means Cluster #3)
DNAm (Beta)
0.50
0.55
0.60
0.65
0.70
Ages (years)
0
20
40
60
80
FDR (Age associated divergence) =0.0952
p(Scalp)=0.00432
p(Dura)=0.238
Scalp
Dura
A
B
C
D
E
F
G
H
I
J
K

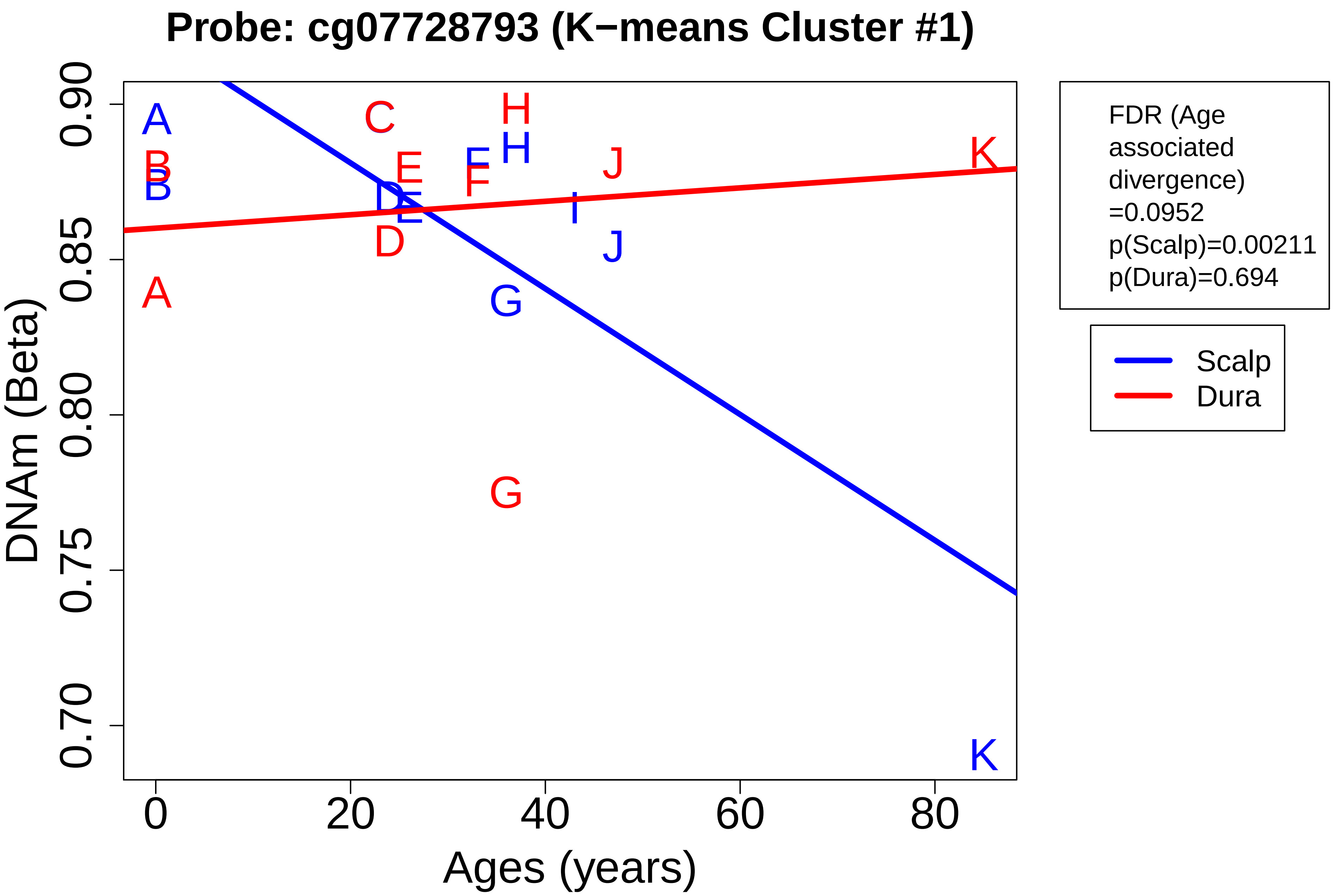

Probe: cg07728793 (K−means Cluster #1)
DNAm (Beta)
0.70
0.75
0.80
0.85
0.90
0
20
40
60
80
Ages (years)
FDR (Age associated divergence) =0.0952
p(Scalp)=0.00211
p(Dura)=0.694
Scalp
Dura

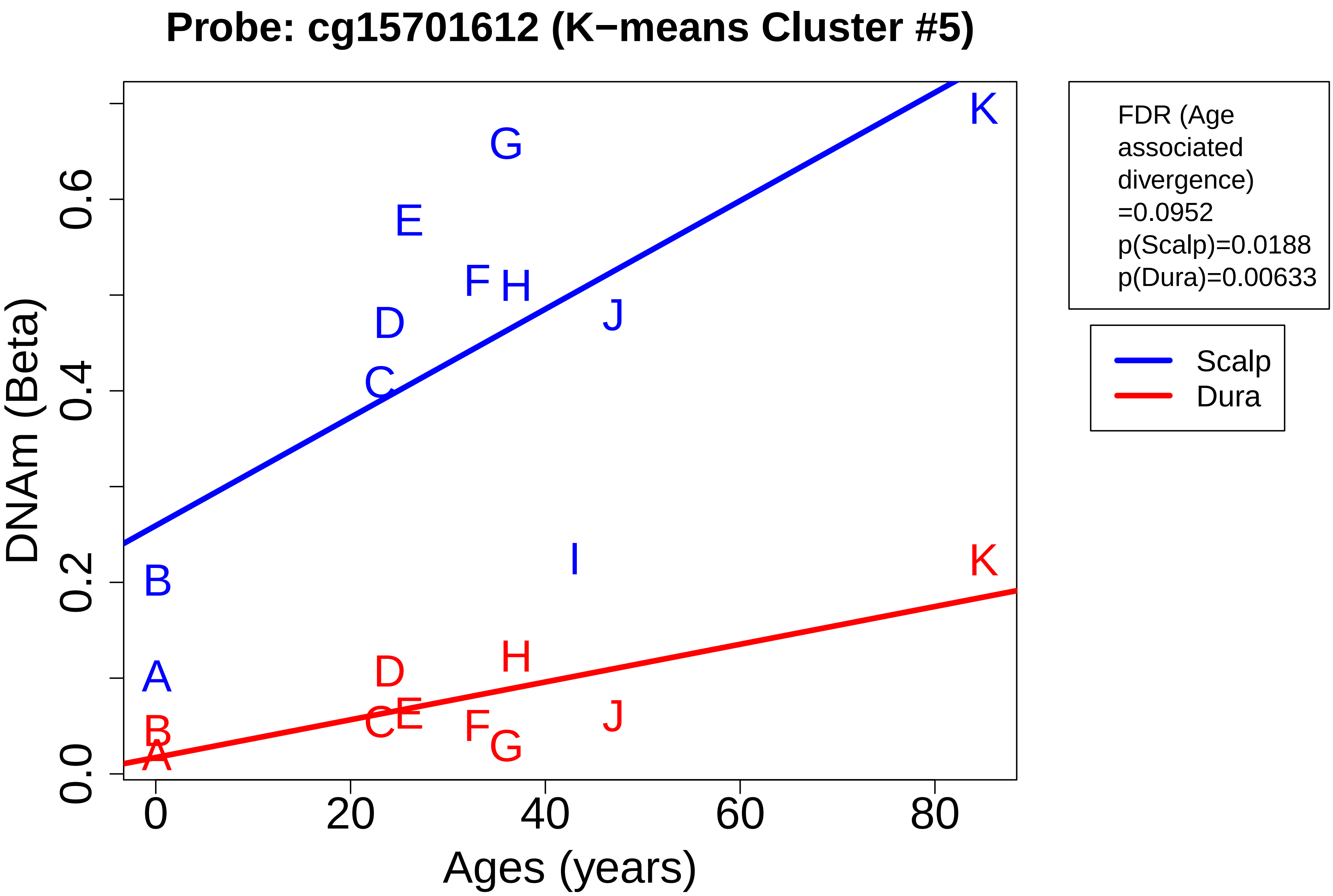

Probe: cg15701612 (K−means Cluster #5)
DNAm (Beta)
0.0
0.2
0.4
0.6
0
20
40
60
80
Ages (years)
A
B
C
D
E
F
G
H
I
J
K
FDR (Age associated divergence) =0.0952
p(Scalp)=0.0188
p(Dura)=0.00633
Scalp
Dura

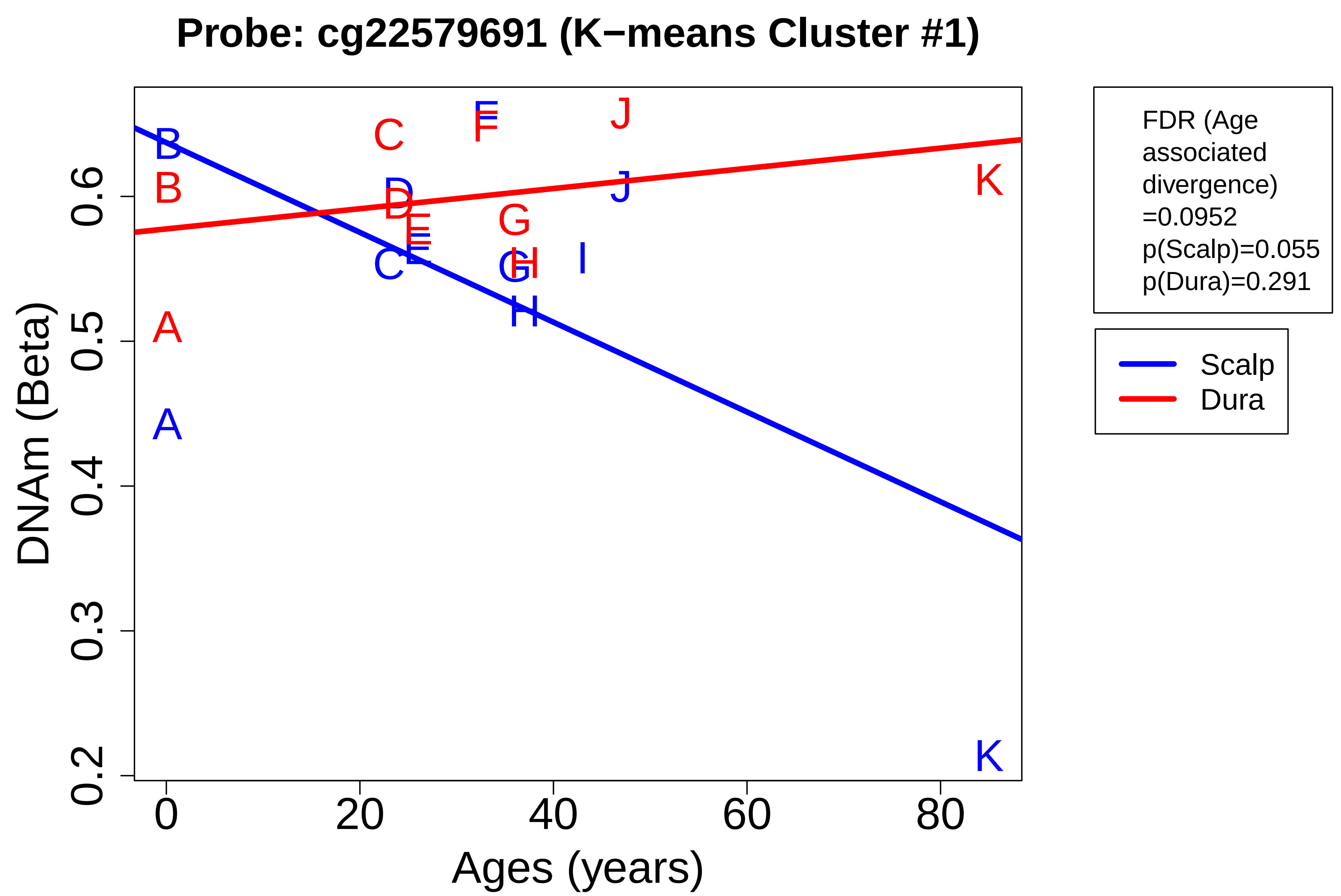

Probe: cg22579691 (K−means Cluster #1)
DNAm (Beta)
Ages (years)
0.2
0.3
0.4
0.5
0.6
0
20
40
60
80
FDR (Age associated divergence) =0.0952
p(Scalp)=0.055
p(Dura)=0.291
Scalp
Dura

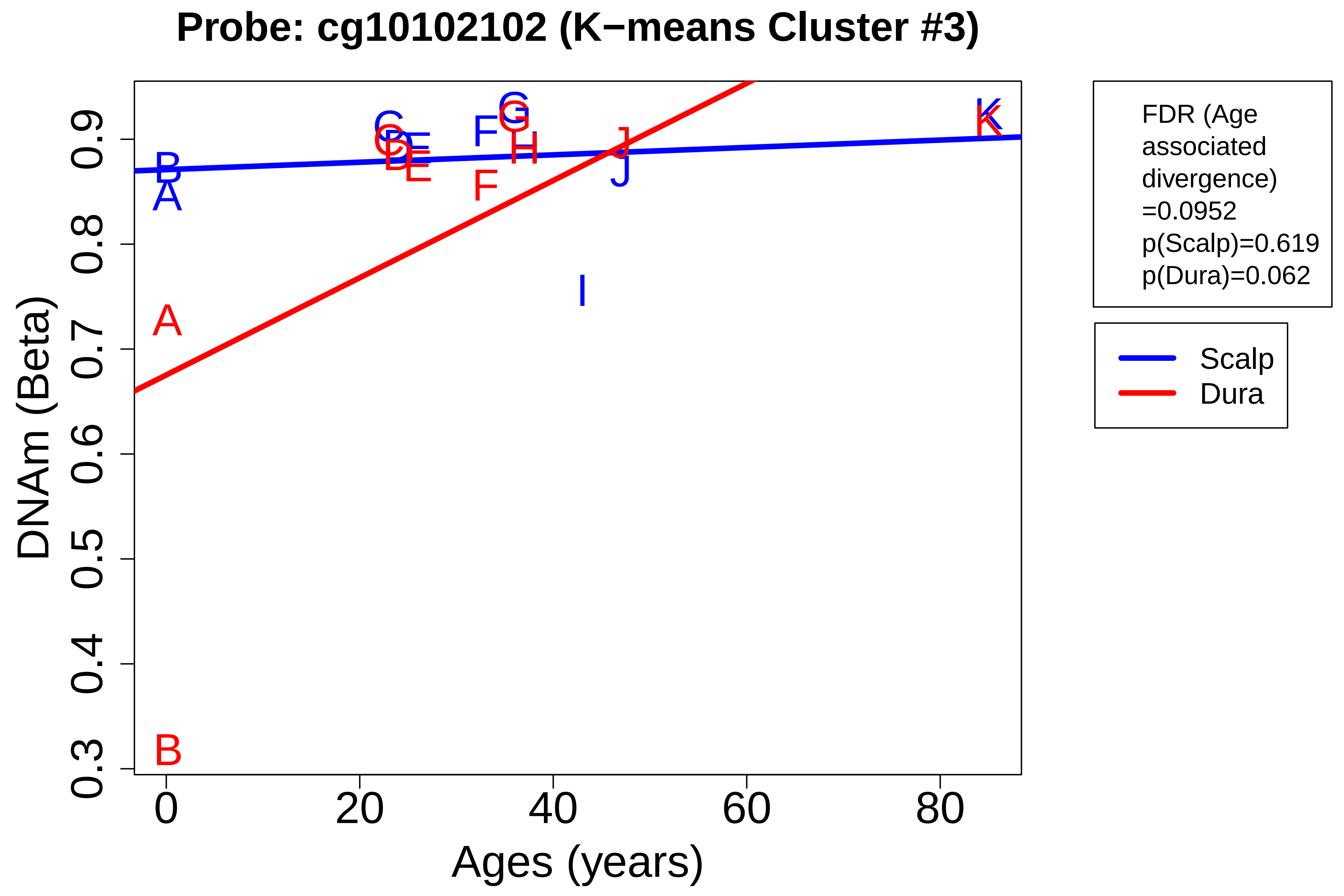
Probe: cg10102102 (K−means Cluster #3)
DNAm (Beta)
0.3
0.4
0.5
0.6
0.7
0.8
0.9
0
20
40
60
80
Ages (years)
FDR (Age associated divergence) =0.0952
p(Scalp)=0.619
p(Dura)=0.062
Scalp
Dura

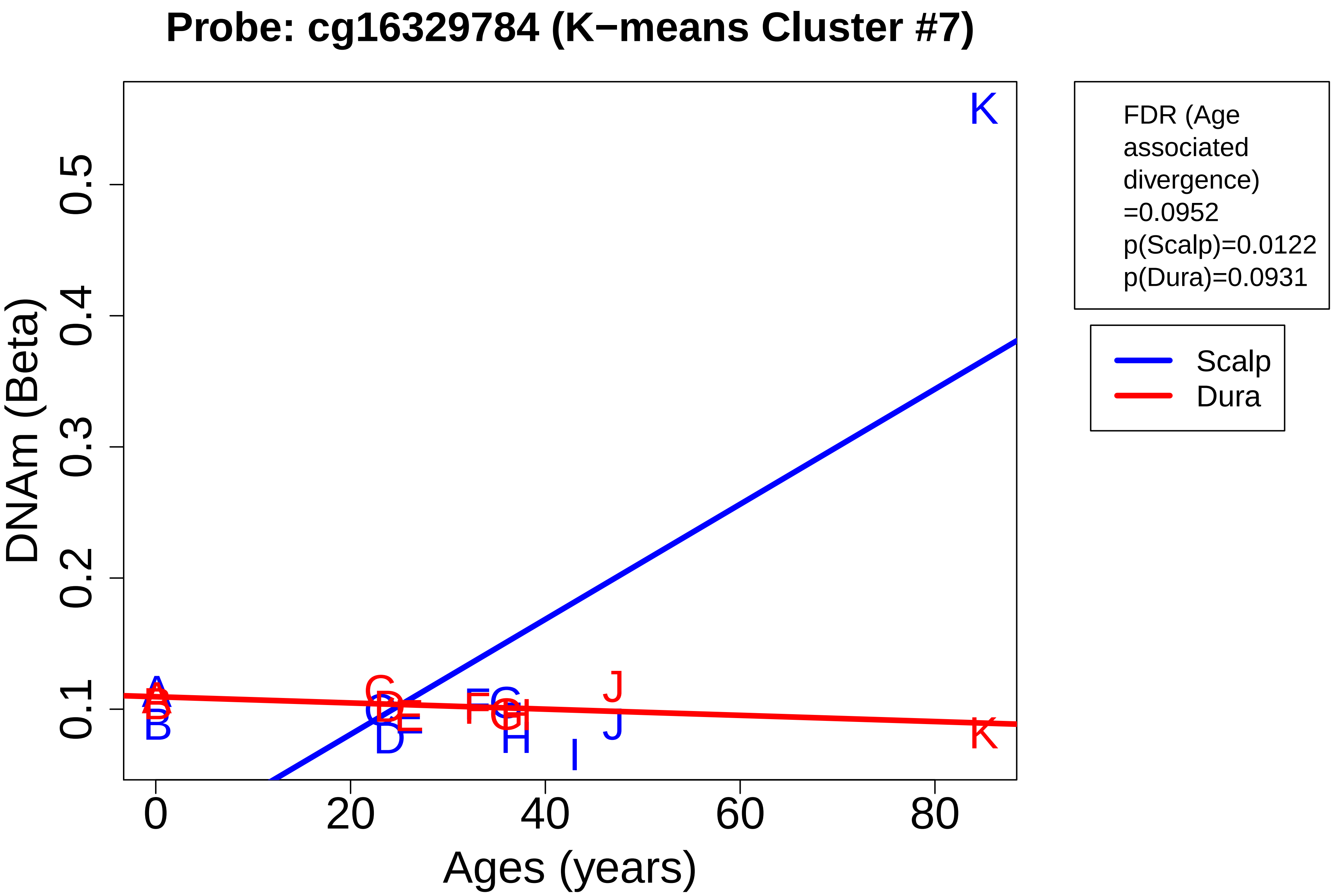

Probe: cg16329784 (K−means Cluster #7)
FDR (Age associated divergence) =0.0952
p(Scalp)=0.0122
p(Dura)=0.0931
Scalp
Dura
DNAm (Beta)
0.1
0.2
0.3
0.4
0.5
Ages (years)
0
20
40
60
80

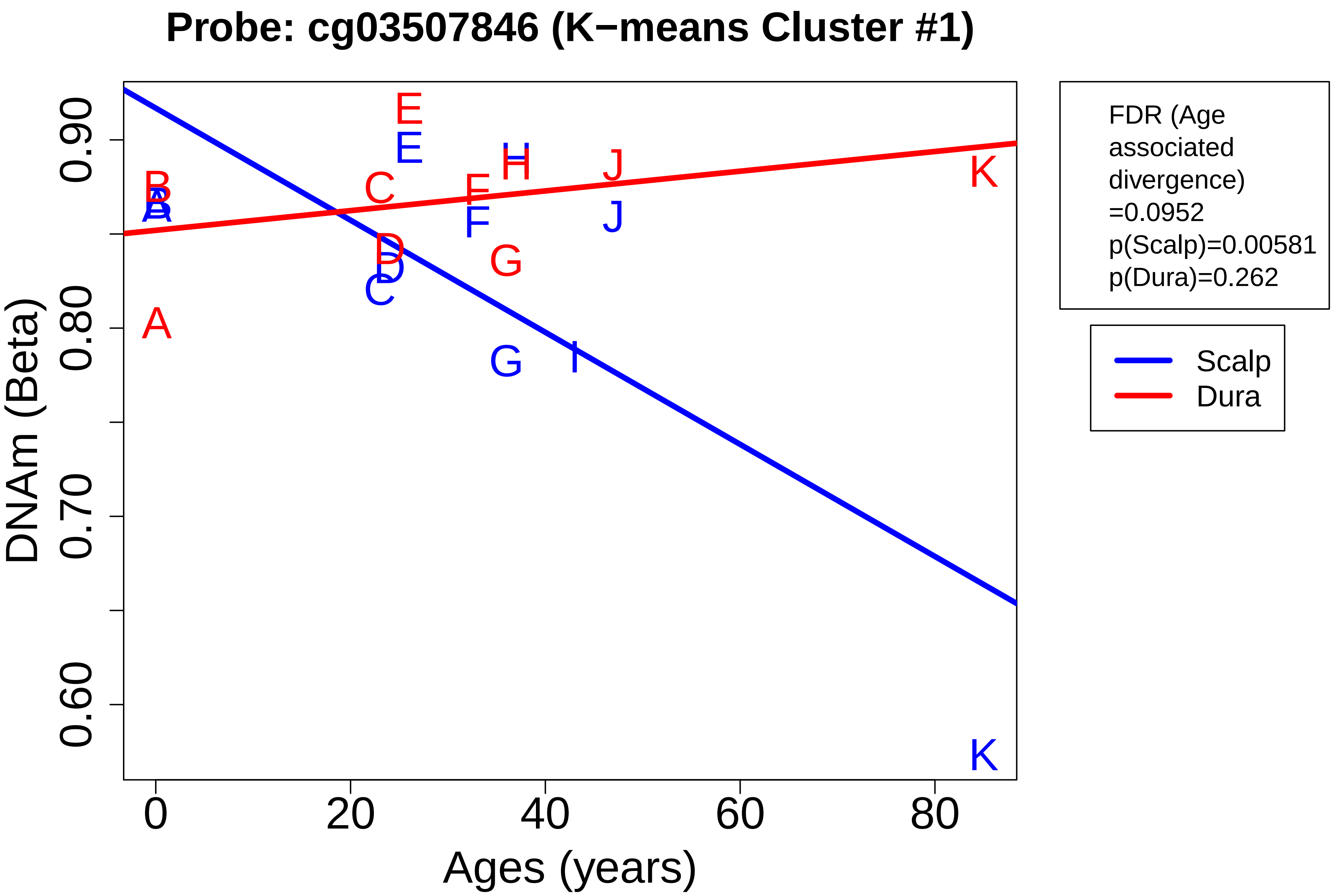

Probe: cg03507846 (K−means Cluster #1)
DNAm (Beta)
0.60
0.70
0.80
0.90
0
20
40
60
80
Ages (years)
FDR (Age associated divergence) =0.0952
p(Scalp)=0.00581
p(Dura)=0.262
Scalp
Dura

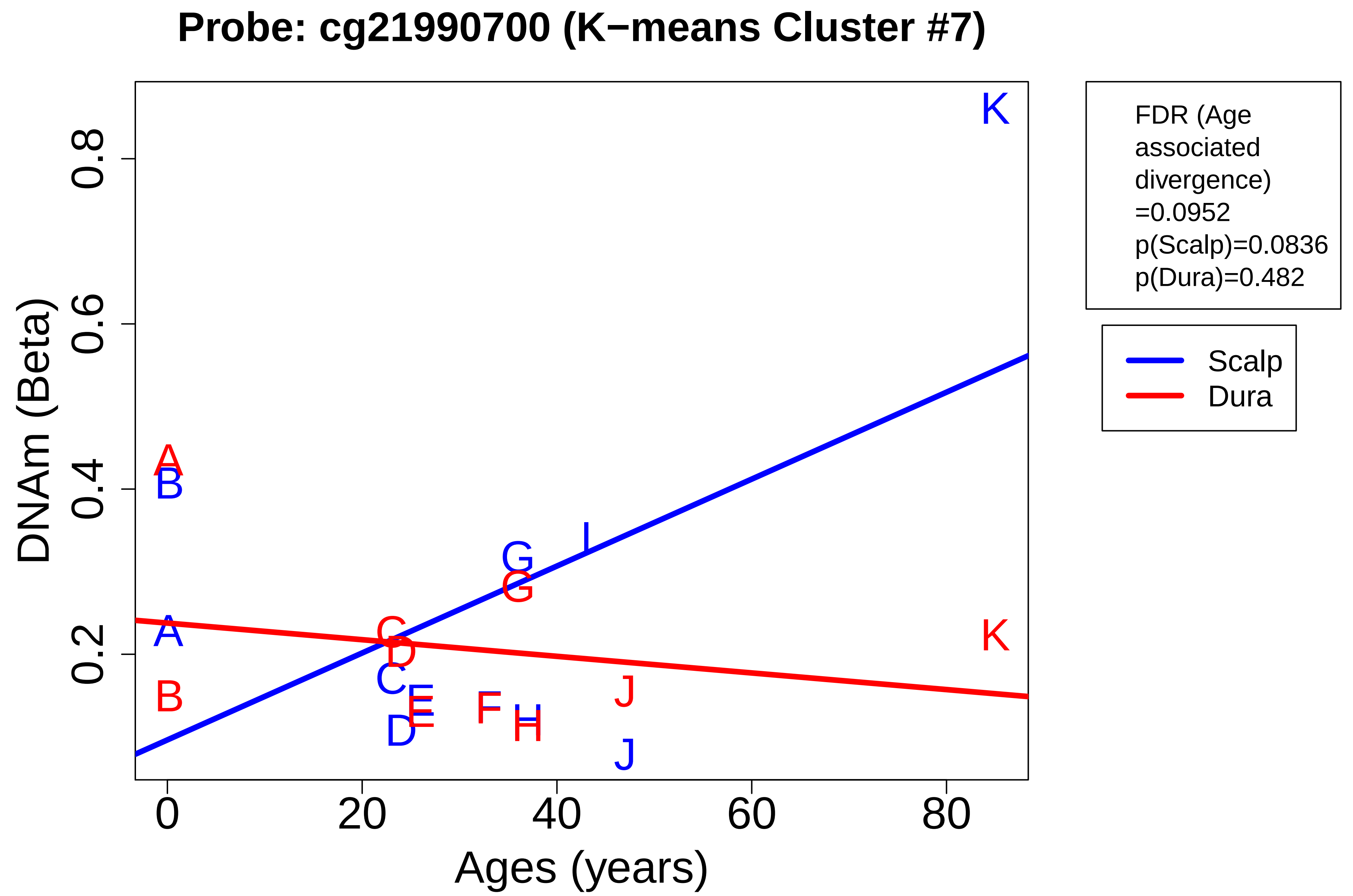

Probe: cg21990700 (K−means Cluster #7)
DNAm (Beta)
0.2
0.4
0.6
0.8
0
20
40
60
80
Ages (years)
A
B
C
D
E
F
G
H
I
J
K
FDR (Age associated divergence) =0.0952
p(Scalp)=0.0836
p(Dura)=0.482
Scalp
Dura

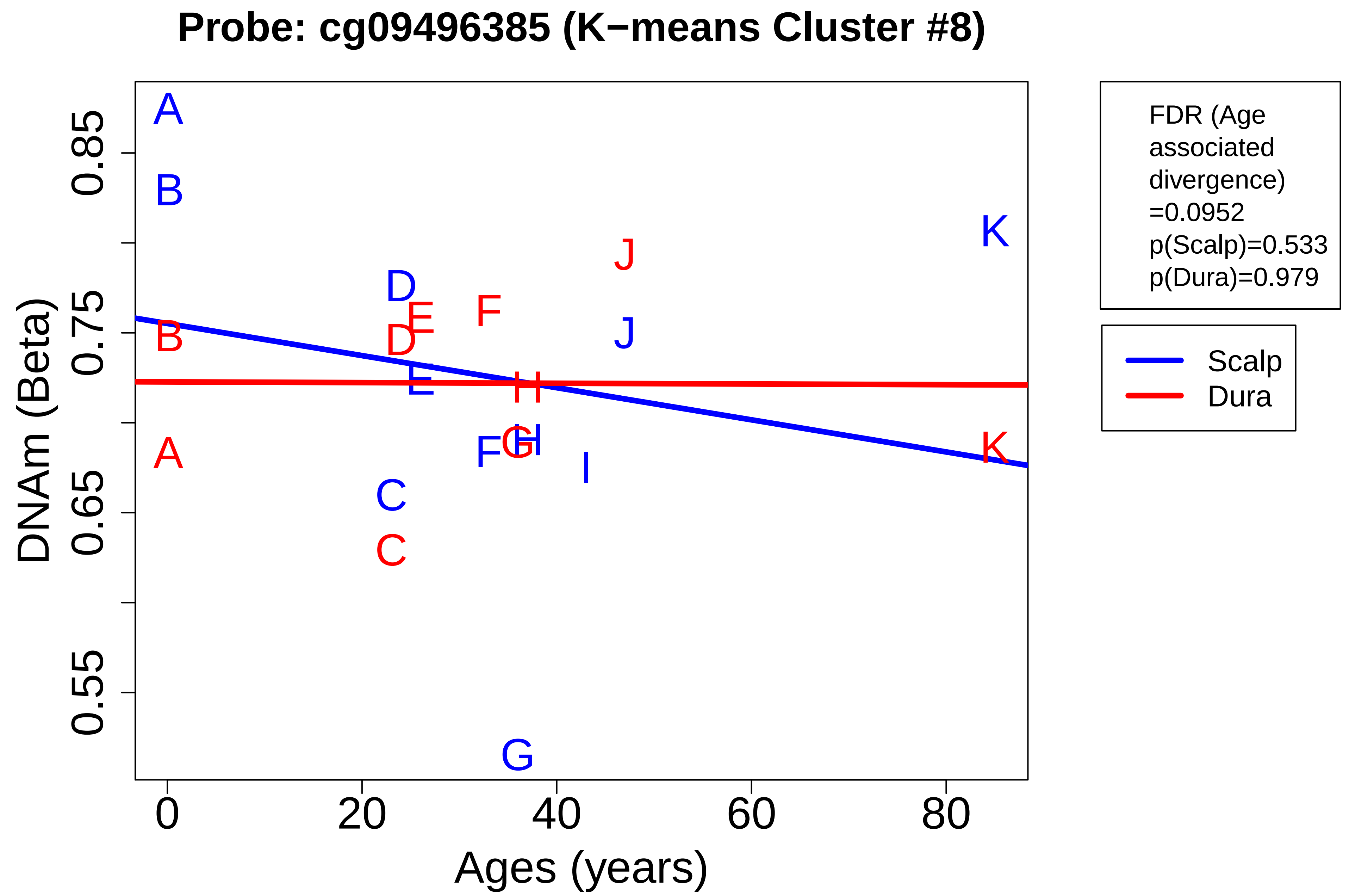

Probe: cg09496385 (K−means Cluster #8)
DNAm (Beta)
0.55
0.65
0.75
0.85
0
20
40
60
80
Ages (years)
A
B
C
D
E
F
G
H
I
J
K
FDR (Age associated divergence) =0.0952
p(Scalp)=0.533
p(Dura)=0.979
Scalp
Dura

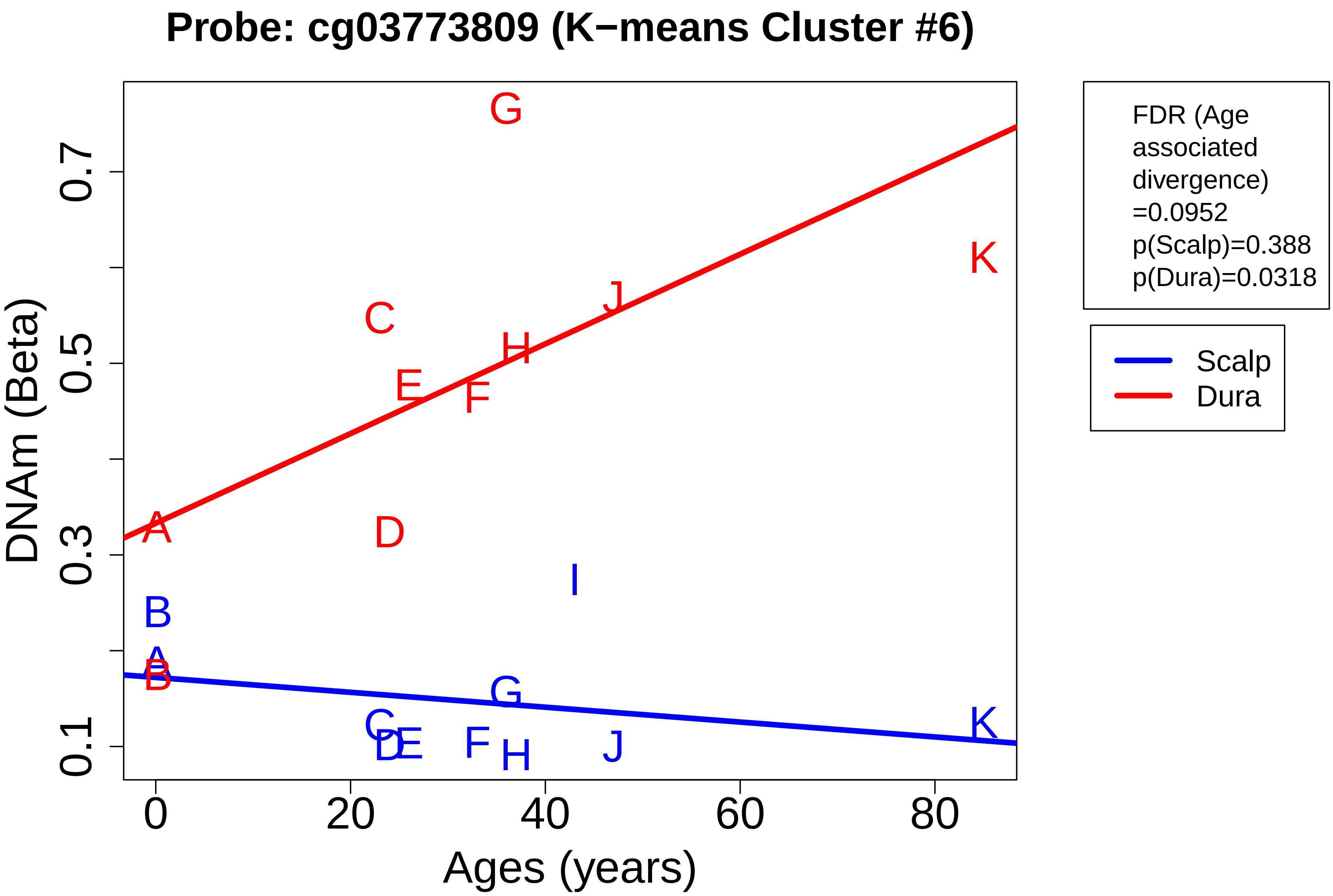

Probe: cg03773809 (K−means Cluster #6)
DNAm (Beta)
0.1
0.3
0.5
0.7
0
20
40
60
80
Ages (years)
FDR (Age associated divergence) =0.0952
p(Scalp)=0.388
p(Dura)=0.0318
Scalp
Dura
A B C D E F G H I J K

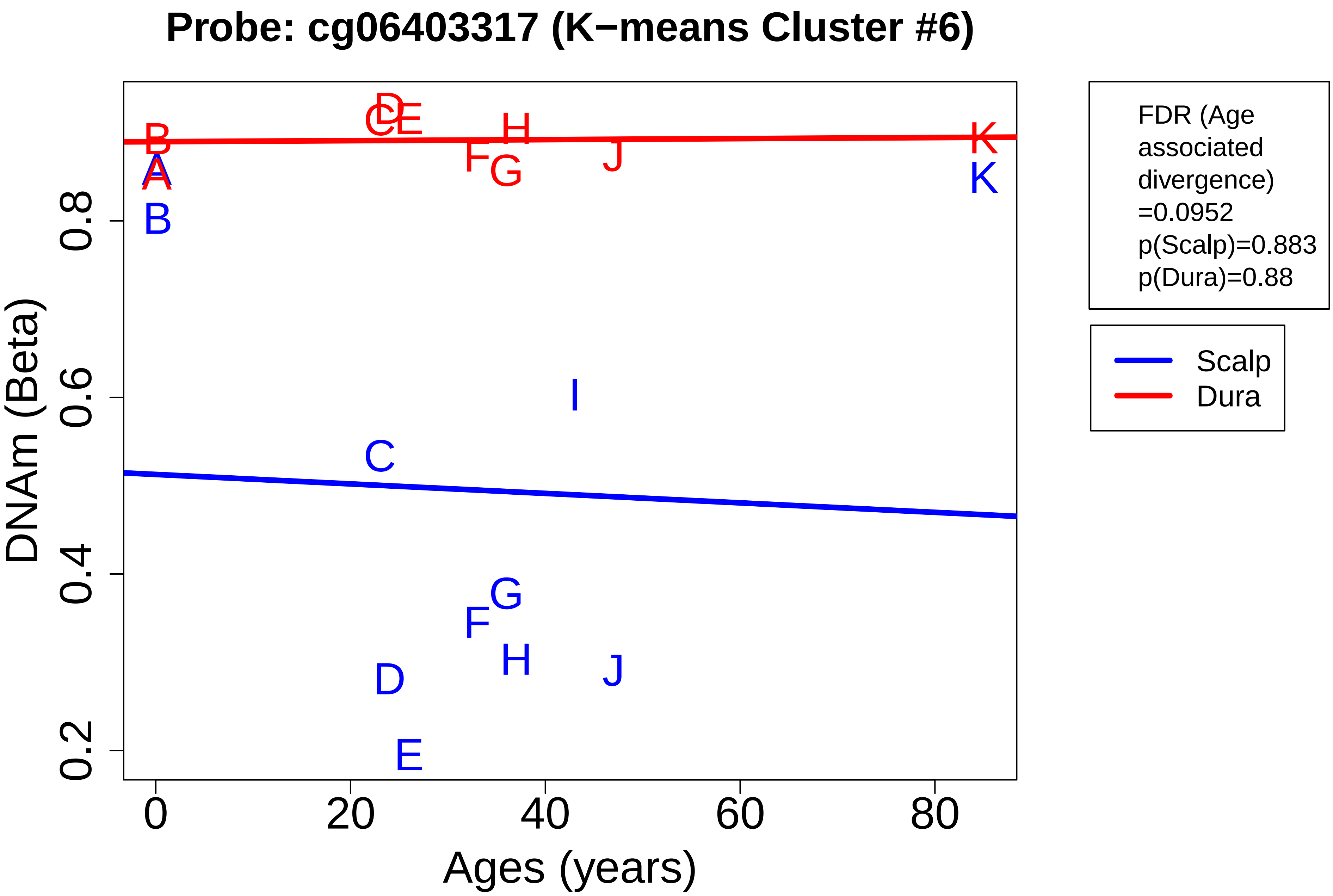

Probe: cg06403317 (K−means Cluster #6)
DNAm (Beta)
0.2
0.4
0.6
0.8
0
20
40
60
80
Ages (years)
FDR (Age associated divergence) =0.0952
p(Scalp)=0.883
p(Dura)=0.88
Scalp
Dura

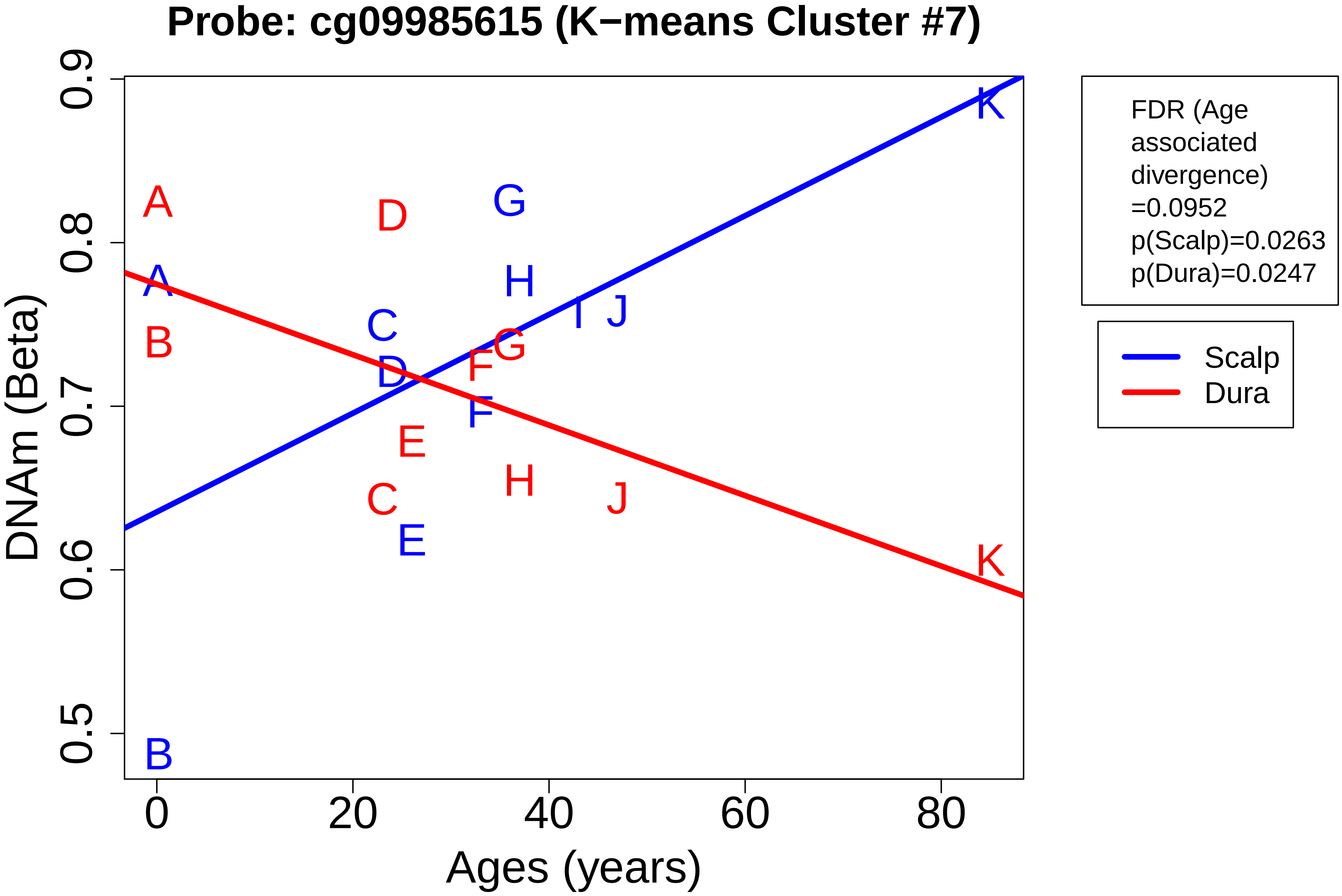
Probe: cg09985615 (K−means Cluster #7)
DNAm (Beta)
0.5
0.6
0.7
0.8
0.9
0
20
40
60
80
Ages (years)
A
B
C
D
E
F
G
H
I
J
K
A
B
C
D
E
F
G
H
J
K
FDR (Age associated divergence) =0.0952
p(Scalp)=0.0263
p(Dura)=0.0247
Scalp
Dura

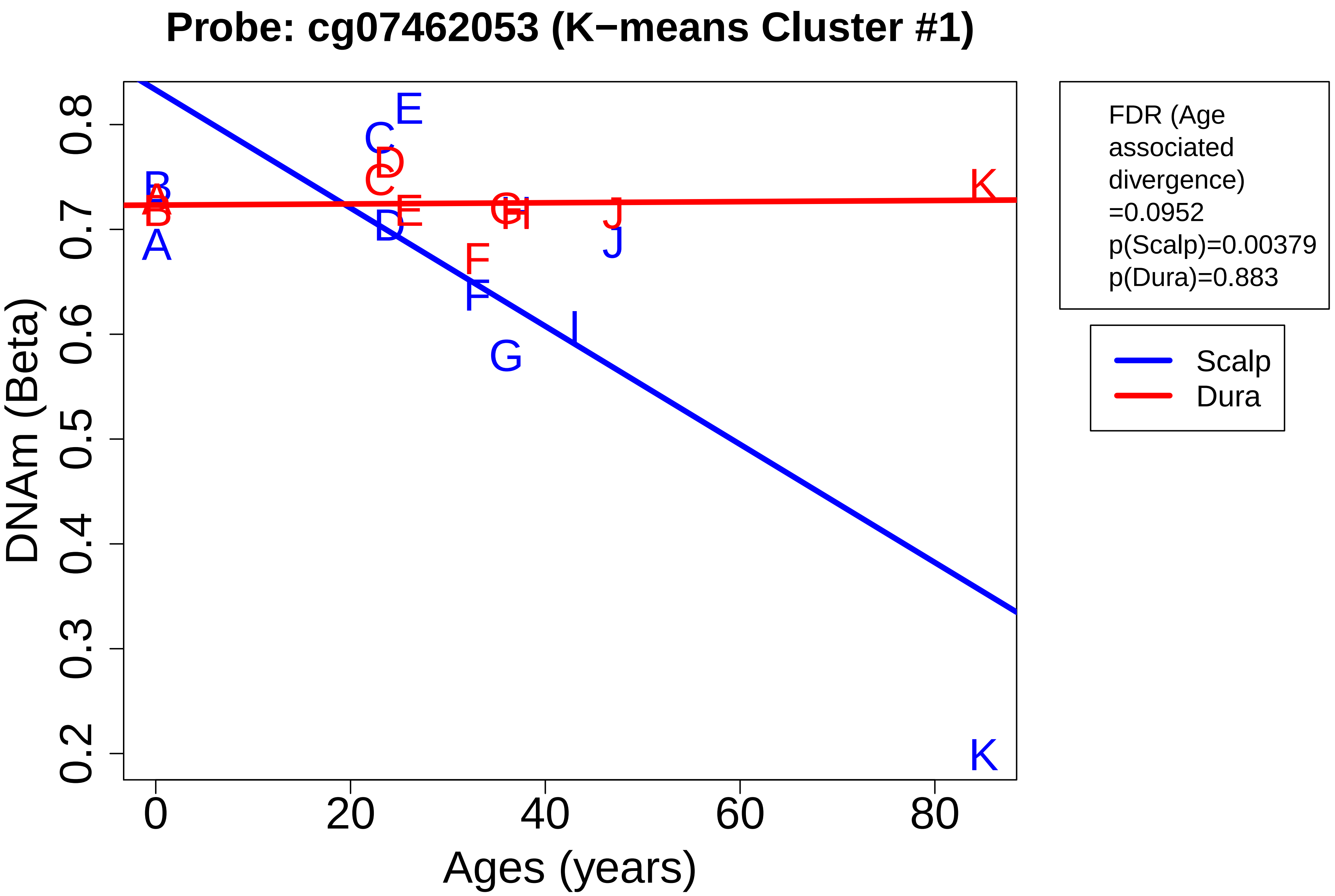

Probe: cg07462053 (K−means Cluster #1)
DNAm (Beta)
Ages (years)
FDR (Age associated divergence) =0.0952
p(Scalp)=0.00379
p(Dura)=0.883
Scalp
Dura

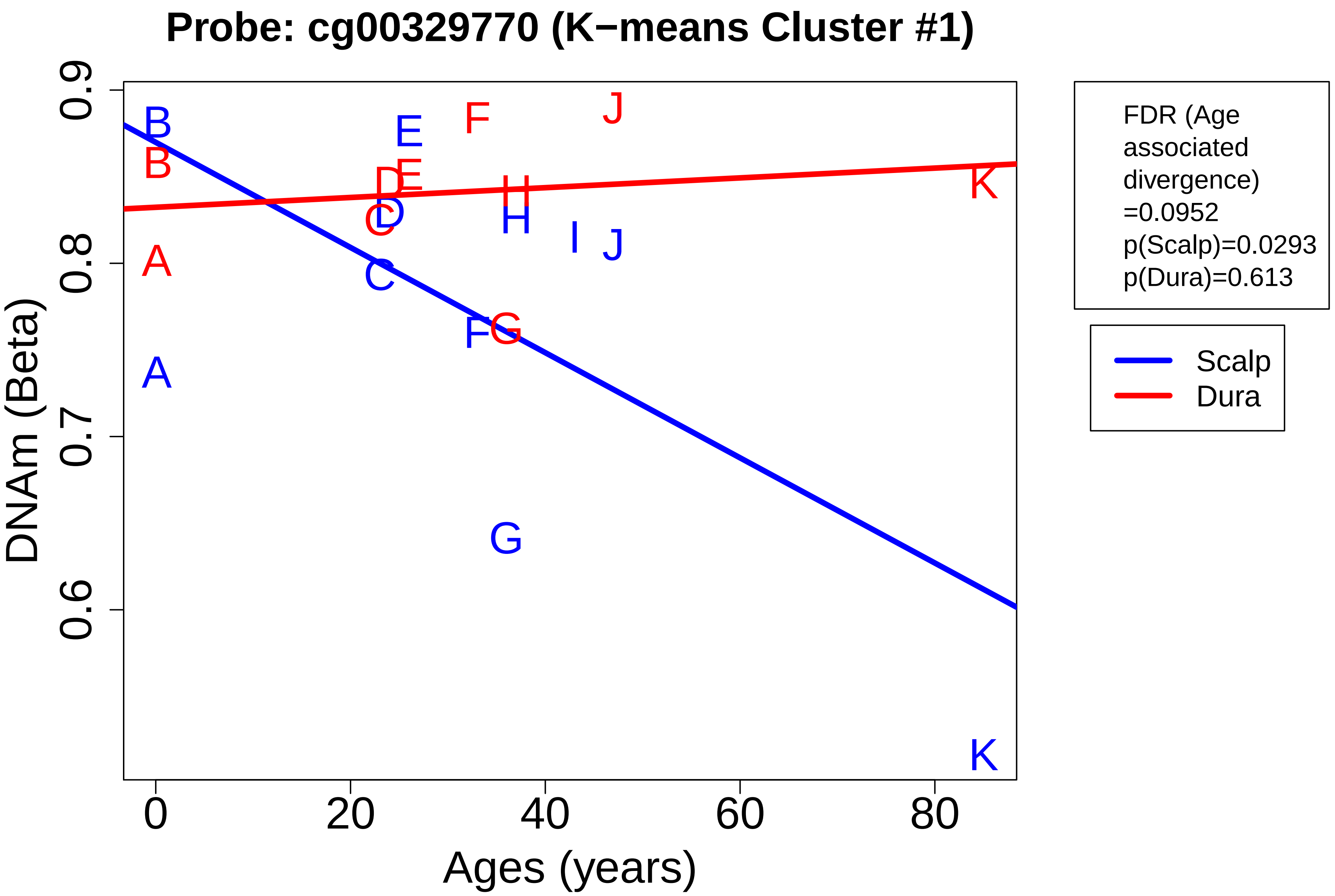

Probe: cg00329770 (K−means Cluster #1)
DNAm (Beta)
0.6
0.7
0.8
0.9
0
20
40
60
80
Ages (years)
FDR (Age associated divergence) =0.0952
p(Scalp)=0.0293
p(Dura)=0.613
Scalp
Dura

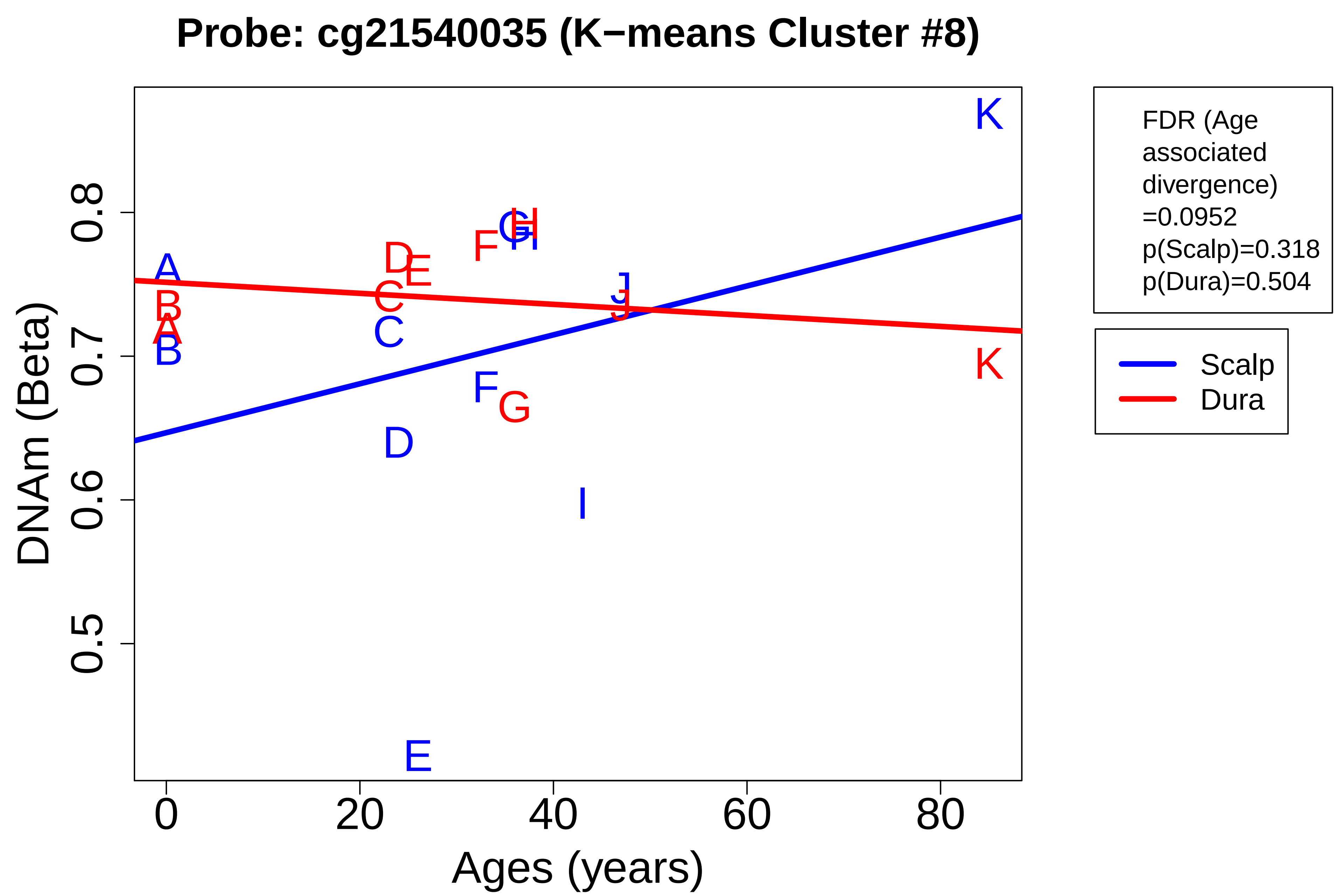

Probe: cg21540035 (K−means Cluster #8)
DNAm (Beta)
0.5
0.6
0.7
0.8
0
20
40
60
80
Ages (years)
FDR (Age associated divergence) =0.0952
p(Scalp)=0.318
p(Dura)=0.504
Scalp
Dura
A
B
C
D
E
F
G
H
I
J
K

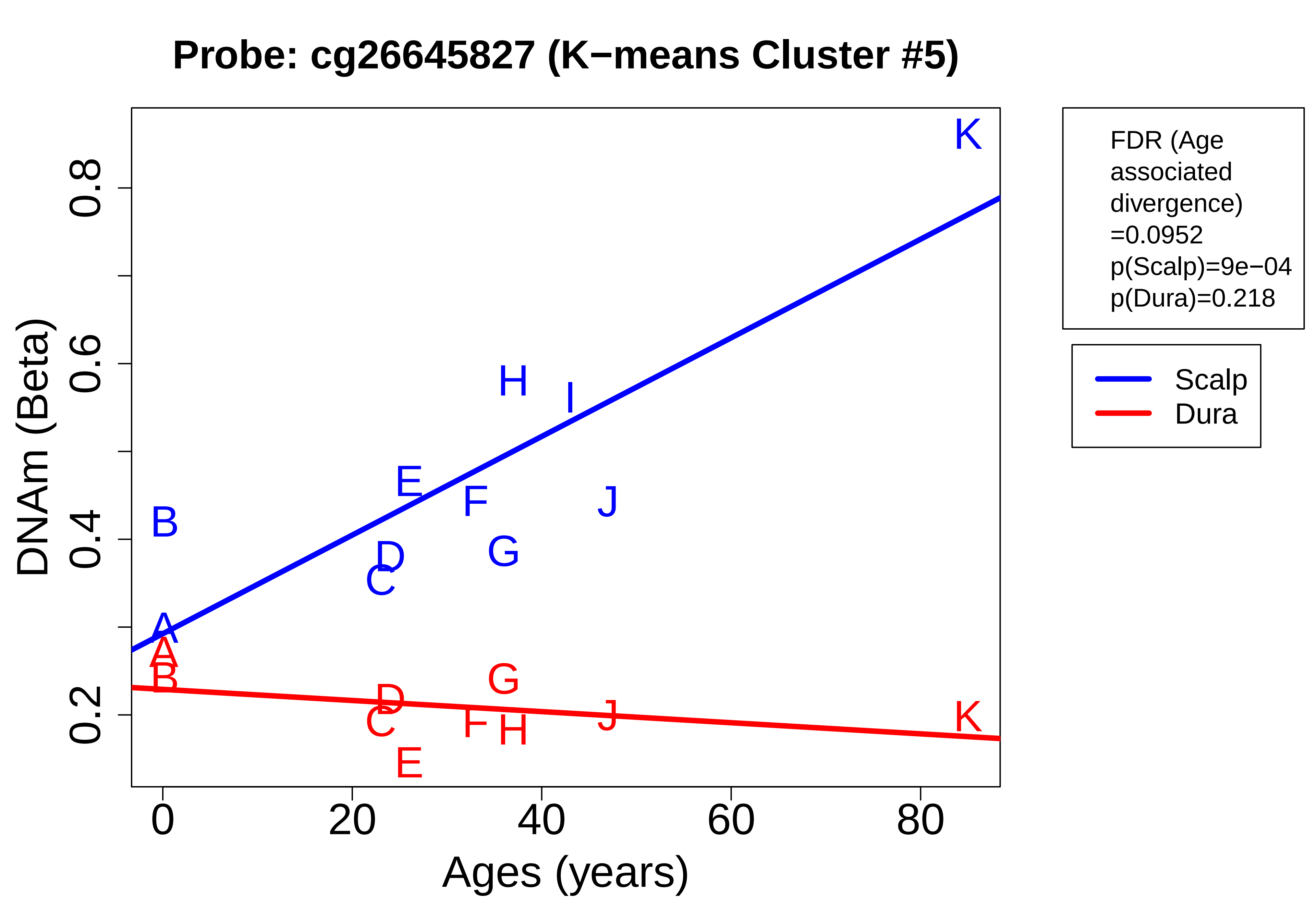

Probe: cg26645827 (K−means Cluster #5)
DNAm (Beta)
0.2
0.4
0.6
0.8
0
20
40
60
80
Ages (years)
FDR (Age associated divergence) =0.0952
p(Scalp)=9e−04
p(Dura)=0.218
Scalp
Dura
A
B
C
D
E
F
G
H
I
J
K

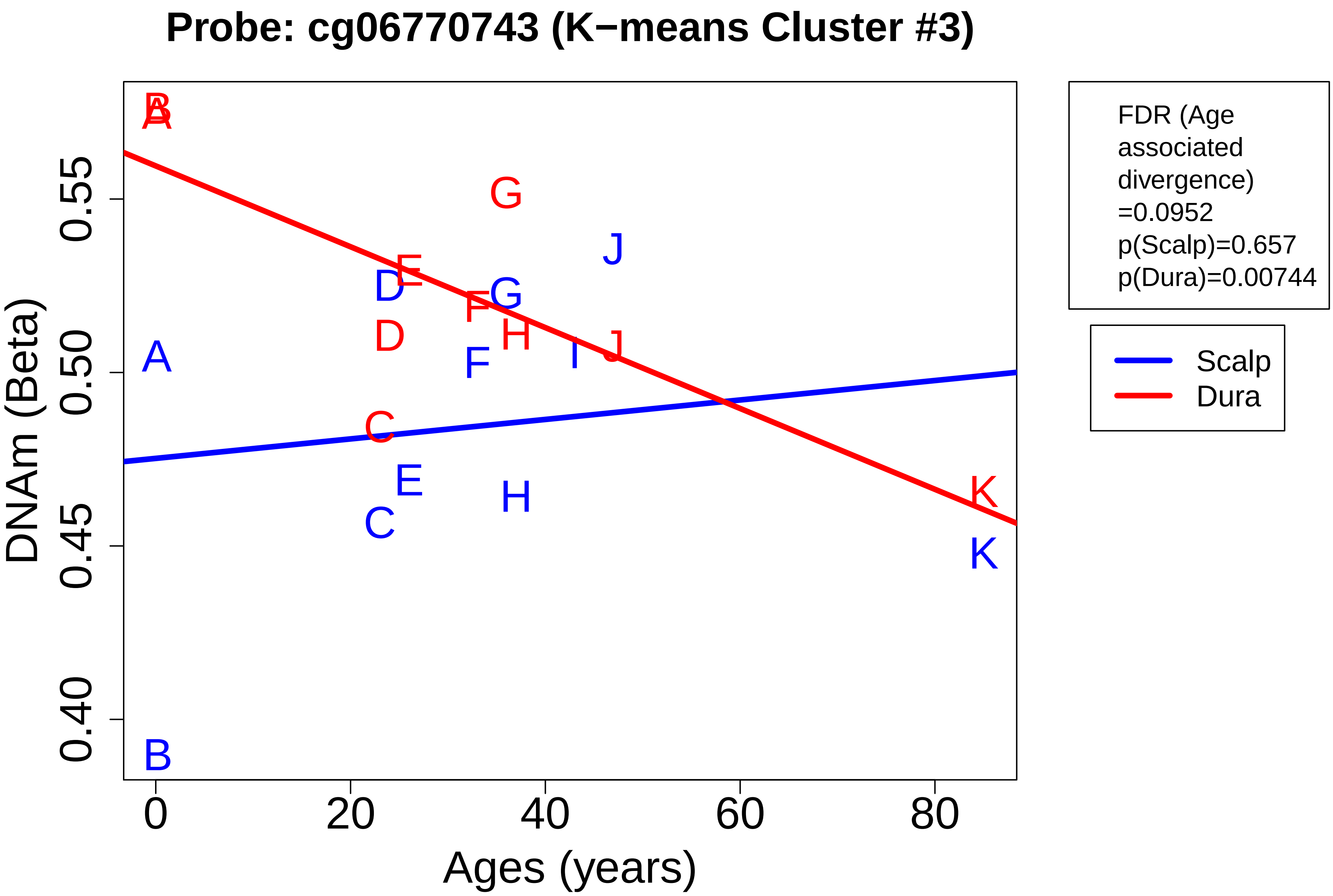

Probe: cg06770743 (K−means Cluster #3)
DNAm (Beta)
0.40
0.45
0.50
0.55
0
20
40
60
80
Ages (years)
FDR (Age associated divergence) =0.0952
p(Scalp)=0.657
p(Dura)=0.00744
Scalp
Dura

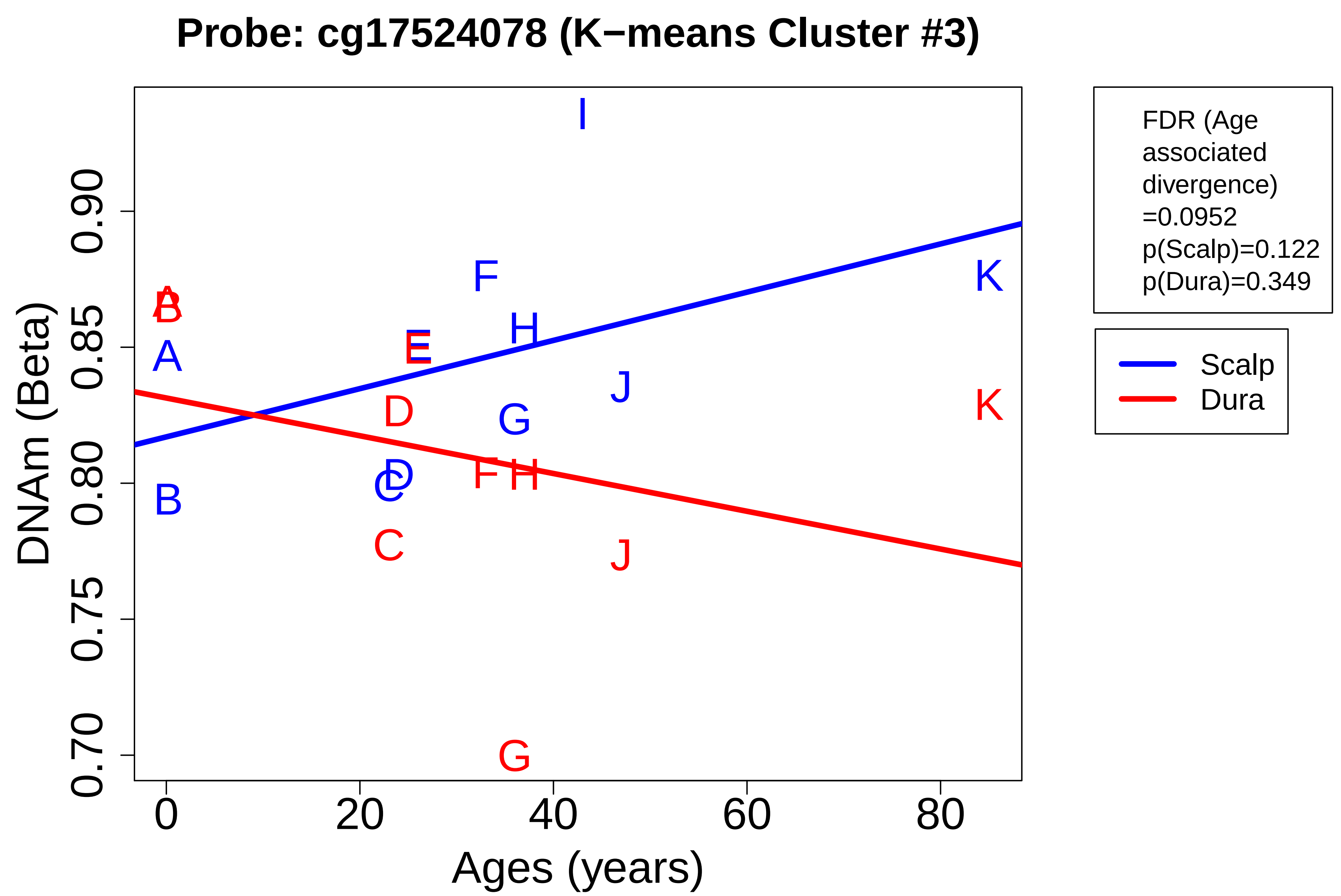

Probe: cg17524078 (K−means Cluster #3)
DNAm (Beta)
0.70
0.75
0.80
0.85
0.90
0
20
40
60
80
Ages (years)
A
B
C
D
E
F
G
H
I
J
K
FDR (Age associated divergence) =0.0952
p(Scalp)=0.122
p(Dura)=0.349
Scalp
Dura

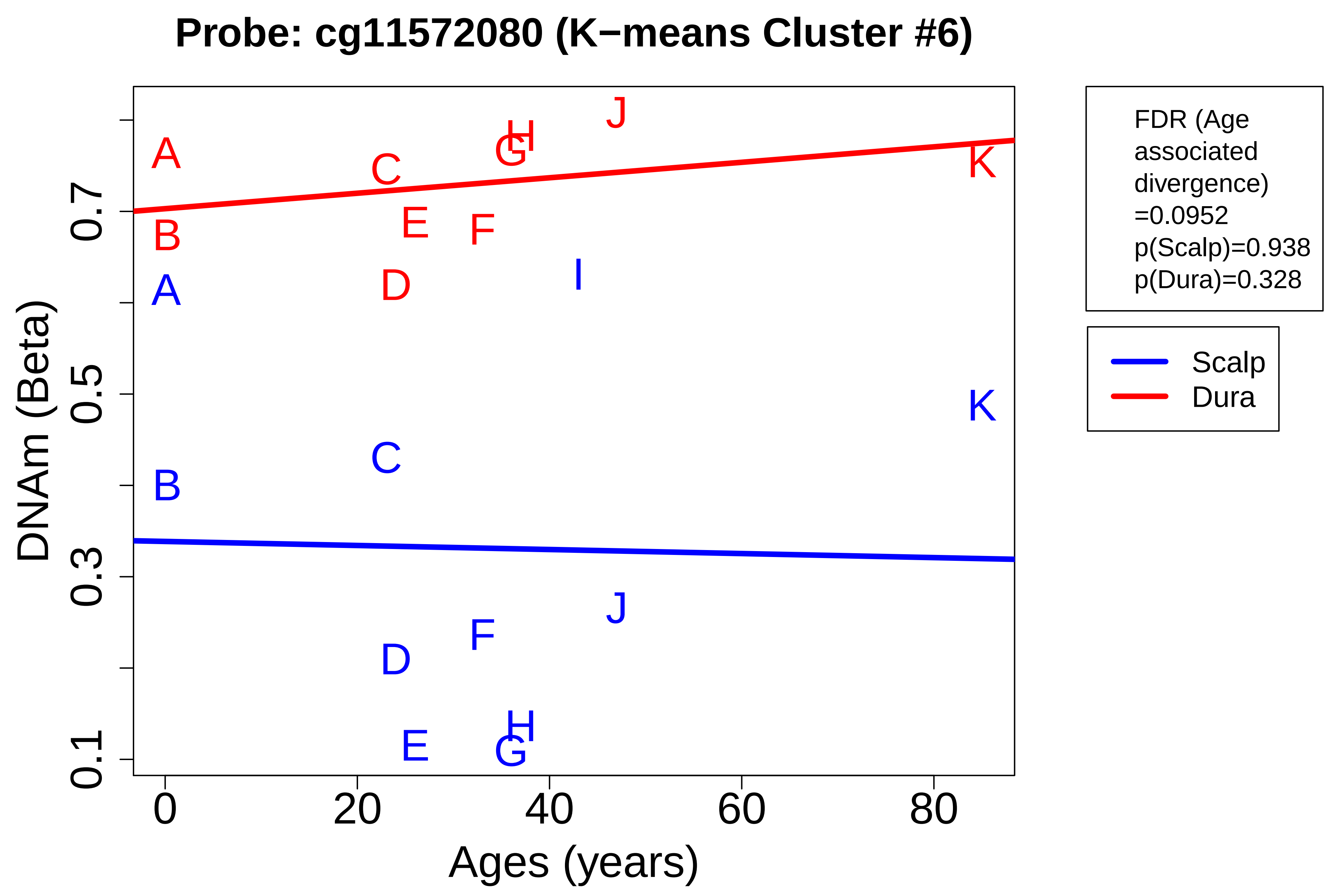

Probe: cg11572080 (K−means Cluster #6)
DNAm (Beta)
0.1
0.3
0.5
0.7
0
20
40
60
80
Ages (years)
A
B
C
D
E
F
G
H
I
J
K
FDR (Age associated divergence) =0.0952
p(Scalp)=0.938
p(Dura)=0.328
Scalp
Dura

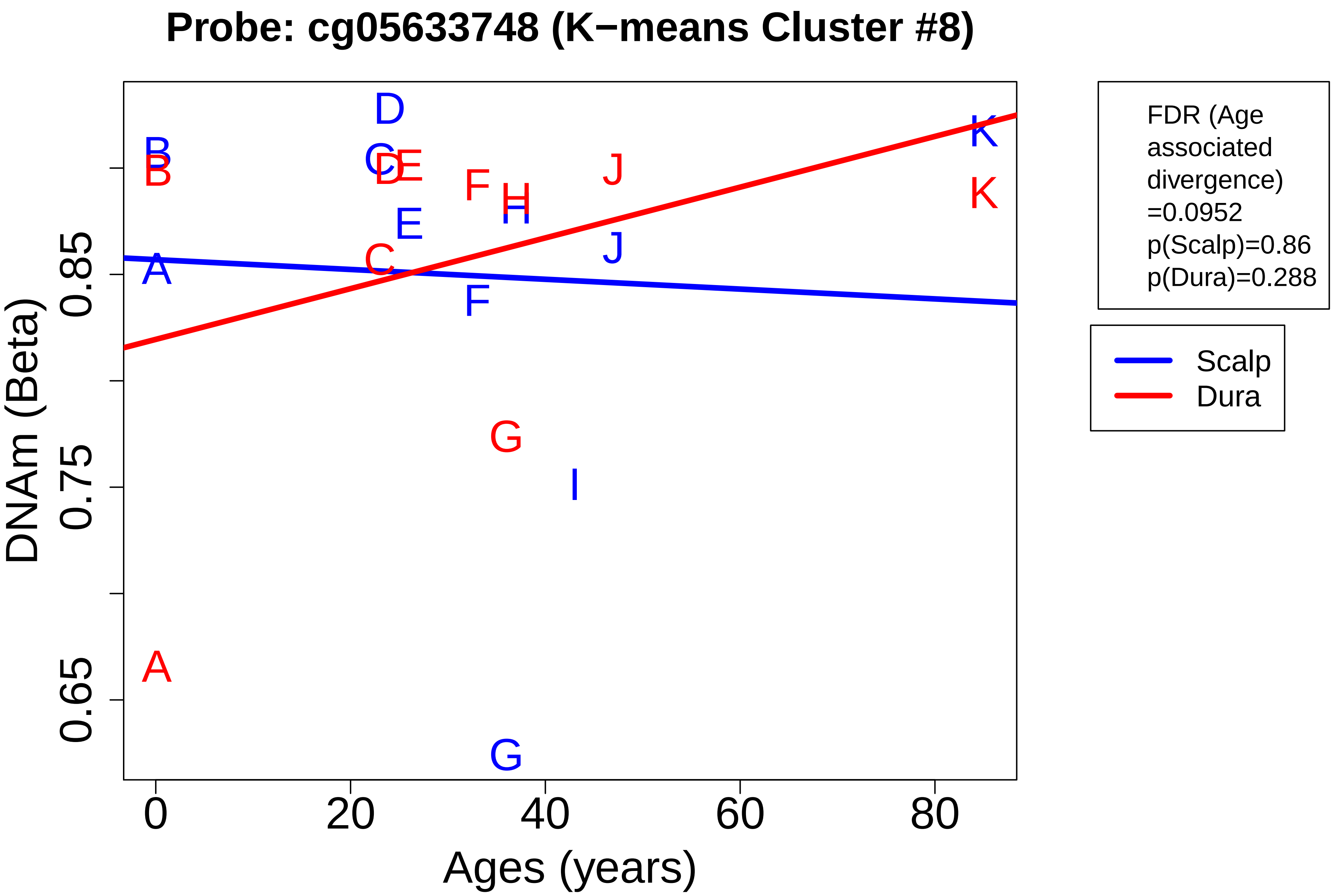

Probe: cg05633748 (K−means Cluster #8)
DNAm (Beta)
0.65
0.75
0.85
Ages (years)
0
20
40
60
80
FDR (Age associated divergence) =0.0952
p(Scalp)=0.86
p(Dura)=0.288
Scalp
Dura

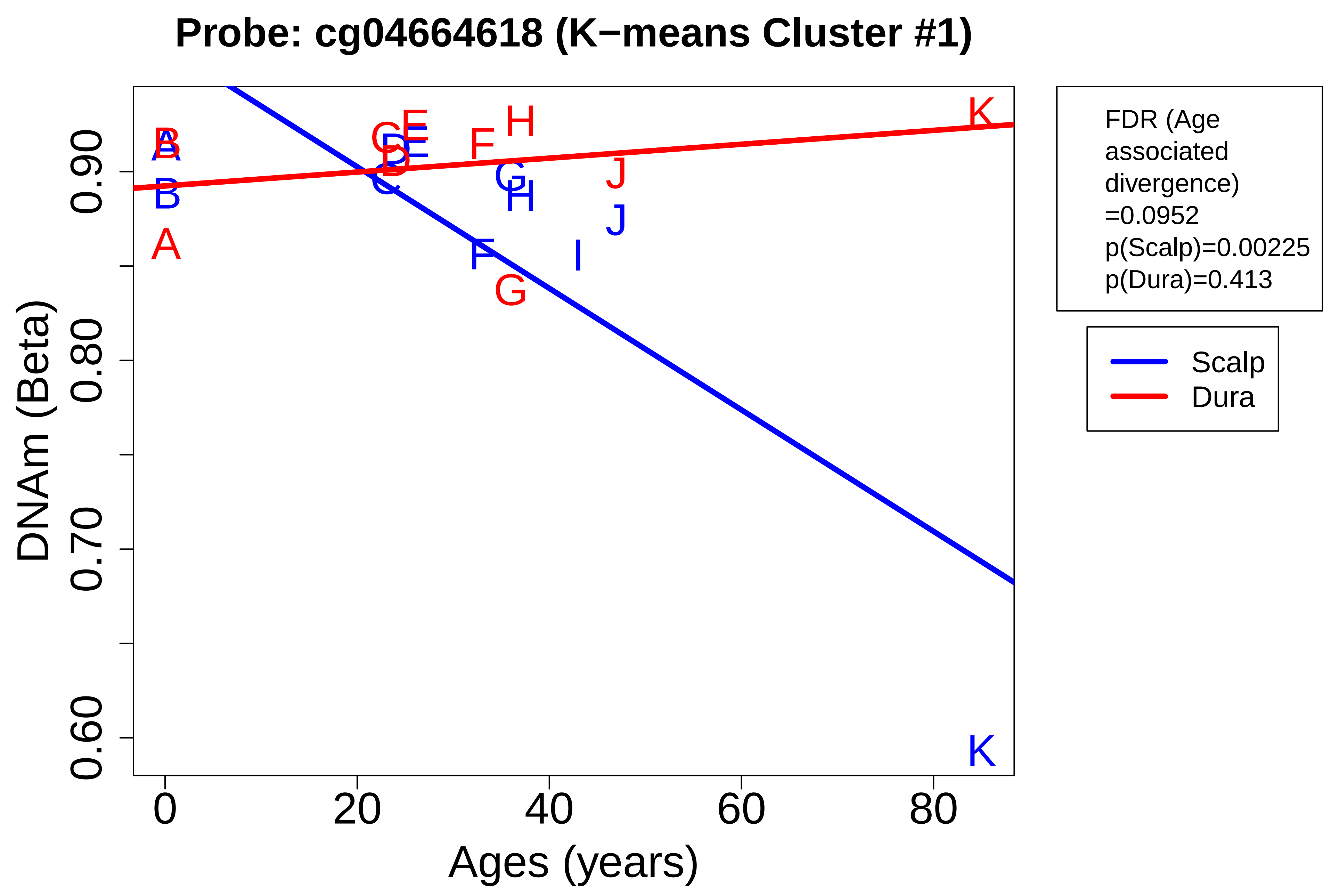

Probe: cg04664618 (K−means Cluster #1)
DNAm (Beta)
0.60
0.70
0.80
0.90
Ages (years)
0
20
40
60
80
A
B
C
D
E
F
G
H
I
J
K
FDR (Age associated divergence) =0.0952
p(Scalp)=0.00225
p(Dura)=0.413
Scalp
Dura

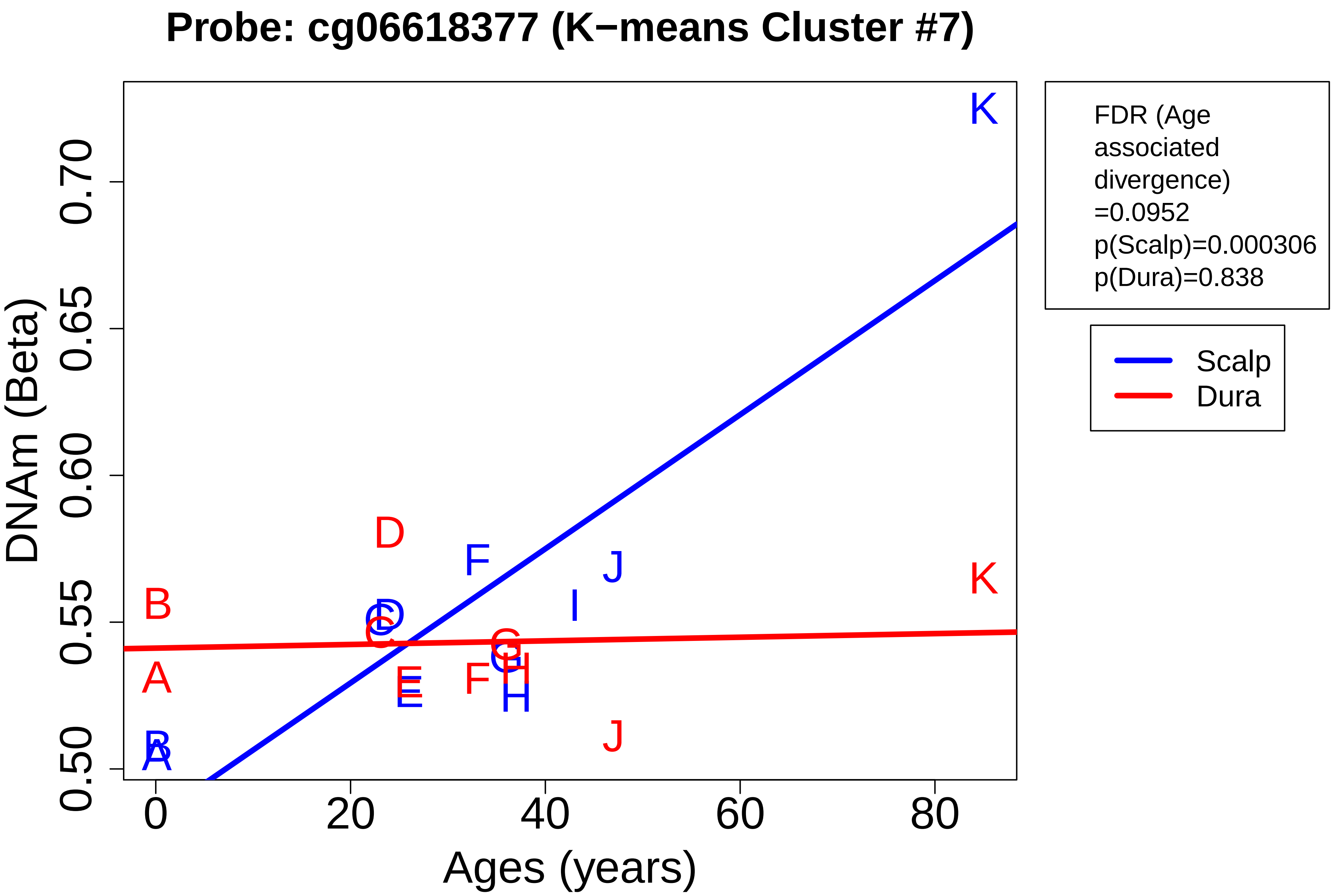
Probe: cg06618377 (K−means Cluster #7)
FDR (Age associated divergence) =0.0952
p(Scalp)=0.000306
p(Dura)=0.838
Scalp
Dura
DNAm (Beta)
0.50
0.55
0.60
0.65
0.70
Ages (years)
0
20
40
60
80

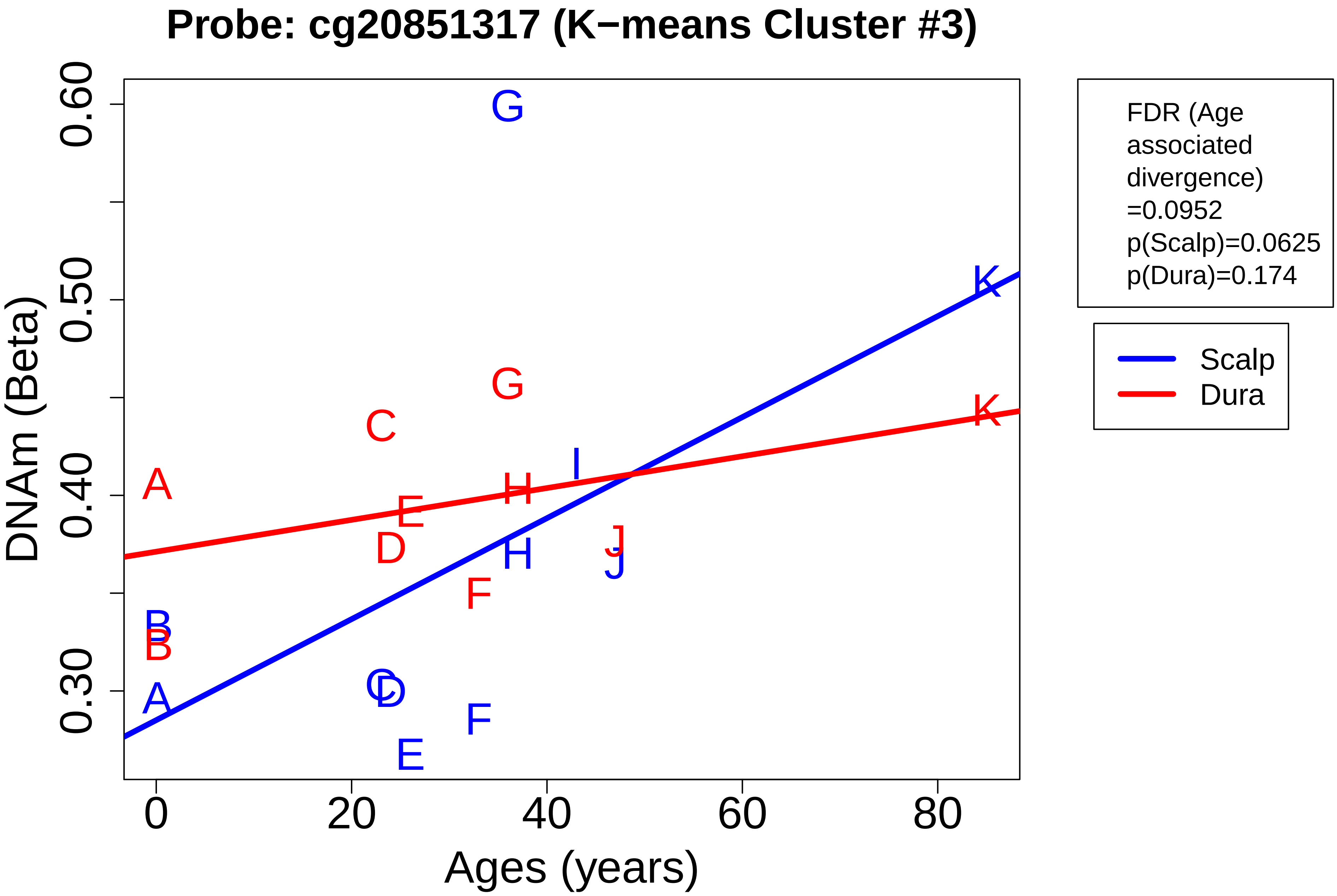

Probe: cg20851317 (K−means Cluster #3)
DNAm (Beta)
0.30
0.40
0.50
0.60
0
20
40
60
80
Ages (years)
FDR (Age associated divergence) =0.0952
p(Scalp)=0.0625
p(Dura)=0.174
Scalp
Dura
A
B
C
D
E
F
G
H
I
J
K

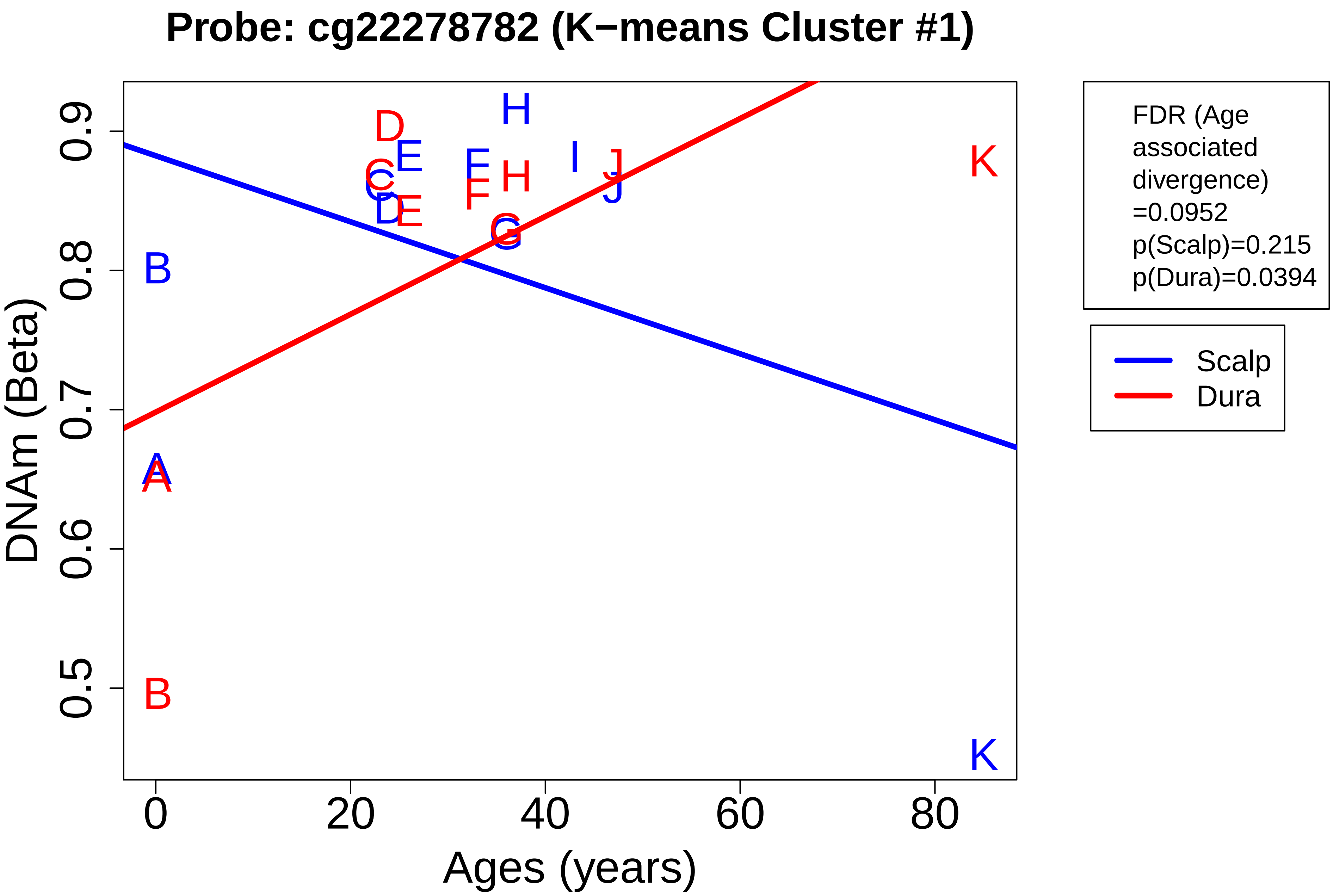

Probe: cg22278782 (K−means Cluster #1)
DNAm (Beta)
0.5
0.6
0.7
0.8
0.9
0
20
40
60
80
Ages (years)
A
A
B
B
C
C
D
D
E
E
F
F
G
G
H
H
I
J
J
K
K
FDR (Age associated divergence) =0.0952
p(Scalp)=0.215
p(Dura)=0.0394
Scalp
Dura

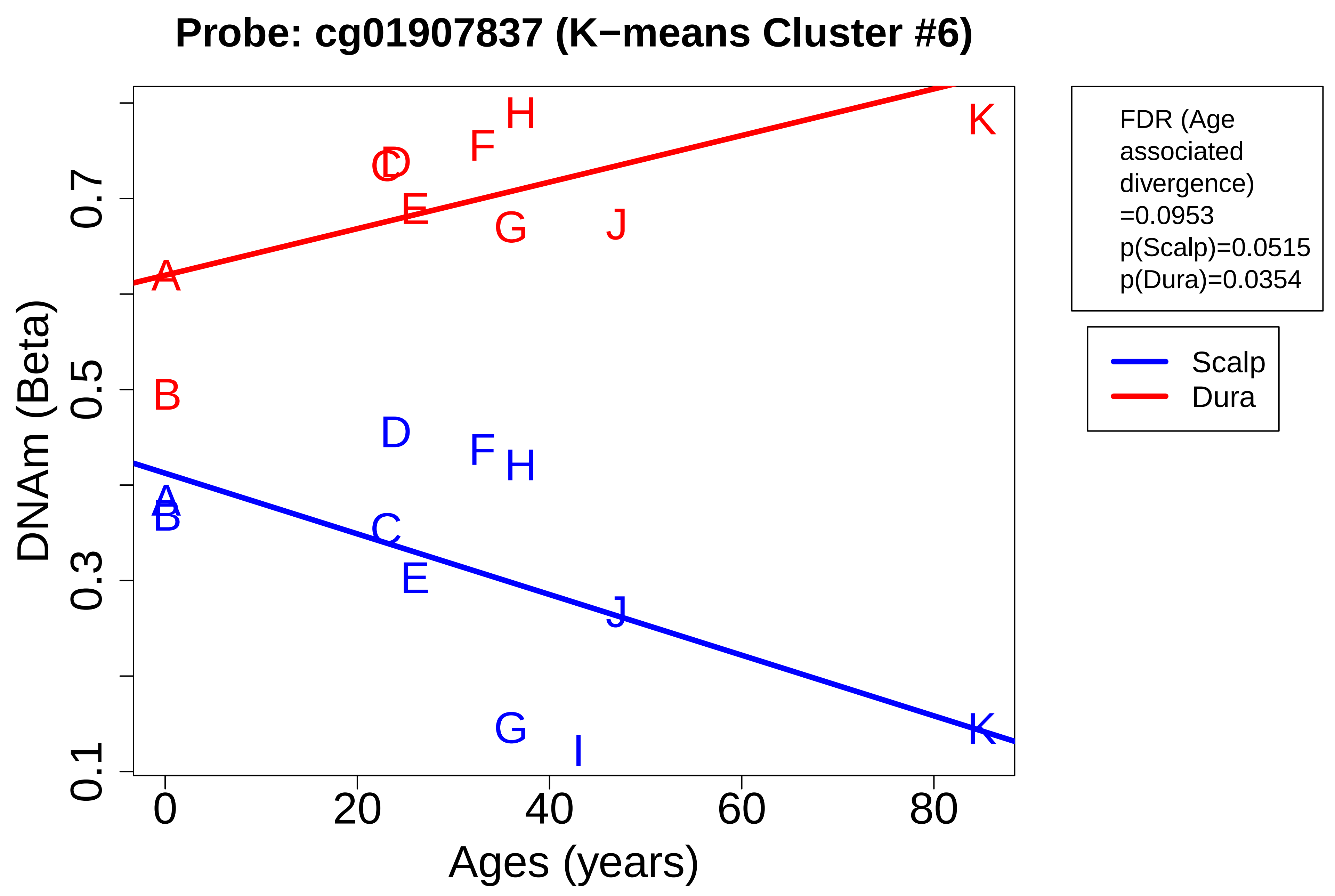

Probe: cg01907837 (K−means Cluster #6)
DNAm (Beta)
0.1
0.3
0.5
0.7
0
20
40
60
80
Ages (years)
A
B
C
D
E
F
G
H
I
J
K
FDR (Age associated divergence) =0.0953
p(Scalp)=0.0515
p(Dura)=0.0354
Scalp
Dura

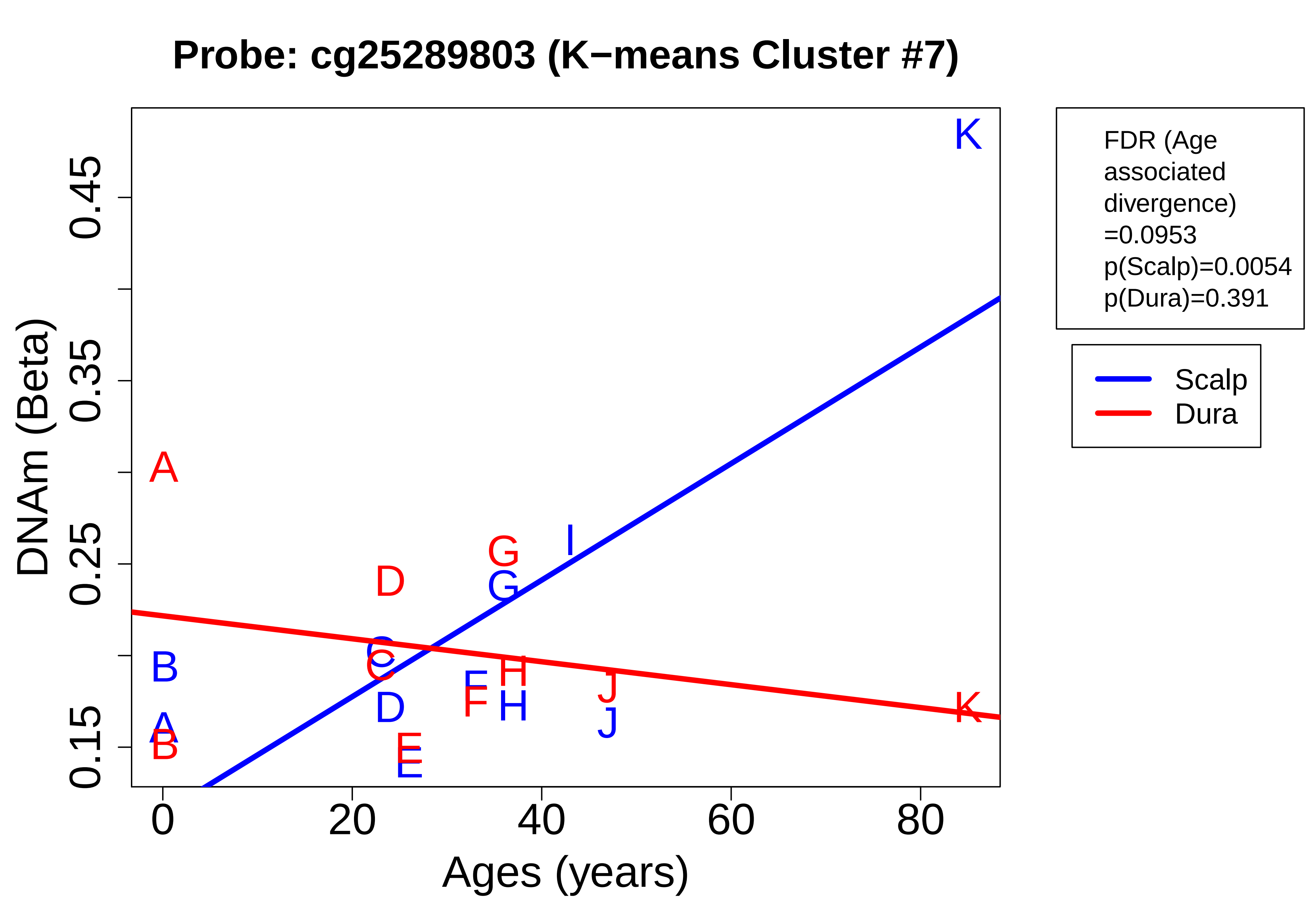

Probe: cg25289803 (K−means Cluster #7)
DNAm (Beta)
0.15
0.25
0.35
0.45
0
20
40
60
80
Ages (years)
FDR (Age associated divergence) =0.0953
p(Scalp)=0.0054
p(Dura)=0.391
Scalp
Dura
A
B
C
D
E
F
G
H
I
J
K

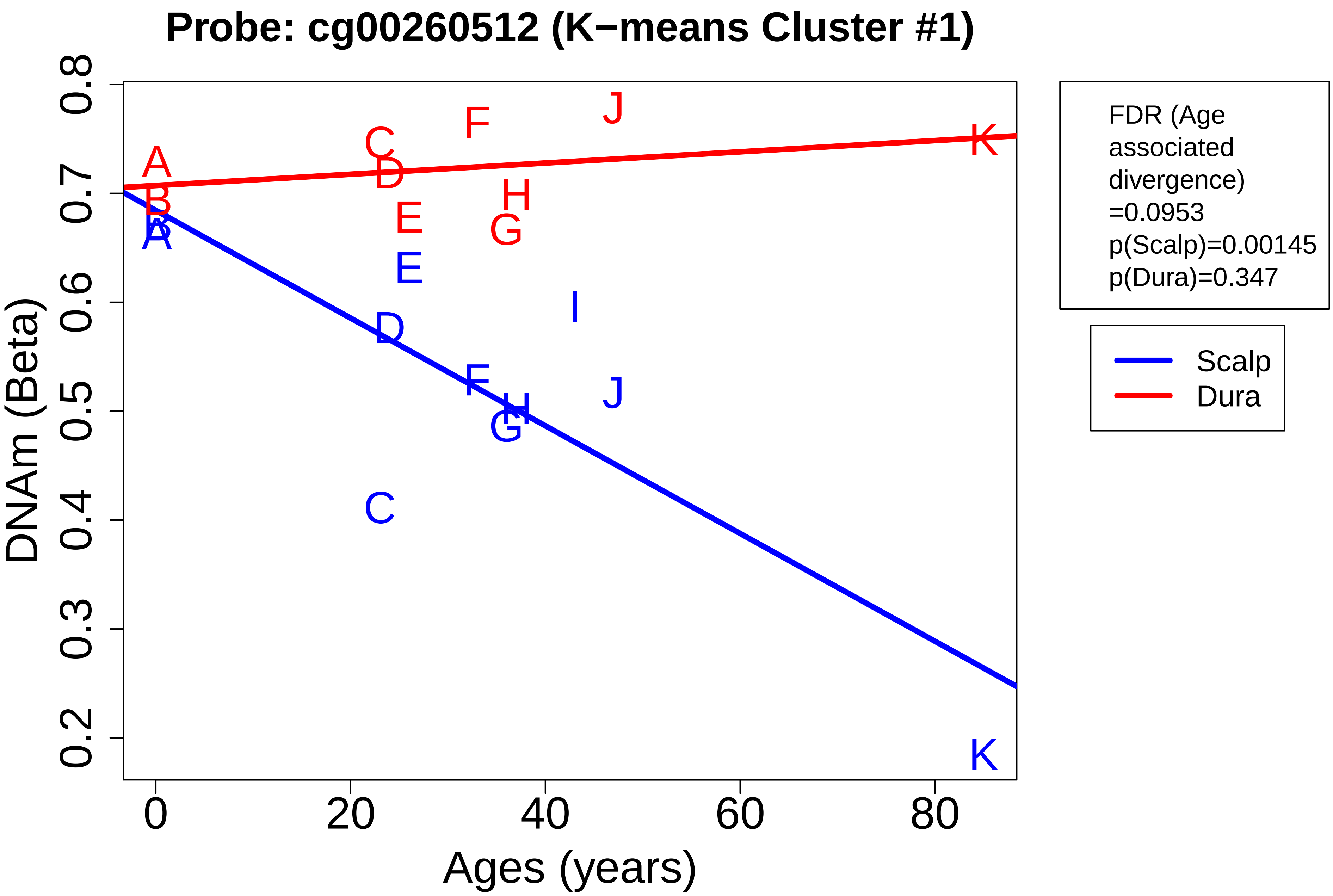

Probe: cg00260512 (K−means Cluster #1)
DNAm (Beta)
Ages (years)
0.2
0.3
0.4
0.5
0.6
0.7
0.8
0
20
40
60
80
FDR (Age associated divergence) =0.0953
p(Scalp)=0.00145
p(Dura)=0.347
Scalp
Dura
A
B
C
D
E
F
G
H
I
J
K

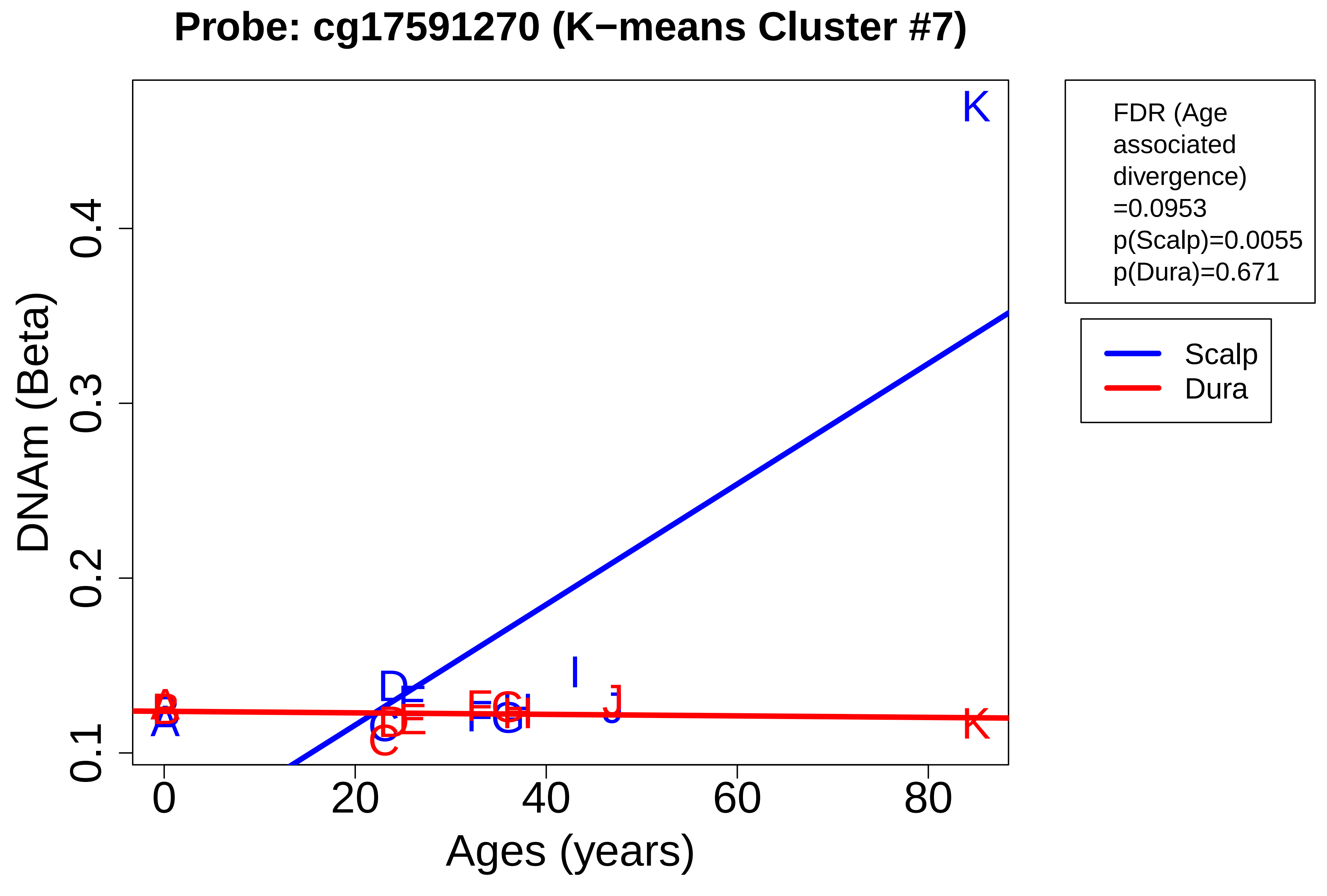

Probe: cg17591270 (K−means Cluster #7)
DNAm (Beta)
0.1
0.2
0.3
0.4
0
20
40
60
80
Ages (years)
FDR (Age associated divergence) =0.0953
p(Scalp)=0.0055
p(Dura)=0.671
Scalp
Dura

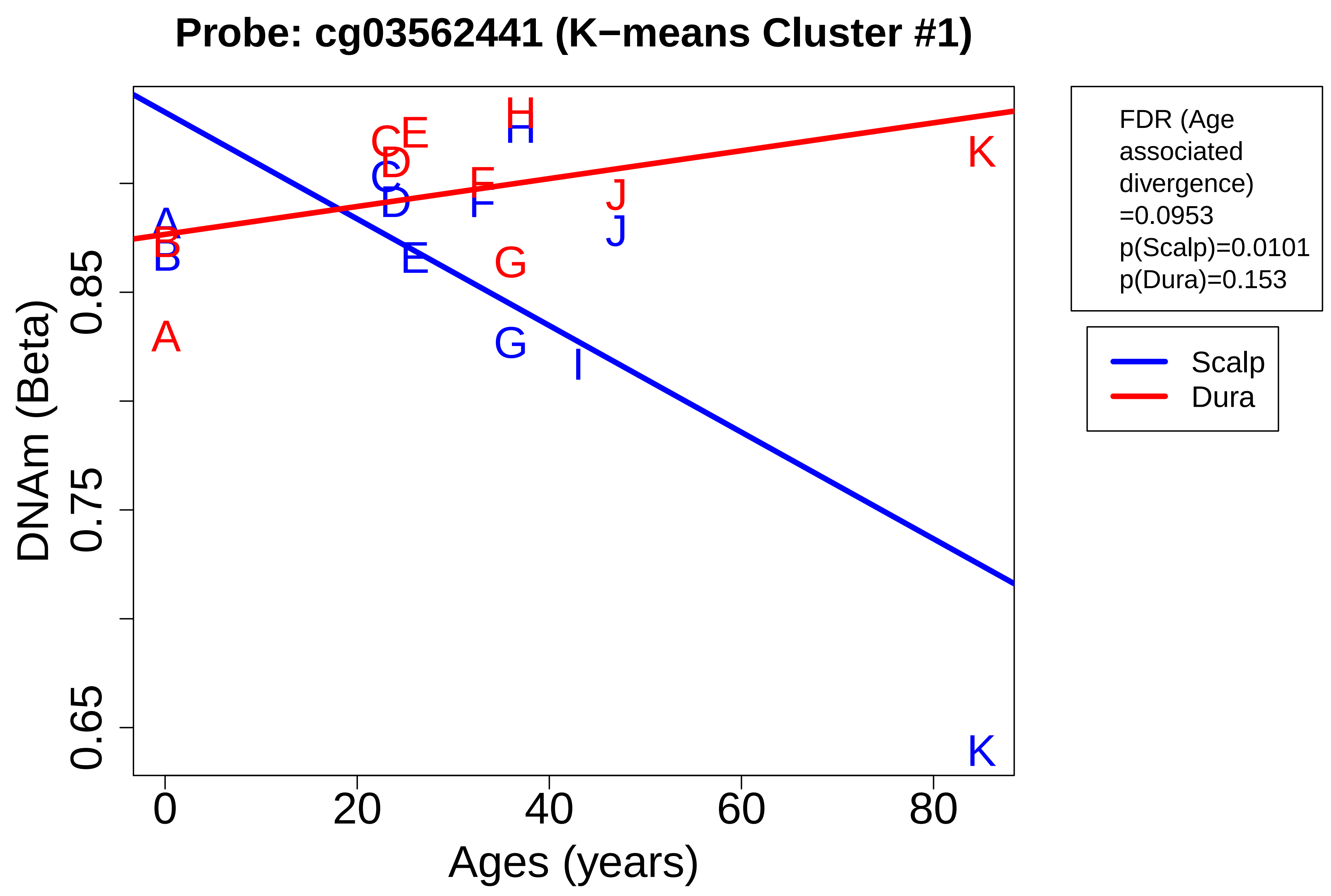

Probe: cg03562441 (K−means Cluster #1)
DNAm (Beta)
0.65
0.75
0.85
0
20
40
60
80
Ages (years)
A
B
C
D
E
F
G
H
I
J
K
FDR (Age associated divergence) =0.0953
p(Scalp)=0.0101
p(Dura)=0.153
Scalp
Dura

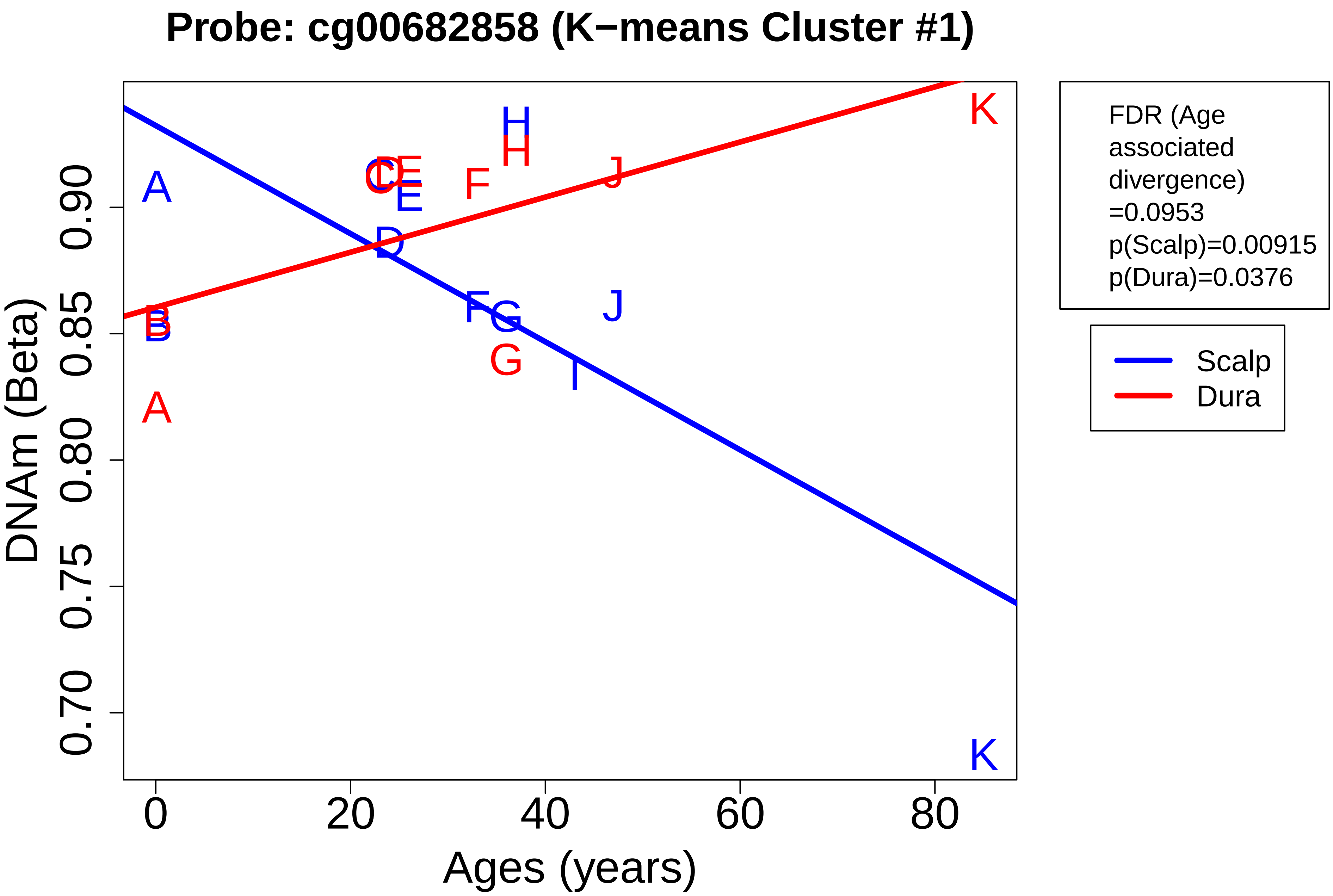

Probe: cg00682858 (K−means Cluster #1)
DNAm (Beta)
0.70
0.75
0.80
0.85
0.90
Ages (years)
0
20
40
60
80
FDR (Age associated divergence) =0.0953
p(Scalp)=0.00915
p(Dura)=0.0376
Scalp
Dura
A
B
C
D
E
F
G
H
I
J
K

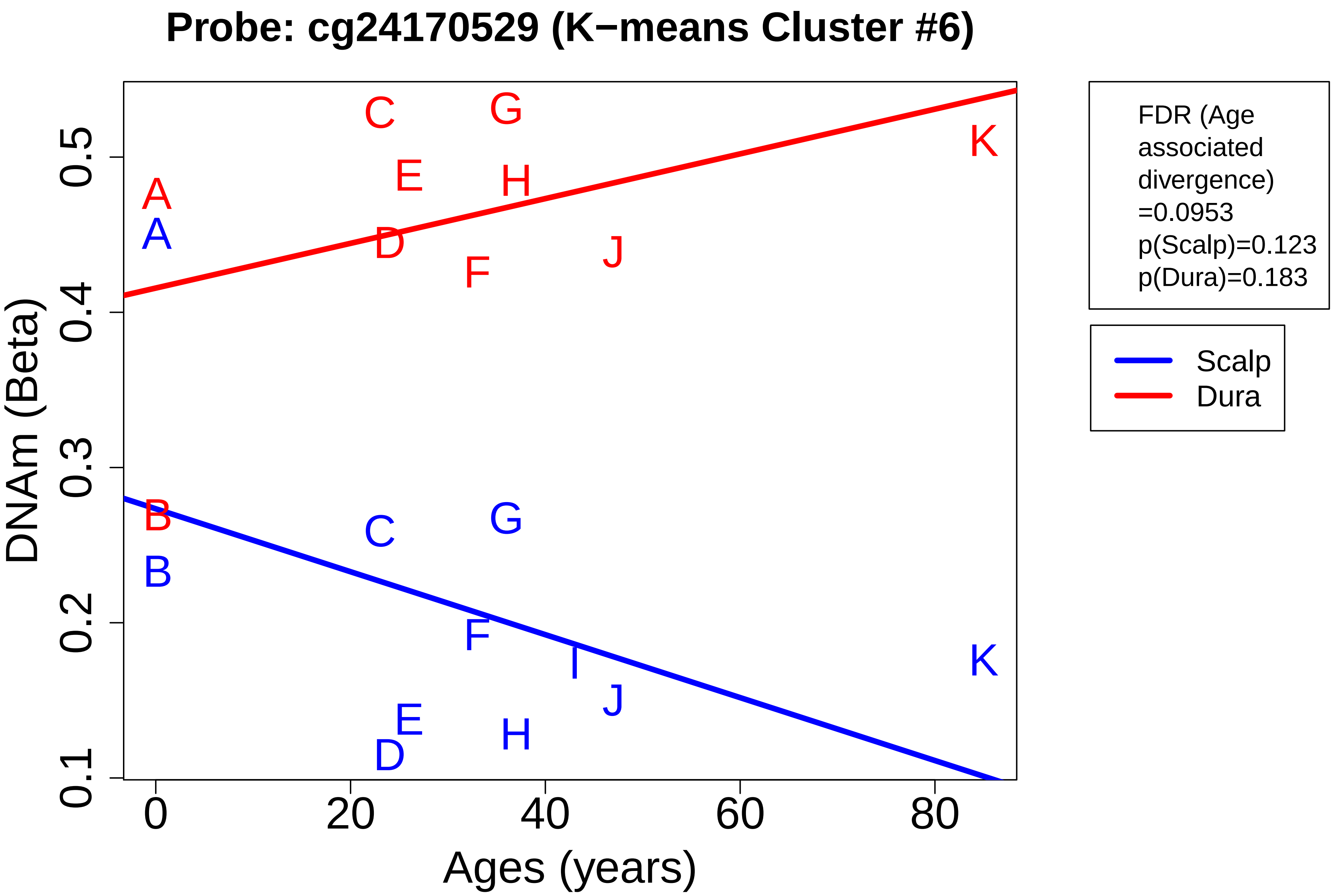

Probe: cg24170529 (K−means Cluster #6)
DNAm (Beta)
0.1
0.2
0.3
0.4
0.5
0
20
40
60
80
Ages (years)
A
B
C
D
E
F
G
H
I
J
K
FDR (Age associated divergence) =0.0953
p(Scalp)=0.123
p(Dura)=0.183
Scalp
Dura

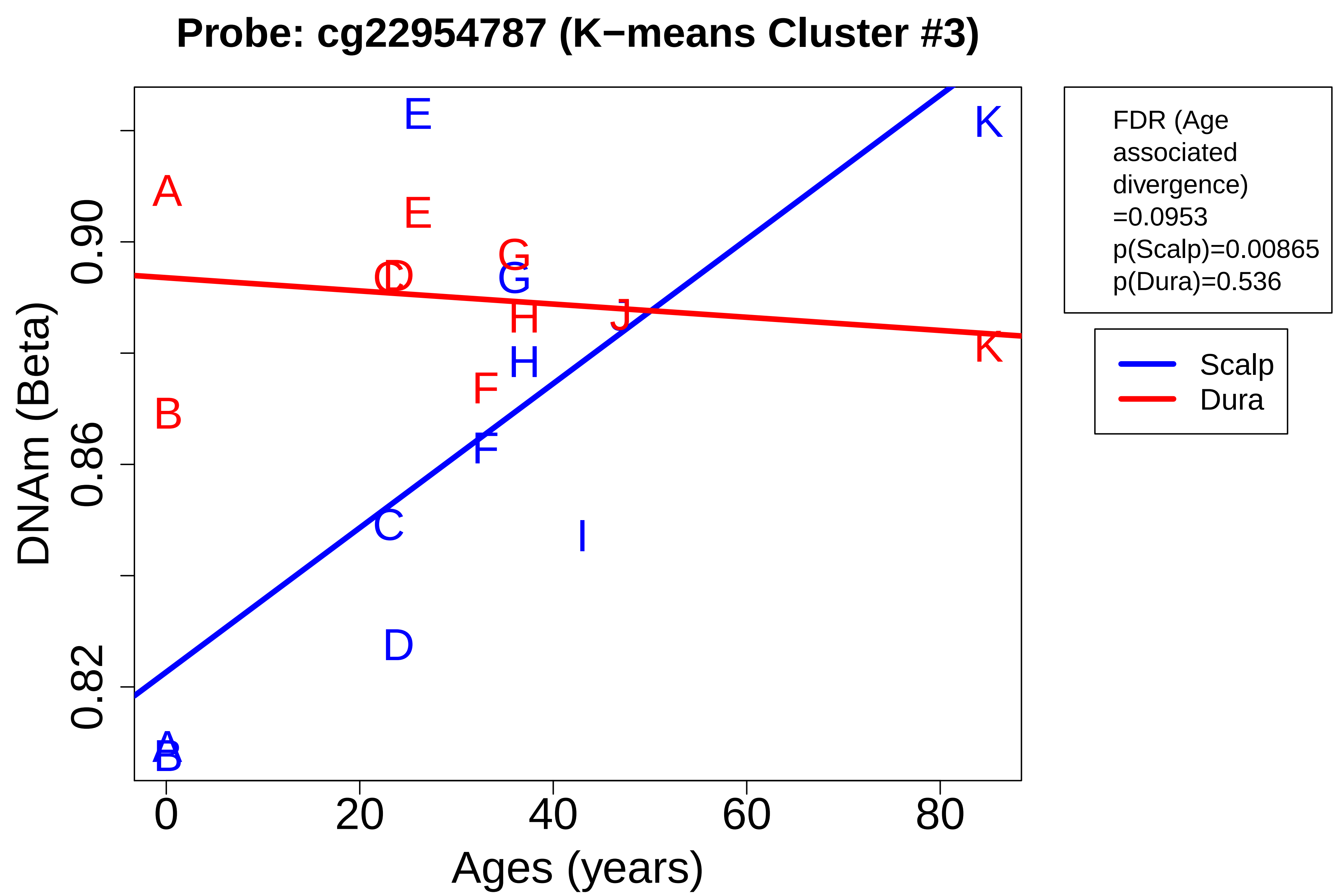
Probe: cg22954787 (K−means Cluster #3)
DNAm (Beta)
0.82
0.86
0.90
Ages (years)
0
20
40
60
80
FDR (Age associated divergence) =0.0953
p(Scalp)=0.00865
p(Dura)=0.536
Scalp
Dura
A
B
C
D
E
F
G
H
I
J
K

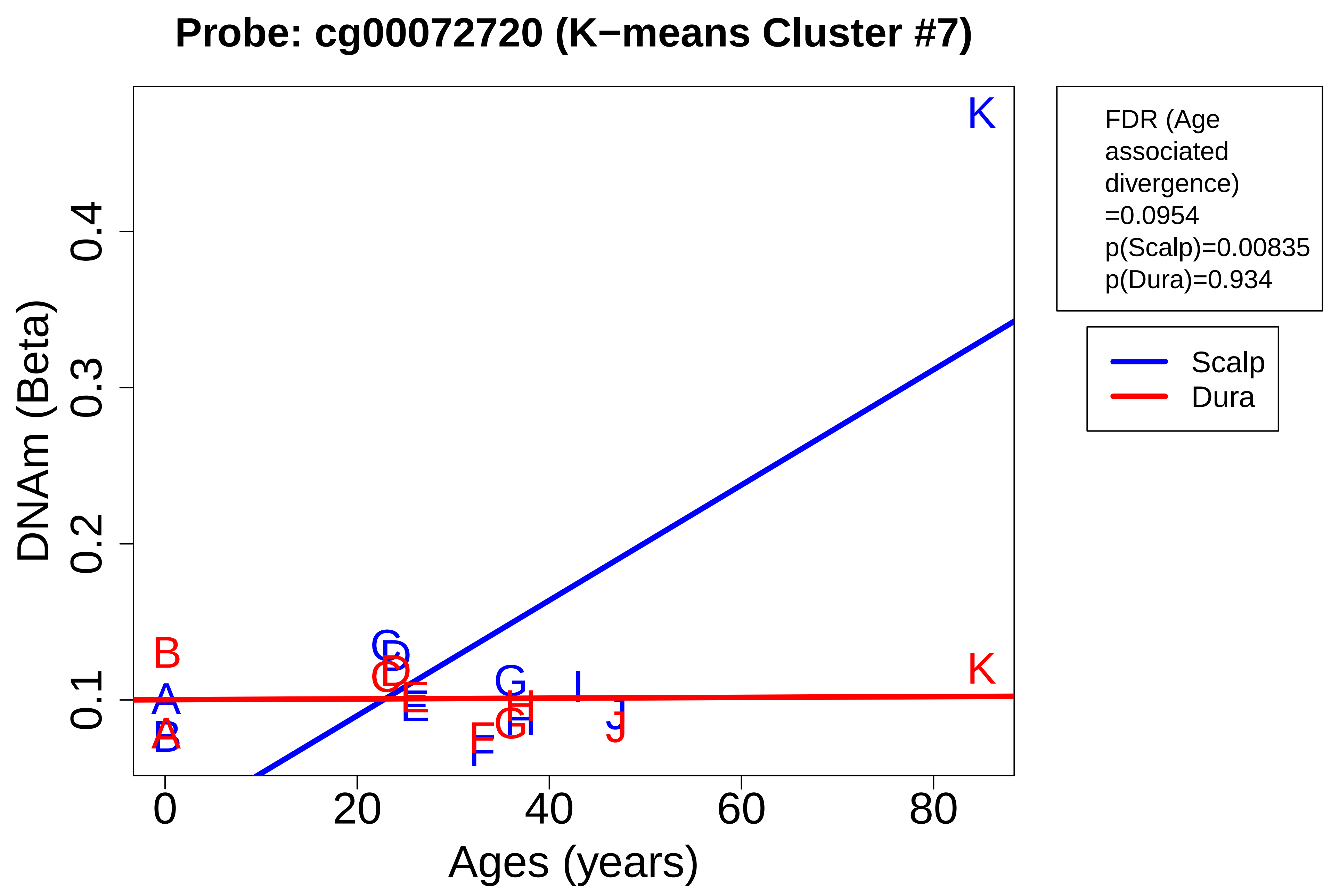
Probe: cg00072720 (K−means Cluster #7)
DNAm (Beta)
0.1
0.2
0.3
0.4
0
20
40
60
80
Ages (years)
A
B
C
D
E
F
G
H
I
J
K
FDR (Age associated divergence) =0.0954
p(Scalp)=0.00835
p(Dura)=0.934
Scalp
Dura

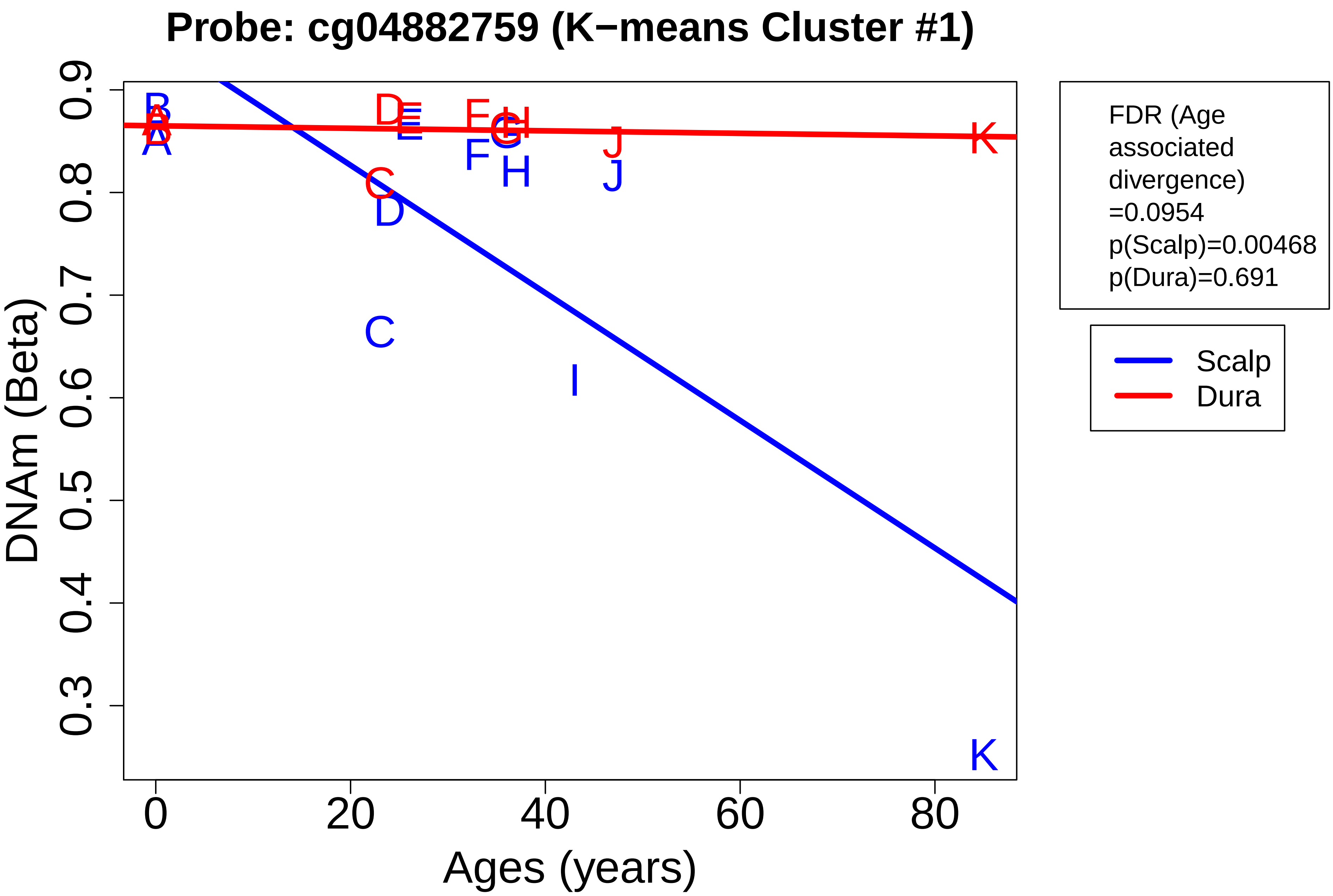

Probe: cg04882759 (K−means Cluster #1)
DNAm (Beta)
Ages (years)
0.3
0.4
0.5
0.6
0.7
0.8
0.9
0
20
40
60
80
FDR (Age associated divergence) =0.0954
p(Scalp)=0.00468
p(Dura)=0.691
Scalp
Dura

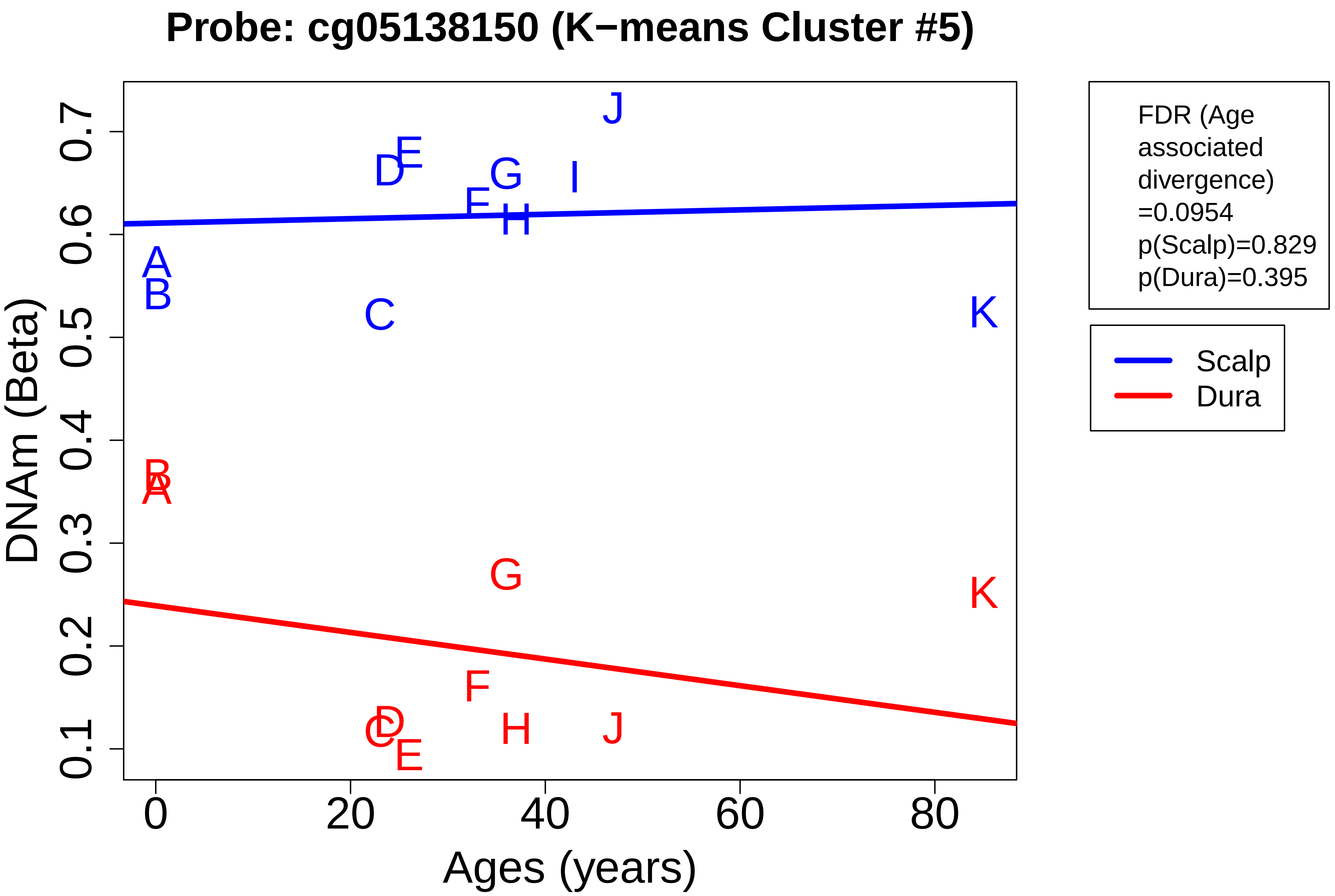

Probe: cg05138150 (K−means Cluster #5)
DNAm (Beta)
0.1
0.2
0.3
0.4
0.5
0.6
0.7
Ages (years)
0
20
40
60
80
FDR (Age associated divergence) =0.0954
p(Scalp)=0.829
p(Dura)=0.395
Scalp
Dura

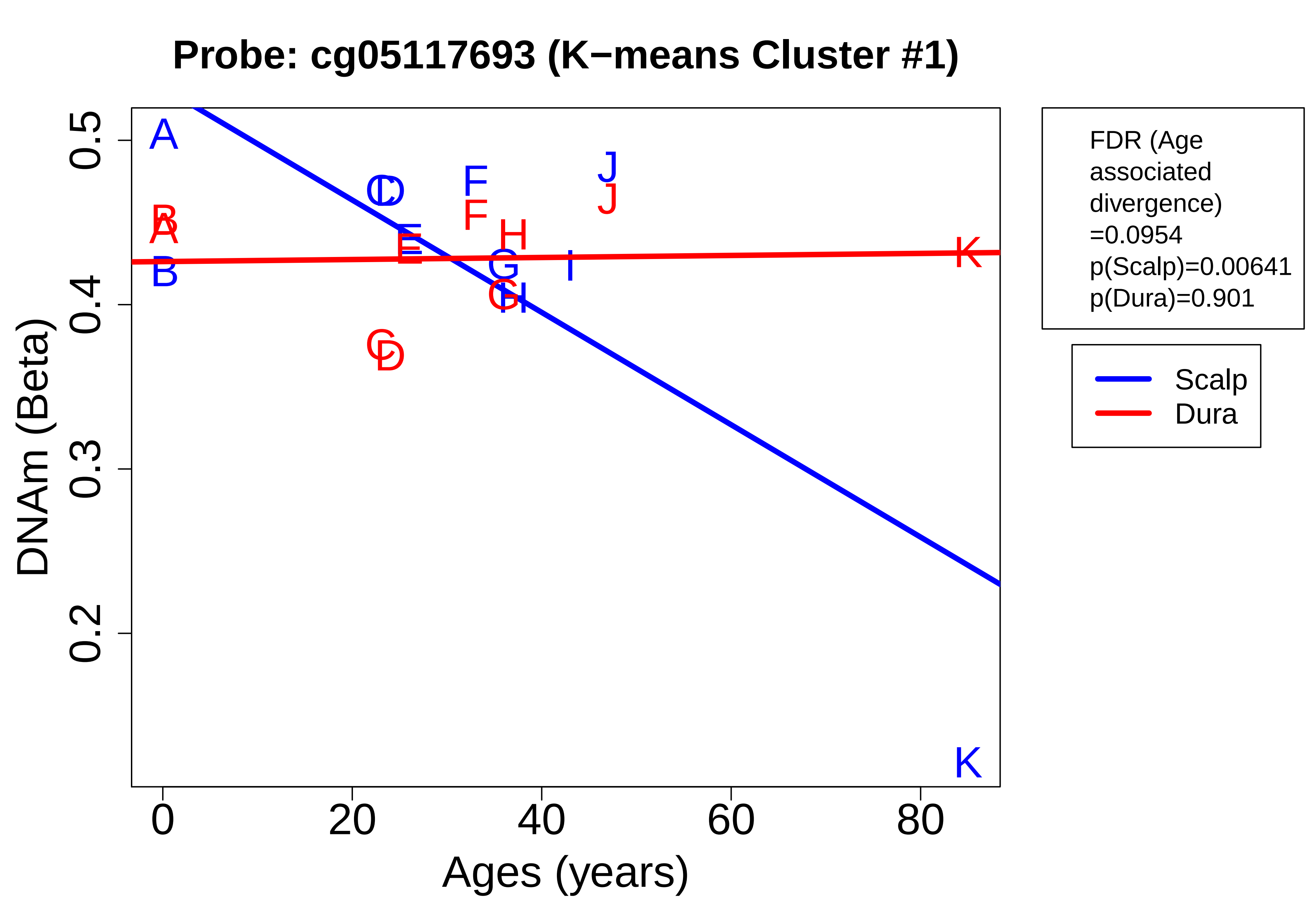
Probe: cg05117693 (K−means Cluster #1)
FDR (Age
associated
divergence)
=0.0954
p(Scalp)=0.00641
p(Dura)=0.901
Scalp
Dura
DNAm (Beta)
0.2
0.3
0.4
0.5
0
20
40
60
80
Ages (years)
A
B
C
D
E
F
G
H
I
J
K

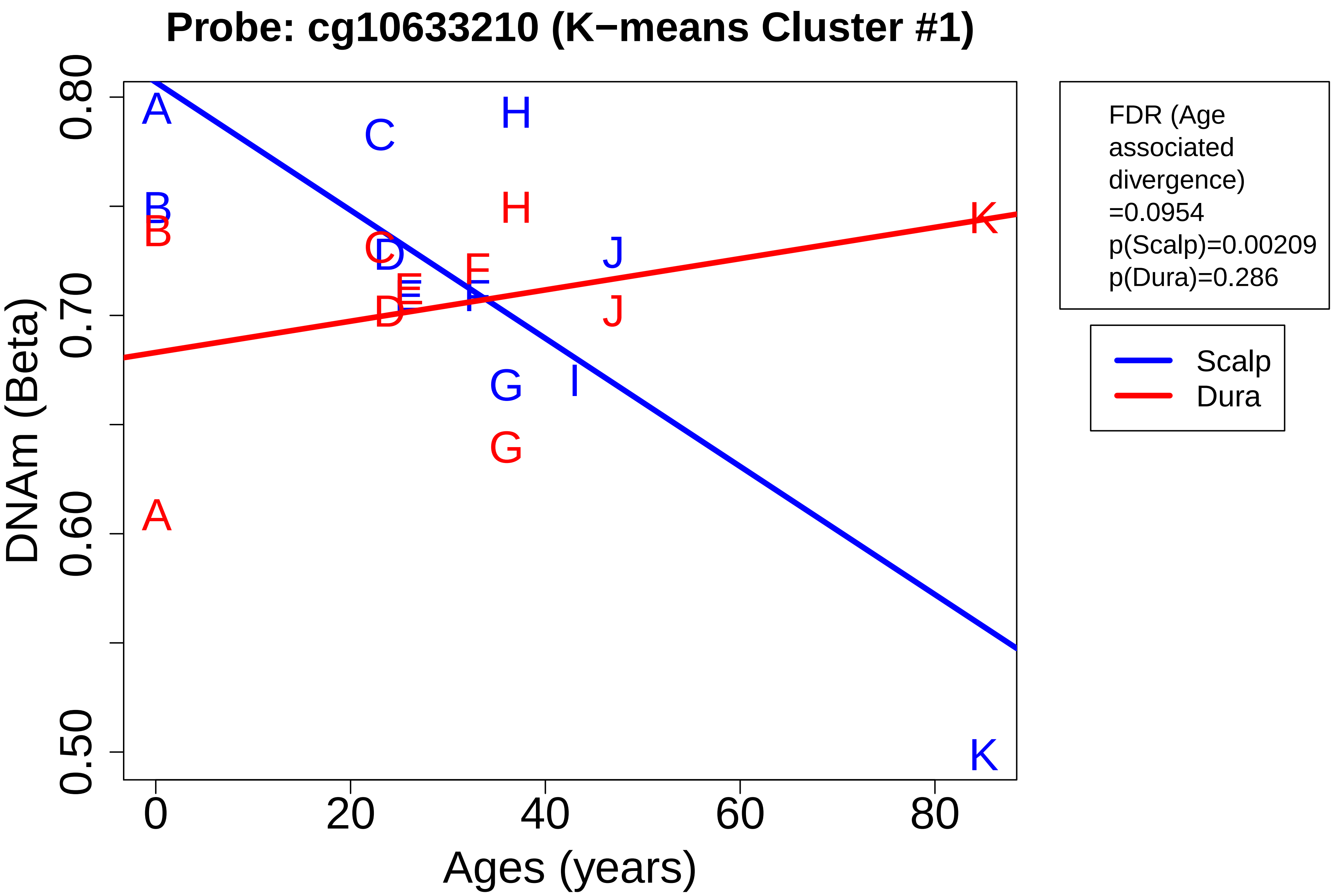

Probe: cg10633210 (K−means Cluster #1)
DNAm (Beta)
0.50
0.60
0.70
0.80
Ages (years)
0
20
40
60
80
FDR (Age associated divergence) =0.0954
p(Scalp)=0.00209
p(Dura)=0.286
Scalp
Dura

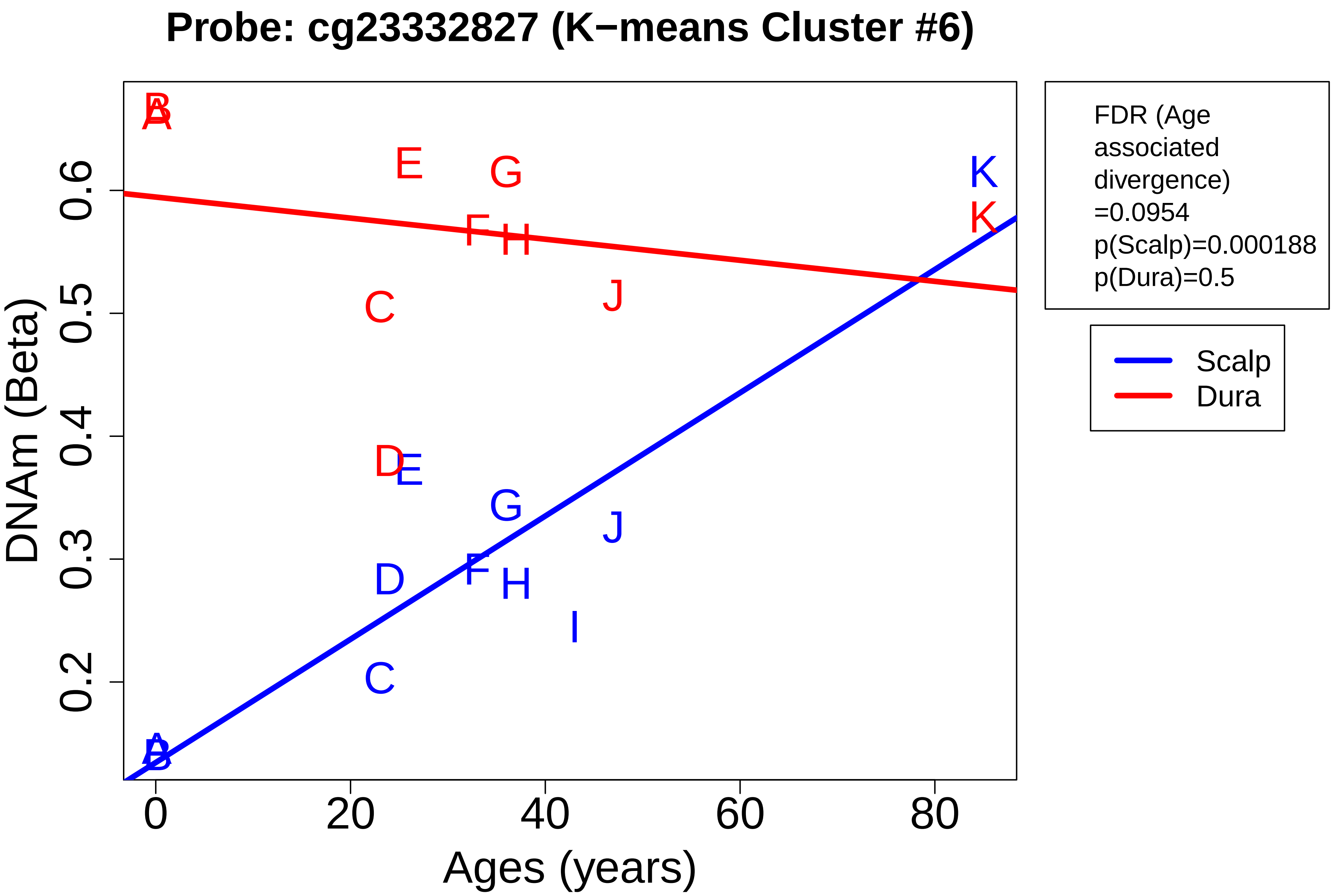

Probe: cg23332827 (K−means Cluster #6)
DNAm (Beta)
0.2
0.3
0.4
0.5
0.6
0
20
40
60
80
Ages (years)
FDR (Age associated divergence) =0.0954
p(Scalp)=0.000188
p(Dura)=0.5
Scalp
Dura

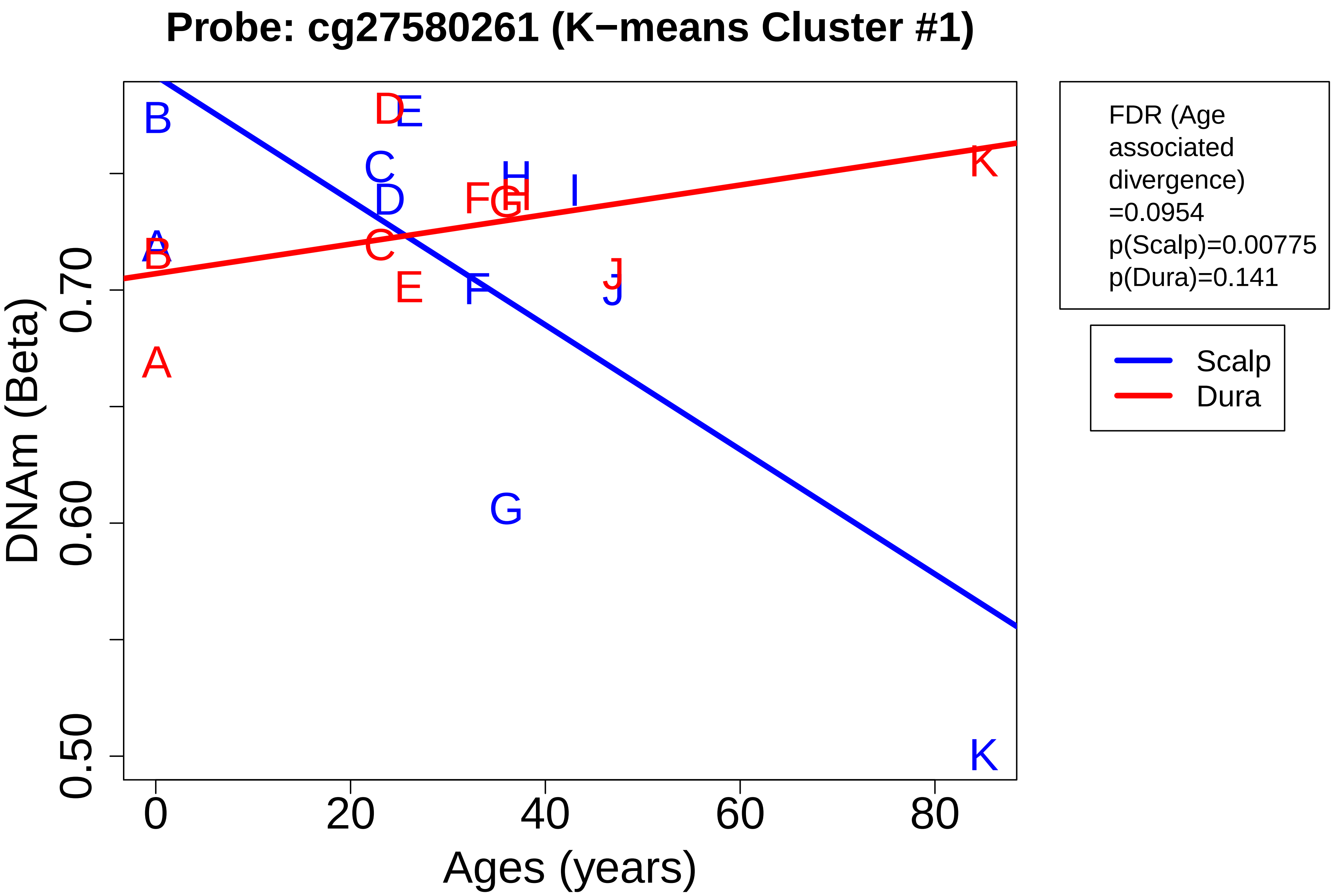

Probe: cg27580261 (K−means Cluster #1)
DNAm (Beta)
0.50
0.60
0.70
Ages (years)
0
20
40
60
80
FDR (Age associated divergence) =0.0954
p(Scalp)=0.00775
p(Dura)=0.141
Scalp
Dura

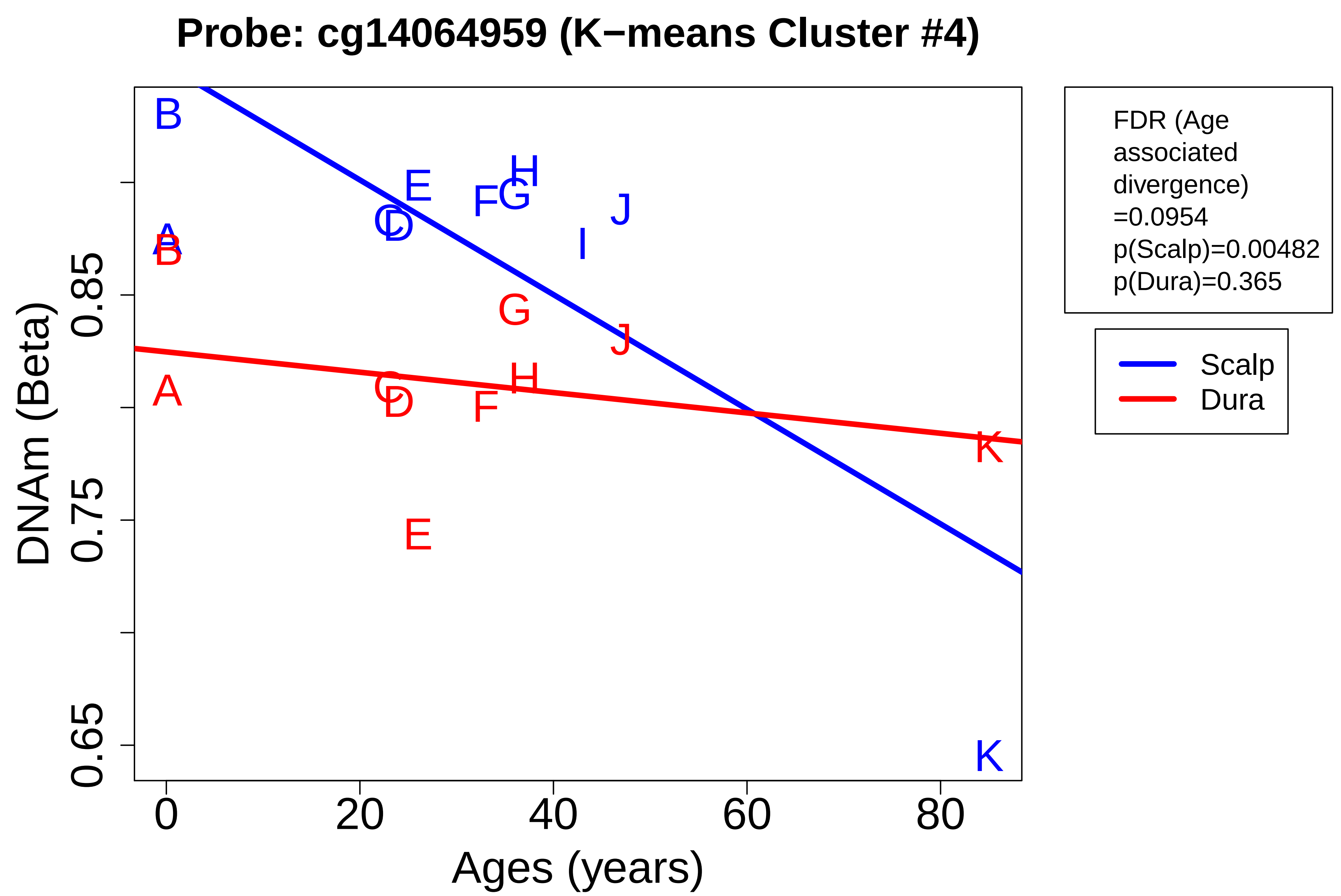

Probe: cg14064959 (K−means Cluster #4)
DNAm (Beta)
0.65
0.75
0.85
Ages (years)
0
20
40
60
80
B
A
C
D
E
F
G
H
I
J
K
FDR (Age associated divergence) =0.0954
p(Scalp)=0.00482
p(Dura)=0.365
Scalp
Dura

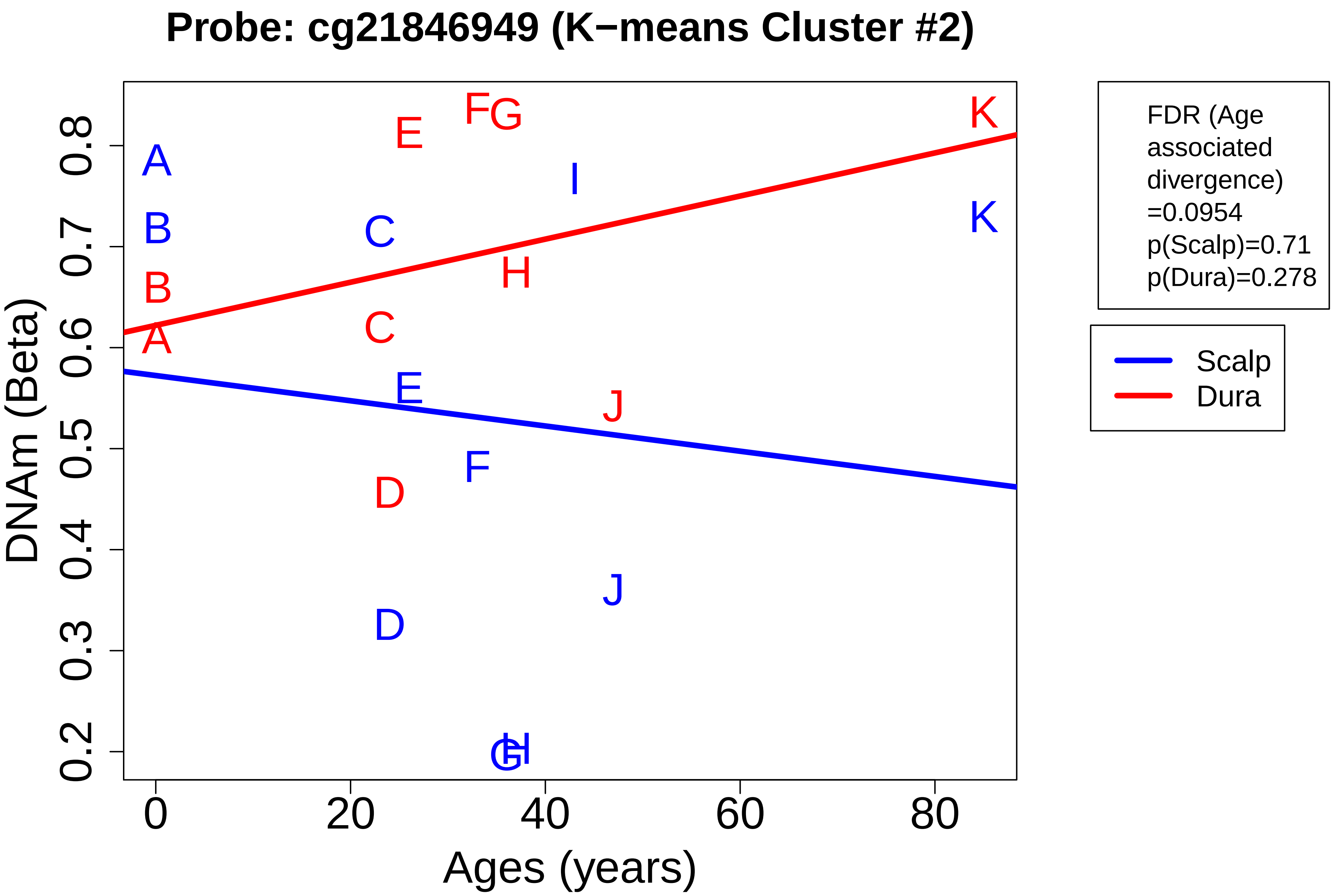
Probe: cg21846949 (K−means Cluster #2)
DNAm (Beta)
0.2
0.3
0.4
0.5
0.6
0.7
0.8
Ages (years)
0
20
40
60
80
FDR (Age associated divergence) =0.0954
p(Scalp)=0.71
p(Dura)=0.278
Scalp
Dura

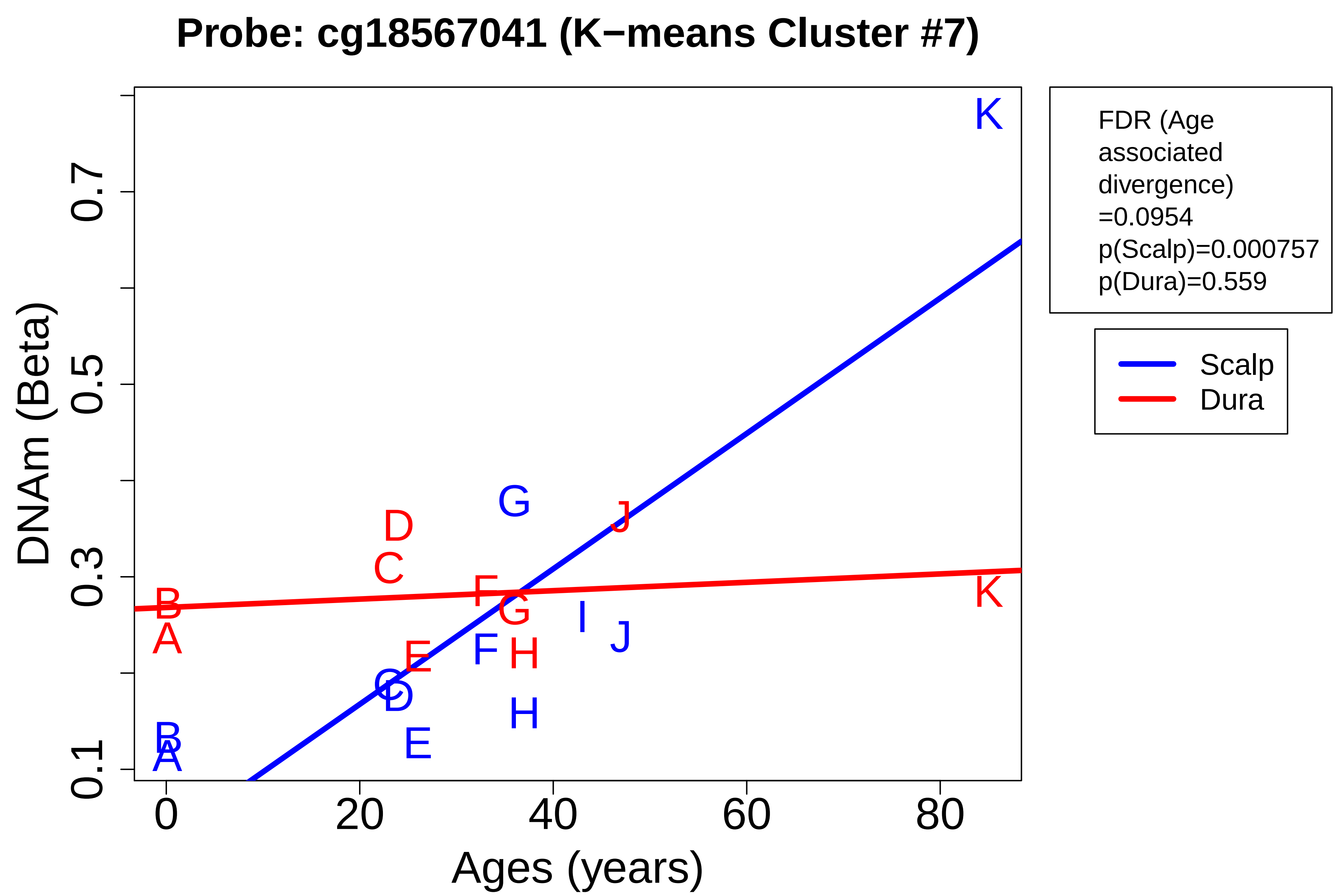

Probe: cg18567041 (K−means Cluster #7)
DNAm (Beta)
0.1
0.3
0.5
0.7
0
20
40
60
80
Ages (years)
K
J
I
H
G
F
E
D
C
B
A
FDR (Age associated divergence) =0.0954
p(Scalp)=0.000757
p(Dura)=0.559
Scalp
Dura

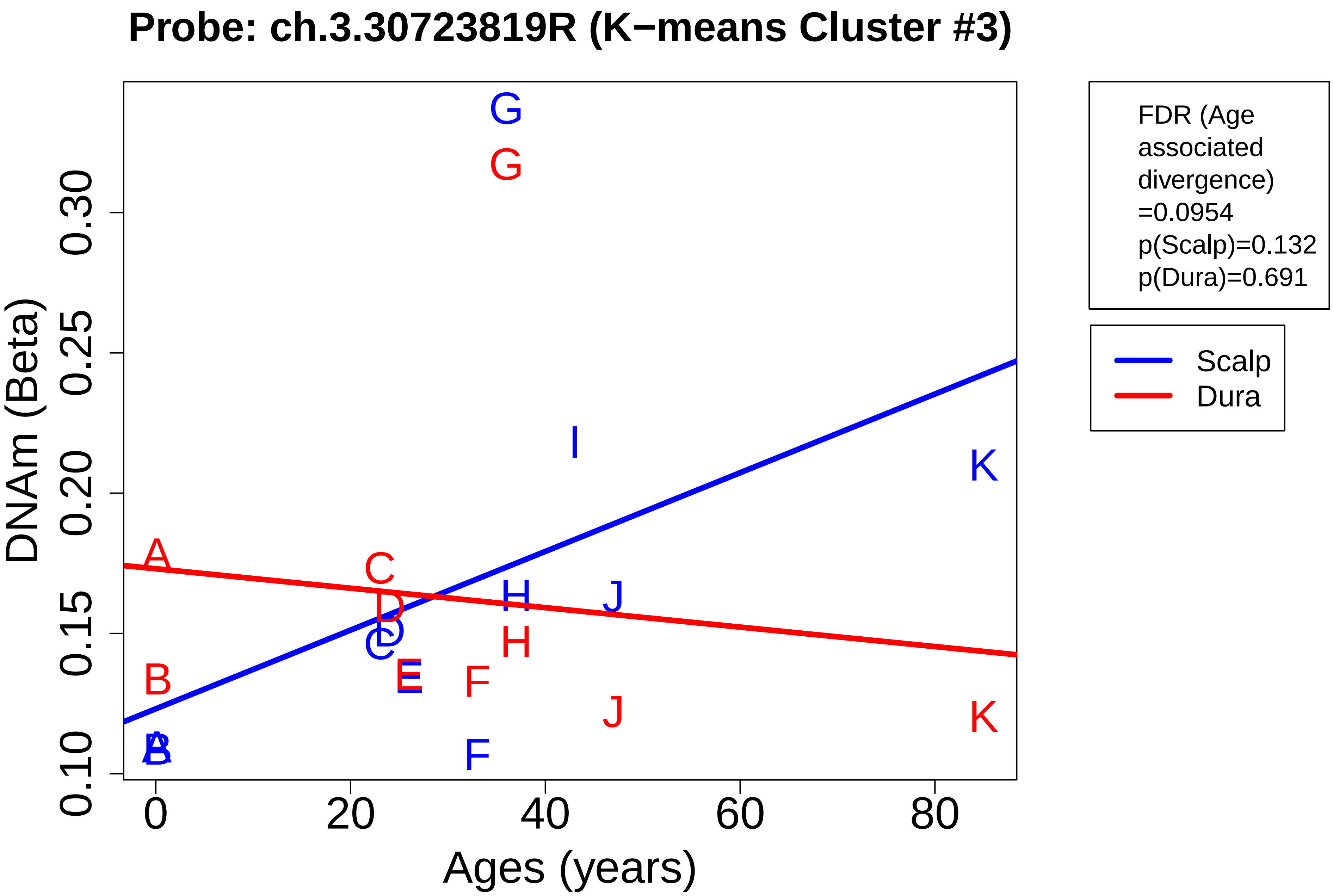

Probe: ch.3.30723819R (K−means Cluster #3)
DNAm (Beta)
0.10
0.15
0.20
0.25
0.30
Ages (years)
0
20
40
60
80
FDR (Age associated divergence) =0.0954
p(Scalp)=0.132
p(Dura)=0.691
Scalp
Dura
A
B
C
D
E
F
G
H
I
J
K

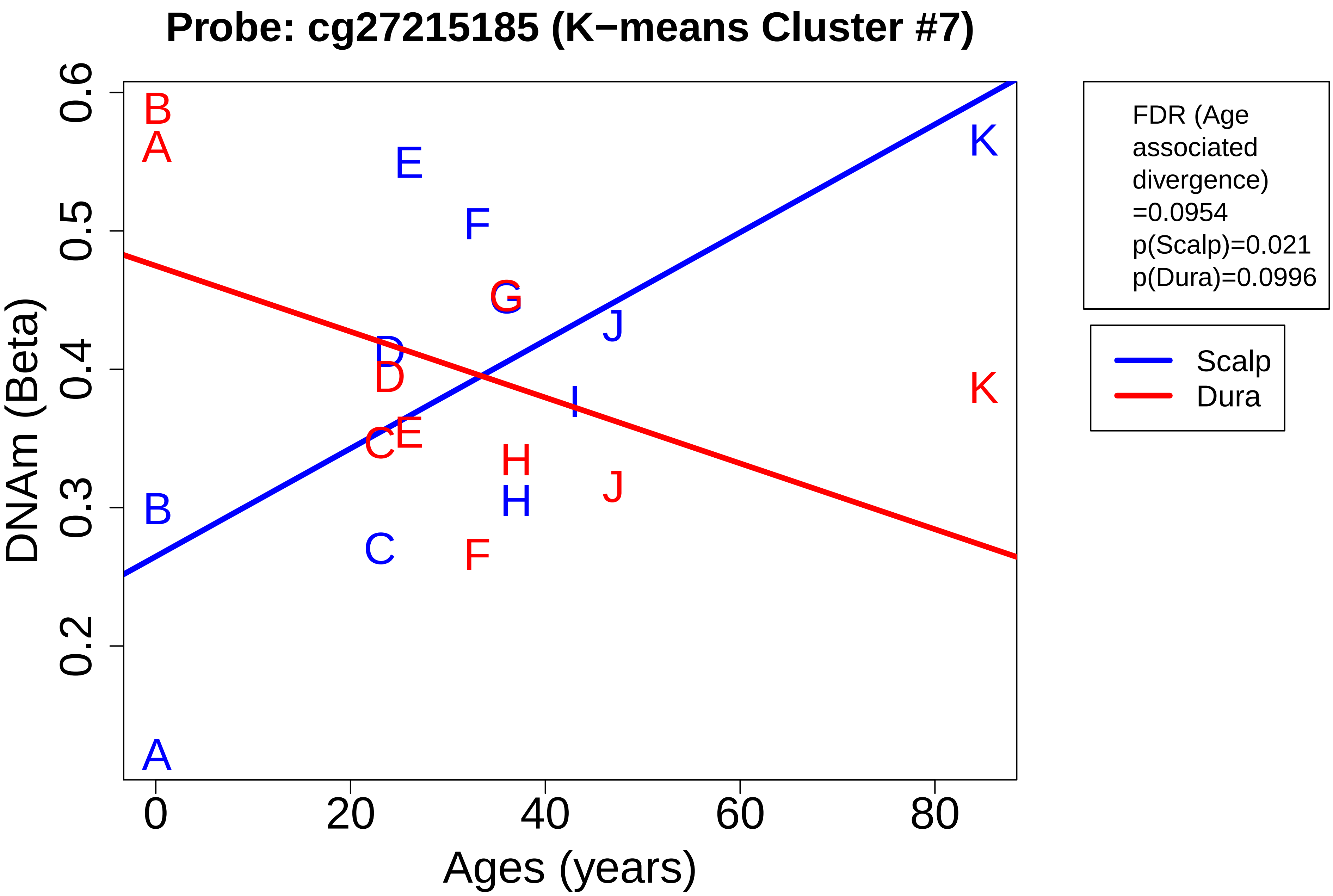

Probe: cg27215185 (K−means Cluster #7)
DNAm (Beta)
0.2
0.3
0.4
0.5
0.6
Ages (years)
0
20
40
60
80
B
A
E
F
G
J
D
D
I
K
K
C
E
H
J
B
H
C
F
A
FDR (Age associated divergence) =0.0954
p(Scalp)=0.021
p(Dura)=0.0996
Scalp
Dura

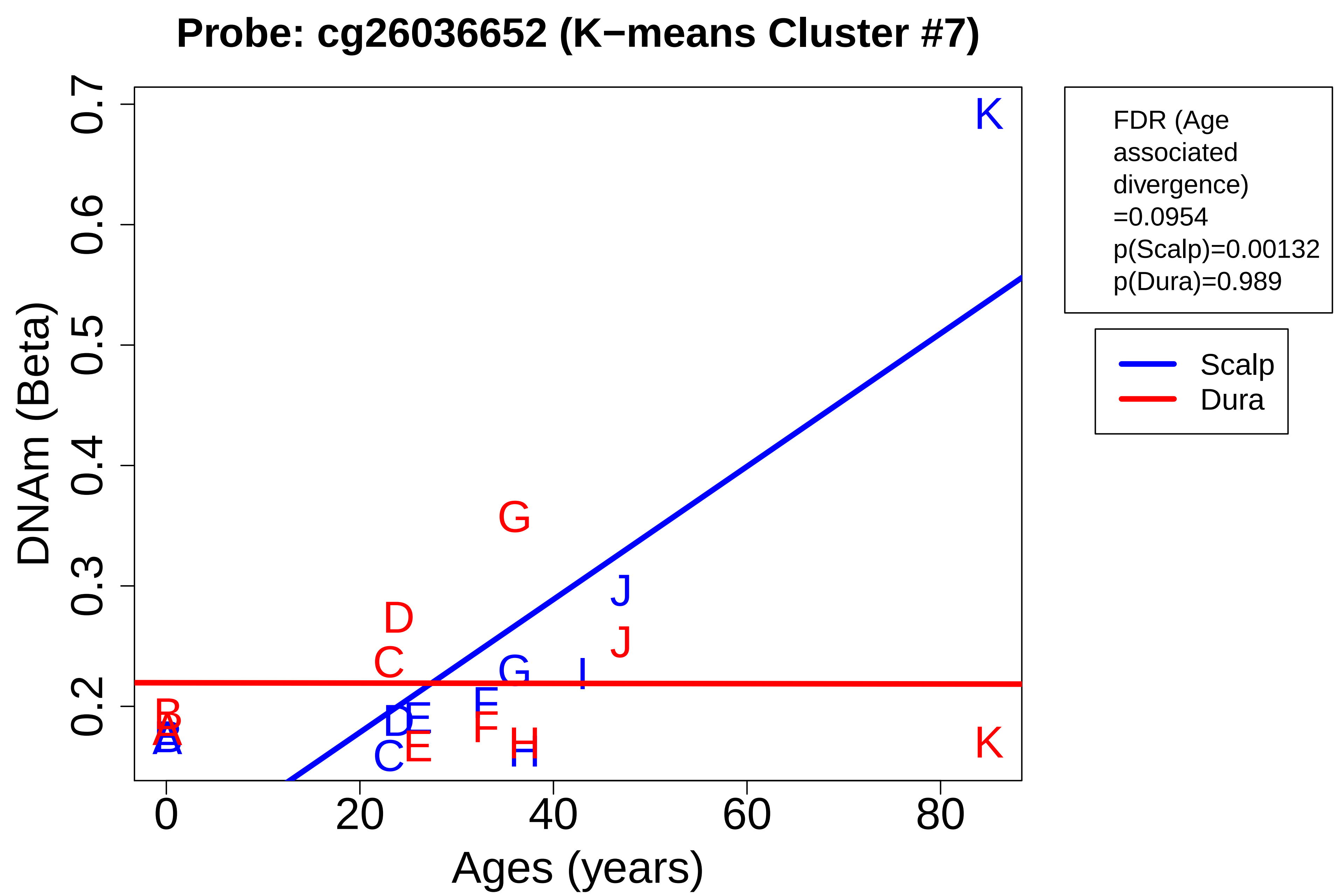

Probe: cg26036652 (K−means Cluster #7)
DNAm (Beta)
Ages (years)
0.2
0.3
0.4
0.5
0.6
0.7
0
20
40
60
80
FDR (Age associated divergence) =0.0954
p(Scalp)=0.00132
p(Dura)=0.989
Scalp
Dura

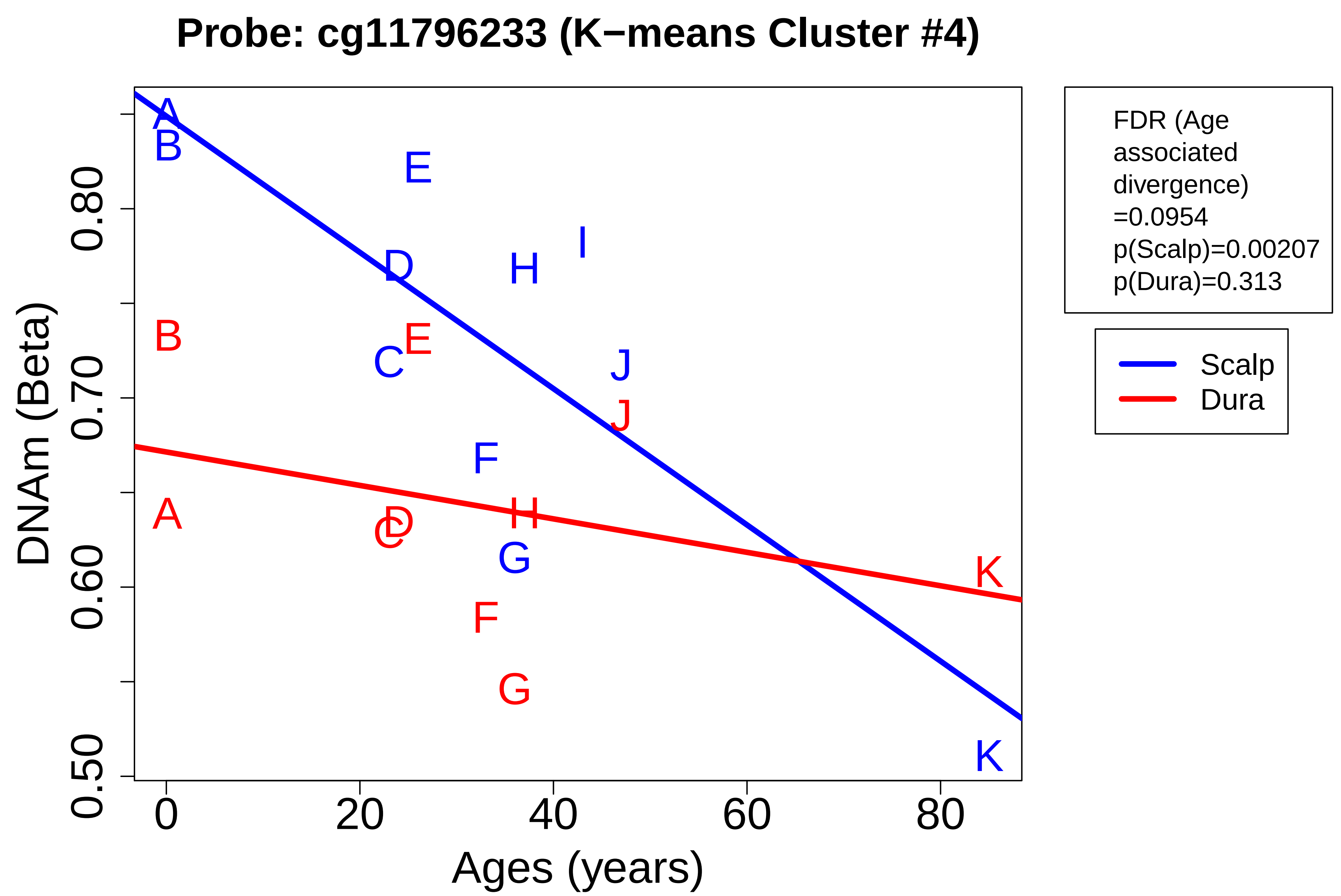

Probe: cg11796233 (K−means Cluster #4)
DNAm (Beta)
0.50
0.60
0.70
0.80
0
20
40
60
80
Ages (years)
A
B
C
D
E
F
G
H
I
J
K
FDR (Age associated divergence) =0.0954
p(Scalp)=0.00207
p(Dura)=0.313
Scalp
Dura

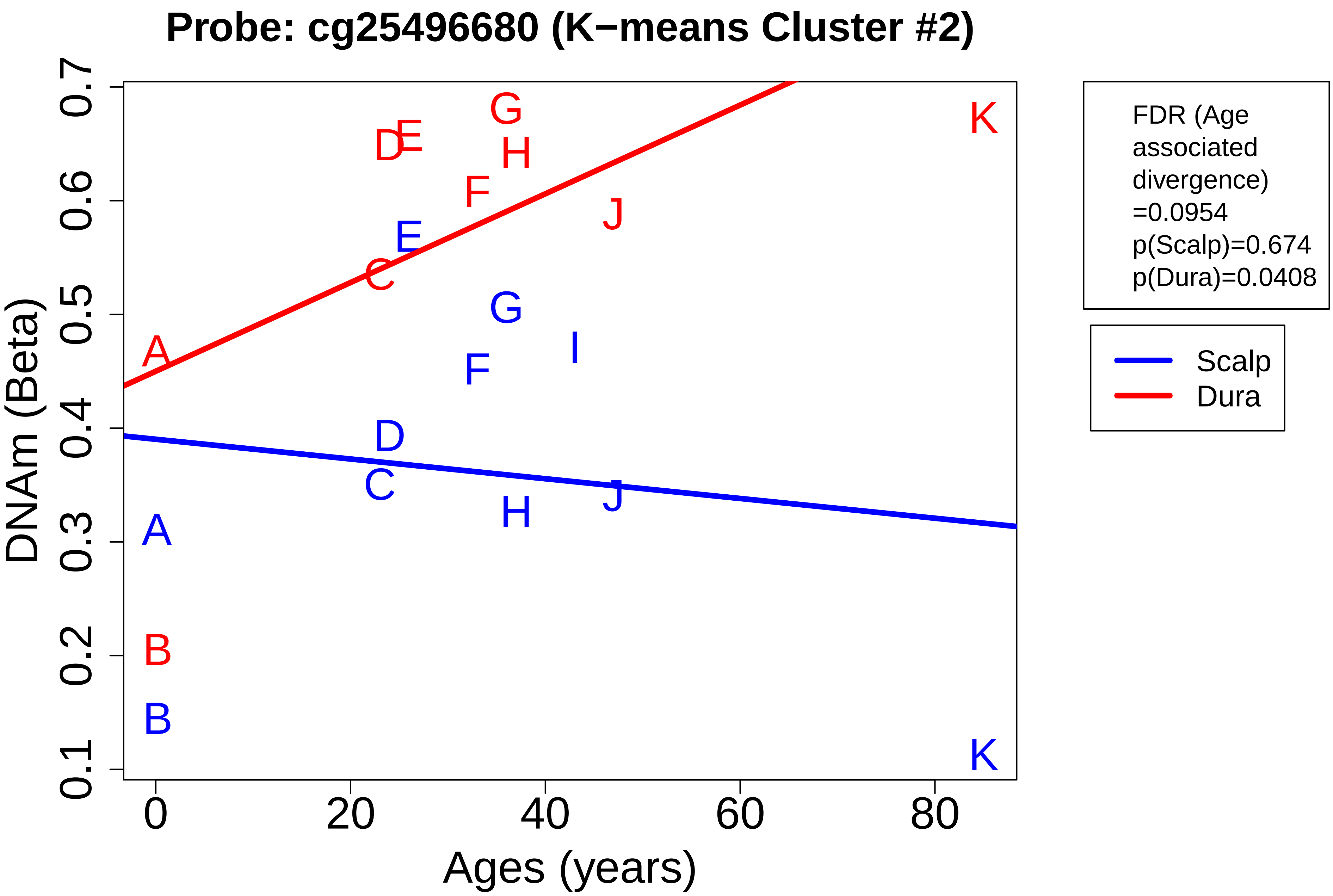

Probe: cg25496680 (K−means Cluster #2)
DNAm (Beta)
0.1
0.2
0.3
0.4
0.5
0.6
0.7
0
20
40
60
80
Ages (years)
A
B
C
D
E
F
G
H
I
J
K
FDR (Age associated divergence) =0.0954
p(Scalp)=0.674
p(Dura)=0.0408
Scalp
Dura

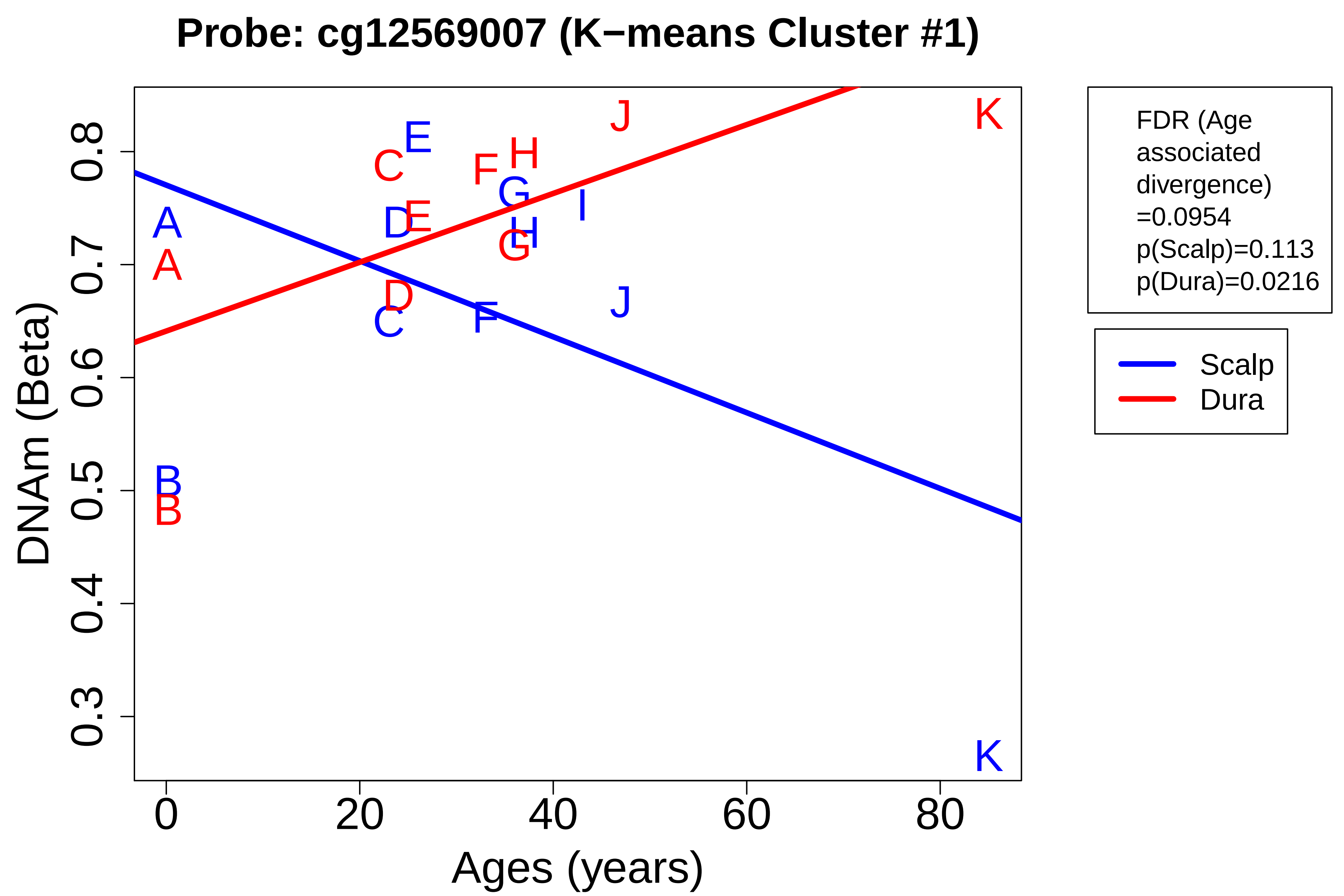

Probe: cg12569007 (K−means Cluster #1)
DNAm (Beta)
0.3
0.4
0.5
0.6
0.7
0.8
Ages (years)
0
20
40
60
80
A
B
C
D
E
F
G
H
I
J
K
FDR (Age associated divergence) =0.0954
p(Scalp)=0.113
p(Dura)=0.0216
Scalp
Dura

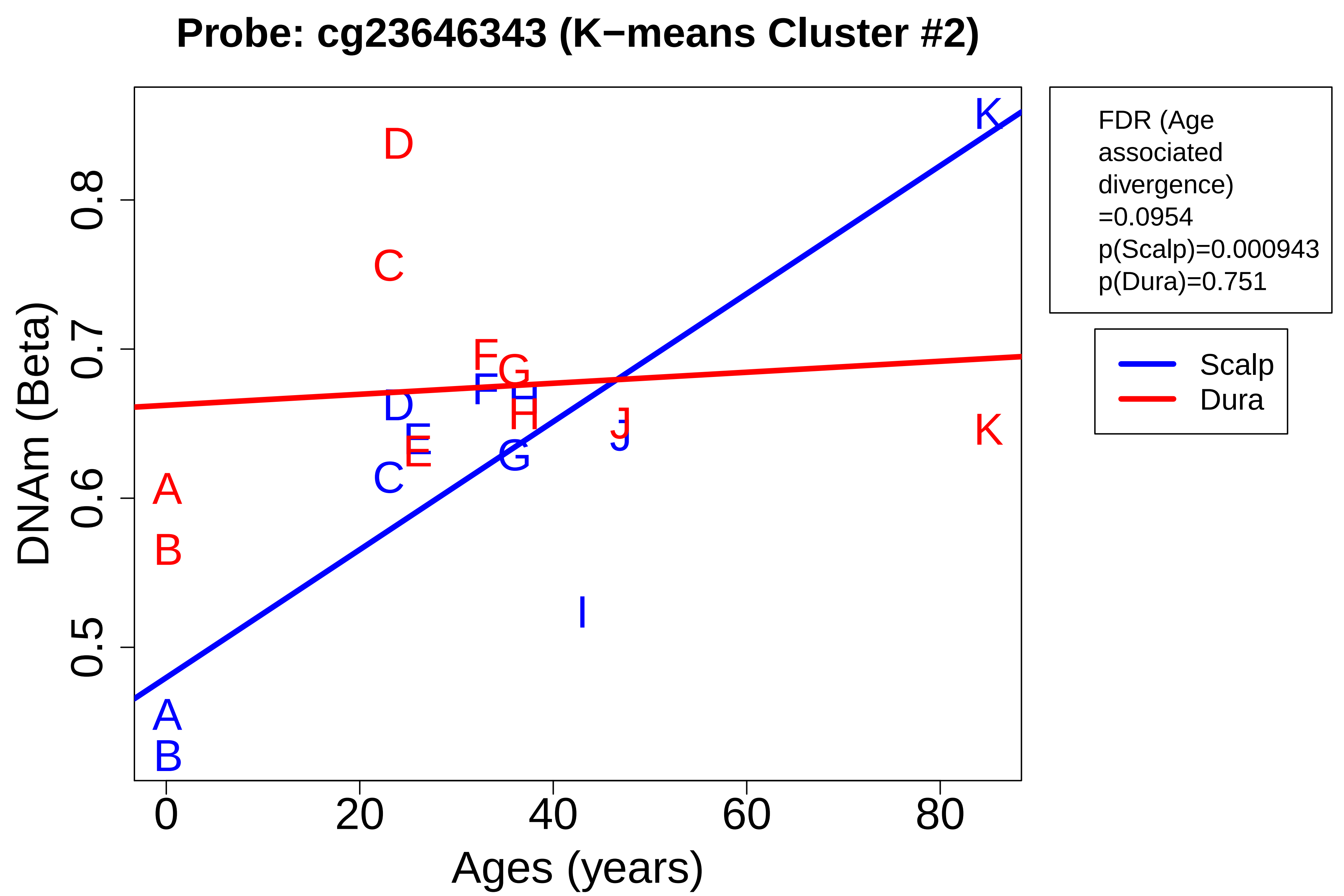

Probe: cg23646343 (K−means Cluster #2)
FDR (Age associated divergence) =0.0954
p(Scalp)=0.000943
p(Dura)=0.751
Scalp
Dura
DNAm (Beta)
0.5
0.6
0.7
0.8
Ages (years)
0
20
40
60
80

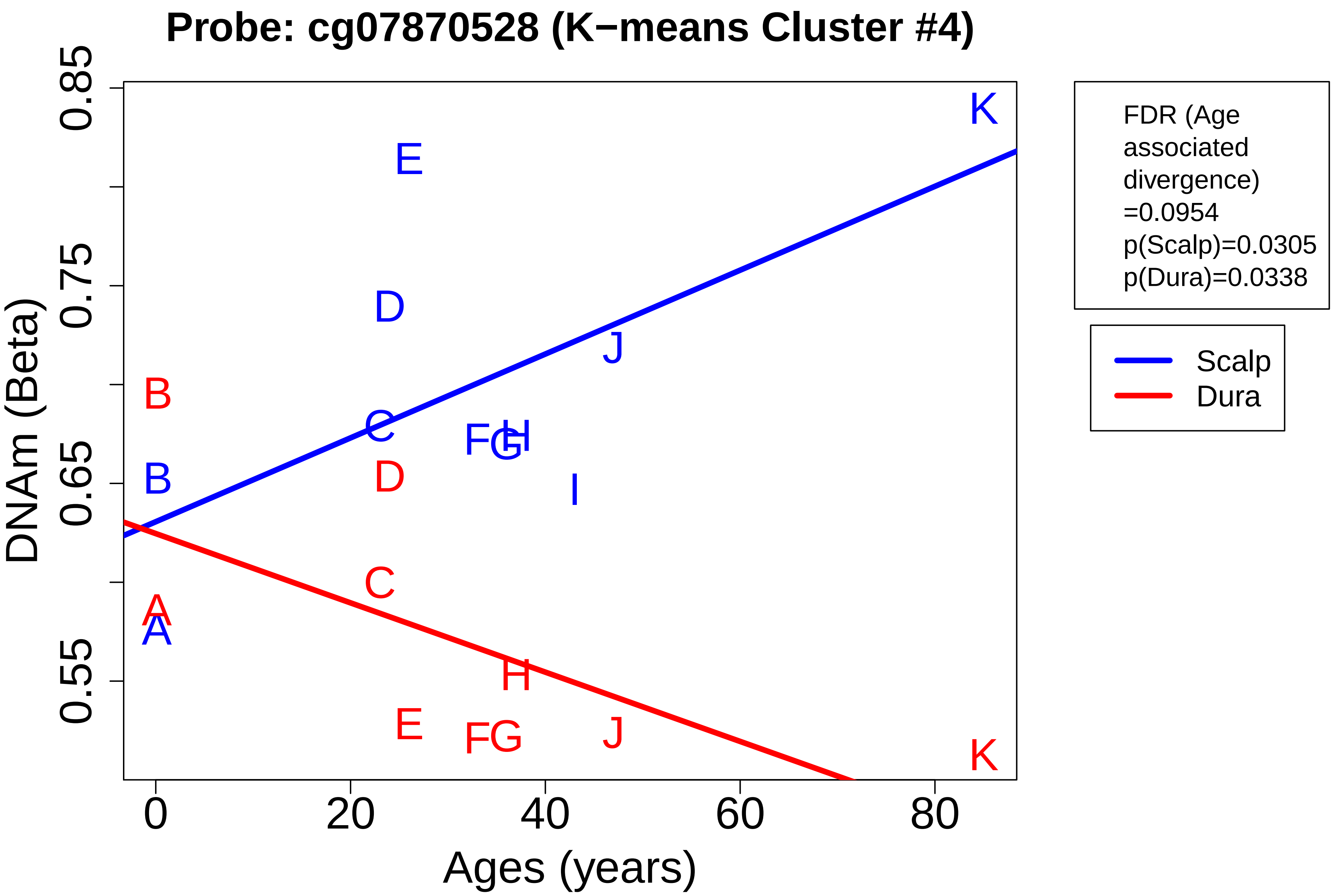
Probe: cg07870528 (K−means Cluster #4)
DNAm (Beta)
0.55
0.65
0.75
0.85
Ages (years)
0
20
40
60
80
FDR (Age associated divergence) =0.0954
p(Scalp)=0.0305
p(Dura)=0.0338
Scalp
Dura
A
B
C
D
E
F
G
H
I
J
K

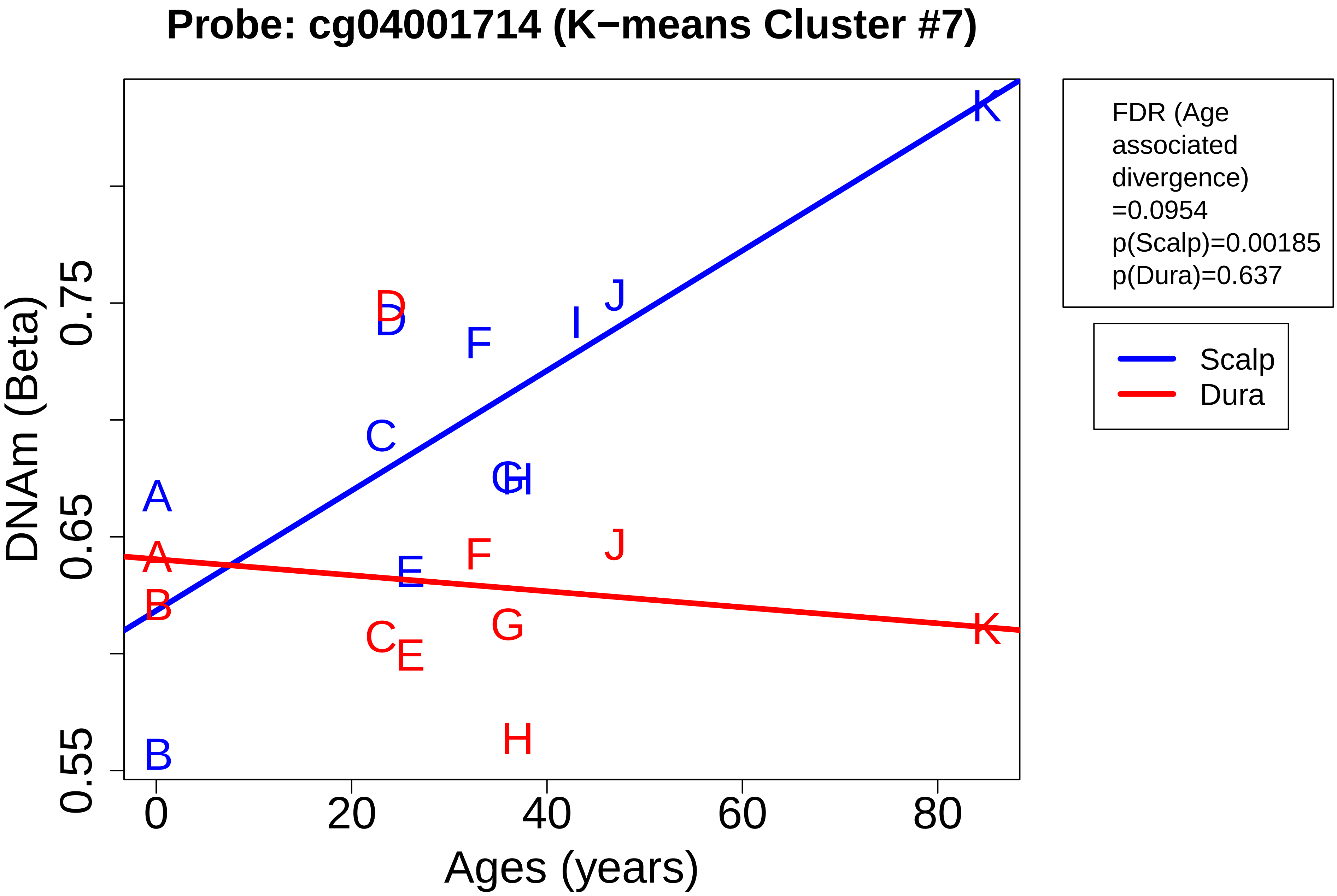

Probe: cg04001714 (K−means Cluster #7)
DNAm (Beta)
0.55
0.65
0.75
0
20
40
60
80
Ages (years)
FDR (Age associated divergence) =0.0954
p(Scalp)=0.00185
p(Dura)=0.637
Scalp
Dura

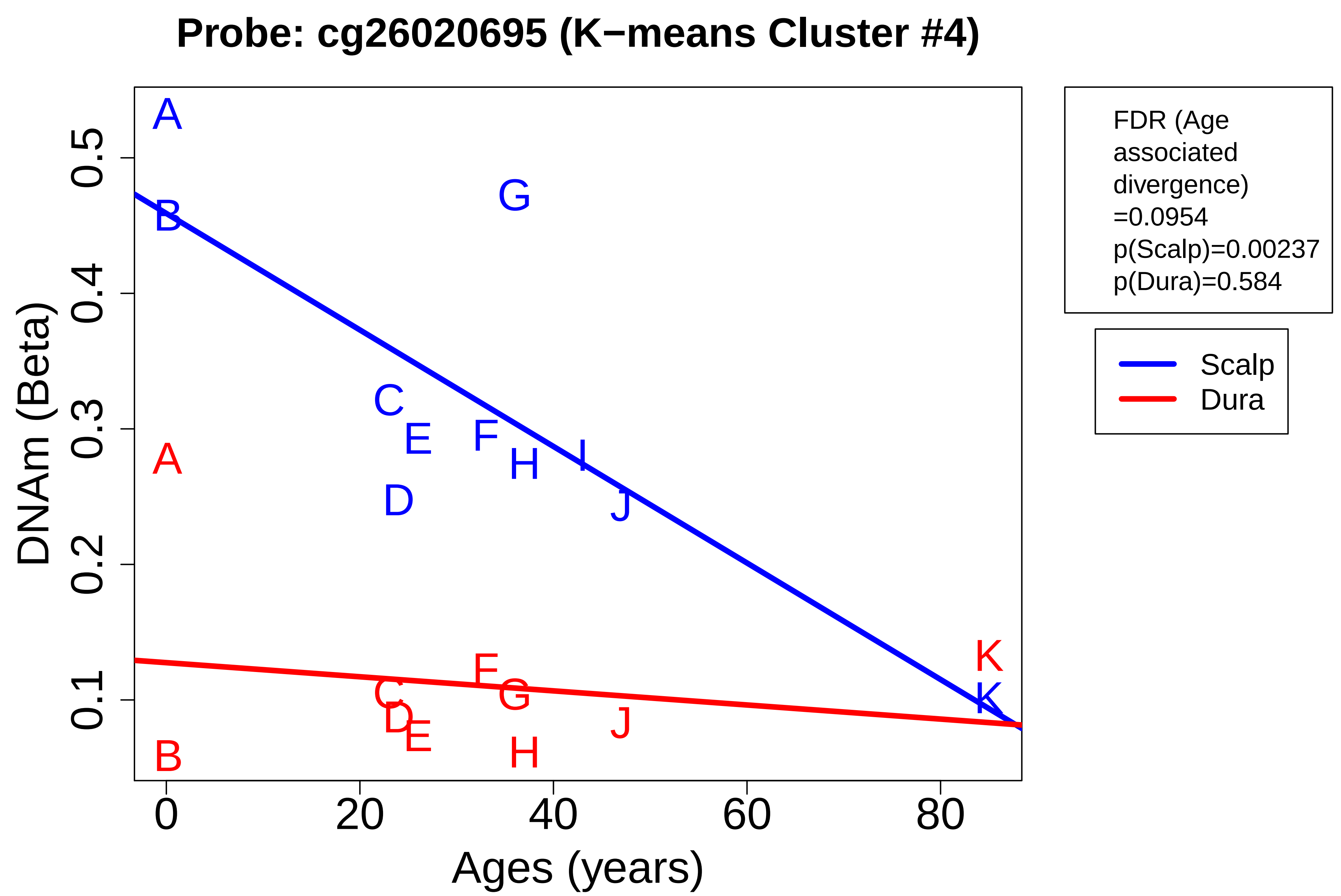

Probe: cg26020695 (K−means Cluster #4)
DNAm (Beta)
Ages (years)
0.1
0.2
0.3
0.4
0.5
0
20
40
60
80
A
B
C
D
E
F
G
H
I
J
K
FDR (Age associated divergence) =0.0954
p(Scalp)=0.00237
p(Dura)=0.584
Scalp
Dura

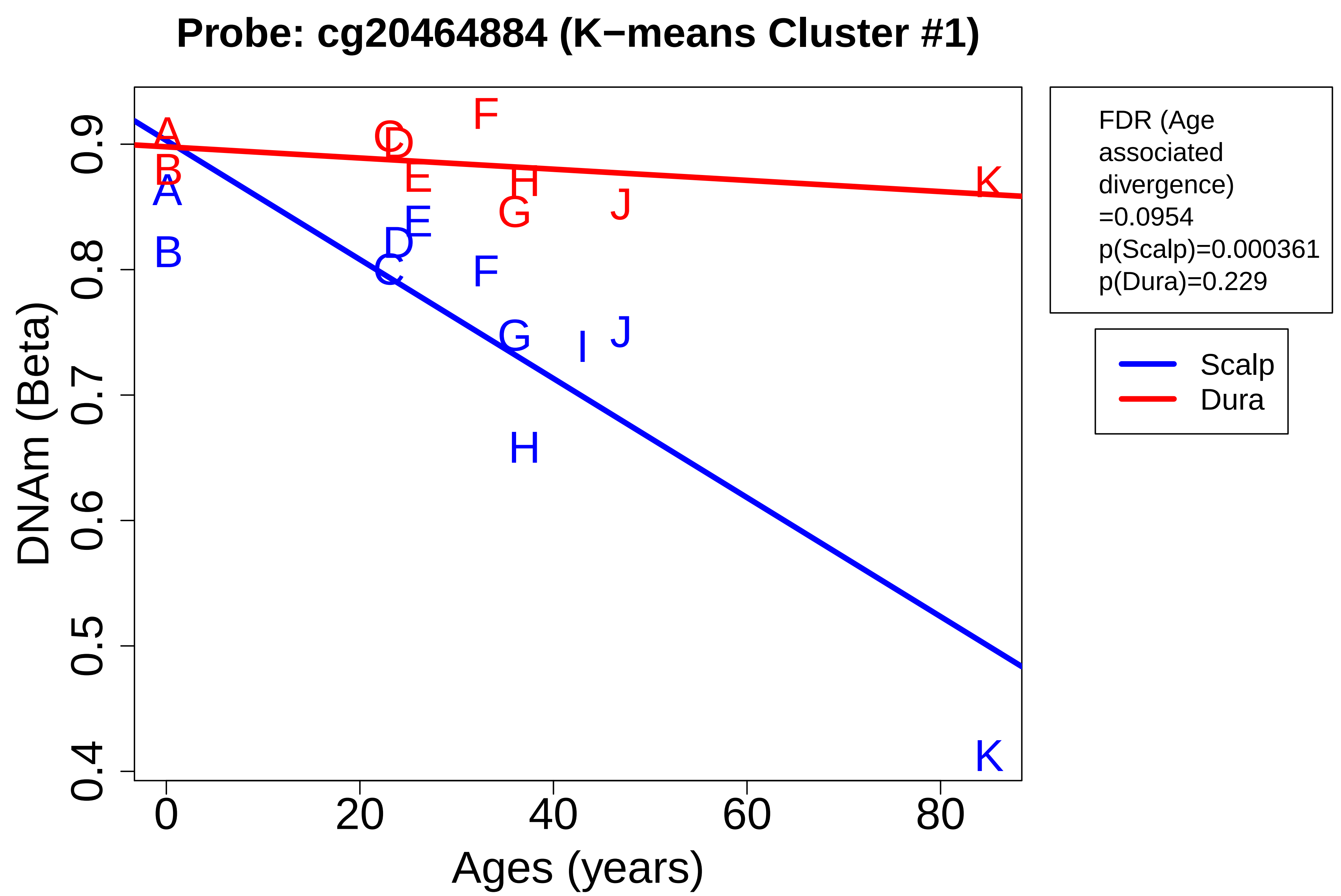

Probe: cg20464884 (K−means Cluster #1)
DNAm (Beta)
0.4
0.5
0.6
0.7
0.8
0.9
0
20
40
60
80
Ages (years)
FDR (Age associated divergence) =0.0954
p(Scalp)=0.000361
p(Dura)=0.229
Scalp
Dura

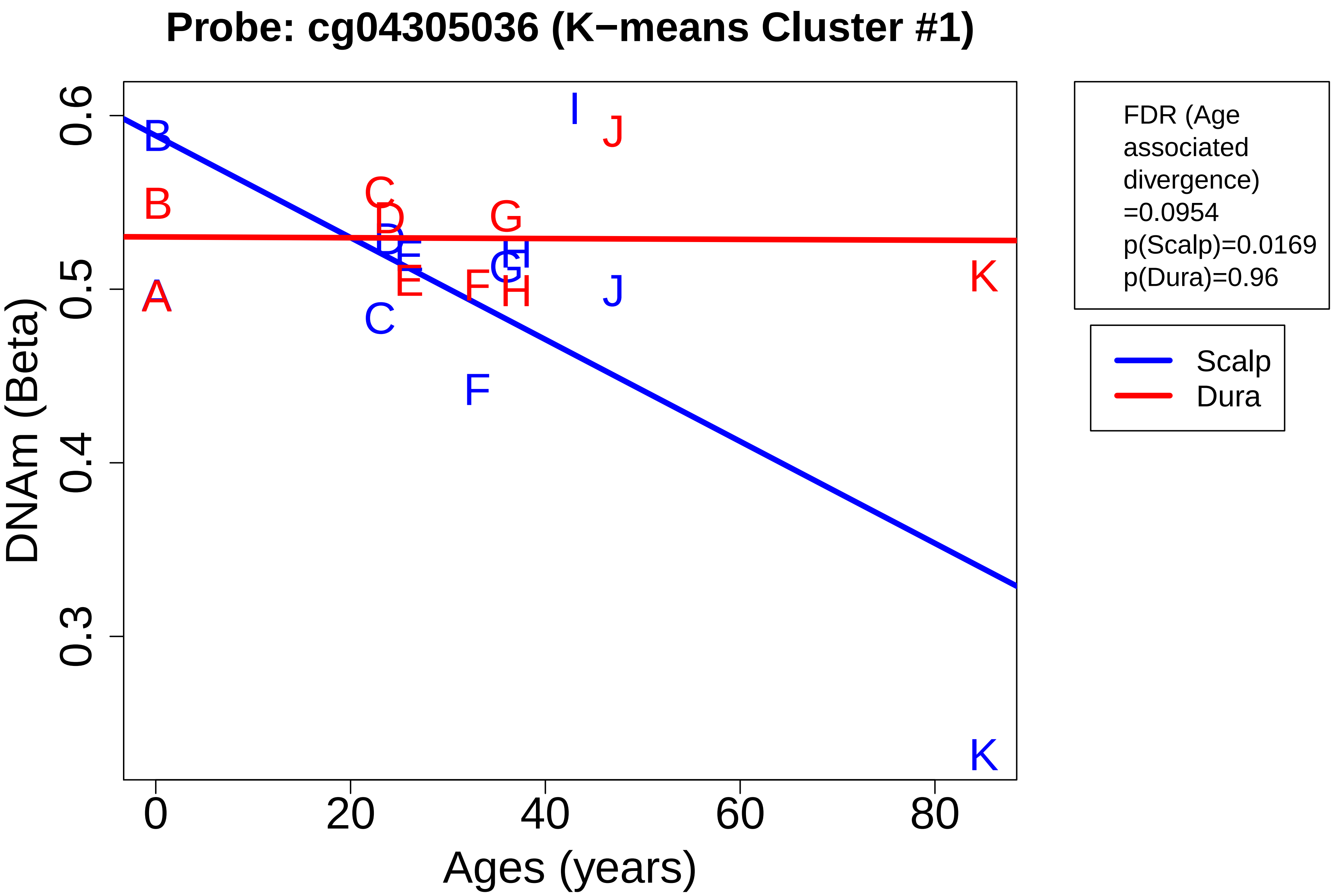

Probe: cg04305036 (K−means Cluster #1)
DNAm (Beta)
0.3
0.4
0.5
0.6
Ages (years)
0
20
40
60
80
FDR (Age associated divergence) =0.0954
p(Scalp)=0.0169
p(Dura)=0.96
Scalp
Dura

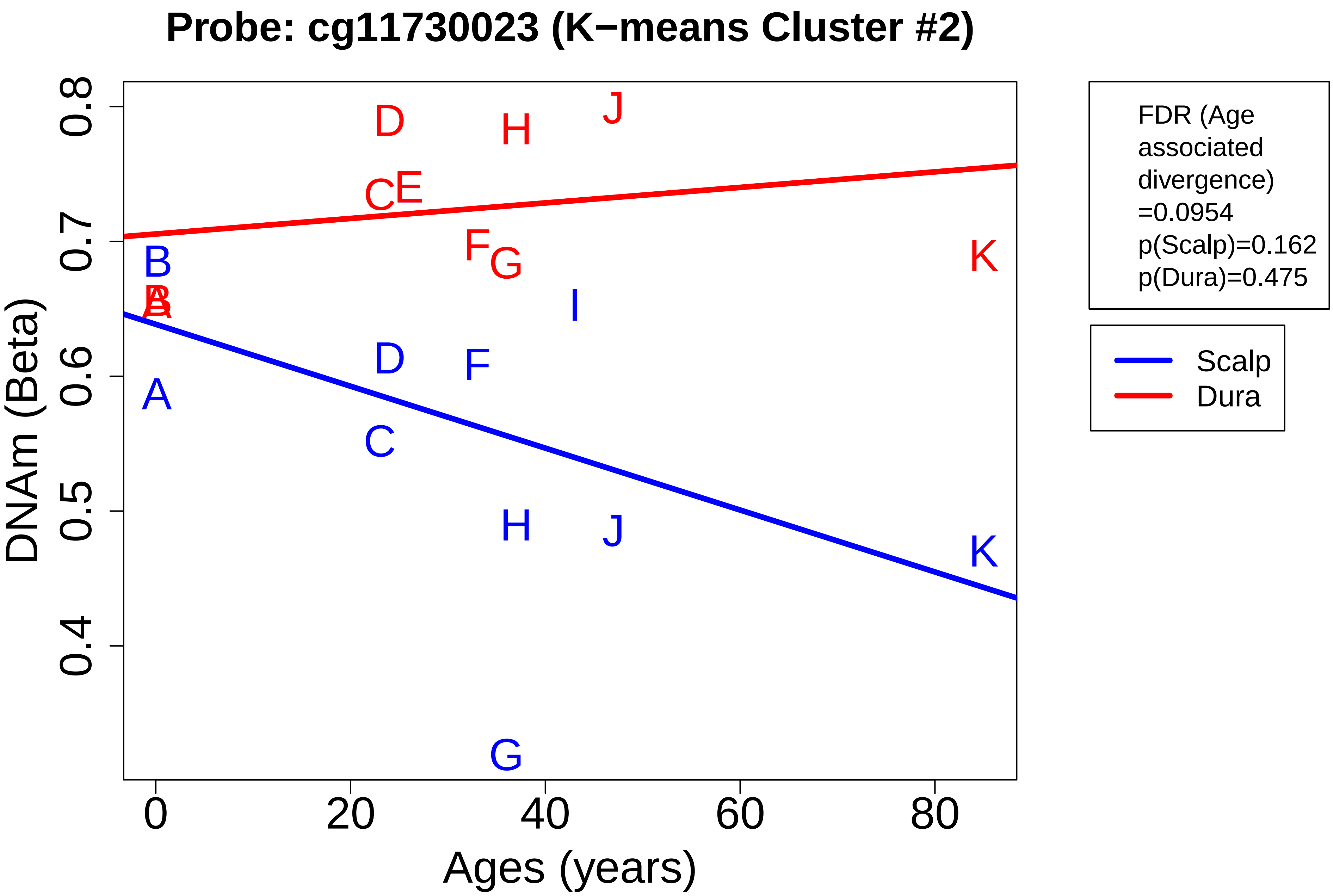

Probe: cg11730023 (K−means Cluster #2)
DNAm (Beta)
0.4
0.5
0.6
0.7
0.8
0
20
40
60
80
Ages (years)
FDR (Age associated divergence) =0.0954
p(Scalp)=0.162
p(Dura)=0.475
Scalp
Dura

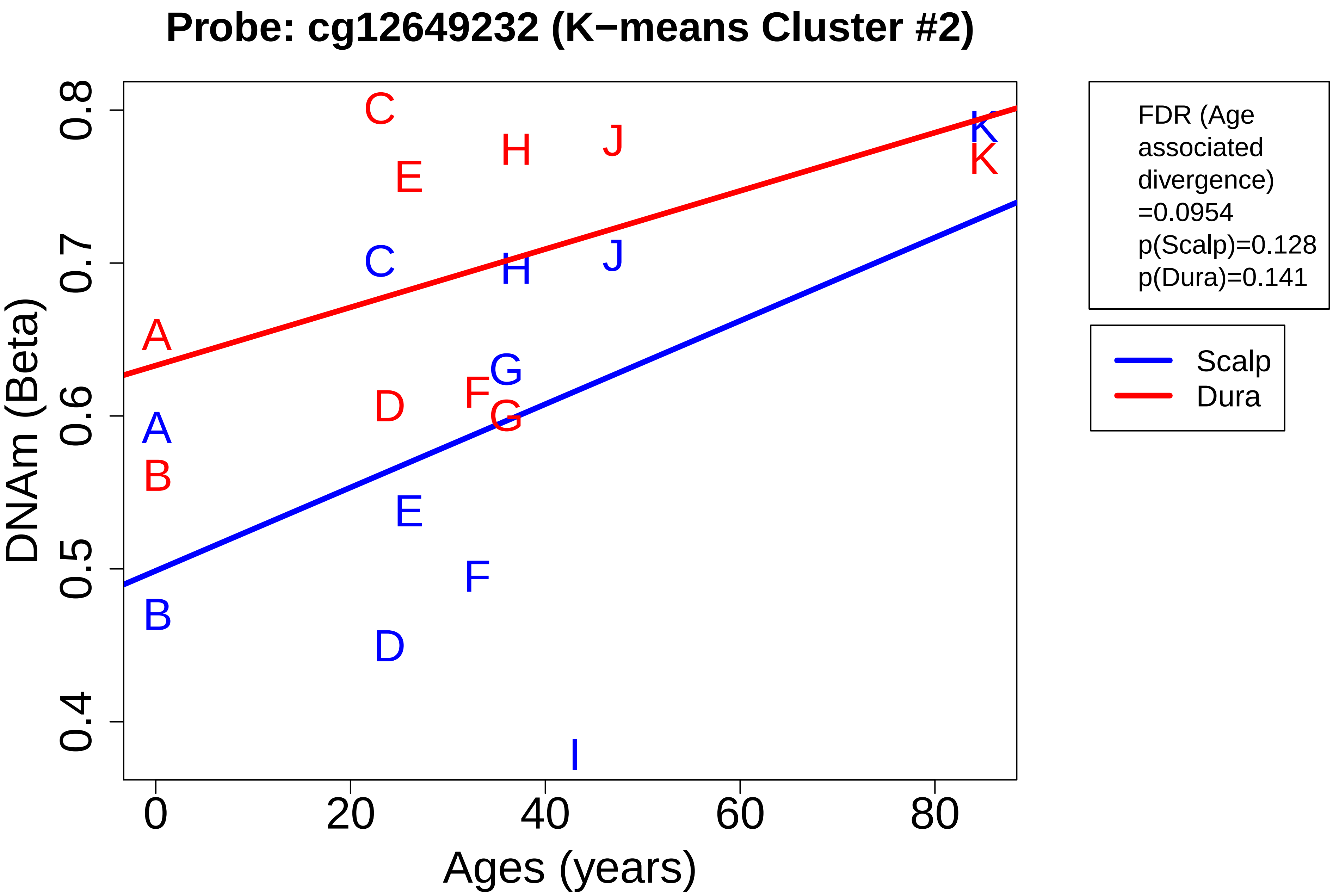

Probe: cg12649232 (K−means Cluster #2)
DNAm (Beta)
Ages (years)
0.4
0.5
0.6
0.7
0.8
0
20
40
60
80
FDR (Age associated divergence) =0.0954
p(Scalp)=0.128
p(Dura)=0.141
Scalp
Dura

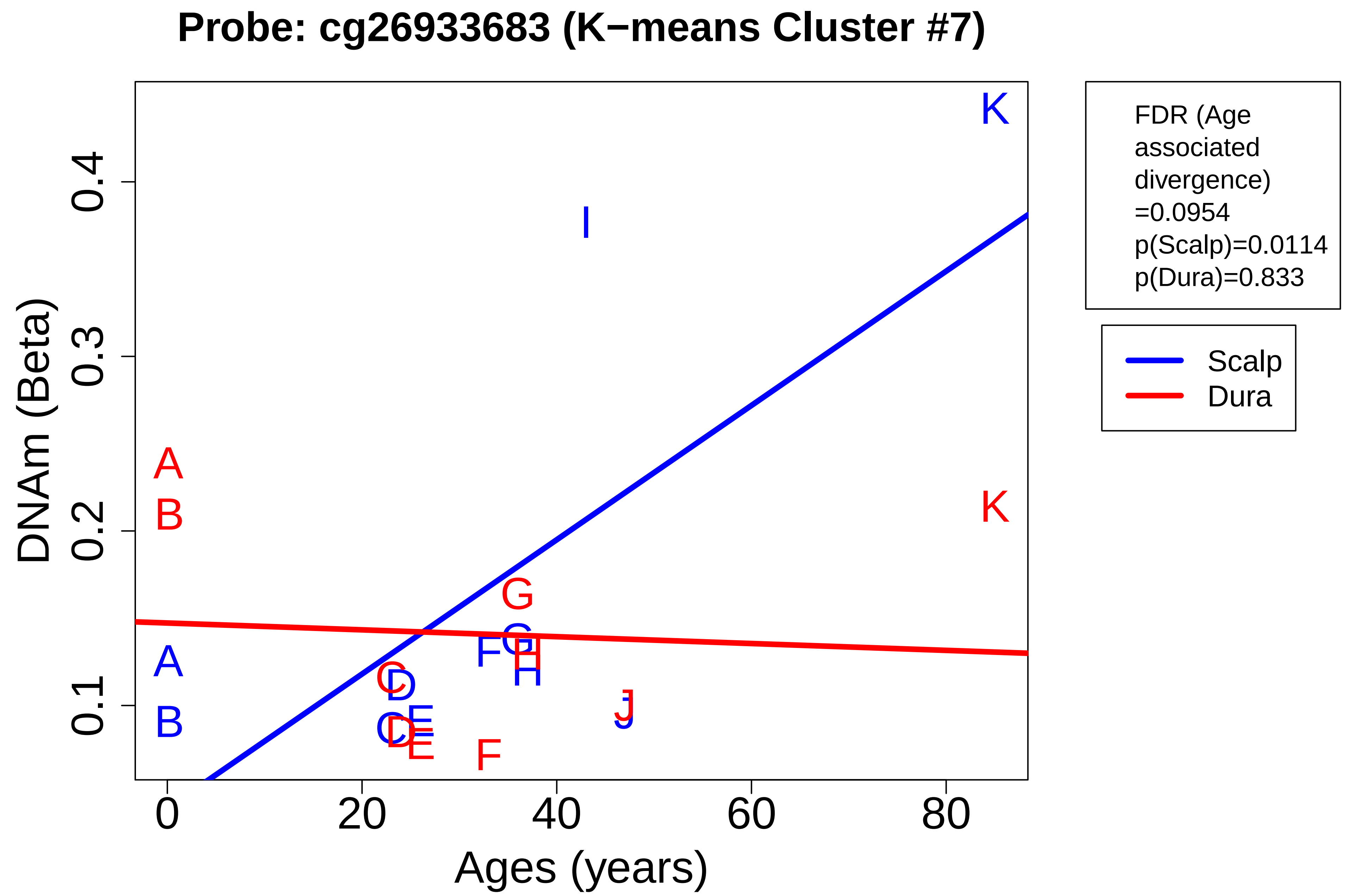

Probe: cg26933683 (K−means Cluster #7)
FDR (Age associated divergence) =0.0954
p(Scalp)=0.0114
p(Dura)=0.833
Scalp
Dura
DNAm (Beta)
0.1
0.2
0.3
0.4
Ages (years)
0
20
40
60
80

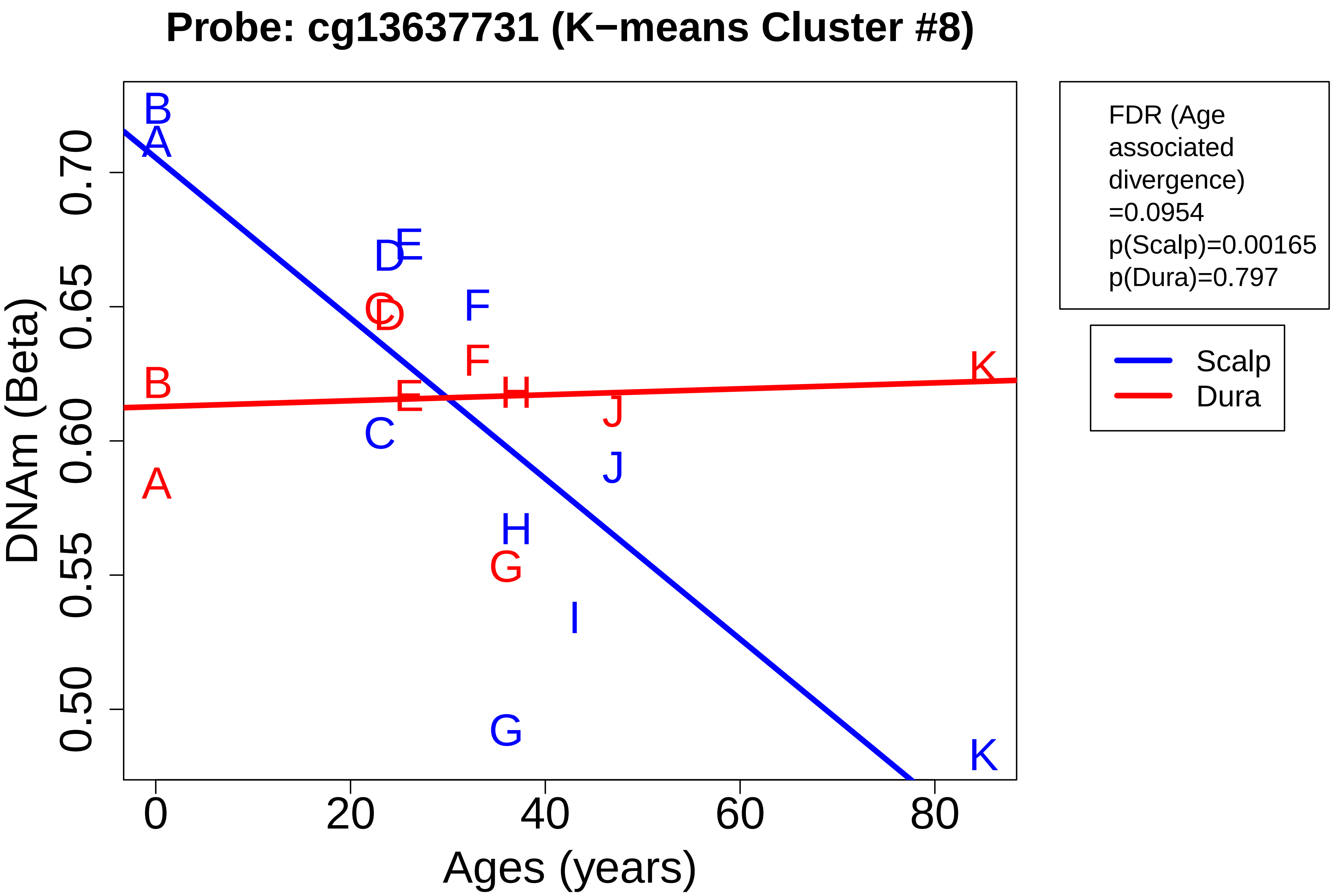

Probe: cg13637731 (K−means Cluster #8)
DNAm (Beta)
0.50
0.55
0.60
0.65
0.70
Ages (years)
0
20
40
60
80
FDR (Age associated divergence) =0.0954
p(Scalp)=0.00165
p(Dura)=0.797
Scalp
Dura

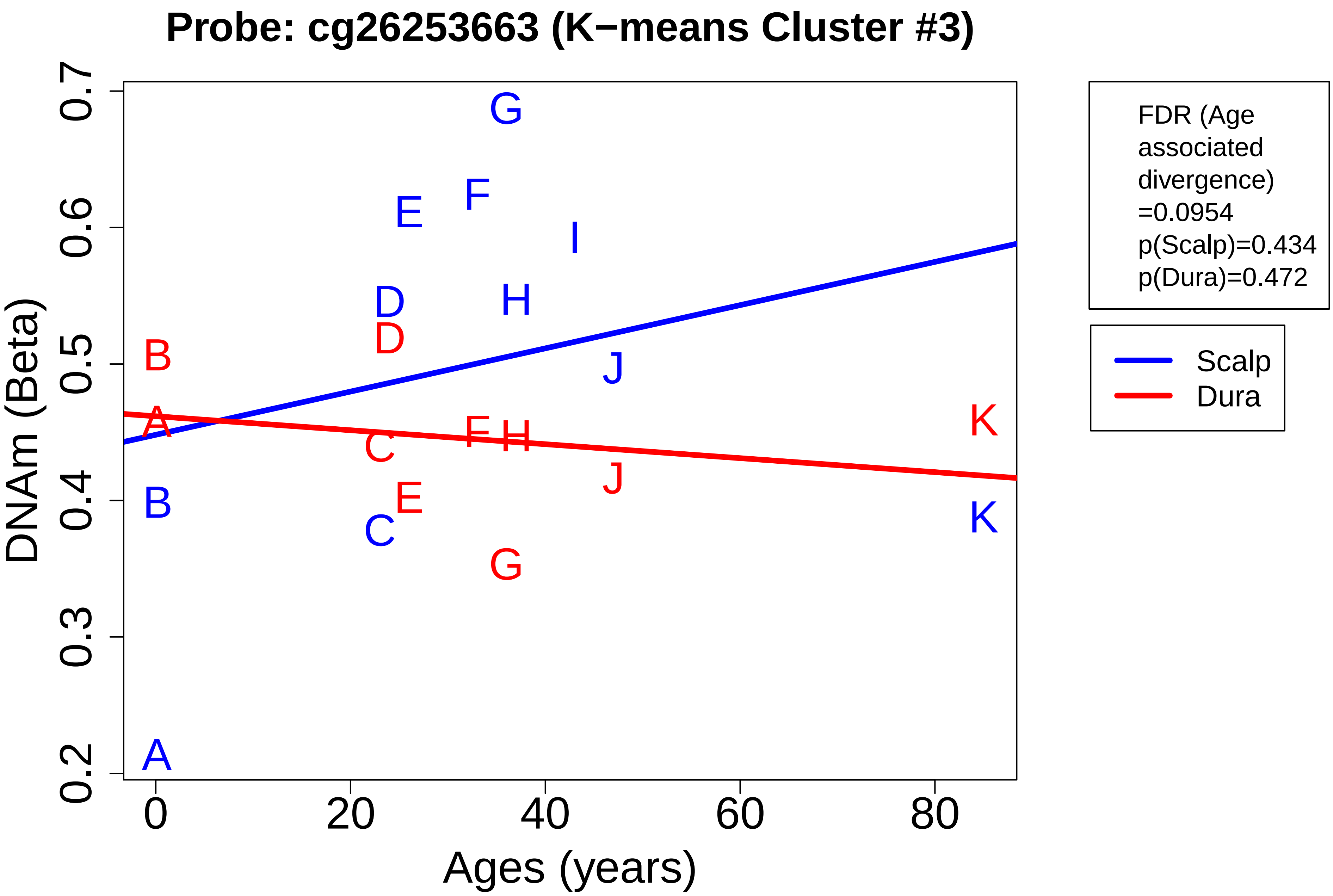

Probe: cg26253663 (K−means Cluster #3)
DNAm (Beta)
0.2
0.3
0.4
0.5
0.6
0.7
Ages (years)
0
20
40
60
80
FDR (Age associated divergence) =0.0954
p(Scalp)=0.434
p(Dura)=0.472
Scalp
Dura
A
B
C
D
E
F
G
H
I
J
K

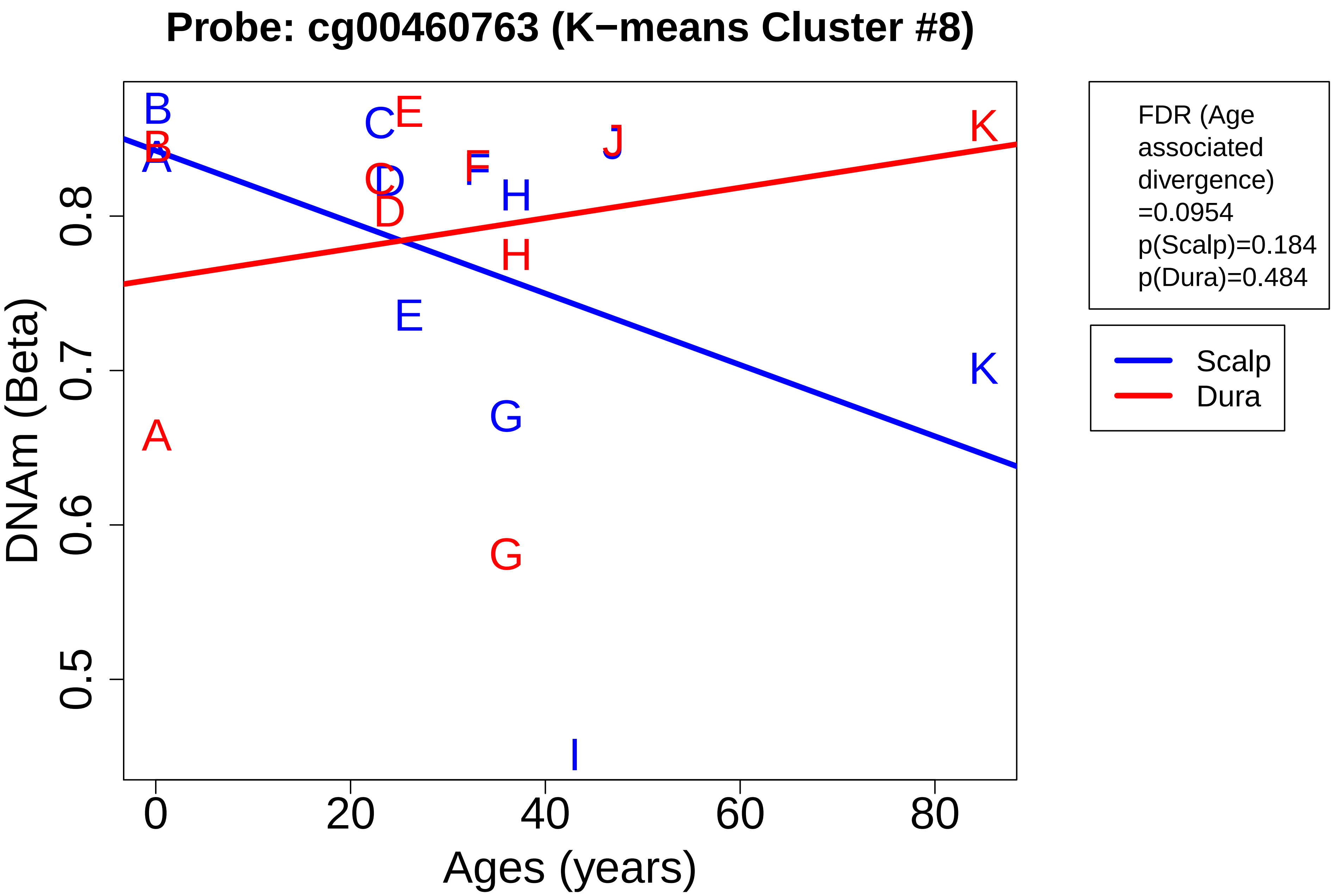

Probe: cg00460763 (K−means Cluster #8)
DNAm (Beta)
0.5
0.6
0.7
0.8
Ages (years)
0
20
40
60
80
B
A
B
A
C
E
C
D
D
F
F
H
H
J
J
K
K
E
G
G
I
FDR (Age associated divergence) =0.0954
p(Scalp)=0.184
p(Dura)=0.484
Scalp
Dura

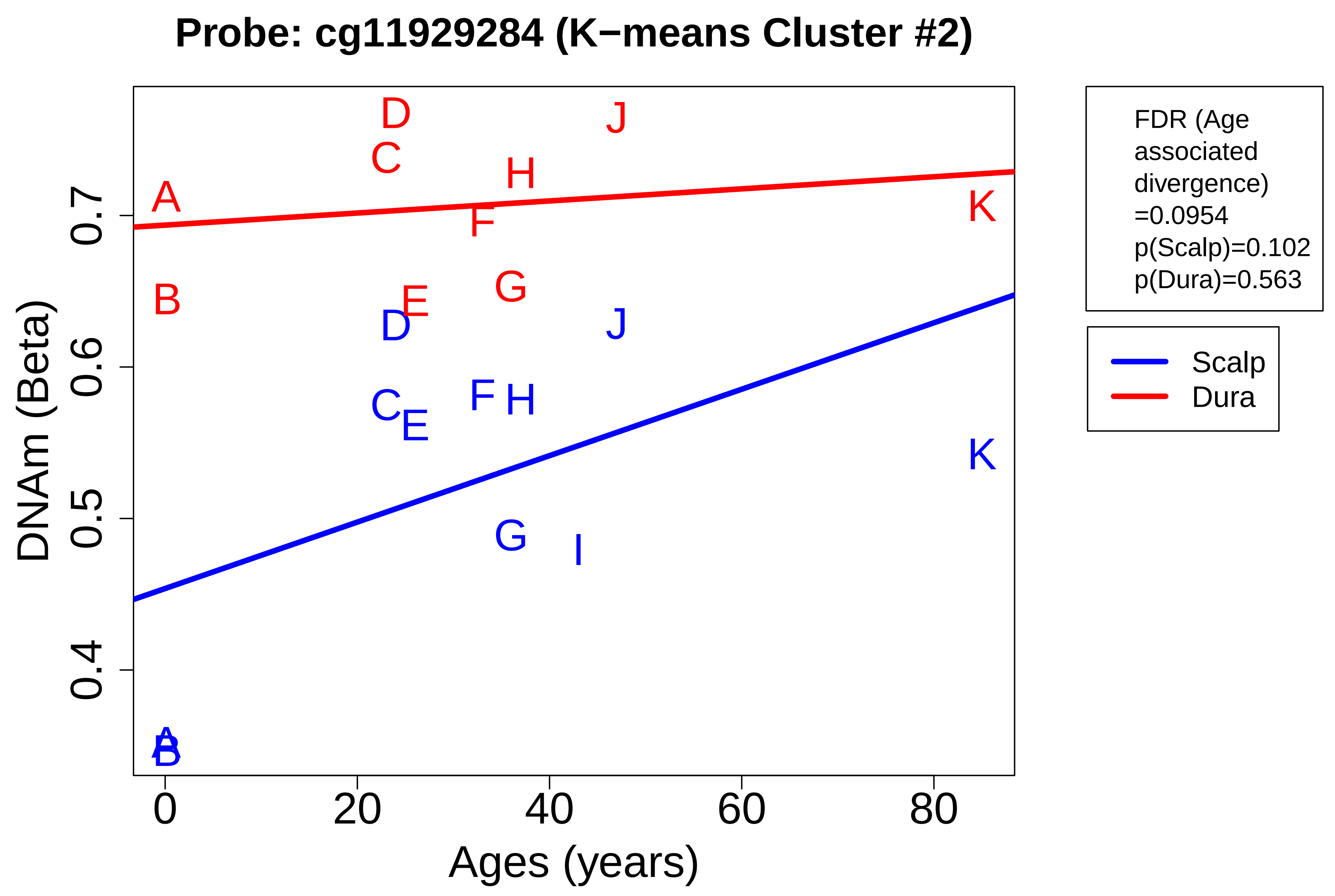

Probe: cg11929284 (K−means Cluster #2)
DNAm (Beta)
0.4
0.5
0.6
0.7
0
20
40
60
80
Ages (years)
FDR (Age associated divergence) =0.0954
p(Scalp)=0.102
p(Dura)=0.563
Scalp
Dura

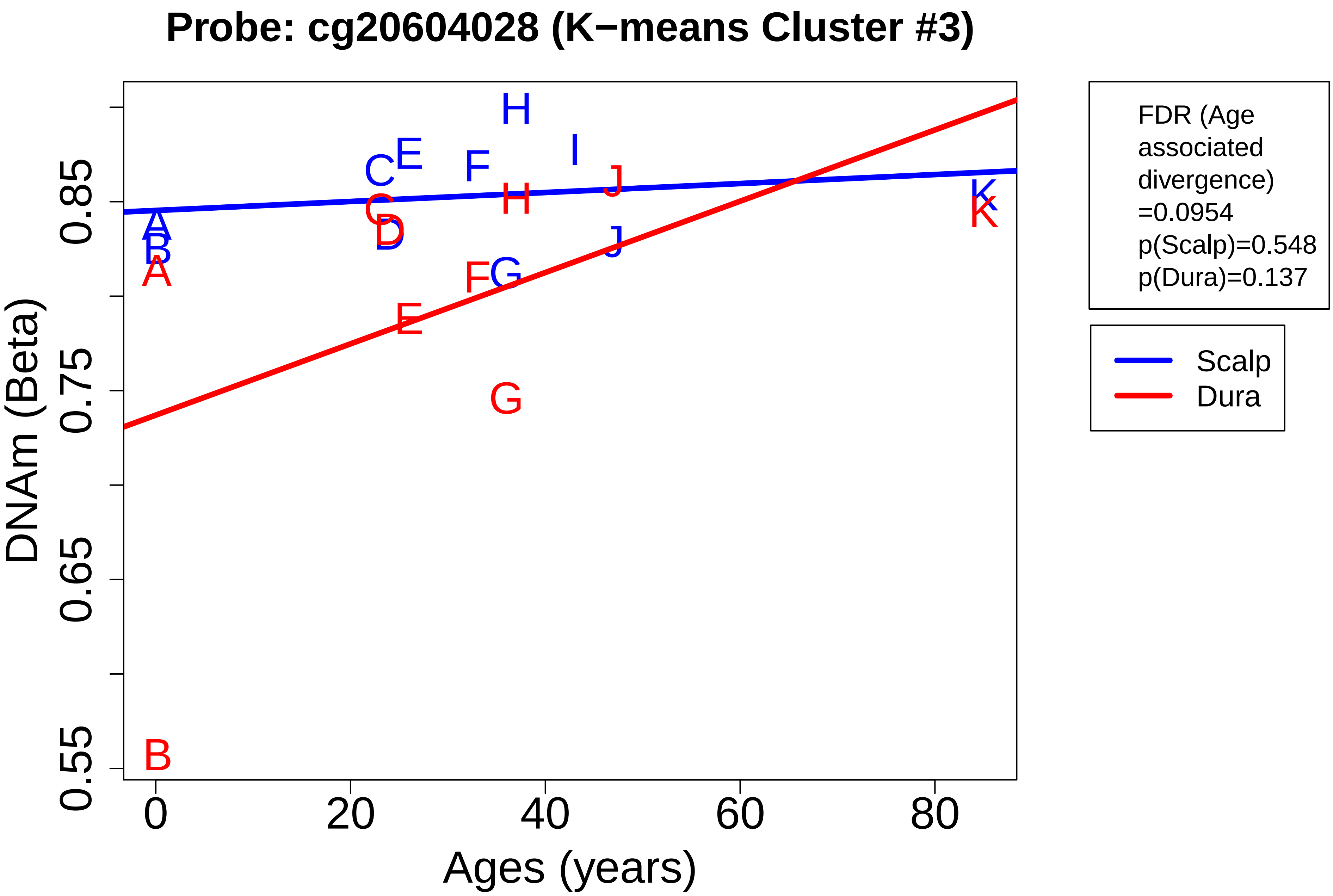

Probe: cg20604028 (K−means Cluster #3)
DNAm (Beta)
0.55
0.65
0.75
0.85
0
20
40
60
80
Ages (years)
FDR (Age associated divergence) =0.0954
p(Scalp)=0.548
p(Dura)=0.137
Scalp
Dura

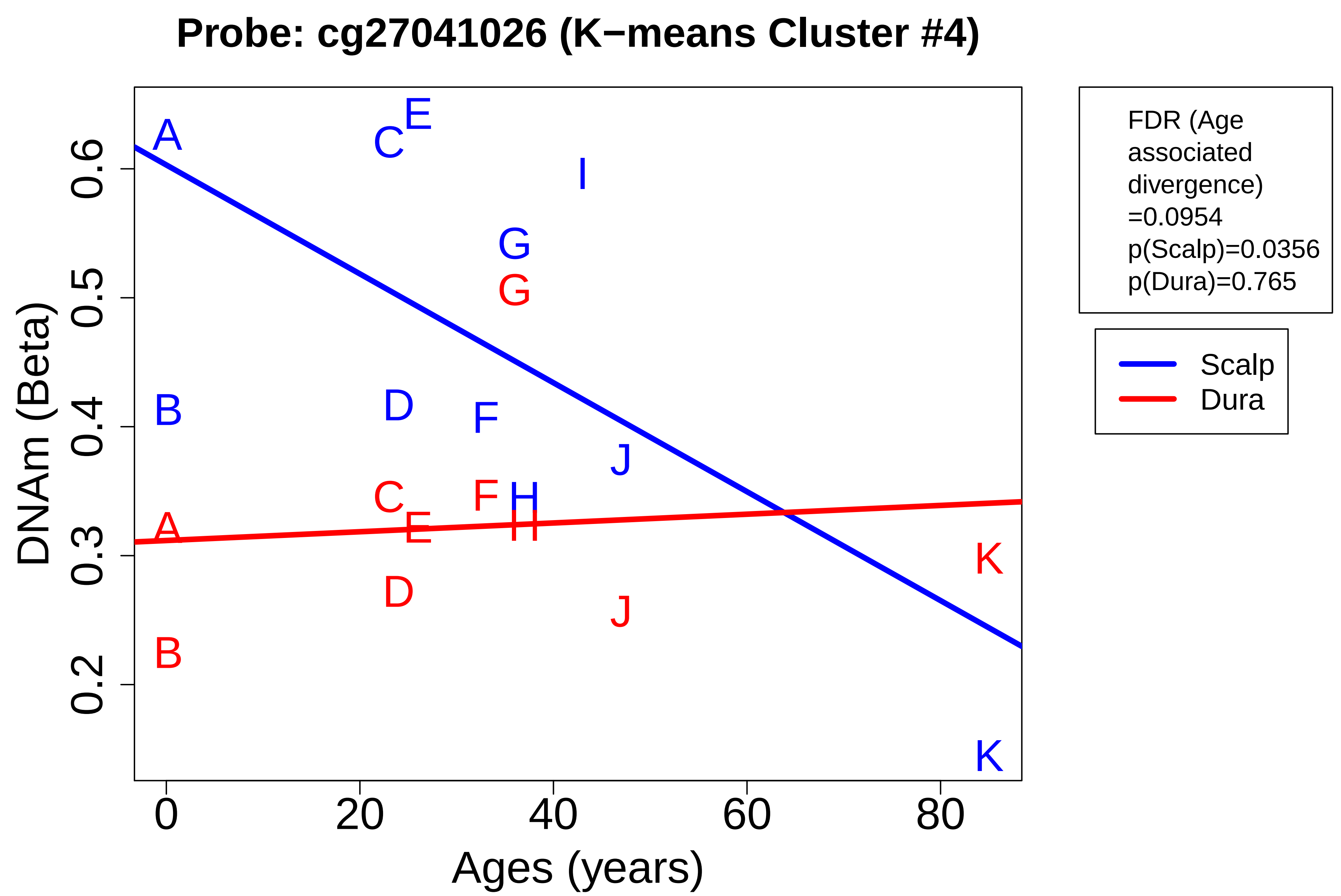
Probe: cg27041026 (K−means Cluster #4)
DNAm (Beta)
0.2
0.3
0.4
0.5
0.6
Ages (years)
0
20
40
60
80
FDR (Age associated divergence) =0.0954
p(Scalp)=0.0356
p(Dura)=0.765
Scalp
Dura
A
B
C
D
E
F
G
H
I
J
K

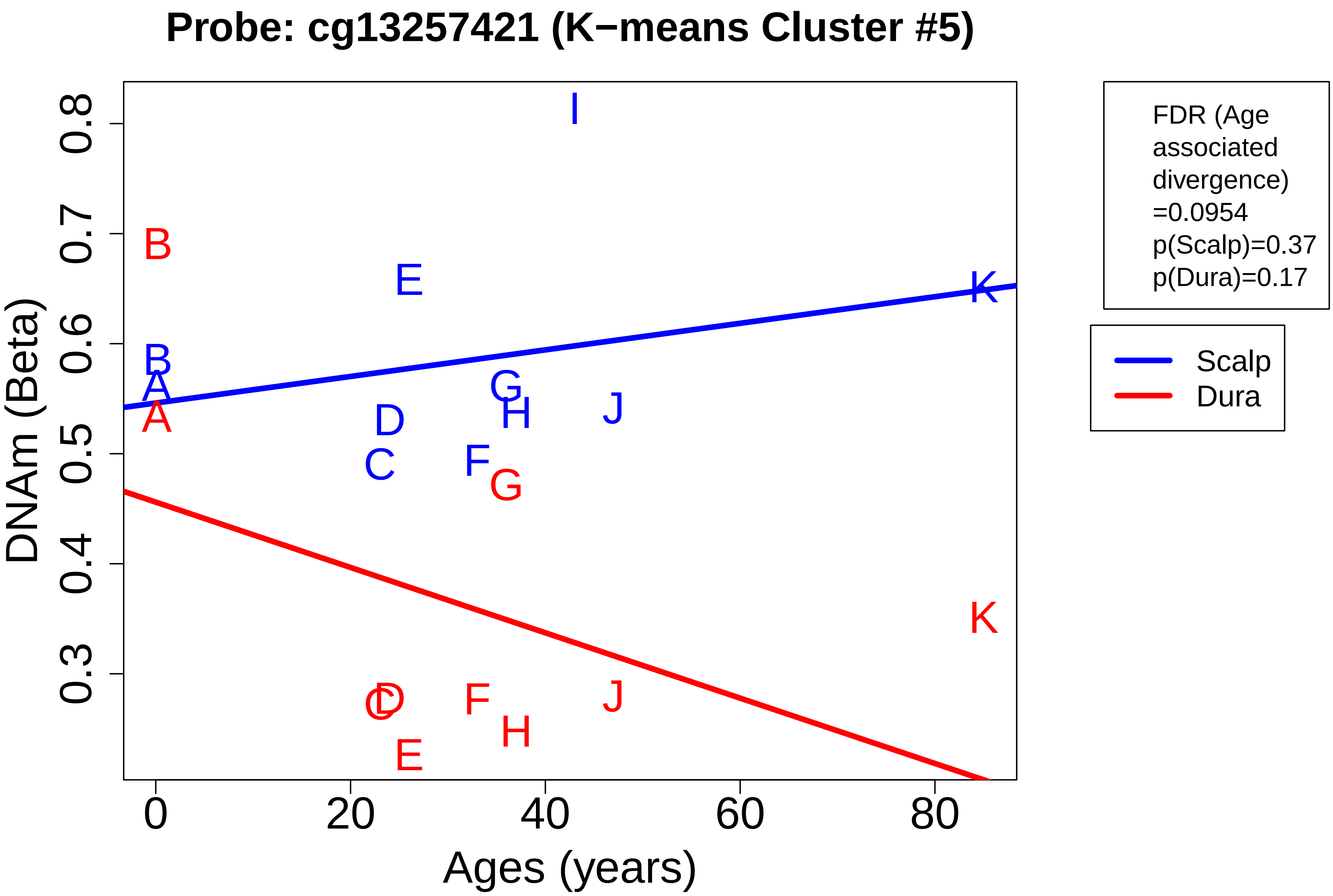

Probe: cg13257421 (K−means Cluster #5)
DNAm (Beta)
0.3
0.4
0.5
0.6
0.7
0.8
0
20
40
60
80
Ages (years)
FDR (Age associated divergence) =0.0954
p(Scalp)=0.37
p(Dura)=0.17
Scalp
Dura

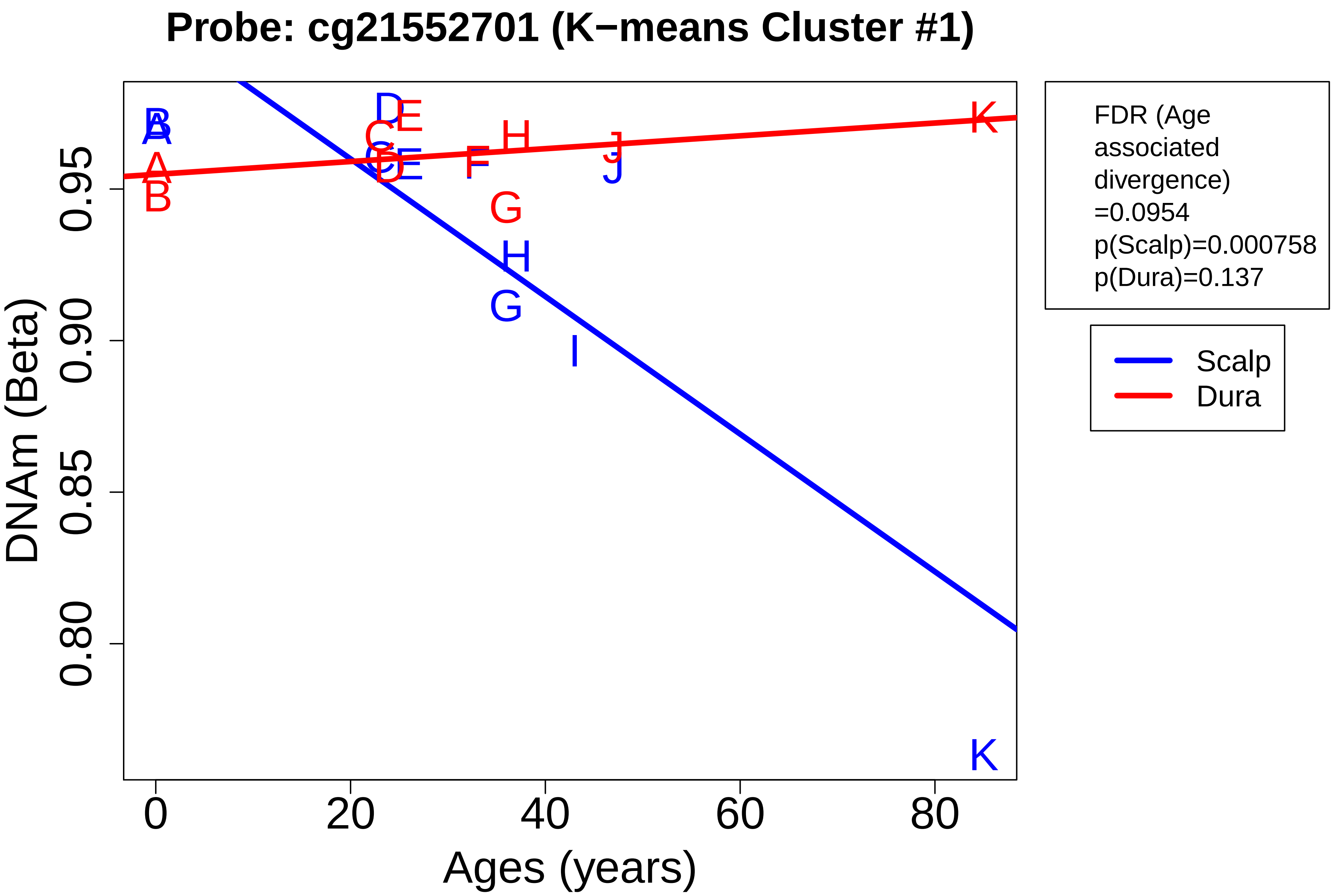
Probe: cg21552701 (K−means Cluster #1)
DNAm (Beta)
0.80
0.85
0.90
0.95
0
20
40
60
80
Ages (years)
FDR (Age associated divergence) =0.0954
p(Scalp)=0.000758
p(Dura)=0.137
Scalp
Dura

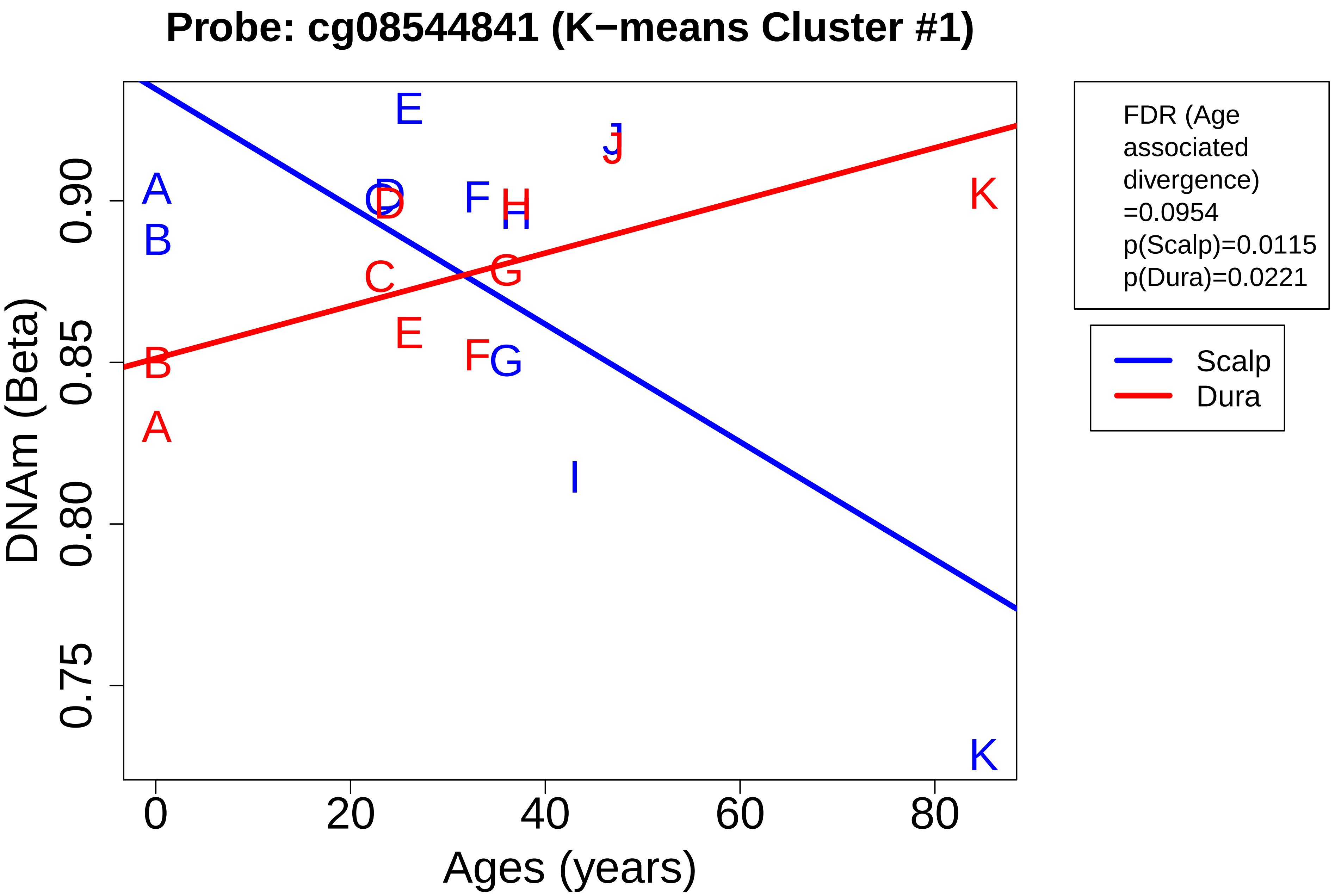

Probe: cg08544841 (K−means Cluster #1)
DNAm (Beta)
0.75
0.80
0.85
0.90
0
20
40
60
80
Ages (years)
A
B
C
D
E
F
G
H
I
J
K
FDR (Age associated divergence) =0.0954
p(Scalp)=0.0115
p(Dura)=0.0221
Scalp
Dura

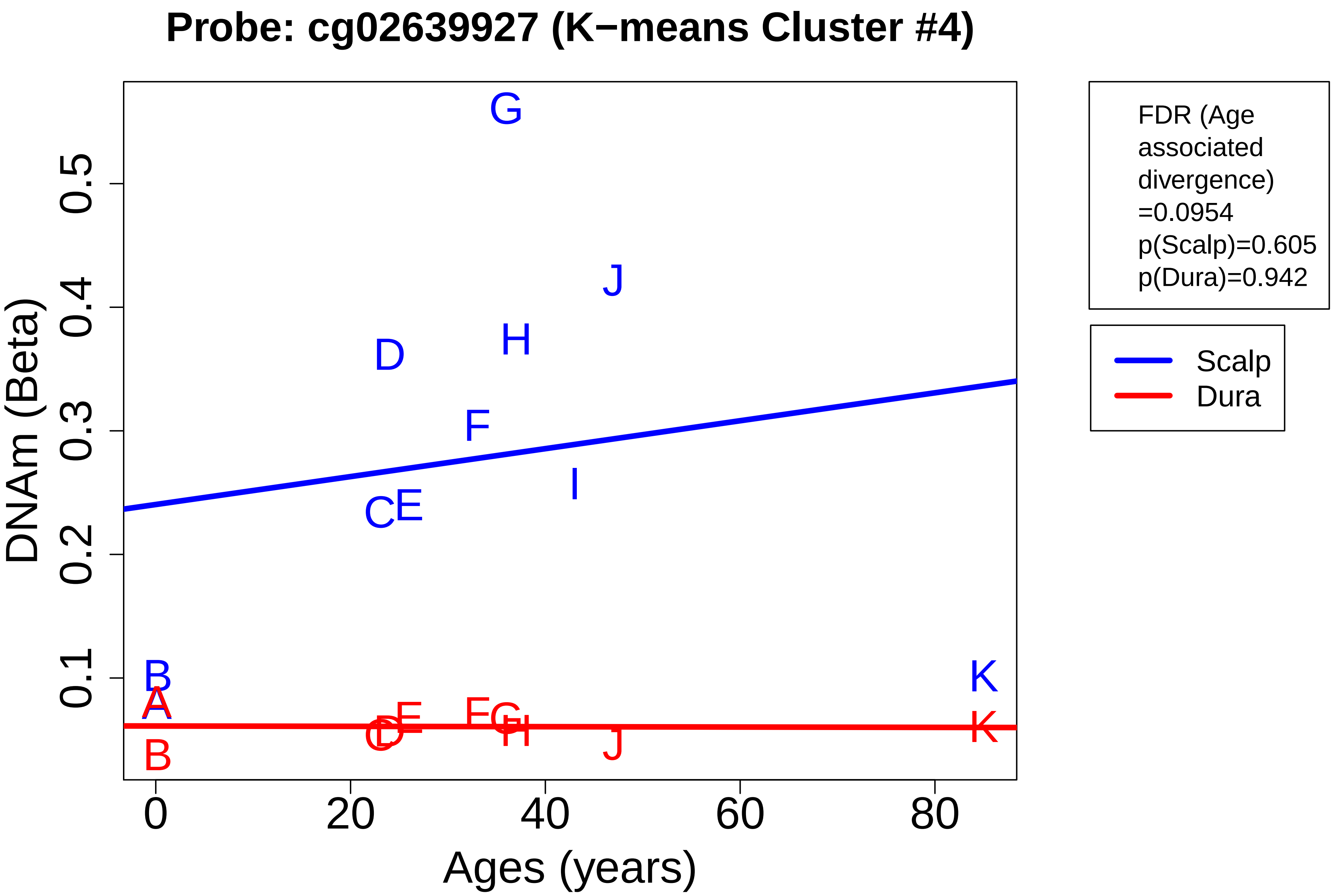
Probe: cg02639927 (K−means Cluster #4)
DNAm (Beta)
0.1
0.2
0.3
0.4
0.5
Ages (years)
0
20
40
60
80
G
J
H
D
F
I
CE
B
A
K
E
FG
H
DC
J
B
K
FDR (Age associated divergence) =0.0954
p(Scalp)=0.605
p(Dura)=0.942
Scalp
Dura

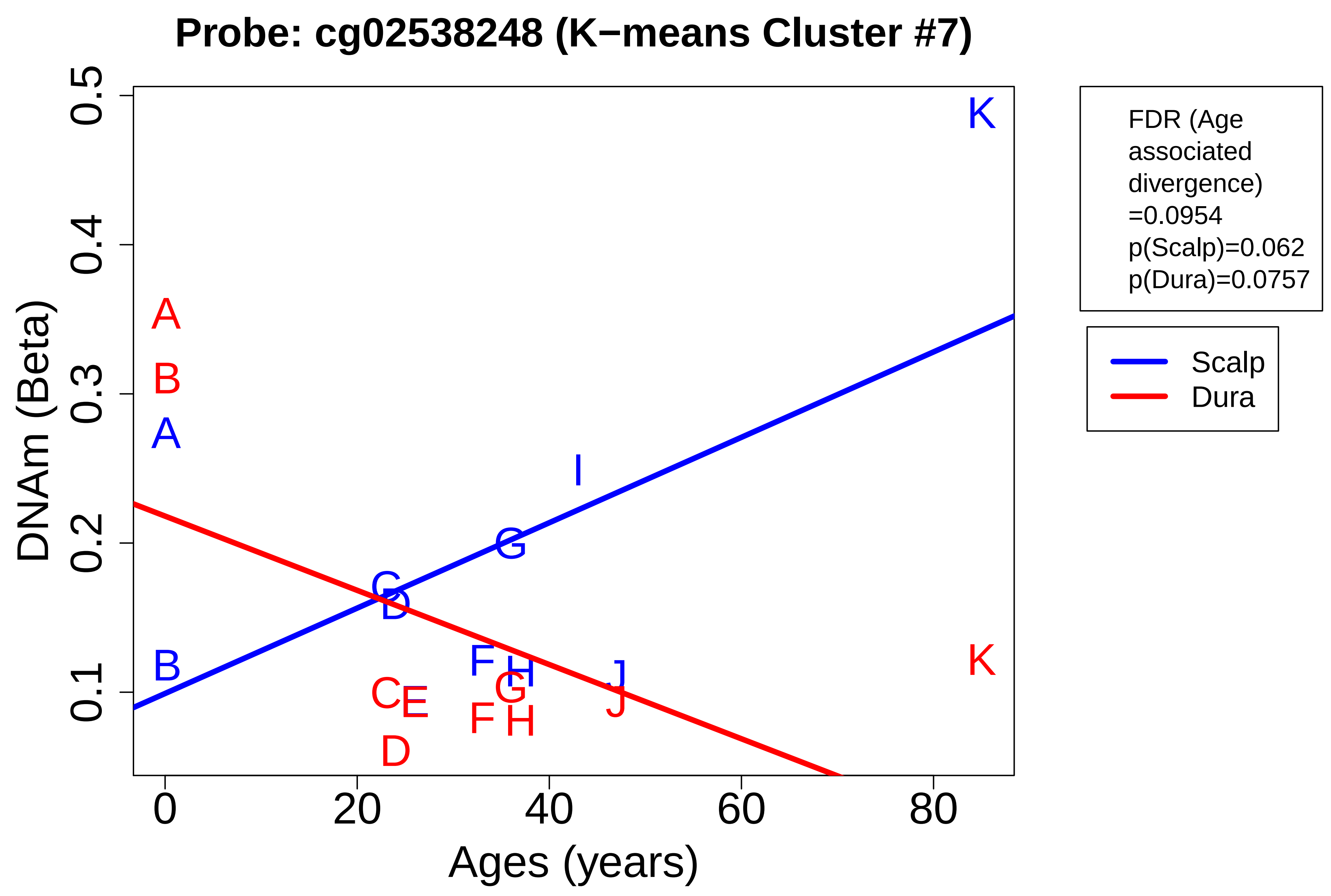

Probe: cg02538248 (K−means Cluster #7)
DNAm (Beta)
0.1
0.2
0.3
0.4
0.5
0
20
40
60
80
Ages (years)
A
B
C
D
E
F
G
H
I
J
K
FDR (Age associated divergence) =0.0954
p(Scalp)=0.062
p(Dura)=0.0757
Scalp
Dura

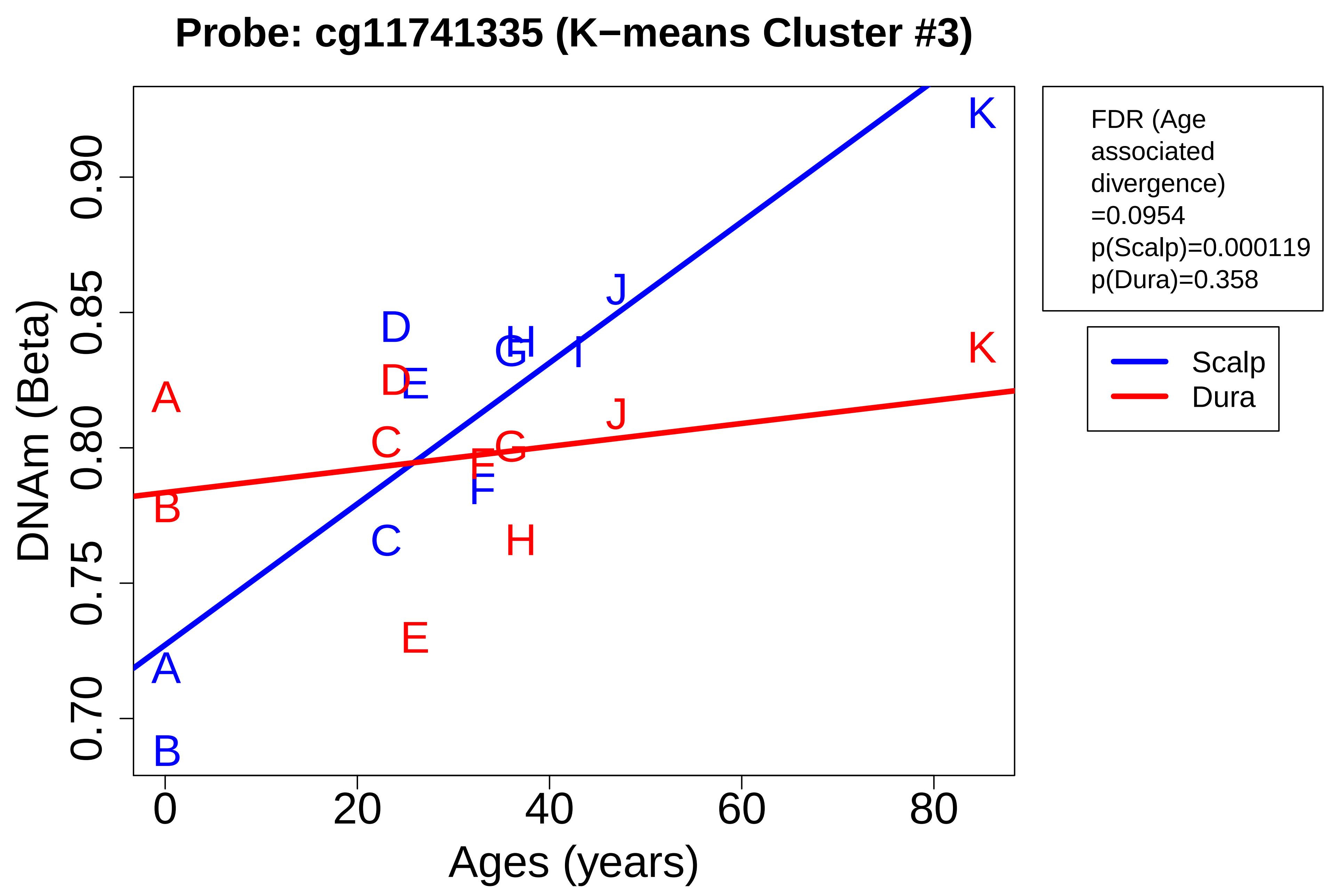

Probe: cg11741335 (K−means Cluster #3)
DNAm (Beta)
0.70
0.75
0.80
0.85
0.90
Ages (years)
0
20
40
60
80
FDR (Age associated divergence) =0.0954
p(Scalp)=0.000119
p(Dura)=0.358
Scalp
Dura

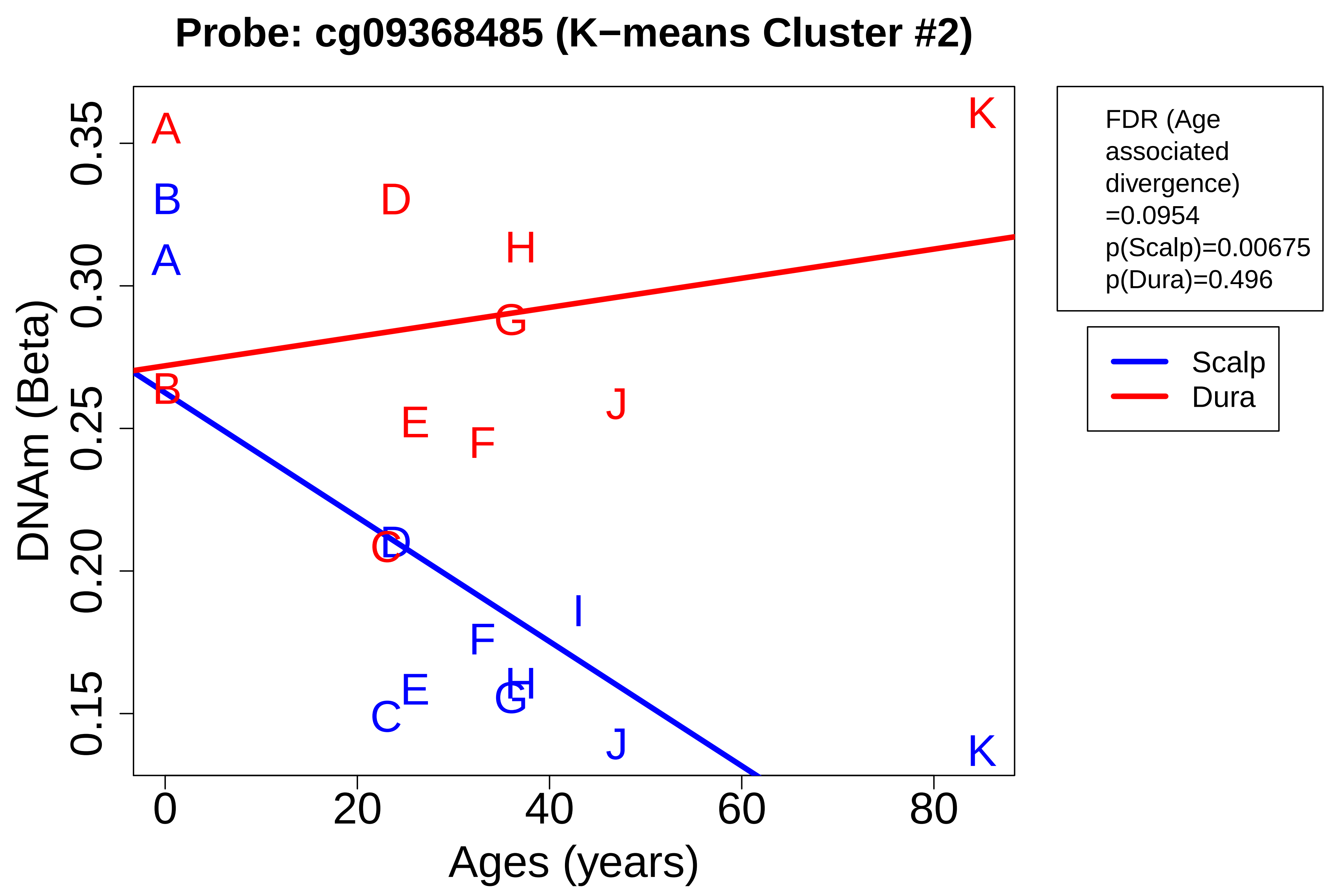
Probe: cg09368485 (K−means Cluster #2)
DNAm (Beta)
0.15
0.20
0.25
0.30
0.35
0
20
40
60
80
Ages (years)
A
B
C
D
E
F
G
H
I
J
K
FDR (Age associated divergence) =0.0954
p(Scalp)=0.00675
p(Dura)=0.496
Scalp
Dura

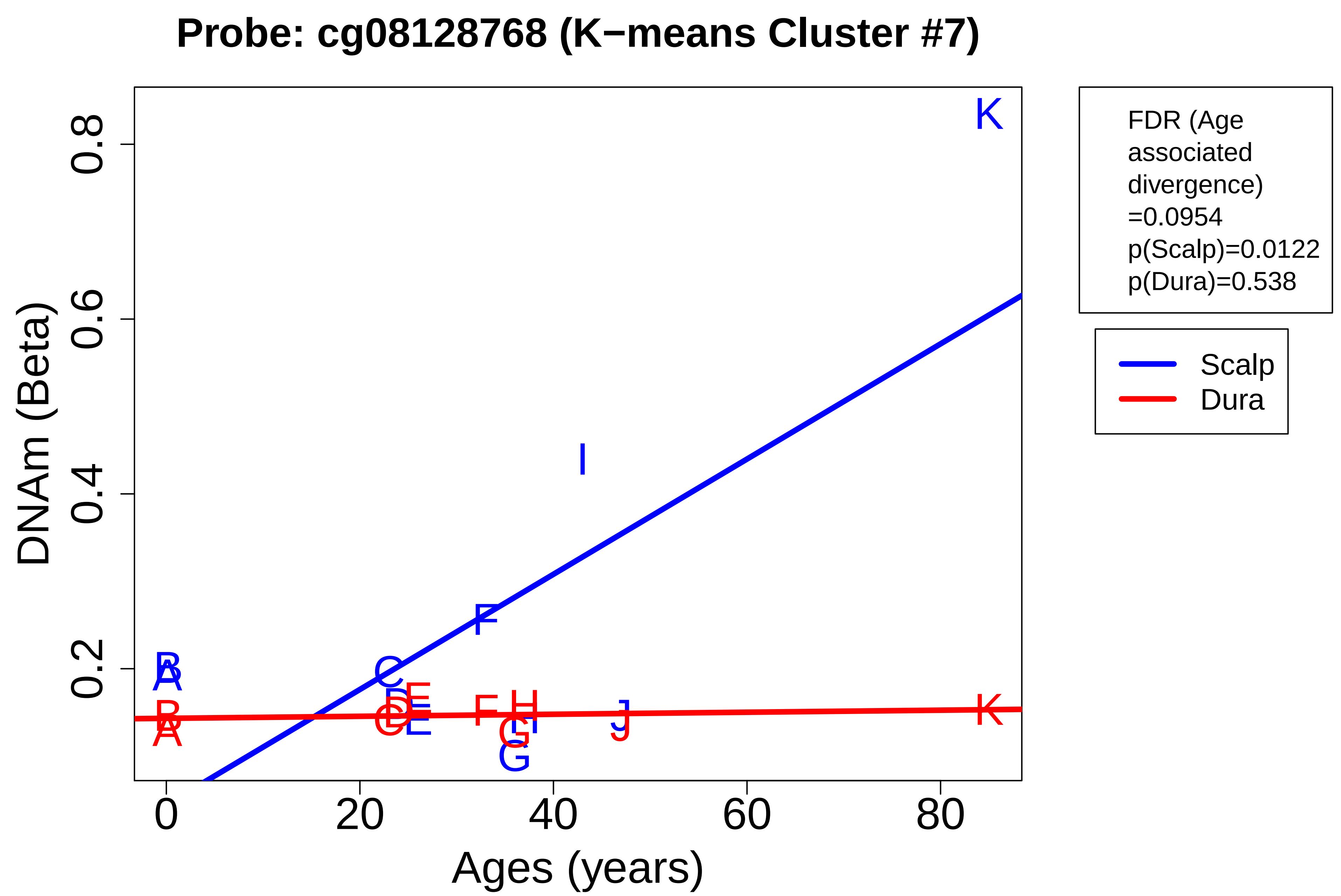

Probe: cg08128768 (K−means Cluster #7)
DNAm (Beta)
0.2
0.4
0.6
0.8
0
20
40
60
80
Ages (years)
A
B
C
D
E
F
G
H
I
J
K
FDR (Age associated divergence) =0.0954
p(Scalp)=0.0122
p(Dura)=0.538
Scalp
Dura

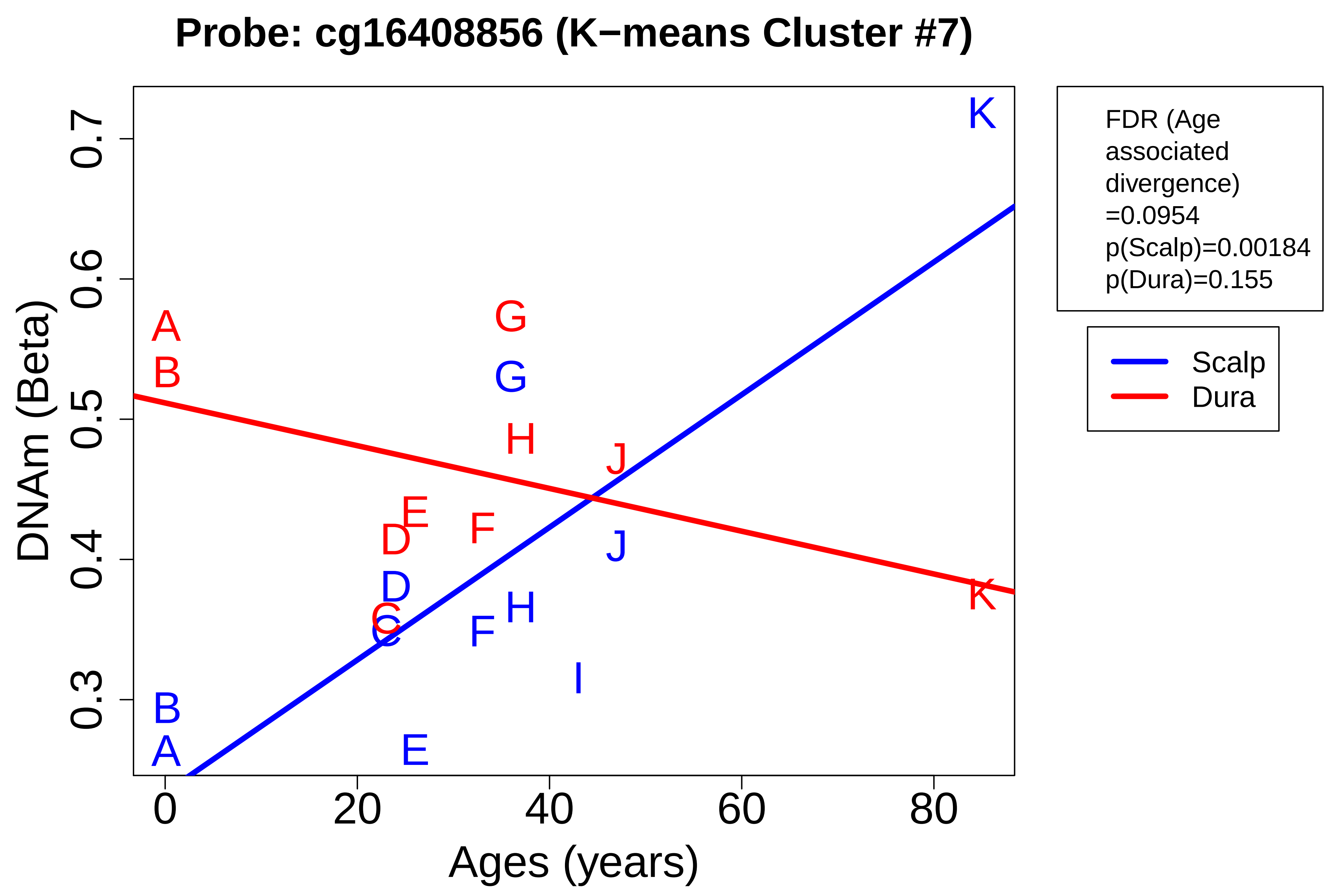

Probe: cg16408856 (K−means Cluster #7)
DNAm (Beta)
0.3
0.4
0.5
0.6
0.7
Ages (years)
0
20
40
60
80
A
B
C
D
E
F
G
H
I
J
K
FDR (Age associated divergence) =0.0954
p(Scalp)=0.00184
p(Dura)=0.155
Scalp
Dura

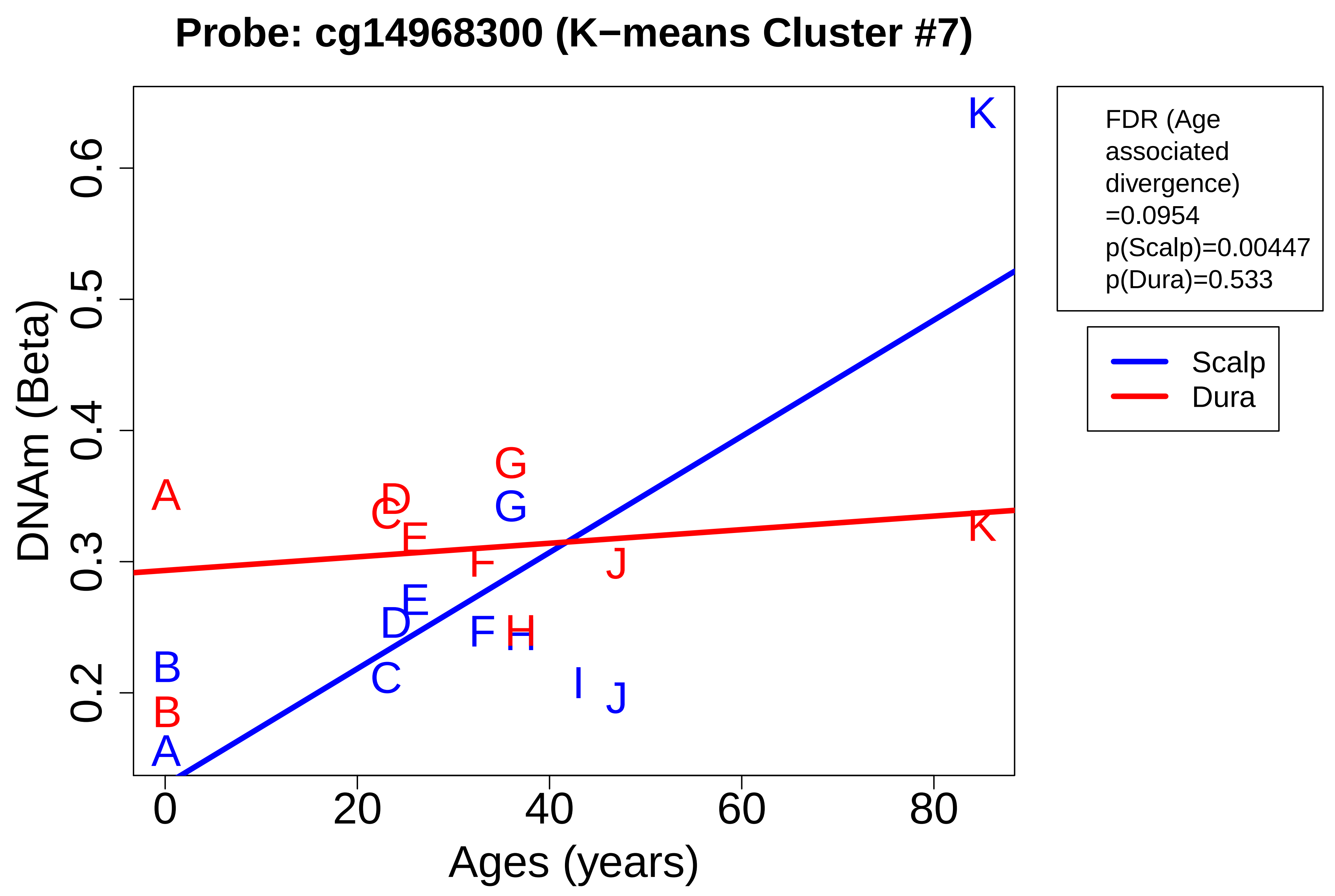
Probe: cg14968300 (K−means Cluster #7)
DNAm (Beta)
Ages (years)
FDR (Age associated divergence) =0.0954
p(Scalp)=0.00447
p(Dura)=0.533
Scalp
Dura

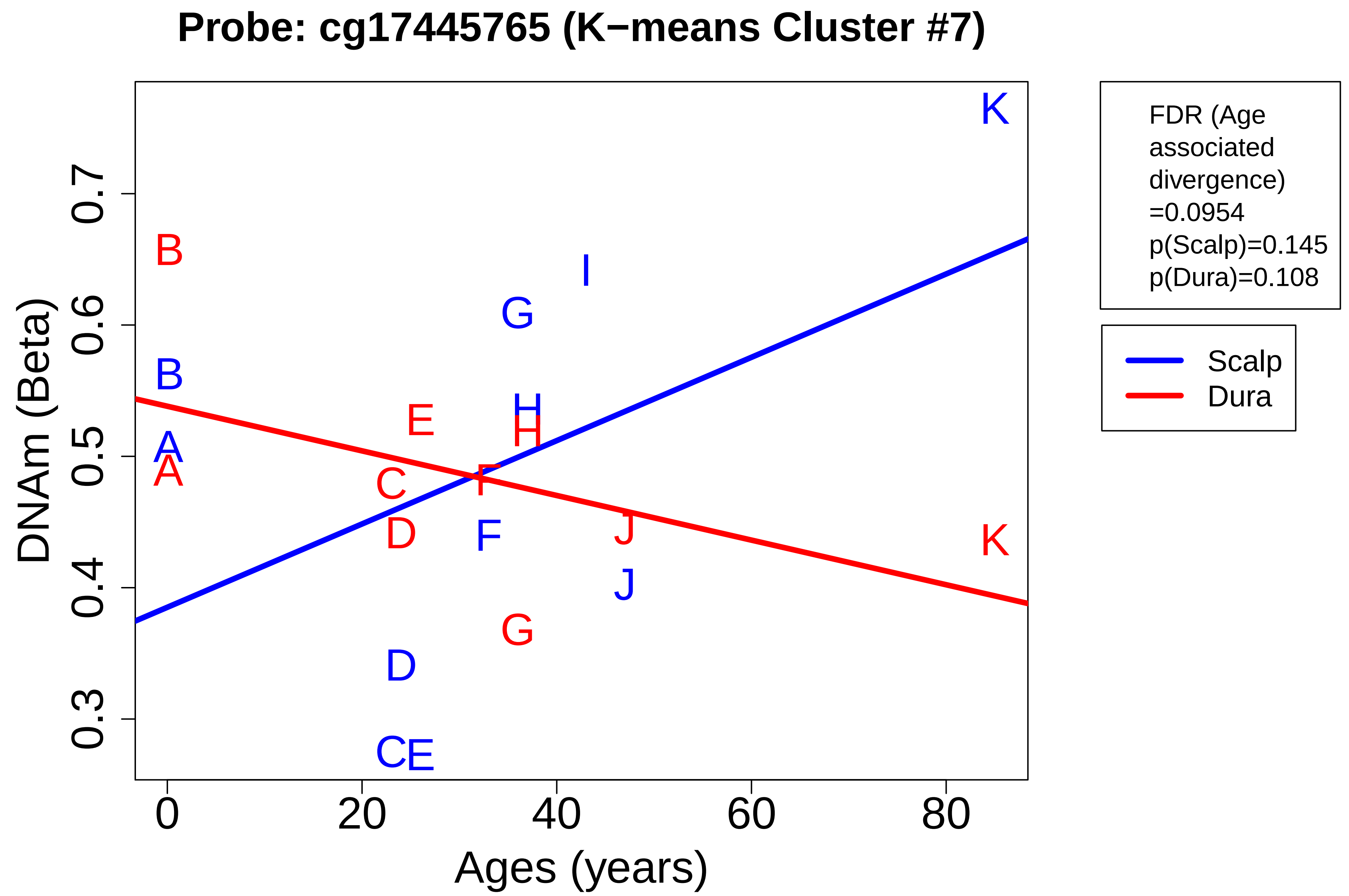

Probe: cg17445765 (K−means Cluster #7)
DNAm (Beta)
0.3
0.4
0.5
0.6
0.7
0
20
40
60
80
Ages (years)
FDR (Age associated divergence) =0.0954
p(Scalp)=0.145
p(Dura)=0.108
Scalp
Dura

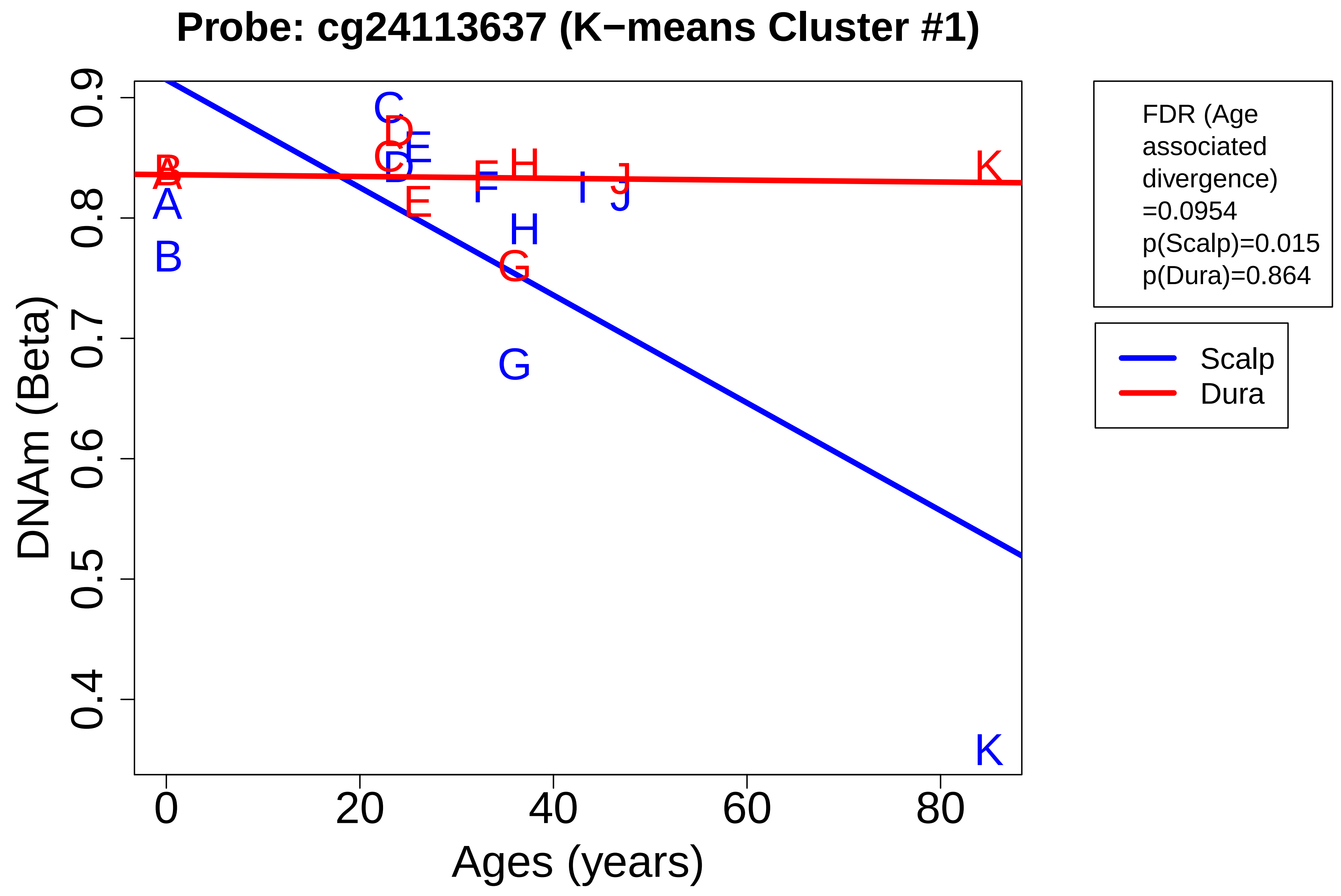

Probe: cg24113637 (K−means Cluster #1)
DNAm (Beta)
0.4
0.5
0.6
0.7
0.8
0.9
0
20
40
60
80
Ages (years)
FDR (Age associated divergence) =0.0954
p(Scalp)=0.015
p(Dura)=0.864
Scalp
Dura

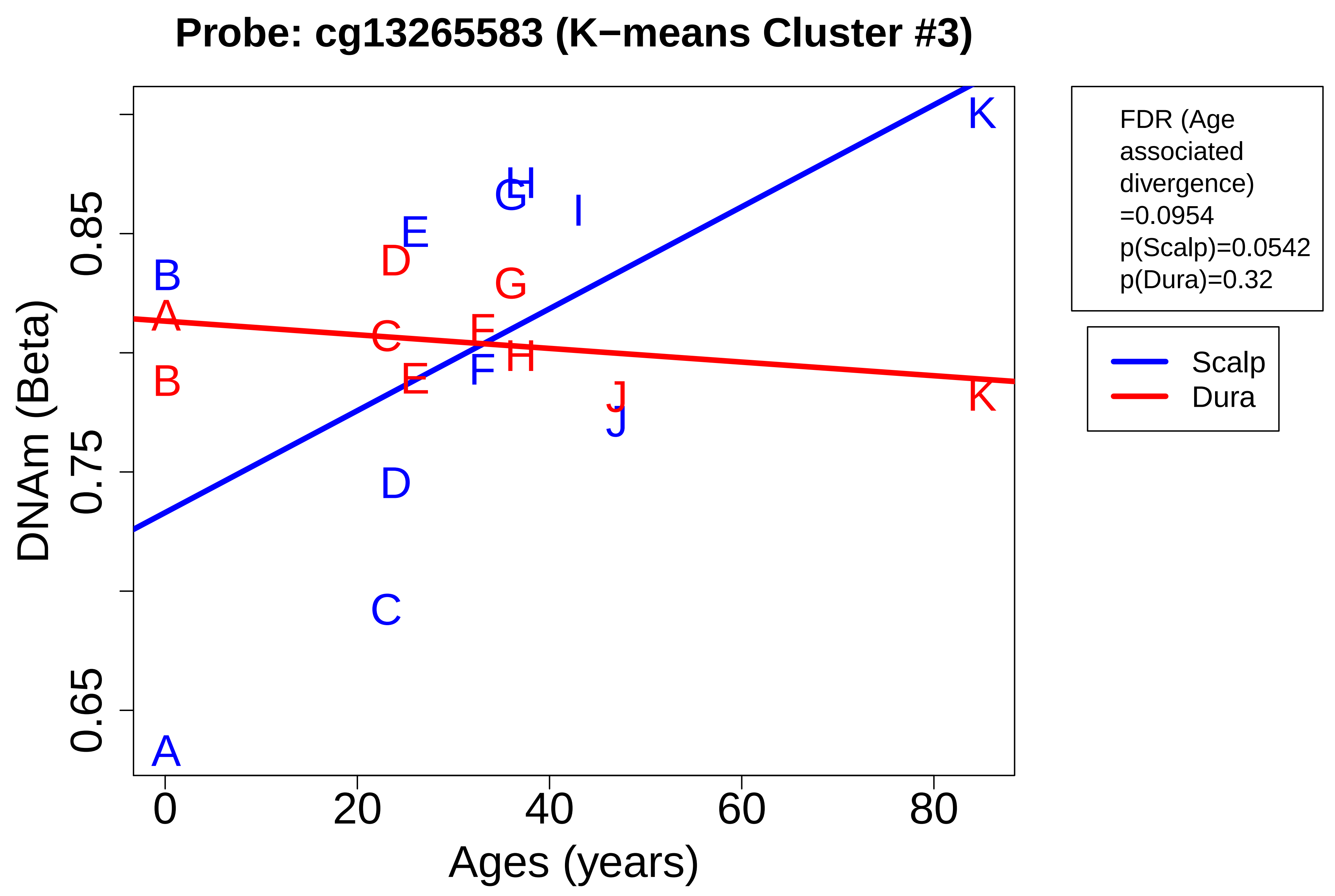

Probe: cg13265583 (K−means Cluster #3)
DNAm (Beta)
0.65
0.75
0.85
0
20
40
60
80
Ages (years)
FDR (Age associated divergence) =0.0954
p(Scalp)=0.0542
p(Dura)=0.32
Scalp
Dura

Probe: cg13666041 (K−means Cluster #1)

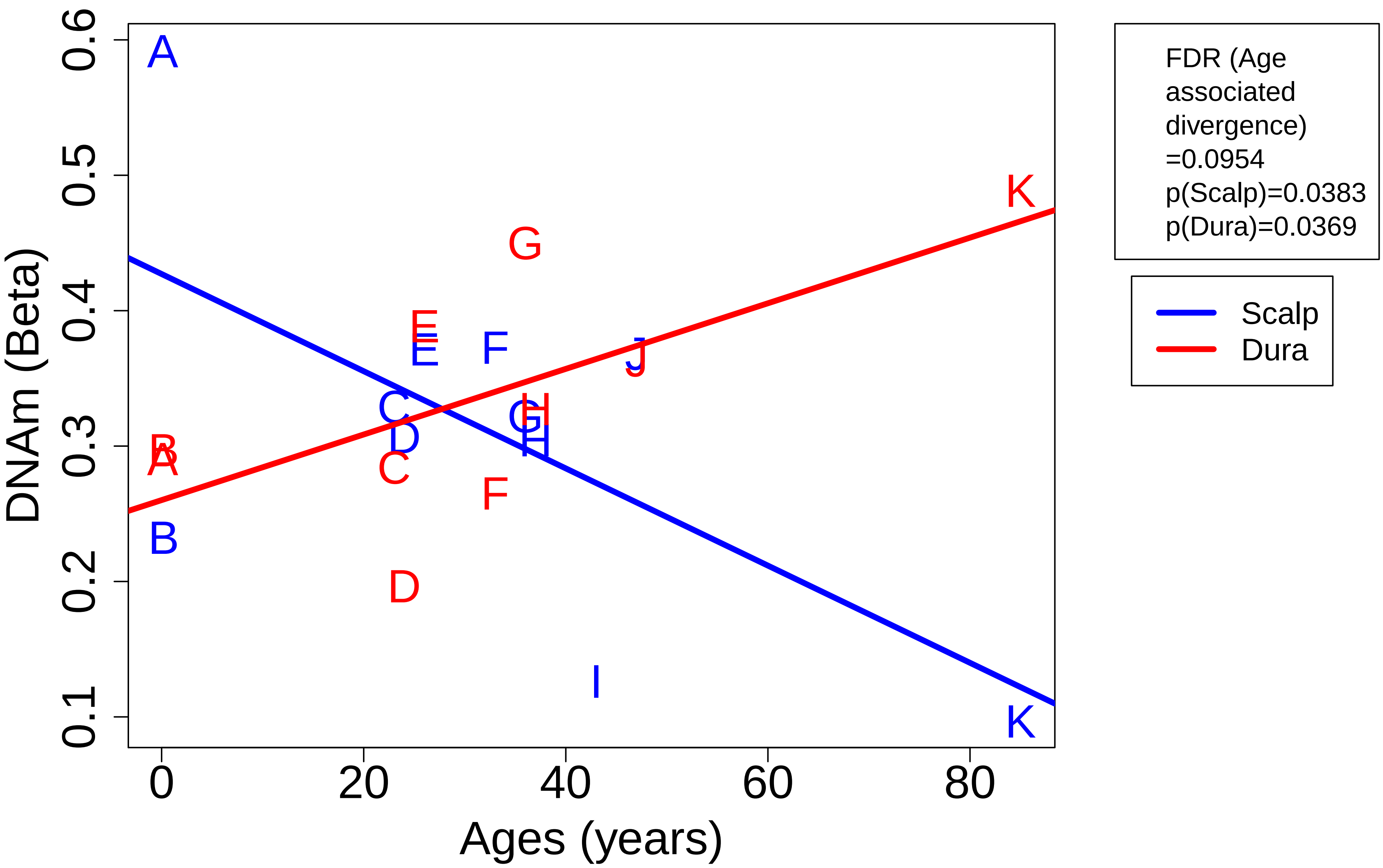

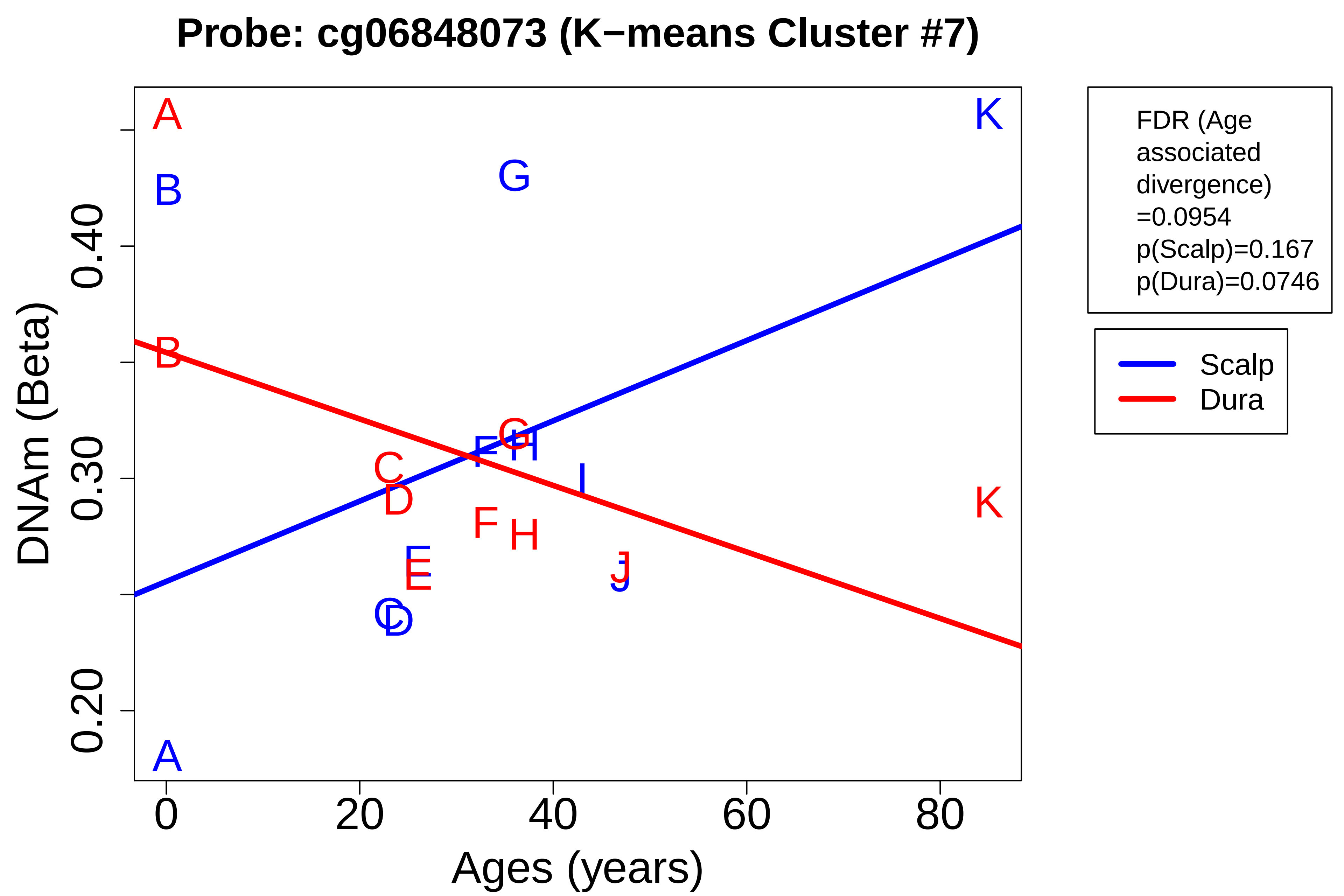

Probe: cg06848073 (K−means Cluster #7)
DNAm (Beta)
0.20
0.30
0.40
0
20
40
60
80
Ages (years)
A
B
C
D
E
F
G
H
I
J
K
FDR (Age associated divergence) =0.0954
p(Scalp)=0.167
p(Dura)=0.0746
Scalp
Dura

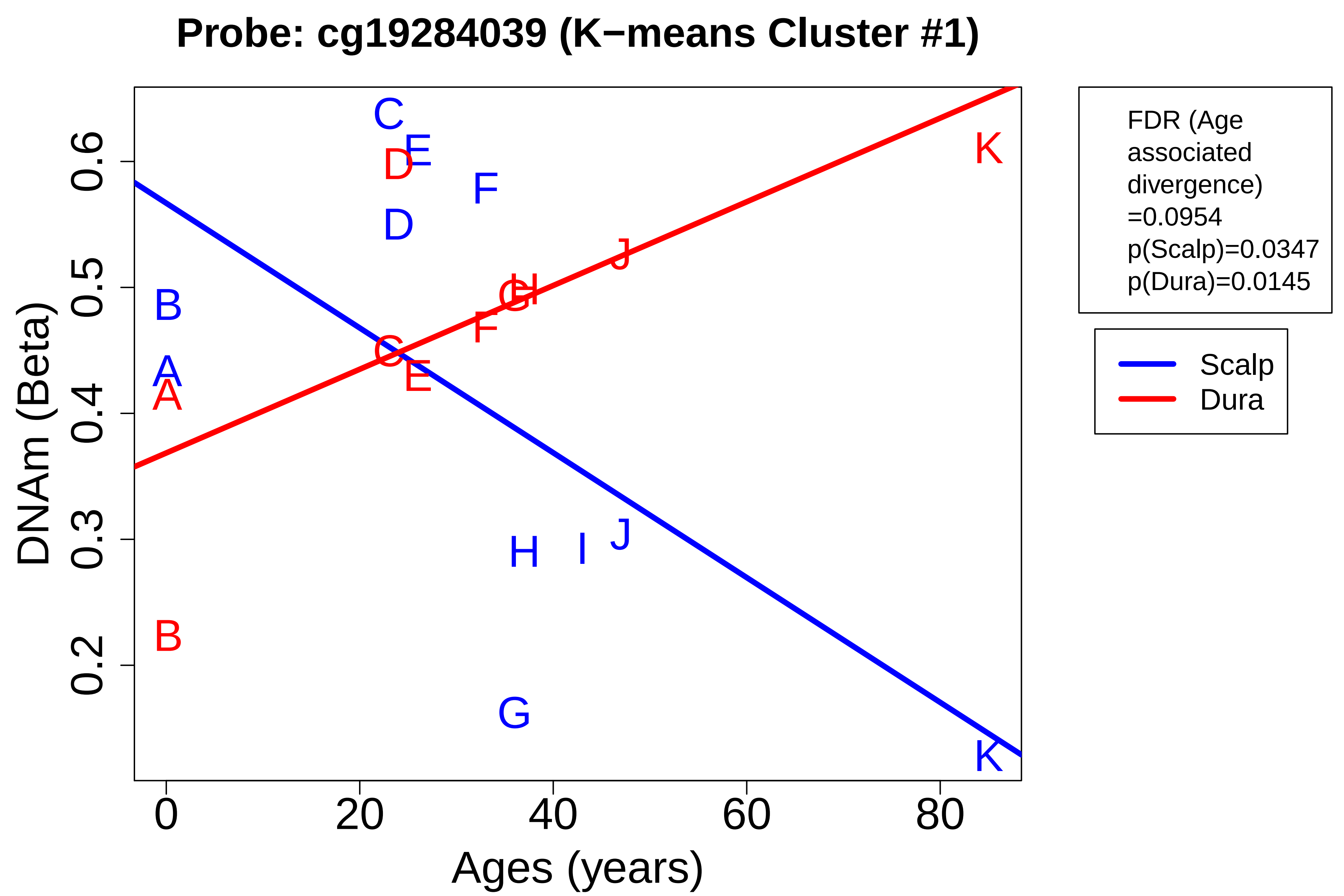

Probe: cg19284039 (K−means Cluster #1)
DNAm (Beta)
Ages (years)
0.2
0.3
0.4
0.5
0.6
0
20
40
60
80
FDR (Age associated divergence) =0.0954
p(Scalp)=0.0347
p(Dura)=0.0145
Scalp
Dura

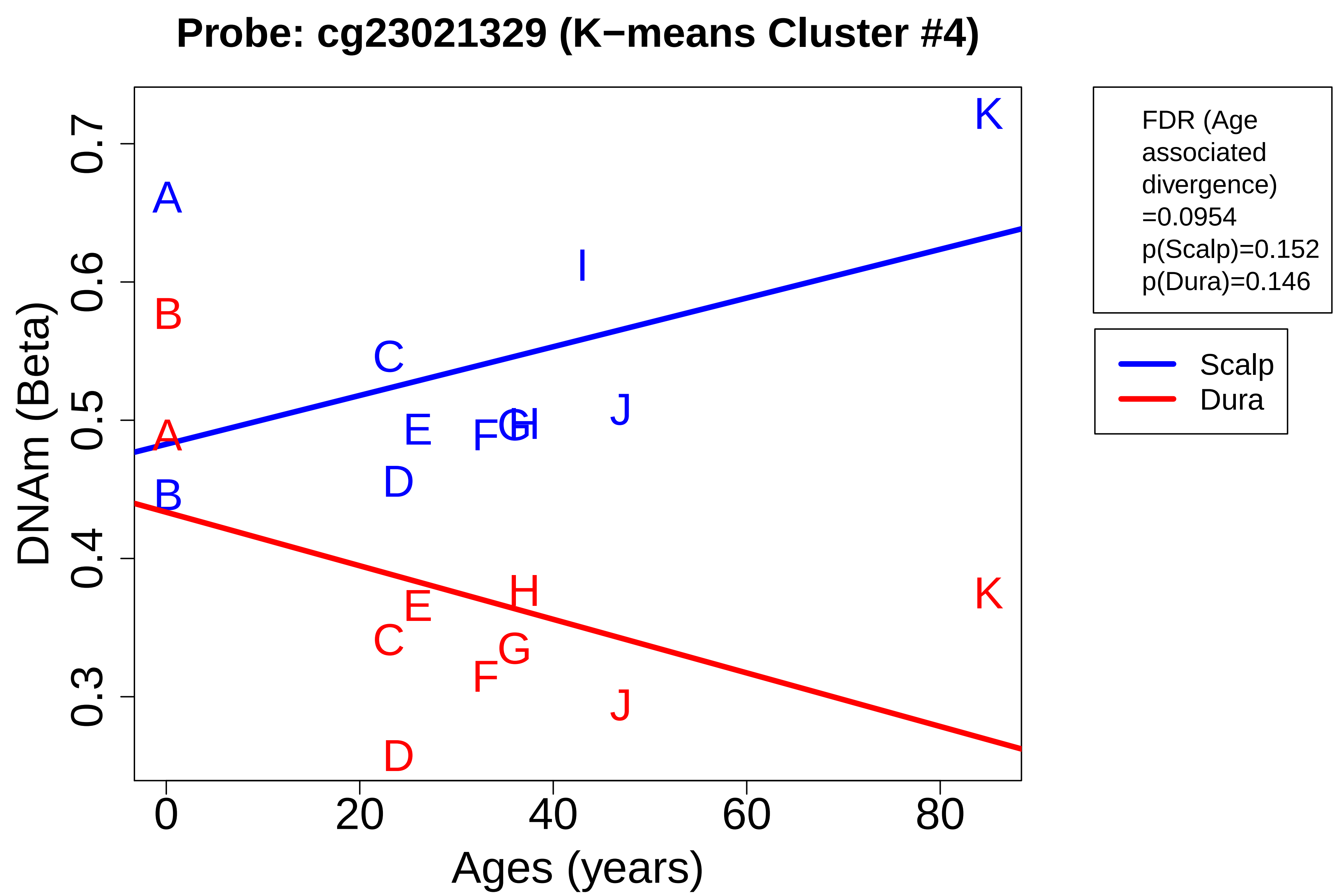

Probe: cg23021329 (K−means Cluster #4)
DNAm (Beta)
0.3
0.4
0.5
0.6
0.7
0
20
40
60
80
Ages (years)
A
B
C
D
E
F
G
H
I
J
K
FDR (Age associated divergence) =0.0954
p(Scalp)=0.152
p(Dura)=0.146
Scalp
Dura

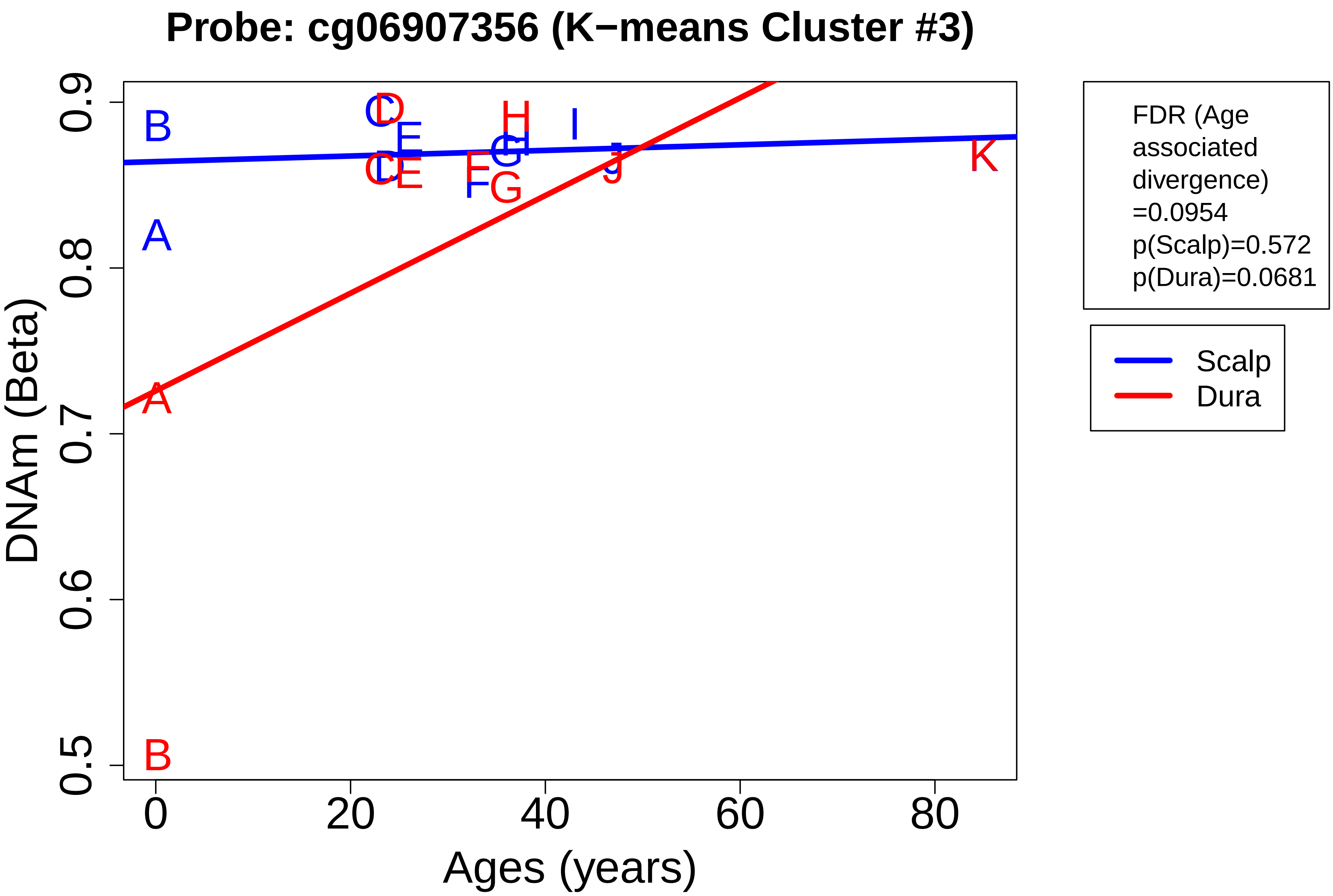

Probe: cg06907356 (K−means Cluster #3)
DNAm (Beta)
0.5
0.6
0.7
0.8
0.9
0
20
40
60
80
Ages (years)
FDR (Age associated divergence) =0.0954
p(Scalp)=0.572
p(Dura)=0.0681
Scalp
Dura

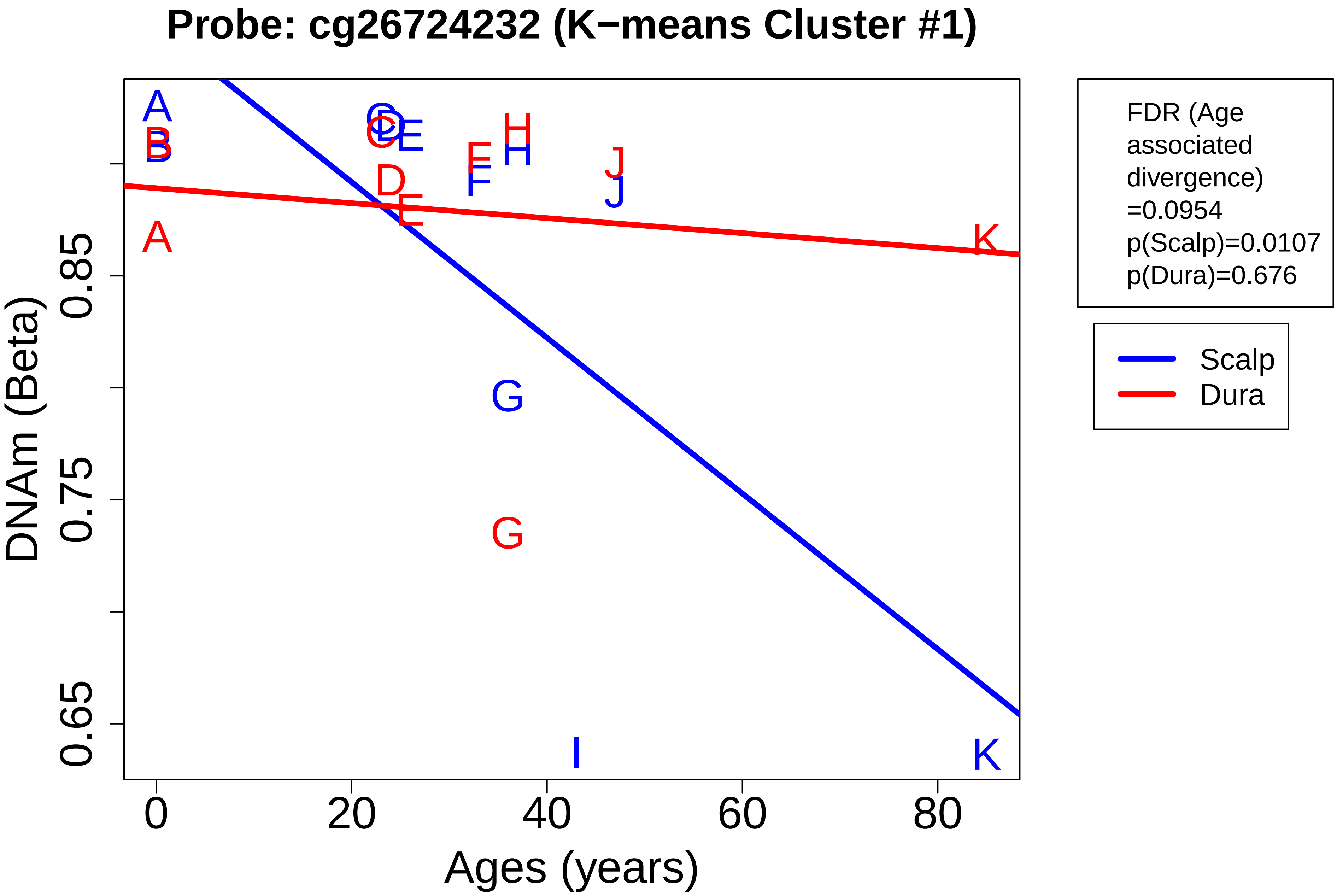

Probe: cg26724232 (K−means Cluster #1)
DNAm (Beta)
0.65
0.75
0.85
Ages (years)
0
20
40
60
80
FDR (Age associated divergence) =0.0954
p(Scalp)=0.0107
p(Dura)=0.676
Scalp
Dura

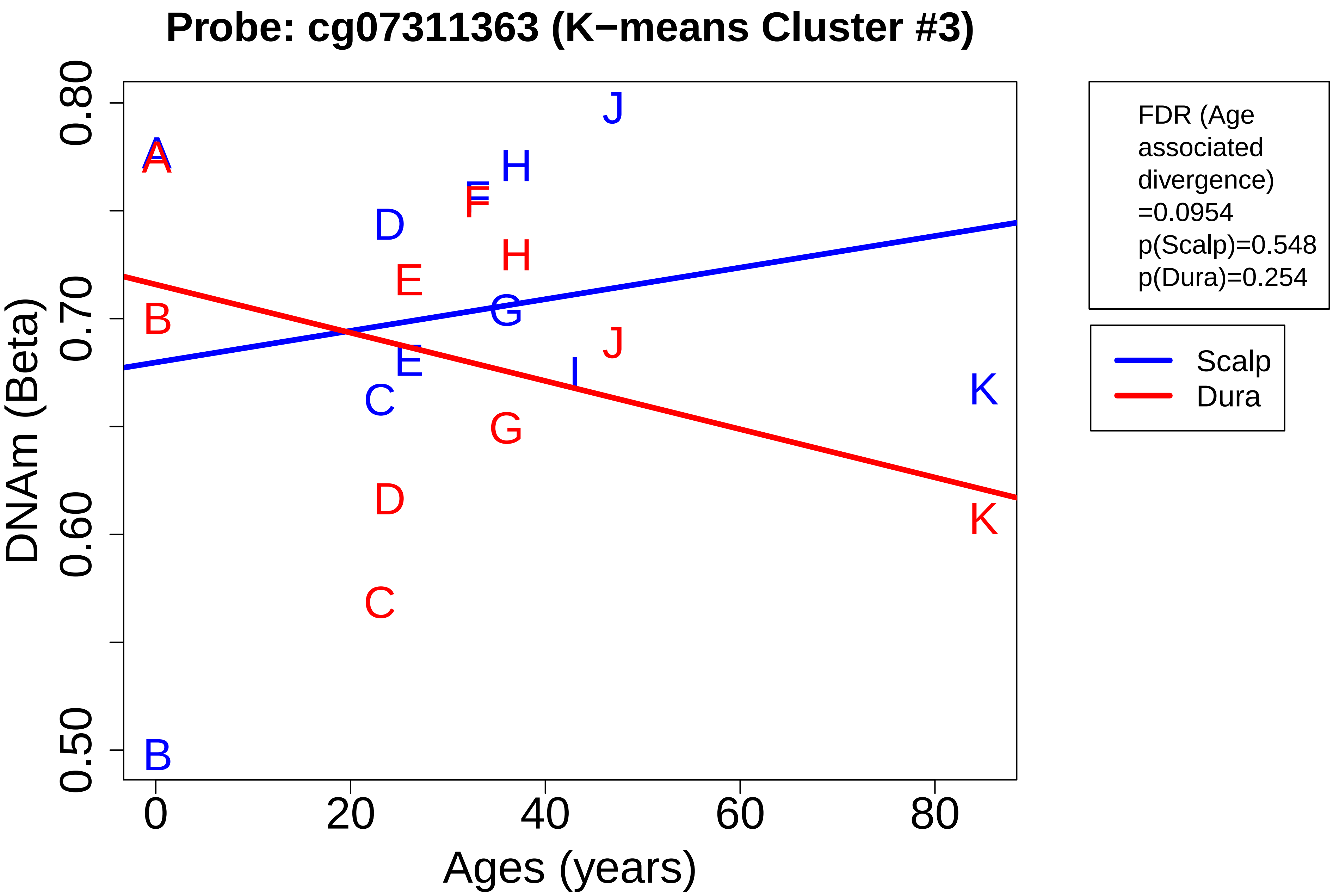

Probe: cg07311363 (K−means Cluster #3)
DNAm (Beta)
0.50
0.60
0.70
0.80
Ages (years)
0
20
40
60
80
A
B
C
D
E
F
G
H
I
J
K
FDR (Age associated divergence) =0.0954
p(Scalp)=0.548
p(Dura)=0.254
Scalp
Dura

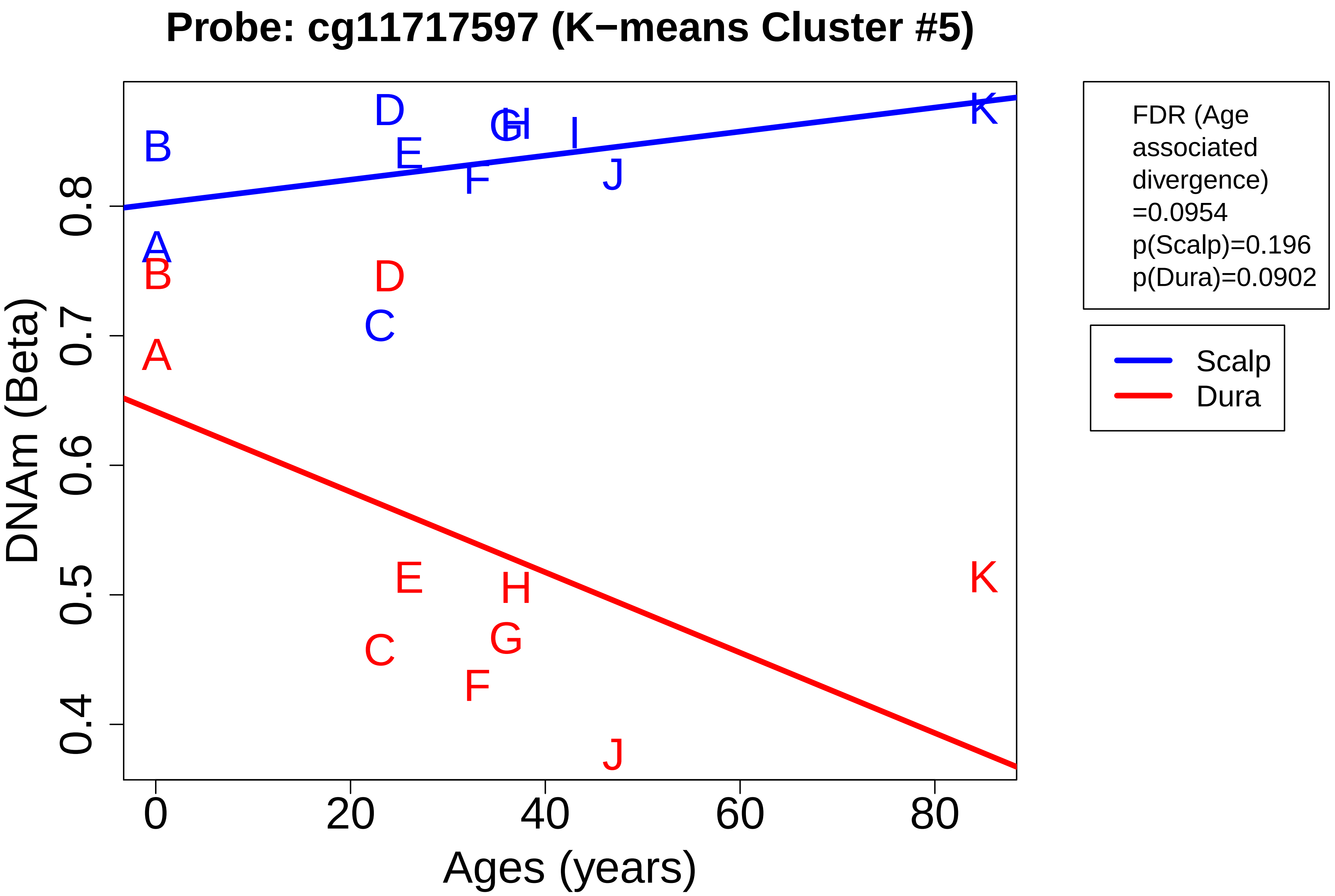

Probe: cg11717597 (K−means Cluster #5)
DNAm (Beta)
0.4
0.5
0.6
0.7
0.8
0
20
40
60
80
Ages (years)
FDR (Age associated divergence) =0.0954
p(Scalp)=0.196
p(Dura)=0.0902
Scalp
Dura

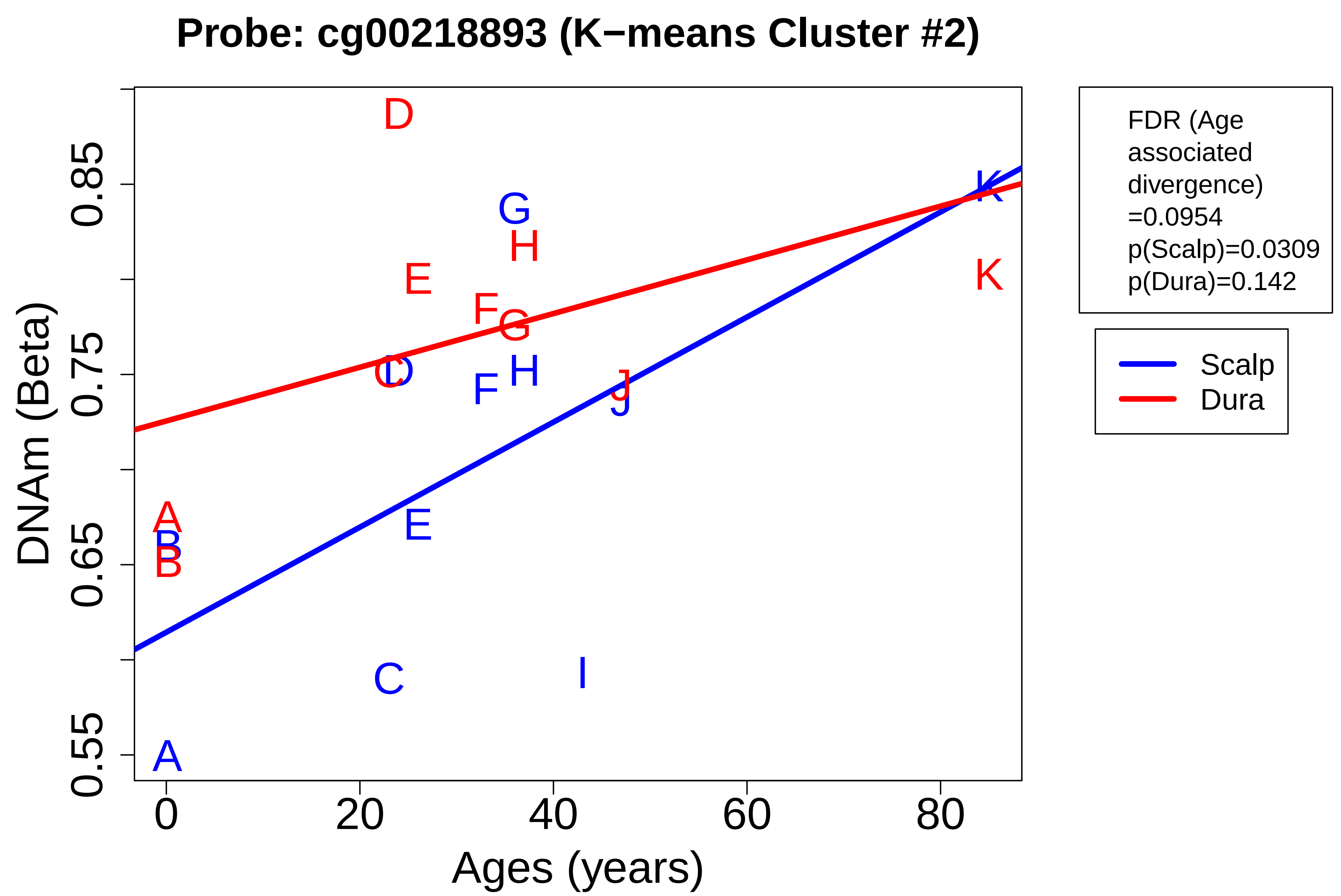

Probe: cg00218893 (K−means Cluster #2)
FDR (Age associated divergence) =0.0954
p(Scalp)=0.0309
p(Dura)=0.142
Scalp
Dura
DNAm (Beta)
0.55
0.65
0.75
0.85
0
20
40
60
80
Ages (years)

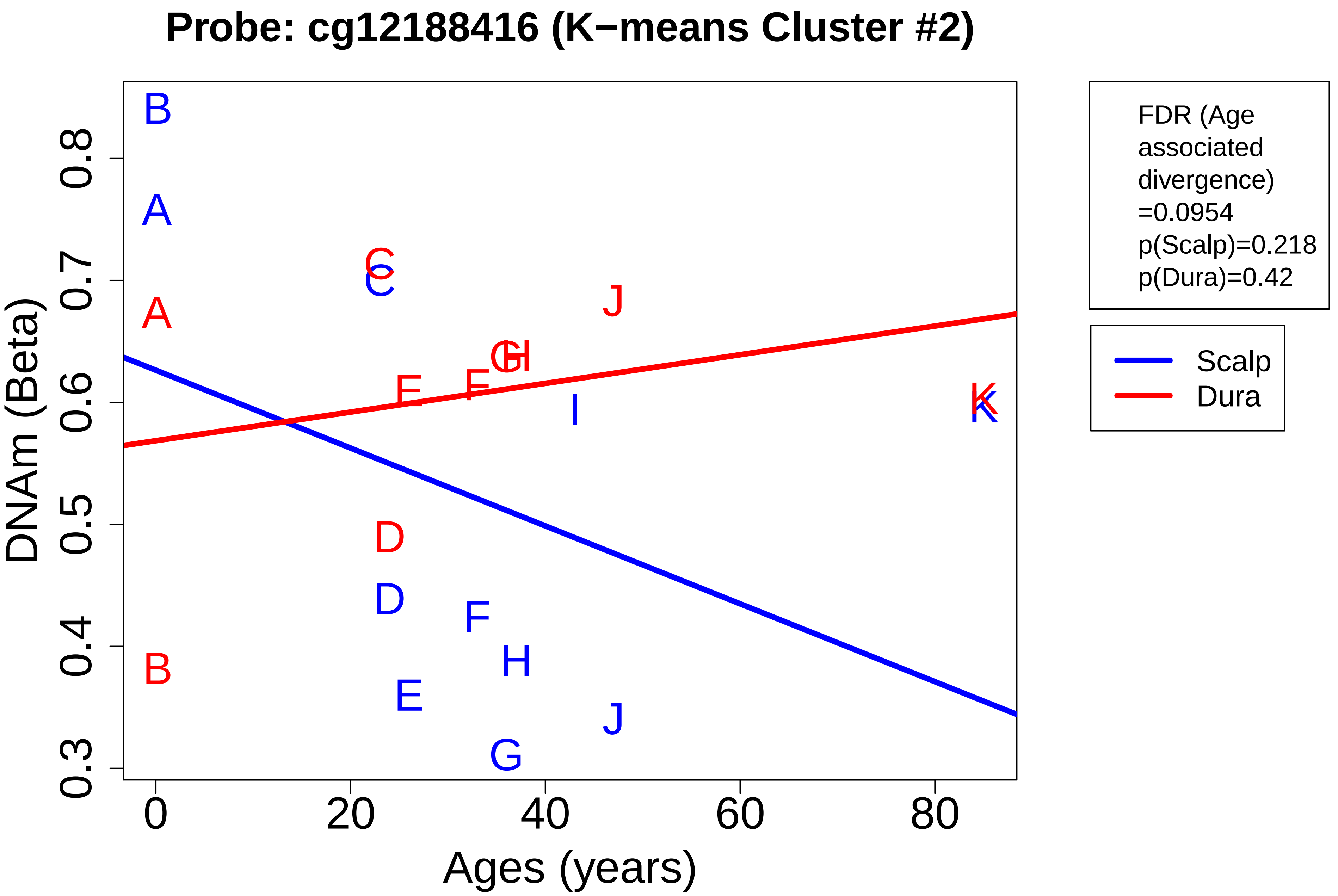
Probe: cg12188416 (K−means Cluster #2)
FDR (Age associated divergence) =0.0954
p(Scalp)=0.218
p(Dura)=0.42
Scalp
Dura
DNAm (Beta)
Ages (years)
0.3
0.4
0.5
0.6
0.7
0.8
0
20
40
60
80

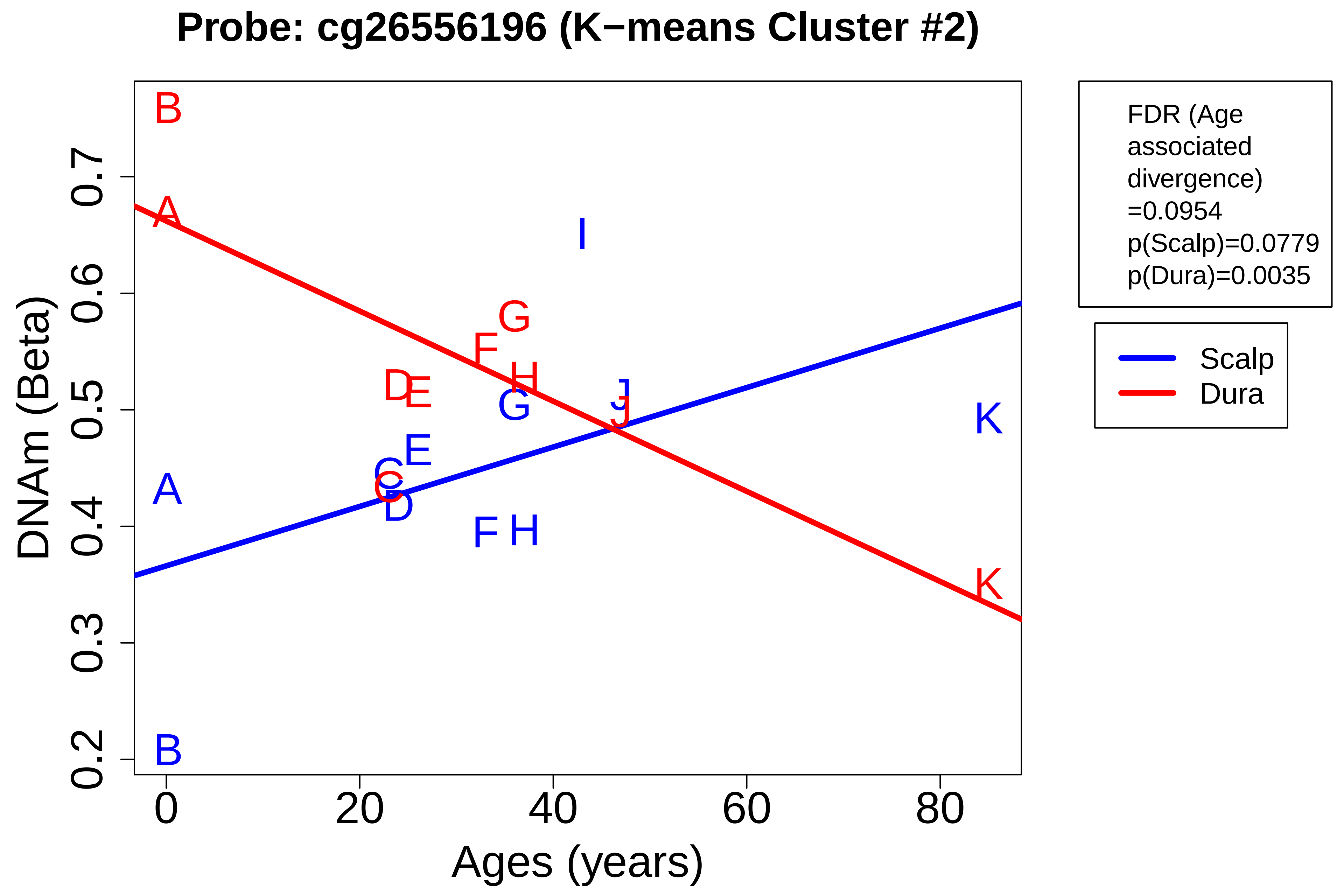

Probe: cg26556196 (K−means Cluster #2)
DNAm (Beta)
0.2
0.3
0.4
0.5
0.6
0.7
0
20
40
60
80
Ages (years)
B
A
G
F
H
D
E
I
J
J
G
K
E
C
C
D
A
F H
K
B
FDR (Age associated divergence) =0.0954
p(Scalp)=0.0779
p(Dura)=0.0035
Scalp
Dura

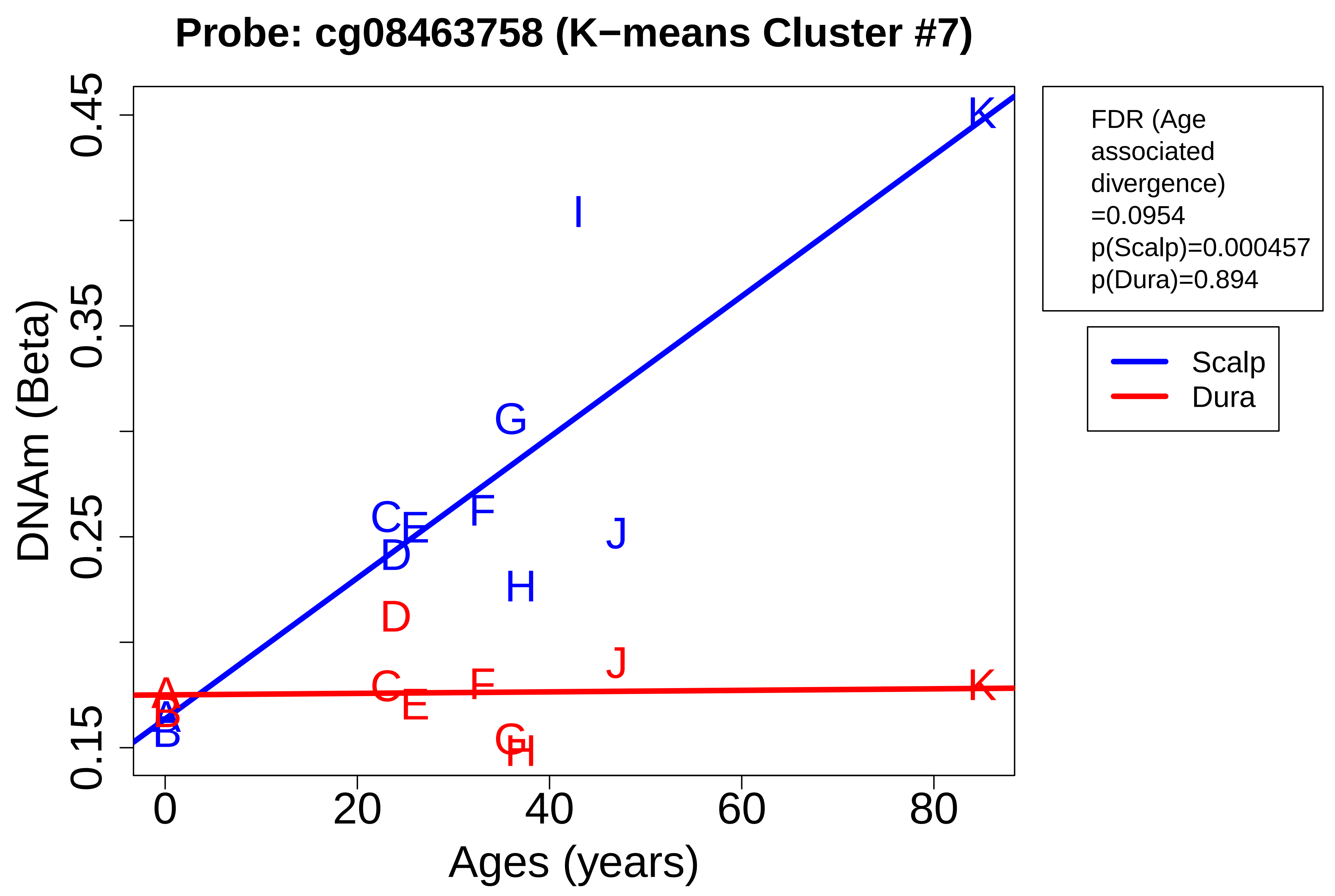

Probe: cg08463758 (K−means Cluster #7)
DNAm (Beta)
0.15
0.25
0.35
0.45
Ages (years)
0
20
40
60
80
FDR (Age associated divergence) =0.0954
p(Scalp)=0.000457
p(Dura)=0.894
Scalp
Dura

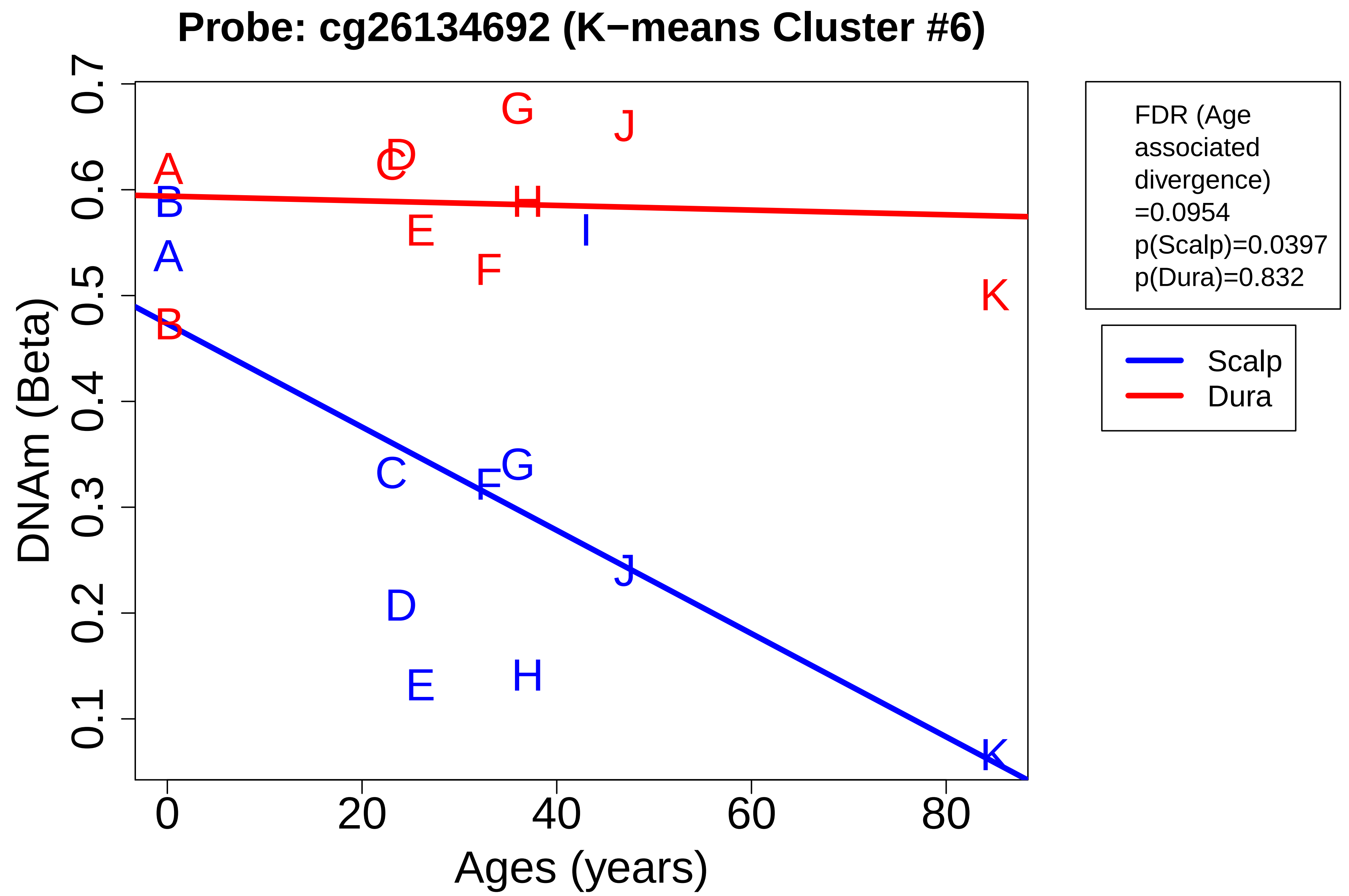

Probe: cg26134692 (K−means Cluster #6)
DNAm (Beta)
0.1
0.2
0.3
0.4
0.5
0.6
0.7
Ages (years)
0
20
40
60
80
FDR (Age associated divergence) =0.0954
p(Scalp)=0.0397
p(Dura)=0.832
Scalp
Dura
A
B
C
D
E
F
G
H
I
J
K

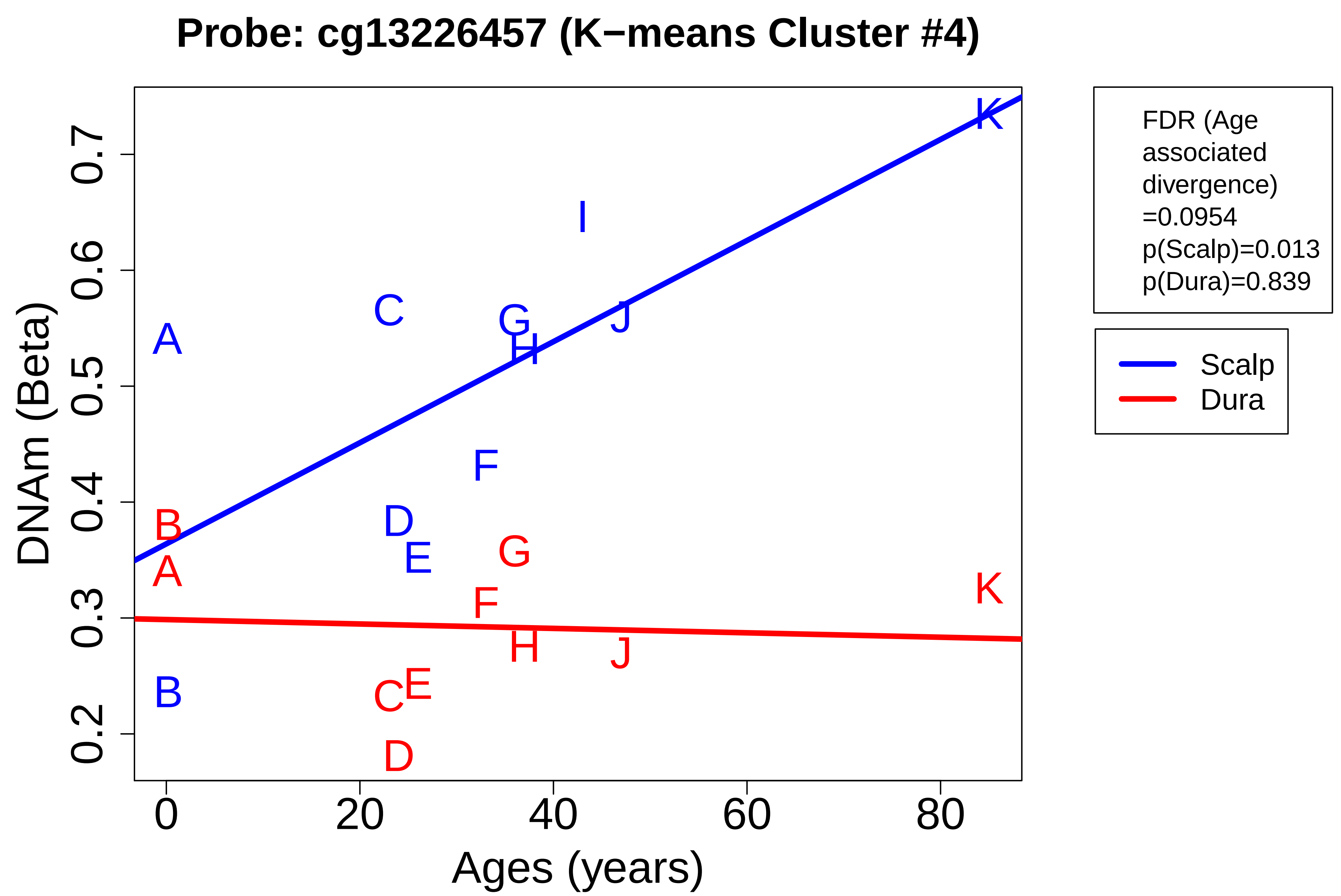

Probe: cg13226457 (K−means Cluster #4)
DNAm (Beta)
0.2
0.3
0.4
0.5
0.6
0.7
0
20
40
60
80
Ages (years)
FDR (Age associated divergence) =0.0954
p(Scalp)=0.013
p(Dura)=0.839
Scalp
Dura
A
B
C
D
E
F
G
H
I
J
K

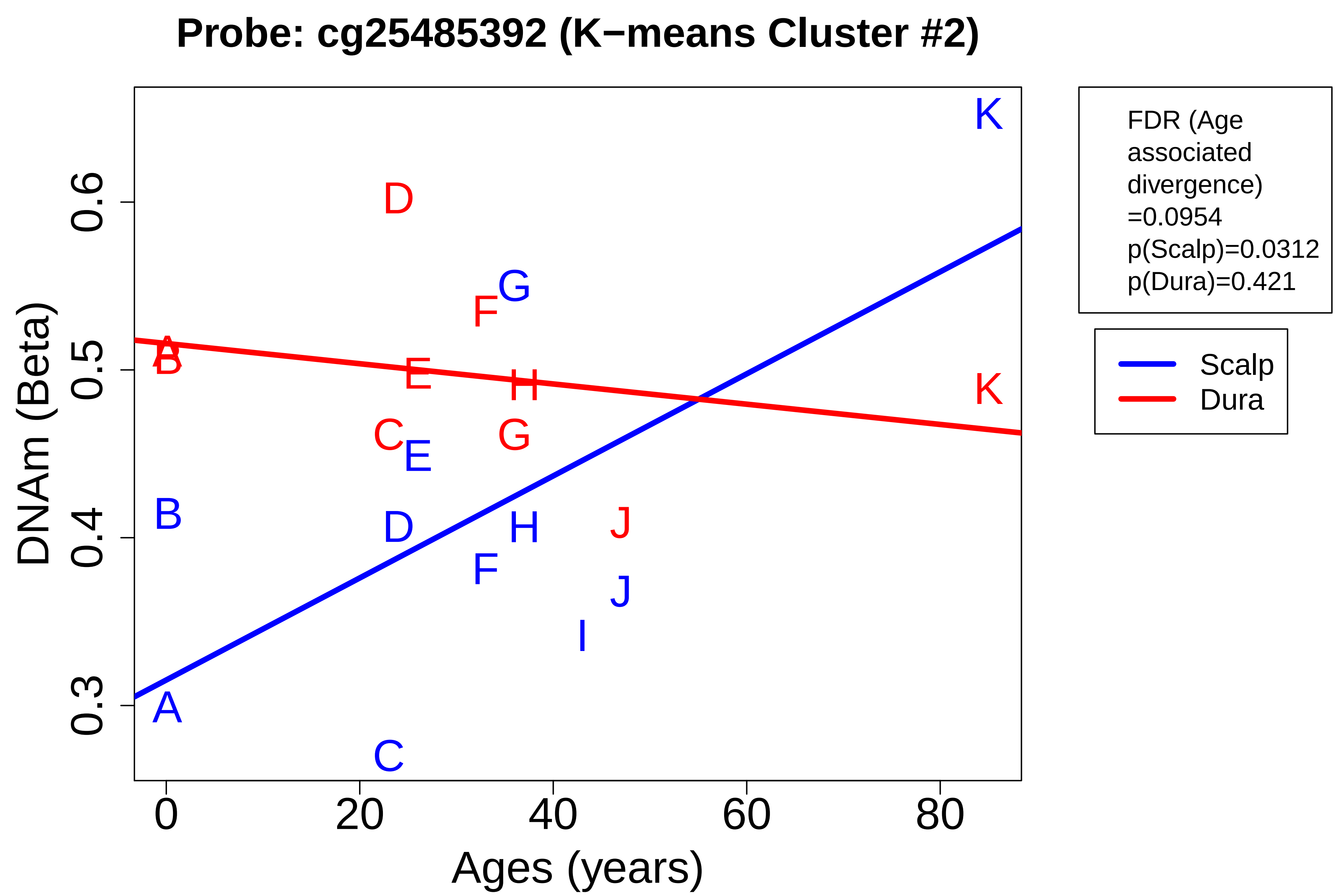

Probe: cg25485392 (K−means Cluster #2)
DNAm (Beta)
0.3
0.4
0.5
0.6
0
20
40
60
80
Ages (years)
FDR (Age associated divergence) =0.0954
p(Scalp)=0.0312
p(Dura)=0.421
Scalp
Dura

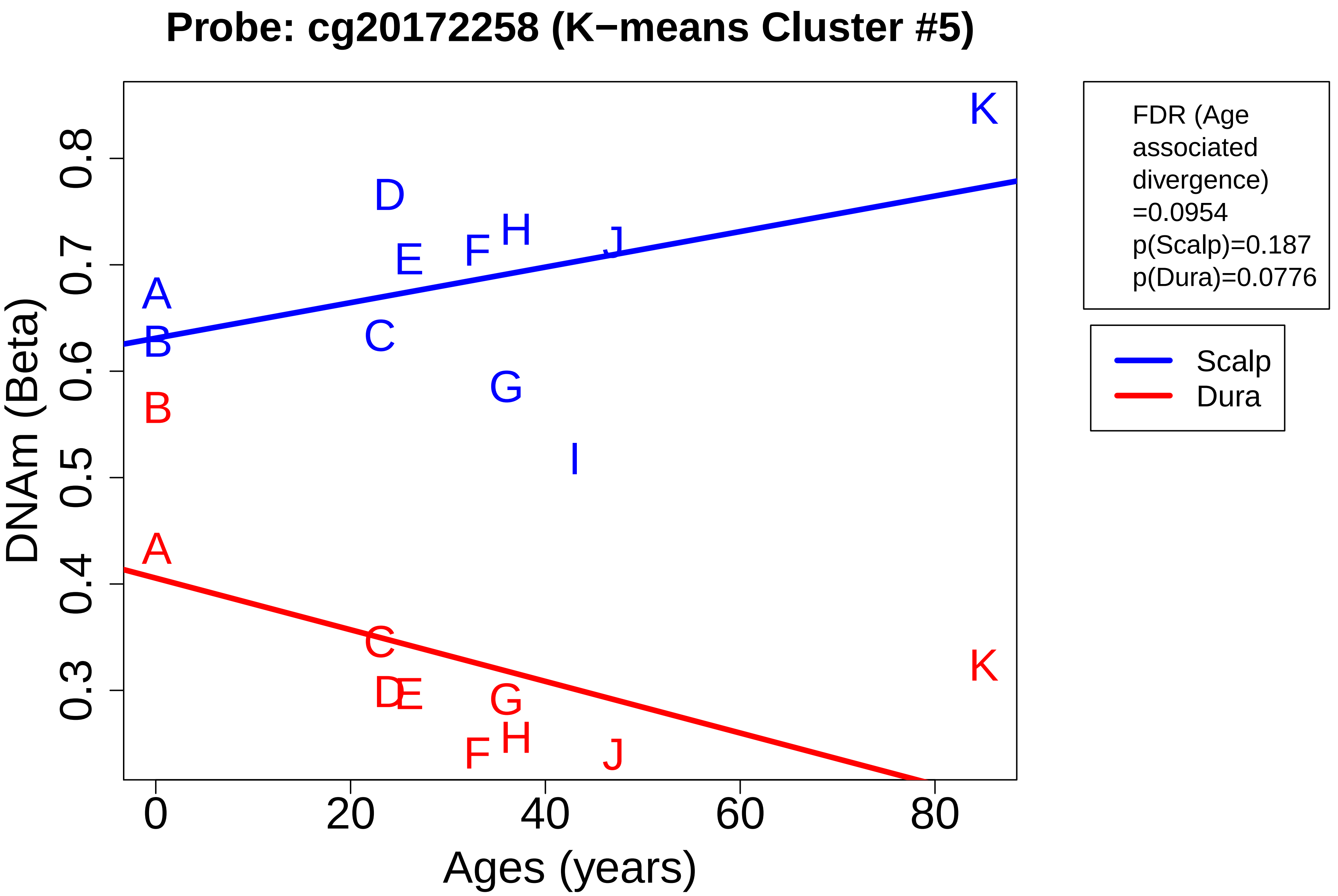
Probe: cg20172258 (K−means Cluster #5)
DNAm (Beta)
0.3
0.4
0.5
0.6
0.7
0.8
0
20
40
60
80
Ages (years)
FDR (Age associated divergence) =0.0954
p(Scalp)=0.187
p(Dura)=0.0776
Scalp
Dura
A
B
C
D
E
F
G
H
I
J
K

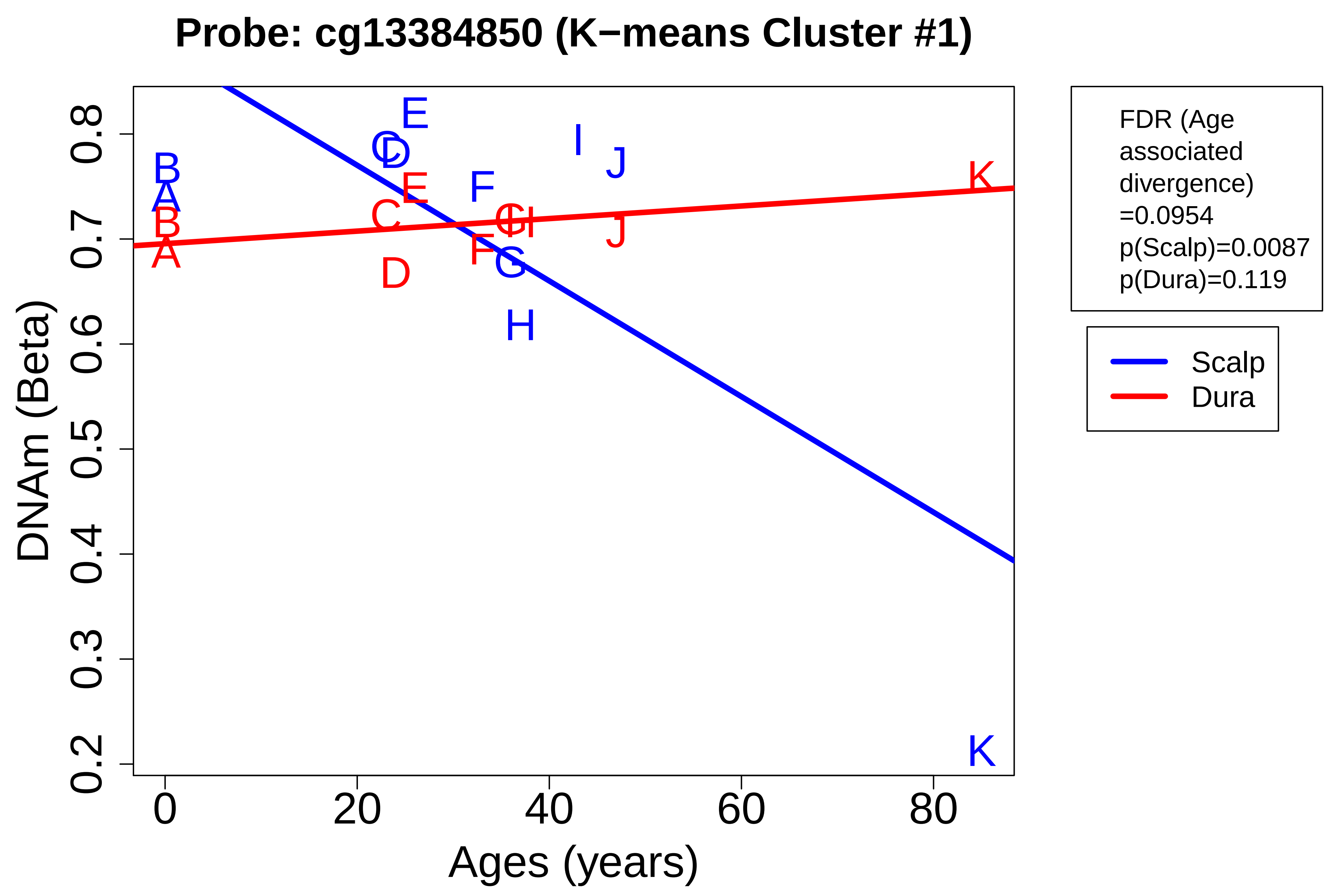

Probe: cg13384850 (K−means Cluster #1)
DNAm (Beta)
0.2
0.3
0.4
0.5
0.6
0.7
0.8
Ages (years)
0
20
40
60
80
FDR (Age associated divergence) =0.0954
p(Scalp)=0.0087
p(Dura)=0.119
Scalp
Dura

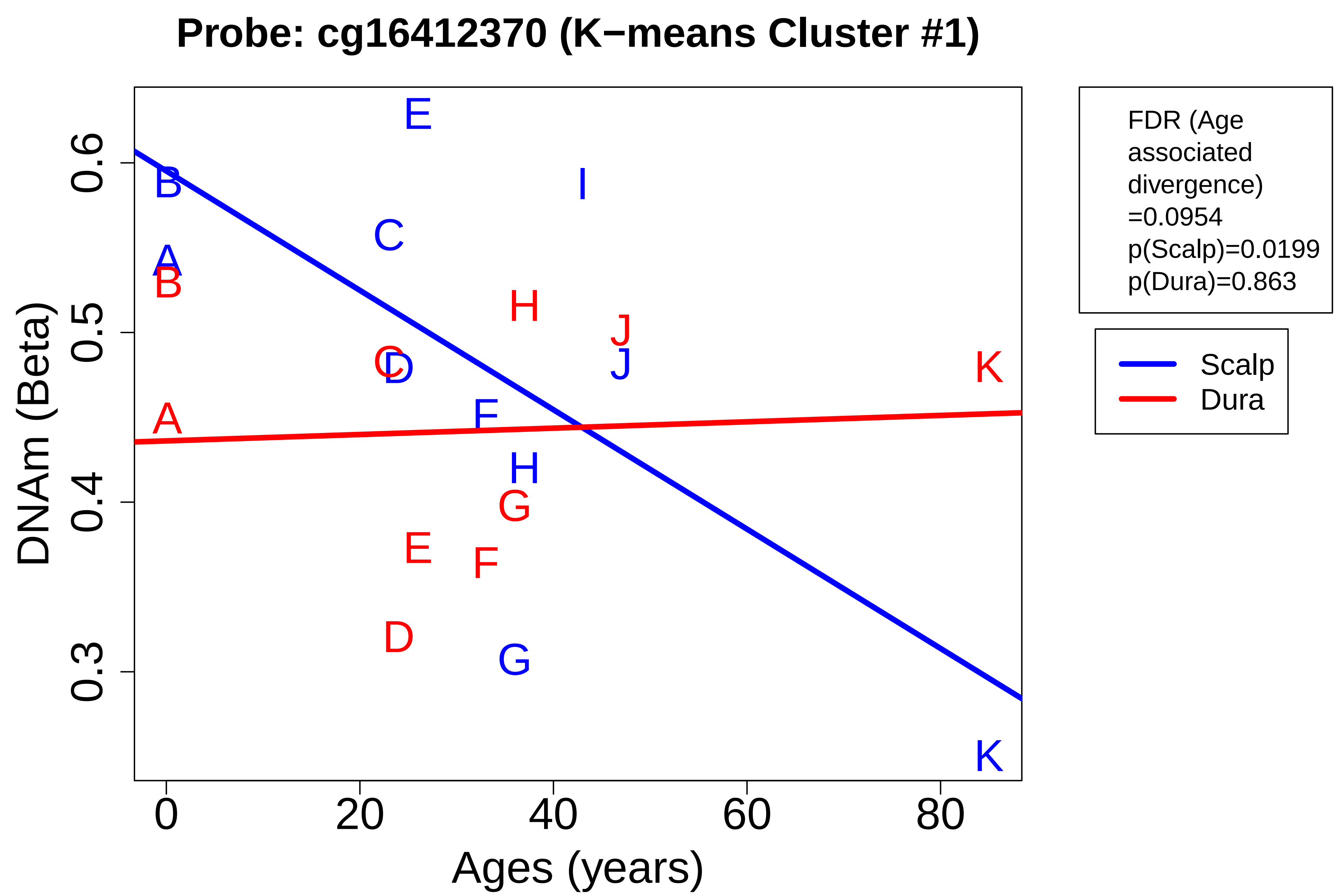

Probe: cg16412370 (K−means Cluster #1)
FDR (Age associated divergence) =0.0954
p(Scalp)=0.0199
p(Dura)=0.863
Scalp
Dura
DNAm (Beta)
0.3
0.4
0.5
0.6
0
20
40
60
80
Ages (years)
A
B
C
D
E
F
G
H
I
J
K

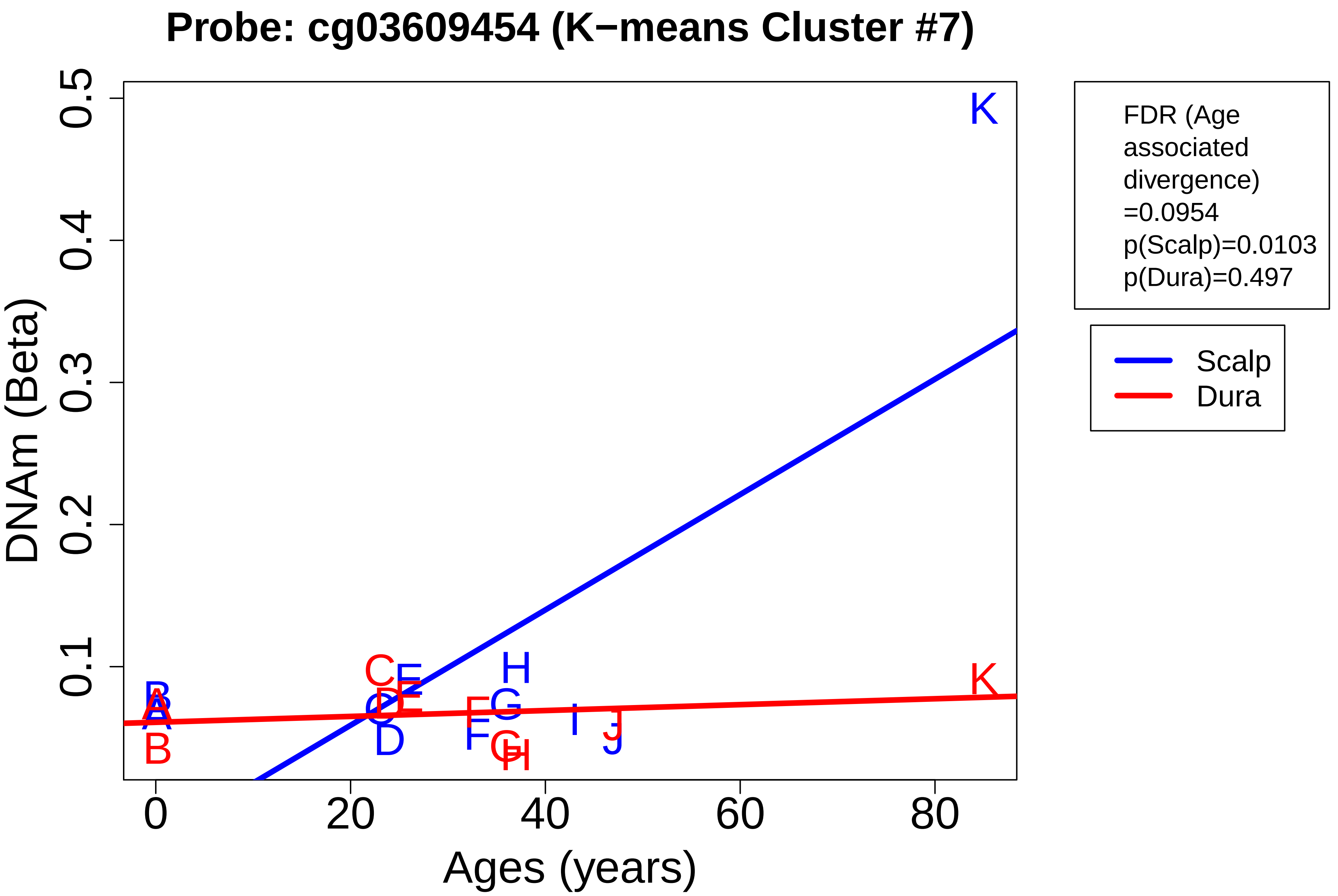
Probe: cg03609454 (K−means Cluster #7)
DNAm (Beta)
0.1
0.2
0.3
0.4
0.5
0
20
40
60
80
Ages (years)
FDR (Age associated divergence) =0.0954
p(Scalp)=0.0103
p(Dura)=0.497
Scalp
Dura

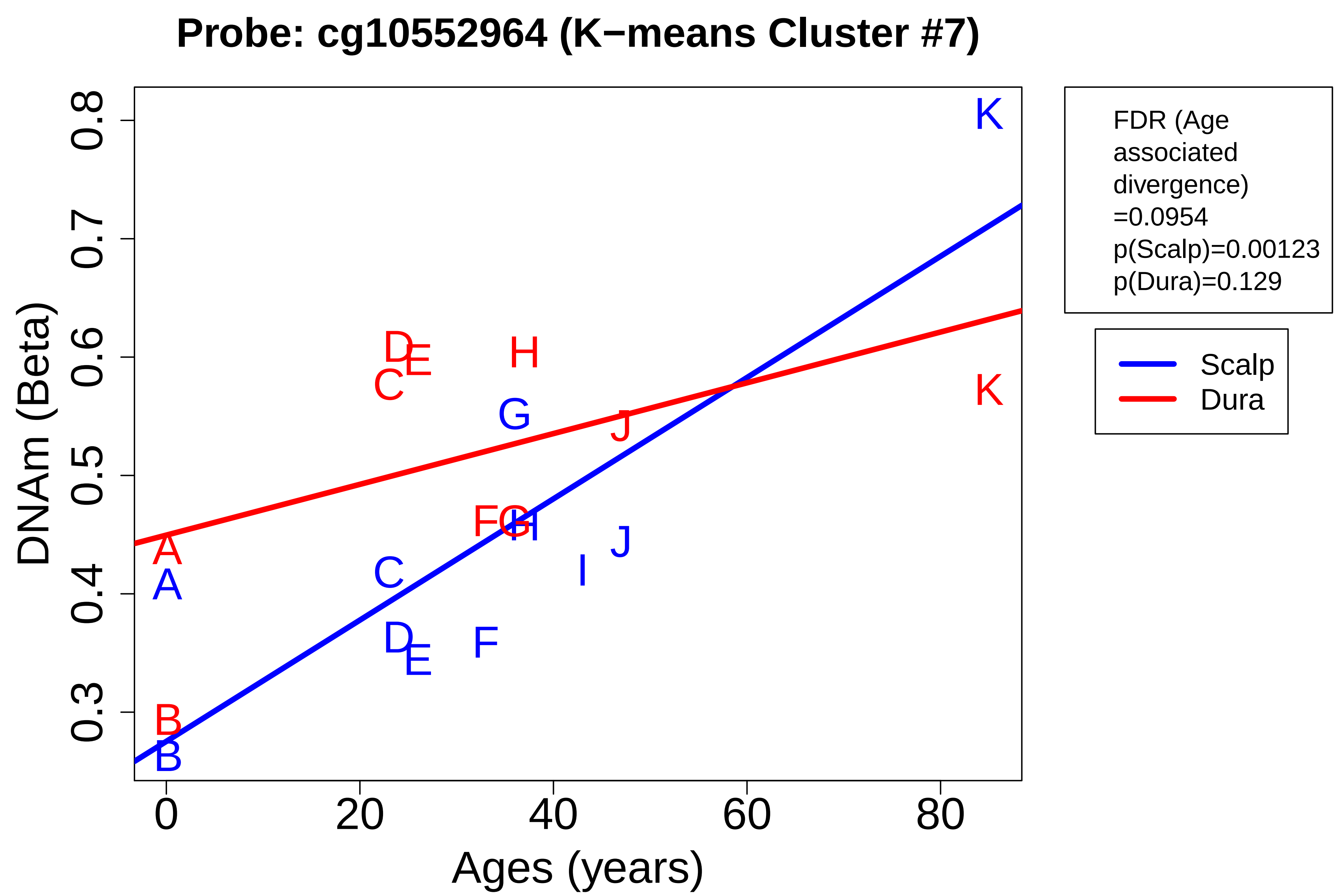

Probe: cg10552964 (K−means Cluster #7)
DNAm (Beta)
Ages (years)
0.3
0.4
0.5
0.6
0.7
0.8
0
20
40
60
80
FDR (Age associated divergence) =0.0954
p(Scalp)=0.00123
p(Dura)=0.129
Scalp
Dura
A
B
C
D
E
F
G
H
I
J
K

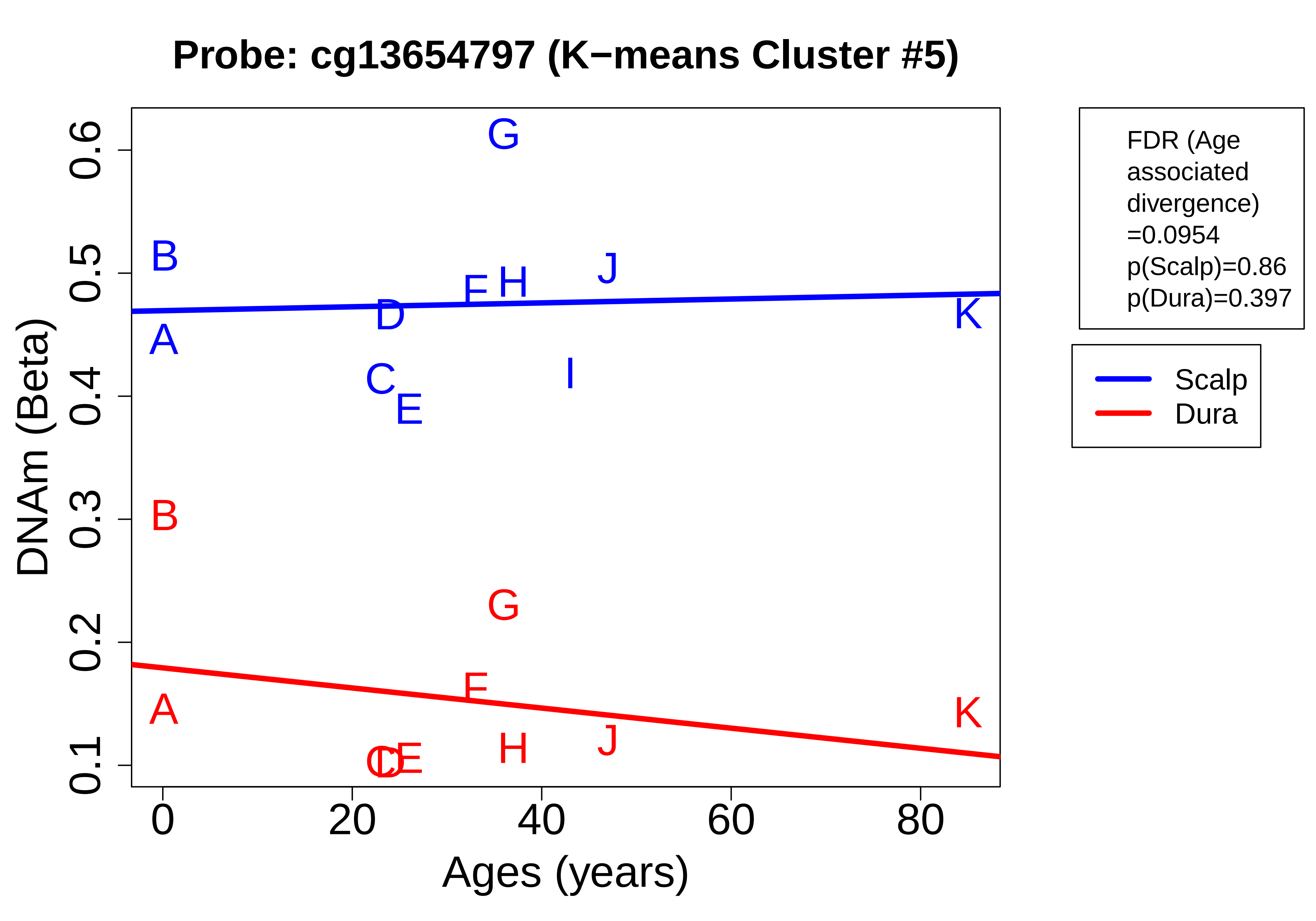

Probe: cg13654797 (K−means Cluster #5)
DNAm (Beta)
Ages (years)
0.1
0.2
0.3
0.4
0.5
0.6
0
20
40
60
80
FDR (Age associated divergence) =0.0954
p(Scalp)=0.86
p(Dura)=0.397
Scalp
Dura

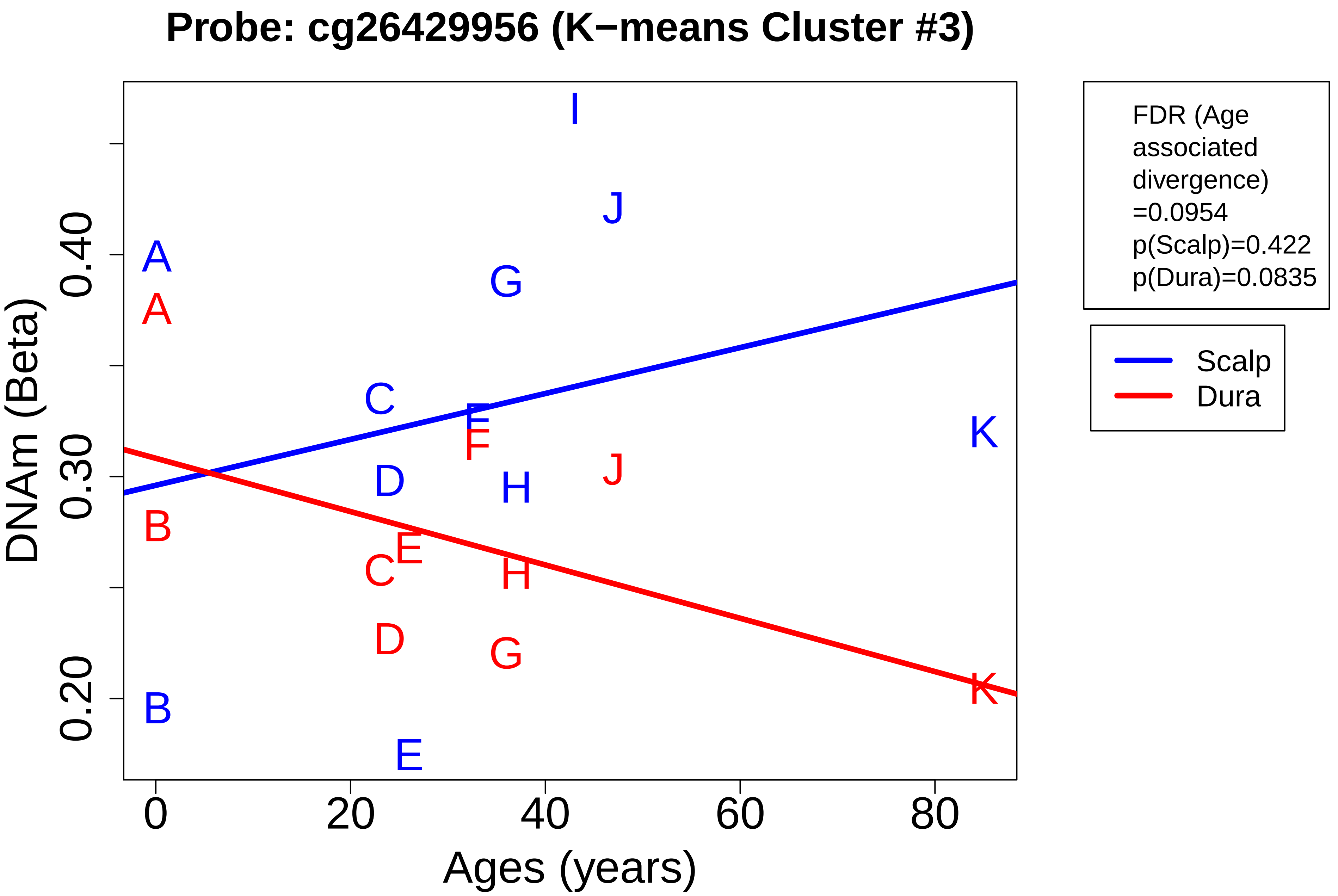

Probe: cg26429956 (K−means Cluster #3)
DNAm (Beta)
0.20
0.30
0.40
Ages (years)
0
20
40
60
80
FDR (Age associated divergence) =0.0954
p(Scalp)=0.422
p(Dura)=0.0835
Scalp
Dura
A
B
C
D
E
F
G
H
I
J
K

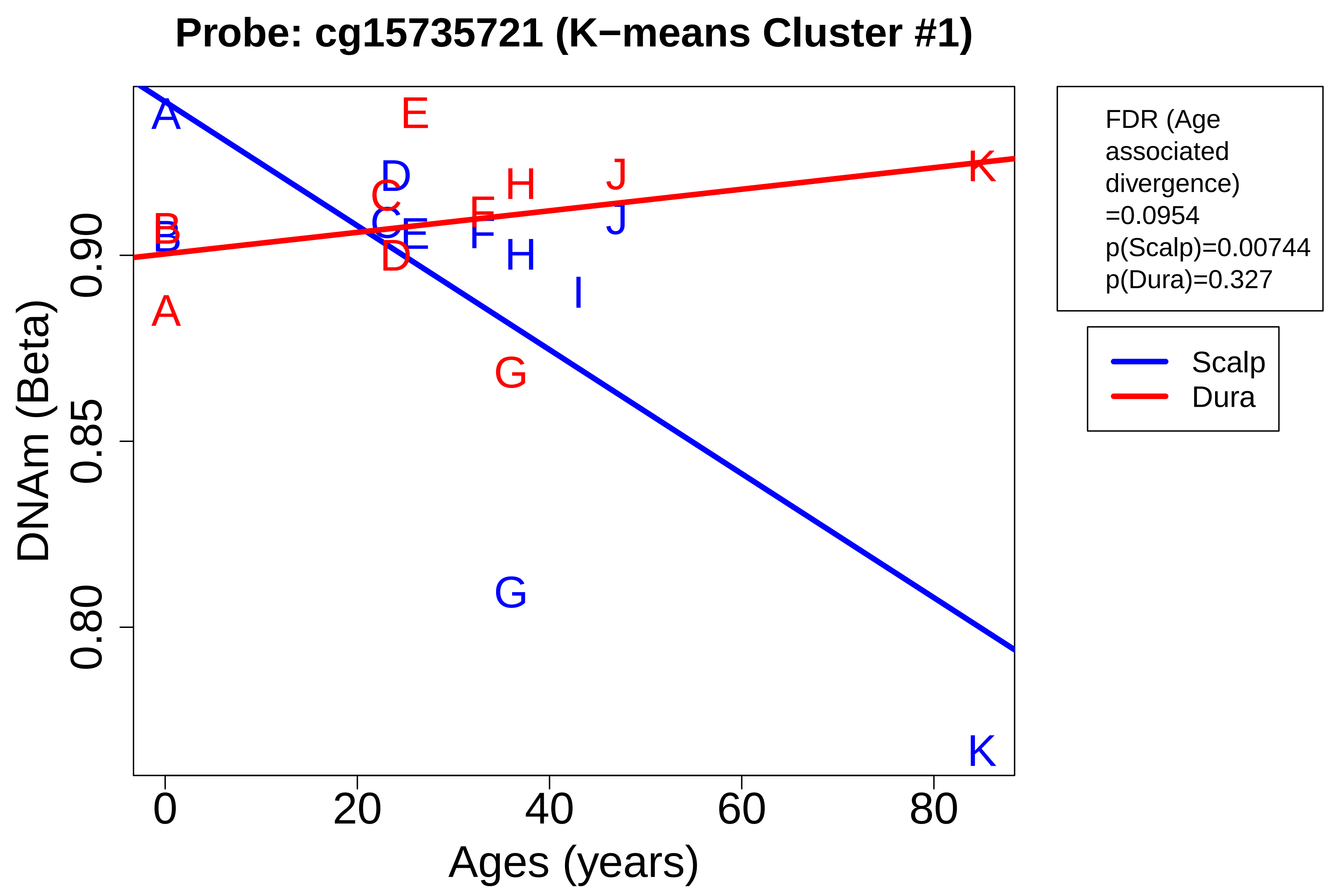

Probe: cg15735721 (K−means Cluster #1)
FDR (Age associated divergence) =0.0954
p(Scalp)=0.00744
p(Dura)=0.327
Scalp
Dura
DNAm (Beta)
0.80
0.85
0.90
Ages (years)
0
20
40
60
80
A
B
C
D
E
F
G
H
I
J
K

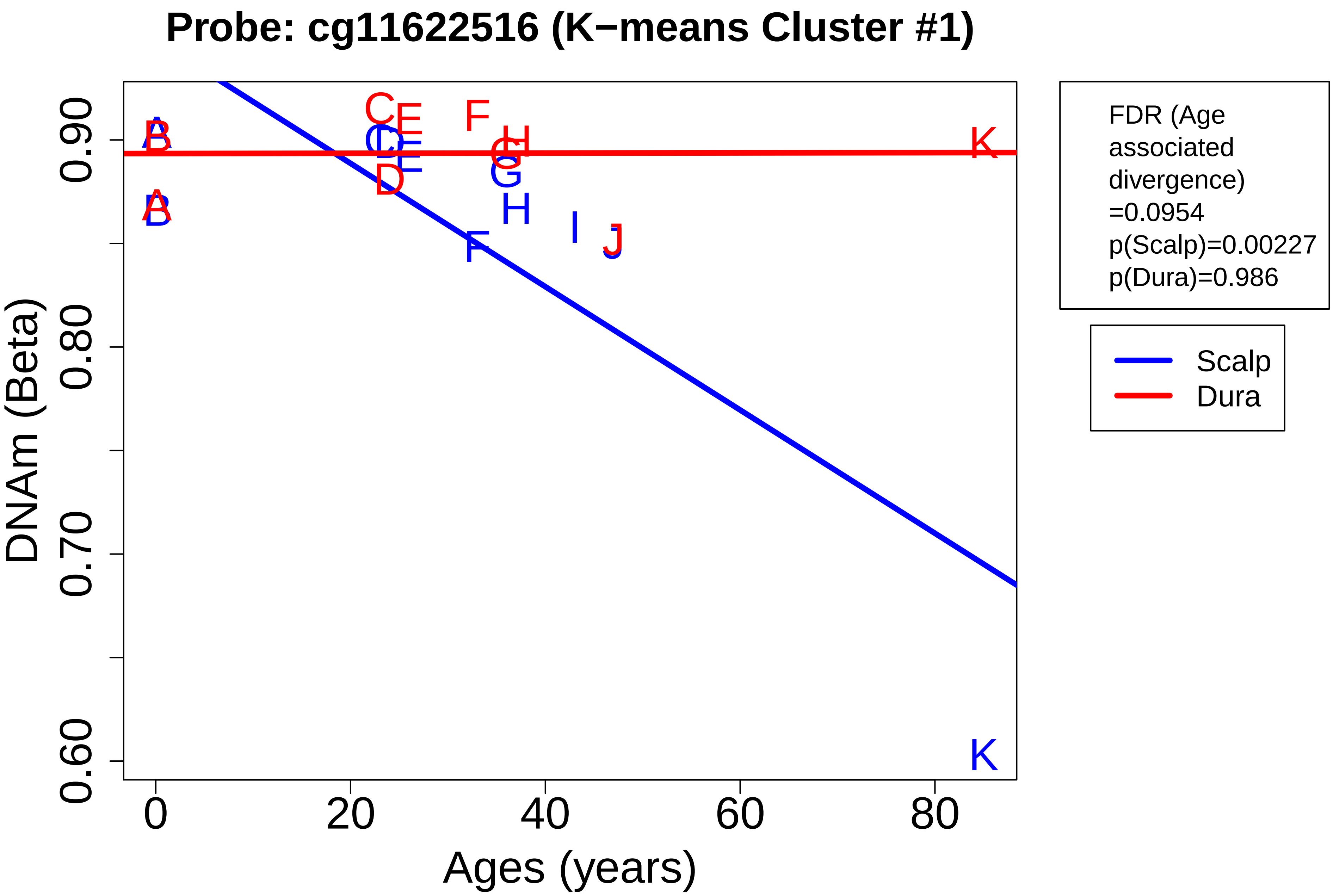

Probe: cg11622516 (K−means Cluster #1)
DNAm (Beta)
0.60
0.70
0.80
0.90
Ages (years)
0
20
40
60
80
FDR (Age associated divergence) =0.0954
p(Scalp)=0.00227
p(Dura)=0.986
Scalp
Dura

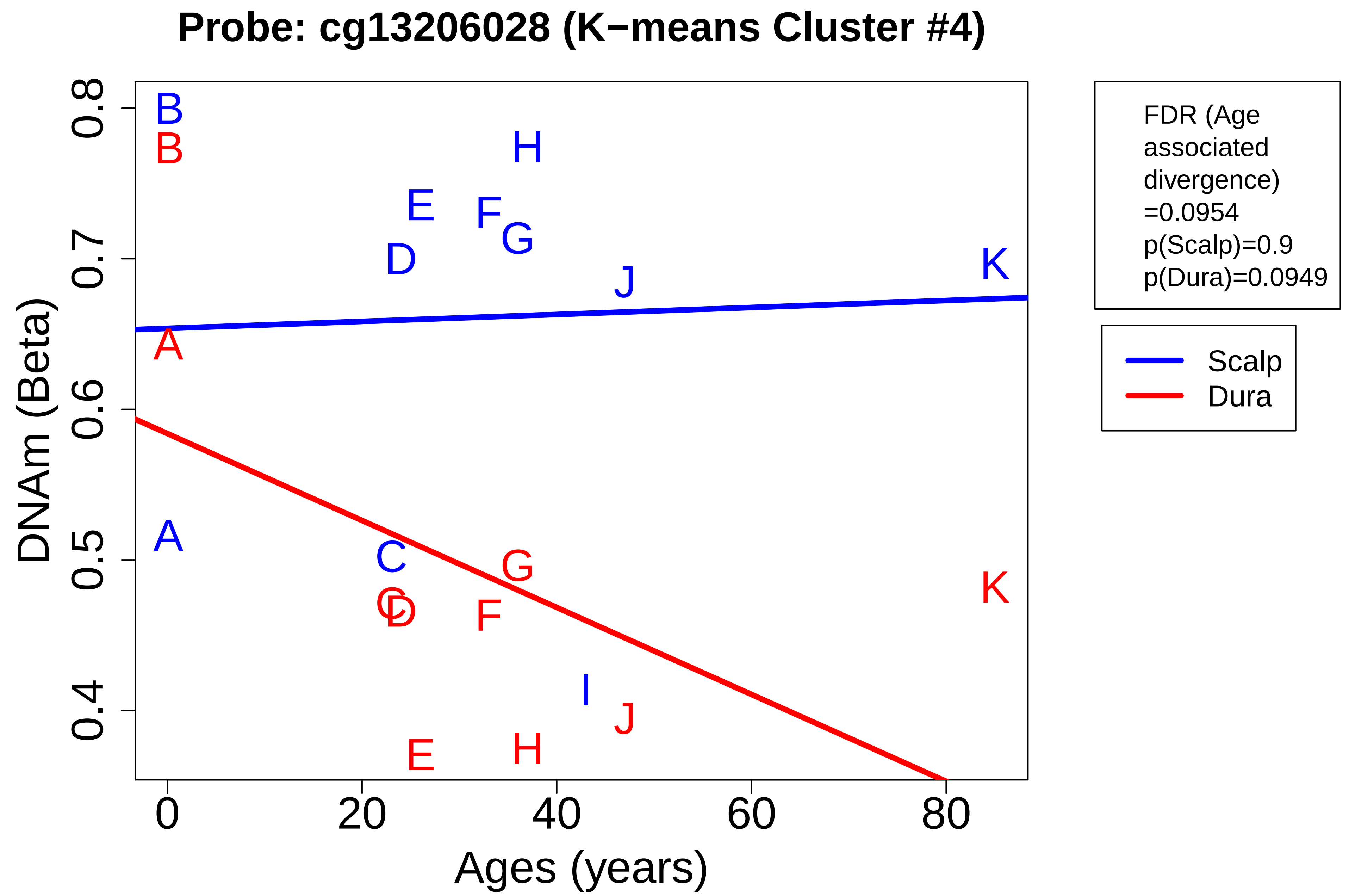

Probe: cg13206028 (K−means Cluster #4)
DNAm (Beta)
0.4
0.5
0.6
0.7
0.8
0
20
40
60
80
Ages (years)
FDR (Age associated divergence) =0.0954
p(Scalp)=0.9
p(Dura)=0.0949
Scalp
Dura

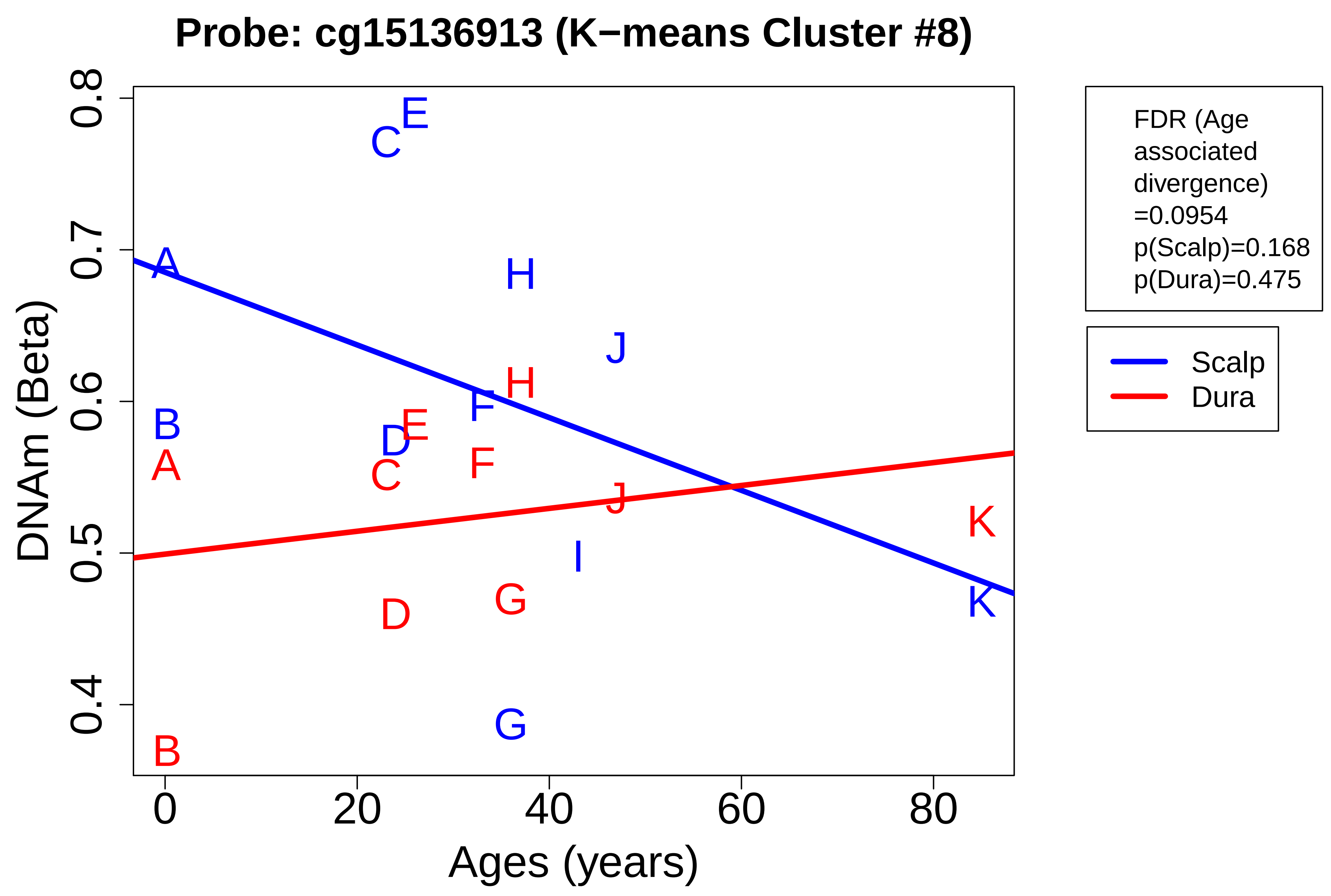

Probe: cg15136913 (K−means Cluster #8)
DNAm (Beta)
0.4
0.5
0.6
0.7
0.8
0
20
40
60
80
Ages (years)
A B C D E F G H I J K
A B C D E F G H J K
FDR (Age associated divergence) =0.0954
p(Scalp)=0.168
p(Dura)=0.475
Scalp
Dura

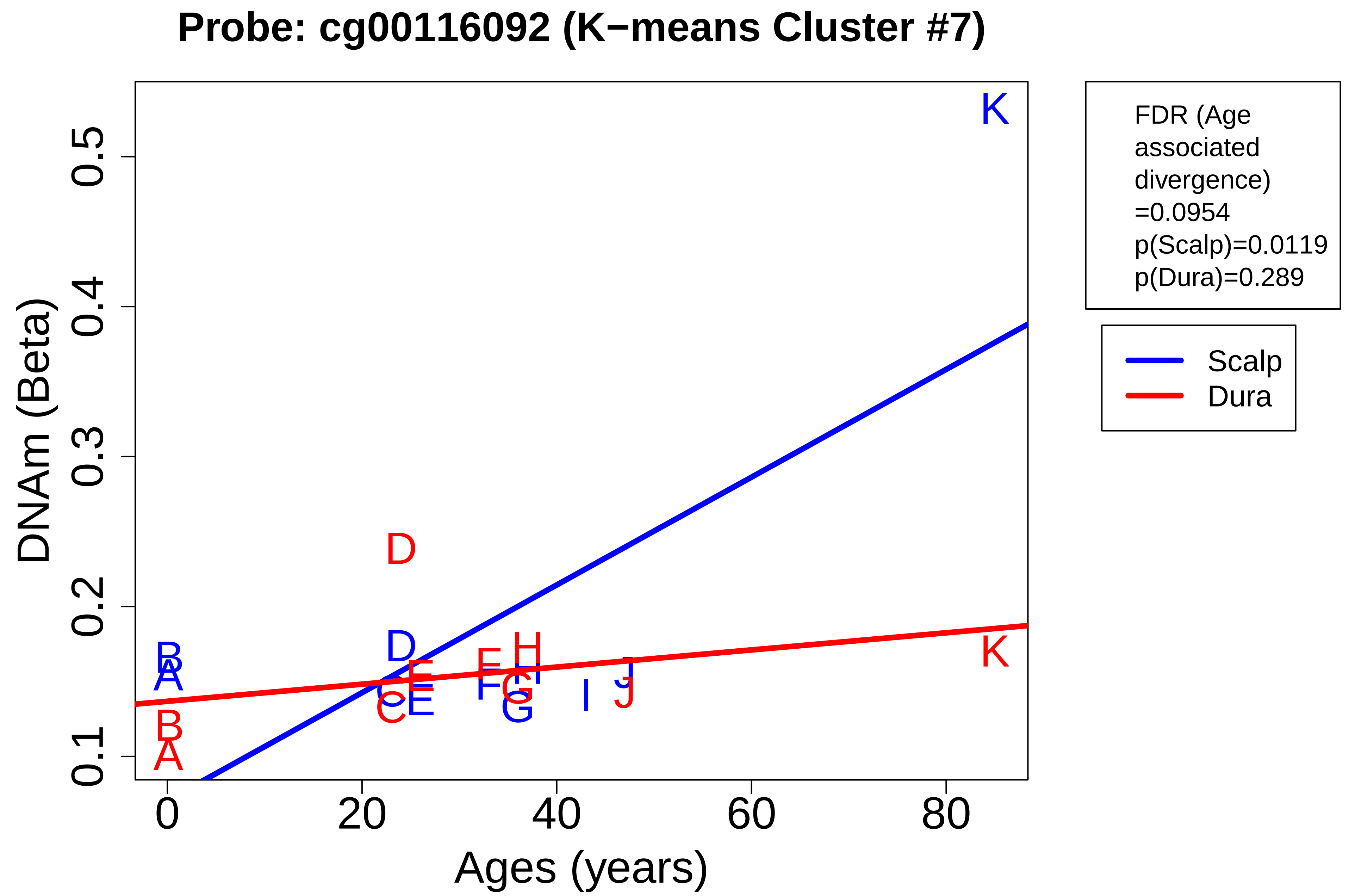
Probe: cg00116092 (K−means Cluster #7)
DNAm (Beta)
0.1
0.2
0.3
0.4
0.5
Ages (years)
0
20
40
60
80
A
B
C
D
E
F
G
H
I
J
K
FDR (Age associated divergence) =0.0954
p(Scalp)=0.0119
p(Dura)=0.289
Scalp
Dura

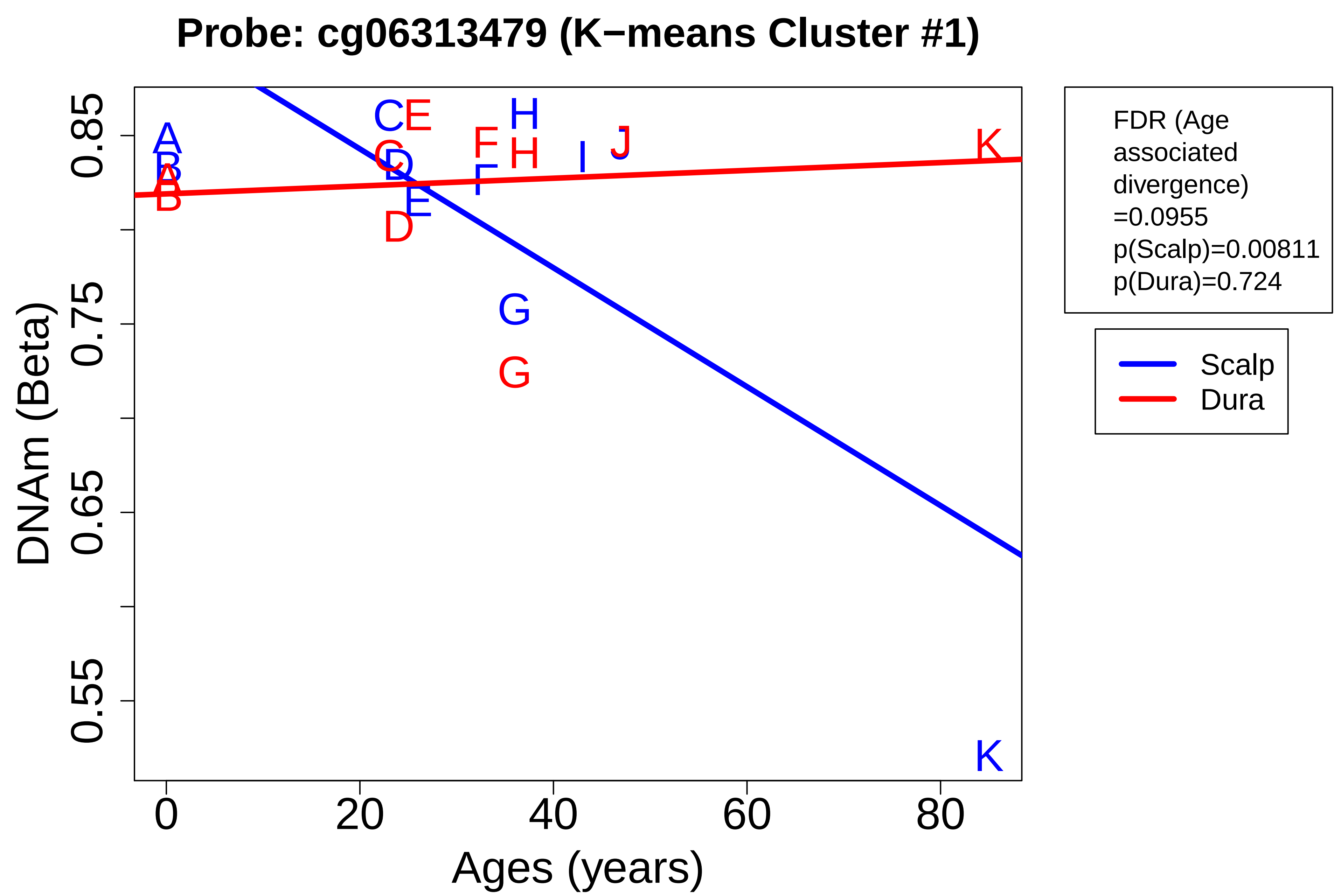

Probe: cg06313479 (K−means Cluster #1)
DNAm (Beta)
0.55
0.65
0.75
0.85
0
20
40
60
80
Ages (years)
A
B
C
D
E
F
G
H
I
J
K
FDR (Age associated divergence) =0.0955
p(Scalp)=0.00811
p(Dura)=0.724
Scalp
Dura

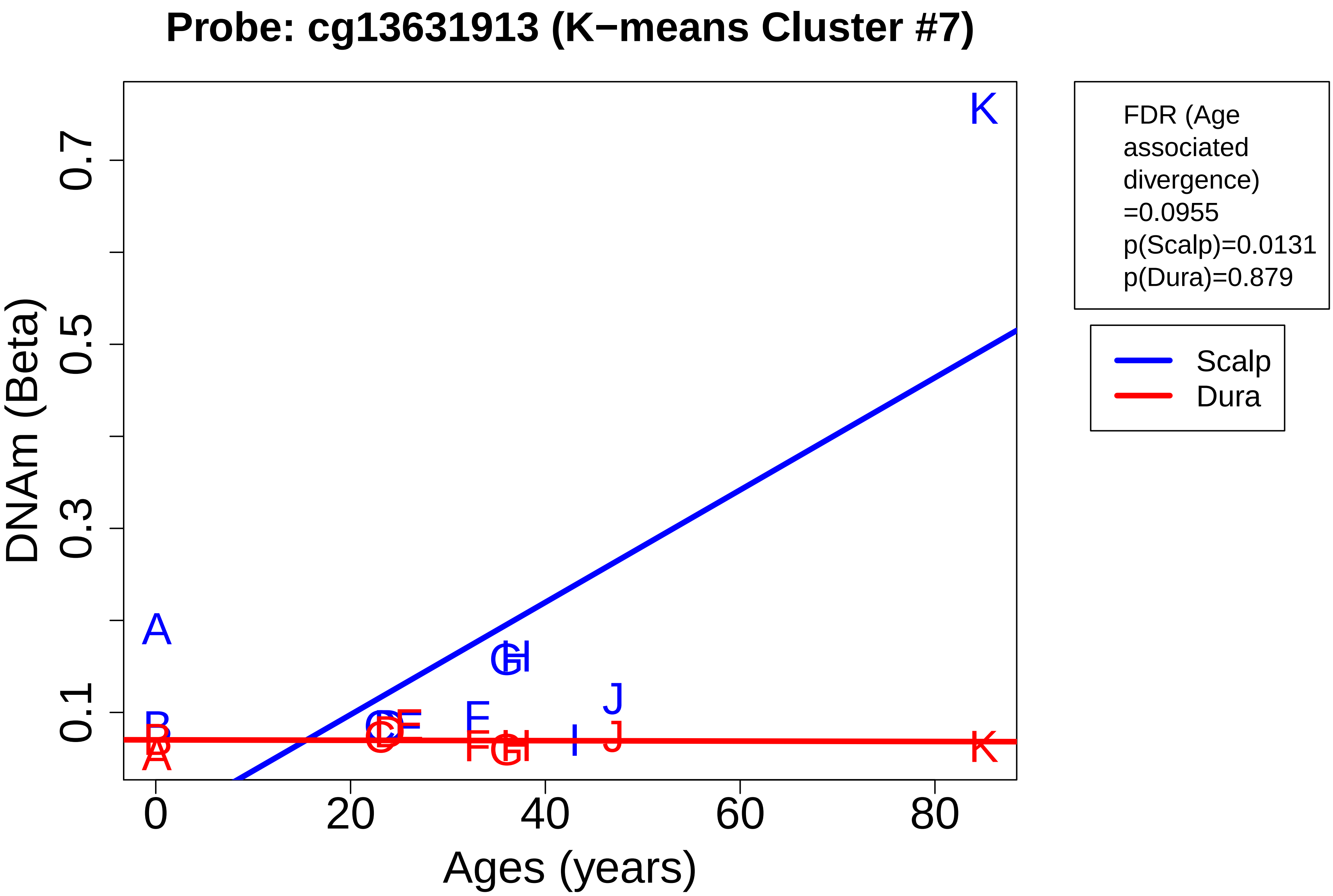

Probe: cg13631913 (K−means Cluster #7)
FDR (Age associated divergence) =0.0955
p(Scalp)=0.0131
p(Dura)=0.879
Scalp
Dura
DNAm (Beta)
0.1
0.3
0.5
0.7
Ages (years)
0
20
40
60
80

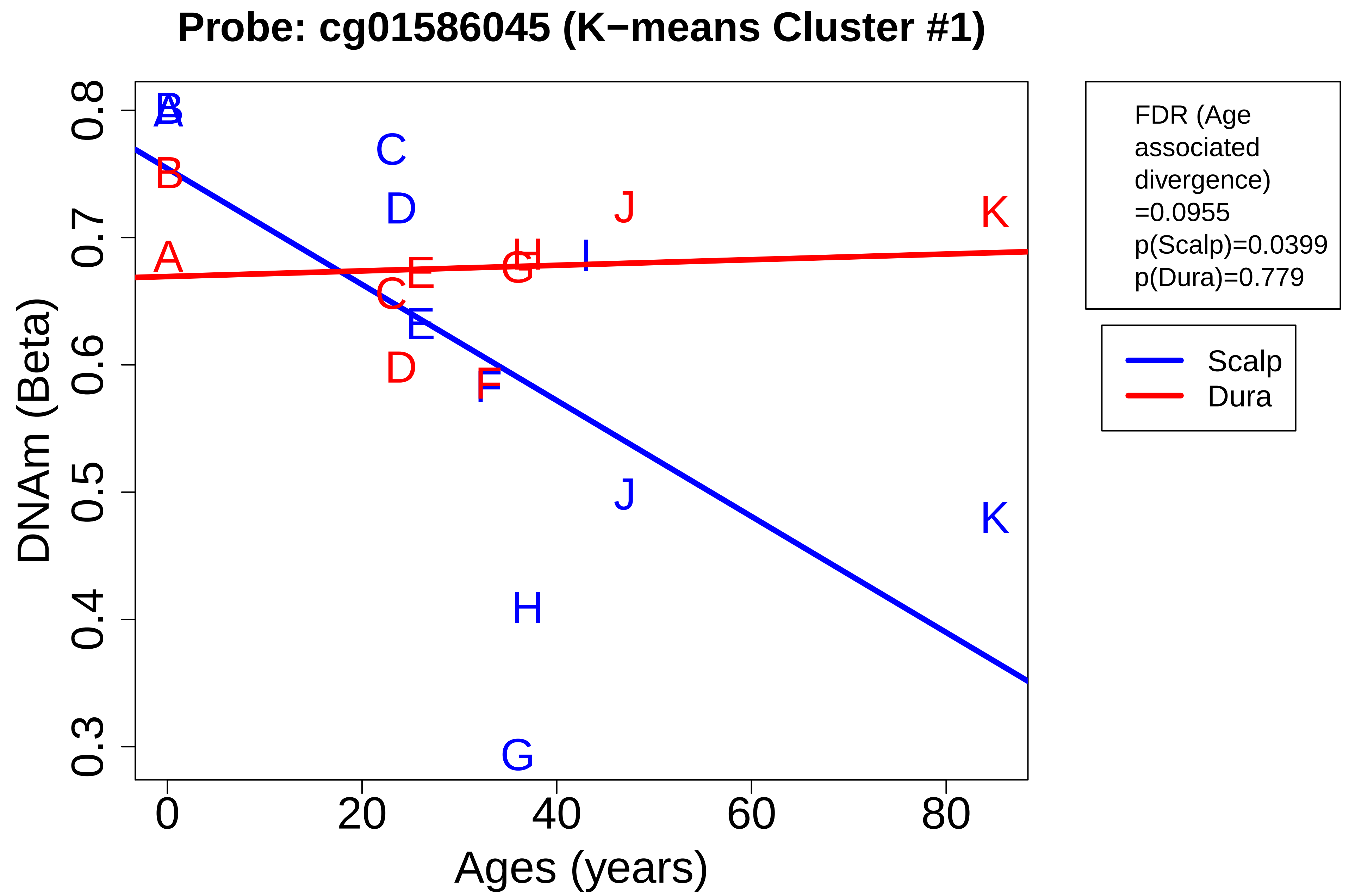

Probe: cg01586045 (K−means Cluster #1)
DNAm (Beta)
0.3
0.4
0.5
0.6
0.7
0.8
Ages (years)
0
20
40
60
80
A
B
C
D
E
F
G
H
I
J
K
FDR (Age associated divergence) =0.0955
p(Scalp)=0.0399
p(Dura)=0.779
Scalp
Dura

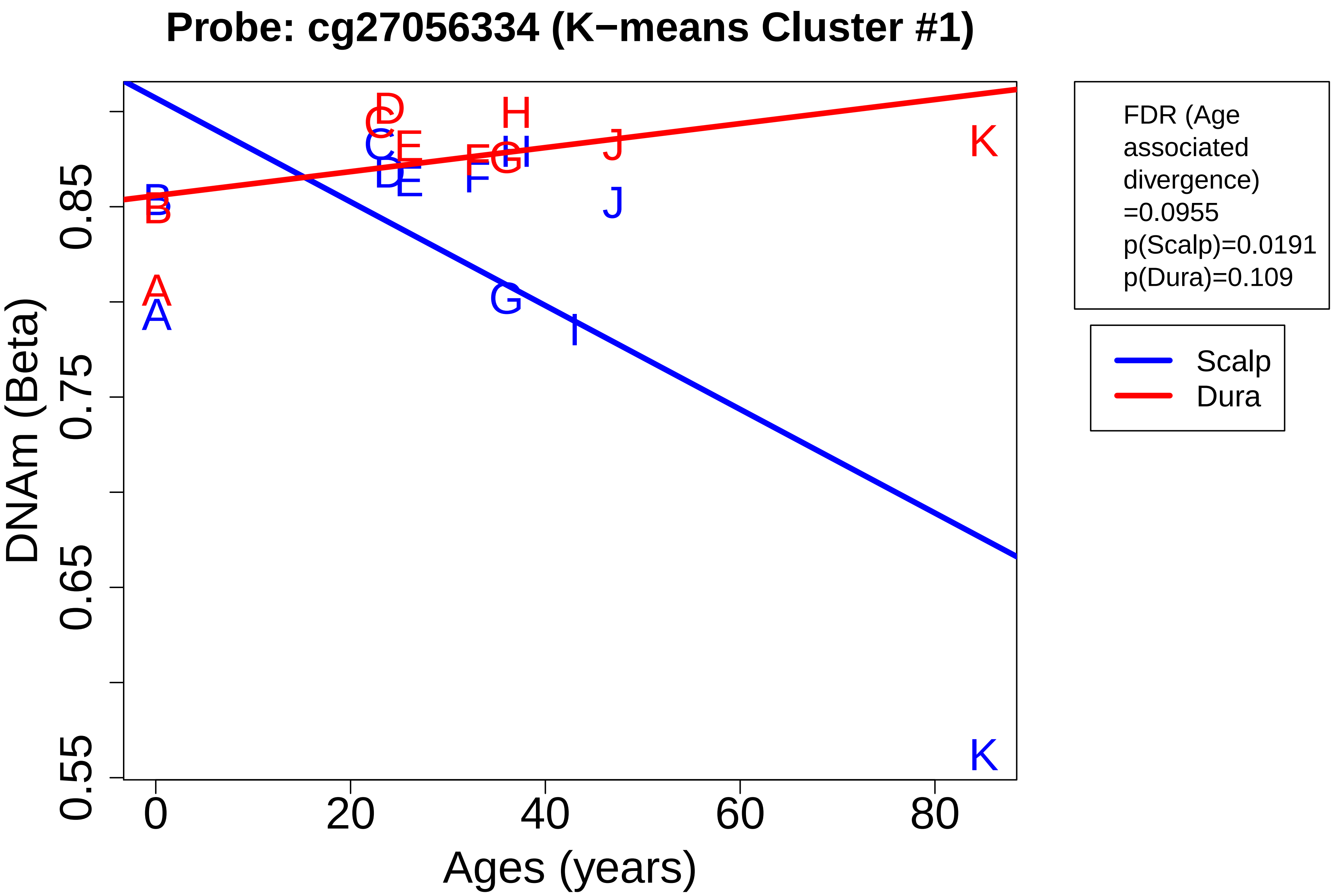

Probe: cg27056334 (K−means Cluster #1)
DNAm (Beta)
0.55
0.65
0.75
0.85
0
20
40
60
80
Ages (years)
FDR (Age associated divergence) =0.0955
p(Scalp)=0.0191
p(Dura)=0.109
Scalp
Dura

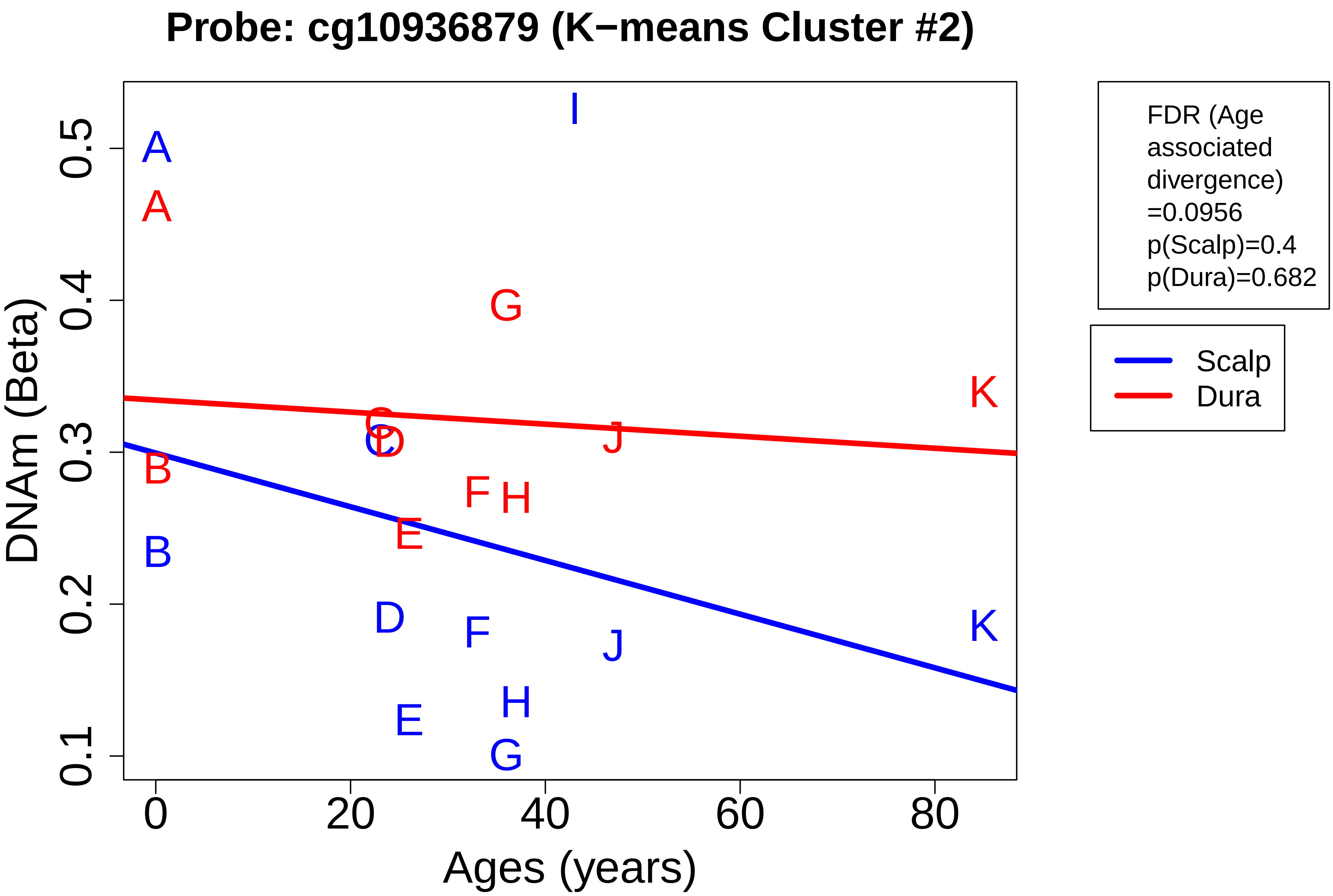

Probe: cg10936879 (K−means Cluster #2)
DNAm (Beta)
0.1
0.2
0.3
0.4
0.5
Ages (years)
0
20
40
60
80
FDR (Age associated divergence) =0.0956
p(Scalp)=0.4
p(Dura)=0.682
Scalp
Dura

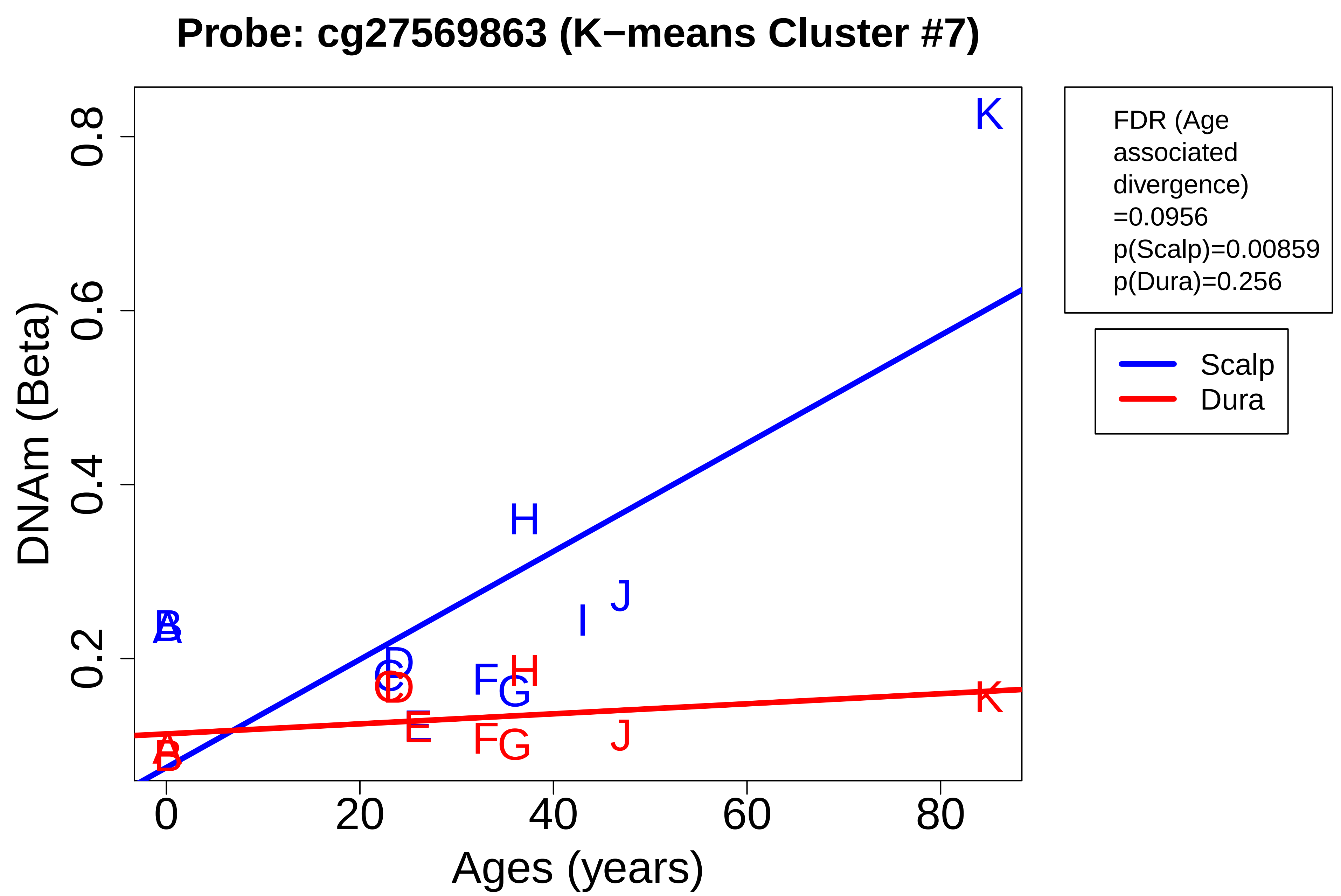

Probe: cg27569863 (K−means Cluster #7)
DNAm (Beta)
0.2
0.4
0.6
0.8
Ages (years)
0
20
40
60
80
FDR (Age associated divergence) =0.0956
p(Scalp)=0.00859
p(Dura)=0.256
Scalp
Dura
A B C D E F G H I J K

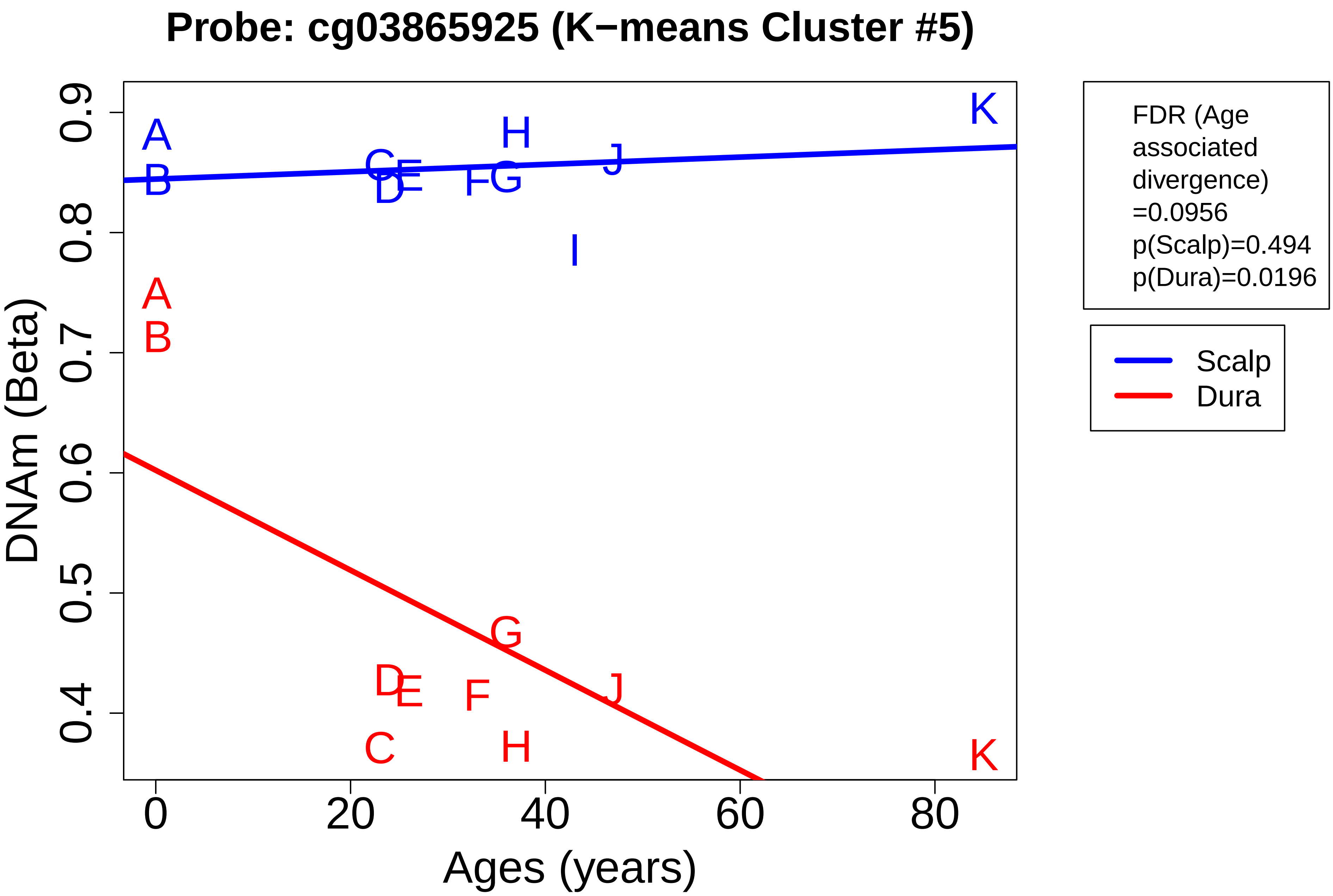

Probe: cg03865925 (K−means Cluster #5)
DNAm (Beta)
0.4
0.5
0.6
0.7
0.8
0.9
0
20
40
60
80
Ages (years)
A
B
C
D
E
F
G
H
I
J
K
FDR (Age associated divergence) =0.0956
p(Scalp)=0.494
p(Dura)=0.0196
Scalp
Dura

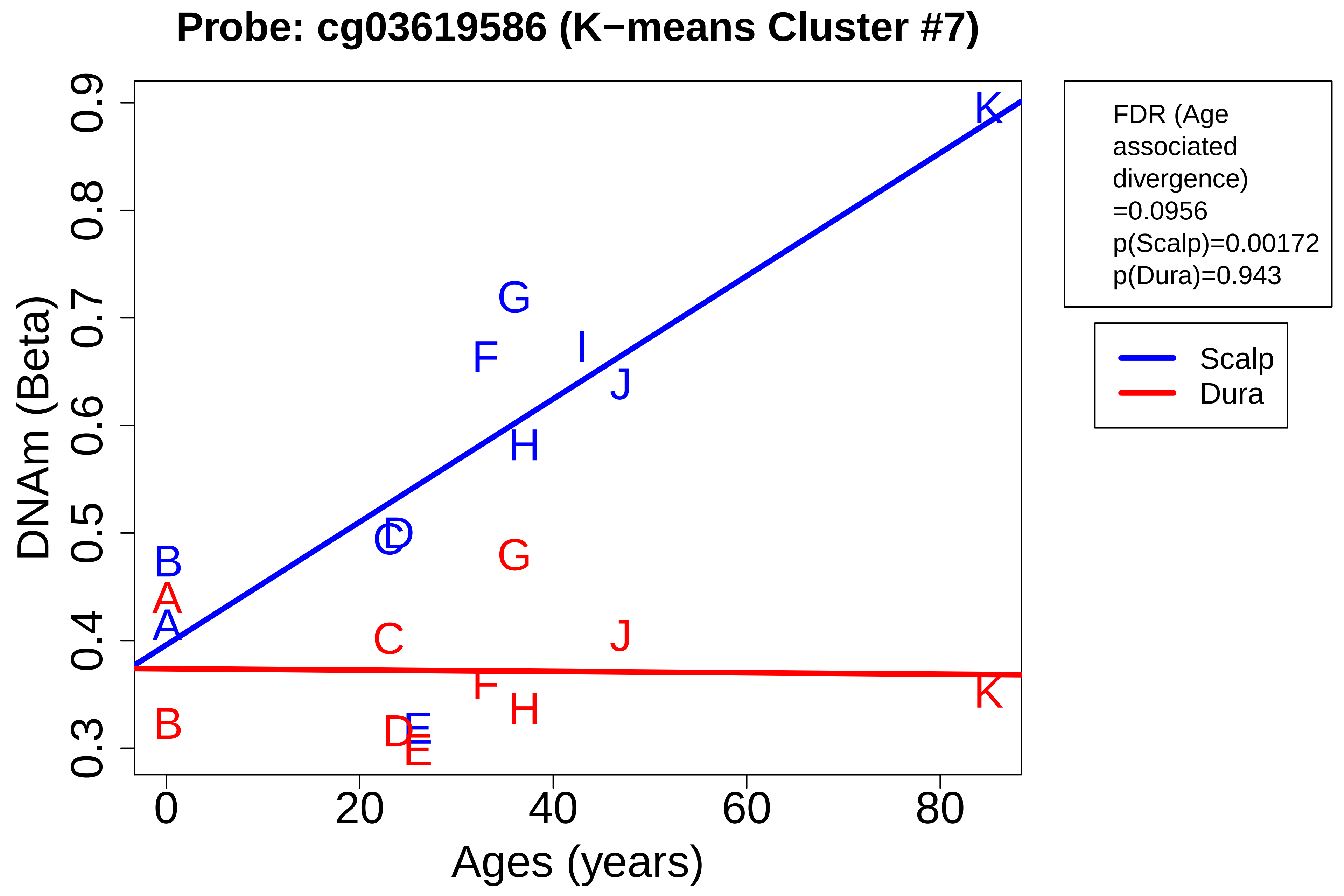
Probe: cg03619586 (K−means Cluster #7)
DNAm (Beta)
0.3
0.4
0.5
0.6
0.7
0.8
0.9
0
20
40
60
80
Ages (years)
FDR (Age associated divergence) =0.0956
p(Scalp)=0.00172
p(Dura)=0.943
Scalp
Dura

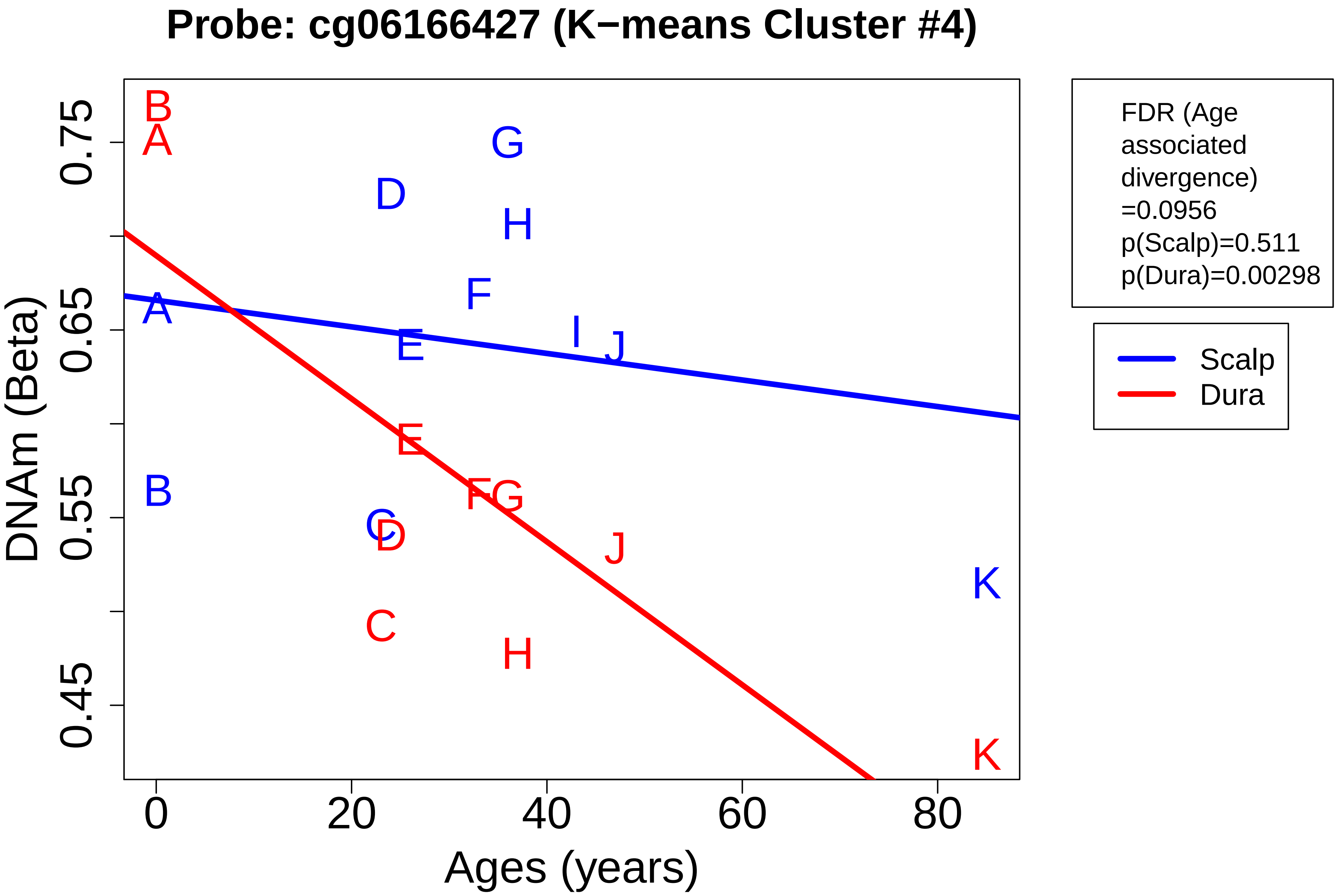

Probe: cg06166427 (K−means Cluster #4)
DNAm (Beta)
0.75
0.65
0.55
0.45
Ages (years)
0
20
40
60
80
FDR (Age associated divergence) =0.0956
p(Scalp)=0.511
p(Dura)=0.00298
Scalp
Dura

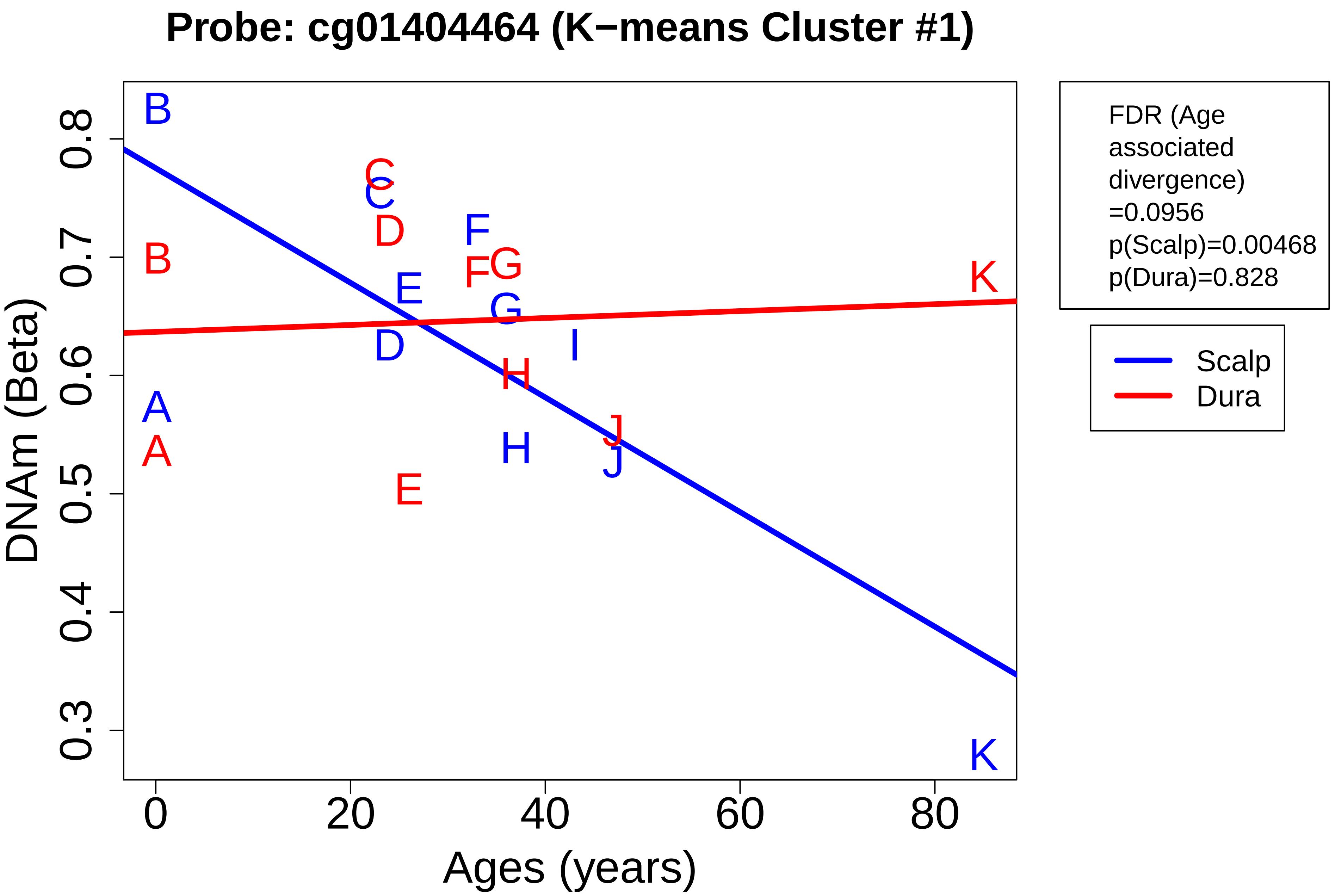

Probe: cg01404464 (K−means Cluster #1)
FDR (Age associated divergence) =0.0956
p(Scalp)=0.00468
p(Dura)=0.828
Scalp
Dura
DNAm (Beta)
Ages (years)
0.3
0.4
0.5
0.6
0.7
0.8
0
20
40
60
80

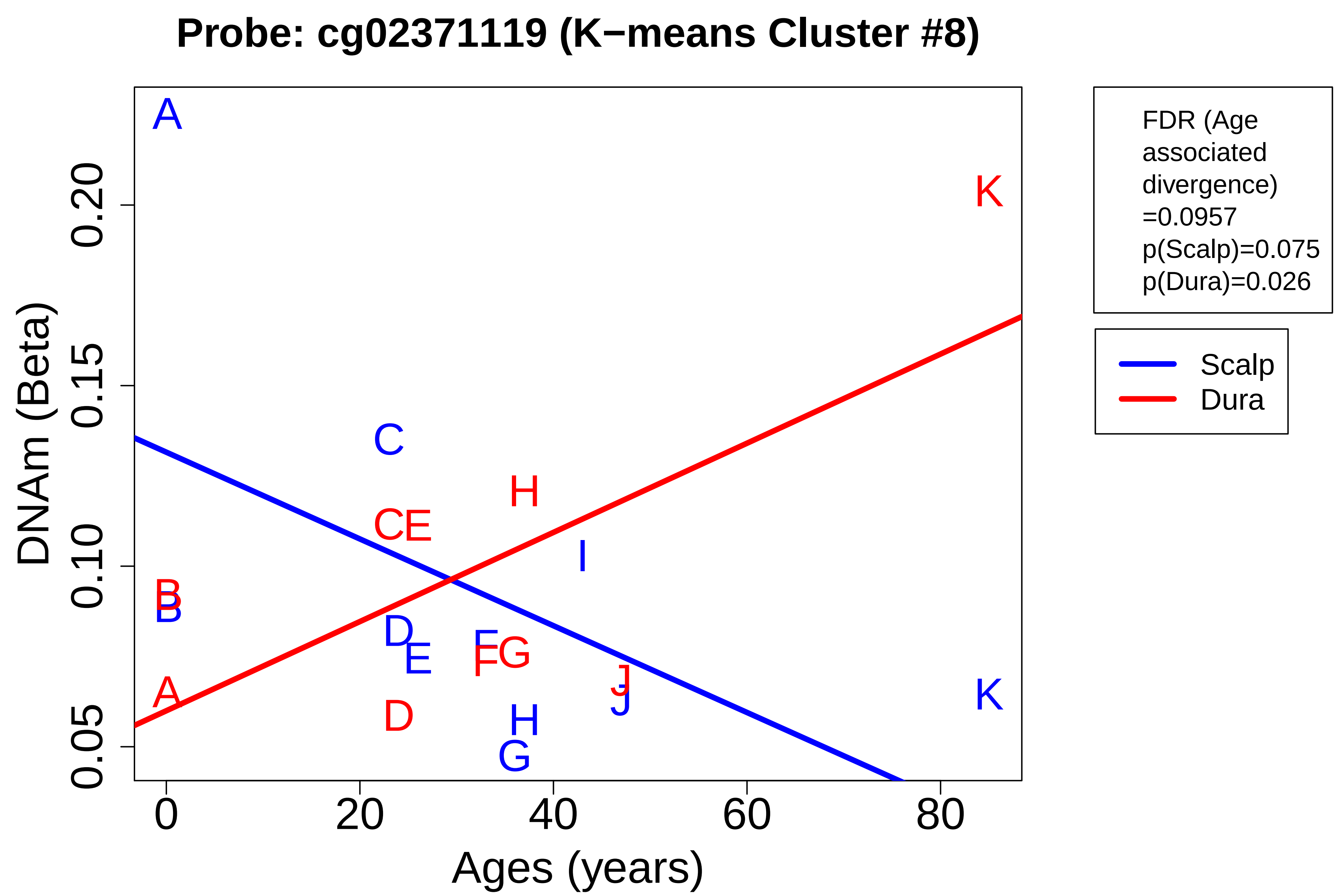

Probe: cg02371119 (K−means Cluster #8)
DNAm (Beta)
0.05
0.10
0.15
0.20
0
20
40
60
80
Ages (years)
A
B
C
D
E
F
G
H
I
J
K
A
B
CE
D
FG
H
J
K
FDR (Age associated divergence) =0.0957
p(Scalp)=0.075
p(Dura)=0.026
Scalp
Dura

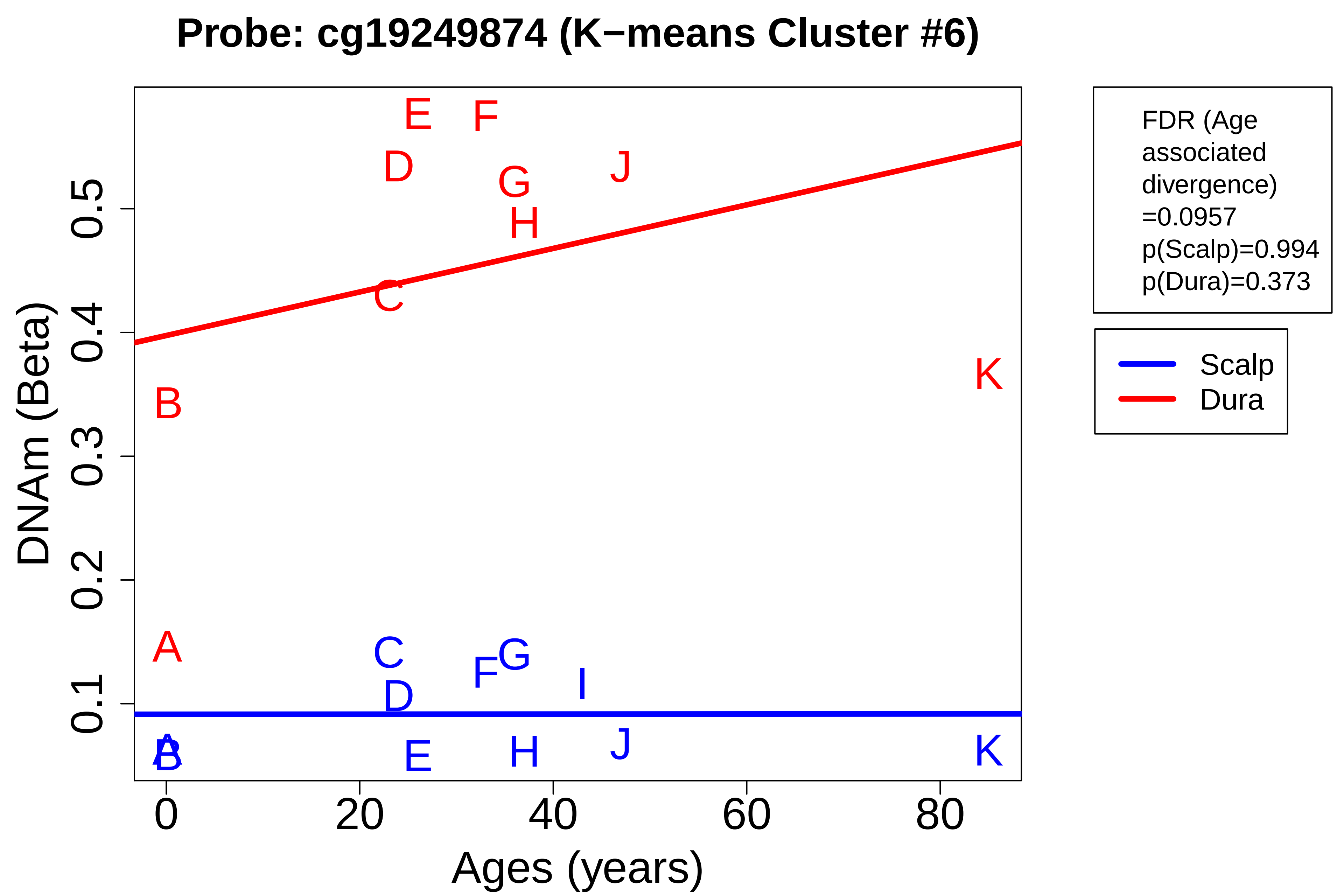

Probe: cg19249874 (K−means Cluster #6)
DNAm (Beta)
0.1
0.2
0.3
0.4
0.5
0
20
40
60
80
Ages (years)
FDR (Age associated divergence) =0.0957
p(Scalp)=0.994
p(Dura)=0.373
Scalp
Dura

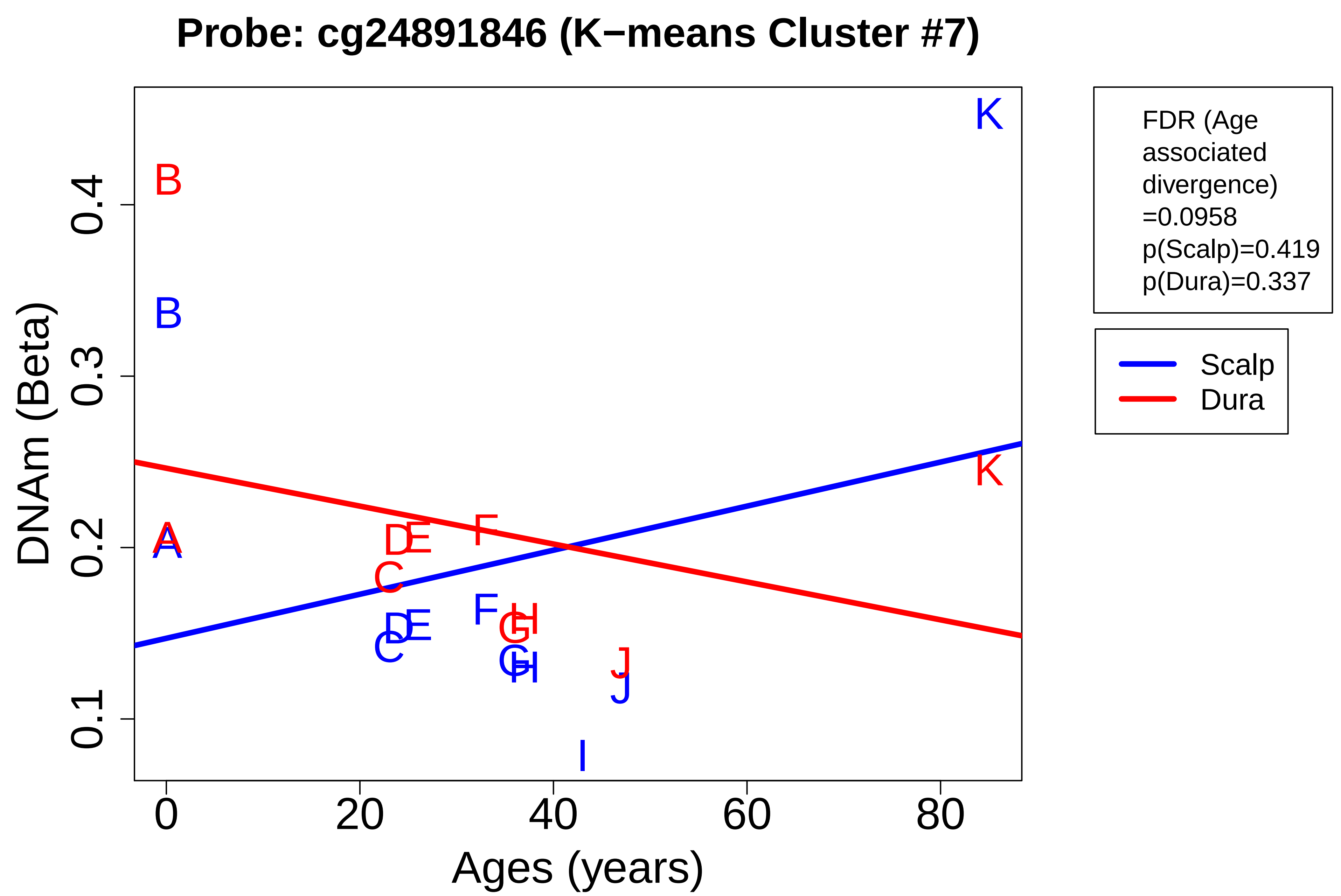

Probe: cg24891846 (K−means Cluster #7)
DNAm (Beta)
0.1
0.2
0.3
0.4
0
20
40
60
80
Ages (years)
A
B
C
D
E
F
G
H
I
J
K
FDR (Age associated divergence) =0.0958
p(Scalp)=0.419
p(Dura)=0.337
Scalp
Dura

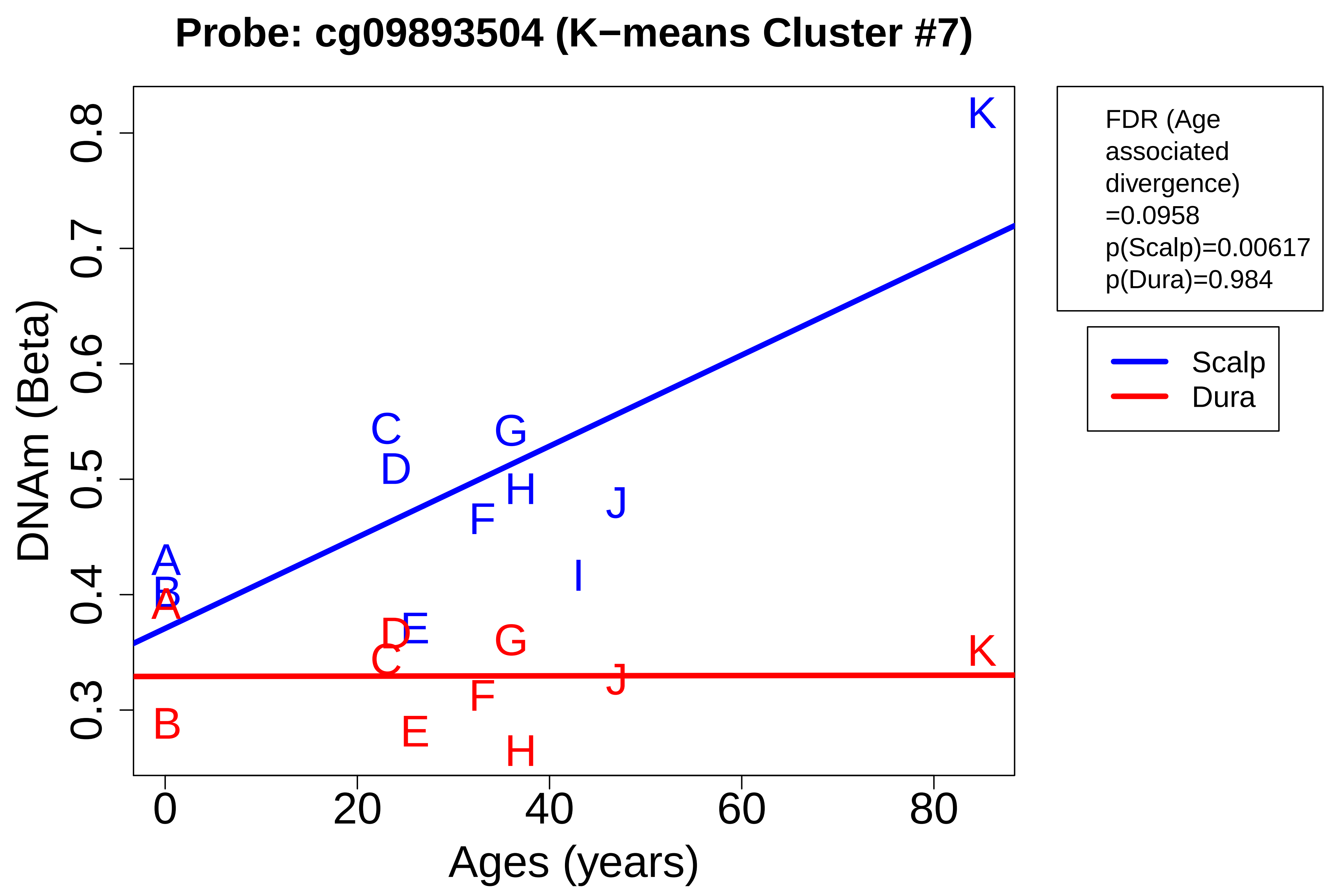
Probe: cg09893504 (K−means Cluster #7)
DNAm (Beta)
0.3
0.4
0.5
0.6
0.7
0.8
Ages (years)
0
20
40
60
80
FDR (Age associated divergence) =0.0958
p(Scalp)=0.00617
p(Dura)=0.984
Scalp
Dura

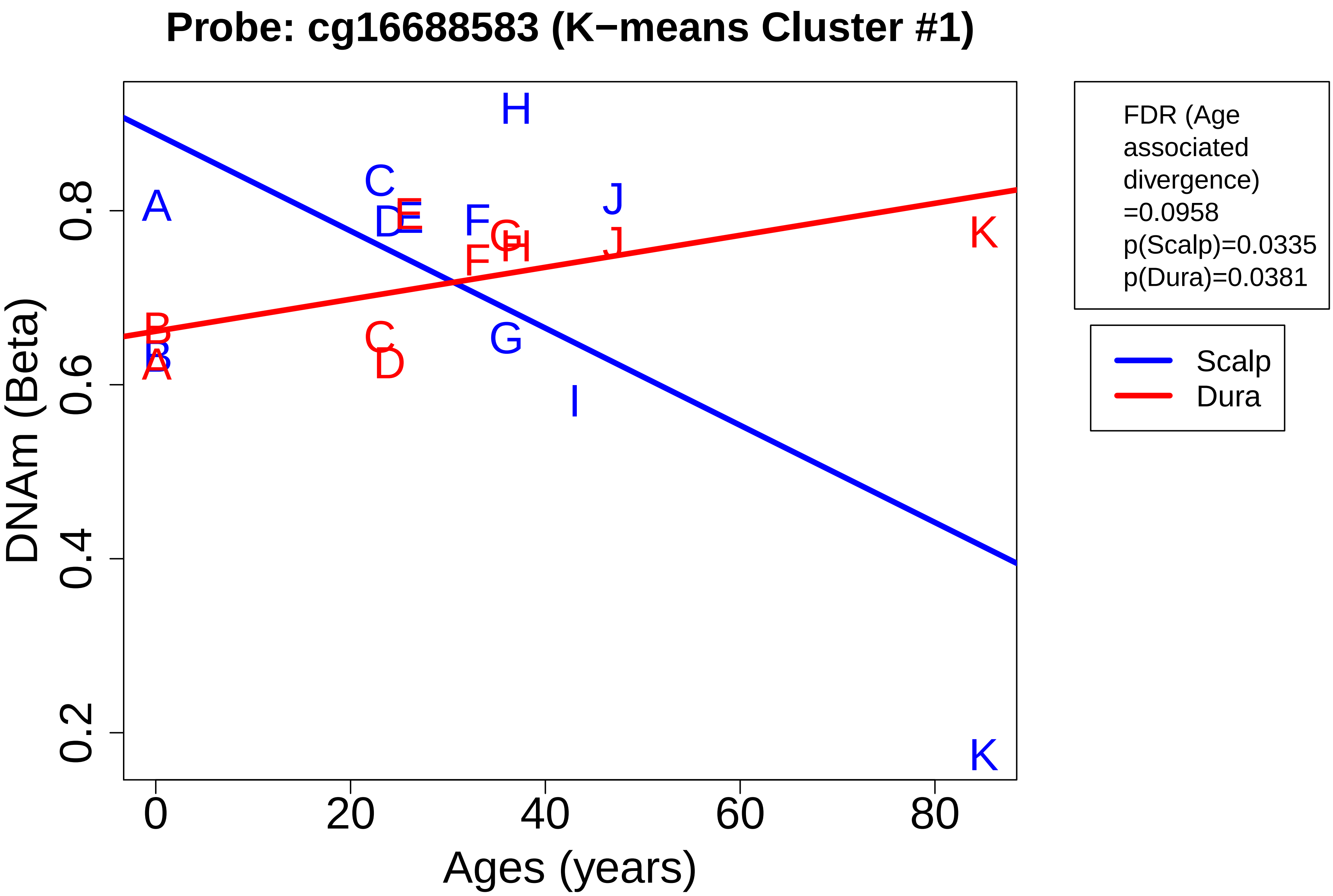

Probe: cg16688583 (K−means Cluster #1)
DNAm (Beta)
0.2
0.4
0.6
0.8
0
20
40
60
80
Ages (years)
FDR (Age associated divergence) =0.0958
p(Scalp)=0.0335
p(Dura)=0.0381
Scalp
Dura

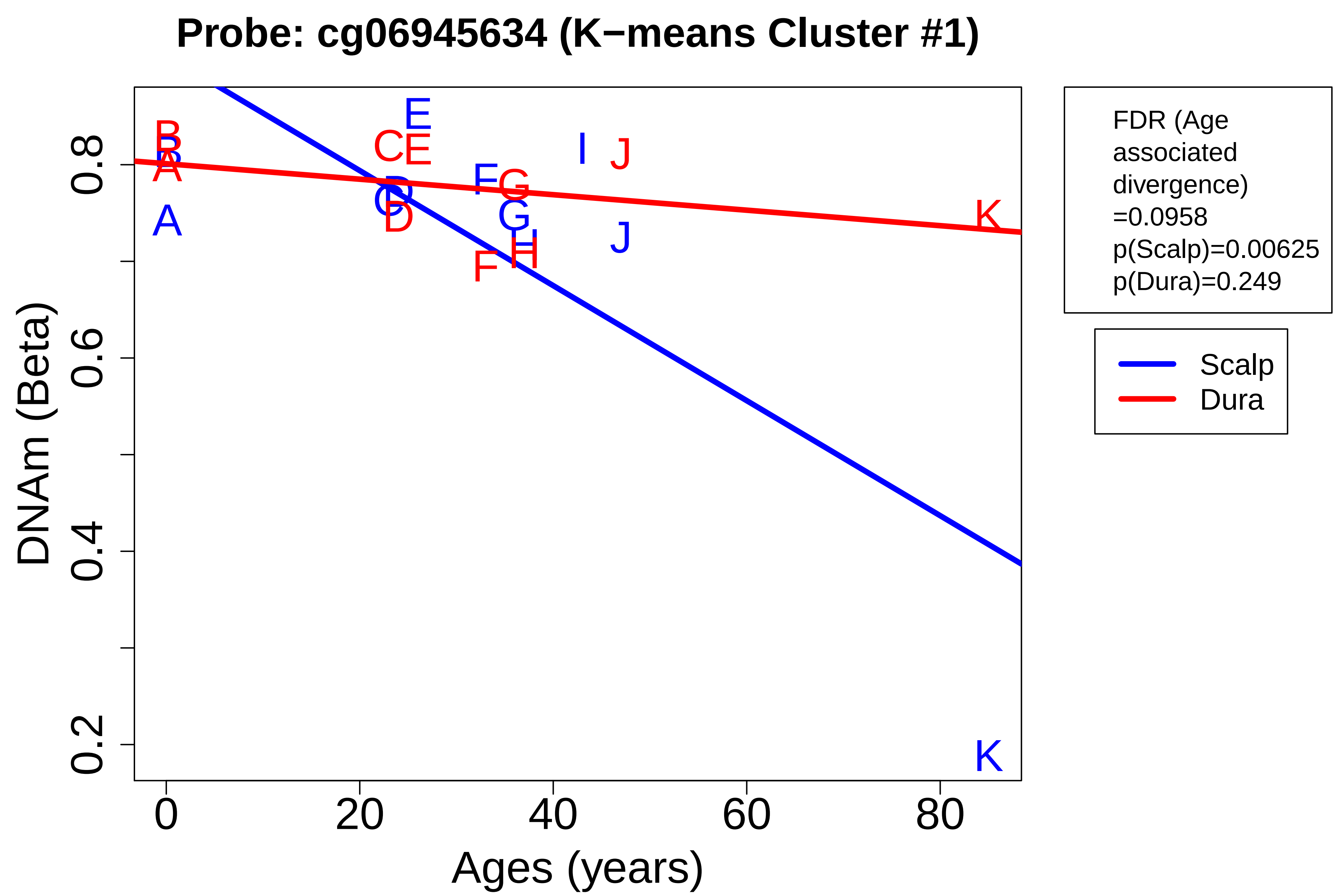

Probe: cg06945634 (K−means Cluster #1)
DNAm (Beta)
0.2
0.4
0.6
0.8
0
20
40
60
80
Ages (years)
FDR (Age associated divergence) =0.0958
p(Scalp)=0.00625
p(Dura)=0.249
Scalp
Dura

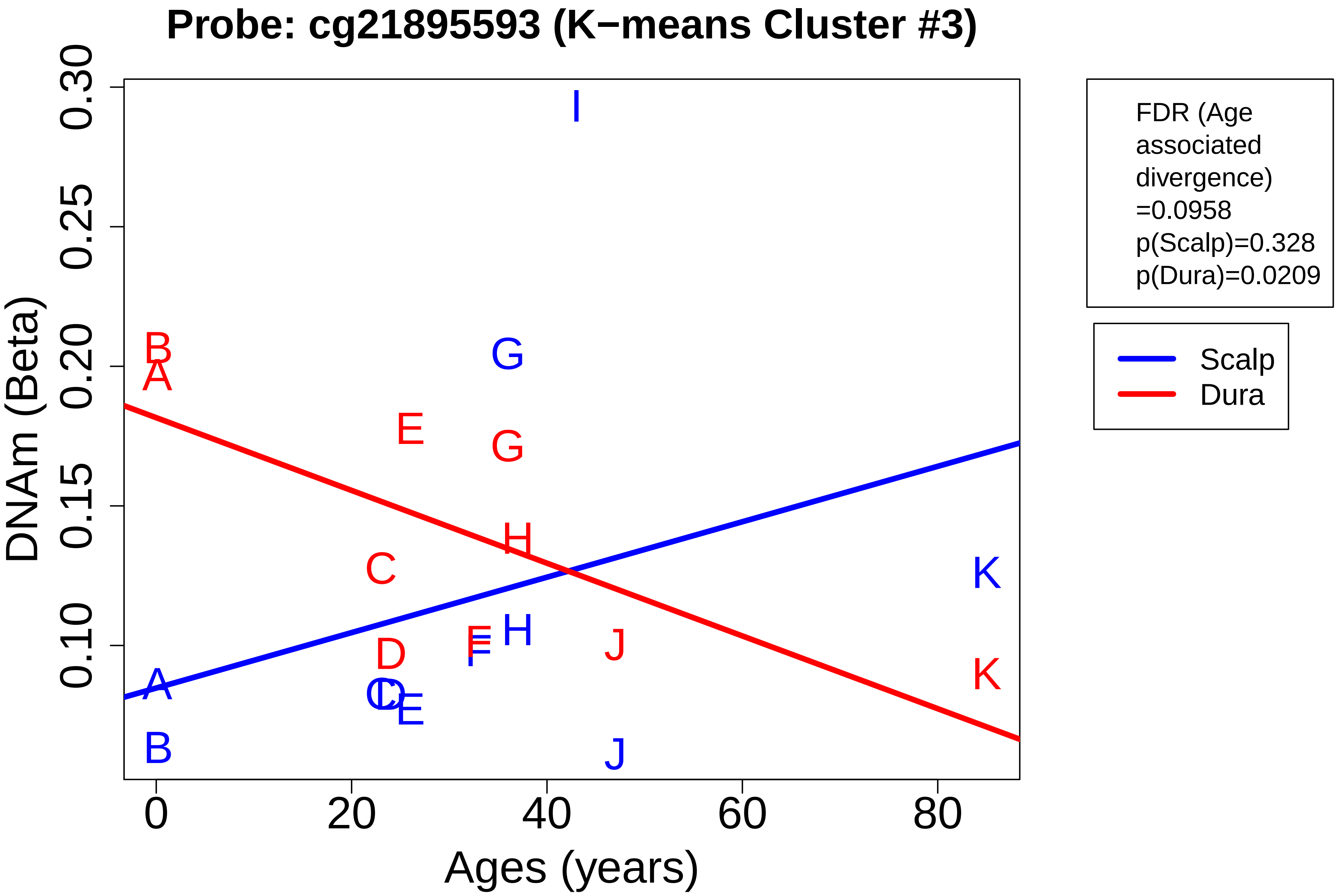

Probe: cg21895593 (K−means Cluster #3)
DNAm (Beta)
0.10
0.15
0.20
0.25
0.30
0
20
40
60
80
Ages (years)
FDR (Age associated divergence) =0.0958
p(Scalp)=0.328
p(Dura)=0.0209
Scalp
Dura

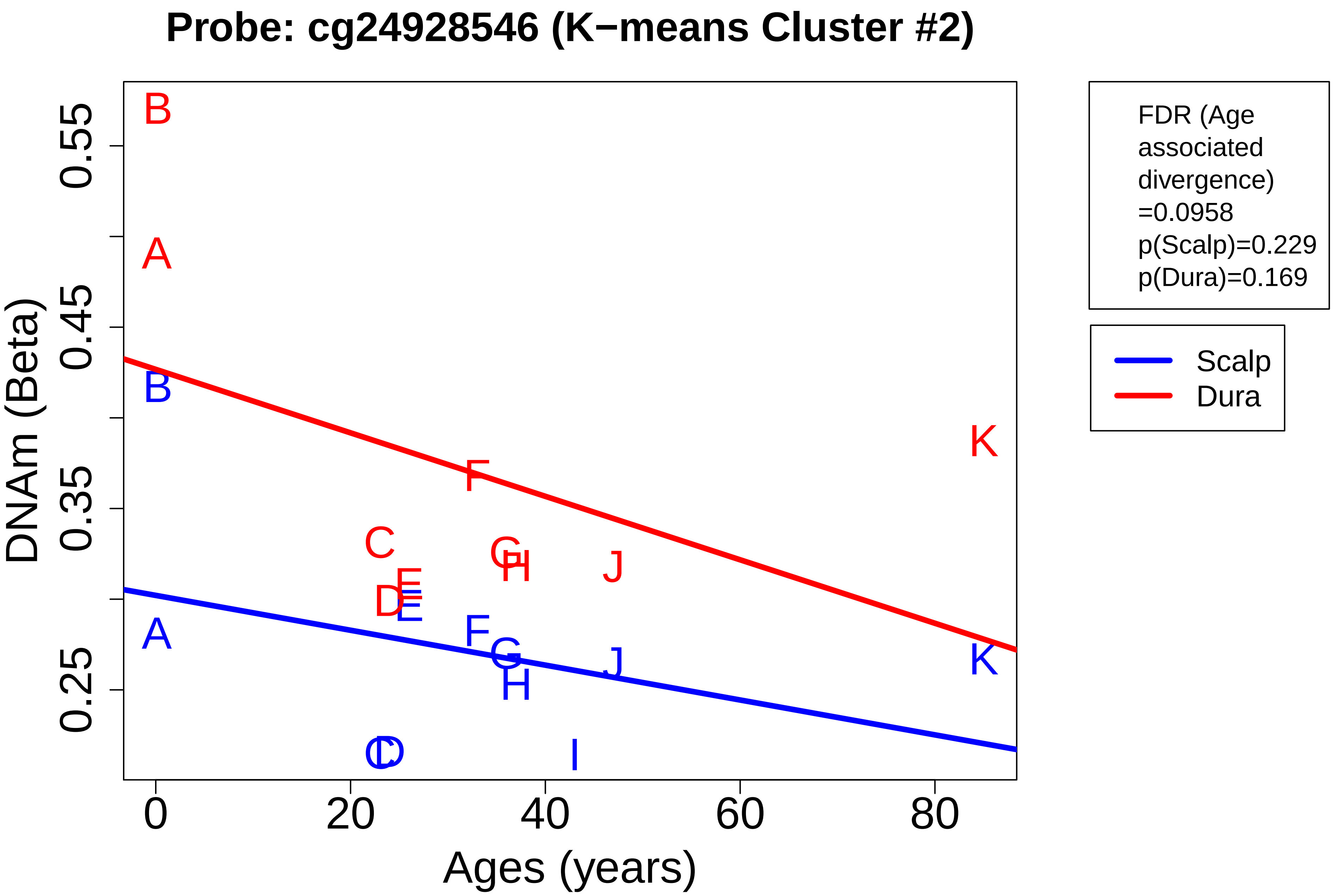

Probe: cg24928546 (K−means Cluster #2)
DNAm (Beta)
0.25
0.35
0.45
0.55
Ages (years)
0
20
40
60
80
FDR (Age associated divergence) =0.0958
p(Scalp)=0.229
p(Dura)=0.169
Scalp
Dura
A
B
C
D
E
F
G
H
I
J
K

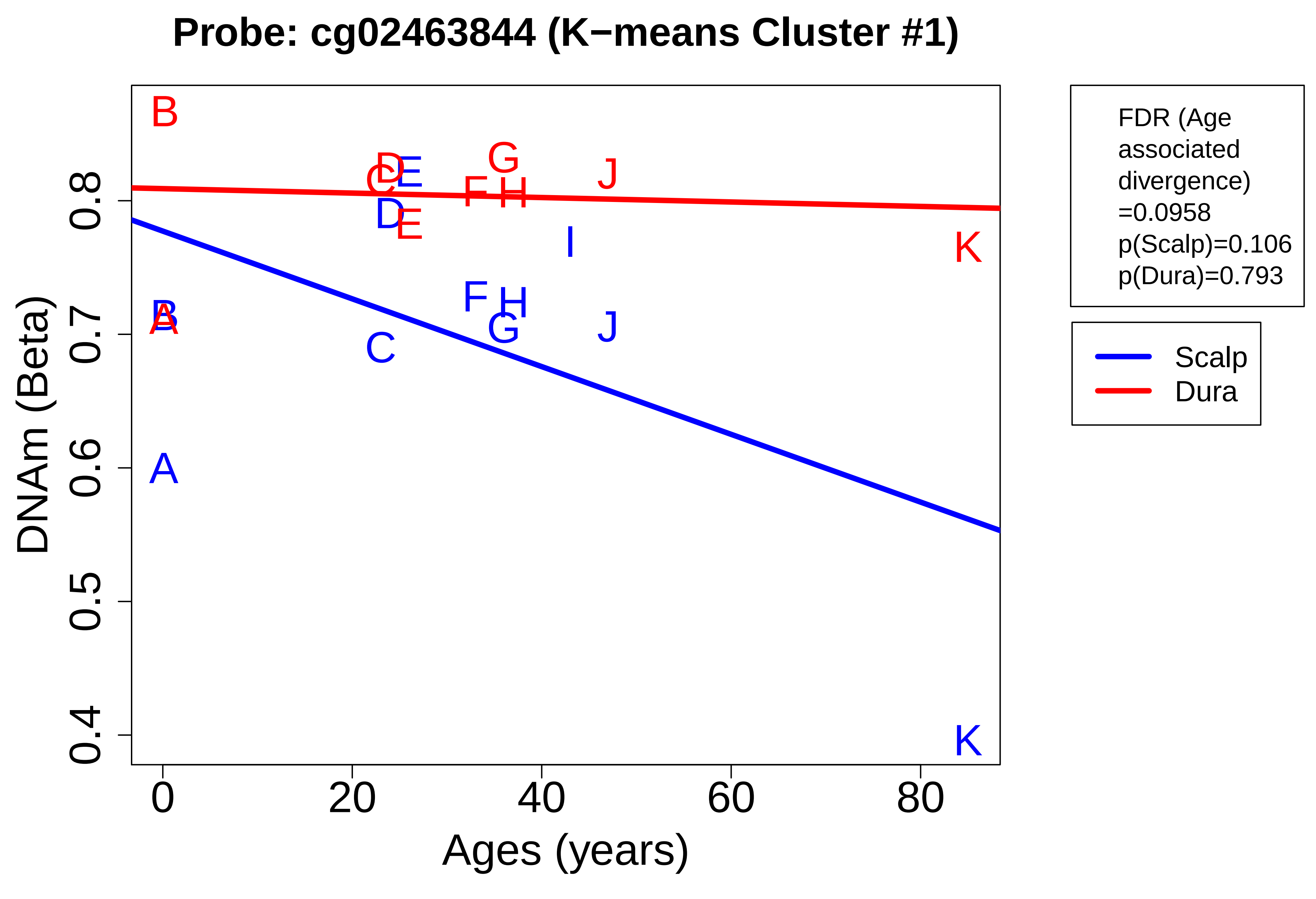

Probe: cg02463844 (K−means Cluster #1)
DNAm (Beta)
0.4
0.5
0.6
0.7
0.8
0
20
40
60
80
Ages (years)
FDR (Age associated divergence) =0.0958
p(Scalp)=0.106
p(Dura)=0.793
Scalp
Dura

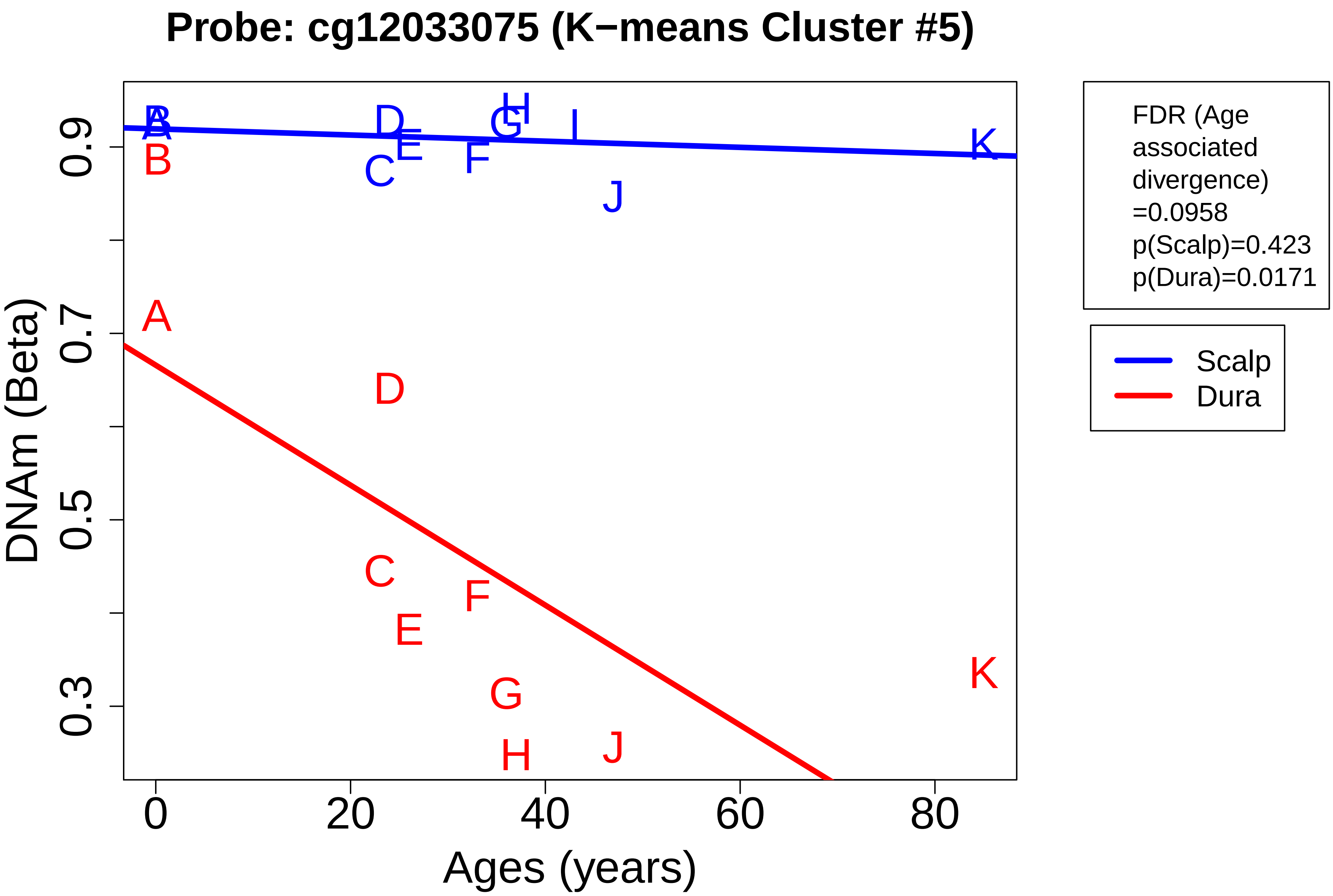

Probe: cg12033075 (K−means Cluster #5)
DNAm (Beta)
0.3
0.5
0.7
0.9
0
20
40
60
80
Ages (years)
FDR (Age associated divergence) =0.0958
p(Scalp)=0.423
p(Dura)=0.0171
Scalp
Dura

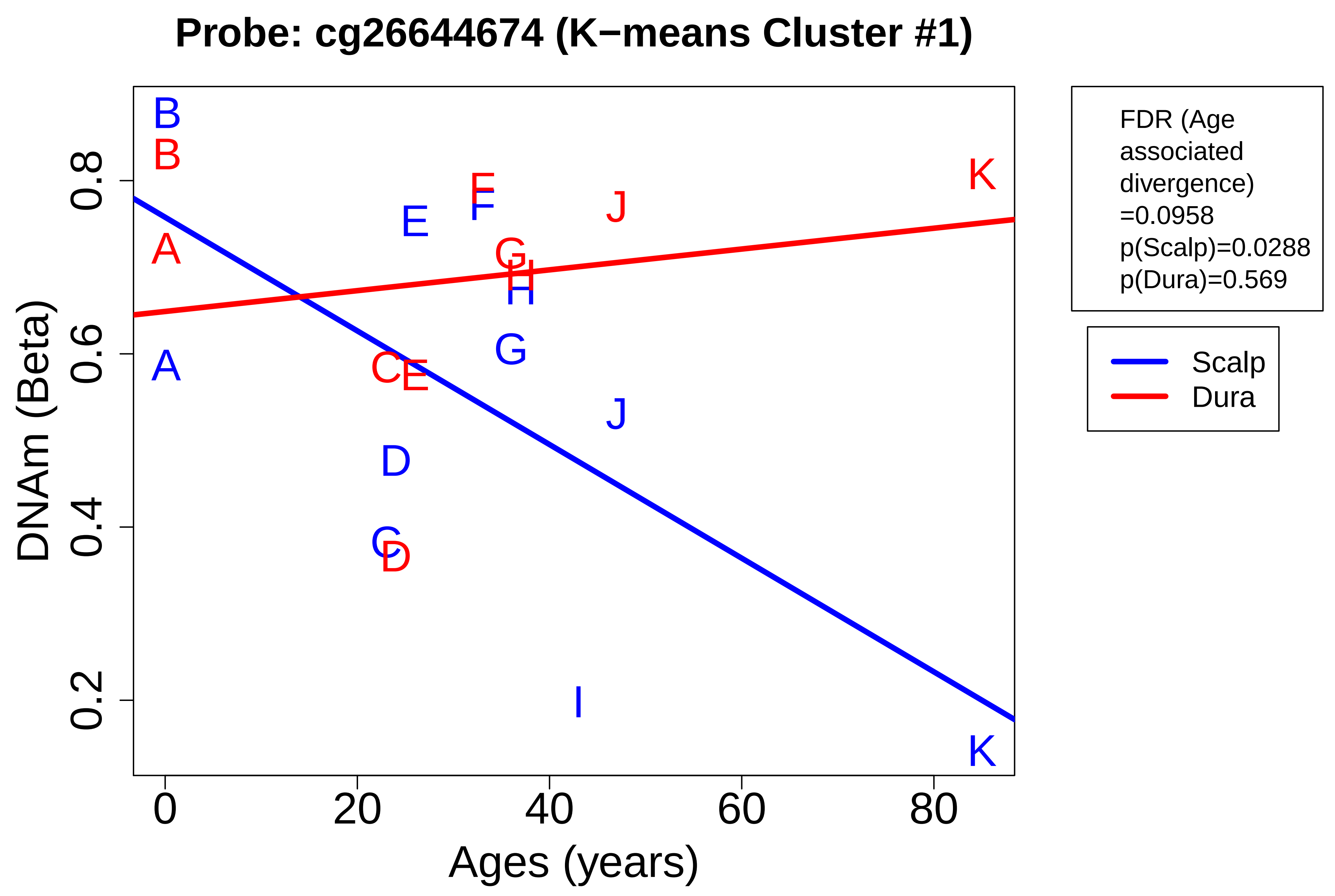

Probe: cg26644674 (K−means Cluster #1)
DNAm (Beta)
0.2
0.4
0.6
0.8
Ages (years)
0
20
40
60
80
FDR (Age associated divergence) =0.0958
p(Scalp)=0.0288
p(Dura)=0.569
Scalp
Dura
A
B
C
D
E
F
G
H
I
J
K

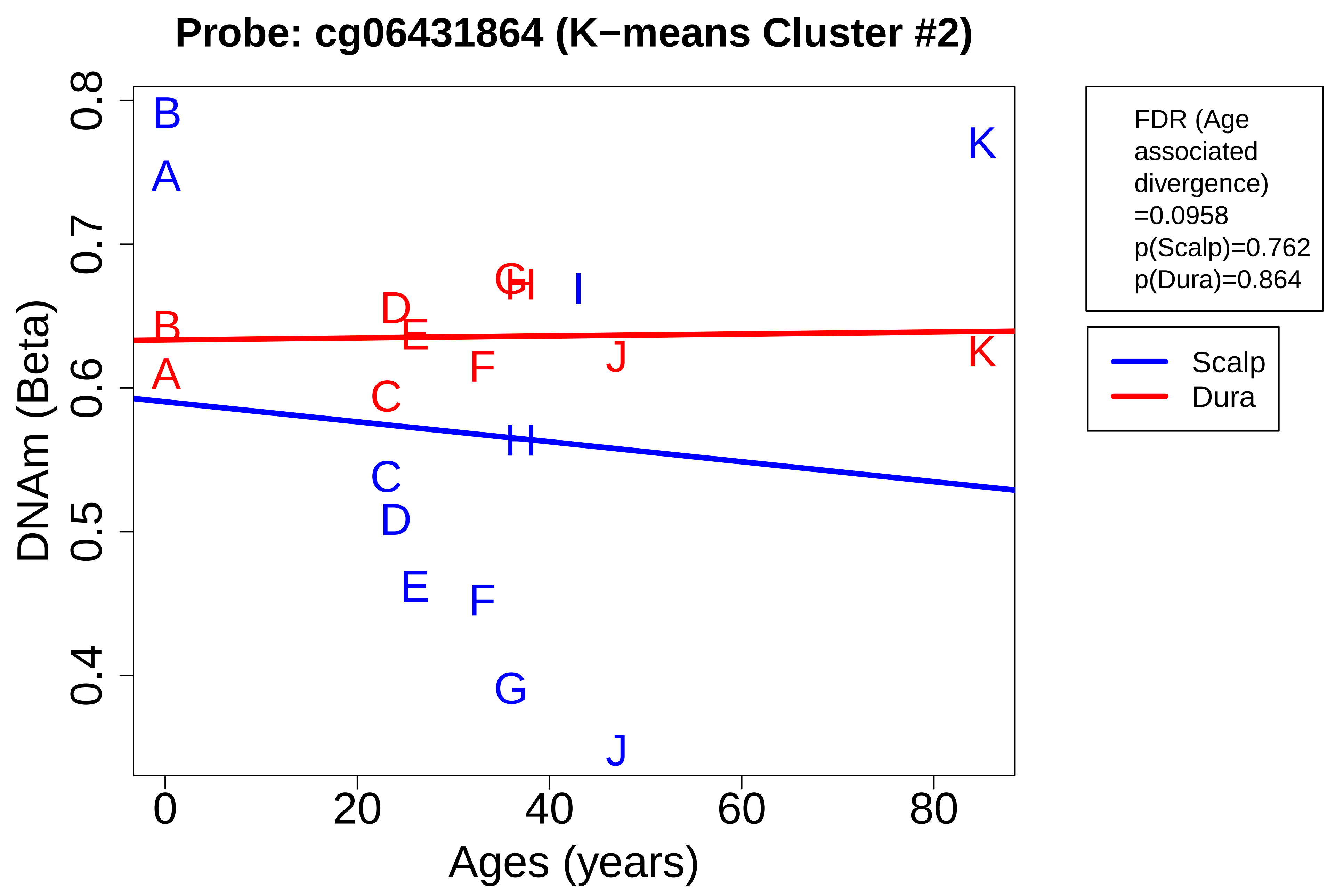
Probe: cg06431864 (K−means Cluster #2)
DNAm (Beta)
0.4
0.5
0.6
0.7
0.8
0
20
40
60
80
Ages (years)
FDR (Age associated divergence) =0.0958
p(Scalp)=0.762
p(Dura)=0.864
Scalp
Dura
A
B
C
D
E
F
G
H
I
J
K

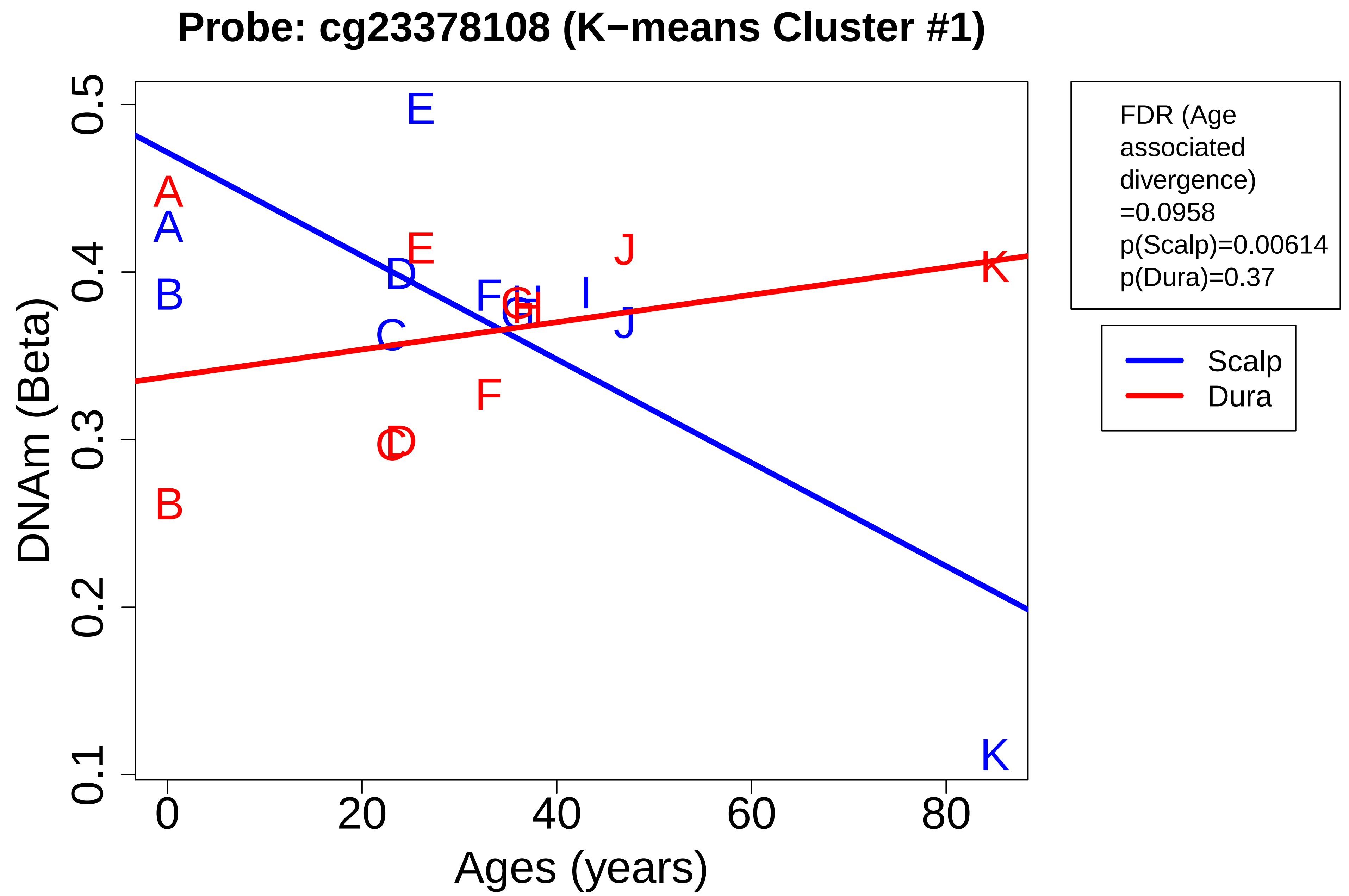

Probe: cg23378108 (K−means Cluster #1)
DNAm (Beta)
0.1
0.2
0.3
0.4
0.5
Ages (years)
0
20
40
60
80
A
B
C
D
E
F
G
H
I
J
K
FDR (Age associated divergence) =0.0958
p(Scalp)=0.00614
p(Dura)=0.37
Scalp
Dura

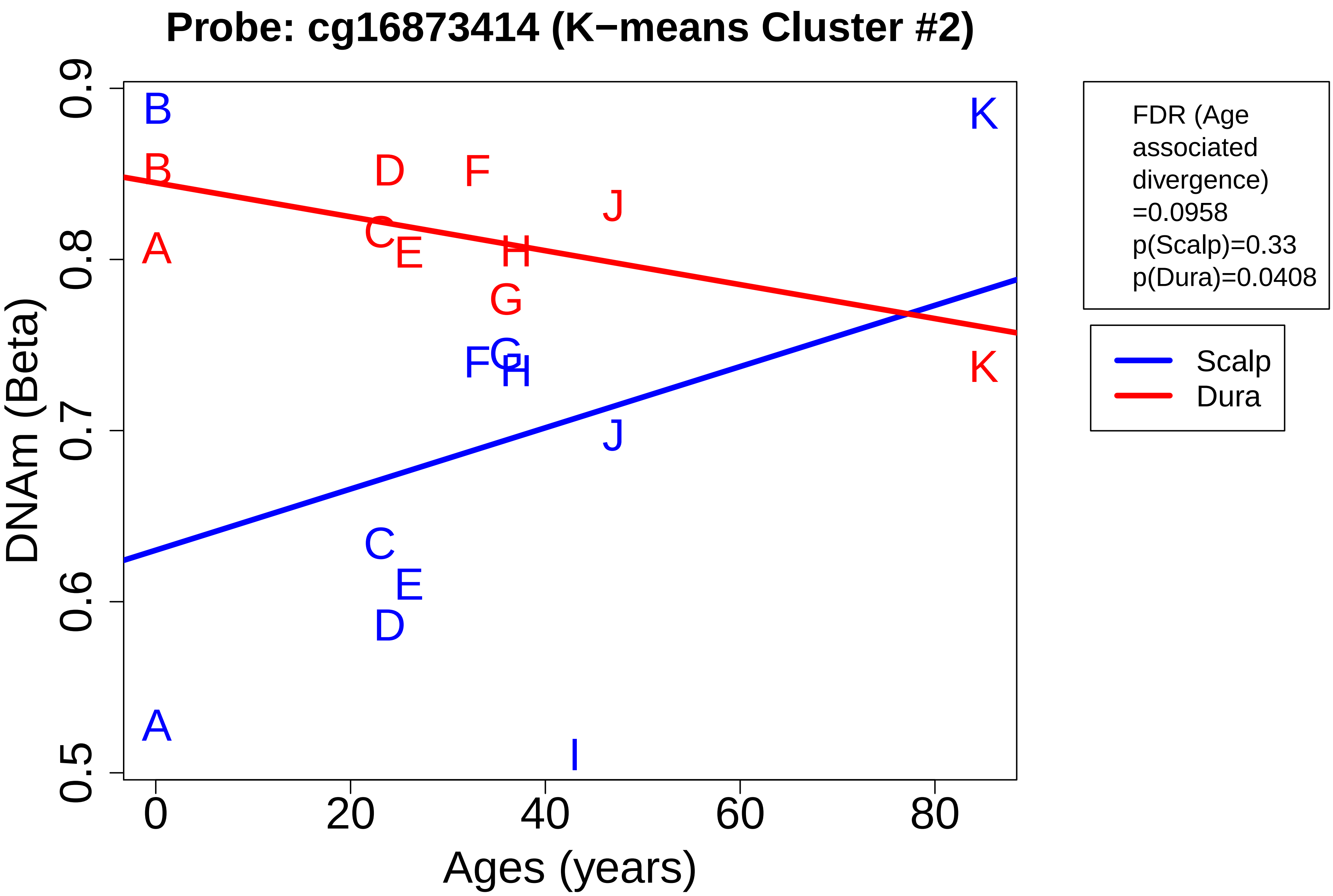

Probe: cg16873414 (K−means Cluster #2)
DNAm (Beta)
0.5
0.6
0.7
0.8
0.9
0
20
40
60
80
Ages (years)
FDR (Age associated divergence) =0.0958
p(Scalp)=0.33
p(Dura)=0.0408
Scalp
Dura

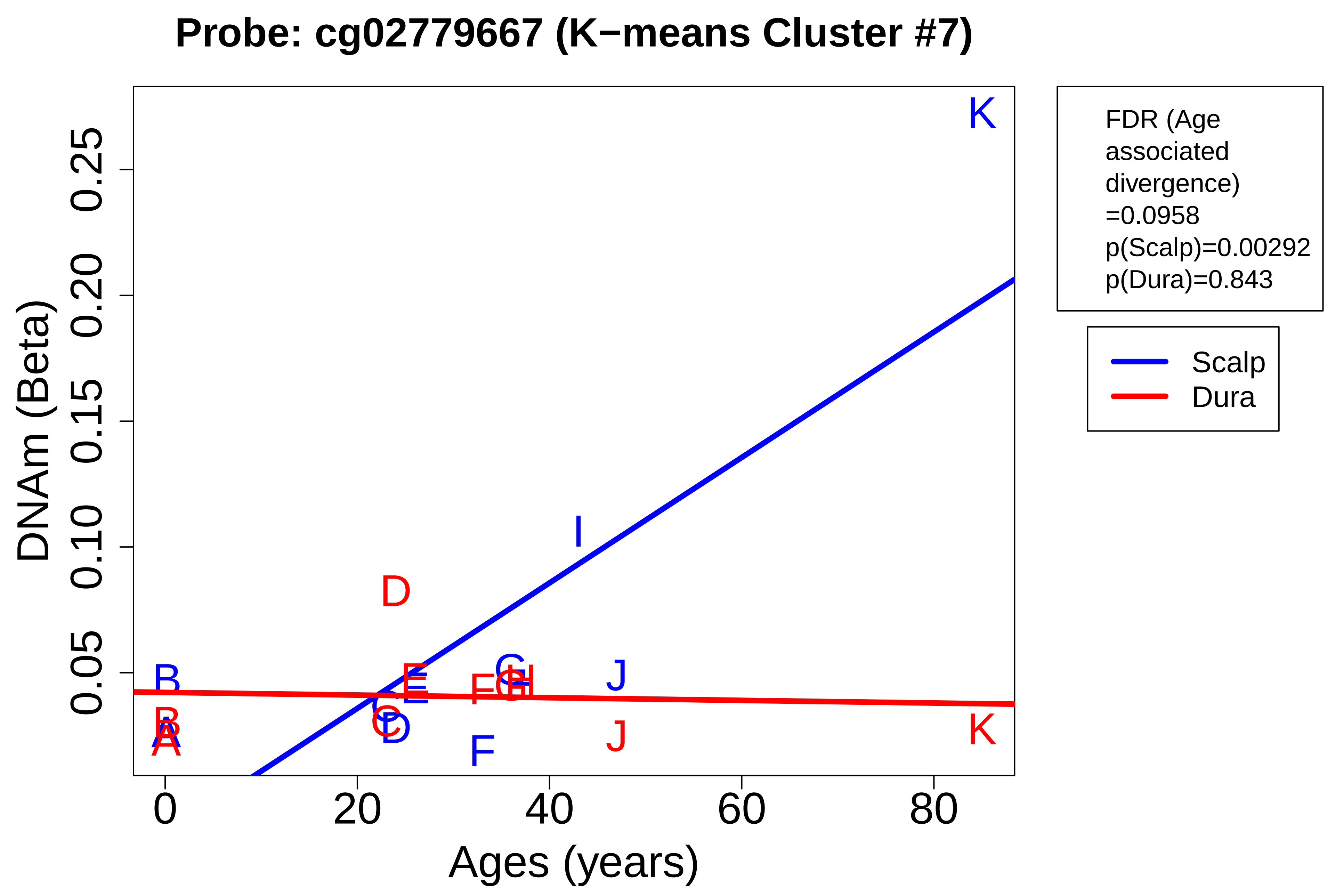

Probe: cg02779667 (K−means Cluster #7)
DNAm (Beta)
0.05
0.10
0.15
0.20
0.25
0
20
40
60
80
Ages (years)
FDR (Age associated divergence) =0.0958
p(Scalp)=0.00292
p(Dura)=0.843
Scalp
Dura

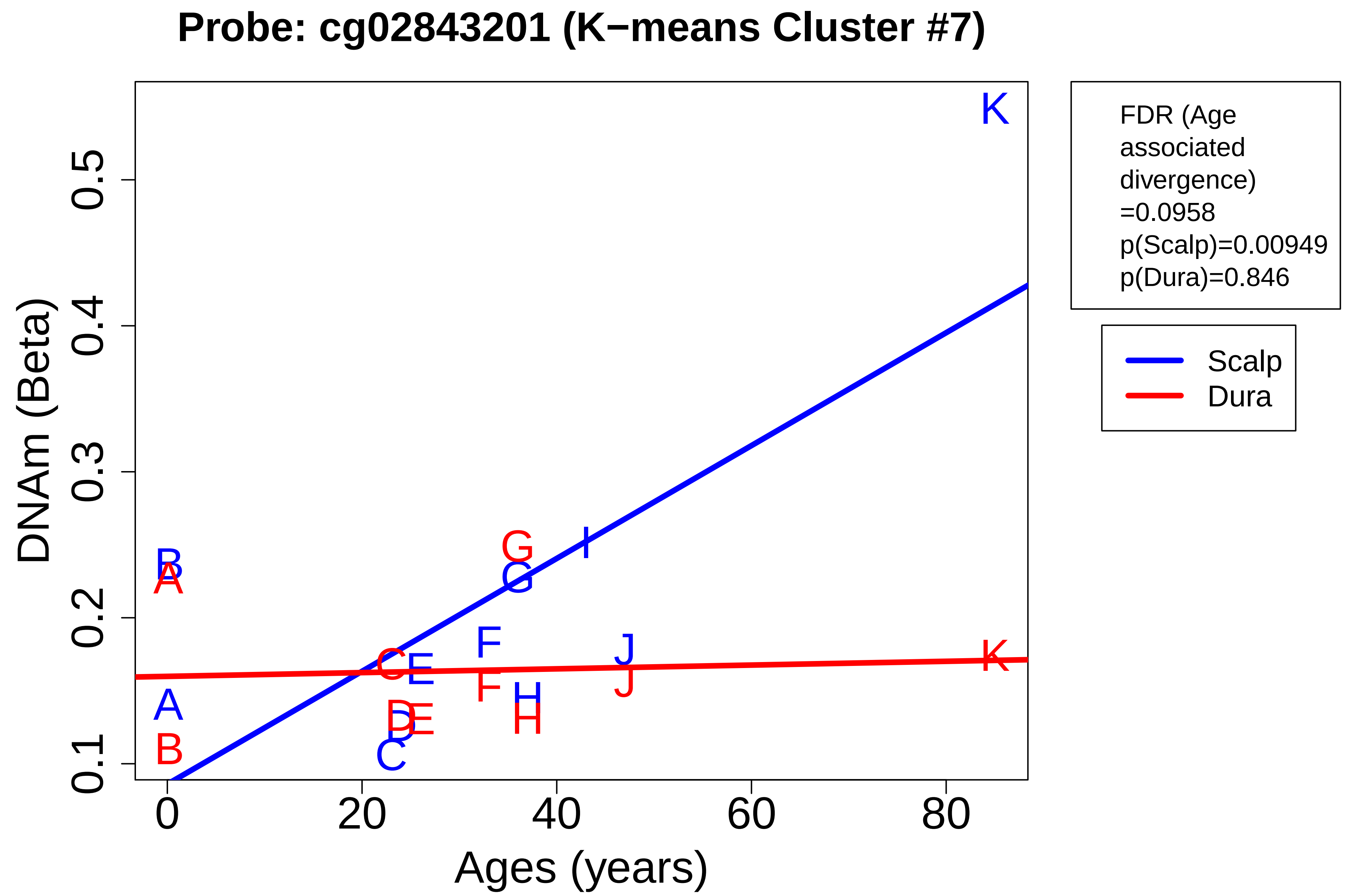

Probe: cg02843201 (K−means Cluster #7)
DNAm (Beta)
0.1
0.2
0.3
0.4
0.5
Ages (years)
0
20
40
60
80
FDR (Age associated divergence) =0.0958
p(Scalp)=0.00949
p(Dura)=0.846
Scalp
Dura

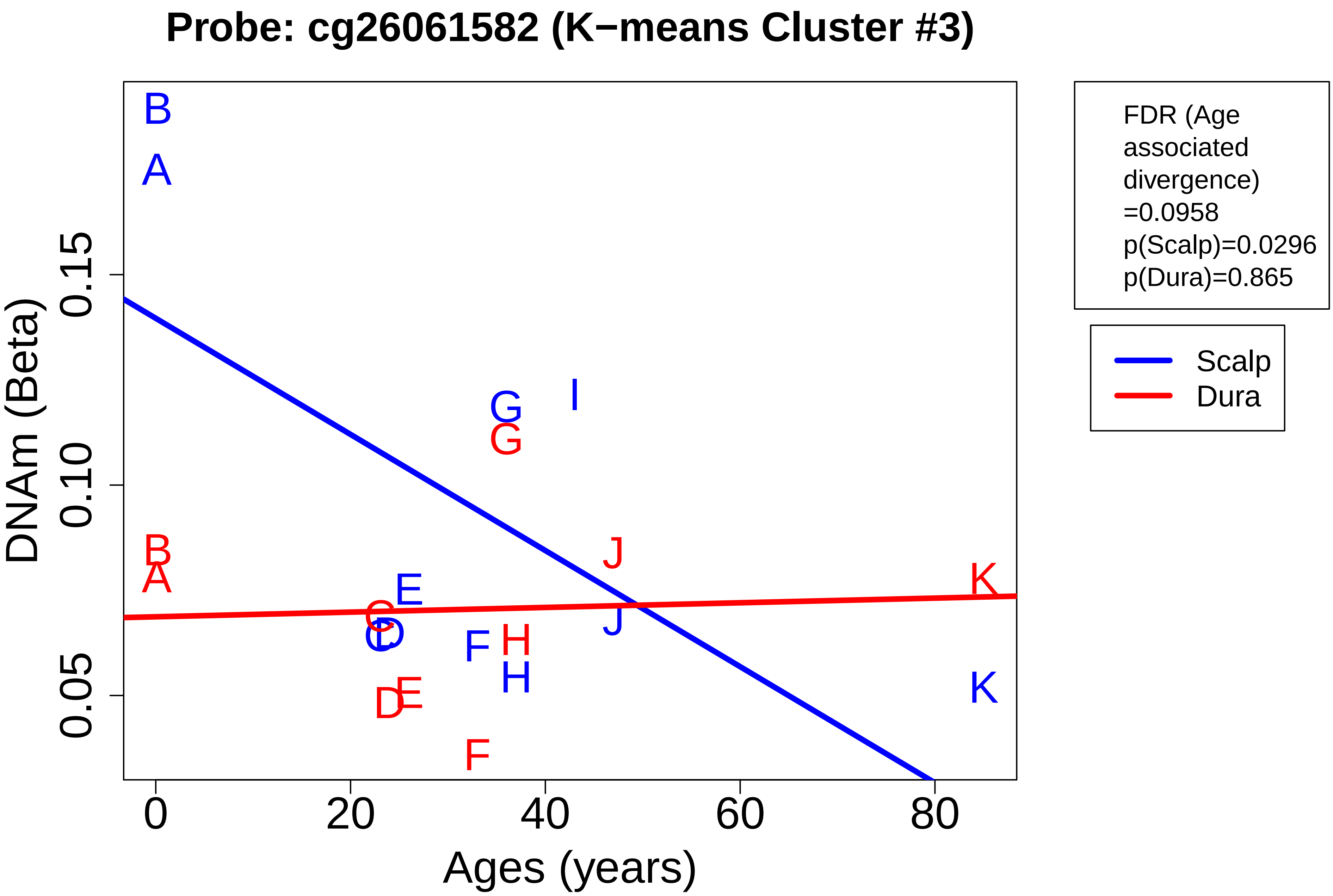

Probe: cg26061582 (K−means Cluster #3)
DNAm (Beta)
0.05
0.10
0.15
Ages (years)
0
20
40
60
80
B
A
G
G
I
B
A
E
C
D
F
H
J
J
K
H
E
D
F
K
FDR (Age associated divergence) =0.0958
p(Scalp)=0.0296
p(Dura)=0.865
Scalp
Dura

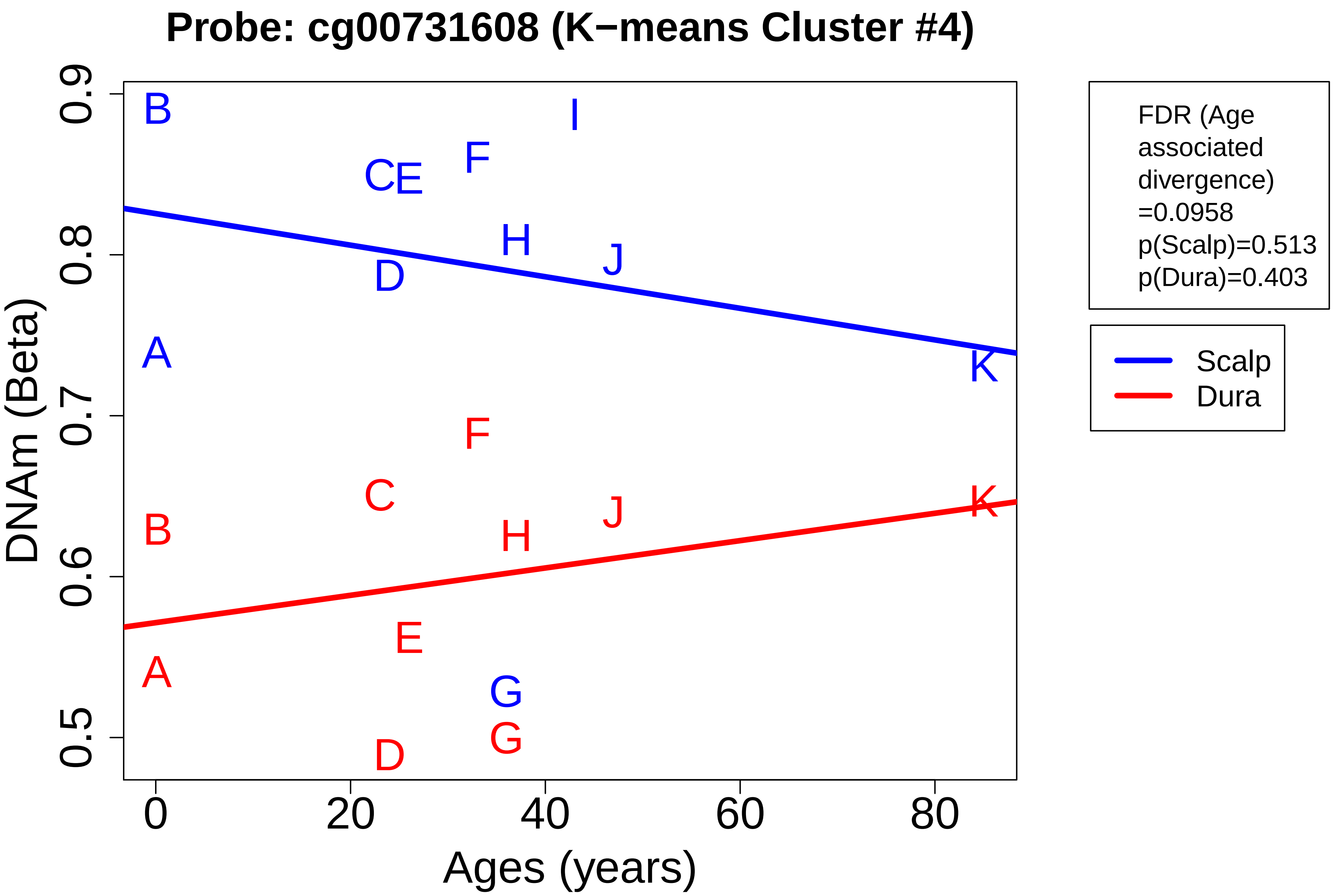

Probe: cg00731608 (K−means Cluster #4)
DNAm (Beta)
0.5
0.6
0.7
0.8
0.9
0
20
40
60
80
Ages (years)
FDR (Age associated divergence) =0.0958
p(Scalp)=0.513
p(Dura)=0.403
Scalp
Dura
A
B
C
D
E
F
G
H
I
J
K

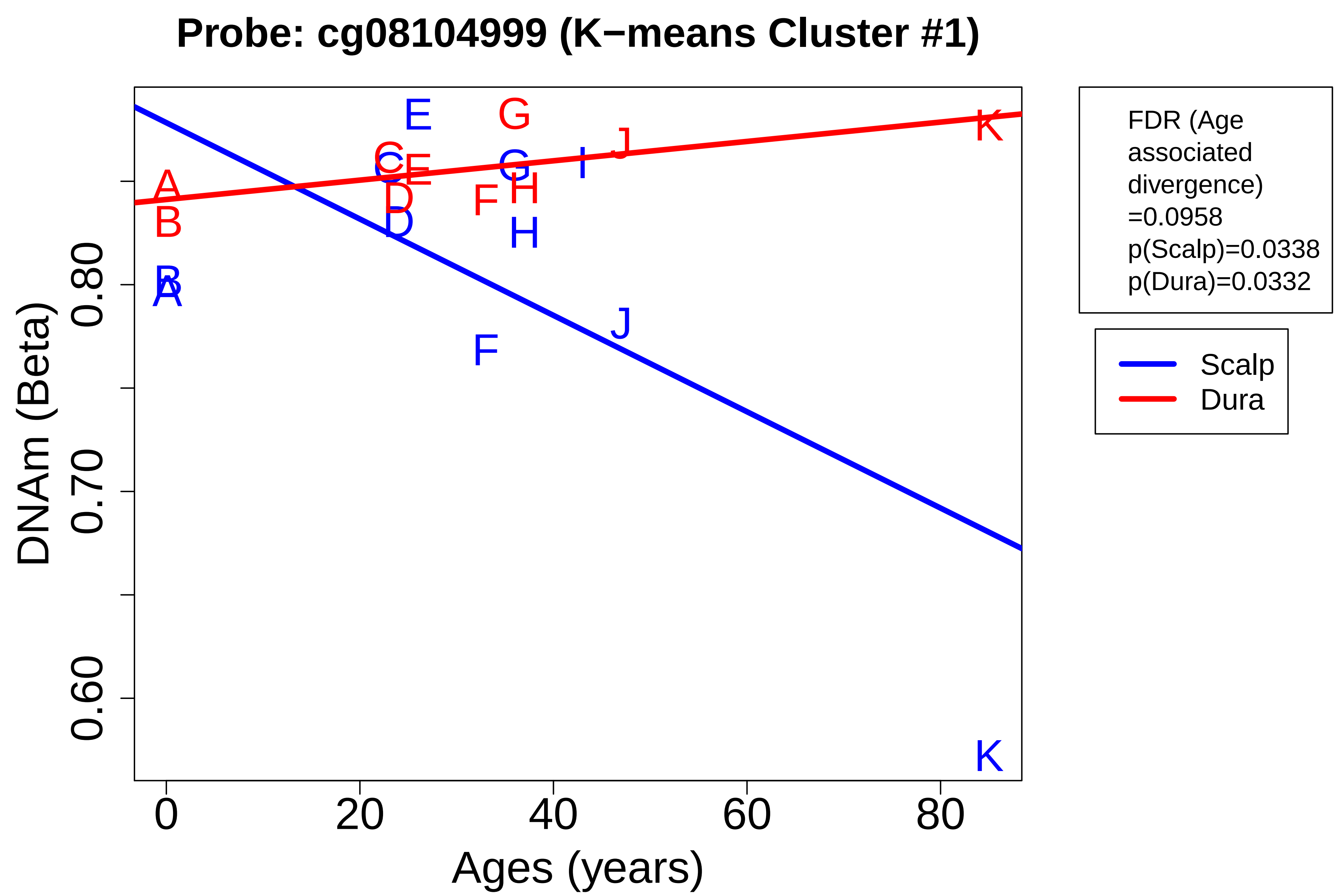

Probe: cg08104999 (K−means Cluster #1)
DNAm (Beta)
0.60
0.70
0.80
Ages (years)
0
20
40
60
80
FDR (Age associated divergence) =0.0958
p(Scalp)=0.0338
p(Dura)=0.0332
Scalp
Dura

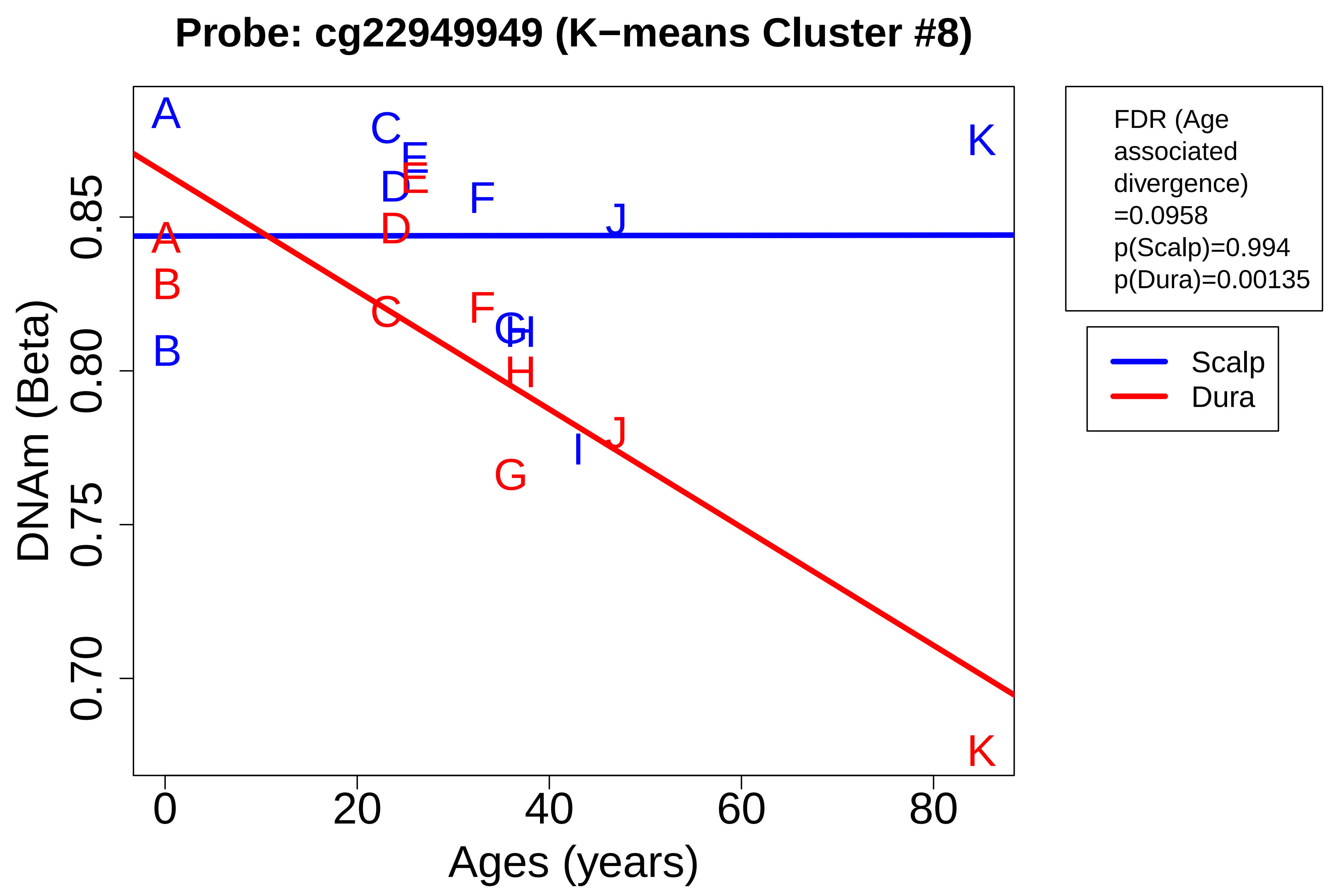

Probe: cg22949949 (K−means Cluster #8)
DNAm (Beta)
0.70
0.75
0.80
0.85
Ages (years)
0
20
40
60
80
FDR (Age associated divergence) =0.0958
p(Scalp)=0.994
p(Dura)=0.00135
Scalp
Dura

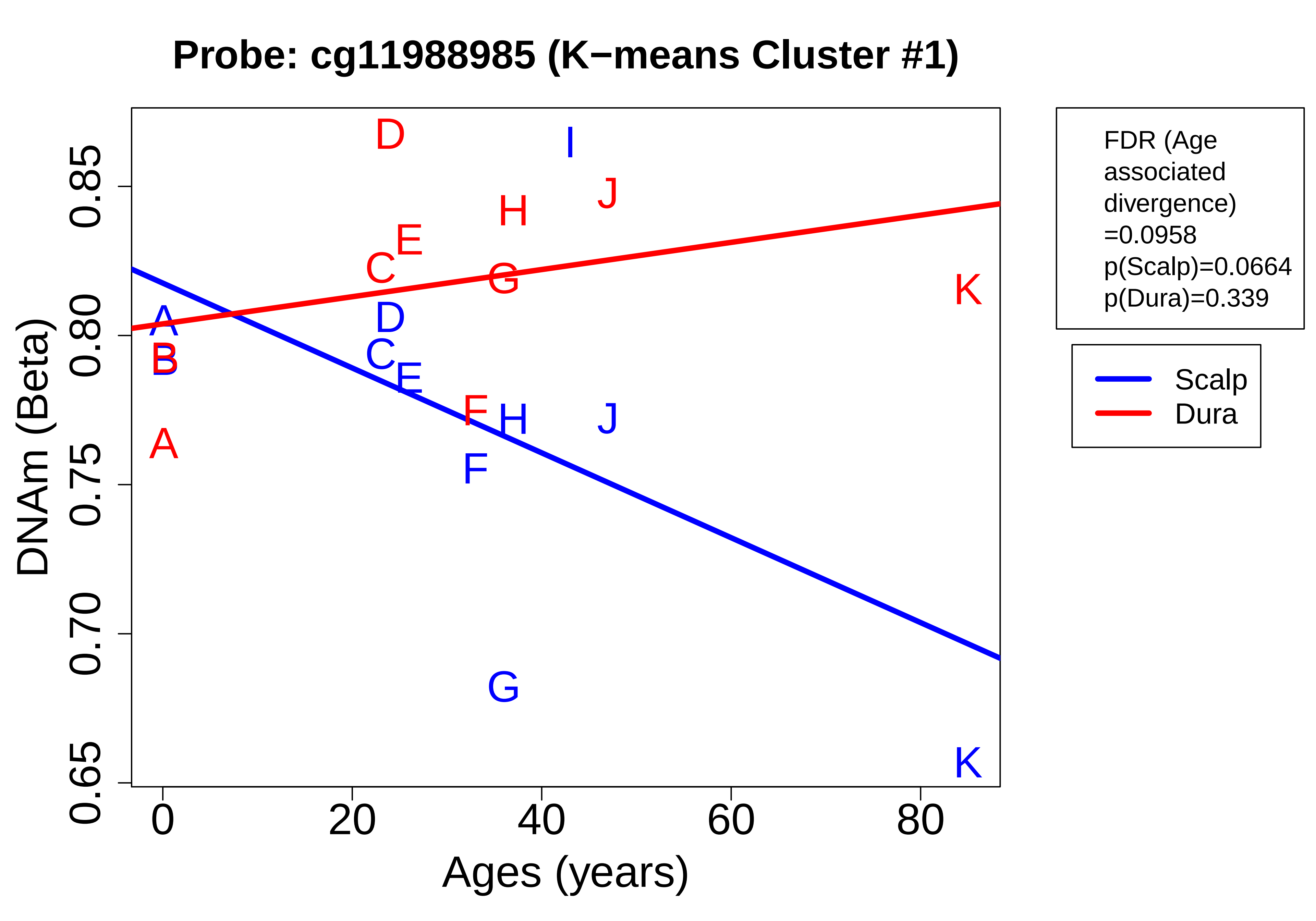
Probe: cg11988985 (K−means Cluster #1)
DNAm (Beta)
0.65
0.70
0.75
0.80
0.85
0
20
40
60
80
Ages (years)
FDR (Age associated divergence) =0.0958
p(Scalp)=0.0664
p(Dura)=0.339
Scalp
Dura
A
B
C
D
E
F
G
H
I
J
K

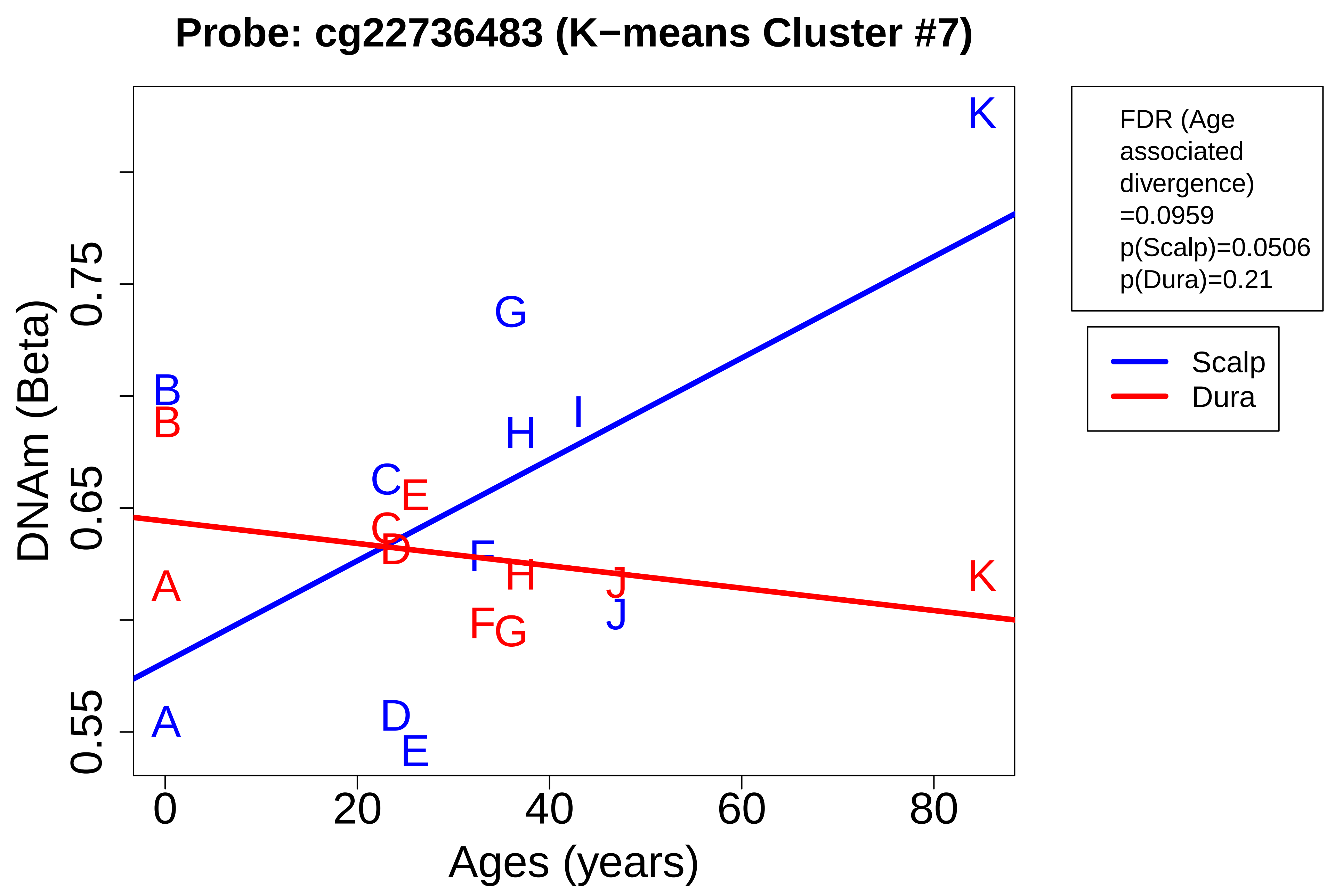
Probe: cg22736483 (K−means Cluster #7)
DNAm (Beta)
0.55
0.65
0.75
Ages (years)
0
20
40
60
80
A
B
C
D
E
F
G
H
I
J
K
A
B
C
D
E
F
G
H
J
K
FDR (Age associated divergence) =0.0959
p(Scalp)=0.0506
p(Dura)=0.21
Scalp
Dura

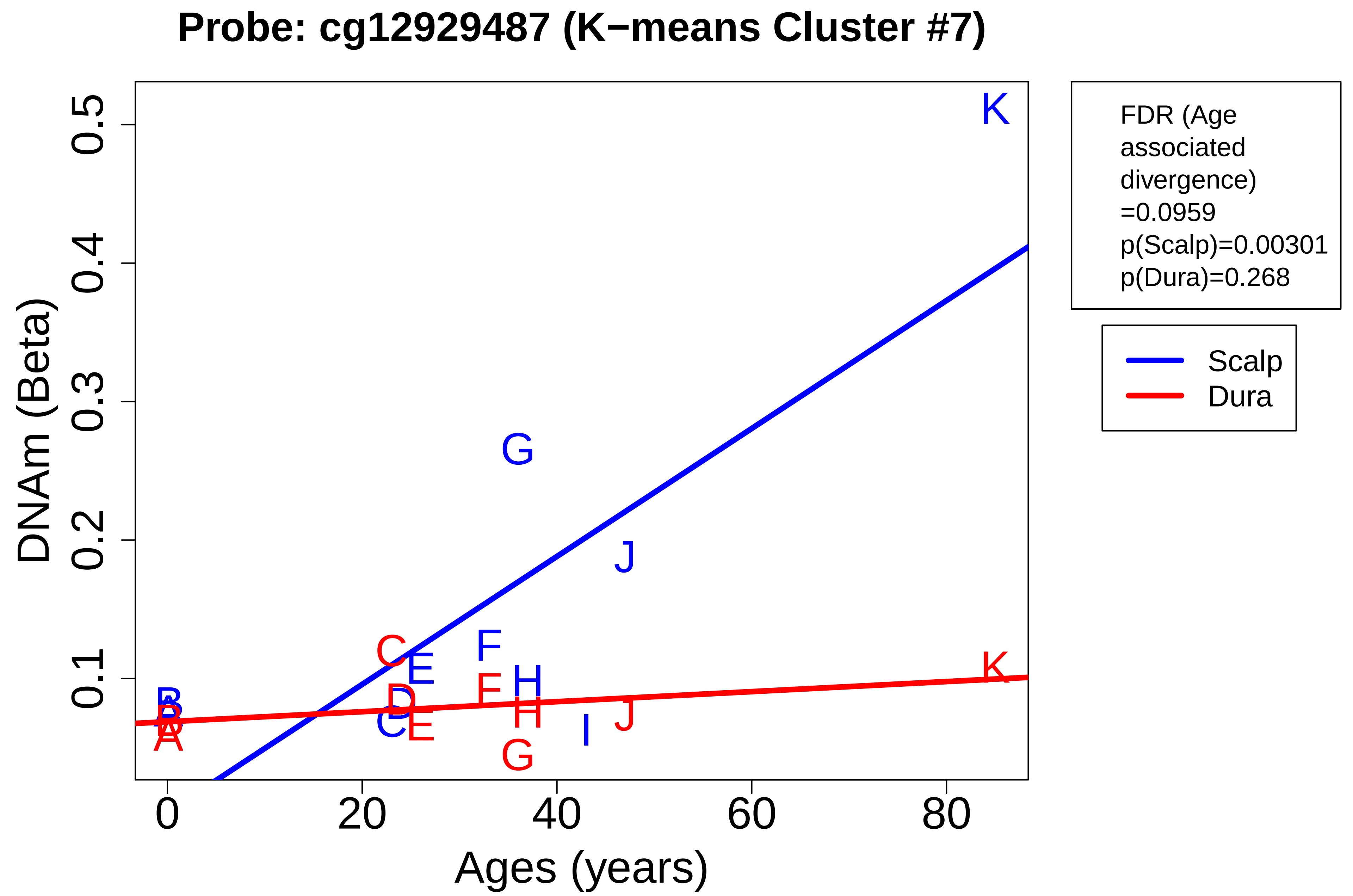
Probe: cg12929487 (K−means Cluster #7)
DNAm (Beta)
0.1
0.2
0.3
0.4
0.5
0
20
40
60
80
Ages (years)
FDR (Age associated divergence) =0.0959
p(Scalp)=0.00301
p(Dura)=0.268
Scalp
Dura
A
B
C
D
E
F
G
H
I
J
K

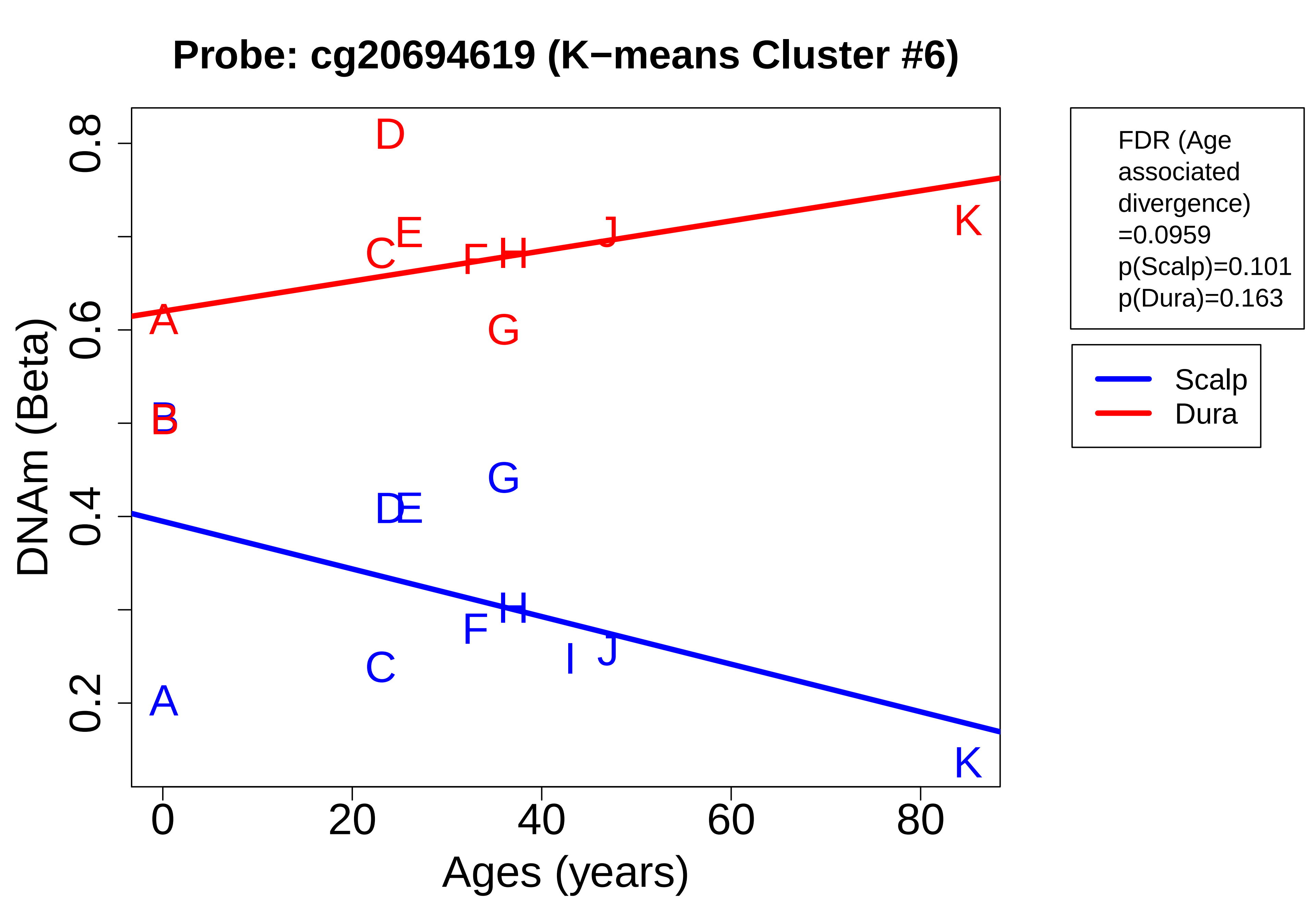

Probe: cg20694619 (K−means Cluster #6)
DNAm (Beta)
0.2
0.4
0.6
0.8
0
20
40
60
80
Ages (years)
FDR (Age associated divergence) =0.0959
p(Scalp)=0.101
p(Dura)=0.163
Scalp
Dura
A
B
C
D
E
F
G
H
I
J
K

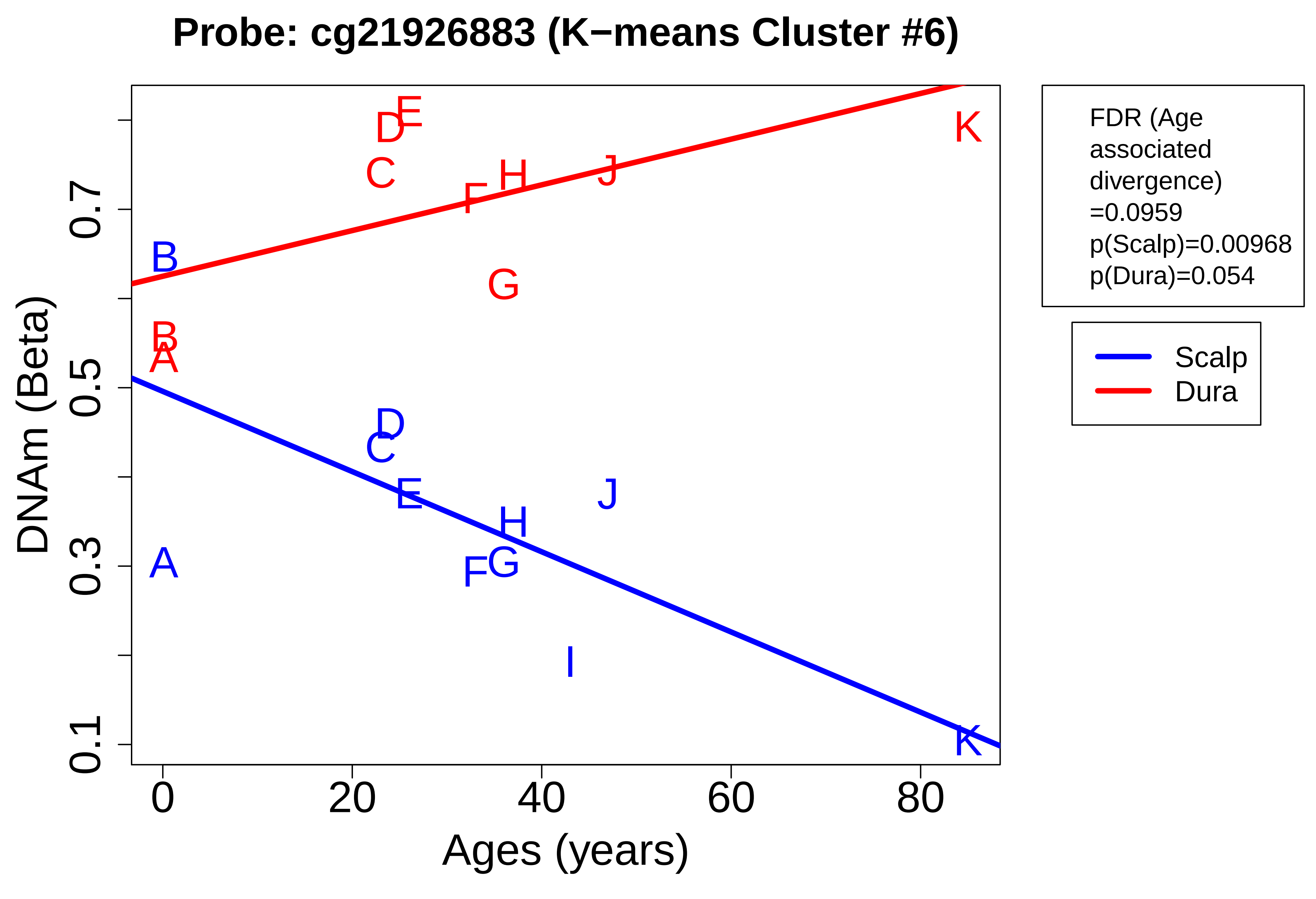

Probe: cg21926883 (K−means Cluster #6)
FDR (Age associated divergence) =0.0959
p(Scalp)=0.00968
p(Dura)=0.054
Scalp
Dura
DNAm (Beta)
0.1
0.3
0.5
0.7
0
20
40
60
80
Ages (years)

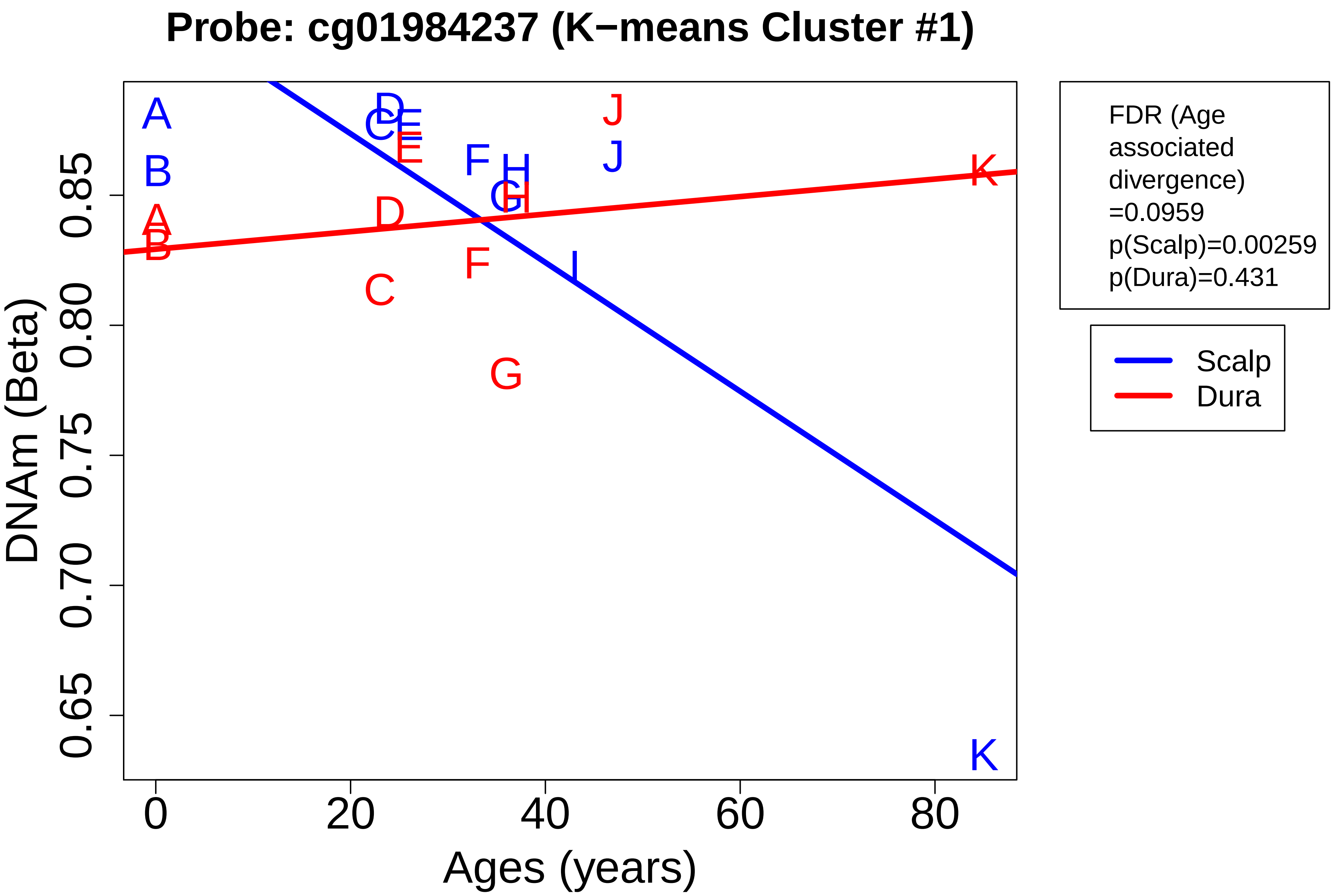

Probe: cg01984237 (K−means Cluster #1)
DNAm (Beta)
Ages (years)
0.65
0.70
0.75
0.80
0.85
0
20
40
60
80
FDR (Age associated divergence) =0.0959
p(Scalp)=0.00259
p(Dura)=0.431
Scalp
Dura
A
B
C
D
E
F
G
H
I
J
K

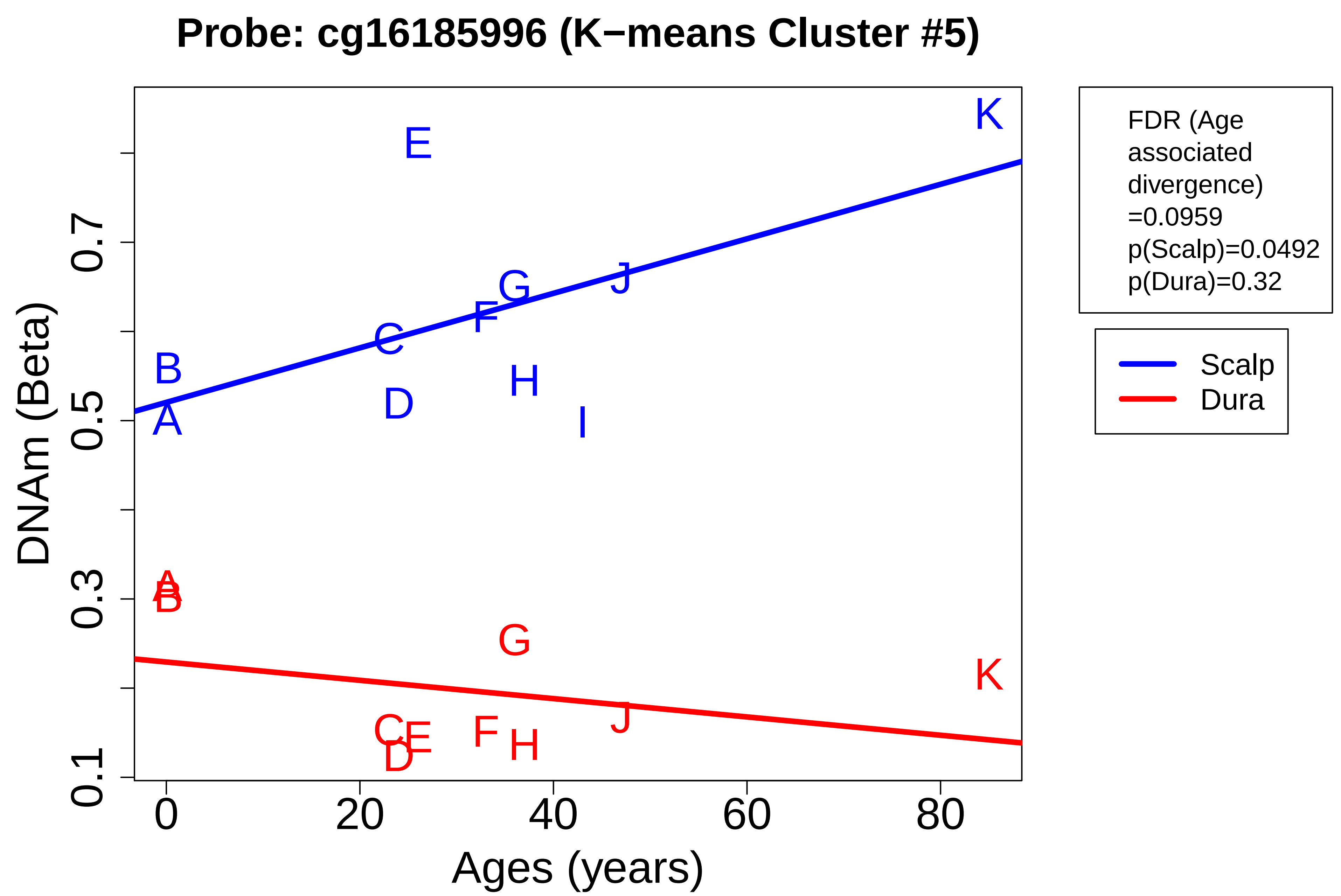

Probe: cg16185996 (K−means Cluster #5)
DNAm (Beta)
Ages (years)
0.1
0.3
0.5
0.7
0
20
40
60
80
A
B
C
D
E
F
G
H
I
J
K
FDR (Age associated divergence) =0.0959
p(Scalp)=0.0492
p(Dura)=0.32
Scalp
Dura

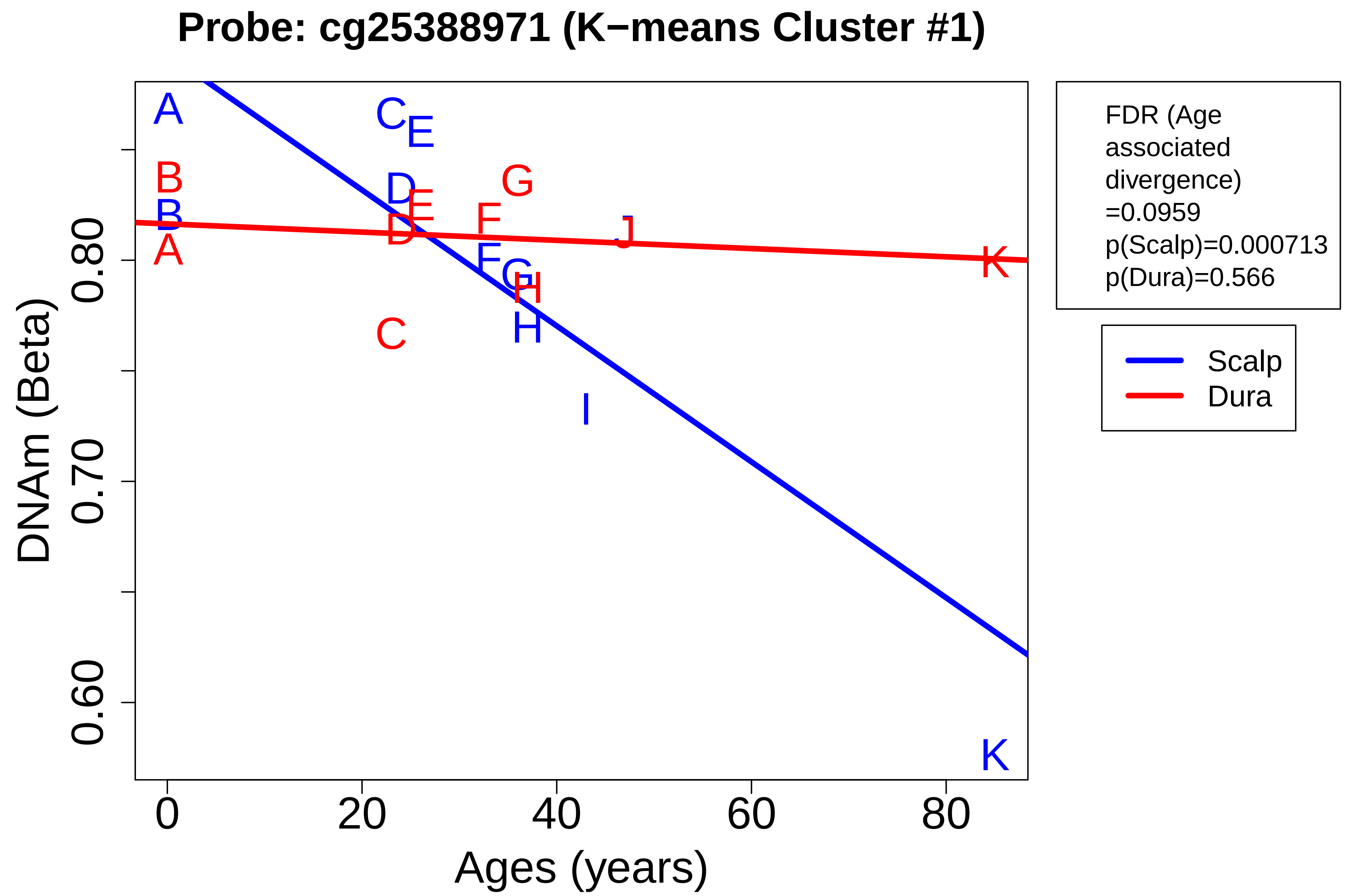

Probe: cg25388971 (K−means Cluster #1)
DNAm (Beta)
0.60
0.70
0.80
Ages (years)
0
20
40
60
80
A
B
C
D
E
F
G
H
I
J
K
FDR (Age associated divergence) =0.0959
p(Scalp)=0.000713
p(Dura)=0.566
Scalp
Dura

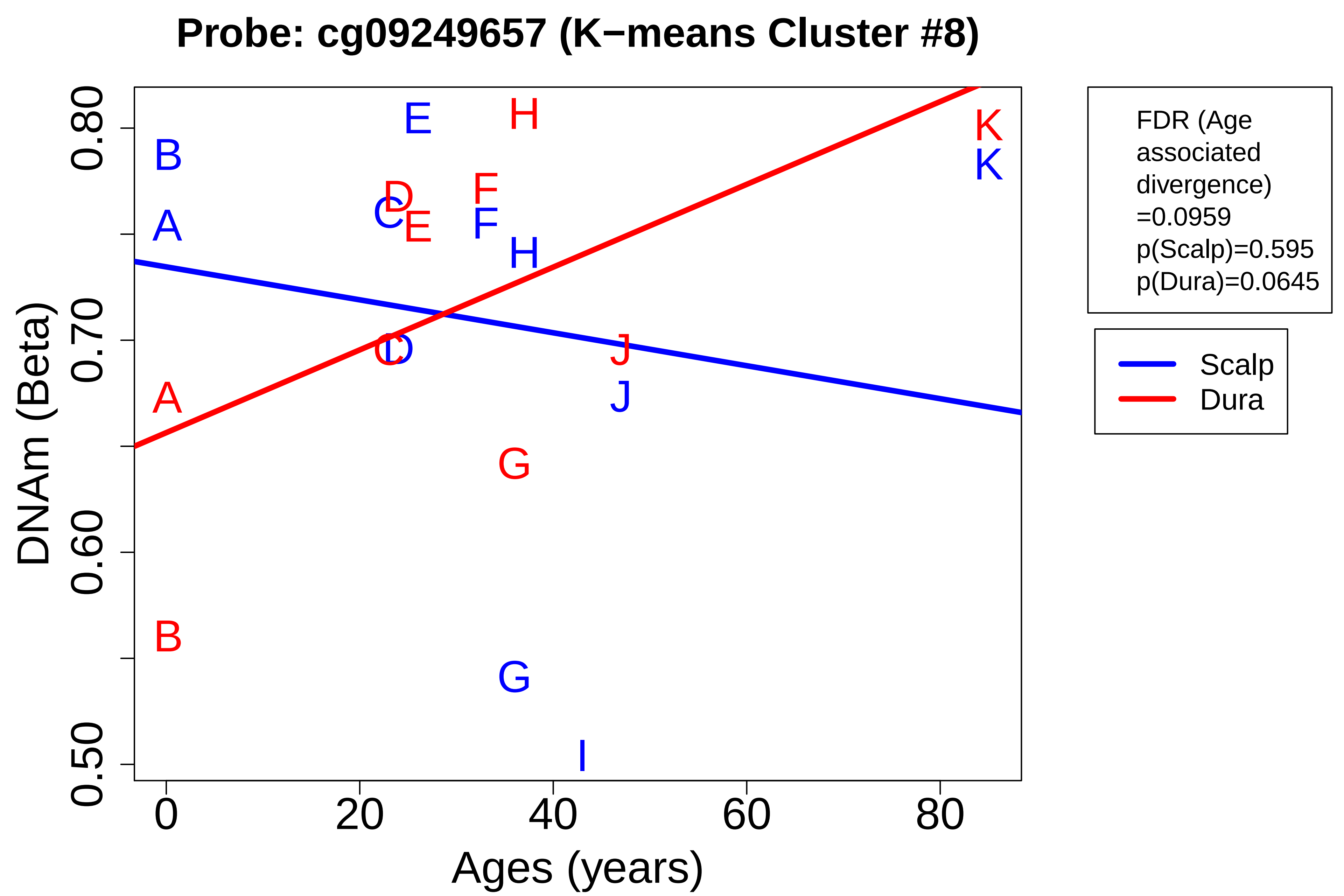

Probe: cg09249657 (K−means Cluster #8)
DNAm (Beta)
0.50
0.60
0.70
0.80
Ages (years)
0
20
40
60
80
A
B
C
D
E
F
G
H
I
J
K
FDR (Age associated divergence) =0.0959
p(Scalp)=0.595
p(Dura)=0.0645
Scalp
Dura

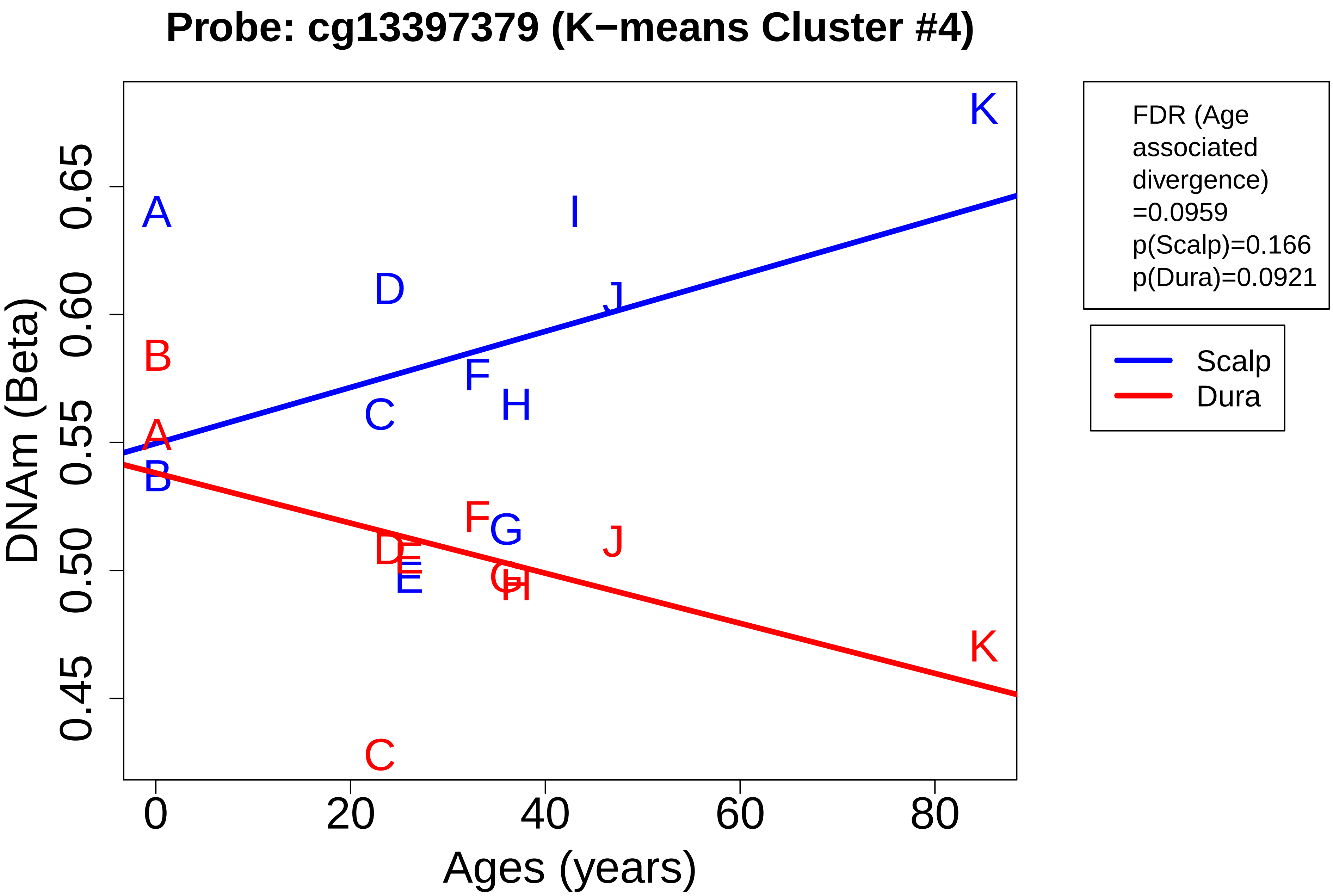
Probe: cg13397379 (K−means Cluster #4)
DNAm (Beta)
Ages (years)
0.45
0.50
0.55
0.60
0.65
0
20
40
60
80
FDR (Age associated divergence) =0.0959
p(Scalp)=0.166
p(Dura)=0.0921
Scalp
Dura
A
B
C
D
E
F
G
H
I
J
K

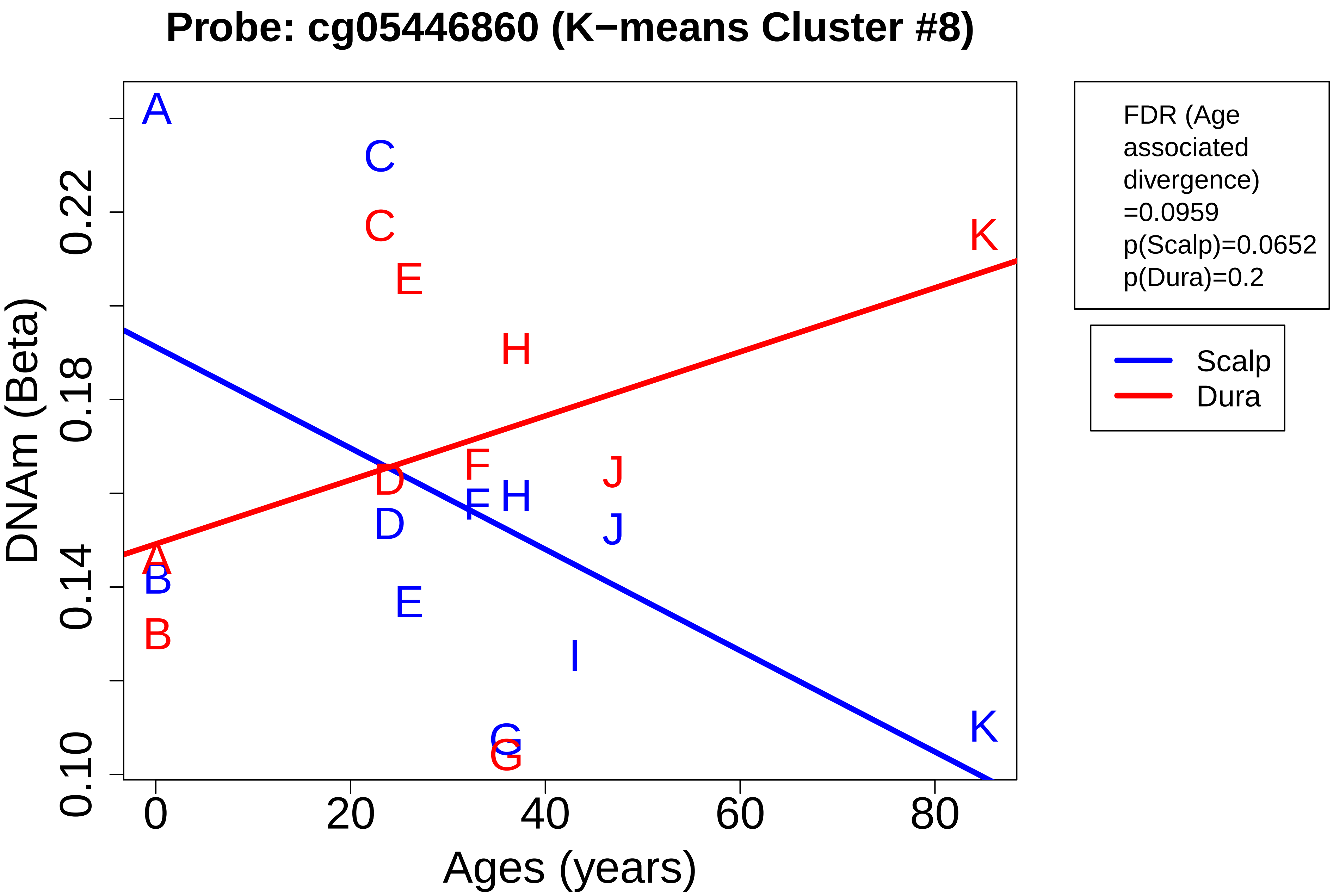

Probe: cg05446860 (K−means Cluster #8)
DNAm (Beta)
0.10
0.14
0.18
0.22
0
20
40
60
80
Ages (years)
A
B
C
D
E
F
G
H
I
J
K
FDR (Age associated divergence) =0.0959
p(Scalp)=0.0652
p(Dura)=0.2
Scalp
Dura

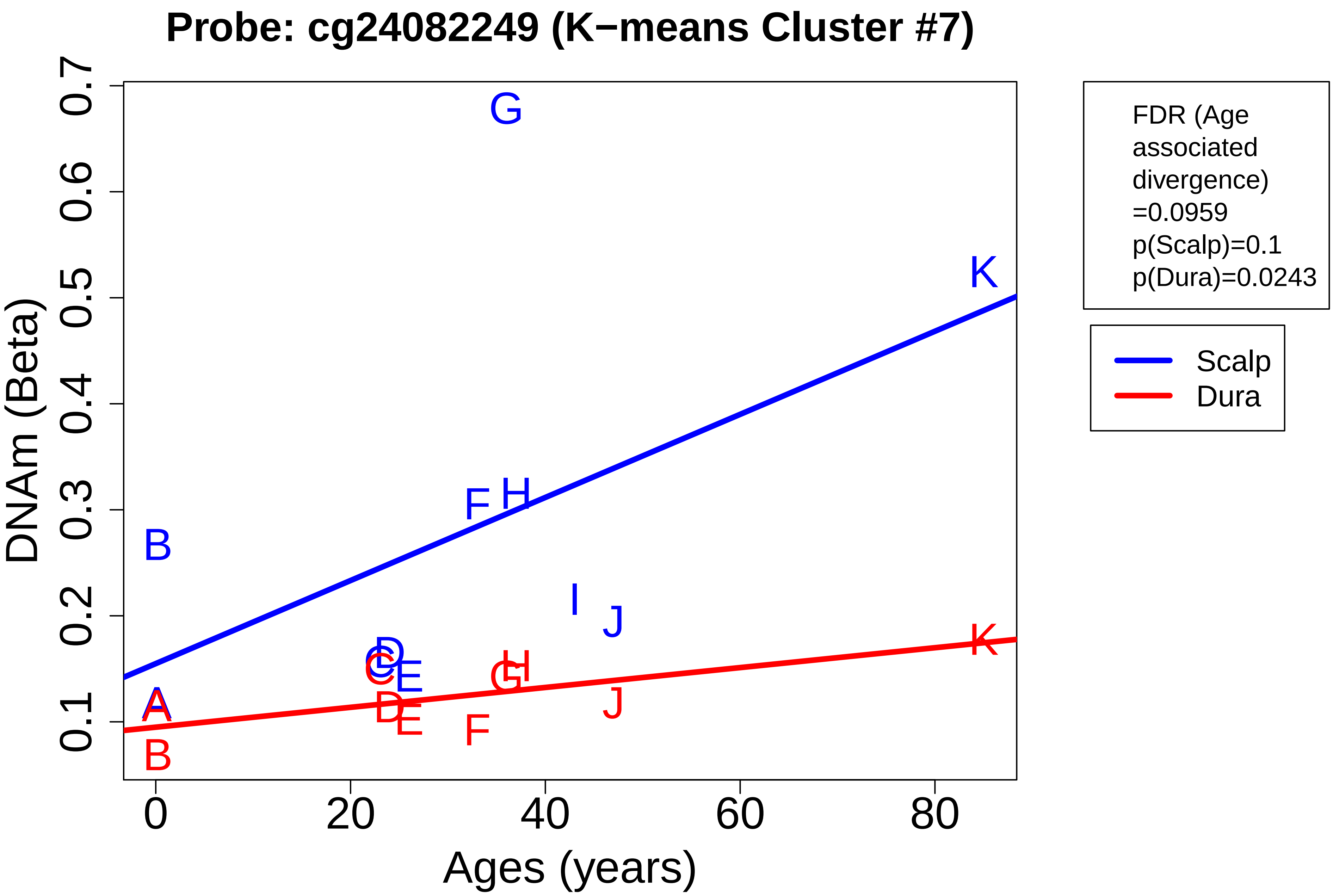

Probe: cg24082249 (K−means Cluster #7)
DNAm (Beta)
0.1
0.2
0.3
0.4
0.5
0.6
0.7
0
20
40
60
80
Ages (years)
FDR (Age associated divergence) =0.0959
p(Scalp)=0.1
p(Dura)=0.0243
Scalp
Dura
A
B
C
D
E
F
G
H
I
J
K

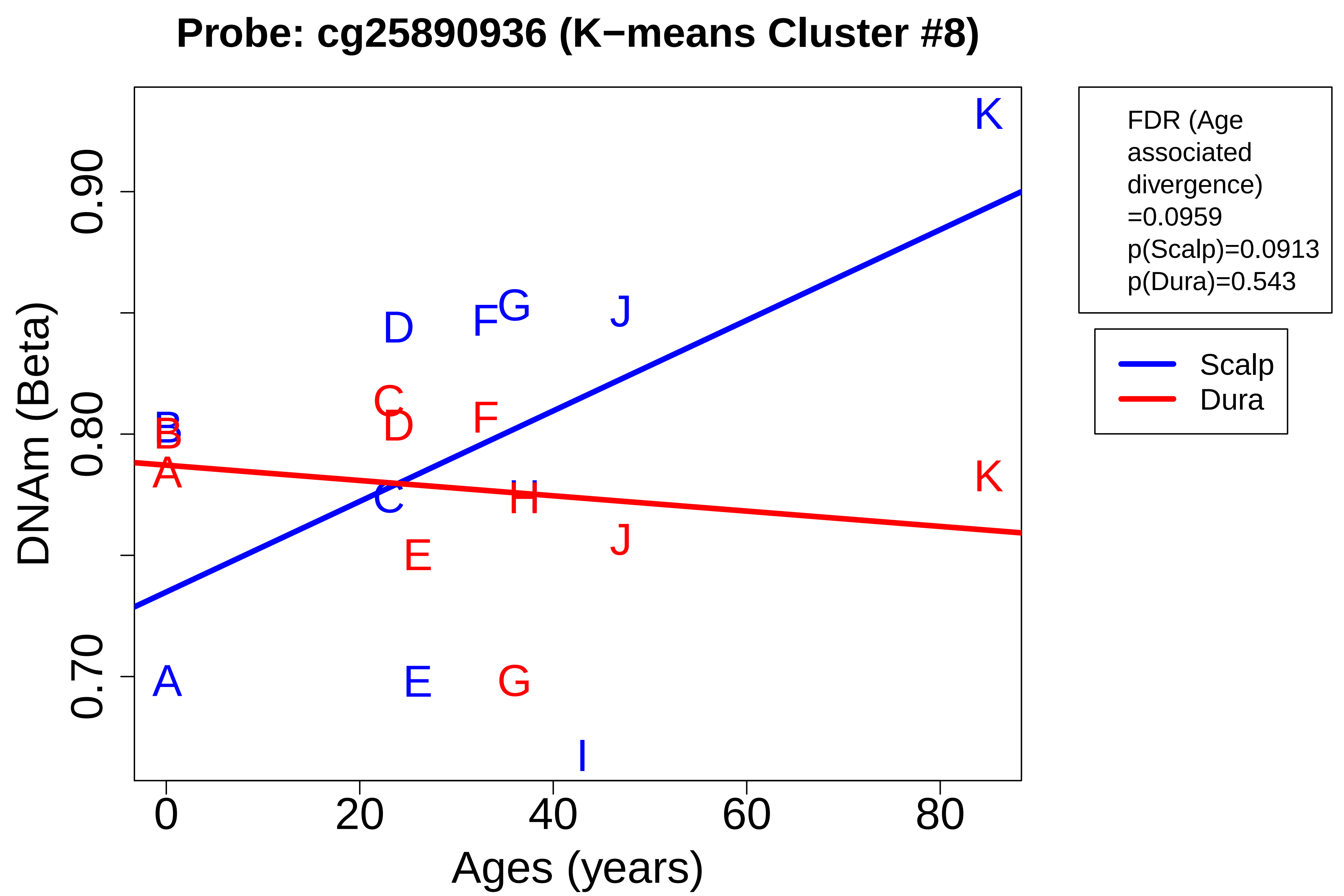

Probe: cg25890936 (K−means Cluster #8)
DNAm (Beta)
0.70
0.80
0.90
Ages (years)
0
20
40
60
80
FDR (Age associated divergence) =0.0959
p(Scalp)=0.0913
p(Dura)=0.543
Scalp
Dura

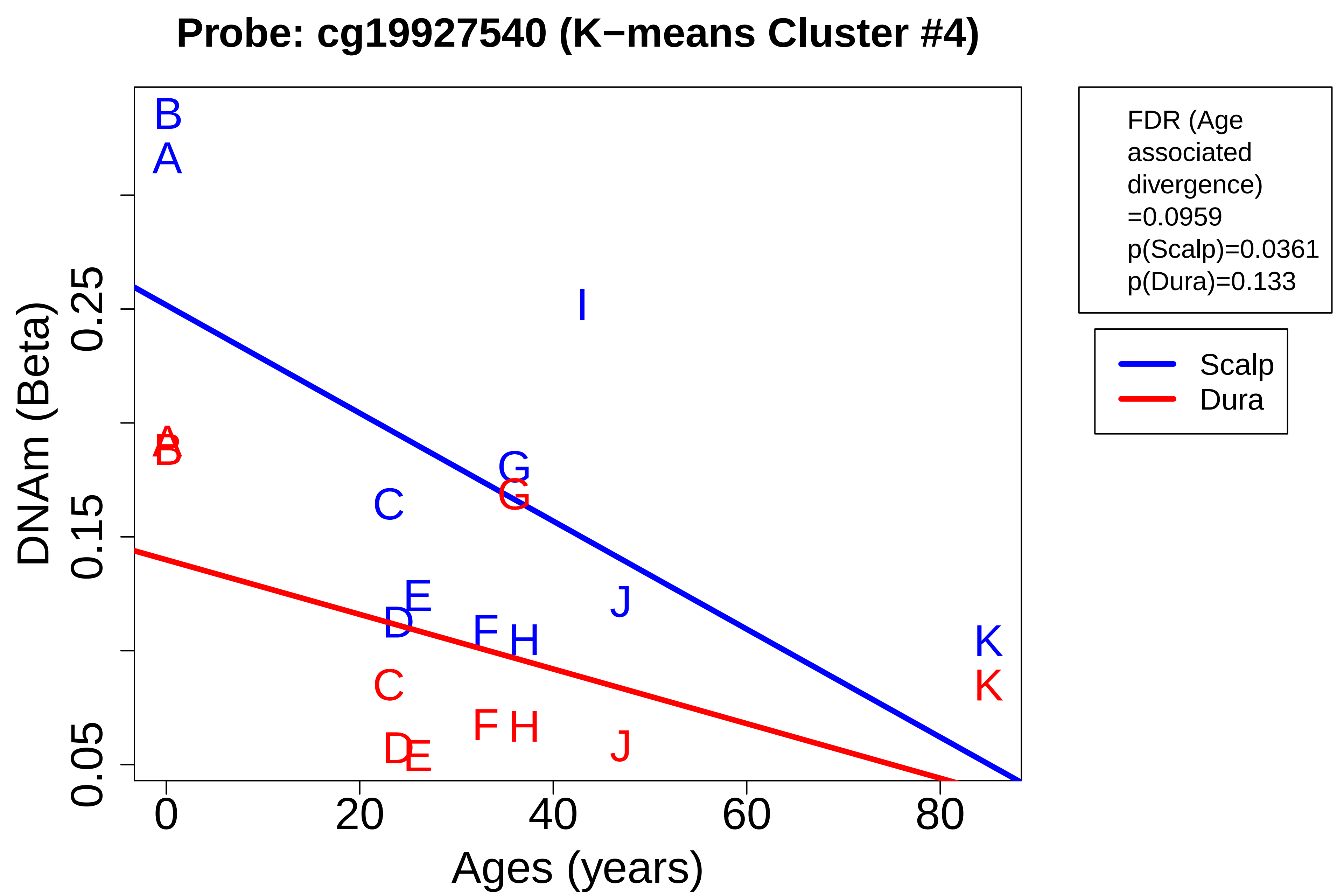

Probe: cg19927540 (K−means Cluster #4)
DNAm (Beta)
0.05
0.15
0.25
Ages (years)
0
20
40
60
80
B
A
I
G
C
E
D
F
H
J
K
C
F
H
J
D
E
K
FDR (Age associated divergence) =0.0959
p(Scalp)=0.0361
p(Dura)=0.133
Scalp
Dura

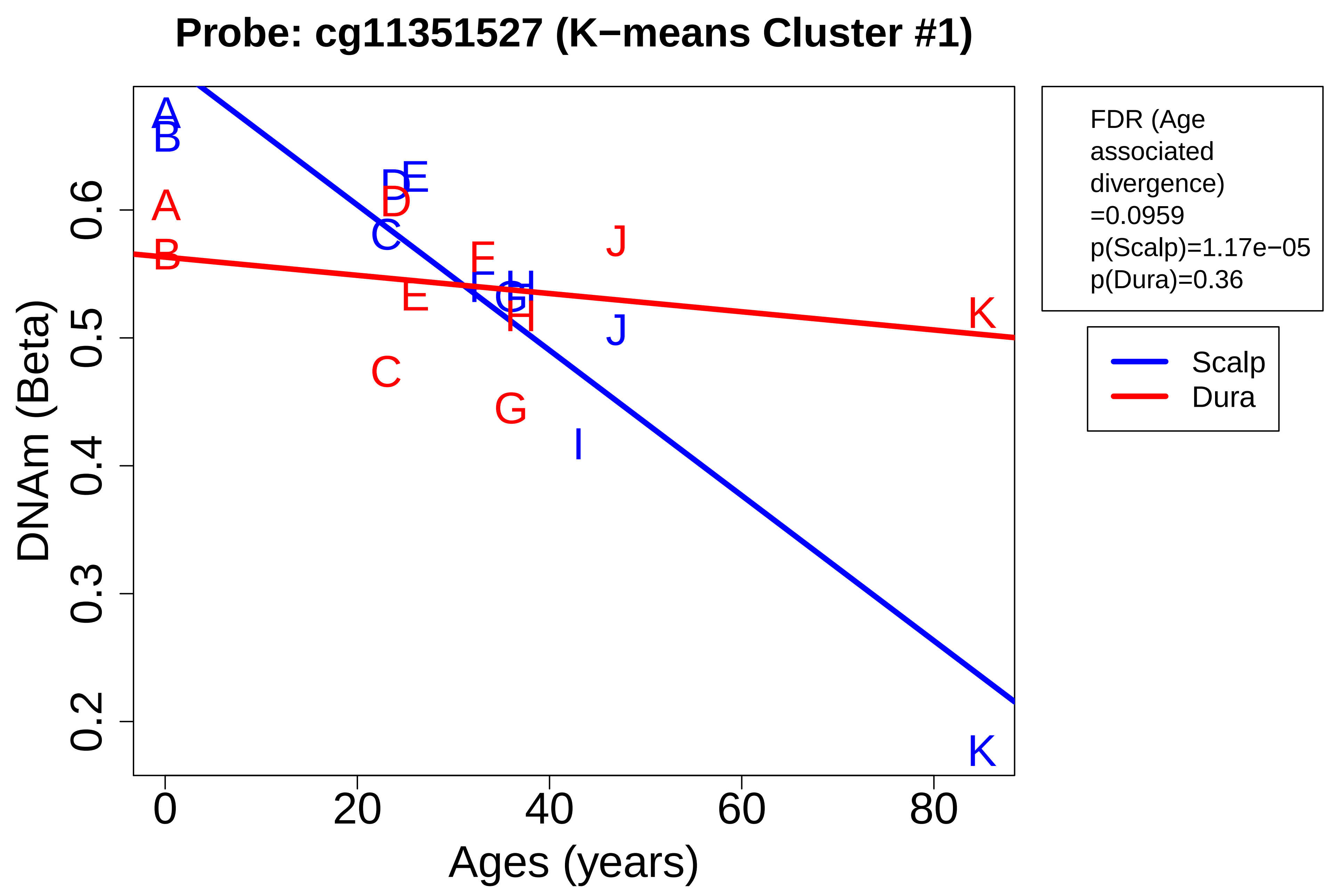

Probe: cg11351527 (K−means Cluster #1)
FDR (Age associated divergence) =0.0959
p(Scalp)=1.17e−05
p(Dura)=0.36
Scalp
Dura
DNAm (Beta)
Ages (years)
0.2
0.3
0.4
0.5
0.6
0
20
40
60
80

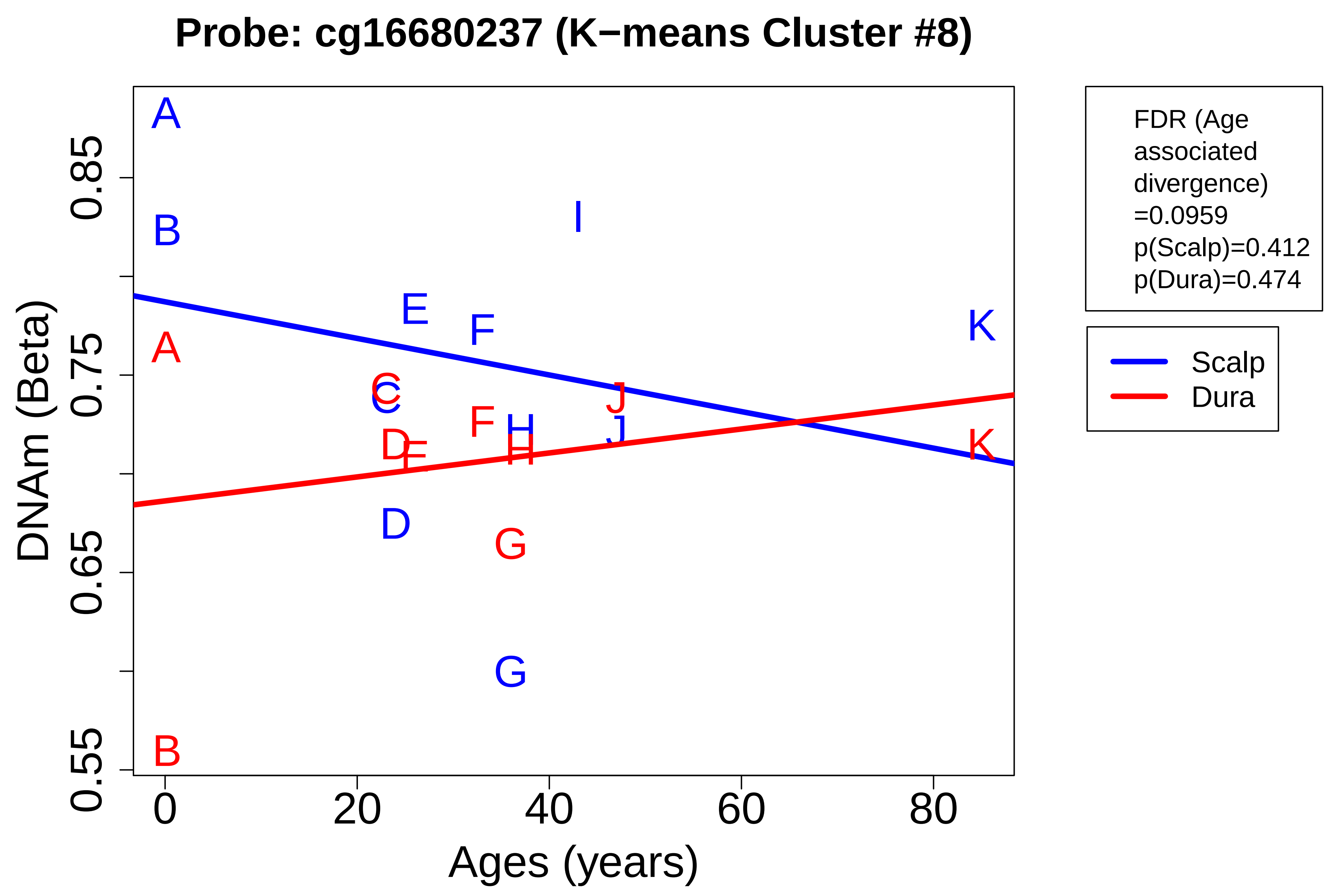

Probe: cg16680237 (K−means Cluster #8)
DNAm (Beta)
0.55
0.65
0.75
0.85
Ages (years)
0
20
40
60
80
A
B
C
D
E
F
G
H
I
J
K
FDR (Age associated divergence) =0.0959
p(Scalp)=0.412
p(Dura)=0.474
Scalp
Dura

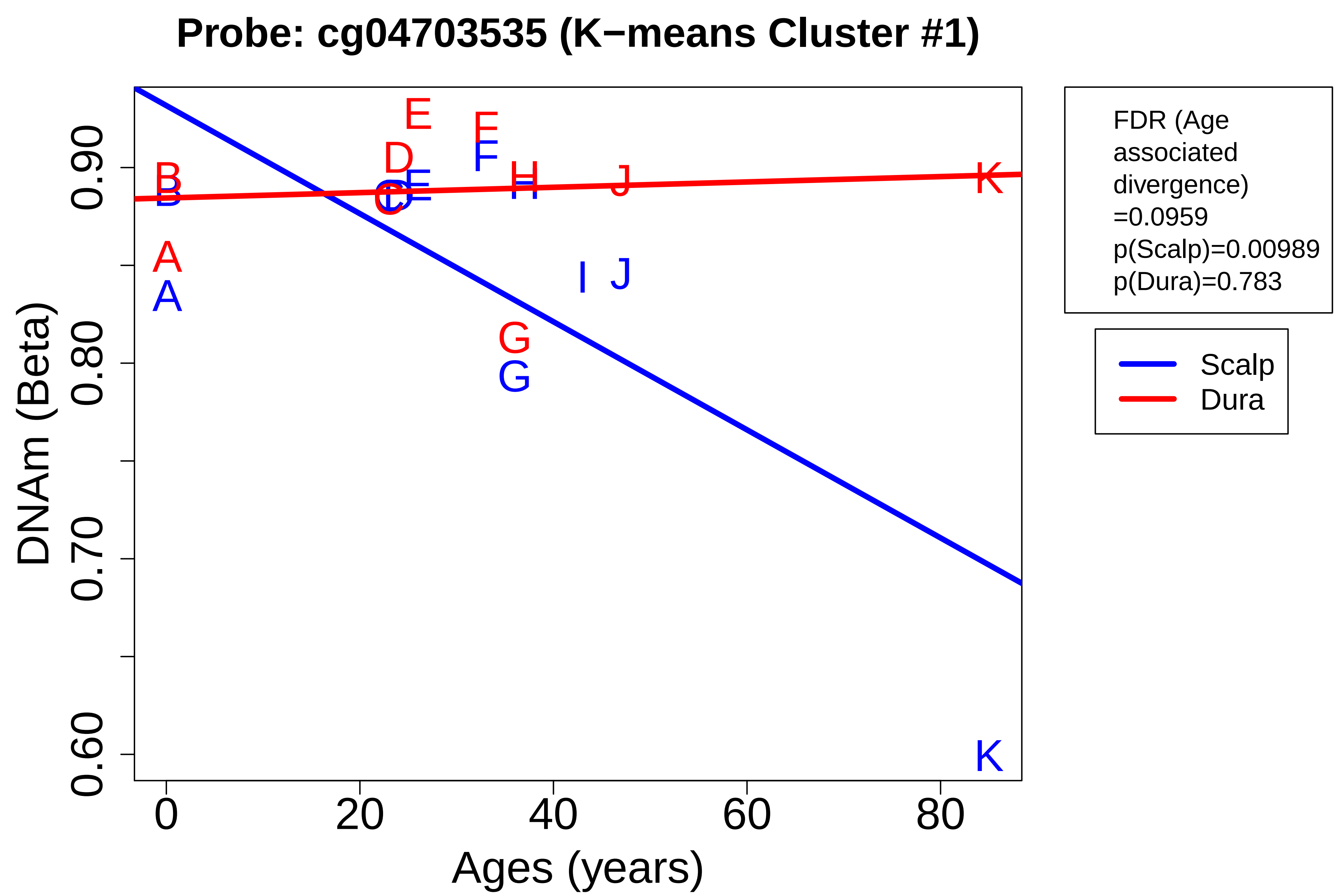
Probe: cg04703535 (K−means Cluster #1)
DNAm (Beta)
0.60
0.70
0.80
0.90
0
20
40
60
80
Ages (years)
A
B
C
D
E
F
G
H
I
J
K
FDR (Age associated divergence) =0.0959
p(Scalp)=0.00989
p(Dura)=0.783
Scalp
Dura

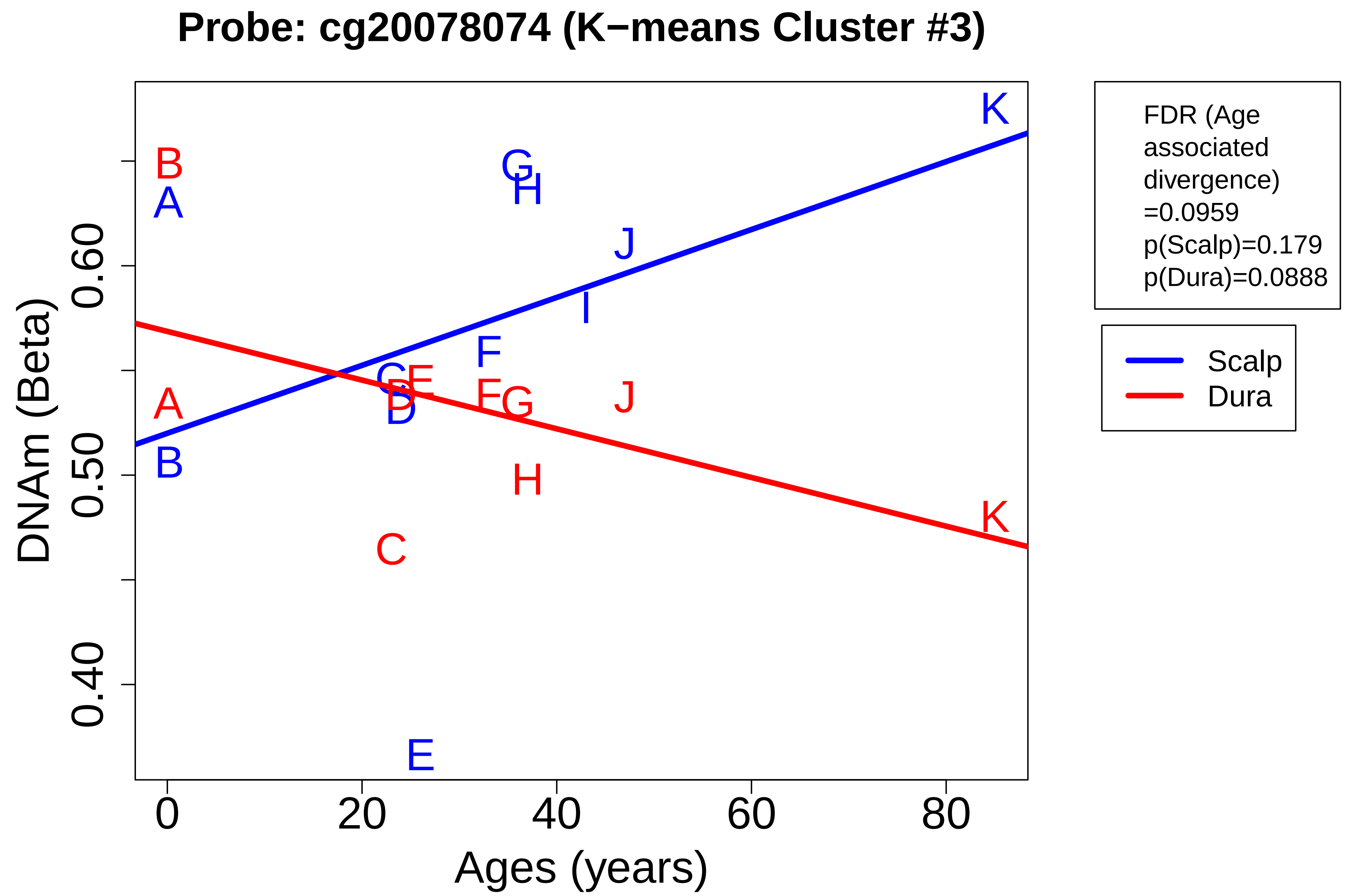

Probe: cg20078074 (K−means Cluster #3)
DNAm (Beta)
0.40
0.50
0.60
0
20
40
60
80
Ages (years)
FDR (Age associated divergence) =0.0959
p(Scalp)=0.179
p(Dura)=0.0888
Scalp
Dura

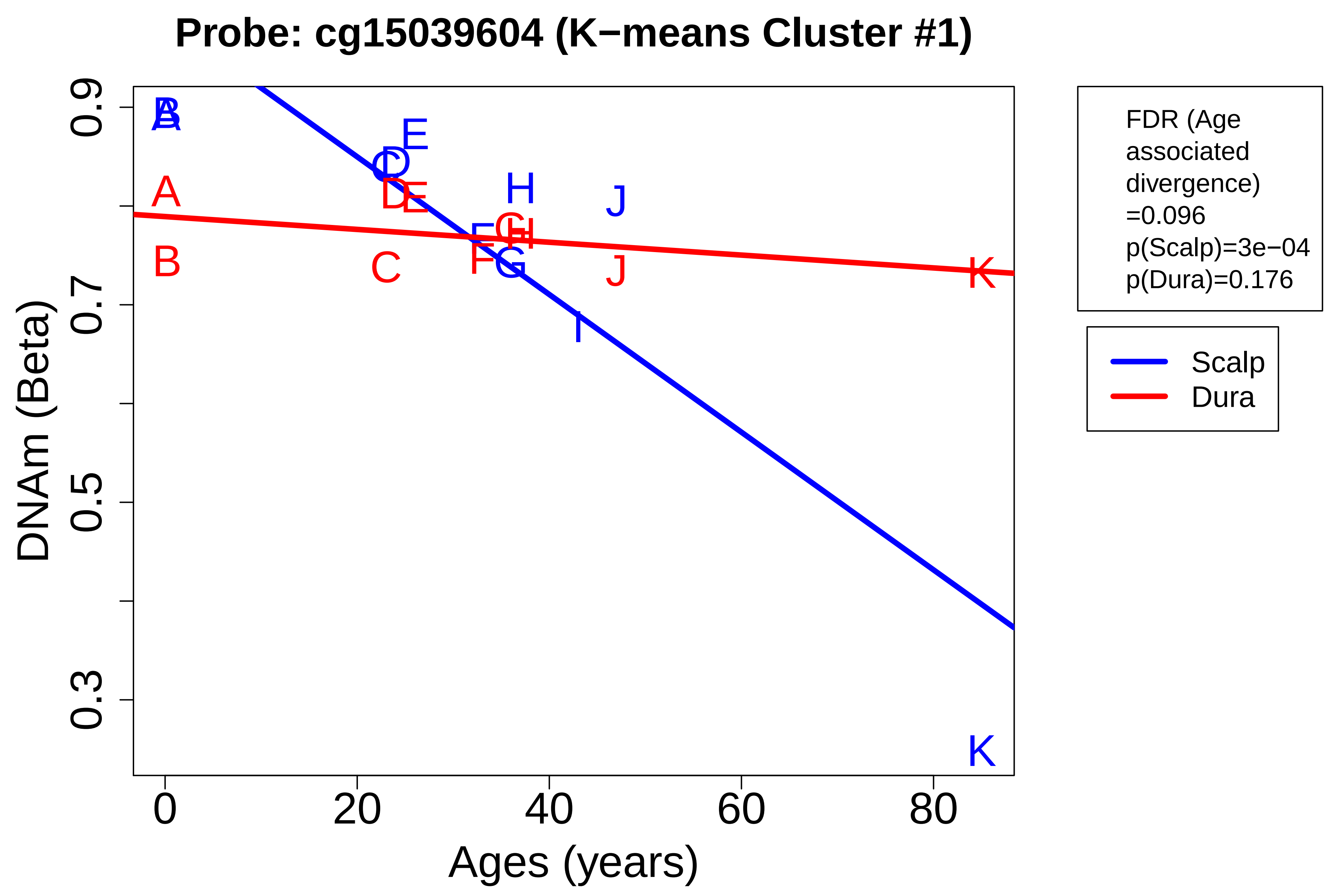

Probe: cg15039604 (K−means Cluster #1)
DNAm (Beta)
0.3
0.5
0.7
0.9
Ages (years)
0
20
40
60
80
FDR (Age associated divergence) =0.096
p(Scalp)=3e−04
p(Dura)=0.176
Scalp
Dura

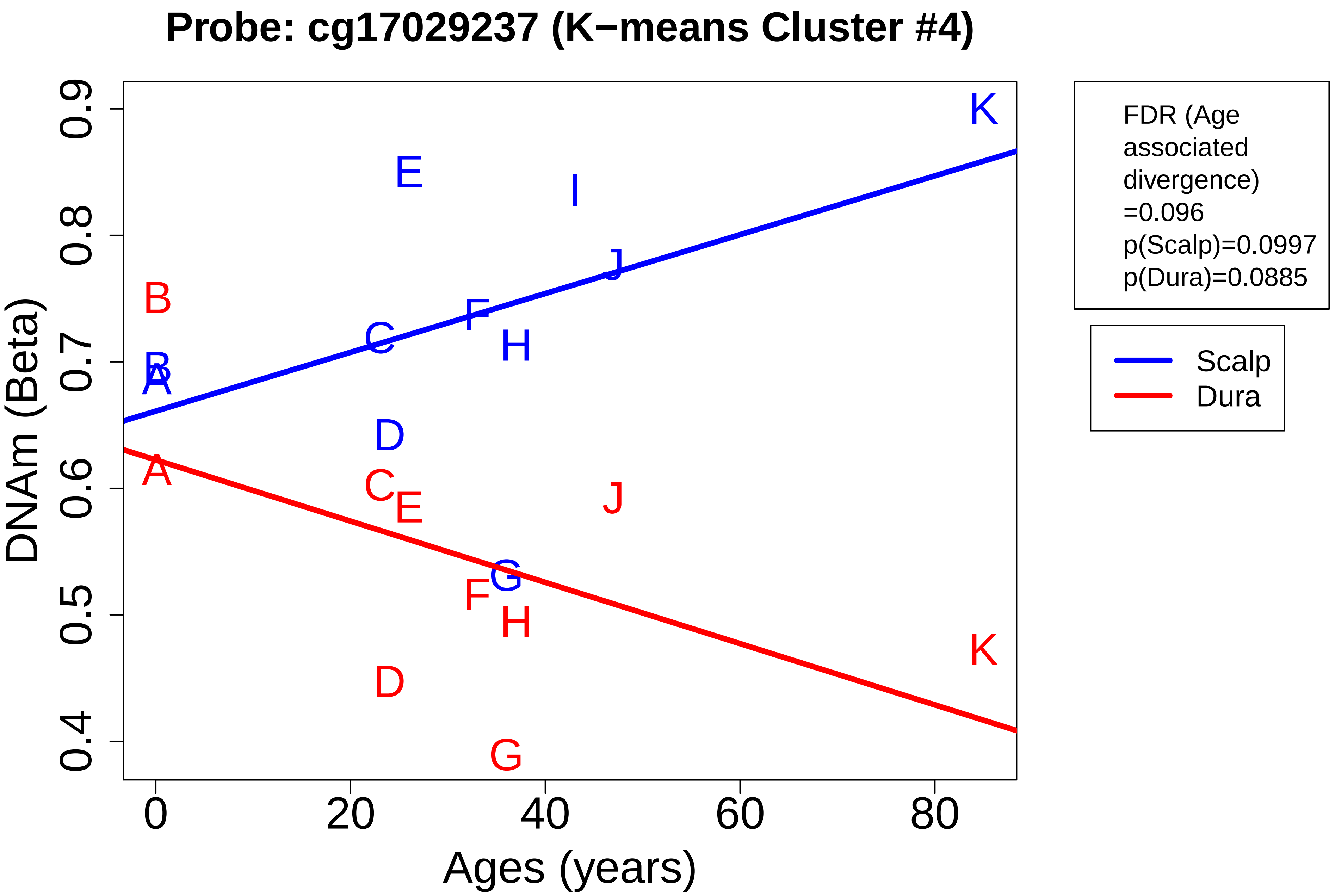

Probe: cg17029237 (K−means Cluster #4)
DNAm (Beta)
Ages (years)
0.4
0.5
0.6
0.7
0.8
0.9
0
20
40
60
80
FDR (Age associated divergence) =0.096
p(Scalp)=0.0997
p(Dura)=0.0885
Scalp
Dura

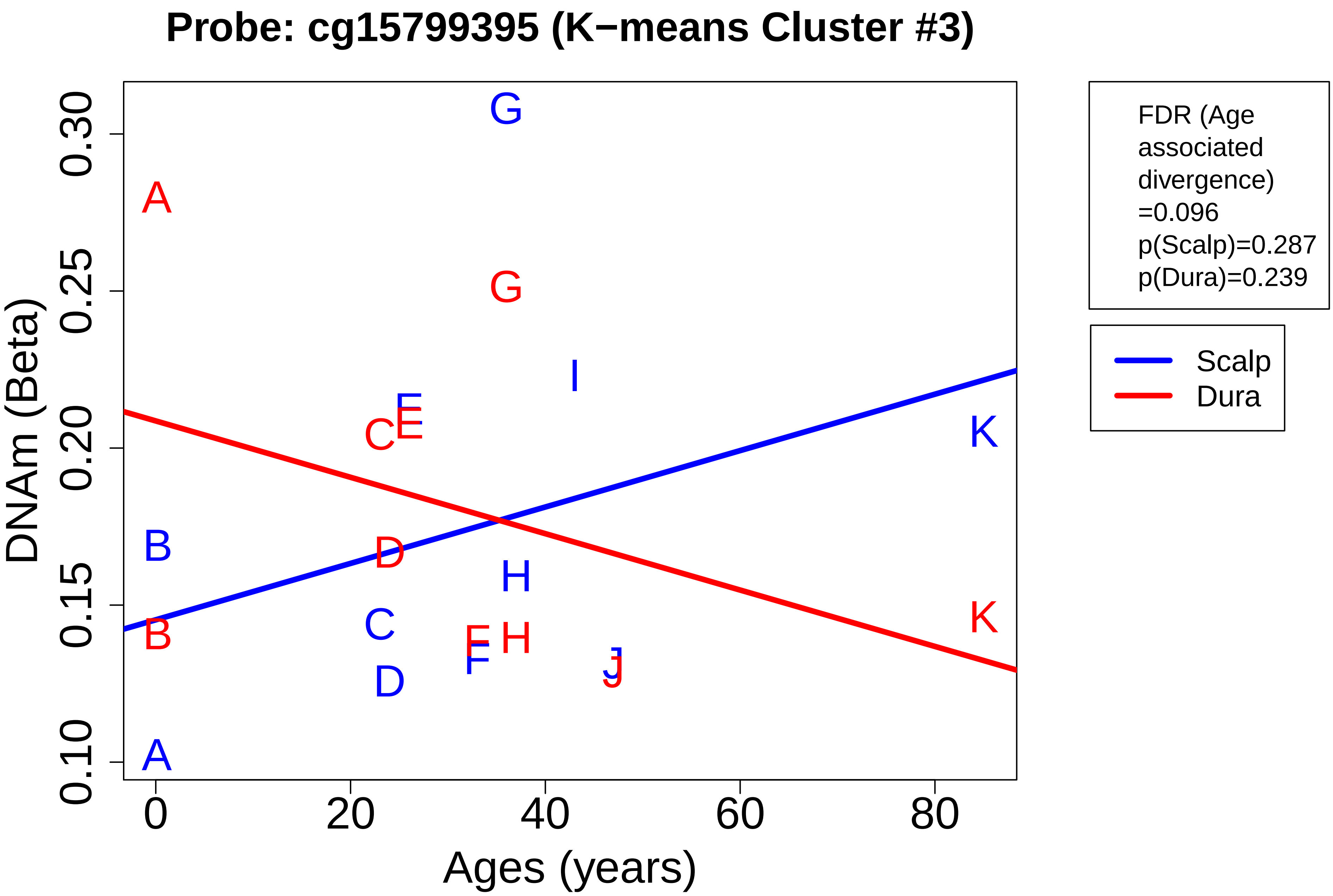

Probe: cg15799395 (K−means Cluster #3)
FDR (Age associated divergence) =0.096
p(Scalp)=0.287
p(Dura)=0.239
Scalp
Dura
DNAm (Beta)
0.10
0.15
0.20
0.25
0.30
0
20
40
60
80
Ages (years)

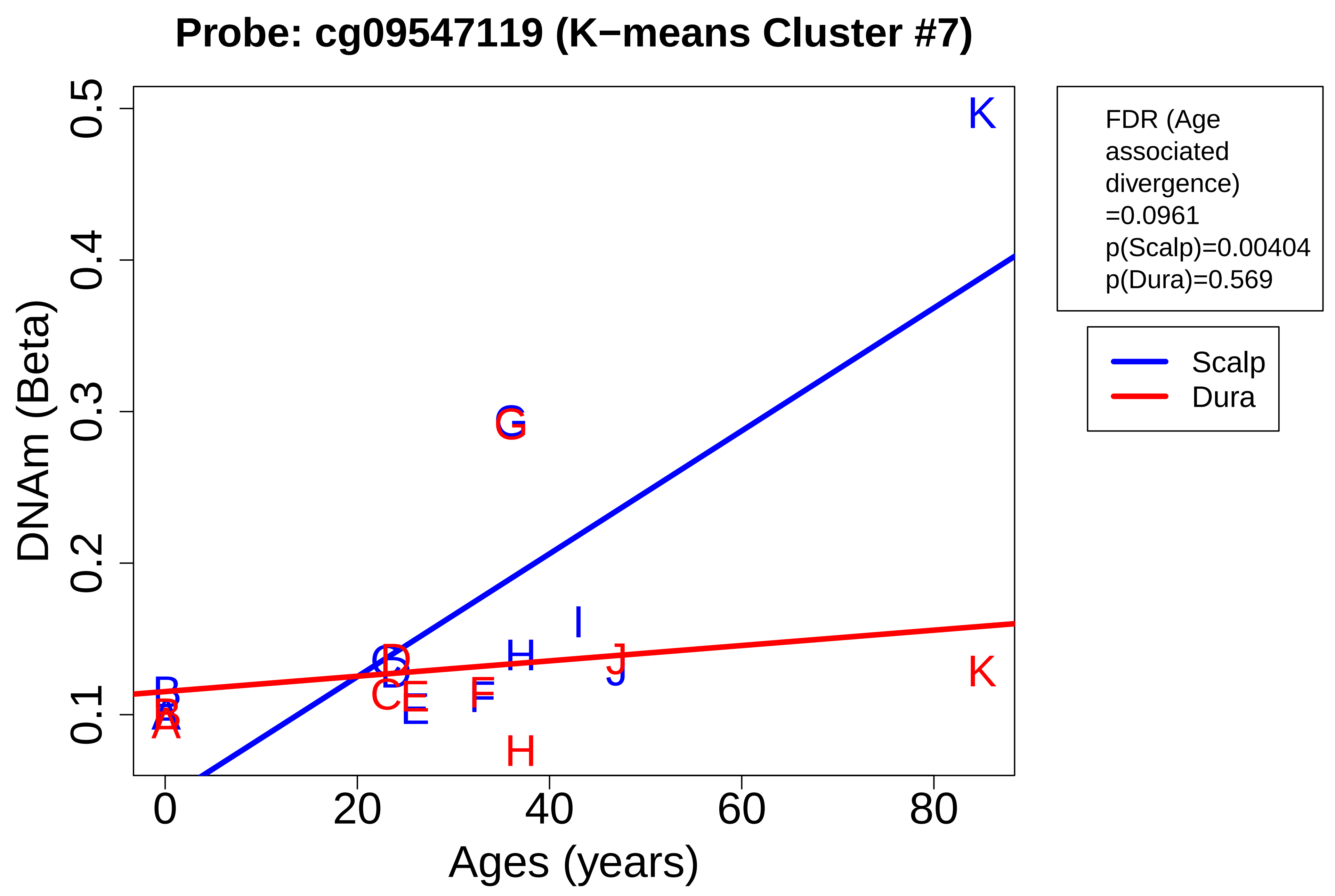

Probe: cg09547119 (K−means Cluster #7)
DNAm (Beta)
0.1
0.2
0.3
0.4
0.5
0
20
40
60
80
Ages (years)
FDR (Age associated divergence) =0.0961
p(Scalp)=0.00404
p(Dura)=0.569
Scalp
Dura

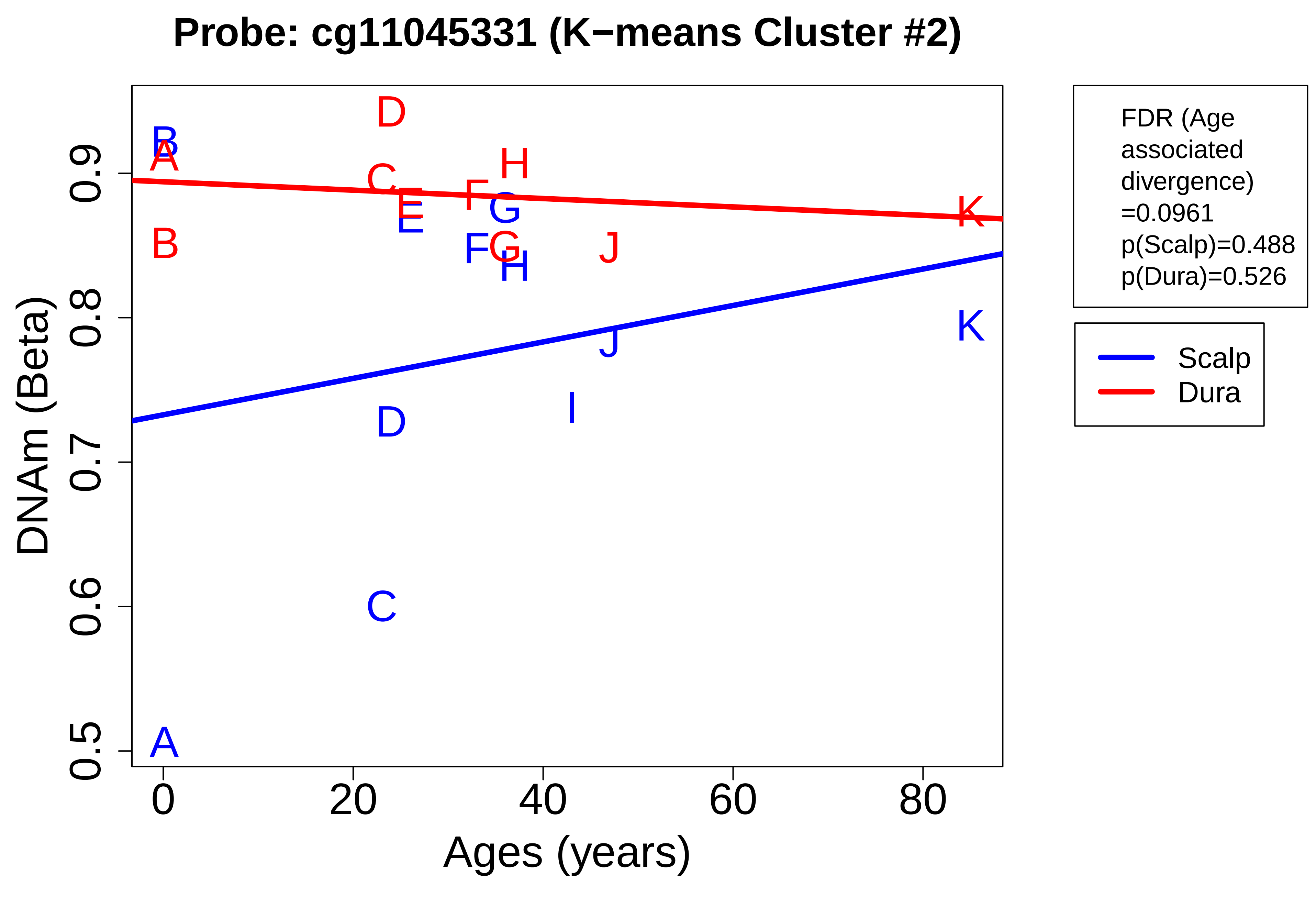

Probe: cg11045331 (K−means Cluster #2)
DNAm (Beta)
0.5
0.6
0.7
0.8
0.9
Ages (years)
0
20
40
60
80
FDR (Age associated divergence) =0.0961
p(Scalp)=0.488
p(Dura)=0.526
Scalp
Dura
A
B
C
D
E
F
G
H
I
J
K

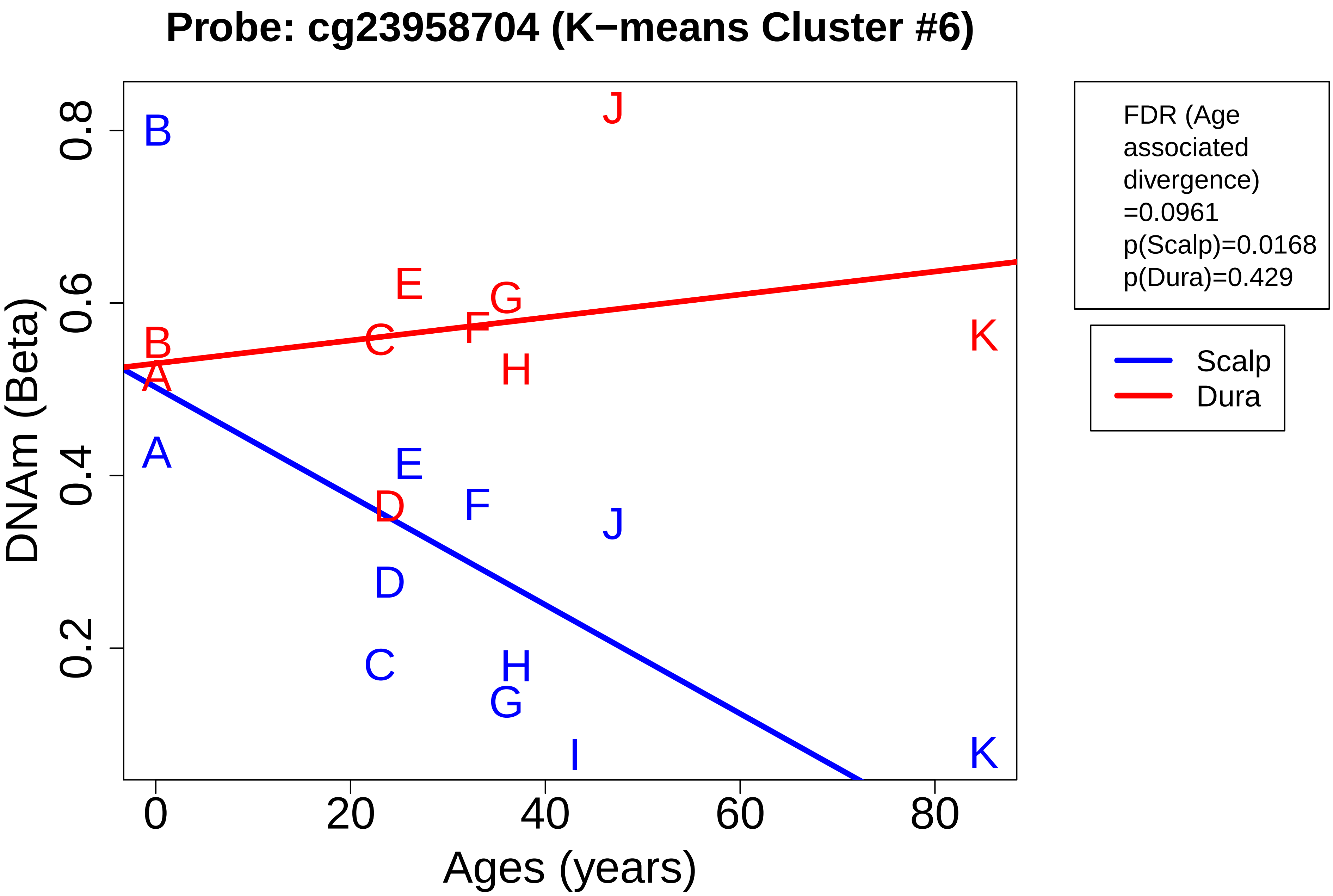

Probe: cg23958704 (K−means Cluster #6)
DNAm (Beta)
0.2
0.4
0.6
0.8
0
20
40
60
80
Ages (years)
B
J
E
G
F
C
B
A
H
K
A
E
D
F
J
D
C
H
G
I
K
FDR (Age associated divergence) =0.0961
p(Scalp)=0.0168
p(Dura)=0.429
Scalp
Dura

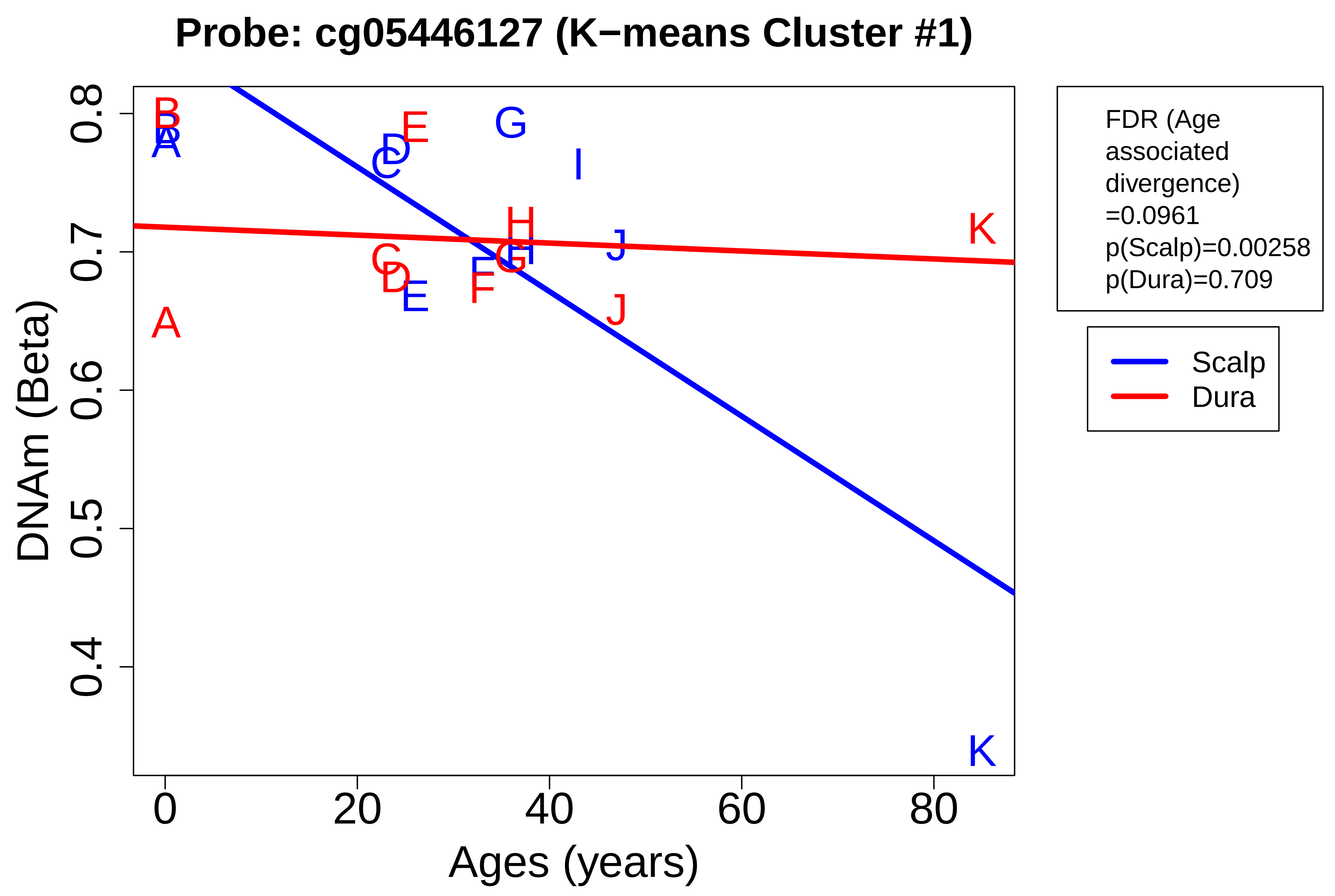

Probe: cg05446127 (K−means Cluster #1)
DNAm (Beta)
0.4
0.5
0.6
0.7
0.8
Ages (years)
0
20
40
60
80
FDR (Age associated divergence) =0.0961
p(Scalp)=0.00258
p(Dura)=0.709
Scalp
Dura

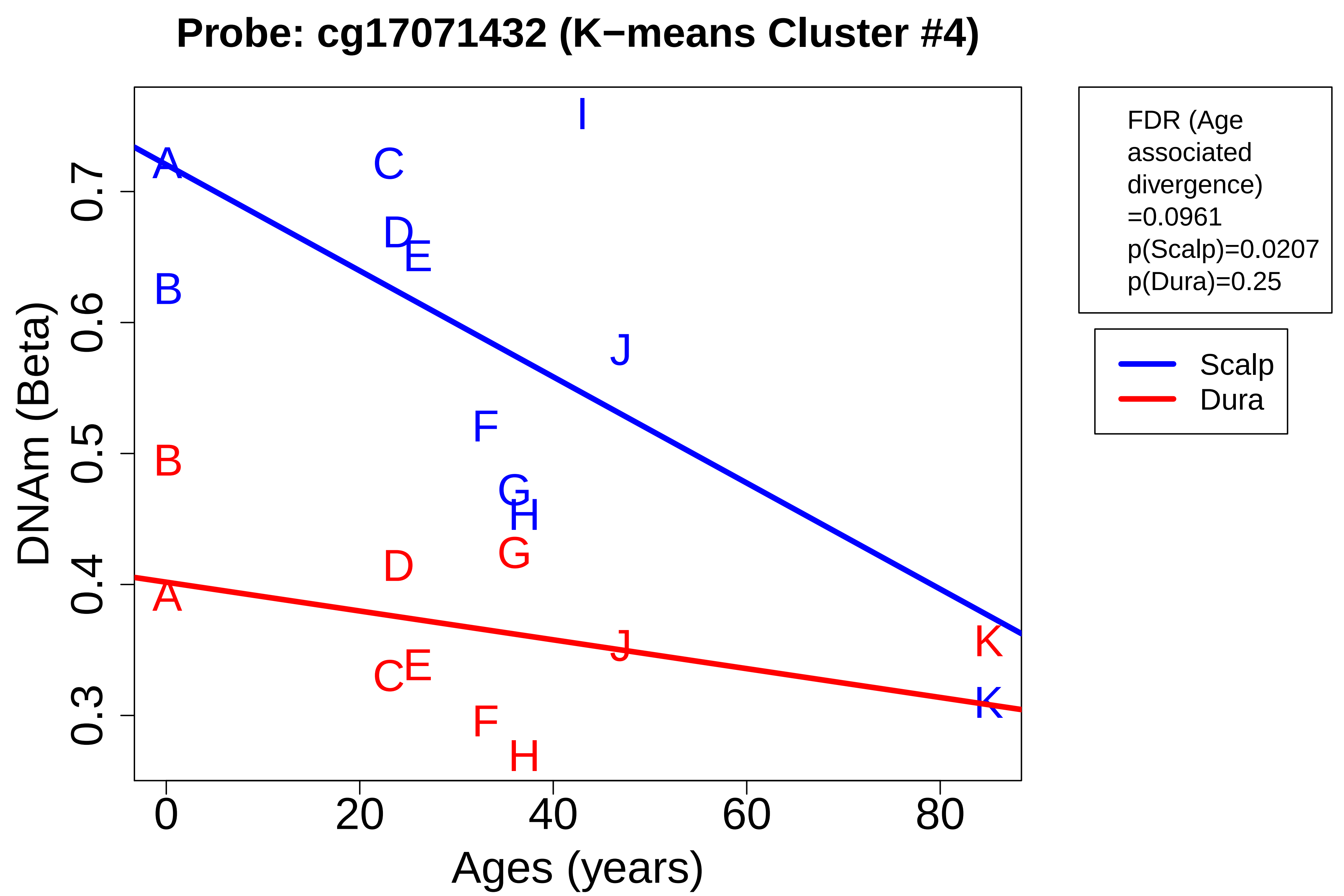

Probe: cg17071432 (K−means Cluster #4)
FDR (Age associated divergence) =0.0961
p(Scalp)=0.0207
p(Dura)=0.25
Scalp
Dura
DNAm (Beta)
0.3
0.4
0.5
0.6
0.7
0
20
40
60
80
Ages (years)
A
B
C
D
E
F
G
H
I
J
K

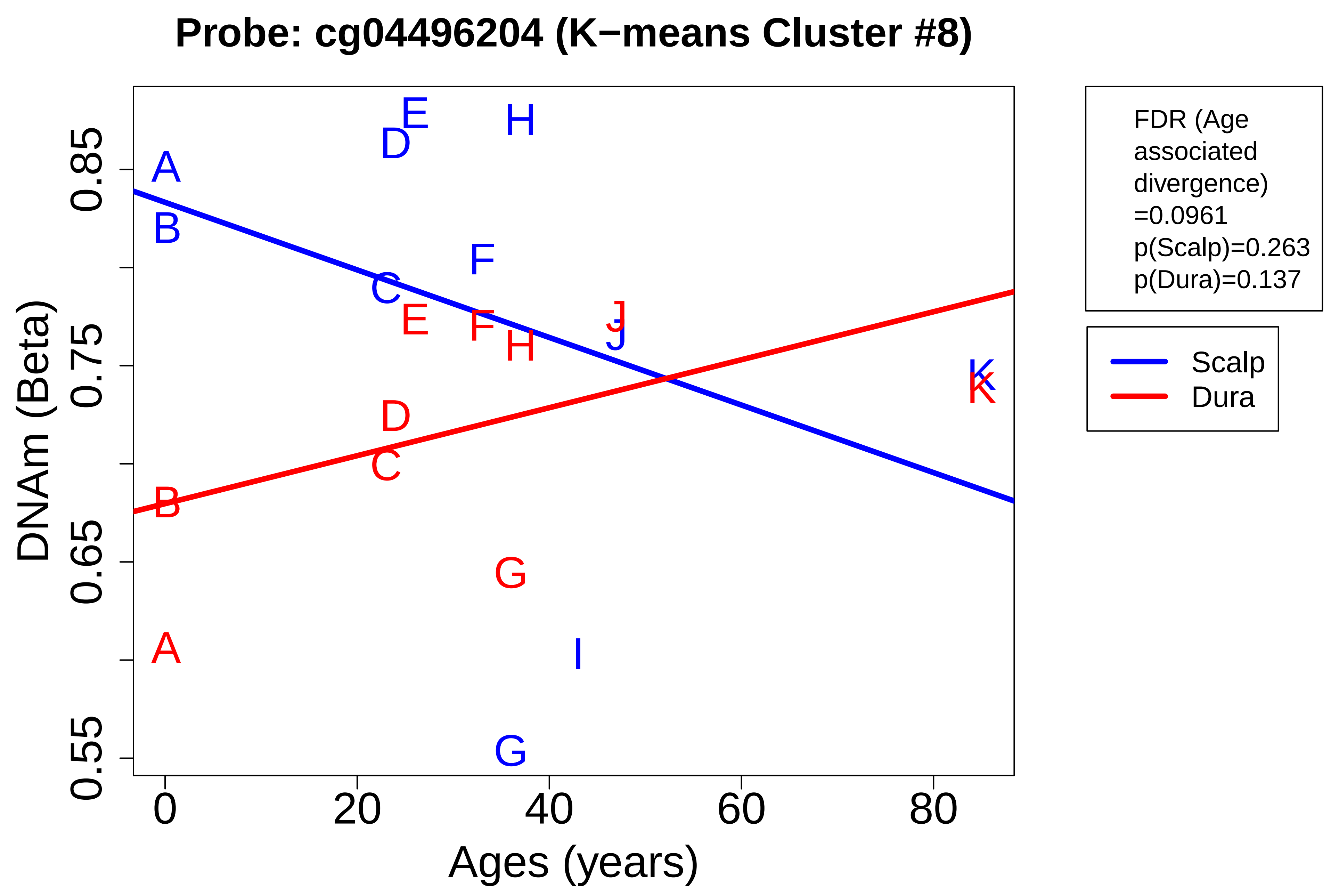

Probe: cg04496204 (K−means Cluster #8)
DNAm (Beta)
0.55
0.65
0.75
0.85
0
20
40
60
80
Ages (years)
FDR (Age associated divergence) =0.0961
p(Scalp)=0.263
p(Dura)=0.137
Scalp
Dura

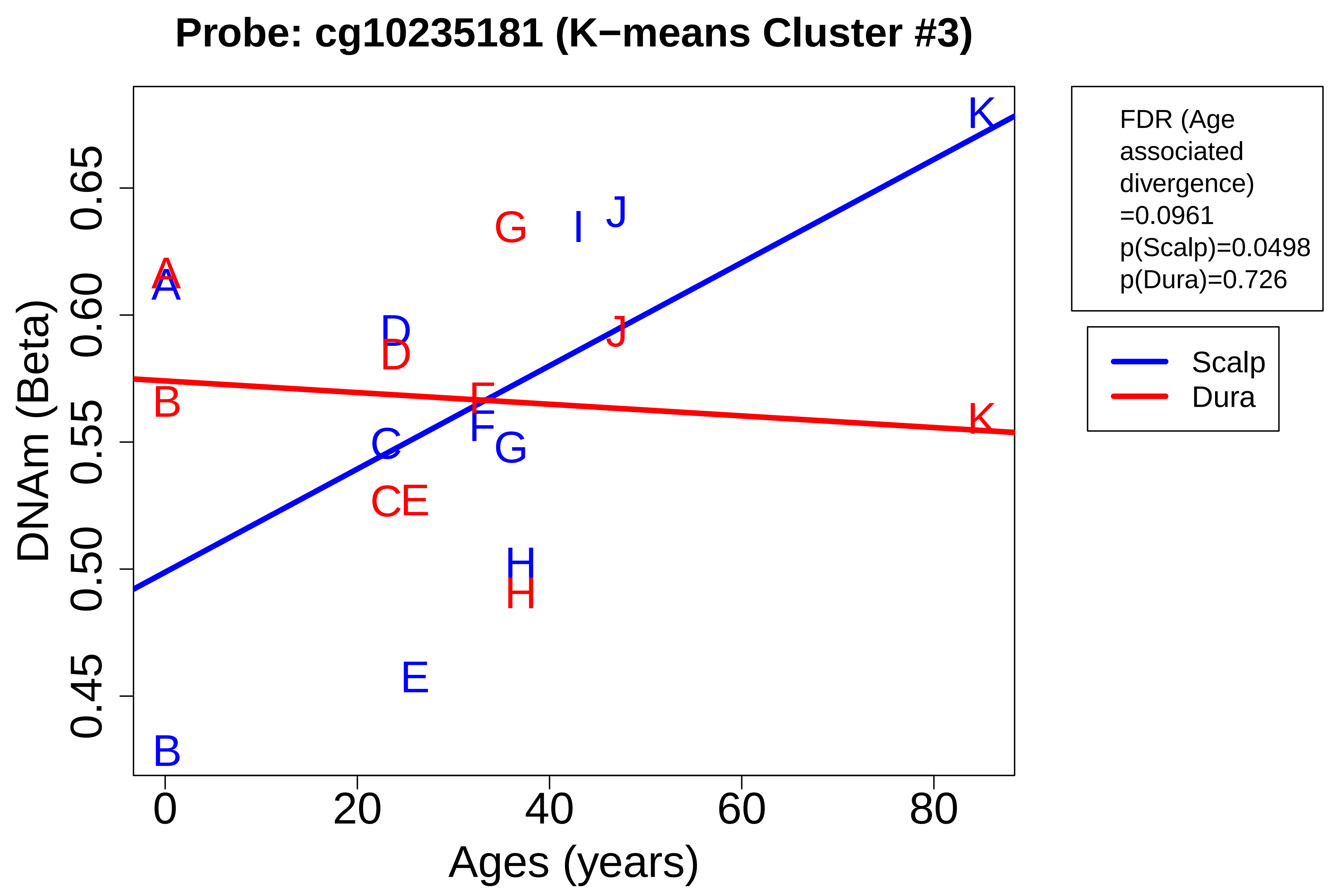
Probe: cg10235181 (K−means Cluster #3)
FDR (Age associated divergence) =0.0961
p(Scalp)=0.0498
p(Dura)=0.726
Scalp
Dura
DNAm (Beta)
0.45
0.50
0.55
0.60
0.65
0
20
40
60
80
Ages (years)
A
B
C
D
E
F
G
H
I
J
K
CE

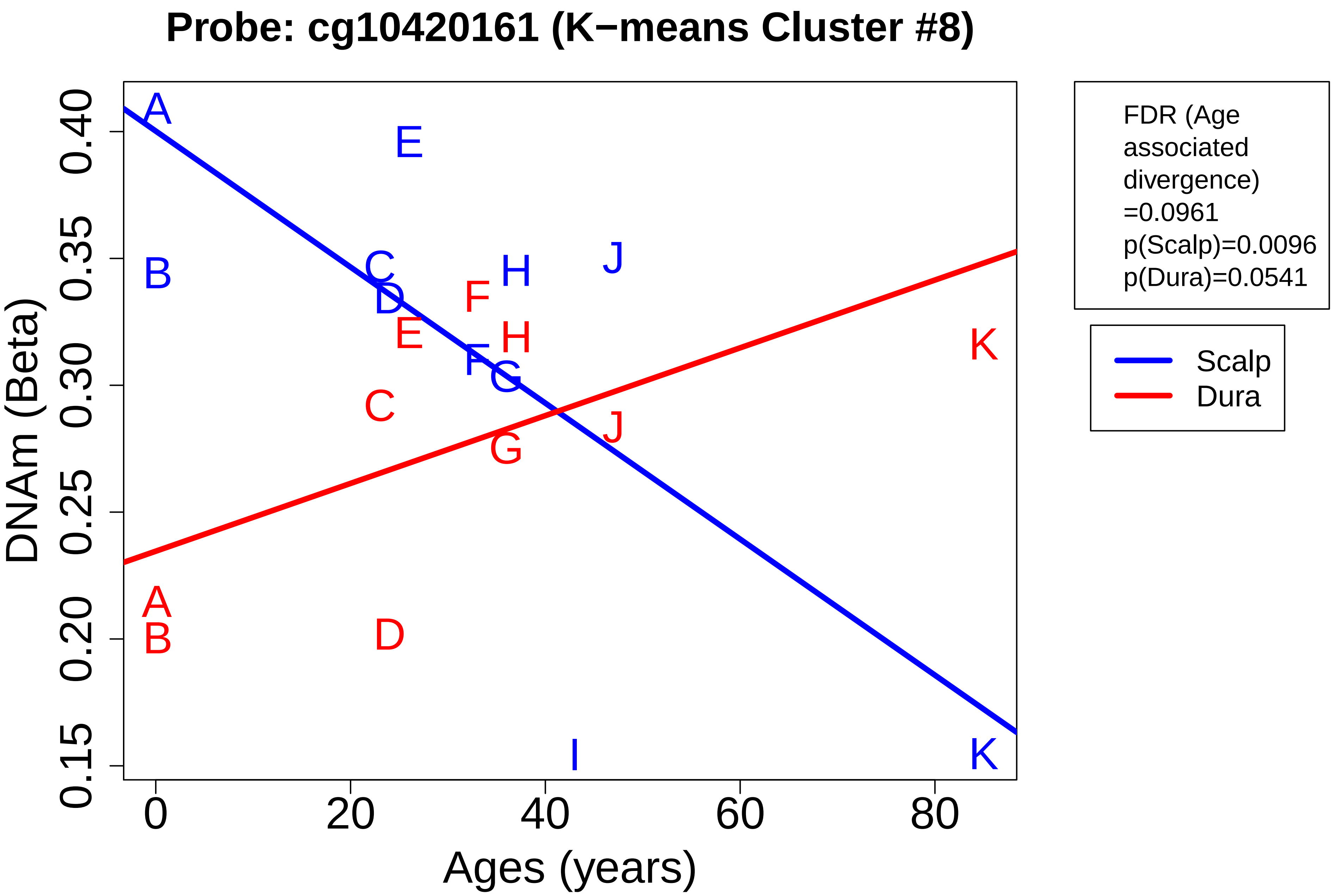

Probe: cg10420161 (K−means Cluster #8)
DNAm (Beta)
0.15
0.20
0.25
0.30
0.35
0.40
Ages (years)
0
20
40
60
80
A
B
C
D
E
F
G
H
I
J
K
A
B
C
D
E
F
G
H
J
K
FDR (Age associated divergence) =0.0961
p(Scalp)=0.0096
p(Dura)=0.0541
Scalp
Dura

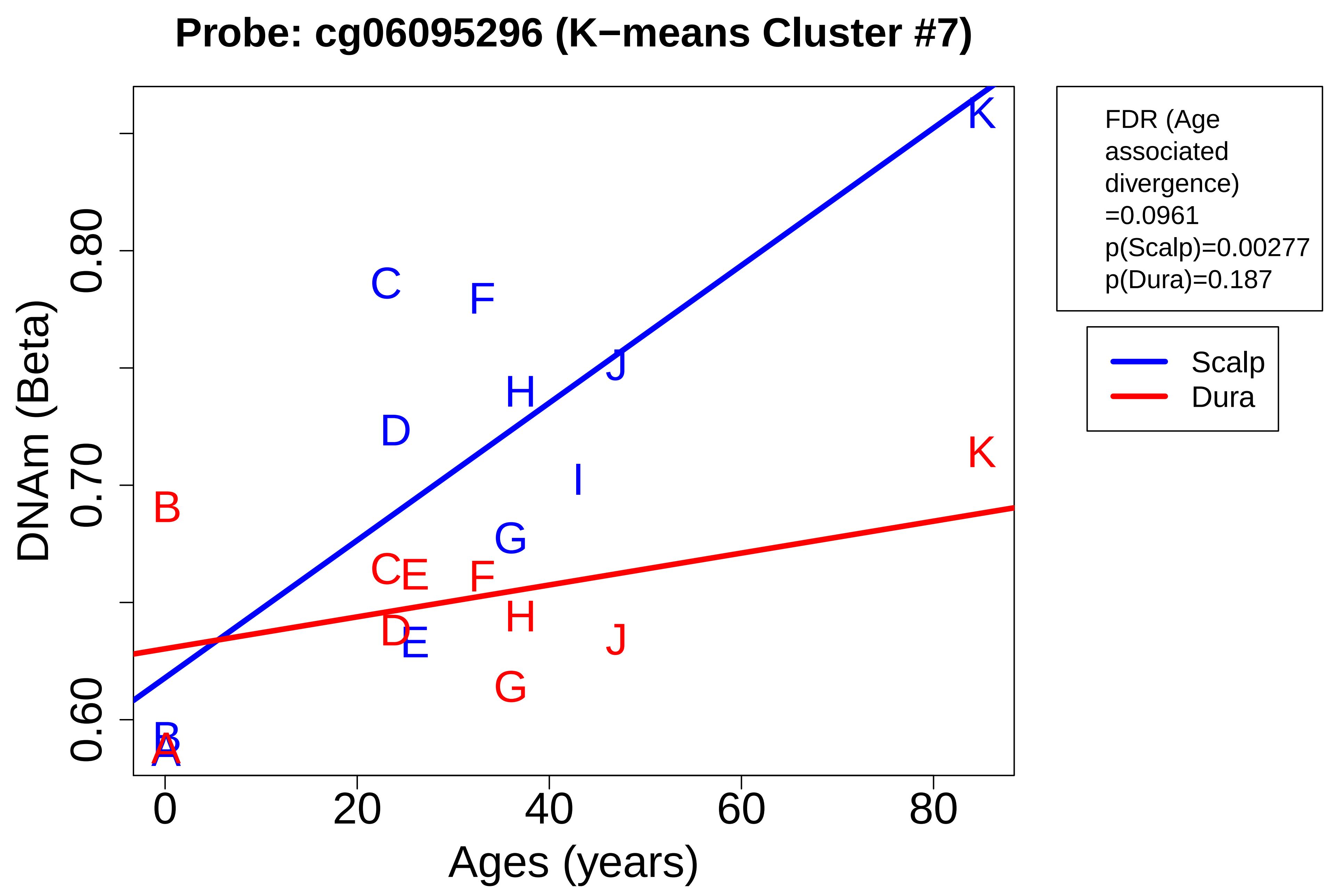

Probe: cg06095296 (K−means Cluster #7)
DNAm (Beta)
0.60
0.70
0.80
0
20
40
60
80
Ages (years)
FDR (Age associated divergence) =0.0961
p(Scalp)=0.00277
p(Dura)=0.187
Scalp
Dura

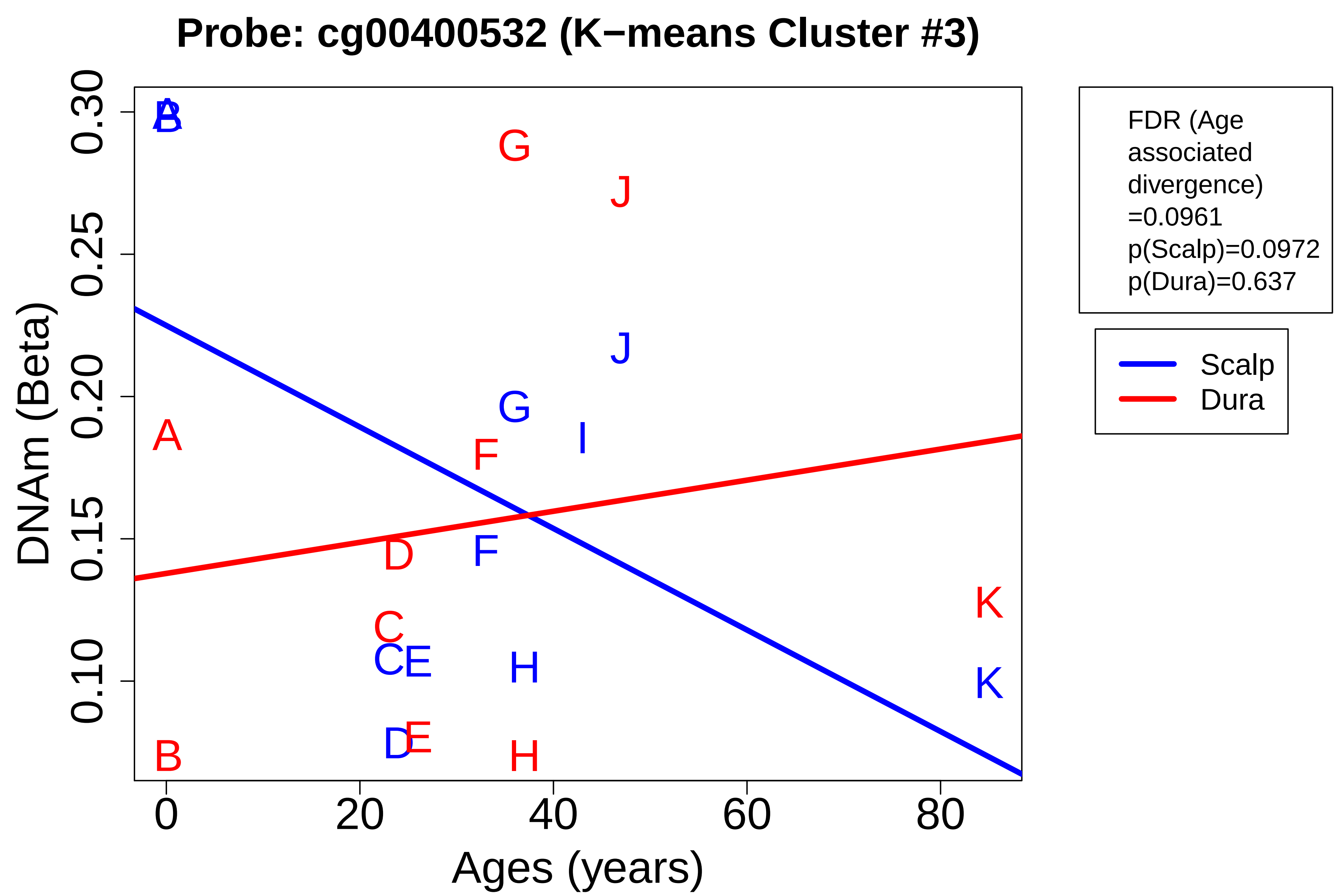

Probe: cg00400532 (K−means Cluster #3)
DNAm (Beta)
0.10
0.15
0.20
0.25
0.30
0
20
40
60
80
Ages (years)
FDR (Age associated divergence) =0.0961
p(Scalp)=0.0972
p(Dura)=0.637
Scalp
Dura

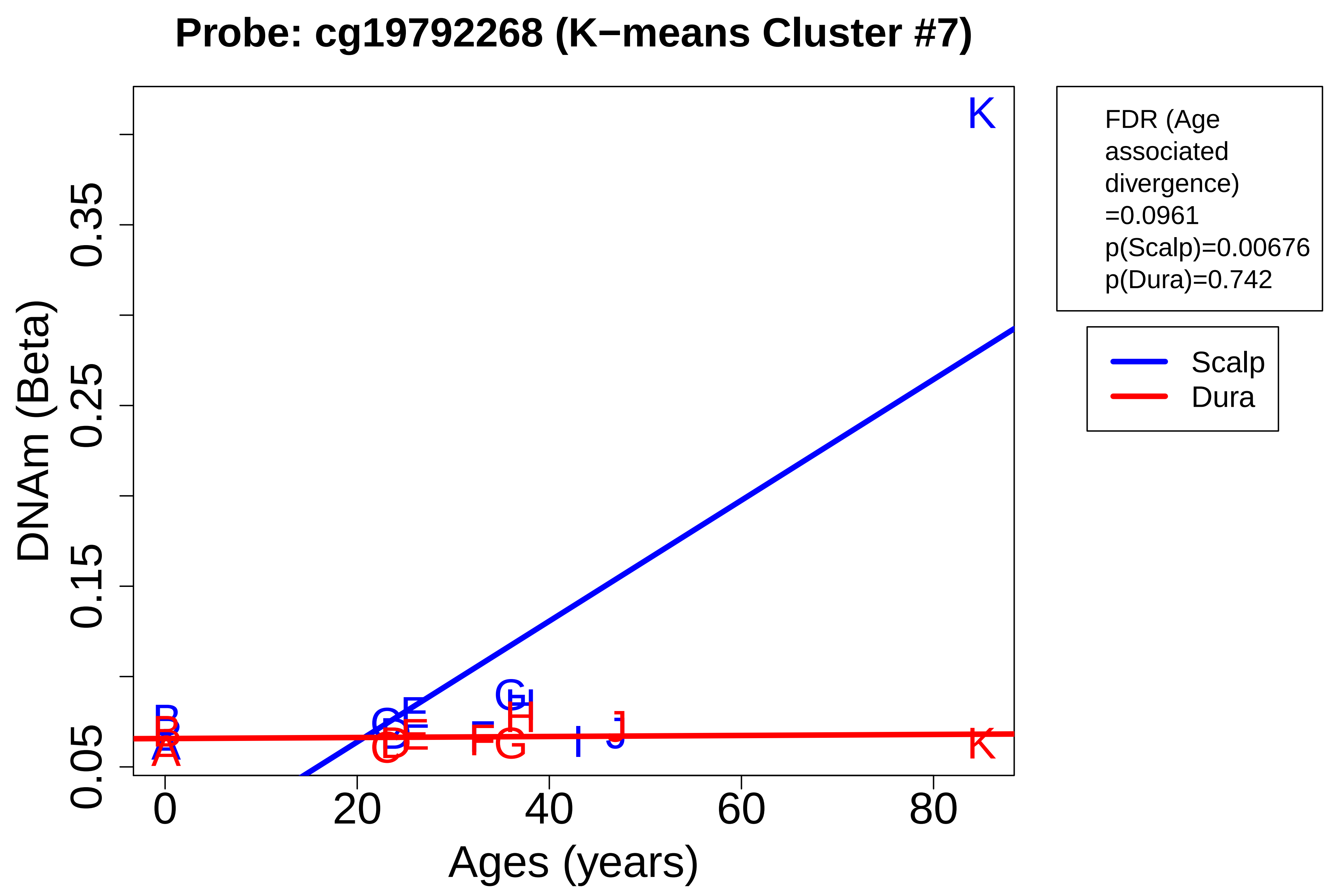

Probe: cg19792268 (K−means Cluster #7)
DNAm (Beta)
0.05
0.15
0.25
0.35
0
20
40
60
80
Ages (years)
FDR (Age associated divergence) =0.0961
p(Scalp)=0.00676
p(Dura)=0.742
Scalp
Dura

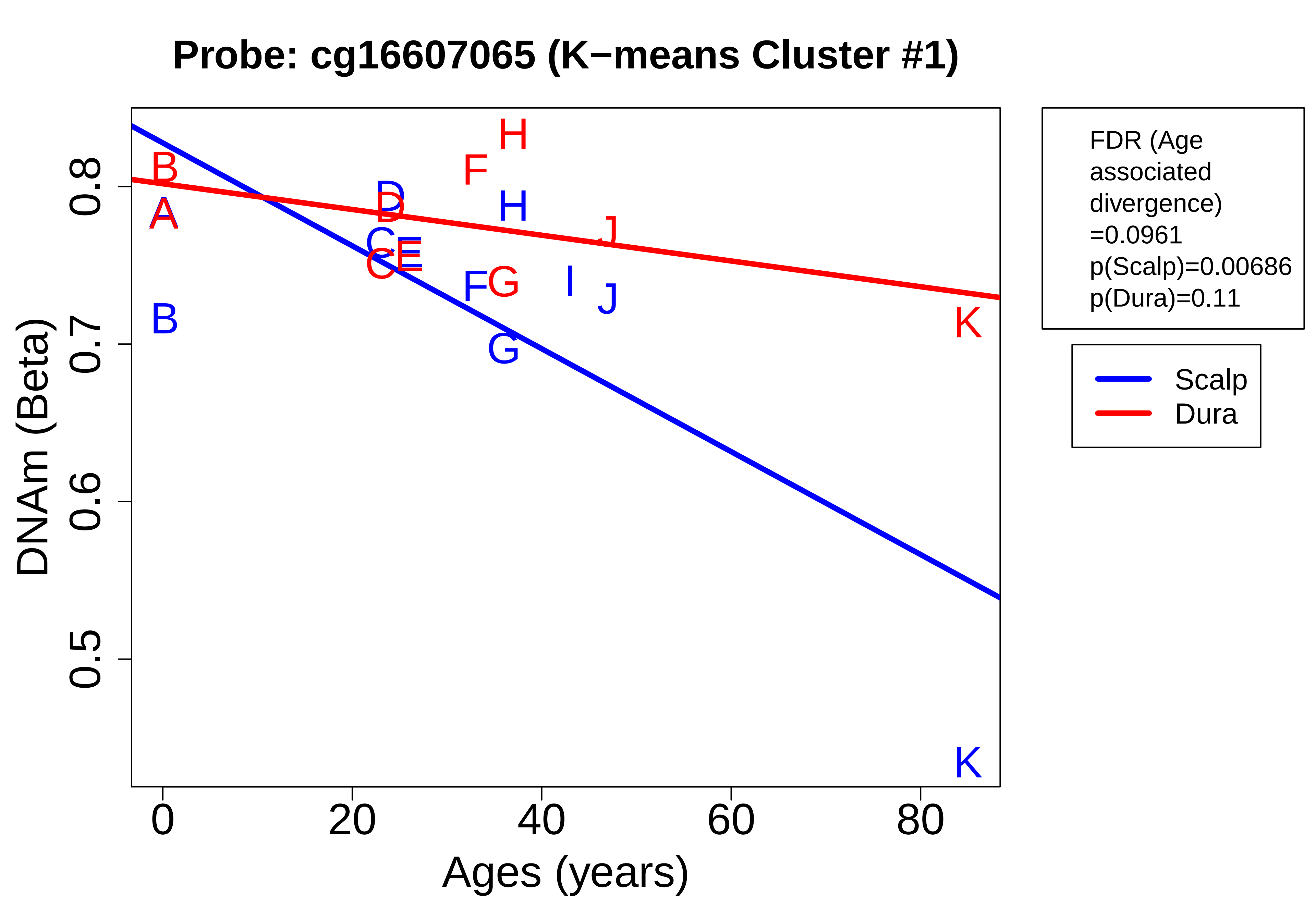
Probe: cg16607065 (K−means Cluster #1)
DNAm (Beta)
0.5
0.6
0.7
0.8
Ages (years)
0
20
40
60
80
FDR (Age associated divergence) =0.0961
p(Scalp)=0.00686
p(Dura)=0.11
Scalp
Dura

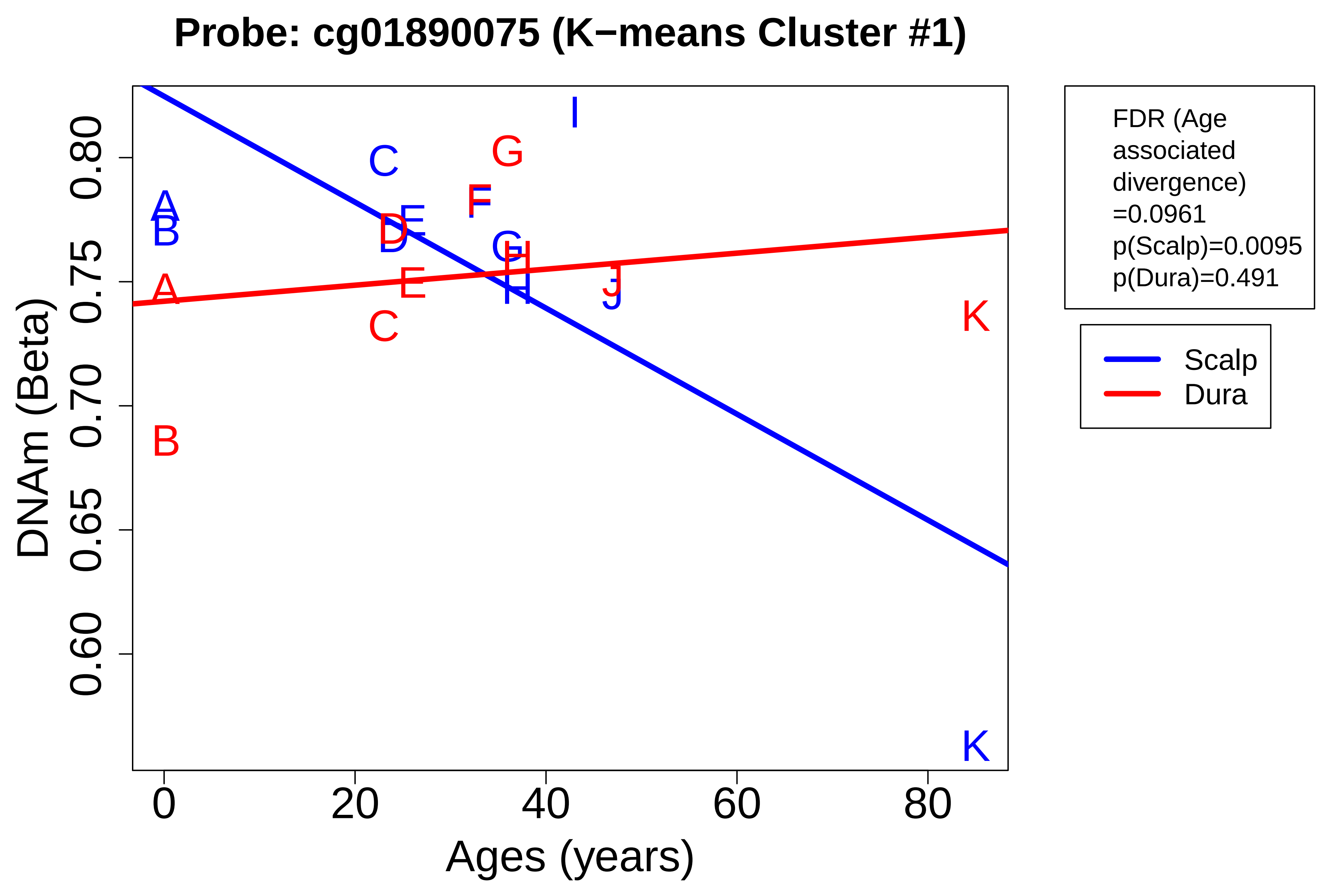

Probe: cg01890075 (K−means Cluster #1)
DNAm (Beta)
0.60
0.65
0.70
0.75
0.80
Ages (years)
0
20
40
60
80
A
B
C
D
E
F
G
H
I
J
K
FDR (Age associated divergence) =0.0961
p(Scalp)=0.0095
p(Dura)=0.491
Scalp
Dura

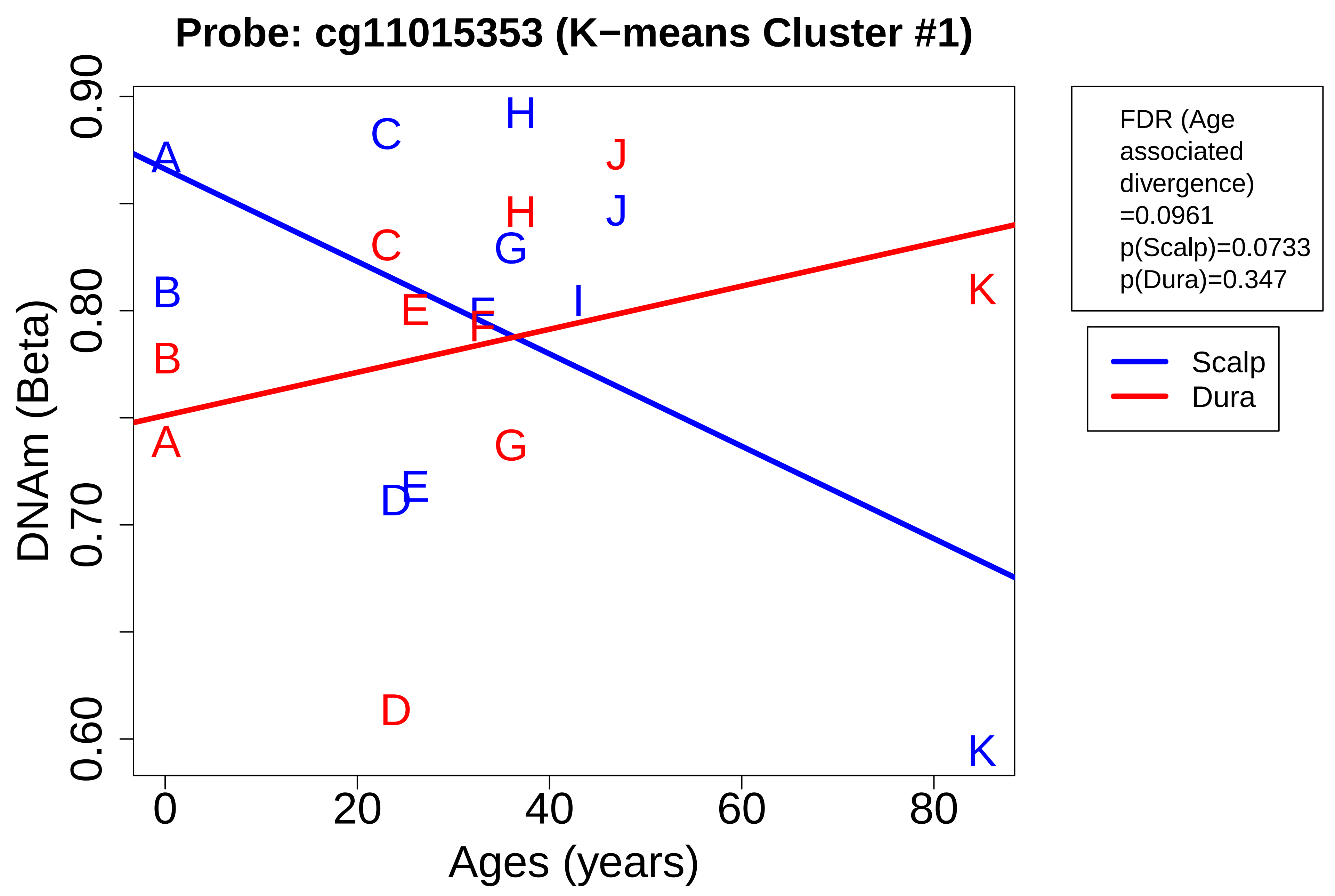

Probe: cg11015353 (K−means Cluster #1)
DNAm (Beta)
0.60
0.70
0.80
0.90
Ages (years)
0
20
40
60
80
A
B
C
D
E
F
G
H
I
J
K
A
B
C
D
E
F
G
H
J
K
FDR (Age associated divergence) =0.0961
p(Scalp)=0.0733
p(Dura)=0.347
Scalp
Dura

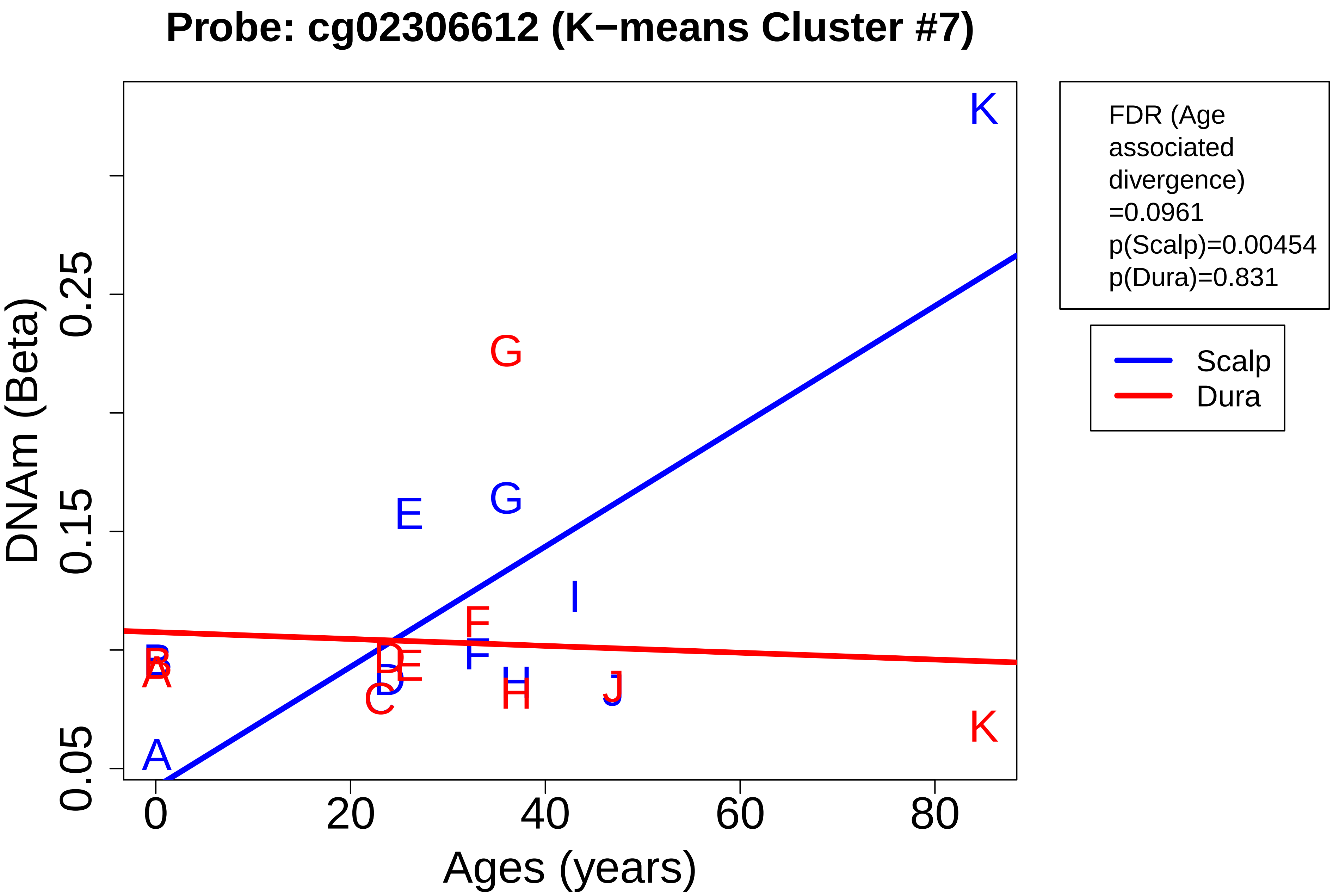

Probe: cg02306612 (K−means Cluster #7)
DNAm (Beta)
0.05
0.15
0.25
0
20
40
60
80
Ages (years)
FDR (Age associated divergence) =0.0961
p(Scalp)=0.00454
p(Dura)=0.831
Scalp
Dura
A
B
C
D
E
F
G
H
I
J
K

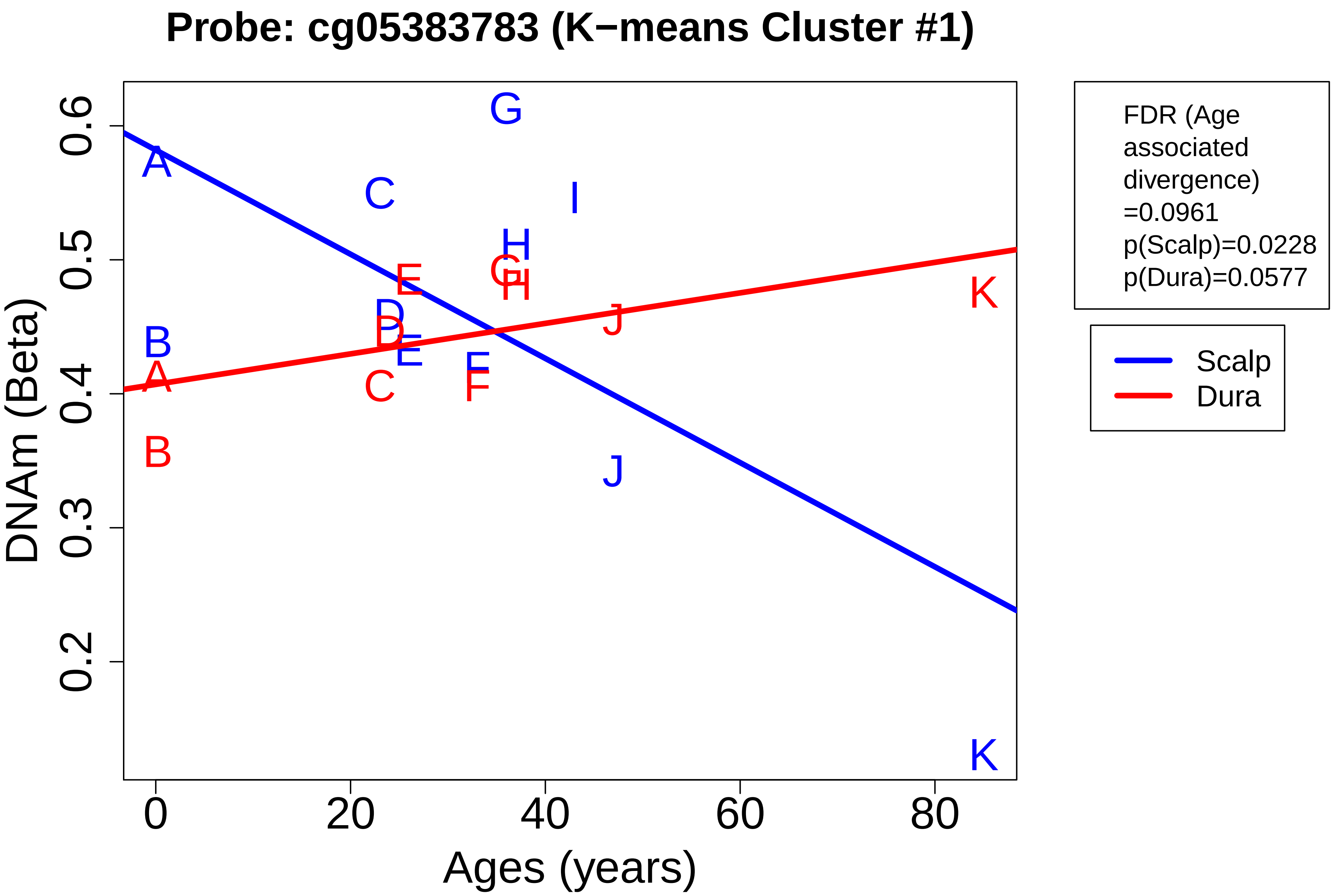

Probe: cg05383783 (K−means Cluster #1)
DNAm (Beta)
0.2
0.3
0.4
0.5
0.6
Ages (years)
0
20
40
60
80
A
B
C
D
E
F
G
H
I
J
K
FDR (Age associated divergence) =0.0961
p(Scalp)=0.0228
p(Dura)=0.0577
Scalp
Dura

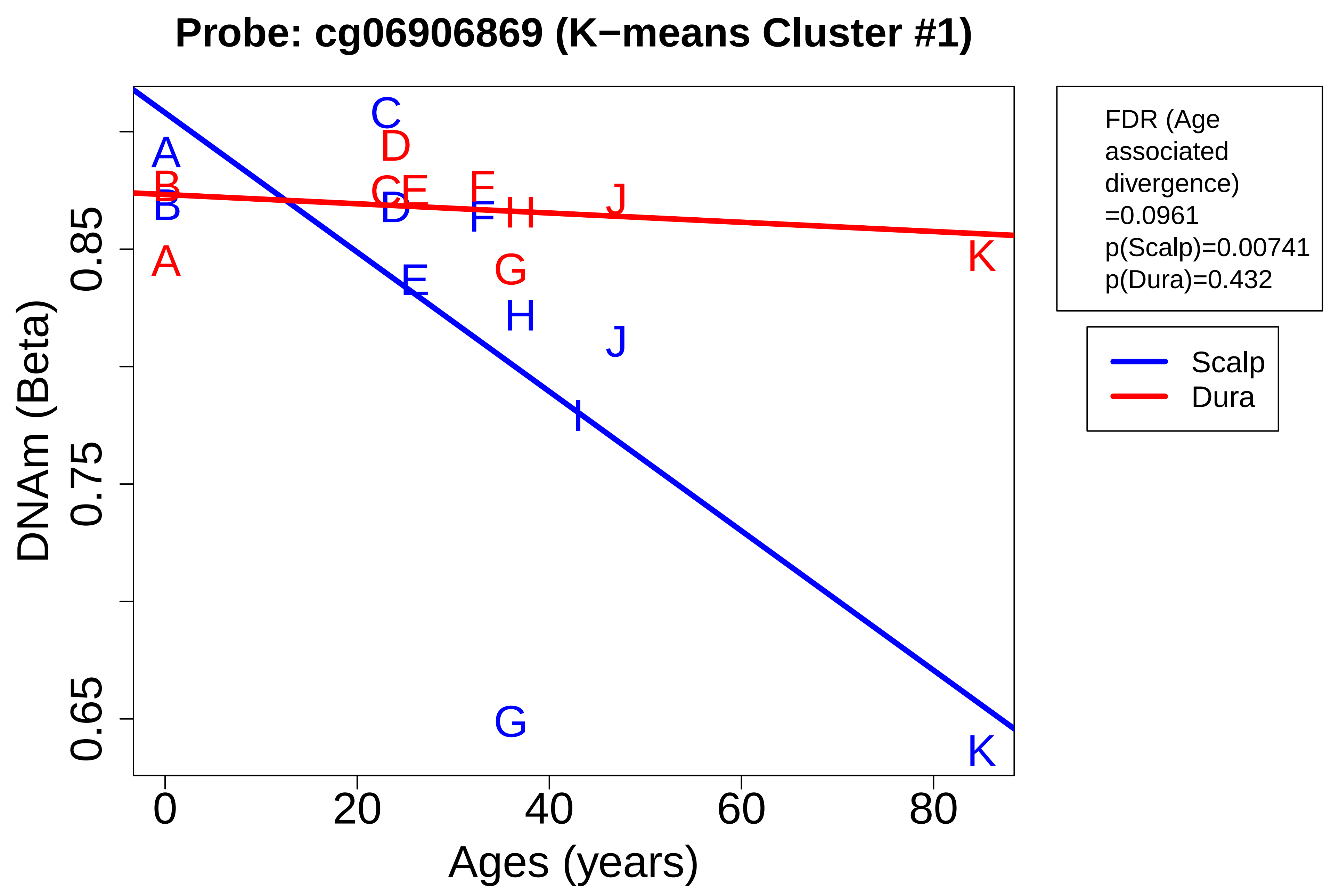

Probe: cg06906869 (K−means Cluster #1)
DNAm (Beta)
0.65
0.75
0.85
Ages (years)
0
20
40
60
80
A
B
C
D
E
F
G
H
I
J
K
FDR (Age associated divergence) =0.0961
p(Scalp)=0.00741
p(Dura)=0.432
Scalp
Dura

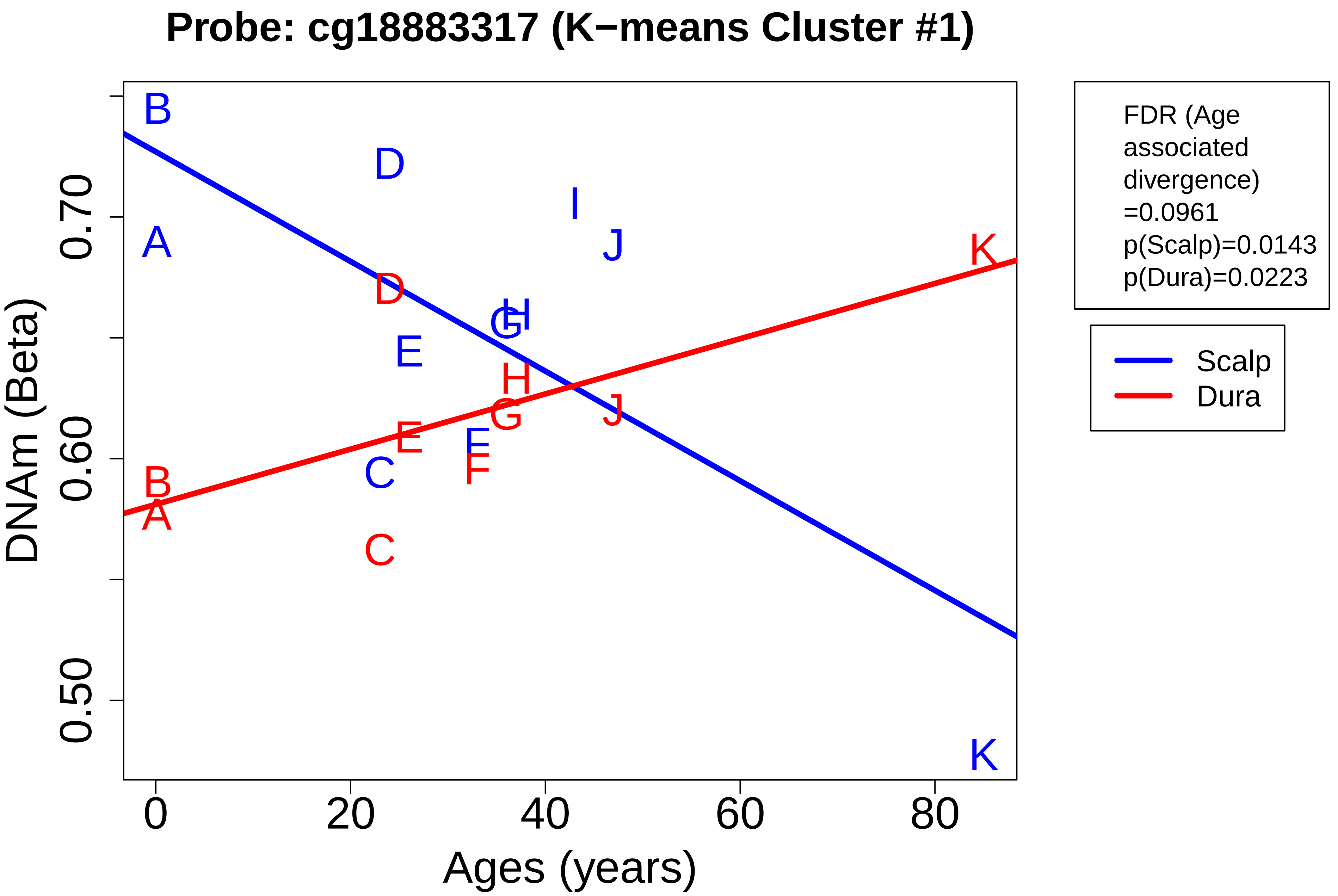

Probe: cg18883317 (K−means Cluster #1)
DNAm (Beta)
0.50
0.60
0.70
Ages (years)
0
20
40
60
80
FDR (Age associated divergence) =0.0961
p(Scalp)=0.0143
p(Dura)=0.0223
Scalp
Dura

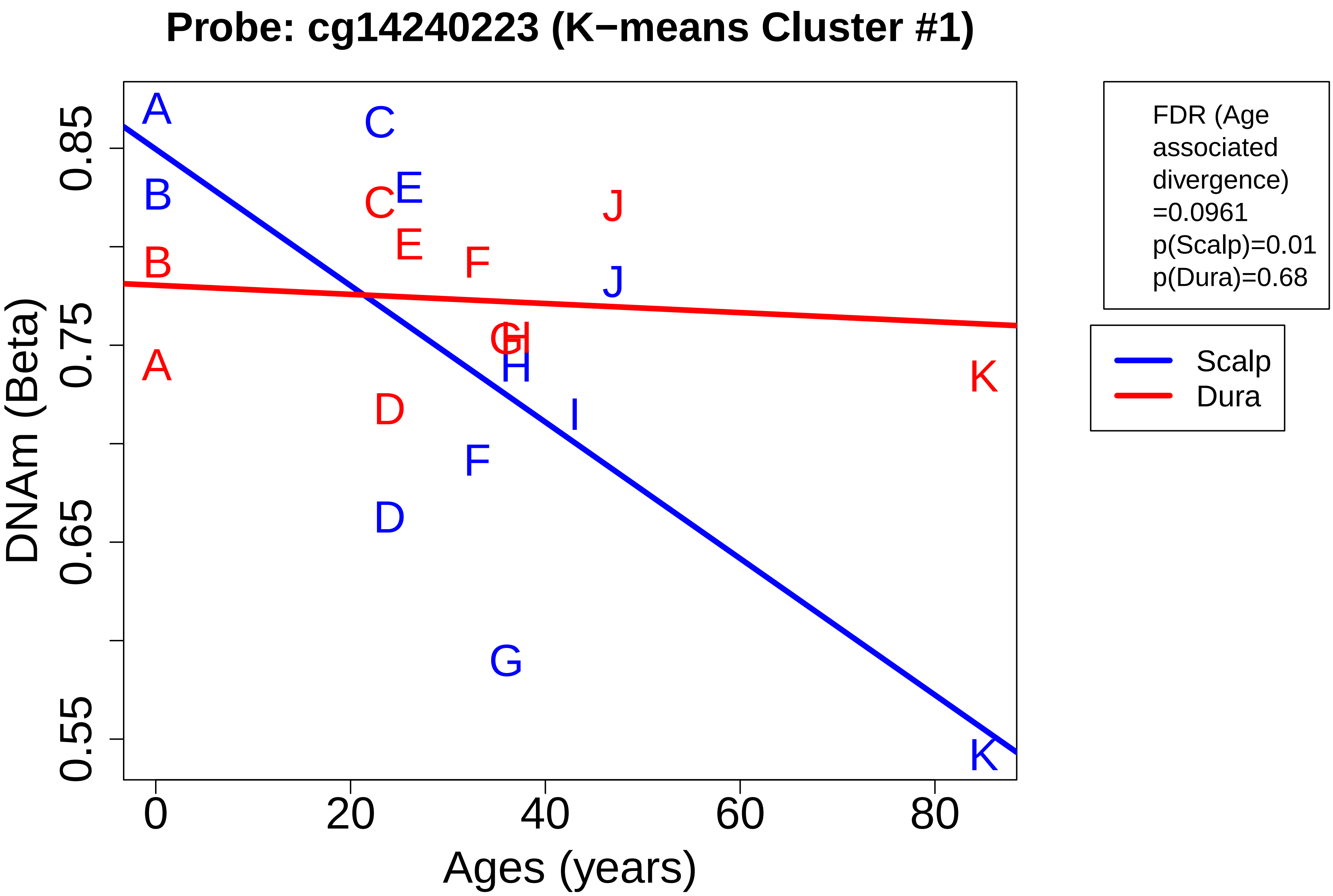

Probe: cg14240223 (K−means Cluster #1)
DNAm (Beta)
0.85
0.75
0.65
0.55
Ages (years)
0
20
40
60
80
FDR (Age associated divergence) =0.0961
p(Scalp)=0.01
p(Dura)=0.68
Scalp
Dura
A
B
C
D
E
F
G
H
I
J
K

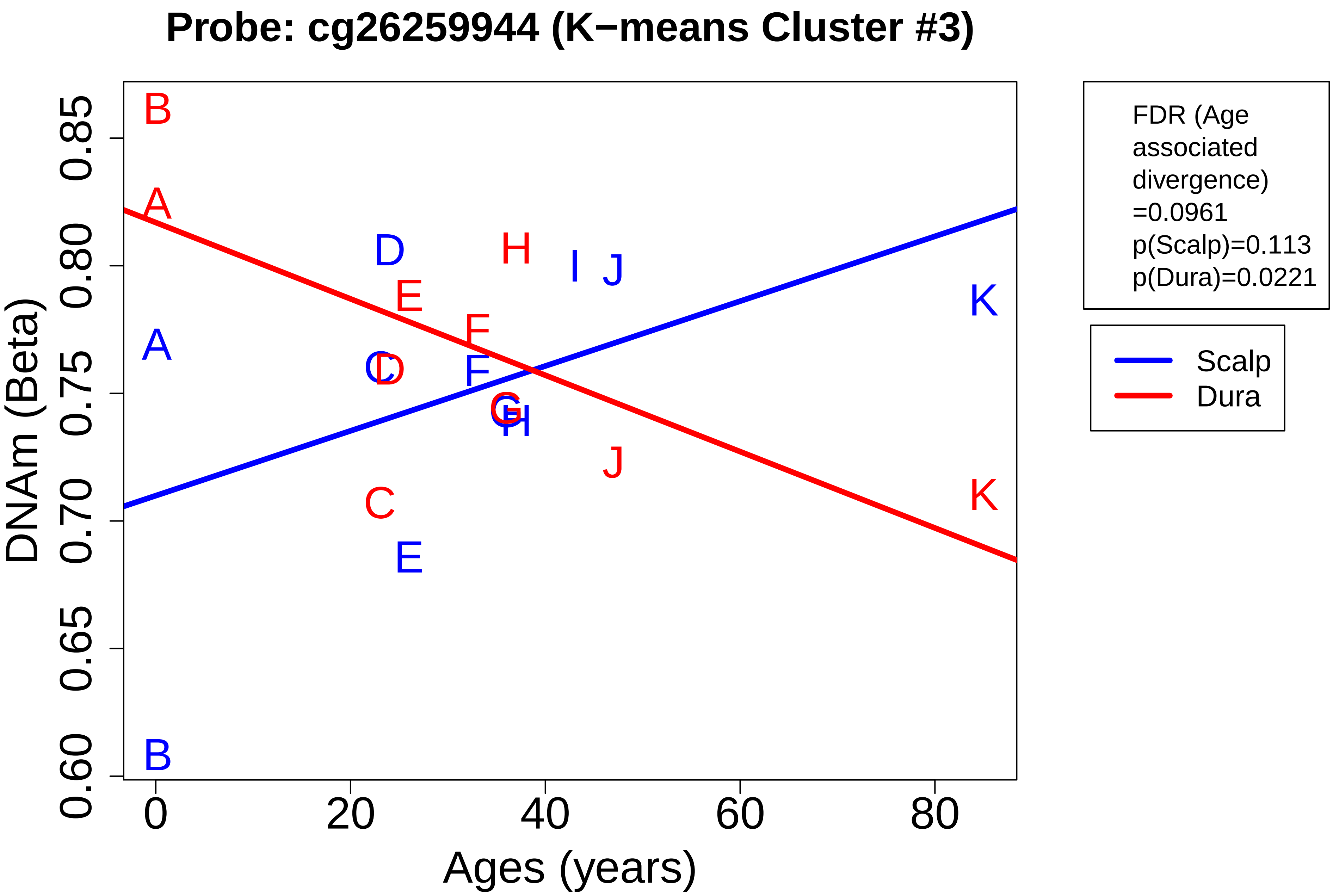
Probe: cg26259944 (K−means Cluster #3)
FDR (Age associated divergence) =0.0961
p(Scalp)=0.113
p(Dura)=0.0221
Scalp
Dura
DNAm (Beta)
Ages (years)
0.60
0.65
0.70
0.75
0.80
0.85
0
20
40
60
80

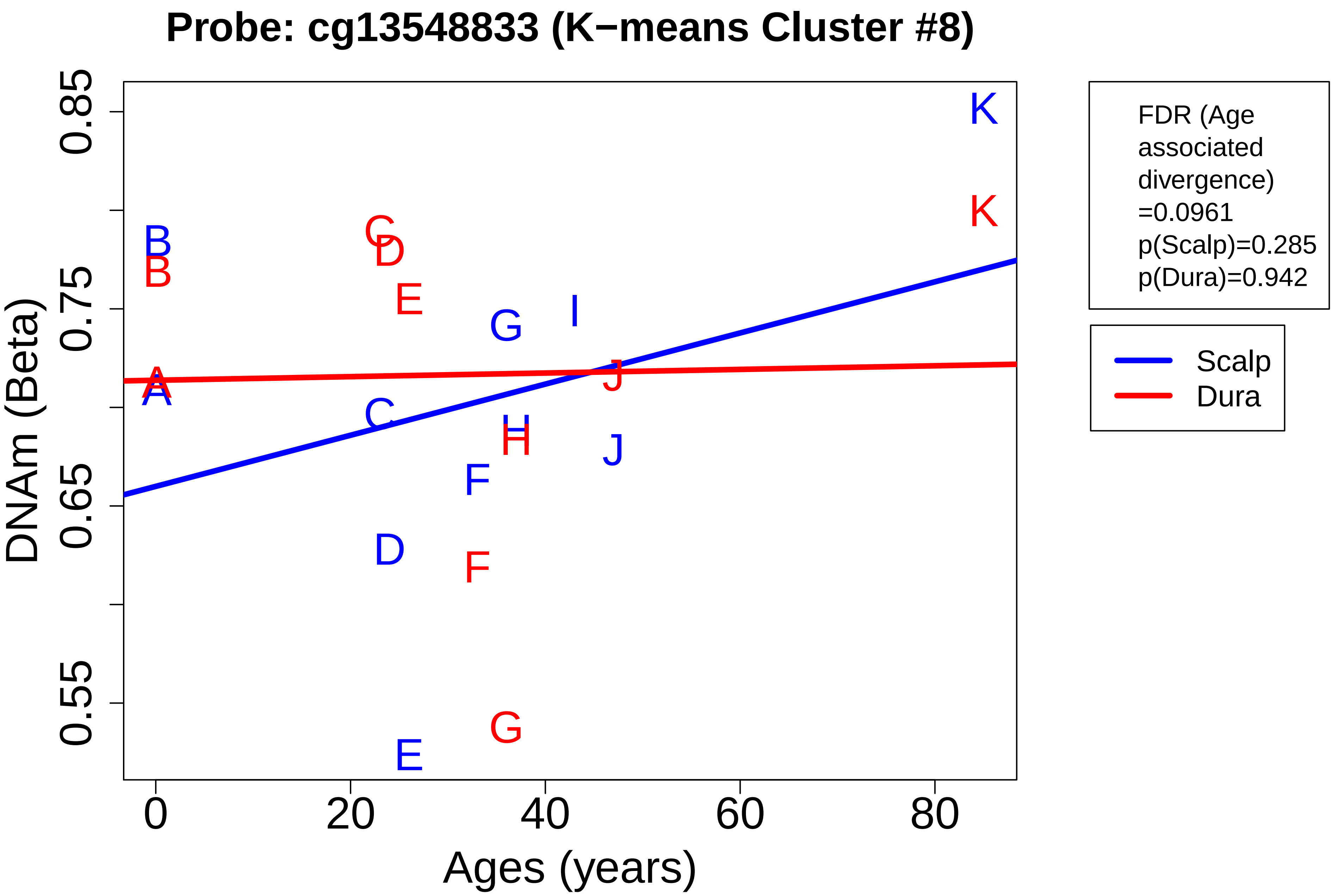

Probe: cg13548833 (K−means Cluster #8)
DNAm (Beta)
0.55
0.65
0.75
0.85
Ages (years)
0
20
40
60
80
FDR (Age associated divergence) =0.0961
p(Scalp)=0.285
p(Dura)=0.942
Scalp
Dura

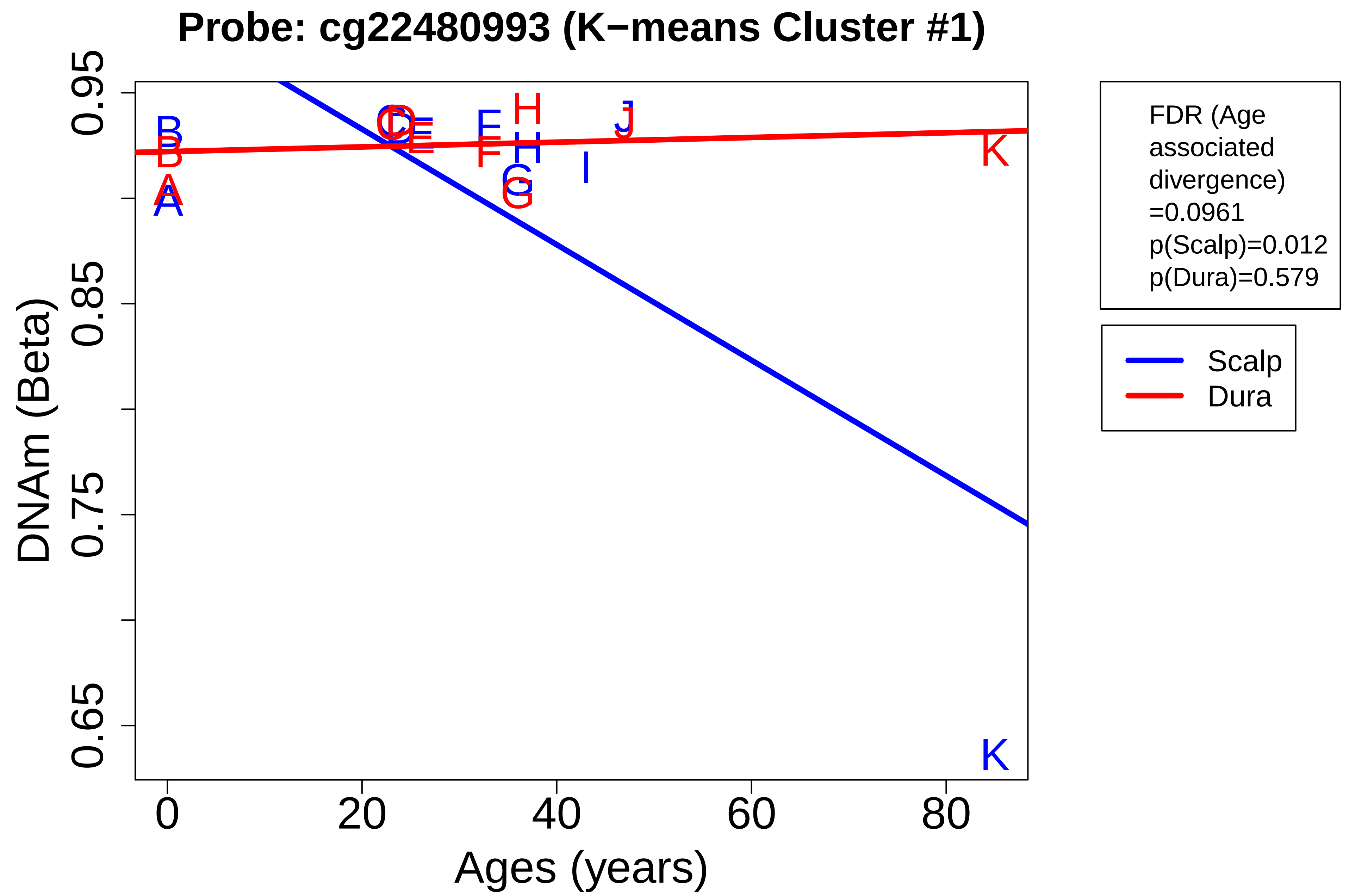

Probe: cg22480993 (K−means Cluster #1)
DNAm (Beta)
0.65
0.75
0.85
0.95
0
20
40
60
80
Ages (years)
A
B
C
D
E
F
G
H
I
J
K
FDR (Age associated divergence) =0.0961
p(Scalp)=0.012
p(Dura)=0.579
Scalp
Dura

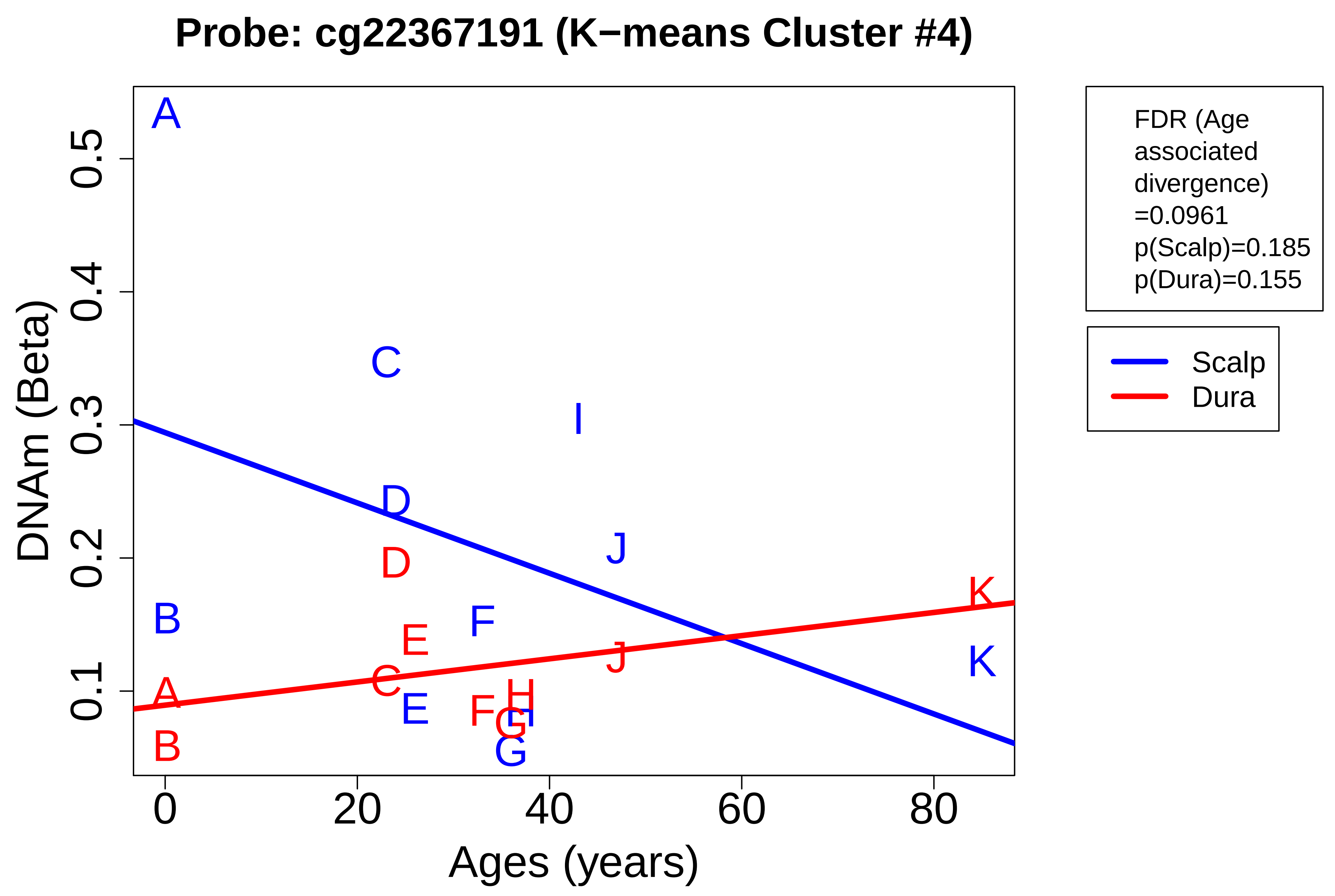
Probe: cg22367191 (K−means Cluster #4)
DNAm (Beta)
0.1
0.2
0.3
0.4
0.5
0
20
40
60
80
Ages (years)
FDR (Age associated divergence) =0.0961
p(Scalp)=0.185
p(Dura)=0.155
Scalp
Dura
A
B
C
D
E
F
G
H
I
J
K

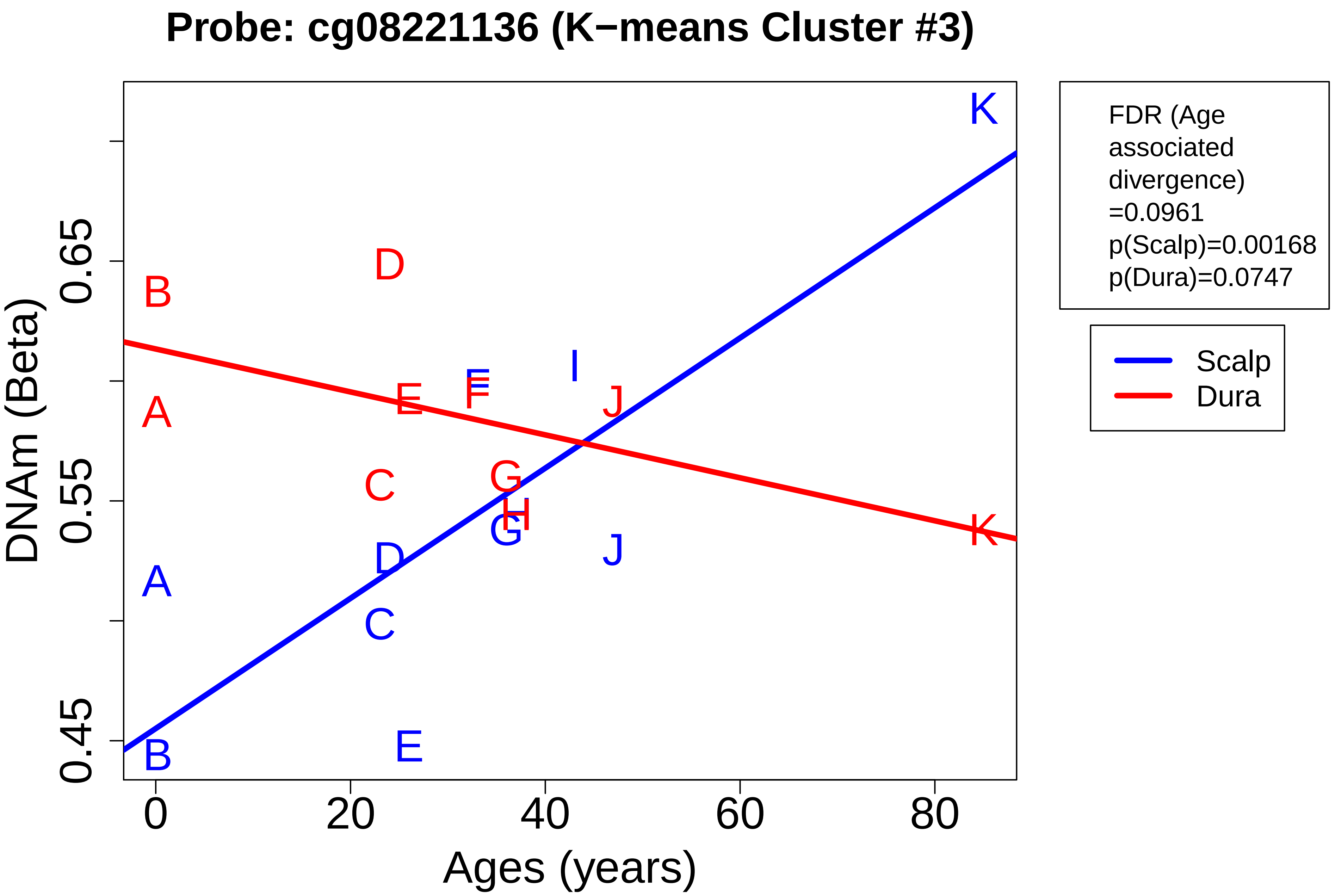

Probe: cg08221136 (K−means Cluster #3)
DNAm (Beta)
0.45
0.55
0.65
0
20
40
60
80
Ages (years)
FDR (Age associated divergence) =0.0961
p(Scalp)=0.00168
p(Dura)=0.0747
Scalp
Dura
A
B
C
D
E
F
G
H
I
J
K

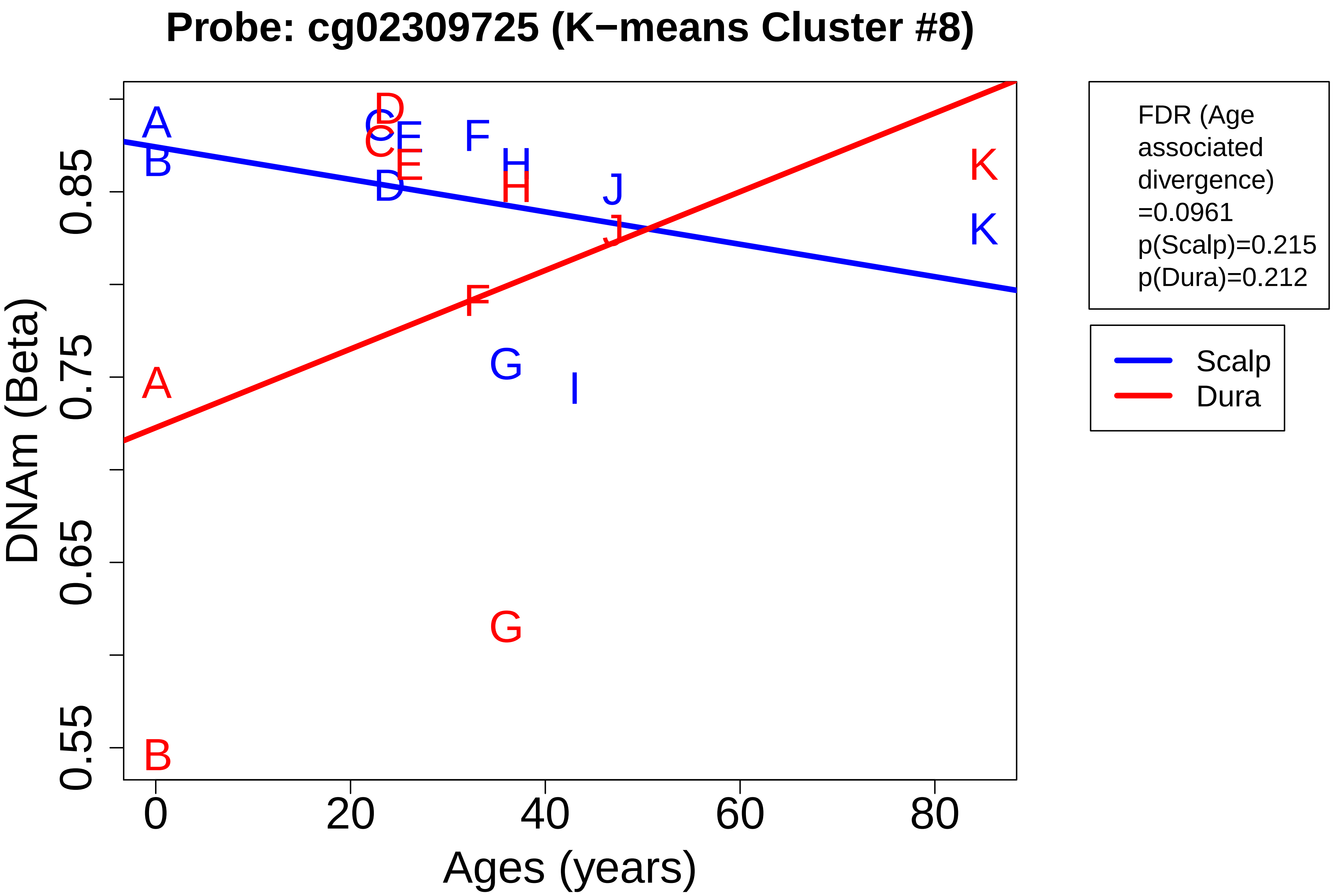

Probe: cg02309725 (K−means Cluster #8)
DNAm (Beta)
0.55
0.65
0.75
0.85
0
20
40
60
80
Ages (years)
FDR (Age associated divergence) =0.0961
p(Scalp)=0.215
p(Dura)=0.212
Scalp
Dura

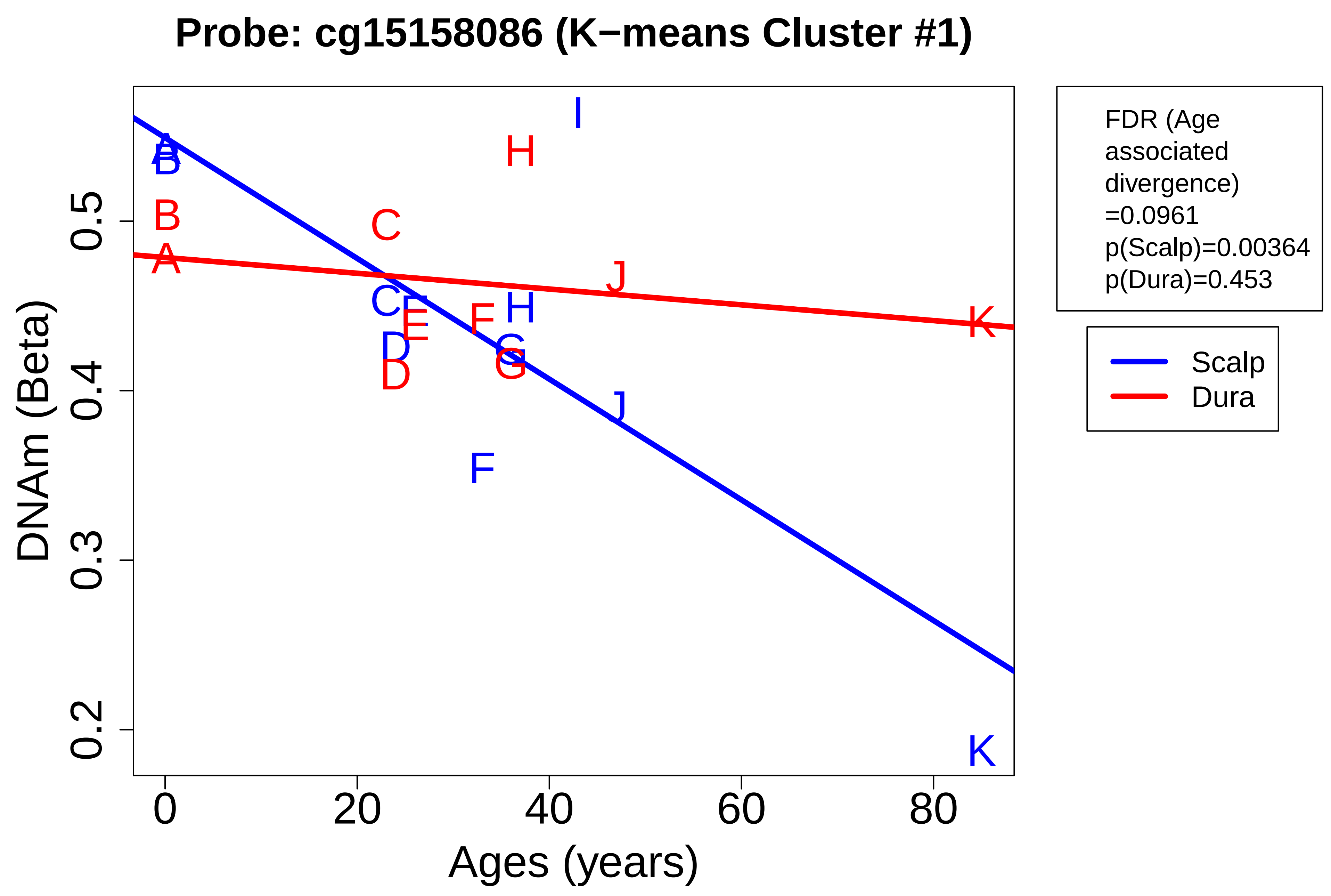

Probe: cg15158086 (K−means Cluster #1)
DNAm (Beta)
0.2
0.3
0.4
0.5
Ages (years)
0
20
40
60
80
A
B
B
A
C
C
D
D
E
E
F
F
G
G
H
H
I
J
J
K
K
FDR (Age associated divergence) =0.0961
p(Scalp)=0.00364
p(Dura)=0.453
Scalp
Dura

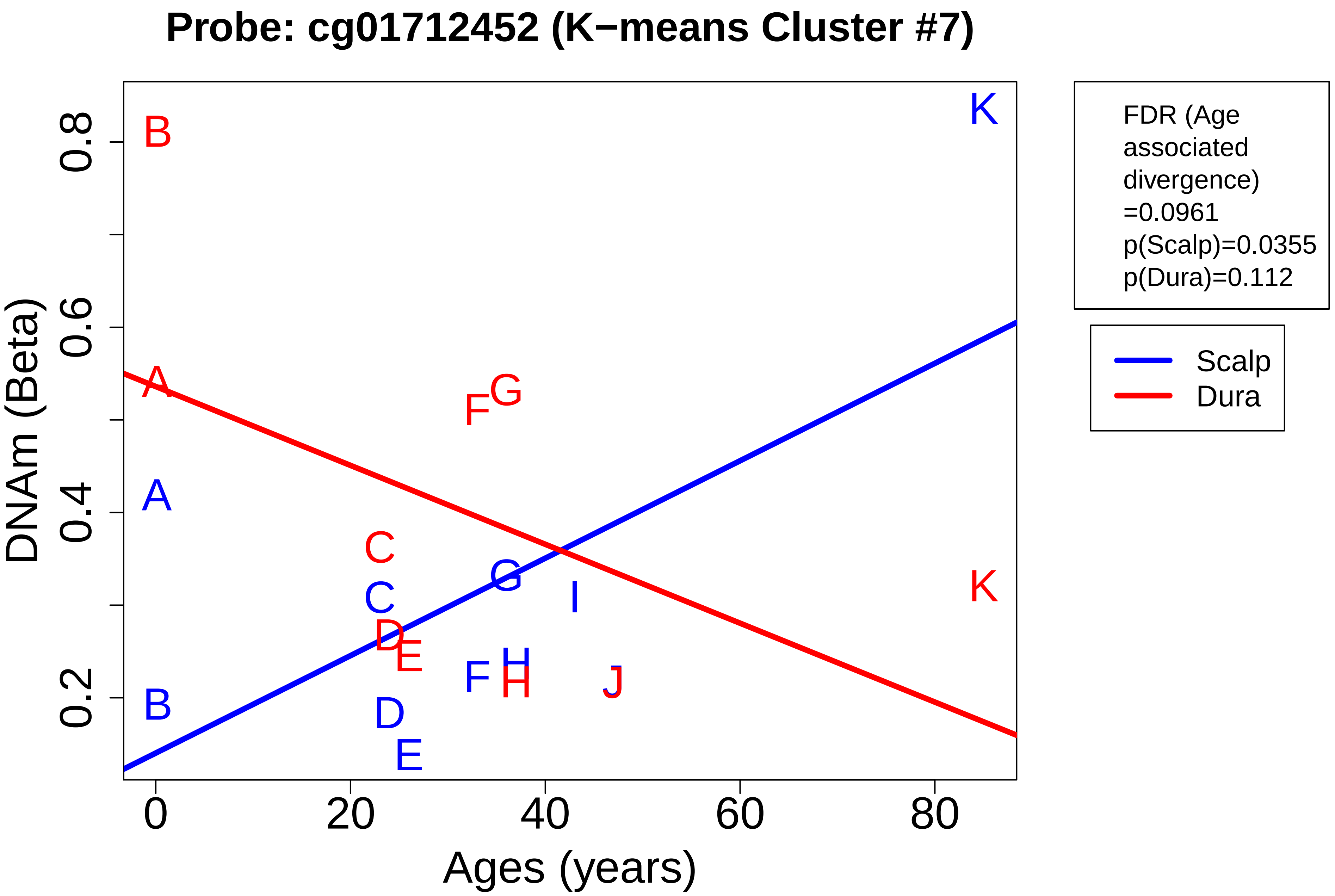

Probe: cg01712452 (K−means Cluster #7)
DNAm (Beta)
0.2
0.4
0.6
0.8
Ages (years)
0
20
40
60
80
FDR (Age associated divergence) =0.0961
p(Scalp)=0.0355
p(Dura)=0.112
Scalp
Dura
A
B
C
D
E
F
G
H
I
J
K

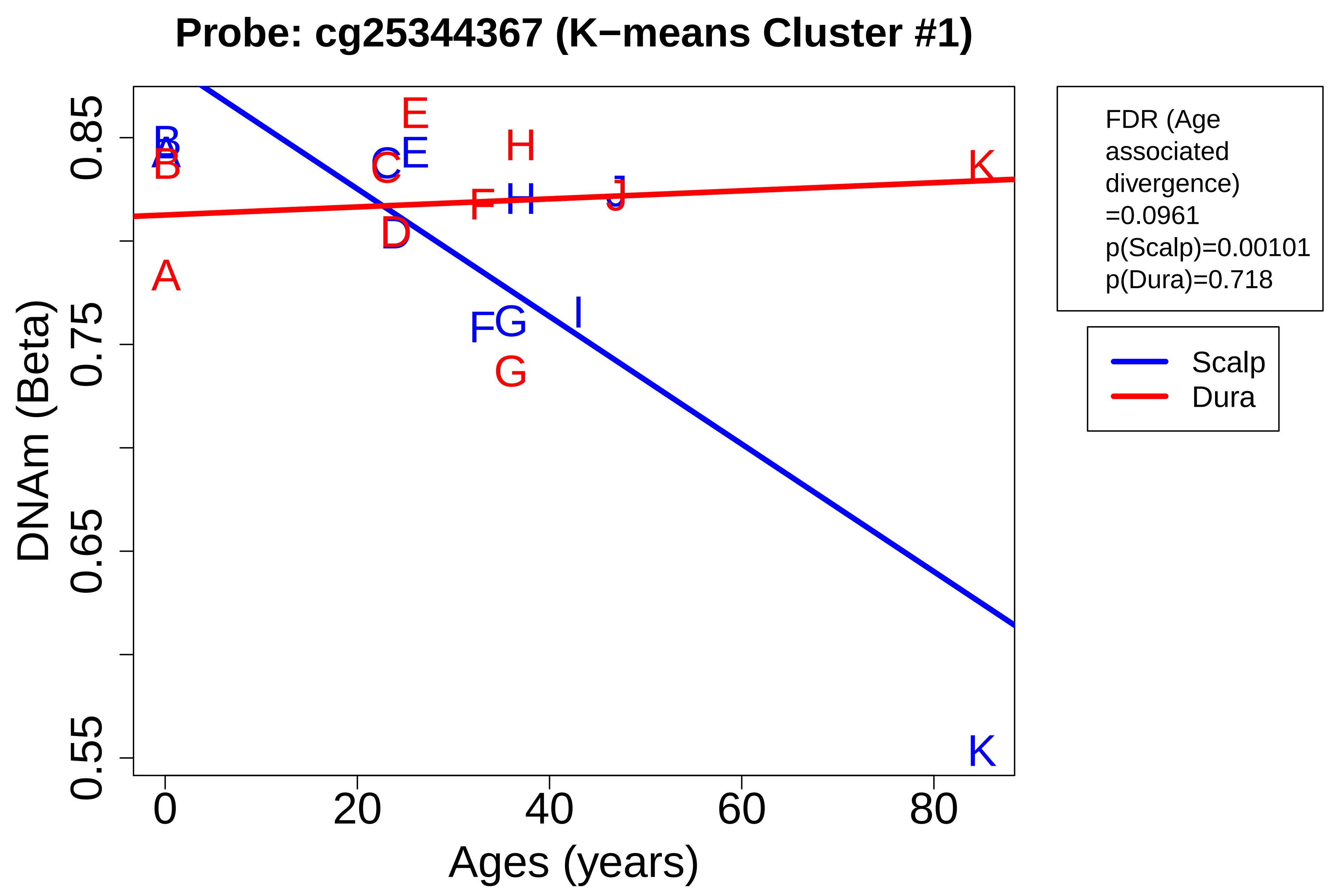

Probe: cg25344367 (K−means Cluster #1)
DNAm (Beta)
0.55
0.65
0.75
0.85
0
20
40
60
80
Ages (years)
FDR (Age associated divergence) =0.0961
p(Scalp)=0.00101
p(Dura)=0.718
Scalp
Dura

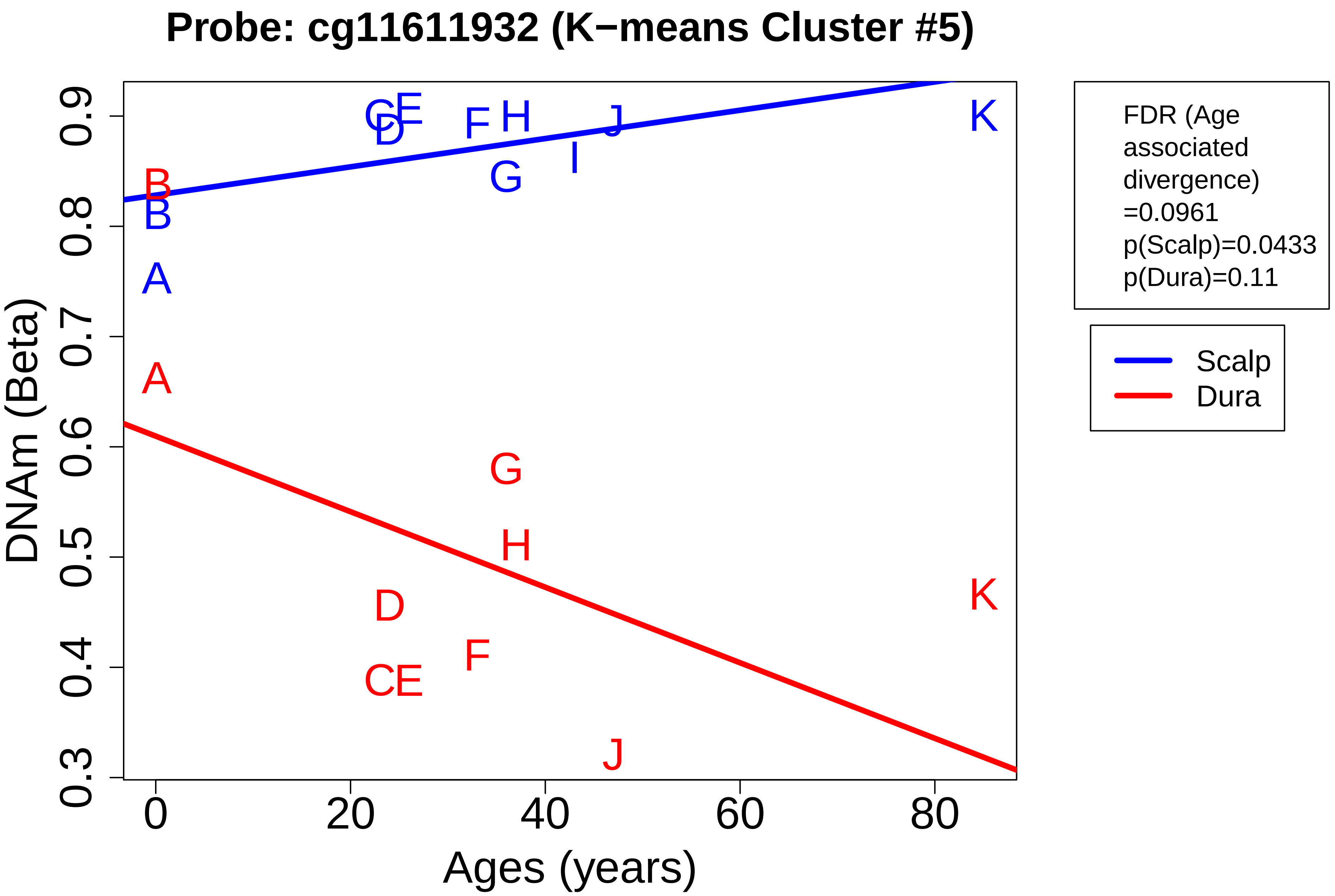

Probe: cg11611932 (K−means Cluster #5)
DNAm (Beta)
0.3
0.4
0.5
0.6
0.7
0.8
0.9
0
20
40
60
80
Ages (years)
FDR (Age associated divergence) =0.0961
p(Scalp)=0.0433
p(Dura)=0.11
Scalp
Dura

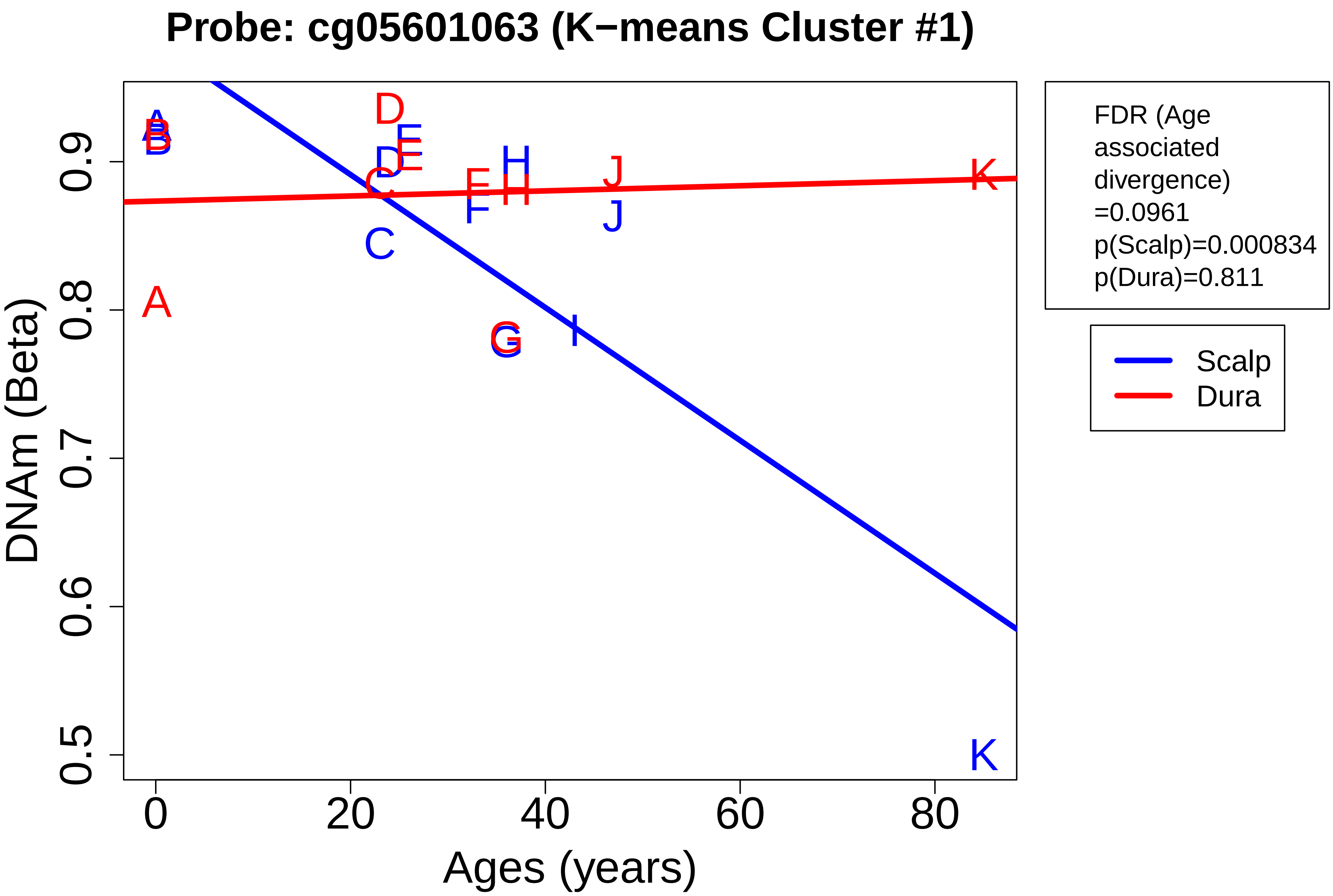

Probe: cg05601063 (K−means Cluster #1)
DNAm (Beta)
0.5
0.6
0.7
0.8
0.9
0
20
40
60
80
Ages (years)
FDR (Age
associated
divergence)
=0.0961
p(Scalp)=0.000834
p(Dura)=0.811
Scalp
Dura

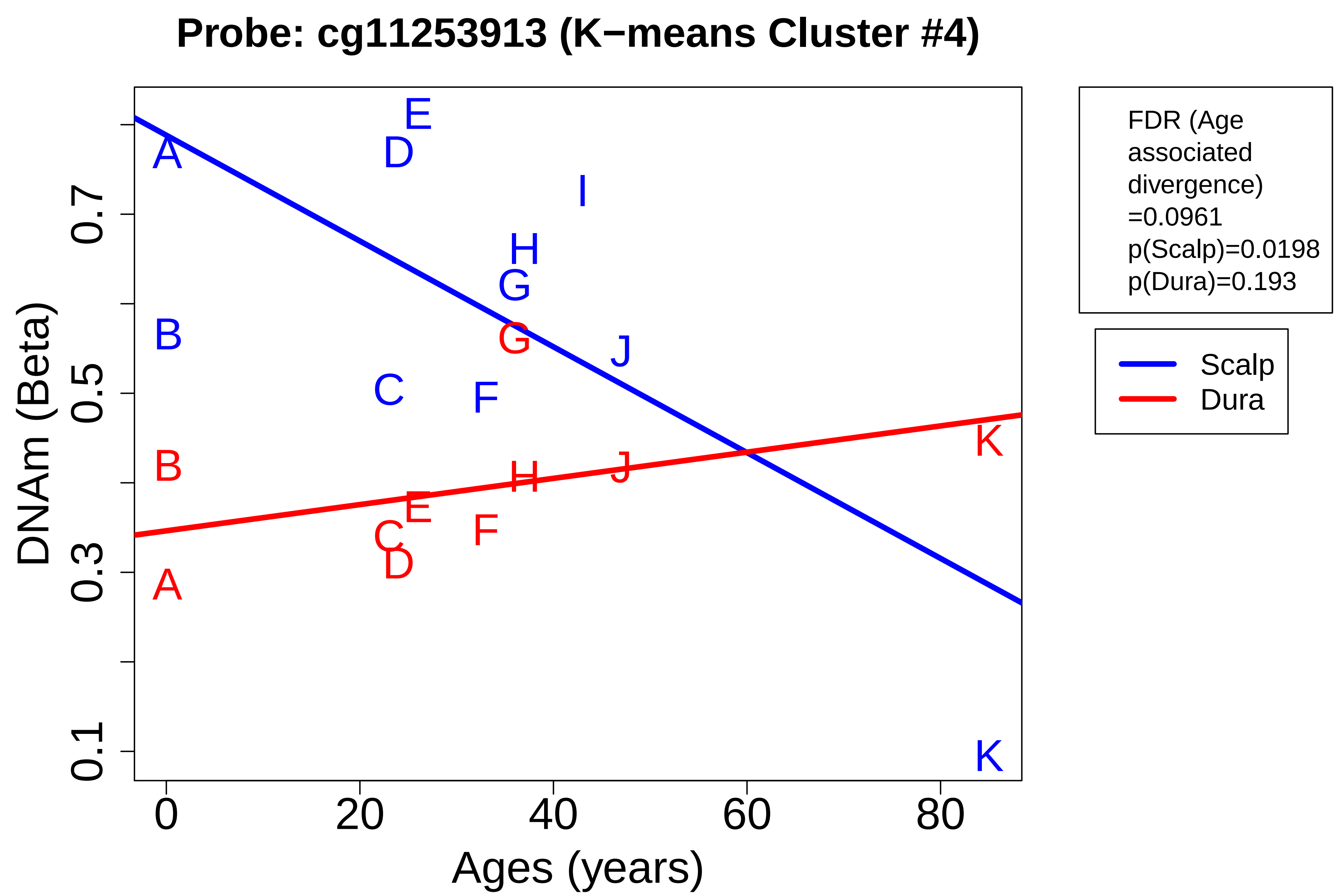

Probe: cg11253913 (K−means Cluster #4)
DNAm (Beta)
0.1
0.3
0.5
0.7
Ages (years)
0
20
40
60
80
A
B
C
D
E
F
G
H
I
J
K
FDR (Age associated divergence) =0.0961
p(Scalp)=0.0198
p(Dura)=0.193
Scalp
Dura

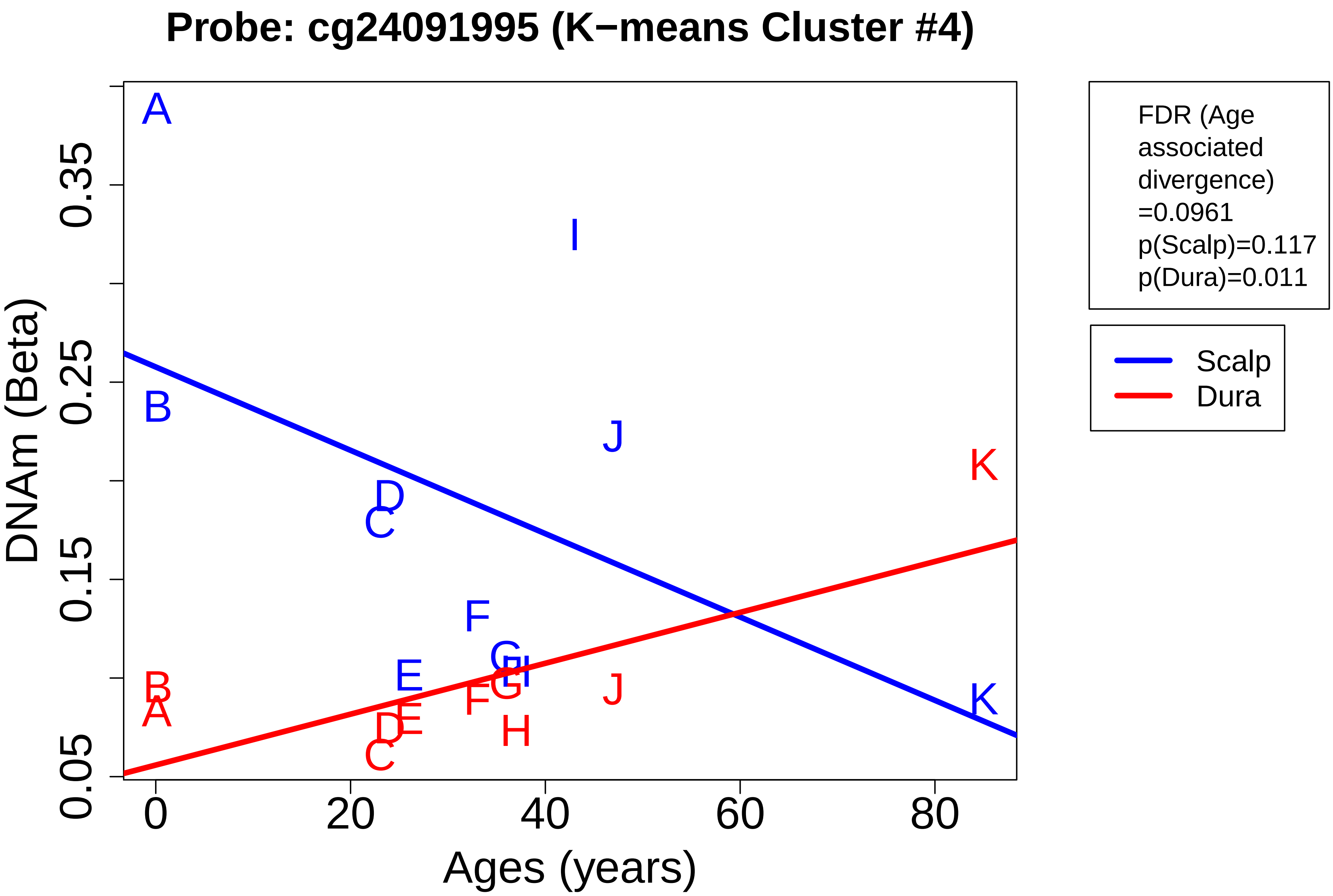
Probe: cg24091995 (K−means Cluster #4)
DNAm (Beta)
0.05
0.15
0.25
0.35
0
20
40
60
80
Ages (years)
FDR (Age associated divergence) =0.0961
p(Scalp)=0.117
p(Dura)=0.011
Scalp
Dura
A
B
C
D
E
F
G
H
I
J
K

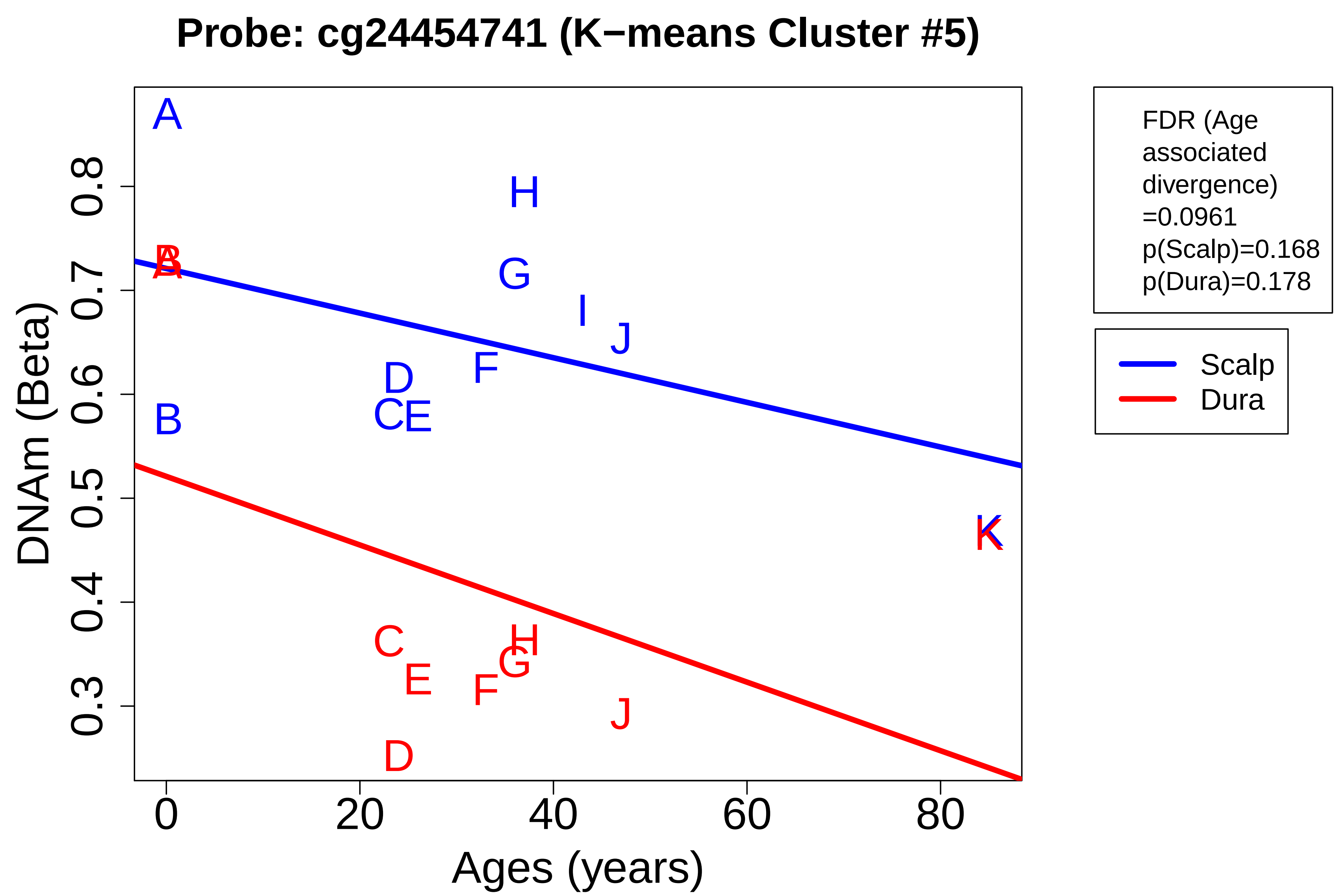

Probe: cg24454741 (K−means Cluster #5)
DNAm (Beta)
0.3
0.4
0.5
0.6
0.7
0.8
Ages (years)
0
20
40
60
80
A
B
C
D
E
F
G
H
I
J
K
FDR (Age associated divergence) =0.0961
p(Scalp)=0.168
p(Dura)=0.178
Scalp
Dura

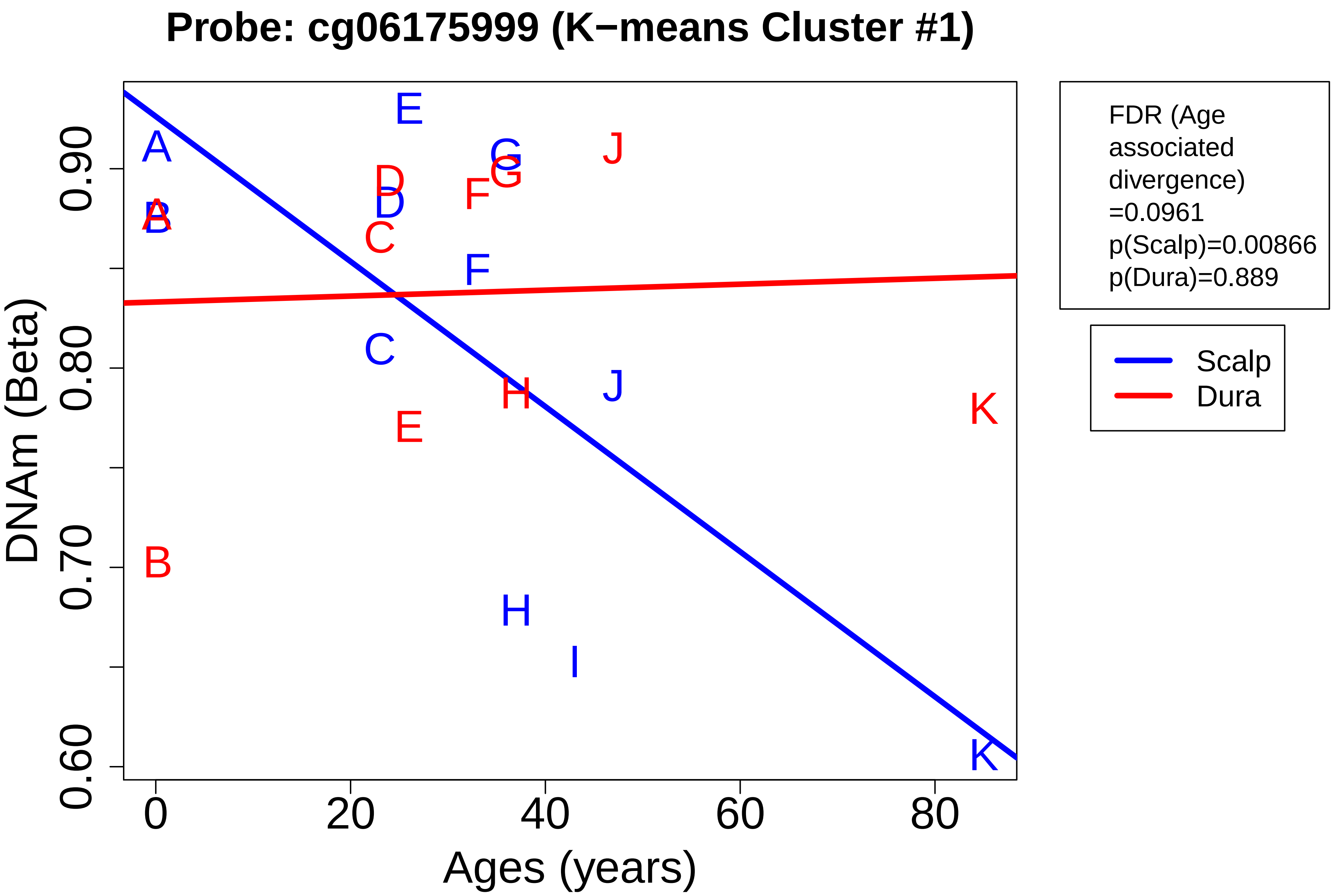
Probe: cg06175999 (K−means Cluster #1)
FDR (Age associated divergence) =0.0961
p(Scalp)=0.00866
p(Dura)=0.889
Scalp
Dura
DNAm (Beta)
0.60
0.70
0.80
0.90
Ages (years)
0
20
40
60
80
A
B
C
D
E
F
G
H
I
J
K

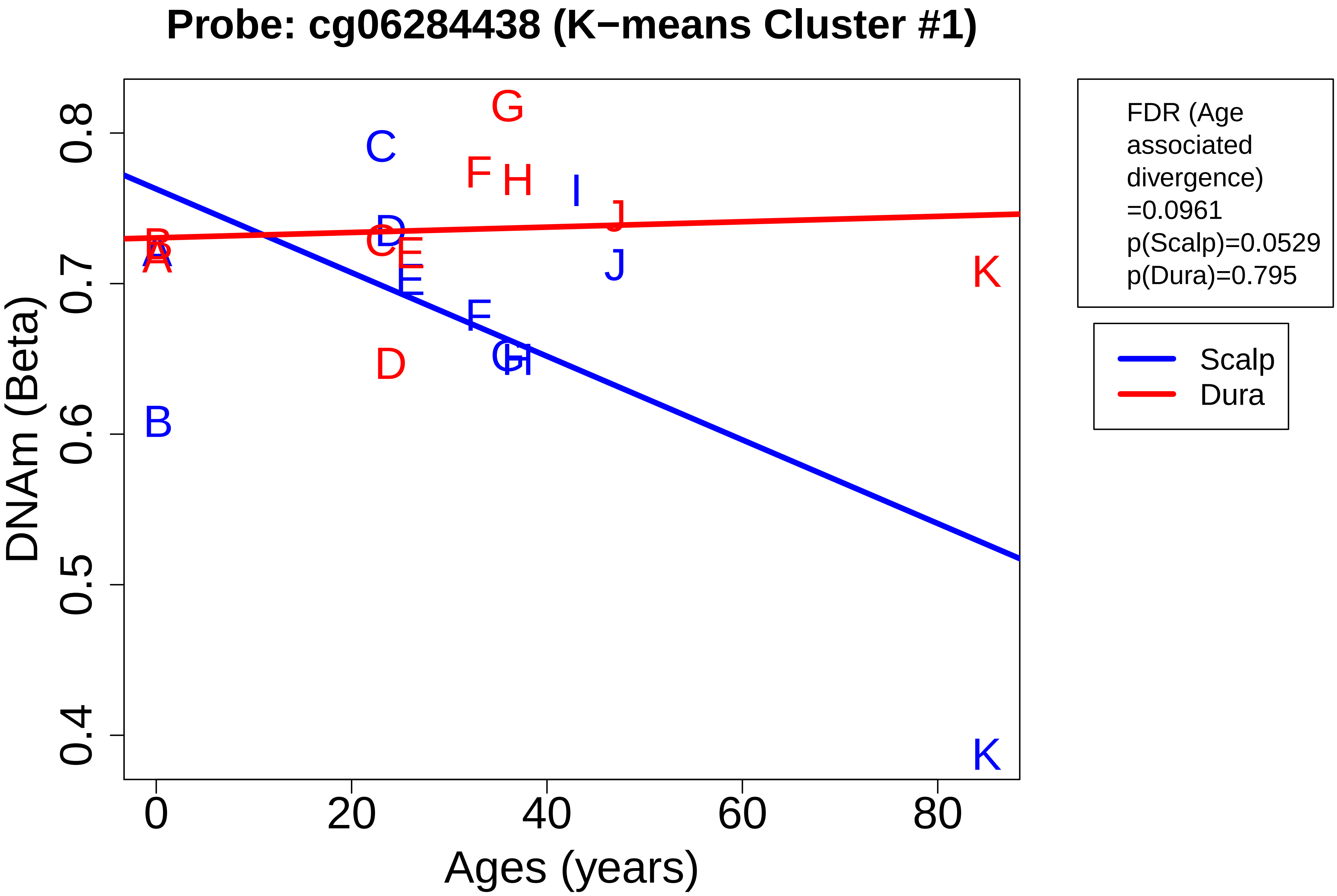

Probe: cg06284438 (K−means Cluster #1)
FDR (Age associated divergence) =0.0961
p(Scalp)=0.0529
p(Dura)=0.795
Scalp
Dura
DNAm (Beta)
0.4
0.5
0.6
0.7
0.8
0
20
40
60
80
Ages (years)

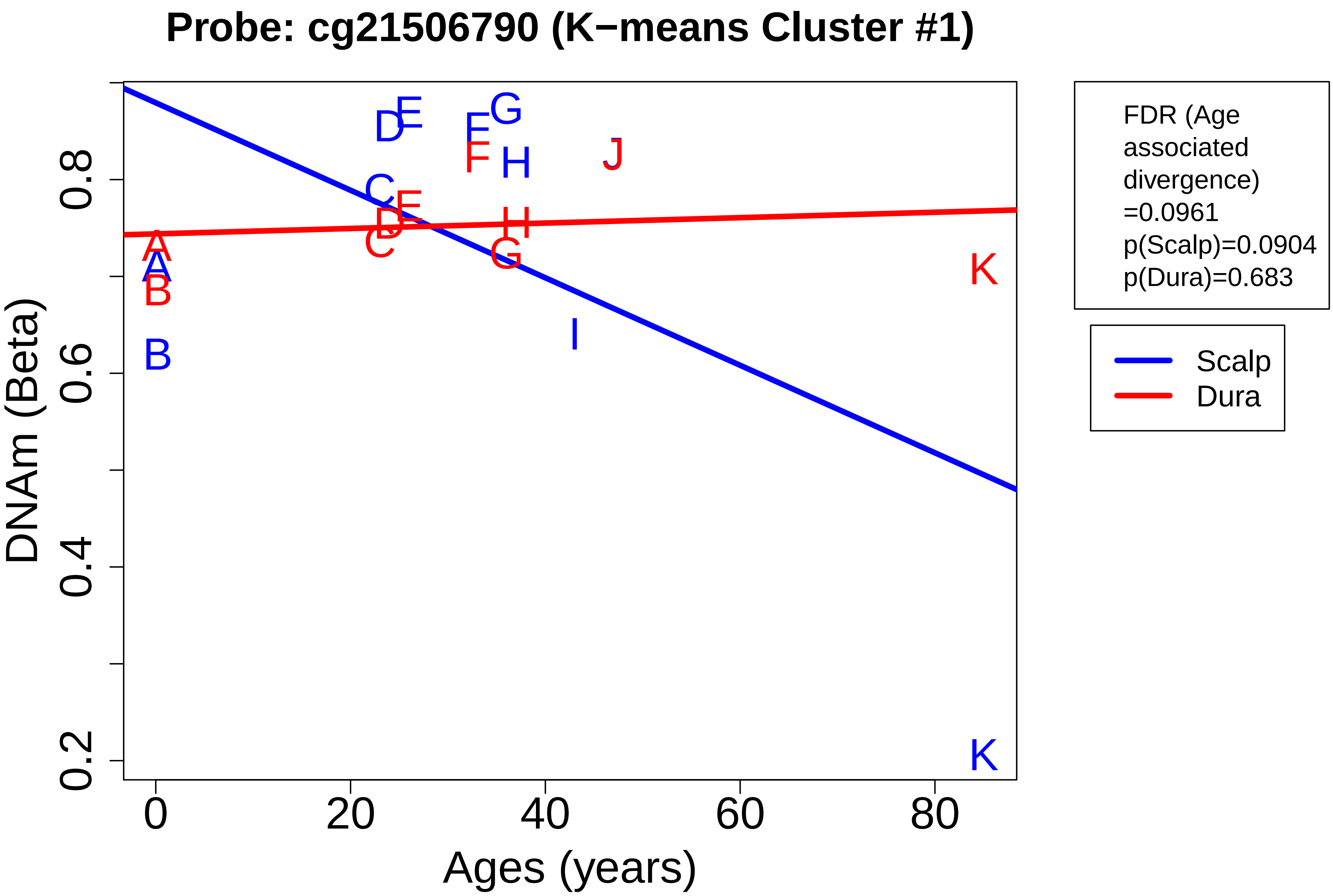

Probe: cg21506790 (K−means Cluster #1)
DNAm (Beta)
0.2
0.4
0.6
0.8
0
20
40
60
80
Ages (years)
FDR (Age associated divergence) =0.0961
p(Scalp)=0.0904
p(Dura)=0.683
Scalp
Dura

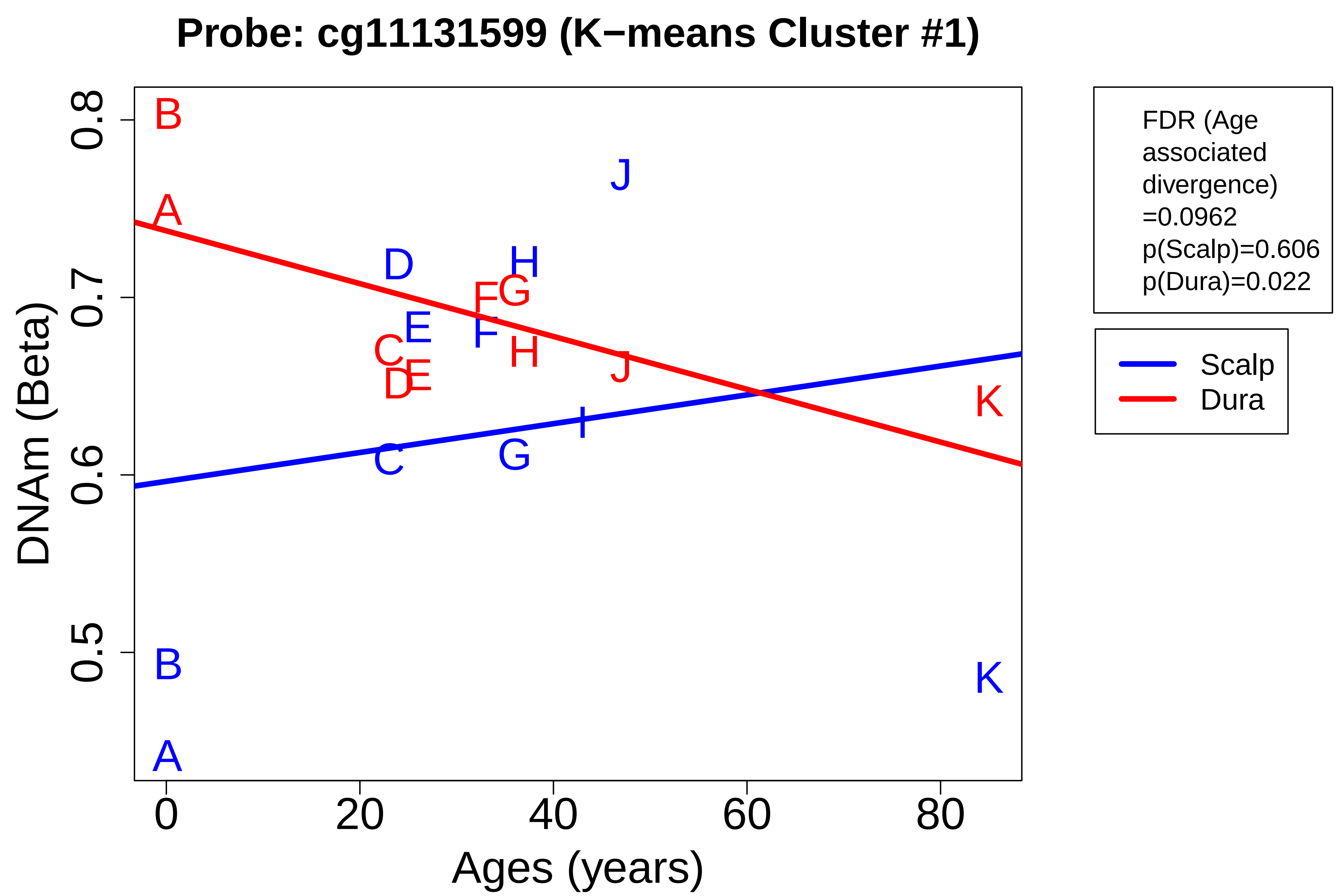

Probe: cg11131599 (K−means Cluster #1)
FDR (Age associated divergence) =0.0962
p(Scalp)=0.606
p(Dura)=0.022
Scalp
Dura
DNAm (Beta)
0.5
0.6
0.7
0.8
Ages (years)
0
20
40
60
80

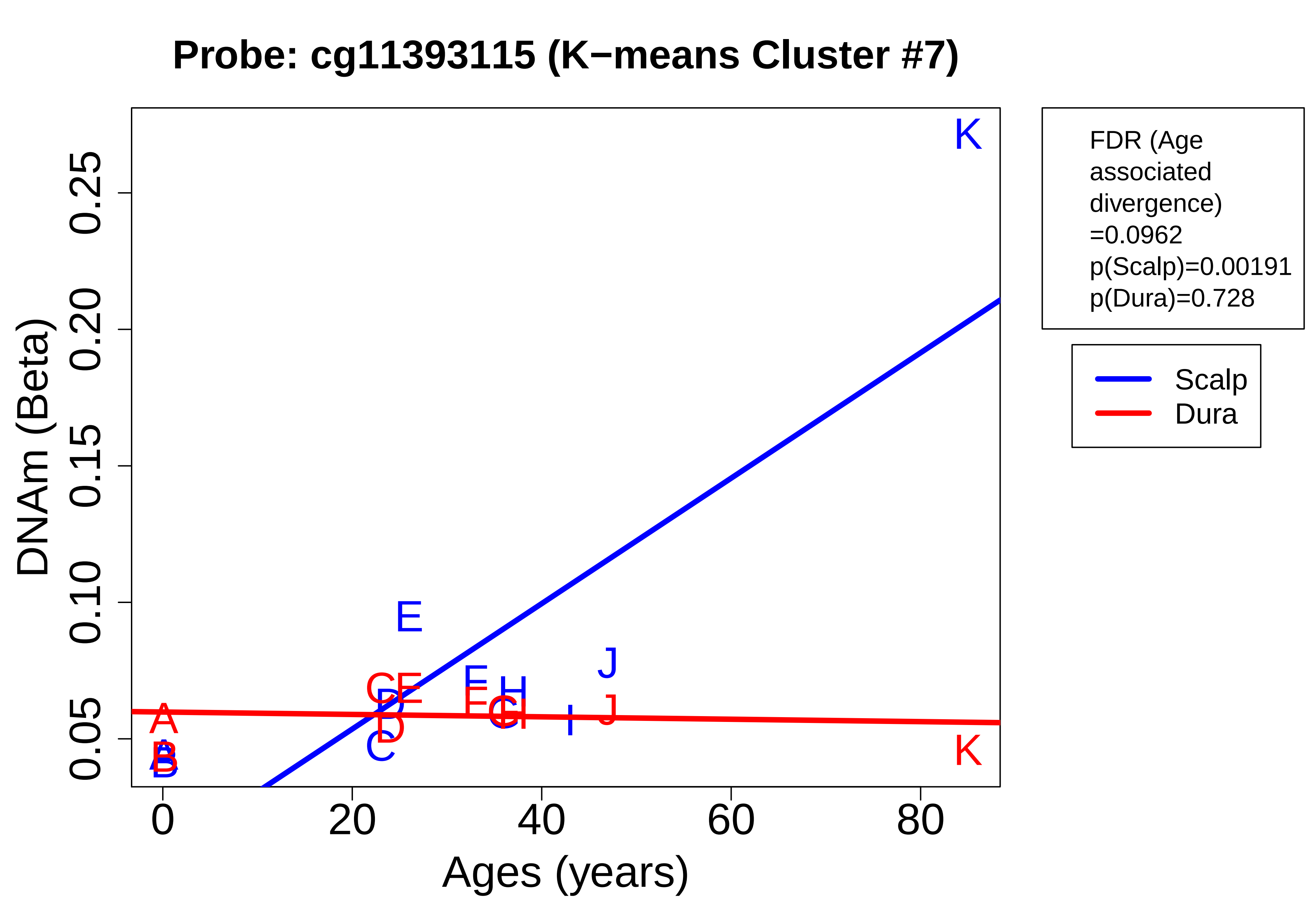

Probe: cg11393115 (K−means Cluster #7)
FDR (Age associated divergence) =0.0962
p(Scalp)=0.00191
p(Dura)=0.728
Scalp
Dura
DNAm (Beta)
0.05
0.10
0.15
0.20
0.25
0
20
40
60
80
Ages (years)

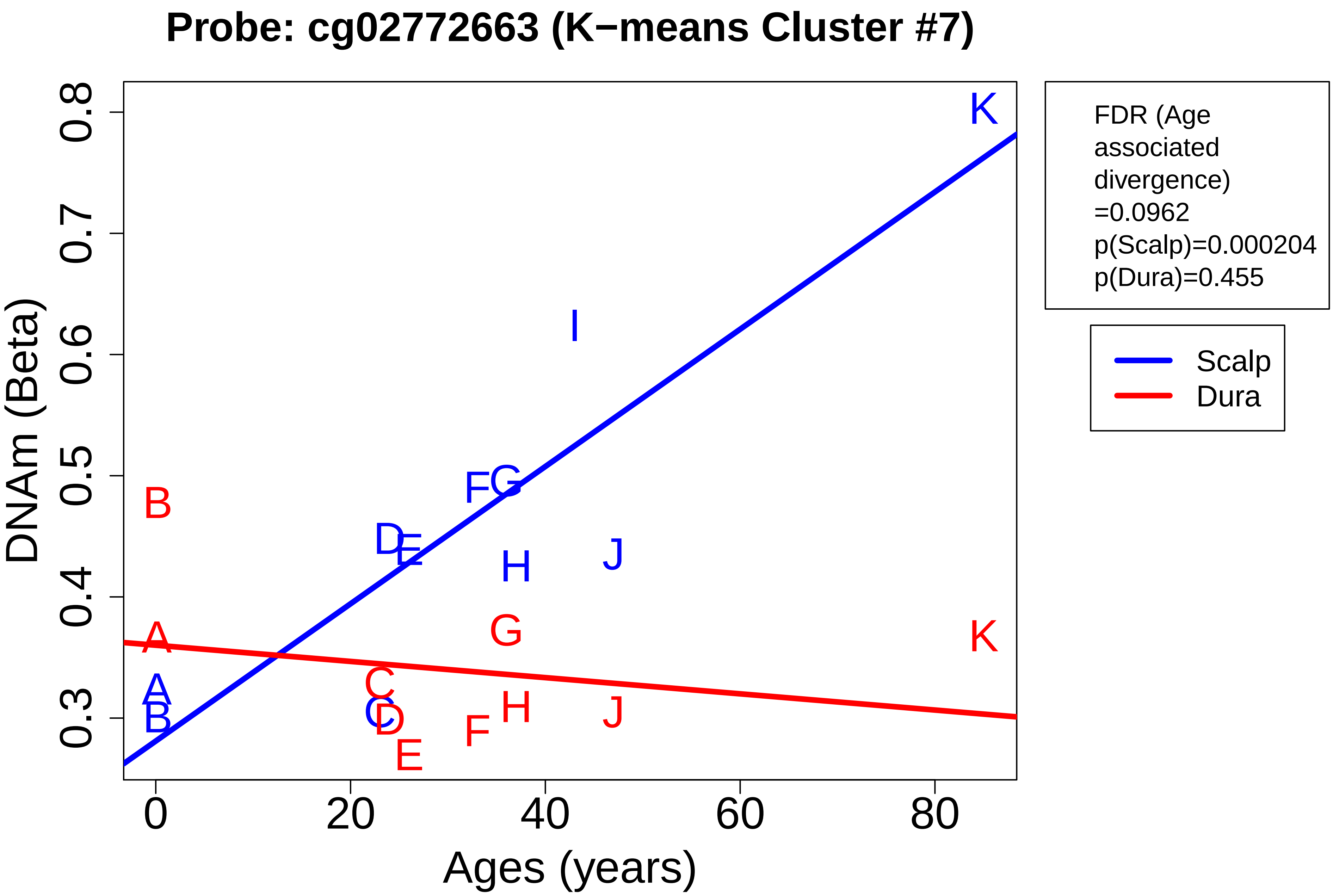

Probe: cg02772663 (K−means Cluster #7)
DNAm (Beta)
0.3
0.4
0.5
0.6
0.7
0.8
Ages (years)
0
20
40
60
80
FDR (Age associated divergence) =0.0962
p(Scalp)=0.000204
p(Dura)=0.455
Scalp
Dura
A
B
C
D
E
F
G
H
I
J
K

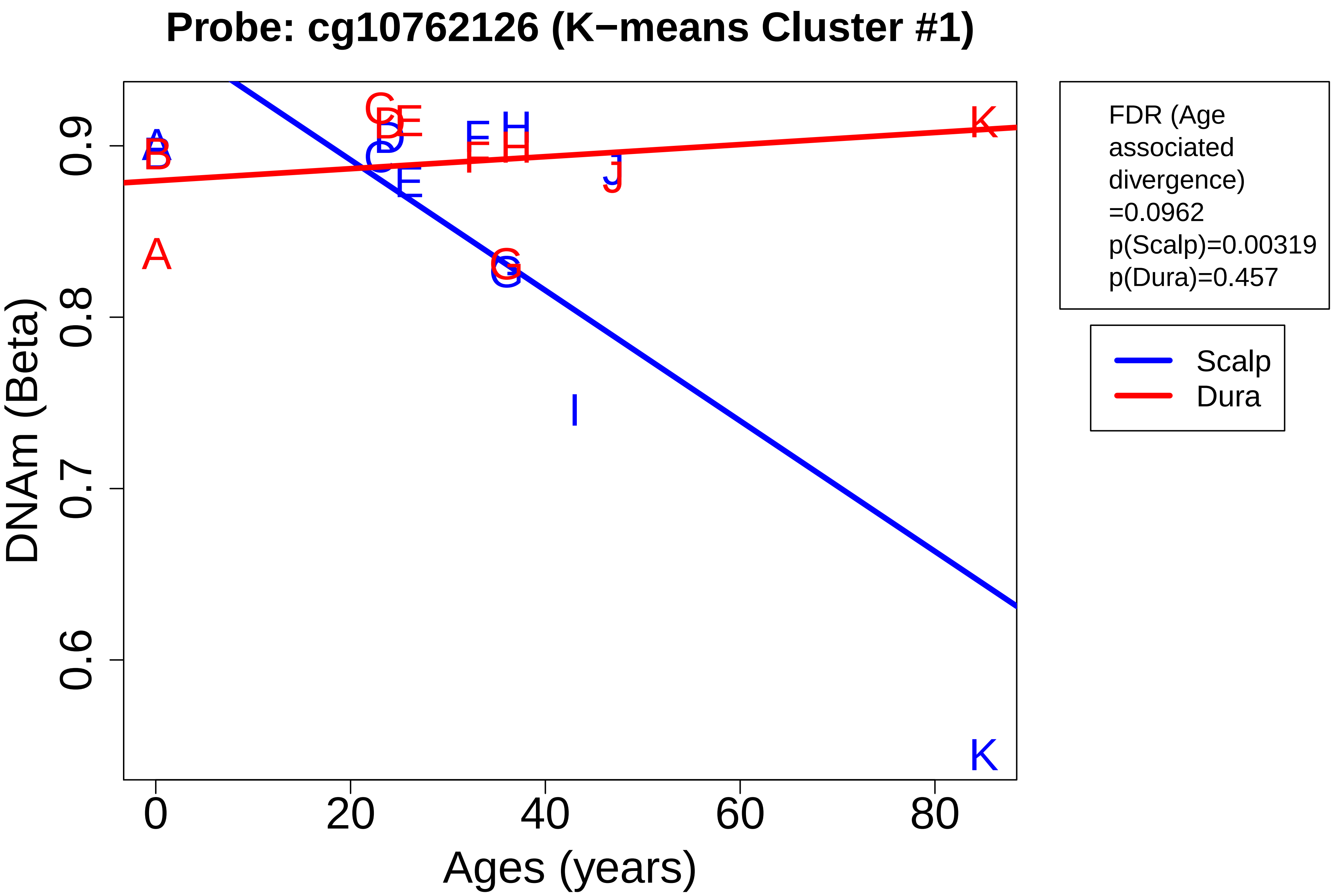

Probe: cg10762126 (K−means Cluster #1)
FDR (Age associated divergence) =0.0962
p(Scalp)=0.00319
p(Dura)=0.457
Scalp
Dura
DNAm (Beta)
Ages (years)
0.6
0.7
0.8
0.9
0
20
40
60
80
A
B
C
D
E
F
G
H
I
J
K

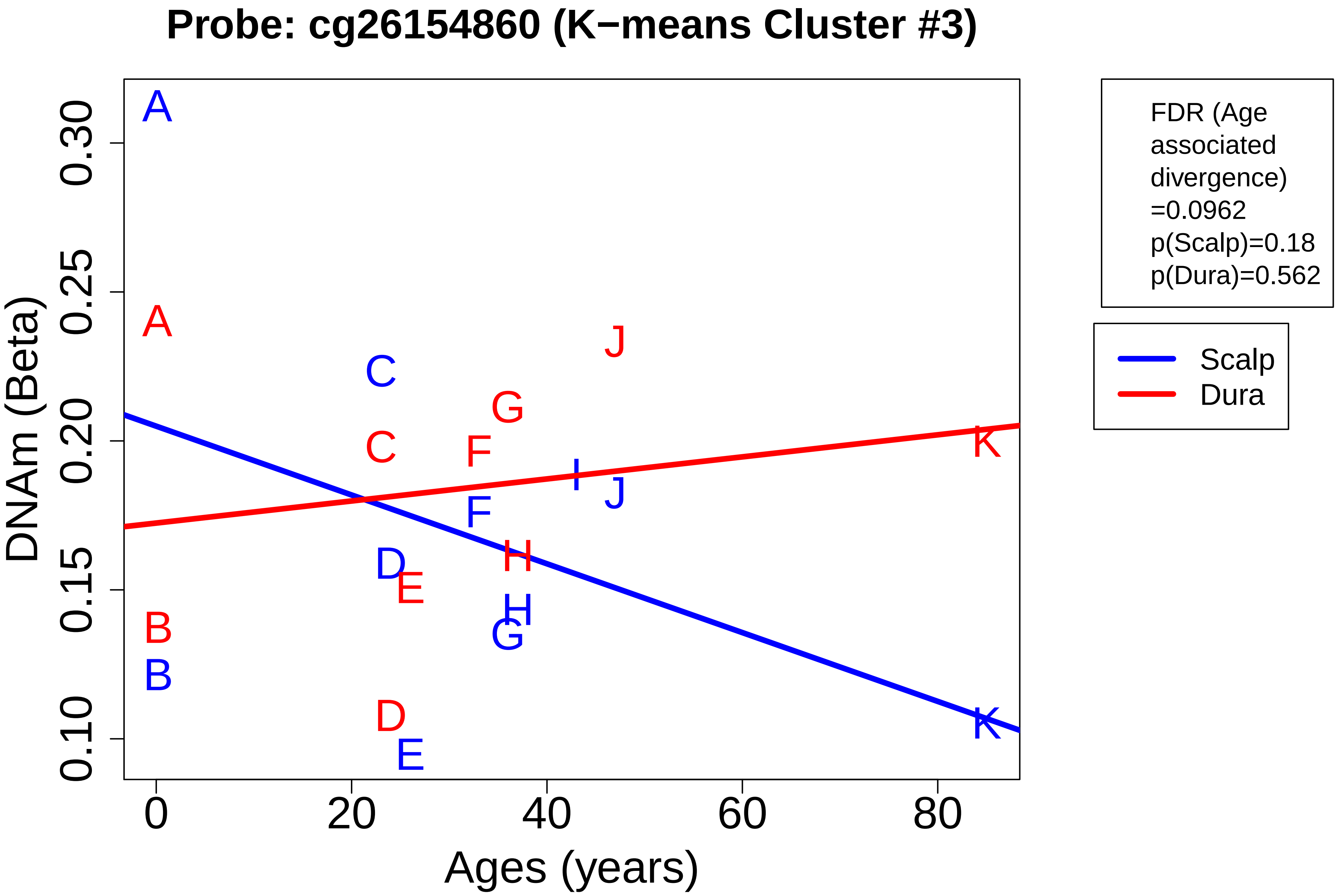

Probe: cg26154860 (K−means Cluster #3)
DNAm (Beta)
0.10
0.15
0.20
0.25
0.30
0
20
40
60
80
Ages (years)
A
B
C
D
E
F
G
H
I
J
K
A
B
C
D
E
F
G
H
J
K
FDR (Age associated divergence) =0.0962
p(Scalp)=0.18
p(Dura)=0.562
Scalp
Dura

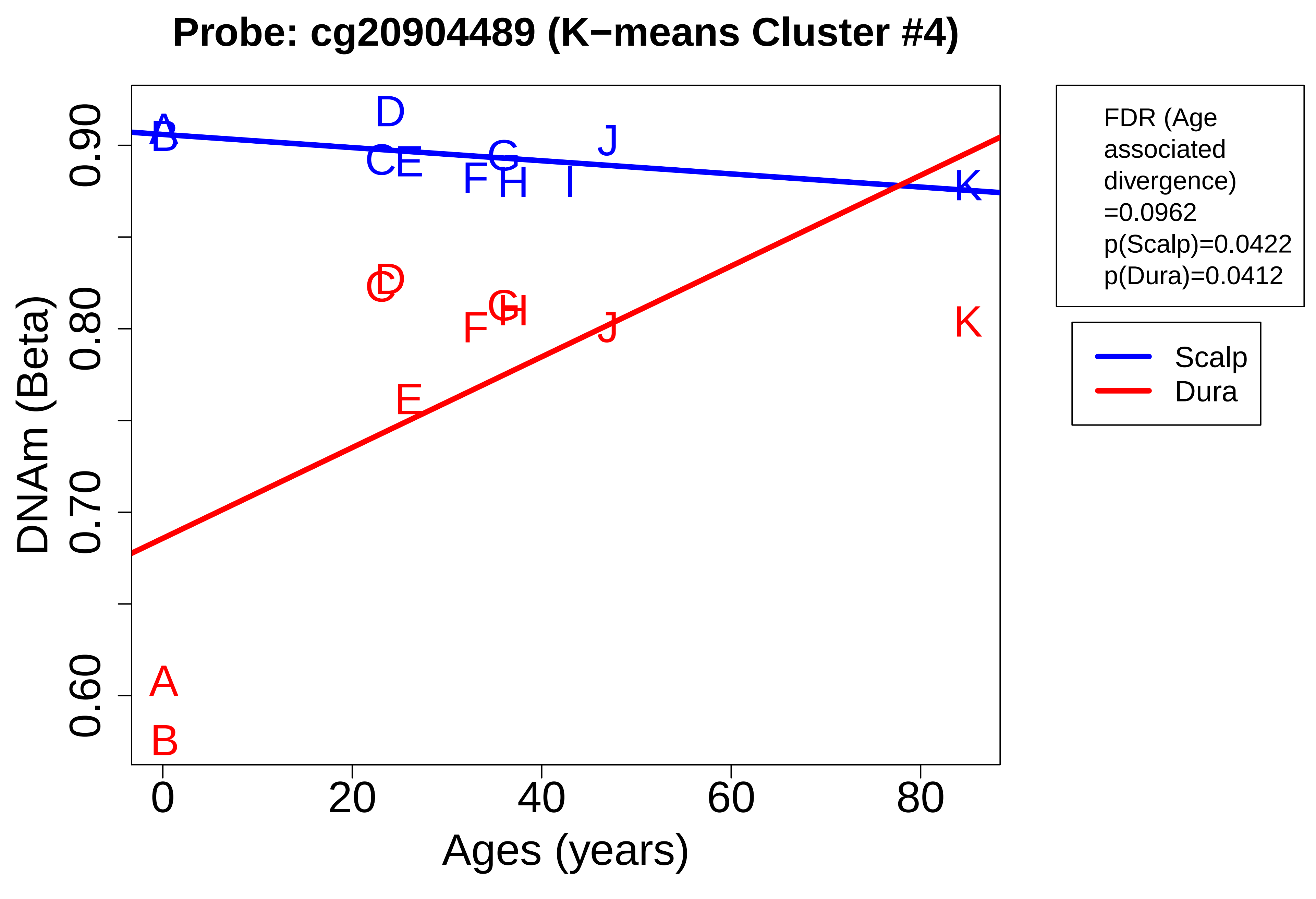

Probe: cg20904489 (K−means Cluster #4)
DNAm (Beta)
0.60
0.70
0.80
0.90
0
20
40
60
80
Ages (years)
A
B
C
D
E
F
G
H
I
J
K
FDR (Age associated divergence) =0.0962
p(Scalp)=0.0422
p(Dura)=0.0412
Scalp
Dura

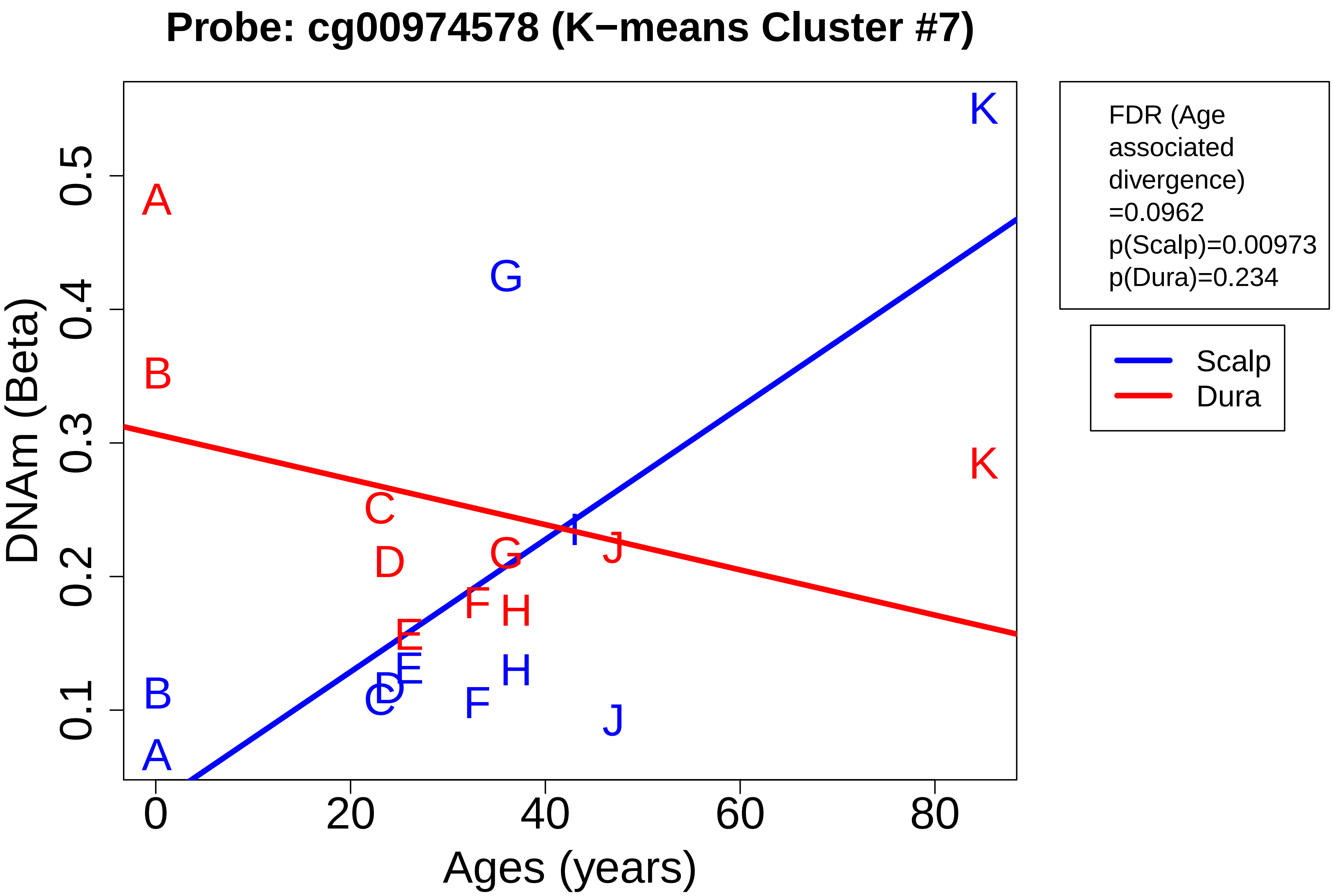
Probe: cg00974578 (K−means Cluster #7)
DNAm (Beta)
Ages (years)
0.1
0.2
0.3
0.4
0.5
0
20
40
60
80
FDR (Age associated divergence) =0.0962
p(Scalp)=0.00973
p(Dura)=0.234
Scalp
Dura
A
B
C
D
E
F
G
H
I
J
K

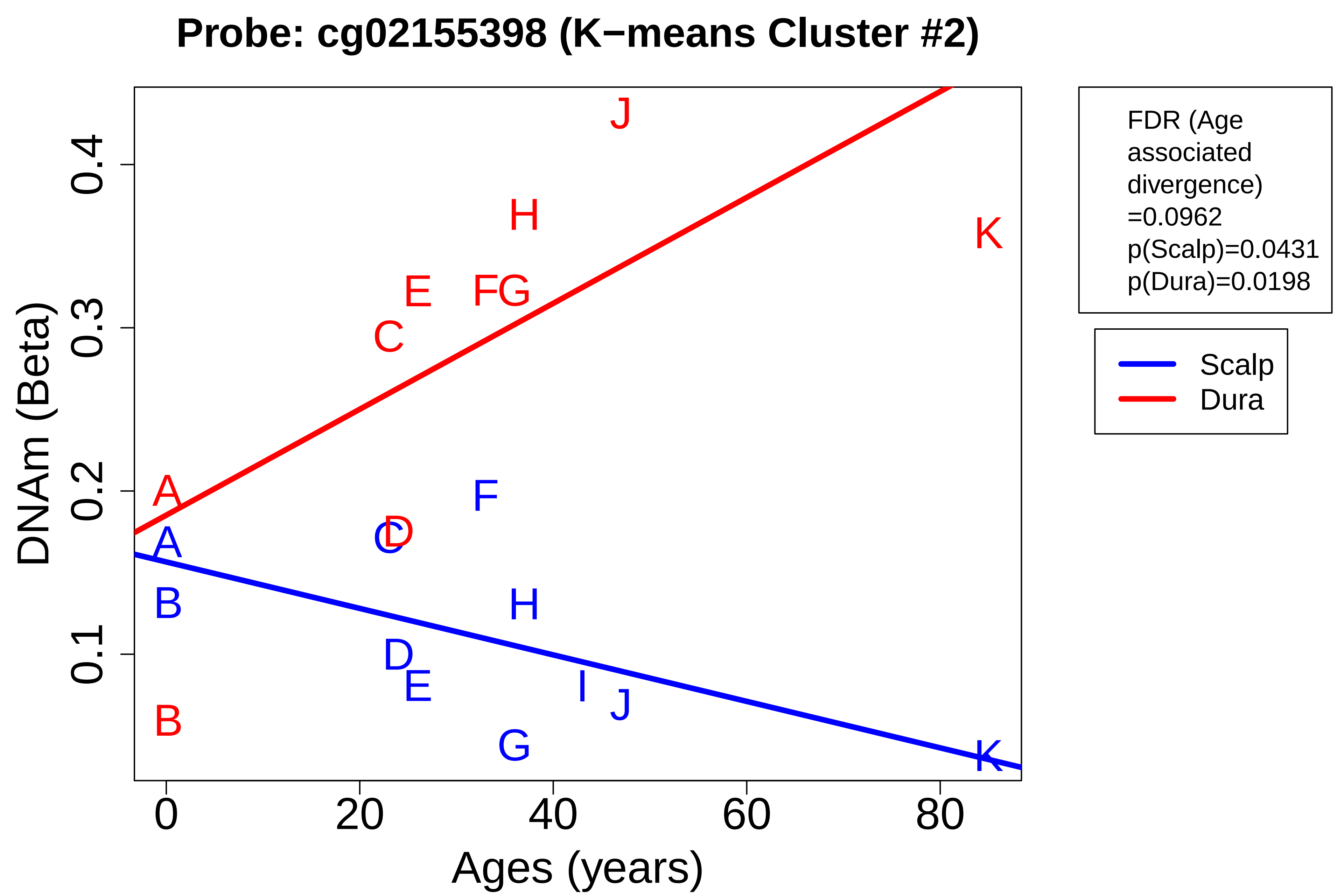

Probe: cg02155398 (K−means Cluster #2)
DNAm (Beta)
0.1
0.2
0.3
0.4
Ages (years)
0
20
40
60
80
FDR (Age associated divergence) =0.0962
p(Scalp)=0.0431
p(Dura)=0.0198
Scalp
Dura
A
B
C
D
E
F
G
H
I
J
K

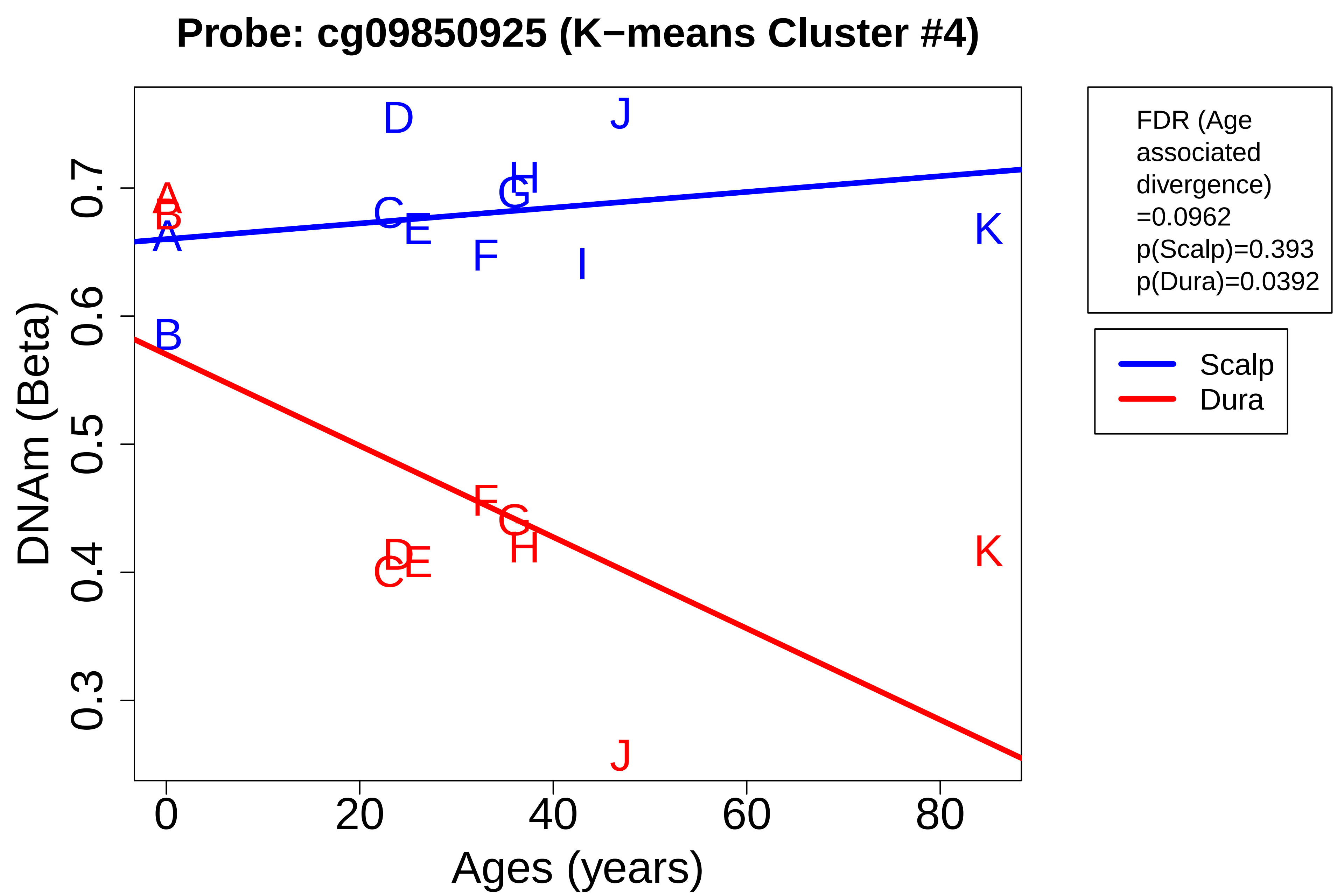

Probe: cg09850925 (K−means Cluster #4)
DNAm (Beta)
0.3
0.4
0.5
0.6
0.7
0
20
40
60
80
Ages (years)
D
J
H
G
C
E
F
I
K
A
B
A
B
F
G
H
D
E
C
K
J
FDR (Age associated divergence) =0.0962
p(Scalp)=0.393
p(Dura)=0.0392
Scalp
Dura

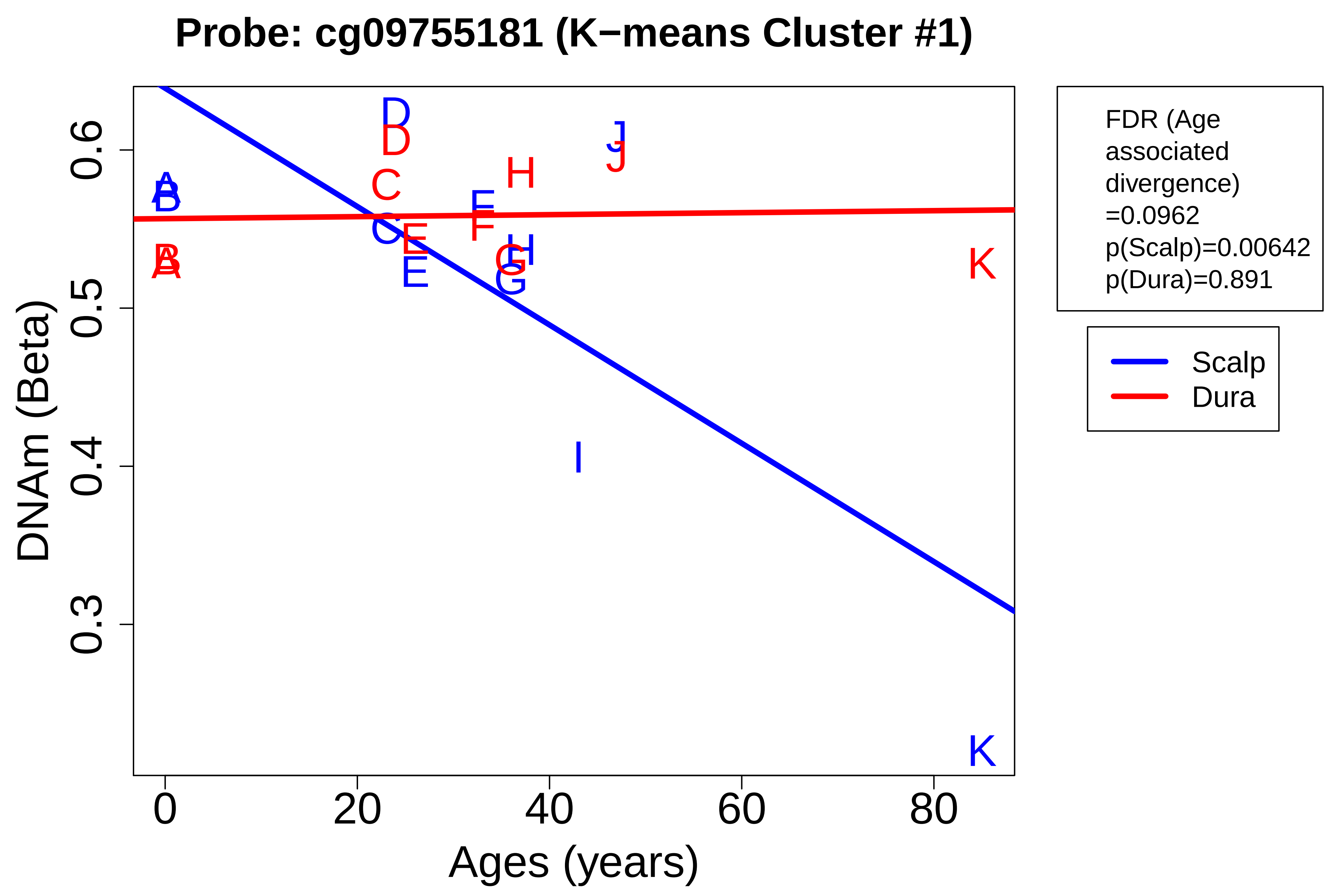

Probe: cg09755181 (K−means Cluster #1)
DNAm (Beta)
0.3
0.4
0.5
0.6
Ages (years)
0
20
40
60
80
FDR (Age associated divergence) =0.0962
p(Scalp)=0.00642
p(Dura)=0.891
Scalp
Dura

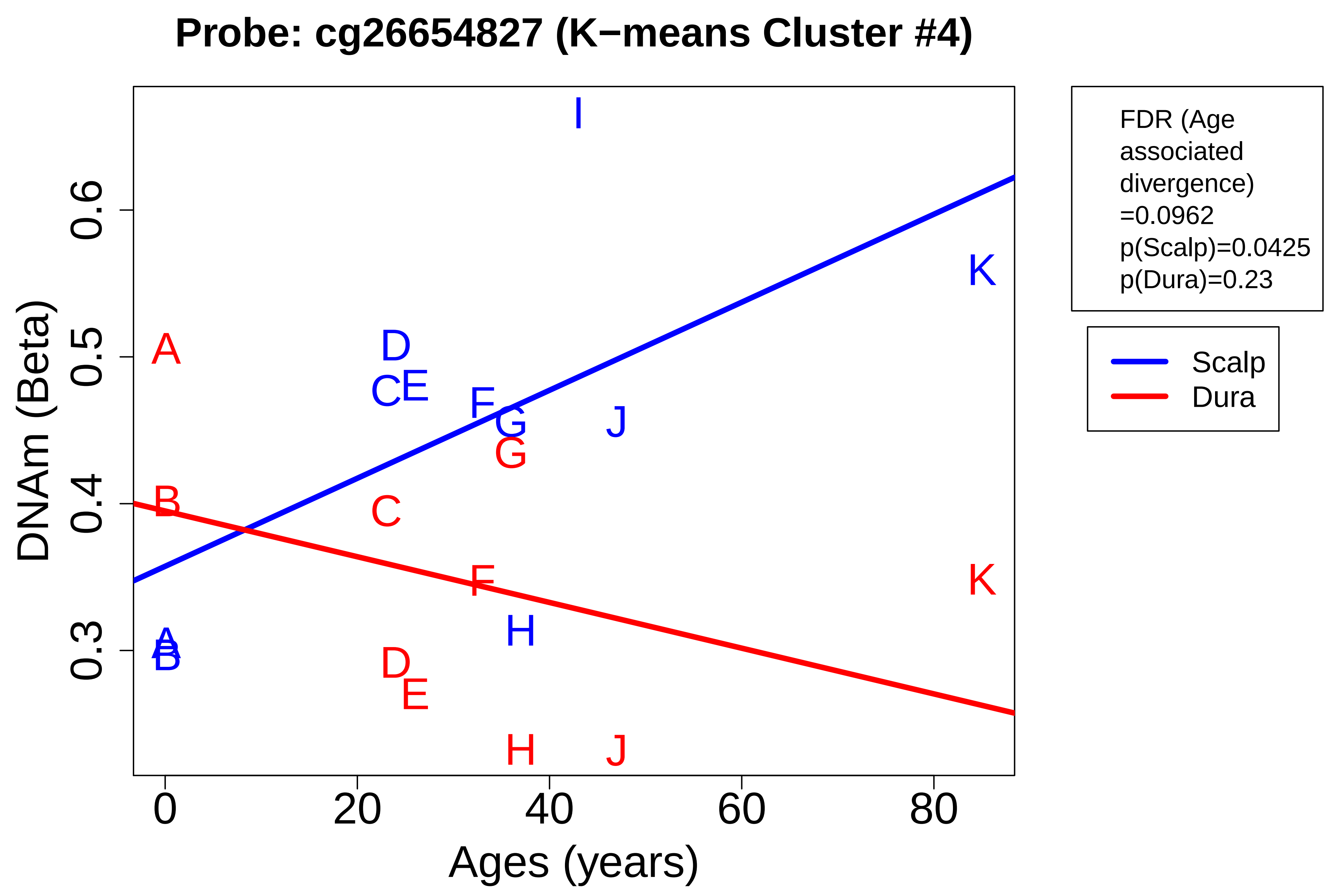

Probe: cg26654827 (K−means Cluster #4)
DNAm (Beta)
0.3
0.4
0.5
0.6
0
20
40
60
80
Ages (years)
FDR (Age associated divergence) =0.0962
p(Scalp)=0.0425
p(Dura)=0.23
Scalp
Dura

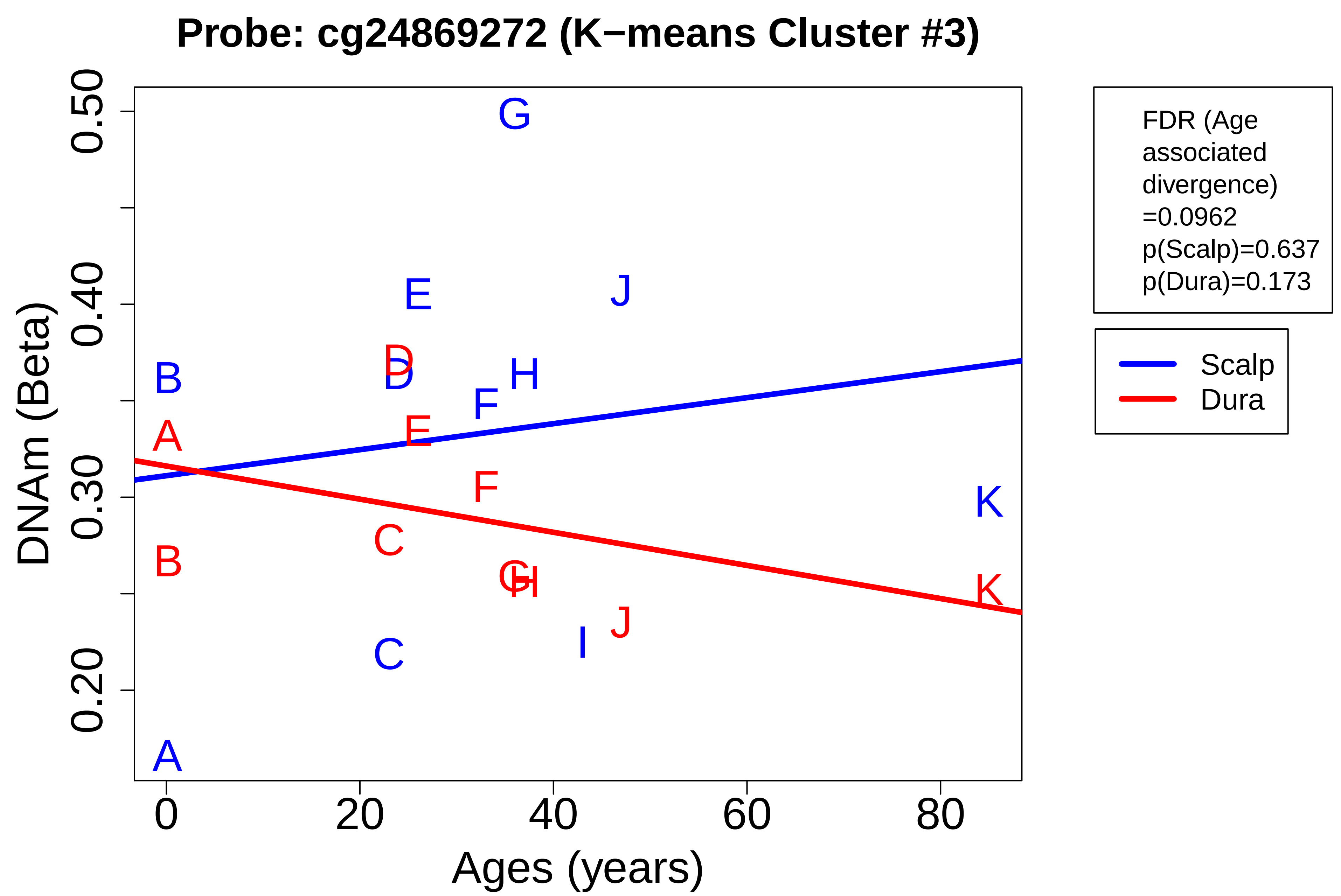

Probe: cg24869272 (K−means Cluster #3)
DNAm (Beta)
0.20
0.30
0.40
0.50
0
20
40
60
80
Ages (years)
FDR (Age associated divergence) =0.0962
p(Scalp)=0.637
p(Dura)=0.173
Scalp
Dura
A
B
C
D
E
F
G
H
I
J
K

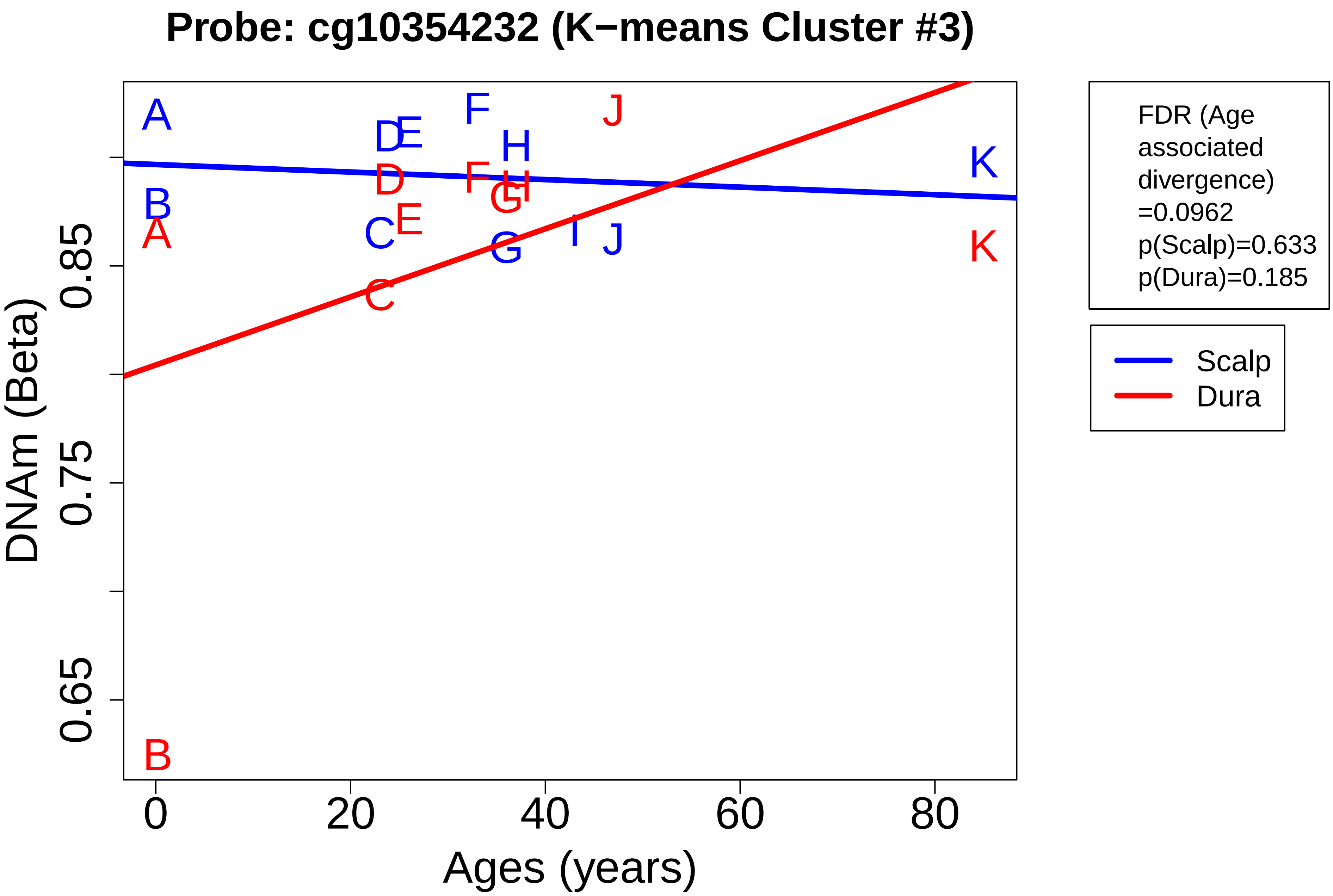

Probe: cg10354232 (K−means Cluster #3)
DNAm (Beta)
0.65
0.75
0.85
Ages (years)
0
20
40
60
80
A
B
C
D
E
F
G
H
I
J
K
FDR (Age associated divergence) =0.0962
p(Scalp)=0.633
p(Dura)=0.185
Scalp
Dura

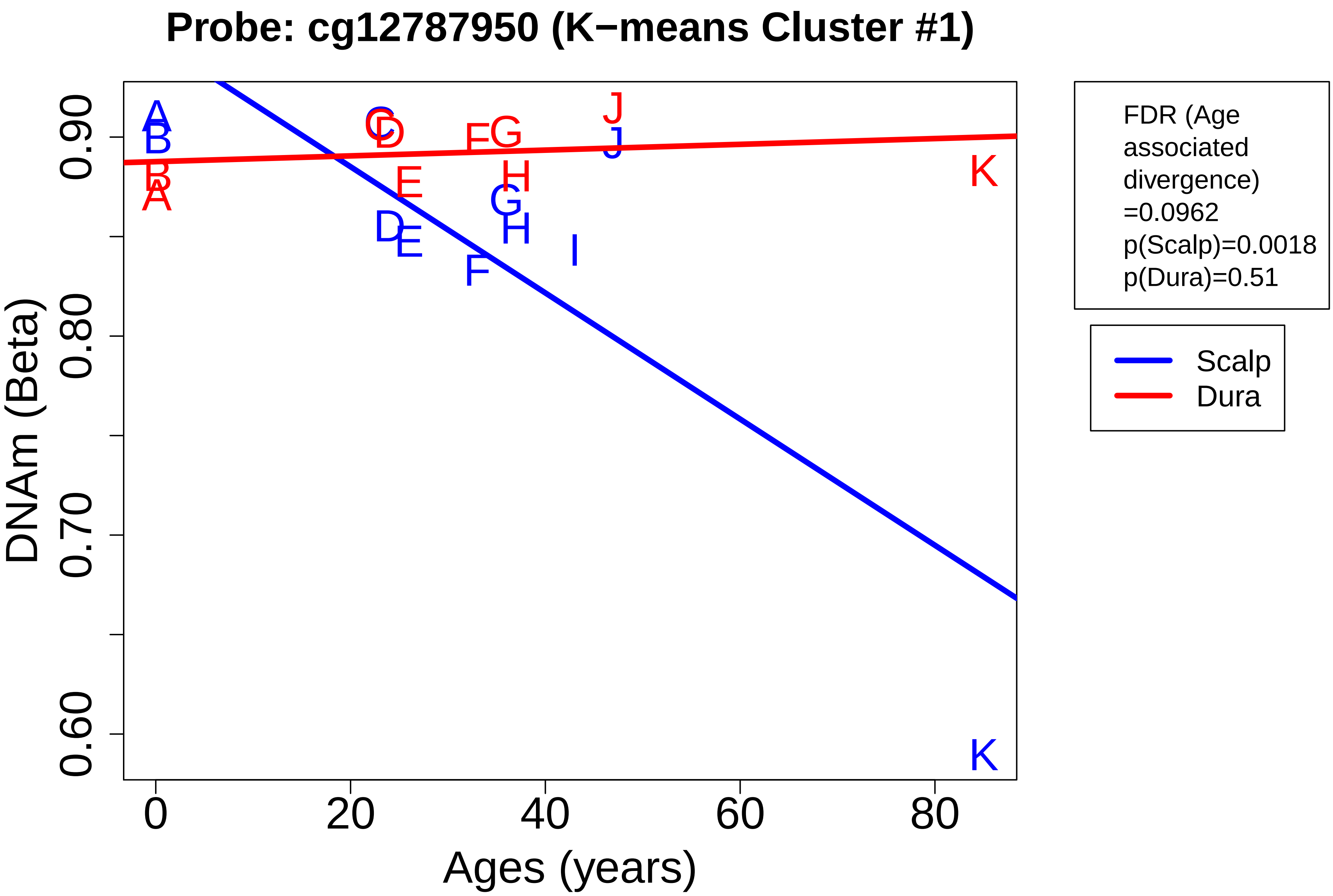

Probe: cg12787950 (K−means Cluster #1)
DNAm (Beta)
0.60
0.70
0.80
0.90
0
20
40
60
80
Ages (years)
A
B
C
D
E
F
G
H
I
J
K
FDR (Age associated divergence) =0.0962
p(Scalp)=0.0018
p(Dura)=0.51
Scalp
Dura

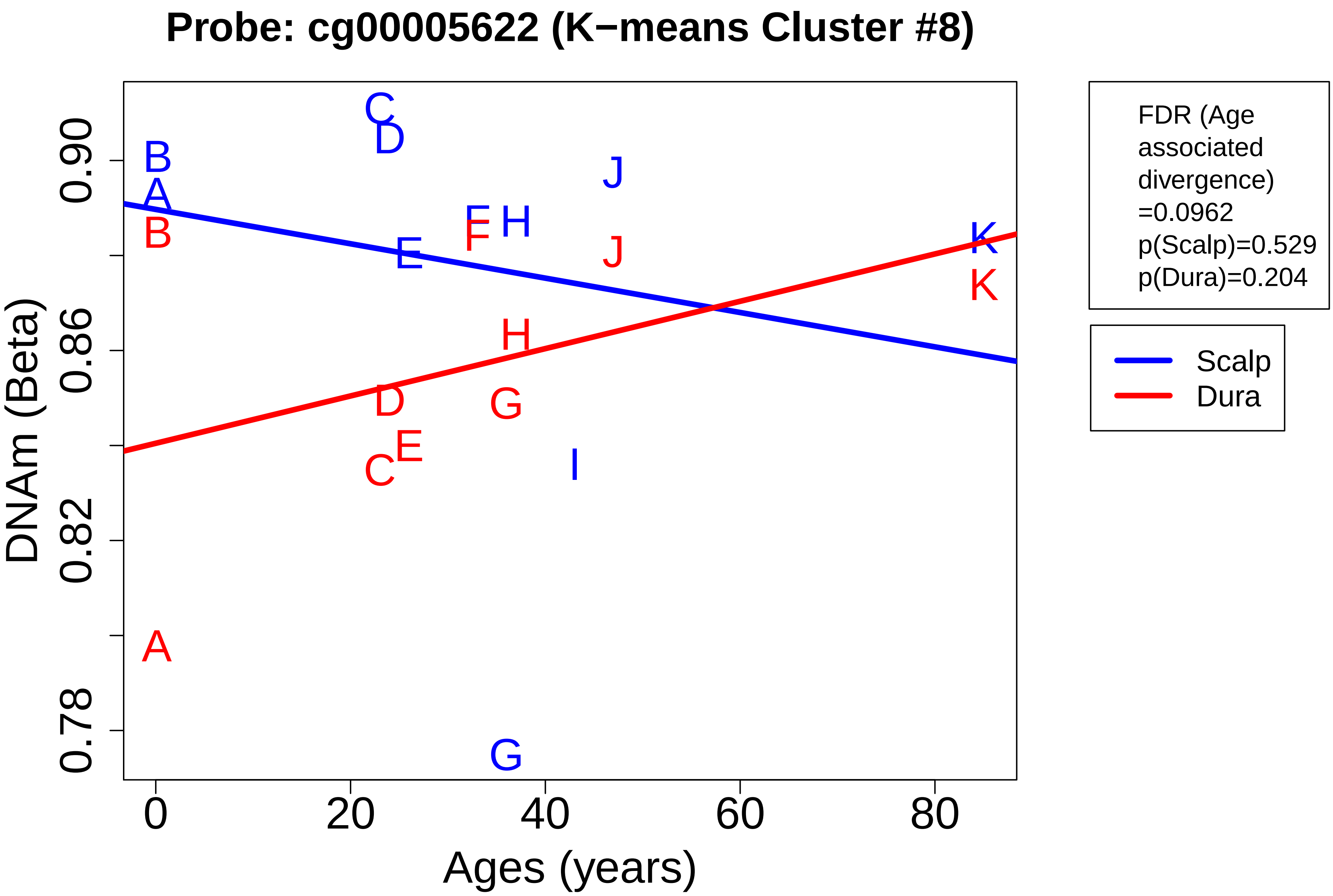
Probe: cg00005622 (K−means Cluster #8)
DNAm (Beta)
Ages (years)
0.78
0.82
0.86
0.90
0
20
40
60
80
FDR (Age associated divergence) =0.0962
p(Scalp)=0.529
p(Dura)=0.204
Scalp
Dura

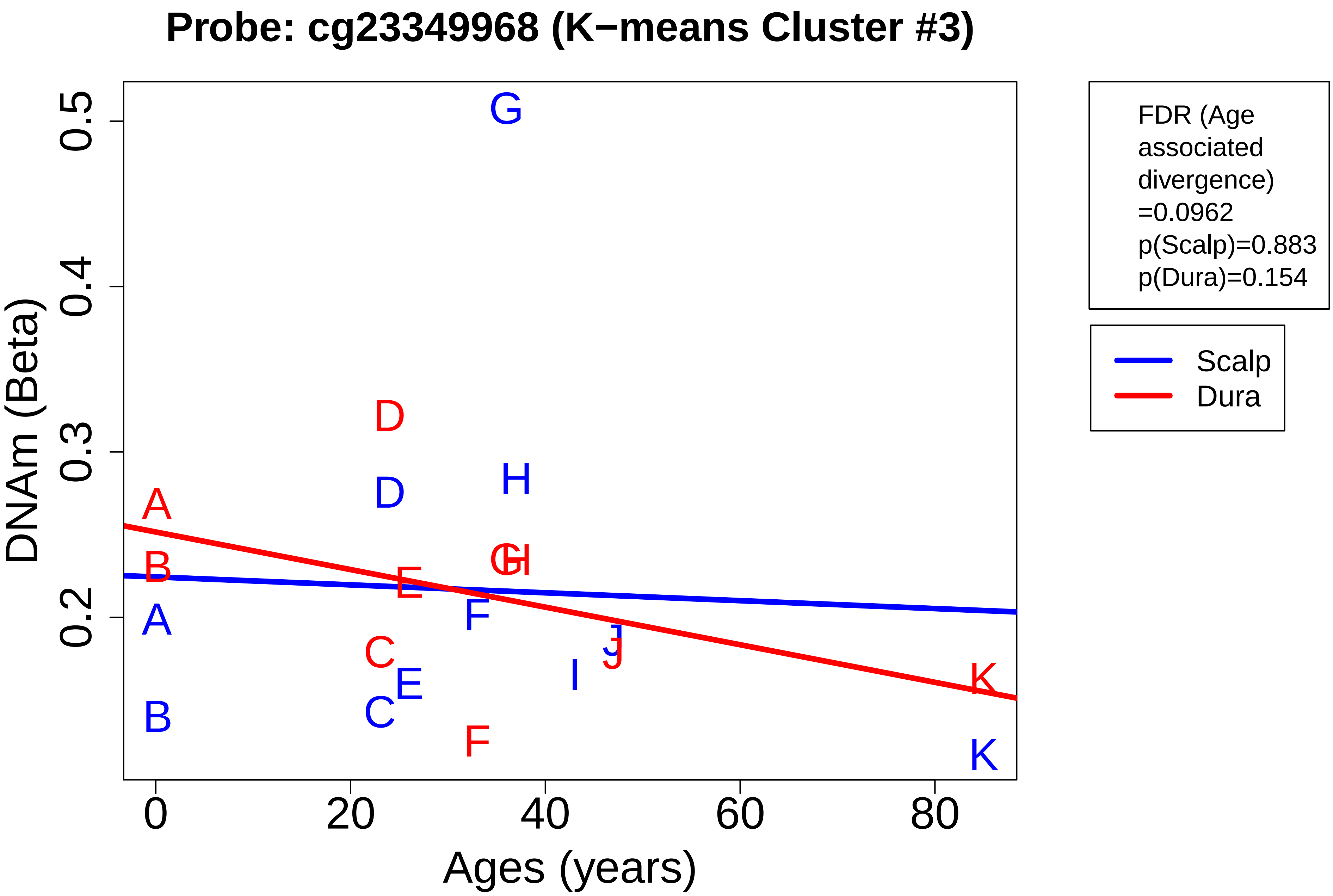
Probe: cg23349968 (K−means Cluster #3)
DNAm (Beta)
0.2
0.3
0.4
0.5
0
20
40
60
80
Ages (years)
A
B
C
D
E
F
G
H
I
J
K
FDR (Age associated divergence) =0.0962
p(Scalp)=0.883
p(Dura)=0.154
Scalp
Dura

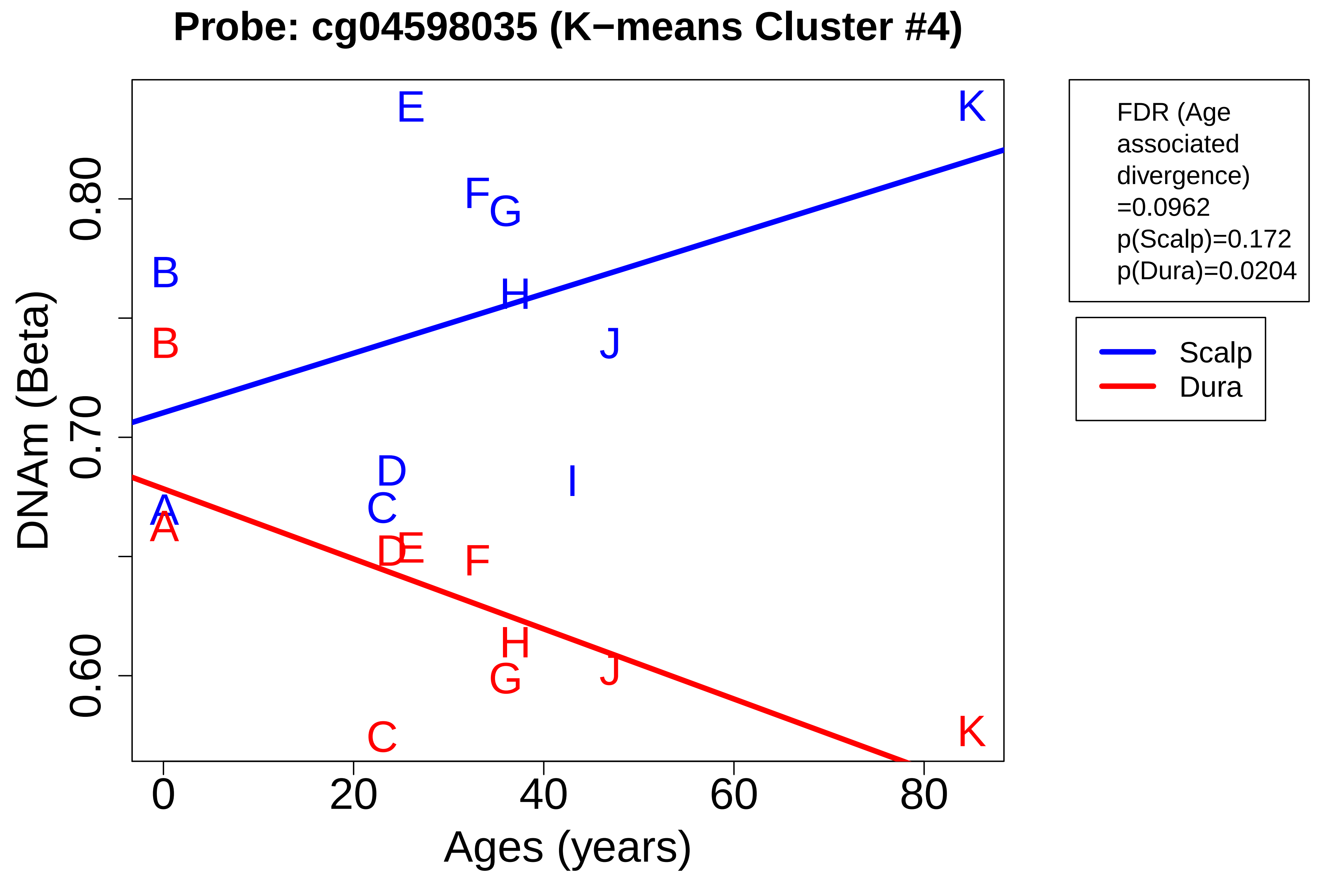
Probe: cg04598035 (K−means Cluster #4)
DNAm (Beta)
0.60
0.70
0.80
Ages (years)
0
20
40
60
80
FDR (Age associated divergence) =0.0962
p(Scalp)=0.172
p(Dura)=0.0204
Scalp
Dura
A
B
C
D
E
F
G
H
I
J
K

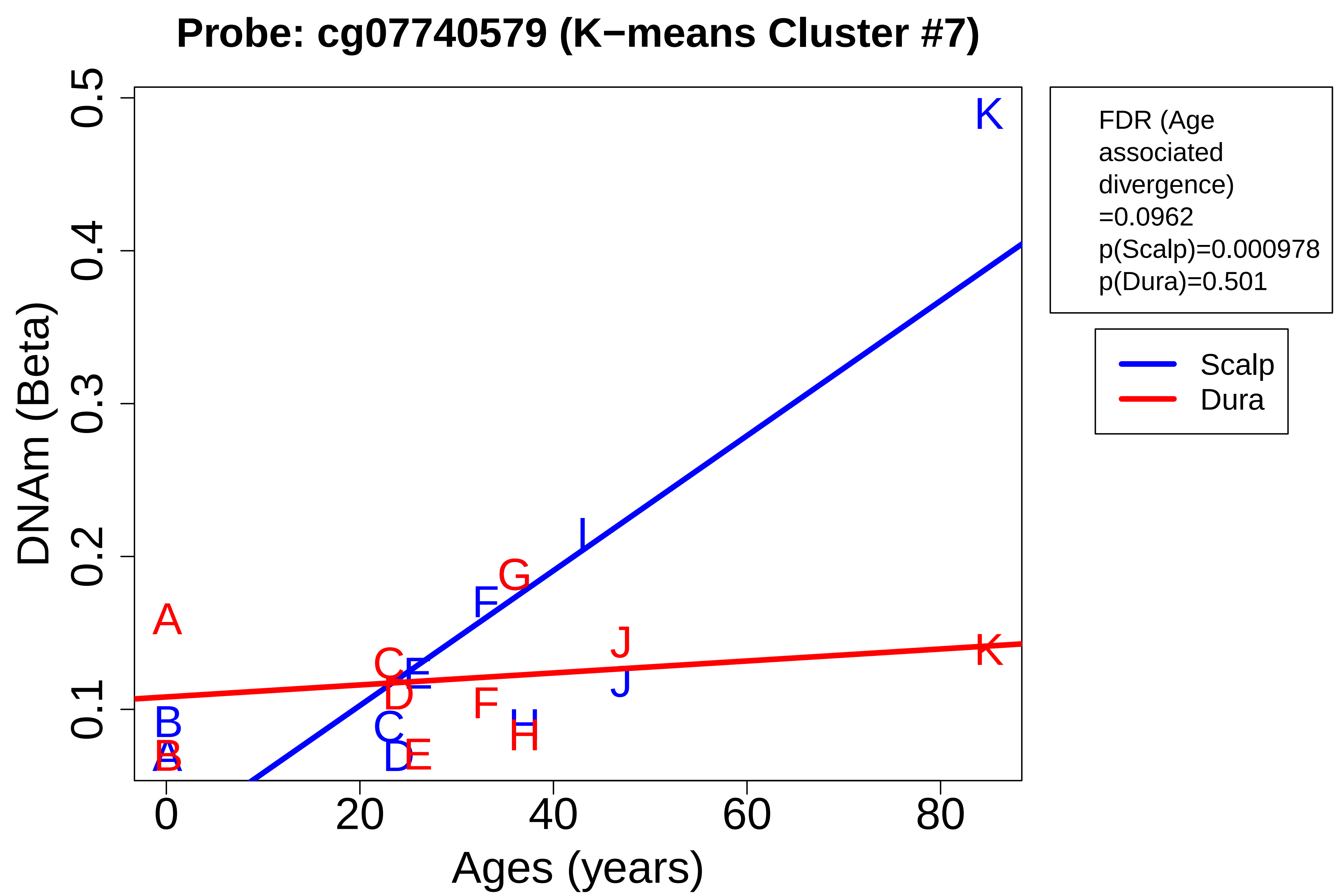

Probe: cg07740579 (K−means Cluster #7)
DNAm (Beta)
0.1
0.2
0.3
0.4
0.5
0
20
40
60
80
Ages (years)
FDR (Age associated divergence) =0.0962
p(Scalp)=0.000978
p(Dura)=0.501
Scalp
Dura

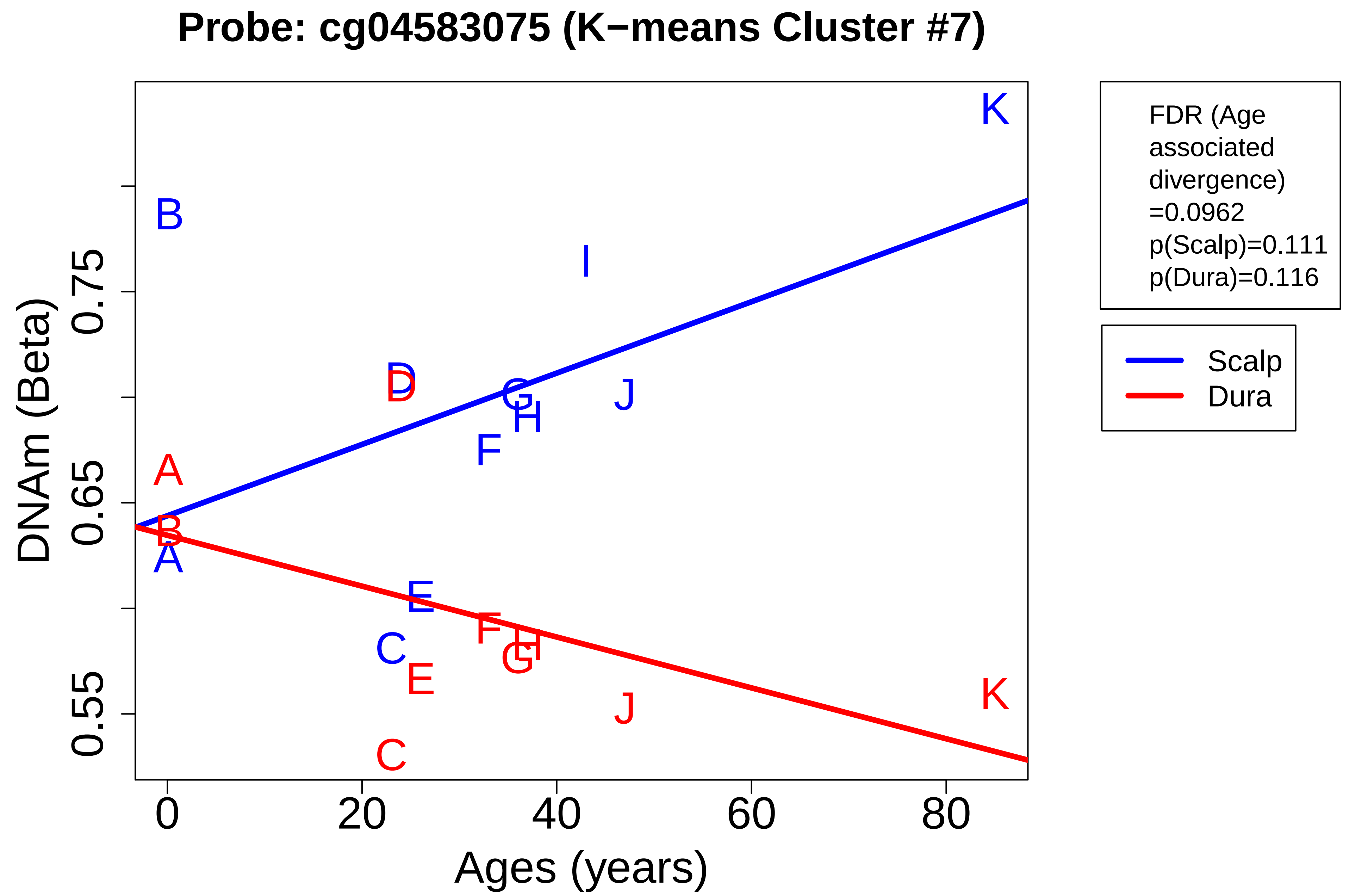
Probe: cg04583075 (K−means Cluster #7)
DNAm (Beta)
0.55
0.65
0.75
Ages (years)
0
20
40
60
80
FDR (Age associated divergence) =0.0962
p(Scalp)=0.111
p(Dura)=0.116
Scalp
Dura
A
B
C
D
E
F
G
H
I
J
K

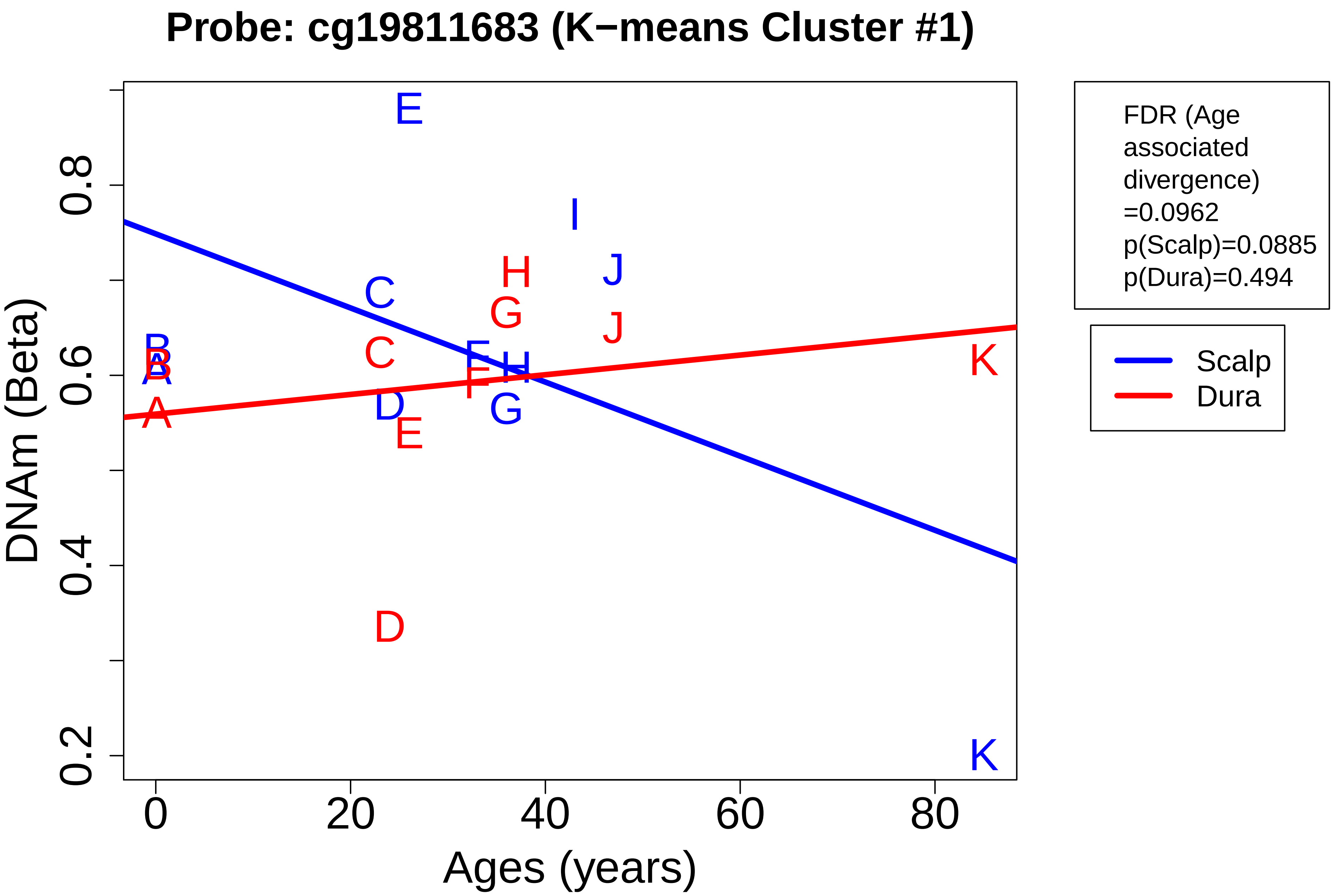

Probe: cg19811683 (K−means Cluster #1)
FDR (Age associated divergence) =0.0962
p(Scalp)=0.0885
p(Dura)=0.494
Scalp
Dura
DNAm (Beta)
0.2
0.4
0.6
0.8
0
20
40
60
80
Ages (years)
A B C D E F G H I J K

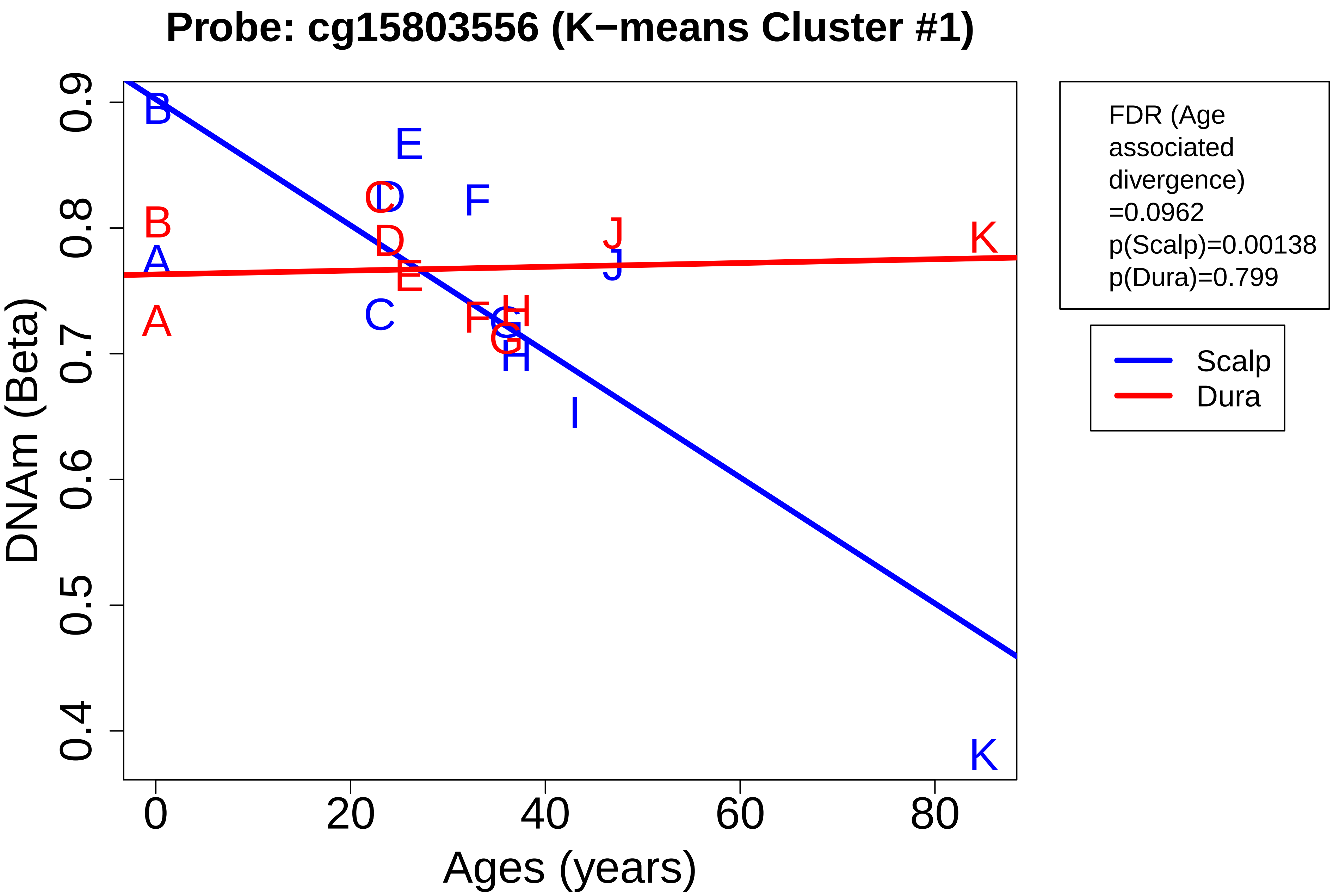

Probe: cg15803556 (K−means Cluster #1)
DNAm (Beta)
0.4
0.5
0.6
0.7
0.8
0.9
Ages (years)
0
20
40
60
80
FDR (Age associated divergence) =0.0962
p(Scalp)=0.00138
p(Dura)=0.799
Scalp
Dura

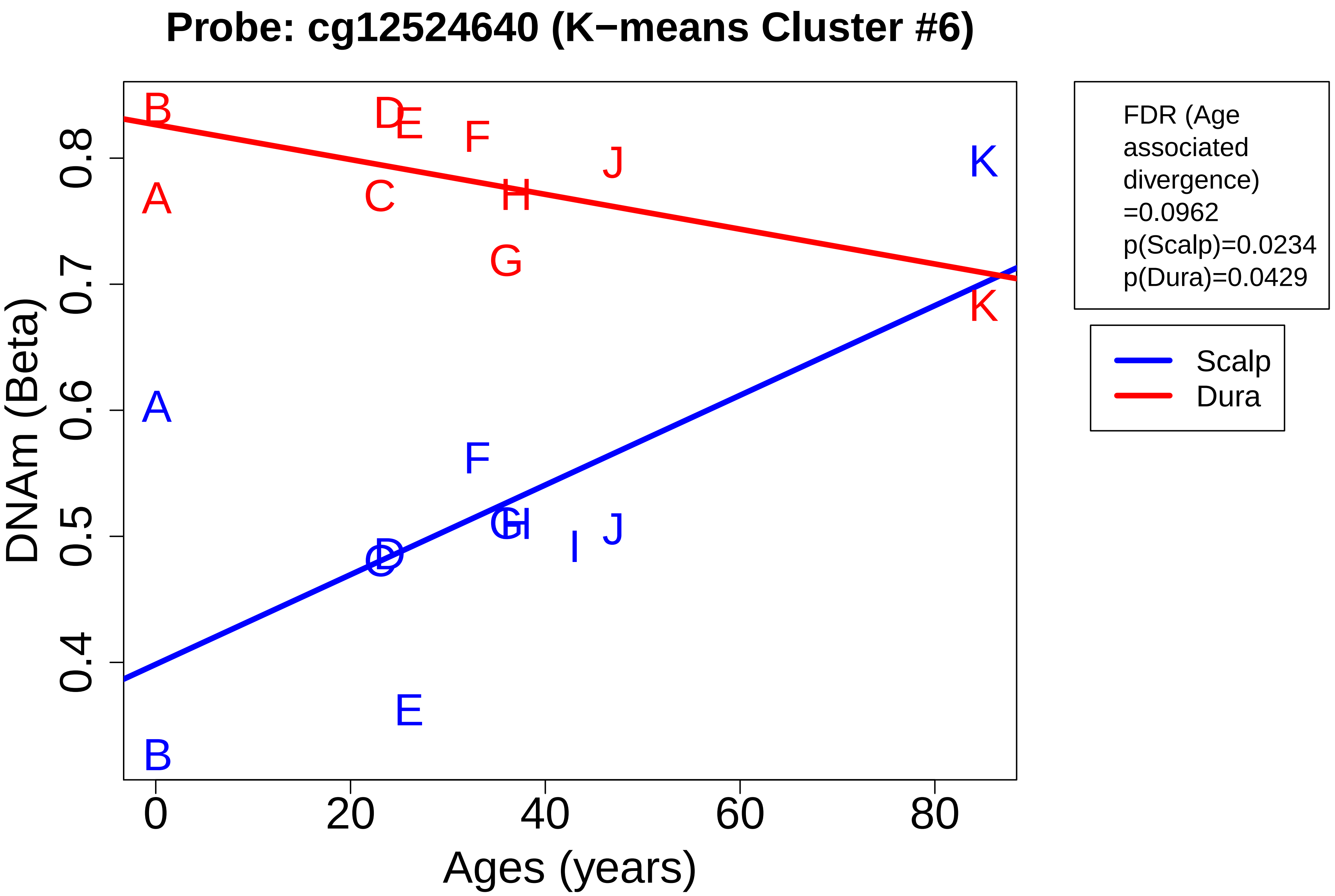

Probe: cg12524640 (K−means Cluster #6)
DNAm (Beta)
0.4
0.5
0.6
0.7
0.8
Ages (years)
0
20
40
60
80
FDR (Age associated divergence) =0.0962
p(Scalp)=0.0234
p(Dura)=0.0429
Scalp
Dura

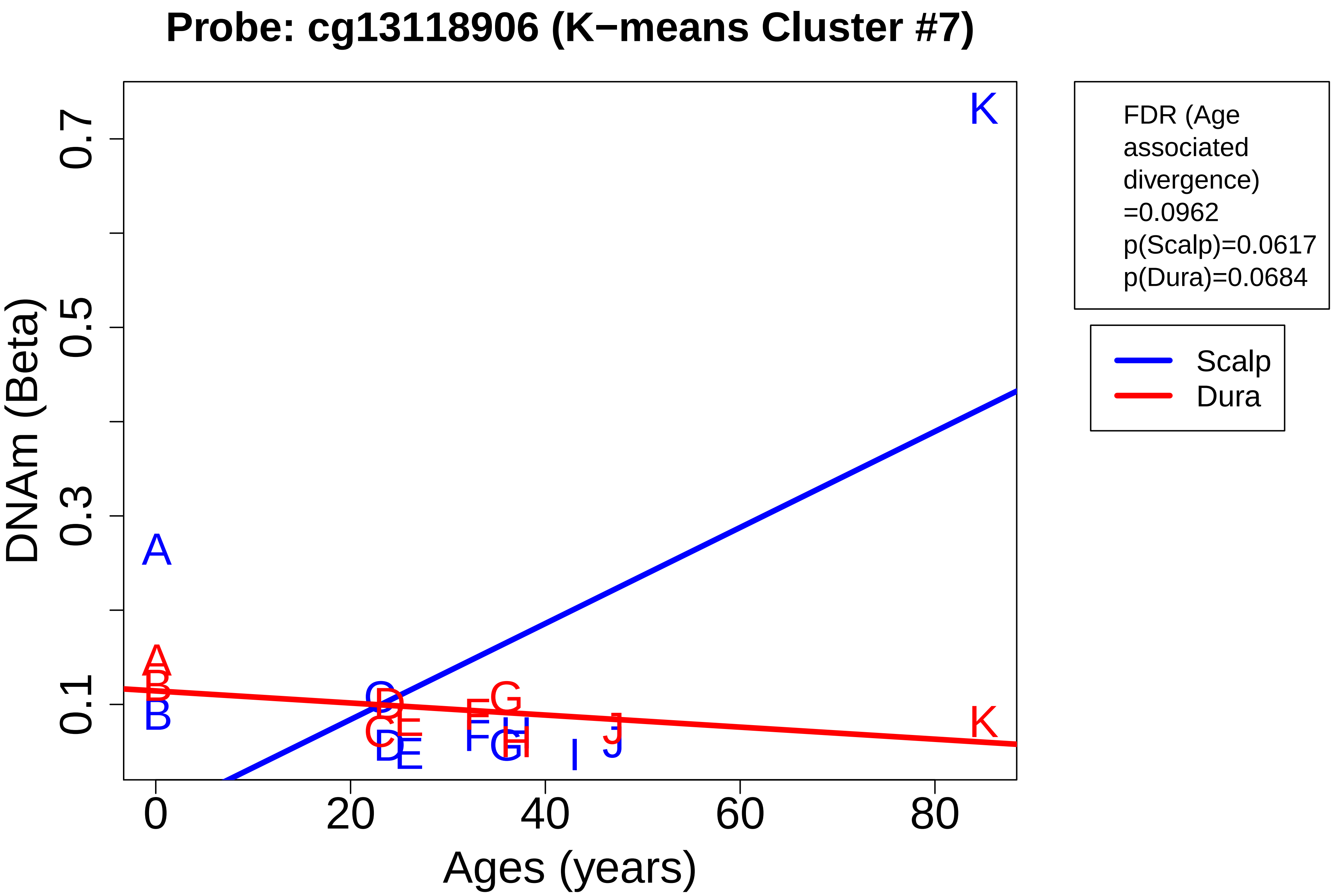

Probe: cg13118906 (K−means Cluster #7)
DNAm (Beta)
0.1
0.3
0.5
0.7
Ages (years)
0
20
40
60
80
FDR (Age associated divergence) =0.0962
p(Scalp)=0.0617
p(Dura)=0.0684
Scalp
Dura

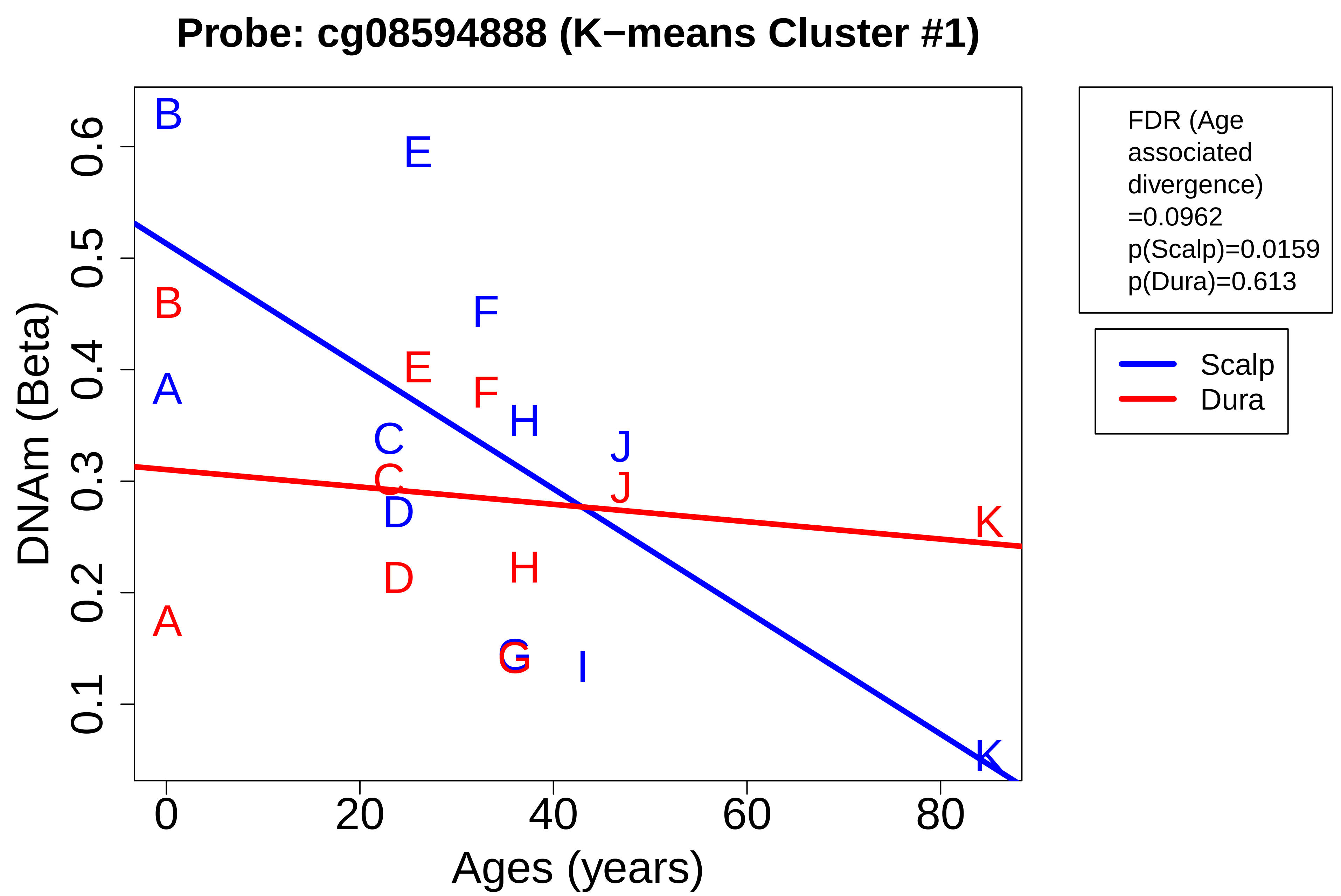
Probe: cg08594888 (K−means Cluster #1)
DNAm (Beta)
Ages (years)
0.1
0.2
0.3
0.4
0.5
0.6
0
20
40
60
80
FDR (Age associated divergence) =0.0962
p(Scalp)=0.0159
p(Dura)=0.613
Scalp
Dura

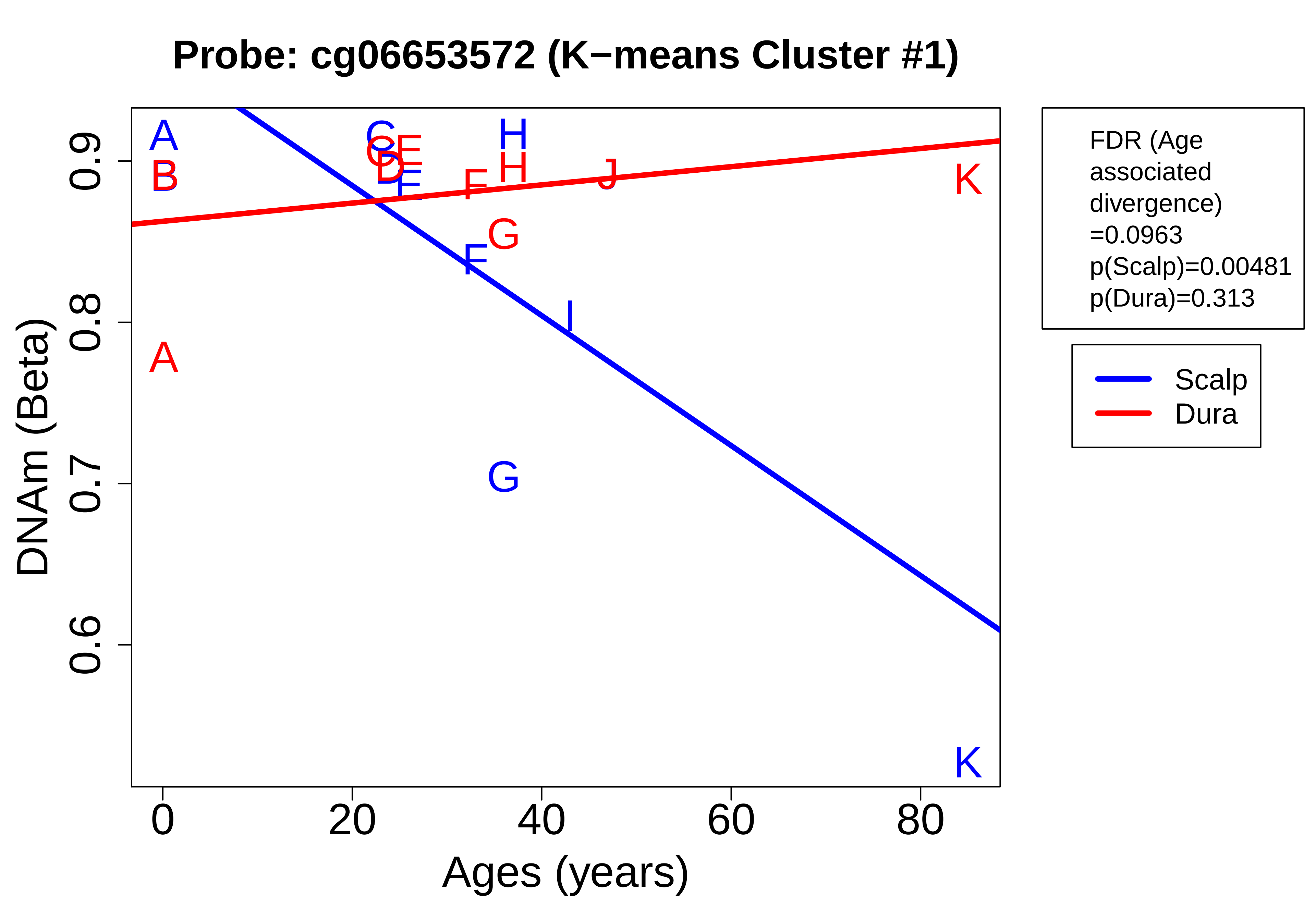

Probe: cg06653572 (K−means Cluster #1)
FDR (Age associated divergence) =0.0963
p(Scalp)=0.00481
p(Dura)=0.313
Scalp
Dura
DNAm (Beta)
0.6
0.7
0.8
0.9
0
20
40
60
80
Ages (years)
A
B
C
D
E
F
G
H
I
J
K

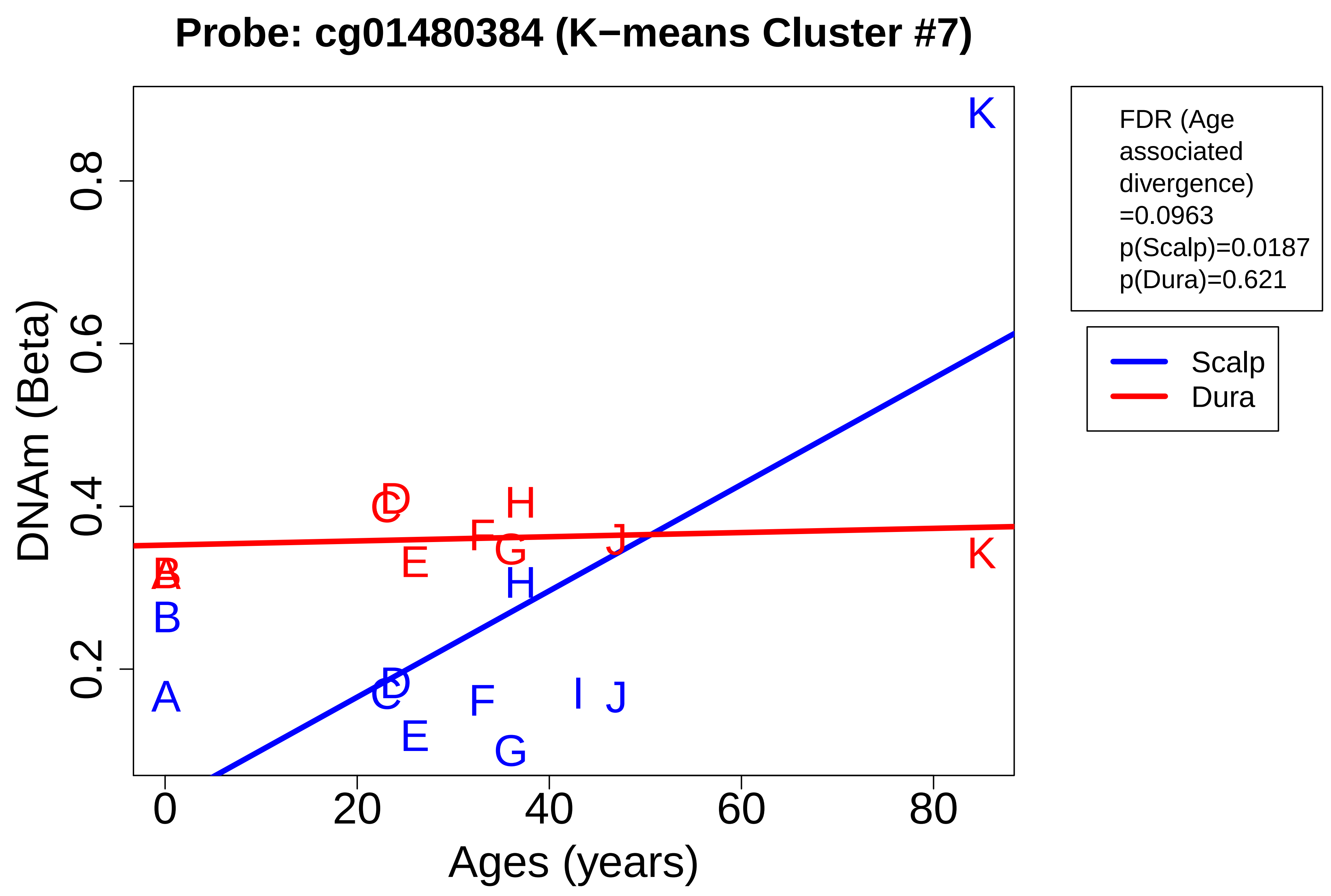

Probe: cg01480384 (K−means Cluster #7)
DNAm (Beta)
0.2
0.4
0.6
0.8
0
20
40
60
80
Ages (years)
FDR (Age associated divergence) =0.0963
p(Scalp)=0.0187
p(Dura)=0.621
Scalp
Dura

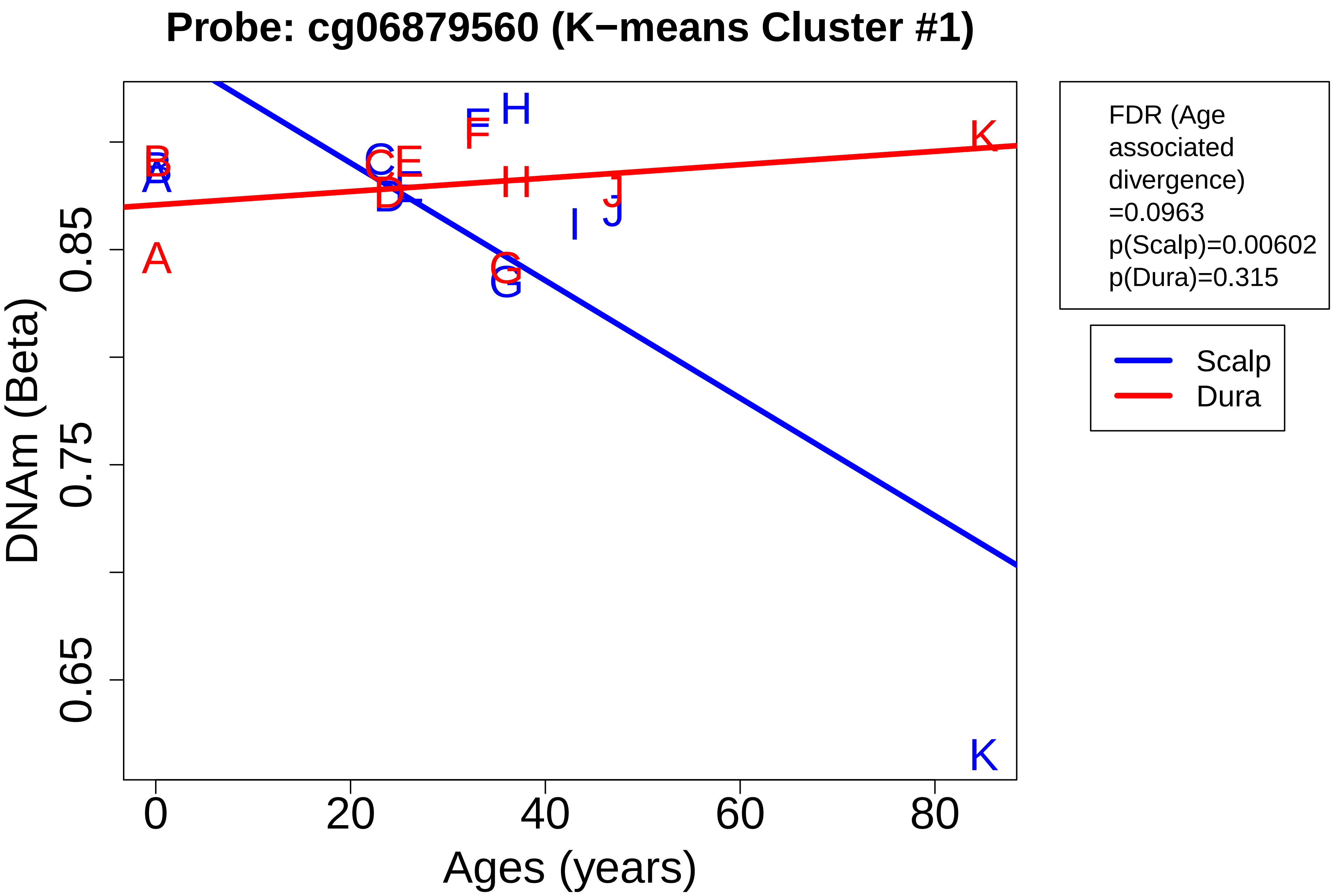

Probe: cg06879560 (K−means Cluster #1)
DNAm (Beta)
0.65
0.75
0.85
0
20
40
60
80
Ages (years)
A
B
C
D
E
F
G
H
I
J
K
FDR (Age associated divergence) =0.0963
p(Scalp)=0.00602
p(Dura)=0.315
Scalp
Dura

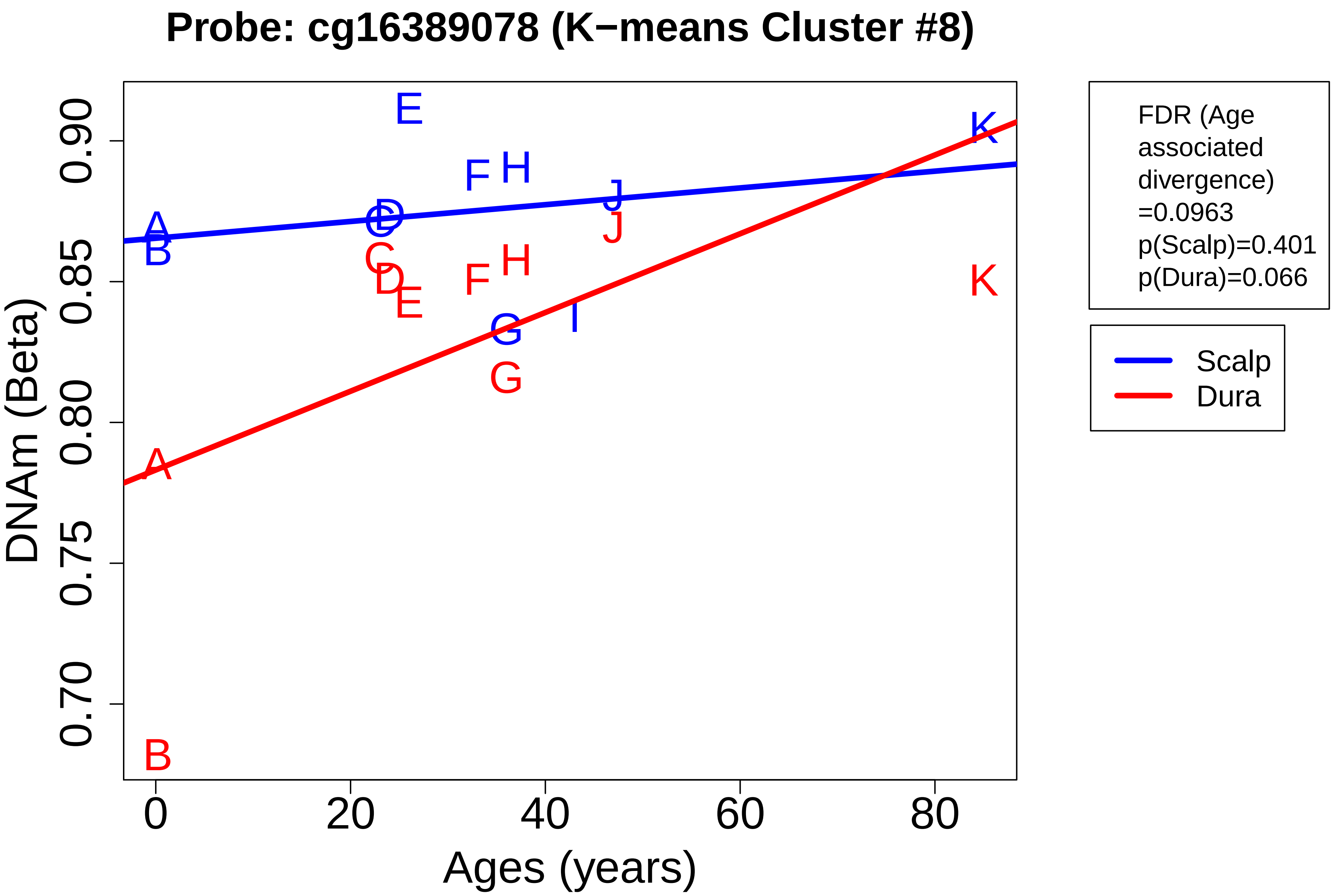

Probe: cg16389078 (K−means Cluster #8)
DNAm (Beta)
0.70
0.75
0.80
0.85
0.90
Ages (years)
0
20
40
60
80
FDR (Age associated divergence) =0.0963
p(Scalp)=0.401
p(Dura)=0.066
Scalp
Dura

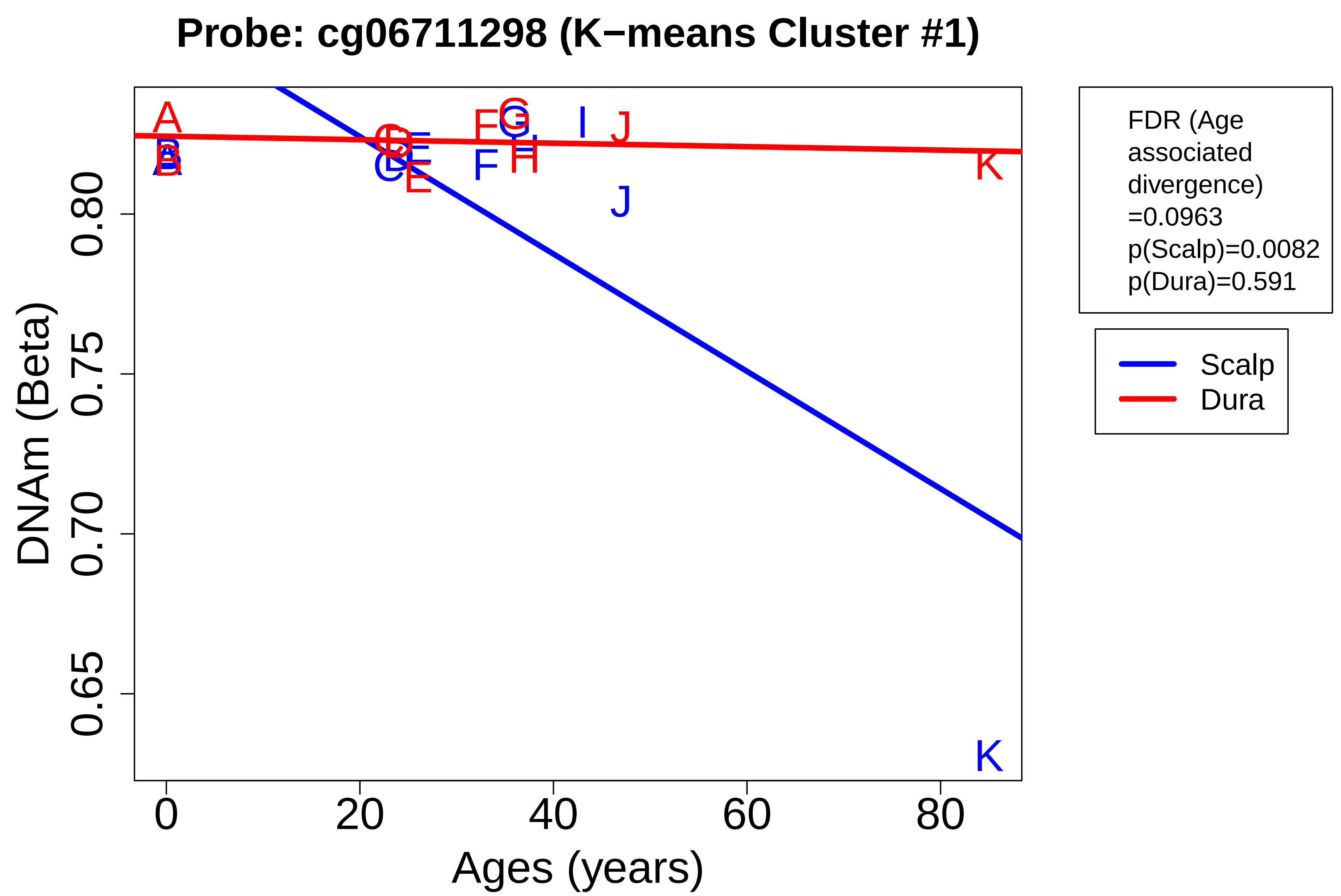
Probe: cg06711298 (K−means Cluster #1)
DNAm (Beta)
0.65
0.70
0.75
0.80
Ages (years)
0
20
40
60
80
A
B
C
D
E
F
G
H
I
J
K
FDR (Age associated divergence) =0.0963
p(Scalp)=0.0082
p(Dura)=0.591
Scalp
Dura

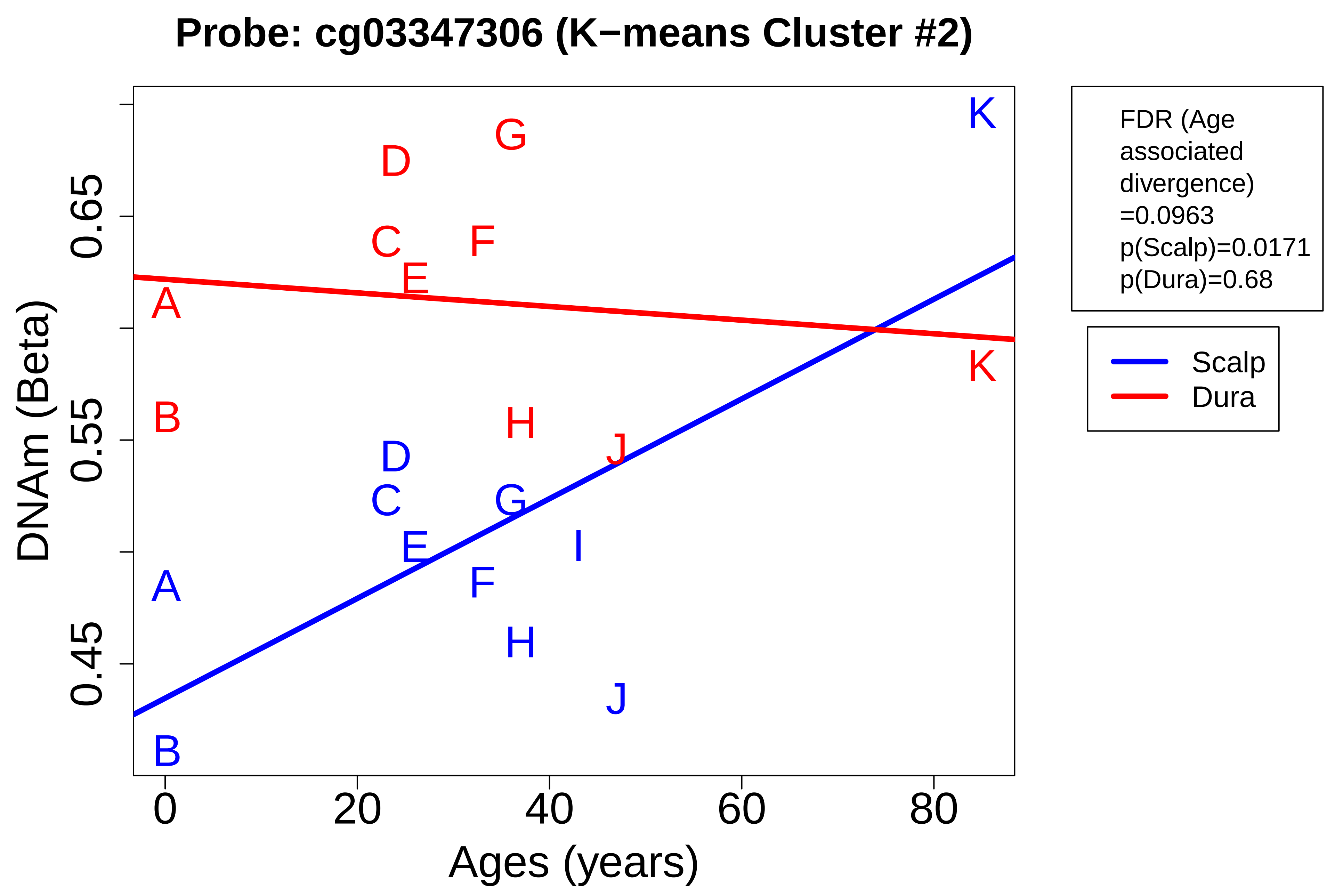

Probe: cg03347306 (K−means Cluster #2)
DNAm (Beta)
0.45
0.55
0.65
Ages (years)
0
20
40
60
80
FDR (Age associated divergence) =0.0963
p(Scalp)=0.0171
p(Dura)=0.68
Scalp
Dura
A
B
C
D
E
F
G
H
I
J
K

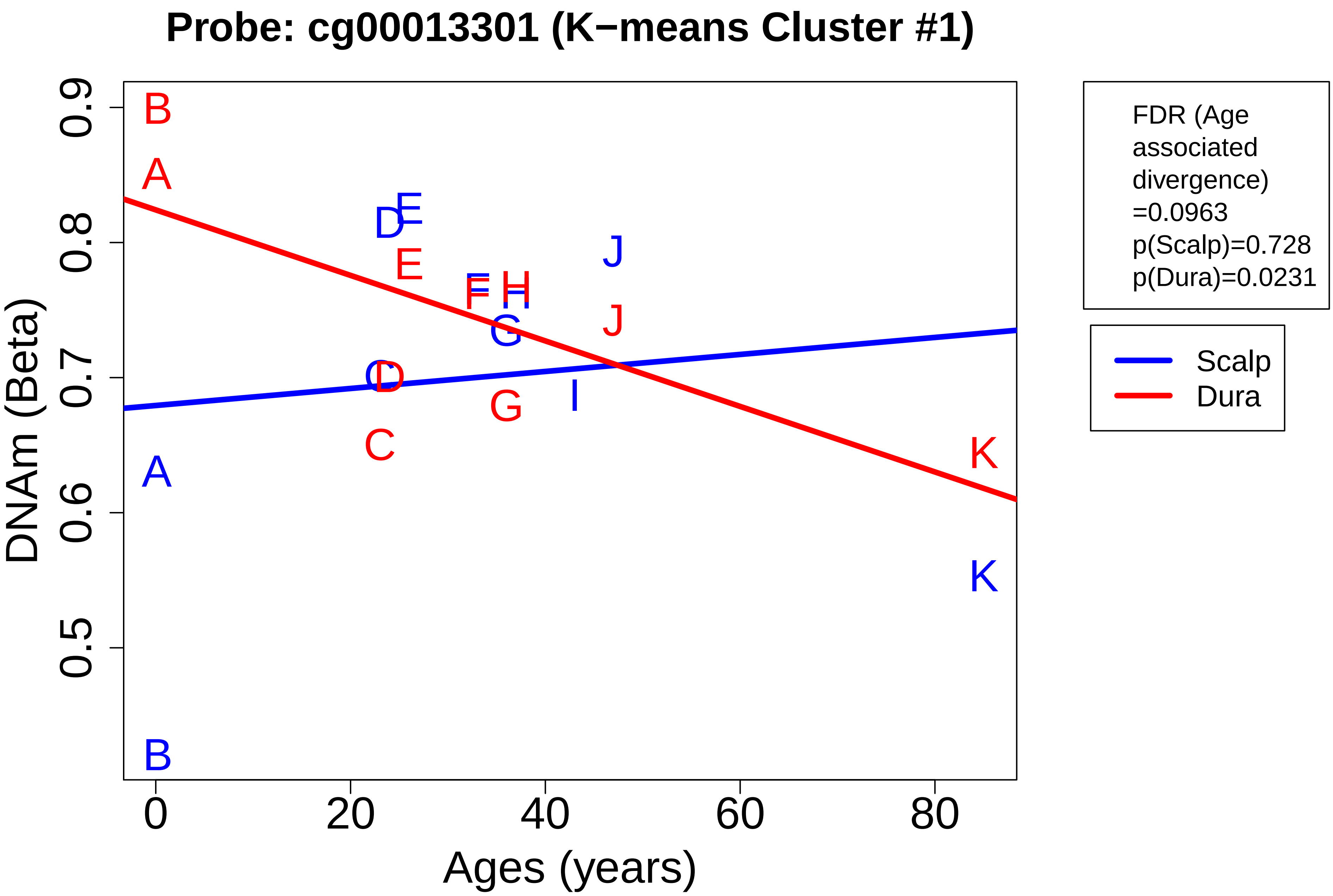

Probe: cg00013301 (K−means Cluster #1)
DNAm (Beta)
Ages (years)
0.5
0.6
0.7
0.8
0.9
0
20
40
60
80
FDR (Age associated divergence) =0.0963
p(Scalp)=0.728
p(Dura)=0.0231
Scalp
Dura

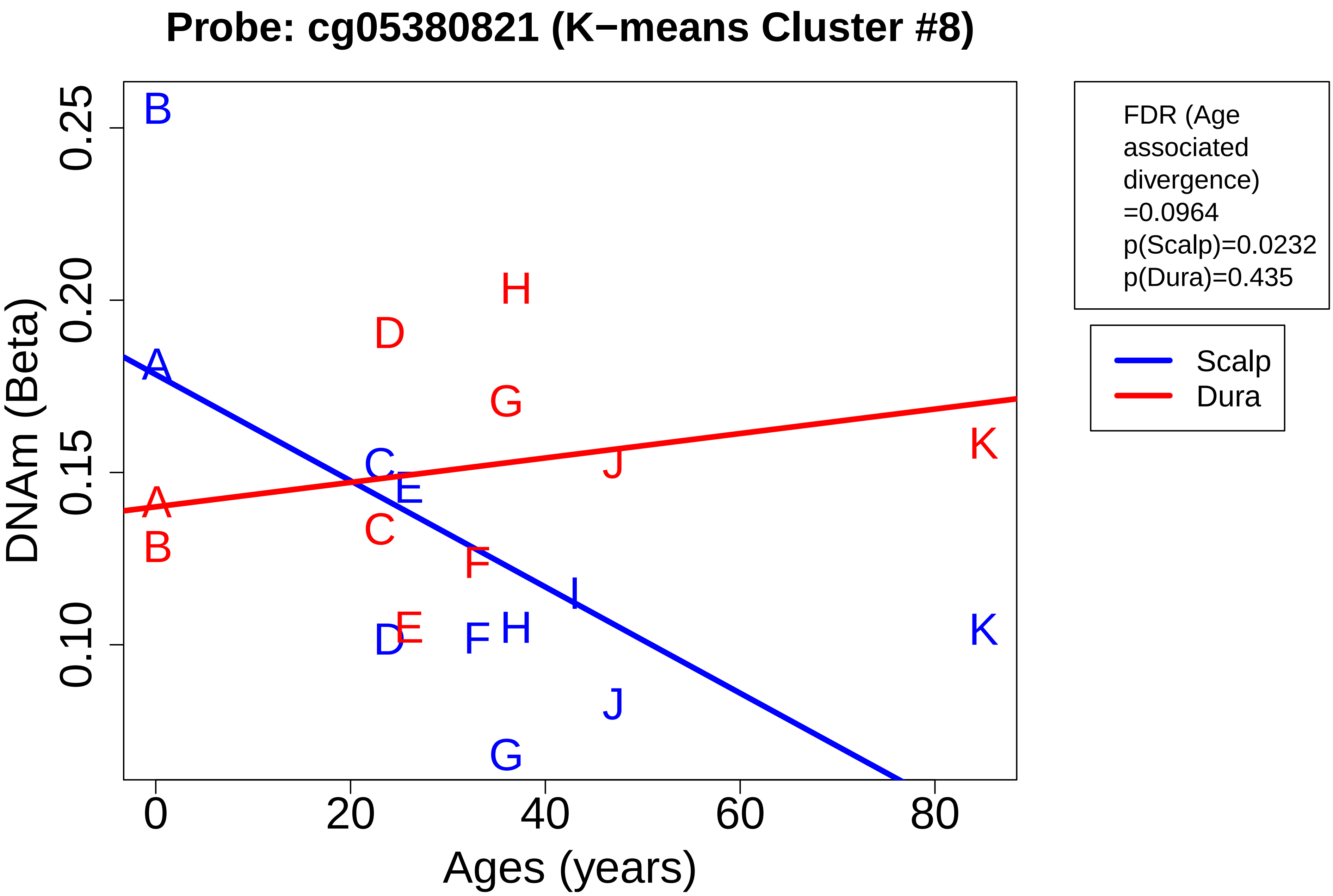

Probe: cg05380821 (K−means Cluster #8)
FDR (Age associated divergence) =0.0964
p(Scalp)=0.0232
p(Dura)=0.435
Scalp
Dura
DNAm (Beta)
0.10
0.15
0.20
0.25
0
20
40
60
80
Ages (years)

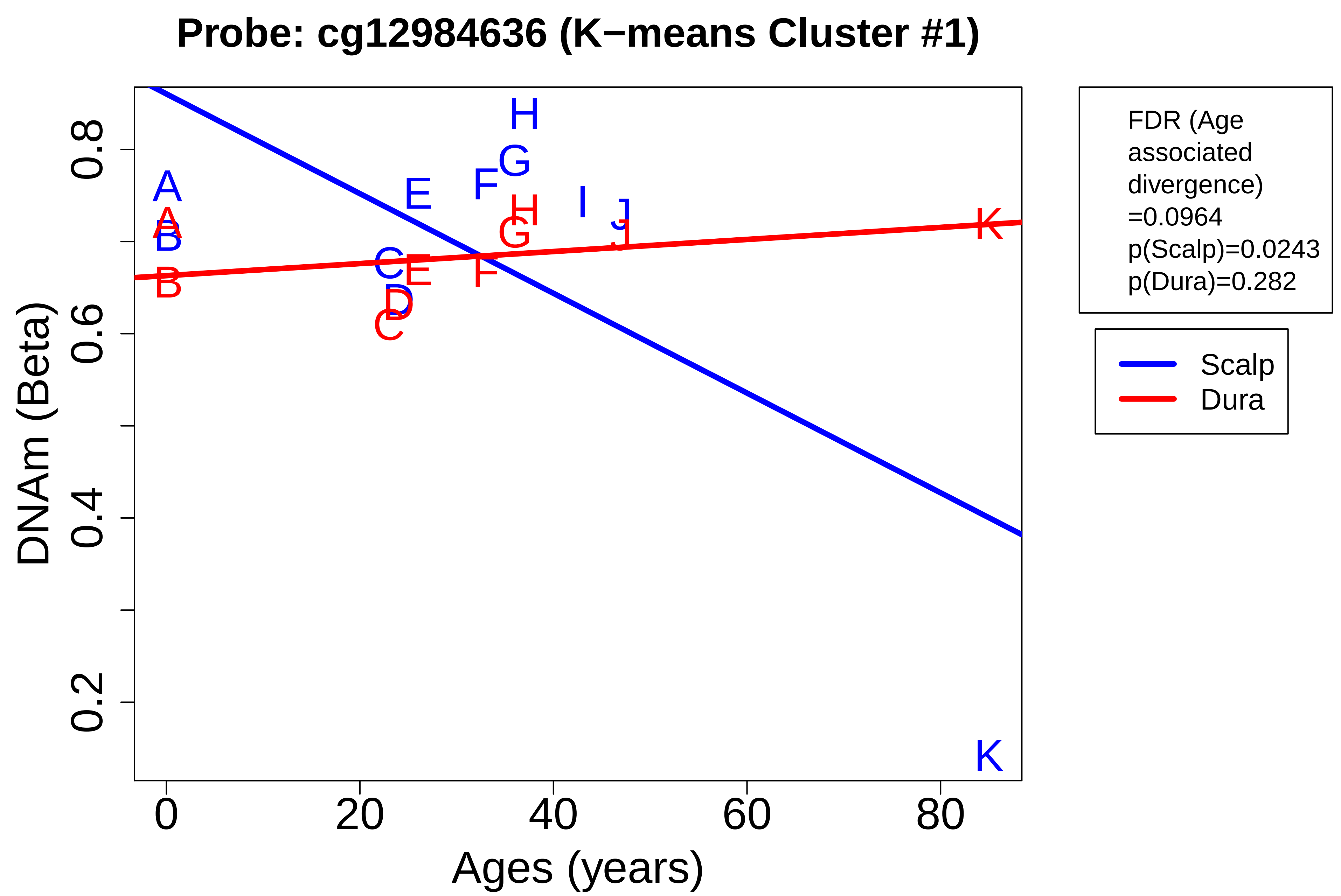

Probe: cg12984636 (K−means Cluster #1)
DNAm (Beta)
0.2
0.4
0.6
0.8
0
20
40
60
80
Ages (years)
FDR (Age associated divergence) =0.0964
p(Scalp)=0.0243
p(Dura)=0.282
Scalp
Dura

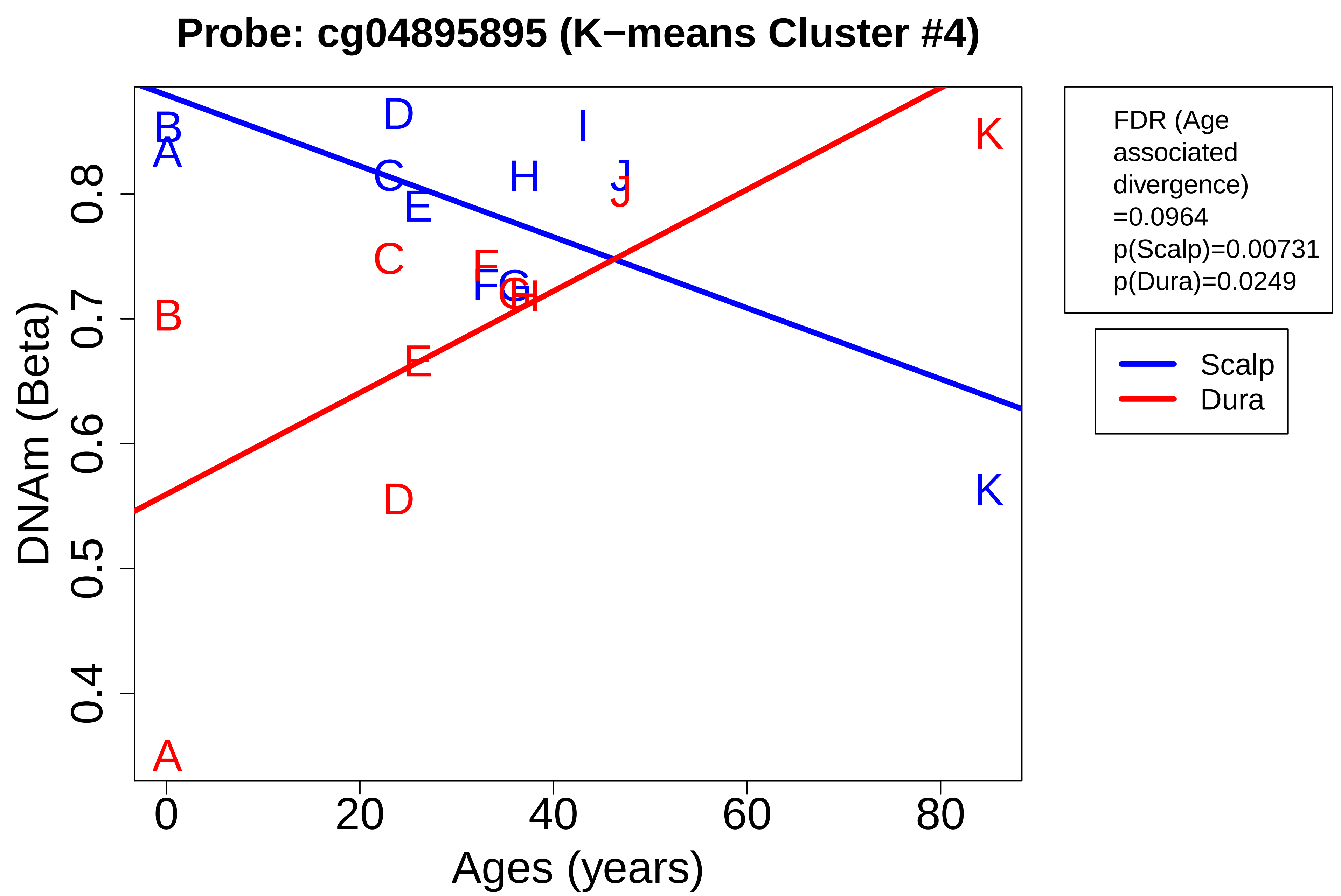

Probe: cg04895895 (K−means Cluster #4)
DNAm (Beta)
0.4
0.5
0.6
0.7
0.8
Ages (years)
0
20
40
60
80
FDR (Age associated divergence) =0.0964
p(Scalp)=0.00731
p(Dura)=0.0249
Scalp
Dura

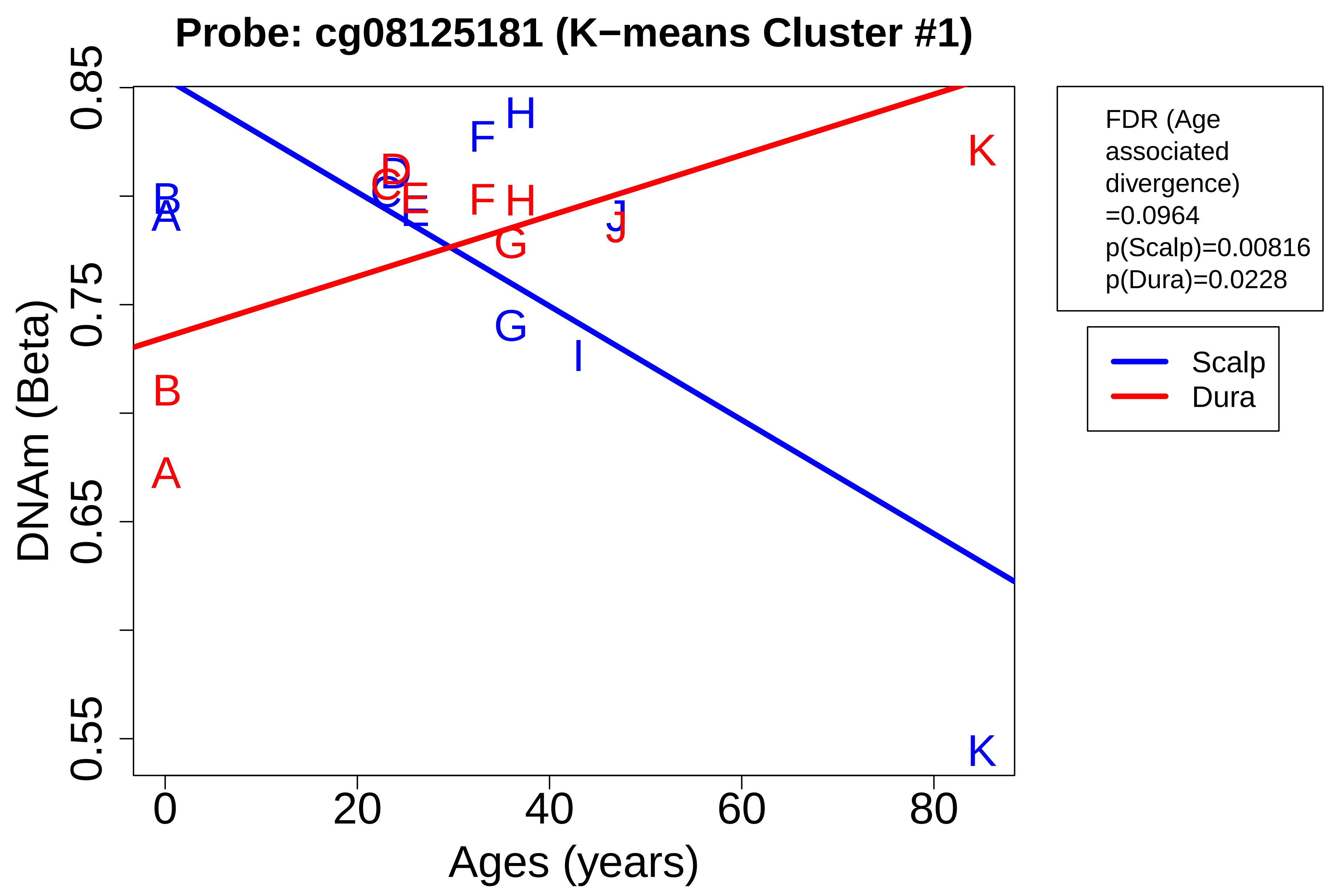

Probe: cg08125181 (K−means Cluster #1)
DNAm (Beta)
0.55
0.65
0.75
0.85
0
20
40
60
80
Ages (years)
A
B
C
D
E
F
G
H
I
J
K
FDR (Age associated divergence) =0.0964
p(Scalp)=0.00816
p(Dura)=0.0228
Scalp
Dura

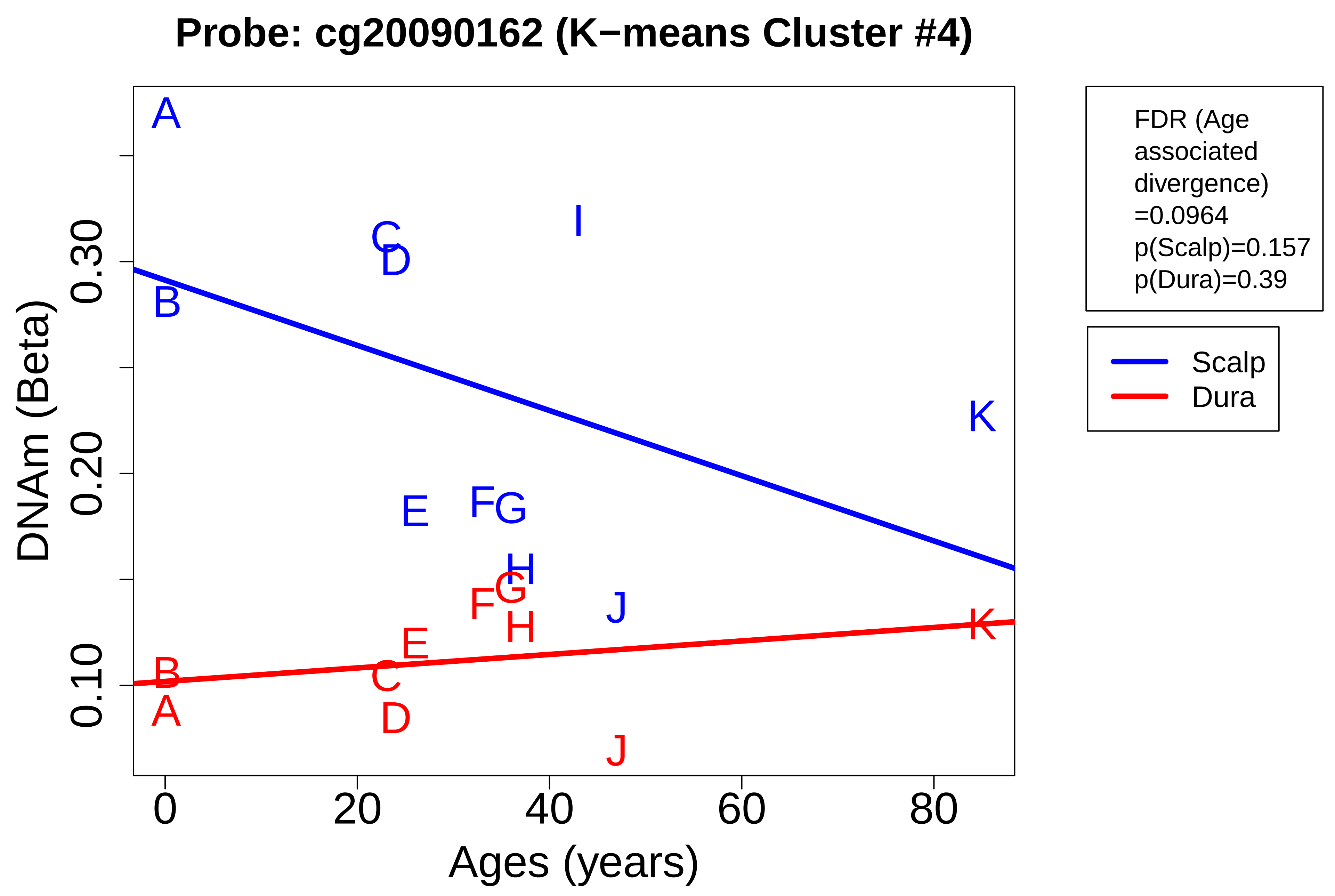

Probe: cg20090162 (K−means Cluster #4)
DNAm (Beta)
0.10
0.20
0.30
Ages (years)
0
20
40
60
80
FDR (Age associated divergence) =0.0964
p(Scalp)=0.157
p(Dura)=0.39
Scalp
Dura
A
B
C
D
E
F
G
H
I
J
K

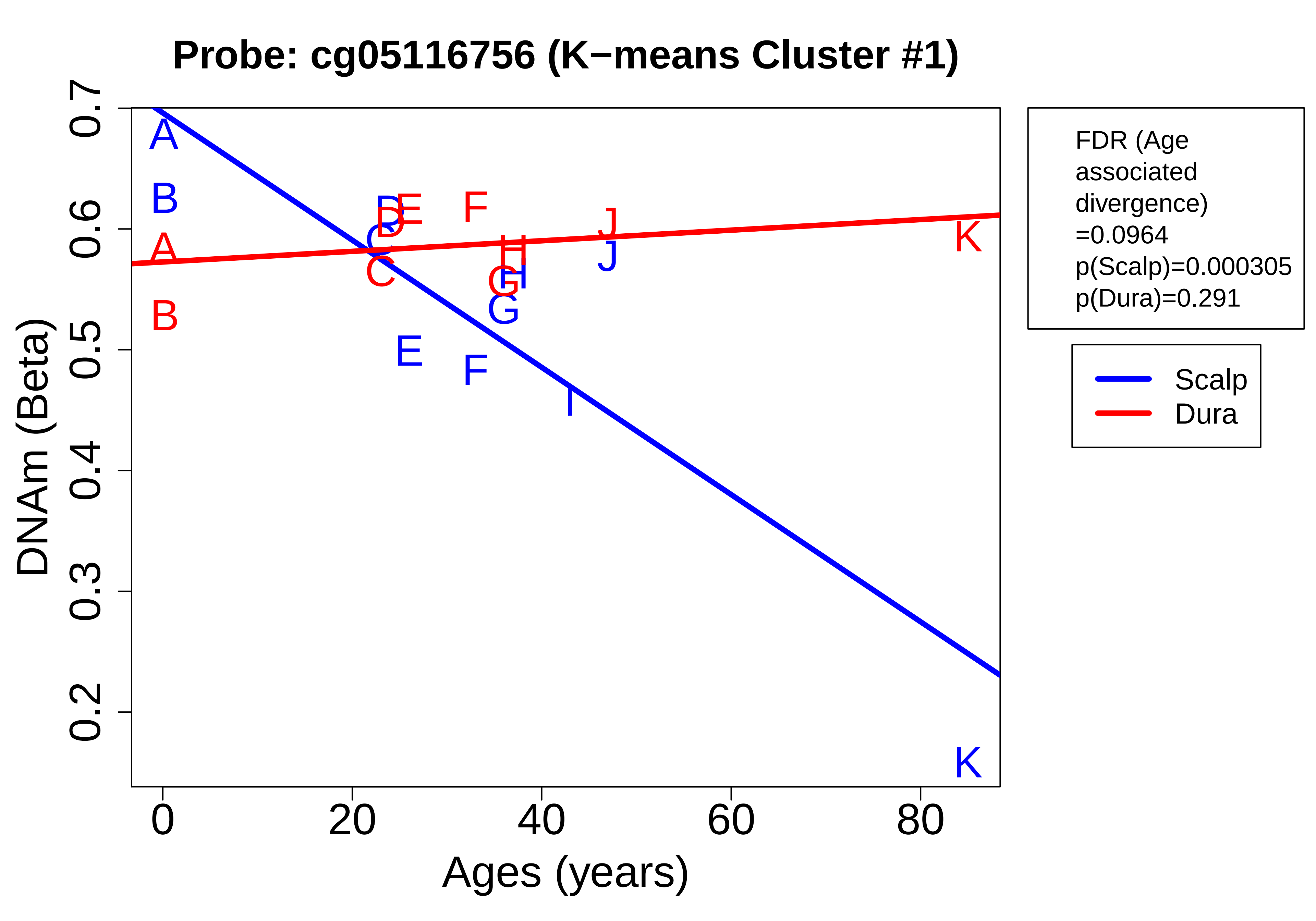

Probe: cg05116756 (K−means Cluster #1)
FDR (Age associated divergence) =0.0964
p(Scalp)=0.000305
p(Dura)=0.291
Scalp
Dura
DNAm (Beta)
0.2
0.3
0.4
0.5
0.6
0.7
Ages (years)
0
20
40
60
80
A
B
C
D
E
F
G
H
I
J
K

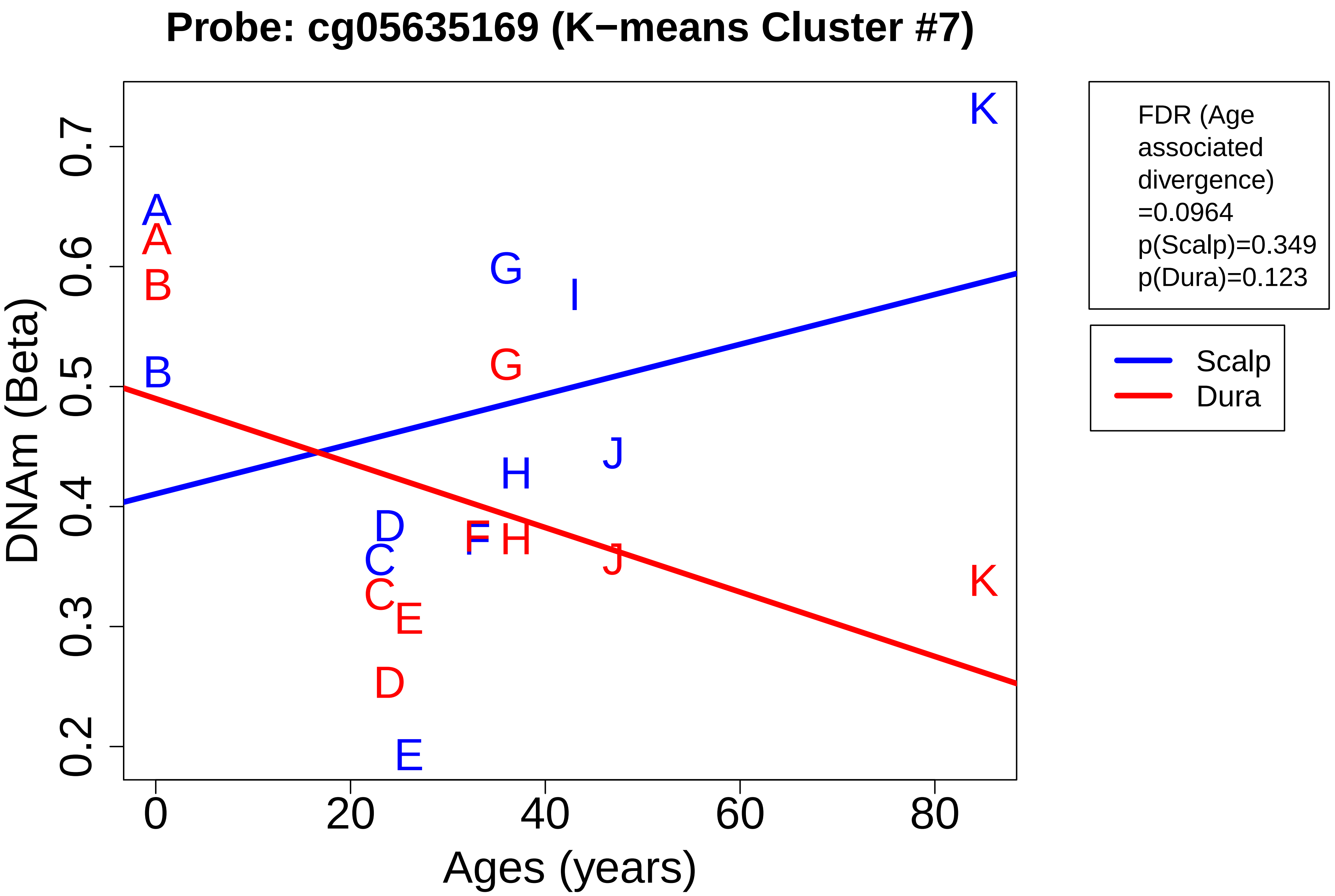
Probe: cg05635169 (K−means Cluster #7)
DNAm (Beta)
Ages (years)
0.2
0.3
0.4
0.5
0.6
0.7
0
20
40
60
80
A
B
C
D
E
F
G
H
I
J
K
FDR (Age associated divergence) =0.0964
p(Scalp)=0.349
p(Dura)=0.123
Scalp
Dura

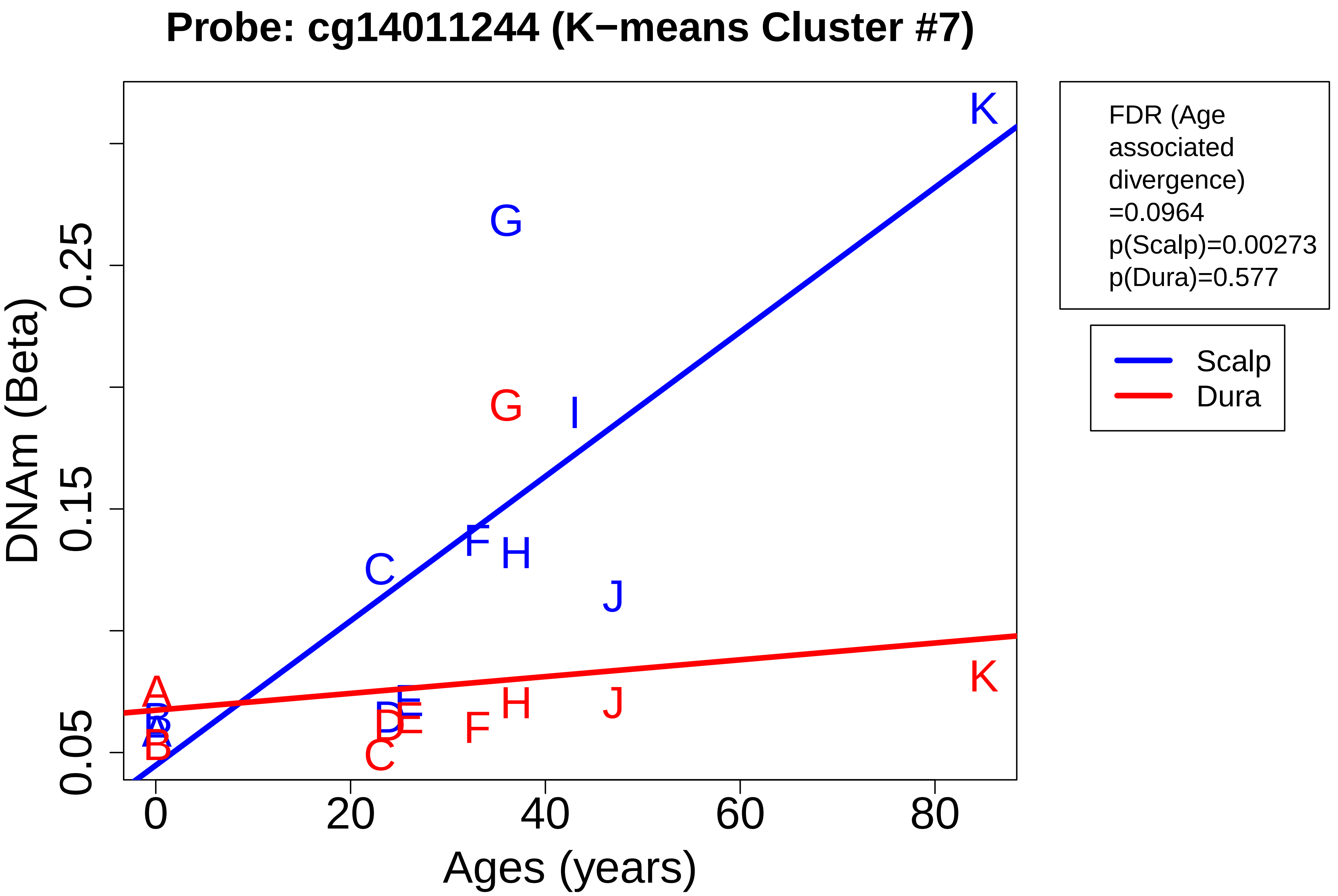

Probe: cg14011244 (K−means Cluster #7)
DNAm (Beta)
Ages (years)
FDR (Age associated divergence) =0.0964
p(Scalp)=0.00273
p(Dura)=0.577
Scalp
Dura

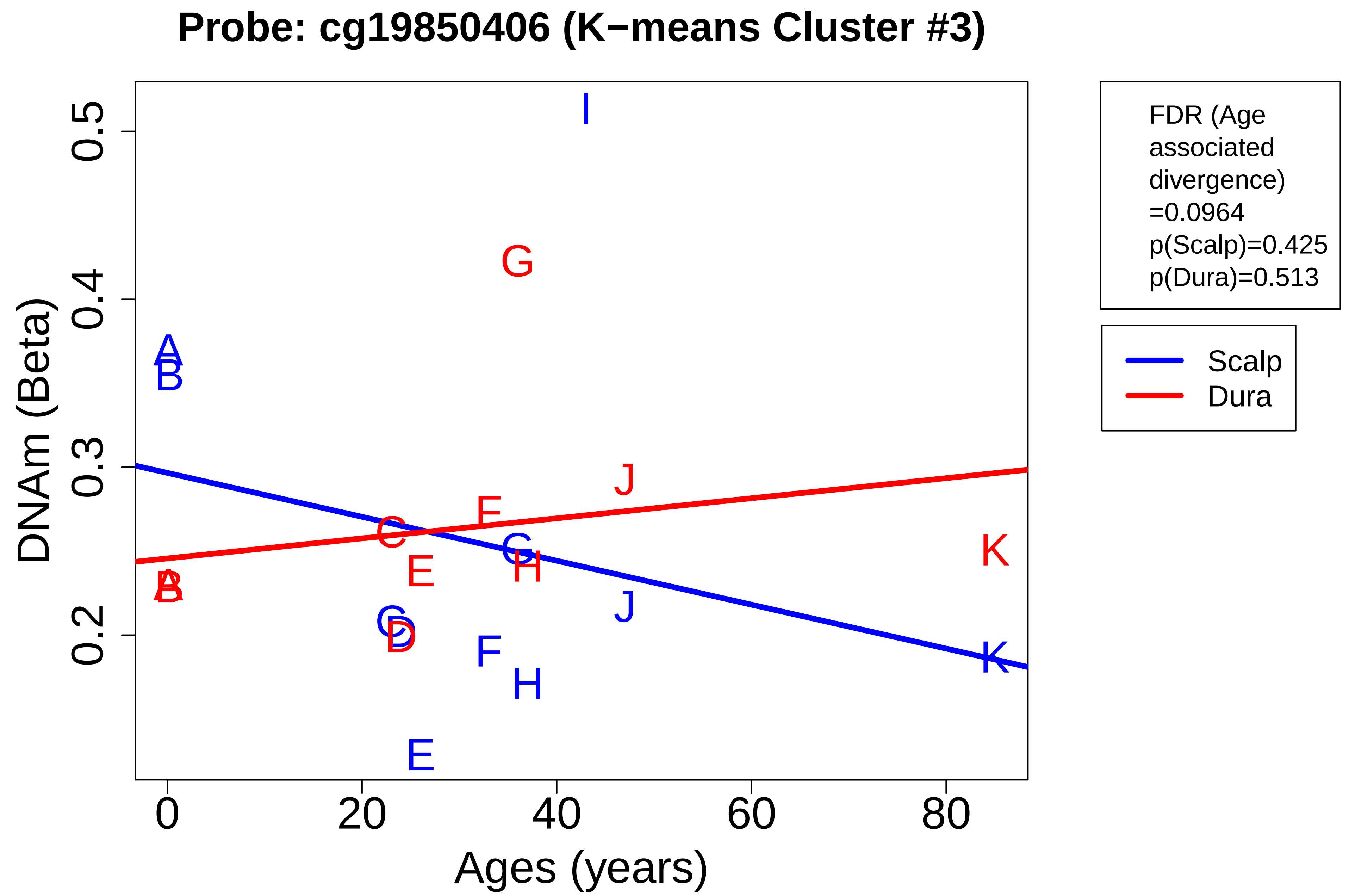

Probe: cg19850406 (K−means Cluster #3)
DNAm (Beta)
0.2
0.3
0.4
0.5
Ages (years)
0
20
40
60
80
FDR (Age associated divergence) =0.0964
p(Scalp)=0.425
p(Dura)=0.513
Scalp
Dura

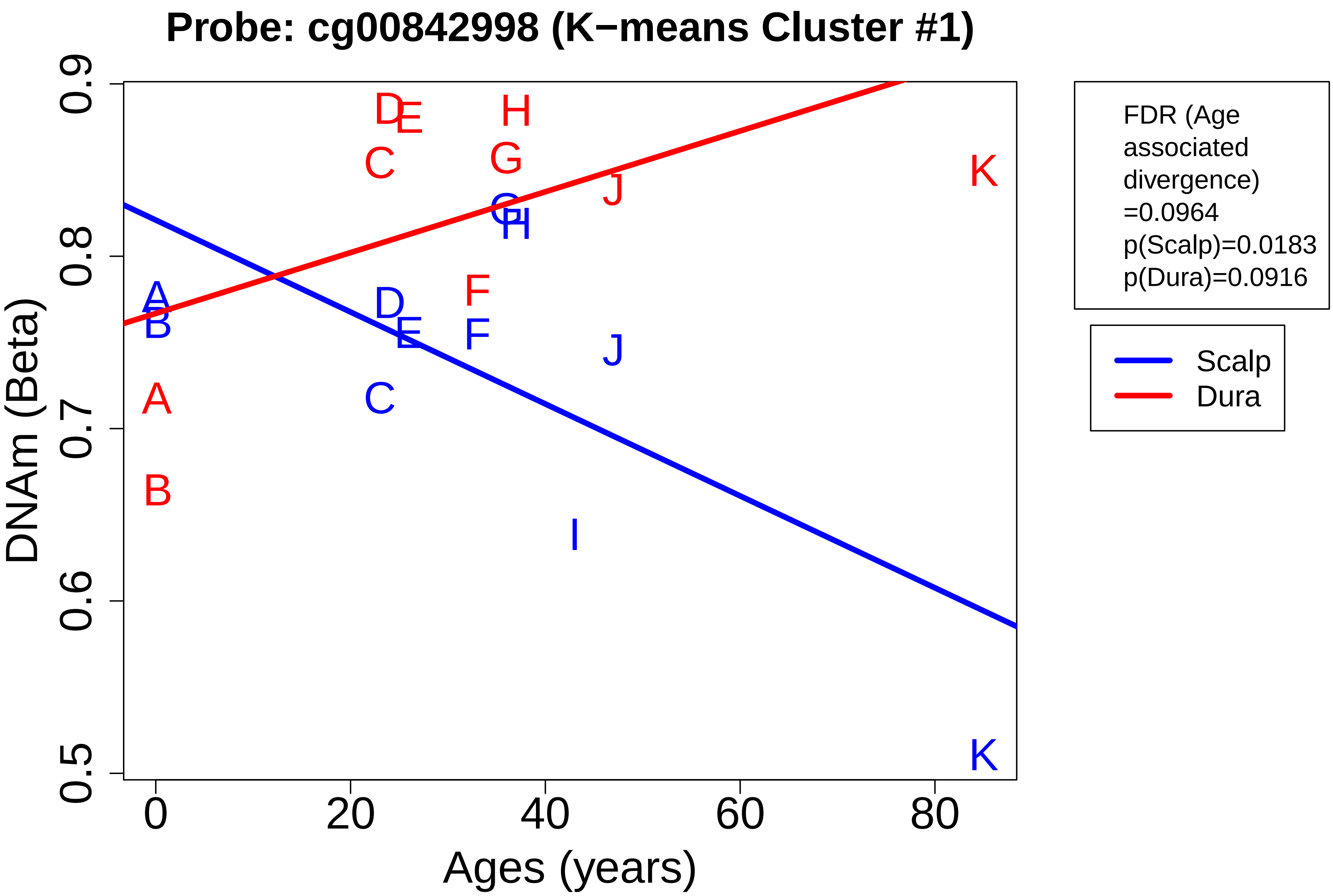

Probe: cg00842998 (K−means Cluster #1)
DNAm (Beta)
0.5
0.6
0.7
0.8
0.9
Ages (years)
0
20
40
60
80
A B C D E F G H I J K
A B C D E F G H J K
FDR (Age associated divergence) =0.0964
p(Scalp)=0.0183
p(Dura)=0.0916
Scalp
Dura

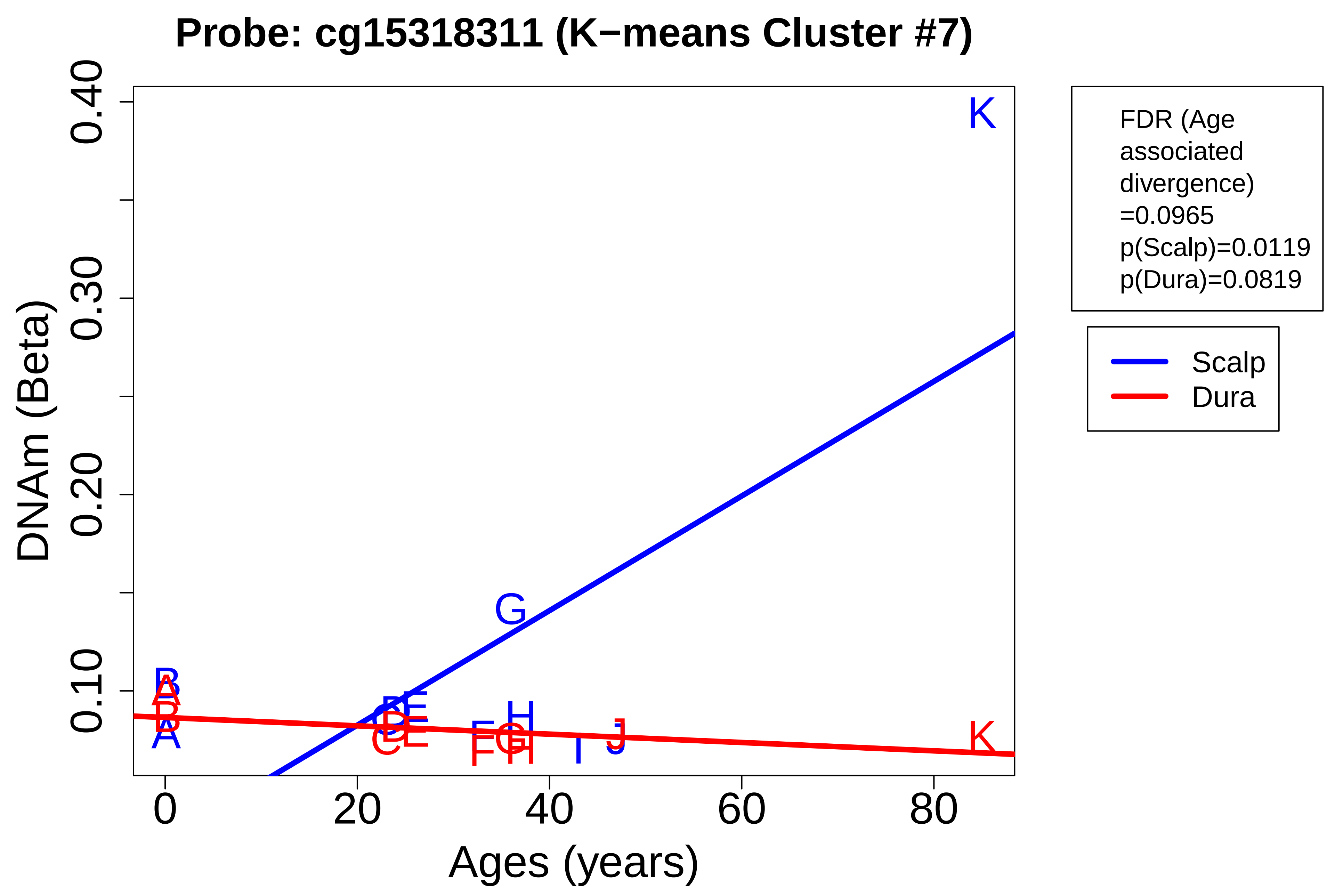

Probe: cg15318311 (K−means Cluster #7)
DNAm (Beta)
0.10
0.20
0.30
0.40
0
20
40
60
80
Ages (years)
FDR (Age associated divergence) =0.0965
p(Scalp)=0.0119
p(Dura)=0.0819
Scalp
Dura

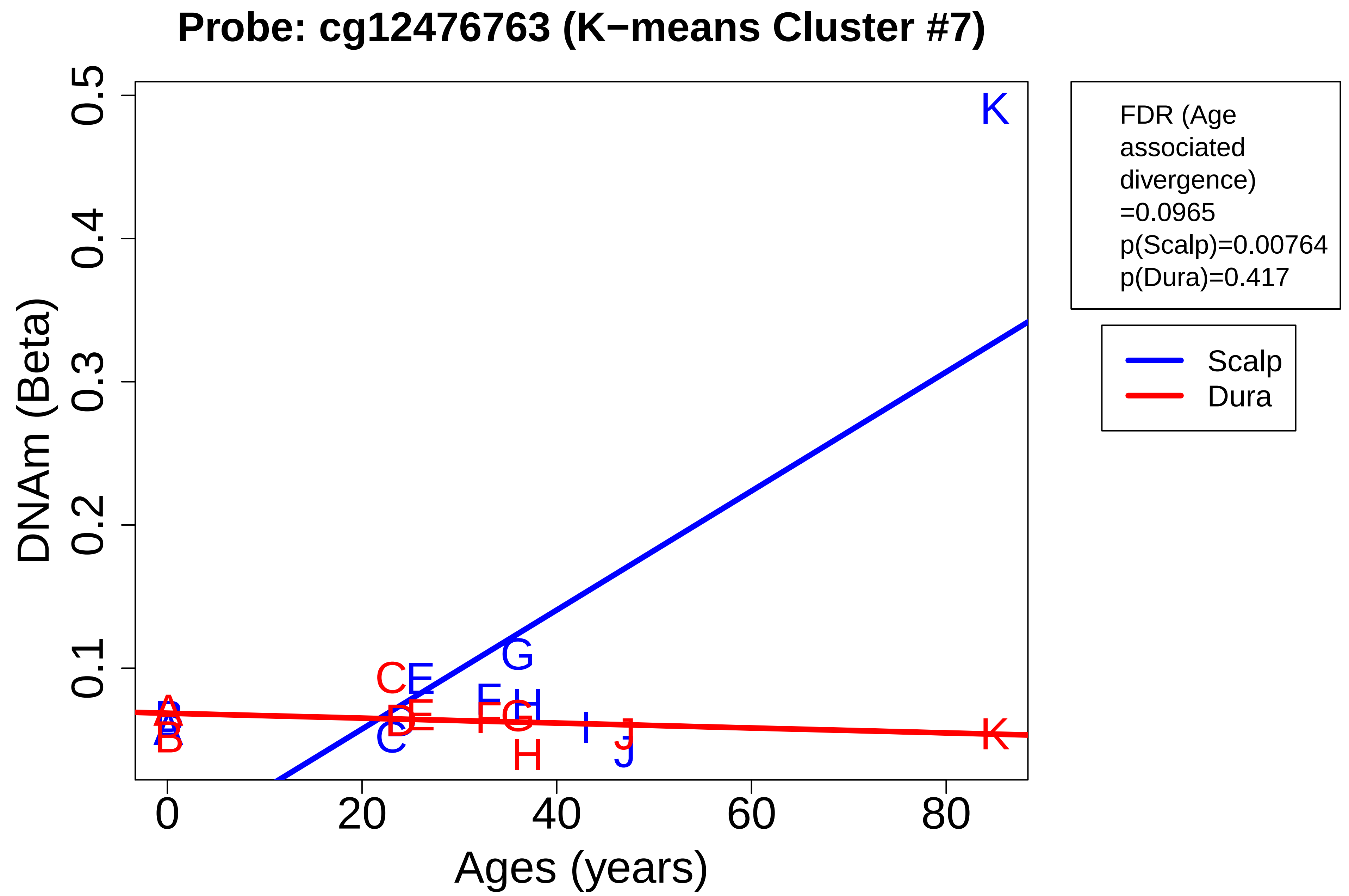

Probe: cg12476763 (K−means Cluster #7)
DNAm (Beta)
0.1
0.2
0.3
0.4
0.5
Ages (years)
0
20
40
60
80
FDR (Age associated divergence) =0.0965
p(Scalp)=0.00764
p(Dura)=0.417
Scalp
Dura

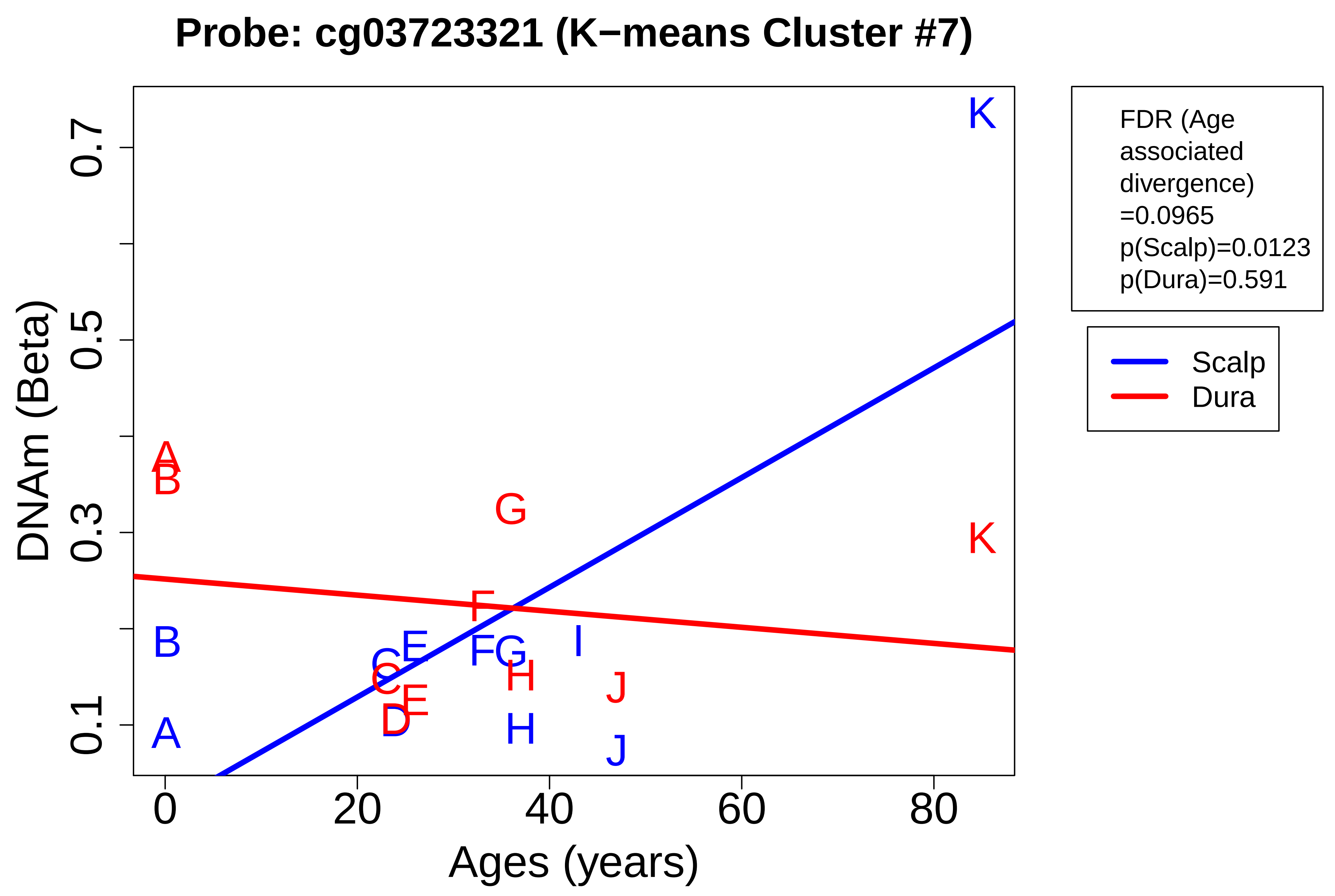

Probe: cg03723321 (K−means Cluster #7)
DNAm (Beta)
0.1
0.3
0.5
0.7
Ages (years)
0
20
40
60
80
FDR (Age associated divergence) =0.0965
p(Scalp)=0.0123
p(Dura)=0.591
Scalp
Dura

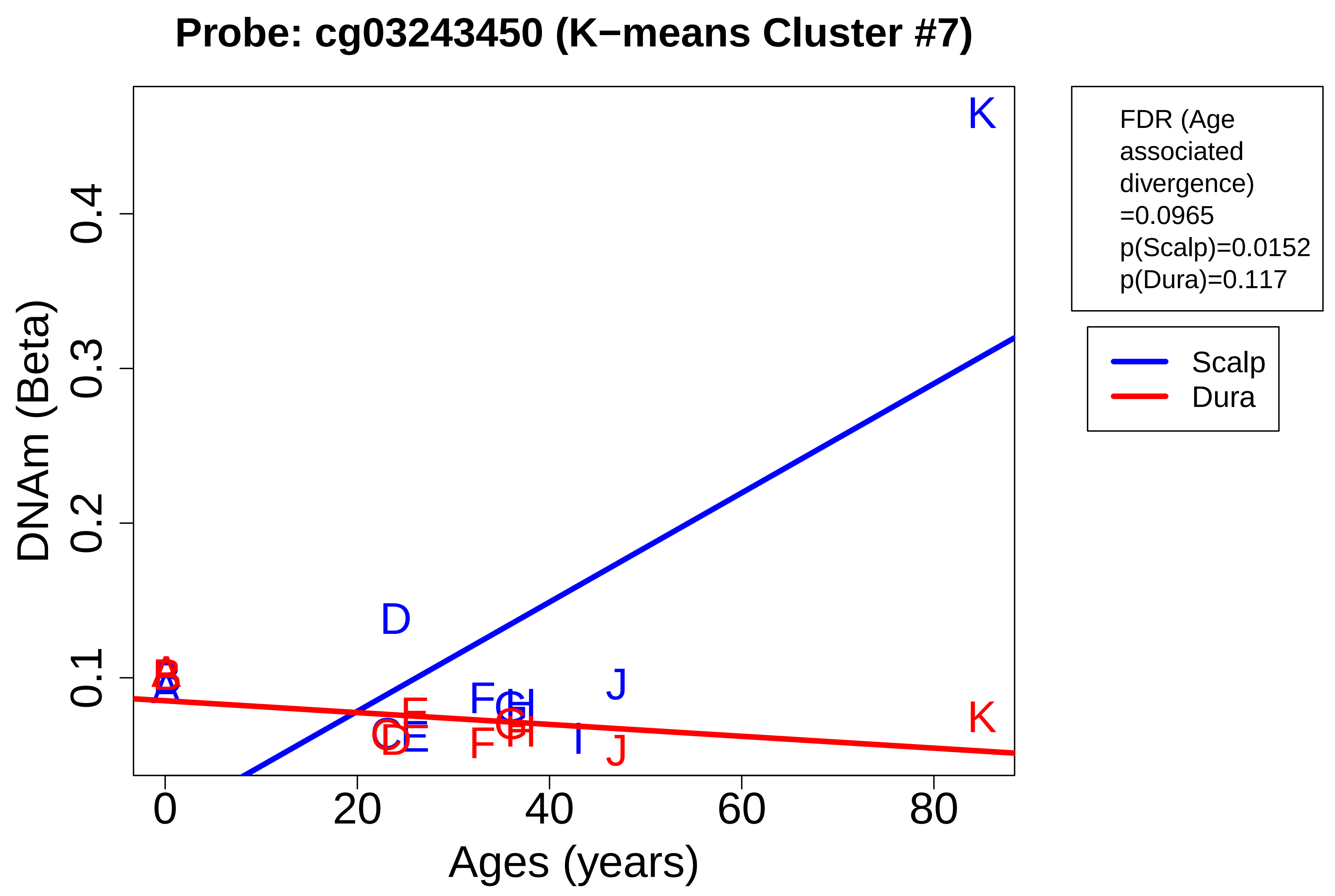

Probe: cg03243450 (K−means Cluster #7)
DNAm (Beta)
0.1
0.2
0.3
0.4
0
20
40
60
80
Ages (years)
FDR (Age associated divergence) =0.0965
p(Scalp)=0.0152
p(Dura)=0.117
Scalp
Dura

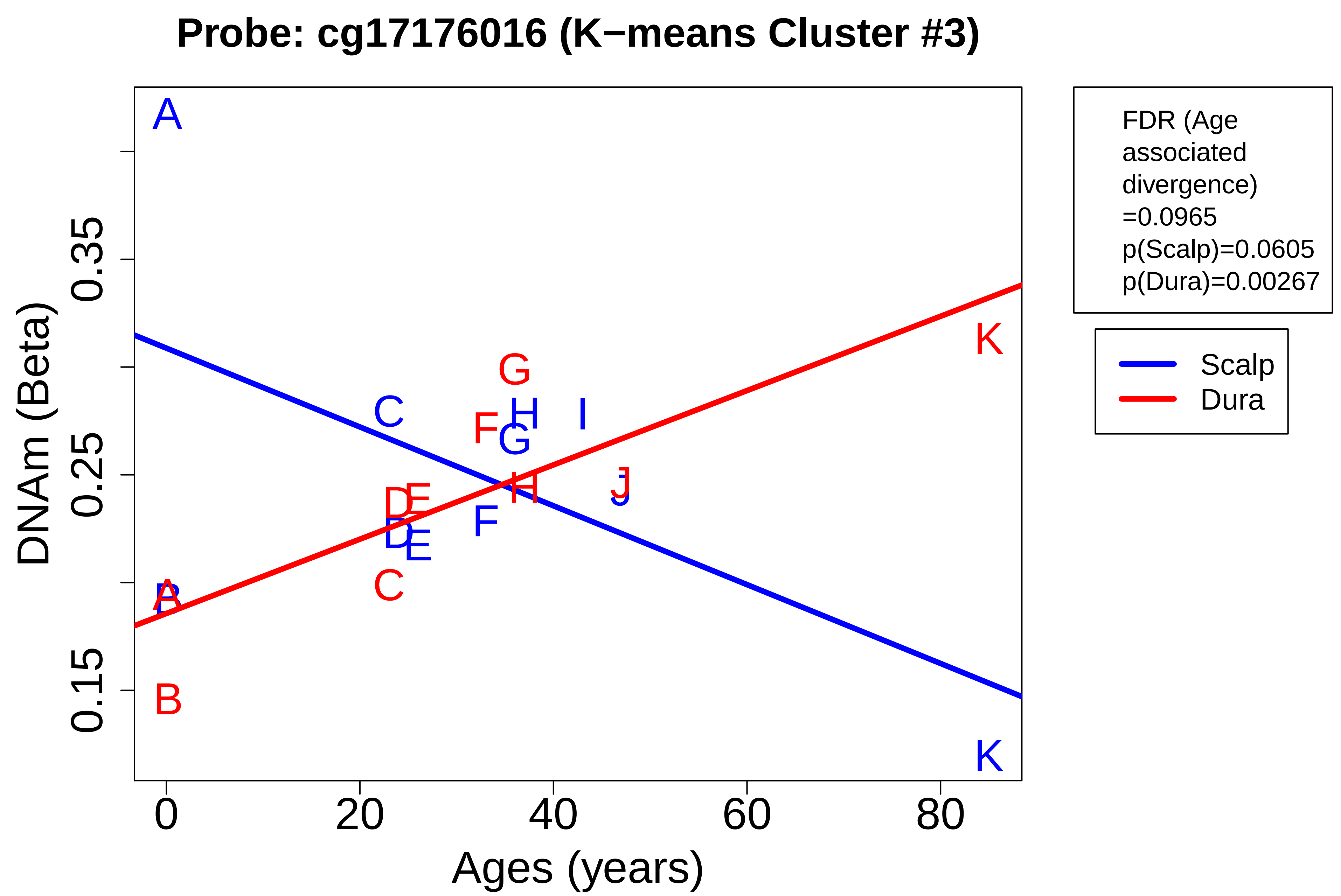

Probe: cg17176016 (K−means Cluster #3)
DNAm (Beta)
0.15
0.25
0.35
Ages (years)
0
20
40
60
80
FDR (Age associated divergence) =0.0965
p(Scalp)=0.0605
p(Dura)=0.00267
Scalp
Dura

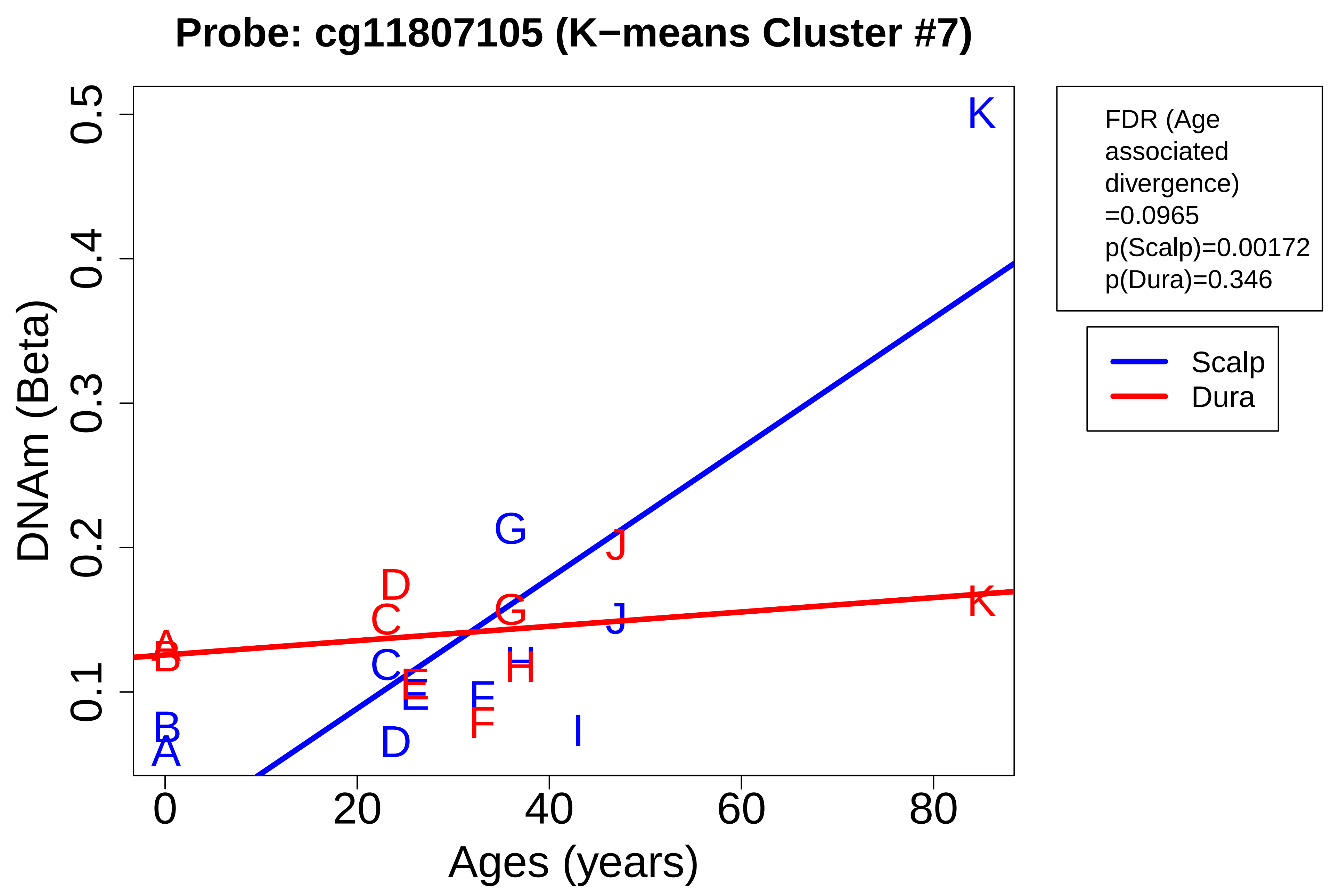

Probe: cg11807105 (K−means Cluster #7)
DNAm (Beta)
0.1
0.2
0.3
0.4
0.5
0
20
40
60
80
Ages (years)
FDR (Age associated divergence) =0.0965
p(Scalp)=0.00172
p(Dura)=0.346
Scalp
Dura

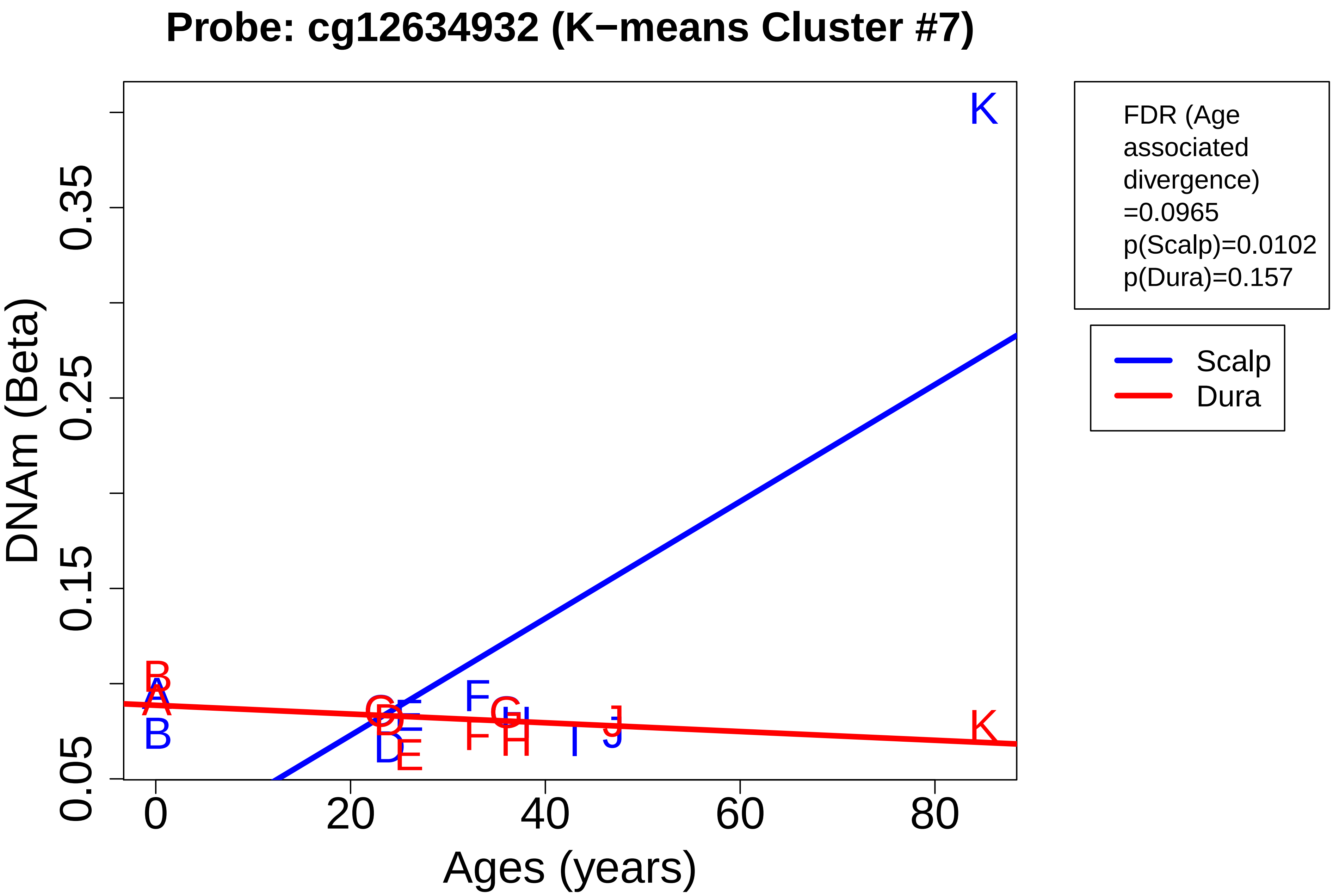

Probe: cg12634932 (K−means Cluster #7)
DNAm (Beta)
0.05
0.15
0.25
0.35
Ages (years)
0
20
40
60
80
FDR (Age associated divergence) =0.0965
p(Scalp)=0.0102
p(Dura)=0.157
Scalp
Dura

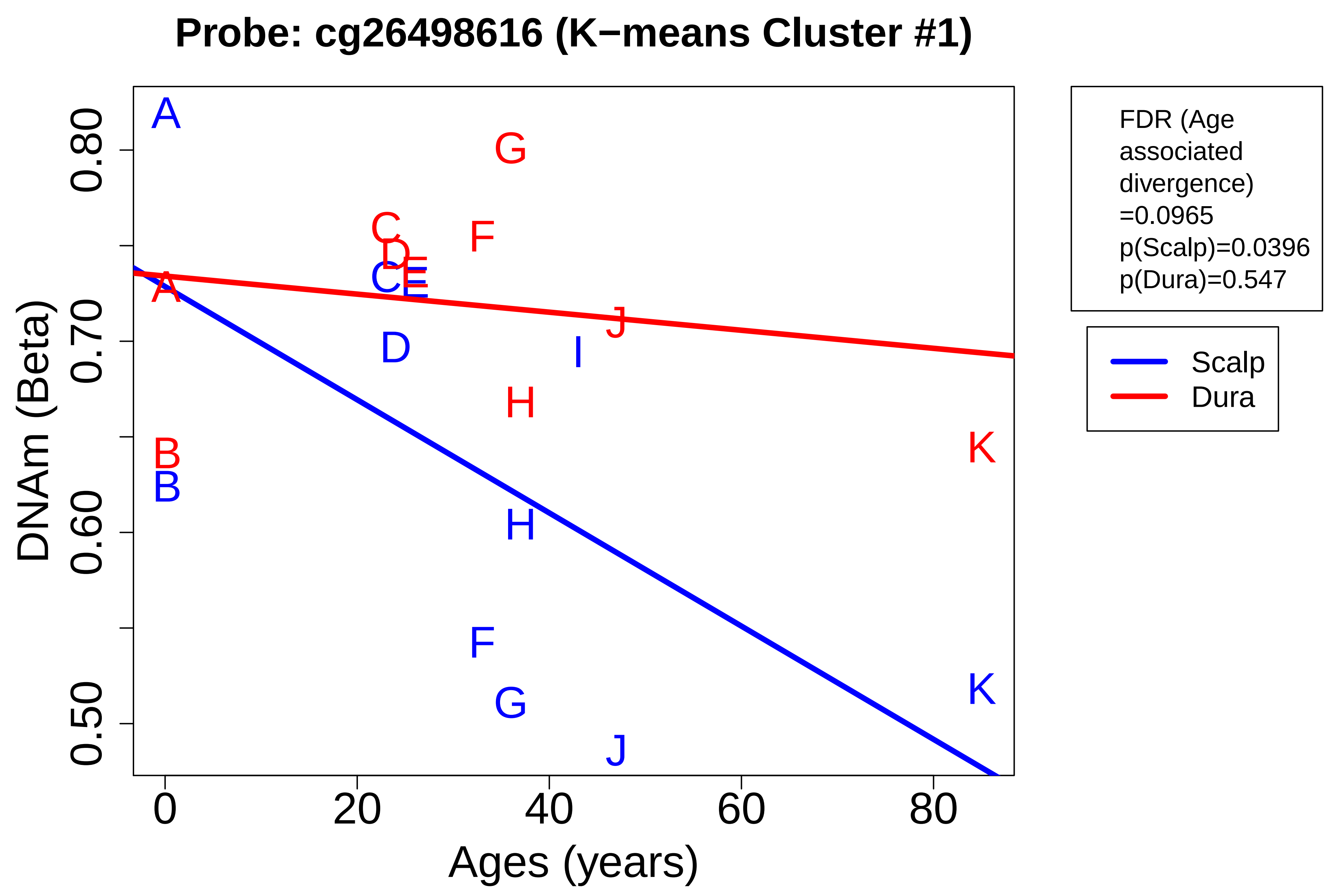

Probe: cg26498616 (K−means Cluster #1)
DNAm (Beta)
0.50
0.60
0.70
0.80
0
20
40
60
80
Ages (years)
A
B
C
D
E
F
G
H
I
J
K
FDR (Age associated divergence) =0.0965
p(Scalp)=0.0396
p(Dura)=0.547
Scalp
Dura

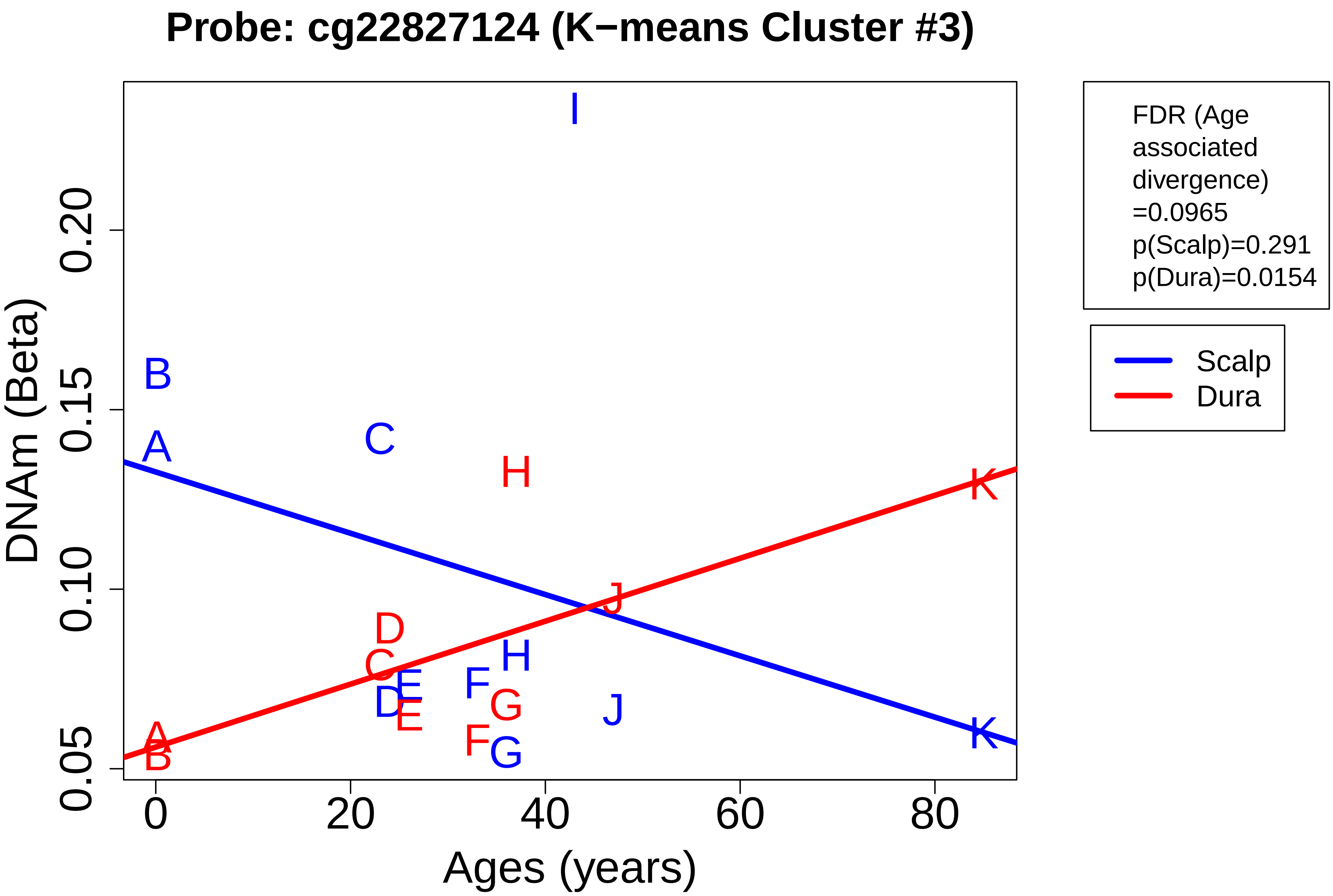

Probe: cg22827124 (K−means Cluster #3)
DNAm (Beta)
0.05
0.10
0.15
0.20
0
20
40
60
80
Ages (years)
FDR (Age associated divergence) =0.0965
p(Scalp)=0.291
p(Dura)=0.0154
Scalp
Dura

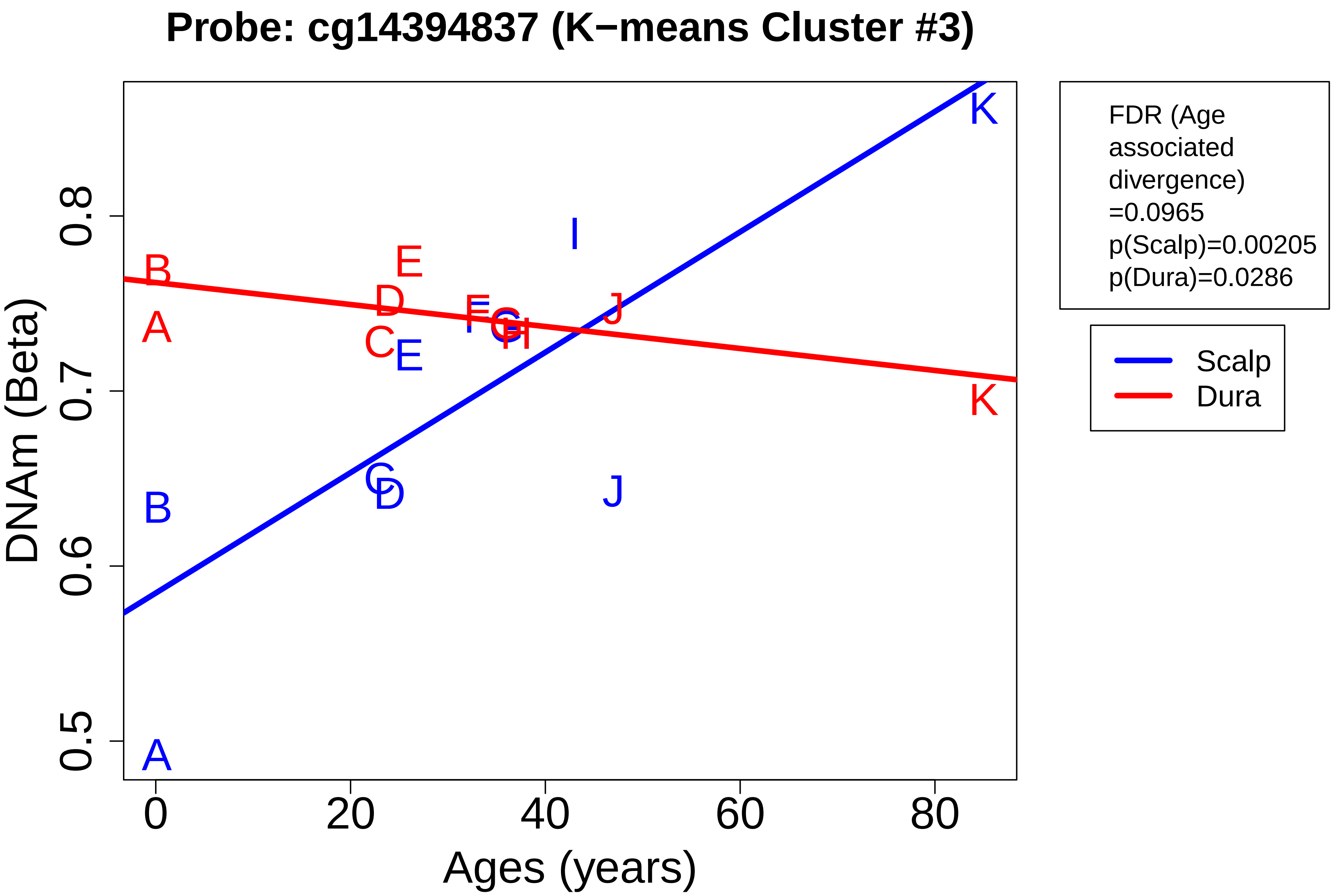

Probe: cg14394837 (K−means Cluster #3)
DNAm (Beta)
0.5
0.6
0.7
0.8
Ages (years)
0
20
40
60
80
FDR (Age associated divergence) =0.0965
p(Scalp)=0.00205
p(Dura)=0.0286
Scalp
Dura

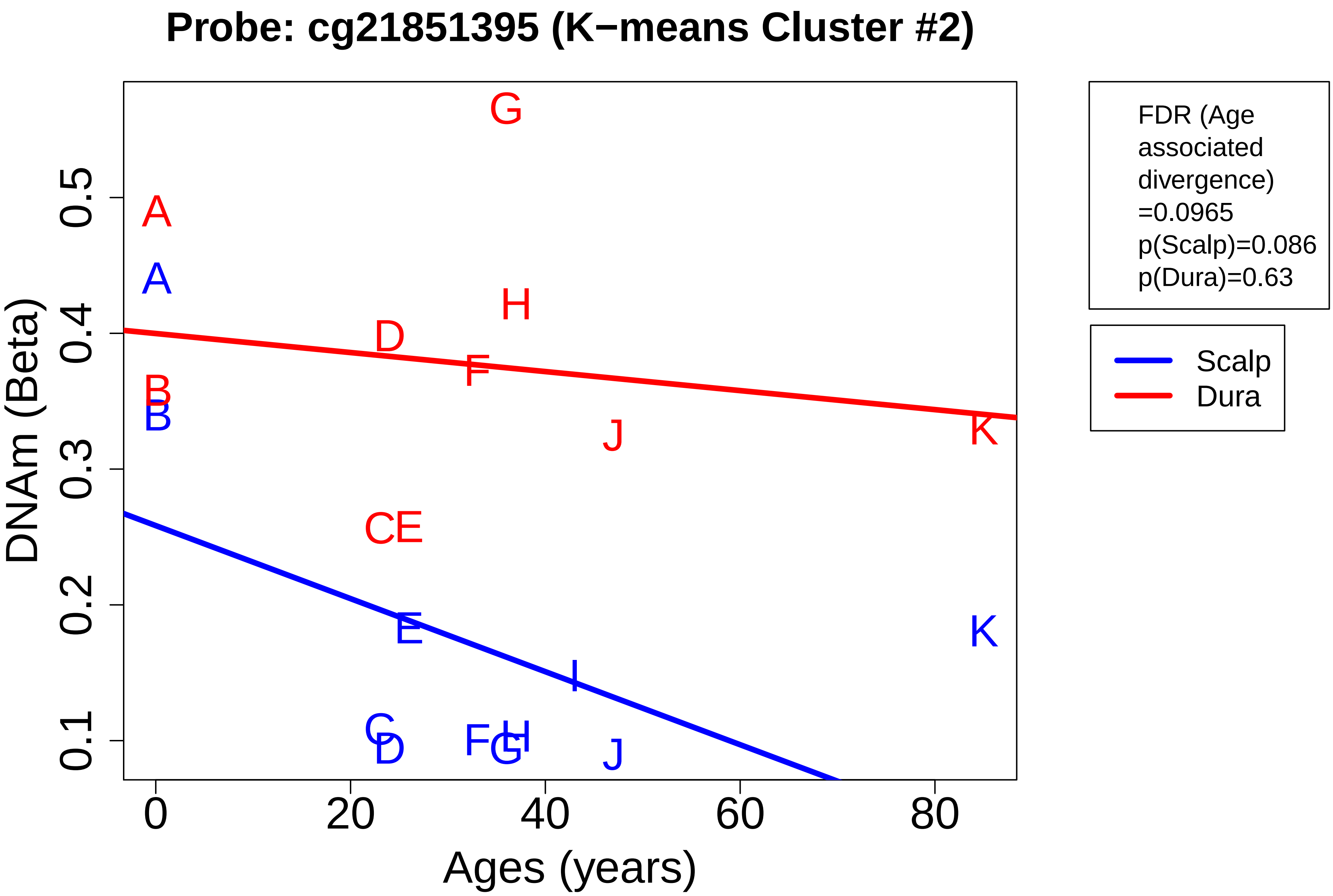

Probe: cg21851395 (K−means Cluster #2)
DNAm (Beta)
0.1
0.2
0.3
0.4
0.5
0
20
40
60
80
Ages (years)
FDR (Age associated divergence) =0.0965
p(Scalp)=0.086
p(Dura)=0.63
Scalp
Dura

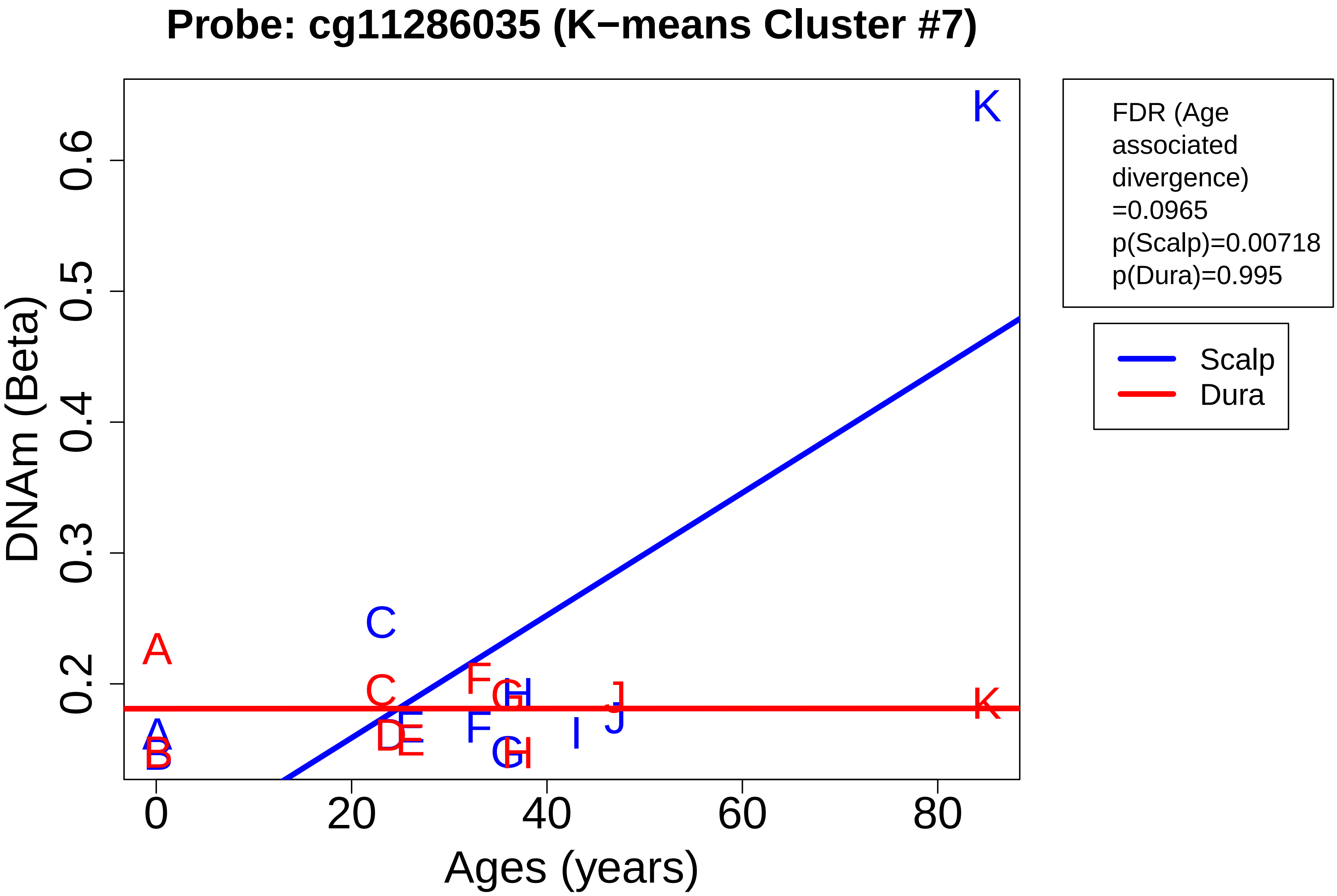

Probe: cg11286035 (K−means Cluster #7)
DNAm (Beta)
0.2
0.3
0.4
0.5
0.6
0
20
40
60
80
Ages (years)
FDR (Age associated divergence) =0.0965
p(Scalp)=0.00718
p(Dura)=0.995
Scalp
Dura

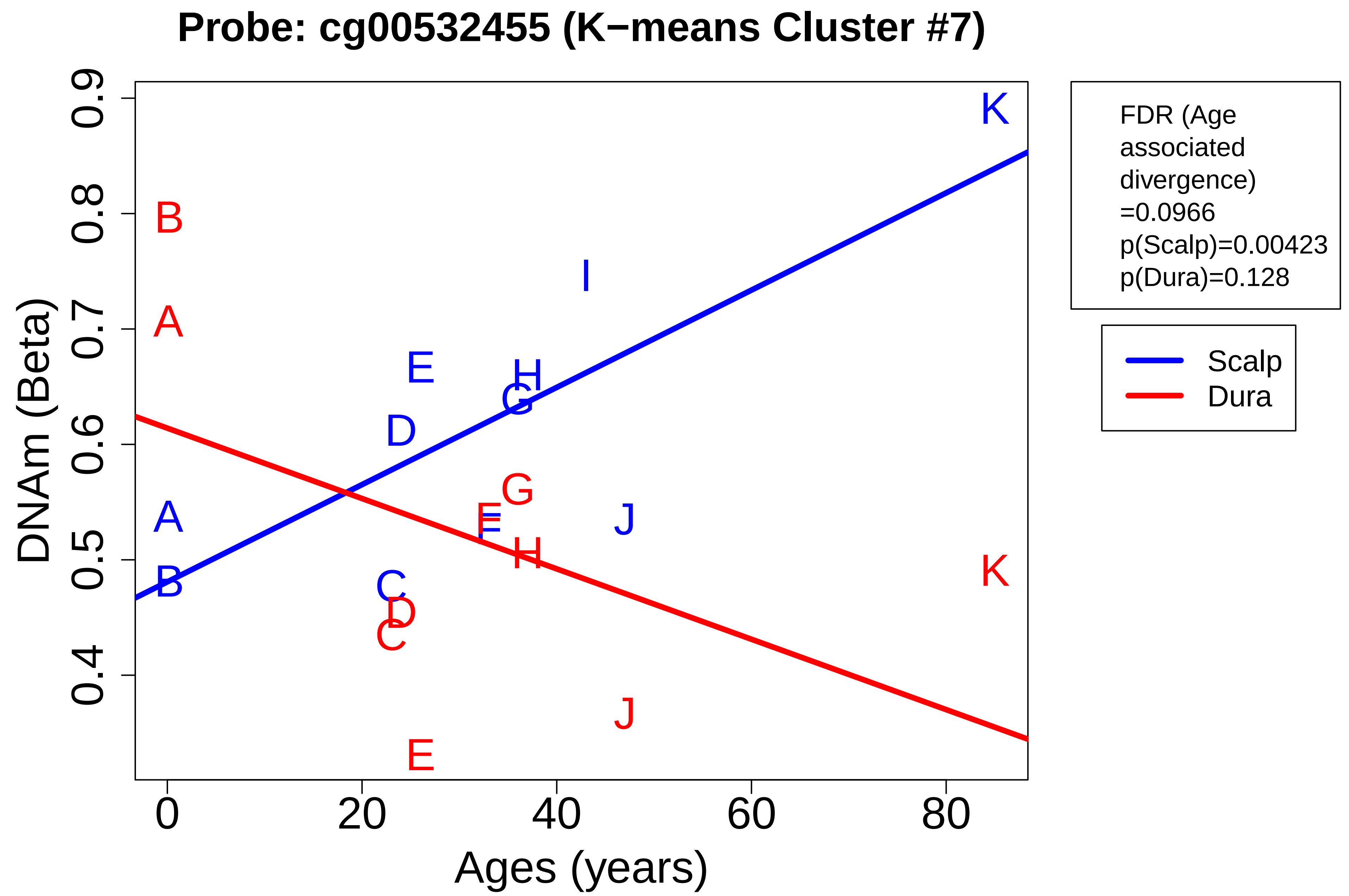

Probe: cg00532455 (K−means Cluster #7)
DNAm (Beta)
0.4
0.5
0.6
0.7
0.8
0.9
Ages (years)
0
20
40
60
80
FDR (Age associated divergence) =0.0966
p(Scalp)=0.00423
p(Dura)=0.128
Scalp
Dura

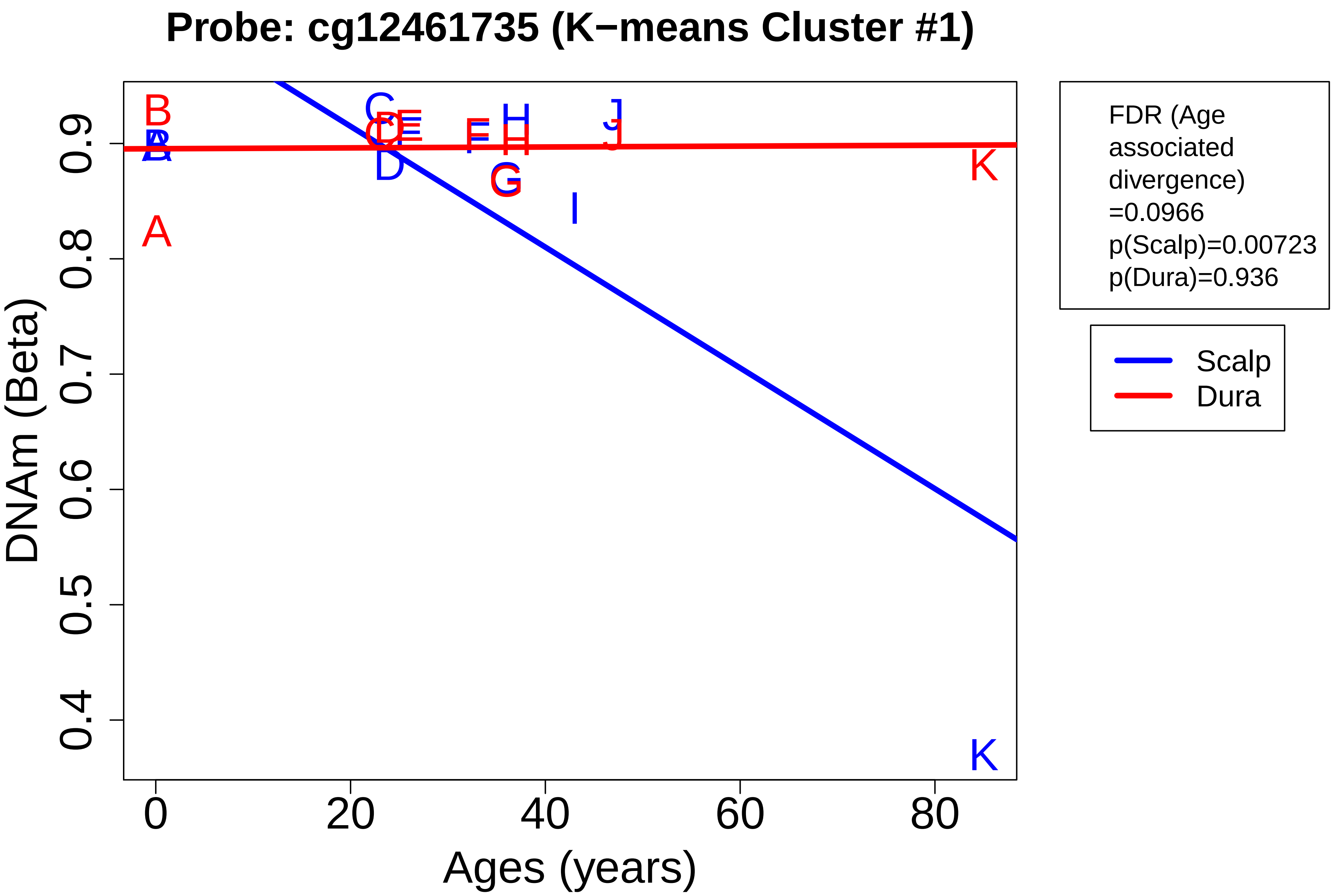

Probe: cg12461735 (K−means Cluster #1)
DNAm (Beta)
0.4
0.5
0.6
0.7
0.8
0.9
Ages (years)
0
20
40
60
80
FDR (Age associated divergence) =0.0966
p(Scalp)=0.00723
p(Dura)=0.936
Scalp
Dura

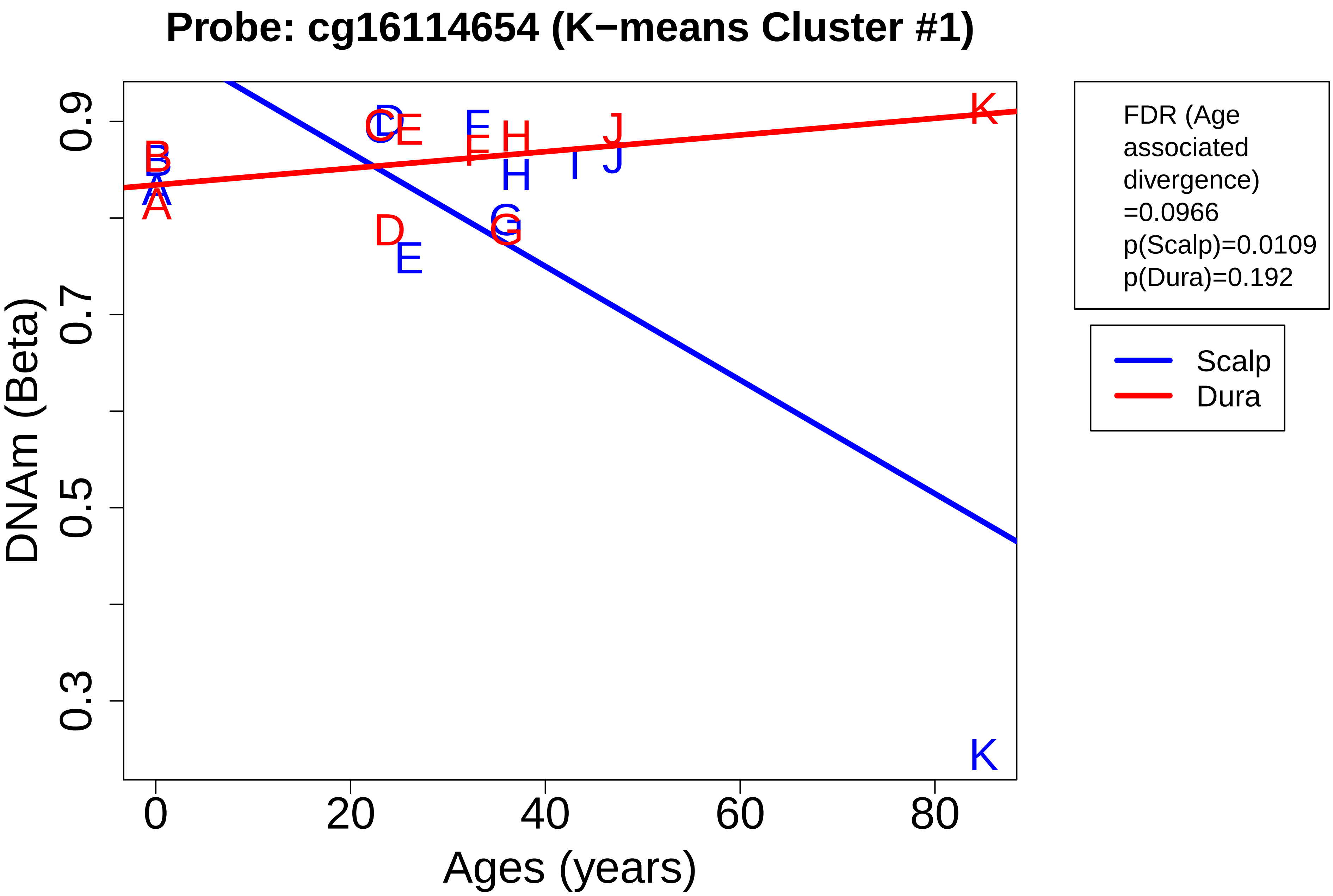
Probe: cg16114654 (K−means Cluster #1)
DNAm (Beta)
0.3
0.5
0.7
0.9
0
20
40
60
80
Ages (years)
FDR (Age associated divergence) =0.0966
p(Scalp)=0.0109
p(Dura)=0.192
Scalp
Dura

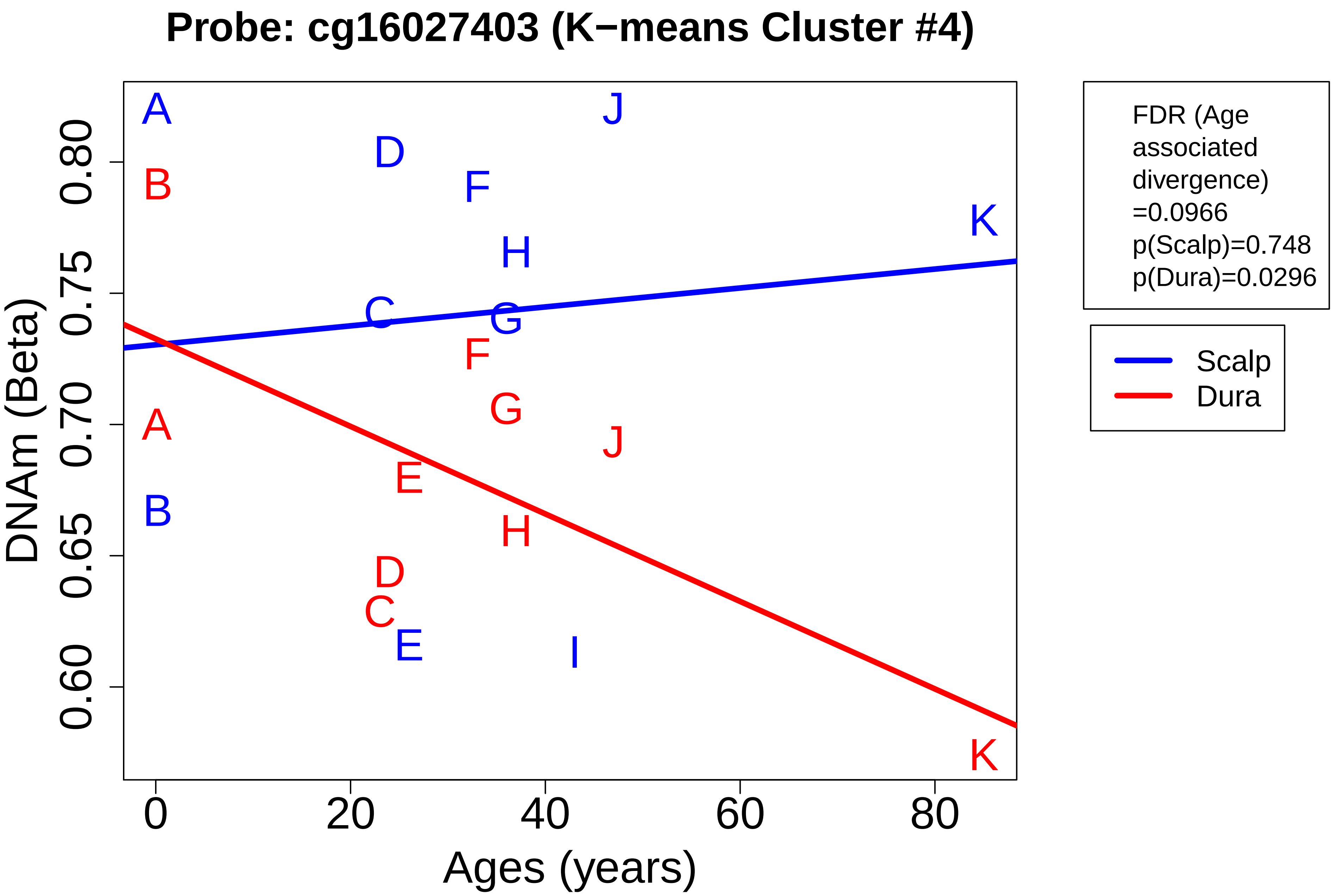

Probe: cg16027403 (K−means Cluster #4)
DNAm (Beta)
0.60
0.65
0.70
0.75
0.80
0
20
40
60
80
Ages (years)
A
B
C
D
E
F
G
H
I
J
K
FDR (Age associated divergence) =0.0966
p(Scalp)=0.748
p(Dura)=0.0296
Scalp
Dura

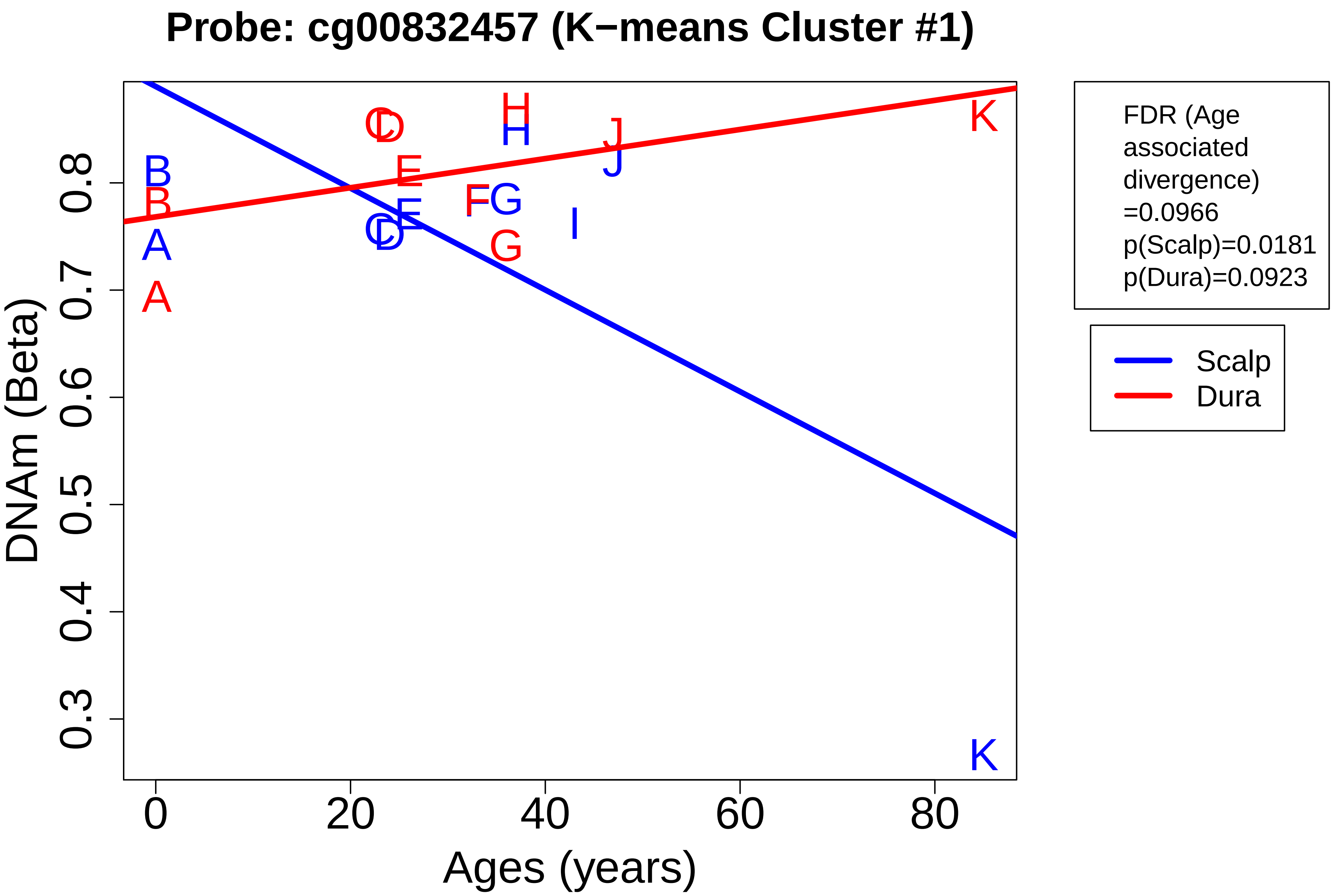

Probe: cg00832457 (K−means Cluster #1)
FDR (Age associated divergence) =0.0966
p(Scalp)=0.0181
p(Dura)=0.0923
Scalp
Dura
DNAm (Beta)
0.3
0.4
0.5
0.6
0.7
0.8
0
20
40
60
80
Ages (years)

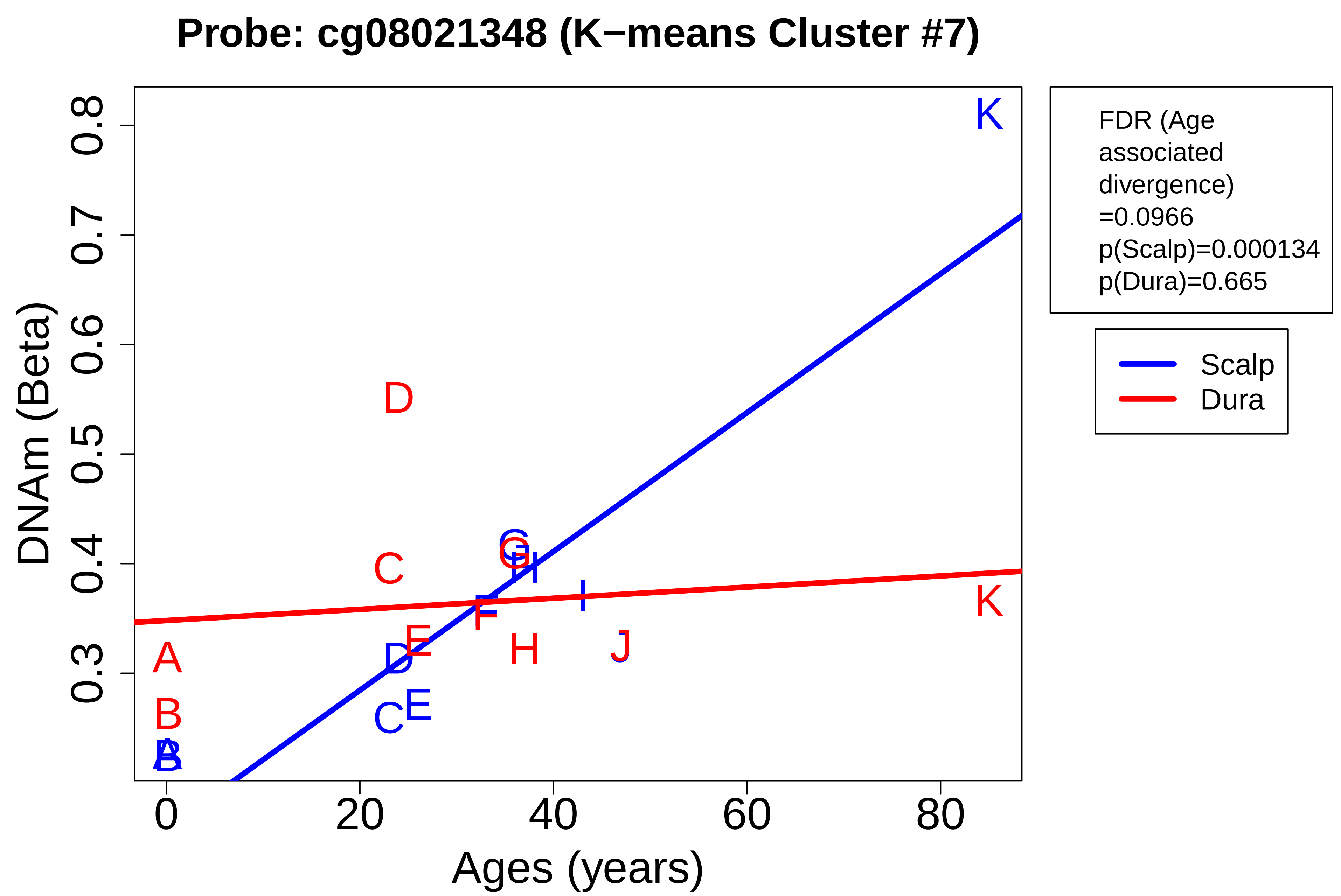

Probe: cg08021348 (K−means Cluster #7)
DNAm (Beta)
0.3
0.4
0.5
0.6
0.7
0.8
0
20
40
60
80
Ages (years)
FDR (Age associated divergence) =0.0966
p(Scalp)=0.000134
p(Dura)=0.665
Scalp
Dura

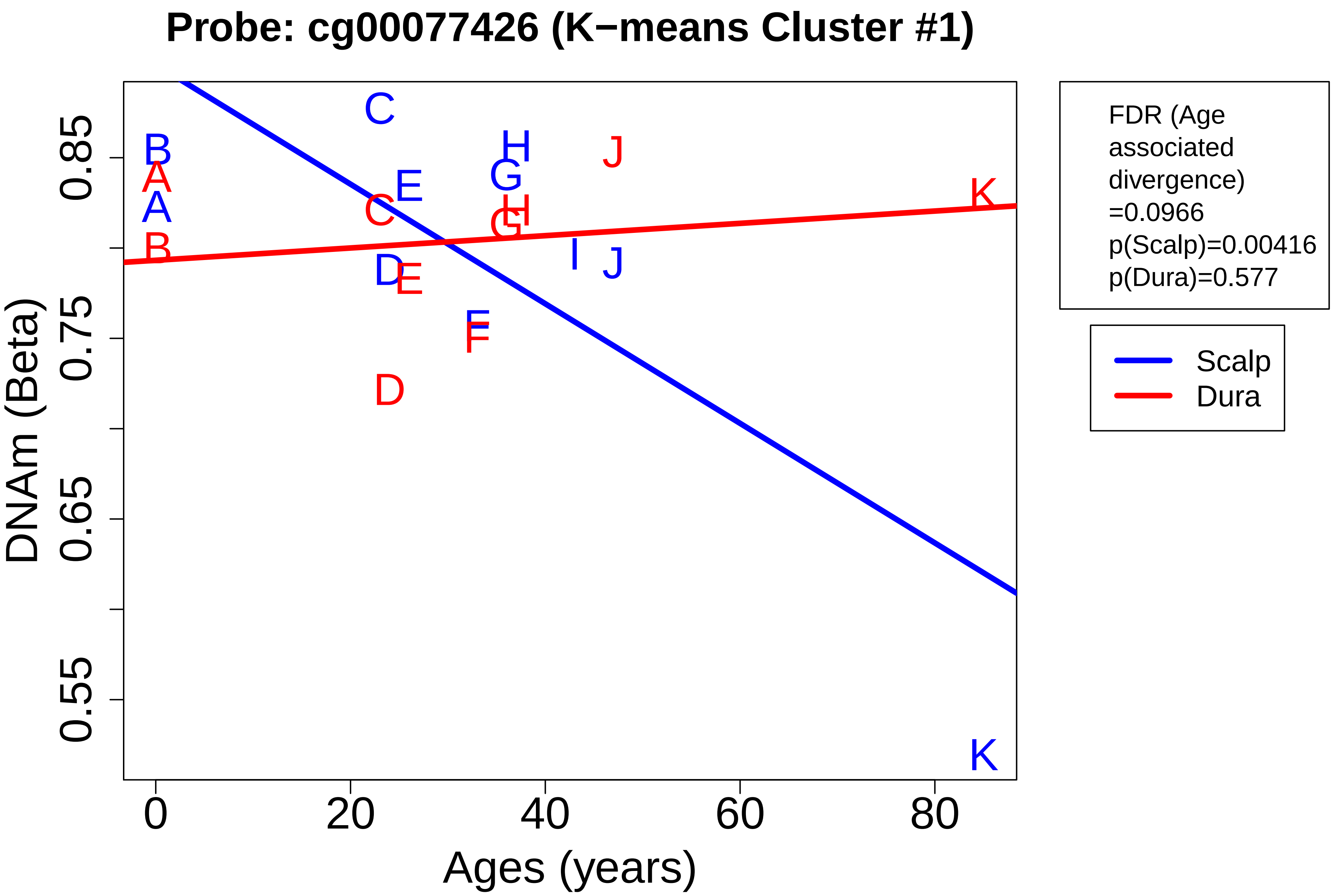

Probe: cg00077426 (K−means Cluster #1)
DNAm (Beta)
0.55
0.65
0.75
0.85
Ages (years)
0
20
40
60
80
FDR (Age associated divergence) =0.0966
p(Scalp)=0.00416
p(Dura)=0.577
Scalp
Dura
A
B
C
D
E
F
G
H
I
J
K

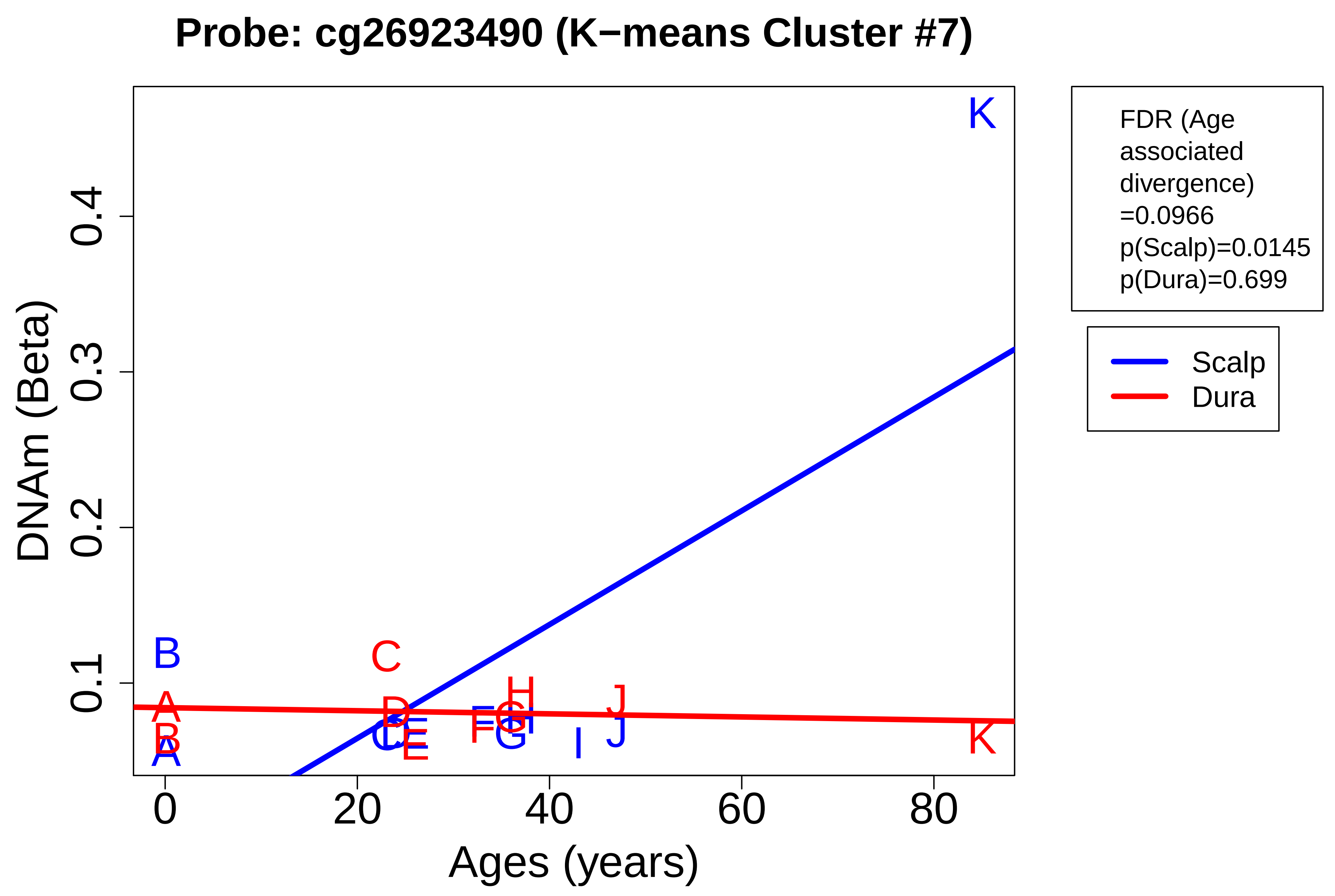

Probe: cg26923490 (K−means Cluster #7)
FDR (Age associated divergence) =0.0966
p(Scalp)=0.0145
p(Dura)=0.699
Scalp
Dura
DNAm (Beta)
0.1
0.2
0.3
0.4
0
20
40
60
80
Ages (years)
A
B
C
D
E
F
G
H
I
J
K

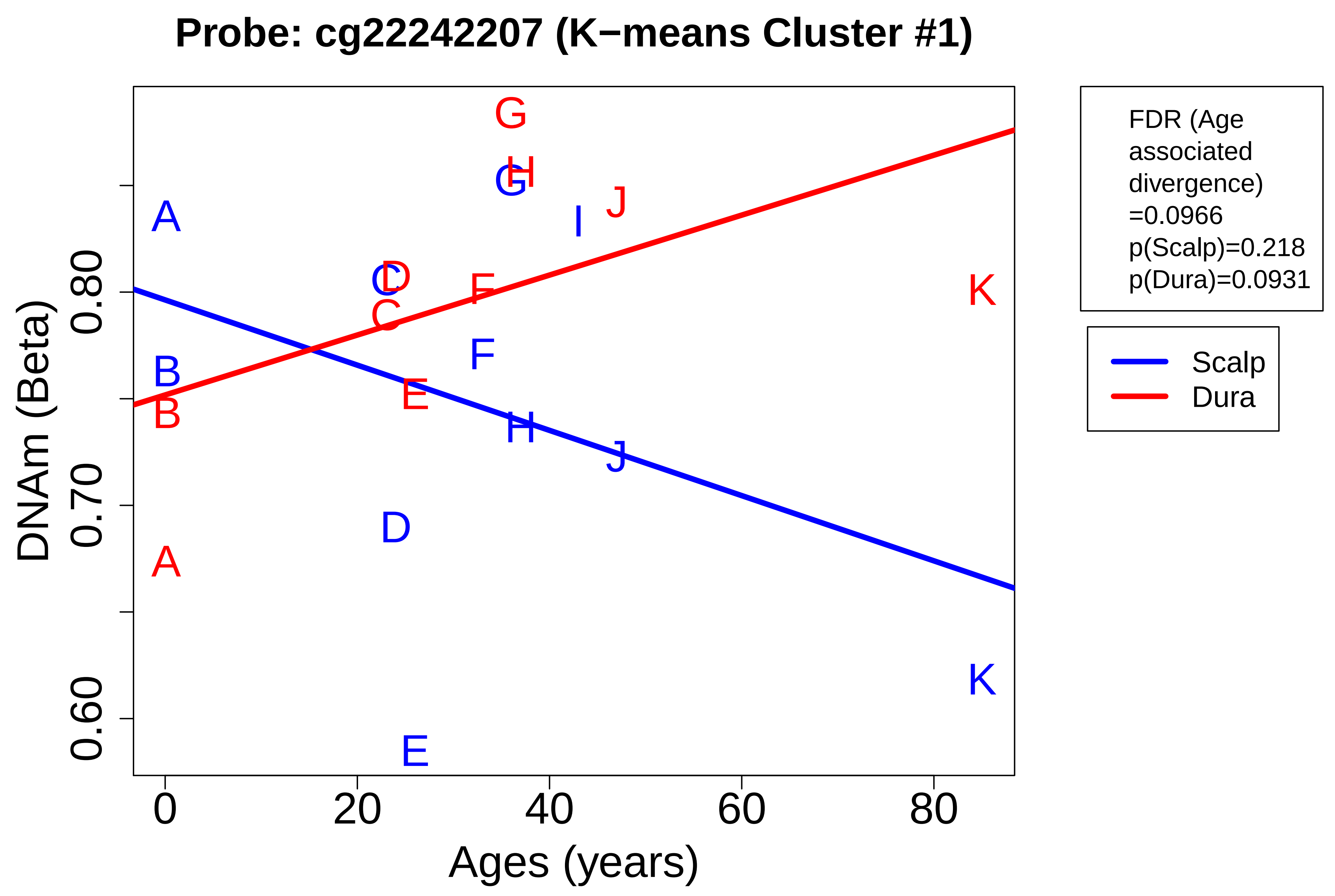

Probe: cg22242207 (K−means Cluster #1)
DNAm (Beta)
0.60
0.70
0.80
0
20
40
60
80
Ages (years)
FDR (Age associated divergence) =0.0966
p(Scalp)=0.218
p(Dura)=0.0931
Scalp
Dura

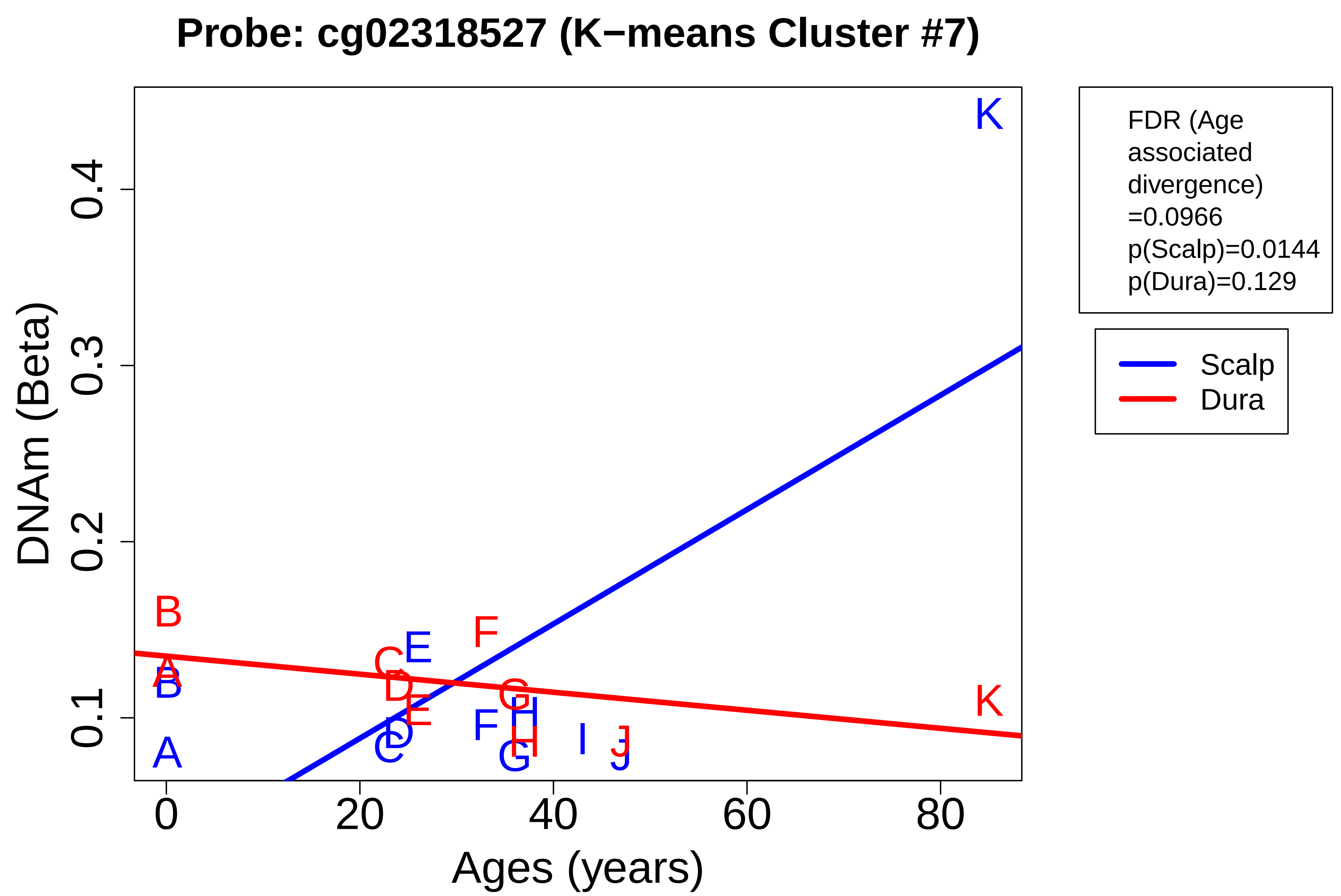

Probe: cg02318527 (K−means Cluster #7)
DNAm (Beta)
0.1
0.2
0.3
0.4
0
20
40
60
80
Ages (years)
A
B
C
D
E
F
G
H
I
J
K
FDR (Age associated divergence) =0.0966
p(Scalp)=0.0144
p(Dura)=0.129
Scalp
Dura

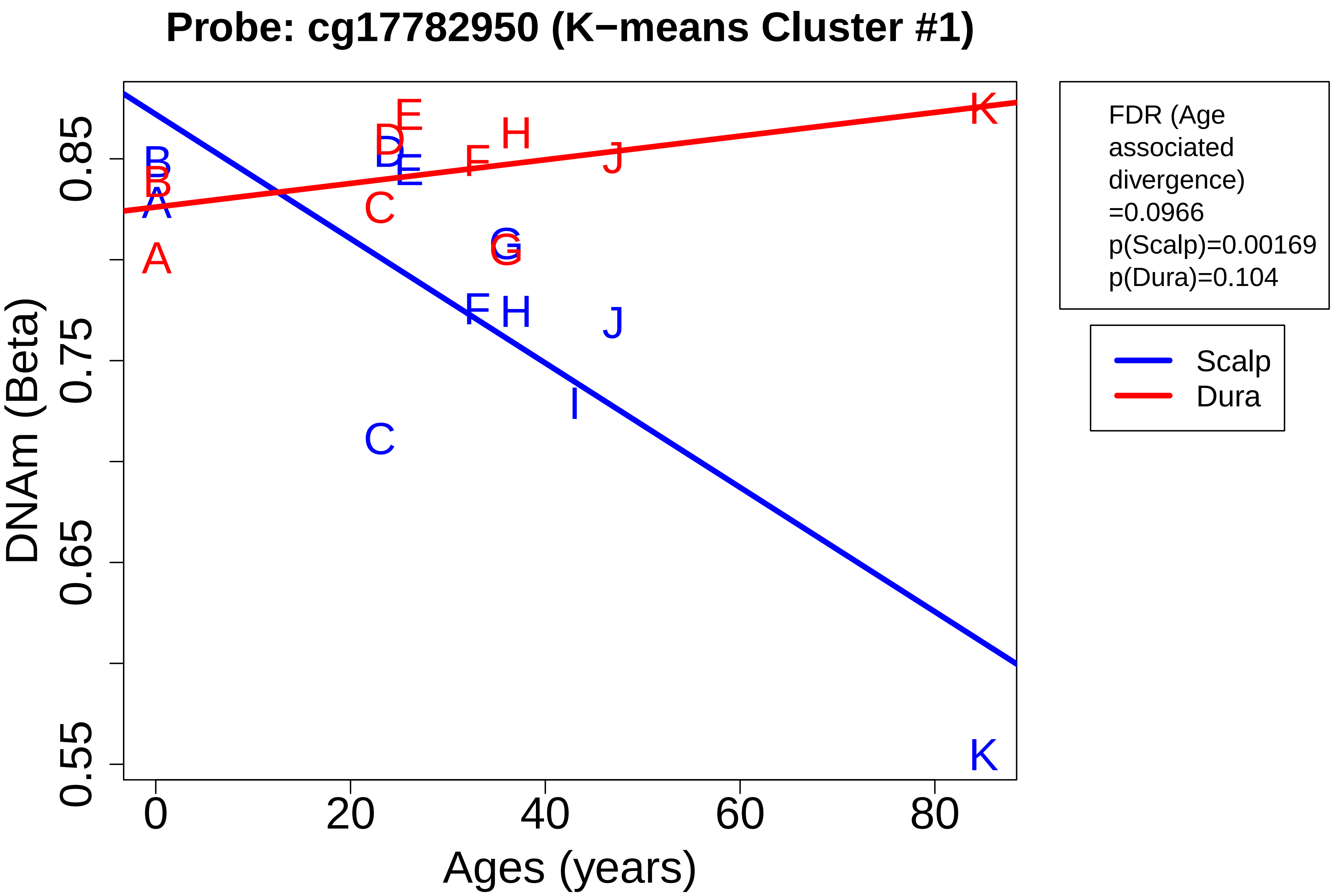

Probe: cg17782950 (K−means Cluster #1)
DNAm (Beta)
0.55
0.65
0.75
0.85
Ages (years)
0
20
40
60
80
FDR (Age associated divergence) =0.0966
p(Scalp)=0.00169
p(Dura)=0.104
Scalp
Dura
A
B
C
D
E
F
G
H
I
J
K

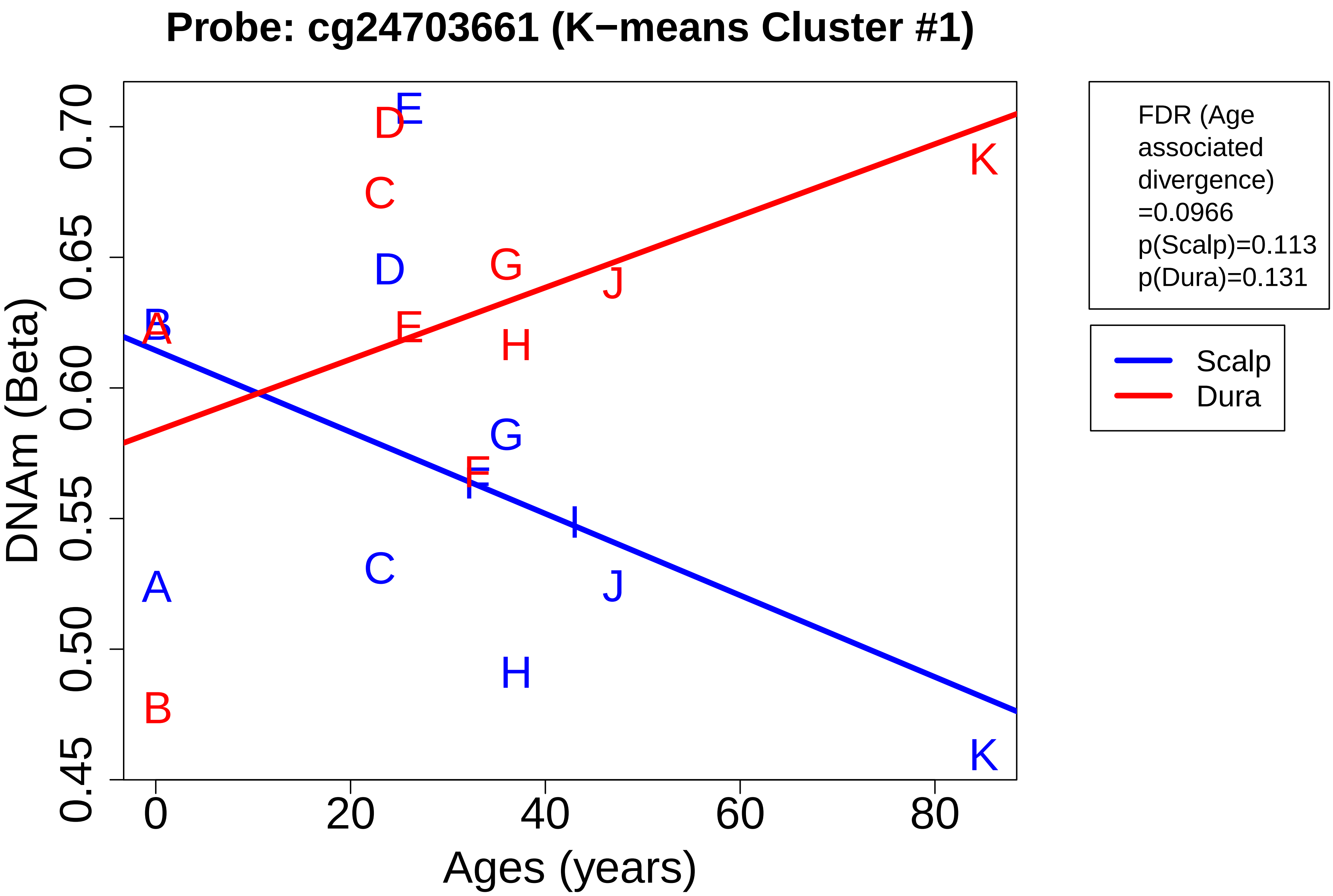

Probe: cg24703661 (K−means Cluster #1)
DNAm (Beta)
0.45
0.50
0.55
0.60
0.65
0.70
0
20
40
60
80
Ages (years)
FDR (Age associated divergence) =0.0966
p(Scalp)=0.113
p(Dura)=0.131
Scalp
Dura

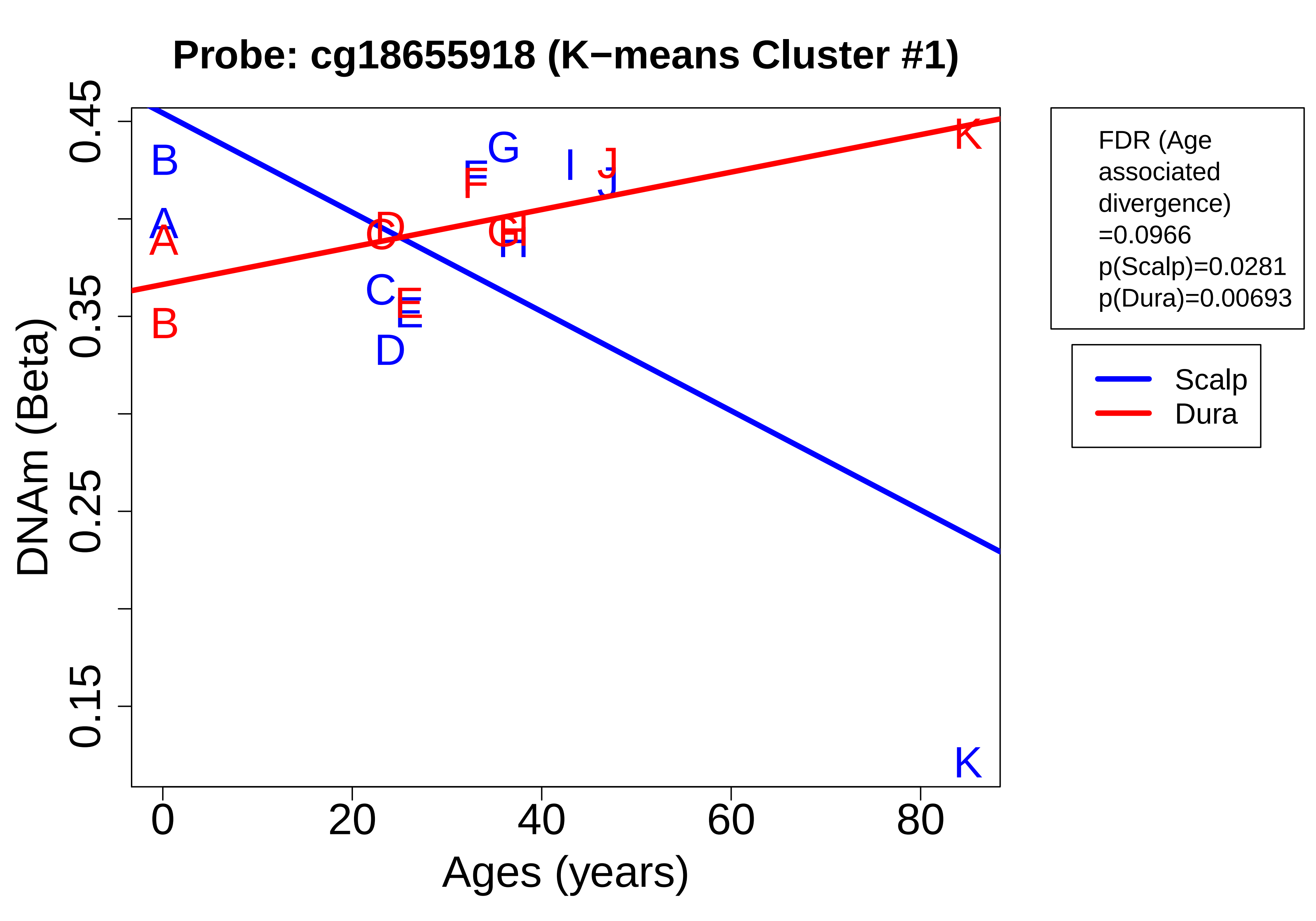

Probe: cg18655918 (K−means Cluster #1)
DNAm (Beta)
0.15
0.25
0.35
0.45
0
20
40
60
80
Ages (years)
FDR (Age associated divergence) =0.0966
p(Scalp)=0.0281
p(Dura)=0.00693
Scalp
Dura

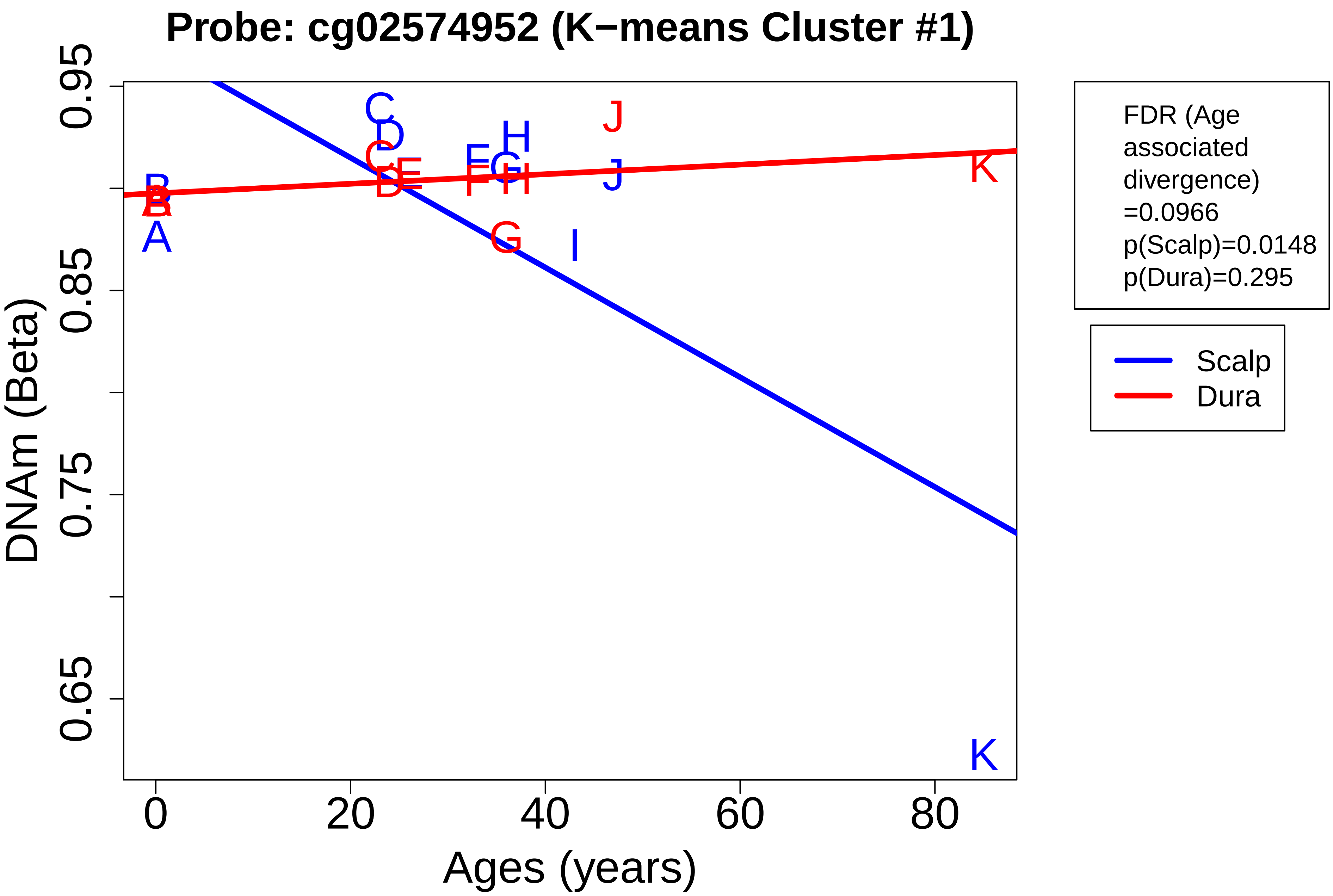

Probe: cg02574952 (K−means Cluster #1)
DNAm (Beta)
0.65
0.75
0.85
0.95
Ages (years)
0
20
40
60
80
FDR (Age associated divergence) =0.0966
p(Scalp)=0.0148
p(Dura)=0.295
Scalp
Dura
A
B
C
D
E
F
G
H
I
J
K

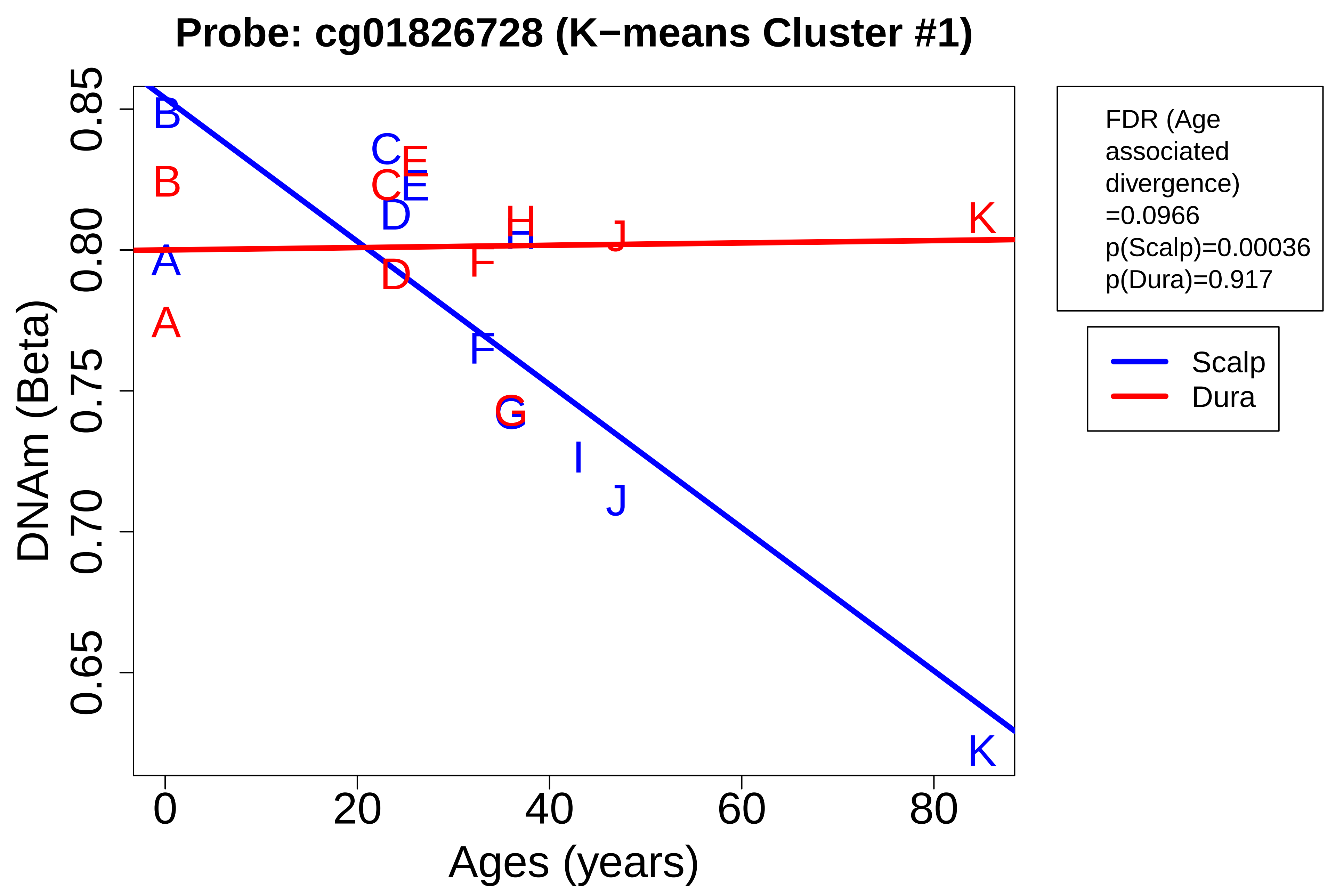

Probe: cg01826728 (K−means Cluster #1)
DNAm (Beta)
0.65
0.70
0.75
0.80
0.85
Ages (years)
0
20
40
60
80
FDR (Age associated divergence) =0.0966
p(Scalp)=0.00036
p(Dura)=0.917
Scalp
Dura

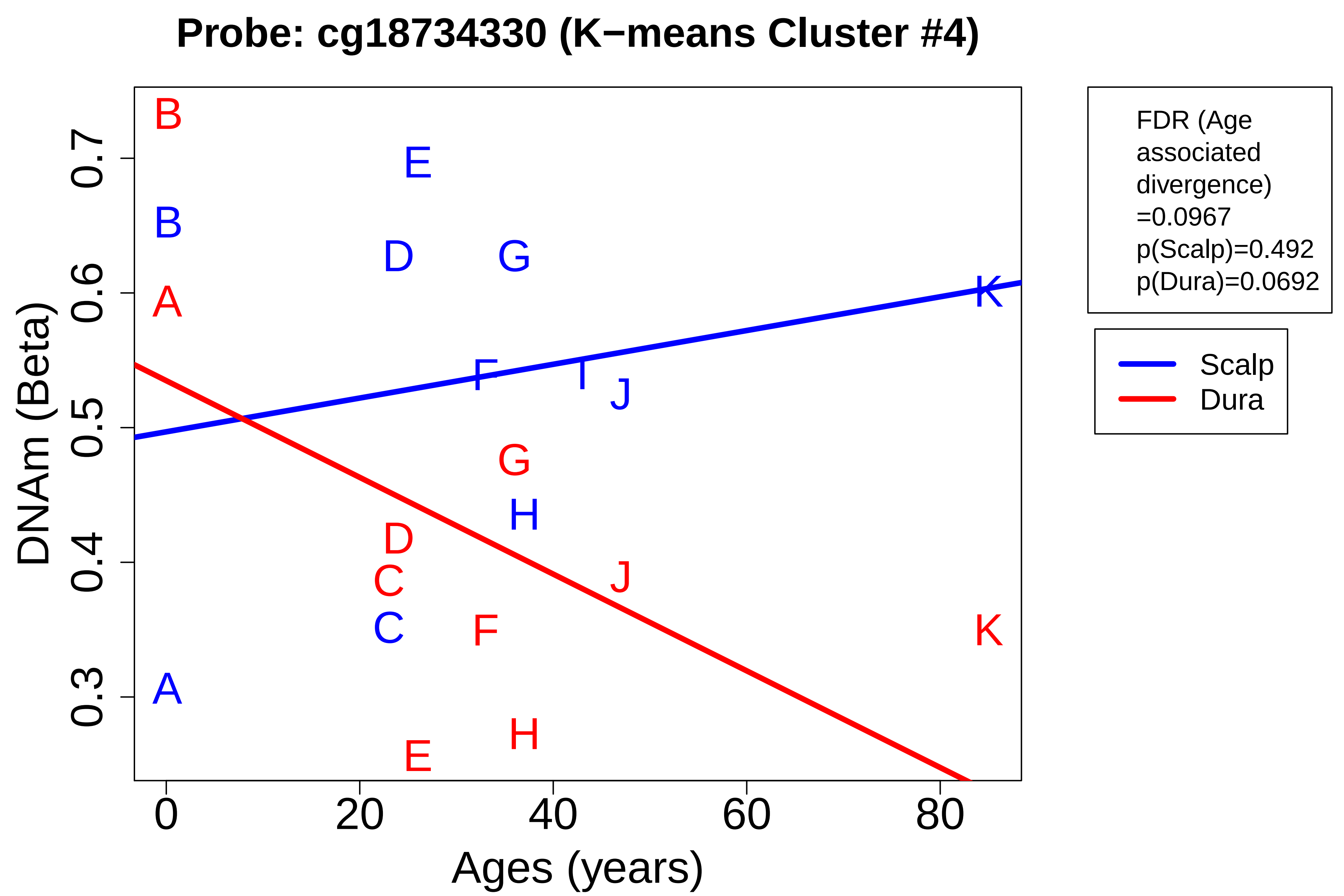
Probe: cg18734330 (K−means Cluster #4)
DNAm (Beta)
0.3
0.4
0.5
0.6
0.7
Ages (years)
0
20
40
60
80
B
E
B
D
G
A
K
F
I
J
G
H
D
C
J
C
F
K
A
H
E
FDR (Age associated divergence) =0.0967
p(Scalp)=0.492
p(Dura)=0.0692
Scalp
Dura

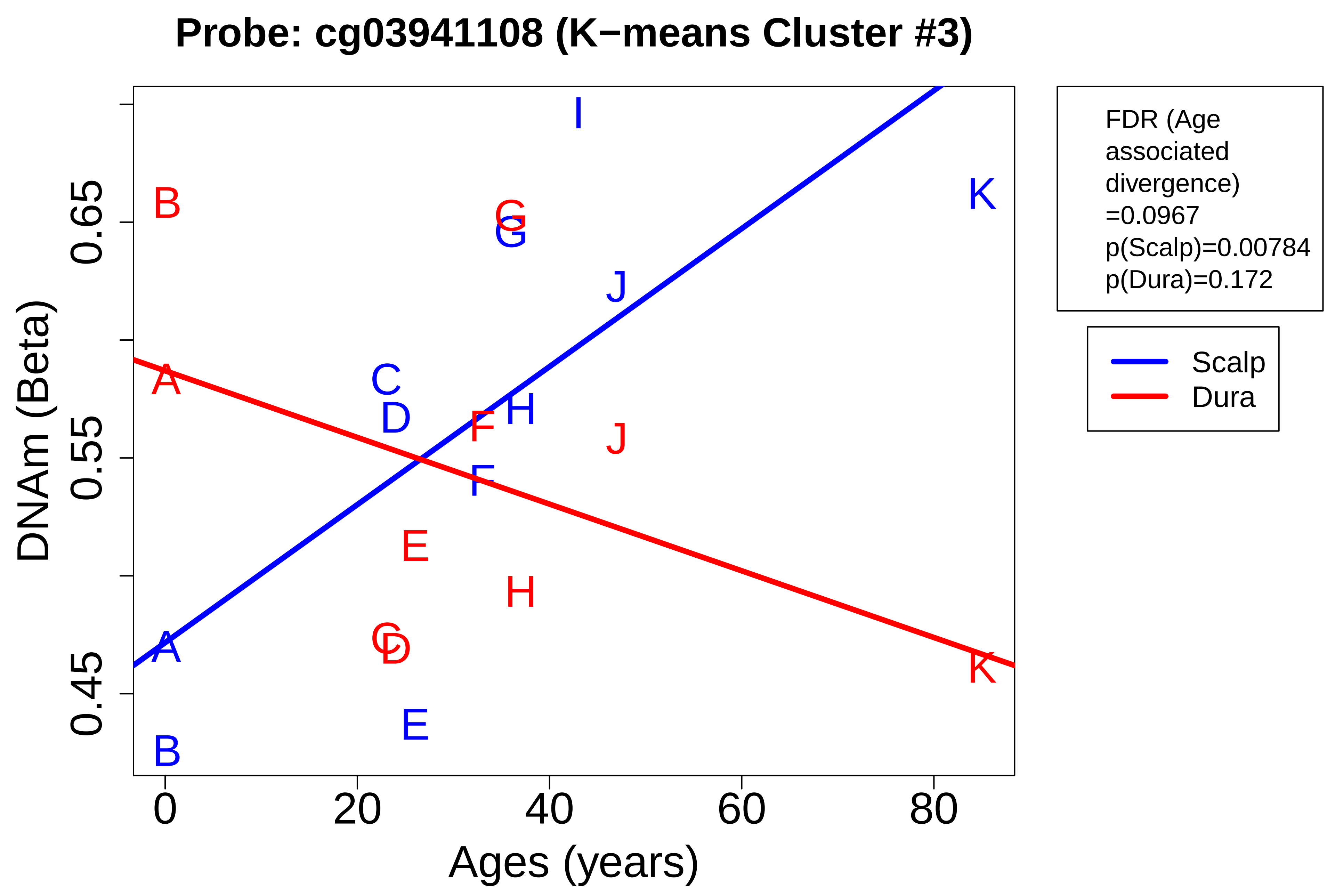
Probe: cg03941108 (K−means Cluster #3)
DNAm (Beta)
0.45
0.55
0.65
Ages (years)
0
20
40
60
80
FDR (Age associated divergence) =0.0967
p(Scalp)=0.00784
p(Dura)=0.172
Scalp
Dura

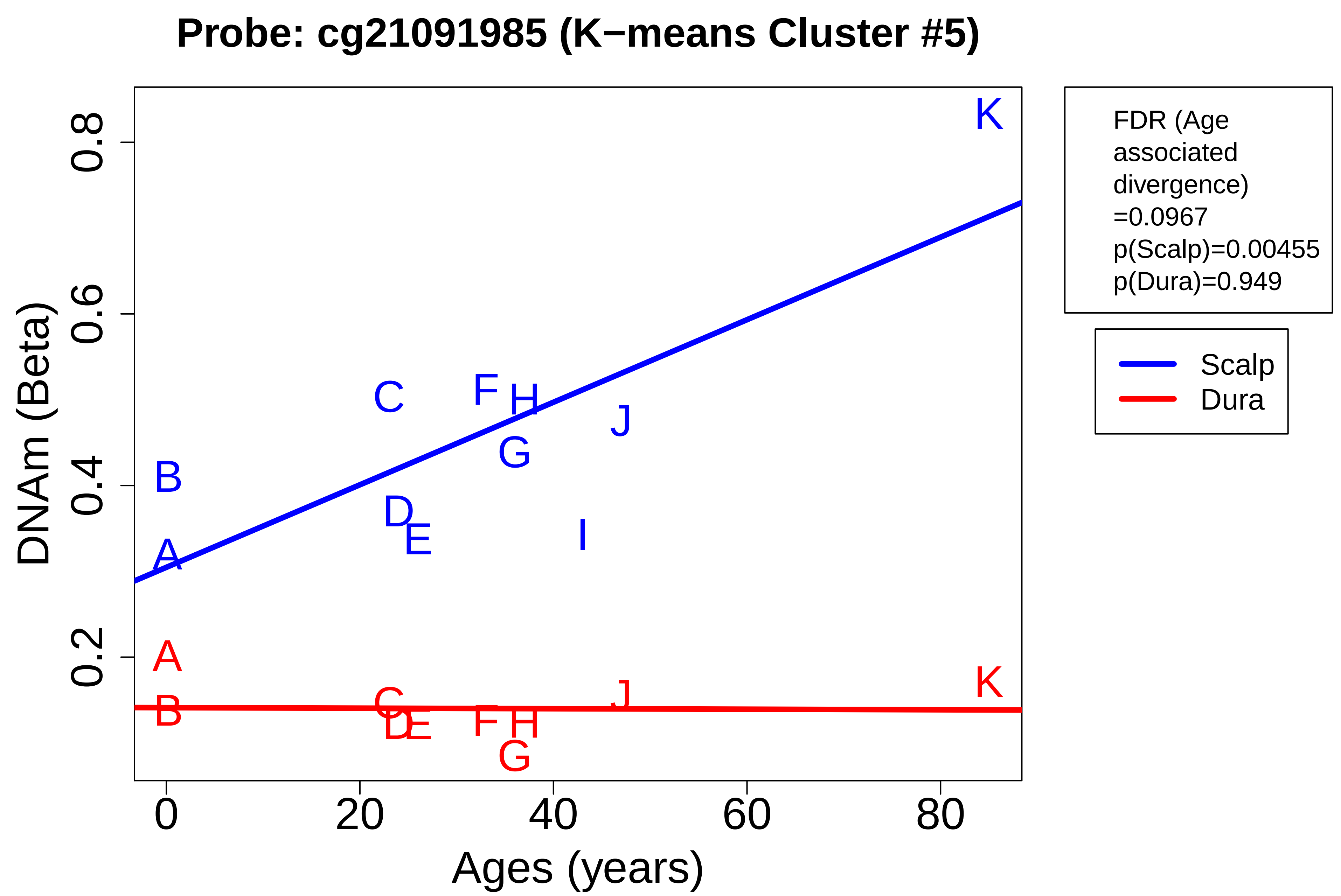

Probe: cg21091985 (K−means Cluster #5)
DNAm (Beta)
0.2
0.4
0.6
0.8
0
20
40
60
80
Ages (years)
A
B
C
D
E
F
G
H
I
J
K
FDR (Age associated divergence) =0.0967
p(Scalp)=0.00455
p(Dura)=0.949
Scalp
Dura

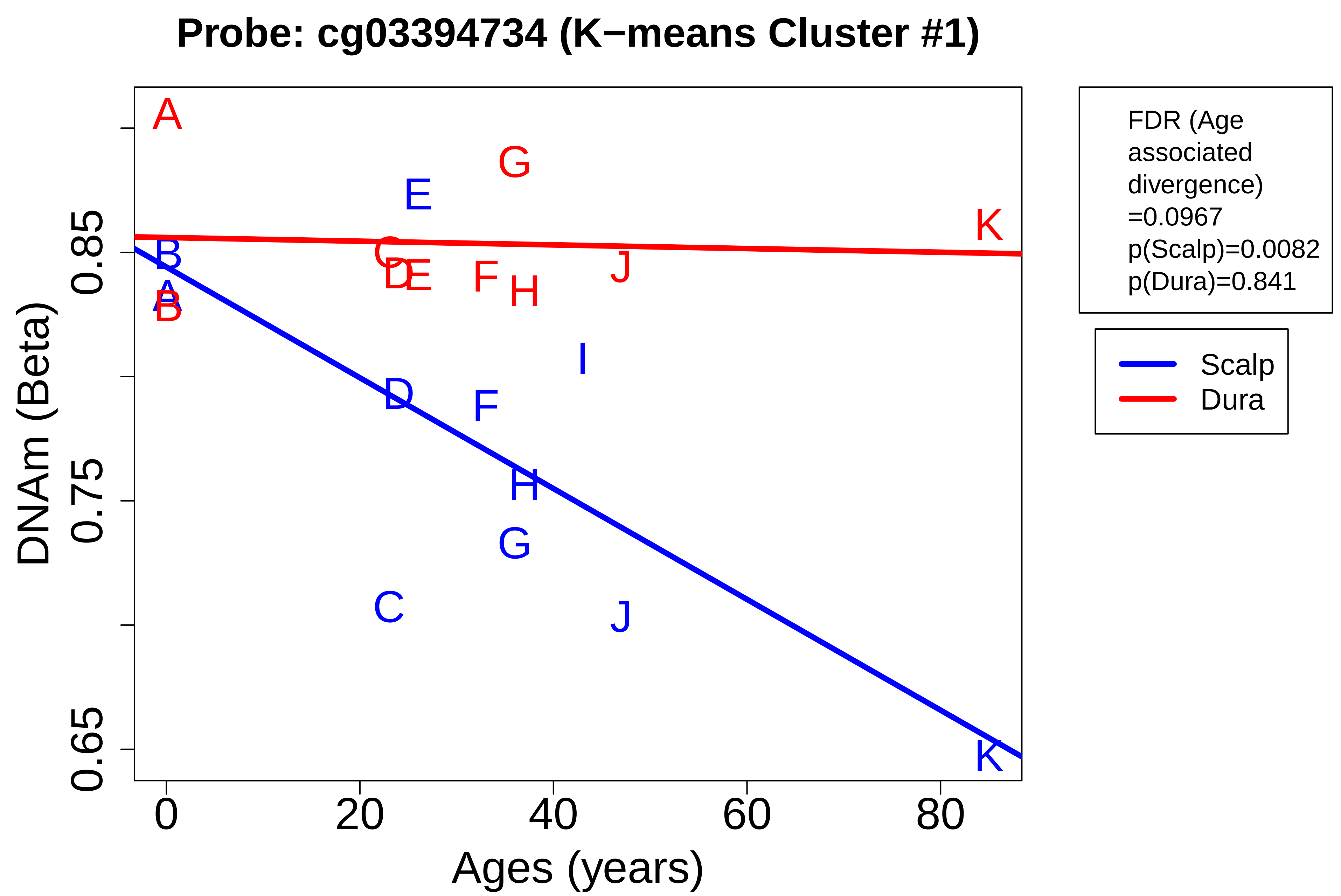

Probe: cg03394734 (K−means Cluster #1)
DNAm (Beta)
0.65
0.75
0.85
Ages (years)
0
20
40
60
80
A
B
C
D
E
F
G
H
I
J
K
FDR (Age associated divergence) =0.0967
p(Scalp)=0.0082
p(Dura)=0.841
Scalp
Dura

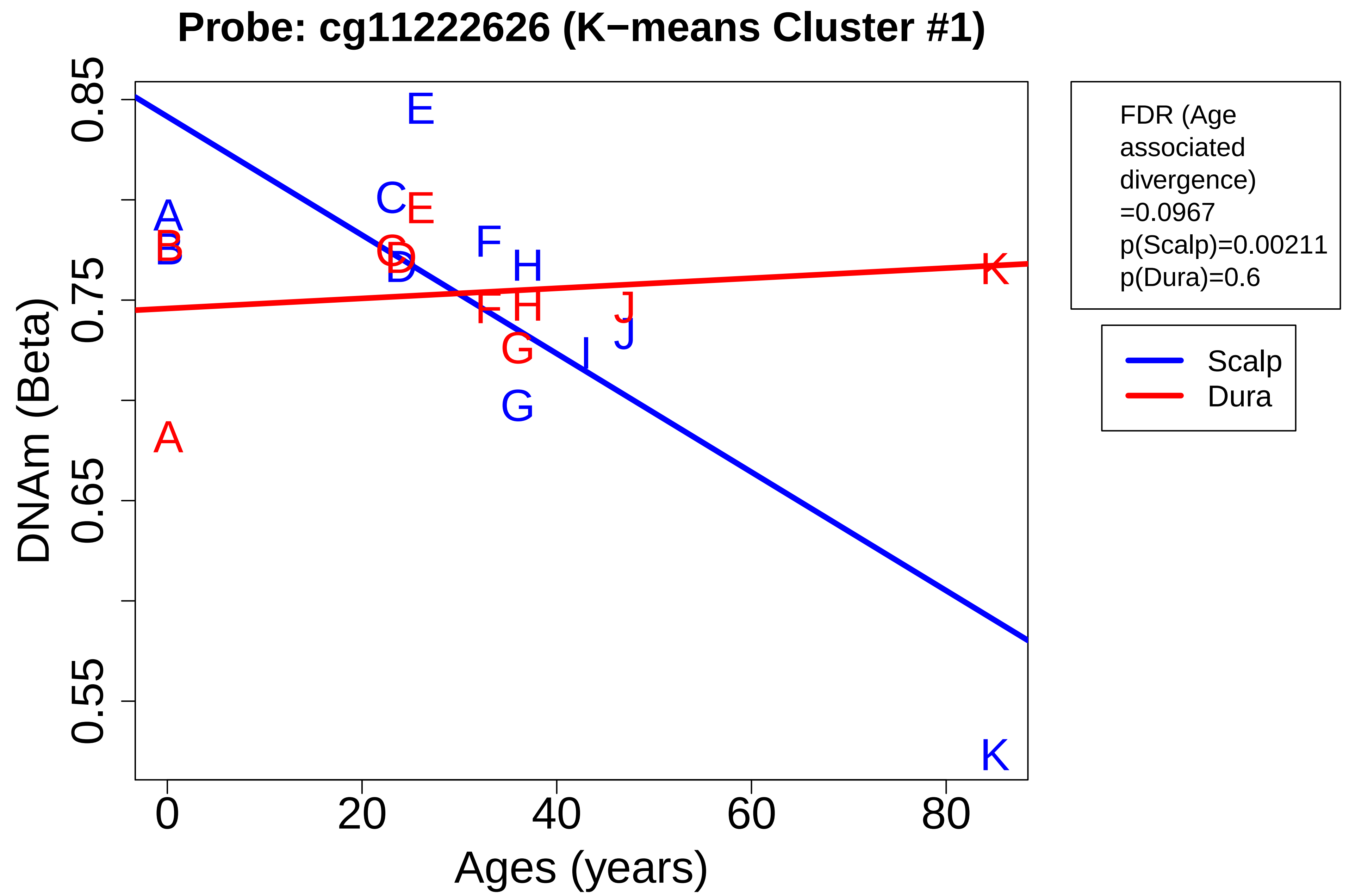

Probe: cg11222626 (K−means Cluster #1)
DNAm (Beta)
0.55
0.65
0.75
0.85
0
20
40
60
80
Ages (years)
A
B
C
D
E
F
G
H
I
J
K
FDR (Age associated divergence) =0.0967
p(Scalp)=0.00211
p(Dura)=0.6
Scalp
Dura

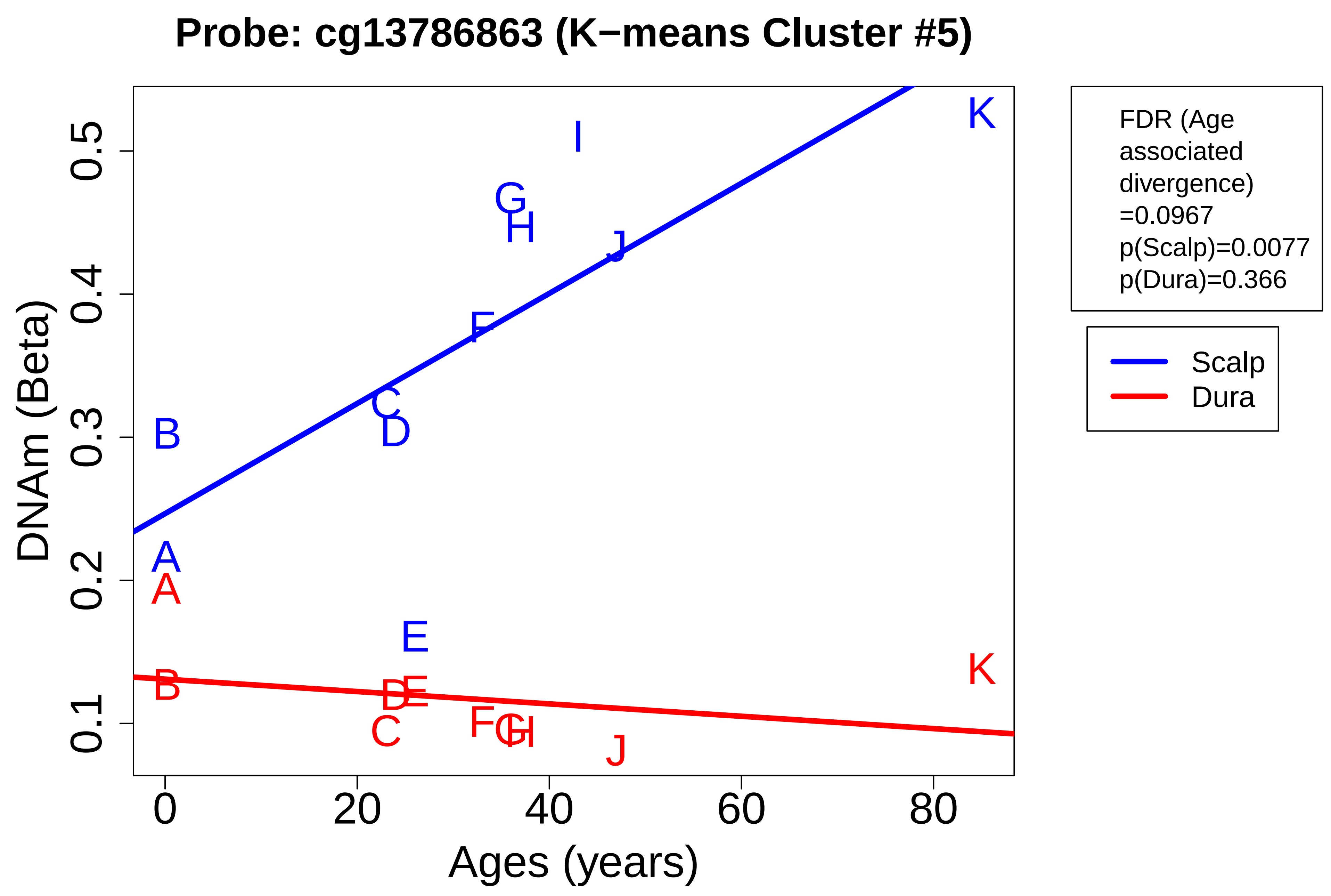

Probe: cg13786863 (K−means Cluster #5)
DNAm (Beta)
0.1
0.2
0.3
0.4
0.5
Ages (years)
0
20
40
60
80
A
B
C
D
E
F
G
H
I
J
K
FDR (Age associated divergence) =0.0967
p(Scalp)=0.0077
p(Dura)=0.366
Scalp
Dura

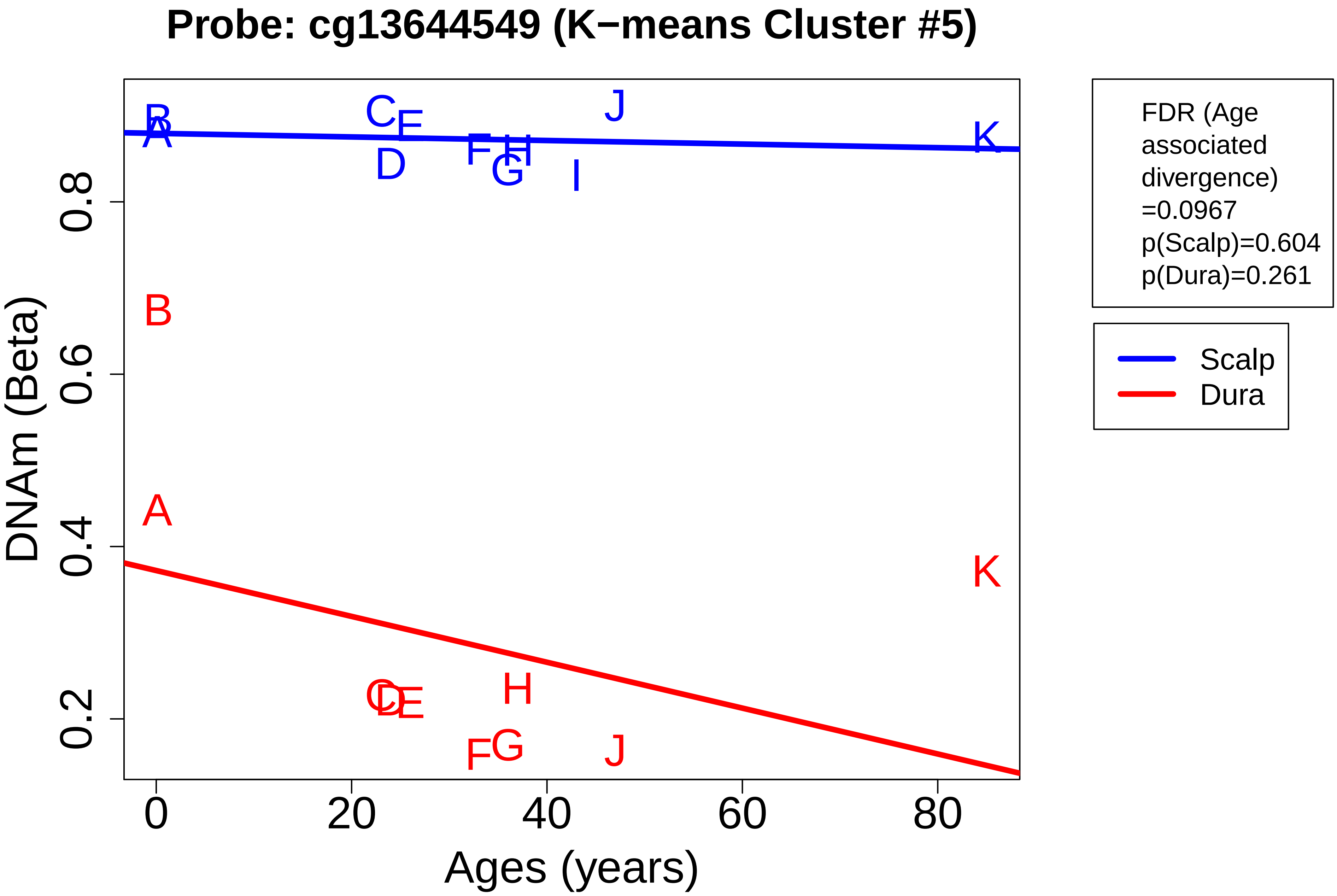

Probe: cg13644549 (K−means Cluster #5)
DNAm (Beta)
0.2
0.4
0.6
0.8
0
20
40
60
80
Ages (years)
A
B
C
D
E
F
G
H
I
J
K
A
B
C
D
E
F
G
H
J
K
FDR (Age associated divergence) =0.0967
p(Scalp)=0.604
p(Dura)=0.261
Scalp
Dura

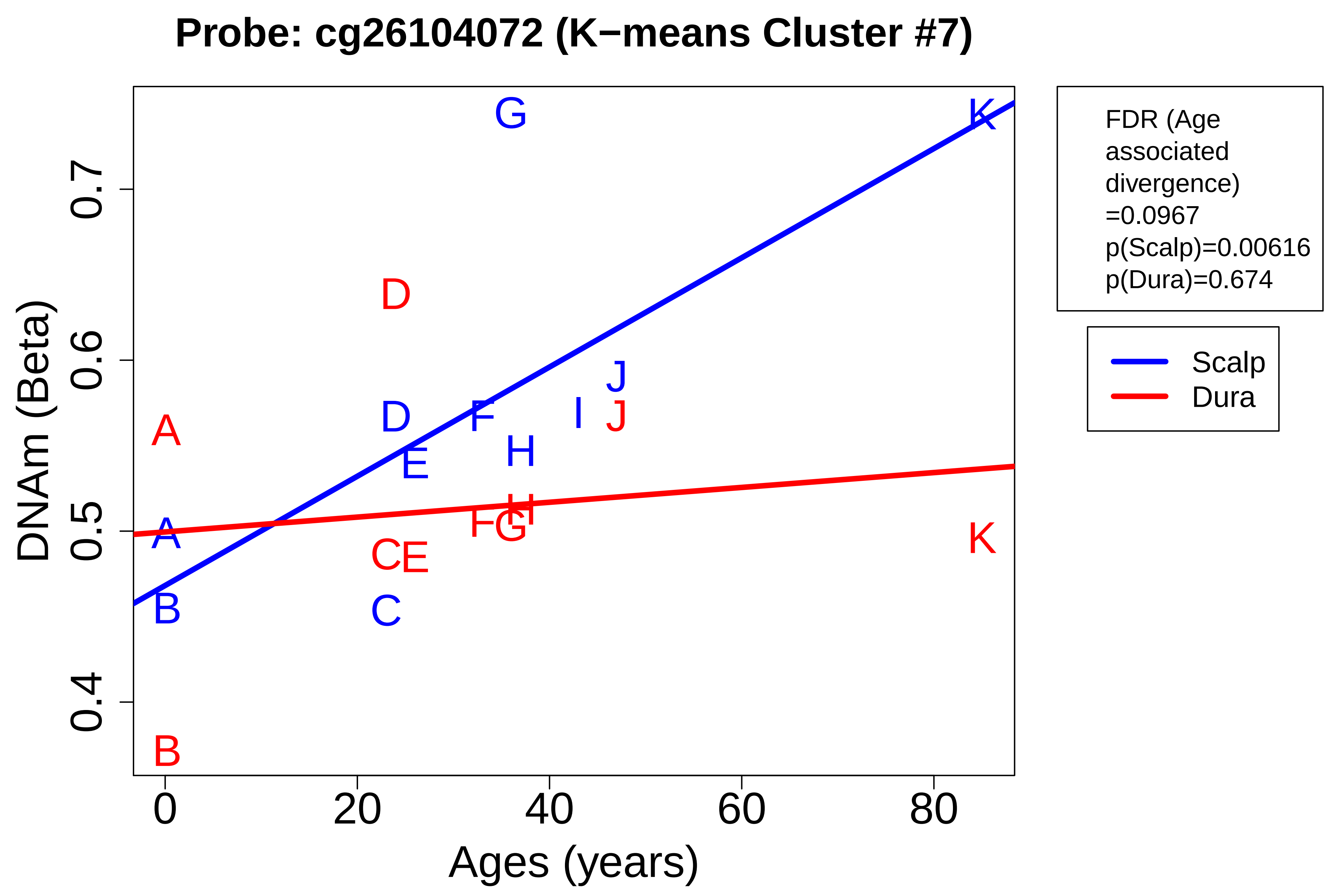

Probe: cg26104072 (K−means Cluster #7)
DNAm (Beta)
0.4
0.5
0.6
0.7
Ages (years)
0
20
40
60
80
FDR (Age associated divergence) =0.0967
p(Scalp)=0.00616
p(Dura)=0.674
Scalp
Dura

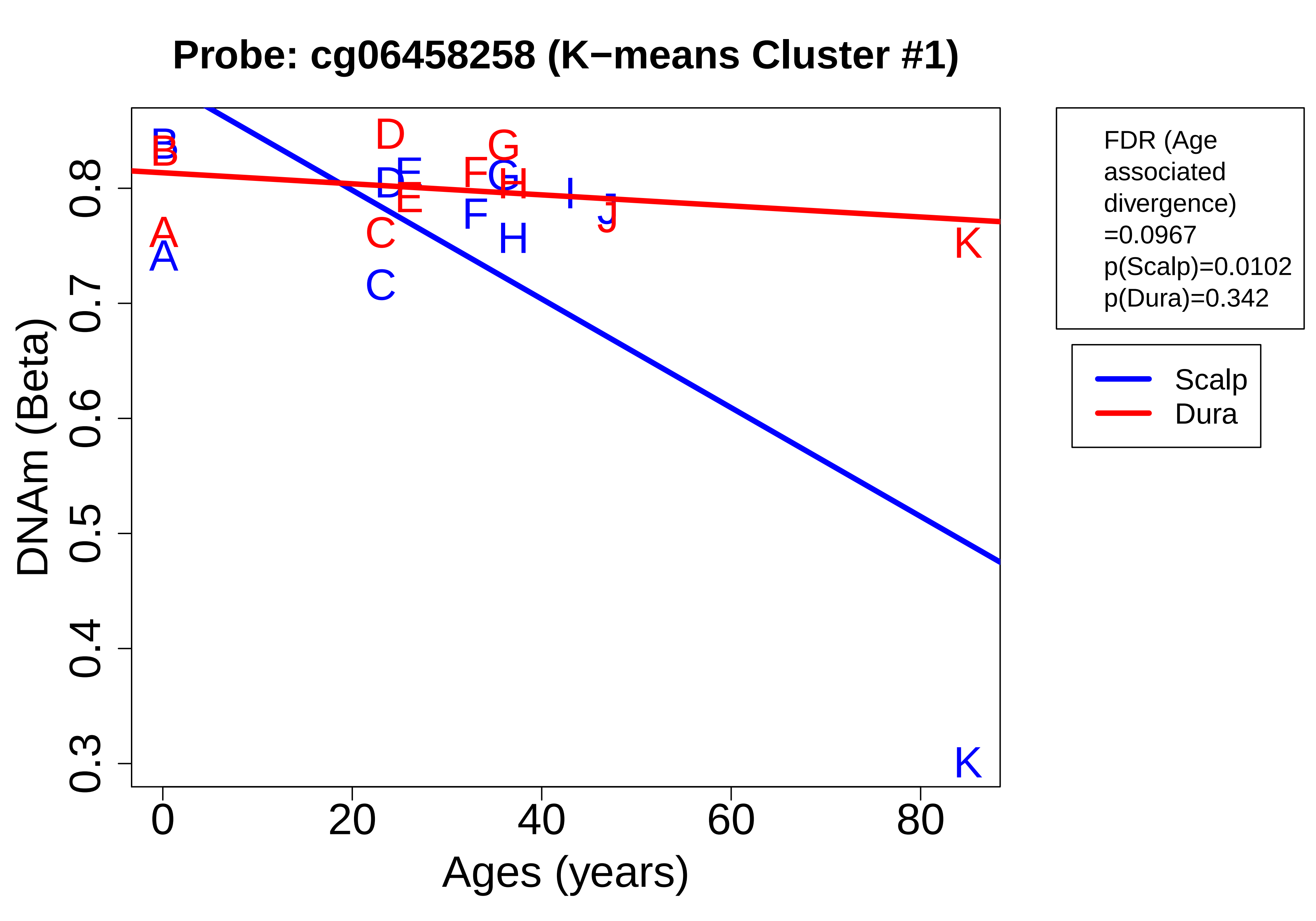

Probe: cg06458258 (K−means Cluster #1)
DNAm (Beta)
0.3
0.4
0.5
0.6
0.7
0.8
0
20
40
60
80
Ages (years)
FDR (Age associated divergence) =0.0967
p(Scalp)=0.0102
p(Dura)=0.342
Scalp
Dura

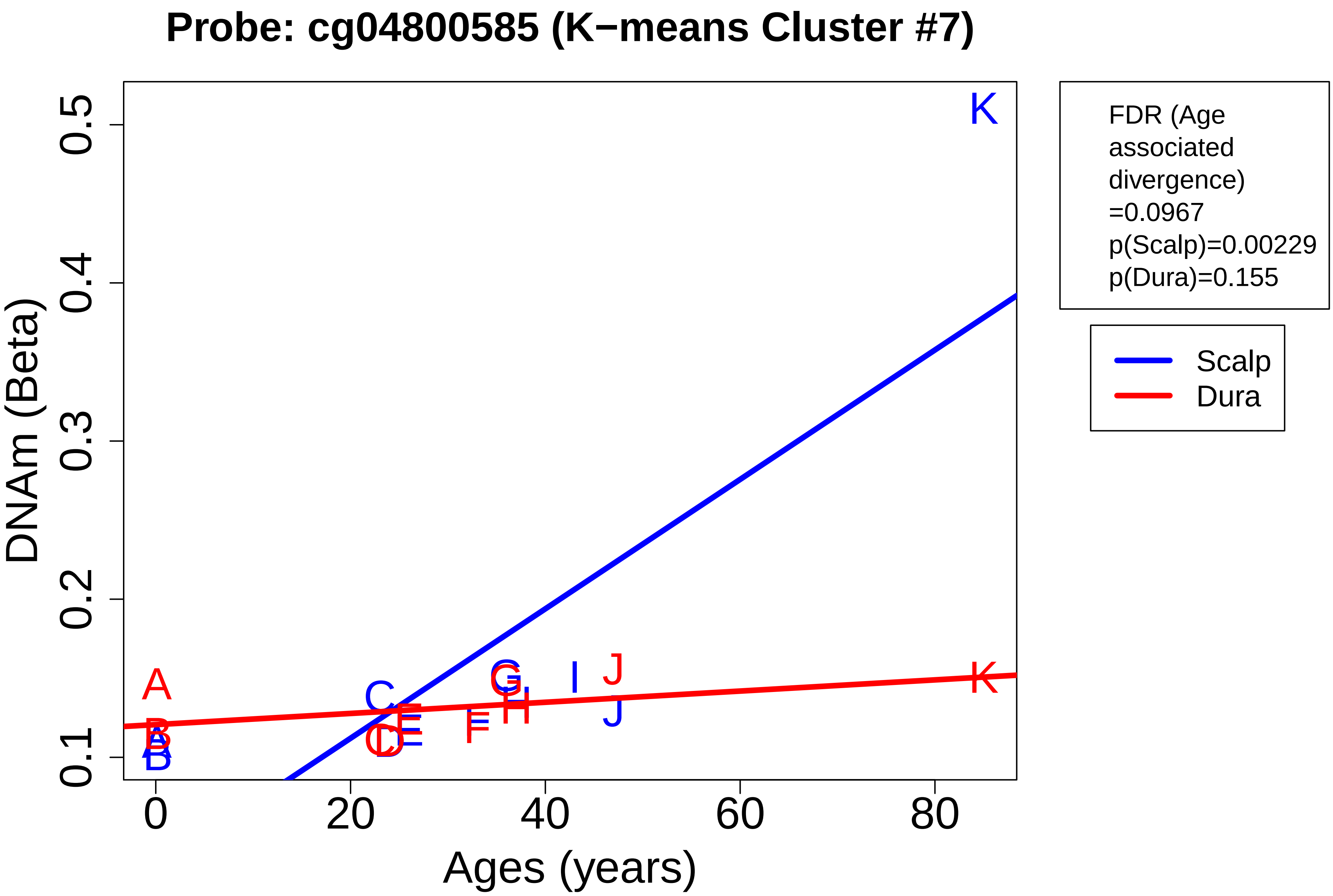

Probe: cg04800585 (K−means Cluster #7)
DNAm (Beta)
Ages (years)
0.1
0.2
0.3
0.4
0.5
0
20
40
60
80
FDR (Age associated divergence) =0.0967
p(Scalp)=0.00229
p(Dura)=0.155
Scalp
Dura

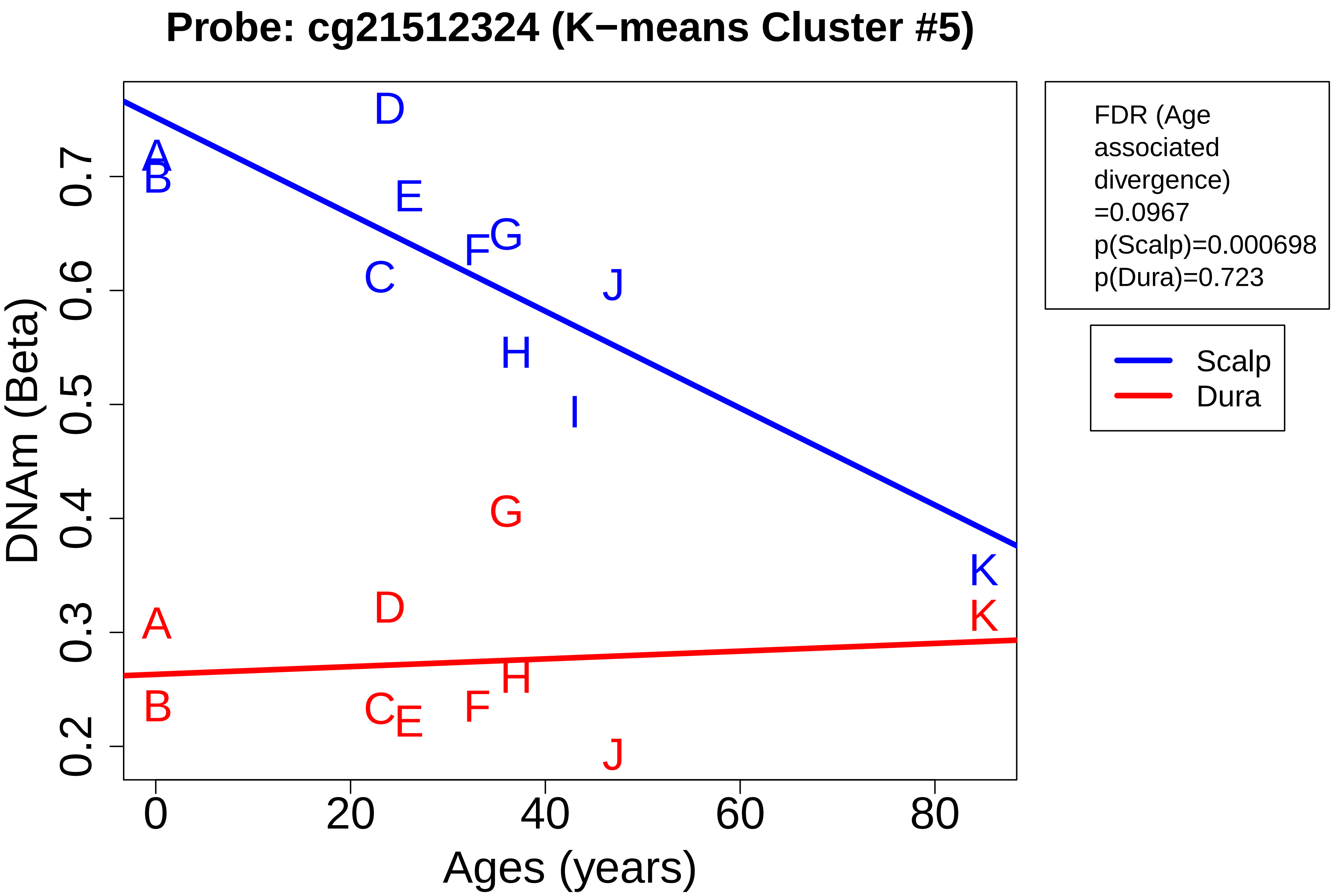

Probe: cg21512324 (K−means Cluster #5)
DNAm (Beta)
0.2
0.3
0.4
0.5
0.6
0.7
0
20
40
60
80
Ages (years)
FDR (Age associated divergence) =0.0967
p(Scalp)=0.000698
p(Dura)=0.723
Scalp
Dura
A
B
C
D
E
F
G
H
I
J
K

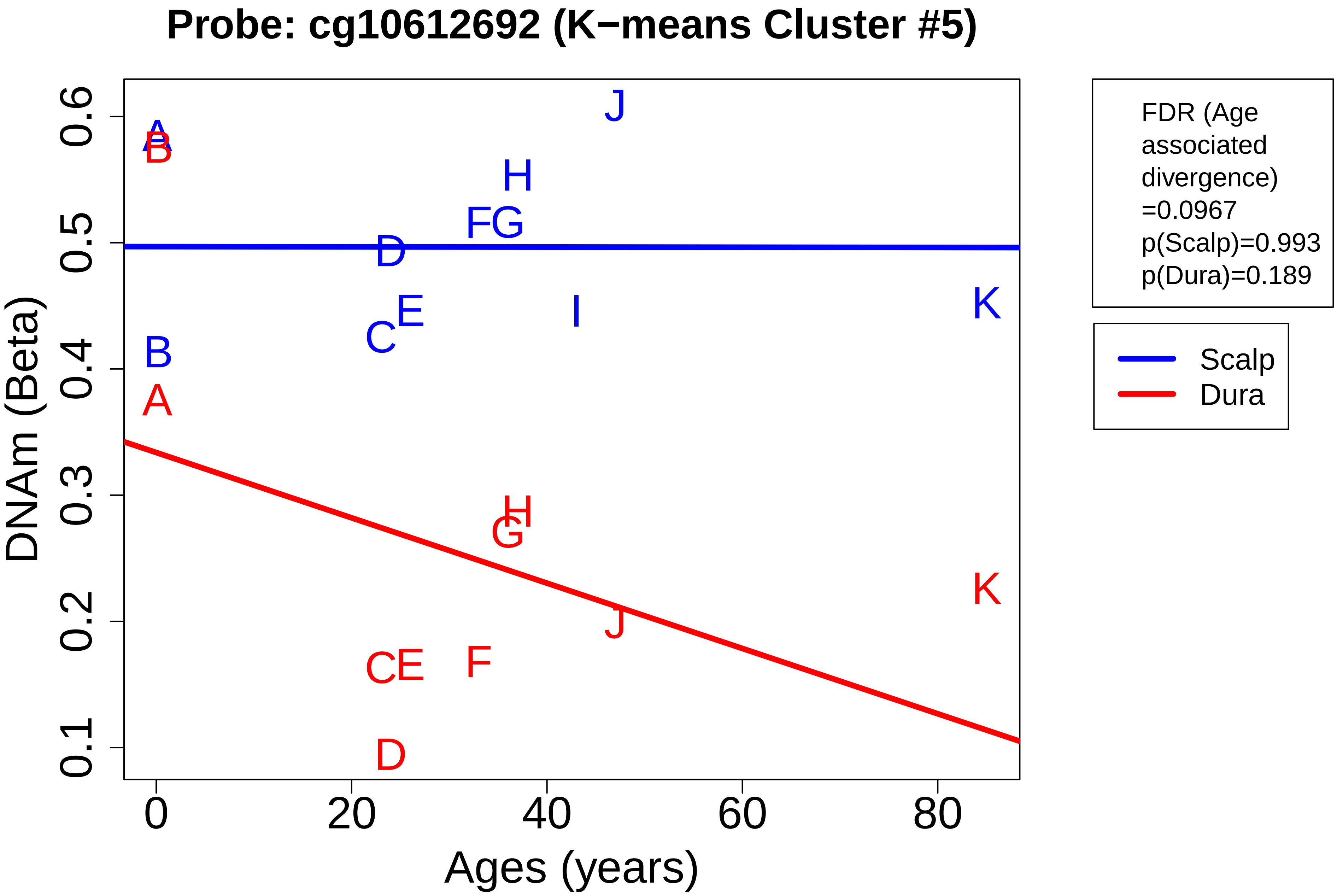

Probe: cg10612692 (K−means Cluster #5)
DNAm (Beta)
0.1
0.2
0.3
0.4
0.5
0.6
0
20
40
60
80
Ages (years)
FDR (Age associated divergence) =0.0967
p(Scalp)=0.993
p(Dura)=0.189
Scalp
Dura
A
B
C
D
E
F
G
H
I
J
K

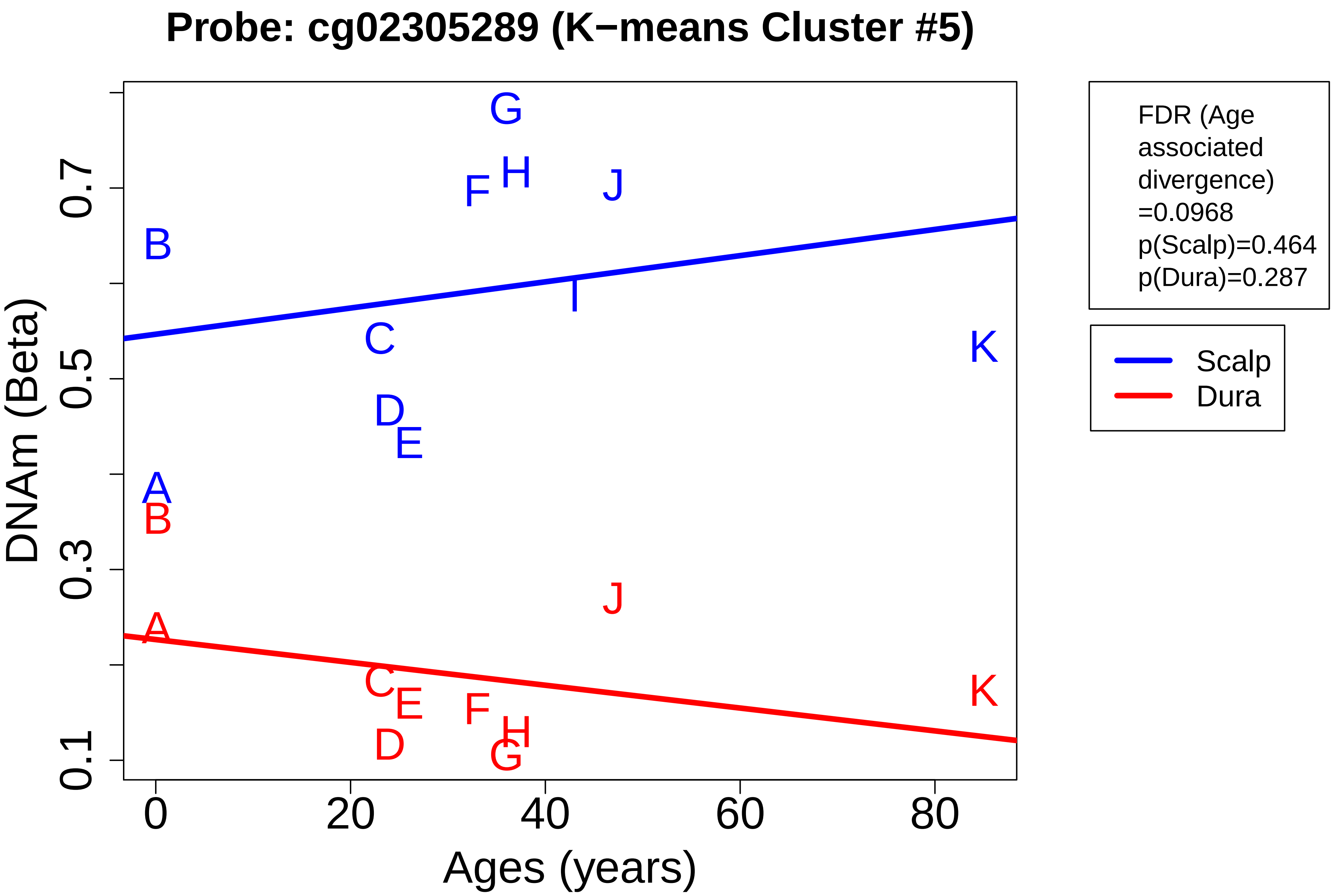

Probe: cg02305289 (K−means Cluster #5)
DNAm (Beta)
0.1
0.3
0.5
0.7
Ages (years)
0
20
40
60
80
FDR (Age associated divergence) =0.0968
p(Scalp)=0.464
p(Dura)=0.287
Scalp
Dura
A
B
C
D
E
F
G
H
I
J
K

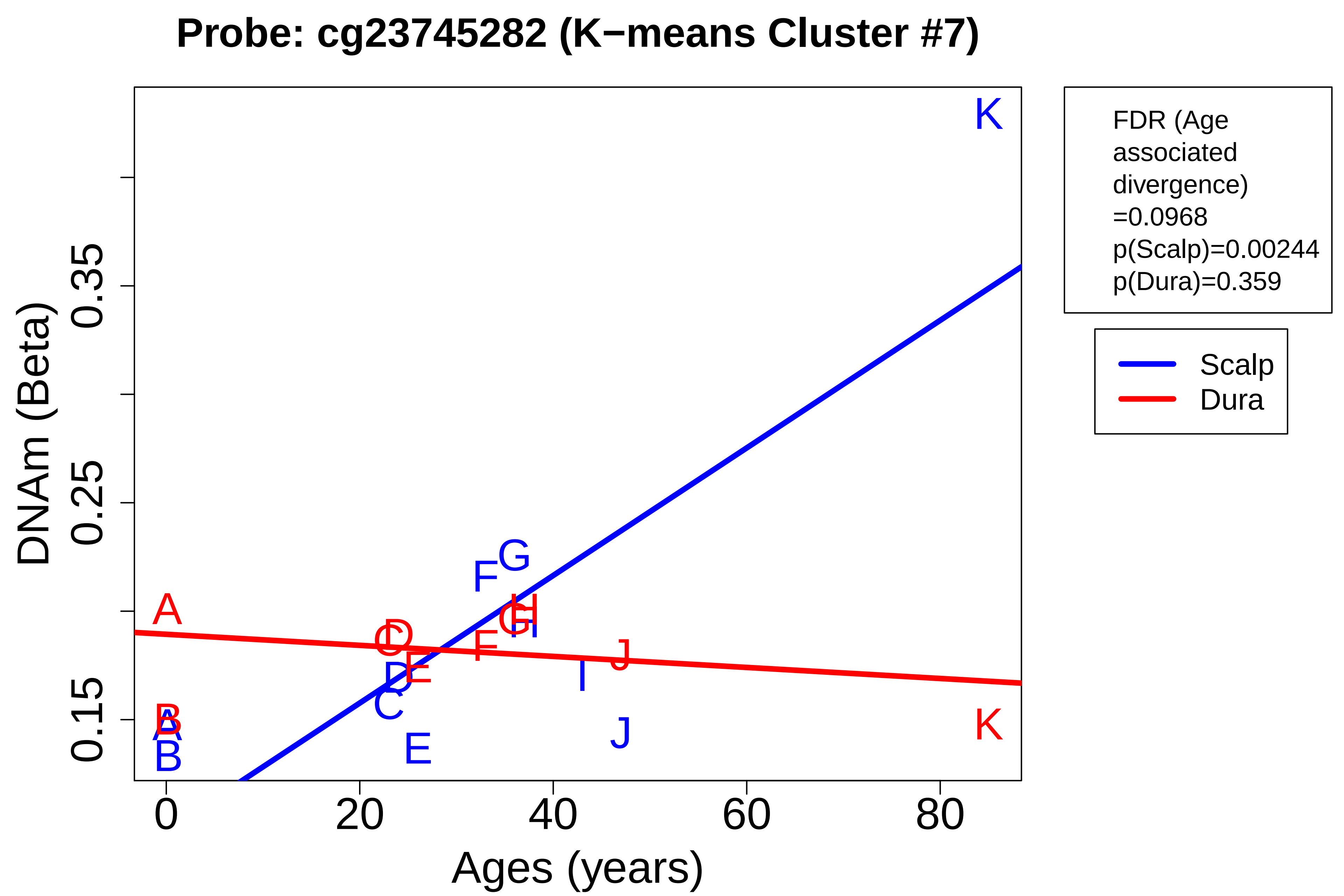
Probe: cg23745282 (K−means Cluster #7)
DNAm (Beta)
0.15
0.25
0.35
0
20
40
60
80
Ages (years)
FDR (Age associated divergence) =0.0968
p(Scalp)=0.00244
p(Dura)=0.359
Scalp
Dura
A
B
C
D
E
F
G
H
I
J
K

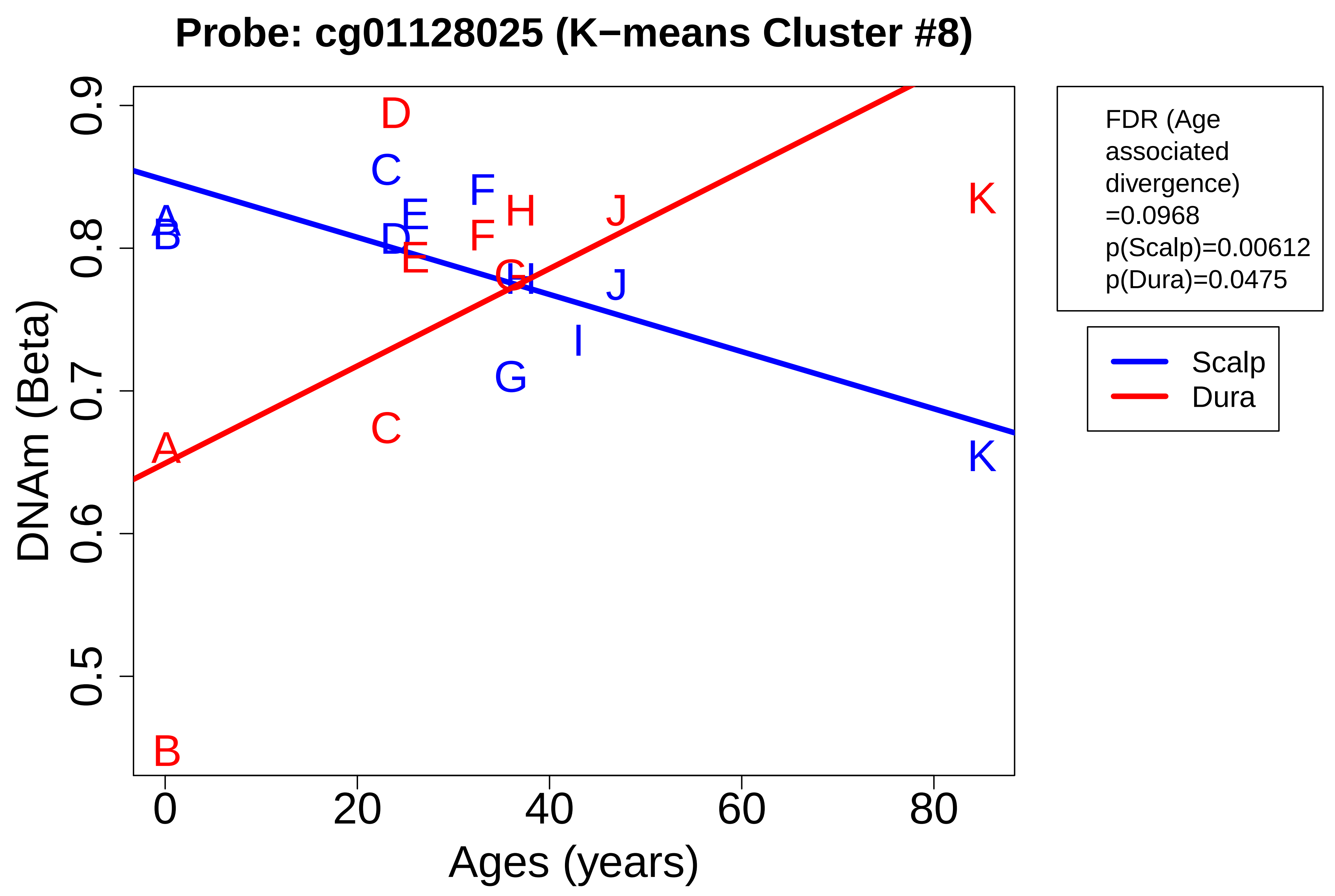

Probe: cg01128025 (K−means Cluster #8)
DNAm (Beta)
0.5
0.6
0.7
0.8
0.9
0
20
40
60
80
Ages (years)
FDR (Age associated divergence) =0.0968
p(Scalp)=0.00612
p(Dura)=0.0475
Scalp
Dura
A
B
C
D
E
F
G
H
I
J
K

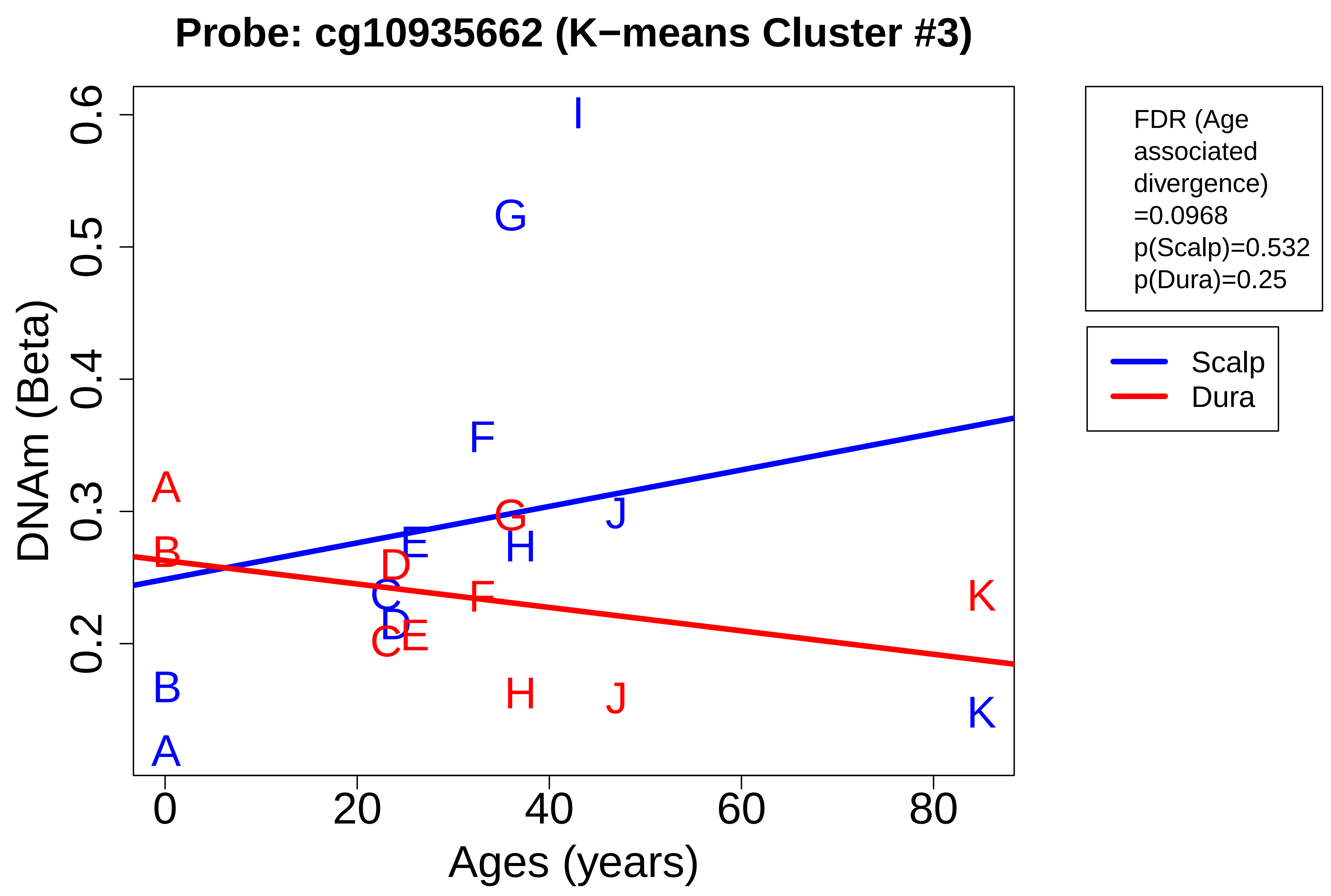

Probe: cg10935662 (K−means Cluster #3)
DNAm (Beta)
0.2
0.3
0.4
0.5
0.6
Ages (years)
0
20
40
60
80
FDR (Age associated divergence) =0.0968
p(Scalp)=0.532
p(Dura)=0.25
Scalp
Dura

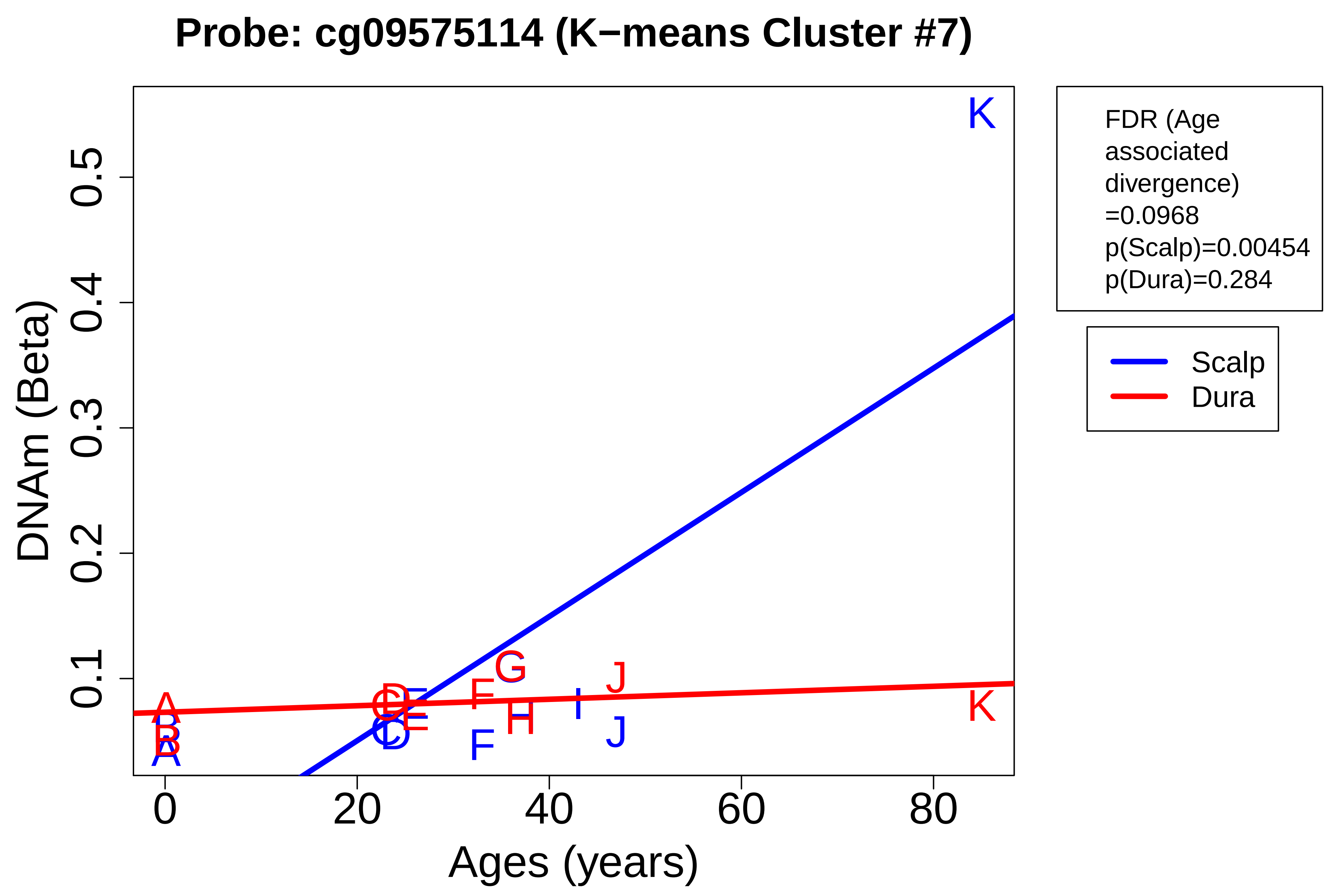

Probe: cg09575114 (K−means Cluster #7)
DNAm (Beta)
0.1
0.2
0.3
0.4
0.5
Ages (years)
0
20
40
60
80
FDR (Age associated divergence) =0.0968
p(Scalp)=0.00454
p(Dura)=0.284
Scalp
Dura

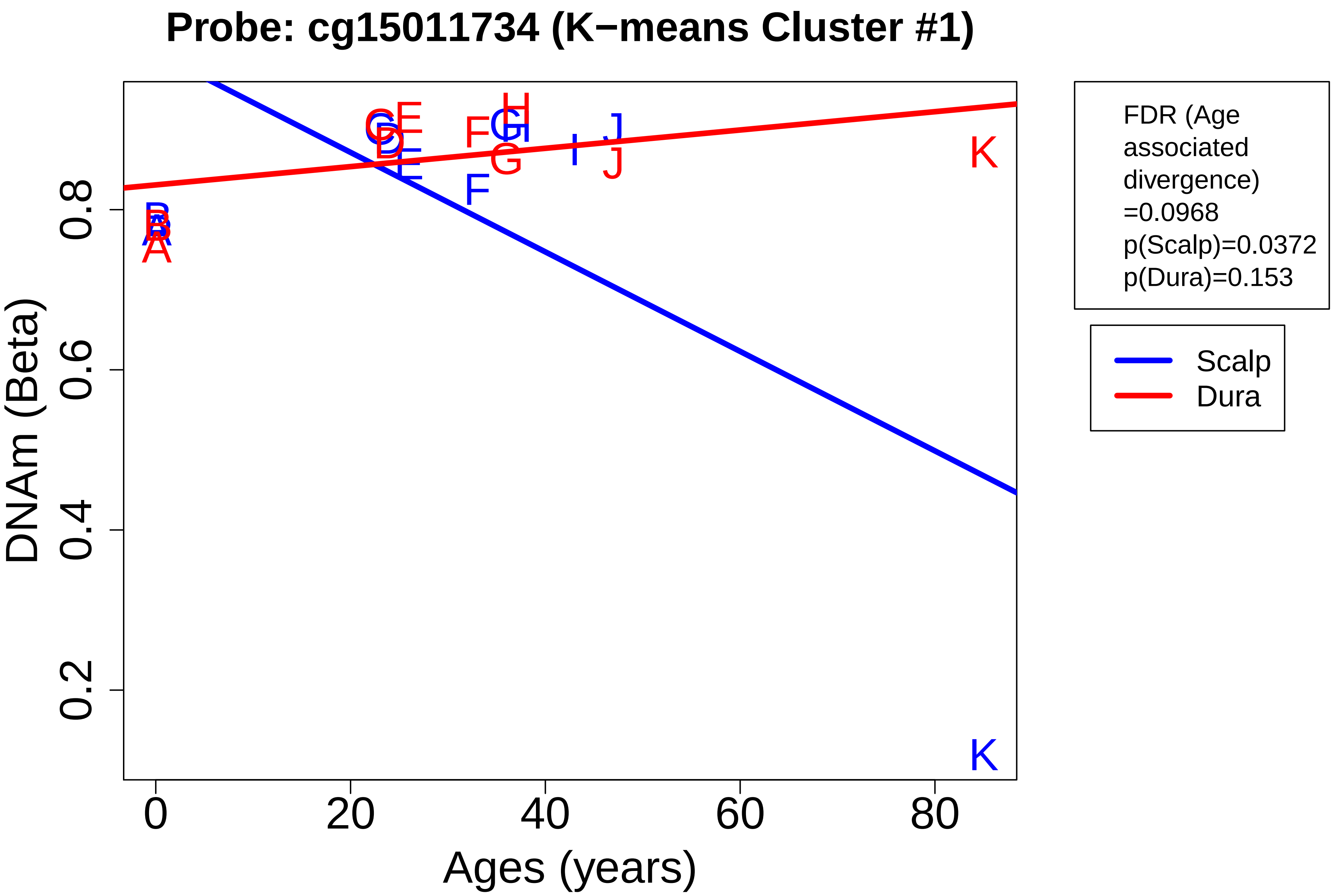

Probe: cg15011734 (K−means Cluster #1)
DNAm (Beta)
0.2
0.4
0.6
0.8
Ages (years)
0
20
40
60
80
FDR (Age associated divergence) =0.0968
p(Scalp)=0.0372
p(Dura)=0.153
Scalp
Dura

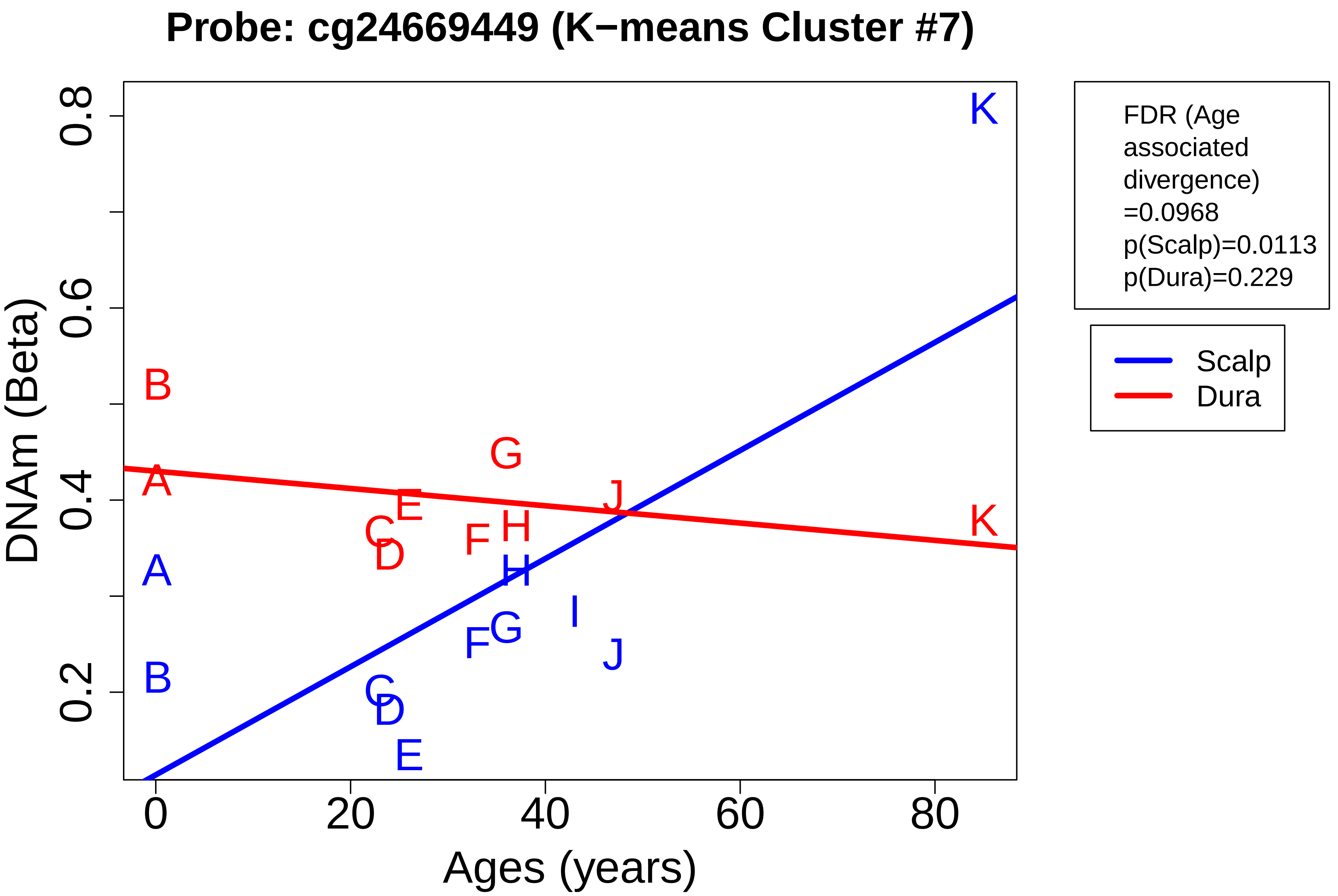

Probe: cg24669449 (K−means Cluster #7)
DNAm (Beta)
0.2
0.4
0.6
0.8
Ages (years)
0
20
40
60
80
A
B
C
D
E
F
G
H
I
J
K
FDR (Age associated divergence) =0.0968
p(Scalp)=0.0113
p(Dura)=0.229
Scalp
Dura

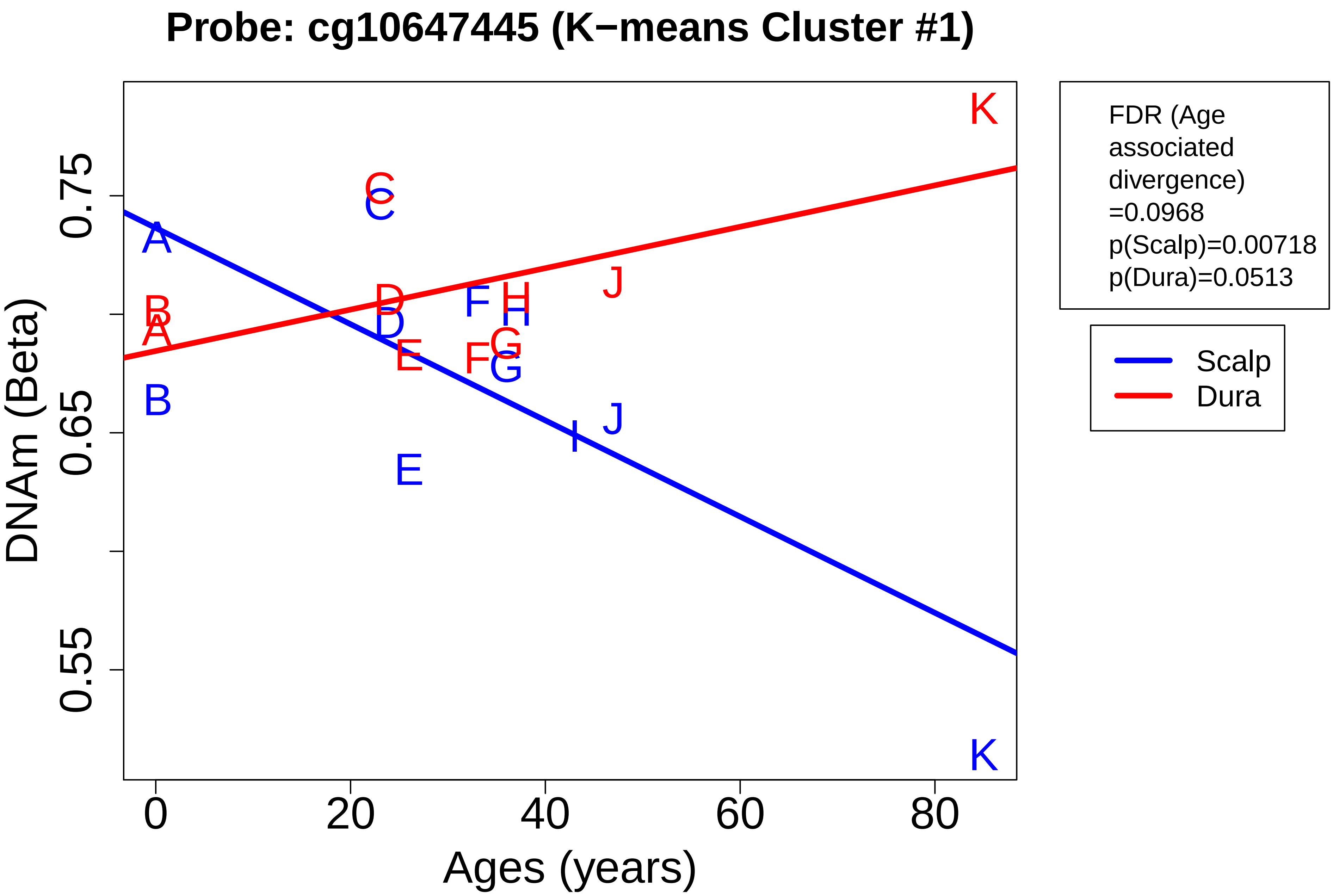

Probe: cg10647445 (K−means Cluster #1)
DNAm (Beta)
0.75
0.65
0.55
Ages (years)
0
20
40
60
80
FDR (Age associated divergence) =0.0968
p(Scalp)=0.00718
p(Dura)=0.0513
Scalp
Dura

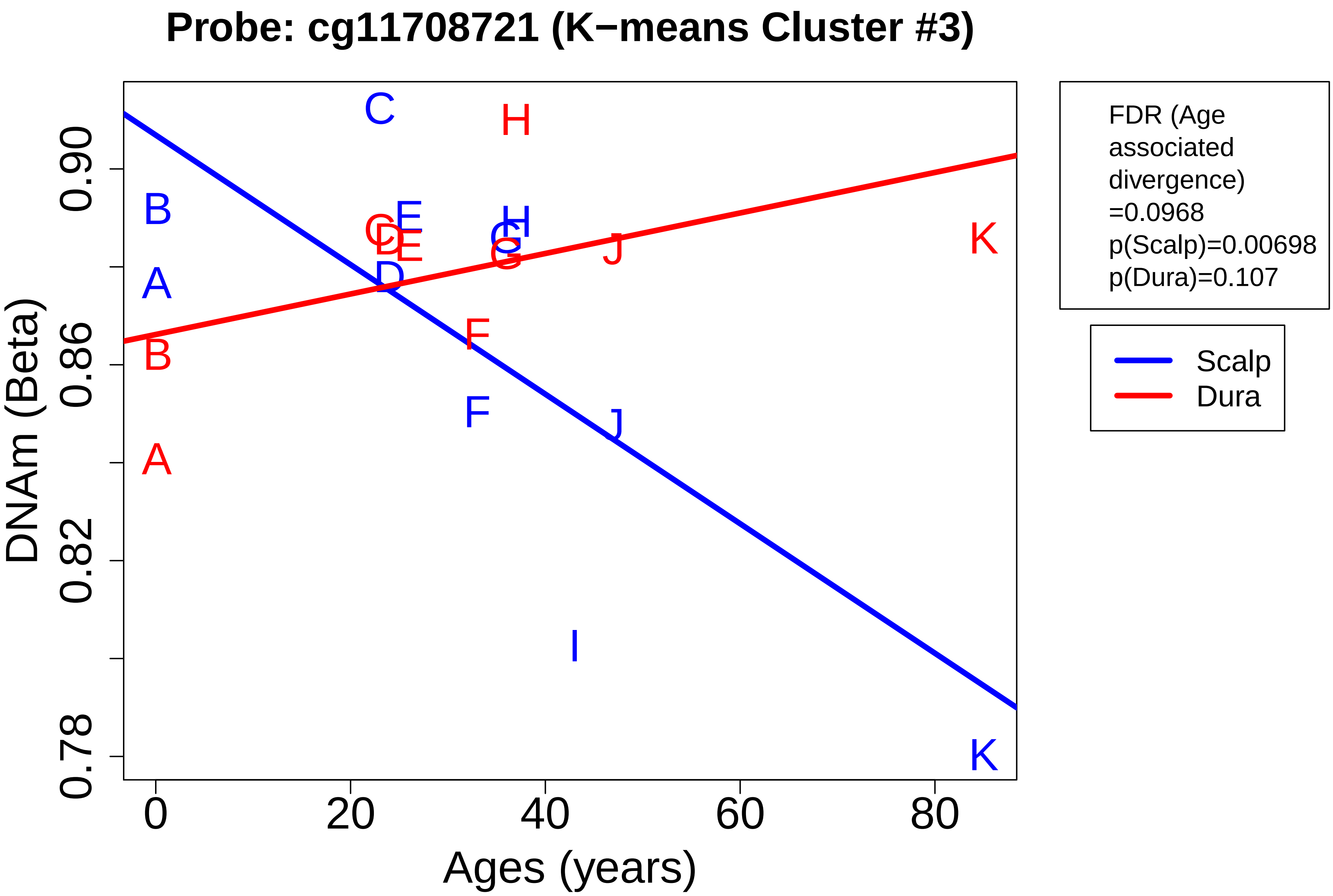

Probe: cg11708721 (K−means Cluster #3)
FDR (Age associated divergence) =0.0968
p(Scalp)=0.00698
p(Dura)=0.107
Scalp
Dura
DNAm (Beta)
0.78
0.82
0.86
0.90
0
20
40
60
80
Ages (years)

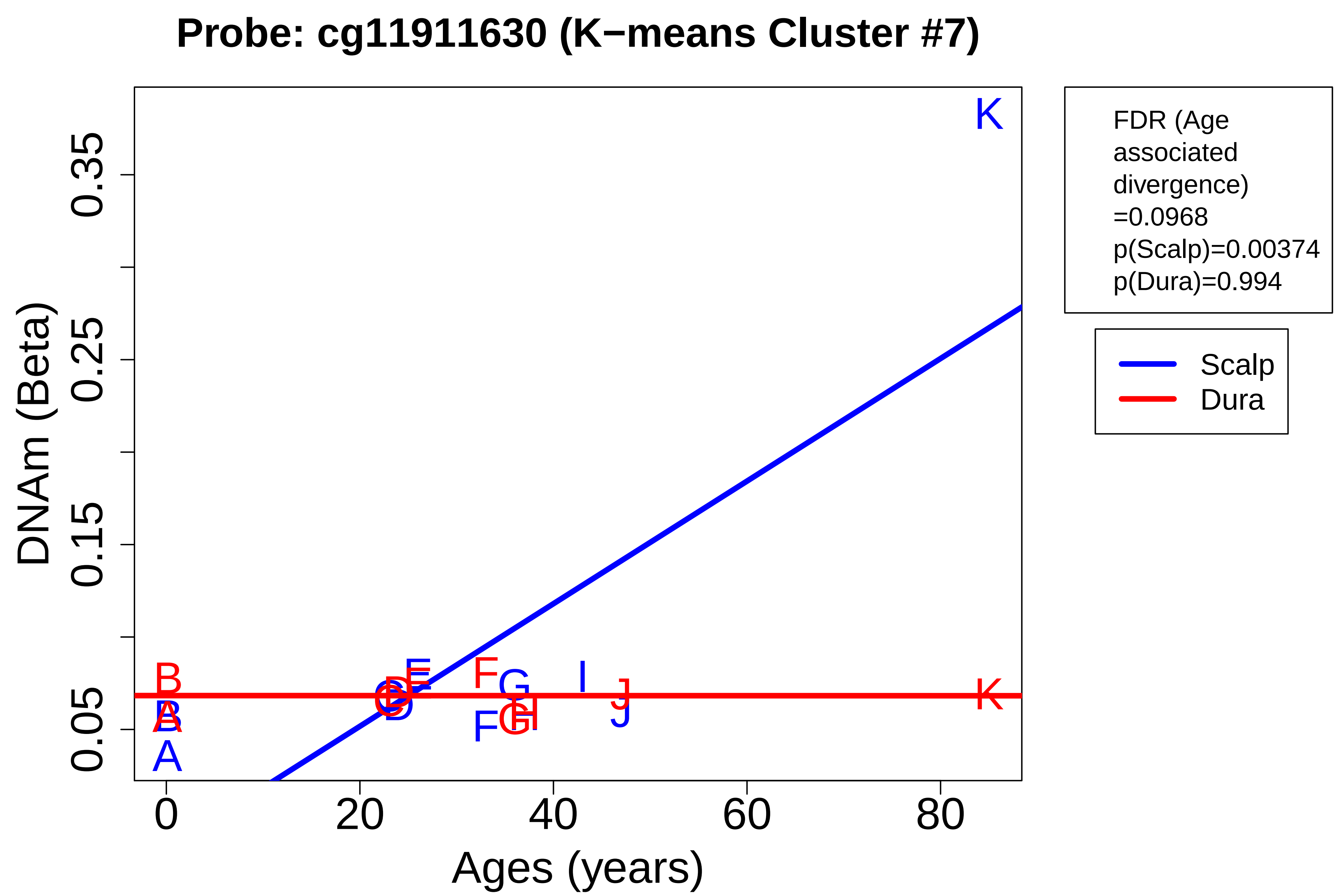

Probe: cg11911630 (K−means Cluster #7)
DNAm (Beta)
0.05
0.15
0.25
0.35
Ages (years)
0
20
40
60
80
FDR (Age associated divergence) =0.0968
p(Scalp)=0.00374
p(Dura)=0.994
Scalp
Dura

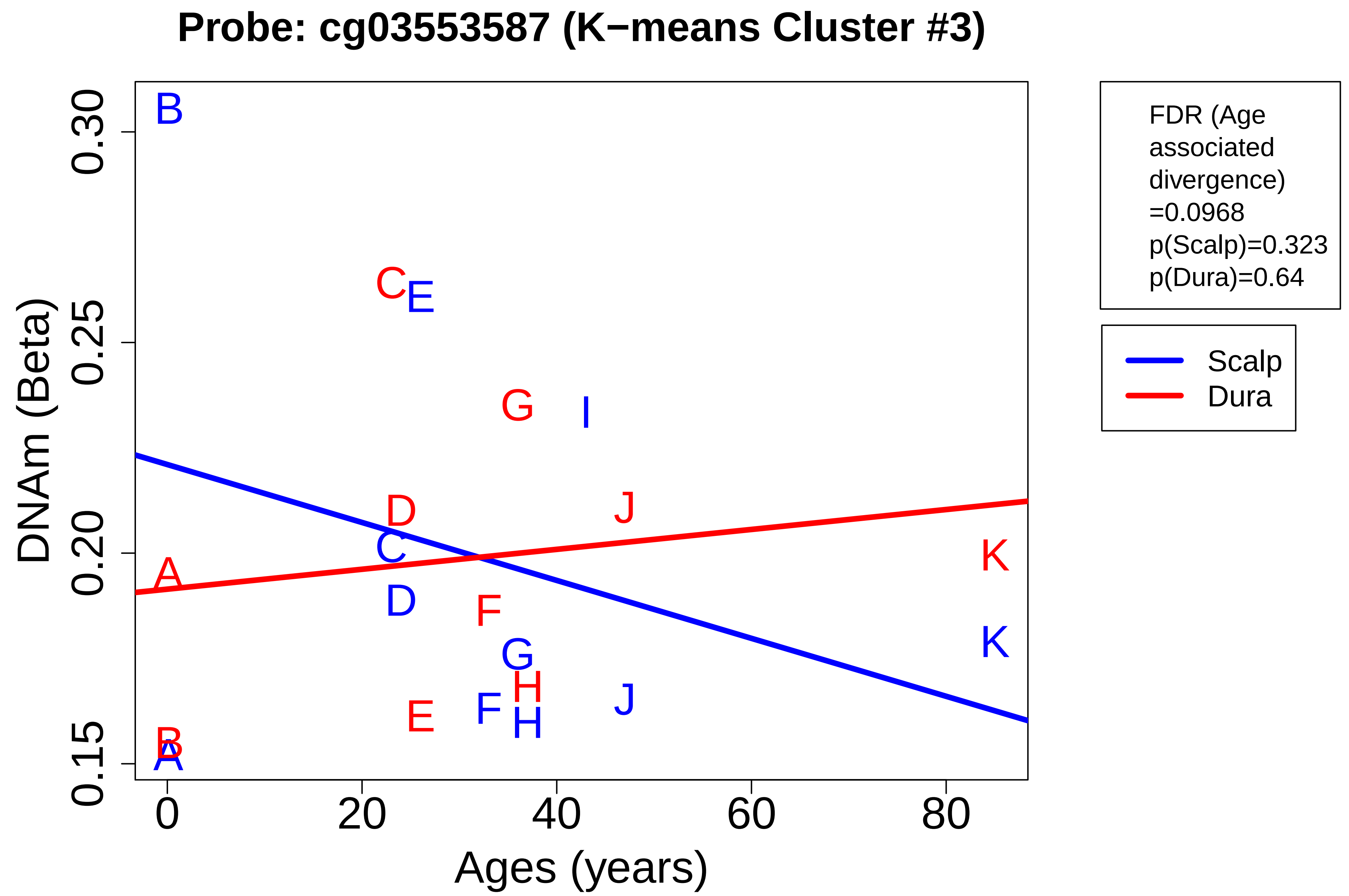
Probe: cg03553587 (K−means Cluster #3)
DNAm (Beta)
0.15
0.20
0.25
0.30
0
20
40
60
80
Ages (years)
FDR (Age associated divergence) =0.0968
p(Scalp)=0.323
p(Dura)=0.64
Scalp
Dura

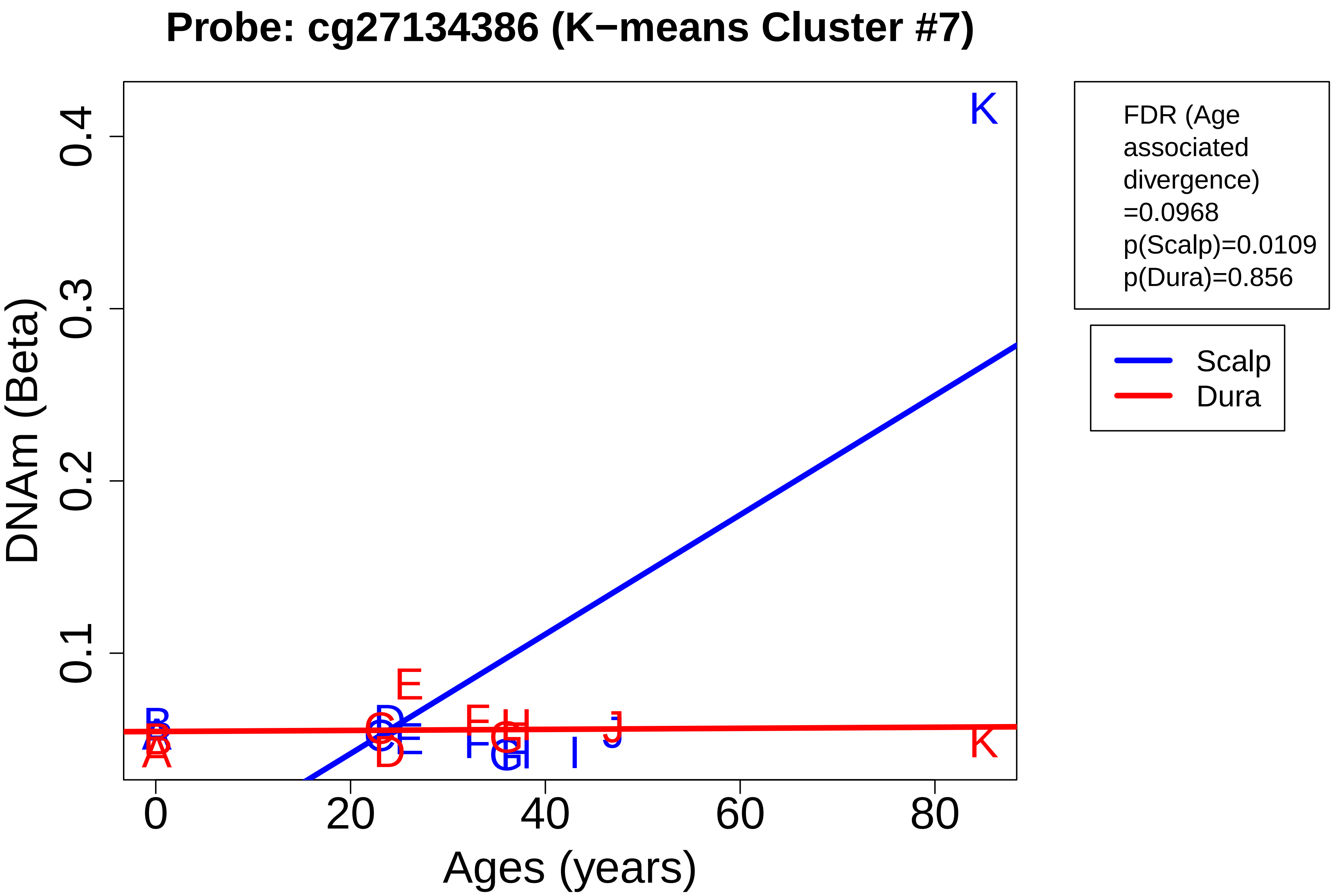
Probe: cg27134386 (K−means Cluster #7)
DNAm (Beta)
Ages (years)
FDR (Age associated divergence) =0.0968
p(Scalp)=0.0109
p(Dura)=0.856
Scalp
Dura

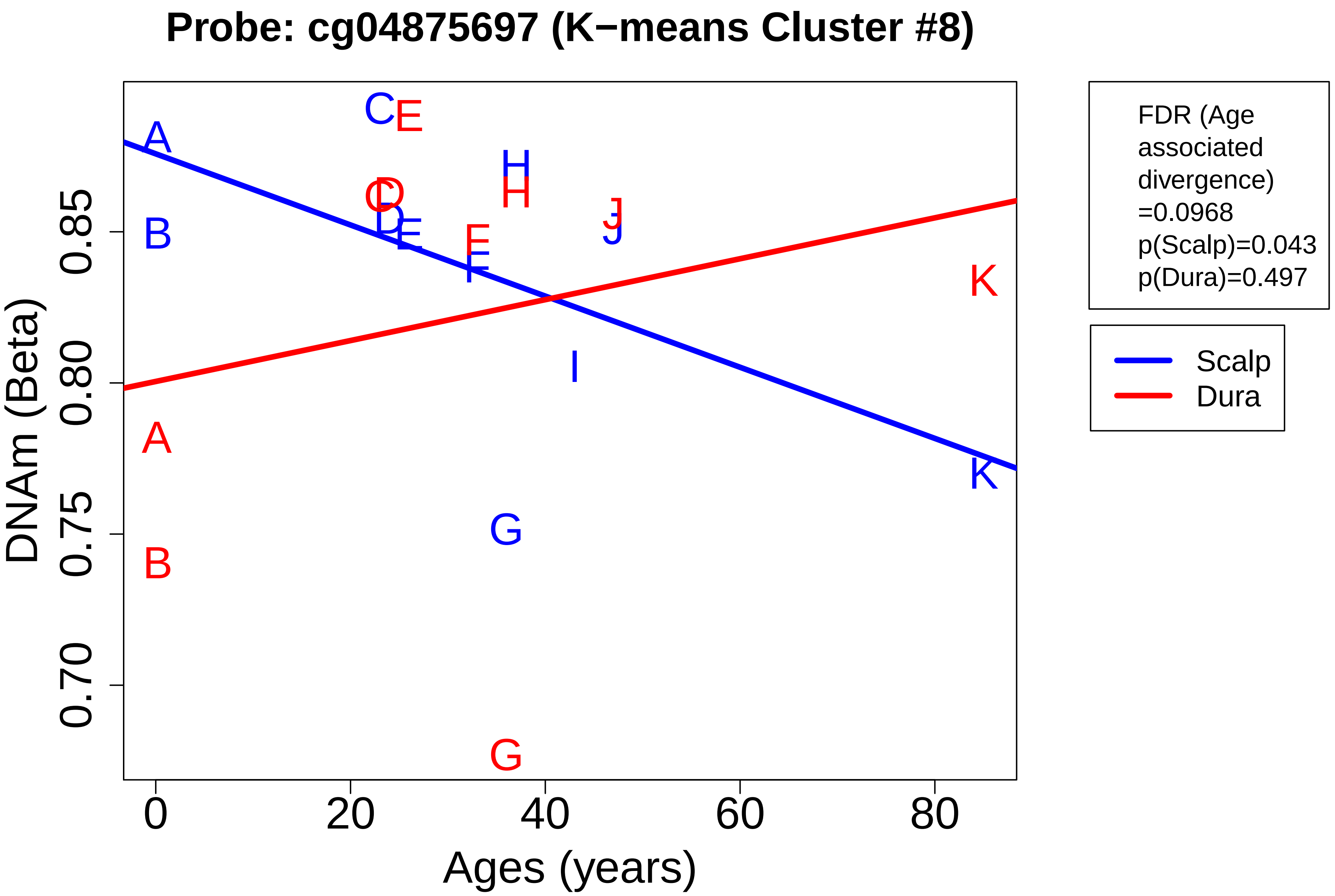
Probe: cg04875697 (K−means Cluster #8)
DNAm (Beta)
0.70
0.75
0.80
0.85
0
20
40
60
80
Ages (years)
FDR (Age associated divergence) =0.0968
p(Scalp)=0.043
p(Dura)=0.497
Scalp
Dura

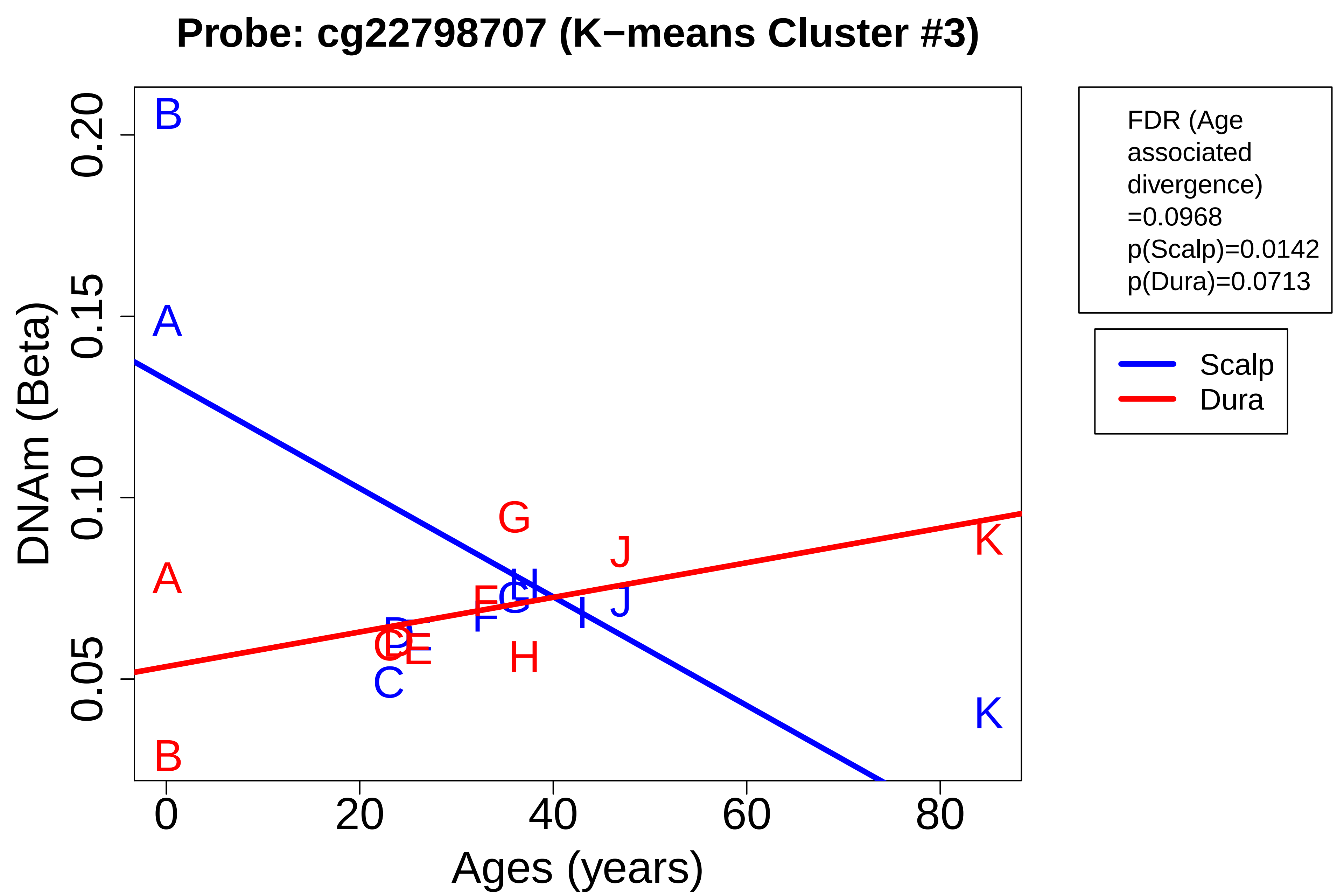

Probe: cg22798707 (K−means Cluster #3)
DNAm (Beta)
0.05
0.10
0.15
0.20
0
20
40
60
80
Ages (years)
FDR (Age associated divergence) =0.0968
p(Scalp)=0.0142
p(Dura)=0.0713
Scalp
Dura

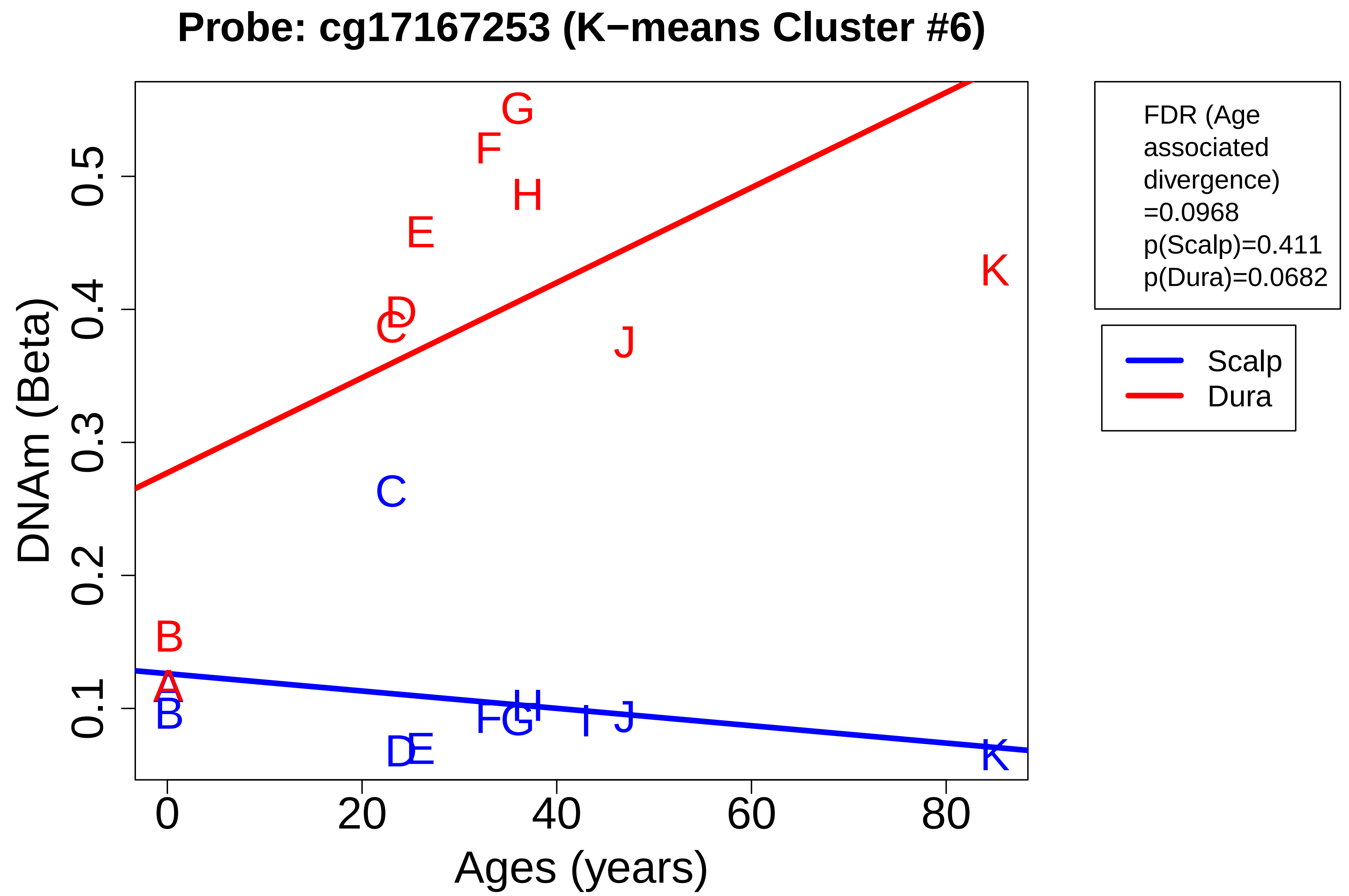
Probe: cg17167253 (K−means Cluster #6)
DNAm (Beta)
Ages (years)
0.1
0.2
0.3
0.4
0.5
0
20
40
60
80
FDR (Age associated divergence) =0.0968
p(Scalp)=0.411
p(Dura)=0.0682
Scalp
Dura

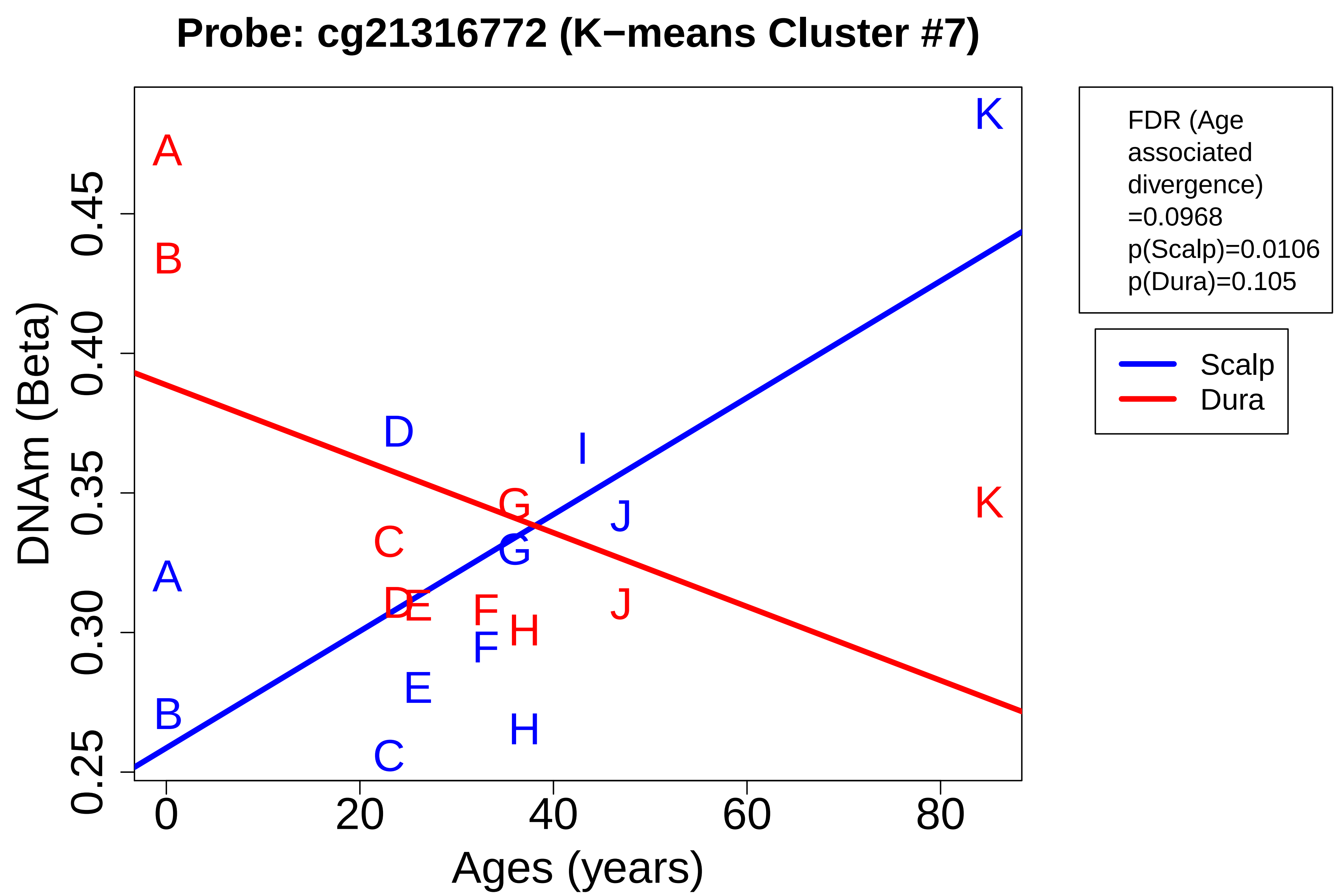

Probe: cg21316772 (K−means Cluster #7)
DNAm (Beta)
0.25
0.30
0.35
0.40
0.45
Ages (years)
0
20
40
60
80
A
B
C
D
E
F
G
H
I
J
K
FDR (Age associated divergence) =0.0968
p(Scalp)=0.0106
p(Dura)=0.105
Scalp
Dura

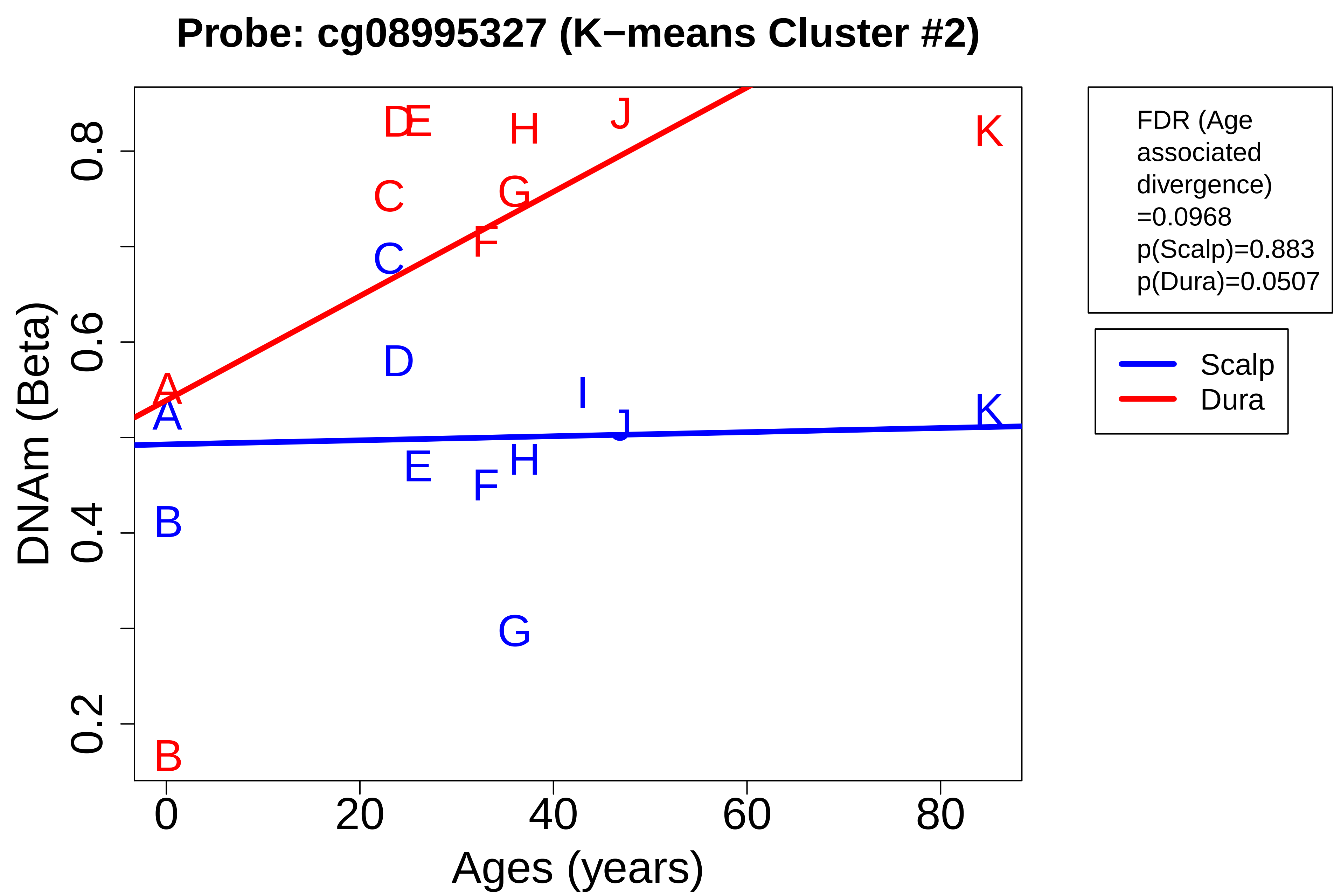
Probe: cg08995327 (K−means Cluster #2)
DNAm (Beta)
0.2
0.4
0.6
0.8
Ages (years)
0
20
40
60
80
FDR (Age associated divergence) =0.0968
p(Scalp)=0.883
p(Dura)=0.0507
Scalp
Dura
A
B
C
D
E
F
G
H
I
J
K

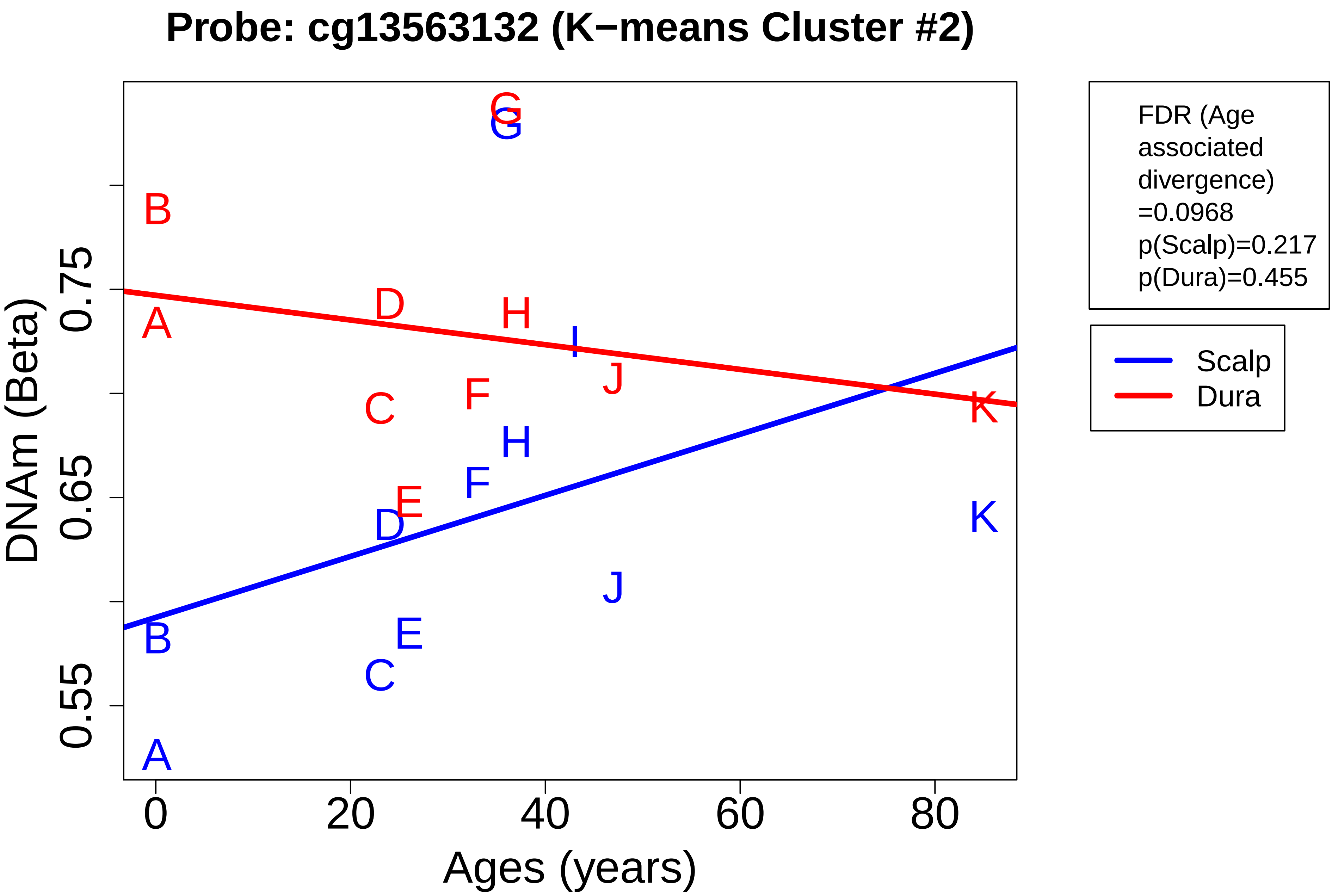
Probe: cg13563132 (K−means Cluster #2)
DNAm (Beta)
0.55
0.65
0.75
Ages (years)
0
20
40
60
80
FDR (Age associated divergence) =0.0968
p(Scalp)=0.217
p(Dura)=0.455
Scalp
Dura

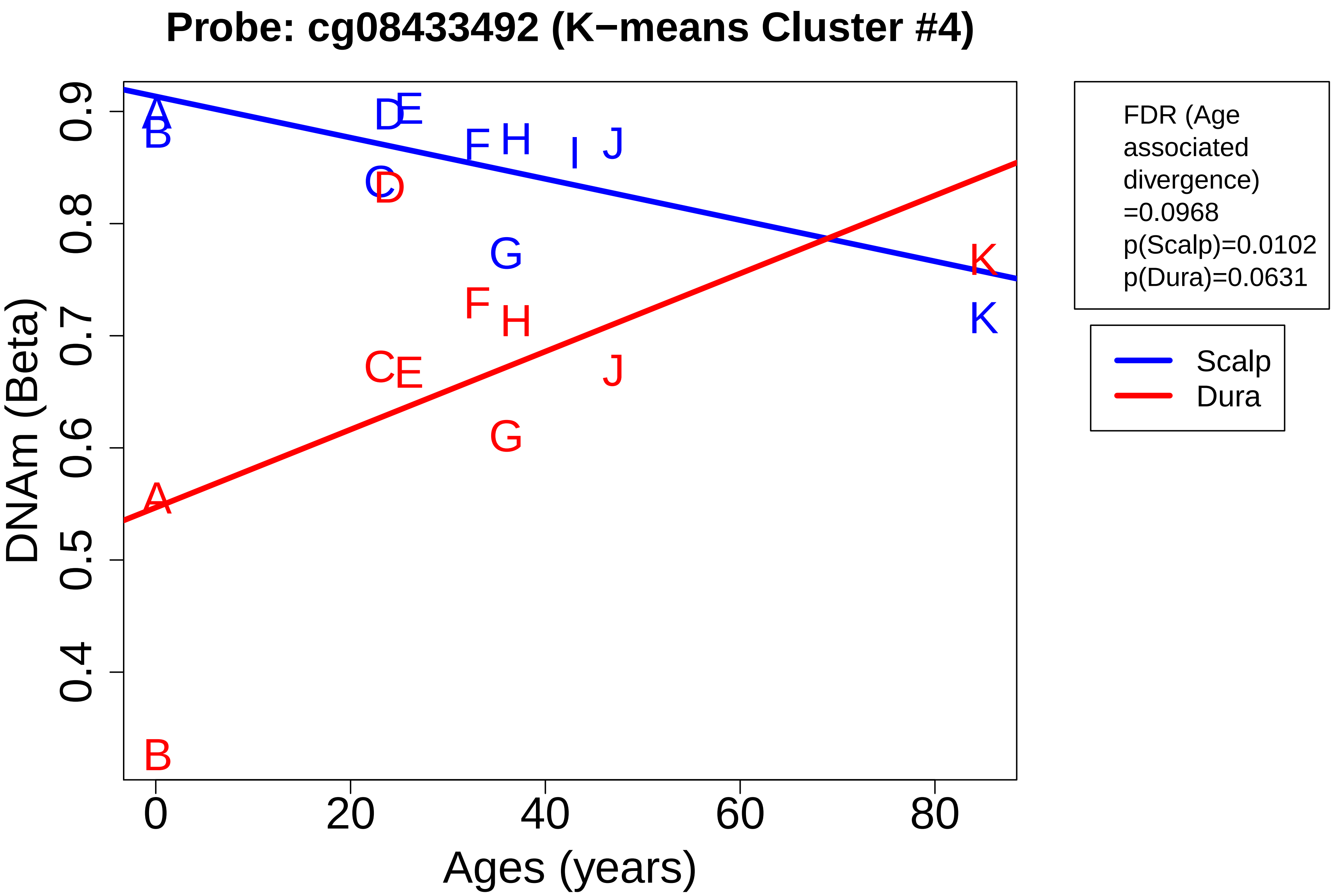
Probe: cg08433492 (K−means Cluster #4)
DNAm (Beta)
0.4
0.5
0.6
0.7
0.8
0.9
0
20
40
60
80
Ages (years)
FDR (Age associated divergence) =0.0968
p(Scalp)=0.0102
p(Dura)=0.0631
Scalp
Dura

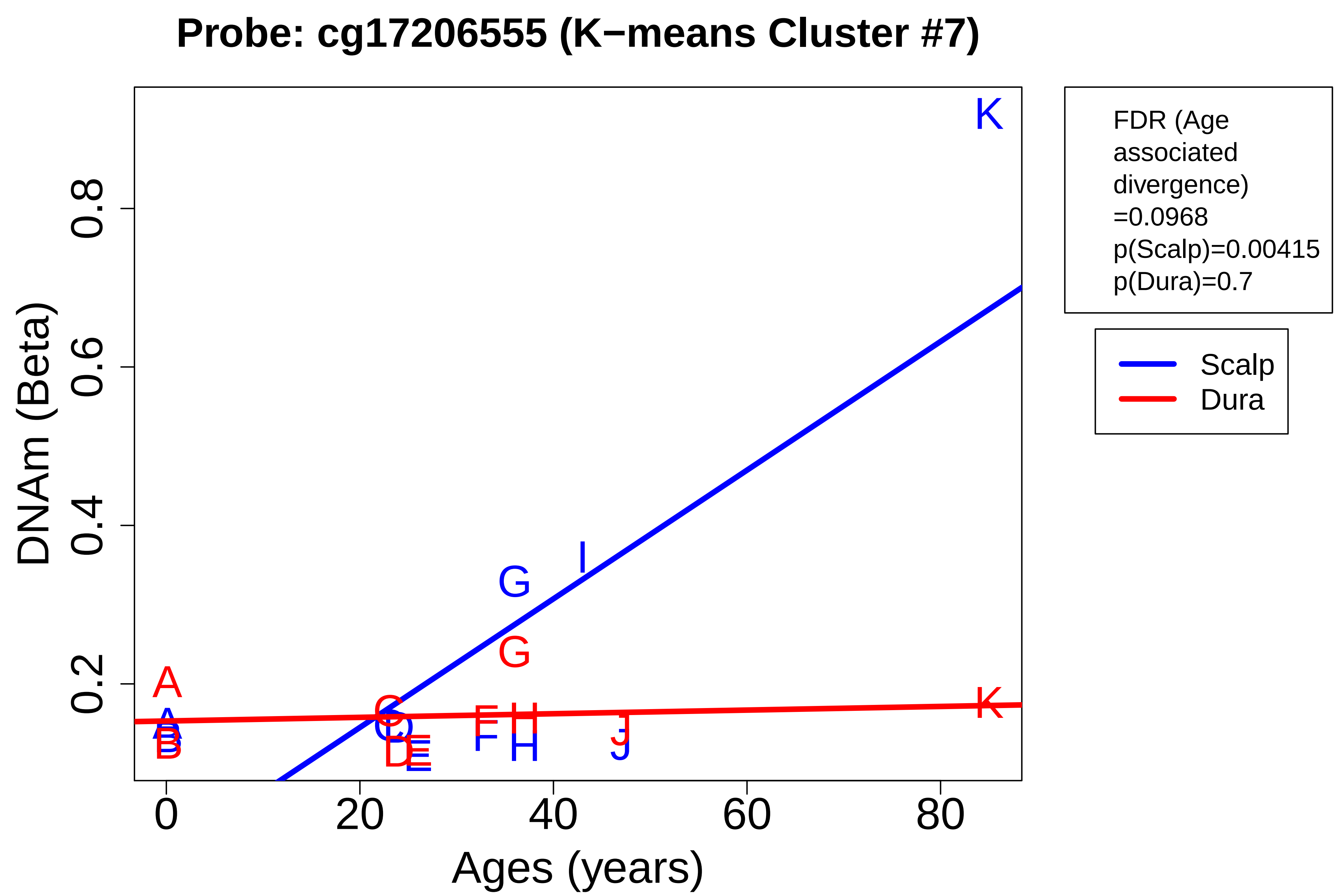
Probe: cg17206555 (K−means Cluster #7)
DNAm (Beta)
0.2
0.4
0.6
0.8
0
20
40
60
80
Ages (years)
A
B
C
D
E
F
G
H
I
J
K
FDR (Age associated divergence) =0.0968
p(Scalp)=0.00415
p(Dura)=0.7
Scalp
Dura

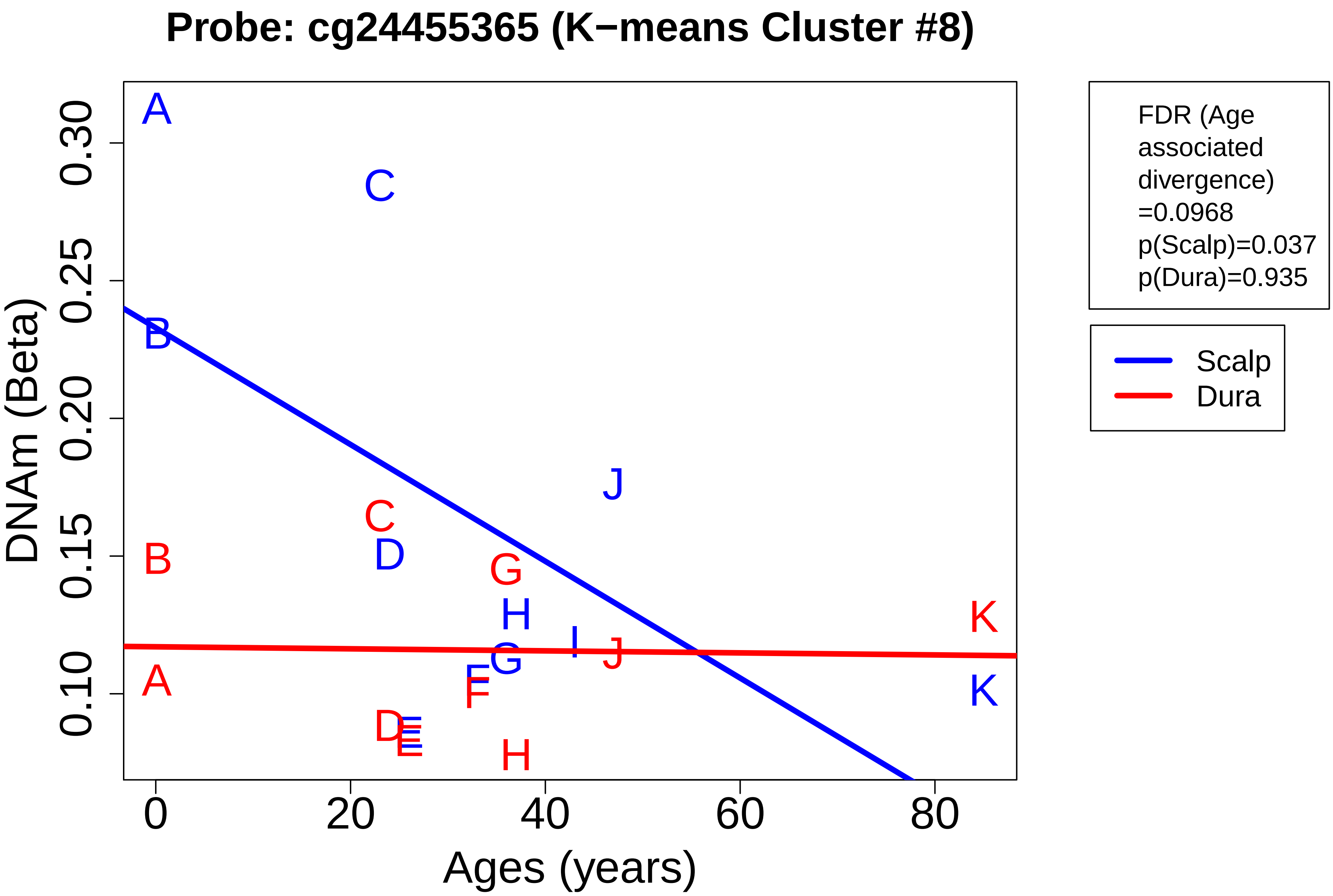

Probe: cg24455365 (K−means Cluster #8)
DNAm (Beta)
0.10
0.15
0.20
0.25
0.30
0
20
40
60
80
Ages (years)
A
B
C
D
E
F
G
H
I
J
K
A
B
C
D
E
F
G
H
J
K
FDR (Age associated divergence) =0.0968
p(Scalp)=0.037
p(Dura)=0.935
Scalp
Dura

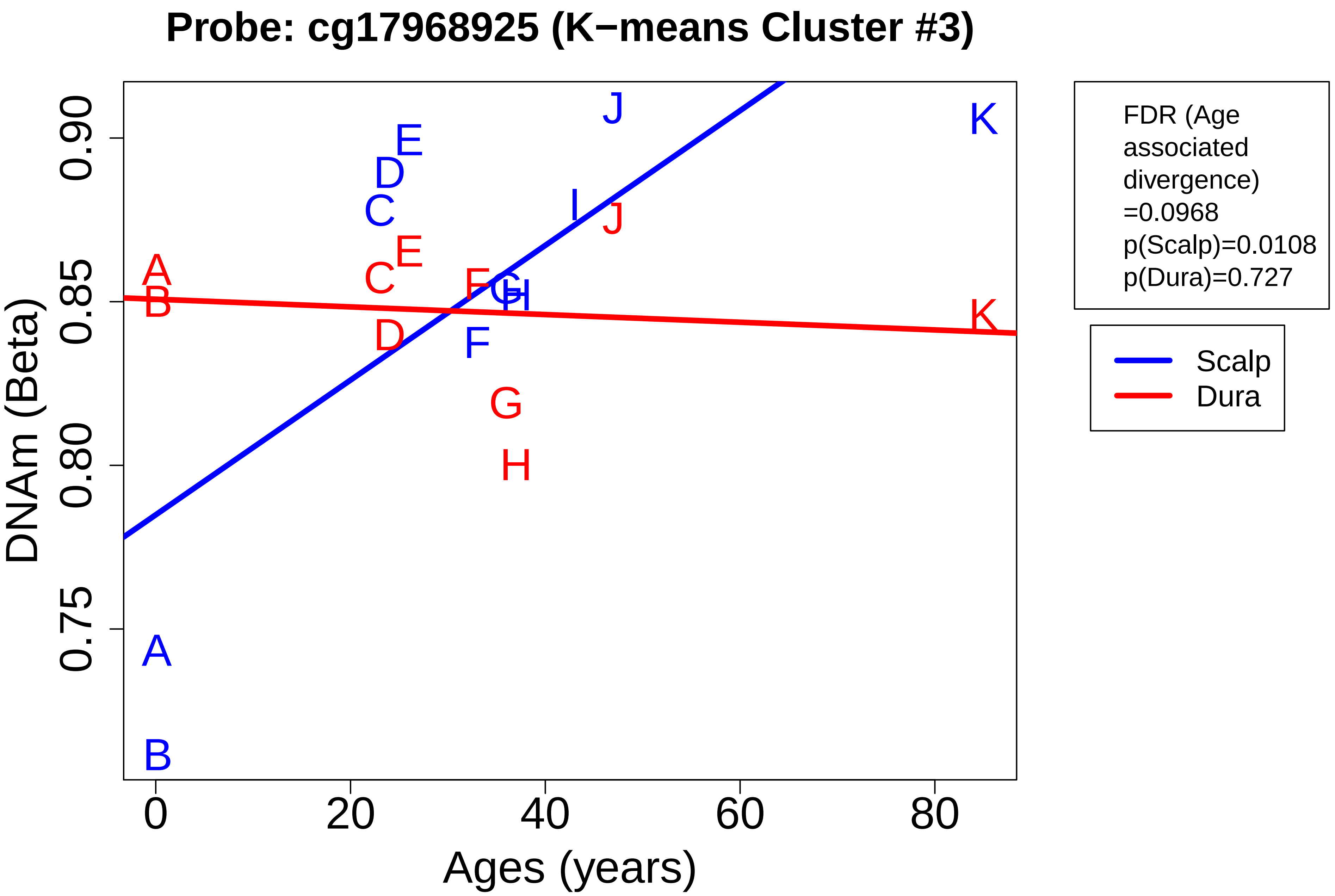

Probe: cg17968925 (K−means Cluster #3)
DNAm (Beta)
Ages (years)
0.75
0.80
0.85
0.90
0
20
40
60
80
FDR (Age associated divergence) =0.0968
p(Scalp)=0.0108
p(Dura)=0.727
Scalp
Dura

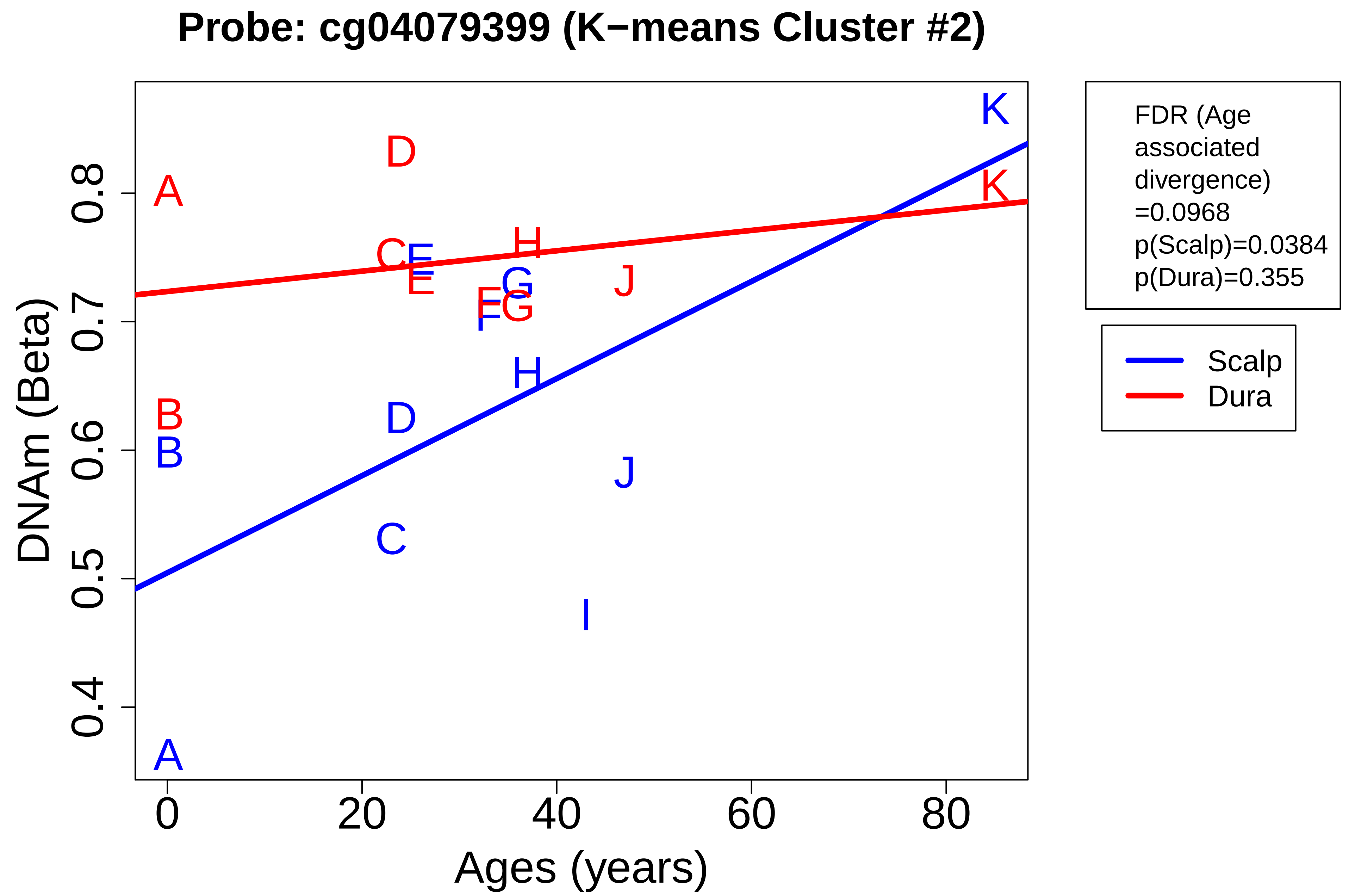

Probe: cg04079399 (K−means Cluster #2)
FDR (Age associated divergence) =0.0968
p(Scalp)=0.0384
p(Dura)=0.355
Scalp
Dura
DNAm (Beta)
0.4
0.5
0.6
0.7
0.8
0
20
40
60
80
Ages (years)
A
B
C
D
E
F
G
H
I
J
K

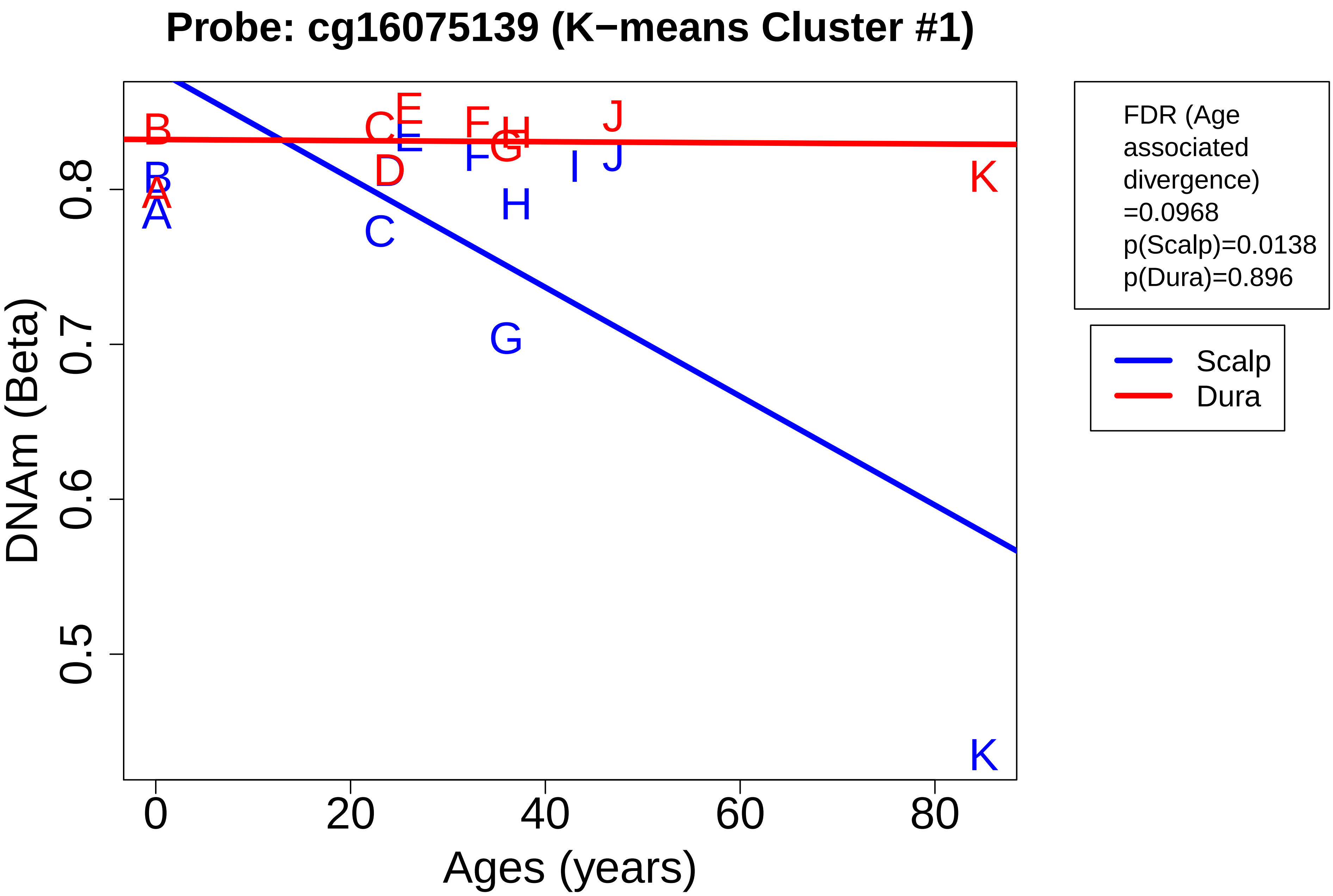

Probe: cg16075139 (K−means Cluster #1)
DNAm (Beta)
0.5
0.6
0.7
0.8
0
20
40
60
80
Ages (years)
A
B
C
D
E
F
G
H
I
J
K
FDR (Age associated divergence) =0.0968
p(Scalp)=0.0138
p(Dura)=0.896
Scalp
Dura

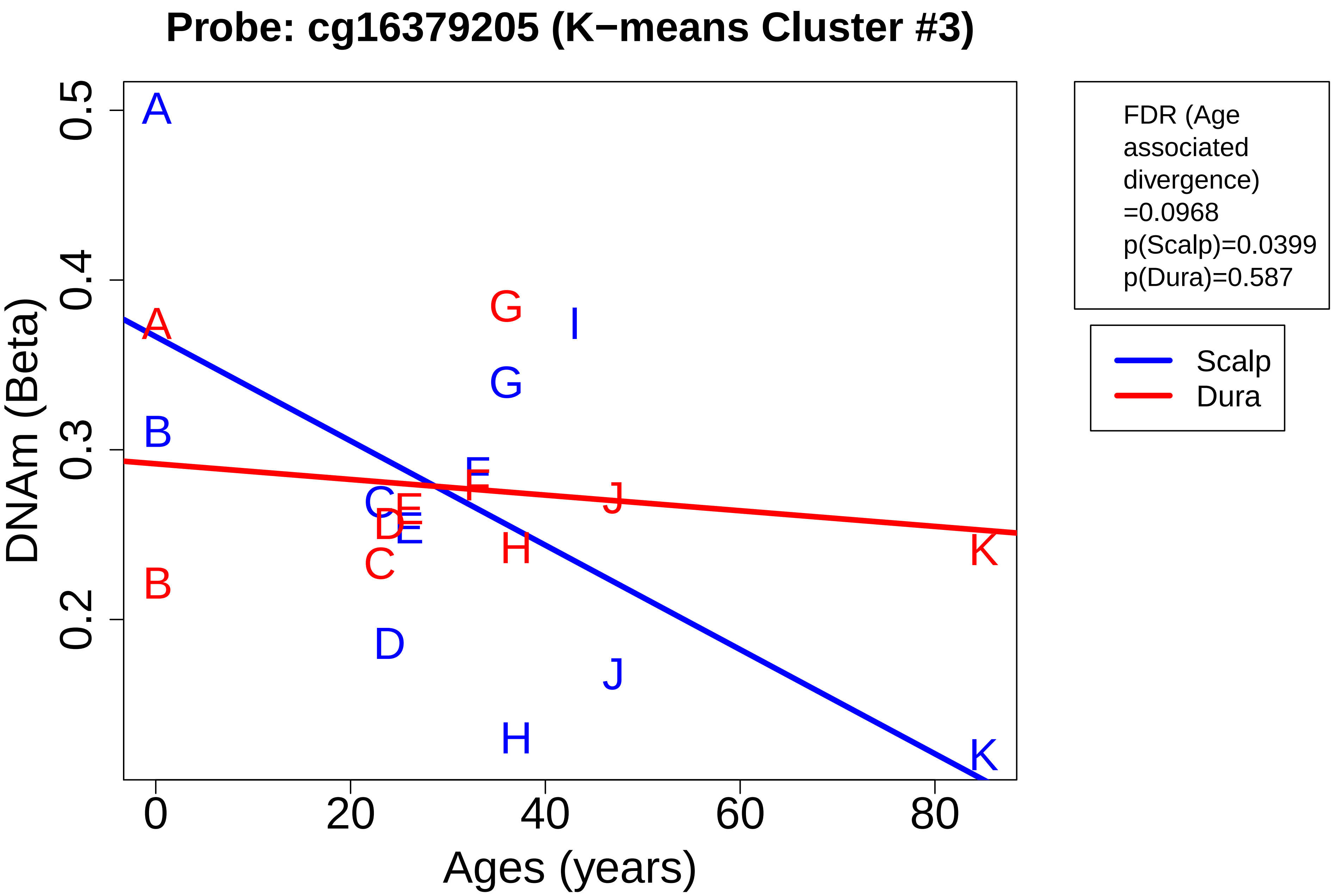

Probe: cg16379205 (K−means Cluster #3)
DNAm (Beta)
0.2
0.3
0.4
0.5
Ages (years)
0
20
40
60
80
FDR (Age associated divergence) =0.0968
p(Scalp)=0.0399
p(Dura)=0.587
Scalp
Dura

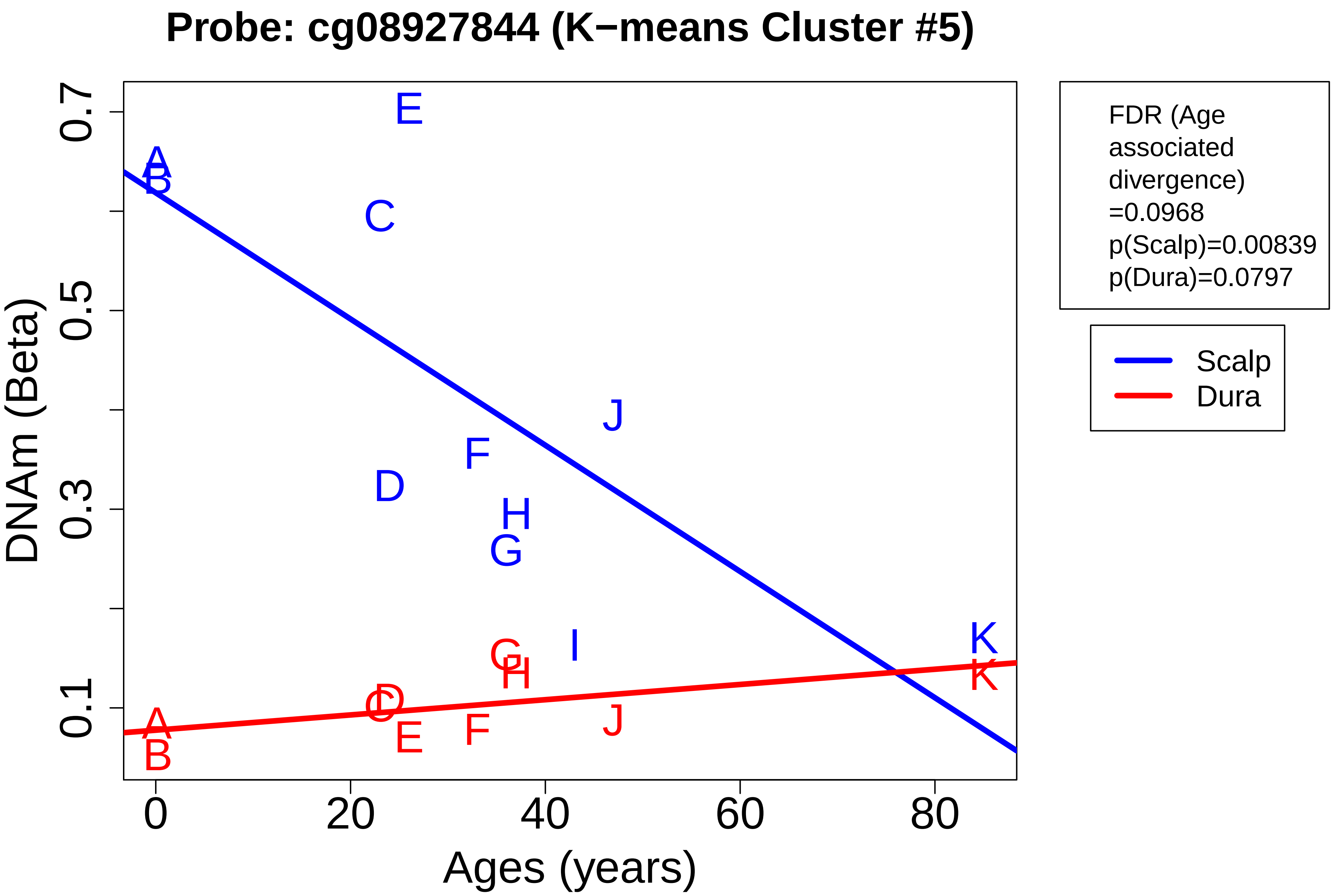

Probe: cg08927844 (K−means Cluster #5)
DNAm (Beta)
0.7
0.5
0.3
0.1
0
20
40
60
80
Ages (years)
FDR (Age associated divergence) =0.0968
p(Scalp)=0.00839
p(Dura)=0.0797
Scalp
Dura
A
B
C
D
E
F
G
H
I
J
K

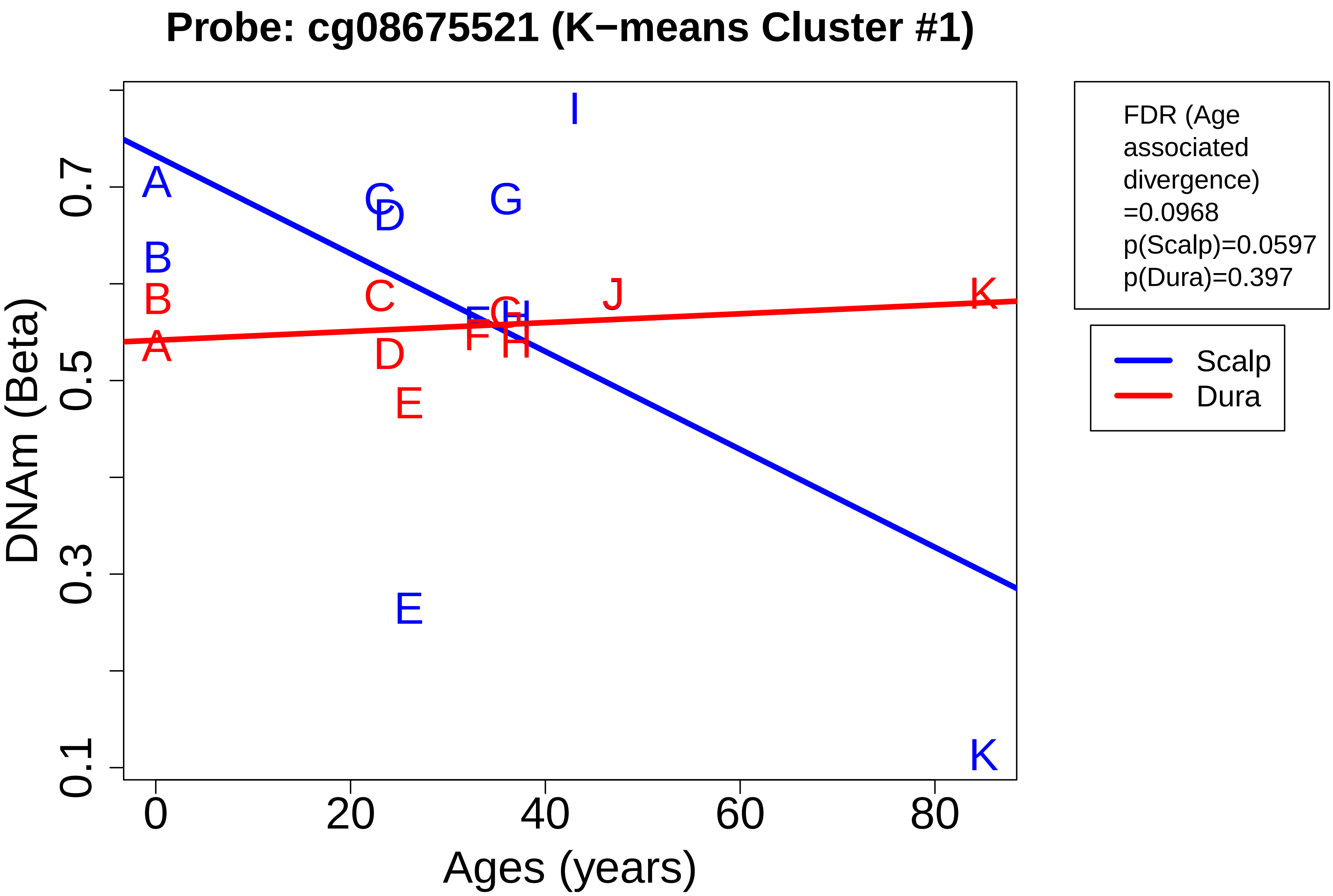

Probe: cg08675521 (K−means Cluster #1)
FDR (Age associated divergence) =0.0968
p(Scalp)=0.0597
p(Dura)=0.397
Scalp
Dura
DNAm (Beta)
0.1
0.3
0.5
0.7
Ages (years)
0
20
40
60
80
A
B
C
D
E
F
G
H
I
J
K

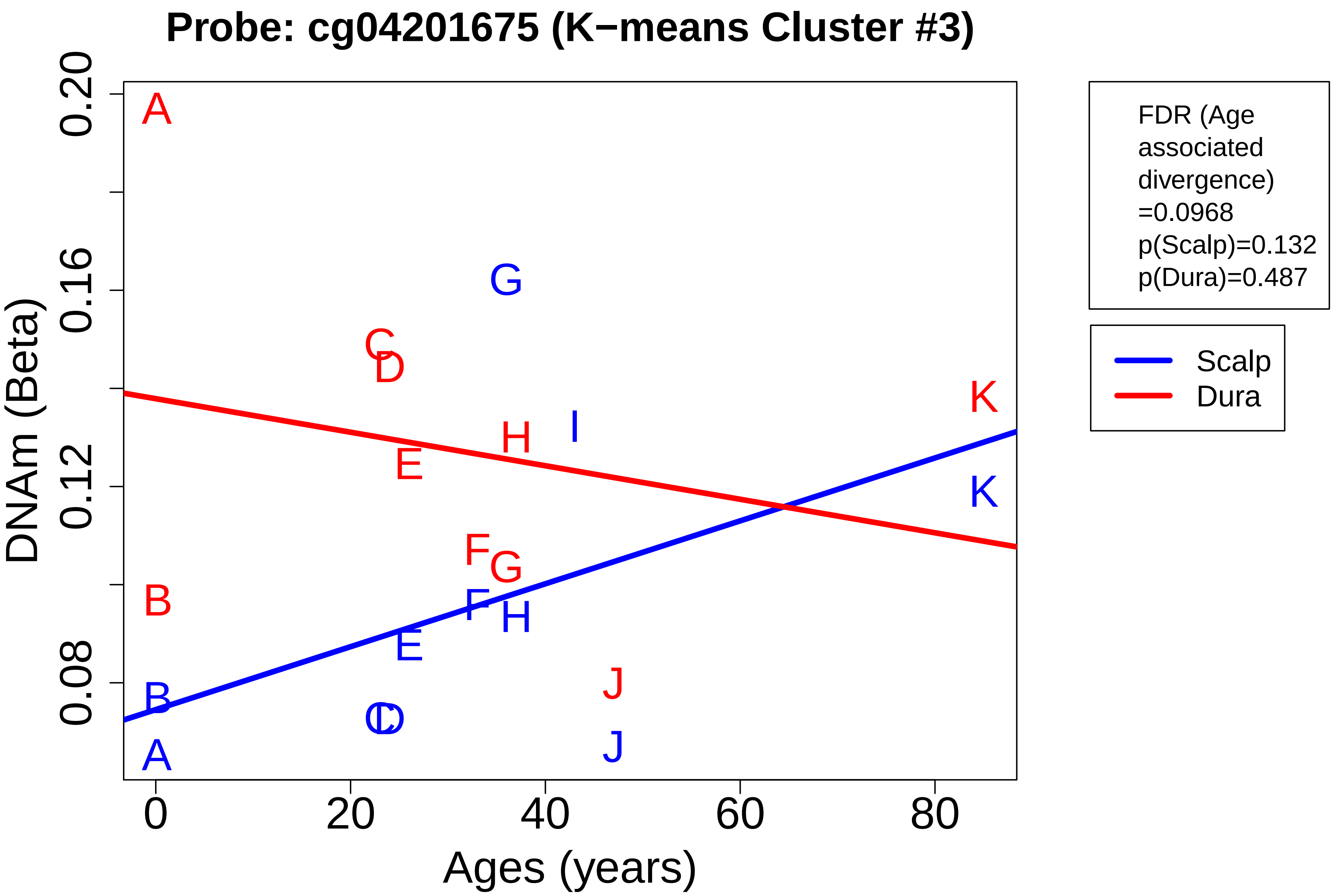

Probe: cg04201675 (K−means Cluster #3)
DNAm (Beta)
0.08
0.12
0.16
0.20
0
20
40
60
80
Ages (years)
FDR (Age associated divergence) =0.0968
p(Scalp)=0.132
p(Dura)=0.487
Scalp
Dura
A
B
C
D
E
F
G
H
I
J
K

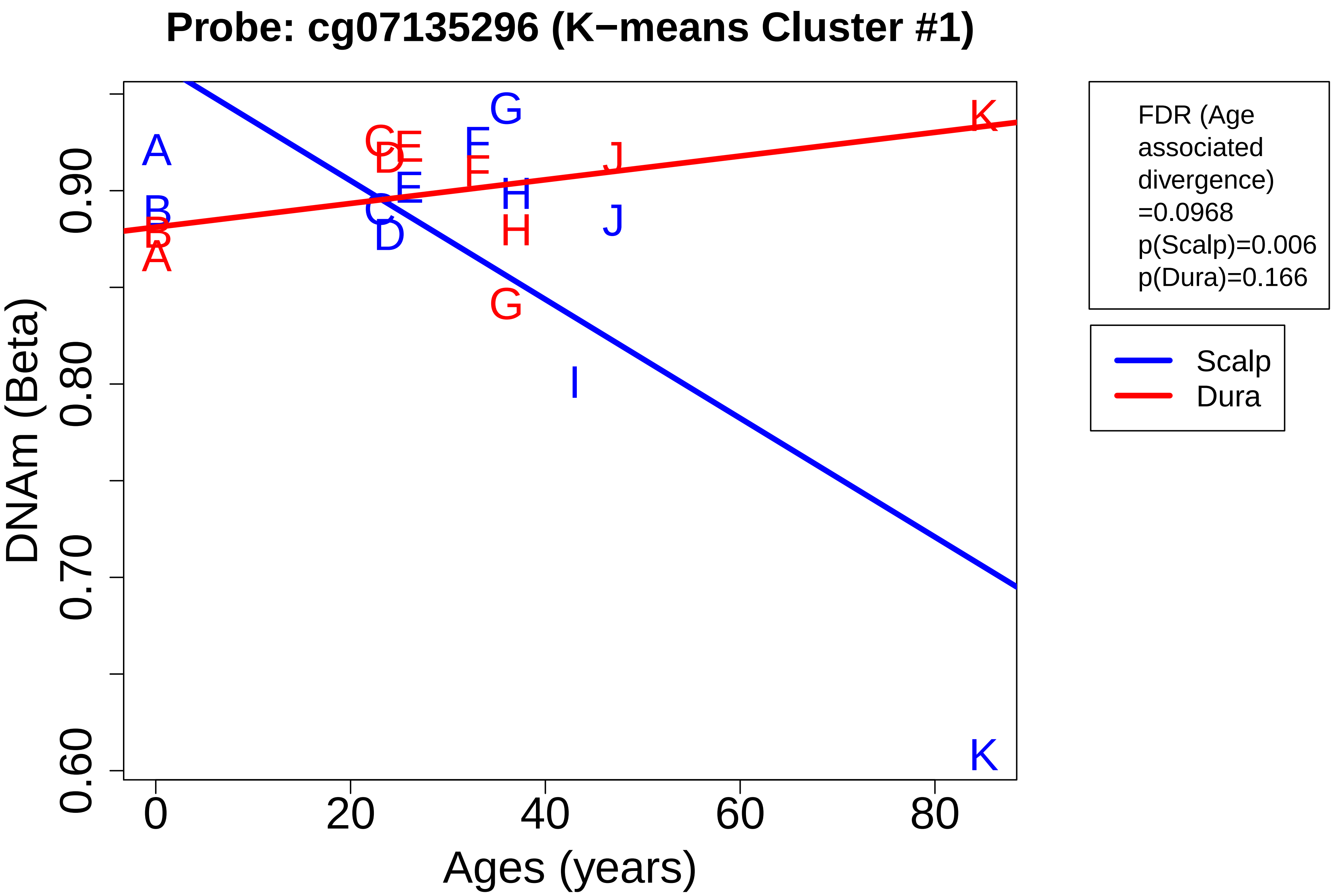

Probe: cg07135296 (K−means Cluster #1)
DNAm (Beta)
0.60
0.70
0.80
0.90
0
20
40
60
80
Ages (years)
FDR (Age associated divergence) =0.0968
p(Scalp)=0.006
p(Dura)=0.166
Scalp
Dura

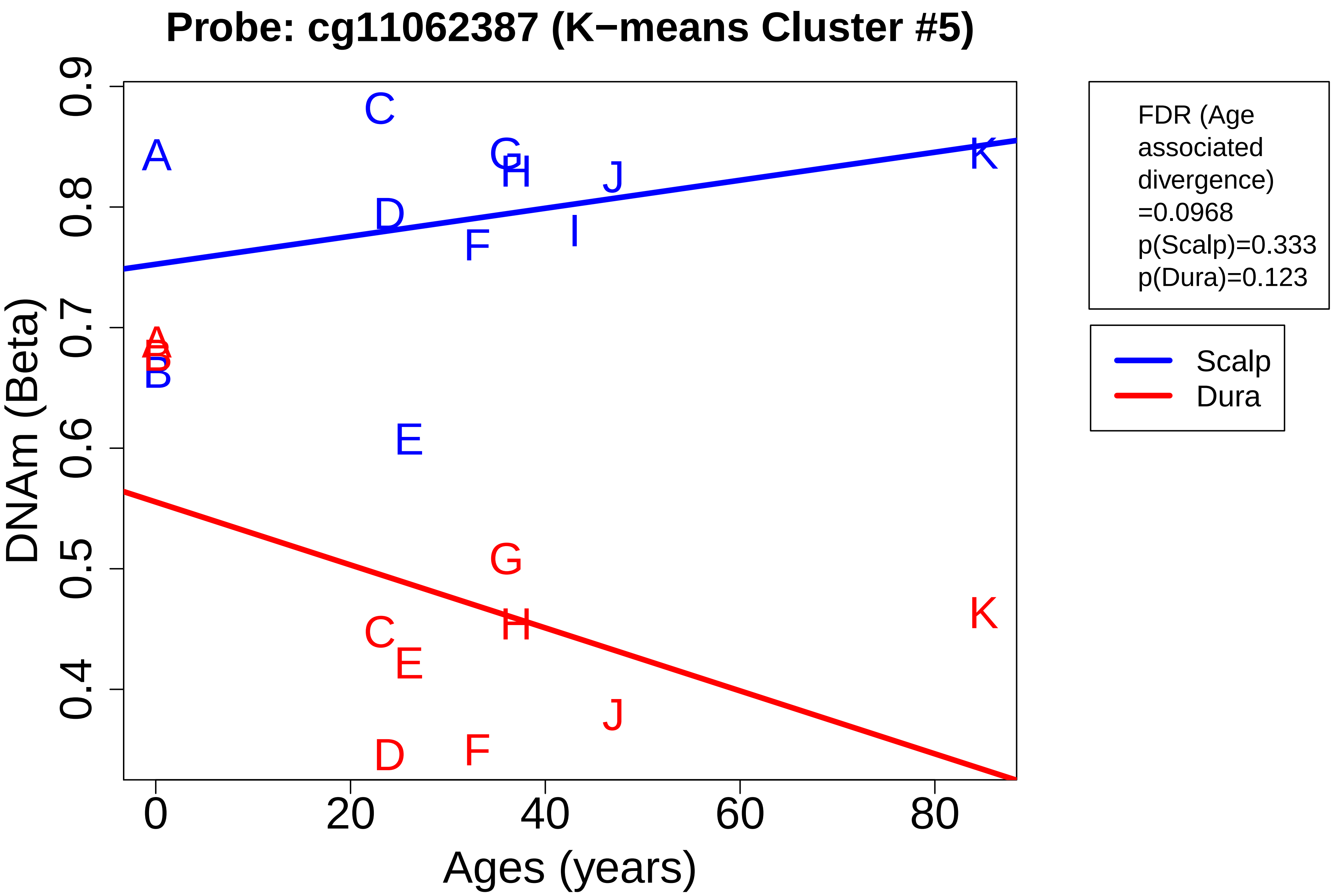

Probe: cg11062387 (K−means Cluster #5)
DNAm (Beta)
0.4
0.5
0.6
0.7
0.8
0.9
0
20
40
60
80
Ages (years)
A
B
C
D
E
F
G
H
I
J
K
FDR (Age associated divergence) =0.0968
p(Scalp)=0.333
p(Dura)=0.123
Scalp
Dura

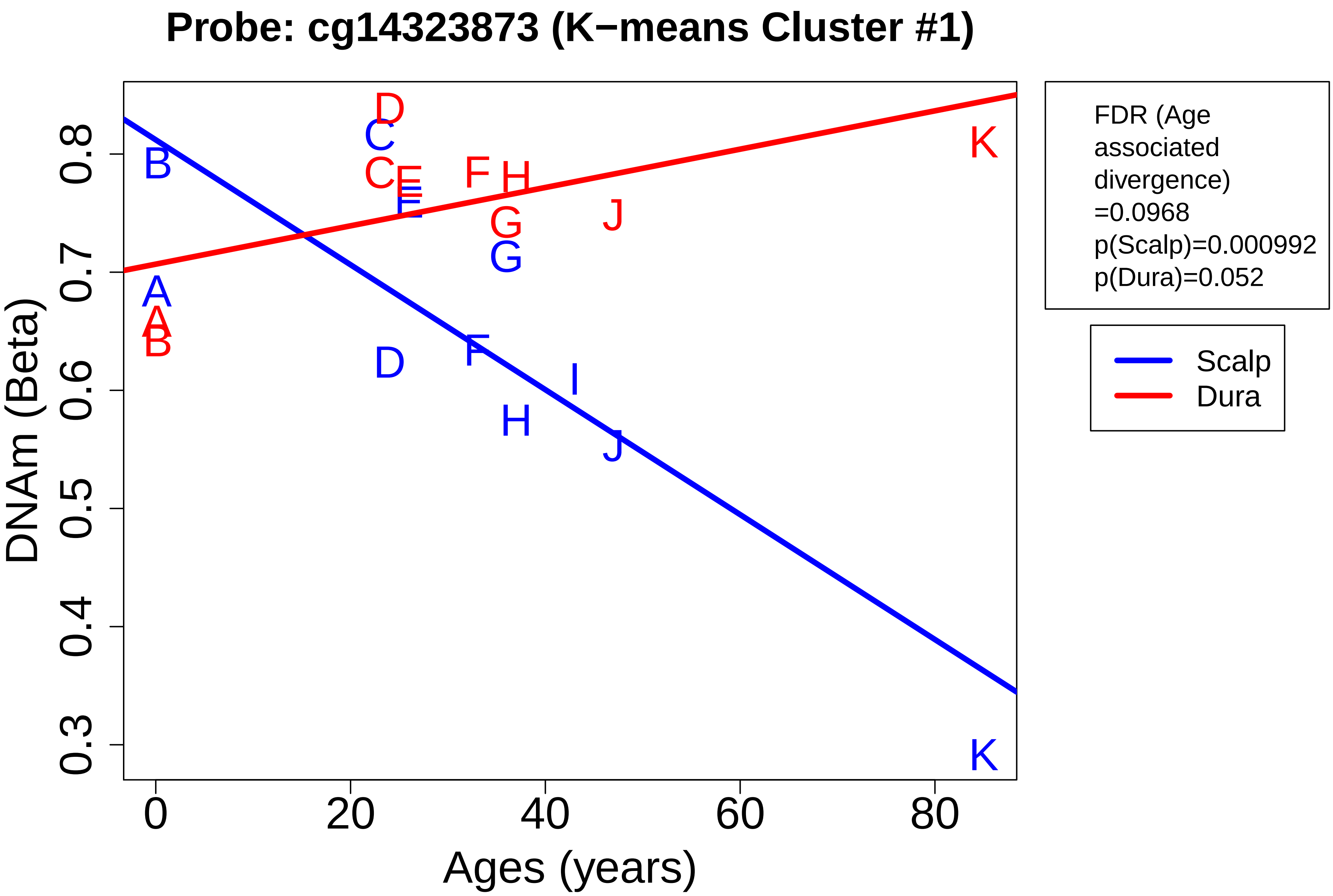

Probe: cg14323873 (K−means Cluster #1)
DNAm (Beta)
0.3
0.4
0.5
0.6
0.7
0.8
0
20
40
60
80
Ages (years)
A
B
C
D
E
F
G
H
I
J
K
FDR (Age associated divergence) =0.0968
p(Scalp)=0.000992
p(Dura)=0.052
Scalp
Dura

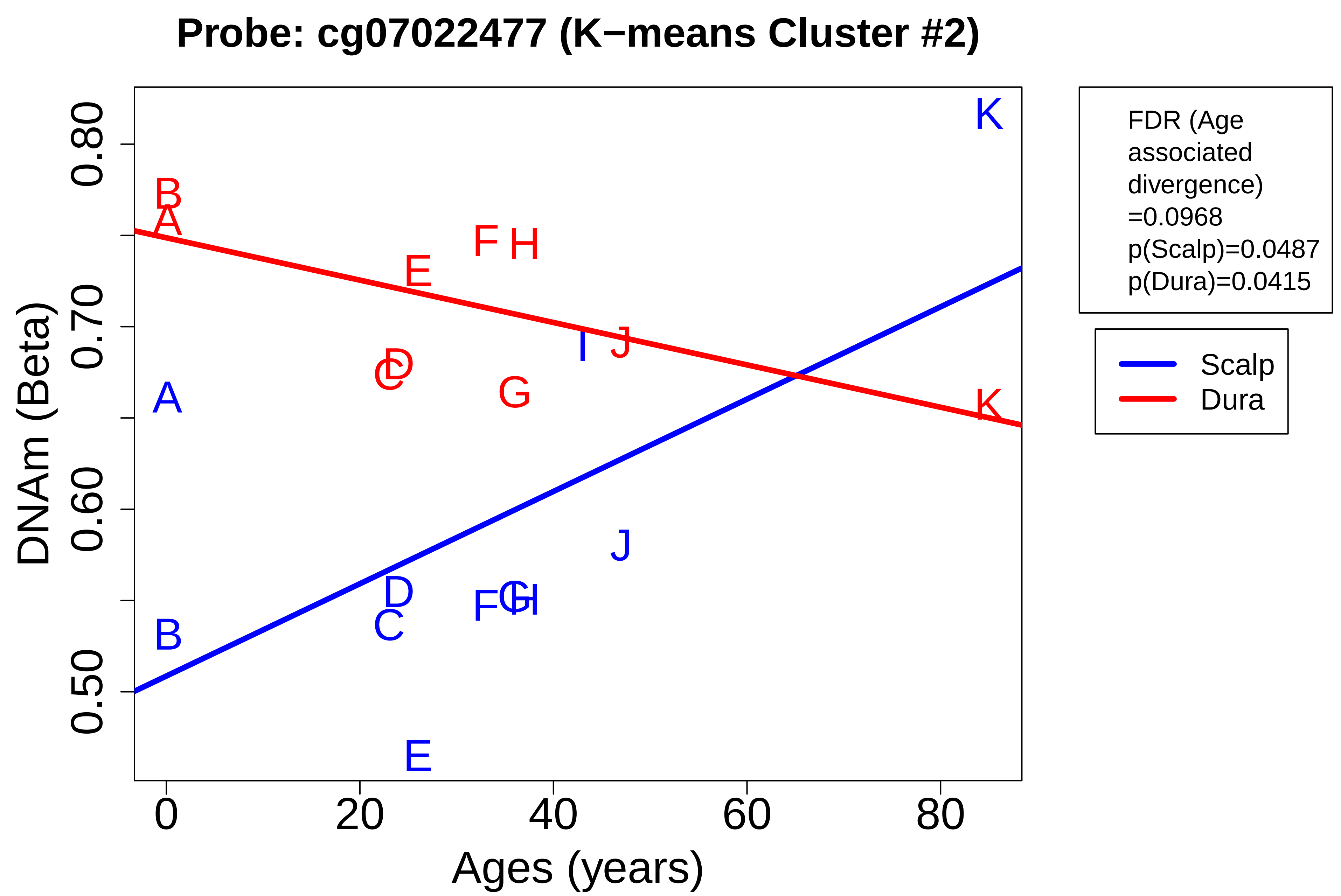
Probe: cg07022477 (K−means Cluster #2)
FDR (Age associated divergence) =0.0968
p(Scalp)=0.0487
p(Dura)=0.0415
Scalp
Dura
DNAm (Beta)
0.50
0.60
0.70
0.80
0
20
40
60
80
Ages (years)
A
B
C
D
E
F
G
H
I
J
K

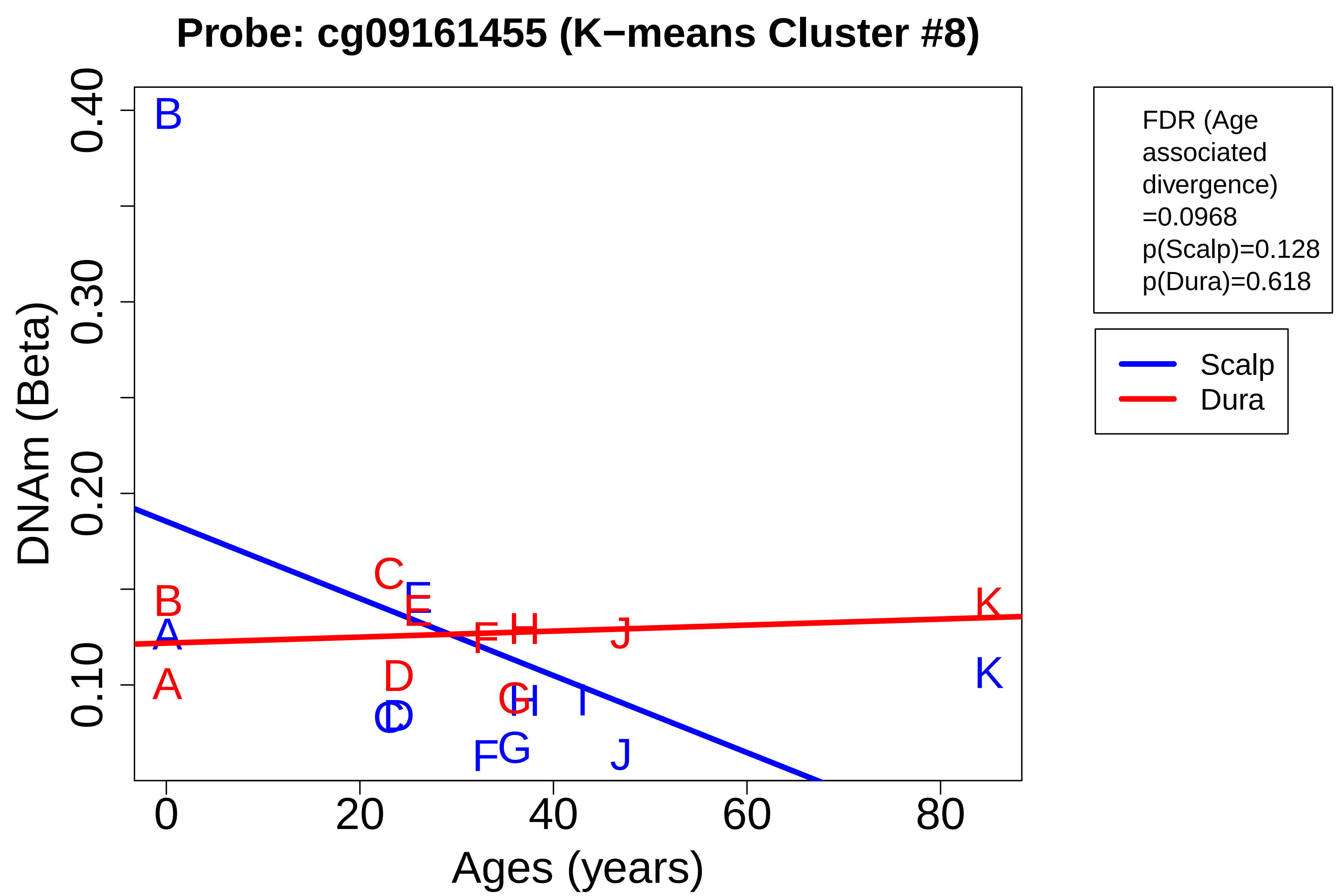

Probe: cg09161455 (K−means Cluster #8)
DNAm (Beta)
0.10
0.20
0.30
0.40
0
20
40
60
80
Ages (years)
A
B
C
D
E
F
G
H
I
J
K
FDR (Age associated divergence) =0.0968
p(Scalp)=0.128
p(Dura)=0.618
Scalp
Dura

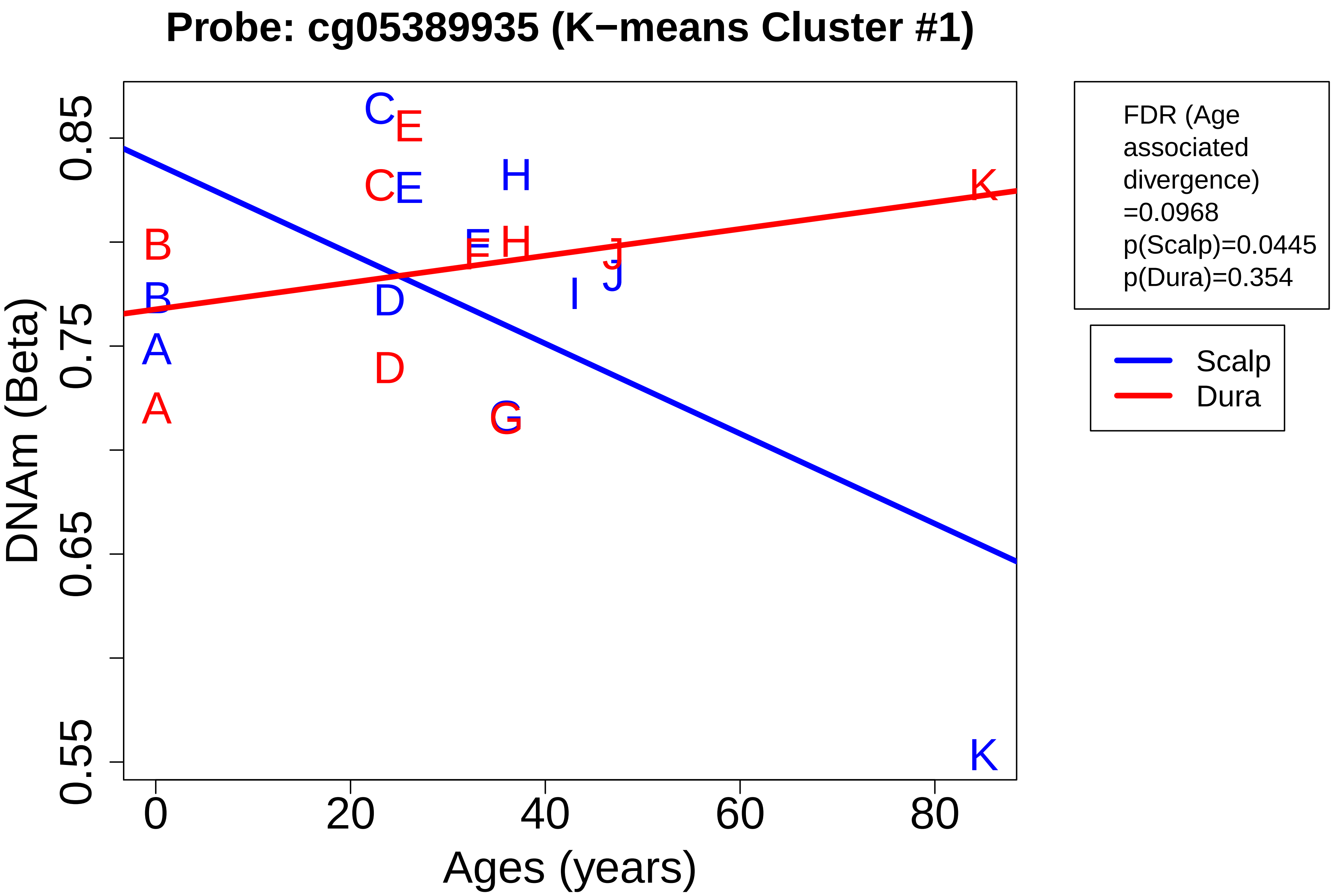

Probe: cg05389935 (K−means Cluster #1)
FDR (Age associated divergence) =0.0968
p(Scalp)=0.0445
p(Dura)=0.354
Scalp
Dura
DNAm (Beta)
0.55
0.65
0.75
0.85
Ages (years)
0
20
40
60
80

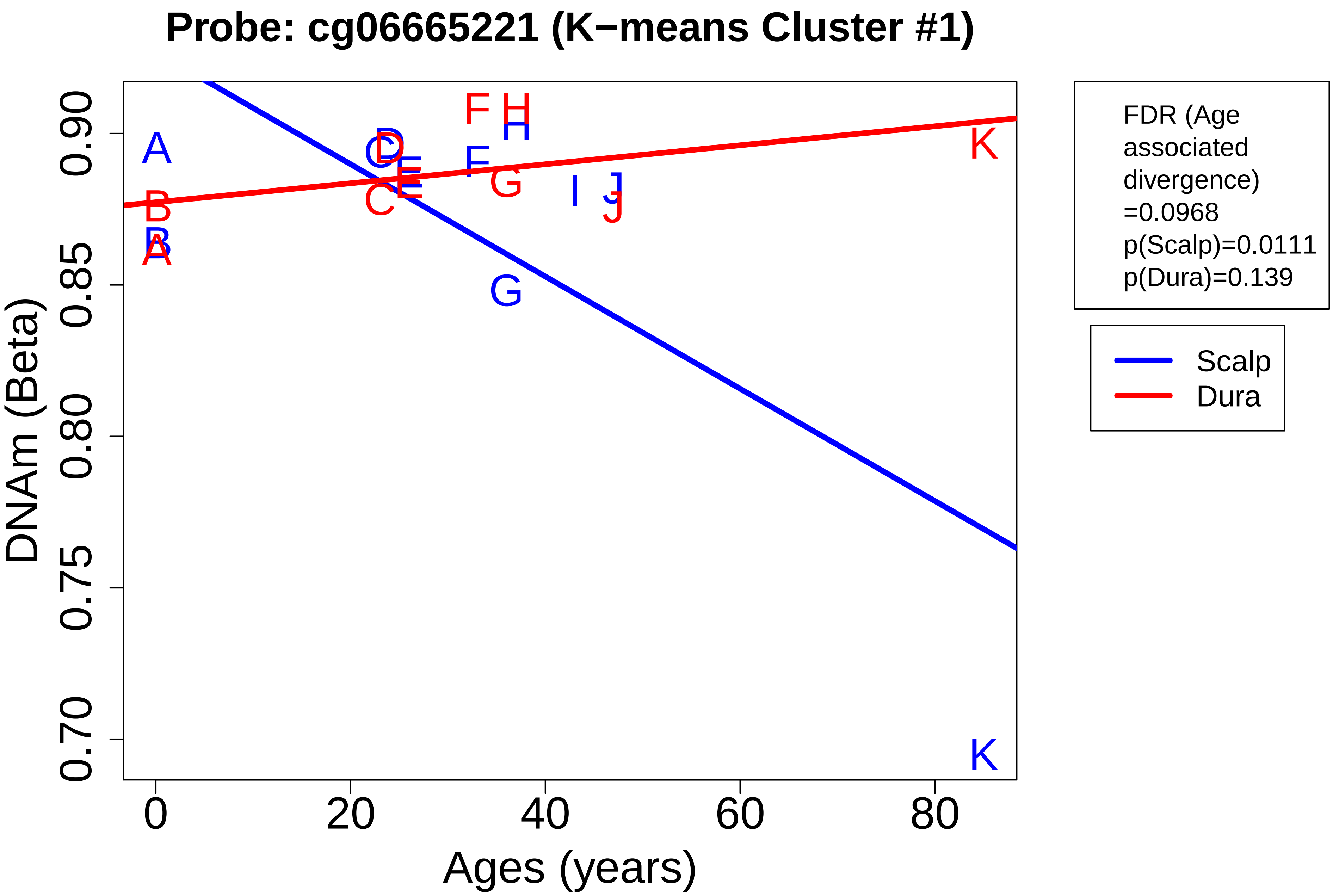
Probe: cg06665221 (K−means Cluster #1)
DNAm (Beta)
0.70
0.75
0.80
0.85
0.90
0
20
40
60
80
Ages (years)
A
B
C
D
E
F
G
H
I
J
K
FDR (Age associated divergence) =0.0968
p(Scalp)=0.0111
p(Dura)=0.139
Scalp
Dura

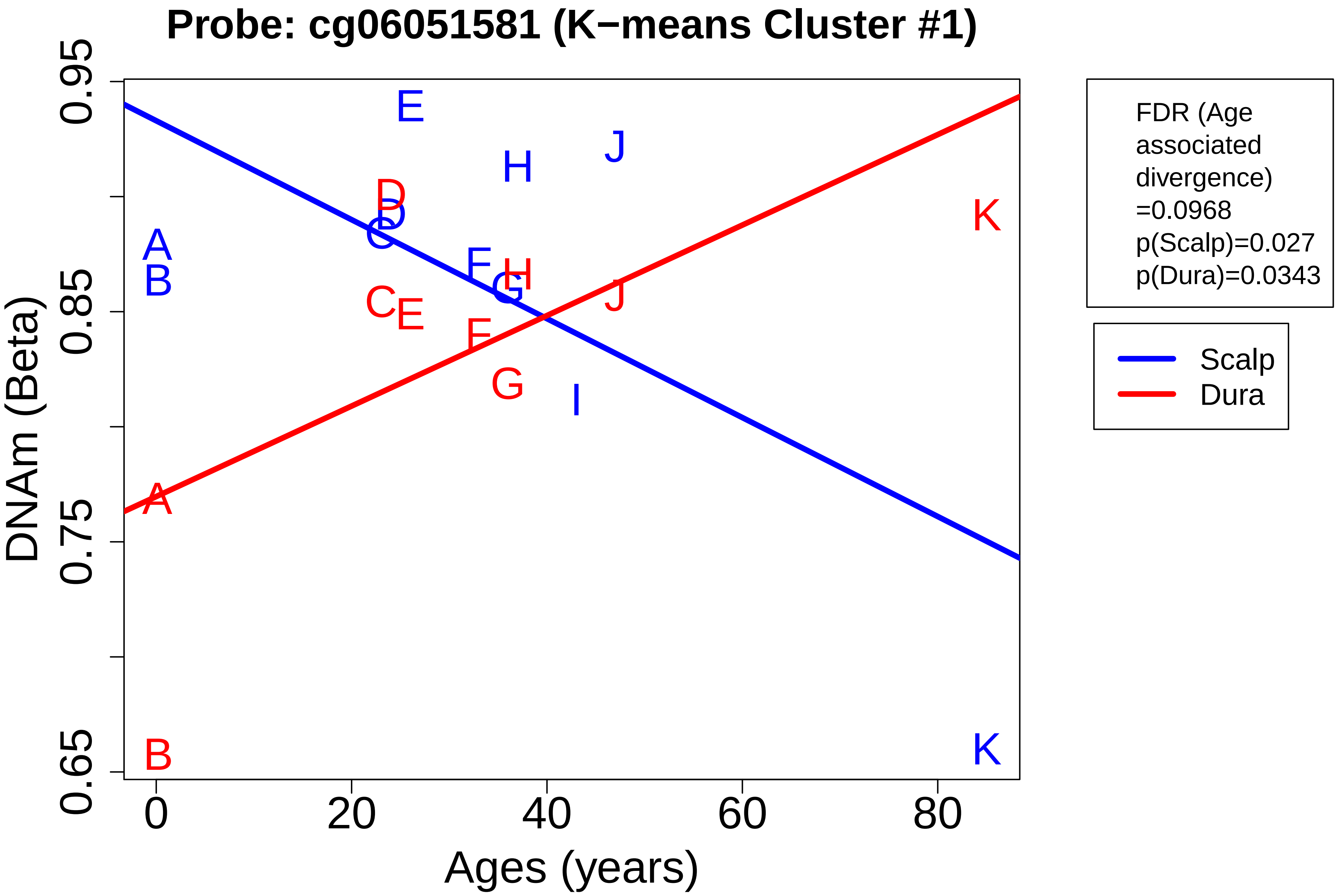

Probe: cg06051581 (K−means Cluster #1)
DNAm (Beta)
0.65
0.75
0.85
0.95
Ages (years)
0
20
40
60
80
FDR (Age associated divergence) =0.0968
p(Scalp)=0.027
p(Dura)=0.0343
Scalp
Dura

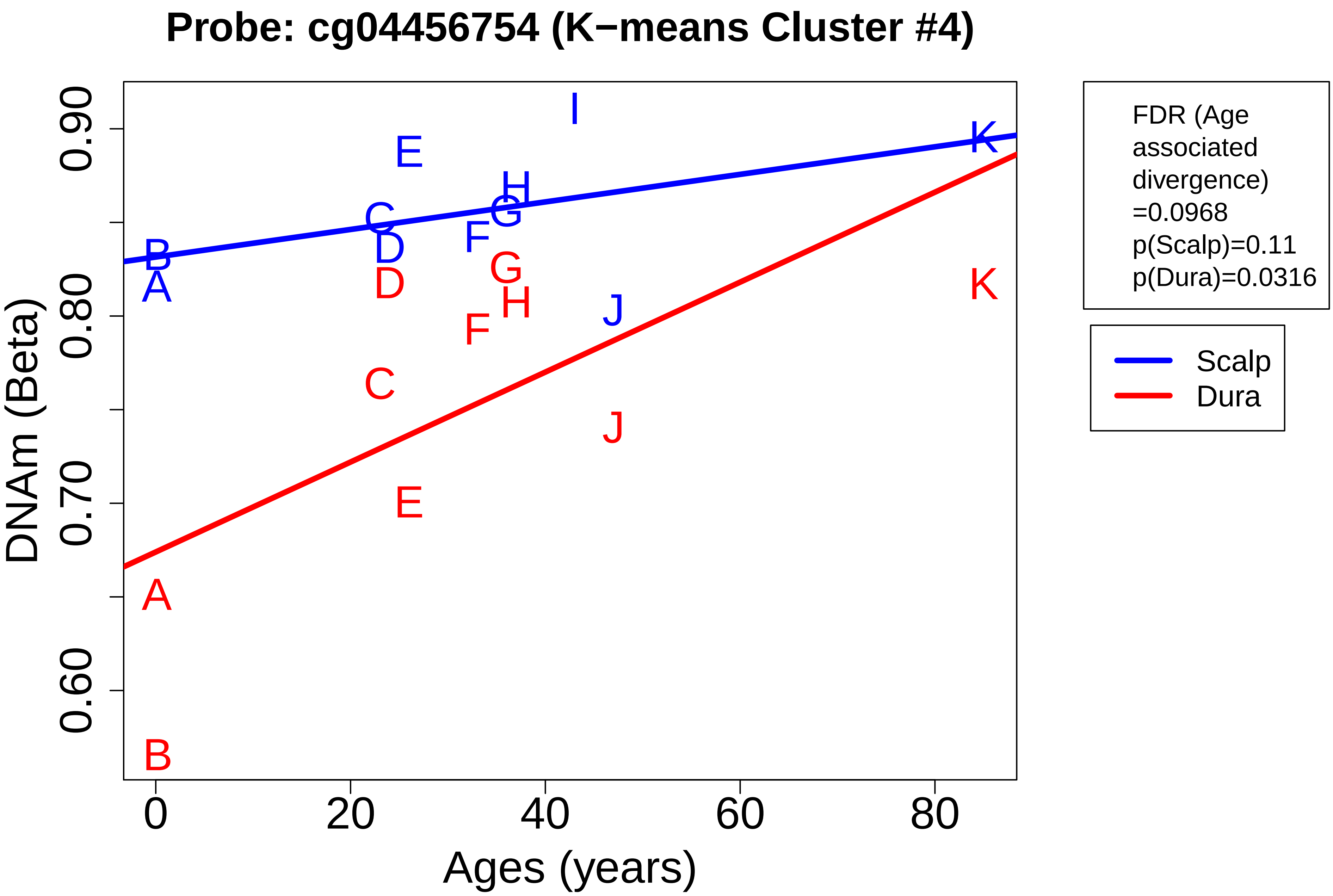

Probe: cg04456754 (K−means Cluster #4)
DNAm (Beta)
0.60
0.70
0.80
0.90
Ages (years)
0
20
40
60
80
A
B
C
D
E
F
G
H
I
J
K
FDR (Age associated divergence) =0.0968
p(Scalp)=0.11
p(Dura)=0.0316
Scalp
Dura

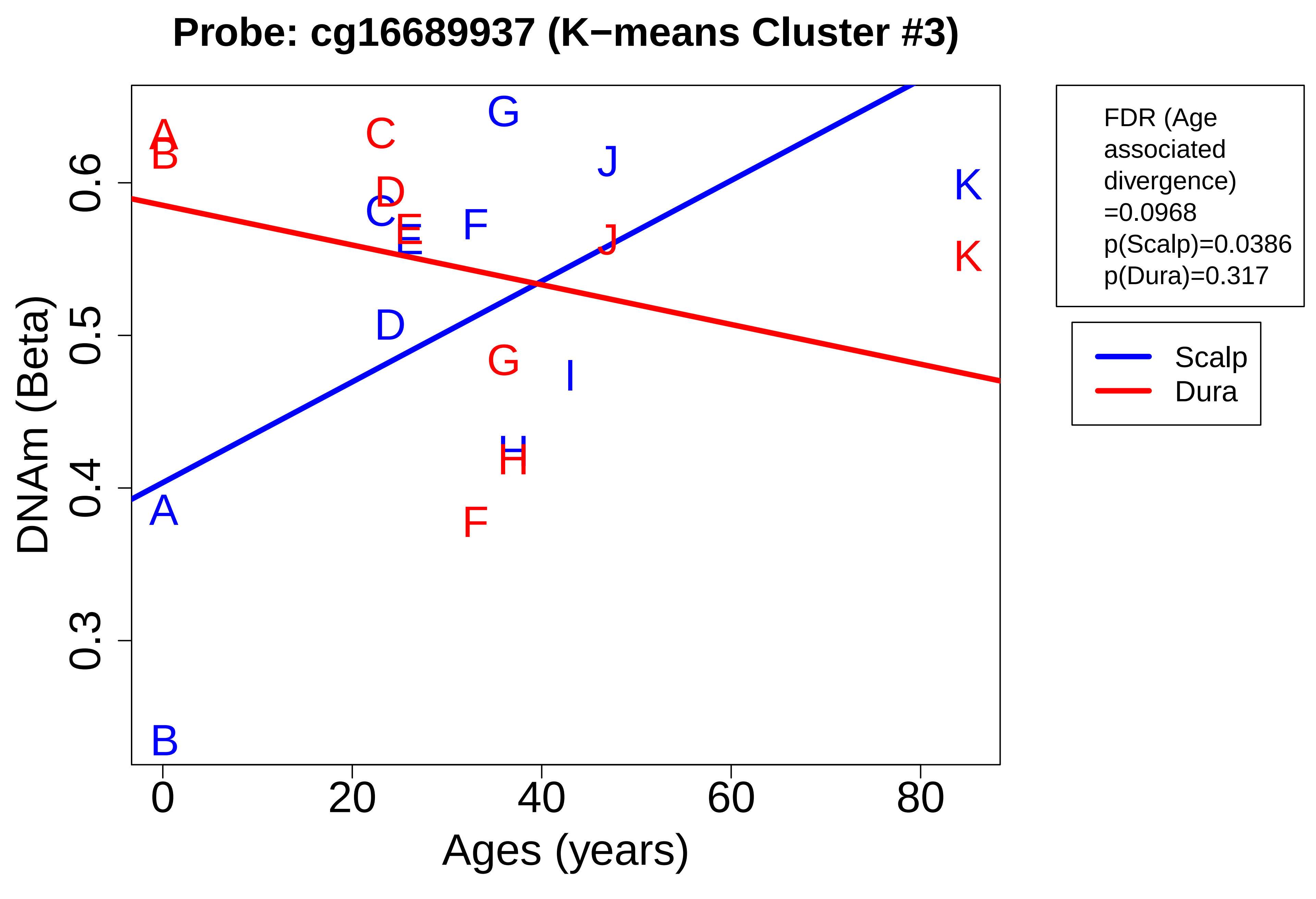

Probe: cg16689937 (K−means Cluster #3)
DNAm (Beta)
0.3
0.4
0.5
0.6
0
20
40
60
80
Ages (years)
A
B
C
D
E
F
G
H
I
J
K
FDR (Age associated divergence) =0.0968
p(Scalp)=0.0386
p(Dura)=0.317
Scalp
Dura

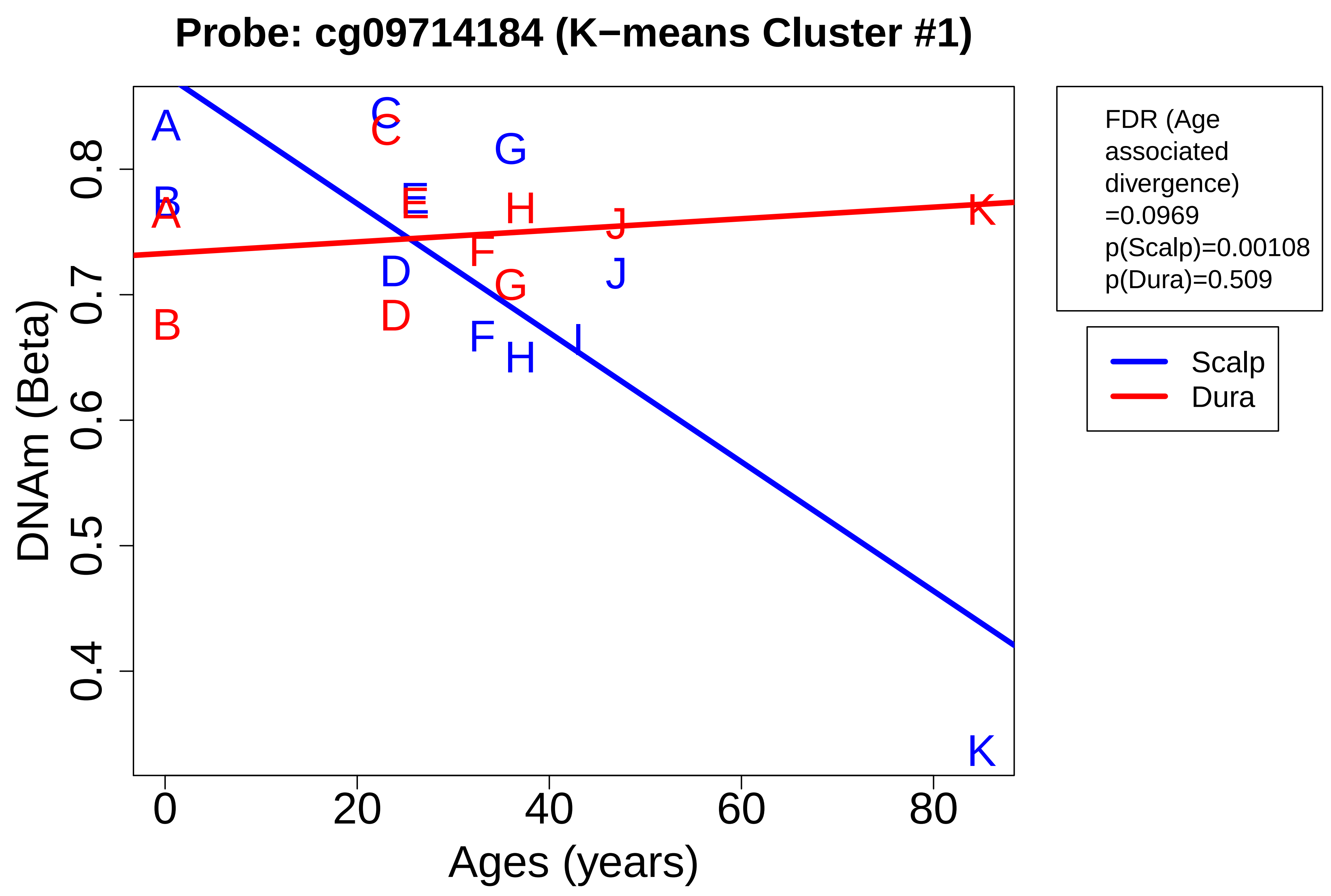

Probe: cg09714184 (K−means Cluster #1)
DNAm (Beta)
0.4
0.5
0.6
0.7
0.8
0
20
40
60
80
Ages (years)
A
B
C
D
E
F
G
H
I
J
K
FDR (Age associated divergence) =0.0969
p(Scalp)=0.00108
p(Dura)=0.509
Scalp
Dura

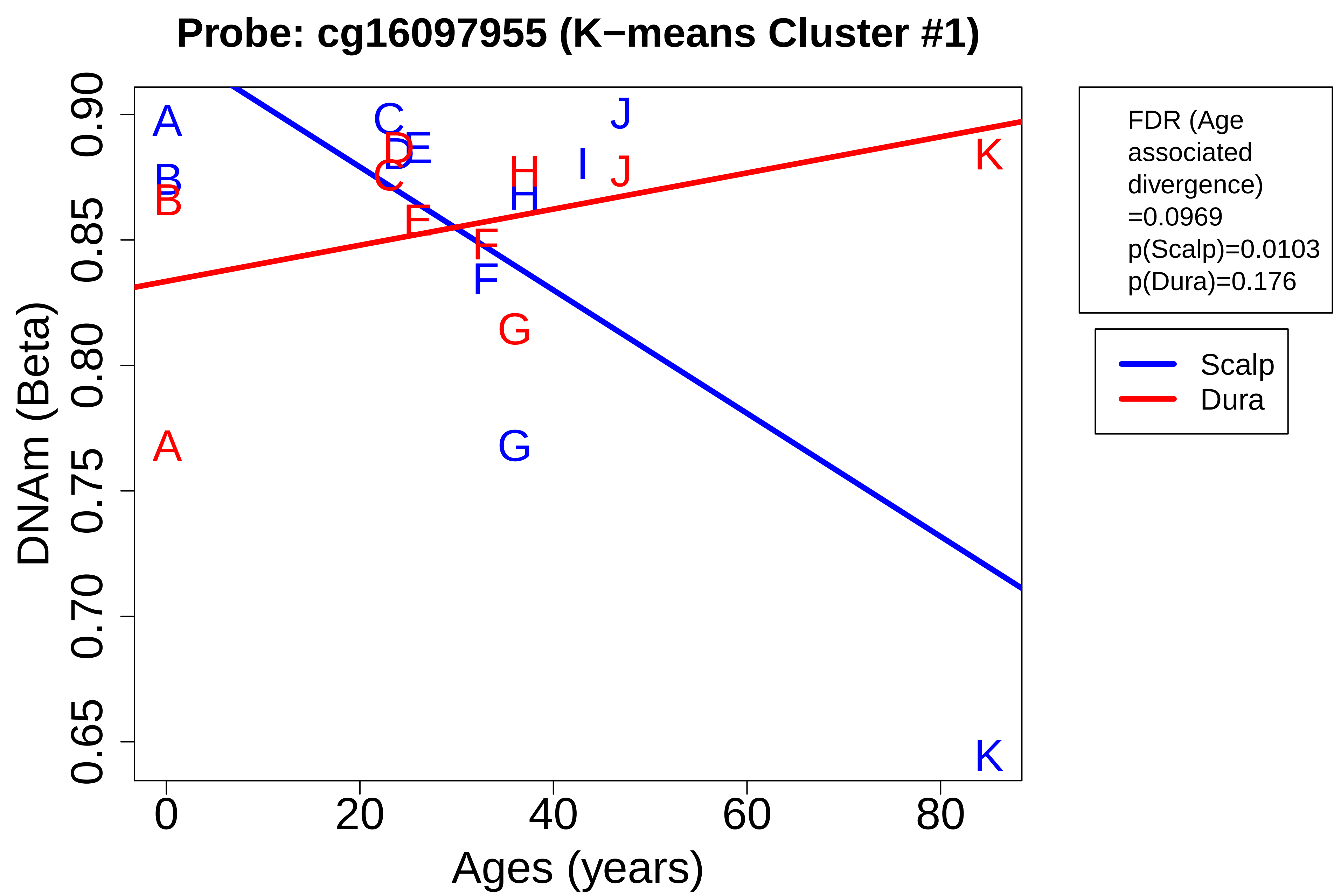
Probe: cg16097955 (K−means Cluster #1)
DNAm (Beta)
0.65
0.70
0.75
0.80
0.85
0.90
0
20
40
60
80
Ages (years)
FDR (Age associated divergence) =0.0969
p(Scalp)=0.0103
p(Dura)=0.176
Scalp
Dura
A
B
C
D
E
F
G
H
I
J
K

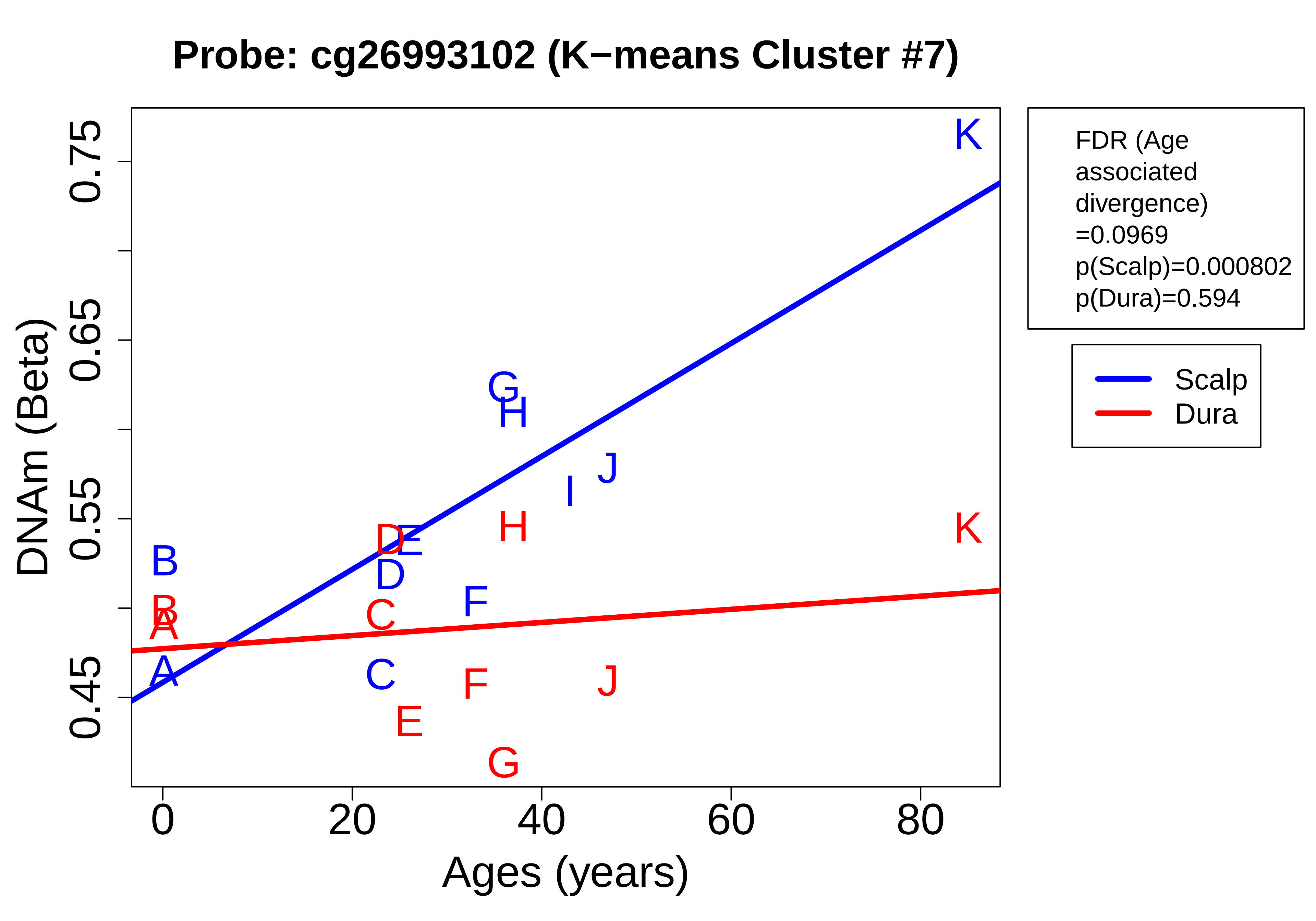

Probe: cg26993102 (K−means Cluster #7)
FDR (Age associated divergence) =0.0969
p(Scalp)=0.000802
p(Dura)=0.594
Scalp
Dura
DNAm (Beta)
0.45
0.55
0.65
0.75
Ages (years)
0
20
40
60
80

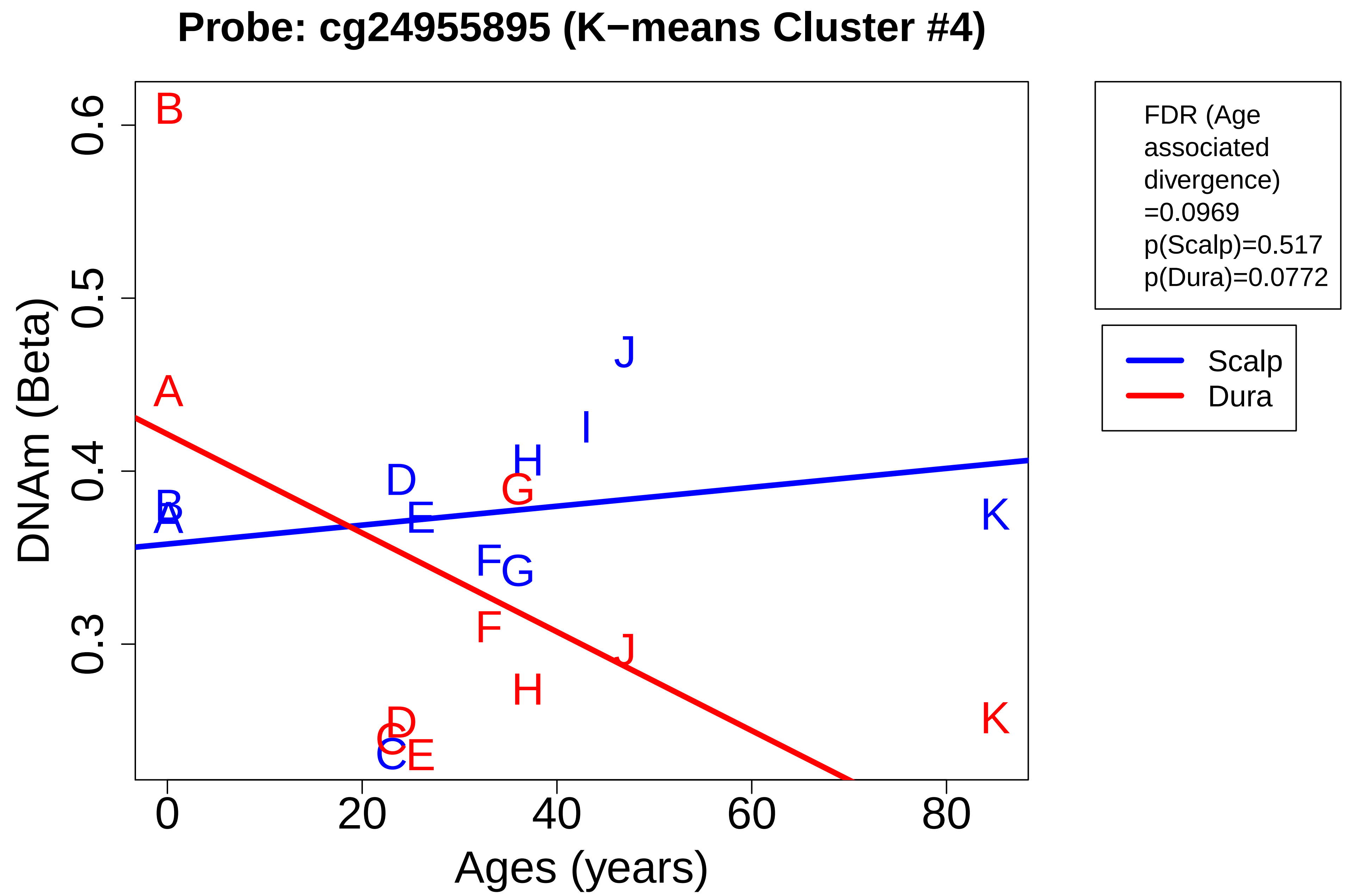

Probe: cg24955895 (K−means Cluster #4)
DNAm (Beta)
0.3
0.4
0.5
0.6
0
20
40
60
80
Ages (years)
FDR (Age associated divergence) =0.0969
p(Scalp)=0.517
p(Dura)=0.0772
Scalp
Dura

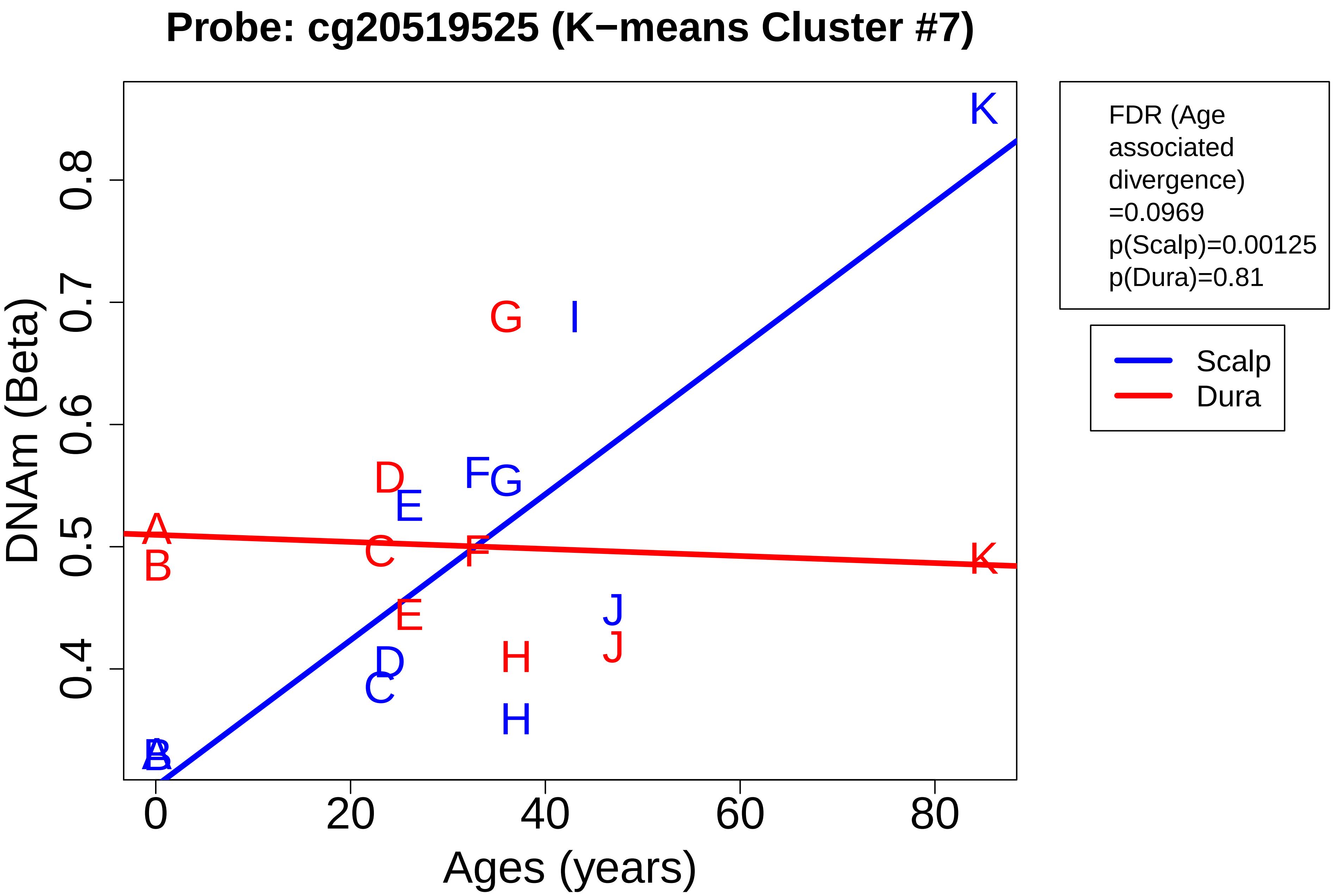
Probe: cg20519525 (K−means Cluster #7)
DNAm (Beta)
Ages (years)
0.4
0.5
0.6
0.7
0.8
0
20
40
60
80
FDR (Age associated divergence) =0.0969
p(Scalp)=0.00125
p(Dura)=0.81
Scalp
Dura

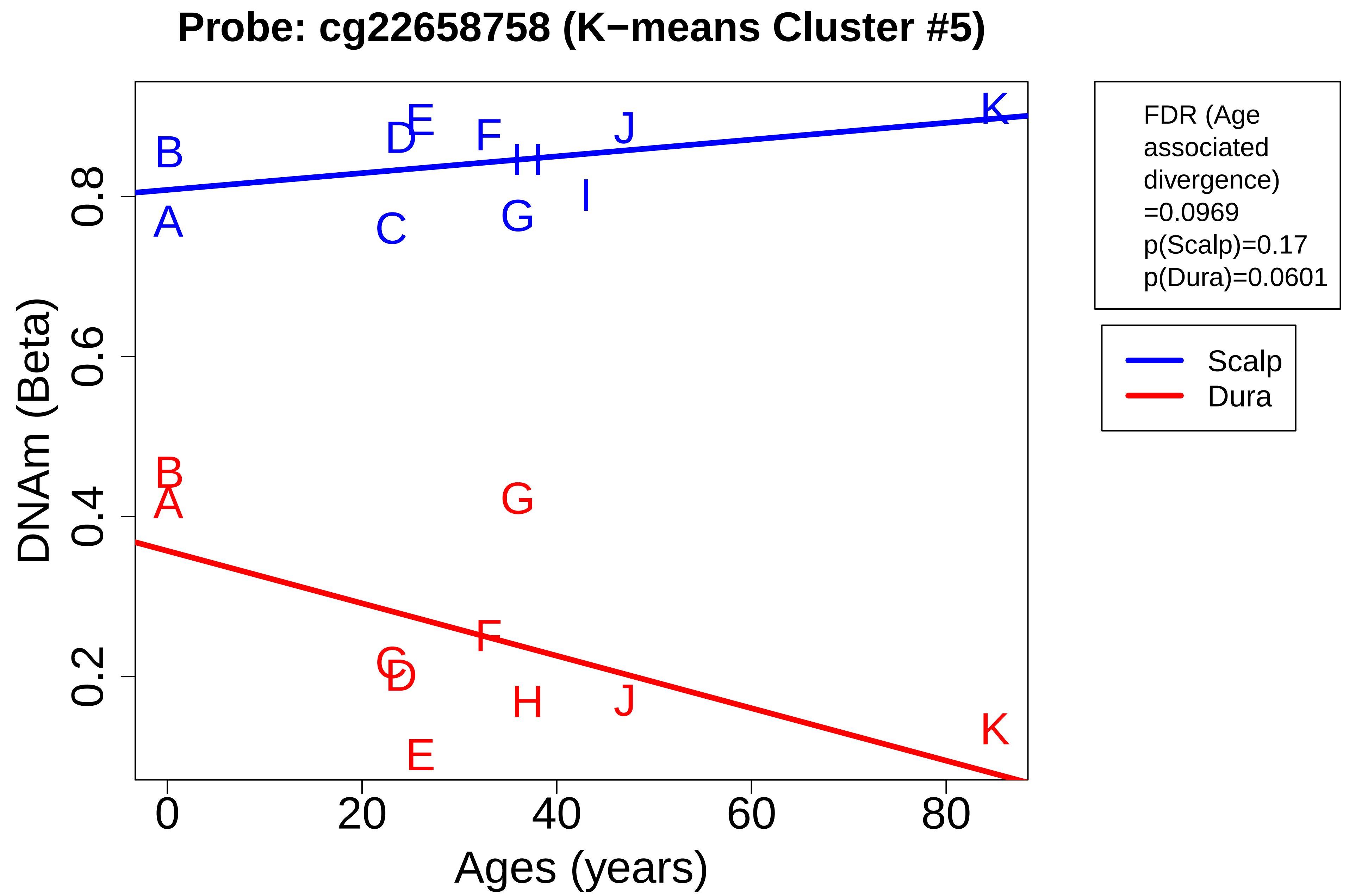

Probe: cg22658758 (K−means Cluster #5)
DNAm (Beta)
0.2
0.4
0.6
0.8
Ages (years)
0
20
40
60
80
A
B
C
D
E
F
G
H
I
J
K
A
B
C
D
E
F
G
H
J
K
FDR (Age associated divergence) =0.0969
p(Scalp)=0.17
p(Dura)=0.0601
Scalp
Dura

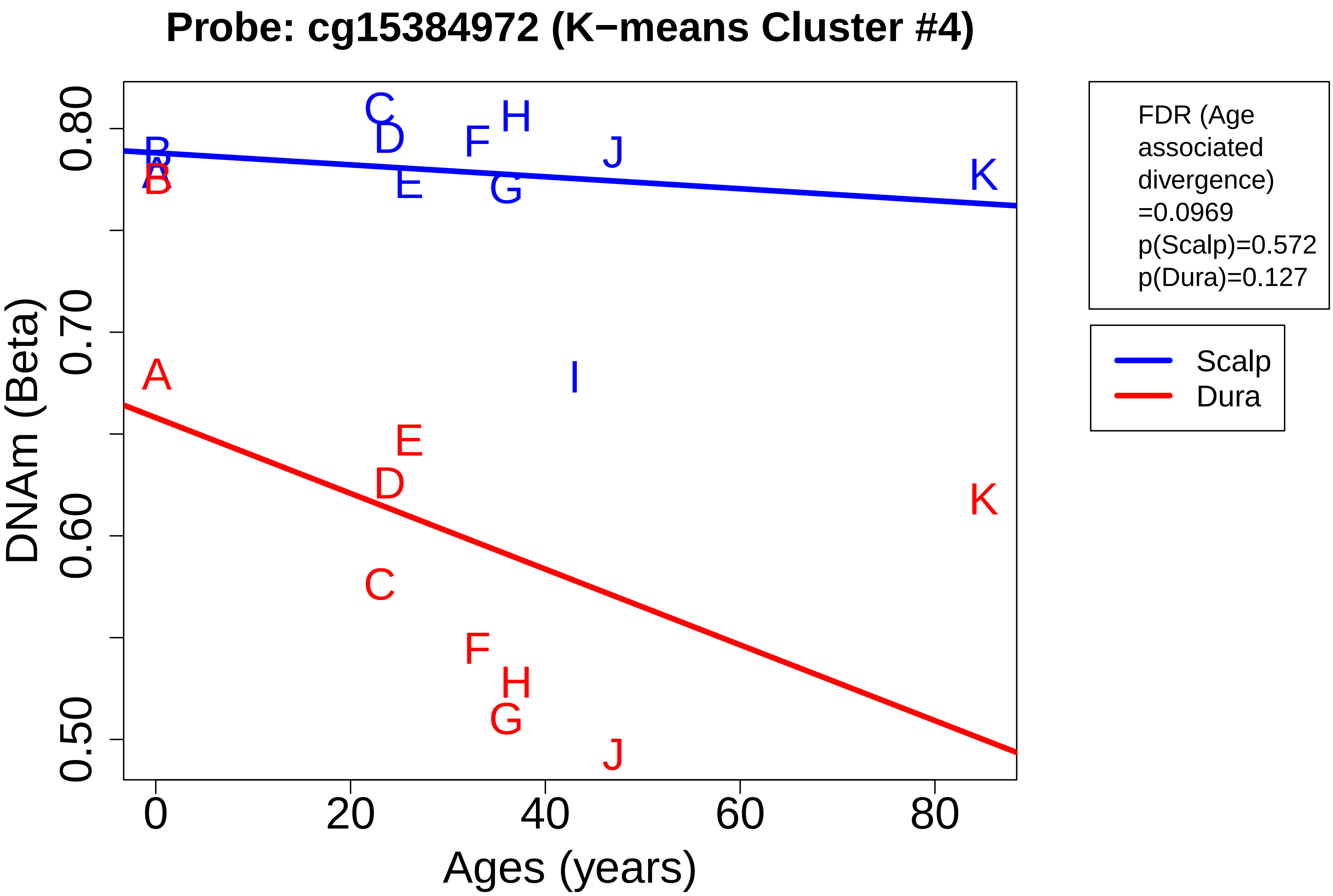

Probe: cg15384972 (K−means Cluster #4)
DNAm (Beta)
0.50
0.60
0.70
0.80
0
20
40
60
80
Ages (years)
A
B
C
D
E
F
G
H
I
J
K
FDR (Age associated divergence) =0.0969
p(Scalp)=0.572
p(Dura)=0.127
Scalp
Dura

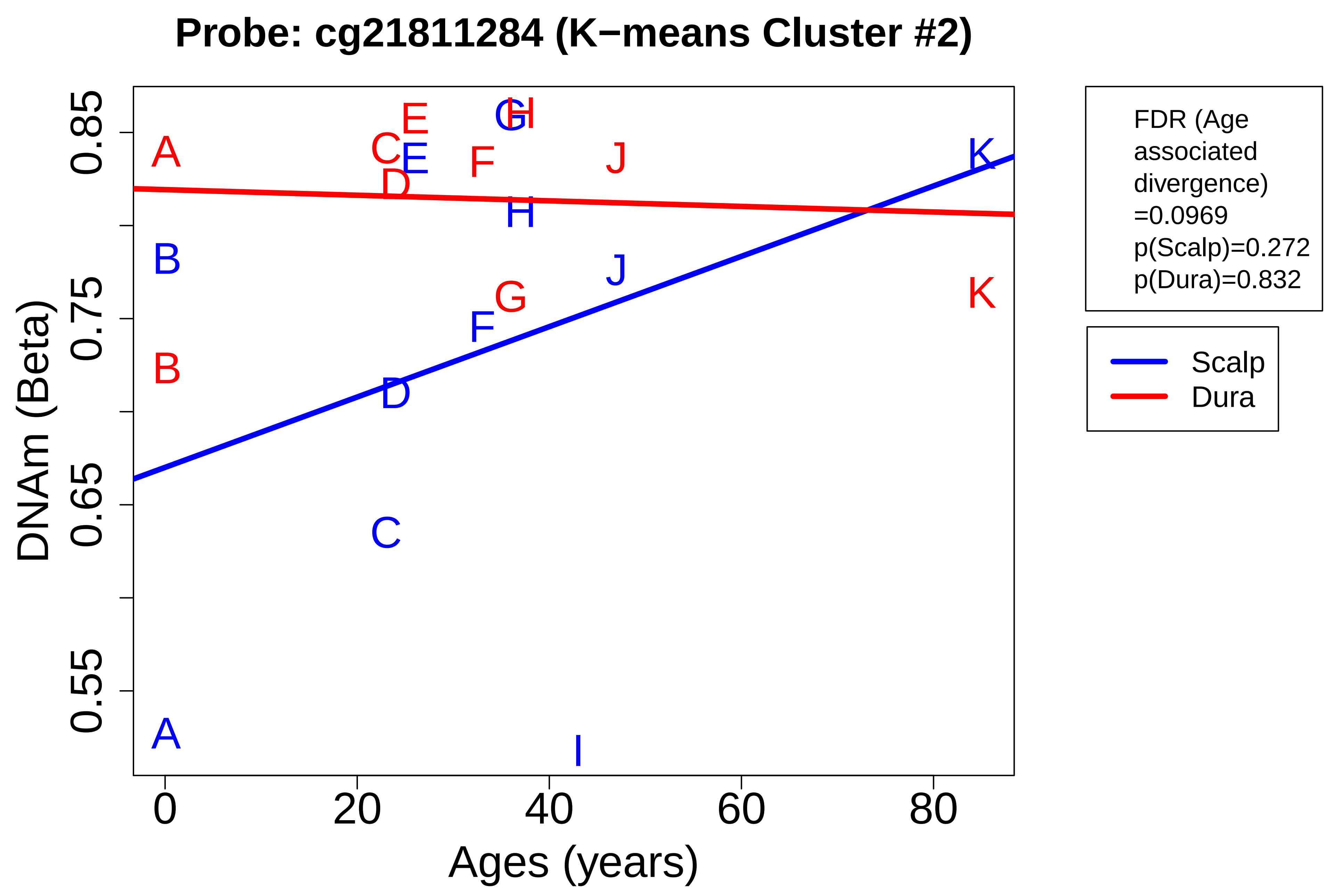

Probe: cg21811284 (K−means Cluster #2)
DNAm (Beta)
0.55
0.65
0.75
0.85
Ages (years)
0
20
40
60
80
FDR (Age associated divergence) =0.0969
p(Scalp)=0.272
p(Dura)=0.832
Scalp
Dura
A
B
C
D
E
F
G
H
I
J
K

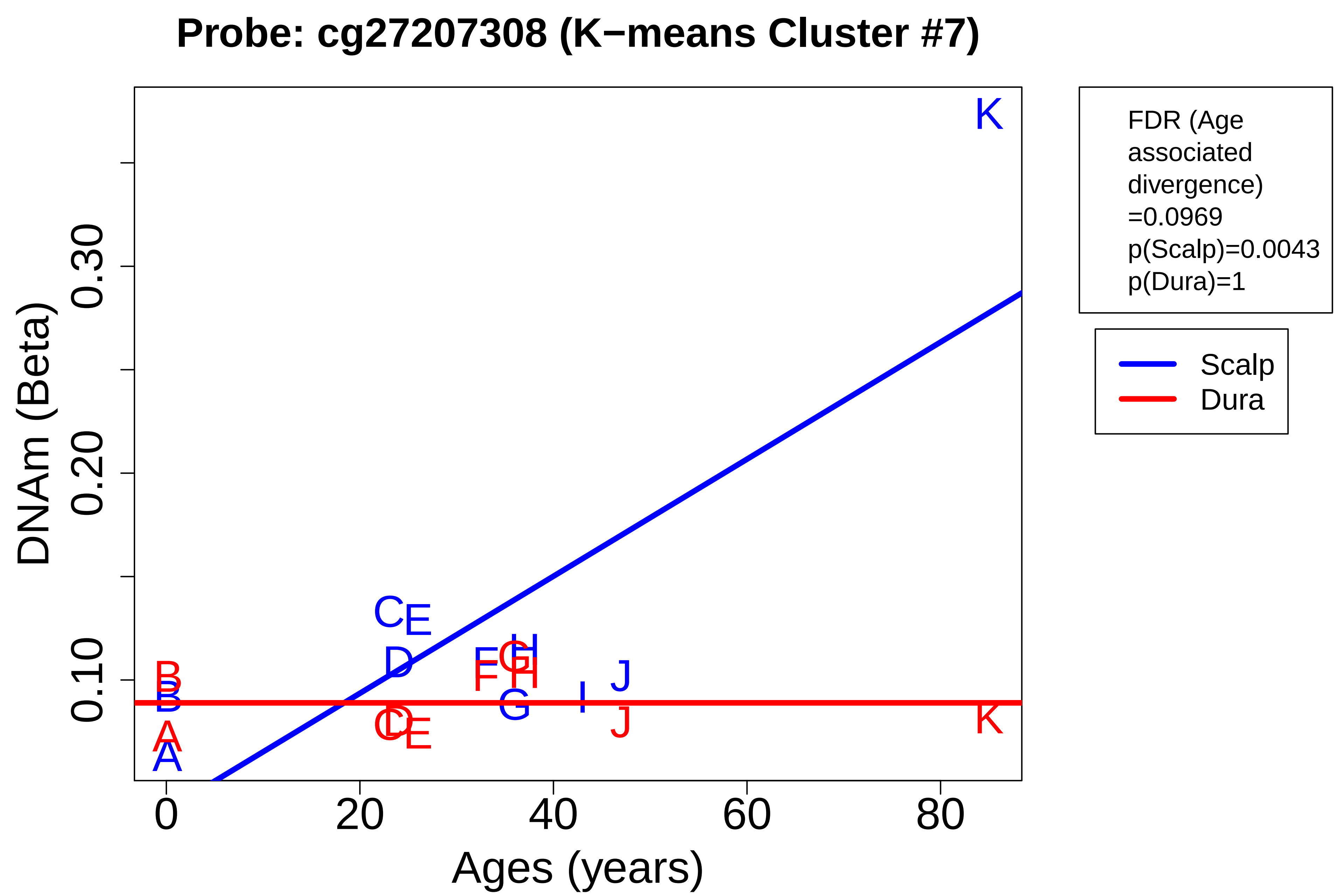

Probe: cg27207308 (K−means Cluster #7)
DNAm (Beta)
0.10
0.20
0.30
0
20
40
60
80
Ages (years)
FDR (Age associated divergence) =0.0969
p(Scalp)=0.0043
p(Dura)=1
Scalp
Dura
A
B
C
D
E
F
G
H
I
J
K

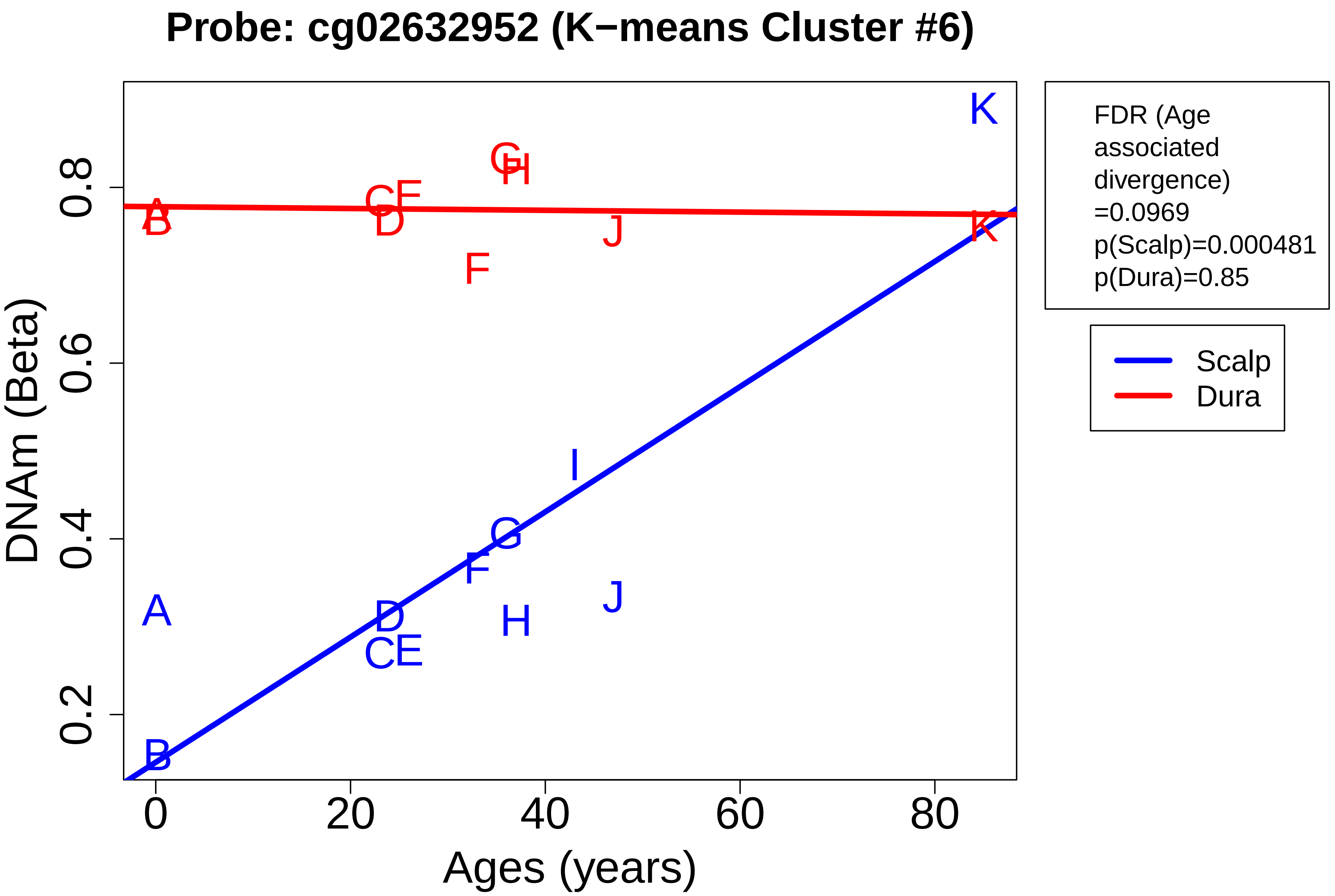

Probe: cg02632952 (K−means Cluster #6)
DNAm (Beta)
0.2
0.4
0.6
0.8
0
20
40
60
80
Ages (years)
A
B
C
D
E
F
G
H
I
J
K
FDR (Age associated divergence) =0.0969
p(Scalp)=0.000481
p(Dura)=0.85
Scalp
Dura

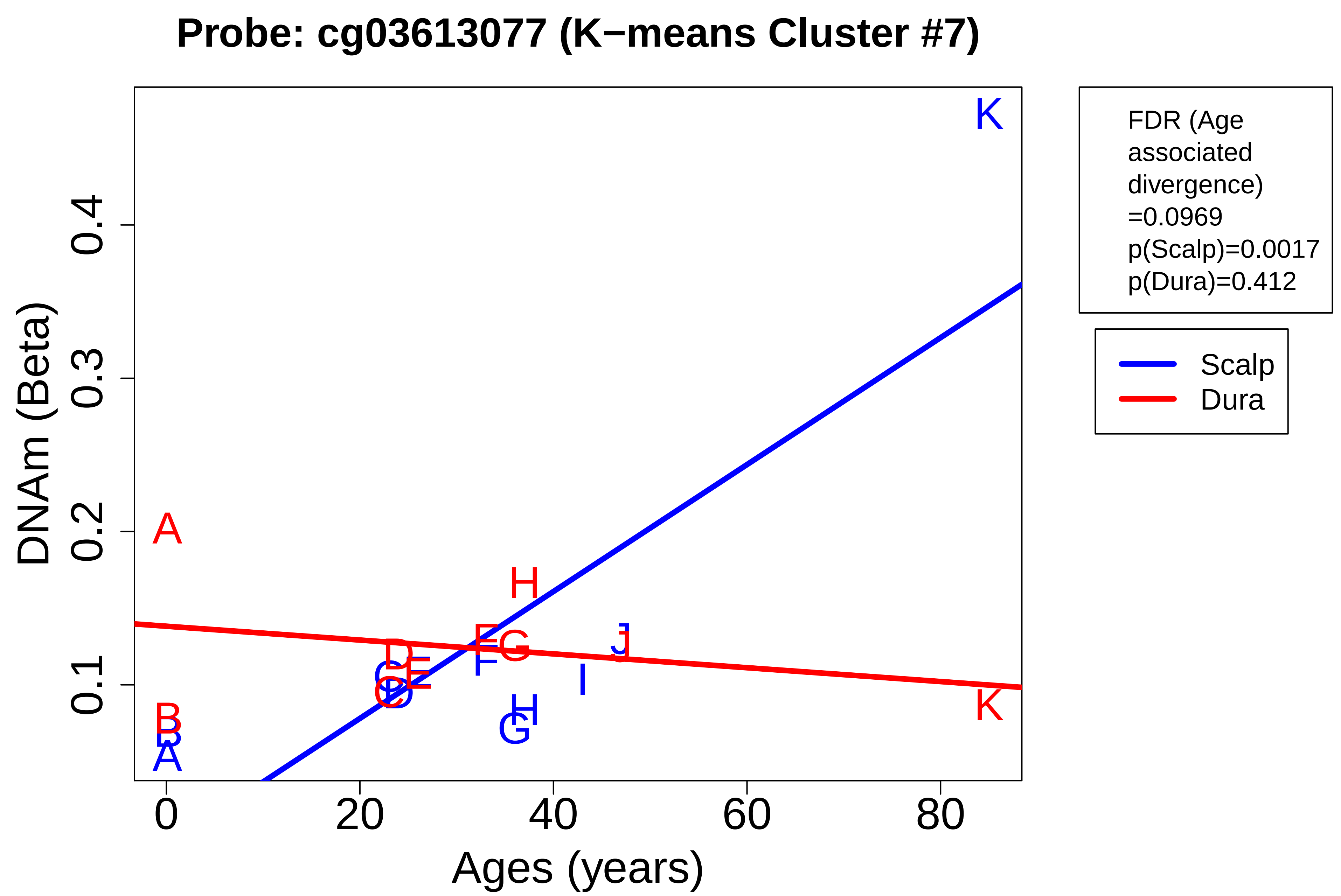

Probe: cg03613077 (K−means Cluster #7)
DNAm (Beta)
0.1
0.2
0.3
0.4
0
20
40
60
80
Ages (years)
FDR (Age associated divergence) =0.0969
p(Scalp)=0.0017
p(Dura)=0.412
Scalp
Dura

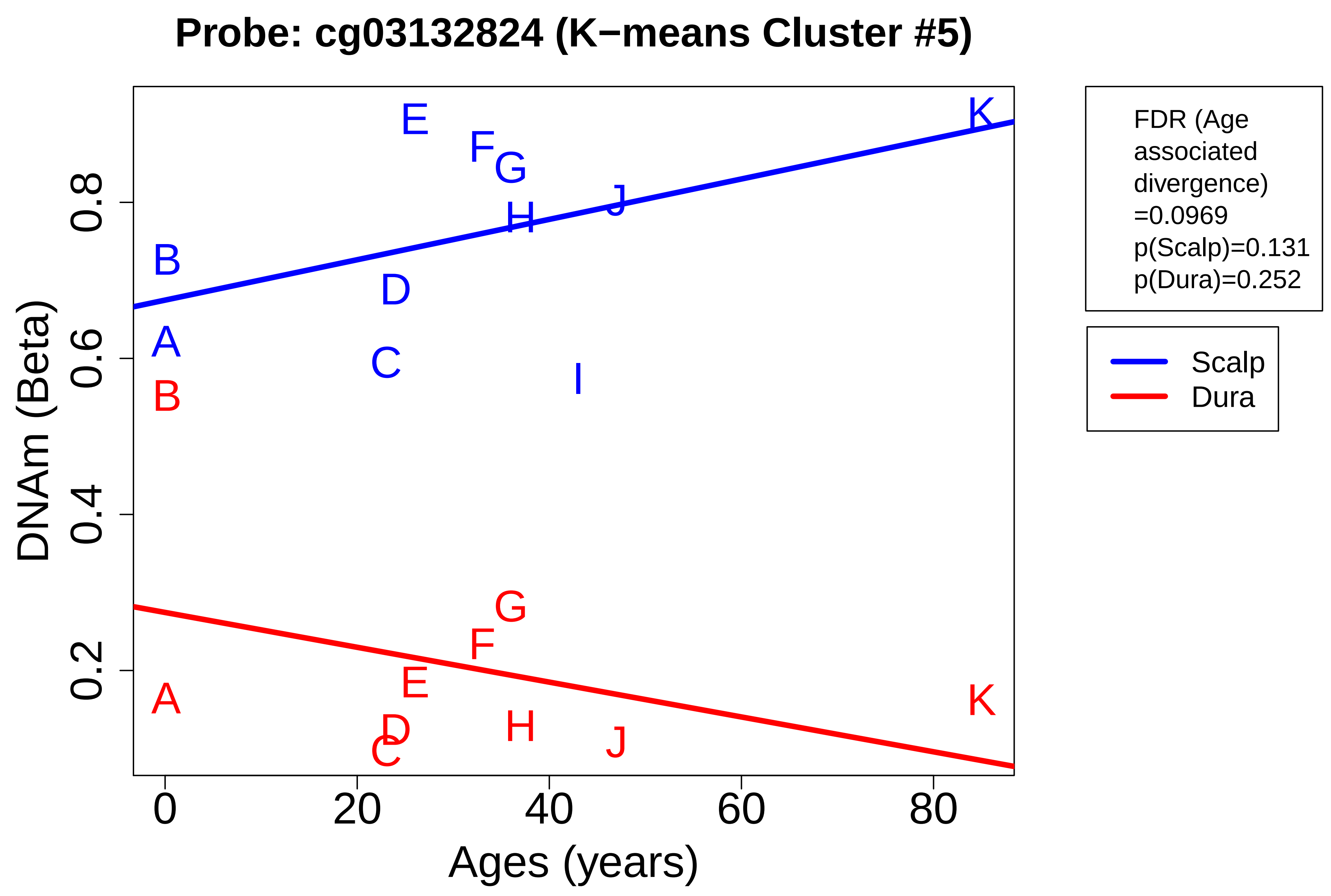

Probe: cg03132824 (K−means Cluster #5)
DNAm (Beta)
0.2
0.4
0.6
0.8
0
20
40
60
80
Ages (years)
FDR (Age associated divergence) =0.0969
p(Scalp)=0.131
p(Dura)=0.252
Scalp
Dura

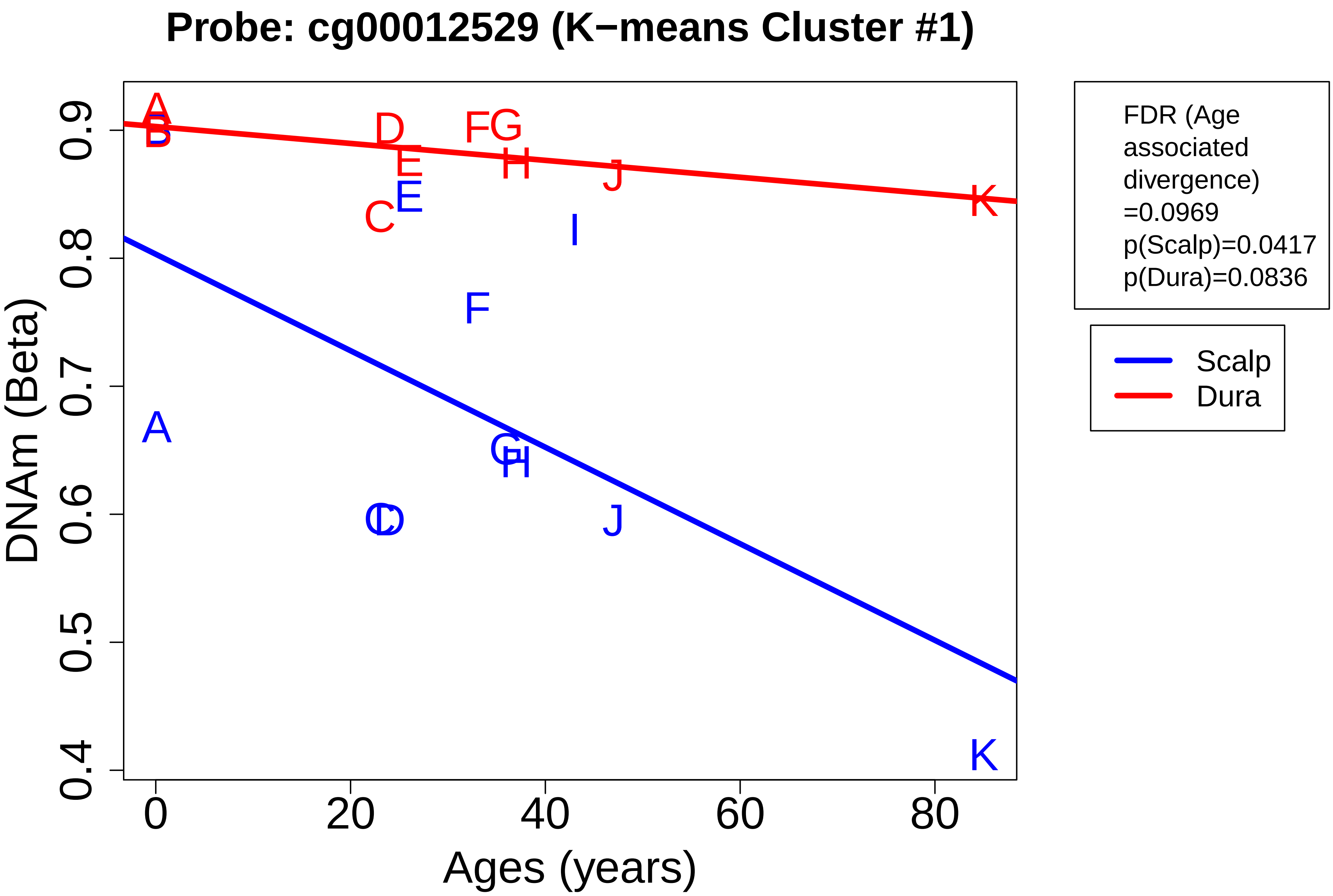

Probe: cg00012529 (K−means Cluster #1)
DNAm (Beta)
0.4
0.5
0.6
0.7
0.8
0.9
0
20
40
60
80
Ages (years)
FDR (Age associated divergence) =0.0969
p(Scalp)=0.0417
p(Dura)=0.0836
Scalp
Dura

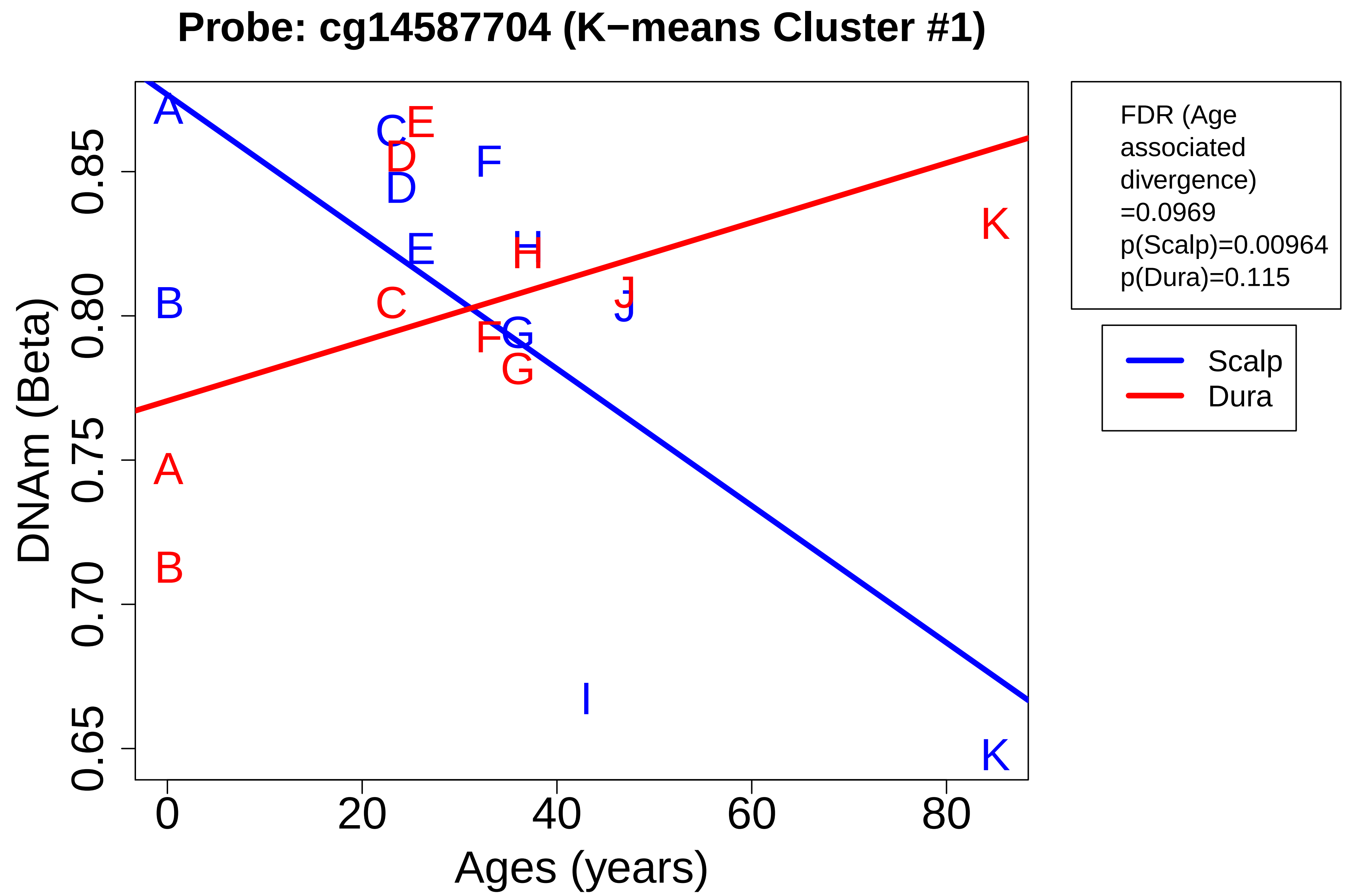

Probe: cg14587704 (K−means Cluster #1)
FDR (Age associated divergence) =0.0969
p(Scalp)=0.00964
p(Dura)=0.115
Scalp
Dura
DNAm (Beta)
0.65
0.70
0.75
0.80
0.85
0
20
40
60
80
Ages (years)
A
B
C
D
E
F
G
H
I
J
K

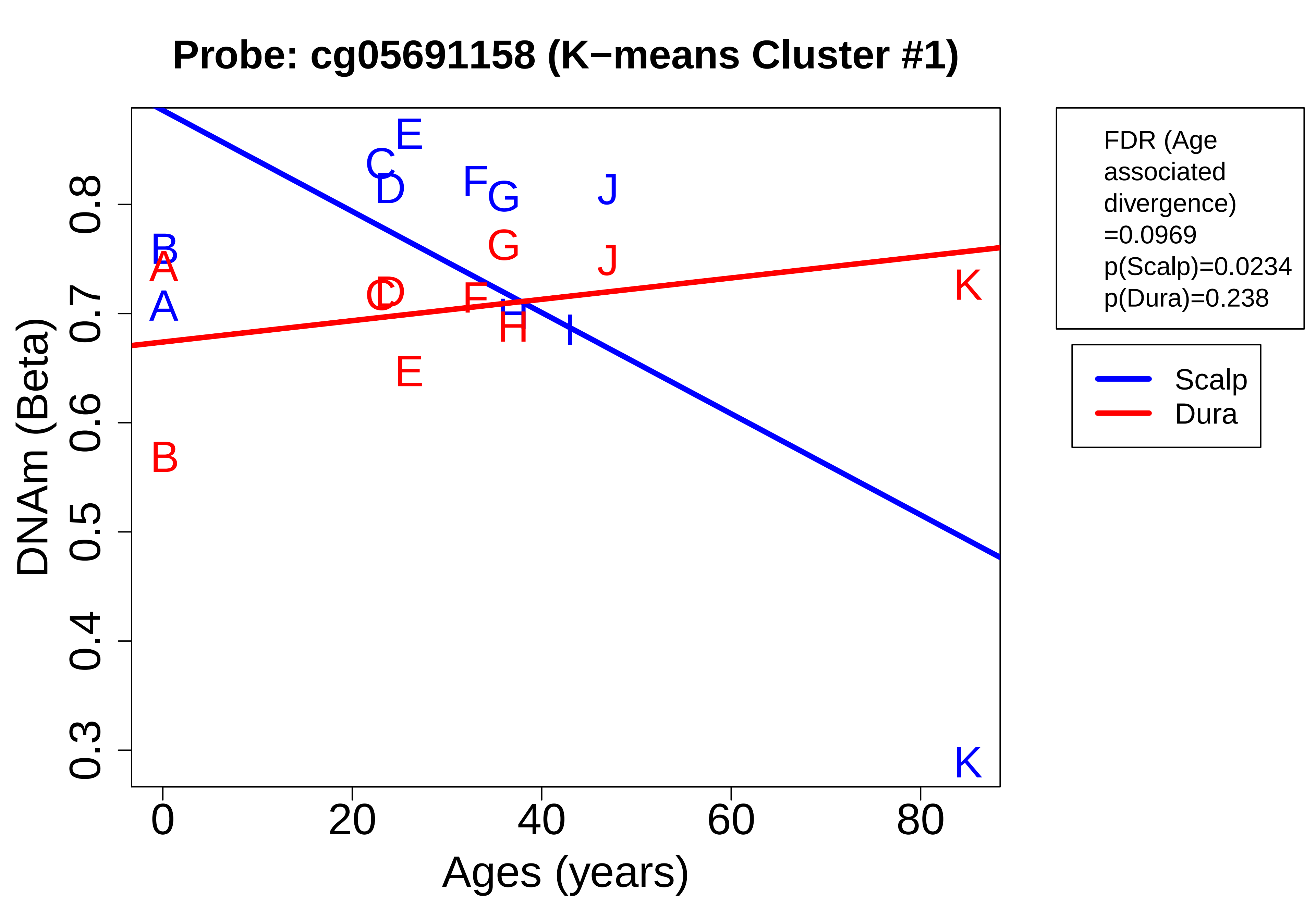

Probe: cg05691158 (K−means Cluster #1)
DNAm (Beta)
0.3
0.4
0.5
0.6
0.7
0.8
0
20
40
60
80
Ages (years)
FDR (Age associated divergence) =0.0969
p(Scalp)=0.0234
p(Dura)=0.238
Scalp
Dura

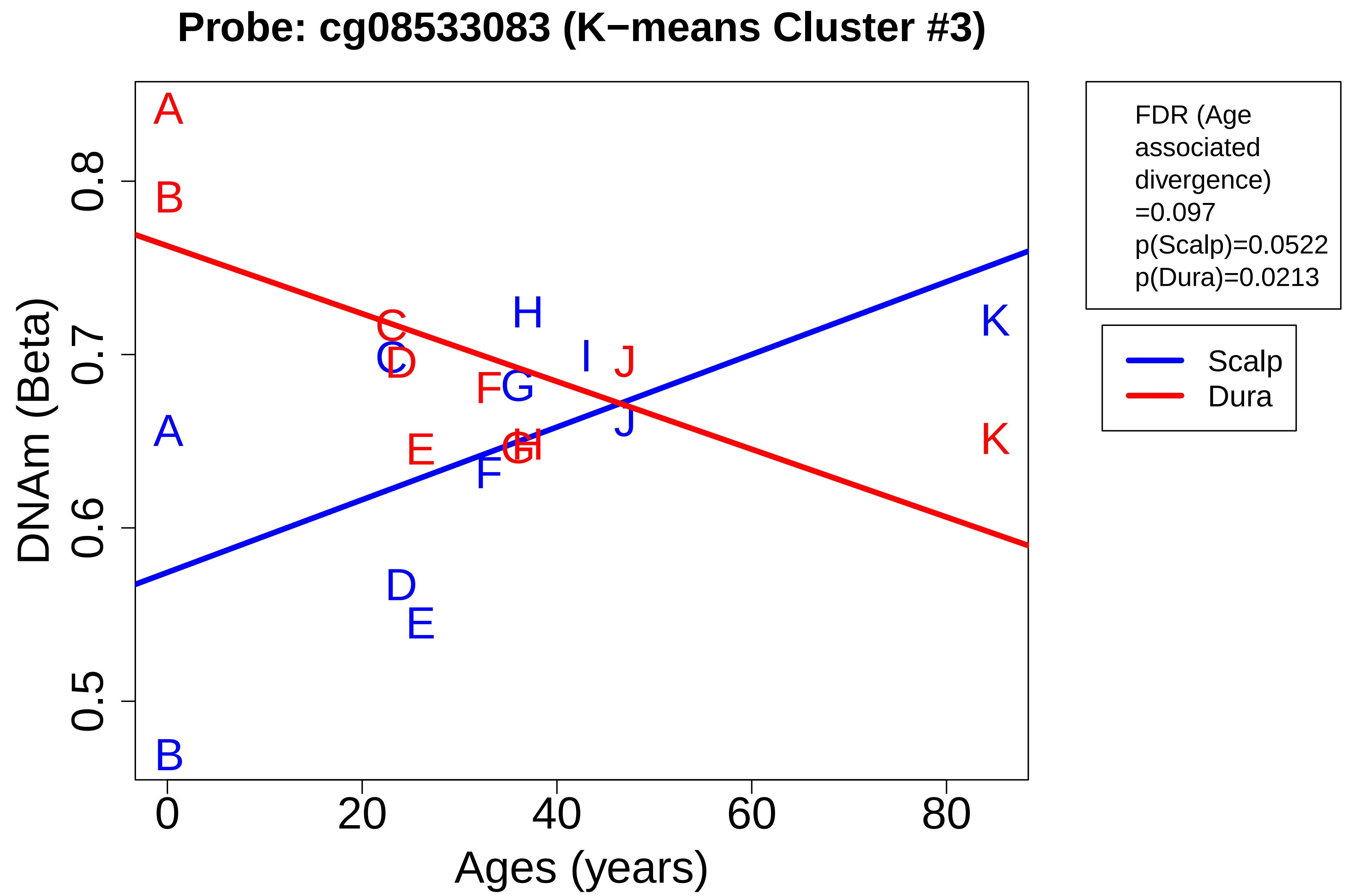

Probe: cg08533083 (K−means Cluster #3)
DNAm (Beta)
0.5
0.6
0.7
0.8
0
20
40
60
80
Ages (years)
A
B
C
D
E
F
G
H
I
J
K
A
B
C
D
E
F
G
H
J
K
FDR (Age associated divergence) =0.097
p(Scalp)=0.0522
p(Dura)=0.0213
Scalp
Dura

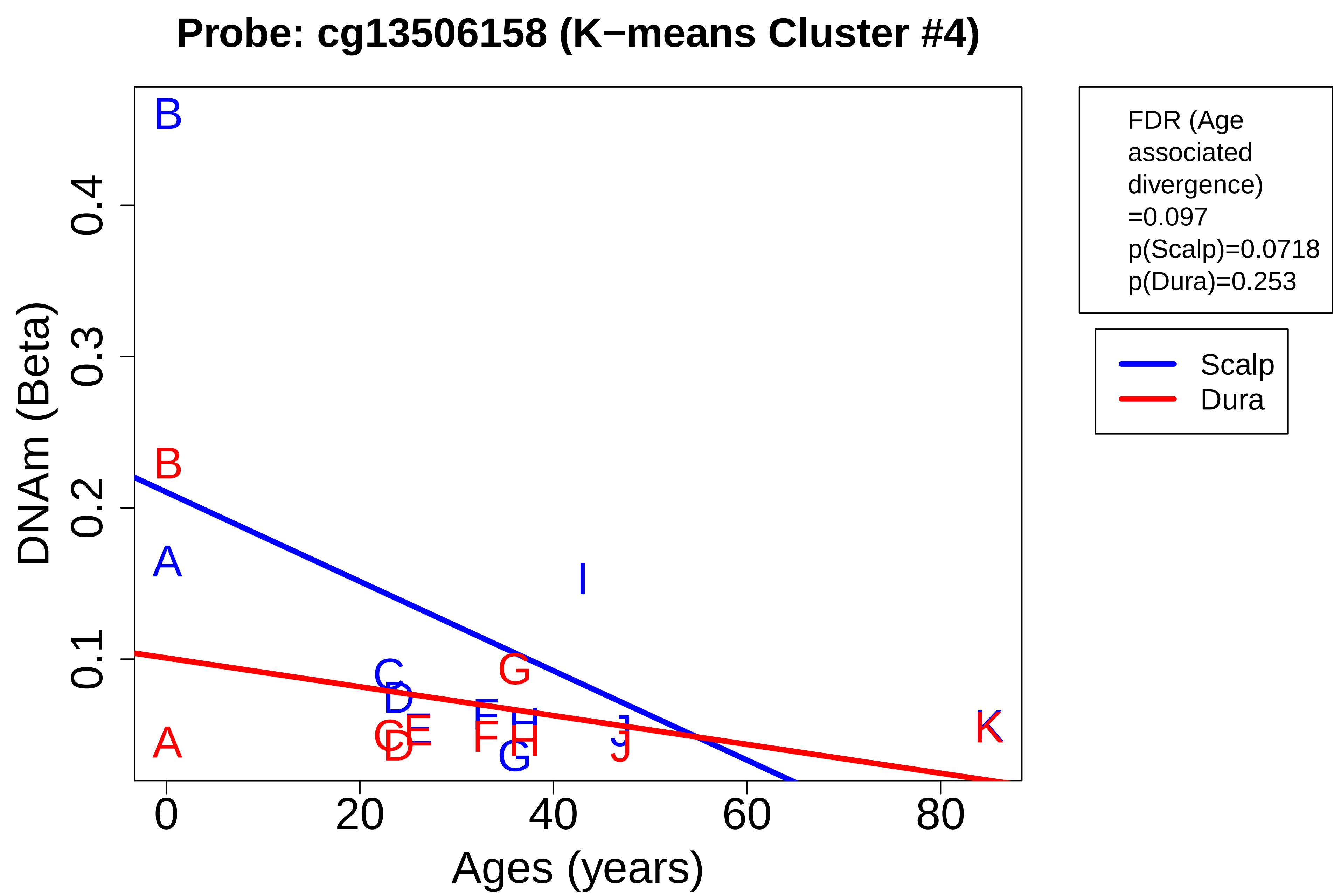

Probe: cg13506158 (K−means Cluster #4)
DNAm (Beta)
0.1
0.2
0.3
0.4
0
20
40
60
80
Ages (years)
FDR (Age associated divergence) =0.097
p(Scalp)=0.0718
p(Dura)=0.253
Scalp
Dura

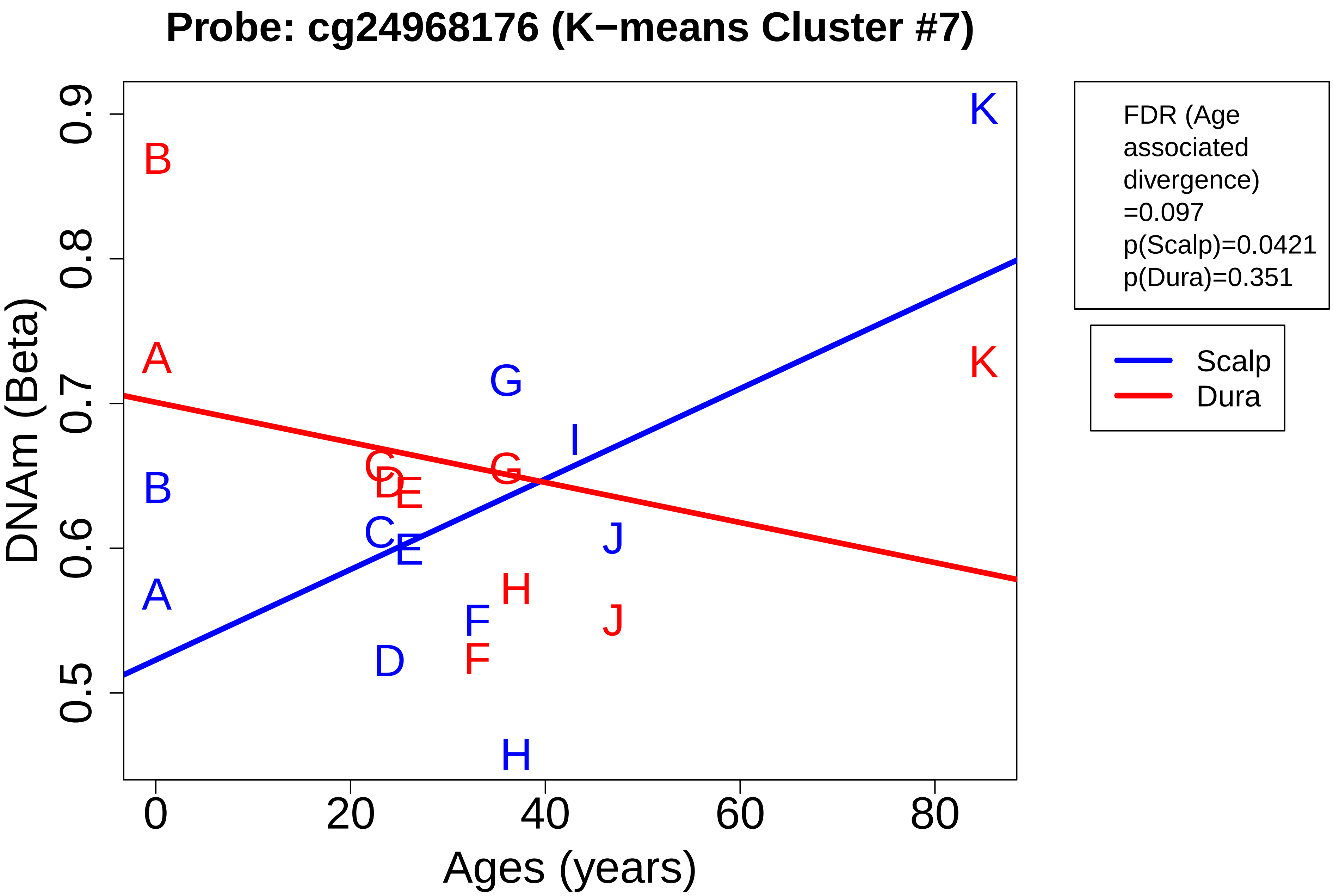

Probe: cg24968176 (K−means Cluster #7)
DNAm (Beta)
0.5
0.6
0.7
0.8
0.9
0
20
40
60
80
Ages (years)
FDR (Age associated divergence) =0.097
p(Scalp)=0.0421
p(Dura)=0.351
Scalp
Dura
A
B
C
D
E
F
G
H
I
J
K

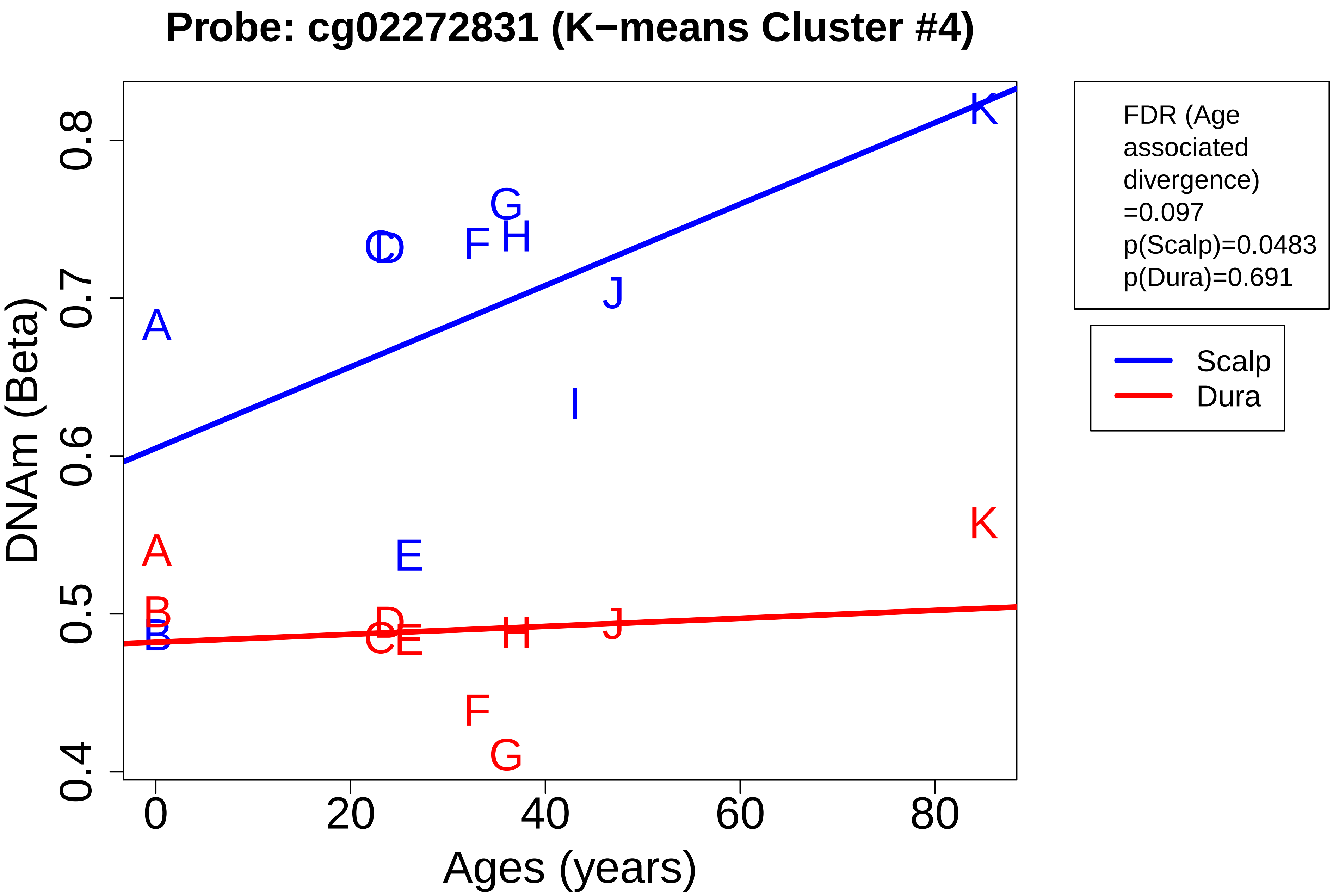

Probe: cg02272831 (K−means Cluster #4)
DNAm (Beta)
0.4
0.5
0.6
0.7
0.8
0
20
40
60
80
Ages (years)
FDR (Age associated divergence) =0.097
p(Scalp)=0.0483
p(Dura)=0.691
Scalp
Dura

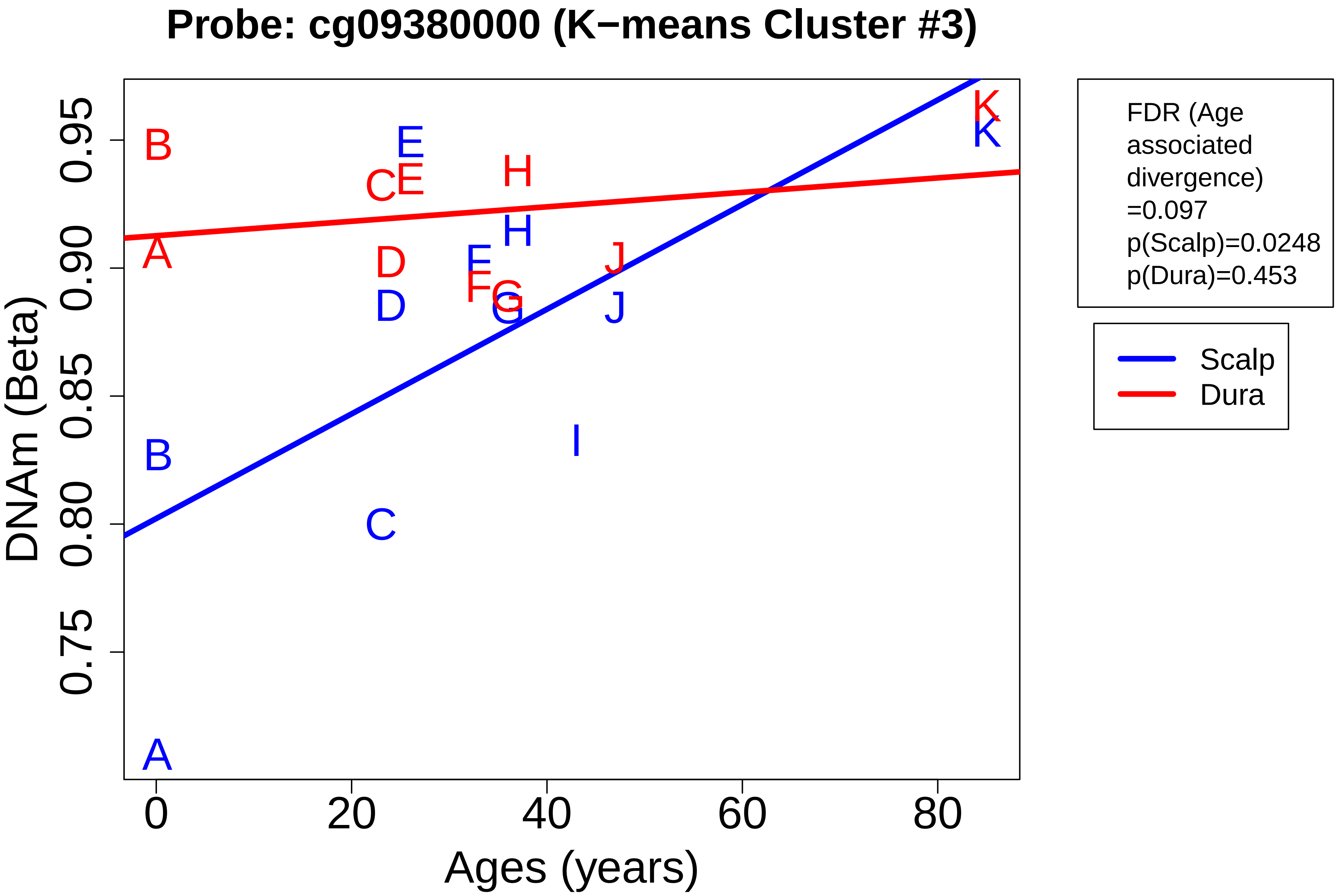

Probe: cg09380000 (K−means Cluster #3)
DNAm (Beta)
0.75
0.80
0.85
0.90
0.95
0
20
40
60
80
Ages (years)
FDR (Age associated divergence) =0.097
p(Scalp)=0.0248
p(Dura)=0.453
Scalp
Dura
A
B
C
D
E
F
G
H
I
J
K

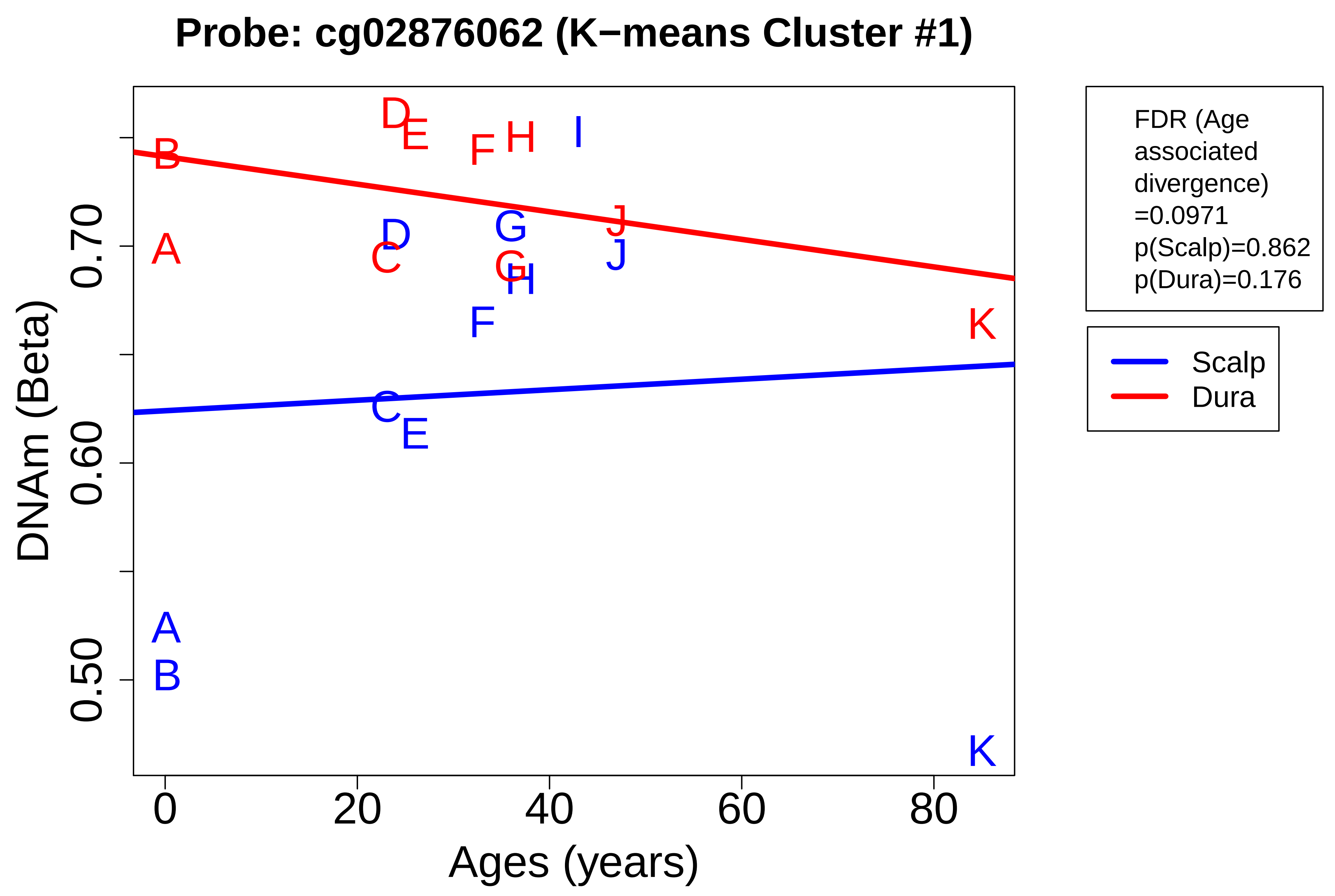
Probe: cg02876062 (K−means Cluster #1)
DNAm (Beta)
0.50
0.60
0.70
0
20
40
60
80
Ages (years)
FDR (Age associated divergence) =0.0971
p(Scalp)=0.862
p(Dura)=0.176
Scalp
Dura
A
B
C
D
E
F
G
H
I
J
K

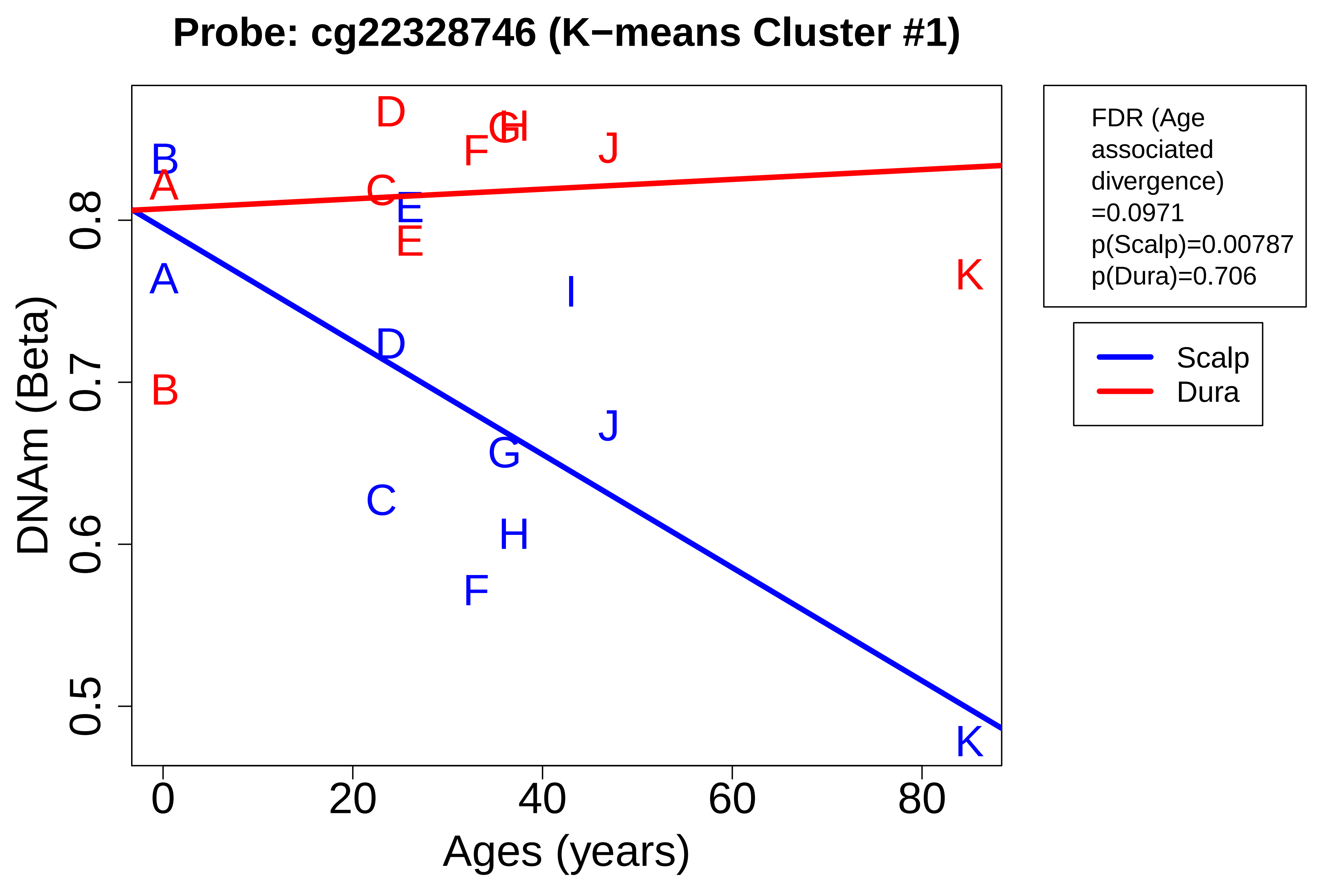

Probe: cg22328746 (K−means Cluster #1)
DNAm (Beta)
0.5
0.6
0.7
0.8
0
20
40
60
80
Ages (years)
FDR (Age associated divergence) =0.0971
p(Scalp)=0.00787
p(Dura)=0.706
Scalp
Dura

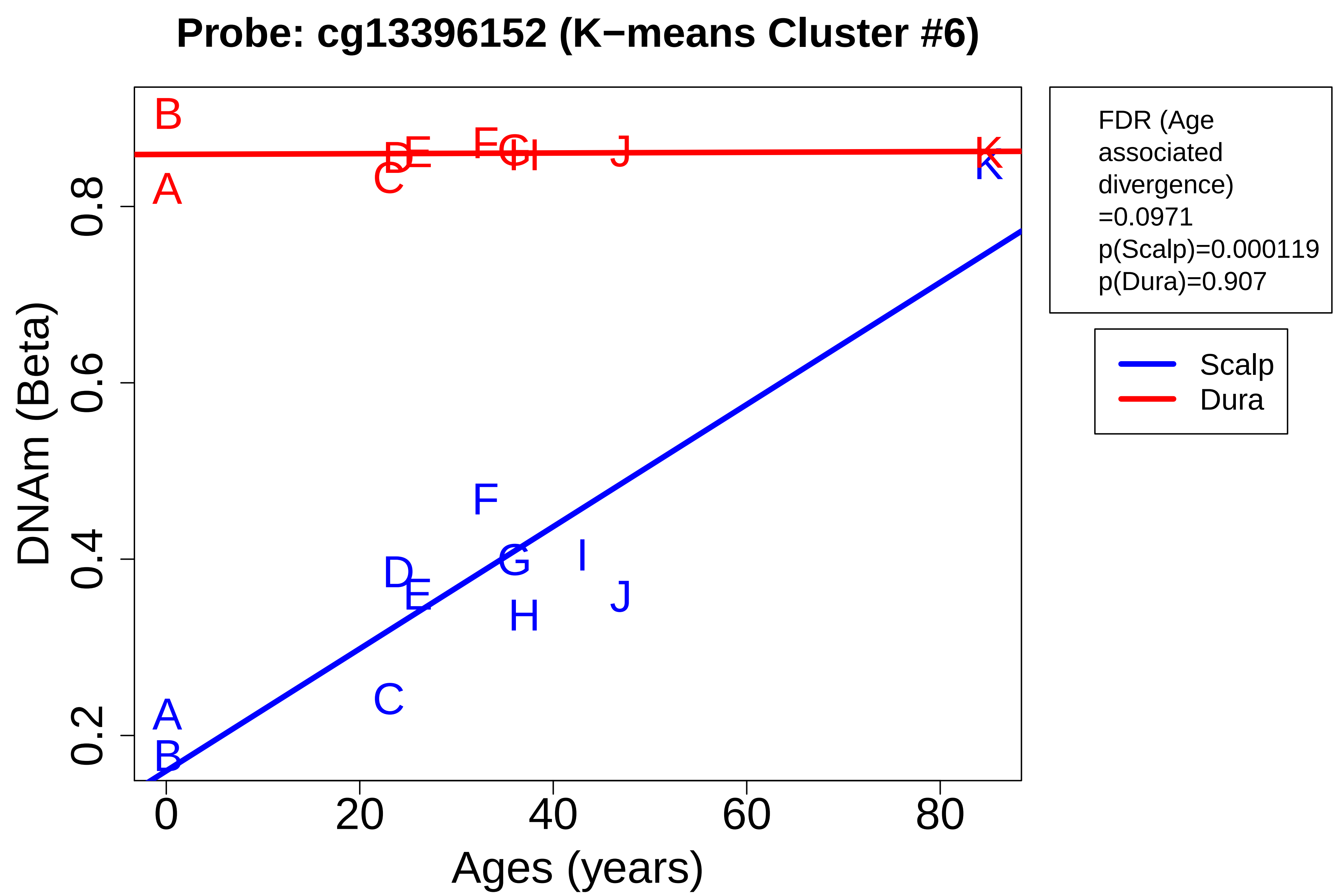

Probe: cg13396152 (K−means Cluster #6)
DNAm (Beta)
0.2
0.4
0.6
0.8
0
20
40
60
80
Ages (years)
FDR (Age associated divergence) =0.0971
p(Scalp)=0.000119
p(Dura)=0.907
Scalp
Dura
A
B
C
D
E
F
G
H
I
J
K

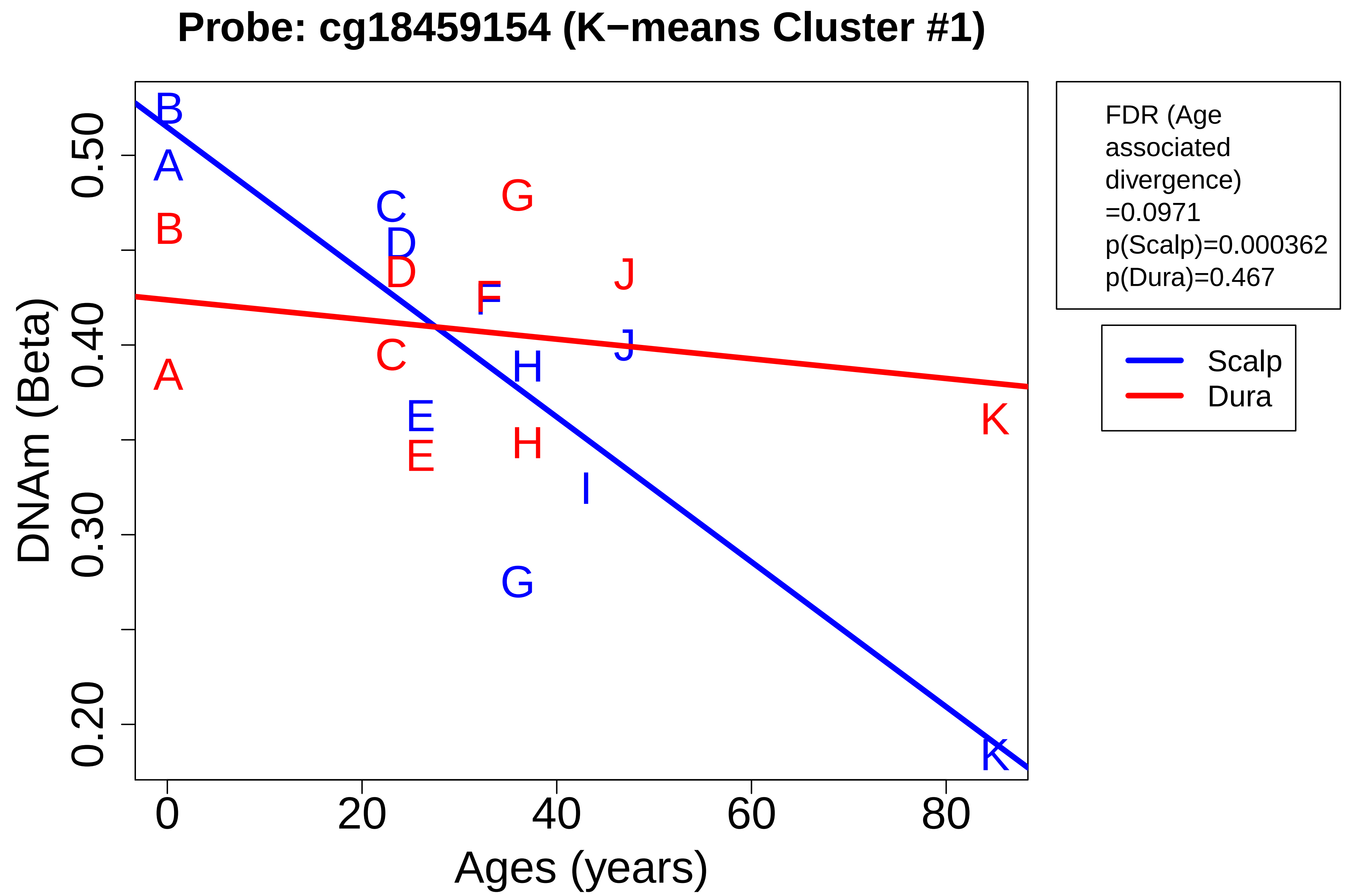

Probe: cg18459154 (K−means Cluster #1)
DNAm (Beta)
0.20
0.30
0.40
0.50
Ages (years)
0
20
40
60
80
B
A
B
C
D
D
G
F
F
J
J
C
A
H
E
E
H
I
G
K
K
FDR (Age associated divergence) =0.0971
p(Scalp)=0.000362
p(Dura)=0.467
Scalp
Dura

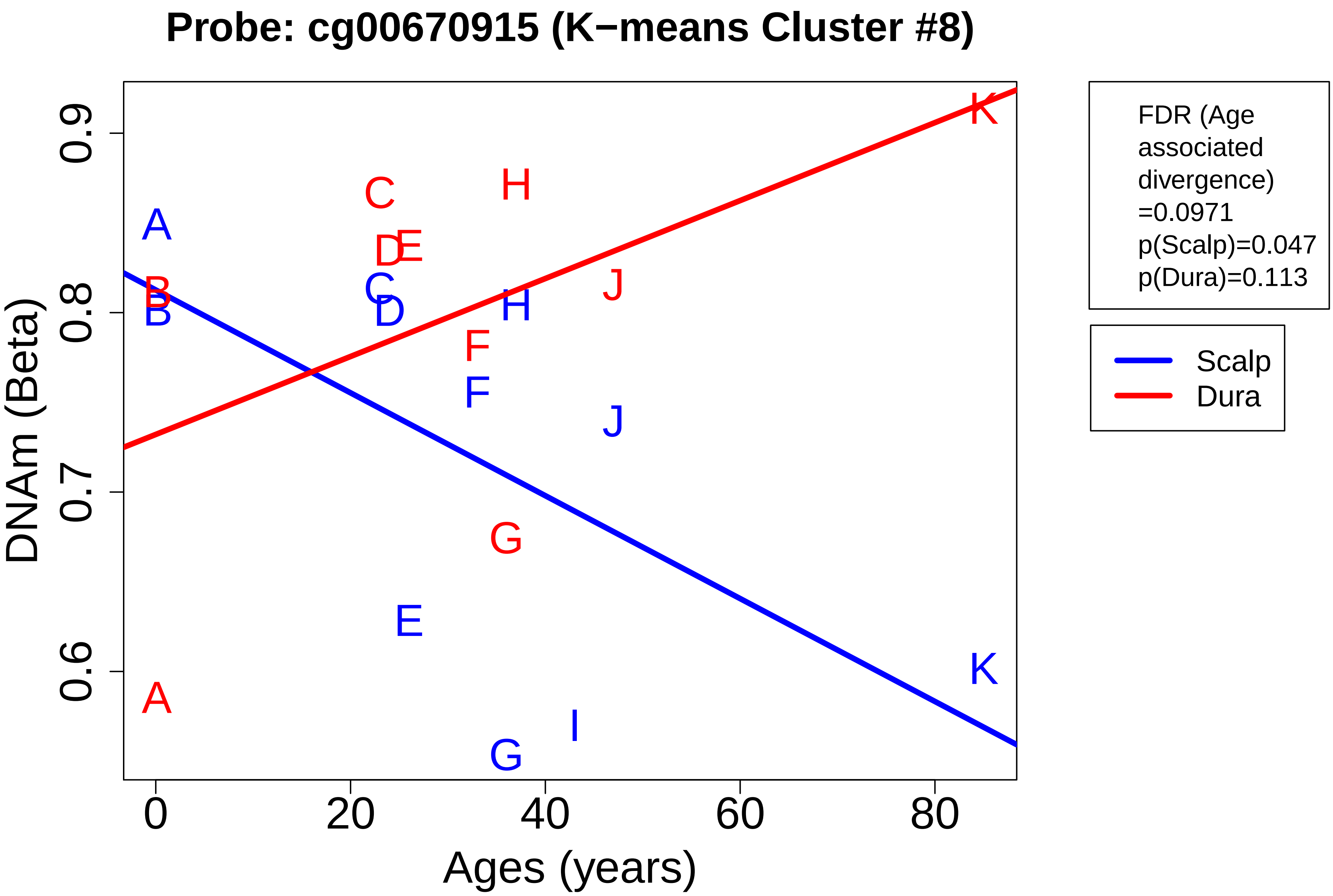
Probe: cg00670915 (K−means Cluster #8)
DNAm (Beta)
0.6
0.7
0.8
0.9
0
20
40
60
80
Ages (years)
A
B
C
D
E
F
G
H
I
J
K
A
B
C
D
E
F
G
H
J
K
FDR (Age associated divergence) =0.0971
p(Scalp)=0.047
p(Dura)=0.113
Scalp
Dura

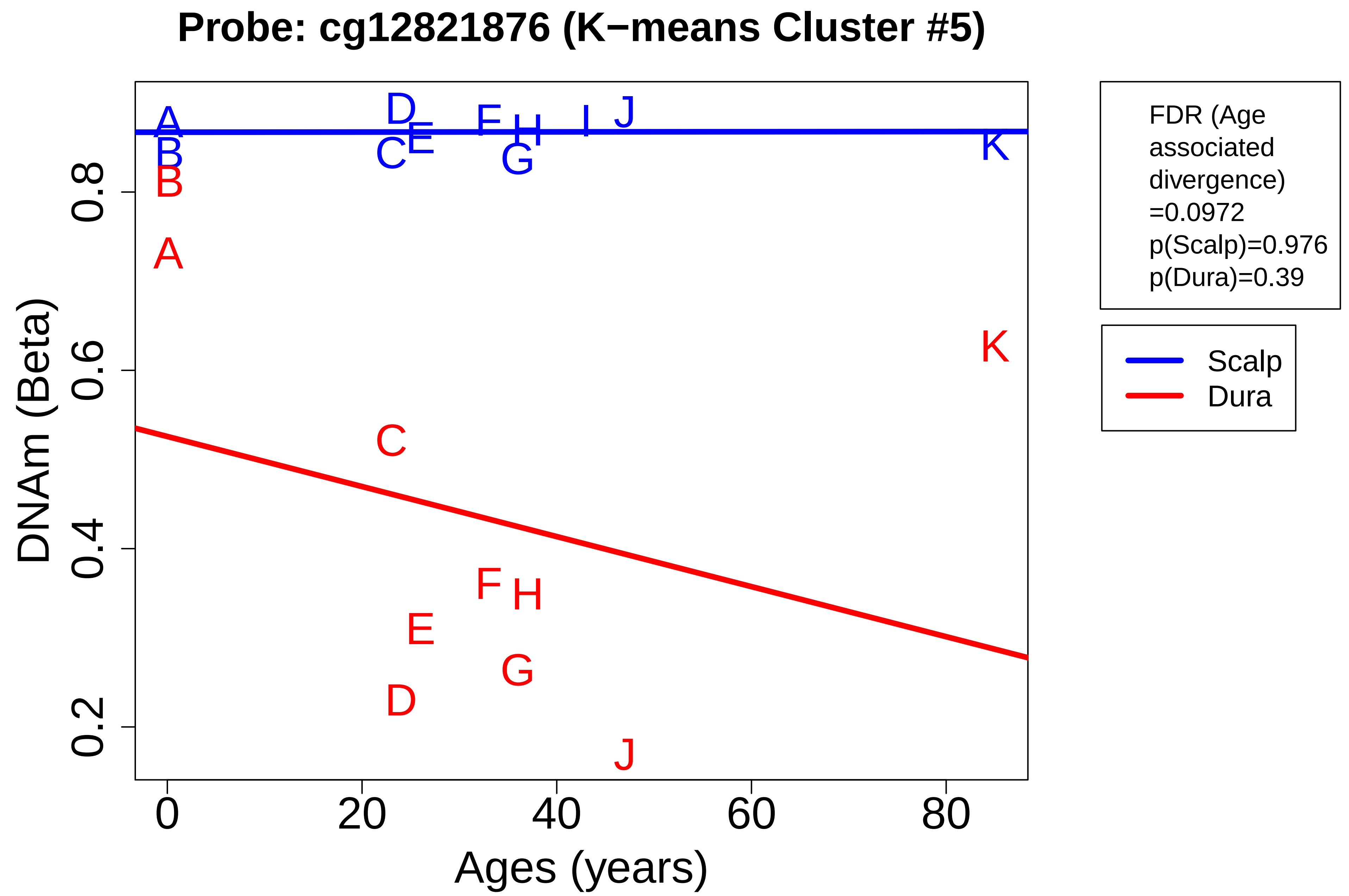

Probe: cg12821876 (K−means Cluster #5)
DNAm (Beta)
0.2
0.4
0.6
0.8
Ages (years)
0
20
40
60
80
A
B
C
D
E
F
G
H
I
J
K
FDR (Age associated divergence) =0.0972
p(Scalp)=0.976
p(Dura)=0.39
Scalp
Dura

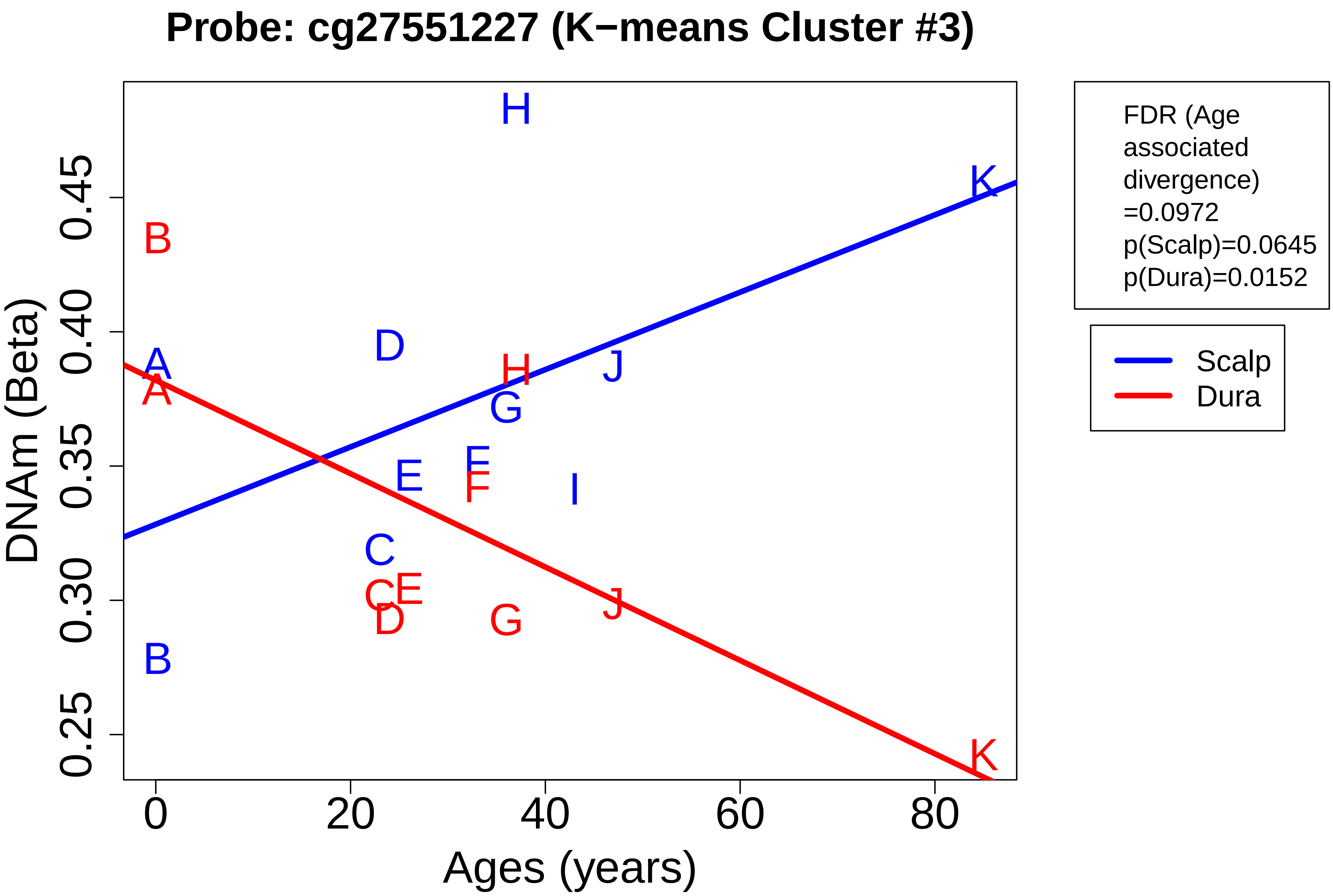

Probe: cg27551227 (K−means Cluster #3)
DNAm (Beta)
0.25
0.30
0.35
0.40
0.45
0
20
40
60
80
Ages (years)
FDR (Age associated divergence) =0.0972
p(Scalp)=0.0645
p(Dura)=0.0152
Scalp
Dura

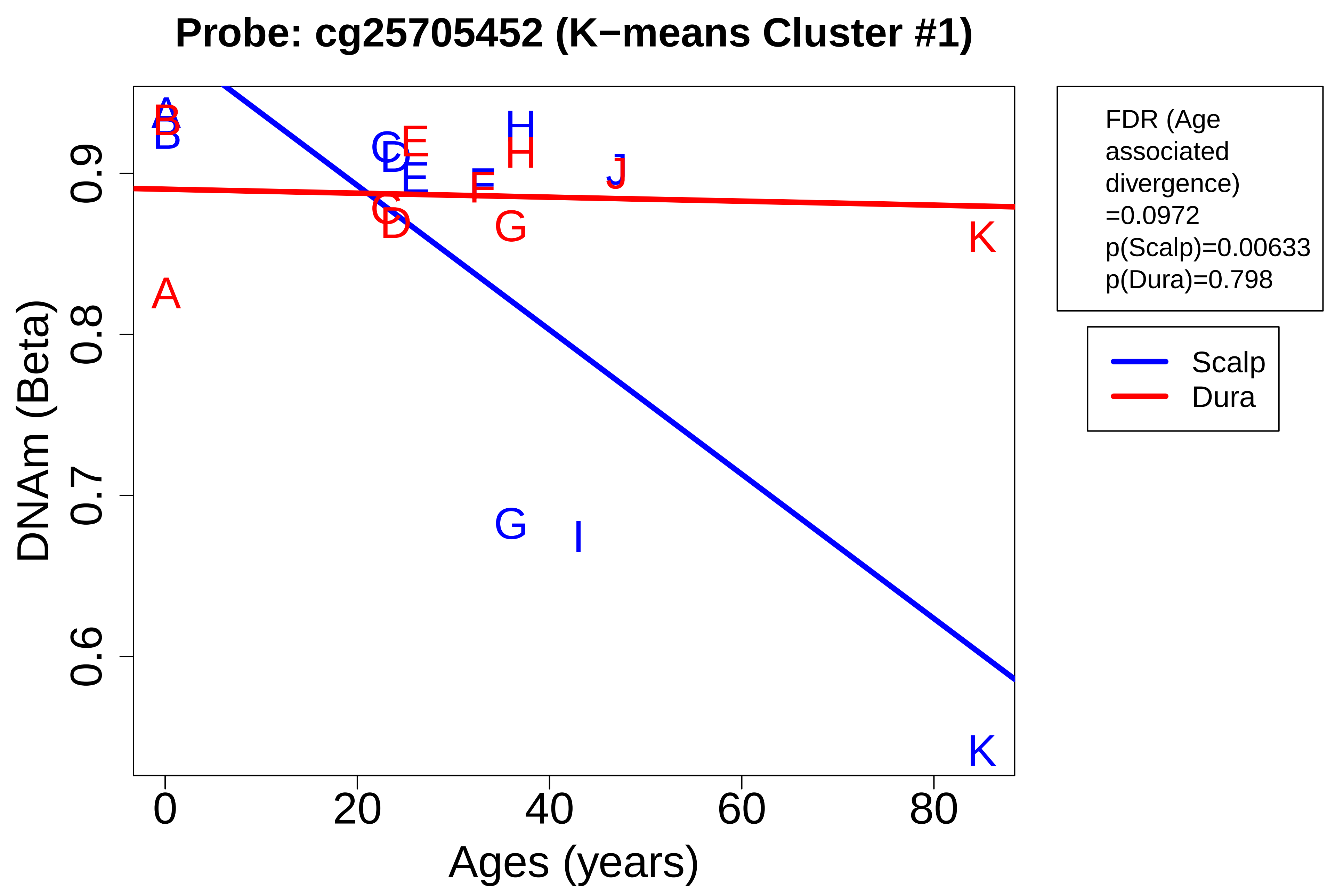

Probe: cg25705452 (K−means Cluster #1)
DNAm (Beta)
0.6
0.7
0.8
0.9
Ages (years)
0
20
40
60
80
FDR (Age associated divergence) =0.0972
p(Scalp)=0.00633
p(Dura)=0.798
Scalp
Dura
A
B
C
D
E
F
G
H
I
J
K

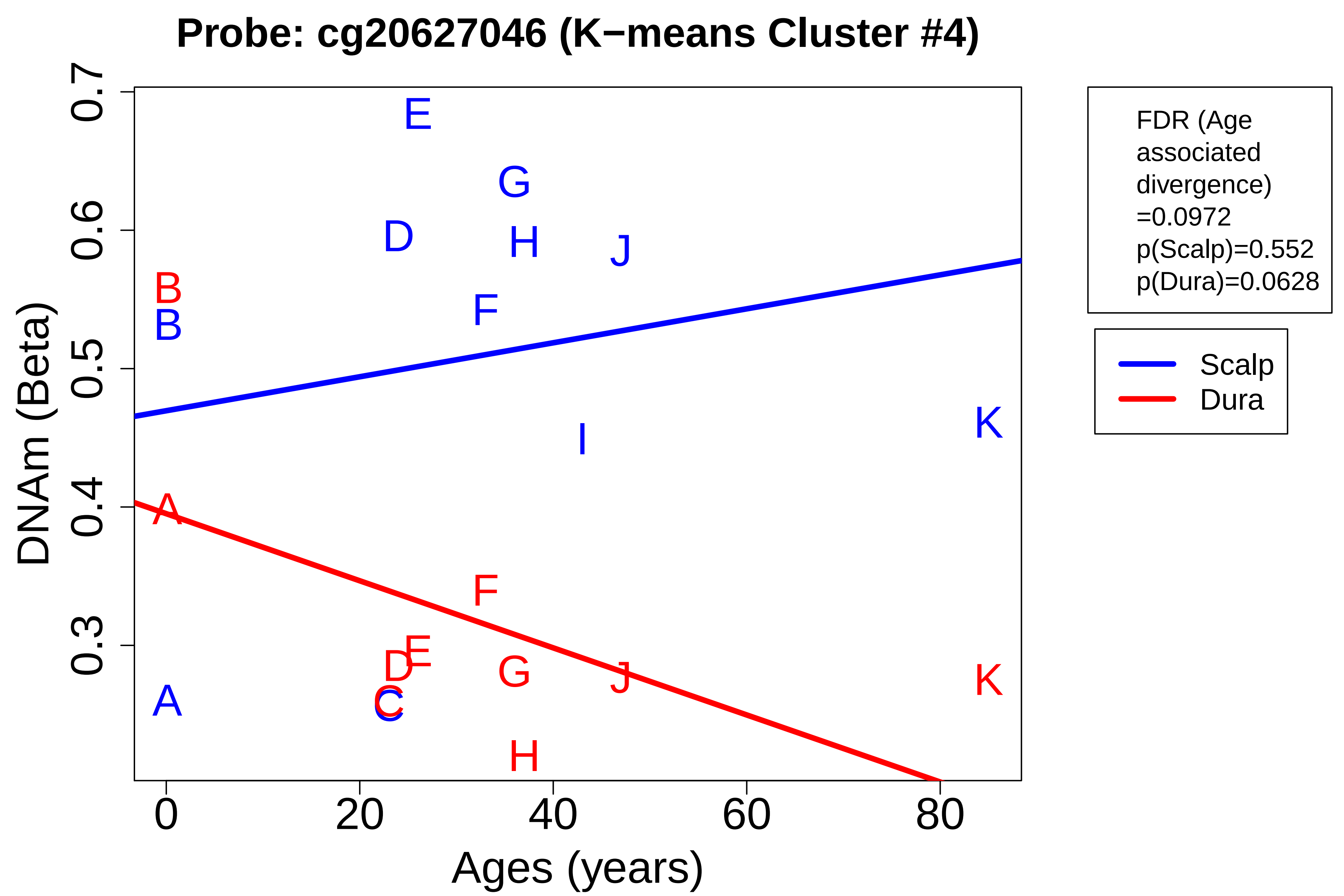

Probe: cg20627046 (K−means Cluster #4)
DNAm (Beta)
0.3
0.4
0.5
0.6
0.7
Ages (years)
0
20
40
60
80
FDR (Age associated divergence) =0.0972
p(Scalp)=0.552
p(Dura)=0.0628
Scalp
Dura
A B C D E F G H I J K

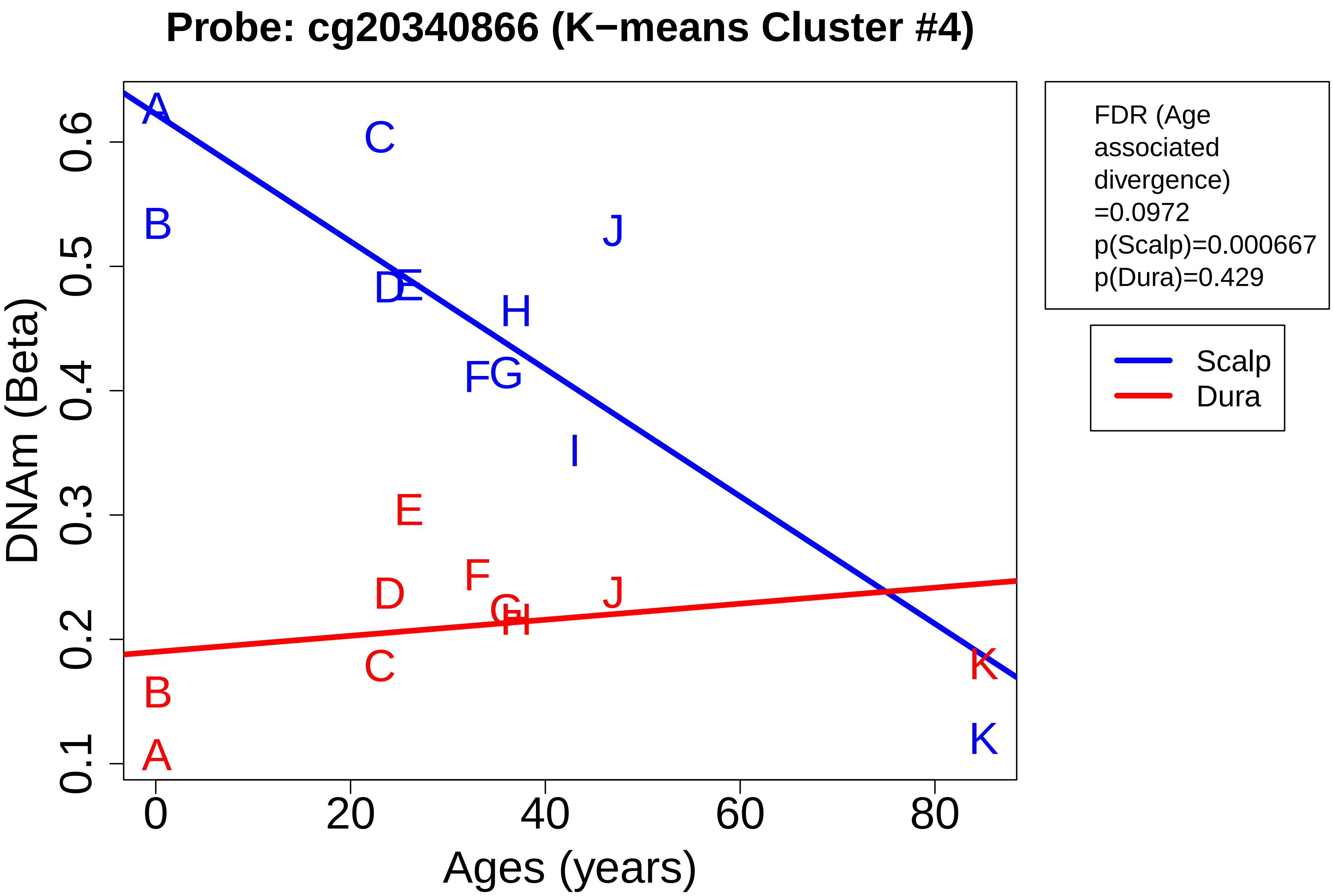

Probe: cg20340866 (K−means Cluster #4)
DNAm (Beta)
Ages (years)
0.1
0.2
0.3
0.4
0.5
0.6
0
20
40
60
80
FDR (Age associated divergence) =0.0972
p(Scalp)=0.000667
p(Dura)=0.429
Scalp
Dura

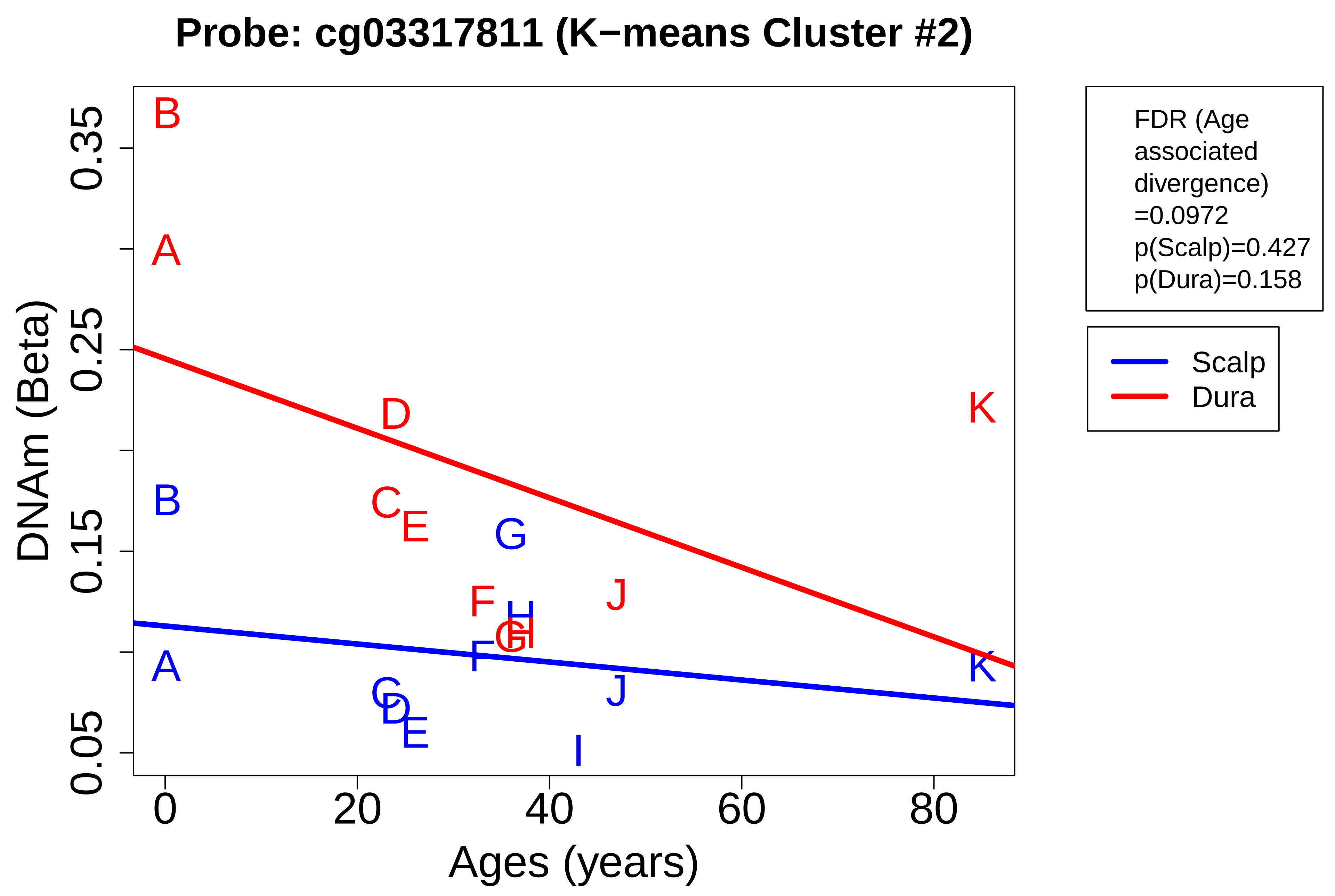

Probe: cg03317811 (K−means Cluster #2)
DNAm (Beta)
0.05
0.15
0.25
0.35
0
20
40
60
80
Ages (years)
FDR (Age associated divergence) =0.0972
p(Scalp)=0.427
p(Dura)=0.158
Scalp
Dura

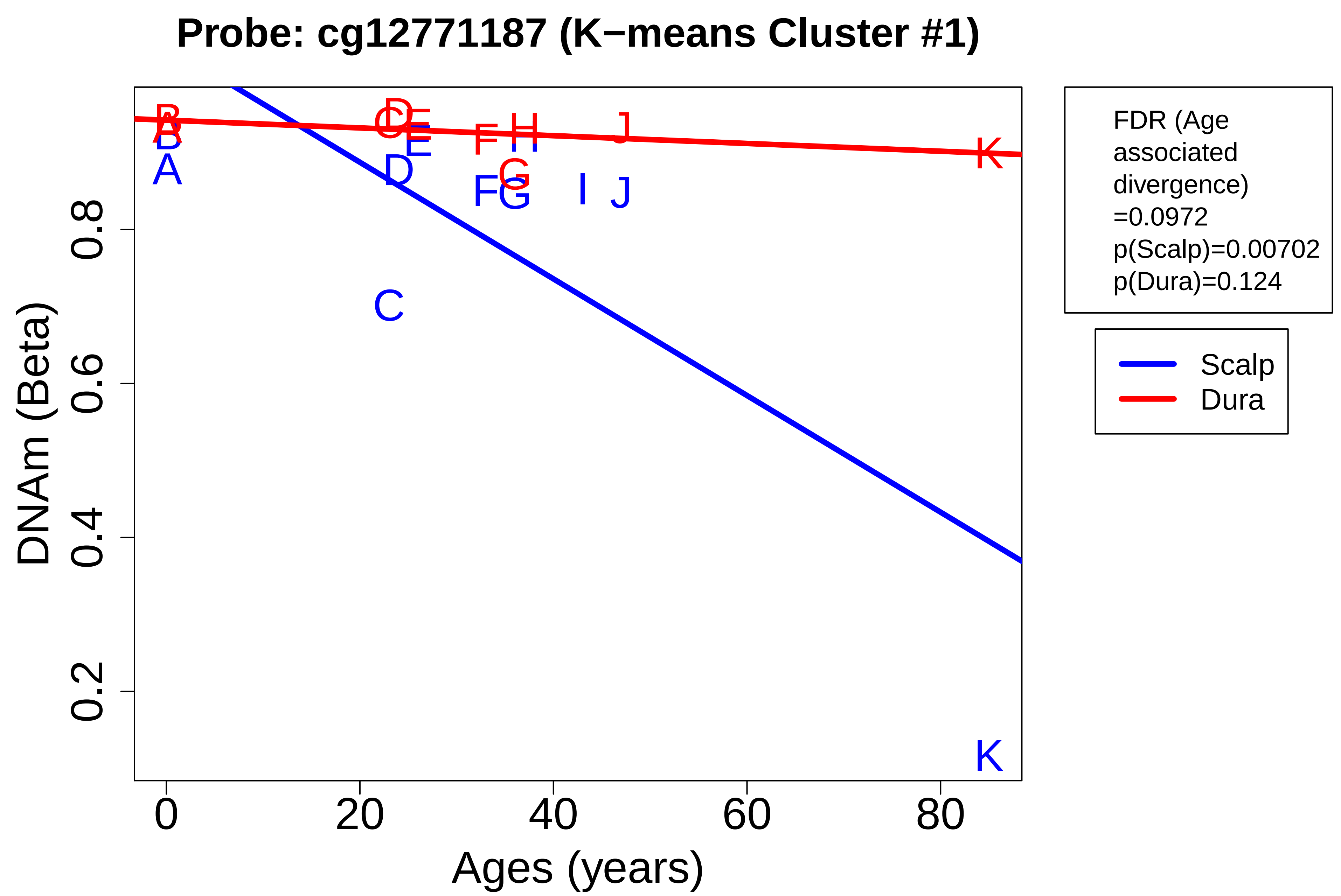

Probe: cg12771187 (K−means Cluster #1)
DNAm (Beta)
0.2
0.4
0.6
0.8
0
20
40
60
80
Ages (years)
A B C D E F G H I J K
FDR (Age associated divergence) =0.0972
p(Scalp)=0.00702
p(Dura)=0.124
Scalp
Dura

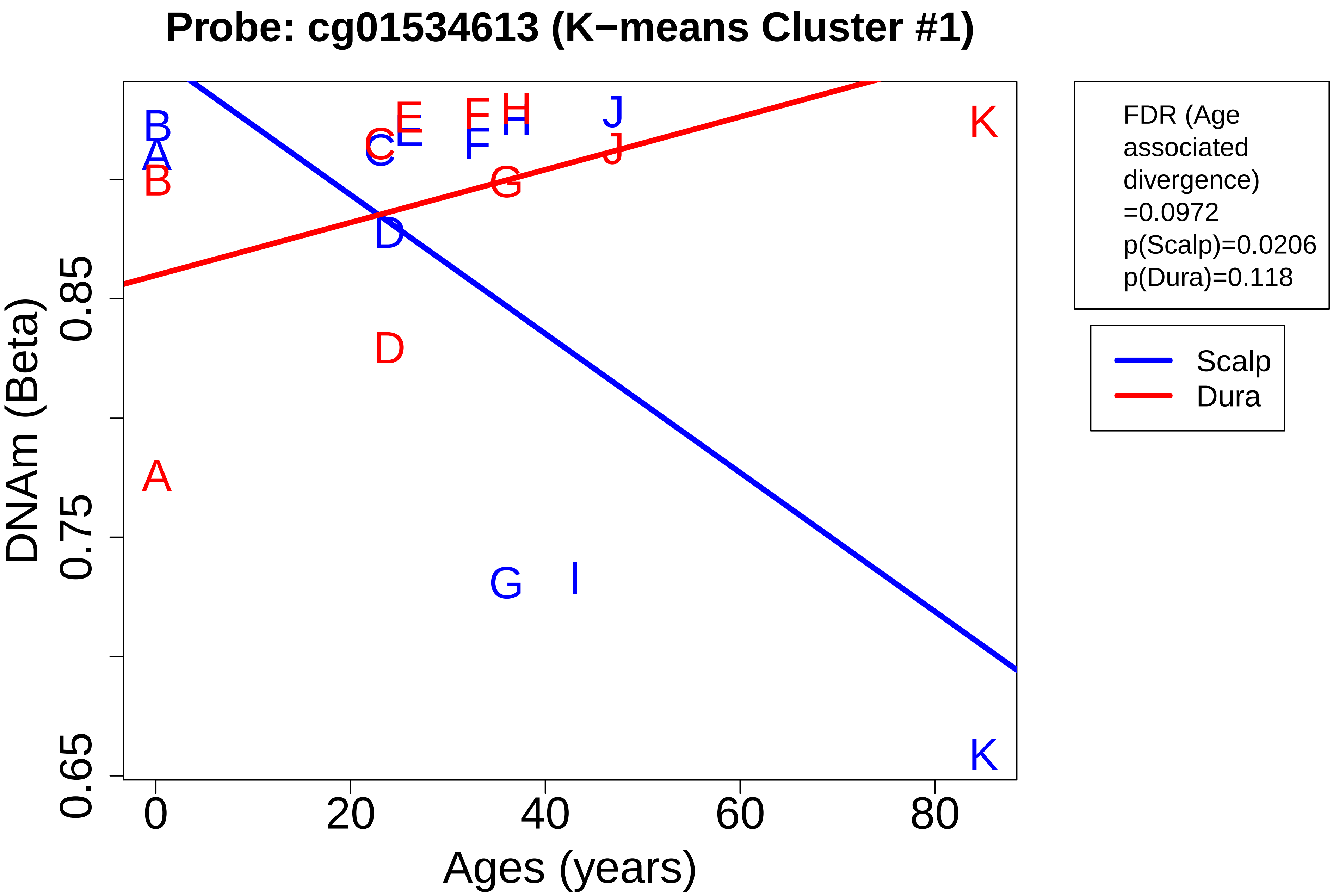

Probe: cg01534613 (K−means Cluster #1)
DNAm (Beta)
0.65
0.75
0.85
Ages (years)
0
20
40
60
80
FDR (Age associated divergence) =0.0972
p(Scalp)=0.0206
p(Dura)=0.118
Scalp
Dura

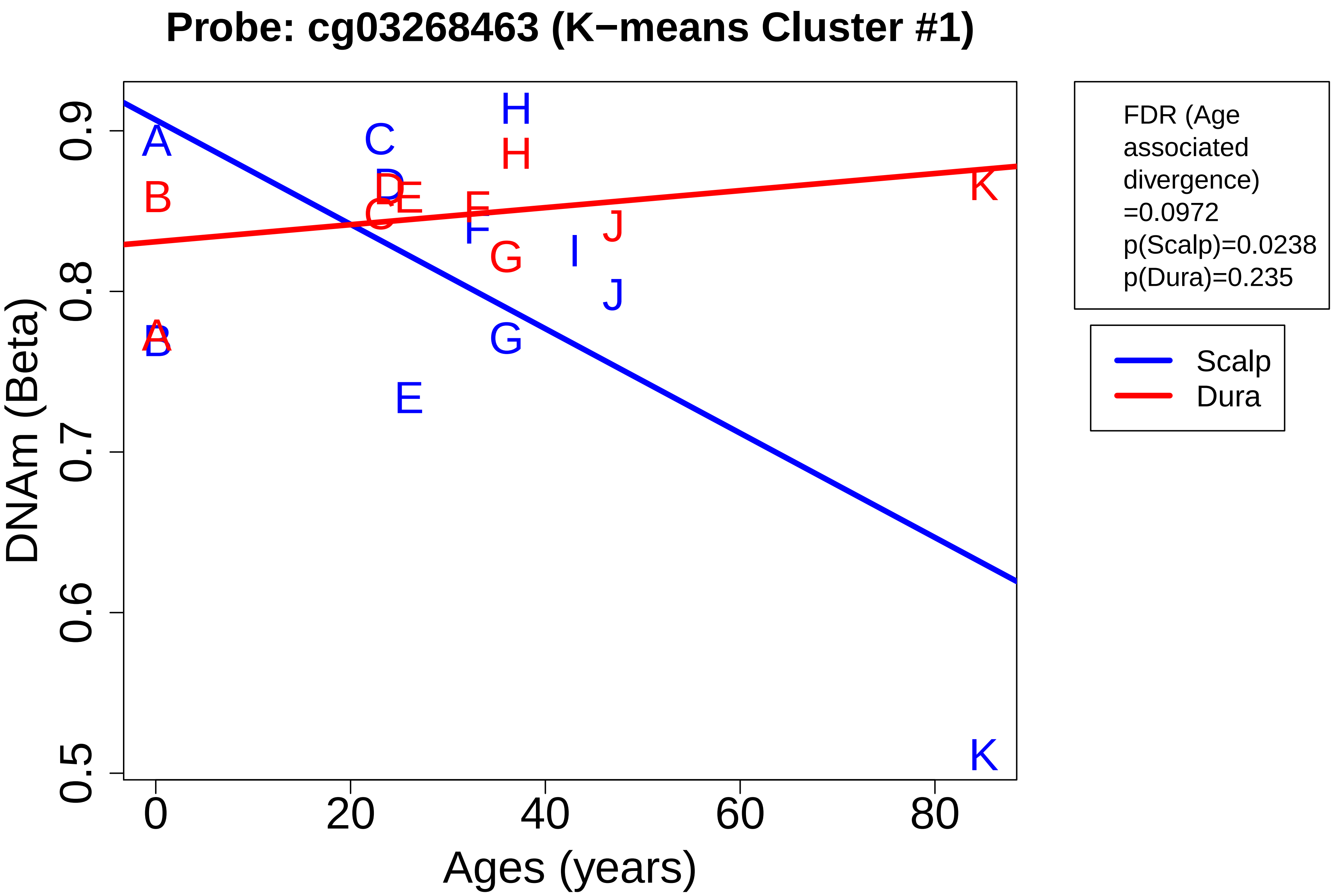

Probe: cg03268463 (K−means Cluster #1)
DNAm (Beta)
0.5
0.6
0.7
0.8
0.9
Ages (years)
0
20
40
60
80
FDR (Age associated divergence) =0.0972
p(Scalp)=0.0238
p(Dura)=0.235
Scalp
Dura
A
B
C
D
E
F
G
H
I
J
K

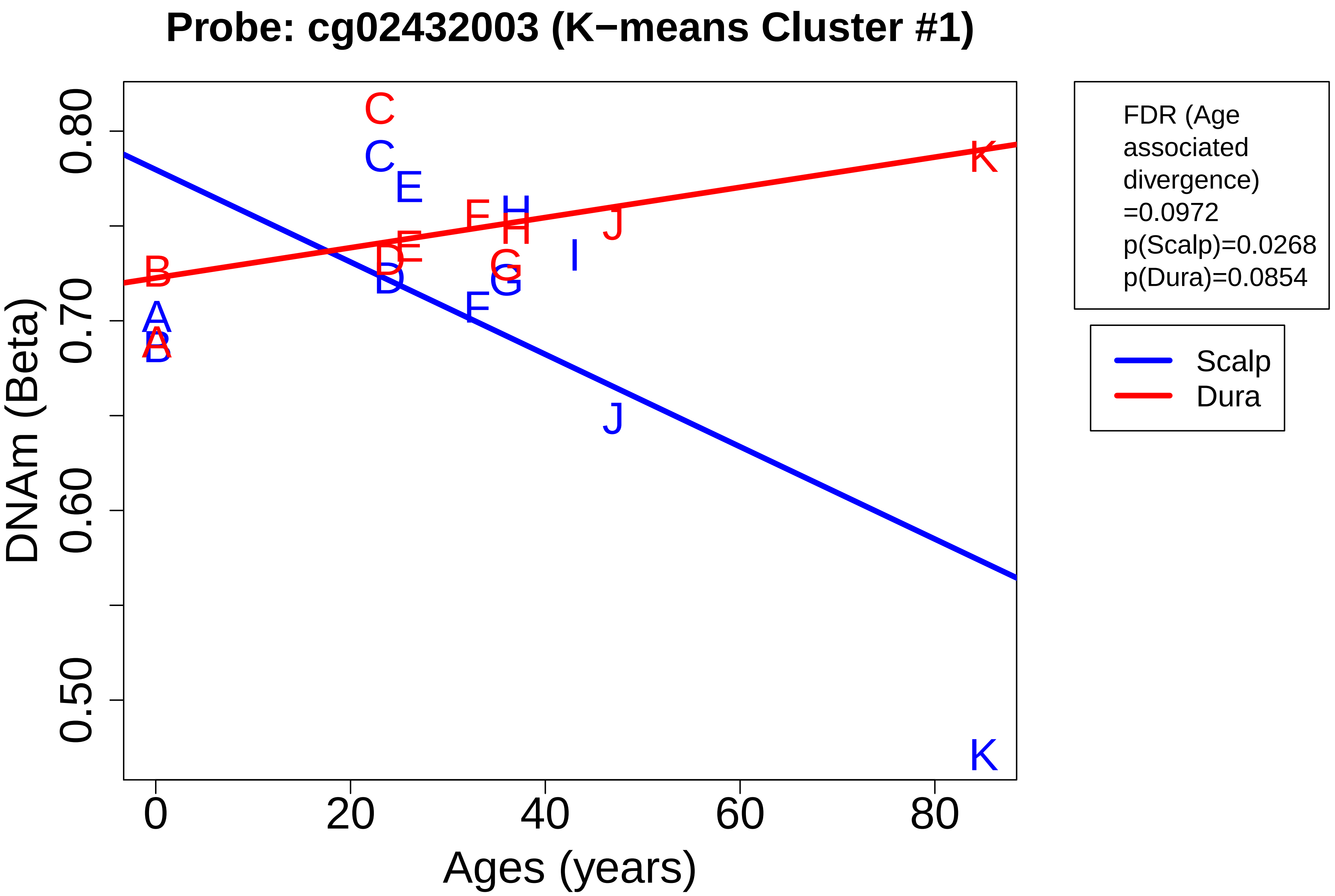

Probe: cg02432003 (K−means Cluster #1)
DNAm (Beta)
0.50
0.60
0.70
0.80
Ages (years)
0
20
40
60
80
A
B
C
D
E
F
G
H
I
J
K
FDR (Age associated divergence) =0.0972
p(Scalp)=0.0268
p(Dura)=0.0854
Scalp
Dura

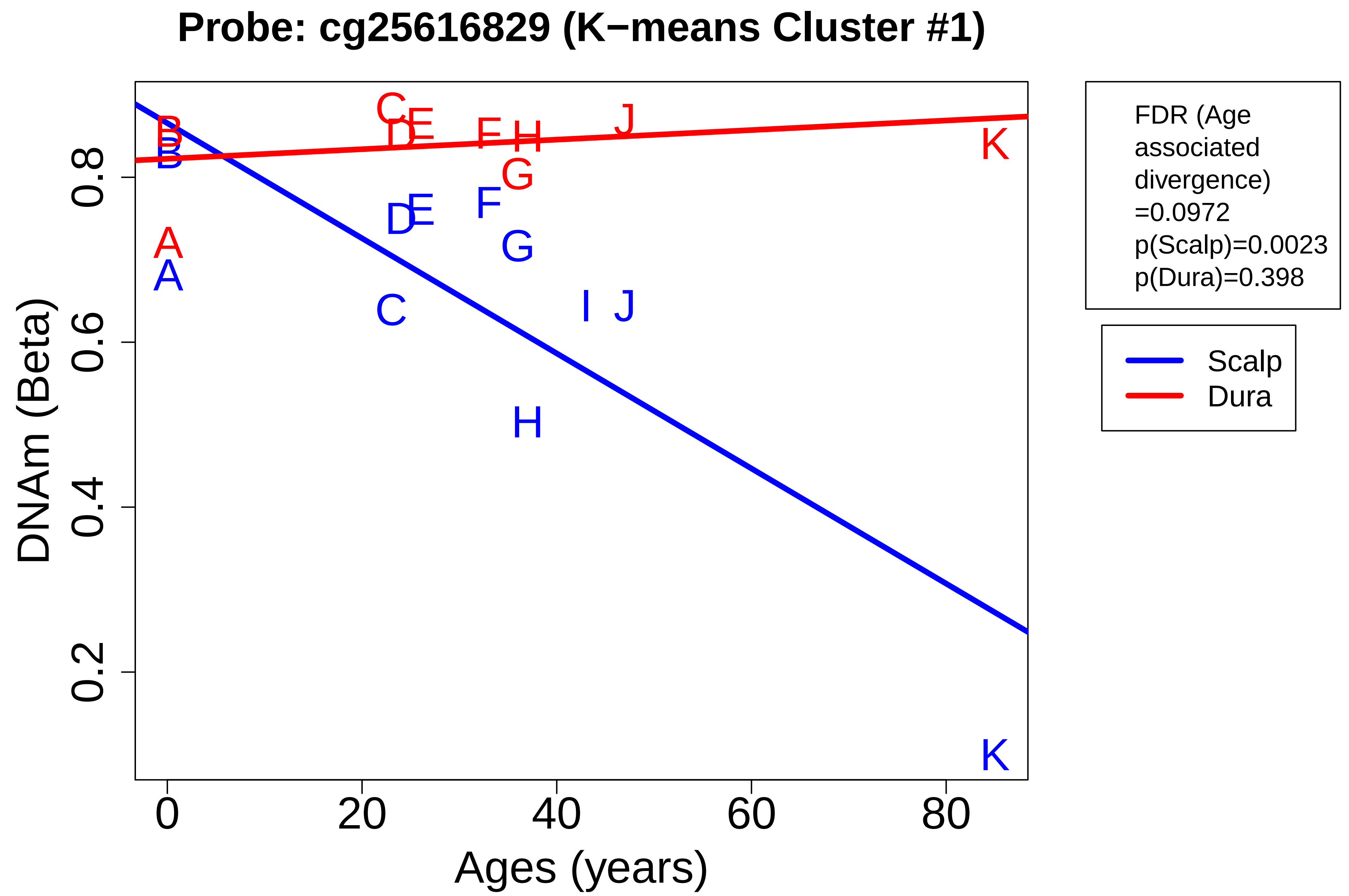

Probe: cg25616829 (K−means Cluster #1)
DNAm (Beta)
0.2
0.4
0.6
0.8
0
20
40
60
80
Ages (years)
FDR (Age associated divergence) =0.0972
p(Scalp)=0.0023
p(Dura)=0.398
Scalp
Dura

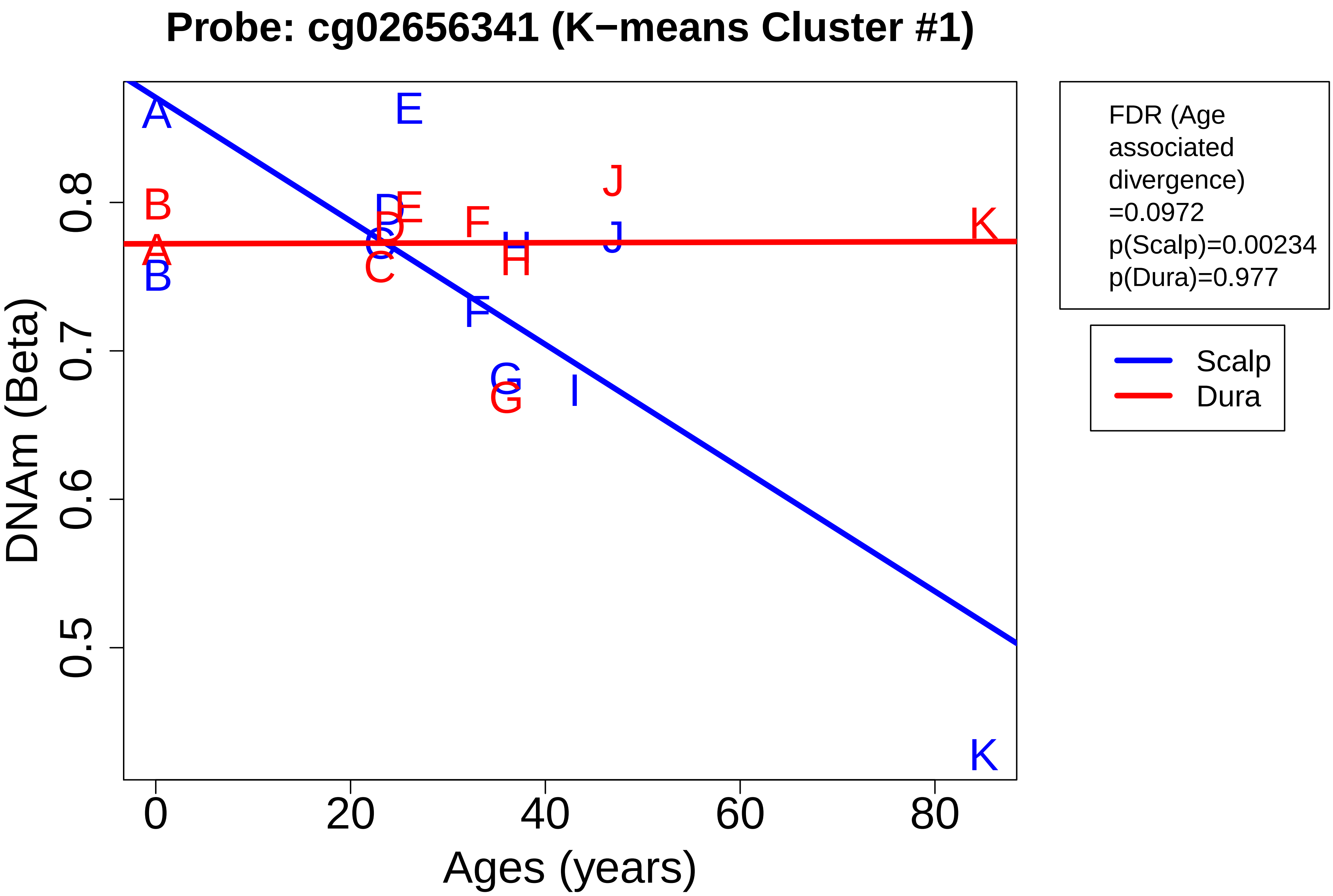

Probe: cg02656341 (K−means Cluster #1)
DNAm (Beta)
0.5
0.6
0.7
0.8
Ages (years)
0
20
40
60
80
A
B
C
D
E
F
G
H
I
J
K
FDR (Age associated divergence) =0.0972
p(Scalp)=0.00234
p(Dura)=0.977
Scalp
Dura

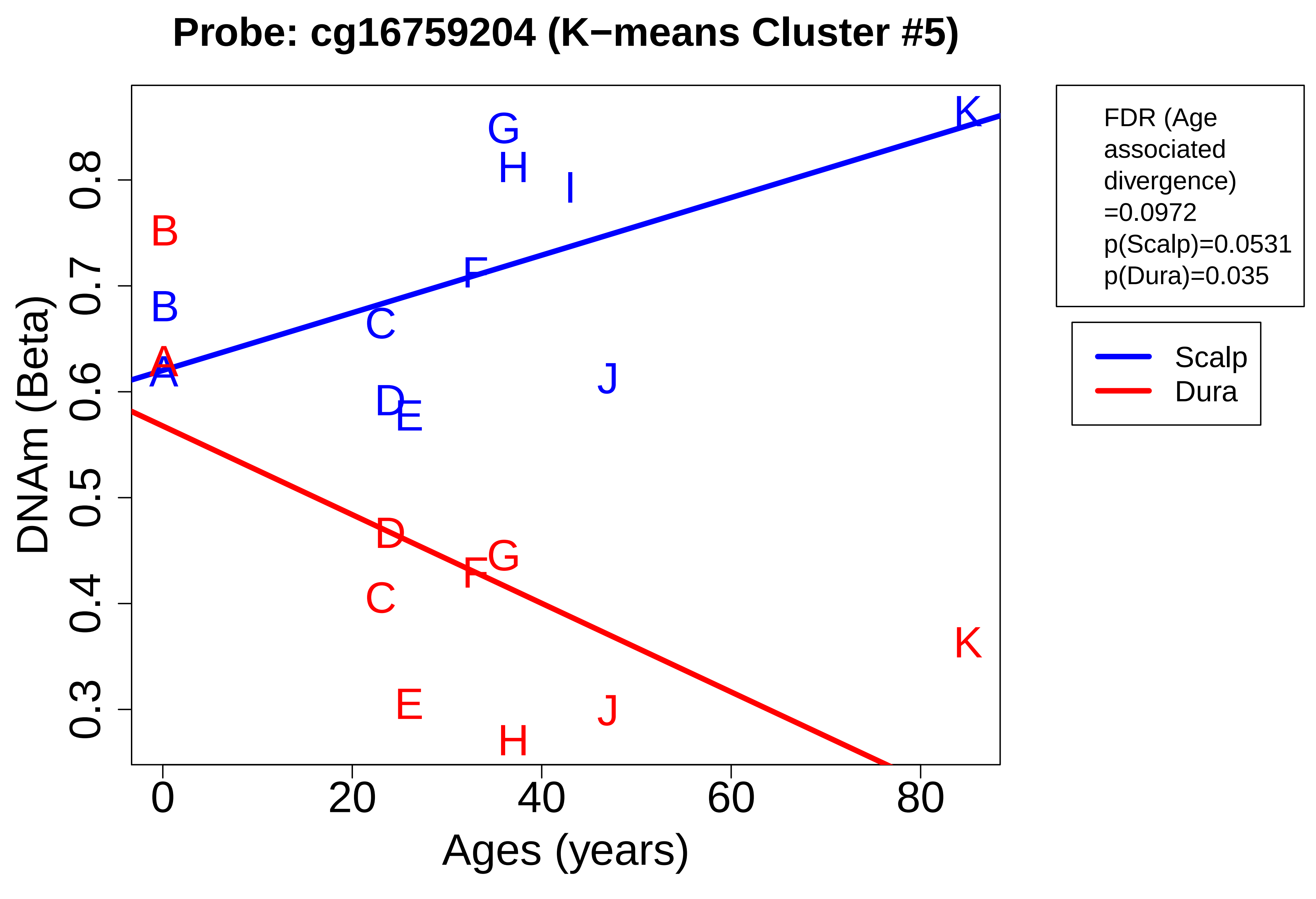

Probe: cg16759204 (K−means Cluster #5)
DNAm (Beta)
0.3
0.4
0.5
0.6
0.7
0.8
Ages (years)
0
20
40
60
80
FDR (Age associated divergence) =0.0972
p(Scalp)=0.0531
p(Dura)=0.035
Scalp
Dura

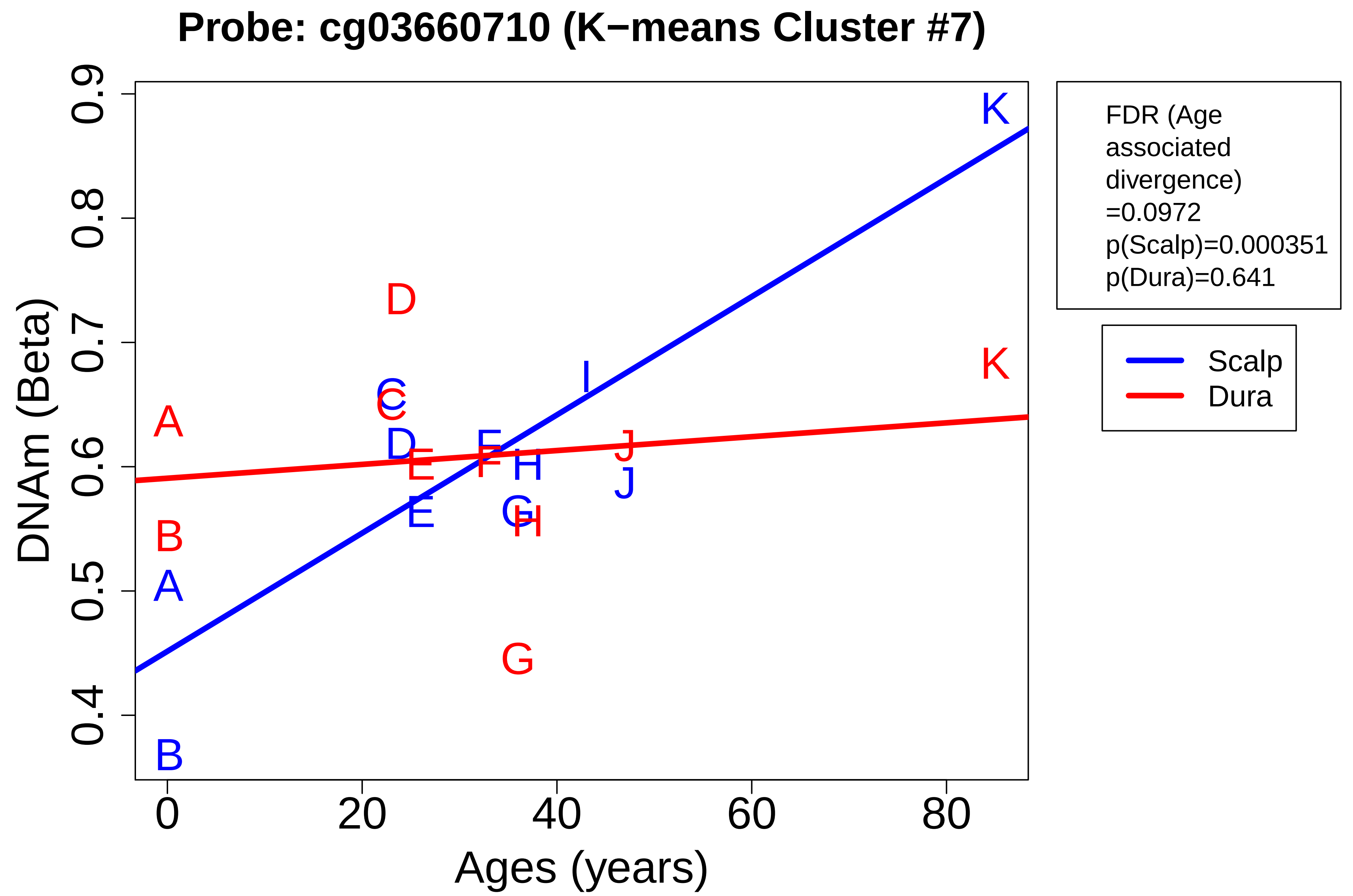

Probe: cg03660710 (K−means Cluster #7)
DNAm (Beta)
0.4
0.5
0.6
0.7
0.8
0.9
0
20
40
60
80
Ages (years)
FDR (Age associated divergence) =0.0972
p(Scalp)=0.000351
p(Dura)=0.641
Scalp
Dura

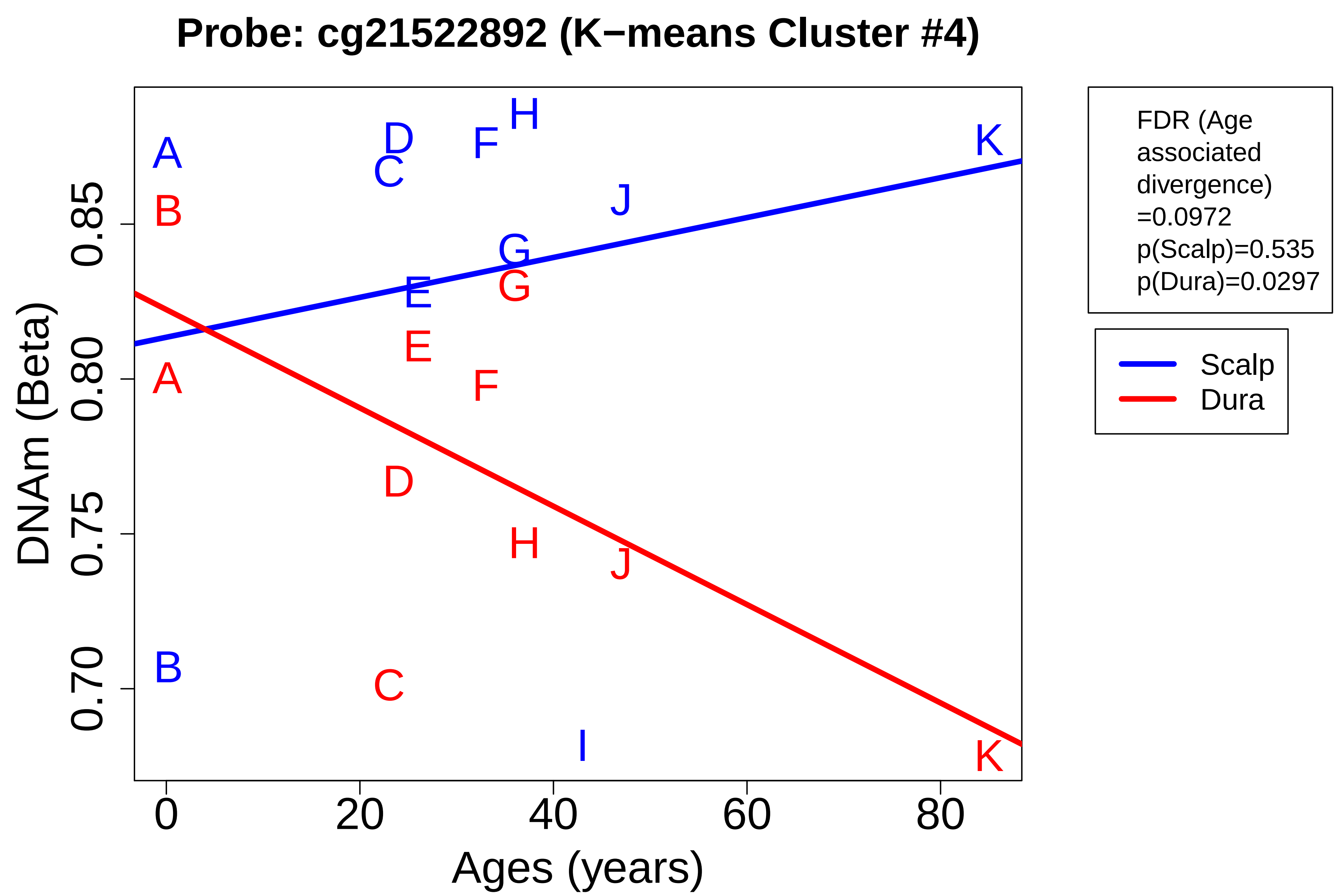
Probe: cg21522892 (K−means Cluster #4)
DNAm (Beta)
0.70
0.75
0.80
0.85
0
20
40
60
80
Ages (years)
A
B
C
D
E
F
G
H
I
J
K
A
B
C
D
E
F
G
H
J
K
FDR (Age associated divergence) =0.0972
p(Scalp)=0.535
p(Dura)=0.0297
Scalp
Dura

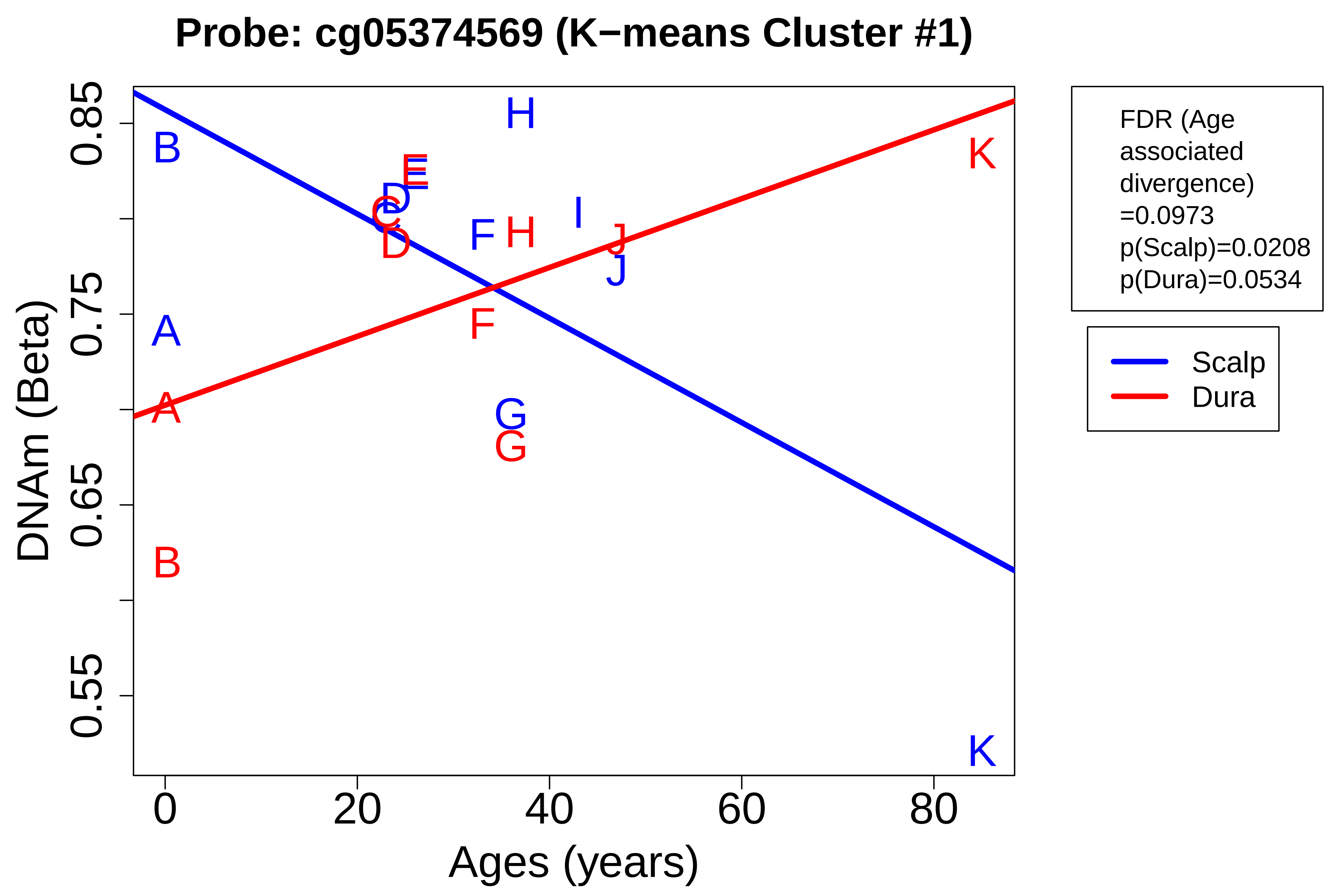

Probe: cg05374569 (K−means Cluster #1)
DNAm (Beta)
0.55
0.65
0.75
0.85
0
20
40
60
80
Ages (years)
A
B
C
D
E
F
G
H
I
J
K
FDR (Age associated divergence) =0.0973
p(Scalp)=0.0208
p(Dura)=0.0534
Scalp
Dura

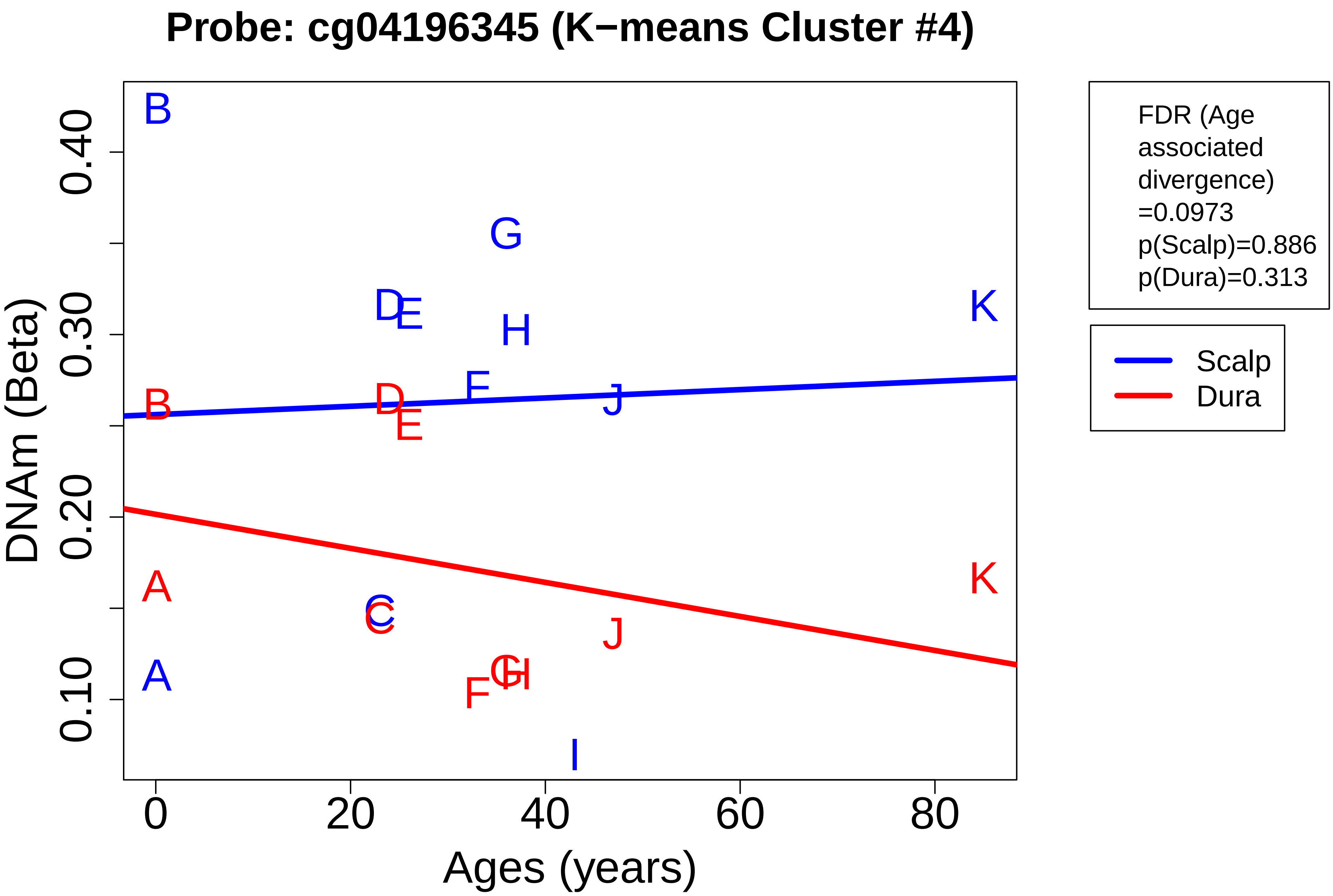

Probe: cg04196345 (K−means Cluster #4)
DNAm (Beta)
0.10
0.20
0.30
0.40
0
20
40
60
80
Ages (years)
FDR (Age associated divergence) =0.0973
p(Scalp)=0.886
p(Dura)=0.313
Scalp
Dura

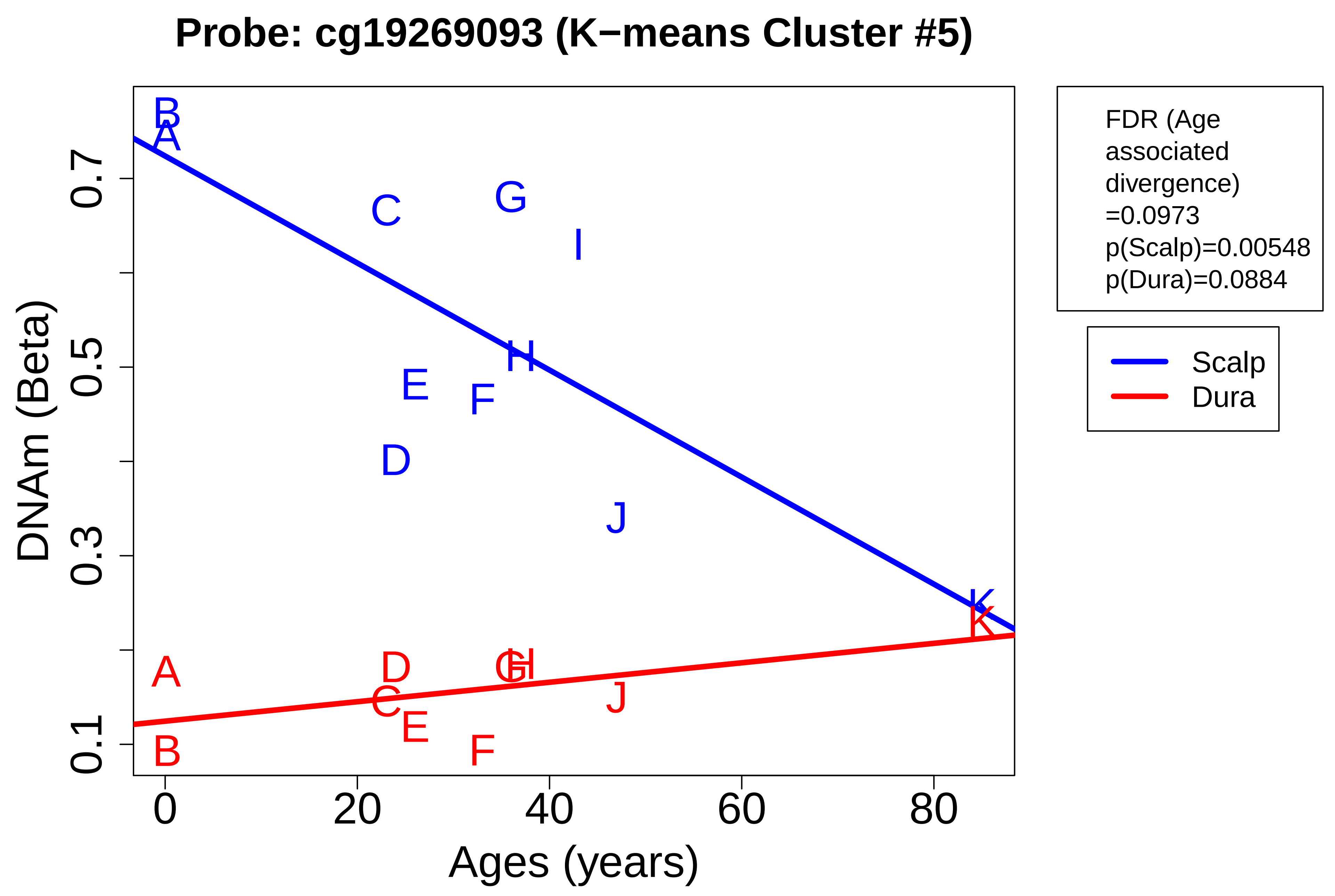

Probe: cg19269093 (K−means Cluster #5)
DNAm (Beta)
0.1
0.3
0.5
0.7
Ages (years)
0
20
40
60
80
A
B
C
D
E
F
G
H
I
J
K
FDR (Age associated divergence) =0.0973
p(Scalp)=0.00548
p(Dura)=0.0884
Scalp
Dura

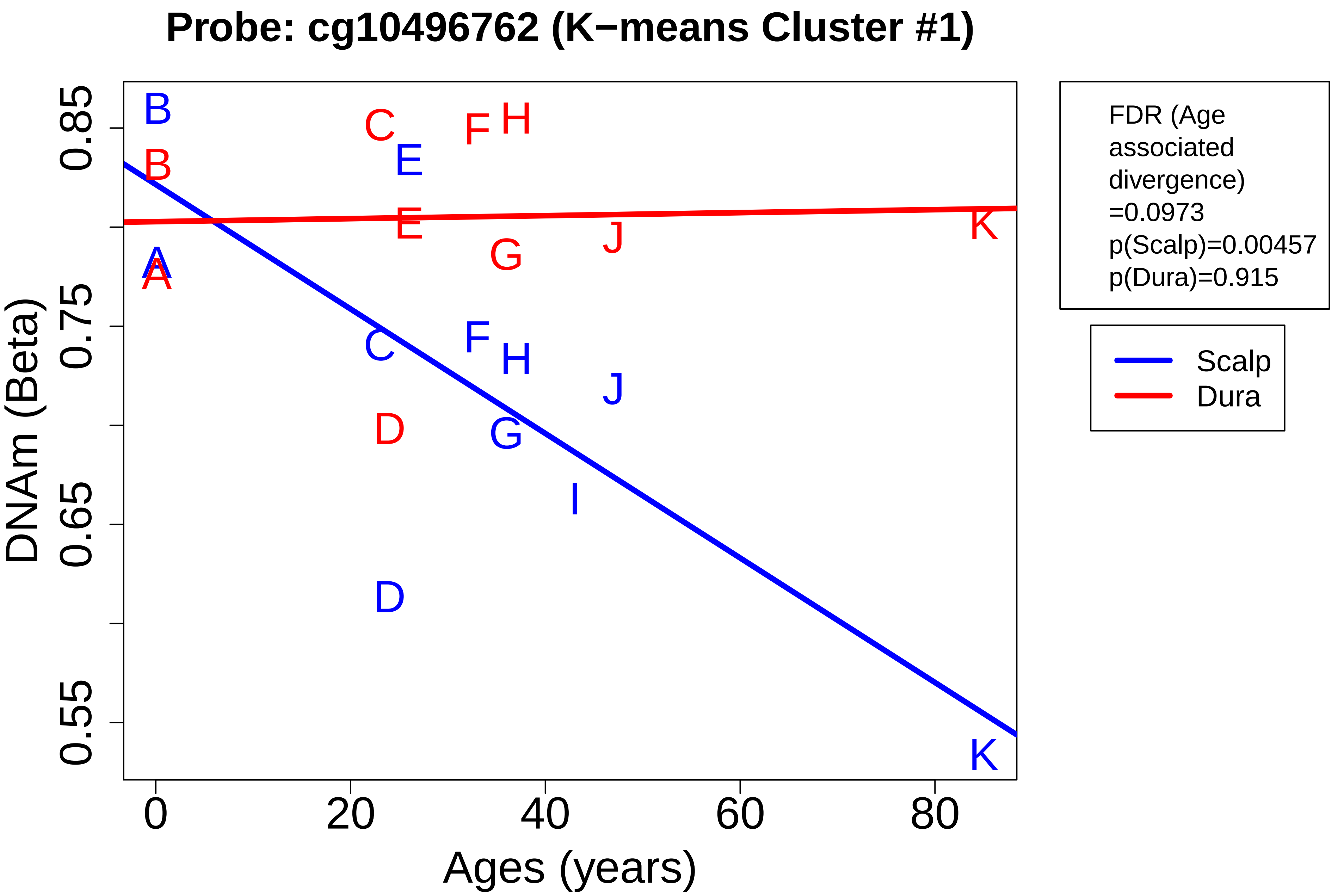
Probe: cg10496762 (K−means Cluster #1)
FDR (Age associated divergence) =0.0973
p(Scalp)=0.00457
p(Dura)=0.915
Scalp
Dura
DNAm (Beta)
0.55
0.65
0.75
0.85
Ages (years)
0
20
40
60
80
A
B
C
D
E
F
G
H
I
J
K

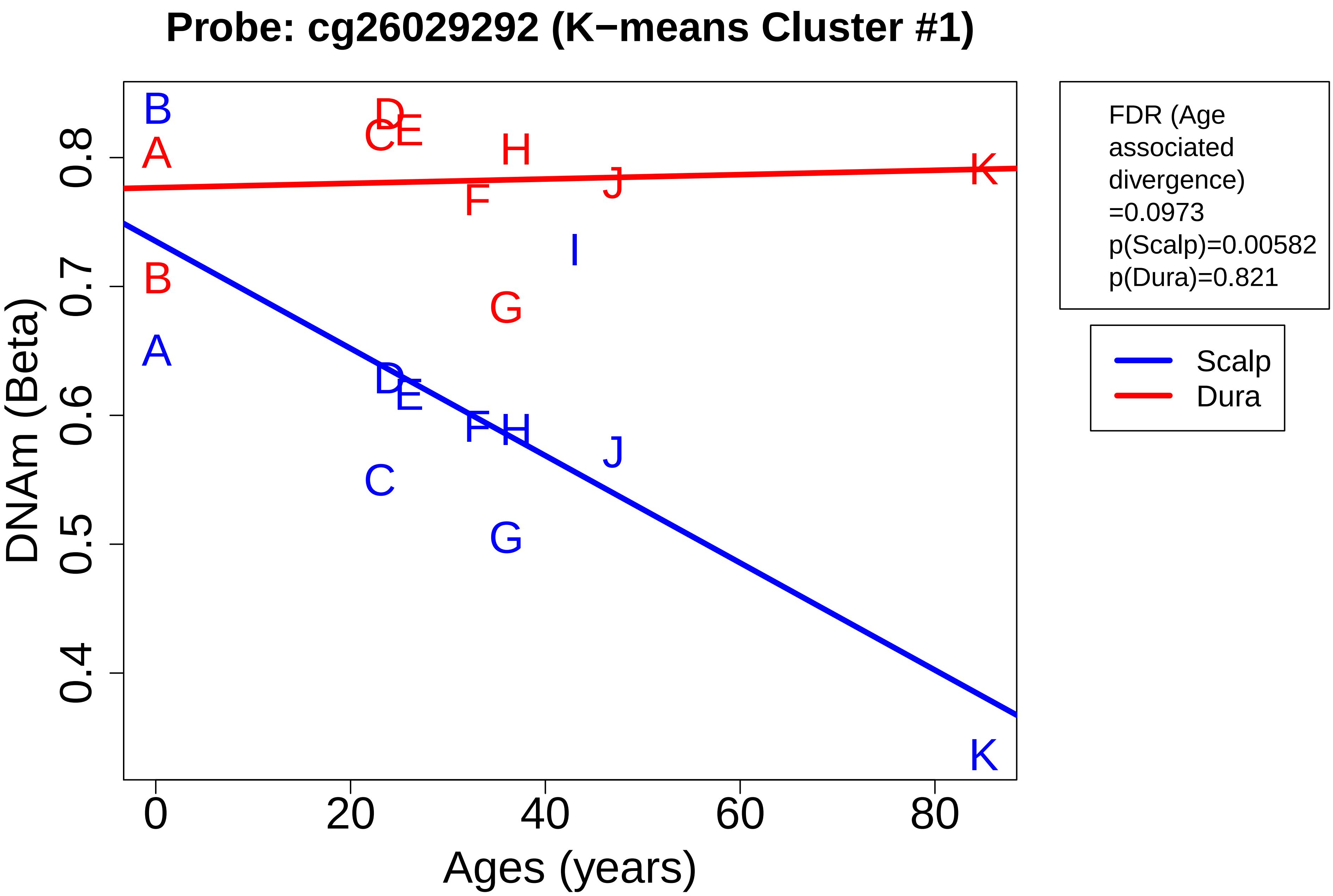

Probe: cg26029292 (K−means Cluster #1)
FDR (Age associated divergence) =0.0973
p(Scalp)=0.00582
p(Dura)=0.821
Scalp
Dura
DNAm (Beta)
0.4
0.5
0.6
0.7
0.8
0
20
40
60
80
Ages (years)
A
B
C
D
E
F
G
H
I
J
K

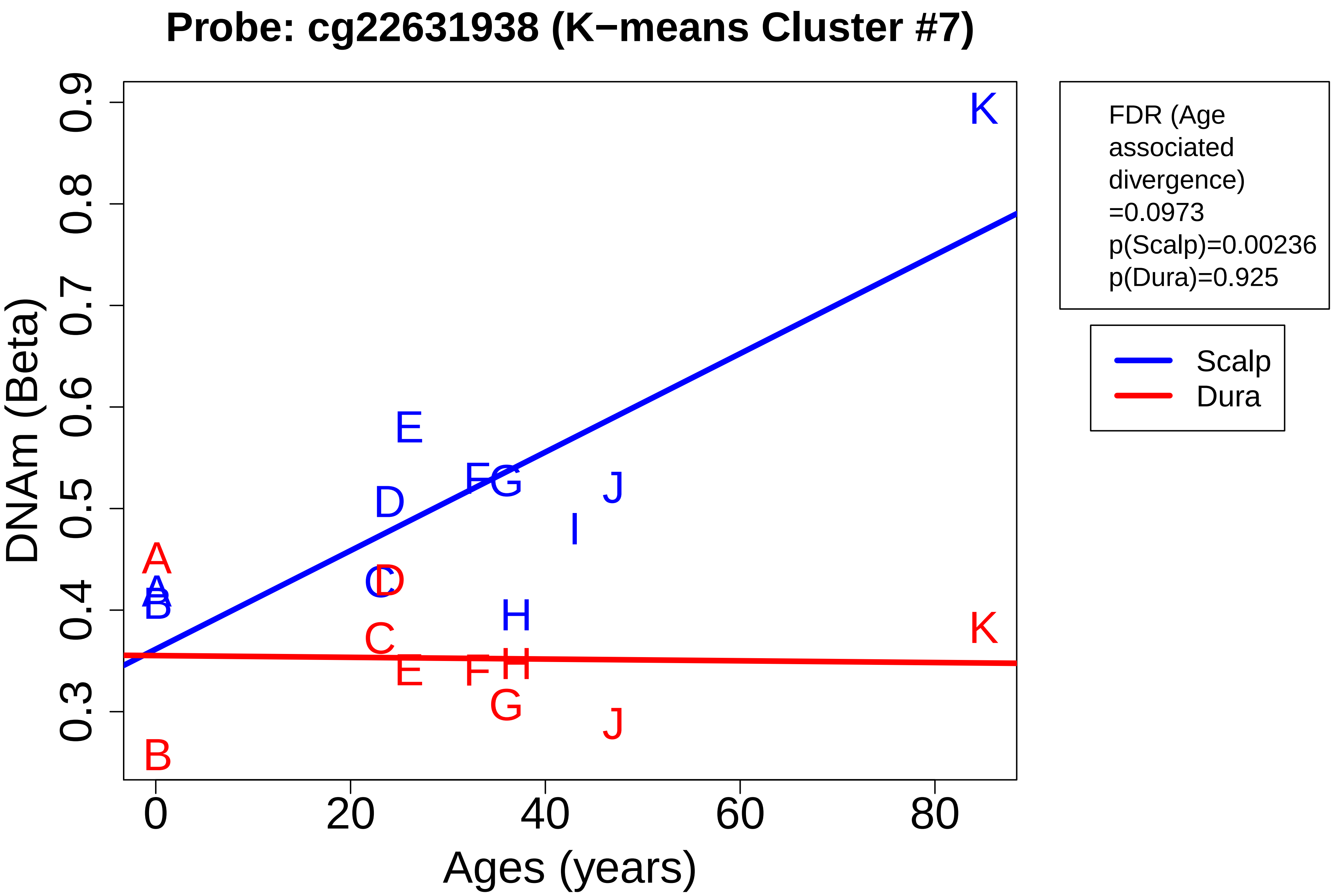

Probe: cg22631938 (K−means Cluster #7)
DNAm (Beta)
0.3
0.4
0.5
0.6
0.7
0.8
0.9
0
20
40
60
80
Ages (years)
FDR (Age associated divergence) =0.0973
p(Scalp)=0.00236
p(Dura)=0.925
Scalp
Dura

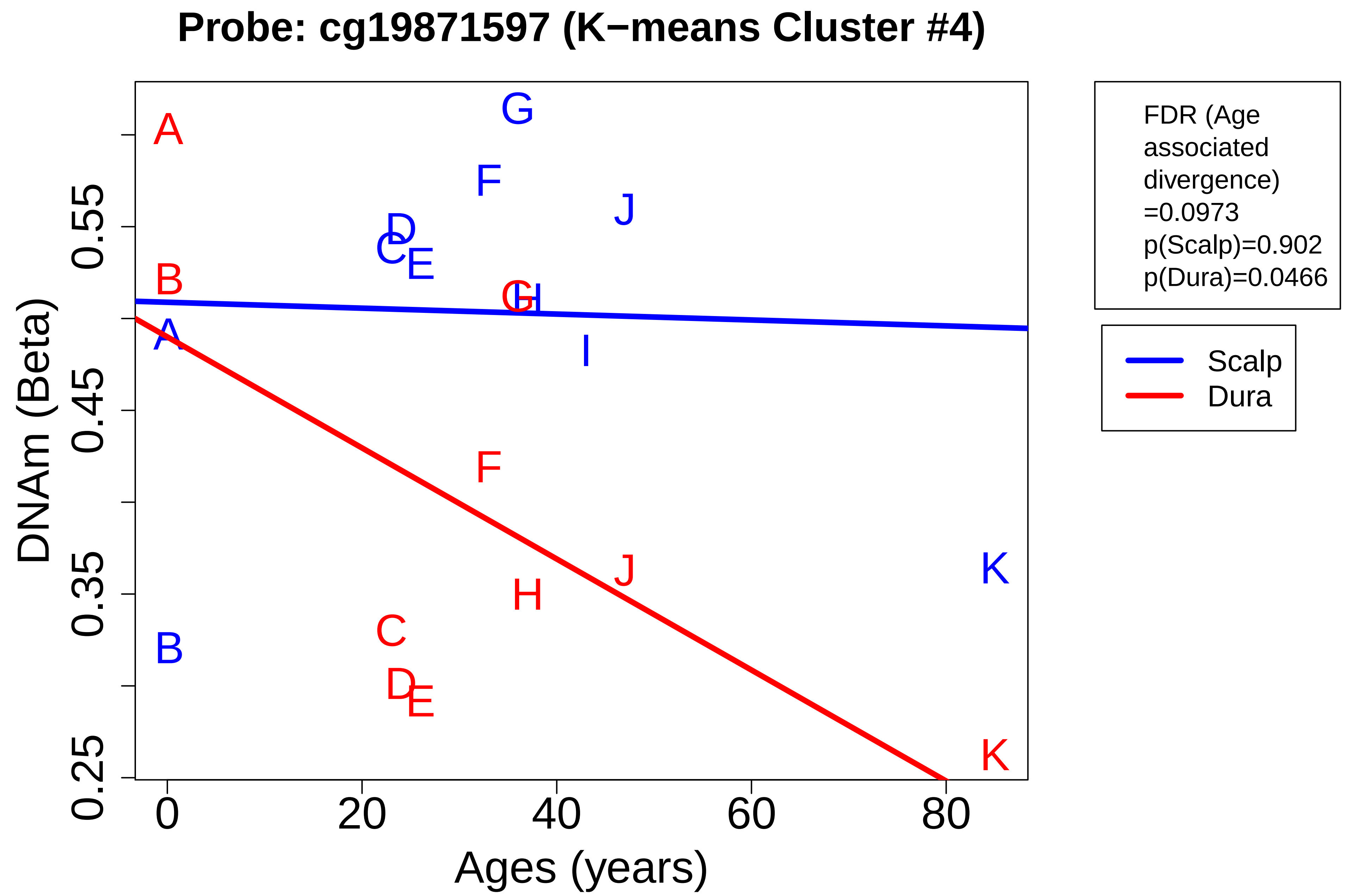

Probe: cg19871597 (K−means Cluster #4)
DNAm (Beta)
0.25
0.35
0.45
0.55
0
20
40
60
80
Ages (years)
A
B
C
D
E
F
G
H
I
J
K
FDR (Age associated divergence) =0.0973
p(Scalp)=0.902
p(Dura)=0.0466
Scalp
Dura

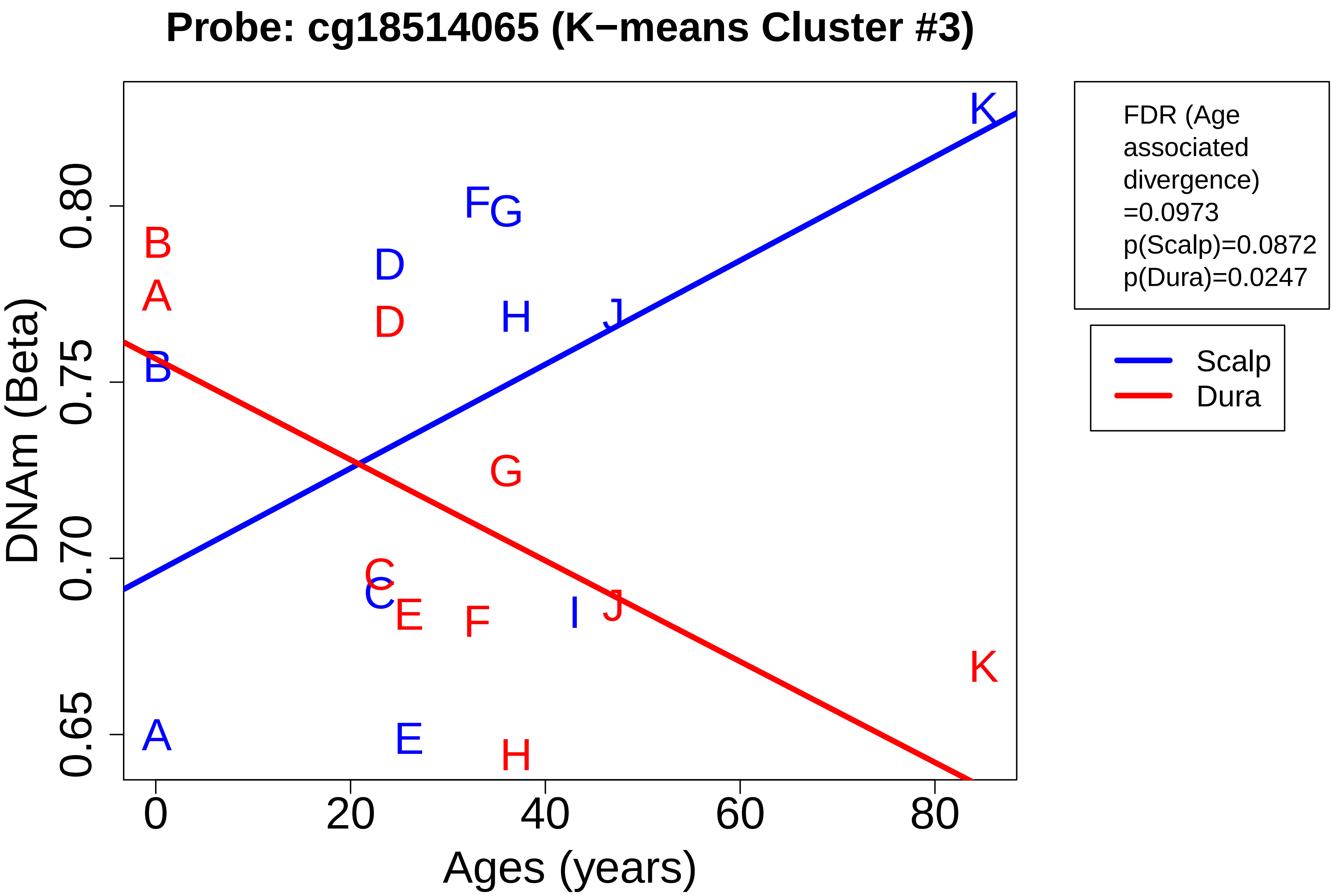

Probe: cg18514065 (K−means Cluster #3)
DNAm (Beta)
0.65
0.70
0.75
0.80
0
20
40
60
80
Ages (years)
FDR (Age associated divergence) =0.0973
p(Scalp)=0.0872
p(Dura)=0.0247
Scalp
Dura
A
B
C
D
E
F
G
H
I
J
K

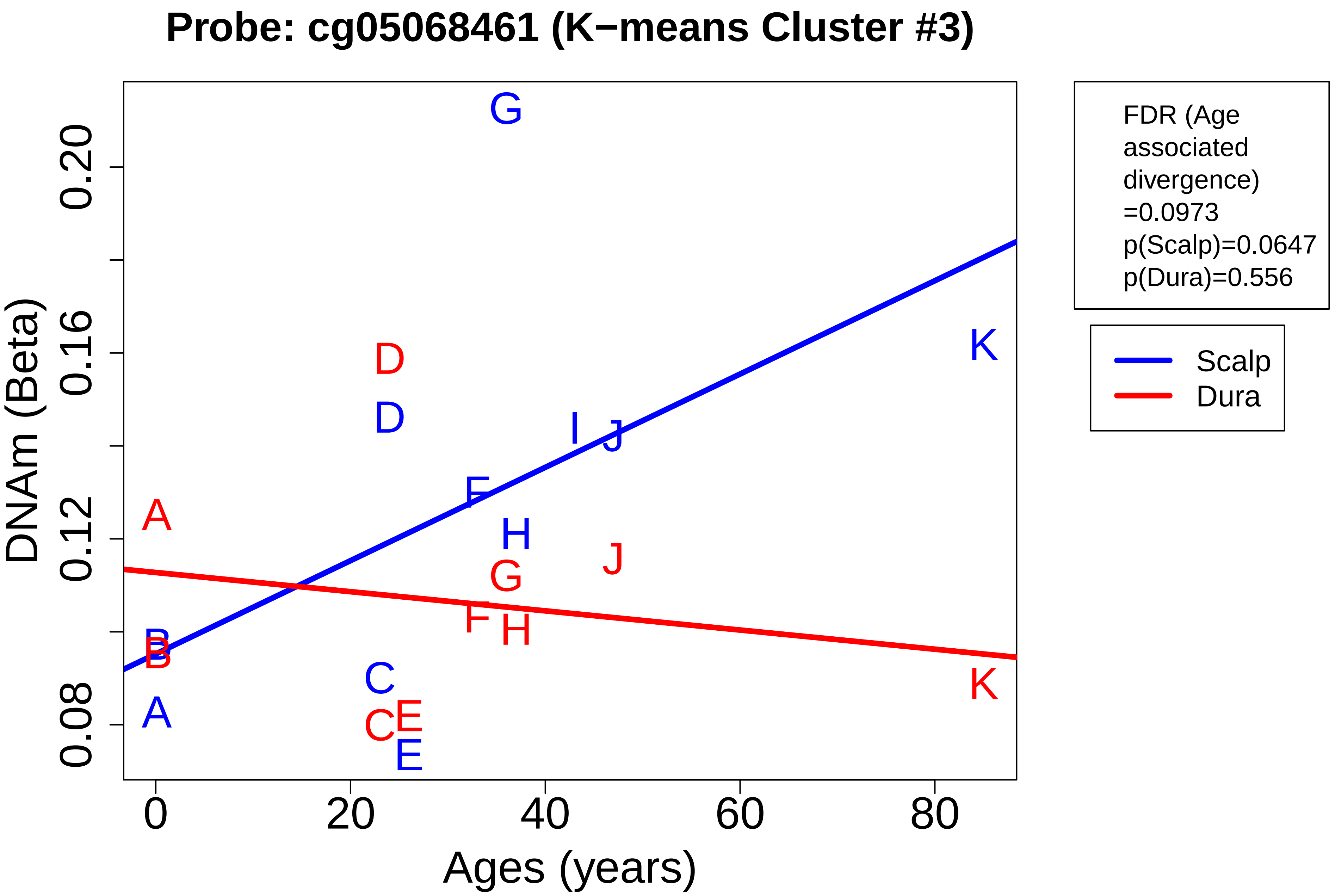
Probe: cg05068461 (K−means Cluster #3)
DNAm (Beta)
0.08
0.12
0.16
0.20
0
20
40
60
80
Ages (years)
FDR (Age associated divergence) =0.0973
p(Scalp)=0.0647
p(Dura)=0.556
Scalp
Dura

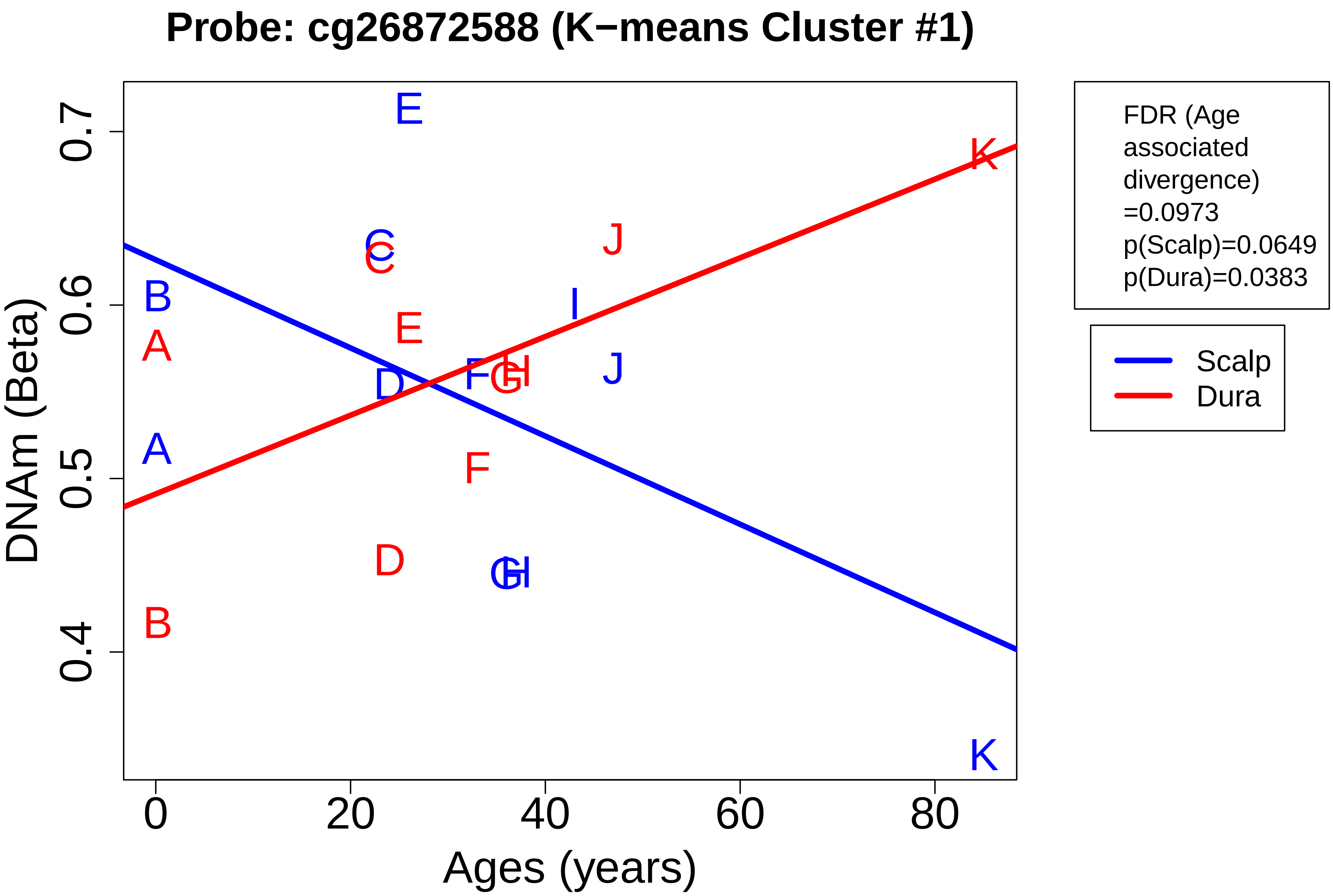
Probe: cg26872588 (K−means Cluster #1)
DNAm (Beta)
Ages (years)
0.4
0.5
0.6
0.7
0
20
40
60
80
FDR (Age associated divergence) =0.0973
p(Scalp)=0.0649
p(Dura)=0.0383
Scalp
Dura

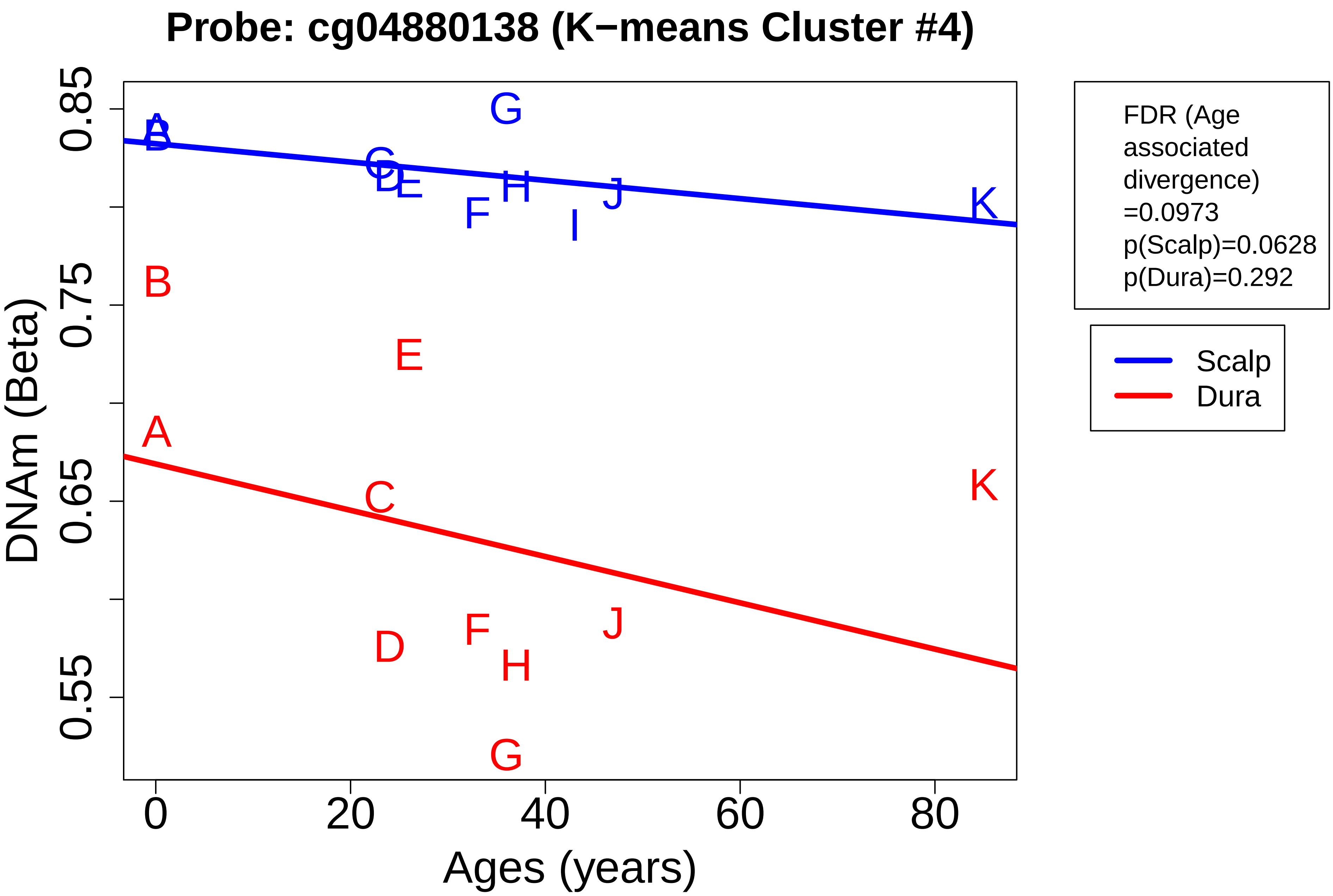

Probe: cg04880138 (K−means Cluster #4)
DNAm (Beta)
0.55
0.65
0.75
0.85
Ages (years)
0
20
40
60
80
FDR (Age associated divergence) =0.0973
p(Scalp)=0.0628
p(Dura)=0.292
Scalp
Dura

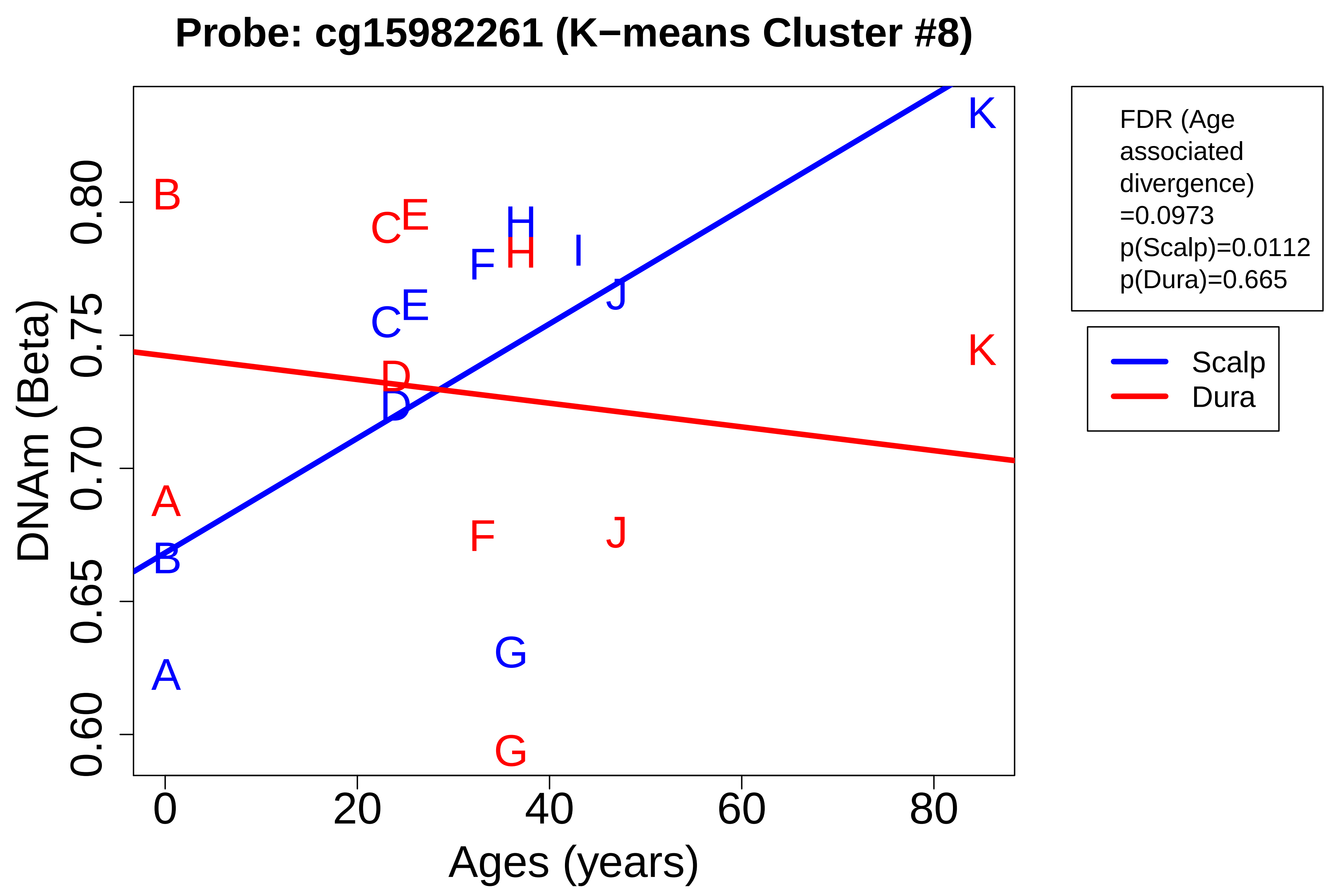

Probe: cg15982261 (K−means Cluster #8)
FDR (Age associated divergence) =0.0973
p(Scalp)=0.0112
p(Dura)=0.665
Scalp
Dura
DNAm (Beta)
0.60
0.65
0.70
0.75
0.80
0
20
40
60
80
Ages (years)
A
B
C
D
E
F
G
H
I
J
K

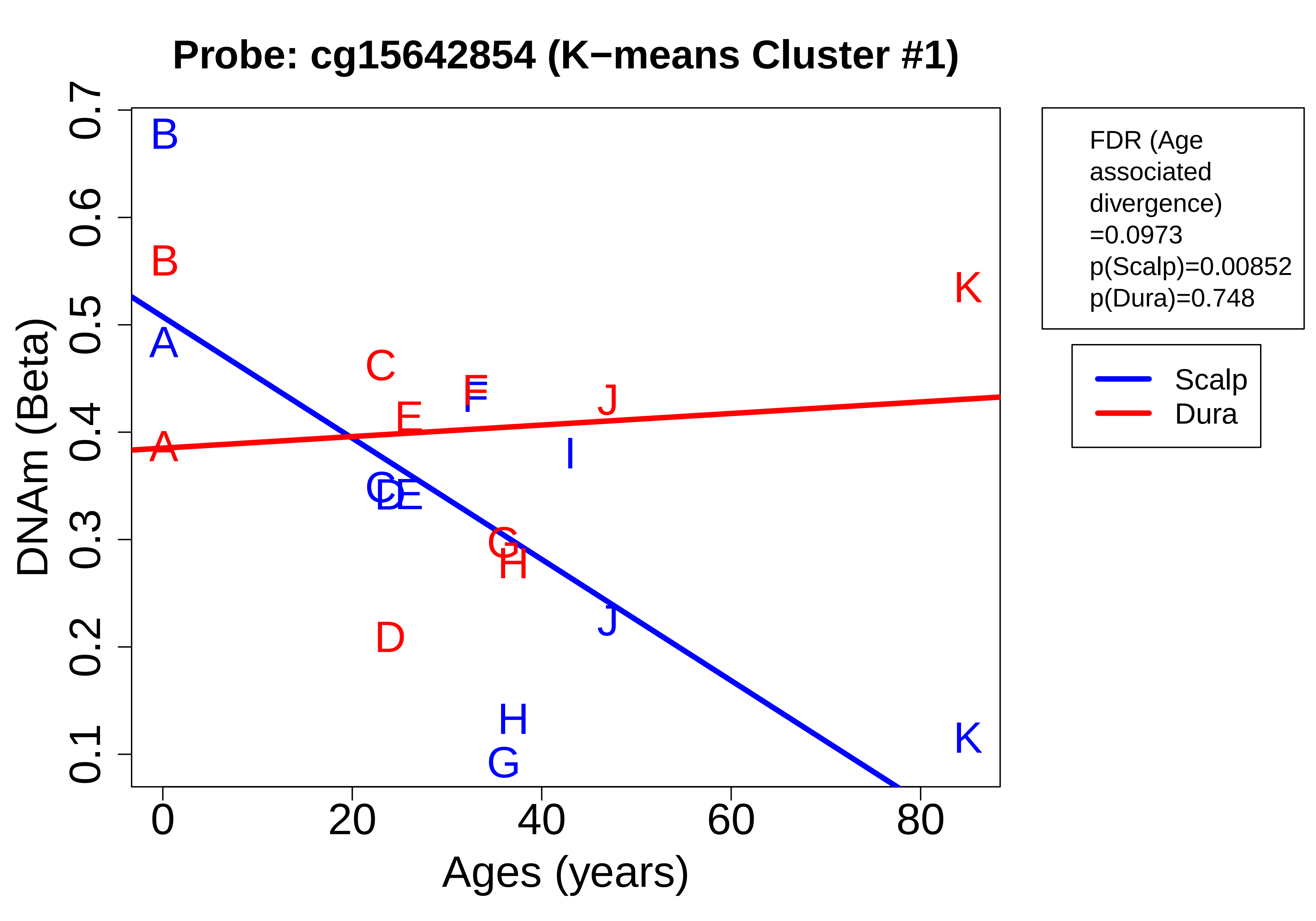
Probe: cg15642854 (K−means Cluster #1)
DNAm (Beta)
0.1
0.2
0.3
0.4
0.5
0.6
0.7
Ages (years)
0
20
40
60
80
FDR (Age associated divergence) =0.0973
p(Scalp)=0.00852
p(Dura)=0.748
Scalp
Dura
A
B
C
D
E
F
G
H
I
J
K

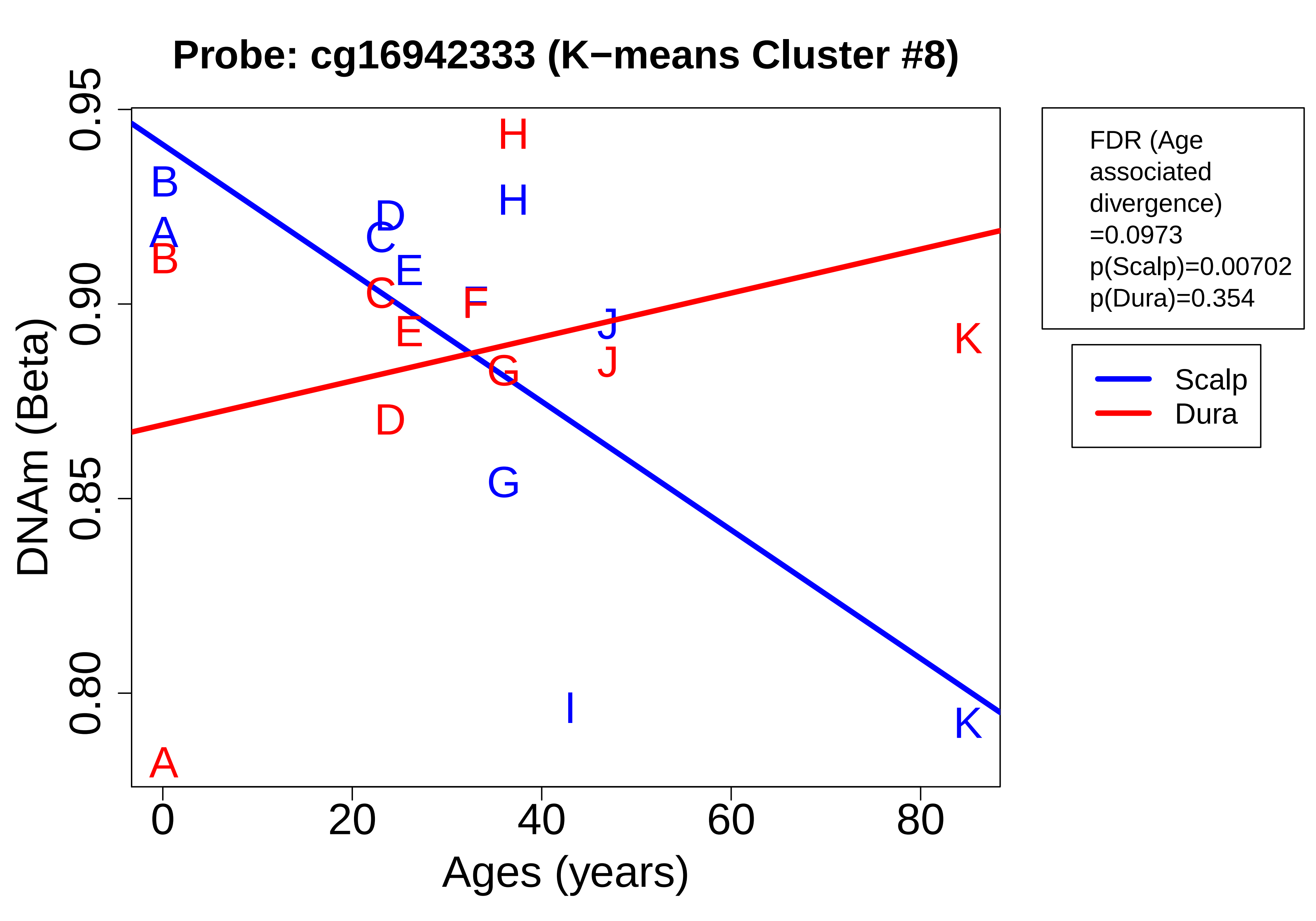

Probe: cg16942333 (K−means Cluster #8)
DNAm (Beta)
0.95
0.90
0.85
0.80
0
20
40
60
80
Ages (years)
A
B
C
D
E
F
G
H
I
J
K
FDR (Age associated divergence) =0.0973
p(Scalp)=0.00702
p(Dura)=0.354
Scalp
Dura

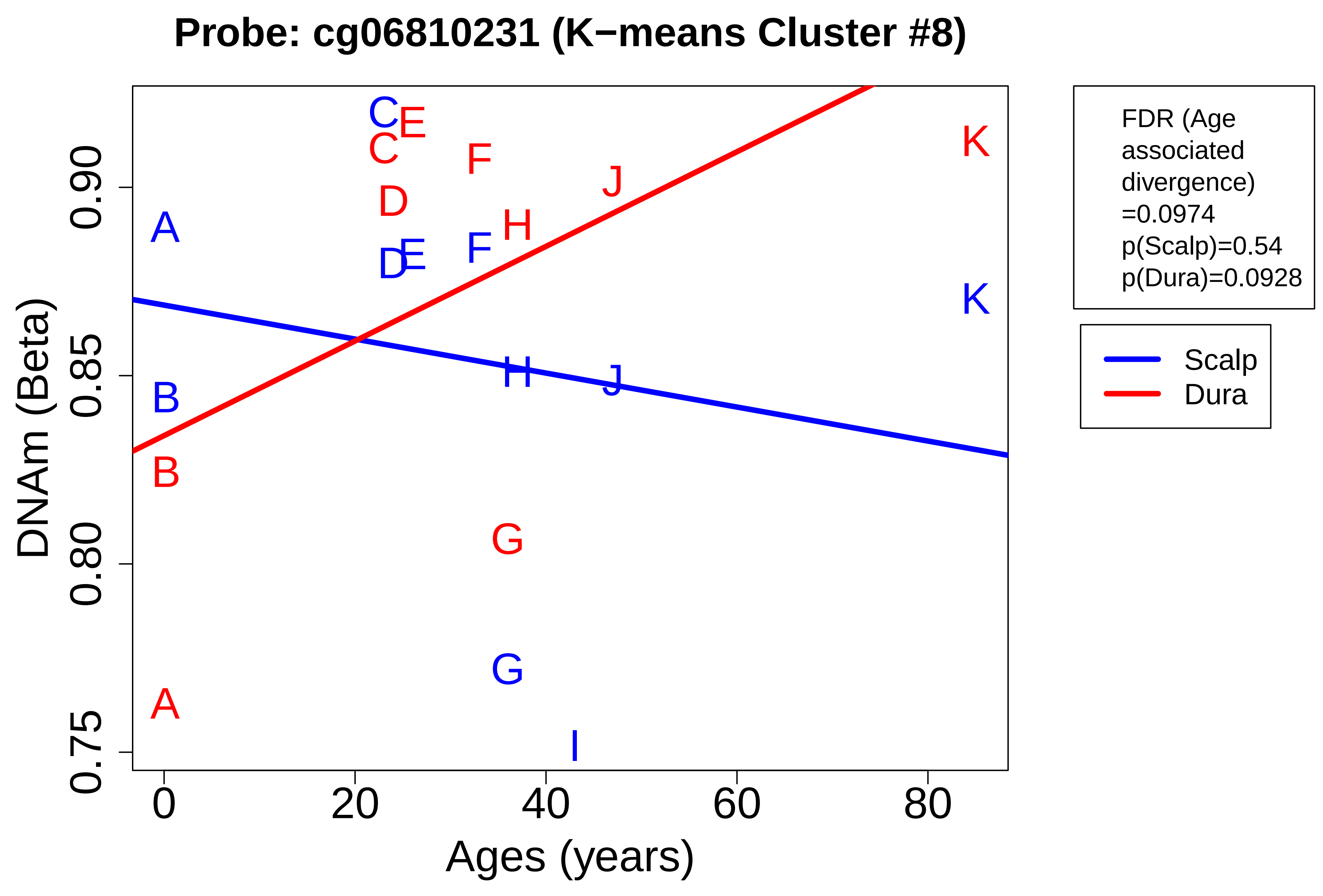

Probe: cg06810231 (K−means Cluster #8)
DNAm (Beta)
0.75
0.80
0.85
0.90
Ages (years)
0
20
40
60
80
A
B
C
D
E
F
G
H
I
J
K
FDR (Age associated divergence) =0.0974
p(Scalp)=0.54
p(Dura)=0.0928
Scalp
Dura

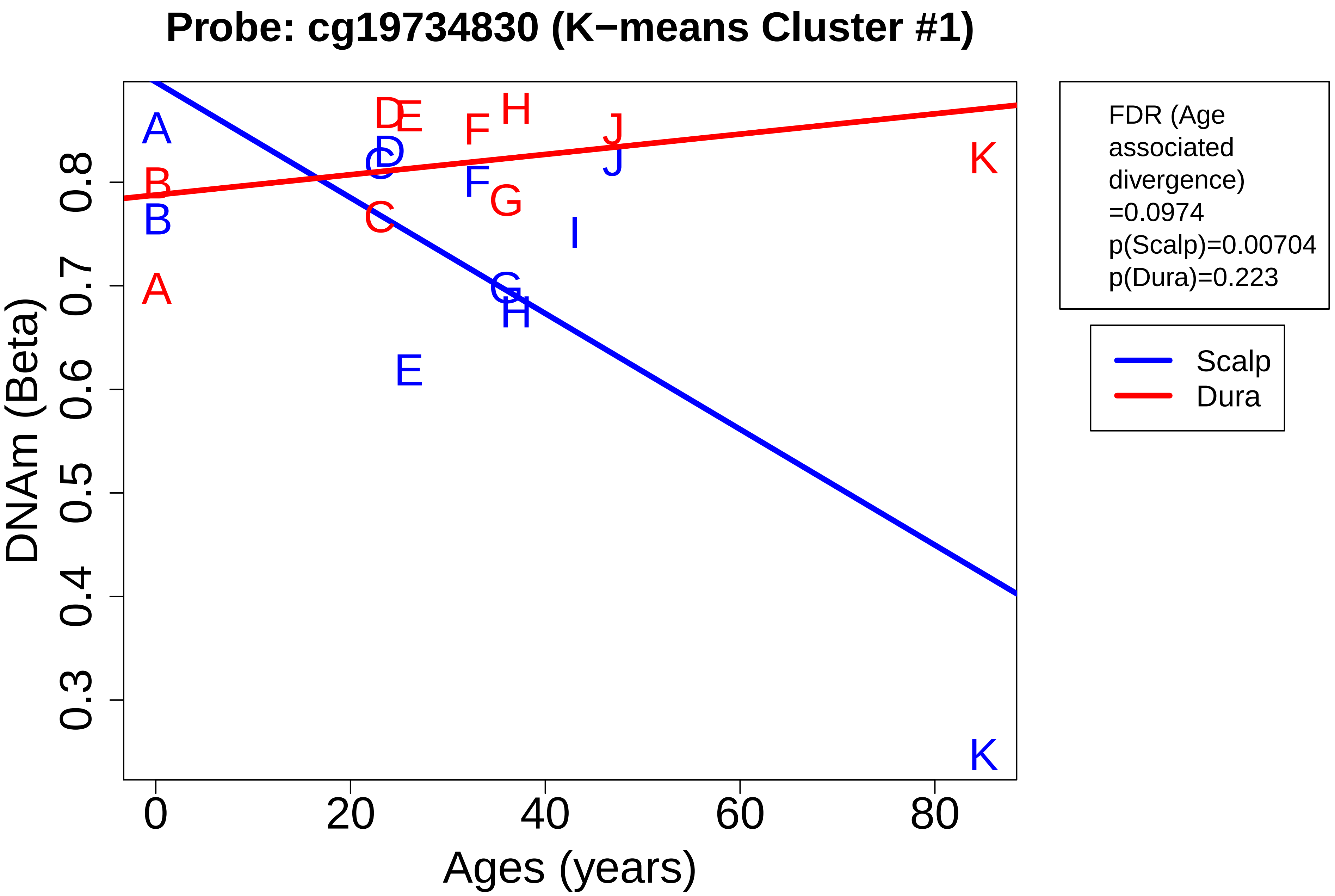

Probe: cg19734830 (K−means Cluster #1)
DNAm (Beta)
0.3
0.4
0.5
0.6
0.7
0.8
0
20
40
60
80
Ages (years)
FDR (Age associated divergence) =0.0974
p(Scalp)=0.00704
p(Dura)=0.223
Scalp
Dura

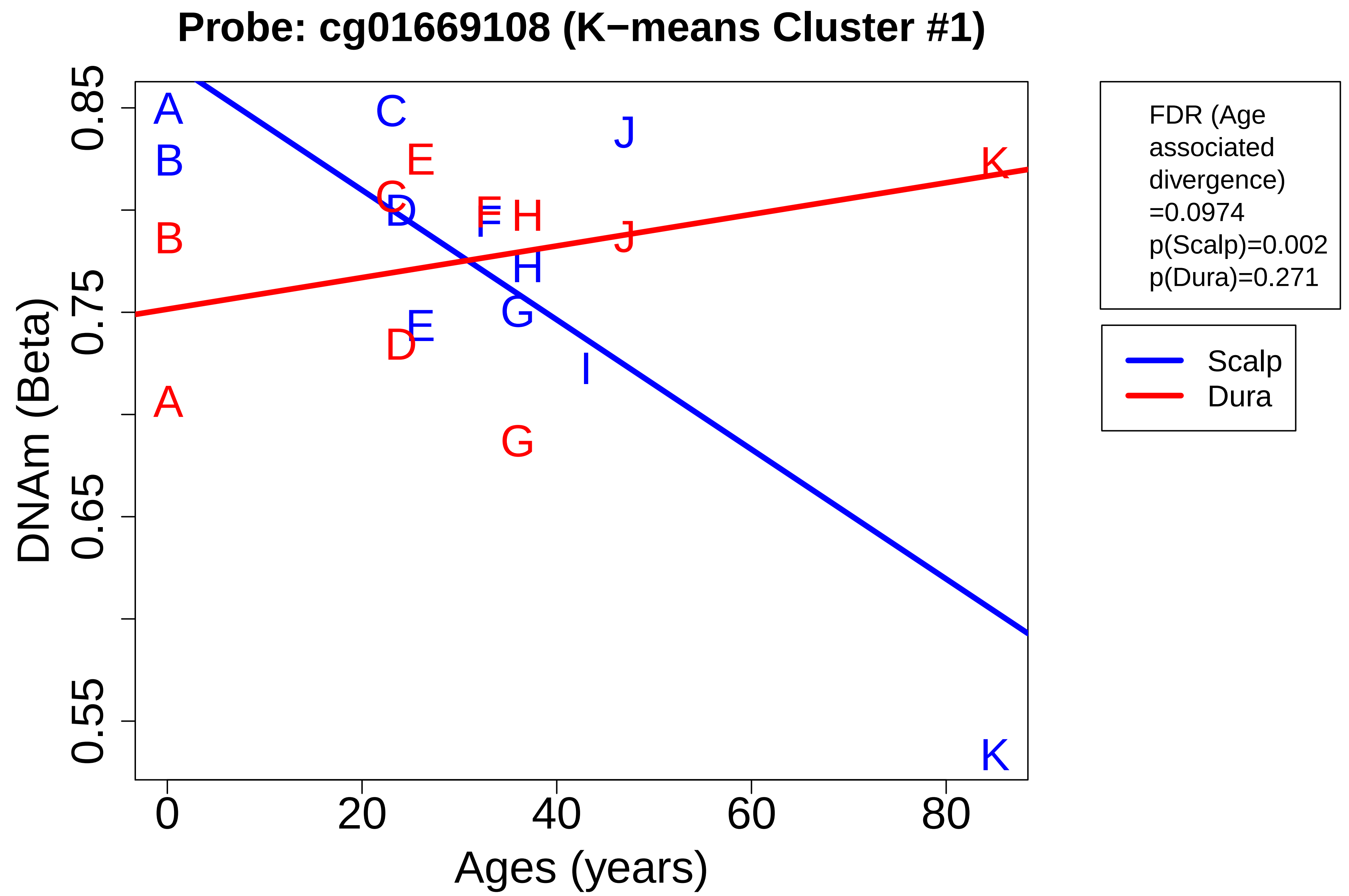

Probe: cg01669108 (K−means Cluster #1)
DNAm (Beta)
0.85
0.75
0.65
0.55
0
20
40
60
80
Ages (years)
A
B
C
D
E
F
G
H
I
J
K
FDR (Age associated divergence) =0.0974
p(Scalp)=0.002
p(Dura)=0.271
Scalp
Dura

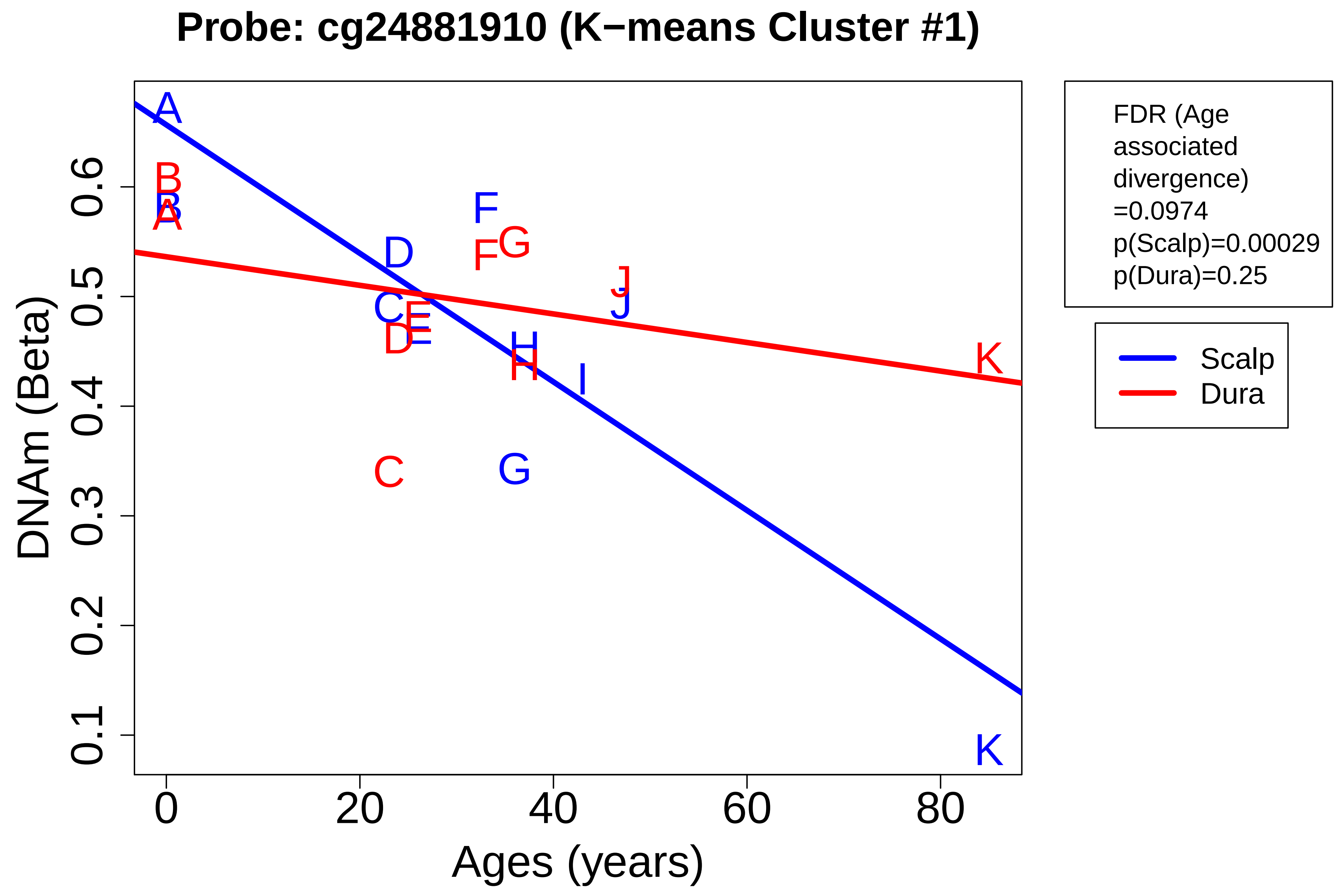

Probe: cg24881910 (K−means Cluster #1)
DNAm (Beta)
0.1
0.2
0.3
0.4
0.5
0.6
Ages (years)
0
20
40
60
80
A
B
B
A
C
D
E
D
F
F
G
C
E
H
H
G
I
J
J
K
K
FDR (Age associated divergence) =0.0974
p(Scalp)=0.00029
p(Dura)=0.25
Scalp
Dura

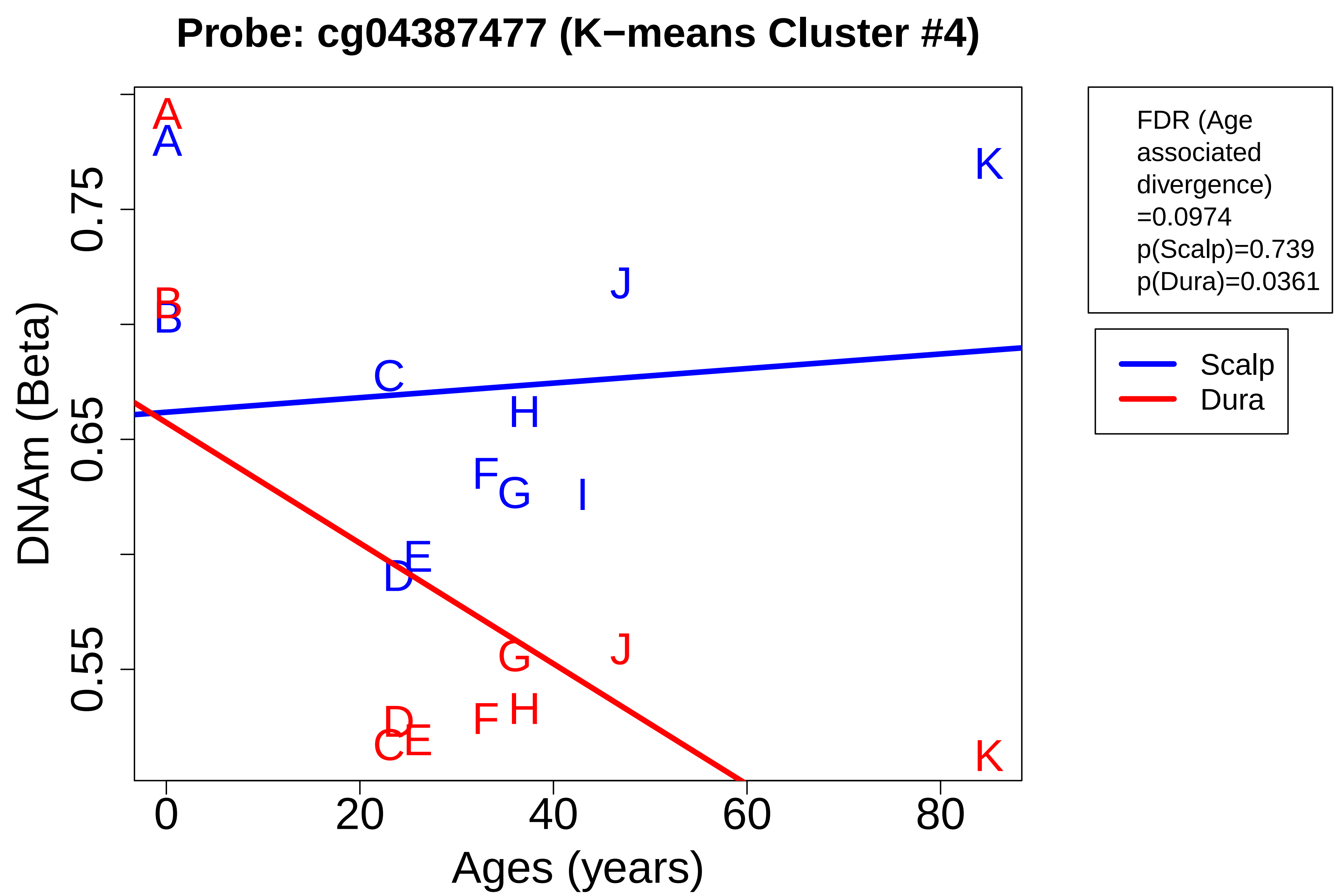

Probe: cg04387477 (K−means Cluster #4)
DNAm (Beta)
0.55
0.65
0.75
0
20
40
60
80
Ages (years)
A
A
B
B
C
C
D
D
E
E
F
F
G
G
H
H
I
J
J
K
K
FDR (Age associated divergence) =0.0974
p(Scalp)=0.739
p(Dura)=0.0361
Scalp
Dura

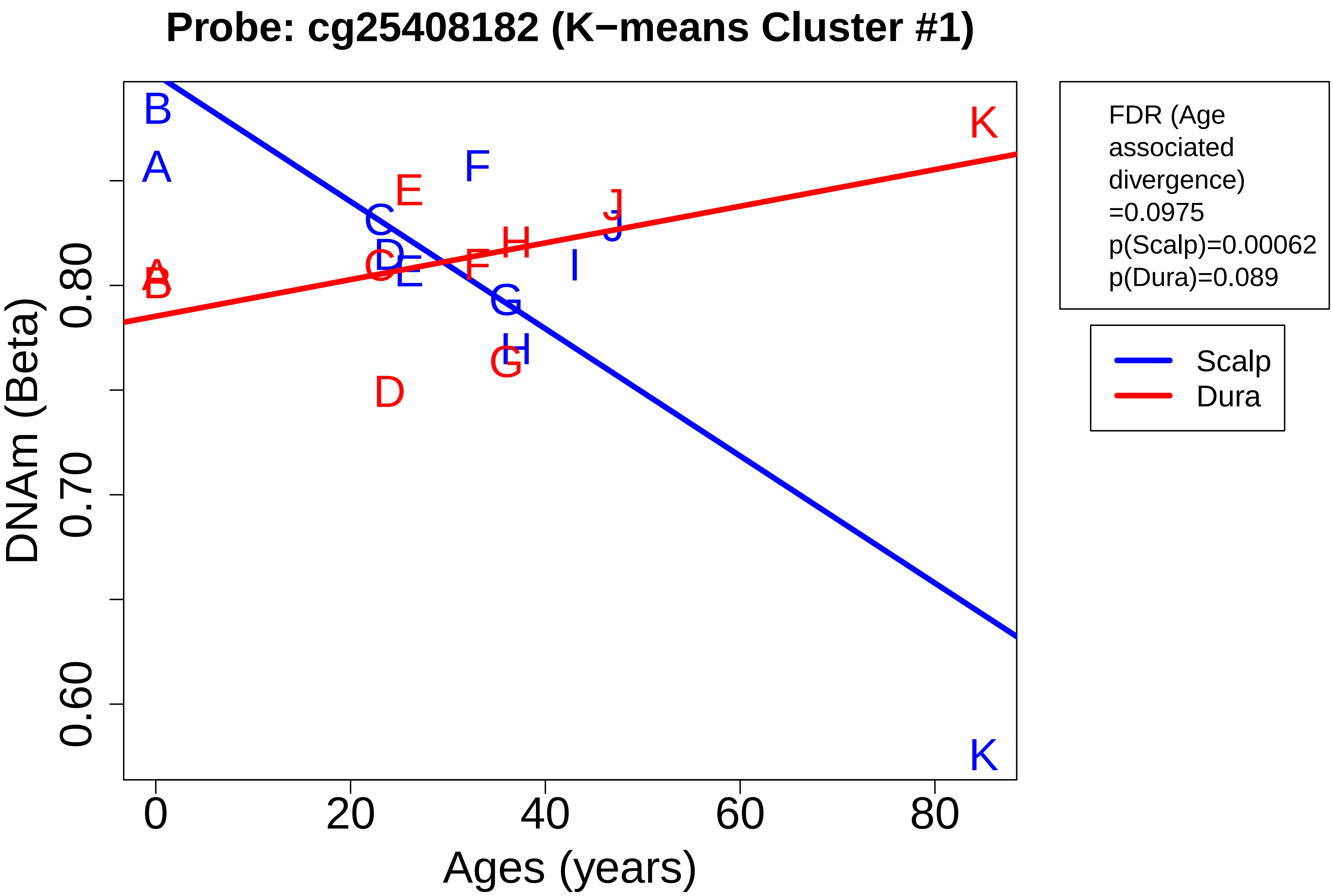

Probe: cg25408182 (K−means Cluster #1)
DNAm (Beta)
0.60
0.70
0.80
Ages (years)
0
20
40
60
80
FDR (Age associated divergence) =0.0975
p(Scalp)=0.00062
p(Dura)=0.089
Scalp
Dura

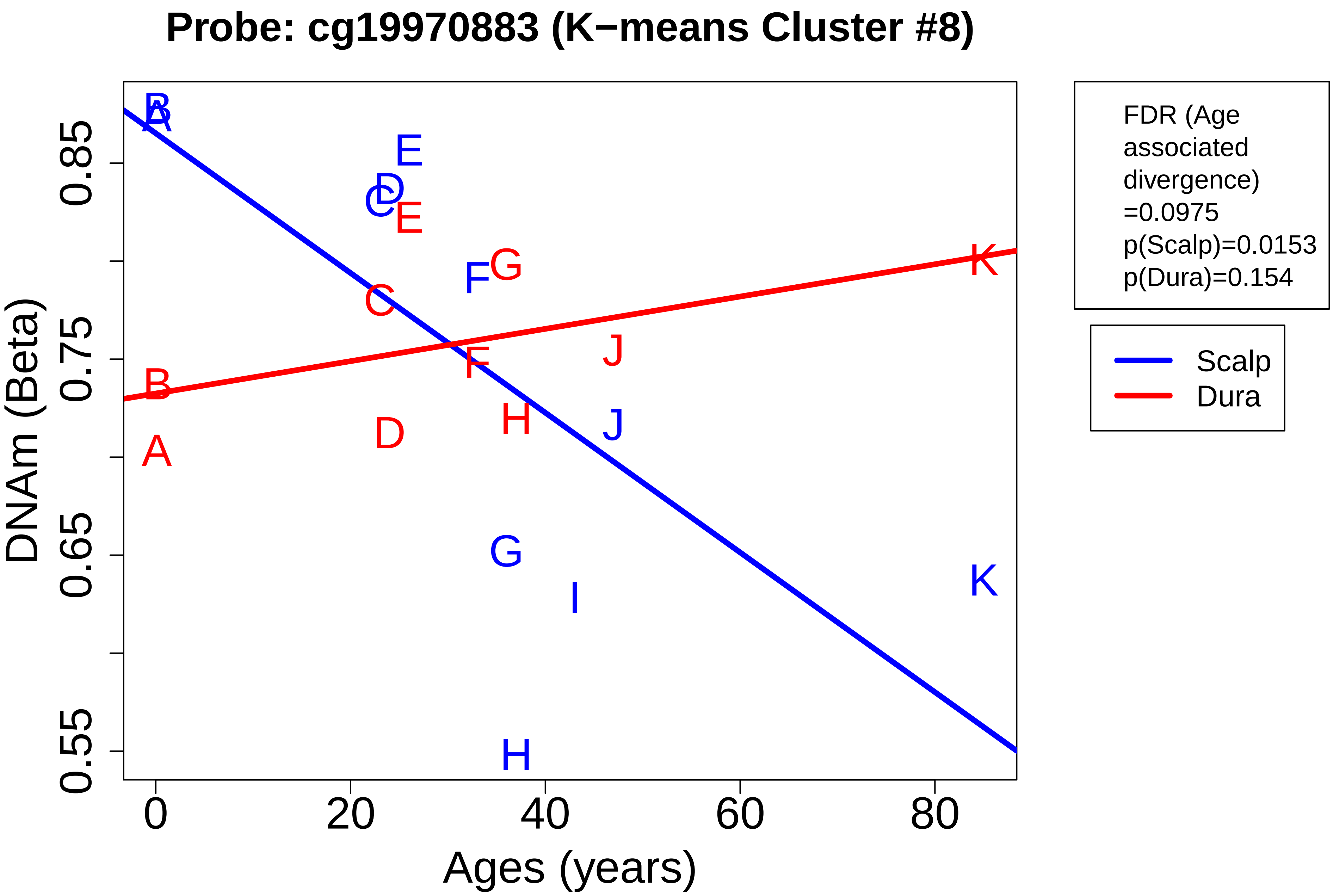

Probe: cg19970883 (K−means Cluster #8)
DNAm (Beta)
0.55
0.65
0.75
0.85
0
20
40
60
80
Ages (years)
FDR (Age associated divergence) =0.0975
p(Scalp)=0.0153
p(Dura)=0.154
Scalp
Dura

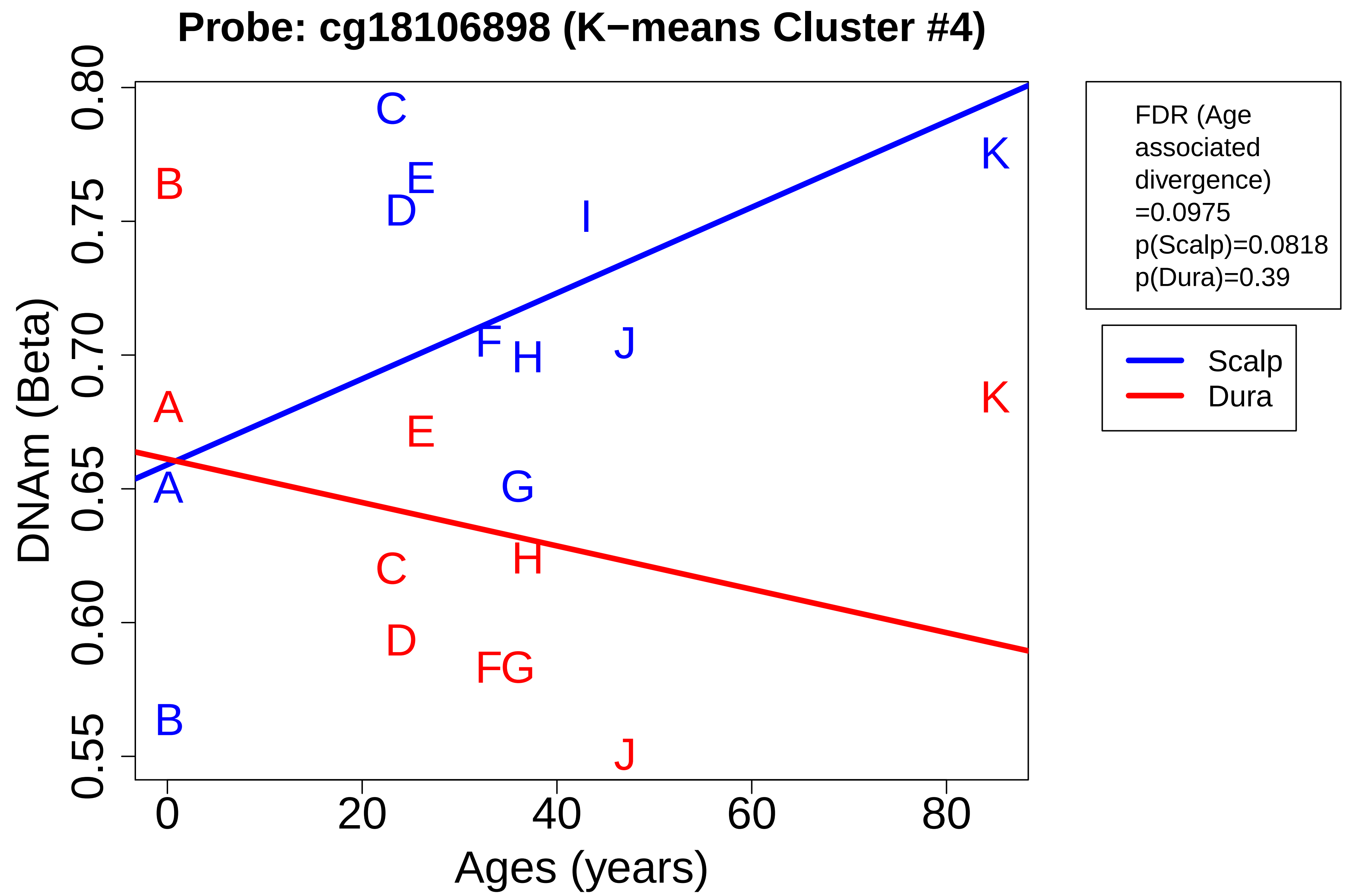
Probe: cg18106898 (K−means Cluster #4)
DNAm (Beta)
0.55
0.60
0.65
0.70
0.75
0.80
0
20
40
60
80
Ages (years)
FDR (Age associated divergence) =0.0975
p(Scalp)=0.0818
p(Dura)=0.39
Scalp
Dura
A
B
C
D
E
F
G
H
I
J
K
A
B
C
D
E
FG
H
J
K

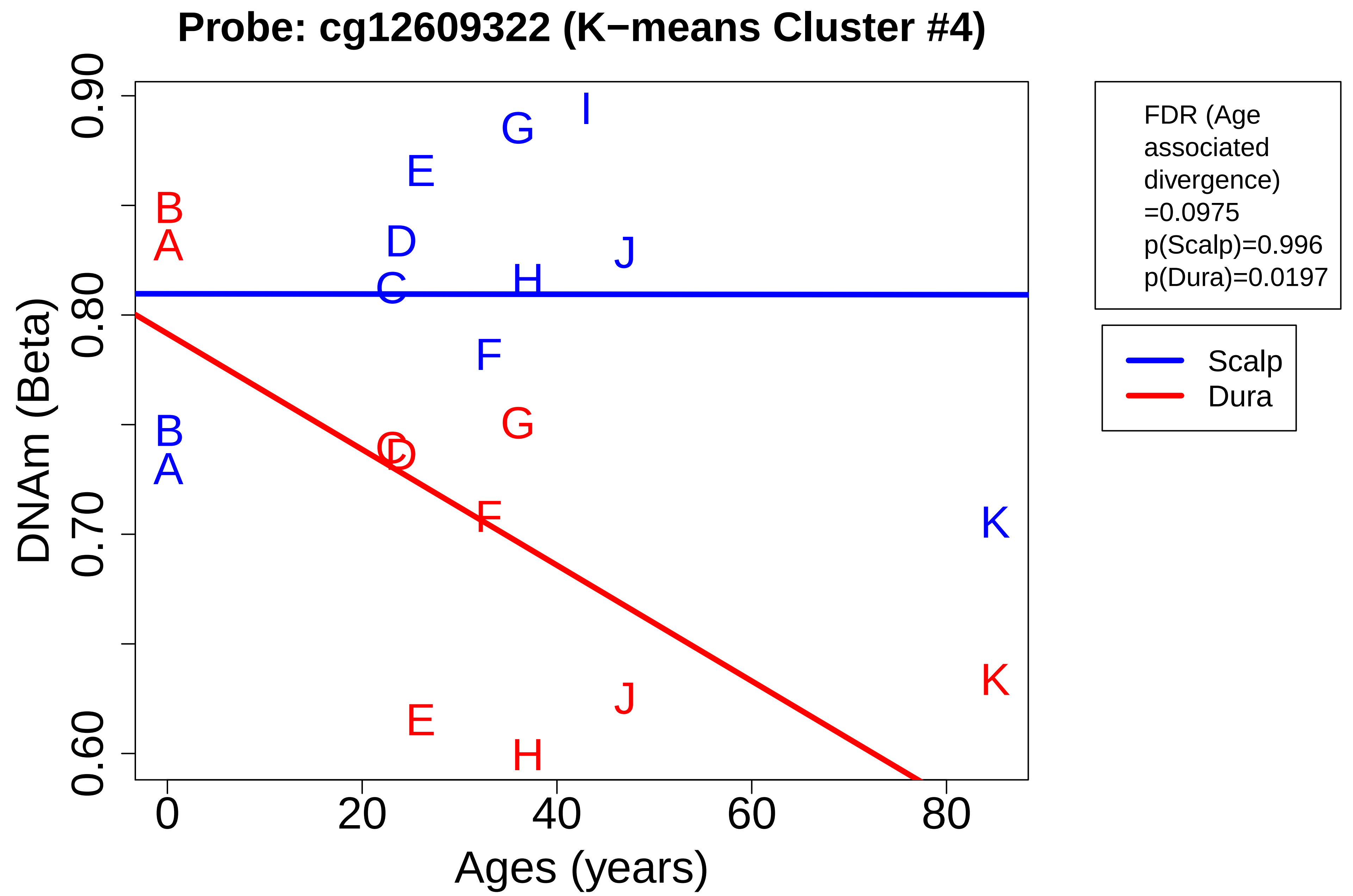
Probe: cg12609322 (K−means Cluster #4)
DNAm (Beta)
0.60
0.70
0.80
0.90
0
20
40
60
80
Ages (years)
FDR (Age associated divergence) =0.0975
p(Scalp)=0.996
p(Dura)=0.0197
Scalp
Dura
A
B
C
D
E
F
G
H
I
J
K

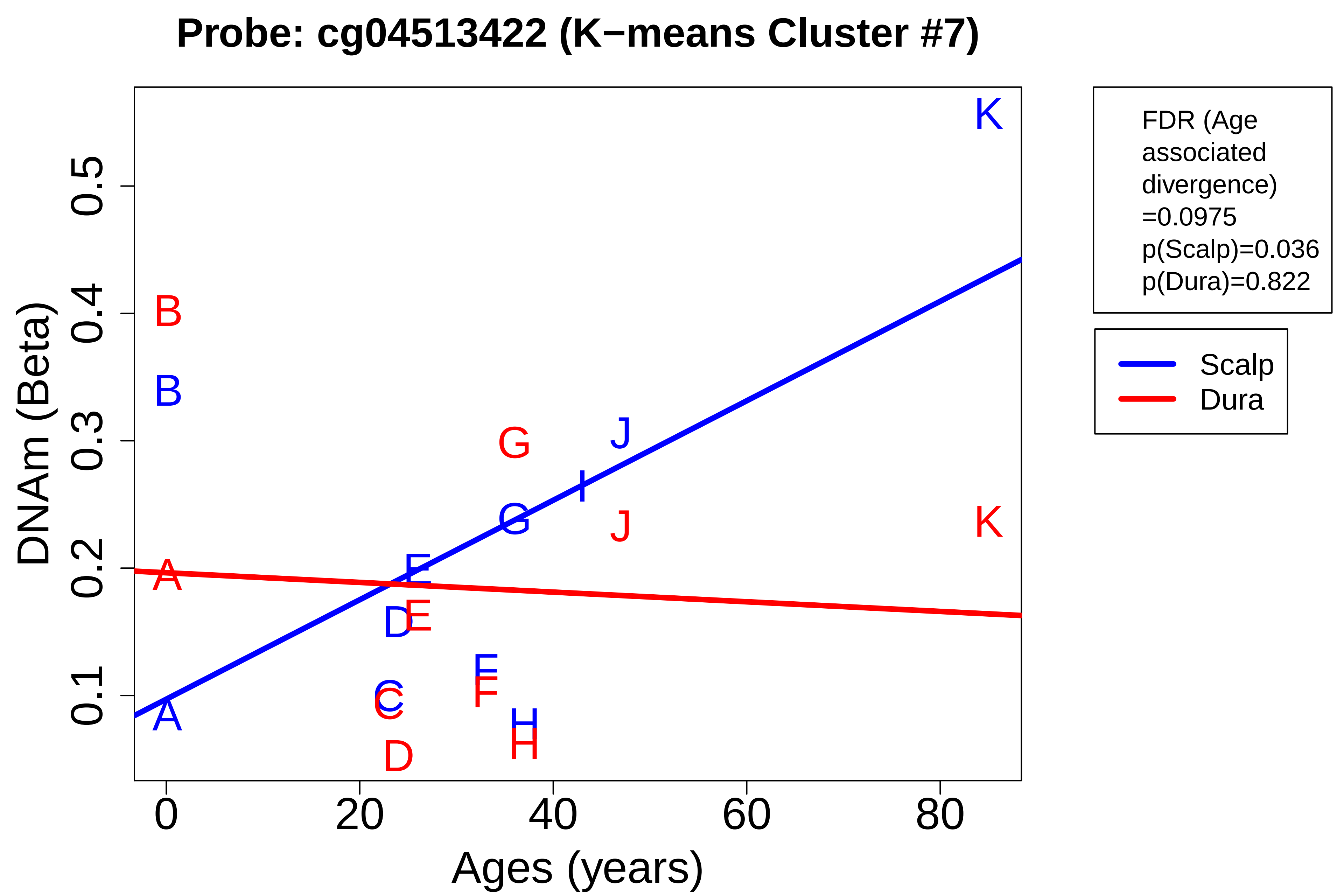

Probe: cg04513422 (K−means Cluster #7)
DNAm (Beta)
0.1
0.2
0.3
0.4
0.5
0
20
40
60
80
Ages (years)
A
B
C
D
E
F
G
H
I
J
K
FDR (Age associated divergence) =0.0975
p(Scalp)=0.036
p(Dura)=0.822
Scalp
Dura

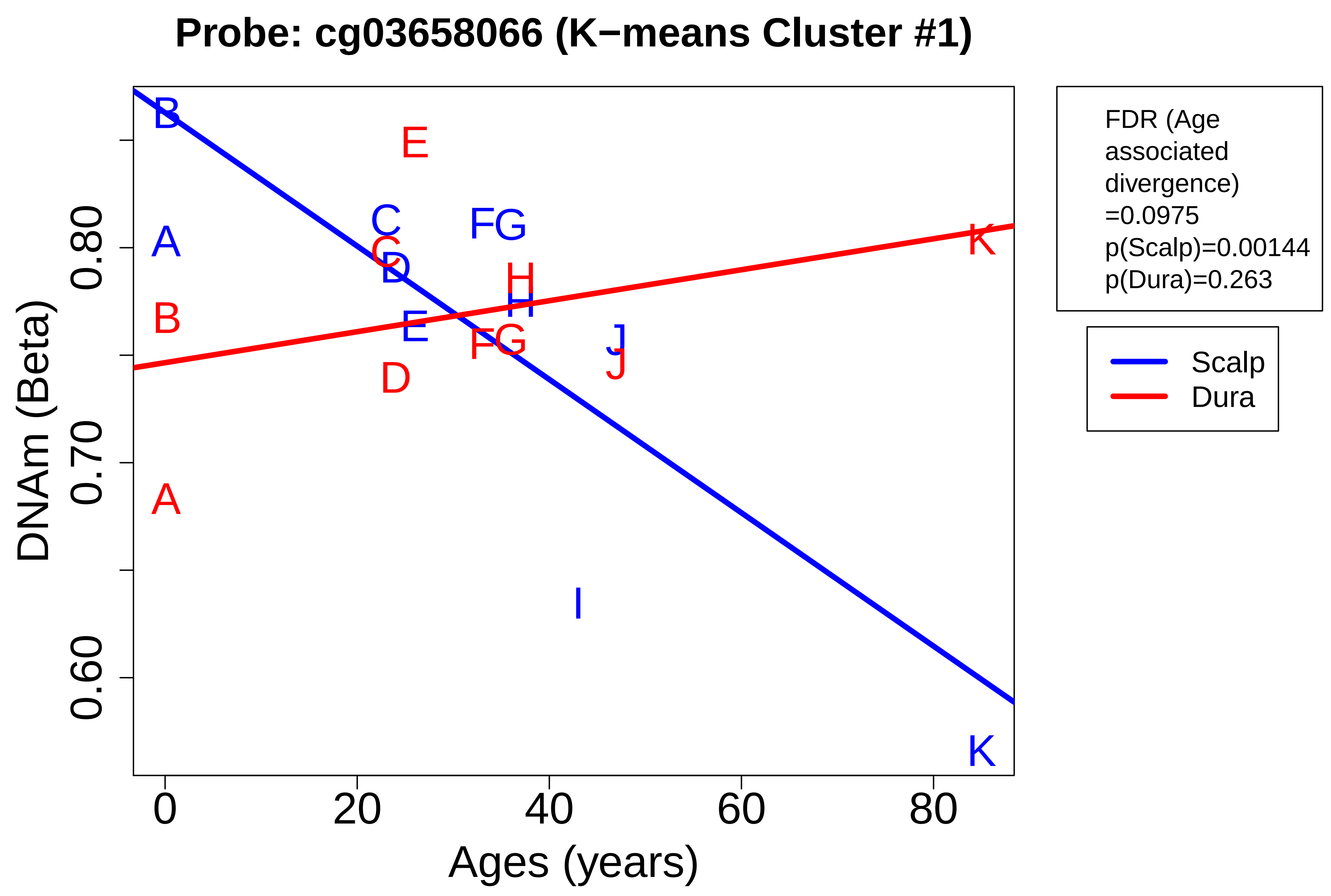

Probe: cg03658066 (K−means Cluster #1)
DNAm (Beta)
0.60
0.70
0.80
0
20
40
60
80
Ages (years)
A
B
C
D
E
F
G
H
I
J
K
FDR (Age associated divergence) =0.0975
p(Scalp)=0.00144
p(Dura)=0.263
Scalp
Dura

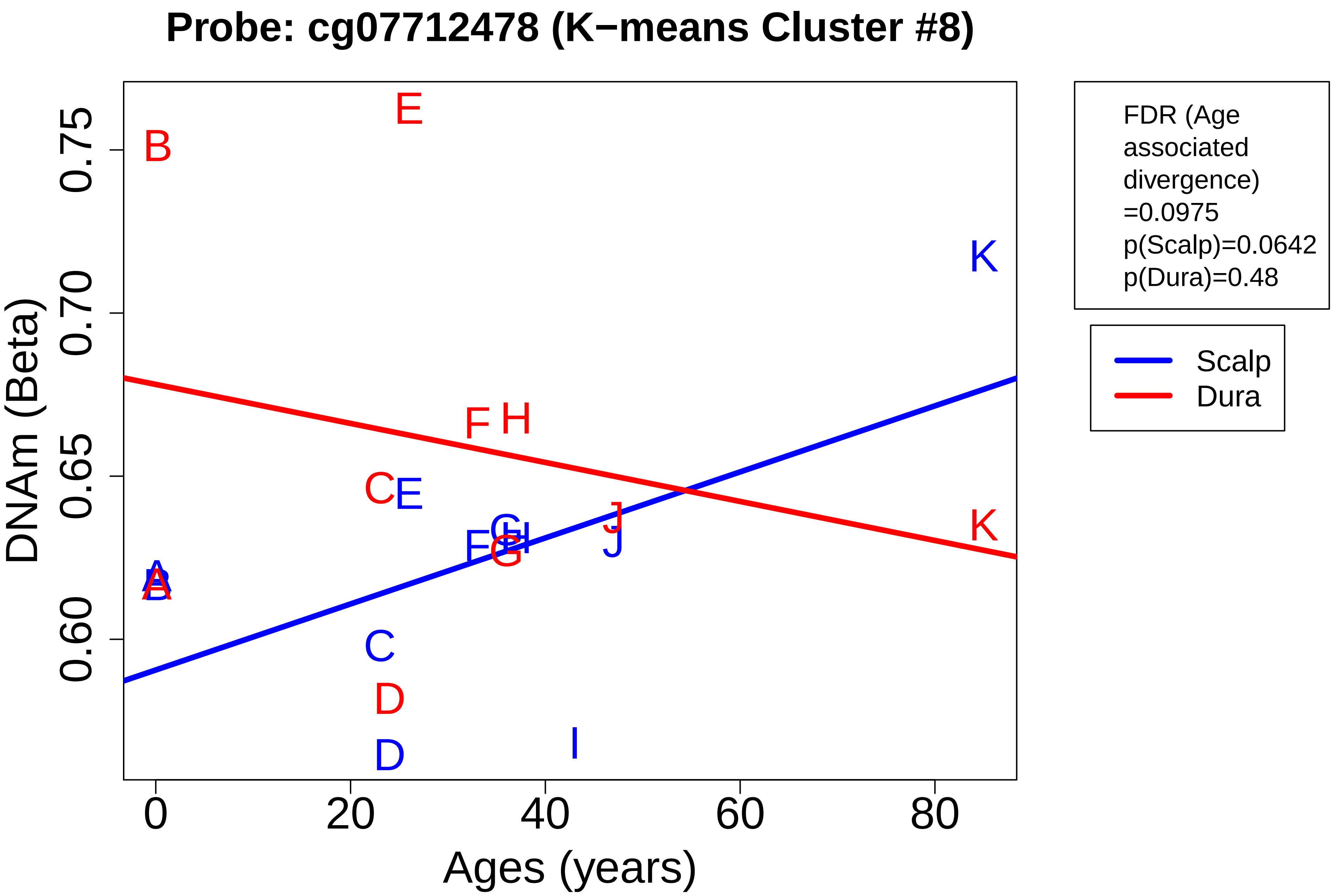

Probe: cg07712478 (K−means Cluster #8)
DNAm (Beta)
0.60
0.65
0.70
0.75
Ages (years)
0
20
40
60
80
FDR (Age associated divergence) =0.0975
p(Scalp)=0.0642
p(Dura)=0.48
Scalp
Dura

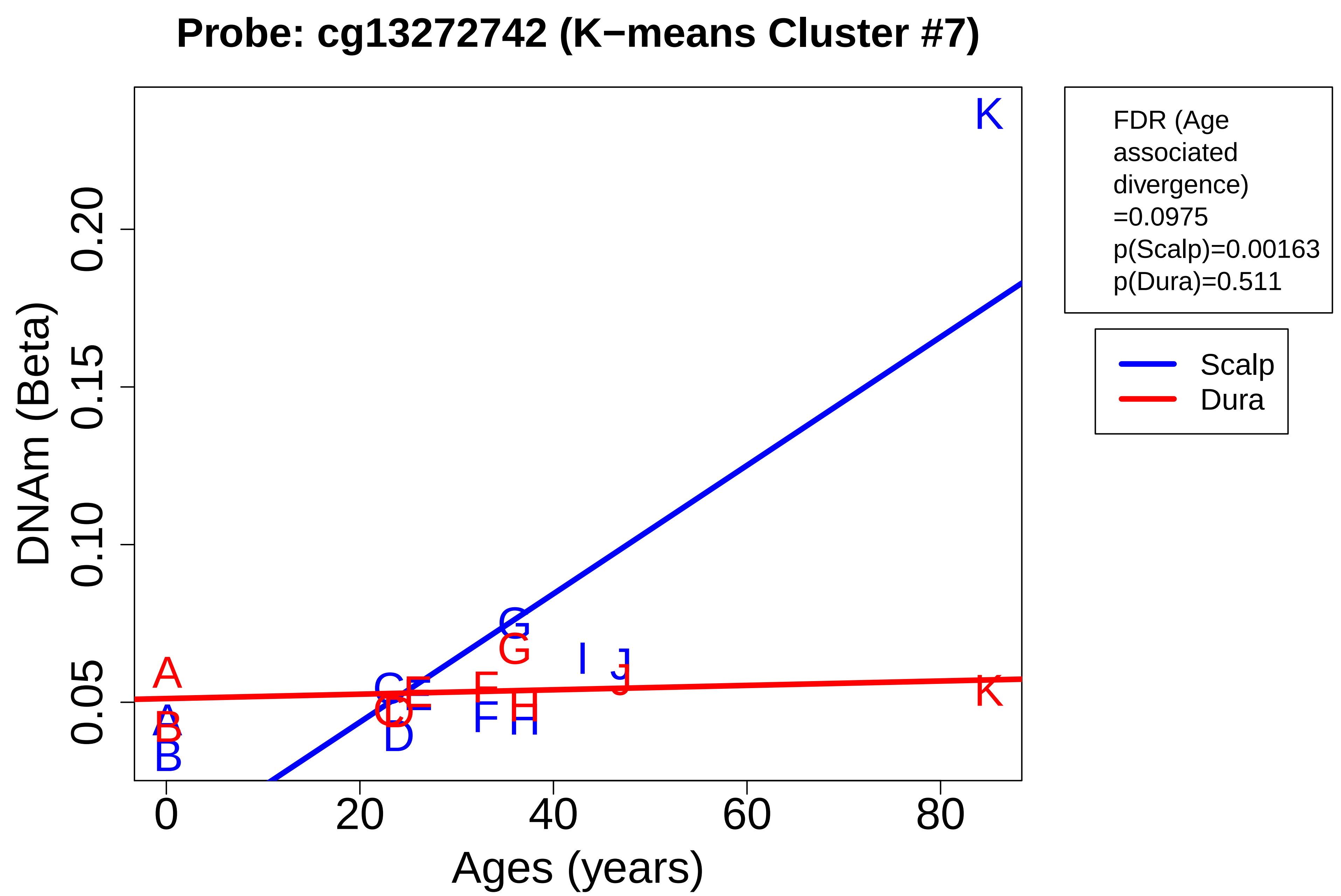
Probe: cg13272742 (K−means Cluster #7)
DNAm (Beta)
0.05
0.10
0.15
0.20
Ages (years)
0
20
40
60
80
FDR (Age associated divergence) =0.0975
p(Scalp)=0.00163
p(Dura)=0.511
Scalp
Dura
A
B
C
D
E
F
G
H
I
J
K

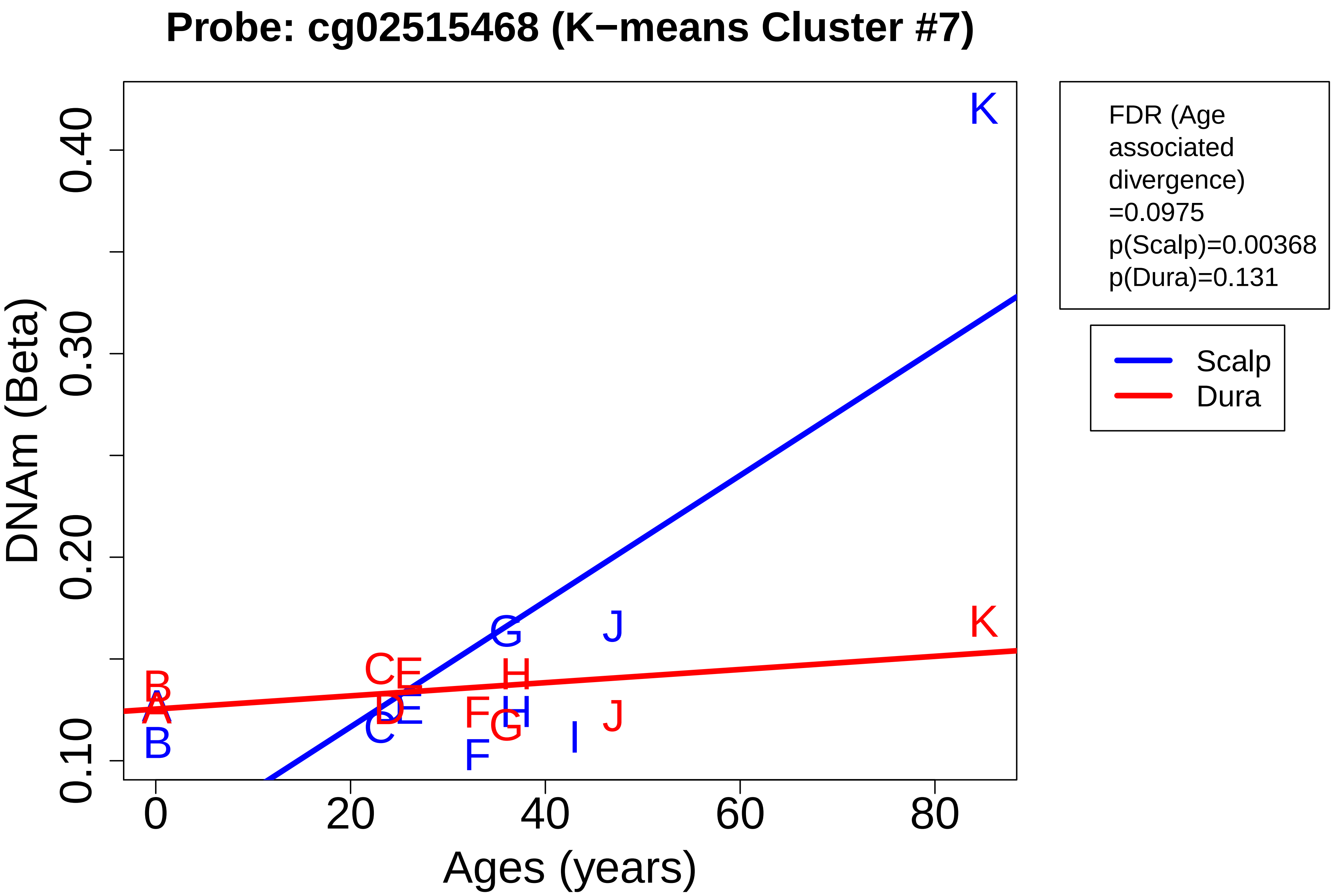
Probe: cg02515468 (K−means Cluster #7)
FDR (Age associated divergence) =0.0975
p(Scalp)=0.00368
p(Dura)=0.131
Scalp
Dura
DNAm (Beta)
0.10
0.20
0.30
0.40
0
20
40
60
80
Ages (years)
A
B
C
D
E
F
G
H
I
J
K

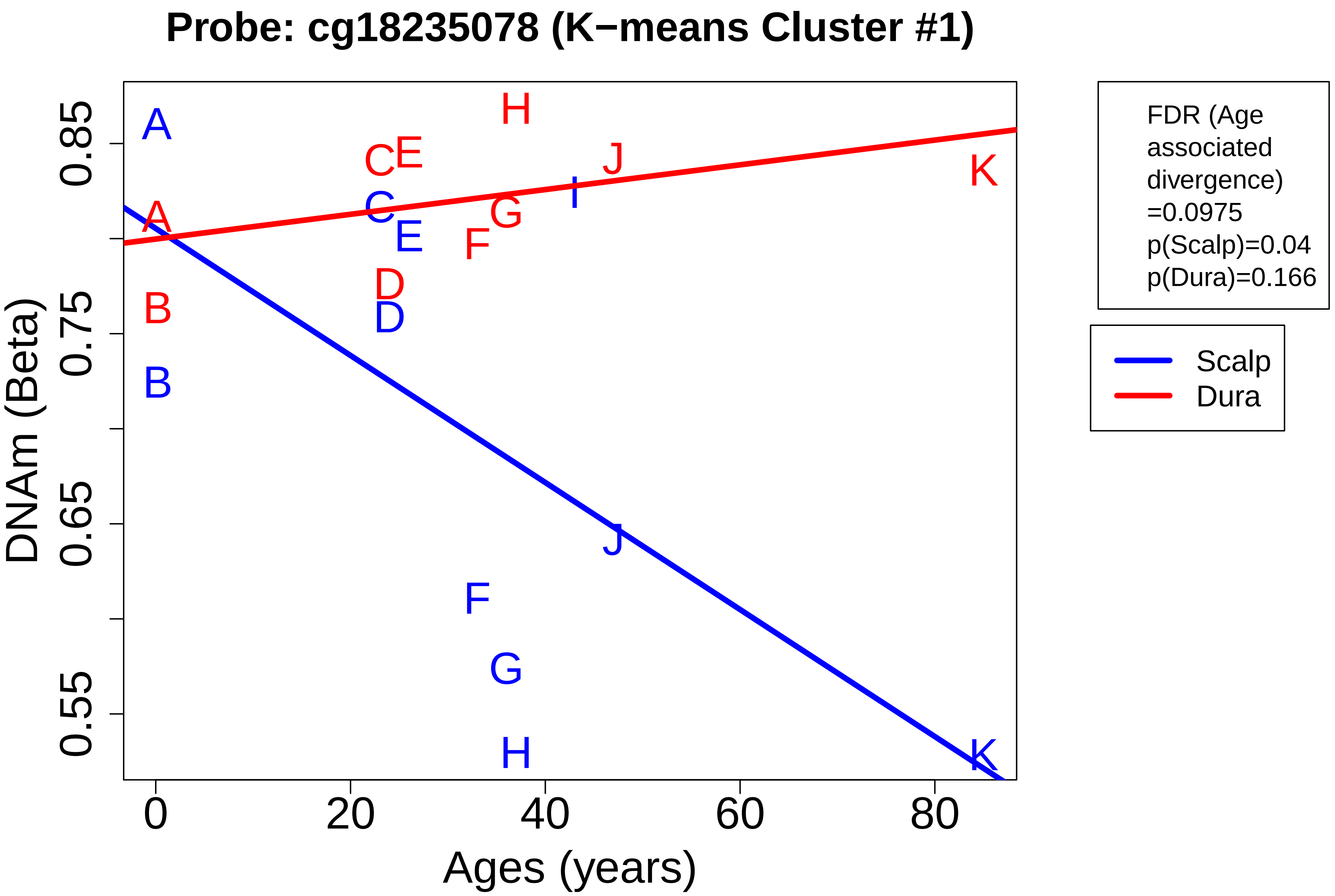
Probe: cg18235078 (K−means Cluster #1)
DNAm (Beta)
0.55
0.65
0.75
0.85
0
20
40
60
80
Ages (years)
FDR (Age associated divergence) =0.0975
p(Scalp)=0.04
p(Dura)=0.166
Scalp
Dura
A
B
C
D
E
F
G
H
I
J
K

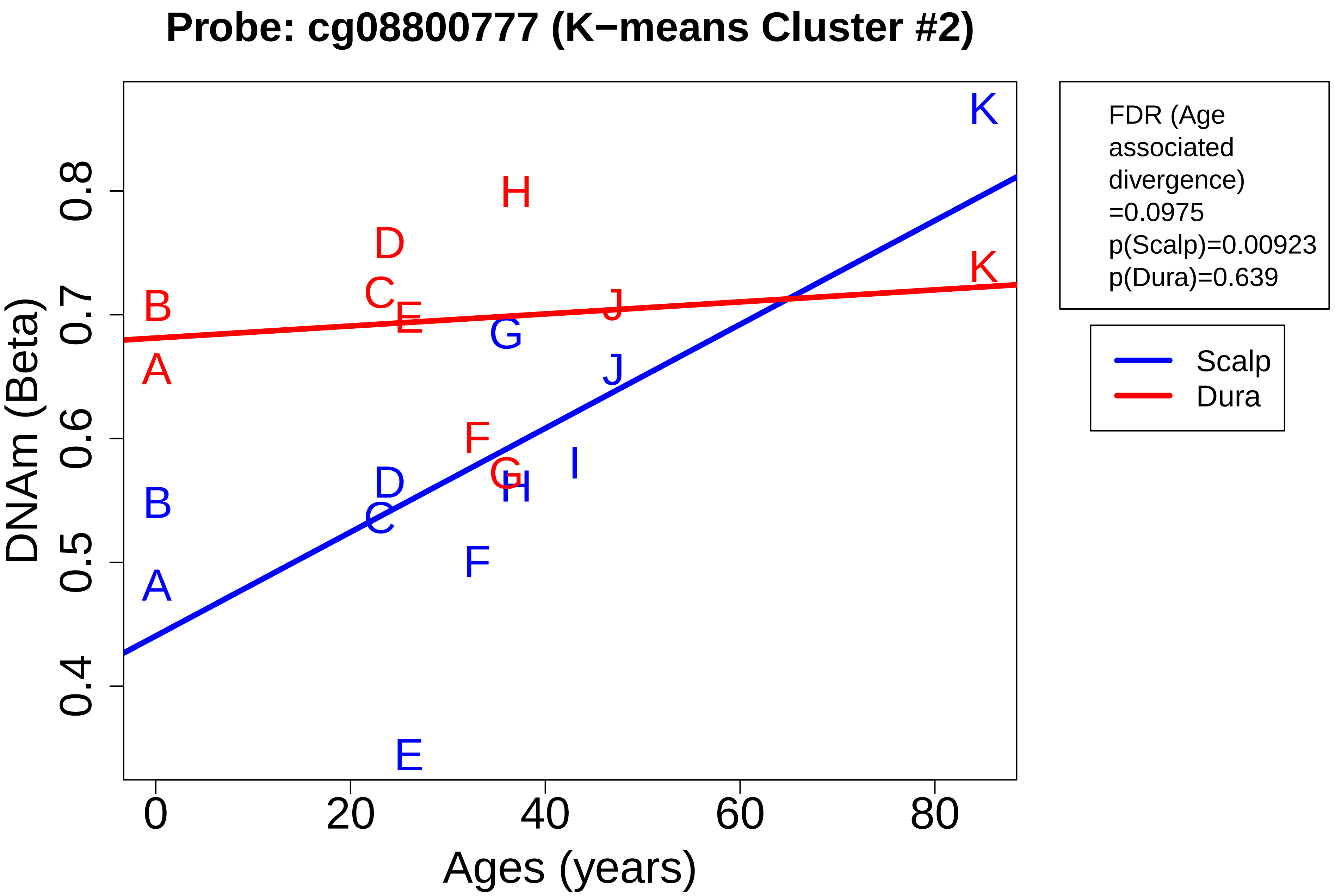

Probe: cg08800777 (K−means Cluster #2)
DNAm (Beta)
0.4
0.5
0.6
0.7
0.8
0
20
40
60
80
Ages (years)
A
B
C
D
E
F
G
H
I
J
K
FDR (Age associated divergence) =0.0975
p(Scalp)=0.00923
p(Dura)=0.639
Scalp
Dura

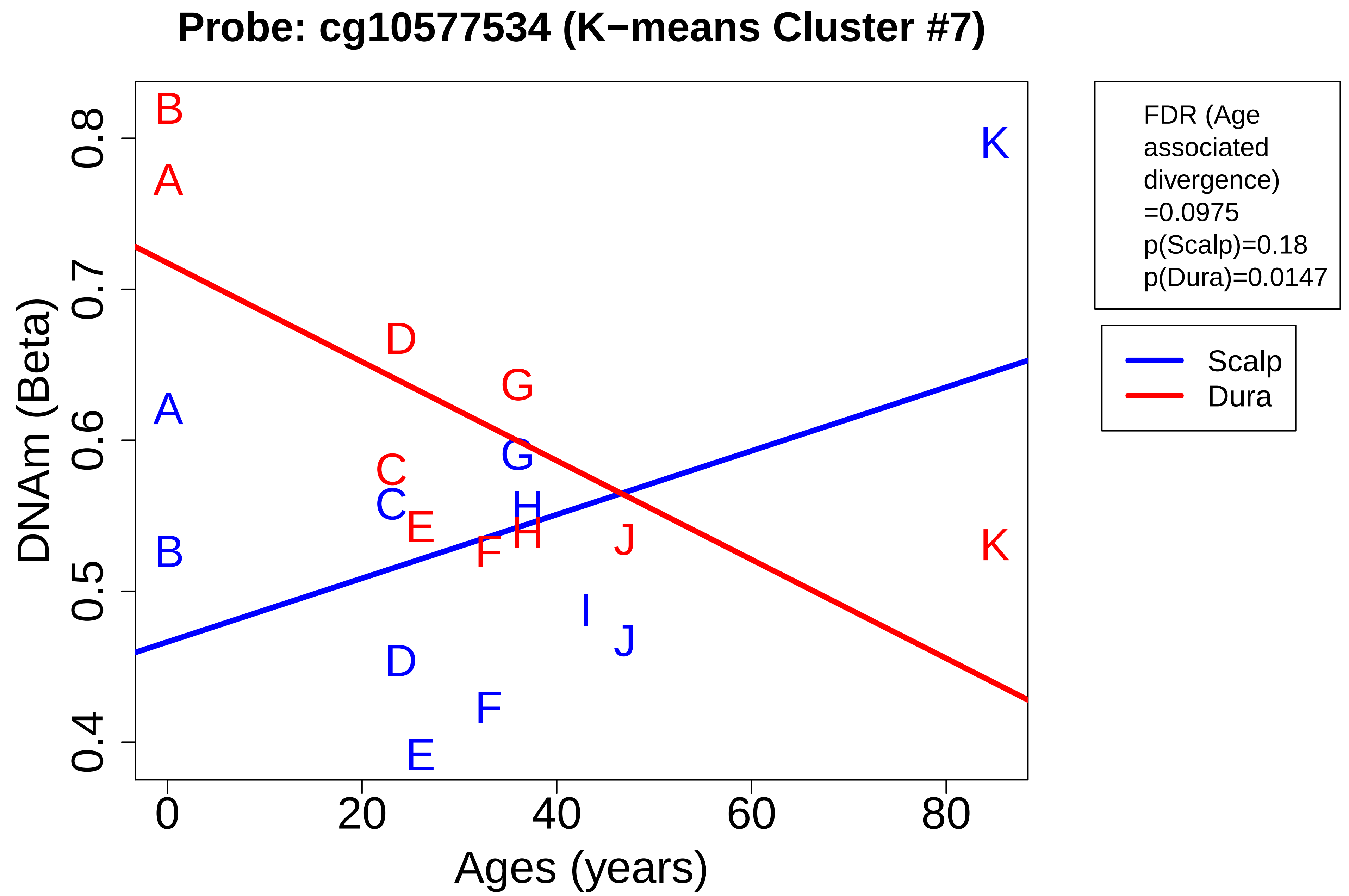

Probe: cg10577534 (K−means Cluster #7)
DNAm (Beta)
0.4
0.5
0.6
0.7
0.8
0
20
40
60
80
Ages (years)
B
A
K
D
G
A
G
C
C
H
E
H
F
J
K
B
I
J
D
F
E
FDR (Age associated divergence) =0.0975
p(Scalp)=0.18
p(Dura)=0.0147
Scalp
Dura

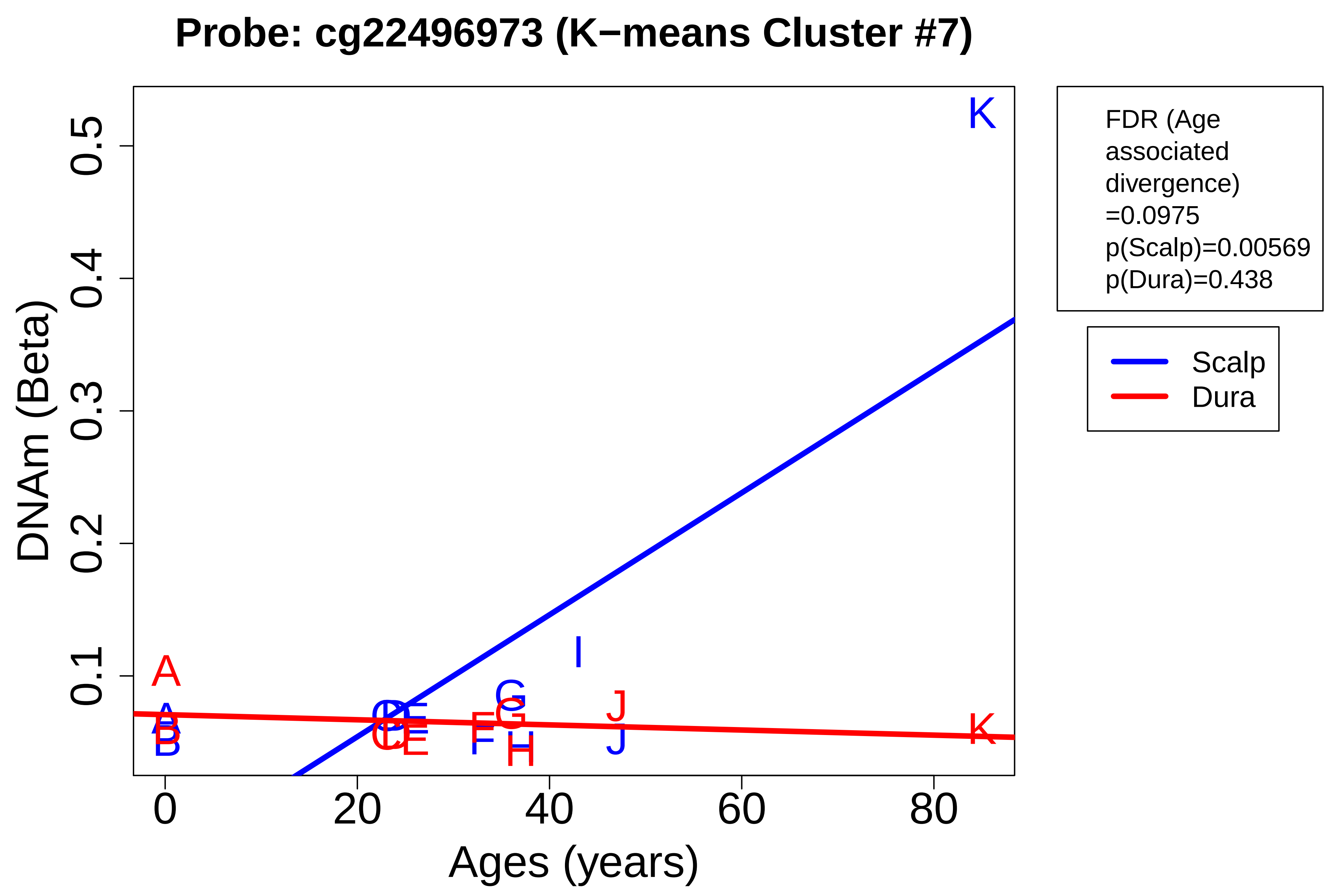
Probe: cg22496973 (K−means Cluster #7)
DNAm (Beta)
Ages (years)
FDR (Age associated divergence) =0.0975
p(Scalp)=0.00569
p(Dura)=0.438
Scalp
Dura
0.1
0.2
0.3
0.4
0.5
0
20
40
60
80

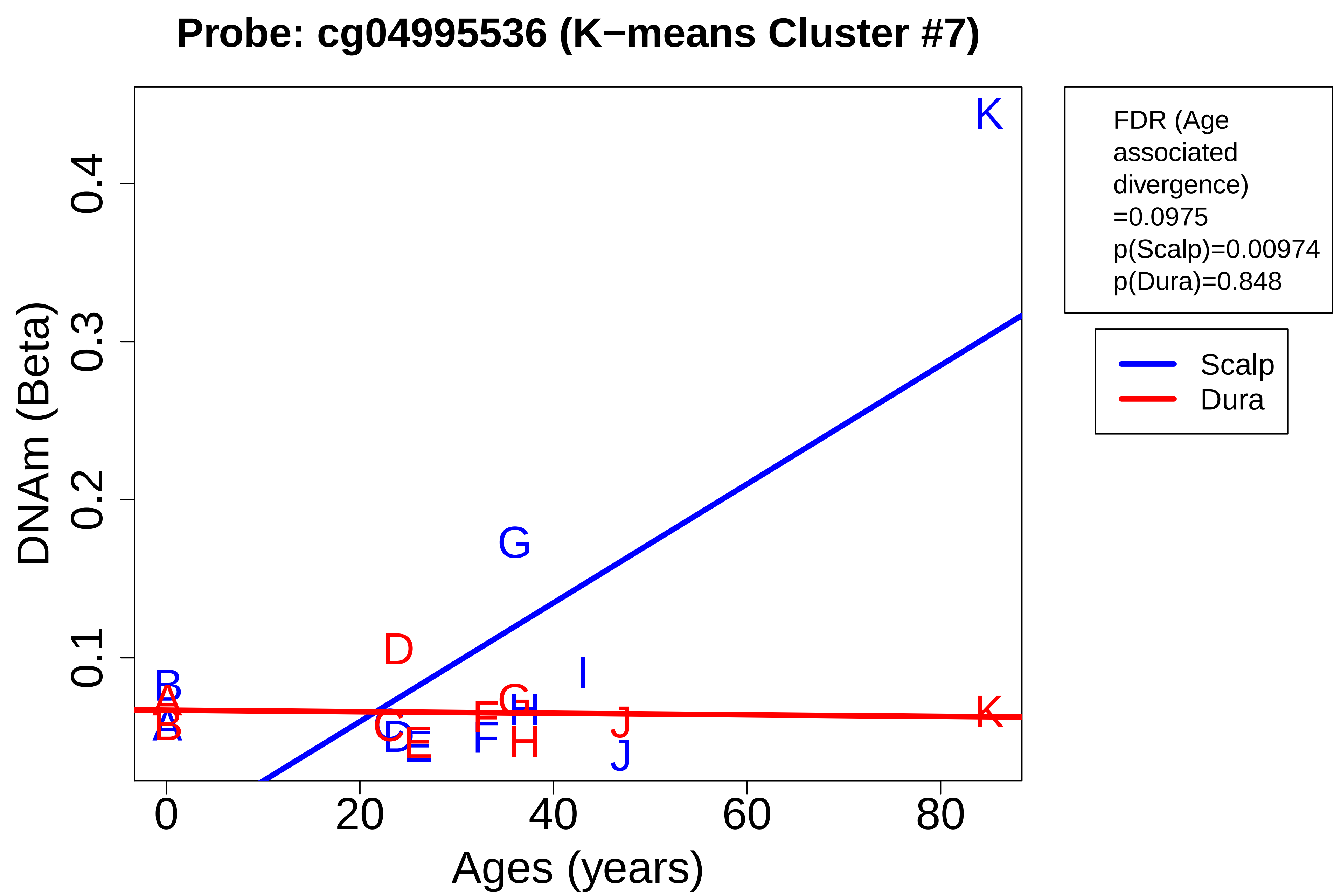

Probe: cg04995536 (K−means Cluster #7)
FDR (Age associated divergence) =0.0975
p(Scalp)=0.00974
p(Dura)=0.848
Scalp
Dura
DNAm (Beta)
0.1
0.2
0.3
0.4
0
20
40
60
80
Ages (years)

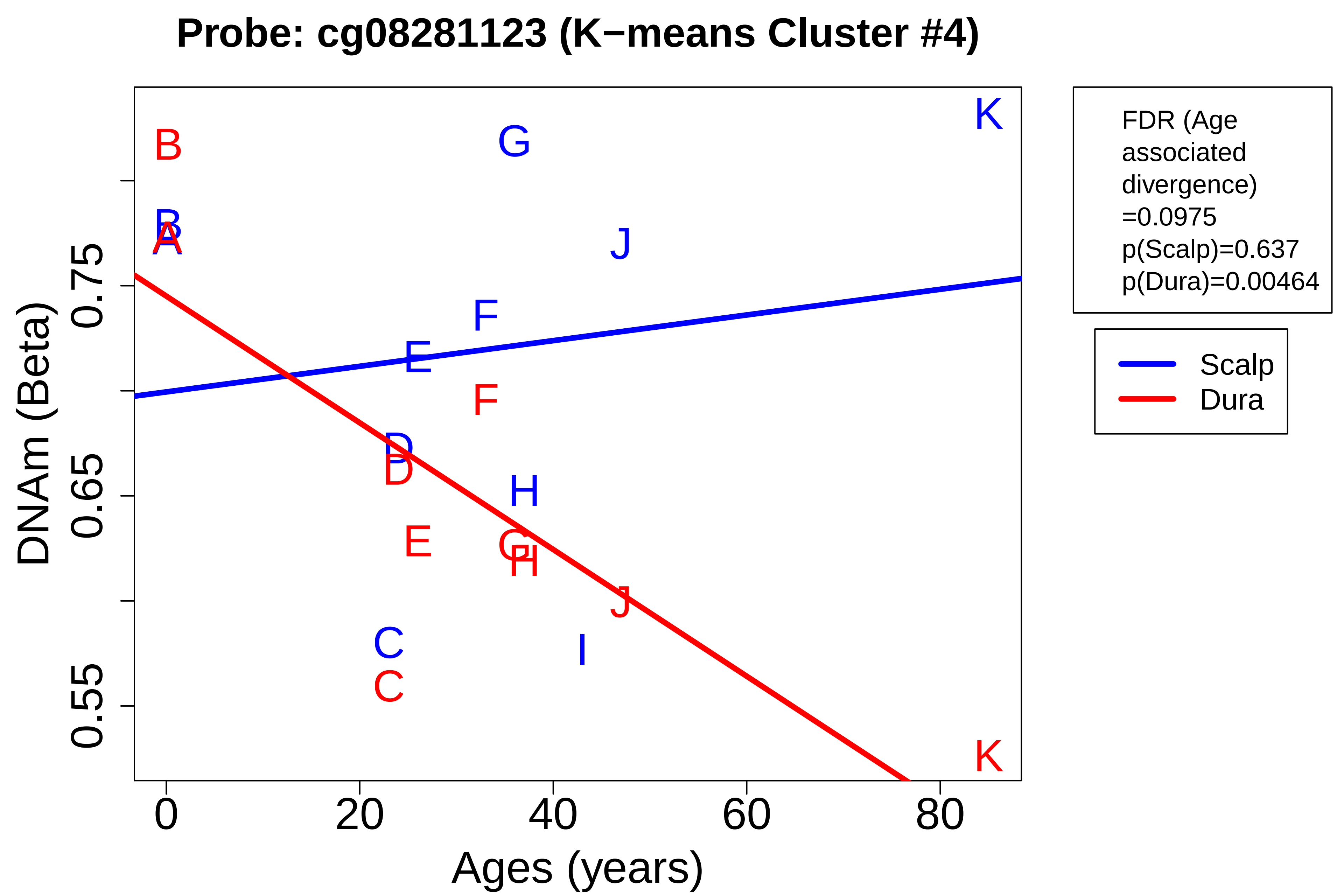

Probe: cg08281123 (K−means Cluster #4)
DNAm (Beta)
0.55
0.65
0.75
0
20
40
60
80
Ages (years)
A
B
C
D
E
F
G
H
I
J
K
FDR (Age associated divergence) =0.0975
p(Scalp)=0.637
p(Dura)=0.00464
Scalp
Dura

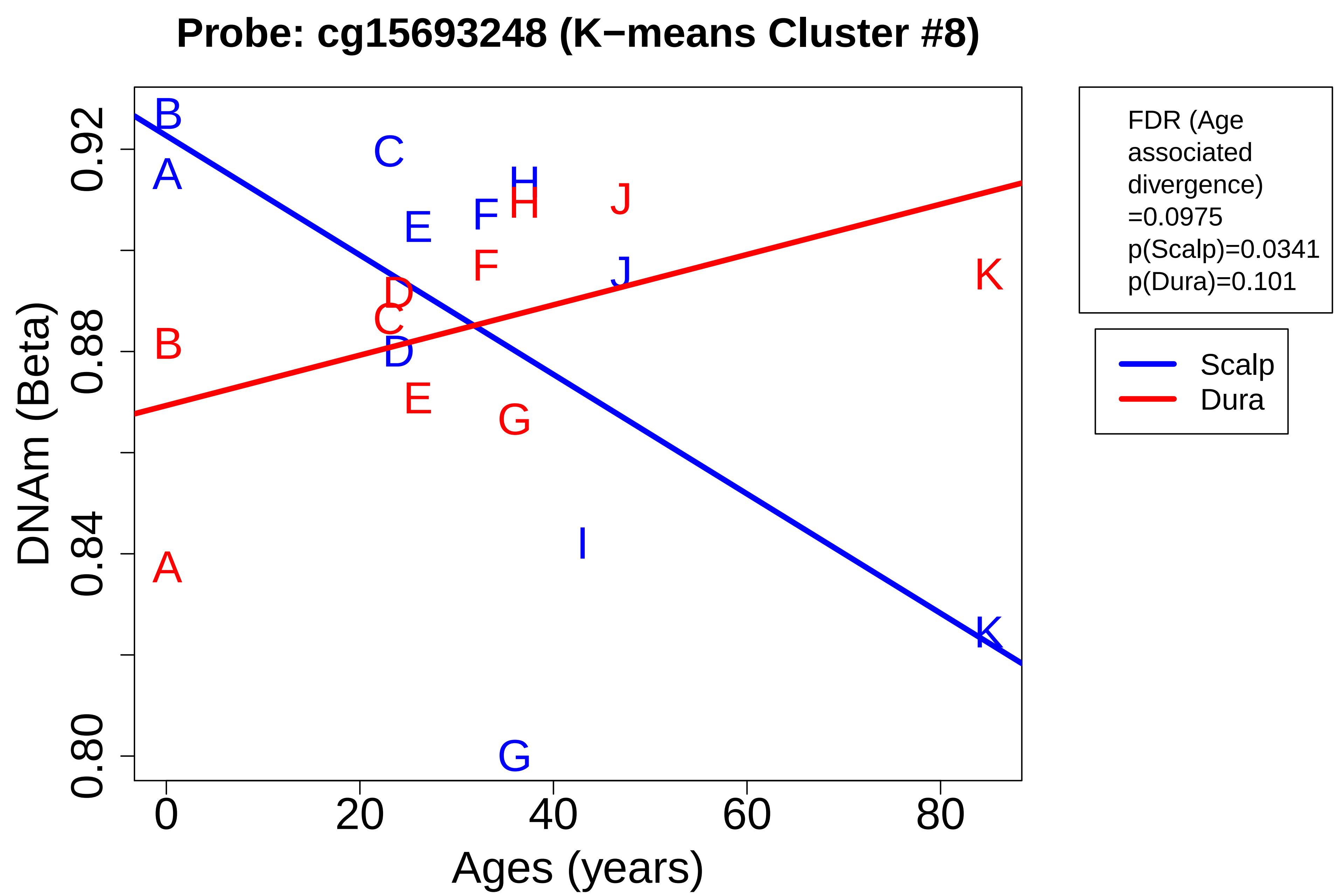

Probe: cg15693248 (K−means Cluster #8)
DNAm (Beta)
0.80
0.84
0.88
0.92
0
20
40
60
80
Ages (years)
FDR (Age associated divergence) =0.0975
p(Scalp)=0.0341
p(Dura)=0.101
Scalp
Dura

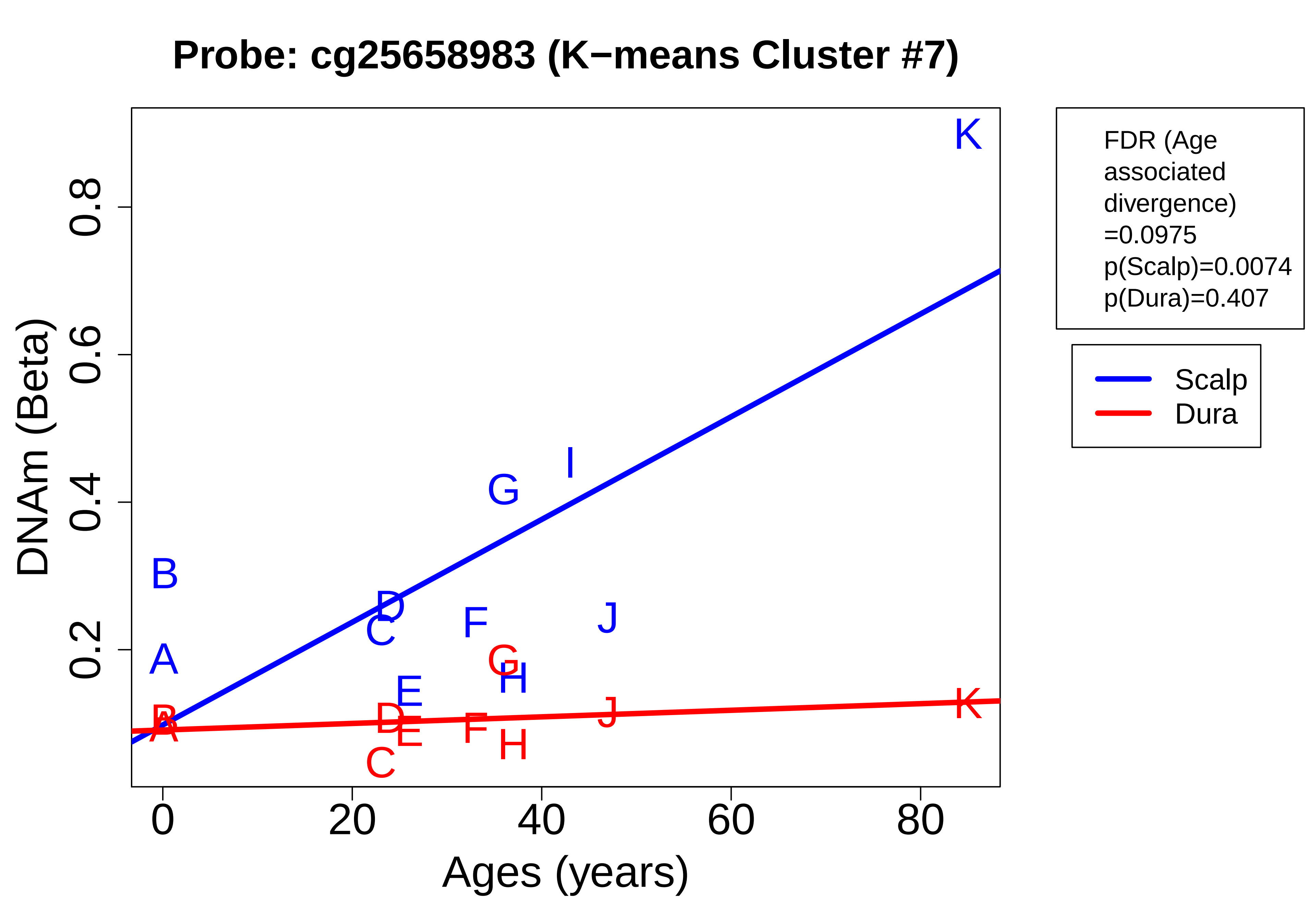
Probe: cg25658983 (K−means Cluster #7)
DNAm (Beta)
0.2
0.4
0.6
0.8
Ages (years)
0
20
40
60
80
FDR (Age associated divergence) =0.0975
p(Scalp)=0.0074
p(Dura)=0.407
Scalp
Dura
A
B
C
D
E
F
G
H
I
J
K

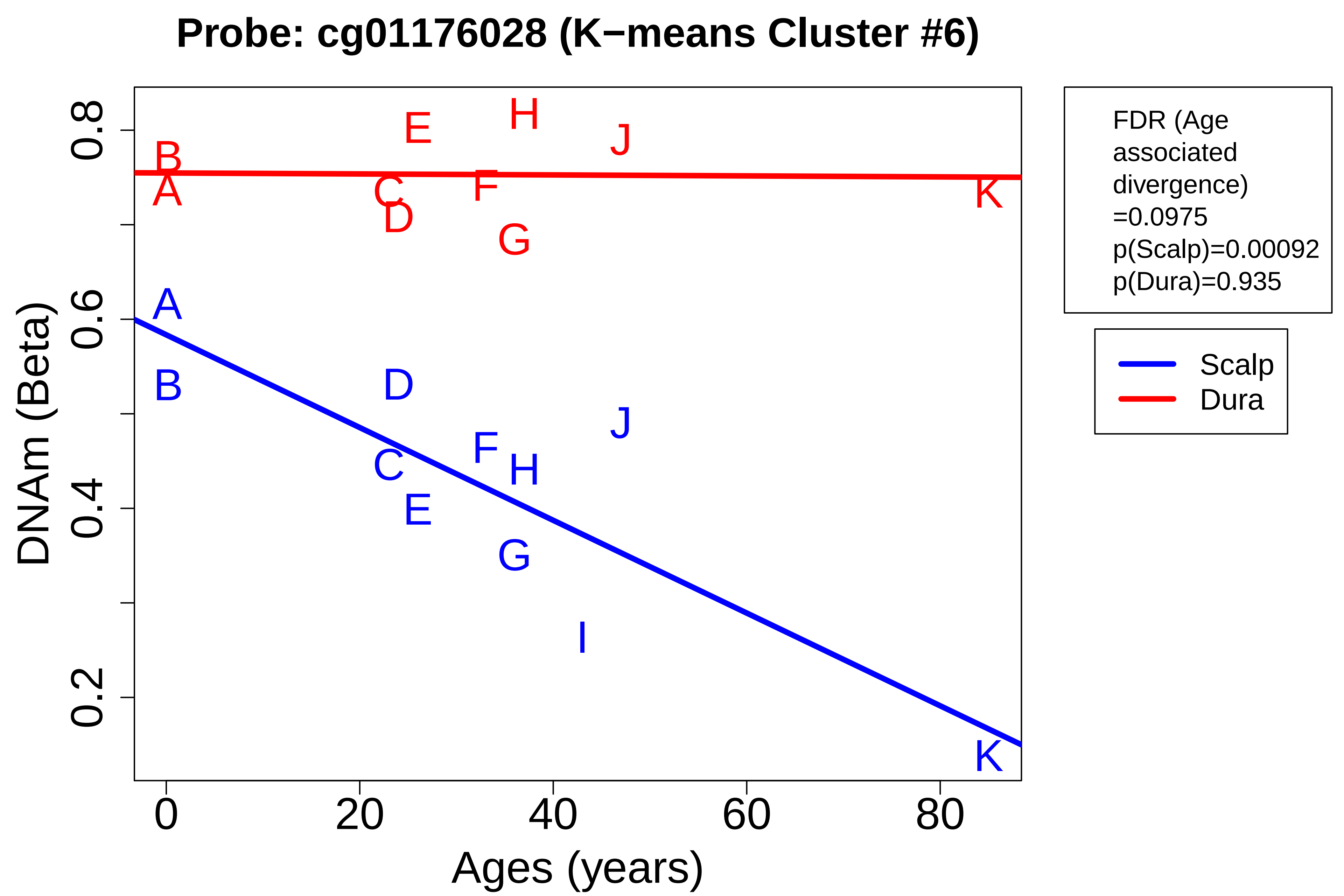

Probe: cg01176028 (K−means Cluster #6)
DNAm (Beta)
0.2
0.4
0.6
0.8
0
20
40
60
80
Ages (years)
A
B
C
D
E
F
G
H
I
J
K
FDR (Age associated divergence) =0.0975
p(Scalp)=0.00092
p(Dura)=0.935
Scalp
Dura

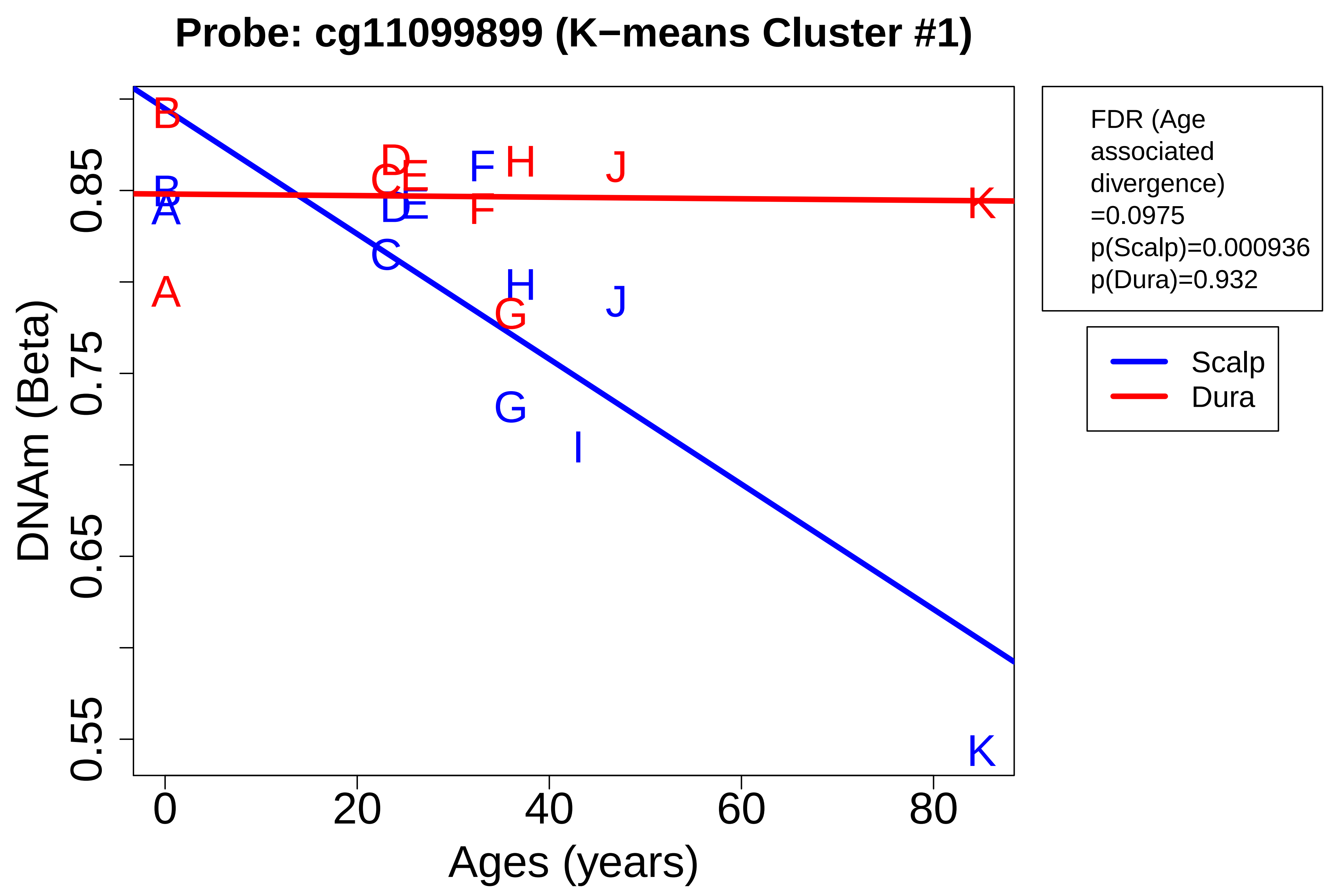
Probe: cg11099899 (K−means Cluster #1)
DNAm (Beta)
0.55
0.65
0.75
0.85
0
20
40
60
80
Ages (years)
FDR (Age associated divergence) =0.0975
p(Scalp)=0.000936
p(Dura)=0.932
Scalp
Dura
A
B
C
D
E
F
G
H
I
J
K

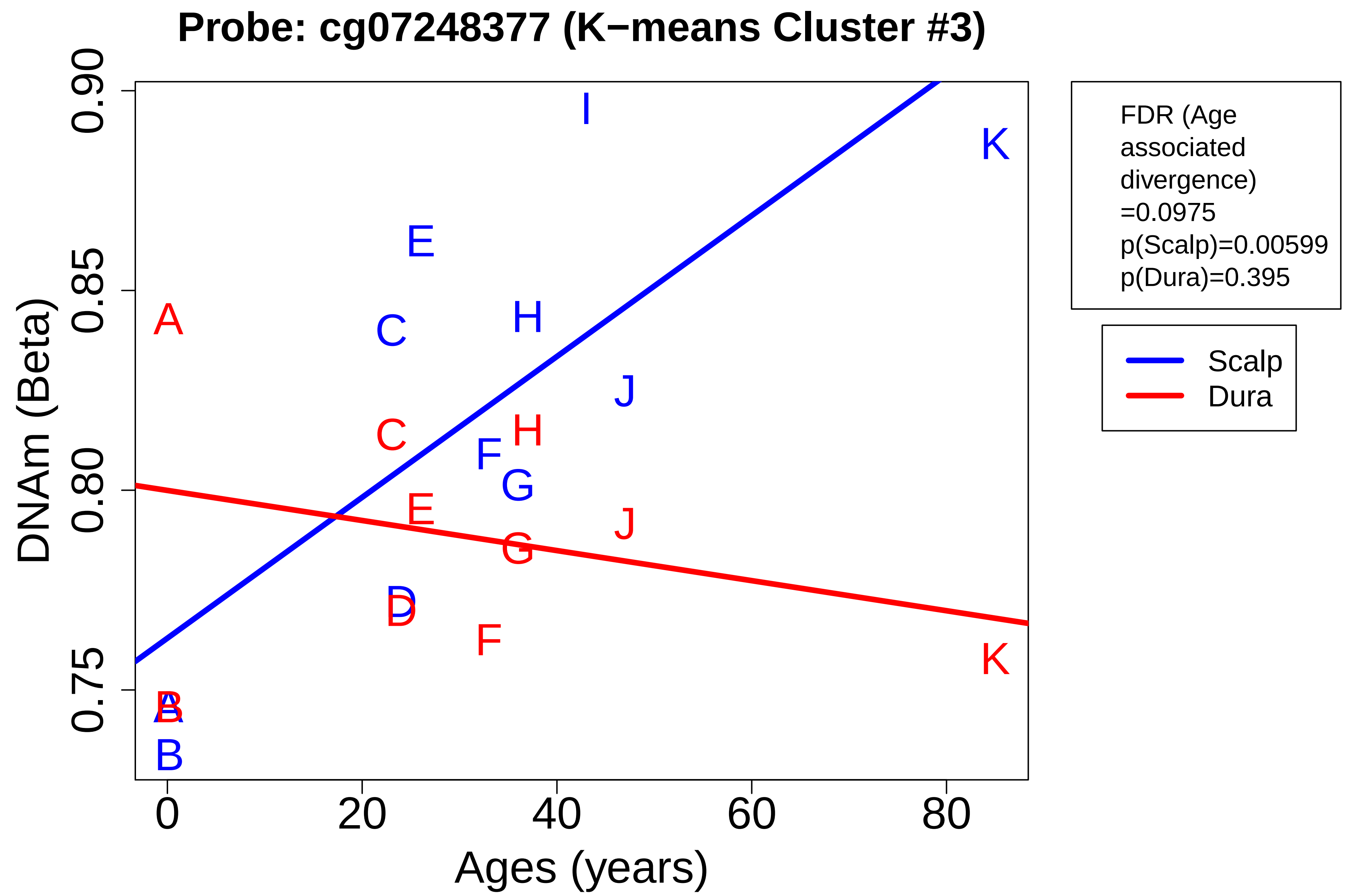

Probe: cg07248377 (K−means Cluster #3)
DNAm (Beta)
0.75
0.80
0.85
0.90
Ages (years)
0
20
40
60
80
FDR (Age associated divergence) =0.0975
p(Scalp)=0.00599
p(Dura)=0.395
Scalp
Dura

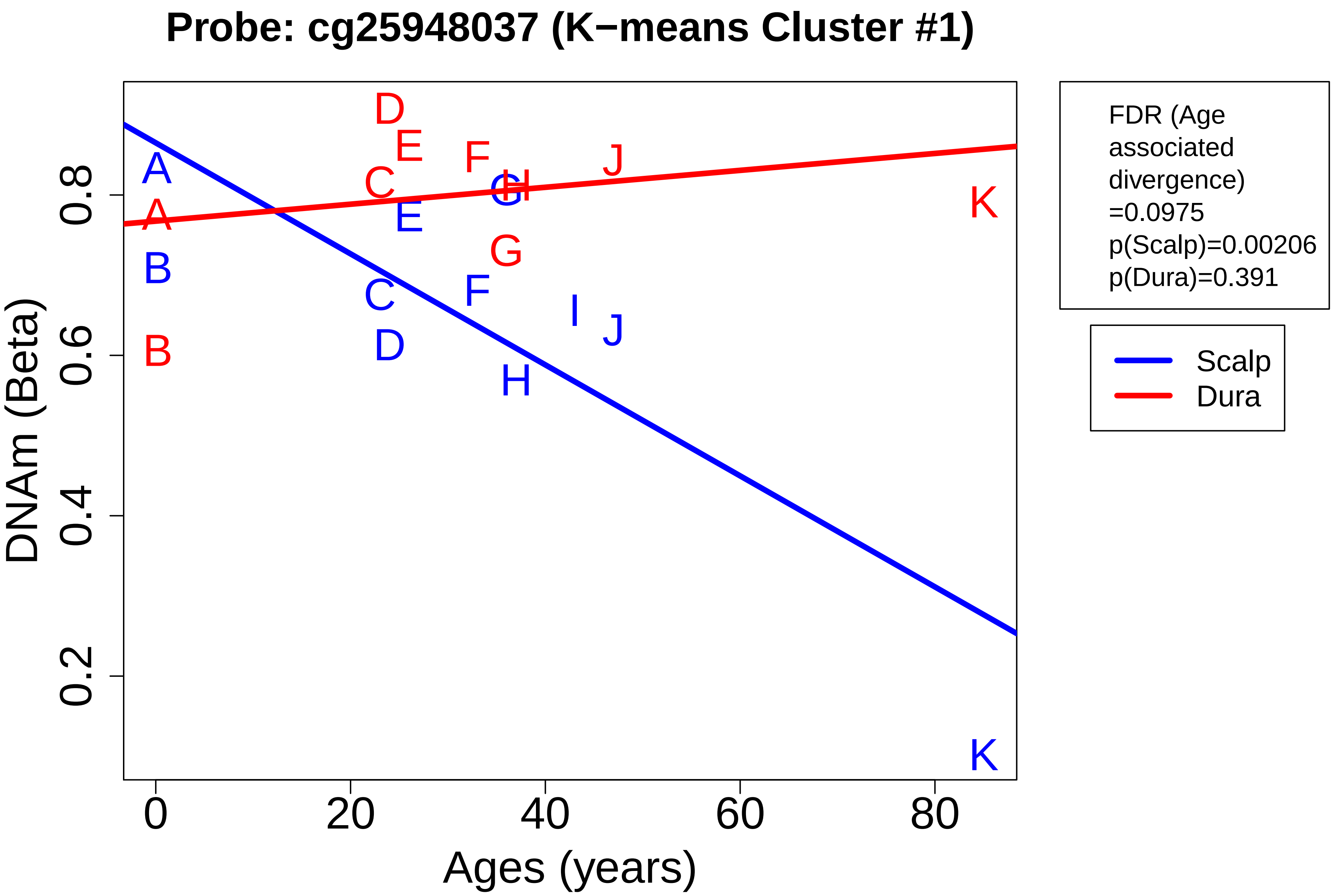

Probe: cg25948037 (K−means Cluster #1)
DNAm (Beta)
0.2
0.4
0.6
0.8
0
20
40
60
80
Ages (years)
FDR (Age associated divergence) =0.0975
p(Scalp)=0.00206
p(Dura)=0.391
Scalp
Dura

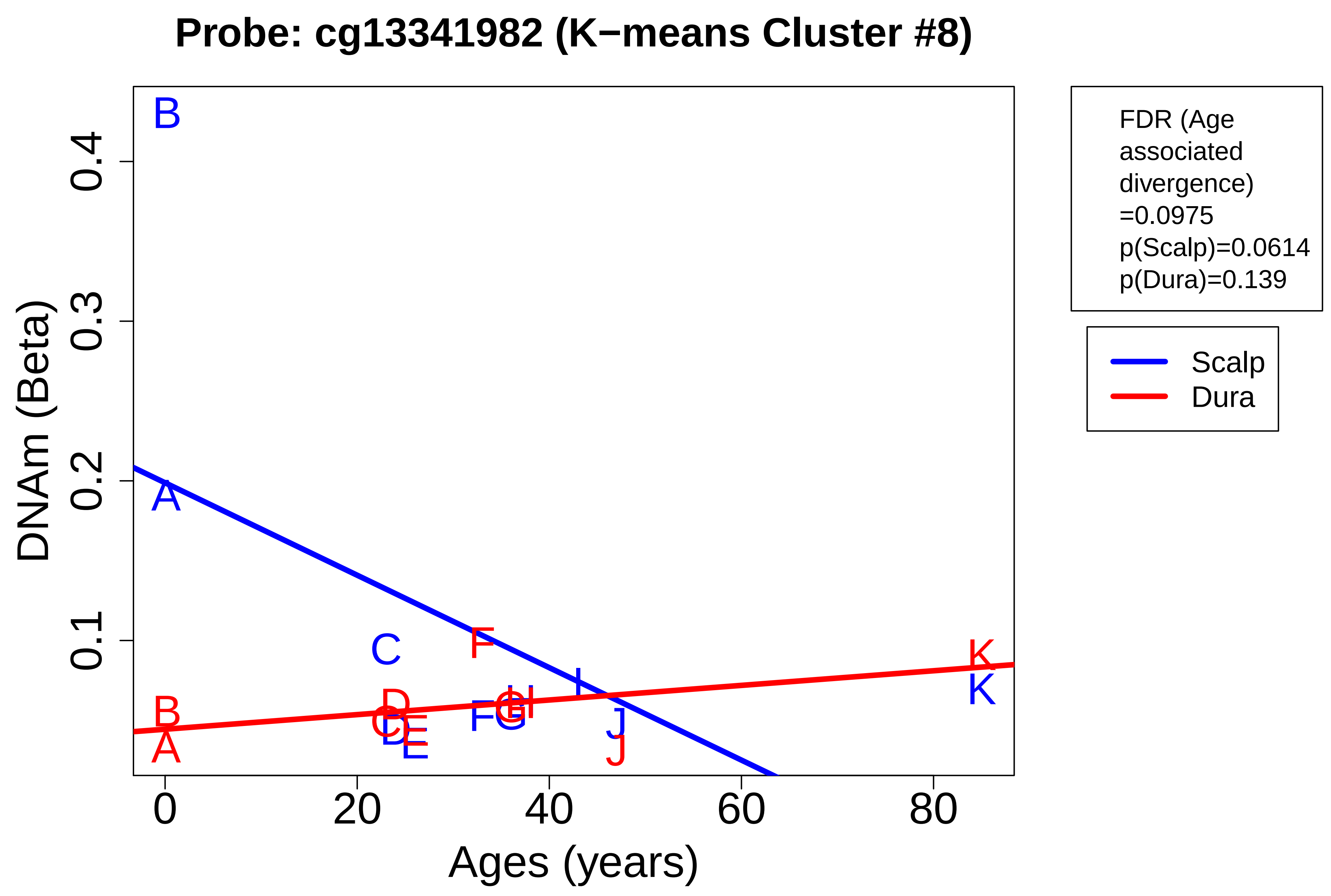

Probe: cg13341982 (K−means Cluster #8)
DNAm (Beta)
0.1
0.2
0.3
0.4
Ages (years)
0
20
40
60
80
FDR (Age associated divergence) =0.0975
p(Scalp)=0.0614
p(Dura)=0.139
Scalp
Dura

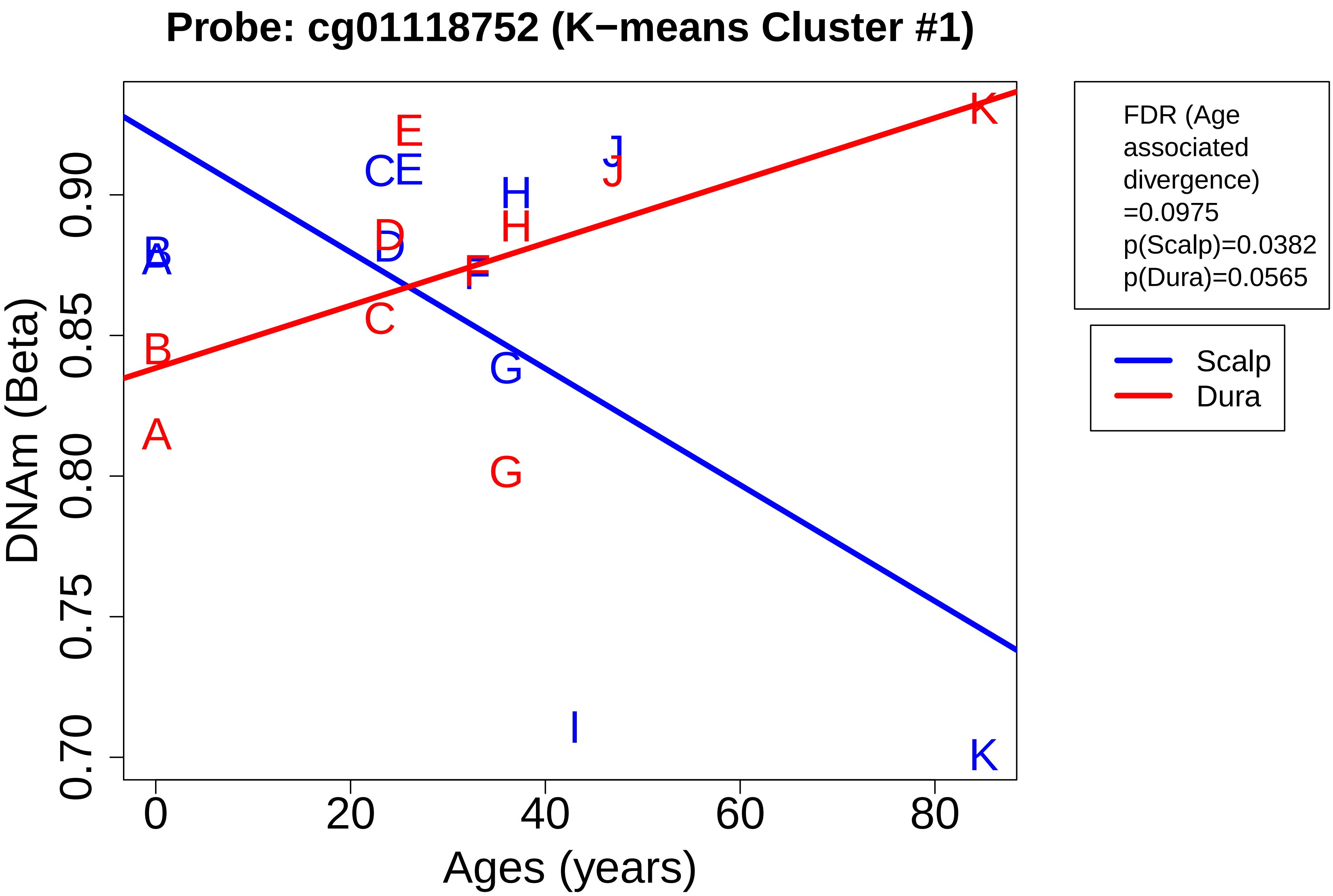

Probe: cg01118752 (K−means Cluster #1)
DNAm (Beta)
0.70
0.75
0.80
0.85
0.90
0
20
40
60
80
Ages (years)
A
B
C
D
E
F
G
H
I
J
K
FDR (Age associated divergence) =0.0975
p(Scalp)=0.0382
p(Dura)=0.0565
Scalp
Dura

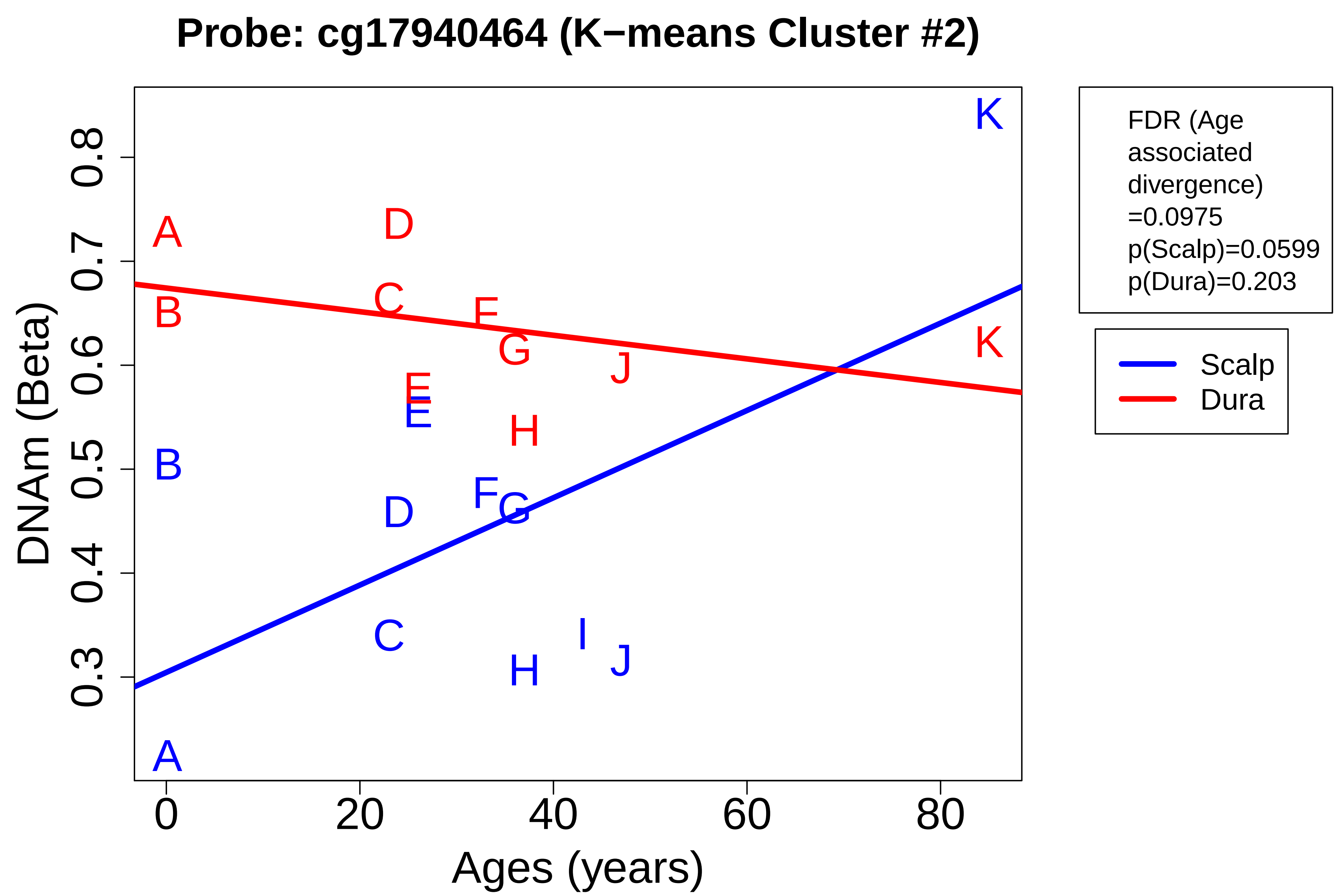

Probe: cg17940464 (K−means Cluster #2)
DNAm (Beta)
0.3
0.4
0.5
0.6
0.7
0.8
0
20
40
60
80
Ages (years)
A
B
C
D
E
F
G
H
I
J
K
FDR (Age associated divergence) =0.0975
p(Scalp)=0.0599
p(Dura)=0.203
Scalp
Dura

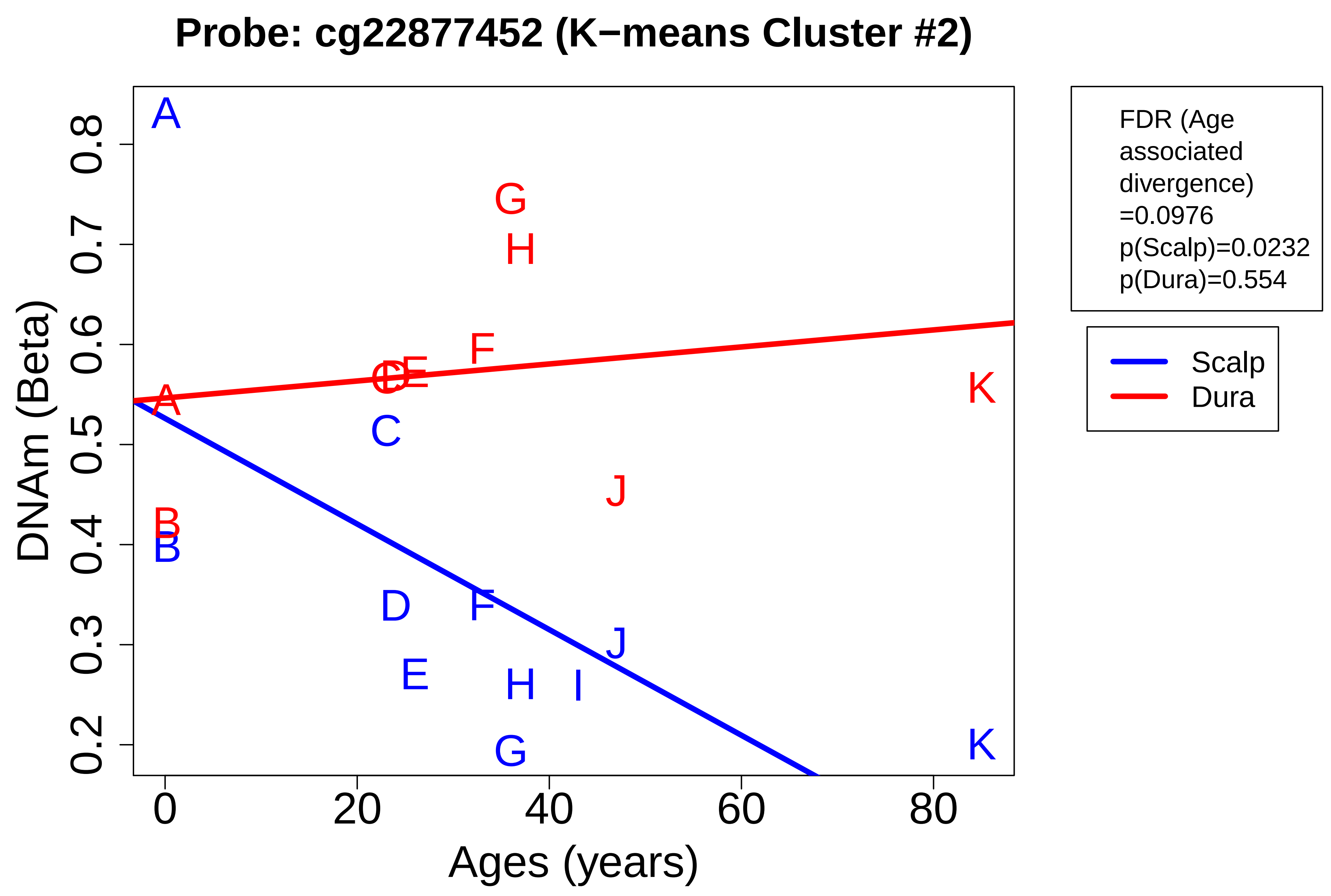

Probe: cg22877452 (K−means Cluster #2)
DNAm (Beta)
0.2
0.3
0.4
0.5
0.6
0.7
0.8
0
20
40
60
80
Ages (years)
FDR (Age associated divergence) =0.0976
p(Scalp)=0.0232
p(Dura)=0.554
Scalp
Dura
A
B
C
D
E
F
G
H
I
J
K

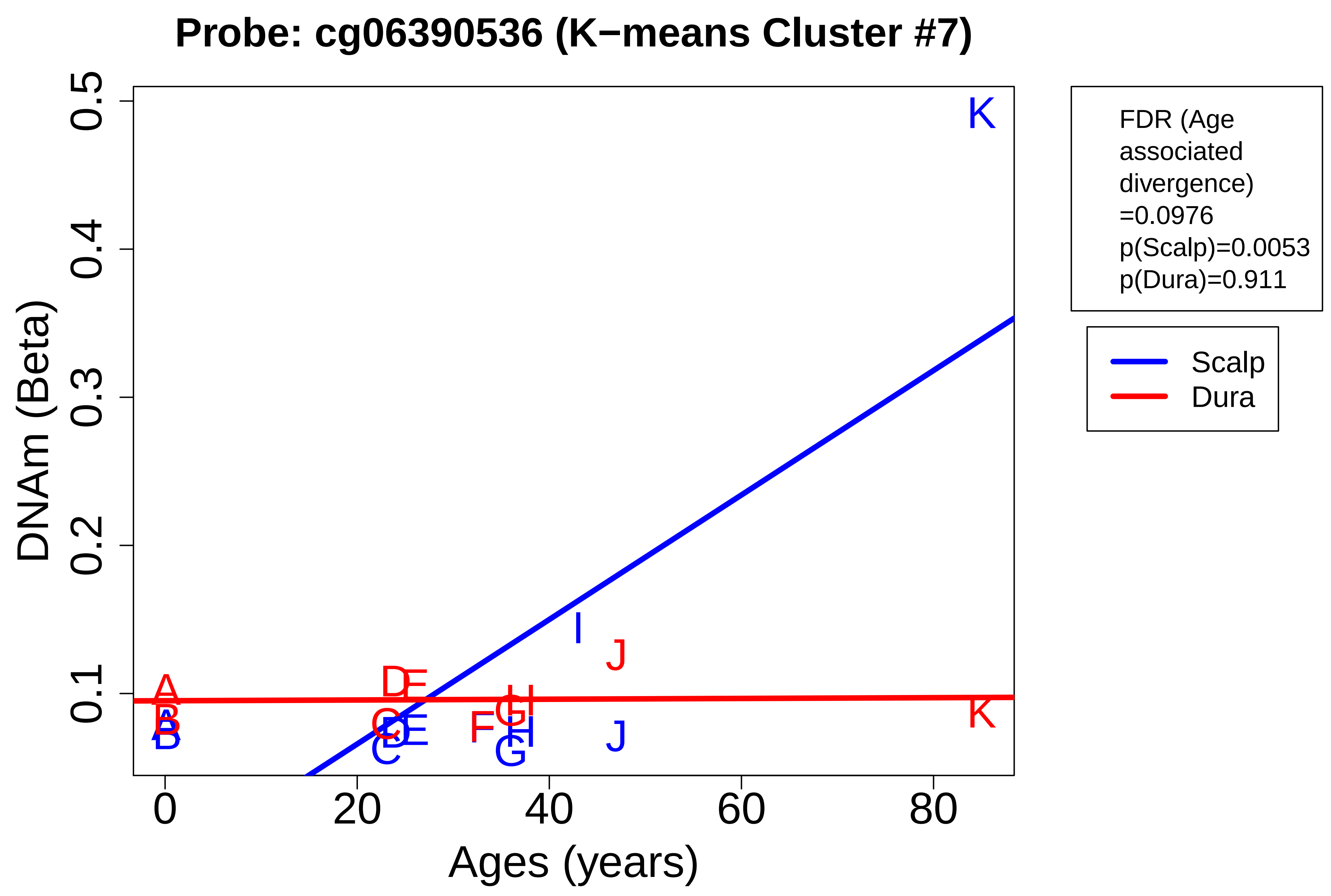
Probe: cg06390536 (K−means Cluster #7)
DNAm (Beta)
0.1
0.2
0.3
0.4
0.5
Ages (years)
0
20
40
60
80
FDR (Age associated divergence) =0.0976
p(Scalp)=0.0053
p(Dura)=0.911
Scalp
Dura
A
B
C
D
E
F
G
H
I
J
K

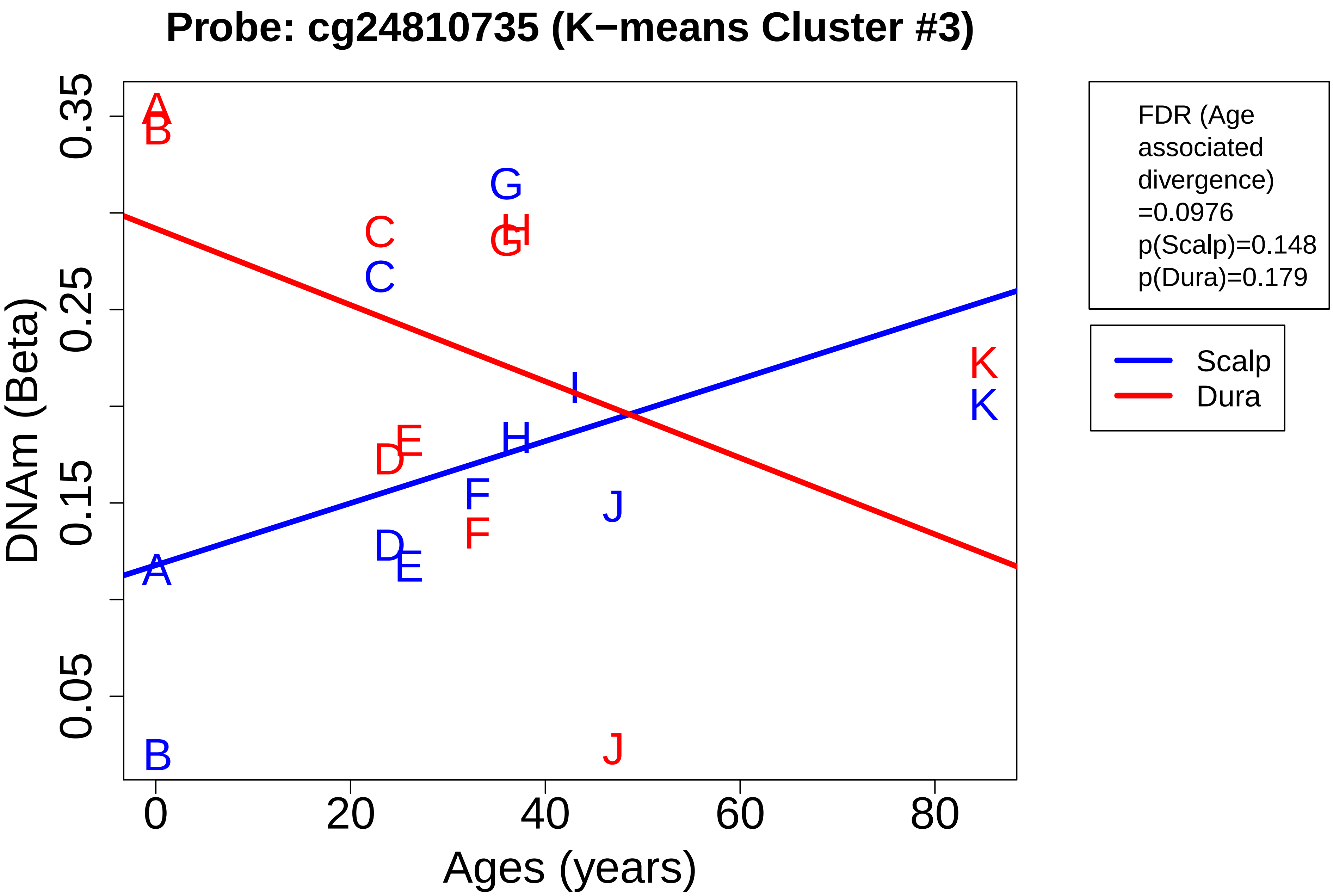

Probe: cg24810735 (K−means Cluster #3)
DNAm (Beta)
0.05
0.15
0.25
0.35
Ages (years)
0
20
40
60
80
A
B
C
D
E
F
G
H
I
J
K
FDR (Age associated divergence) =0.0976
p(Scalp)=0.148
p(Dura)=0.179
Scalp
Dura

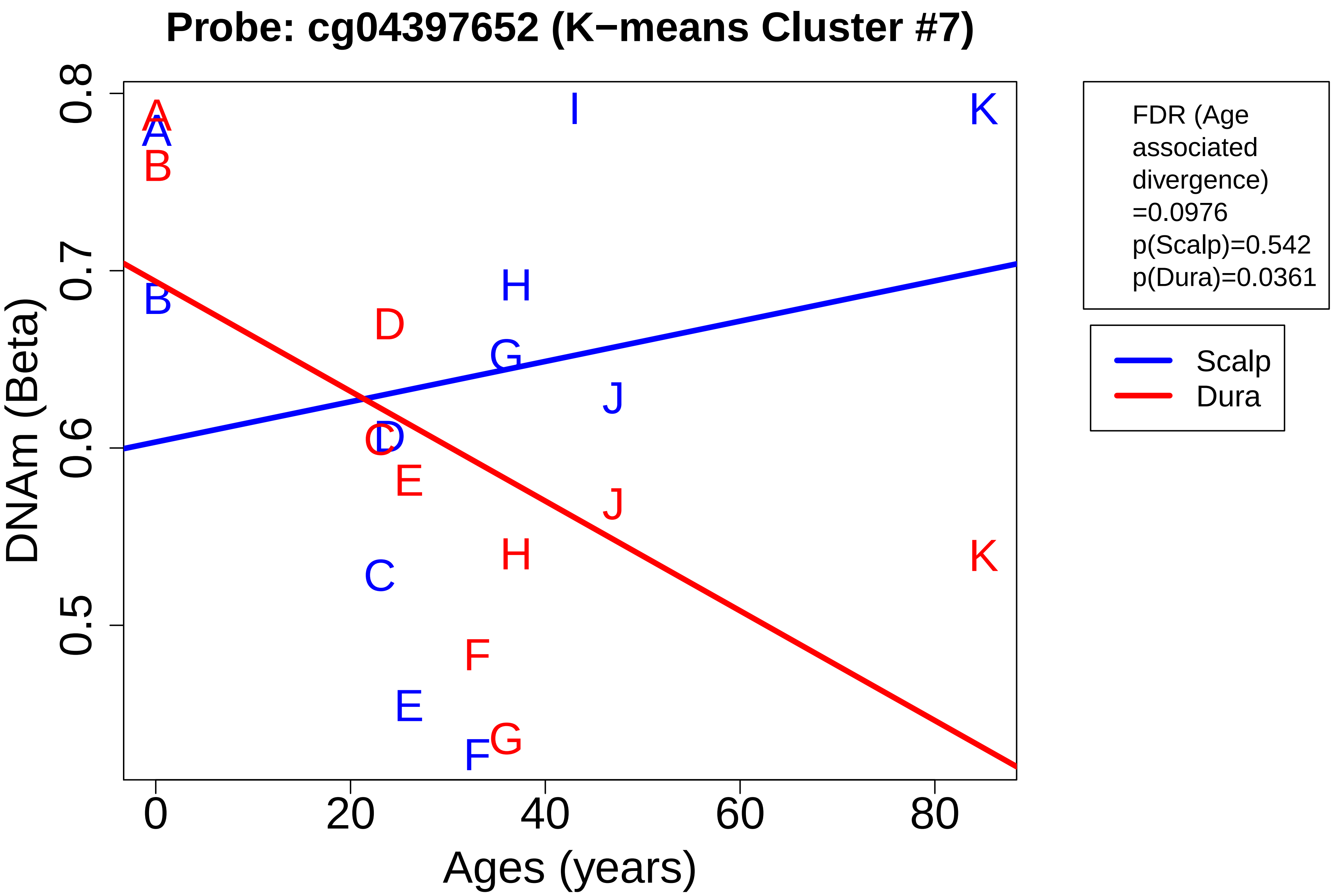

Probe: cg04397652 (K−means Cluster #7)
DNAm (Beta)
0.5
0.6
0.7
0.8
0
20
40
60
80
Ages (years)
FDR (Age associated divergence) =0.0976
p(Scalp)=0.542
p(Dura)=0.0361
Scalp
Dura

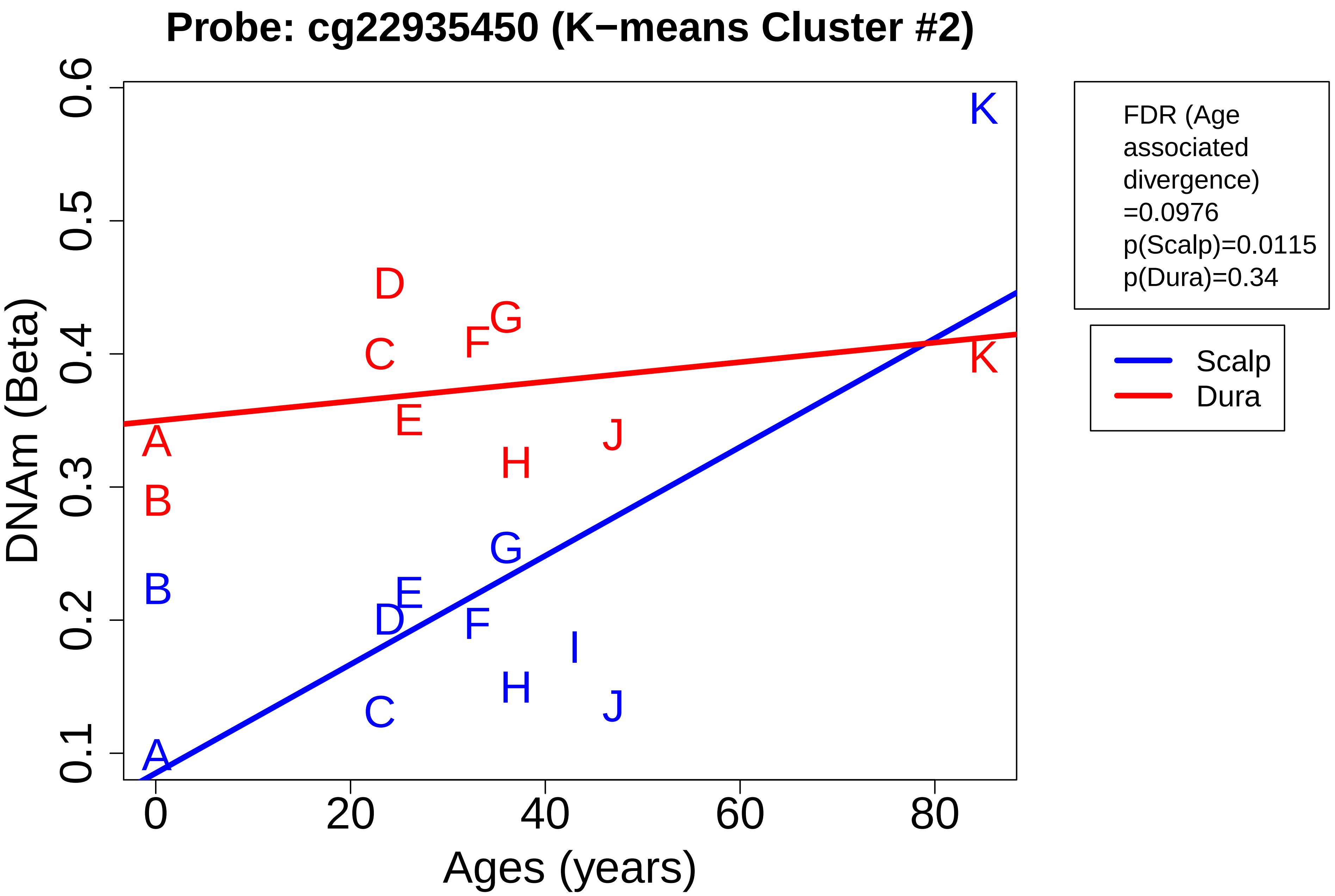

Probe: cg22935450 (K−means Cluster #2)
DNAm (Beta)
0.1
0.2
0.3
0.4
0.5
0.6
Ages (years)
0
20
40
60
80
FDR (Age associated divergence) =0.0976
p(Scalp)=0.0115
p(Dura)=0.34
Scalp
Dura
A
B
C
D
E
F
G
H
I
J
K

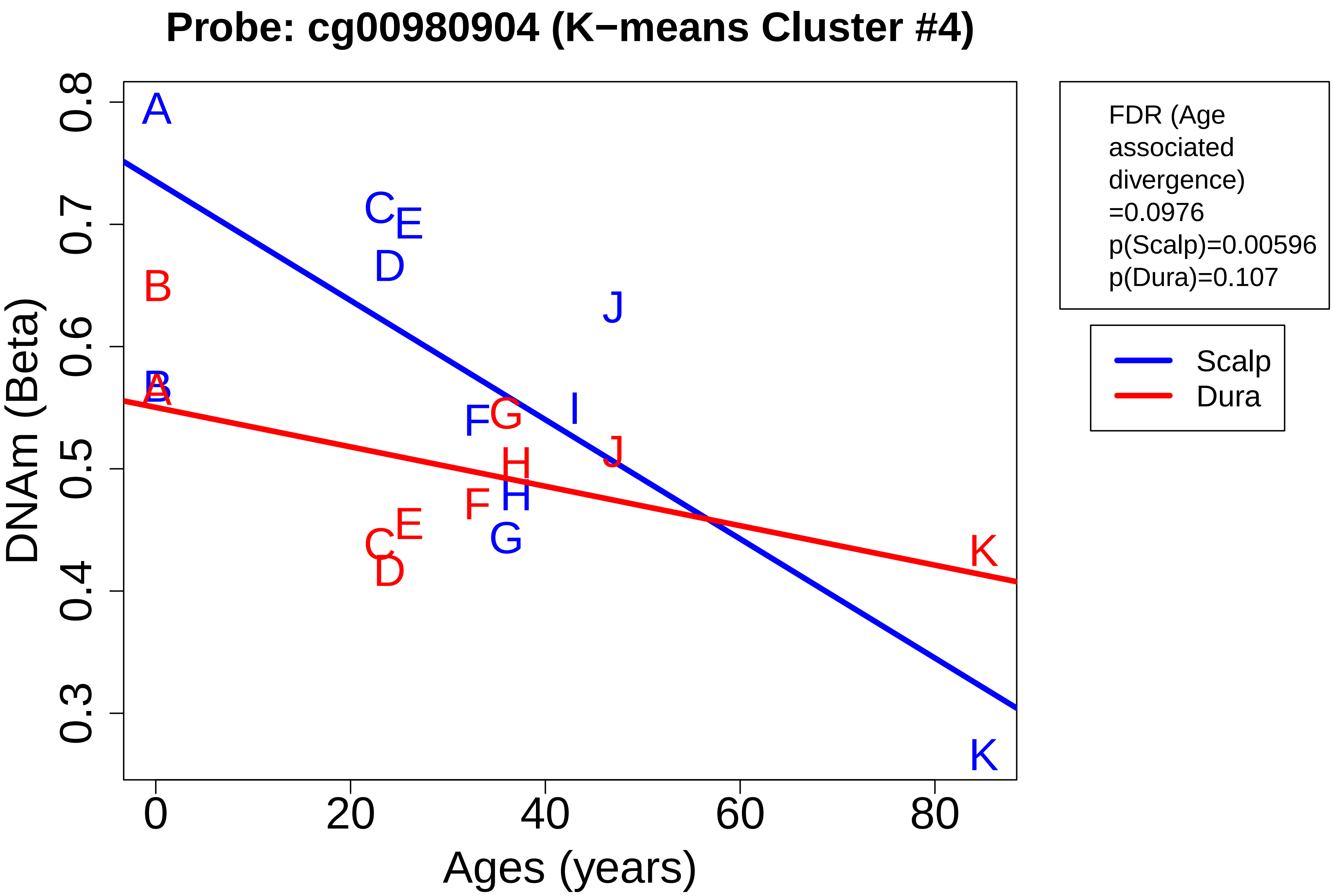

Probe: cg00980904 (K−means Cluster #4)
DNAm (Beta)
0.3
0.4
0.5
0.6
0.7
0.8
0
20
40
60
80
Ages (years)
FDR (Age associated divergence) =0.0976
p(Scalp)=0.00596
p(Dura)=0.107
Scalp
Dura

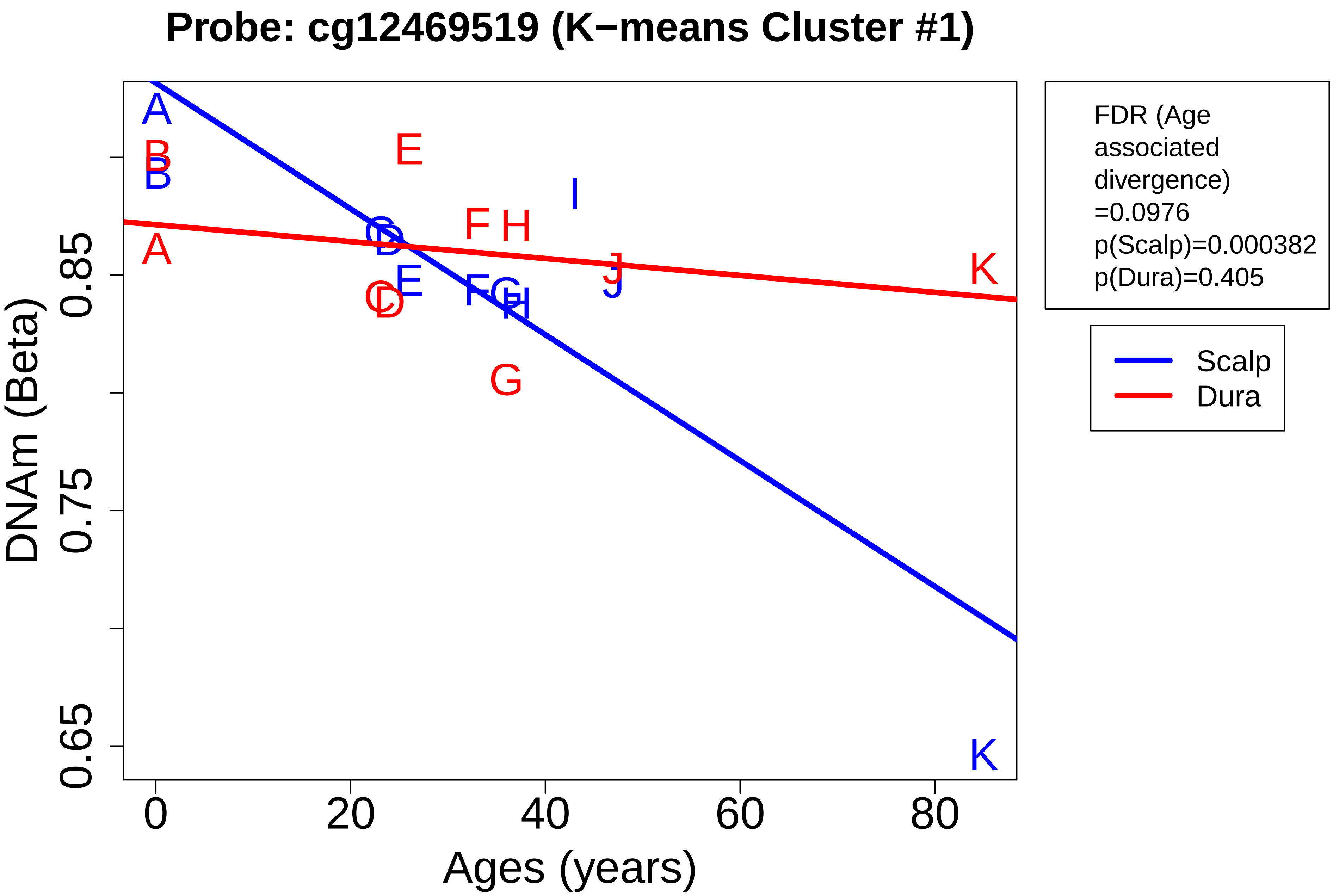

Probe: cg12469519 (K−means Cluster #1)
DNAm (Beta)
0.65
0.75
0.85
Ages (years)
0
20
40
60
80
A
B
B
E
I
C
D
F
H
A
J
K
E
C
D
F
G
H
J
G
K
FDR (Age associated divergence) =0.0976
p(Scalp)=0.000382
p(Dura)=0.405
Scalp
Dura

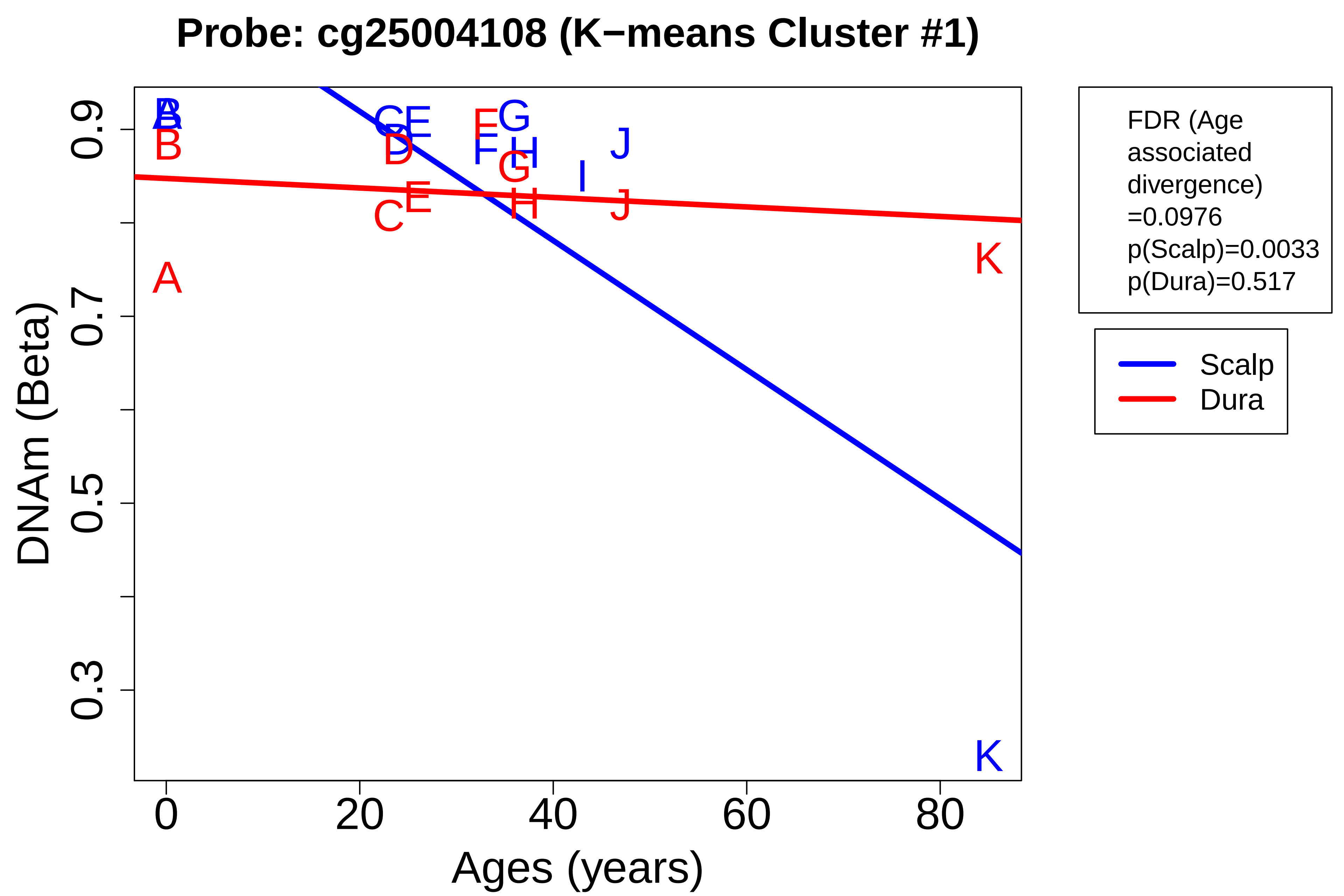

Probe: cg25004108 (K−means Cluster #1)
DNAm (Beta)
0.3
0.5
0.7
0.9
0
20
40
60
80
Ages (years)
A
B
C
D
E
F
G
H
I
J
K
FDR (Age associated divergence) =0.0976
p(Scalp)=0.0033
p(Dura)=0.517
Scalp
Dura

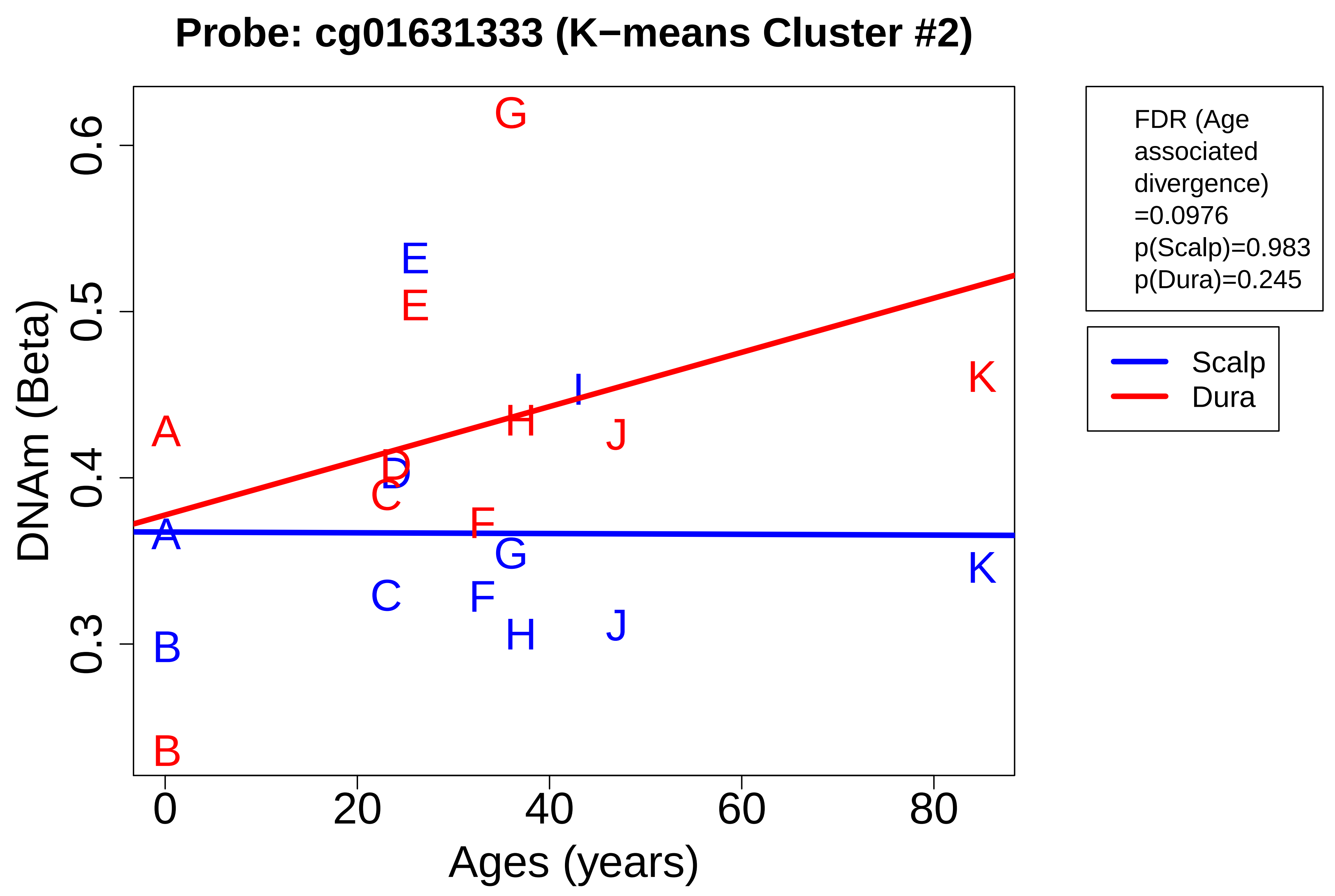

Probe: cg01631333 (K−means Cluster #2)
FDR (Age associated divergence) =0.0976
p(Scalp)=0.983
p(Dura)=0.245
Scalp
Dura
DNAm (Beta)
Ages (years)
0.3
0.4
0.5
0.6
0
20
40
60
80

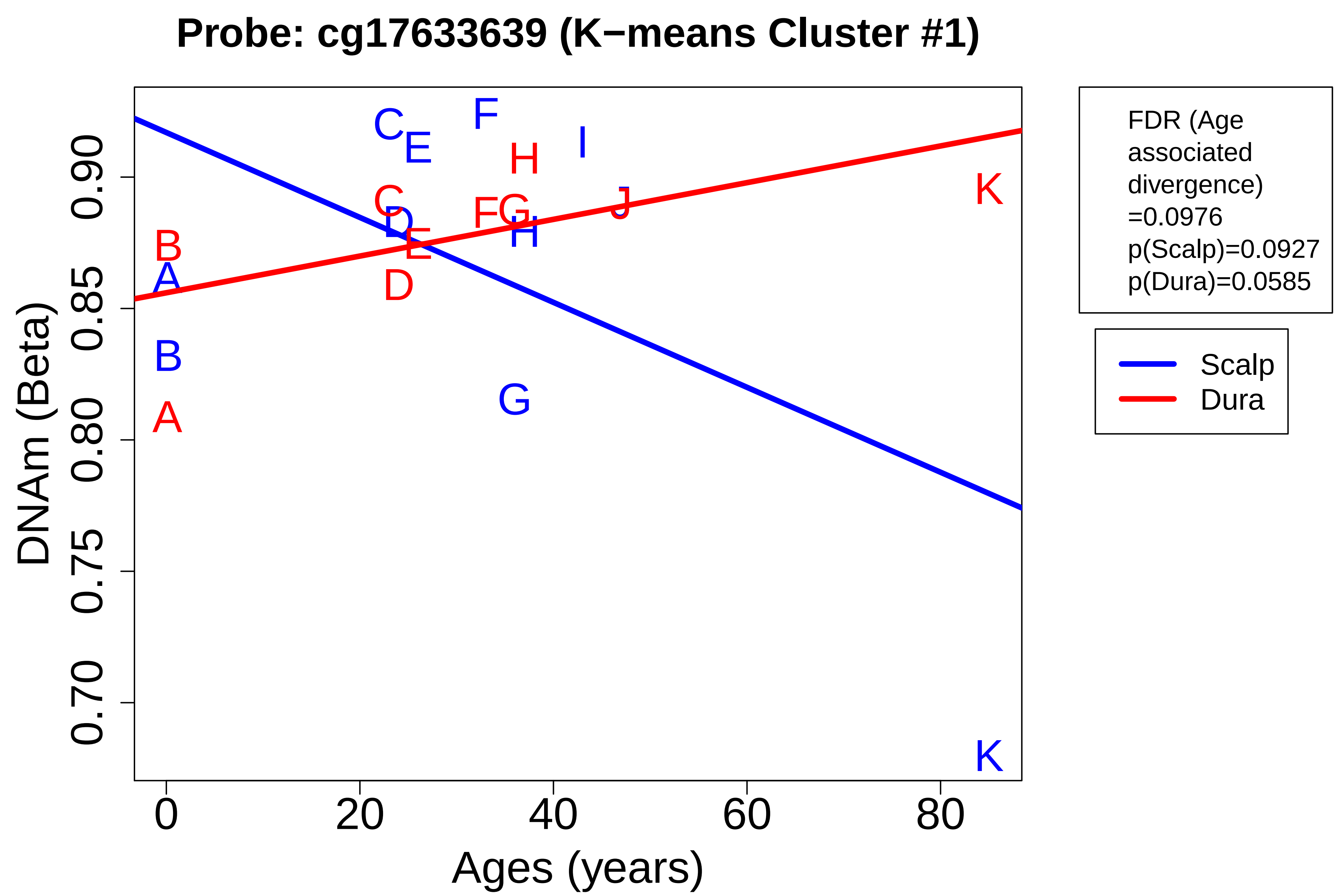

Probe: cg17633639 (K−means Cluster #1)
DNAm (Beta)
0.70
0.75
0.80
0.85
0.90
0
20
40
60
80
Ages (years)
A
B
C
D
E
F
G
H
I
J
K
FDR (Age associated divergence) =0.0976
p(Scalp)=0.0927
p(Dura)=0.0585
Scalp
Dura

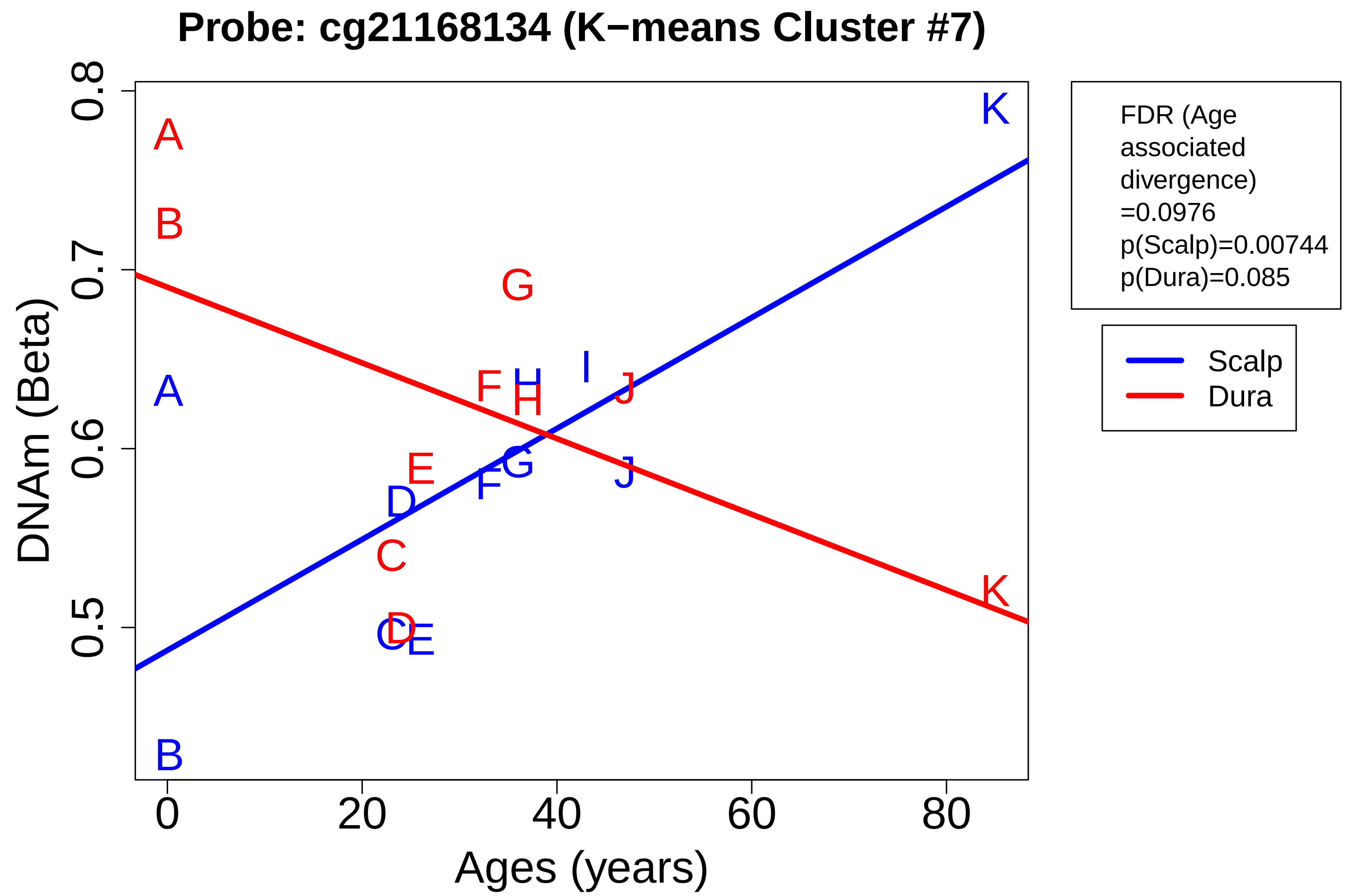

Probe: cg21168134 (K−means Cluster #7)
DNAm (Beta)
0.5
0.6
0.7
0.8
0
20
40
60
80
Ages (years)
A
B
C
D
E
F
G
H
I
J
K
FDR (Age associated divergence) =0.0976
p(Scalp)=0.00744
p(Dura)=0.085
Scalp
Dura

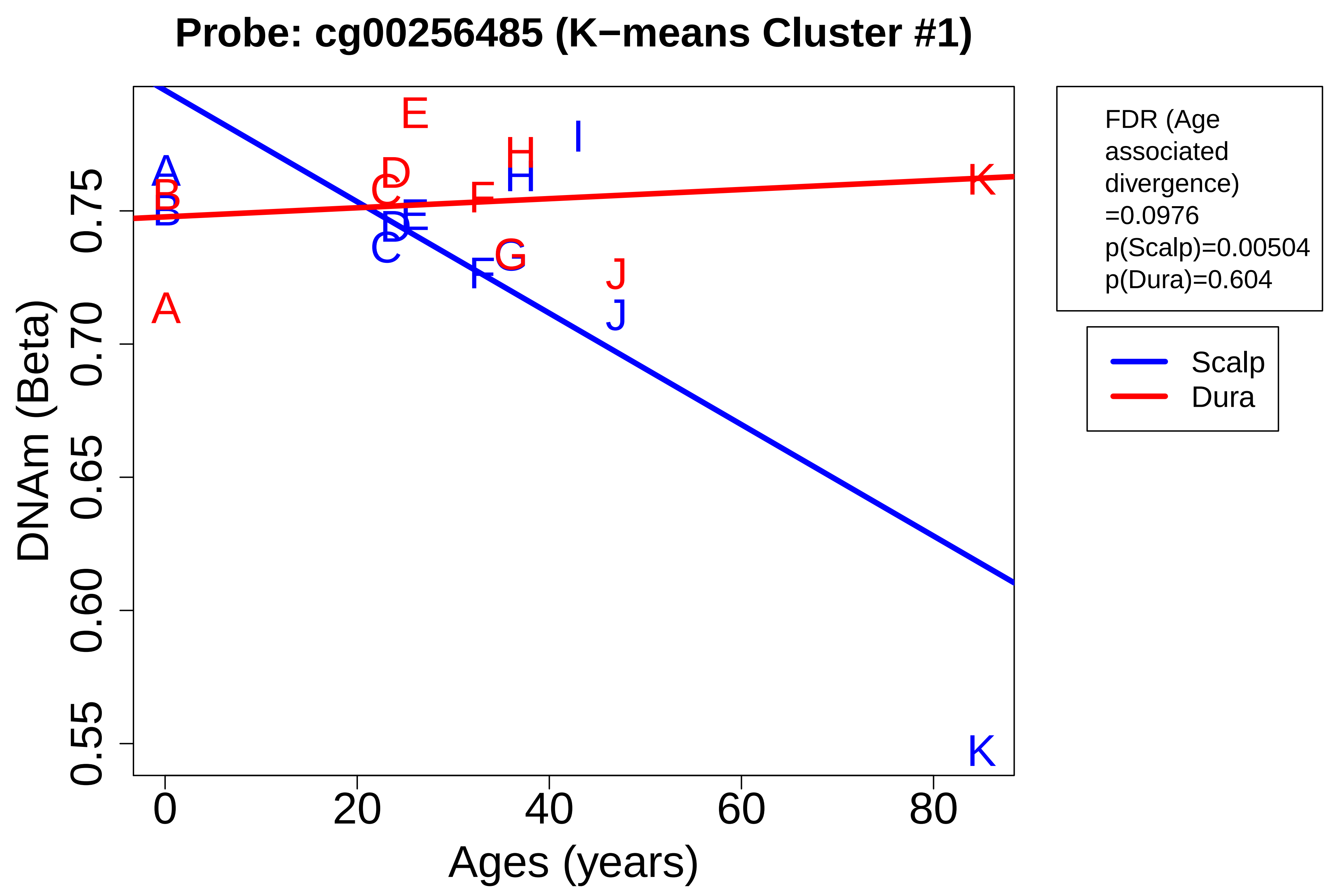

Probe: cg00256485 (K−means Cluster #1)
DNAm (Beta)
0.55
0.60
0.65
0.70
0.75
0
20
40
60
80
Ages (years)
FDR (Age associated divergence) =0.0976
p(Scalp)=0.00504
p(Dura)=0.604
Scalp
Dura
A
B
C
D
E
F
G
H
I
J
K

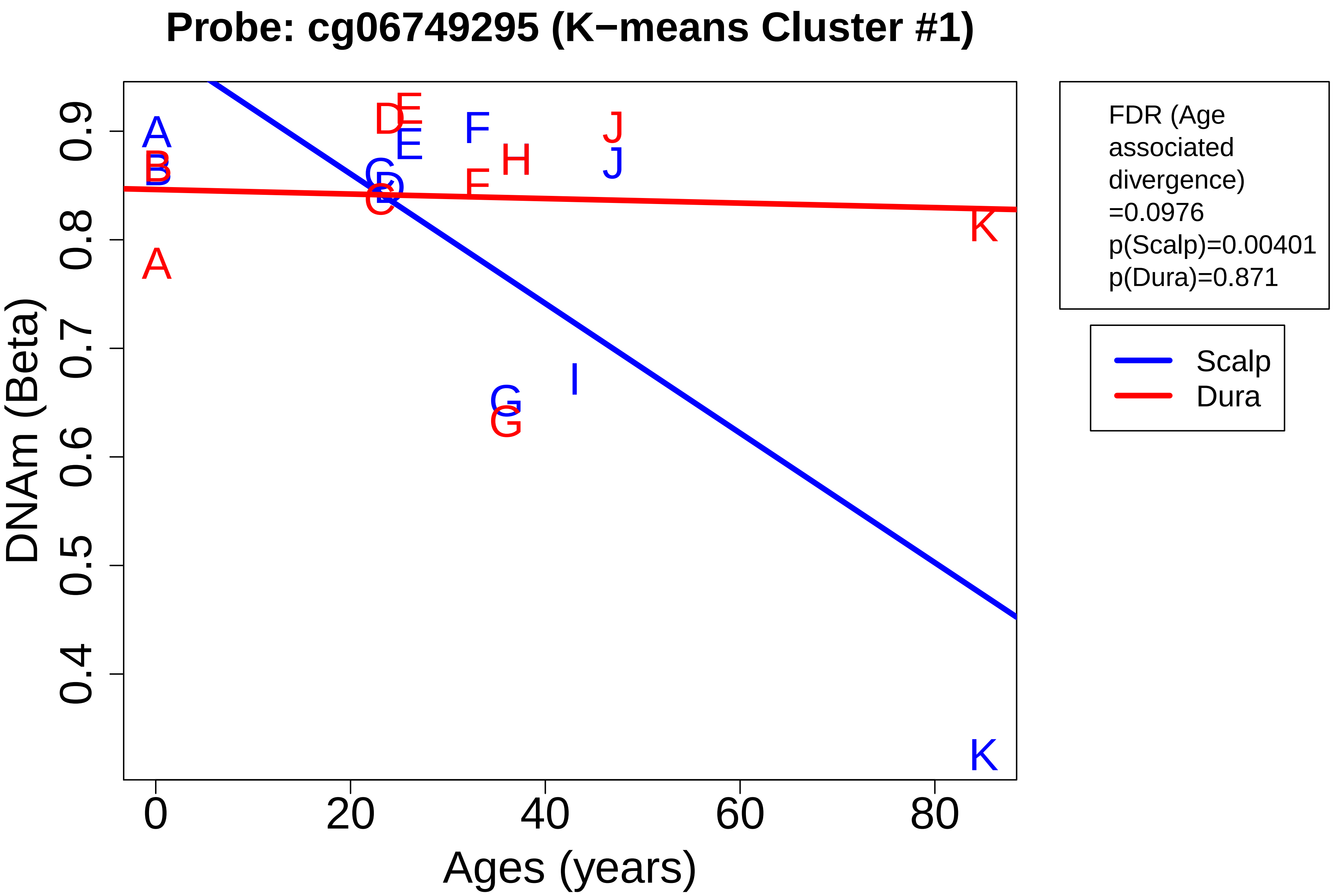

Probe: cg06749295 (K−means Cluster #1)
DNAm (Beta)
0.4
0.5
0.6
0.7
0.8
0.9
0
20
40
60
80
Ages (years)
FDR (Age associated divergence) =0.0976
p(Scalp)=0.00401
p(Dura)=0.871
Scalp
Dura
A
B
C
D
E
F
G
H
I
J
K

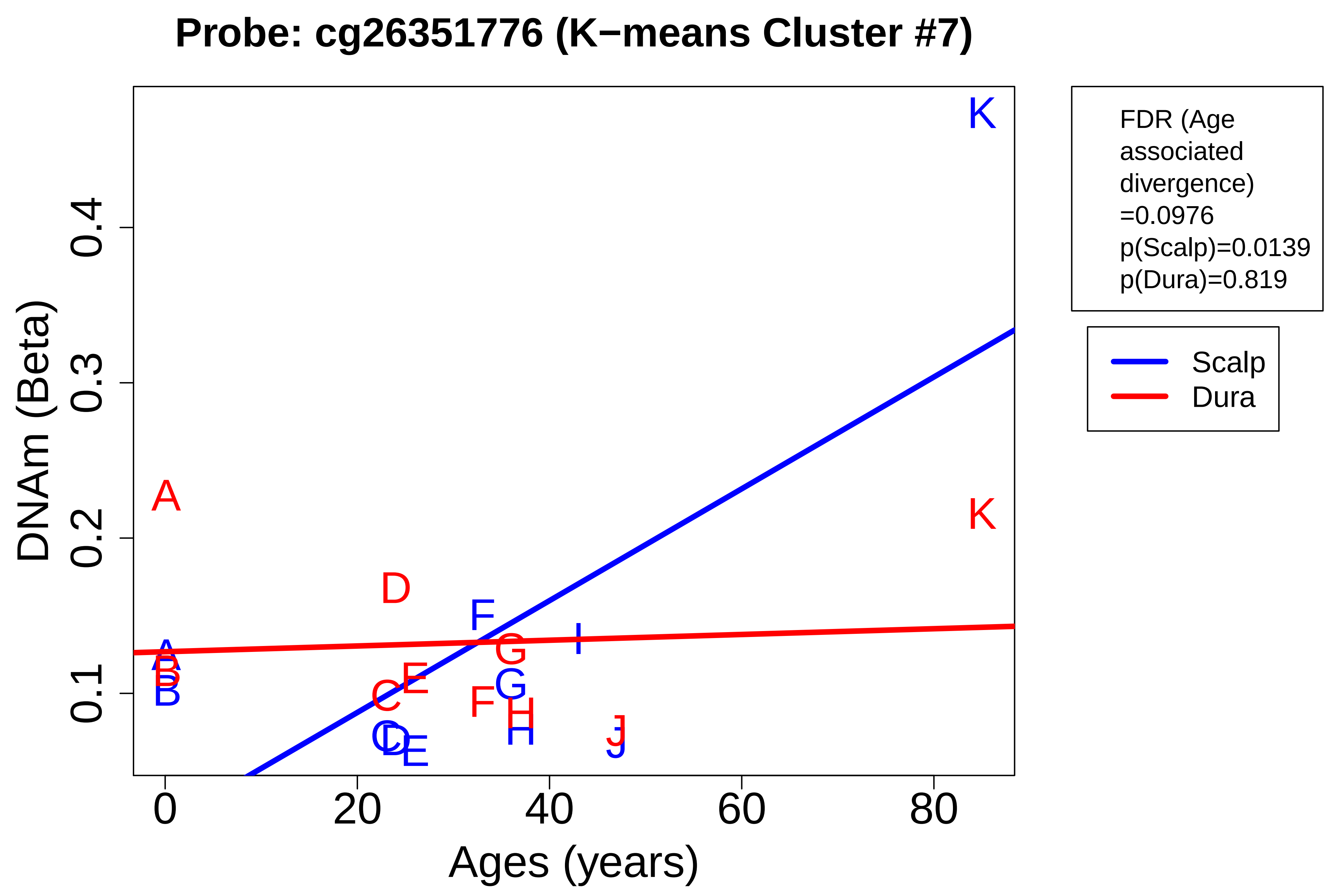

Probe: cg26351776 (K−means Cluster #7)
DNAm (Beta)
0.1
0.2
0.3
0.4
0
20
40
60
80
Ages (years)
A
B
C
D
E
F
G
H
I
J
K
FDR (Age associated divergence) =0.0976
p(Scalp)=0.0139
p(Dura)=0.819
Scalp
Dura

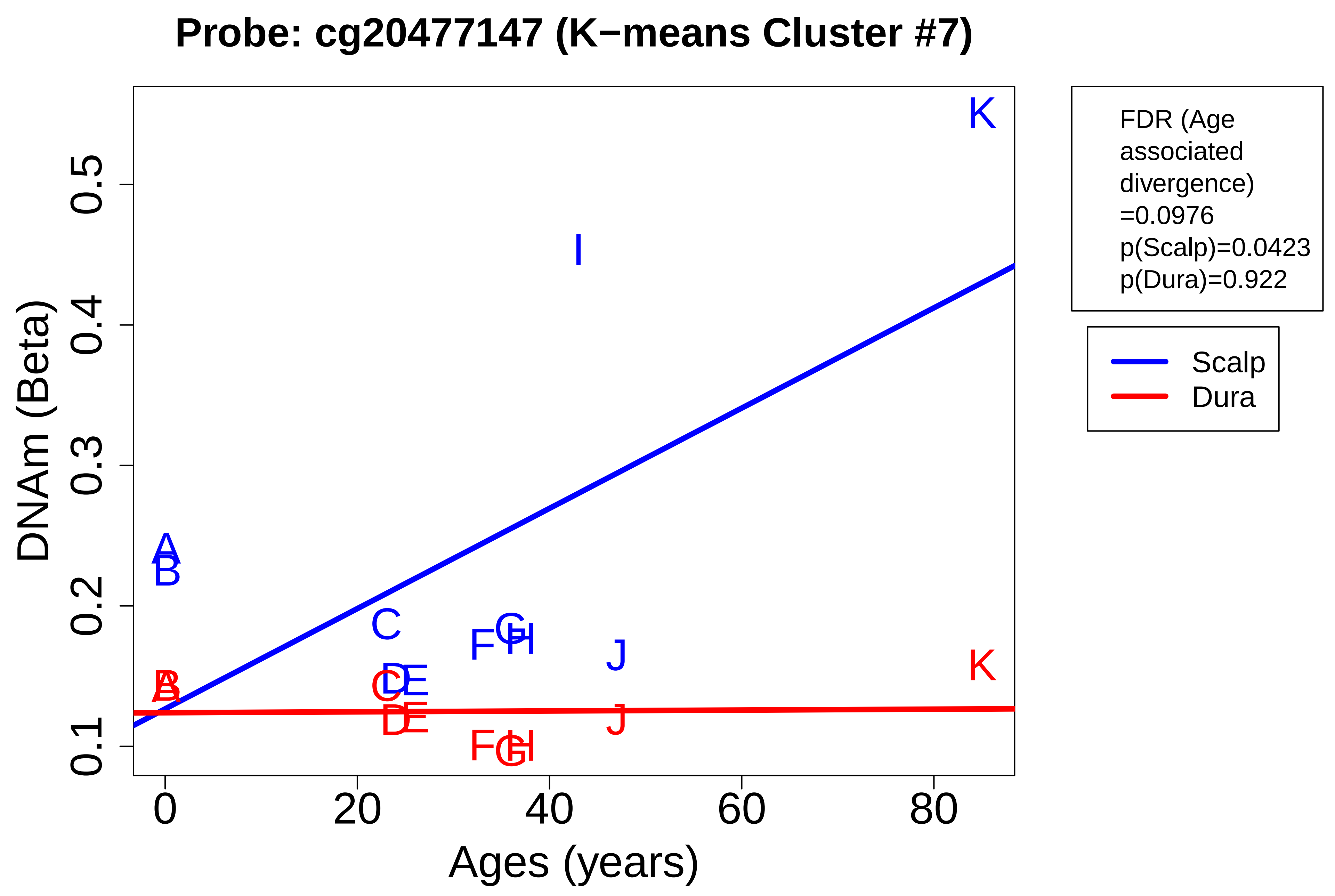

Probe: cg20477147 (K−means Cluster #7)
DNAm (Beta)
0.1
0.2
0.3
0.4
0.5
Ages (years)
0
20
40
60
80
A
B
C
D
E
F
G
H
I
J
K
FDR (Age associated divergence) =0.0976
p(Scalp)=0.0423
p(Dura)=0.922
Scalp
Dura

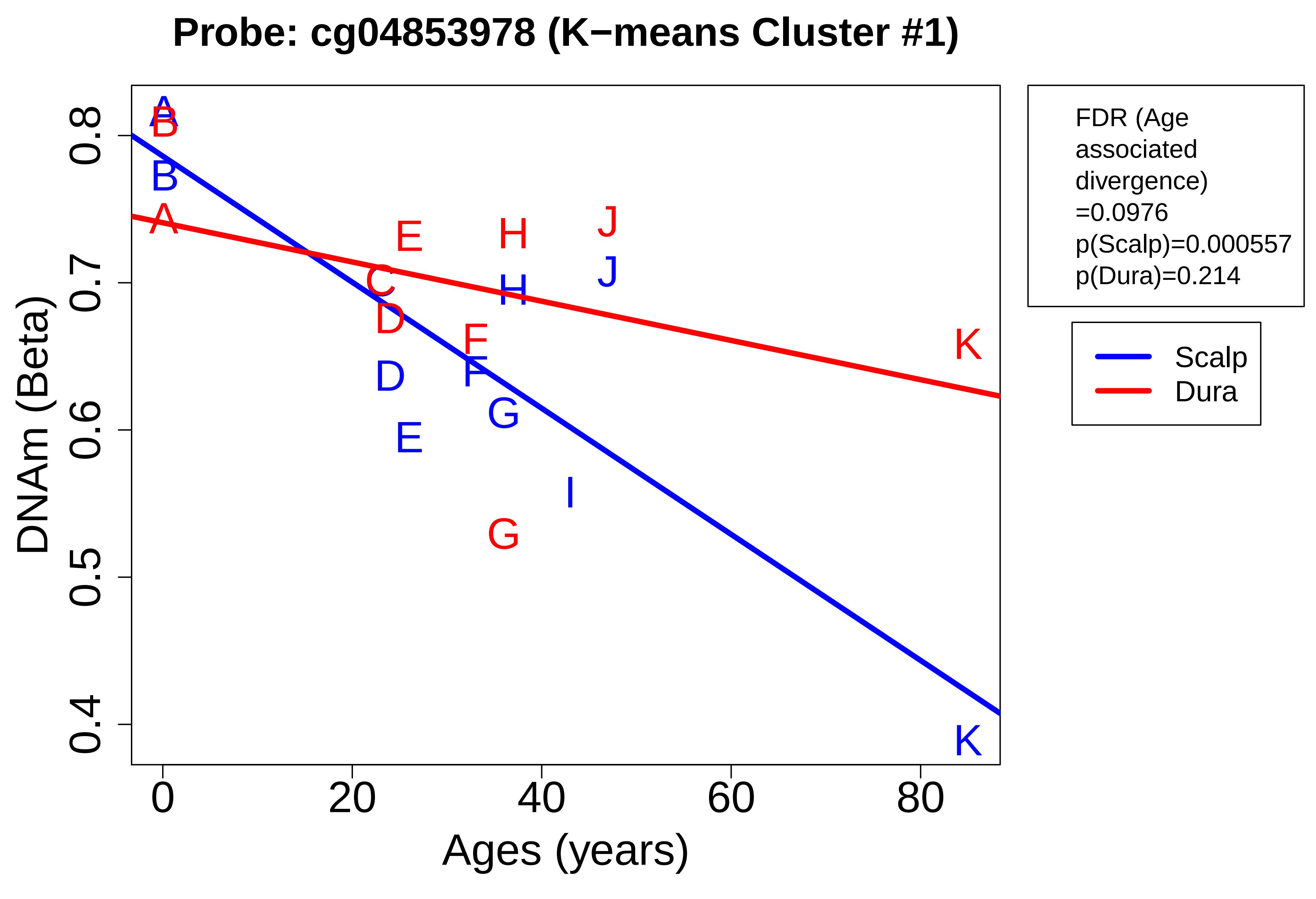

Probe: cg04853978 (K−means Cluster #1)
DNAm (Beta)
0.4
0.5
0.6
0.7
0.8
Ages (years)
0
20
40
60
80
FDR (Age associated divergence) =0.0976
p(Scalp)=0.000557
p(Dura)=0.214
Scalp
Dura
A
B
C
D
E
F
G
H
I
J
K

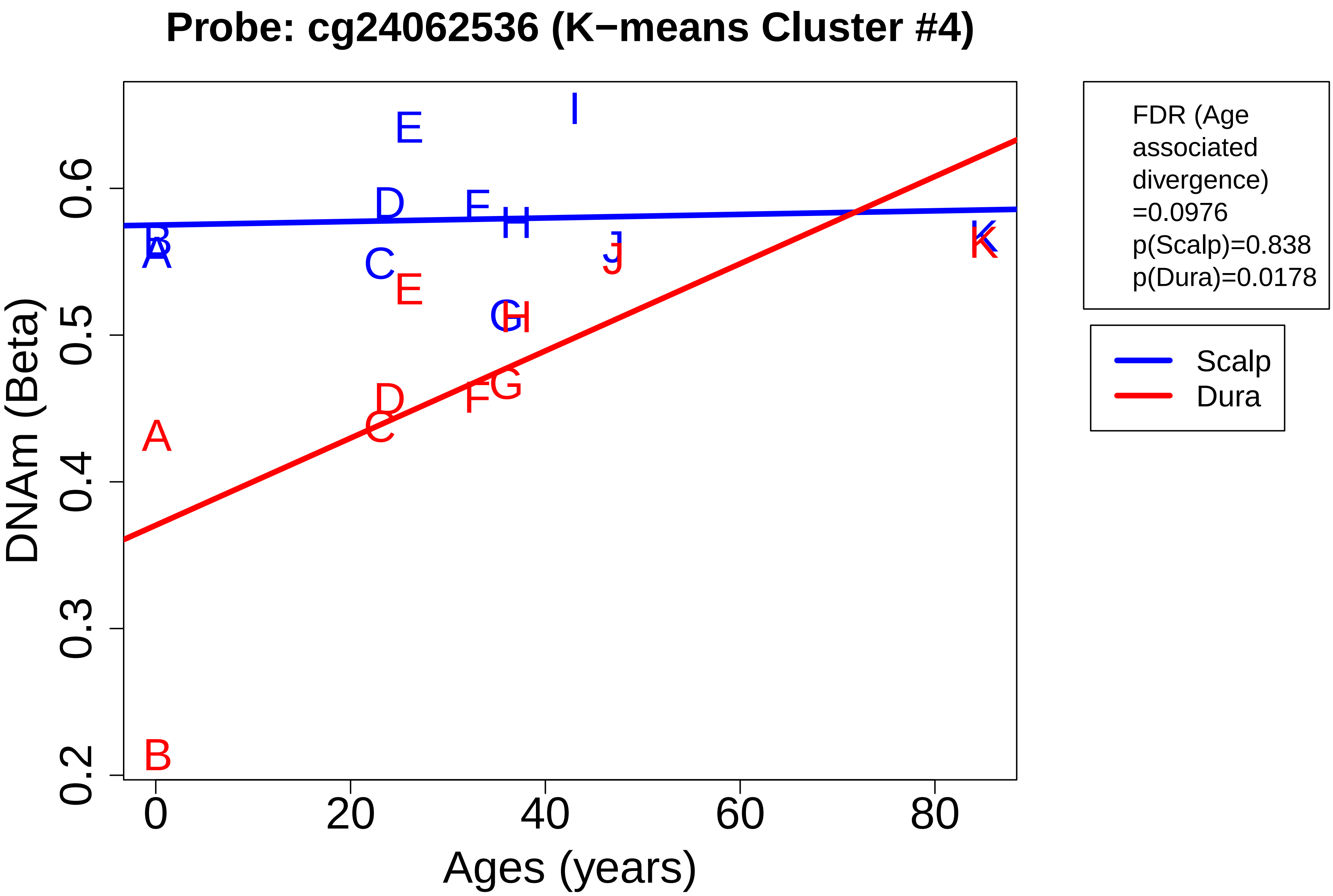
Probe: cg24062536 (K−means Cluster #4)
DNAm (Beta)
0.2
0.3
0.4
0.5
0.6
0
20
40
60
80
Ages (years)
FDR (Age associated divergence) =0.0976
p(Scalp)=0.838
p(Dura)=0.0178
Scalp
Dura

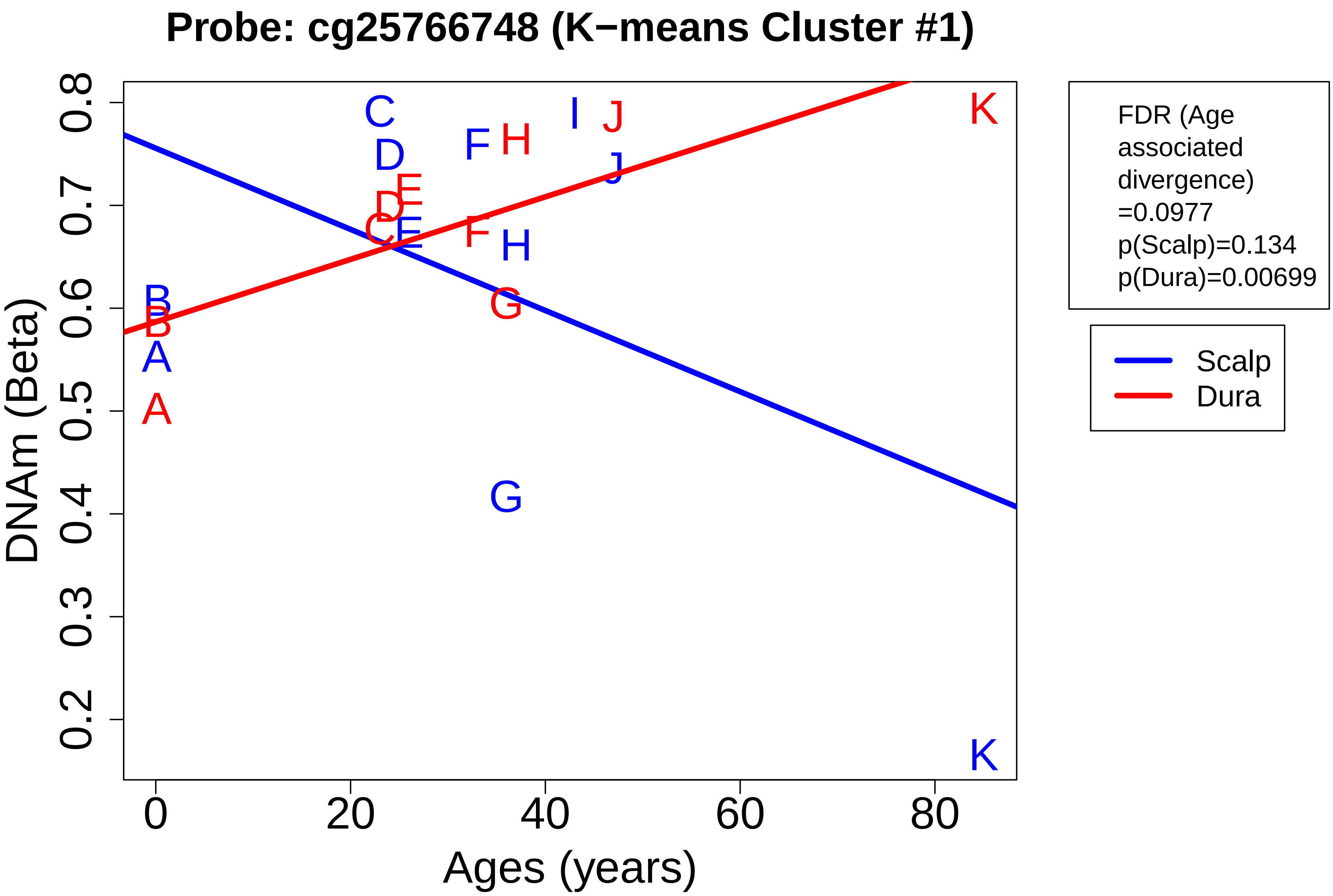

Probe: cg25766748 (K−means Cluster #1)
DNAm (Beta)
0.2
0.3
0.4
0.5
0.6
0.7
0.8
0
20
40
60
80
Ages (years)
FDR (Age associated divergence) =0.0977
p(Scalp)=0.134
p(Dura)=0.00699
Scalp
Dura

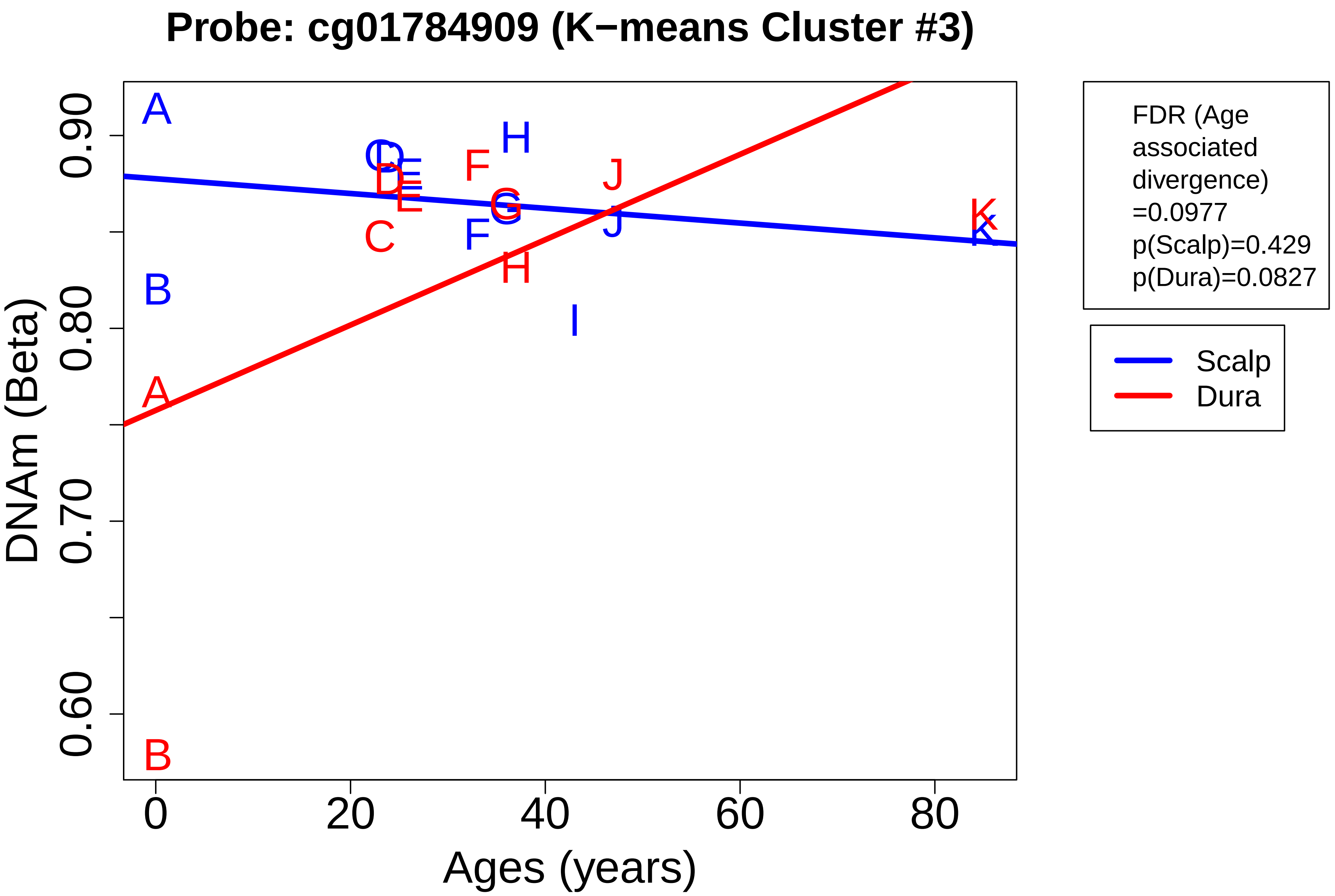

Probe: cg01784909 (K−means Cluster #3)
DNAm (Beta)
0.60
0.70
0.80
0.90
0
20
40
60
80
Ages (years)
FDR (Age associated divergence) =0.0977
p(Scalp)=0.429
p(Dura)=0.0827
Scalp
Dura

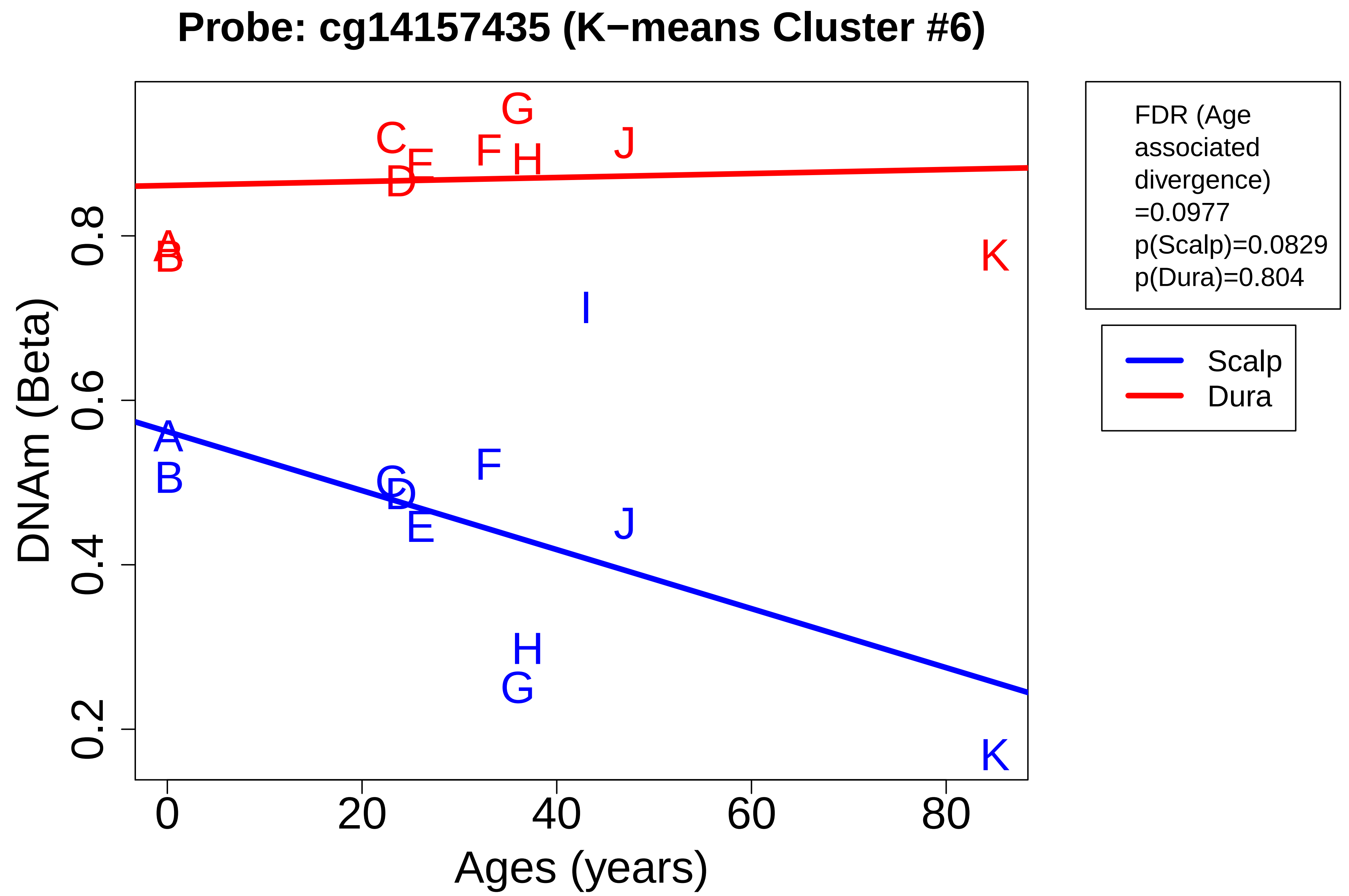

Probe: cg14157435 (K−means Cluster #6)
DNAm (Beta)
0.2
0.4
0.6
0.8
0
20
40
60
80
Ages (years)
FDR (Age associated divergence) =0.0977
p(Scalp)=0.0829
p(Dura)=0.804
Scalp
Dura
A
B
C
D
E
F
G
H
I
J
K

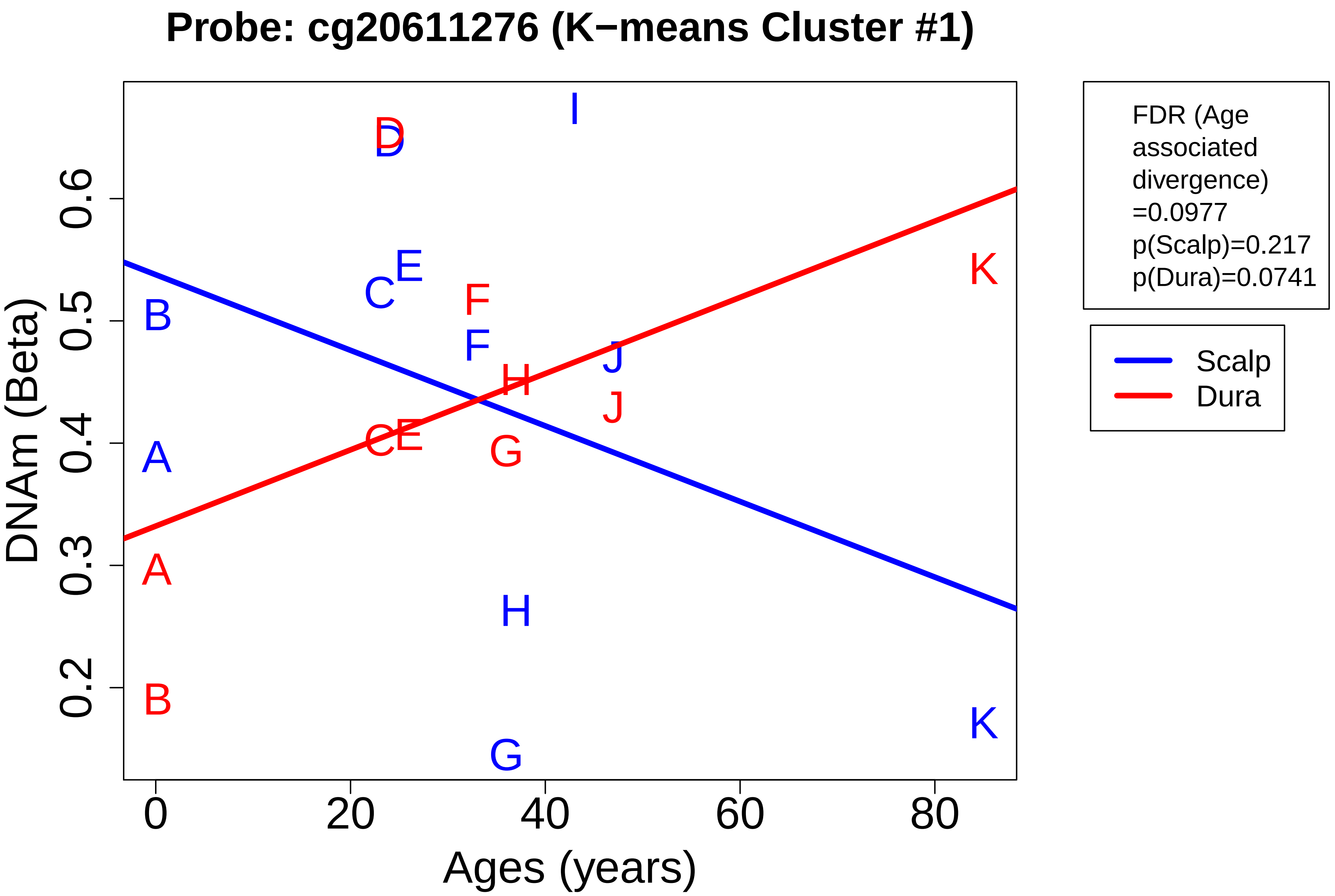
Probe: cg20611276 (K−means Cluster #1)
DNAm (Beta)
0.2
0.3
0.4
0.5
0.6
Ages (years)
0
20
40
60
80
FDR (Age associated divergence) =0.0977
p(Scalp)=0.217
p(Dura)=0.0741
Scalp
Dura
A
B
C
D
E
F
G
H
I
J
K

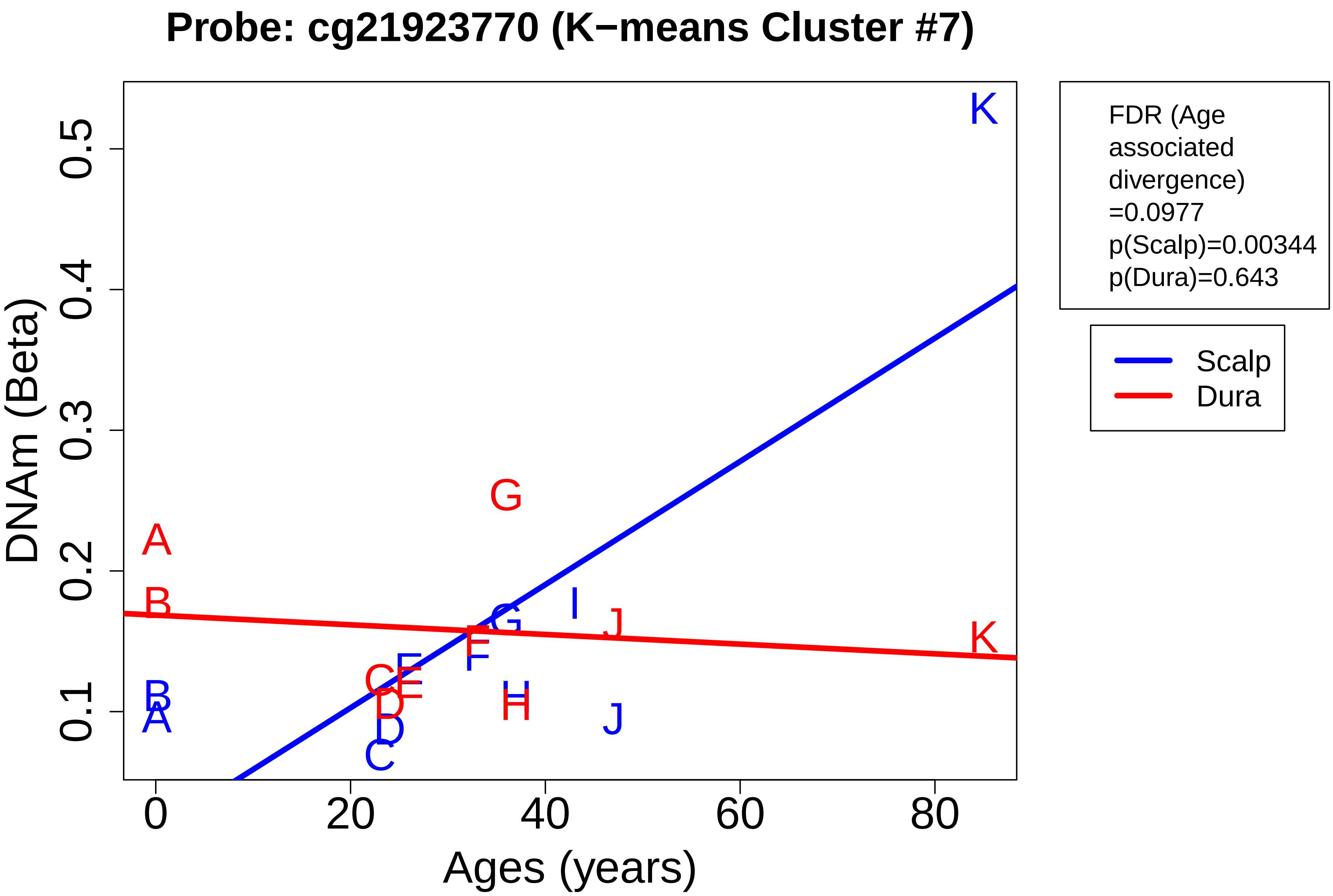

Probe: cg21923770 (K−means Cluster #7)
DNAm (Beta)
0.1
0.2
0.3
0.4
0.5
0
20
40
60
80
Ages (years)
A
B
C
D
E
F
G
H
I
J
K
FDR (Age associated divergence) =0.0977
p(Scalp)=0.00344
p(Dura)=0.643
Scalp
Dura

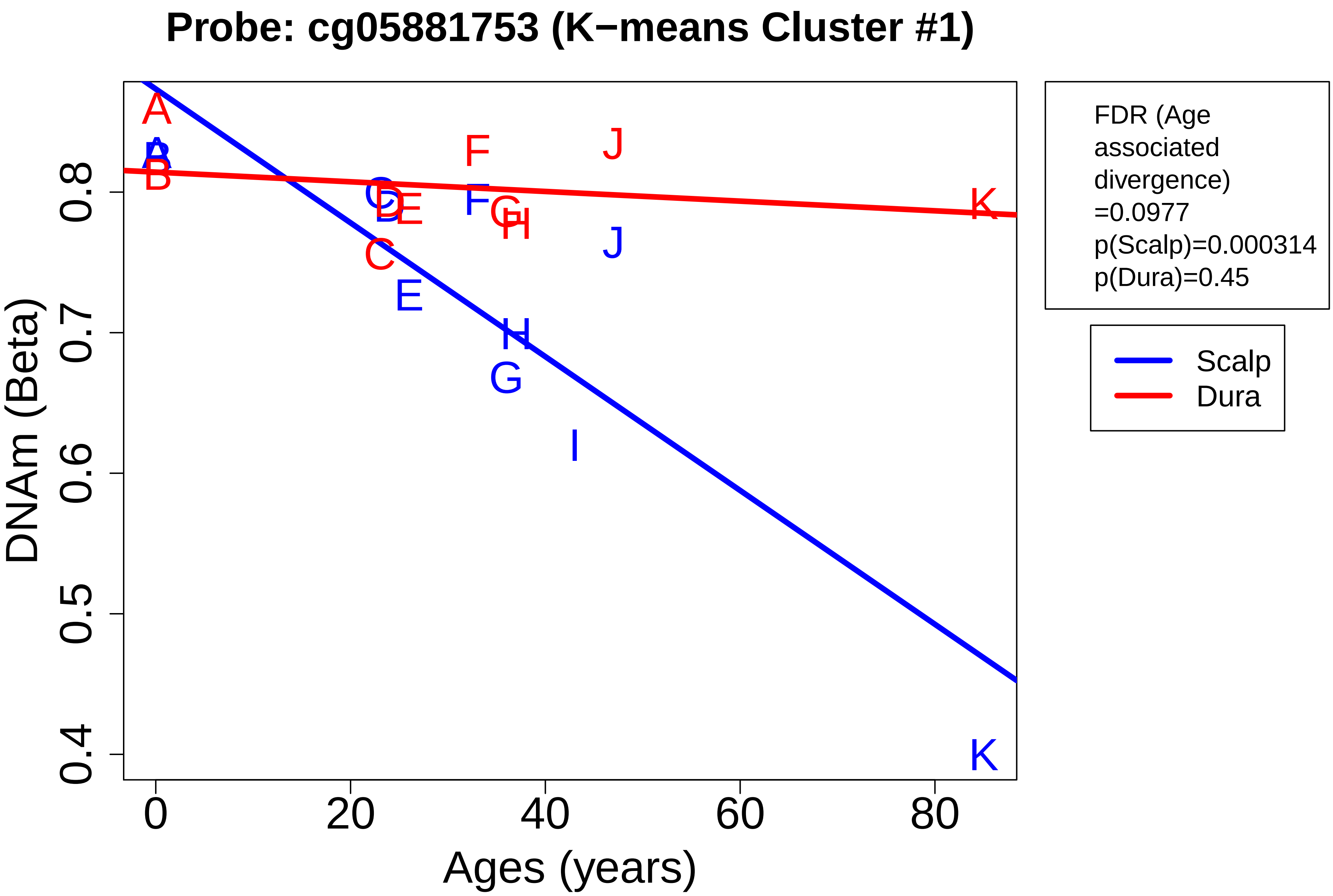

Probe: cg05881753 (K−means Cluster #1)
FDR (Age associated divergence) =0.0977
p(Scalp)=0.000314
p(Dura)=0.45
Scalp
Dura
DNAm (Beta)
Ages (years)
0.4
0.5
0.6
0.7
0.8
0
20
40
60
80

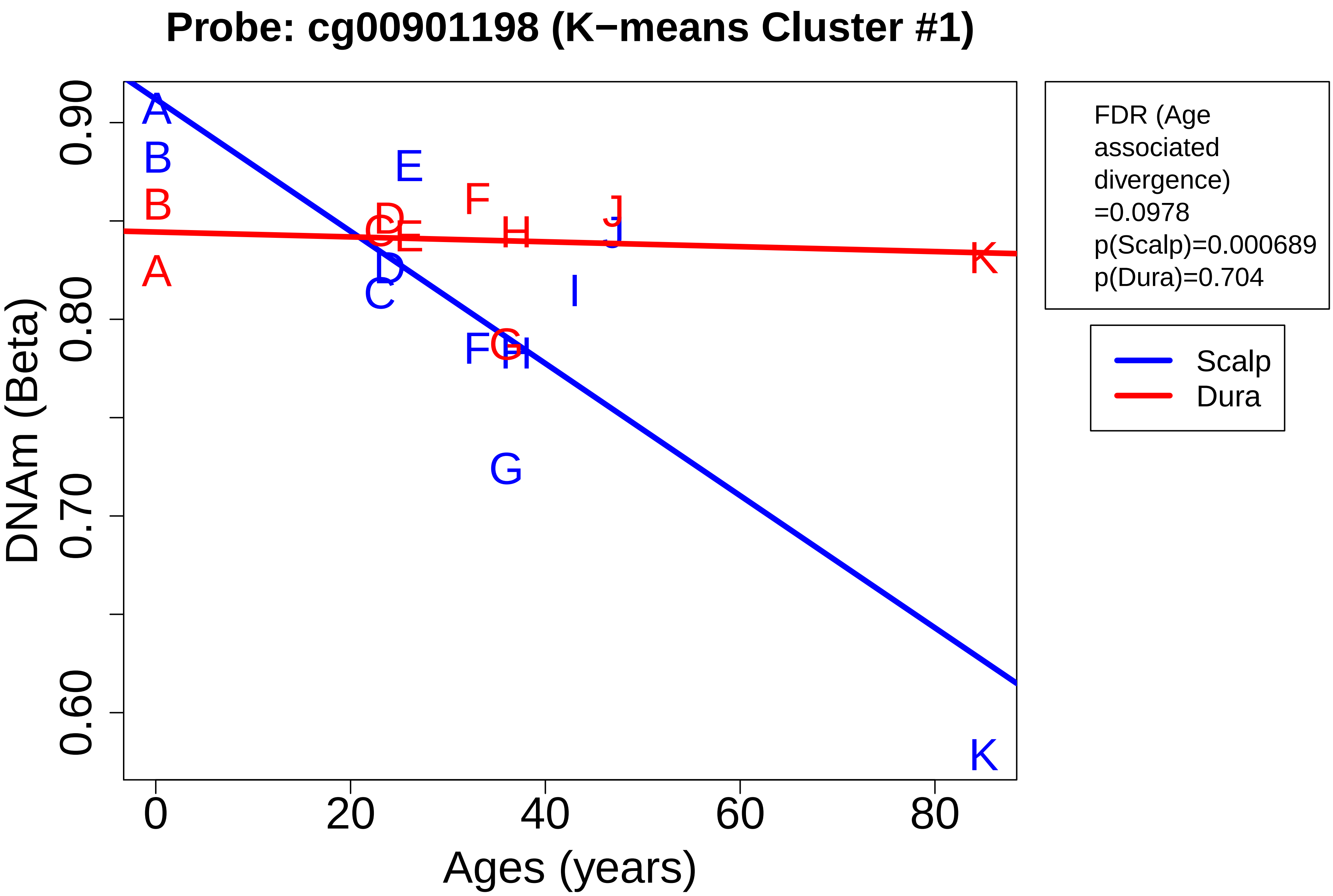

Probe: cg00901198 (K−means Cluster #1)
DNAm (Beta)
0.60
0.70
0.80
0.90
Ages (years)
0
20
40
60
80
FDR (Age associated divergence) =0.0978
p(Scalp)=0.000689
p(Dura)=0.704
Scalp
Dura

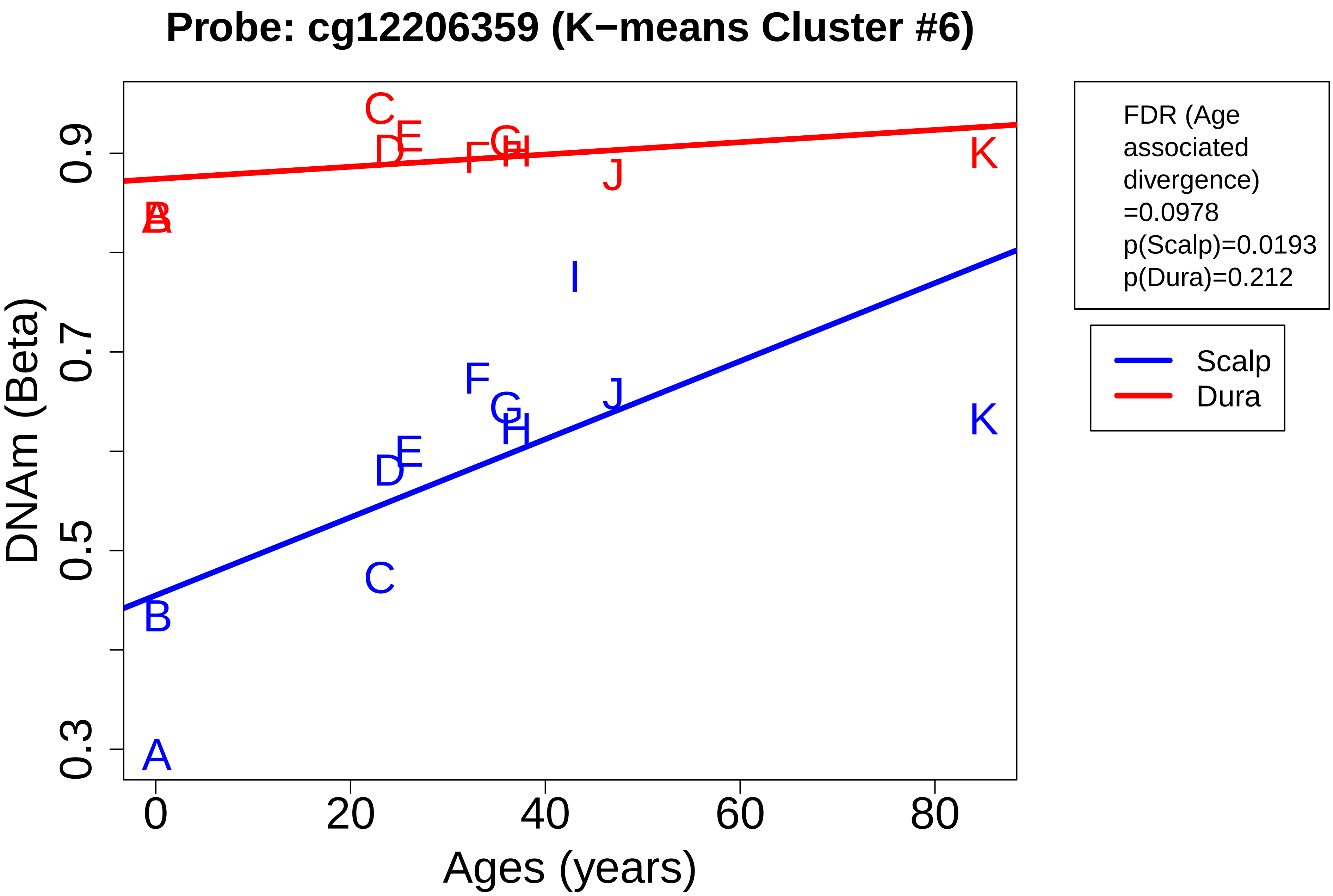

Probe: cg12206359 (K−means Cluster #6)
DNAm (Beta)
Ages (years)
0.3
0.5
0.7
0.9
0
20
40
60
80
FDR (Age associated divergence) =0.0978
p(Scalp)=0.0193
p(Dura)=0.212
Scalp
Dura

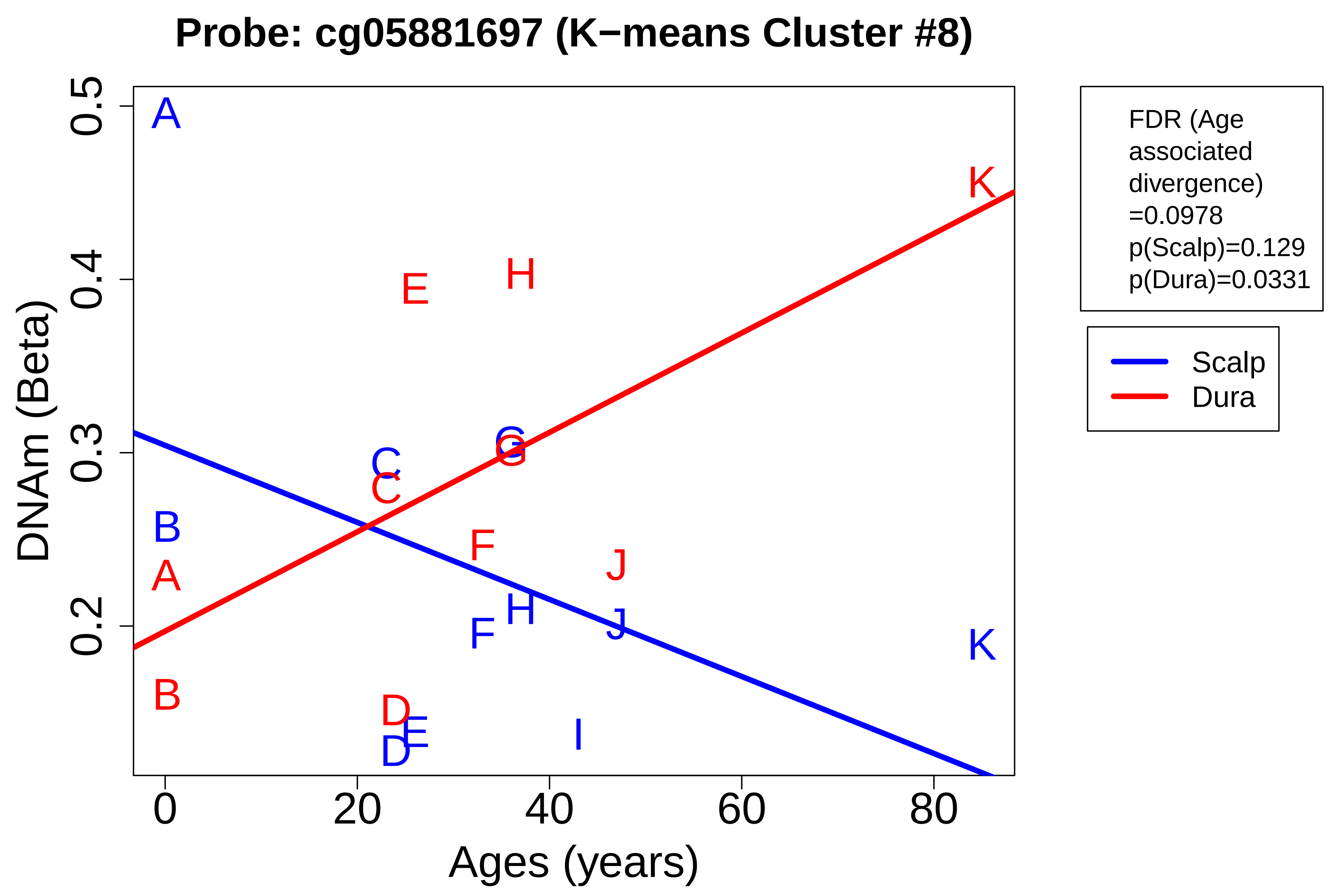

Probe: cg05881697 (K−means Cluster #8)
DNAm (Beta)
0.2
0.3
0.4
0.5
0
20
40
60
80
Ages (years)
FDR (Age associated divergence) =0.0978
p(Scalp)=0.129
p(Dura)=0.0331
Scalp
Dura

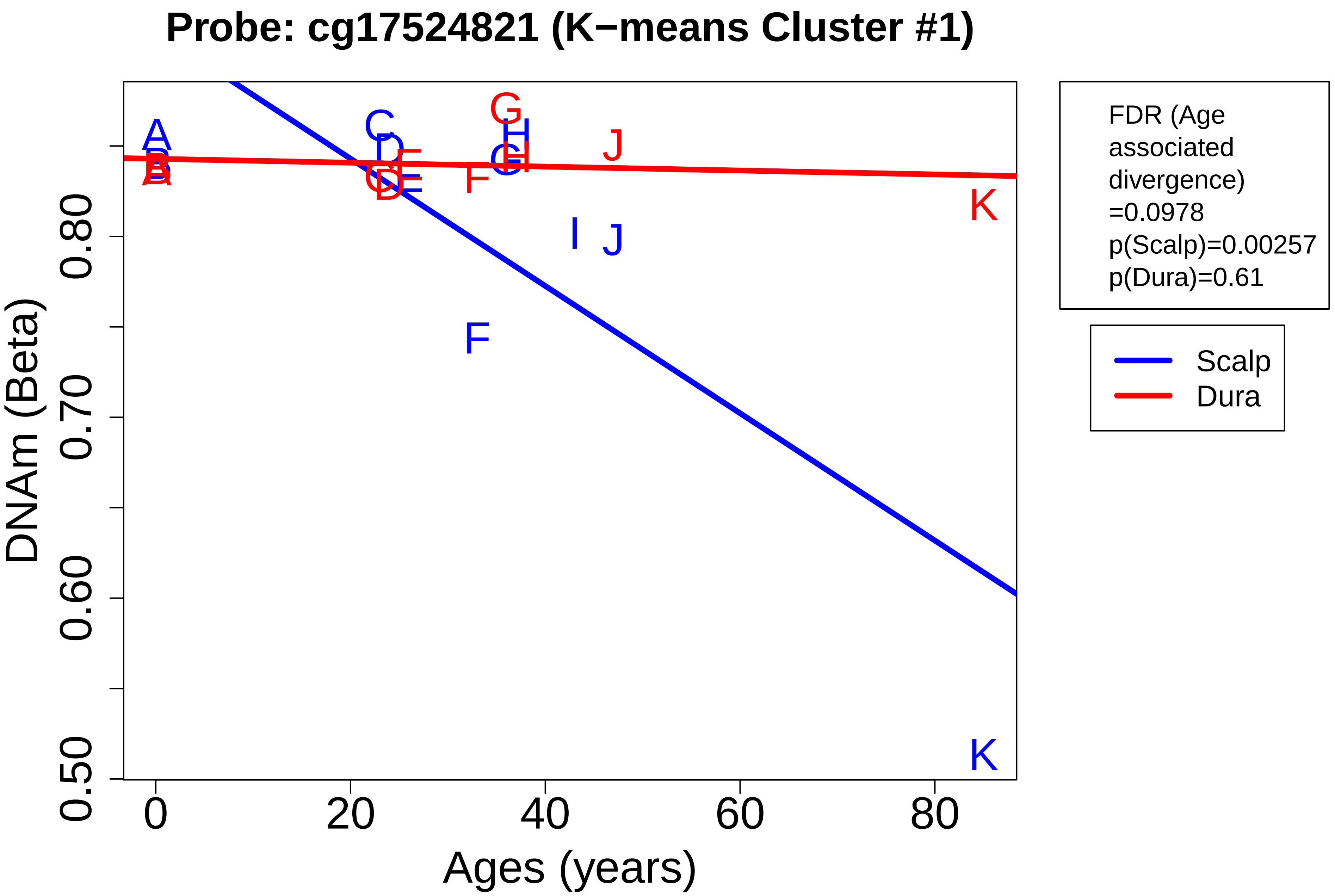
Probe: cg17524821 (K−means Cluster #1)
FDR (Age associated divergence) =0.0978
p(Scalp)=0.00257
p(Dura)=0.61
Scalp
Dura
DNAm (Beta)
0.50
0.60
0.70
0.80
0
20
40
60
80
Ages (years)
A
B
C
D
E
F
G
H
I
J
K

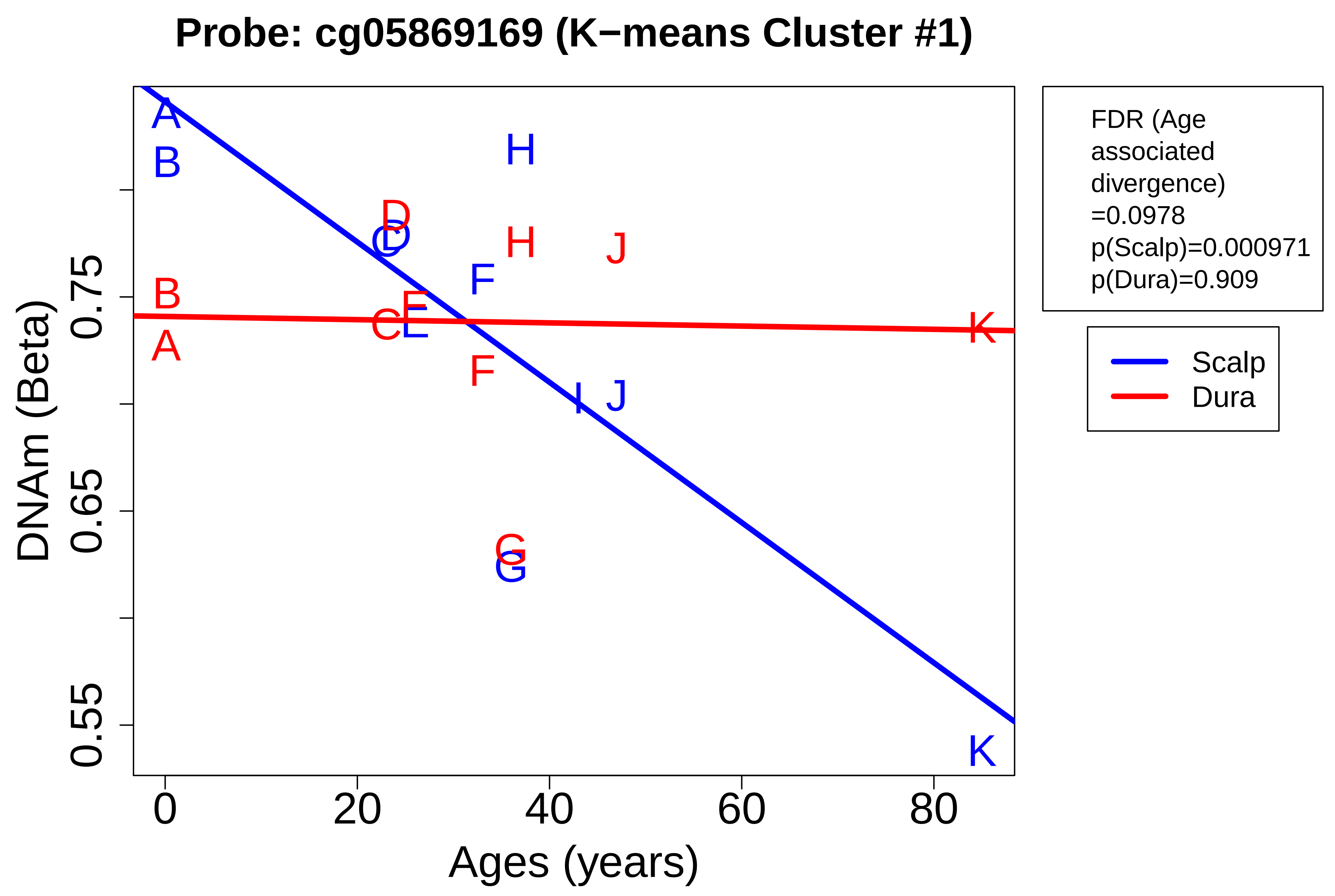

Probe: cg05869169 (K−means Cluster #1)
DNAm (Beta)
0.55
0.65
0.75
Ages (years)
0
20
40
60
80
A
B
C
D
E
F
G
H
I
J
K
FDR (Age associated divergence) =0.0978
p(Scalp)=0.000971
p(Dura)=0.909
Scalp
Dura

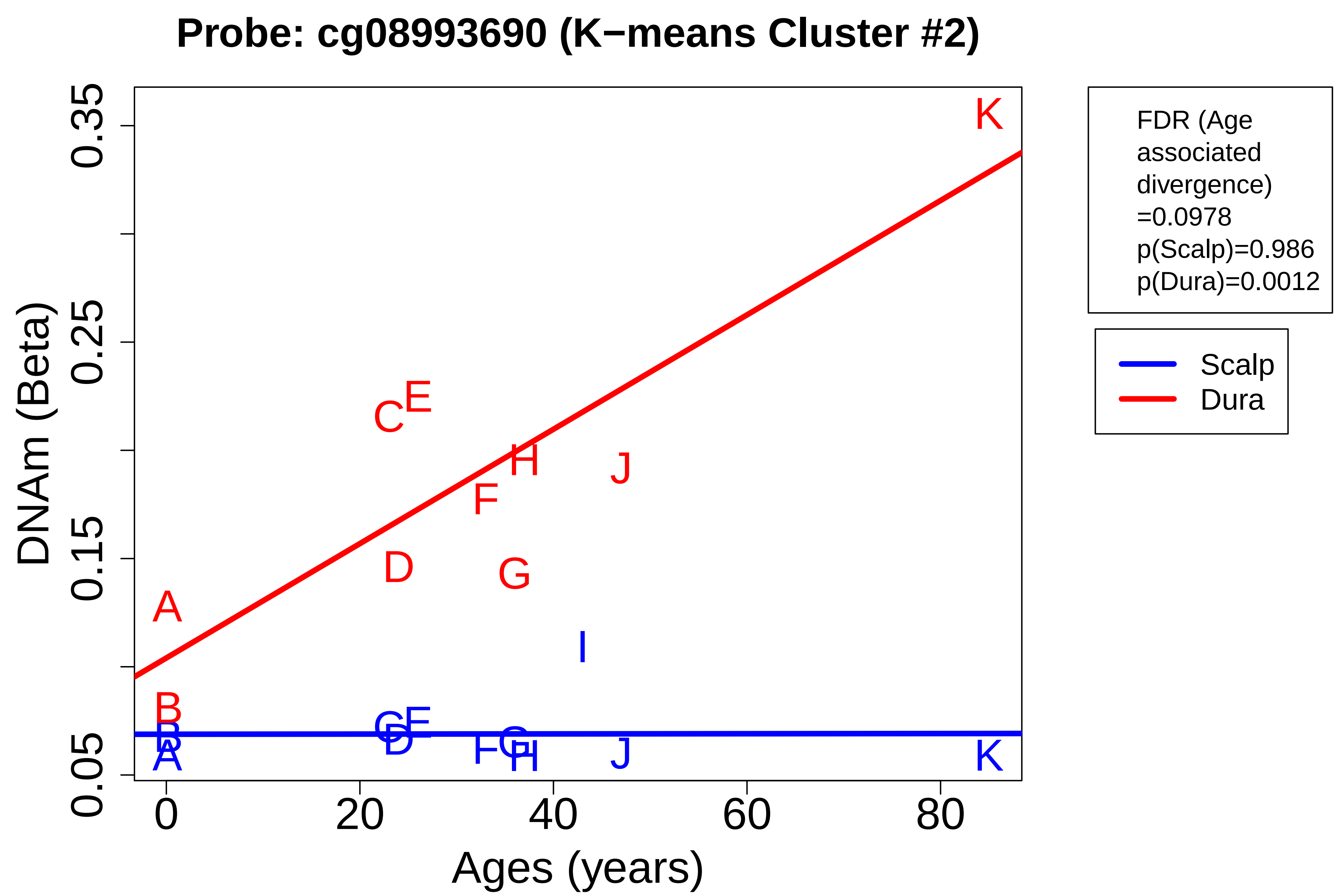

Probe: cg08993690 (K−means Cluster #2)
DNAm (Beta)
0.05
0.15
0.25
0.35
Ages (years)
0
20
40
60
80
FDR (Age associated divergence) =0.0978
p(Scalp)=0.986
p(Dura)=0.0012
Scalp
Dura

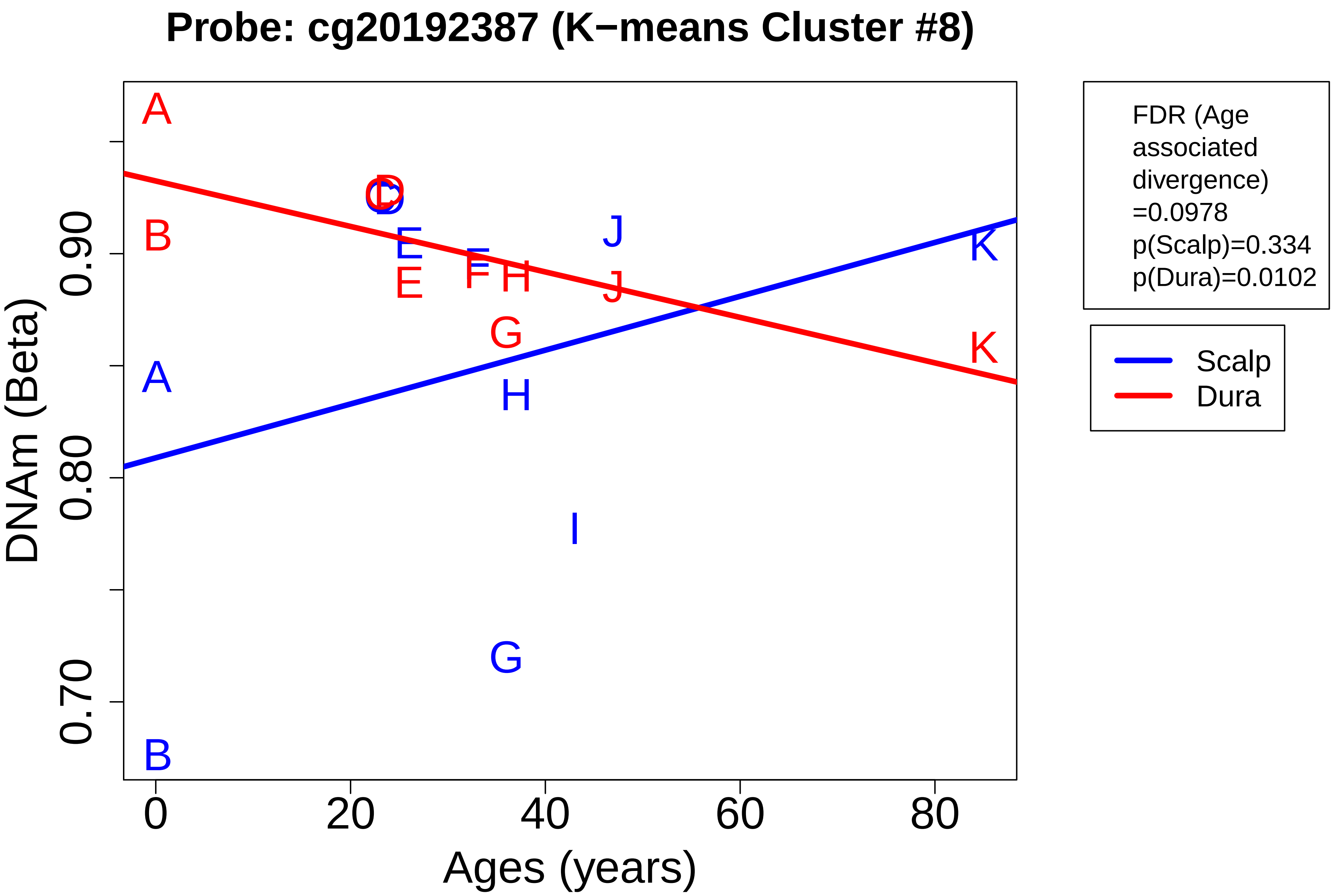

Probe: cg20192387 (K−means Cluster #8)
DNAm (Beta)
0.70
0.80
0.90
Ages (years)
0
20
40
60
80
A
B
C
D
E
F
G
H
I
J
K
FDR (Age associated divergence) =0.0978
p(Scalp)=0.334
p(Dura)=0.0102
Scalp
Dura

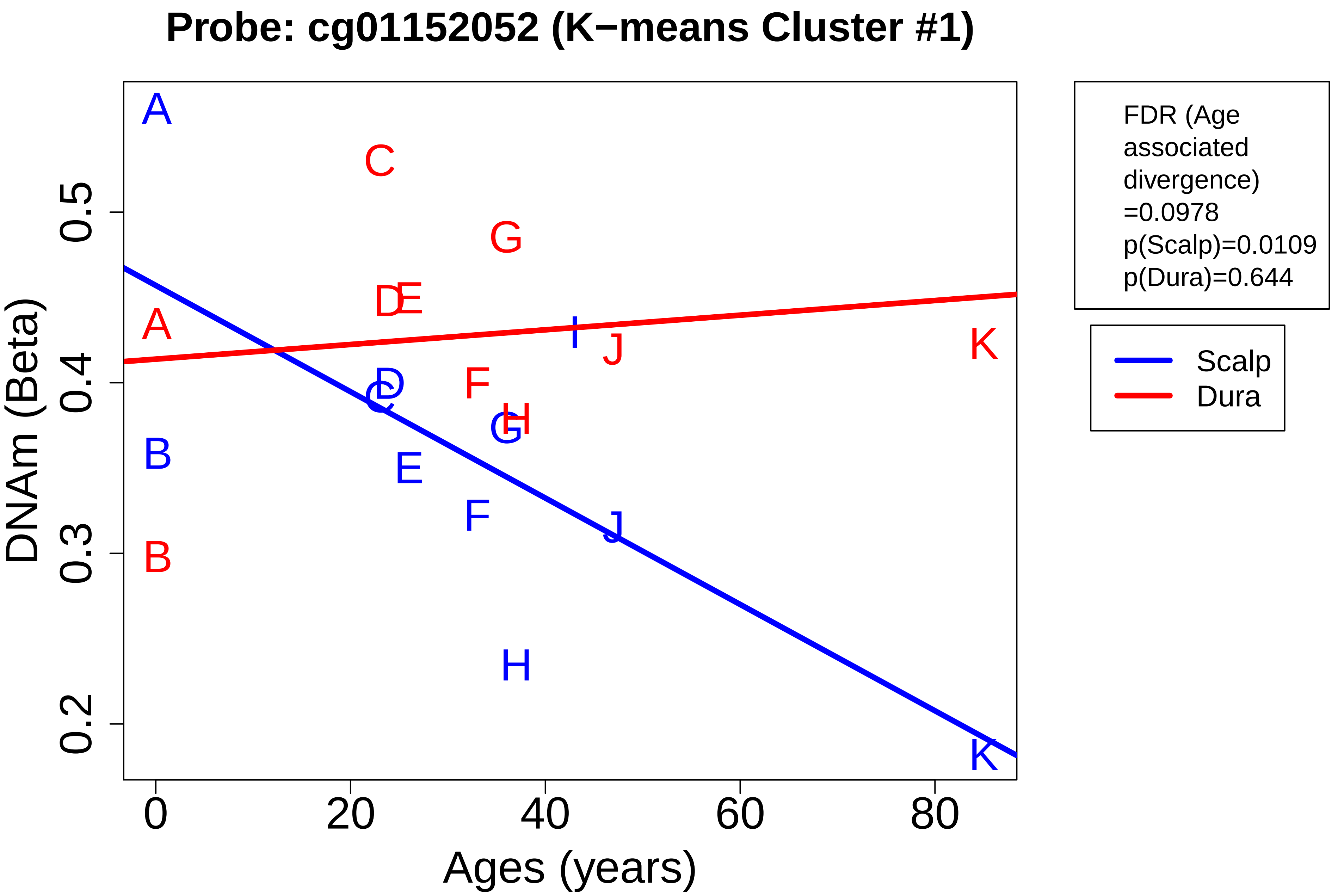

Probe: cg01152052 (K−means Cluster #1)
DNAm (Beta)
0.2
0.3
0.4
0.5
0
20
40
60
80
Ages (years)
FDR (Age associated divergence) =0.0978
p(Scalp)=0.0109
p(Dura)=0.644
Scalp
Dura
A
B
C
D
E
F
G
H
I
J
K

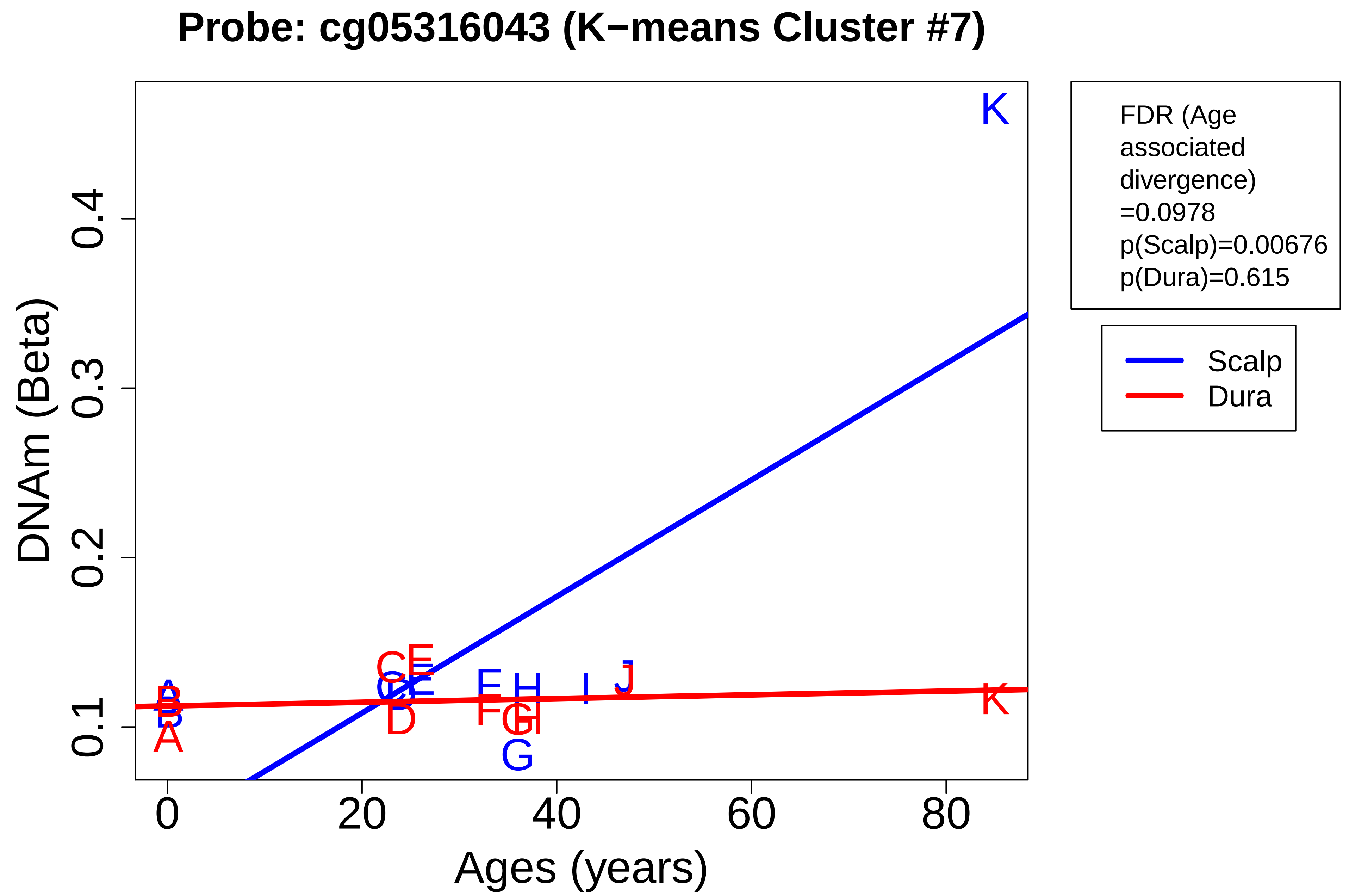
Probe: cg05316043 (K−means Cluster #7)
DNAm (Beta)
0.1
0.2
0.3
0.4
0
20
40
60
80
Ages (years)
FDR (Age associated divergence) =0.0978
p(Scalp)=0.00676
p(Dura)=0.615
Scalp
Dura

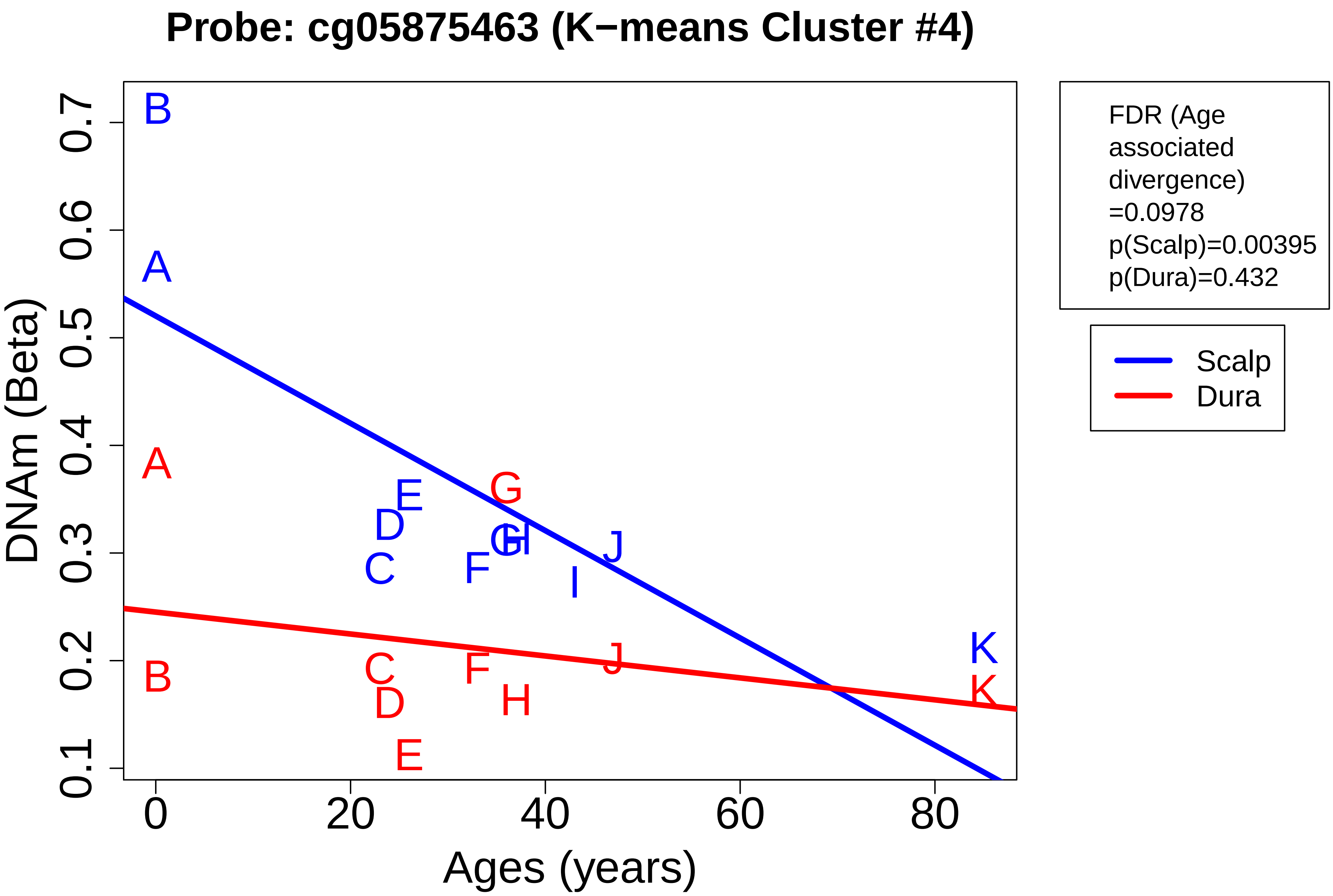

Probe: cg05875463 (K−means Cluster #4)
DNAm (Beta)
0.1
0.2
0.3
0.4
0.5
0.6
0.7
Ages (years)
0
20
40
60
80
B
A
A
E
D
C
G
GH
F
J
I
B
C
D
E
F
H
J
K
K
FDR (Age associated divergence) =0.0978
p(Scalp)=0.00395
p(Dura)=0.432
Scalp
Dura

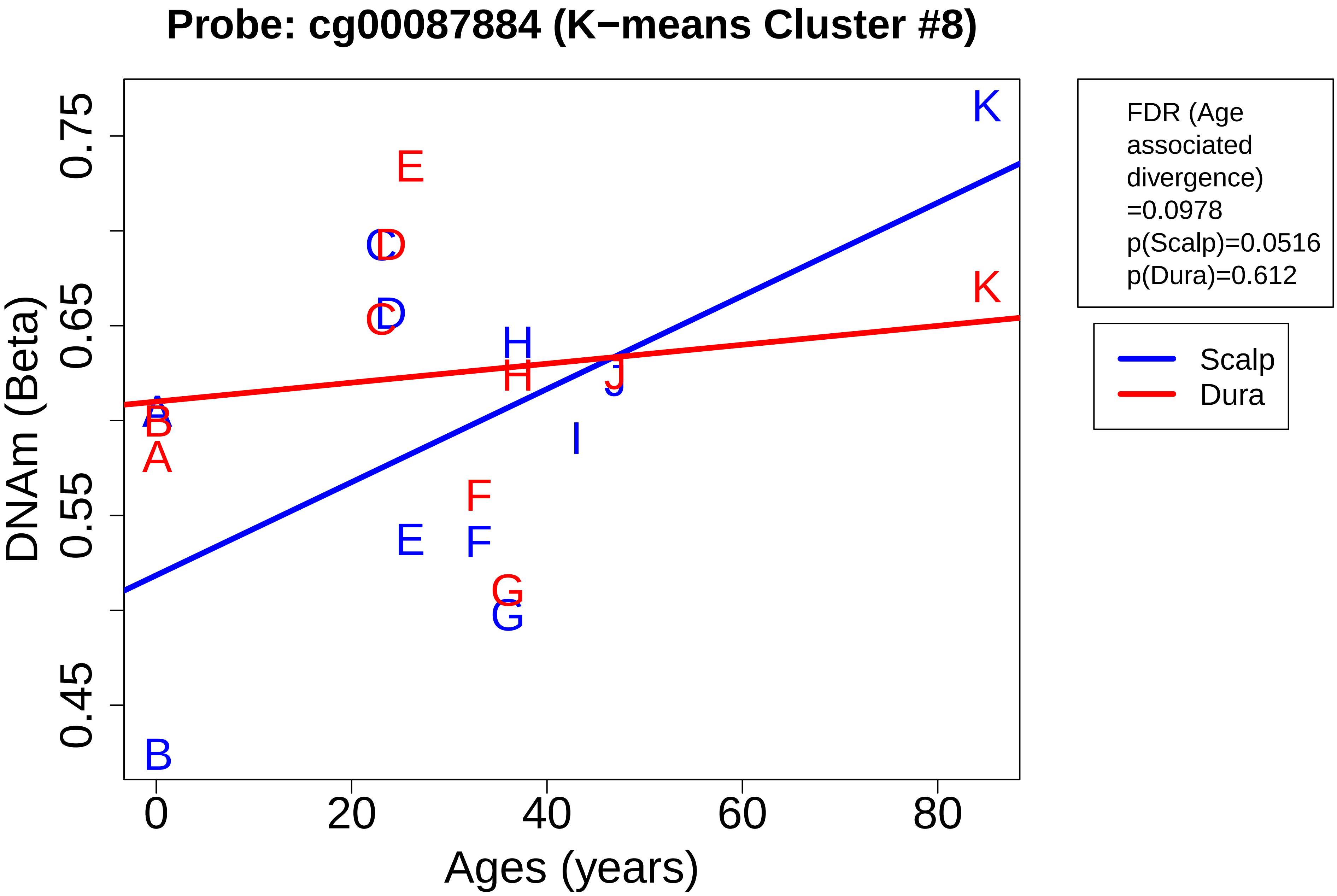
Probe: cg00087884 (K−means Cluster #8)
DNAm (Beta)
Ages (years)
0.45
0.55
0.65
0.75
0
20
40
60
80
FDR (Age associated divergence) =0.0978
p(Scalp)=0.0516
p(Dura)=0.612
Scalp
Dura

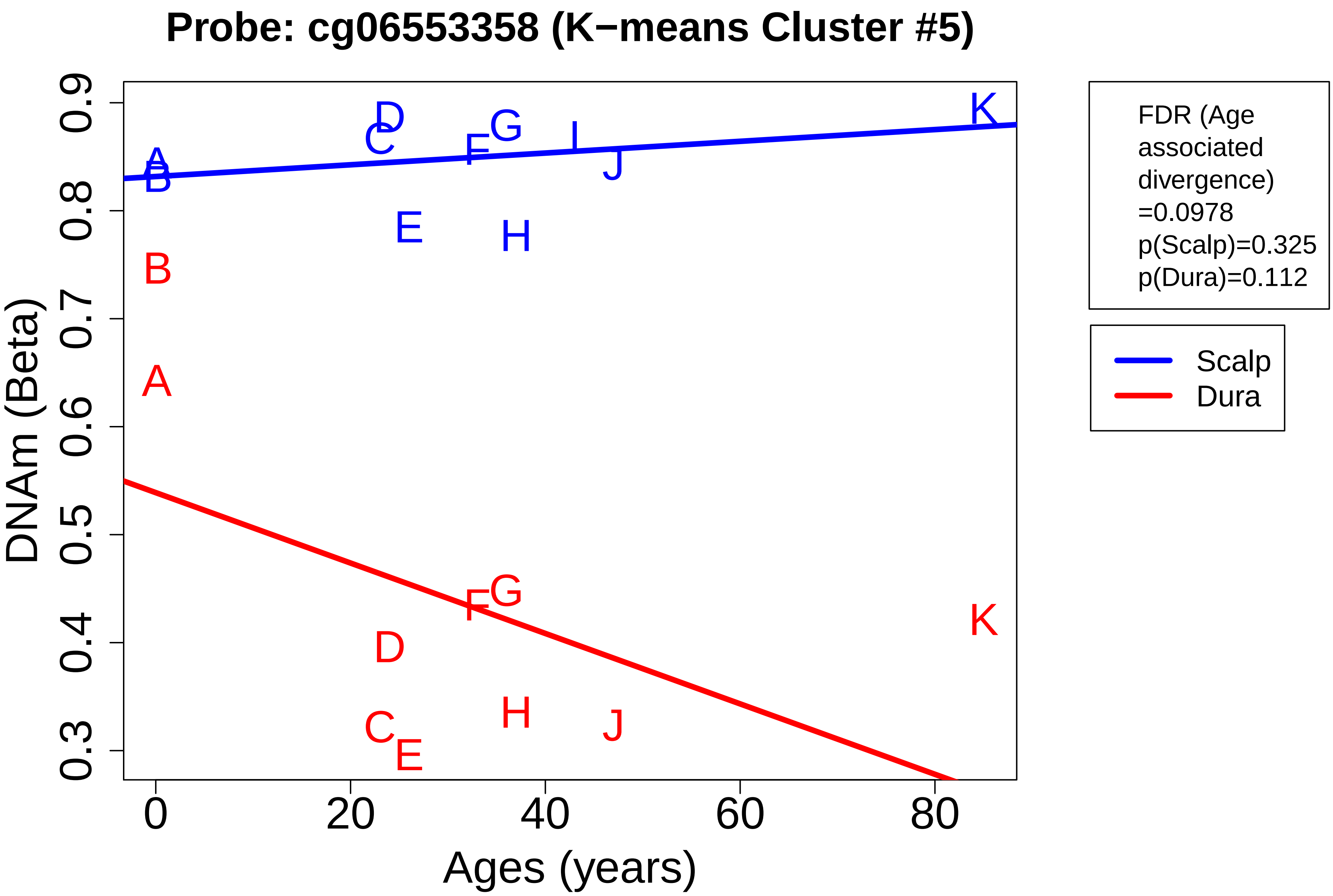
Probe: cg06553358 (K−means Cluster #5)
DNAm (Beta)
Ages (years)
0.3
0.4
0.5
0.6
0.7
0.8
0.9
0
20
40
60
80
A
B
C
D
E
F
G
H
I
J
K
FDR (Age associated divergence) =0.0978
p(Scalp)=0.325
p(Dura)=0.112
Scalp
Dura

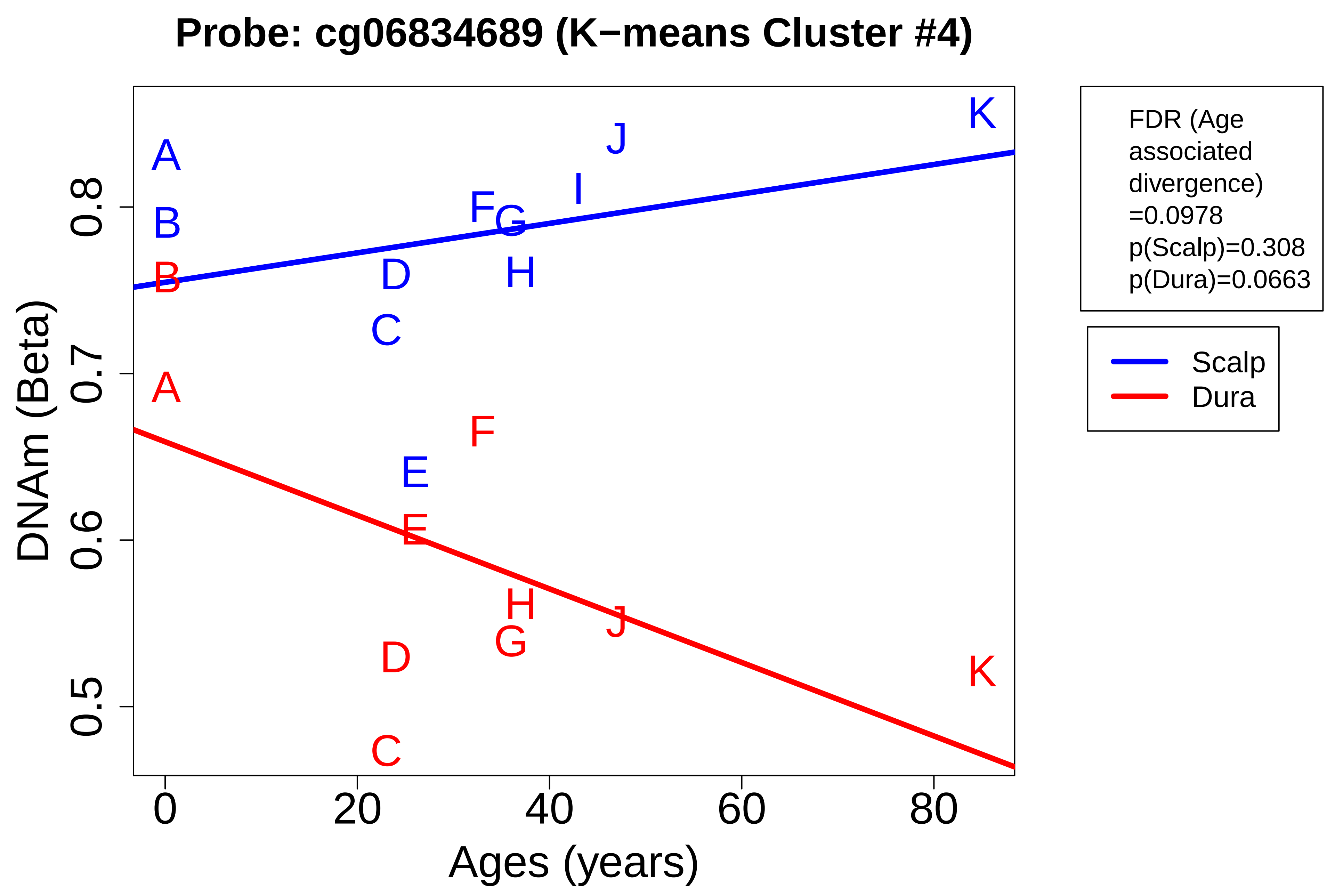

Probe: cg06834689 (K−means Cluster #4)
DNAm (Beta)
0.5
0.6
0.7
0.8
0
20
40
60
80
Ages (years)
A
B
C
D
E
F
G
H
I
J
K
FDR (Age associated divergence) =0.0978
p(Scalp)=0.308
p(Dura)=0.0663
Scalp
Dura

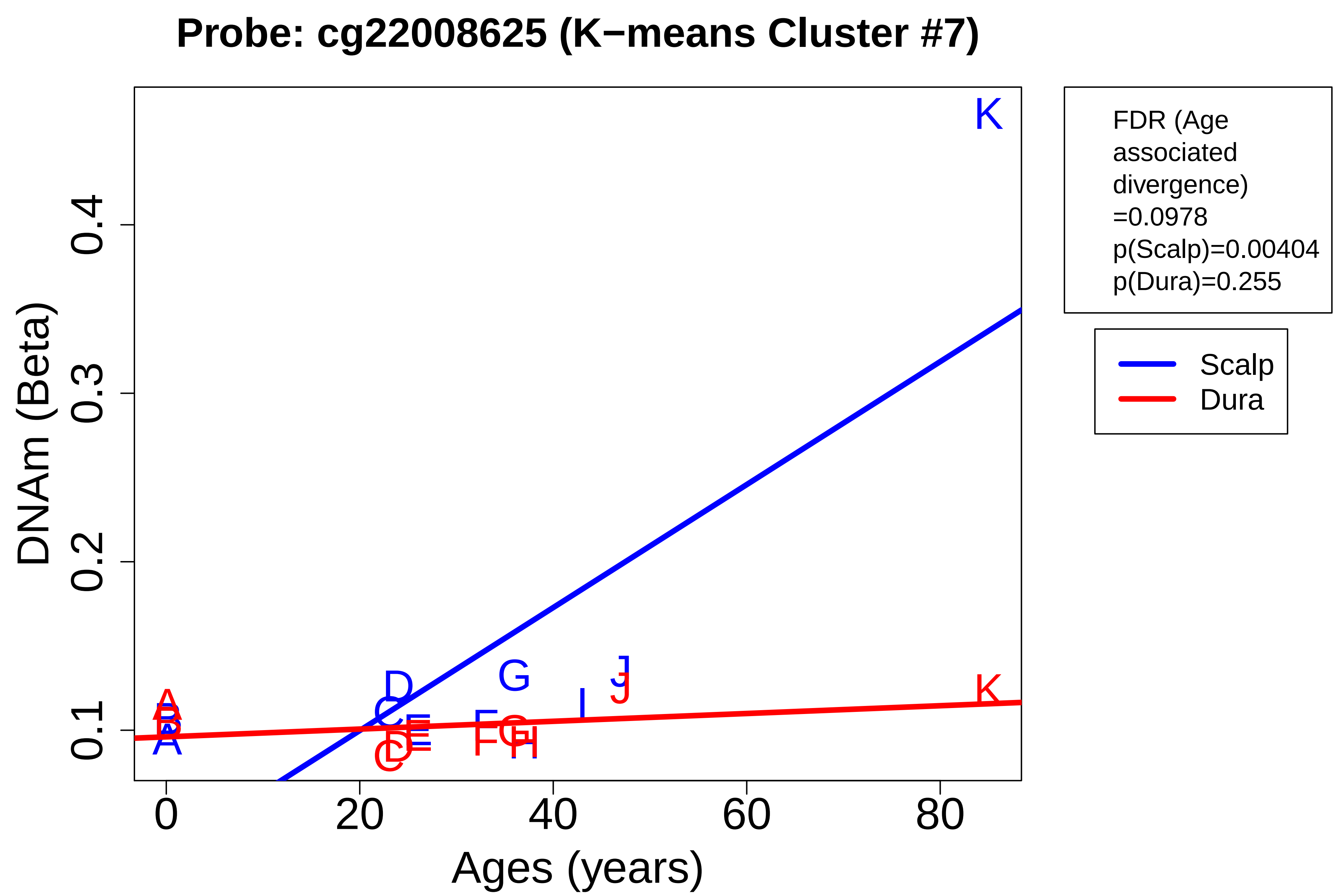

Probe: cg22008625 (K−means Cluster #7)
DNAm (Beta)
0.1
0.2
0.3
0.4
0
20
40
60
80
Ages (years)
FDR (Age associated divergence) =0.0978
p(Scalp)=0.00404
p(Dura)=0.255
Scalp
Dura

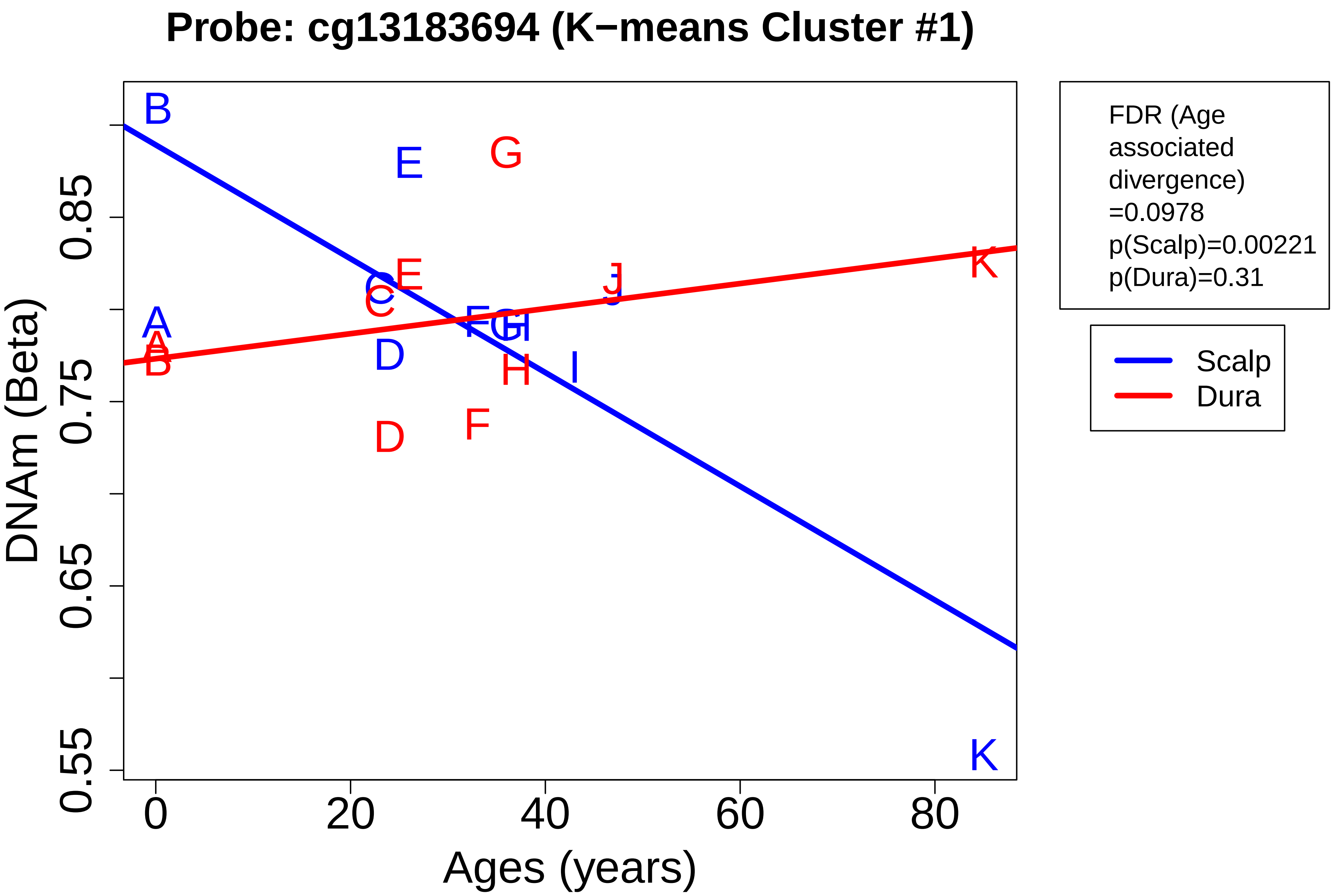
Probe: cg13183694 (K−means Cluster #1)
FDR (Age associated divergence) =0.0978
p(Scalp)=0.00221
p(Dura)=0.31
Scalp
Dura
DNAm (Beta)
0.55
0.65
0.75
0.85
0
20
40
60
80
Ages (years)
A
B
C
D
E
F
G
H
I
J
K

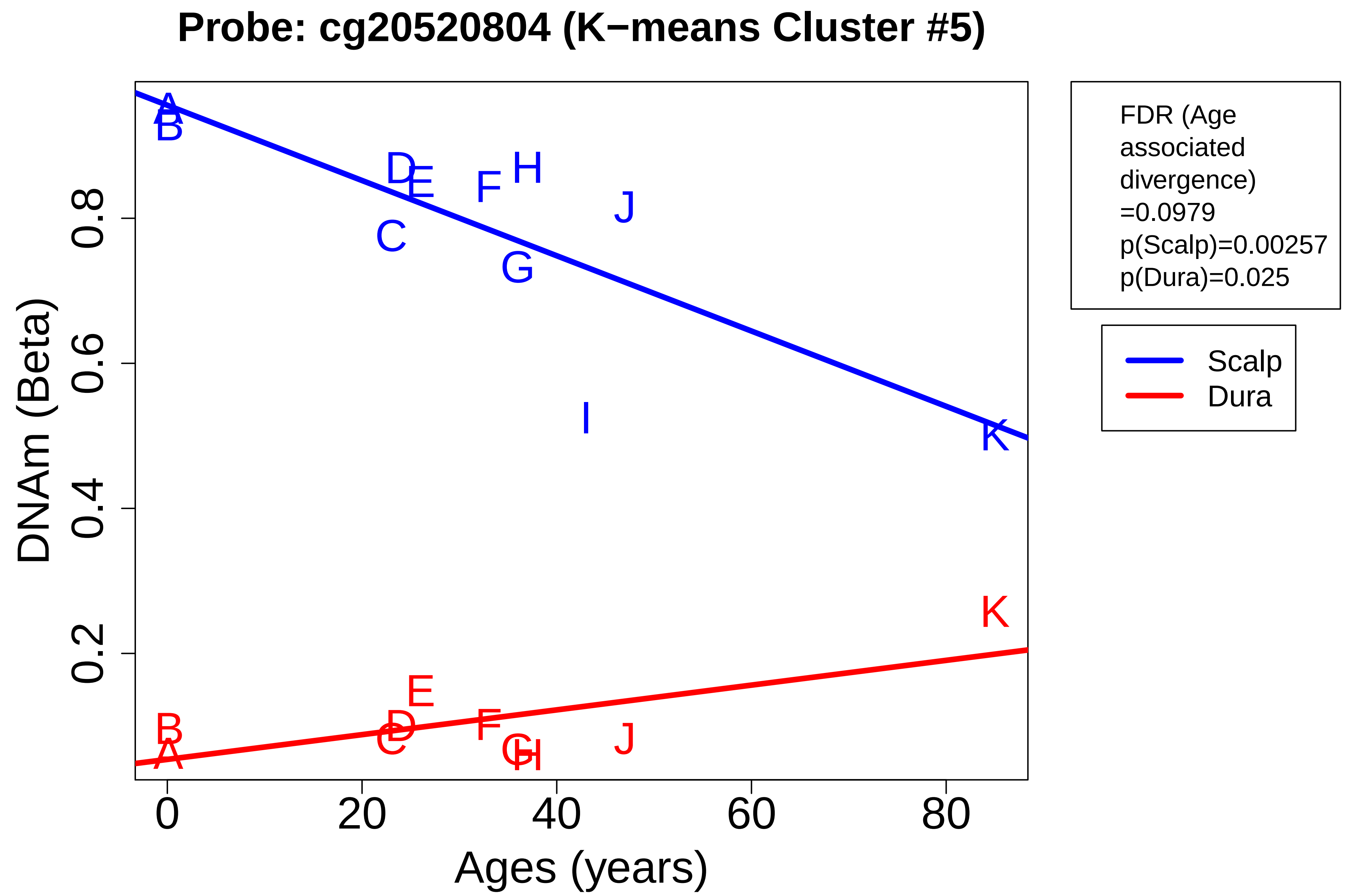
Probe: cg20520804 (K−means Cluster #5)
DNAm (Beta)
0.2
0.4
0.6
0.8
Ages (years)
0
20
40
60
80
A
B
C
D
E
F
G
H
I
J
K
FDR (Age associated divergence) =0.0979
p(Scalp)=0.00257
p(Dura)=0.025
Scalp
Dura

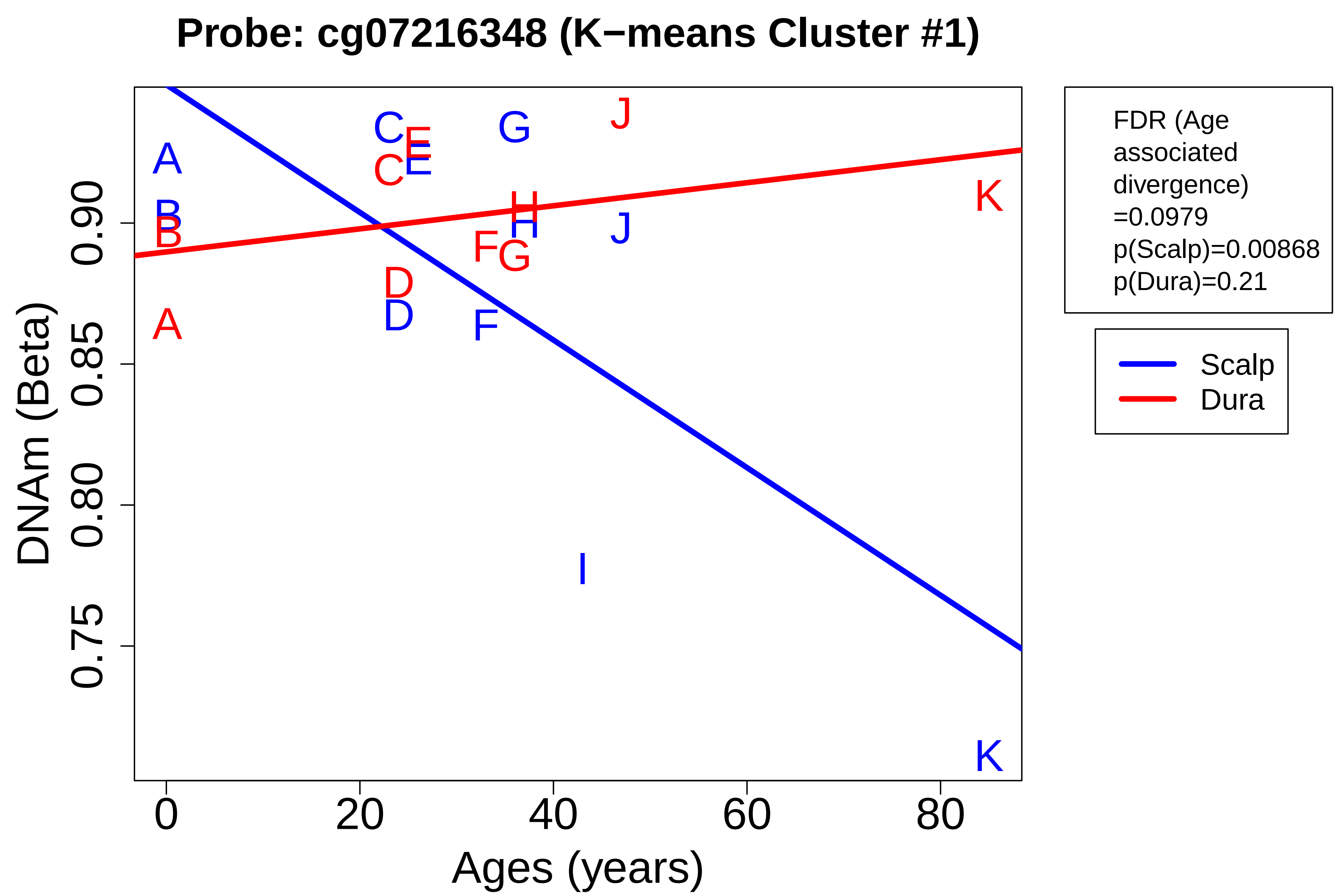

Probe: cg07216348 (K−means Cluster #1)
DNAm (Beta)
0.75
0.80
0.85
0.90
Ages (years)
0
20
40
60
80
A
B
C
D
E
F
G
H
I
J
K
FDR (Age associated divergence) =0.0979
p(Scalp)=0.00868
p(Dura)=0.21
Scalp
Dura

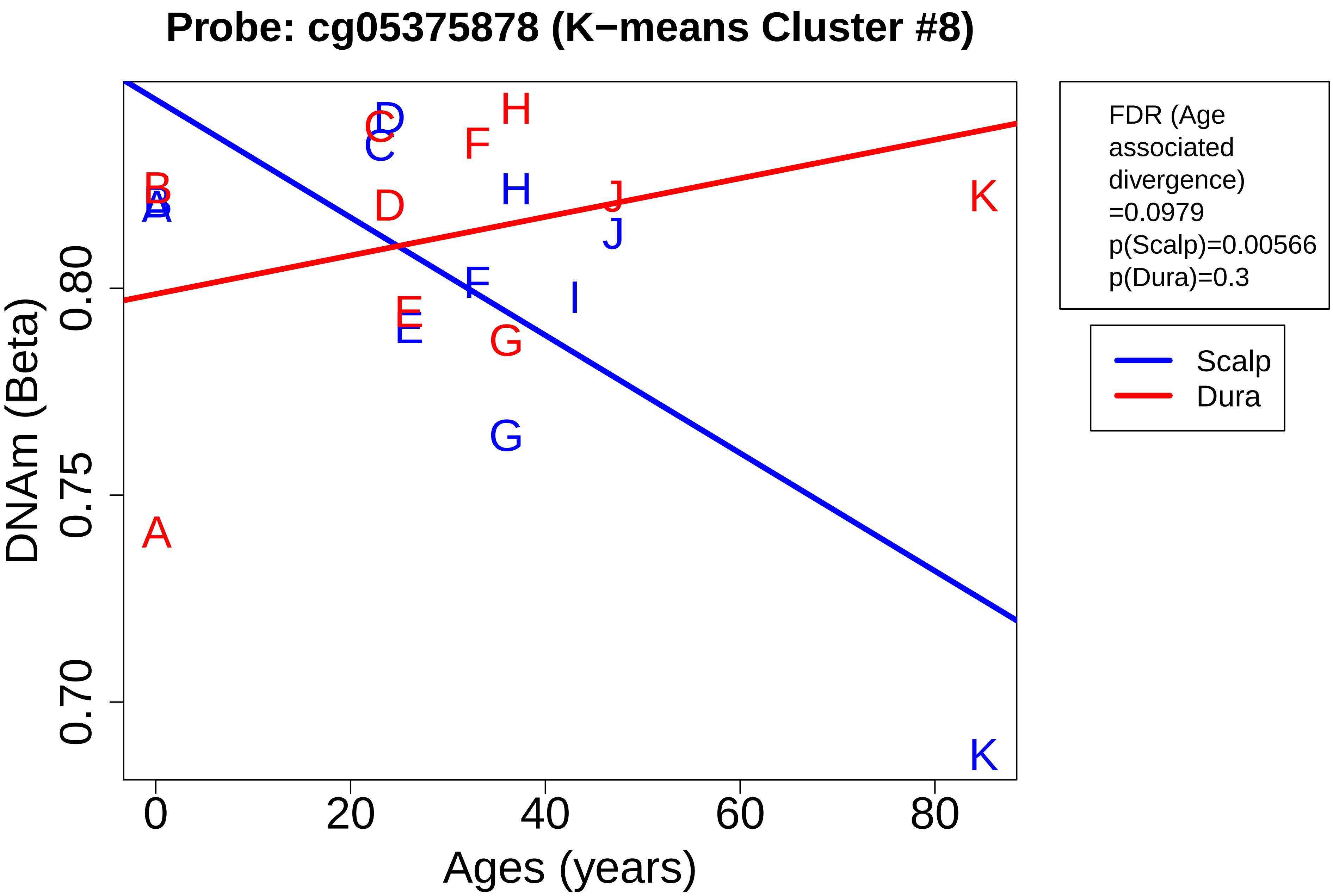
Probe: cg05375878 (K−means Cluster #8)
DNAm (Beta)
0.70
0.75
0.80
0
20
40
60
80
Ages (years)
FDR (Age associated divergence) =0.0979
p(Scalp)=0.00566
p(Dura)=0.3
Scalp
Dura

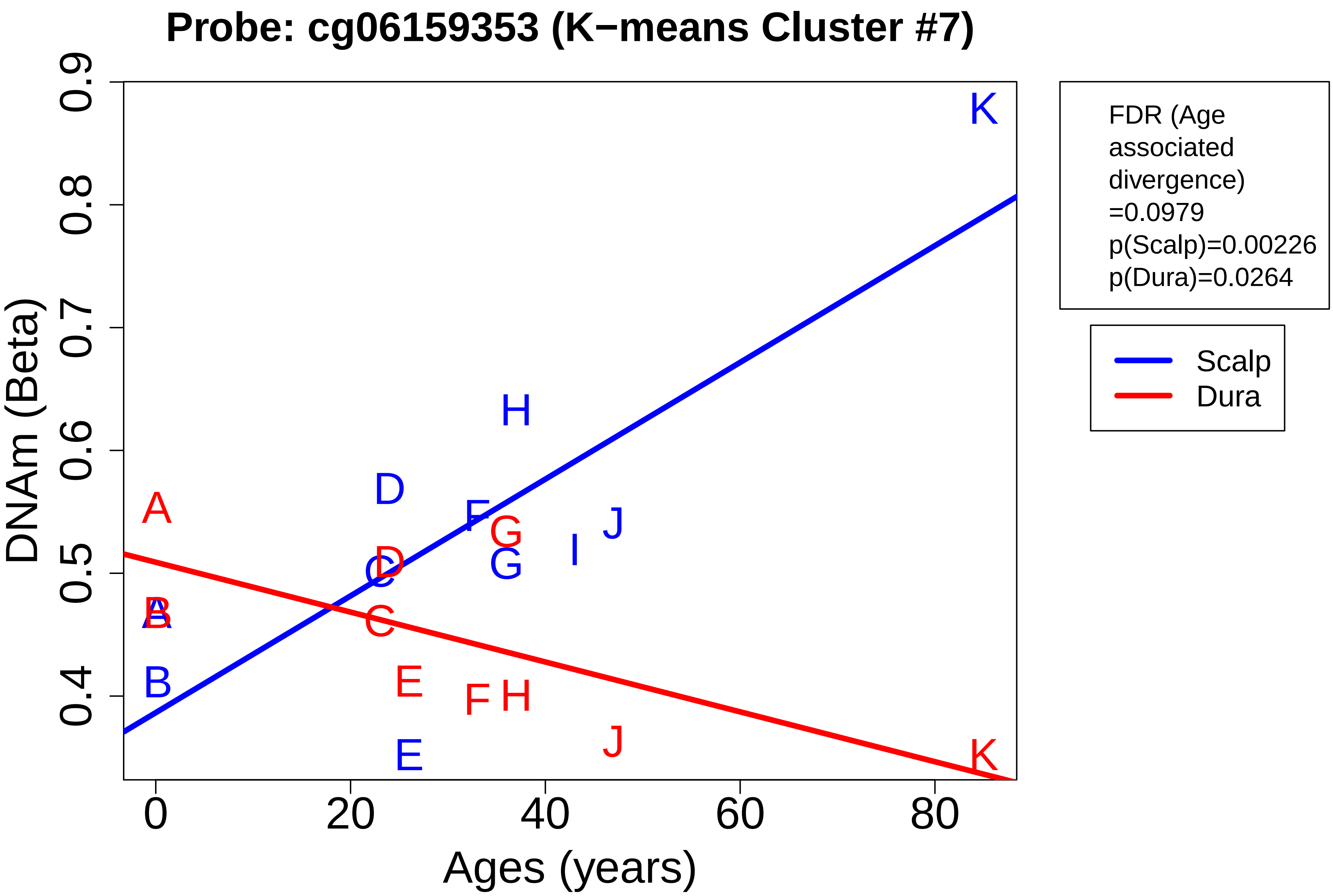

Probe: cg06159353 (K−means Cluster #7)
DNAm (Beta)
0.4
0.5
0.6
0.7
0.8
0.9
Ages (years)
0
20
40
60
80
FDR (Age associated divergence) =0.0979
p(Scalp)=0.00226
p(Dura)=0.0264
Scalp
Dura

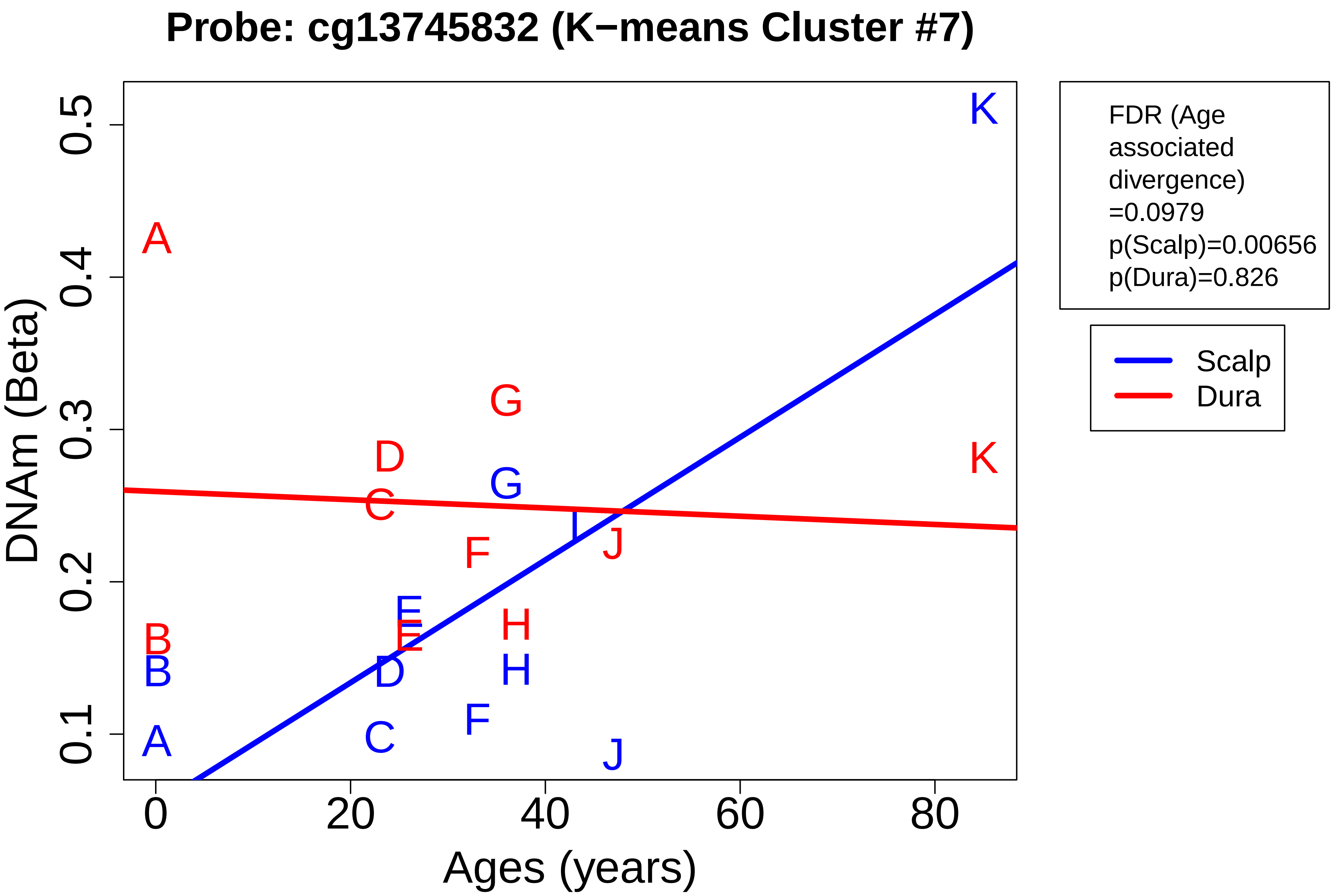
Probe: cg13745832 (K−means Cluster #7)
DNAm (Beta)
0.1
0.2
0.3
0.4
0.5
Ages (years)
0
20
40
60
80
A
B
C
D
E
F
G
H
I
J
K
FDR (Age associated divergence) =0.0979
p(Scalp)=0.00656
p(Dura)=0.826
Scalp
Dura

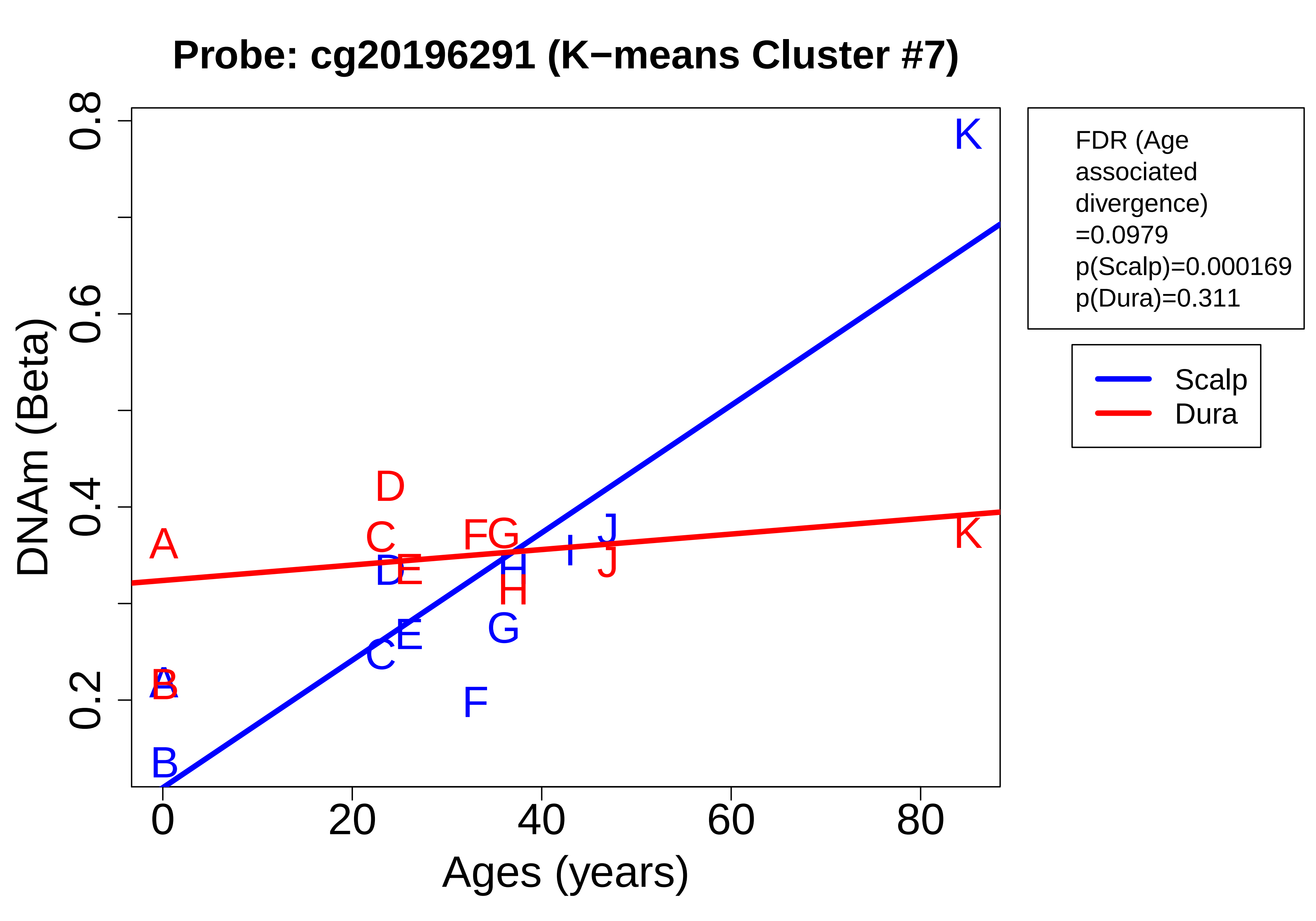

Probe: cg20196291 (K−means Cluster #7)
DNAm (Beta)
0.2
0.4
0.6
0.8
Ages (years)
0
20
40
60
80
A
B
C
D
E
F
G
H
I
J
K
FDR (Age associated divergence) =0.0979
p(Scalp)=0.000169
p(Dura)=0.311
Scalp
Dura

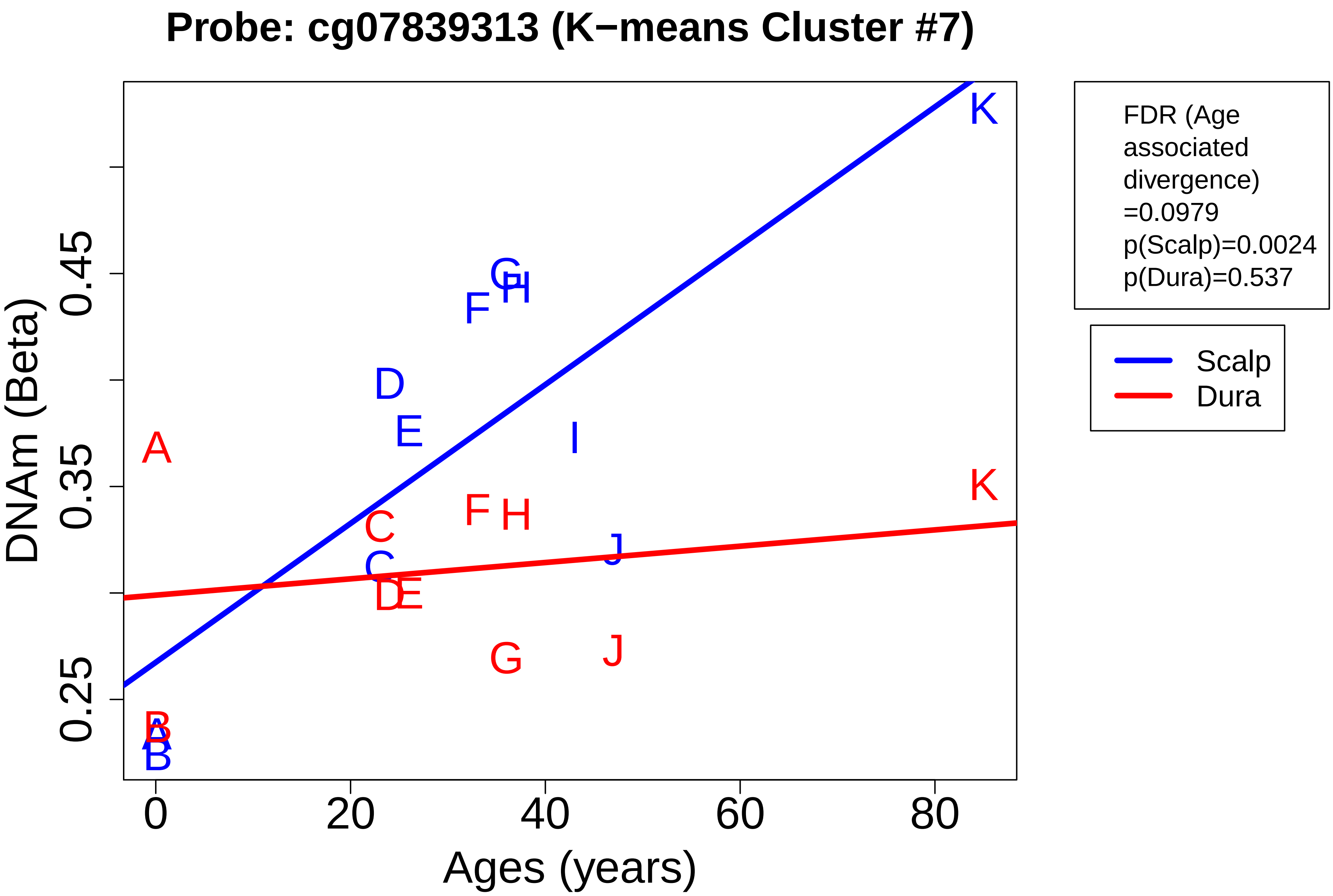

Probe: cg07839313 (K−means Cluster #7)
DNAm (Beta)
0.25
0.35
0.45
Ages (years)
0
20
40
60
80
FDR (Age associated divergence) =0.0979
p(Scalp)=0.0024
p(Dura)=0.537
Scalp
Dura
A
B
C
D
E
F
G
H
I
J
K

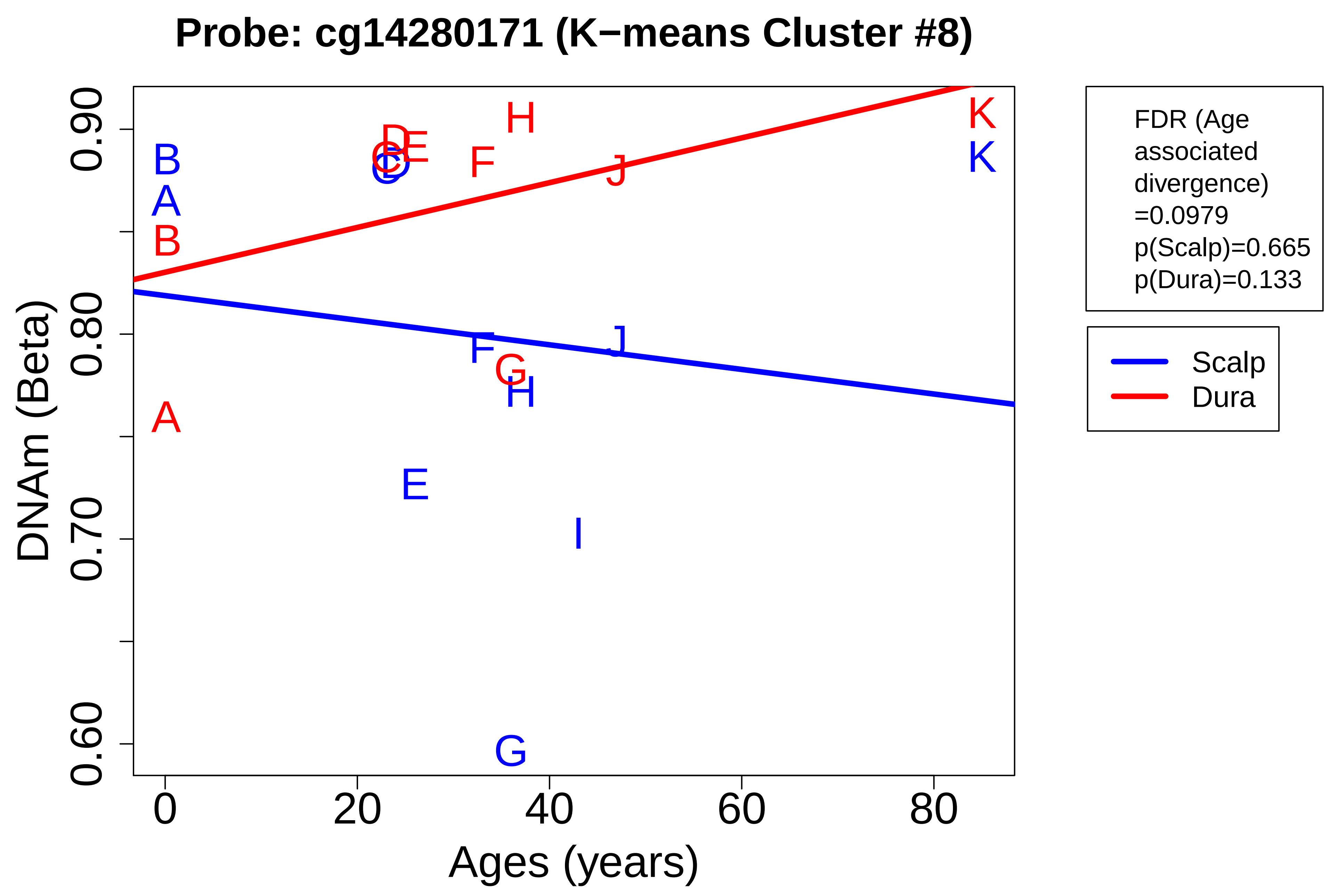

Probe: cg14280171 (K−means Cluster #8)
DNAm (Beta)
0.60
0.70
0.80
0.90
0
20
40
60
80
Ages (years)
FDR (Age associated divergence) =0.0979
p(Scalp)=0.665
p(Dura)=0.133
Scalp
Dura

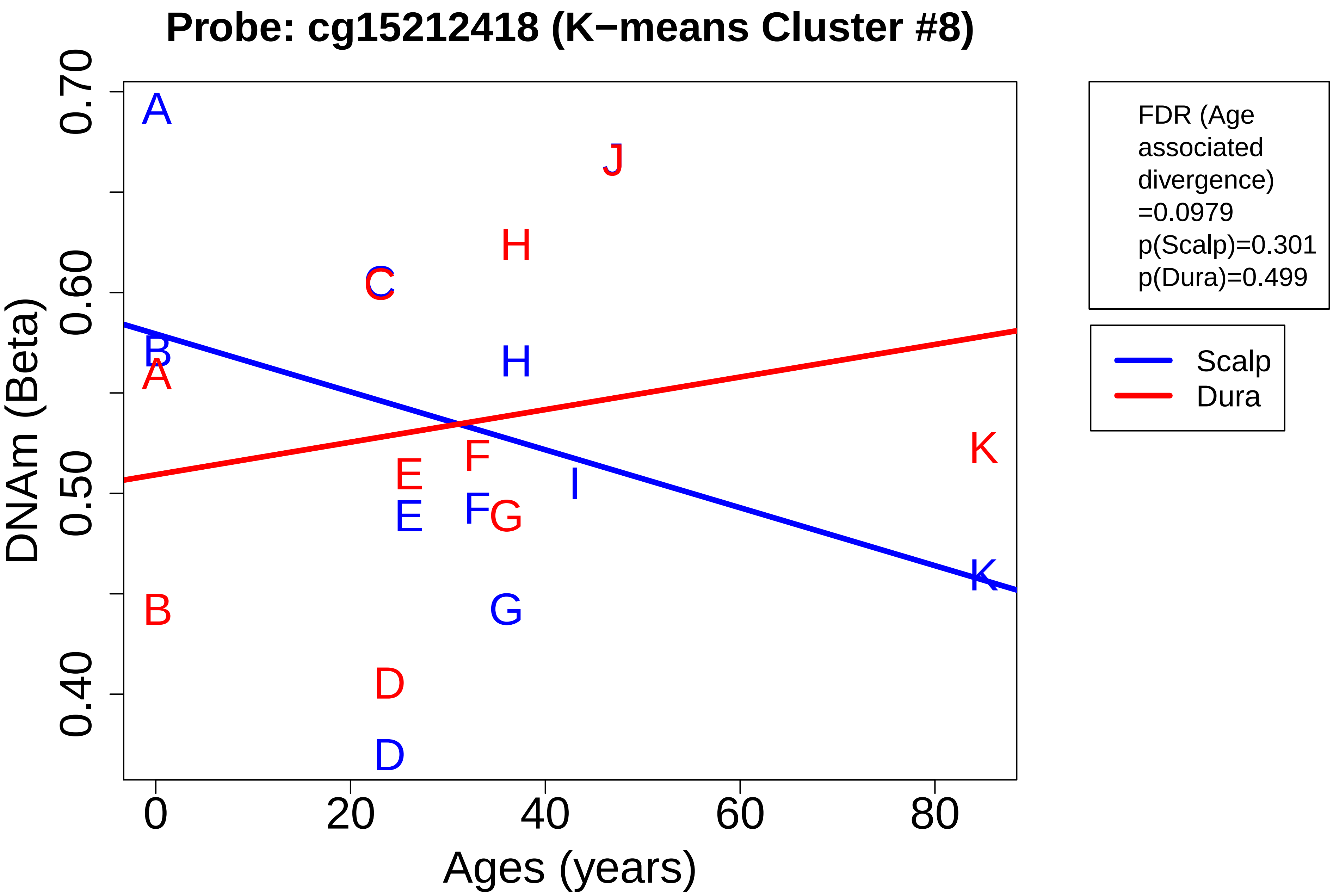
Probe: cg15212418 (K−means Cluster #8)
DNAm (Beta)
0.40
0.50
0.60
0.70
Ages (years)
0
20
40
60
80
A
B
C
D
E
F
G
H
I
J
K
FDR (Age associated divergence) =0.0979
p(Scalp)=0.301
p(Dura)=0.499
Scalp
Dura

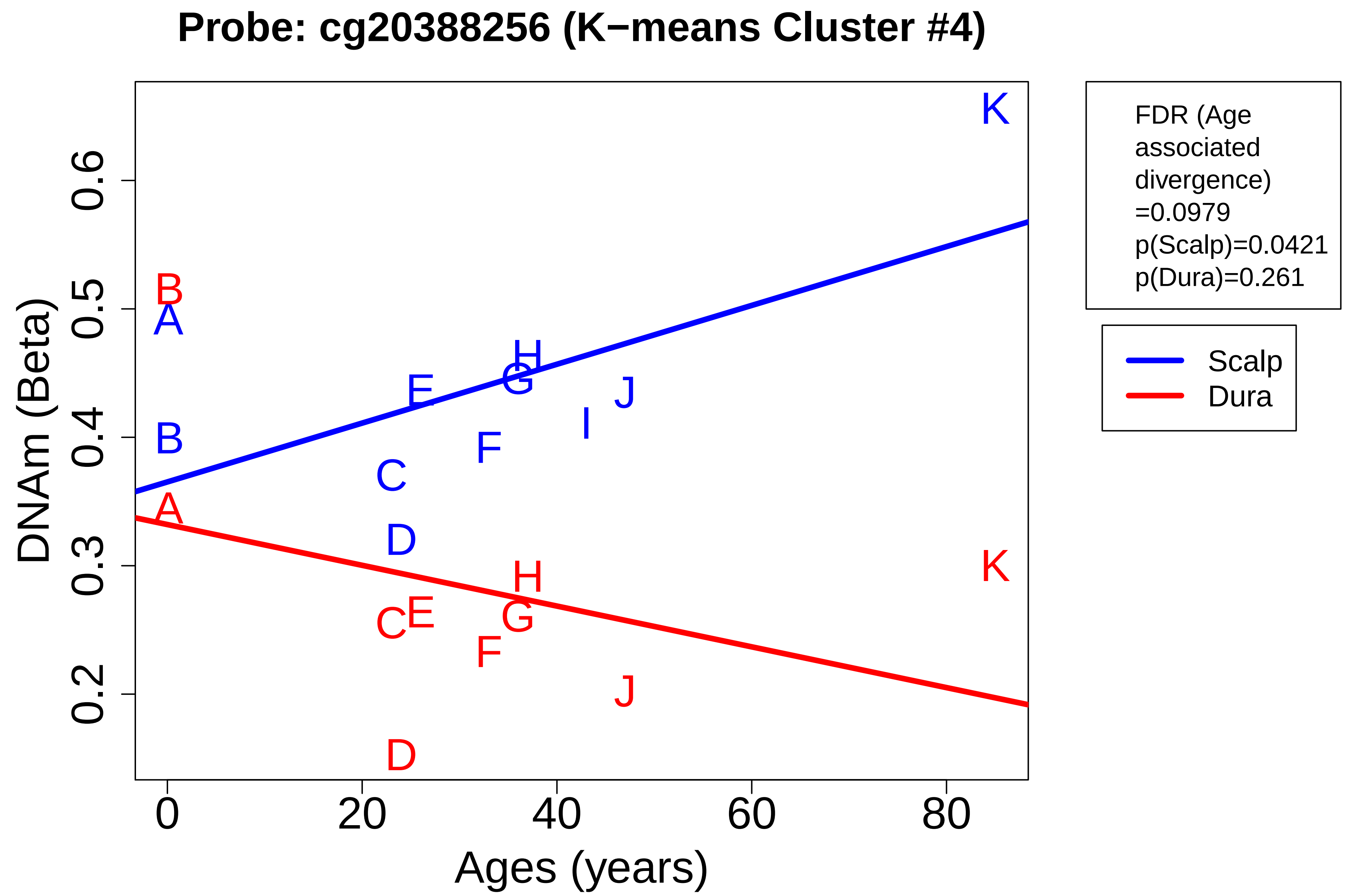

Probe: cg20388256 (K−means Cluster #4)
DNAm (Beta)
0.2
0.3
0.4
0.5
0.6
0
20
40
60
80
Ages (years)
K
B
A
H
E
G
J
I
B
F
C
A
D
H
K
C
E
G
F
J
D
FDR (Age associated divergence) =0.0979
p(Scalp)=0.0421
p(Dura)=0.261
Scalp
Dura

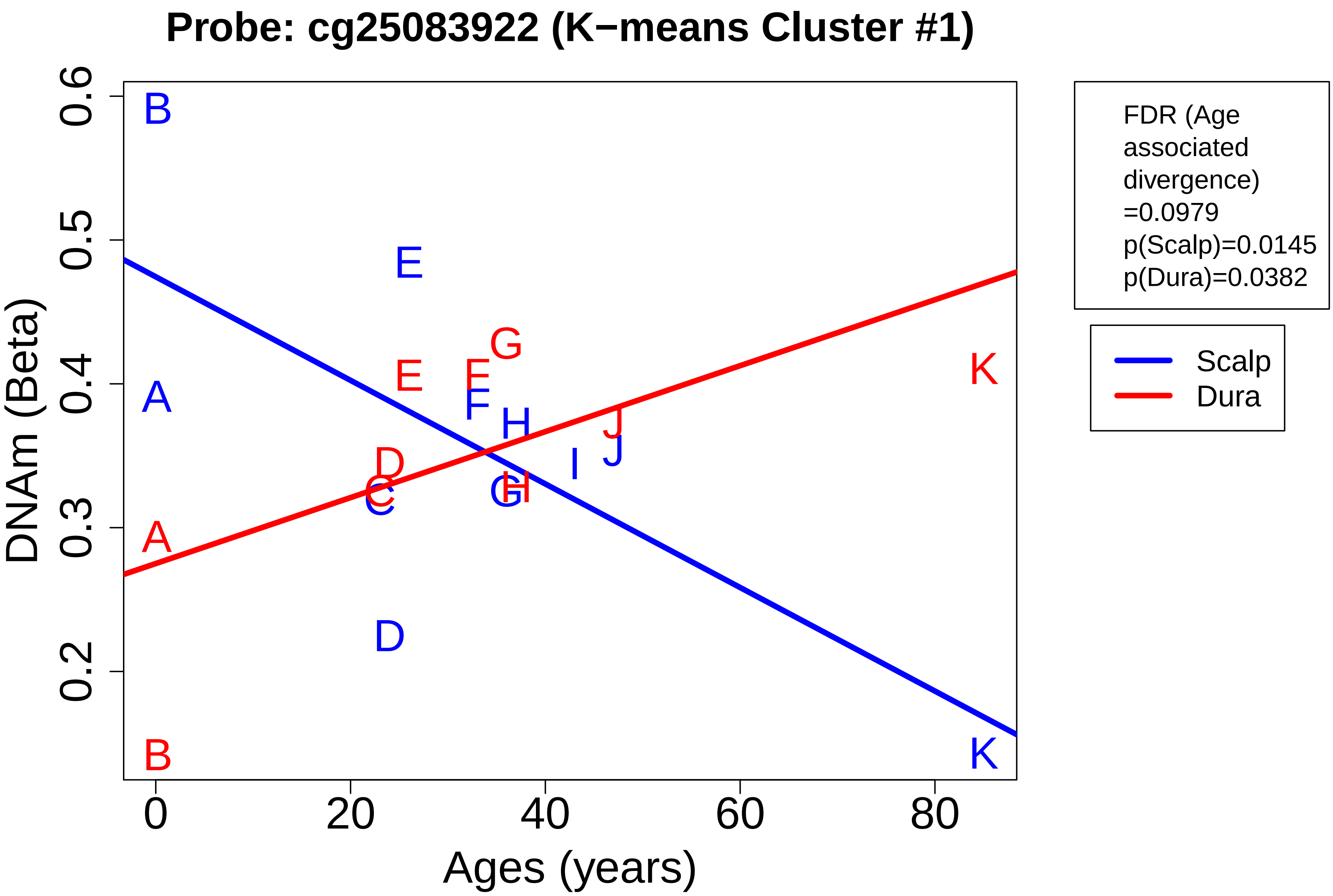

Probe: cg25083922 (K−means Cluster #1)
DNAm (Beta)
0.2
0.3
0.4
0.5
0.6
Ages (years)
0
20
40
60
80
FDR (Age associated divergence) =0.0979
p(Scalp)=0.0145
p(Dura)=0.0382
Scalp
Dura
A
B
C
D
E
F
G
H
I
J
K

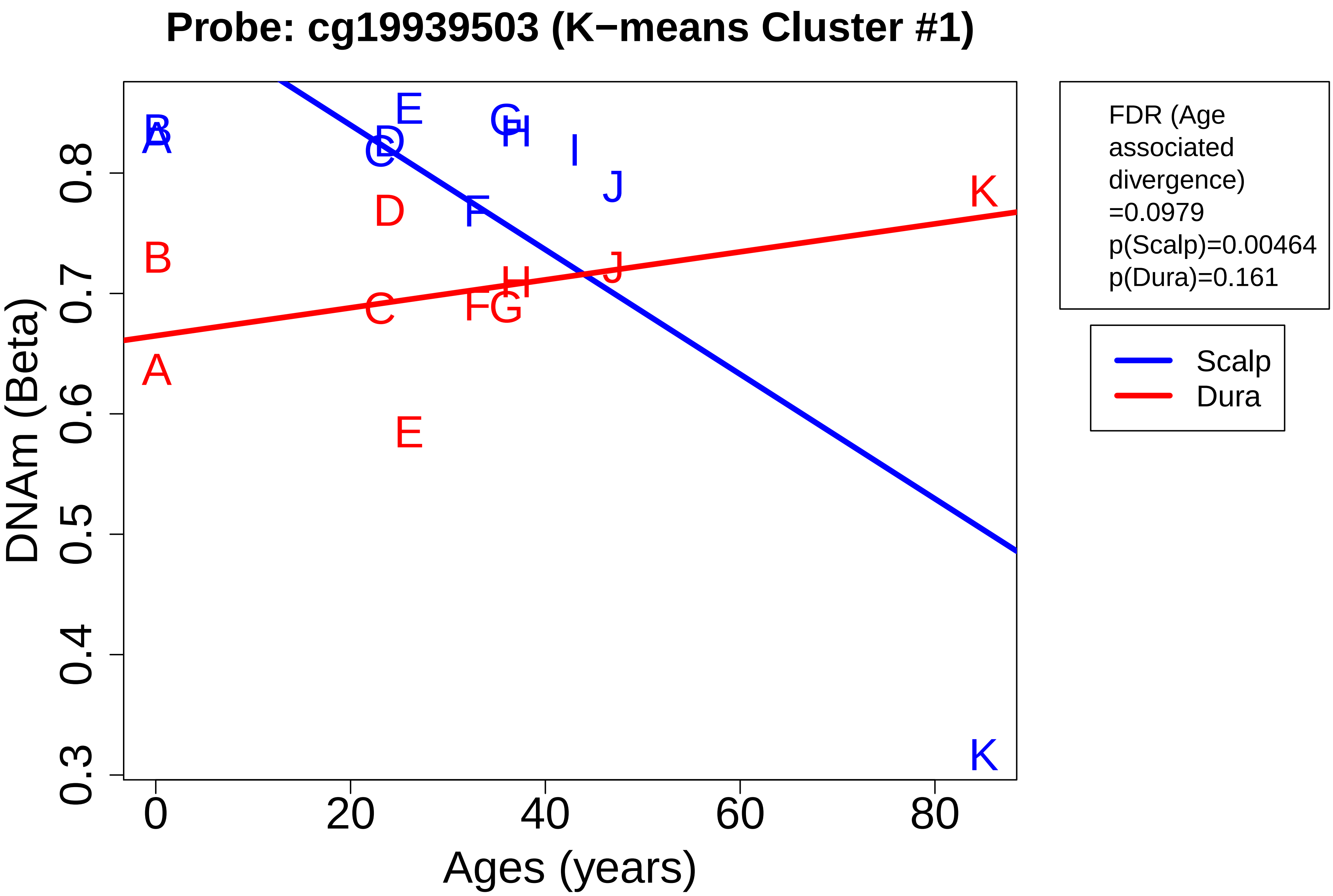
Probe: cg19939503 (K−means Cluster #1)
DNAm (Beta)
0.3
0.4
0.5
0.6
0.7
0.8
0
20
40
60
80
Ages (years)
A
B
C
D
E
F
G
H
I
J
K
FDR (Age associated divergence) =0.0979
p(Scalp)=0.00464
p(Dura)=0.161
Scalp
Dura

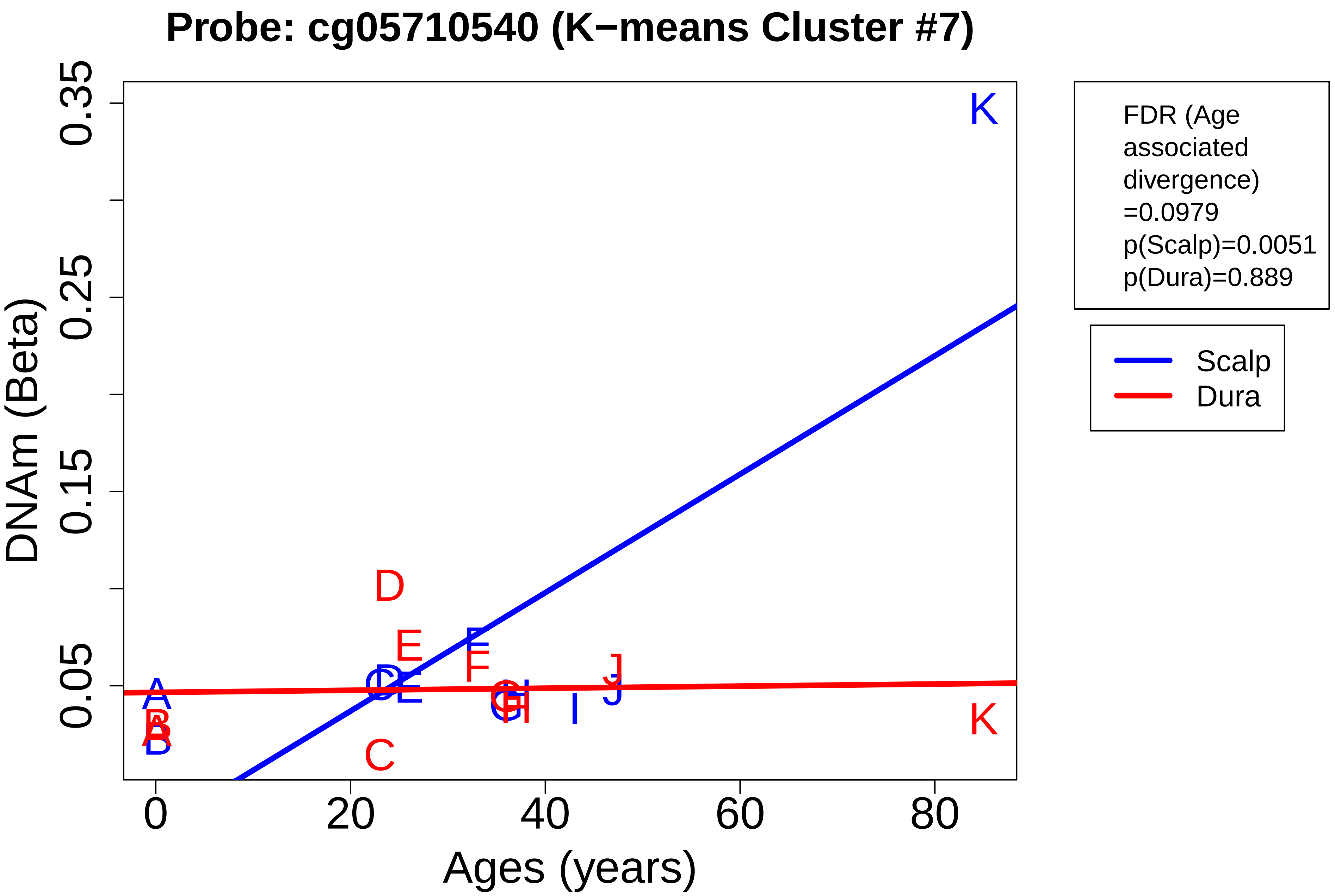

Probe: cg05710540 (K−means Cluster #7)
DNAm (Beta)
0.05
0.15
0.25
0.35
Ages (years)
0
20
40
60
80
A
B
C
D
E
F
G
H
I
J
K
FDR (Age associated divergence) =0.0979
p(Scalp)=0.0051
p(Dura)=0.889
Scalp
Dura

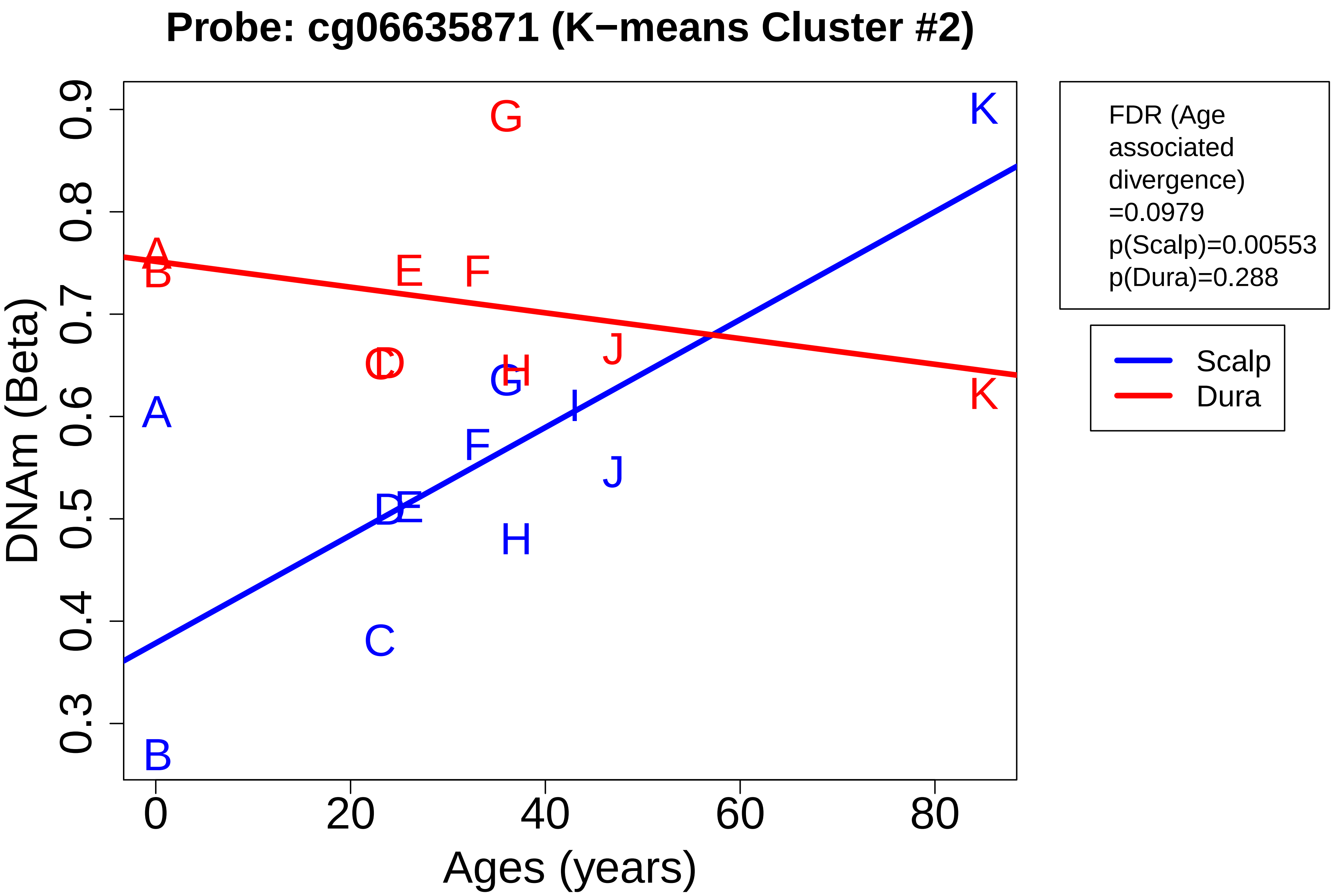

Probe: cg06635871 (K−means Cluster #2)
DNAm (Beta)
0.3
0.4
0.5
0.6
0.7
0.8
0.9
0
20
40
60
80
Ages (years)
FDR (Age associated divergence) =0.0979
p(Scalp)=0.00553
p(Dura)=0.288
Scalp
Dura

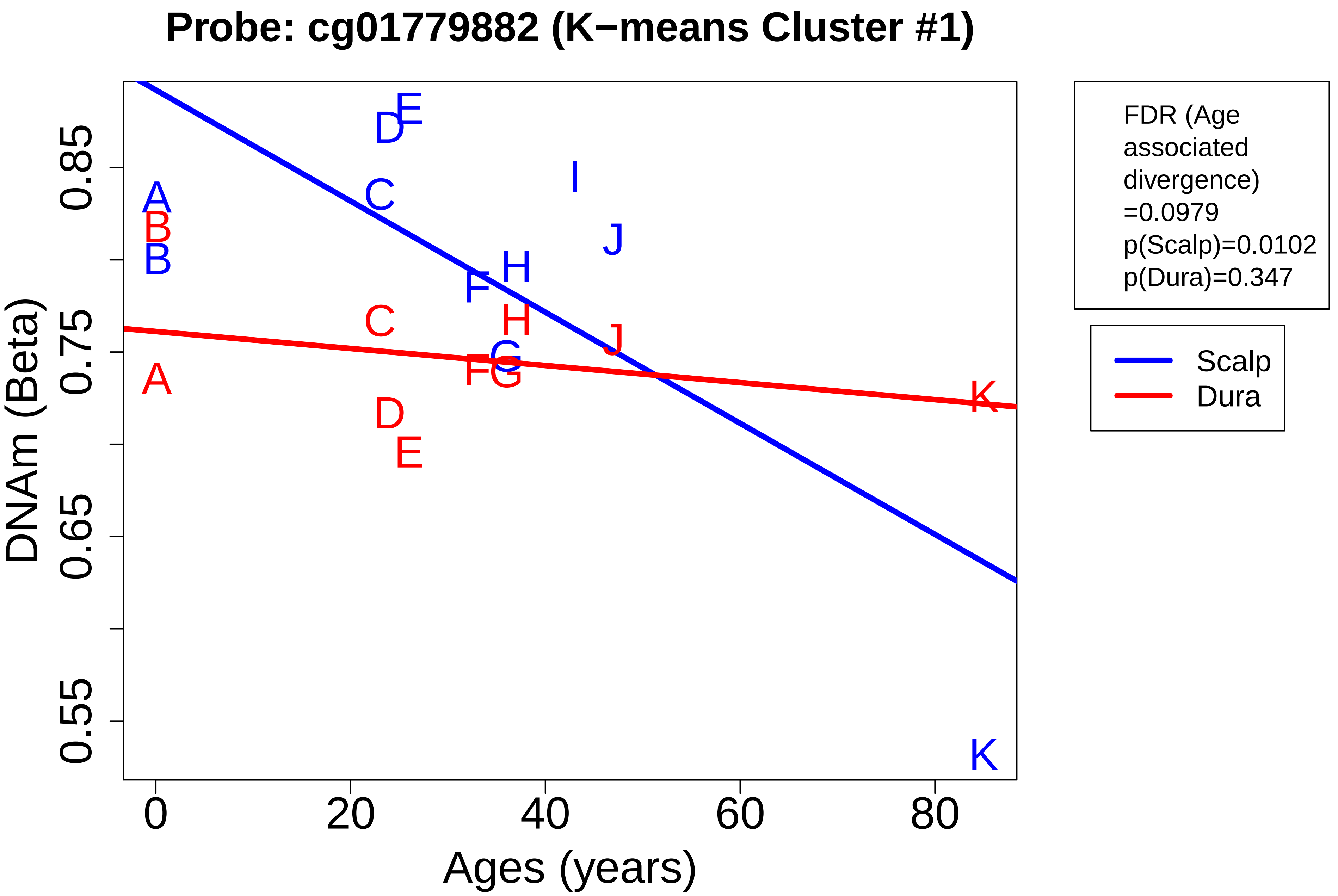
Probe: cg01779882 (K−means Cluster #1)
DNAm (Beta)
0.55
0.65
0.75
0.85
0
20
40
60
80
Ages (years)
FDR (Age associated divergence) =0.0979
p(Scalp)=0.0102
p(Dura)=0.347
Scalp
Dura
A
B
C
D
E
F
G
H
I
J
K

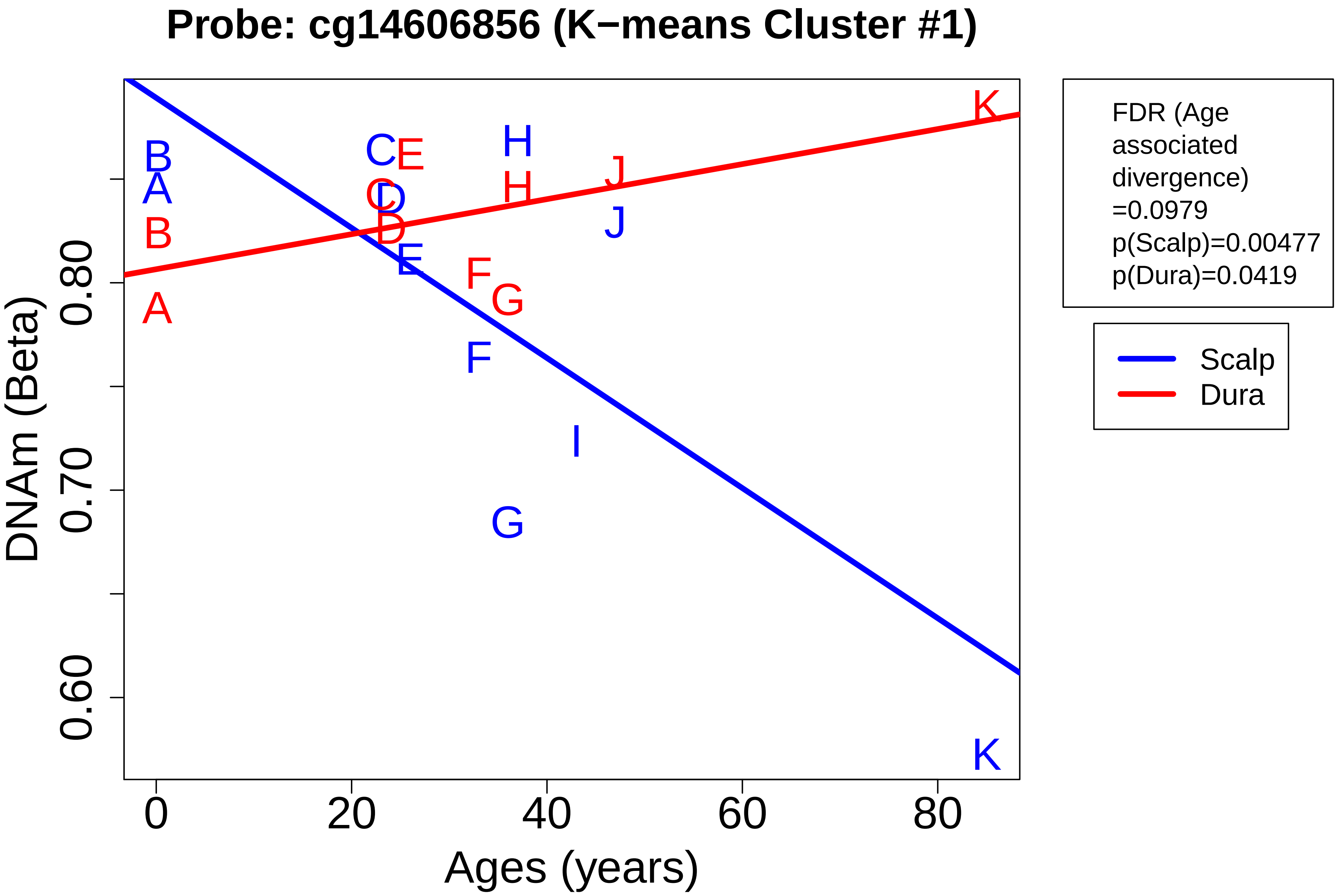

Probe: cg14606856 (K−means Cluster #1)
DNAm (Beta)
0.60
0.70
0.80
Ages (years)
0
20
40
60
80
FDR (Age associated divergence) =0.0979
p(Scalp)=0.00477
p(Dura)=0.0419
Scalp
Dura

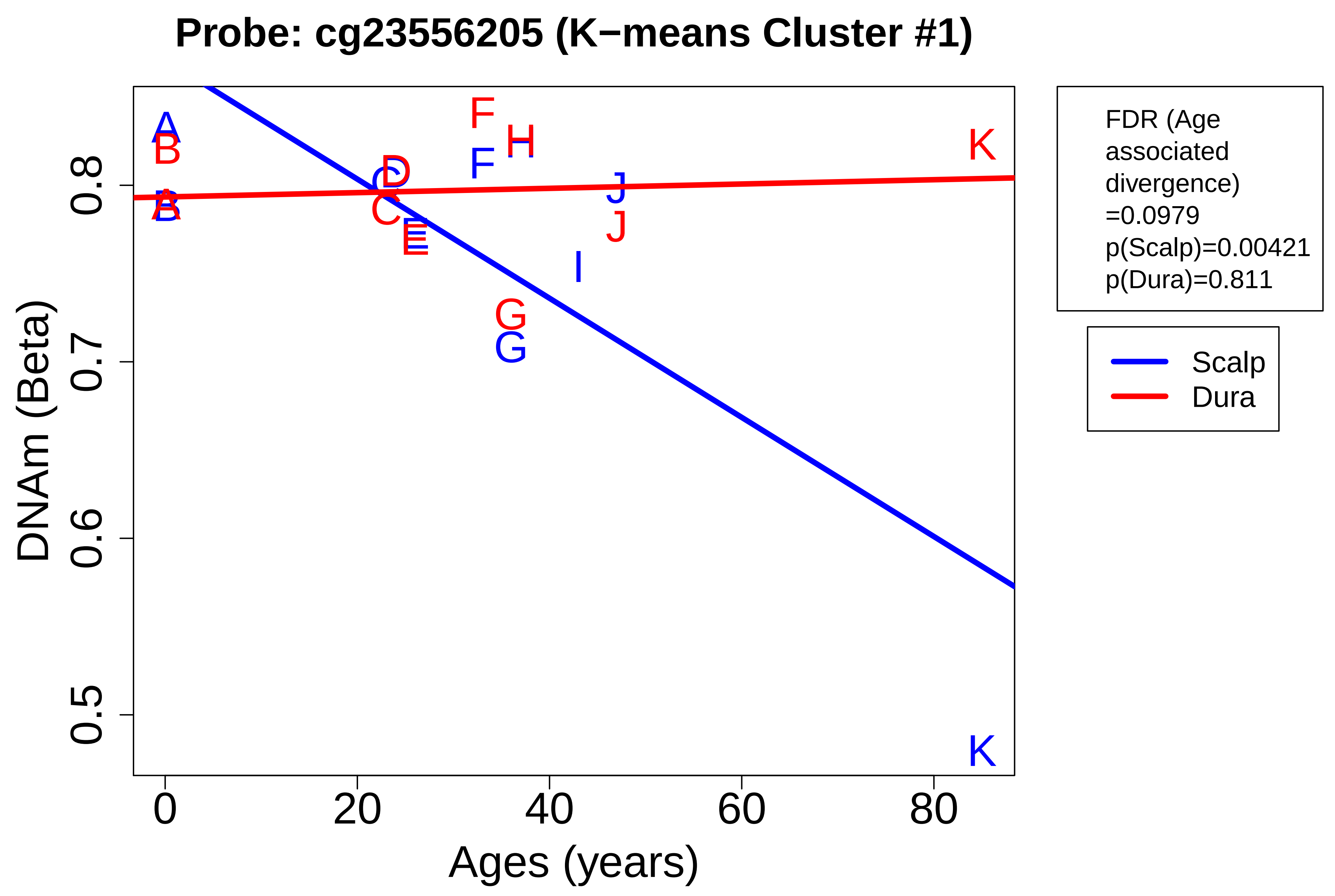

Probe: cg23556205 (K−means Cluster #1)
DNAm (Beta)
0.5
0.6
0.7
0.8
Ages (years)
0
20
40
60
80
FDR (Age associated divergence) =0.0979
p(Scalp)=0.00421
p(Dura)=0.811
Scalp
Dura
A
B
C
D
E
F
G
H
I
J
K

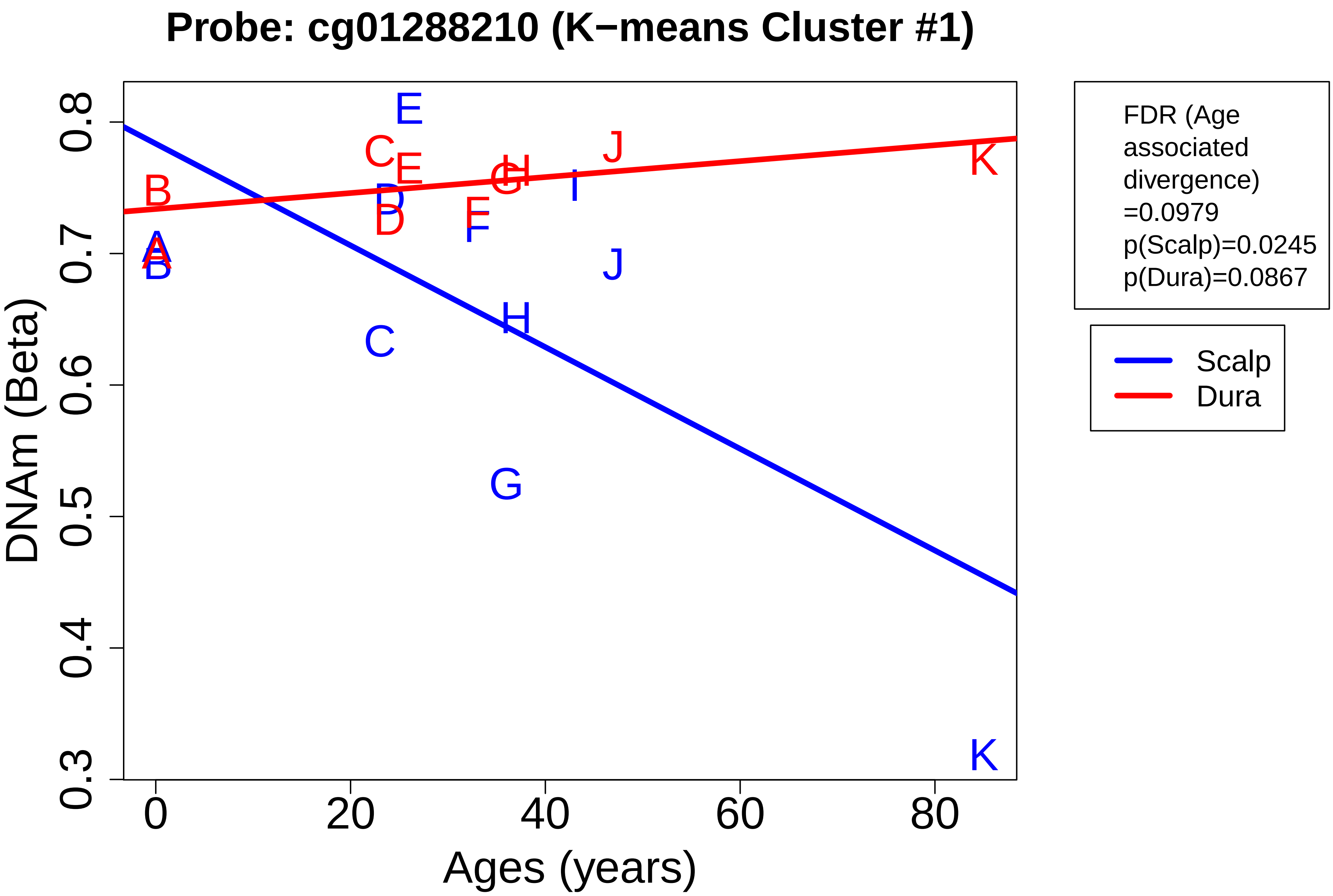
Probe: cg01288210 (K−means Cluster #1)
DNAm (Beta)
Ages (years)
0.3
0.4
0.5
0.6
0.7
0.8
0
20
40
60
80
FDR (Age associated divergence) =0.0979
p(Scalp)=0.0245
p(Dura)=0.0867
Scalp
Dura

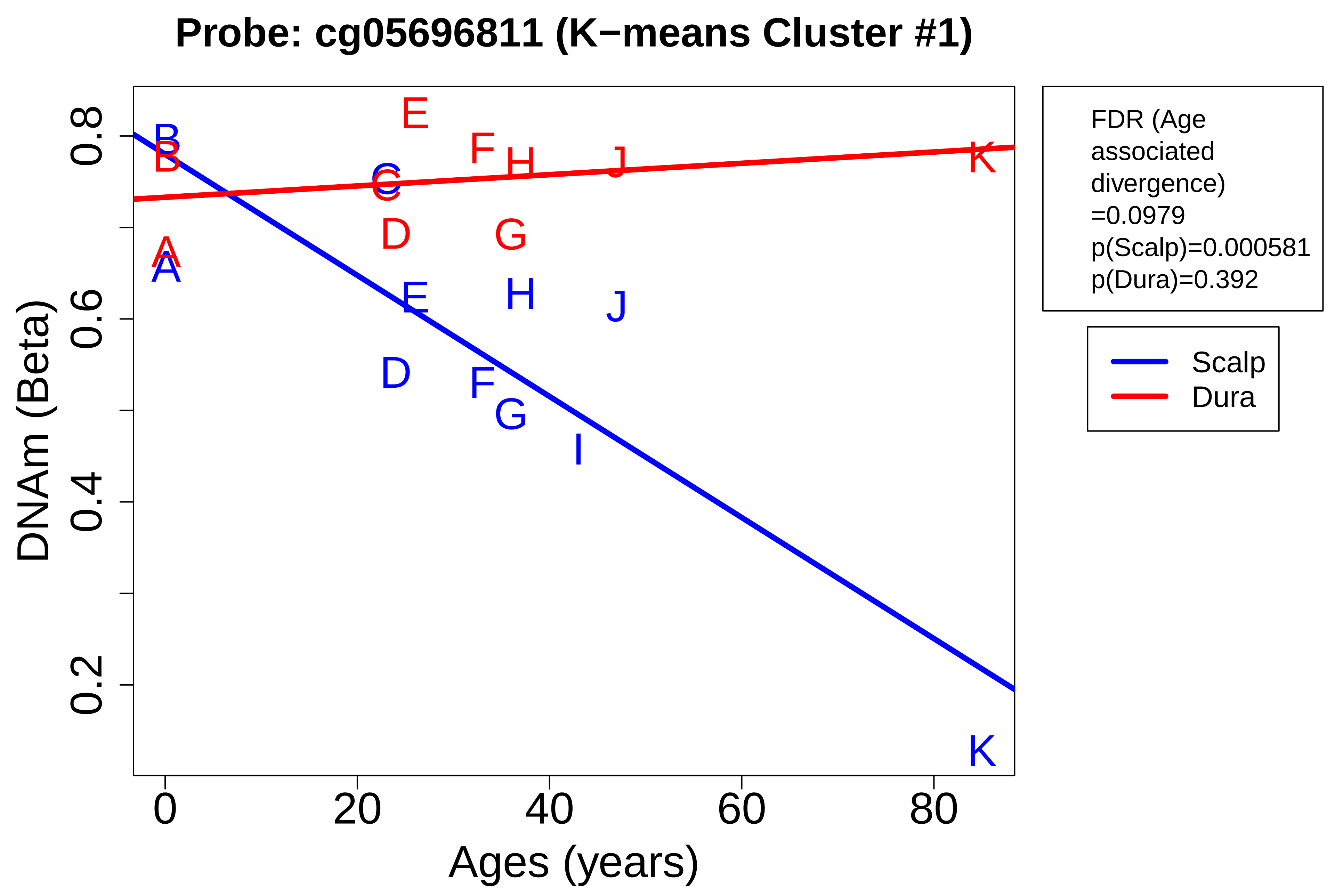
Probe: cg05696811 (K−means Cluster #1)
DNAm (Beta)
0.2
0.4
0.6
0.8
Ages (years)
0
20
40
60
80
A B C D E F G H I J K
FDR (Age associated divergence) =0.0979
p(Scalp)=0.000581
p(Dura)=0.392
Scalp
Dura

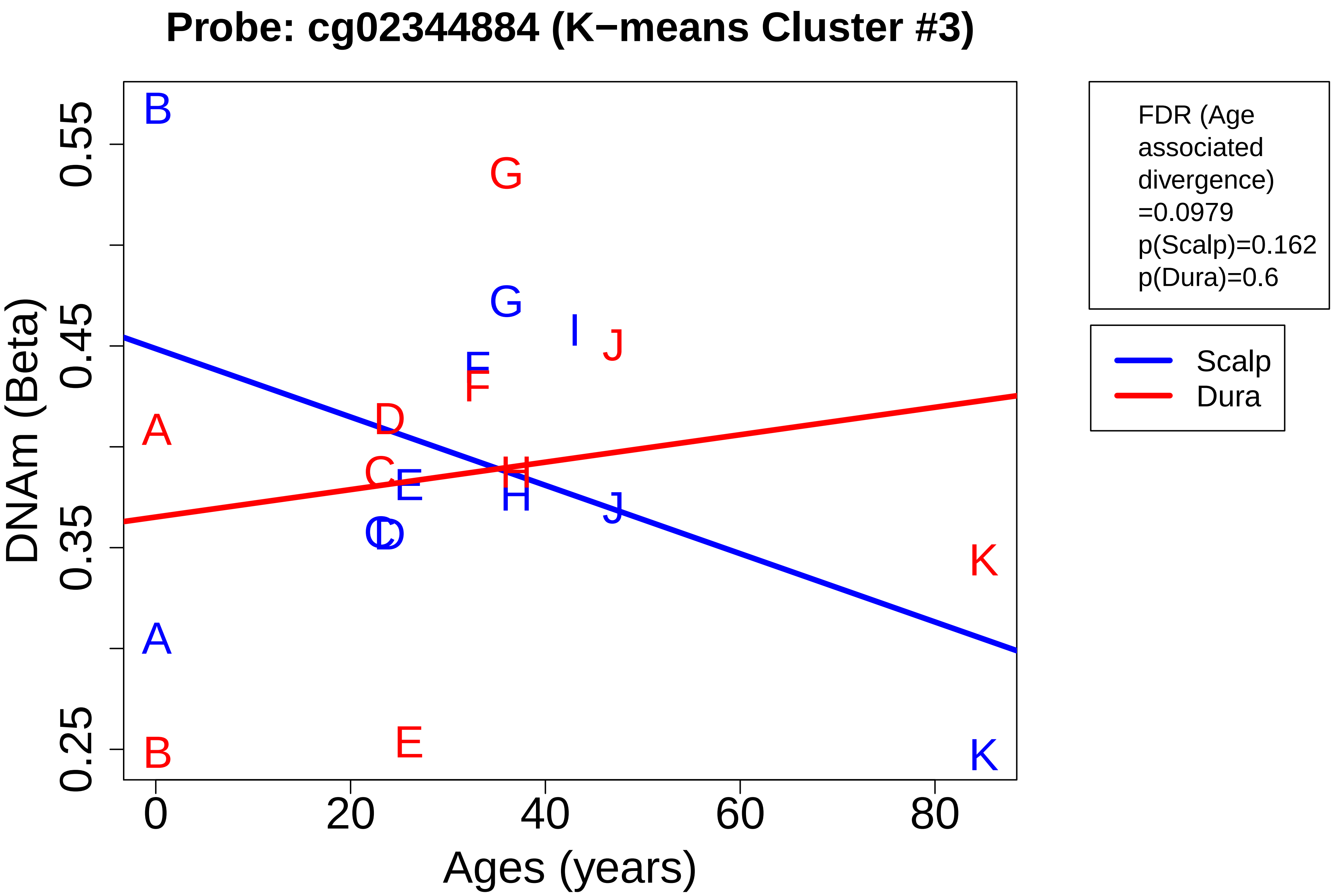

Probe: cg02344884 (K−means Cluster #3)
DNAm (Beta)
0.25
0.35
0.45
0.55
Ages (years)
0
20
40
60
80
FDR (Age associated divergence) =0.0979
p(Scalp)=0.162
p(Dura)=0.6
Scalp
Dura
A
B
C
D
E
F
G
H
I
J
K

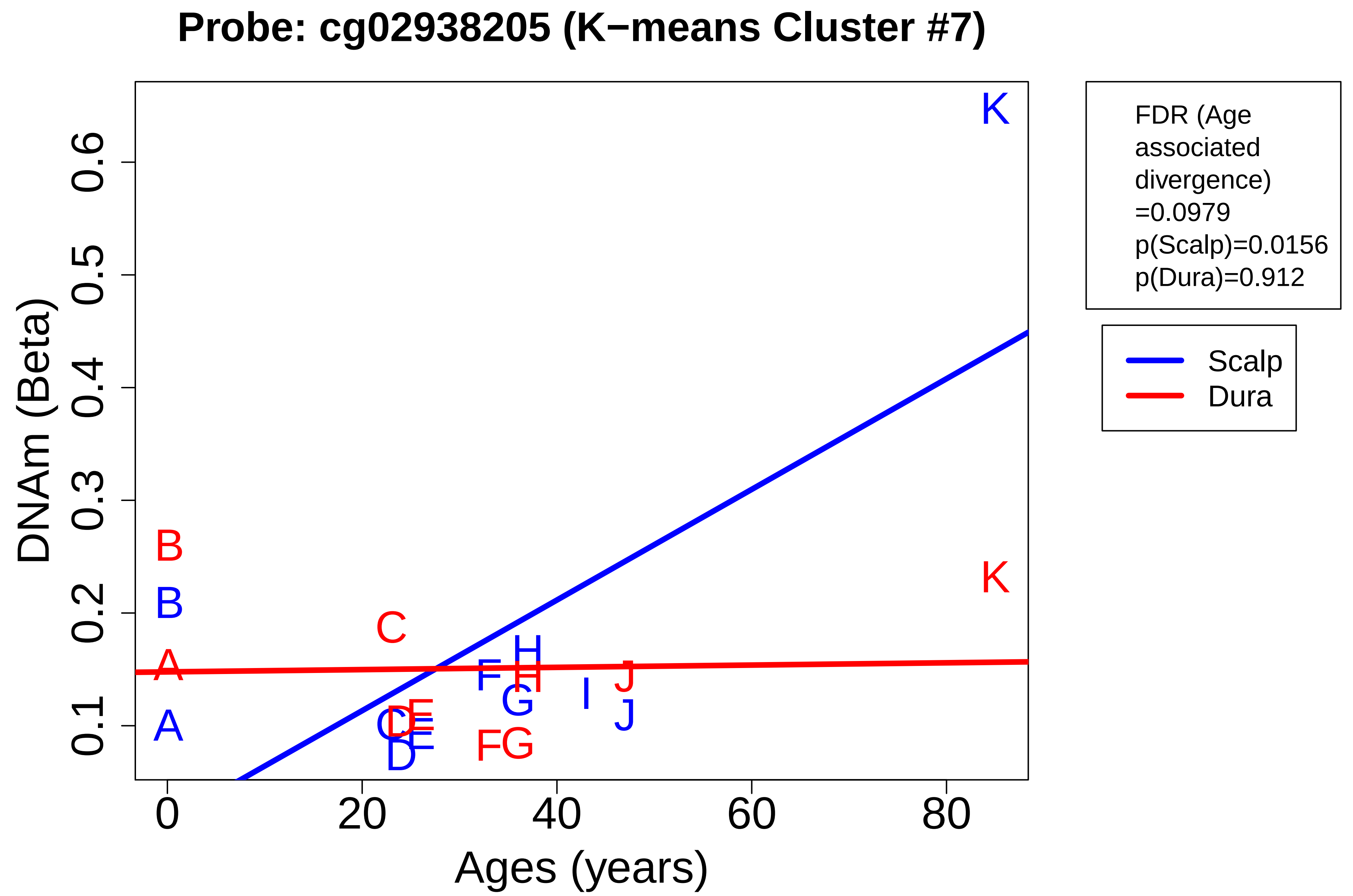

Probe: cg02938205 (K−means Cluster #7)
DNAm (Beta)
Ages (years)
FDR (Age associated divergence) =0.0979
p(Scalp)=0.0156
p(Dura)=0.912
Scalp
Dura

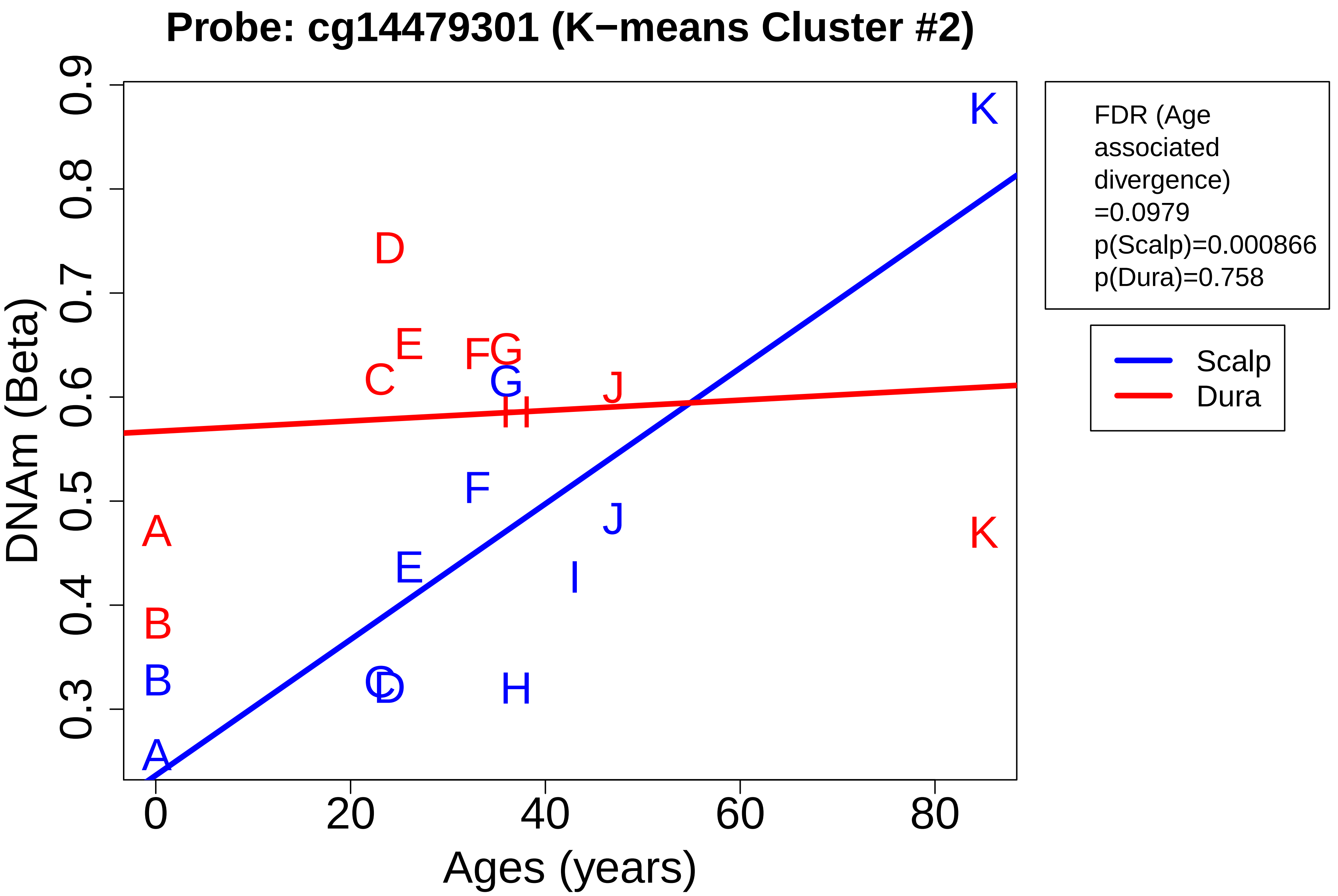

Probe: cg14479301 (K−means Cluster #2)
DNAm (Beta)
0.3
0.4
0.5
0.6
0.7
0.8
0.9
Ages (years)
0
20
40
60
80
A
B
C
D
E
F
G
H
I
J
K
FDR (Age associated divergence) =0.0979
p(Scalp)=0.000866
p(Dura)=0.758
Scalp
Dura

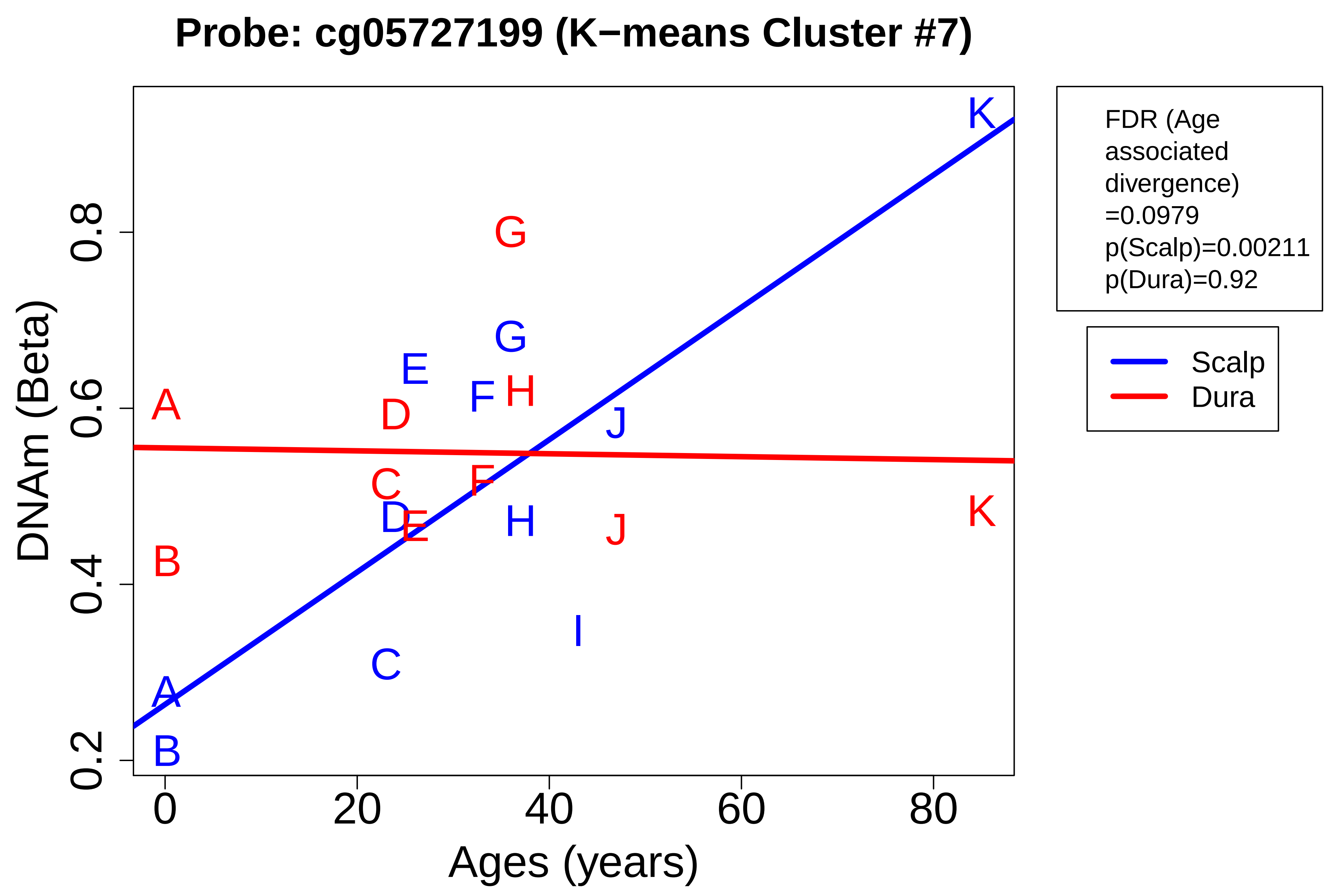

Probe: cg05727199 (K−means Cluster #7)
DNAm (Beta)
0.2
0.4
0.6
0.8
0
20
40
60
80
Ages (years)
FDR (Age associated divergence) =0.0979
p(Scalp)=0.00211
p(Dura)=0.92
Scalp
Dura
A
B
C
D
E
F
G
H
I
J
K

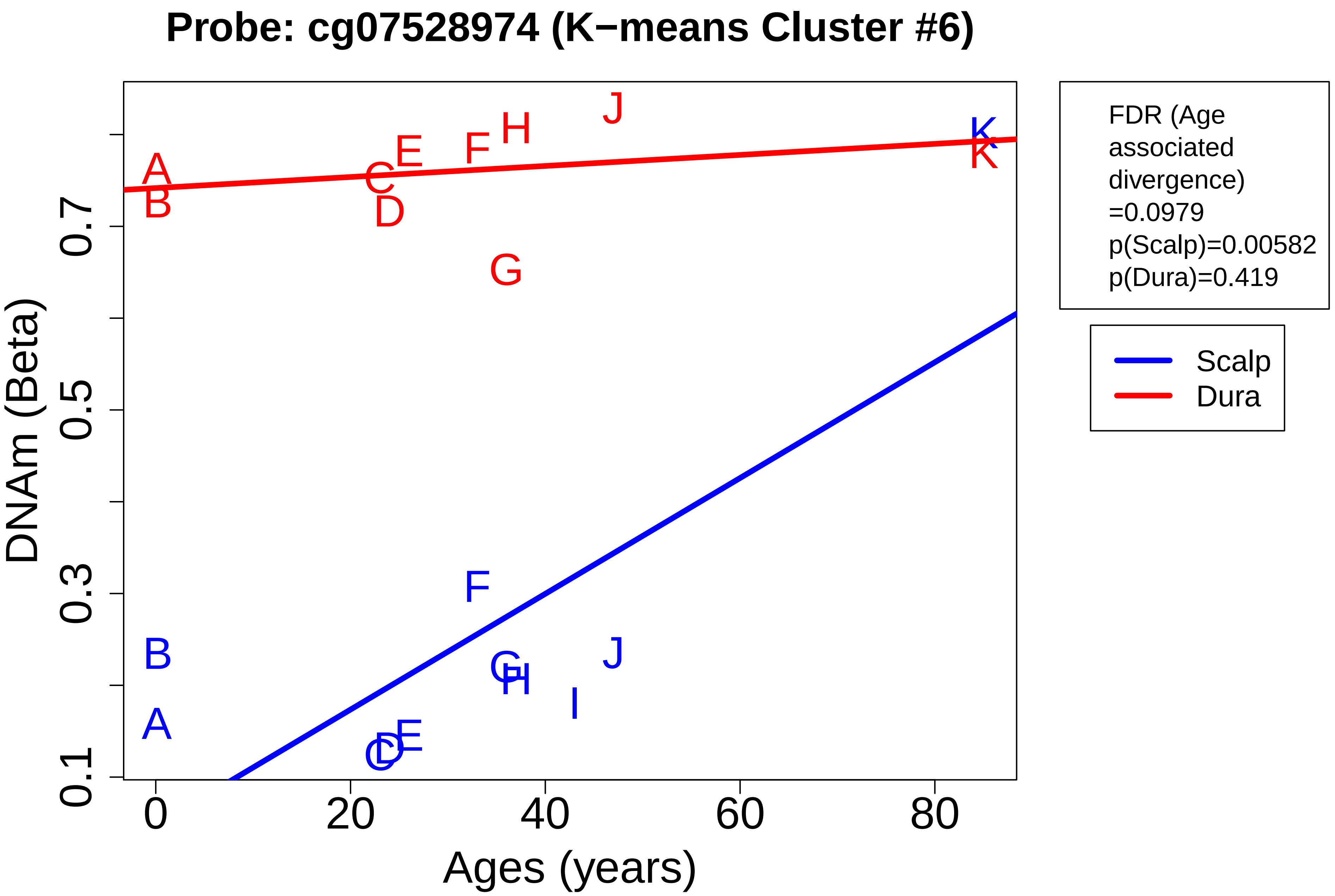

Probe: cg07528974 (K−means Cluster #6)
DNAm (Beta)
0.1
0.3
0.5
0.7
0
20
40
60
80
Ages (years)
FDR (Age associated divergence) =0.0979
p(Scalp)=0.00582
p(Dura)=0.419
Scalp
Dura

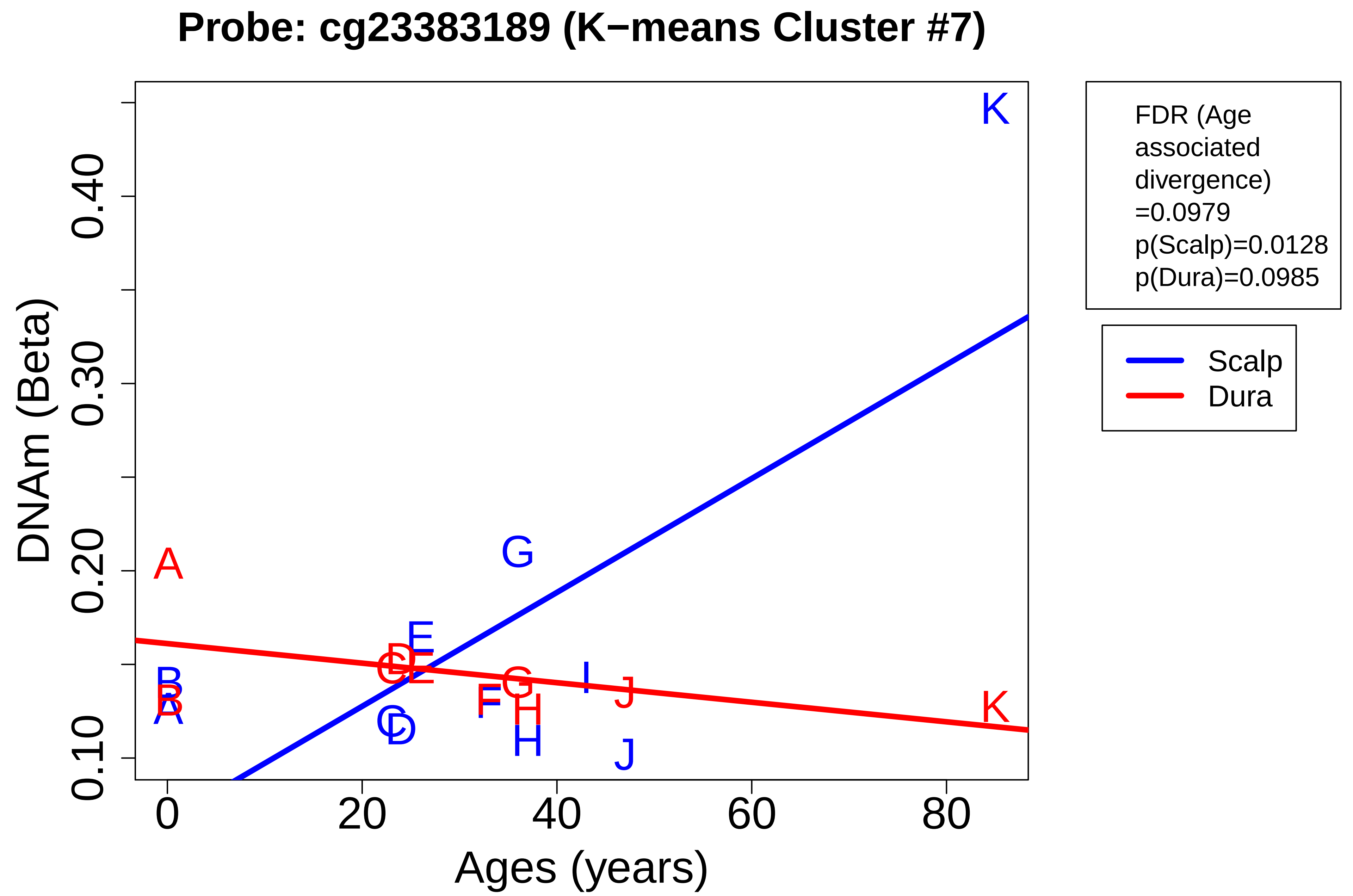
Probe: cg23383189 (K−means Cluster #7)
DNAm (Beta)
0.10
0.20
0.30
0.40
0
20
40
60
80
Ages (years)
FDR (Age associated divergence) =0.0979
p(Scalp)=0.0128
p(Dura)=0.0985
Scalp
Dura
A
B
C
D
E
F
G
H
I
J
K

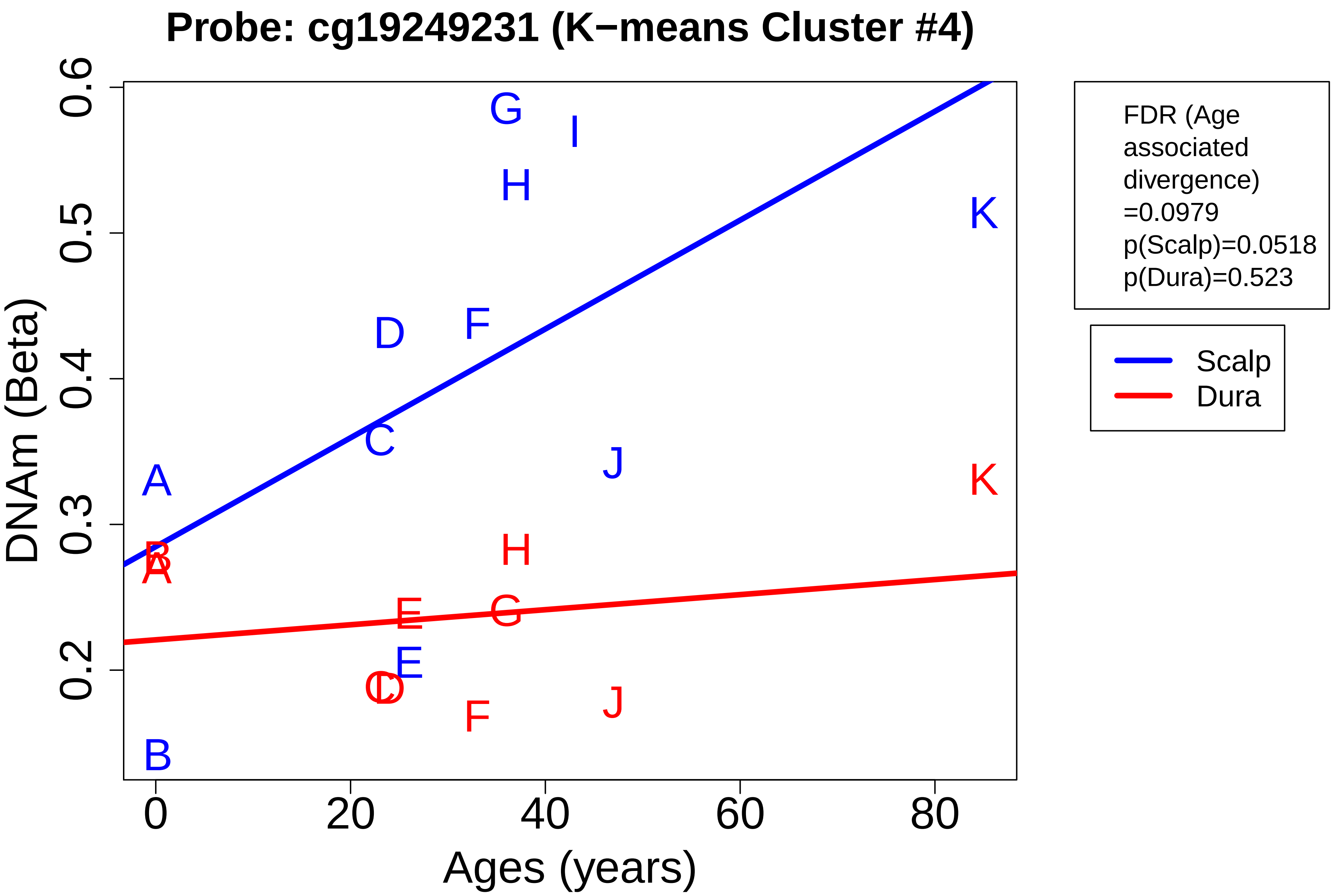

Probe: cg19249231 (K−means Cluster #4)
DNAm (Beta)
0.2
0.3
0.4
0.5
0.6
Ages (years)
0
20
40
60
80
FDR (Age associated divergence) =0.0979
p(Scalp)=0.0518
p(Dura)=0.523
Scalp
Dura

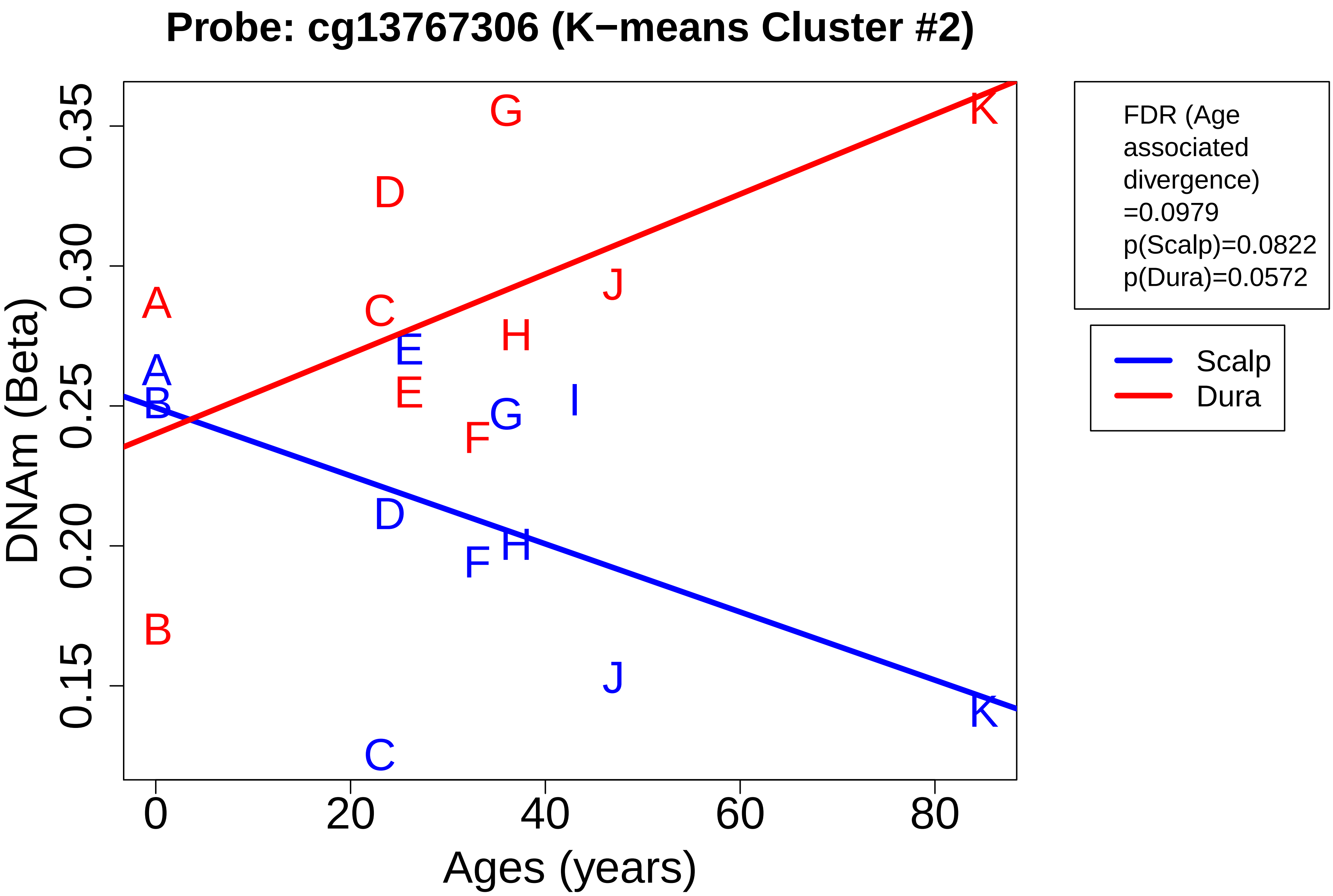

Probe: cg13767306 (K−means Cluster #2)
DNAm (Beta)
0.15
0.20
0.25
0.30
0.35
Ages (years)
0
20
40
60
80
FDR (Age associated divergence) =0.0979
p(Scalp)=0.0822
p(Dura)=0.0572
Scalp
Dura
A
B
C
D
E
F
G
H
I
J
K

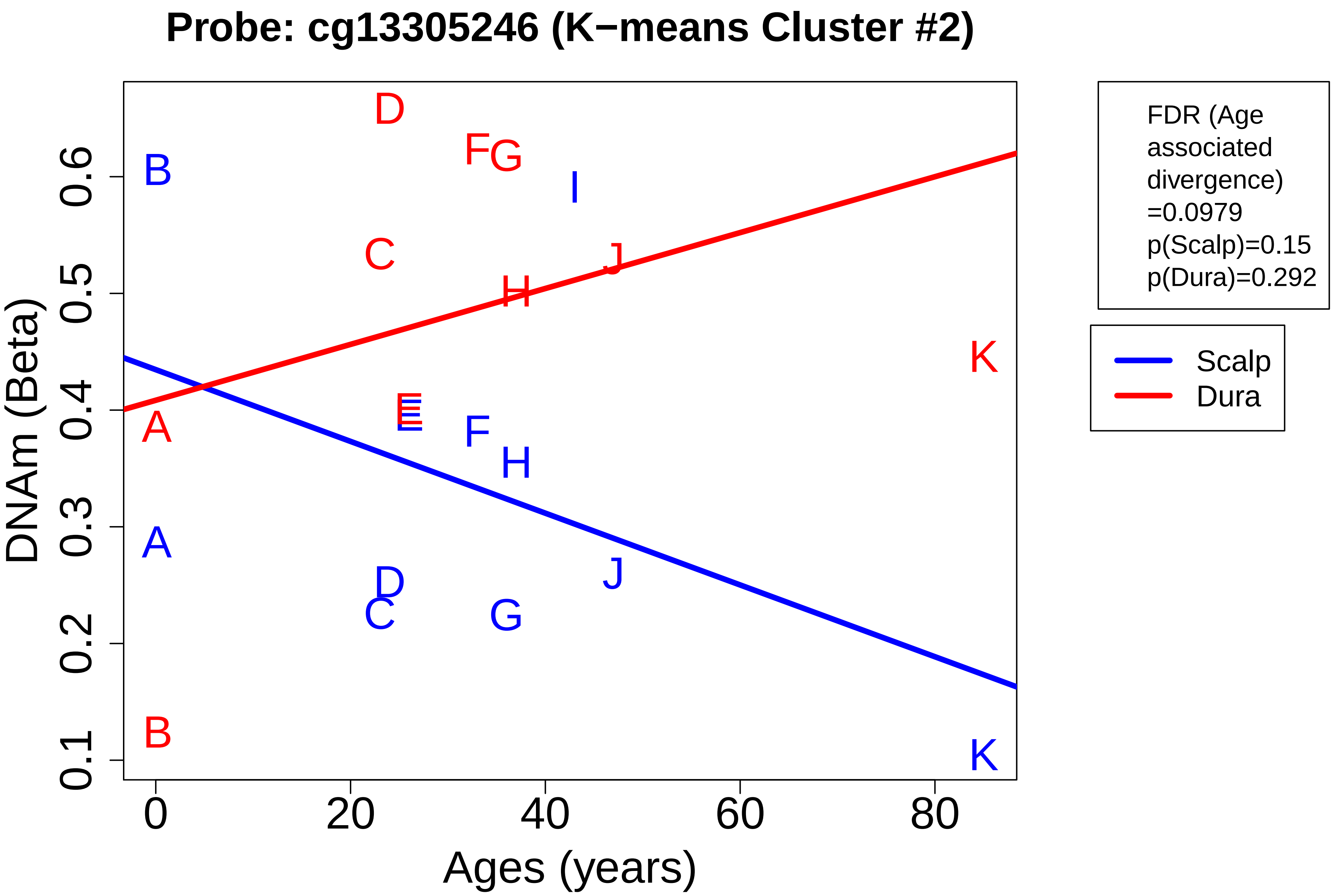

Probe: cg13305246 (K−means Cluster #2)
DNAm (Beta)
0.1
0.2
0.3
0.4
0.5
0.6
Ages (years)
0
20
40
60
80
FDR (Age associated divergence) =0.0979
p(Scalp)=0.15
p(Dura)=0.292
Scalp
Dura
A B C D E F G H I J K

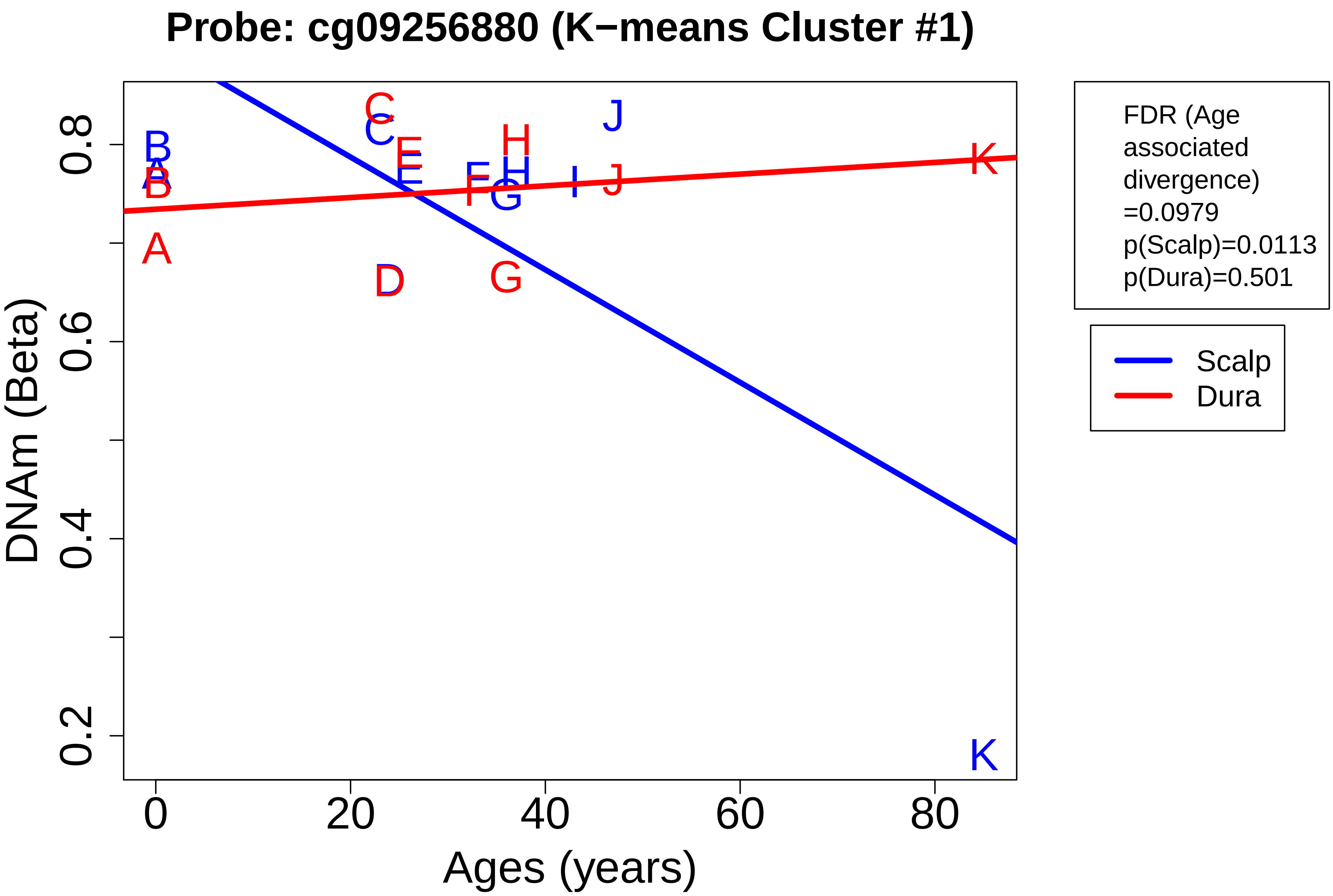

Probe: cg09256880 (K−means Cluster #1)
DNAm (Beta)
0.2
0.4
0.6
0.8
Ages (years)
0
20
40
60
80
A
B
C
D
E
F
G
H
I
J
K
FDR (Age associated divergence) =0.0979
p(Scalp)=0.0113
p(Dura)=0.501
Scalp
Dura

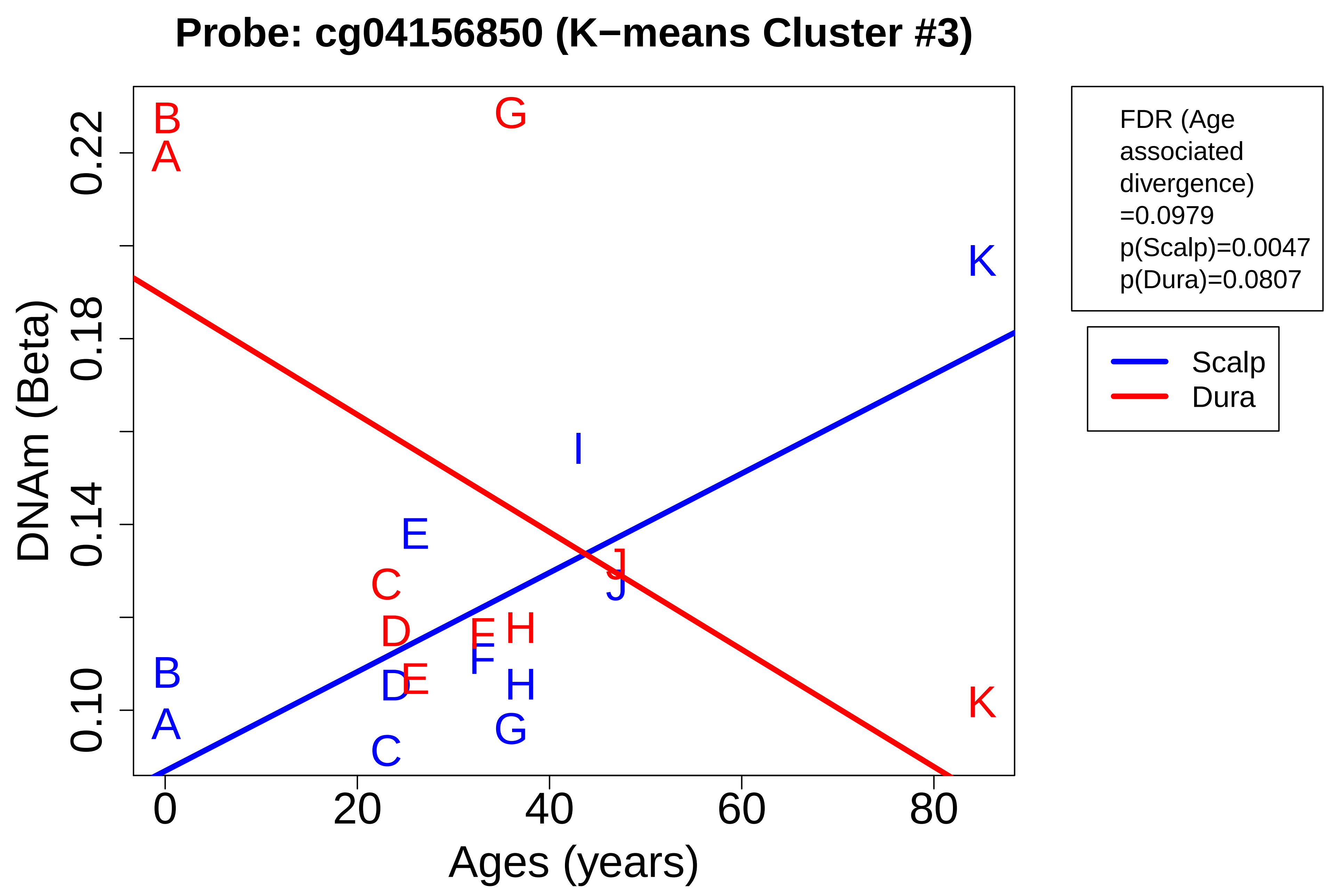

Probe: cg04156850 (K−means Cluster #3)
DNAm (Beta)
0.10
0.14
0.18
0.22
Ages (years)
0
20
40
60
80
FDR (Age associated divergence) =0.0979
p(Scalp)=0.0047
p(Dura)=0.0807
Scalp
Dura

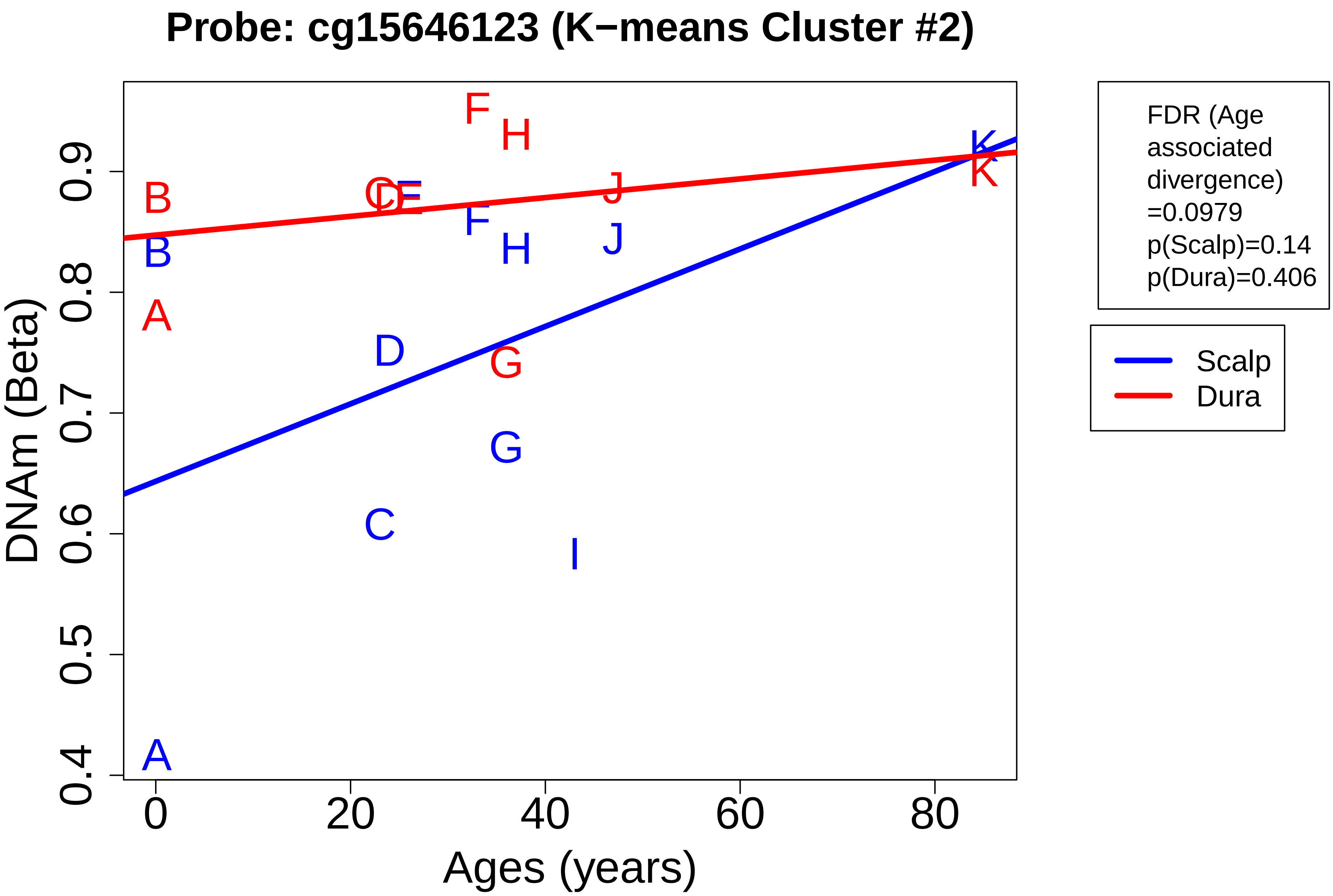

Probe: cg15646123 (K−means Cluster #2)
DNAm (Beta)
0.4
0.5
0.6
0.7
0.8
0.9
0
20
40
60
80
Ages (years)
FDR (Age associated divergence) =0.0979
p(Scalp)=0.14
p(Dura)=0.406
Scalp
Dura

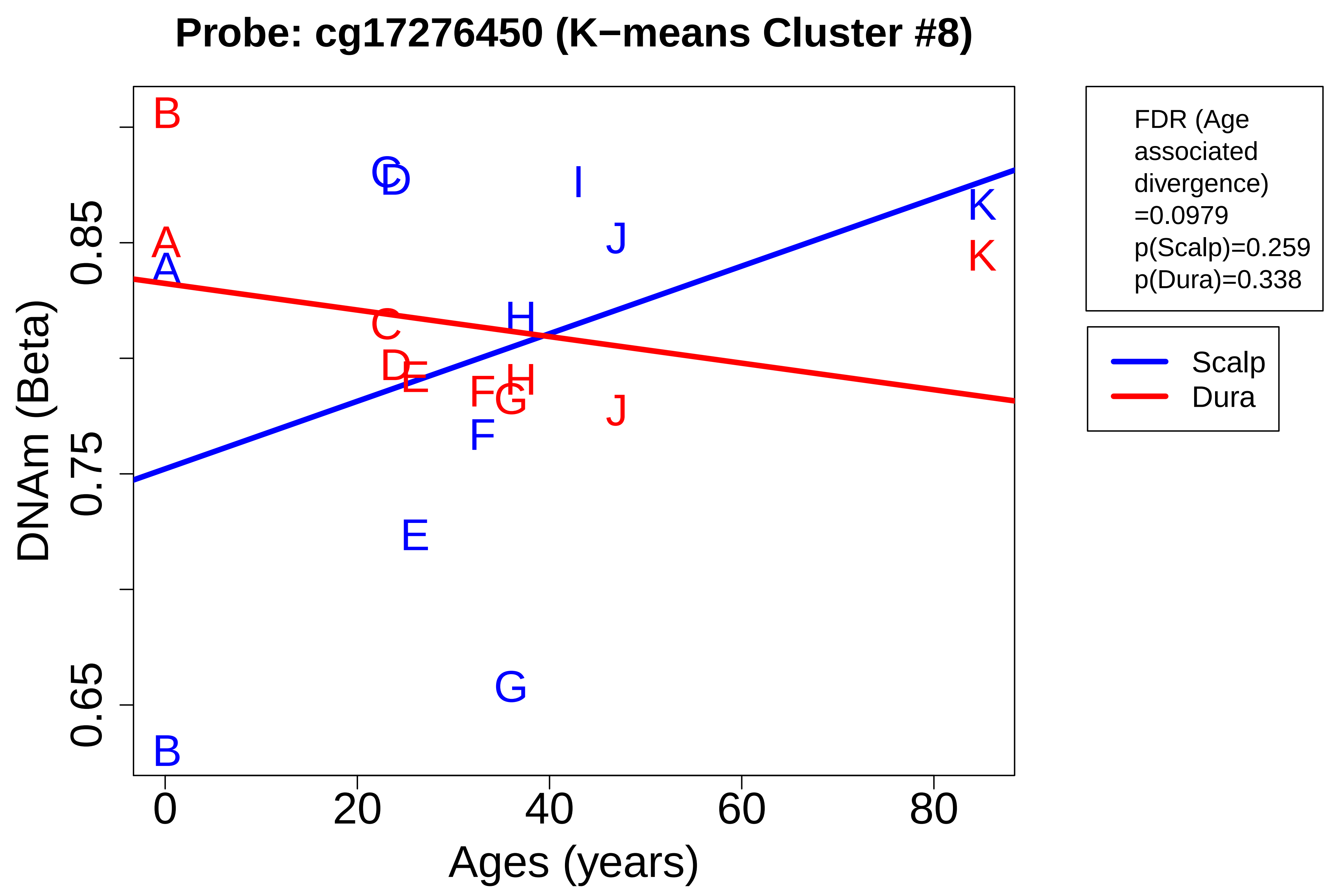
Probe: cg17276450 (K−means Cluster #8)
DNAm (Beta)
0.65
0.75
0.85
Ages (years)
0
20
40
60
80
FDR (Age associated divergence) =0.0979
p(Scalp)=0.259
p(Dura)=0.338
Scalp
Dura

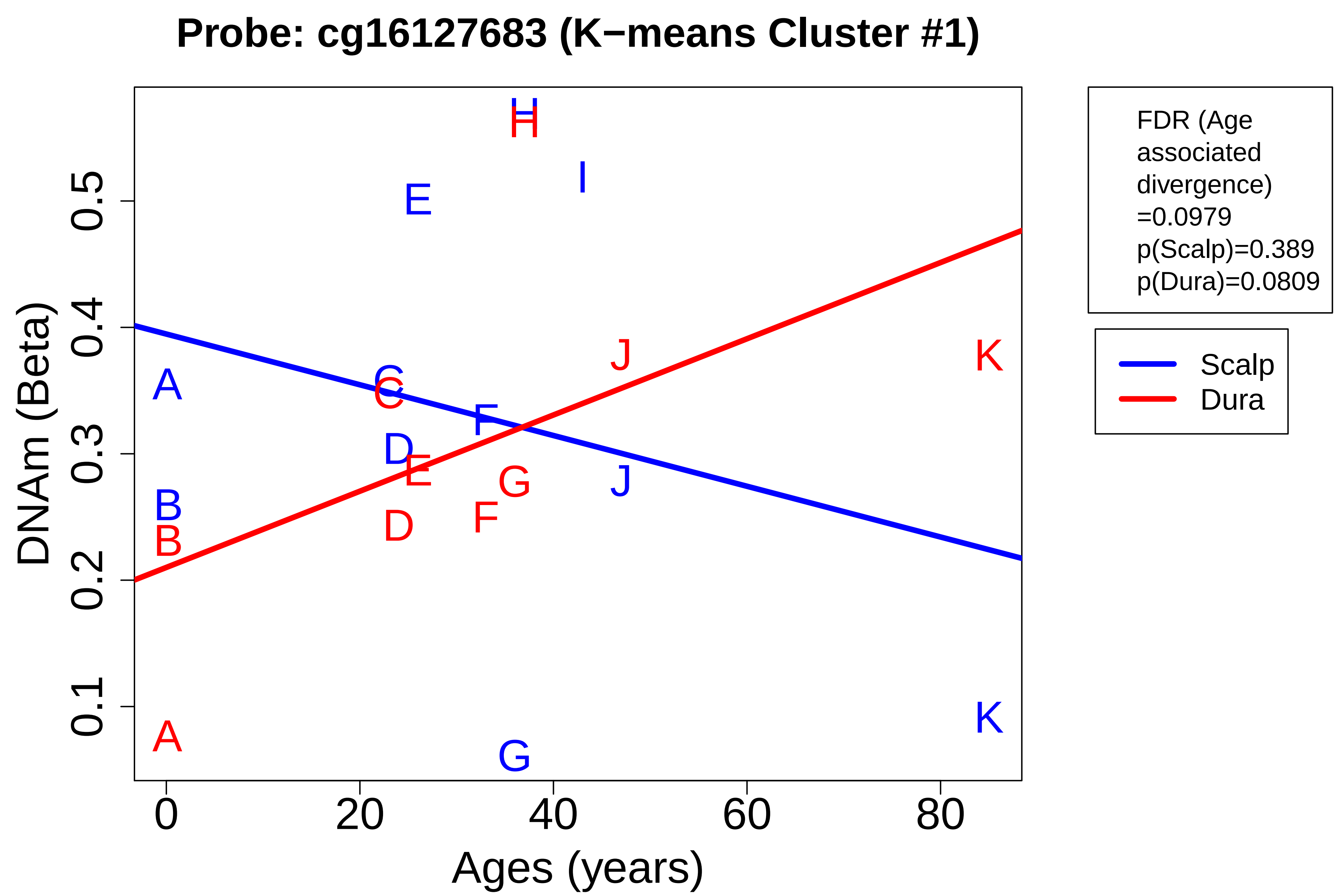

Probe: cg16127683 (K−means Cluster #1)
DNAm (Beta)
0.1
0.2
0.3
0.4
0.5
0
20
40
60
80
Ages (years)
A
B
C
D
E
F
G
H
I
J
K
FDR (Age associated divergence) =0.0979
p(Scalp)=0.389
p(Dura)=0.0809
Scalp
Dura

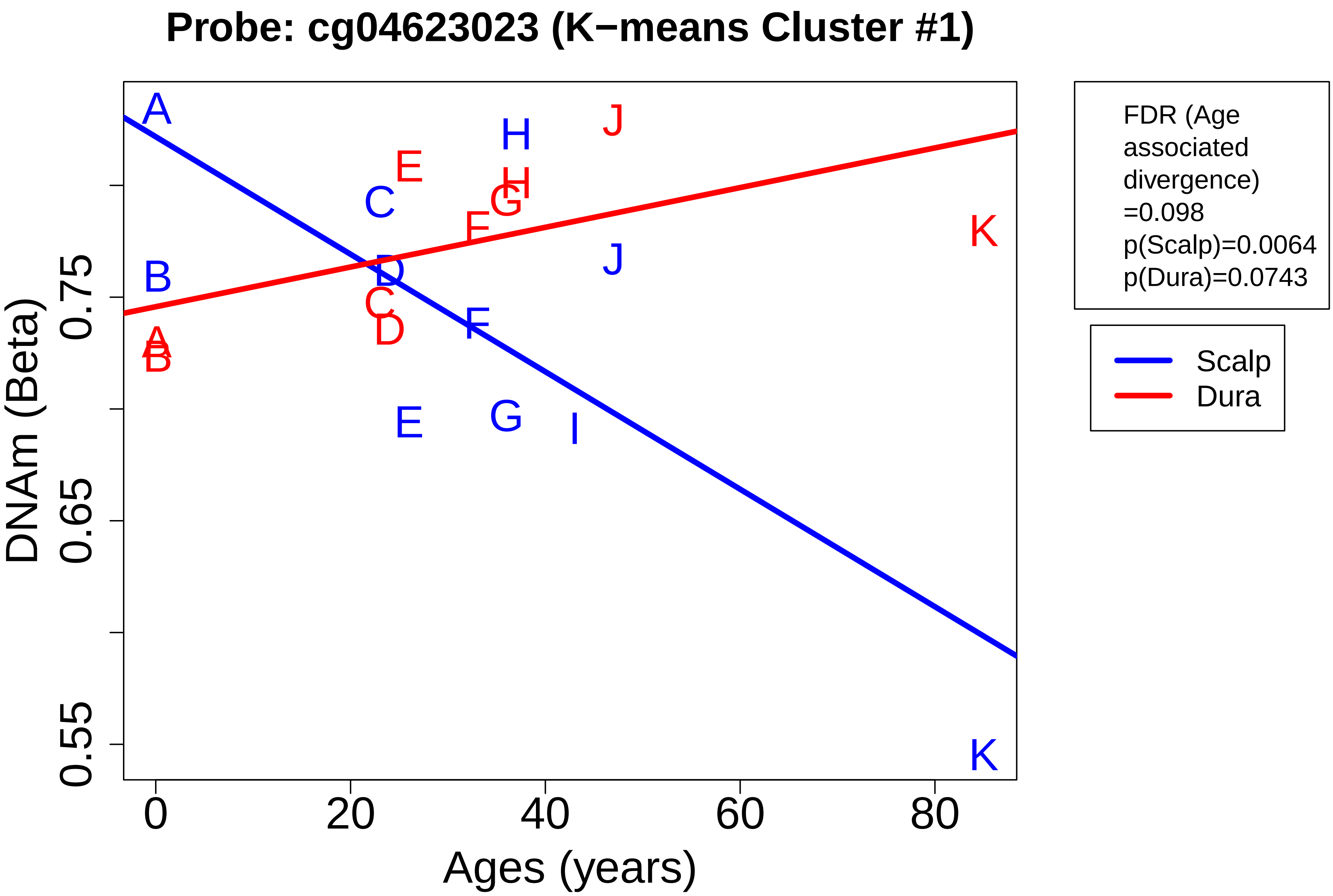
Probe: cg04623023 (K−means Cluster #1)
DNAm (Beta)
0.55
0.65
0.75
Ages (years)
0
20
40
60
80
A
B
C
D
E
F
G
H
I
J
K
FDR (Age associated divergence) =0.098
p(Scalp)=0.0064
p(Dura)=0.0743
Scalp
Dura

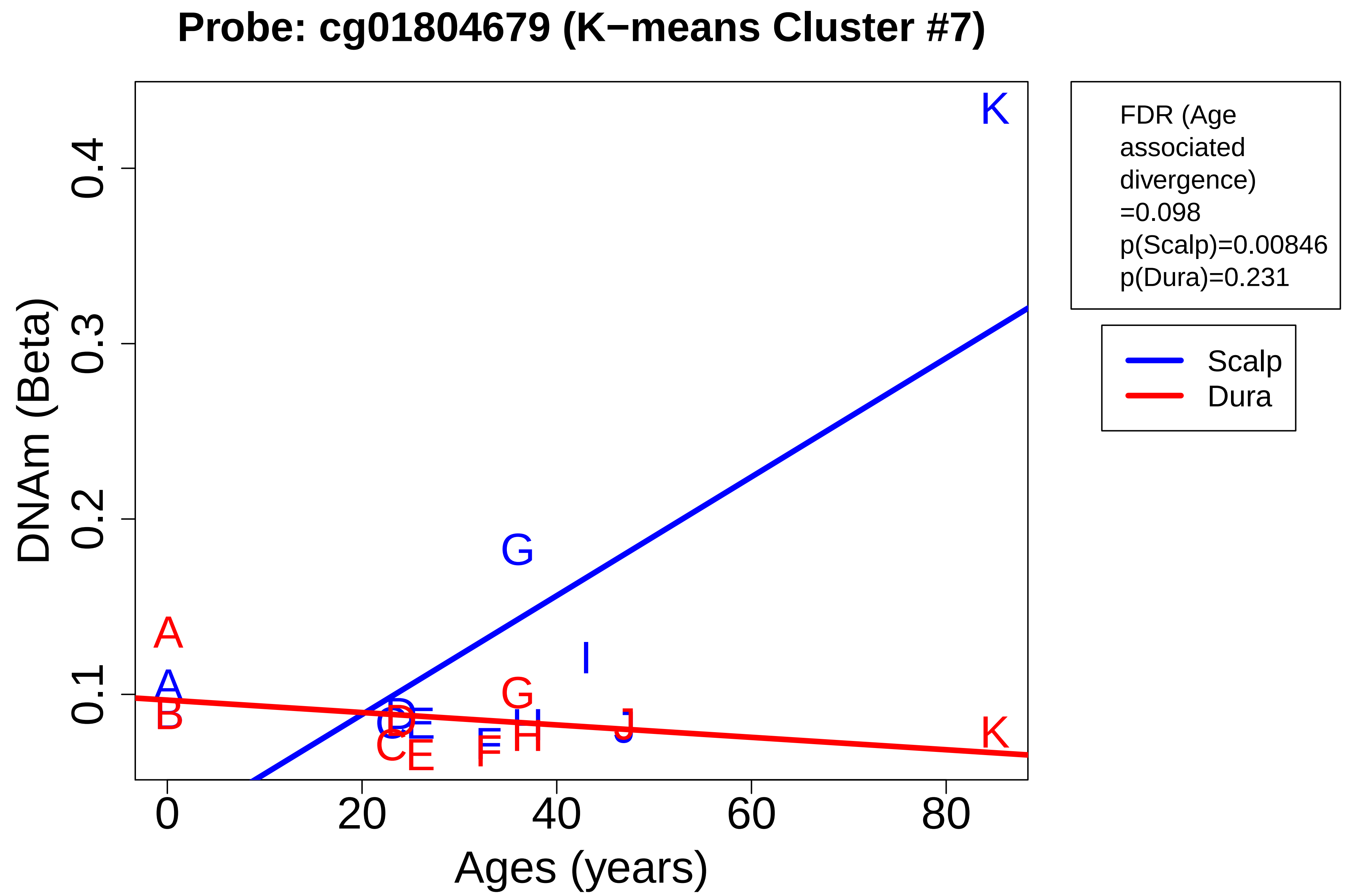

Probe: cg01804679 (K−means Cluster #7)
DNAm (Beta)
0.1
0.2
0.3
0.4
0
20
40
60
80
Ages (years)
FDR (Age associated divergence) =0.098
p(Scalp)=0.00846
p(Dura)=0.231
Scalp
Dura

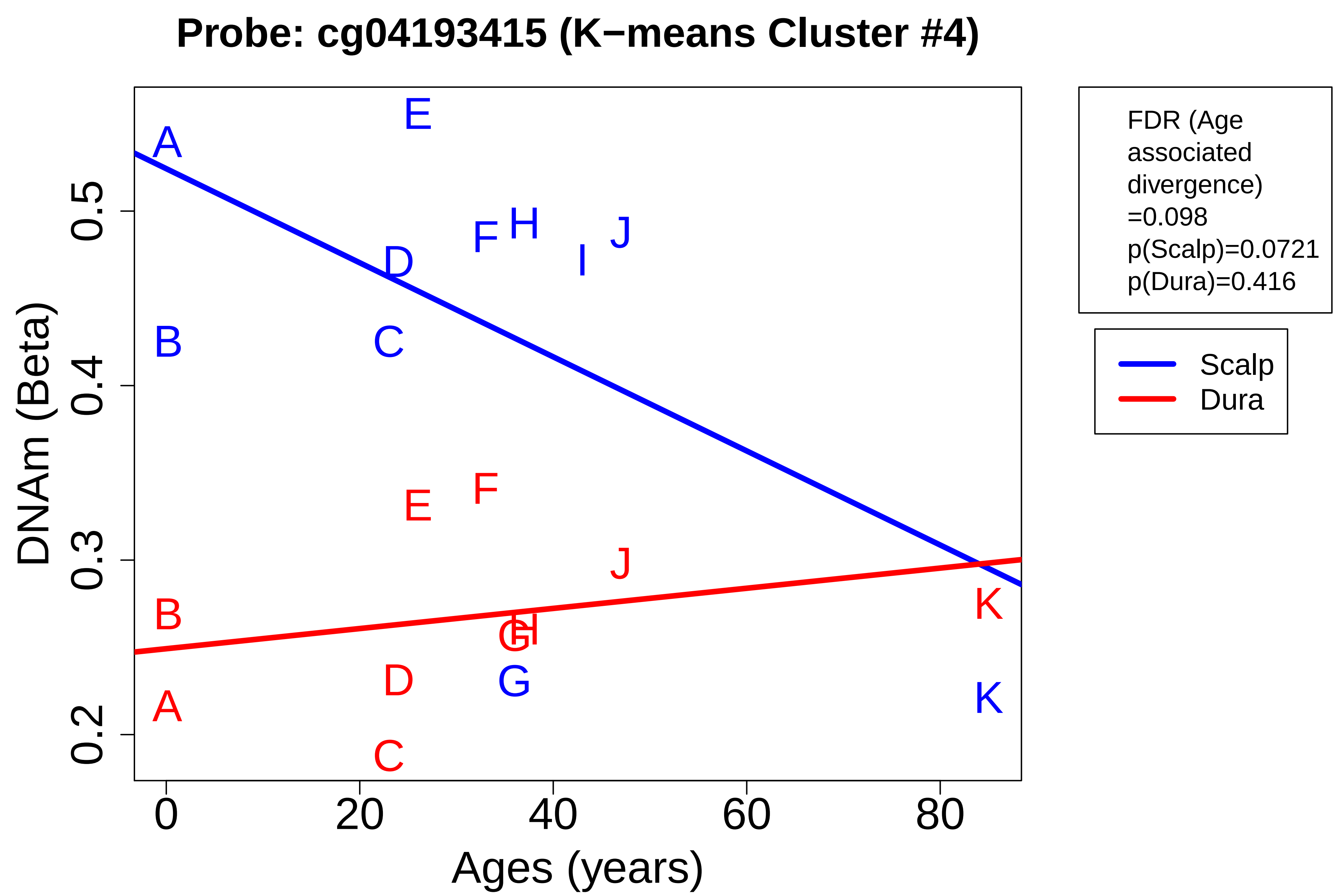

Probe: cg04193415 (K−means Cluster #4)
DNAm (Beta)
0.2
0.3
0.4
0.5
0
20
40
60
80
Ages (years)
A
B
C
D
E
F
G
H
I
J
K
FDR (Age associated divergence) =0.098
p(Scalp)=0.0721
p(Dura)=0.416
Scalp
Dura

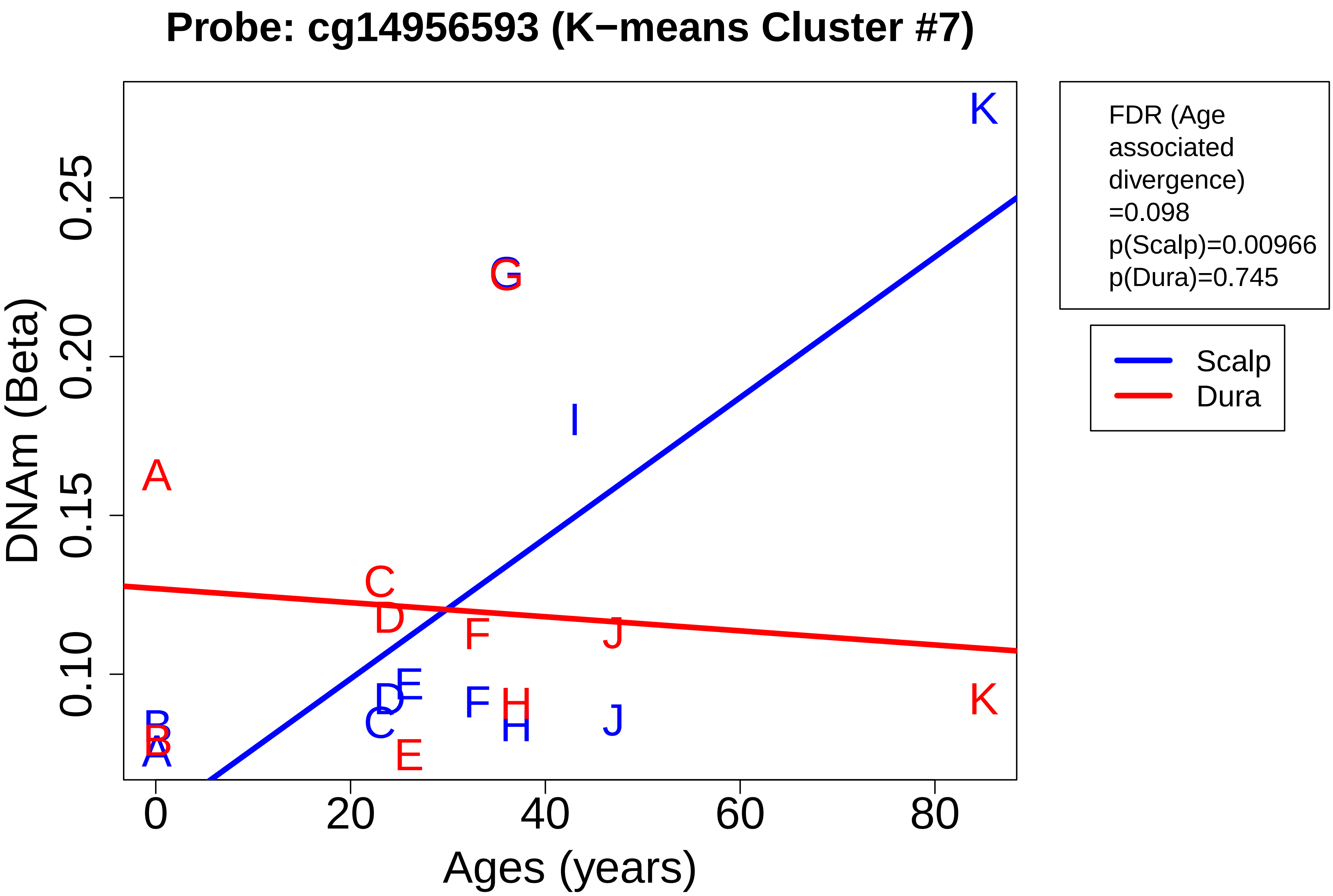

Probe: cg14956593 (K−means Cluster #7)
DNAm (Beta)
0.10
0.15
0.20
0.25
Ages (years)
0
20
40
60
80
FDR (Age associated divergence) =0.098
p(Scalp)=0.00966
p(Dura)=0.745
Scalp
Dura

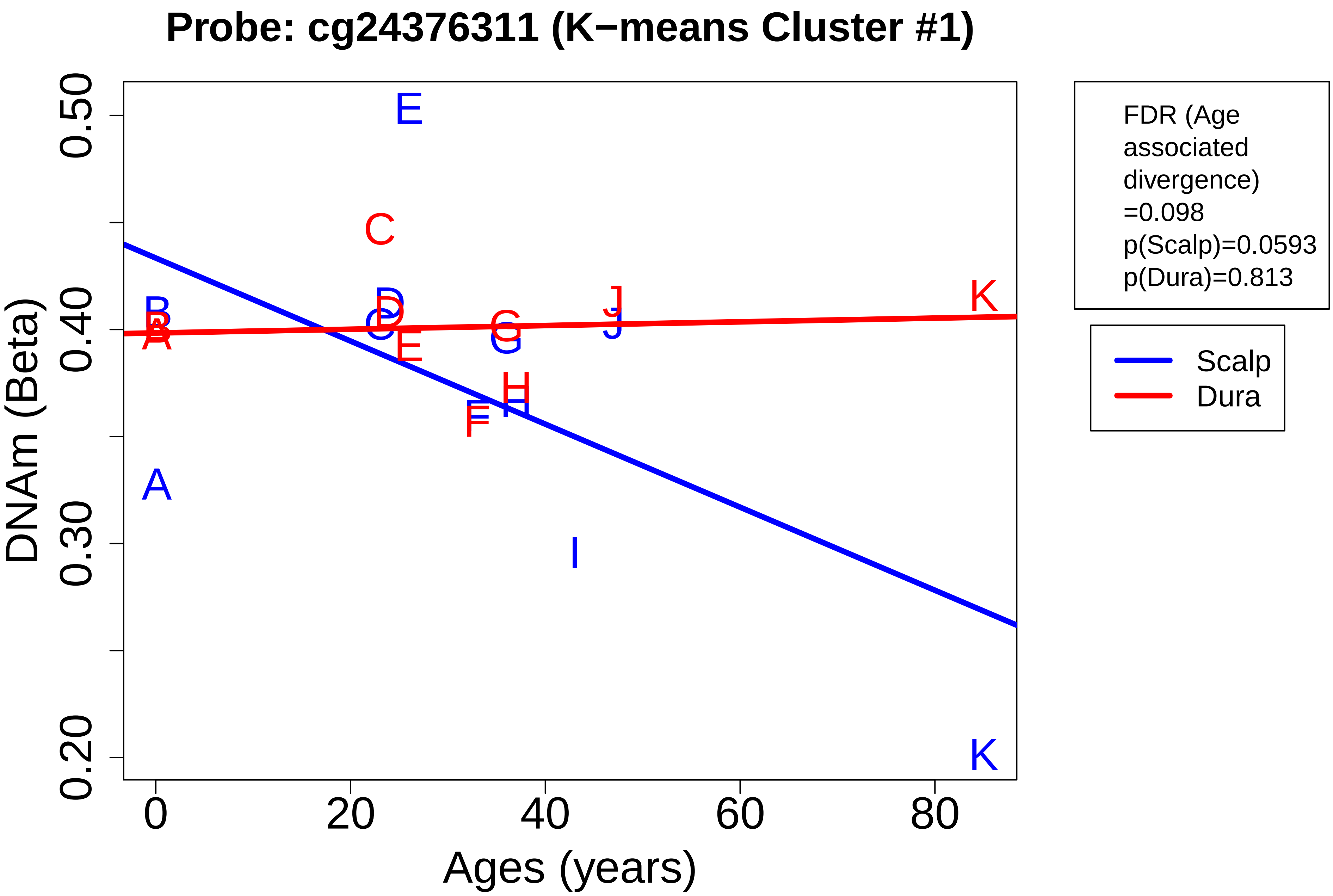

Probe: cg24376311 (K−means Cluster #1)
DNAm (Beta)
0.20
0.30
0.40
0.50
0
20
40
60
80
Ages (years)
FDR (Age associated divergence) =0.098
p(Scalp)=0.0593
p(Dura)=0.813
Scalp
Dura

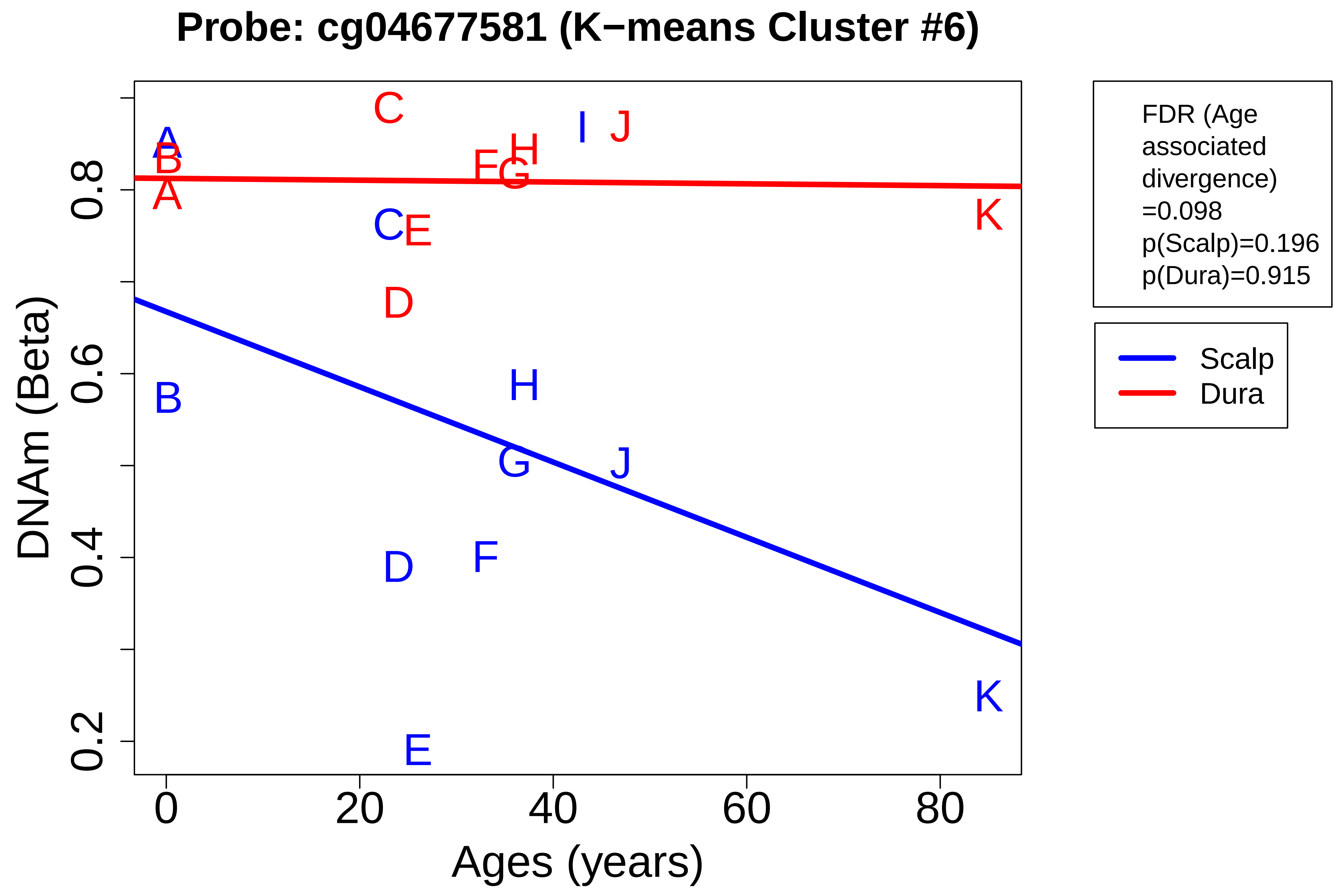

Probe: cg04677581 (K−means Cluster #6)
DNAm (Beta)
0.2
0.4
0.6
0.8
0
20
40
60
80
Ages (years)
FDR (Age associated divergence) =0.098
p(Scalp)=0.196
p(Dura)=0.915
Scalp
Dura
A
B
C
D
E
F
G
H
I
J
K

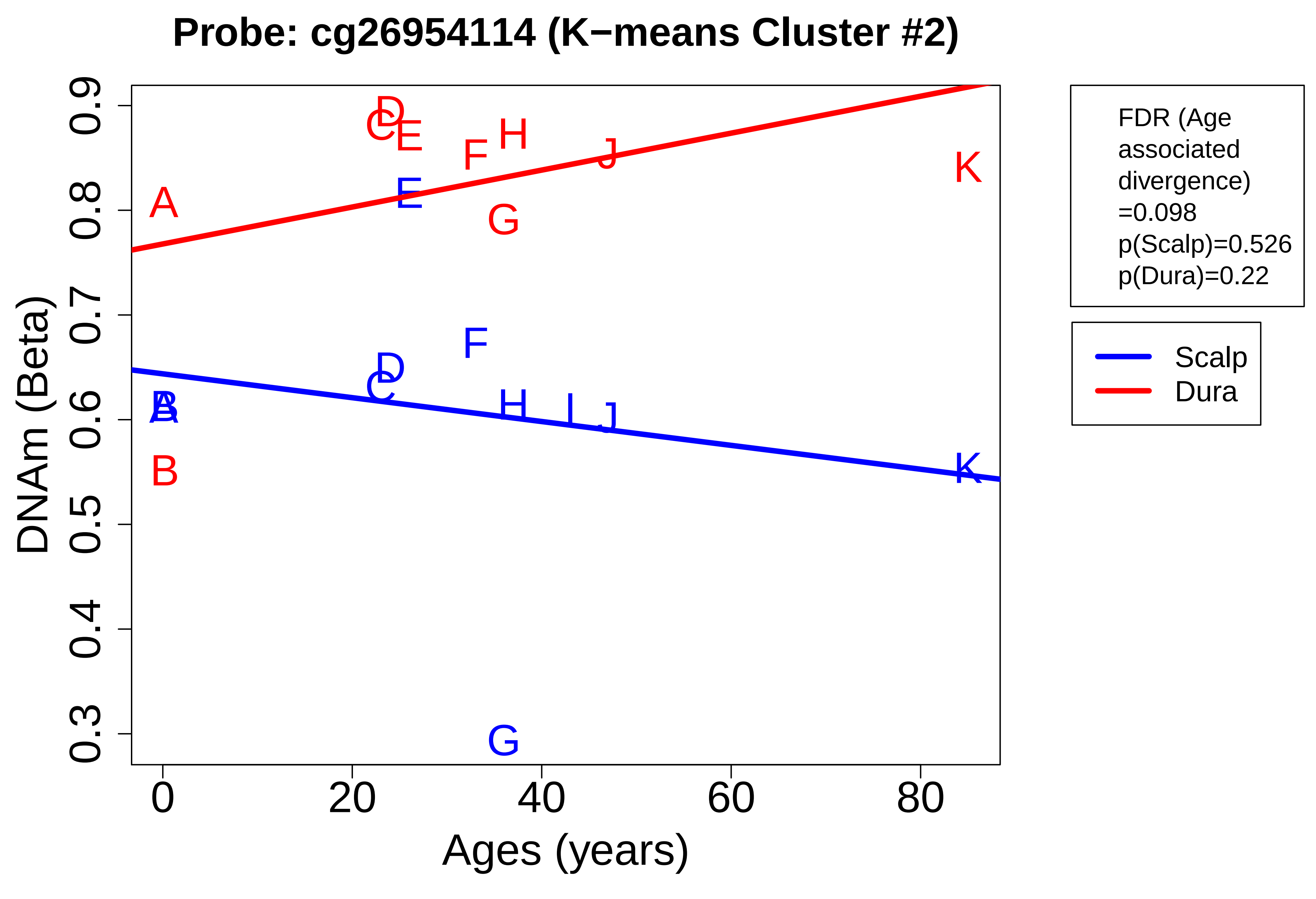

Probe: cg26954114 (K−means Cluster #2)
DNAm (Beta)
Ages (years)
0.3
0.4
0.5
0.6
0.7
0.8
0.9
0
20
40
60
80
FDR (Age associated divergence) =0.098
p(Scalp)=0.526
p(Dura)=0.22
Scalp
Dura

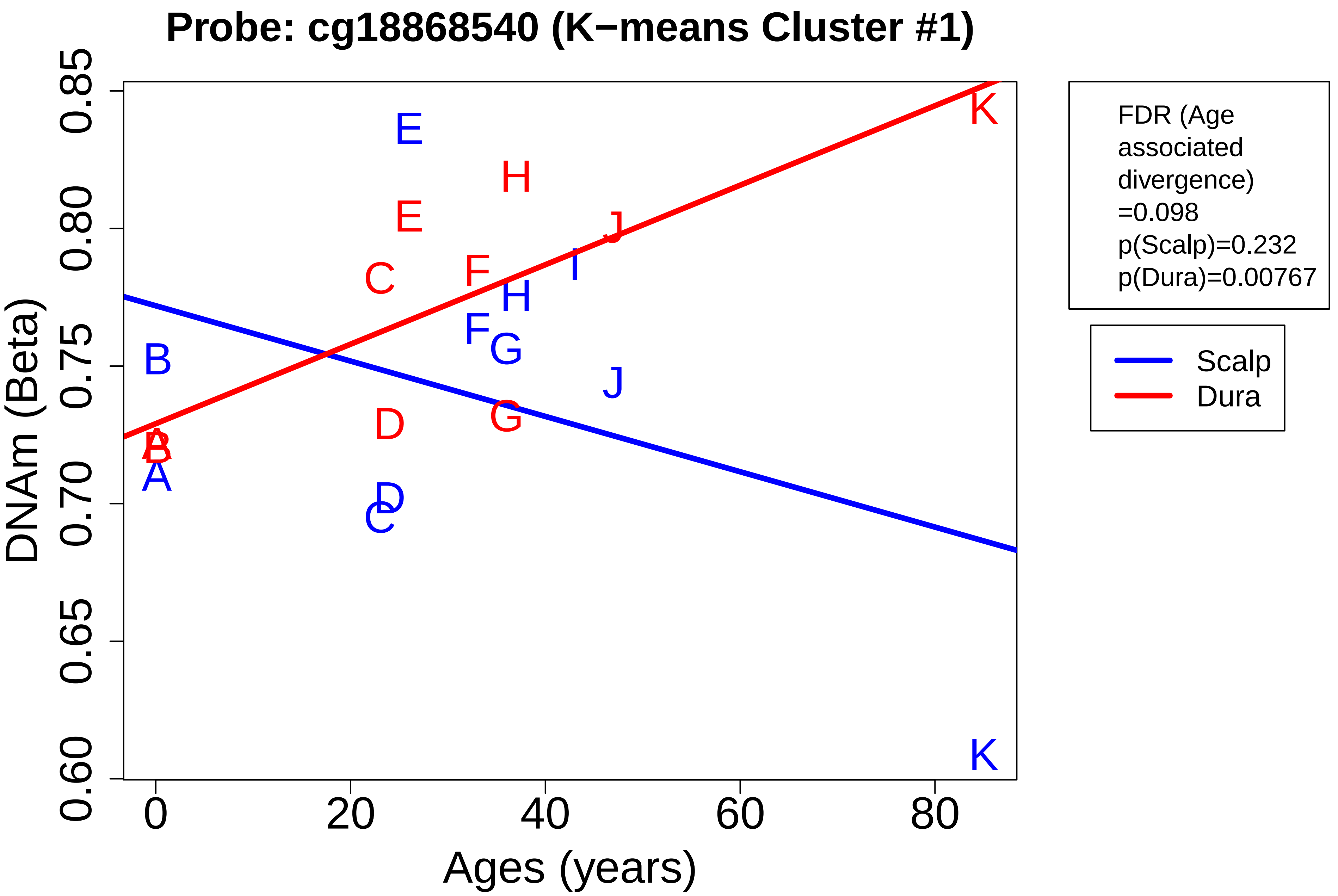
Probe: cg18868540 (K−means Cluster #1)
DNAm (Beta)
0.60
0.65
0.70
0.75
0.80
0.85
0
20
40
60
80
Ages (years)
A
B
C
D
E
F
G
H
I
J
K
FDR (Age associated divergence) =0.098
p(Scalp)=0.232
p(Dura)=0.00767
Scalp
Dura

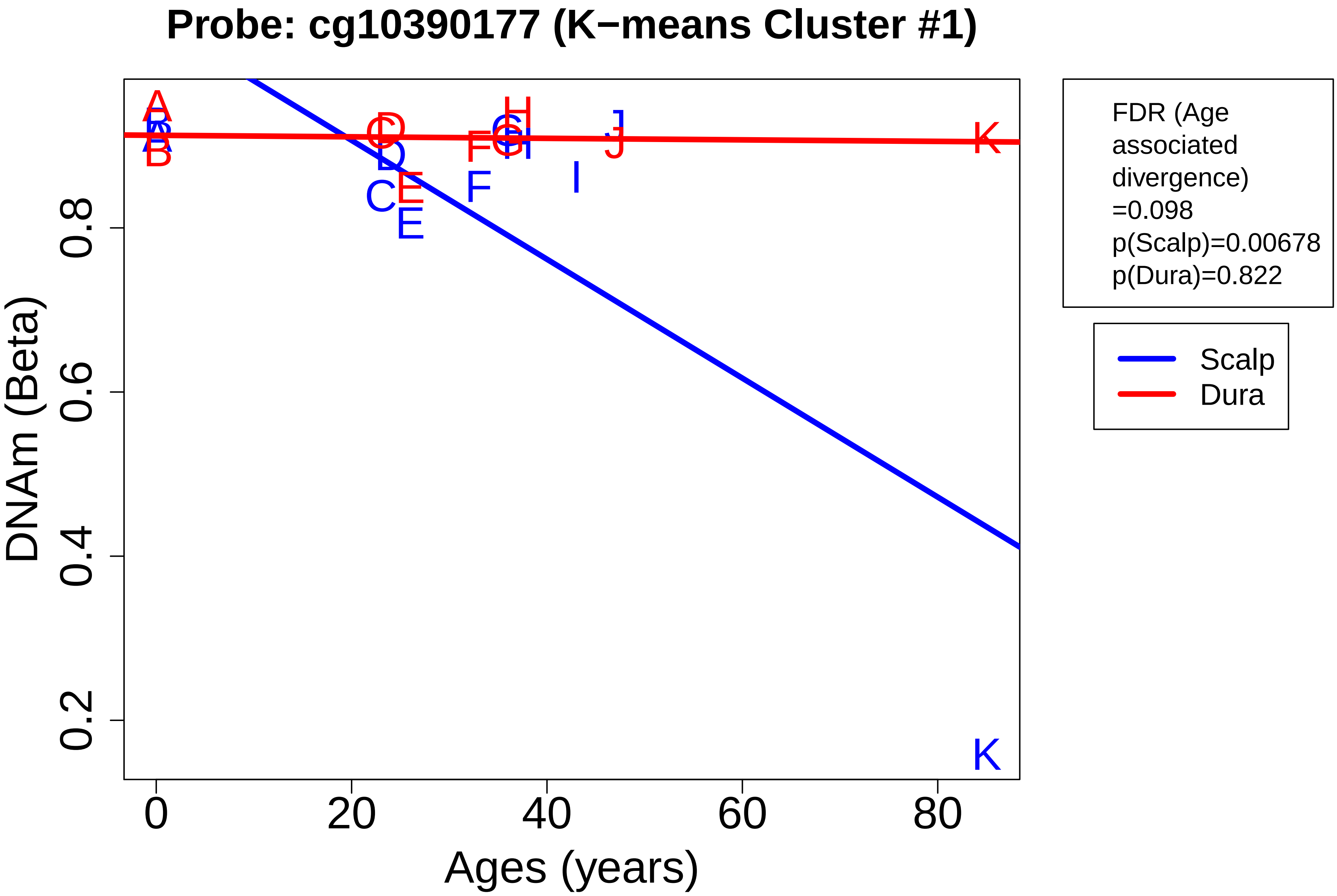

Probe: cg10390177 (K−means Cluster #1)
DNAm (Beta)
Ages (years)
0.2
0.4
0.6
0.8
0
20
40
60
80
FDR (Age associated divergence) =0.098
p(Scalp)=0.00678
p(Dura)=0.822
Scalp
Dura

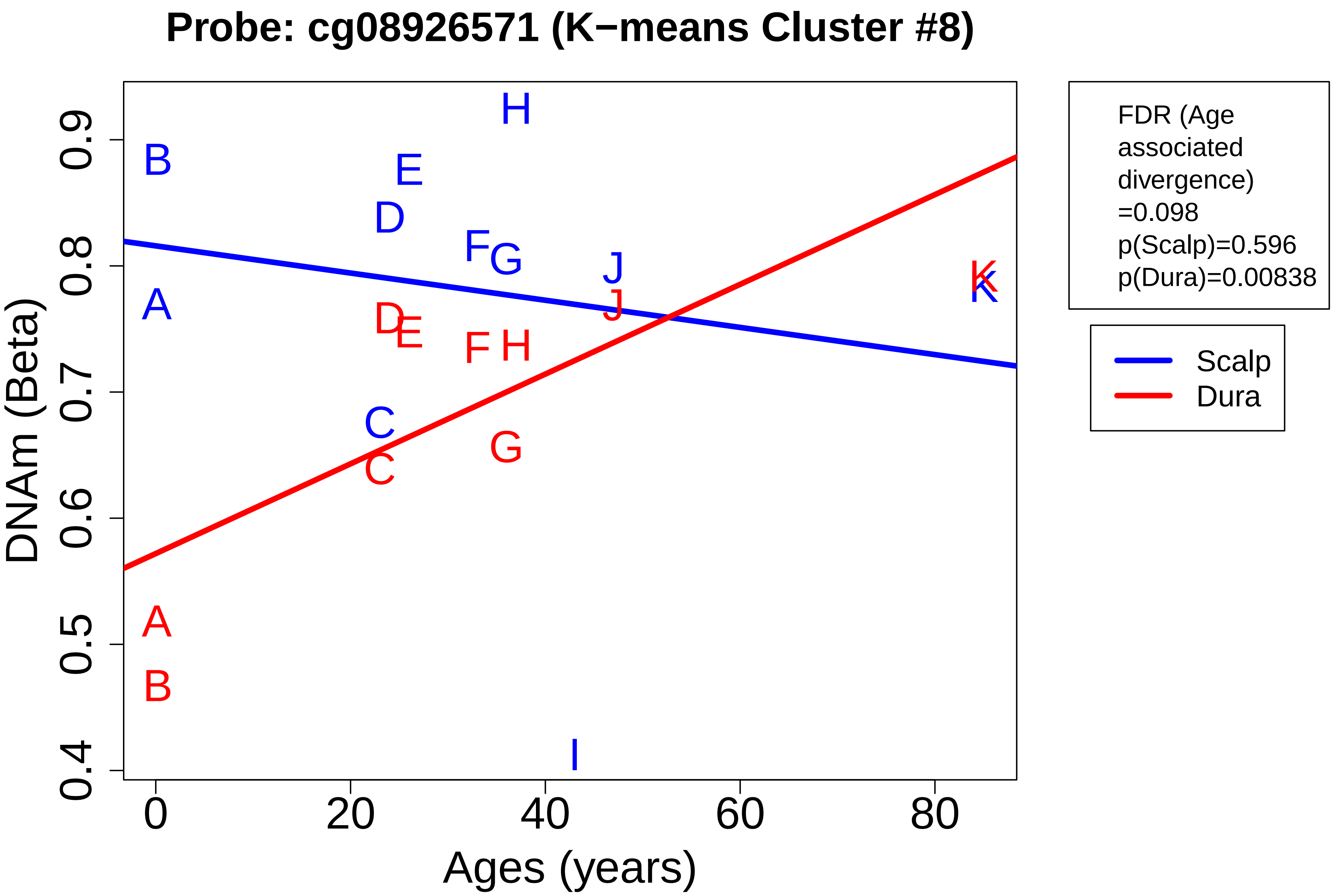

Probe: cg08926571 (K−means Cluster #8)
DNAm (Beta)
0.4
0.5
0.6
0.7
0.8
0.9
0
20
40
60
80
Ages (years)
FDR (Age associated divergence) =0.098
p(Scalp)=0.596
p(Dura)=0.00838
Scalp
Dura

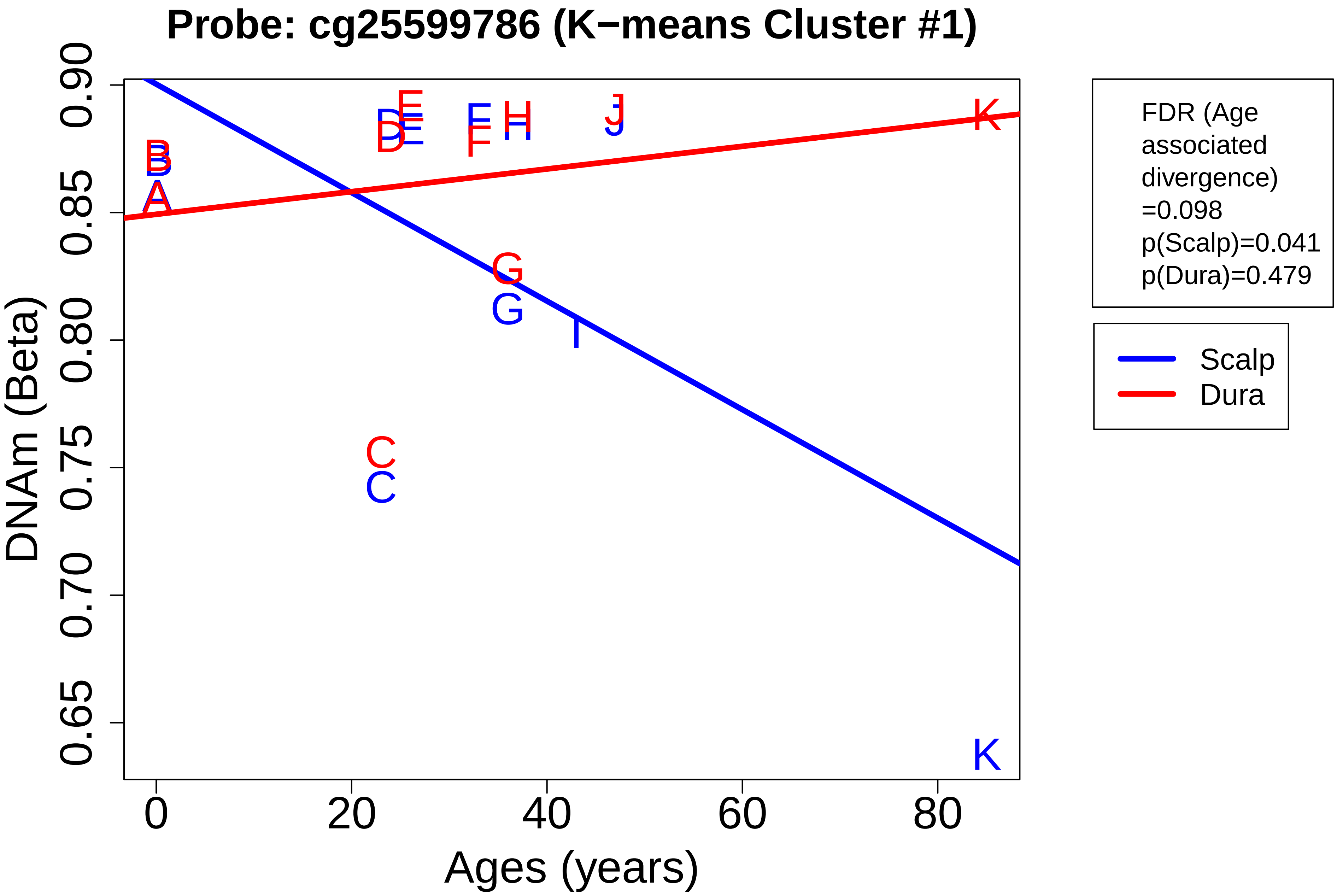

Probe: cg25599786 (K−means Cluster #1)
DNAm (Beta)
0.65
0.70
0.75
0.80
0.85
0.90
0
20
40
60
80
Ages (years)
FDR (Age associated divergence) =0.098
p(Scalp)=0.041
p(Dura)=0.479
Scalp
Dura

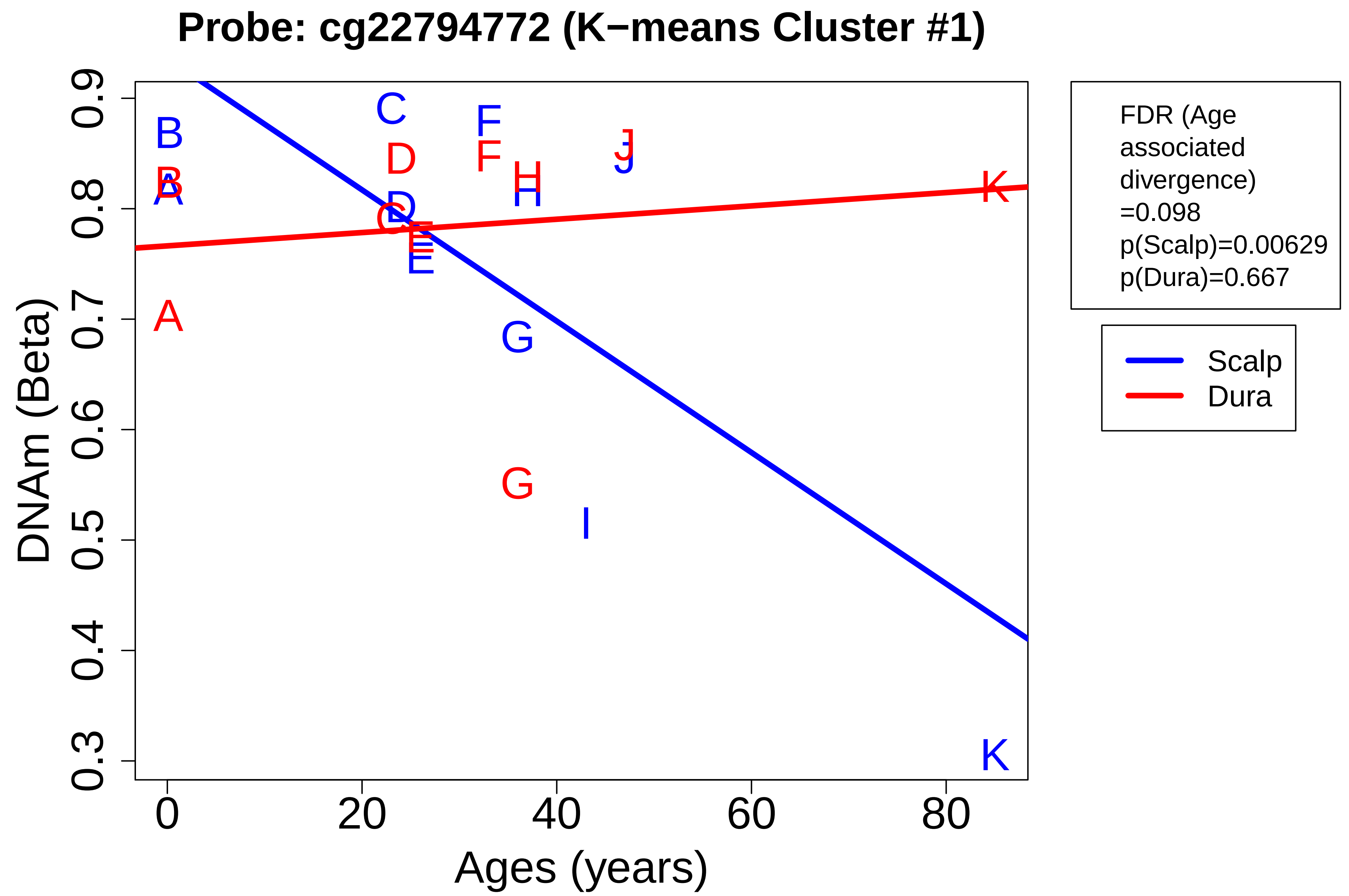

Probe: cg22794772 (K−means Cluster #1)
DNAm (Beta)
0.3
0.4
0.5
0.6
0.7
0.8
0.9
0
20
40
60
80
Ages (years)
FDR (Age associated divergence) =0.098
p(Scalp)=0.00629
p(Dura)=0.667
Scalp
Dura
A
B
C
D
E
F
G
H
I
J
K

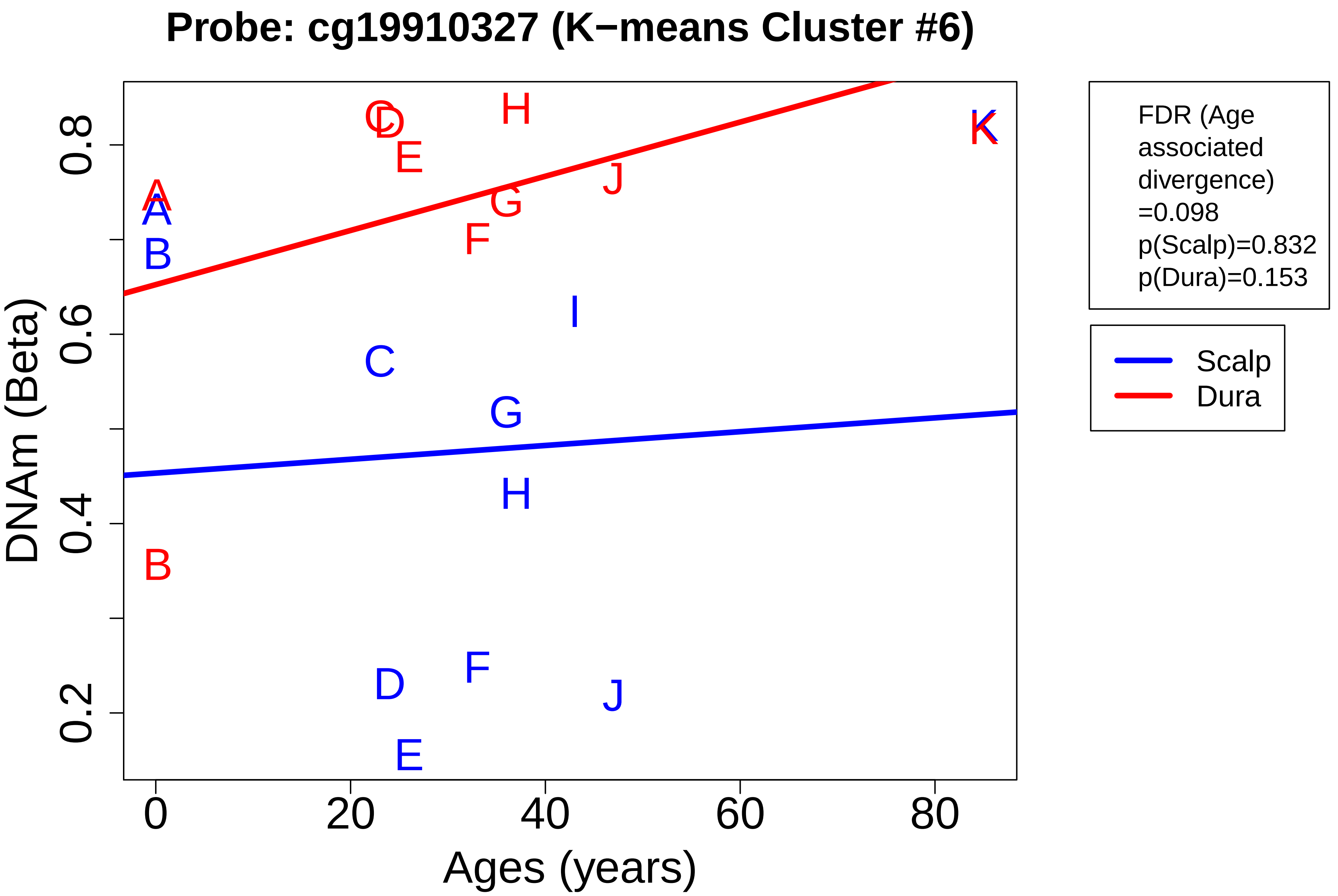
Probe: cg19910327 (K−means Cluster #6)
DNAm (Beta)
0.2
0.4
0.6
0.8
0
20
40
60
80
Ages (years)
FDR (Age associated divergence) =0.098
p(Scalp)=0.832
p(Dura)=0.153
Scalp
Dura

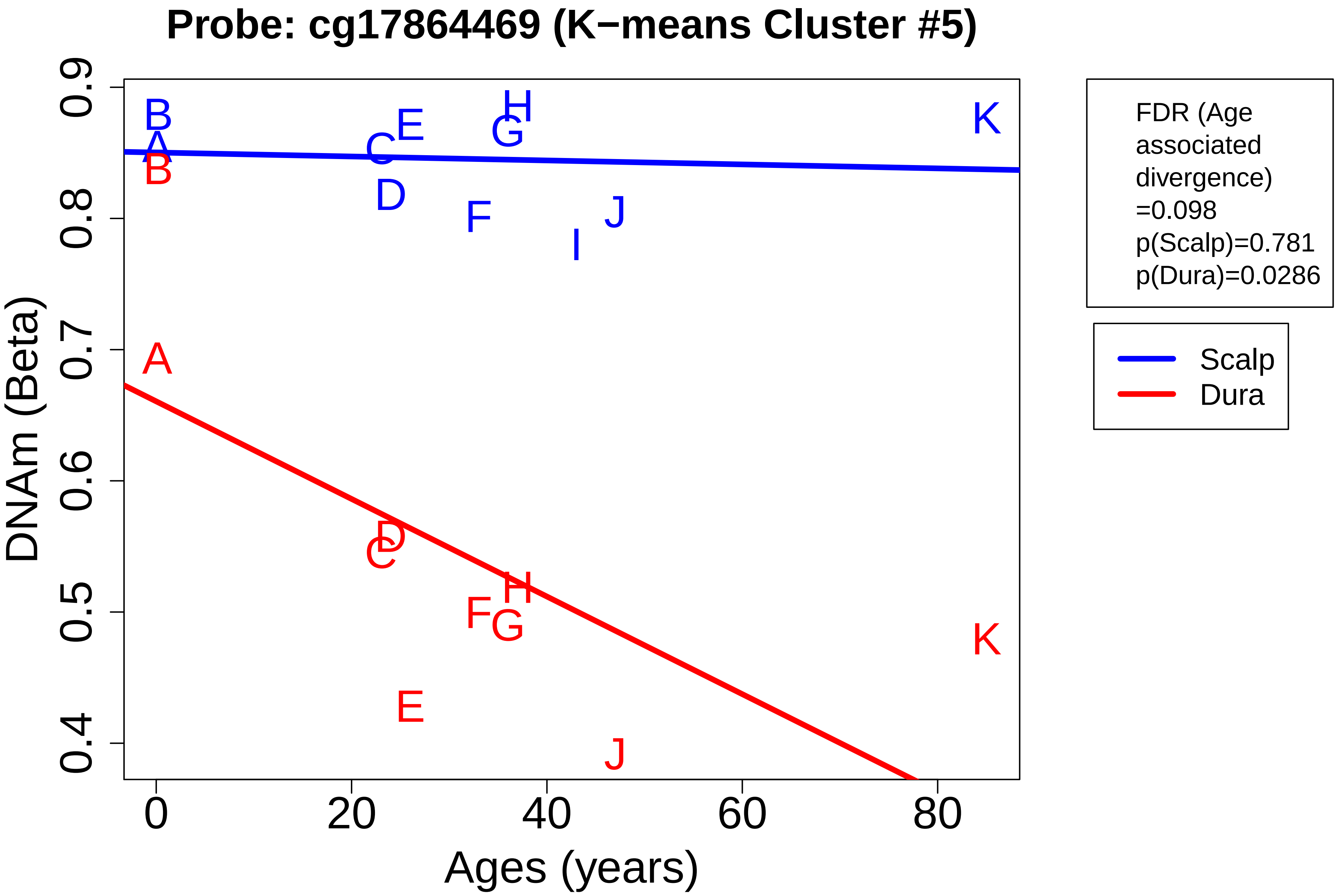

Probe: cg17864469 (K−means Cluster #5)
DNAm (Beta)
0.4
0.5
0.6
0.7
0.8
0.9
0
20
40
60
80
Ages (years)
FDR (Age associated divergence) =0.098
p(Scalp)=0.781
p(Dura)=0.0286
Scalp
Dura

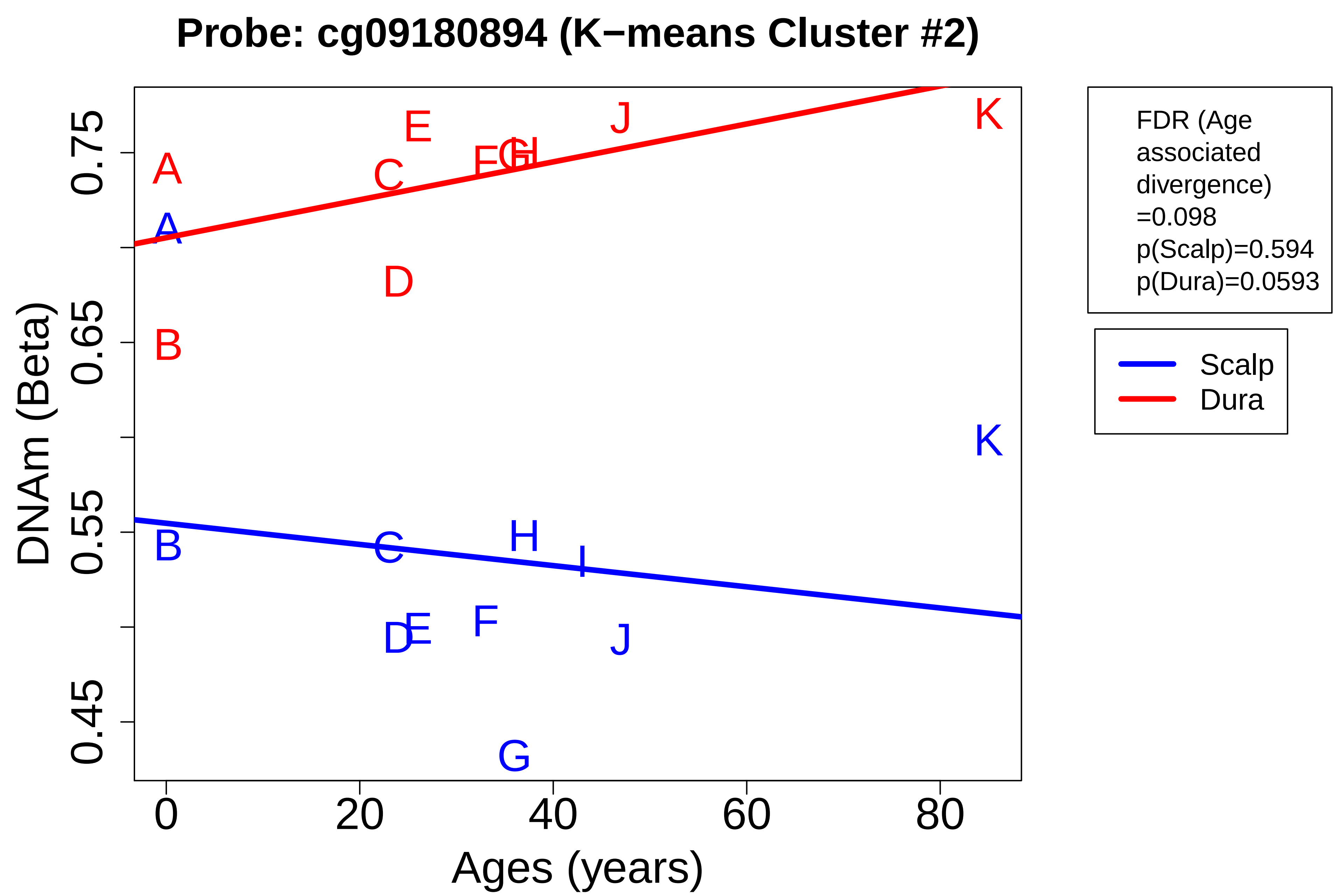

Probe: cg09180894 (K−means Cluster #2)
FDR (Age associated divergence) =0.098
p(Scalp)=0.594
p(Dura)=0.0593
Scalp
Dura
DNAm (Beta)
0.45
0.55
0.65
0.75
0
20
40
60
80
Ages (years)
A
B
C
D
E
F
G
H
I
J
K

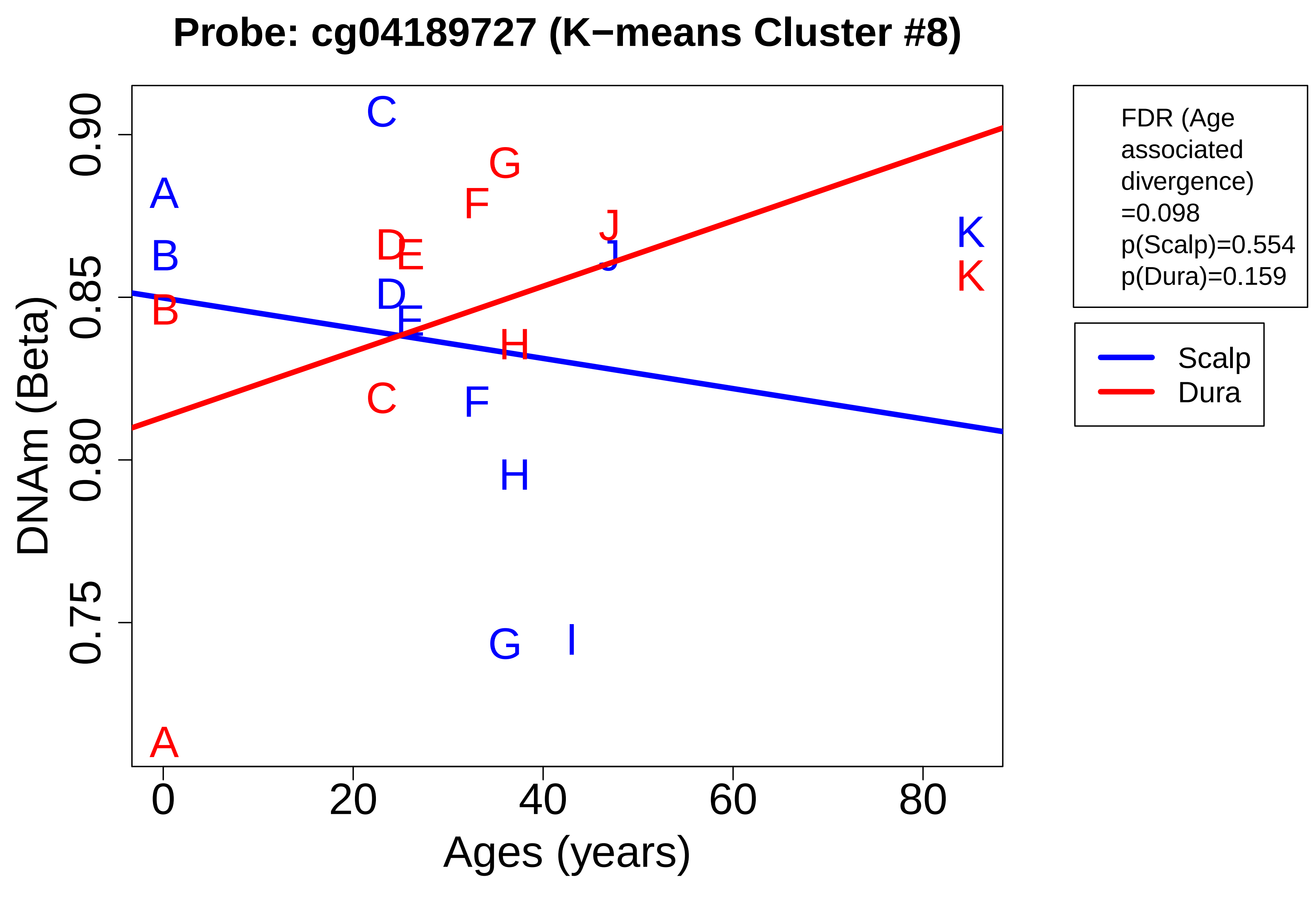
Probe: cg04189727 (K−means Cluster #8)
DNAm (Beta)
Ages (years)
0.75
0.80
0.85
0.90
0
20
40
60
80
FDR (Age associated divergence) =0.098
p(Scalp)=0.554
p(Dura)=0.159
Scalp
Dura
A
B
C
D
E
F
G
H
I
J
K

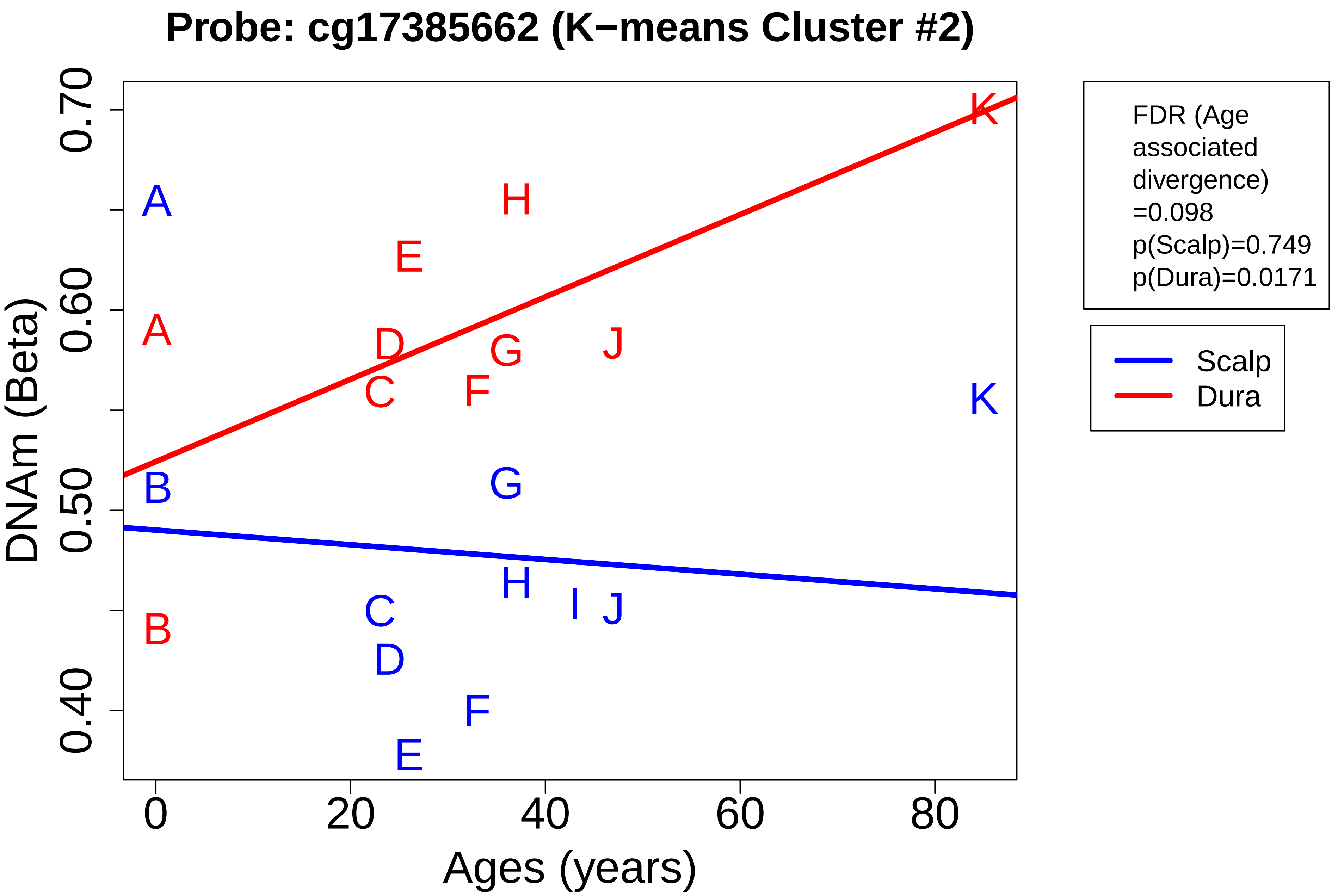

Probe: cg17385662 (K−means Cluster #2)
DNAm (Beta)
0.40
0.50
0.60
0.70
Ages (years)
0
20
40
60
80
A
B
C
D
E
F
G
H
I
J
K
FDR (Age associated divergence) =0.098
p(Scalp)=0.749
p(Dura)=0.0171
Scalp
Dura

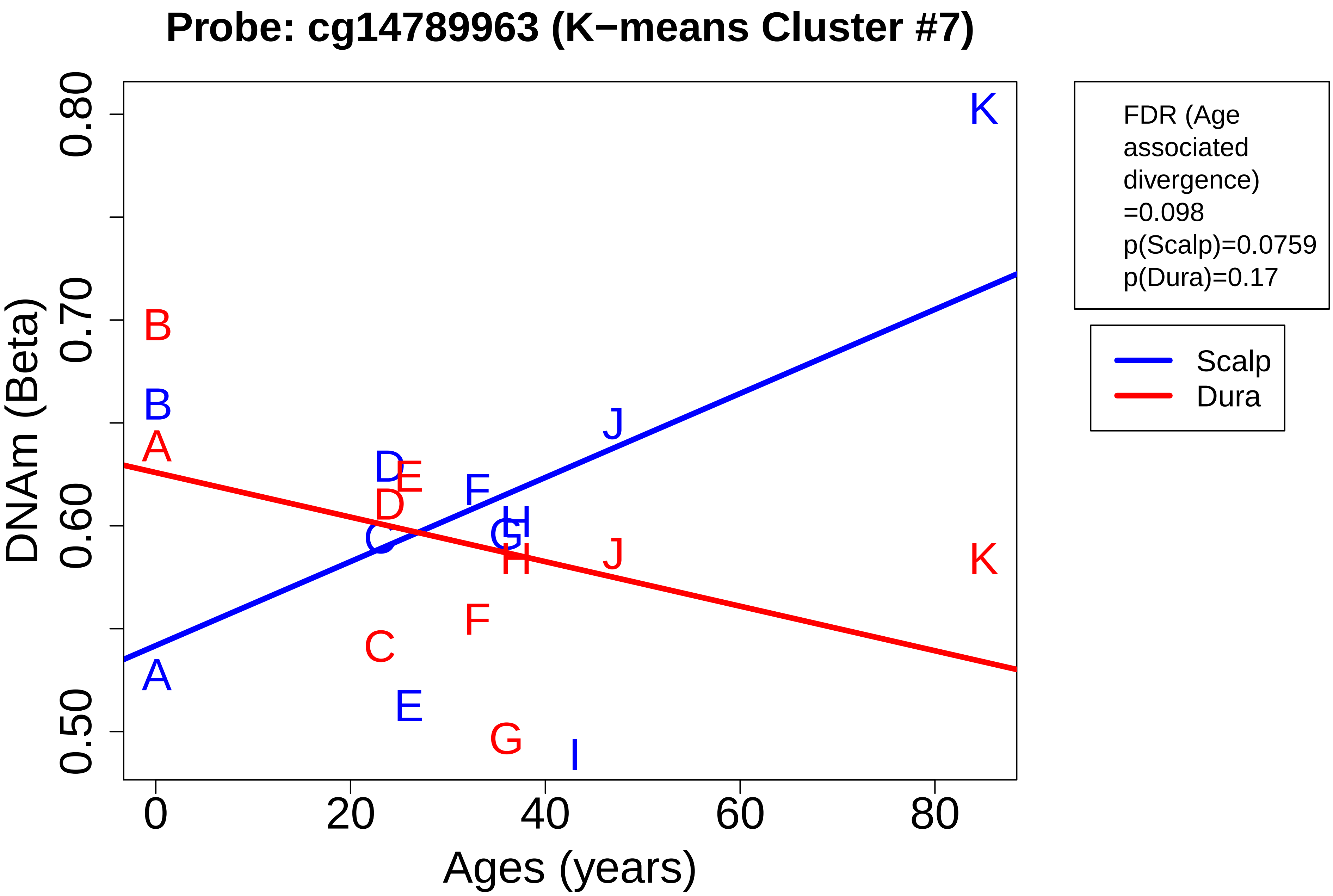

Probe: cg14789963 (K−means Cluster #7)
DNAm (Beta)
0.50
0.60
0.70
0.80
Ages (years)
0
20
40
60
80
A
B
C
D
E
F
G
H
I
J
K
FDR (Age associated divergence) =0.098
p(Scalp)=0.0759
p(Dura)=0.17
Scalp
Dura

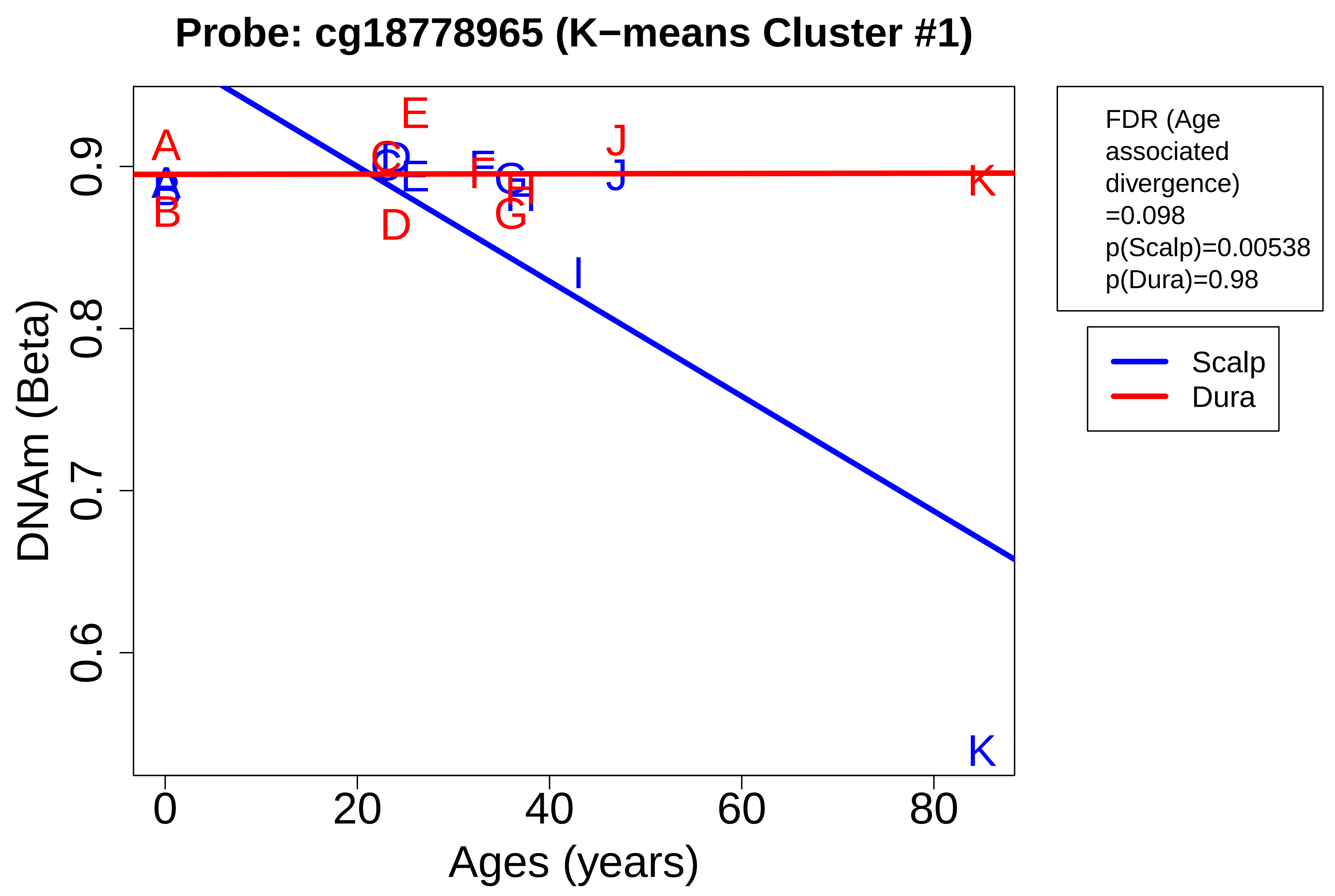

Probe: cg18778965 (K−means Cluster #1)
DNAm (Beta)
0.6
0.7
0.8
0.9
Ages (years)
0
20
40
60
80
FDR (Age associated divergence) =0.098
p(Scalp)=0.00538
p(Dura)=0.98
Scalp
Dura
A
B
C
D
E
F
G
H
I
J
K

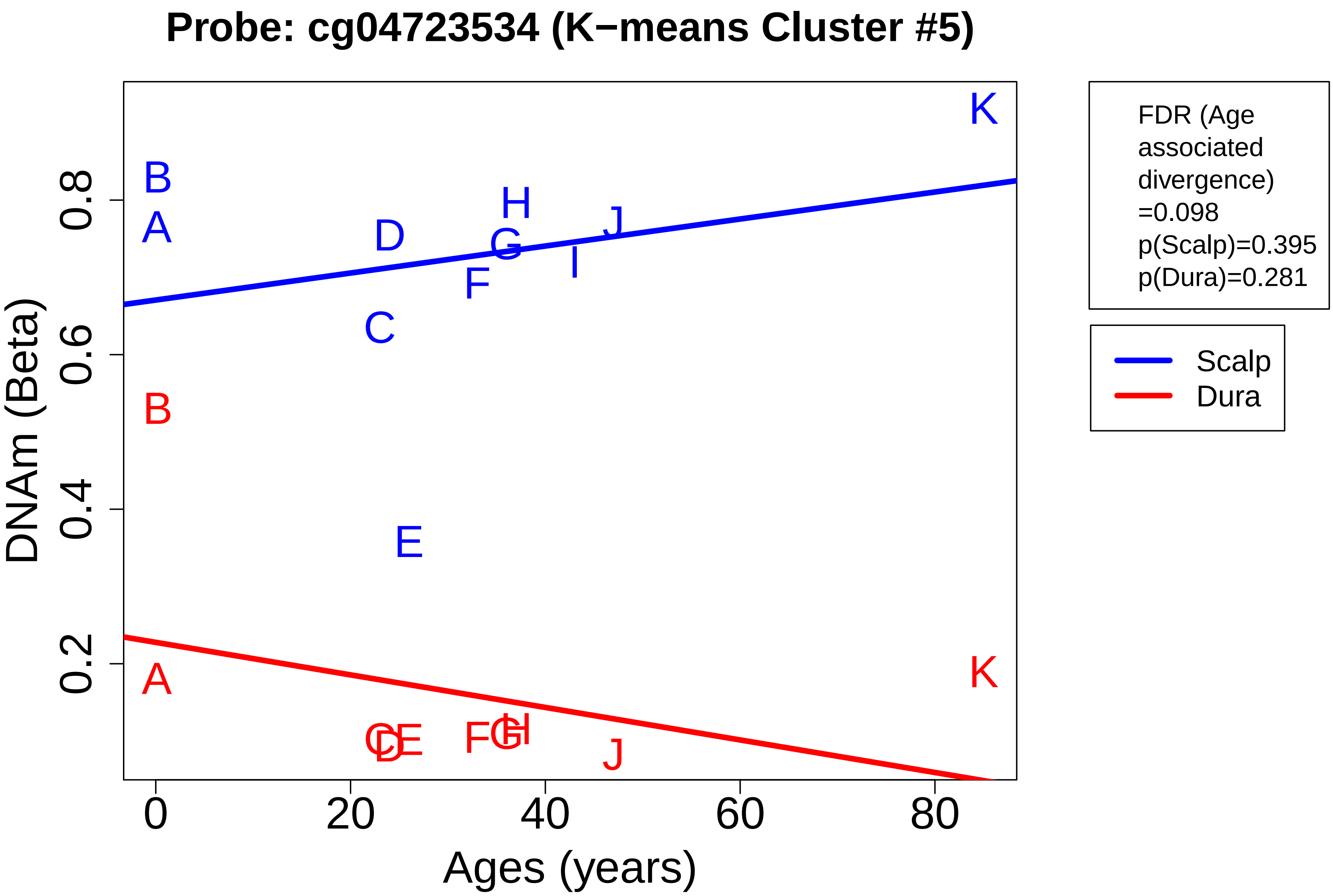

Probe: cg04723534 (K−means Cluster #5)
DNAm (Beta)
0.2
0.4
0.6
0.8
Ages (years)
0
20
40
60
80
FDR (Age associated divergence) =0.098
p(Scalp)=0.395
p(Dura)=0.281
Scalp
Dura
A
B
C
D
E
F
G
H
I
J
K

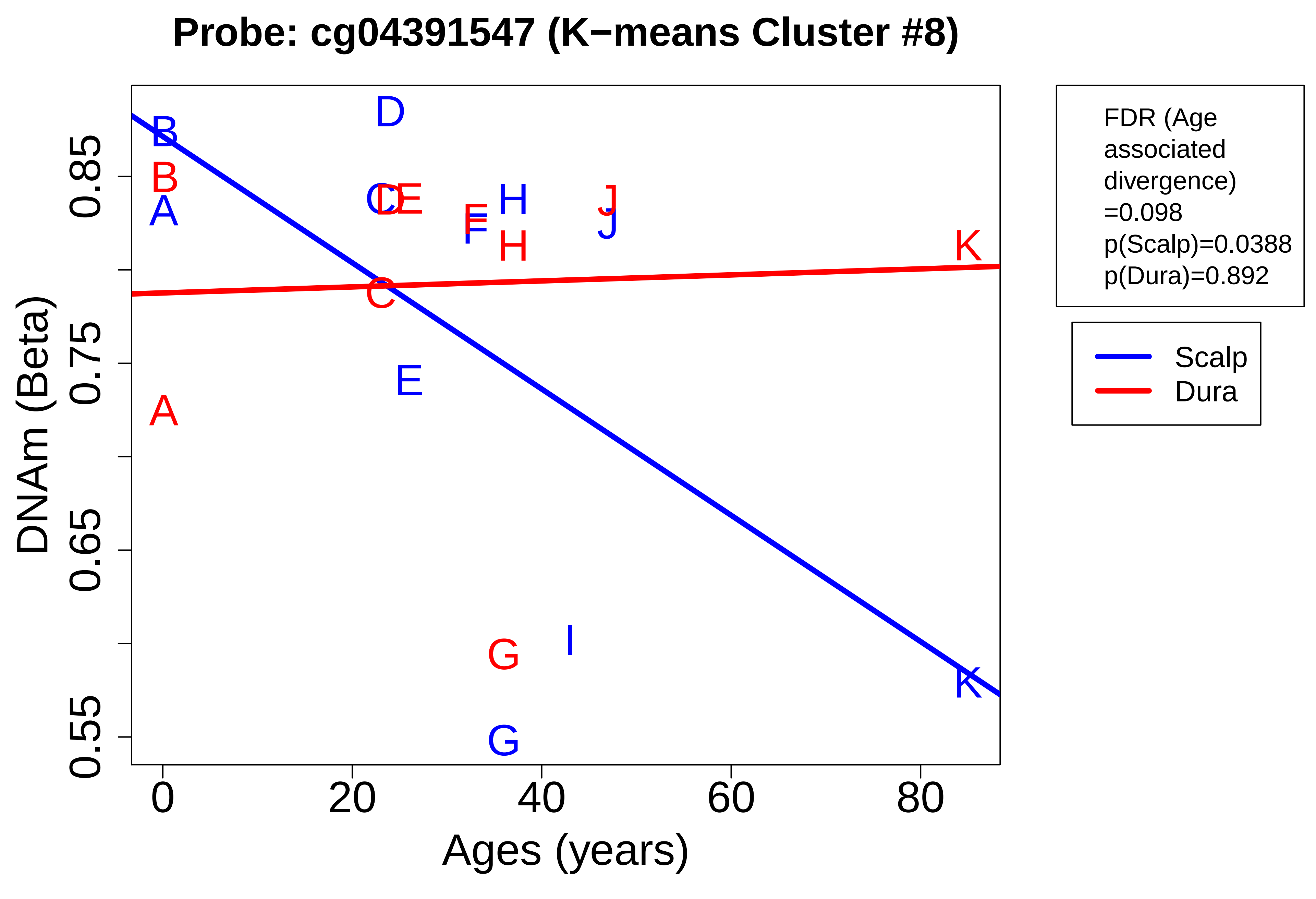

Probe: cg04391547 (K−means Cluster #8)
DNAm (Beta)
0.55
0.65
0.75
0.85
Ages (years)
0
20
40
60
80
FDR (Age associated divergence) =0.098
p(Scalp)=0.0388
p(Dura)=0.892
Scalp
Dura
A
B
C
D
E
F
G
H
I
J
K

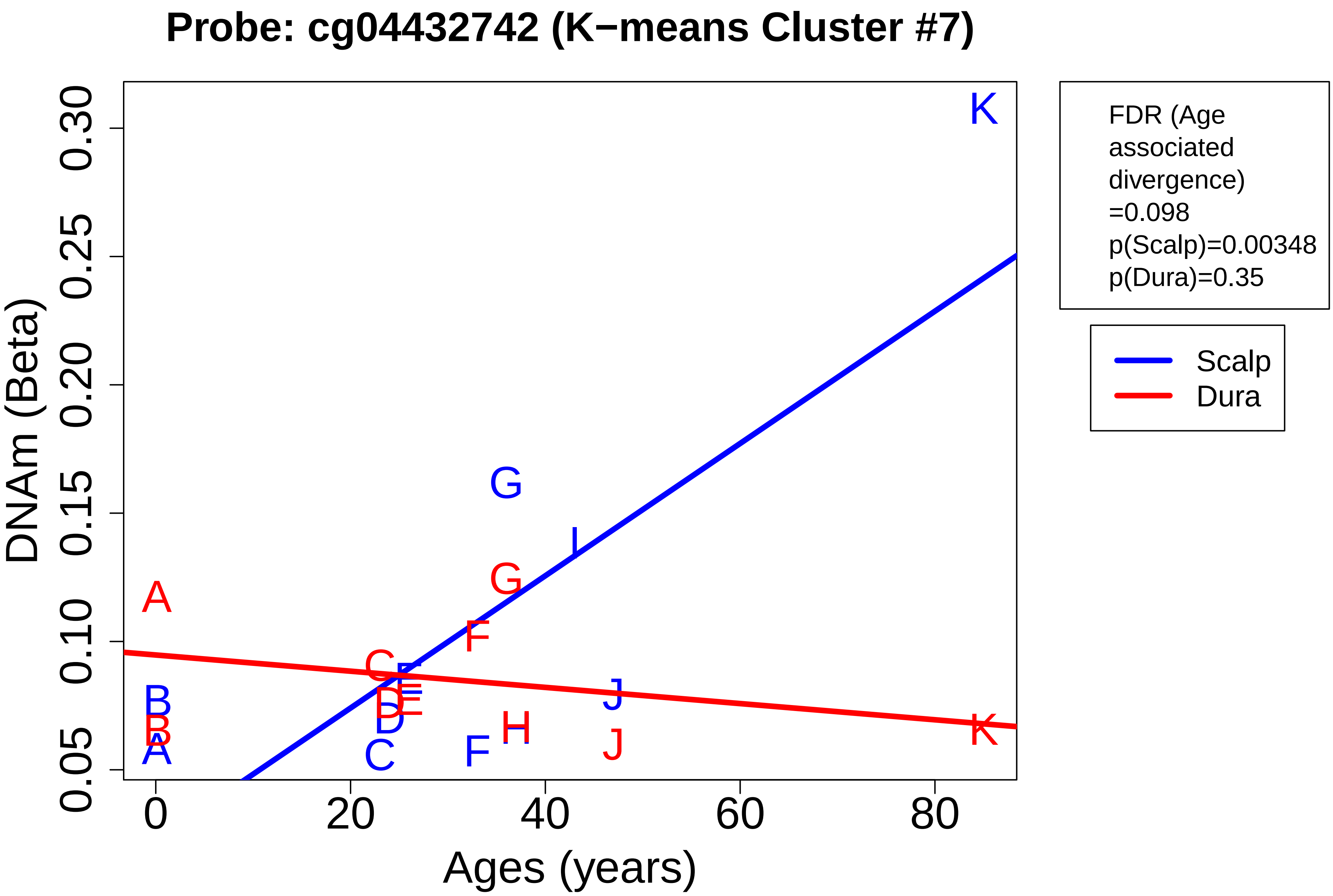

Probe: cg04432742 (K−means Cluster #7)
DNAm (Beta)
0.05
0.10
0.15
0.20
0.25
0.30
0
20
40
60
80
Ages (years)
FDR (Age associated divergence) =0.098
p(Scalp)=0.00348
p(Dura)=0.35
Scalp
Dura

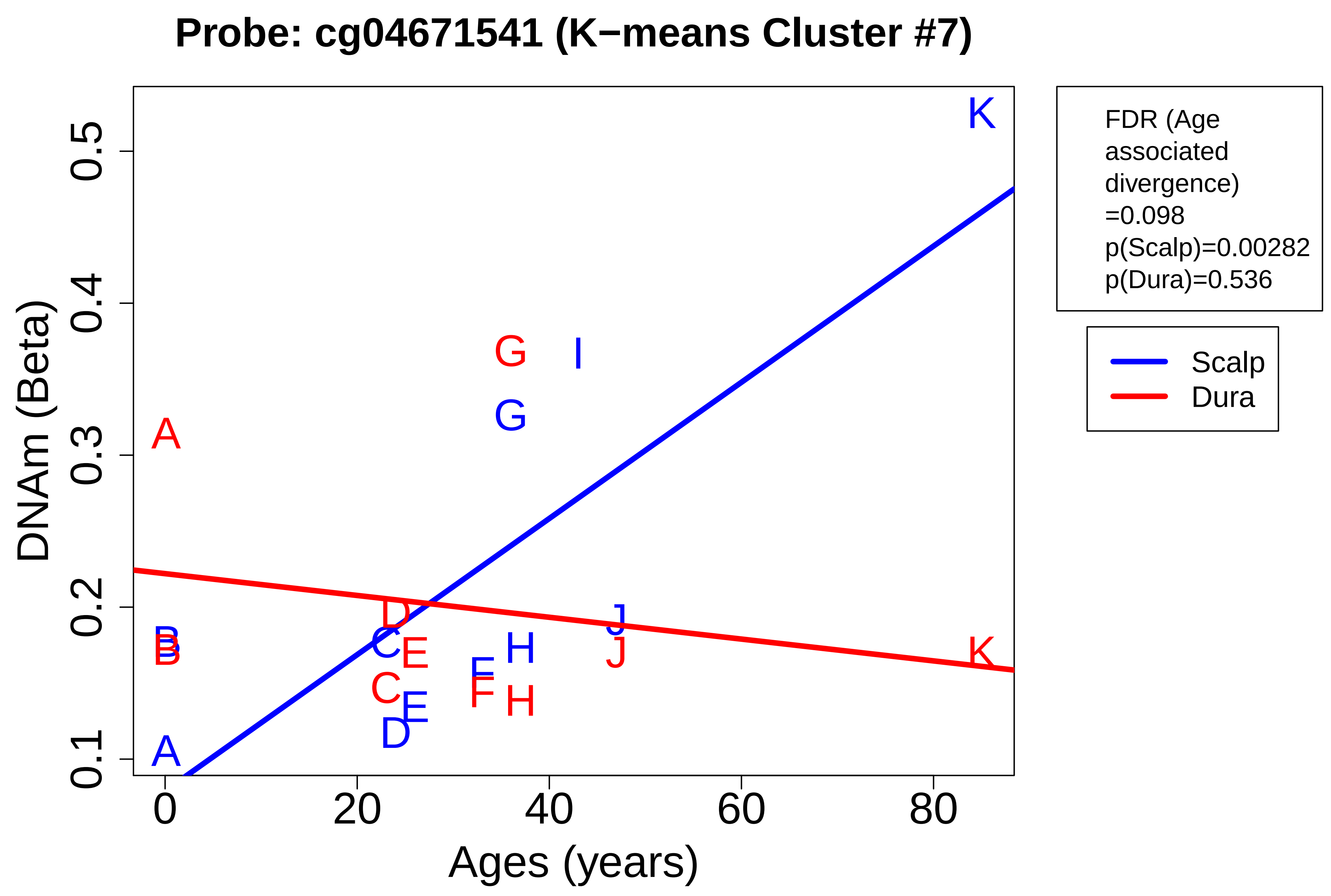
Probe: cg04671541 (K−means Cluster #7)
DNAm (Beta)
0.1
0.2
0.3
0.4
0.5
0
20
40
60
80
Ages (years)
FDR (Age associated divergence) =0.098
p(Scalp)=0.00282
p(Dura)=0.536
Scalp
Dura
A B C D E F G H I J K

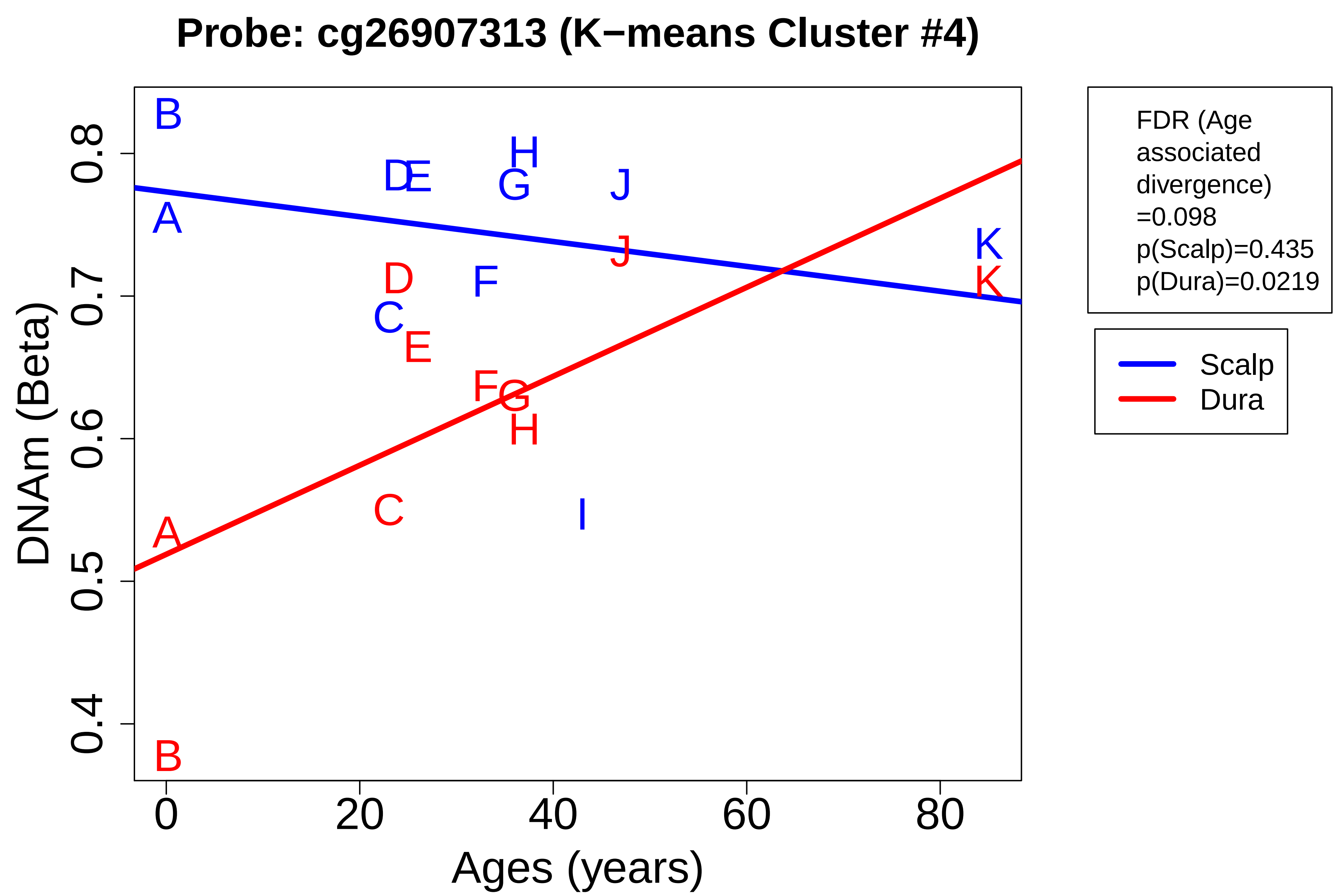
Probe: cg26907313 (K−means Cluster #4)
DNAm (Beta)
Ages (years)
0.4
0.5
0.6
0.7
0.8
0
20
40
60
80
FDR (Age associated divergence) =0.098
p(Scalp)=0.435
p(Dura)=0.0219
Scalp
Dura
A
B
C
D
E
F
G
H
I
J
K

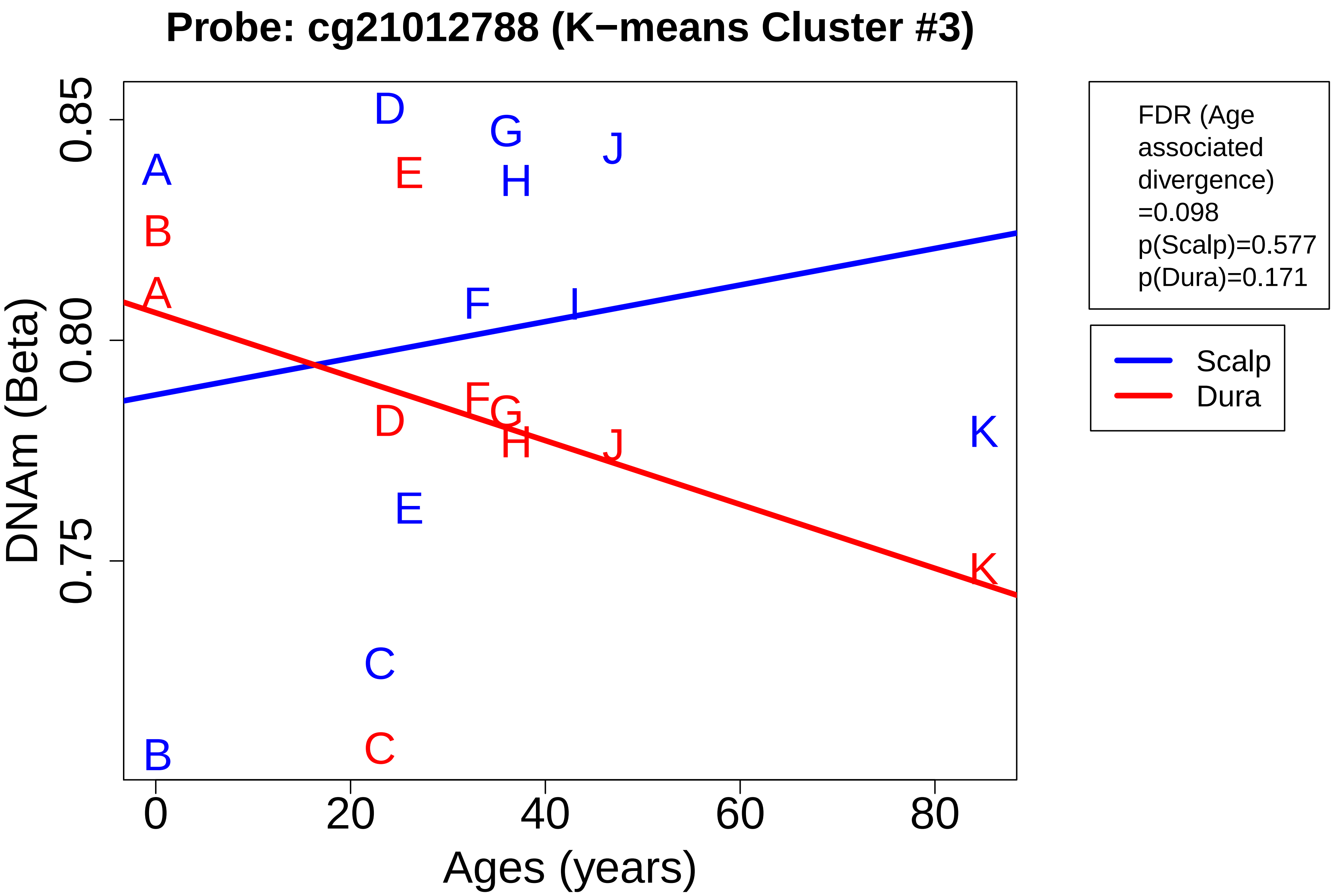

Probe: cg21012788 (K−means Cluster #3)
DNAm (Beta)
0.75
0.80
0.85
Ages (years)
0
20
40
60
80
A
B
C
D
E
F
G
H
I
J
K
A
B
C
D
E
F
G
H
J
K
FDR (Age associated divergence) =0.098
p(Scalp)=0.577
p(Dura)=0.171
Scalp
Dura

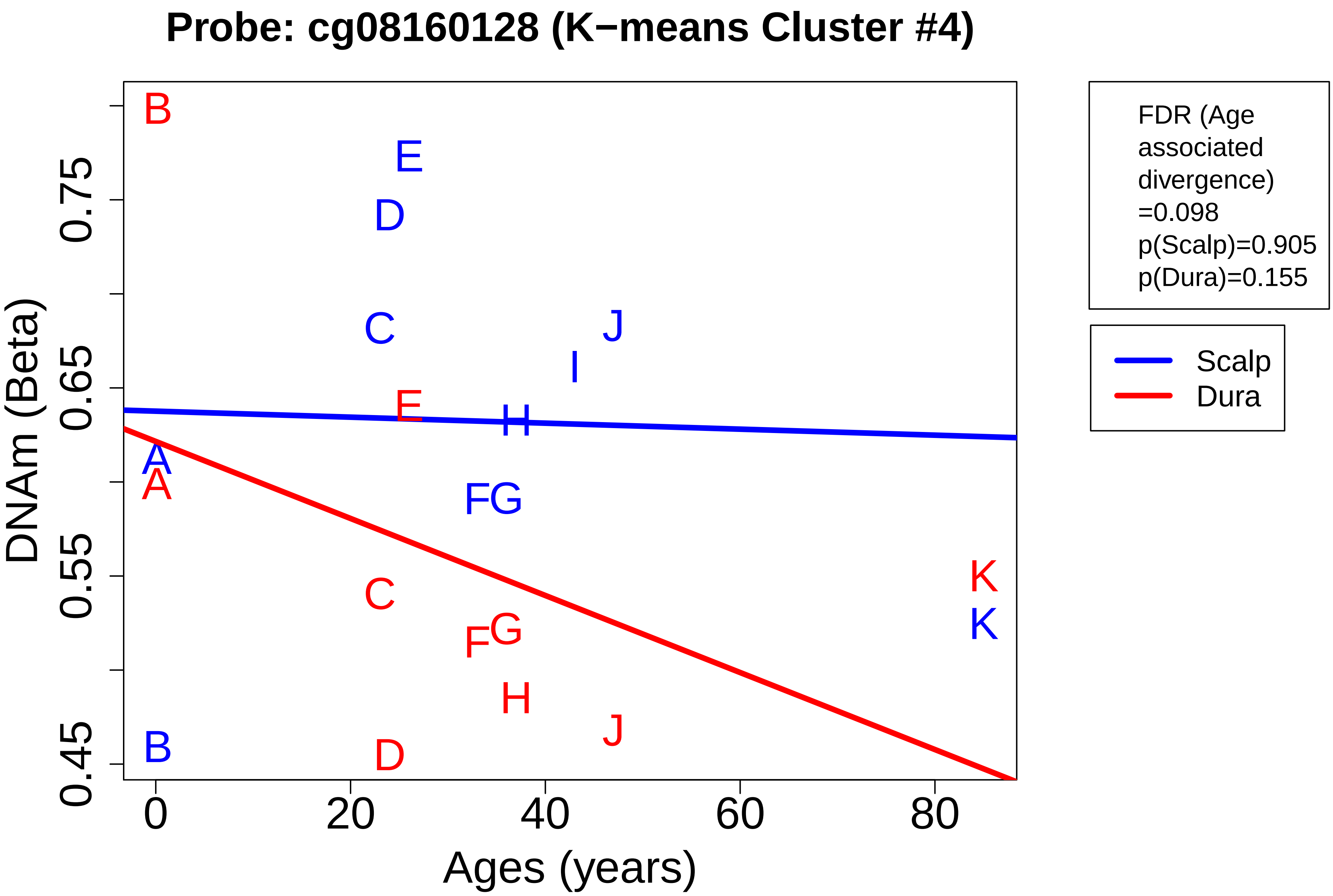

Probe: cg08160128 (K−means Cluster #4)
FDR (Age associated divergence) =0.098
p(Scalp)=0.905
p(Dura)=0.155
Scalp
Dura
DNAm (Beta)
0.45
0.55
0.65
0.75
Ages (years)
0
20
40
60
80

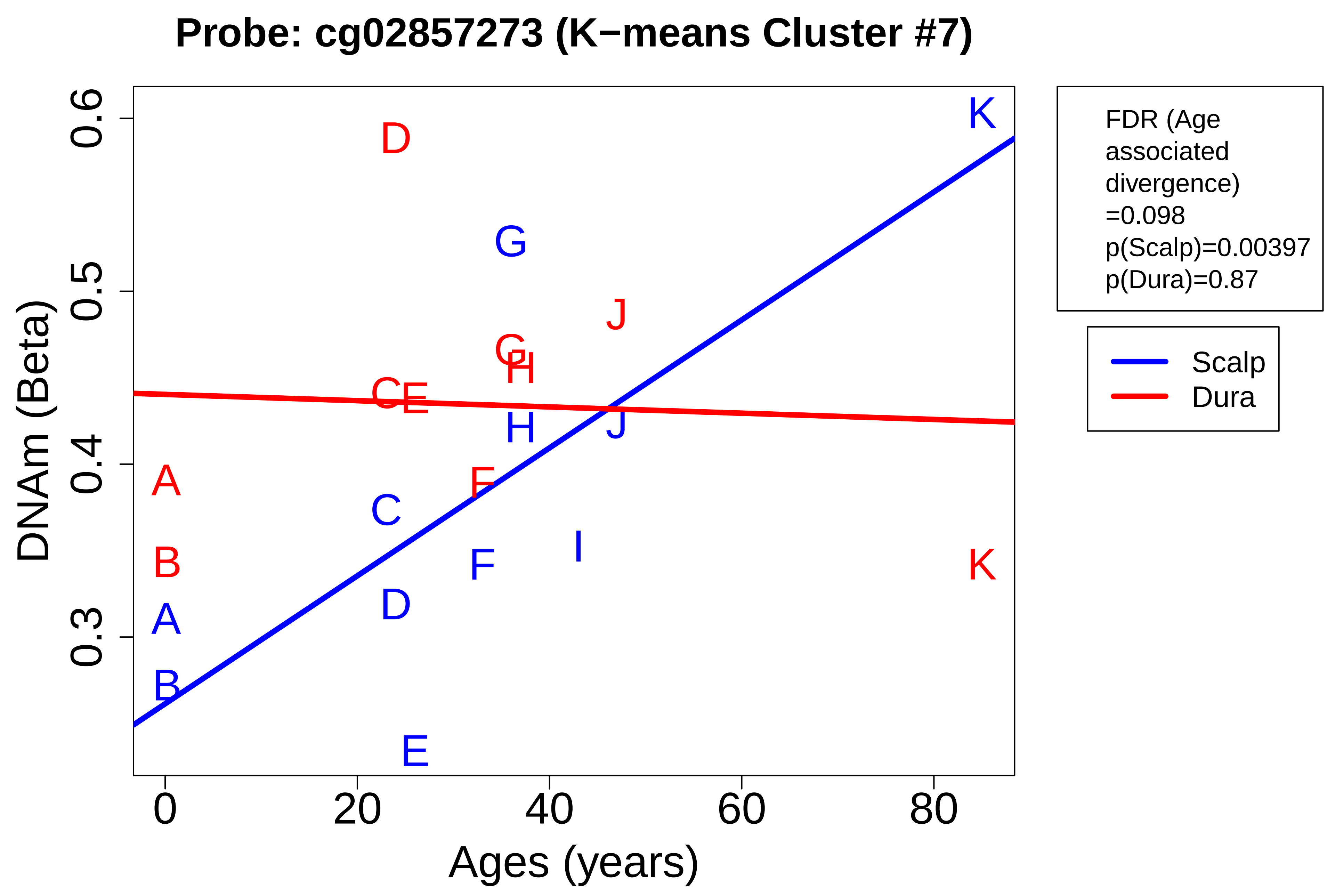
Probe: cg02857273 (K−means Cluster #7)
FDR (Age associated divergence) =0.098
p(Scalp)=0.00397
p(Dura)=0.87
Scalp
Dura
DNAm (Beta)
Ages (years)
0.3
0.4
0.5
0.6
0
20
40
60
80

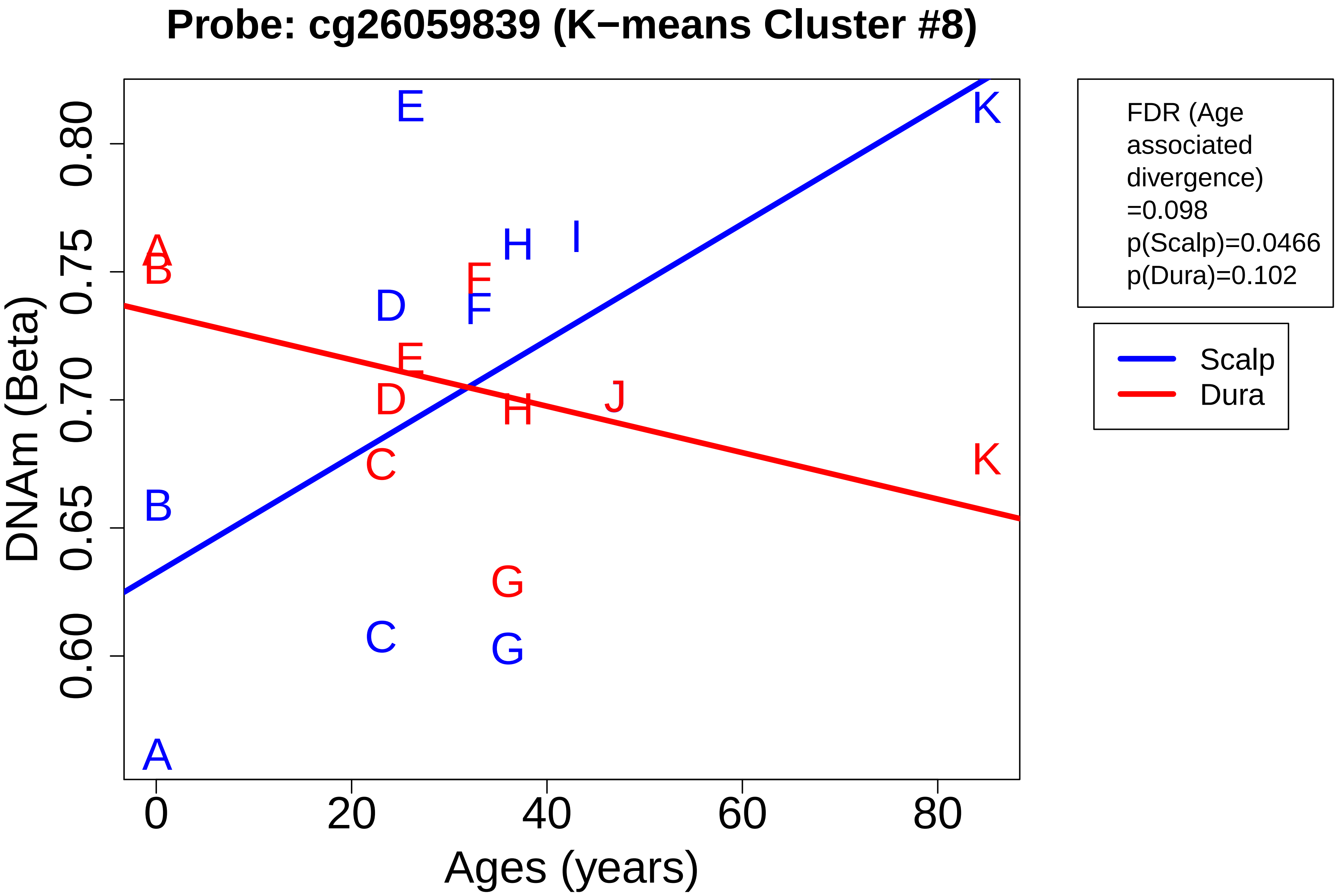

Probe: cg26059839 (K−means Cluster #8)
DNAm (Beta)
0.60
0.65
0.70
0.75
0.80
0
20
40
60
80
Ages (years)
E
K
H
I
A
B
E
D
F
E
D
H
J
C
K
B
G
C
G
A
FDR (Age associated divergence) =0.098
p(Scalp)=0.0466
p(Dura)=0.102
Scalp
Dura

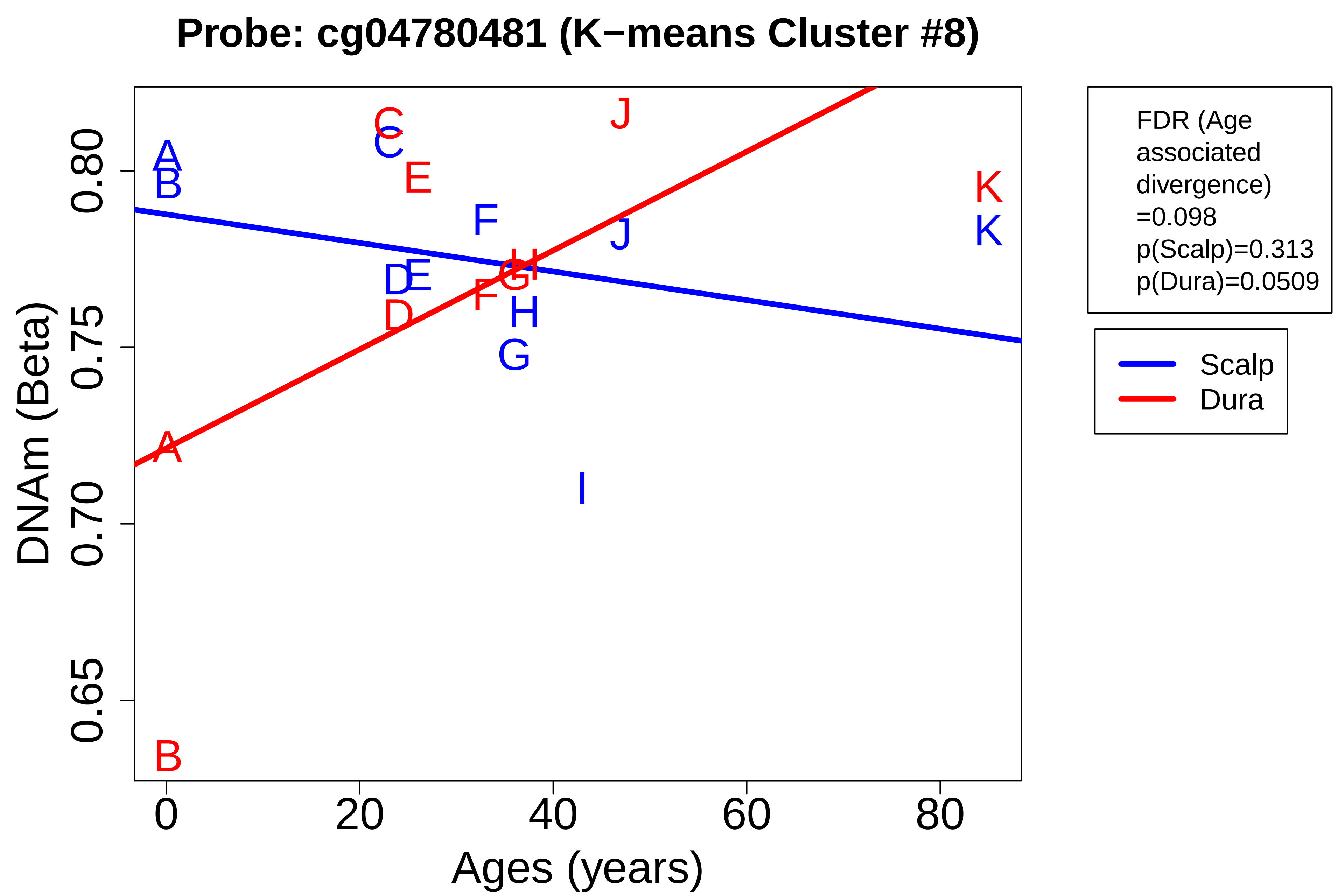
Probe: cg04780481 (K−means Cluster #8)
DNAm (Beta)
0.65
0.70
0.75
0.80
0
20
40
60
80
Ages (years)
A
B
C
D
E
F
G
H
I
J
K
FDR (Age associated divergence) =0.098
p(Scalp)=0.313
p(Dura)=0.0509
Scalp
Dura

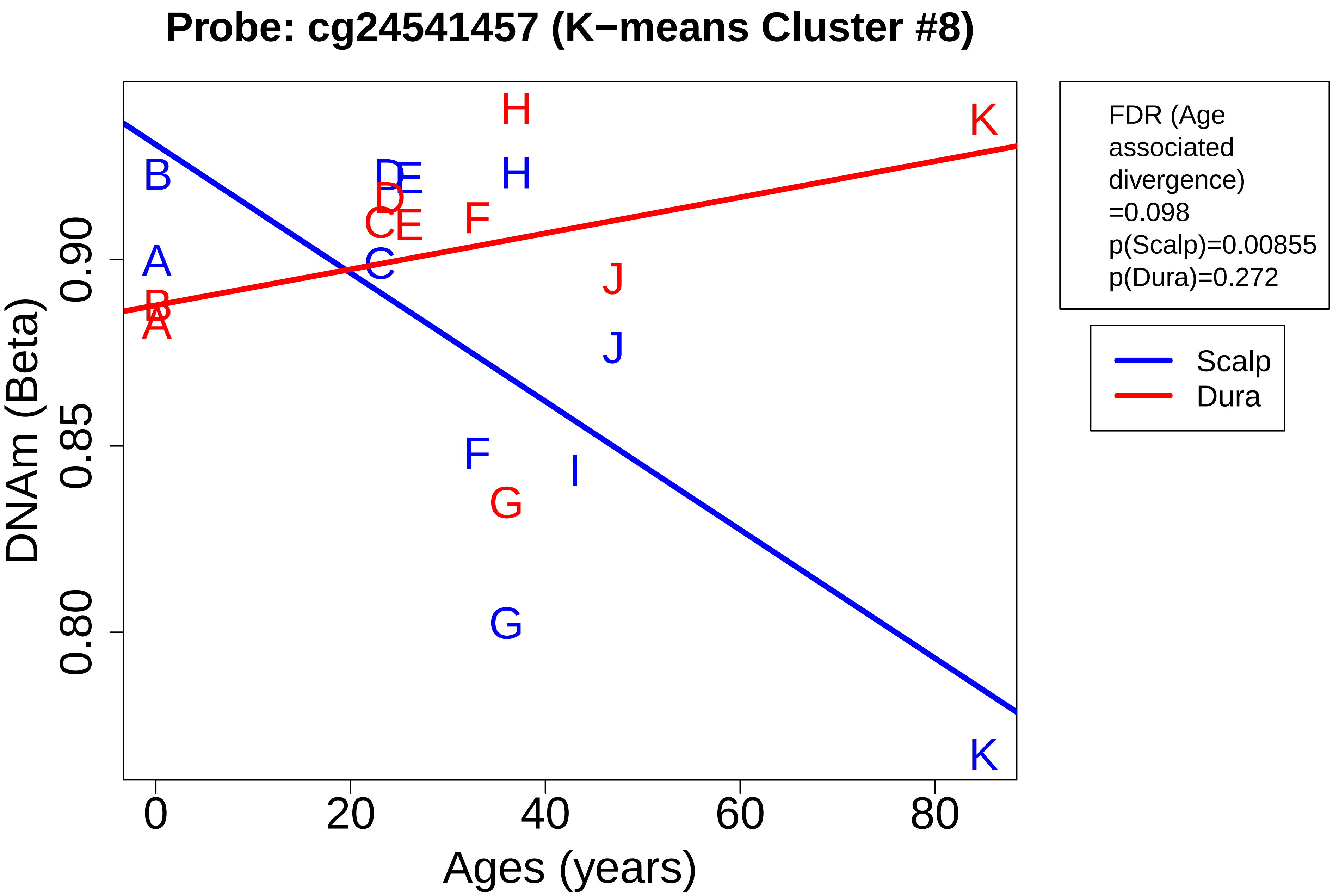

Probe: cg24541457 (K−means Cluster #8)
DNAm (Beta)
0.80
0.85
0.90
Ages (years)
0
20
40
60
80
FDR (Age associated divergence) =0.098
p(Scalp)=0.00855
p(Dura)=0.272
Scalp
Dura

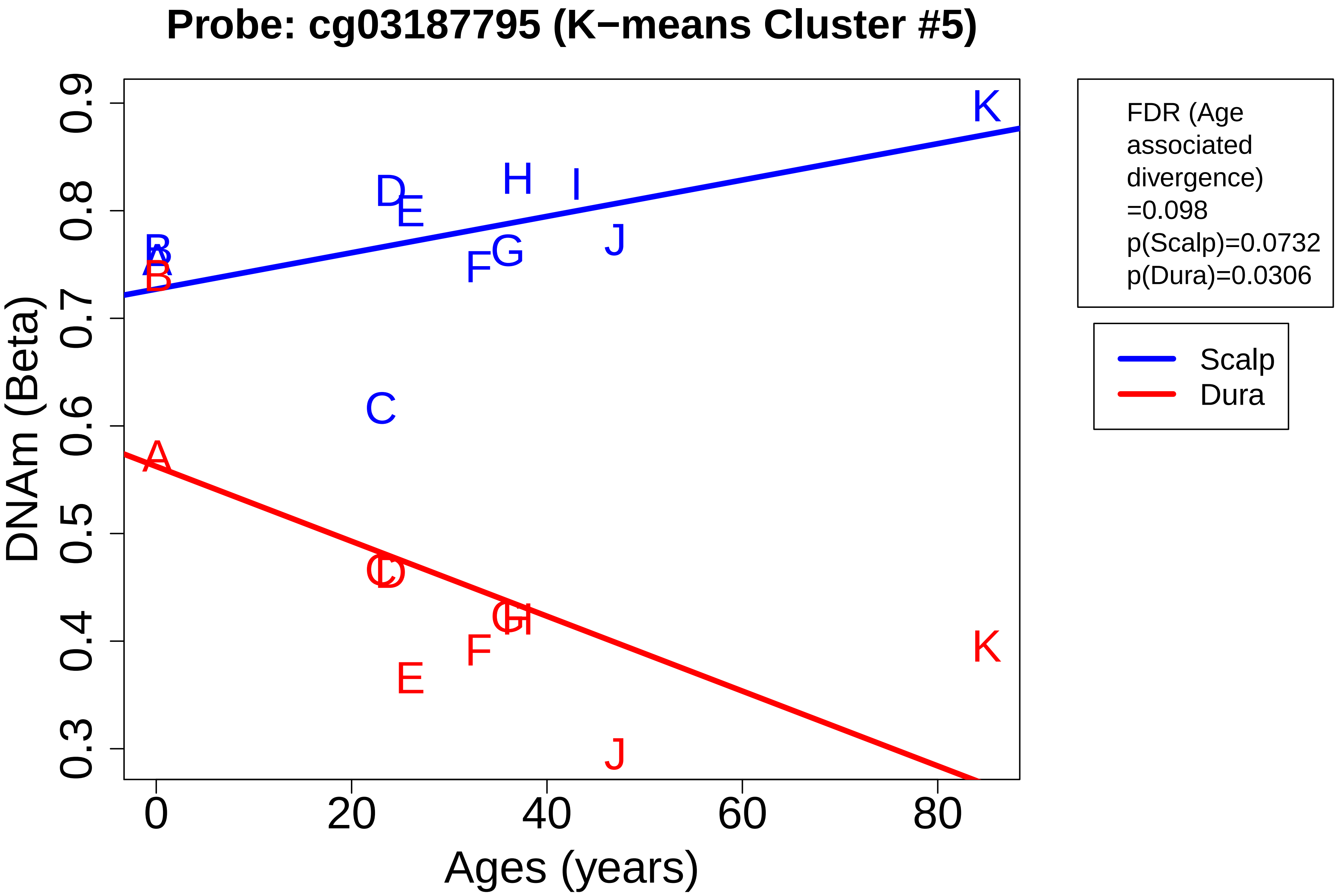

Probe: cg03187795 (K−means Cluster #5)
DNAm (Beta)
0.3
0.4
0.5
0.6
0.7
0.8
0.9
0
20
40
60
80
Ages (years)
FDR (Age associated divergence) =0.098
p(Scalp)=0.0732
p(Dura)=0.0306
Scalp
Dura

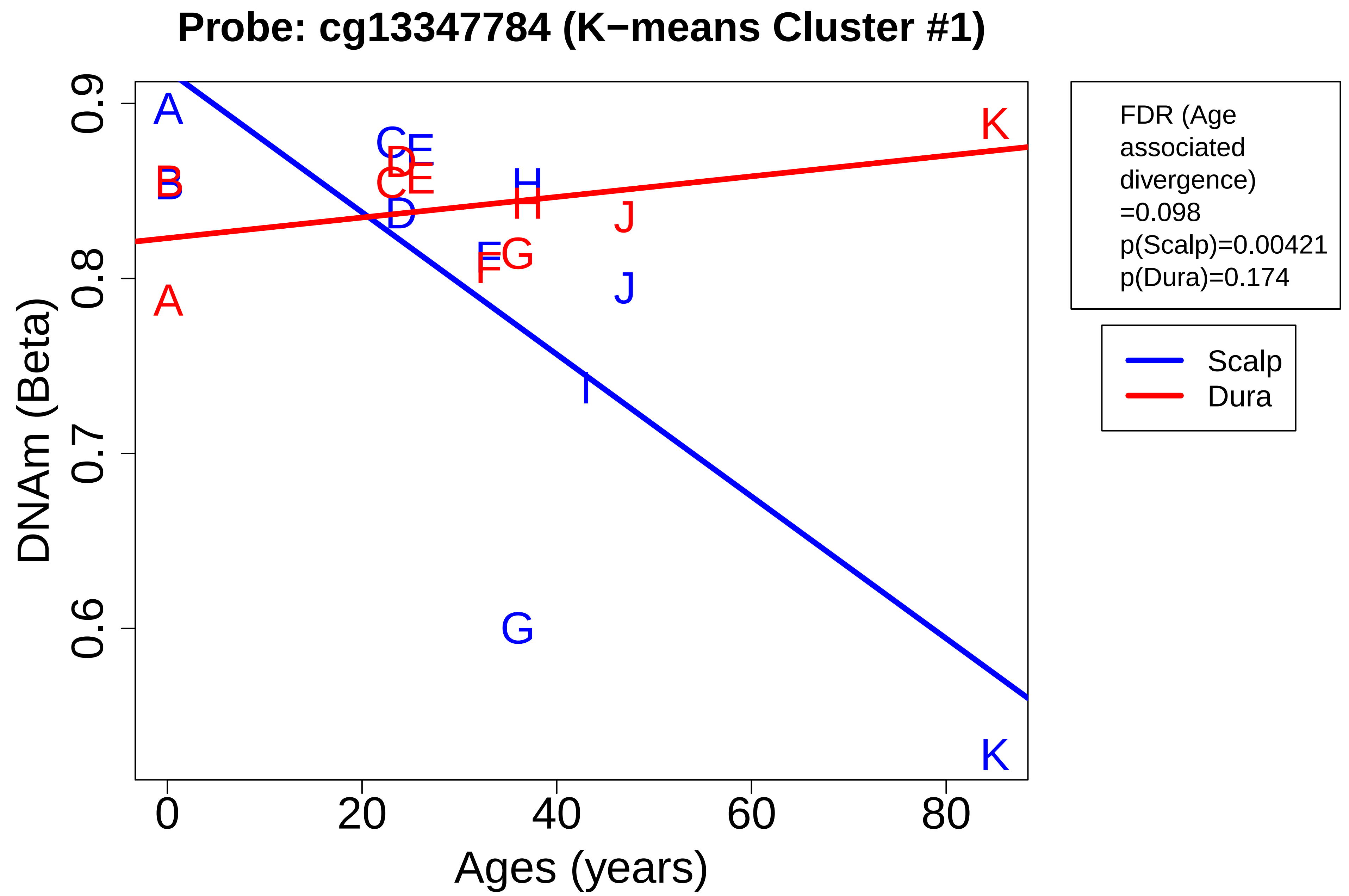

Probe: cg13347784 (K−means Cluster #1)
DNAm (Beta)
0.6
0.7
0.8
0.9
0
20
40
60
80
Ages (years)
A
B
C
D
E
F
G
H
I
J
K
FDR (Age associated divergence) =0.098
p(Scalp)=0.00421
p(Dura)=0.174
Scalp
Dura

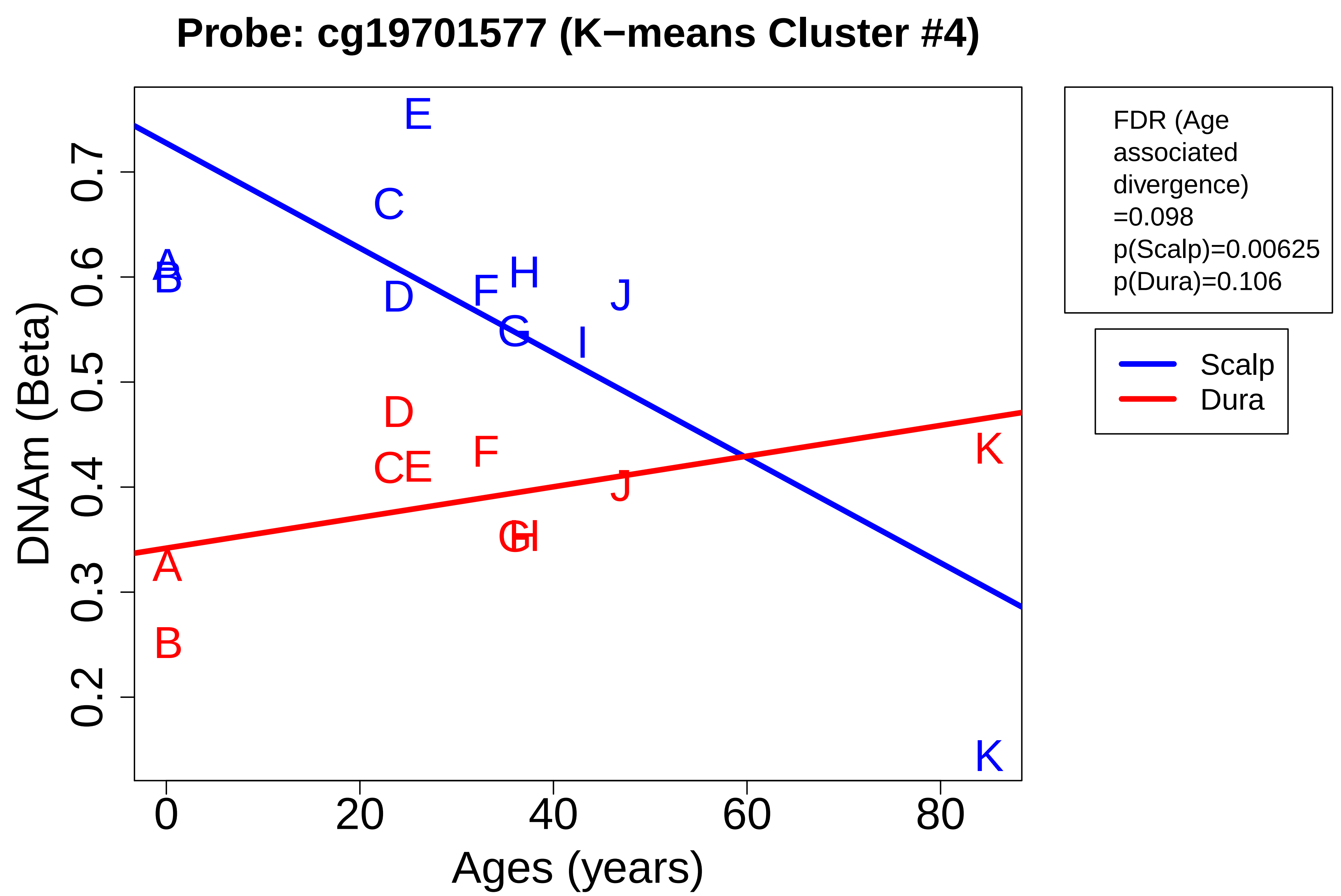

Probe: cg19701577 (K−means Cluster #4)
DNAm (Beta)
0.2
0.3
0.4
0.5
0.6
0.7
Ages (years)
0
20
40
60
80
E
C
A
B
D
F
H
J
G
I
D
CE
F
J
K
GH
A
B
K
FDR (Age associated divergence) =0.098
p(Scalp)=0.00625
p(Dura)=0.106
Scalp
Dura

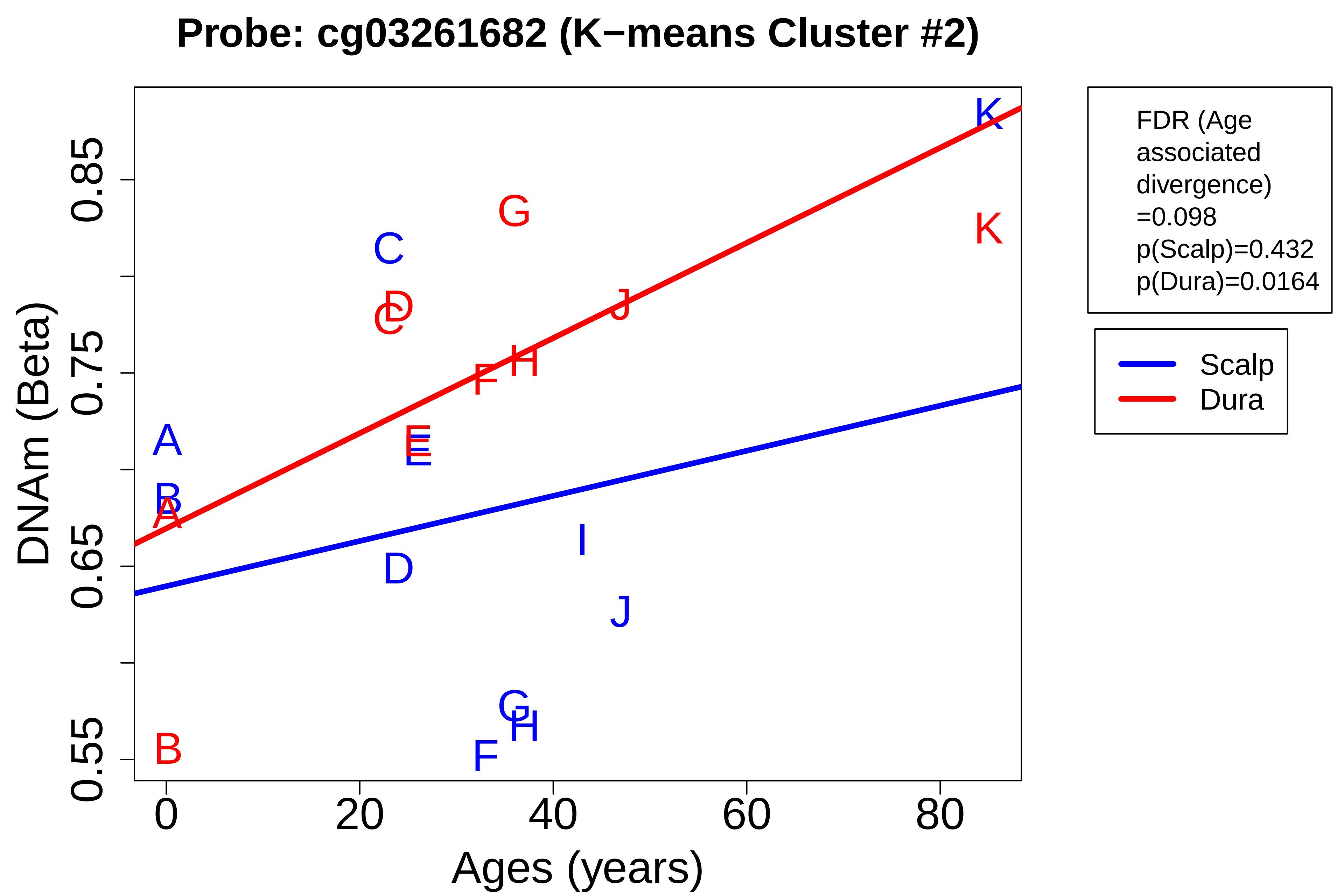

Probe: cg03261682 (K−means Cluster #2)
DNAm (Beta)
0.55
0.65
0.75
0.85
0
20
40
60
80
Ages (years)
FDR (Age associated divergence) =0.098
p(Scalp)=0.432
p(Dura)=0.0164
Scalp
Dura

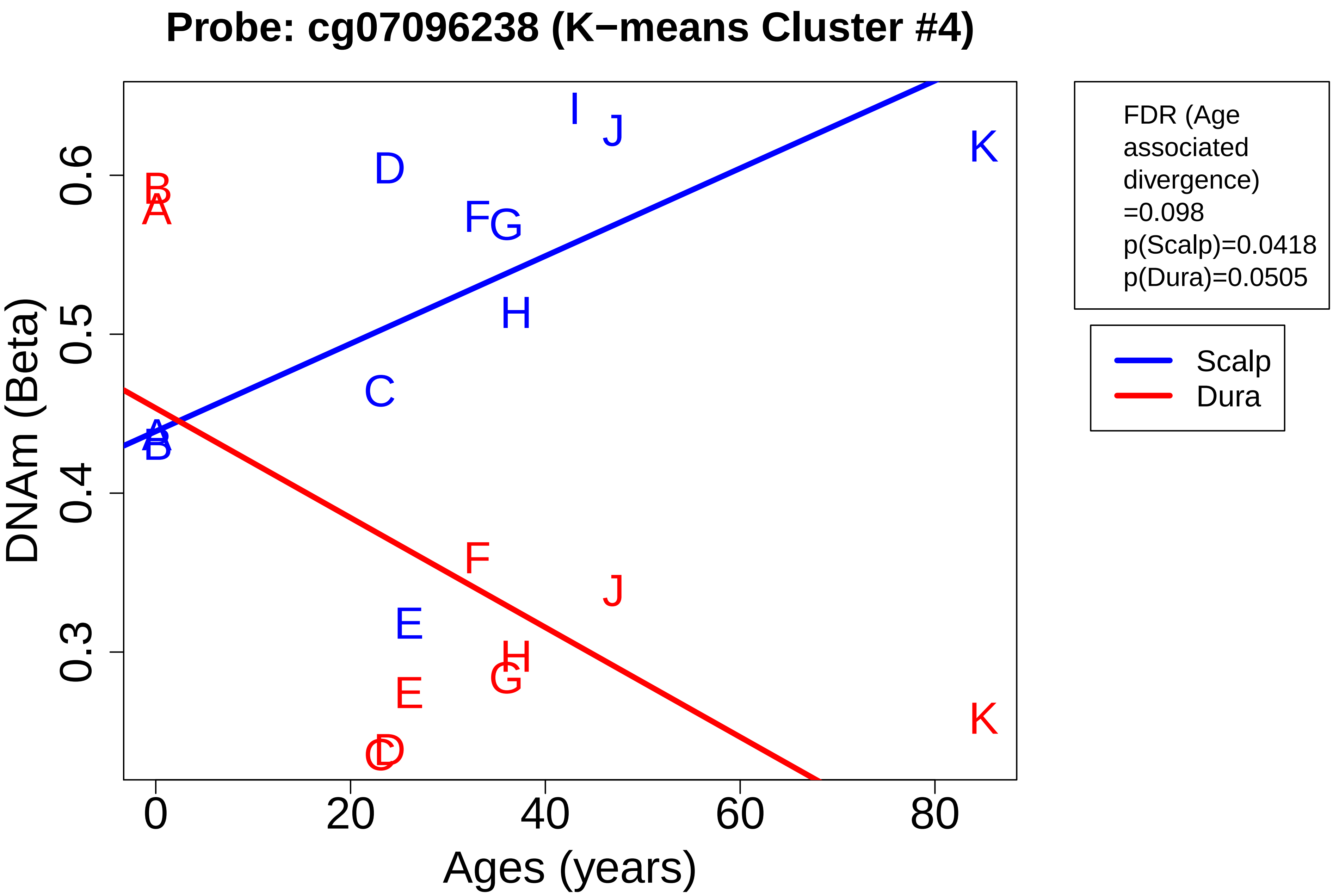
Probe: cg07096238 (K−means Cluster #4)
DNAm (Beta)
0.3
0.4
0.5
0.6
Ages (years)
0
20
40
60
80
FDR (Age associated divergence) =0.098
p(Scalp)=0.0418
p(Dura)=0.0505
Scalp
Dura

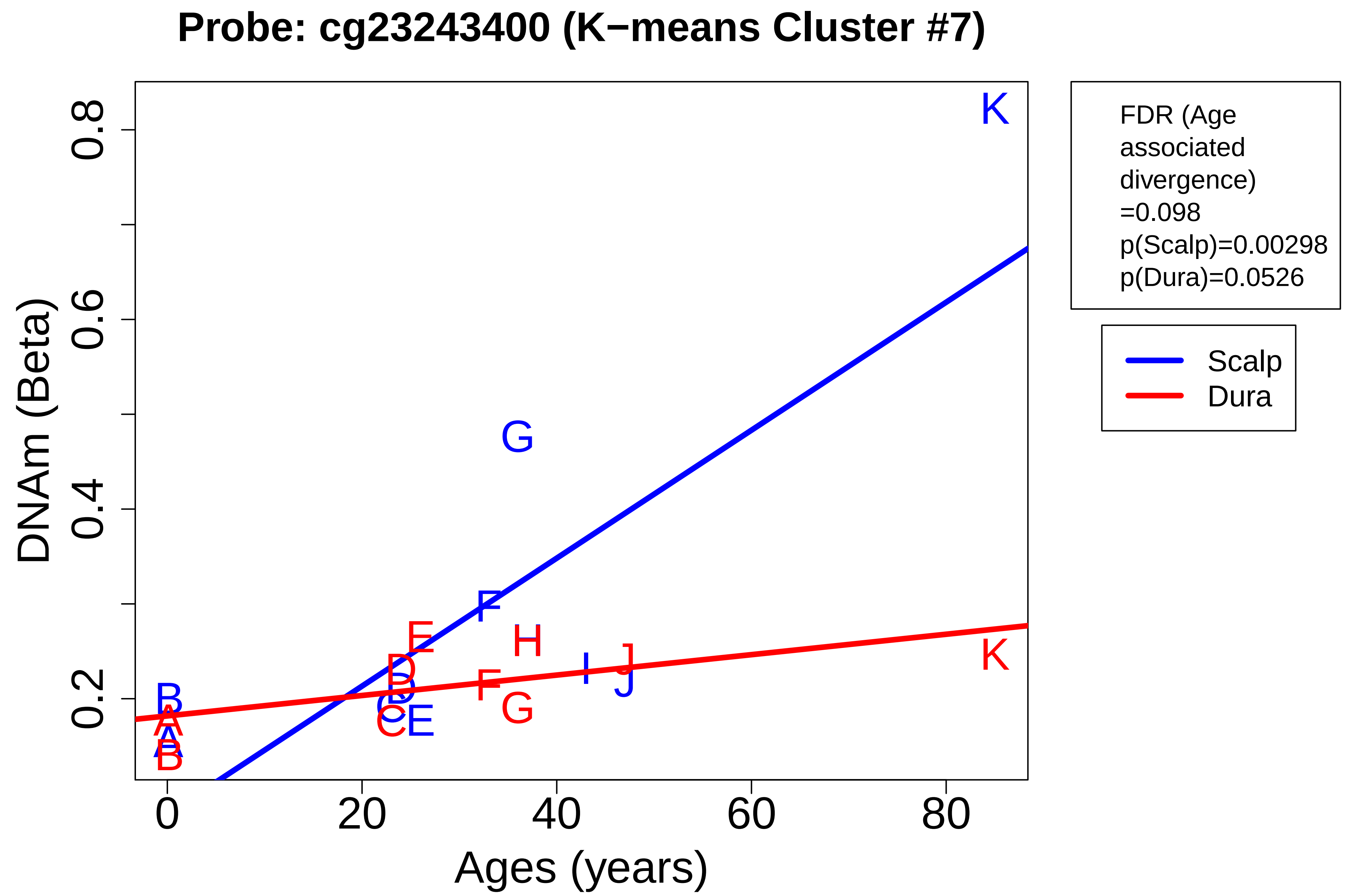

Probe: cg23243400 (K−means Cluster #7)
DNAm (Beta)
0.2
0.4
0.6
0.8
0
20
40
60
80
Ages (years)
FDR (Age associated divergence) =0.098
p(Scalp)=0.00298
p(Dura)=0.0526
Scalp
Dura

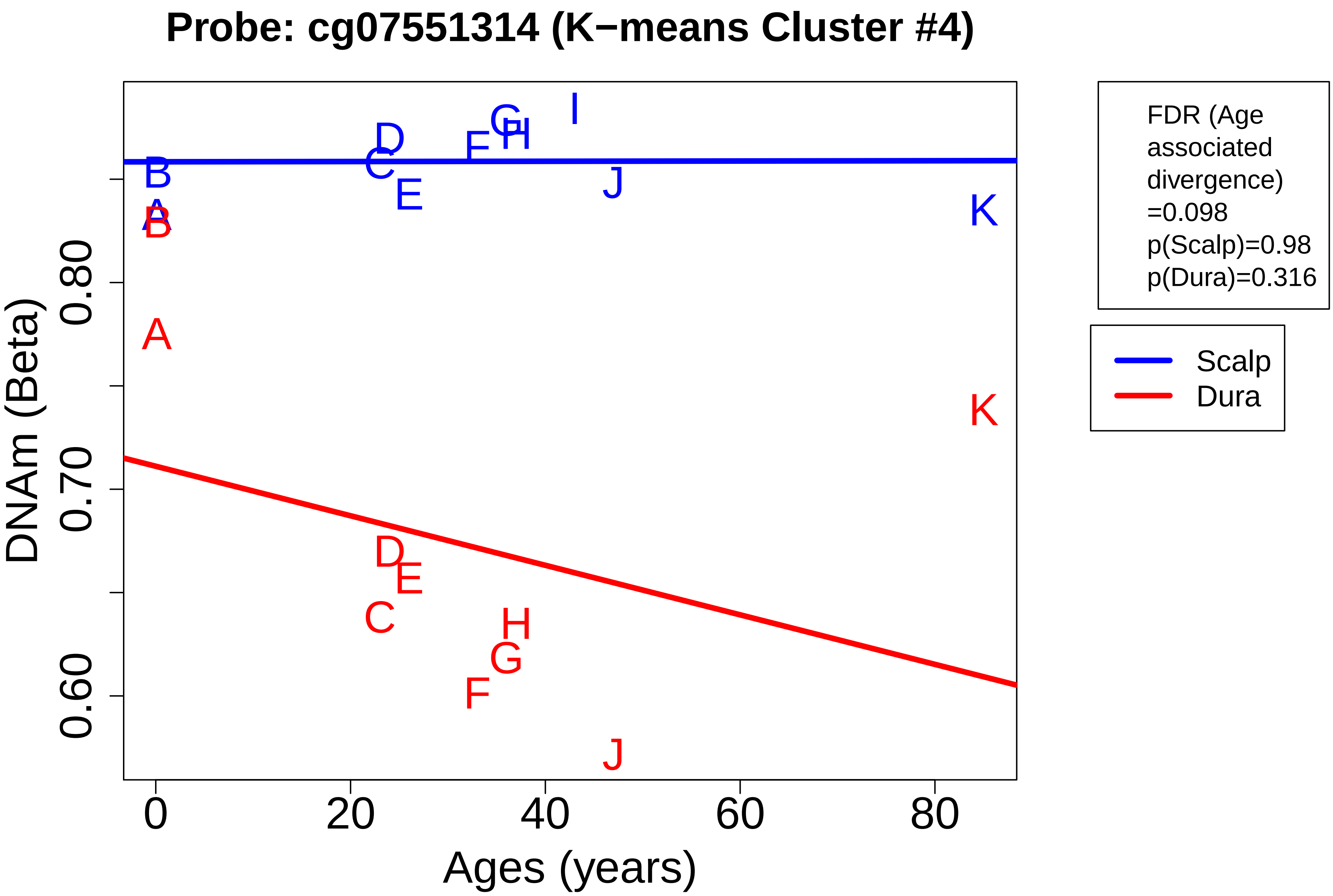

Probe: cg07551314 (K−means Cluster #4)
DNAm (Beta)
0.60
0.70
0.80
Ages (years)
0
20
40
60
80
FDR (Age associated divergence) =0.098
p(Scalp)=0.98
p(Dura)=0.316
Scalp
Dura
A
B
C
D
E
F
G
H
I
J
K

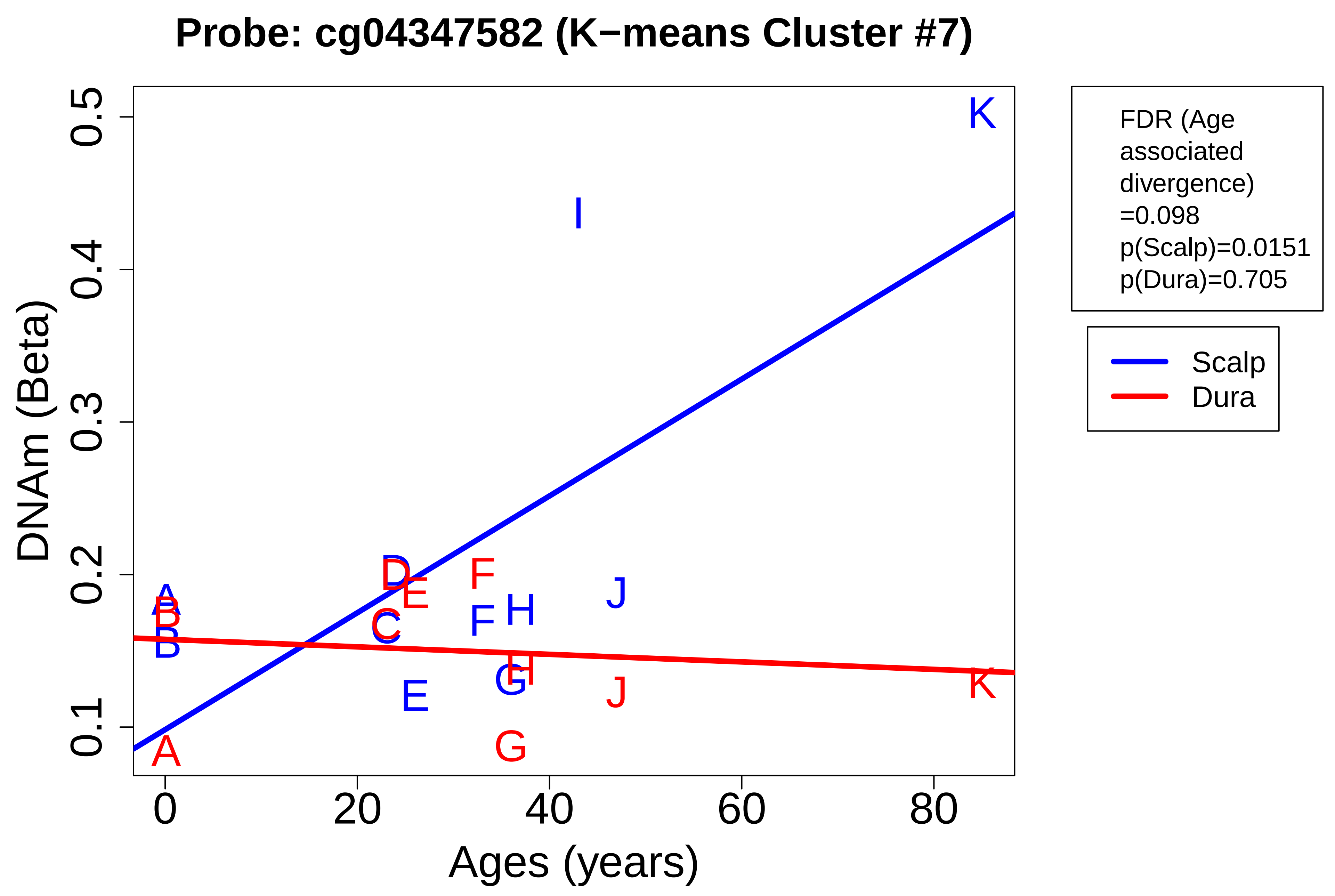
Probe: cg04347582 (K−means Cluster #7)
DNAm (Beta)
Ages (years)
0.1
0.2
0.3
0.4
0.5
0
20
40
60
80
FDR (Age associated divergence) =0.098
p(Scalp)=0.0151
p(Dura)=0.705
Scalp
Dura

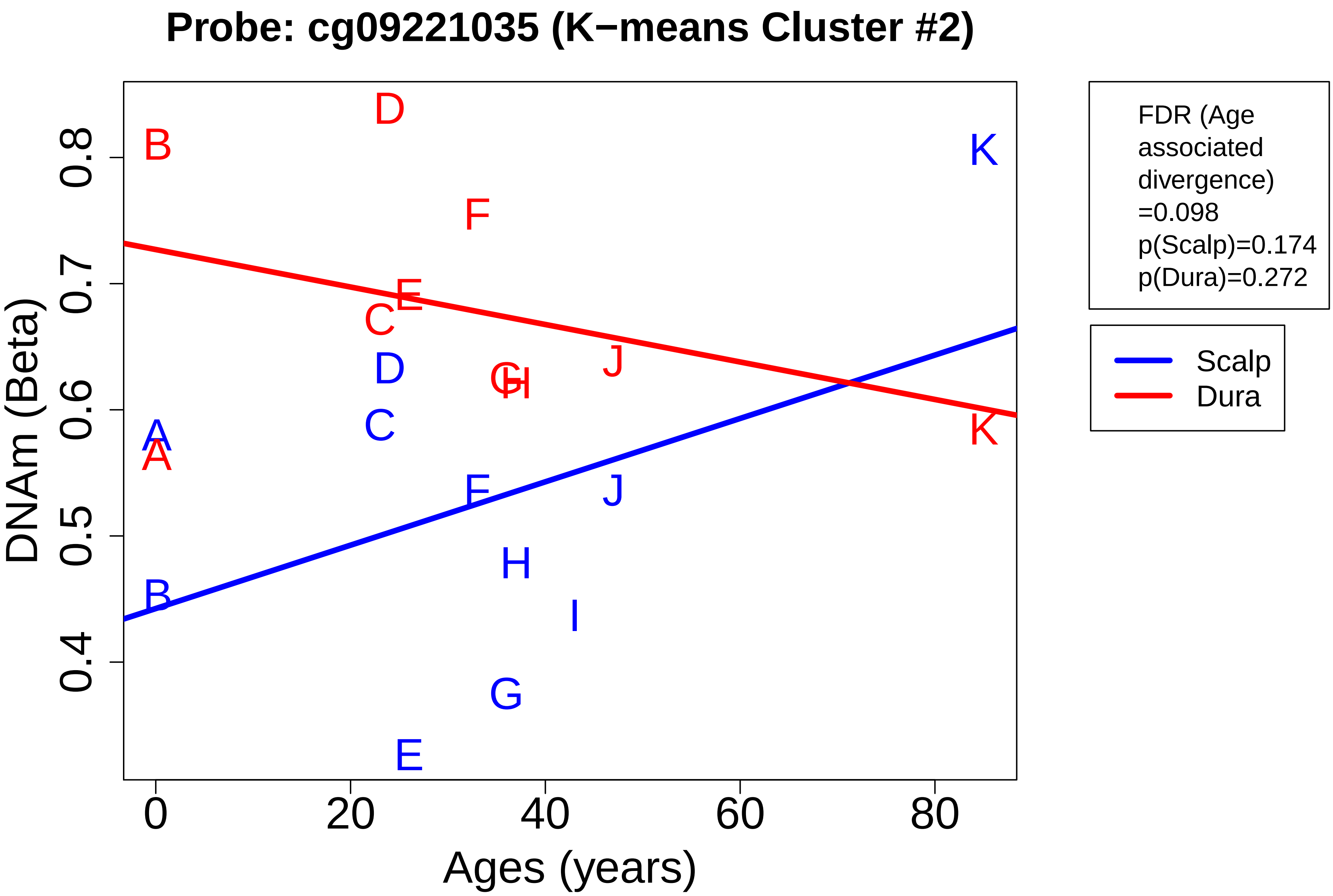

Probe: cg09221035 (K−means Cluster #2)
DNAm (Beta)
0.4
0.5
0.6
0.7
0.8
0
20
40
60
80
Ages (years)
FDR (Age associated divergence) =0.098
p(Scalp)=0.174
p(Dura)=0.272
Scalp
Dura

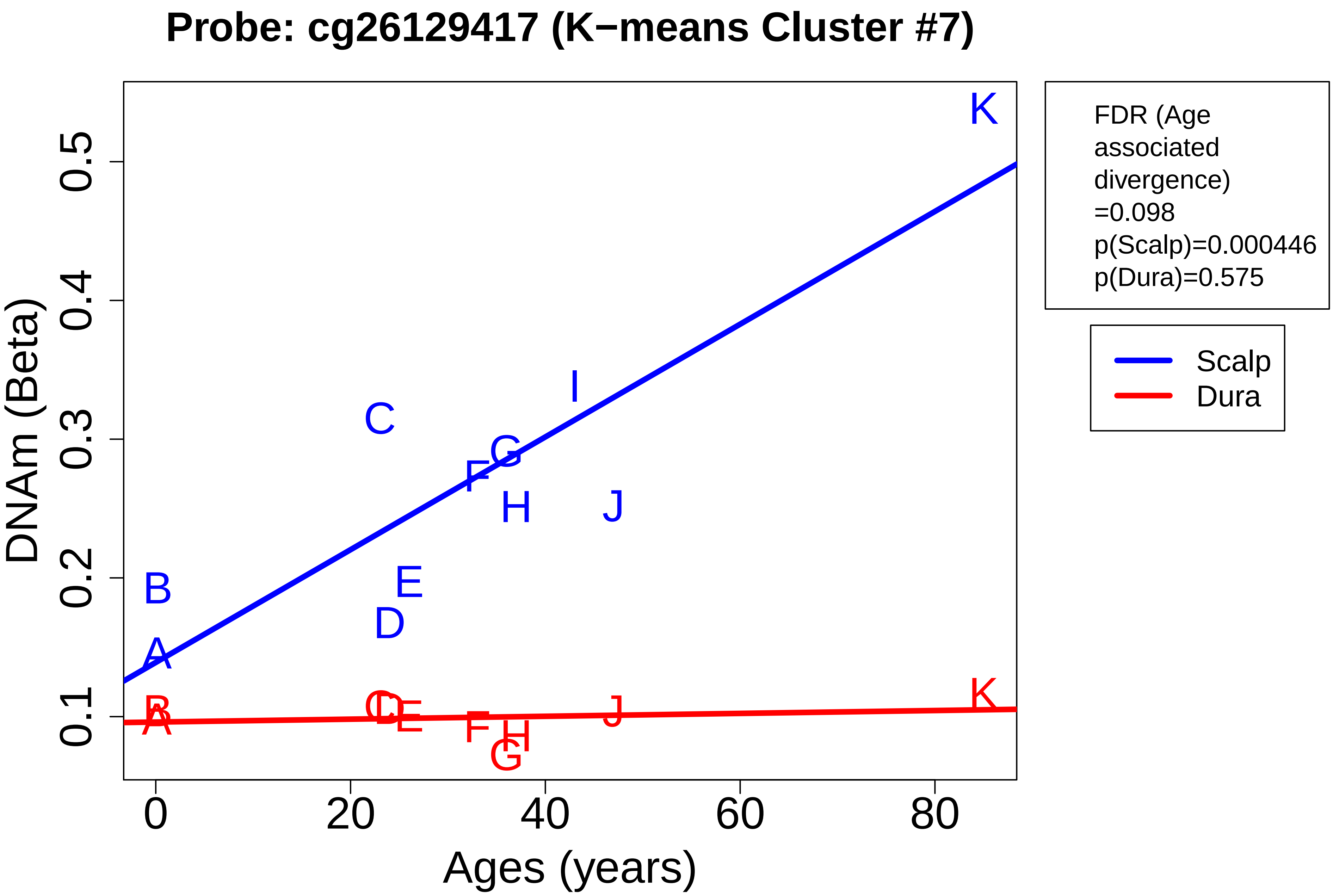

Probe: cg26129417 (K−means Cluster #7)
DNAm (Beta)
0.1
0.2
0.3
0.4
0.5
0
20
40
60
80
Ages (years)
A
B
C
D
E
F
G
H
I
J
K
FDR (Age associated divergence) =0.098
p(Scalp)=0.000446
p(Dura)=0.575
Scalp
Dura

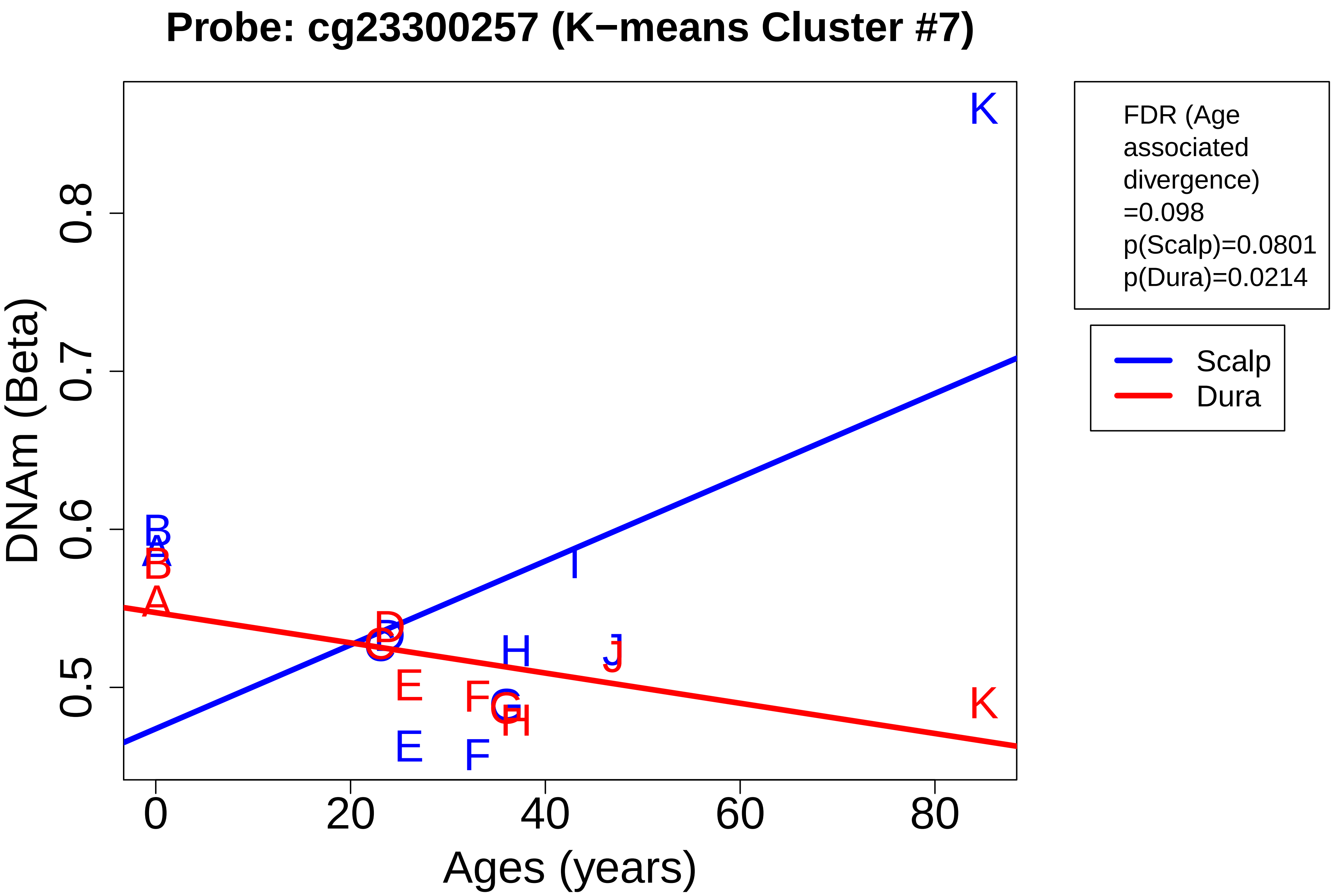
Probe: cg23300257 (K−means Cluster #7)
DNAm (Beta)
0.5
0.6
0.7
0.8
Ages (years)
0
20
40
60
80
FDR (Age associated divergence) =0.098
p(Scalp)=0.0801
p(Dura)=0.0214
Scalp
Dura
A
B
C
D
E
F
G
H
I
J
K

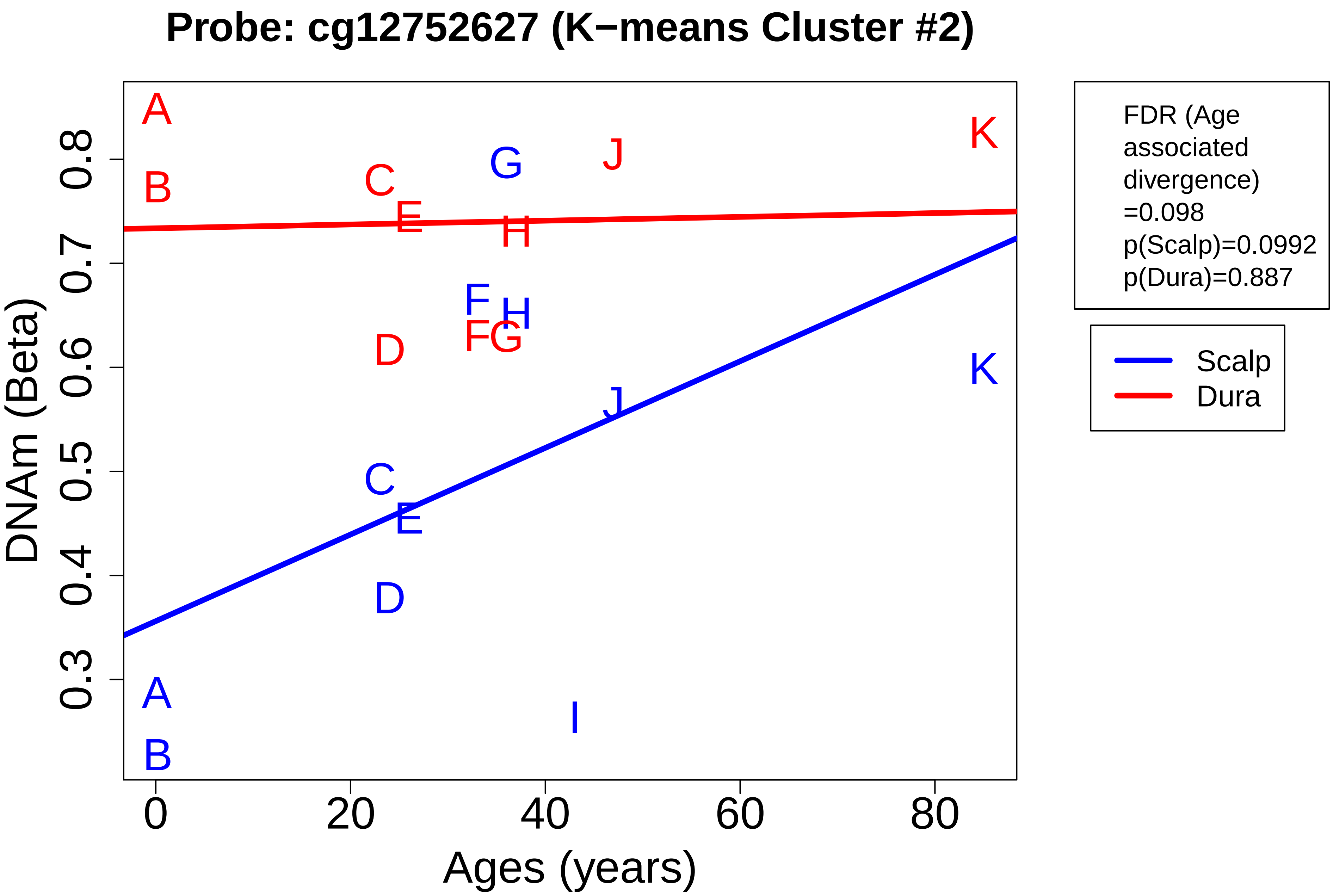

Probe: cg12752627 (K−means Cluster #2)
DNAm (Beta)
0.3
0.4
0.5
0.6
0.7
0.8
0
20
40
60
80
Ages (years)
A
B
C
D
E
F
G
H
I
J
K
A
B
C
D
E
FG
H
J
K
FDR (Age associated divergence) =0.098
p(Scalp)=0.0992
p(Dura)=0.887
Scalp
Dura

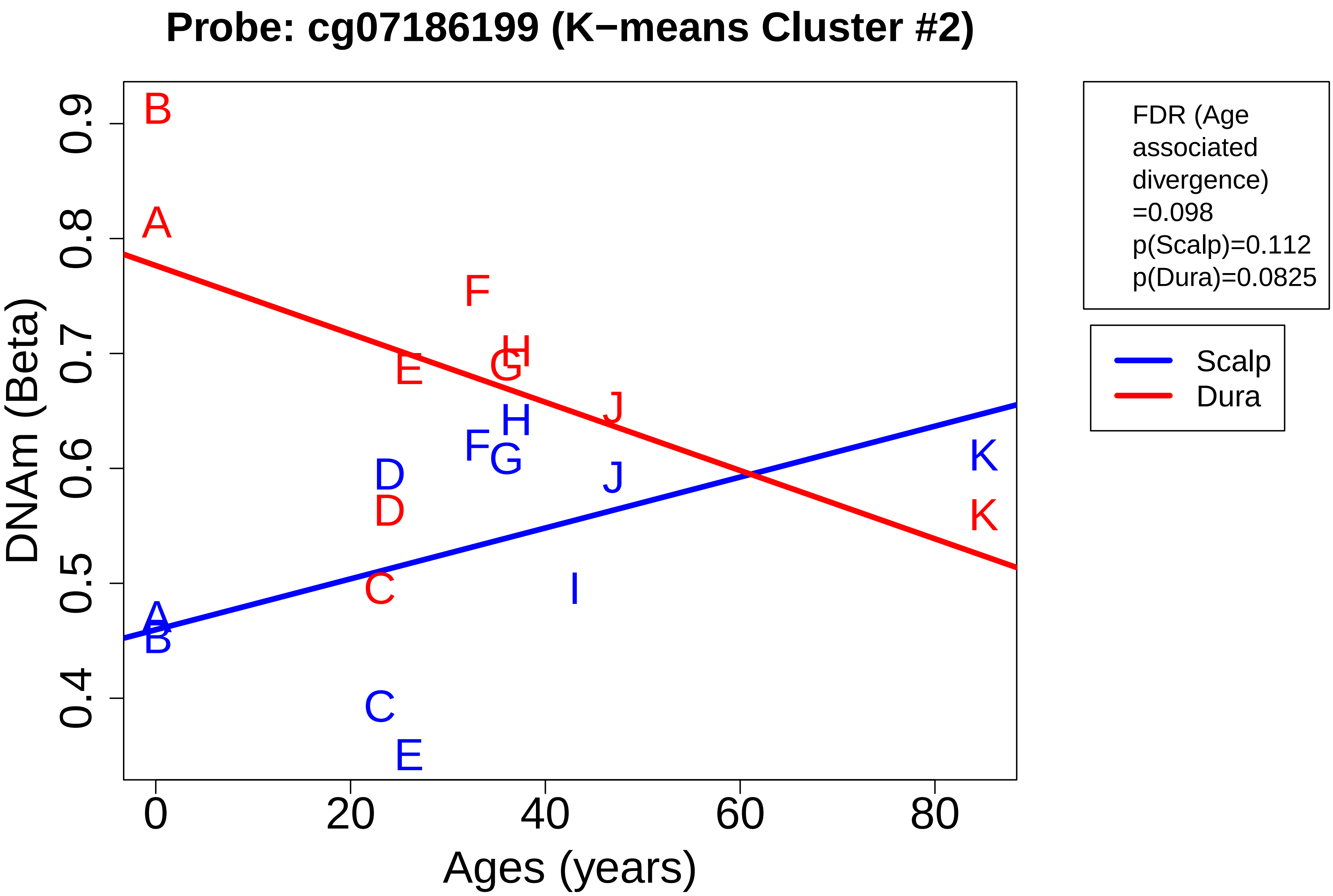
Probe: cg07186199 (K−means Cluster #2)
DNAm (Beta)
0.4
0.5
0.6
0.7
0.8
0.9
0
20
40
60
80
Ages (years)
FDR (Age associated divergence) =0.098
p(Scalp)=0.112
p(Dura)=0.0825
Scalp
Dura

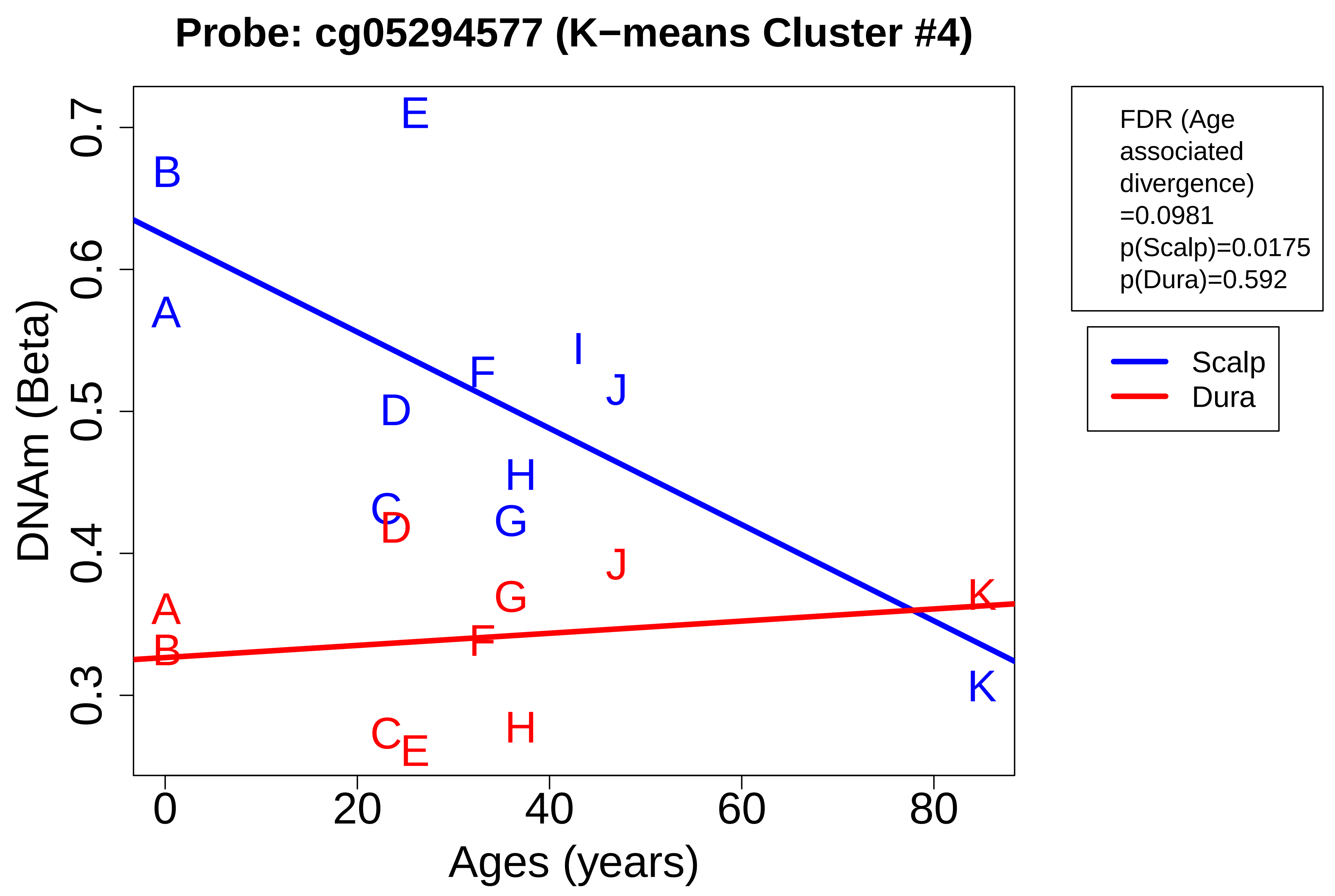

Probe: cg05294577 (K−means Cluster #4)
FDR (Age associated divergence) =0.0981
p(Scalp)=0.0175
p(Dura)=0.592
Scalp
Dura
DNAm (Beta)
0.3
0.4
0.5
0.6
0.7
Ages (years)
0
20
40
60
80
A
B
C
D
E
F
G
H
I
J
K

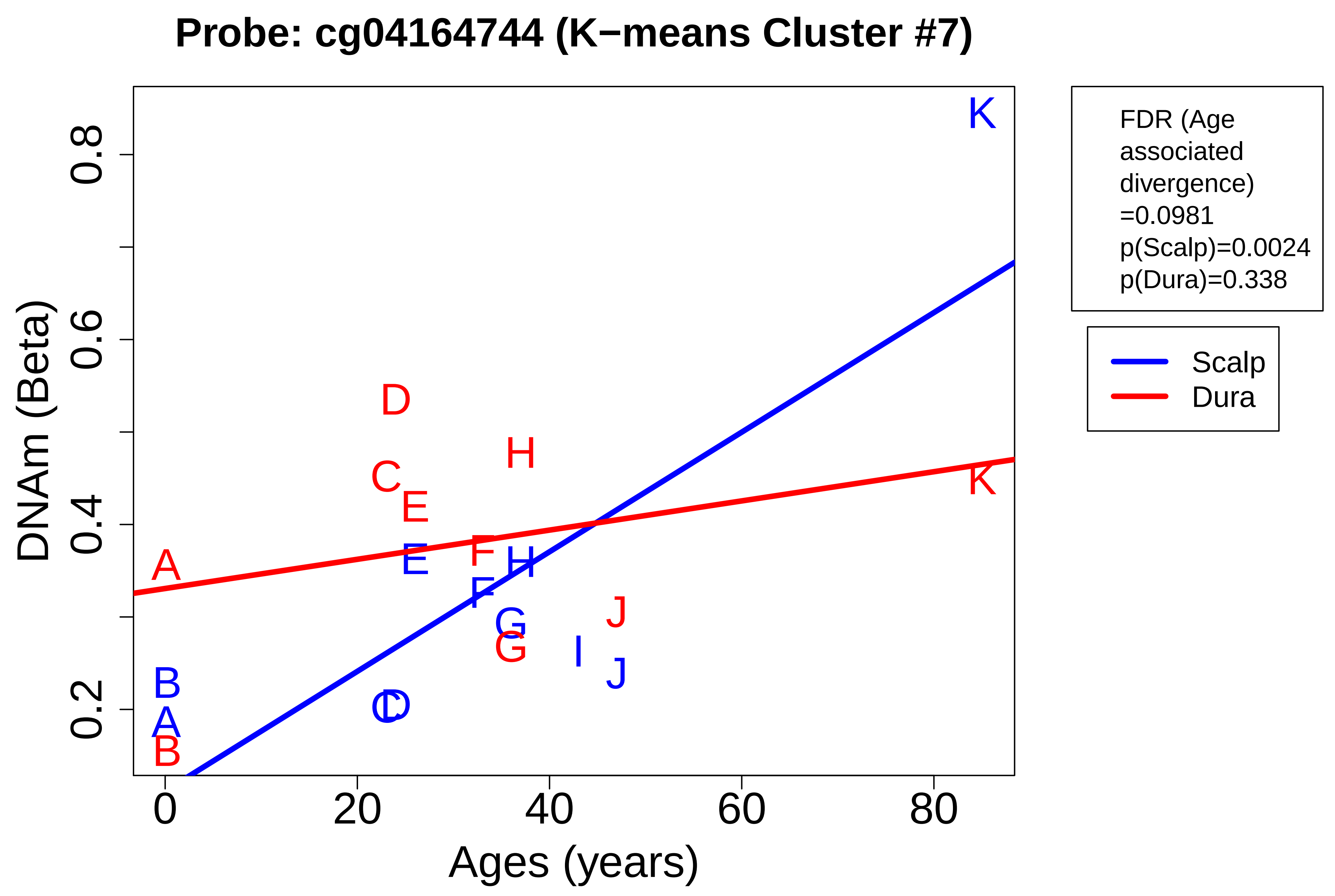

Probe: cg04164744 (K−means Cluster #7)
FDR (Age associated divergence) =0.0981
p(Scalp)=0.0024
p(Dura)=0.338
Scalp
Dura
DNAm (Beta)
0.2
0.4
0.6
0.8
0
20
40
60
80
Ages (years)
A
B
C
D
E
F
G
H
I
J
K

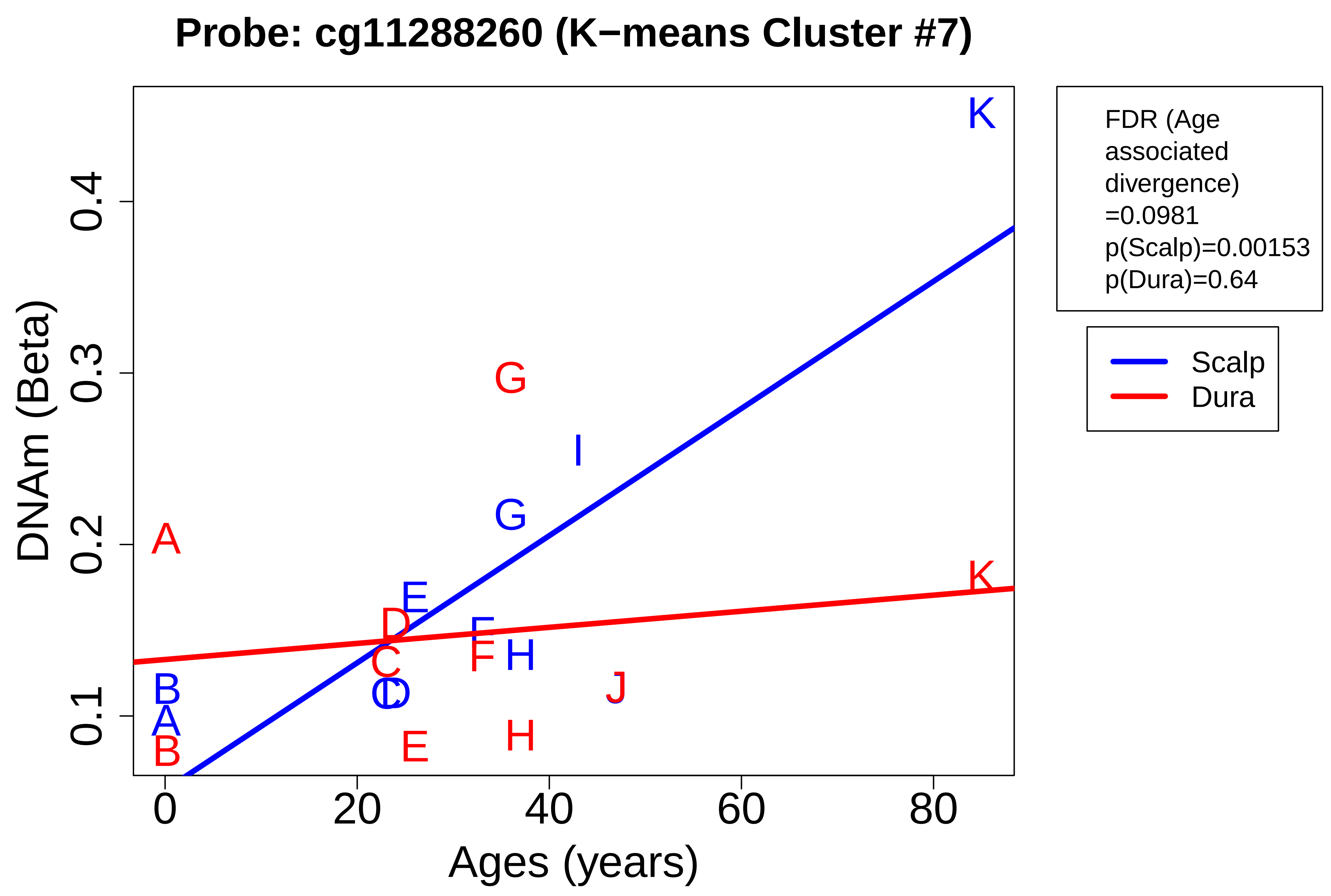

Probe: cg11288260 (K−means Cluster #7)
DNAm (Beta)
0.1
0.2
0.3
0.4
0
20
40
60
80
Ages (years)
A
B
C
D
E
F
G
H
I
J
K
FDR (Age associated divergence) =0.0981
p(Scalp)=0.00153
p(Dura)=0.64
Scalp
Dura

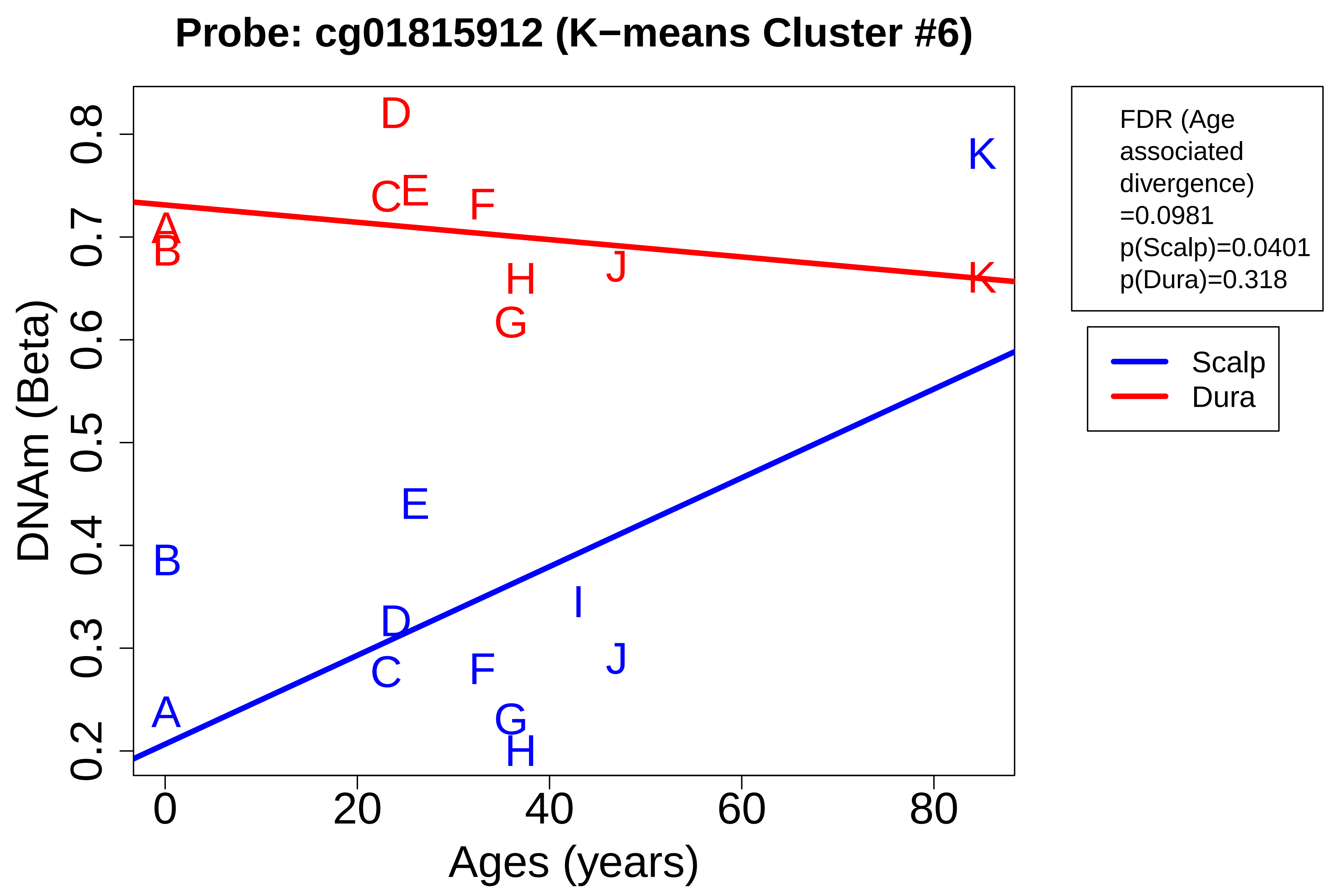

Probe: cg01815912 (K−means Cluster #6)
DNAm (Beta)
0.2
0.3
0.4
0.5
0.6
0.7
0.8
Ages (years)
0
20
40
60
80
D
CE
F
A
B
H
J
G
K
K
E
B
I
D
C
F
J
A
G
H
FDR (Age associated divergence) =0.0981
p(Scalp)=0.0401
p(Dura)=0.318
Scalp
Dura

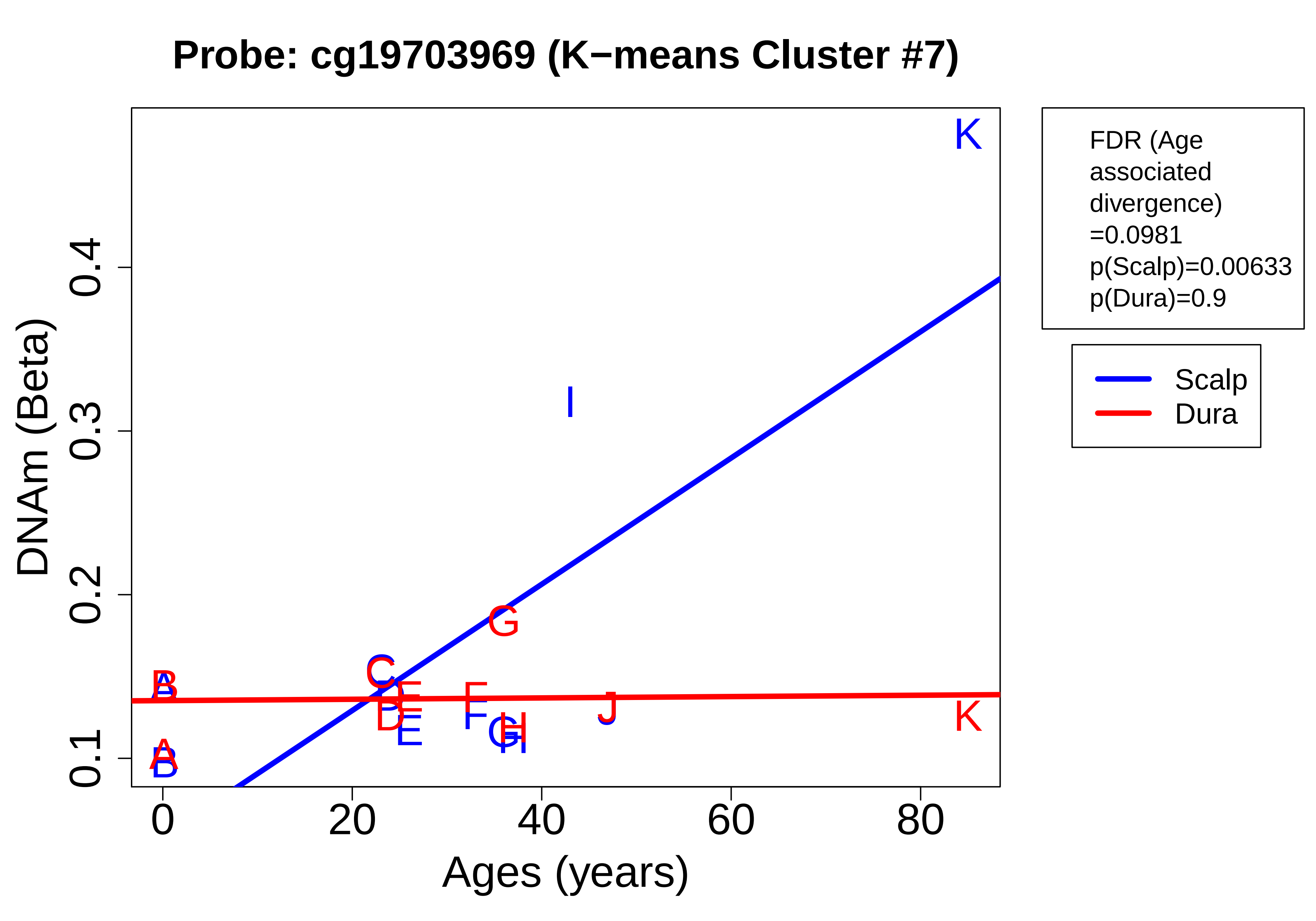

Probe: cg19703969 (K−means Cluster #7)
FDR (Age associated divergence) =0.0981
p(Scalp)=0.00633
p(Dura)=0.9
Scalp
Dura
DNAm (Beta)
0.1
0.2
0.3
0.4
0
20
40
60
80
Ages (years)
A
B
C
D
E
F
G
H
I
J
K

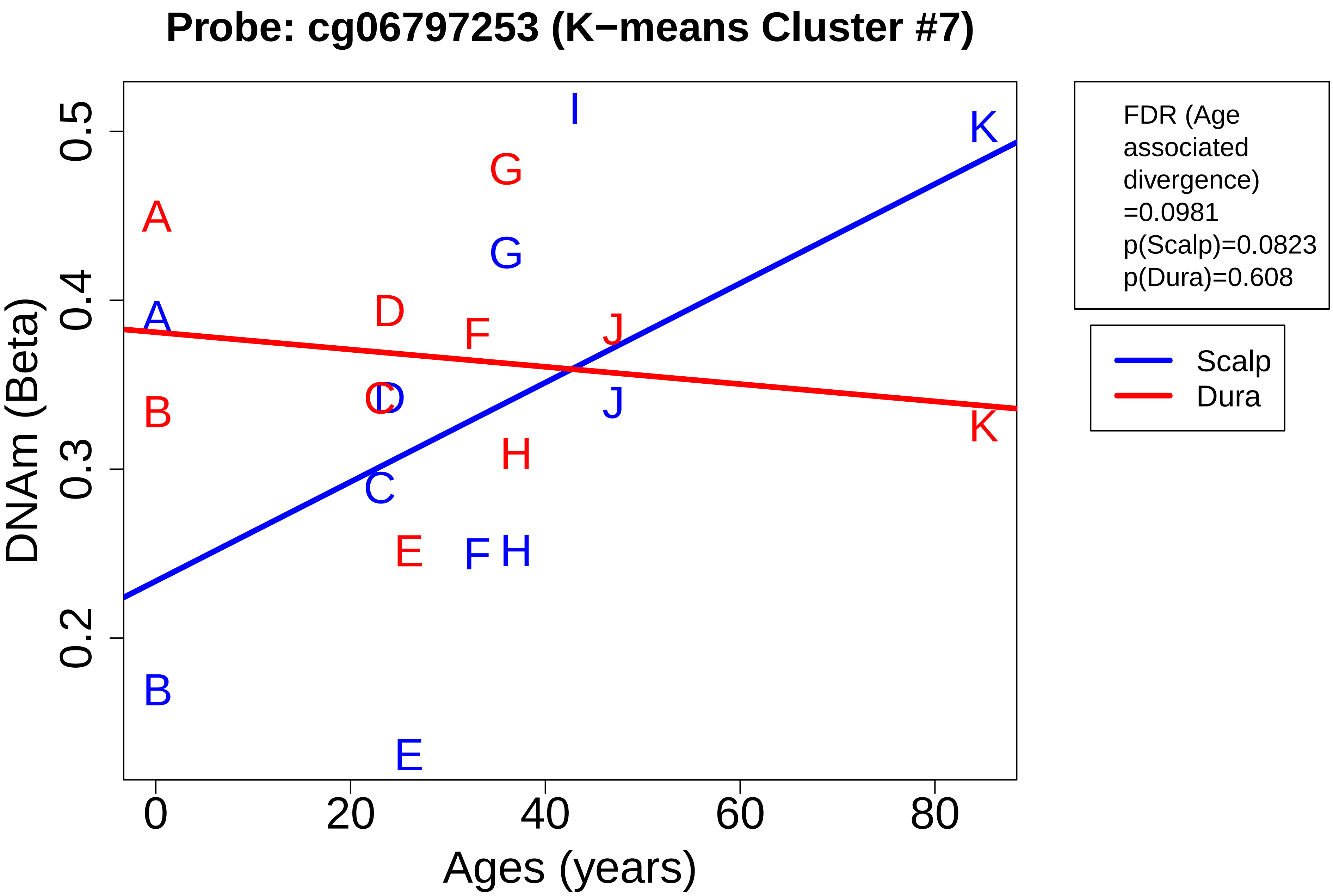

Probe: cg06797253 (K−means Cluster #7)
DNAm (Beta)
0.2
0.3
0.4
0.5
0
20
40
60
80
Ages (years)
A
B
C
D
E
F
G
H
I
J
K
FDR (Age associated divergence) =0.0981
p(Scalp)=0.0823
p(Dura)=0.608
Scalp
Dura

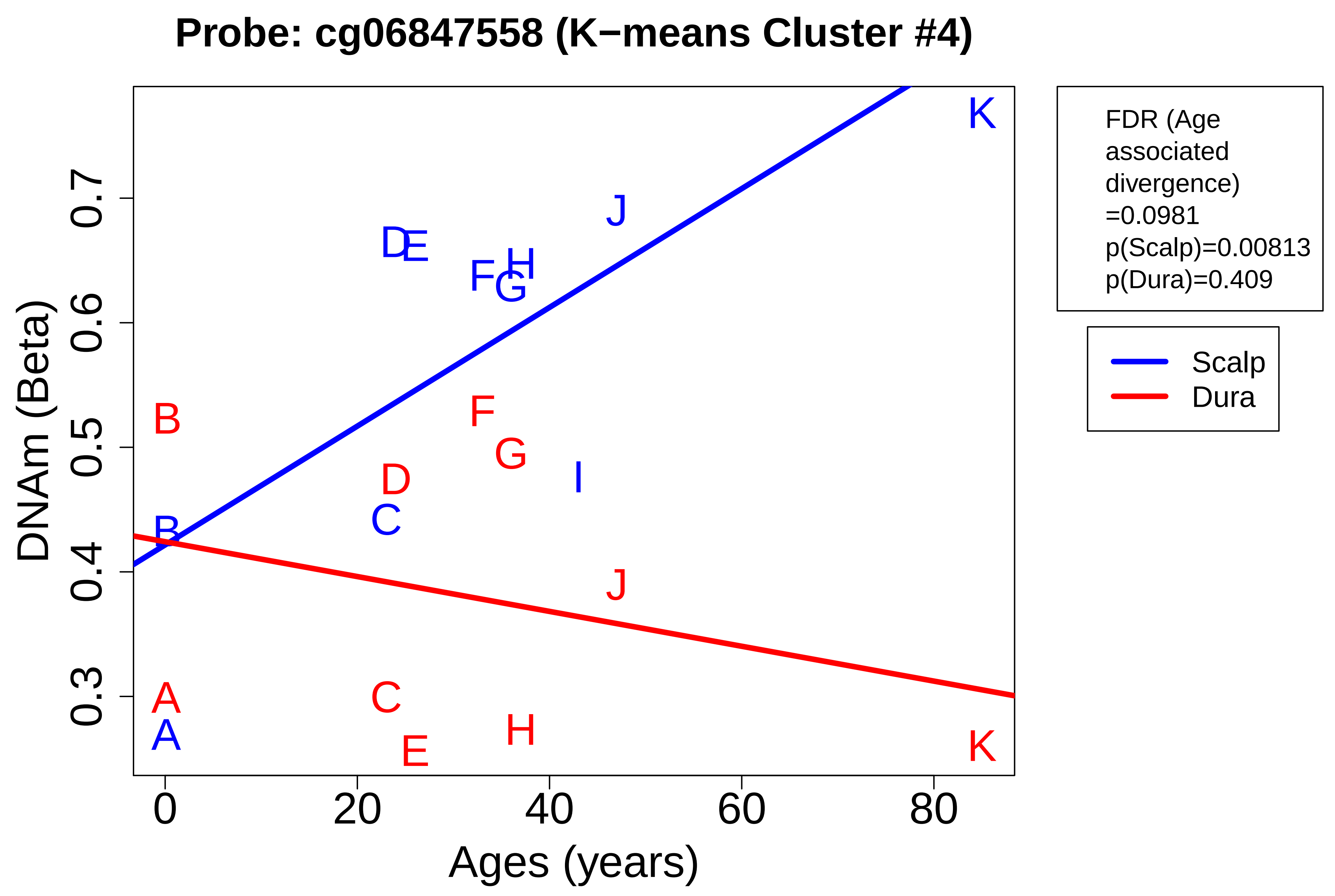

Probe: cg06847558 (K−means Cluster #4)
DNAm (Beta)
0.3
0.4
0.5
0.6
0.7
Ages (years)
0
20
40
60
80
A
B
C
D
E
F
G
H
I
J
K
FDR (Age associated divergence) =0.0981
p(Scalp)=0.00813
p(Dura)=0.409
Scalp
Dura

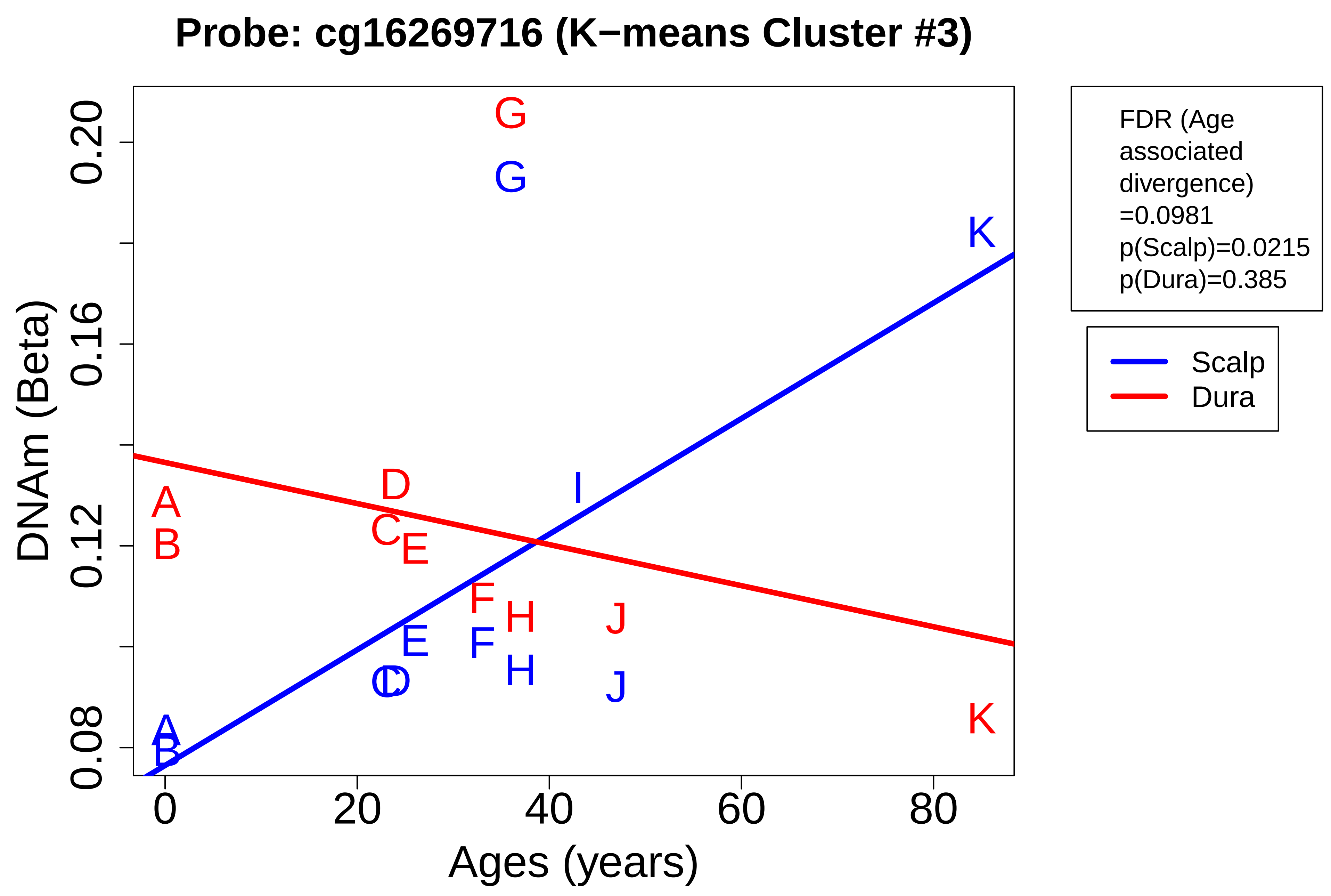

Probe: cg16269716 (K−means Cluster #3)
DNAm (Beta)
0.08
0.12
0.16
0.20
Ages (years)
0
20
40
60
80
A
B
C
D
E
F
G
H
I
J
K
FDR (Age associated divergence) =0.0981
p(Scalp)=0.0215
p(Dura)=0.385
Scalp
Dura

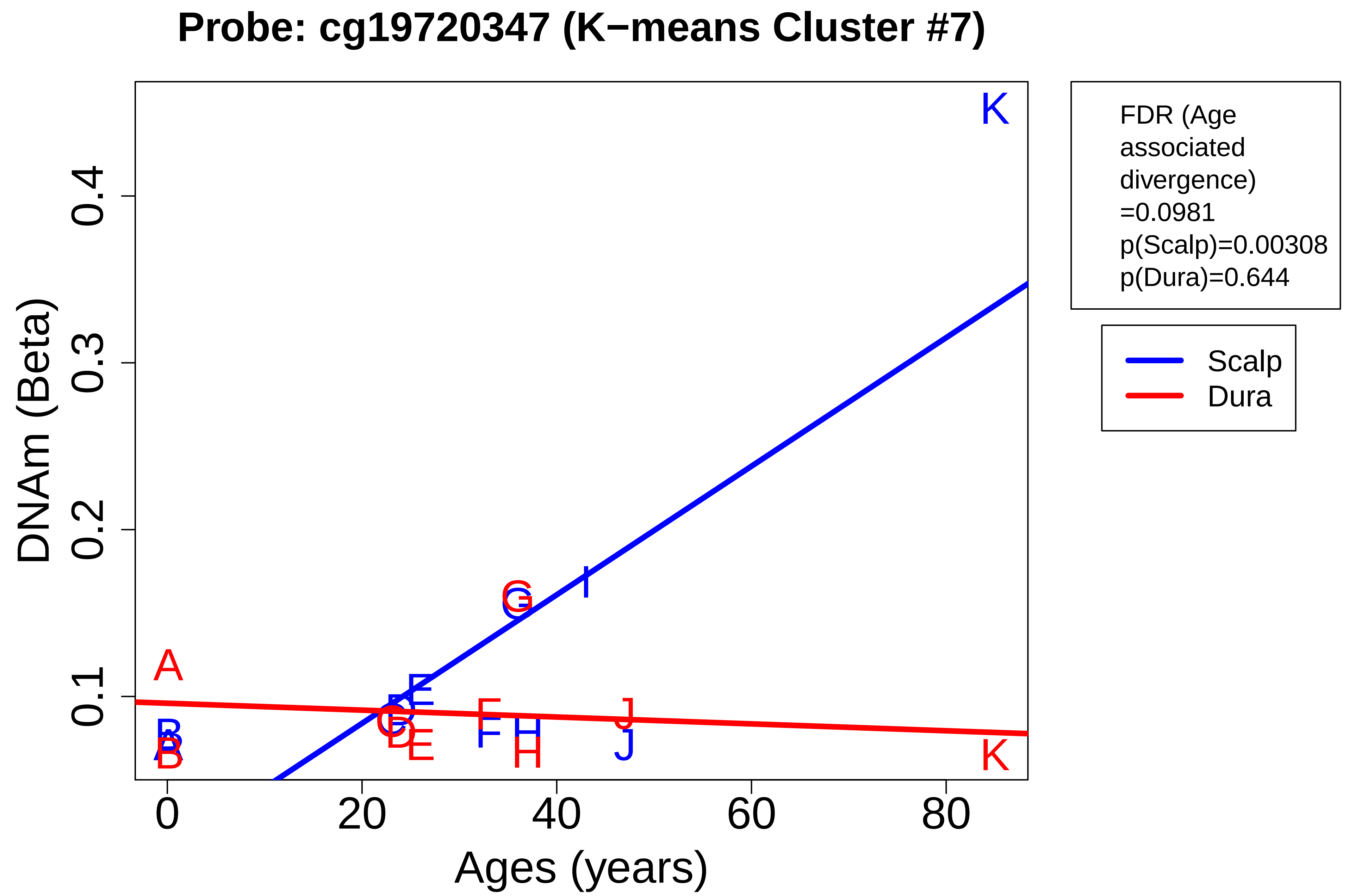

Probe: cg19720347 (K−means Cluster #7)
DNAm (Beta)
0.1
0.2
0.3
0.4
Ages (years)
0
20
40
60
80
FDR (Age associated divergence) =0.0981
p(Scalp)=0.00308
p(Dura)=0.644
Scalp
Dura

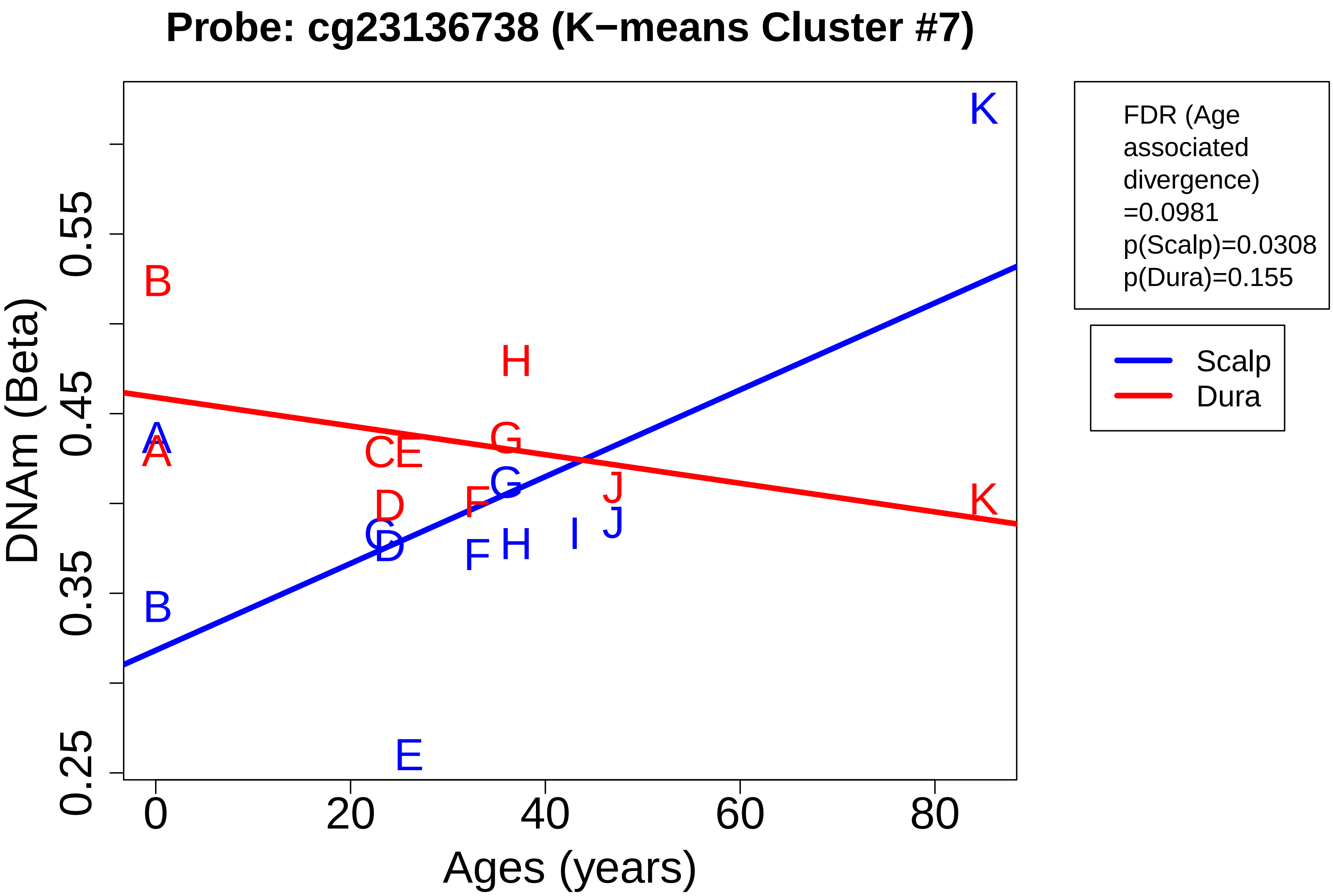

Probe: cg23136738 (K−means Cluster #7)
DNAm (Beta)
0.25
0.35
0.45
0.55
Ages (years)
0
20
40
60
80
FDR (Age associated divergence) =0.0981
p(Scalp)=0.0308
p(Dura)=0.155
Scalp
Dura
A
B
C
D
E
F
G
H
I
J
K

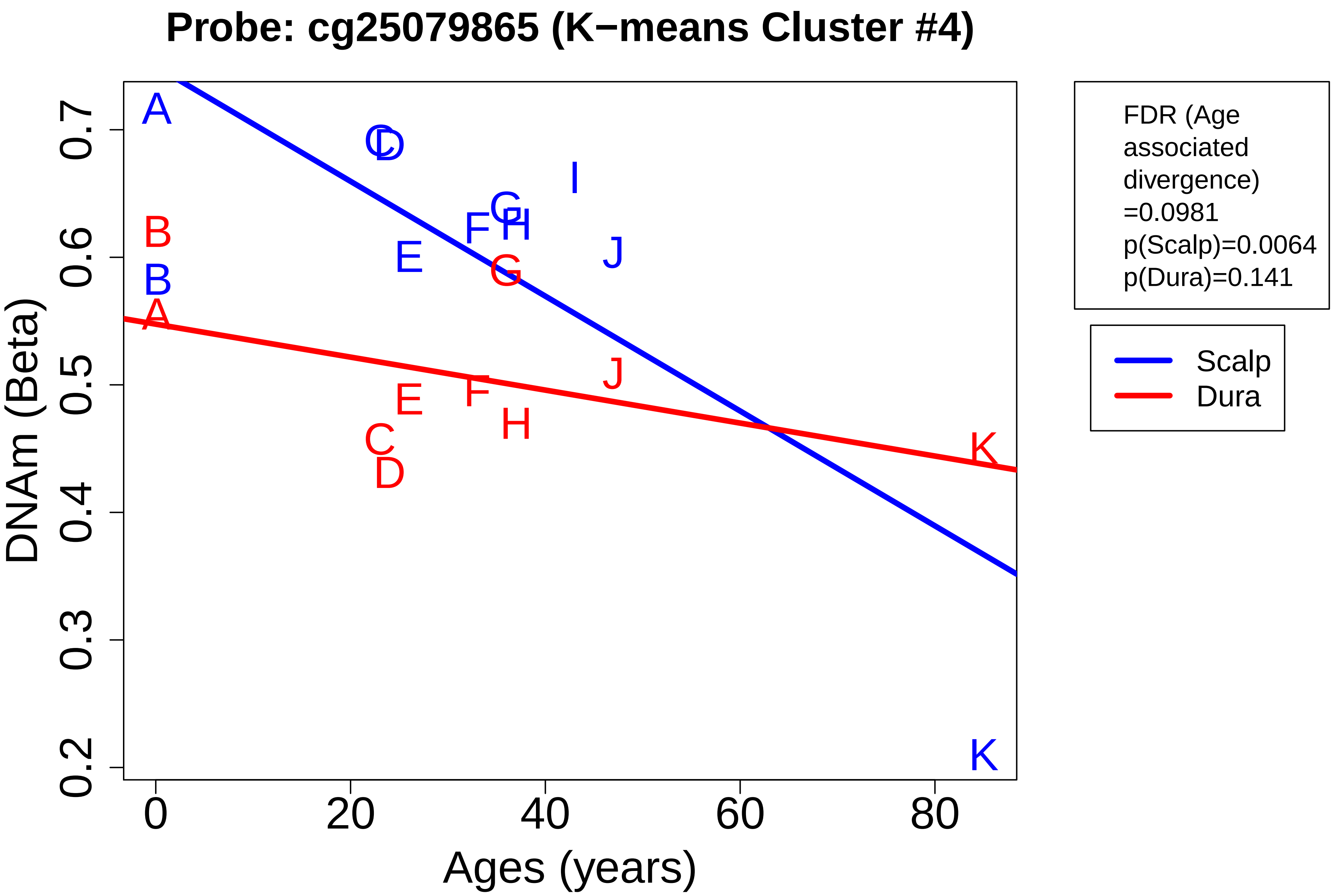

Probe: cg25079865 (K−means Cluster #4)
DNAm (Beta)
Ages (years)
0.2
0.3
0.4
0.5
0.6
0.7
0
20
40
60
80
FDR (Age associated divergence) =0.0981
p(Scalp)=0.0064
p(Dura)=0.141
Scalp
Dura

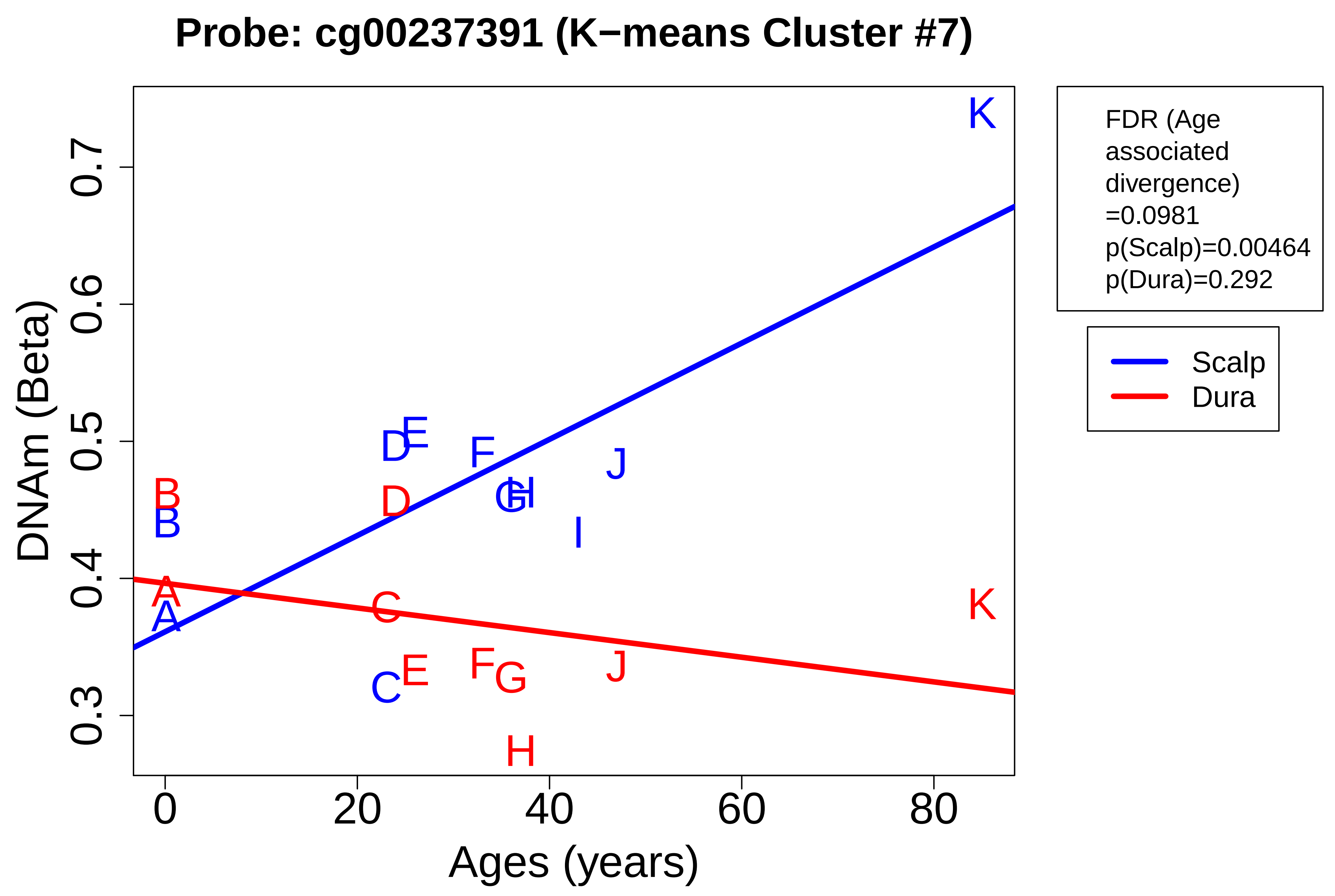
Probe: cg00237391 (K−means Cluster #7)
DNAm (Beta)
0.3
0.4
0.5
0.6
0.7
Ages (years)
0
20
40
60
80
A
B
C
D
E
F
G
H
I
J
K
FDR (Age associated divergence) =0.0981
p(Scalp)=0.00464
p(Dura)=0.292
Scalp
Dura

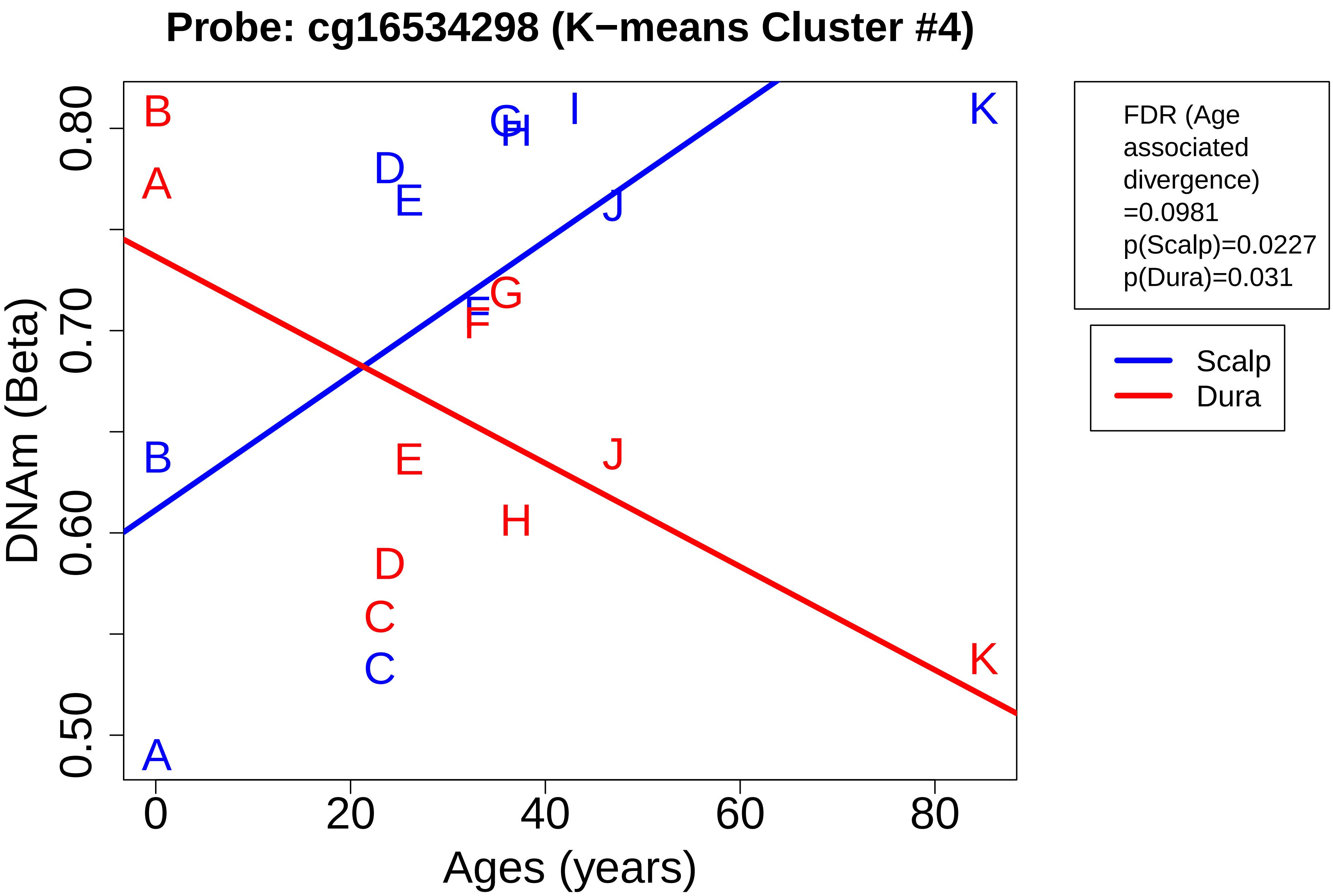
Probe: cg16534298 (K−means Cluster #4)
DNAm (Beta)
0.50
0.60
0.70
0.80
Ages (years)
0
20
40
60
80
A
B
C
D
E
F
G
H
I
J
K
A
B
C
D
E
F
G
H
J
K
FDR (Age associated divergence) =0.0981
p(Scalp)=0.0227
p(Dura)=0.031
Scalp
Dura

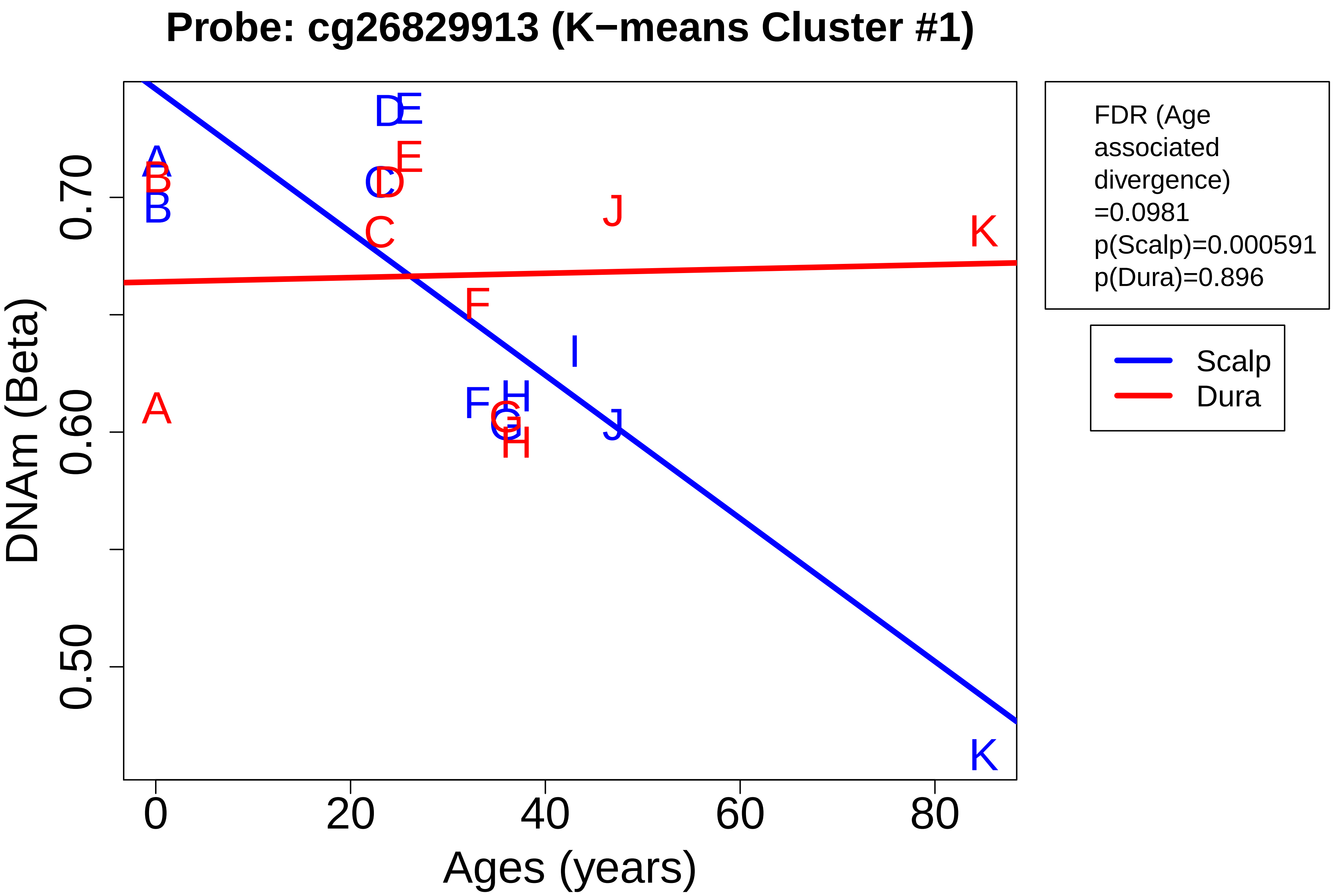
Probe: cg26829913 (K−means Cluster #1)
DNAm (Beta)
0.50
0.60
0.70
0
20
40
60
80
Ages (years)
A
B
C
D
E
F
G
H
I
J
K
FDR (Age associated divergence) =0.0981
p(Scalp)=0.000591
p(Dura)=0.896
Scalp
Dura

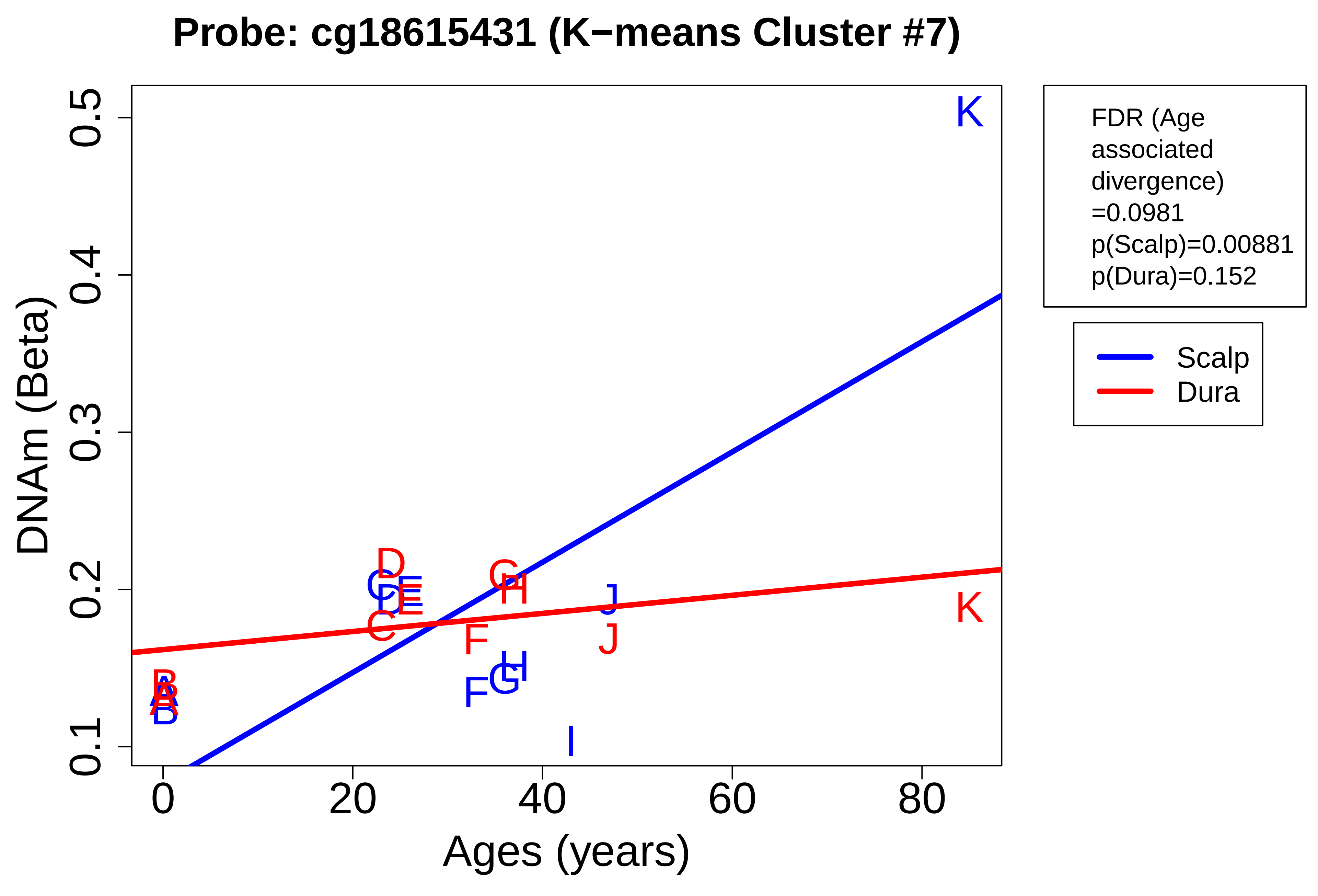
Probe: cg18615431 (K−means Cluster #7)
DNAm (Beta)
Ages (years)
FDR (Age associated divergence) =0.0981
p(Scalp)=0.00881
p(Dura)=0.152
Scalp
Dura

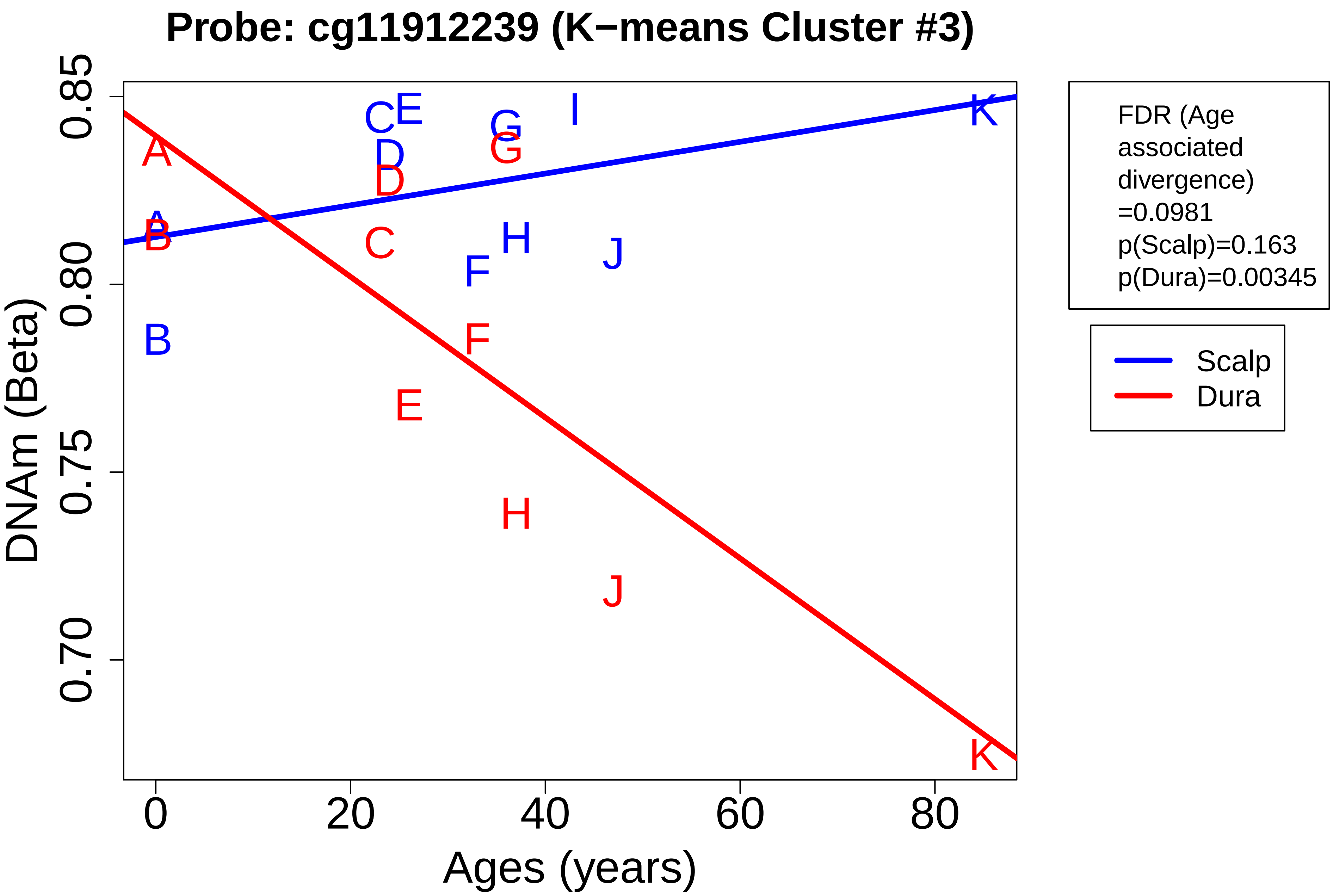

Probe: cg11912239 (K−means Cluster #3)
DNAm (Beta)
0.70
0.75
0.80
0.85
0
20
40
60
80
Ages (years)
FDR (Age associated divergence) =0.0981
p(Scalp)=0.163
p(Dura)=0.00345
Scalp
Dura
A
B
C
D
E
F
G
H
I
J
K

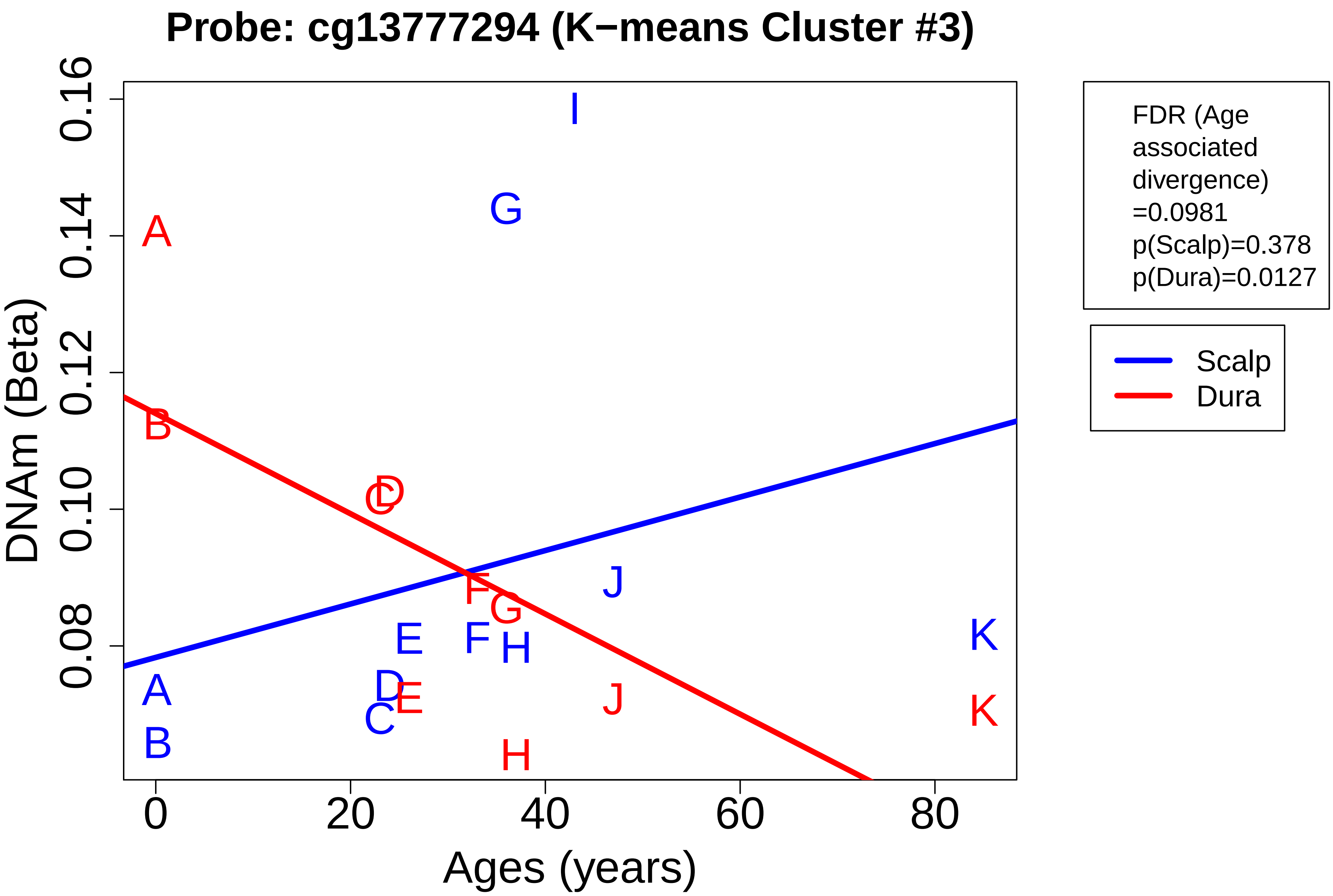
Probe: cg13777294 (K−means Cluster #3)
DNAm (Beta)
0.08
0.10
0.12
0.14
0.16
Ages (years)
0
20
40
60
80
FDR (Age associated divergence) =0.0981
p(Scalp)=0.378
p(Dura)=0.0127
Scalp
Dura
A
B
C
D
E
F
G
H
I
J
K

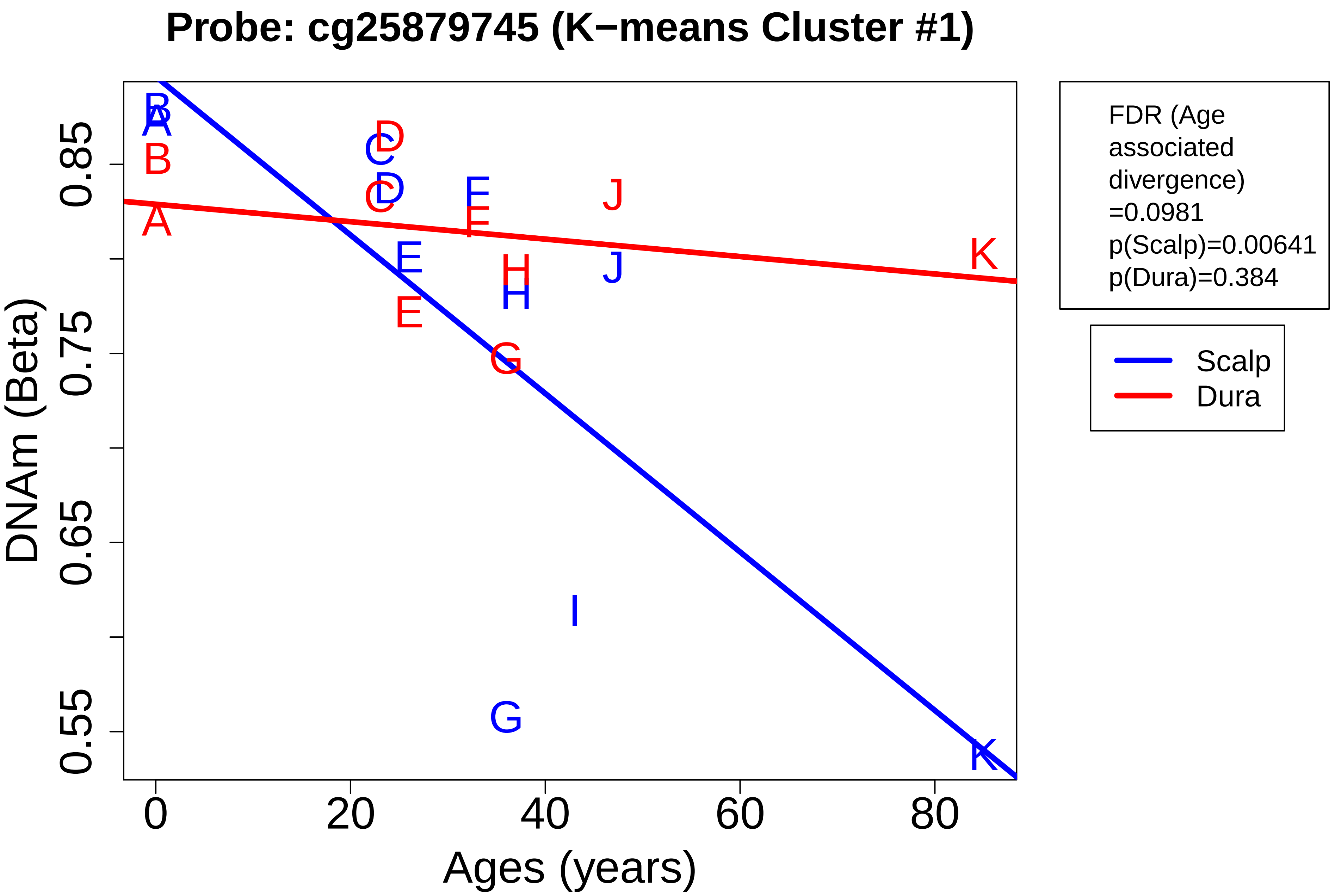
Probe: cg25879745 (K−means Cluster #1)
DNAm (Beta)
0.55
0.65
0.75
0.85
Ages (years)
0
20
40
60
80
FDR (Age associated divergence) =0.0981
p(Scalp)=0.00641
p(Dura)=0.384
Scalp
Dura

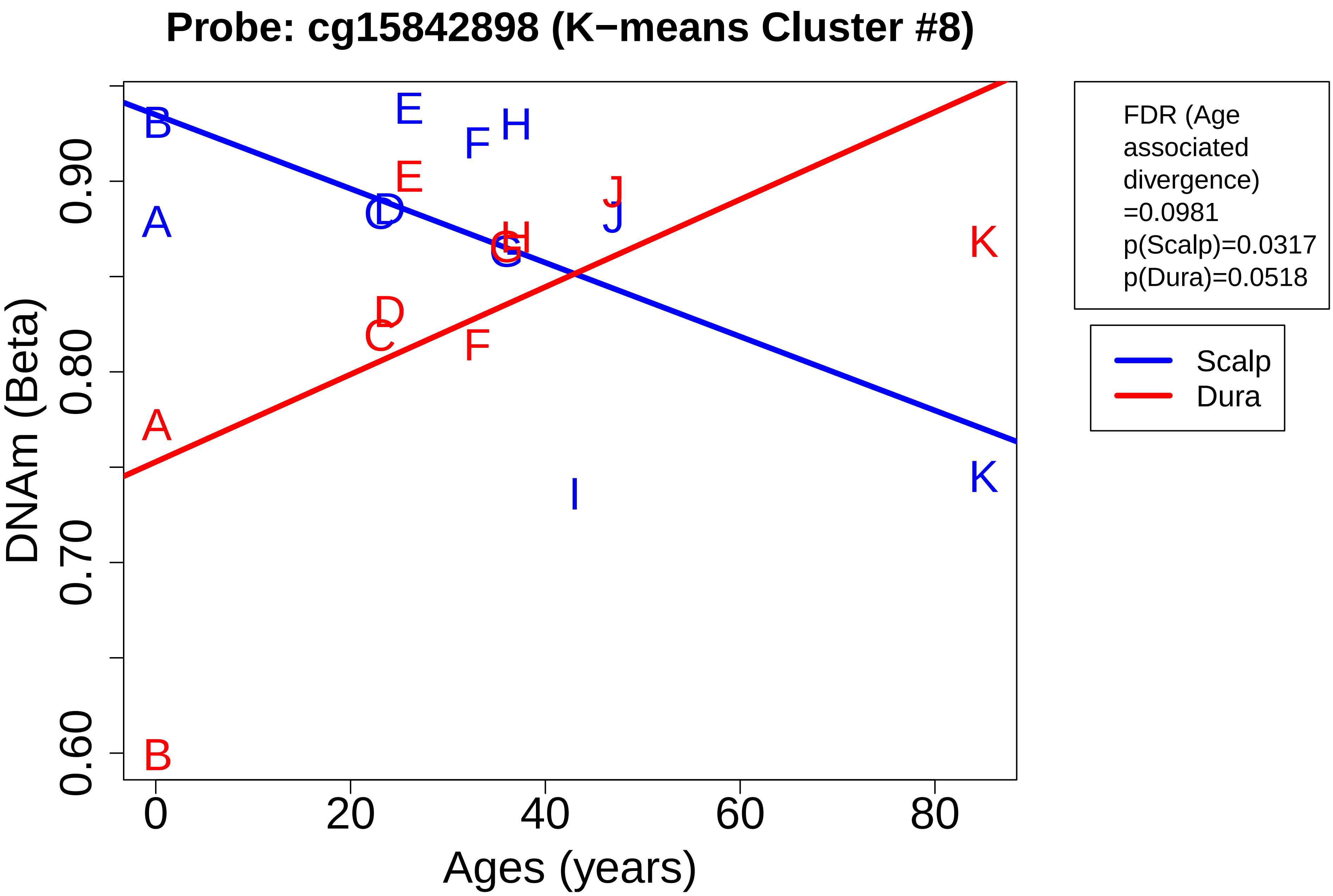

Probe: cg15842898 (K−means Cluster #8)
DNAm (Beta)
0.60
0.70
0.80
0.90
0
20
40
60
80
Ages (years)
FDR (Age associated divergence) =0.0981
p(Scalp)=0.0317
p(Dura)=0.0518
Scalp
Dura
A
B
C
D
E
F
G
H
I
J
K

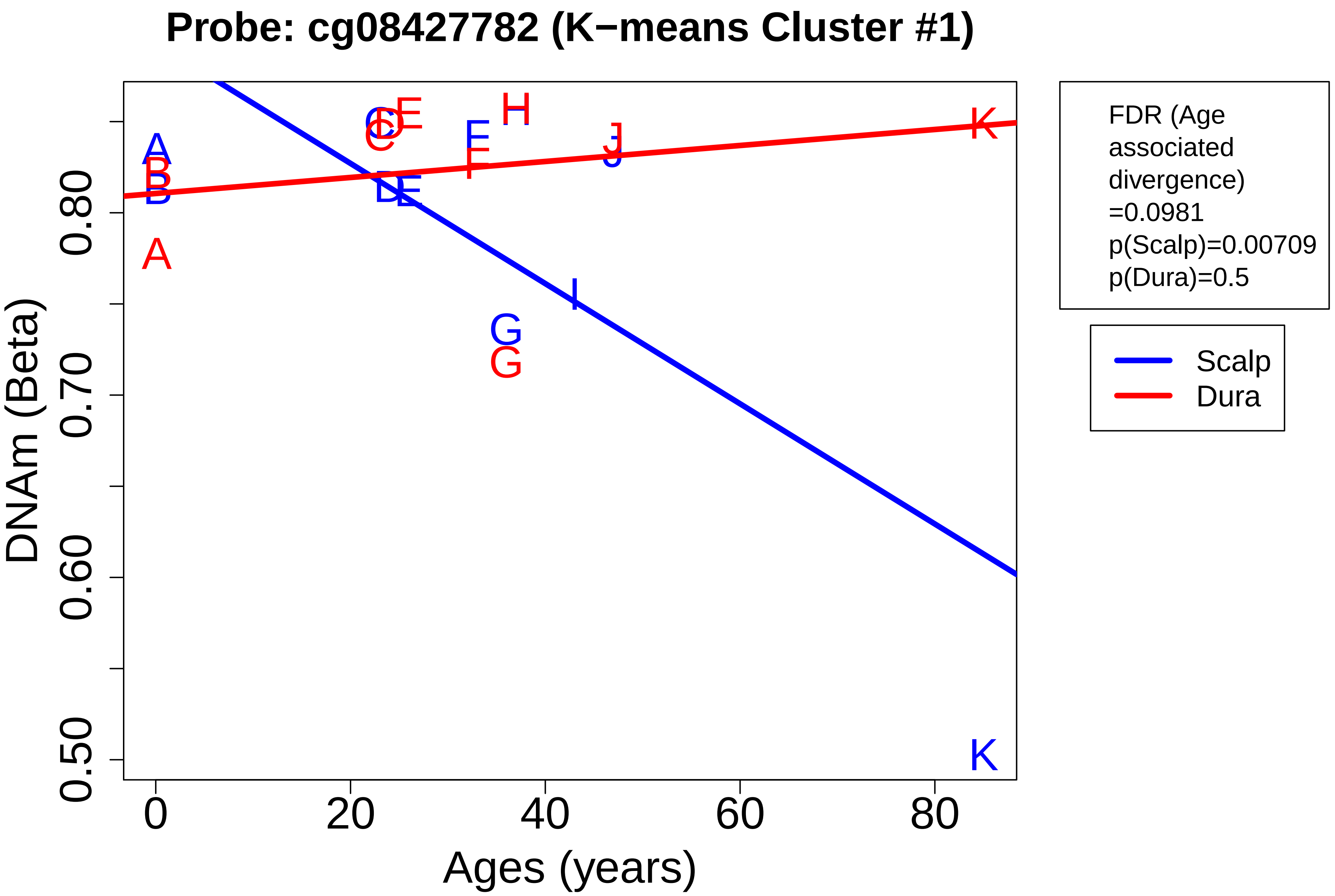

Probe: cg08427782 (K−means Cluster #1)
DNAm (Beta)
0.50
0.60
0.70
0.80
0
20
40
60
80
Ages (years)
FDR (Age associated divergence) =0.0981
p(Scalp)=0.00709
p(Dura)=0.5
Scalp
Dura
A
B
C
D
E
F
G
H
I
J
K

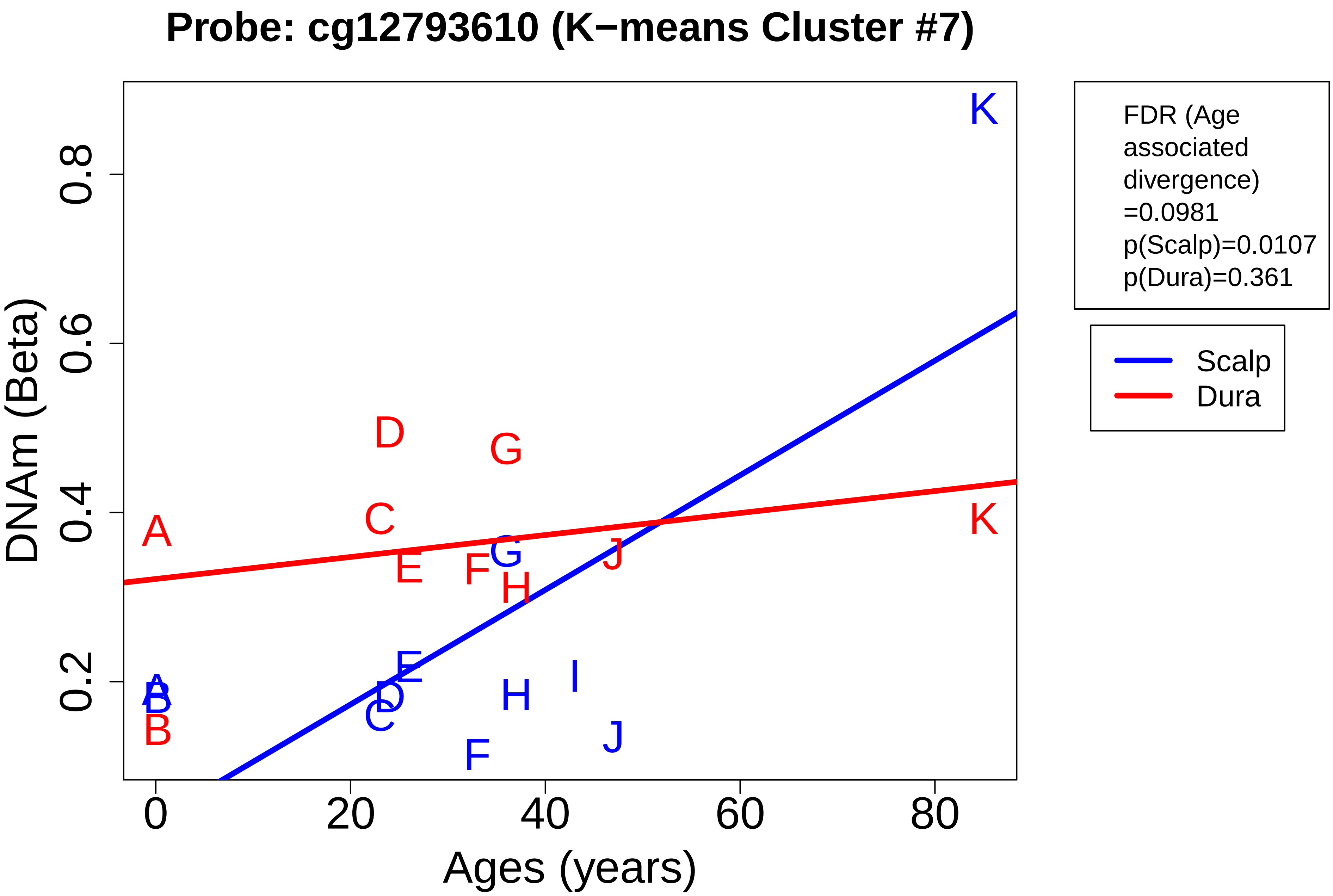

Probe: cg12793610 (K−means Cluster #7)
DNAm (Beta)
0.2
0.4
0.6
0.8
0
20
40
60
80
Ages (years)
FDR (Age associated divergence) =0.0981
p(Scalp)=0.0107
p(Dura)=0.361
Scalp
Dura
A
B
C
D
E
F
G
H
I
J
K

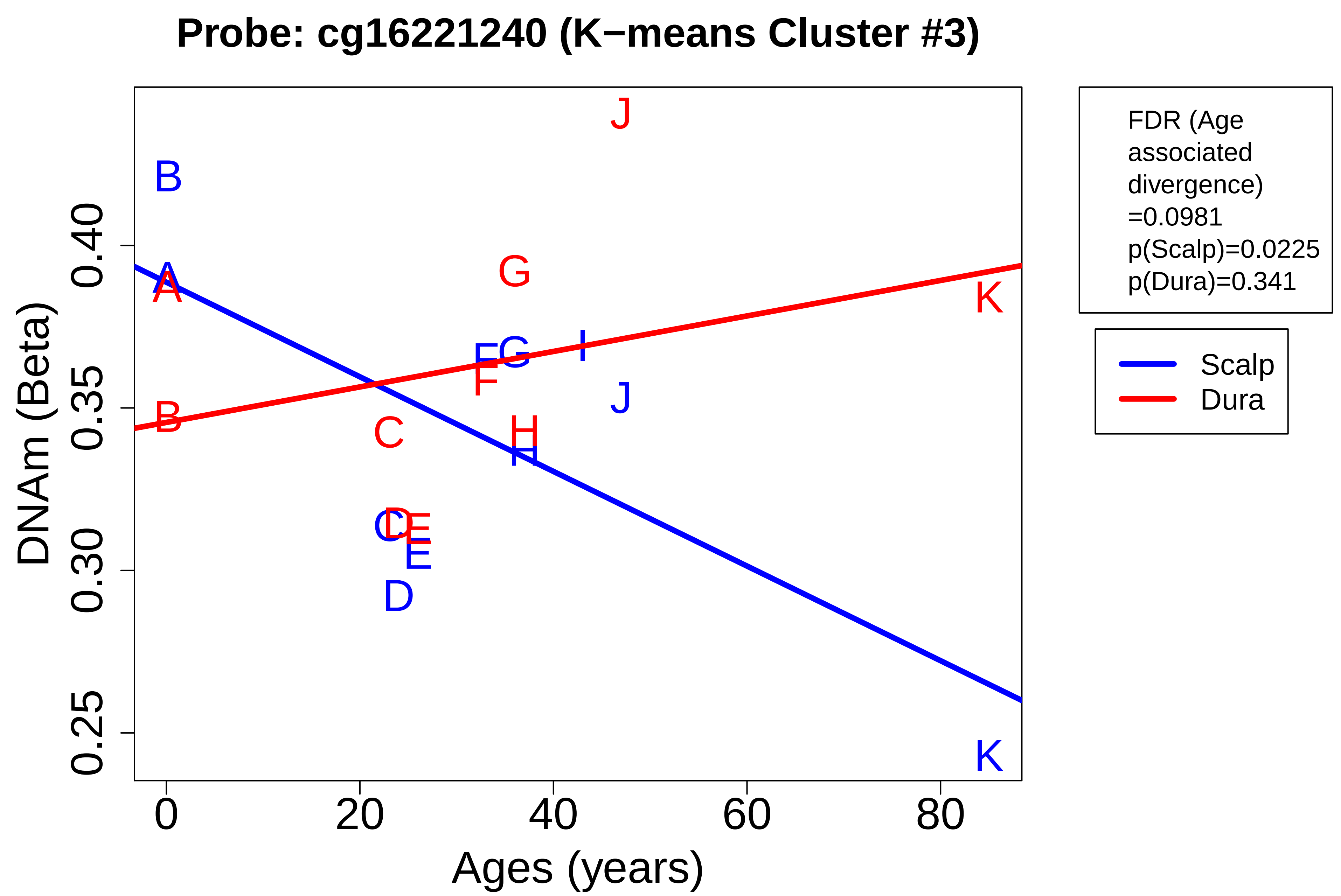
Probe: cg16221240 (K−means Cluster #3)
DNAm (Beta)
0.25
0.30
0.35
0.40
Ages (years)
0
20
40
60
80
FDR (Age associated divergence) =0.0981
p(Scalp)=0.0225
p(Dura)=0.341
Scalp
Dura
A
B
C
D
E
F
G
H
I
J
K

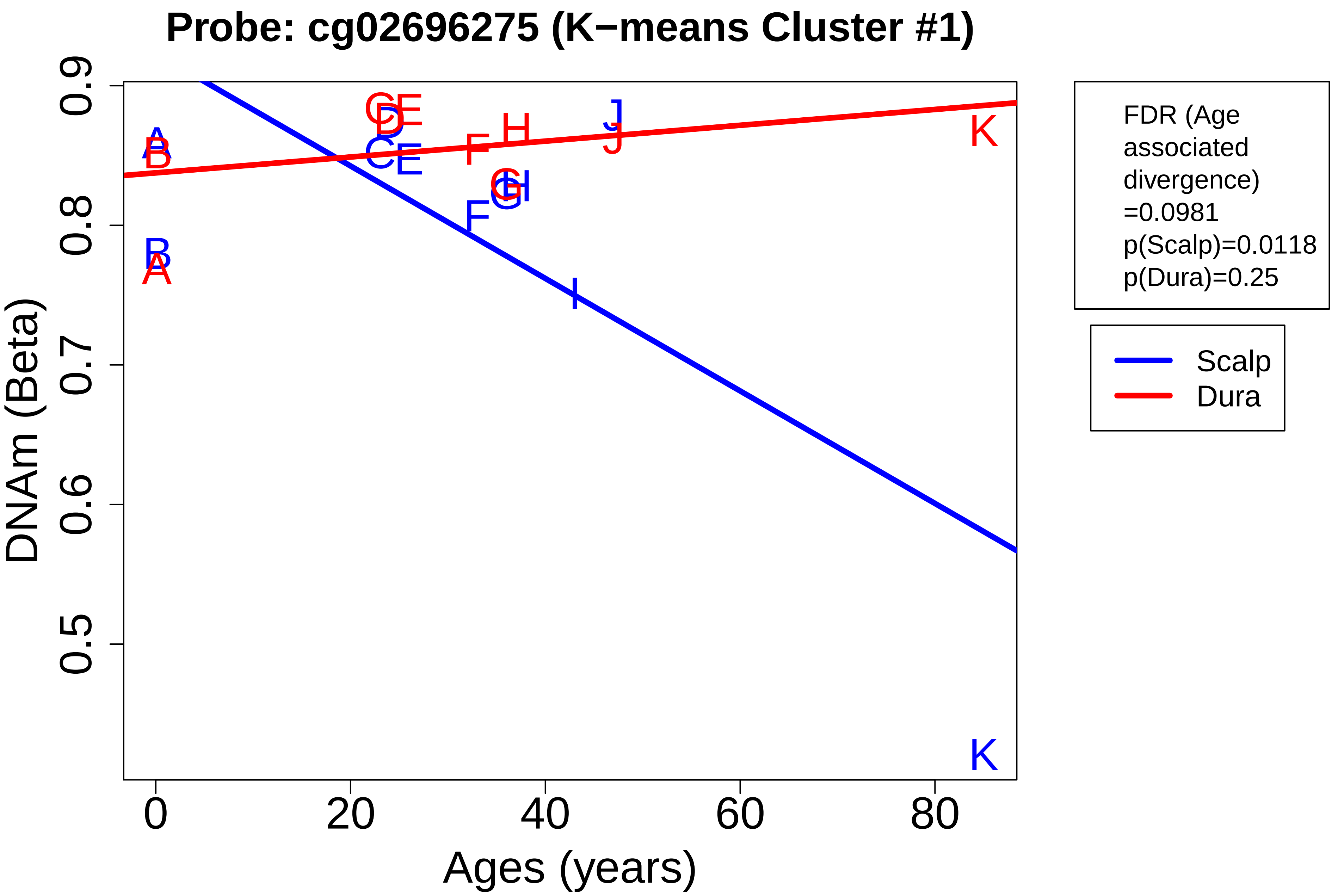

Probe: cg02696275 (K−means Cluster #1)
DNAm (Beta)
0.5
0.6
0.7
0.8
0.9
Ages (years)
0
20
40
60
80
FDR (Age associated divergence) =0.0981
p(Scalp)=0.0118
p(Dura)=0.25
Scalp
Dura
A
B
C
D
E
F
G
H
I
J
K

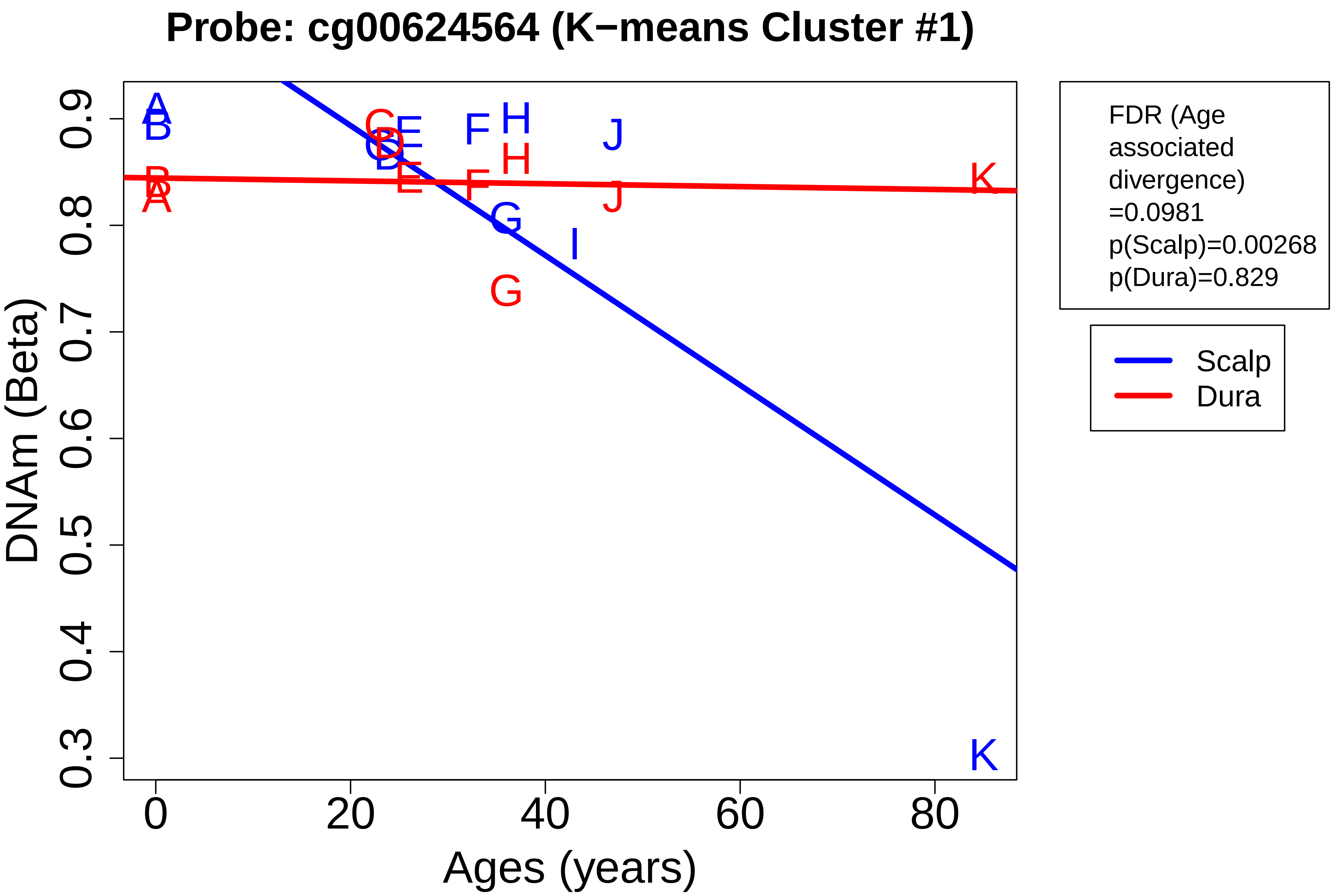

Probe: cg00624564 (K−means Cluster #1)
DNAm (Beta)
Ages (years)
0.3
0.4
0.5
0.6
0.7
0.8
0.9
0
20
40
60
80
FDR (Age associated divergence) =0.0981
p(Scalp)=0.00268
p(Dura)=0.829
Scalp
Dura

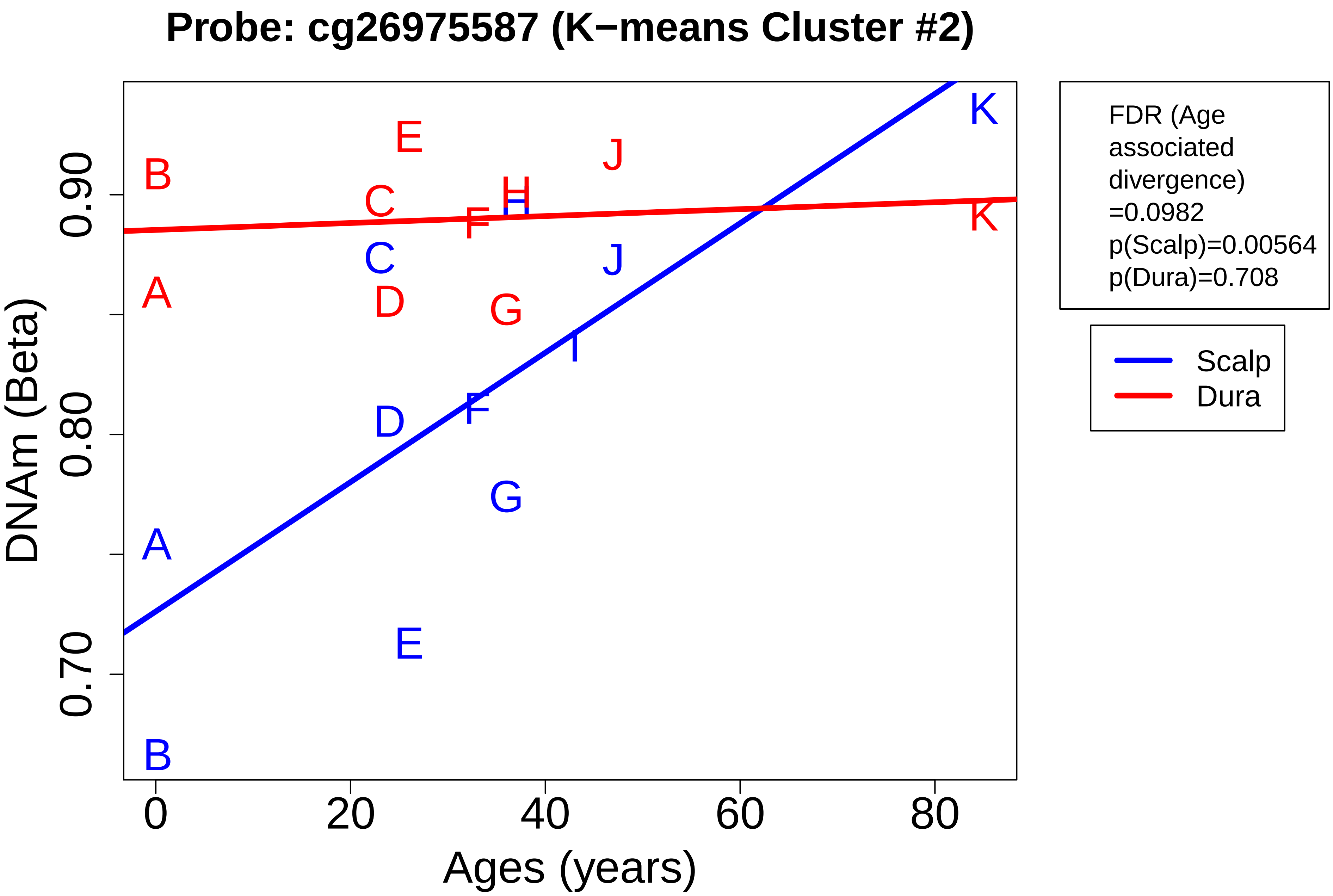

Probe: cg26975587 (K−means Cluster #2)
FDR (Age associated divergence) =0.0982
p(Scalp)=0.00564
p(Dura)=0.708
Scalp
Dura
DNAm (Beta)
0.70
0.80
0.90
Ages (years)
0
20
40
60
80
A
B
C
D
E
F
G
H
I
J
K

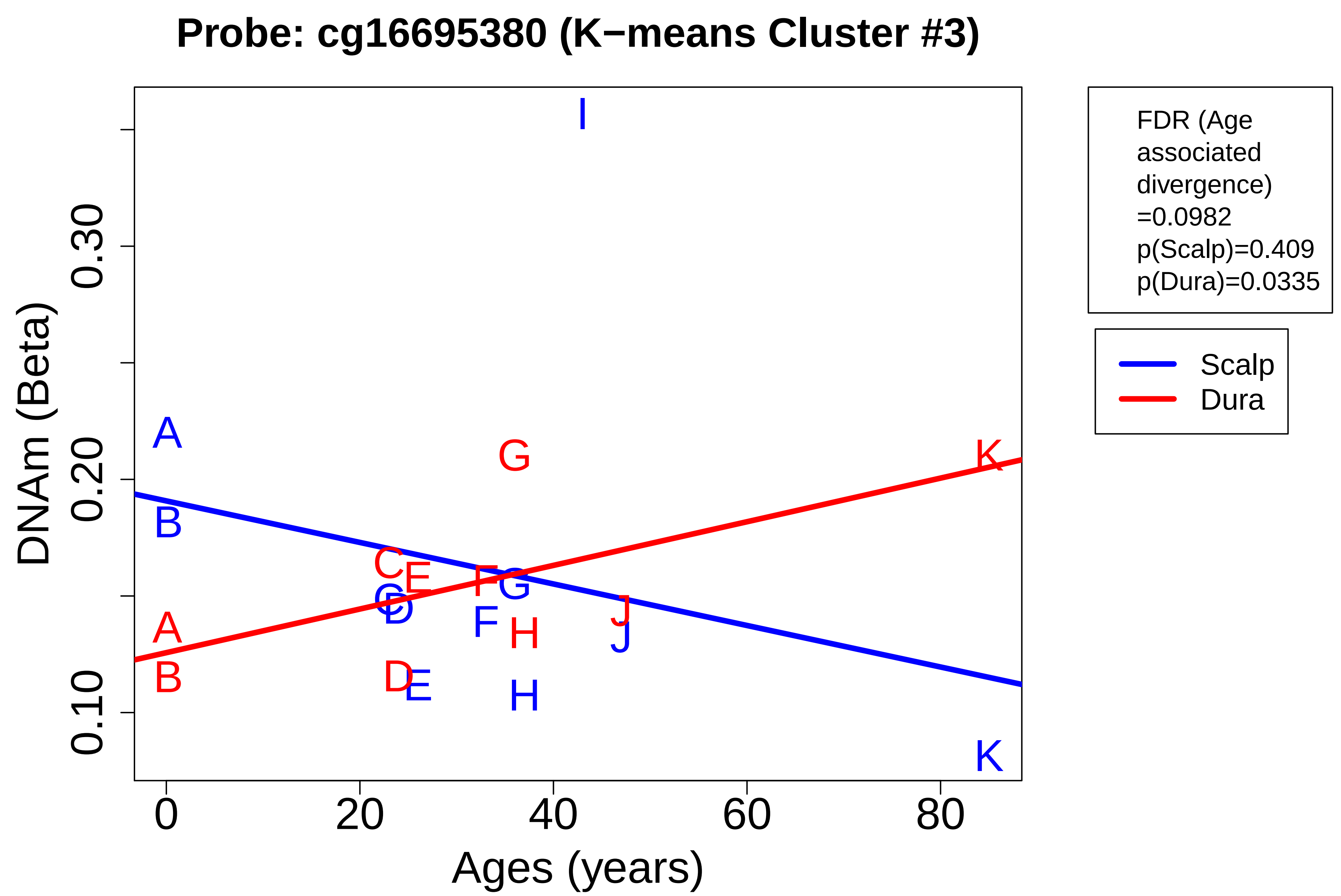

Probe: cg16695380 (K−means Cluster #3)
FDR (Age associated divergence) =0.0982
p(Scalp)=0.409
p(Dura)=0.0335
Scalp
Dura
DNAm (Beta)
0.10
0.20
0.30
Ages (years)
0
20
40
60
80
A
B
C
D
E
F
G
H
I
J
K

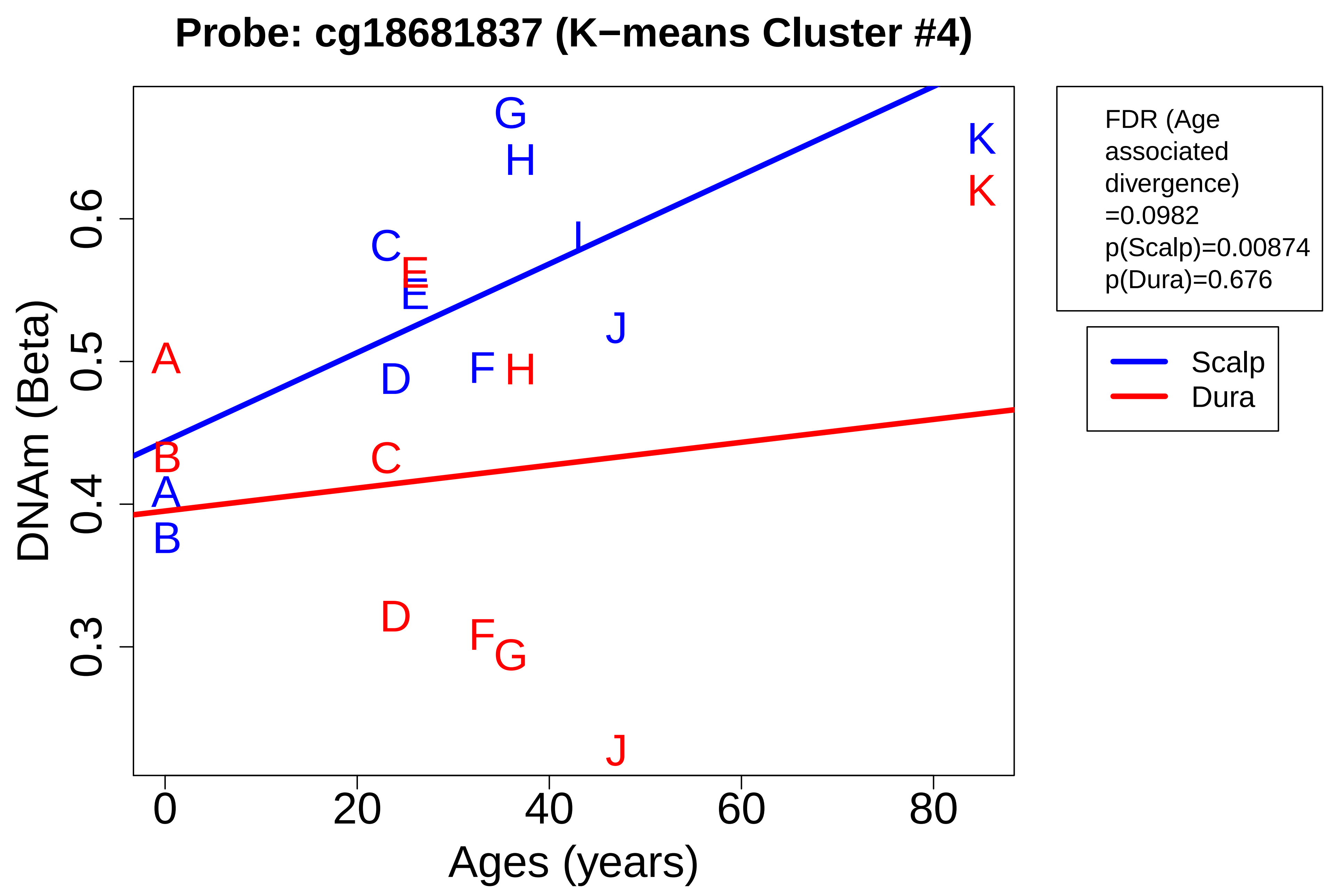

Probe: cg18681837 (K−means Cluster #4)
FDR (Age associated divergence) =0.0982
p(Scalp)=0.00874
p(Dura)=0.676
Scalp
Dura
DNAm (Beta)
0.3
0.4
0.5
0.6
Ages (years)
0
20
40
60
80
A
B
C
D
E
F
G
H
I
J
K

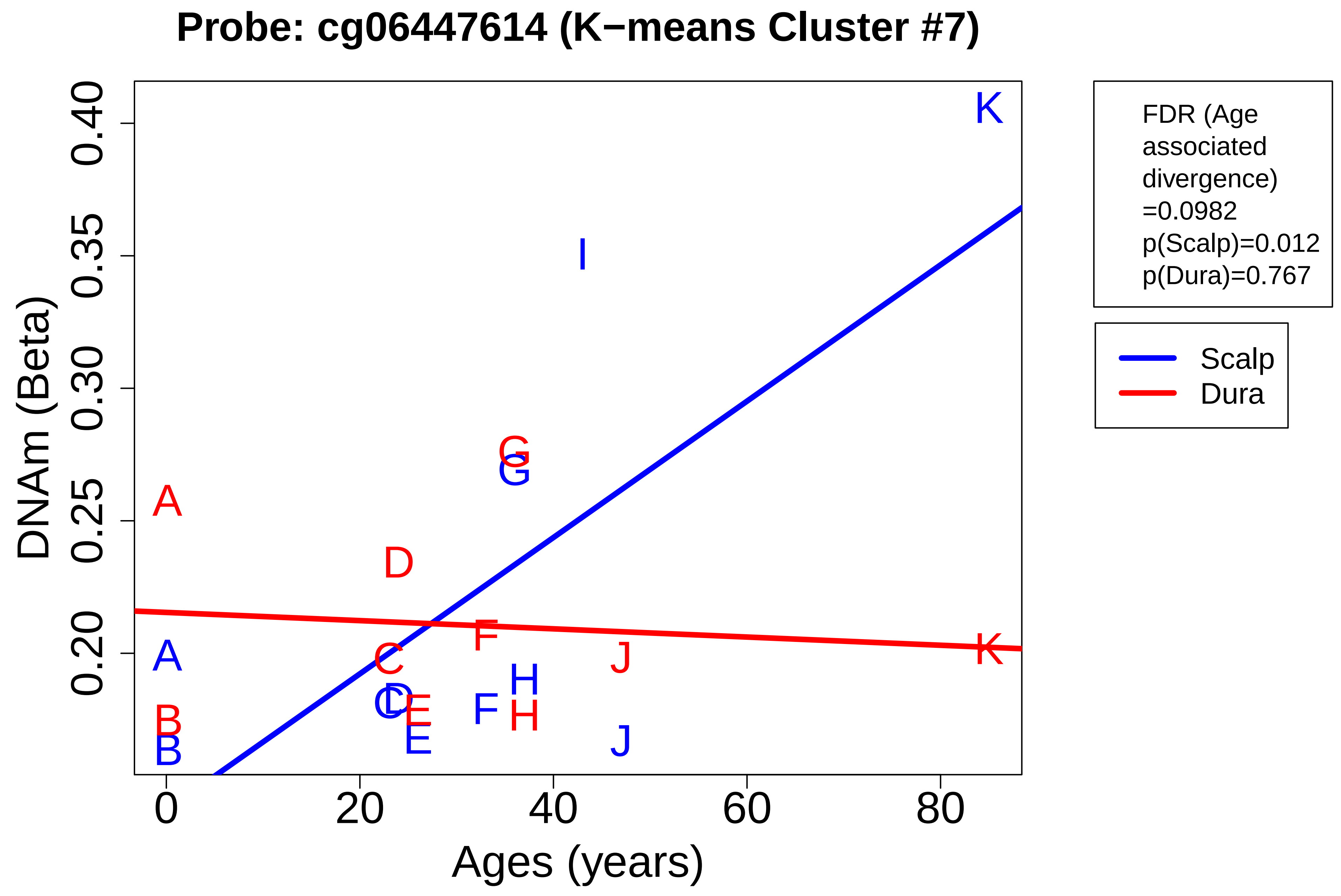

Probe: cg06447614 (K−means Cluster #7)
DNAm (Beta)
0.20
0.25
0.30
0.35
0.40
0
20
40
60
80
Ages (years)
A
B
C
D
E
F
G
H
I
J
K
A
B
C
D
E
F
G
H
J
K
FDR (Age associated divergence) =0.0982
p(Scalp)=0.012
p(Dura)=0.767
Scalp
Dura

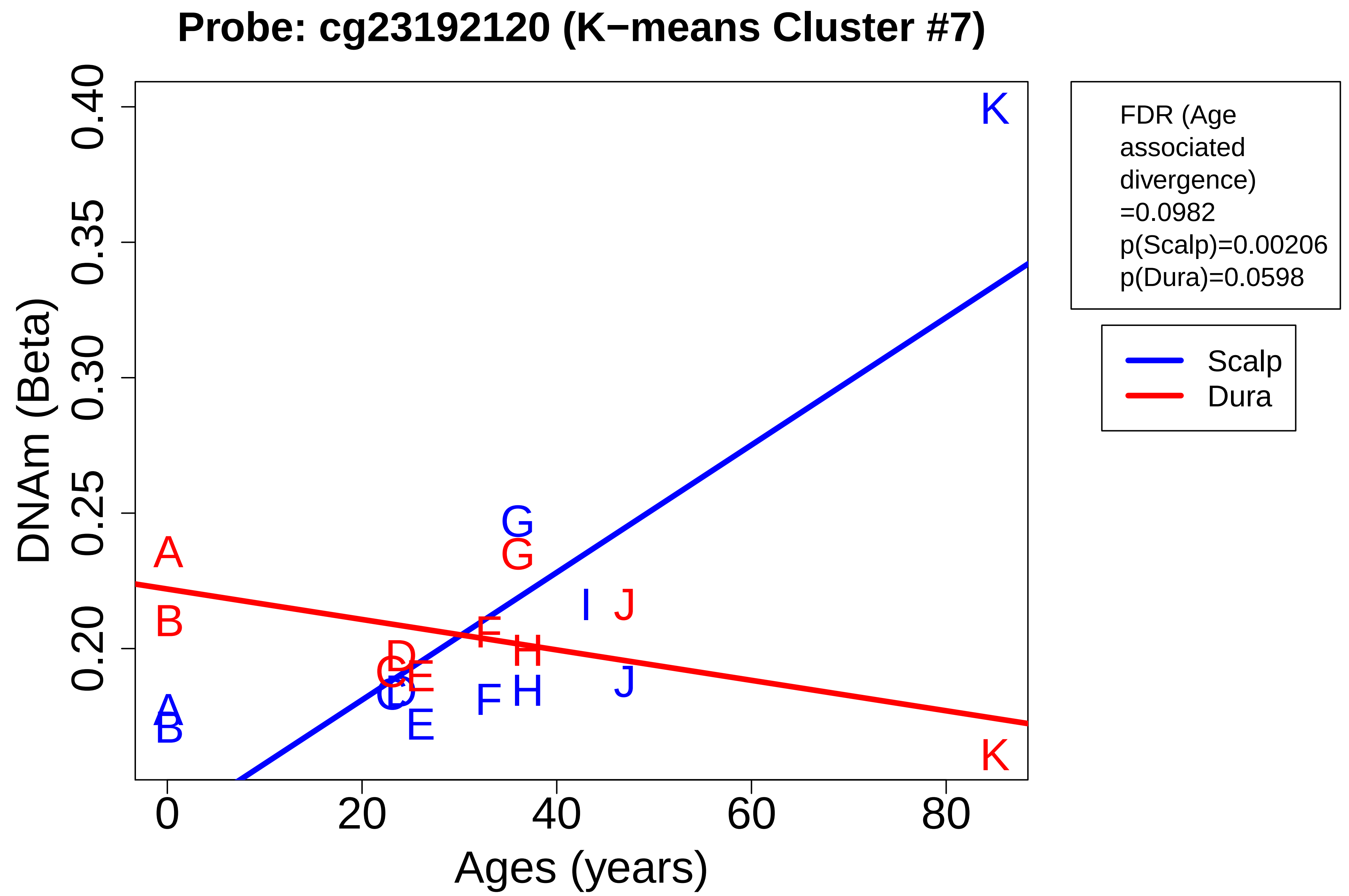

Probe: cg23192120 (K−means Cluster #7)
DNAm (Beta)
0.20
0.25
0.30
0.35
0.40
Ages (years)
0
20
40
60
80
FDR (Age associated divergence) =0.0982
p(Scalp)=0.00206
p(Dura)=0.0598
Scalp
Dura

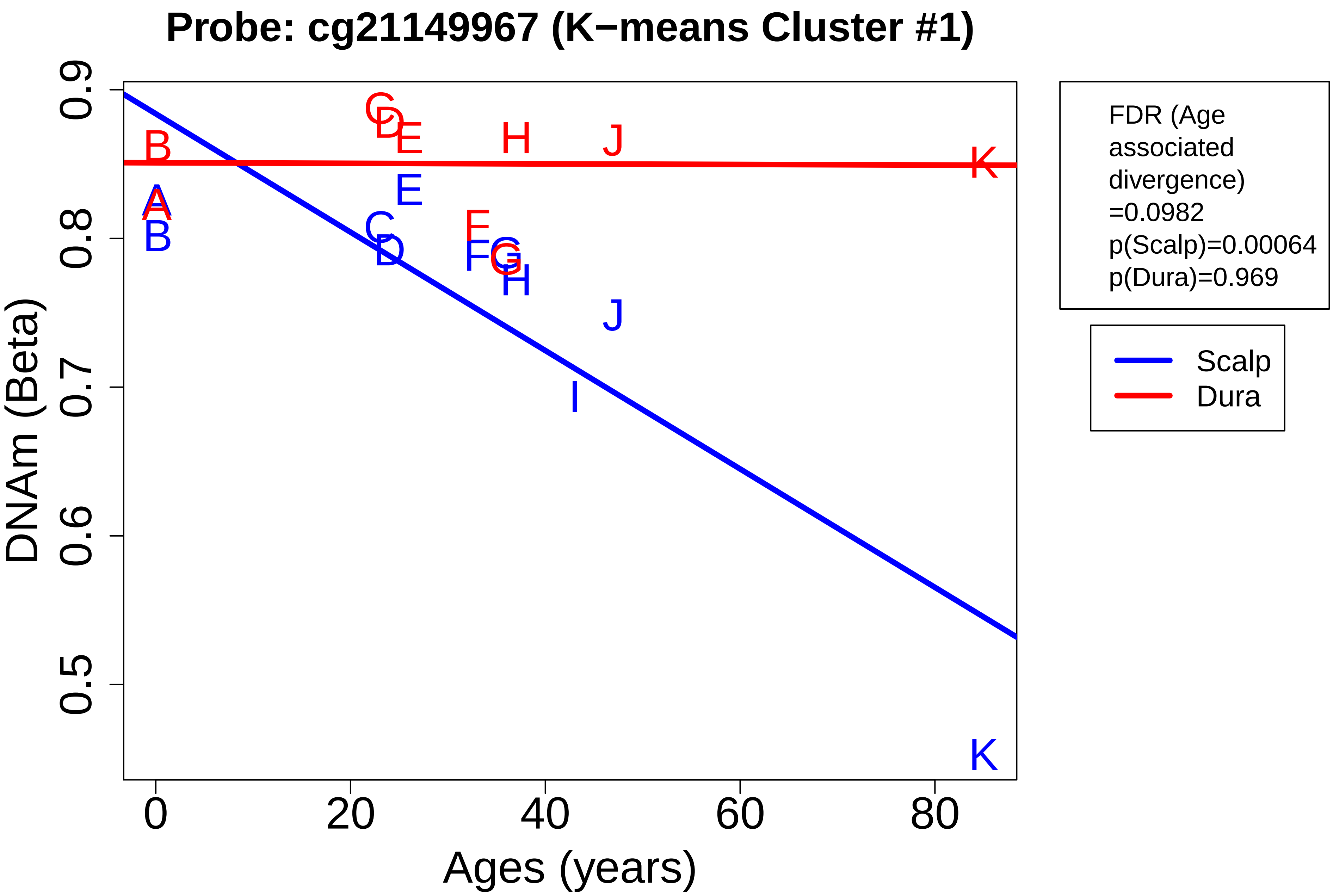

Probe: cg21149967 (K−means Cluster #1)
DNAm (Beta)
0.5
0.6
0.7
0.8
0.9
0
20
40
60
80
Ages (years)
A
B
C
D
E
F
G
H
I
J
K
FDR (Age associated divergence) =0.0982
p(Scalp)=0.00064
p(Dura)=0.969
Scalp
Dura

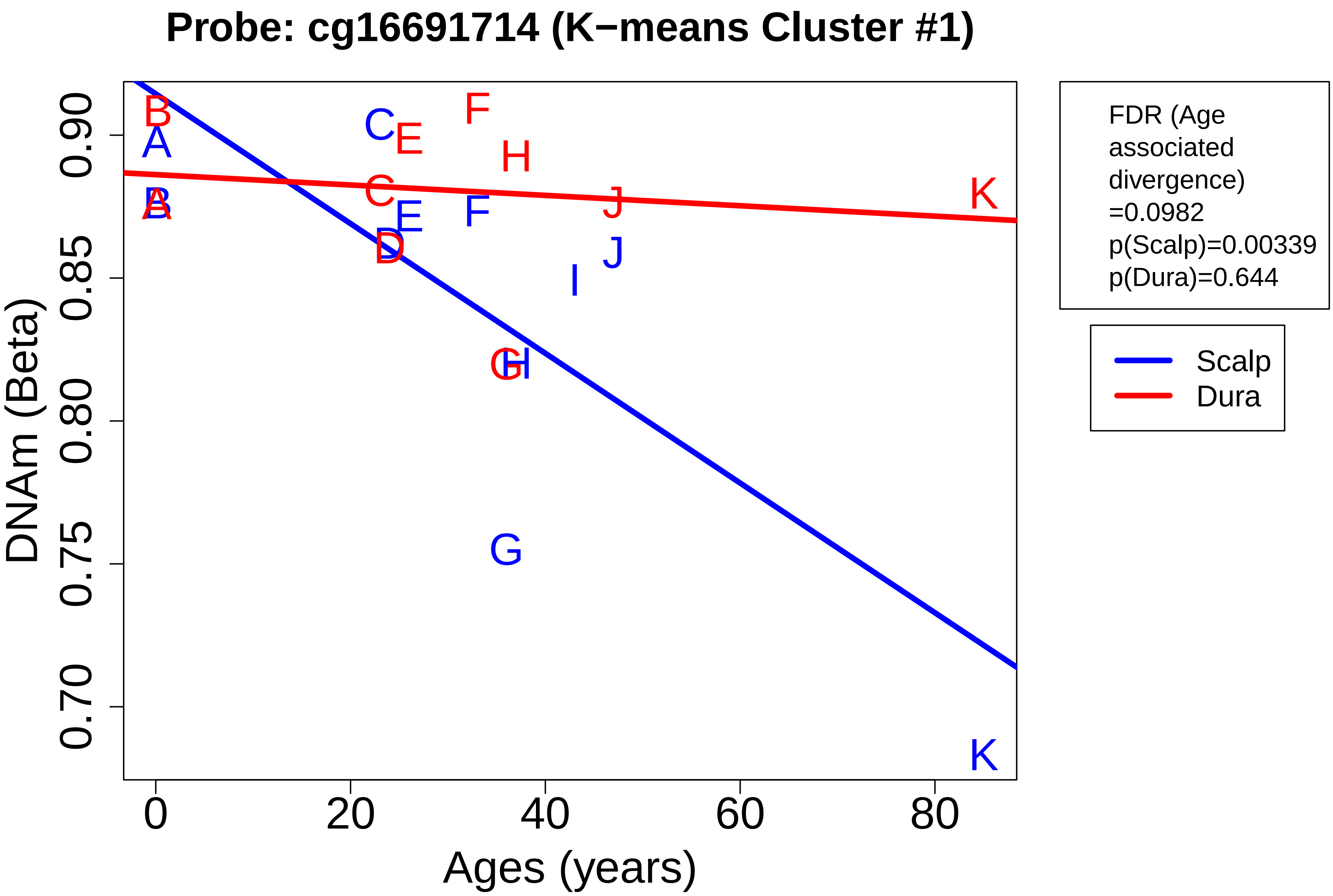

Probe: cg16691714 (K−means Cluster #1)
DNAm (Beta)
0.70
0.75
0.80
0.85
0.90
0
20
40
60
80
Ages (years)
FDR (Age associated divergence) =0.0982
p(Scalp)=0.00339
p(Dura)=0.644
Scalp
Dura

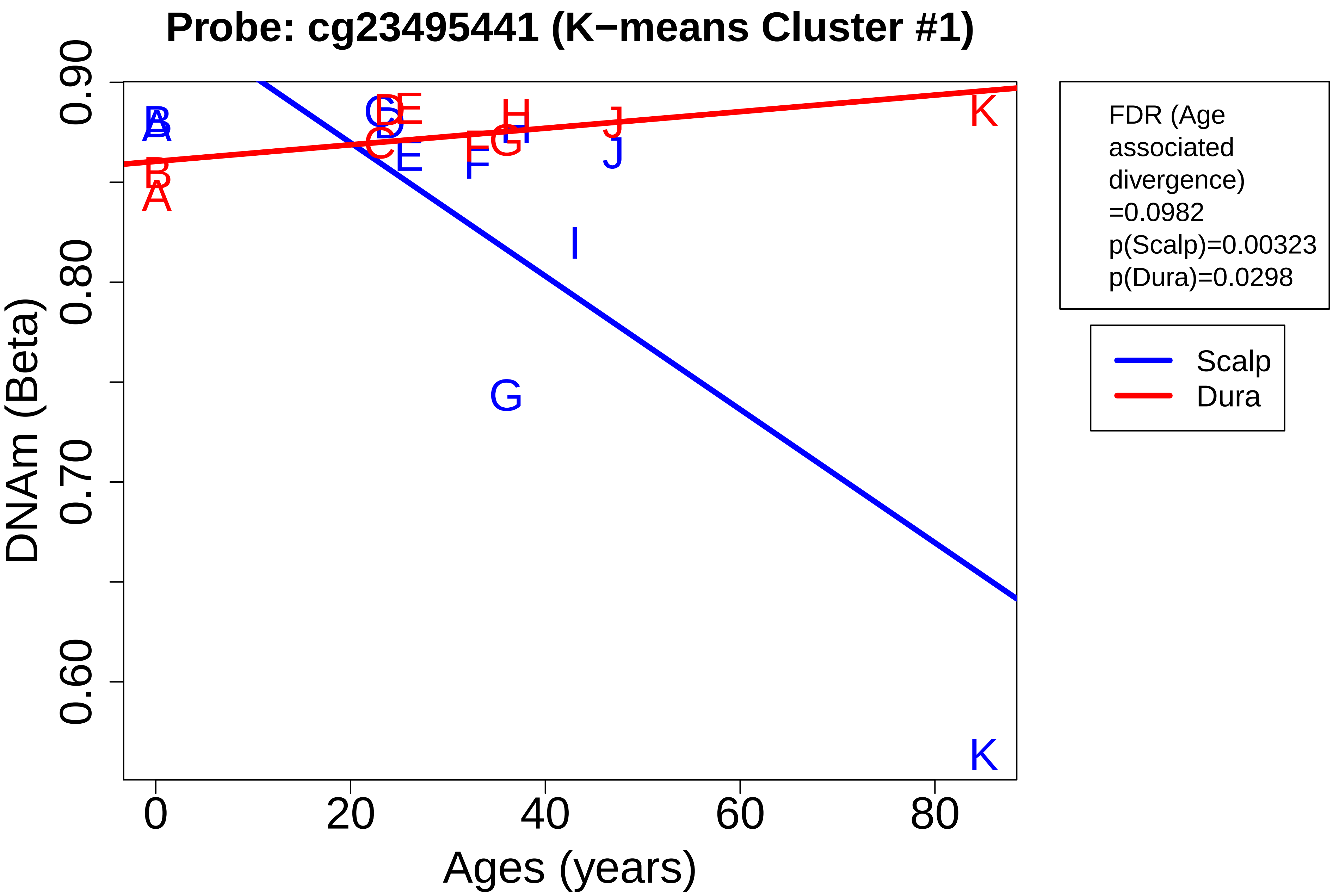

Probe: cg23495441 (K−means Cluster #1)
DNAm (Beta)
0.60
0.70
0.80
0.90
Ages (years)
0
20
40
60
80
FDR (Age associated divergence) =0.0982
p(Scalp)=0.00323
p(Dura)=0.0298
Scalp
Dura

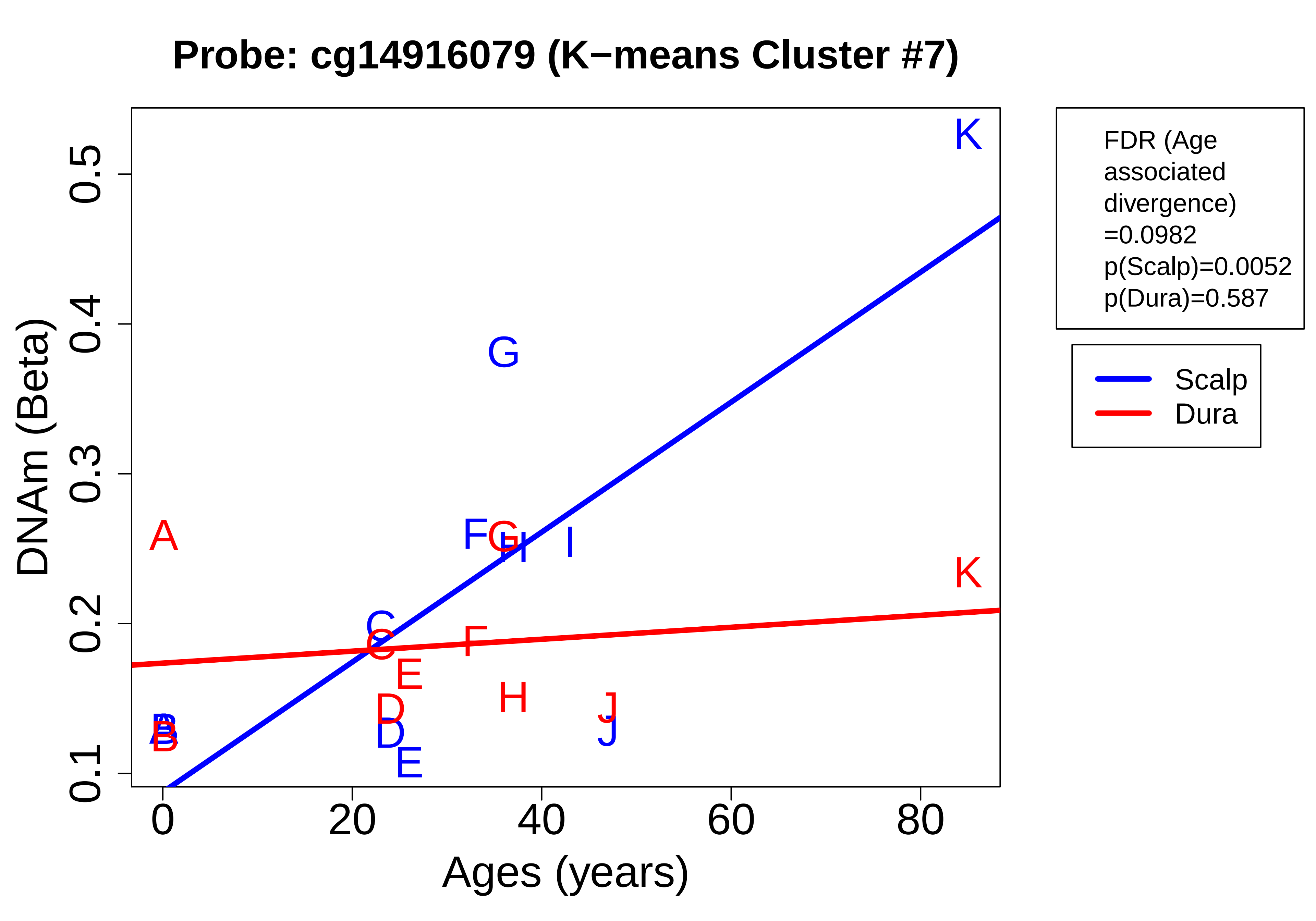

Probe: cg14916079 (K−means Cluster #7)
DNAm (Beta)
Ages (years)
0.1
0.2
0.3
0.4
0.5
0
20
40
60
80
FDR (Age associated divergence) =0.0982
p(Scalp)=0.0052
p(Dura)=0.587
Scalp
Dura

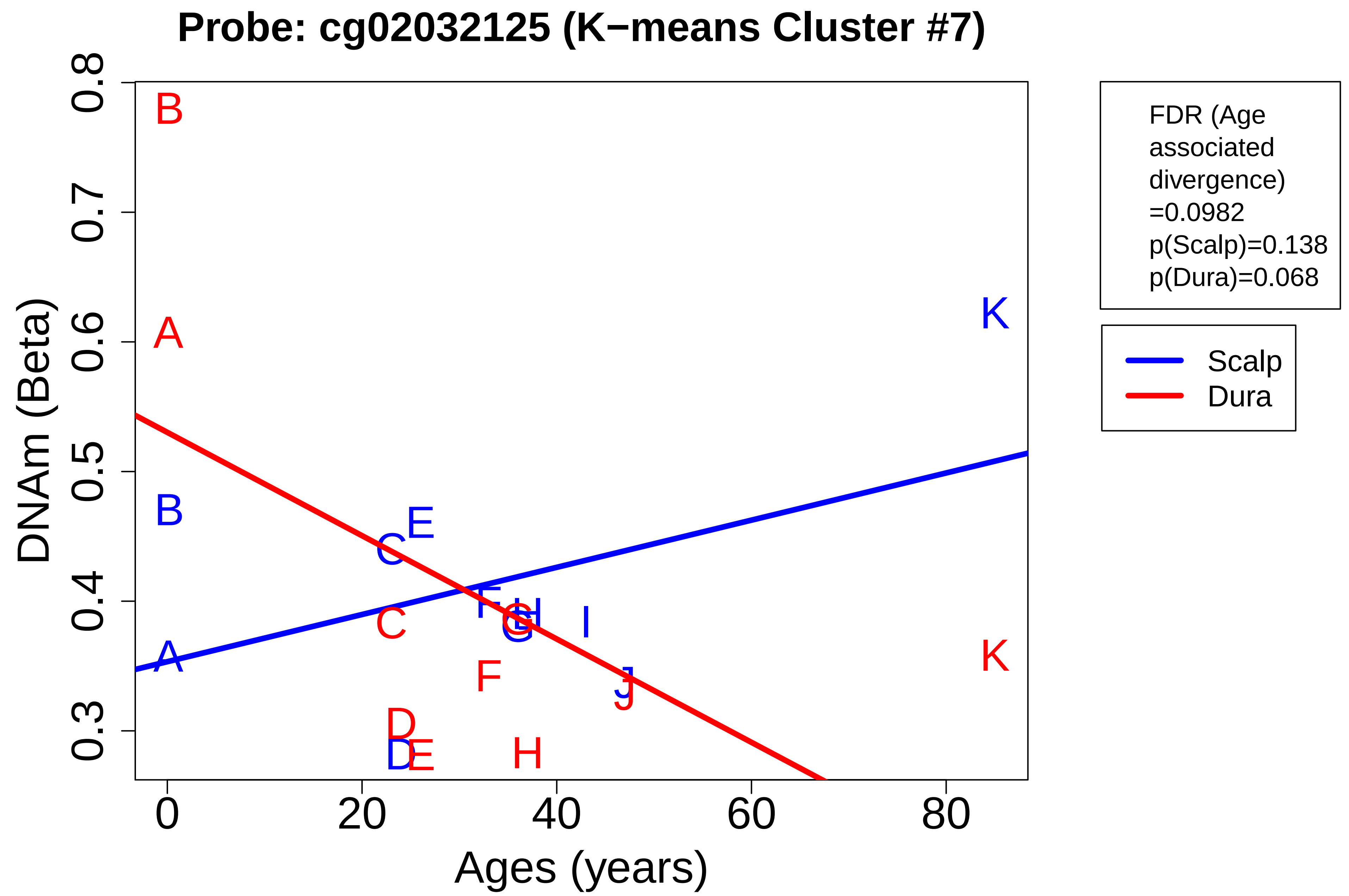

Probe: cg02032125 (K−means Cluster #7)
DNAm (Beta)
0.3
0.4
0.5
0.6
0.7
0.8
0
20
40
60
80
Ages (years)
FDR (Age associated divergence) =0.0982
p(Scalp)=0.138
p(Dura)=0.068
Scalp
Dura

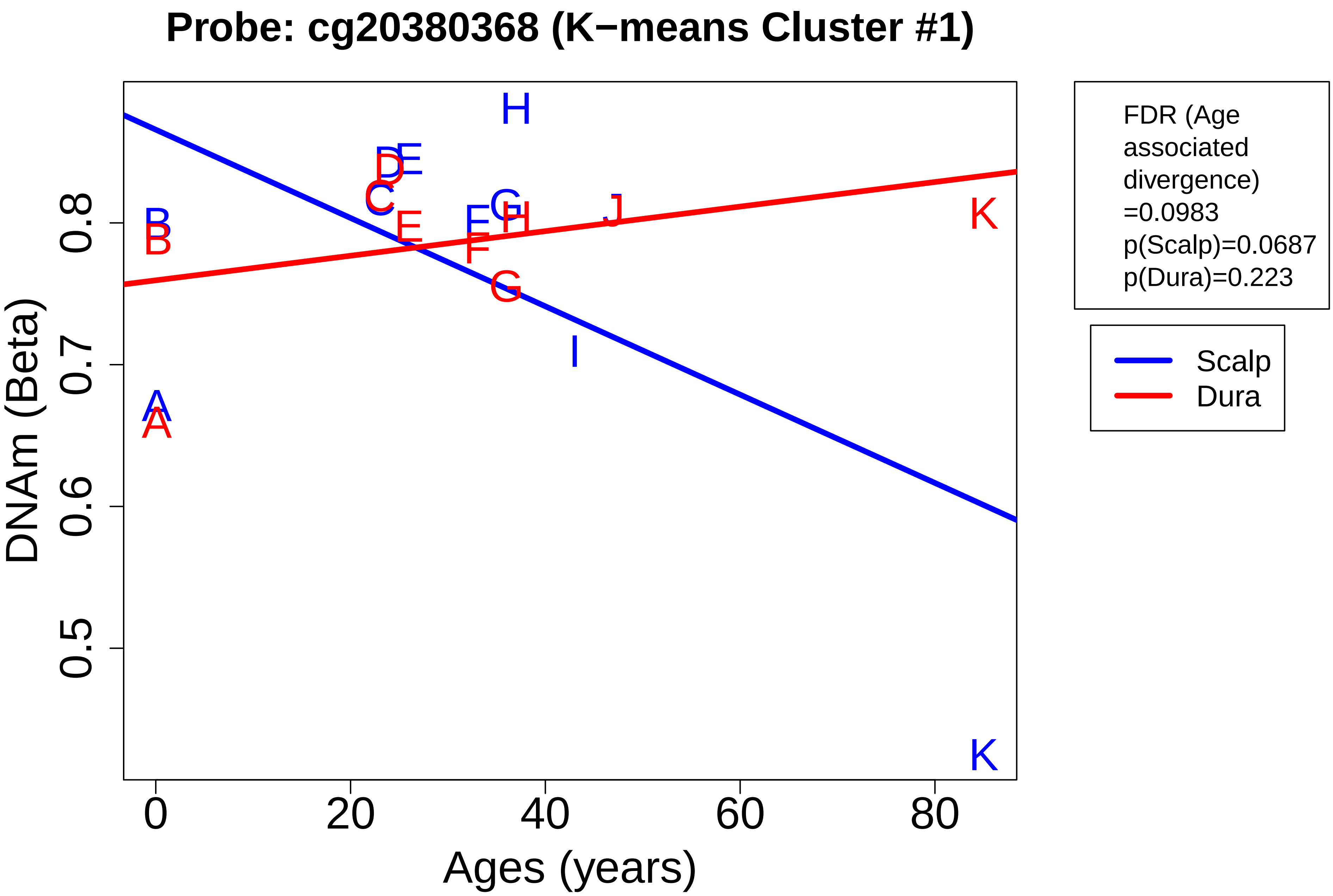

Probe: cg20380368 (K−means Cluster #1)
DNAm (Beta)
0.5
0.6
0.7
0.8
0
20
40
60
80
Ages (years)
FDR (Age associated divergence) =0.0983
p(Scalp)=0.0687
p(Dura)=0.223
Scalp
Dura

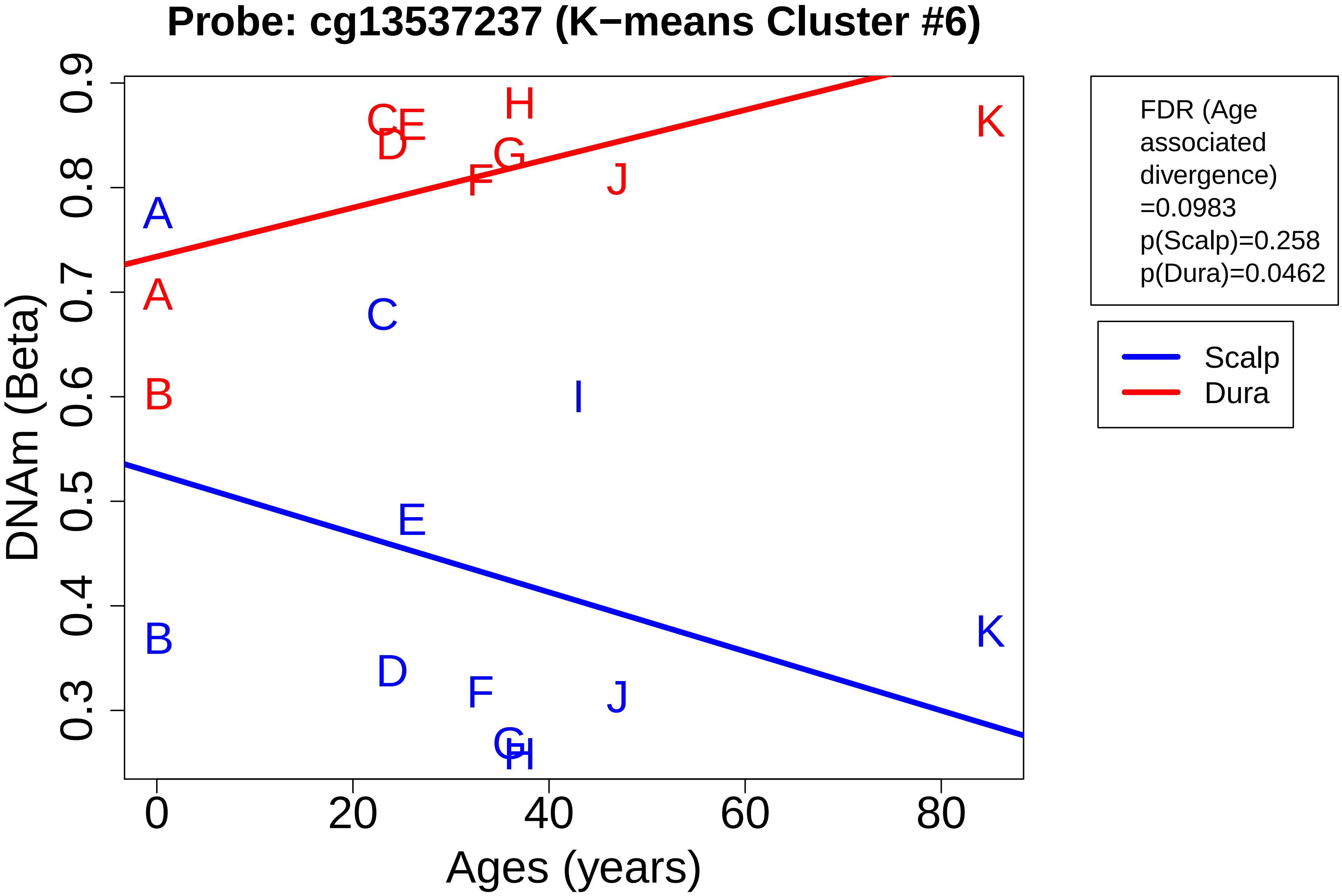

Probe: cg13537237 (K−means Cluster #6)
DNAm (Beta)
0.3
0.4
0.5
0.6
0.7
0.8
0.9
0
20
40
60
80
Ages (years)
A
B
C
D
E
F
G
H
I
J
K
FDR (Age associated divergence) =0.0983
p(Scalp)=0.258
p(Dura)=0.0462
Scalp
Dura

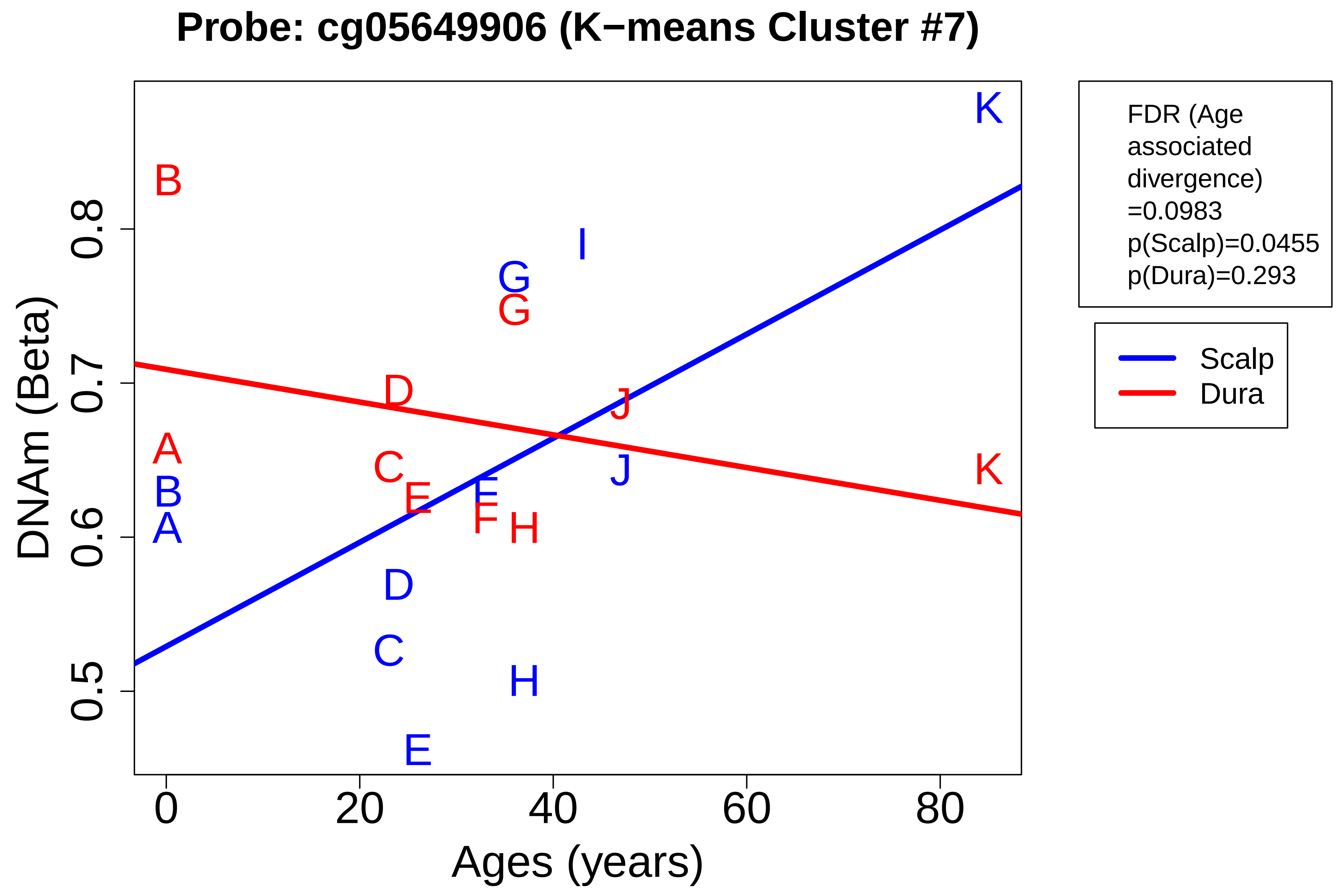

Probe: cg05649906 (K−means Cluster #7)
DNAm (Beta)
0.5
0.6
0.7
0.8
0
20
40
60
80
Ages (years)
FDR (Age associated divergence) =0.0983
p(Scalp)=0.0455
p(Dura)=0.293
Scalp
Dura
A
B
C
D
E
F
G
H
I
J
K

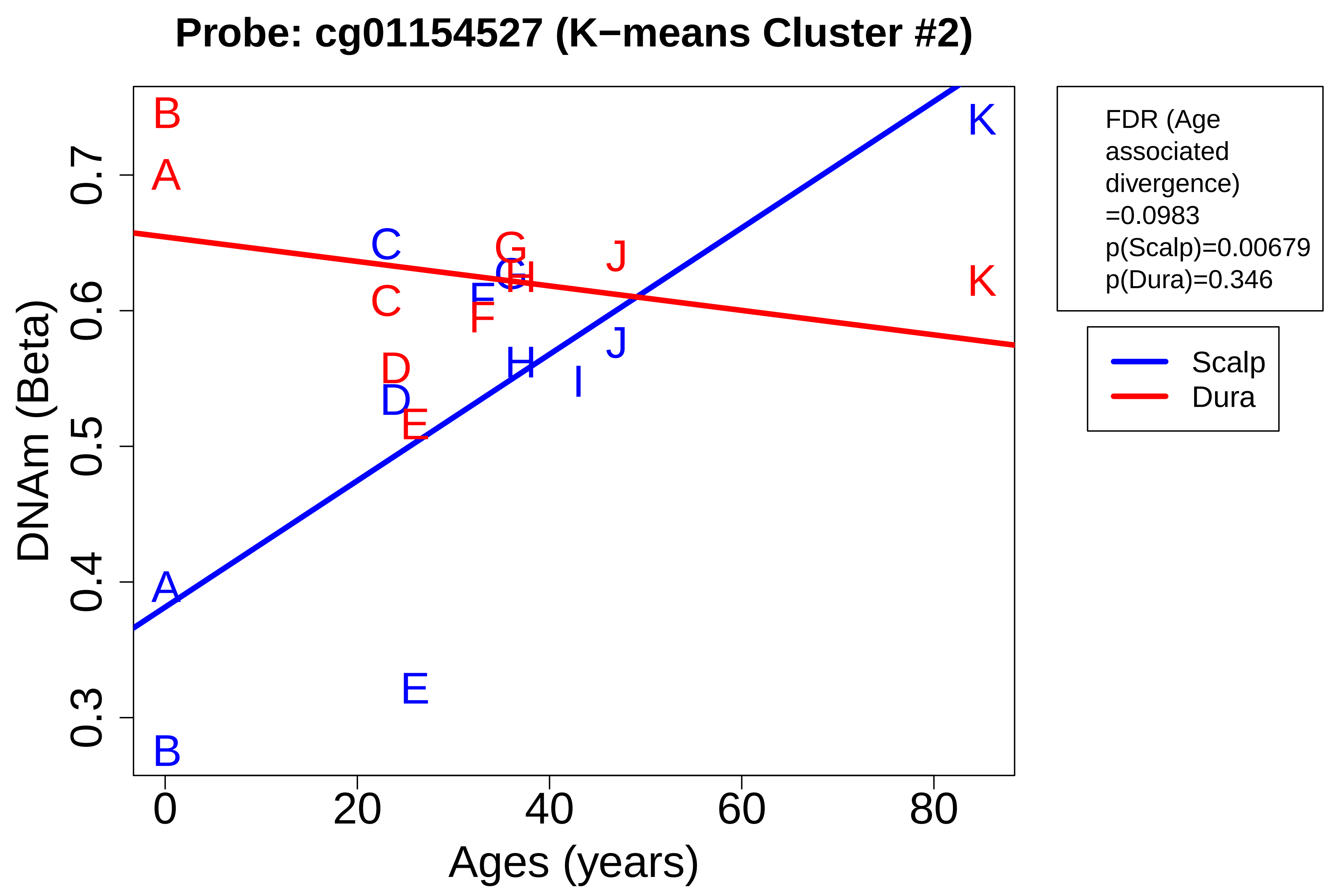

Probe: cg01154527 (K−means Cluster #2)
DNAm (Beta)
0.3
0.4
0.5
0.6
0.7
0
20
40
60
80
Ages (years)
FDR (Age associated divergence) =0.0983
p(Scalp)=0.00679
p(Dura)=0.346
Scalp
Dura
A
B
C
D
E
F
G
H
I
J
K

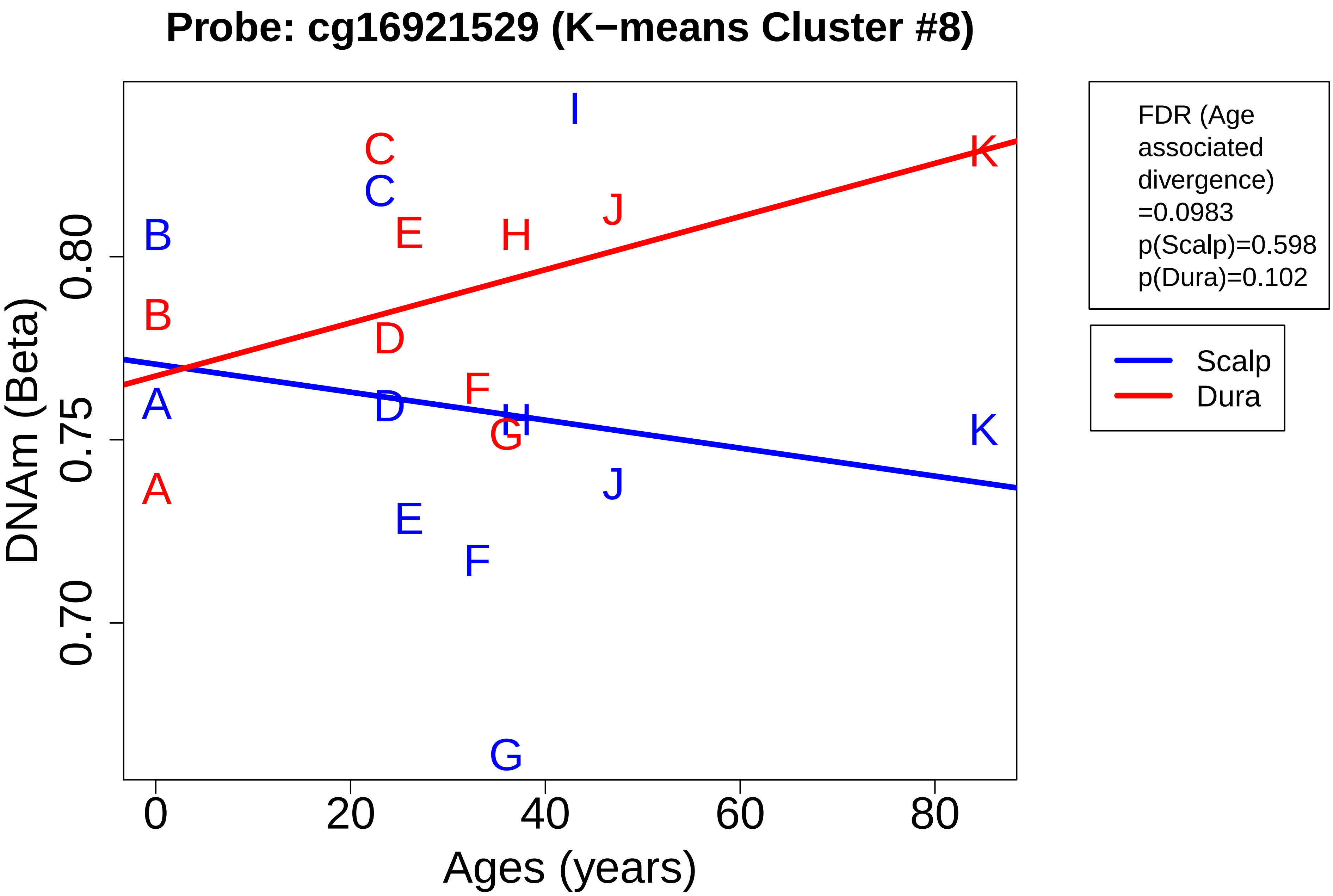

Probe: cg16921529 (K−means Cluster #8)
DNAm (Beta)
0.70
0.75
0.80
Ages (years)
0
20
40
60
80
FDR (Age associated divergence) =0.0983
p(Scalp)=0.598
p(Dura)=0.102
Scalp
Dura

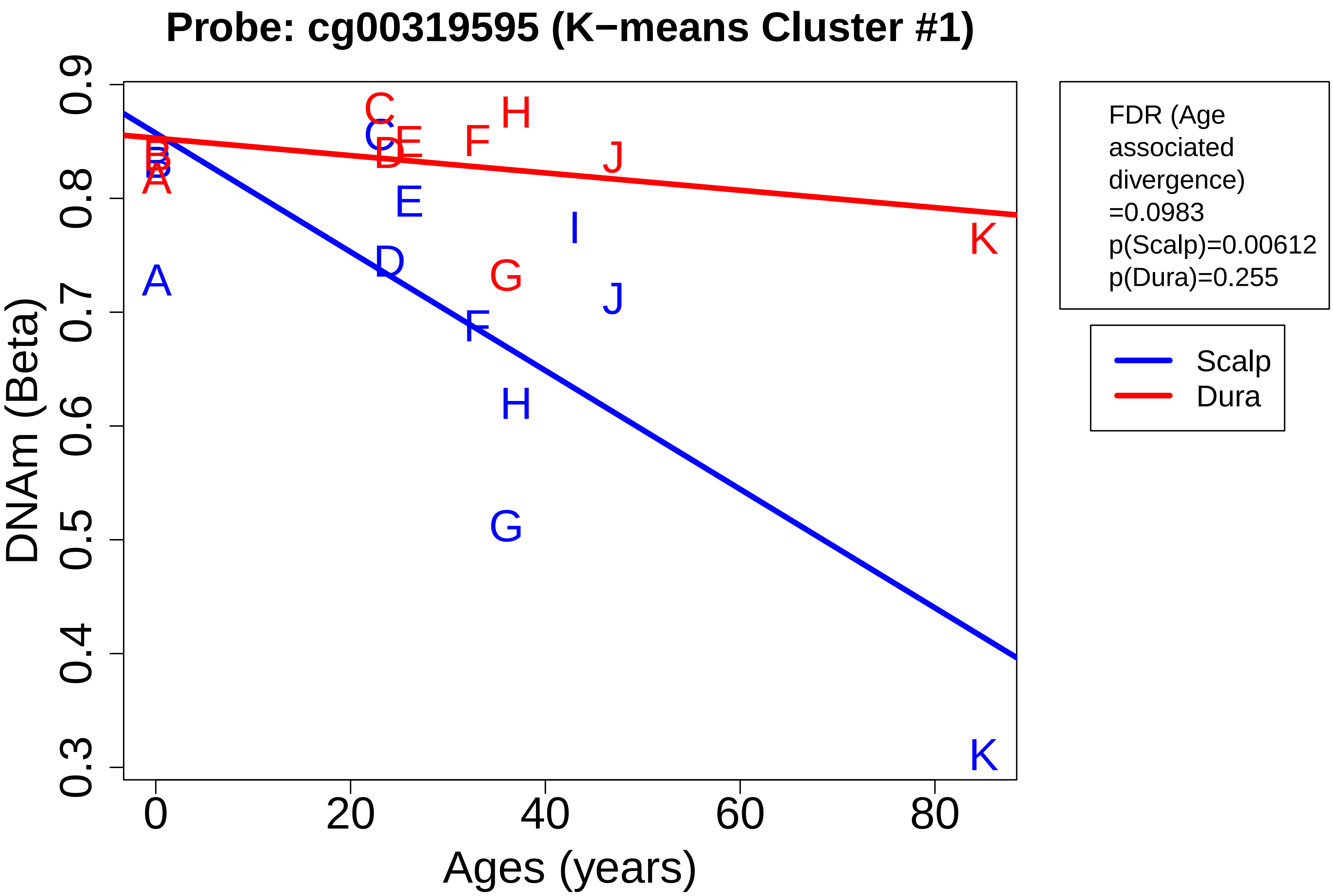
Probe: cg00319595 (K−means Cluster #1)
DNAm (Beta)
0.3
0.4
0.5
0.6
0.7
0.8
0.9
0
20
40
60
80
Ages (years)
A
B
C
D
E
F
G
H
I
J
K
FDR (Age associated divergence) =0.0983
p(Scalp)=0.00612
p(Dura)=0.255
Scalp
Dura

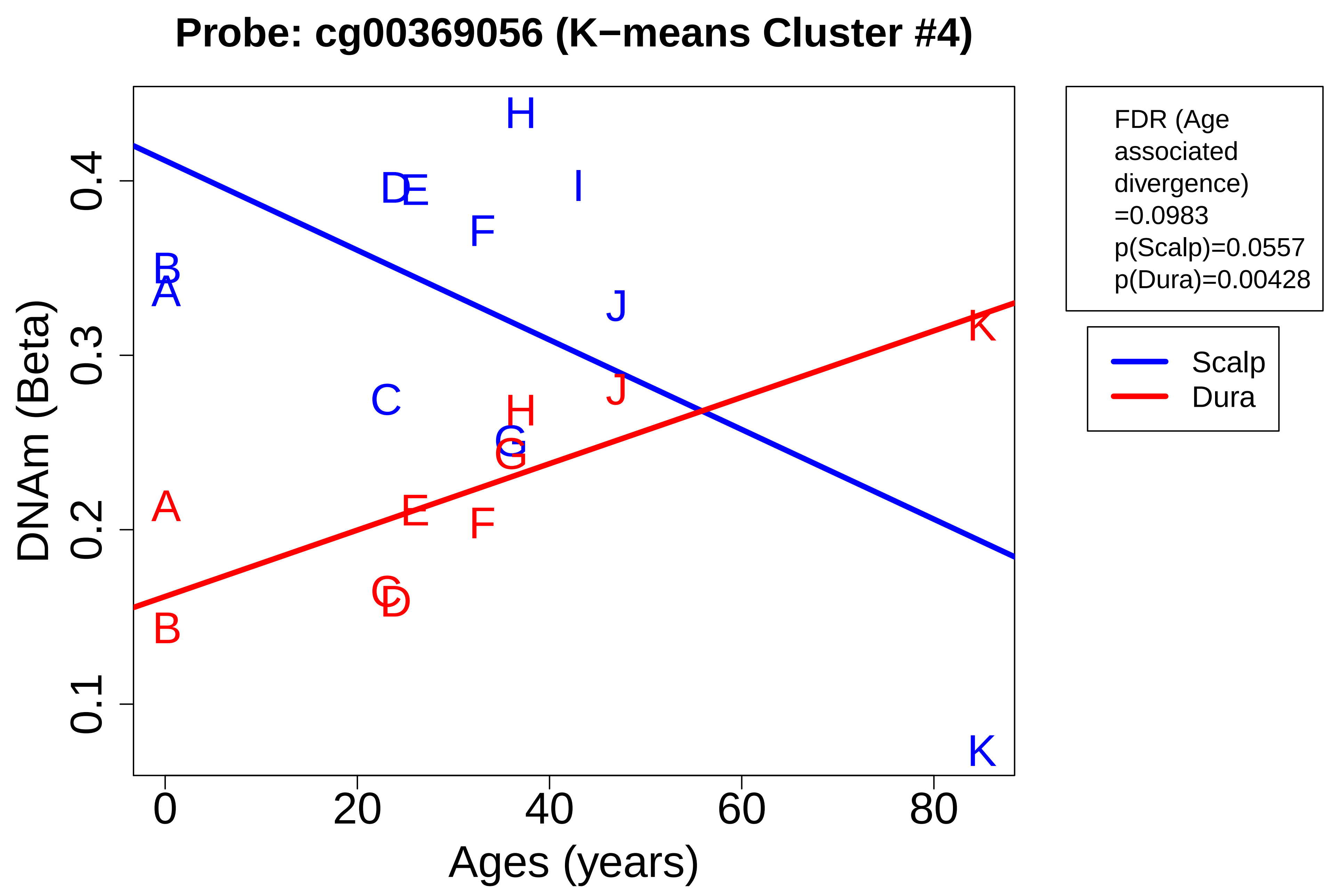

Probe: cg00369056 (K−means Cluster #4)
DNAm (Beta)
0.1
0.2
0.3
0.4
Ages (years)
0
20
40
60
80
H
D
E
I
F
B
A
J
K
C
J
H
G
G
A
E
F
C
D
B
K
FDR (Age associated divergence) =0.0983
p(Scalp)=0.0557
p(Dura)=0.00428
Scalp
Dura

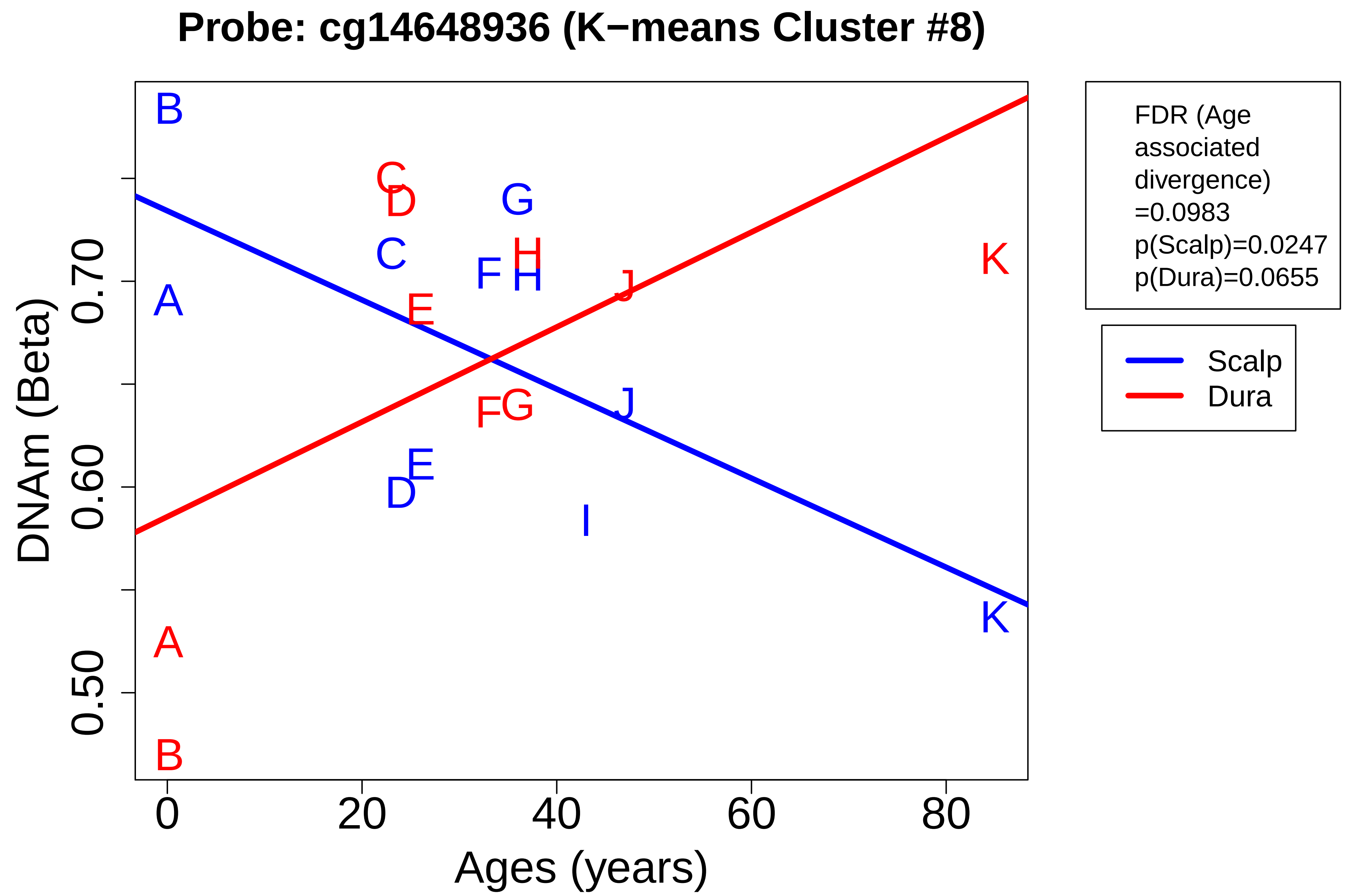

Probe: cg14648936 (K−means Cluster #8)
DNAm (Beta)
0.50
0.60
0.70
Ages (years)
0
20
40
60
80
B
C
D
G
C
H
F
H
J
K
A
E
FG
J
E
D
I
K
A
B
FDR (Age associated divergence) =0.0983
p(Scalp)=0.0247
p(Dura)=0.0655
Scalp
Dura

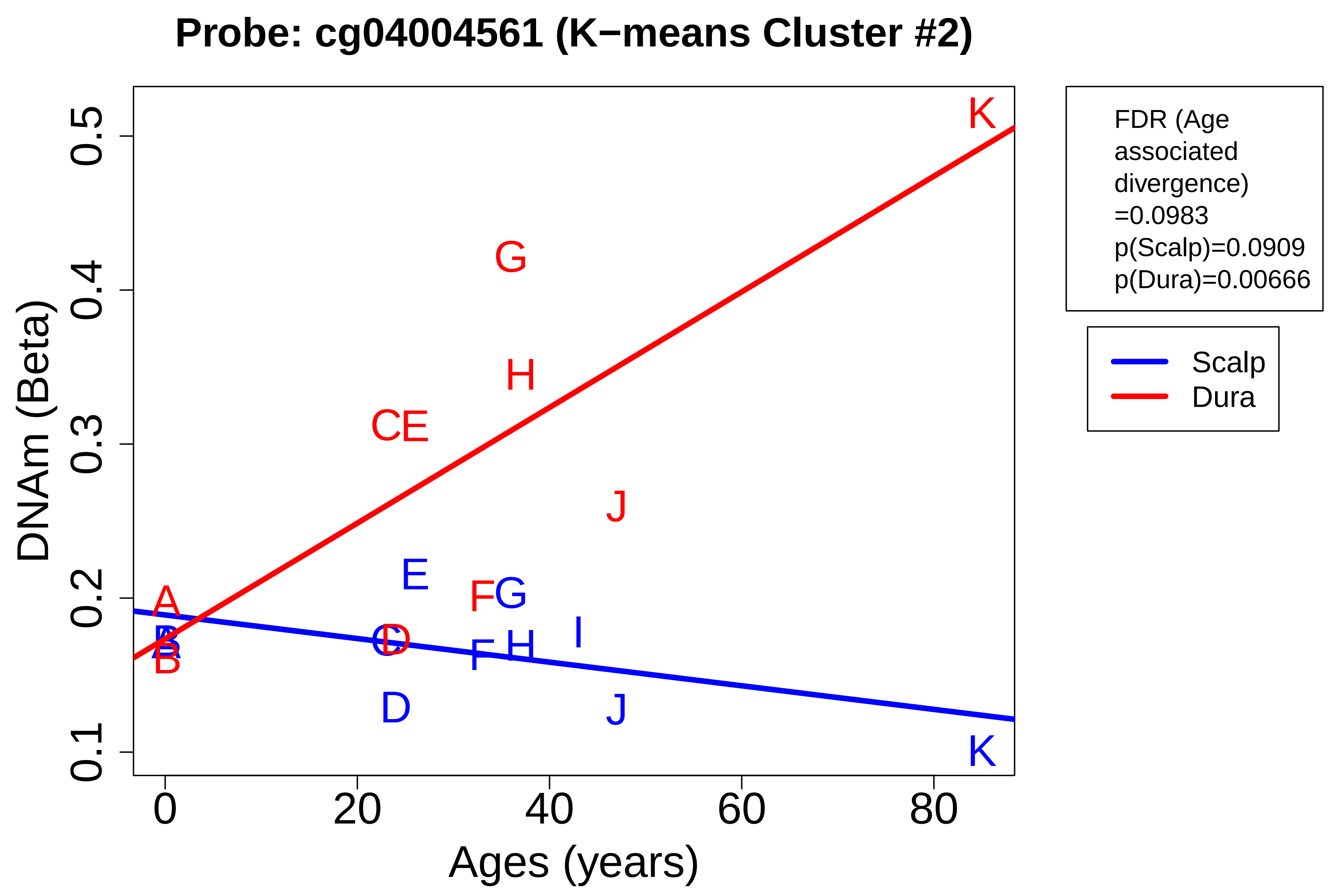
Probe: cg04004561 (K−means Cluster #2)
DNAm (Beta)
Ages (years)
0.1
0.2
0.3
0.4
0.5
0
20
40
60
80
FDR (Age associated divergence) =0.0983
p(Scalp)=0.0909
p(Dura)=0.00666
Scalp
Dura

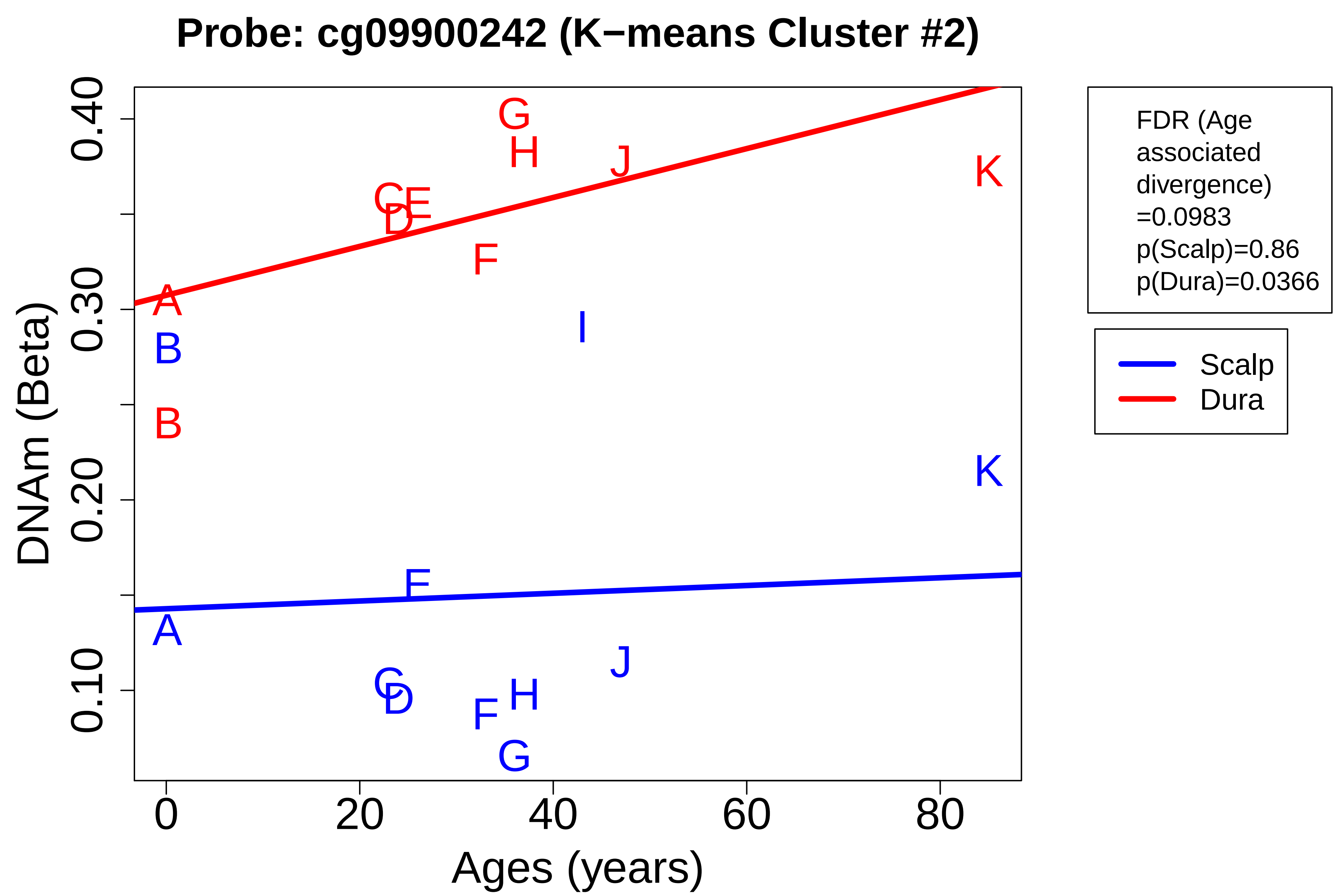

Probe: cg09900242 (K−means Cluster #2)
DNAm (Beta)
0.10
0.20
0.30
0.40
Ages (years)
0
20
40
60
80
FDR (Age associated divergence) =0.0983
p(Scalp)=0.86
p(Dura)=0.0366
Scalp
Dura
A
B
C
D
E
F
G
H
I
J
K

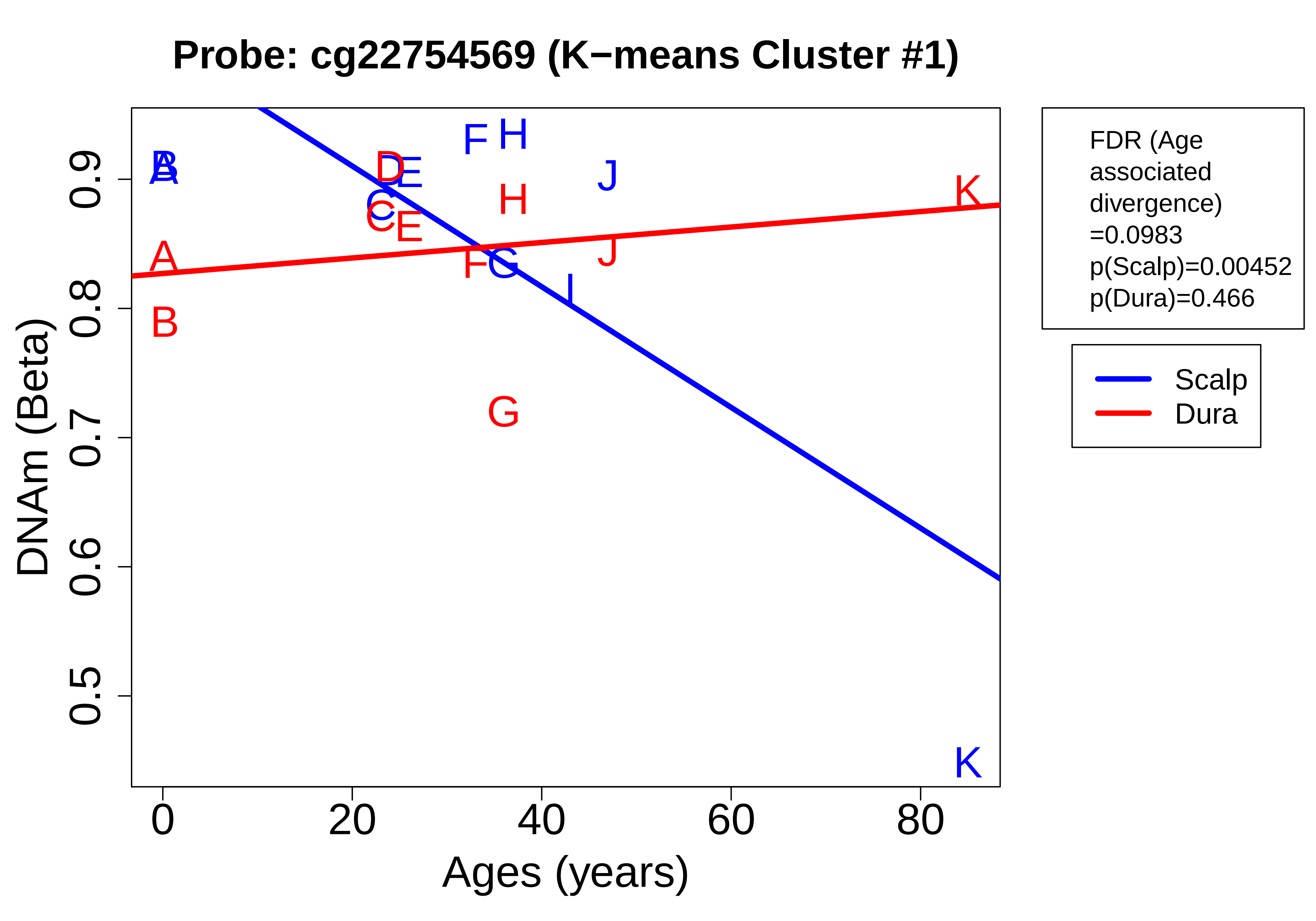
Probe: cg22754569 (K−means Cluster #1)
DNAm (Beta)
0.5
0.6
0.7
0.8
0.9
0
20
40
60
80
Ages (years)
FDR (Age associated divergence) =0.0983
p(Scalp)=0.00452
p(Dura)=0.466
Scalp
Dura

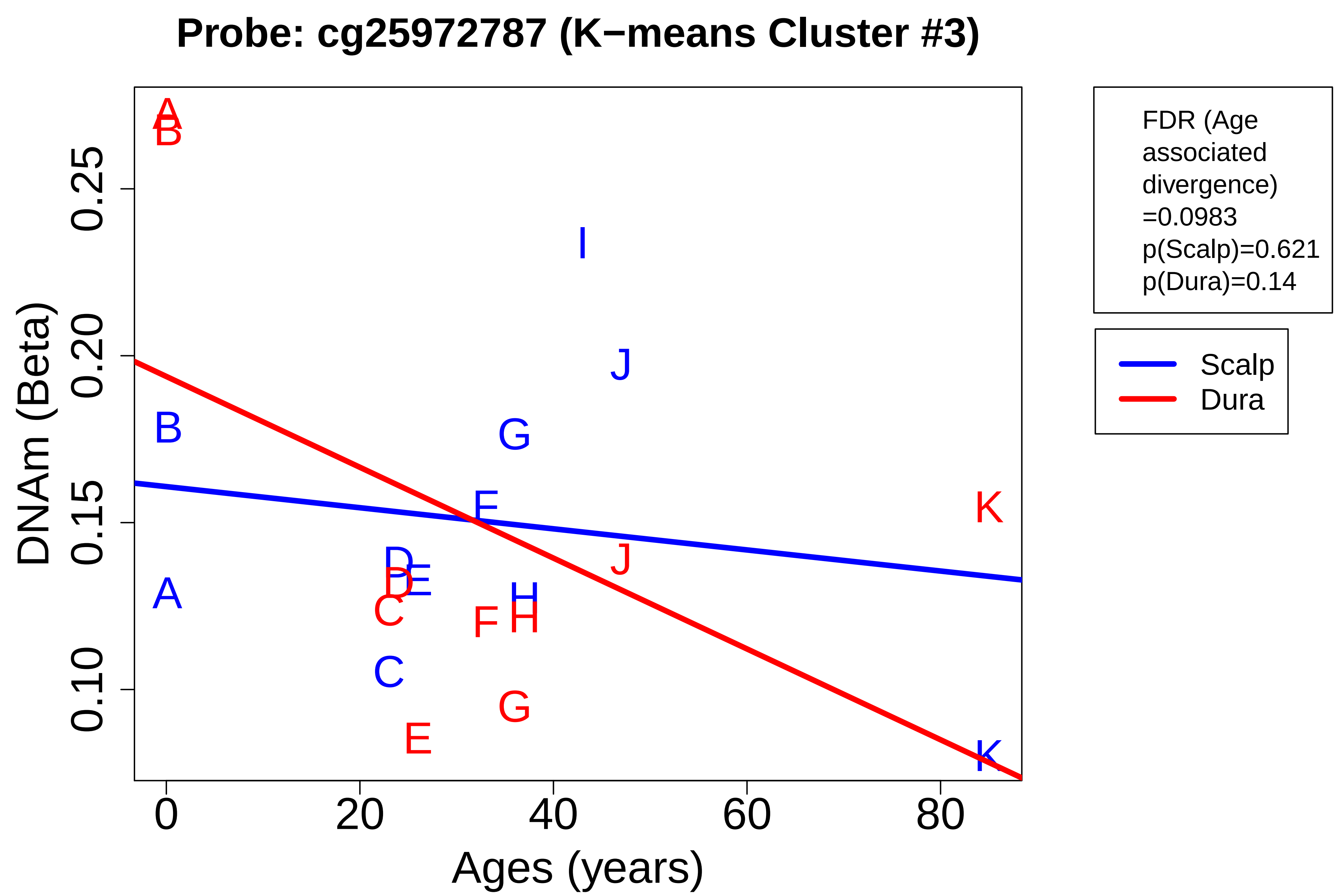
Probe: cg25972787 (K−means Cluster #3)
DNAm (Beta)
0.10
0.15
0.20
0.25
0
20
40
60
80
Ages (years)
FDR (Age associated divergence) =0.0983
p(Scalp)=0.621
p(Dura)=0.14
Scalp
Dura
A
B
C
D
E
F
G
H
I
J
K

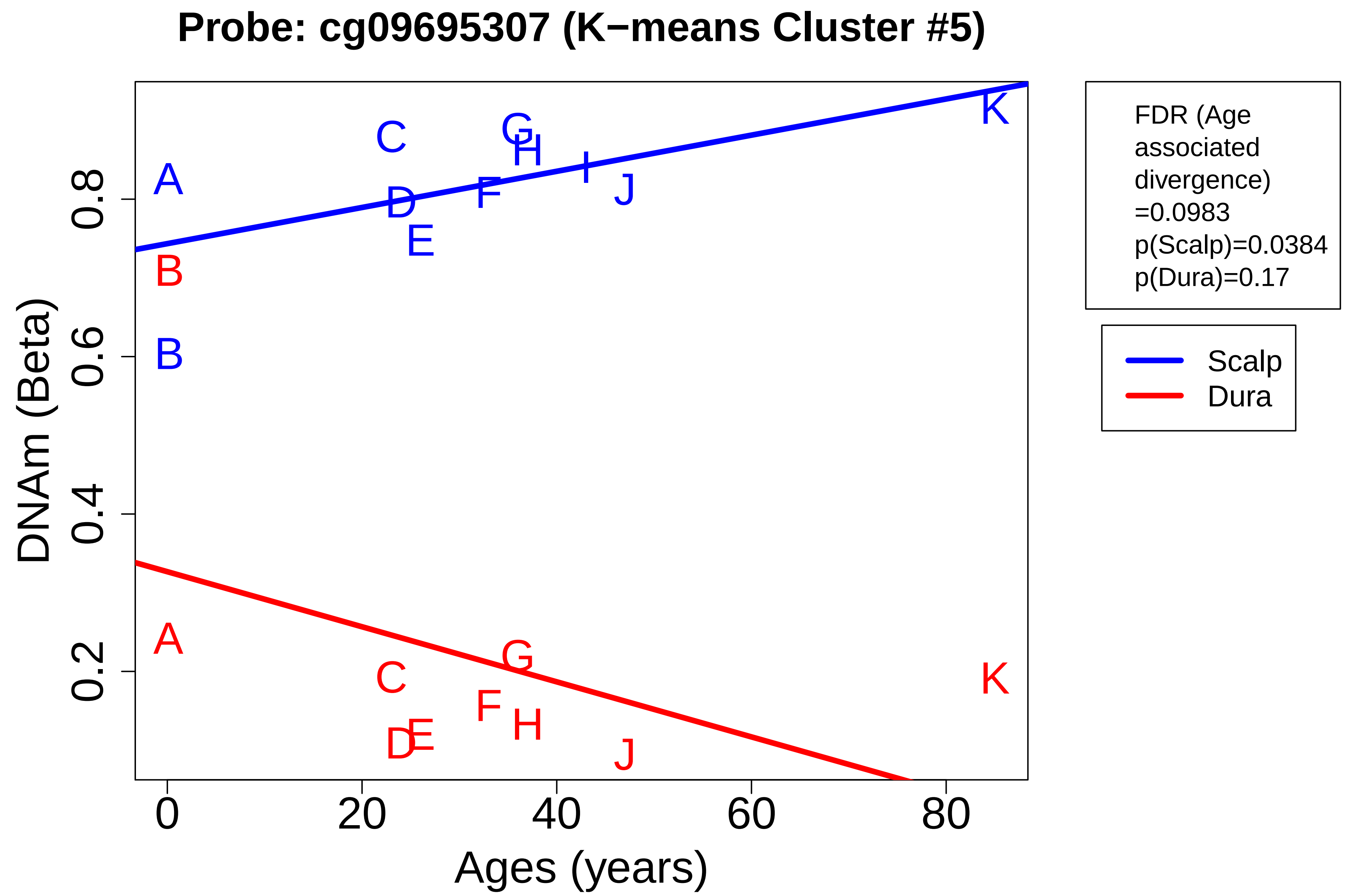

Probe: cg09695307 (K−means Cluster #5)
DNAm (Beta)
0.2
0.4
0.6
0.8
0
20
40
60
80
Ages (years)
A
B
C
D
E
F
G
H
I
J
K
A
B
C
D
E
F
G
H
J
K
FDR (Age associated divergence) =0.0983
p(Scalp)=0.0384
p(Dura)=0.17
Scalp
Dura

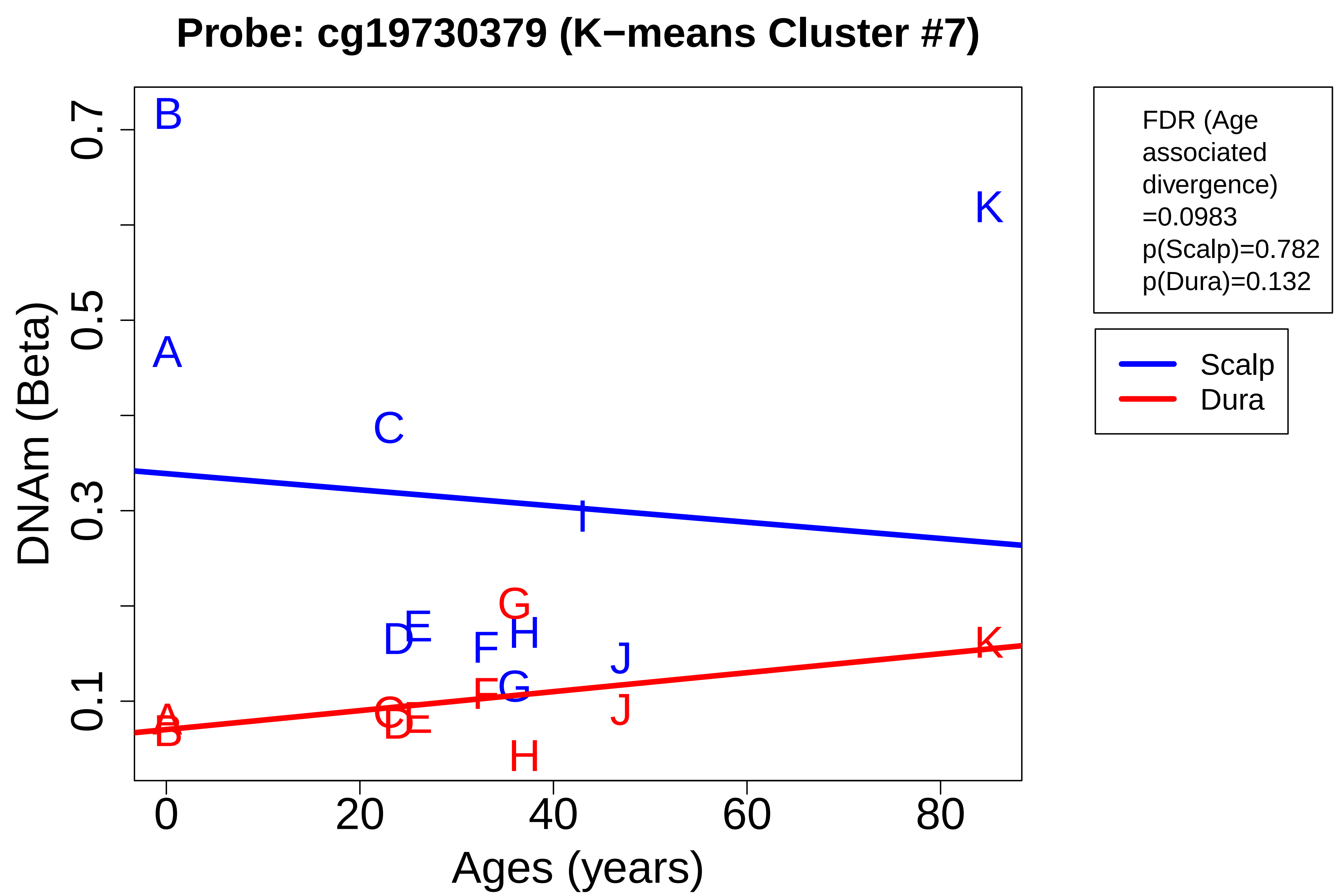

Probe: cg19730379 (K−means Cluster #7)
DNAm (Beta)
0.1
0.3
0.5
0.7
0
20
40
60
80
Ages (years)
FDR (Age associated divergence) =0.0983
p(Scalp)=0.782
p(Dura)=0.132
Scalp
Dura

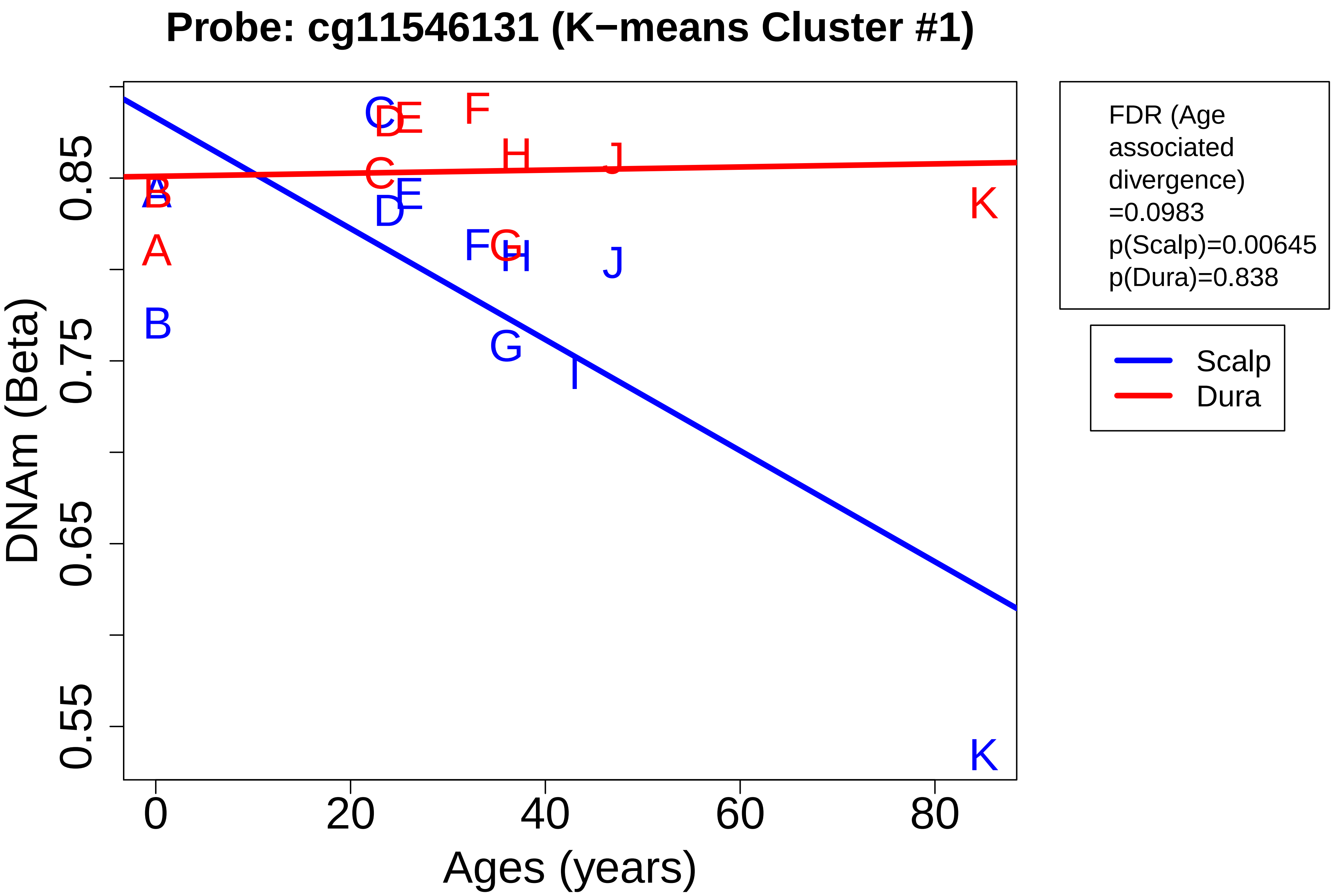

Probe: cg11546131 (K−means Cluster #1)
DNAm (Beta)
0.55
0.65
0.75
0.85
Ages (years)
0
20
40
60
80
FDR (Age associated divergence) =0.0983
p(Scalp)=0.00645
p(Dura)=0.838
Scalp
Dura

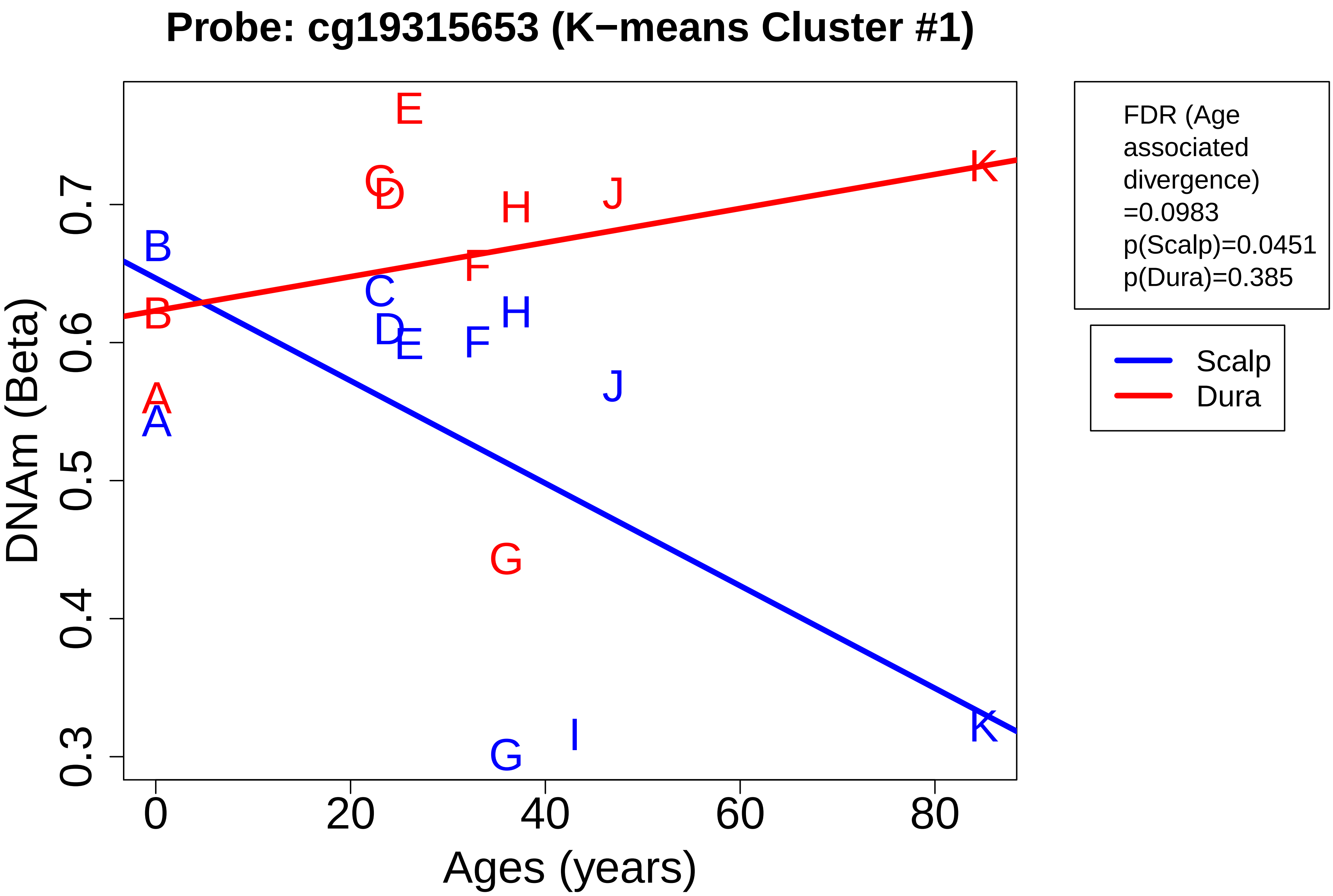
Probe: cg19315653 (K−means Cluster #1)
DNAm (Beta)
0.3
0.4
0.5
0.6
0.7
0
20
40
60
80
Ages (years)
FDR (Age associated divergence) =0.0983
p(Scalp)=0.0451
p(Dura)=0.385
Scalp
Dura

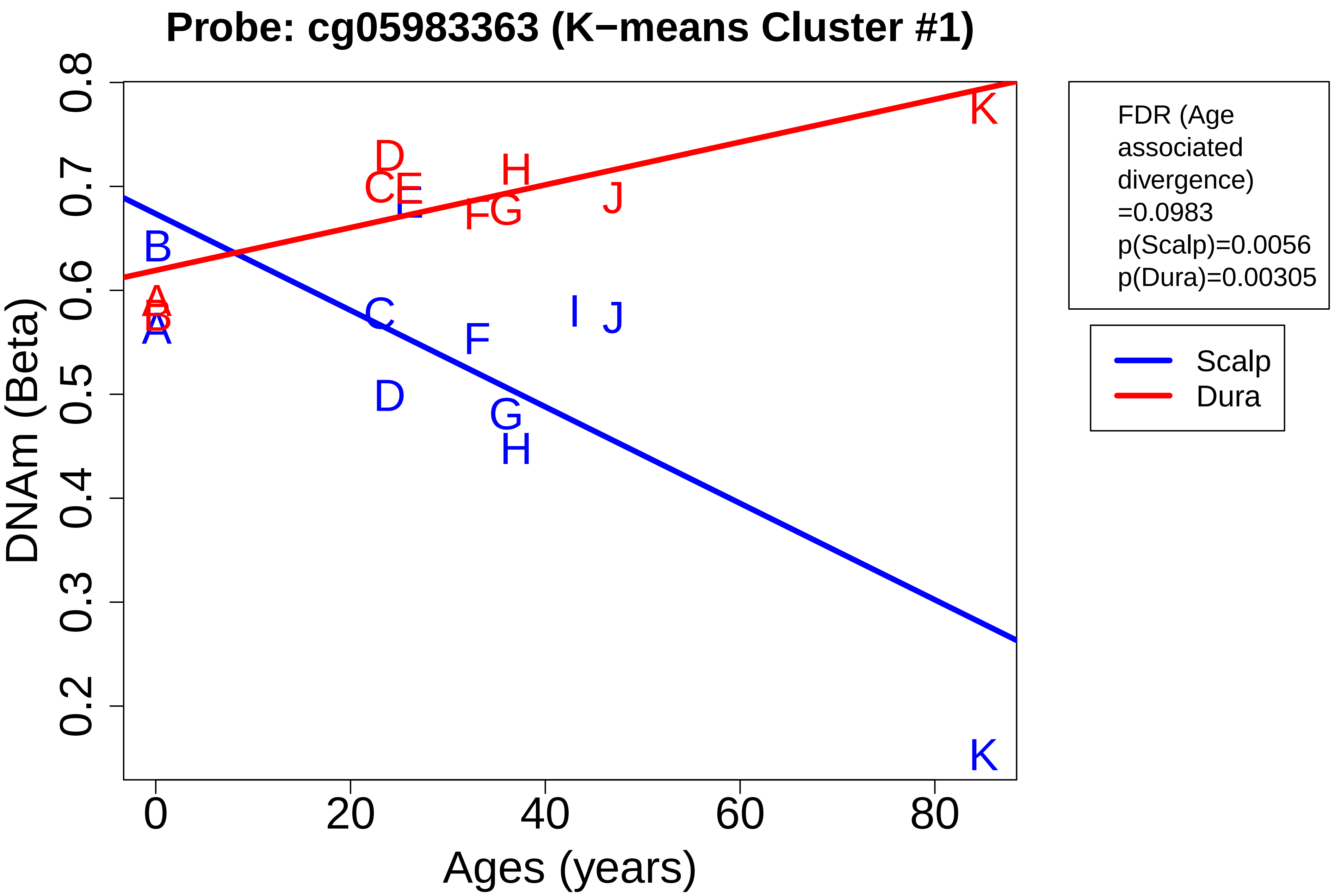

Probe: cg05983363 (K−means Cluster #1)
DNAm (Beta)
0.2
0.3
0.4
0.5
0.6
0.7
0.8
0
20
40
60
80
Ages (years)
FDR (Age associated divergence) =0.0983
p(Scalp)=0.0056
p(Dura)=0.00305
Scalp
Dura
A
B
C
D
E
F
G
H
I
J
K

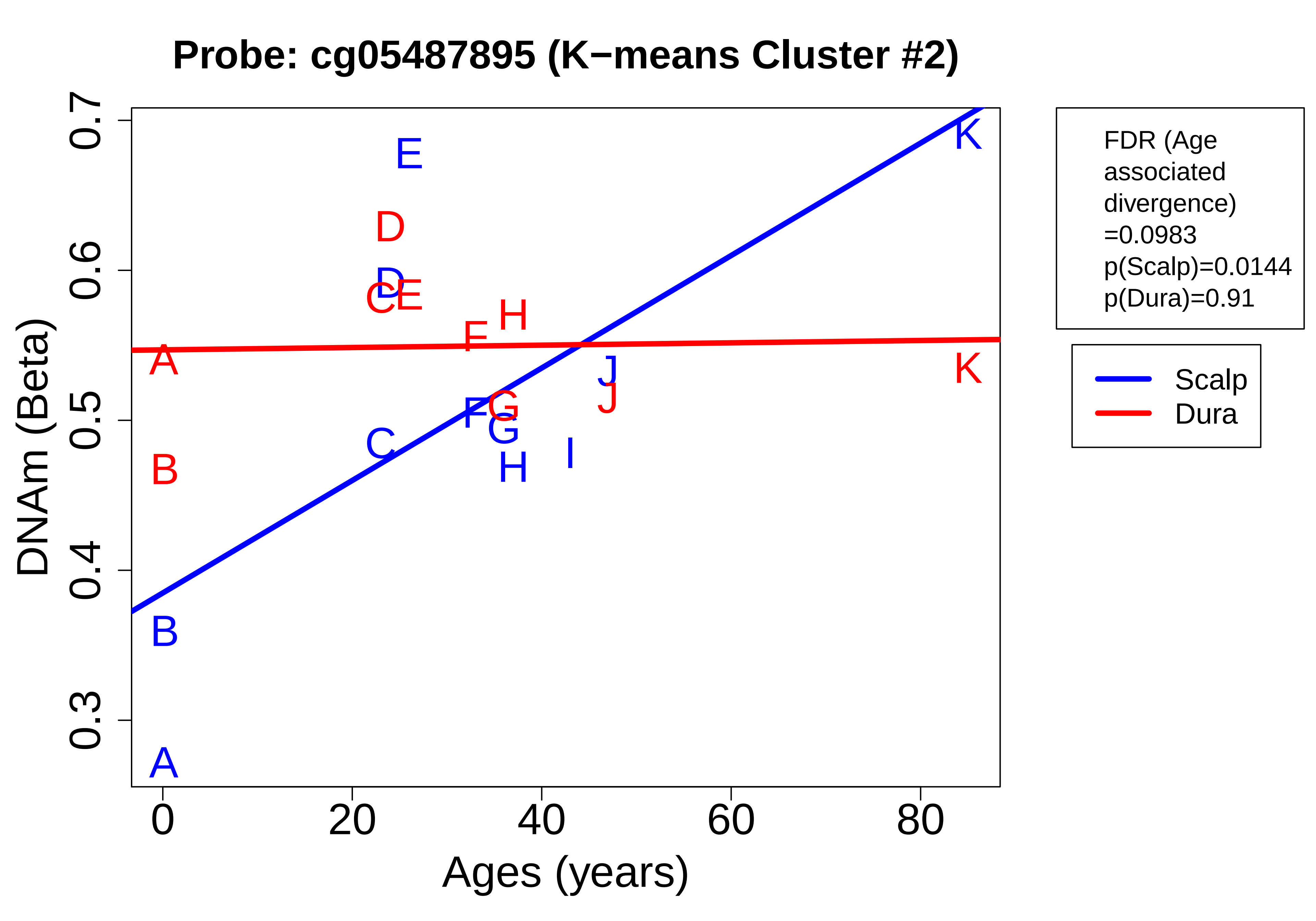

Probe: cg05487895 (K−means Cluster #2)
DNAm (Beta)
0.3
0.4
0.5
0.6
0.7
Ages (years)
0
20
40
60
80
A
B
C
D
E
F
G
H
I
J
K
FDR (Age associated divergence) =0.0983
p(Scalp)=0.0144
p(Dura)=0.91
Scalp
Dura

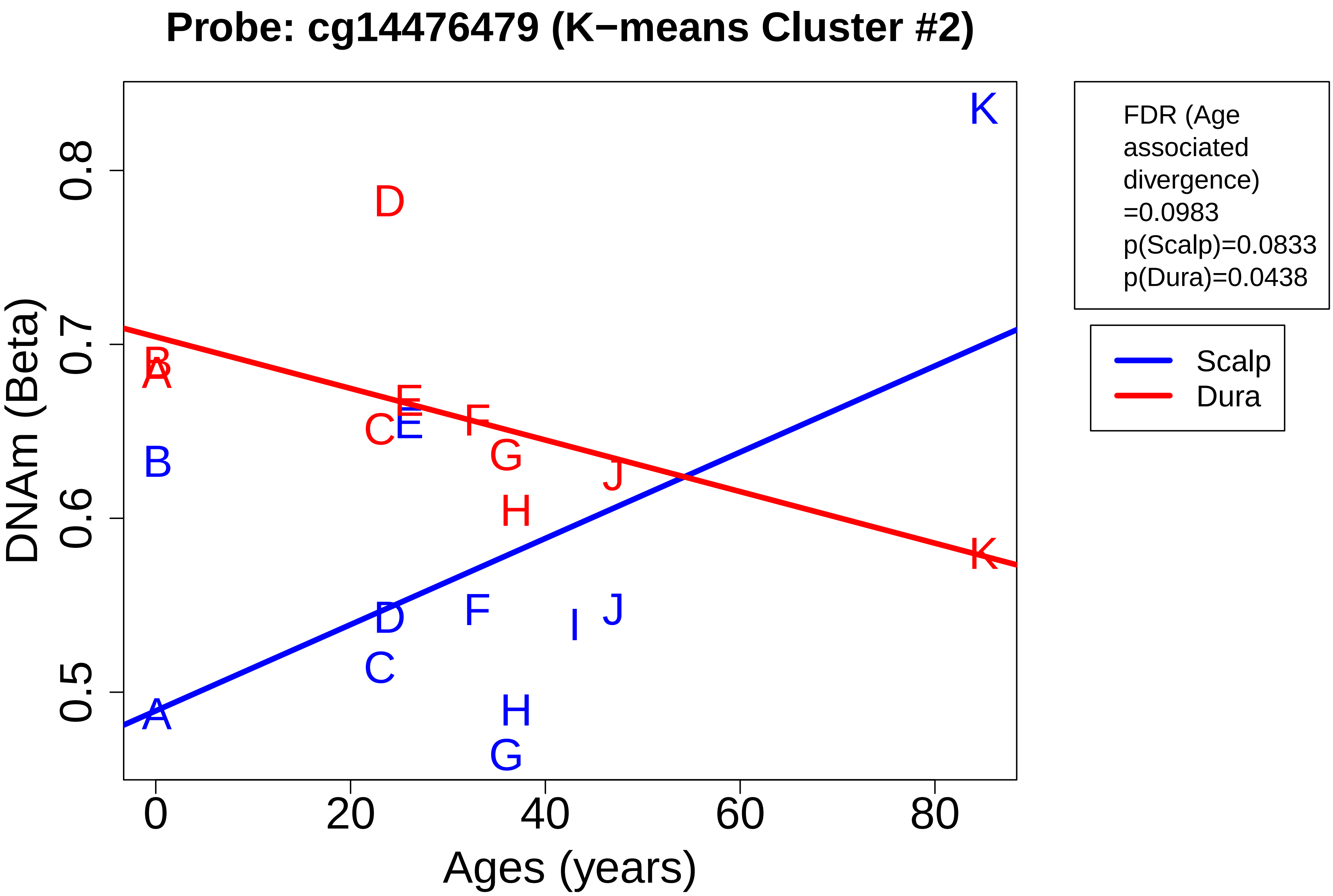

Probe: cg14476479 (K−means Cluster #2)
DNAm (Beta)
0.5
0.6
0.7
0.8
Ages (years)
0
20
40
60
80
FDR (Age associated divergence) =0.0983
p(Scalp)=0.0833
p(Dura)=0.0438
Scalp
Dura

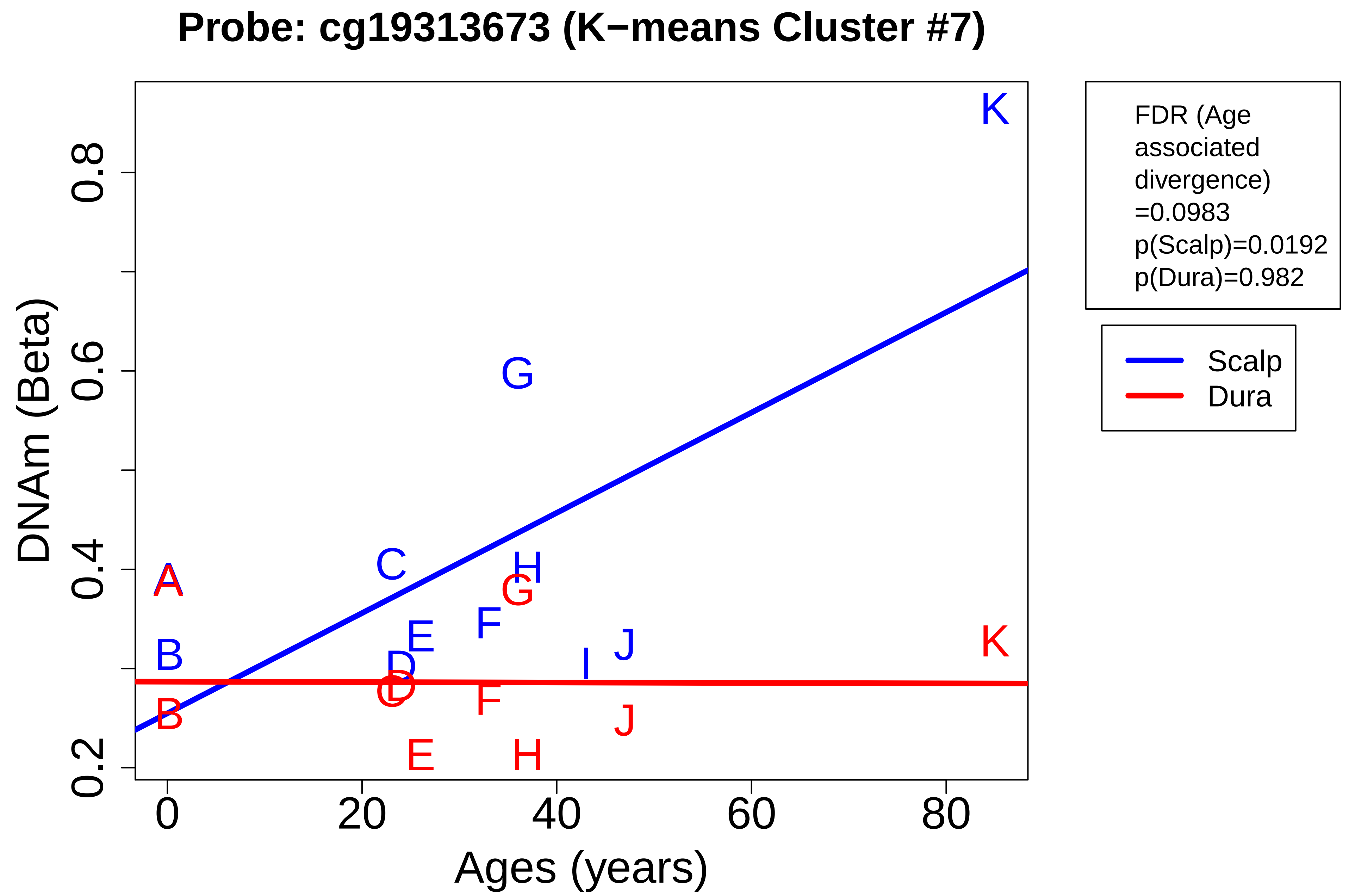

Probe: cg19313673 (K−means Cluster #7)
DNAm (Beta)
0.2
0.4
0.6
0.8
0
20
40
60
80
Ages (years)
FDR (Age associated divergence) =0.0983
p(Scalp)=0.0192
p(Dura)=0.982
Scalp
Dura
A
B
C
D
E
F
G
H
I
J
K

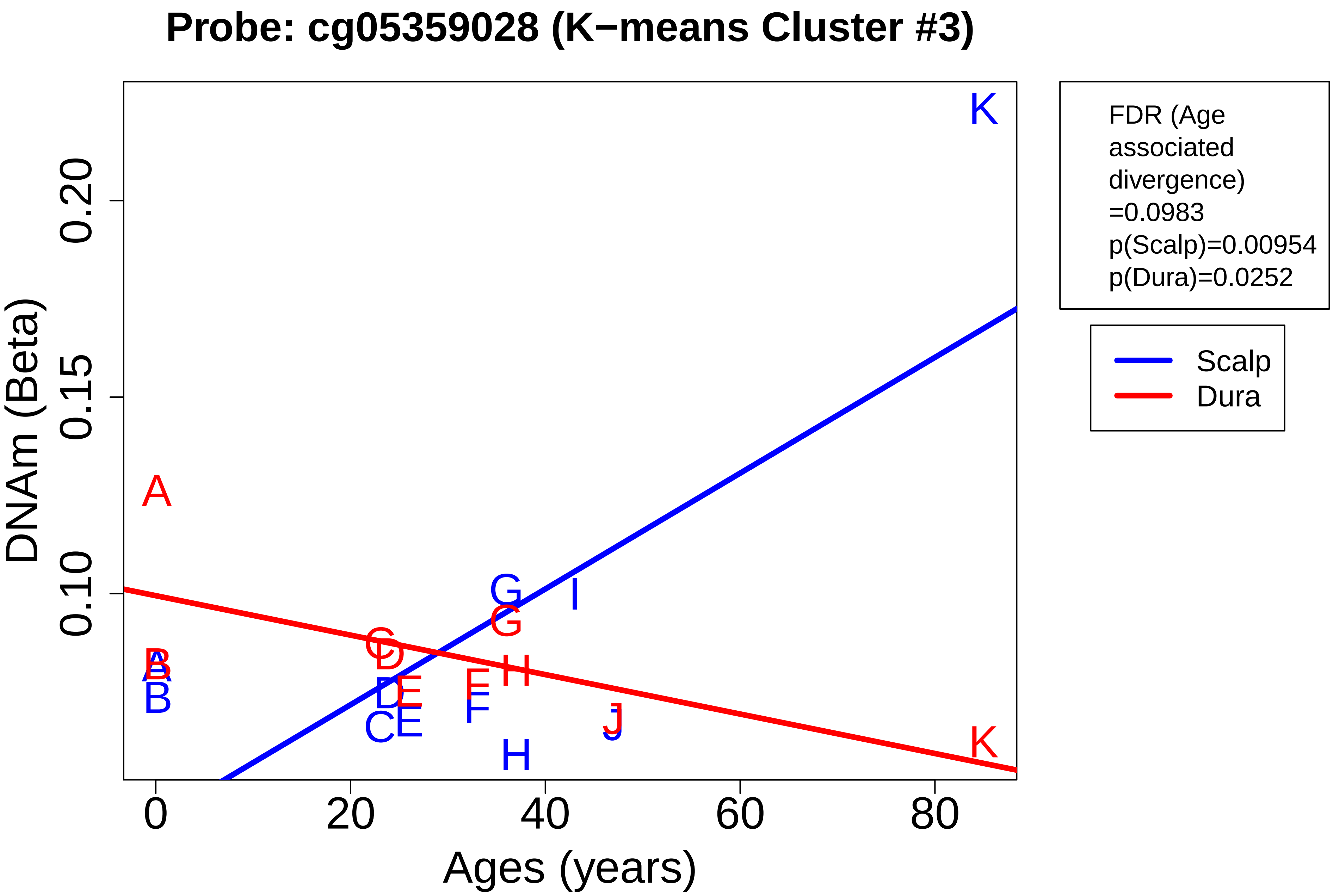

Probe: cg05359028 (K−means Cluster #3)
DNAm (Beta)
0.10
0.15
0.20
Ages (years)
0
20
40
60
80
FDR (Age associated divergence) =0.0983
p(Scalp)=0.00954
p(Dura)=0.0252
Scalp
Dura
A
B
C
D
E
F
G
H
I
J
K

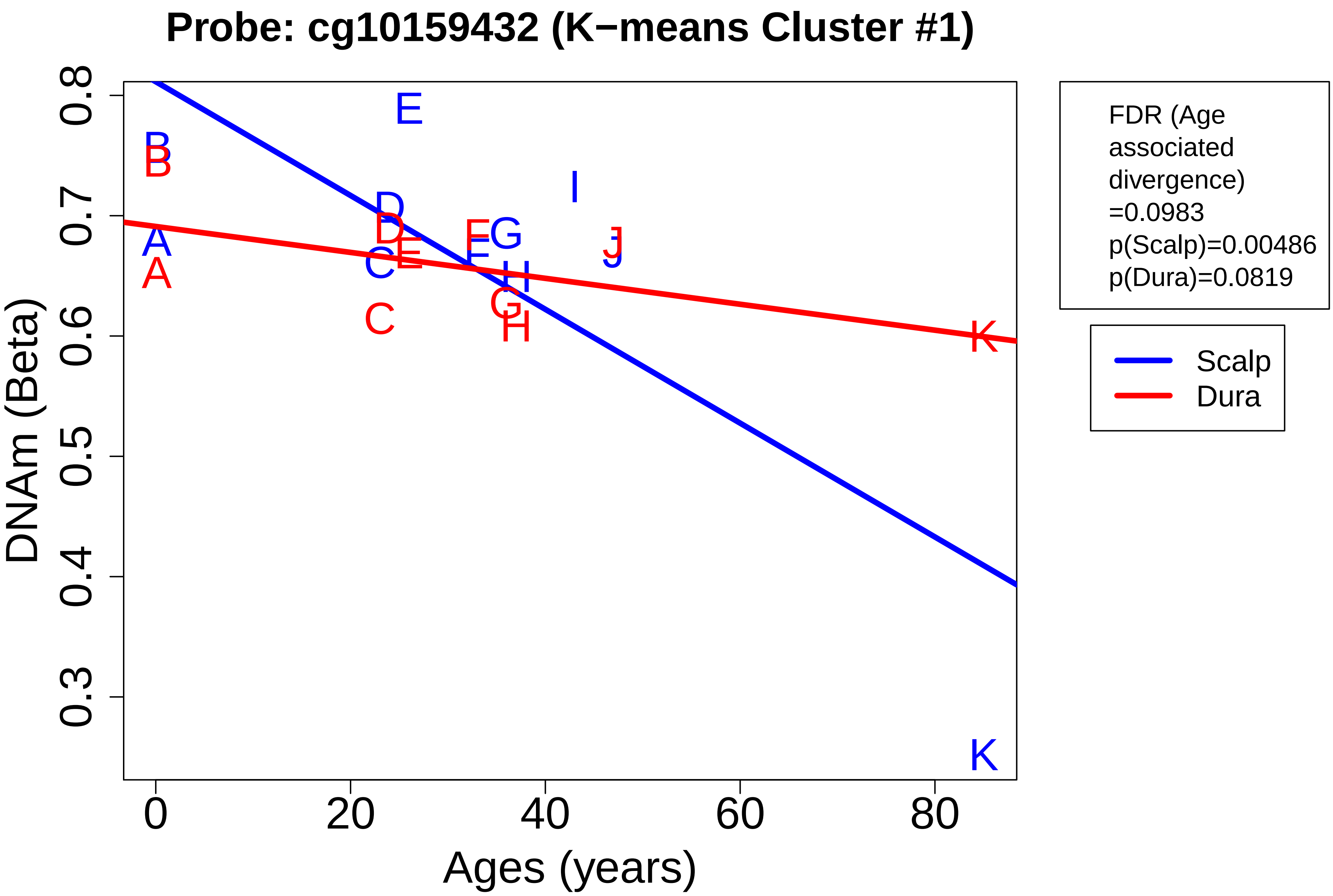

Probe: cg10159432 (K−means Cluster #1)
DNAm (Beta)
0.3
0.4
0.5
0.6
0.7
0.8
Ages (years)
0
20
40
60
80
A
B
C
D
E
F
G
H
I
J
K
FDR (Age associated divergence) =0.0983
p(Scalp)=0.00486
p(Dura)=0.0819
Scalp
Dura

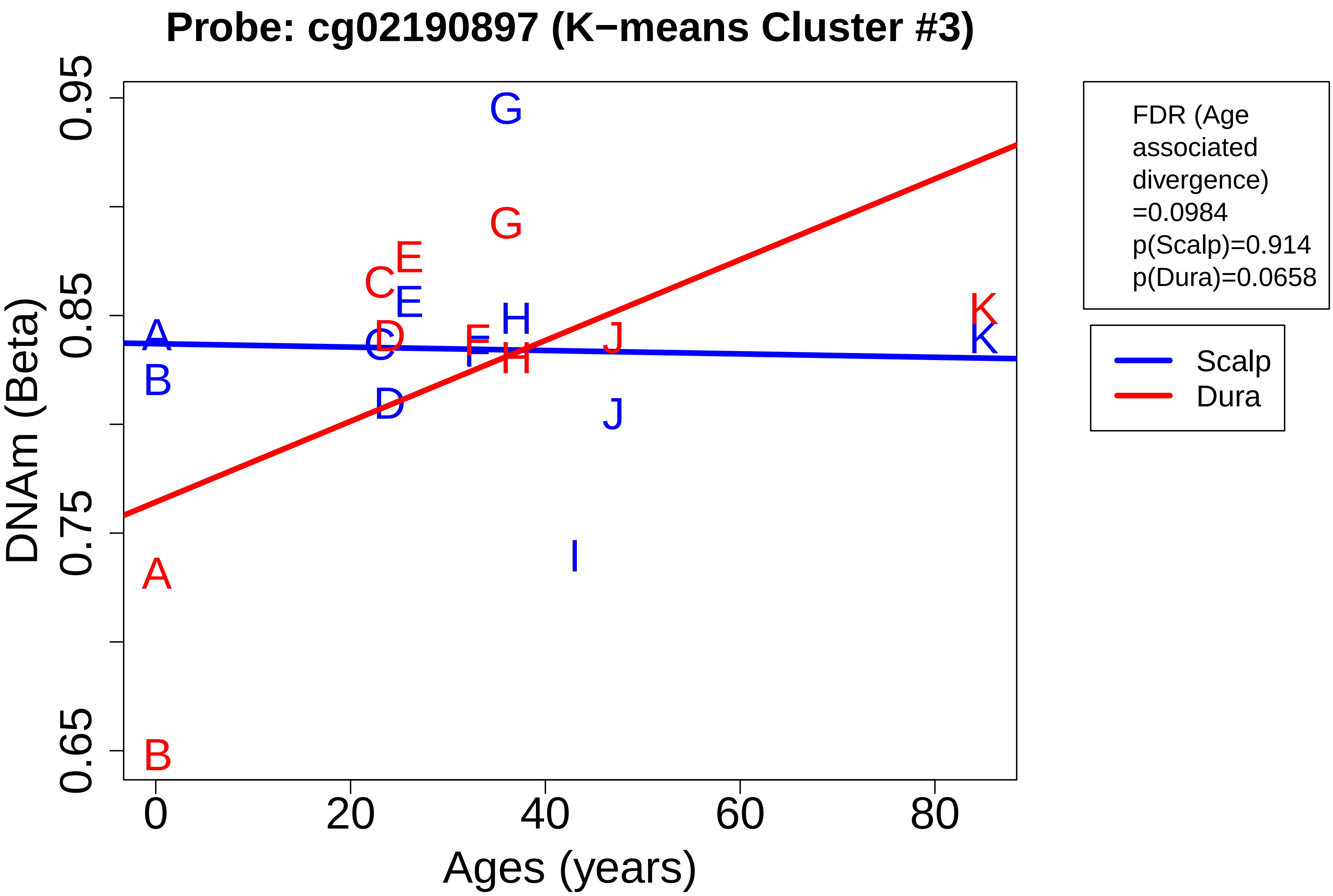

Probe: cg02190897 (K−means Cluster #3)
DNAm (Beta)
0.65
0.75
0.85
0.95
0
20
40
60
80
Ages (years)
FDR (Age associated divergence) =0.0984
p(Scalp)=0.914
p(Dura)=0.0658
Scalp
Dura

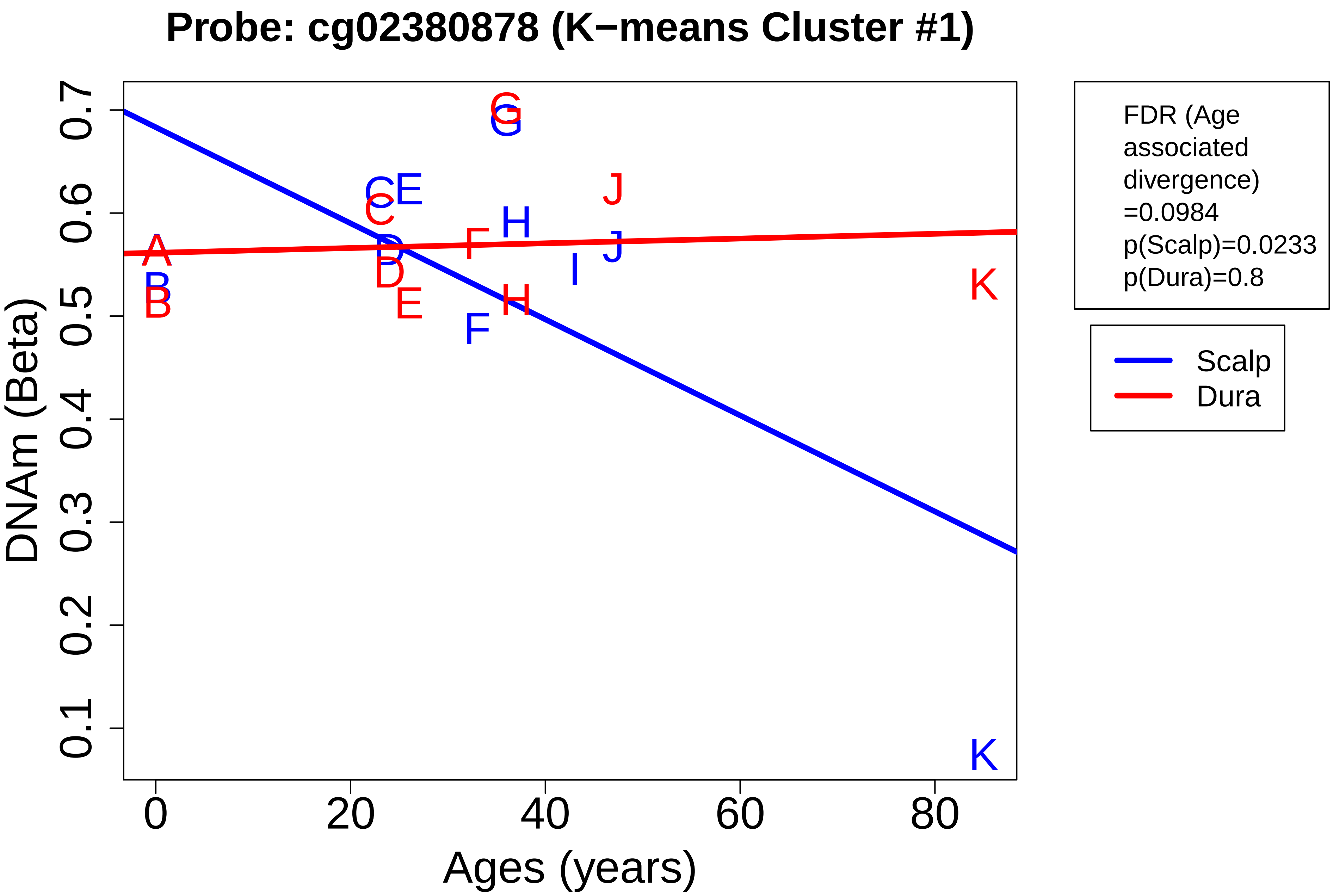

Probe: cg02380878 (K−means Cluster #1)
DNAm (Beta)
Ages (years)
0.1
0.2
0.3
0.4
0.5
0.6
0.7
0
20
40
60
80
A
B
C
D
E
F
G
H
I
J
K
FDR (Age associated divergence) =0.0984
p(Scalp)=0.0233
p(Dura)=0.8
Scalp
Dura

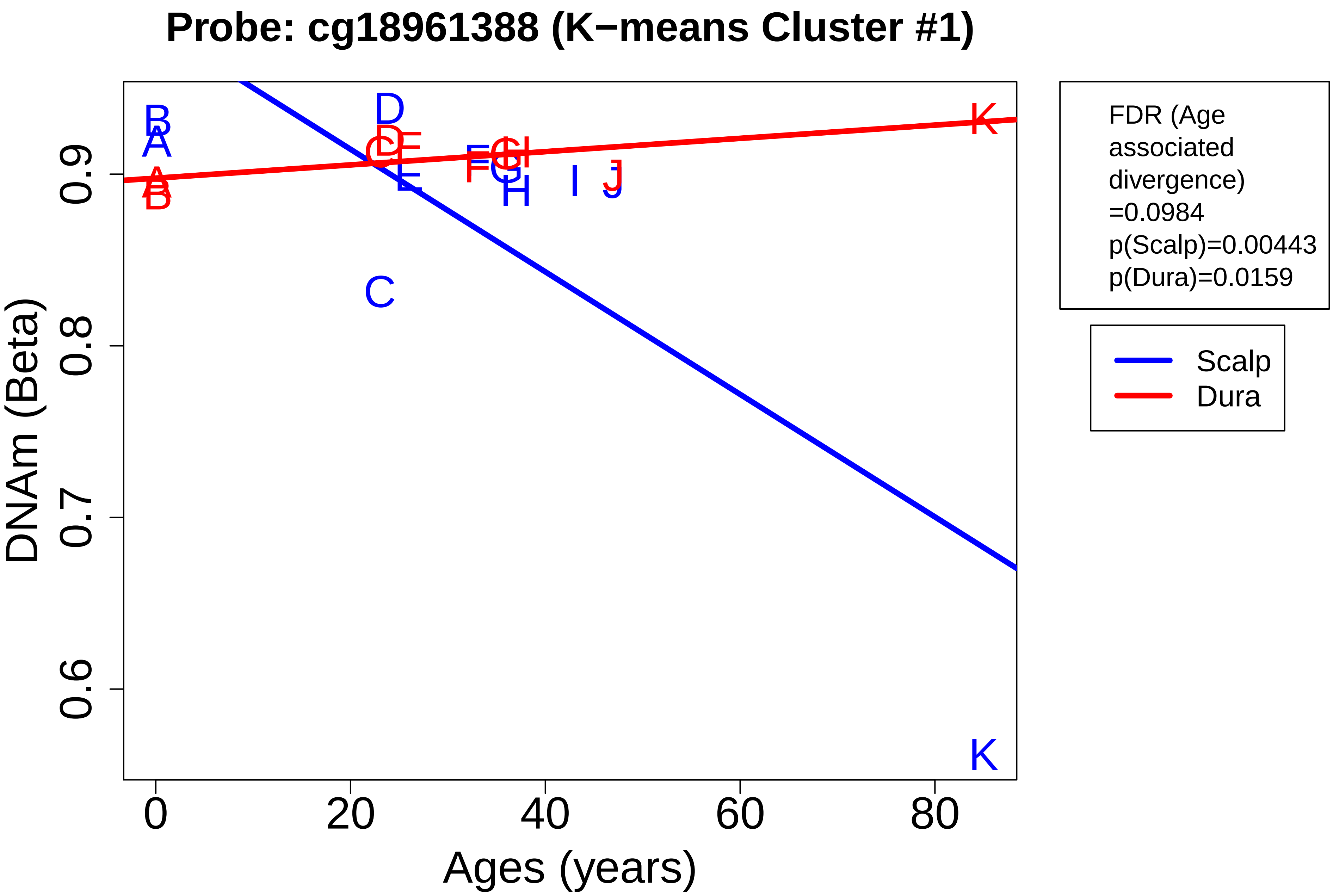
Probe: cg18961388 (K−means Cluster #1)
DNAm (Beta)
0.6
0.7
0.8
0.9
Ages (years)
0
20
40
60
80
FDR (Age associated divergence) =0.0984
p(Scalp)=0.00443
p(Dura)=0.0159
Scalp
Dura

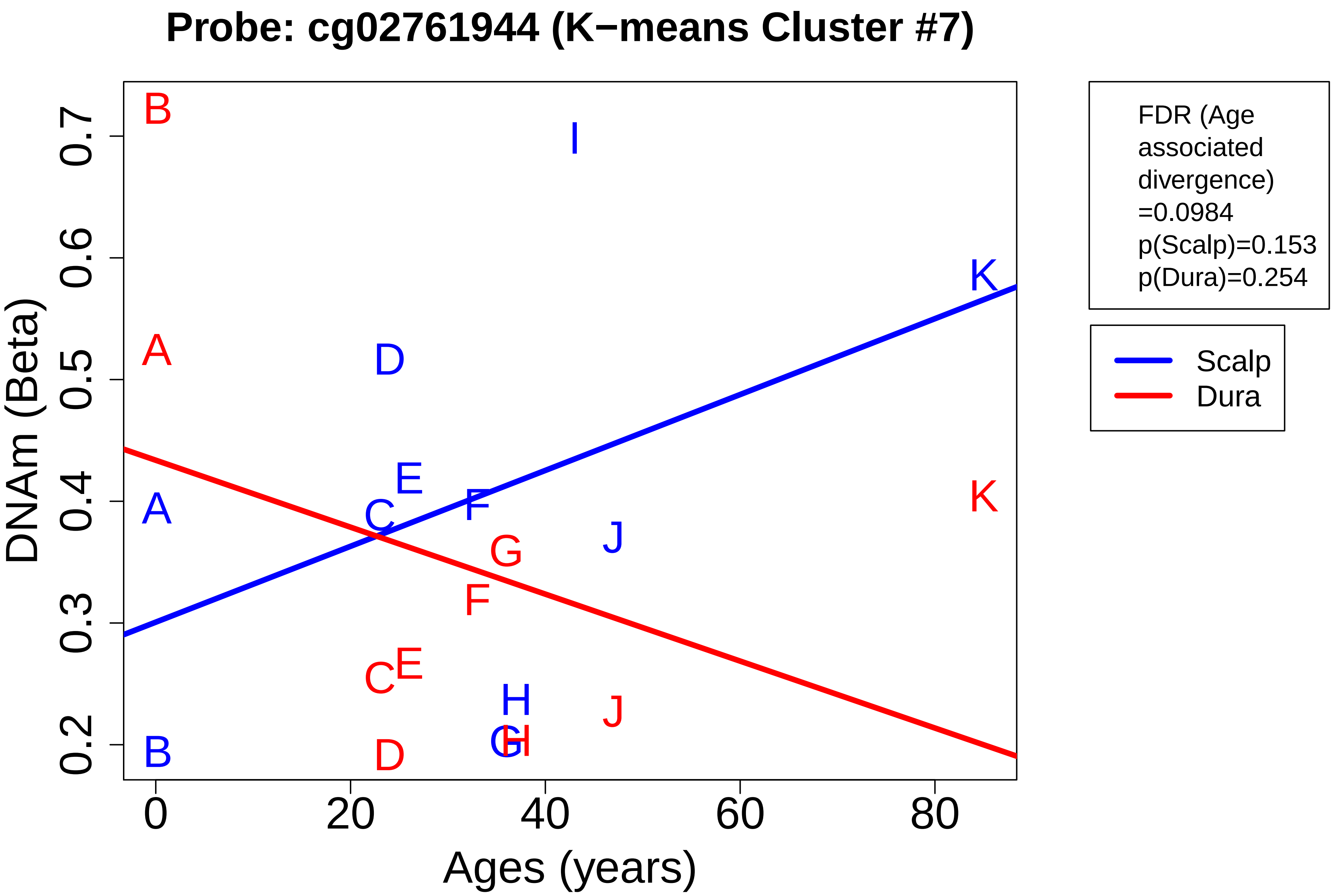

Probe: cg02761944 (K−means Cluster #7)
FDR (Age
associated
divergence)
=0.0984
p(Scalp)=0.153
p(Dura)=0.254
Scalp
Dura
DNAm (Beta)
0.2
0.3
0.4
0.5
0.6
0.7
0
20
40
60
80
Ages (years)

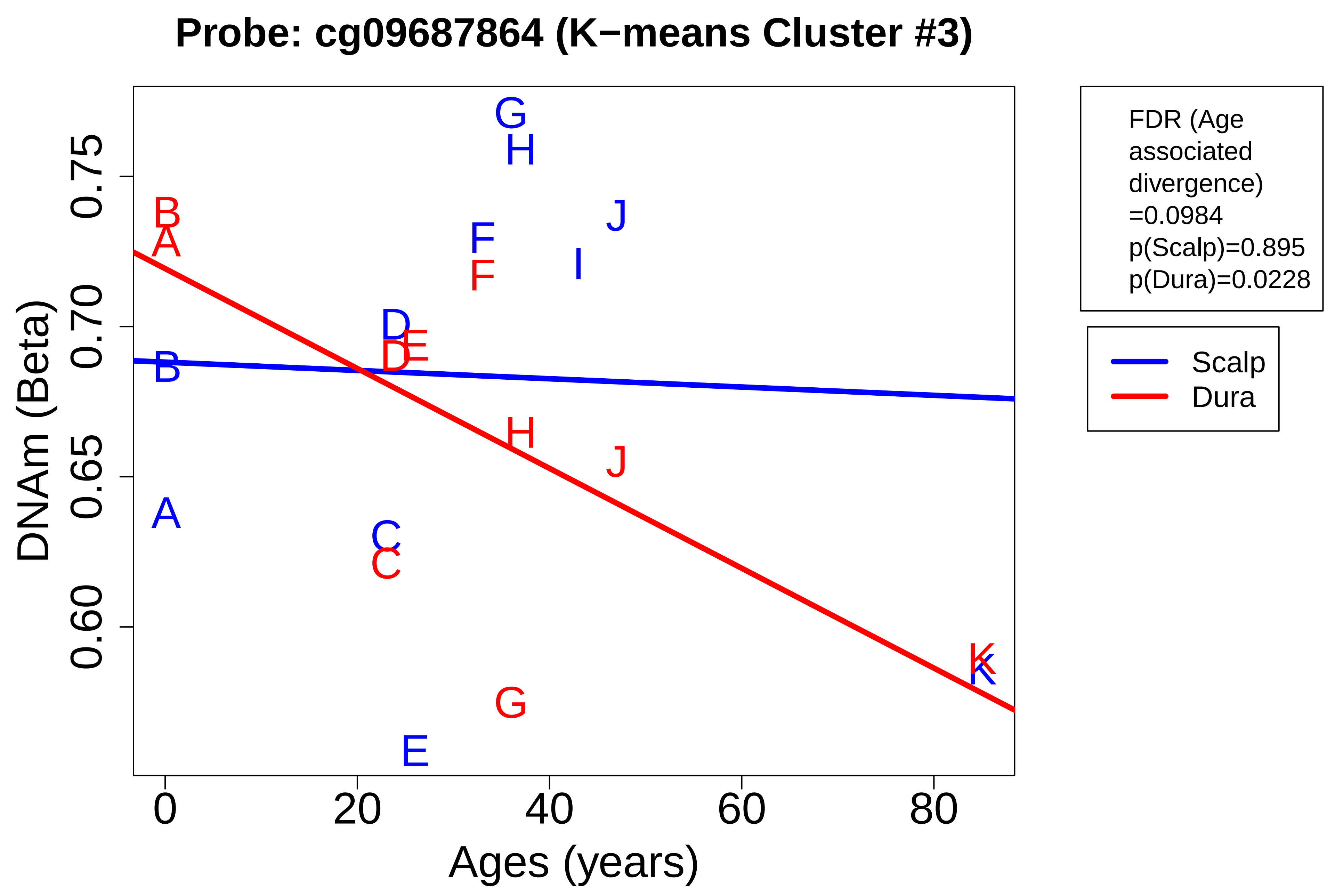

Probe: cg09687864 (K−means Cluster #3)
DNAm (Beta)
0.60
0.65
0.70
0.75
0
20
40
60
80
Ages (years)
FDR (Age associated divergence) =0.0984
p(Scalp)=0.895
p(Dura)=0.0228
Scalp
Dura

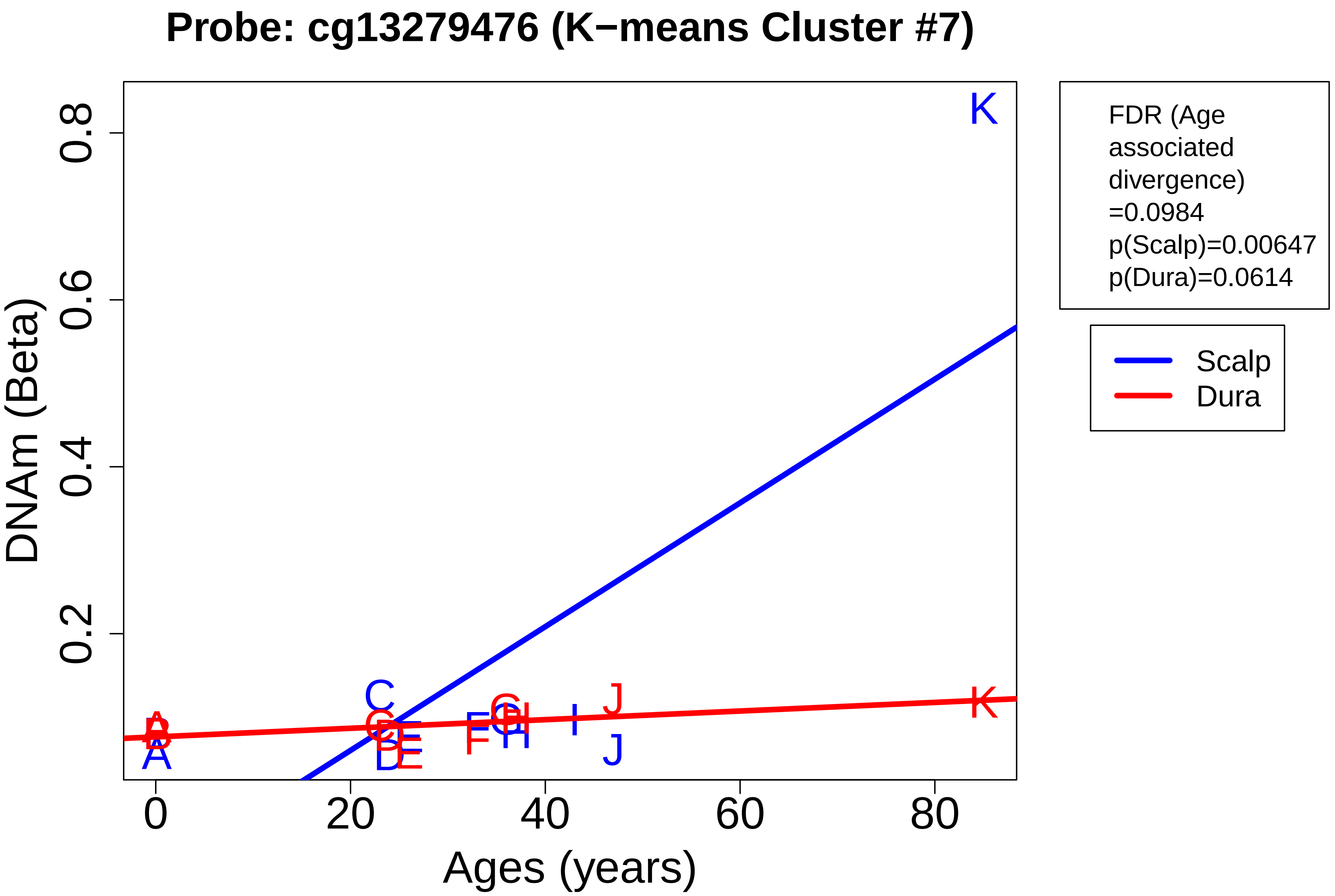

Probe: cg13279476 (K−means Cluster #7)
DNAm (Beta)
Ages (years)
0.2
0.4
0.6
0.8
0
20
40
60
80
FDR (Age associated divergence) =0.0984
p(Scalp)=0.00647
p(Dura)=0.0614
Scalp
Dura
A
B
C
D
E
F
G
H
I
J
K

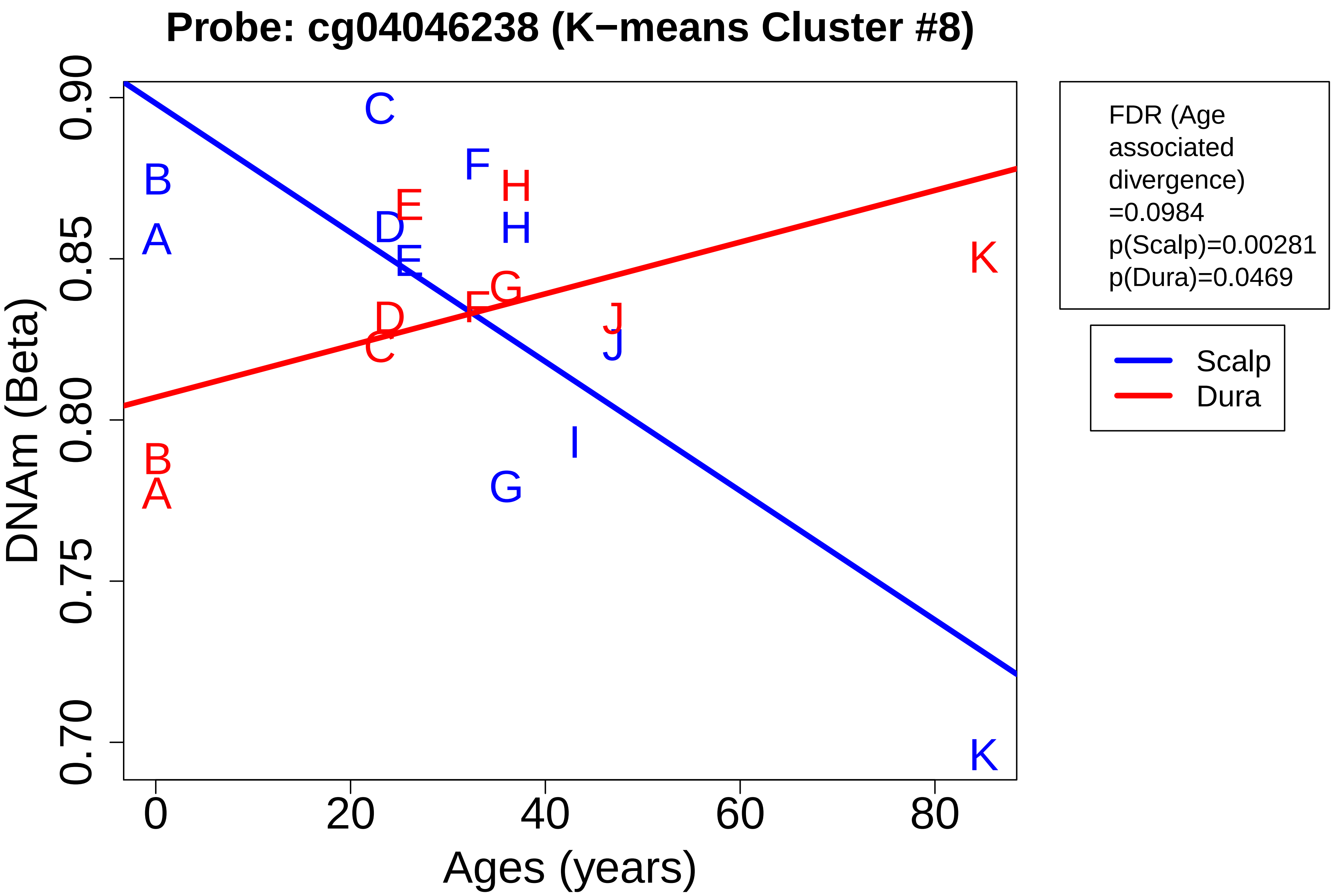

Probe: cg04046238 (K−means Cluster #8)
DNAm (Beta)
0.70
0.75
0.80
0.85
0.90
0
20
40
60
80
Ages (years)
FDR (Age associated divergence) =0.0984
p(Scalp)=0.00281
p(Dura)=0.0469
Scalp
Dura

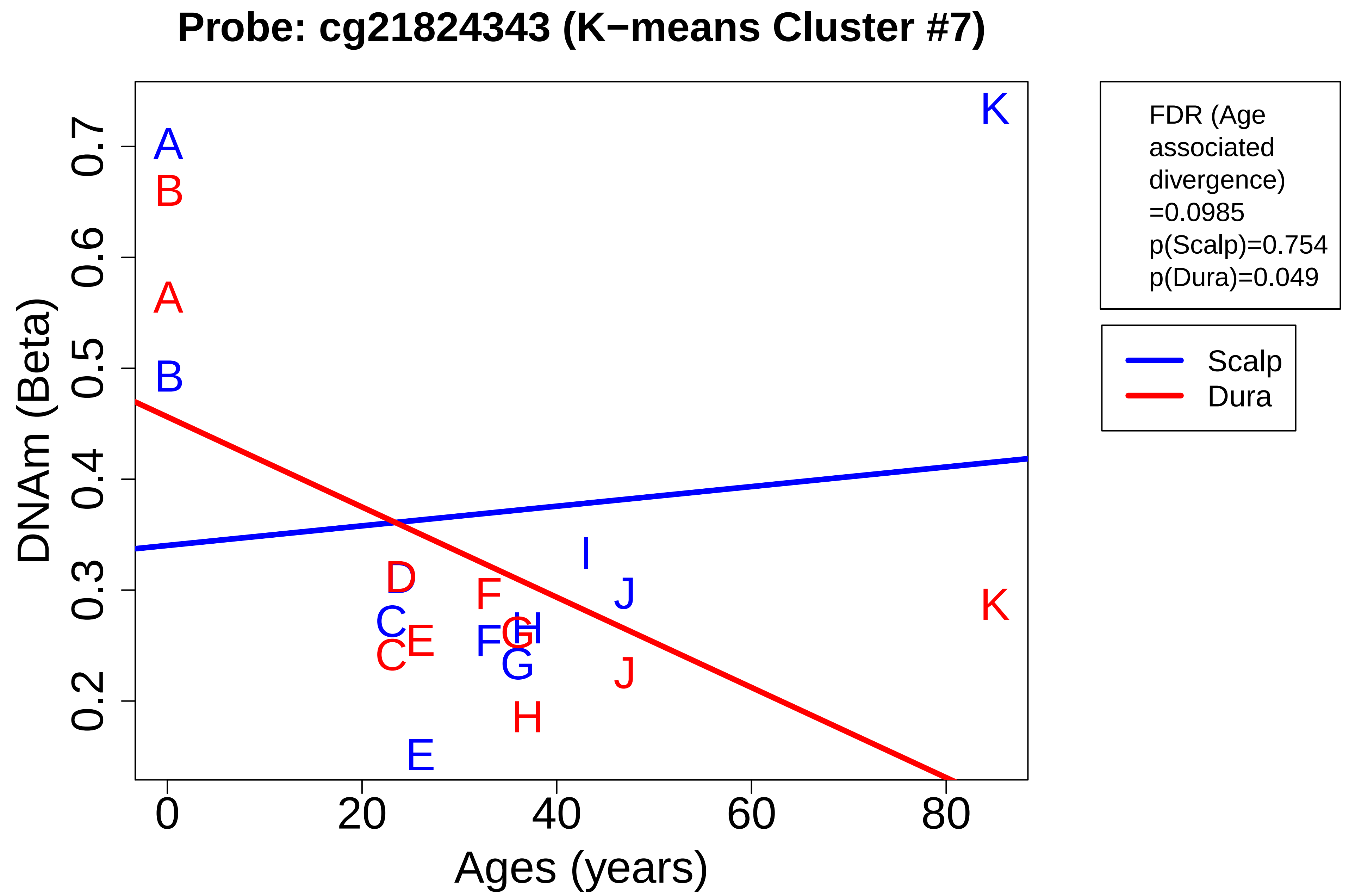
Probe: cg21824343 (K−means Cluster #7)
DNAm (Beta)
0.2
0.3
0.4
0.5
0.6
0.7
0
20
40
60
80
Ages (years)
A
B
C
D
E
F
G
H
I
J
K
FDR (Age associated divergence) =0.0985
p(Scalp)=0.754
p(Dura)=0.049
Scalp
Dura

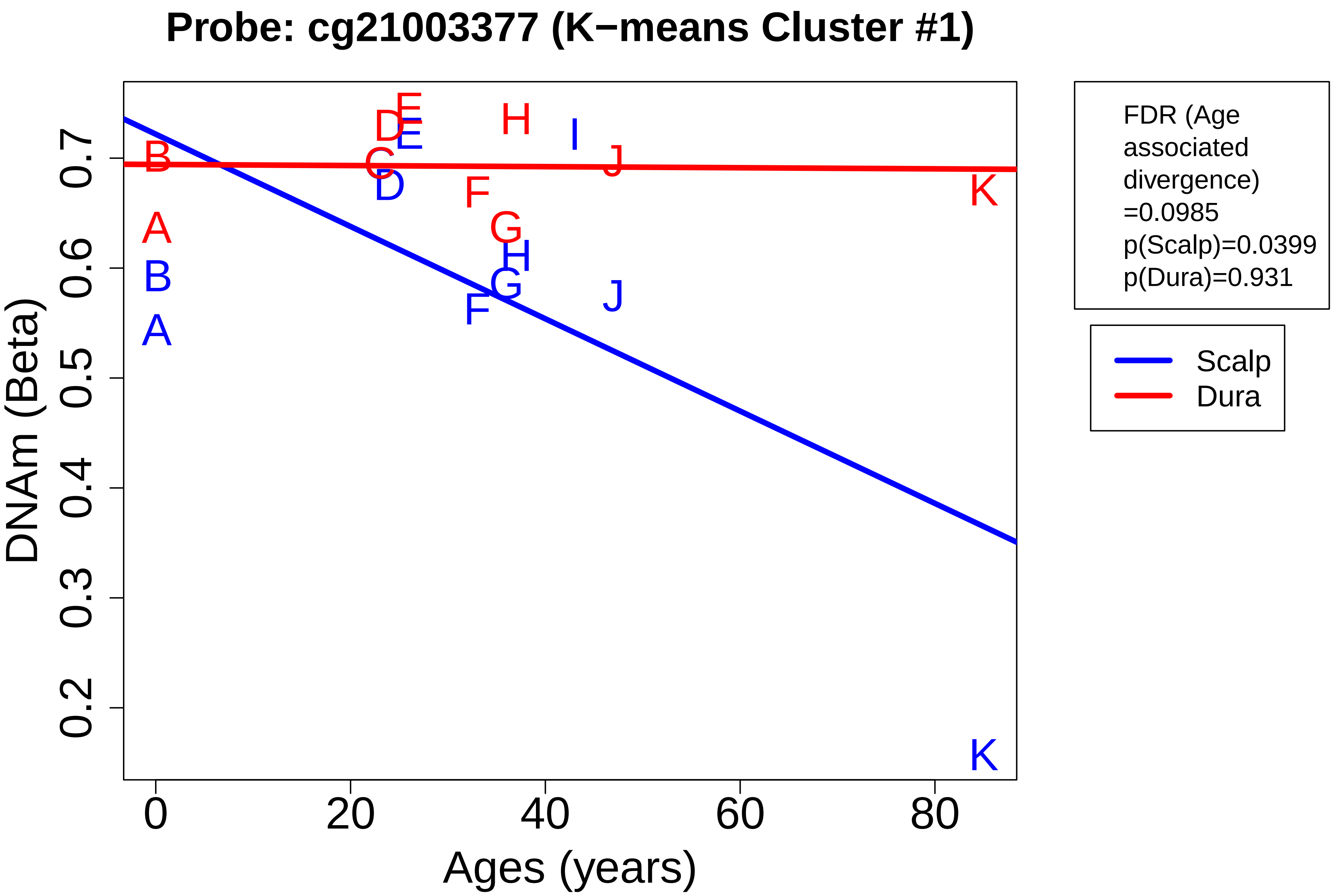

Probe: cg21003377 (K−means Cluster #1)
DNAm (Beta)
0.2
0.3
0.4
0.5
0.6
0.7
Ages (years)
0
20
40
60
80
A
B
D
E
G
F
H
I
J
K
FDR (Age associated divergence) =0.0985
p(Scalp)=0.0399
p(Dura)=0.931
Scalp
Dura

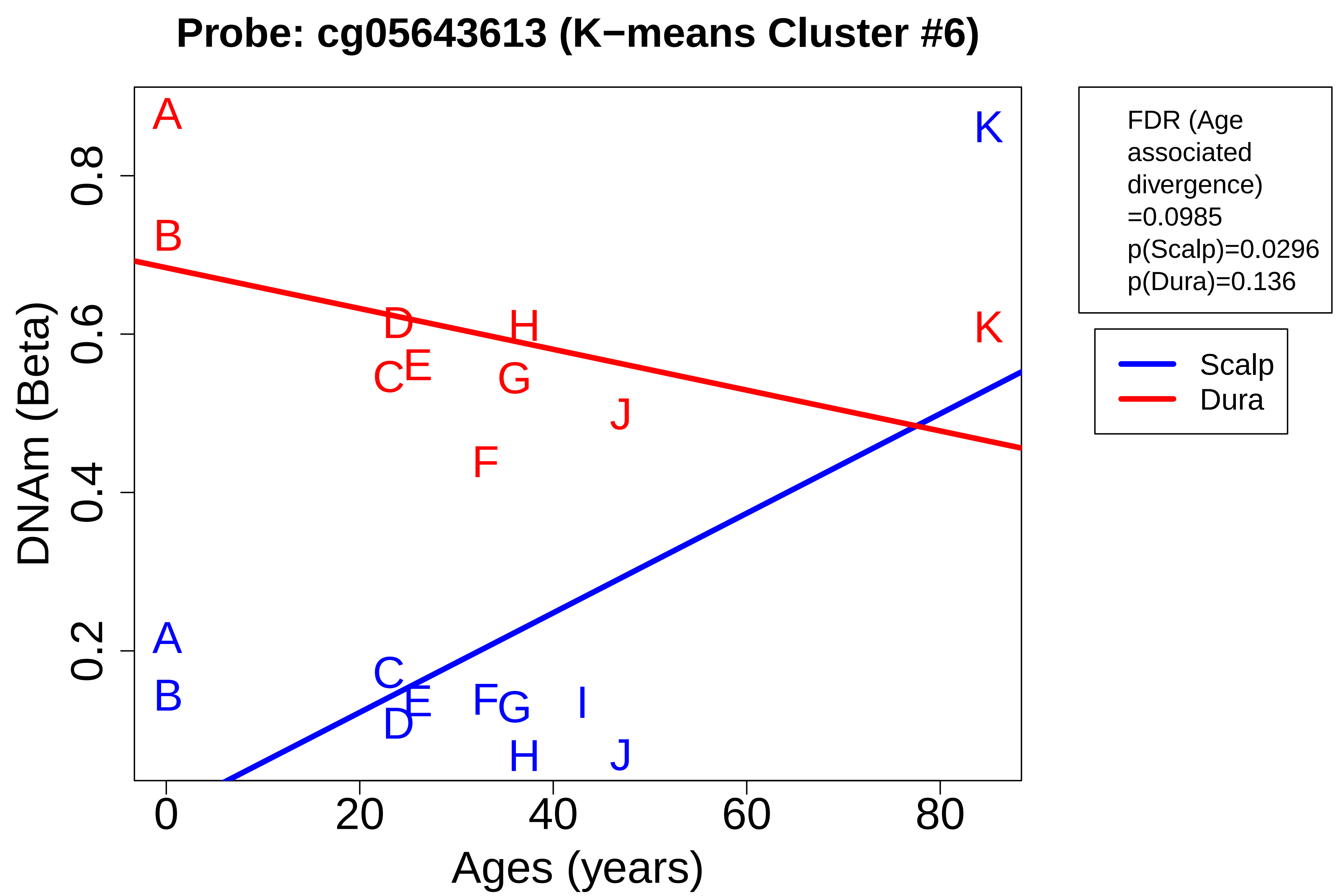

Probe: cg05643613 (K−means Cluster #6)
FDR (Age associated divergence) =0.0985
p(Scalp)=0.0296
p(Dura)=0.136
Scalp
Dura
DNAm (Beta)
0.2
0.4
0.6
0.8
Ages (years)
0
20
40
60
80
A
B
C
D
E
F
G
H
I
J
K

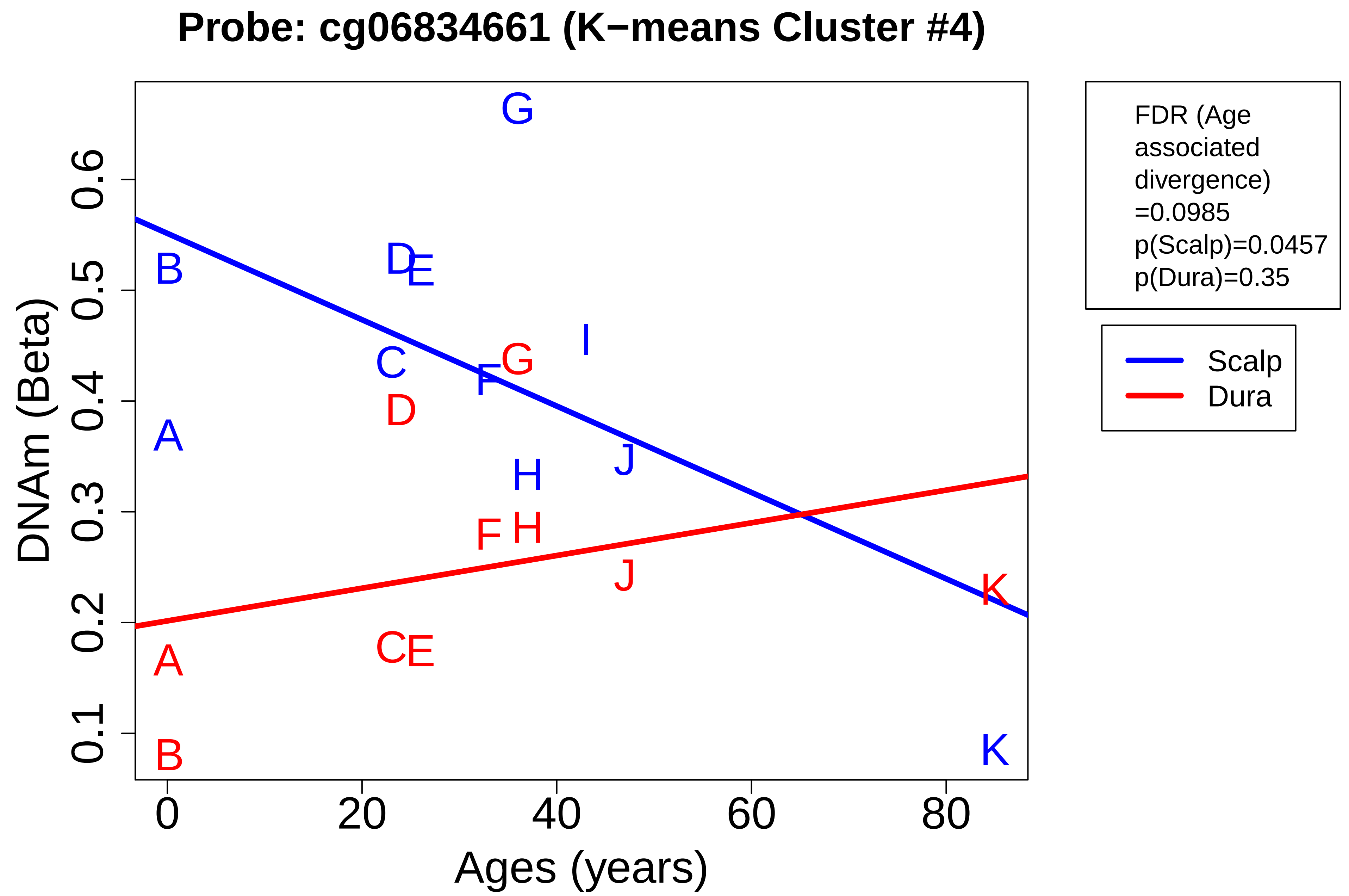

Probe: cg06834661 (K−means Cluster #4)
DNAm (Beta)
Ages (years)
FDR (Age associated divergence) =0.0985
p(Scalp)=0.0457
p(Dura)=0.35
Scalp
Dura

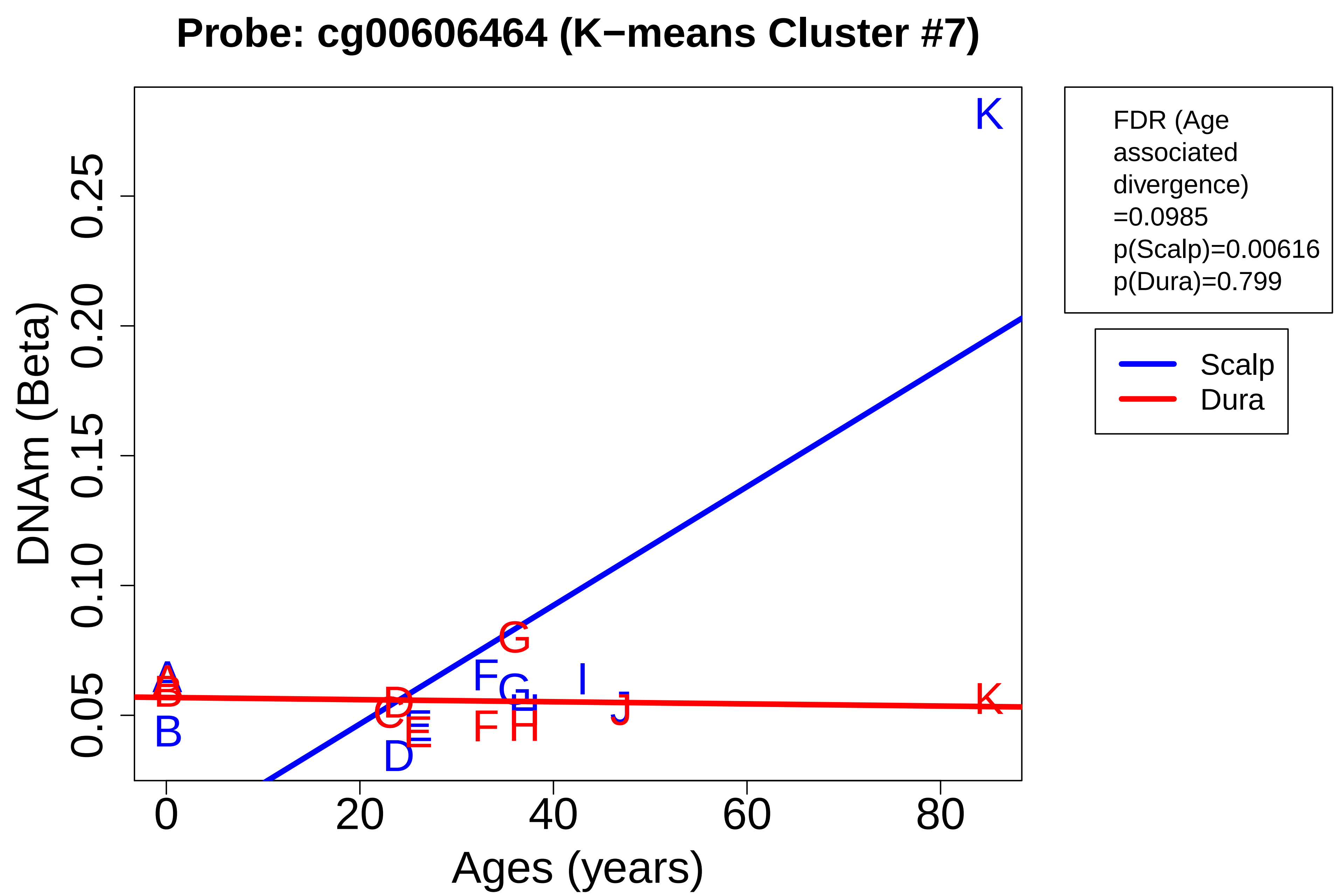
Probe: cg00606464 (K−means Cluster #7)
DNAm (Beta)
0.05
0.10
0.15
0.20
0.25
0
20
40
60
80
Ages (years)
A
B
C
D
E
F
G
H
I
J
K
FDR (Age associated divergence) =0.0985
p(Scalp)=0.00616
p(Dura)=0.799
Scalp
Dura

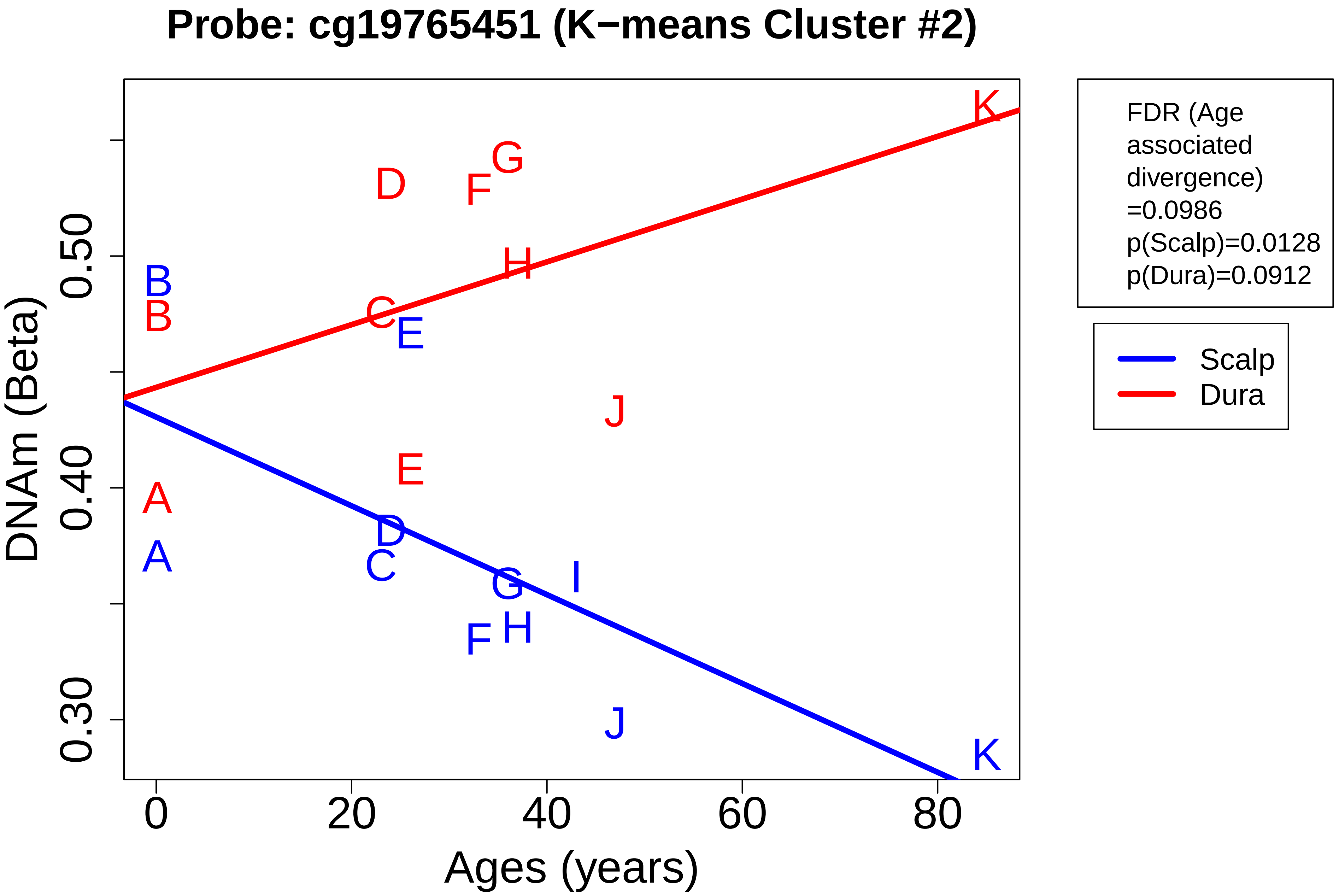

Probe: cg19765451 (K−means Cluster #2)
FDR (Age associated divergence) =0.0986
p(Scalp)=0.0128
p(Dura)=0.0912
Scalp
Dura
DNAm (Beta)
0.30
0.40
0.50
0
20
40
60
80
Ages (years)
A
B
C
D
E
F
G
H
I
J
K

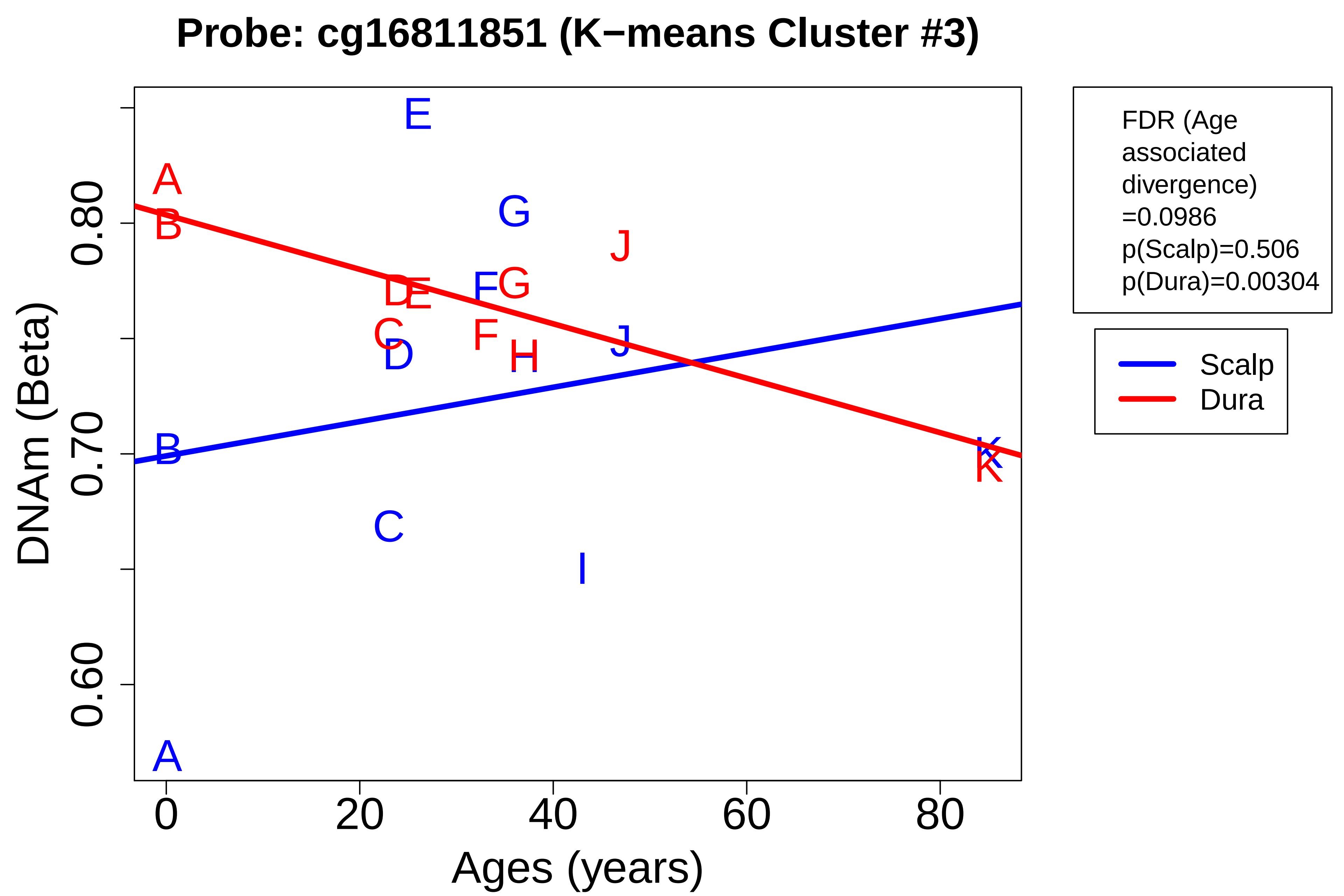

Probe: cg16811851 (K−means Cluster #3)
FDR (Age associated divergence) =0.0986
p(Scalp)=0.506
p(Dura)=0.00304
Scalp
Dura
DNAm (Beta)
0.60
0.70
0.80
0
20
40
60
80
Ages (years)
A
B
C
D
E
F
G
H
I
J
K

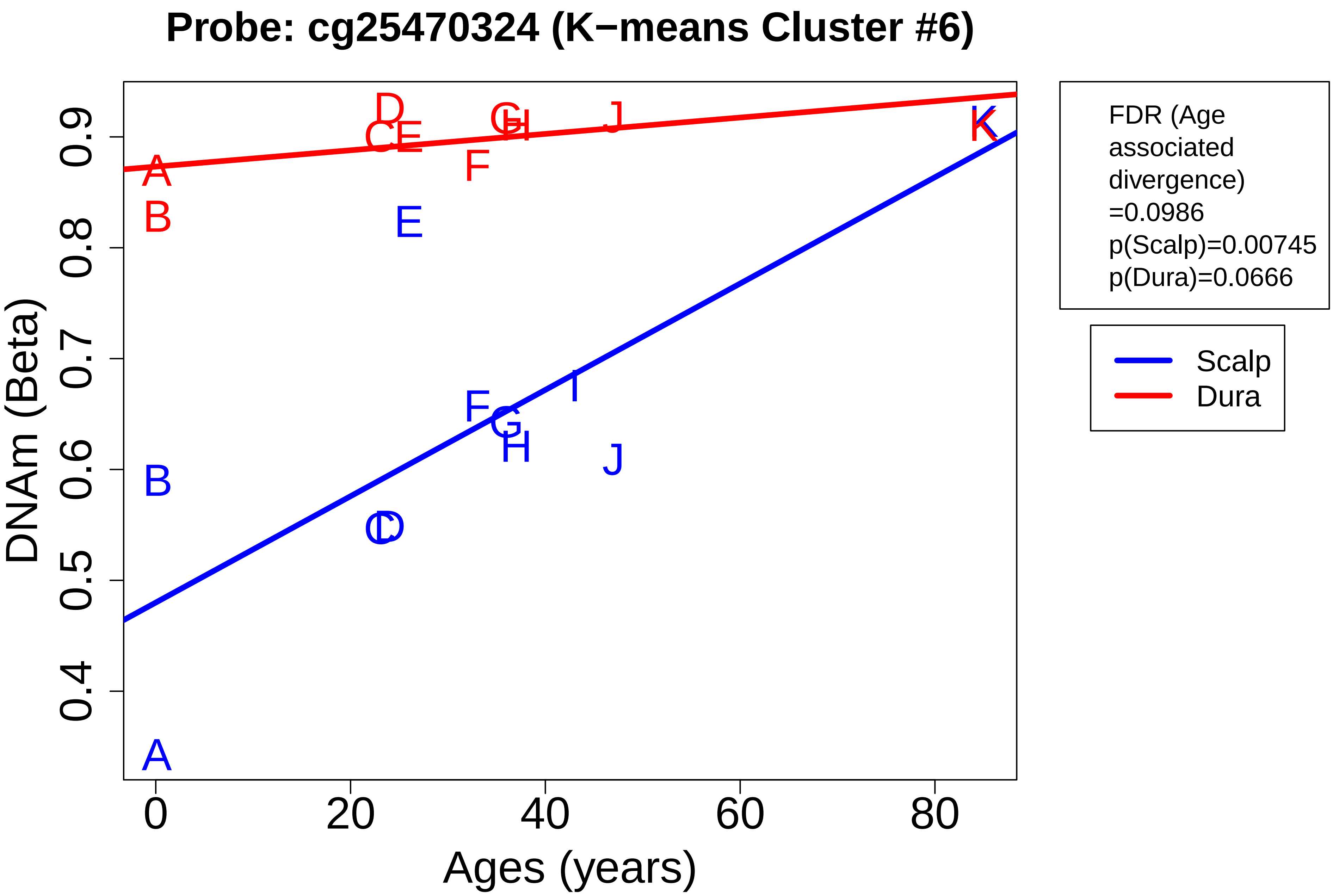

Probe: cg25470324 (K−means Cluster #6)
DNAm (Beta)
0.4
0.5
0.6
0.7
0.8
0.9
0
20
40
60
80
Ages (years)
FDR (Age associated divergence) =0.0986
p(Scalp)=0.00745
p(Dura)=0.0666
Scalp
Dura

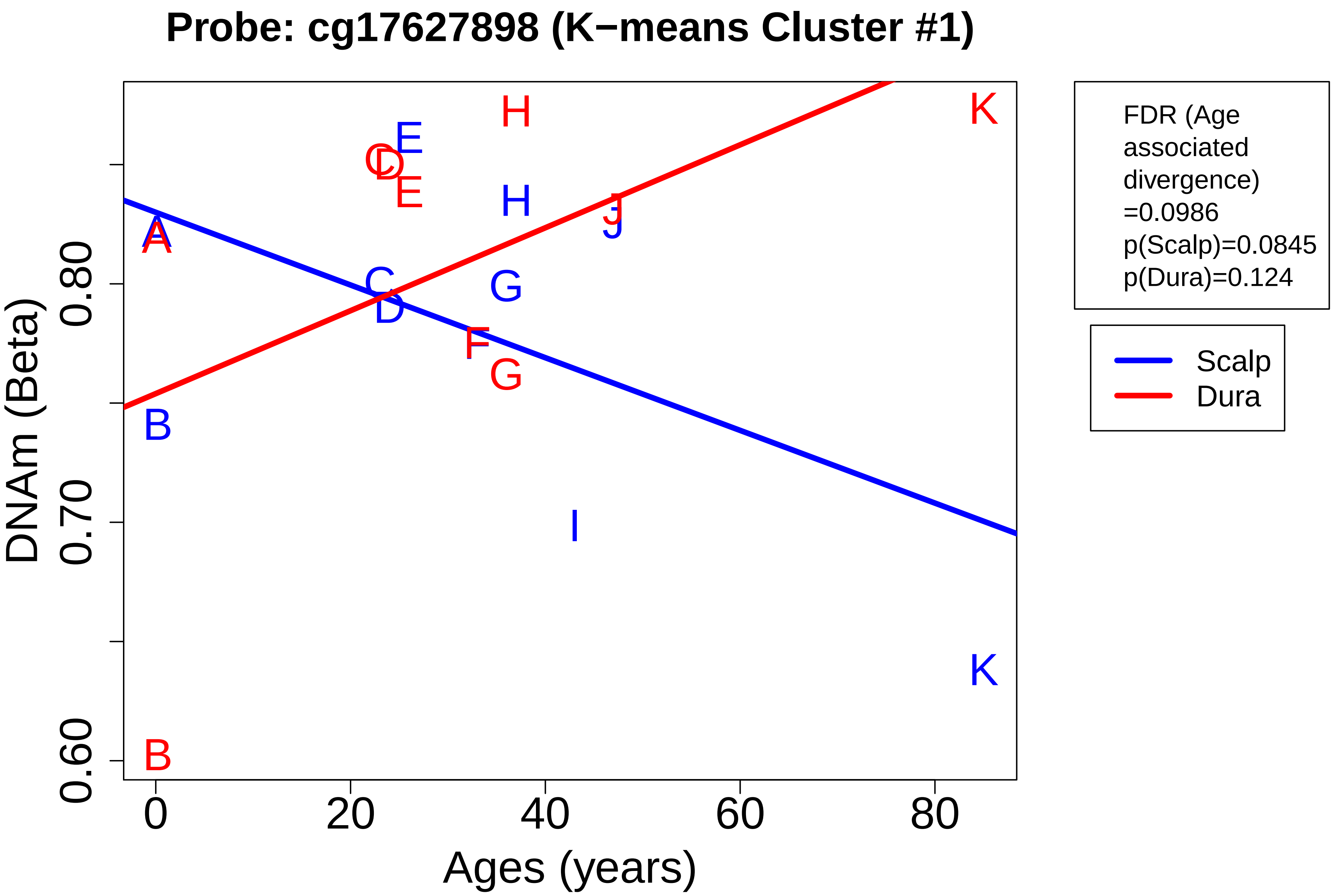
Probe: cg17627898 (K−means Cluster #1)
FDR (Age associated divergence) =0.0986
p(Scalp)=0.0845
p(Dura)=0.124
Scalp
Dura
DNAm (Beta)
0.60
0.70
0.80
Ages (years)
0
20
40
60
80
A
B
C
D
E
F
G
H
I
J
K

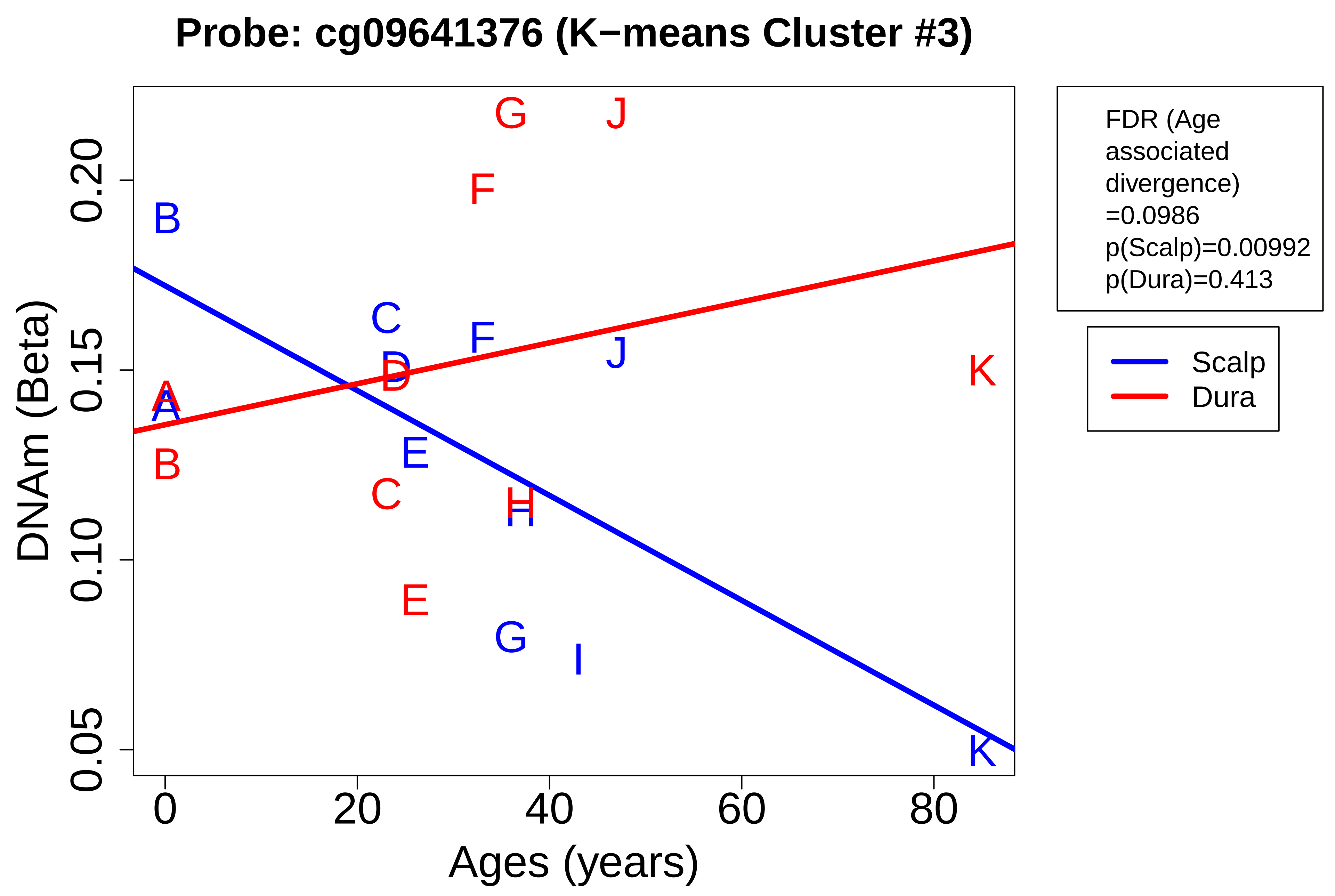

Probe: cg09641376 (K−means Cluster #3)
DNAm (Beta)
Ages (years)
0.05
0.10
0.15
0.20
0
20
40
60
80
A
B
C
D
E
F
G
H
I
J
K
FDR (Age associated divergence) =0.0986
p(Scalp)=0.00992
p(Dura)=0.413
Scalp
Dura

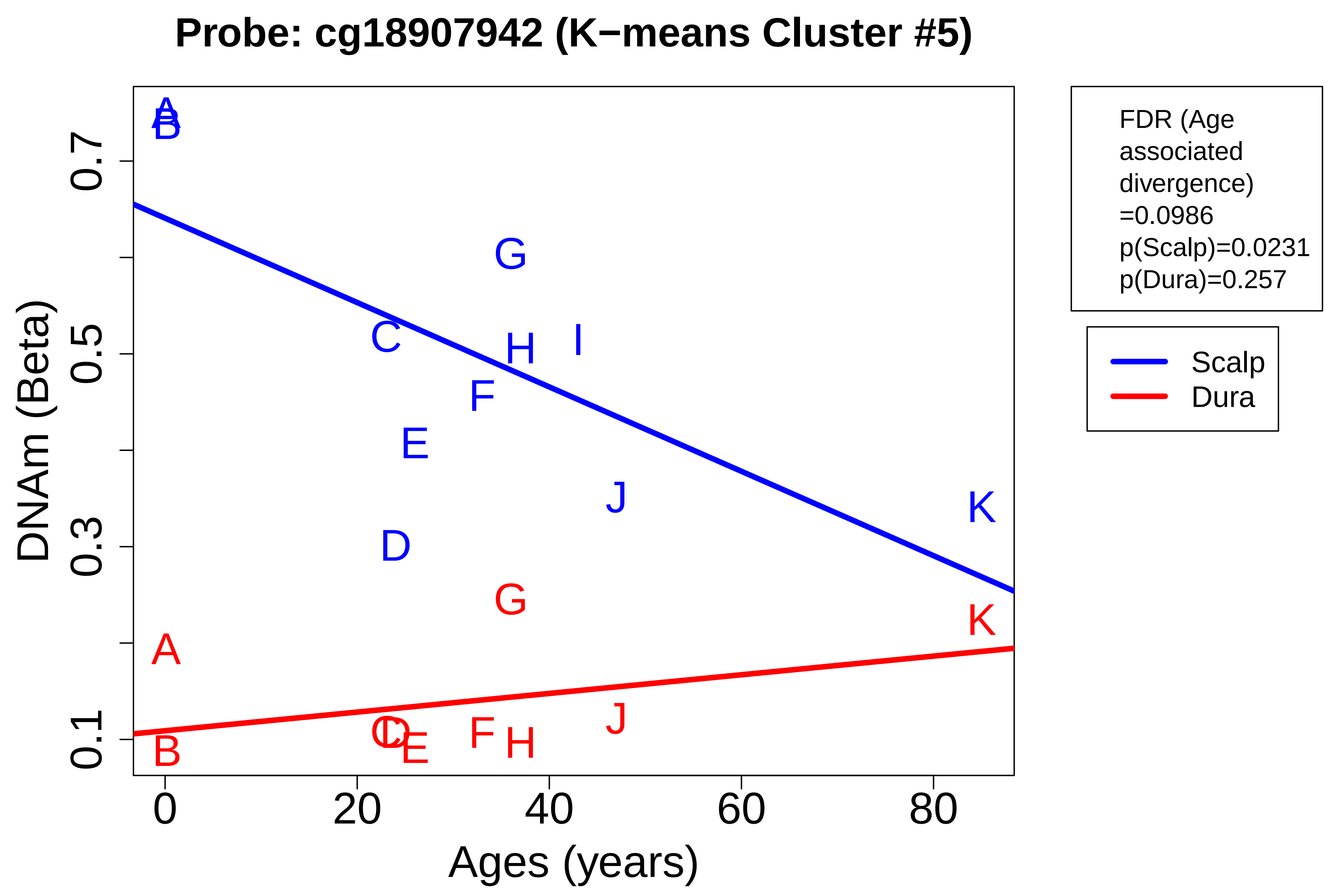

Probe: cg18907942 (K−means Cluster #5)
DNAm (Beta)
0.1
0.3
0.5
0.7
Ages (years)
0
20
40
60
80
A
B
C
D
E
F
G
H
I
J
K
FDR (Age associated divergence) =0.0986
p(Scalp)=0.0231
p(Dura)=0.257
Scalp
Dura

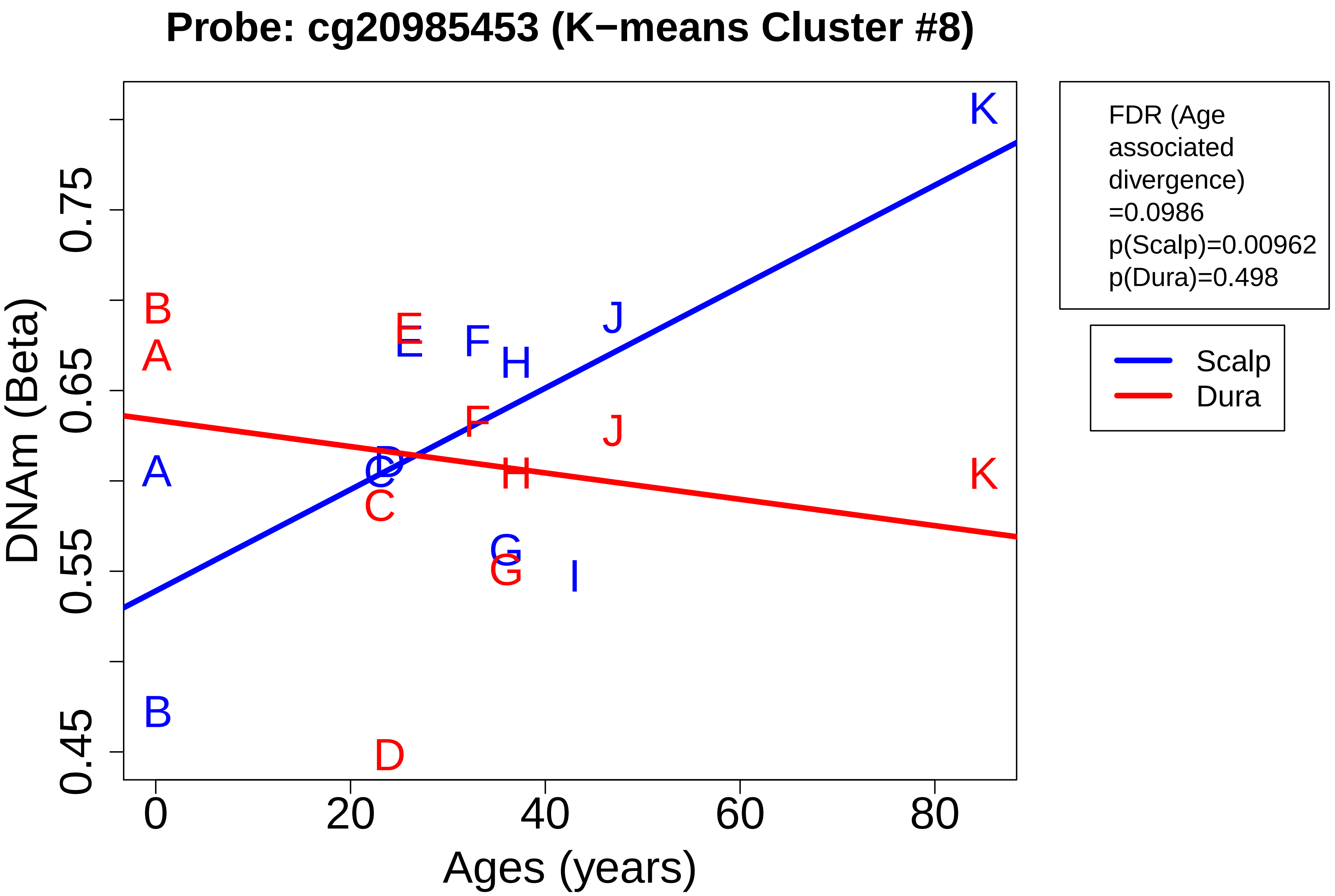

Probe: cg20985453 (K−means Cluster #8)
DNAm (Beta)
Ages (years)
0.45
0.55
0.65
0.75
0
20
40
60
80
FDR (Age associated divergence) =0.0986
p(Scalp)=0.00962
p(Dura)=0.498
Scalp
Dura

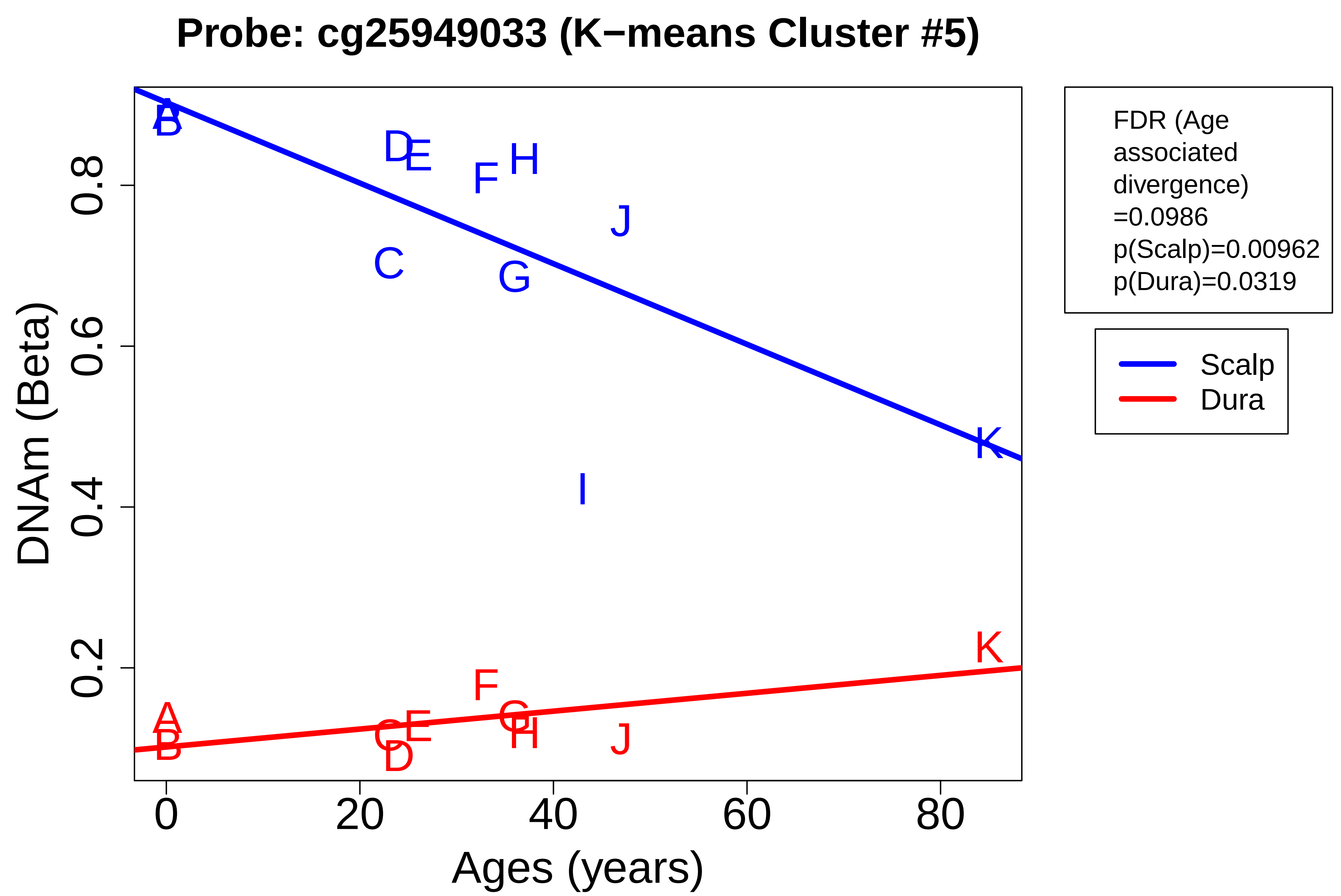

Probe: cg25949033 (K−means Cluster #5)
DNAm (Beta)
0.2
0.4
0.6
0.8
0
20
40
60
80
Ages (years)
A B C D E F G H I J K
FDR (Age associated divergence) =0.0986
p(Scalp)=0.00962
p(Dura)=0.0319
Scalp
Dura

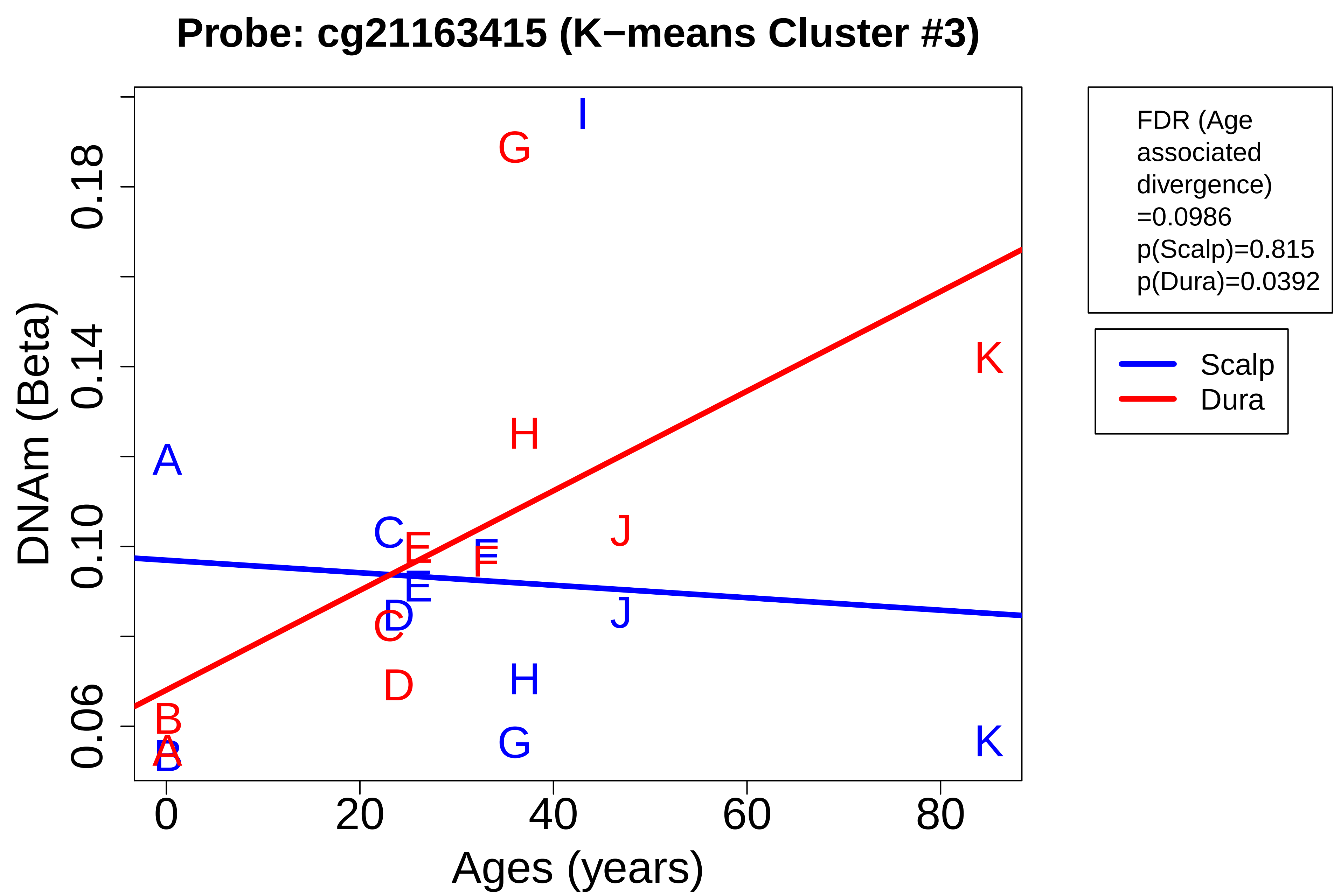

Probe: cg21163415 (K−means Cluster #3)
DNAm (Beta)
0.06
0.10
0.14
0.18
0
20
40
60
80
Ages (years)
FDR (Age associated divergence) =0.0986
p(Scalp)=0.815
p(Dura)=0.0392
Scalp
Dura
A
B
C
D
E
F
G
H
I
J
K

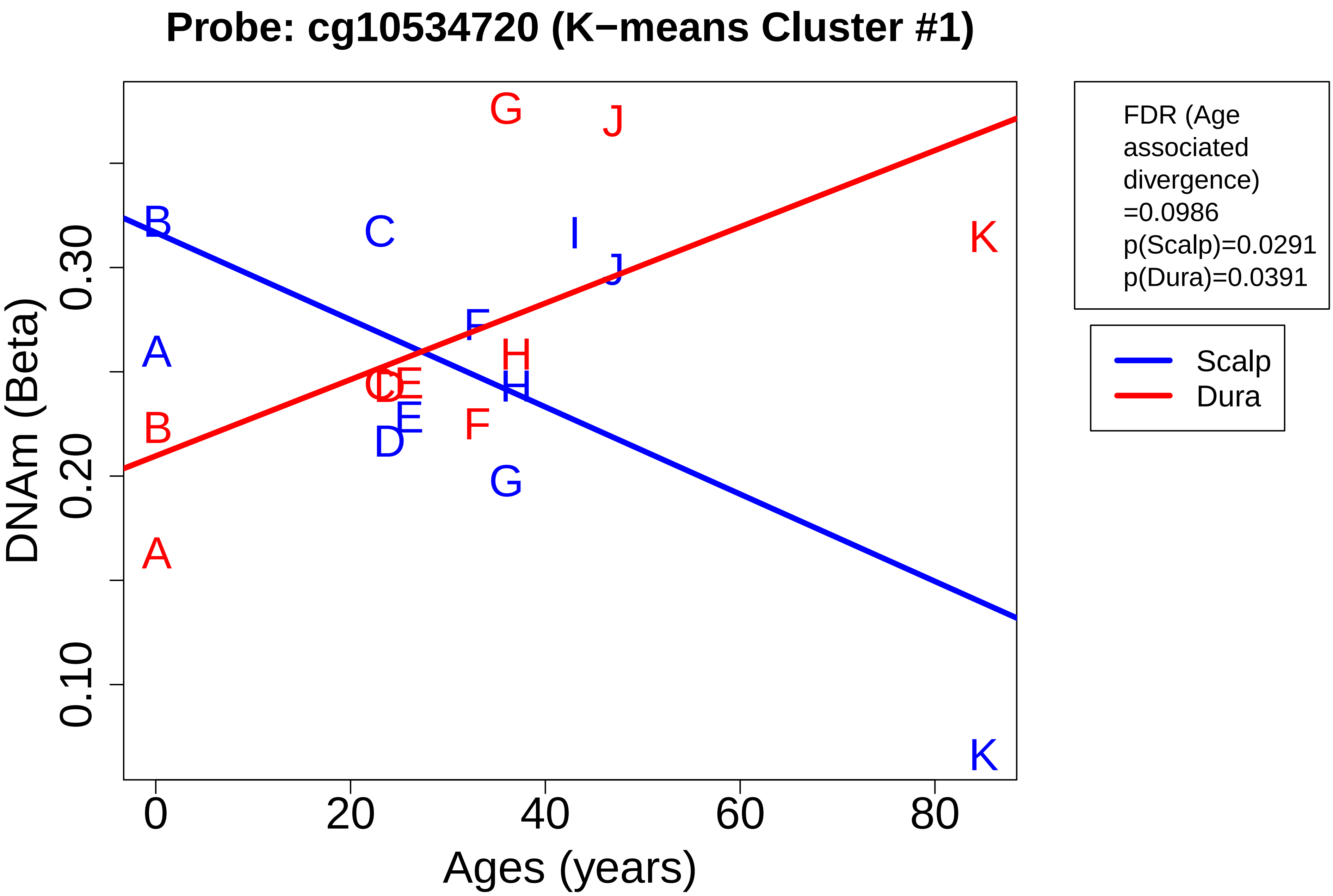

Probe: cg10534720 (K−means Cluster #1)
FDR (Age associated divergence) =0.0986
p(Scalp)=0.0291
p(Dura)=0.0391
Scalp
Dura
DNAm (Beta)
0.10
0.20
0.30
0
20
40
60
80
Ages (years)

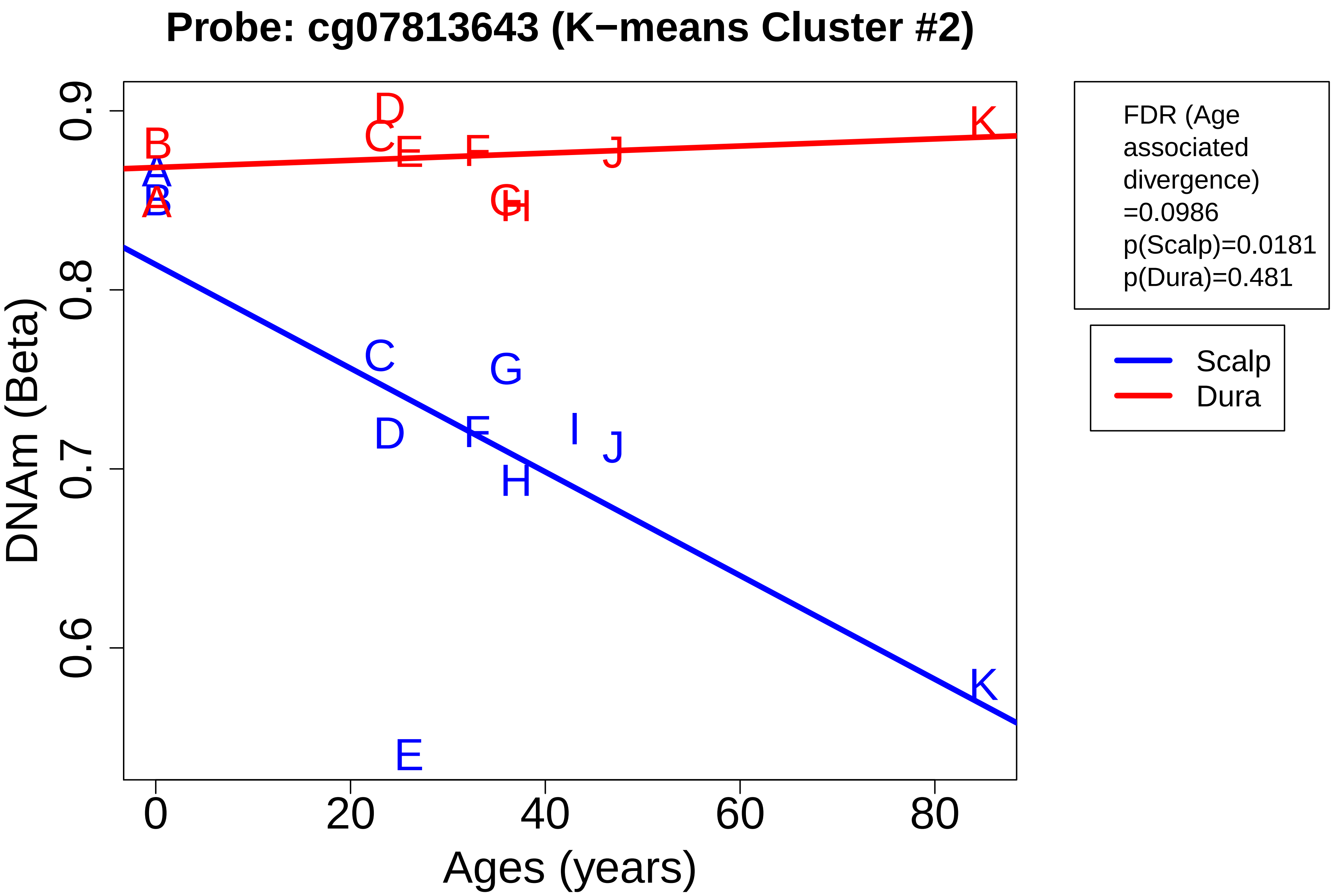

Probe: cg07813643 (K−means Cluster #2)
DNAm (Beta)
0.6
0.7
0.8
0.9
0
20
40
60
80
Ages (years)
A
B
C
D
E
F
G
H
I
J
K
FDR (Age associated divergence) =0.0986
p(Scalp)=0.0181
p(Dura)=0.481
Scalp
Dura

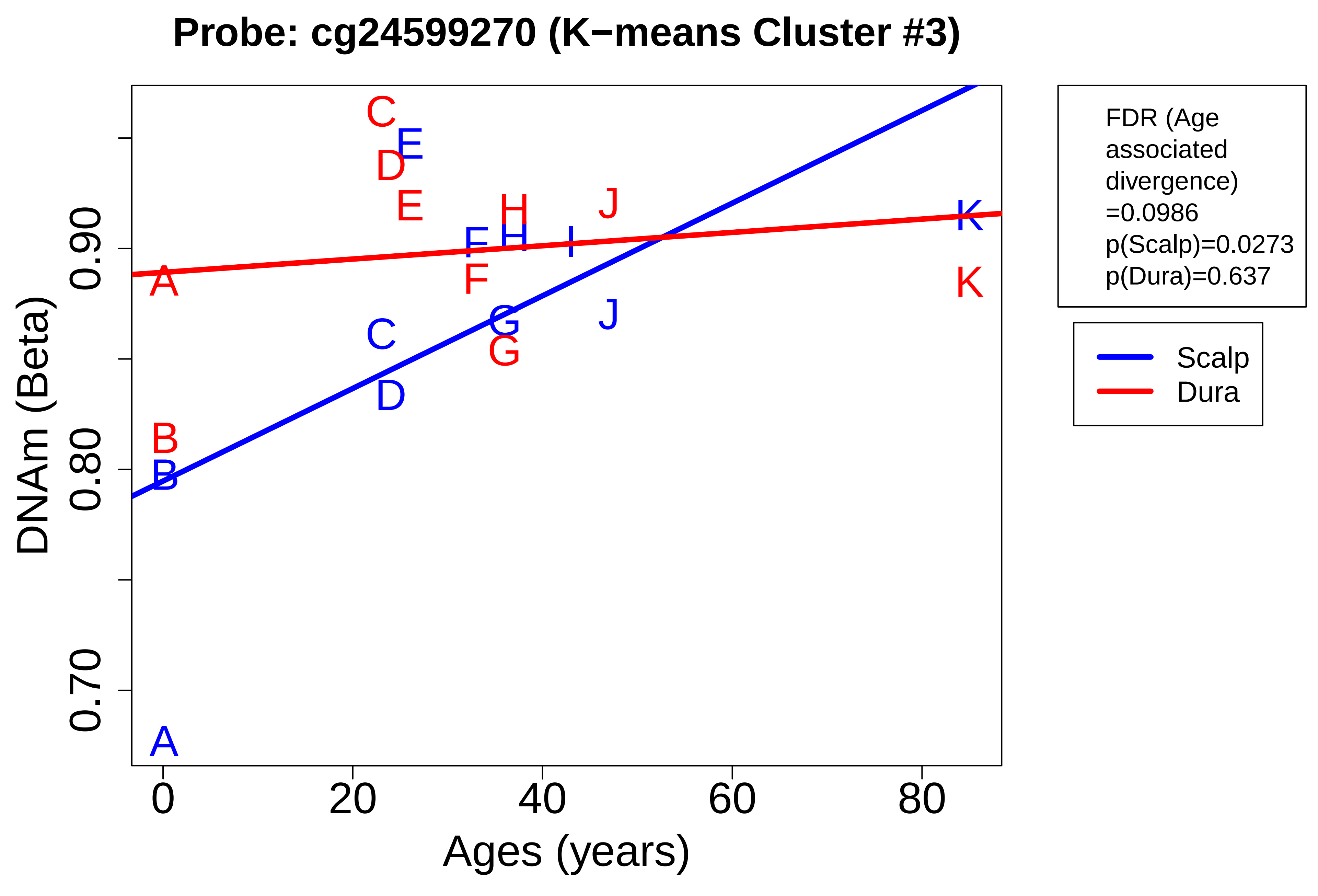

Probe: cg24599270 (K−means Cluster #3)
DNAm (Beta)
0.70
0.80
0.90
0
20
40
60
80
Ages (years)
FDR (Age associated divergence) =0.0986
p(Scalp)=0.0273
p(Dura)=0.637
Scalp
Dura

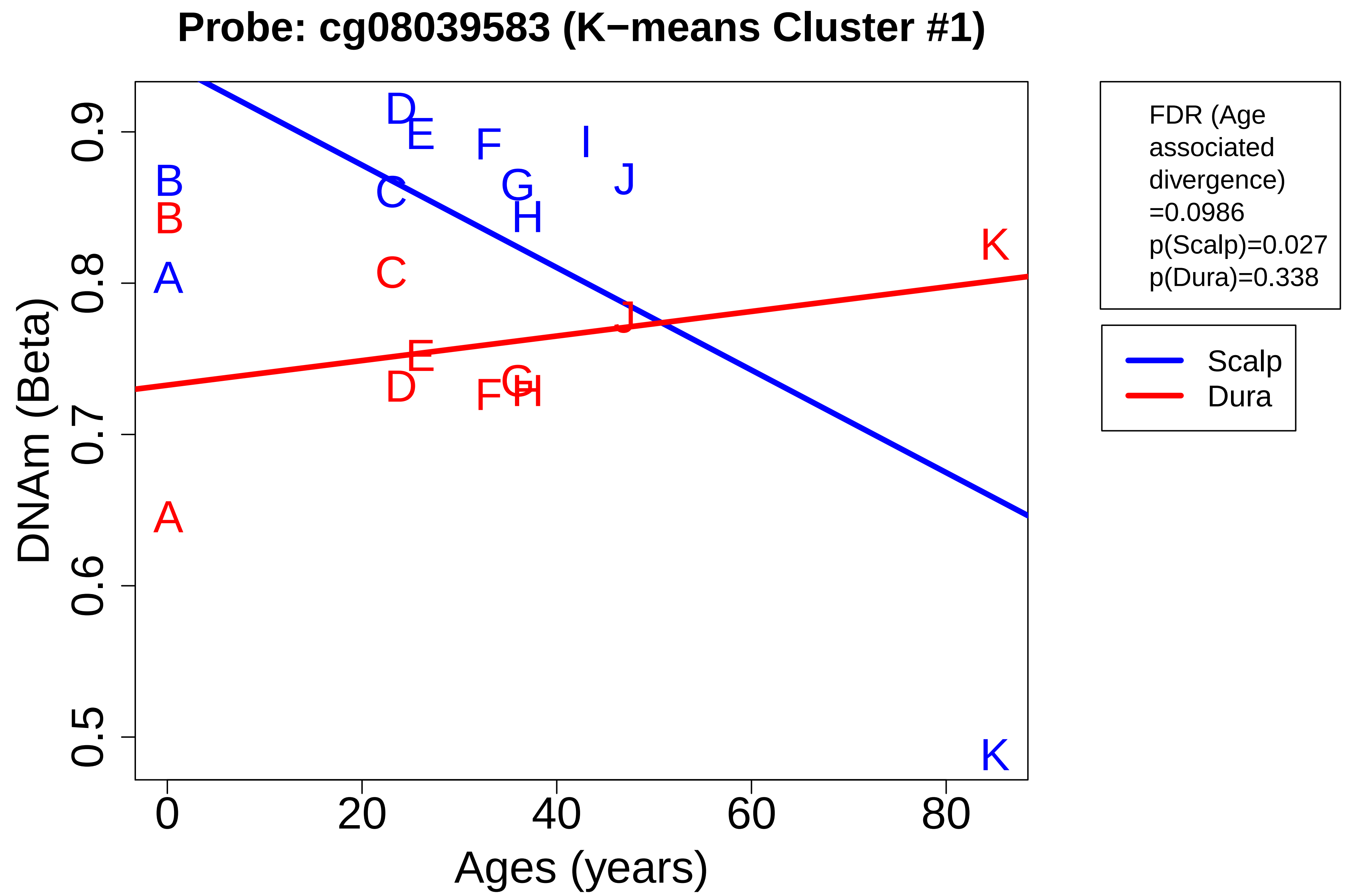

Probe: cg08039583 (K−means Cluster #1)
DNAm (Beta)
0.5
0.6
0.7
0.8
0.9
0
20
40
60
80
Ages (years)
A
B
C
D
E
F
G
H
I
J
K
FDR (Age associated divergence) =0.0986
p(Scalp)=0.027
p(Dura)=0.338
Scalp
Dura

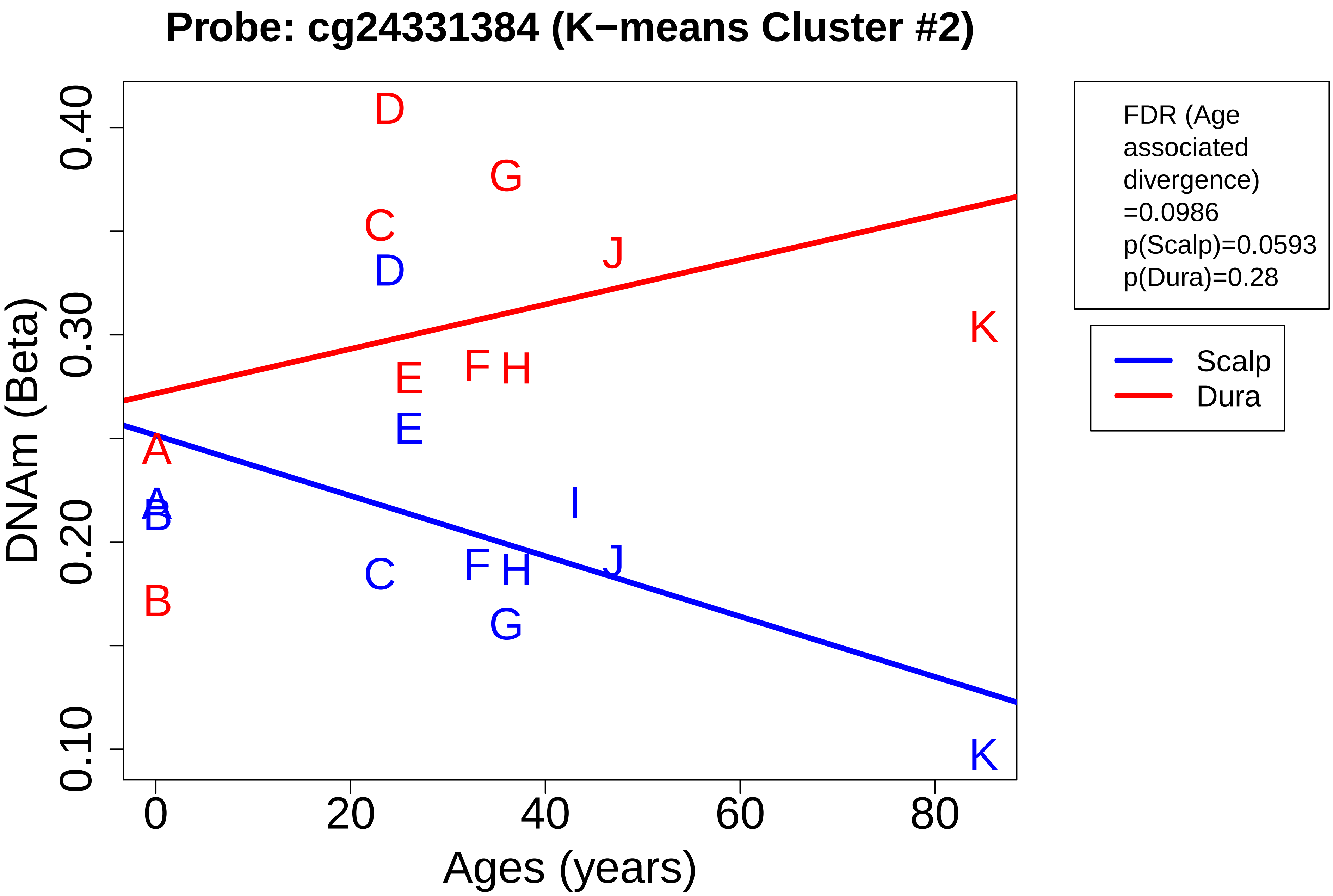

Probe: cg24331384 (K−means Cluster #2)
FDR (Age associated divergence) =0.0986
p(Scalp)=0.0593
p(Dura)=0.28
Scalp
Dura
DNAm (Beta)
0.10
0.20
0.30
0.40
Ages (years)
0
20
40
60
80

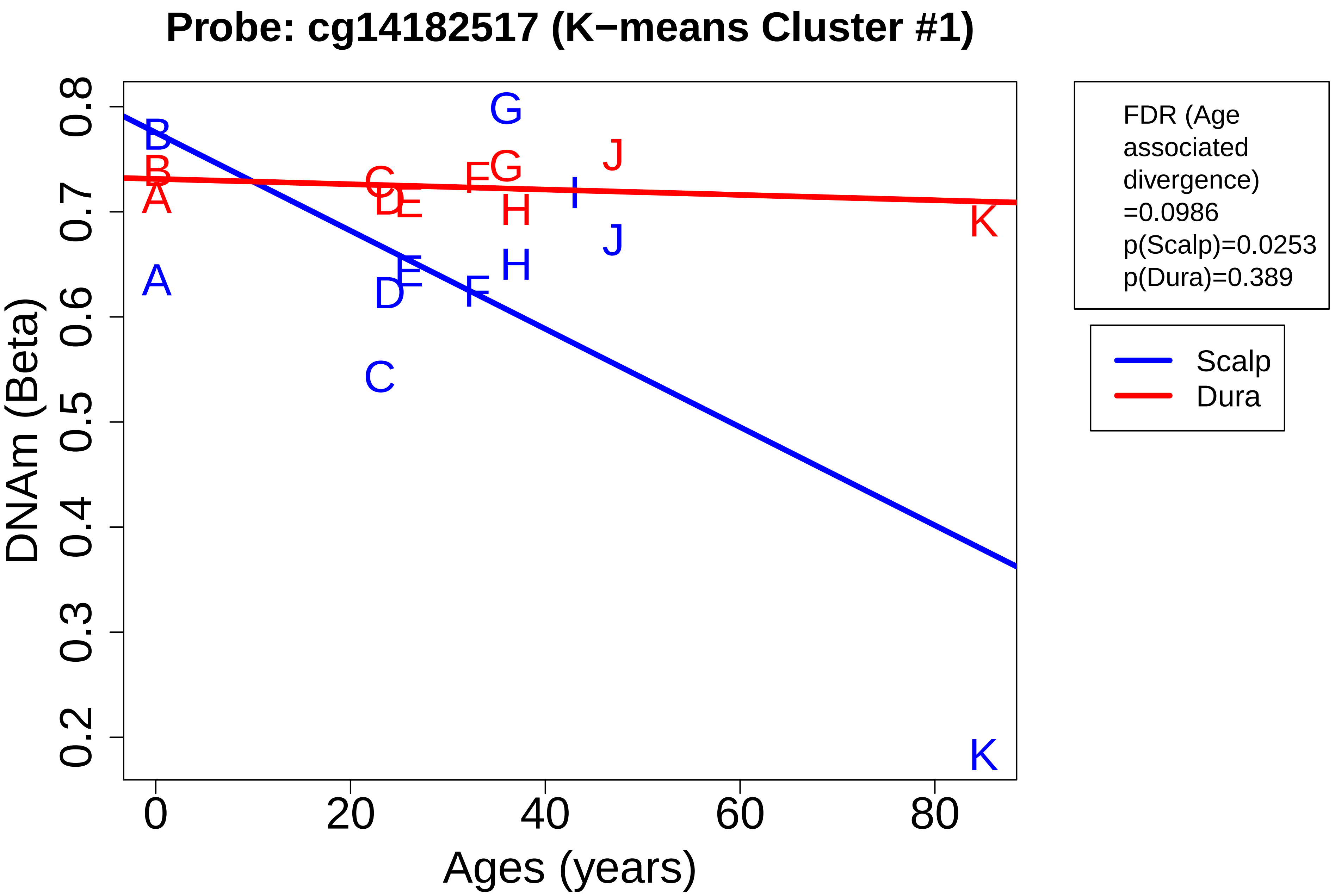

Probe: cg14182517 (K−means Cluster #1)
DNAm (Beta)
0.2
0.3
0.4
0.5
0.6
0.7
0.8
0
20
40
60
80
Ages (years)
A
B
C
D
E
F
G
H
I
J
K
FDR (Age associated divergence) =0.0986
p(Scalp)=0.0253
p(Dura)=0.389
Scalp
Dura

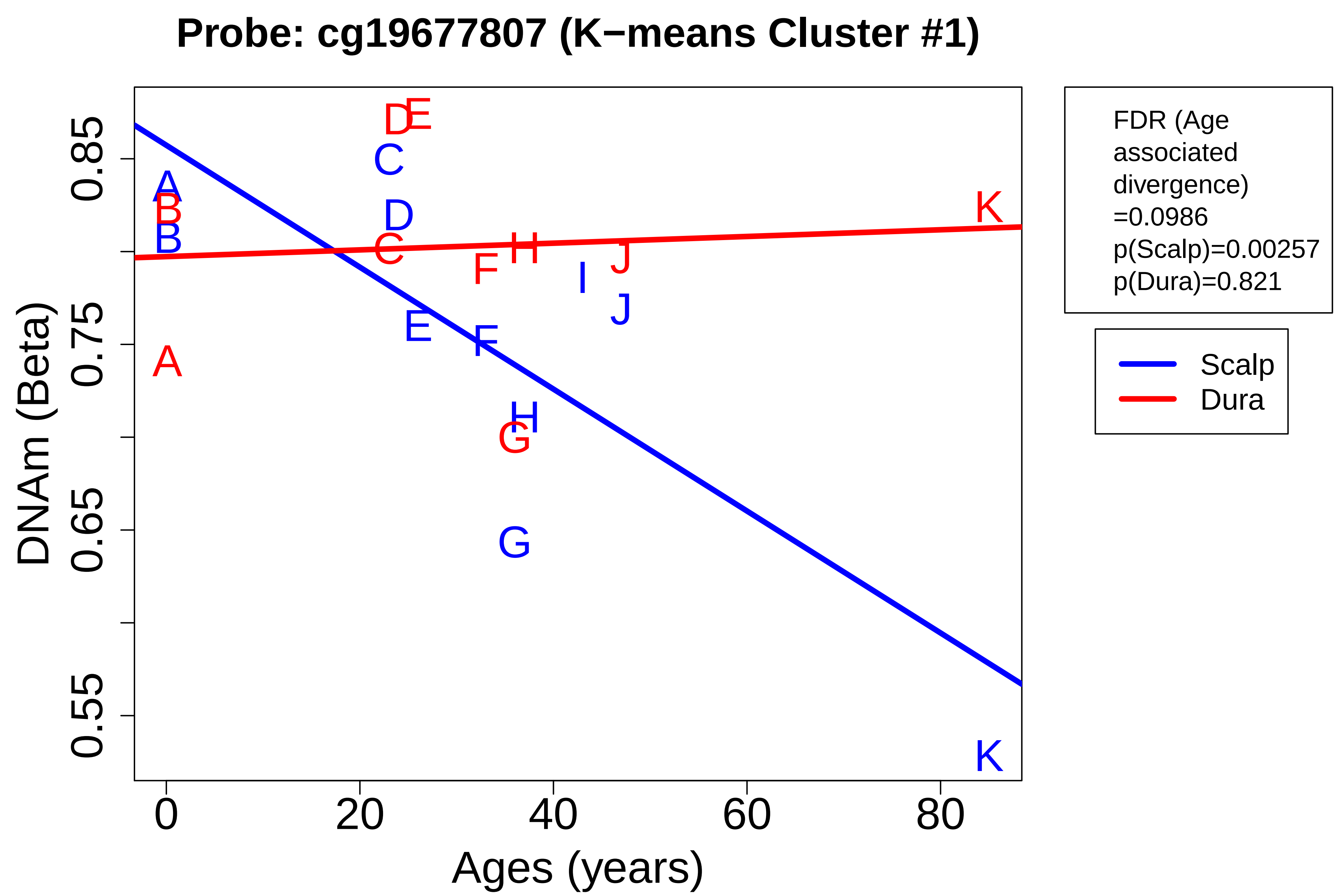

Probe: cg19677807 (K−means Cluster #1)
DNAm (Beta)
0.55
0.65
0.75
0.85
0
20
40
60
80
Ages (years)
A
B
C
D
E
F
G
H
I
J
K
FDR (Age associated divergence) =0.0986
p(Scalp)=0.00257
p(Dura)=0.821
Scalp
Dura

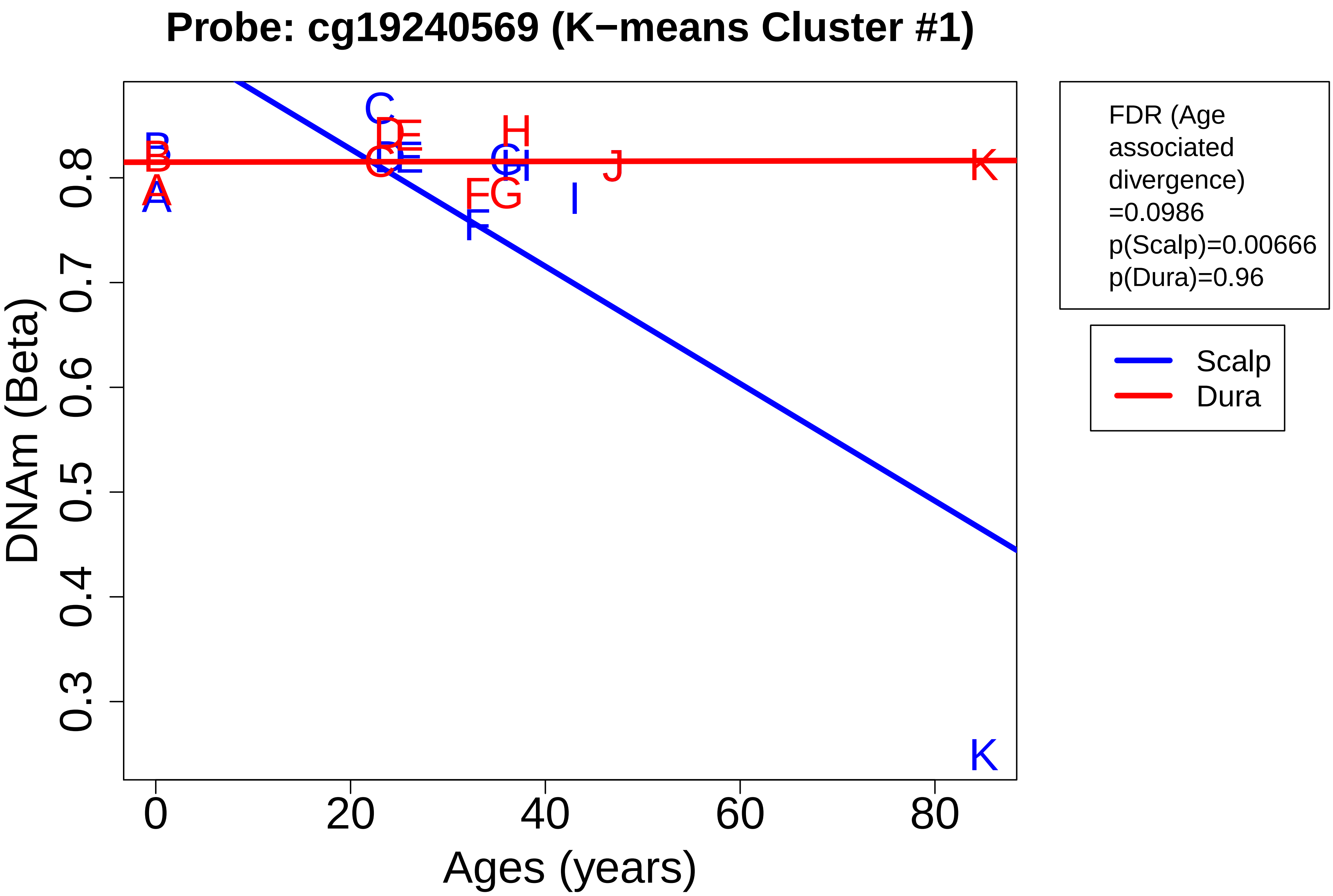

Probe: cg19240569 (K−means Cluster #1)
DNAm (Beta)
0.3
0.4
0.5
0.6
0.7
0.8
0
20
40
60
80
Ages (years)
FDR (Age associated divergence) =0.0986
p(Scalp)=0.00666
p(Dura)=0.96
Scalp
Dura

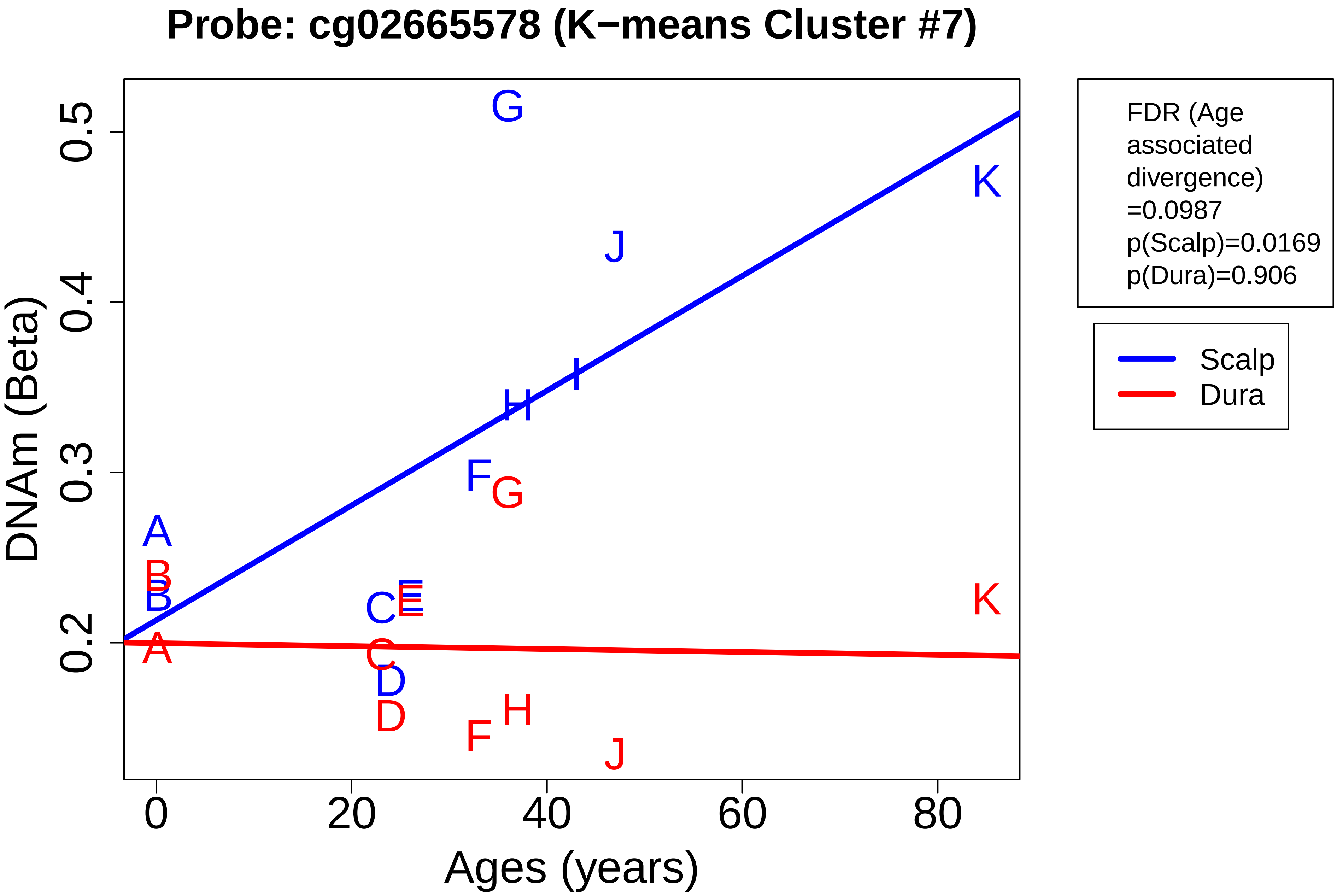

Probe: cg02665578 (K−means Cluster #7)
FDR (Age associated divergence) =0.0987
p(Scalp)=0.0169
p(Dura)=0.906
Scalp
Dura
DNAm (Beta)
0.2
0.3
0.4
0.5
Ages (years)
0
20
40
60
80

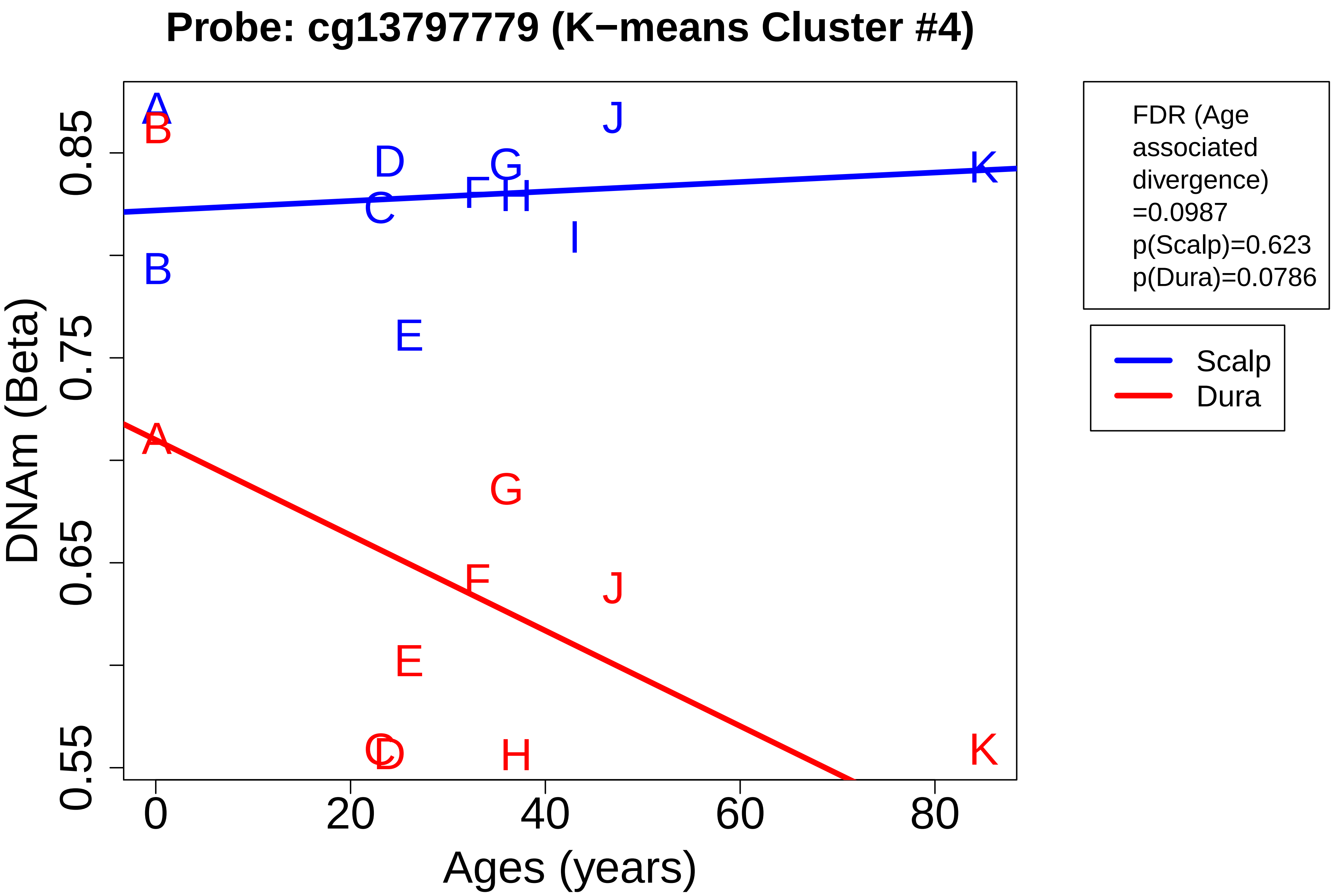
Probe: cg13797779 (K−means Cluster #4)
DNAm (Beta)
0.55
0.65
0.75
0.85
Ages (years)
0
20
40
60
80
FDR (Age associated divergence) =0.0987
p(Scalp)=0.623
p(Dura)=0.0786
Scalp
Dura
A
B
C
D
E
F
G
H
I
J
K

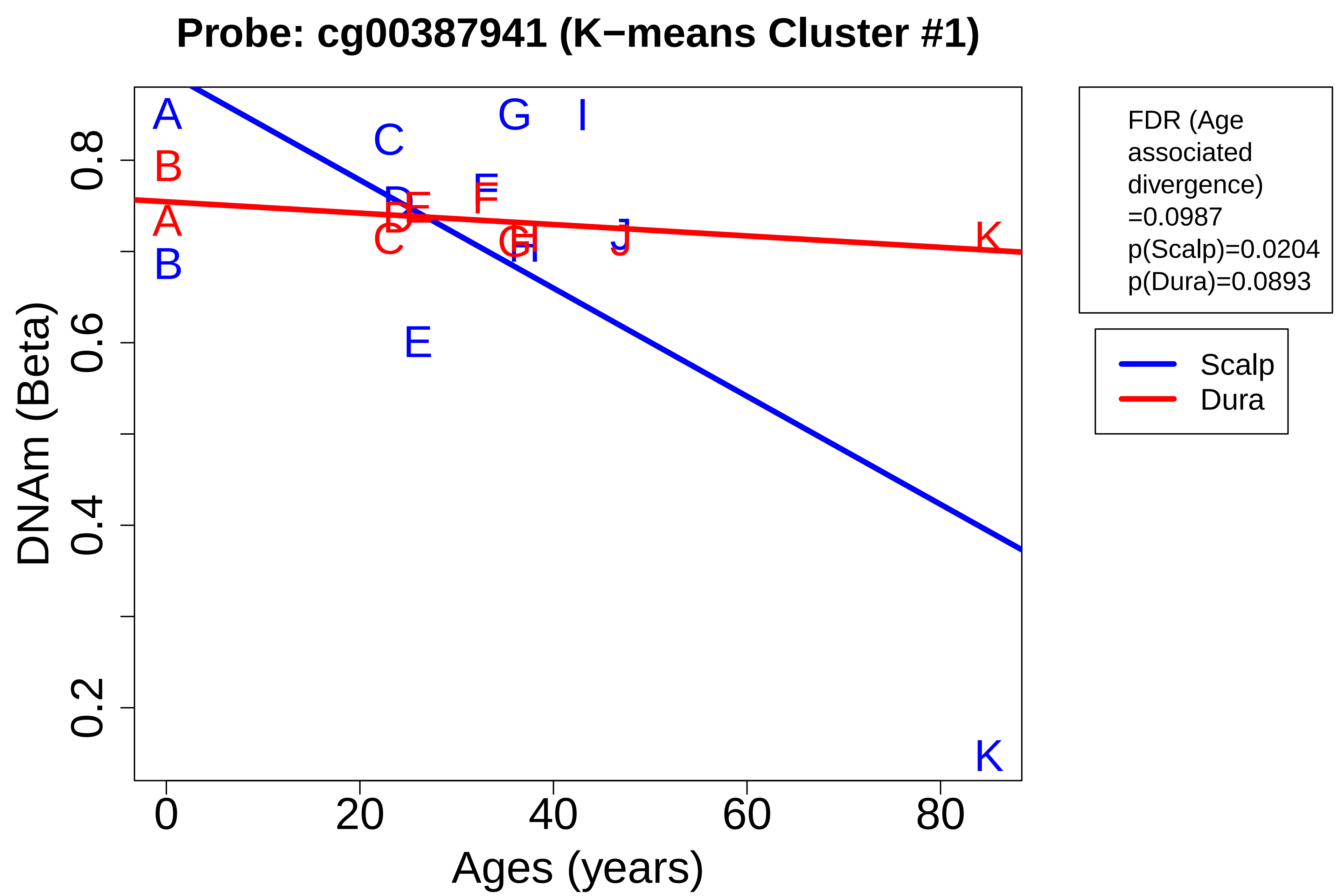

Probe: cg00387941 (K−means Cluster #1)
DNAm (Beta)
0.2
0.4
0.6
0.8
0
20
40
60
80
Ages (years)
A
B
C
D
E
F
G
H
I
J
K
FDR (Age associated divergence) =0.0987
p(Scalp)=0.0204
p(Dura)=0.0893
Scalp
Dura

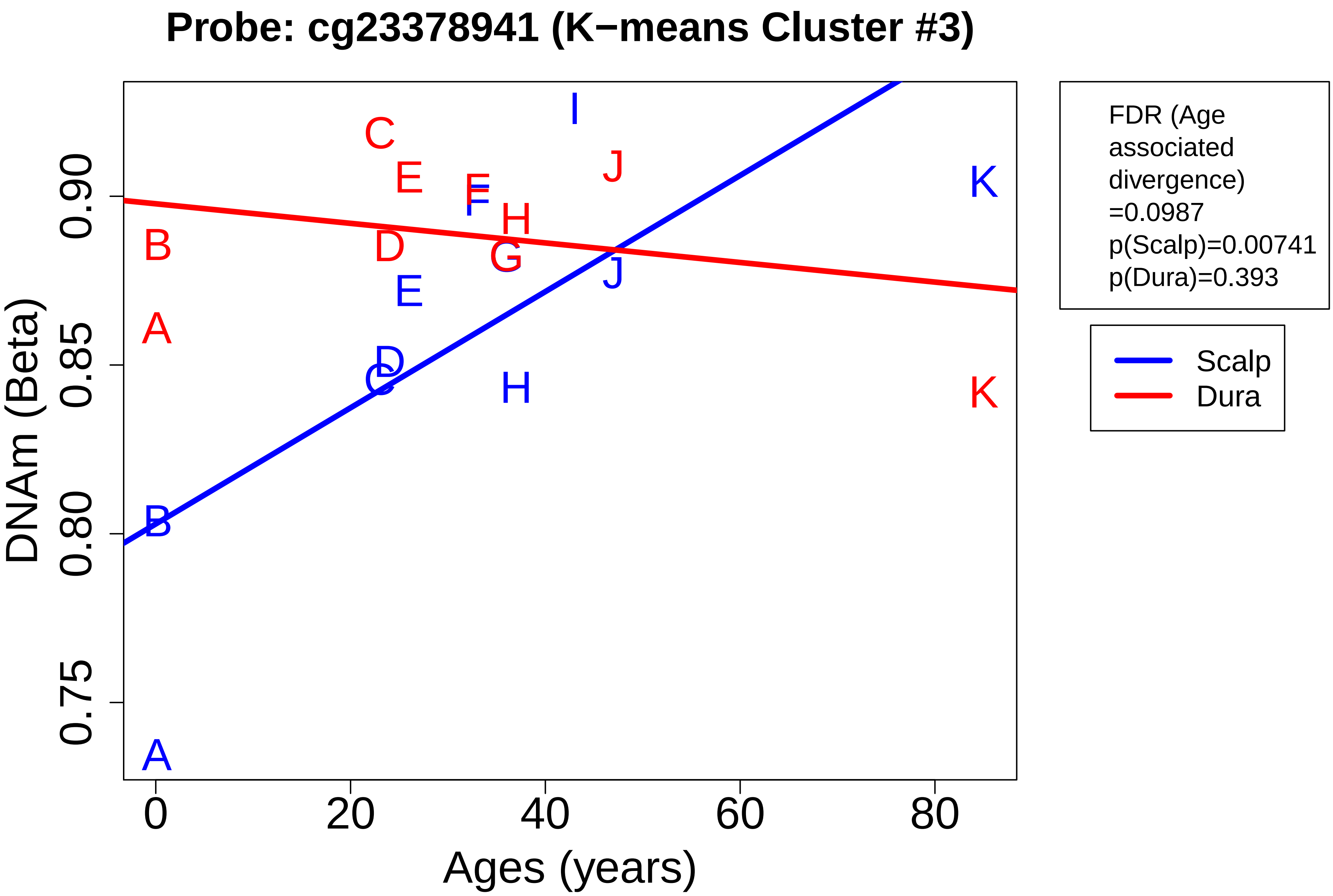

Probe: cg23378941 (K−means Cluster #3)
DNAm (Beta)
0.75
0.80
0.85
0.90
Ages (years)
0
20
40
60
80
FDR (Age associated divergence) =0.0987
p(Scalp)=0.00741
p(Dura)=0.393
Scalp
Dura

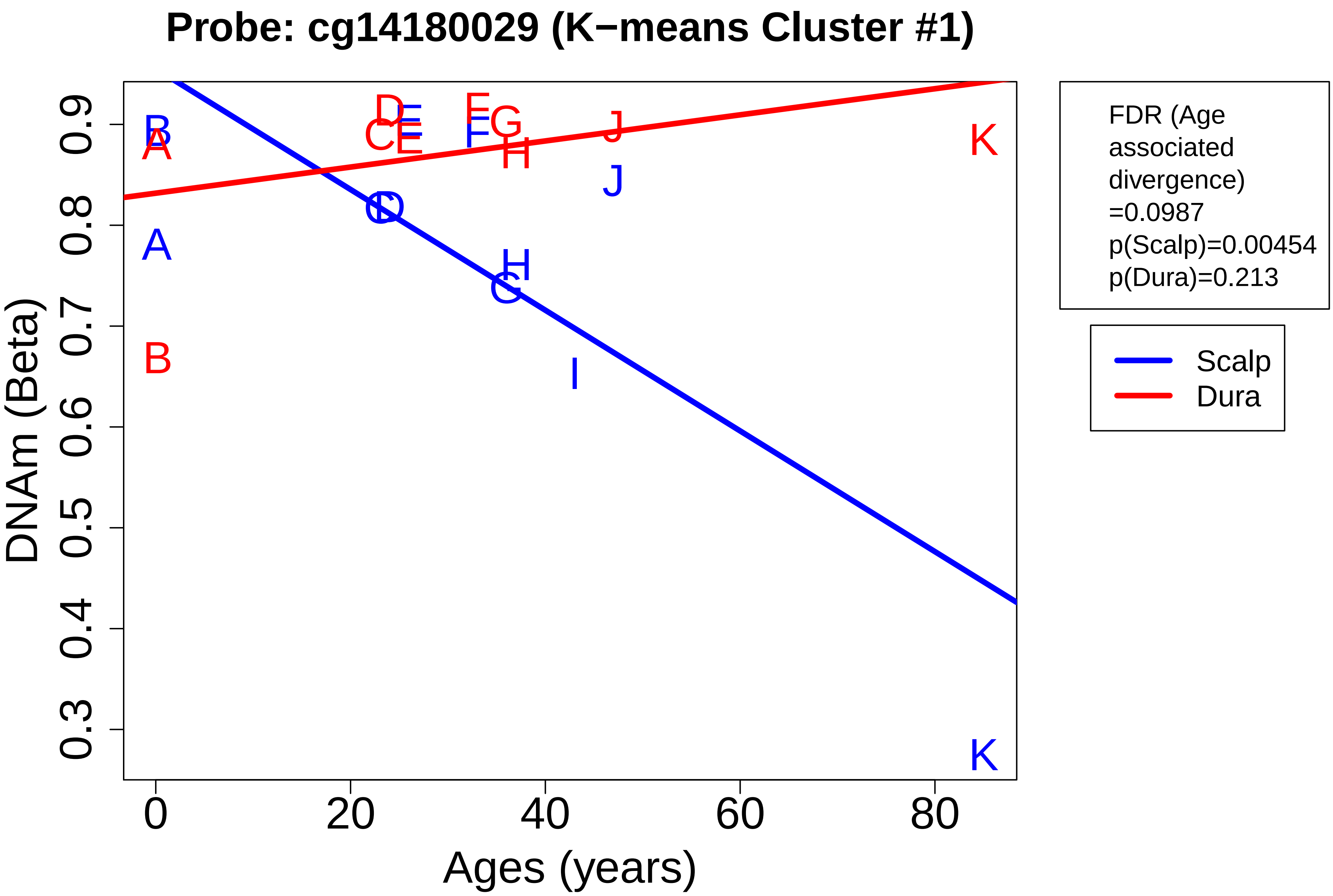
Probe: cg14180029 (K−means Cluster #1)
DNAm (Beta)
Ages (years)
0.3
0.4
0.5
0.6
0.7
0.8
0.9
0
20
40
60
80
FDR (Age associated divergence) =0.0987
p(Scalp)=0.00454
p(Dura)=0.213
Scalp
Dura

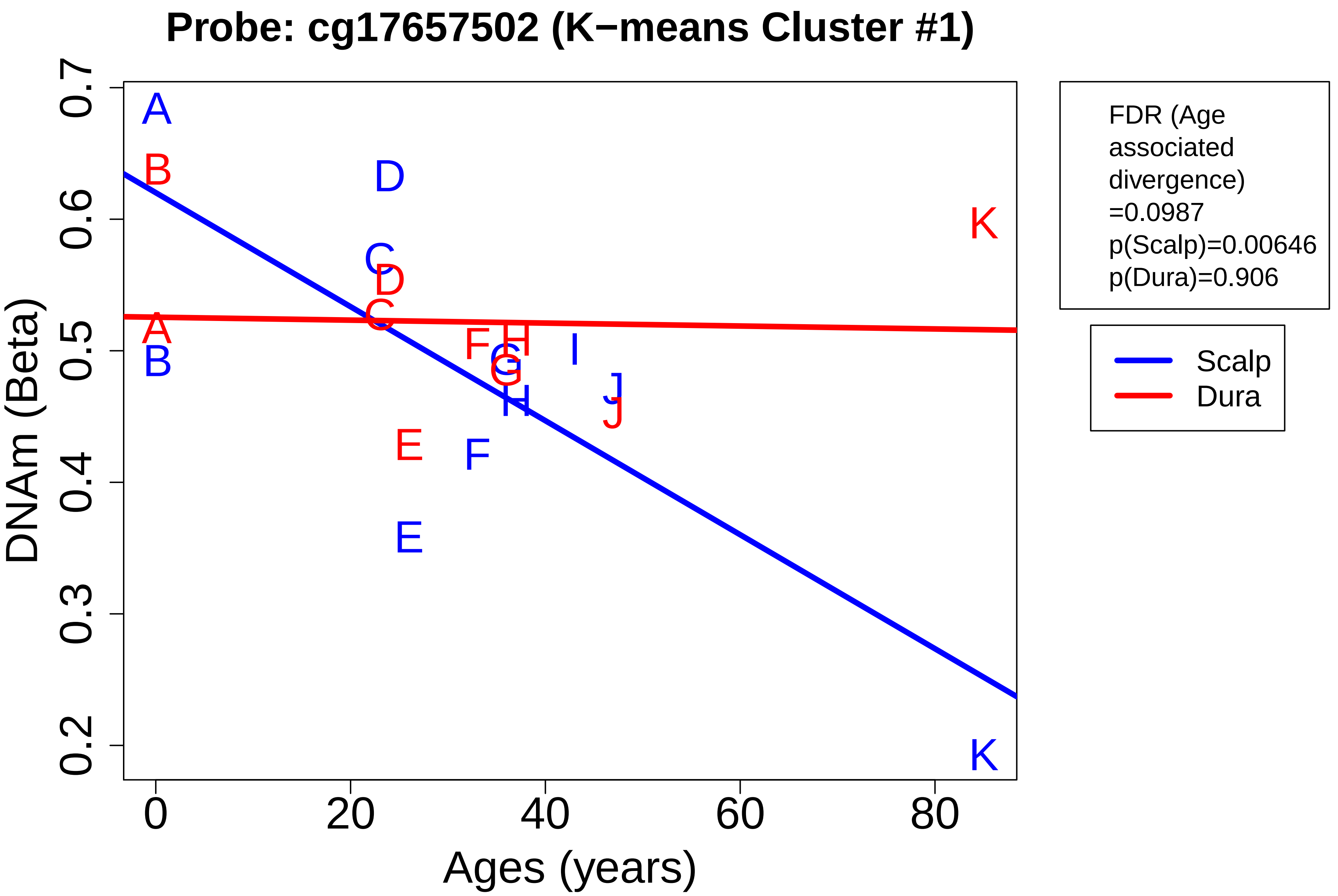
Probe: cg17657502 (K−means Cluster #1)
DNAm (Beta)
0.2
0.3
0.4
0.5
0.6
0.7
Ages (years)
0
20
40
60
80
A
B
C
D
E
F
G
H
I
J
K
FDR (Age associated divergence) =0.0987
p(Scalp)=0.00646
p(Dura)=0.906
Scalp
Dura

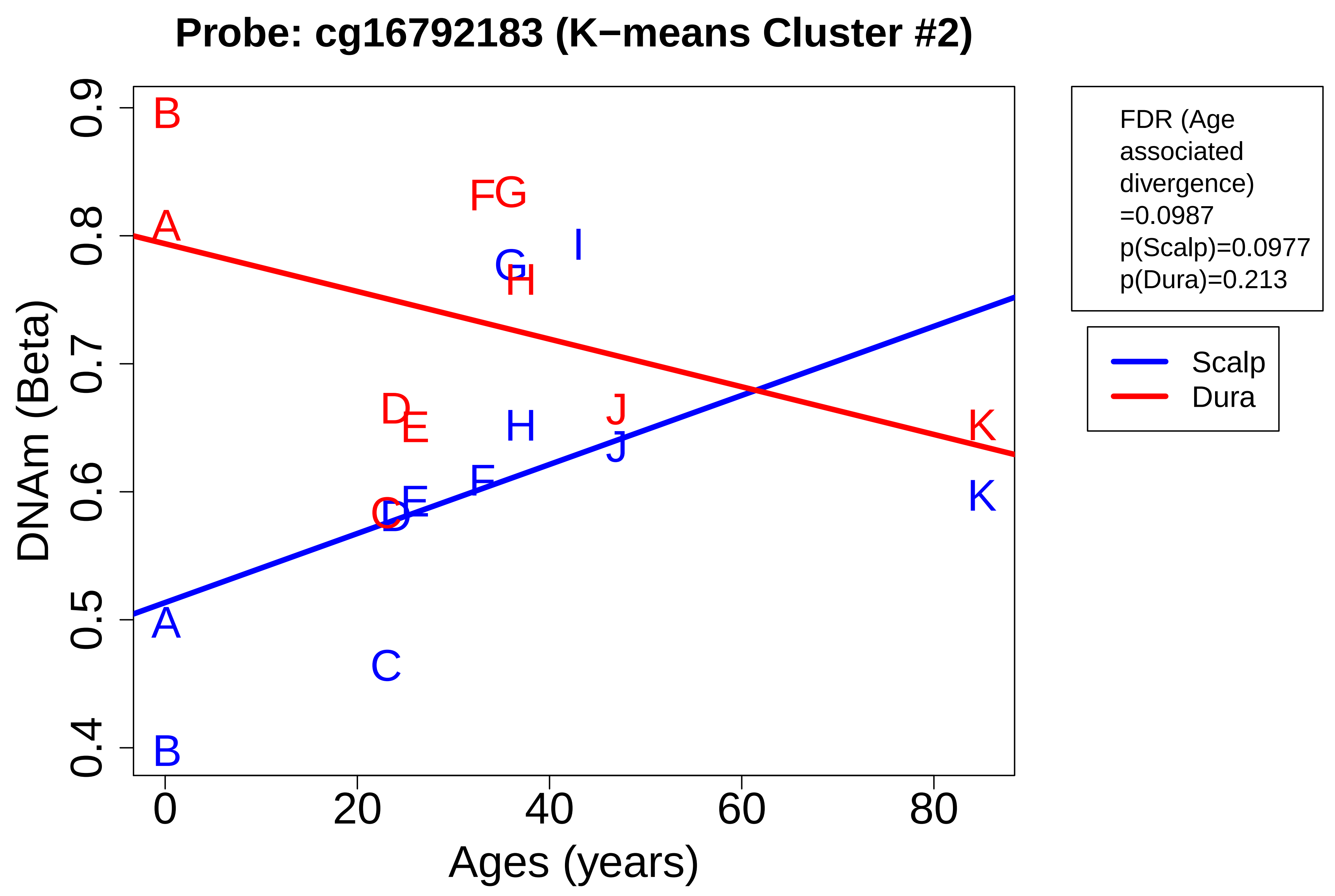

Probe: cg16792183 (K−means Cluster #2)
DNAm (Beta)
Ages (years)
0.4
0.5
0.6
0.7
0.8
0.9
0
20
40
60
80
FDR (Age associated divergence) =0.0987
p(Scalp)=0.0977
p(Dura)=0.213
Scalp
Dura

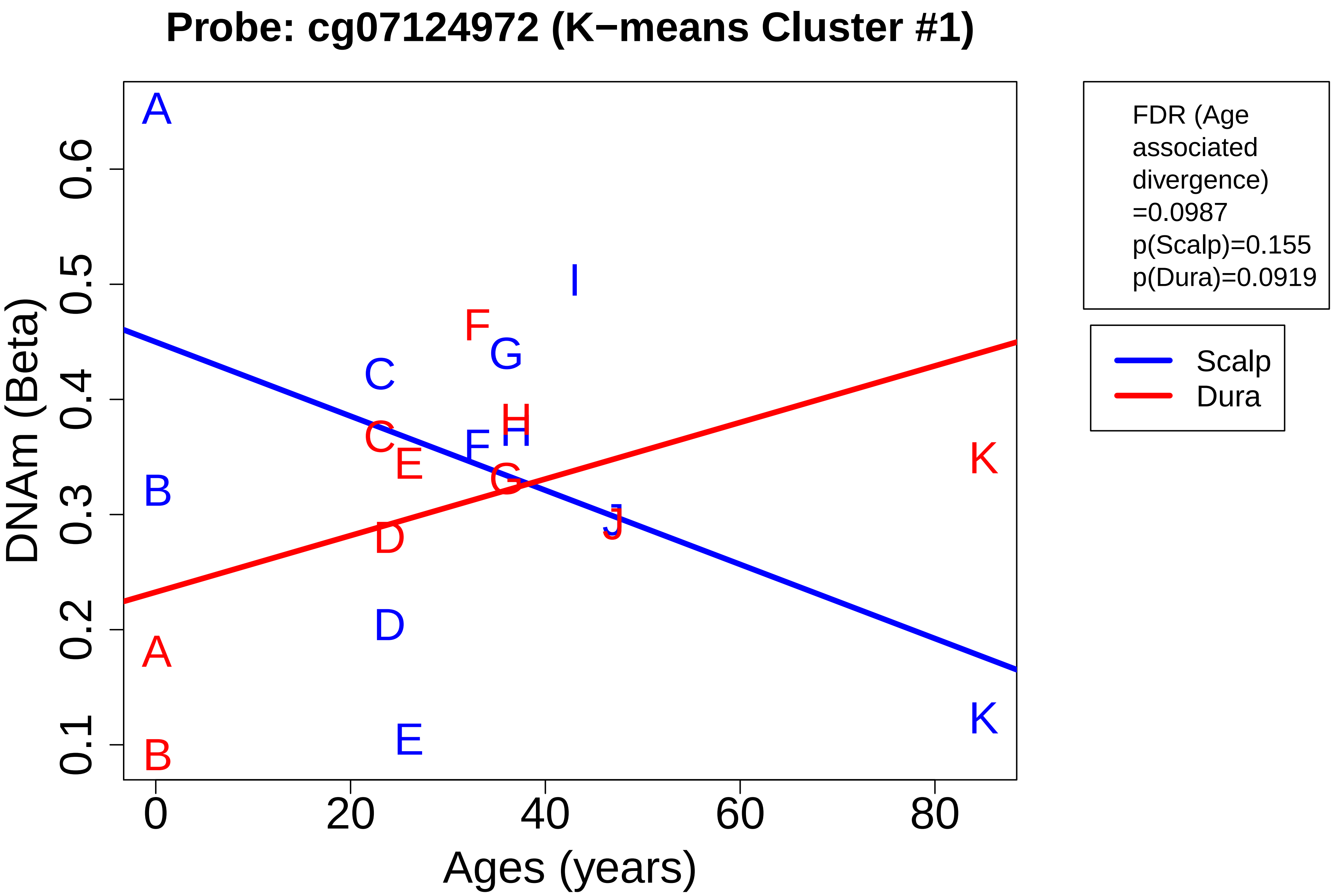

Probe: cg07124972 (K−means Cluster #1)
DNAm (Beta)
Ages (years)
0.1
0.2
0.3
0.4
0.5
0.6
0
20
40
60
80
FDR (Age associated divergence) =0.0987
p(Scalp)=0.155
p(Dura)=0.0919
Scalp
Dura

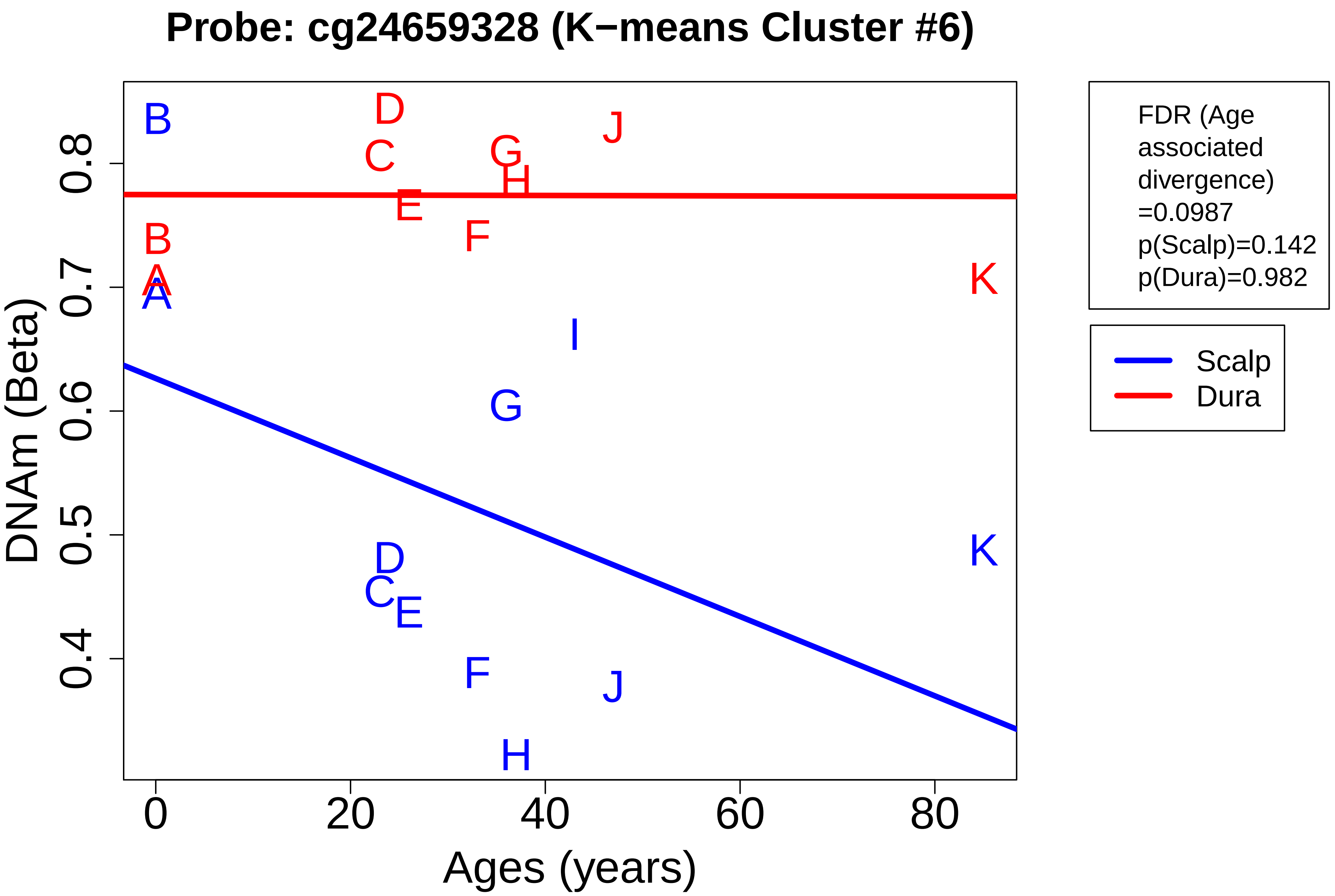

Probe: cg24659328 (K−means Cluster #6)
DNAm (Beta)
0.4
0.5
0.6
0.7
0.8
0
20
40
60
80
Ages (years)
FDR (Age associated divergence) =0.0987
p(Scalp)=0.142
p(Dura)=0.982
Scalp
Dura

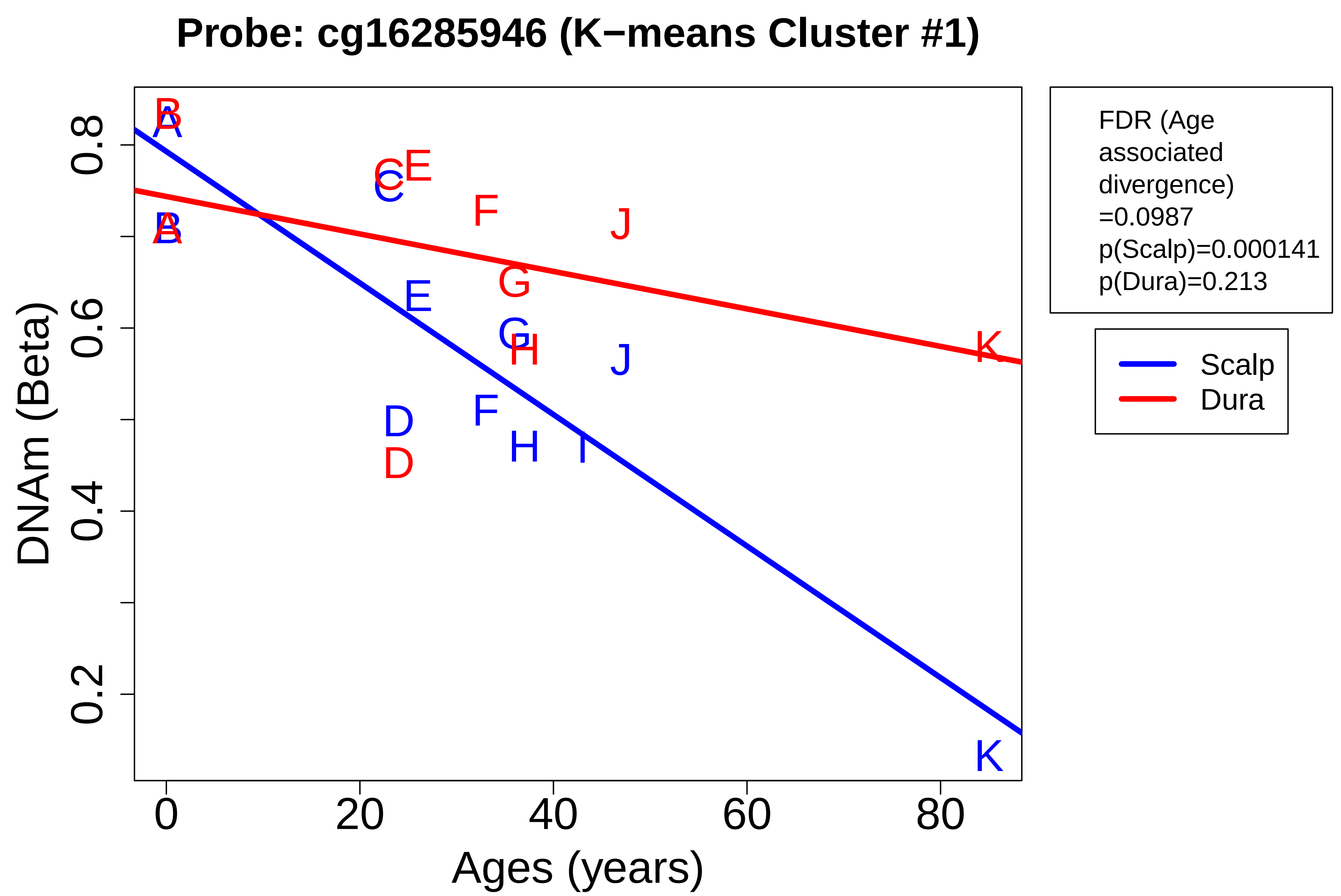

Probe: cg16285946 (K−means Cluster #1)
DNAm (Beta)
0.2
0.4
0.6
0.8
0
20
40
60
80
Ages (years)
FDR (Age associated divergence) =0.0987
p(Scalp)=0.000141
p(Dura)=0.213
Scalp
Dura

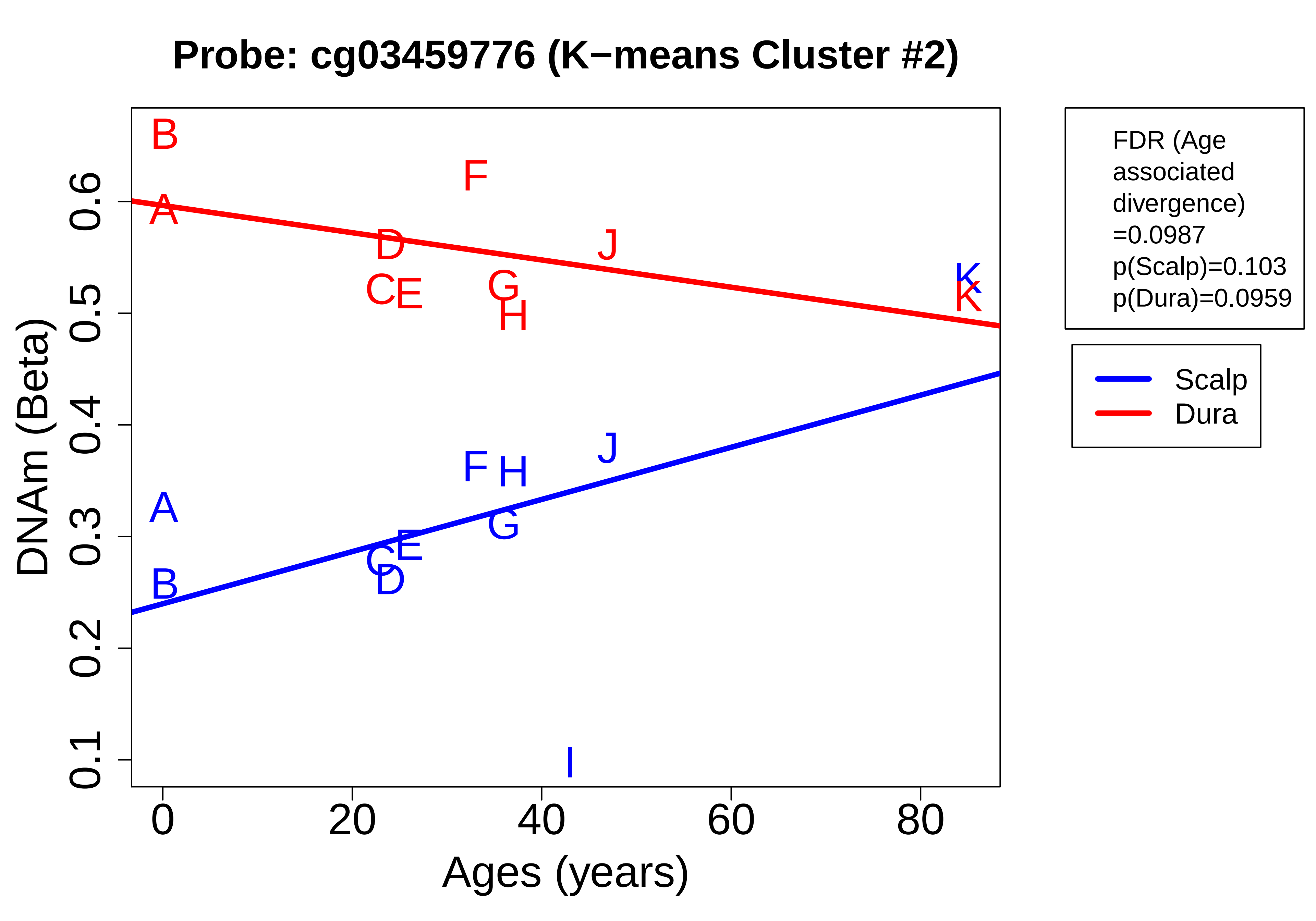

Probe: cg03459776 (K−means Cluster #2)
DNAm (Beta)
Ages (years)
0.1
0.2
0.3
0.4
0.5
0.6
0
20
40
60
80
FDR (Age associated divergence) =0.0987
p(Scalp)=0.103
p(Dura)=0.0959
Scalp
Dura

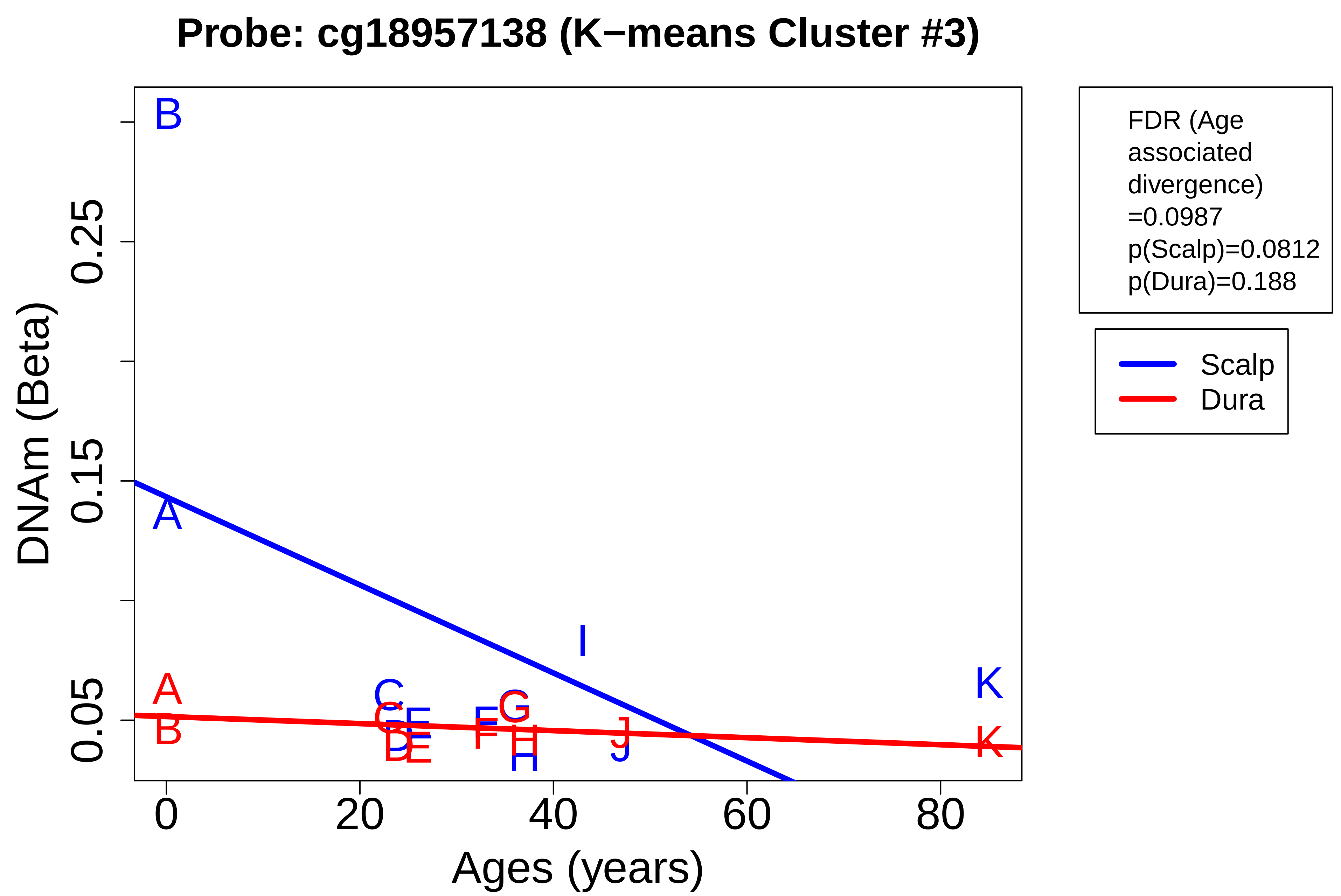
Probe: cg18957138 (K−means Cluster #3)
DNAm (Beta)
0.05
0.15
0.25
0
20
40
60
80
Ages (years)
FDR (Age associated divergence) =0.0987
p(Scalp)=0.0812
p(Dura)=0.188
Scalp
Dura
A
B
C
D
E
F
G
H
I
J
K

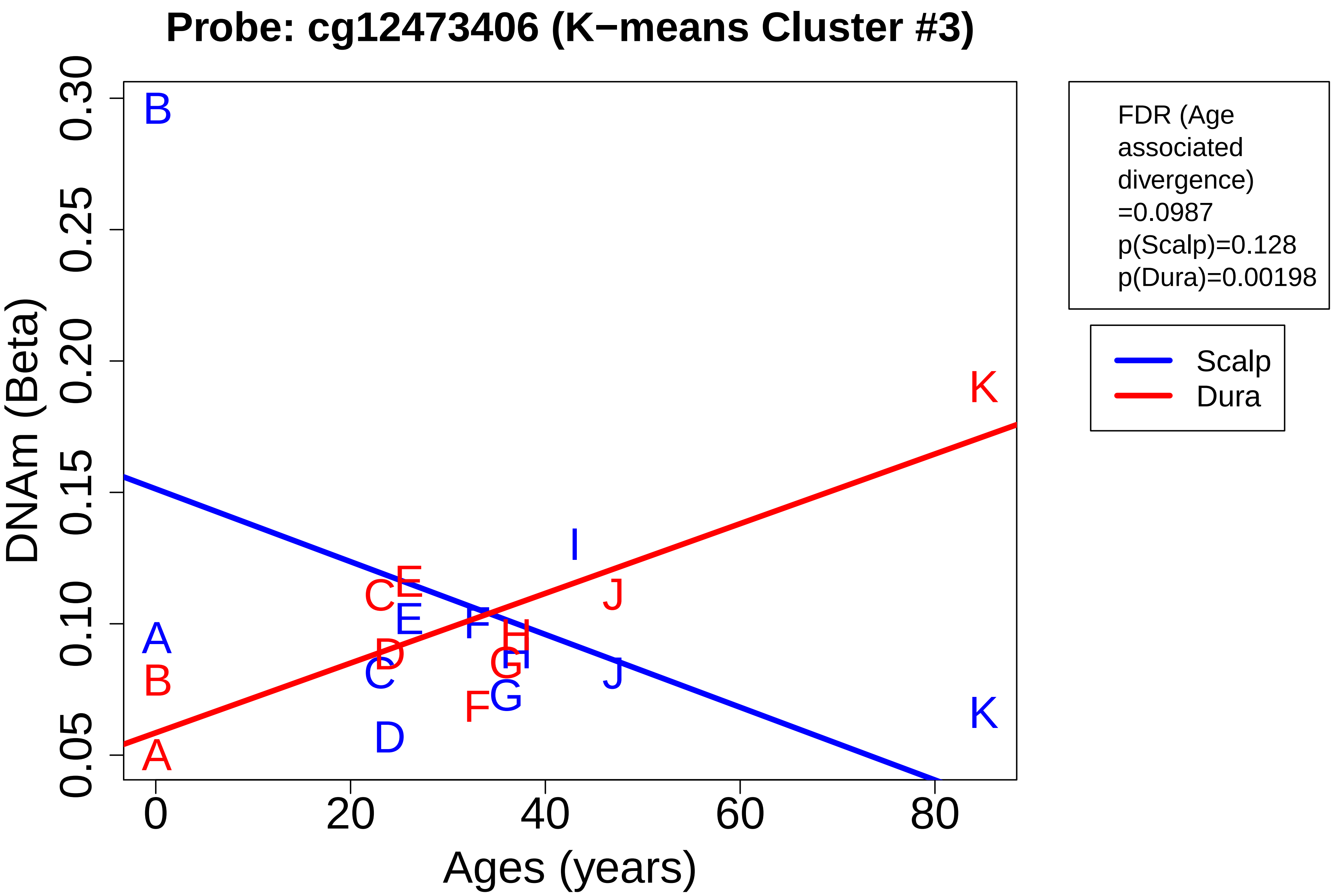
Probe: cg12473406 (K−means Cluster #3)
DNAm (Beta)
0.05
0.10
0.15
0.20
0.25
0.30
0
20
40
60
80
Ages (years)
A
B
C
D
E
F
G
H
I
J
K
FDR (Age associated divergence) =0.0987
p(Scalp)=0.128
p(Dura)=0.00198
Scalp
Dura

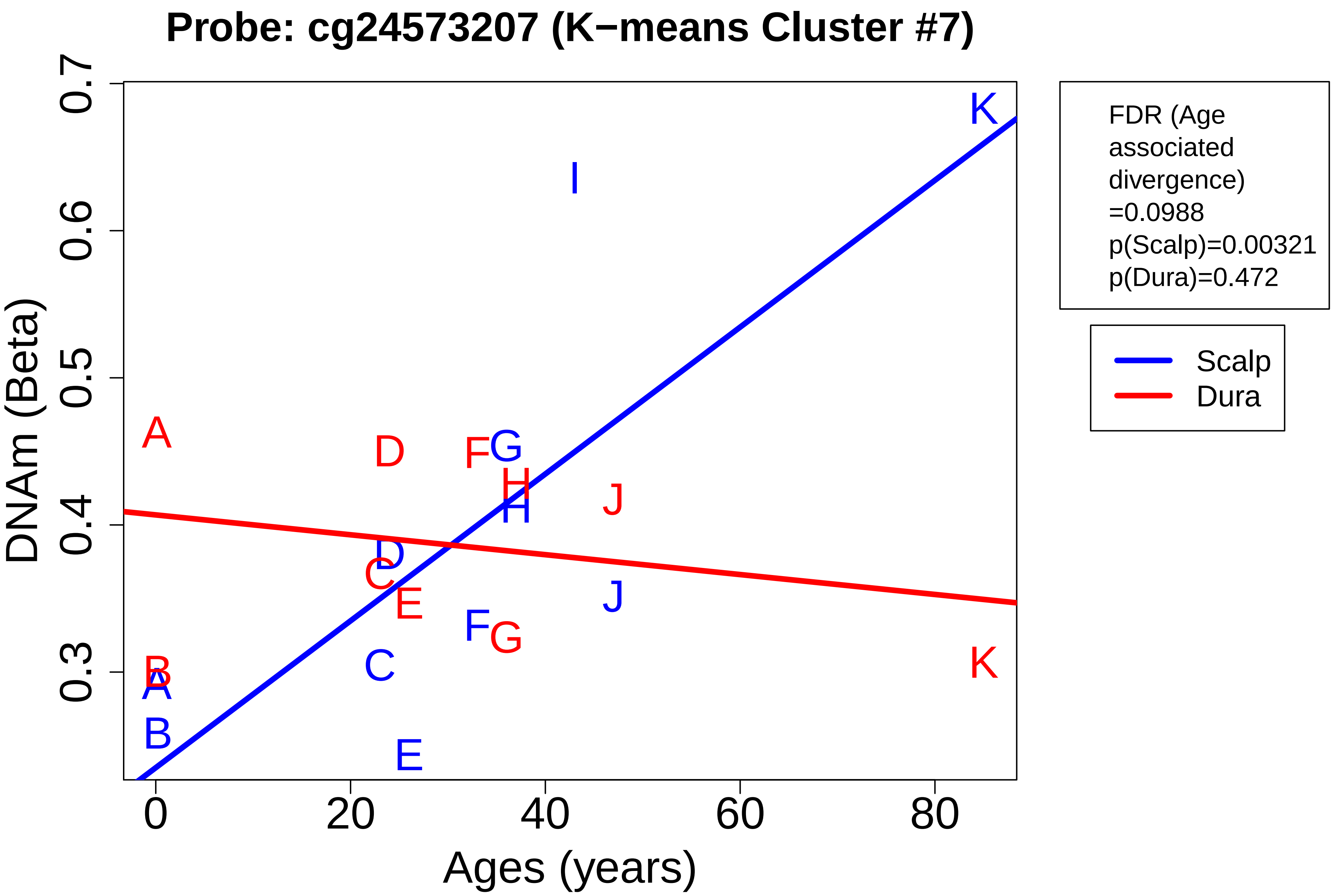
Probe: cg24573207 (K−means Cluster #7)
DNAm (Beta)
Ages (years)
FDR (Age associated divergence) =0.0988
p(Scalp)=0.00321
p(Dura)=0.472
Scalp
Dura

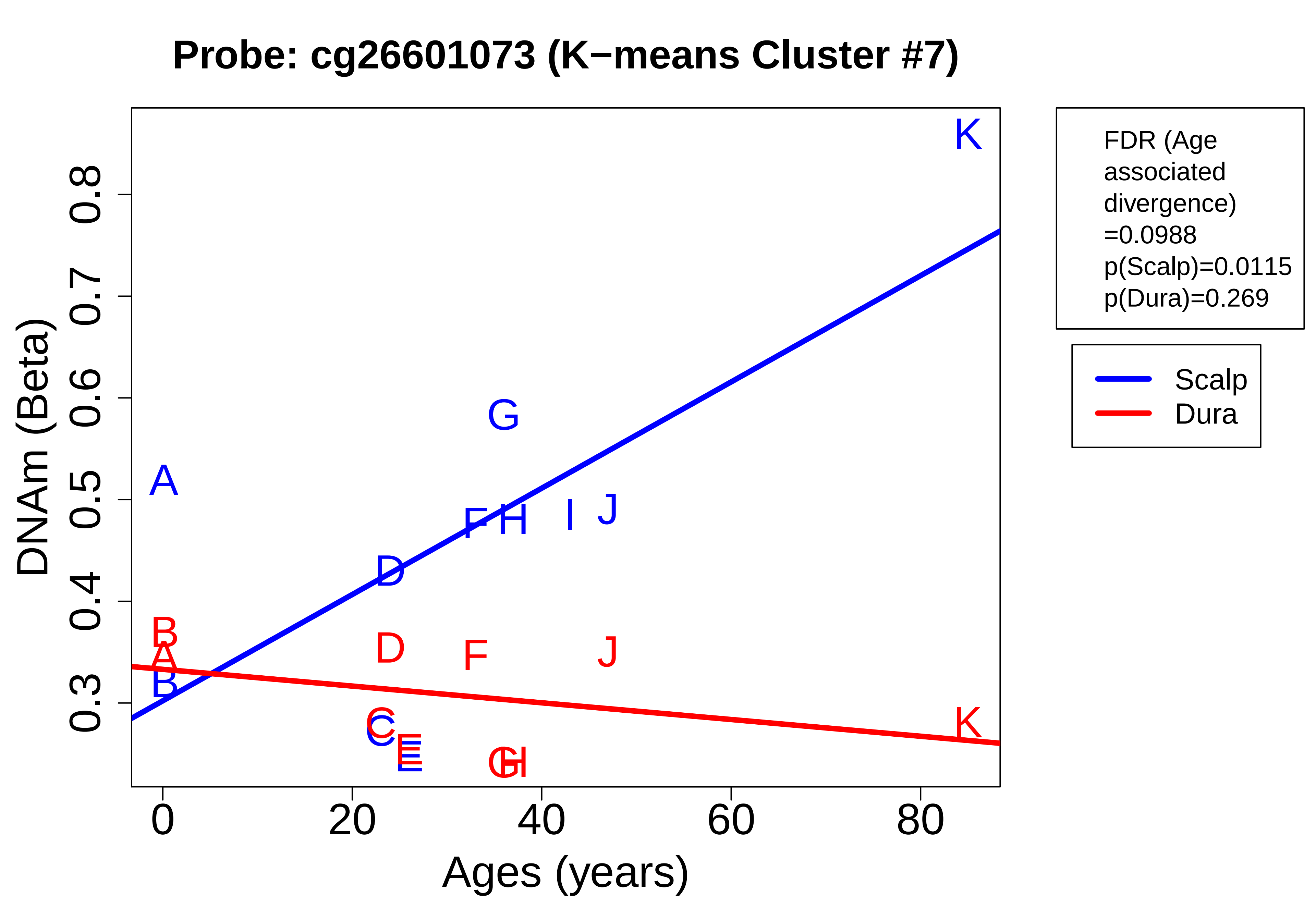

Probe: cg26601073 (K−means Cluster #7)
DNAm (Beta)
0.3
0.4
0.5
0.6
0.7
0.8
0
20
40
60
80
Ages (years)
FDR (Age associated divergence) =0.0988
p(Scalp)=0.0115
p(Dura)=0.269
Scalp
Dura

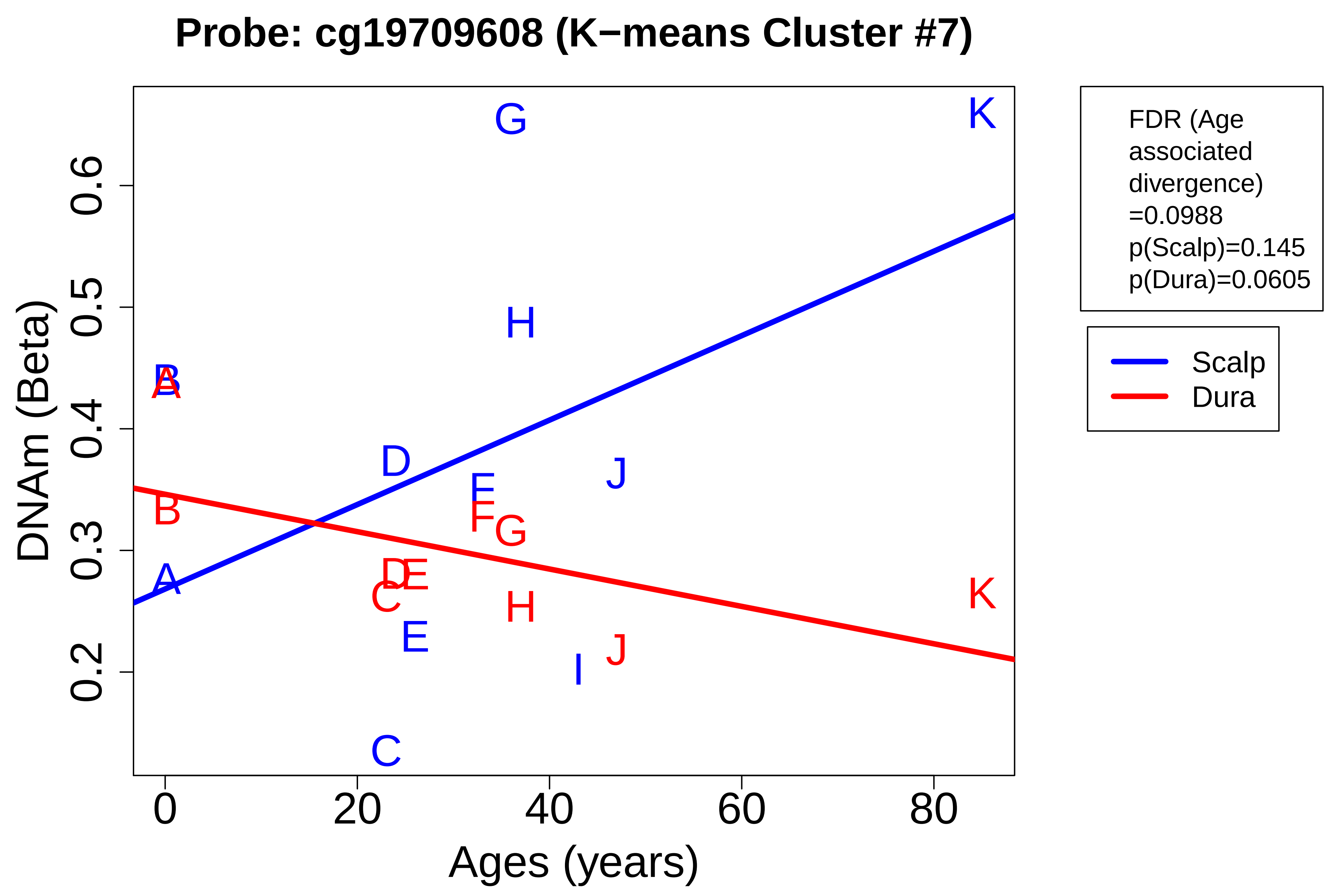

Probe: cg19709608 (K−means Cluster #7)
FDR (Age associated divergence) =0.0988
p(Scalp)=0.145
p(Dura)=0.0605
Scalp
Dura
DNAm (Beta)
0.2
0.3
0.4
0.5
0.6
0
20
40
60
80
Ages (years)

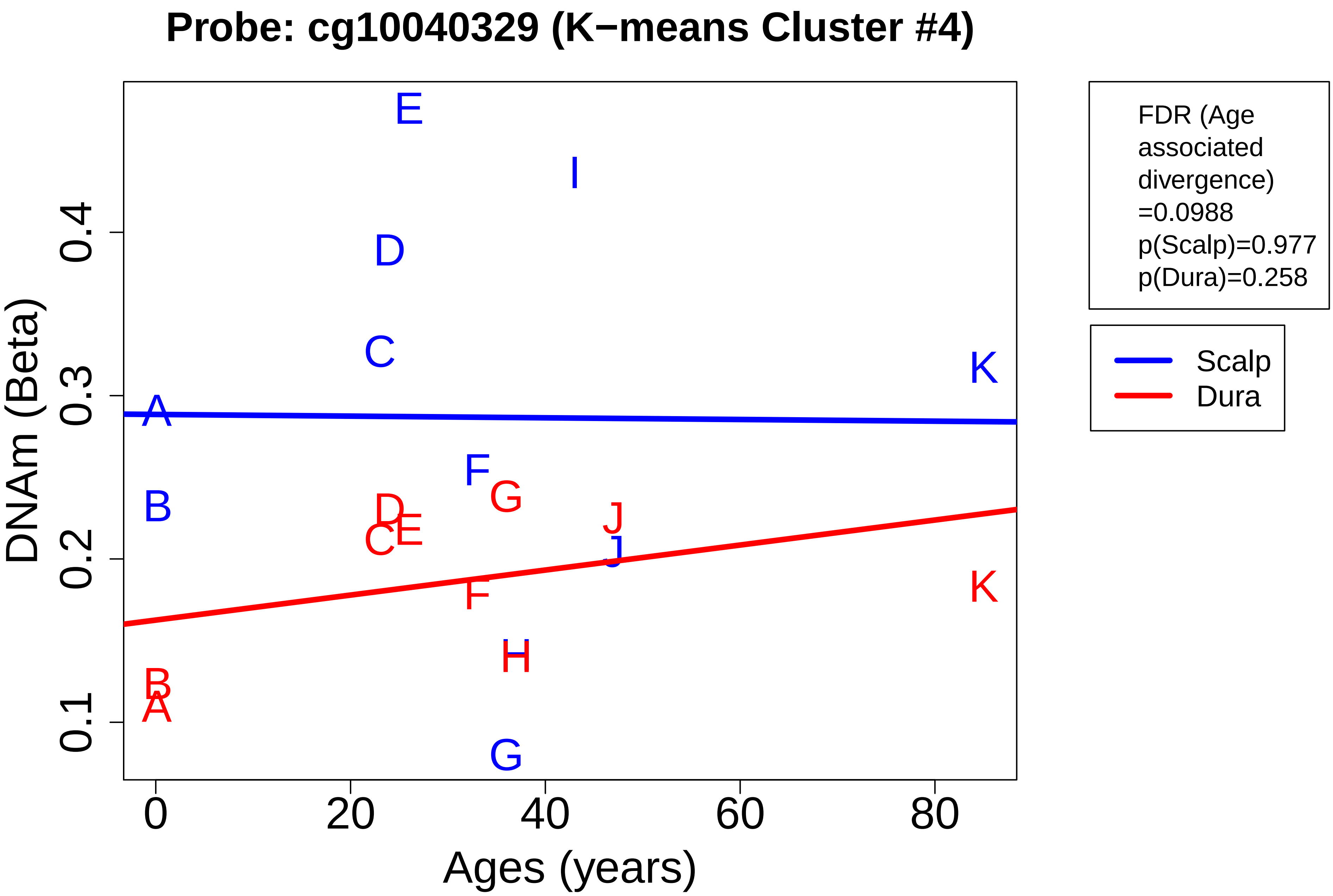

Probe: cg10040329 (K−means Cluster #4)
DNAm (Beta)
0.1
0.2
0.3
0.4
0
20
40
60
80
Ages (years)
FDR (Age associated divergence) =0.0988
p(Scalp)=0.977
p(Dura)=0.258
Scalp
Dura

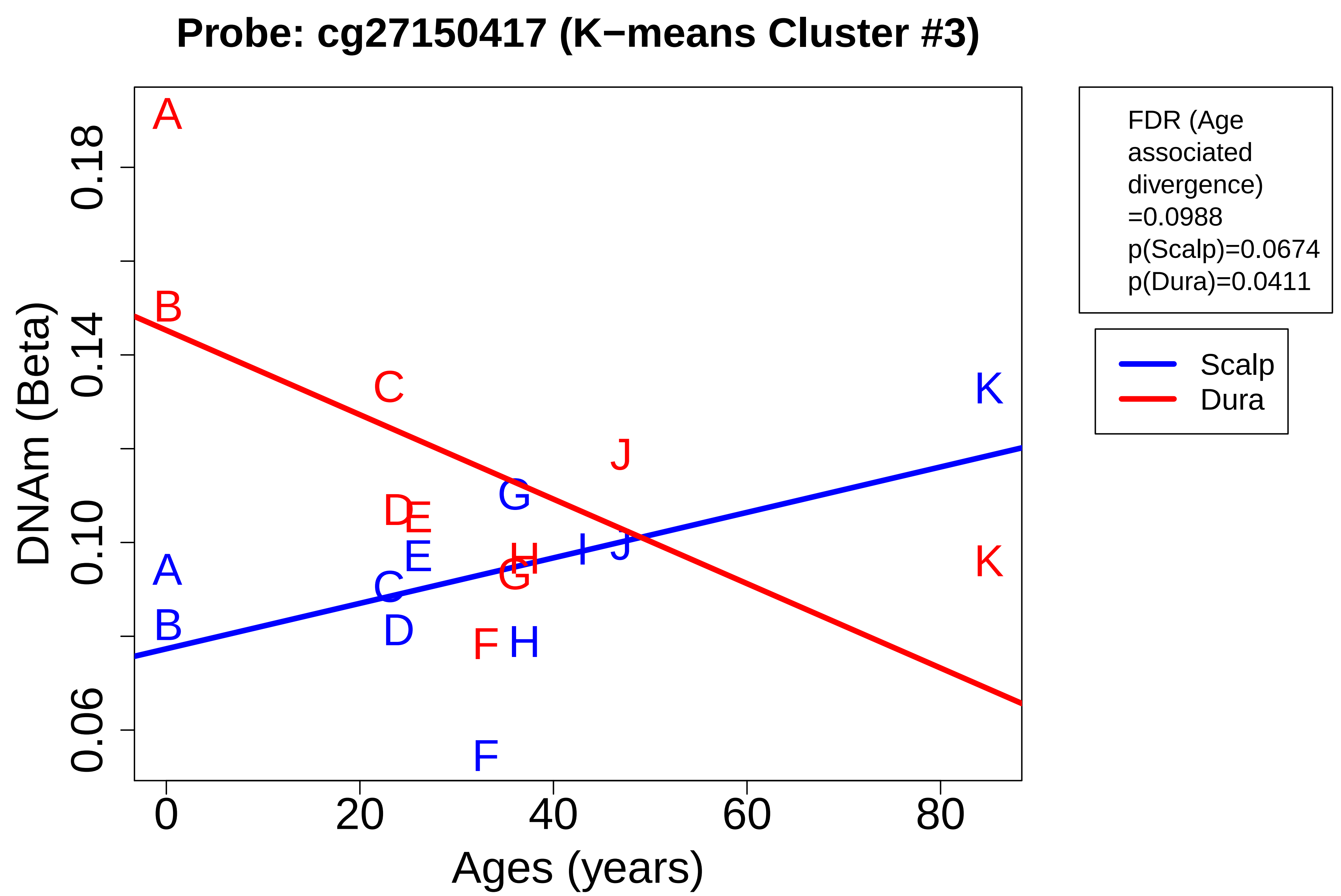

Probe: cg27150417 (K−means Cluster #3)
DNAm (Beta)
0.06
0.10
0.14
0.18
0
20
40
60
80
Ages (years)
A
B
C
D
E
F
G
H
I
J
K
FDR (Age associated divergence) =0.0988
p(Scalp)=0.0674
p(Dura)=0.0411
Scalp
Dura

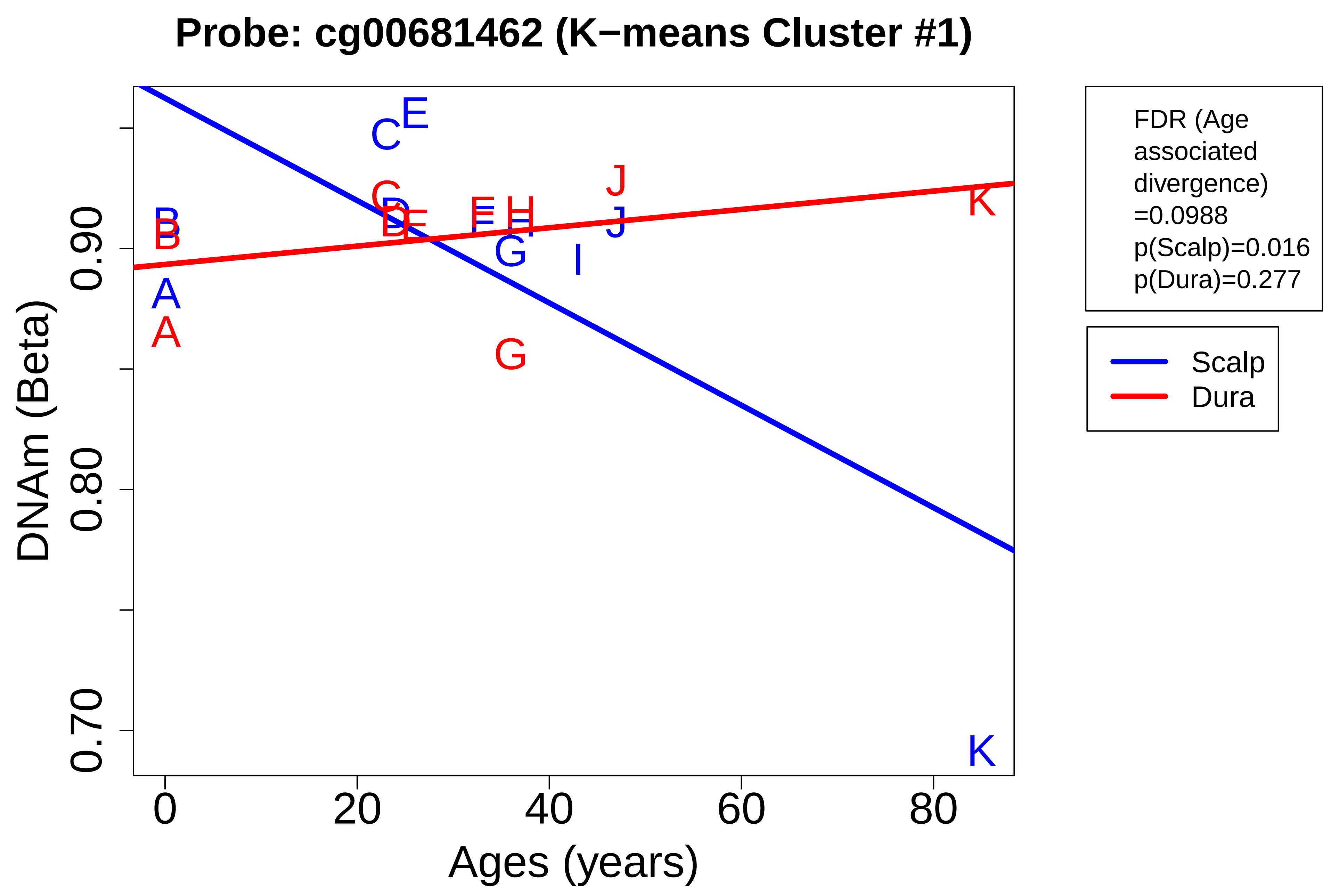

Probe: cg00681462 (K−means Cluster #1)
DNAm (Beta)
0.70
0.80
0.90
0
20
40
60
80
Ages (years)
A
B
C
D
E
F
G
H
I
J
K
FDR (Age associated divergence) =0.0988
p(Scalp)=0.016
p(Dura)=0.277
Scalp
Dura

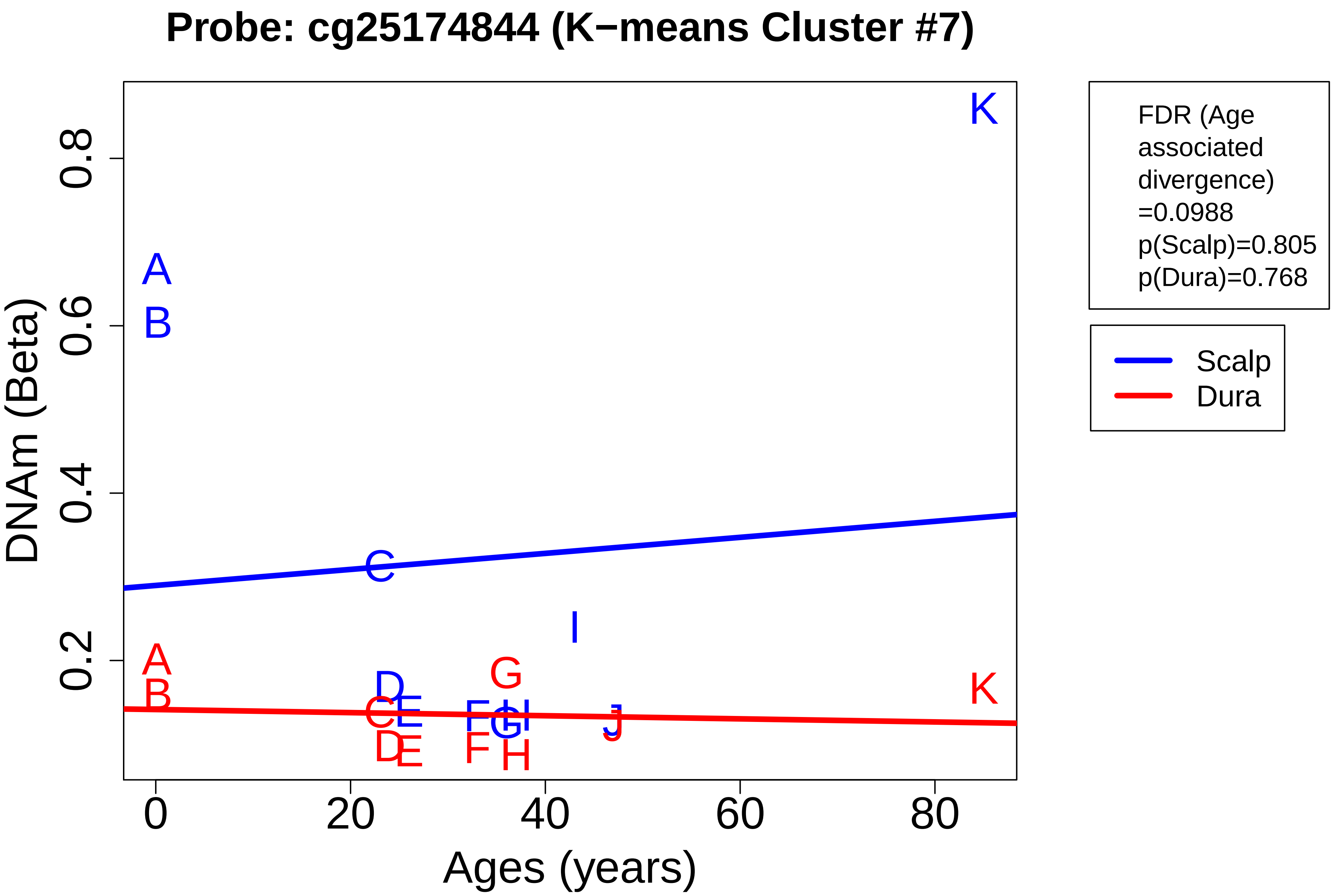

Probe: cg25174844 (K−means Cluster #7)
DNAm (Beta)
0.2
0.4
0.6
0.8
0
20
40
60
80
Ages (years)
FDR (Age associated divergence) =0.0988
p(Scalp)=0.805
p(Dura)=0.768
Scalp
Dura

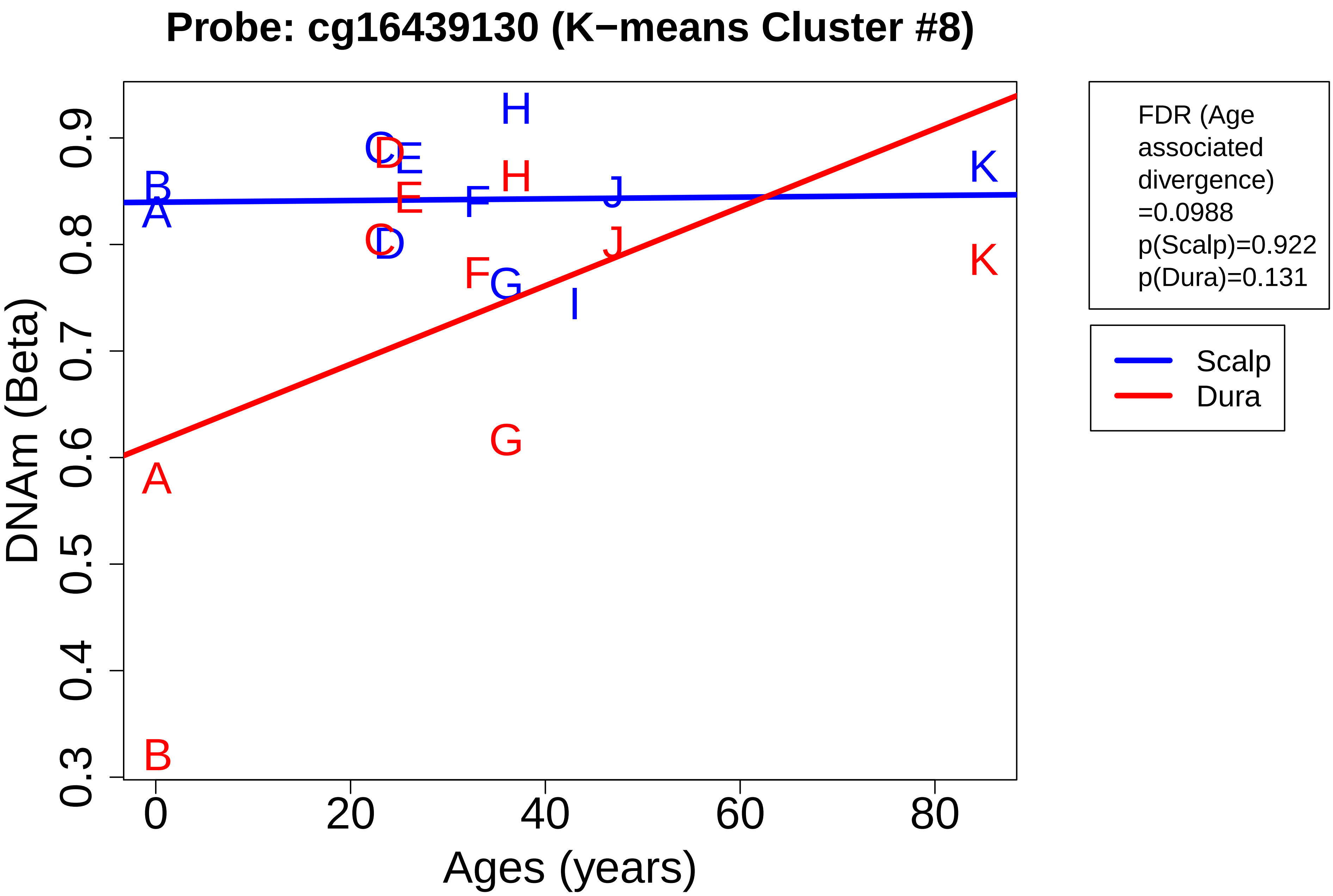

Probe: cg16439130 (K−means Cluster #8)
DNAm (Beta)
0.3
0.4
0.5
0.6
0.7
0.8
0.9
0
20
40
60
80
Ages (years)
FDR (Age associated divergence) =0.0988
p(Scalp)=0.922
p(Dura)=0.131
Scalp
Dura

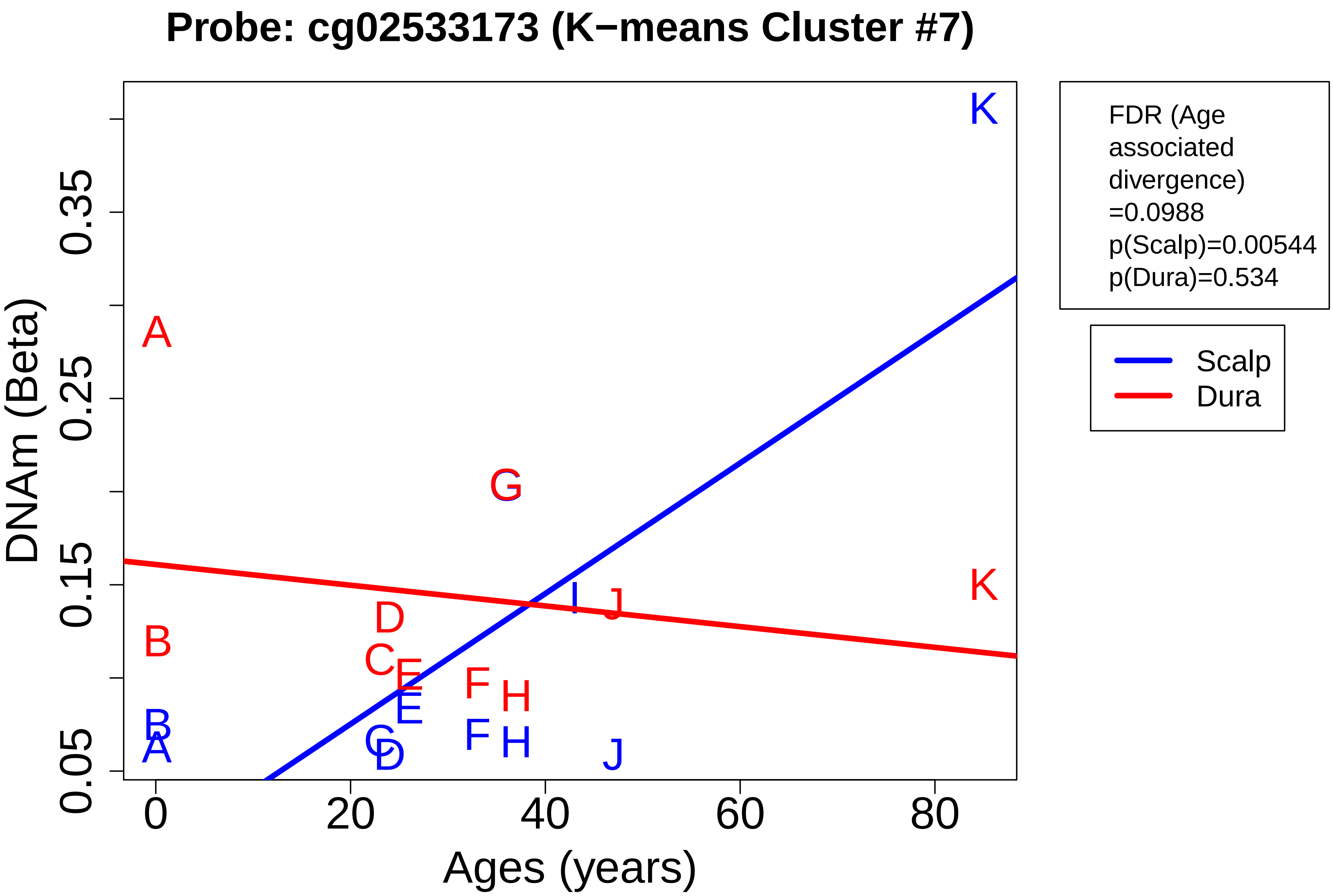

Probe: cg02533173 (K−means Cluster #7)
DNAm (Beta)
0.05
0.15
0.25
0.35
0
20
40
60
80
Ages (years)
FDR (Age associated divergence) =0.0988
p(Scalp)=0.00544
p(Dura)=0.534
Scalp
Dura
A
B
C
D
E
F
G
H
I
J
K

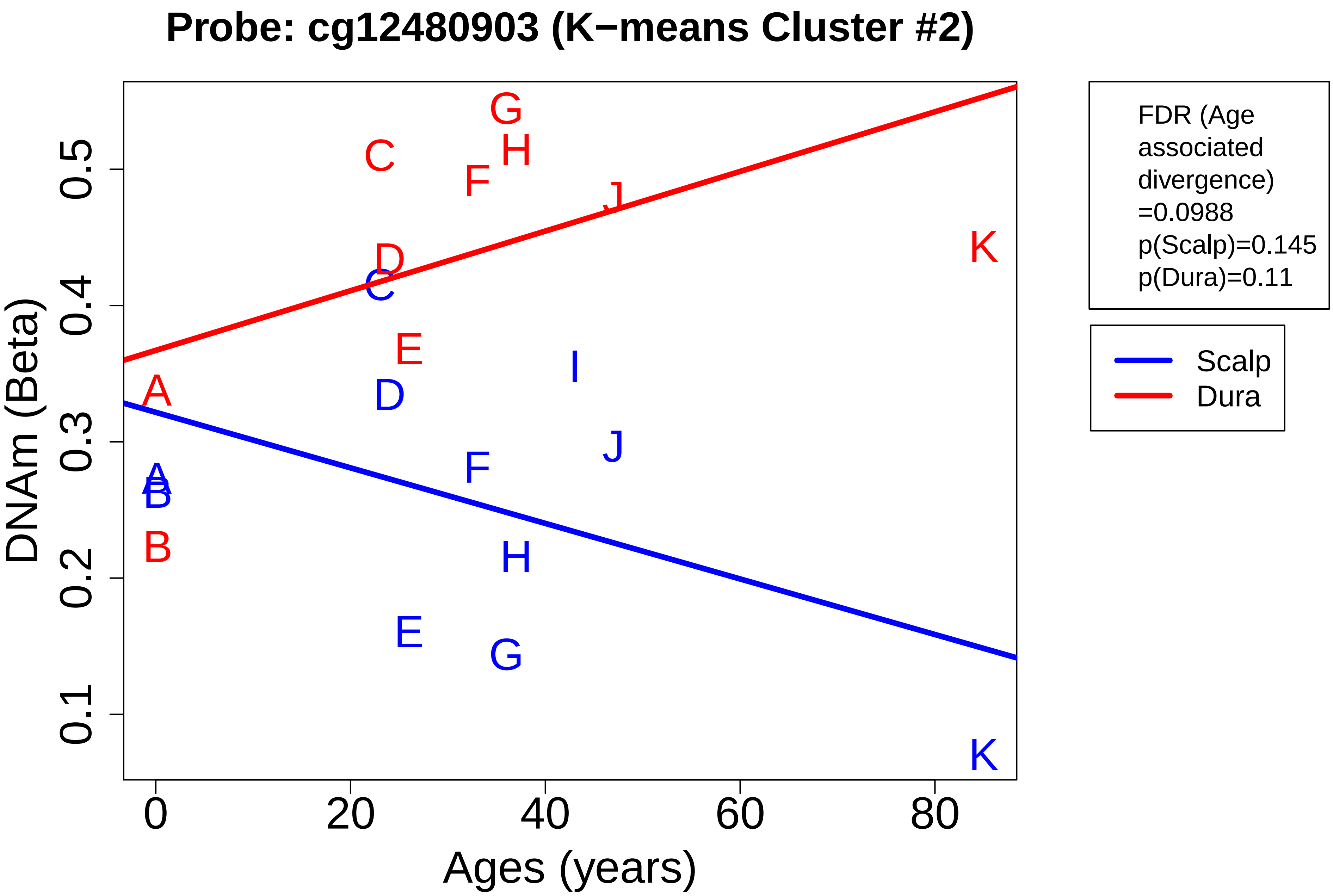
Probe: cg12480903 (K−means Cluster #2)
DNAm (Beta)
0.1
0.2
0.3
0.4
0.5
Ages (years)
0
20
40
60
80
FDR (Age associated divergence) =0.0988
p(Scalp)=0.145
p(Dura)=0.11
Scalp
Dura
A
B
C
D
E
F
G
H
I
J
K

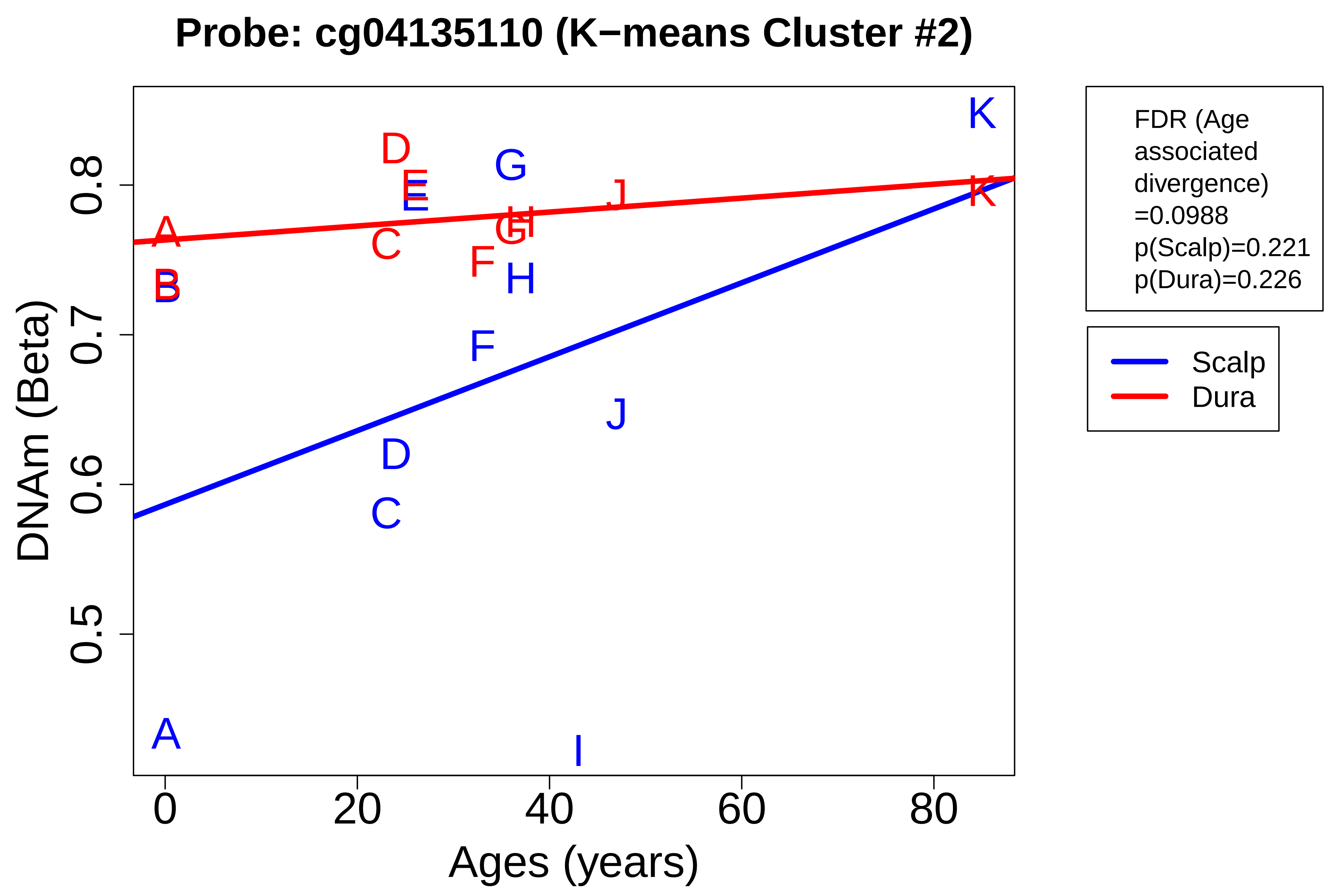

Probe: cg04135110 (K−means Cluster #2)
DNAm (Beta)
0.5
0.6
0.7
0.8
Ages (years)
0
20
40
60
80
A
B
C
D
E
F
G
H
I
J
K
FDR (Age associated divergence) =0.0988
p(Scalp)=0.221
p(Dura)=0.226
Scalp
Dura

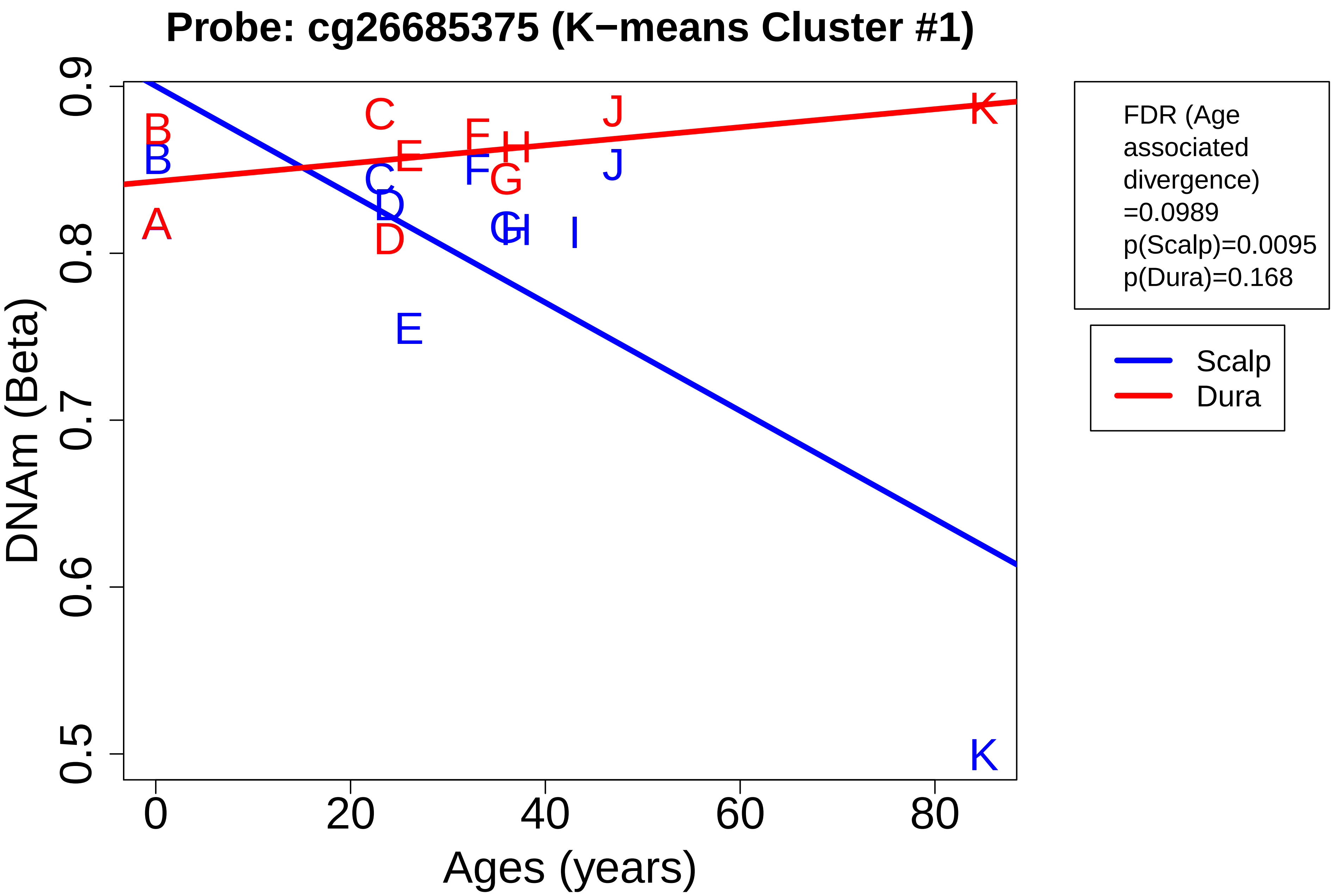

Probe: cg26685375 (K−means Cluster #1)
DNAm (Beta)
0.9
0.8
0.7
0.6
0.5
0
20
40
60
80
Ages (years)
A
B
B
C
C
D
D
E
E
F
F
G
G
H
H
I
J
J
K
K
FDR (Age associated divergence) =0.0989
p(Scalp)=0.0095
p(Dura)=0.168
Scalp
Dura

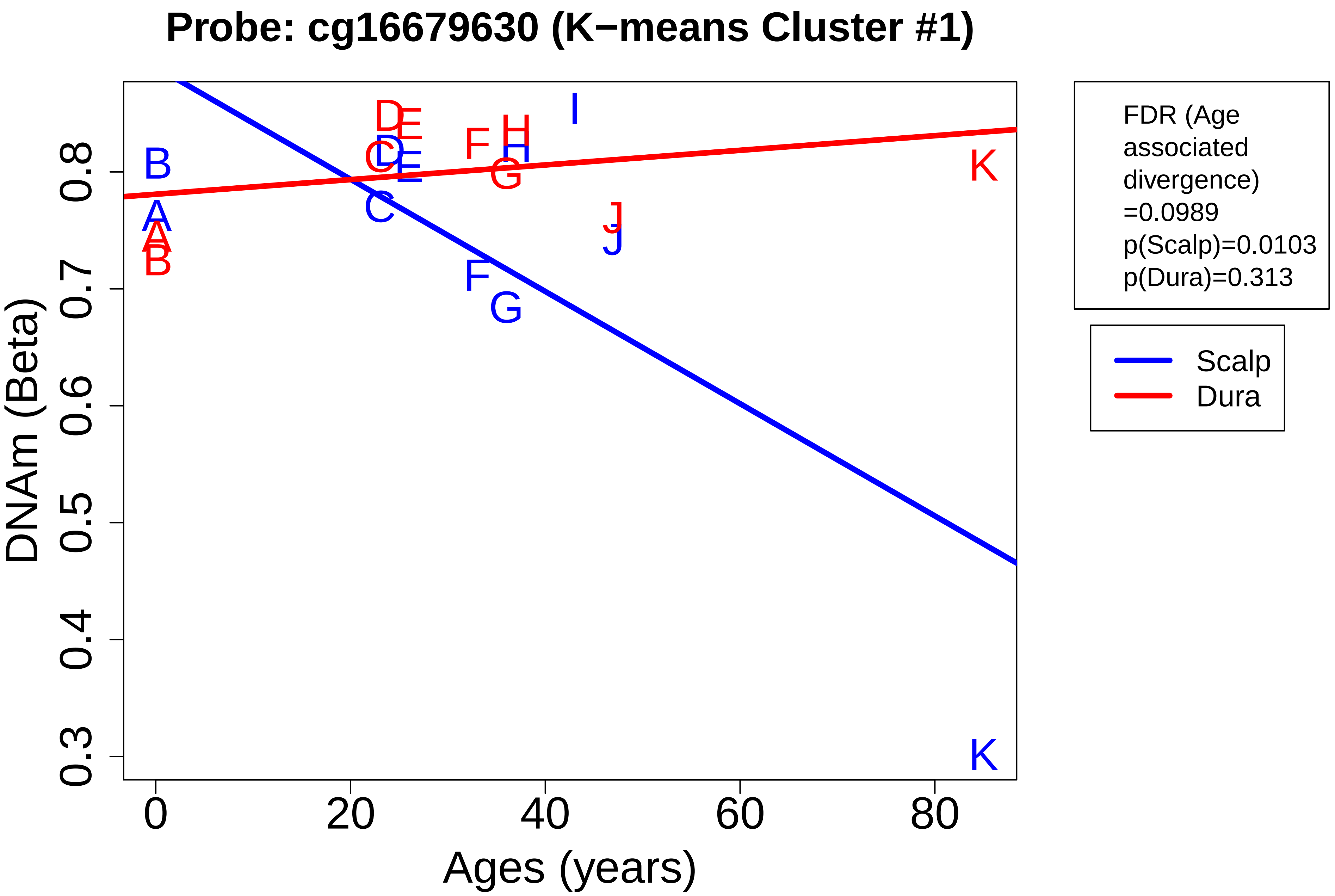

Probe: cg16679630 (K−means Cluster #1)
DNAm (Beta)
0.3
0.4
0.5
0.6
0.7
0.8
0
20
40
60
80
Ages (years)
FDR (Age associated divergence) =0.0989
p(Scalp)=0.0103
p(Dura)=0.313
Scalp
Dura

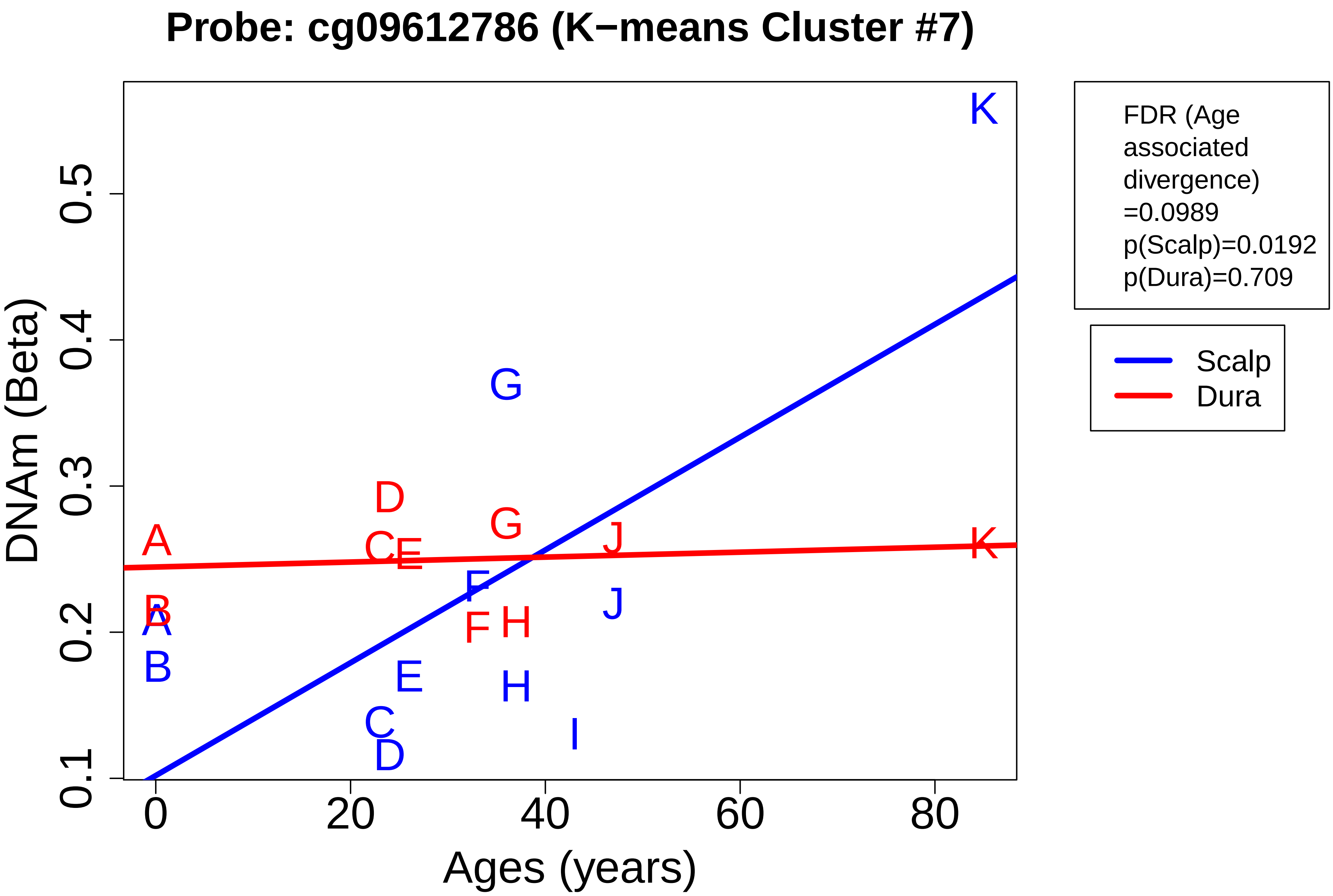
Probe: cg09612786 (K−means Cluster #7)
DNAm (Beta)
Ages (years)
0.1
0.2
0.3
0.4
0.5
0
20
40
60
80
FDR (Age associated divergence) =0.0989
p(Scalp)=0.0192
p(Dura)=0.709
Scalp
Dura

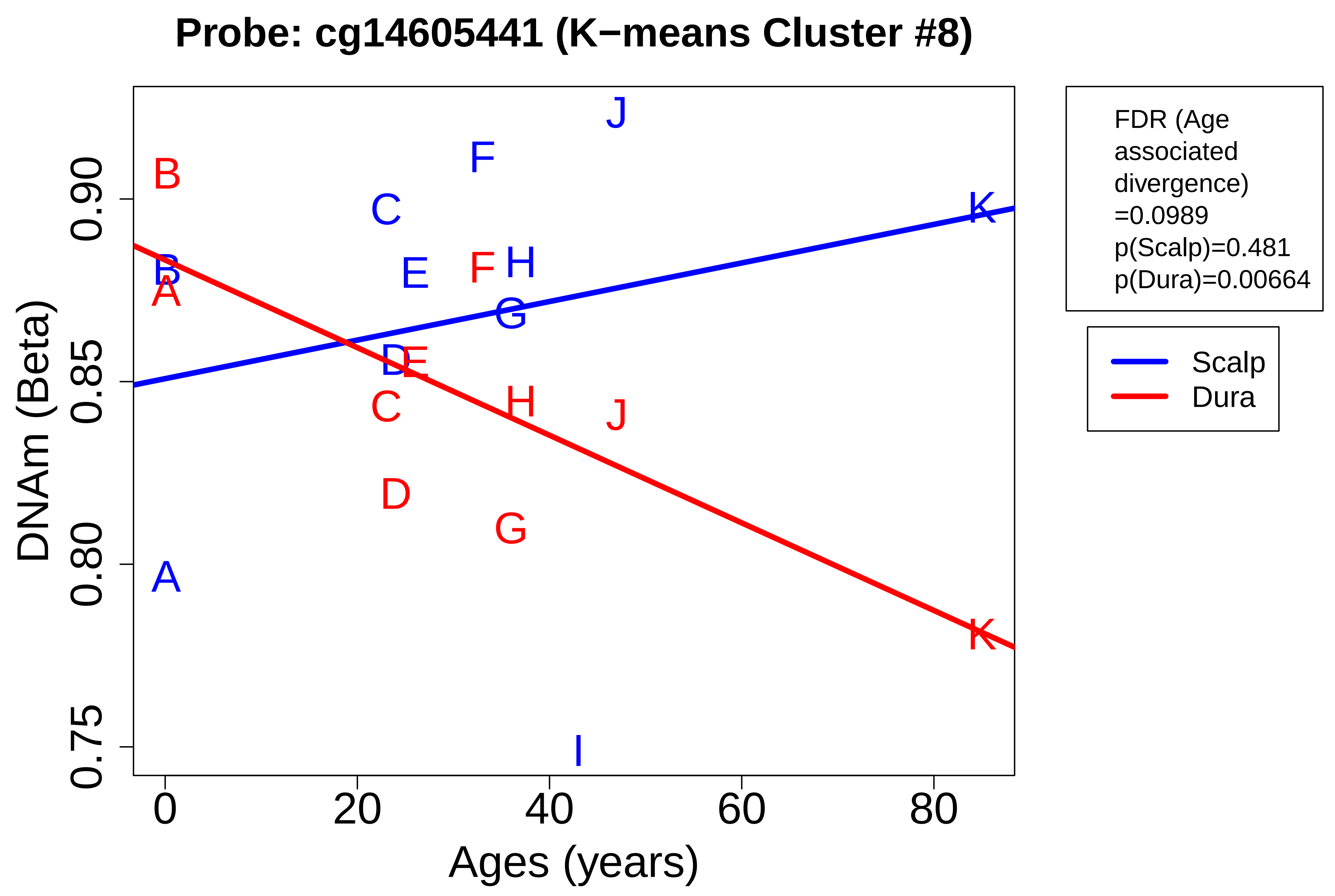

Probe: cg14605441 (K−means Cluster #8)
DNAm (Beta)
0.75
0.80
0.85
0.90
0
20
40
60
80
Ages (years)
A
B
C
D
E
F
G
H
I
J
K
FDR (Age associated divergence) =0.0989
p(Scalp)=0.481
p(Dura)=0.00664
Scalp
Dura

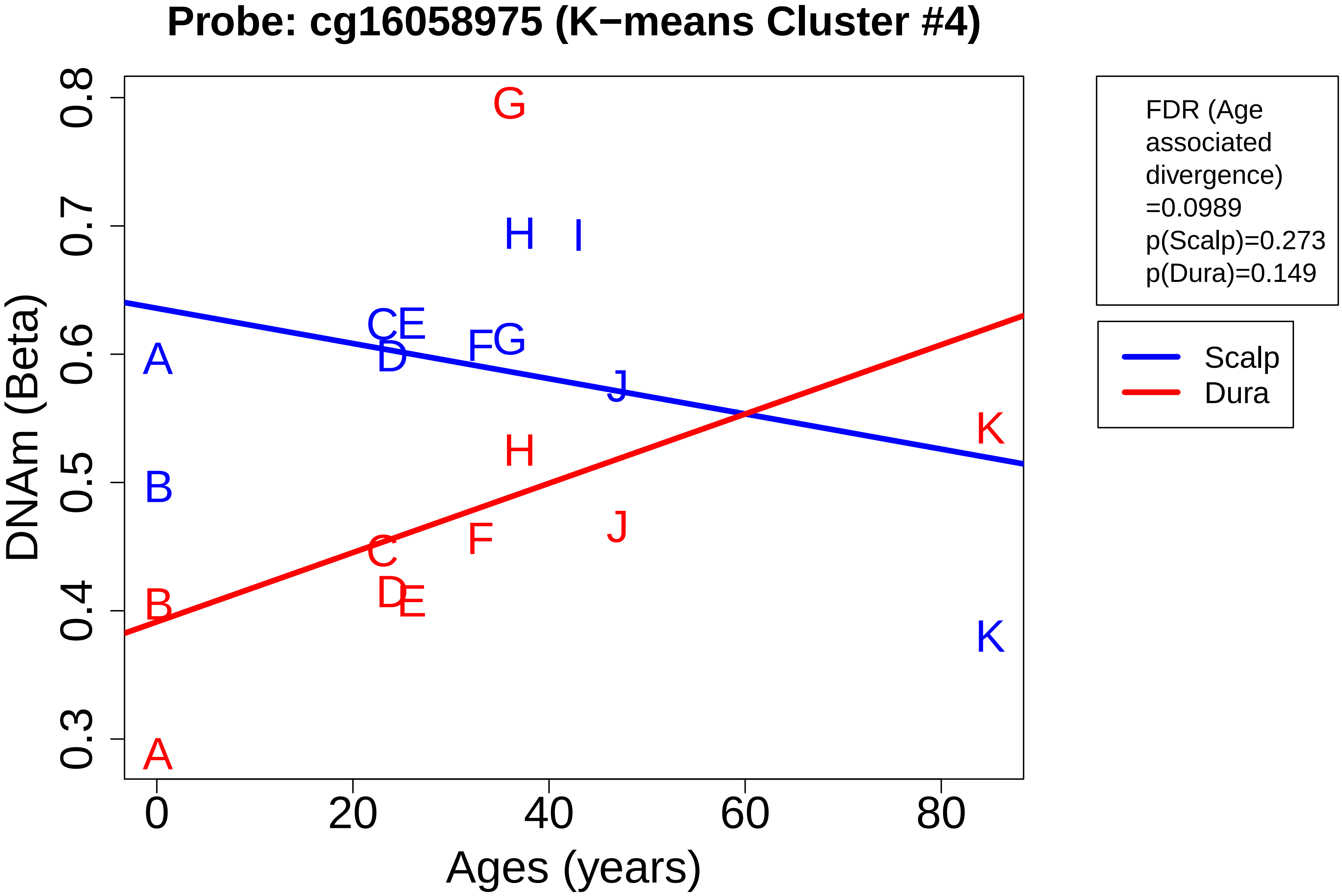

Probe: cg16058975 (K−means Cluster #4)
DNAm (Beta)
Ages (years)
0.3
0.4
0.5
0.6
0.7
0.8
0
20
40
60
80
FDR (Age associated divergence) =0.0989
p(Scalp)=0.273
p(Dura)=0.149
Scalp
Dura

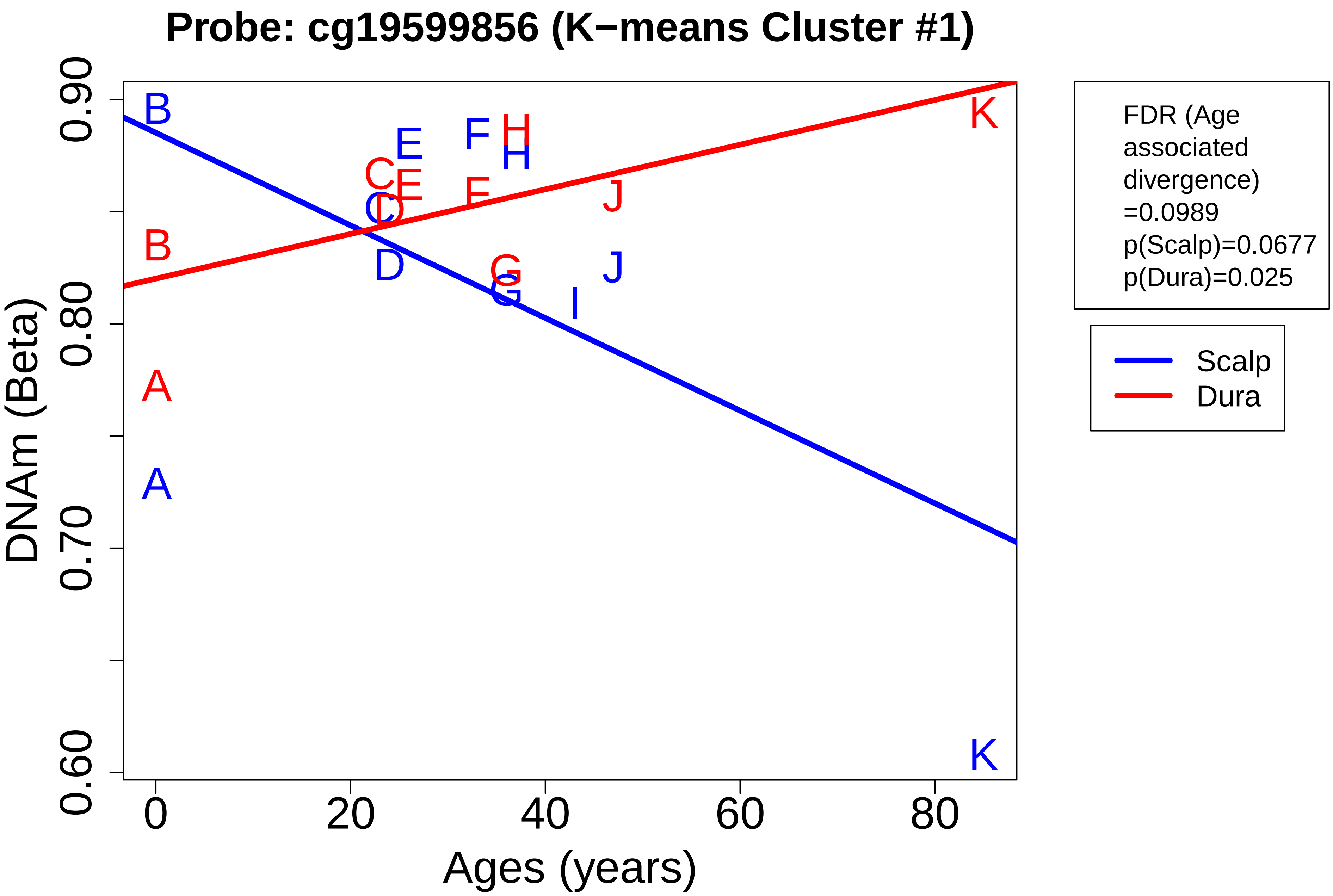

Probe: cg19599856 (K−means Cluster #1)
DNAm (Beta)
0.60
0.70
0.80
0.90
0
20
40
60
80
Ages (years)
A
B
C
D
E
F
G
H
I
J
K
FDR (Age associated divergence) =0.0989
p(Scalp)=0.0677
p(Dura)=0.025
Scalp
Dura

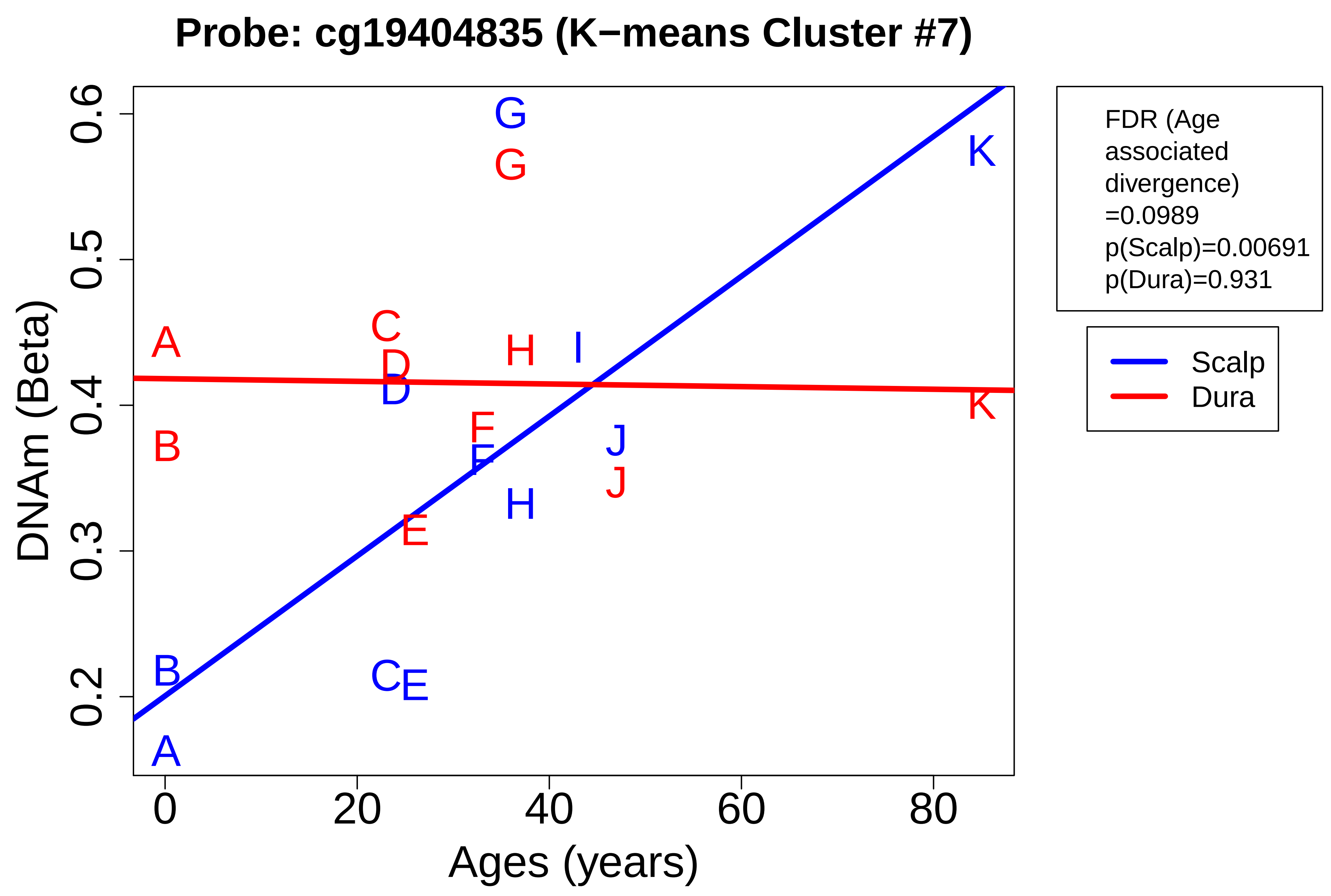

Probe: cg19404835 (K−means Cluster #7)
DNAm (Beta)
Ages (years)
0.2
0.3
0.4
0.5
0.6
0
20
40
60
80
FDR (Age associated divergence) =0.0989
p(Scalp)=0.00691
p(Dura)=0.931
Scalp
Dura

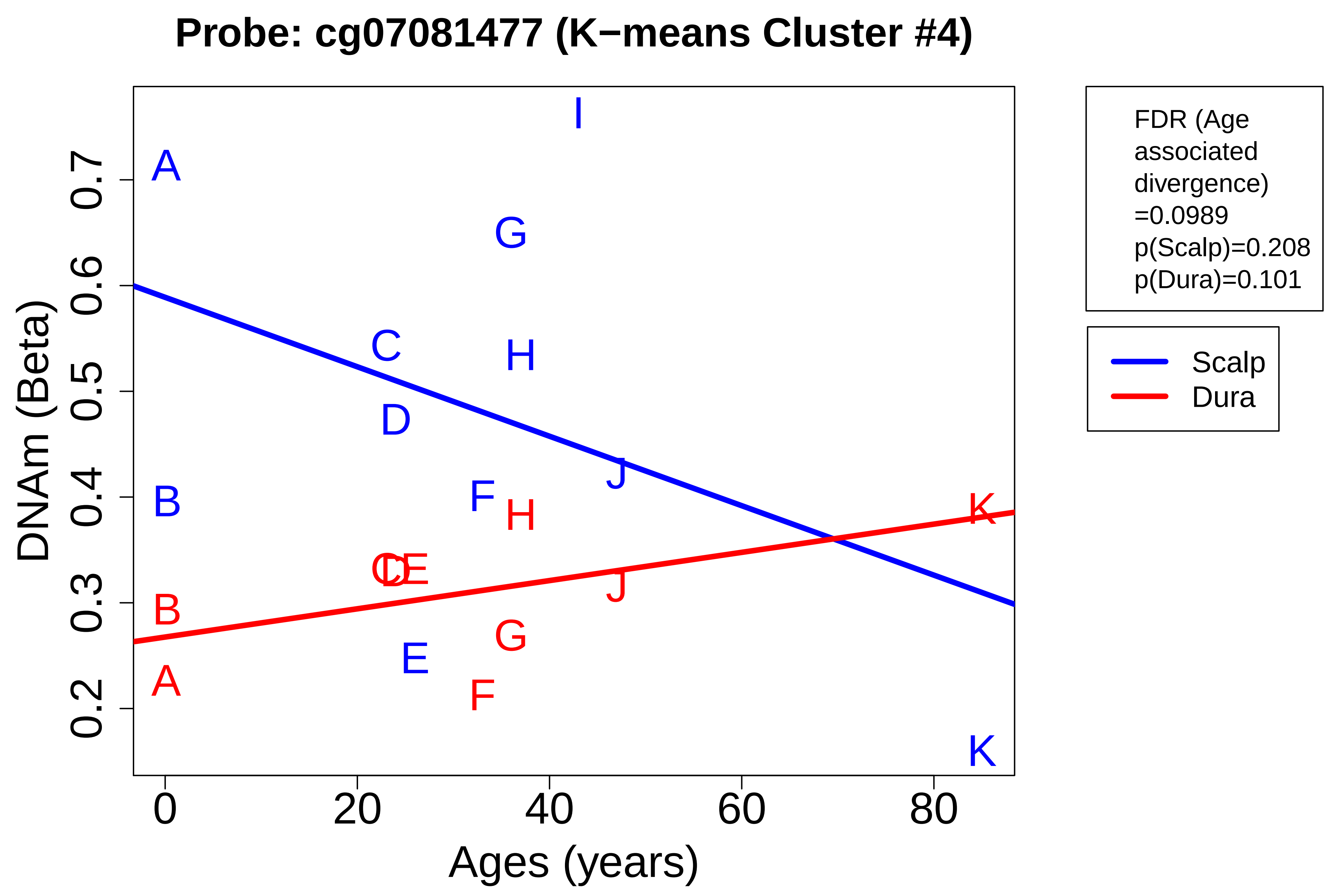

Probe: cg07081477 (K−means Cluster #4)
DNAm (Beta)
0.2
0.3
0.4
0.5
0.6
0.7
Ages (years)
0
20
40
60
80
A
B
C
D
E
F
G
H
I
J
K
FDR (Age associated divergence) =0.0989
p(Scalp)=0.208
p(Dura)=0.101
Scalp
Dura

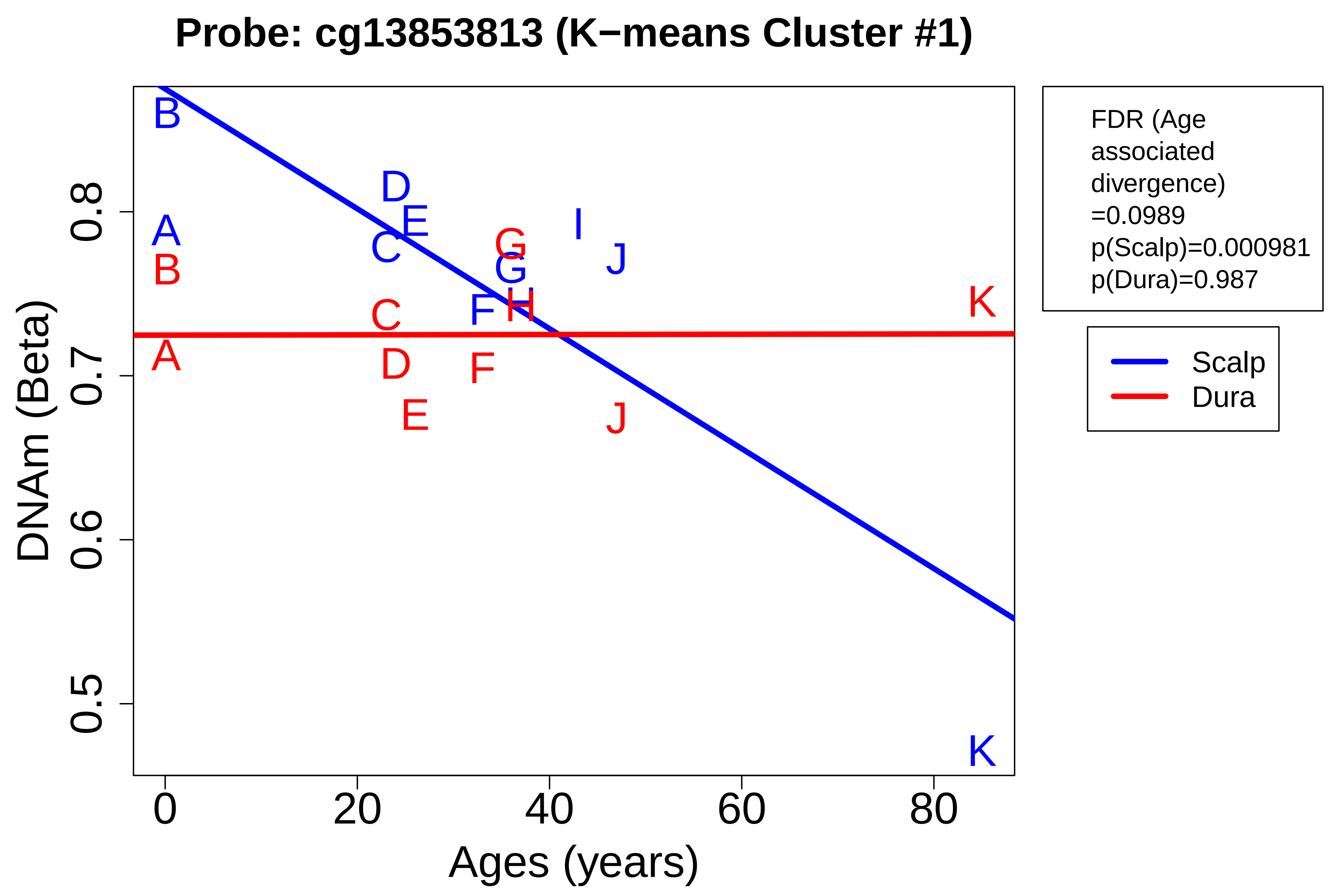

Probe: cg13853813 (K−means Cluster #1)
DNAm (Beta)
0.5
0.6
0.7
0.8
0
20
40
60
80
Ages (years)
FDR (Age associated divergence) =0.0989
p(Scalp)=0.000981
p(Dura)=0.987
Scalp
Dura

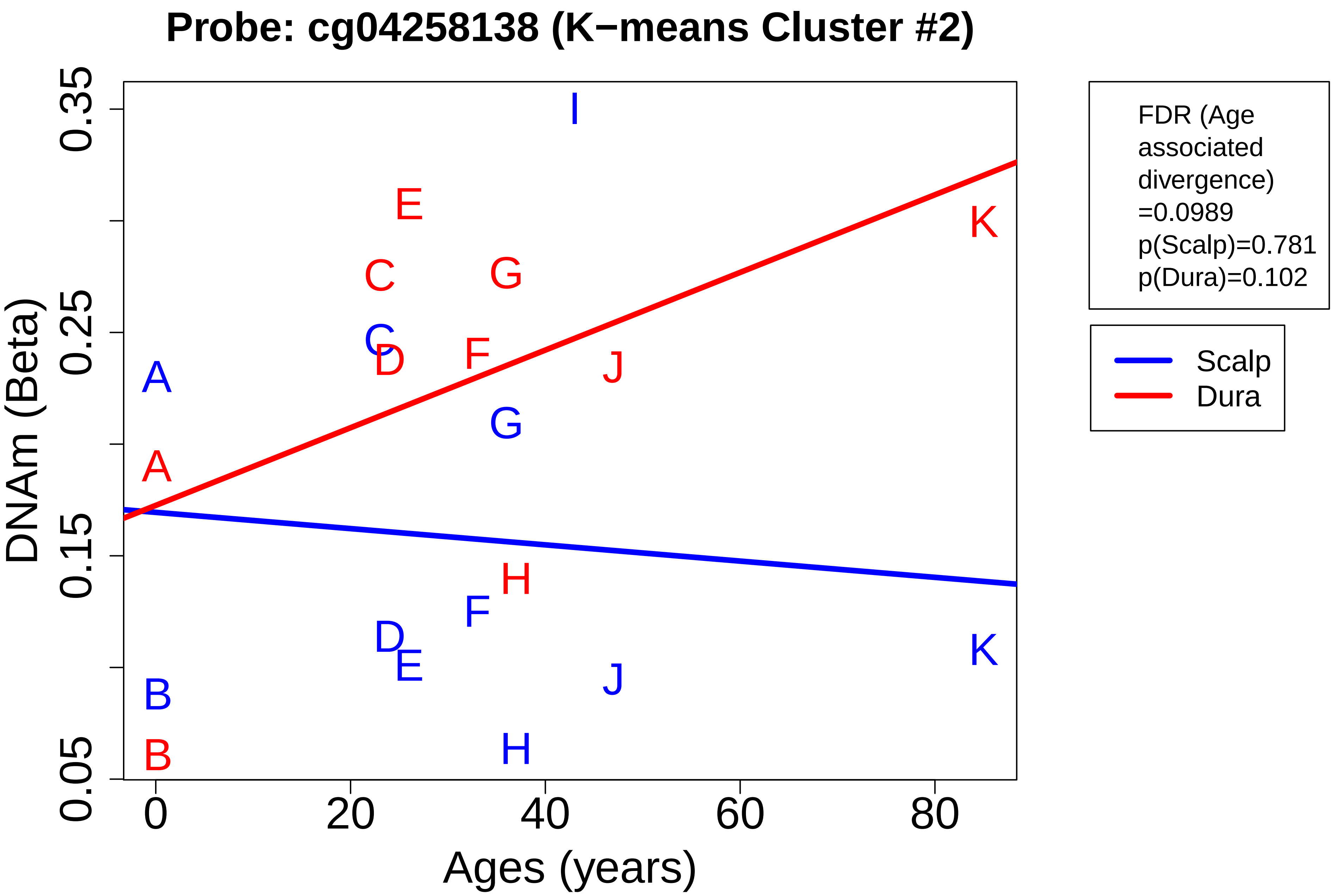
Probe: cg04258138 (K−means Cluster #2)
DNAm (Beta)
0.05
0.15
0.25
0.35
0
20
40
60
80
Ages (years)
FDR (Age associated divergence) =0.0989
p(Scalp)=0.781
p(Dura)=0.102
Scalp
Dura

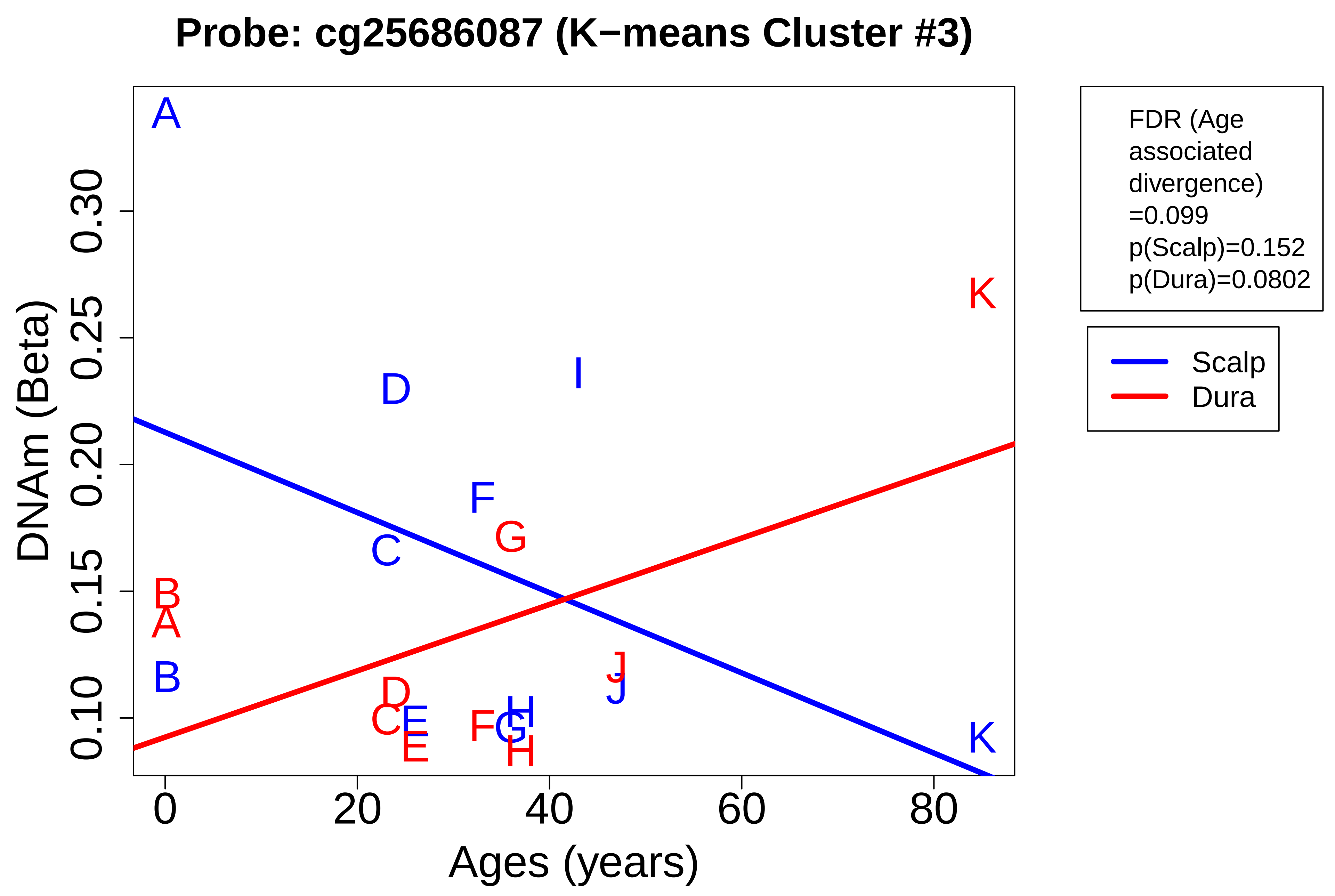

Probe: cg25686087 (K−means Cluster #3)
DNAm (Beta)
0.10
0.15
0.20
0.25
0.30
Ages (years)
0
20
40
60
80
A
B
C
D
E
F
G
H
I
J
K
A
B
C
D
E
F
G
H
J
K
FDR (Age associated divergence) =0.099
p(Scalp)=0.152
p(Dura)=0.0802
Scalp
Dura

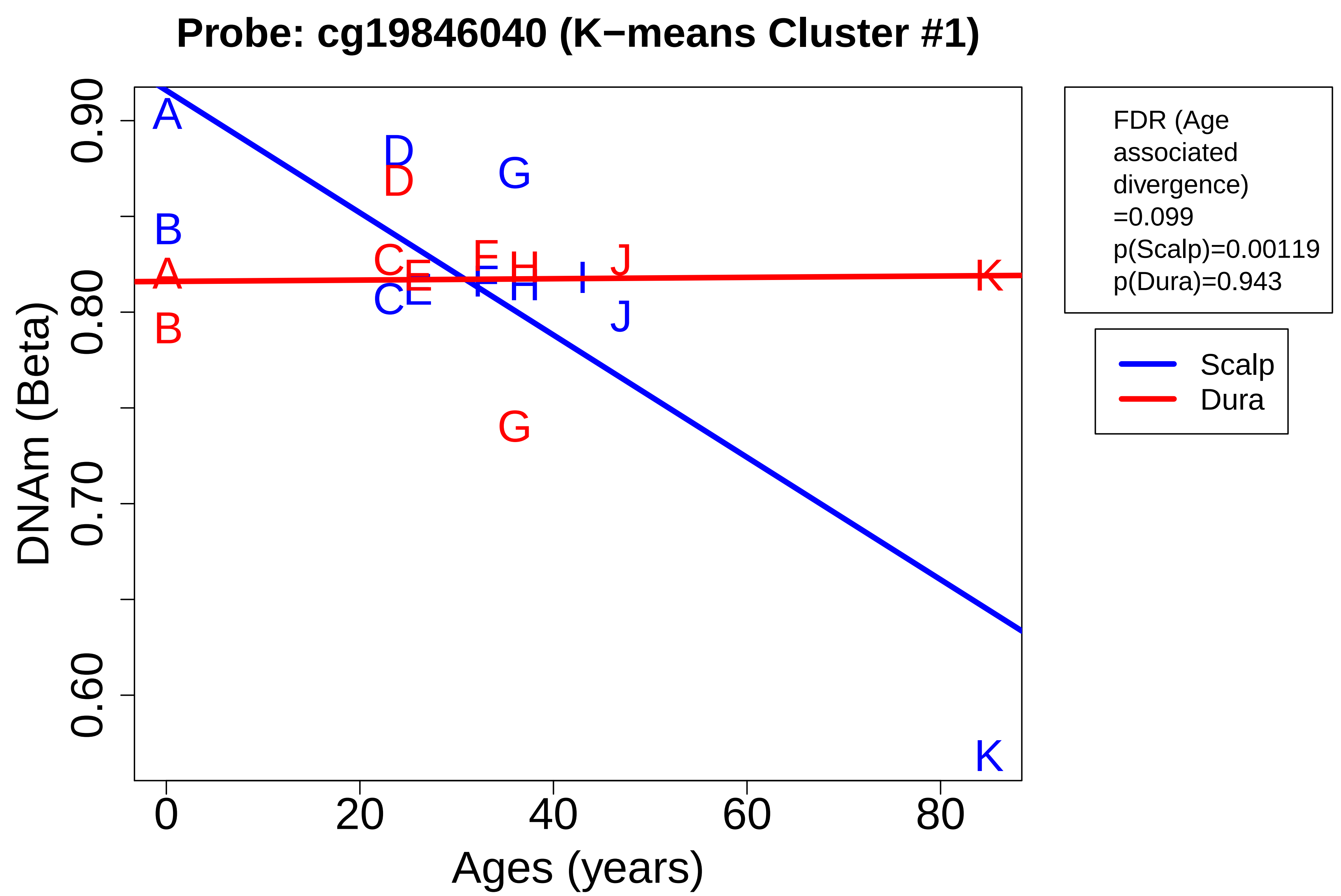

Probe: cg19846040 (K−means Cluster #1)
DNAm (Beta)
0.60
0.70
0.80
0.90
Ages (years)
0
20
40
60
80
A
B
C
D
E
G
H
I
J
K
FDR (Age associated divergence) =0.099
p(Scalp)=0.00119
p(Dura)=0.943
Scalp
Dura

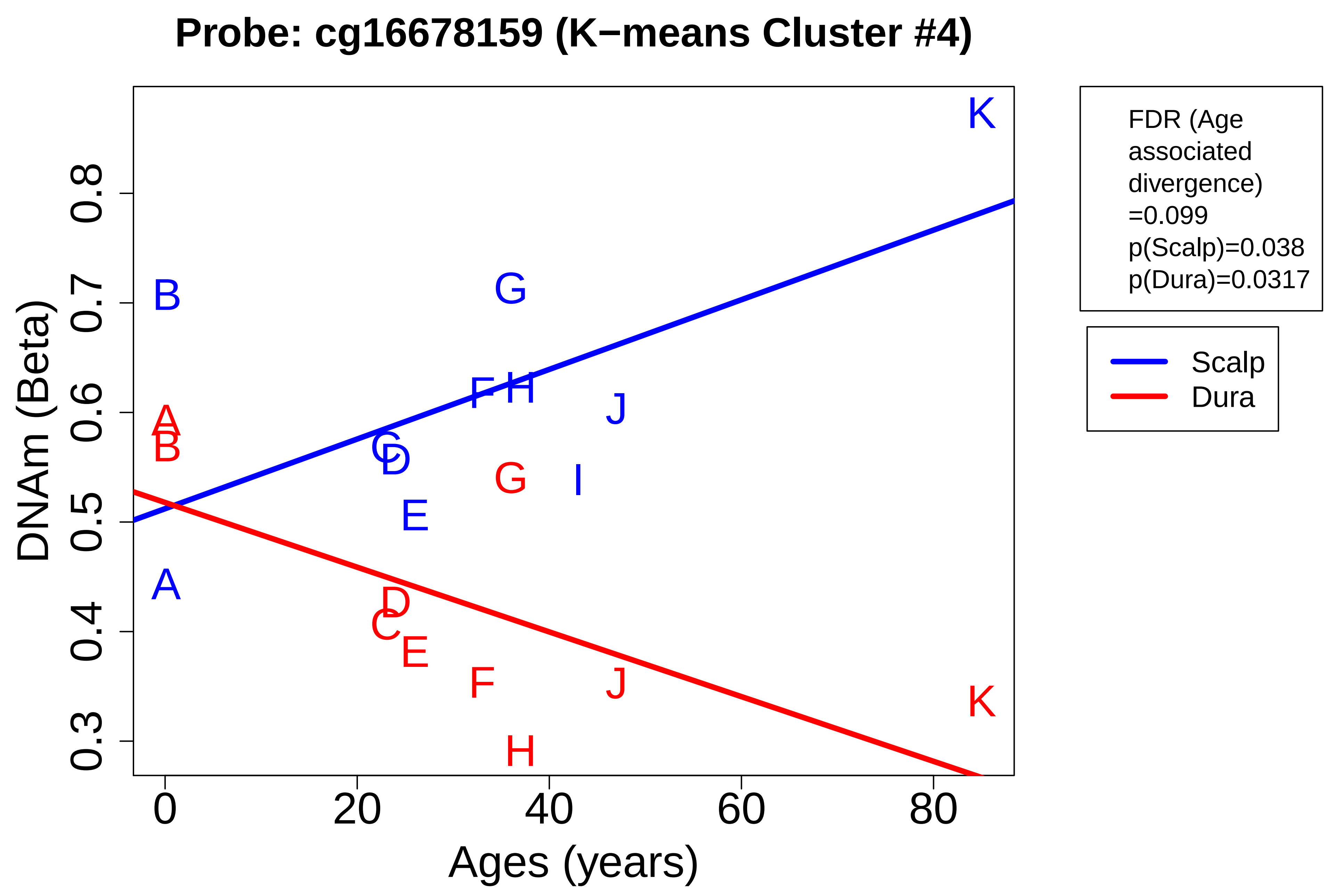

Probe: cg16678159 (K−means Cluster #4)
DNAm (Beta)
0.3
0.4
0.5
0.6
0.7
0.8
0
20
40
60
80
Ages (years)
A
B
C
D
E
F
G
H
I
J
K
FDR (Age associated divergence) =0.099
p(Scalp)=0.038
p(Dura)=0.0317
Scalp
Dura

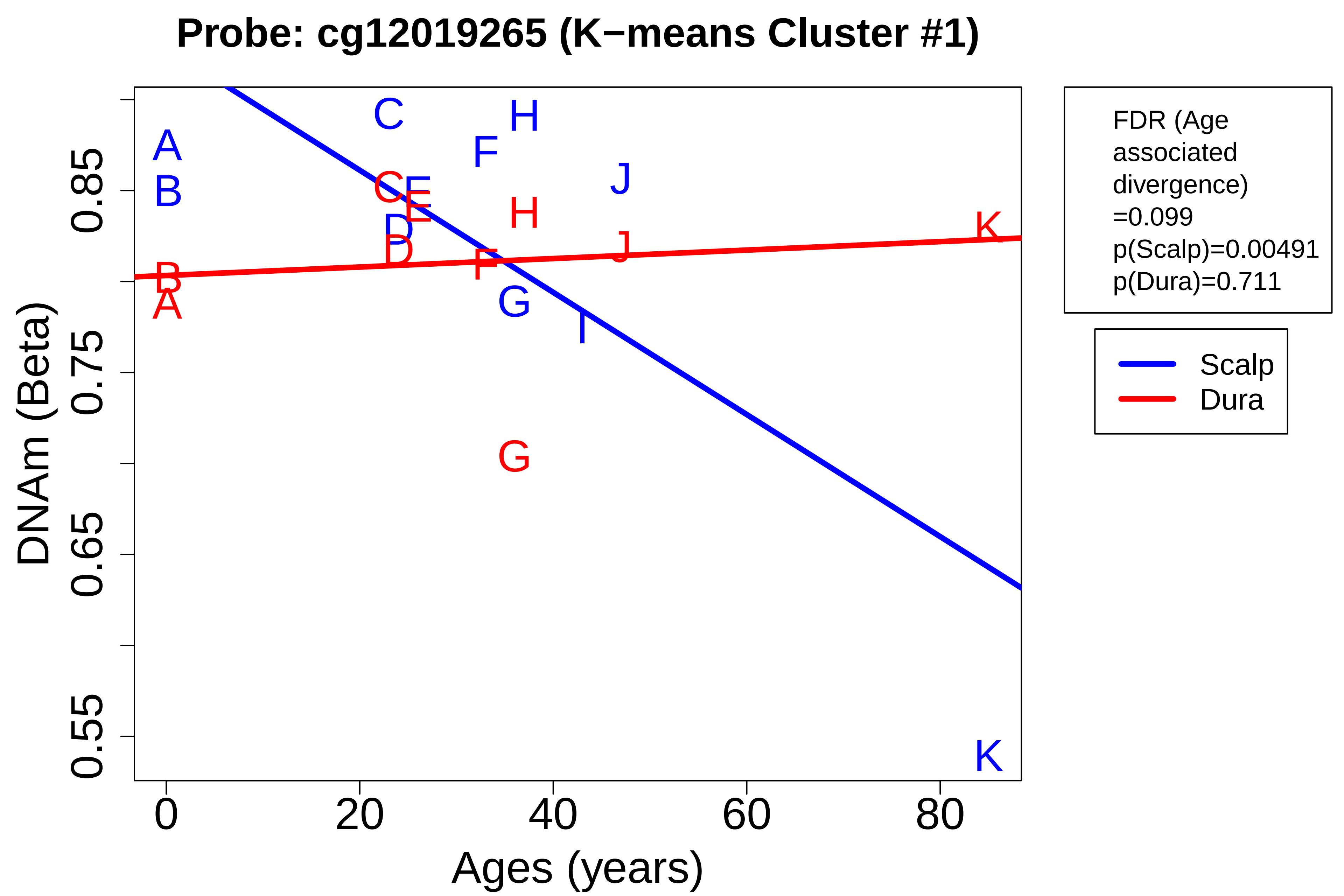

Probe: cg12019265 (K−means Cluster #1)
DNAm (Beta)
0.55
0.65
0.75
0.85
0
20
40
60
80
Ages (years)
FDR (Age associated divergence) =0.099
p(Scalp)=0.00491
p(Dura)=0.711
Scalp
Dura

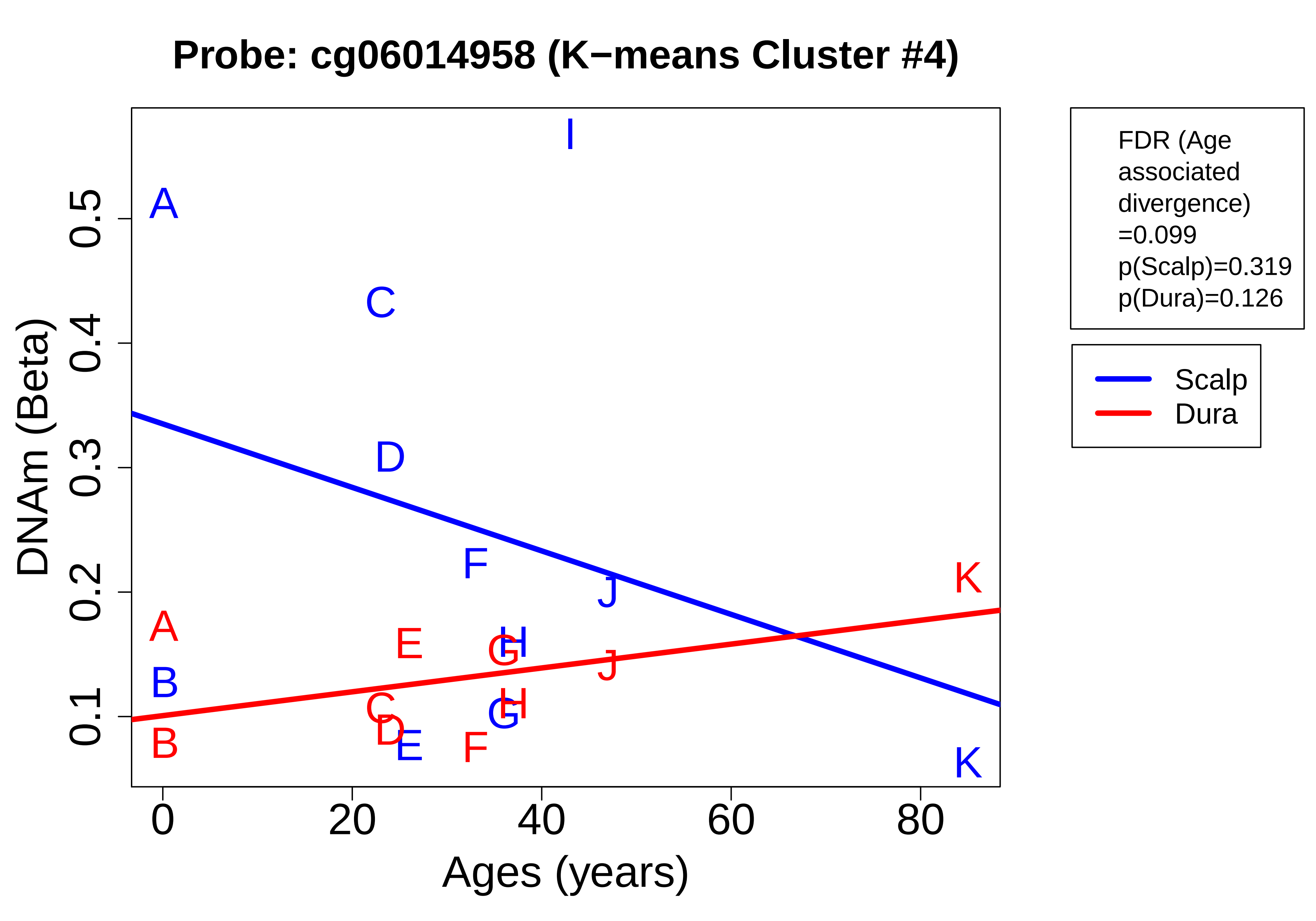

Probe: cg06014958 (K−means Cluster #4)
DNAm (Beta)
0.1
0.2
0.3
0.4
0.5
Ages (years)
0
20
40
60
80
FDR (Age associated divergence) =0.099
p(Scalp)=0.319
p(Dura)=0.126
Scalp
Dura
A
B
C
D
E
F
G
H
I
J
K

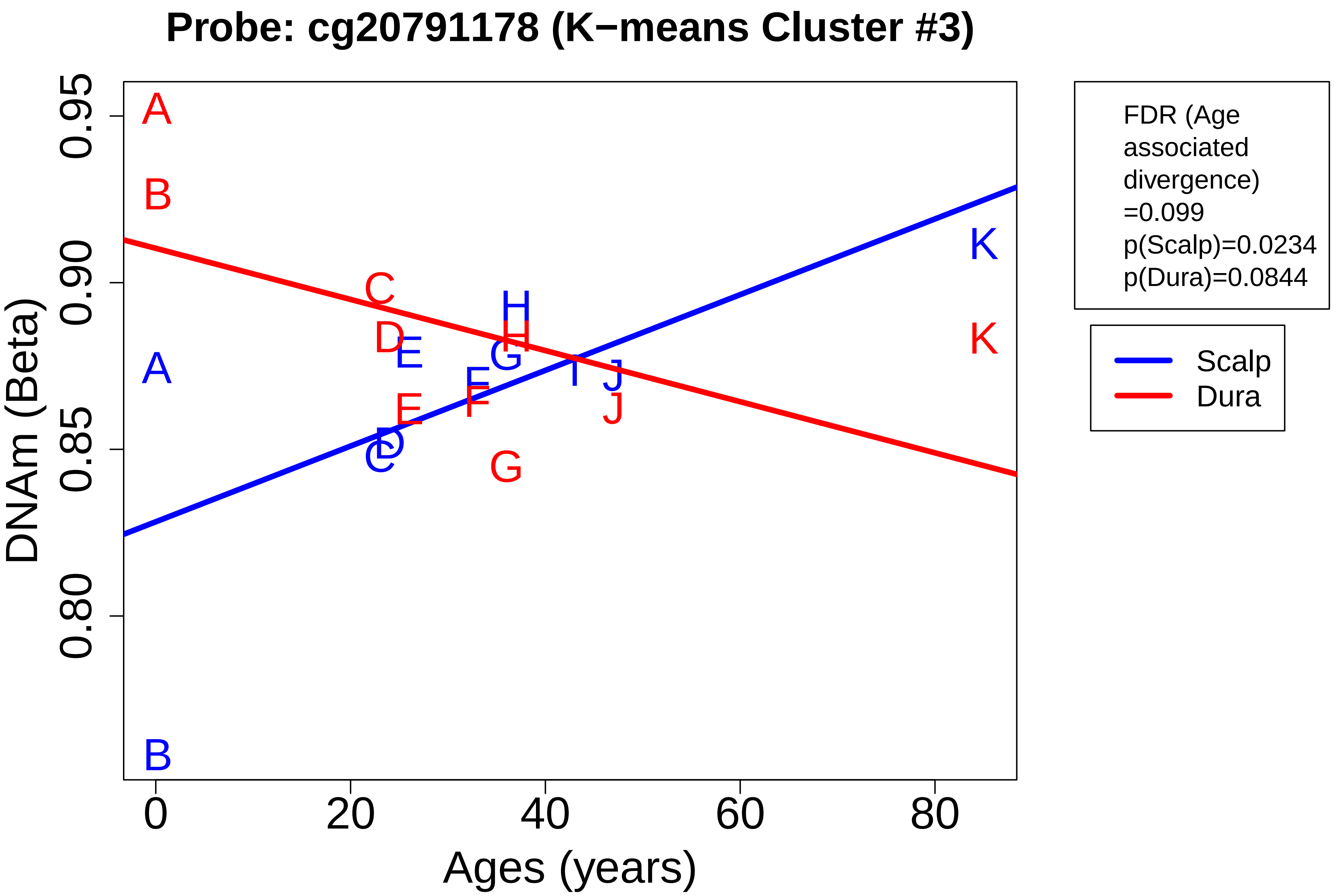

Probe: cg20791178 (K−means Cluster #3)
DNAm (Beta)
0.80
0.85
0.90
0.95
Ages (years)
0
20
40
60
80
A
B
C
D
E
F
G
H
I
J
K
A
B
C
D
E
F
G
H
J
K
FDR (Age associated divergence) =0.099
p(Scalp)=0.0234
p(Dura)=0.0844
Scalp
Dura

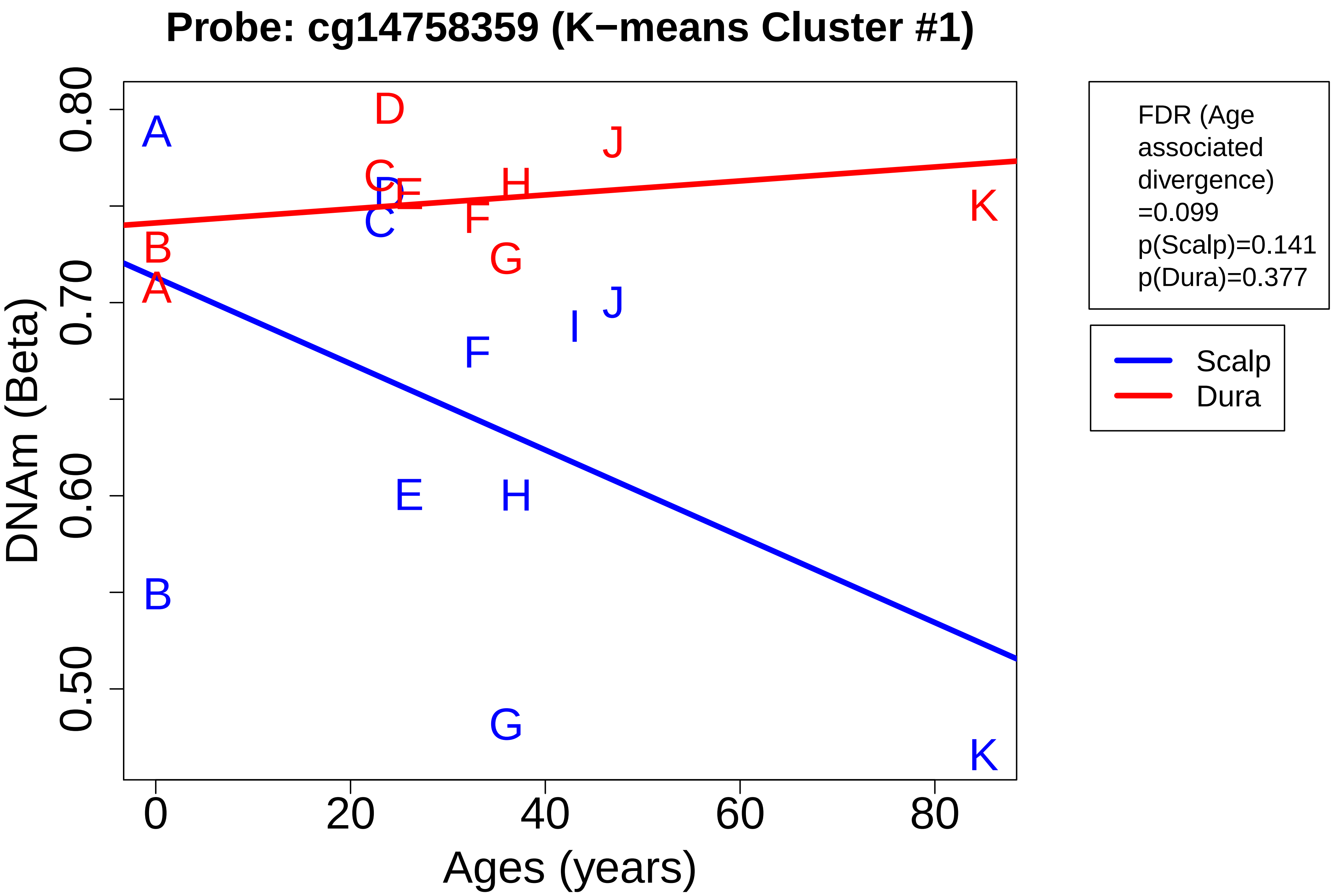

Probe: cg14758359 (K−means Cluster #1)
DNAm (Beta)
0.50
0.60
0.70
0.80
Ages (years)
0
20
40
60
80
A
B
C
D
E
F
G
H
I
J
K
FDR (Age associated divergence) =0.099
p(Scalp)=0.141
p(Dura)=0.377
Scalp
Dura

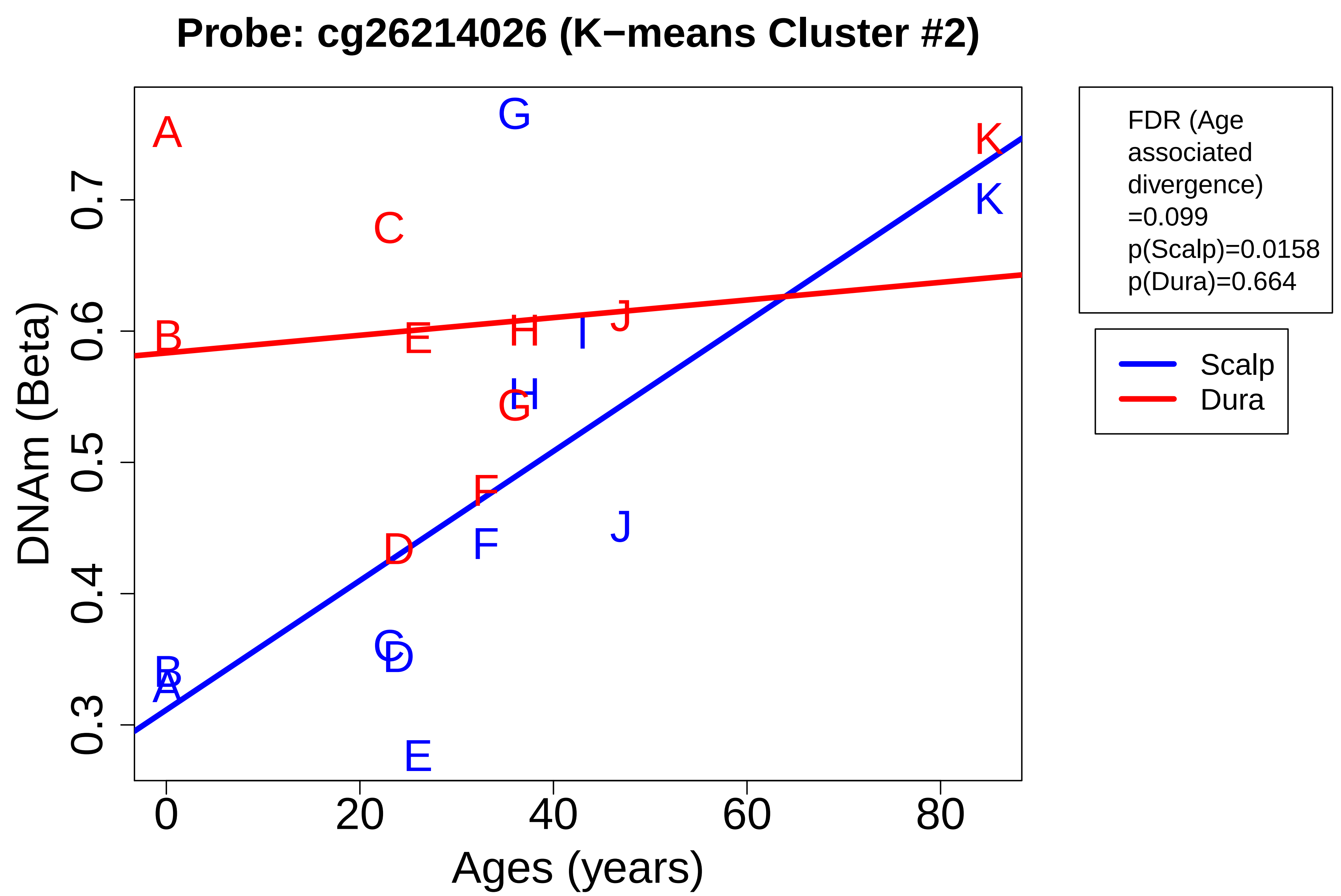

Probe: cg26214026 (K−means Cluster #2)
DNAm (Beta)
0.3
0.4
0.5
0.6
0.7
Ages (years)
0
20
40
60
80
A
B
C
D
E
F
G
H
I
J
K
FDR (Age associated divergence) =0.099
p(Scalp)=0.0158
p(Dura)=0.664
Scalp
Dura

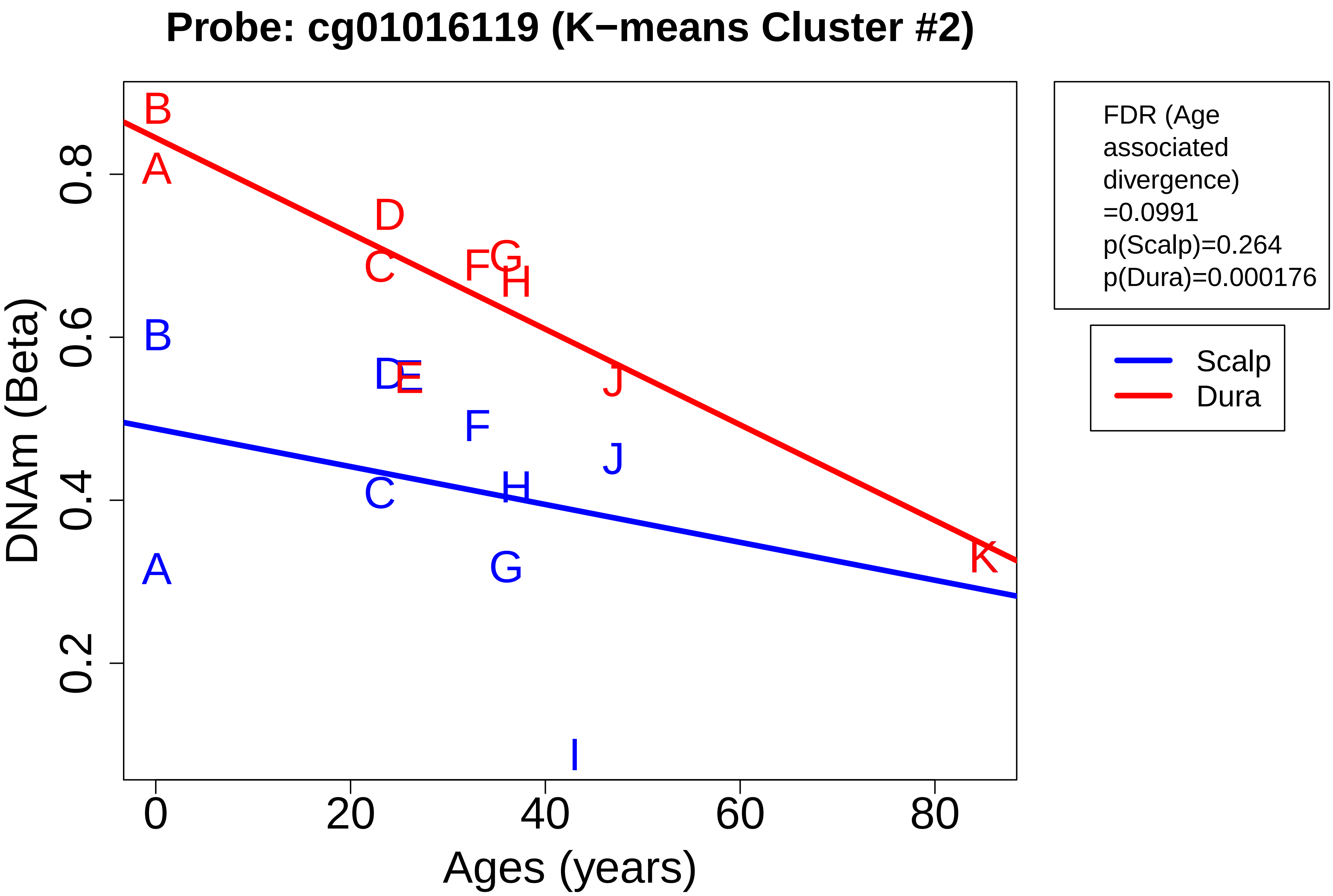
Probe: cg01016119 (K−means Cluster #2)
DNAm (Beta)
0.2
0.4
0.6
0.8
0
20
40
60
80
Ages (years)
B
A
D
C
F
G
H
B
D
E
J
F
J
C
H
A
G
K
I
FDR (Age associated divergence) =0.0991
p(Scalp)=0.264
p(Dura)=0.000176
Scalp
Dura

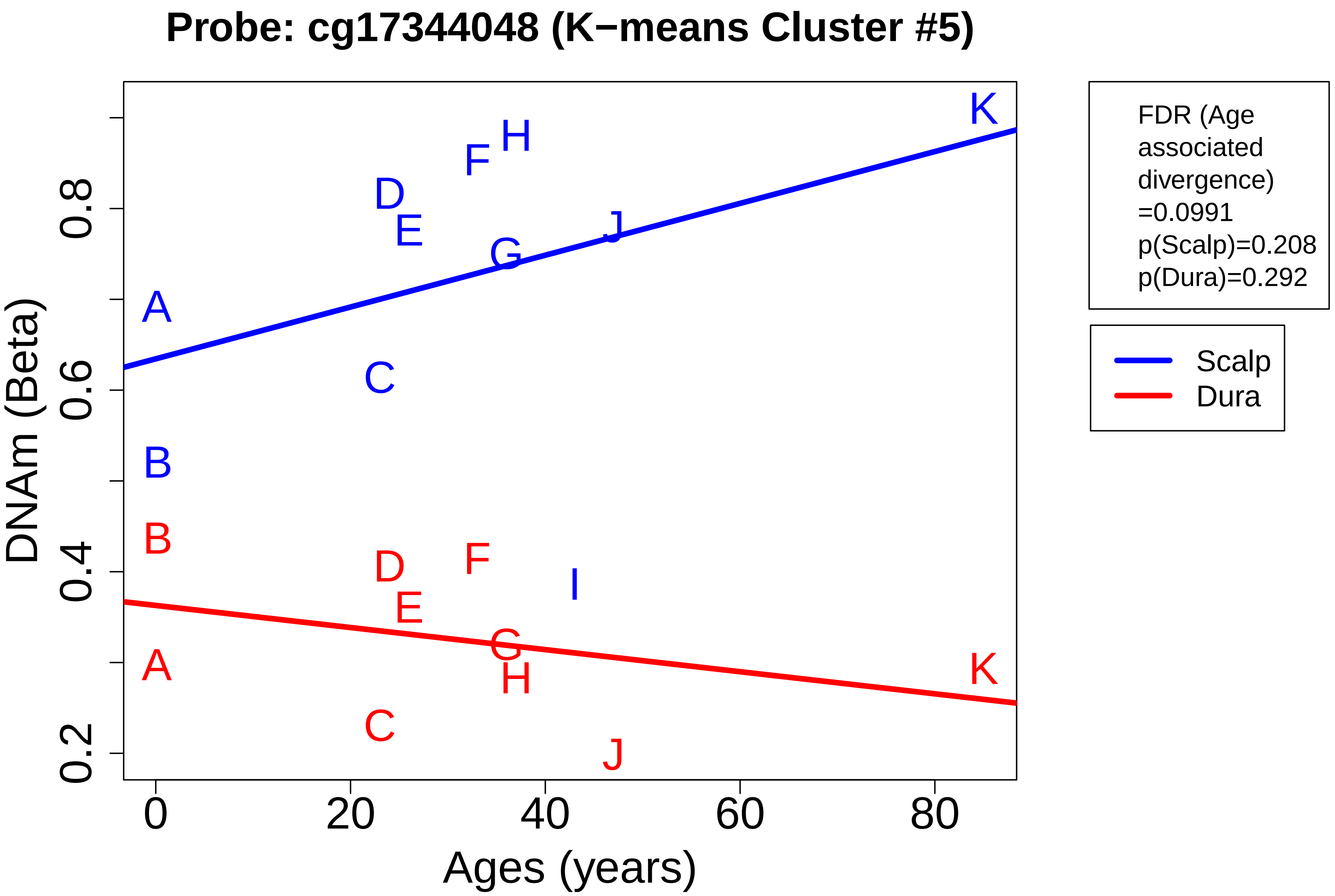
Probe: cg17344048 (K−means Cluster #5)
DNAm (Beta)
0.2
0.4
0.6
0.8
0
20
40
60
80
Ages (years)
A
B
C
D
E
F
G
H
I
J
K
FDR (Age associated divergence) =0.0991
p(Scalp)=0.208
p(Dura)=0.292
Scalp
Dura

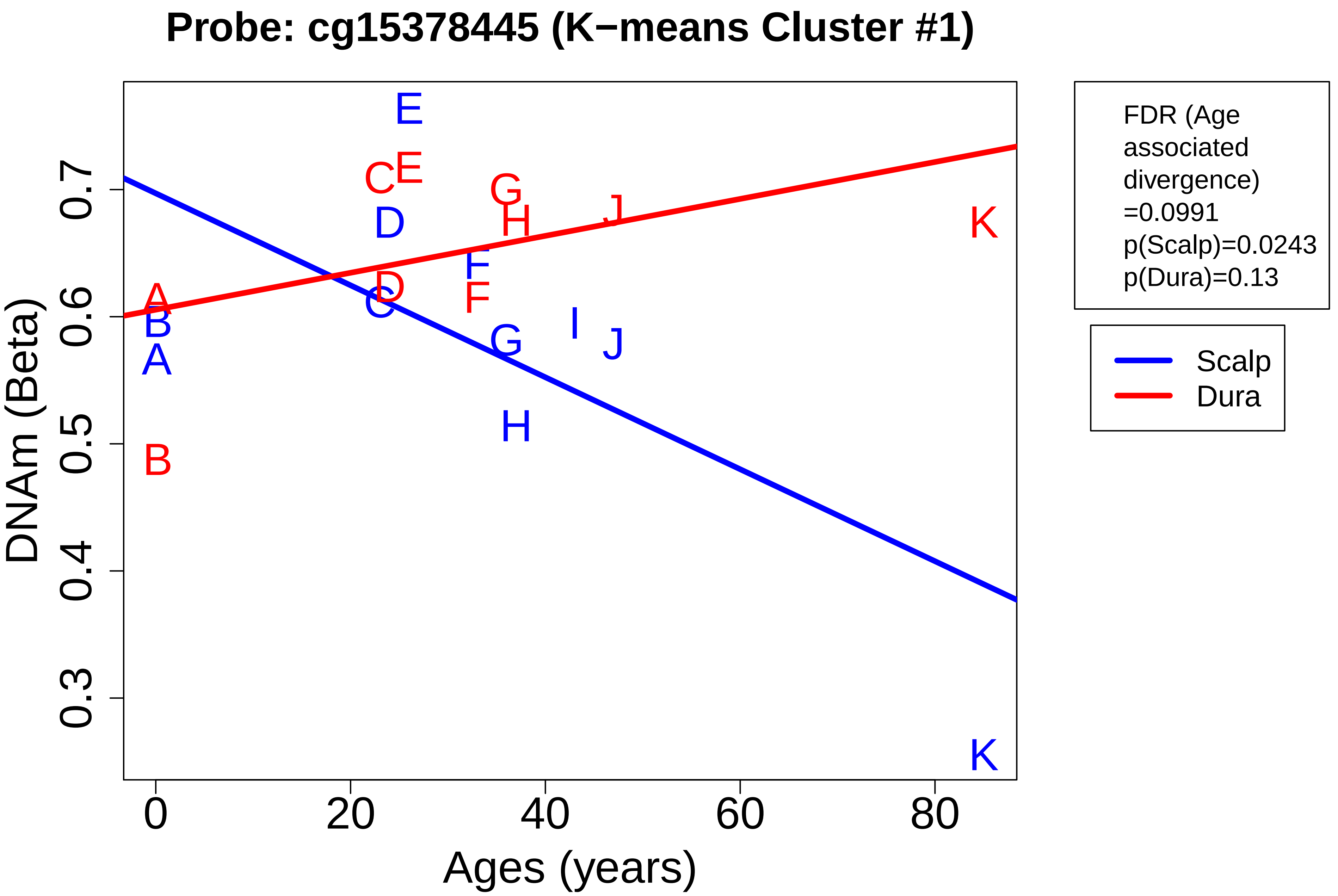

Probe: cg15378445 (K−means Cluster #1)
DNAm (Beta)
0.3
0.4
0.5
0.6
0.7
0
20
40
60
80
Ages (years)
FDR (Age associated divergence) =0.0991
p(Scalp)=0.0243
p(Dura)=0.13
Scalp
Dura
A B C D E F G H I J K

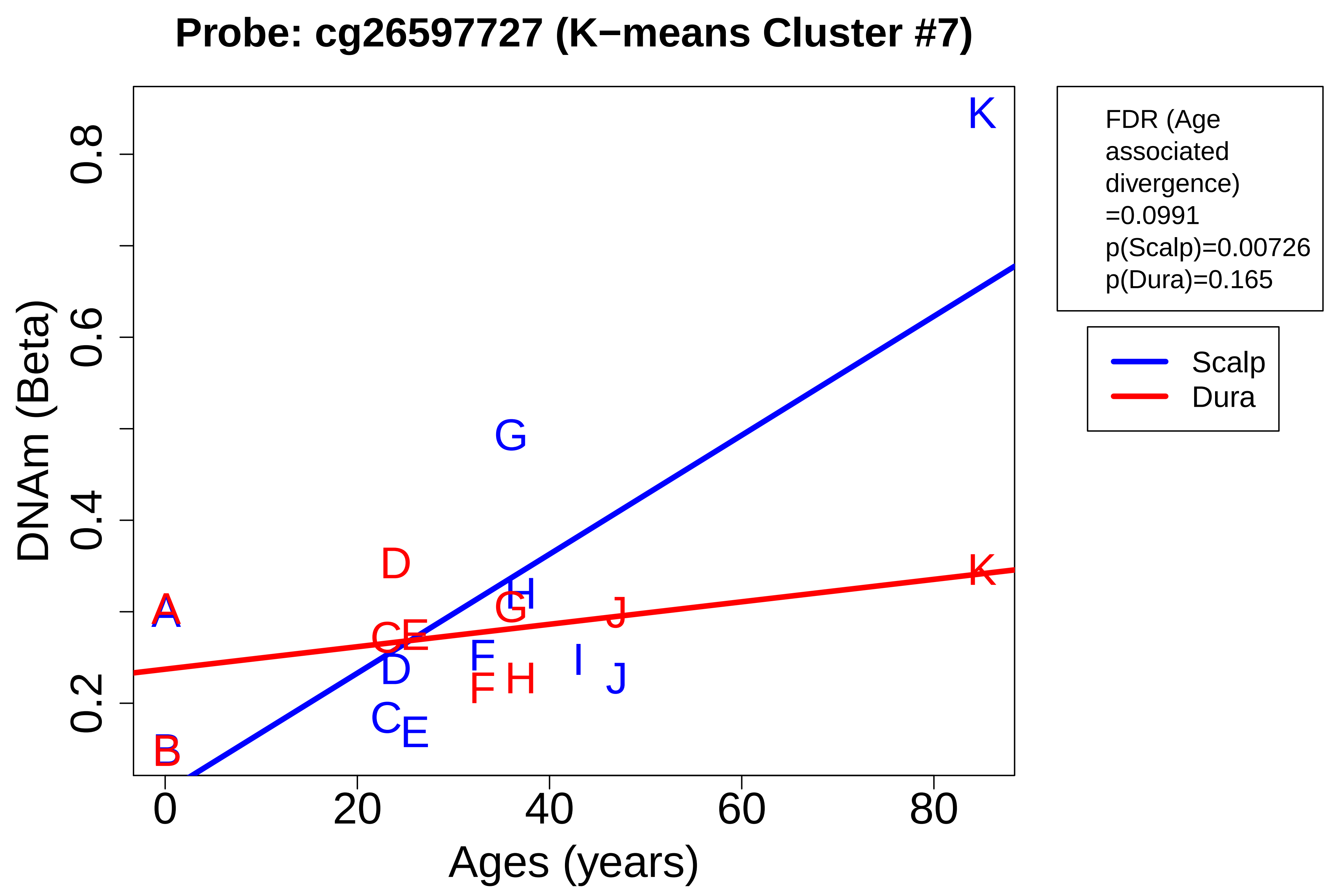

Probe: cg26597727 (K−means Cluster #7)
DNAm (Beta)
0.2
0.4
0.6
0.8
0
20
40
60
80
Ages (years)
FDR (Age associated divergence) =0.0991
p(Scalp)=0.00726
p(Dura)=0.165
Scalp
Dura

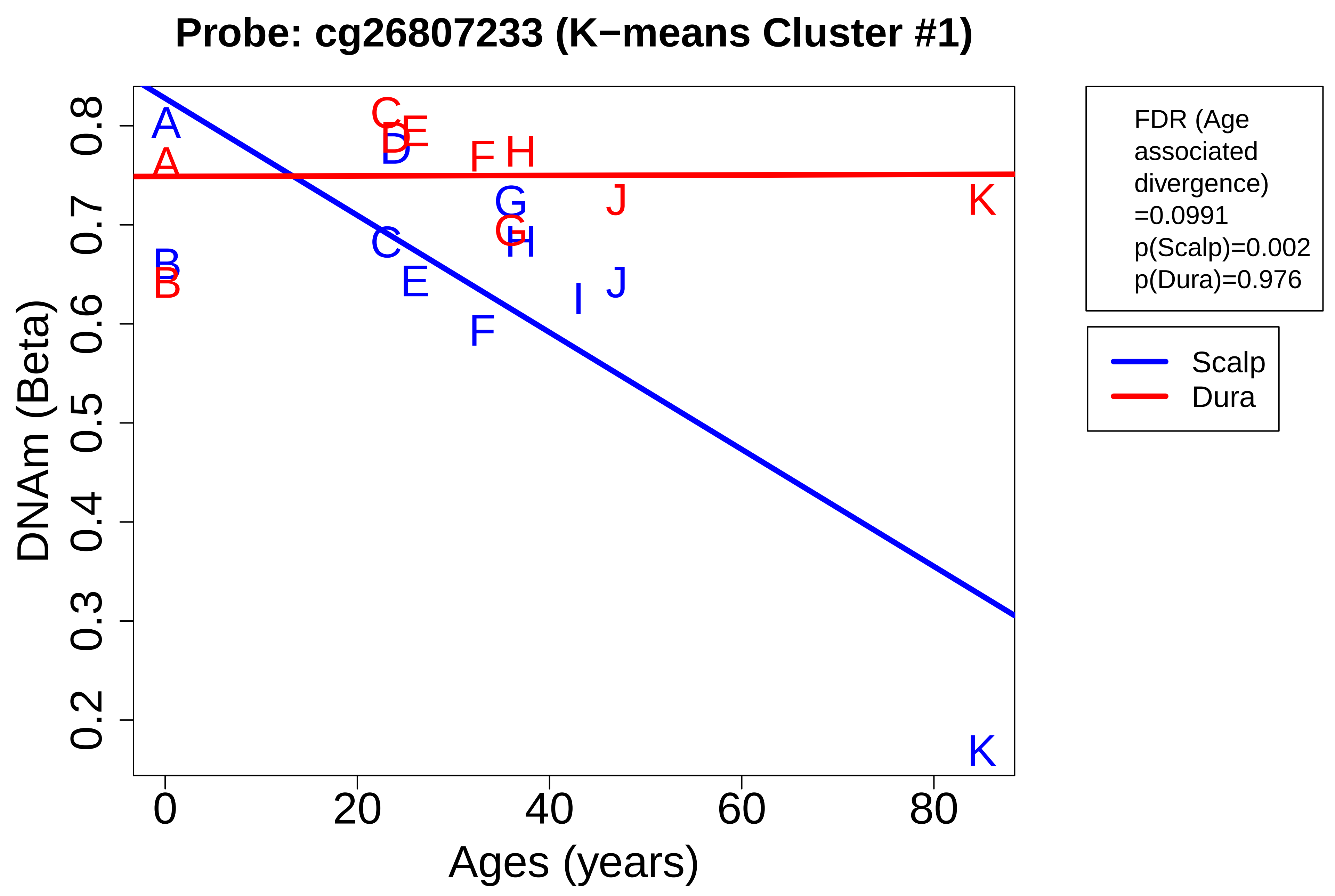

Probe: cg26807233 (K−means Cluster #1)
DNAm (Beta)
0.2
0.3
0.4
0.5
0.6
0.7
0.8
0
20
40
60
80
Ages (years)
A
B
C
D
E
F
G
H
I
J
K
FDR (Age associated divergence) =0.0991
p(Scalp)=0.002
p(Dura)=0.976
Scalp
Dura

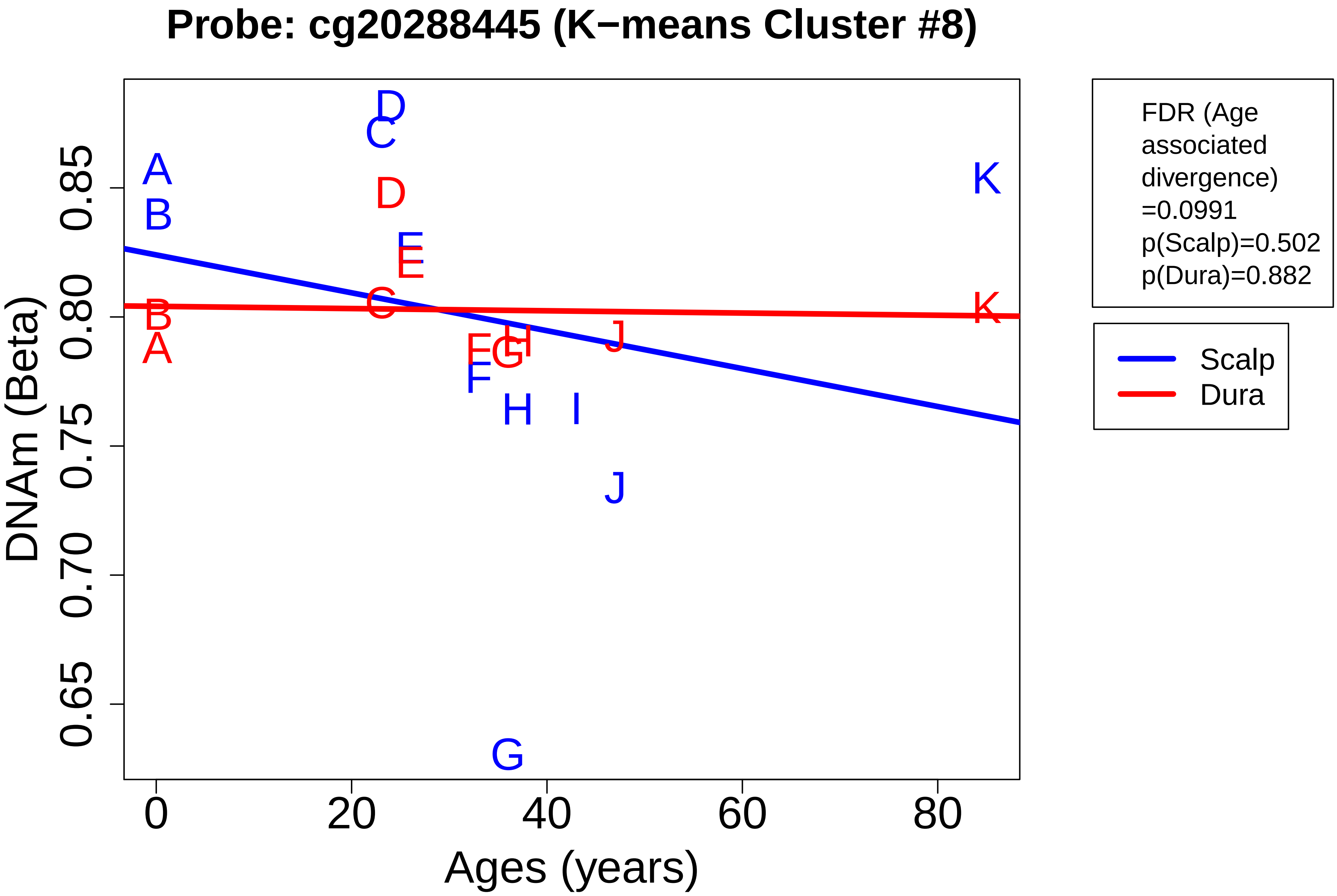

Probe: cg20288445 (K−means Cluster #8)
DNAm (Beta)
0.65
0.70
0.75
0.80
0.85
0
20
40
60
80
Ages (years)
A
B
C
D
E
F
G
H
I
J
K
FDR (Age associated divergence) =0.0991
p(Scalp)=0.502
p(Dura)=0.882
Scalp
Dura

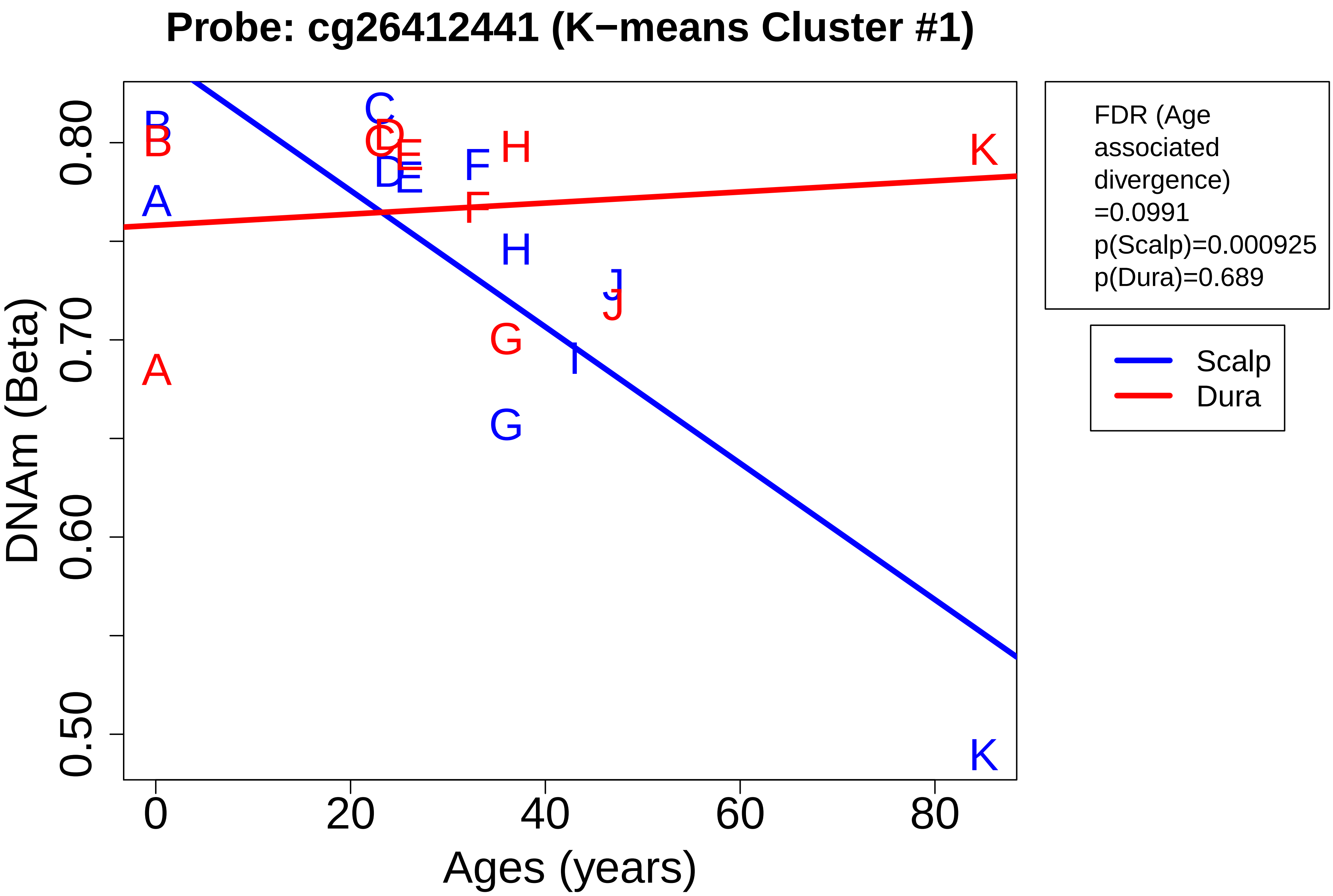
Probe: cg26412441 (K−means Cluster #1)
FDR (Age associated divergence) =0.0991
p(Scalp)=0.000925
p(Dura)=0.689
Scalp
Dura
DNAm (Beta)
0.50
0.60
0.70
0.80
0
20
40
60
80
Ages (years)
A
B
C
D
E
F
G
H
I
J
K

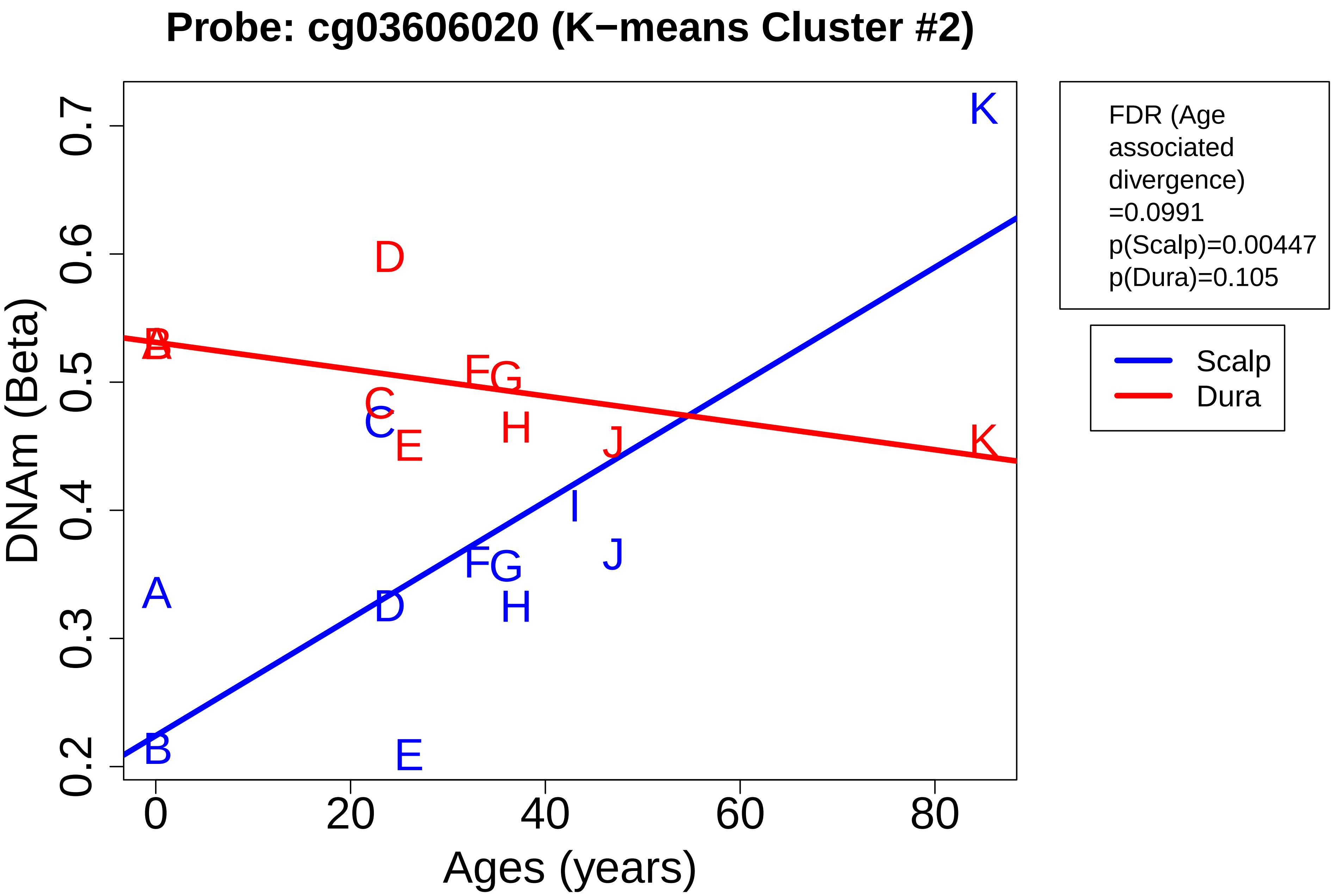
Probe: cg03606020 (K−means Cluster #2)
DNAm (Beta)
0.2
0.3
0.4
0.5
0.6
0.7
Ages (years)
0
20
40
60
80
A
B
C
D
E
F
G
H
I
J
K
FDR (Age associated divergence) =0.0991
p(Scalp)=0.00447
p(Dura)=0.105
Scalp
Dura

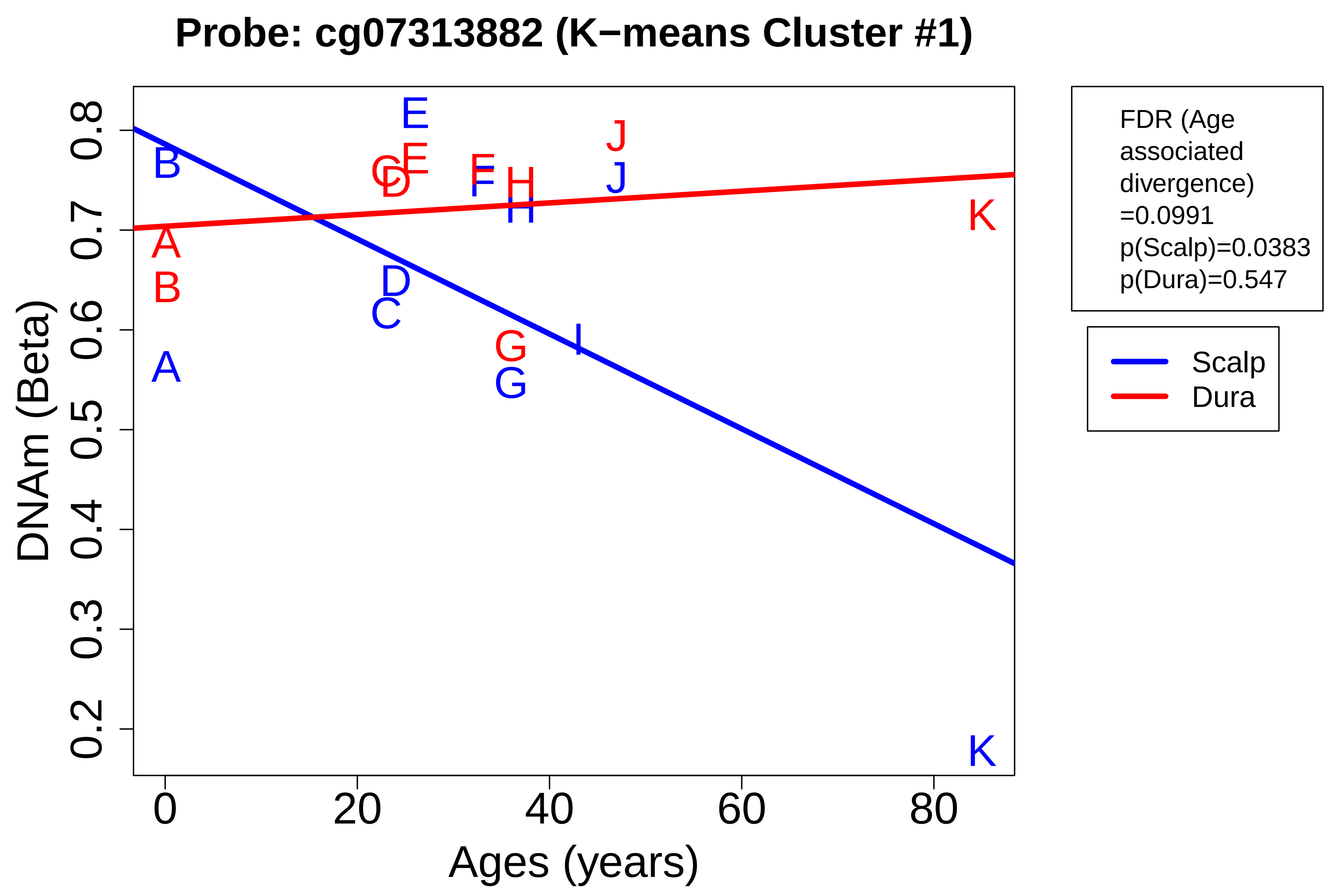

Probe: cg07313882 (K−means Cluster #1)
DNAm (Beta)
0.2
0.3
0.4
0.5
0.6
0.7
0.8
0
20
40
60
80
Ages (years)
FDR (Age associated divergence) =0.0991
p(Scalp)=0.0383
p(Dura)=0.547
Scalp
Dura

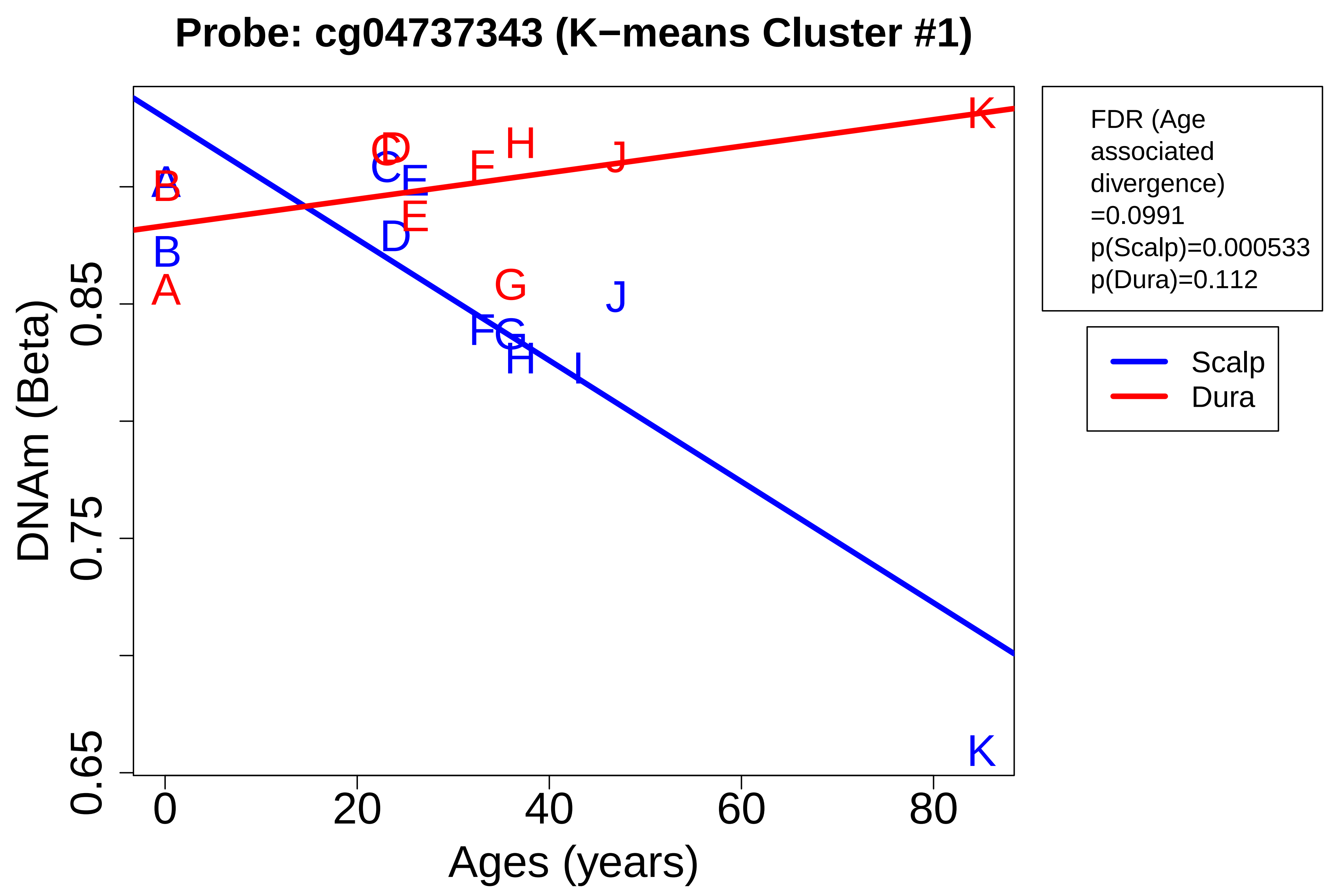

Probe: cg04737343 (K−means Cluster #1)
DNAm (Beta)
0.65
0.75
0.85
Ages (years)
0
20
40
60
80
FDR (Age associated divergence) =0.0991
p(Scalp)=0.000533
p(Dura)=0.112
Scalp
Dura

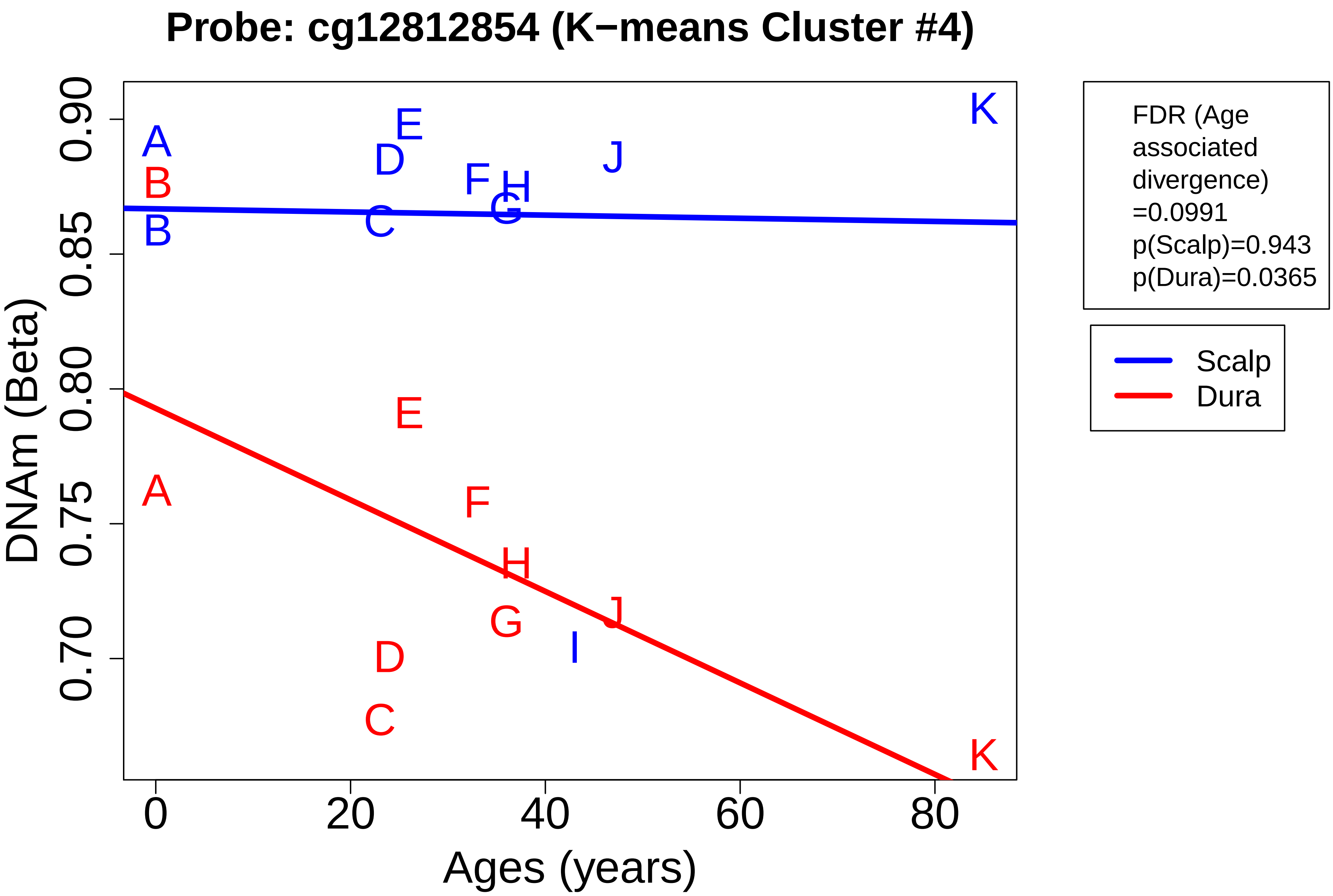
Probe: cg12812854 (K−means Cluster #4)
DNAm (Beta)
0.70
0.75
0.80
0.85
0.90
Ages (years)
0
20
40
60
80
FDR (Age associated divergence) =0.0991
p(Scalp)=0.943
p(Dura)=0.0365
Scalp
Dura

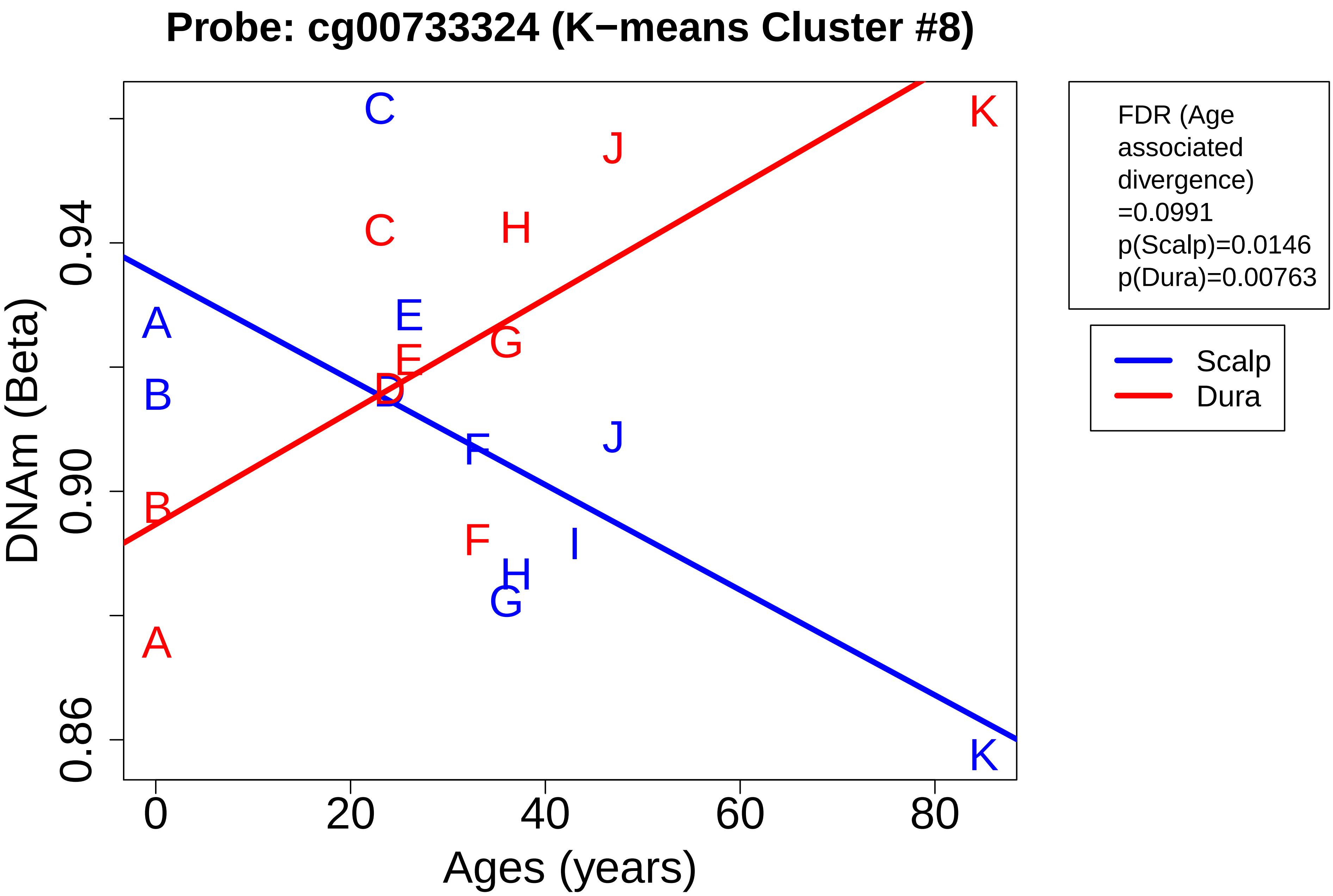

Probe: cg00733324 (K−means Cluster #8)
DNAm (Beta)
0.86
0.90
0.94
0
20
40
60
80
Ages (years)
A
B
C
D
E
F
G
H
I
J
K
FDR (Age associated divergence) =0.0991
p(Scalp)=0.0146
p(Dura)=0.00763
Scalp
Dura

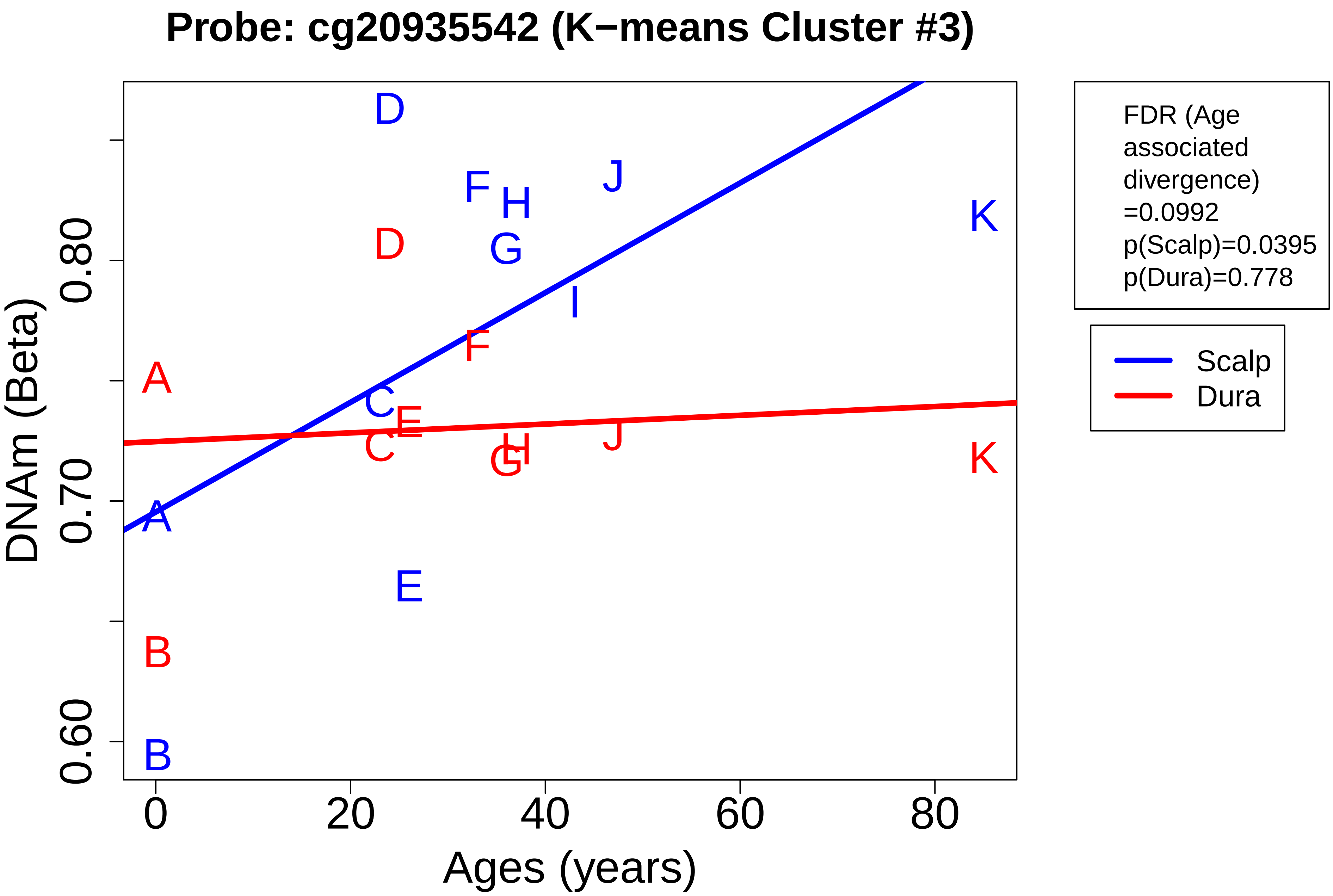
Probe: cg20935542 (K−means Cluster #3)
DNAm (Beta)
0.60
0.70
0.80
0
20
40
60
80
Ages (years)
FDR (Age associated divergence) =0.0992
p(Scalp)=0.0395
p(Dura)=0.778
Scalp
Dura
A
B
C
D
E
F
G
H
I
J
K

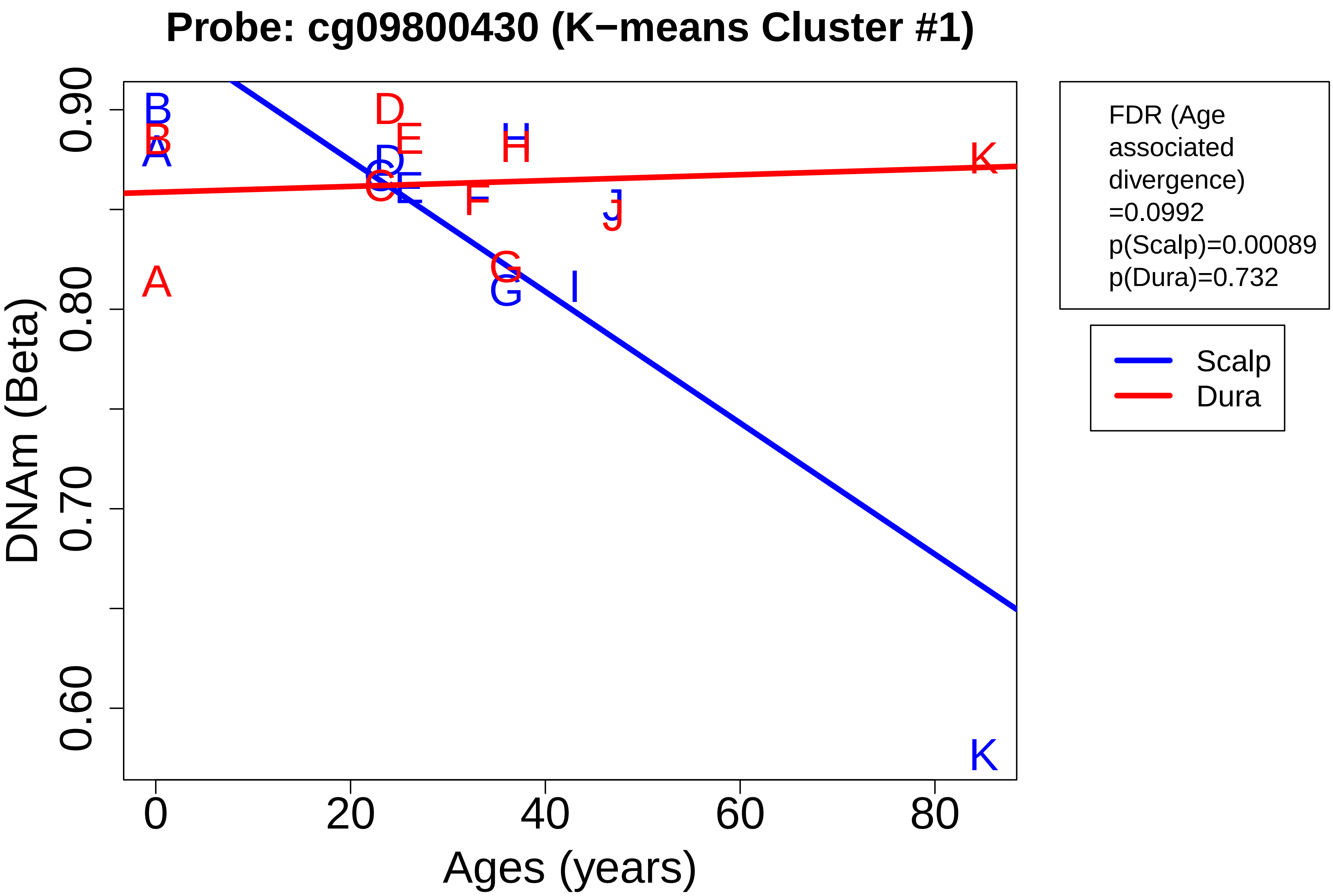

Probe: cg09800430 (K−means Cluster #1)
DNAm (Beta)
0.60
0.70
0.80
0.90
0
20
40
60
80
Ages (years)
FDR (Age associated divergence) =0.0992
p(Scalp)=0.00089
p(Dura)=0.732
Scalp
Dura

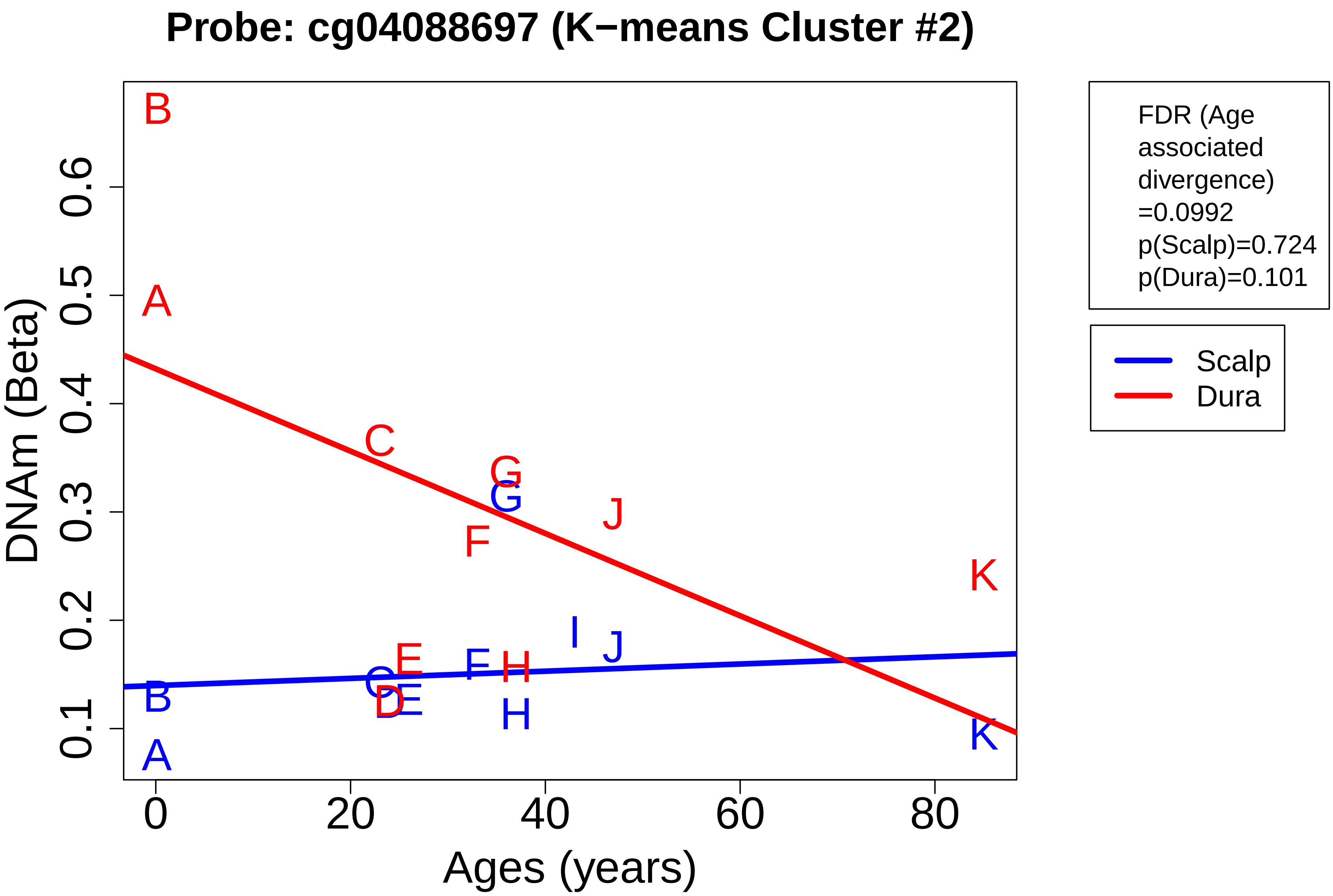

Probe: cg04088697 (K−means Cluster #2)
DNAm (Beta)
0.1
0.2
0.3
0.4
0.5
0.6
0
20
40
60
80
Ages (years)
FDR (Age associated divergence) =0.0992
p(Scalp)=0.724
p(Dura)=0.101
Scalp
Dura

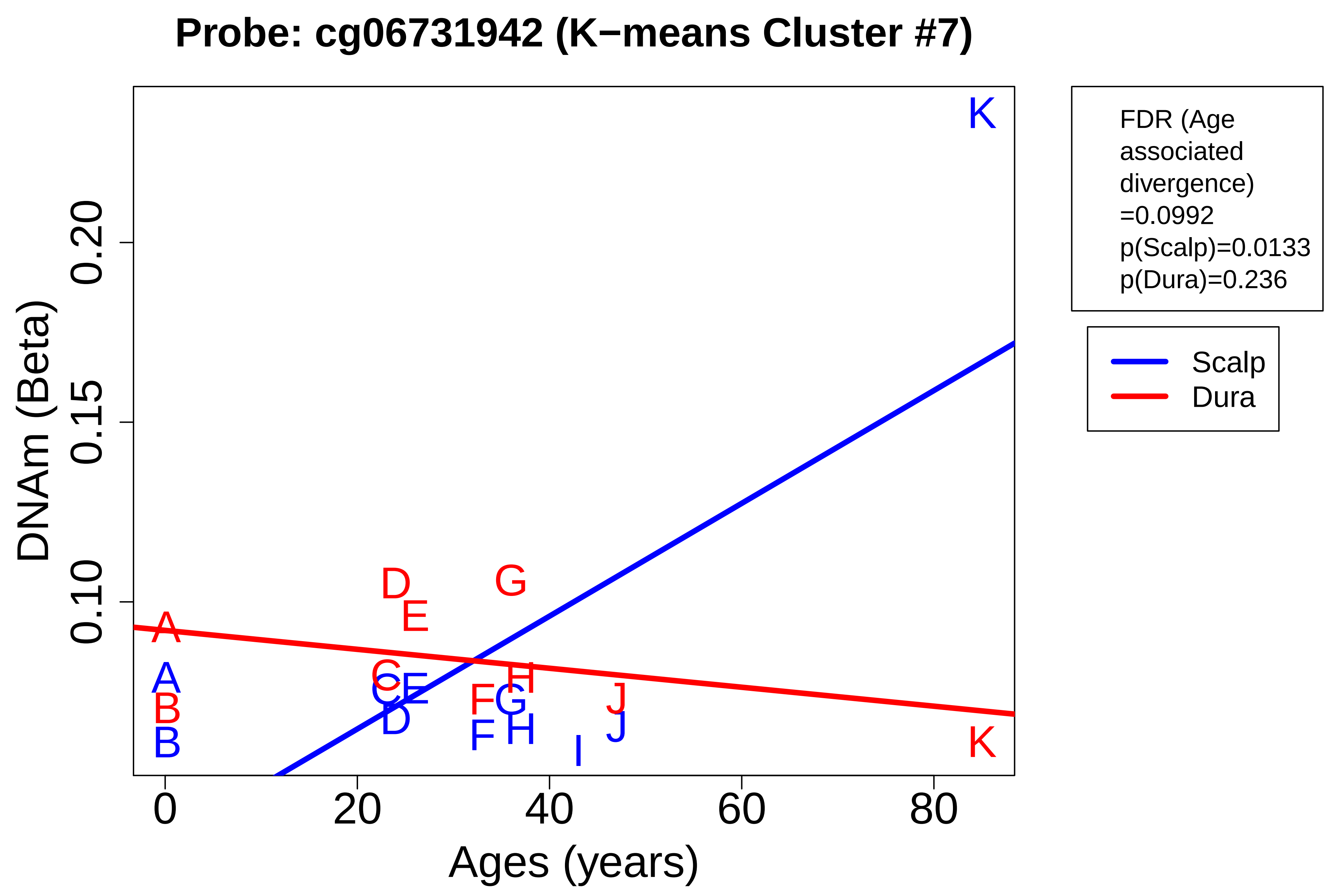

Probe: cg06731942 (K−means Cluster #7)
DNAm (Beta)
0.10
0.15
0.20
Ages (years)
0
20
40
60
80
FDR (Age associated divergence) =0.0992
p(Scalp)=0.0133
p(Dura)=0.236
Scalp
Dura

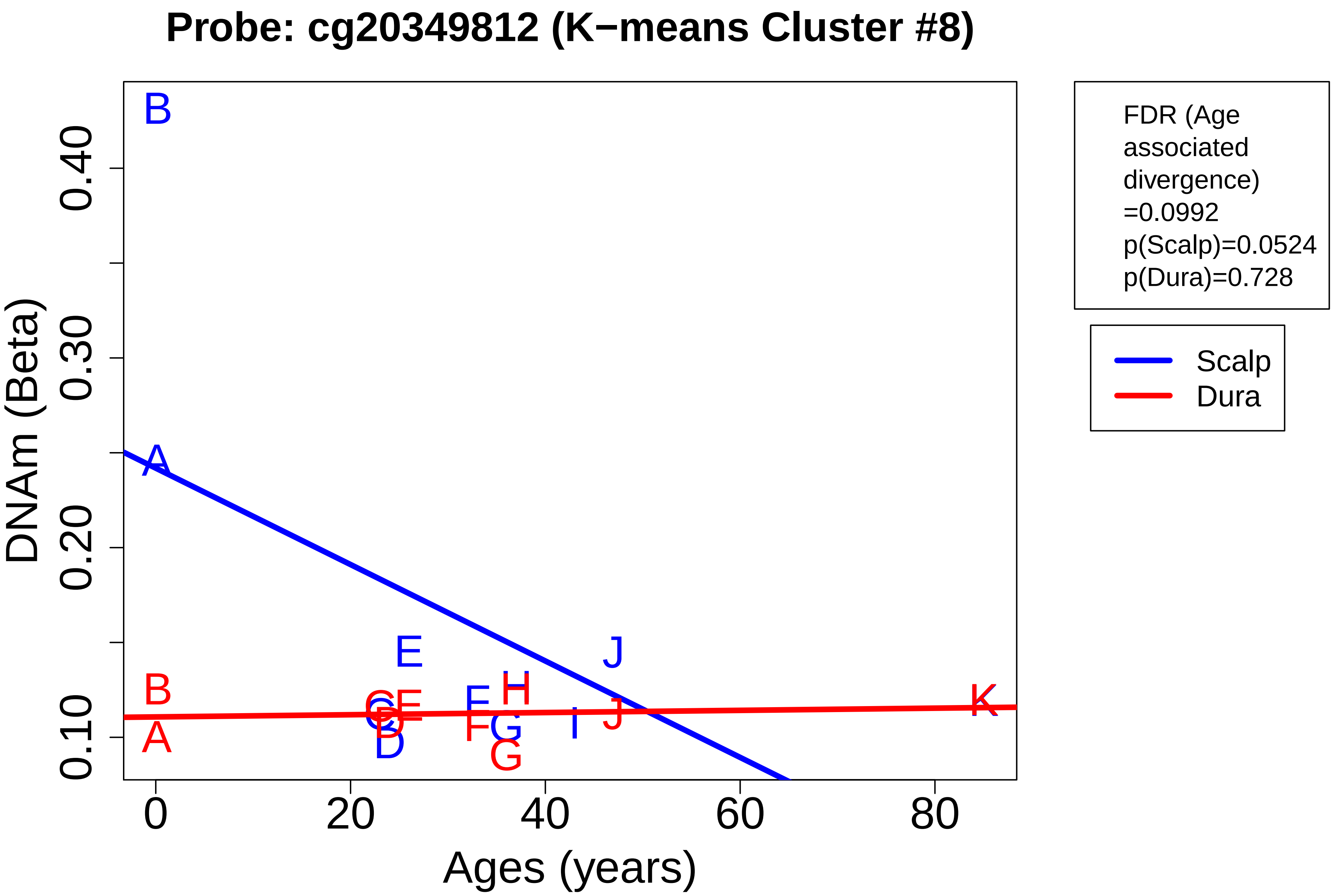
Probe: cg20349812 (K−means Cluster #8)
DNAm (Beta)
0.10
0.20
0.30
0.40
0
20
40
60
80
Ages (years)
FDR (Age associated divergence) =0.0992
p(Scalp)=0.0524
p(Dura)=0.728
Scalp
Dura

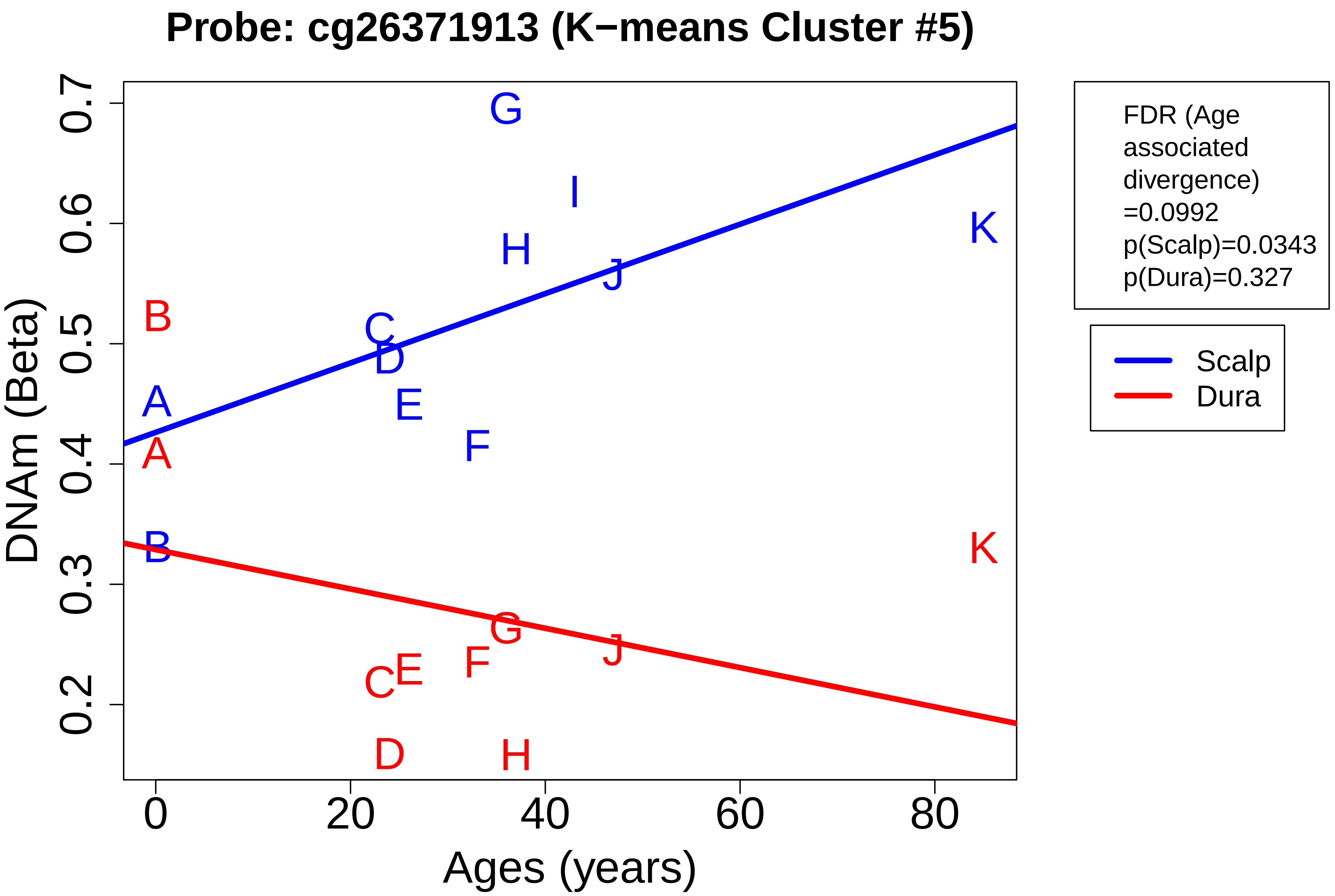

Probe: cg26371913 (K−means Cluster #5)
DNAm (Beta)
0.2
0.3
0.4
0.5
0.6
0.7
Ages (years)
0
20
40
60
80
A
B
C
D
E
F
G
H
I
J
K
A
B
C
D
E
F
G
H
J
K
FDR (Age associated divergence) =0.0992
p(Scalp)=0.0343
p(Dura)=0.327
Scalp
Dura

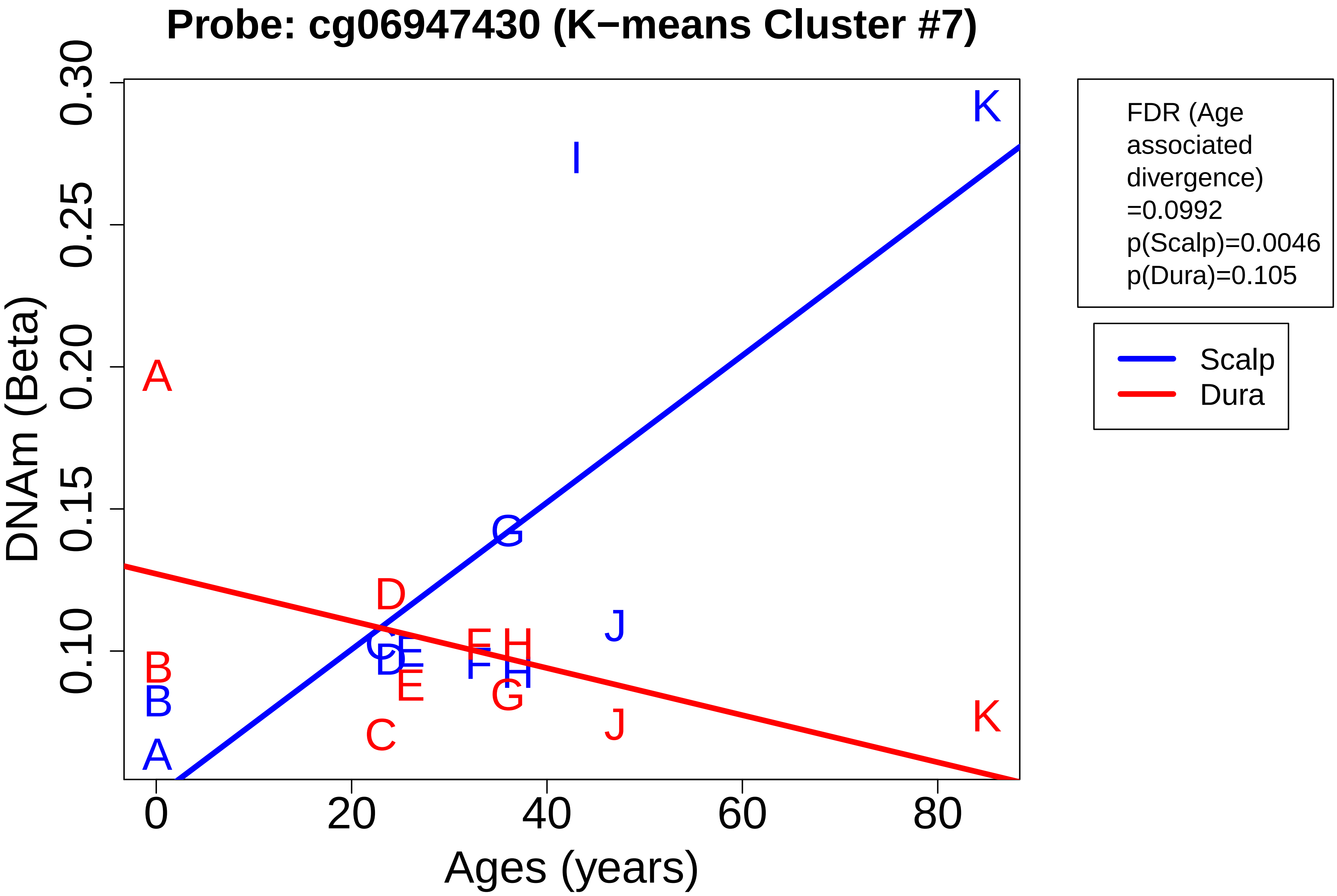

Probe: cg06947430 (K−means Cluster #7)
DNAm (Beta)
0.10
0.15
0.20
0.25
0.30
Ages (years)
0
20
40
60
80
A
B
C
D
E
F
G
H
I
J
K
FDR (Age associated divergence) =0.0992
p(Scalp)=0.0046
p(Dura)=0.105
Scalp
Dura

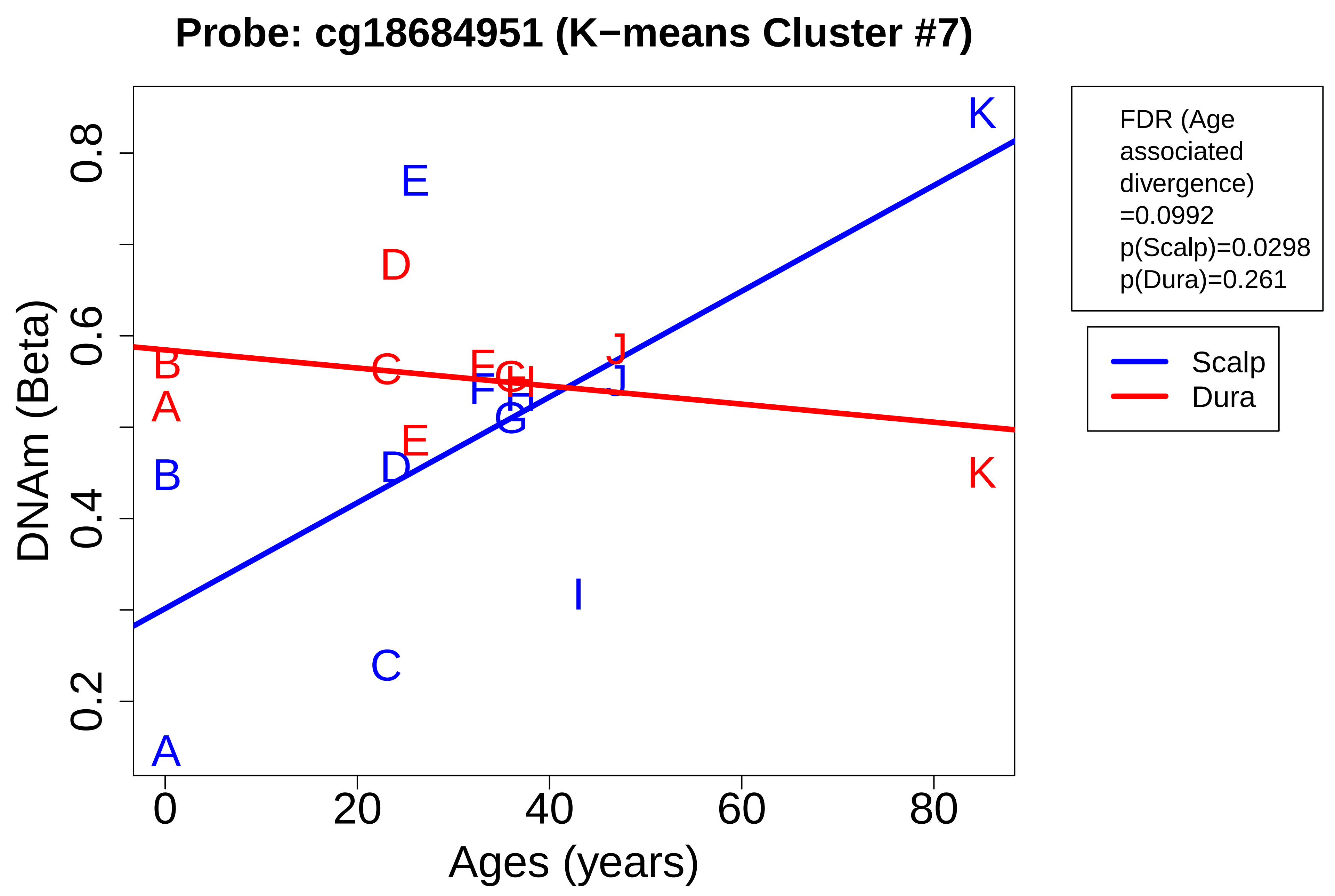

Probe: cg18684951 (K−means Cluster #7)
DNAm (Beta)
0.2
0.4
0.6
0.8
0
20
40
60
80
Ages (years)
FDR (Age associated divergence) =0.0992
p(Scalp)=0.0298
p(Dura)=0.261
Scalp
Dura

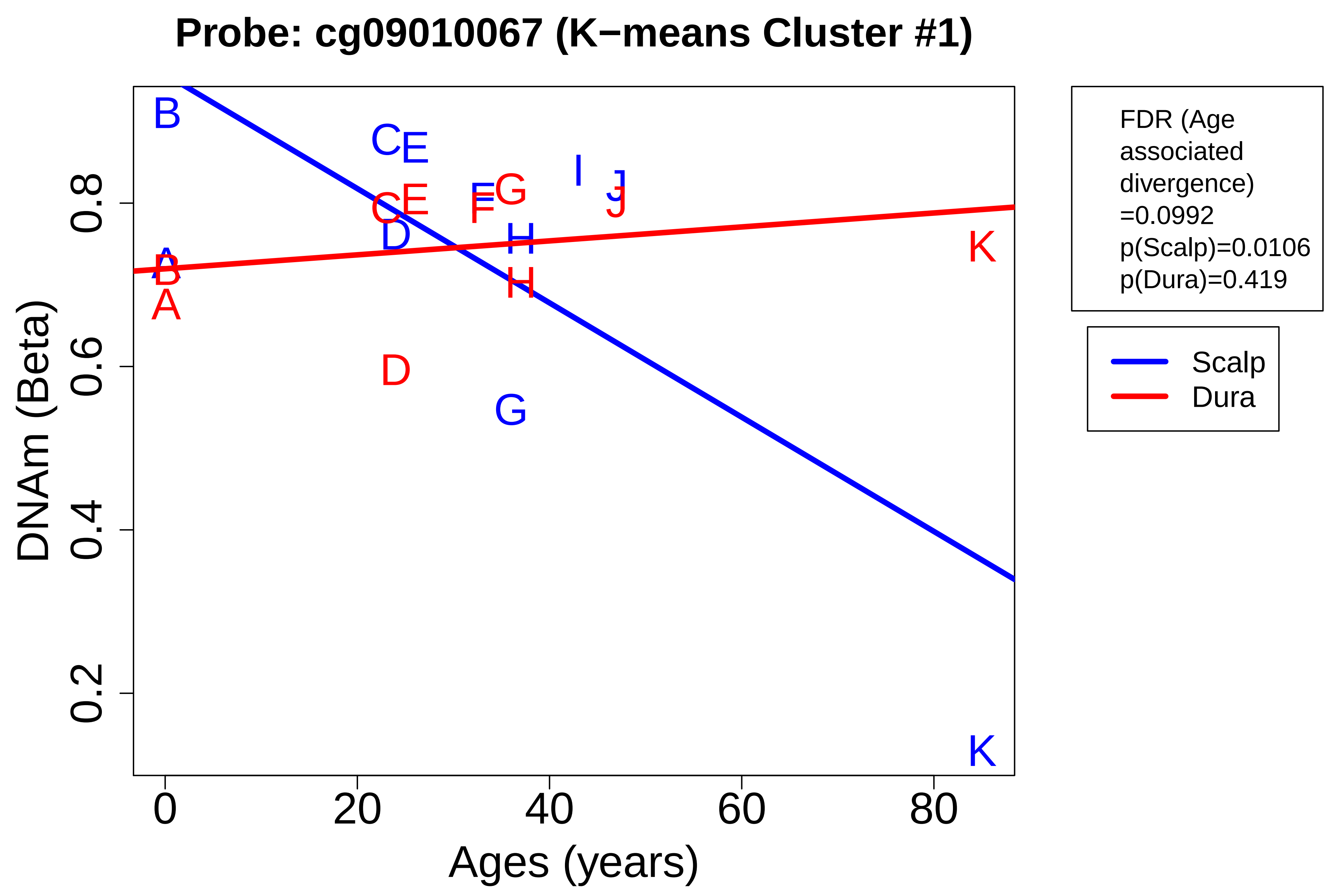

Probe: cg09010067 (K−means Cluster #1)
DNAm (Beta)
0.2
0.4
0.6
0.8
0
20
40
60
80
Ages (years)
FDR (Age associated divergence) =0.0992
p(Scalp)=0.0106
p(Dura)=0.419
Scalp
Dura
A
B
C
D
E
F
G
H
I
J
K

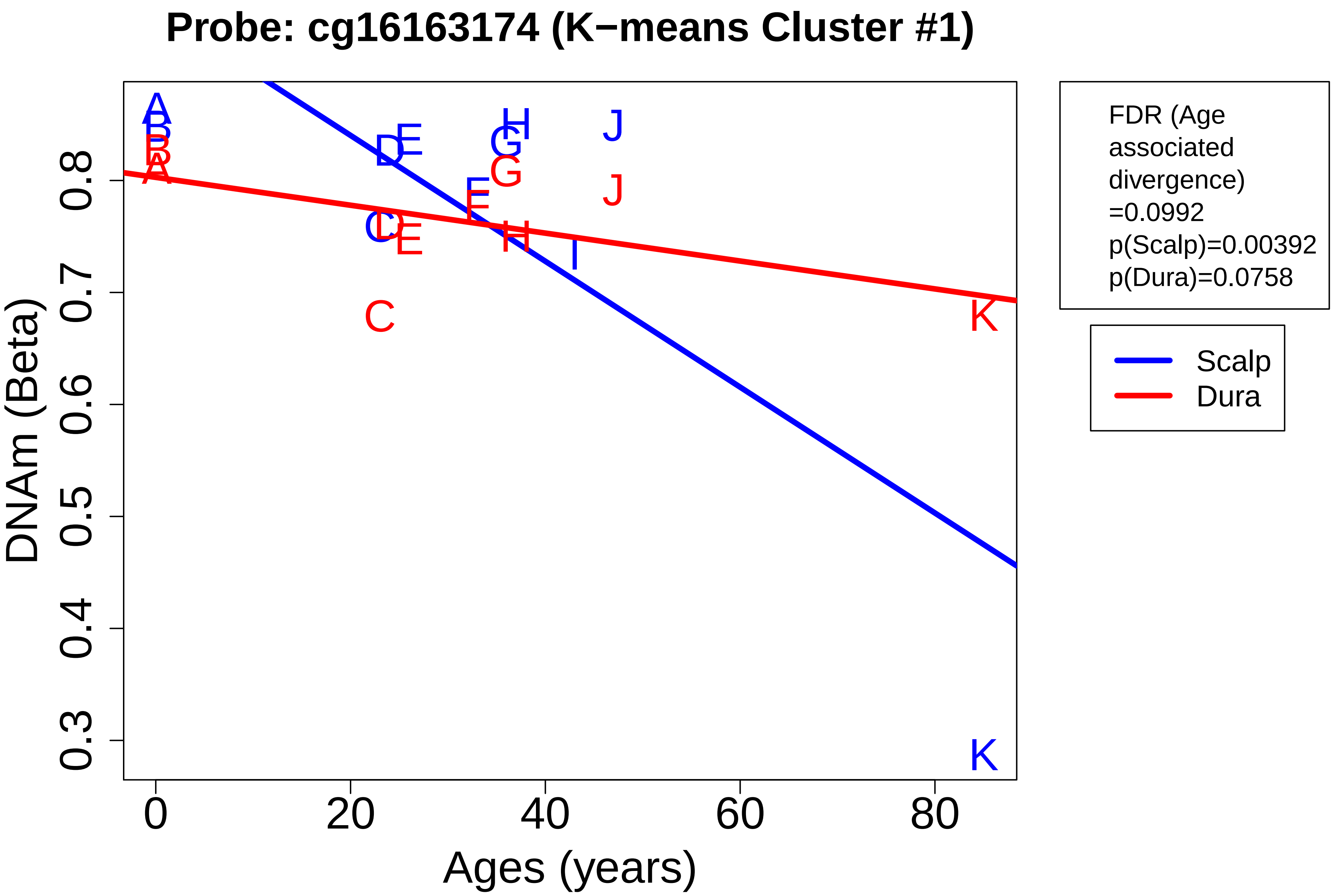

Probe: cg16163174 (K−means Cluster #1)
DNAm (Beta)
0.3
0.4
0.5
0.6
0.7
0.8
0
20
40
60
80
Ages (years)
A
B
C
D
E
F
G
H
I
J
K
FDR (Age associated divergence) =0.0992
p(Scalp)=0.00392
p(Dura)=0.0758
Scalp
Dura

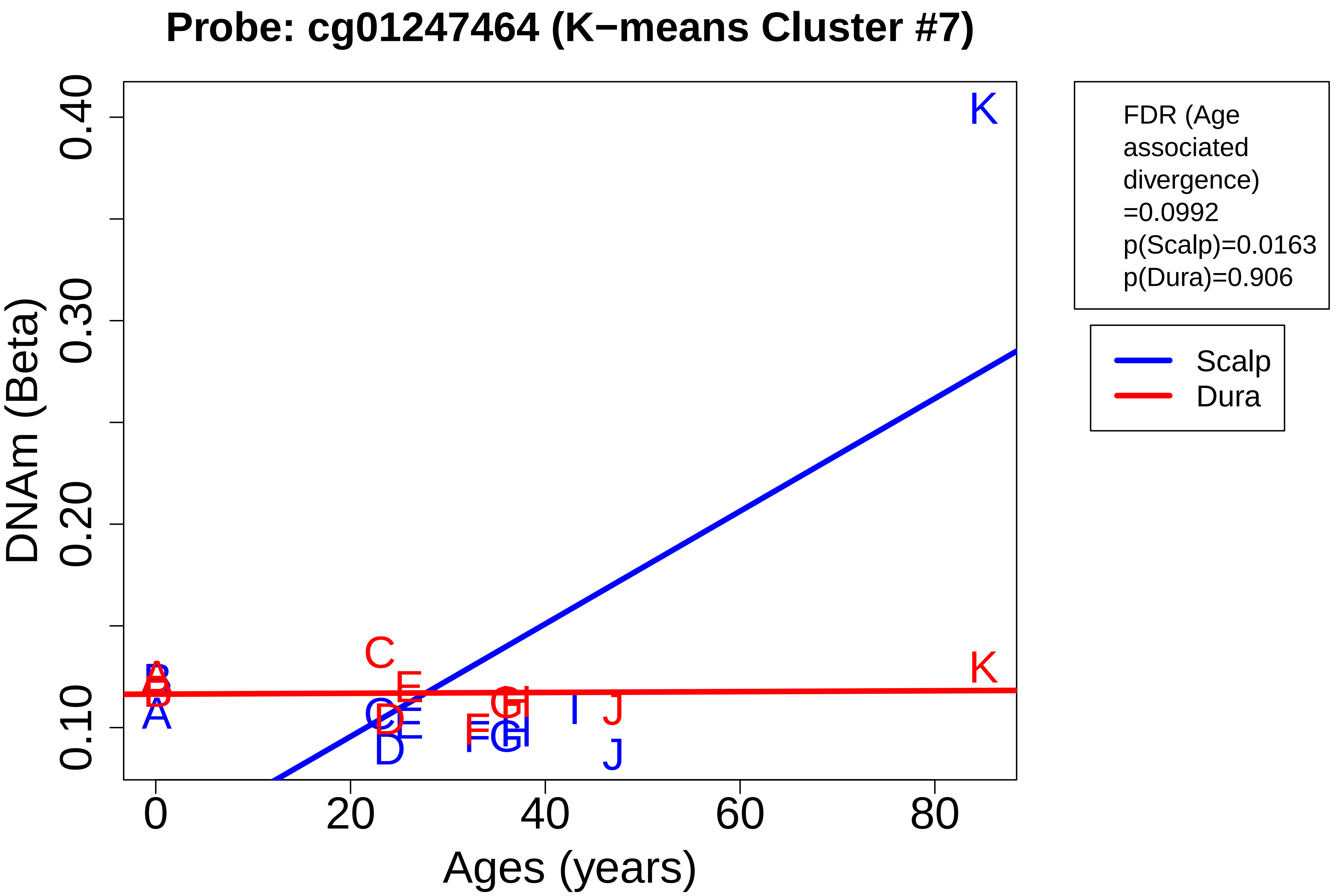

Probe: cg01247464 (K−means Cluster #7)
DNAm (Beta)
0.10
0.20
0.30
0.40
Ages (years)
0
20
40
60
80
FDR (Age associated divergence) =0.0992
p(Scalp)=0.0163
p(Dura)=0.906
Scalp
Dura

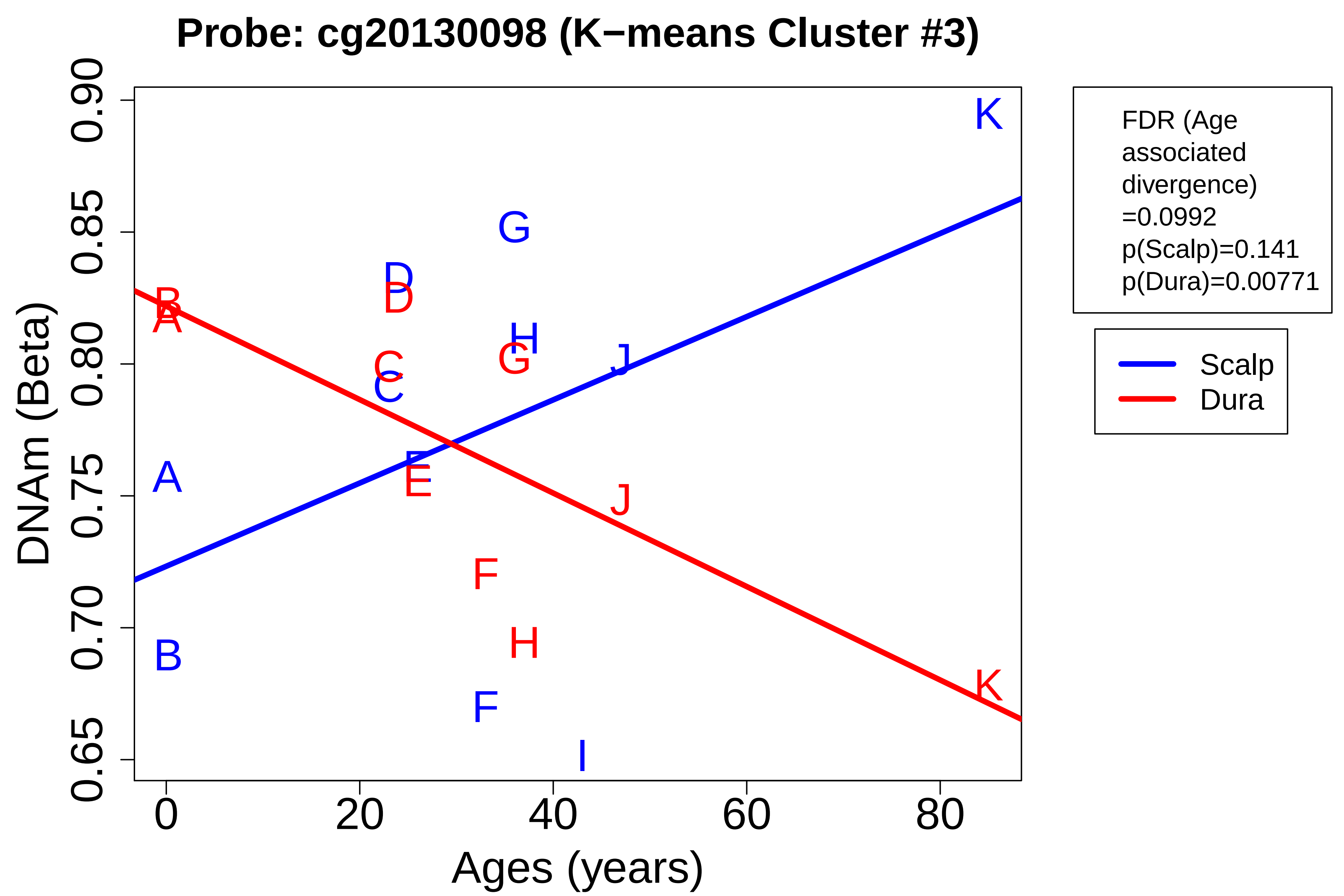

Probe: cg20130098 (K−means Cluster #3)
DNAm (Beta)
0.65
0.70
0.75
0.80
0.85
0.90
0
20
40
60
80
Ages (years)
FDR (Age associated divergence) =0.0992
p(Scalp)=0.141
p(Dura)=0.00771
Scalp
Dura
A
B
C
D
E
F
G
H
I
J
K

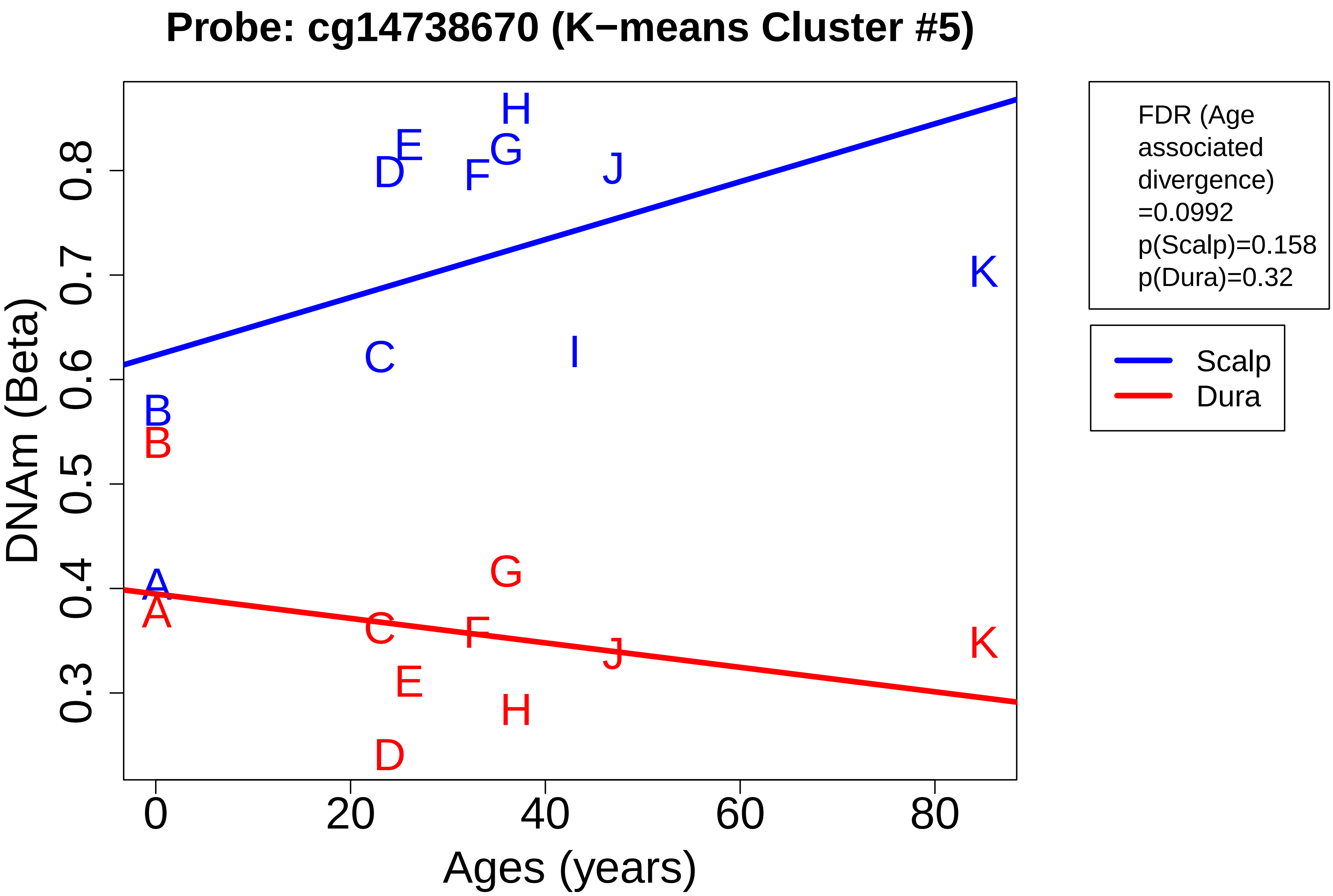

Probe: cg14738670 (K−means Cluster #5)
DNAm (Beta)
0.3
0.4
0.5
0.6
0.7
0.8
0
20
40
60
80
Ages (years)
FDR (Age associated divergence) =0.0992
p(Scalp)=0.158
p(Dura)=0.32
Scalp
Dura
A
B
C
D
E
F
G
H
I
J
K

Probe: cg10291119 (K−means Cluster #2)

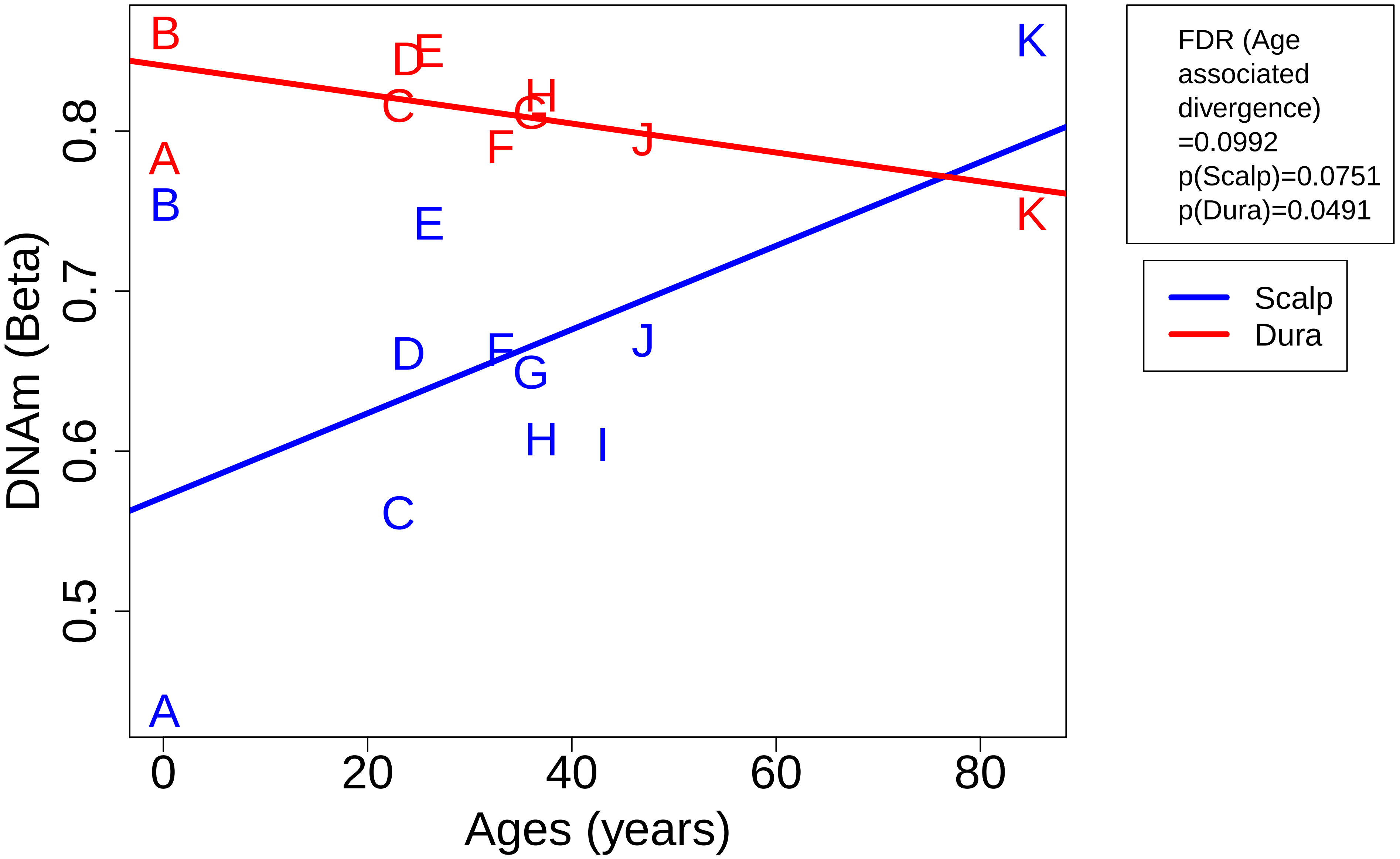

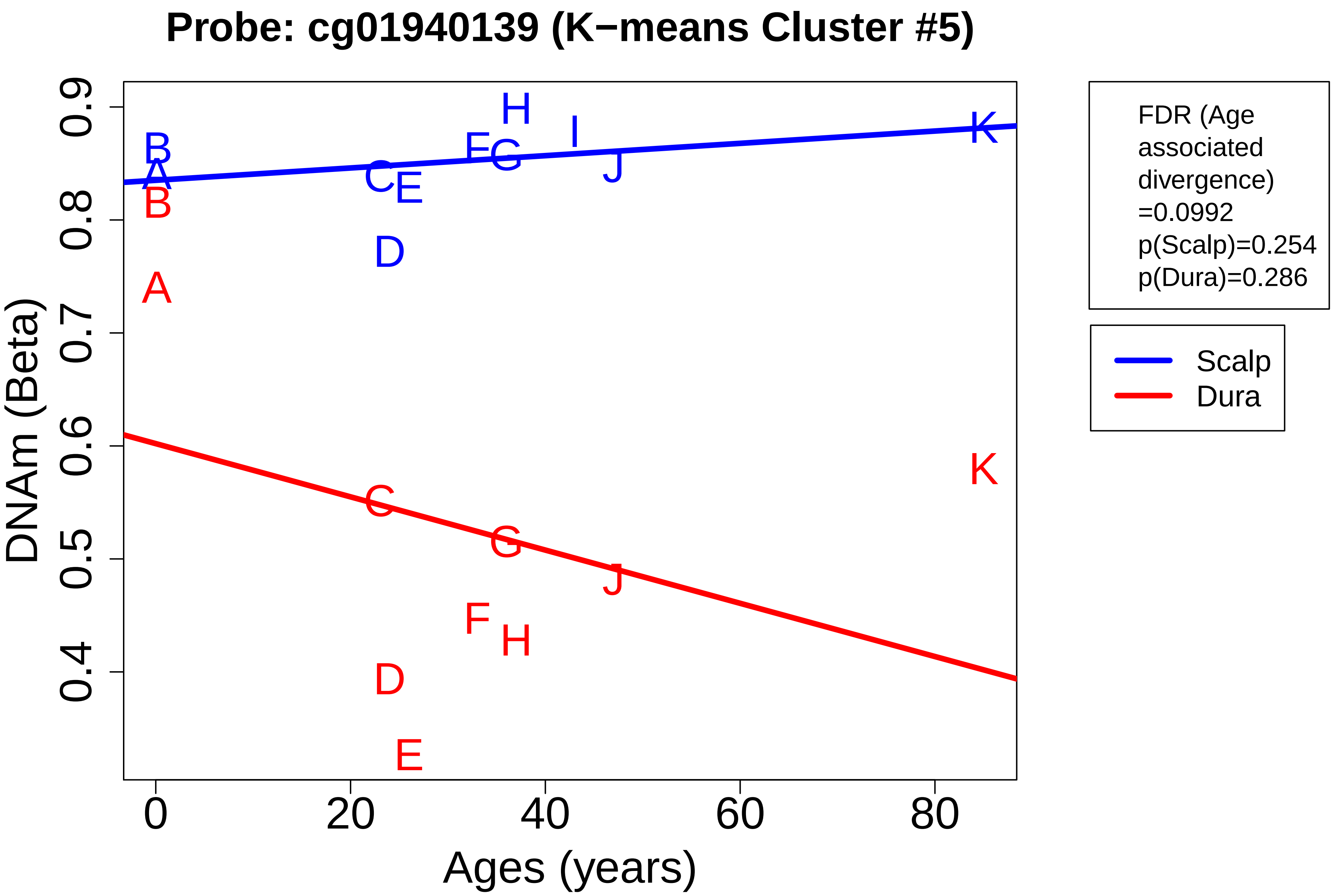

Probe: cg01940139 (K−means Cluster #5)
DNAm (Beta)
0.4
0.5
0.6
0.7
0.8
0.9
0
20
40
60
80
Ages (years)
FDR (Age associated divergence) =0.0992
p(Scalp)=0.254
p(Dura)=0.286
Scalp
Dura

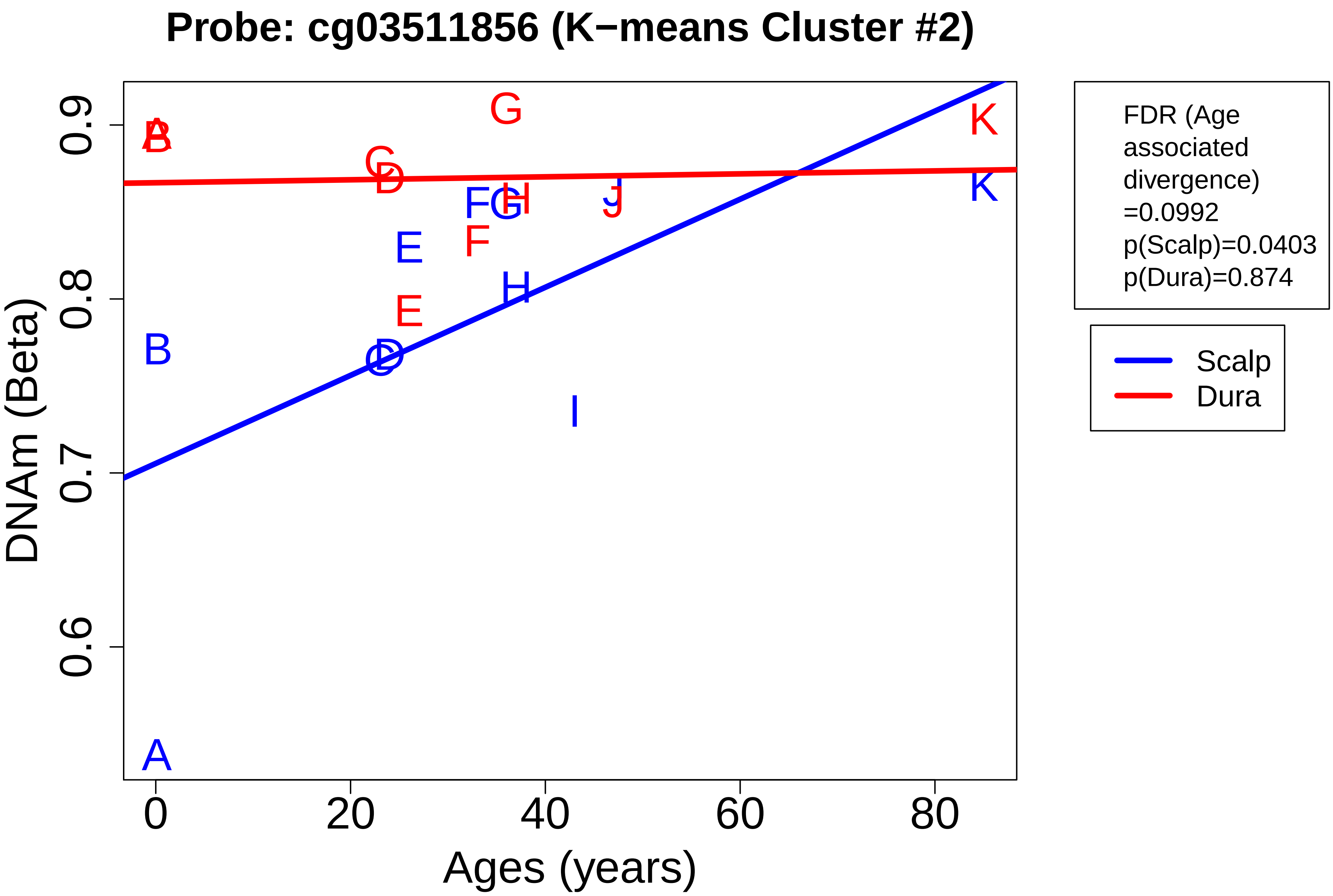

Probe: cg03511856 (K−means Cluster #2)
DNAm (Beta)
0.6
0.7
0.8
0.9
0
20
40
60
80
Ages (years)
FDR (Age associated divergence) =0.0992
p(Scalp)=0.0403
p(Dura)=0.874
Scalp
Dura

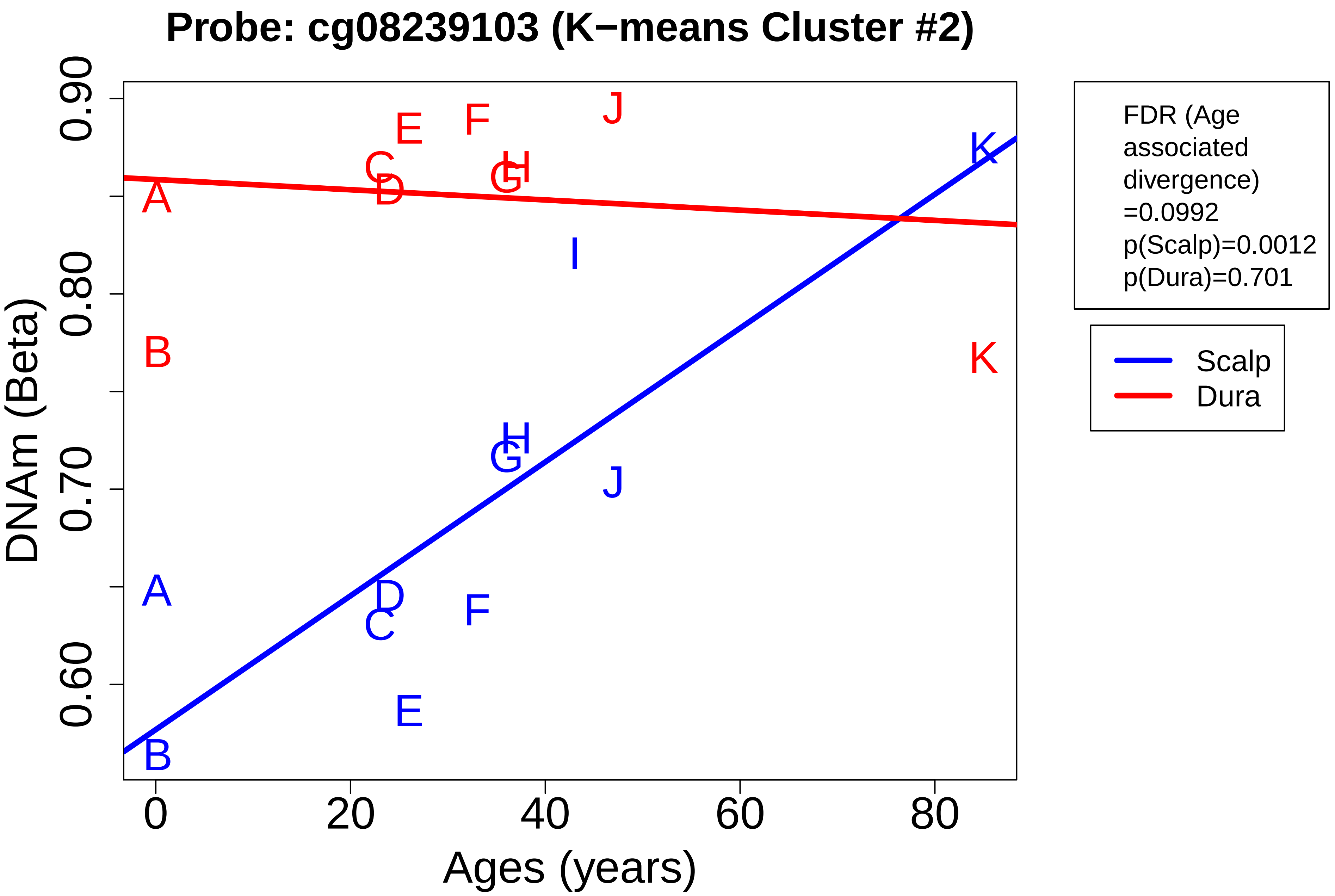
Probe: cg08239103 (K−means Cluster #2)
DNAm (Beta)
0.60
0.70
0.80
0.90
0
20
40
60
80
Ages (years)
FDR (Age associated divergence) =0.0992
p(Scalp)=0.0012
p(Dura)=0.701
Scalp
Dura
A
B
C
D
E
F
G
H
I
J
K

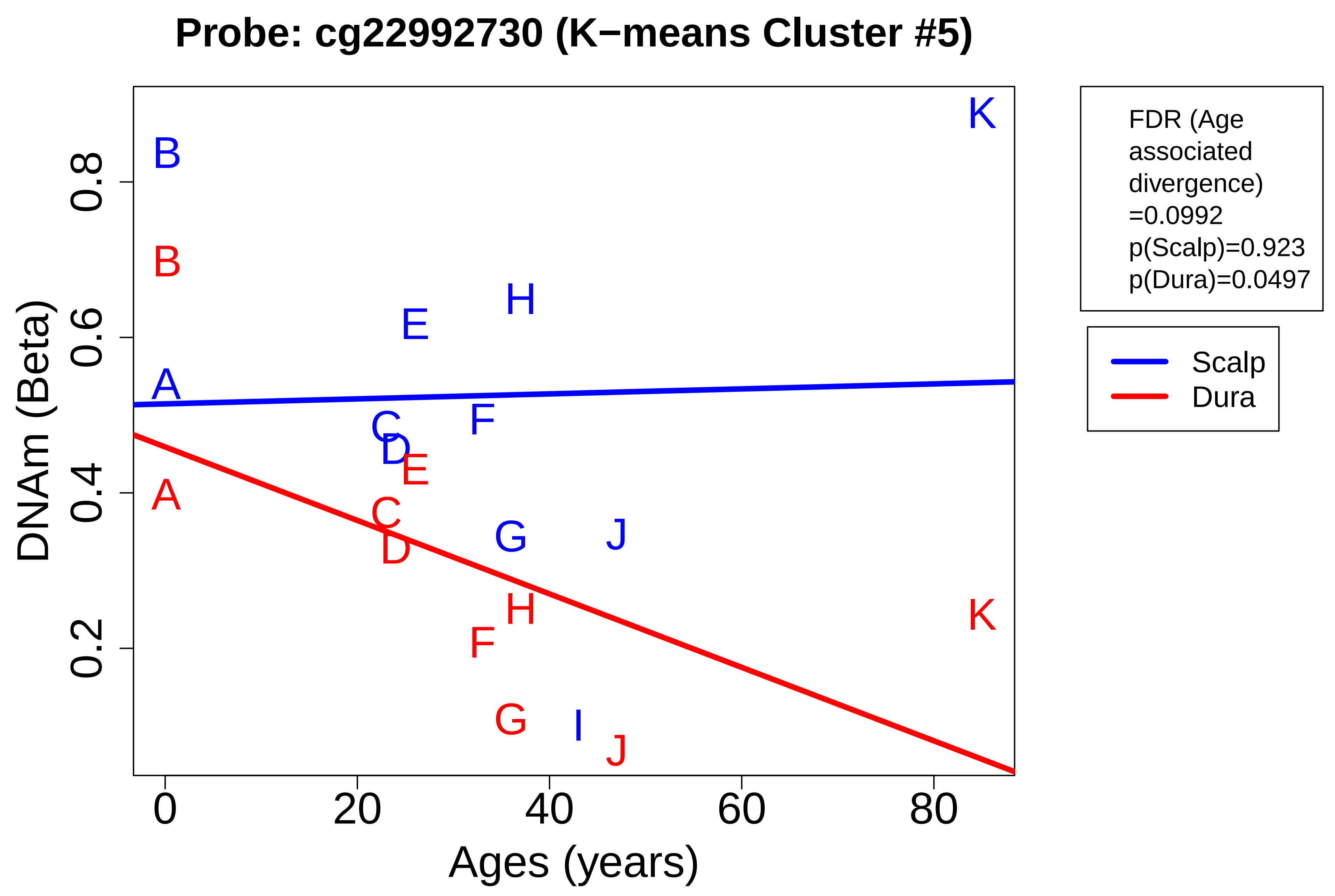

Probe: cg22992730 (K−means Cluster #5)
DNAm (Beta)
0.2
0.4
0.6
0.8
0
20
40
60
80
Ages (years)
FDR (Age associated divergence) =0.0992
p(Scalp)=0.923
p(Dura)=0.0497
Scalp
Dura
A
B
C
D
E
F
G
H
I
J
K

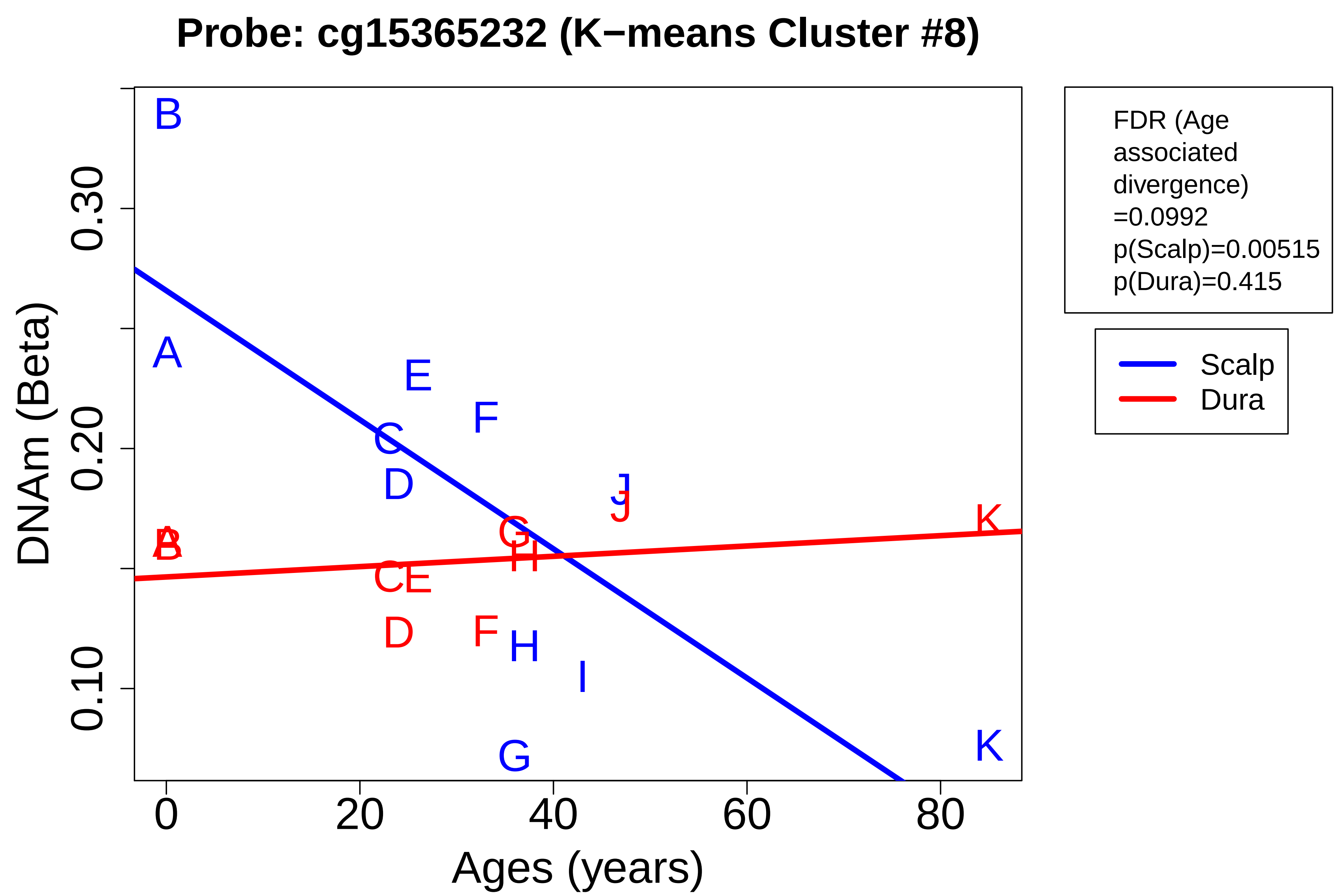
Probe: cg15365232 (K−means Cluster #8)
DNAm (Beta)
0.10
0.20
0.30
0
20
40
60
80
Ages (years)
FDR (Age associated divergence) =0.0992
p(Scalp)=0.00515
p(Dura)=0.415
Scalp
Dura
A
B
C
D
E
F
G
H
I
J
K

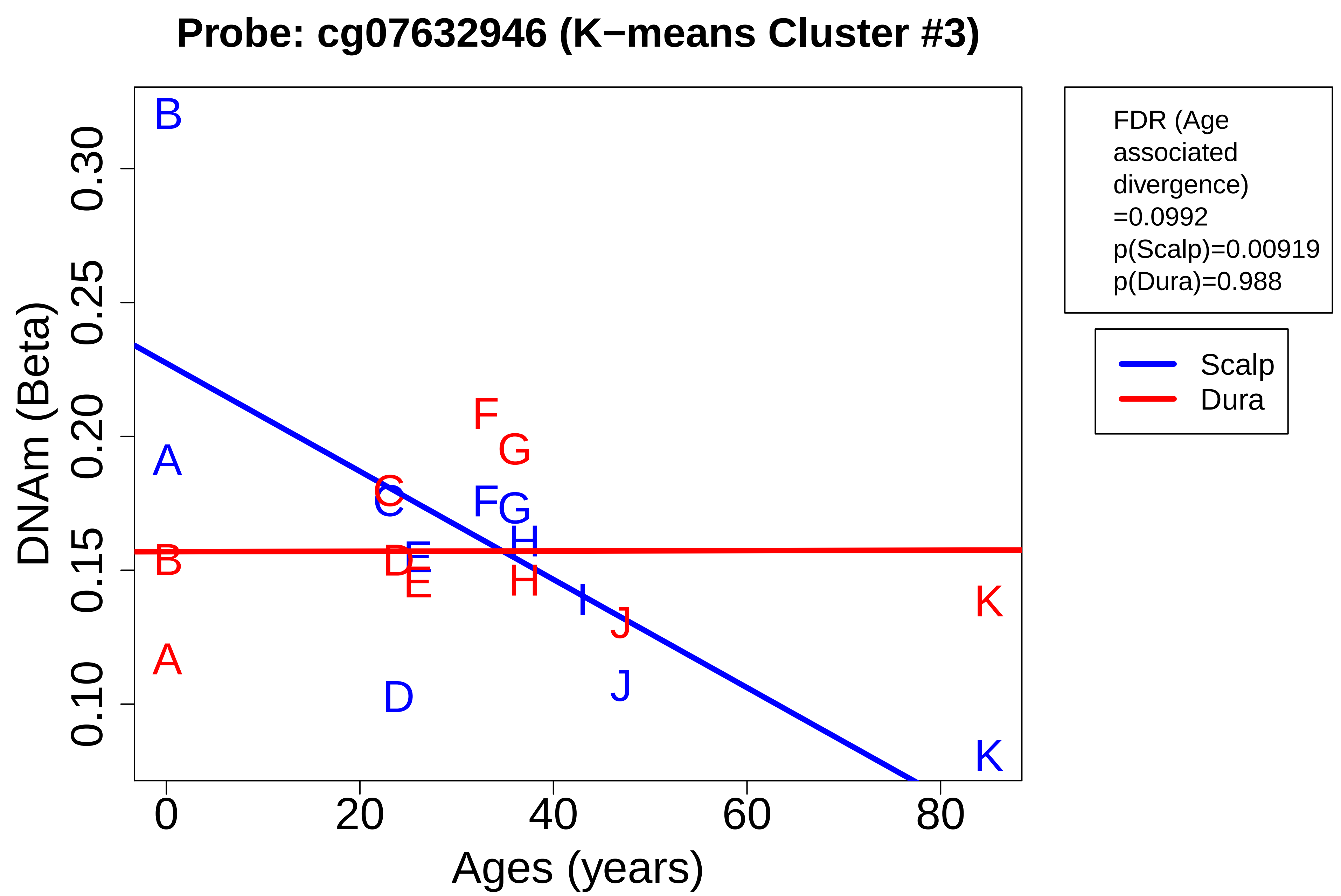

Probe: cg07632946 (K−means Cluster #3)
DNAm (Beta)
0.10
0.15
0.20
0.25
0.30
Ages (years)
0
20
40
60
80
FDR (Age associated divergence) =0.0992
p(Scalp)=0.00919
p(Dura)=0.988
Scalp
Dura

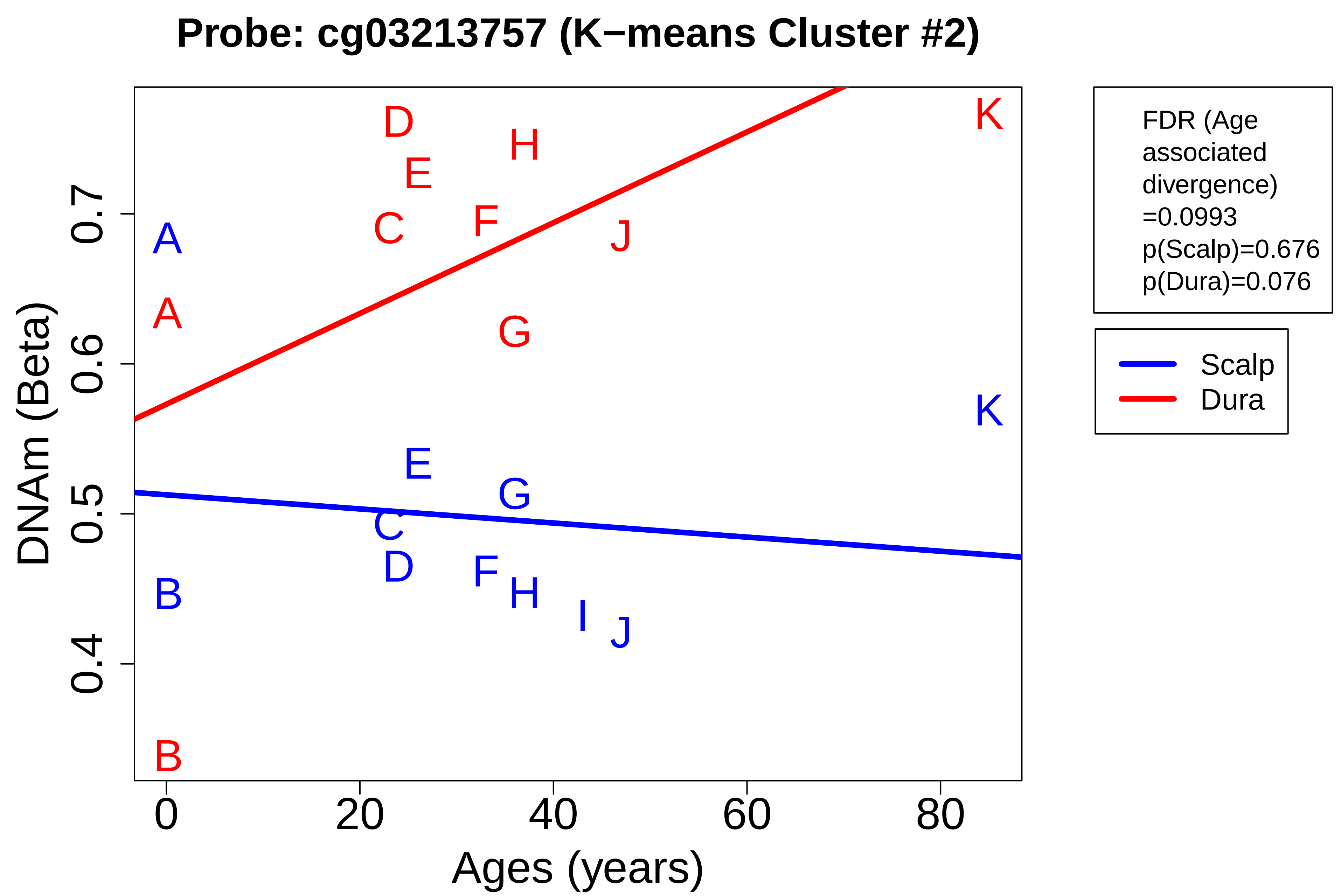

Probe: cg03213757 (K−means Cluster #2)
DNAm (Beta)
0.4
0.5
0.6
0.7
Ages (years)
0
20
40
60
80
A
B
C
D
E
F
G
H
I
J
K
FDR (Age associated divergence) =0.0993
p(Scalp)=0.676
p(Dura)=0.076
Scalp
Dura

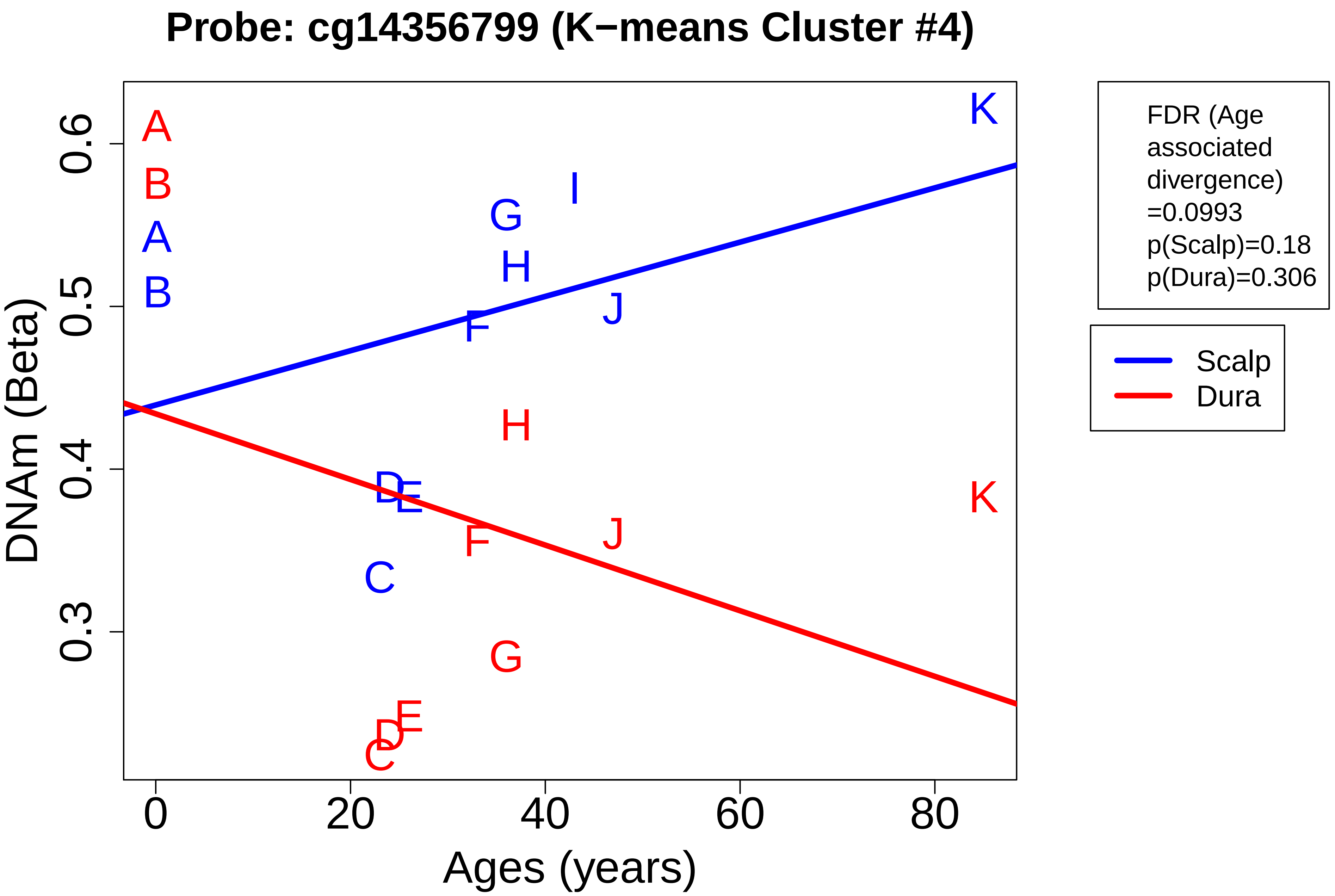

Probe: cg14356799 (K−means Cluster #4)
DNAm (Beta)
0.3
0.4
0.5
0.6
0
20
40
60
80
Ages (years)
A
B
A
B
C
D
E
F
G
H
I
J
K
C
D
E
F
G
H
J
K
FDR (Age associated divergence) =0.0993
p(Scalp)=0.18
p(Dura)=0.306
Scalp
Dura

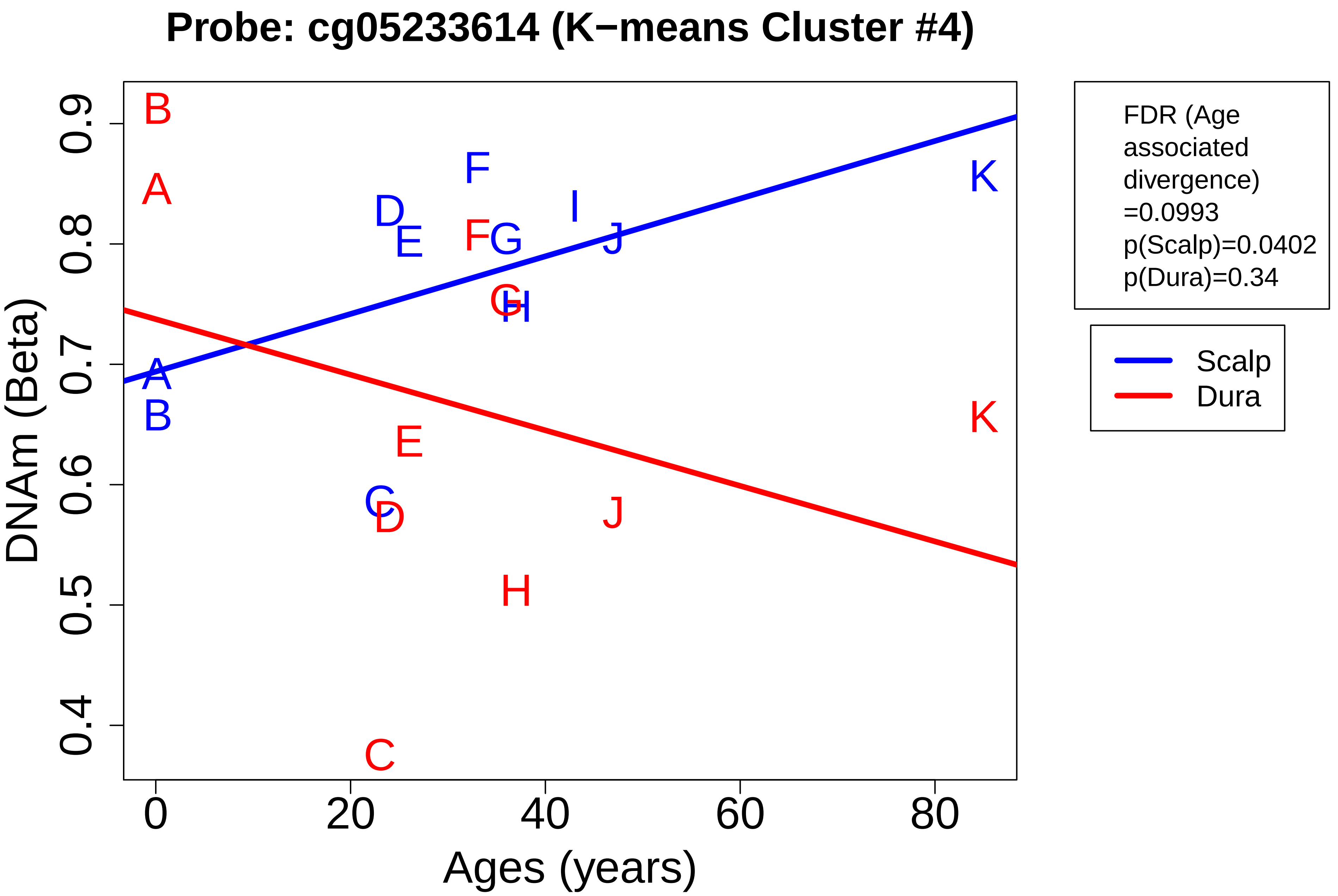

Probe: cg05233614 (K−means Cluster #4)
DNAm (Beta)
Ages (years)
0.4
0.5
0.6
0.7
0.8
0.9
0
20
40
60
80
FDR (Age associated divergence) =0.0993
p(Scalp)=0.0402
p(Dura)=0.34
Scalp
Dura

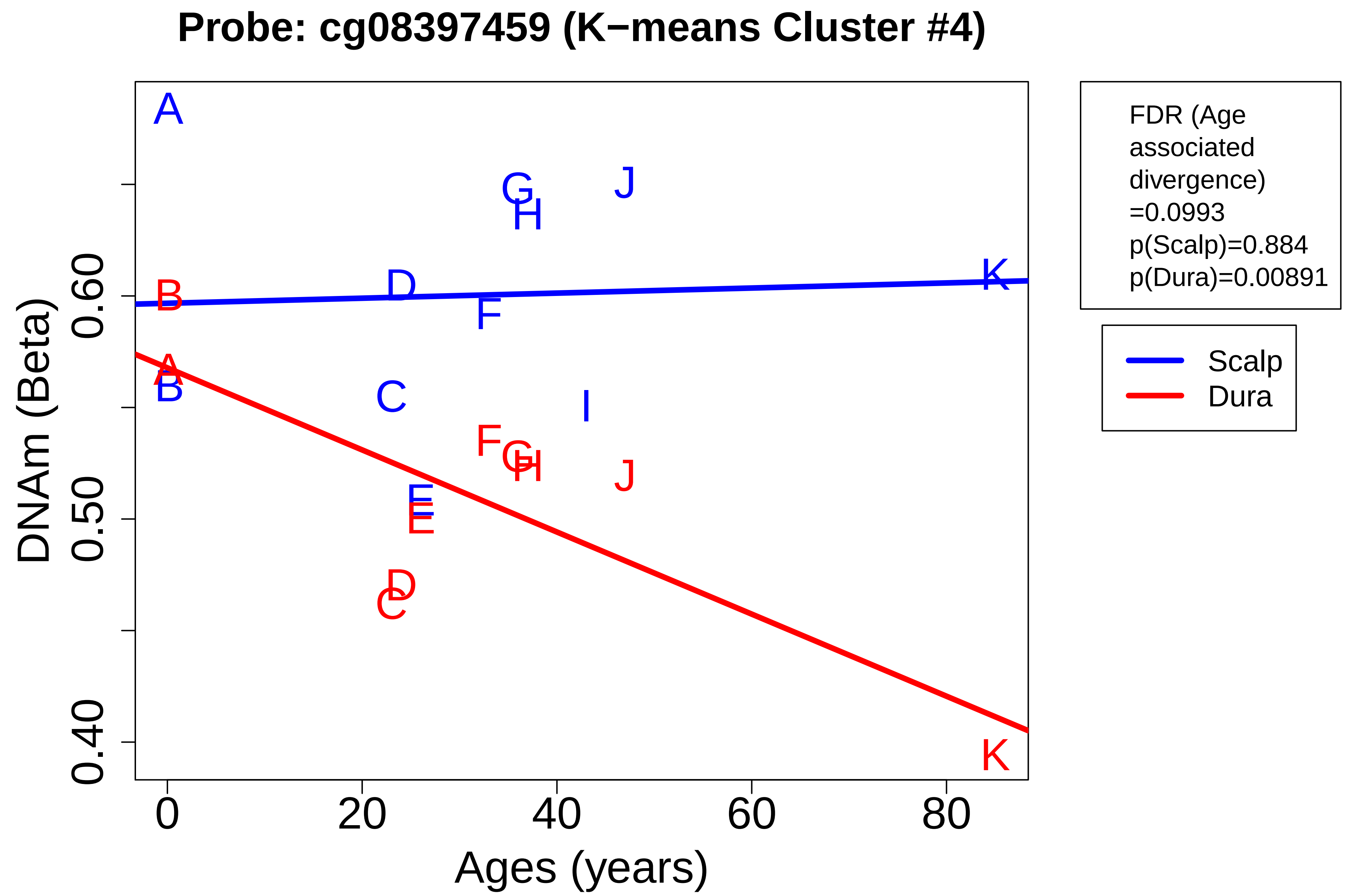

Probe: cg08397459 (K−means Cluster #4)
DNAm (Beta)
0.40
0.50
0.60
0
20
40
60
80
Ages (years)
A
B
C
D
E
F
G
H
I
J
K
FDR (Age associated divergence) =0.0993
p(Scalp)=0.884
p(Dura)=0.00891
Scalp
Dura

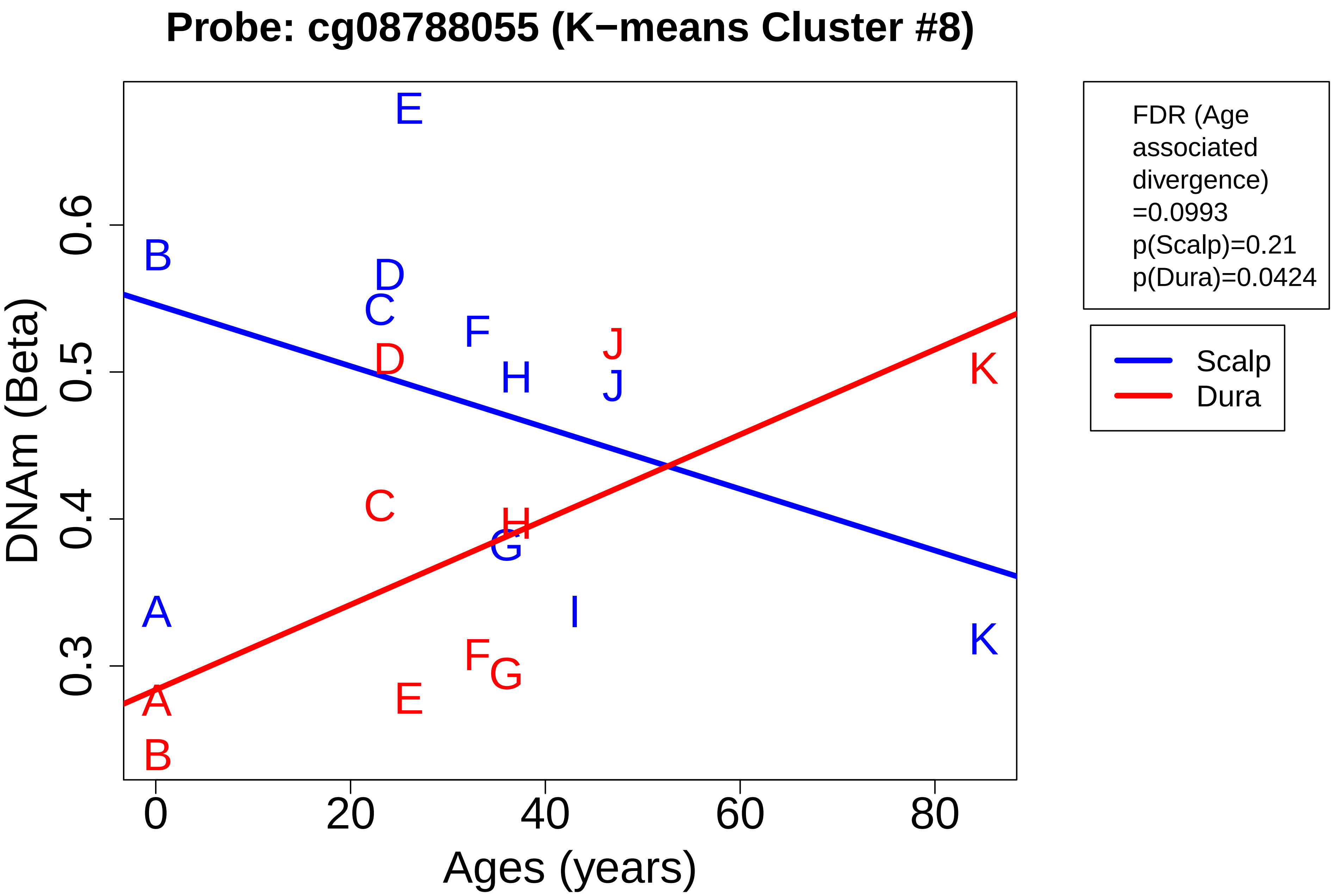
Probe: cg08788055 (K−means Cluster #8)
DNAm (Beta)
0.3
0.4
0.5
0.6
0
20
40
60
80
Ages (years)
FDR (Age associated divergence) =0.0993
p(Scalp)=0.21
p(Dura)=0.0424
Scalp
Dura

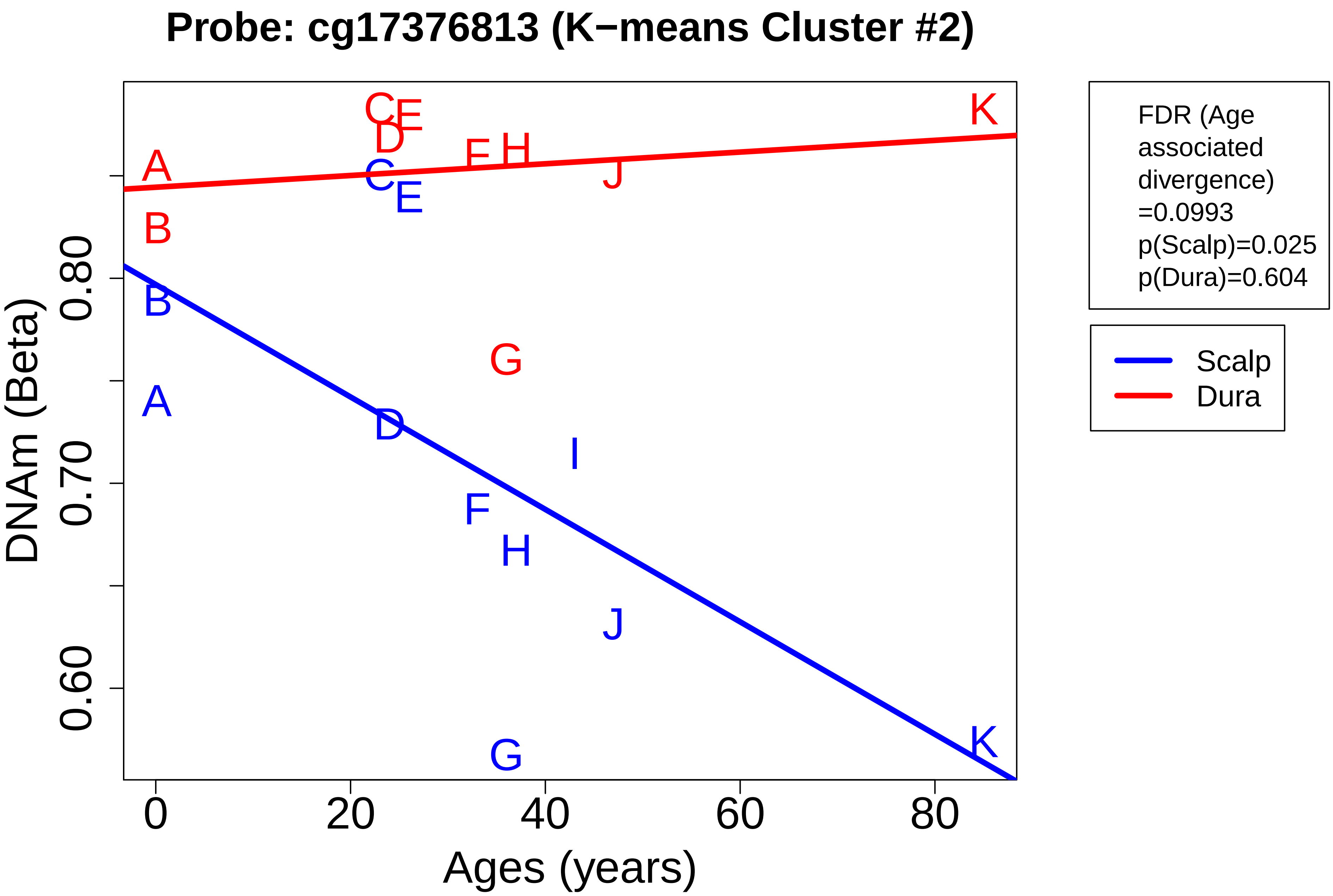

Probe: cg17376813 (K−means Cluster #2)
DNAm (Beta)
0.60
0.70
0.80
0
20
40
60
80
Ages (years)
FDR (Age associated divergence) =0.0993
p(Scalp)=0.025
p(Dura)=0.604
Scalp
Dura

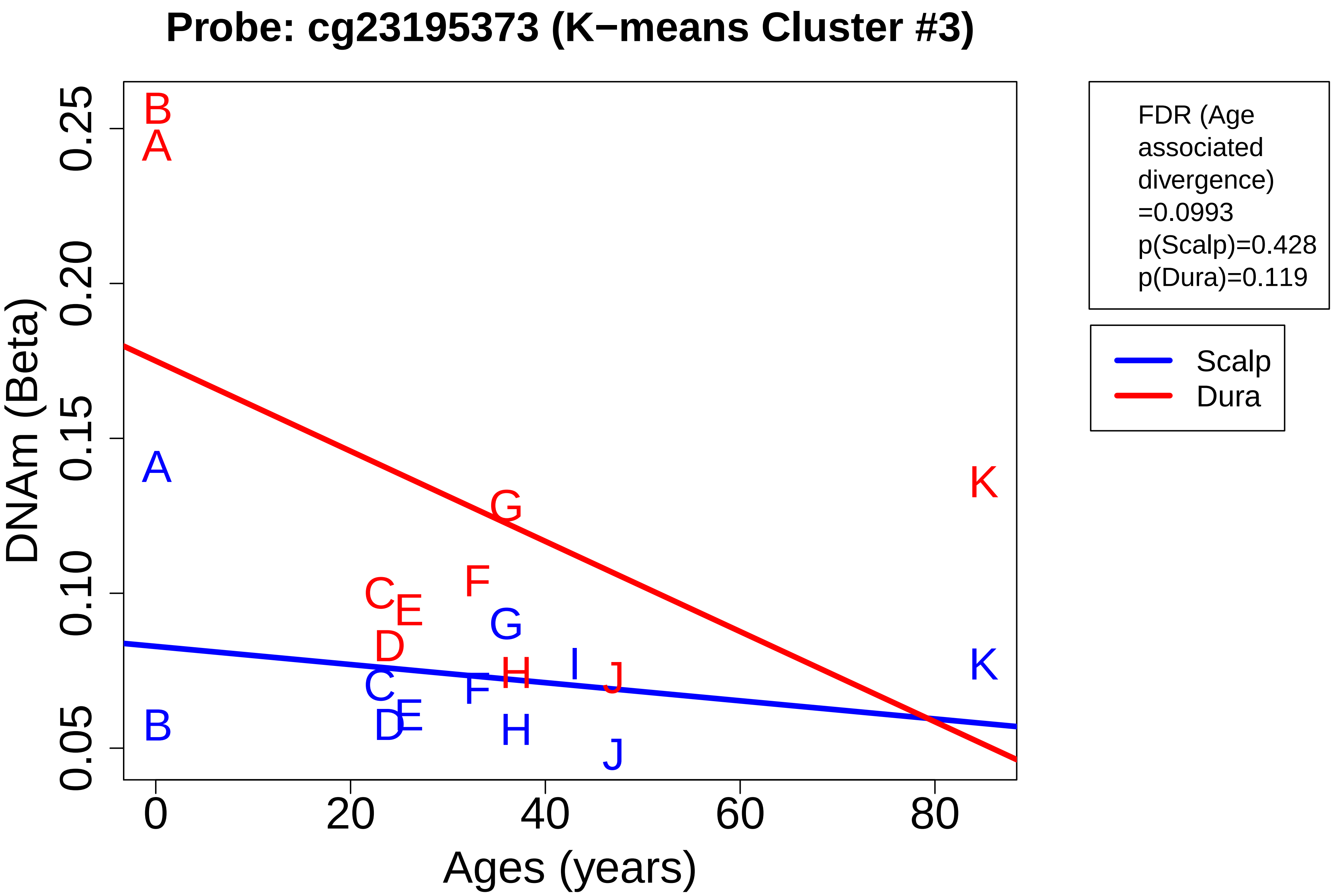

Probe: cg23195373 (K−means Cluster #3)
DNAm (Beta)
0.05
0.10
0.15
0.20
0.25
Ages (years)
0
20
40
60
80
FDR (Age associated divergence) =0.0993
p(Scalp)=0.428
p(Dura)=0.119
Scalp
Dura

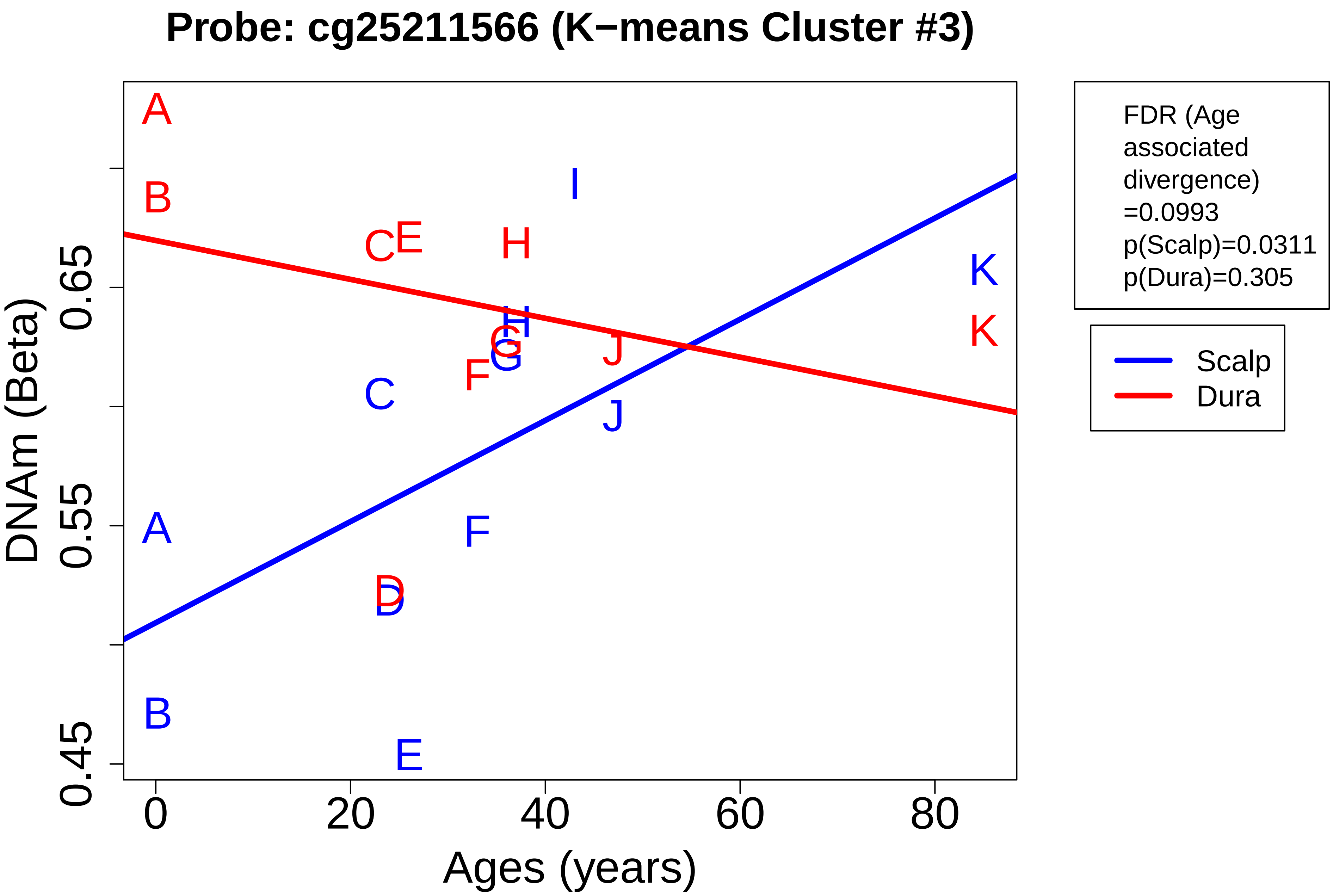

Probe: cg25211566 (K−means Cluster #3)
DNAm (Beta)
0.45
0.55
0.65
Ages (years)
0
20
40
60
80
A
B
C
D
E
F
G
H
I
J
K
FDR (Age associated divergence) =0.0993
p(Scalp)=0.0311
p(Dura)=0.305
Scalp
Dura

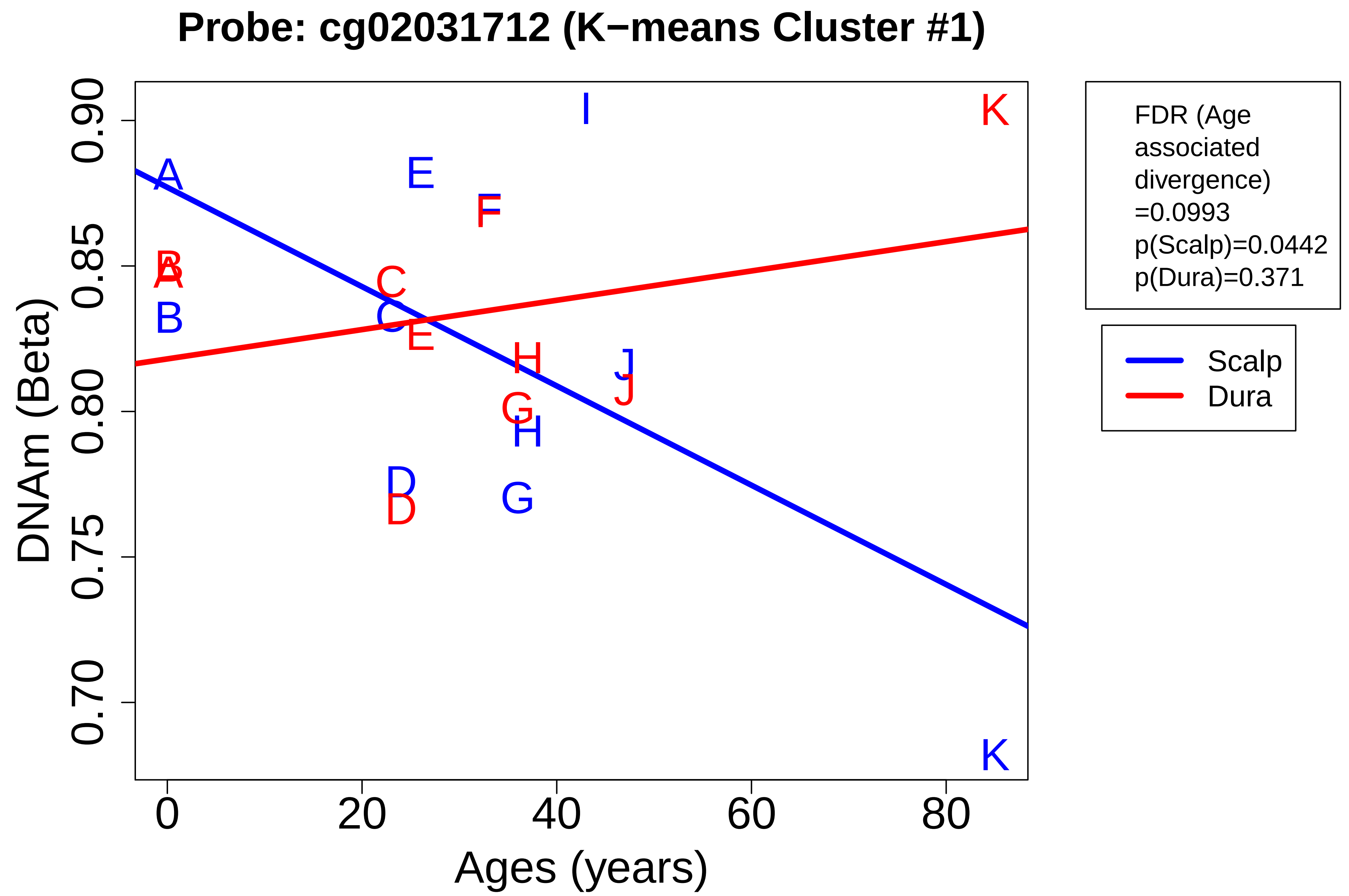

Probe: cg02031712 (K−means Cluster #1)
DNAm (Beta)
0.70
0.75
0.80
0.85
0.90
0
20
40
60
80
Ages (years)
FDR (Age associated divergence) =0.0993
p(Scalp)=0.0442
p(Dura)=0.371
Scalp
Dura

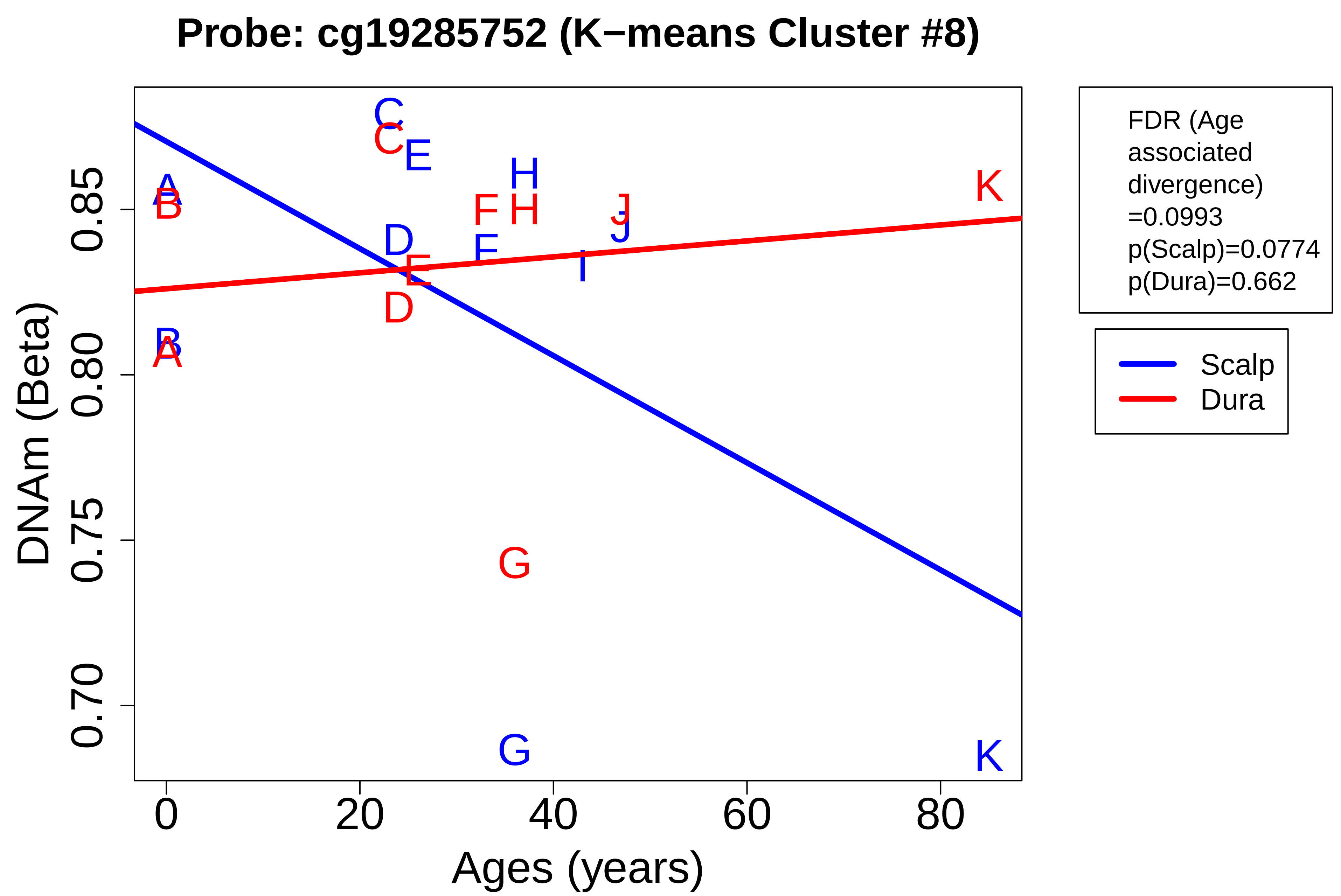
Probe: cg19285752 (K−means Cluster #8)
DNAm (Beta)
0.70
0.75
0.80
0.85
0
20
40
60
80
Ages (years)
A
B
C
D
E
F
G
H
I
J
K
FDR (Age associated divergence) =0.0993
p(Scalp)=0.0774
p(Dura)=0.662
Scalp
Dura

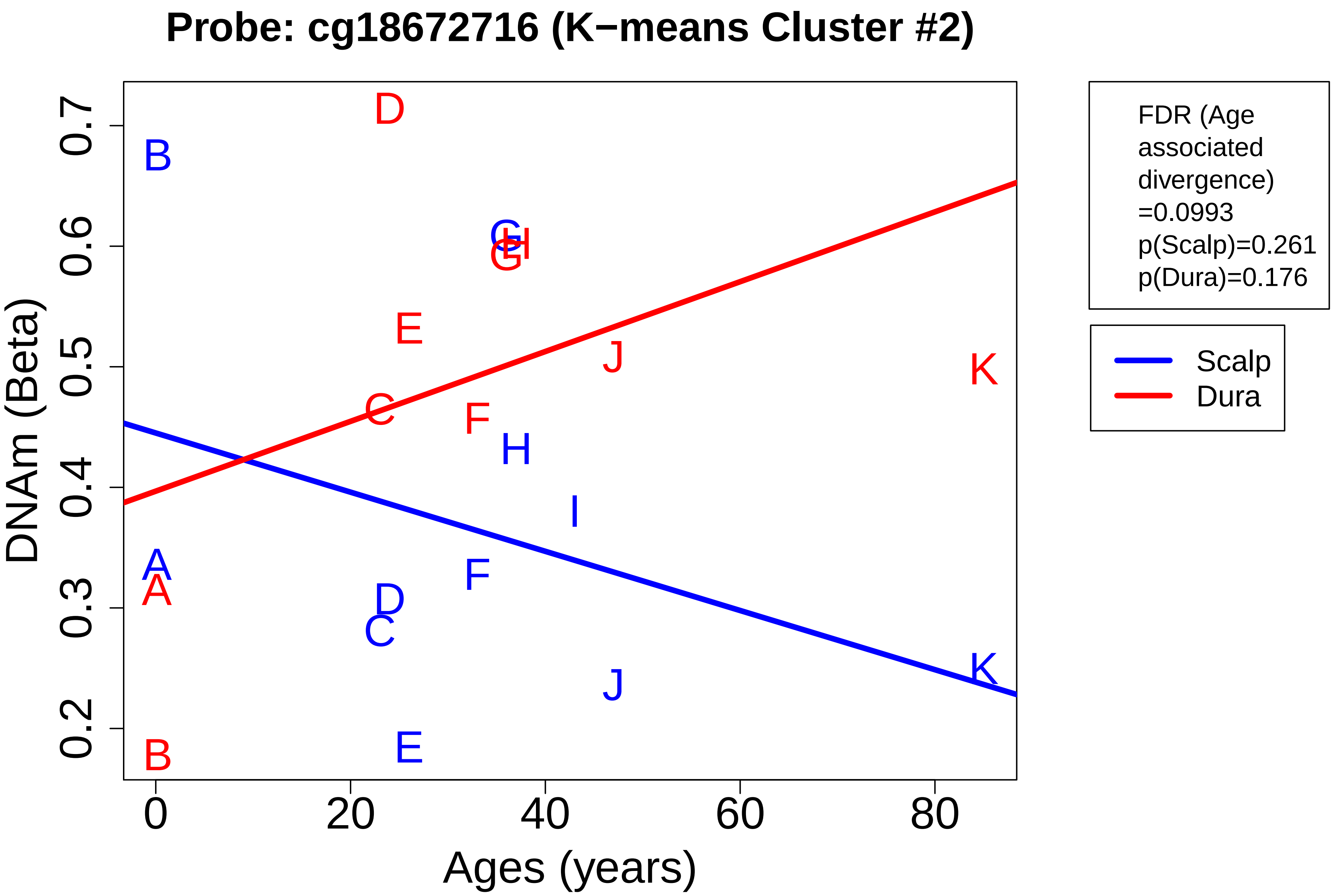

Probe: cg18672716 (K−means Cluster #2)
DNAm (Beta)
0.2
0.3
0.4
0.5
0.6
0.7
0
20
40
60
80
Ages (years)
FDR (Age associated divergence) =0.0993
p(Scalp)=0.261
p(Dura)=0.176
Scalp
Dura
A
B
C
D
E
F
G
H
I
J
K

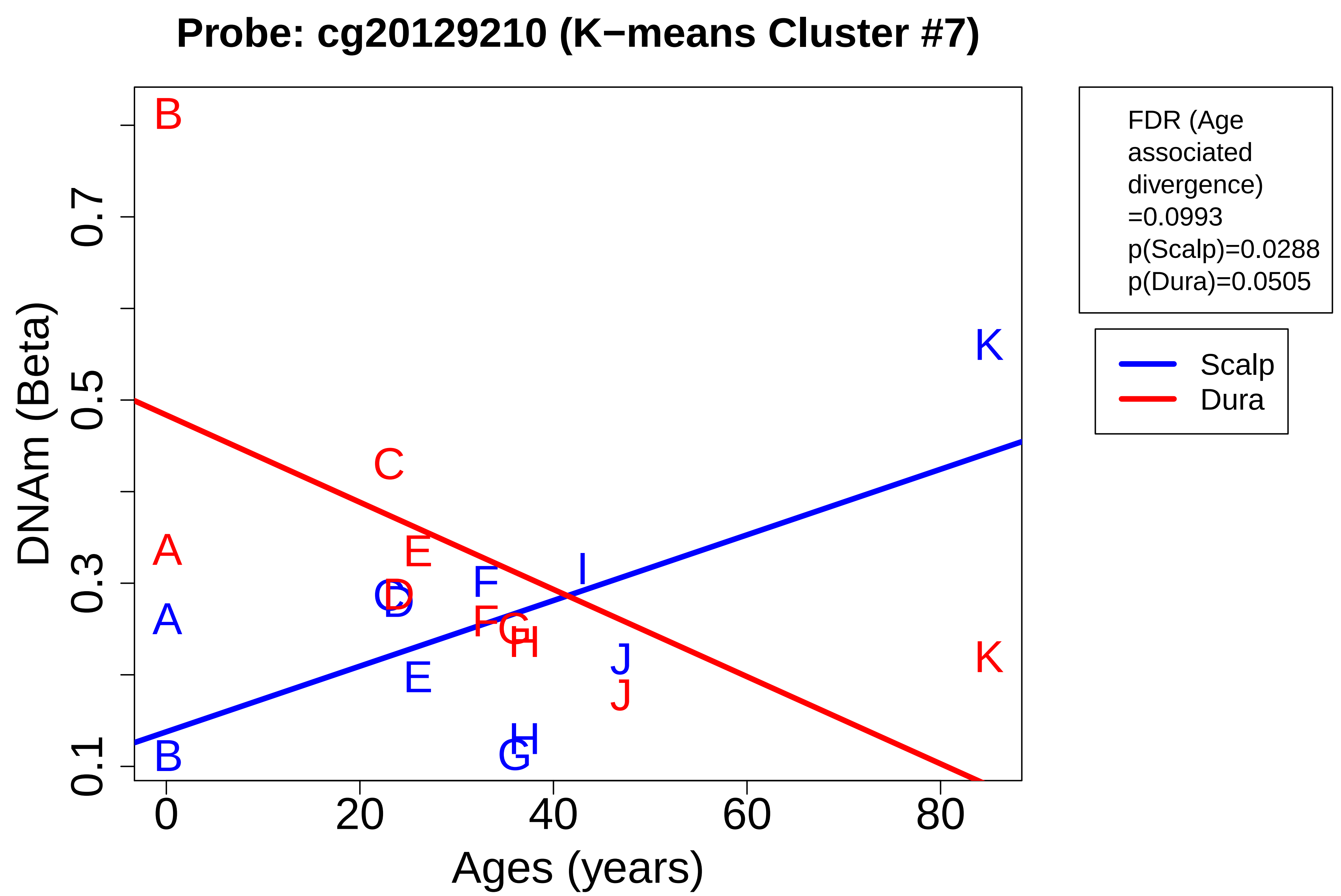

Probe: cg20129210 (K−means Cluster #7)
DNAm (Beta)
0.1
0.3
0.5
0.7
0
20
40
60
80
Ages (years)
FDR (Age associated divergence) =0.0993
p(Scalp)=0.0288
p(Dura)=0.0505
Scalp
Dura

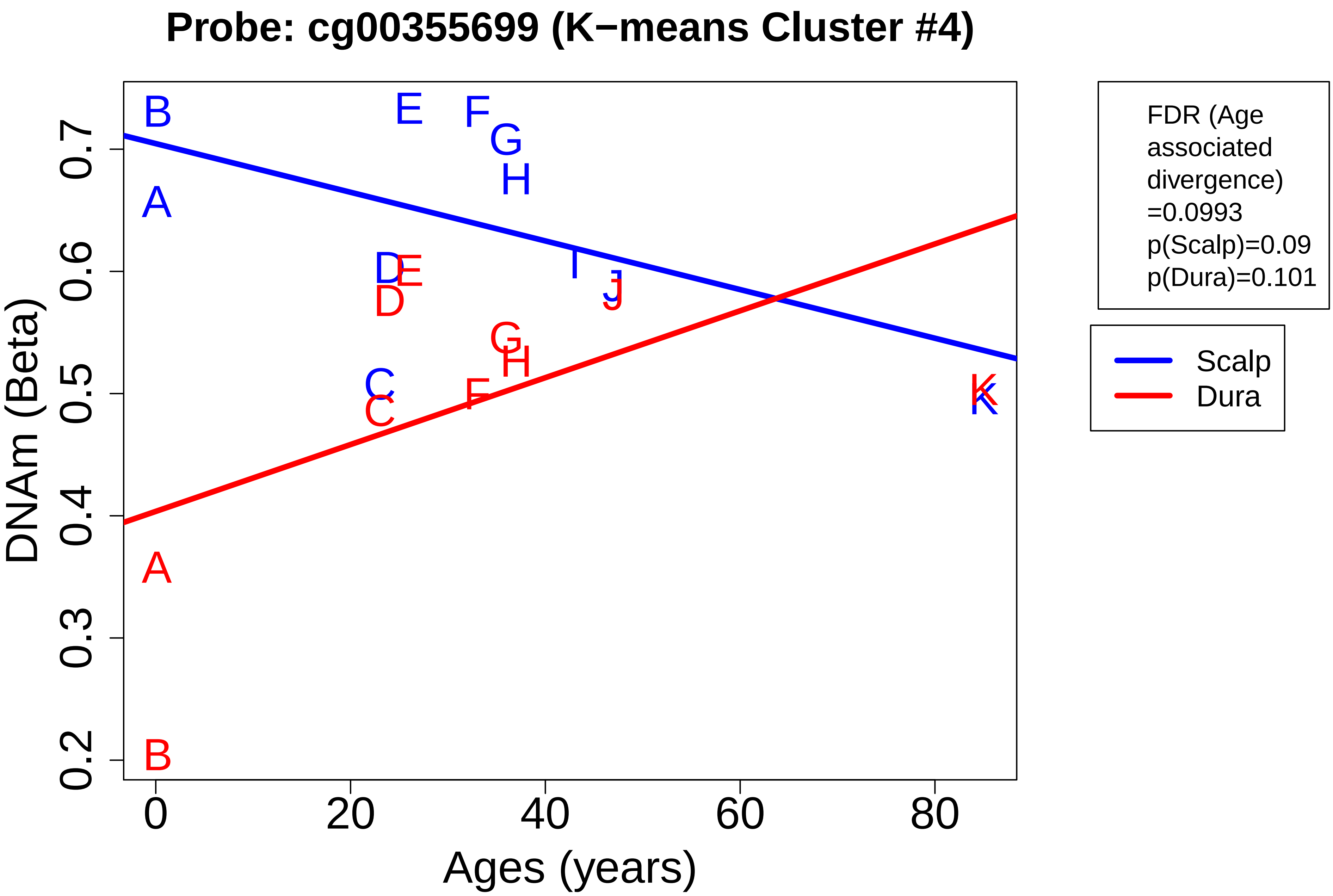

Probe: cg00355699 (K−means Cluster #4)
DNAm (Beta)
0.2
0.3
0.4
0.5
0.6
0.7
0
20
40
60
80
Ages (years)
FDR (Age associated divergence) =0.0993
p(Scalp)=0.09
p(Dura)=0.101
Scalp
Dura

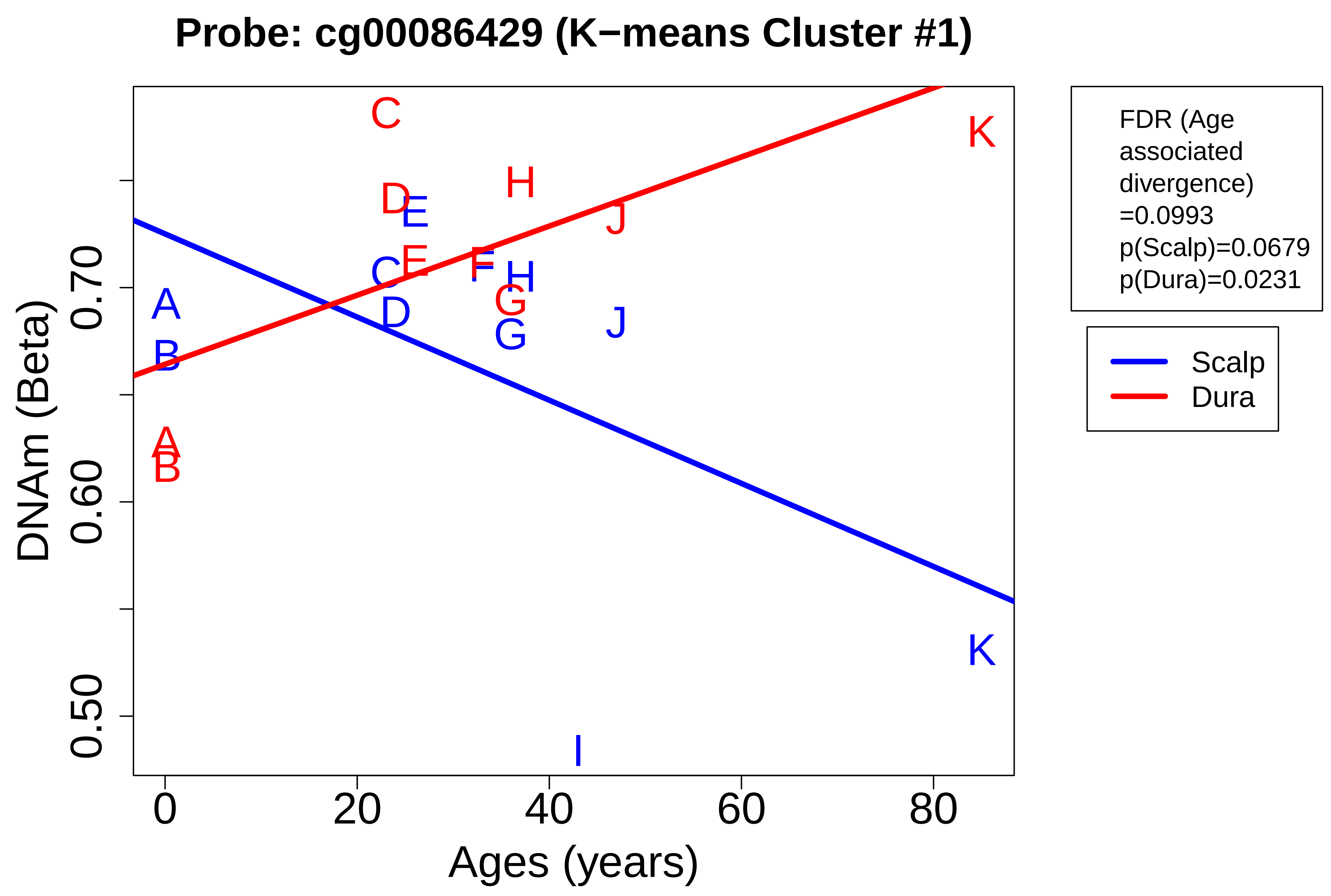

Probe: cg00086429 (K−means Cluster #1)
FDR (Age associated divergence) =0.0993
p(Scalp)=0.0679
p(Dura)=0.0231
Scalp
Dura
DNAm (Beta)
0.50
0.60
0.70
0
20
40
60
80
Ages (years)

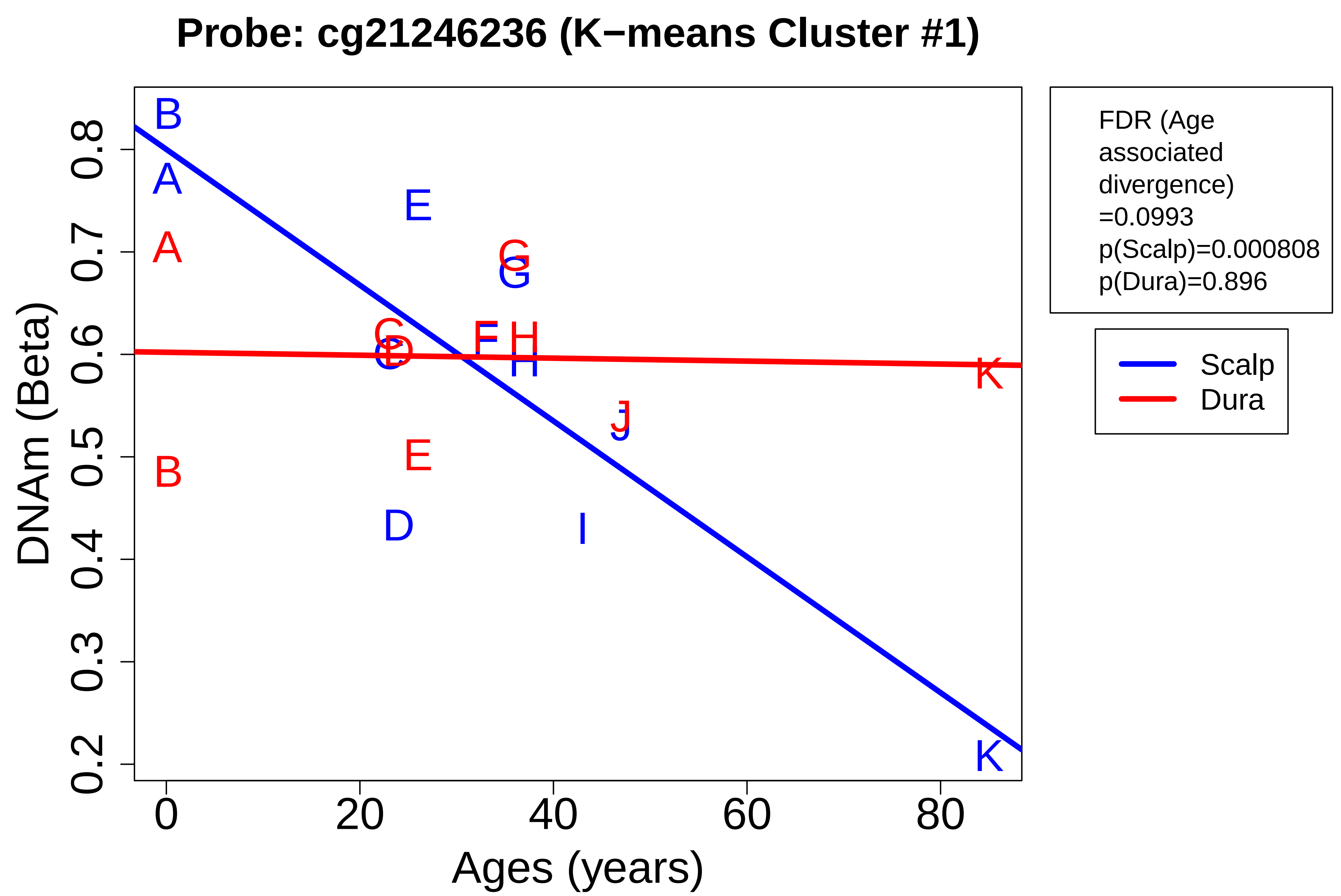

Probe: cg21246236 (K−means Cluster #1)
FDR (Age associated divergence) =0.0993
p(Scalp)=0.000808
p(Dura)=0.896
Scalp
Dura
DNAm (Beta)
0.2
0.3
0.4
0.5
0.6
0.7
0.8
Ages (years)
0
20
40
60
80

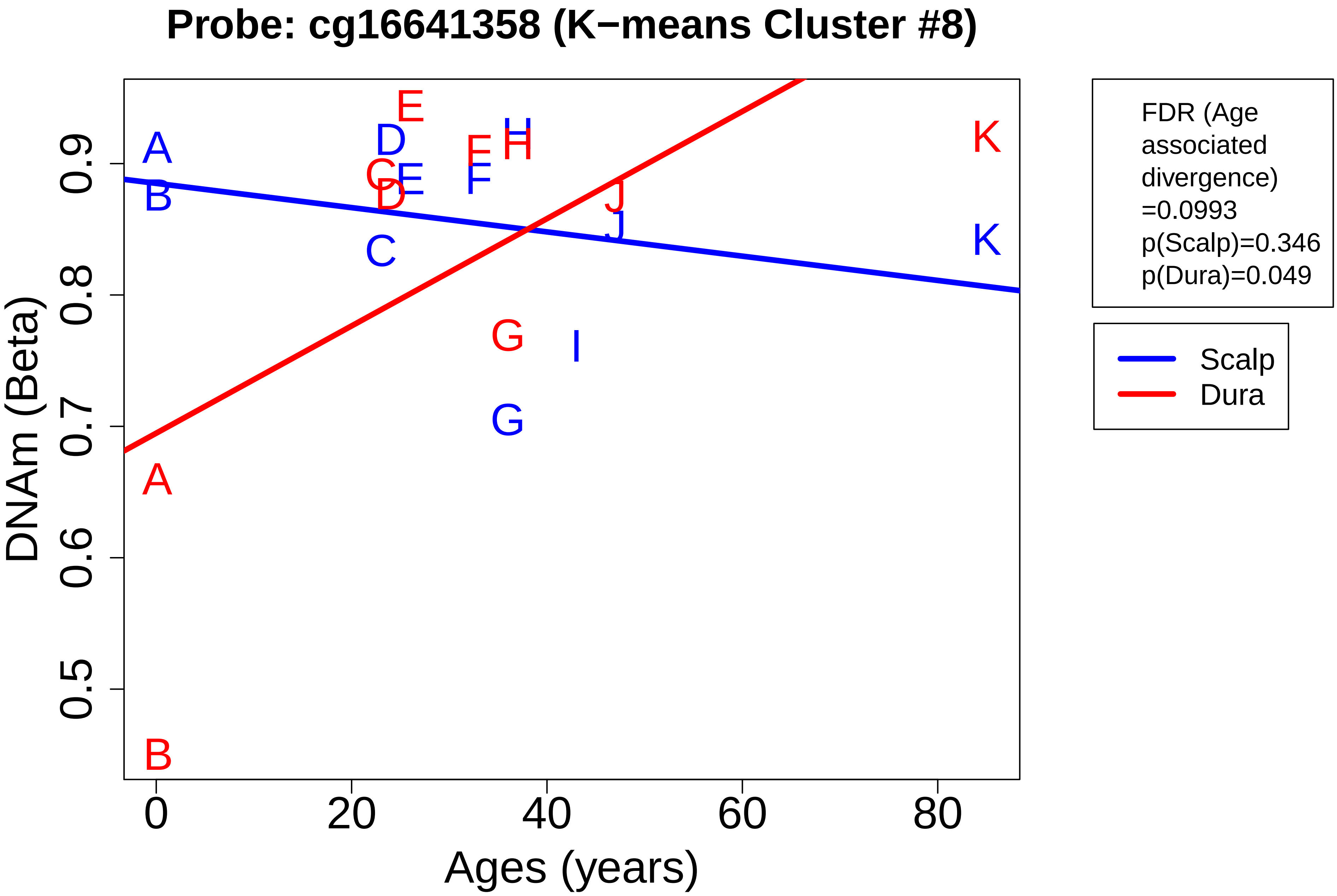
Probe: cg16641358 (K−means Cluster #8)
DNAm (Beta)
Ages (years)
0.5
0.6
0.7
0.8
0.9
0
20
40
60
80
A
B
C
D
E
F
G
H
I
J
K
FDR (Age associated divergence) =0.0993
p(Scalp)=0.346
p(Dura)=0.049
Scalp
Dura

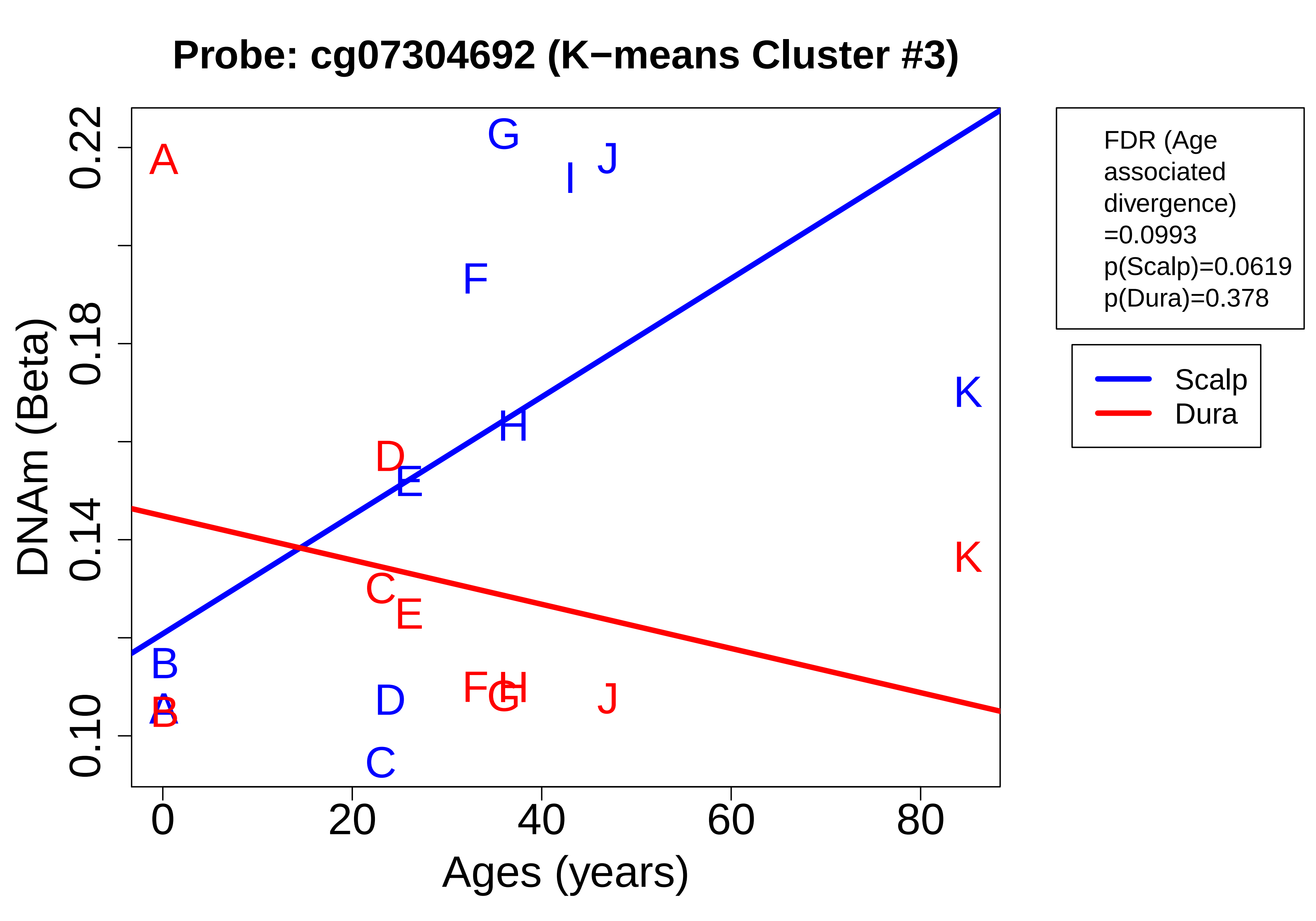

Probe: cg07304692 (K−means Cluster #3)
DNAm (Beta)
0.10
0.14
0.18
0.22
Ages (years)
0
20
40
60
80
A
B
C
D
E
F
G
H
I
J
K
FDR (Age associated divergence) =0.0993
p(Scalp)=0.0619
p(Dura)=0.378
Scalp
Dura

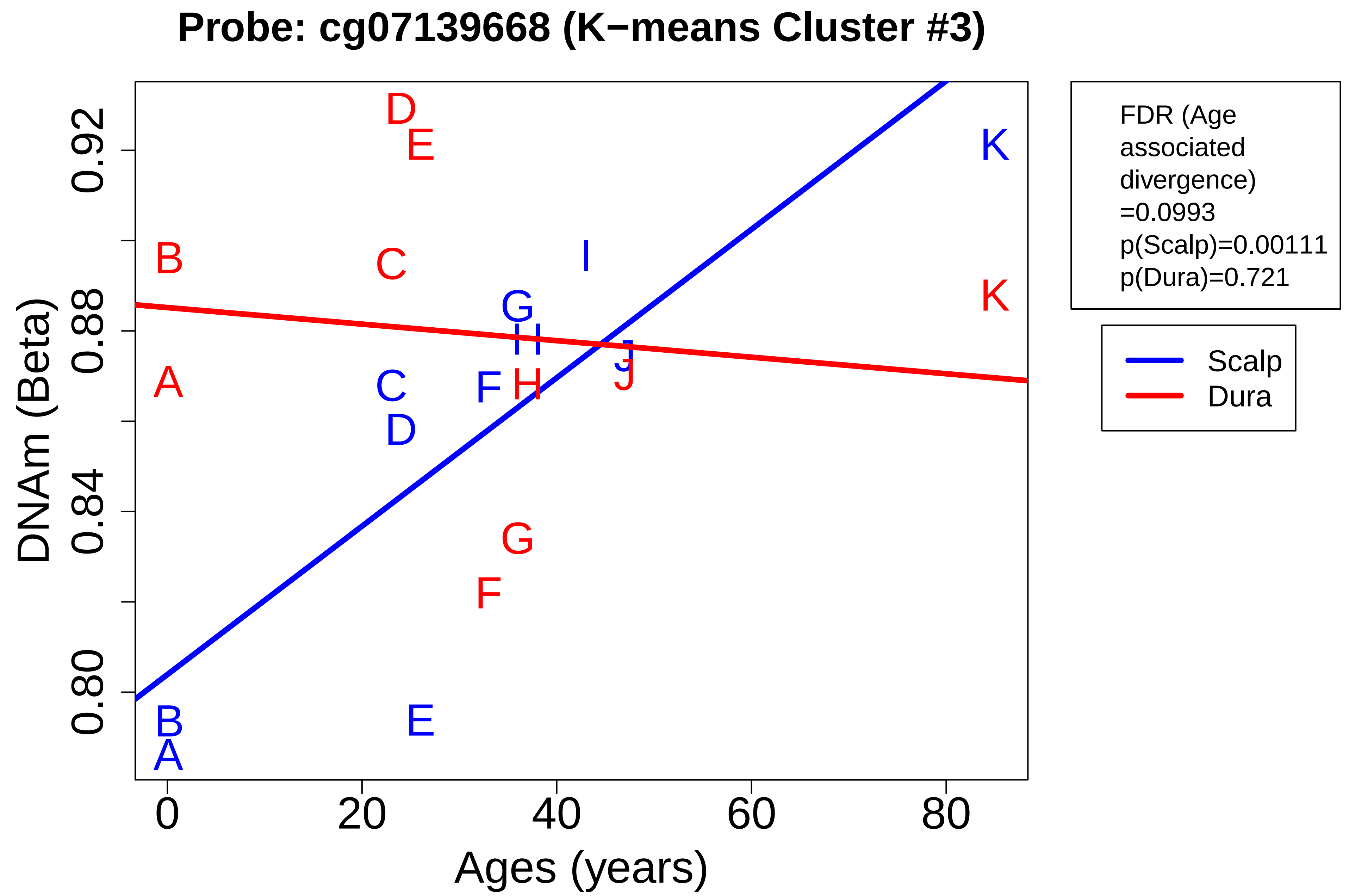

Probe: cg07139668 (K−means Cluster #3)
DNAm (Beta)
0.80
0.84
0.88
0.92
Ages (years)
0
20
40
60
80
A
B
C
D
E
F
G
H
I
J
K
FDR (Age associated divergence) =0.0993
p(Scalp)=0.00111
p(Dura)=0.721
Scalp
Dura

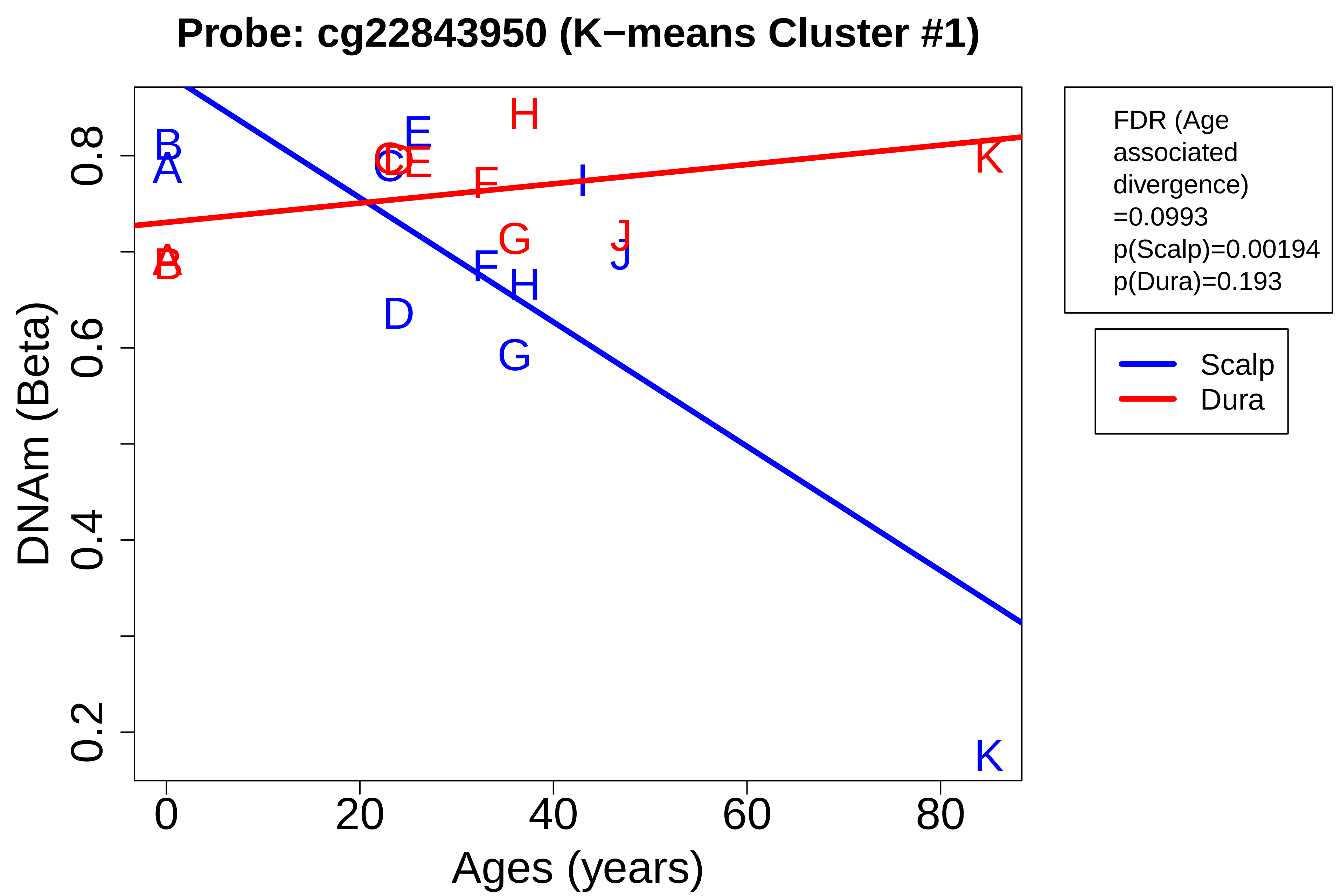

Probe: cg22843950 (K−means Cluster #1)
DNAm (Beta)
0.2
0.4
0.6
0.8
0
20
40
60
80
Ages (years)
FDR (Age associated divergence) =0.0993
p(Scalp)=0.00194
p(Dura)=0.193
Scalp
Dura

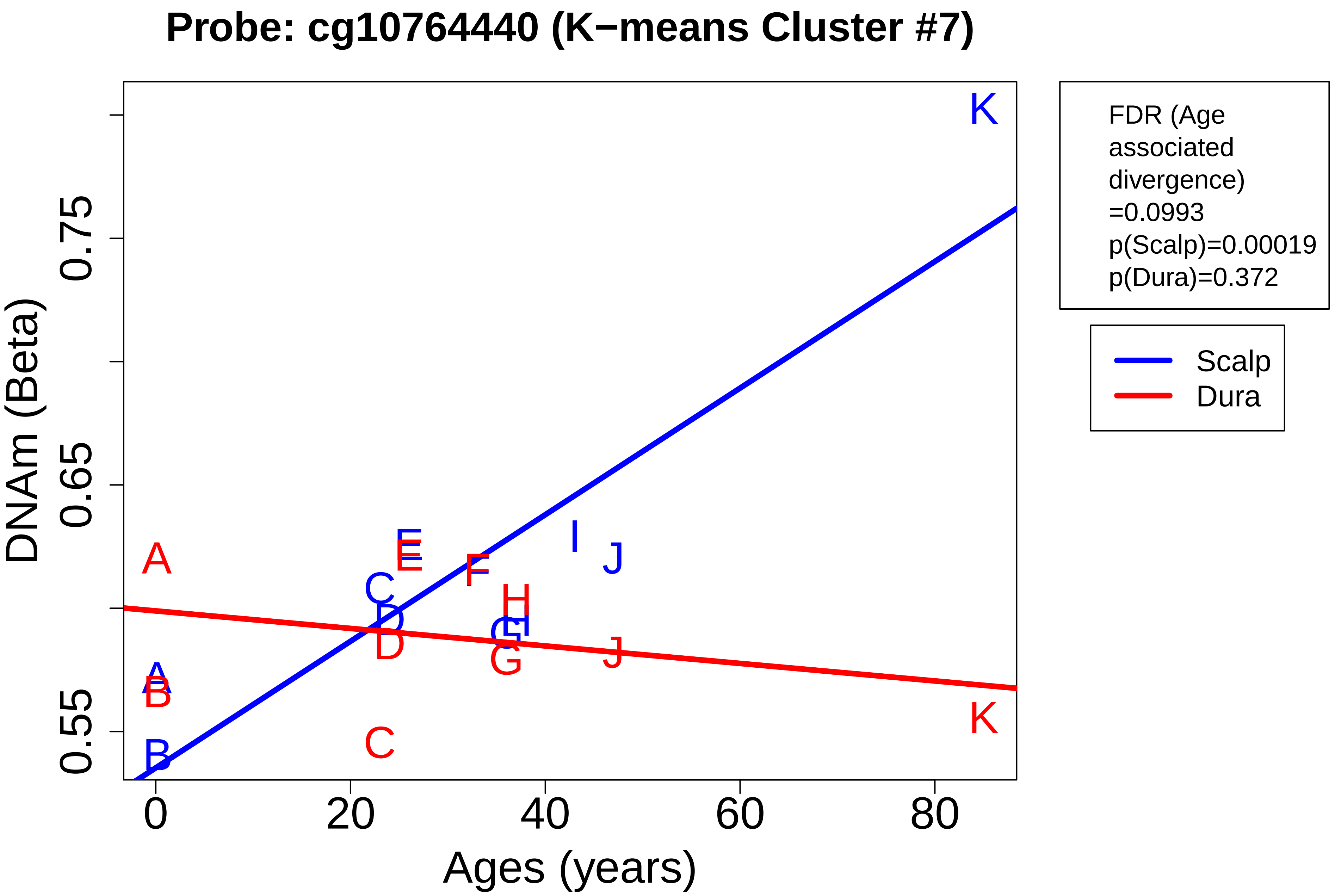

Probe: cg10764440 (K−means Cluster #7)
DNAm (Beta)
0.55
0.65
0.75
0
20
40
60
80
Ages (years)
A
B
C
D
E
F
G
H
I
J
K
FDR (Age associated divergence) =0.0993
p(Scalp)=0.00019
p(Dura)=0.372
Scalp
Dura

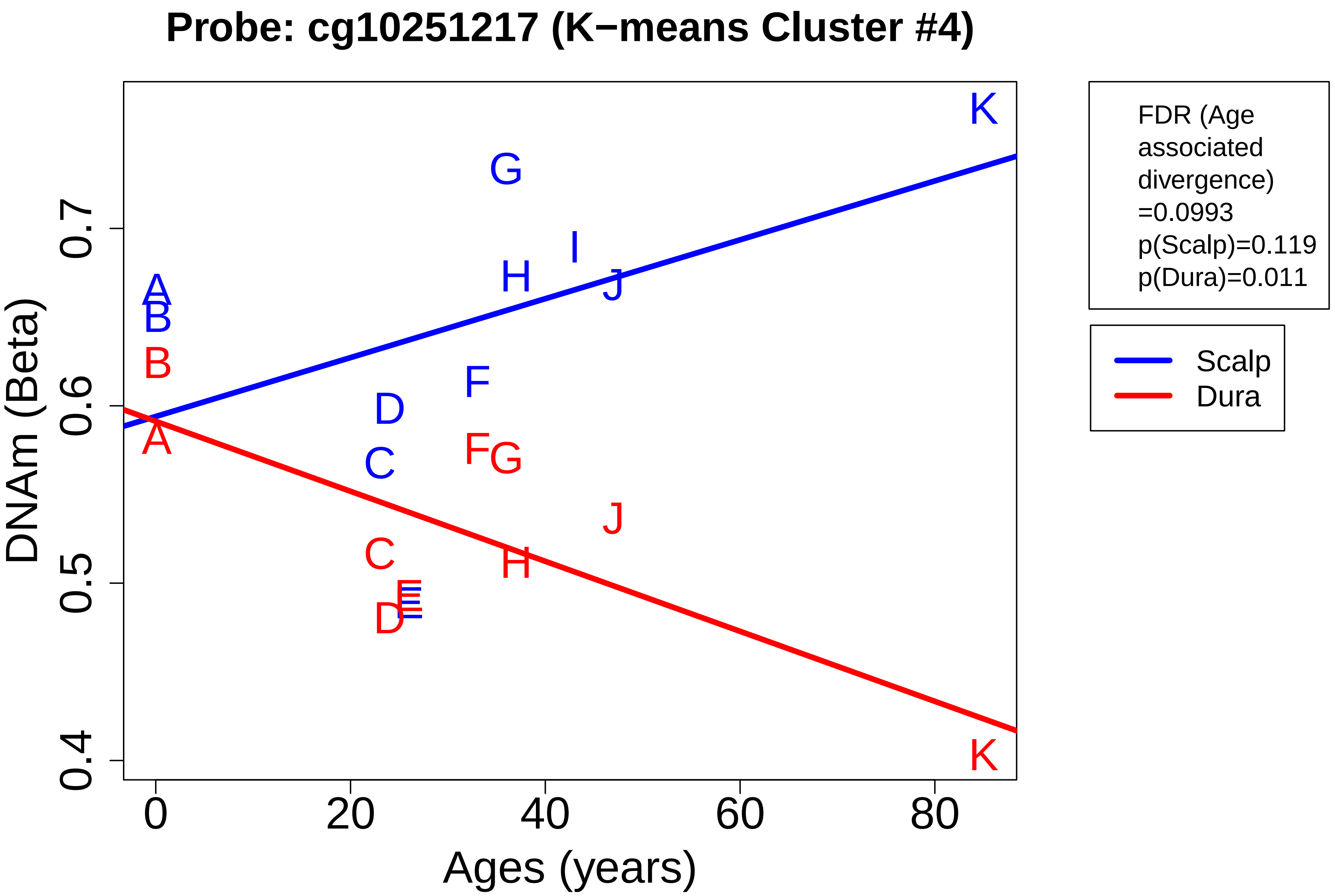

Probe: cg10251217 (K−means Cluster #4)
DNAm (Beta)
Ages (years)
0.4
0.5
0.6
0.7
0
20
40
60
80
FDR (Age associated divergence) =0.0993
p(Scalp)=0.119
p(Dura)=0.011
Scalp
Dura
A
B
C
D
E
F
G
H
I
J
K

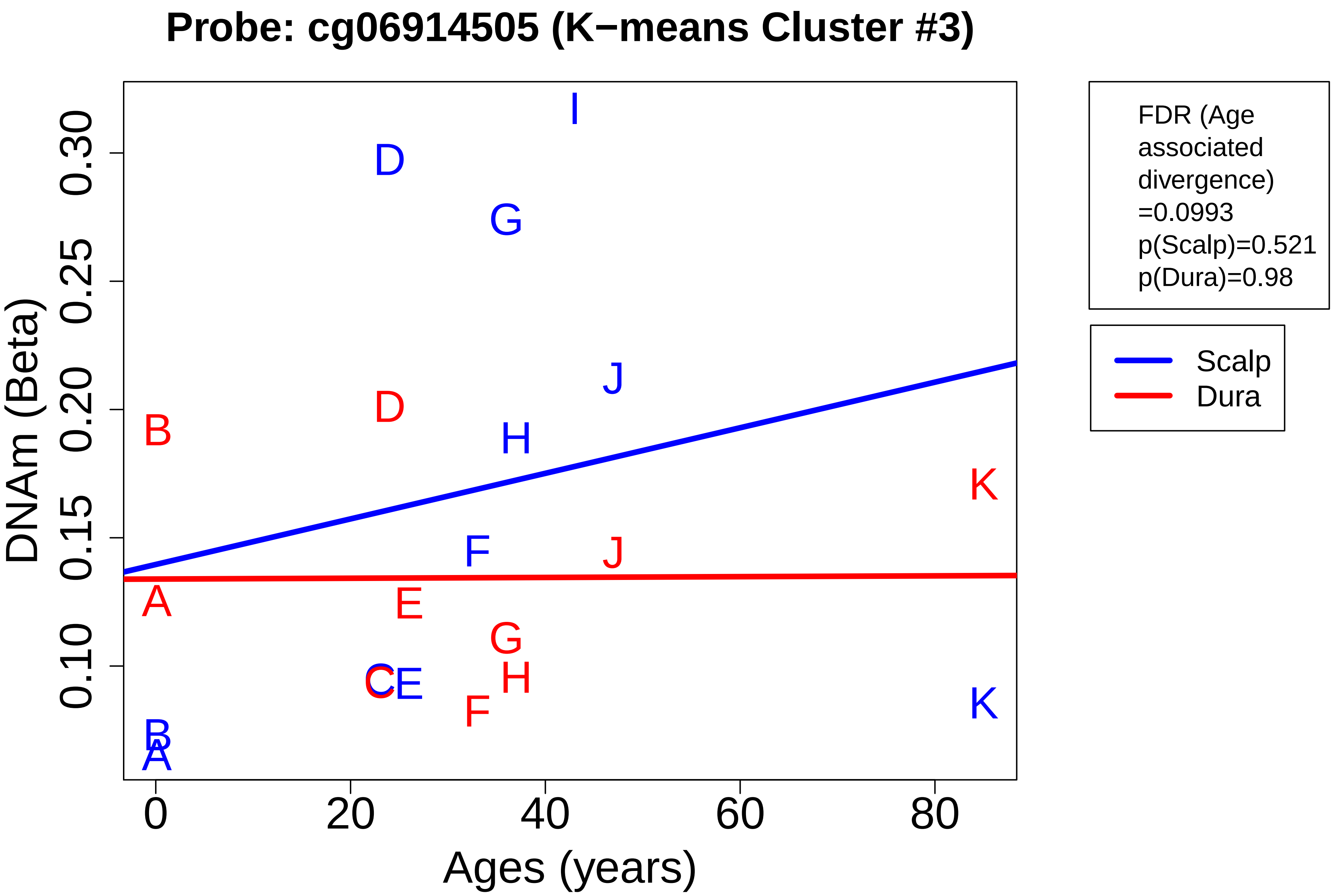

Probe: cg06914505 (K−means Cluster #3)
DNAm (Beta)
0.10
0.15
0.20
0.25
0.30
0
20
40
60
80
Ages (years)
FDR (Age associated divergence) =0.0993
p(Scalp)=0.521
p(Dura)=0.98
Scalp
Dura

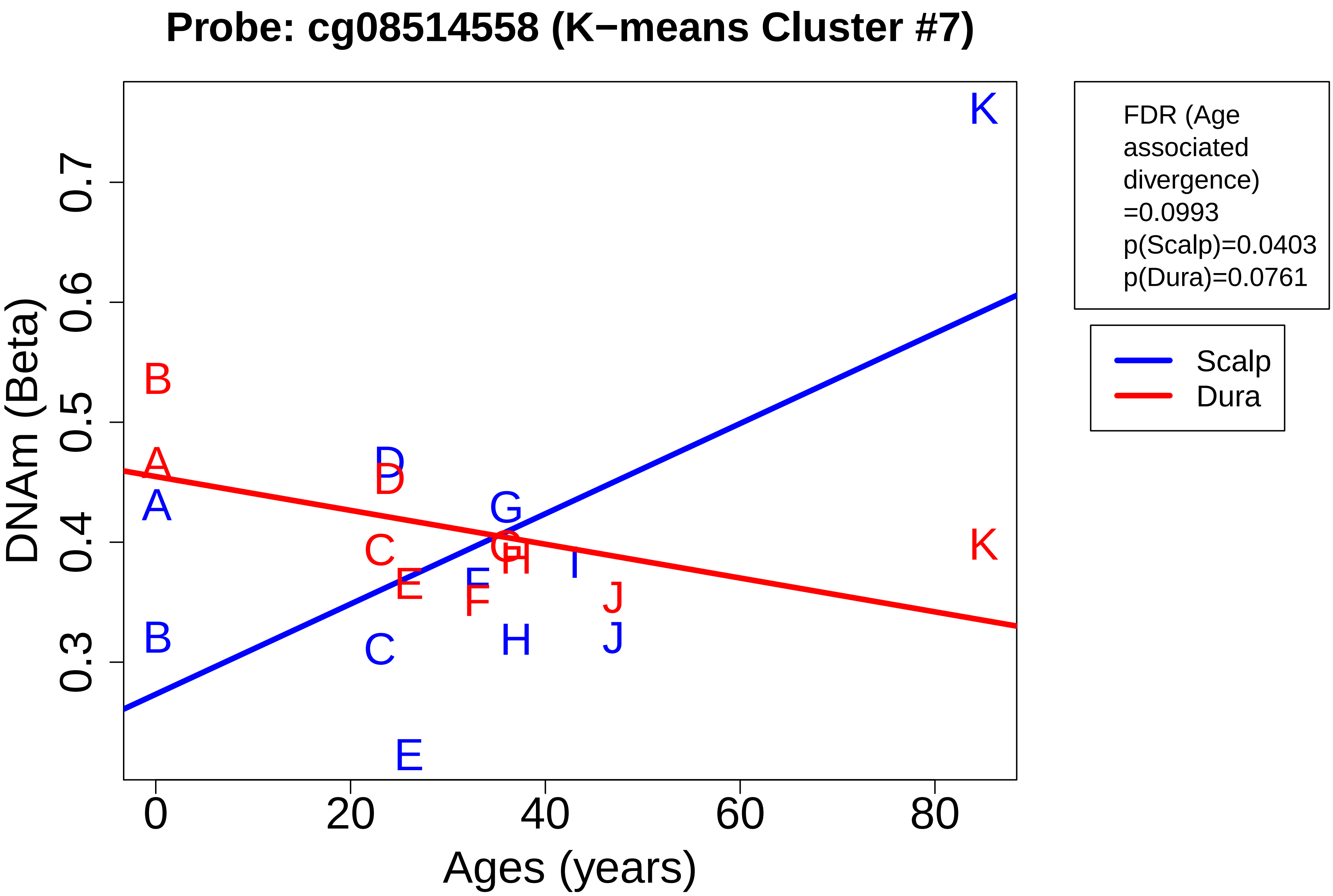
Probe: cg08514558 (K−means Cluster #7)
FDR (Age associated divergence) =0.0993
p(Scalp)=0.0403
p(Dura)=0.0761
Scalp
Dura
DNAm (Beta)
0.3
0.4
0.5
0.6
0.7
0
20
40
60
80
Ages (years)
A
B
C
D
E
F
G
H
I
J
K

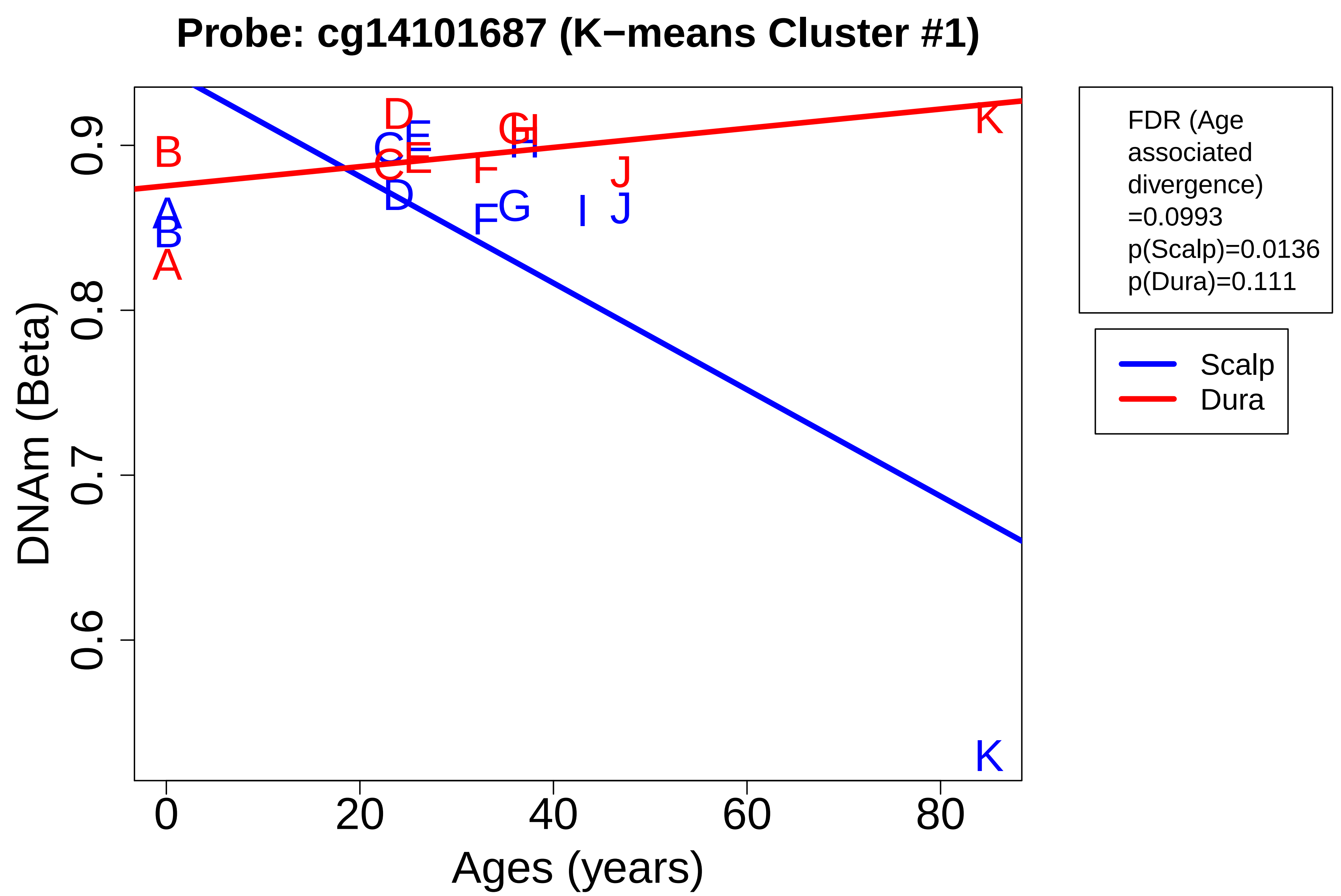

Probe: cg14101687 (K−means Cluster #1)
FDR (Age associated divergence) =0.0993
p(Scalp)=0.0136
p(Dura)=0.111
Scalp
Dura
DNAm (Beta)
Ages (years)
0.6
0.7
0.8
0.9
0
20
40
60
80

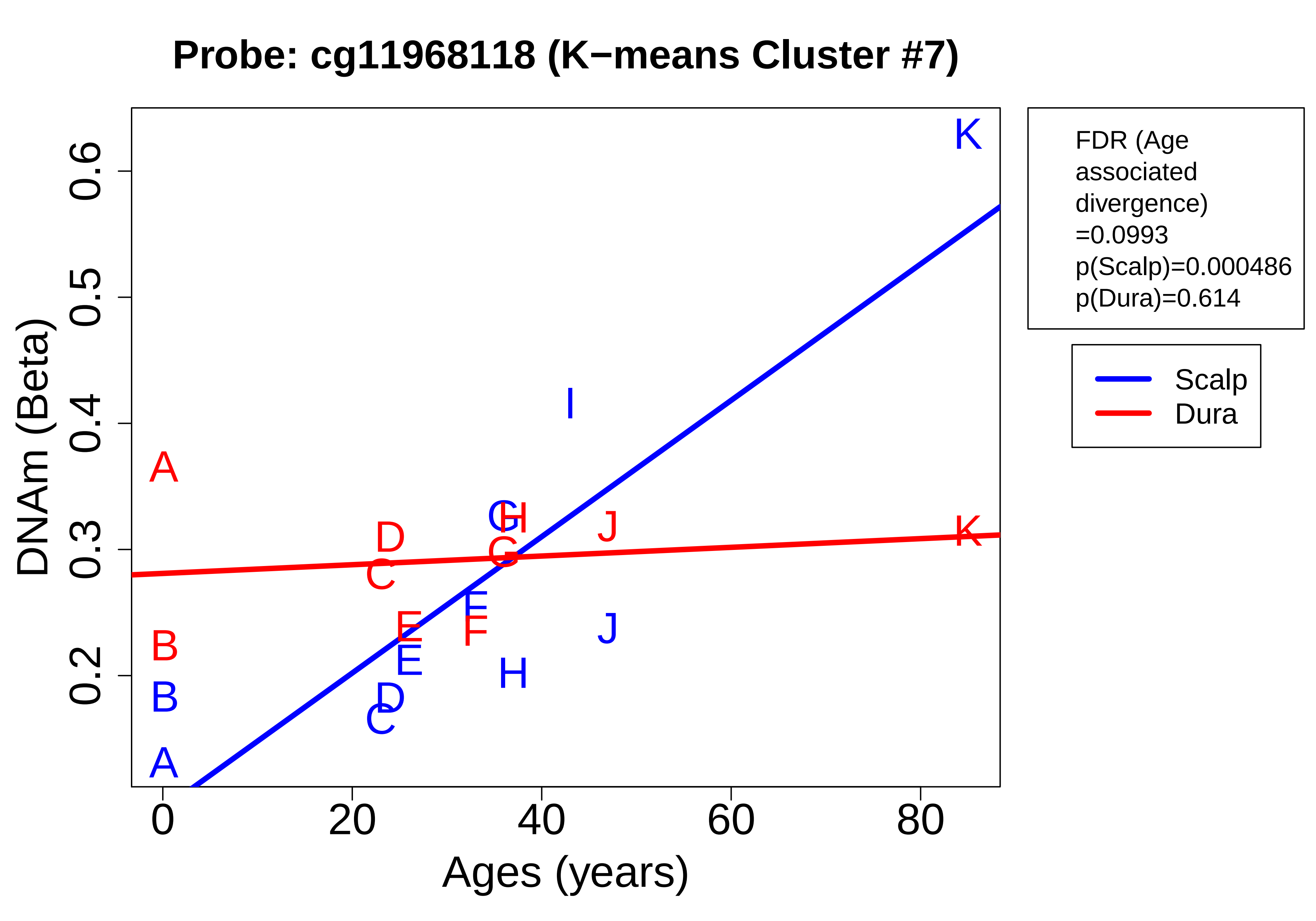
Probe: cg11968118 (K−means Cluster #7)
DNAm (Beta)
0.2
0.3
0.4
0.5
0.6
Ages (years)
0
20
40
60
80
FDR (Age associated divergence) =0.0993
p(Scalp)=0.000486
p(Dura)=0.614
Scalp
Dura
A
B
C
D
E
F
G
H
I
J
K

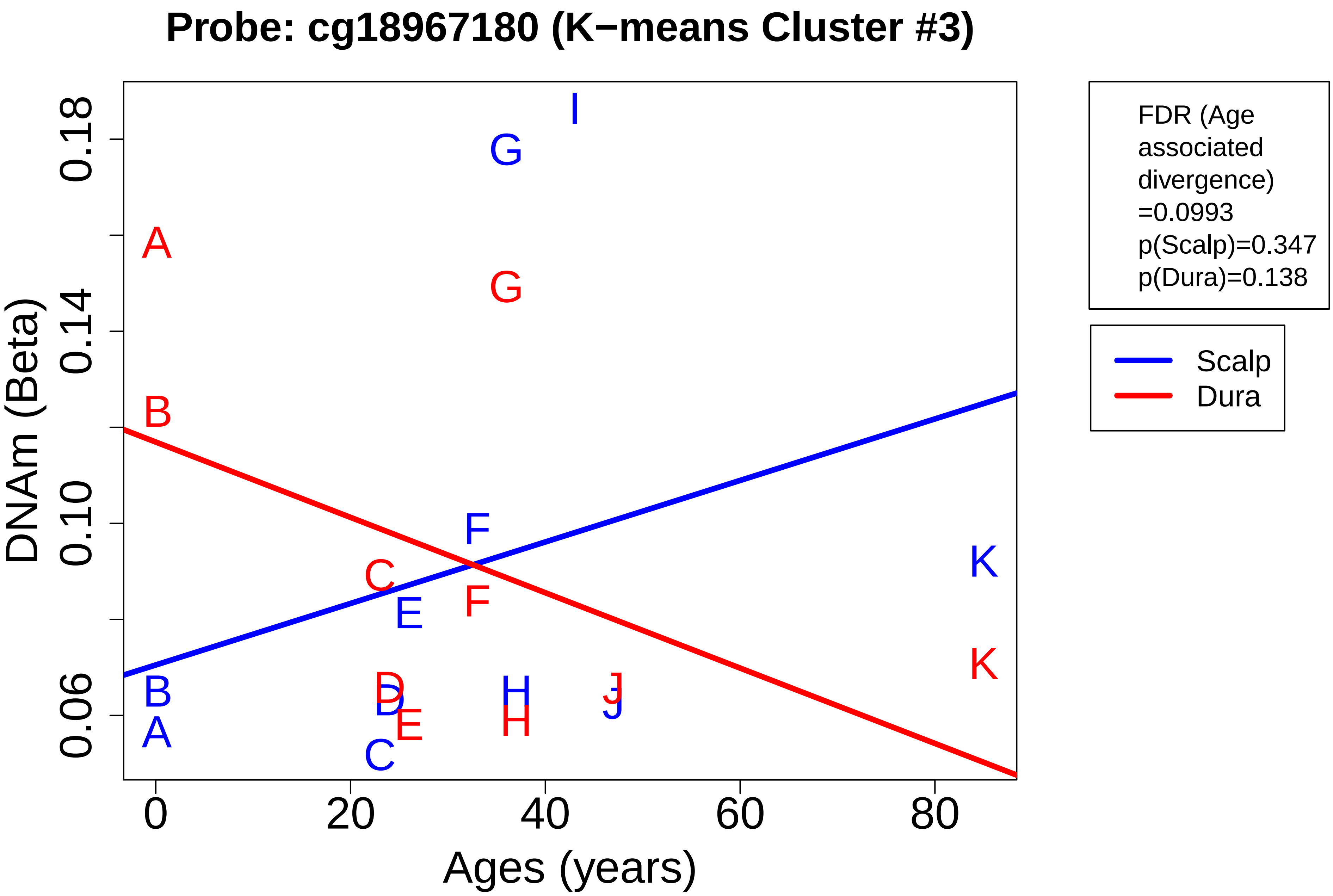

Probe: cg18967180 (K−means Cluster #3)
DNAm (Beta)
0.06
0.10
0.14
0.18
0
20
40
60
80
Ages (years)
A
B
C
D
E
F
G
H
I
J
K
FDR (Age associated divergence) =0.0993
p(Scalp)=0.347
p(Dura)=0.138
Scalp
Dura

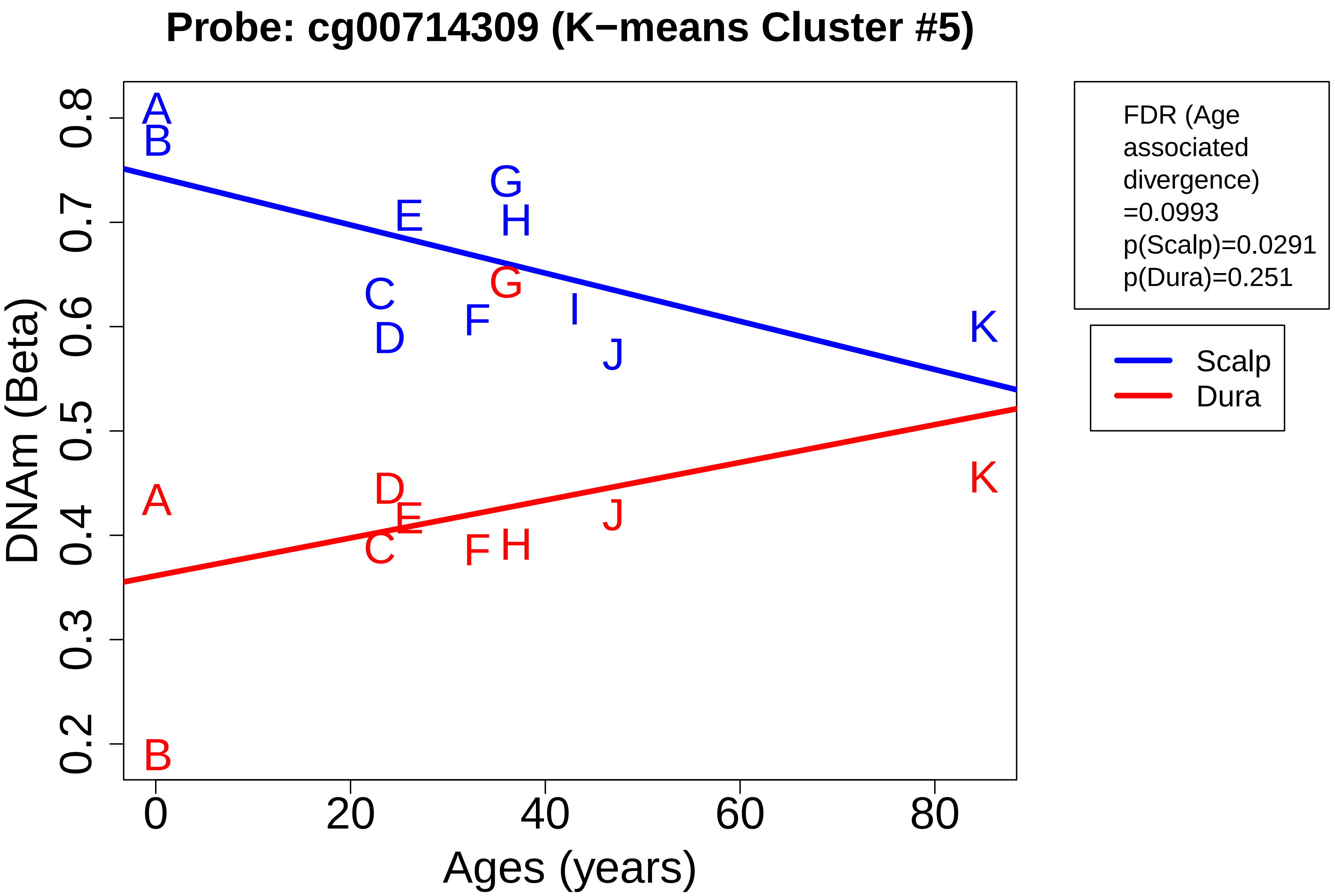
Probe: cg00714309 (K−means Cluster #5)
DNAm (Beta)
0.2
0.3
0.4
0.5
0.6
0.7
0.8
0
20
40
60
80
Ages (years)
A
B
C
D
E
F
G
H
I
J
K
FDR (Age associated divergence) =0.0993
p(Scalp)=0.0291
p(Dura)=0.251
Scalp
Dura

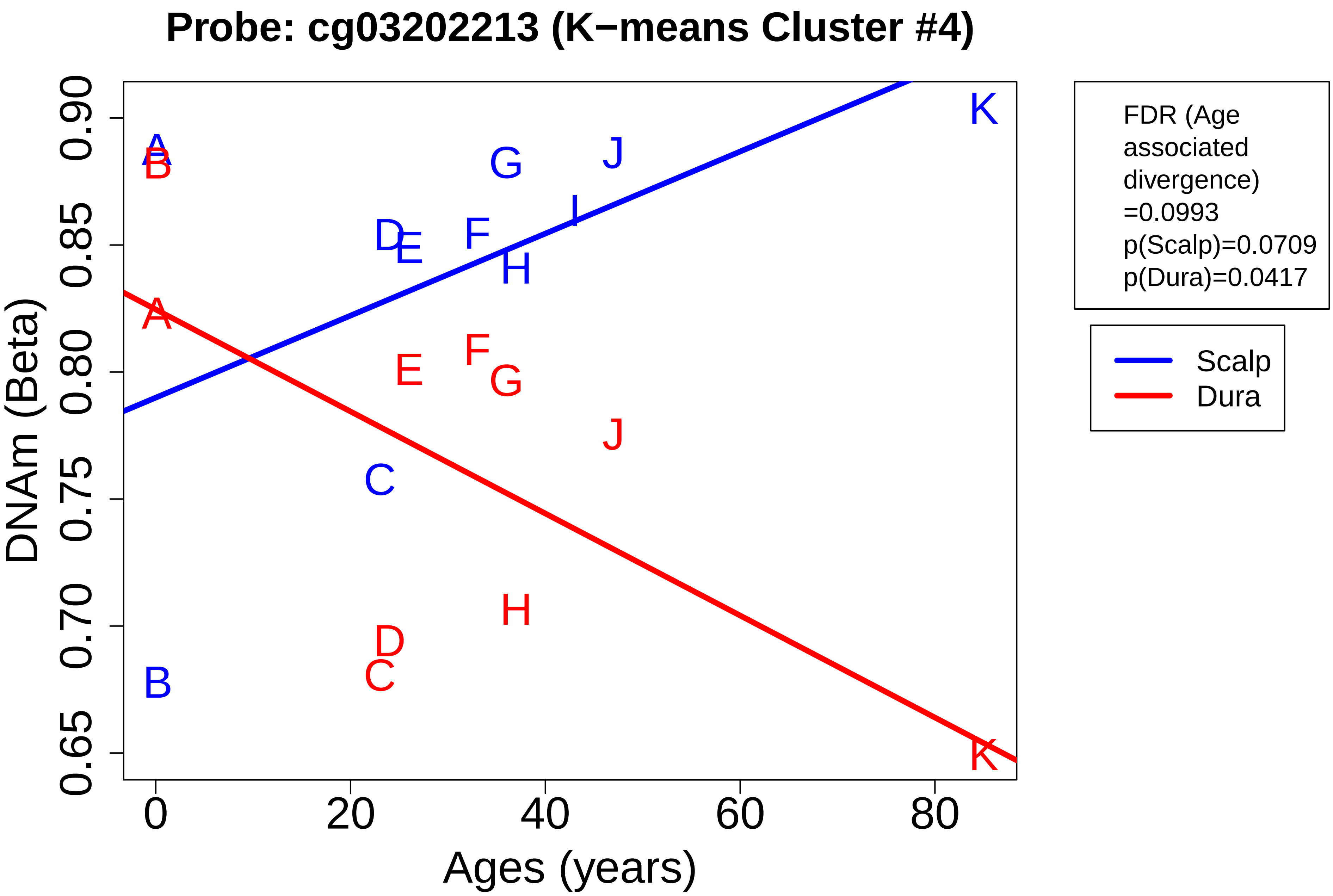
Probe: cg03202213 (K−means Cluster #4)
DNAm (Beta)
0.65
0.70
0.75
0.80
0.85
0.90
0
20
40
60
80
Ages (years)
FDR (Age associated divergence) =0.0993
p(Scalp)=0.0709
p(Dura)=0.0417
Scalp
Dura
A
B
C
D
E
F
G
H
I
J
K

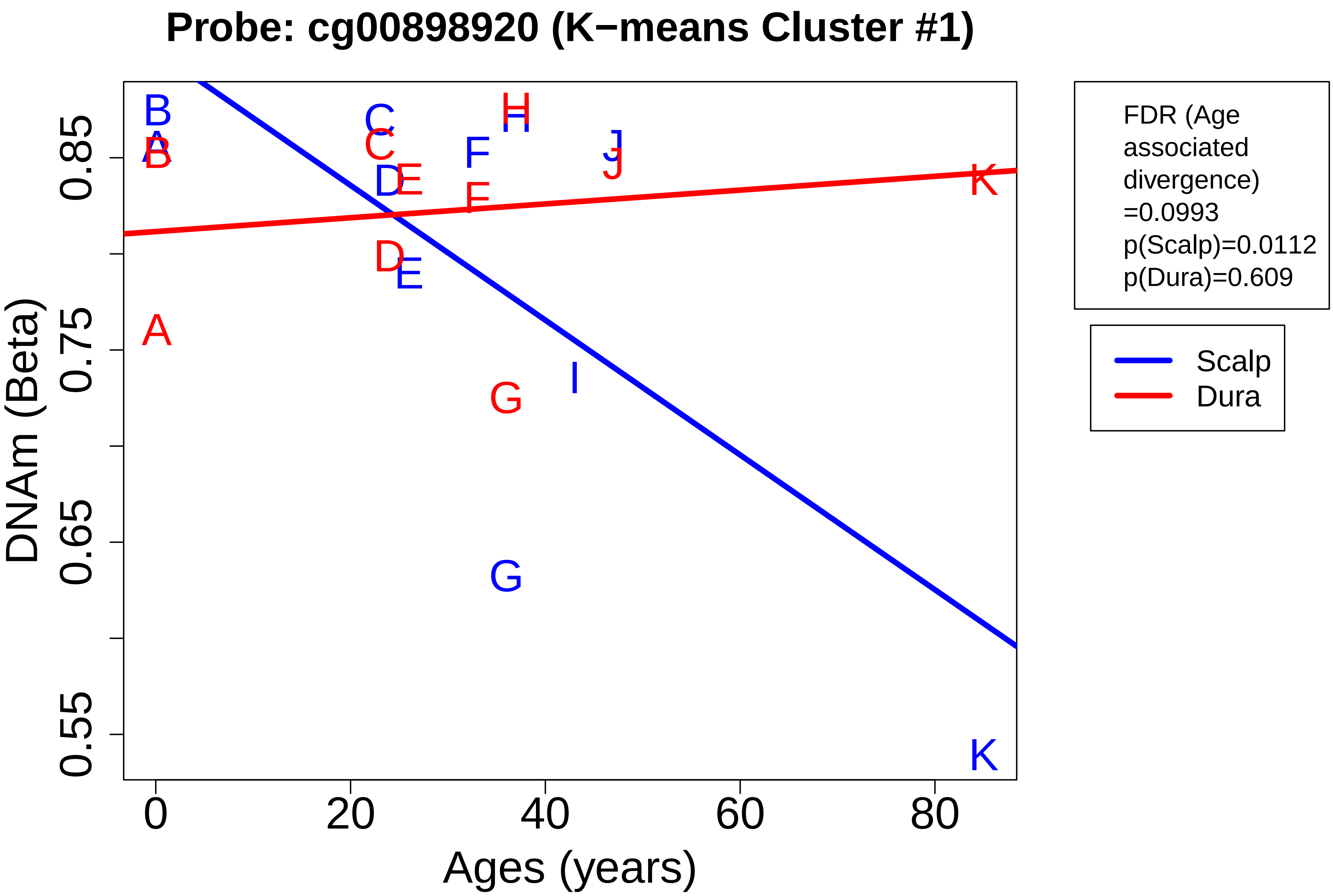

Probe: cg00898920 (K−means Cluster #1)
DNAm (Beta)
0.55
0.65
0.75
0.85
0
20
40
60
80
Ages (years)
FDR (Age associated divergence) =0.0993
p(Scalp)=0.0112
p(Dura)=0.609
Scalp
Dura

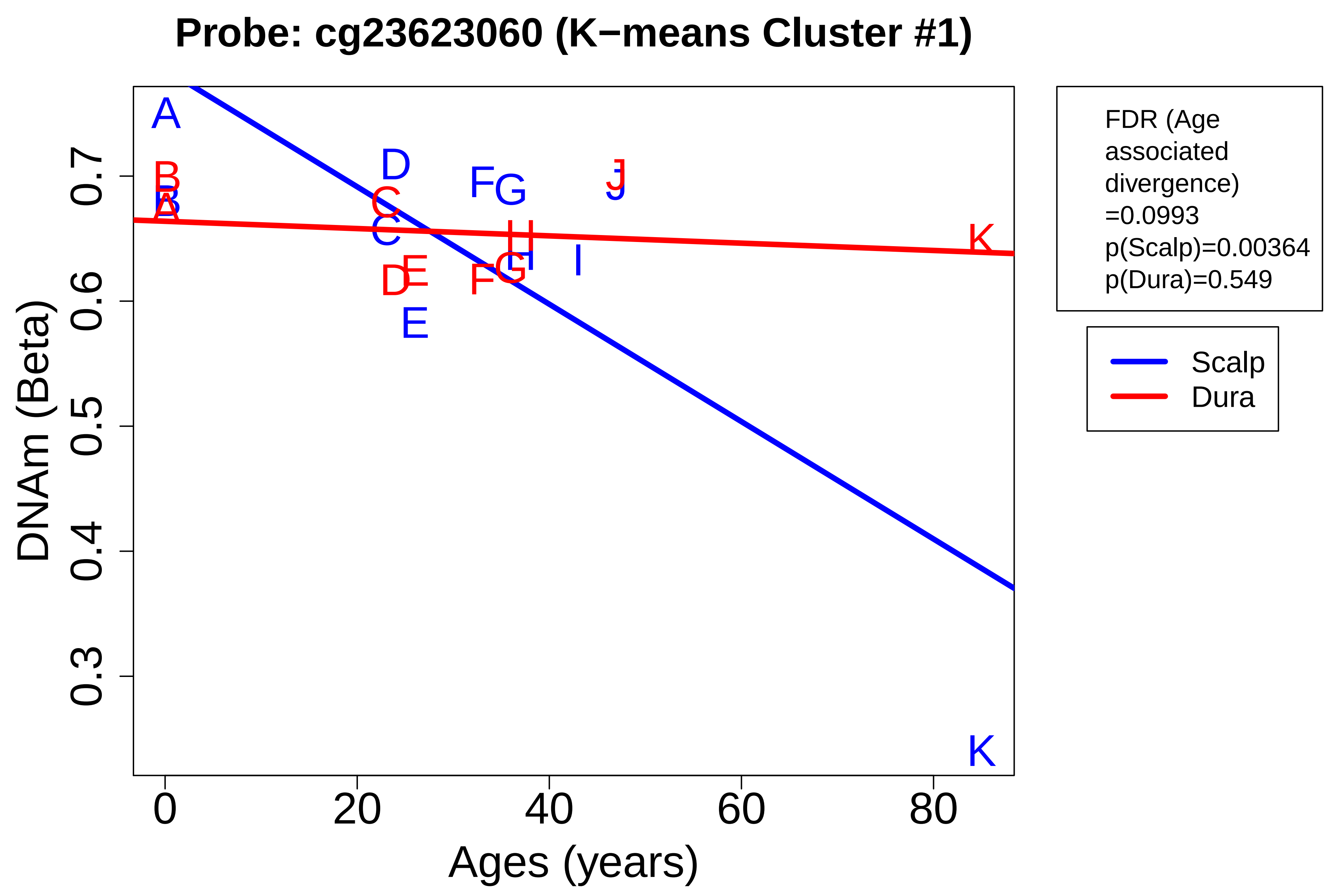

Probe: cg23623060 (K−means Cluster #1)
DNAm (Beta)
0.3
0.4
0.5
0.6
0.7
0
20
40
60
80
Ages (years)
FDR (Age associated divergence) =0.0993
p(Scalp)=0.00364
p(Dura)=0.549
Scalp
Dura
A
B
C
D
E
F
G
H
I
J
K

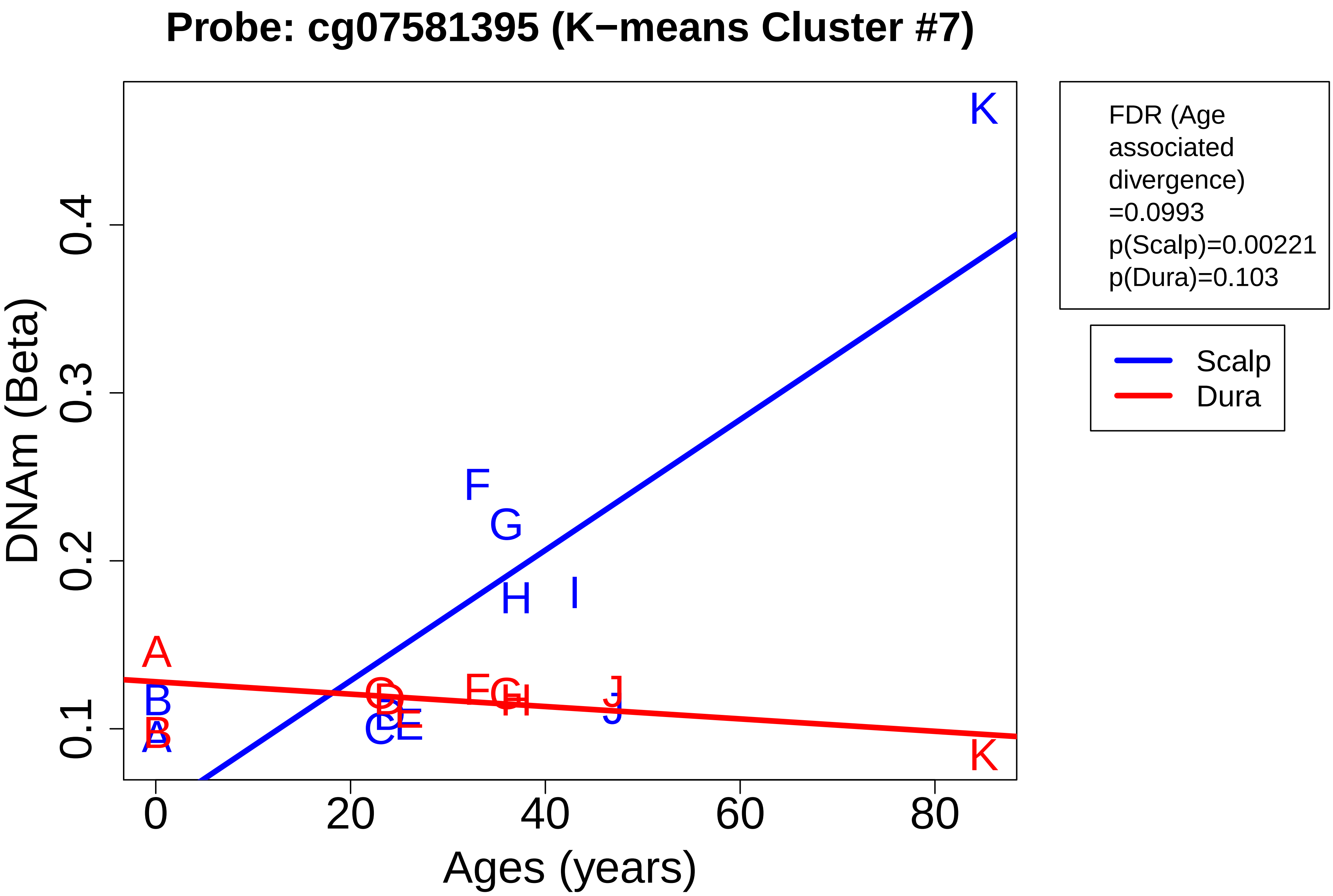

Probe: cg07581395 (K−means Cluster #7)
DNAm (Beta)
0.1
0.2
0.3
0.4
0
20
40
60
80
Ages (years)
FDR (Age associated divergence) =0.0993
p(Scalp)=0.00221
p(Dura)=0.103
Scalp
Dura

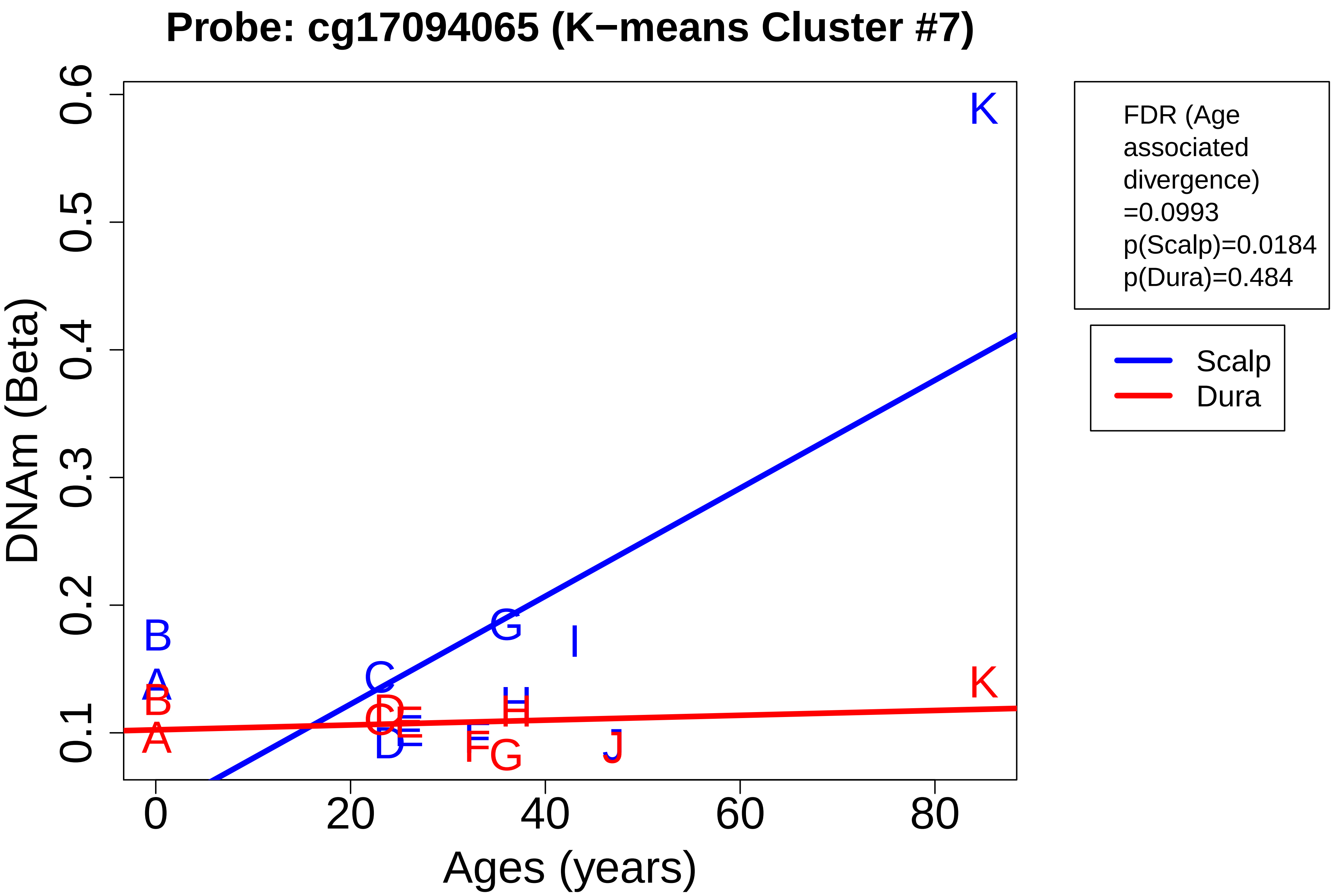

Probe: cg17094065 (K−means Cluster #7)
DNAm (Beta)
0.1
0.2
0.3
0.4
0.5
0.6
0
20
40
60
80
Ages (years)
FDR (Age associated divergence) =0.0993
p(Scalp)=0.0184
p(Dura)=0.484
Scalp
Dura

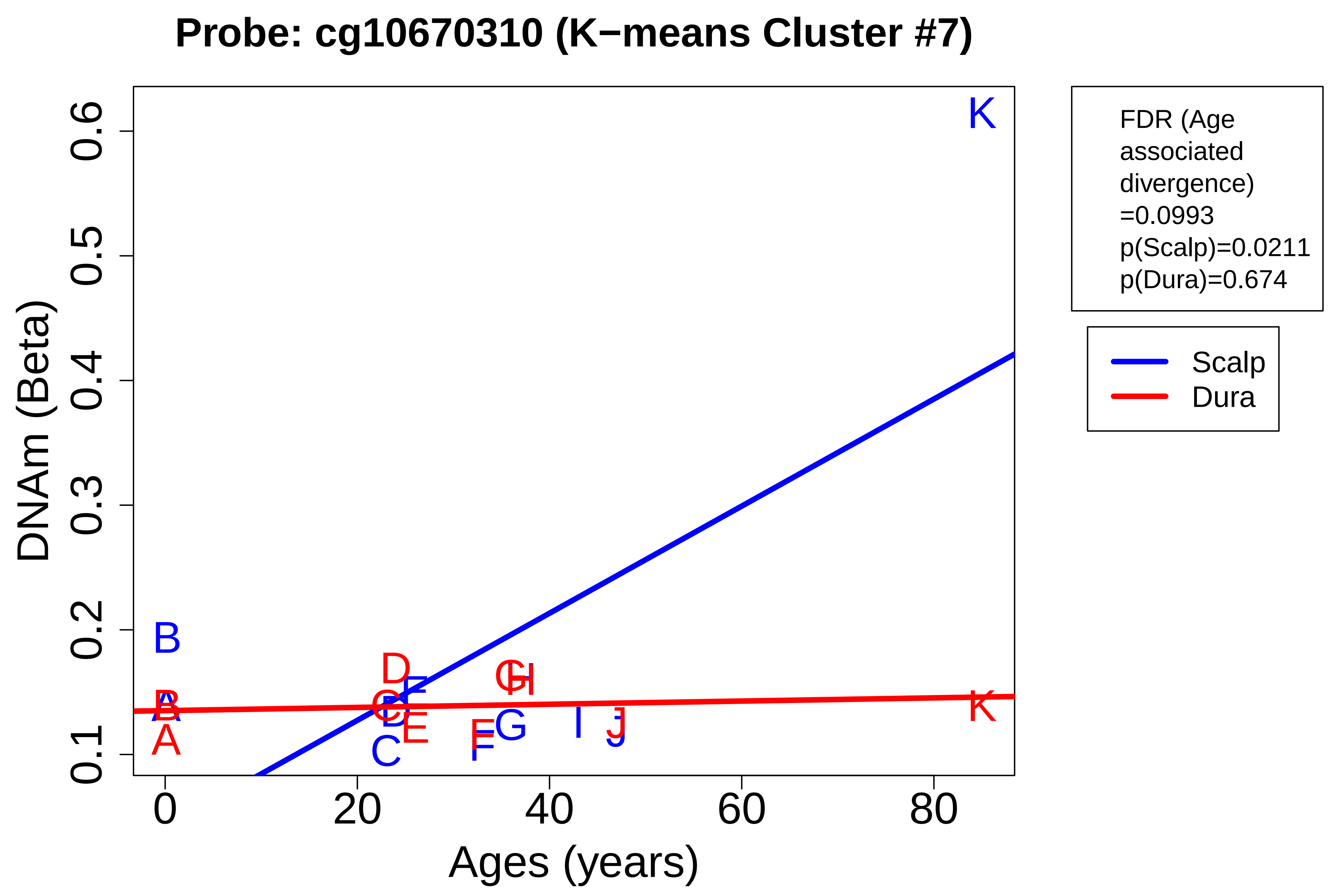

Probe: cg10670310 (K−means Cluster #7)
DNAm (Beta)
0.1
0.2
0.3
0.4
0.5
0.6
0
20
40
60
80
Ages (years)
FDR (Age associated divergence) =0.0993
p(Scalp)=0.0211
p(Dura)=0.674
Scalp
Dura

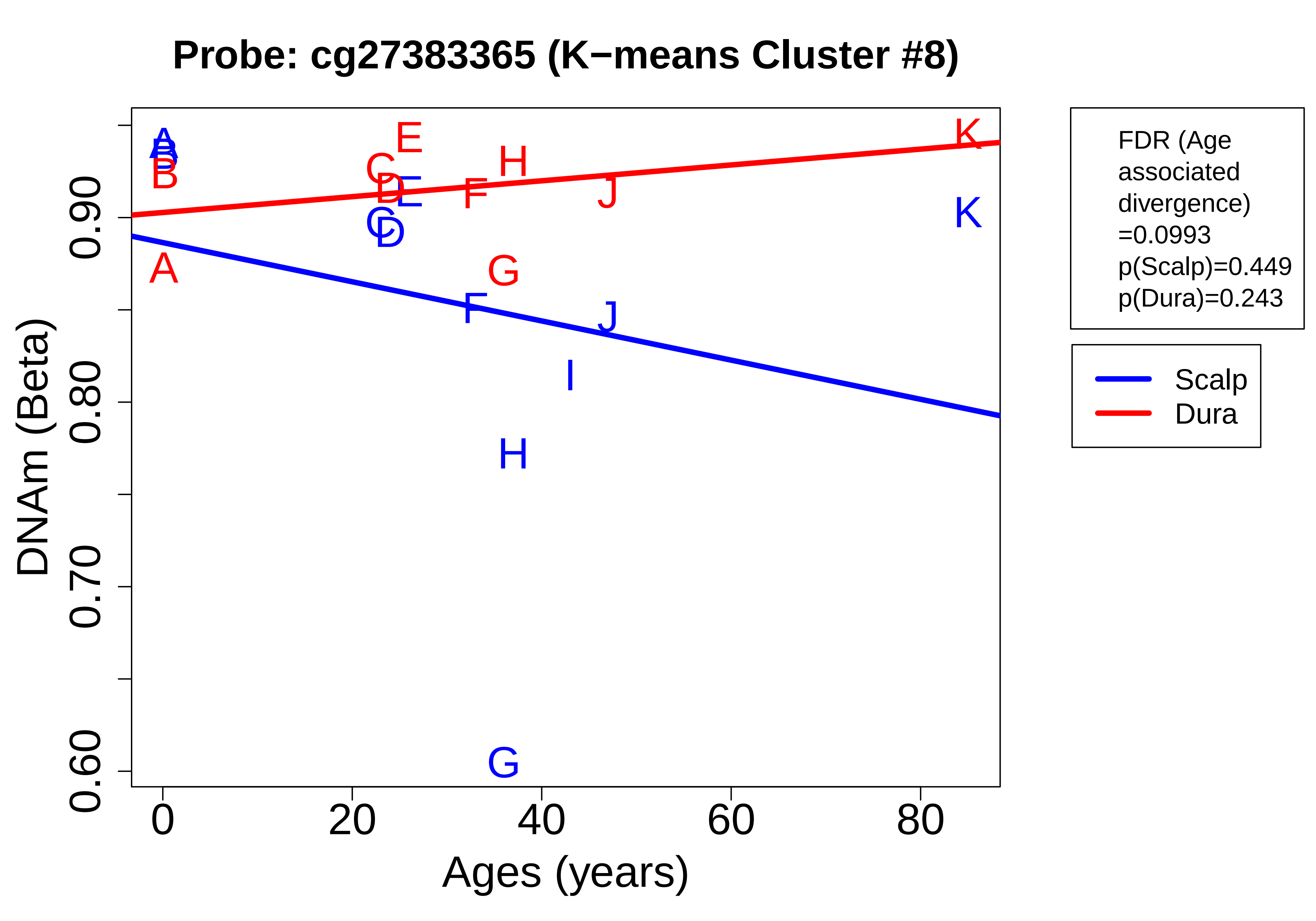

Probe: cg27383365 (K−means Cluster #8)
DNAm (Beta)
0.60
0.70
0.80
0.90
0
20
40
60
80
Ages (years)
FDR (Age associated divergence) =0.0993
p(Scalp)=0.449
p(Dura)=0.243
Scalp
Dura

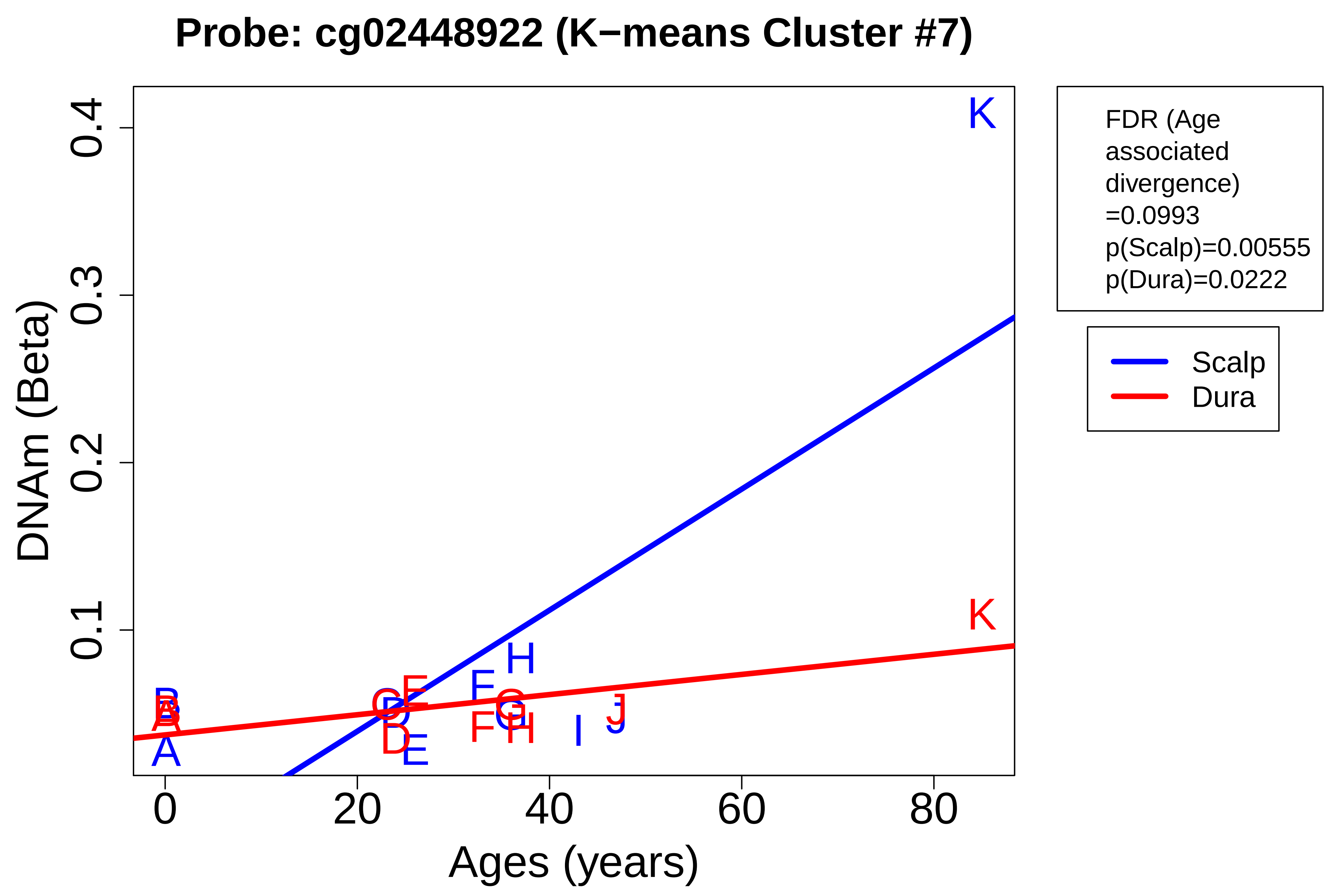

Probe: cg02448922 (K−means Cluster #7)
DNAm (Beta)
0.1
0.2
0.3
0.4
0
20
40
60
80
Ages (years)
FDR (Age associated divergence) =0.0993
p(Scalp)=0.00555
p(Dura)=0.0222
Scalp
Dura
A
B
C
D
E
F
G
H
I
J
K

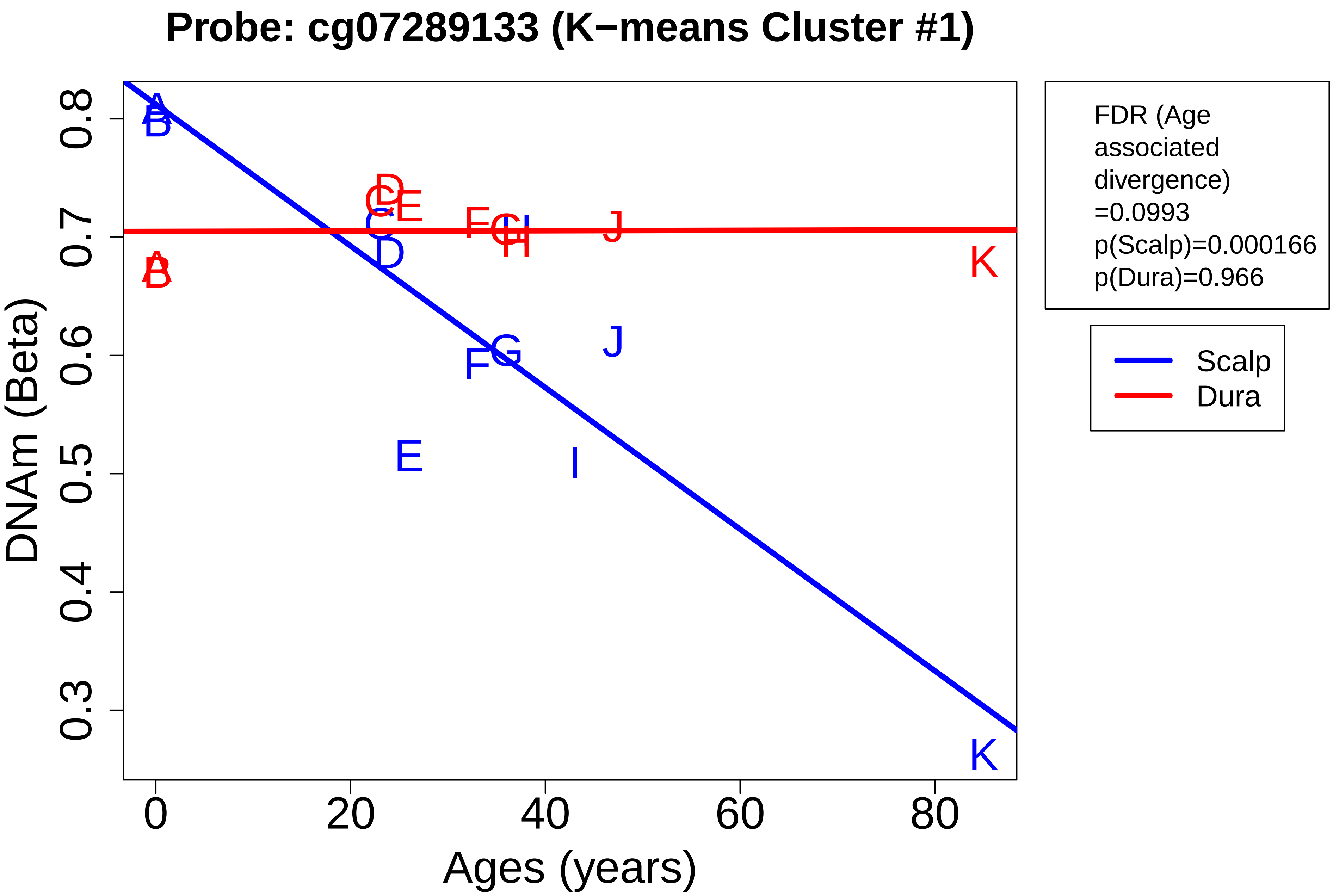

Probe: cg07289133 (K−means Cluster #1)
DNAm (Beta)
0.3
0.4
0.5
0.6
0.7
0.8
Ages (years)
0
20
40
60
80
FDR (Age associated divergence) =0.0993
p(Scalp)=0.000166
p(Dura)=0.966
Scalp
Dura

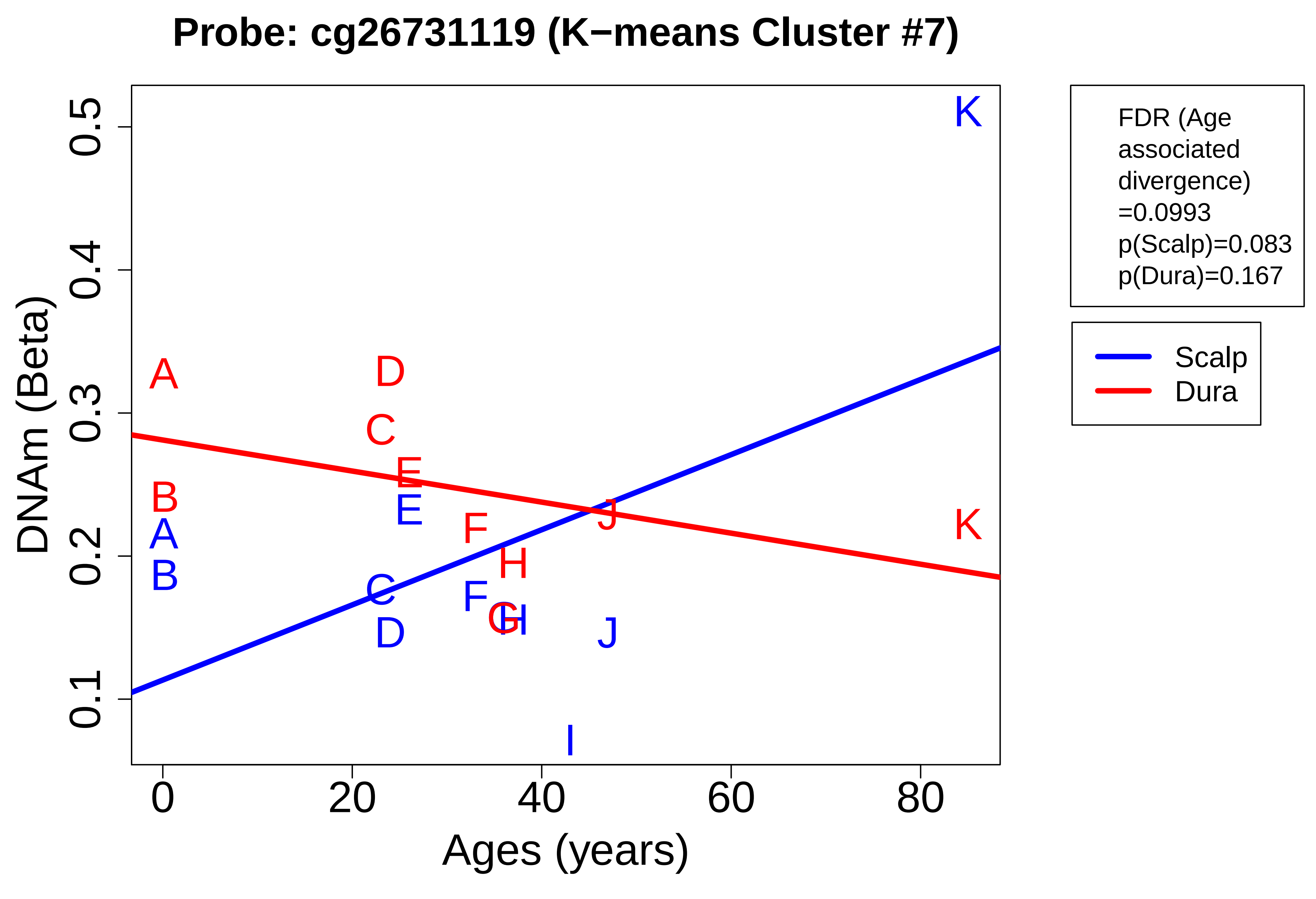

Probe: cg26731119 (K−means Cluster #7)
DNAm (Beta)
0.1
0.2
0.3
0.4
0.5
0
20
40
60
80
Ages (years)
FDR (Age associated divergence) =0.0993
p(Scalp)=0.083
p(Dura)=0.167
Scalp
Dura

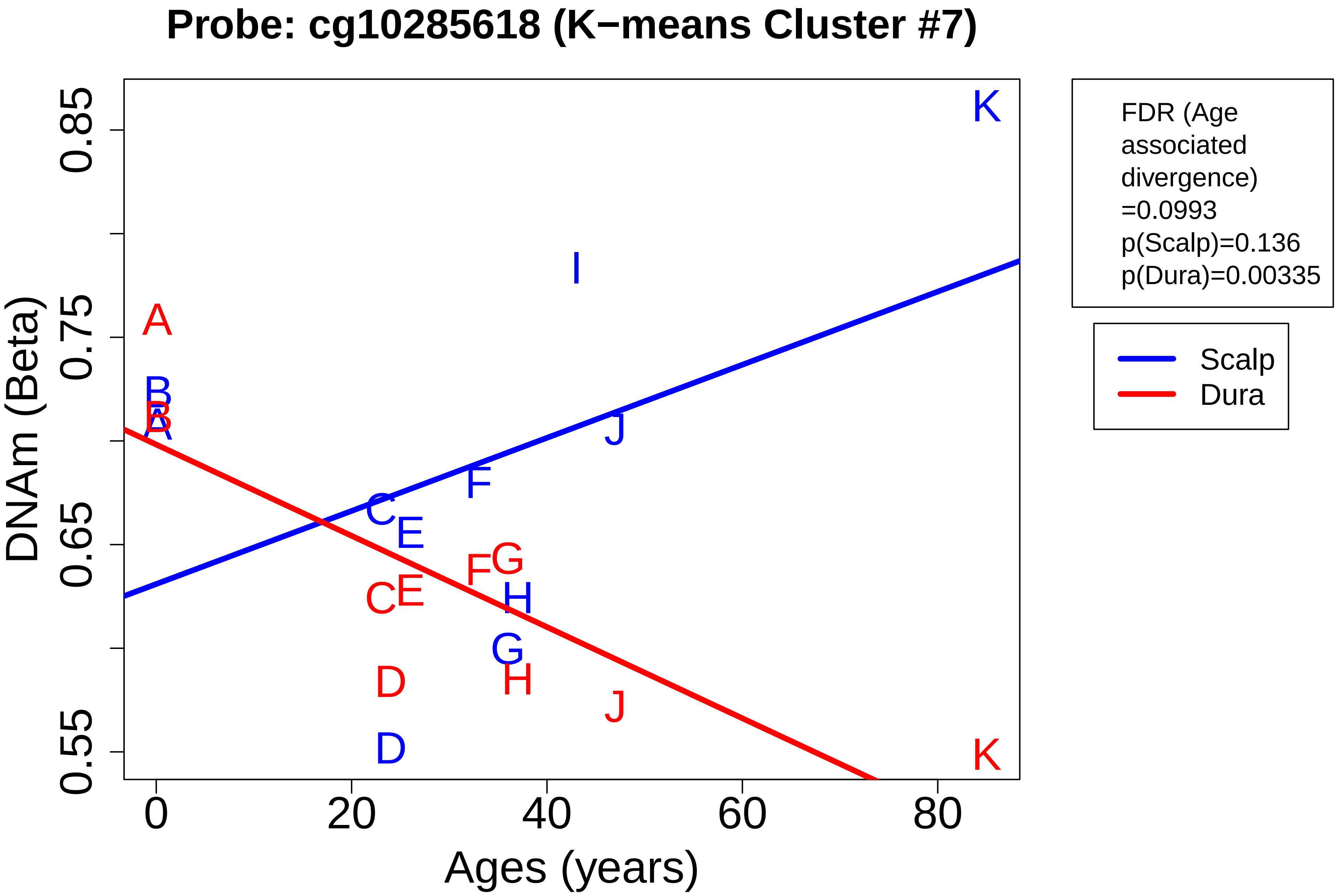

Probe: cg10285618 (K−means Cluster #7)
DNAm (Beta)
Ages (years)
0.55
0.65
0.75
0.85
0
20
40
60
80
FDR (Age associated divergence) =0.0993
p(Scalp)=0.136
p(Dura)=0.00335
Scalp
Dura

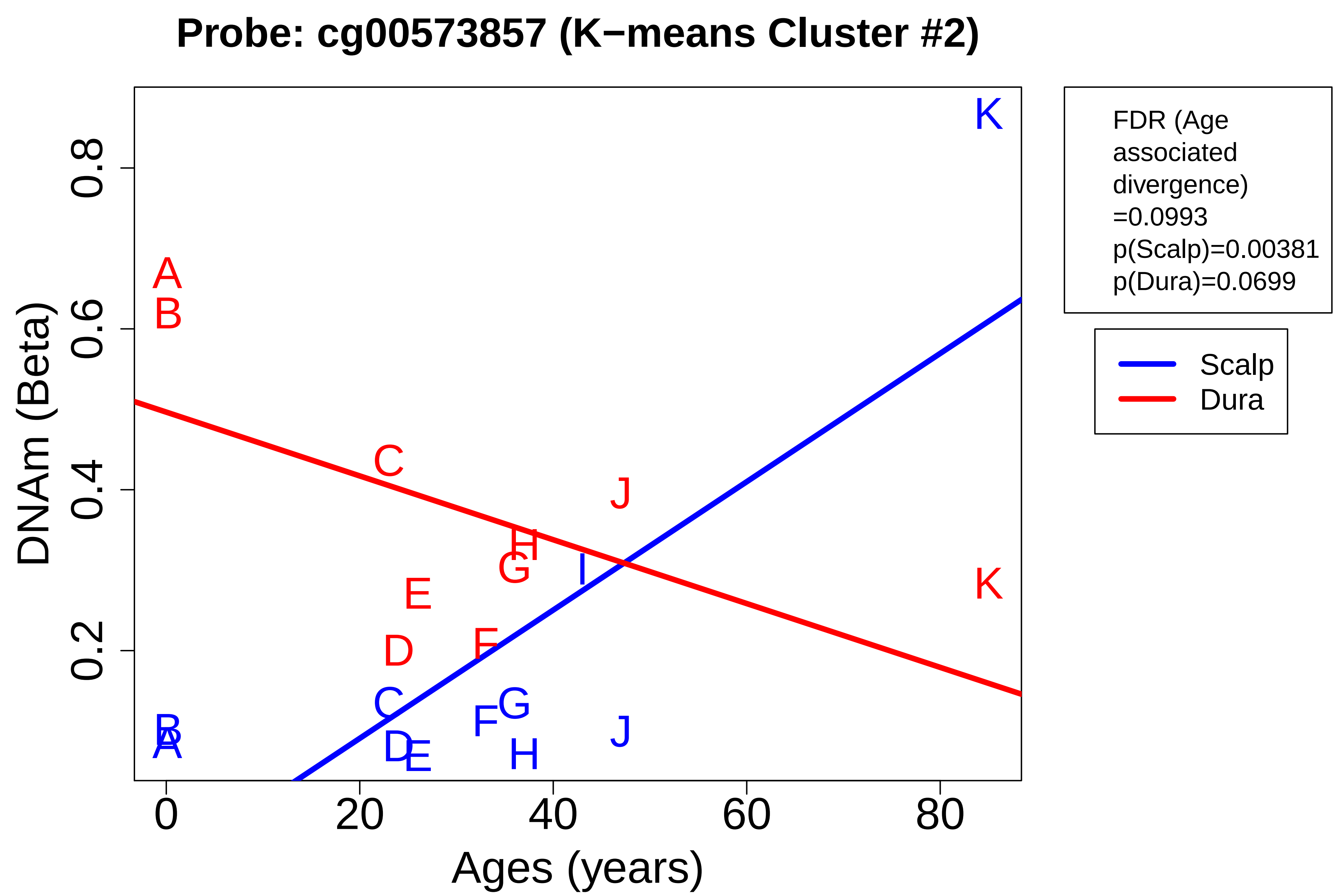
Probe: cg00573857 (K−means Cluster #2)
DNAm (Beta)
0.2
0.4
0.6
0.8
Ages (years)
0
20
40
60
80
A
B
C
D
E
F
G
H
I
J
K
FDR (Age associated divergence) =0.0993
p(Scalp)=0.00381
p(Dura)=0.0699
Scalp
Dura

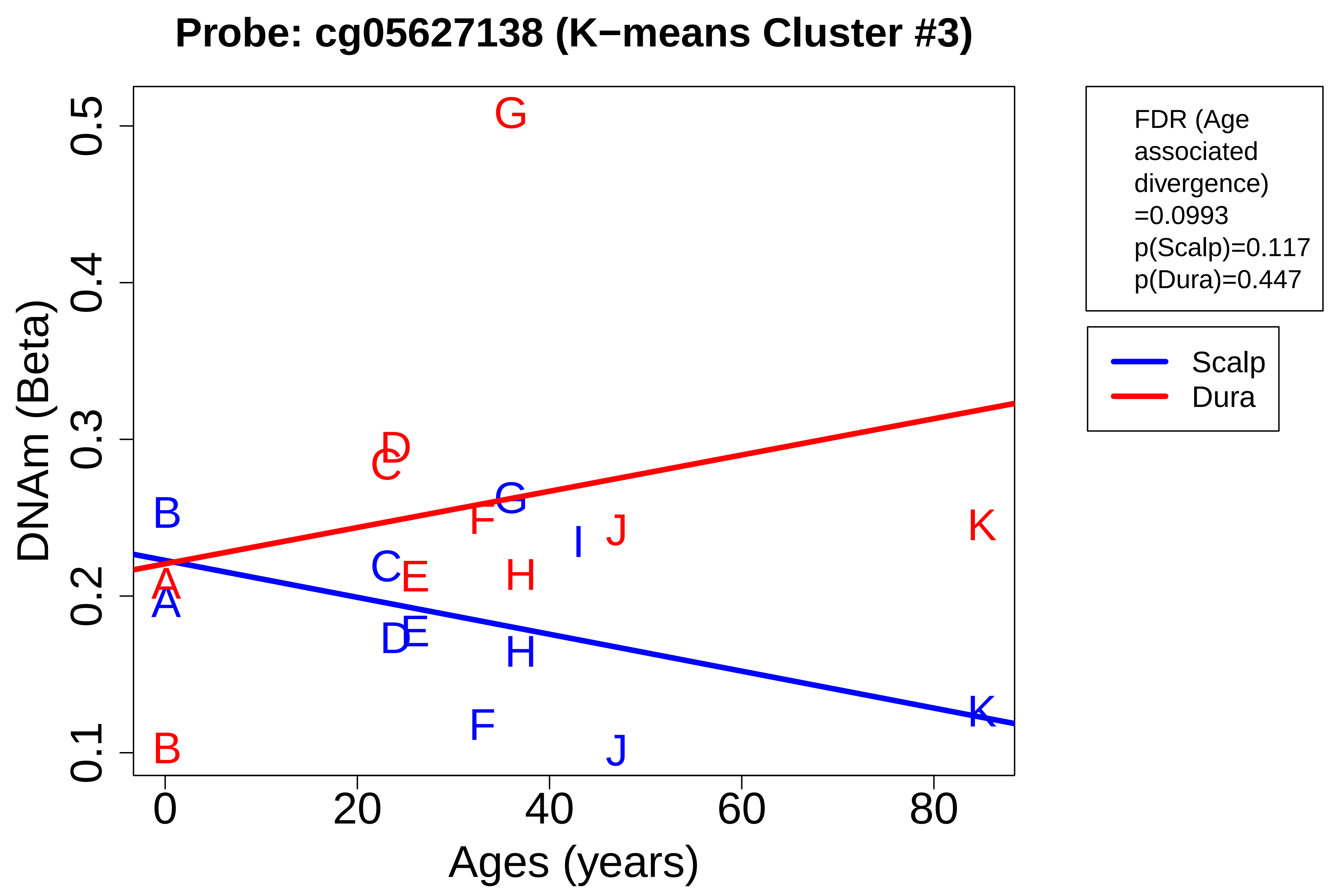

Probe: cg05627138 (K−means Cluster #3)
DNAm (Beta)
0.1
0.2
0.3
0.4
0.5
0
20
40
60
80
Ages (years)
FDR (Age associated divergence) =0.0993
p(Scalp)=0.117
p(Dura)=0.447
Scalp
Dura

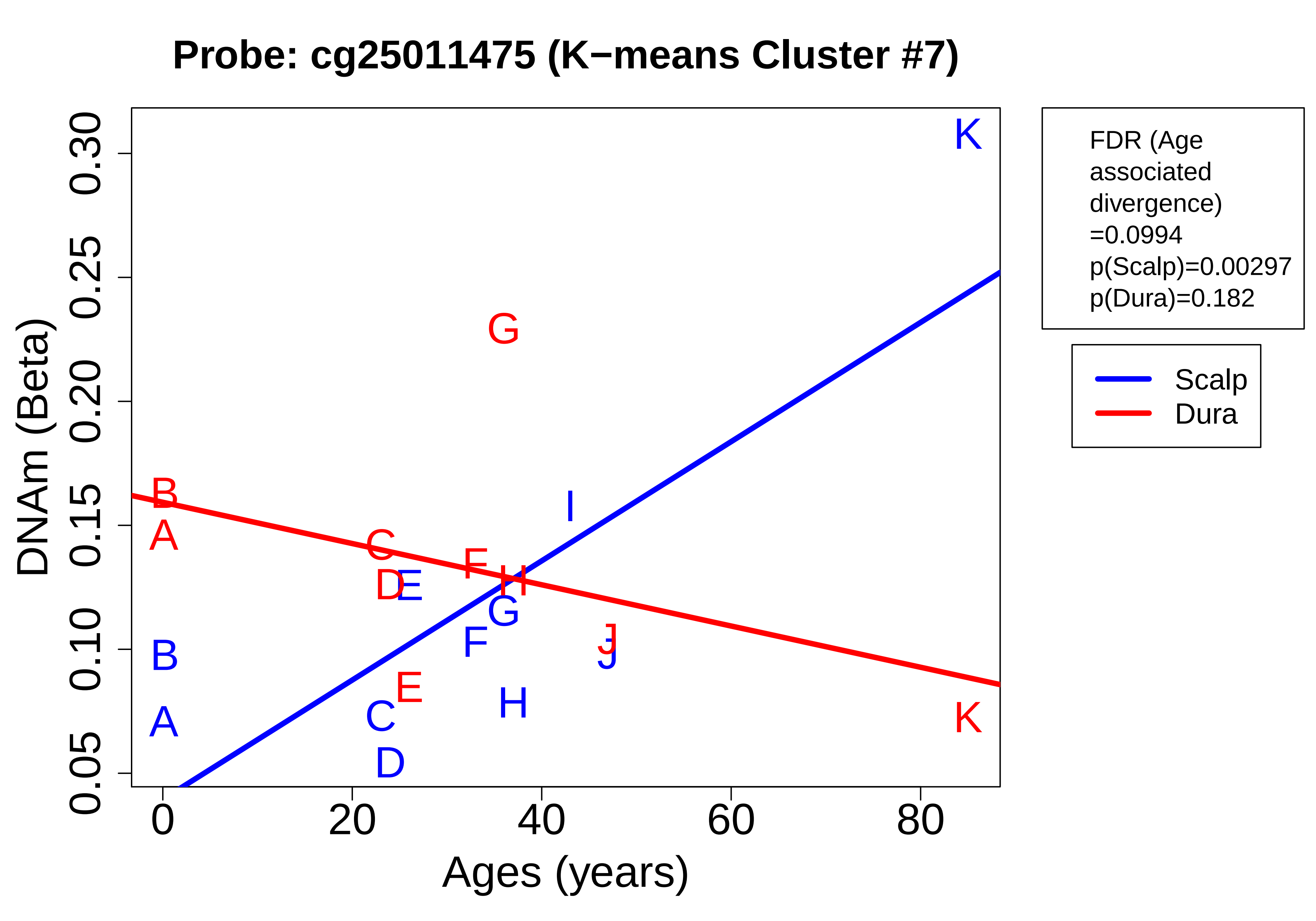
Probe: cg25011475 (K−means Cluster #7)
DNAm (Beta)
0.05
0.10
0.15
0.20
0.25
0.30
0
20
40
60
80
Ages (years)
FDR (Age associated divergence) =0.0994
p(Scalp)=0.00297
p(Dura)=0.182
Scalp
Dura
A
B
C
D
E
F
G
H
I
J
K

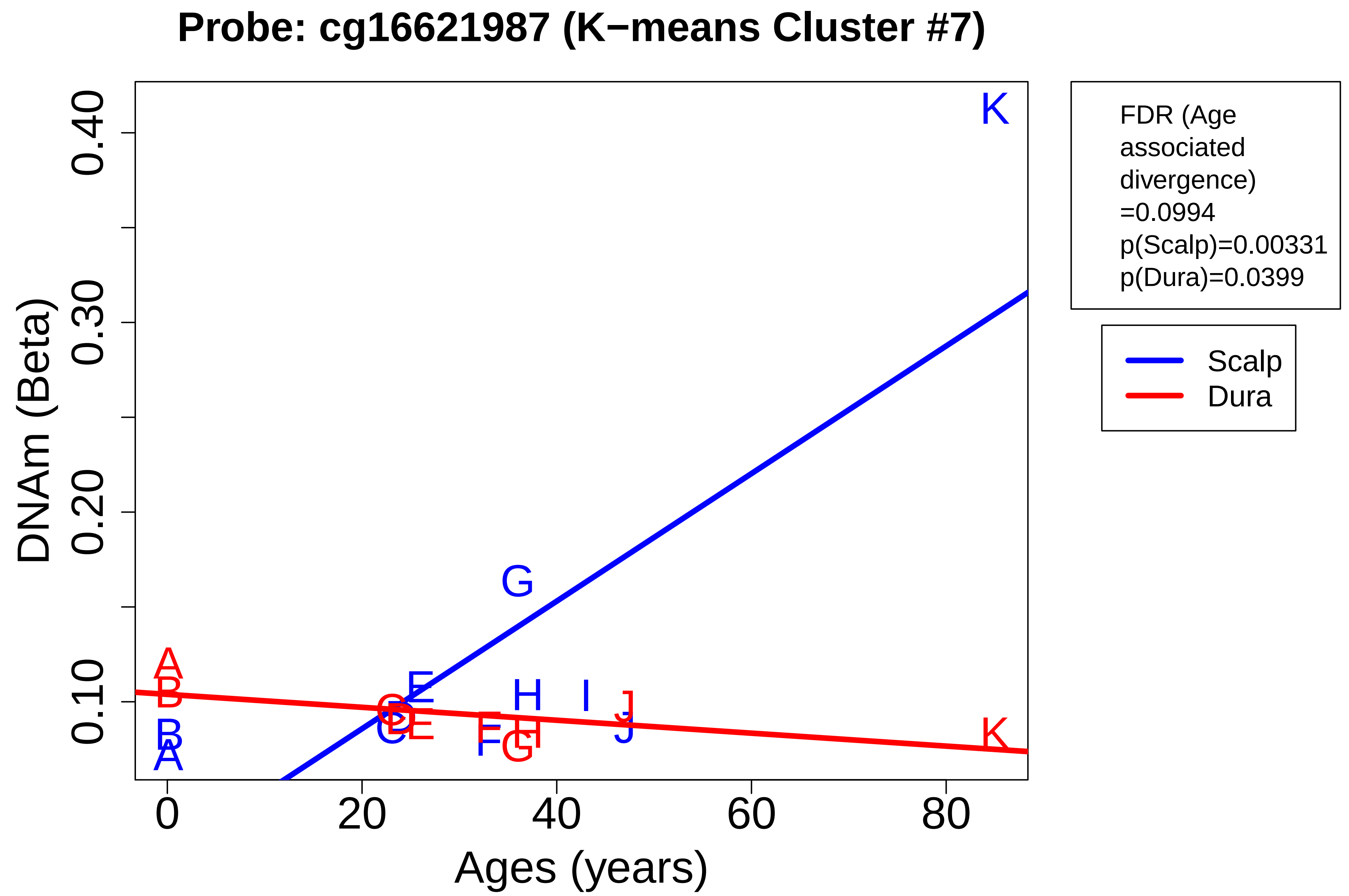

Probe: cg16621987 (K−means Cluster #7)
DNAm (Beta)
0.10
0.20
0.30
0.40
0
20
40
60
80
Ages (years)
FDR (Age associated divergence) =0.0994
p(Scalp)=0.00331
p(Dura)=0.0399
Scalp
Dura

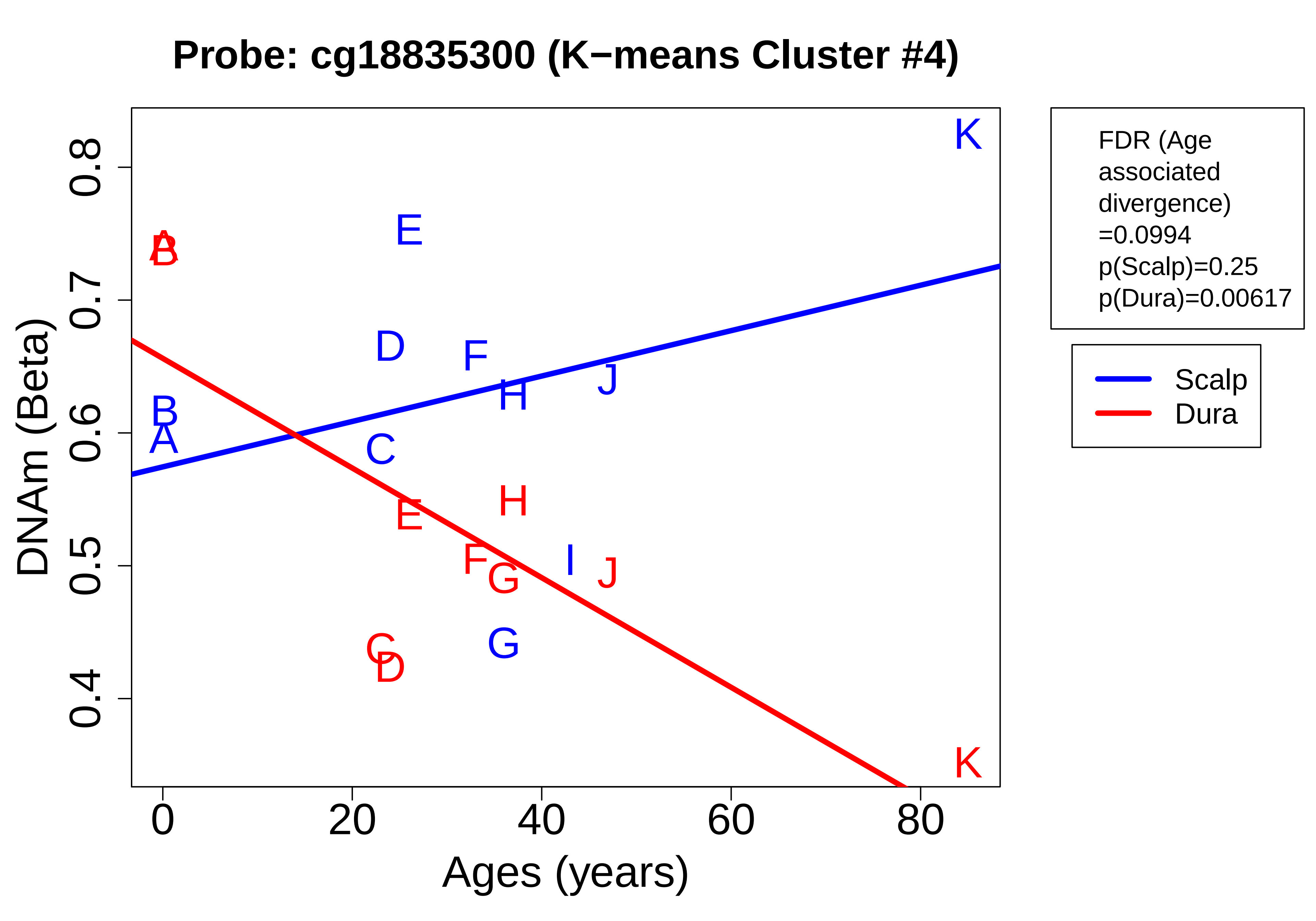

Probe: cg18835300 (K−means Cluster #4)
DNAm (Beta)
0.4
0.5
0.6
0.7
0.8
0
20
40
60
80
Ages (years)
FDR (Age associated divergence) =0.0994
p(Scalp)=0.25
p(Dura)=0.00617
Scalp
Dura
A
B
C
D
E
F
G
H
I
J
K

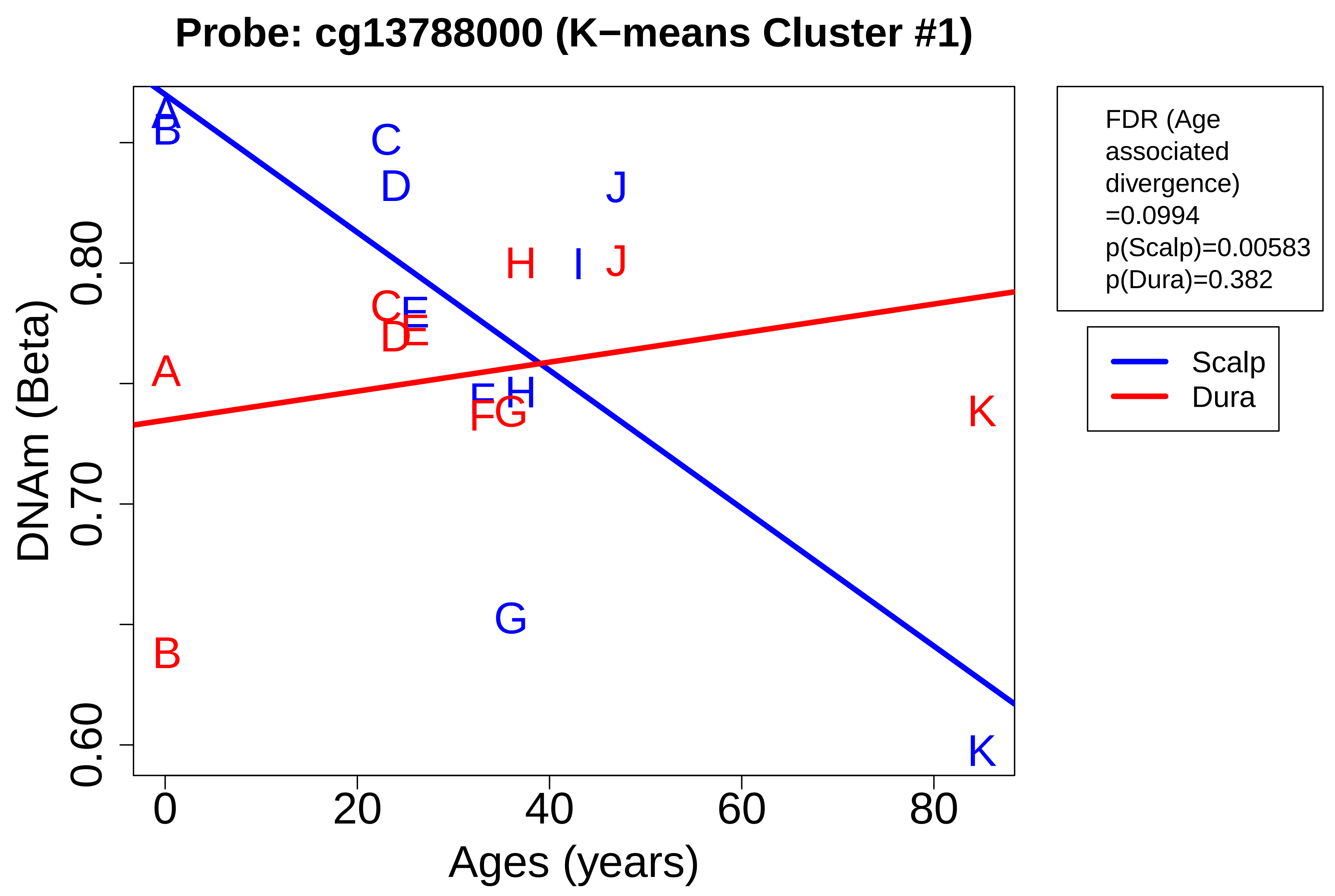

Probe: cg13788000 (K−means Cluster #1)
DNAm (Beta)
0.60
0.70
0.80
Ages (years)
0
20
40
60
80
A
B
C
D
E
F
G
H
I
J
K
FDR (Age associated divergence) =0.0994
p(Scalp)=0.00583
p(Dura)=0.382
Scalp
Dura

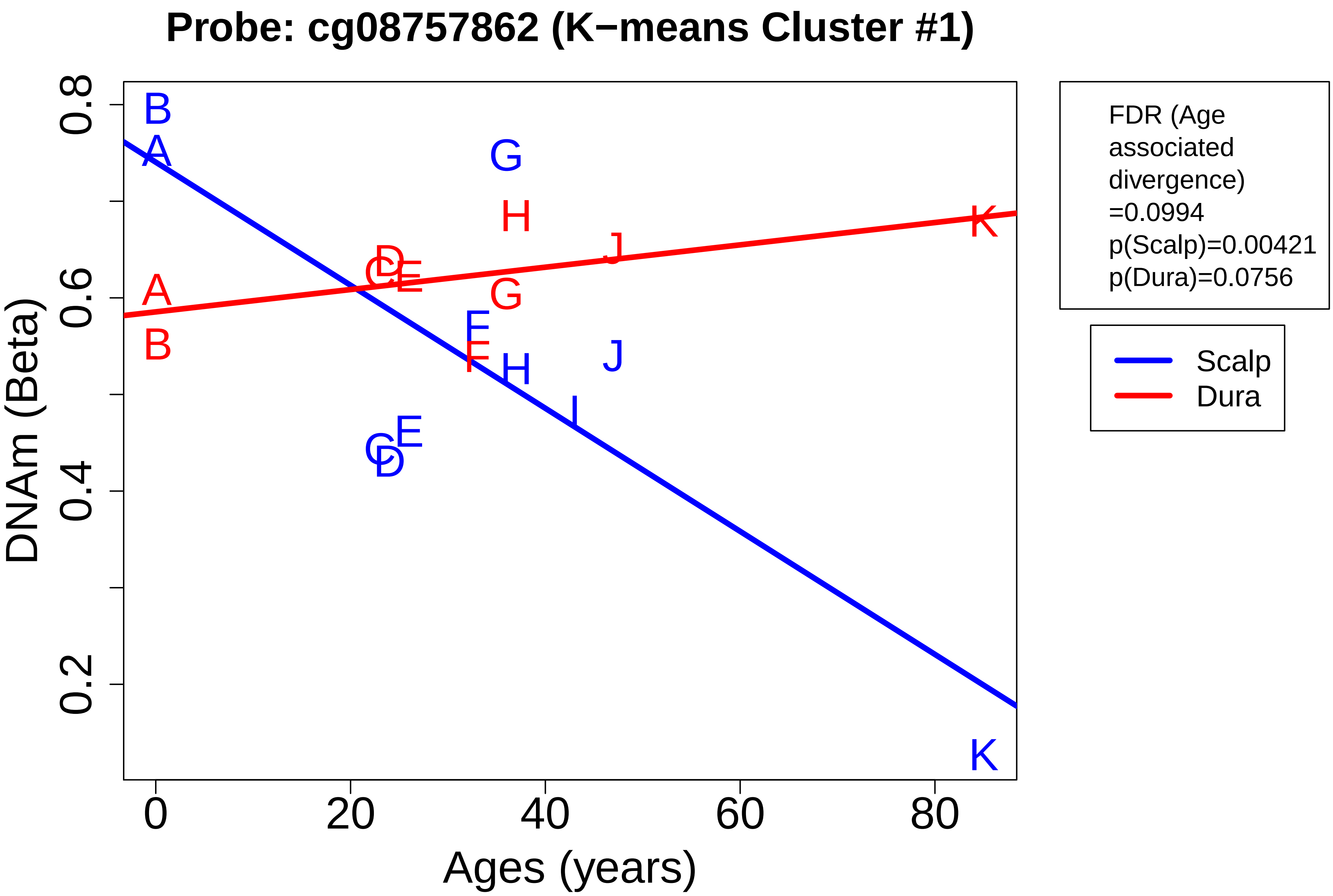

Probe: cg08757862 (K−means Cluster #1)
DNAm (Beta)
0.2
0.4
0.6
0.8
0
20
40
60
80
Ages (years)
FDR (Age associated divergence) =0.0994
p(Scalp)=0.00421
p(Dura)=0.0756
Scalp
Dura

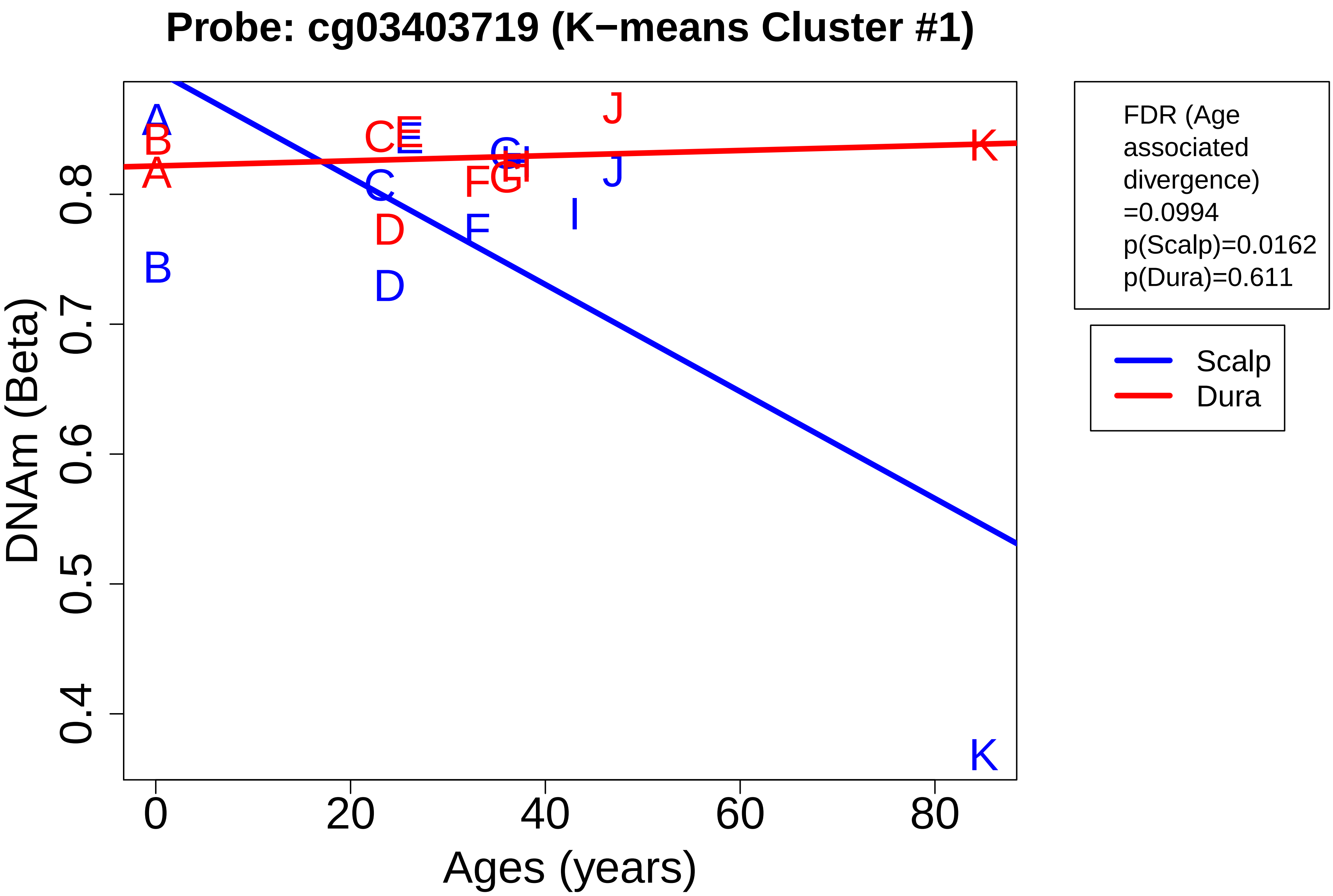

Probe: cg03403719 (K−means Cluster #1)
DNAm (Beta)
0.4
0.5
0.6
0.7
0.8
0
20
40
60
80
Ages (years)
FDR (Age associated divergence) =0.0994
p(Scalp)=0.0162
p(Dura)=0.611
Scalp
Dura

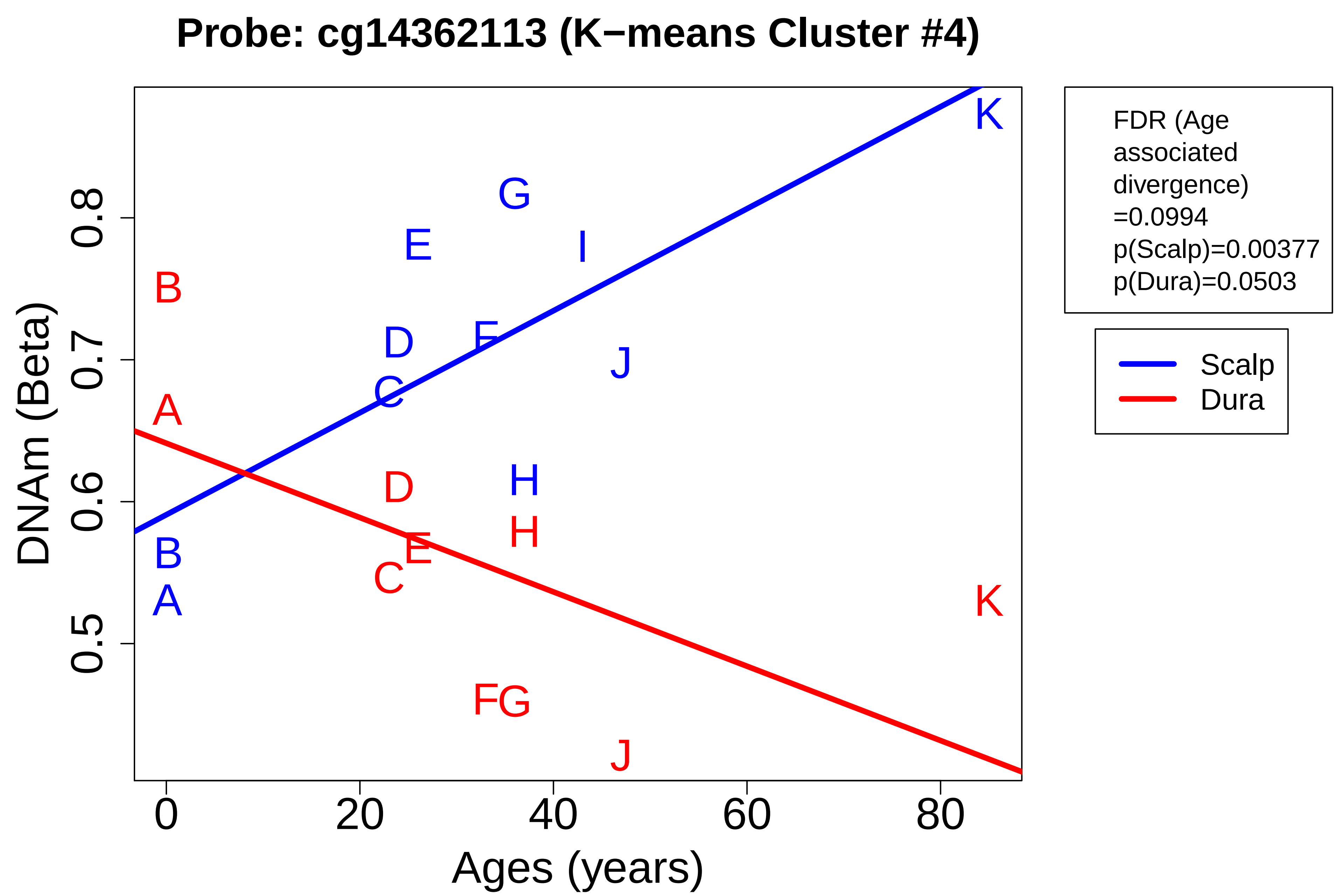

Probe: cg14362113 (K−means Cluster #4)
DNAm (Beta)
0.5
0.6
0.7
0.8
Ages (years)
0
20
40
60
80
FDR (Age associated divergence) =0.0994
p(Scalp)=0.00377
p(Dura)=0.0503
Scalp
Dura
A
B
C
D
E
F
G
H
I
J
K

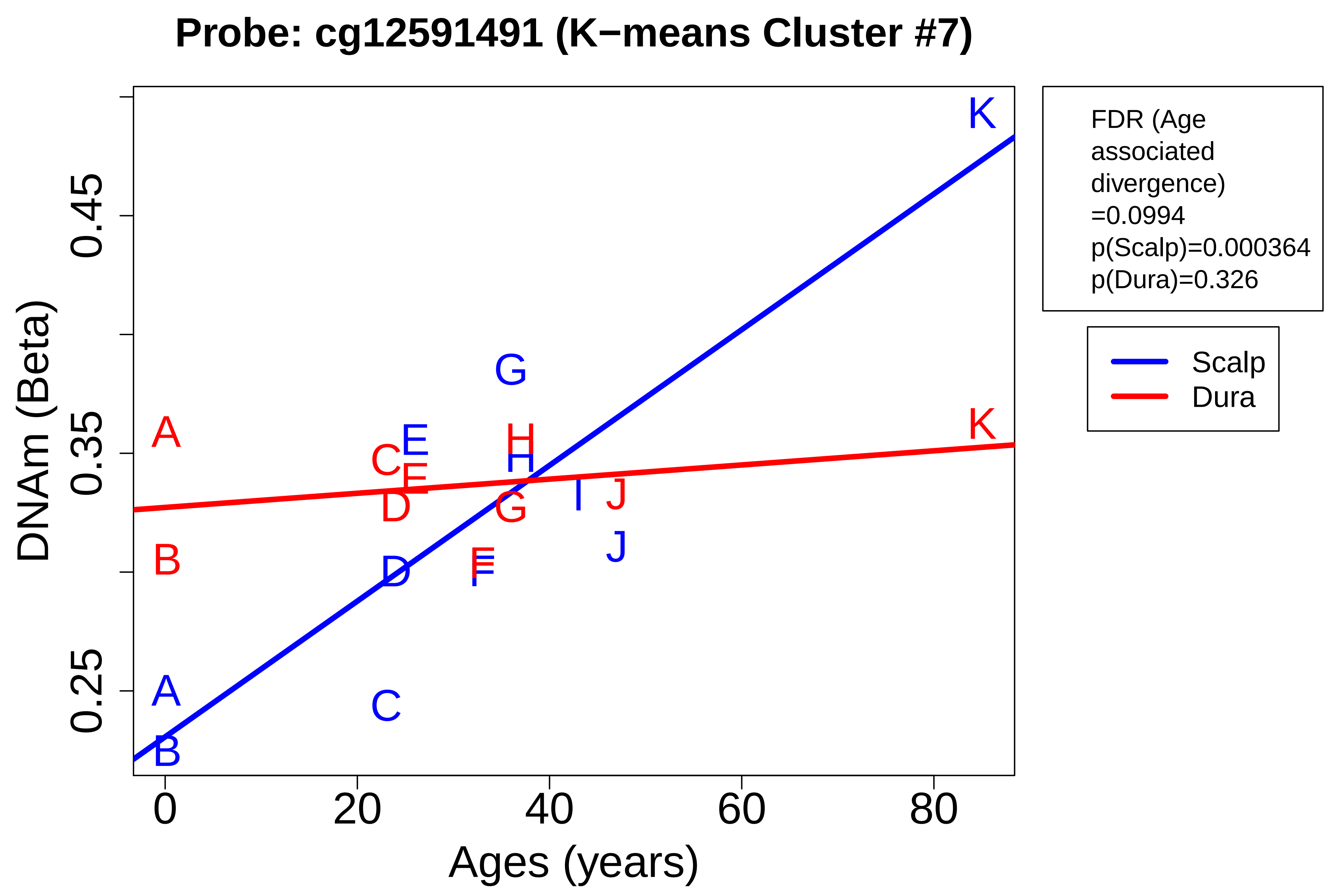

Probe: cg12591491 (K−means Cluster #7)
DNAm (Beta)
0.25
0.35
0.45
Ages (years)
0
20
40
60
80
A
B
C
D
E
F
G
H
I
J
K
FDR (Age associated divergence) =0.0994
p(Scalp)=0.000364
p(Dura)=0.326
Scalp
Dura

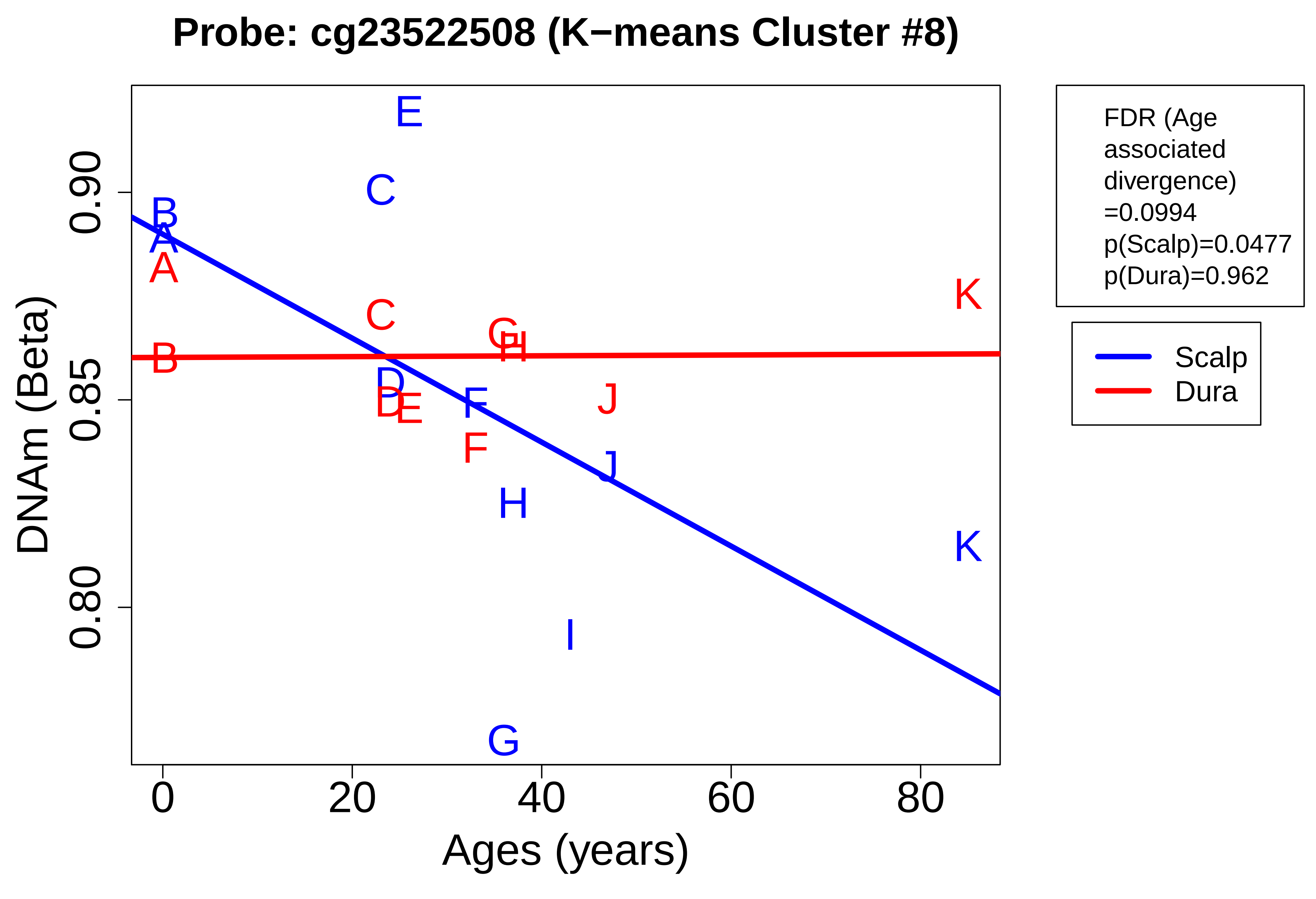

Probe: cg23522508 (K−means Cluster #8)
DNAm (Beta)
0.80
0.85
0.90
Ages (years)
0
20
40
60
80
FDR (Age associated divergence) =0.0994
p(Scalp)=0.0477
p(Dura)=0.962
Scalp
Dura

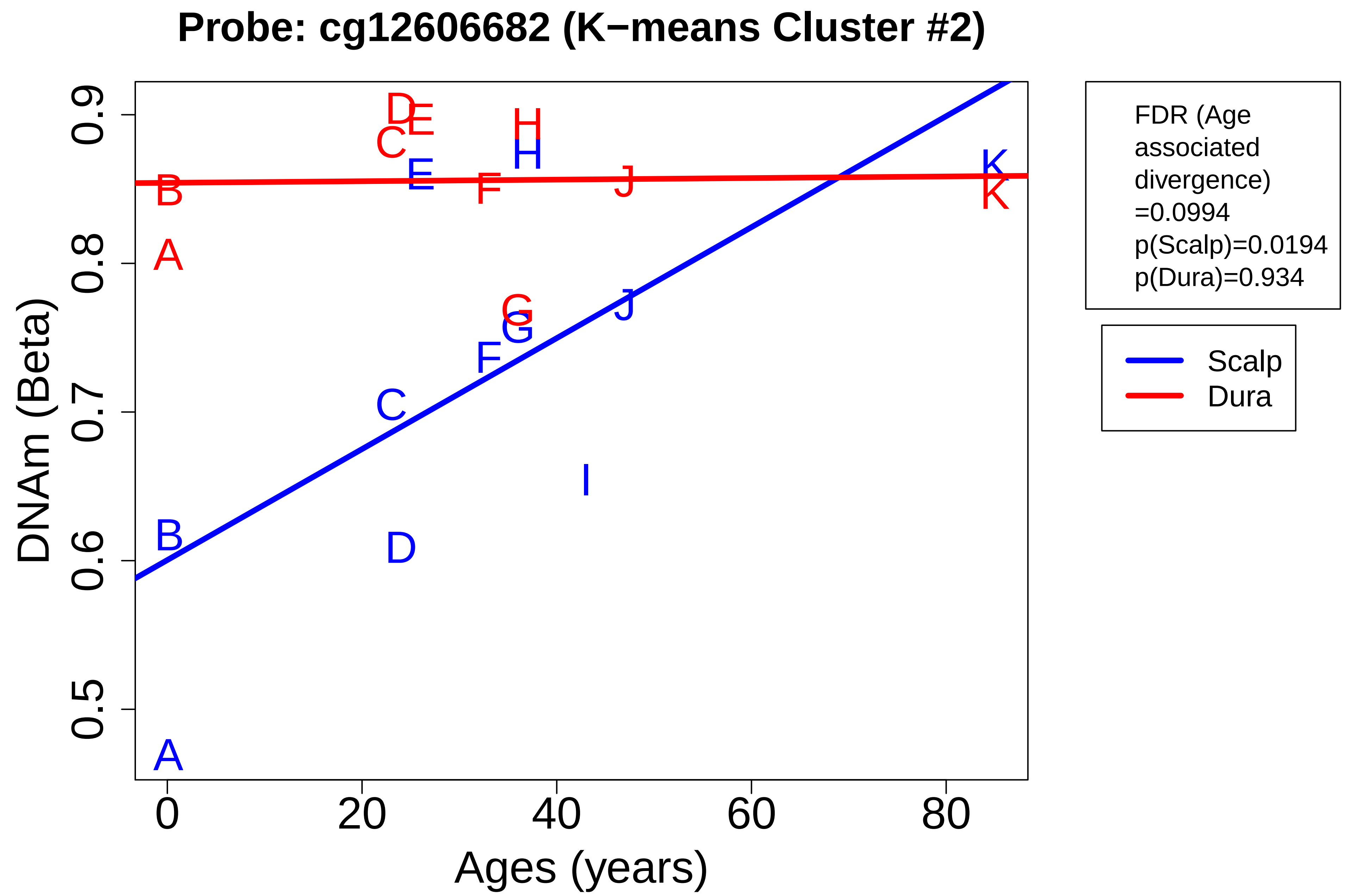
Probe: cg12606682 (K−means Cluster #2)
DNAm (Beta)
0.5
0.6
0.7
0.8
0.9
Ages (years)
0
20
40
60
80
A
B
C
D
E
F
G
H
I
J
K
FDR (Age associated divergence) =0.0994
p(Scalp)=0.0194
p(Dura)=0.934
Scalp
Dura

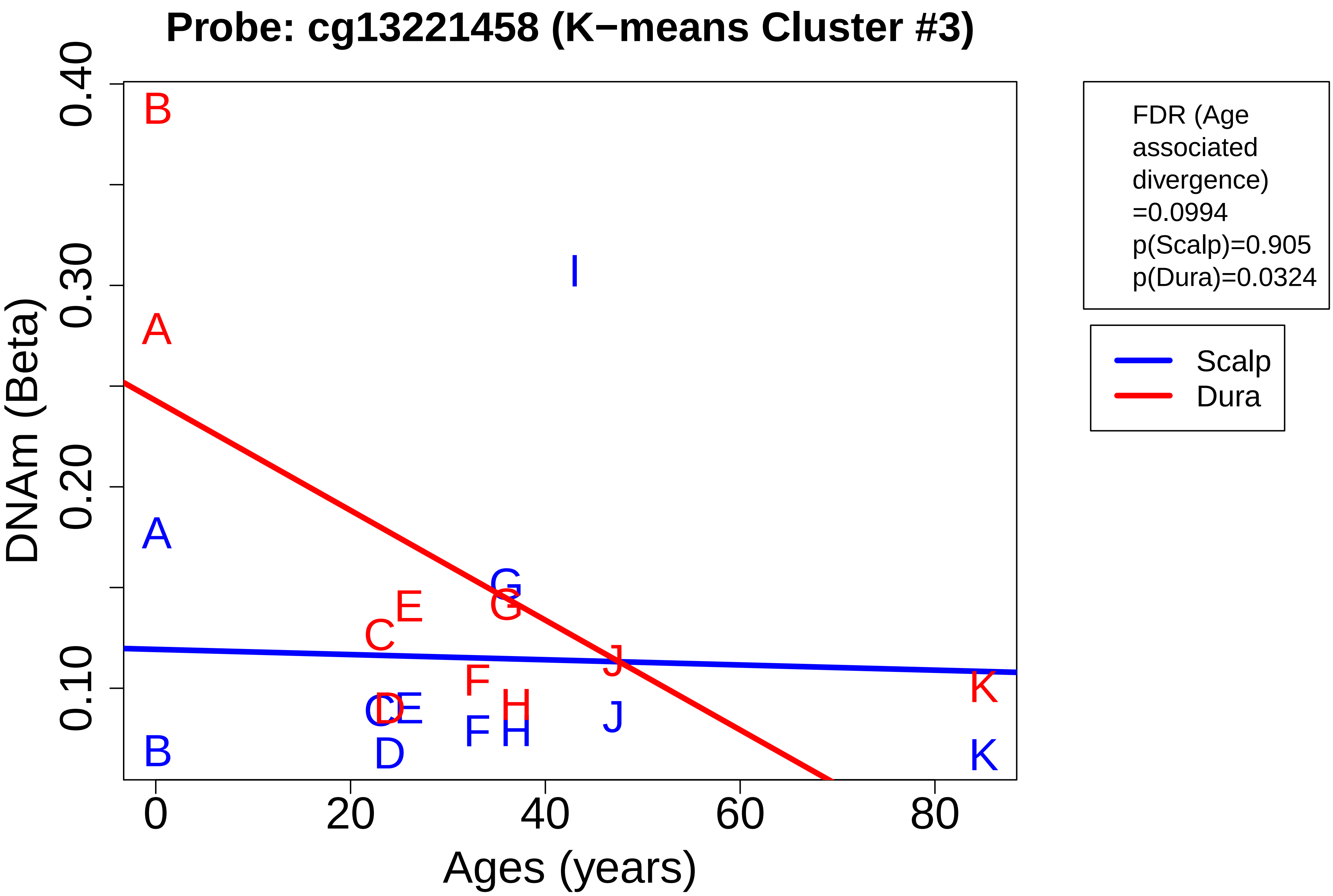

Probe: cg13221458 (K−means Cluster #3)
DNAm (Beta)
0.10
0.20
0.30
0.40
Ages (years)
0
20
40
60
80
FDR (Age associated divergence) =0.0994
p(Scalp)=0.905
p(Dura)=0.0324
Scalp
Dura

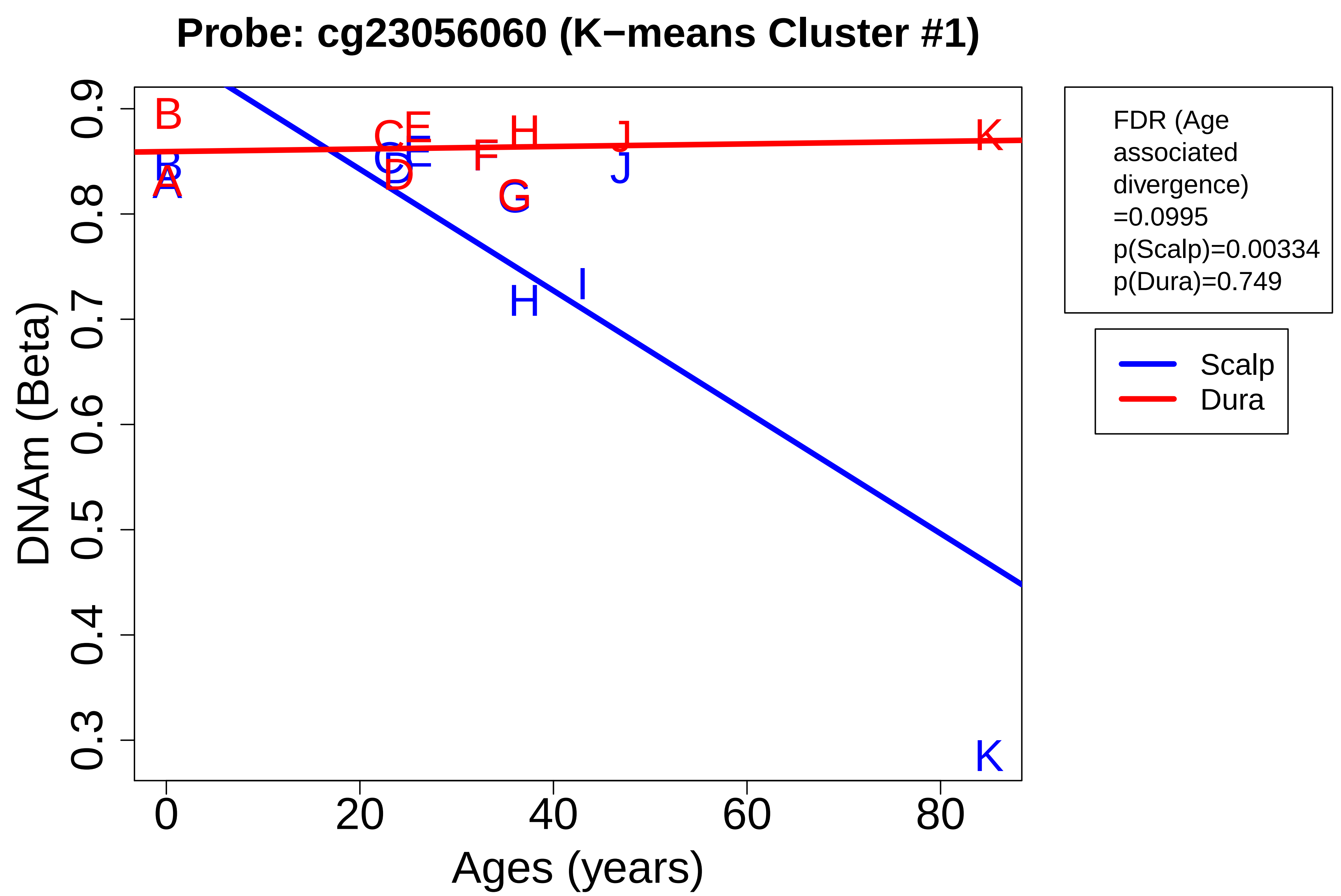

Probe: cg23056060 (K−means Cluster #1)
DNAm (Beta)
0.3
0.4
0.5
0.6
0.7
0.8
0.9
0
20
40
60
80
Ages (years)
FDR (Age associated divergence) =0.0995
p(Scalp)=0.00334
p(Dura)=0.749
Scalp
Dura

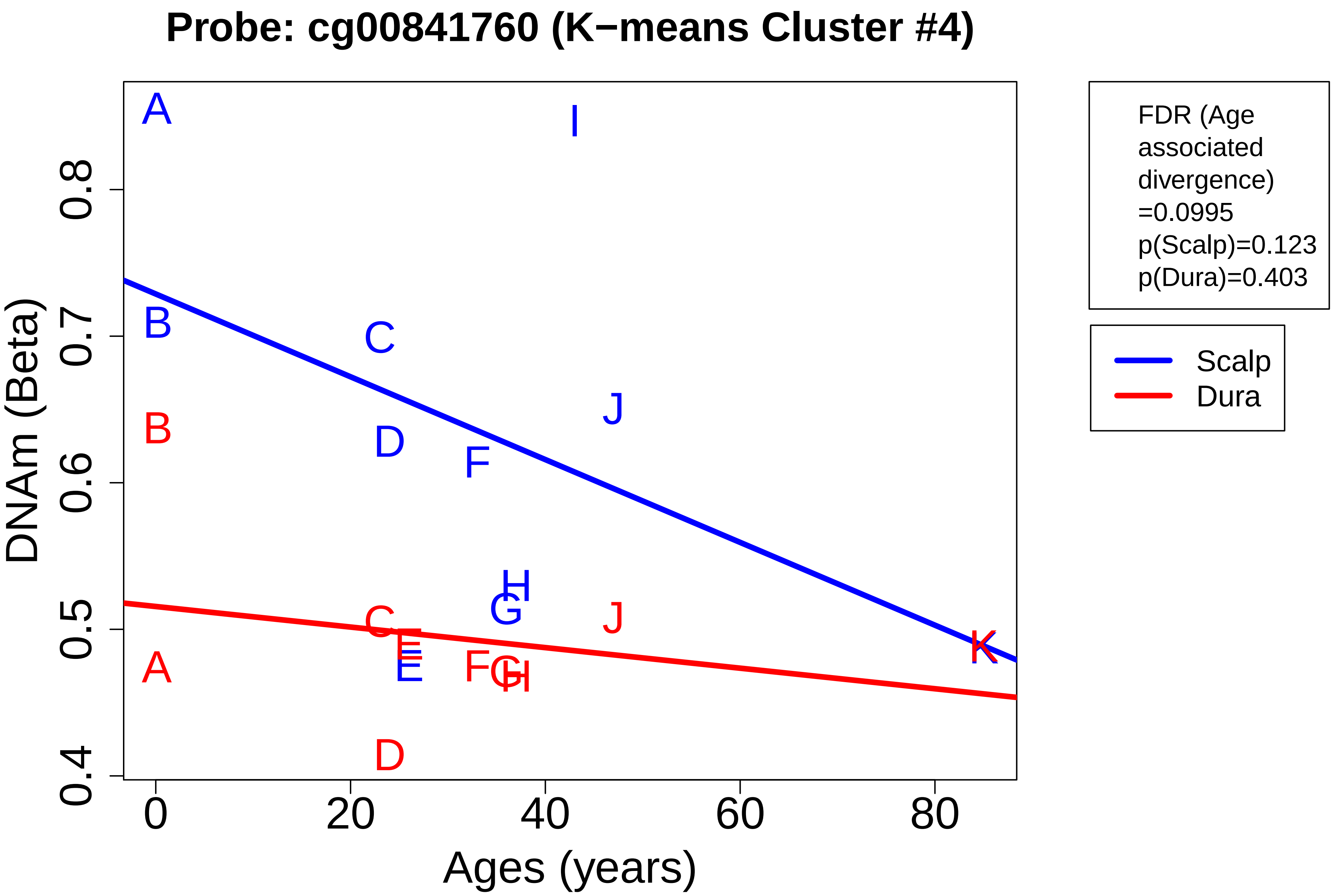

Probe: cg00841760 (K−means Cluster #4)
DNAm (Beta)
0.4
0.5
0.6
0.7
0.8
0
20
40
60
80
Ages (years)
FDR (Age associated divergence) =0.0995
p(Scalp)=0.123
p(Dura)=0.403
Scalp
Dura

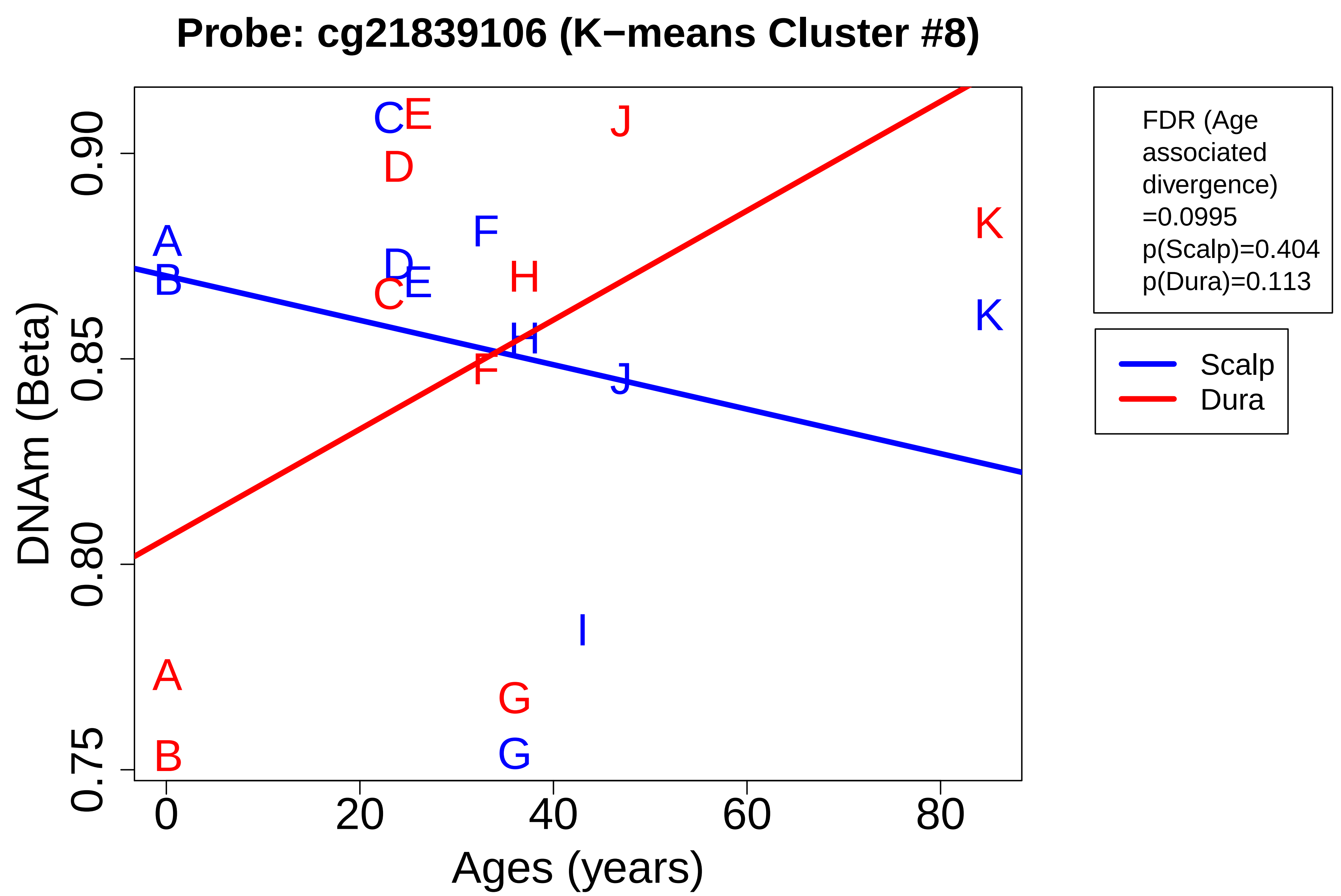

Probe: cg21839106 (K−means Cluster #8)
DNAm (Beta)
0.75
0.80
0.85
0.90
0
20
40
60
80
Ages (years)
FDR (Age associated divergence) =0.0995
p(Scalp)=0.404
p(Dura)=0.113
Scalp
Dura

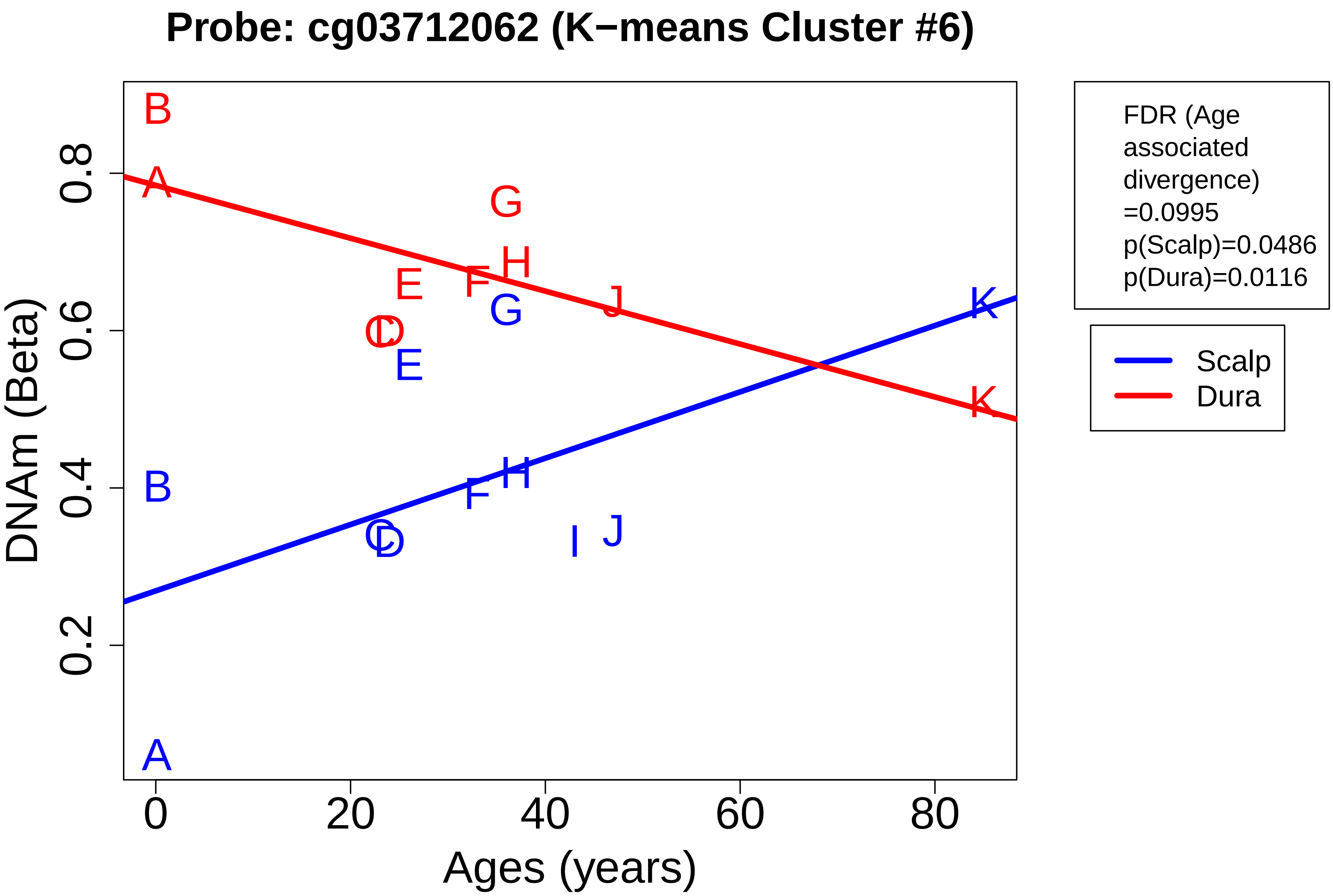

Probe: cg03712062 (K−means Cluster #6)
FDR (Age associated divergence) =0.0995
p(Scalp)=0.0486
p(Dura)=0.0116
Scalp
Dura
DNAm (Beta)
0.2
0.4
0.6
0.8
0
20
40
60
80
Ages (years)
A
B
C
D
E
F
G
H
I
J
K

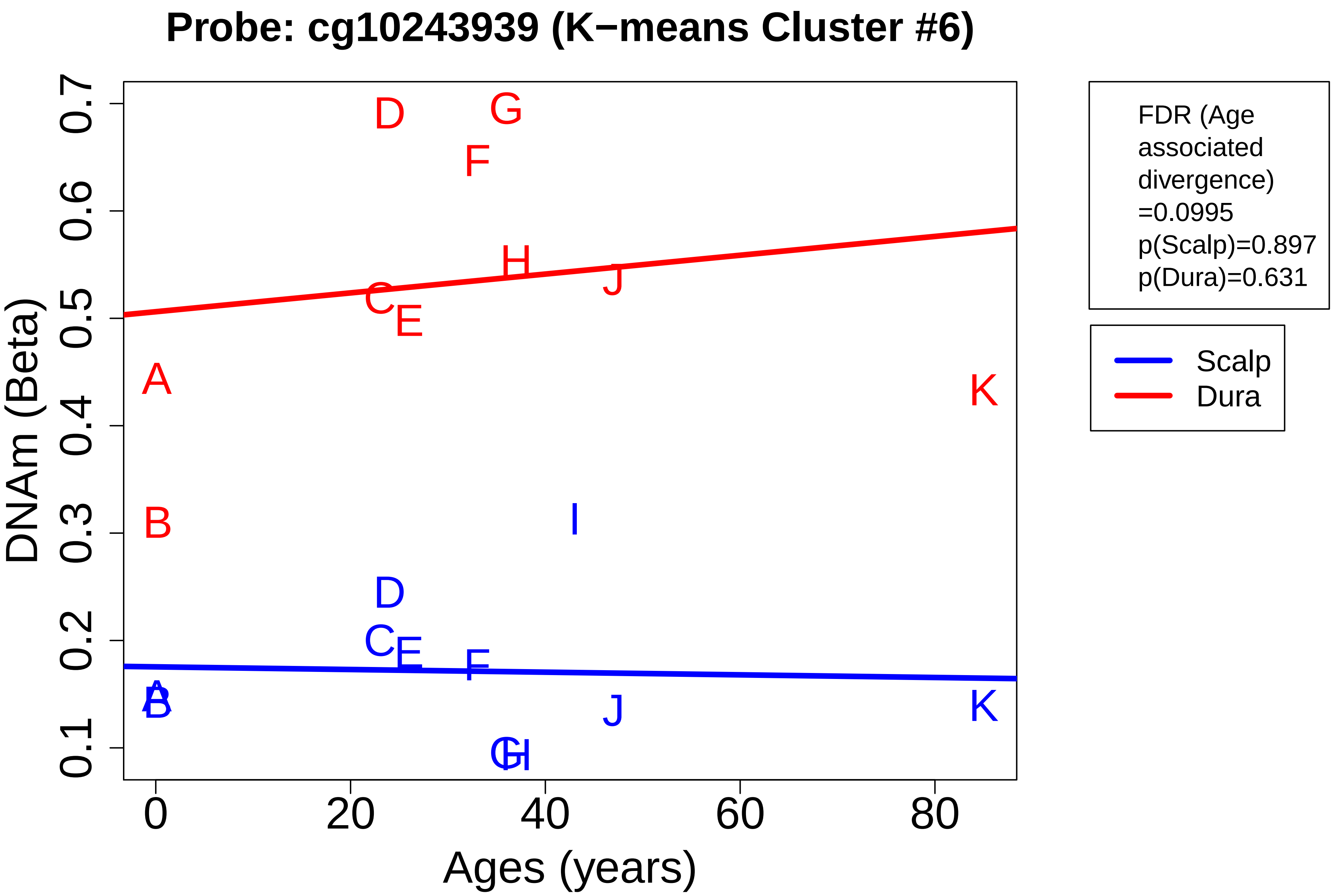

Probe: cg10243939 (K−means Cluster #6)
DNAm (Beta)
0.1
0.2
0.3
0.4
0.5
0.6
0.7
0
20
40
60
80
Ages (years)
FDR (Age
associated
divergence)
=0.0995
p(Scalp)=0.897
p(Dura)=0.631
Scalp
Dura

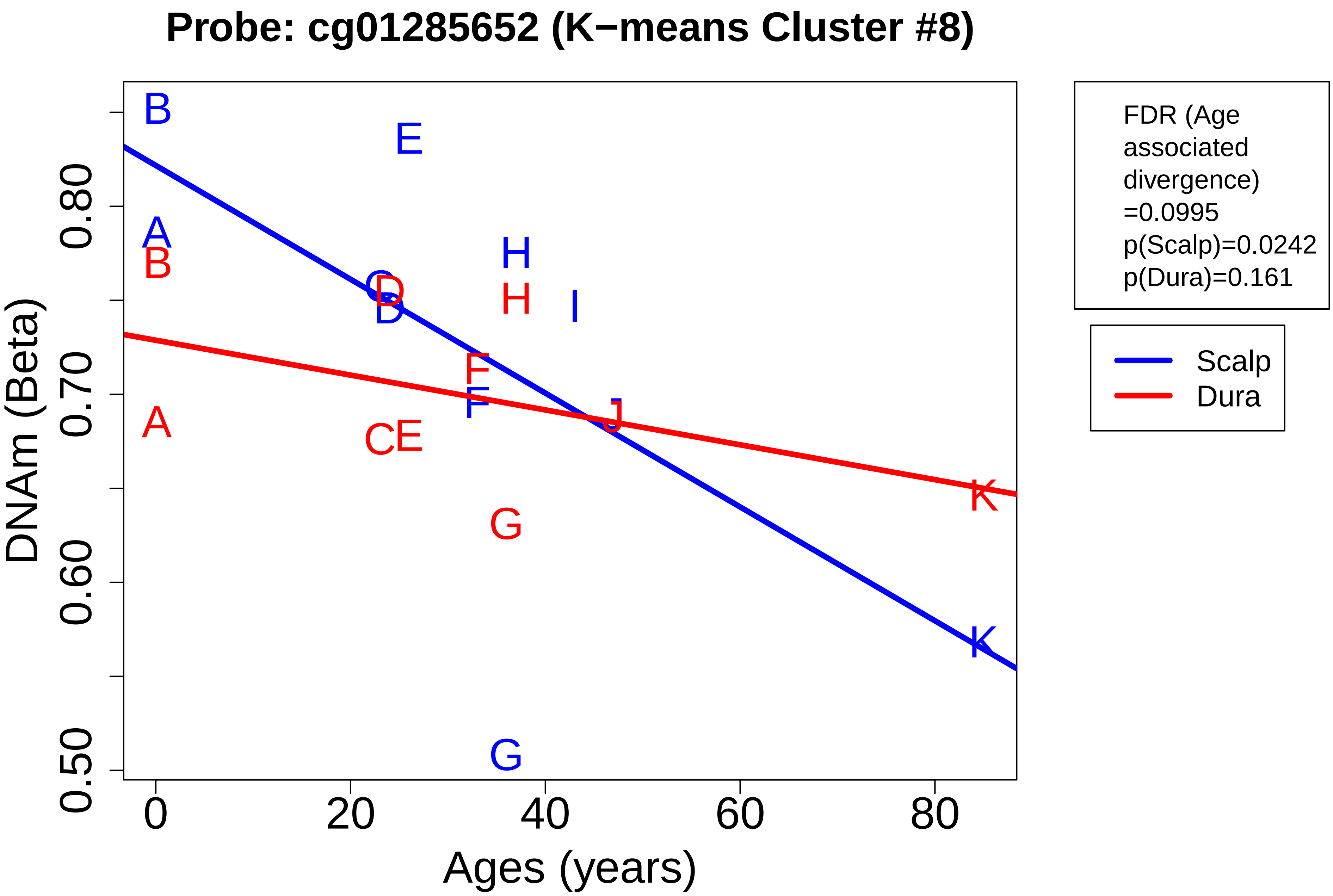

Probe: cg01285652 (K−means Cluster #8)
DNAm (Beta)
0.50
0.60
0.70
0.80
0
20
40
60
80
Ages (years)
FDR (Age associated divergence) =0.0995
p(Scalp)=0.0242
p(Dura)=0.161
Scalp
Dura

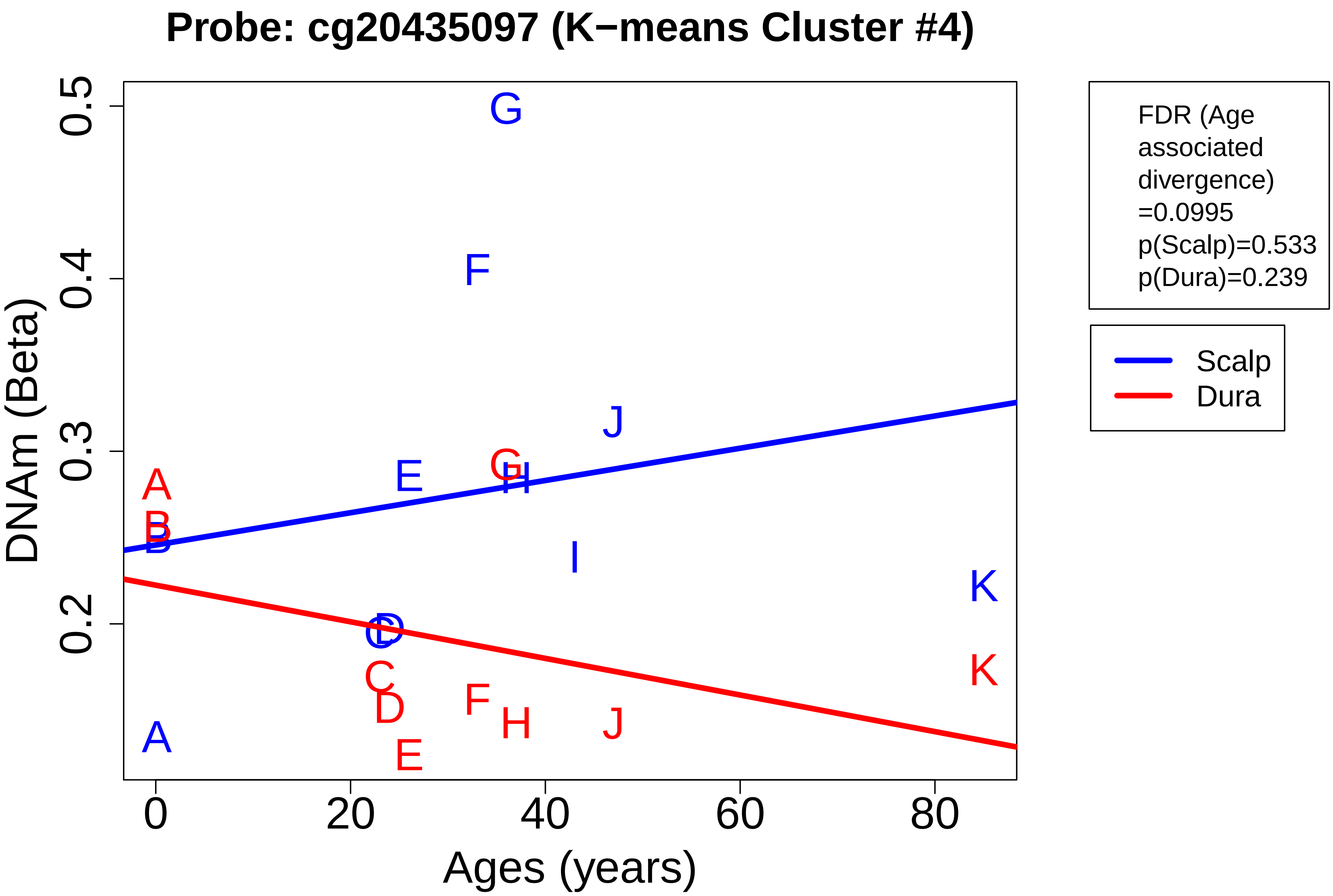
Probe: cg20435097 (K−means Cluster #4)
DNAm (Beta)
0.2
0.3
0.4
0.5
Ages (years)
0
20
40
60
80
FDR (Age associated divergence) =0.0995
p(Scalp)=0.533
p(Dura)=0.239
Scalp
Dura
A
B
C
D
E
F
G
H
I
J
K

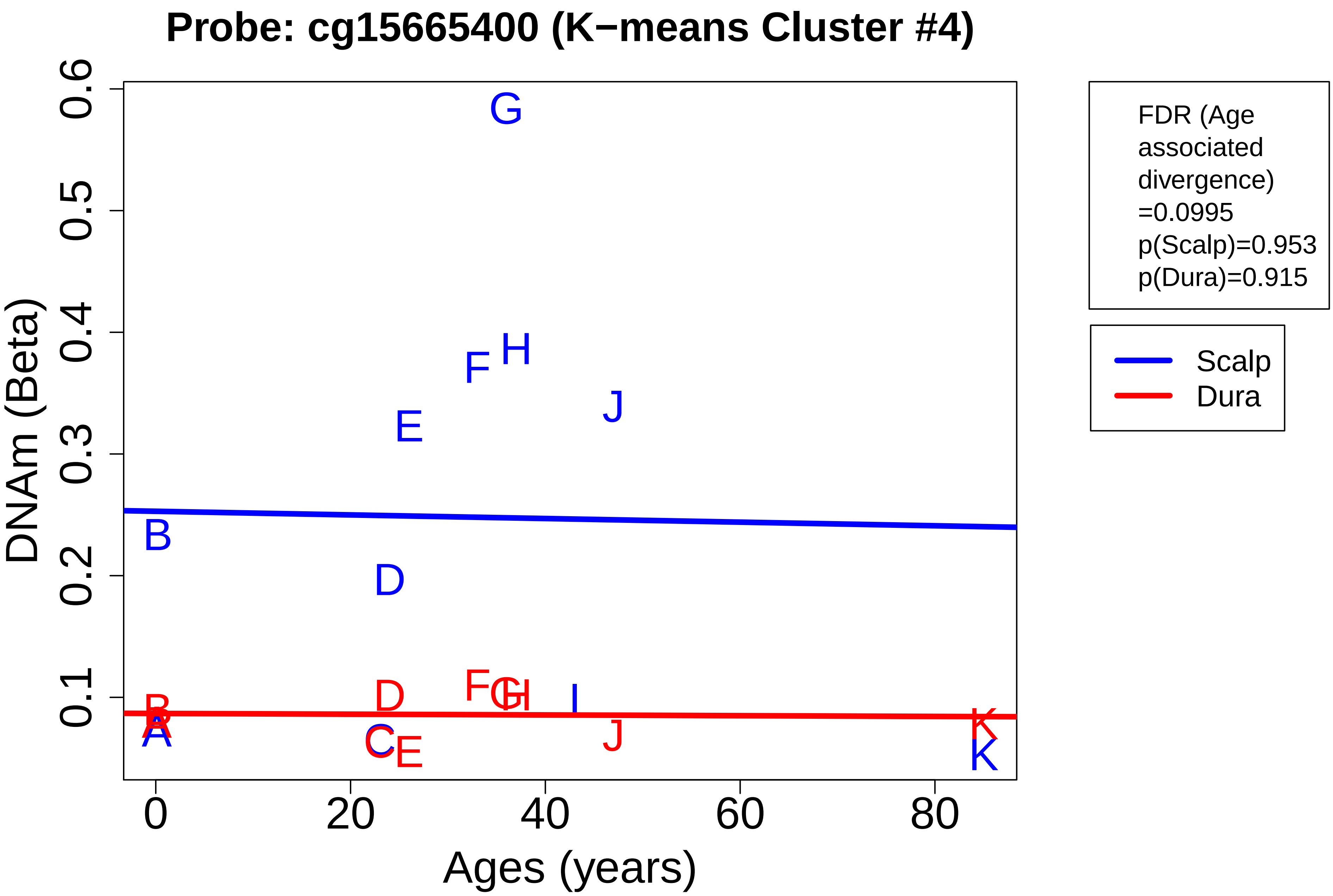

Probe: cg15665400 (K−means Cluster #4)
DNAm (Beta)
0.1
0.2
0.3
0.4
0.5
0.6
0
20
40
60
80
Ages (years)
FDR (Age associated divergence) =0.0995
p(Scalp)=0.953
p(Dura)=0.915
Scalp
Dura

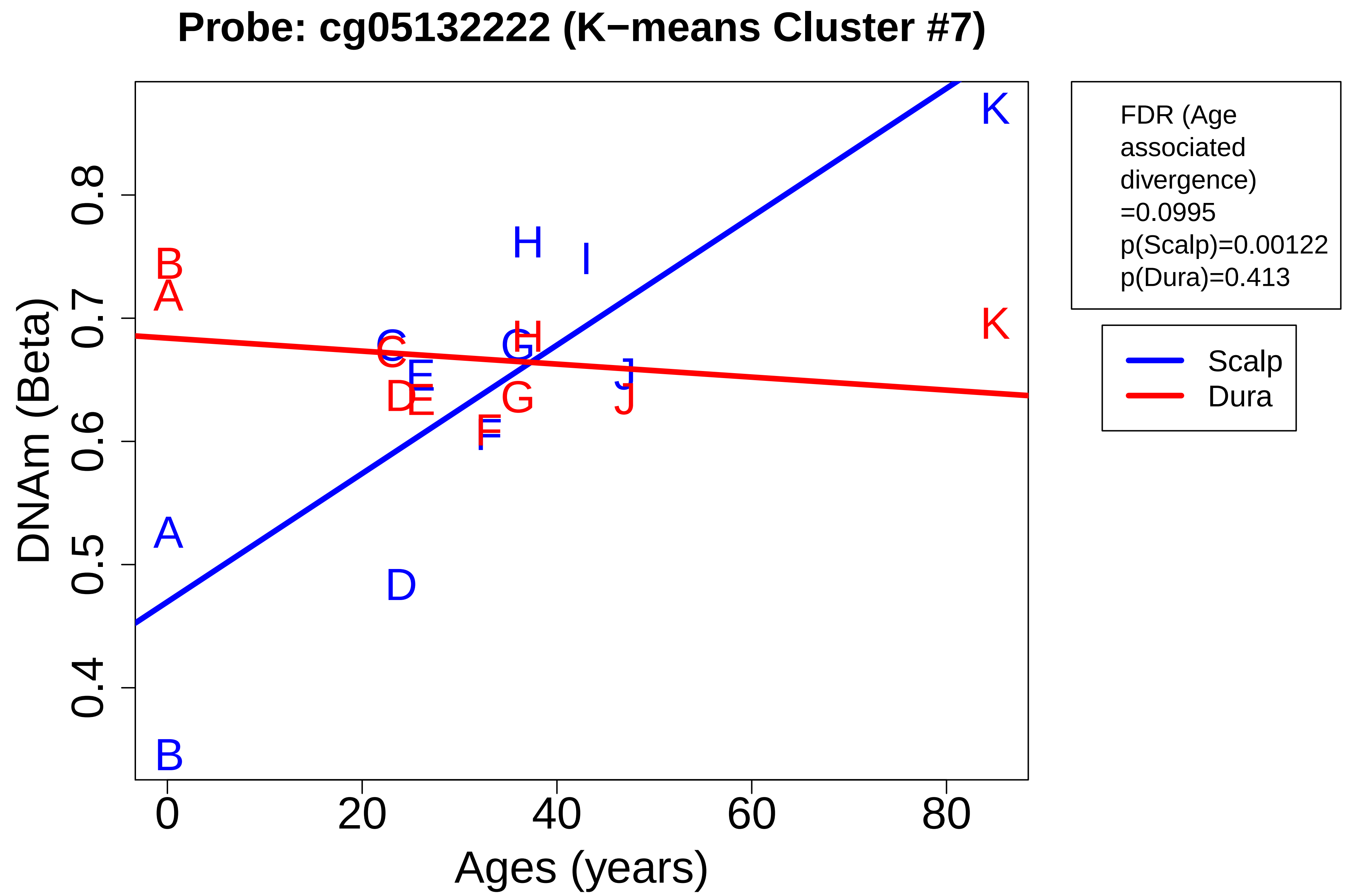

Probe: cg05132222 (K−means Cluster #7)
DNAm (Beta)
Ages (years)
0.4
0.5
0.6
0.7
0.8
0
20
40
60
80
FDR (Age associated divergence) =0.0995
p(Scalp)=0.00122
p(Dura)=0.413
Scalp
Dura
A B C D E F G H I J K

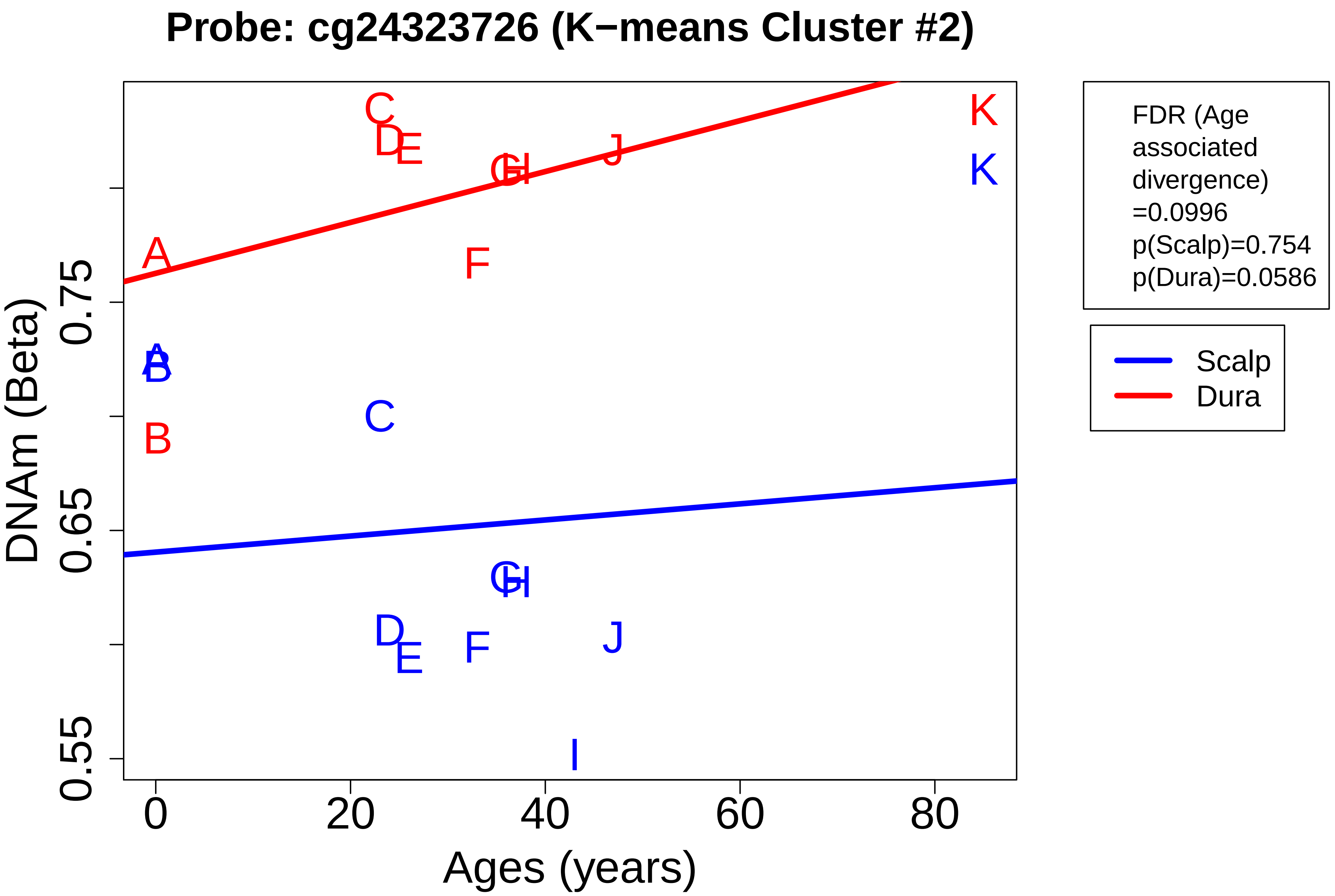

Probe: cg24323726 (K−means Cluster #2)
DNAm (Beta)
0.55
0.65
0.75
Ages (years)
0
20
40
60
80
FDR (Age associated divergence) =0.0996
p(Scalp)=0.754
p(Dura)=0.0586
Scalp
Dura

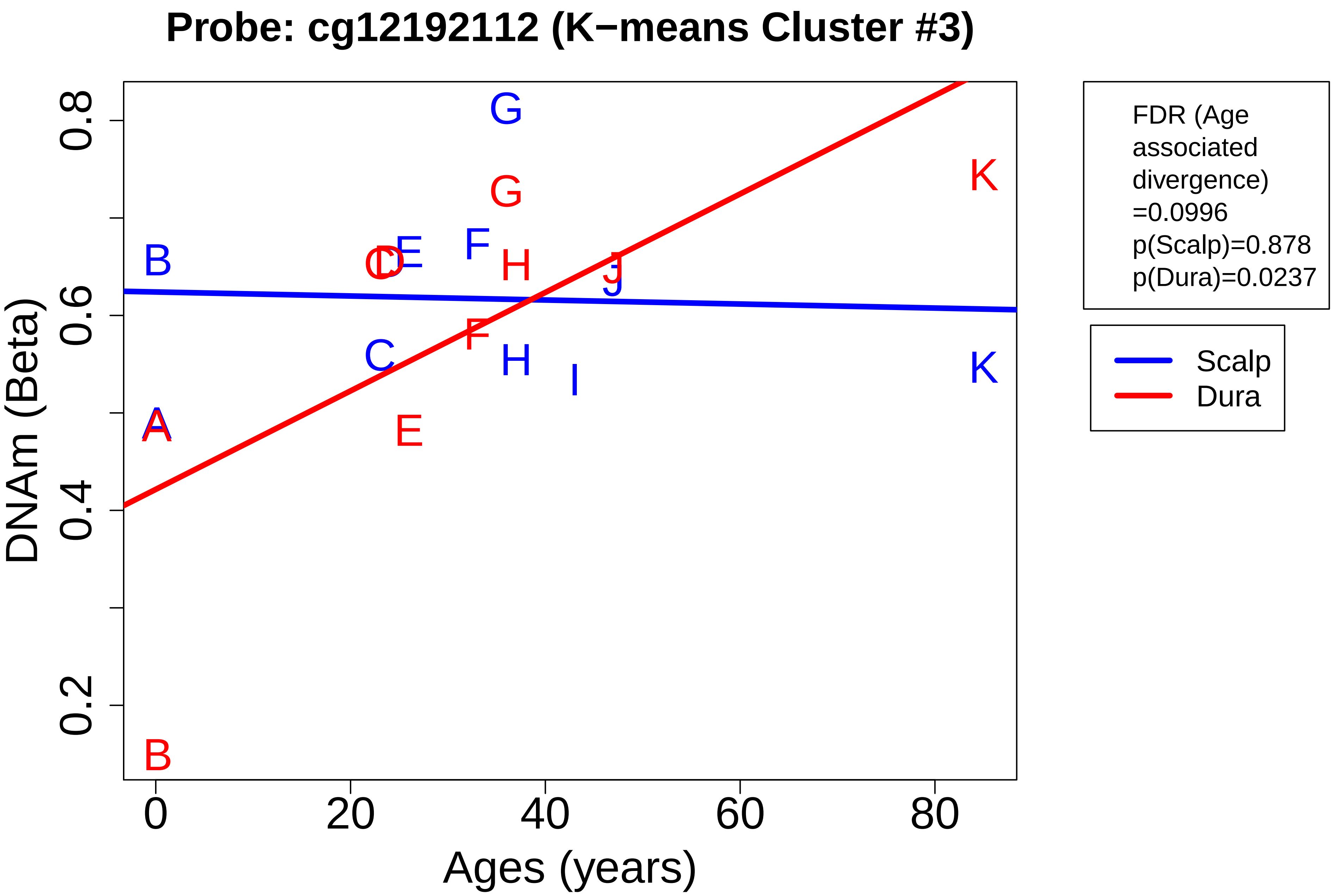

Probe: cg12192112 (K−means Cluster #3)
FDR (Age associated divergence) =0.0996
p(Scalp)=0.878
p(Dura)=0.0237
Scalp
Dura
DNAm (Beta)
Ages (years)
0.2
0.4
0.6
0.8
0
20
40
60
80
A
B
C
D
E
F
G
H
I
J
K

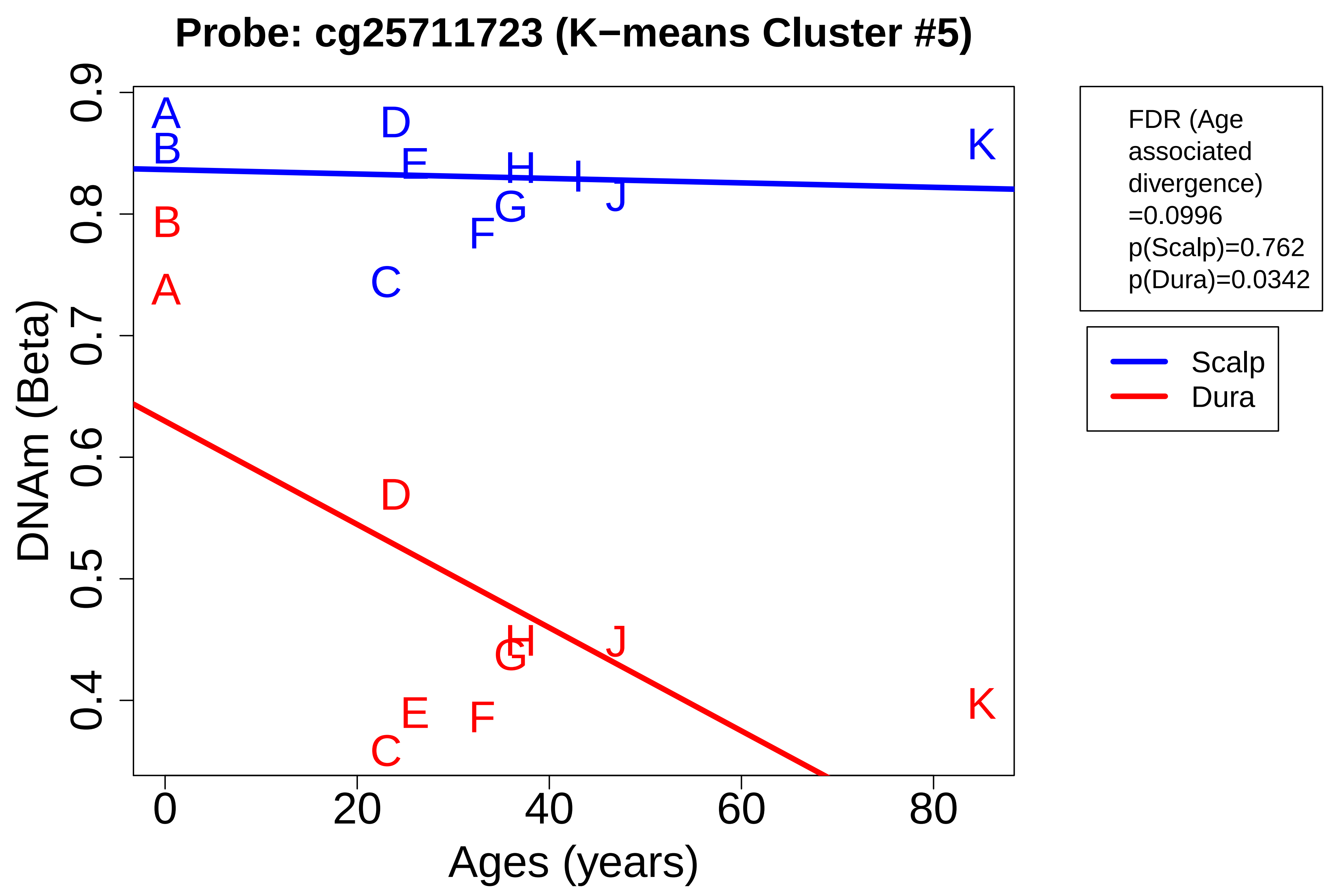

Probe: cg25711723 (K−means Cluster #5)
DNAm (Beta)
0.4
0.5
0.6
0.7
0.8
0.9
Ages (years)
0
20
40
60
80
FDR (Age associated divergence) =0.0996
p(Scalp)=0.762
p(Dura)=0.0342
Scalp
Dura
A
B
C
D
E
F
G
H
I
J
K

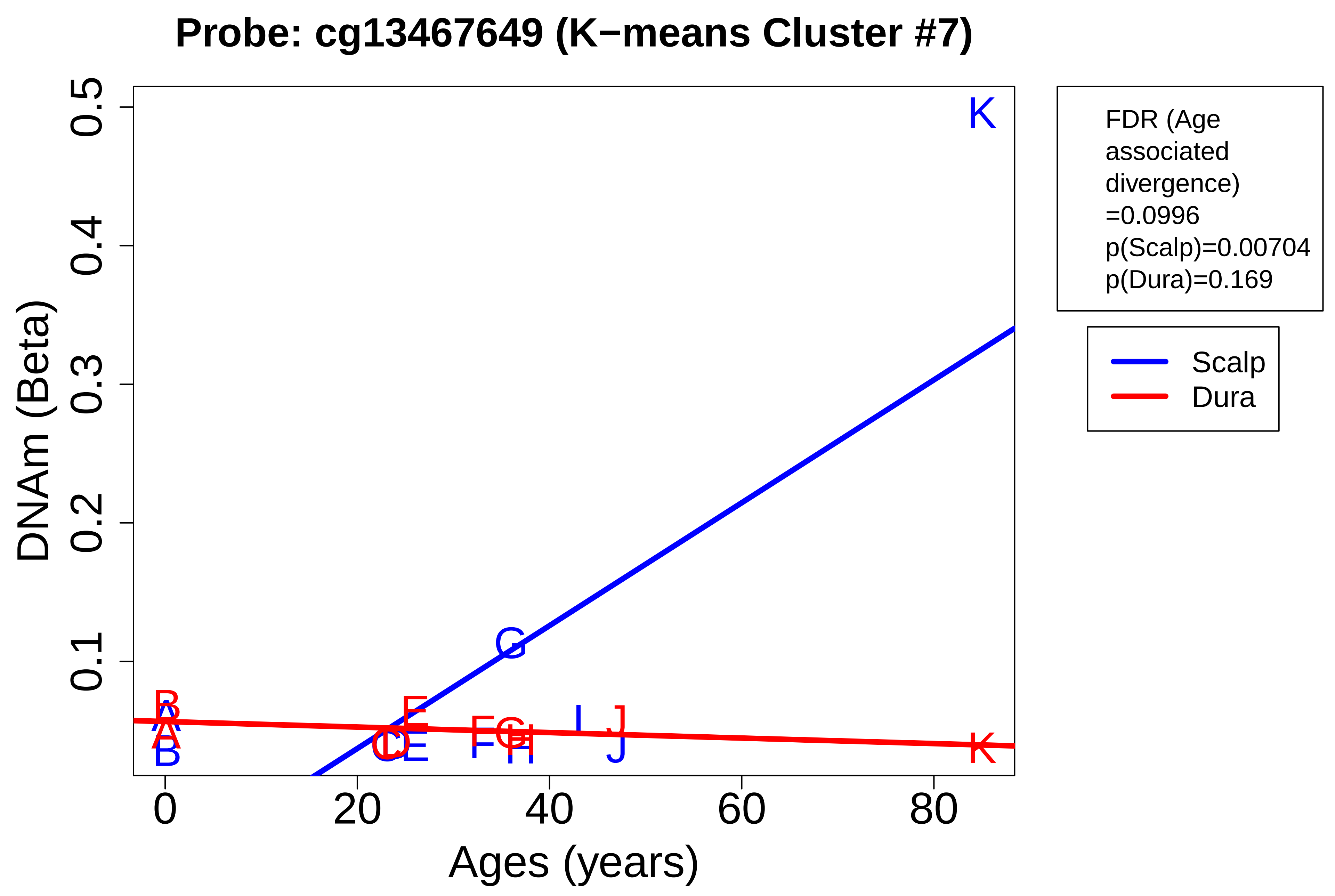

Probe: cg13467649 (K−means Cluster #7)
DNAm (Beta)
0.1
0.2
0.3
0.4
0.5
0
20
40
60
80
Ages (years)
FDR (Age associated divergence) =0.0996
p(Scalp)=0.00704
p(Dura)=0.169
Scalp
Dura

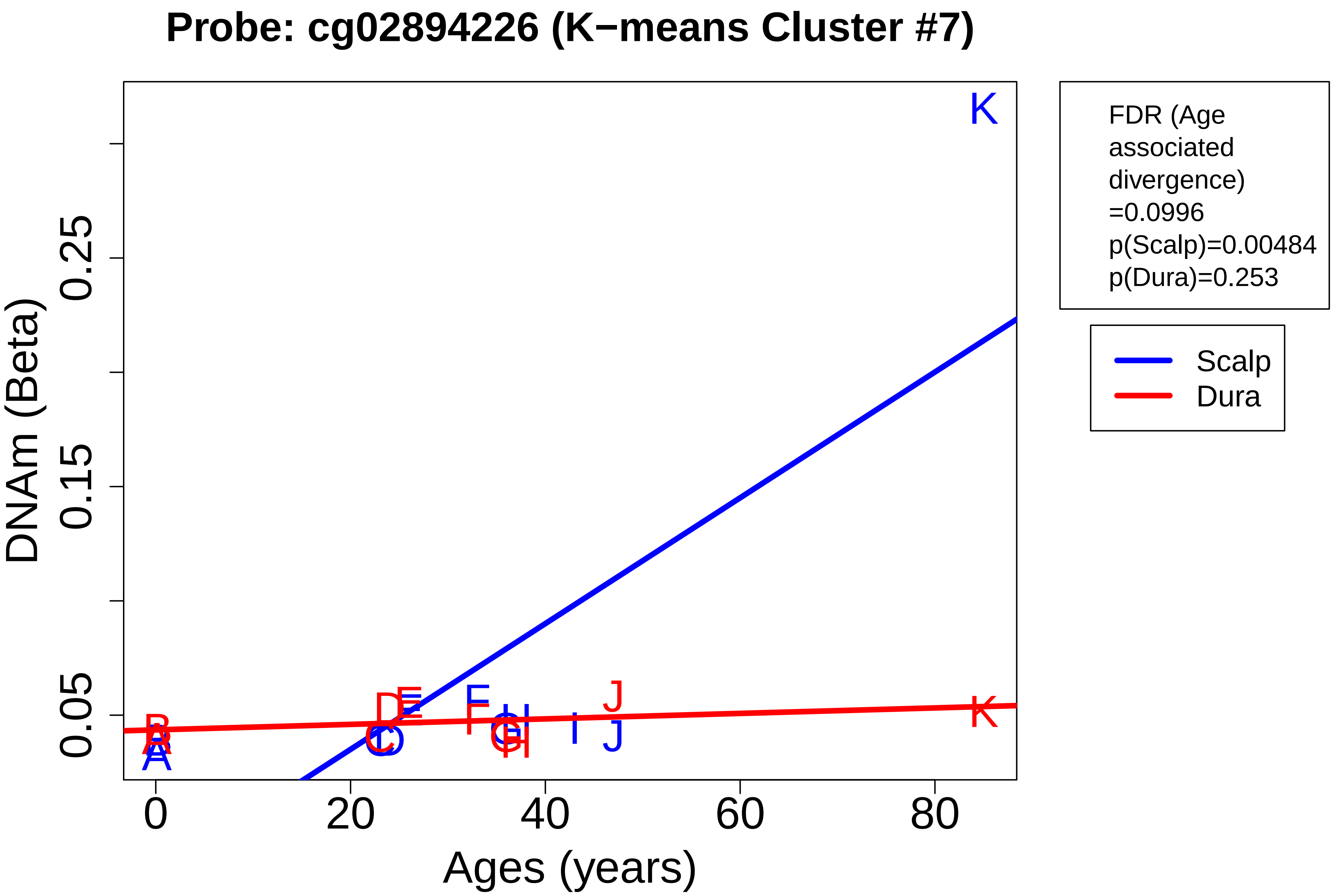
Probe: cg02894226 (K−means Cluster #7)
DNAm (Beta)
0.05
0.15
0.25
0
20
40
60
80
Ages (years)
FDR (Age associated divergence) =0.0996
p(Scalp)=0.00484
p(Dura)=0.253
Scalp
Dura

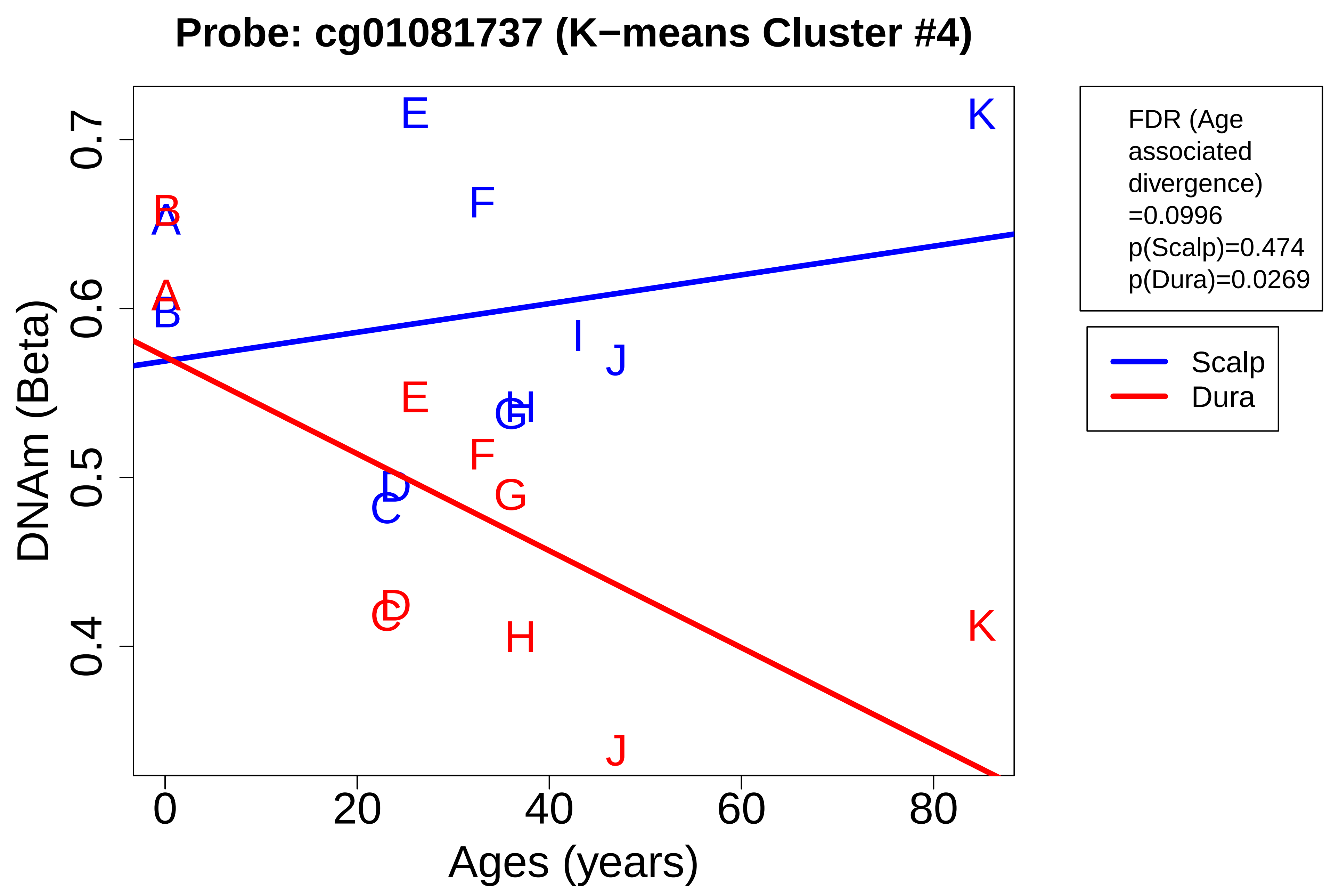

Probe: cg01081737 (K−means Cluster #4)
DNAm (Beta)
0.4
0.5
0.6
0.7
Ages (years)
0
20
40
60
80
E
K
F
B
A
A
B
I
J
E
H
G
F
D
C
G
D
C
H
K
J
FDR (Age associated divergence) =0.0996
p(Scalp)=0.474
p(Dura)=0.0269
Scalp
Dura

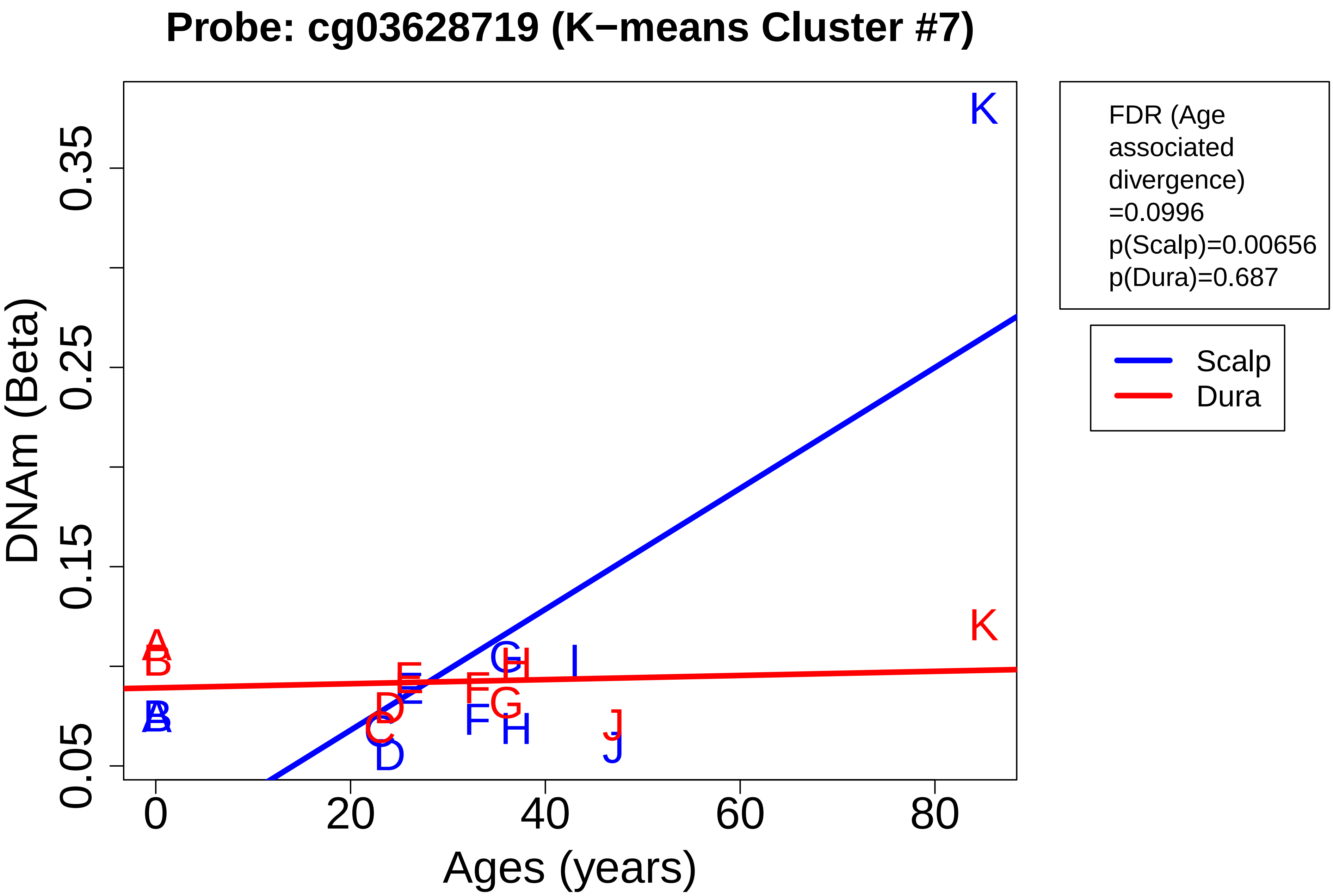
Probe: cg03628719 (K−means Cluster #7)
DNAm (Beta)
0.05
0.15
0.25
0.35
0
20
40
60
80
Ages (years)
FDR (Age associated divergence) =0.0996
p(Scalp)=0.00656
p(Dura)=0.687
Scalp
Dura

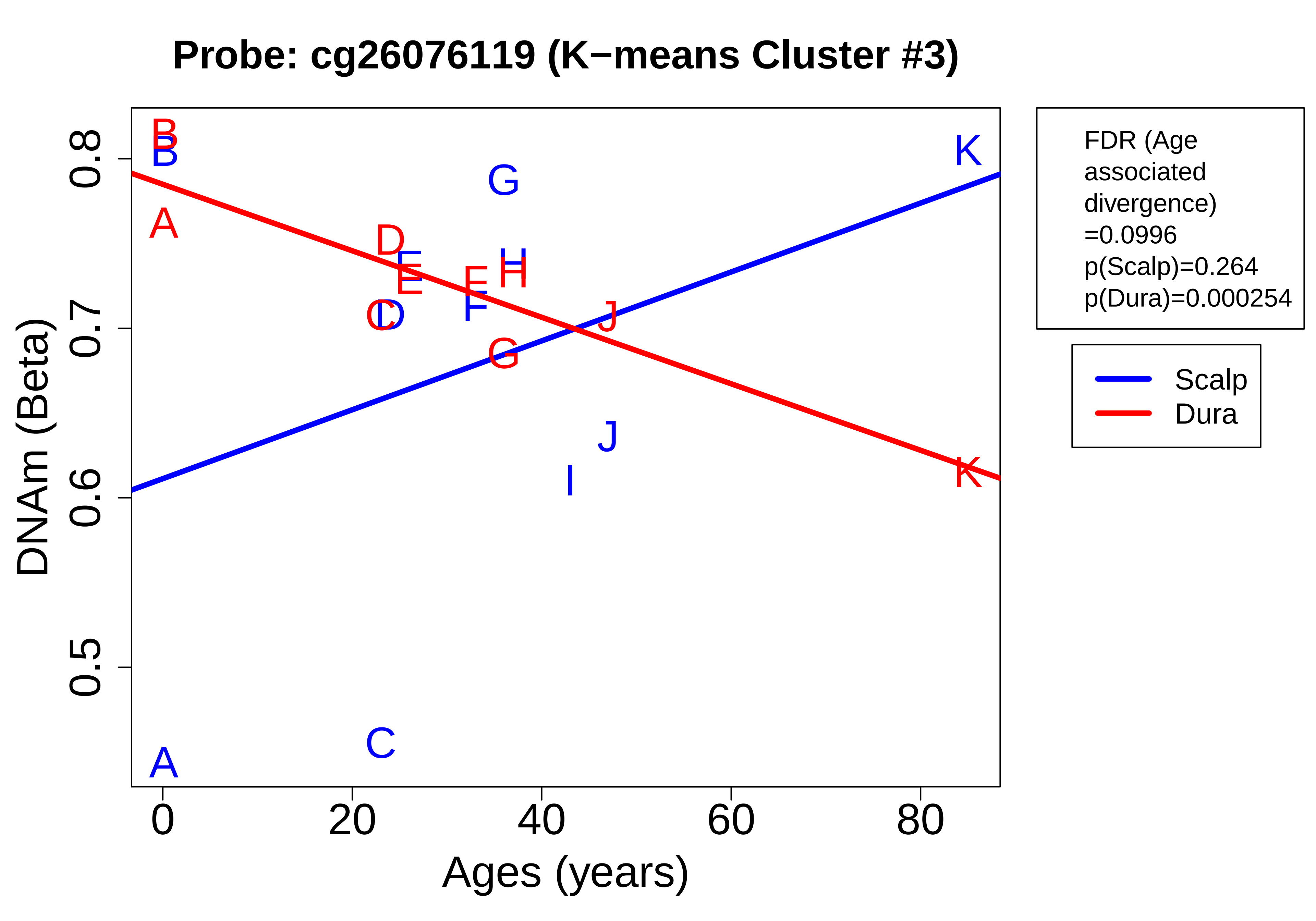

Probe: cg26076119 (K−means Cluster #3)
DNAm (Beta)
0.5
0.6
0.7
0.8
0
20
40
60
80
Ages (years)
A
B
C
D
E
F
G
H
I
J
K
FDR (Age associated divergence) =0.0996
p(Scalp)=0.264
p(Dura)=0.000254
Scalp
Dura

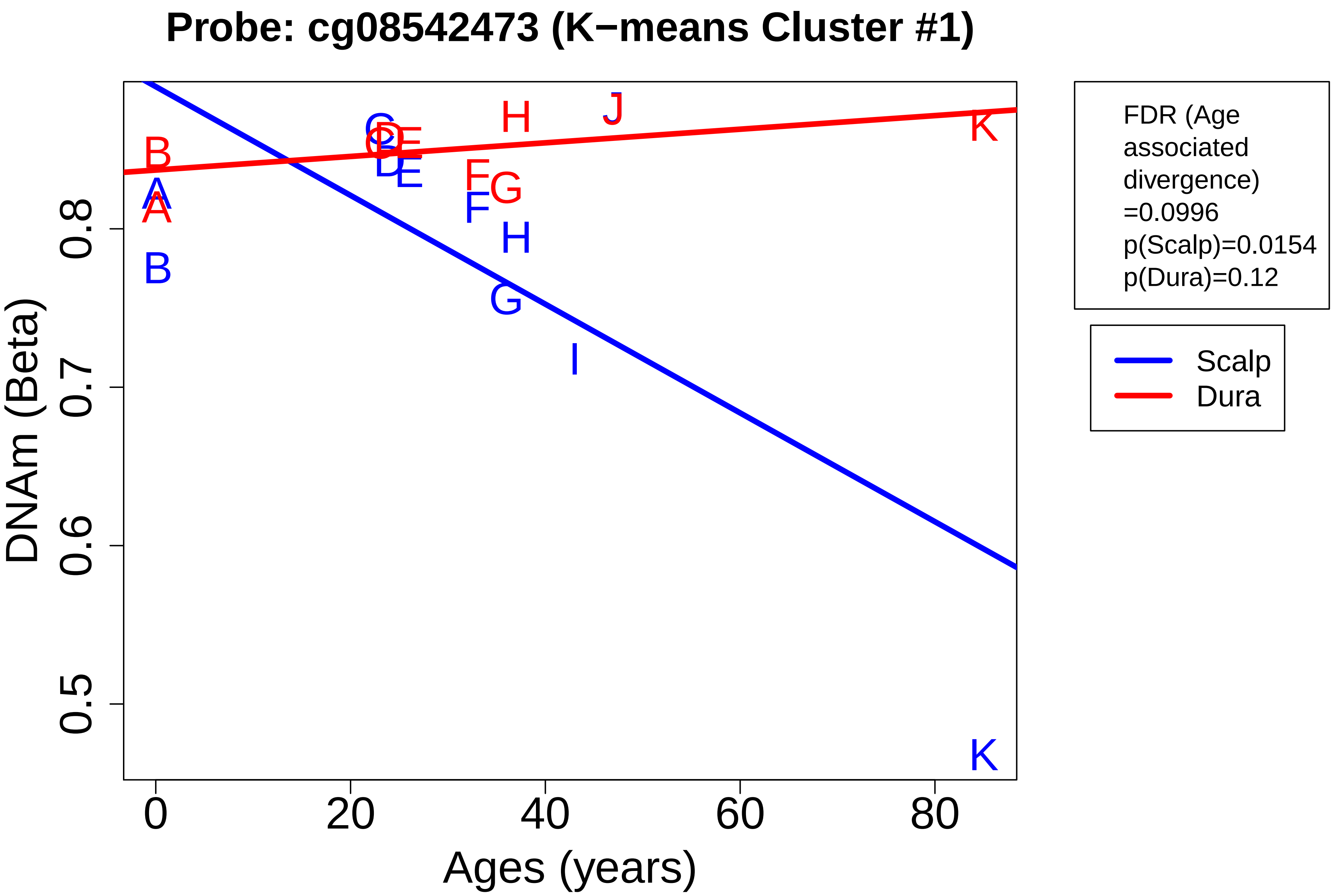

Probe: cg08542473 (K−means Cluster #1)
DNAm (Beta)
0.5
0.6
0.7
0.8
0
20
40
60
80
Ages (years)
FDR (Age associated divergence) =0.0996
p(Scalp)=0.0154
p(Dura)=0.12
Scalp
Dura

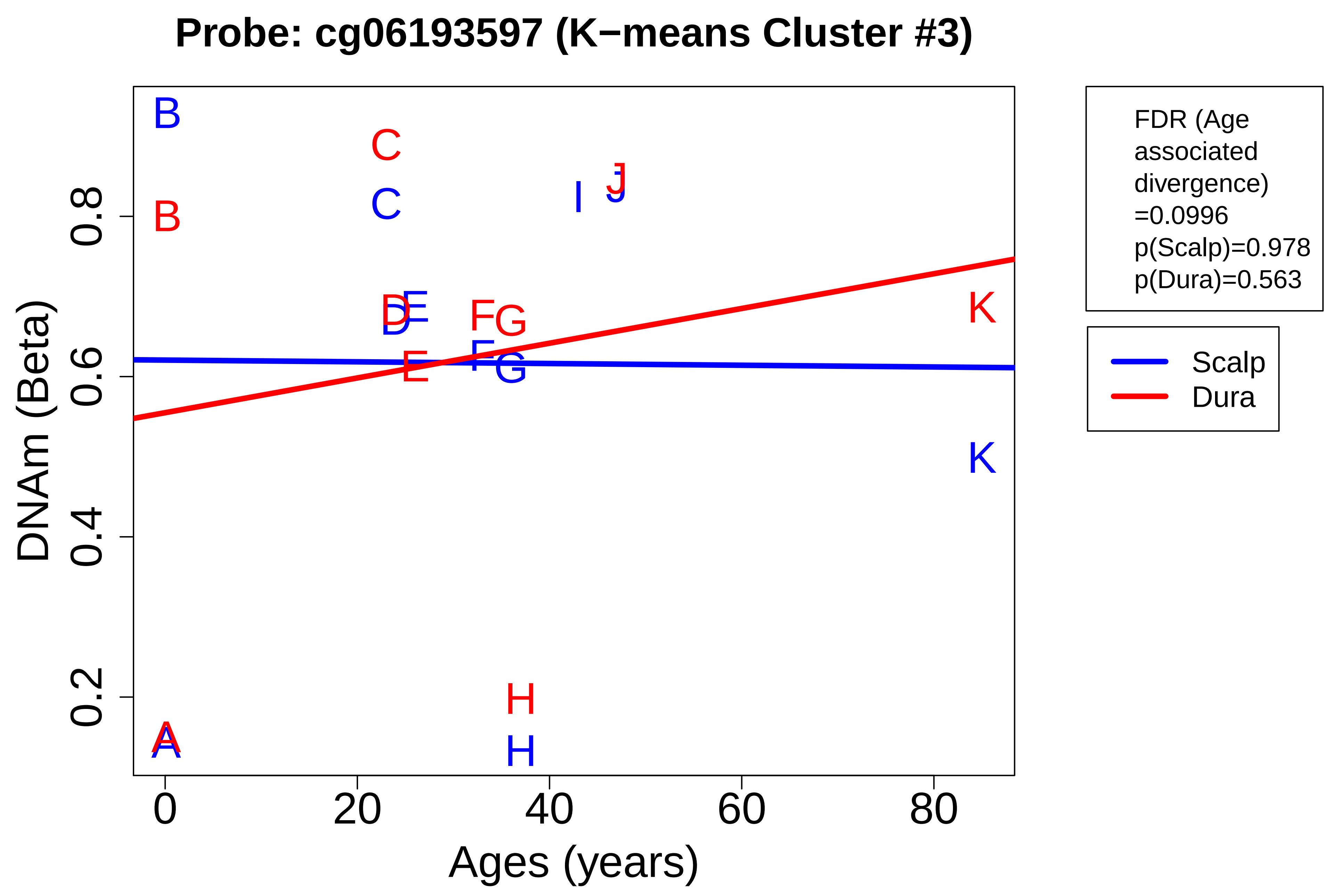

Probe: cg06193597 (K−means Cluster #3)
DNAm (Beta)
0.2
0.4
0.6
0.8
0
20
40
60
80
Ages (years)
FDR (Age associated divergence) =0.0996
p(Scalp)=0.978
p(Dura)=0.563
Scalp
Dura

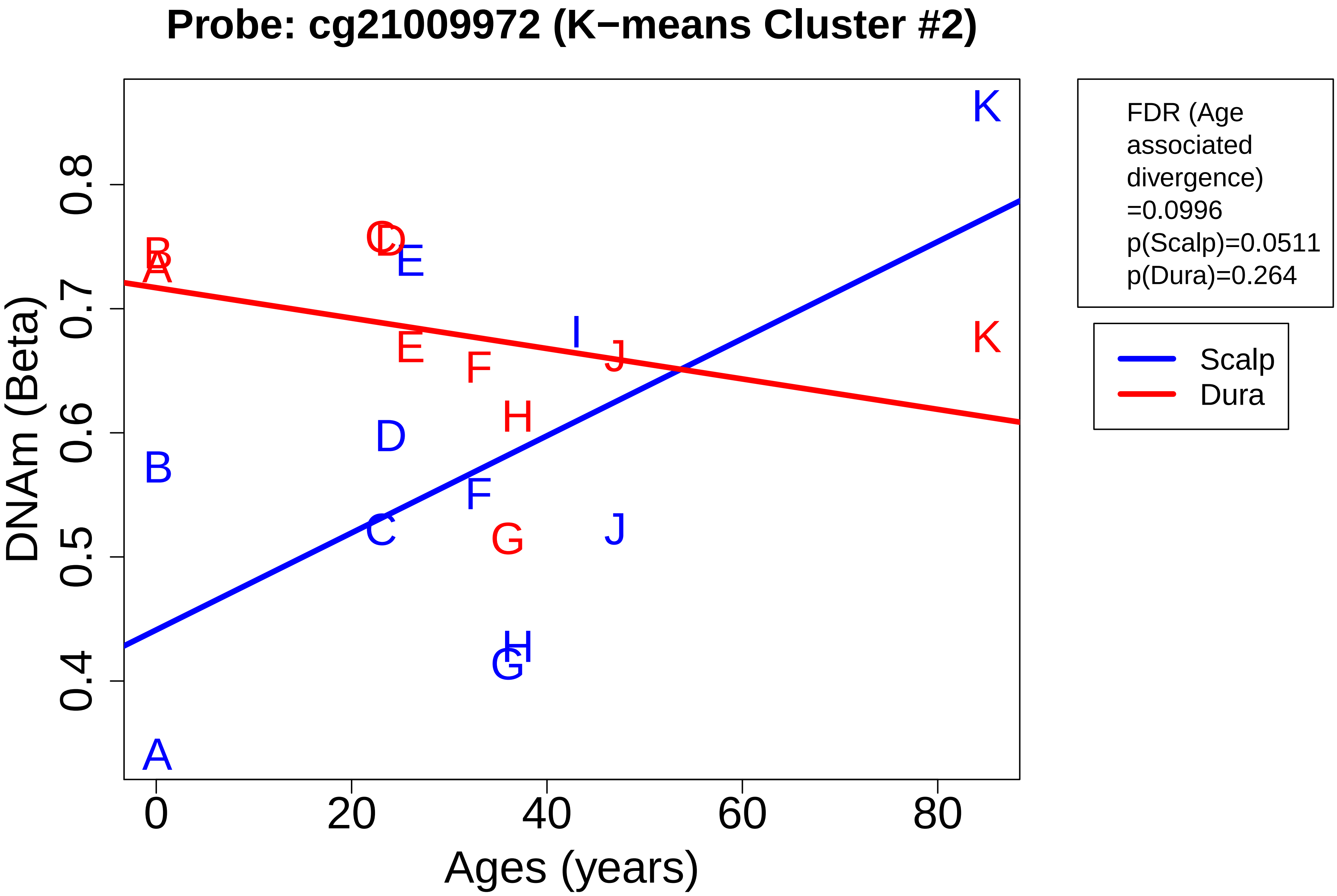

Probe: cg21009972 (K−means Cluster #2)
DNAm (Beta)
0.4
0.5
0.6
0.7
0.8
Ages (years)
0
20
40
60
80
A
B
C
D
E
F
G
H
I
J
K
FDR (Age associated divergence) =0.0996
p(Scalp)=0.0511
p(Dura)=0.264
Scalp
Dura

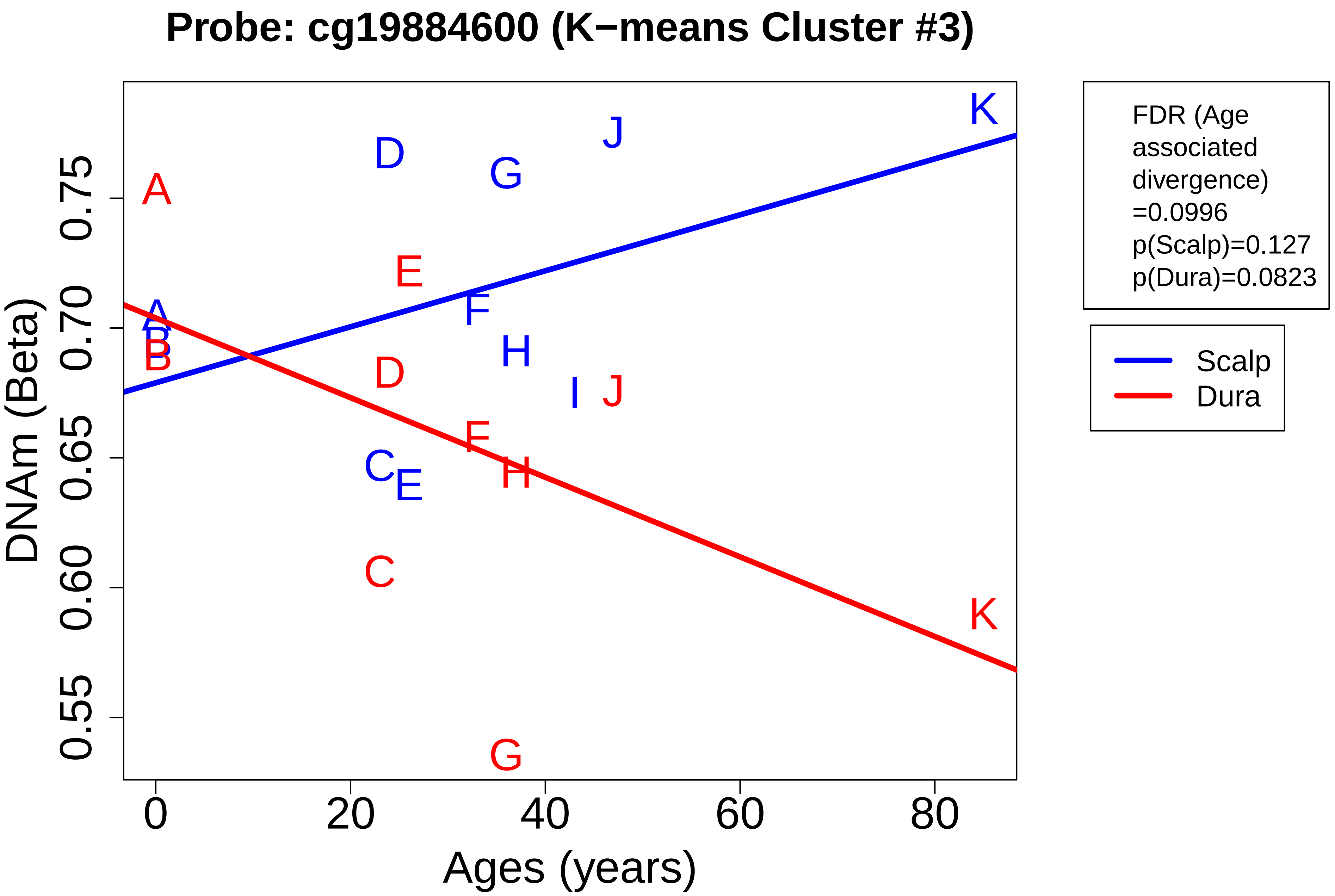

Probe: cg19884600 (K−means Cluster #3)
DNAm (Beta)
0.55
0.60
0.65
0.70
0.75
Ages (years)
0
20
40
60
80
A
B
C
D
E
F
G
H
I
J
K
FDR (Age associated divergence) =0.0996
p(Scalp)=0.127
p(Dura)=0.0823
Scalp
Dura

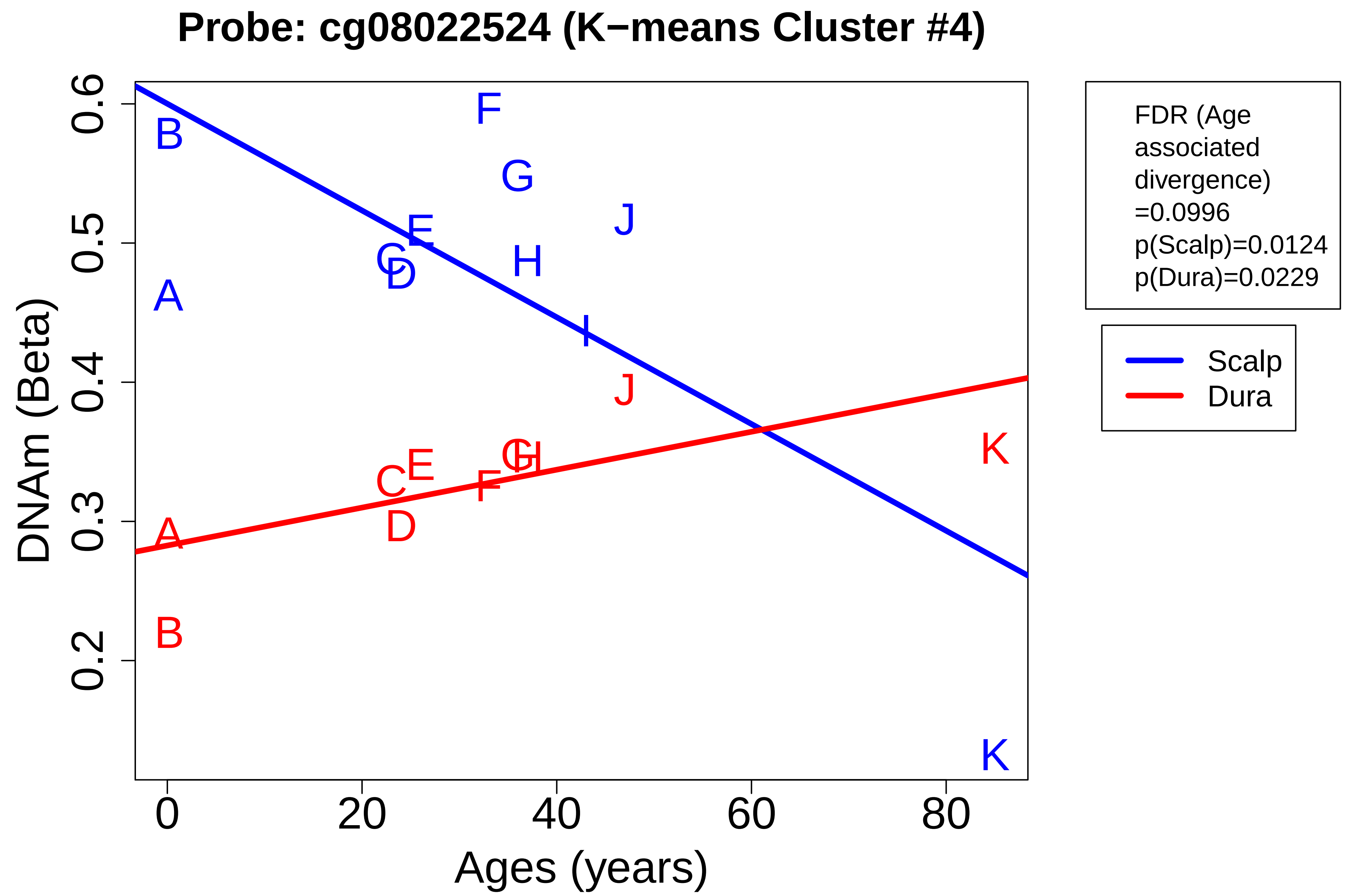

Probe: cg08022524 (K−means Cluster #4)
DNAm (Beta)
0.2
0.3
0.4
0.5
0.6
Ages (years)
0
20
40
60
80
FDR (Age associated divergence) =0.0996
p(Scalp)=0.0124
p(Dura)=0.0229
Scalp
Dura

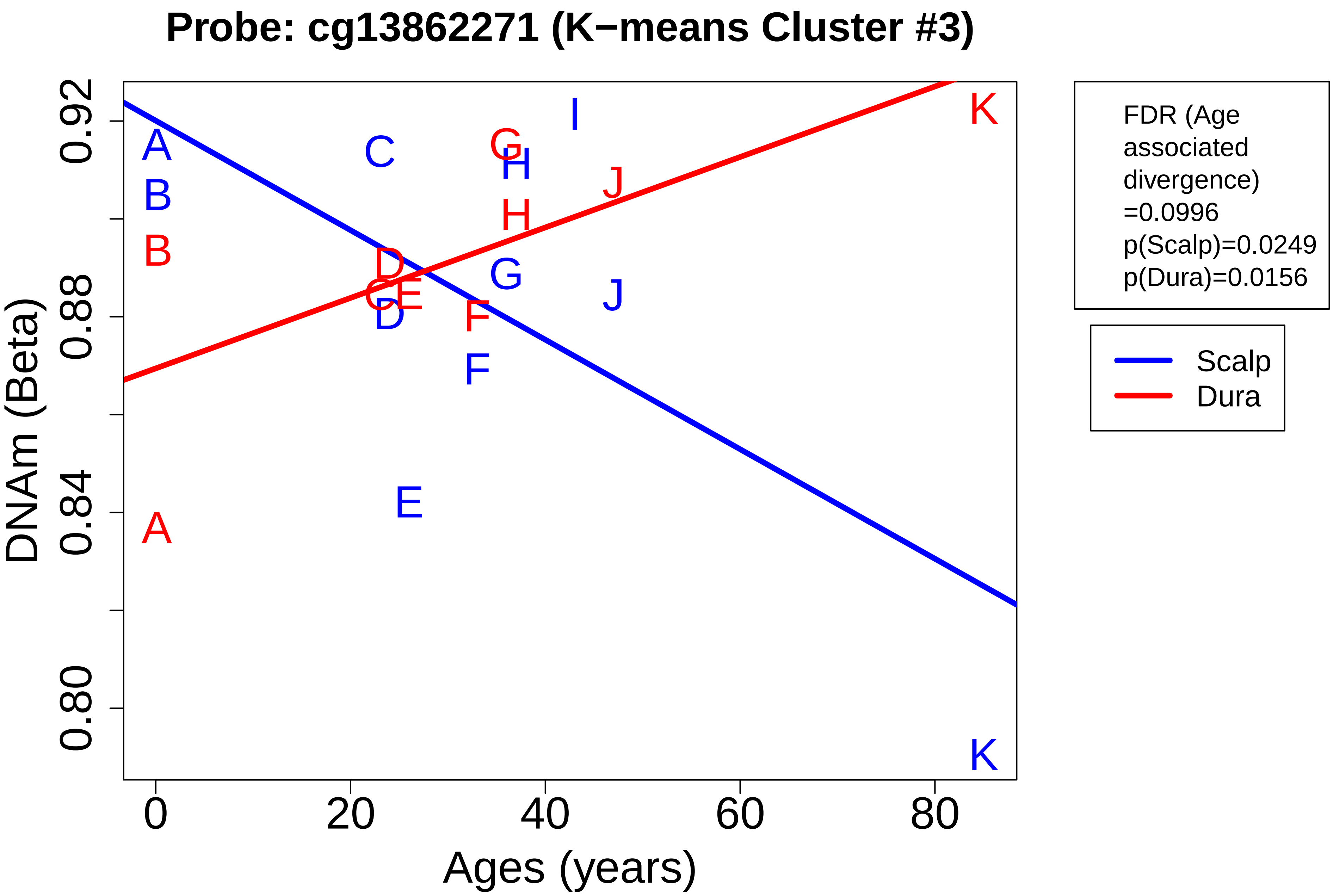

Probe: cg13862271 (K−means Cluster #3)
DNAm (Beta)
0.80
0.84
0.88
0.92
Ages (years)
0
20
40
60
80
FDR (Age associated divergence) =0.0996
p(Scalp)=0.0249
p(Dura)=0.0156
Scalp
Dura

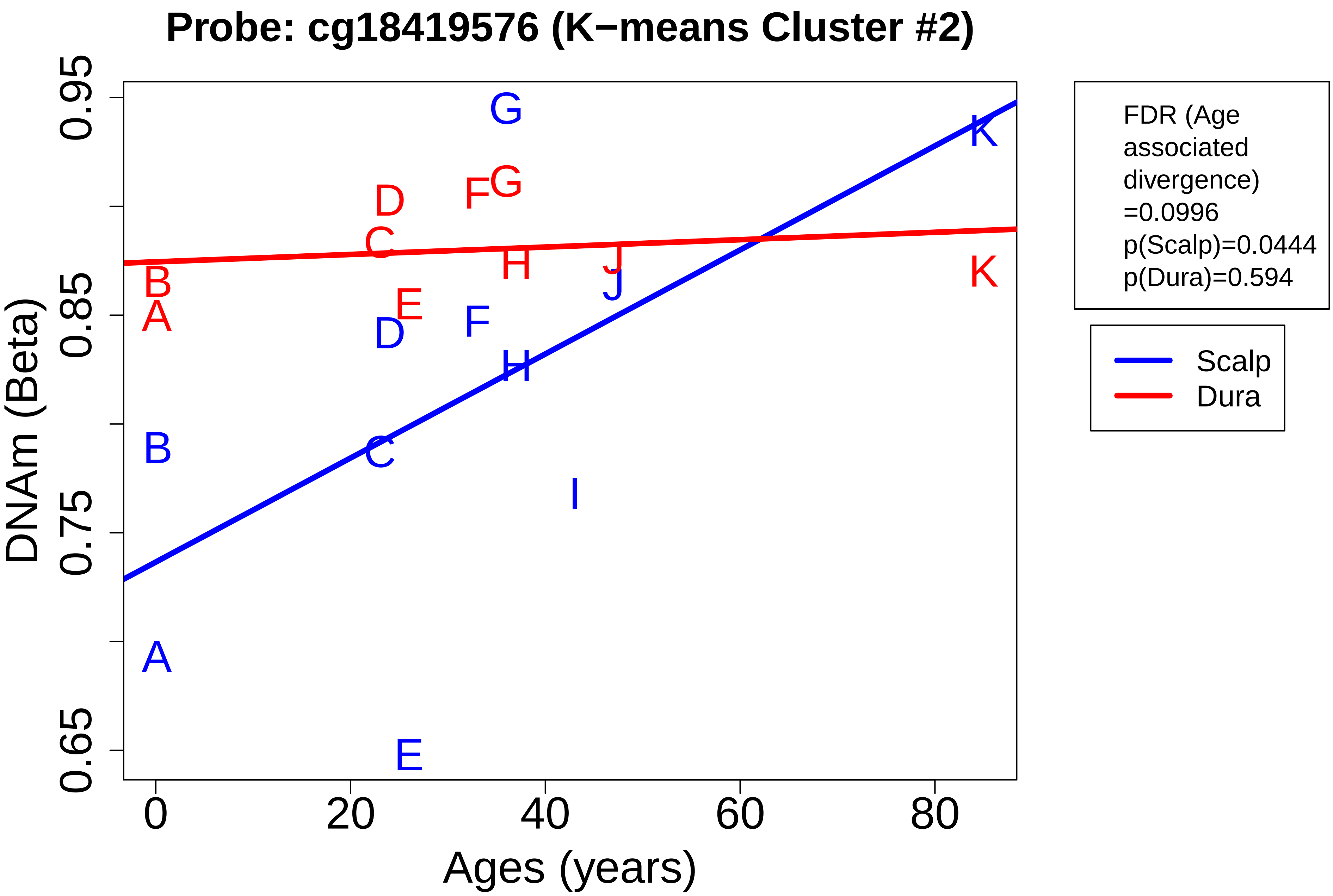

Probe: cg18419576 (K−means Cluster #2)
DNAm (Beta)
0.65
0.75
0.85
0.95
Ages (years)
0
20
40
60
80
A
B
C
D
E
F
G
H
I
J
K
FDR (Age associated divergence) =0.0996
p(Scalp)=0.0444
p(Dura)=0.594
Scalp
Dura

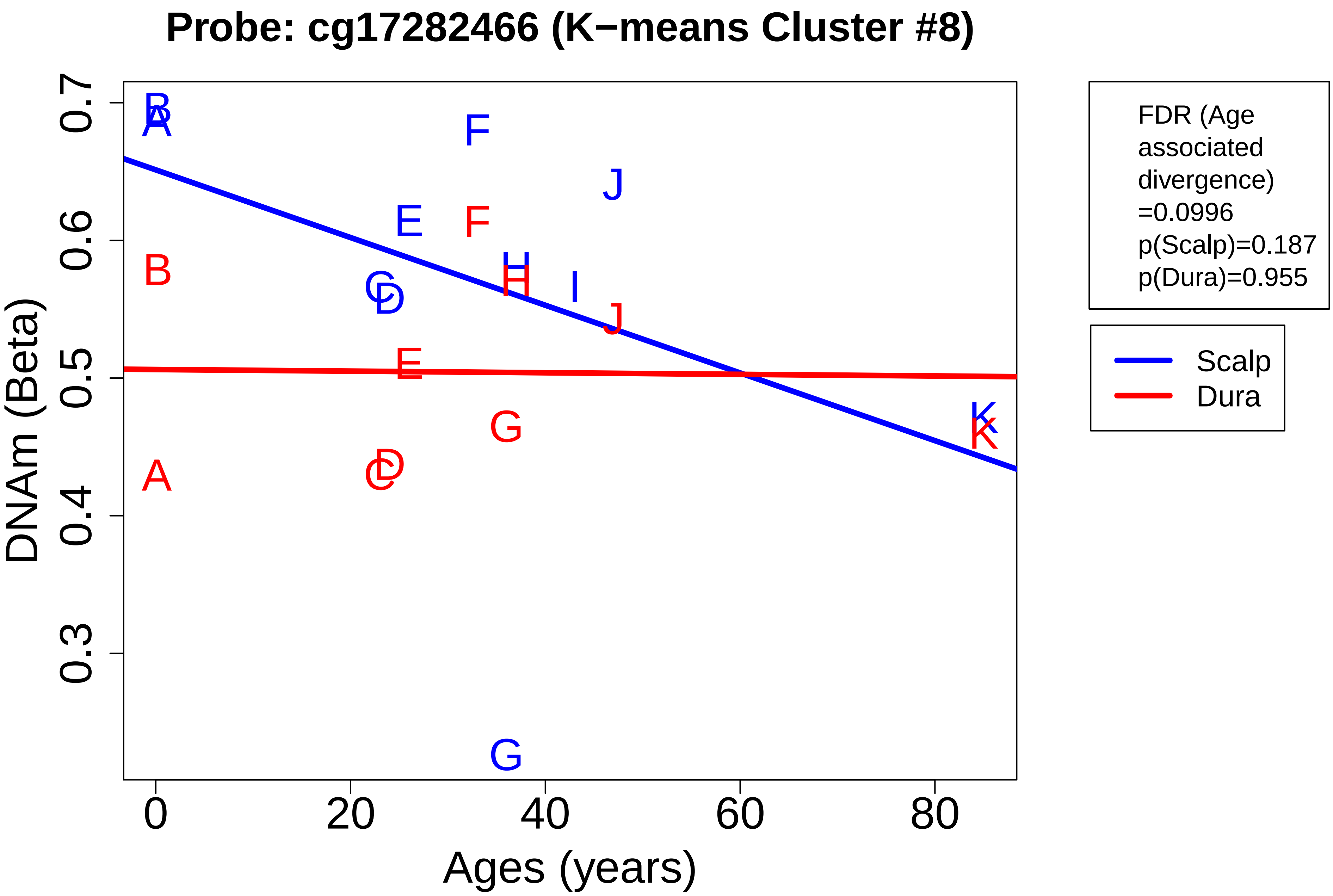

Probe: cg17282466 (K−means Cluster #8)
DNAm (Beta)
Ages (years)
0.3
0.4
0.5
0.6
0.7
0
20
40
60
80
FDR (Age associated divergence) =0.0996
p(Scalp)=0.187
p(Dura)=0.955
Scalp
Dura

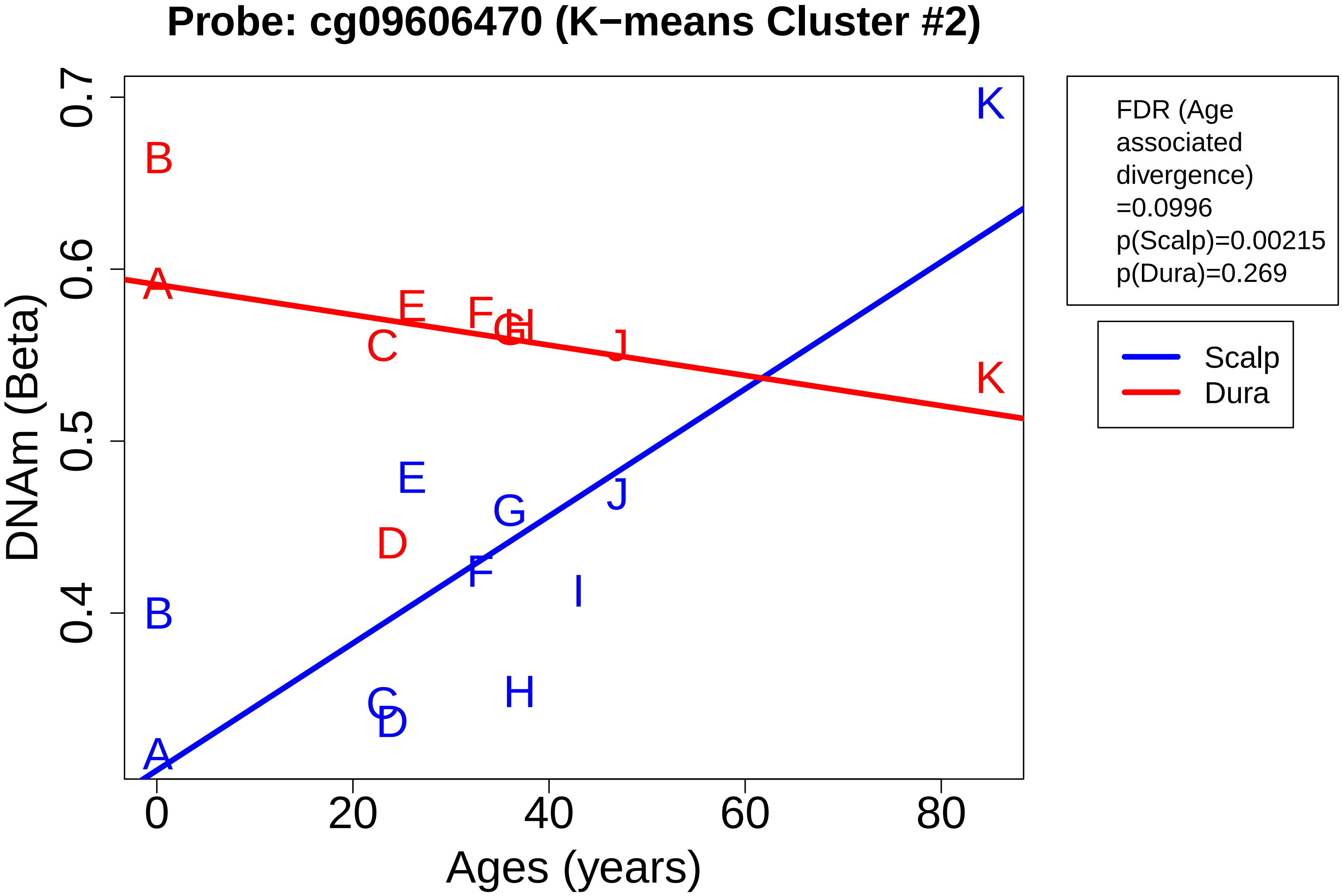

Probe: cg09606470 (K−means Cluster #2)
DNAm (Beta)
0.4
0.5
0.6
0.7
0
20
40
60
80
Ages (years)
FDR (Age associated divergence) =0.0996
p(Scalp)=0.00215
p(Dura)=0.269
Scalp
Dura

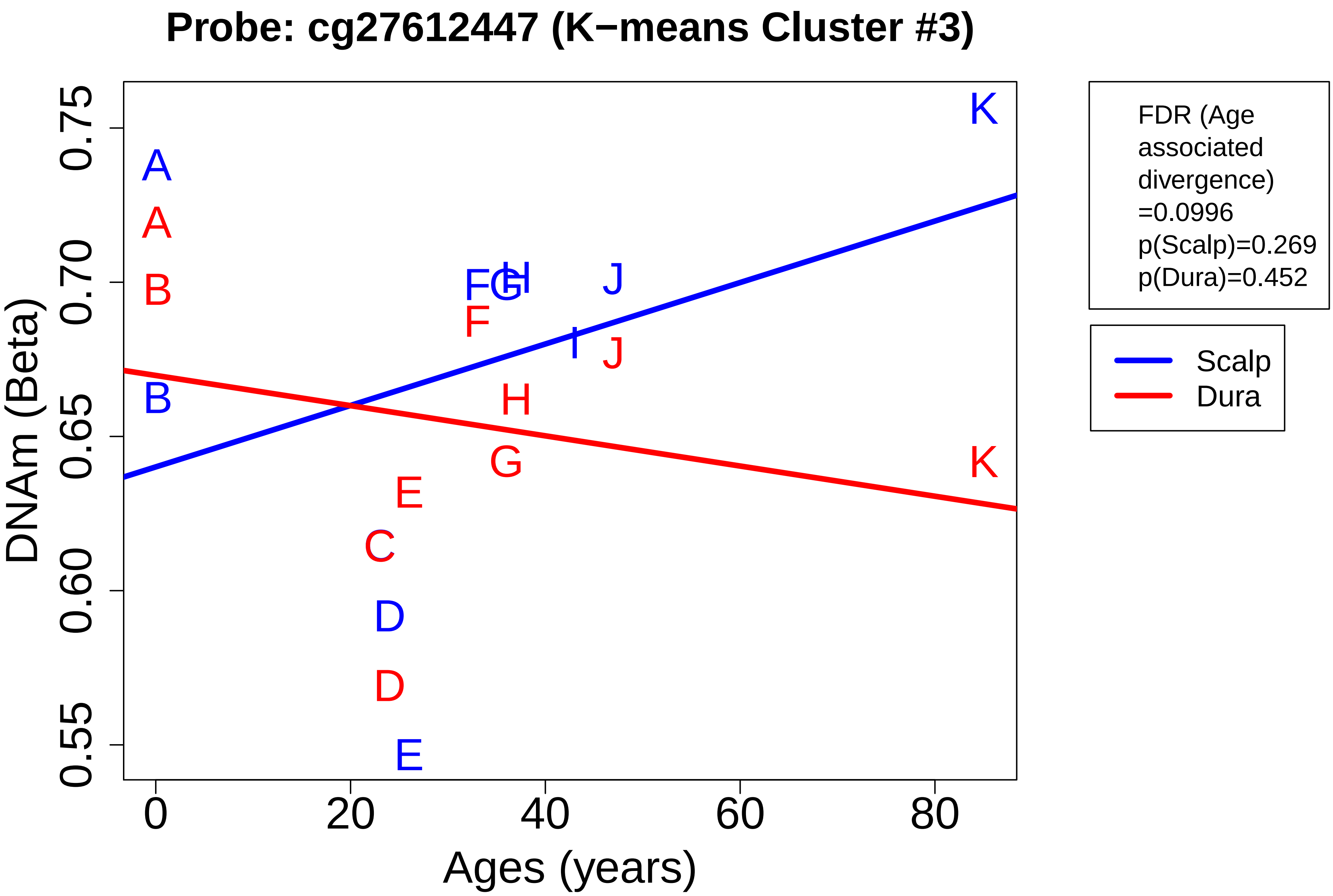

Probe: cg27612447 (K−means Cluster #3)
FDR (Age associated divergence) =0.0996
p(Scalp)=0.269
p(Dura)=0.452
Scalp
Dura
DNAm (Beta)
0.55
0.60
0.65
0.70
0.75
0
20
40
60
80
Ages (years)
A
A
B
B
C
D
D
E
E
F
F
G
G
H
H
I
J
J
K
K

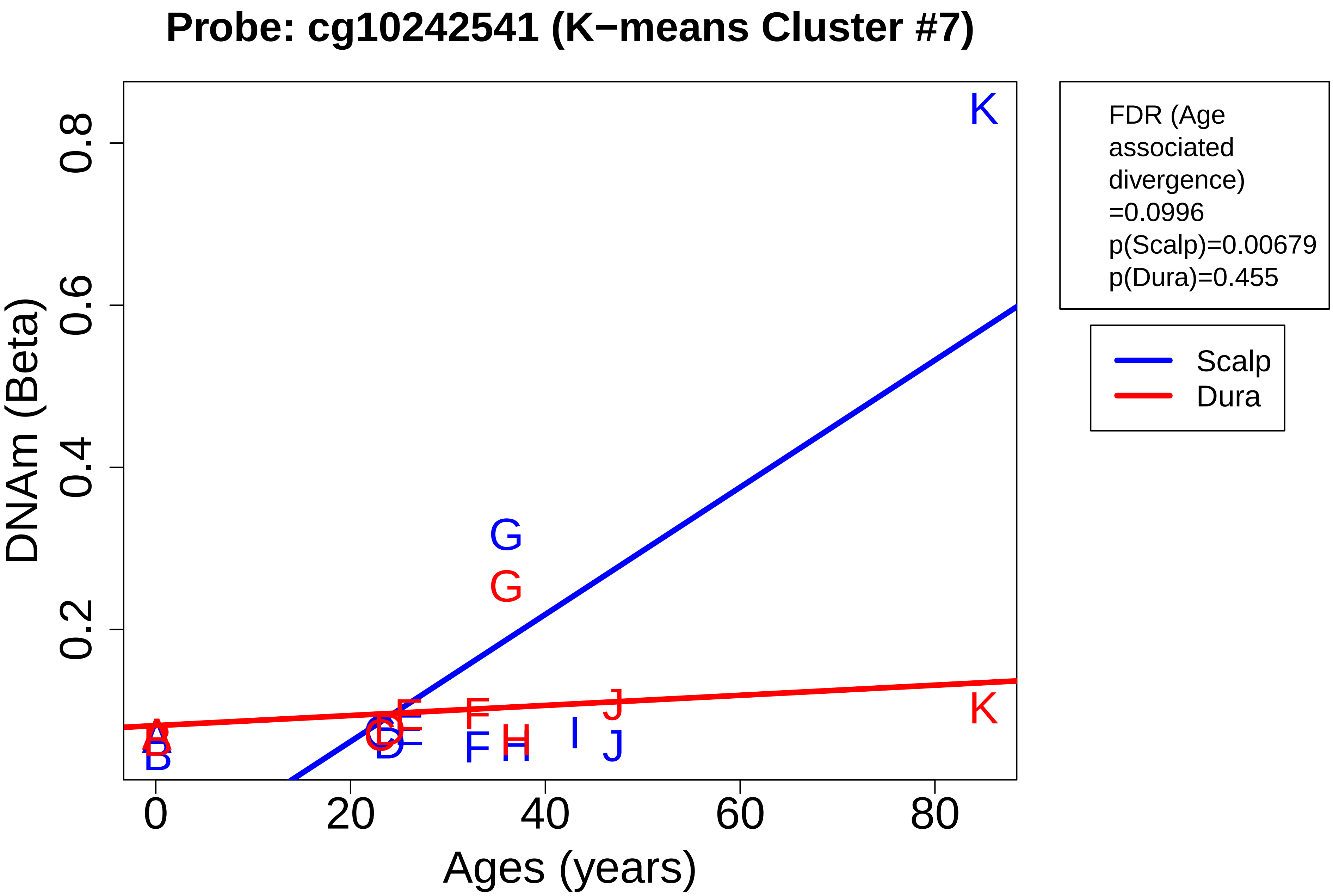

Probe: cg10242541 (K−means Cluster #7)
DNAm (Beta)
0.2
0.4
0.6
0.8
Ages (years)
0
20
40
60
80
K
G
G
A
B
C
D
E
F
F
H
H
I
J
J
K
FDR (Age associated divergence) =0.0996
p(Scalp)=0.00679
p(Dura)=0.455
Scalp
Dura

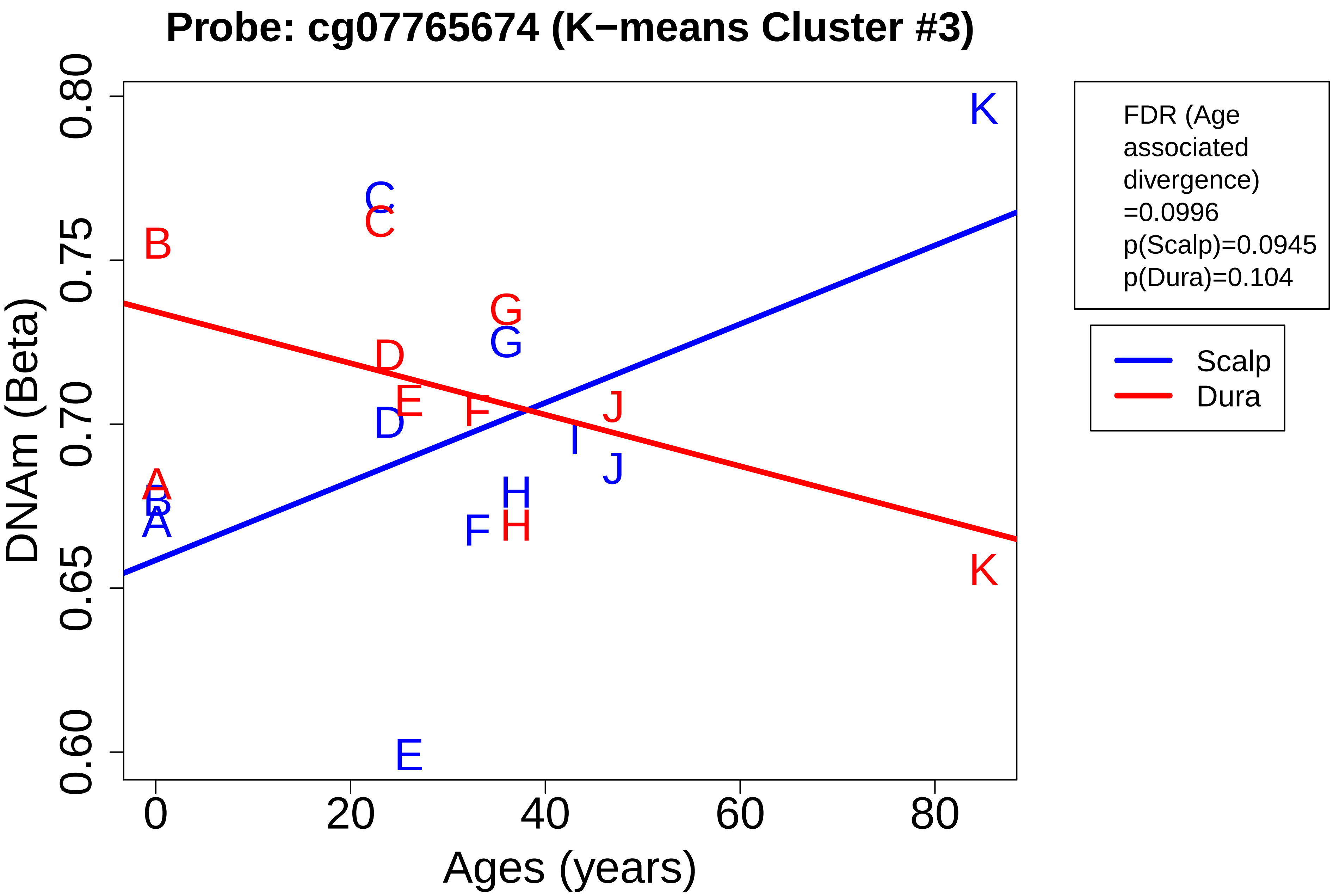
Probe: cg07765674 (K−means Cluster #3)
DNAm (Beta)
0.60
0.65
0.70
0.75
0.80
0
20
40
60
80
Ages (years)
FDR (Age associated divergence) =0.0996
p(Scalp)=0.0945
p(Dura)=0.104
Scalp
Dura
A
B
C
D
E
F
G
H
I
J
K

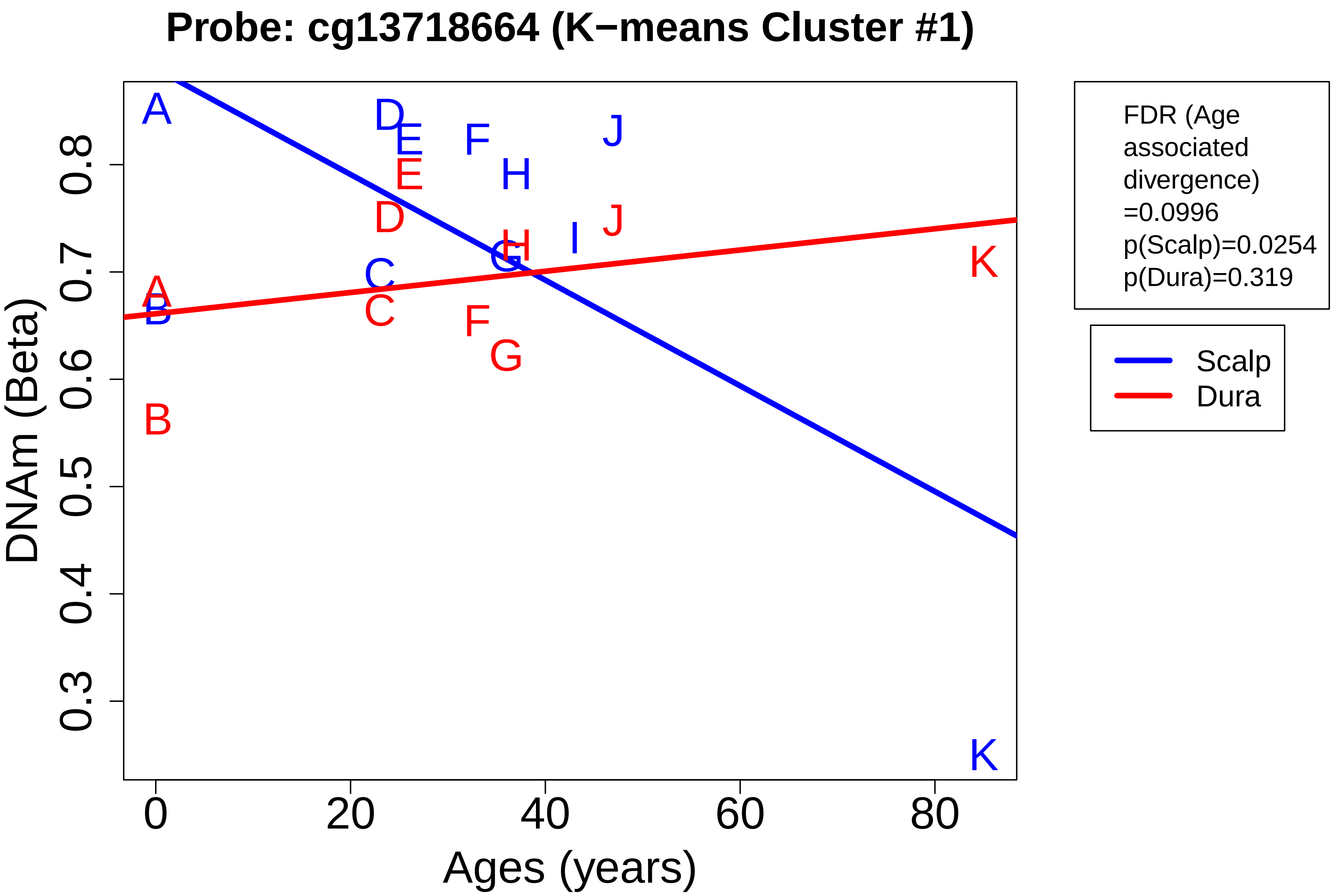

Probe: cg13718664 (K−means Cluster #1)
DNAm (Beta)
0.3
0.4
0.5
0.6
0.7
0.8
Ages (years)
0
20
40
60
80
A
B
C
D
E
F
G
H
I
J
K
FDR (Age associated divergence) =0.0996
p(Scalp)=0.0254
p(Dura)=0.319
Scalp
Dura

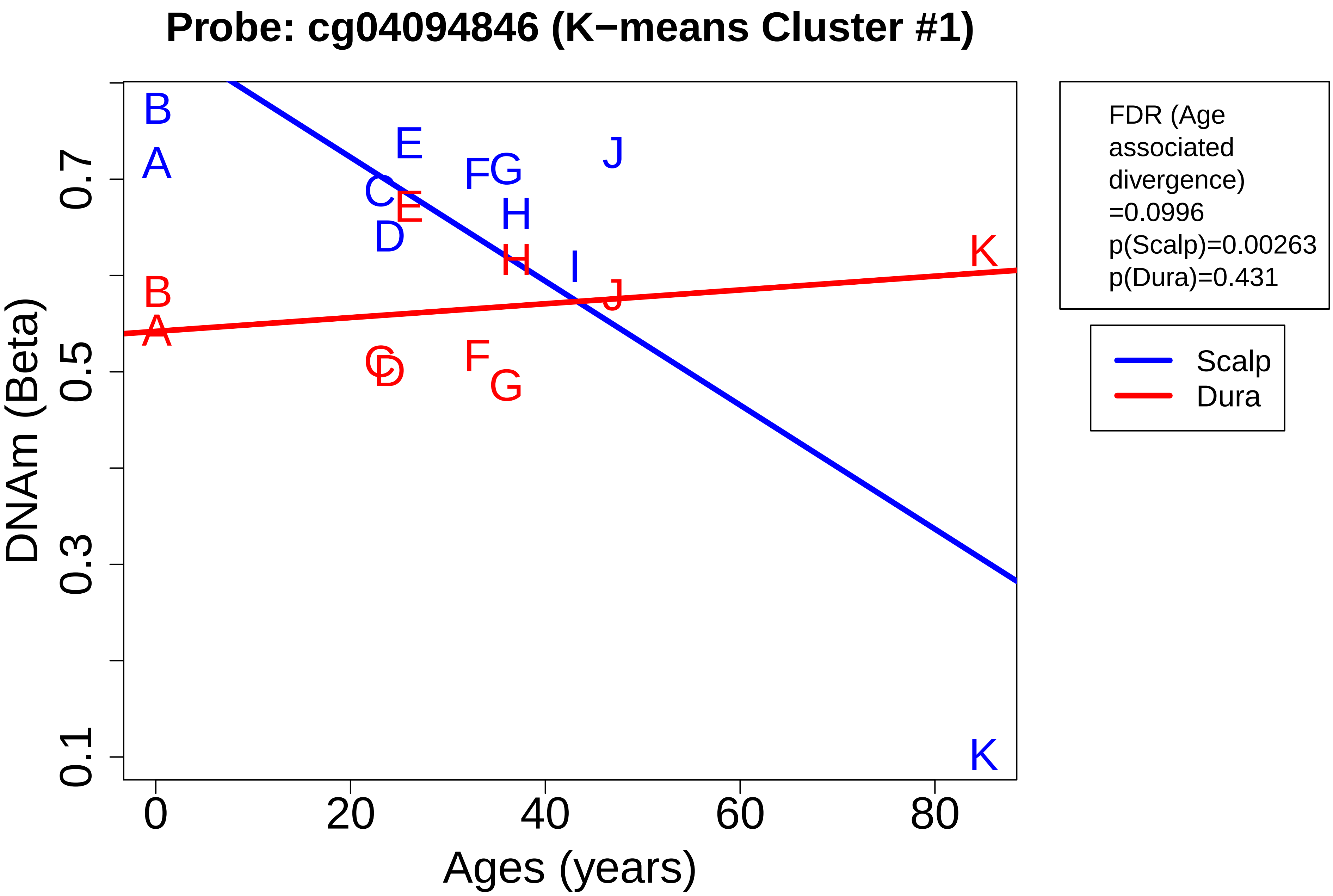

Probe: cg04094846 (K−means Cluster #1)
DNAm (Beta)
0.1
0.3
0.5
0.7
Ages (years)
0
20
40
60
80
A
B
C
D
E
F
G
H
I
J
K
FDR (Age associated divergence) =0.0996
p(Scalp)=0.00263
p(Dura)=0.431
Scalp
Dura

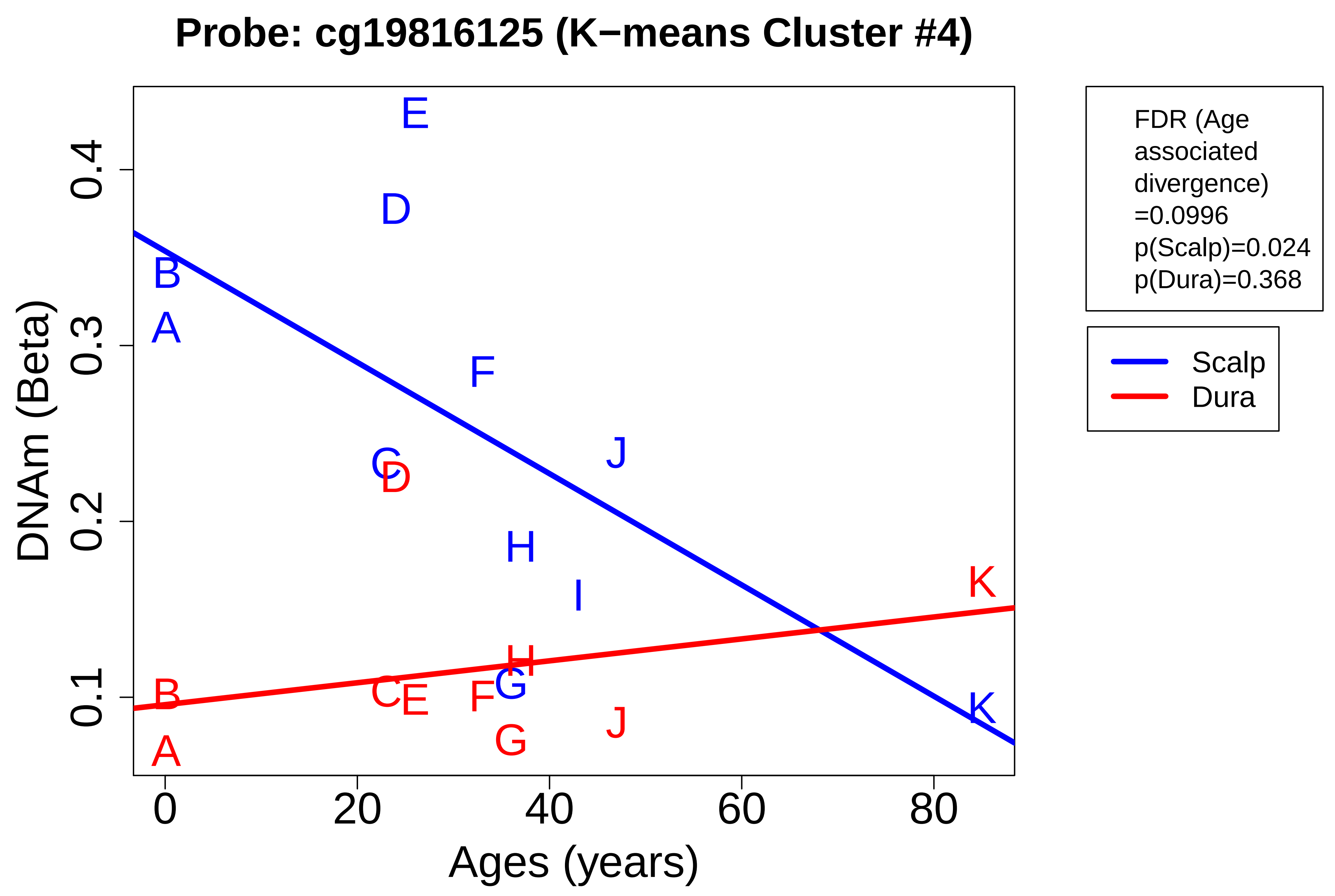

Probe: cg19816125 (K−means Cluster #4)
DNAm (Beta)
0.1
0.2
0.3
0.4
0
20
40
60
80
Ages (years)
FDR (Age associated divergence) =0.0996
p(Scalp)=0.024
p(Dura)=0.368
Scalp
Dura

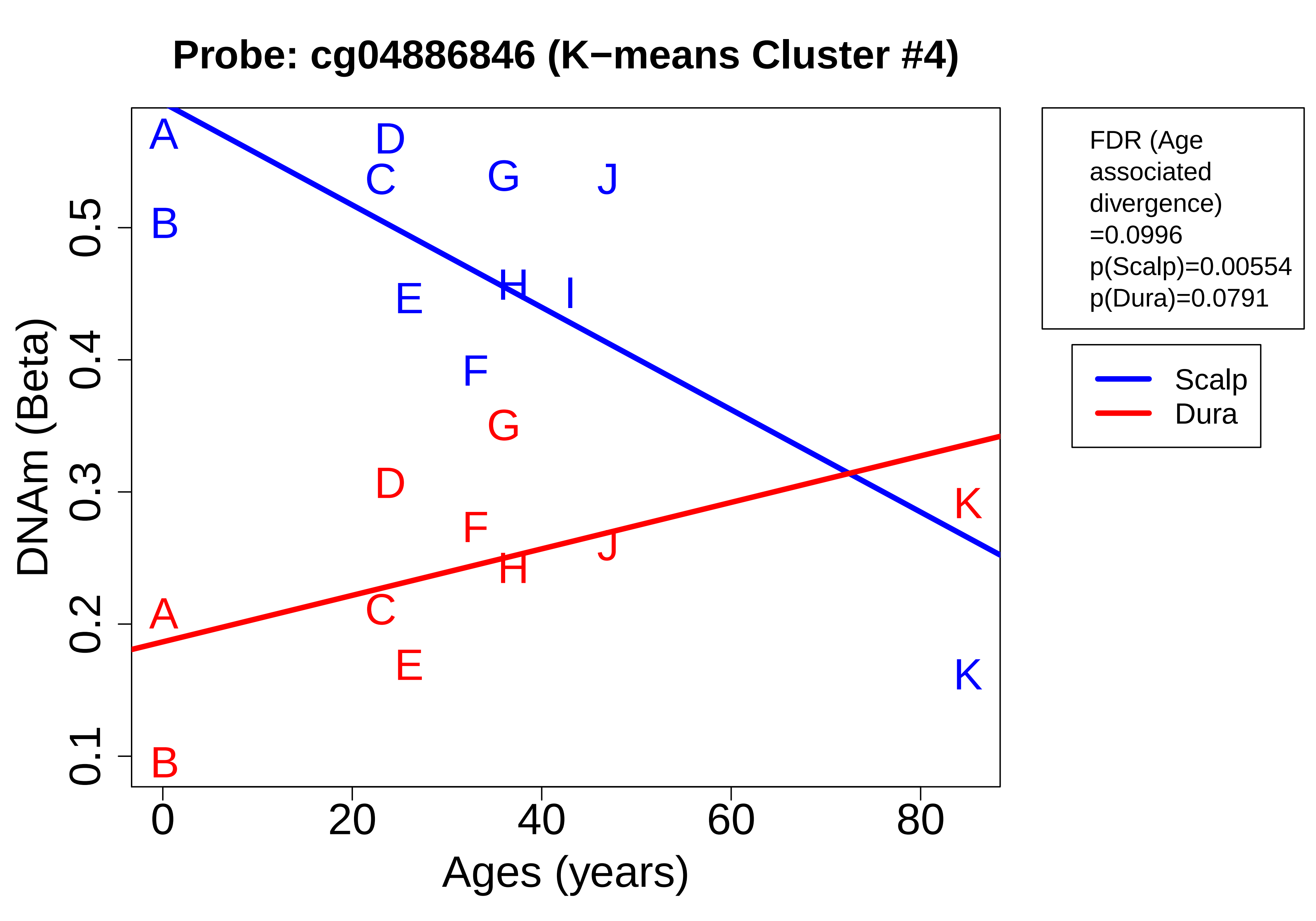
Probe: cg04886846 (K−means Cluster #4)
DNAm (Beta)
Ages (years)
FDR (Age associated divergence) =0.0996
p(Scalp)=0.00554
p(Dura)=0.0791
Scalp
Dura
0.1
0.2
0.3
0.4
0.5
0
20
40
60
80
A
B
C
D
E
F
G
H
I
J
K

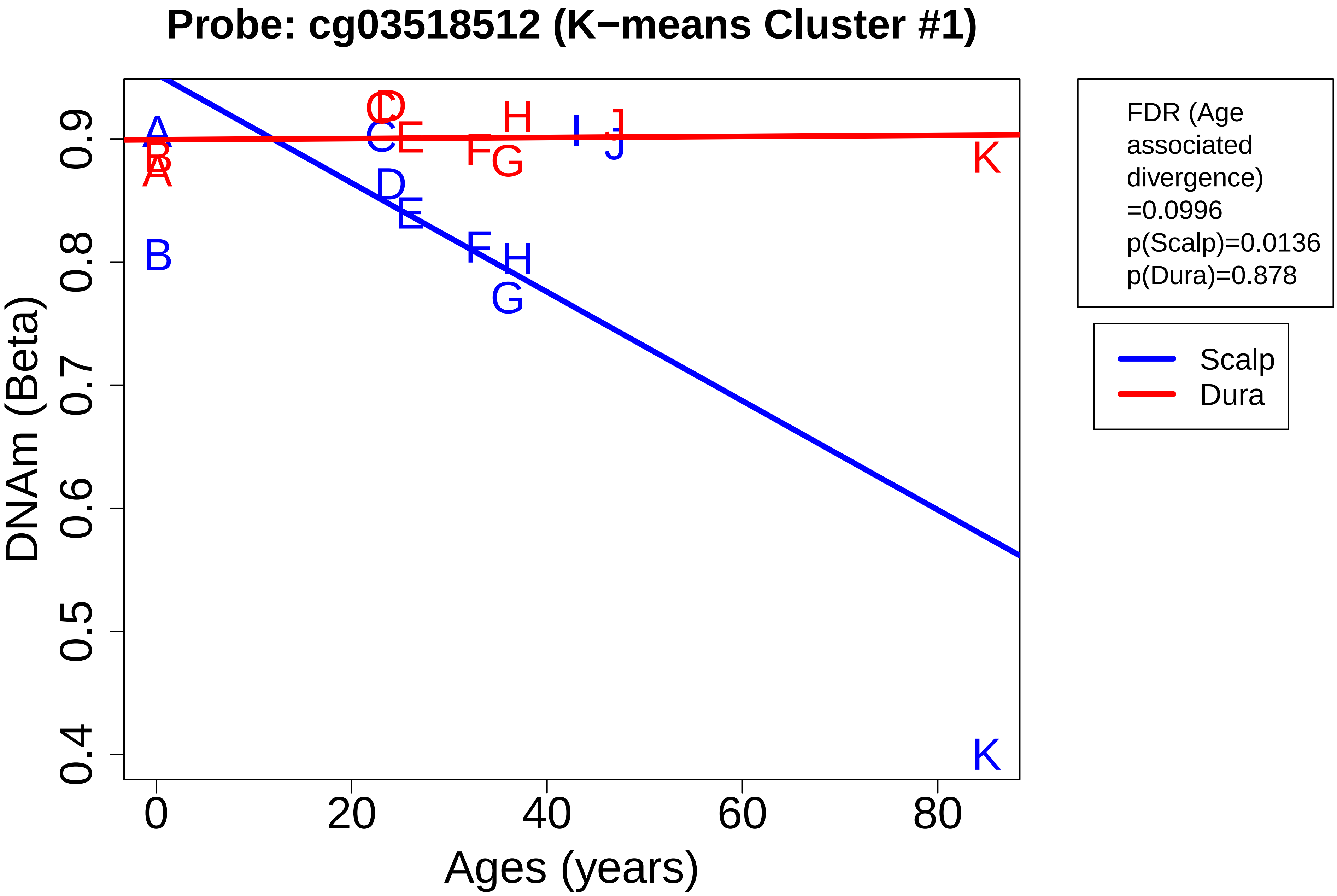

Probe: cg03518512 (K−means Cluster #1)
DNAm (Beta)
Ages (years)
0.4
0.5
0.6
0.7
0.8
0.9
0
20
40
60
80
FDR (Age associated divergence) =0.0996
p(Scalp)=0.0136
p(Dura)=0.878
Scalp
Dura

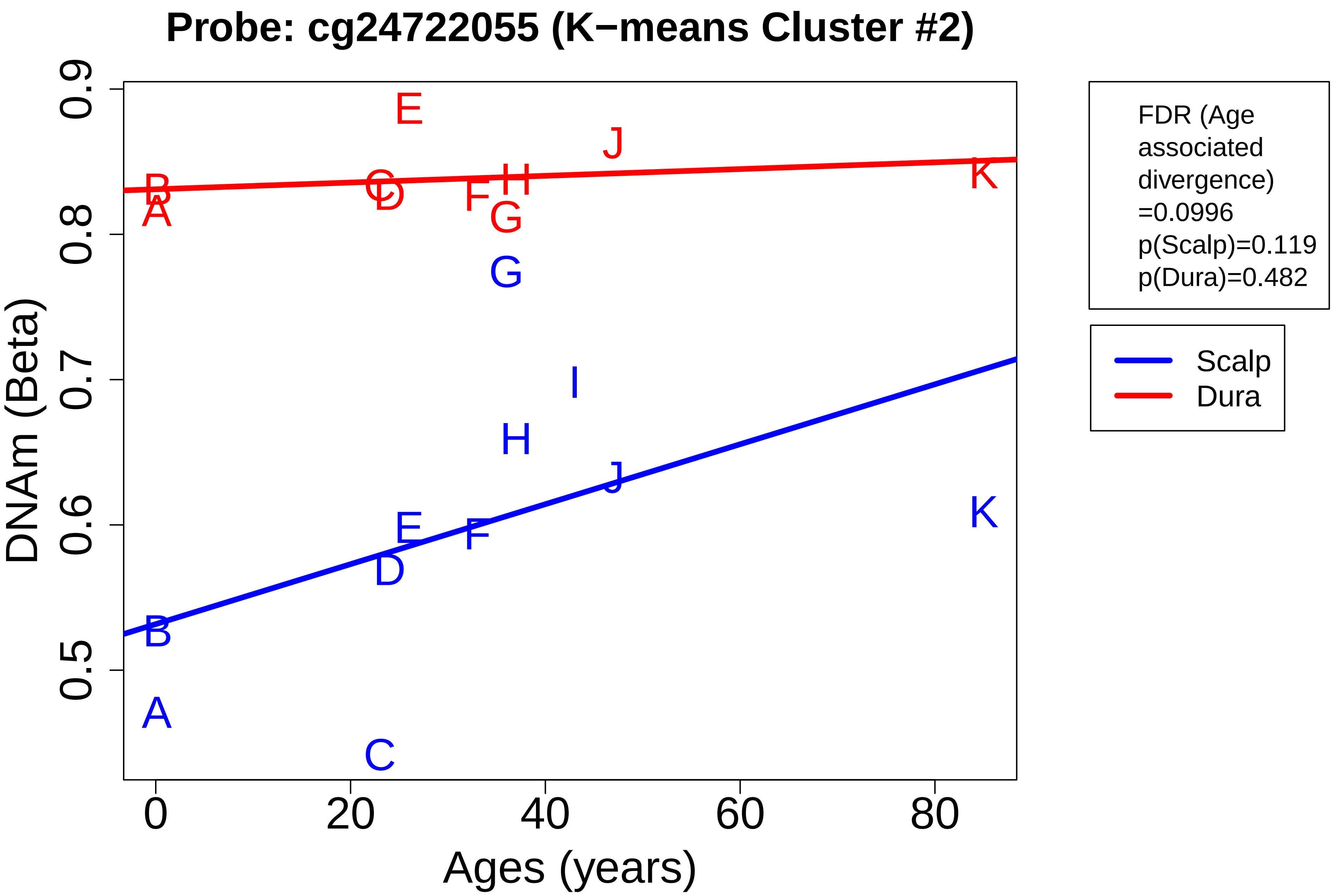

Probe: cg24722055 (K−means Cluster #2)
DNAm (Beta)
0.5
0.6
0.7
0.8
0.9
0
20
40
60
80
Ages (years)
FDR (Age associated divergence) =0.0996
p(Scalp)=0.119
p(Dura)=0.482
Scalp
Dura

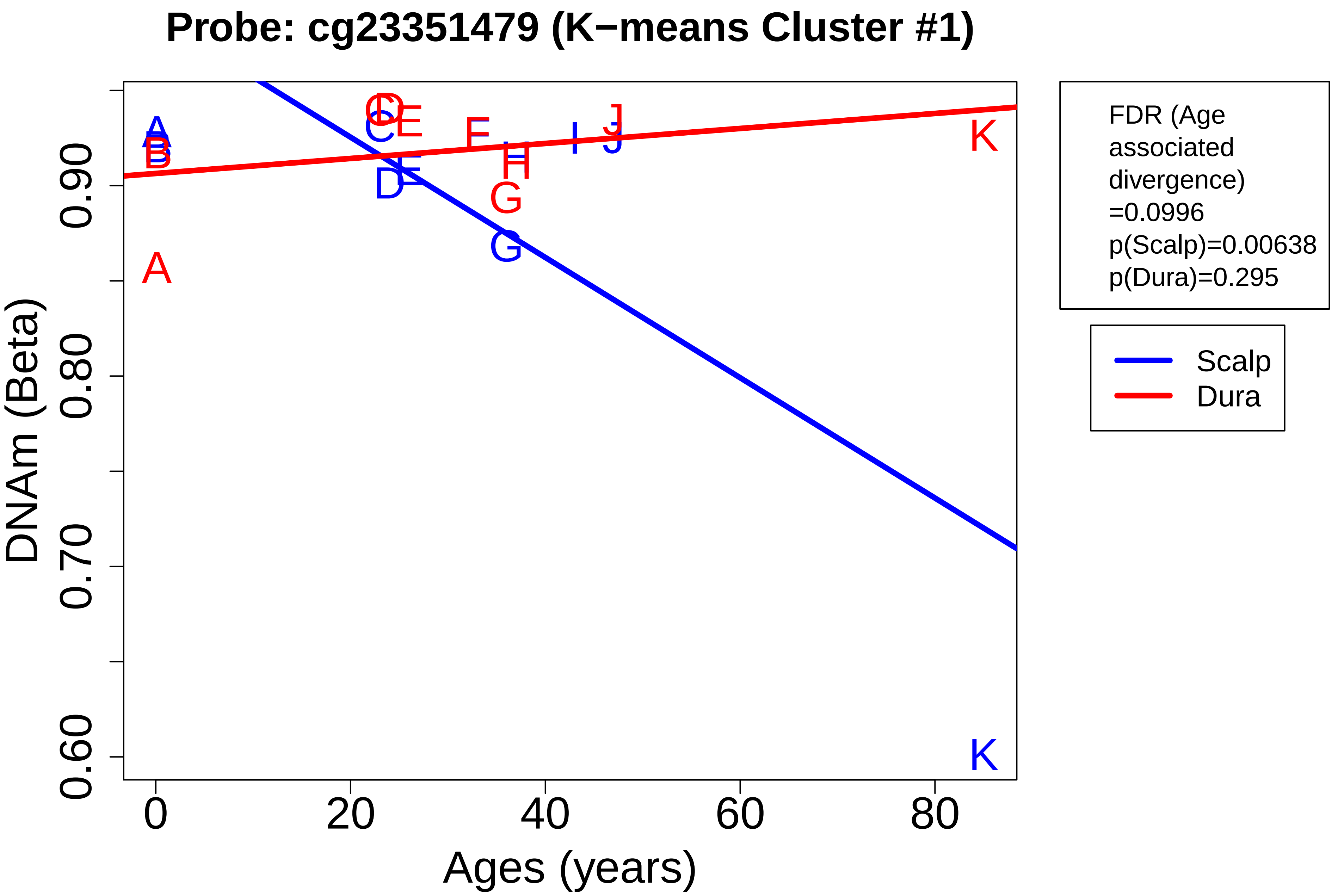

Probe: cg23351479 (K−means Cluster #1)
DNAm (Beta)
Ages (years)
0.60
0.70
0.80
0.90
0
20
40
60
80
FDR (Age associated divergence) =0.0996
p(Scalp)=0.00638
p(Dura)=0.295
Scalp
Dura

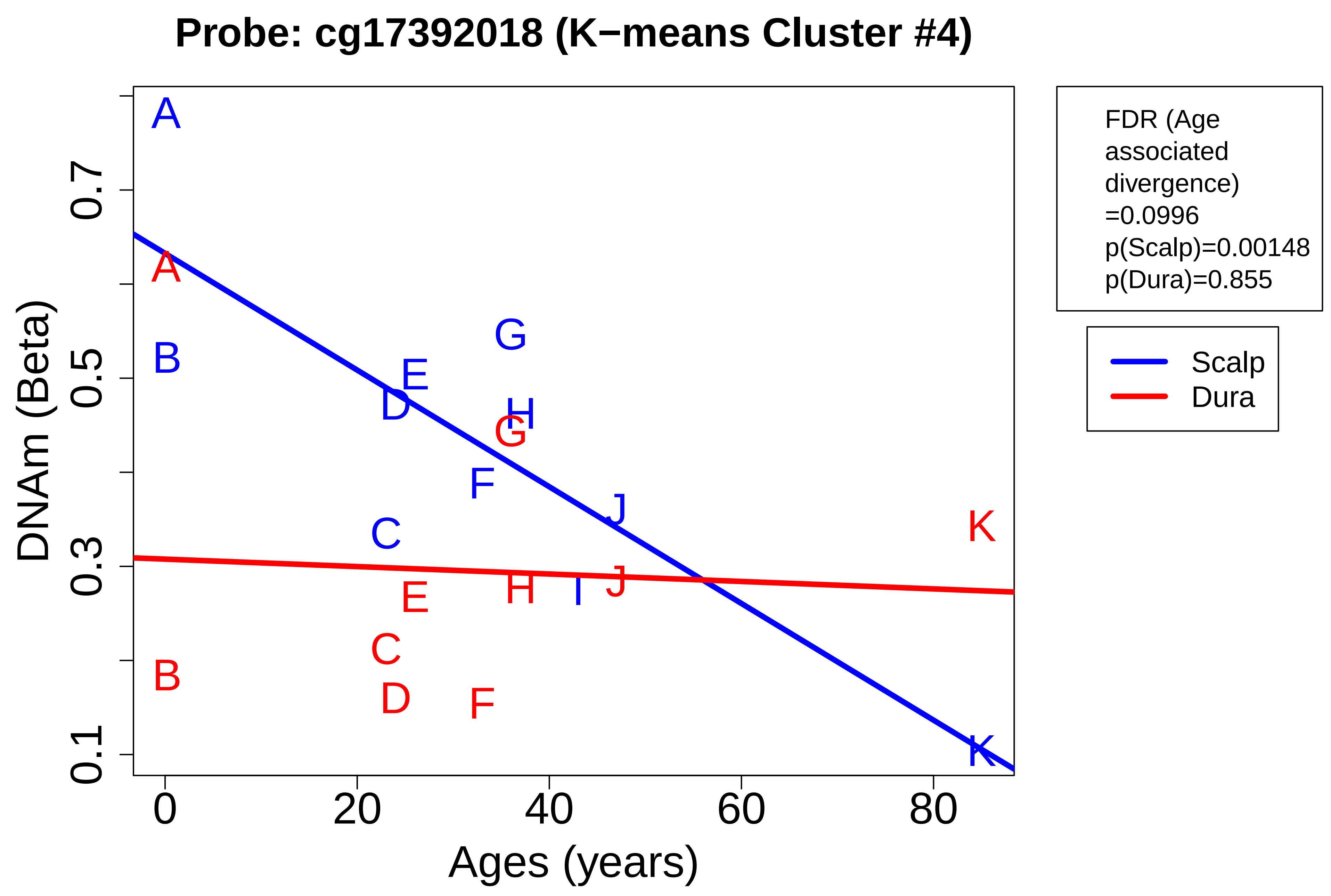

Probe: cg17392018 (K−means Cluster #4)
DNAm (Beta)
0.1
0.3
0.5
0.7
0
20
40
60
80
Ages (years)
A
B
C
D
E
F
G
H
I
J
K
FDR (Age associated divergence) =0.0996
p(Scalp)=0.00148
p(Dura)=0.855
Scalp
Dura

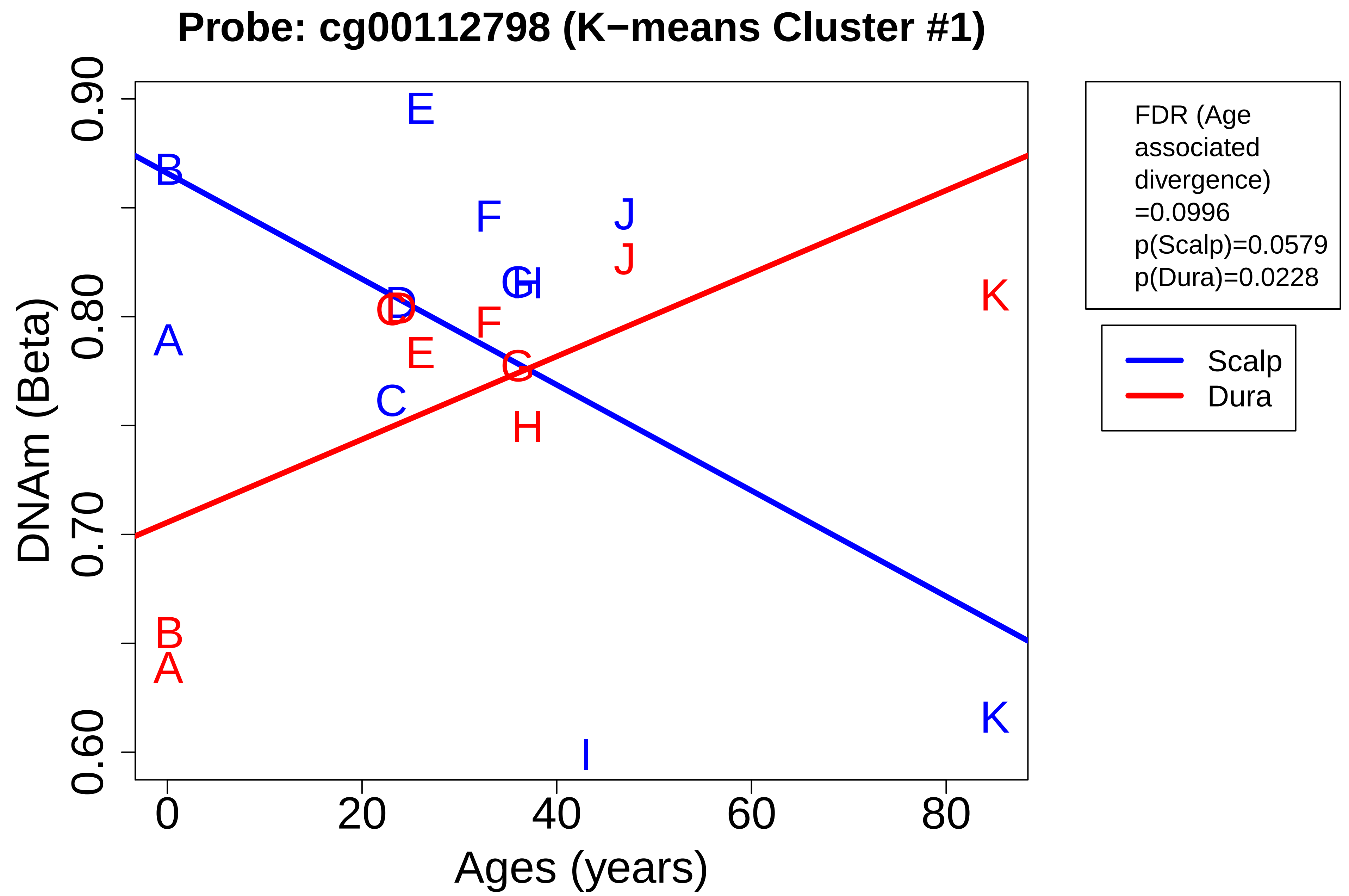

Probe: cg00112798 (K−means Cluster #1)
DNAm (Beta)
0.60
0.70
0.80
0.90
Ages (years)
0
20
40
60
80
FDR (Age associated divergence) =0.0996
p(Scalp)=0.0579
p(Dura)=0.0228
Scalp
Dura

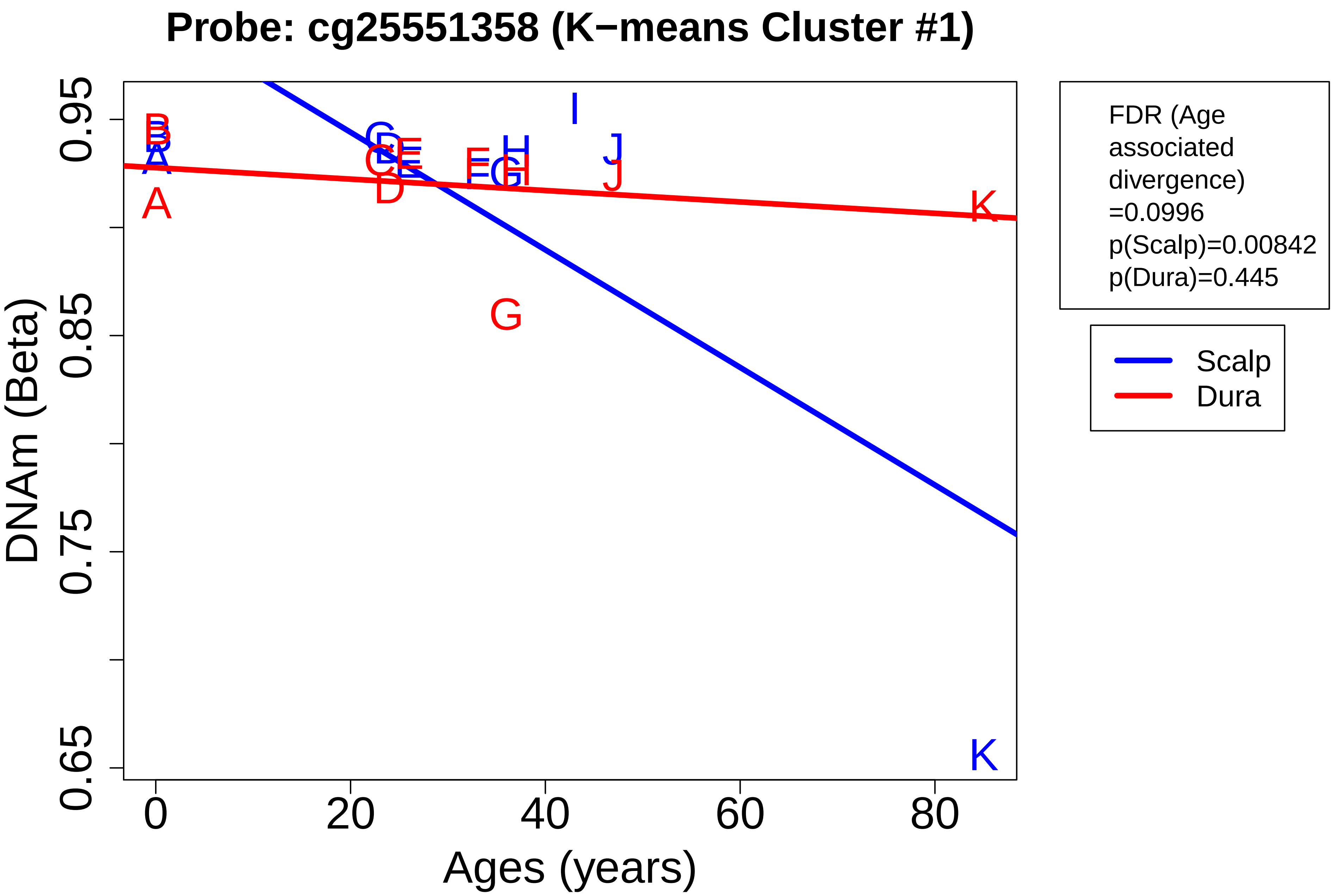

Probe: cg25551358 (K−means Cluster #1)
DNAm (Beta)
0.65
0.75
0.85
0.95
0
20
40
60
80
Ages (years)
FDR (Age associated divergence) =0.0996
p(Scalp)=0.00842
p(Dura)=0.445
Scalp
Dura

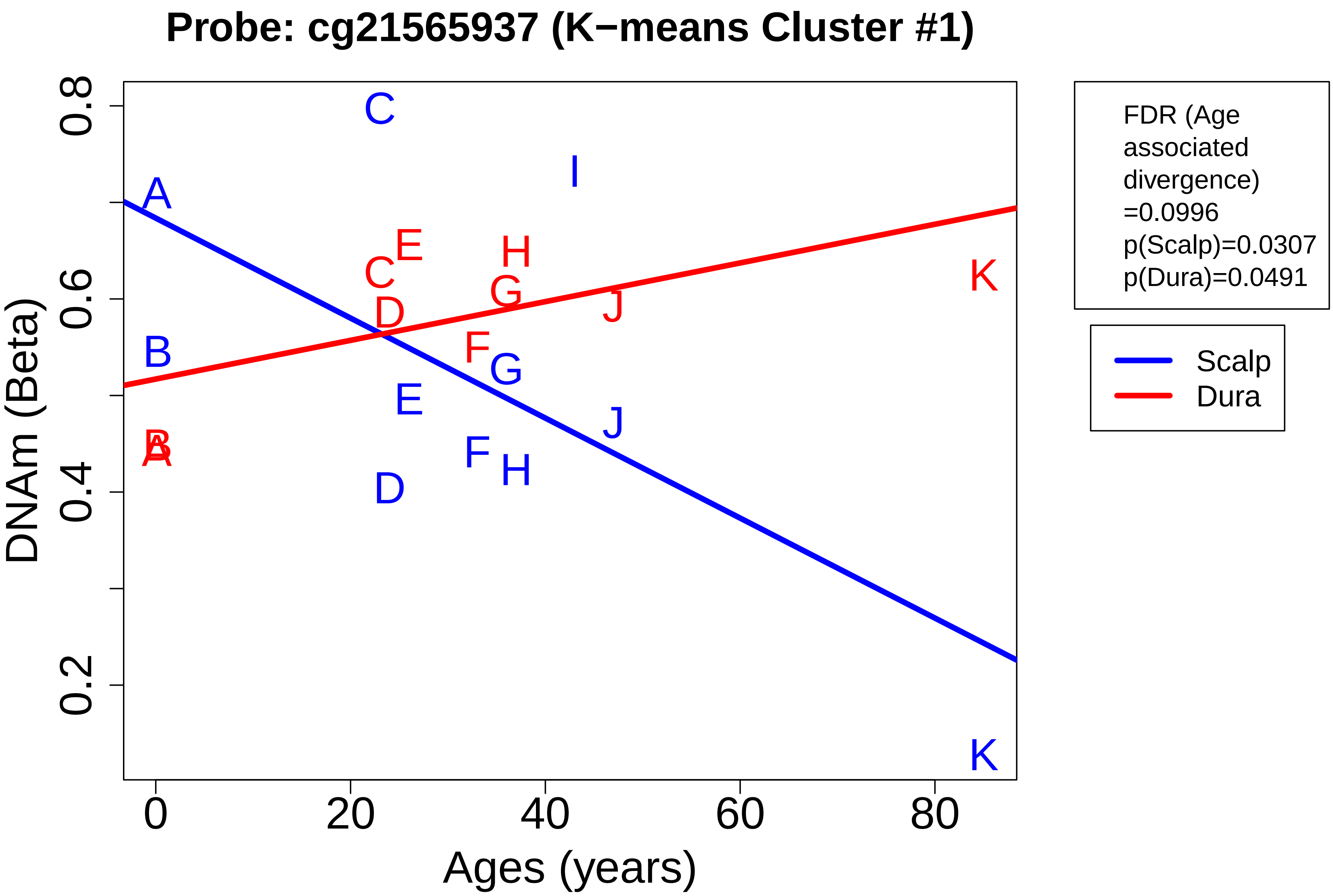

Probe: cg21565937 (K−means Cluster #1)
DNAm (Beta)
0.2
0.4
0.6
0.8
0
20
40
60
80
Ages (years)
FDR (Age associated divergence) =0.0996
p(Scalp)=0.0307
p(Dura)=0.0491
Scalp
Dura

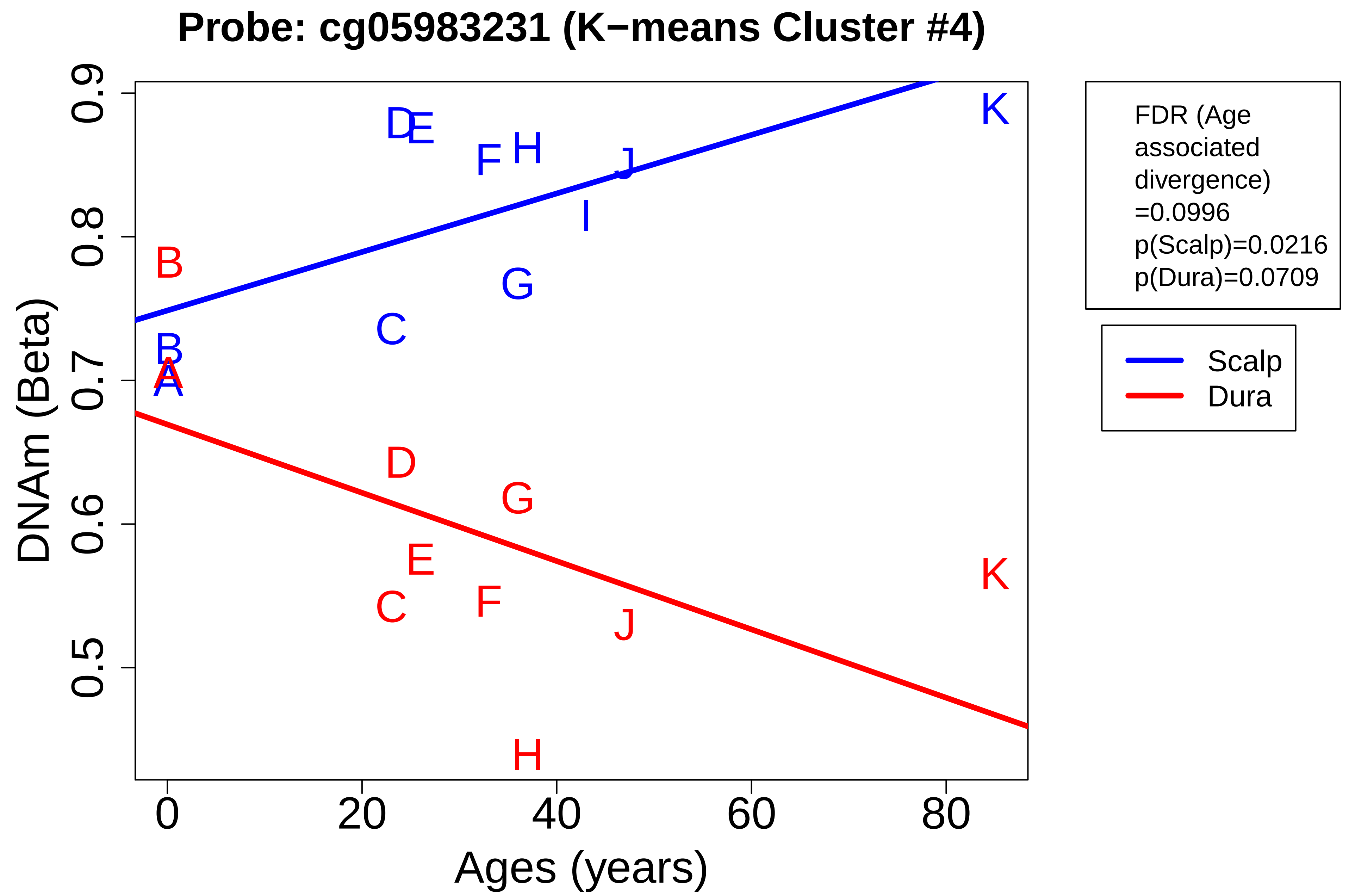

Probe: cg05983231 (K−means Cluster #4)
DNAm (Beta)
0.5
0.6
0.7
0.8
0.9
0
20
40
60
80
Ages (years)
A
B
C
D
E
F
G
H
I
J
K
A
B
C
D
E
F
G
H
J
K
FDR (Age associated divergence) =0.0996
p(Scalp)=0.0216
p(Dura)=0.0709
Scalp
Dura

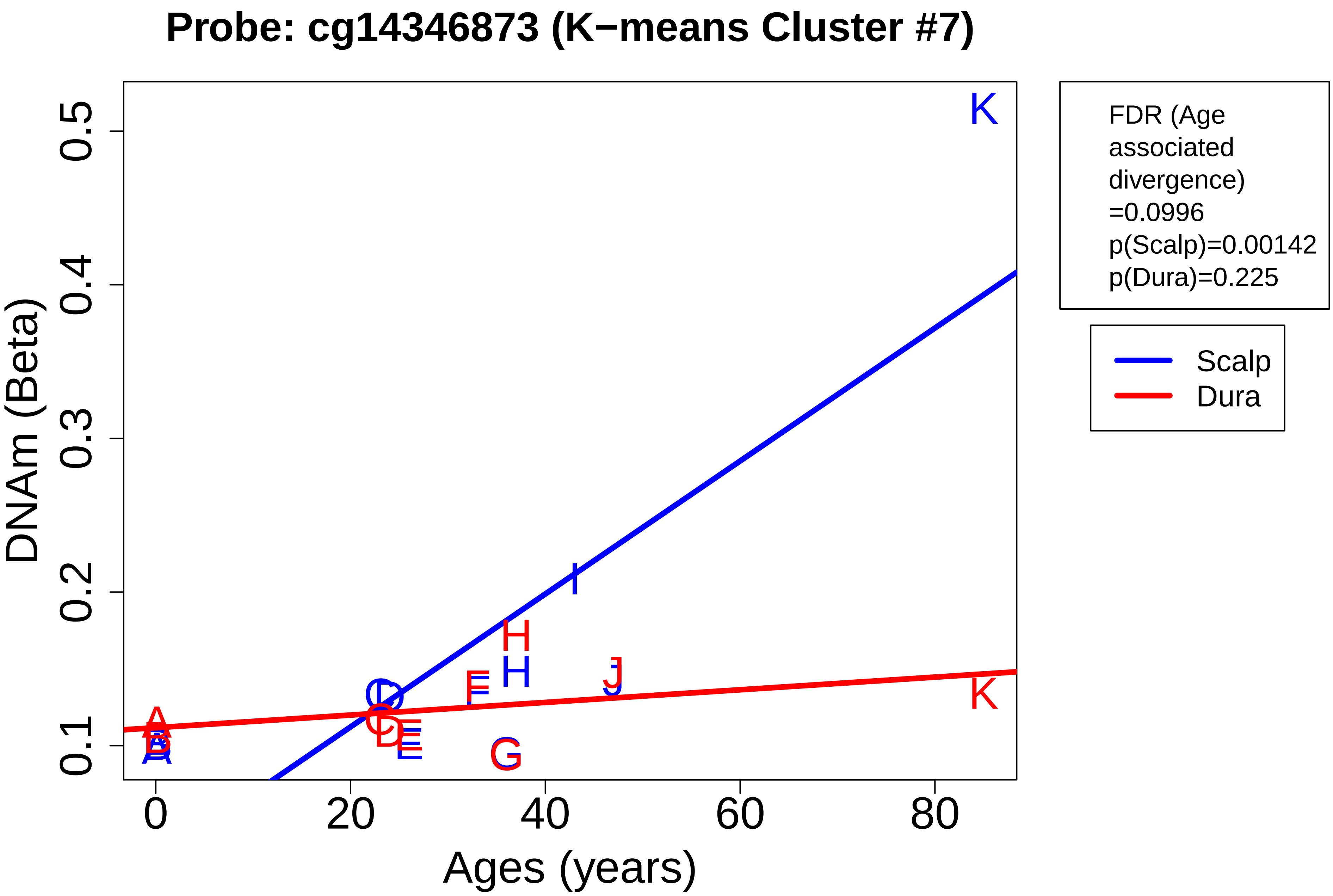
Probe: cg14346873 (K−means Cluster #7)
DNAm (Beta)
Ages (years)
0.1
0.2
0.3
0.4
0.5
0
20
40
60
80
FDR (Age associated divergence) =0.0996
p(Scalp)=0.00142
p(Dura)=0.225
Scalp
Dura

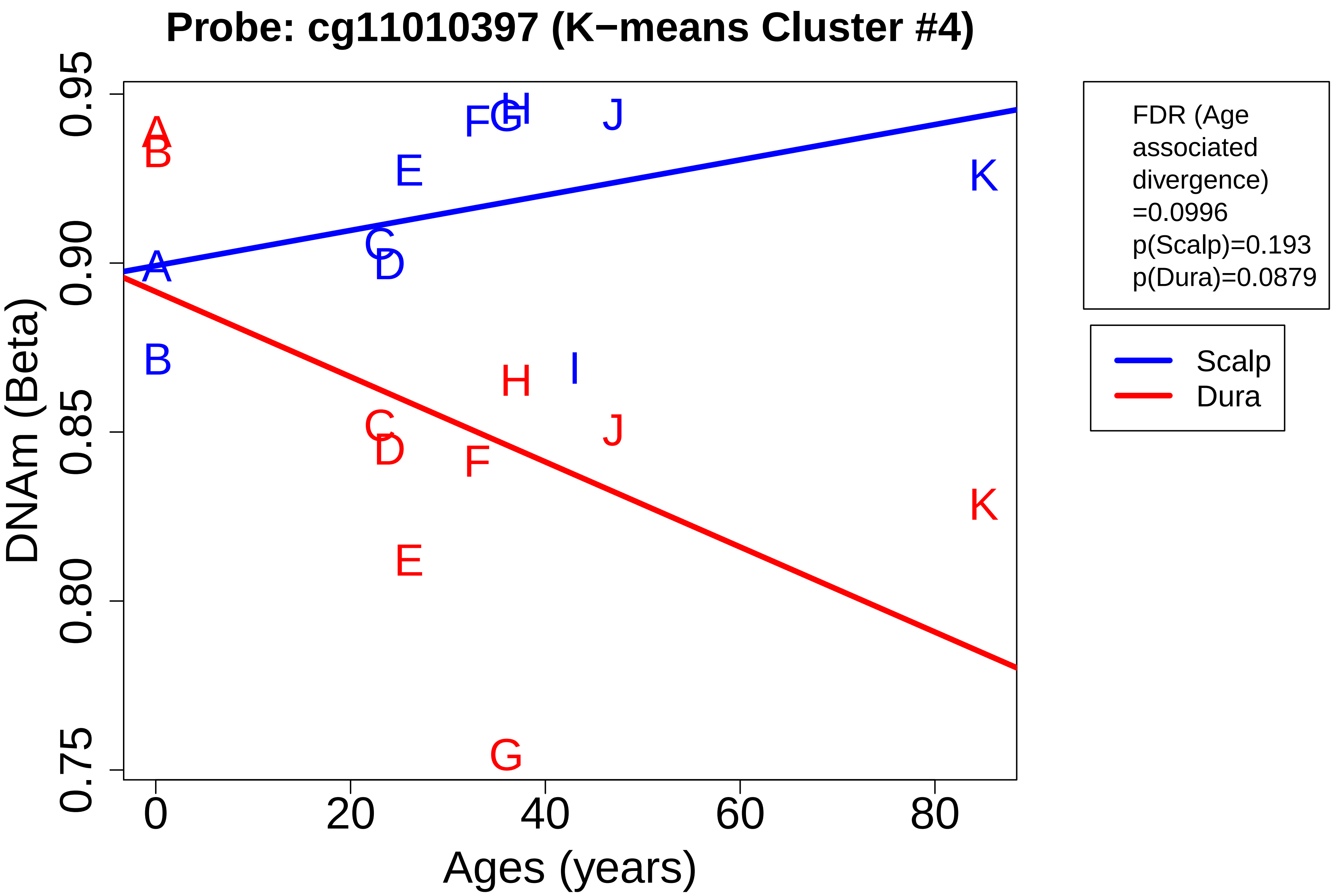

Probe: cg11010397 (K−means Cluster #4)
DNAm (Beta)
0.75
0.80
0.85
0.90
0.95
0
20
40
60
80
Ages (years)
FDR (Age associated divergence) =0.0996
p(Scalp)=0.193
p(Dura)=0.0879
Scalp
Dura

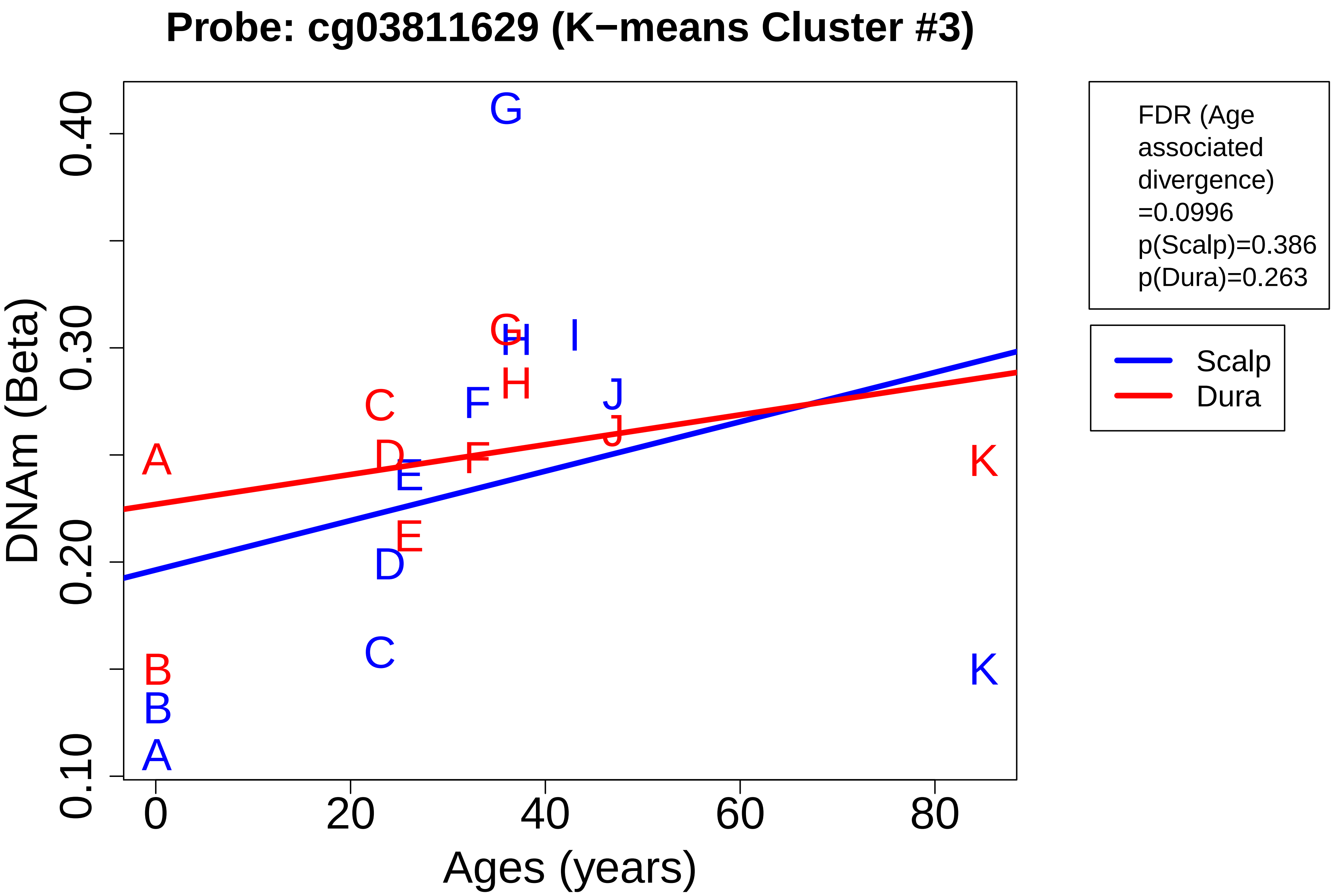

Probe: cg03811629 (K−means Cluster #3)
DNAm (Beta)
0.10
0.20
0.30
0.40
0
20
40
60
80
Ages (years)
FDR (Age associated divergence) =0.0996
p(Scalp)=0.386
p(Dura)=0.263
Scalp
Dura

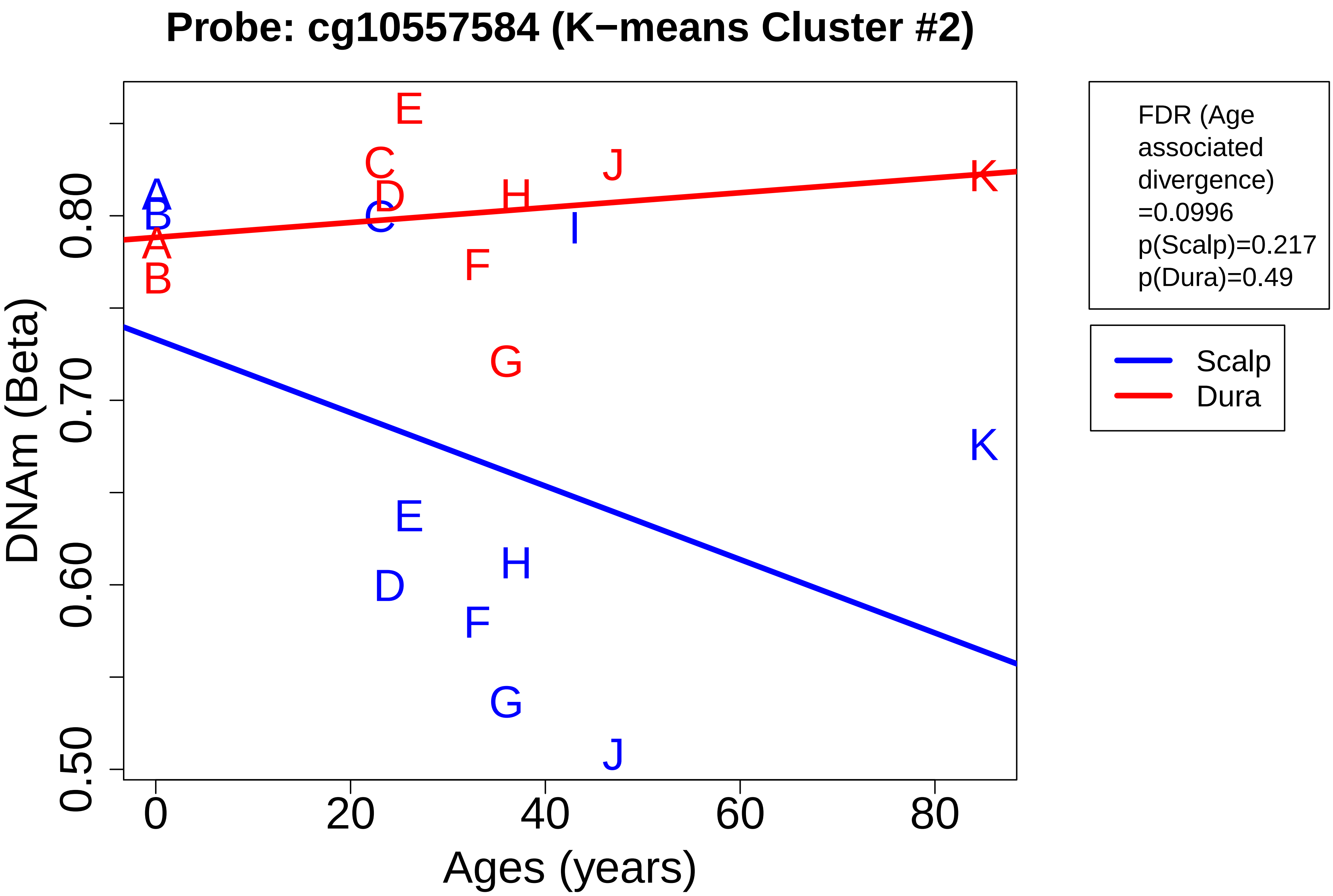

Probe: cg10557584 (K−means Cluster #2)
DNAm (Beta)
0.50
0.60
0.70
0.80
Ages (years)
0
20
40
60
80
FDR (Age associated divergence) =0.0996
p(Scalp)=0.217
p(Dura)=0.49
Scalp
Dura

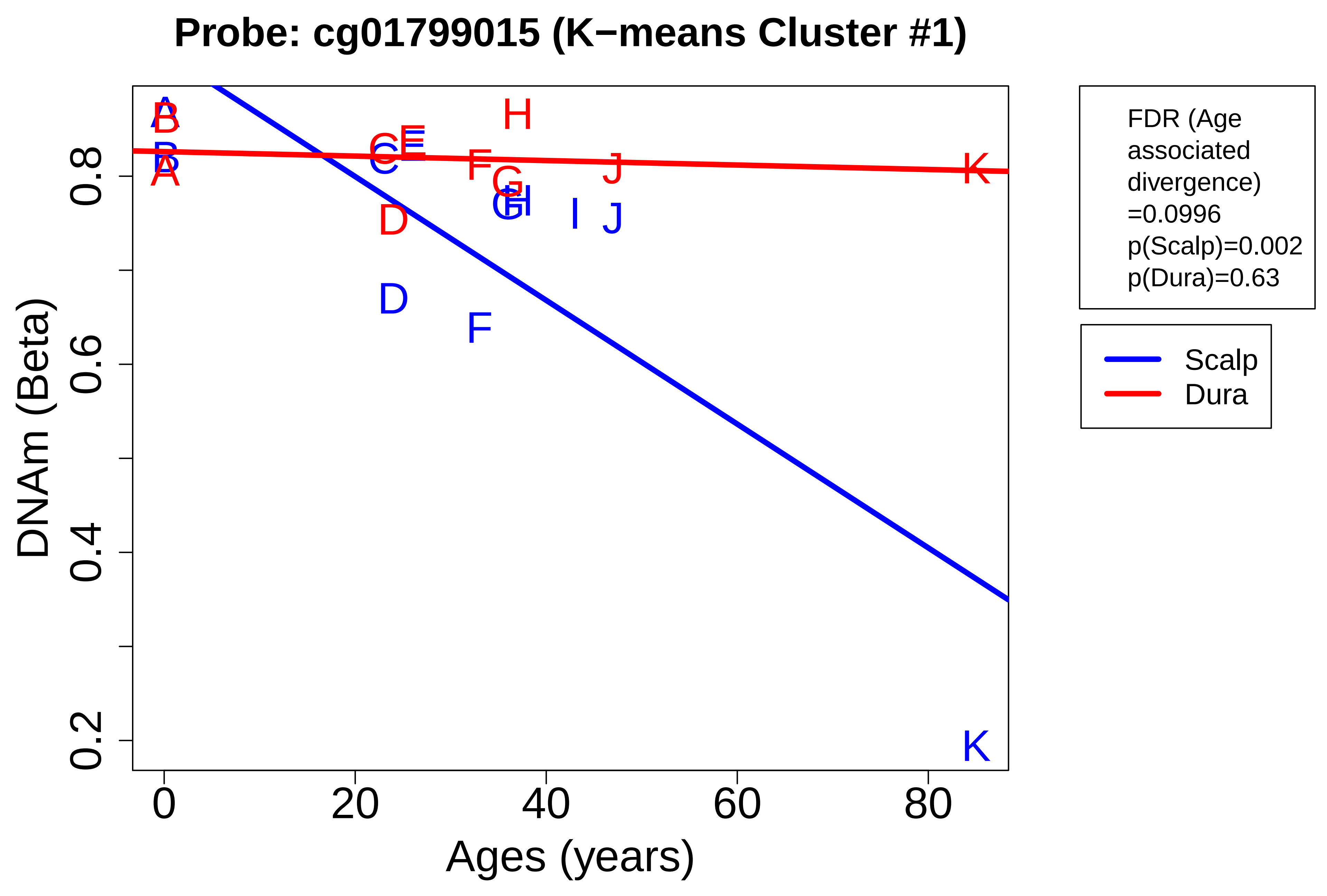

Probe: cg01799015 (K−means Cluster #1)
DNAm (Beta)
0.2
0.4
0.6
0.8
0
20
40
60
80
Ages (years)
FDR (Age associated divergence) =0.0996
p(Scalp)=0.002
p(Dura)=0.63
Scalp
Dura
A
B
C
D
E
F
G
H
I
J
K

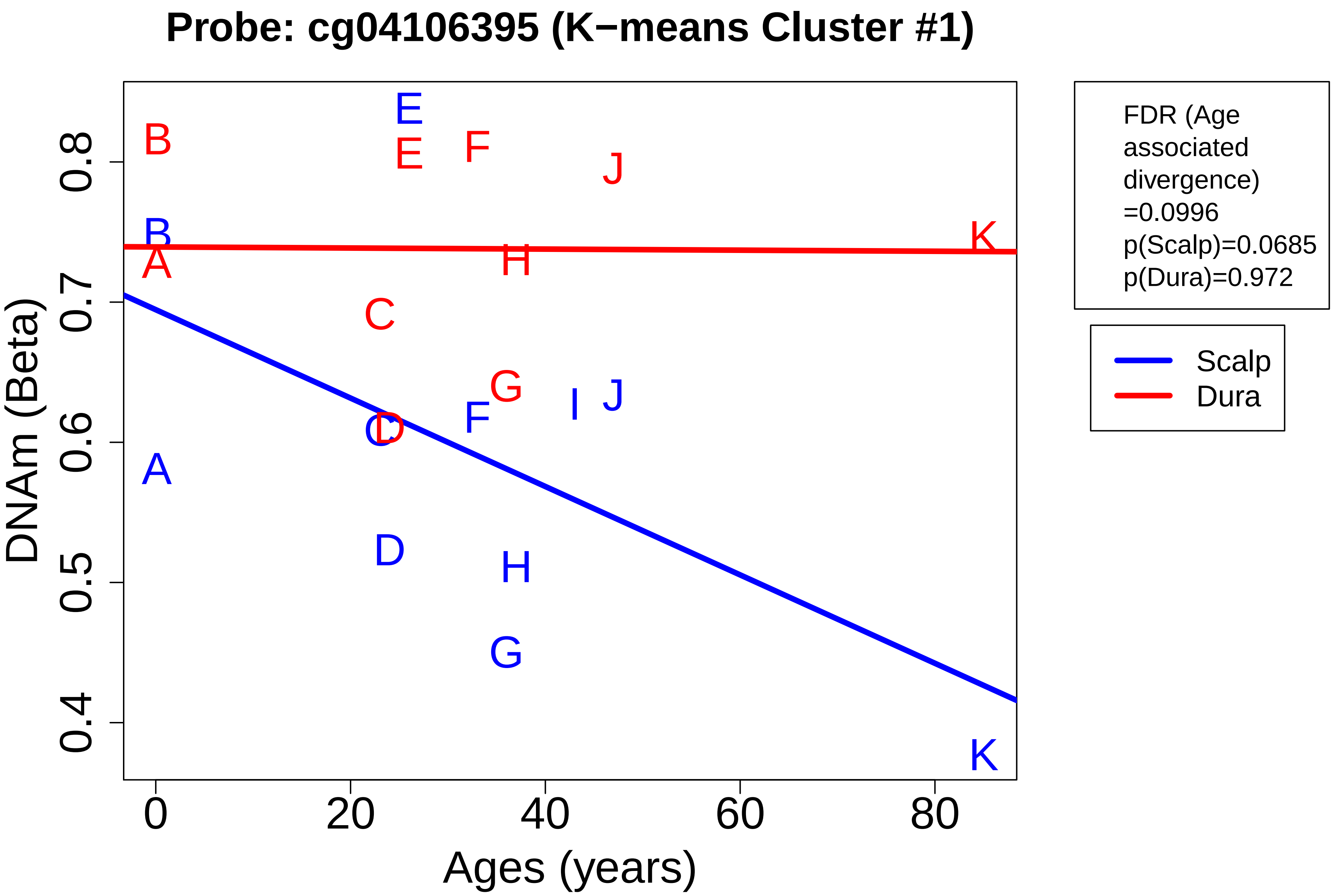

Probe: cg04106395 (K−means Cluster #1)
DNAm (Beta)
0.4
0.5
0.6
0.7
0.8
Ages (years)
0
20
40
60
80
FDR (Age associated divergence) =0.0996
p(Scalp)=0.0685
p(Dura)=0.972
Scalp
Dura
A
B
C
D
E
F
G
H
I
J
K

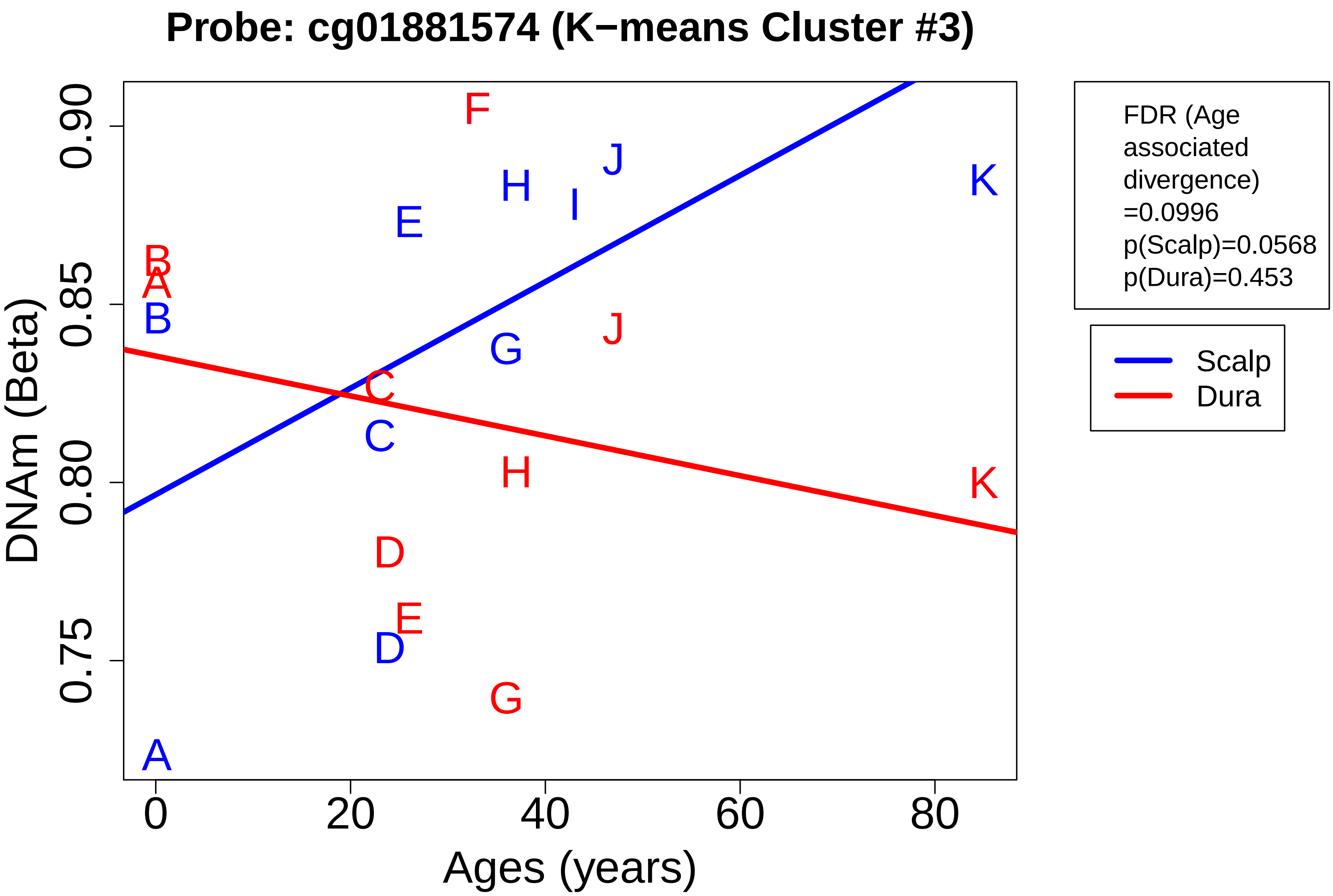

Probe: cg01881574 (K−means Cluster #3)
DNAm (Beta)
0.75
0.80
0.85
0.90
Ages (years)
0
20
40
60
80
FDR (Age associated divergence) =0.0996
p(Scalp)=0.0568
p(Dura)=0.453
Scalp
Dura
A
B
C
D
E
F
G
H
I
J
K

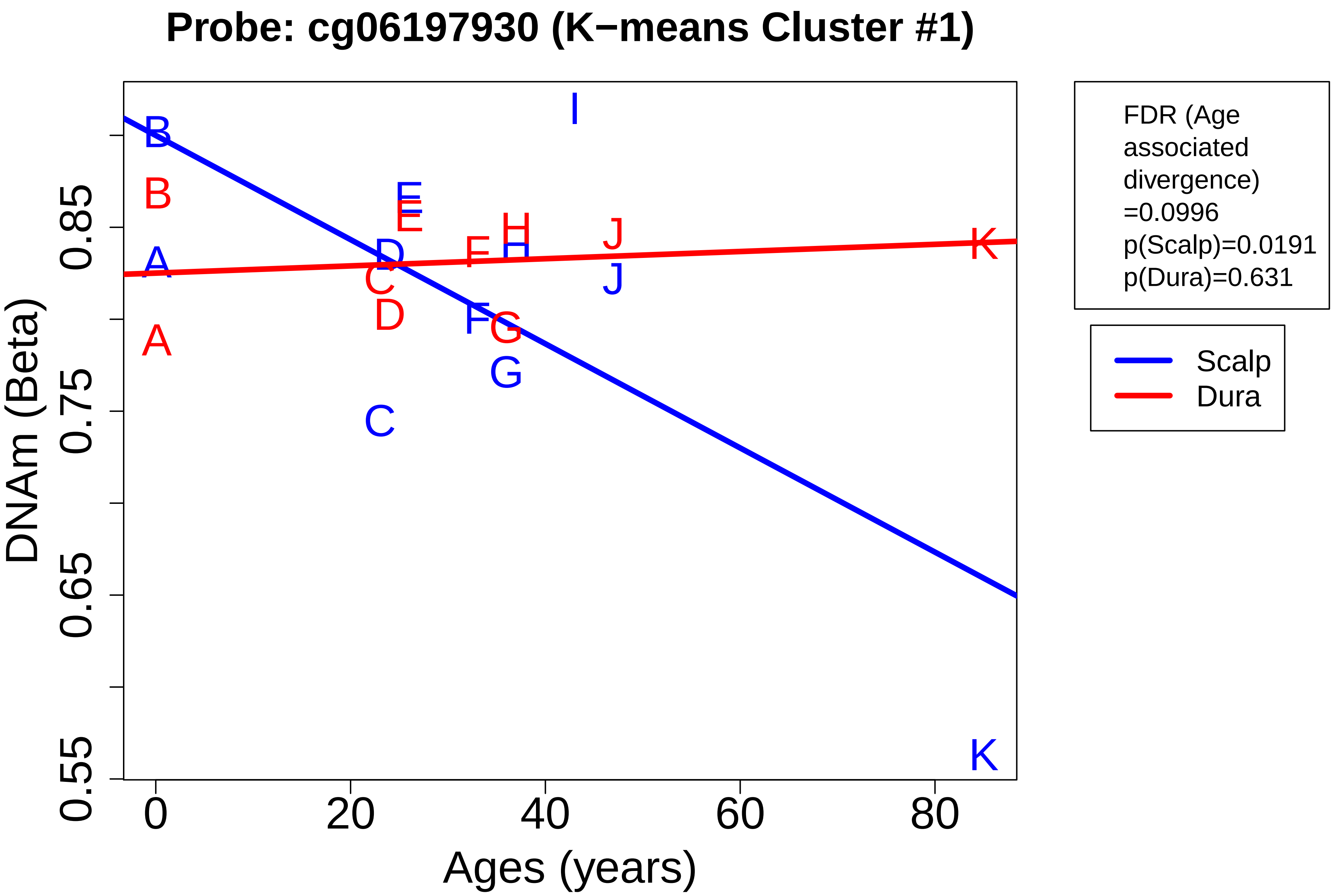

Probe: cg06197930 (K−means Cluster #1)
DNAm (Beta)
0.55
0.65
0.75
0.85
0
20
40
60
80
Ages (years)
FDR (Age associated divergence) =0.0996
p(Scalp)=0.0191
p(Dura)=0.631
Scalp
Dura

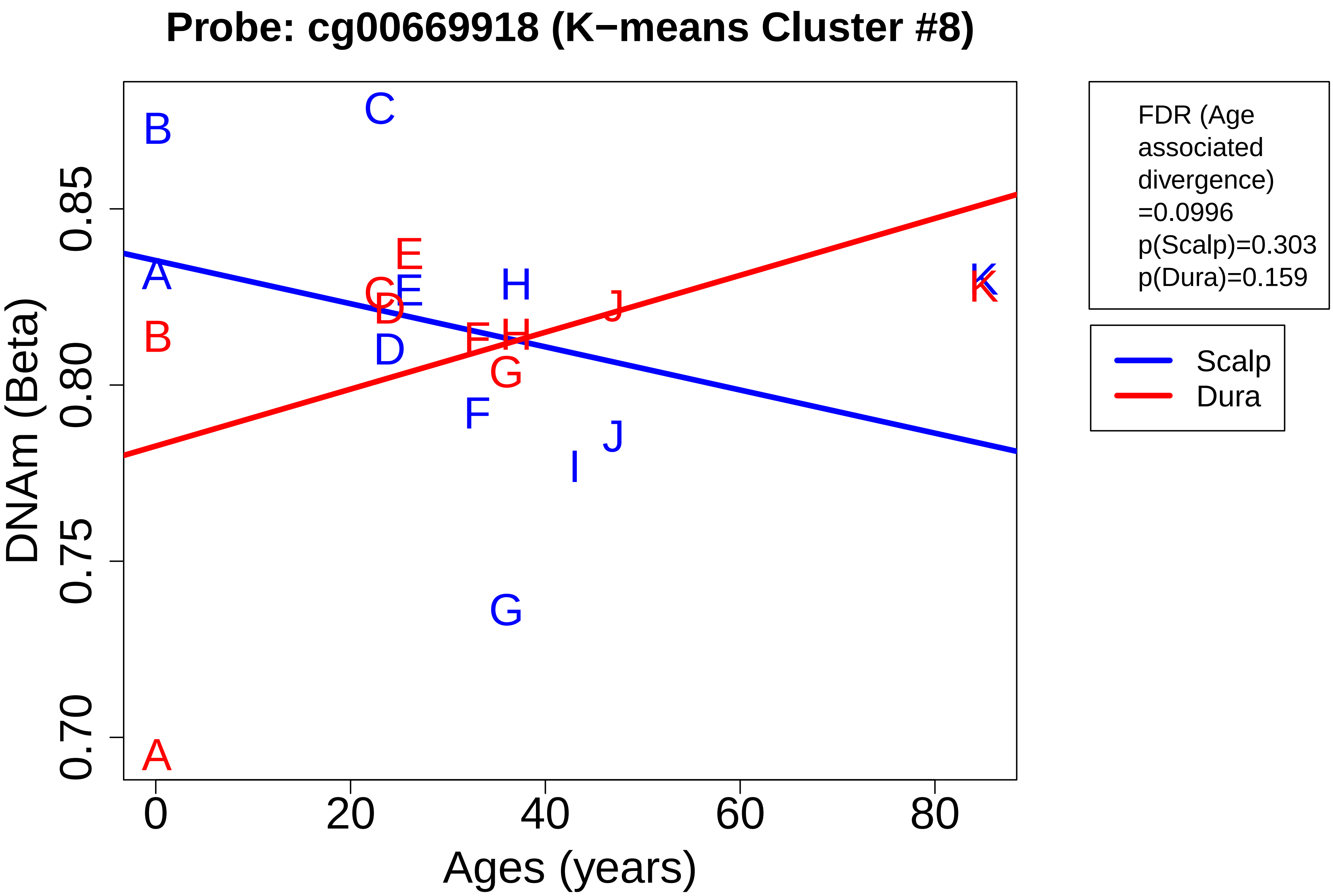

Probe: cg00669918 (K−means Cluster #8)
DNAm (Beta)
0.70
0.75
0.80
0.85
0
20
40
60
80
Ages (years)
FDR (Age associated divergence) =0.0996
p(Scalp)=0.303
p(Dura)=0.159
Scalp
Dura

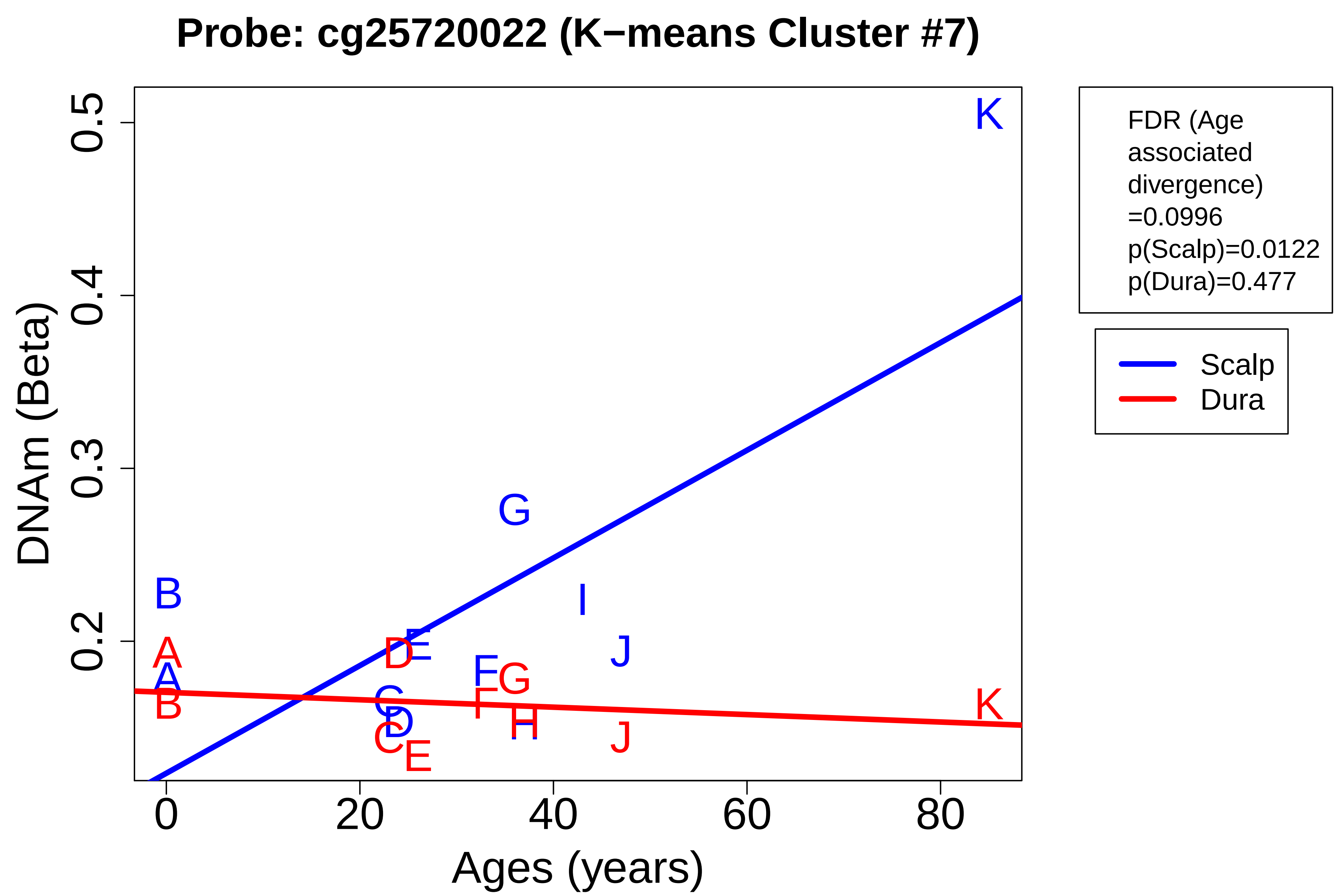
Probe: cg25720022 (K−means Cluster #7)
DNAm (Beta)
0.2
0.3
0.4
0.5
Ages (years)
0
20
40
60
80
FDR (Age associated divergence) =0.0996
p(Scalp)=0.0122
p(Dura)=0.477
Scalp
Dura
A
B
C
D
E
F
G
H
I
J
K

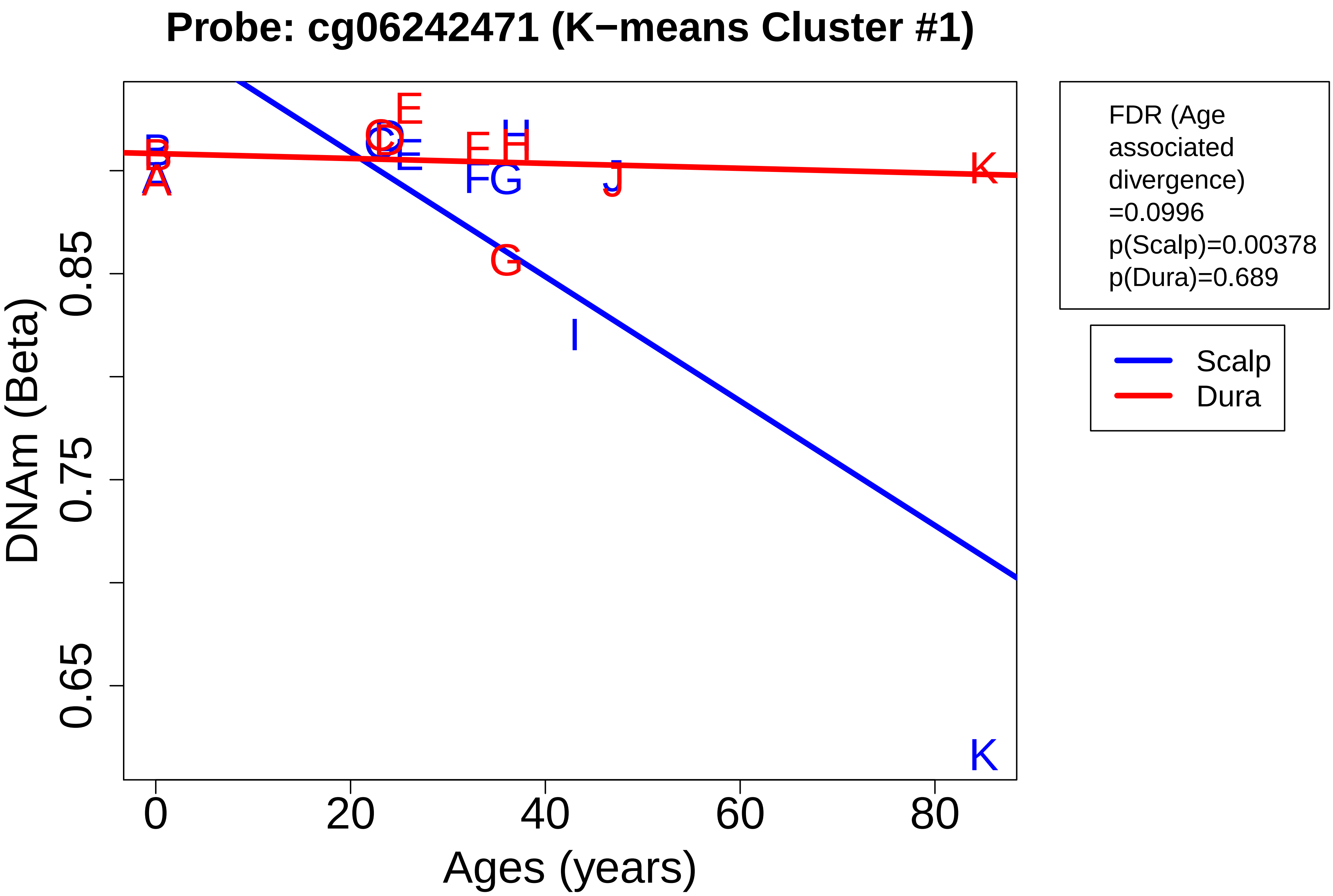
Probe: cg06242471 (K−means Cluster #1)
DNAm (Beta)
0.65
0.75
0.85
Ages (years)
0
20
40
60
80
FDR (Age associated divergence) =0.0996
p(Scalp)=0.00378
p(Dura)=0.689
Scalp
Dura

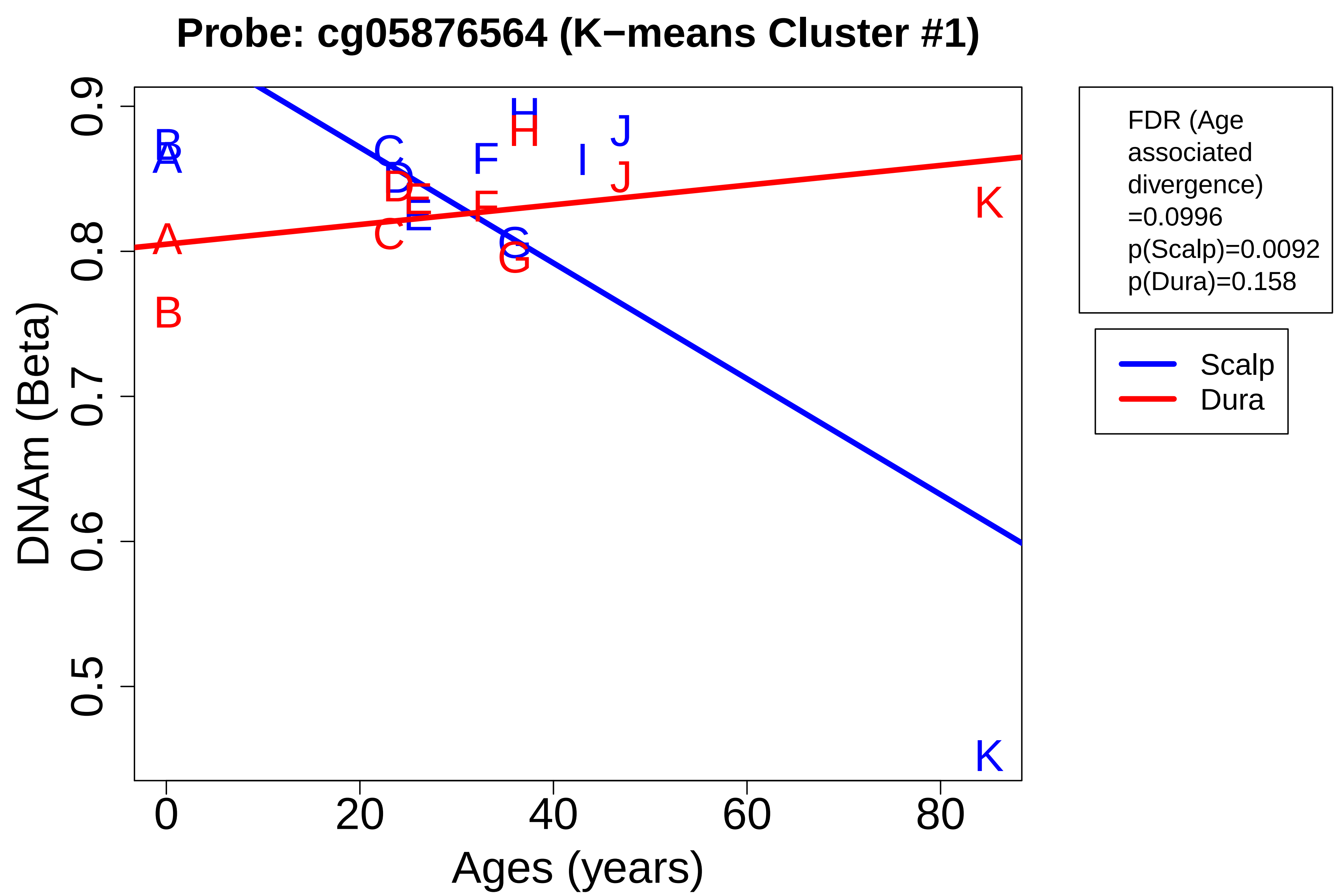

Probe: cg05876564 (K−means Cluster #1)
DNAm (Beta)
0.5
0.6
0.7
0.8
0.9
0
20
40
60
80
Ages (years)
A
B
C
D
E
F
G
H
I
J
K
FDR (Age associated divergence) =0.0996
p(Scalp)=0.0092
p(Dura)=0.158
Scalp
Dura

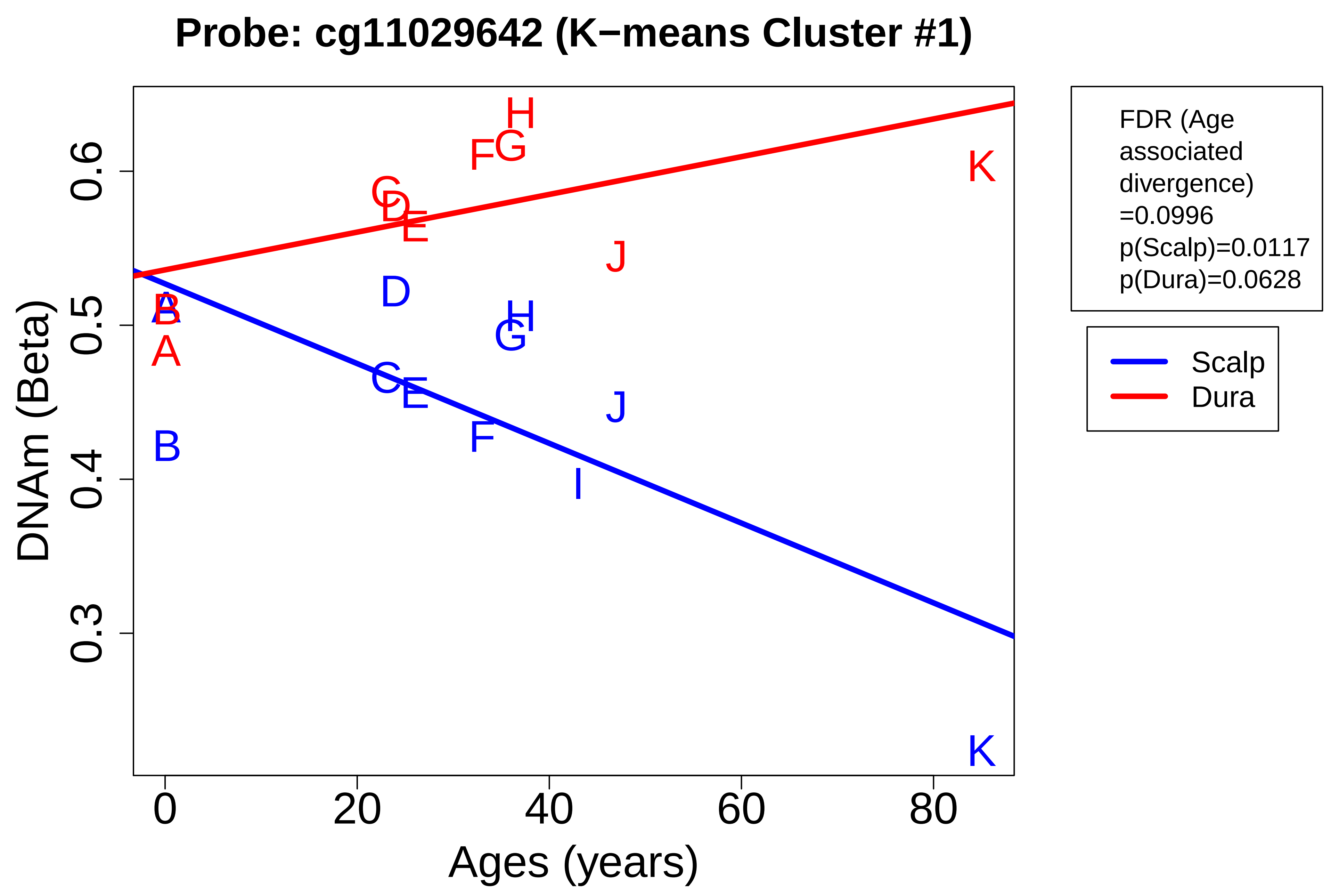
Probe: cg11029642 (K−means Cluster #1)
FDR (Age associated divergence) =0.0996
p(Scalp)=0.0117
p(Dura)=0.0628
Scalp
Dura
DNAm (Beta)
Ages (years)
0.3
0.4
0.5
0.6
0
20
40
60
80
A
B
C
D
E
F
G
H
I
J
K

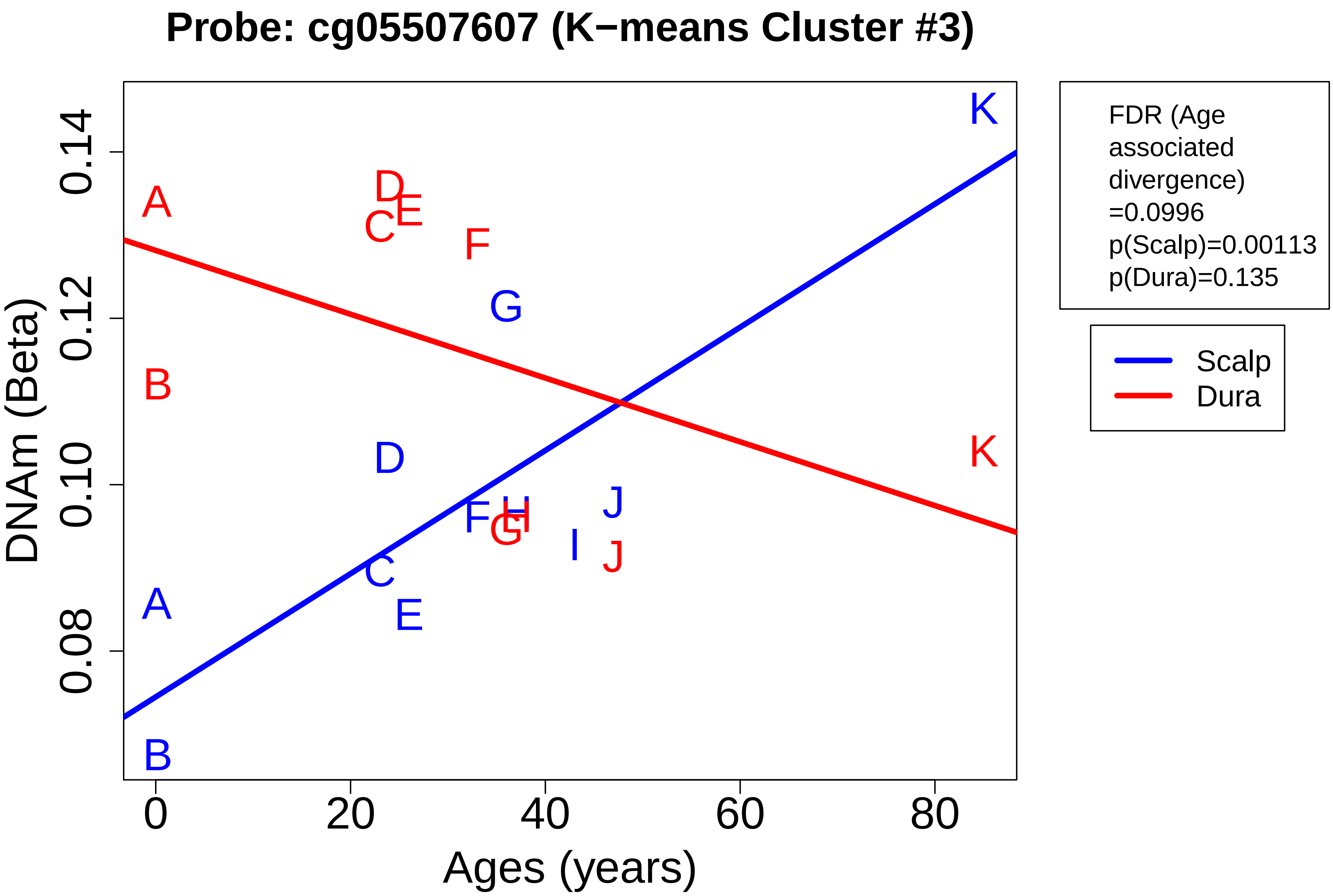

Probe: cg05507607 (K−means Cluster #3)
DNAm (Beta)
0.08
0.10
0.12
0.14
0
20
40
60
80
Ages (years)
FDR (Age associated divergence) =0.0996
p(Scalp)=0.00113
p(Dura)=0.135
Scalp
Dura
A
B
C
D
E
F
G
H
I
J
K

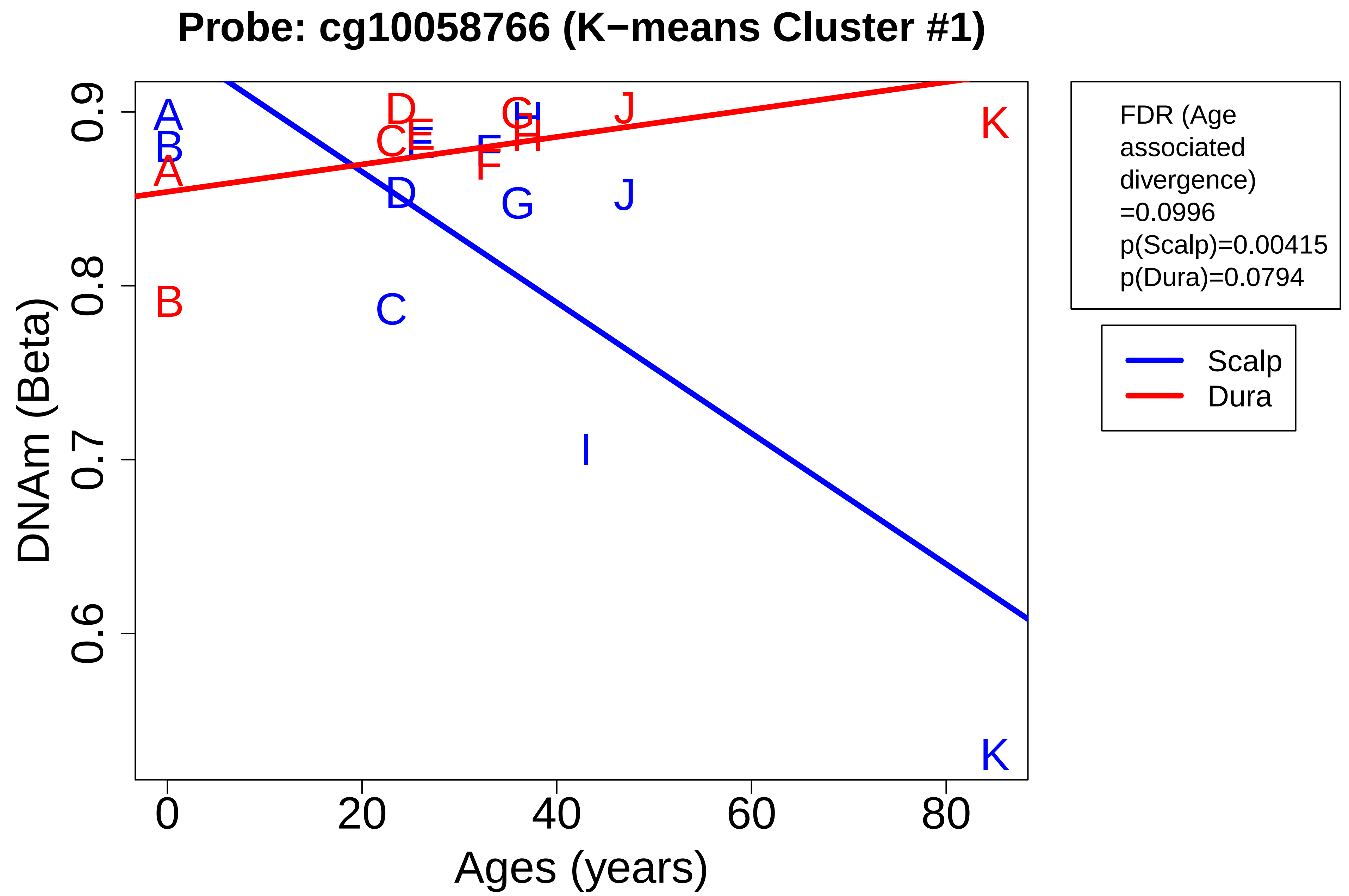

Probe: cg10058766 (K−means Cluster #1)
DNAm (Beta)
0.6
0.7
0.8
0.9
Ages (years)
0
20
40
60
80
FDR (Age associated divergence) =0.0996
p(Scalp)=0.00415
p(Dura)=0.0794
Scalp
Dura

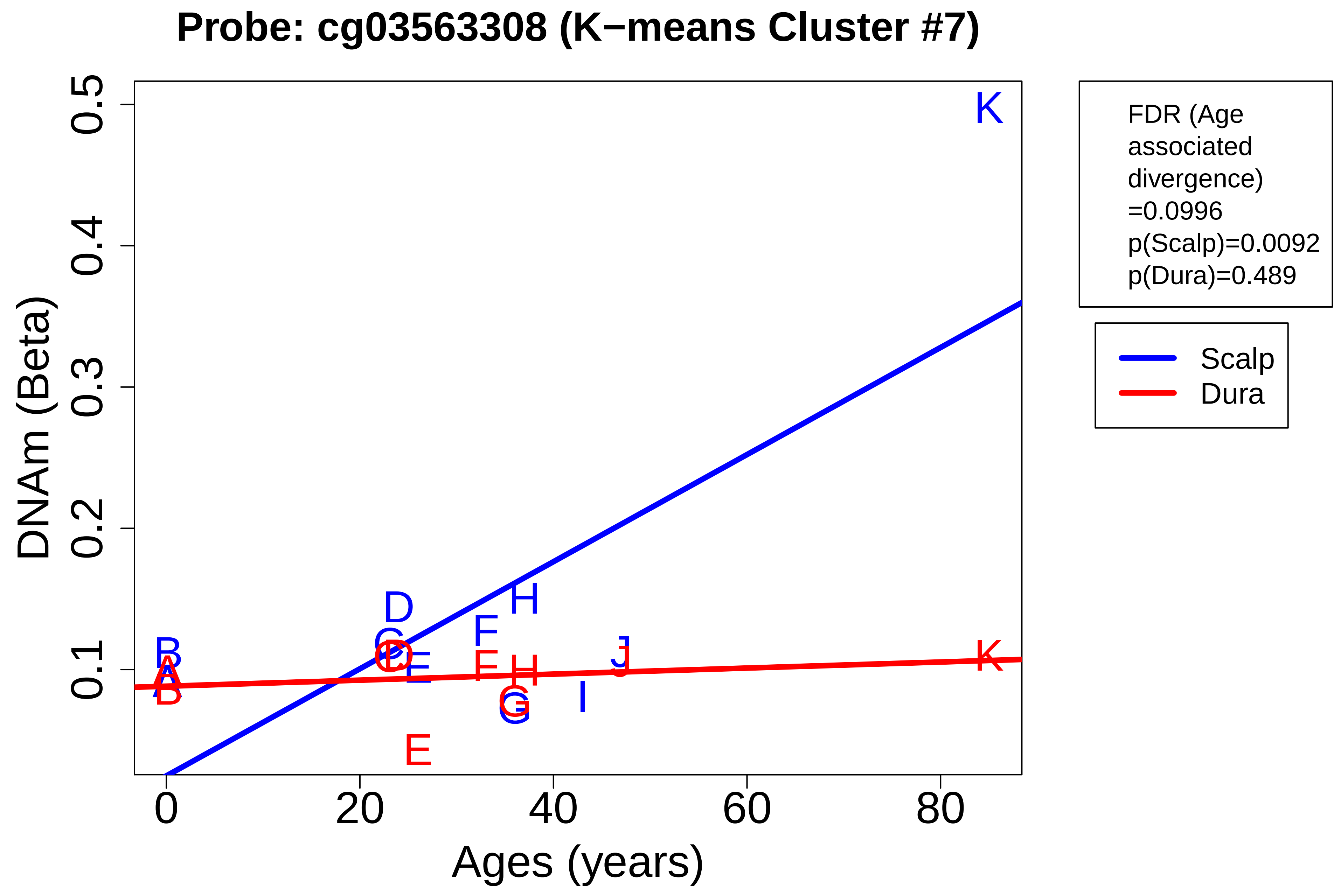

Probe: cg03563308 (K−means Cluster #7)
DNAm (Beta)
0.1
0.2
0.3
0.4
0.5
0
20
40
60
80
Ages (years)
FDR (Age associated divergence) =0.0996
p(Scalp)=0.0092
p(Dura)=0.489
Scalp
Dura

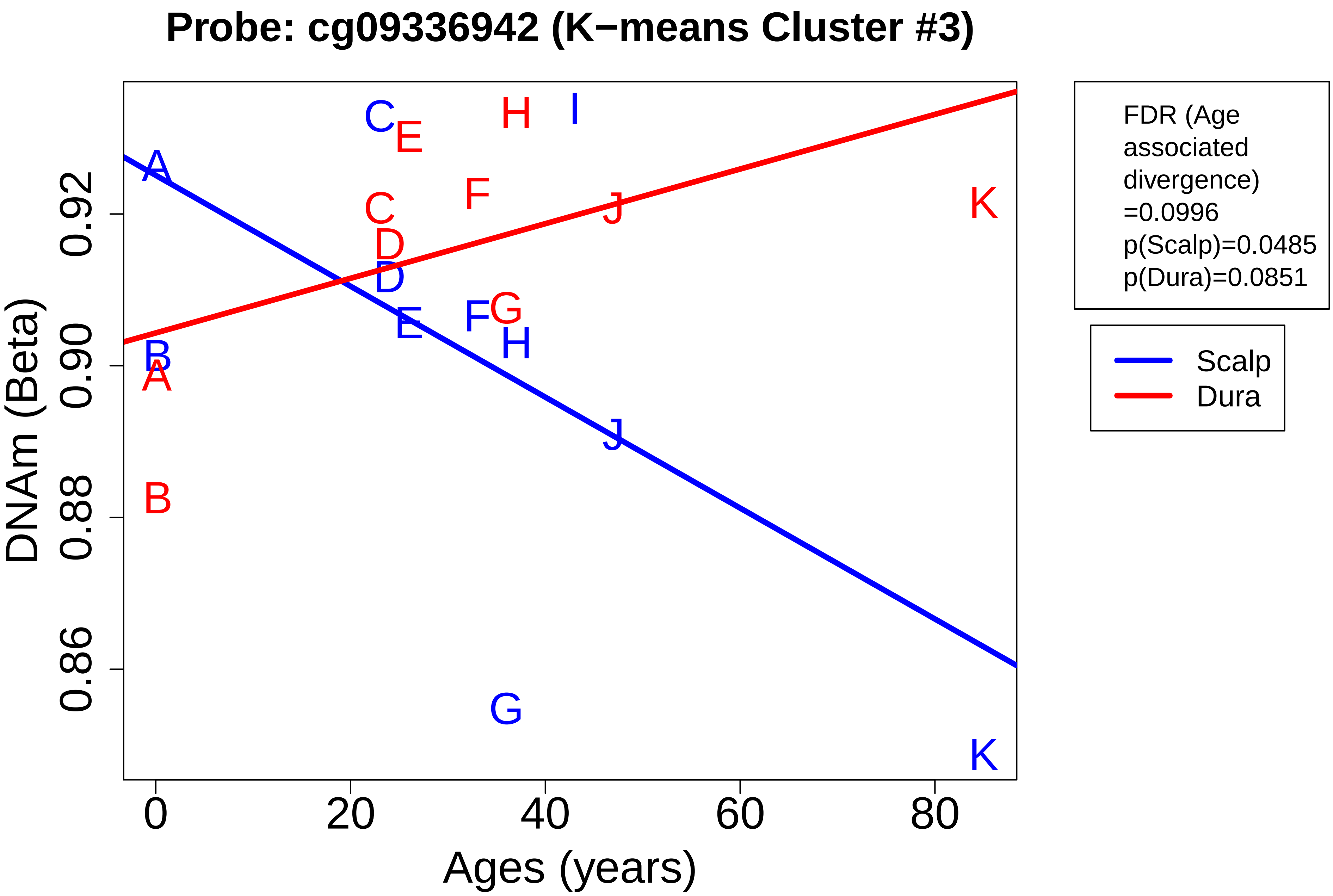

Probe: cg09336942 (K−means Cluster #3)
DNAm (Beta)
0.86
0.88
0.90
0.92
Ages (years)
0
20
40
60
80
A
B
C
D
E
F
G
H
I
J
K
FDR (Age associated divergence) =0.0996
p(Scalp)=0.0485
p(Dura)=0.0851
Scalp
Dura

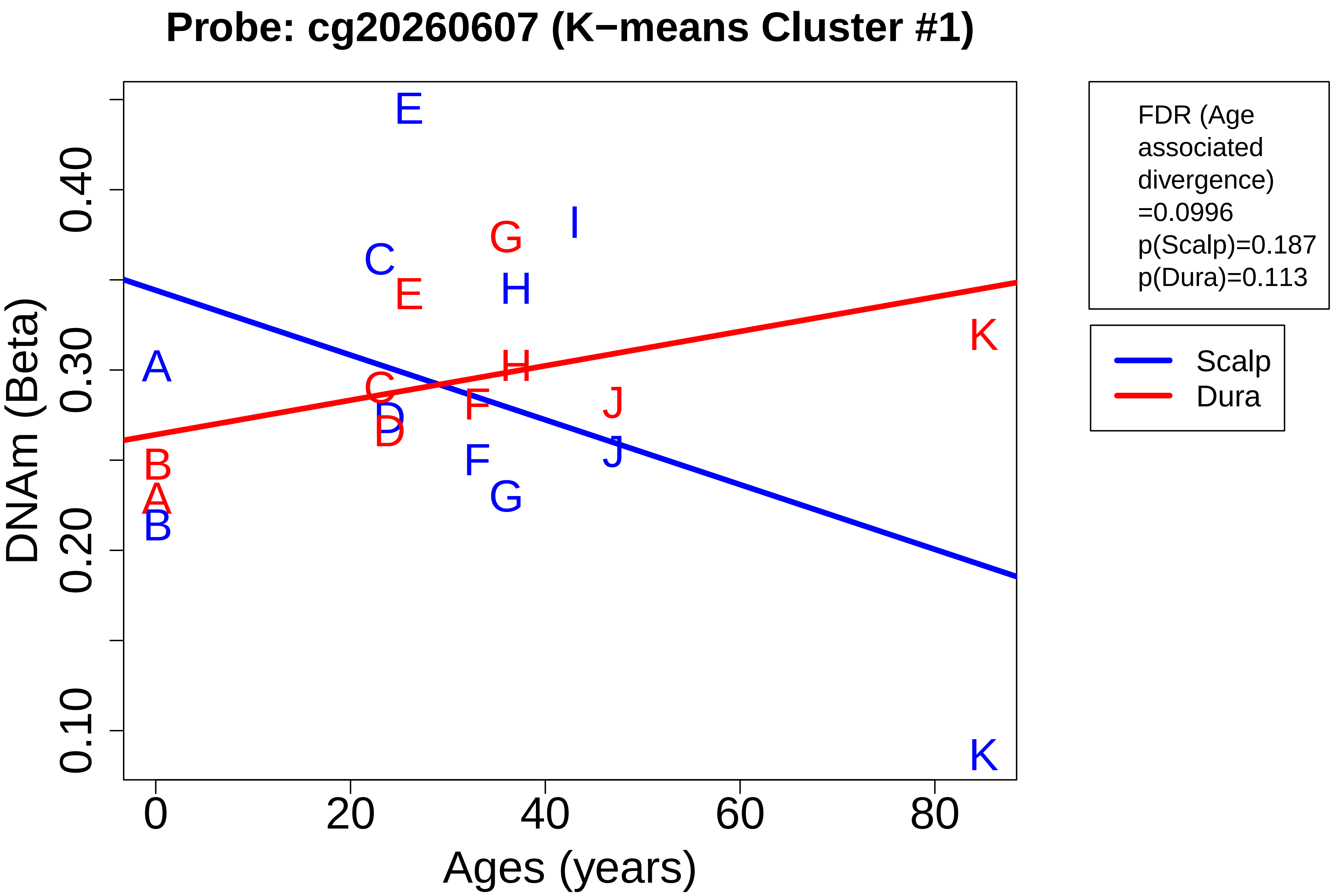
Probe: cg20260607 (K−means Cluster #1)
DNAm (Beta)
0.10
0.20
0.30
0.40
0
20
40
60
80
Ages (years)
A
B
C
D
E
F
G
H
I
J
K
FDR (Age associated divergence) =0.0996
p(Scalp)=0.187
p(Dura)=0.113
Scalp
Dura

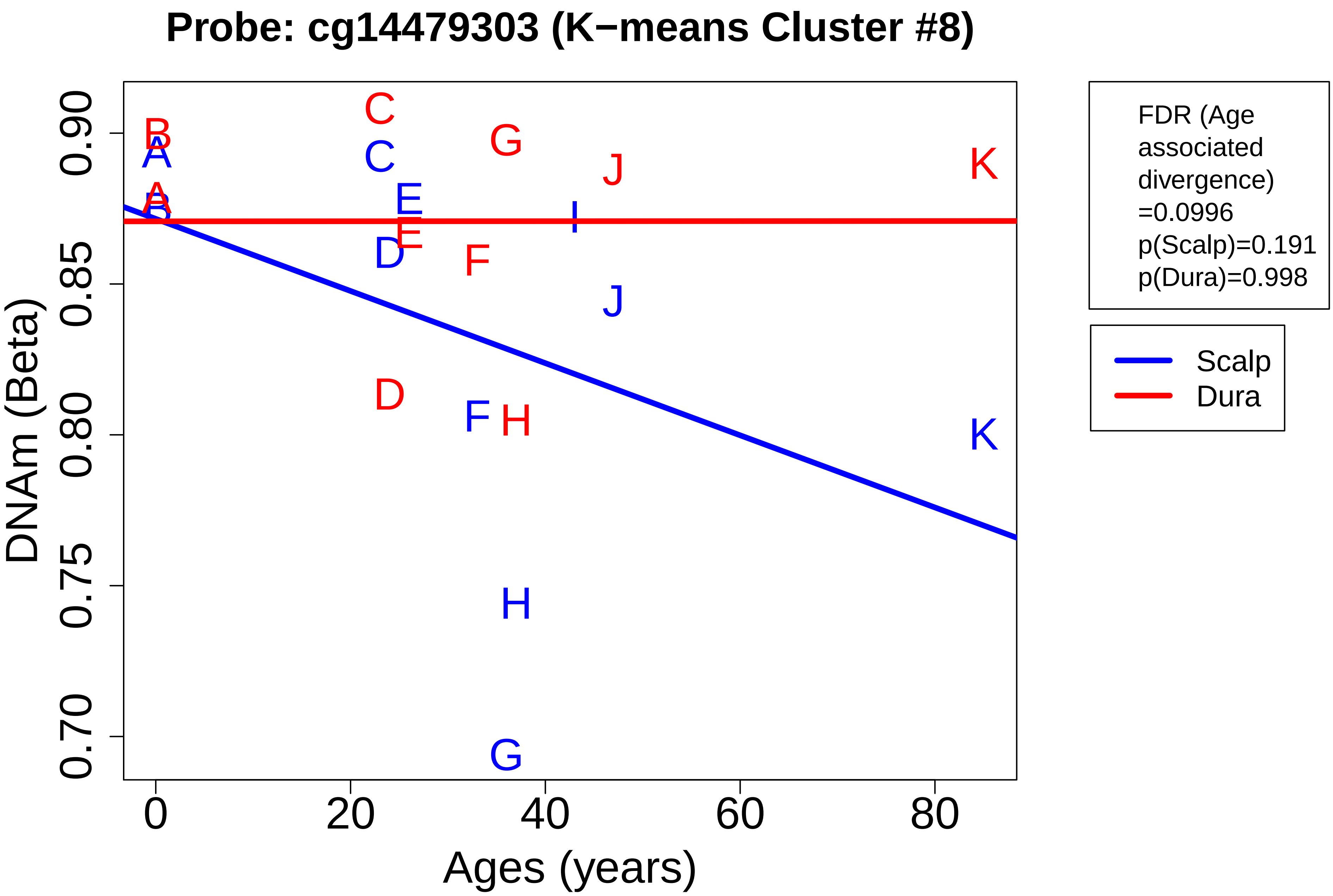

Probe: cg14479303 (K−means Cluster #8)
DNAm (Beta)
0.70
0.75
0.80
0.85
0.90
0
20
40
60
80
Ages (years)
FDR (Age associated divergence) =0.0996
p(Scalp)=0.191
p(Dura)=0.998
Scalp
Dura

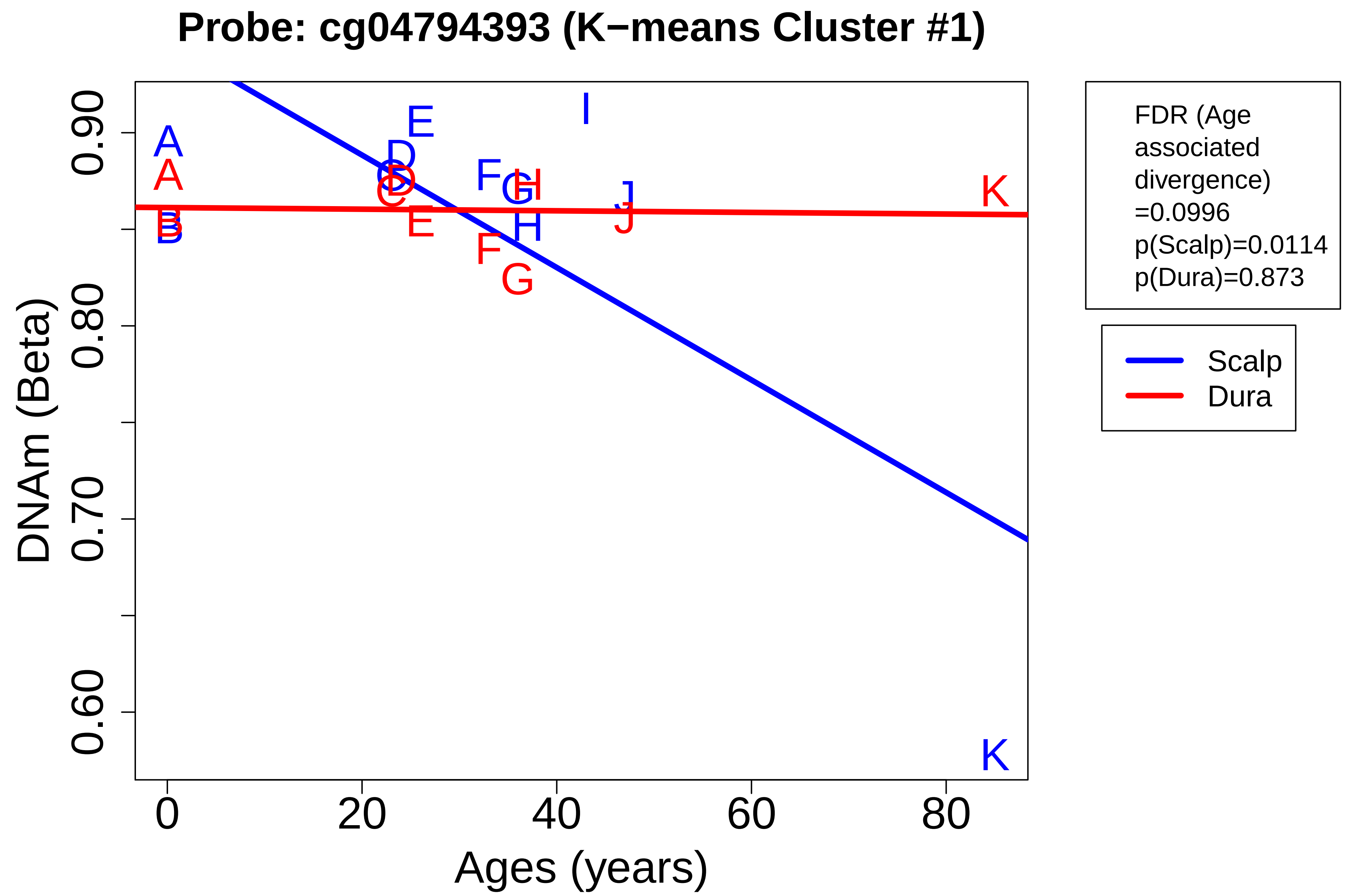

Probe: cg04794393 (K−means Cluster #1)
DNAm (Beta)
0.60
0.70
0.80
0.90
Ages (years)
0
20
40
60
80
A
B
C
D
E
F
G
H
I
J
K
FDR (Age associated divergence) =0.0996
p(Scalp)=0.0114
p(Dura)=0.873
Scalp
Dura

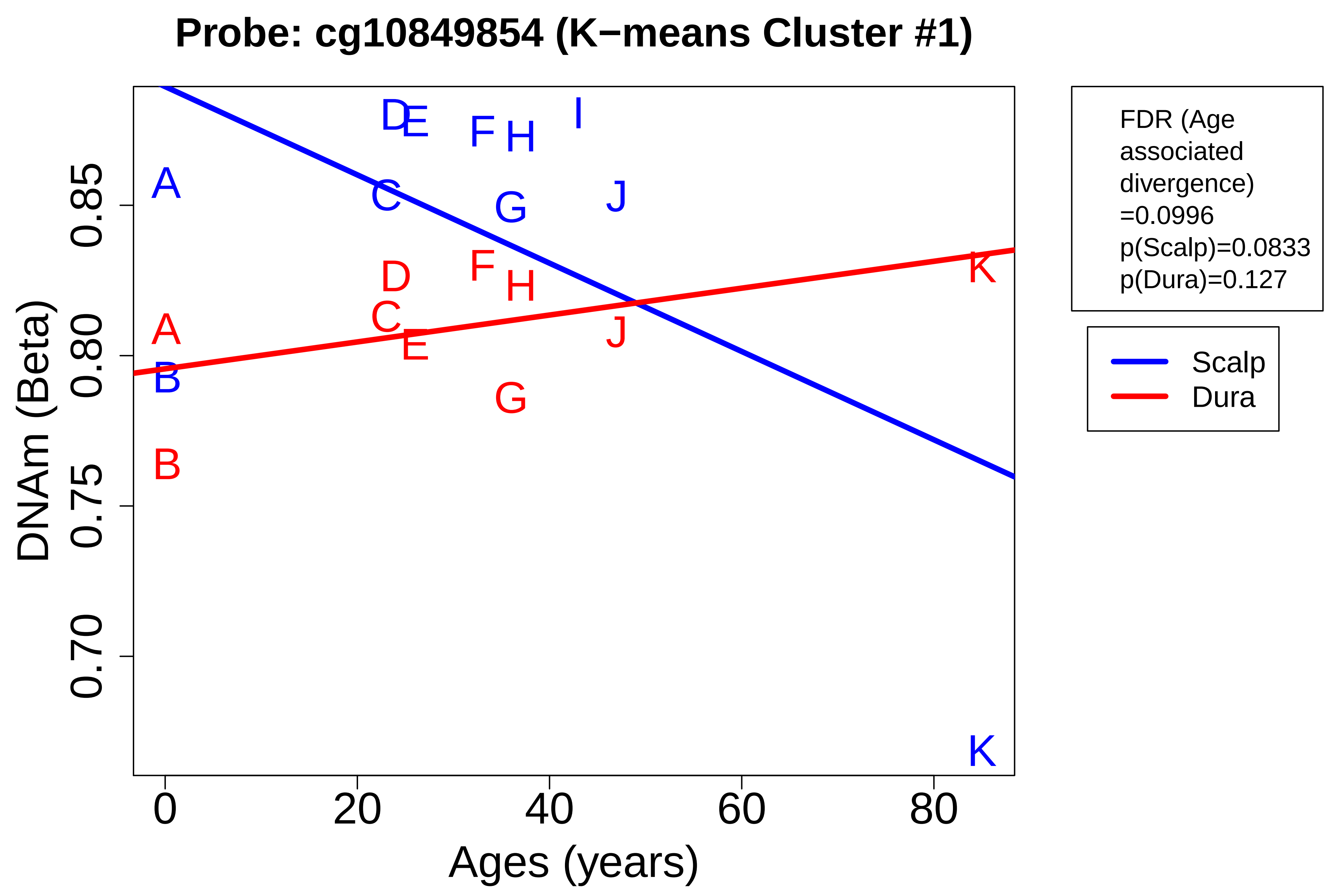

Probe: cg10849854 (K−means Cluster #1)
DNAm (Beta)
0.70
0.75
0.80
0.85
Ages (years)
0
20
40
60
80
A
B
C
D
E
F
G
H
I
J
K
FDR (Age associated divergence) =0.0996
p(Scalp)=0.0833
p(Dura)=0.127
Scalp
Dura

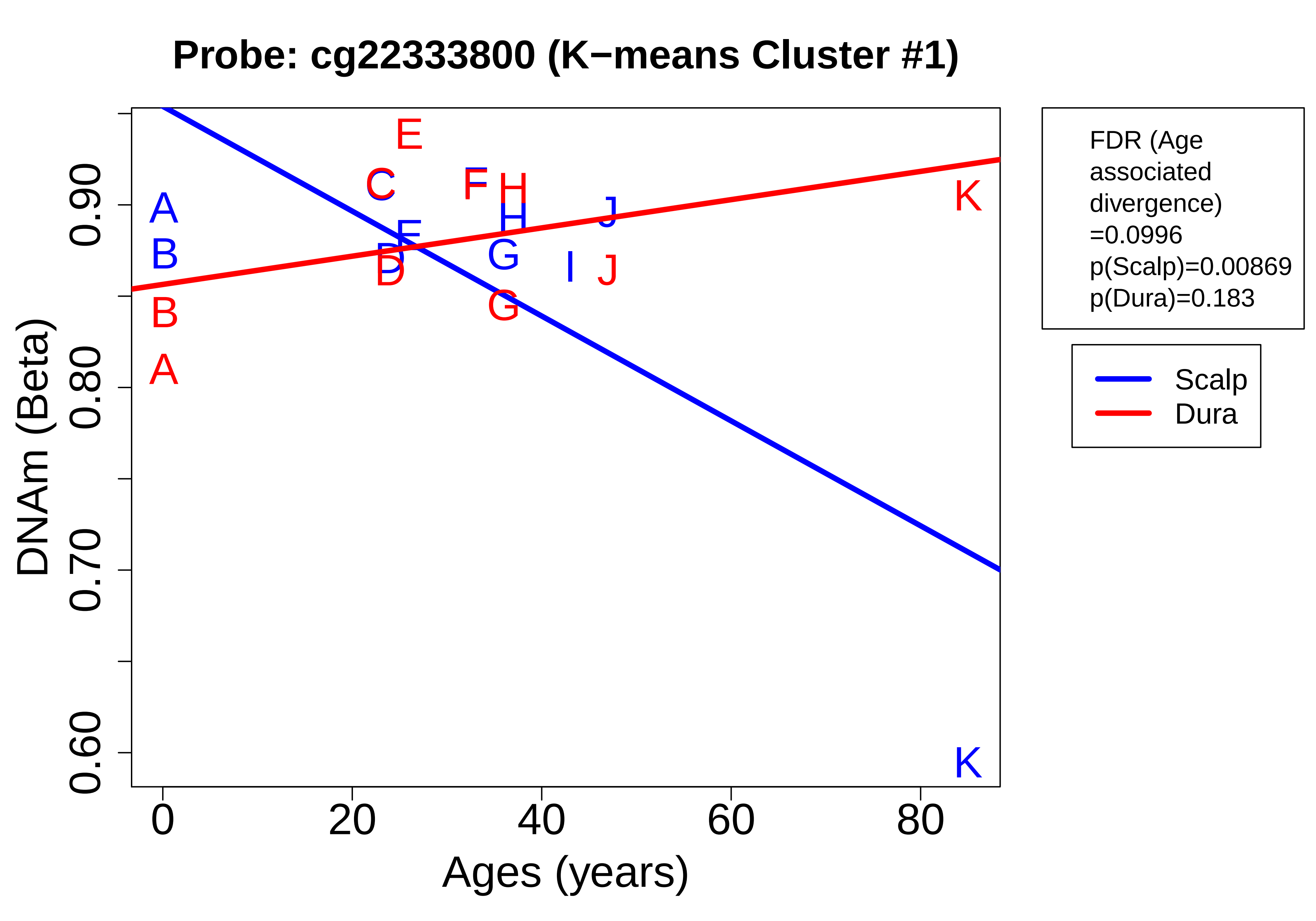

Probe: cg22333800 (K−means Cluster #1)
DNAm (Beta)
0.60
0.70
0.80
0.90
0
20
40
60
80
Ages (years)
A
B
C
D
E
F
G
H
I
J
K
FDR (Age associated divergence) =0.0996
p(Scalp)=0.00869
p(Dura)=0.183
Scalp
Dura

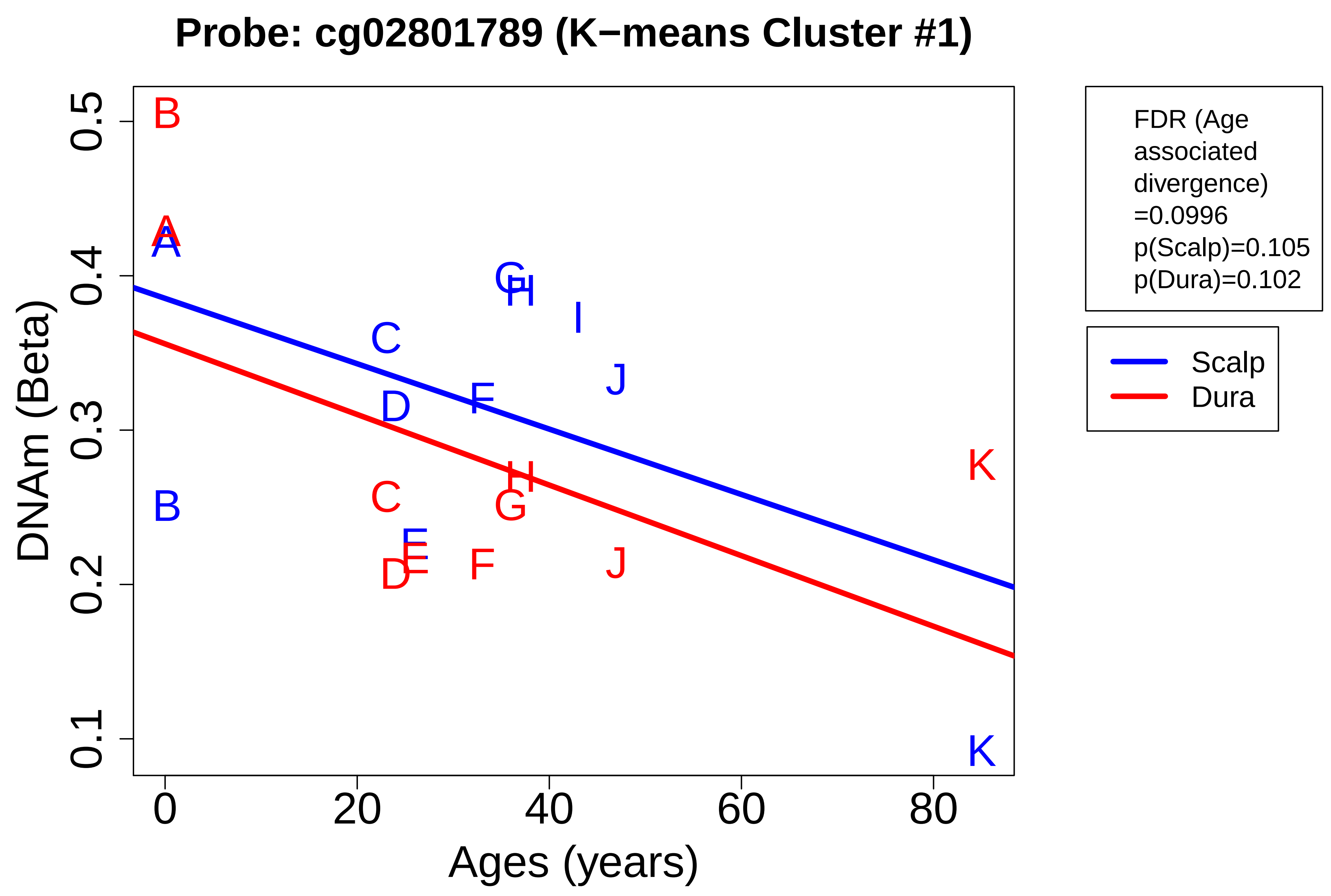

Probe: cg02801789 (K−means Cluster #1)
DNAm (Beta)
0.1
0.2
0.3
0.4
0.5
0
20
40
60
80
Ages (years)
FDR (Age associated divergence) =0.0996
p(Scalp)=0.105
p(Dura)=0.102
Scalp
Dura

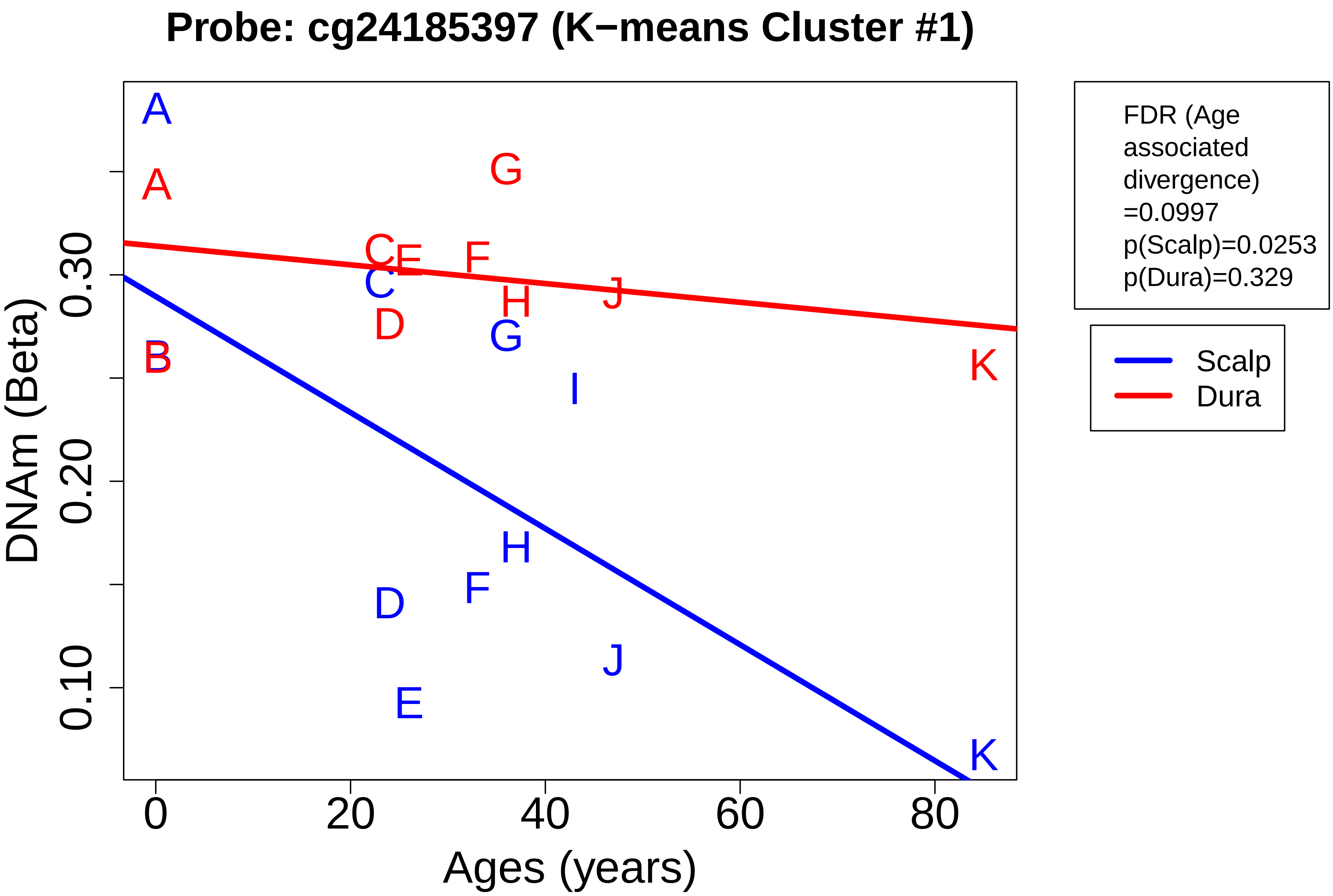

Probe: cg24185397 (K−means Cluster #1)
DNAm (Beta)
0.10
0.20
0.30
Ages (years)
0
20
40
60
80
FDR (Age associated divergence) =0.0997
p(Scalp)=0.0253
p(Dura)=0.329
Scalp
Dura
A
B
C
D
E
F
G
H
I
J
K

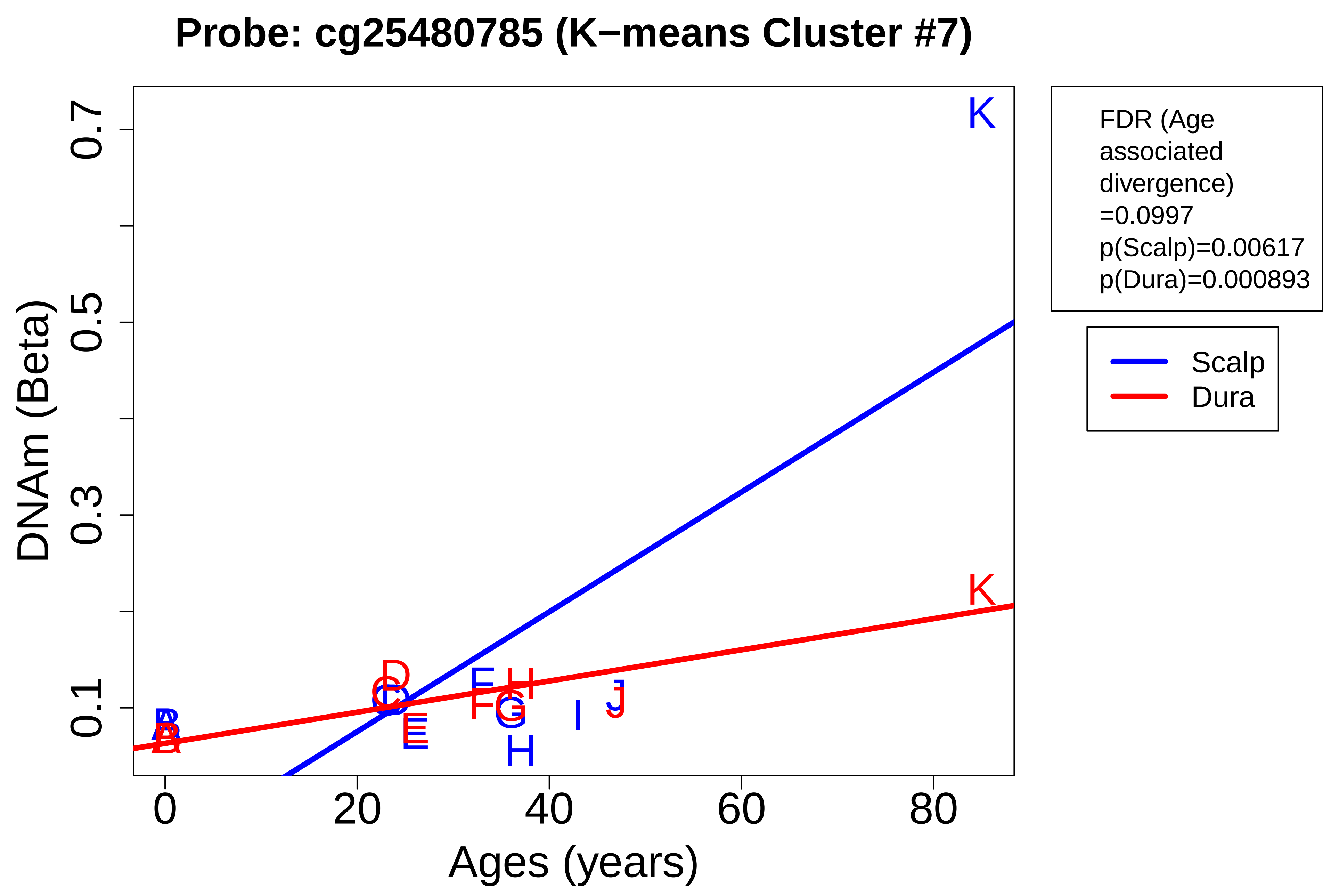

Probe: cg25480785 (K−means Cluster #7)
DNAm (Beta)
0.1
0.3
0.5
0.7
0
20
40
60
80
Ages (years)
FDR (Age associated divergence) =0.0997
p(Scalp)=0.00617
p(Dura)=0.000893
Scalp
Dura

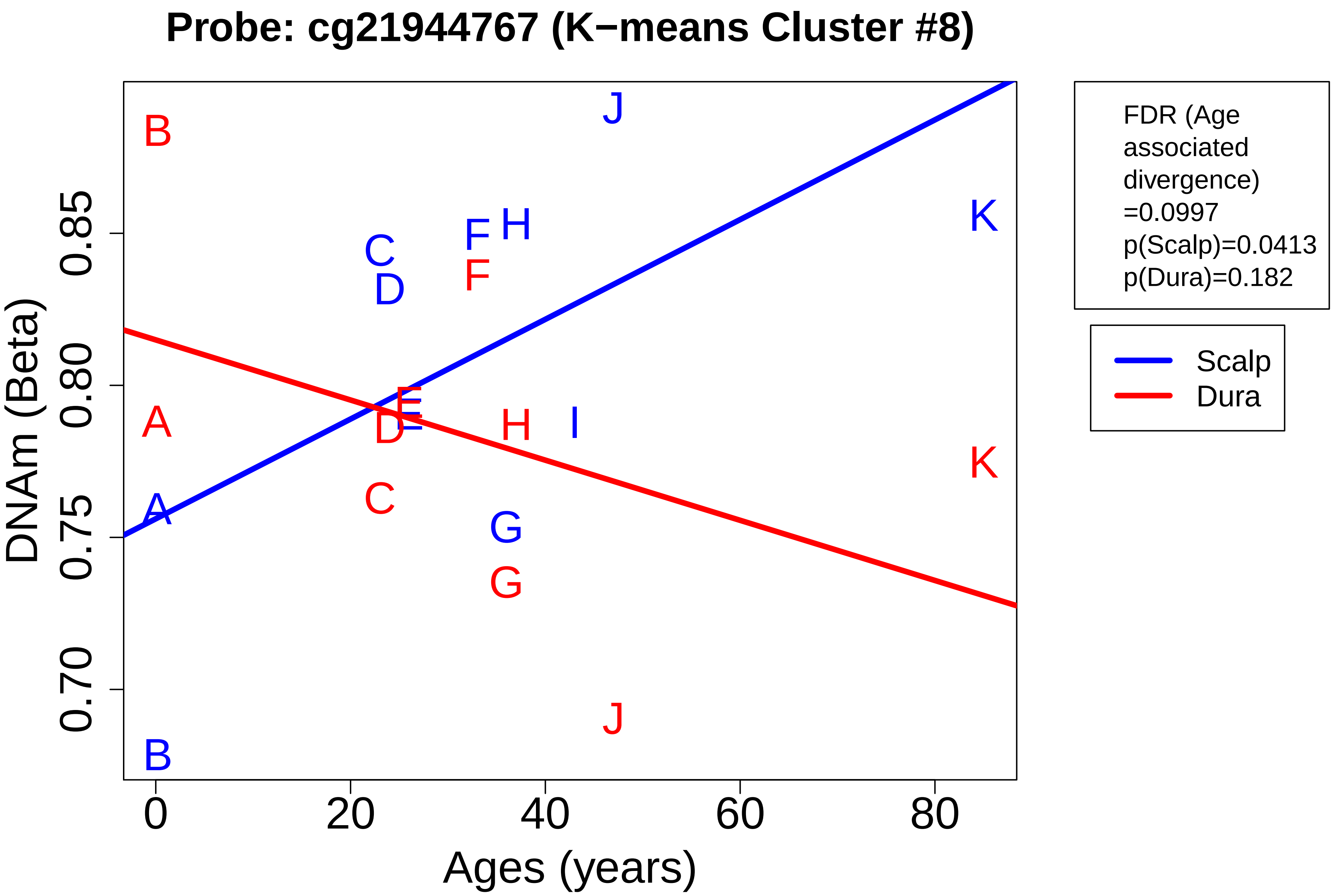

Probe: cg21944767 (K−means Cluster #8)
DNAm (Beta)
0.70
0.75
0.80
0.85
0
20
40
60
80
Ages (years)
FDR (Age associated divergence) =0.0997
p(Scalp)=0.0413
p(Dura)=0.182
Scalp
Dura
A
B
C
D
E
F
G
H
I
J
K

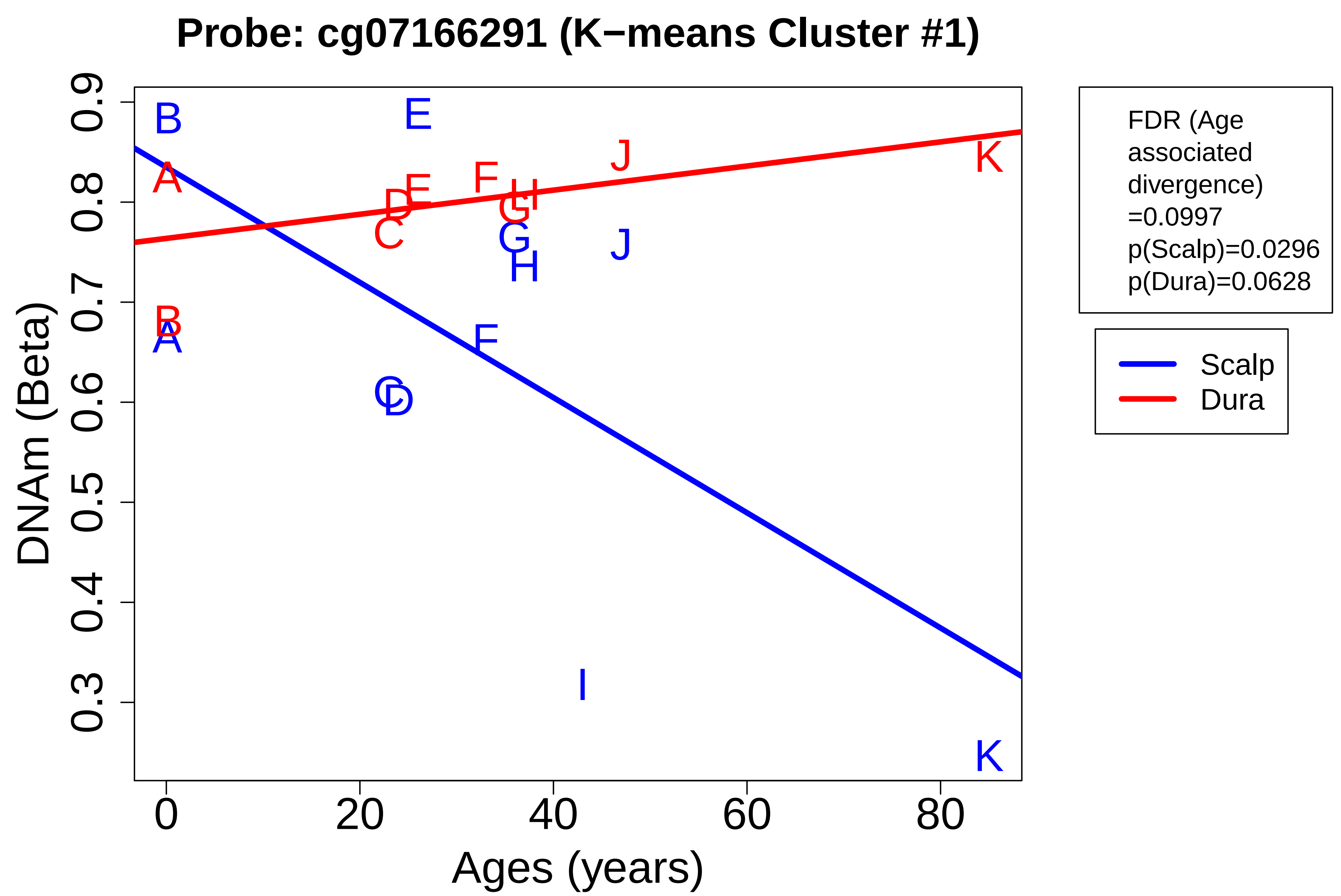
Probe: cg07166291 (K−means Cluster #1)
DNAm (Beta)
Ages (years)
0.3
0.4
0.5
0.6
0.7
0.8
0.9
0
20
40
60
80
FDR (Age associated divergence) =0.0997
p(Scalp)=0.0296
p(Dura)=0.0628
Scalp
Dura
A
B
C
D
E
F
G
H
I
J
K

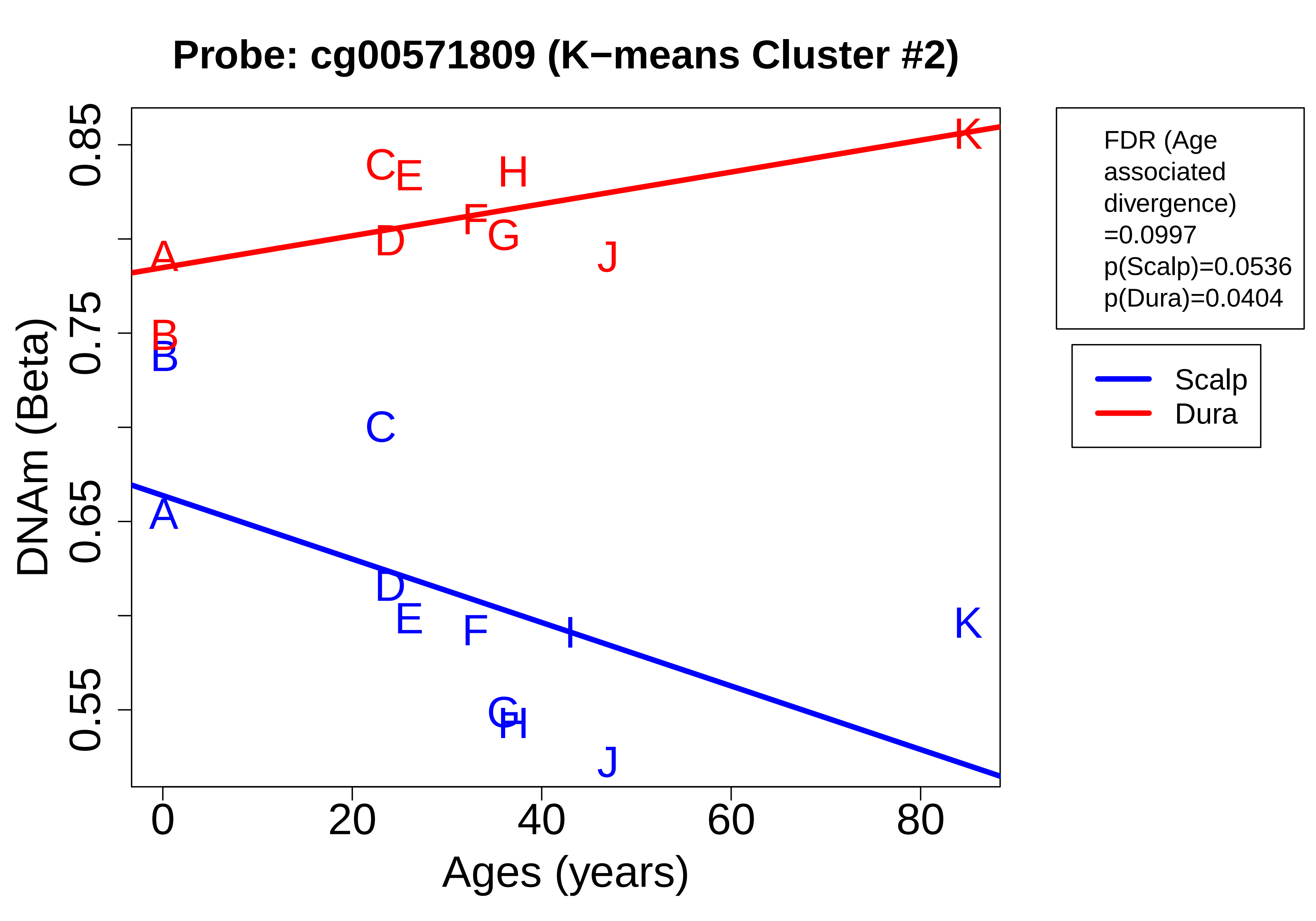

Probe: cg00571809 (K−means Cluster #2)
DNAm (Beta)
0.55
0.65
0.75
0.85
Ages (years)
0
20
40
60
80
FDR (Age associated divergence) =0.0997
p(Scalp)=0.0536
p(Dura)=0.0404
Scalp
Dura
A
B
C
D
E
F
G
H
I
J
K

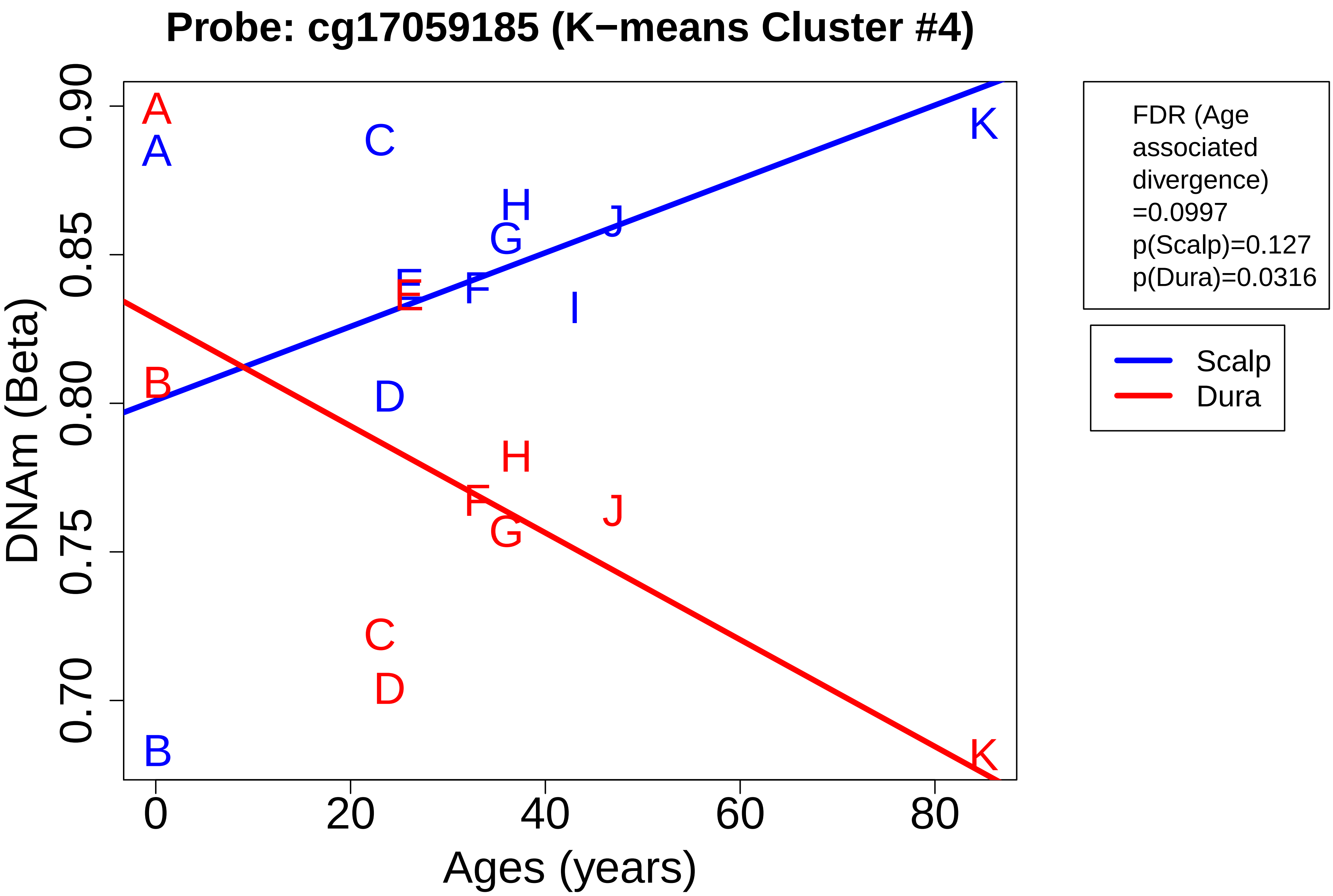

Probe: cg17059185 (K−means Cluster #4)
DNAm (Beta)
0.70
0.75
0.80
0.85
0.90
Ages (years)
0
20
40
60
80
A
B
C
D
E
F
G
H
I
J
K
FDR (Age associated divergence) =0.0997
p(Scalp)=0.127
p(Dura)=0.0316
Scalp
Dura

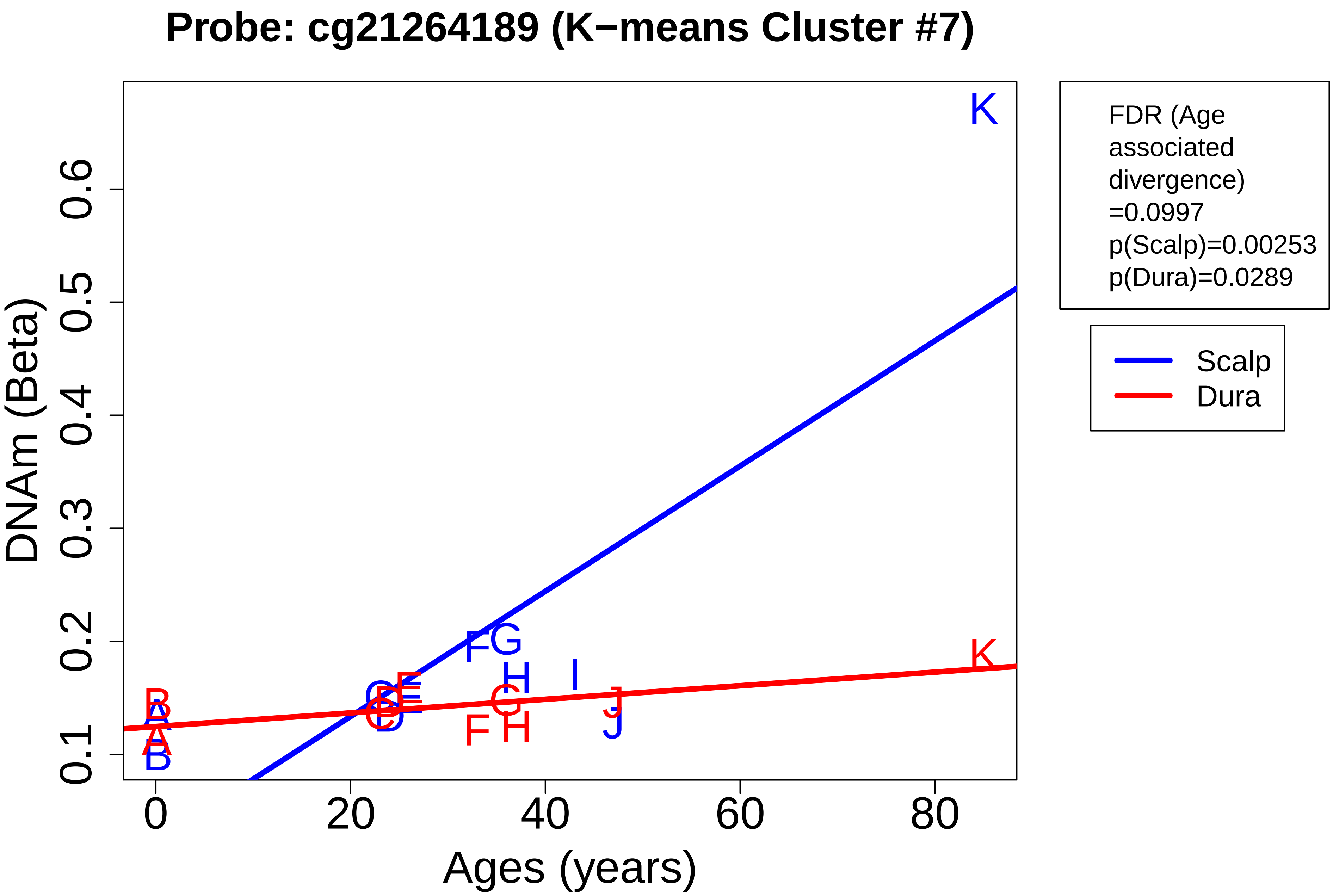

Probe: cg21264189 (K−means Cluster #7)
DNAm (Beta)
Ages (years)
0.1
0.2
0.3
0.4
0.5
0.6
0
20
40
60
80
FDR (Age associated divergence) =0.0997
p(Scalp)=0.00253
p(Dura)=0.0289
Scalp
Dura

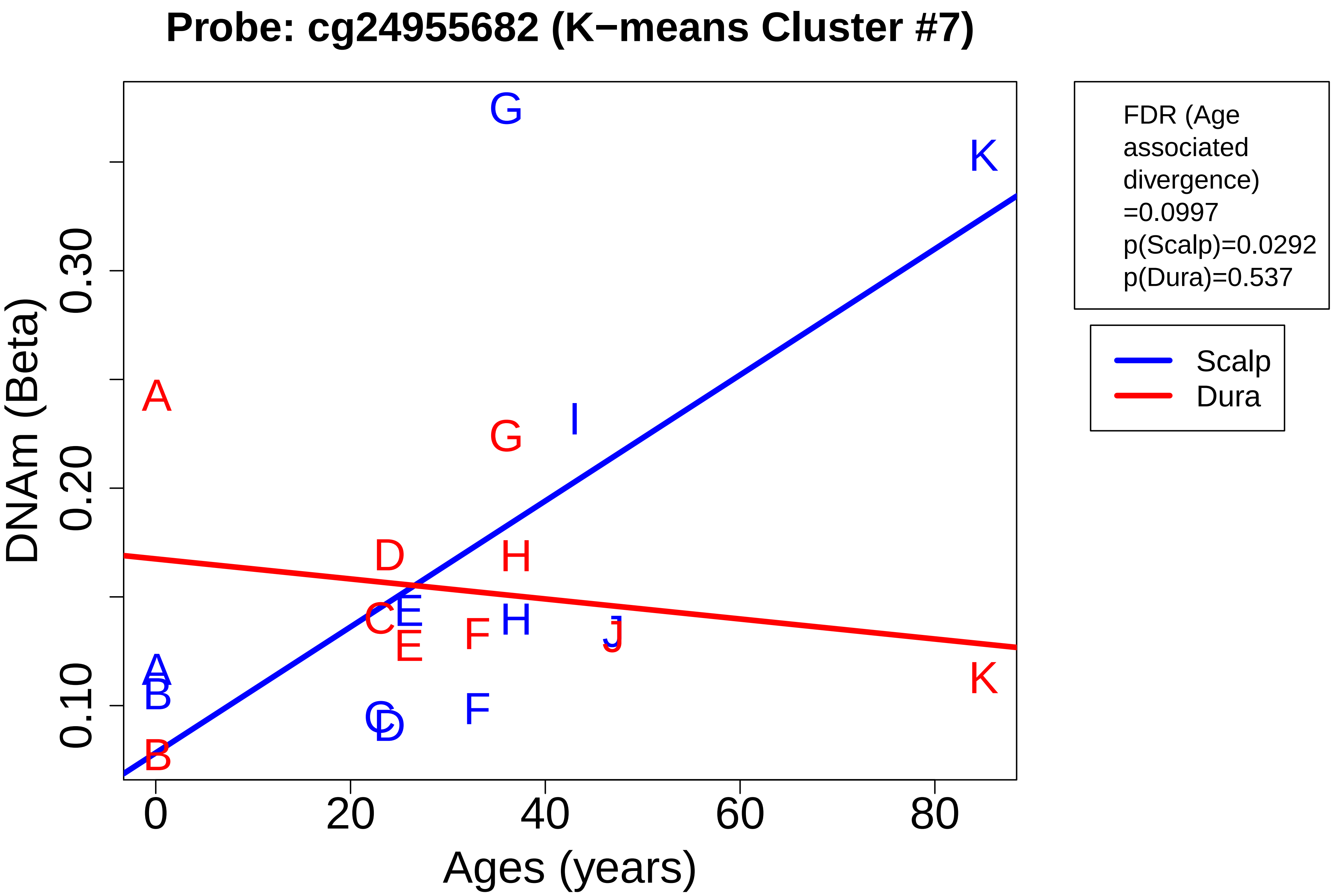

Probe: cg24955682 (K−means Cluster #7)
FDR (Age associated divergence) =0.0997
p(Scalp)=0.0292
p(Dura)=0.537
Scalp
Dura
DNAm (Beta)
0.10
0.20
0.30
0
20
40
60
80
Ages (years)
A
B
C
D
E
F
G
H
I
J
K

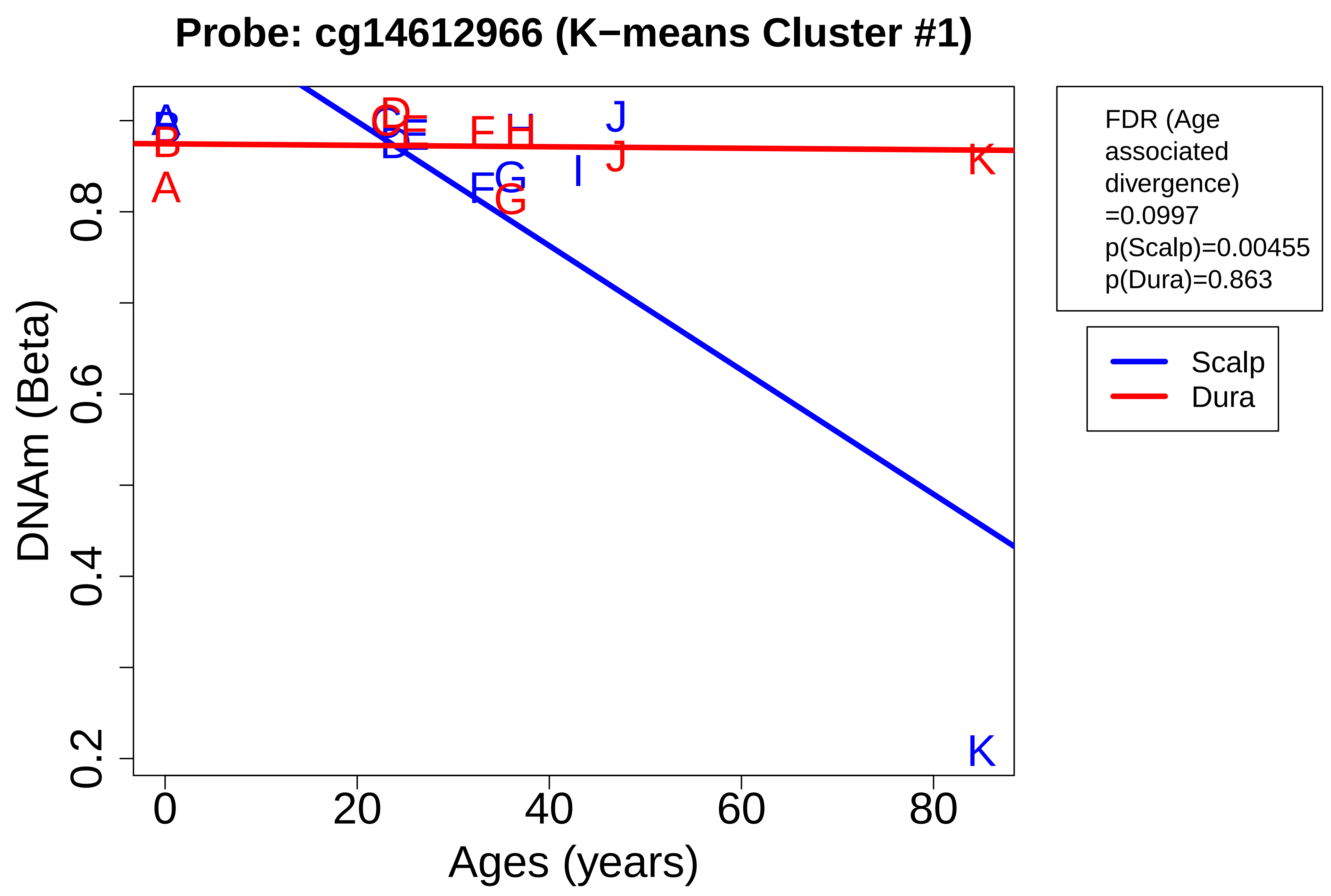

Probe: cg14612966 (K−means Cluster #1)
DNAm (Beta)
0.2
0.4
0.6
0.8
0
20
40
60
80
Ages (years)
FDR (Age associated divergence) =0.0997
p(Scalp)=0.00455
p(Dura)=0.863
Scalp
Dura

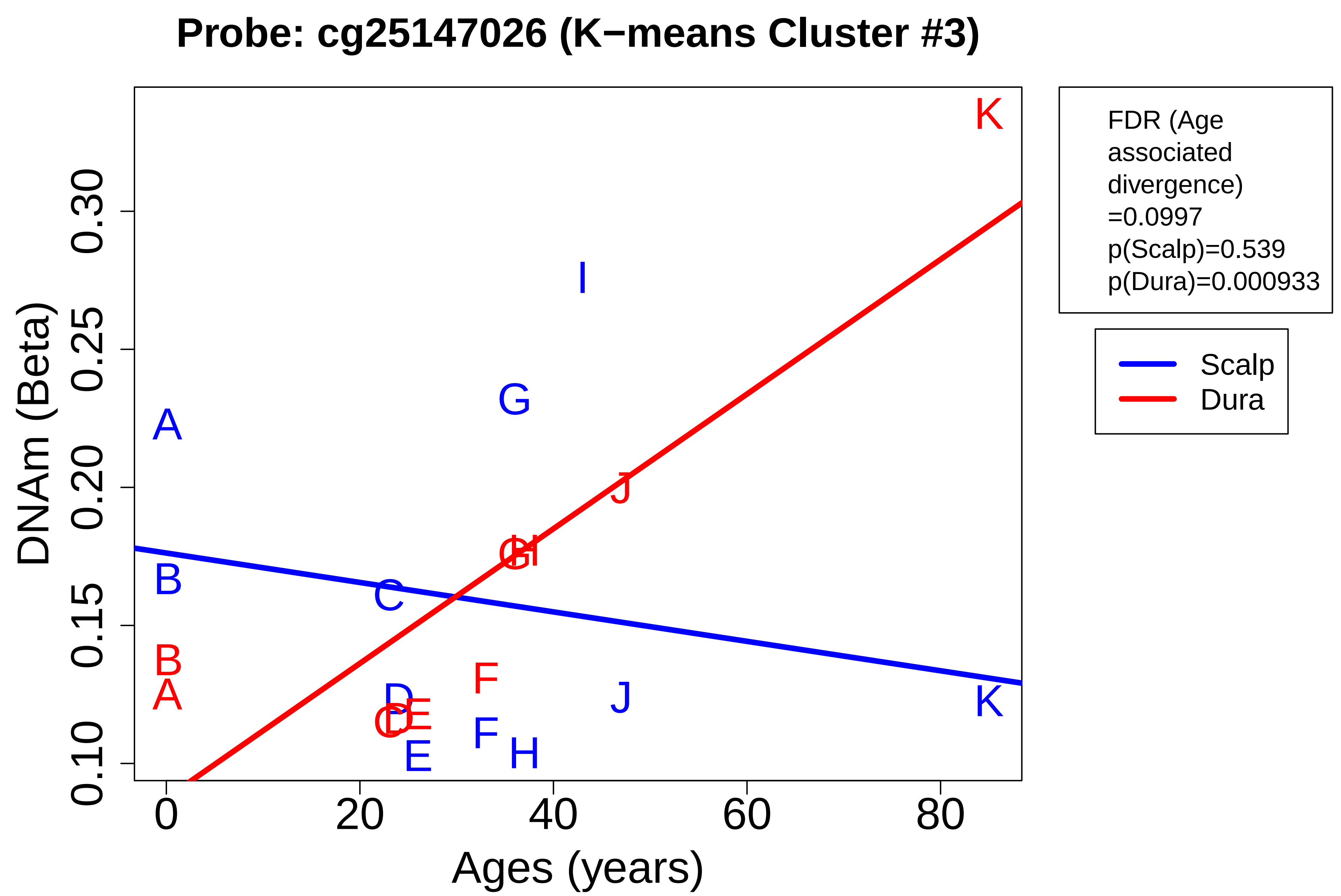

Probe: cg25147026 (K−means Cluster #3)
FDR (Age associated divergence) =0.0997
p(Scalp)=0.539
p(Dura)=0.000933
Scalp
Dura
DNAm (Beta)
0.10
0.15
0.20
0.25
0.30
0
20
40
60
80
Ages (years)
A
B
C
D
E
F
G
H
I
J
K

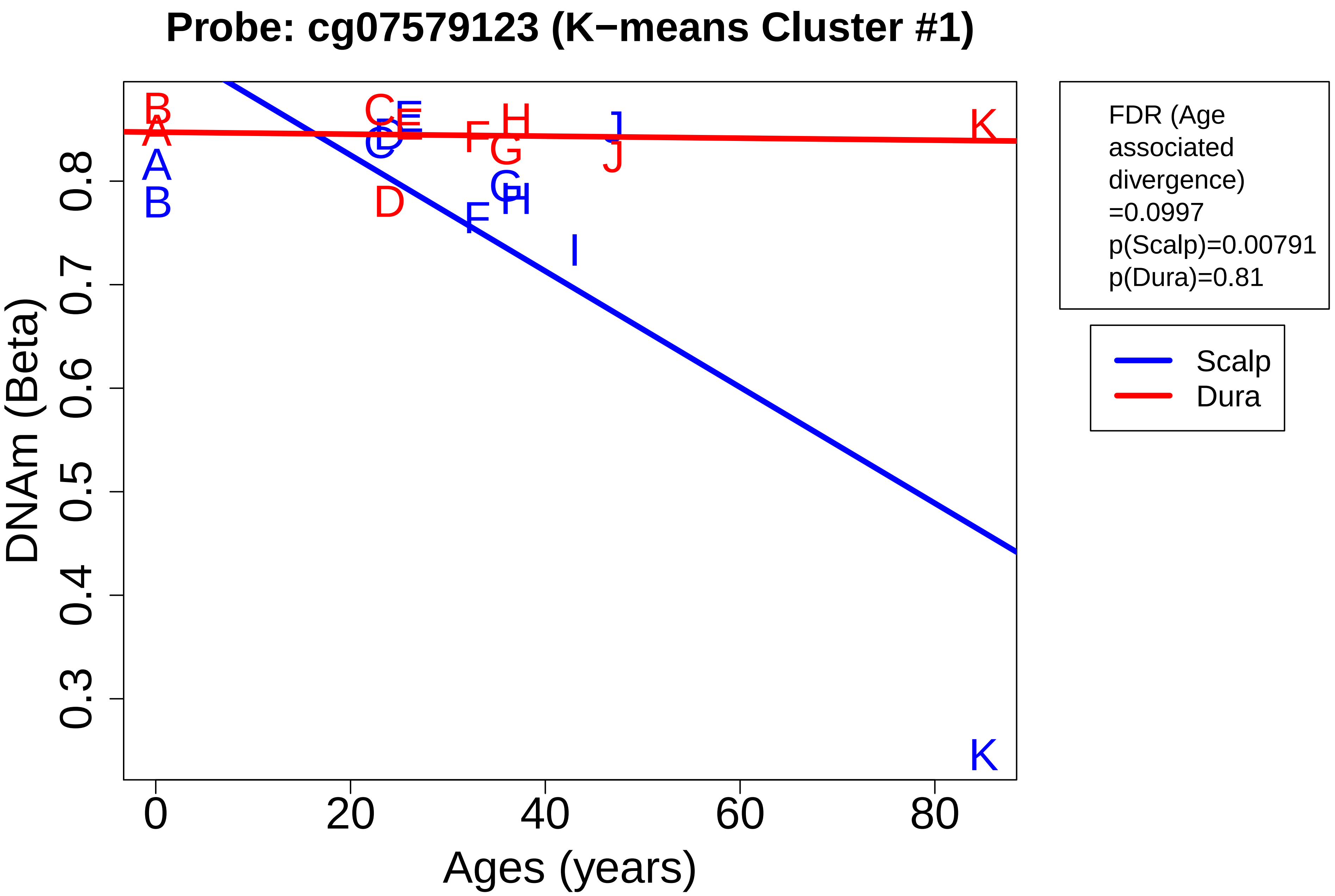

Probe: cg07579123 (K−means Cluster #1)
DNAm (Beta)
0.3
0.4
0.5
0.6
0.7
0.8
Ages (years)
0
20
40
60
80
FDR (Age associated divergence) =0.0997
p(Scalp)=0.00791
p(Dura)=0.81
Scalp
Dura
A
B
C
D
E
F
G
H
I
J
K

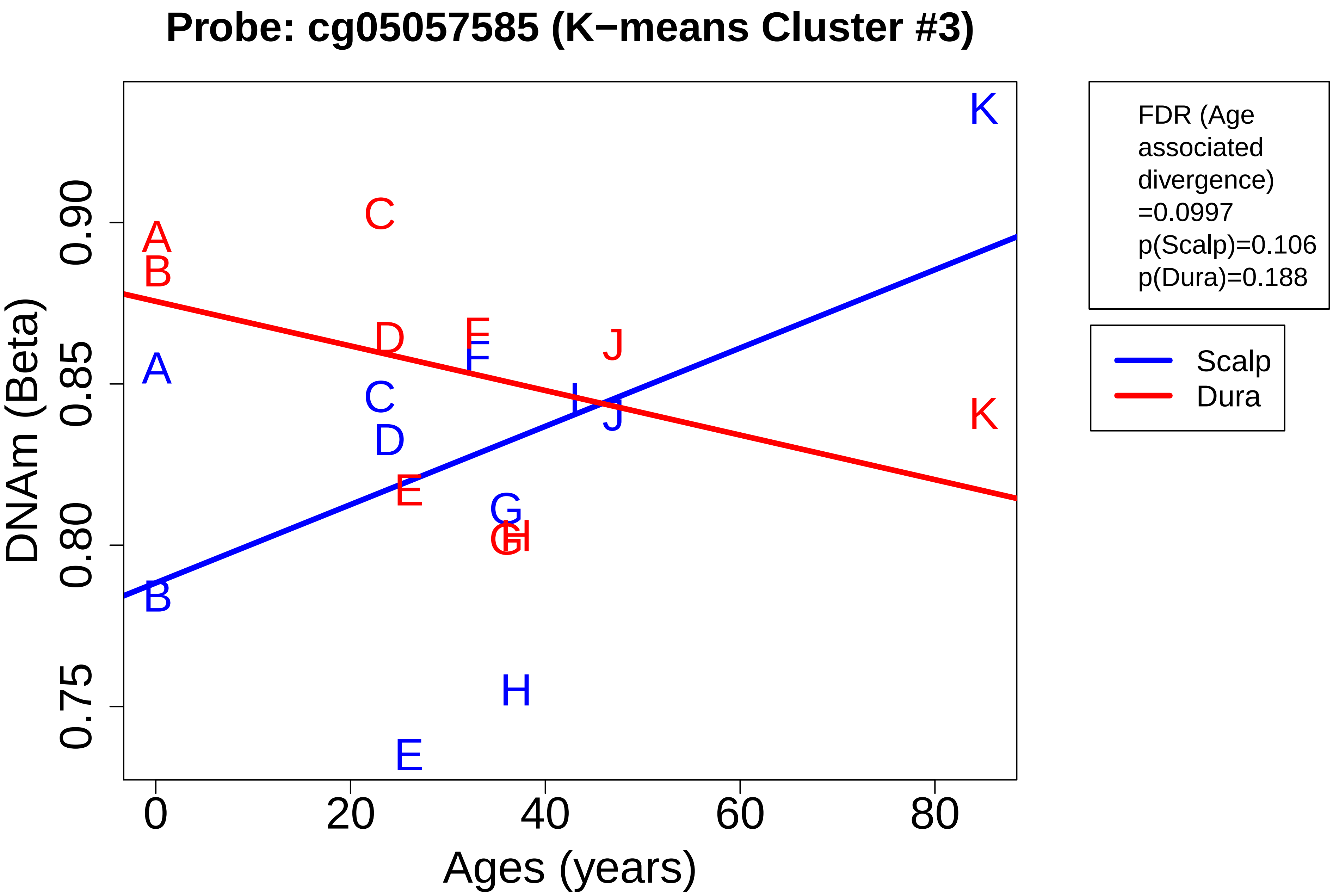

Probe: cg05057585 (K−means Cluster #3)
DNAm (Beta)
Ages (years)
0.75
0.80
0.85
0.90
0
20
40
60
80
FDR (Age associated divergence) =0.0997
p(Scalp)=0.106
p(Dura)=0.188
Scalp
Dura

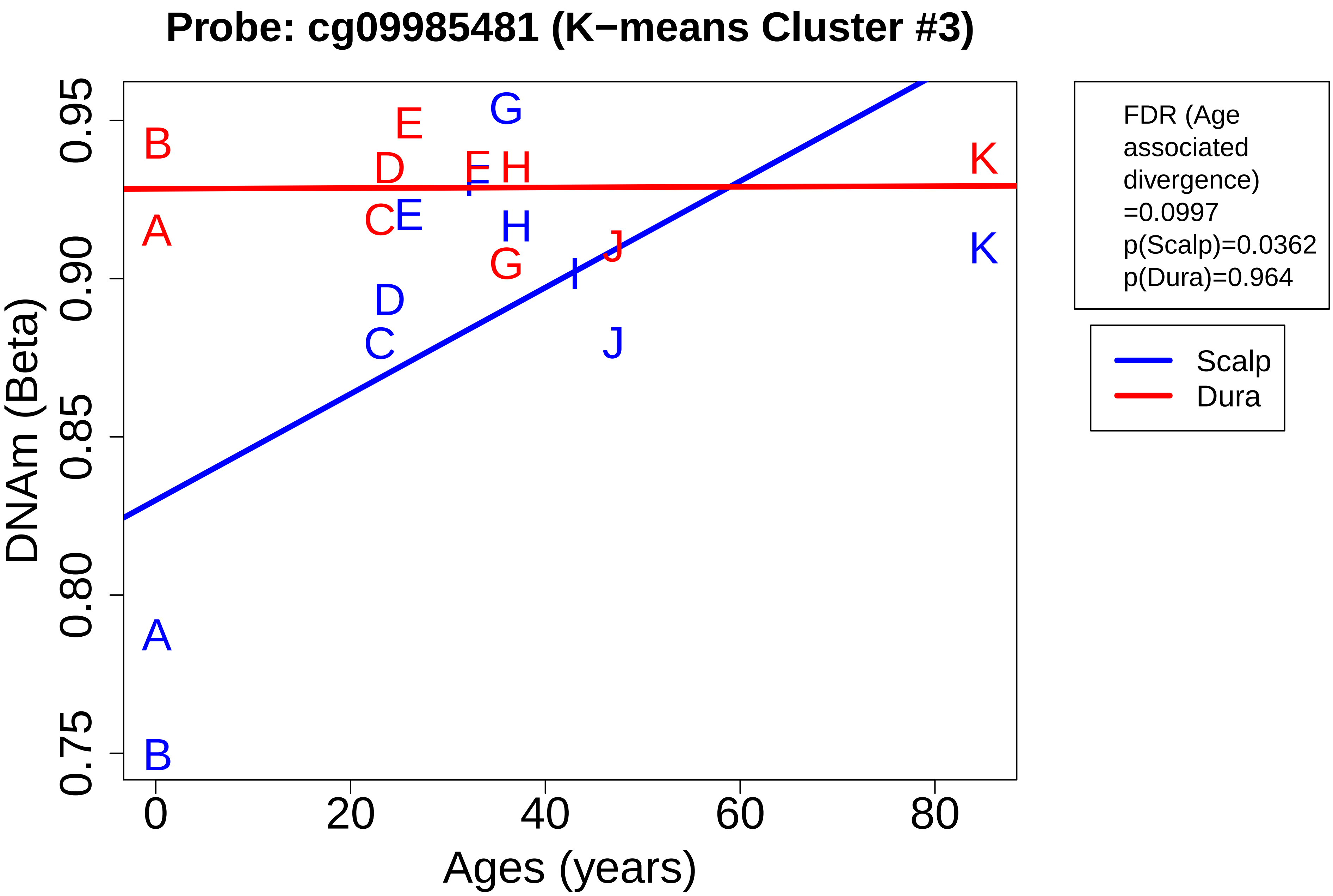
Probe: cg09985481 (K−means Cluster #3)
DNAm (Beta)
0.75
0.80
0.85
0.90
0.95
0
20
40
60
80
Ages (years)
A
B
C
D
E
F
G
H
I
J
K
A
B
C
D
E
F
G
H
J
K
FDR (Age
associated
divergence)
=0.0997
p(Scalp)=0.0362
p(Dura)=0.964
Scalp
Dura

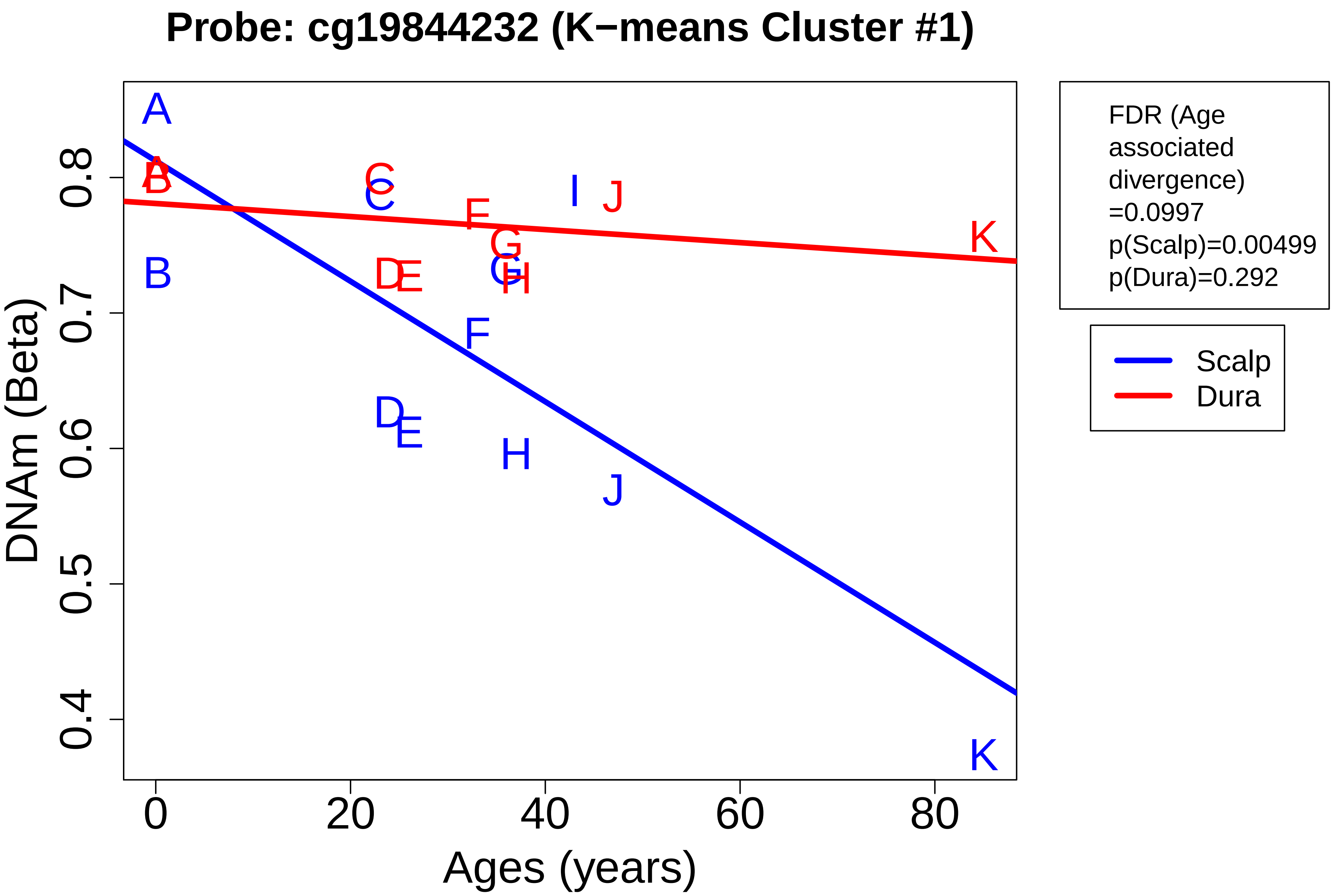

Probe: cg19844232 (K−means Cluster #1)
DNAm (Beta)
Ages (years)
0.4
0.5
0.6
0.7
0.8
0
20
40
60
80
FDR (Age associated divergence) =0.0997
p(Scalp)=0.00499
p(Dura)=0.292
Scalp
Dura

Probe: cg26583474 (K−means Cluster #1)

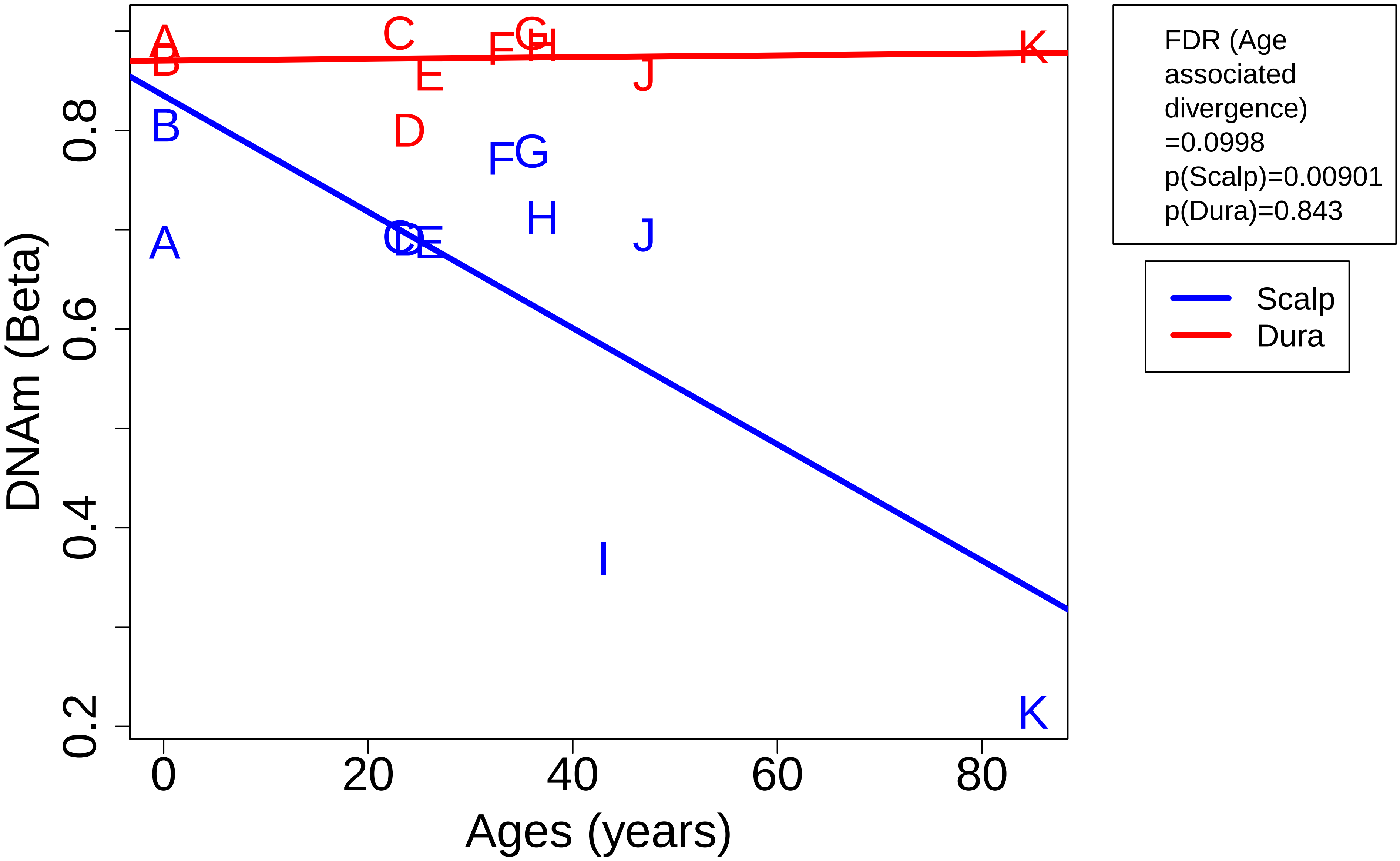

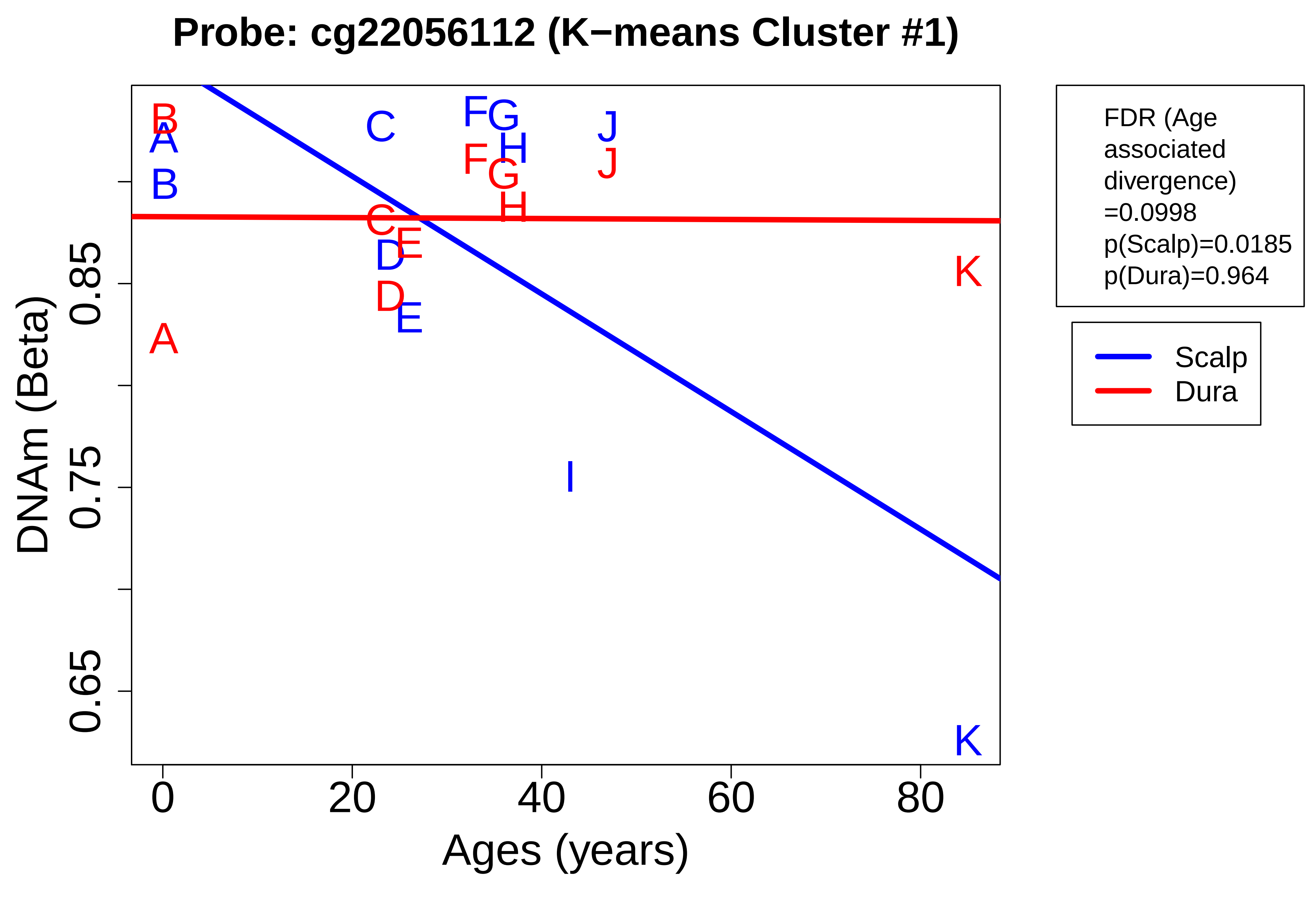

Probe: cg22056112 (K−means Cluster #1)
DNAm (Beta)
0.65
0.75
0.85
Ages (years)
0
20
40
60
80
A
B
C
D
E
F
G
H
I
J
K
FDR (Age associated divergence) =0.0998
p(Scalp)=0.0185
p(Dura)=0.964
Scalp
Dura

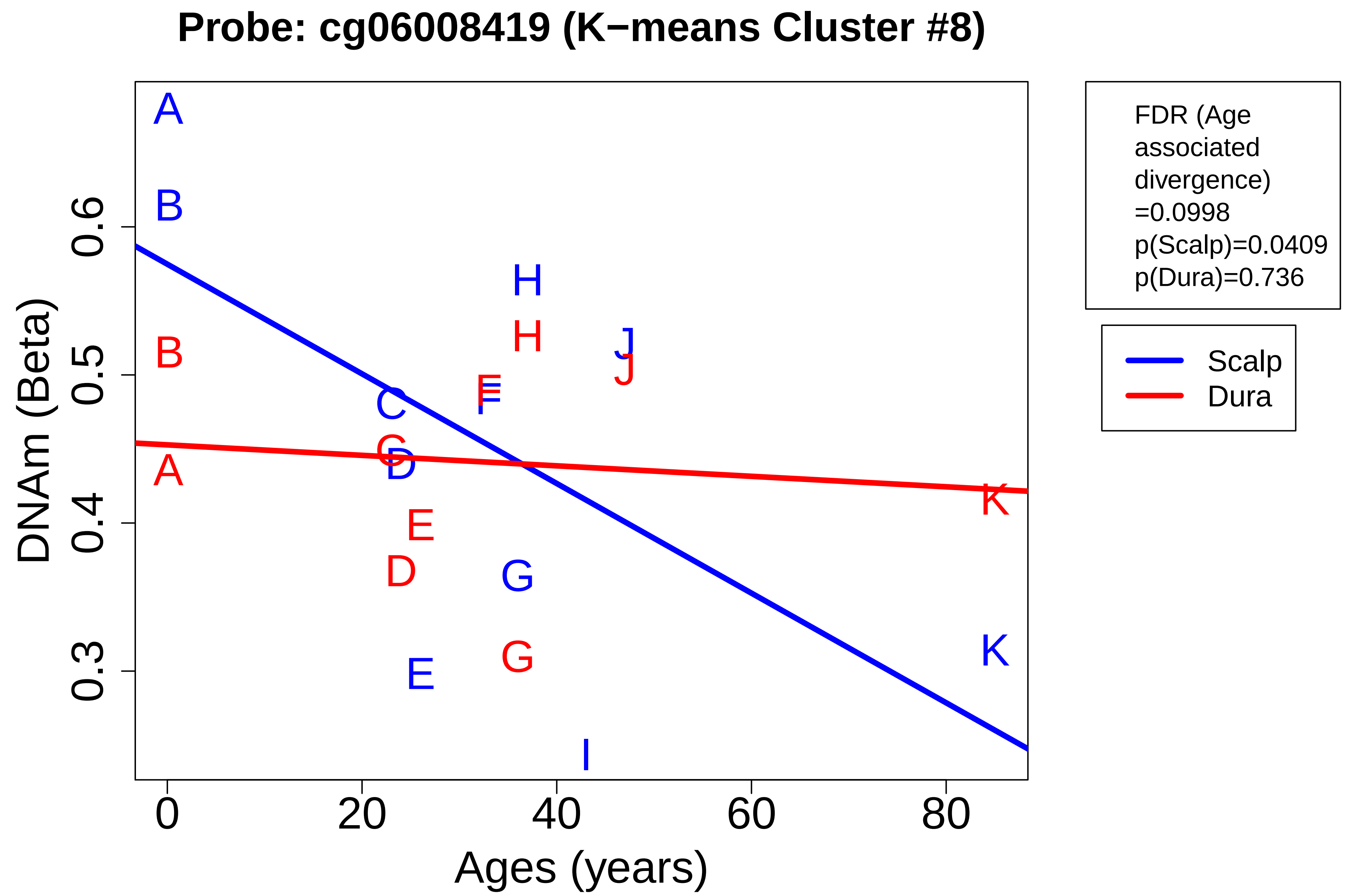

Probe: cg06008419 (K−means Cluster #8)
DNAm (Beta)
0.3
0.4
0.5
0.6
0
20
40
60
80
Ages (years)
FDR (Age associated divergence) =0.0998
p(Scalp)=0.0409
p(Dura)=0.736
Scalp
Dura

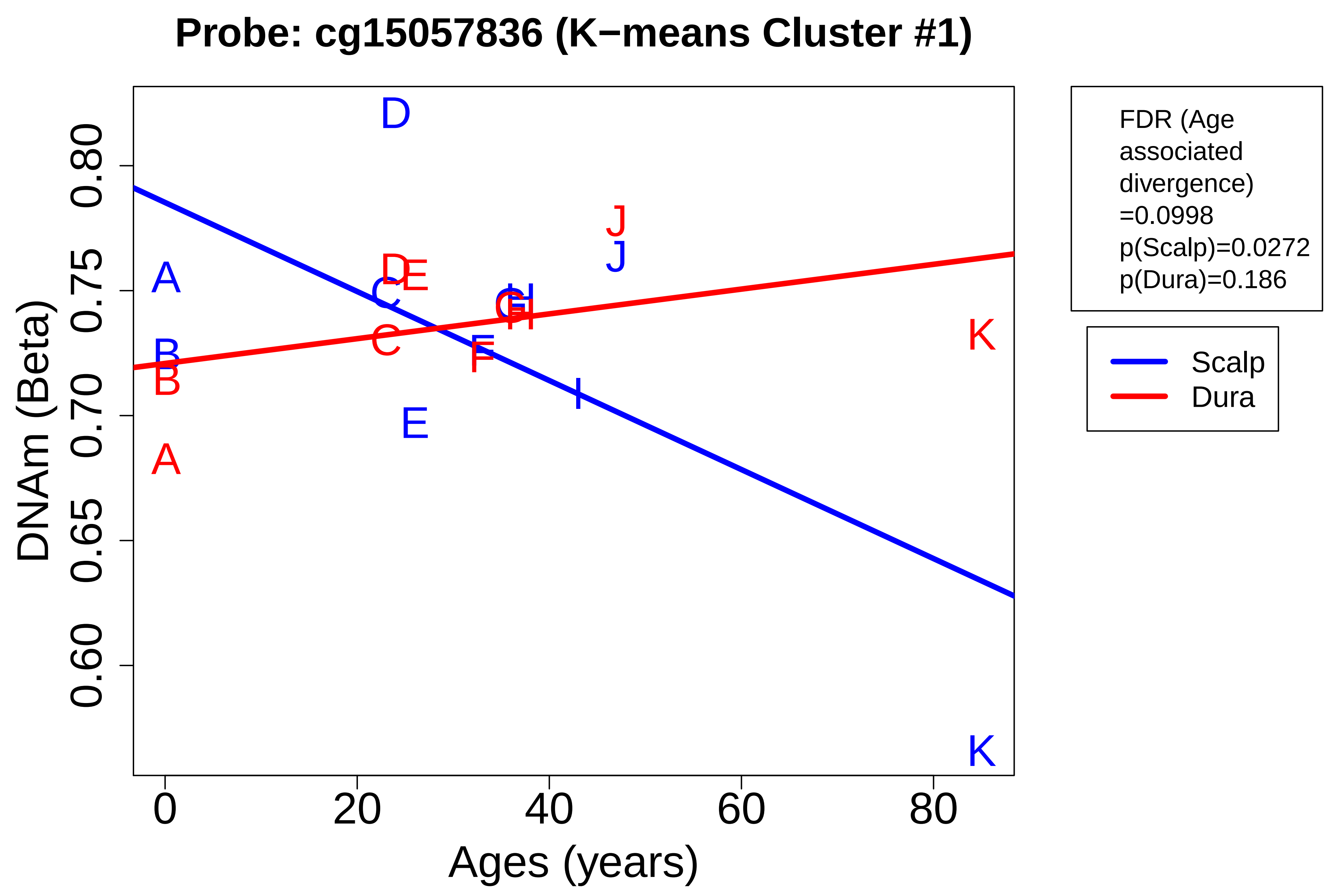

Probe: cg15057836 (K−means Cluster #1)
DNAm (Beta)
0.60
0.65
0.70
0.75
0.80
Ages (years)
0
20
40
60
80
FDR (Age associated divergence) =0.0998
p(Scalp)=0.0272
p(Dura)=0.186
Scalp
Dura
A
B
C
D
E
F
G
H
I
J
K

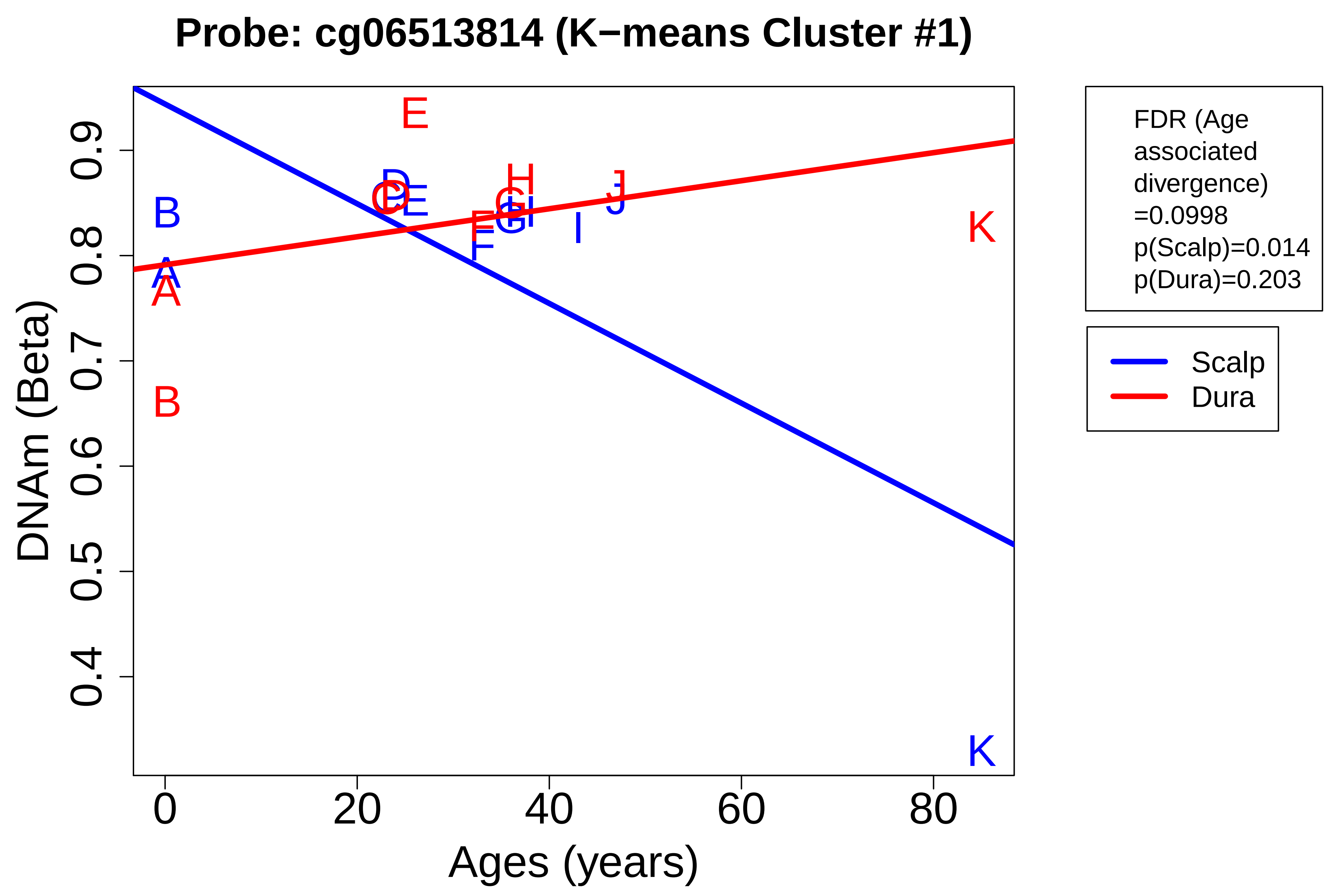

Probe: cg06513814 (K−means Cluster #1)
DNAm (Beta)
0.4
0.5
0.6
0.7
0.8
0.9
0
20
40
60
80
Ages (years)
FDR (Age associated divergence) =0.0998
p(Scalp)=0.014
p(Dura)=0.203
Scalp
Dura

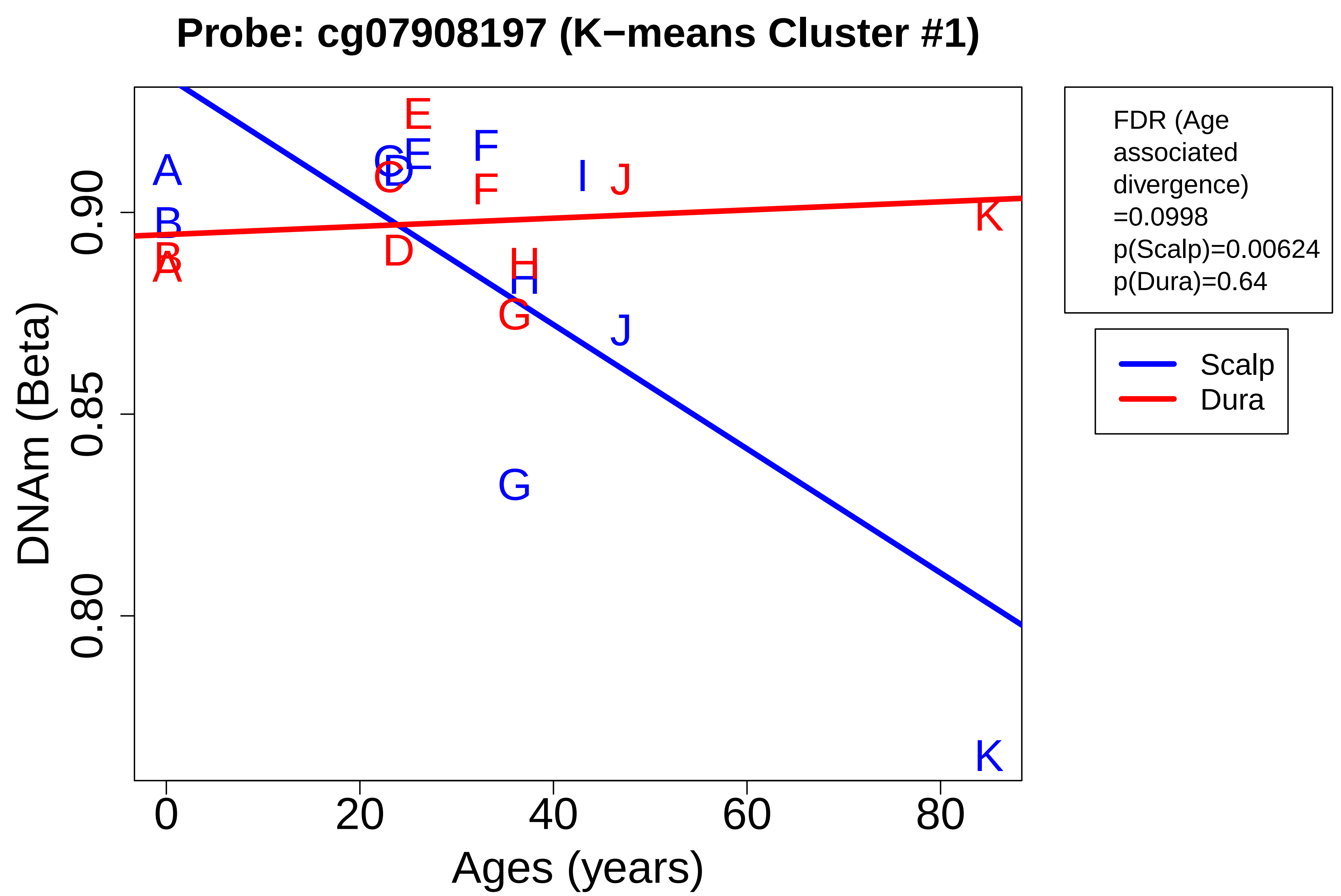
Probe: cg07908197 (K−means Cluster #1)
DNAm (Beta)
0.80
0.85
0.90
0
20
40
60
80
Ages (years)
FDR (Age associated divergence) =0.0998
p(Scalp)=0.00624
p(Dura)=0.64
Scalp
Dura

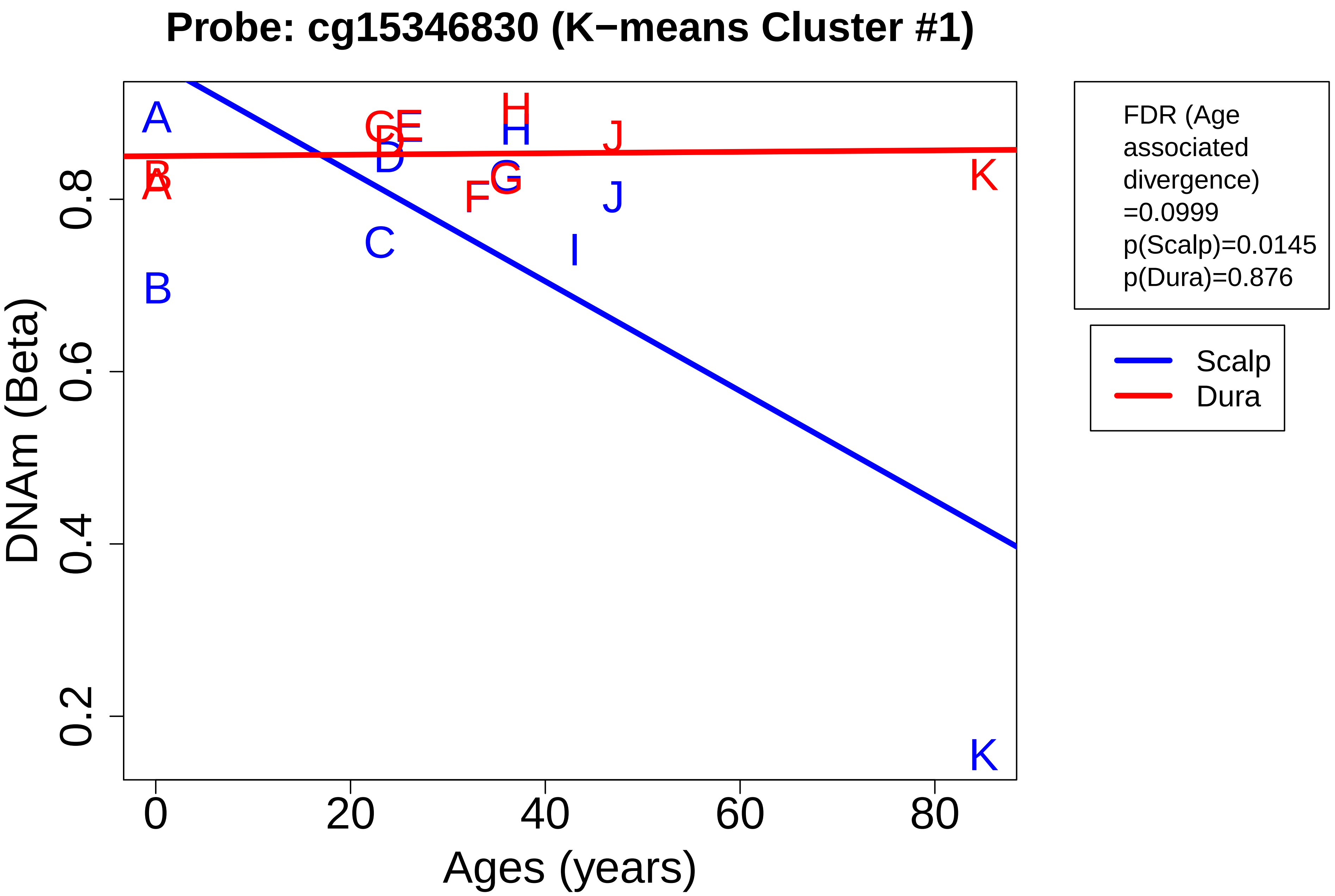

Probe: cg15346830 (K−means Cluster #1)
DNAm (Beta)
0.2
0.4
0.6
0.8
0
20
40
60
80
Ages (years)
A
B
C
D
E
F
G
H
I
J
K
FDR (Age associated divergence) =0.0999
p(Scalp)=0.0145
p(Dura)=0.876
Scalp
Dura

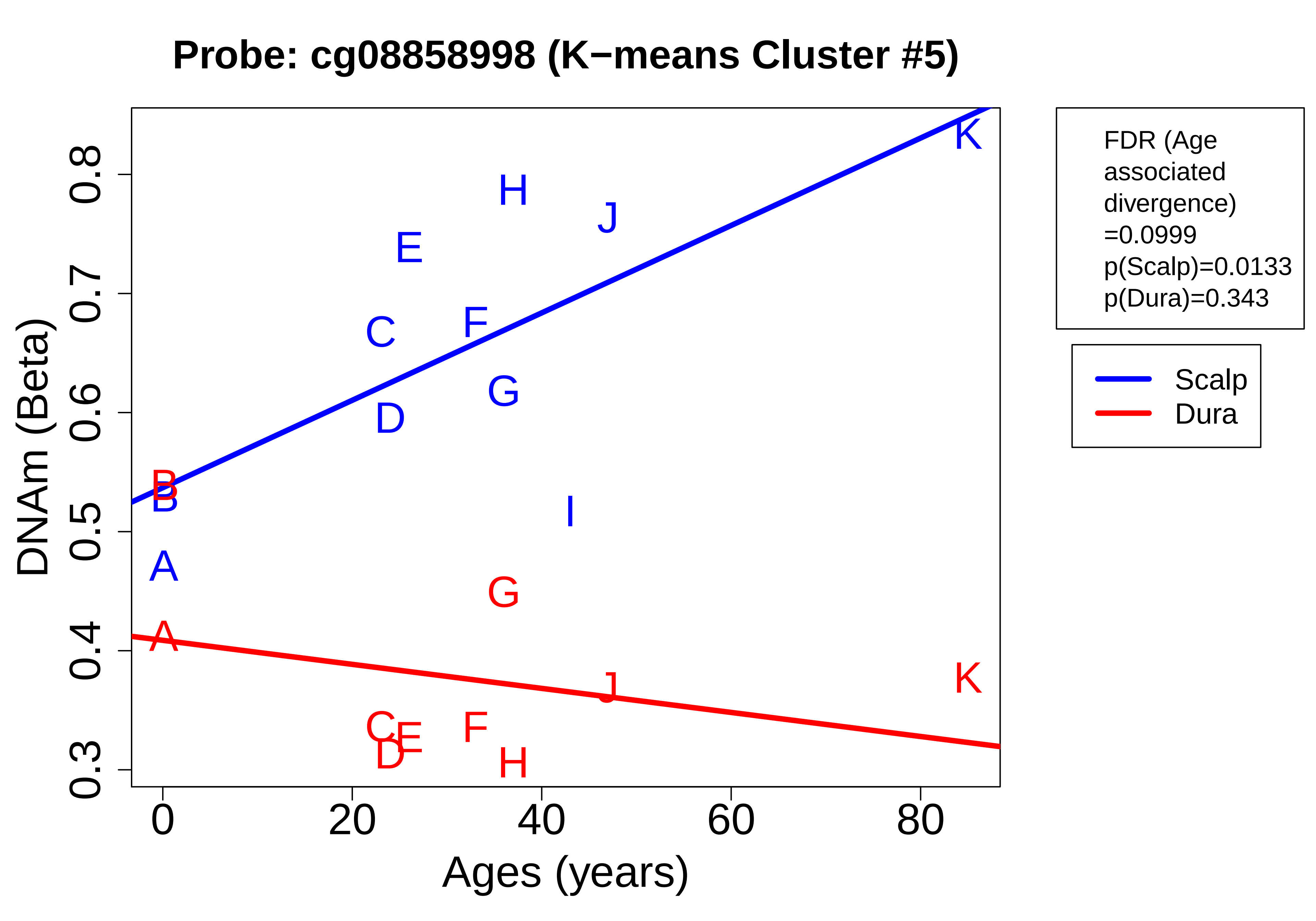

Probe: cg08858998 (K−means Cluster #5)
DNAm (Beta)
0.3
0.4
0.5
0.6
0.7
0.8
0
20
40
60
80
Ages (years)
FDR (Age associated divergence) =0.0999
p(Scalp)=0.0133
p(Dura)=0.343
Scalp
Dura
A
B
C
D
E
F
G
H
I
J
K

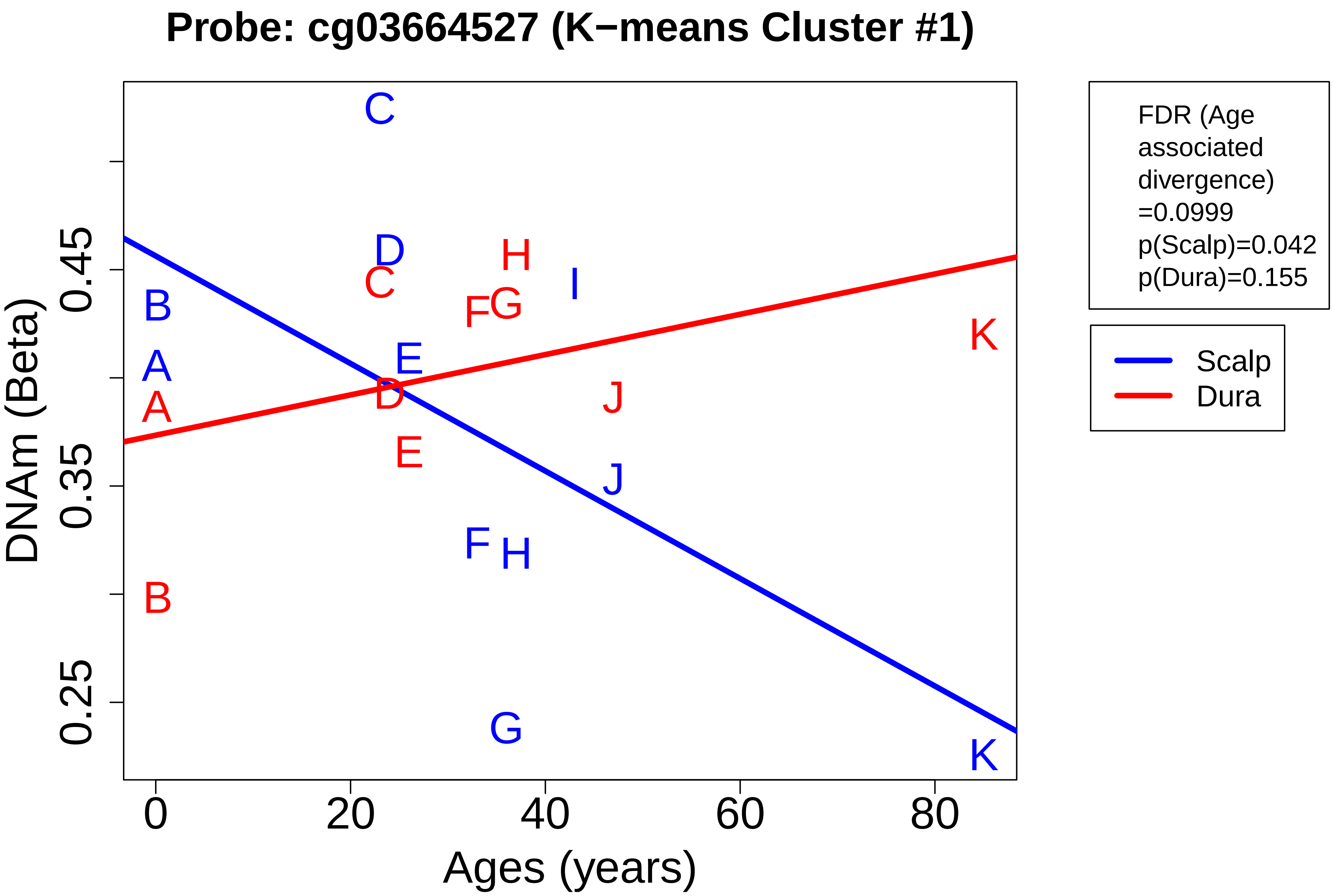

Probe: cg03664527 (K−means Cluster #1)
DNAm (Beta)
0.25
0.35
0.45
Ages (years)
0
20
40
60
80
FDR (Age associated divergence) =0.0999
p(Scalp)=0.042
p(Dura)=0.155
Scalp
Dura

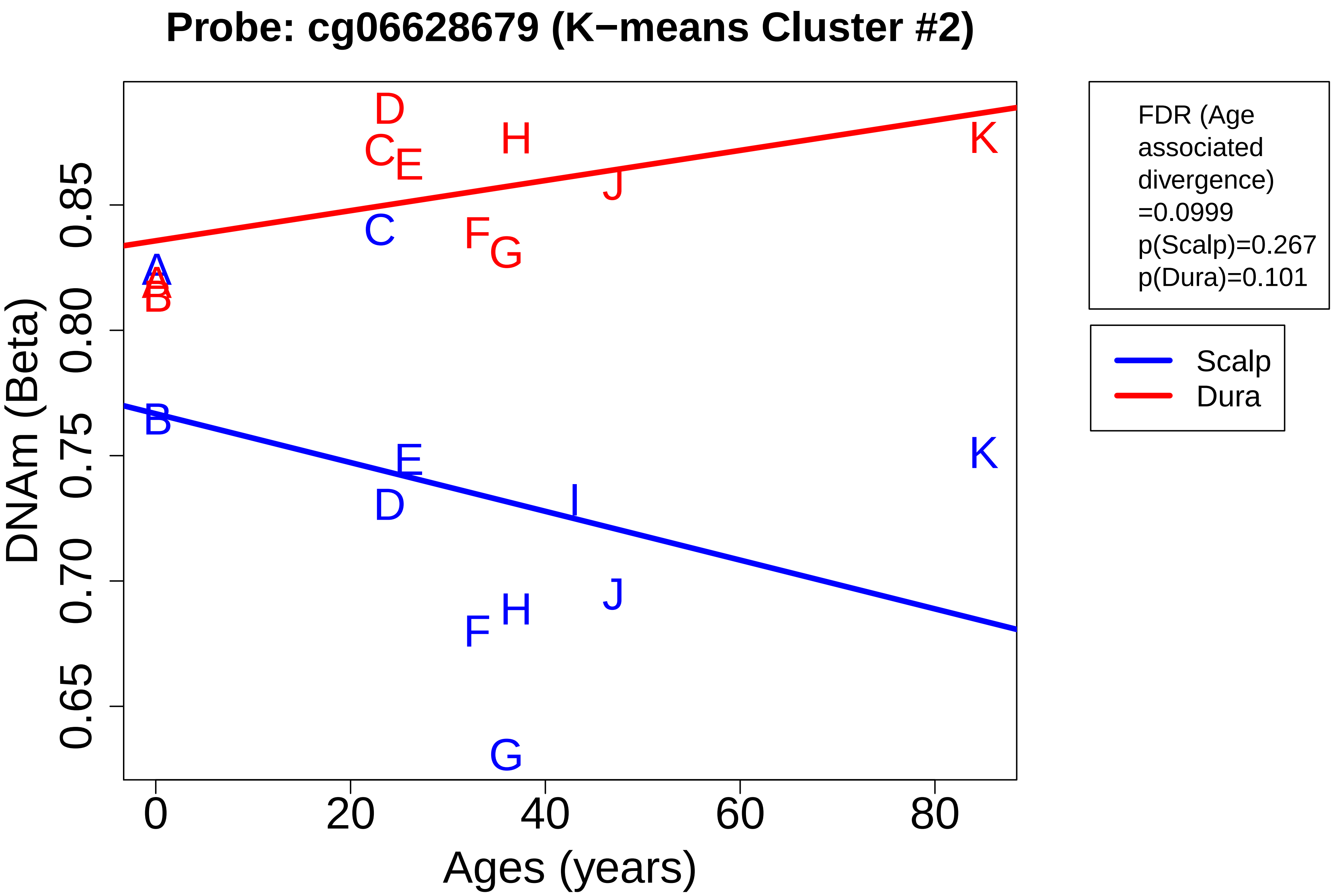

Probe: cg06628679 (K−means Cluster #2)
FDR (Age associated divergence) =0.0999
p(Scalp)=0.267
p(Dura)=0.101
Scalp
Dura
DNAm (Beta)
0.65
0.70
0.75
0.80
0.85
0
20
40
60
80
Ages (years)

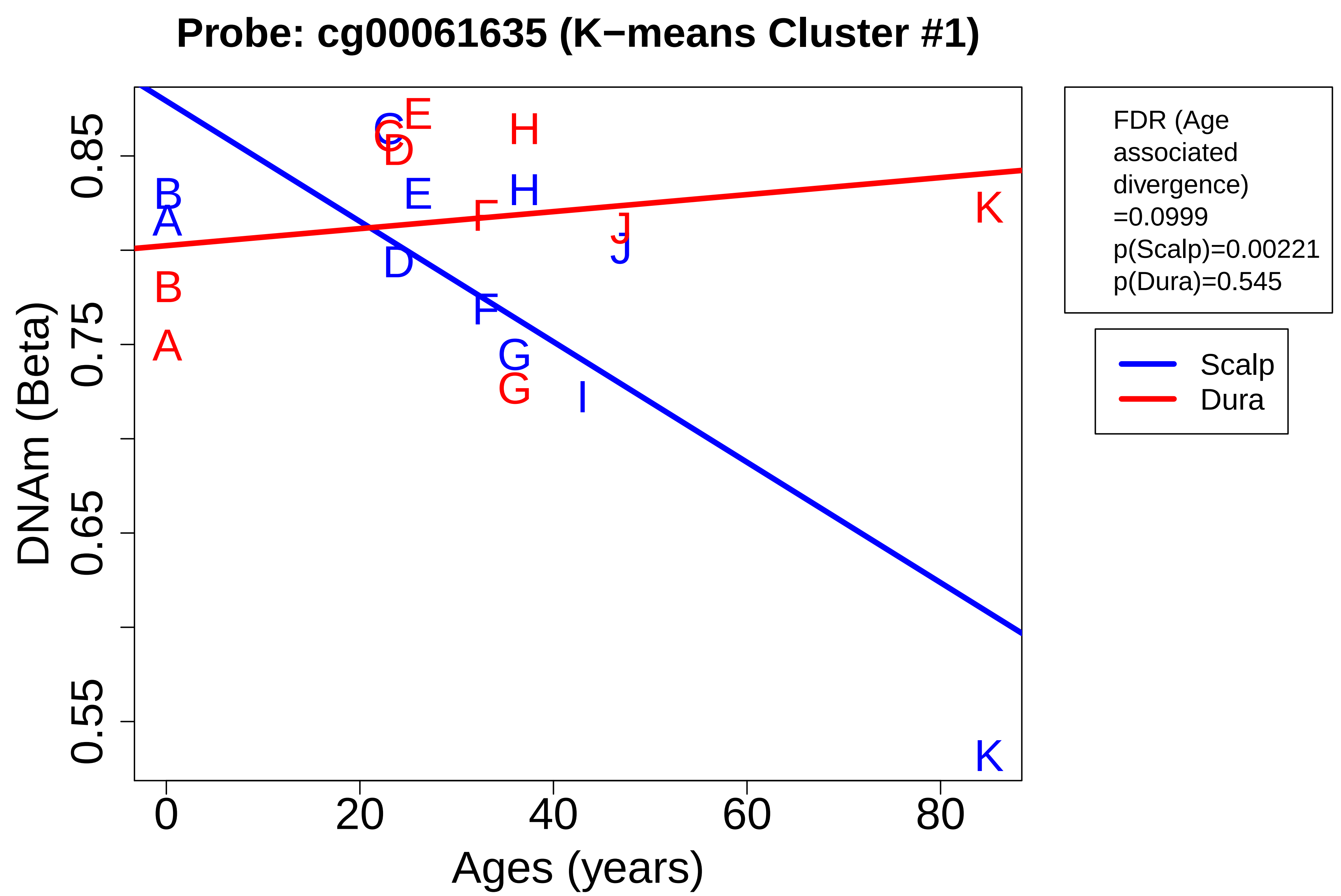

Probe: cg00061635 (K−means Cluster #1)
DNAm (Beta)
0.55
0.65
0.75
0.85
0
20
40
60
80
Ages (years)
FDR (Age associated divergence) =0.0999
p(Scalp)=0.00221
p(Dura)=0.545
Scalp
Dura

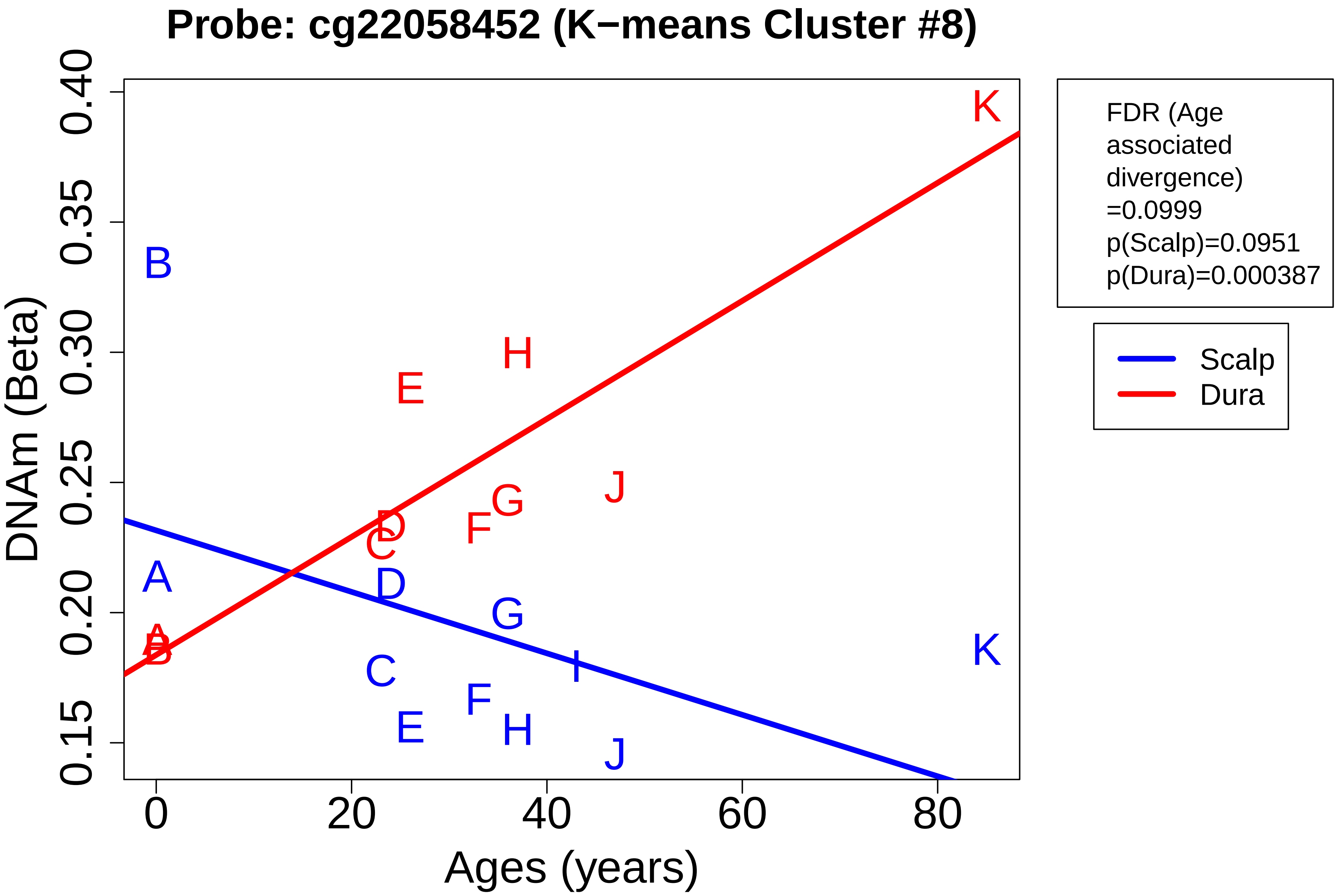

Probe: cg22058452 (K−means Cluster #8)
DNAm (Beta)
0.15
0.20
0.25
0.30
0.35
0.40
Ages (years)
0
20
40
60
80
FDR (Age associated divergence) =0.0999
p(Scalp)=0.0951
p(Dura)=0.000387
Scalp
Dura

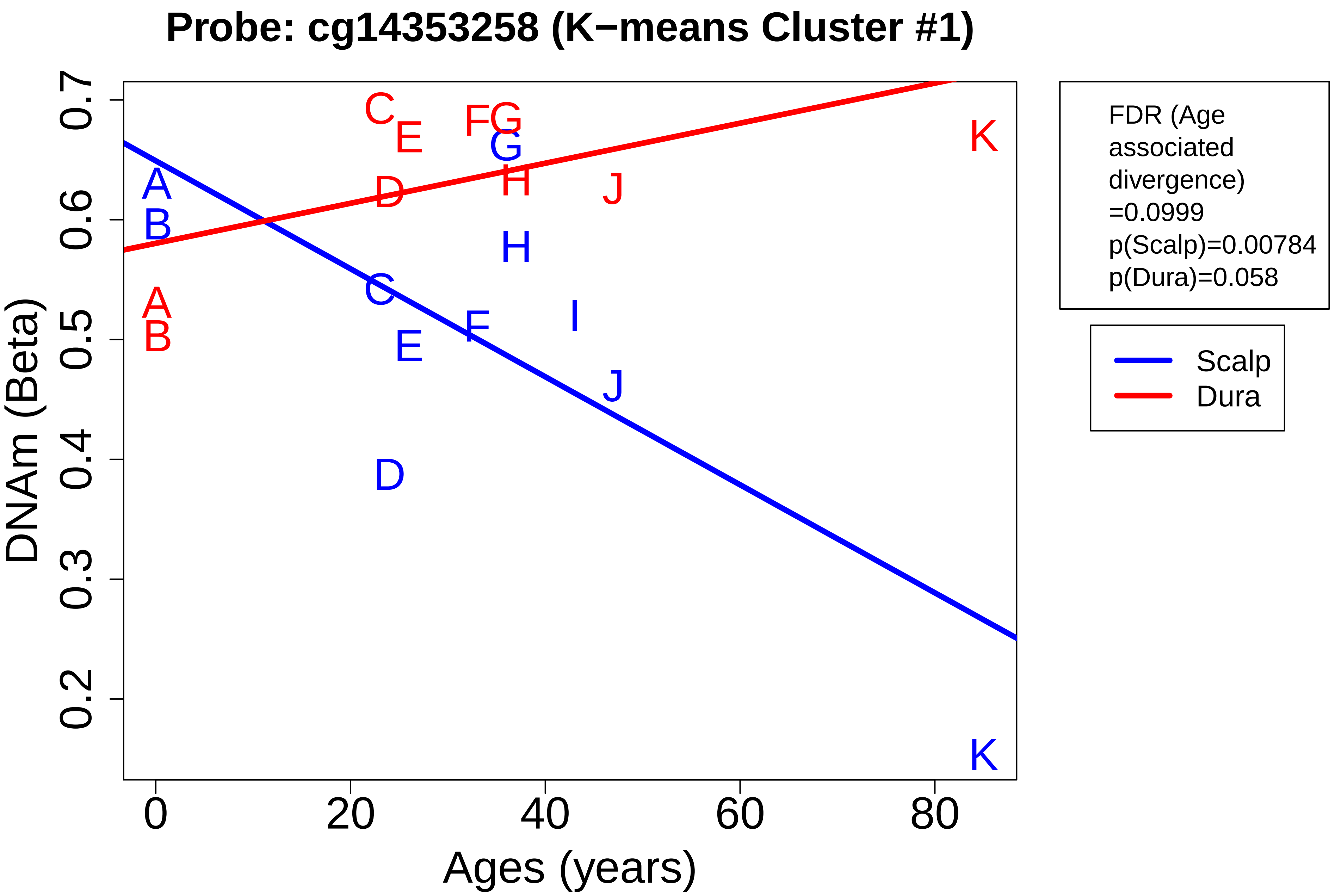

Probe: cg14353258 (K−means Cluster #1)
DNAm (Beta)
0.2
0.3
0.4
0.5
0.6
0.7
0
20
40
60
80
Ages (years)
FDR (Age associated divergence) =0.0999
p(Scalp)=0.00784
p(Dura)=0.058
Scalp
Dura
A
B
C
D
E
F
G
H
I
J
K

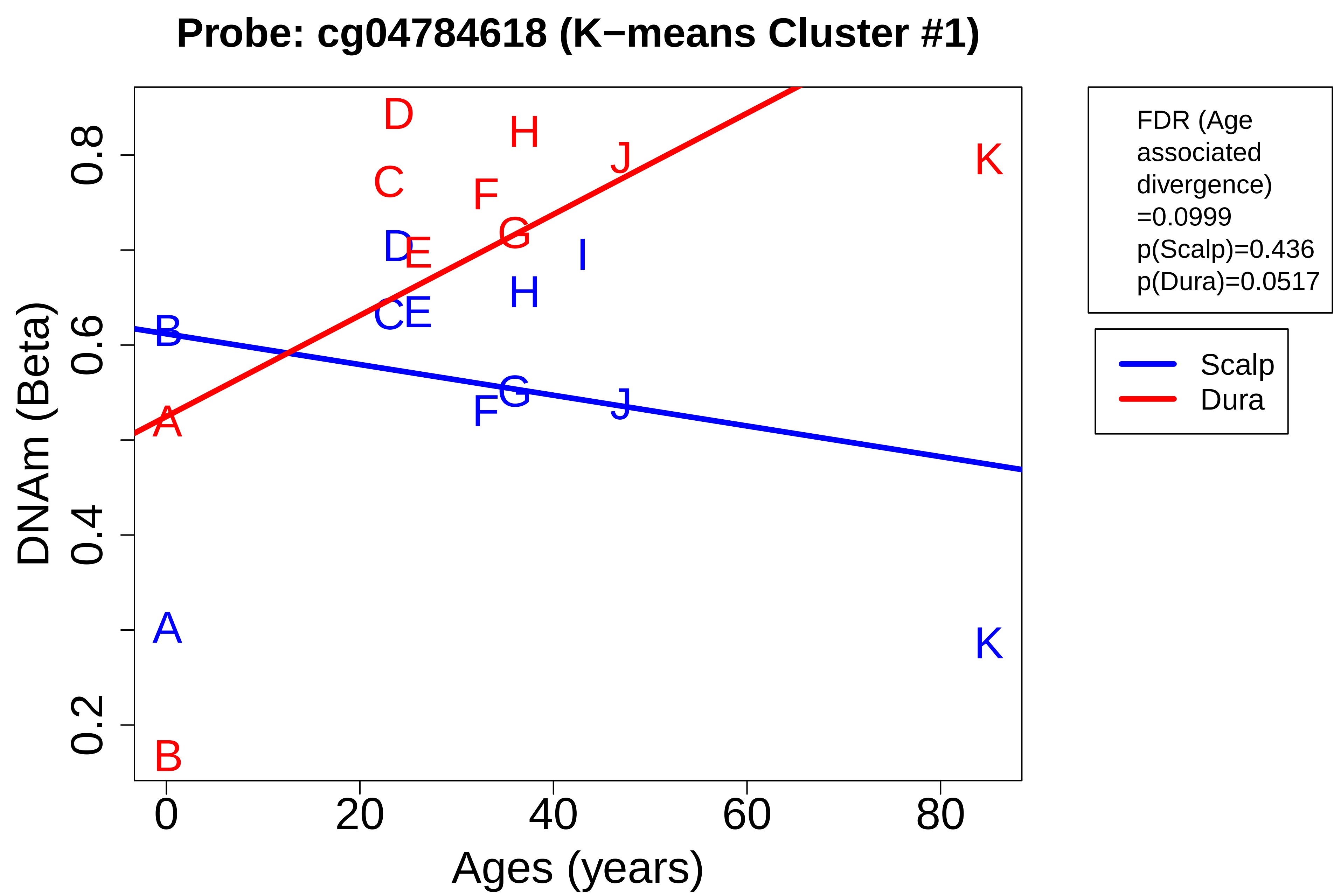

Probe: cg04784618 (K−means Cluster #1)
DNAm (Beta)
0.2
0.4
0.6
0.8
0
20
40
60
80
Ages (years)
FDR (Age associated divergence) =0.0999
p(Scalp)=0.436
p(Dura)=0.0517
Scalp
Dura

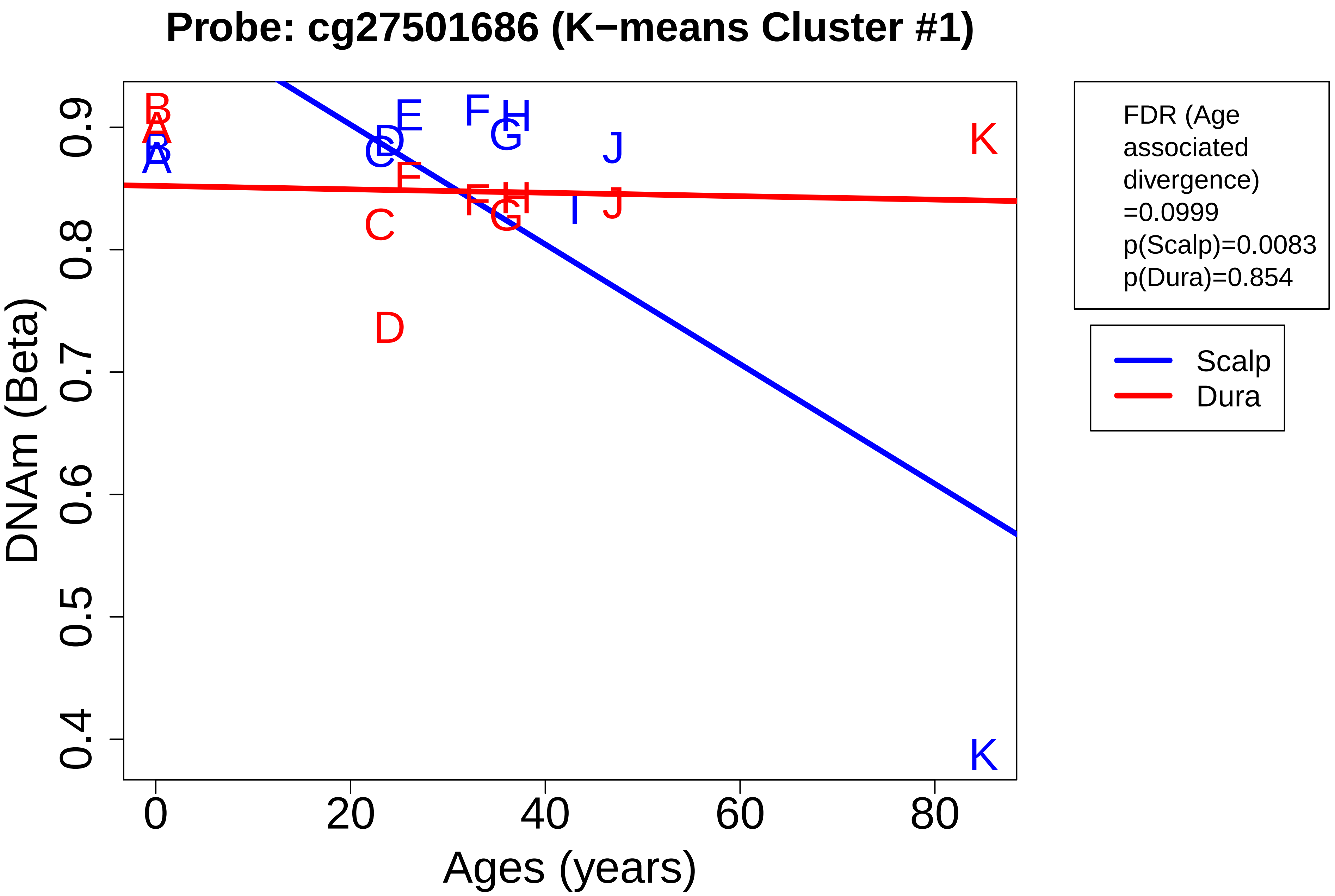

Probe: cg27501686 (K−means Cluster #1)
DNAm (Beta)
0.4
0.5
0.6
0.7
0.8
0.9
0
20
40
60
80
Ages (years)
FDR (Age associated divergence) =0.0999
p(Scalp)=0.0083
p(Dura)=0.854
Scalp
Dura
A
B
C
D
E
F
G
H
I
J
K

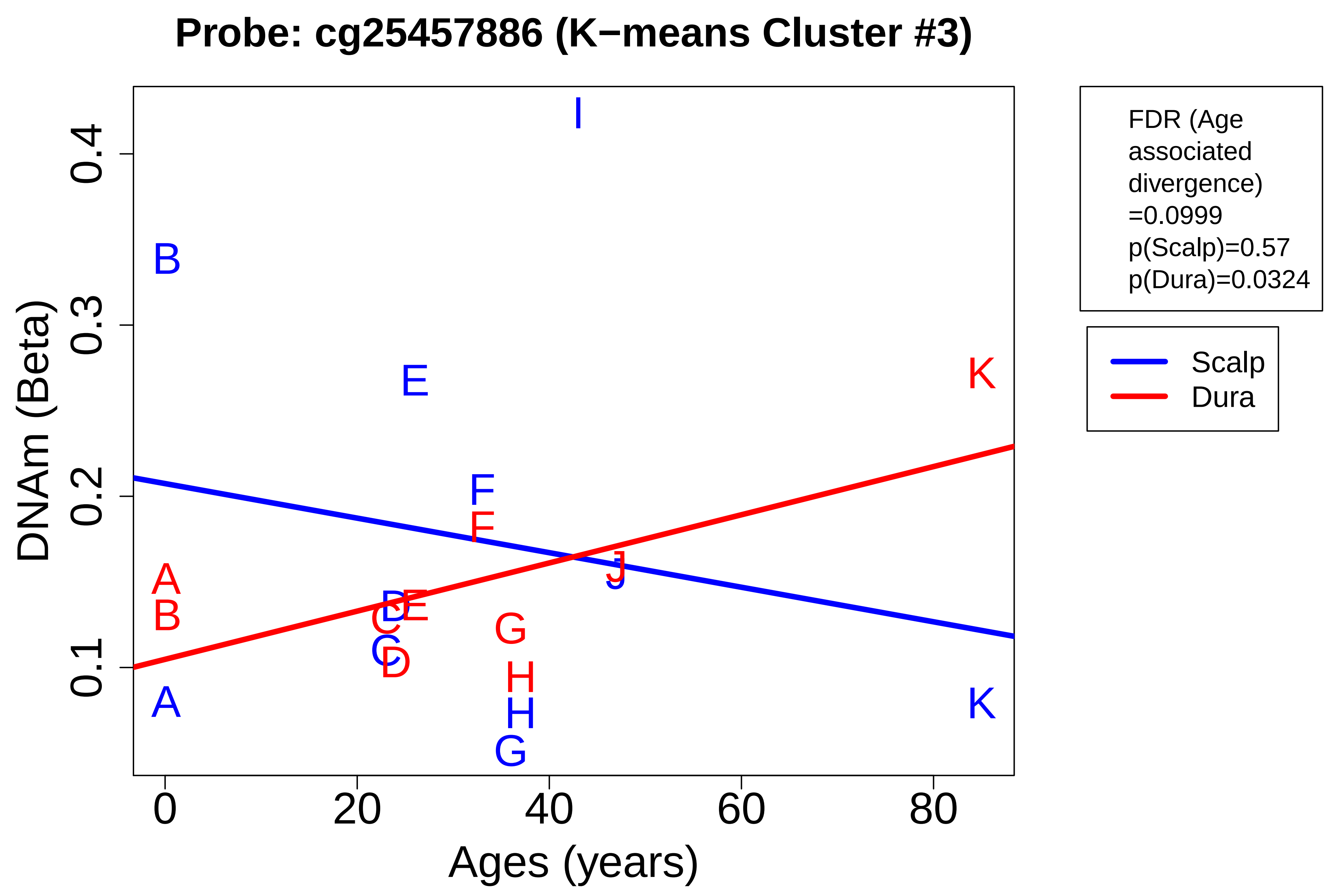
Probe: cg25457886 (K−means Cluster #3)
DNAm (Beta)
0.1
0.2
0.3
0.4
0
20
40
60
80
Ages (years)
FDR (Age associated divergence) =0.0999
p(Scalp)=0.57
p(Dura)=0.0324
Scalp
Dura
A
B
C
D
E
F
G
H
I
J
K

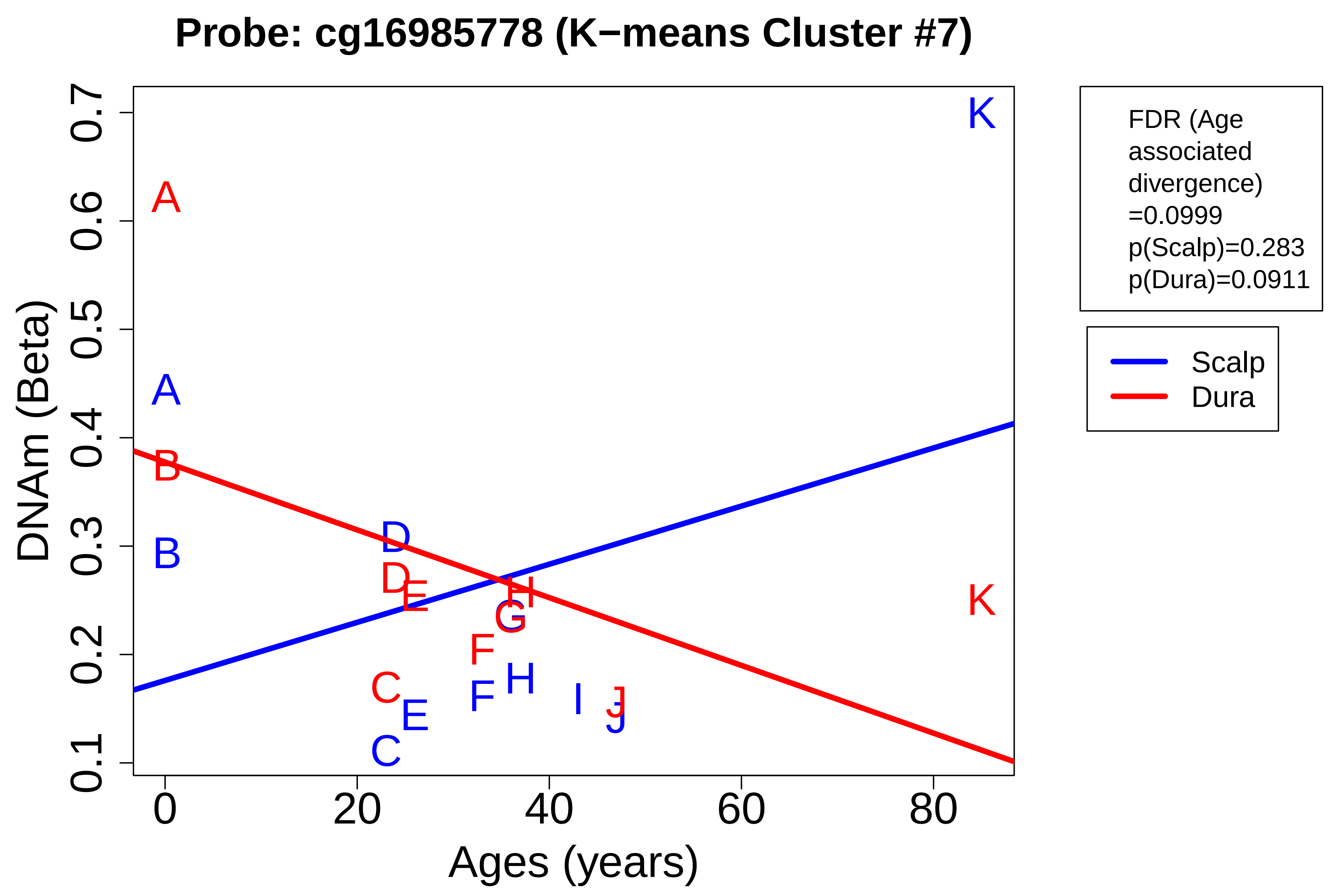

Probe: cg16985778 (K−means Cluster #7)
DNAm (Beta)
0.1
0.2
0.3
0.4
0.5
0.6
0.7
Ages (years)
0
20
40
60
80
A
A
B
B
C
C
D
D
E
E
F
F
G
H
H
I
J
J
K
K
FDR (Age associated divergence) =0.0999
p(Scalp)=0.283
p(Dura)=0.0911
Scalp
Dura

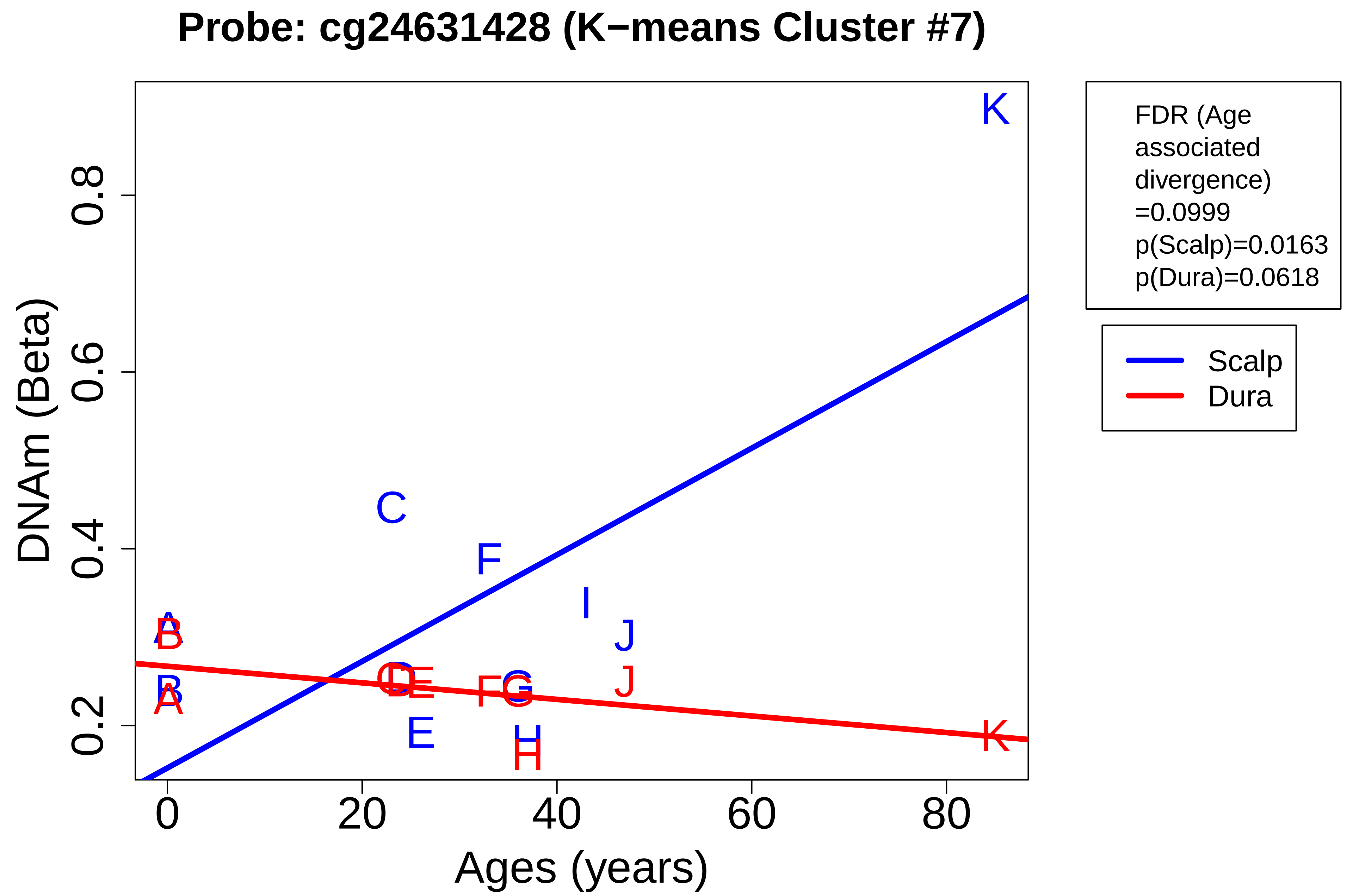

Probe: cg24631428 (K−means Cluster #7)
FDR (Age associated divergence) =0.0999
p(Scalp)=0.0163
p(Dura)=0.0618
Scalp
Dura
DNAm (Beta)
0.2
0.4
0.6
0.8
0
20
40
60
80
Ages (years)
A
B
C
D
E
F
G
H
I
J
K

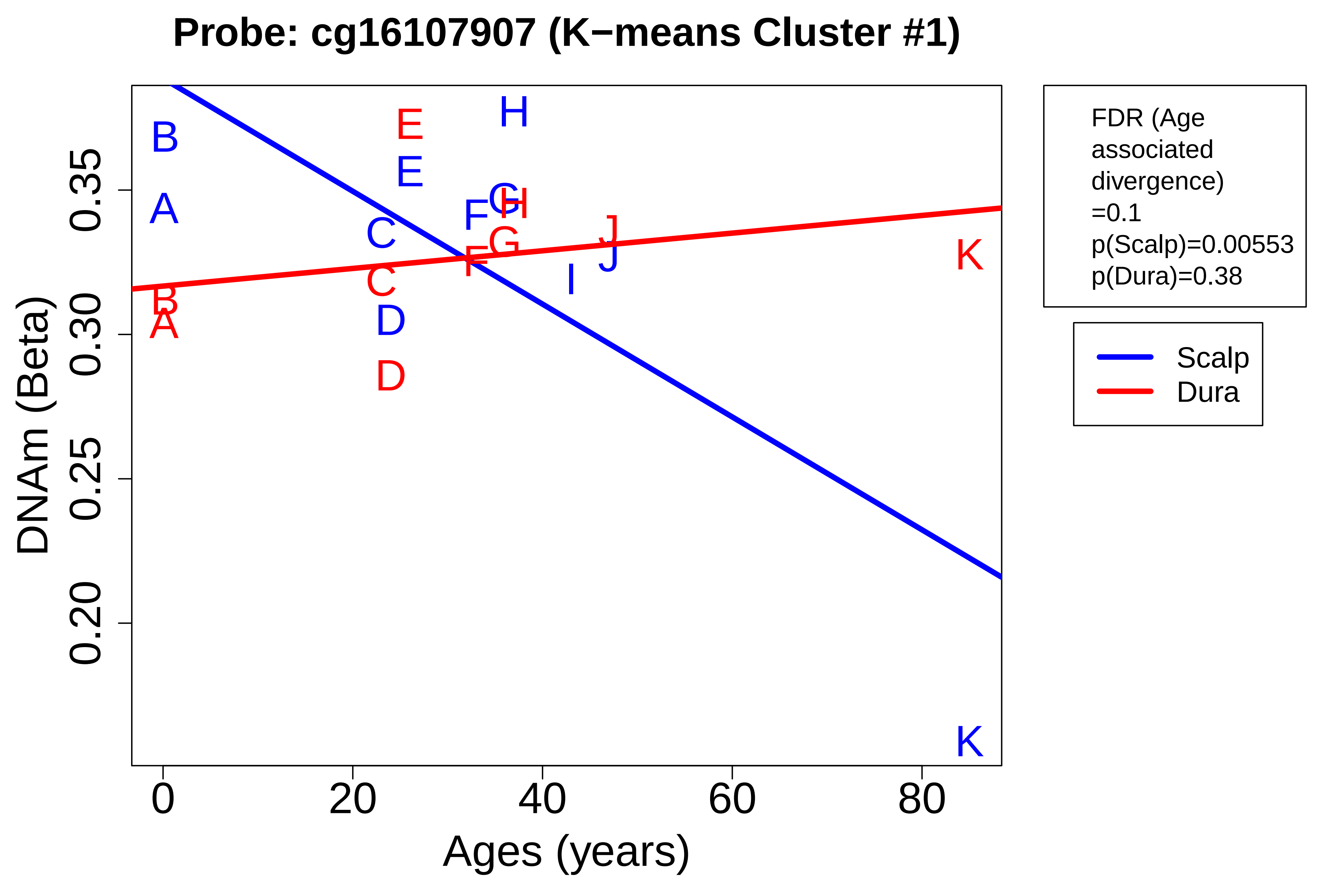

Probe: cg16107907 (K−means Cluster #1)
DNAm (Beta)
0.20
0.25
0.30
0.35
0
20
40
60
80
Ages (years)
FDR (Age associated divergence) =0.1
p(Scalp)=0.00553
p(Dura)=0.38
Scalp
Dura

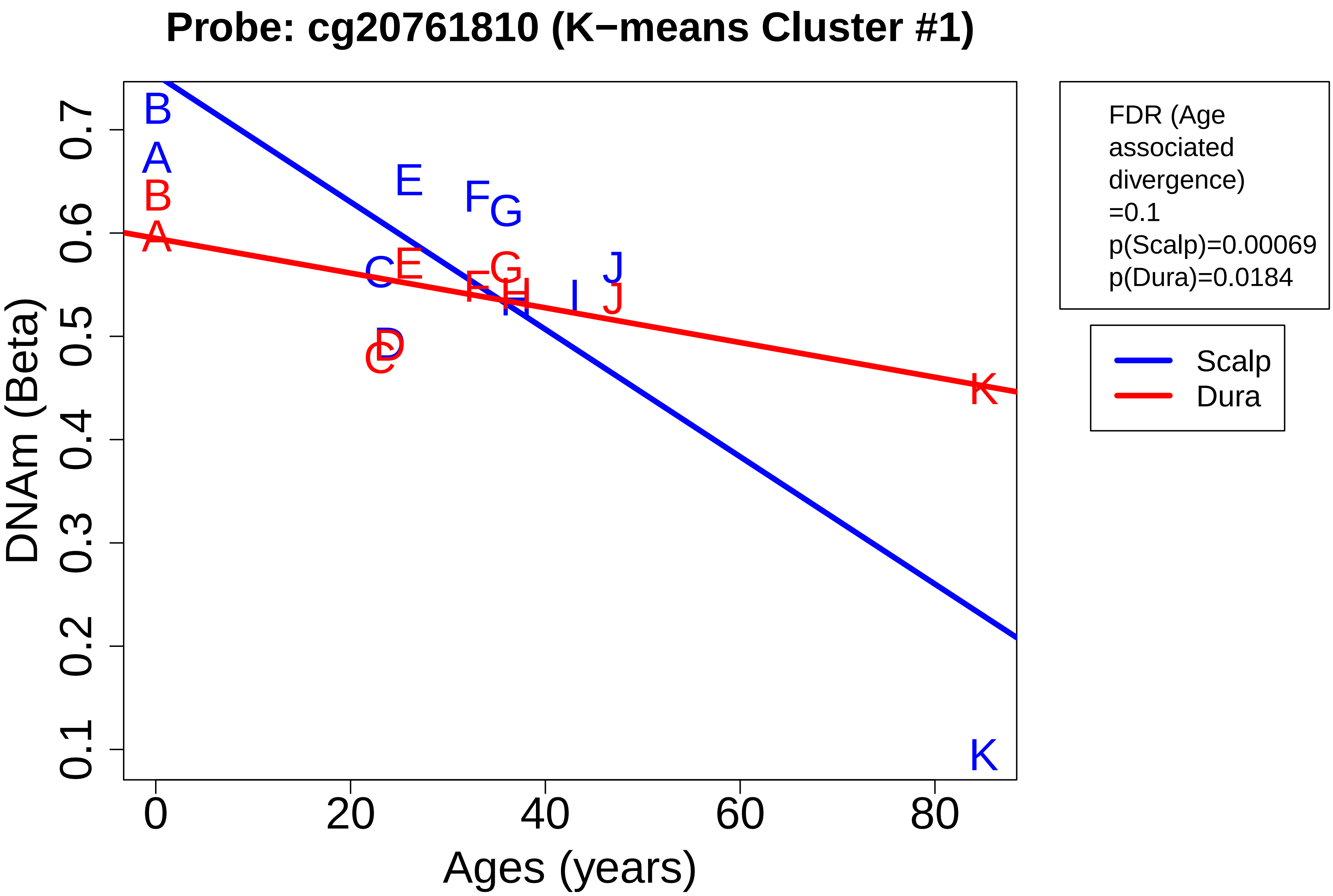

Probe: cg20761810 (K−means Cluster #1)
DNAm (Beta)
Ages (years)
0.1
0.2
0.3
0.4
0.5
0.6
0.7
0
20
40
60
80
FDR (Age associated divergence) =0.1
p(Scalp)=0.00069
p(Dura)=0.0184
Scalp
Dura

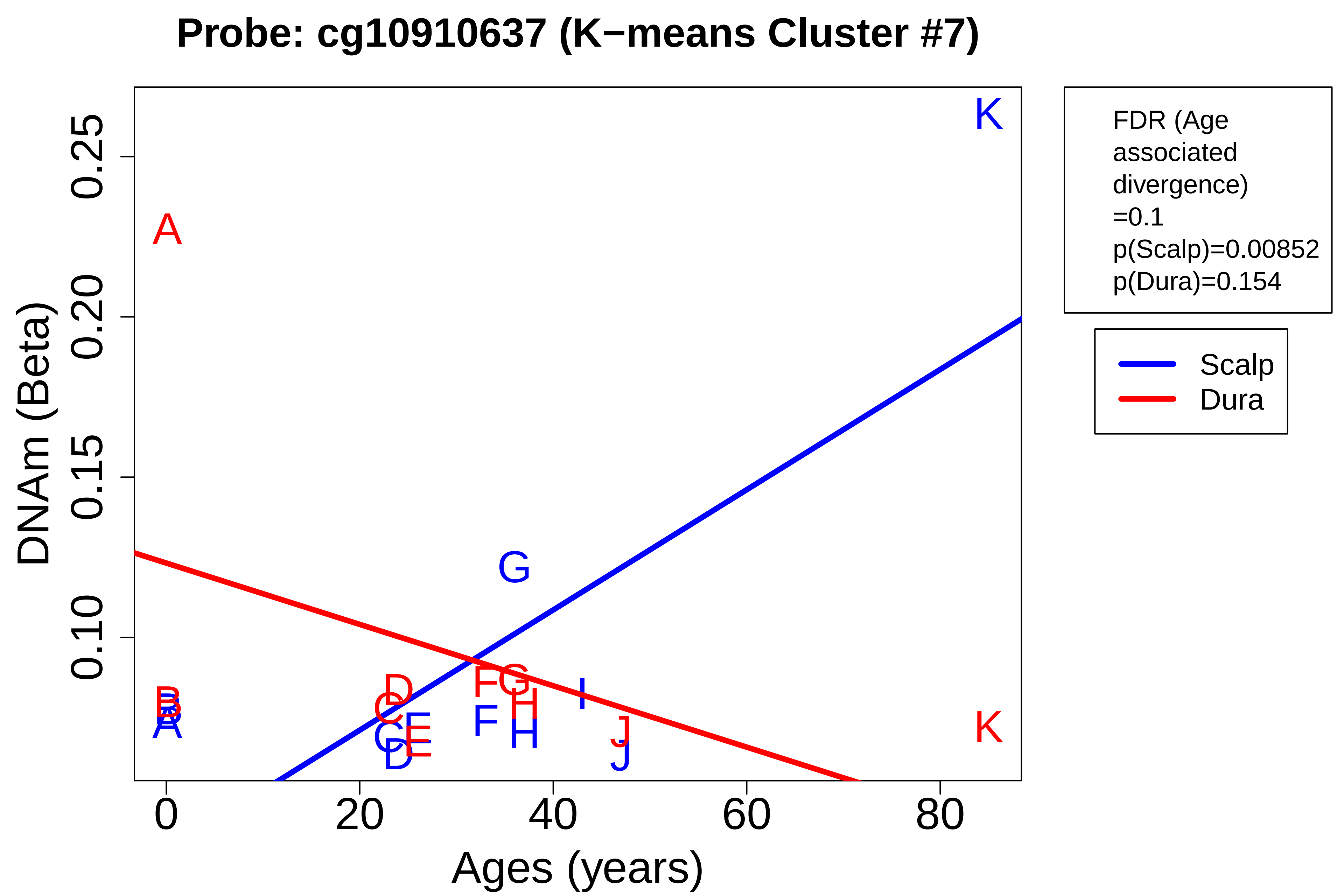

Probe: cg10910637 (K−means Cluster #7)
FDR (Age associated divergence) =0.1
p(Scalp)=0.00852
p(Dura)=0.154
Scalp
Dura
DNAm (Beta)
0.10
0.15
0.20
0.25
0
20
40
60
80
Ages (years)

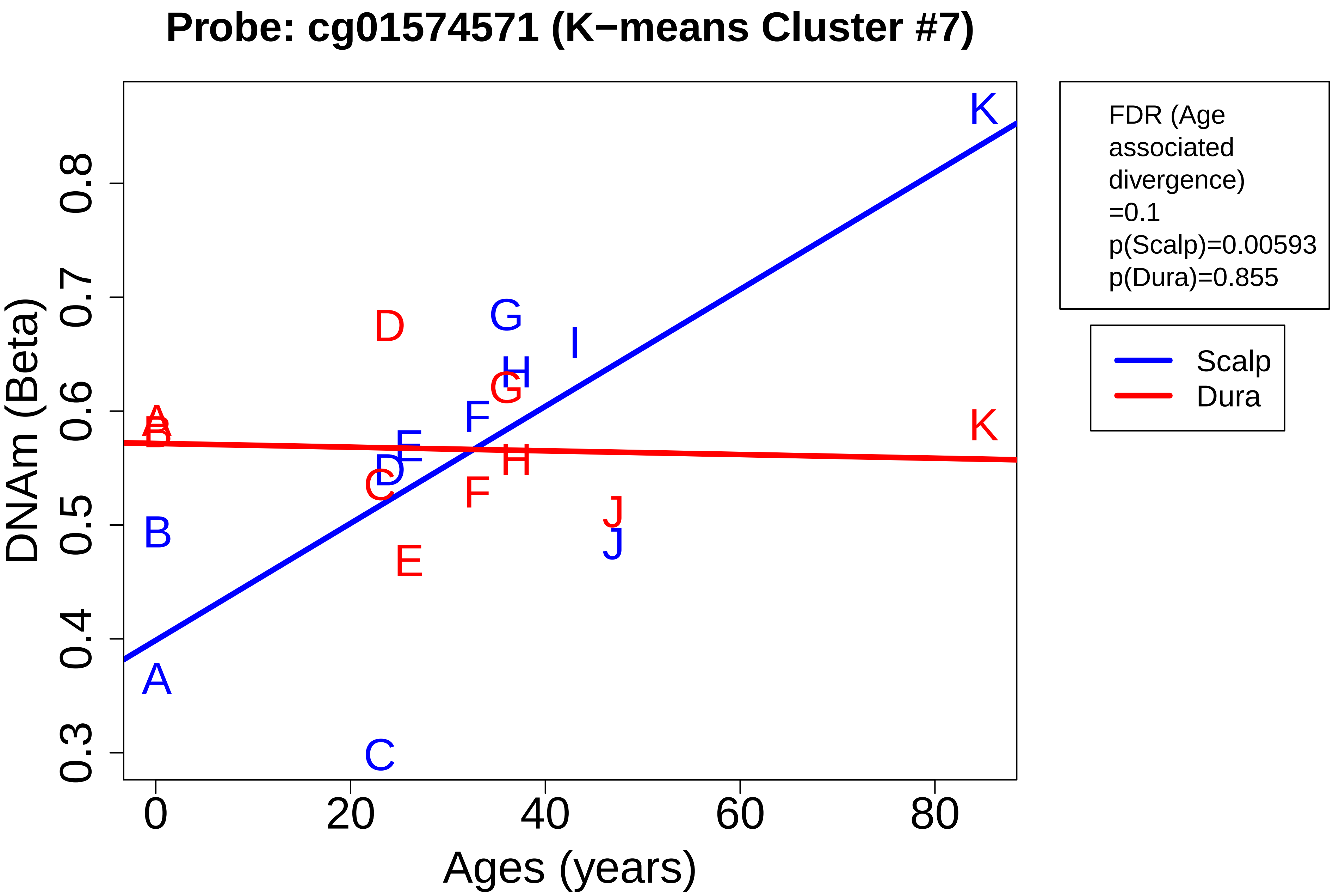

Probe: cg01574571 (K−means Cluster #7)
DNAm (Beta)
0.3
0.4
0.5
0.6
0.7
0.8
0
20
40
60
80
Ages (years)
FDR (Age associated divergence) =0.1
p(Scalp)=0.00593
p(Dura)=0.855
Scalp
Dura
